# Global burden of 87 risk factors in 204 countries and territories, 1990–2019: a systematic analysis for the Global Burden of Disease Study 2019

**DOI:** 10.1016/S0140-6736(20)30752-2

**Published:** 2020-10-17

**Authors:** GBD 2019 Risk Factors Collaborators, Christopher J L Murray, Aleksandr Y Aravkin, Peng Zheng, Cristiana Abbafati, Kaja M Abbas, Mohsen Abbasi-Kangevari, Foad Abd-Allah, Ahmed Abdelalim, Mohammad Abdollahi, Ibrahim Abdollahpour, Kedir Hussein Abegaz, Hassan Abolhassani, Victor Aboyans, Lucas Guimarães Abreu, Michael R M Abrigo, Ahmed Abualhasan, Laith Jamal Abu-Raddad, Abdelrahman I Abushouk, Maryam Adabi, Victor Adekanmbi, Abiodun Moshood Adeoye, Olatunji O Adetokunboh, Davoud Adham, Shailesh M Advani, Gina Agarwal, Seyed Mohammad Kazem Aghamir, Anurag Agrawal, Tauseef Ahmad, Keivan Ahmadi, Mehdi Ahmadi, Hamid Ahmadieh, Muktar Beshir Ahmed, Temesgen Yihunie Akalu, Rufus Olusola Akinyemi, Tomi Akinyemiju, Blessing Akombi, Chisom Joyqueenet Akunna, Fares Alahdab, Ziyad Al-Aly, Khurshid Alam, Samiah Alam, Tahiya Alam, Fahad Mashhour Alanezi, Turki M Alanzi, Biresaw wassihun Alemu, Khalid F Alhabib, Muhammad Ali, Saqib Ali, Gianfranco Alicandro, Cyrus Alinia, Vahid Alipour, Hesam Alizade, Syed Mohamed Aljunid, François Alla, Peter Allebeck, Amir Almasi-Hashiani, Hesham M Al-Mekhlafi, Jordi Alonso, Khalid A Altirkawi, Mostafa Amini-Rarani, Fatemeh Amiri, Dickson A Amugsi, Robert Ancuceanu, Deanna Anderlini, Jason A Anderson, Catalina Liliana Andrei, Tudorel Andrei, Colin Angus, Mina Anjomshoa, Fereshteh Ansari, Alireza Ansari-Moghaddam, Ippazio Cosimo Antonazzo, Carl Abelardo T Antonio, Catherine M Antony, Ernoiz Antriyandarti, Davood Anvari, Razique Anwer, Seth Christopher Yaw Appiah, Jalal Arabloo, Morteza Arab-Zozani, Filippo Ariani, Bahram Armoon, Johan Ärnlöv, Afsaneh Arzani, Mehran Asadi-Aliabadi, Ali A Asadi-Pooya, Charlie Ashbaugh, Michael Assmus, Zahra Atafar, Desta Debalkie Atnafu, Maha Moh'd Wahbi Atout, Floriane Ausloos, Marcel Ausloos, Beatriz Paulina Ayala Quintanilla, Getinet Ayano, Martin Amogre Ayanore, Samad Azari, Ghasem Azarian, Zelalem Nigussie Azene, Alaa Badawi, Ashish D Badiye, Mohammad Amin Bahrami, Mohammad Hossein Bakhshaei, Ahad Bakhtiari, Shankar M Bakkannavar, Alberto Baldasseroni, Kylie Ball, Shoshana H Ballew, Daniela Balzi, Maciej Banach, Srikanta K Banerjee, Agegnehu Bante Bante, Adhanom Gebreegziabher Baraki, Suzanne Lyn Barker-Collo, Till Winfried Bärnighausen, Lope H Barrero, Celine M Barthelemy, Lingkan Barua, Sanjay Basu, Bernhard T Baune, Mohsen Bayati, Jacob S Becker, Neeraj Bedi, Ettore Beghi, Yannick Béjot, Michellr L Bell, Fiona B Bennitt, Isabela M Bensenor, Kidanemaryam Berhe, Adam E Berman, Akshaya Srikanth Bhagavathula, Reshmi Bhageerathy, Neeraj Bhala, Dinesh Bhandari, Krittika Bhattacharyya, Zulfiqar A Bhutta, Ali Bijani, Boris Bikbov, Muhammad Shahdaat Bin Sayeed, Antonio Biondi, Binyam Minuye Birihane, Catherine Bisignano, Raaj Kishore Biswas, Helen Bitew, Somayeh Bohlouli, Mehdi Bohluli, Alexandra S Boon-Dooley, Guilherme Borges, Antonio Maria Borzì, Shiva Borzouei, Cristina Bosetti, Soufiane Boufous, Dejana Braithwaite, Nicholas J K Breitborde, Susanne Breitner, Hermann Brenner, Paul Svitil Briant, Andrey Nikolaevich Briko, Nikolay Ivanovich Briko, Gabrielle B Britton, Dana Bryazka, Blair R Bumgarner, Katrin Burkart, Richard Thomas Burnett, Sharath Burugina Nagaraja, Zahid A Butt, Florentino Luciano Caetano dos Santos, Leah E Cahill, Luis LA Alberto Cámera, Ismael R Campos-Nonato, Rosario Cárdenas, Giulia Carreras, Juan J Carrero, Felix Carvalho, Joao Mauricio Castaldelli-Maia, Carlos A Castañeda-Orjuela, Giulio Castelpietra, Franz Castro, Kate Causey, Christopher R Cederroth, Kelly M Cercy, Ester Cerin, Joht Singh Chandan, Kai-Lan Chang, Fiona J Charlson, Vijay Kumar Chattu, Sarika Chaturvedi, Nicolas Cherbuin, Odgerel Chimed-Ochir, Daniel Youngwhan Cho, Jee-Young Jasmine Choi, Hanne Christensen, Dinh-Toi Chu, Michael T Chung, Sheng-Chia Chung, Flavia M Cicuttini, Liliana G Ciobanu, Massimo Cirillo, Thomas Khaled Dwayne Classen, Aaron J Cohen, Kelly Compton, Owen R Cooper, Vera Marisa Costa, Ewerton Cousin, Richard G Cowden, Di H Cross, Jessica A Cruz, Saad M A Dahlawi, Albertino Antonio Moura Damasceno, Giovanni Damiani, Lalit Dandona, Rakhi Dandona, William James Dangel, Anna-Karin Danielsson, Paul I Dargan, Aso Mohammad Darwesh, Ahmad Daryani, Jai K Das, Rajat Das Gupta, José das Neves, Claudio Alberto Dávila-Cervantes, Dragos Virgil Davitoiu, Diego De Leo, Louisa Degenhardt, Marissa DeLang, Robert Paul Dellavalle, Feleke Mekonnen Demeke, Gebre Teklemariam Demoz, Desalegn Getnet Demsie, Edgar Denova-Gutiérrez, Nikolaos Dervenis, Govinda Prasad Dhungana, Mostafa Dianatinasab, Diana Dias da Silva, Daniel Diaz, Zahra Sadat Dibaji Forooshani, Shirin Djalalinia, Hoa Thi Do, Klara Dokova, Fariba Dorostkar, Leila Doshmangir, Tim Robert Driscoll, Bruce B Duncan, Andre Rodrigues Duraes, Arielle Wilder Eagan, David Edvardsson, Nevine El Nahas, Iman El Sayed, Maha El Tantawi, Iffat Elbarazi, Islam Y Elgendy, Shaimaa I El-Jaafary, Iqbal RF Elyazar, Sophia Emmons-Bell, Holly E Erskine, Sharareh Eskandarieh, Saman Esmaeilnejad, Alireza Esteghamati, Kara Estep, Arash Etemadi, Atkilt Esaiyas Etisso, Jessica Fanzo, Mohammad Farahmand, Mohammad Fareed, Roghiyeh Faridnia, Andrea Farioli, Andre Faro, Mithila Faruque, Farshad Farzadfar, Nazir Fattahi, Mehdi Fazlzadeh, Valery L Feigin, Rachel Feldman, Seyed-Mohammad Fereshtehnejad, Eduarda Fernandes, Giannina Ferrara, Alize J Ferrari, Manuela L Ferreira, Irina Filip, Florian Fischer, James L Fisher, Luisa Sorio Flor, Nataliya A Foigt, Morenike Oluwatoyin Folayan, Artem Alekseevich Fomenkov, Lisa M Force, Masoud Foroutan, Richard Charles Franklin, Marisa Freitas, Weijia Fu, Takeshi Fukumoto, João M Furtado, Mohamed M Gad, Emmanuela Gakidou, Silvano Gallus, Alberto L Garcia-Basteiro, William M Gardner, Biniyam Sahiledengle Geberemariyam, Assefa Ayalew Ayalew Ayalew Gebreslassie, Abraham Geremew, Anna Gershberg Hayoon, Peter W Gething, Maryam Ghadimi, Keyghobad Ghadiri, Fatemeh Ghaffarifar, Mansour Ghafourifard, Farhad Ghamari, Ahmad Ghashghaee, Hesam Ghiasvand, Nermin Ghith, Asadollah Gholamian, Rakesh Ghosh, Paramjit Singh Gill, Themba G G Ginindza, Giorgia Giussani, Elena V Gnedovskaya, Salime Goharinezhad, Sameer Vali Gopalani, Giuseppe Gorini, Houman Goudarzi, Alessandra C Goulart, Felix Greaves, Michal Grivna, Giuseppe Grosso, Mohammed Ibrahim Mohialdeen Gubari, Harish Chander Gugnani, Rafael Alves Guimarães, Rashid Abdi Guled, Gaorui Guo, Yuming Guo, Rajeev Gupta, Tarun Gupta, Beatrix Haddock, Nima Hafezi-Nejad, Abdul Hafiz, Arvin Haj-Mirzaian, Arya Haj-Mirzaian, Brian J Hall, Iman Halvaei, Randah R Hamadeh, Samer Hamidi, Melanie S Hammer, Graeme J Hankey, Hamidreza Haririan, Josep Maria Haro, Ahmed I Hasaballah, Md Mehedi Hasan, Edris Hasanpoor, Abdiwahab Hashi, Soheil Hassanipour, Hadi Hassankhani, Rasmus J Havmoeller, Simon I Hay, Khezar Hayat, Golnaz Heidari, Reza Heidari-Soureshjani, Hannah J Henrikson, Molly E Herbert, Claudiu Herteliu, Fatemeh Heydarpour, Thomas R Hird, Hans W Hoek, Ramesh Holla, Praveen Hoogar, H Dean Hosgood, Naznin Hossain, Mostafa Hosseini, Mehdi Hosseinzadeh, Mihaela Hostiuc, Sorin Hostiuc, Mowafa Househ, Mohamed Hsairi, Vivian Chia-rong Hsieh, Guoqing Hu, Kejia Hu, Tanvir M Huda, Ayesha Humayun, Chantal K Huynh, Bing-Fang Hwang, Vincent C Iannucci, Segun Emmanuel Ibitoye, Nayu Ikeda, Kevin S Ikuta, Olayinka Stephen Ilesanmi, Irena M Ilic, Milena D Ilic, Leeberk Raja Inbaraj, Helen Ippolito, Usman Iqbal, Seyed Sina Naghibi Irvani, Caleb Mackay Salpeter Irvine, M Mofizul Islam, Sheikh Mohammed Shariful Islam, Hiroyasu Iso, Rebecca Q Ivers, Chidozie C D Iwu, Chinwe Juliana Iwu, Ihoghosa Osamuyi Iyamu, Jalil Jaafari, Kathryn H Jacobsen, Hussain Jafari, Morteza Jafarinia, Mohammad Ali Jahani, Mihajlo Jakovljevic, Farzad Jalilian, Spencer L James, Hosna Janjani, Tahereh Javaheri, Javad Javidnia, Panniyammakal Jeemon, Ensiyeh Jenabi, Ravi Prakash Jha, Vivekanand Jha, John S Ji, Lars Johansson, Oommen John, Yetunde O John-Akinola, Catherine Owens Johnson, Jost B Jonas, Farahnaz Joukar, Jacek Jerzy Jozwiak, Mikk Jürisson, Ali Kabir, Zubair Kabir, Hamed Kalani, Rizwan Kalani, Leila R Kalankesh, Rohollah Kalhor, Tanuj Kanchan, Neeti Kapoor, Behzad Karami Matin, André Karch, Mohd Anisul Karim, Getachew Mullu Kassa, Srinivasa Vittal Katikireddi, Gbenga A Kayode, Ali Kazemi Karyani, Peter Njenga Keiyoro, Cathleen Keller, Laura Kemmer, Parkes J Kendrick, Nauman Khalid, Mohammad Khammarnia, Ejaz Ahmad Khan, Maseer Khan, Khaled Khatab, Mona M Khater, Mahalaqua Nazli Khatib, Maryam Khayamzadeh, Salman Khazaei, Christian Kieling, Yun Jin Kim, Ruth W Kimokoti, Adnan Kisa, Sezer Kisa, Mika Kivimäki, Luke D Knibbs, Ann Kristin Skrindo Knudsen, Jonathan M Kocarnik, Sonali Kochhar, Jacek A Kopec, Vladimir Andreevich Korshunov, Parvaiz A Koul, Ai Koyanagi, Moritz U G Kraemer, Kewal Krishan, Kris J Krohn, Hans Kromhout, Barthelemy Kuate Defo, G Anil Kumar, Vivek Kumar, Om P Kurmi, Dian Kusuma, Carlo La Vecchia, Ben Lacey, Dharmesh Kumar Lal, Ratilal Lalloo, Tea Lallukka, Faris Hasan Lami, Iván Landires, Justin J Lang, Sinéad M Langan, Anders O Larsson, Savita Lasrado, Paolo Lauriola, Jeffrey V Lazarus, Paul H Lee, Shaun Wen Huey Lee, Kate E LeGrand, James Leigh, Matilde Leonardi, Haley Lescinsky, Janni Leung, Miriam Levi, Shanshan Li, Lee-Ling Lim, Shai Linn, Shiwei Liu, Simin Liu, Yang Liu, Justin Lo, Alan D Lopez, Jaifred Christian F Lopez, Platon D Lopukhov, Stefan Lorkowski, Paulo A Lotufo, Alton Lu, Alessandra Lugo, Emilie R Maddison, Phetole Walter Mahasha, Mokhtar Mahdavi Mahdavi, Morteza Mahmoudi, Azeem Majeed, Afshin Maleki, Shokofeh Maleki, Reza Malekzadeh, Deborah Carvalho Malta, Abdullah A Mamun, Ana Laura Manda, Helena Manguerra, Fariborz Mansour-Ghanaei, Borhan Mansouri, Mohammad Ali Mansournia, Ana M Mantilla Herrera, Joemer C Maravilla, Ashley Marks, Randall V Martin, Santi Martini, Francisco Rogerlândio Martins-Melo, Anthony Masaka, Seyedeh Zahra Masoumi, Manu Raj Mathur, Kunihiro Matsushita, Pallab K Maulik, Colm McAlinden, John J McGrath, Martin McKee, Man Mohan Mehndiratta, Fereshteh Mehri, Kala M Mehta, Ziad A Memish, Walter Mendoza, Ritesh G Menezes, Endalkachew Worku Mengesha, Alibek Mereke, Seid Tiku Mereta, Atte Meretoja, Tuomo J Meretoja, Tomislav Mestrovic, Bartosz Miazgowski, Tomasz Miazgowski, Irmina Maria Michalek, Ted R Miller, Edward J Mills, GK Mini, Mohammad Miri, Andreea Mirica, Erkin M Mirrakhimov, Hamed Mirzaei, Maryam Mirzaei, Roya Mirzaei, Mehdi Mirzaei-Alavijeh, Awoke Temesgen Misganaw, Prasanna Mithra, Babak Moazen, Dara K Mohammad, Yousef Mohammad, Naser Mohammad Gholi Mezerji, Abdollah Mohammadian-Hafshejani, Noushin Mohammadifard, Reza Mohammadpourhodki, Ammas Siraj Mohammed, Hussen Mohammed, Jemal Abdu Mohammed, Shafiu Mohammed, Ali H Mokdad, Mariam Molokhia, Lorenzo Monasta, Meghan D Mooney, Ghobad Moradi, Masoud Moradi, Maziar Moradi-Lakeh, Rahmatollah Moradzadeh, Paula Moraga, Lidia Morawska, Joana Morgado-da-Costa, Shane Douglas Morrison, Abbas Mosapour, Jonathan F Mosser, Simin Mouodi, Seyyed Meysam Mousavi, Amin Mousavi Khaneghah, Ulrich Otto Mueller, Satinath Mukhopadhyay, Erin C Mullany, Kamarul Imran Musa, Saravanan Muthupandian, Ashraf F Nabhan, Mehdi Naderi, Ahamarshan Jayaraman Nagarajan, Gabriele Nagel, Mohsen Naghavi, Behshad Naghshtabrizi, Mukhammad David Naimzada, Farid Najafi, Vinay Nangia, Jobert Richie Nansseu, Morteza Naserbakht, Vinod C Nayak, Ionut Negoi, Josephine W Ngunjiri, Cuong Tat Nguyen, Huong Lan Thi Nguyen, Minh Nguyen, Yeshambel T Nigatu, Rajan Nikbakhsh, Molly R Nixon, Chukwudi A Nnaji, Shuhei Nomura, Bo Norrving, Jean Jacques Noubiap, Christoph Nowak, Virginia Nunez-Samudio, Adrian Oţoiu, Bogdan Oancea, Christopher M Odell, Felix Akpojene Ogbo, In-Hwan Oh, Emmanuel Wandera Okunga, Morteza Oladnabi, Andrew T Olagunju, Bolajoko Olubukunola Olusanya, Jacob Olusegun Olusanya, Muktar Omer Omer, Kanyin L Ong, Obinna E Onwujekwe, Heather M Orpana, Alberto Ortiz, Osayomwanbo Osarenotor, Frank B Osei, Samuel M Ostroff, Nikita Otstavnov, Stanislav S Otstavnov, Simon Øverland, Mayowa O Owolabi, Mahesh P A, Jagadish Rao Padubidri, Raffaele Palladino, Songhomitra Panda-Jonas, Anamika Pandey, Charles D H Parry, Maja Pasovic, Deepak Kumar Pasupula, Sangram Kishor Patel, Mona Pathak, Scott B Patten, George C Patton, Hamidreza Pazoki Toroudi, Amy E Peden, Alyssa Pennini, Veincent Christian Filipino Pepito, Emmanuel K Peprah, David M Pereira, Konrad Pesudovs, Hai Quang Pham, Michael R Phillips, Cristiano Piccinelli, Tessa M Pilz, Michael A Piradov, Meghdad Pirsaheb, Dietrich Plass, Suzanne Polinder, Kevan R Polkinghorne, Constance Dimity Pond, Maarten J Postma, Hadi Pourjafar, Farshad Pourmalek, Anna Poznańska, Sergio I Prada, V Prakash, Dimas Ria Angga Pribadi, Elisabetta Pupillo, Zahiruddin Quazi Syed, Mohammad Rabiee, Navid Rabiee, Amir Radfar, Ata Rafiee, Alberto Raggi, Muhammad Aziz Rahman, Ali Rajabpour-Sanati, Fatemeh Rajati, Ivo Rakovac, Pradhum Ram, Kiana Ramezanzadeh, Chhabi Lal Ranabhat, Puja C Rao, Sowmya J Rao, Vahid Rashedi, Priya Rathi, David Laith Rawaf, Salman Rawaf, Lal Rawal, Reza Rawassizadeh, Ramu Rawat, Christian Razo, Sofia Boston Redford, Robert C Reiner, Marissa Bettay Reitsma, Giuseppe Remuzzi, Vishnu Renjith, Andre M N Renzaho, Serge Resnikoff, Negar Rezaei, Nima Rezaei, Aziz Rezapour, Phoebe-Anne Rhinehart, Seyed Mohammad Riahi, Daniel Cury Ribeiro, Daniela Ribeiro, Jennifer Rickard, Juan A Rivera, Nicholas L S Roberts, Sonia Rodríguez-Ramírez, Leonardo Roever, Luca Ronfani, Robin Room, Gholamreza Roshandel, Gregory A Roth, Dietrich Rothenbacher, Enrico Rubagotti, Godfrey M Rwegerera, Siamak Sabour, Perminder S Sachdev, Basema Saddik, Ehsan Sadeghi, Masoumeh Sadeghi, Reza Saeedi, Sahar Saeedi Moghaddam, Yahya Safari, Sare Safi, Saeid Safiri, Rajesh Sagar, Amirhossein Sahebkar, S Mohammad Sajadi, Nasir Salam, Payman Salamati, Hosni Salem, Marwa R Rashad Salem, Hamideh Salimzadeh, Omar Mukhtar Salman, Joshua A Salomon, Zainab Samad, Hossein Samadi Kafil, Evanson Zondani Sambala, Abdallah M Samy, Juan Sanabria, Tania G Sánchez-Pimienta, Damian Francesco Santomauro, Itamar S Santos, João Vasco Santos, Milena M Santric-Milicevic, Sivan Yegnanarayana Iyer Saraswathy, Rodrigo Sarmiento-Suárez, Nizal Sarrafzadegan, Benn Sartorius, Arash Sarveazad, Brijesh Sathian, Thirunavukkarasu Sathish, Davide Sattin, Sonia Saxena, Lauren E Schaeffer, Silvia Schiavolin, Markus P Schlaich, Maria Inês Schmidt, Aletta Elisabeth Schutte, David C Schwebel, Falk Schwendicke, Anbissa Muleta Senbeta, Subramanian Senthilkumaran, Sadaf G Sepanlou, Berrin Serdar, Marc L Serre, Jamileh Shadid, Omid Shafaat, Saeed Shahabi, Amira A Shaheen, Masood Ali Shaikh, Ali S Shalash, Mehran Shams-Beyranvand, Morteza Shamsizadeh, Kiomars Sharafi, Aziz Sheikh, Abbas Sheikhtaheri, Kenji Shibuya, Kevin David Shield, Mika Shigematsu, Jae Il Shin, Min-Jeong Shin, Rahman Shiri, Reza Shirkoohi, Kerem Shuval, Soraya Siabani, Radoslaw Sierpinski, Inga Dora Sigfusdottir, Rannveig Sigurvinsdottir, João Pedro Silva, Kyle E Simpson, Jasvinder A Singh, Pushpendra Singh, Eirini Skiadaresi, Søren T Skou Skou, Valentin Yurievich Skryabin, Emma U R Smith, Amin Soheili, Shahin Soltani, Moslem Soofi, Reed J D Sorensen, Joan B Soriano, Muluken Bekele Sorrie, Sergey Soshnikov, Ireneous N Soyiri, Cory N Spencer, Adel Spotin, Chandrashekhar T Sreeramareddy, Vinay Srinivasan, Jeffrey D Stanaway, Caroline Stein, Dan J Stein, Caitlyn Steiner, Leo Stockfelt, Mark A Stokes, Kurt Straif, Jacob L Stubbs, Mu'awiyyah Babale Sufiyan, Hafiz Ansar Rasul Suleria, Rizwan Suliankatchi Abdulkader, Gerhard Sulo, Iyad Sultan, Łukasz Szumowski, Rafael Tabarés-Seisdedos, Karen M Tabb, Takahiro Tabuchi, Amir Taherkhani, Masih Tajdini, Ken Takahashi, Jukka S Takala, Animut Tagele Tamiru, Nuno Taveira, Arash Tehrani-Banihashemi, Mohamad-Hani Temsah, Getayeneh Antehunegn Tesema, Zemenu Tadesse Tessema, George D Thurston, Mariya Vladimirovna Titova, Hamid Reza Tohidinik, Marcello Tonelli, Roman Topor-Madry, Fotis Topouzis, Anna E Torre, Mathilde Touvier, Marcos Roberto Roberto Tovani-Palone, Bach Xuan Tran, Ravensara Travillian, Aristidis Tsatsakis, Lorainne Tudor Car, Stefanos Tyrovolas, Riaz Uddin, Chukwuma David Umeokonkwo, Bhaskaran Unnikrishnan, Era Upadhyay, Marco Vacante, Pascual R Valdez, Aaron van Donkelaar, Tommi Juhani Vasankari, Yasser Vasseghian, Yousef Veisani, Narayanaswamy Venketasubramanian, Francesco S Violante, Vasily Vlassov, Stein Emil Vollset, Theo Vos, Rade Vukovic, Yasir Waheed, Mitchell Taylor Wallin, Yafeng Wang, Yuan-Pang Wang, Alexandrea Watson, Jingkai Wei, Melissa Y Wei Wei, Robert G Weintraub, Jordan Weiss, Andrea Werdecker, J Jason West, Ronny Westerman, Joanna L Whisnant, Harvey A Whiteford, Kirsten E Wiens, Charles D A Wolfe, Sarah S Wozniak, Ai-Min Wu, Junjie Wu, Sarah Wulf Hanson, Gelin Xu, Rixing Xu, Simon Yadgir, Seyed Hossein Yahyazadeh Jabbari, Kazumasa Yamagishi, Mousa Yaminfirooz, Yuichiro Yano, Sanni Yaya, Vahid Yazdi-Feyzabadi, Tomas Y Yeheyis, Christopher Sabo Yilgwan, Mekdes Tigistu Yilma, Paul Yip, Naohiro Yonemoto, Mustafa Z Younis, Theodore Patrick Younker, Bahman Yousefi, Zabihollah Yousefi, Taraneh Yousefinezhadi, Abdilahi Yousuf Yousuf, Chuanhua Yu, Hasan Yusefzadeh, Telma Zahirian Moghadam, Mohammad Zamani, Maryam Zamanian, Hamed Zandian, Mikhail Sergeevich Zastrozhin, Yunquan Zhang, Zhi-Jiang Zhang, Jeff T Zhao, Xiu-Ju George Zhao, Yingxi Zhao, Maigeng Zhou, Arash Ziapour, Stephanie R M Zimsen, Michael Brauer, Ashkan Afshin, Stephen S Lim

## Abstract

**Background:**

Rigorous analysis of levels and trends in exposure to leading risk factors and quantification of their effect on human health are important to identify where public health is making progress and in which cases current efforts are inadequate. The Global Burden of Diseases, Injuries, and Risk Factors Study (GBD) 2019 provides a standardised and comprehensive assessment of the magnitude of risk factor exposure, relative risk, and attributable burden of disease.

**Methods:**

GBD 2019 estimated attributable mortality, years of life lost (YLLs), years of life lived with disability (YLDs), and disability-adjusted life-years (DALYs) for 87 risk factors and combinations of risk factors, at the global level, regionally, and for 204 countries and territories. GBD uses a hierarchical list of risk factors so that specific risk factors (eg, sodium intake), and related aggregates (eg, diet quality), are both evaluated. This method has six analytical steps. (1) We included 560 risk–outcome pairs that met criteria for convincing or probable evidence on the basis of research studies. 12 risk–outcome pairs included in GBD 2017 no longer met inclusion criteria and 47 risk–outcome pairs for risks already included in GBD 2017 were added based on new evidence. (2) Relative risks were estimated as a function of exposure based on published systematic reviews, 81 systematic reviews done for GBD 2019, and meta-regression. (3) Levels of exposure in each age-sex-location-year included in the study were estimated based on all available data sources using spatiotemporal Gaussian process regression, DisMod-MR 2.1, a Bayesian meta-regression method, or alternative methods. (4) We determined, from published trials or cohort studies, the level of exposure associated with minimum risk, called the theoretical minimum risk exposure level. (5) Attributable deaths, YLLs, YLDs, and DALYs were computed by multiplying population attributable fractions (PAFs) by the relevant outcome quantity for each age-sex-location-year. (6) PAFs and attributable burden for combinations of risk factors were estimated taking into account mediation of different risk factors through other risk factors. Across all six analytical steps, 30 652 distinct data sources were used in the analysis. Uncertainty in each step of the analysis was propagated into the final estimates of attributable burden. Exposure levels for dichotomous, polytomous, and continuous risk factors were summarised with use of the summary exposure value to facilitate comparisons over time, across location, and across risks. Because the entire time series from 1990 to 2019 has been re-estimated with use of consistent data and methods, these results supersede previously published GBD estimates of attributable burden.

**Findings:**

The largest declines in risk exposure from 2010 to 2019 were among a set of risks that are strongly linked to social and economic development, including household air pollution; unsafe water, sanitation, and handwashing; and child growth failure. Global declines also occurred for tobacco smoking and lead exposure. The largest increases in risk exposure were for ambient particulate matter pollution, drug use, high fasting plasma glucose, and high body-mass index. In 2019, the leading Level 2 risk factor globally for attributable deaths was high systolic blood pressure, which accounted for 10·8 million (95% uncertainty interval [UI] 9·51–12·1) deaths (19·2% [16·9–21·3] of all deaths in 2019), followed by tobacco (smoked, second-hand, and chewing), which accounted for 8·71 million (8·12–9·31) deaths (15·4% [14·6–16·2] of all deaths in 2019). The leading Level 2 risk factor for attributable DALYs globally in 2019 was child and maternal malnutrition, which largely affects health in the youngest age groups and accounted for 295 million (253–350) DALYs (11·6% [10·3–13·1] of all global DALYs that year). The risk factor burden varied considerably in 2019 between age groups and locations. Among children aged 0–9 years, the three leading detailed risk factors for attributable DALYs were all related to malnutrition. Iron deficiency was the leading risk factor for those aged 10–24 years, alcohol use for those aged 25–49 years, and high systolic blood pressure for those aged 50–74 years and 75 years and older.

**Interpretation:**

Overall, the record for reducing exposure to harmful risks over the past three decades is poor. Success with reducing smoking and lead exposure through regulatory policy might point the way for a stronger role for public policy on other risks in addition to continued efforts to provide information on risk factor harm to the general public.

**Funding:**

Bill & Melinda Gates Foundation.

**Research in context**

**Evidence before this study**

The Global Burden of Diseases, Injuries, and Risk Factors Study (GBD) 2017 provided the most recent assessment of deaths, years of life lost because of premature mortality, years of life lived with disability, and disability-adjusted life-years attributable to metabolic, environmental and occupational, and behavioural risk factors. GBD 2017 provided estimates from 1990 to 2017 for 195 countries and territories. Many reports explore the burden of disease that can be attributed to a specific risk factor in a specific country or territory, region, or globally, but none attempts to assess an extensive list of risk factors in all countries and regions.

**Added value of this study**

GBD 2019 advances the technical quantification of attributable burden in 12 ways. (1) In support of the agreement between GBD and WHO, nine new countries have been added to the analysis: Cook Islands, Monaco, San Marino, Nauru, Niue, Palau, Saint Kitts and Nevis, Tokelau, and Tuvalu. (2) Subnational assessments for Italy, Nigeria, Pakistan, the Philippines, and Poland have been added to GBD 2019. (3) High and low non-optimal temperatures have been added as risk factors (54 new risk–outcome pairs). (4) For 81 risk–outcome pairs, new systematic reviews have been done as part of GBD 2019. (5) For 139 risk–outcome pairs, dose–response meta-regressions have been done to evaluate if the relationship between exposure and relative risk might not be adequately captured by assuming a log-linear relationship between risk and per unit increase in exposure. (6) On the basis of the systematic reviews and dose–response meta-regression, 12 risk–outcome pairs have been excluded from GBD 2019 because they no longer met inclusion criteria. (7) On the basis of the systematic reviews and meta-regressions, 47 new risk–outcome pairs have been included for risks that were previously included. This includes outcomes linked to low birthweight and short gestational age as intermediate outcomes linked to particulate matter with a diameter smaller than 2·5 μm (PM_2·5_), which has increased the burden attributable to PM_2·5_. (8) New cohorts, trials, and case-control studies have been added for the assessment of risk functions. (9) New sources have been added to the analysis of risk factor exposure by age, sex, and location. (10) Corrections for non-reference method exposure measurements have been revised using network or related meta-regression. (11) For dietary risks, the theoretical minimum risk exposure level (TMREL) has been revised based on the new systematic reviews. (12) The distribution of alcohol use across individuals has been revised to better capture the asymmetric nature of the distribution. In addition to the technical improvements in each step of the quantification of risk factor exposure, relative risk, TMREL, and attributable burden, in this study we have focused attention on the broad trends in risk exposure by computing summary exposure values for aggregations of risk factors. Isolating the long-term global and national trends in risk exposure reveals in which cases the world has been successful in reducing exposure to harmful risks.

**Implications of all the available evidence**

Improved analysis of risk exposure and burden attributable to risk factors at the national, regional, and global level can help to focus attention on risks for which exposure is increasing and in which locations. This quantification is an essential input into public health prioritisation and evaluation of programme success.

## Introduction

The mechanism for much of disease and injury prevention is through modifying environmental, occupational, behavioural, and metabolic risk factors. Other pathways, such as vaccination or addressing social determinants of health, are crucially important, but a substantial component of public health has targeted modifying the aforementioned risk factors. Whether the risk factor is targeted through public policy such as taxation or regulation, through programmes such as water supply improvement, or primary care advice and pharmacological intervention, it is essential to track progress on risk exposure. Which risk factors are declining, stagnating, or even increasing gives insights into where current efforts are working or are insufficient. Understanding where the promise of prevention is being realised might generate lessons that can be applied to other risks in which progress is slow. Tracking the burden attributable to risk exposure, measured by deaths, years of life lost (YLLs), years lived with disability (YLDs), or disability-adjusted life-years (DALYs), can also help governments, donor agencies, international organisations, and civil society organisations to identify new priorities.^1^, ^2^, ^3^

To help track risk exposures and the burden attributable to these exposures, many studies are published each year on the burden of specific risks, often in specific countries or regions.^4^, ^5^, ^6^, ^7^ To our knowledge, the only effort to examine attributable burden with standardised methods across a wide set of risk factors spanning all countries is the Global Burden of Diseases, Injuries, and Risk Factors Study (GBD).^8^, ^9^, ^10^, ^11^, ^12^ Many choices go into the comparable quantification of the burden of risk factors; GBD provides a rules-based approach to evidence synthesis that follows the Guidelines on Accurate and Transparent Health Estimates Reporting.^13^ Comparable quantification across risks over time and across populations facilitates identifying relative importance and how population health risks are evolving over time. GBD also provides a framework to understand both the trends in risk exposure and the trends in burden attributable to risks. Quantifying and reporting both exposure and attributable burden is important because exposure might be increasing and attributable burden decreasing if other drivers of the underlying outcomes are declining at a fast enough rate.

In this study, we present new or updated results for the quantification of 560 risk–outcome pairs including updated data for exposure, updated data for relative risks, methods innovation in evaluating risk-exposure functions, and the addition of two new risk factors—high and low non-optimal temperatures. In addition to providing quantification of exposure and attributable burden in 204 locations over the period 1990–2019, we used summary exposure values (SEVs) for aggregates of risk factors to understand where public health is making progress tackling the major environmental, occupational, behavioural, and metabolic risk factors, and where it is not.

## Methods

### Overview

The GBD 2019 estimation of attributable burden followed the general framework established for comparative risk assessment (CRA)^14^, ^15^ used in GBD since 2002. Here, we provide a general overview and details on major innovations since GBD 2017. More detailed methods are available in appendix 1. CRA can be divided into six key steps: inclusion of risk–outcome pairs in the analysis; estimation of relative risk as a function of exposure; estimation of exposure levels and distributions; determination of the counterfactual level of exposure, the level of exposure with minimum risk called the theoretical minimum risk exposure level (TMREL); computation of population attributable fractions and attributable burden; and estimation of mediation of different risk factors through other risk factors such as high body-mass index (BMI) and ischaemic heart disease, mediated through elevated systolic blood pressure (SBP), elevated fasting plasma glucose (FPG), and elevated LDL cholesterol, to compute the burden attributable to various combinations of risk factors.^10^

### Geographical units, age groups, and time periods

GBD 2019 estimated prevalence of exposure and attributable deaths, YLLs, YLDs, and DALYs for 23 age groups; males, females, and both sexes combined; and 204 countries and territories that were grouped into 21 regions and seven super-regions. GBD 2019 includes subnational analyses for Italy, Nigeria, Pakistan, the Philippines, and Poland, and 16 countries previously estimated at subnational levels (Brazil, China, Ethiopia, India, Indonesia, Iran, Japan, Kenya, Mexico, New Zealand, Norway, Russia, South Africa, Sweden, the UK, and the USA). All subnational analyses are at the first level of administrative organisation within each country except for New Zealand (by Māori ethnicity), Sweden (by Stockholm and non-Stockholm), the UK (by local government authorities), and the Philippines (by province). In this publication, we present subnational estimates for Brazil, India, Indonesia, Japan, Kenya, Mexico, Sweden, the UK, and the USA; given space constraints, these results are presented in appendix 2. For this cycle, nine countries and territories (Cook Islands, Monaco, San Marino, Nauru, Niue, Palau, Saint Kitts and Nevis, Tokelau, and Tuvalu) were added, such that the GBD location hierarchy now includes all WHO member states. These new locations were previously included in regional totals by assuming that age-specific rates were equal to the regional rates. At the most detailed level, we generated estimates for 990 locations. The GBD diseases and injuries analytical framework generated estimates for every year from 1990 to 2019.

### GBD risk factor hierarchy

Individual risk factors such as low birthweight or ambient ozone pollution are evaluated in the GBD CRA. In addition, there has been policy interest in groups of risk factors such as household air pollution combined with ambient particulate matter. To accommodate these diverse interests, the GBD CRA has a risk factor hierarchy. Level 1 risk factors are behavioural, environmental and occupational, and metabolic; Level 2 risk factors include 20 risks or clusters of risks; Level 3 includes 52 risk factors or clusters of risks; and Level 4 includes 69 specific risk factors. Counting all specific risk factors and aggregates computed in GBD 2019 yields 87 risks or clusters of risks. For a full list of risk factors by level, see appendix 1 (section 5, table S2).

### Determining the inclusion of risk–outcome pairs in GBD

Since GBD 2010, we have used the World Cancer Research Fund criteria for convincing or probable evidence of risk–outcome pairs.^16^ For GBD 2019, we completely updated our systematic reviews for 81 risk–outcome pairs. Preferred Reporting Items for Systematic Reviews and Meta-Analyses flowcharts on these reviews are available in appendix 1 (section 4). Convincing evidence requires more than one study type, at least two cohorts, no substantial unexplained heterogeneity across studies, good-quality studies to exclude the risk of confounding and selection bias, and biologically plausible dose–response gradients. For GBD, for a newly proposed or evaluated risk–outcome pair, we additionally required that there was a significant association (p<0·05) after taking into account sources of potential bias. To avoid risk–outcome pairs repetitively entering and leaving the analysis with each cycle of GBD, the criteria for exclusion requires that with the available studies the association has a p value greater than 0·1. On the basis of these reviews and meta-regressions, 12 risk–outcome pairs included in GBD 2017 were excluded from GBD 2019: vitamin A deficiency and lower respiratory infections; zinc deficiency and lower respiratory infections; diet low in fruits and four outcomes: lip and oral cavity cancer, nasopharynx cancer, other pharynx cancer, and larynx cancer; diet low in whole grains and two outcomes: intracerebral haemorrhage and subarachnoid haemorrhage; intimate partner violence and maternal abortion and miscarriage; and high FPG and three outcomes: chronic kidney disease due to hypertension, chronic kidney disease due to glomerulonephritis, and chronic kidney disease due to other and unspecified causes. In addition, on the basis of multiple requests to begin capturing important dimensions of climate change into GBD, we evaluated the direct relationship between high and low non-optimal temperatures on all GBD disease and injury outcomes. Rather than rely on a heterogeneous literature with a small number of studies examining relationships with specific diseases and injuries, we analysed individual-level cause of death data for all locations with available information on daily temperature, location, and International Classification of Diseases-coded cause of death. These data totalled 58·9 million deaths covering eight countries. On the basis of this analysis, 27 GBD cause Level 3 outcomes met the inclusion criteria for each non-optimal risk factor (appendix 1 section 2.2.1) and were included in this analysis. Other climate-related relationships, such as between precipitation or humidity and health outcomes, have not yet been evaluated.

### Estimating relative risk as a function of exposure for each risk–outcome pair

In GBD, we use published systematic reviews and for GBD 2019, we updated these where necessary to include any new studies that became available before Dec 31, 2019. We did meta-analyses of relative risks from these studies as a function of exposure (appendix 1 sections 2.2.2, 4). For GBD 2019, 81 new systematic reviews were done, including for 44 diet risk–outcome pairs. To allow for risk functions that might not be log-linear, we relaxed the meta-regression assumptions to allow for monotonically increasing or decreasing but potentially non-linear functions for 147 risk–outcome pairs. Appendix 1 (section 2) provides the mathematical and computational details for how we implemented this approach for meta-regression. 218 risk–outcome pairs were estimated assuming log-linear relationships. For 126 risk–outcome pairs, exposure was dichotomous or polytomous. For 37 risk–outcome pairs, the population attributable fractions were assumed by definition to be 100% (eg, 100% of diabetes is assumed to be, by definition, related to elevated FPG). For 32 risk–outcome pairs, other approaches were used that reflected the nature of the evidence that has been collected for those risks (appendix 1 section 4). For risks that affect cardiovascular outcomes, we adjusted relative risks by age such that they follow the empirical pattern of attenuation seen in published studies for elevated SBP, FPG, and LDL cholesterol.

### Estimation of the distribution of exposure for each risk by age-sex-location-year

For each risk factor, we systematically searched for published studies, household surveys, censuses, administrative data, ground monitor data, or remote sensing data that could inform estimates of risk exposure. To estimate mean levels of exposure by age-sex-location-year, specific methods varied across risk factors (appendix 1 sections 2.1, 4). For many risk factors, exposure data were modelled using either spatiotemporal Gaussian process regression or DisMod-MR 2.1,^17^, ^18^ which are Bayesian statistical models developed over the past 12 years for GBD analyses. For most risk factors, the distribution of exposure across individuals was estimated by modelling a measure of dispersion, usually the SD, and fitting an ensemble of parametric distributions to the predicted mean and SD. Ensemble distributions for each risk were estimated based on individual-level data. Details for each risk factor modelling for mean, SD, and ensemble distribution are available in appendix 1 (section 4). Because of the strong dependency between birthweight and gestational age, exposure for these risks was modelled as a joint distribution using the copula method.^19^

In many cases, exposure data were available for the reference method of ascertainment and for alternative methods, such as tobacco surveys reporting daily smoking versus total smoking; in these cases, we estimated the statistical relationship between the reference and alternative methods of ascertainment using network meta-regression and corrected the alternative data using this relationship.

### Determining the TMREL

For harmful risk factors with monotonically increasing risk functions, the theoretical minimum risk level was set to 0. For risk factors with J-shaped or U-shaped risk functions, such as for sodium and ischaemic heart disease or BMI and ischaemic heart disease, the TMREL was determined as the low point of the risk function. When the bottom of the risk function was flat or poorly determined, the TMREL uncertainty interval (UI) captured the range over which risks are indistinguishable. For protective risks with monotonically declining risk functions with exposure, namely risk factors where exposure lowers the risk of an outcome, the challenge is selecting the level of exposure with the lowest level of risk strongly supported by the available data. Projecting beyond the level of exposure supported by the available studies could exaggerate the attributable burden for a risk factor. In these cases, for each risk–outcome pair, we determined the exposure level at the 85th percentile of exposure in the cohorts or trials used in the risk meta-regression. We then generated the TMREL by weighting each risk–outcome pair by the relative global magnitude of each outcome. Appendix 1 (section 2.4 and 4) provides details on the TMREL estimation for each risk.

**Table:** Global age-standardised SEVs for both sexes combined in 1990, 2010, and 2019, and annualised rate of change between 1990 and 2019 and 2010 and 2019

			**SEV 1990**	**SEV 2010**	**SEV 2019**	**ARC 1990–2019**	**ARC 2010–19**
**All risk factors**			**23·09 (20·22 to 25·67)**	**21·21 (18·04 to 24·26)**	**21·22 (18·05 to 24·42)**	−0·29% (−0·46 to −0·15)*	**0·00% (−0·18 to 0·20)**
**Environmental and occupational risks**			**52·55 (48·66 to 55·92)**	**48·50 (44·44 to 52·15)**	**45·36 (41·16 to 49·19)**	−0·51% (−0·62 to −0·40)*	−0·74% (−0·88 to −0·61)*
	**Unsafe water, sanitation, and handwashing**		**55·40 (54·39 to 56·61)**	**49·70 (48·99 to 50·47)**	**47·13 (46·51 to 47·84)**	−0·56% (−0·61 to −0·51)*	−0·59% (−0·67 to −0·52)*
	Unsafe water source		42·78 (41·06 to 44·39)	36·29 (34·57 to 37·92)	32·74 (30·82 to 34·41)	−0·92% (−1·08 to −0·76)*	−1·14% (−1·52 to −0·77)*
	Unsafe sanitation		56·28 (54·14 to 58·38)	38·21 (35·98 to 40·80)	28·93 (26·81 to 31·24)	−2·29% (−2·52 to −2·07)*	−3·09% (−3·68 to −2·47)*
	No access to handwashing facility		36·77 (36·54 to 37·03)	34·05 (33·80 to 34·32)	32·19 (31·92 to 32·48)	−0·46% (−0·50 to −0·42)*	−0·63% (−0·70 to −0·56)*
	**Air pollution**		**45·11 (32·85 to 56·03)**	**38·36 (28·33 to 48·55)**	**34·68 (25·76 to 44·37)**	−0·91% (−1·21 to −0·60)*	−1·12% (−1·48 to −0·81)*
	Particulate matter pollution		44·22 (31·97 to 55·06)	37·56 (27·57 to 47·75)	33·94 (25·11 to 43·56)	−0·91% (−1·24 to −0·61)*	−1·13% (−1·48 to −0·81)*
		Ambient particulate matter pollution	15·65 (10·62 to 21·58)	22·98 (18·28 to 27·62)	26·22 (21·57 to 30·50)	1·78% (0·95 to 2·71)*	1·46% (0·81 to 2·10)*
		Household air pollution from solid fuels	27·08 (16·20 to 38·13)	16·33 (9·59 to 24·52)	11·71 (6·64 to 18·27)	−2·89% (−3·60 to −2·25)*	−3·70% (−4·64 to −2·88)*
	Ambient ozone pollution		47·56 (22·76 to 60·54)	54·34 (29·48 to 65·36)	55·06 (32·21 to 67·16)	0·51% (0·27 to 1·24)*	0·15% (−0·10 to 1·08)
	**Non-optimal temperature**		**29·57 (26·06 to 33·72)**	**30·21 (26·17 to 34·83)**	**29·53 (25·41 to 34·26)**	**0·00% (−0·13 to 0·11)**	−0·25% (−0·39 to −0·13)*
	High temperature		25·98 (22·07 to 30·21)	29·25 (24·92 to 33·82)	29·59 (25·16 to 34·26)	0·45% (0·29 to 0·59)*	0·13% (−0·01 to 0·26)
	Low temperature		33·21 (29·24 to 37·58)	33·47 (29·06 to 38·25)	32·92 (28·44 to 37·82)	−0·03% (−0·13 to 0·06)	−0·18% (−0·31 to −0·07)*
	**Other environmental risks**		**50·81 (40·53 to 59·86)**	**45·11 (34·46 to 55·29)**	**39·67 (29·01 to 50·86)**	−0·85% (−1·18 to −0·55)*	−1·43% (−1·95 to −0·93)*
	Residential radon		18·54 (12·37 to 25·82)	18·20 (12·23 to 25·41)	18·12 (12·17 to 25·43)	−0·08% (−0·27 to 0·10)	−0·05% (−0·25 to 0·14)
	Lead exposure		68·52 (53·18 to 80·97)	59·82 (43·52 to 74·40)	51·26 (35·09 to 67·32)	−1·00% (−1·43 to −0·63)*	−1·72% (−2·40 to −1·09)*
	**Occupational risks**		**3·36 (2·99 to 3·90)**	**3·33 (2·97 to 3·89)**	**3·32 (2·96 to 3·87)**	**−0·05% (−0·15 to 0·05)**	**−0·05% (−0·22 to 0·13)**
**Behavioural risks**			**16·80 (14·82 to 19·05)**	**15·38 (13·28 to 17·72)**	**15·09 (12·96 to 17·43)**	−0·37% (−0·50 to −0·25)*	−0·21% (−0·36 to −0·07)*
	**Child and maternal malnutrition**		**20·05 (19·06 to 21·19)**	**17·77 (16·61 to 19·07)**	**17·23 (15·98 to 18·55)**	−0·52% (−0·67 to −0·40)*	−0·34% (−0·51 to −0·18)*
	Suboptimal breastfeeding		21·66 (20·28 to 22·96)	20·05 (18·26 to 21·34)	19·34 (17·42 to 20·68)	−0·39% (−0·55 to −0·31)*	−0·40% (−0·61 to −0·21)*
		Non-exclusive breastfeeding	21·34 (14·67 to 29·82)	19·40 (13·38 to 27·18)	18·39 (12·91 to 25·53)	−0·51% (−0·61 to −0·40)*	−0·59% (−0·83 to −0·31)*
		Discontinued breastfeeding	12·33 (12·04 to 12·65)	10·73 (10·50 to 10·99)	10·24 (9·96 to 10·54)	−0·64% (−0·77 to −0·52)*	−0·52% (−0·87 to −0·17)*
	Child growth failure		4·93 (4·41 to 5·57)	4·21 (3·70 to 4·78)	3·53 (3·01 to 4·10)	−1·15% (−1·43 to −0·83)*	−1·95% (−2·37 to −1·50)*
		Child underweight	13·32 (11·73 to 14·71)	10·51 (8·98 to 11·97)	8·13 (6·50 to 9·68)	−1·70% (−2·05 to −1·45)*	−2·86% (−3·54 to −2·37)*
		Child wasting	5·28 (4·50 to 5·98)	5·23 (4·41 to 5·97)	4·89 (4·08 to 5·61)	−0·26% (−0·34 to −0·21)*	−0·74% (−0·88 to −0·64)*
		Child stunting	24·07 (16·71 to 26·41)	19·65 (13·76 to 22·01)	16·24 (11·45 to 18·72)	−1·36% (−1·63 to −1·17)*	−2·11% (−2·68 to −1·74)*
	Low birthweight and short gestation		11·92 (10·66 to 13·44)	11·32 (10·15 to 12·67)	11·10 (9·99 to 12·42)	−0·25% (−0·46 to −0·10)*	−0·21% (−0·49 to 0·02)
		Short gestation	13·88 (12·81 to 15·20)	13·04 (12·19 to 13·96)	13·17 (12·30 to 14·13)	−0·18% (−0·43 to −0·01)*	0·11% (−0·22 to 0·39)
		Low birthweight	11·03 (10·41 to 11·81)	10·11 (9·68 to 10·52)	9·69 (9·28 to 10·14)	−0·45% (−0·69 to −0·28)*	−0·47% (−0·76 to −0·21)*
	Iron deficiency		22·65 (21·51 to 23·98)	20·11 (18·78 to 21·59)	19·57 (18·11 to 21·12)	−0·50% (−0·65 to −0·38)*	−0·30% (−0·47 to −0·14)*
	Vitamin A deficiency		33·42 (30·78 to 36·10)	22·00 (19·70 to 24·45)	15·01 (13·55 to 16·86)	−2·76% (−3·13 to −2·30)*	−4·25% (−5·02 to −3·47)*
	Zinc deficiency		13·84 (5·91 to 24·06)	11·88 (4·96 to 21·34)	8·78 (2·89 to 17·60)	−1·57% (−2·57 to −1·07)*	−3·35% (−6·44 to −2·04)*
	**Tobacco**		**30·54 (29·08 to 32·10)**	**25·32 (24·00 to 26·80)**	**24·03 (22·75 to 25·44)**	−0·83% (−0·89 to −0·77)*	−0·58% (−0·69 to −0·47)*
	Smoking		14·85 (13·27 to 16·56)	12·41 (11·08 to 13·94)	11·14 (9·93 to 12·54)	−0·99% (−1·04 to −0·94)*	−1·20% (−1·29 to −1·11)*
	Chewing tobacco		4·58 (4·18 to 4·98)	4·95 (4·71 to 5·20)	5·11 (4·80 to 5·44)	0·37% (0·03 to 0·76)*	0·36% (−0·32 to 1·05)
	Secondhand smoke		43·20 (42·80 to 43·62)	37·76 (37·32 to 38·19)	37·51 (37·00 to 38·09)	−0·49% (−0·54 to −0·43)*	−0·07% (−0·20 to 0·06)
	**Alcohol use**		**6·50 (4·62 to 8·84)**	**6·68 (4·81 to 9·02)**	**6·99 (4·98 to 9·41)**	**0·25% (0·00 to 0·56)**	0·50% (0·05 to 0·95)*
	**Drug use**		**0·18 (0·12 to 0·28)**	**0·18 (0·13 to 0·27)**	**0·19 (0·14 to 0·27)**	**0·28% (−0·19 to 0·69)**	0·53% (0·06 to 0·97)*
	**Dietary risks**		**51·31 (40·44 to 62·42)**	**48·28 (36·60 to 60·37)**	**47·10 (35·39 to 59·62)**	−0·30% (−0·50 to −0·15)*	−0·28% (−0·50 to −0·10)*
	Diet low in fruits		66·70 (59·36 to 75·08)	59·09 (51·17 to 67·81)	56·86 (49·36 to 65·37)	−0·55% (−0·71 to −0·42)*	−0·43% (−0·58 to −0·29)*
	Diet low in vegetables		51·32 (38·33 to 65·78)	40·29 (29·88 to 52·52)	40·24 (29·59 to 52·46)	−0·84% (−0·93 to −0·74)*	−0·02% (−0·14 to 0·10)
	Diet low in legumes		69·46 (36·73 to 91·69)	61·20 (28·89 to 84·10)	59·67 (27·55 to 83·28)	−0·52% (−1·08 to −0·32)*	−0·28% (−0·67 to 0·00)
	Diet low in whole grains		79·92 (72·52 to 87·44)	79·57 (72·09 to 87·12)	78·81 (71·06 to 86·78)	−0·05% (−0·07 to −0·03)*	−0·11% (−0·17 to −0·06)*
	Diet low in nuts and seeds		57·76 (29·48 to 73·08)	50·13 (25·10 to 68·03)	47·47 (23·73 to 66·35)	−0·68% (−0·92 to −0·29)*	−0·61% (−0·91 to −0·26)*
	Diet low in milk		80·09 (68·47 to 89·10)	80·81 (70·31 to 89·37)	82·54 (71·88 to 91·12)	0·10% (0·05 to 0·18)*	0·23% (0·16 to 0·33)*
	Diet high in red meat		40·50 (33·75 to 47·06)	43·15 (36·95 to 49·10)	43·94 (38·03 to 49·58)	0·28% (0·15 to 0·47)*	0·20% (−0·04 to 0·50)
	Diet high in processed meat		30·95 (20·80 to 42·39)	30·56 (20·13 to 43·05)	29·81 (19·04 to 43·32)	−0·13% (−0·39 to 0·12)	−0·27% (−0·69 to 0·10)
	Diet high in sugar-sweetened beverages		29·97 (22·97 to 42·54)	29·35 (21·94 to 41·88)	30·36 (22·71 to 43·05)	0·04% (−0·43 to 0·37)	0·38% (−0·22 to 0·76)
	Diet low in fiber		36·87 (25·93 to 47·86)	31·43 (21·20 to 41·62)	27·62 (18·60 to 36·95)	−1·00% (−1·23 to −0·81)*	−1·43% (−1·78 to −1·11)*
	Diet low in calcium		52·64 (43·62 to 64·79)	48·63 (38·79 to 62·22)	46·02 (35·93 to 60·32)	−0·46% (−0·68 to −0·23)*	−0·61% (−0·89 to −0·31)*
	Diet low in seafood omega-3 fatty acids		96·35 (93·21 to 99·89)	93·13 (89·11 to 98·47)	93·52 (88·71 to 99·41)	−0·10% (−0·18 to −0·01)*	0·05% (−0·07 to 0·15)
	Diet low in polyunsaturated fatty acids		69·53 (49·68 to 82·70)	62·66 (37·55 to 79·83)	61·86 (35·56 to 80·13)	−0·40% (−1·08 to −0·08)*	−0·14% (−0·50 to 0·14)
	Diet high in trans fatty acids		50·54 (43·82 to 63·48)	45·22 (38·20 to 58·98)	44·67 (37·57 to 58·75)	−0·43% (−0·58 to −0·17)*	−0·14% (−0·41 to 0·08)
	Diet high in sodium		48·42 (32·26 to 64·13)	46·04 (28·63 to 62·81)	44·97 (27·44 to 62·14)	−0·25% (−0·59 to −0·09)*	−0·26% (−0·60 to −0·07)*
	**Intimate partner violence**		**22·48 (13·03 to 30·15)**	**22·17 (13·13 to 29·08)**	**22·98 (13·31 to 30·37)**	**0·07% (0·00 to 0·16)**	**0·40% (0·00 to 0·73)**
	**Childhood sexual abuse and bullying**		**7·55 (4·99 to 11·23)**	**8·46 (5·63 to 12·84)**	**9·10 (6·04 to 13·85)**	0·65% (0·49 to 0·79)*	0·81% (0·64 to 0·96)*
	Childhood sexual abuse		8·68 (6·85 to 10·90)	8·65 (6·89 to 10·78)	9·36 (7·40 to 11·79)	0·26% (0·18 to 0·33)*	0·87% (0·60 to 1·15)*
	Bullying victimisation		5·51 (2·34 to 11·04)	6·83 (3·04 to 13·36)	7·31 (3·25 to 14·34)	0·98% (0·82 to 1·28)*	0·76% (0·54 to 0·90)*
	**Unsafe sex**		**..**	**..**	**..**	**..**	**..**
	**Low physical activity**		**3·34 (1·79 to 6·00)**	**3·43 (1·90 to 6·08)**	**3·54 (1·95 to 6·26)**	0·20% (0·06 to 0·41)*	**0·37% (−0·13 to 0·87)**
**Metabolic risks**			**14·90 (12·02 to 18·55)**	**19·40 (16·12 to 23·38)**	**22·14 (18·63 to 26·36)**	1·37% (1·17 to 1·56)*	1·46% (1·26 to 1·69)*
	**High fasting plasma glucose**		**7·88 (6·96 to 8·85)**	**10·41 (9·43 to 11·42)**	**11·72 (10·56 to 12·94)**	1·37% (1·27 to 1·46)*	1·32% (1·01 to 1·64)*
	**High LDL cholesterol**		**35·68 (32·92 to 38·73)**	**32·67 (29·73 to 35·84)**	**32·44 (29·49 to 35·57)**	−0·33% (−0·38 to −0·28)*	−0·08% (−0·12 to −0·05)*
	**High systolic blood pressure**		**27·12 (25·51 to 28·87)**	**26·50 (24·51 to 28·46)**	**27·74 (25·70 to 29·72)**	**0·08% (−0·12 to 0·28)**	0·51% (0·04 to 1·00)*
	**High body-mass index**		**11·09 (7·96 to 15·23)**	**16·46 (12·79 to 21·04)**	**19·45 (15·57 to 24·39)**	1·94% (1·56 to 2·35)*	1·86% (1·55 to 2·19)*
	**Low bone mineral density**		**17·06 (12·11 to 23·39)**	**16·42 (11·66 to 22·72)**	**16·26 (11·41 to 22·60)**	−0·16% (−0·25 to −0·10)*	**−0·10% (−0·34 to 0·09)**
	**Kidney dysfunction**		**20·56 (14·29 to 27·97)**	**22·35 (15·82 to 29·79)**	**22·74 (16·24 to 30·25)**	0·35% (0·26 to 0·47)*	0·19% (0·13 to 0·28)*

Data in parentheses are 95% uncertainty intervals. SEVs are measured on a 0 to 100 scale, in which 100 is when the entire population is exposed to maximum risk and 0 is when the entire population is at minimum risk. SEVs are shown for all levels of the risk factor hierarchy. ARC=annualised rate of change. SEVs=summary exposure values.

* Statistically significant increase or decrease.

### Estimation of the population attributable fraction and attributable burden

For each risk factor *j*, we computed the population attributable fraction (PAF) by age-sex-location-year using the following general formula for a continuous risk:

$${\text{PAF}}_{joasgt}=\frac{\int_{x=l}^{u}{\text{RR}}_{joasg}\left(x\right)P_{jasgt}\left(x\right)dx-{\text{RR}}_{joasg}\left({\text{TMREL}}_{jas}\right)}{\int_{x=l}^{u}{\text{RR}}_{joasg}\left(x\right)P_{jasgt}\left(x\right)dx}$$

where PAF_joasgt_ is the PAF for cause *o,* for age group *a*, sex *s*, location *g*, and year *t*; RR_joasg_(*x*) is the relative risk as a function of exposure level *x* for risk factor *j*, for cause *o* controlled for confounding, age group *a*, sex *s,* and location *g* with the lowest level of observed exposure as *l* and the highest as *u*; *P*_jasgt_*(x)* is the distribution of exposure at *x* for age group *a*, sex *s*, location *g*, and year *t*; and TMREL_jas_ is the TMREL for risk factor *j*, age group *a*, and sex *s*. Where risk exposure is dichotomous or polytomous, this formula simplifies to the discrete form of the equation.

Estimation of the PAF took into account the risk function and the distribution of exposure across individuals in each age-sex-location-year. By drawing 1000 samples from the risk function, 1000 distributions of exposure for each age-sex-location-year, and 1000 samples from the TMREL, we propagated all of these sources of uncertainty into the PAF distributions. PAFs were also applied at the draw level to the uncertainty distributions of each associated outcome for that age-sex-location-year.

### Estimating the PAF and attributable burden for combinations of risk factors

For the estimation of each specific risk factor, the counterfactual distribution of exposure is the TMREL for that specific risk with no change in other risk factors. Thus, the sum of these risk-specific estimates of attributable burden can exceed 100% for some causes, such as cardiovascular diseases. It is also useful to assess the PAF and attributable burden for combinations of risk factors, such as all diet components together or household air and ambient particulate matter pollution. To estimate the combined effects of risk factors, we should take into account how one risk factor might be mediated through another (eg, the effect of fruit intake might be partly mediated through fibre intake). We used the mediation matrix as developed in GBD 2017^12^ to try to correct for overestimation of the PAF and the attributable burden for combinations of risks if we were to simply assume independence without any mediation. Appendix 1 (section 5, table S6) provides the estimated mediation matrix.

### Summary exposure value

As in previous rounds of GBD, we summarised exposure distributions for dichotomous, polytomous, and continuous risk factors using the SEV. The SEV compares the distribution of excess risk times exposure level to a population where everyone is at maximum risk.

$${\text{SEV}}_{rc}=\frac{\int_{x=l}^{u}P\left(x\right)\text{RR}\left(x\right)\text{d}x-1}{{\text{RR}}_{\max}-1}$$

For a given risk *r* and outcome *c* pair where RR_max_ is the relative risk at the 99th percentile of the global distribution of exposure. We then averaged across outcomes to compute the SEV for a given risk as

$${\text{SEV}}_{r}=\frac{1}{N\left(c\right)}\underset{c}{\Sigma }{\text{SEV}}_{rc}$$

where *N*(*c*) is the total number of outcomes for a risk.

The SEV is effectively excess risk-weighted prevalence, which allows for comparisons across different types of exposures. Maximum risk in the denominator of the SEV is determined by the relative risk at the 99th percentile of the global distribution of exposure. The SEV is on a 0–100 scale where 100 means the entire population is at maximum risk and 0 means everyone in the population is at minimum risk. We computed age-standardised SEVs by age-standardising age-specific SEVs across the age groups in which that risk factor was evaluated; this method is a change from GBD 2017 in which age-standardisation included age groups in which the risk was not evaluated. For example, the SEV for low birthweight is now age-standardised across age groups 0–6 days to 7–27 days.

To estimate SEVs for groups of risk factors, we first estimated the value of RR_2_ without mediation through risk 1 (RR_2/1_).

$${\text{RR}}_{2/1}={\text{MF}}_{2/1}\left({\text{RR}}_{2}-1\right)+1$$

where RR_2_ is the relative risk of risk factor 2 and MF_2/1_ is the mediation factor, or the proportion of the risk of risk factor 2 that is mediated through risk factor 1. We then computed the PAF using the non-mediated relative risk (RR_1/2_) and computed the joint PAF as

$${\text{PAF}}_{1..j}=1-\prod_{j=1}^{n}\left(1-{\text{PAF}}_{j}\right).$$

We cannot simply multiply RR_max_ values used for the SEV of each component risk as this would exaggerate the joint RR_max_. We approximated the 99th percentile of risk for the combination of risk factors by taking the geometric mean of the ratio between the individual risk maximum risk and the individual risk global mean risk and multiplied that by the global mean joint risk. Formally,

$$\prod_{r}{\text{RR}}_{\text{global mean}}\prod_{r}\frac{{\text{RR}}_{\max}\frac{1}{N\left(r\right)}}{{\text{RR}}_{\text{global mean}}}$$

where *N*(*r*) is the total number of risks.

### Risk-deleted death rates

We computed risk-deleted death rates as the death rates that would be observed if all risk factors were set to their respective TMRELs. This was calculated as the death rate in each age-sex group multiplied by 1 minus the all-risk PAF for that age-sex group in each location.

### Role of the funding source

The funders of the study had no role in study design, data collection, data analysis, data interpretation, or writing of the report. The corresponding author had full access to all the data in the study and had final responsibility for the decision to submit for publication.

## Results

### Global exposure to risks

The table shows the trends in risk exposure for each risk factor at the global level over two time intervals: the full duration of the study, 1990–2019, and the past decade, 2010–19. On the basis of this table, we can divide risks into three groups based on the percentage change in the global SEV from 2010 to 2019: substantial declines (annual rate of change larger than −0·5%), substantial increases (annual rate of change greater than 0·5%) and the remainder of risks with either non-significant rates of change or significant rates of change between −0·5% and 0·5%. The declining risks fall into two categories. First, a set of risks that are strongly linked to social and economic development, measured by the Socio-demographic Index (SDI): household air pollution; unsafe water, sanitation, and handwashing; child growth failure; vitamin A deficiency; and zinc deficiency. The second set of declining risks includes tobacco smoking and lead, which historically have not been negatively correlated with SDI. These risks could in fact increase as countries and territories increase SDI, at least for a phase in the development process. For a long list of risk factors, including some large risks, the annual rate of change was either statistically insignificant (p>0·05) or the annual rate of change was between −0·5% and 0·5% per year: ambient ozone pollution, high temperature, low temperature, residential radon, occupational risks, sub-optimal breastfeeding, short gestation, low birthweight, iron deficiency, chewing tobacco, dietary risks as a group, intimate partner violence, low physical activity, high LDL cholesterol, low bone mineral density, and kidney dysfunction. Many of these stagnating risks have been or are targets of concerted public health efforts spanning public policy, targeted programmes, and primary care intervention.

**Figure 1:**
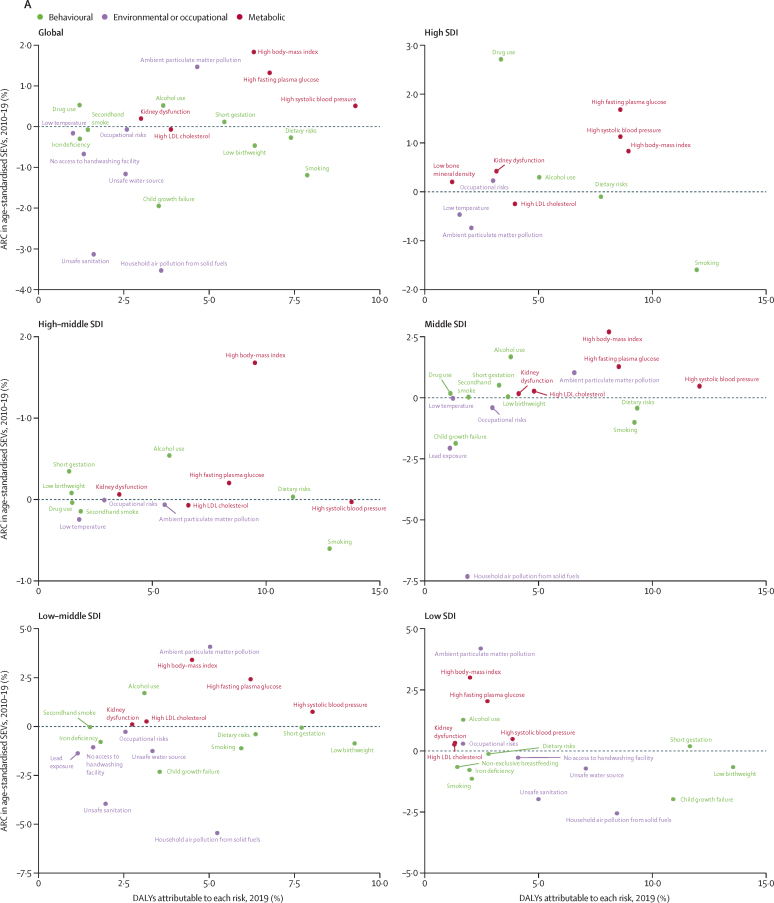

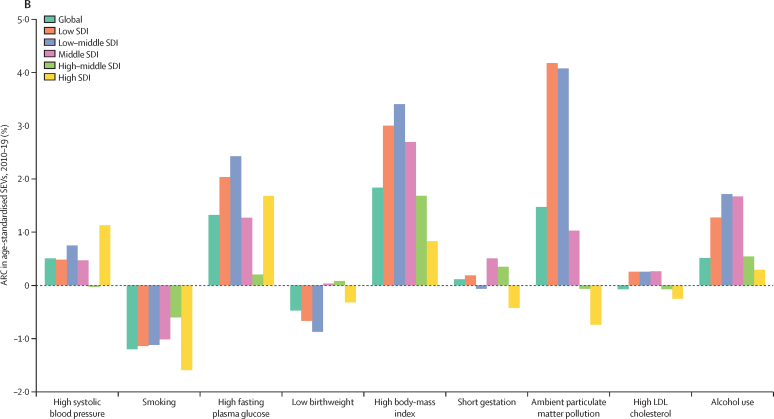
ARC in age-standardised SEVs, globally and by SDI quintile, 2010–19 (A) Level 4 risks and occupational risks, dietary risks, and child growth failure, compared with percentage of DALYs attributable to each risk. (B) Top nine Level 4 risks by attributable DALYs. Only risk factors causing more than 1% of DALYs are shown in panel A. SEVs are measured on a 0–100 scale in which 100 is when the entire population is exposed to maximum risk and 0 is when the entire population is at minimum risk. ARC=annualised rate of change. DALYs=disability-adjusted life-years. SDI=Socio-demographic Index. SEVs=summary exposure values.

Concerning for both current and future health are the exposures that are increasing at more than 0·5% per year. This list includes ambient particulate matter pollution, alcohol use, drug use, childhood sexual abuse, bullying victimisation, high FPG, high SBP, and high BMI. Many of the increasing risks are metabolic risk factors; in fact, taken together, the exposure to metabolic risks increased 1·37% per year (95% UI 1·17–1·56) from 1990 to 2019 and 1·46% per year (1·26–1·69) from 2010 to 2019. Figure 1A, which shows the trends in the age-standardised SEV for each risk factor compared with the fraction of global DALYs attributable to each risk factor, further emphasises these patterns. In 2019, there were three risks that accounted for more than 1% of DALYs and were increasing in age-standardised SEVs by more than 1% per year, dominating the figure: high FPG, high BMI, and ambient particulate matter pollution. Reductions in risks that currently still have large attributable burden are almost exclusively those inversely associated with rising SDI, except smoking. It might be assumed that effective efforts to reduce risk exposure have been concentrated on the world's largest risk factors, but we see no discernible pattern between trends in exposure and attributable burden. The global trends shown in the table and figure 1 give a high-level view of how well the world is managing exposure to an extensive list of harmful risks, but regional and country trends can be markedly variable. Figure 1B shows trends for the largest risks in terms of global attributable age-standardised DALY rates for countries grouped into quintiles of SDI in 2019. There is considerable variation across quintiles in trends in exposure. Notably, ambient particulate matter pollution exposure is increasing in the low SDI up to middle SDI quintiles but decreasing in the high SDI quintile. High FPG and high BMI are increasing in all quintiles, as is alcohol use. Smoking is declining in all SDI quintiles. Regional and national trends in SEVs are available in appendix 2 (table S1).

**Figure 2:**
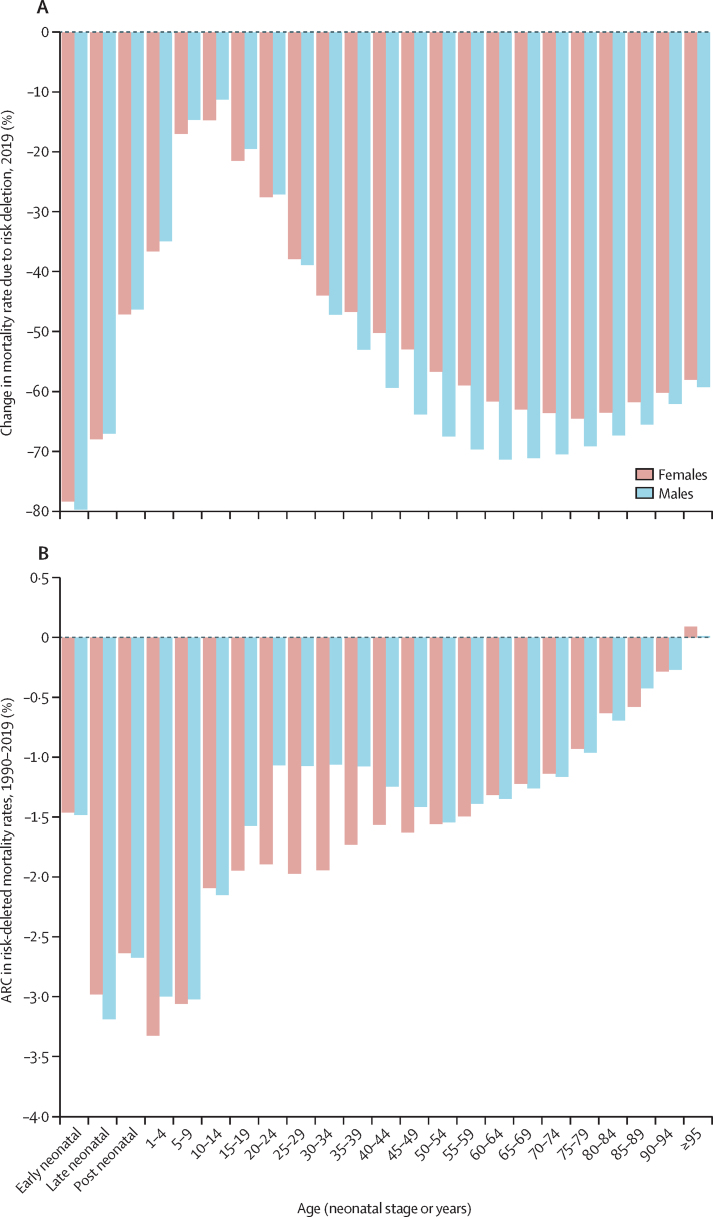
Change in global mortality rates after risk deletion, by age group and sex (A) Percentage change in age-specific mortality rates in 2019 after removing the effect of all risk factors in this study. (B) ARC in risk-deleted mortality rates from 1990 to 2019. ARC=annualised rate of change.

Figure 2A provides an alternative way to consider the link between risk exposures and overall trends in mortality. Removing the effect of all risk factors included in this study leads to large percentage reductions in mortality in those younger than 5 years and in the middle and older age groups. Risk reduction can have a slightly larger effect on male mortality than female mortality; in other words, some of the difference between male and female life expectancy can be explained by risk exposures. The percentage of age-specific mortality explained by all risk factors combined in 1990 is very similar to the share shown in figure 2A (appendix 2 table S3). Figure 2B shows the annualised rate of decline in risk-deleted age-specific mortality from 1990 to 2019. Risk-deleted mortality rates declined from 1990 to 2019 in all age groups other than in those aged 95 years and older, declining between 1·0% and 3·3% per year for all the age groups younger than 75 years, and at lower rates for those aged 75 years and older. The substantial declines in risk-deleted mortality rates are likely to be related to reductions in risks not included in our assessment, reductions in case-fatality rates, or other factors. The observed rates of decline for all-cause mortality for ages younger than 10 years and older than 65 years have been faster than the risk-deleted rates, suggesting reduction of risks included in our analysis has played a role in progress in these age groups, particularly in those younger than 5 years. Notably, risk-deleted death rates have declined faster than observed rates, particularly for women aged between 25 and 59 years, implying that risk exposure has increased in those age groups.

### Global attributable burden

**Figure 3:**
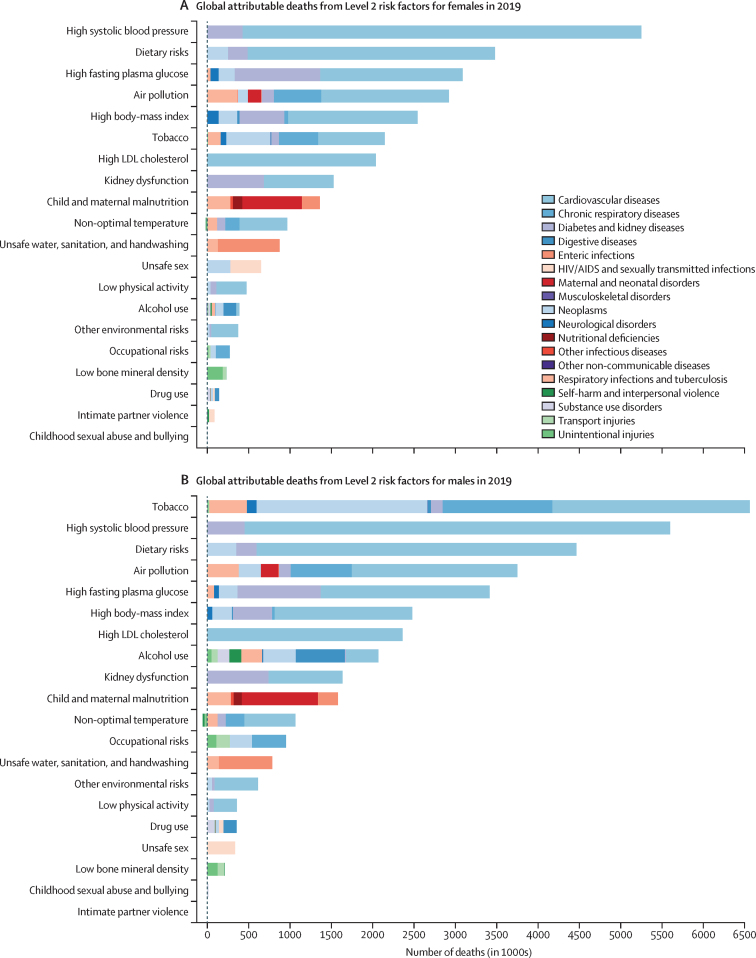

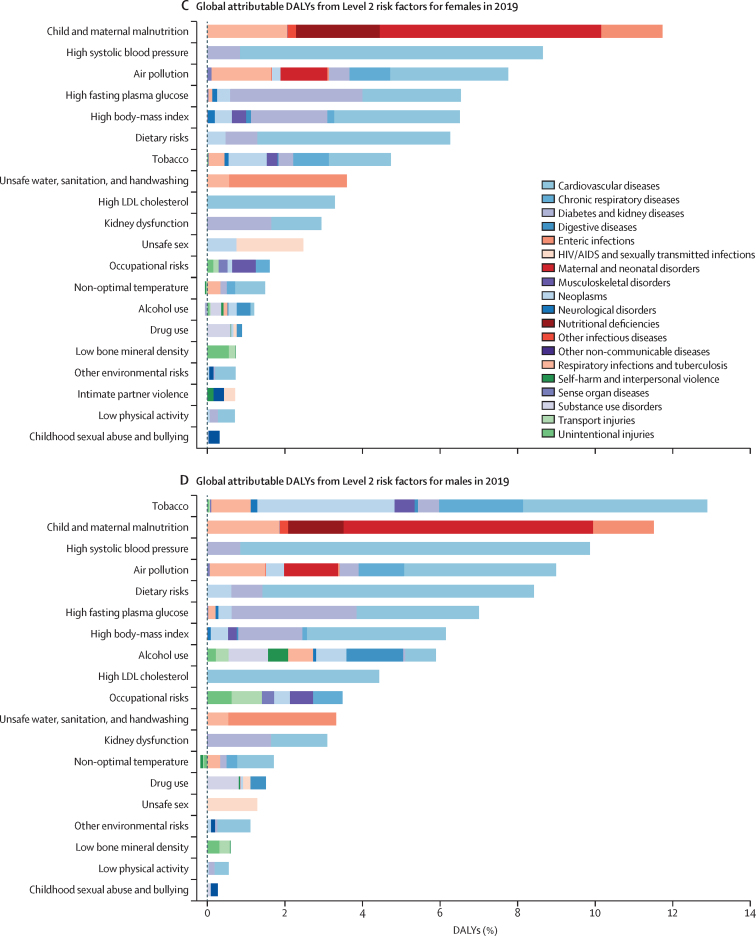
Global number of deaths and percentage of DALYs attributable to Level 2 risk factors, by cause and sex, 2019 DALYs=disability-adjusted life-years.

Figure 3A and 3B show global attributable deaths for females and males in 2019 for the 20 risk factors at Level 2 of the risk factor hierarchy (appendix 2 table S3) The top five risks for attributable deaths for females were high SBP (5·25 million [95% UI 4·49–6·00] deaths, or 20·3% [17·5–22·9] of all female deaths in 2019), dietary risks (3·48 million [2·78–4·37] deaths, or 13·5% [10·8–16·7] of all female deaths in 2019), high FPG (3·09 million [2·40–3·98] deaths, or 11·9% [9·4–15·3] of all female deaths in 2019), air pollution (2·92 million [2·53–3·33] deaths or 11·3% [10·0–12·6] of all female deaths in 2019), and high BMI (2·54 million [1·68–3·56] deaths or 9·8% [6·5–13·7] of all female deaths in 2019). For males, the top five risks differed slightly. In 2019, the leading Level 2 risk factor for attributable deaths globally in males was tobacco (smoked, second-hand, and chewing), which accounted for 6·56 million (95% UI 6·02–7·10) deaths (21·4% [20·5–22·3] of all male deaths in 2019), followed by high SBP, which accounted for 5·60 million (4·90–6·29) deaths (18·2% [16·2–20·1] of all male deaths in 2019). The third largest Level 2 risk factor for attributable deaths among males in 2019 was dietary risks (4·47 million [3·65–5·45] deaths, or 14·6% [12·0–17·6] of all male deaths in 2019) followed by air pollution (ambient particulate matter and ambient ozone pollution, accounting for 3·75 million [3·31–4·24] deaths (12·2% [11·0–13·4] of all male deaths in 2019), and then high FPG (3·14 million [2·70–4·34] deaths, or 11·1% [8·9–14·1] of all male deaths in 2019). Outside of the top five, there were large differences between attributable deaths in males and females due to alcohol use, which accounted for 2·07 million (1·79–2·37) deaths in males and 0·374 million (0·298–0·461) deaths in females in 2019. Newly included in GBD 2019, non-optimal temperature accounted for 1·01 million (0·880–1·15) deaths in males and 0·946 million (0·812–1·09) deaths in females. For both sexes combined, the leading Level 2 risk factor for deaths was high SBP, accounting for 10·8 million (9·51–12·1) deaths in 2019 (19·2% [16·9–21·3] of all deaths that year), followed by tobacco, with 8·71 million (8·12–9·31) deaths (15·4% [14·6–16·2] of all deaths that year).

When viewed in terms of DALYs (figure 3C, D), the ranking of Level 2 risk factors reflects the differential ages of death and the contribution of non-fatal disease burden. Most notably, child and maternal malnutrition (including low birthweight, short gestation, child growth failure, non-optimal breastfeeding, and low intake of micronutrients), which has predominant health effects among the young, was the second leading Level 2 risk factor for males and leading risk factor for females in 2019, accounting for 11·5% (95% UI 10·1–13·1) of DALYs for males and 11·7% (10·5–13·2) of DALYs for females. Tobacco was ranked first for males and seventh for females in terms of attributable DALYs. For both sexes combined, the leading Level 2 risk factor globally for attributable DALYs was child and maternal malnutrition, at 295 million (95% UI 253–350) DALYs in 2019 (11·6% [10·3–13·1] of all DALYs that year).

**Figure 4:**
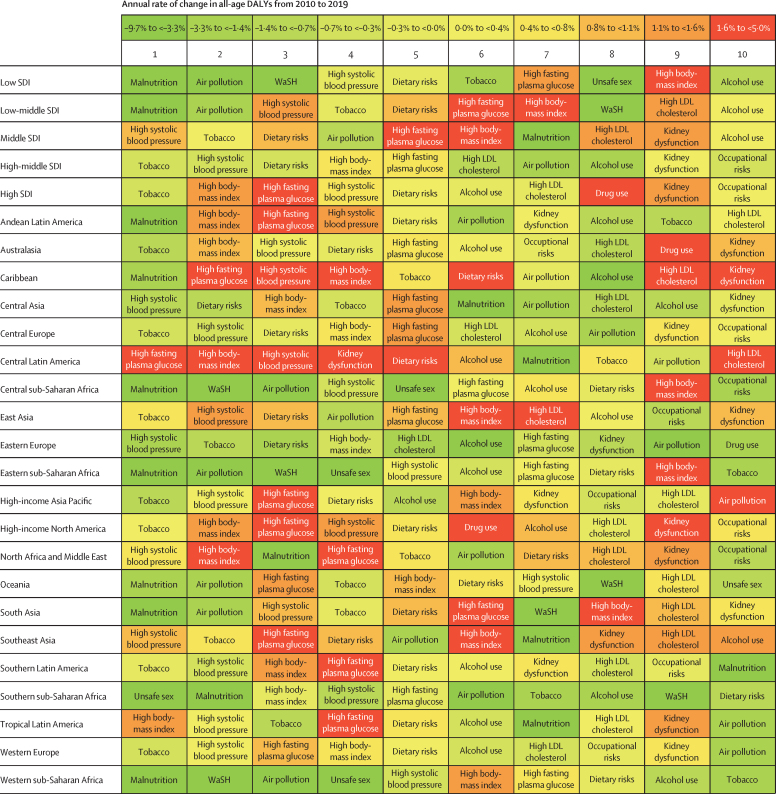
Leading ten Level 2 risk factors for attributable DALYs by GBD region and SDI quintile, 2019 For each region and SDI quintile, Level 2 risks are ranked by attributable DALYs from left (first) to right (tenth). Risks are coloured by their annualised rate of change in all-age DALY rates from 2010 to 2019. GBD=Global Burden of Diseases, Injuries, and Risk Factors Study. Malnutrition=child and maternal malnutrition. SDI=Socio-demographic Index. WaSH=water, sanitation, and handwashing.

Figure 4 shows the ranking of Level 2 risk factors by attributable DALYs, both for SDI quintiles and the 21 GBD regions. Risk factors are shaded by the trend in the attributable DALY rates over the past decade. In the low SDI quintile, the most important risk factors were malnutrition; air pollution; and water, sanitation, and handwashing. In the low-middle SDI quintile, malnutrition and air pollution were still the leading risk factors for attributable DALYs, but high SBP rose to third. In the middle to high SDI quintiles, the leading risks transitioned to tobacco, high SBP, dietary risks, high BMI, and high FPG. The risk transition is evident across quintiles. Select regional patterns stand out. In the Caribbean and central Latin America, large increases were seen in attributable burden for high FPG, high BMI, high SBP, kidney dysfunction, dietary risks, and high LDL cholesterol. In seven regions, child and maternal malnutrition is the leading risk factor, and in another seven regions, tobacco is the leading risk factor. In the remainder of regions, the leading risk factor is high SBP (four regions), high FPG (one region), high BMI (one region), and unsafe sex (one region). The largest rates of increase in attributable DALYs have been seen for high FPG in ten of 21 regions and for high BMI in ten of 21 regions.

### Attributable burden by age group

**Figure 5:**
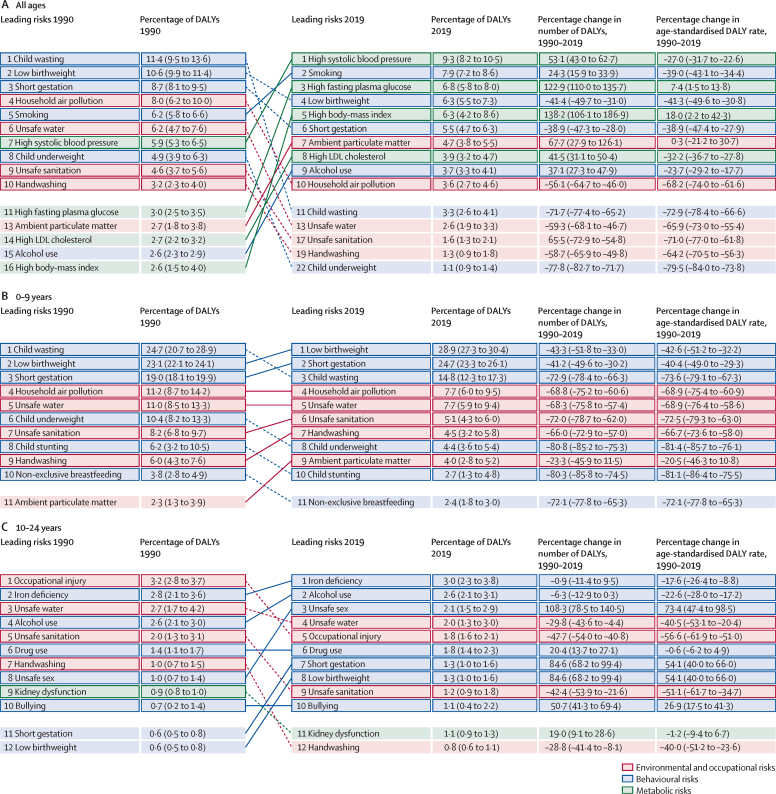

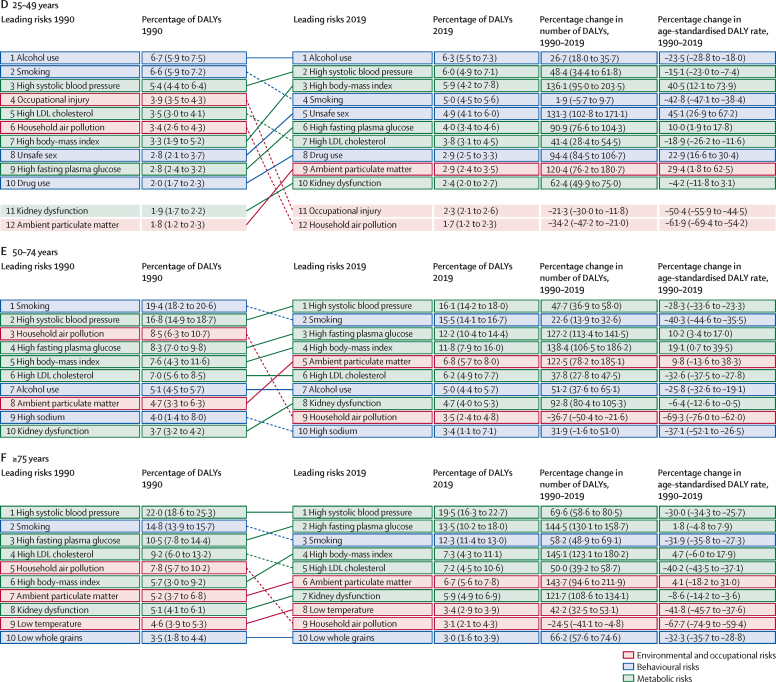
Leading ten Level 4 risks by attributable DALYs, 1990–2019 For all ages (A), 0–9 years (B), 10–24 years (C), 25–49 years (D), 50–74 years (E), and 75 years and older (F). DALYs=disability-adjusted life-years.

The pattern of risk-factor-attributable burden varied considerably by age and over time, as shown in figure 5, which includes arrows plots for all age groups combined and for five broad age groups (0–9, 10–24, 25–49, 50–74, and 75 years and older). These figures show specific risk factors at Level 4 of the risk factor hierarchy. Figure 5A shows how risk exposure trends, underlying changes in disease rates, and rising mean age of populations have led to marked changes in risk rankings from 1990 to 2019. In 1990, the leading risk factors were child wasting, low birthweight, short gestation, and household air pollution, all of which have dropped substantially in magnitude in terms of percentage of attributable DALYs and rank by 2019. The leading risks in 2019 were high SBP, smoking, high FPG, low birthweight, and high BMI. Other notable shifts include the large increase in percentage of attributable DALYs and rank for ambient particulate matter pollution, high LDL cholesterol, and alcohol use. Among the youngest age group (0–9 years), shown in figure 5B, the leading Level 4 risk factors were composed exclusively of malnutrition and environmental risk factors. Over the 1990–2019 period, there were substantial reductions in the burden attributable to these risk factors in both absolute numbers and rates. The largest declines among the leading ten risks were for child growth failure (child underweight, stunting, and wasting); water, sanitation, and handwashing; and household air pollution. Large but more moderate declines in attributable burden occurred for short gestation and low birthweight, with the smallest reduction observed for ambient particulate matter pollution.

Among adolescents and young adults (aged 10–24 years; figure 5C), the pattern of risk factor burden was notably different from the 0–9 years age group, with iron deficiency, alcohol use, and unsafe sex ranking first to third for attributable DALYs in this age group in 2019. There were declines in unsafe sex DALYs in the second half of the study period, but due to rapid increases from 1990 to 2004, there was still a 108·3% (95% UI 78·5–140·5) increase in unsafe sex DALYs from 1990 to 2019. The long-term consequences of short gestation and low birthweight also increased in importance.

In the 25–49 years age group (figure 5D), alcohol use was the leading Level 4 risk factor for attributable burden, followed by high SBP and then high BMI, smoking, unsafe sex, and high FPG. The number of DALYs increased for all the top ten risks, but age-standardised attributable DALY rates increased only for high BMI, unsafe sex, high FPG, drug use, and ambient particulate matter pollution. In the two oldest age groups, the set of leading risks are quite similar to one another, dominated by high SBP at the top, and followed by other metabolic risk factors including high FPG, high BMI, high LDL cholesterol, and kidney dysfunction. Smoking also contributed substantially to the risk attributable burden in these age groups, ranked second in ages 50–74 years (figure 5E) and third in ages 75 years and older (figure 5F). In the oldest age group, low temperature was also one of the top ten risks, although age-standardised attributable DALY rates declined from 1990 to 2019. Sex-specific rankings by age group are available in appendix 2 (figures S4, S5).

### National findings

**Figure 6:**
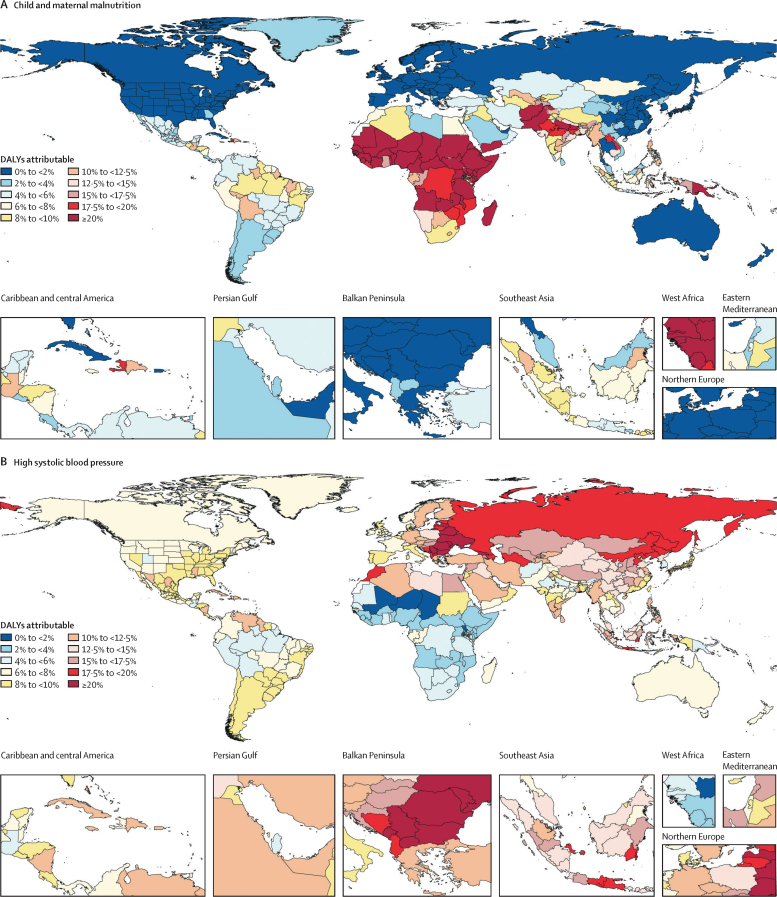

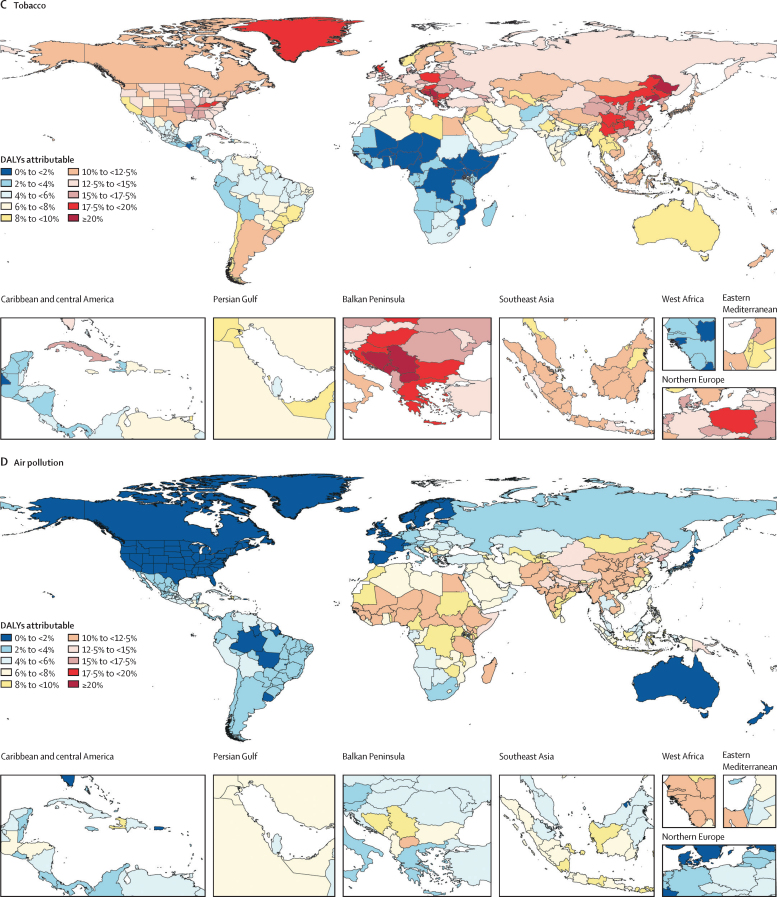

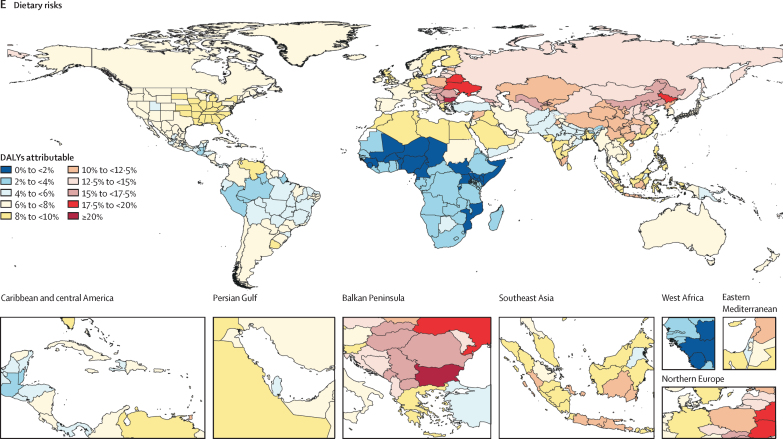
Percentage of all DALYs attributable to the five leading Level 2 risk factors, 2019 DALYs attributable to child and maternal malnutrition (A), high systolic blood pressure (B), tobacco (C), air pollution (D), and dietary risks (E). DALYs=disability-adjusted life-years.

The leading risk factors for attributable DALYs had highly varied geographical patterns, as shown in figure 6, which presents maps of the percentage of burden attributable to the top five Level 2 risk factors globally in 2019. The highest proportions (greater than 20%) of burden attributable to the leading Level 2 risk factor in 2019, child and maternal malnutrition, were seen in most of western, central, and eastern sub-Saharan African regions (figure 6A). In addition, rates greater than 20% were seen in Afghanistan, Pakistan, states in northern India, Yemen, and Papua New Guinea. Rates between 10% and 20% were seen in a diverse set of central American countries, states in Brazil, Tajikistan, Uzbekistan, Myanmar, regions of the Philippines, and some Indonesian provinces.

Figure 6B shows the burden attributable to the second leading Level 2 risk factor in 2019, high SBP. Locations with more than 20% of DALYs attributable to high SBP included Georgia and most of central and eastern Europe. Most countries in north Africa and the Middle East had between 10% and 20% of DALYs attributable to high SBP as did states in southern India and many parts of southeast Asia. The only countries with less than 2% of all-age DALYs attributable to high SBP were in western sub-Saharan Africa.

The third leading Level 2 risk factor, tobacco, is shown in figure 6C. Locations with more than 20% of DALYs attributable in 2019 include countries in the Balkan Peninsula and two provinces in China—Liaoning and Heilongjiang. Most countries in Europe had between 10% and 20% of DALYs attributable to smoking; Canada, most states in the USA, Russia, the rest of China, and many parts of southeast Asia were also in this category. Attributable burden remains less than 6% in most of Mexico, central America, and Andean Latin America. The burden attributable to tobacco is less than 2% in much of western and eastern sub-Saharan Africa.

Figure 6D shows the burden attributable to air pollution (ambient particulate matter, household air pollution, and ambient ozone pollution). No location had more than 20% of DALYs attributable to air pollution. But a wide range of countries in western and eastern sub-Saharan Africa had attributable burden percentages between 10% and 15%. Similarly, nearly all locations in south Asia, many parts of southeast Asia, and most provinces in China also had the same levels of attributable burden. The spatial patterns of the constituent risks included in air pollution—particularly ambient particulate matter and household air pollution—were quite different (see GBD Compare for data), with ambient particulate matter pollution playing a much greater role in Asia than in Africa.

The fifth most important Level 2 risk factor was the dietary risks that are based on the joint effects of 15 diet quality components (figure 6E). In Bulgaria, dietary risks accounted for more than 20% of attributable DALYs. However, diet accounts for more than 10% of DALYs in many locations in central and eastern Europe, central Asia, and most of China. The lowest shares of DALYs attributed to dietary risks are in sub-Saharan Africa, particularly countries in the Sahel.

### Risk-specific trends

Two-page risk-specific summaries provide detailed results on attributable deaths, YLLs, YLDs, and DALYs for a selection of the 87 risk factors in the GBD risk hierarchy. These summaries include 2019 counts, age-standardised rates, and rankings for attributable burden; the composition of attributable burden for leading causes; patterns of attributable burden over time and age; and age-standardised SEVs by location and SDI. They were written to increase the accessibility to and transparency of GBD estimates for each risk factor. Summaries for select risk factors are highlighted in print (pp S216–319); summaries for all risk factors can be found online.

## Discussion

### Main findings

Our analysis of risk-attributable burden using 30 652 sources for exposure, relative risk, and the TMREL showed that in 2019, 47·8% (95% UI 45·3–50·1) of global DALYs were attributed to present and past exposure for the 87 environmental, occupational, behavioural, and metabolic risk factors and combinations of risk factors included in this analysis. Overall, combined global exposure to the risks included in this study has remained remarkably constant over the past 30 years. Risk-deleted mortality rates over the same period have declined, ranging from a 3·3% decline per year in females aged 1–4 years to a 0·3% decline per year in males aged 90–94 years. Despite this overall pattern, reductions in key risks highly correlated with SDI—unsafe water, sanitation, and handwashing; household air pollution; child growth failure; and vitamin A and zinc deficiencies—have contributed to reductions in global child death rates. Among the most detailed major non-communicable disease risks, only tobacco smoking has declined steadily. At the global level in 2019, there were three risk factors that accounted for more than 1% of DALYs and were increasing in exposure by more than 1% per year: high BMI, ambient particulate matter pollution, and high FPG. There is large scope for public regulatory policy, community programmes, and primary care interventions on risks to have a greater effect on prevention. These broad global patterns mask considerable heterogeneity in risk levels and trends at the country level, reinforcing the need for country assessments and country-specific prevention planning.

### Important risk factor trends

Some risk exposures are highly correlated with social and economic development, as measured by SDI. As countries and territories increase SDI through higher levels of education, particularly among women; increased GDP per capita; and improved access to modern contraceptives, we should expect progress on these risks. The incremental effect of campaigns, policies, and programmes on top of this social and economic development process is yet to be established in this analysis. Bending the development curve is possible, as evidenced by the abrupt accelerations in the decline in some risk factors, such as the recent decline in unsafe sanitation in India.^20^ Even for risks that are historically highly correlated with SDI, intervention can accelerate progress. The range of policy initiatives to accelerate the transition to cleaner cooking fuels is another example of this effort.^21^, ^22^ Analysis of exemplars, countries with lower SEVs for these risks for their level of SDI or faster progress than expected for the change in SDI, could yield further insights.

Two risk factors that have not been highly correlated with SDI in the past have also seen declines in exposure at nearly 1% per year over the study period: tobacco smoking and lead exposure. Progress on exposure to these risks stands out compared with the increases in exposure to many metabolic risks and no substantial change for others such as diet quality. In both of these cases, government action through taxation and regulatory policy for tobacco smoking,^23^ including advertising bans and clean air legislation, and regulation of lead content,^24^, ^25^ have had a major effect. Tobacco interventions highlight how regulatory policy can lead to behaviour change. International efforts for tobacco control have also been bolstered by the Framework Convention on Tobacco Control.^26^ Despite the more than 1% per year decline in age-standardised tobacco smoking exposure between 2010 and 2019, tobacco remains the third leading risk factor for attributable DALYs among Level 2 risks. For the three major and rapidly increasing risks, the role of taxation and regulatory policy should be examined. For ambient particulate matter pollution, regulation can clearly have a direct impact.^27^, ^28^ For the nexus of high FPG and high BMI, regulatory strategies are less clear. We are failing to deal with these risks, and concerted research and policy efforts are needed to reverse the trends.

The marked rise of metabolic risks as a group, in particular high FPG and high BMI, and their large contribution to attributable burden is perhaps most disturbing. During this period of rising metabolic risk exposure, global cardiovascular disease age-standardised mortality has been declining as documented in GBD 2019 for diseases and injuries.^29^ The seeming paradox could, to a large extent, be explained by the effect of access to care,^30^ social determinants of health, cohort effects, and other behavioural, occupational, and environmental risks not quantified here. Rising metabolic risks might at some point overwhelm these other drivers and eventually lead to rising cardiovascular mortality in the future. This situation might have arrived in some high-income countries in which age-standardised cardiovascular disease mortality has plateaued or increased since 2017.^31^ If year-on-year declines in cardiovascular disease mortality come to an end, the effect on mortality and longevity at the global level could be massive. While high BMI and high FPG have steadily increased, high LDL cholesterol has remained constant over the past decade despite the expected correlation with BMI; this finding warrants further investigation and could be related to changes in diet quality, pharmacological intervention, or other factors. Although not increasing at the rate of high BMI or high FPG, high SBP has become the leading risk factor for disease burden at the global level, among the most detailed risks in this analysis. A range of strategies including primary care management and reductions in sodium intake are known to be potentially effective in reducing the burden of this critical risk factor.^32^, ^33^

The rise of high BMI and its probable role in increasing high FPG needs further examination. Increased BMI can be traced to the combination of physical inactivity, excess caloric intake, and diet quality.^34^ At the global level, we find that high BMI is rising considerably faster than low physical activity and poor diet quality. Diet quality on its own is the fifth leading Level 2 risk factor for attributable DALYs. The effect of diet on human health goes beyond diet quality and should include the contribution of diet intake above energy requirements. Some studies suggest that certain diet components are more likely to contribute to increased BMI than others; the mechanism of these effects can be complex and include effects on appetite, absorption, and displacement of other foods.^35^ It is currently hard to understand the role of physical inactivity, excess caloric intake, and diet quality in driving the increase in BMI. The large combined burden of diet quality, physical inactivity, and high BMI (11·9% [95% UI 9·6–14·5] of all DALYs in 2019) indicates just how profoundly important the nexus of diet and physical activity can be to current and future health. The setting for understanding the potential of changes in overall diet is to use future health scenarios to trace how public policies such as subsidies, taxes, information campaigns, and improving accessibility can affect health in each country. In this study, no country or territory has had a significant decline in the proportion of the population with high BMI between 1990 to 2019 or in the past decade. The complete failure to reduce BMI at the national level implies that efforts to modify the nexus of physical inactivity, diet quality, and excess energy intake might be very challenging. Tackling this diet quality and excess energy intake will not only be important for human health but has important ramifications for environmental sustainability.^36^

The two types of exposure to particulate matter with a diameter of less than 2·5 μm (PM_2·5_) have profoundly different relationships with socio-demographic development: household air pollution is strongly related to SDI and tends to decrease steadily with socio-demographic development. By contrast, ambient particulate matter pollution tends to increase with industrialisation and then decline with air-quality management at higher levels of SDI.^37^, ^38^ The global increases in ambient particulate matter pollution exposure are being driven by the middle SDI quintiles, as seen in figure 1B. Studies have shown that for ambient particulate matter pollution (ambient PM_2·5_), the main sources of exposure are residential energy use, industry, and power generation.^39^ The concentration of PM_2·5_ burden in south Asia highlights how the absence of national policy actions can have a major effect. Among the large risk factors in which exposure is increasing, ambient particulate matter pollution stands out because exposure is declining in countries with a higher SDI. Like tobacco and lead, regulation can have a profound effect on exposure to and health effects of ambient particulate matter pollution and does not require individual action.^40^, ^41^ There is a clear role for global organisations to encourage regulatory change in middle SDI countries with large and increasing exposure to ambient particulate matter pollution. This agenda is all the more urgent because of the direct linkage to global climate change.

Because of profound global interest in the potential health effects of climate change, we have included high and low non-optimal temperatures in GBD 2019. Climate change will have impacts on human health through many mechanisms: direct effects of temperature rise, humidity changes, sea-level rise, extreme weather events, and reduced agricultural yields and increased rural poverty.^42^, ^43^ We have so far included only one of these pathways in the GBD analysis, namely the direct effects of ambient temperature on different disease outcomes. Our analysis showed that the TMREL varies as a function of mean annual temperature. Locations where mean temperature is higher tend to have higher optimal temperatures, probably through physical and social adaptation. In 2019, the burden (as measured by percentage of total DALYs) attributable to low temperature was 2·2 times greater than the burden attributable to high temperature. This balance does not, however, hold true when looking at specific locations or regions. While for high SDI countries, the cold-related burden is 15·4 times greater than the heat-related burden, this relationship is switched for other regions, such as south Asia where we observed a 1·7 times greater heat-related burden and sub-Saharan Africa where we observed a 3·6 times greater heat-related burden. Rising temperature will probably have a substantial effect in locations with less capacity to adapt to increased temperature, potentially exacerbating health inequalities across countries. The social capacity to adapt is also probably tied to economic development: for example, air conditioners in the USA have mitigated the impact of heat waves over the past 50 years.^44^ In terms of trends, there was a marked increase in exposure to high temperature from 1990 to 2010 and then a slight decline from 2010 to 2019; there are major annual fluctuations in temperature exposure on top of long-term warming trends, and 2010 stood out as a year with high temperatures in many regions. Our analysis does not provide a basis for understanding the full effects of future climate change, which will operate through many different pathways in addition to the direct effects of temperature.

In the GBD CRA work to date, we have estimated the burden attributable to past exposure in a given year. The CRA framework also laid out the important utility for policy making of estimating how changes in current and future exposure can change future levels of health; this concept is called avoidable burden. Most CRA work to date has focused on estimating attributable burden, even though avoidable burden is arguably more relevant to policy prioritisation. The dominance of work on attributable burden is founded on two premises: it is very difficult to estimate avoidable burden as this estimate requires a comprehensive future health scenarios framework; and attributable burden is likely to be highly correlated with avoidable burden. With the availability of a GBD-informed future health scenarios platform,^45^, ^46^ the possibility of estimating avoidable burden is much more tractable. Future work on avoidable burden for each GBD risk factor might allow us to examine the true relationship between the two approaches to CRA. The relationships between avoidable and attributable burden can vary across countries; in high mortality settings, competing risks might mean that avoidable burden will be systematically smaller than attributable burden.

For GBD 2019 and all previous GBD CRA efforts, our inclusion criteria for a risk–outcome pair were based on the World Cancer Research Fund criteria for convincing or probable evidence. We also required that published studies, when meta-analysed together, yielded a significant (p<0·05) relative risk for any risk–outcome pair meeting these criteria. To avoid risk–outcome pairs on the cusp of statistical significance coming in and out of GBD with different cycles, we introduced a threshold of p>0·1 to exclude a risk–outcome pair that has previously been included in GBD. Among the included risk–outcome pairs, the consistency of the evidence and risk of bias varies considerably. The evidence linking smoking to lung cancer is clearly far stronger than the evidence on omega-3 and ischaemic heart disease. The UI of the mean effect does not fully capture this difference in the consistency of evidence or the risk of bias. A more robust measure needs to take into account various risks of bias and the unexplained variation in effect after taking into account these risks of bias as well as the magnitude of the effect size. The relative risk of lung cancer from smoking is very high across levels of exposure, with a relative risk of 3·4 at ten pack-years of smoking and a relative risk of 6·5 at 20 pack-years of smoking, which makes it far more likely to be causal than an exposure with a relative risk of 1·1. If we included the unexplained heterogeneity across studies after adjusting for risk of bias in the UI of the relative risks, several risk–outcome pairs might not meet inclusion criteria. We are working on developing an evidence scoring system that quantifies consistency and risk of bias for GBD and would allow readers to understand that not all risk–outcome pairs have the same evidence base. It would, however, be misleading and potentially harmful to argue that we should only examine the GBD risk–outcome pairs with the highest grade of evidence. The precautionary principle for public policy implies that governments have a duty to act on risk factors that are probably or potentially harmful and not only those that have overwhelming evidence.^47^ Relying only on effects on the basis of the highest degree of evidence will very seriously delay public recognition and proactive policies, which in turn would result in perpetuating preventable burden. We hope to inform both individuals and public policy makers with quantification of burden and strength of evidence so they are empowered to make sense of the available data.

Analysing relative risks to inform choices by individuals and choices by population health decision makers might legitimately have different perspectives. Some guidelines on systematic reviews^48^, ^49^ recommend reporting absolute risk levels related to exposure; this method is perhaps appropriate for informing individual choice. For public policy, attributable burden might be more relevant. If a risk factor is related to 1000 deaths, from a public policy perspective, the concentration of the risk in a smaller or larger group of individuals might matter less than it does to individuals. To provide a synthesis of the evidence for different users, we included estimation of the all-cause mortality relative risk associated with exposure levels of each risk factor using the global distribution of burden across outcomes. However, risks that cause relatively modest increases for individuals but are highly prevalent, such as air pollution, are, nevertheless, legitimate targets for public policy.

### Substantial changes compared with GBD 2017

Compared with GBD 2017, our GBD 2019 estimates of the burden (as measured by percentage of total DALYs) attributable to diet quality in 2017 were 29·7% lower. These reductions stem from three major sources: changes in the crosswalks between alternative and reference methods for estimating diet intake, new systematic reviews and meta-regressions, and more empirical standardised methods for selecting the TMREL for protective factors. Although there were changes in the overall burden of diet, there were larger changes in the diet components themselves, particularly the substantial increase in the attributable burden from red meat and the decline in the burden attributable to low vegetable intake. The sources of the changes were the same as for diet quality overall. One of the most important insights from this enriched analysis is that for many harmful and protective factors, the relative risk functions tend to flatten out at higher exposure levels; the previous practice of imposing a log-linear functional form on the risk equation—widely used in the scientific literature—might have led to overestimation. For protective diet components (whole grains, fruit, fibre, nuts and seeds, omega-3, polyunsaturated fatty acids, vegetables, milk, and calcium), we set the TMREL to the 85th percentile of levels of exposure included in the published cohort studies or randomised controlled trials. With further study of individuals with higher levels of intake, it is possible that the level of intake associated with the lowest risk is in fact higher than the TMREL set for protective diet components in GBD 2019. 12 diet risk–outcome pairs from GBD 2017 were excluded from GBD 2019 because our re-analysis with updated data suggested that the effects were no longer significant. Some risk–outcome pairs, such as omega-3 and ischaemic heart disease, which remained in the analysis as the result of the new meta-regression of 21 trials and 27 cohort studies, met inclusion criteria but future studies could shift the balance of the evidence to be excluded.

Particulate matter pollution burden in 2017 was 44·6% higher in GBD 2019 than in GBD 2017. The increase was due to the inclusion of low birthweight and short gestation as risk factors that are themselves affected by PM_2·5_, as well as increases in the relative risk curve for cardiovascular diseases, particularly stroke, due to newly added data and changes in fitting the exposure-response curves. Given the very large burden of low birthweight and short gestation on neonatal mortality, the inclusion of these intermediates has been an important change in our assessment. The burden of stroke and ischaemic heart disease attributable to kidney dysfunction increased between GBD 2017 and GBD 2019 after updating the relative risks with new data from 44 cohorts. For instance, the comparable estimate of the proportion of cardiovascular DALYs due to kidney dysfunction in 2010 increased from 6·8% (95% UI 6·0–7·6) to 8·5% (6·8–10·3).

### Limitations

In GBD 2019, we undertook a reassessment of dose–response relationships and relaxed previous assumptions that the risk curve is log-linear. This reassessment was limited, however, to dietary risks, kidney dysfunction, and air pollution. Future reassessments of other continuous risk factors that currently assume a log-linear relationship could materially change risk factor rankings in the future and could also lead to exclusion of other risk–outcome pairs.

Assessment of the joint effects of risk factors depends on two critical factors: the correlation of risk exposure and the estimation of the joint effects of groups of risks together. For exposure, we assumed that for each age-sex-location-year, the estimates of the prevalence of exposure were independent. Previous simulation analyses undertaken for GBD 2010^8^ with use of US data from the National Health and Nutrition Examination Survey suggested this assumption did not materially bias our findings. To assess the joint effects of risk factors, we assumed in general that relative risks are multiplicative. This simple assumption has been modified to take into account known pathways in which one risk factor, such as fruit consumption, is mediated through another risk factor such as fibre intake. To avoid over-estimation of the joint effects, we computed the non-mediated relative risks and then assumed that non-mediated relative risks are multiplicative. This approach does not capture potential synergy between relative risks in which some combinations might be super-multiplicative. For some areas such as diet, the joint estimation is very important for public policy. Further, more detailed work is needed to strengthen the evidence base for understanding mediation. In particular, mediation implies necessarily that exposure between mediated risks is correlated. Factoring in that implied correlation into risk exposure estimation could strengthen estimates in the future.

The main limitation of our estimates of risk-attributable burden is the availability and quality of primary data that underpin the analysis. Data for risk relationships of several risk factors, such as ambient ozone pollution, residential radon, occupational risks, childhood sexual abuse, intimate partner violence, bullying victimisation, and child growth failure are sparse. For exposure measurement, patterns of data availability are non-uniform across geography and over time and, where available, might be based on less reliable modes of data collection such as self-report. In GBD 2019, we implemented more explicit corrections for bias associated with non-reference methods of exposure measurement that improved the estimation of risk exposure. Furthermore, these assessments can be used to guide future data collection efforts by identifying those populations with not only sparse but low-quality data based on the collection mode.

Our analysis, particularly the overall assessment of burden attributable to all risks combined and risk-deleted mortality, is limited by several potentially important risk factors not included in this analysis. The most important set is likely to be social determinants of health such as educational attainment, poverty, or social exclusion. We are currently doing systematic reviews on educational attainment, which will be the first social determinant to be incorporated into future rounds of the GBD CRA. There is also a wide range of other risk factors not yet included such as nitrous oxide, heavy metals, environmental noise, sleep, stress, UV radiation, among others. Future rounds of GBD might evaluate whether these risk factors meet inclusion criteria.

To date, GBD has not included Mendelian randomisation studies in meta-regression. These studies could provide new insights on the causal connections between risks and outcomes.^50^ Not all Mendelian randomisation studies are appropriate for inclusion.^51^, ^52^, ^53^ Future rounds of GBD will give careful consideration to including these studies for some risk–outcome pairs.

For harmful risks with monotonically increasing risk functions, we have generally assumed that the TMREL is 0. For protective risks such as fruit or whole grain intake, selecting the level of exposure that is minimum risk is more challenging. Extrapolating the risk function beyond where the available cohort studies or trials support the protective effect could easily lead to both exaggerated estimates of attributable burden and implausible recommendations on consumption. To avoid this exaggeration, we set the TMREL for protective risks to be equal to the 85th percentile of exposure in the available cohorts and trials. The 85th percentile is arbitrary, but sensitivity analysis did not suggest major changes if we selected the 90th or 80th percentiles.

Lastly, in most cases, we assume that relative risks as a function of exposure are universal and apply in all locations and time periods. Exceptions include temperature, in which the risk functions clearly depend on the annual mean temperature, and the relative risks for high BMI for breast cancer that differ in Asian and non-Asian populations. Our rules require that there is evidence of significant differences in the relative risk for different subgroups; to date, few cases have met this standard. As evidence accumulates, more location-specific or subgroup relative risks might be identified.

### Conclusion

Using the most up-to-date assessment of the data for exposure and relative risk, we found that global exposure to harmful environmental risks has been declining, with the notable exception of ambient particulate matter pollution. Environmental risk reduction is making an important contribution to reductions in child mortality. In aggregate, there has been no real progress reducing exposure to behavioural risks, while metabolic risks are, on average, increasing every year. As a world, we are failing to change some behaviours, particularly those related to diet quality, caloric intake, and physical activity. Progress on reducing harm from one crucial behaviour, tobacco smoking, shows the power of taxation and regulation. The promise of prevention through risk modification is not being realised in adult populations around the world. Urgent attention on more successful strategies to reduce risks is needed.

Correspondence to: Prof Christopher J L Murray, Institute for Health Metrics and Evaluation, University of Washington, Seattle, WA 98195, USA cjlm@uw.edu

## Data sharing

To download the data used in these analyses, please visit the Global Health Data Exchange GBD 2019 website.
