# Supplementary material for: Global burden of 87 risk factors in 204 countries and territories, 1990–2019: a systematic analysis for the Global Burden of Disease Study 2019
Source: Lancet. 2020 Oct 17;396(10258):1223–49. doi: 10.1016/S0140-6736(20)30752-2 (PMC7566194; doi:10.1016/S0140-6736(20)30752-2)
Supplement: Supplementary appendix 2 [file mmc2a.pdf]

THE LANCET

Supplementary appendix 2a (Part 1 of 2)

This appendix formed part of the original submission and has been peer reviewed. We post it as supplied by the authors.

Supplement to: GBD 2019 Risk Factors Collaborators. Global burden of 87 risk factors in 204 countries and territories, 1990–2019: a systematic analysis for the Global Burden of Disease Study 2019. *Lancet* 2020; **396:** 1223–49.

Appendix 2A: Supplementary results appendix for “Global burden of 87 risk factors in 204 countries and territories, 1990–2019: a systematic analysis for the Global Burden of Disease Study 2019”

This appendix provides supplemental figures and more detailed results for "Global burden of 87 risk factors in 204 countries and territories, 1990–2019: a systematic analysis for the Global Burden of Disease Study 2019."

List of supplementary results appendix figures and tables

Appendix 2A

Figure S1 a-j: Leading 20 Level 4 risk factors for DALYs by SDI level and sex, 1990, 2010,and 2019. . . . 1

Figure S2: Leading 10 Level 3 risk factors for deaths in 2019 by location. 11

Figure S3: Leading 10 Level 3 risk factors for DALYs in 2019 by location. 32

Table S1: Age-standardised SEVs for Level 4 risk factors and percentage change, by sex, 1990, 2010, and 2019, by geography 53

Appendix 2B

Table S2: All-age PAFs attributable to the joint distribution of all risk factors, both sexes combined for each GBD Level 1, Level 2, and Level 3 cause and all causes in 2019, by geography. 1193

Table S3: All-age deaths, death PAFs, DALYs, and DALY PAFs, and percentage change for each risk factor and outcome for both sexes combined in 2010 and 2019, by geography. 3873

Figure S1a: Leading 20 risk factors for DALYs for females in Low SDI countries, 1990, 2010 and 2019.

Leading risks 1990	Leading risks 2010	Mean % change number of YLLs 1990-2010	Mean % change all-age YLL rate 1990-2010	Mean % change age-standardised YLL rate 1990-2010
1 Child wasting	1 Child wasting	-32%	-60.2%	-56.7%
2 Low birth weight	2 Low birth weight	9.9%	-35.7%	-25.1%
3 Unsafe water	3 Short gestation	12.2%	-34.4%	-23.6%
4 Household air pollution	4 Household air pollution	-12.2%	-48.6%	-36.9%
5 Short gestation	5 Unsafe water	-23.3%	-55.1%	-51.9%
6 Child underweight	6 Unsafe sanitation	-25%	-56.2%	-53%
7 Unsafe sanitation	7 Unsafe sex	143.2%	42.2%	42.6%
8 Handwashing	8 Child underweight	-46.5%	-68.7%	-65.6%
9 Child stunting	9 Handwashing	-21.7%	-54.2%	-49.7%
10 Non-exclusive breastfeeding	10 Child stunting	-45.9%	-68.4%	-65.9%
11 Unsafe sex	11 High blood pressure	53.7%	-10.1%	-8.1%
12 High blood pressure	12 Iron deficiency	38.1%	-19.2%	-20.7%
13 Vitamin A deficiency	13 Non-exclusive breastfeeding	-21.2%	-53.9%	-47.5%
14 Iron deficiency	14 High fasting plasma glucose	85.2%	8.4%	11.8%
15 Ambient particulate matter	15 Ambient particulate matter	67.8%	-1.9%	25.2%
16 High fasting plasma glucose	16 High body-mass index	109%	22.2%	25%
17 Second-hand smoke	17 Intimate partner violence	106%	20.5%	24.6%
18 High temperature	18 Kidney dysfunction	55.2%	-9.2%	-3.7%
19 High body-mass index	19 High LDL	64.8%	-3.6%	-2.8%
20 Kidney dysfunction	20 High temperature	8.9%	-36.3%	-17.9%
21 Intimate partner violence	21 Second-hand smoke			
22 High LDL	22 Vitamin A deficiency			
	23 Smoking			

Leading risks 2019	Mean % change number of YLLs 2010-2019	Mean % change all-age YLL rate 2010-2019	Mean % change age-standardised YLL rate 2010-2019
1 Low birth weight	-12.8%	-30.6%	-19.7%
2 Short gestation	-10.5%	-28.8%	-17.6%
3 Child wasting	-29.7%	-44.1%	-36.1%
4 Household air pollution	-17.2%	-34.1%	-25.3%
5 Unsafe water	-24.1%	-39.7%	-31.4%
6 Unsafe sanitation	-28%	-42.7%	-36%
7 Handwashing	-22.7%	-38.5%	-30%
8 High blood pressure	26.5%	0.6%	-6.7%
9 Child underweight	-36.8%	-49.7%	-42%
10 Unsafe sex	-40.4%	-52.6%	-52.9%
11 High fasting plasma glucose	42.2%	13.1%	6.2%
12 Iron deficiency	11.2%	-11.6%	-12.4%
13 High body-mass index	59.9%	27.2%	19%
14 Ambient particulate matter	32.4%	5.3%	24.9%
15 Child stunting	-38.4%	-51%	-44.3%
16 Non-exclusive breastfeeding	-30.2%	-44.5%	-35.9%
17 Kidney dysfunction	28.4%	2.1%	-0.5%
18 High LDL	30.4%	3.7%	-3%
19 Smoking	16.2%	-7.6%	-12.4%
20 Second-hand smoke	2.7%	-18.3%	-7.5%
21 Intimate partner violence			
22 High temperature			

Legend:
Environmental
Behavioural
Metabolic

Figure S1b: Leading 20 risk factors for DALYs for females in Low-middle SDI countries, 1990, 2010 and 2019.

Leading risks 1990
1 Child wasting
2 Low birth weight
3 Short gestation
4 Household air pollution
5 Unsafe water
6 Child underweight
7 Unsafe sanitation
8 Handwashing
9 Child stunting
10 High blood pressure
11 Non-exclusive breastfeeding
12 Iron deficiency
13 Second-hand smoke
14 High fasting plasma glucose
15 Ambient particulate matter
16 High LDL
17 Kidney dysfunction
18 Smoking
19 High body-mass index
20 Vitamin A deficiency
21 Unsafe sex
24 Low fruit

Leading risks 2010	Mean % change number of YLLs 1990-2010	Mean % change all-age YLL rate 1990-2010	Mean % change age-standardised YLL rate 1990-2010
1 Low birth weight	**-29.1%**	**-49.3%**	**-28.3%**
2 Short gestation	**-27.1%**	**-47.9%**	**-26.3%**
3 Household air pollution	**-40.2%**	**-57.3%**	**-50.1%**
4 Child wasting	**-63%**	**-73.6%**	**-64.8%**
5 Unsafe water	**-49.3%**	**-63.8%**	**-57.7%**
6 High blood pressure	**55.2%**	**10.9%**	**-13.3%**
7 Unsafe sanitation	**-53.2%**	**-66.5%**	**-61%**
8 Unsafe sex	**289%**	**178%**	**130.4%**
9 High fasting plasma glucose	**95.4%**	**39.6%**	**11.9%**
10 Ambient particulate matter	**56.4%**	11.8%	34%
11 High body-mass index	**159.7%**	**85.6%**	**49.4%**
12 Handwashing	**-49.3%**	**-63.8%**	**-56%**
13 Child underweight	**-72.7%**	**-80.5%**	**-74.3%**
14 Iron deficiency	6.7%	**-23.8%**	**-24.3%**
15 Kidney dysfunction	**62.1%**	**15.9%**	-1.9%
16 High LDL	**56.2%**	**11.6%**	**-10.5%**
17 Second-hand smoke	**-27.5%**	**-48.2%**	**-39.8%**
18 Child stunting	**-68.8%**	**-77.7%**	**-70.2%**
19 Smoking	**25.5%**	-10.3%	**-28.7%**
20 Non-exclusive breastfeeding	**-56.5%**	**-68.9%**	**-56.8%**
22 Low fruit			
25 Low whole grains			
40 Vitamin A deficiency			

Leading risks 2019	Mean % change number of YLLs 2010-2019	Mean % change all-age YLL rate 2010-2019	Mean % change age-standardised YLL rate 2010-2019
1 Low birth weight	**-29.1%**	**-37.4%**	**-25.7%**
2 High blood pressure	**29.7%**	**14.5%**	-3.1%
3 Short gestation	**-27.6%**	**-36.1%**	**-24.3%**
4 High fasting plasma glucose	**48.9%**	**31.4%**	**11.6%**
5 Household air pollution	**-30.8%**	**-38.9%**	**-38.9%**
6 High body-mass index	**56.6%**	**38.2%**	**19.8%**
7 Ambient particulate matter	**40.5%**	**24%**	**24.1%**
8 Unsafe water	**-36.9%**	**-44.3%**	**-40.7%**
9 Child wasting	**-51.7%**	**-57.4%**	**-49.8%**
10 Unsafe sex	**-23.9%**	**-32.8%**	**-35.7%**
11 High LDL	**34.5%**	**18.7%**	1.5%
12 Kidney dysfunction	**29.3%**	**14.1%**	0.5%
13 Iron deficiency	-1.5%	**-13%**	**-12.7%**
14 Unsafe sanitation	**-47.4%**	**-53.5%**	**-50.6%**
15 Smoking	**18.5%**	4.6%	**-11.7%**
16 Second-hand smoke	5.9%	-6.5%	**-11%**
17 Handwashing	**-40.2%**	**-47.2%**	**-42.5%**
18 Child underweight	**-56.5%**	**-61.6%**	**-55.2%**
19 Low fruit	**28%**	**12.9%**	-2.6%
20 Low whole grains	**33.3%**	**17.7%**	0.5%
32 Non-exclusive breastfeeding			
33 Child stunting			

Legend:
Environmental
Behavioural
Metabolic

Figure S1c: Leading 20 risk factors for DALYs for females in Middle SDI countries, 1990, 2010 and 2019.

Leading risks 1990	Leading risks 2010	Mean % change number of YLLs 1990-2010	Mean % change all-age YLL rate 1990-2010	Mean % change age-standardised YLL rate 1990-2010	Leading risks 2019	Mean % change number of YLLs 2010-2019	Mean % change all-age YLL rate 2010-2019	Mean % change age-standardised YLL rate 2010-2019
1 Child wasting	1 High blood pressure	**53.8%**	**19.1%**	**-15.4%**	1 High blood pressure	**19.3%**	9.2%	**-12.1%**
2 Low birth weight	2 High fasting plasma glucose	**91.5%**	**48.2%**	7.3%	2 High fasting plasma glucose	**32.3%**	**21.2%**	-2.5%
3 Short gestation	3 High body-mass index	**121.1%**	**71.1%**	**24.3%**	3 High body-mass index	**38.5%**	**26.8%**	5.7%
4 Household air pollution	4 Low birth weight	**-41.1%**	**-54.4%**	**-33.4%**	4 Ambient particulate matter	**18.2%**	8.3%	-8.7%
5 High blood pressure	5 Ambient particulate matter	**47.5%**	14.2%	7.8%	5 Kidney dysfunction	**21.2%**	**10.9%**	**-7.3%**
6 Unsafe water	6 Short gestation	**-39.6%**	**-53.2%**	**-31.8%**	6 High LDL	**23.3%**	**12.9%**	-7.7%
7 High fasting plasma glucose	7 Unsafe sex	**412.2%**	**296.4%**	**185.8%**	7 Low birth weight	**-32%**	**-37.8%**	**-30.4%**
8 Ambient particulate matter	8 Kidney dysfunction	**68.6%**	**30.5%**	0.7%	8 Short gestation	**-31.1%**	**-36.9%**	**-29.6%**
9 Unsafe sanitation	9 High LDL	**62.9%**	**26.1%**	**-9.1%**	9 Unsafe sex	**-25.8%**	**-32.1%**	**-34.5%**
10 Second-hand smoke	10 Household air pollution	**-57.2%**	**-66.8%**	**-70%**	10 Smoking	5.1%	-3.7%	**-23.3%**
11 High body-mass index	11 Child wasting	**-72.1%**	**-78.4%**	**-69.1%**	11 Second-hand smoke	9.6%	0.3%	**-16.4%**
12 Child underweight	12 Smoking	**36.6%**	5.7%	**-24.5%**	12 High sodium	13.3%	3.8%	**-15.9%**
13 High LDL	13 Second-hand smoke	**-23.9%**	**-41.1%**	**-46.8%**	13 Household air pollution	**-37.5%**	**-42.7%**	**-52.1%**
14 Kidney dysfunction	14 High sodium	12.3%	-13.1%	**-37.3%**	14 Low whole grains	**23.2%**	**12.8%**	-8%
15 Child stunting	15 Unsafe water	**-60.7%**	**-69.6%**	**-62.3%**	15 Iron deficiency	-1.8%	**-10.1%**	**-9.8%**
16 Handwashing	16 Iron deficiency	**-11.7%**	**-31.7%**	**-31.8%**	16 Child wasting	**-45%**	**-49.7%**	**-45.9%**
17 Smoking	17 Low whole grains	**64.7%**	**27.5%**	**-8.1%**	17 Low temperature	10.7%	1.4%	**-20.5%**
18 Non-exclusive breastfeeding	18 Low temperature	**-19.6%**	**-37.8%**	**-48%**	18 Low fruit	6.4%	-2.6%	**-19.9%**
19 Iron deficiency	19 Low fruit	13.6%	**-12.1%**	**-36.1%**	19 High red meat	**26.2%**	**15.6%**	-4.8%
20 High sodium	20 Intimate partner violence	**146.6%**	**90.9%**	**69.4%**	20 Unsafe water	**-33%**	**-38.6%**	**-41%**
21 Low temperature	21 Unsafe sanitation				26 Intimate partner violence			
22 Low fruit	22 High red meat				33 Unsafe sanitation			
23 Low whole grains	25 Child underweight							
24 Unsafe sex	31 Handwashing							
35 Intimate partner violence	34 Non-exclusive breastfeeding							
	37 Child stunting							

Legend:
Environmental
Behavioural
Metabolic

Figure S1d: Leading 20 risk factors for DALYs for females in High-middle SDI countries, 1990, 2010 and 2019.

Leading risks 1990
1 High blood pressure
2 High body-mass index
3 High fasting plasma glucose
4 High LDL
5 Smoking
6 Low birth weight
7 Short gestation
8 Ambient particulate matter
9 Household air pollution
10 Child wasting
11 Second-hand smoke
12 Kidney dysfunction
13 Low temperature
14 Low whole grains
15 High sodium
16 Alcohol use
17 High red meat
18 Iron deficiency
19 Low fruit
20 Low legumes
22 Unsafe sex
24 Low physical activity

Leading risks 2010	Mean % change number of YLLs 1990-2010	Mean % change all-age YLL rate 1990-2010	Mean % change age-standardised YLL rate 1990-2010
1 High blood pressure	**13.4%**	-3.6%	**-26.3%**
2 High body-mass index	**36.9%**	**16.4%**	-7.3%
3 High fasting plasma glucose	**50.4%**	**27.8%**	-0.7%
4 High LDL	**15.2%**	-2.1%	**-25.8%**
5 Smoking	**11%**	-5.6%	**-25.2%**
6 Ambient particulate matter	22.8%	4.3%	-15.1%
7 Kidney dysfunction	**30.6%**	**11%**	**-13%**
8 Alcohol use	**41.9%**	**20.6%**	1.1%
9 Low whole grains	**10.7%**	**-6%**	**-28.1%**
10 Second-hand smoke	**-17.8%**	**-30.1%**	**-44.9%**
11 Low birth weight	**-54.8%**	**-61.6%**	**-46%**
12 High sodium	0.4%	**-14.7%**	**-33.5%**
13 Low temperature	**-14.8%**	**-27.6%**	**-47.4%**
14 Short gestation	**-53.7%**	**-60.6%**	**-45%**
15 High red meat	**12.2%**	-4.6%	**-25.2%**
16 Low legumes	5.5%	**-10.3%**	**-32%**
17 Unsafe sex	**57.5%**	**33.9%**	12.9%
18 Low fruit	-4%	**-18.4%**	**-36.4%**
19 Household air pollution	**-68.6%**	**-73.3%**	**-78.8%**
20 Low physical activity	**38.1%**	**17.4%**	**-14.1%**
22 Iron deficiency			
23 Low bone mineral density			
27 Child wasting			

Leading risks 2019	Mean % change number of YLLs 2010-2019	Mean % change all-age YLL rate 2010-2019	Mean % change age-standardised YLL rate 2010-2019
1 High blood pressure	0.7%	-4.3%	**-19.8%**
2 High body-mass index	**11.4%**	5.9%	**-9.2%**
3 High fasting plasma glucose	**10.5%**	5.1%	**-11.2%**
4 High LDL	0.5%	-4.5%	**-19.9%**
5 Smoking	**7.2%**	1.9%	**-12.9%**
6 Ambient particulate matter	-0.2%	-5.1%	**-19.7%**
7 Kidney dysfunction	**7.6%**	2.3%	**-12.6%**
8 Low whole grains	1.4%	-3.6%	**-19%**
9 Second-hand smoke	0.2%	-4.7%	**-18.8%**
10 High sodium	3.1%	-2%	**-17.3%**
11 Alcohol use	**-20.5%**	**-24.4%**	**-28.6%**
12 Low temperature	-2.2%	-7%	**-24.1%**
13 High red meat	5.8%	0.6%	**-13.9%**
14 Low birth weight	**-30%**	**-33.5%**	**-31.5%**
15 Short gestation	**-29%**	**-32.5%**	**-30.7%**
16 Low legumes	-2.8%	**-7.6%**	**-22.9%**
17 Unsafe sex	-3.1%	**-7.9%**	**-13.8%**
18 Low physical activity	**12.9%**	7.3%	**-12%**
19 Low fruit	-7.1%	**-11.7%**	**-24.5%**
20 Low bone mineral density	**21.8%**	**15.8%**	-3.1%
22 Iron deficiency			
25 Household air pollution			

Legend:
Environmental
Behavioural
Metabolic

Figure S1e: Leading 20 risk factors for DALYs for females in High SDI countries, 1990, 2010 and 2019.

Leading risks 1990
1 High blood pressure
2 Smoking
3 High body-mass index
4 High fasting plasma glucose
5 High LDL
6 Alcohol use
7 Ambient particulate matter
8 Kidney dysfunction
9 Low whole grains
10 Low temperature
11 Low birth weight
12 Short gestation
13 High red meat
14 Low legumes
15 Low physical activity
16 High sodium
17 Second-hand smoke
18 Low fruit
19 Low bone mineral density
20 Drug use
23 High processed meat
25 Occupational ergonomic

Leading risks 2010	Mean % change number of YLLs 1990-2010	Mean % change all-age YLL rate 1990-2010	Mean % change age-standardised YLL rate 1990-2010
1 Smoking	**-9.3%**	**-21.3%**	**-33.6%**
2 High body-mass index	**21.4%**	5.3%	-10.6%
3 High blood pressure	**-29.4%**	**-38.7%**	**-52.4%**
4 High fasting plasma glucose	**20.5%**	4.6%	**-13.9%**
5 High LDL	**-39%**	**-47%**	**-58%**
6 Kidney dysfunction	**11.1%**	-3.6%	**-22.2%**
7 Alcohol use	4.3%	**-9.5%**	**-19.3%**
8 Drug use	**108.4%**	**80.8%**	**82.2%**
9 Ambient particulate matter	-26.3%	**-36%**	**-45.2%**
10 Low temperature	**-11.3%**	**-23%**	**-42.6%**
11 Low whole grains	**-18.4%**	**-29.2%**	**-42.6%**
12 Low bone mineral density	**37.4%**	**19.2%**	**-8.9%**
13 High red meat	**-15.3%**	**-26.5%**	**-38.2%**
14 Low physical activity	-3.1%	-15.9%	**-33.8%**
15 Low birth weight	**-32.7%**	**-41.6%**	**-34.2%**
16 Short gestation	**-31.9%**	**-40.9%**	**-33.5%**
17 Second-hand smoke	**-21.8%**	**-32.2%**	**-43.7%**
18 High processed meat	2.8%	-10.7%	**-25.4%**
19 Occupational ergonomic	**20.9%**	4.9%	-3.1%
20 Low legumes	**-33.5%**	**-42.3%**	**-54.5%**
21 High sodium			
22 Low fruit			

Leading risks 2019	Mean % change number of YLLs 2010-2019	Mean % change all-age YLL rate 2010-2019	Mean % change age-standardised YLL rate 2010-2019
1 Smoking	**3.4%**	**-2.1%**	**-11.8%**
2 High body-mass index	**17.9%**	**11.6%**	2.2%
3 High fasting plasma glucose	**20.9%**	**14.4%**	3.2%
4 High blood pressure	**11.4%**	**5.5%**	**-6%**
5 High LDL	**3.4%**	-2.1%	**-12.3%**
6 Kidney dysfunction	**16.1%**	**10%**	-1.6%
7 Alcohol use	4.1%	-1.5%	**-6.2%**
8 Drug use	**41.9%**	**34.4%**	**38%**
9 Ambient particulate matter	-4.9%	**-10%**	**-17.8%**
10 Low temperature	**6.5%**	0.8%	**-12.2%**
11 Low bone mineral density	**24.9%**	**18.3%**	**4.8%**
12 Low whole grains	**8%**	2.3%	**-7.8%**
13 High red meat	**11.5%**	**5.6%**	-3.7%
14 Low physical activity	**8.6%**	2.8%	-5.6%
15 Low birth weight	**-12.7%**	**-17.3%**	**-13.7%**
16 High processed meat	**11.1%**	**5.2%**	-4.3%
17 Second-hand smoke	**8.7%**	2.9%	**-5.2%**
18 Short gestation	**-12.5%**	**-17.2%**	**-13.6%**
19 Occupational ergonomic	**4.9%**	-0.7%	-0.8%
20 Low legumes	**4%**	-1.5%	**-12.1%**
21 High sodium			

Legend:
Environmental
Behavioural
Metabolic

Figure S1f: Leading 20 risk factors for DALYs for males in Low SDI countries, 1990, 2010 and 2019.

Leading risks 1990
1 Child wasting
2 Low birth weight
3 Household air pollution
4 Short gestation
5 Unsafe water
6 Child underweight
7 Unsafe sanitation
8 Handwashing
9 Child stunting
10 Non-exclusive breastfeeding
11 Smoking
12 Vitamin A deficiency
13 High blood pressure
14 Alcohol use
15 High fasting plasma glucose
16 Ambient particulate matter
17 Unsafe sex
18 Occupational injury
19 Second-hand smoke
20 High temperature
21 Iron deficiency
22 High LDL
23 Kidney dysfunction
24 High body-mass index

Leading risks 2010	Mean % change number of YLLs 1990-2010	Mean % change all-age YLL rate 1990-2010	Mean % change age-standardised YLL rate 1990-2010
1 Low birth weight	**15.1%**	**-32.4%**	**-21.8%**
2 Child wasting	**-32.8%**	**-60.5%**	**-57.2%**
3 Short gestation	**17.7%**	**-30.9%**	**-20%**
4 Household air pollution	**-13.2%**	**-49.1%**	**-37.8%**
5 Unsafe water	**-19.5%**	**-52.7%**	**-46.3%**
6 Unsafe sanitation	**-21.2%**	**-53.7%**	**-47.5%**
7 Handwashing	**-20.7%**	**-53.4%**	**-46%**
8 Child underweight	**-46.9%**	**-68.8%**	**-65.7%**
9 High blood pressure	**59.5%**	-6.3%	-0.9%
10 Child stunting	**-45.3%**	**-67.9%**	**-65.7%**
11 Smoking	**23.9%**	**-27.2%**	**-22.4%**
12 Unsafe sex	**141.1%**	**41.6%**	**39.9%**
13 Alcohol use	**44.8%**	**-15%**	**-16.1%**
14 High fasting plasma glucose	**72.6%**	1.3%	8.7%
15 Non-exclusive breastfeeding	**-23.9%**	**-55.3%**	**-49.4%**
16 Ambient particulate matter	**72.7%**	1.4%	29.6%
17 Occupational injury	**26.5%**	**-25.8%**	**-27.7%**
18 Iron deficiency	**55.4%**	**-8.7%**	**-9.2%**
19 High LDL	**65.9%**	-2.6%	0.8%
20 Kidney dysfunction	**59.6%**	-6.3%	-0.5%
21 High body-mass index			
22 High temperature			
23 Vitamin A deficiency			
24 Second-hand smoke			

Leading risks 2019	Mean % change number of YLLs 2010-2019	Mean % change all-age YLL rate 2010-2019	Mean % change age-standardised YLL rate 2010-2019
1 Low birth weight	-12.3%	**-29.7%**	**-19.2%**
2 Short gestation	-10.4%	**-28.2%**	**-17.4%**
3 Child wasting	**-30.7%**	**-44.4%**	**-37.2%**
4 Household air pollution	**-20.2%**	**-36.1%**	**-29%**
5 Unsafe water	**-23.6%**	**-38.7%**	**-31.2%**
6 Unsafe sanitation	**-26.7%**	**-41.3%**	**-34.8%**
7 Handwashing	**-23%**	**-38.3%**	**-30%**
8 High blood pressure	**23.7%**	-0.9%	-7.2%
9 Smoking	**13.2%**	-9.3%	**-14.2%**
10 Child underweight	**-39.5%**	**-51.5%**	**-44.7%**
11 High fasting plasma glucose	**37.3%**	**10%**	3.6%
12 Alcohol use	**12.8%**	-9.6%	**-12.4%**
13 Ambient particulate matter	**31.8%**	5.6%	18.4%
14 Unsafe sex	**-31.6%**	**-45.2%**	**-47.8%**
15 Child stunting	**-37.9%**	**-50.2%**	**-43.9%**
16 High body-mass index	**60.4%**	**28.6%**	**20.4%**
17 High LDL	**26%**	1%	-5%
18 Iron deficiency	**16.1%**	**-7%**	**-4.8%**
19 Non-exclusive breastfeeding	**-30.1%**	**-44%**	**-35.9%**
20 Kidney dysfunction	**24.2%**	-0.5%	-4.6%
21 Occupational injury			

Legend:
Environmental
Behavioural
Metabolic

Figure S1g: Leading 20 risk factors for DALYs for males in Low-middle SDI countries, 1990, 2010 and 2019.

Leading risks 1990
1 Low birth weight
2 Child wasting
3 Short gestation
4 Household air pollution
5 Unsafe water
6 Child underweight
7 Unsafe sanitation
8 Smoking
9 Handwashing
10 High blood pressure
11 Child stunting
12 Alcohol use
13 Non-exclusive breastfeeding
14 Ambient particulate matter
15 High fasting plasma glucose
16 Occupational injury
17 Second-hand smoke
18 High LDL
19 Kidney dysfunction
20 Vitamin A deficiency
23 High body-mass index
26 High sodium
27 Low whole grains
37 Unsafe sex

Leading risks 2010	Mean % change number of YLLs 1990-2010	Mean % change all-age YLL rate 1990-2010	Mean % change age-standardised YLL rate 1990-2010
1 Low birth weight	**-28.4%**	**-47.9%**	**-28%**
2 Short gestation	**-26.3%**	**-46.4%**	**-25.9%**
3 Smoking	**23.4%**	**-10.2%**	**-25.2%**
4 Household air pollution	**-40.1%**	**-56.4%**	**-48.9%**
5 High blood pressure	**72.6%**	**25.6%**	3.5%
6 Child wasting	**-63.8%**	**-73.7%**	**-65.6%**
7 Unsafe water	**-50.8%**	**-64.2%**	**-56.8%**
8 High fasting plasma glucose	**98.6%**	**44.4%**	**20.9%**
9 Alcohol use	**60.1%**	**16.5%**	0.3%
10 Ambient particulate matter	**75.2%**	27.5%	44.9%
11 Unsafe sanitation	**-54.4%**	**-66.8%**	**-60%**
12 High LDL	**72.1%**	**25.2%**	4.5%
13 High body-mass index	**192.7%**	**112.9%**	**77.9%**
14 Handwashing	**-50.1%**	**-63.7%**	**-54.6%**
15 Kidney dysfunction	**70.9%**	**24.3%**	**9%**
16 Child underweight	**-73.6%**	**-80.8%**	**-75%**
17 Unsafe sex	**555%**	**376.4%**	**327.7%**
18 Occupational injury	-12.2%	**-36.1%**	**-41.7%**
19 Low whole grains	**70%**	**23.7%**	2.1%
20 High sodium	**56.5%**	13.8%	-4.8%
21 Second-hand smoke			
24 Child stunting			
25 Low fruit			
26 Non-exclusive breastfeeding			
42 Vitamin A deficiency			

Leading risks 2019	Mean % change number of YLLs 2010-2019	Mean % change all-age YLL rate 2010-2019	Mean % change age-standardised YLL rate 2010-2019
1 Low birth weight	**-29.8%**	**-37.3%**	**-26.4%**
2 Smoking	**10.9%**	-0.9%	**-13.5%**
3 High blood pressure	**21%**	8.1%	-5.8%
4 Short gestation	**-28.2%**	**-35.8%**	**-24.7%**
5 High fasting plasma glucose	**44.3%**	**28.9%**	**11.2%**
6 Ambient particulate matter	**35.2%**	**20.8%**	17.3%
7 Alcohol use	**16.2%**	3.8%	-4.4%
8 Household air pollution	**-36.8%**	**-43.6%**	**-43.4%**
9 High body-mass index	**59.7%**	**42.7%**	**25.7%**
10 High LDL	**22%**	9%	-3.5%
11 Child wasting	**-50.9%**	**-56.1%**	**-48.9%**
12 Unsafe water	**-41.3%**	**-47.6%**	**-43.8%**
13 Kidney dysfunction	**18.7%**	6%	-5.3%
14 Unsafe sanitation	**-49.9%**	**-55.2%**	**-52.2%**
15 Low whole grains	**22.5%**	9.4%	-3.7%
16 Occupational injury	-10.8%	**-20.3%**	**-24.2%**
17 High sodium	15.7%	3.4%	-9.7%
18 Handwashing	**-41.6%**	**-47.8%**	**-43%**
19 Unsafe sex	**-25.8%**	**-33.7%**	**-37.2%**
20 Low fruit	**16.4%**	4%	-8.2%
22 Second-hand smoke			
26 Child underweight			

Legend:
Environmental
Behavioural
Metabolic

Figure S1h: Leading 20 risk factors for DALYs for males in Middle SDI countries, 1990, 2010 and 2019.

Leading risks 1990	Leading risks 2010	Mean % change number of YLLs 1990-2010	Mean % change all-age YLL rate 1990-2010	Mean % change age-standardised YLL rate 1990-2010	Leading risks 2019	Mean % change number of YLLs 2010-2019	Mean % change all-age YLL rate 2010-2019	Mean % change age-standardised YLL rate 2010-2019
1 Low birth weight	1 Smoking	**41.2%**	**10.7%**	**-21.4%**	1 Smoking	11.2%	2.7%	**-15.2%**
2 Smoking	2 High blood pressure	**78.8%**	**40.1%**	-0.8%	2 High blood pressure	**19.8%**	10.7%	-8%
3 Short gestation	3 Ambient particulate matter	**67.3%**	**31.1%**	18.2%	3 High fasting plasma glucose	**36.1%**	**25.7%**	2.6%
4 Child wasting	4 High fasting plasma glucose	**113.2%**	**67.1%**	**20.3%**	4 High body-mass index	**45.5%**	**34.4%**	**15.2%**
5 Household air pollution	5 Low birth weight	**-41.6%**	**-54.2%**	**-34.3%**	5 Ambient particulate matter	**14.5%**	5.7%	-9.4%
6 High blood pressure	6 Alcohol use	**38.5%**	8.6%	**-13.5%**	6 Alcohol use	**11.1%**	2.7%	-6.6%
7 Alcohol use	7 High body-mass index	**155.5%**	**100.2%**	**45.9%**	7 High LDL	**20.4%**	**11.2%**	-5.1%
8 Ambient particulate matter	8 Short gestation	**-39.9%**	**-52.9%**	**-32.5%**	8 Kidney dysfunction	**22.1%**	**12.7%**	-4.1%
9 Unsafe water	9 High LDL	**79.7%**	**40.8%**	1.8%	9 Low birth weight	**-34.1%**	**-39.1%**	**-31.9%**
10 High fasting plasma glucose	10 Kidney dysfunction	**85.9%**	**45.7%**	**11.4%**	10 High sodium	14.2%	5.4%	-12.4%
11 Unsafe sanitation	11 Household air pollution	**-56%**	**-65.5%**	**-67.9%**	11 Short gestation	**-33%**	**-38.1%**	**-30.9%**
12 High LDL	12 High sodium	**44.6%**	13.3%	**-18.7%**	12 Low whole grains	**20.8%**	**11.6%**	-5.6%
13 Child underweight	13 Child wasting	**-72%**	**-78.1%**	**-69.4%**	13 Household air pollution	**-41%**	**-45.5%**	**-53%**
14 Occupational injury	14 Unsafe sex	**1119.8%**	**855.9%**	**736.6%**	14 Second-hand smoke	8.1%	-0.2%	**-15.1%**
15 High body-mass index	15 Low whole grains	**81.8%**	**42.4%**	2.8%	15 Unsafe sex	**-23.7%**	**-29.5%**	**-30.5%**
16 High sodium	16 Occupational injury	**-28.6%**	**-44.1%**	**-49.8%**	16 Occupational injury	-10.7%	**-17.6%**	**-18.3%**
17 Second-hand smoke	17 Second-hand smoke	**-26.7%**	**-42.6%**	**-41.4%**	17 Low fruit	5.7%	-2.3%	**-17.2%**
18 Kidney dysfunction	18 Unsafe water	**-63.7%**	**-71.6%**	**-64.8%**	18 Drug use	**8%**	-0.2%	-3.6%
19 Handwashing	19 Low fruit	**32.4%**	3.7%	**-24.3%**	19 Lead	2.6%	-5.2%	**-19.4%**
20 Child stunting	20 Lead	**42.1%**	11.4%	**-13.2%**	20 High red meat	**26.7%**	**17%**	-1.1%
22 Low whole grains	21 Drug use				23 Child wasting			
23 Low fruit	24 High red meat				24 Unsafe water			
25 Lead	26 Unsafe sanitation							
26 Drug use	28 Child underweight							
44 Unsafe sex	32 Handwashing							
	42 Child stunting							

Legend:
Environmental
Behavioural
Metabolic

Figure S1i: Leading 20 risk factors for DALYs for males in High-middle SDI countries, 1990, 2010 and 2019.

Leading risks 1990
1 Smoking
2 High blood pressure
3 Alcohol use
4 High LDL
5 Low birth weight
6 High body-mass index
7 Ambient particulate matter
8 Short gestation
9 High fasting plasma glucose
10 Household air pollution
11 High sodium
12 Low whole grains
13 Child wasting
14 Kidney dysfunction
15 Occupational injury
16 Second-hand smoke
17 Low legumes
18 Low temperature
19 Low fruit
20 High red meat
21 Drug use
24 Lead

Leading risks 2010	Mean % change number of YLLs 1990-2010	Mean % change all-age YLL rate 1990-2010	Mean % change age-standardised YLL rate 1990-2010
1 Smoking	**12.6%**	-4.6%	**-27.6%**
2 High blood pressure	**30.6%**	**10.7%**	**-17.5%**
3 Alcohol use	**30.4%**	**10.4%**	**-9.5%**
4 High body-mass index	**61.8%**	**37.1%**	6.1%
5 High fasting plasma glucose	**68.1%**	**42.4%**	6.9%
6 High LDL	**22.4%**	3.7%	**-21.9%**
7 Ambient particulate matter	**31%**	11%	-11.6%
8 High sodium	17.2%	-0.7%	**-25.2%**
9 Kidney dysfunction	**40.5%**	**19%**	**-8.7%**
10 Low whole grains	**20.4%**	2%	**-23.1%**
11 Low birth weight	**-57.9%**	**-64.3%**	**-49.2%**
12 Drug use	**49.1%**	**26.4%**	**11.5%**
13 Short gestation	**-56.9%**	**-63.5%**	**-48.4%**
14 High red meat	**28%**	8.5%	**-17%**
15 Low legumes	10.6%	-6.3%	**-29.1%**
16 Second-hand smoke	**-15.5%**	**-28.4%**	**-41.7%**
17 Low temperature	5.7%	-10.4%	**-39.4%**
18 Low fruit	5.6%	**-10.6%**	**-31.2%**
19 Occupational injury	**-32%**	**-42.3%**	**-47.9%**
20 Household air pollution	**-69.4%**	**-74.1%**	**-79.2%**
22 Lead			
30 Child wasting			

Leading risks 2019	Mean % change number of YLLs 2010-2019	Mean % change all-age YLL rate 2010-2019	Mean % change age-standardised YLL rate 2010-2019
1 Smoking	0.2%	-5.3%	**-19.7%**
2 High blood pressure	0.6%	-5%	**-19.4%**
3 High body-mass index	**13.7%**	7.4%	-7%
4 Alcohol use	**-10.4%**	**-15.3%**	**-21.2%**
5 High fasting plasma glucose	**9.1%**	3.1%	**-13.8%**
6 High LDL	-3.3%	**-8.6%**	**-21%**
7 Ambient particulate matter	-1.6%	-7%	**-21%**
8 High sodium	2.8%	-2.9%	**-17.5%**
9 Kidney dysfunction	5.8%	0%	**-14.5%**
10 Low whole grains	-0.2%	-5.7%	**-19.2%**
11 Drug use	-4.2%	**-9.5%**	**-10.6%**
12 High red meat	5.6%	-0.2%	**-13.8%**
13 Low temperature	6.3%	0.4%	**-22.1%**
14 Low legumes	-5.3%	**-10.5%**	**-22.9%**
15 Second-hand smoke	-0.3%	-5.8%	**-20.2%**
16 Low birth weight	**-32.7%**	**-36.4%**	**-33.2%**
17 Low fruit	-7.5%	**-12.6%**	**-24.3%**
18 Occupational injury	-8.9%	**-13.9%**	**-13.3%**
19 Short gestation	**-31.4%**	**-35.2%**	**-32.2%**
20 Lead	-6.5%	**-11.6%**	**-24.7%**
28 Household air pollution			

Legend:
Environmental
Behavioural
Metabolic

Figure S1j: Leading 20 risk factors for DALYs for males in High SDI countries, 1990, 2010 and 2019.

Leading risks 1990
1 Smoking
2 High blood pressure
3 High LDL
4 Alcohol use
5 High body-mass index
6 High fasting plasma glucose
7 Ambient particulate matter
8 Kidney dysfunction
9 Low whole grains
10 Low legumes
11 Low birth weight
12 Drug use
13 High sodium
14 Short gestation
15 Occupational injury
16 High red meat
17 Low temperature
18 Second-hand smoke
19 High trans fat
20 Low fruit
21 Occupational asbestos
22 High processed meat

Leading risks 2010	Mean % change number of YLLs 1990-2010	Mean % change all-age YLL rate 1990-2010	Mean % change age-standardised YLL rate 1990-2010
1 Smoking	**-11.5%**	**-24.6%**	**-41.3%**
2 High blood pressure	**-25.2%**	**-36.3%**	**-51.2%**
3 High body-mass index	**33.4%**	**13.7%**	-8.4%
4 High fasting plasma glucose	**31.4%**	**12%**	**-14.5%**
5 Alcohol use	-0.3%	**-15%**	**-26.4%**
6 High LDL	**-35.5%**	**-45%**	**-57.3%**
7 Drug use	**76.5%**	**50.4%**	**50.4%**
8 Kidney dysfunction	**16.6%**	-0.6%	**-25%**
9 Ambient particulate matter	-23.2%	**-34.5%**	**-47.8%**
10 Low whole grains	**-14.2%**	**-26.8%**	**-43.2%**
11 High sodium	**-15%**	**-27.5%**	**-44%**
12 Low temperature	-3.9%	**-18%**	**-45%**
13 High red meat	-11.6%	**-24.6%**	**-40.3%**
14 Low legumes	**-29.5%**	**-39.9%**	**-53.2%**
15 Occupational asbestos	**8.3%**	**-7.6%**	**-30.6%**
16 Low birth weight	**-37.8%**	**-47%**	**-37.8%**
17 Short gestation	**-37%**	**-46.3%**	**-37.1%**
18 High processed meat	7.2%	-8.6%	-28.6%
19 Occupational injury	**-36%**	**-45.4%**	**-46.3%**
20 Second-hand smoke	**-27.1%**	**-37.8%**	**-50.7%**
21 Low fruit			
23 High trans fat			
24 Low bone mineral density			

Leading risks 2019	Mean % change number of YLLs 2010-2019	Mean % change all-age YLL rate 2010-2019	Mean % change age-standardised YLL rate 2010-2019
1 Smoking	1.4%	**-4.8%**	**-16.1%**
2 High blood pressure	**12.4%**	**5.6%**	**-7.7%**
3 High fasting plasma glucose	**24.2%**	**16.6%**	1.7%
4 High body-mass index	**19.7%**	**12.4%**	1.7%
5 Alcohol use	**4.5%**	-1.8%	**-7.7%**
6 High LDL	**3.8%**	-2.6%	**-12.8%**
7 Drug use	**43.1%**	**34.4%**	**38.3%**
8 Kidney dysfunction	**20.1%**	**12.8%**	**-3.1%**
9 Ambient particulate matter	-2.2%	**-8.2%**	**-17.5%**
10 Low whole grains	**9.4%**	2.7%	**-8.9%**
11 Low temperature	**13%**	**6.1%**	**-13.3%**
12 High red meat	**12.8%**	**6%**	-5.1%
13 High sodium	7%	0.5%	**-12%**
14 Low legumes	**5.4%**	-1%	**-12.4%**
15 Occupational asbestos	5.6%	-0.9%	**-17.1%**
16 High processed meat	**10.9%**	**4.2%**	**-7.4%**
17 Second-hand smoke	**6.9%**	0.4%	**-9.8%**
18 Occupational injury	0.5%	-5.6%	-4.4%
19 Low fruit	4.1%	-2.3%	**-12.6%**
20 Low bone mineral density	**22.1%**	**14.7%**	-0.7%
21 Low birth weight			
22 Short gestation			

Legend:
Environmental
Behavioural
Metabolic

Figure S2. Leading 10 level 3 risk factors for deaths in 2019 by location. Each risk is colored by the percentage change in all-age deaths from 2010 to 2019.

	Percentage change in all-age deaths from 2010 to 2019									
	-0.800 to -0.062	-0.062 to 0.007	0.007 to 0.052	0.052 to 0.093	0.093 to 0.132	0.132 to 0.174	0.174 to 0.223	0.223 to 0.287	0.287 to 0.372	0.372 to 1.738
	1	2	3	4	5	6	7	8	9	10
**Global**	High blood pressure	Smoking	High fasting plasma glucose	Particulate matter	High body-mass index	High LDL	Kidney dysfunction	Alcohol use	High sodium	Low whole grains
**Low SDI**	Particulate matter	Low birth weight & short gestation	High blood pressure	Child growth failure	Unsafe water	High fasting plasma glucose	Unsafe sanitation	Smoking	Handwashing	High body-mass index
**Low-middle SDI**	High blood pressure	Particulate matter	Smoking	High fasting plasma glucose	High body-mass index	High LDL	Low birth weight & short gestation	Kidney dysfunction	Unsafe water	Alcohol use
**Middle SDI**	High blood pressure	Smoking	High fasting plasma glucose	Particulate matter	High body-mass index	High LDL	Kidney dysfunction	High sodium	Alcohol use	Low whole grains
**High-middle SDI**	High blood pressure	Smoking	High fasting plasma glucose	High LDL	High body-mass index	Particulate matter	Kidney dysfunction	Alcohol use	Low whole grains	High sodium
**High SDI**	High blood pressure	Smoking	High fasting plasma glucose	High body-mass index	High LDL	Kidney dysfunction	Alcohol use	Low temperature	Low whole grains	Particulate matter
**Central Europe, eastern Europe, and central Asia**	High blood pressure	High LDL	Smoking	High body-mass index	High fasting plasma glucose	Alcohol use	Low whole grains	Particulate matter	Kidney dysfunction	Low temperature
**Central Asia**	High blood pressure	High fasting plasma glucose	High body-mass index	High LDL	Smoking	Particulate matter	Low whole grains	Kidney dysfunction	Low legumes	Alcohol use
Armenia	High blood pressure	High fasting plasma glucose	Smoking	High body-mass index	High LDL	Particulate matter	Low whole grains	Kidney dysfunction	Low legumes	Low temperature
Azerbaijan	High blood pressure	High fasting plasma glucose	High body-mass index	Smoking	High LDL	Particulate matter	Low whole grains	Kidney dysfunction	Low legumes	Low temperature
Georgia	High blood pressure	High fasting plasma glucose	High body-mass index	Smoking	High LDL	Particulate matter	Low whole grains	Kidney dysfunction	Low temperature	High sodium
Kazakhstan	High blood pressure	High body-mass index	High fasting plasma glucose	Smoking	High LDL	Particulate matter	Alcohol use	Low whole grains	Kidney dysfunction	Low temperature
Kyrgyzstan	High blood pressure	Smoking	High LDL	High body-mass index	Particulate matter	Low whole grains	Alcohol use	High fasting plasma glucose	Kidney dysfunction	Low temperature
Mongolia	High blood pressure	Smoking	High body-mass index	Alcohol use	Particulate matter	High LDL	High red meat	Kidney dysfunction	Low whole grains	Low fruit
Tajikistan	High blood pressure	High fasting plasma glucose	Particulate matter	High LDL	High body-mass index	Smoking	Low whole grains	Low temperature	Low legumes	Kidney dysfunction
Turkmenistan	High blood pressure	High body-mass index	High LDL	High fasting plasma glucose	Smoking	Particulate matter	Low whole grains	Kidney dysfunction	Alcohol use	Low legumes
Uzbekistan	High blood pressure	High fasting plasma glucose	High body-mass index	High LDL	Particulate matter	Smoking	Low whole grains	Kidney dysfunction	Low legumes	Low temperature
**Central Europe**	High blood pressure	Smoking	High fasting plasma glucose	High body-mass index	High LDL	High sodium	Particulate matter	Alcohol use	Kidney dysfunction	Low temperature
Albania	High blood pressure	Smoking	High body-mass index	High LDL	High sodium	Particulate matter	High fasting plasma glucose	Low whole grains	Kidney dysfunction	Low temperature
Bosnia and Herzegovina	High blood pressure	High fasting plasma glucose	Smoking	High body-mass index	High LDL	Particulate matter	High sodium	Kidney dysfunction	Low temperature	Low whole grains
Bulgaria	High blood pressure	High body-mass index	High fasting plasma glucose	Smoking	High LDL	High sodium	Particulate matter	Kidney dysfunction	Low temperature	Alcohol use
Croatia	High blood pressure	Smoking	High fasting plasma glucose	High body-mass index	High LDL	High sodium	Kidney dysfunction	Particulate matter	Alcohol use	Low temperature
Czech Republic	High blood pressure	High fasting plasma glucose	Smoking	High body-mass index	High LDL	High sodium	Alcohol use	Kidney dysfunction	Particulate matter	Low temperature
Hungary	High blood pressure	Smoking	High fasting plasma glucose	High body-mass index	High LDL	High sodium	Alcohol use	Particulate matter	Kidney dysfunction	Low whole grains
Montenegro	High blood pressure	Smoking	High fasting plasma glucose	High body-mass index	High LDL	Particulate matter	High sodium	Kidney dysfunction	Alcohol use	Low temperature
North Macedonia	High blood pressure	High fasting plasma glucose	Smoking	High body-mass index	Particulate matter	High LDL	High sodium	Kidney dysfunction	Low temperature	Low whole grains
Poland	High blood pressure	Smoking	High fasting plasma glucose	High body-mass index	High LDL	Particulate matter	Alcohol use	High sodium	Low whole grains	Kidney dysfunction
Romania	High blood pressure	High body-mass index	High LDL	Smoking	High sodium	High fasting plasma glucose	Alcohol use	Particulate matter	Low temperature	Kidney dysfunction
Serbia	High blood pressure	High fasting plasma glucose	Smoking	High body-mass index	High LDL	Particulate matter	High sodium	Kidney dysfunction	Low temperature	Low whole grains
Slovakia	High blood pressure	High LDL	High body-mass index	Smoking	High fasting plasma glucose	High sodium	Particulate matter	Kidney dysfunction	Alcohol use	Low whole grains
Slovenia	High blood pressure	Smoking	High body-mass index	High fasting plasma glucose	High LDL	High sodium	Alcohol use	Particulate matter	Low temperature	Kidney dysfunction
**Eastern Europe**	High blood pressure	High LDL	Smoking	High body-mass index	High fasting plasma glucose	Alcohol use	Low whole grains	Kidney dysfunction	Low legumes	Particulate matter

Figure S2. Leading 10 level 3 risk factors for deaths in 2019 by location. Each risk is colored by the percentage change in all-age deaths from 2010 to 2019.

	Percentage change in all-age deaths from 2010 to 2019									
	-0.800 to -0.062	-0.062 to 0.007	0.007 to 0.052	0.052 to 0.093	0.093 to 0.132	0.132 to 0.174	0.174 to 0.223	0.223 to 0.287	0.287 to 0.372	0.372 to 1.738
	**1**	**2**	**3**	**4**	**5**	**6**	**7**	**8**	**9**	**10**
Belarus	High blood pressure	High LDL	Smoking	High body-mass index	Low whole grains	High fasting plasma glucose	Low legumes	Alcohol use	Particulate matter	Kidney dysfunction
Estonia	High blood pressure	High body-mass index	High LDL	Smoking	High fasting plasma glucose	Alcohol use	Kidney dysfunction	Low whole grains	Low legumes	Low temperature
Latvia	High blood pressure	High LDL	High body-mass index	Smoking	High fasting plasma glucose	Low whole grains	Kidney dysfunction	Alcohol use	Low legumes	Low temperature
Lithuania	High blood pressure	High LDL	High body-mass index	Smoking	High fasting plasma glucose	Low whole grains	Alcohol use	Kidney dysfunction	Low legumes	Low temperature
Moldova	High blood pressure	High body-mass index	High LDL	Smoking	High fasting plasma glucose	Alcohol use	Low whole grains	Low legumes	Particulate matter	Kidney dysfunction
Russia	High blood pressure	High LDL	Smoking	High body-mass index	High fasting plasma glucose	Alcohol use	Low whole grains	Kidney dysfunction	Low temperature	Low legumes
Ukraine	High blood pressure	High LDL	High body-mass index	Smoking	High fasting plasma glucose	Low whole grains	Low legumes	Particulate matter	Alcohol use	Kidney dysfunction
**High income**	High blood pressure	Smoking	High fasting plasma glucose	High body-mass index	High LDL	Kidney dysfunction	Alcohol use	Low temperature	Low whole grains	Particulate matter
**Australasia**	High blood pressure	Smoking	High body-mass index	High fasting plasma glucose	High LDL	Kidney dysfunction	Alcohol use	Low whole grains	High red meat	Low temperature
Australia	High blood pressure	Smoking	High body-mass index	High fasting plasma glucose	High LDL	Kidney dysfunction	Alcohol use	High red meat	Low whole grains	Occupational carcinogens
New Zealand	High blood pressure	Smoking	High body-mass index	High fasting plasma glucose	High LDL	Kidney dysfunction	Low temperature	Alcohol use	Low whole grains	High red meat
**High-income Asia Pacific**	Smoking	High blood pressure	High fasting plasma glucose	Kidney dysfunction	High LDL	Alcohol use	High body-mass index	Low temperature	Particulate matter	High sodium
Brunei	High fasting plasma glucose	High blood pressure	Smoking	High body-mass index	High LDL	Kidney dysfunction	High sodium	Low whole grains	Low fruit	Low fibre
Japan	Smoking	High blood pressure	High fasting plasma glucose	Kidney dysfunction	High LDL	Low temperature	High body-mass index	Alcohol use	Particulate matter	High sodium
Aichi	Smoking	High blood pressure	High fasting plasma glucose	Kidney dysfunction	High LDL	High body-mass index	Alcohol use	Low temperature	Particulate matter	High sodium
Akita	High blood pressure	Smoking	High fasting plasma glucose	Kidney dysfunction	High LDL	Low temperature	High body-mass index	High sodium	Alcohol use	Low whole grains
Aomori	High blood pressure	Smoking	High fasting plasma glucose	Kidney dysfunction	High LDL	Low temperature	High body-mass index	Alcohol use	High sodium	Low whole grains
Chiba	Smoking	High blood pressure	High fasting plasma glucose	Kidney dysfunction	High LDL	Low temperature	Alcohol use	High body-mass index	Particulate matter	High sodium
Ehime	High blood pressure	Smoking	High fasting plasma glucose	Kidney dysfunction	High LDL	Low temperature	High body-mass index	Alcohol use	High sodium	Particulate matter
Fukui	High blood pressure	Smoking	High fasting plasma glucose	Kidney dysfunction	High LDL	Low temperature	High body-mass index	High sodium	Alcohol use	Particulate matter
Fukuoka	Smoking	High blood pressure	High fasting plasma glucose	Kidney dysfunction	High LDL	Alcohol use	High body-mass index	Particulate matter	Low temperature	High sodium
Fukushima	High blood pressure	Smoking	High fasting plasma glucose	High LDL	Kidney dysfunction	Low temperature	High body-mass index	High sodium	Alcohol use	Particulate matter
Gifu	High blood pressure	Smoking	High fasting plasma glucose	Kidney dysfunction	High LDL	Low temperature	High body-mass index	Alcohol use	High sodium	Particulate matter
Gunma	High blood pressure	Smoking	High fasting plasma glucose	High LDL	Kidney dysfunction	Low temperature	High body-mass index	Alcohol use	High sodium	Particulate matter
Hiroshima	High blood pressure	Smoking	High fasting plasma glucose	Kidney dysfunction	High LDL	Low temperature	High body-mass index	Alcohol use	High sodium	Particulate matter
Hokkaidō	Smoking	High blood pressure	High fasting plasma glucose	Kidney dysfunction	High LDL	Low temperature	High body-mass index	Alcohol use	High sodium	Low whole grains
Hyōgo	Smoking	High blood pressure	High fasting plasma glucose	Kidney dysfunction	High LDL	Low temperature	Alcohol use	High body-mass index	Particulate matter	High sodium
Ibaraki	High blood pressure	Smoking	High fasting plasma glucose	Kidney dysfunction	High LDL	Low temperature	High body-mass index	Alcohol use	High sodium	Particulate matter
Ishikawa	High blood pressure	Smoking	High fasting plasma glucose	High LDL	Kidney dysfunction	Low temperature	High body-mass index	Alcohol use	High sodium	Particulate matter
Iwate	High blood pressure	Smoking	High fasting plasma glucose	Kidney dysfunction	High LDL	Low temperature	High body-mass index	High sodium	Alcohol use	Low whole grains
Kagawa	High blood pressure	Smoking	High fasting plasma glucose	Kidney dysfunction	High LDL	Low temperature	High body-mass index	Alcohol use	Particulate matter	High sodium
Kagoshima	High blood pressure	Smoking	High fasting plasma glucose	Kidney dysfunction	High LDL	High body-mass index	Particulate matter	Alcohol use	Low temperature	High sodium

Figure S2. Leading 10 level 3 risk factors for deaths in 2019 by location. Each risk is colored by the percentage change in all-age deaths from 2010 to 2019.

	**Percentage change in all-age deaths from 2010 to 2019**									
	-0.800 to -0.062	-0.062 to 0.007	0.007 to 0.052	0.052 to 0.093	0.093 to 0.132	0.132 to 0.174	0.174 to 0.223	0.223 to 0.287	0.287 to 0.372	0.372 to 1.738
	**1**	**2**	**3**	**4**	**5**	**6**	**7**	**8**	**9**	**10**
Kanagawa	Smoking	High blood pressure	High fasting plasma glucose	Kidney dysfunction	High LDL	Alcohol use	High body-mass index	Low temperature	Particulate matter	High sodium
Kōchi	High blood pressure	Smoking	High fasting plasma glucose	Kidney dysfunction	High LDL	Low temperature	High body-mass index	Particulate matter	Alcohol use	High sodium
Kumamoto	High blood pressure	Smoking	High fasting plasma glucose	Kidney dysfunction	High LDL	Low temperature	High body-mass index	Particulate matter	Alcohol use	High sodium
Kyōto	High blood pressure	Smoking	High fasting plasma glucose	Kidney dysfunction	High LDL	Low temperature	High body-mass index	Alcohol use	High sodium	Particulate matter
Mie	High blood pressure	Smoking	High fasting plasma glucose	Kidney dysfunction	High LDL	Low temperature	High body-mass index	Alcohol use	High sodium	Particulate matter
Miyagi	High blood pressure	Smoking	High fasting plasma glucose	High LDL	Kidney dysfunction	Low temperature	High body-mass index	Alcohol use	High sodium	Low whole grains
Miyazaki	High blood pressure	Smoking	High fasting plasma glucose	Kidney dysfunction	High LDL	Low temperature	High body-mass index	Particulate matter	Alcohol use	High sodium
Nagano	High blood pressure	Smoking	High fasting plasma glucose	High LDL	Kidney dysfunction	Low temperature	High body-mass index	High sodium	Alcohol use	Particulate matter
Nagasaki	High blood pressure	Smoking	High fasting plasma glucose	Kidney dysfunction	High LDL	Particulate matter	High body-mass index	Low temperature	Alcohol use	High sodium
Nara	Smoking	High blood pressure	High fasting plasma glucose	Kidney dysfunction	High LDL	Low temperature	High body-mass index	Alcohol use	Particulate matter	High sodium
Niigata	Smoking	High blood pressure	High fasting plasma glucose	High LDL	Kidney dysfunction	Low temperature	High body-mass index	High sodium	Alcohol use	Particulate matter
Ōita	High blood pressure	Smoking	High fasting plasma glucose	Kidney dysfunction	High LDL	High body-mass index	Low temperature	Alcohol use	Particulate matter	High sodium
Okayama	High blood pressure	Smoking	High fasting plasma glucose	Kidney dysfunction	High LDL	Low temperature	High body-mass index	Alcohol use	Particulate matter	High sodium
Okinawa	Smoking	High blood pressure	High fasting plasma glucose	Kidney dysfunction	High LDL	High body-mass index	Alcohol use	Particulate matter	High sodium	Low whole grains
Ōsaka	Smoking	High blood pressure	High fasting plasma glucose	Kidney dysfunction	High LDL	Alcohol use	High body-mass index	Low temperature	Particulate matter	High sodium
Saga	High blood pressure	Smoking	High fasting plasma glucose	Kidney dysfunction	High LDL	Low temperature	High body-mass index	Particulate matter	Alcohol use	High sodium
Saitama	Smoking	High blood pressure	High fasting plasma glucose	High LDL	Kidney dysfunction	Low temperature	Particulate matter	High body-mass index	Alcohol use	High sodium
Shiga	High blood pressure	Smoking	High fasting plasma glucose	Kidney dysfunction	High LDL	Low temperature	High body-mass index	Alcohol use	High sodium	Particulate matter
Shimane	High blood pressure	Smoking	High fasting plasma glucose	Kidney dysfunction	High LDL	Low temperature	High body-mass index	Alcohol use	High sodium	Particulate matter
Shizuoka	High blood pressure	Smoking	High fasting plasma glucose	Kidney dysfunction	High LDL	Low temperature	High body-mass index	Alcohol use	High sodium	Particulate matter
Tochigi	High blood pressure	Smoking	High fasting plasma glucose	High LDL	Kidney dysfunction	Low temperature	High body-mass index	Alcohol use	Particulate matter	High sodium
Tokushima	High blood pressure	Smoking	High fasting plasma glucose	Kidney dysfunction	High LDL	Low temperature	High body-mass index	Alcohol use	Particulate matter	High sodium
Tōkyō	Smoking	High blood pressure	High fasting plasma glucose	High LDL	Kidney dysfunction	Alcohol use	High body-mass index	Low temperature	Particulate matter	High sodium
Tottori	High blood pressure	Smoking	High fasting plasma glucose	High LDL	Kidney dysfunction	Low temperature	High body-mass index	Particulate matter	High sodium	Alcohol use
Toyama	High blood pressure	Smoking	High fasting plasma glucose	High LDL	Kidney dysfunction	Low temperature	High body-mass index	Alcohol use	High sodium	Particulate matter
Wakayama	High blood pressure	Smoking	High fasting plasma glucose	Kidney dysfunction	High LDL	Low temperature	High body-mass index	Alcohol use	Particulate matter	High sodium
Yamagata	High blood pressure	Smoking	High fasting plasma glucose	High LDL	Kidney dysfunction	Low temperature	High body-mass index	High sodium	Alcohol use	Low whole grains
Yamaguchi	High blood pressure	Smoking	High fasting plasma glucose	Kidney dysfunction	High LDL	Low temperature	High body-mass index	Alcohol use	High sodium	Particulate matter
Yamanashi	High blood pressure	Smoking	High fasting plasma glucose	Kidney dysfunction	High LDL	Low temperature	High body-mass index	Alcohol use	High sodium	Particulate matter
South Korea	Smoking	High fasting plasma glucose	High blood pressure	Alcohol use	Particulate matter	High body-mass index	High LDL	Kidney dysfunction	Low temperature	High sodium
Singapore	High blood pressure	Smoking	High fasting plasma glucose	High LDL	High body-mass index	Kidney dysfunction	Particulate matter	High sodium	Low whole grains	High red meat
**High-income North America**	Smoking	High blood pressure	High fasting plasma glucose	High body-mass index	High LDL	Kidney dysfunction	Alcohol use	Low temperature	Low whole grains	Drug use

Figure S2. Leading 10 level 3 risk factors for deaths in 2019 by location. Each risk is colored by the percentage change in all-age deaths from 2010 to 2019.

Percentage change in all-age deaths from 2010 to 2019

-0.800 to -0.062	-0.062 to 0.007	0.007 to 0.052	0.052 to 0.093	0.093 to 0.132	0.132 to 0.174	0.174 to 0.223	0.223 to 0.287	0.287 to 0.372	0.372 to 1.738

	1	2	3	4	5	6	7	8	9	10
Canada	Smoking	High blood pressure	High fasting plasma glucose	High body-mass index	High LDL	Kidney dysfunction	Alcohol use	Low temperature	Low whole grains	Occupational carcinogens
Greenland	Smoking	High blood pressure	High body-mass index	High fasting plasma glucose	Alcohol use	High LDL	Kidney dysfunction	Low temperature	Low whole grains	Occupational carcinogens
USA	Smoking	High blood pressure	High fasting plasma glucose	High body-mass index	High LDL	Kidney dysfunction	Alcohol use	Low temperature	Drug use	Low whole grains
Alabama	Smoking	High blood pressure	High fasting plasma glucose	High body-mass index	High LDL	Kidney dysfunction	Alcohol use	Drug use	Low temperature	Low whole grains
Alaska	Smoking	High blood pressure	High body-mass index	High fasting plasma glucose	Alcohol use	High LDL	Drug use	Kidney dysfunction	Low temperature	Low whole grains
Arizona	Smoking	High blood pressure	High fasting plasma glucose	High body-mass index	High LDL	Kidney dysfunction	Alcohol use	Drug use	Low temperature	Low whole grains
Arkansas	Smoking	High blood pressure	High fasting plasma glucose	High body-mass index	High LDL	Kidney dysfunction	Low temperature	Low whole grains	Alcohol use	Low legumes
California	High blood pressure	Smoking	High fasting plasma glucose	High body-mass index	High LDL	Kidney dysfunction	Alcohol use	Low temperature	Low whole grains	Drug use
Colorado	Smoking	High blood pressure	High fasting plasma glucose	High body-mass index	High LDL	Alcohol use	Kidney dysfunction	Low temperature	Drug use	Low whole grains
Connecticut	Smoking	High blood pressure	High fasting plasma glucose	High body-mass index	High LDL	Kidney dysfunction	Alcohol use	Low temperature	Drug use	Low whole grains
Delaware	Smoking	High blood pressure	High fasting plasma glucose	High body-mass index	High LDL	Kidney dysfunction	Low temperature	Alcohol use	Drug use	Low whole grains
Washington, DC	High blood pressure	High body-mass index	Smoking	High fasting plasma glucose	High LDL	Kidney dysfunction	Alcohol use	Drug use	Low temperature	Low whole grains
Florida	Smoking	High blood pressure	High fasting plasma glucose	High body-mass index	High LDL	Kidney dysfunction	Alcohol use	Low whole grains	Drug use	Low legumes
Georgia	Smoking	High blood pressure	High fasting plasma glucose	High body-mass index	Kidney dysfunction	High LDL	Alcohol use	Low whole grains	Low temperature	Drug use
Hawaii	High blood pressure	Smoking	High fasting plasma glucose	High body-mass index	Kidney dysfunction	High LDL	Alcohol use	Low whole grains	Drug use	High red meat
Idaho	Smoking	High blood pressure	High fasting plasma glucose	High body-mass index	High LDL	Kidney dysfunction	Low temperature	Alcohol use	Low whole grains	Drug use
Illinois	Smoking	High blood pressure	High fasting plasma glucose	High body-mass index	Kidney dysfunction	High LDL	Low temperature	Alcohol use	Low whole grains	Drug use
Indiana	Smoking	High blood pressure	High fasting plasma glucose	High body-mass index	Kidney dysfunction	High LDL	Low temperature	Alcohol use	Low whole grains	Drug use
Iowa	Smoking	High blood pressure	High fasting plasma glucose	High body-mass index	High LDL	Kidney dysfunction	Low temperature	Low whole grains	Alcohol use	High red meat
Kansas	Smoking	High blood pressure	High fasting plasma glucose	High body-mass index	High LDL	Kidney dysfunction	Low temperature	Alcohol use	Low whole grains	Drug use
Kentucky	Smoking	High blood pressure	High fasting plasma glucose	High body-mass index	High LDL	Kidney dysfunction	Drug use	Low temperature	Alcohol use	Low whole grains
Louisiana	Smoking	High blood pressure	High fasting plasma glucose	High body-mass index	Kidney dysfunction	High LDL	Alcohol use	Drug use	Low whole grains	Low temperature
Maine	Smoking	High blood pressure	High fasting plasma glucose	High body-mass index	High LDL	Kidney dysfunction	Low temperature	Alcohol use	Drug use	Low whole grains
Maryland	High blood pressure	Smoking	High fasting plasma glucose	High body-mass index	High LDL	Kidney dysfunction	Alcohol use	Low temperature	Low whole grains	Drug use
Massachusetts	Smoking	High blood pressure	High fasting plasma glucose	High body-mass index	Kidney dysfunction	High LDL	Alcohol use	Low temperature	Drug use	Low whole grains
Michigan	Smoking	High blood pressure	High fasting plasma glucose	High body-mass index	High LDL	Kidney dysfunction	Low temperature	Alcohol use	Drug use	Low whole grains
Minnesota	Smoking	High blood pressure	High fasting plasma glucose	High body-mass index	Kidney dysfunction	High LDL	Alcohol use	Low temperature	Low whole grains	Drug use
Mississippi	Smoking	High blood pressure	High fasting plasma glucose	High body-mass index	Kidney dysfunction	High LDL	Alcohol use	Low temperature	Low whole grains	Drug use
Missouri	Smoking	High blood pressure	High fasting plasma glucose	High body-mass index	High LDL	Kidney dysfunction	Low temperature	Alcohol use	Low whole grains	Drug use
Montana	Smoking	High blood pressure	High fasting plasma glucose	High body-mass index	High LDL	Kidney dysfunction	Alcohol use	Low temperature	Low whole grains	Drug use
Nebraska	Smoking	High blood pressure	High fasting plasma glucose	High body-mass index	Kidney dysfunction	High LDL	Low temperature	Alcohol use	Low whole grains	High red meat
Nevada	Smoking	High blood pressure	High fasting plasma glucose	High body-mass index	High LDL	Kidney dysfunction	Alcohol use	Low temperature	Drug use	Low whole grains

Figure S2. Leading 10 level 3 risk factors for deaths in 2019 by location. Each risk is colored by the percentage change in all-age deaths from 2010 to 2019.

Percentage change in all-age deaths from 2010 to 2019

-0.800 to -0.062	-0.062 to 0.007	0.007 to 0.052	0.052 to 0.093	0.093 to 0.132	0.132 to 0.174	0.174 to 0.223	0.223 to 0.287	0.287 to 0.372	0.372 to 1.738

	1	2	3	4	5	6	7	8	9	10
New Hampshire	Smoking	High blood pressure	High fasting plasma glucose	High body-mass index	High LDL	Kidney dysfunction	Alcohol use	Drug use	Low temperature	Low whole grains
New Jersey	High blood pressure	Smoking	High fasting plasma glucose	High body-mass index	High LDL	Kidney dysfunction	Alcohol use	Low temperature	Low whole grains	Drug use
New Mexico	Smoking	High fasting plasma glucose	High blood pressure	High body-mass index	High LDL	Kidney dysfunction	Alcohol use	Drug use	Low temperature	Low whole grains
New York	High blood pressure	Smoking	High fasting plasma glucose	High body-mass index	High LDL	Kidney dysfunction	Low temperature	Alcohol use	Low whole grains	Low legumes
North Carolina	Smoking	High blood pressure	High fasting plasma glucose	High body-mass index	Kidney dysfunction	High LDL	Alcohol use	Low temperature	Drug use	Low whole grains
North Dakota	Smoking	High blood pressure	High fasting plasma glucose	High body-mass index	High LDL	Kidney dysfunction	Alcohol use	Low temperature	Low whole grains	High red meat
Ohio	Smoking	High blood pressure	High fasting plasma glucose	High body-mass index	High LDL	Kidney dysfunction	Low temperature	Drug use	Alcohol use	Low whole grains
Oklahoma	Smoking	High blood pressure	High fasting plasma glucose	High body-mass index	High LDL	Kidney dysfunction	Low temperature	Drug use	Alcohol use	Low whole grains
Oregon	Smoking	High blood pressure	High fasting plasma glucose	High body-mass index	Kidney dysfunction	High LDL	Alcohol use	Low temperature	Drug use	Low whole grains
Pennsylvania	Smoking	High blood pressure	High fasting plasma glucose	High body-mass index	High LDL	Kidney dysfunction	Low temperature	Alcohol use	Drug use	Low whole grains
Rhode Island	Smoking	High blood pressure	High fasting plasma glucose	High body-mass index	High LDL	Kidney dysfunction	Low temperature	Alcohol use	Drug use	Low whole grains
South Carolina	Smoking	High blood pressure	High fasting plasma glucose	High body-mass index	Kidney dysfunction	High LDL	Alcohol use	Drug use	Low temperature	Low whole grains
South Dakota	Smoking	High blood pressure	High fasting plasma glucose	High body-mass index	High LDL	Kidney dysfunction	Low temperature	Alcohol use	Low whole grains	Low legumes
Tennessee	Smoking	High blood pressure	High fasting plasma glucose	High body-mass index	High LDL	Kidney dysfunction	Drug use	Low temperature	Alcohol use	Low whole grains
Texas	Smoking	High blood pressure	High fasting plasma glucose	High body-mass index	High LDL	Kidney dysfunction	Alcohol use	Low temperature	Low whole grains	Drug use
Utah	High blood pressure	High fasting plasma glucose	High body-mass index	Smoking	Kidney dysfunction	High LDL	Drug use	Alcohol use	Low temperature	Low whole grains
Vermont	Smoking	High blood pressure	High fasting plasma glucose	High body-mass index	High LDL	Kidney dysfunction	Alcohol use	Low temperature	Low whole grains	Drug use
Virginia	Smoking	High blood pressure	High fasting plasma glucose	High body-mass index	Kidney dysfunction	High LDL	Alcohol use	Low temperature	Low whole grains	Drug use
Washington	Smoking	High blood pressure	High fasting plasma glucose	High body-mass index	High LDL	Kidney dysfunction	Alcohol use	Low temperature	Drug use	Low whole grains
West Virginia	Smoking	High fasting plasma glucose	High blood pressure	High body-mass index	High LDL	Kidney dysfunction	Drug use	Low temperature	Low whole grains	Alcohol use
Wisconsin	Smoking	High blood pressure	High fasting plasma glucose	High body-mass index	Kidney dysfunction	High LDL	Alcohol use	Low temperature	Low whole grains	Drug use
Wyoming	Smoking	High blood pressure	High fasting plasma glucose	High body-mass index	High LDL	Kidney dysfunction	Alcohol use	Low temperature	Drug use	Low whole grains
**Southern Latin America**	High blood pressure	Smoking	High fasting plasma glucose	High body-mass index	Kidney dysfunction	High LDL	Alcohol use	Low temperature	Particulate matter	Low whole grains
Argentina	High blood pressure	Smoking	High fasting plasma glucose	High body-mass index	Kidney dysfunction	High LDL	Alcohol use	Low temperature	Particulate matter	Low whole grains
Chile	High blood pressure	High fasting plasma glucose	High body-mass index	Smoking	Alcohol use	Kidney dysfunction	High LDL	Particulate matter	Low temperature	Low whole grains
Uruguay	High blood pressure	Smoking	High fasting plasma glucose	High body-mass index	High LDL	Kidney dysfunction	Alcohol use	Low whole grains	Low temperature	High red meat
**Western Europe**	High blood pressure	Smoking	High fasting plasma glucose	High body-mass index	High LDL	Alcohol use	Kidney dysfunction	Low temperature	Low whole grains	Particulate matter
Andorra	Smoking	High blood pressure	High body-mass index	High fasting plasma glucose	High LDL	Alcohol use	Kidney dysfunction	Low temperature	Occupational carcinogens	Low whole grains
Austria	High blood pressure	Smoking	High fasting plasma glucose	High LDL	High body-mass index	Kidney dysfunction	Alcohol use	Low temperature	Low whole grains	Low legumes
Belgium	Smoking	High blood pressure	High fasting plasma glucose	High body-mass index	High LDL	Alcohol use	Kidney dysfunction	Low temperature	Particulate matter	Low whole grains
Cyprus	High blood pressure	High fasting plasma glucose	Smoking	High LDL	High body-mass index	Kidney dysfunction	Particulate matter	Low whole grains	Alcohol use	Low temperature
Denmark	Smoking	High blood pressure	High fasting plasma glucose	High body-mass index	High LDL	Alcohol use	Low temperature	Kidney dysfunction	Low whole grains	Occupational carcinogens

Figure S2. Leading 10 level 3 risk factors for deaths in 2019 by location. Each risk is colored by the percentage change in all-age deaths from 2010 to 2019.

	Percentage change in all-age deaths from 2010 to 2019									
	-0.800 to -0.062	-0.062 to 0.007	0.007 to 0.052	0.052 to 0.093	0.093 to 0.132	0.132 to 0.174	0.174 to 0.223	0.223 to 0.287	0.287 to 0.372	0.372 to 1.738
	1	2	3	4	5	6	7	8	9	10
Finland	High blood pressure	High fasting plasma glucose	High LDL	Smoking	High body-mass index	Kidney dysfunction	Alcohol use	Low whole grains	Low temperature	Low legumes
France	High blood pressure	Smoking	High body-mass index	Alcohol use	High fasting plasma glucose	High LDL	Kidney dysfunction	Low temperature	Low whole grains	Occupational carcinogens
Germany	High blood pressure	High fasting plasma glucose	Smoking	High body-mass index	High LDL	Alcohol use	Kidney dysfunction	Low temperature	Low whole grains	Low legumes
Greece	High blood pressure	Smoking	High fasting plasma glucose	High LDL	High body-mass index	Kidney dysfunction	Low whole grains	Low temperature	Particulate matter	Alcohol use
Iceland	High blood pressure	Smoking	High LDL	High fasting plasma glucose	High body-mass index	Low temperature	Kidney dysfunction	Low whole grains	Alcohol use	Low legumes
Ireland	Smoking	High blood pressure	High fasting plasma glucose	High body-mass index	High LDL	Low temperature	Kidney dysfunction	Alcohol use	Low physical activity	High red meat
Israel	High blood pressure	High fasting plasma glucose	Smoking	High body-mass index	Kidney dysfunction	High LDL	Particulate matter	Low whole grains	Low physical activity	Low temperature
Italy	High blood pressure	High fasting plasma glucose	Smoking	High body-mass index	High LDL	Kidney dysfunction	Low whole grains	Alcohol use	Low temperature	Particulate matter
Luxembourg	High blood pressure	Smoking	High fasting plasma glucose	High body-mass index	High LDL	Alcohol use	Kidney dysfunction	Low temperature	Low whole grains	High red meat
Malta	High blood pressure	High fasting plasma glucose	Smoking	High LDL	High body-mass index	Kidney dysfunction	Low whole grains	Low physical activity	Particulate matter	Low temperature
Monaco	Smoking	High blood pressure	High body-mass index	High fasting plasma glucose	High LDL	Kidney dysfunction	Low temperature	Occupational carcinogens	Low whole grains	Alcohol use
Netherlands	Smoking	High blood pressure	High fasting plasma glucose	High body-mass index	High LDL	Alcohol use	Low temperature	Kidney dysfunction	Occupational carcinogens	Particulate matter
Norway	High blood pressure	Smoking	High fasting plasma glucose	High LDL	High body-mass index	Low temperature	Kidney dysfunction	Low whole grains	Alcohol use	Low physical activity
Portugal	High blood pressure	High fasting plasma glucose	Smoking	High body-mass index	High LDL	Alcohol use	Kidney dysfunction	Low temperature	Low whole grains	Low physical activity
San Marino	High blood pressure	Smoking	High fasting plasma glucose	High body-mass index	High LDL	Alcohol use	Kidney dysfunction	Low temperature	Low whole grains	Low physical activity
Spain	High blood pressure	Smoking	High fasting plasma glucose	High body-mass index	High LDL	Kidney dysfunction	Low temperature	Alcohol use	Low whole grains	Low physical activity
Sweden	High blood pressure	Smoking	High fasting plasma glucose	High LDL	High body-mass index	Kidney dysfunction	Alcohol use	Low temperature	Low whole grains	Low legumes
Stockholm	High blood pressure	Smoking	High fasting plasma glucose	High LDL	High body-mass index	Alcohol use	Low whole grains	Kidney dysfunction	Low temperature	Low legumes
Sweden except Stockholm	High blood pressure	Smoking	High fasting plasma glucose	High LDL	High body-mass index	Kidney dysfunction	Low temperature	Alcohol use	Low whole grains	Low legumes
Switzerland	High blood pressure	Smoking	High fasting plasma glucose	High LDL	High body-mass index	Kidney dysfunction	Alcohol use	Low temperature	Low whole grains	Low physical activity
UK	Smoking	High blood pressure	High fasting plasma glucose	High body-mass index	High LDL	Low temperature	Alcohol use	Kidney dysfunction	Low whole grains	Occupational carcinogens
England	Smoking	High blood pressure	High fasting plasma glucose	High body-mass index	High LDL	Low temperature	Alcohol use	Kidney dysfunction	Low whole grains	Occupational carcinogens
East Midlands	Smoking	High blood pressure	High fasting plasma glucose	High body-mass index	High LDL	Low temperature	Kidney dysfunction	Alcohol use	Low whole grains	Occupational carcinogens
Derby	Smoking	High blood pressure	High fasting plasma glucose	High body-mass index	High LDL	Low temperature	Alcohol use	Kidney dysfunction	Low whole grains	Occupational carcinogens
Derbyshire	Smoking	High blood pressure	High fasting plasma glucose	High body-mass index	High LDL	Low temperature	Kidney dysfunction	Low whole grains	Alcohol use	Occupational carcinogens
Leicester	Smoking	High blood pressure	High fasting plasma glucose	High body-mass index	High LDL	Low temperature	Alcohol use	Low whole grains	Kidney dysfunction	Particulate matter
Leicestershire	Smoking	High blood pressure	High fasting plasma glucose	High body-mass index	High LDL	Low temperature	Kidney dysfunction	Alcohol use	Low whole grains	Occupational carcinogens
Lincolnshire	Smoking	High blood pressure	High fasting plasma glucose	High body-mass index	High LDL	Low temperature	Kidney dysfunction	Low whole grains	Alcohol use	Occupational carcinogens
Northamptonshire	Smoking	High blood pressure	High fasting plasma glucose	High body-mass index	High LDL	Low temperature	Alcohol use	Kidney dysfunction	Low whole grains	Occupational carcinogens
Nottingham	Smoking	High blood pressure	High fasting plasma glucose	High body-mass index	High LDL	Low temperature	Alcohol use	Kidney dysfunction	Low whole grains	Particulate matter
Nottinghamshire	Smoking	High blood pressure	High fasting plasma glucose	High body-mass index	High LDL	Low temperature	Kidney dysfunction	Low whole grains	Alcohol use	Occupational carcinogens
Rutland	Smoking	High blood pressure	High fasting plasma glucose	High body-mass index	High LDL	Low temperature	Kidney dysfunction	Low whole grains	Alcohol use	Occupational carcinogens

Figure S2. Leading 10 level 3 risk factors for deaths in 2019 by location. Each risk is colored by the percentage change in all-age deaths from 2010 to 2019.

	Percentage change in all-age deaths from 2010 to 2019									
	-0.800 to -0.062	-0.062 to 0.007	0.007 to 0.052	0.052 to 0.093	0.093 to 0.132	0.132 to 0.174	0.174 to 0.223	0.223 to 0.287	0.287 to 0.372	0.372 to 1.738
	1	2	3	4	5	6	7	8	9	10
East of England	Smoking	High blood pressure	High fasting plasma glucose	High body-mass index	High LDL	Low temperature	Alcohol use	Kidney dysfunction	Low whole grains	Occupational carcinogens
Bedford	Smoking	High blood pressure	High fasting plasma glucose	High body-mass index	High LDL	Low temperature	Alcohol use	Kidney dysfunction	Low whole grains	Occupational carcinogens
Cambridgeshire	Smoking	High blood pressure	High fasting plasma glucose	High body-mass index	High LDL	Low temperature	Alcohol use	Kidney dysfunction	Low whole grains	Occupational carcinogens
Central Bedfordshire	Smoking	High blood pressure	High fasting plasma glucose	High body-mass index	High LDL	Low temperature	Alcohol use	Kidney dysfunction	Low whole grains	Occupational carcinogens
Essex	Smoking	High blood pressure	High fasting plasma glucose	High body-mass index	High LDL	Low temperature	Kidney dysfunction	Occupational carcinogens	Alcohol use	Low whole grains
Hertfordshire	Smoking	High blood pressure	High fasting plasma glucose	High body-mass index	High LDL	Low temperature	Alcohol use	Kidney dysfunction	Low whole grains	Occupational carcinogens
Luton	Smoking	High blood pressure	High fasting plasma glucose	High body-mass index	High LDL	Low temperature	Alcohol use	Kidney dysfunction	Low whole grains	Occupational carcinogens
Norfolk	Smoking	High blood pressure	High fasting plasma glucose	High body-mass index	High LDL	Low temperature	Kidney dysfunction	Low whole grains	Alcohol use	Occupational carcinogens
Peterborough	Smoking	High blood pressure	High fasting plasma glucose	High body-mass index	High LDL	Low temperature	Alcohol use	Kidney dysfunction	Low whole grains	Occupational carcinogens
Southend-on-Sea	Smoking	High blood pressure	High fasting plasma glucose	High body-mass index	High LDL	Low temperature	Alcohol use	Kidney dysfunction	Low whole grains	Occupational carcinogens
Suffolk	Smoking	High blood pressure	High fasting plasma glucose	High body-mass index	High LDL	Low temperature	Kidney dysfunction	Low whole grains	Alcohol use	Occupational carcinogens
Thurrock	Smoking	High blood pressure	High fasting plasma glucose	High body-mass index	High LDL	Low temperature	Occupational carcinogens	Kidney dysfunction	Alcohol use	Low whole grains
Greater London	Smoking	High blood pressure	High fasting plasma glucose	High body-mass index	High LDL	Low temperature	Alcohol use	Kidney dysfunction	Particulate matter	Low whole grains
Barking and Dagenham	Smoking	High blood pressure	High fasting plasma glucose	High body-mass index	High LDL	Low temperature	Alcohol use	Occupational carcinogens	Particulate matter	Low whole grains
Barnet	Smoking	High blood pressure	High fasting plasma glucose	High body-mass index	High LDL	Low temperature	Alcohol use	Kidney dysfunction	Low whole grains	Particulate matter
Bexley	Smoking	High blood pressure	High fasting plasma glucose	High body-mass index	High LDL	Low temperature	Alcohol use	Kidney dysfunction	Occupational carcinogens	Low whole grains
Brent	Smoking	High blood pressure	High fasting plasma glucose	High body-mass index	High LDL	Alcohol use	Low temperature	Particulate matter	Kidney dysfunction	Low whole grains
Bromley	Smoking	High blood pressure	High fasting plasma glucose	High body-mass index	High LDL	Low temperature	Alcohol use	Kidney dysfunction	Low whole grains	Particulate matter
Camden	Smoking	High blood pressure	High fasting plasma glucose	High body-mass index	Alcohol use	High LDL	Low temperature	Particulate matter	Kidney dysfunction	Occupational carcinogens
Croydon	Smoking	High blood pressure	High fasting plasma glucose	High body-mass index	High LDL	Low temperature	Alcohol use	Kidney dysfunction	Low whole grains	Particulate matter
Ealing	Smoking	High blood pressure	High fasting plasma glucose	High body-mass index	High LDL	Alcohol use	Low temperature	Kidney dysfunction	Particulate matter	Low whole grains
Enfield	Smoking	High blood pressure	High fasting plasma glucose	High body-mass index	High LDL	Low temperature	Alcohol use	Kidney dysfunction	Low whole grains	Particulate matter
Greenwich	Smoking	High blood pressure	High fasting plasma glucose	High body-mass index	High LDL	Low temperature	Alcohol use	Occupational carcinogens	Particulate matter	Kidney dysfunction
Hackney	Smoking	High blood pressure	High fasting plasma glucose	High body-mass index	High LDL	Alcohol use	Low temperature	Particulate matter	Kidney dysfunction	Low whole grains
Hammersmith and Fulham	Smoking	High blood pressure	High fasting plasma glucose	High body-mass index	Alcohol use	High LDL	Low temperature	Kidney dysfunction	Particulate matter	Low whole grains
Haringey	Smoking	High blood pressure	High fasting plasma glucose	High body-mass index	High LDL	Alcohol use	Low temperature	Particulate matter	Kidney dysfunction	Low whole grains
Harrow	Smoking	High blood pressure	High fasting plasma glucose	High body-mass index	High LDL	Low temperature	Alcohol use	Kidney dysfunction	Low whole grains	Particulate matter
Havering	Smoking	High blood pressure	High fasting plasma glucose	High body-mass index	High LDL	Low temperature	Occupational carcinogens	Alcohol use	Kidney dysfunction	Low whole grains
Hillingdon	Smoking	High blood pressure	High fasting plasma glucose	High body-mass index	High LDL	Low temperature	Alcohol use	Kidney dysfunction	Low whole grains	Occupational carcinogens
Hounslow	Smoking	High blood pressure	High fasting plasma glucose	High body-mass index	High LDL	Alcohol use	Low temperature	Kidney dysfunction	Low whole grains	Particulate matter
Islington	Smoking	High blood pressure	High fasting plasma glucose	High body-mass index	Alcohol use	High LDL	Low temperature	Particulate matter	Kidney dysfunction	Low whole grains
Kensington and Chelsea	Smoking	High blood pressure	High fasting plasma glucose	High body-mass index	Alcohol use	High LDL	Low temperature	Kidney dysfunction	Particulate matter	Low whole grains

Figure S2. Leading 10 level 3 risk factors for deaths in 2019 by location. Each risk is colored by the percentage change in all-age deaths from 2010 to 2019.

Percentage change in all-age deaths from 2010 to 2019

	-0.800 to -0.062	-0.062 to 0.007	0.007 to 0.052	0.052 to 0.093	0.093 to 0.132	0.132 to 0.174	0.174 to 0.223	0.223 to 0.287	0.287 to 0.372	0.372 to 1.738
	1	2	3	4	5	6	7	8	9	10
Kingston upon Thames	Smoking	High blood pressure	High fasting plasma glucose	High body-mass index	High LDL	Low temperature	Alcohol use	Kidney dysfunction	Low whole grains	Occupational carcinogens
Lambeth	Smoking	High blood pressure	High fasting plasma glucose	High body-mass index	Alcohol use	High LDL	Low temperature	Kidney dysfunction	Low whole grains	Occupational carcinogens
Lewisham	Smoking	High blood pressure	High fasting plasma glucose	High body-mass index	High LDL	Low temperature	Alcohol use	Particulate matter	Low whole grains	Kidney dysfunction
Merton	Smoking	High blood pressure	High fasting plasma glucose	High body-mass index	High LDL	Low temperature	Alcohol use	Kidney dysfunction	Low whole grains	Particulate matter
Newham	Smoking	High blood pressure	High fasting plasma glucose	High body-mass index	High LDL	Alcohol use	Low temperature	Particulate matter	Kidney dysfunction	Occupational carcinogens
Redbridge	Smoking	High blood pressure	High fasting plasma glucose	High body-mass index	High LDL	Low temperature	Alcohol use	Kidney dysfunction	Particulate matter	Low whole grains
Richmond upon Thames	Smoking	High blood pressure	High fasting plasma glucose	High body-mass index	High LDL	Alcohol use	Low temperature	Kidney dysfunction	Low whole grains	Particulate matter
Southwark	Smoking	High blood pressure	High fasting plasma glucose	High body-mass index	Alcohol use	High LDL	Low temperature	Particulate matter	Kidney dysfunction	Occupational carcinogens
Sutton	Smoking	High blood pressure	High fasting plasma glucose	High body-mass index	High LDL	Low temperature	Alcohol use	Occupational carcinogens	Low whole grains	Kidney dysfunction
Tower Hamlets	Smoking	High blood pressure	High fasting plasma glucose	High body-mass index	Alcohol use	High LDL	Low temperature	Particulate matter	Occupational carcinogens	Kidney dysfunction
Waltham Forest	Smoking	High blood pressure	High fasting plasma glucose	High body-mass index	High LDL	Low temperature	Alcohol use	Particulate matter	Kidney dysfunction	Low whole grains
Wandsworth	Smoking	High blood pressure	High fasting plasma glucose	High body-mass index	High LDL	Alcohol use	Low temperature	Kidney dysfunction	Low whole grains	Particulate matter
Westminster	Smoking	High blood pressure	High fasting plasma glucose	High body-mass index	Alcohol use	High LDL	Low temperature	Particulate matter	Kidney dysfunction	Low whole grains
North East England	Smoking	High blood pressure	High fasting plasma glucose	High body-mass index	High LDL	Low temperature	Occupational carcinogens	Alcohol use	Kidney dysfunction	Low whole grains
County Durham	Smoking	High blood pressure	High fasting plasma glucose	High body-mass index	High LDL	Low temperature	Occupational carcinogens	Alcohol use	Kidney dysfunction	Low whole grains
Darlington	Smoking	High blood pressure	High fasting plasma glucose	High body-mass index	High LDL	Low temperature	Alcohol use	Occupational carcinogens	Low whole grains	Kidney dysfunction
Gateshead	Smoking	High blood pressure	High fasting plasma glucose	High body-mass index	High LDL	Low temperature	Occupational carcinogens	Alcohol use	Low whole grains	Kidney dysfunction
Hartlepool	Smoking	High blood pressure	High fasting plasma glucose	High body-mass index	High LDL	Low temperature	Occupational carcinogens	Kidney dysfunction	Alcohol use	Low whole grains
Middlesbrough	Smoking	High blood pressure	High fasting plasma glucose	High body-mass index	High LDL	Low temperature	Occupational carcinogens	Alcohol use	Kidney dysfunction	Low whole grains
Newcastle upon Tyne	Smoking	High blood pressure	High fasting plasma glucose	High body-mass index	High LDL	Low temperature	Alcohol use	Occupational carcinogens	Low whole grains	Kidney dysfunction
North Tyneside	Smoking	High blood pressure	High fasting plasma glucose	High body-mass index	High LDL	Low temperature	Occupational carcinogens	Alcohol use	Low whole grains	Kidney dysfunction
Northumberland	Smoking	High blood pressure	High fasting plasma glucose	High body-mass index	High LDL	Low temperature	Occupational carcinogens	Alcohol use	Low whole grains	Kidney dysfunction
Redcar and Cleveland	Smoking	High blood pressure	High fasting plasma glucose	High body-mass index	High LDL	Low temperature	Occupational carcinogens	Kidney dysfunction	Low whole grains	Alcohol use
South Tyneside	Smoking	High blood pressure	High fasting plasma glucose	High body-mass index	High LDL	Low temperature	Occupational carcinogens	Alcohol use	Kidney dysfunction	Low whole grains
Stockton-on-Tees	Smoking	High blood pressure	High fasting plasma glucose	High body-mass index	High LDL	Low temperature	Occupational carcinogens	Alcohol use	Kidney dysfunction	Low whole grains
Sunderland	Smoking	High blood pressure	High fasting plasma glucose	High body-mass index	High LDL	Low temperature	Occupational carcinogens	Alcohol use	Low whole grains	Kidney dysfunction
North West England	Smoking	High blood pressure	High fasting plasma glucose	High body-mass index	High LDL	Low temperature	Alcohol use	Low whole grains	Kidney dysfunction	Occupational carcinogens
Blackburn with Darwen	Smoking	High blood pressure	High fasting plasma glucose	High body-mass index	High LDL	Low temperature	Low whole grains	Kidney dysfunction	Alcohol use	Occupational carcinogens
Blackpool	Smoking	High blood pressure	High fasting plasma glucose	High LDL	High body-mass index	Low temperature	Alcohol use	Low whole grains	Occupational carcinogens	Kidney dysfunction
Bolton	Smoking	High blood pressure	High fasting plasma glucose	High body-mass index	High LDL	Low temperature	Alcohol use	Low whole grains	Occupational carcinogens	Kidney dysfunction
Bury	Smoking	High blood pressure	High fasting plasma glucose	High body-mass index	High LDL	Low temperature	Kidney dysfunction	Low whole grains	Alcohol use	Occupational carcinogens
Cheshire East	Smoking	High blood pressure	High fasting plasma glucose	High body-mass index	High LDL	Low temperature	Alcohol use	Kidney dysfunction	Low whole grains	Occupational carcinogens

Figure S2. Leading 10 level 3 risk factors for deaths in 2019 by location. Each risk is colored by the percentage change in all-age deaths from 2010 to 2019.

Percentage change in all-age deaths from 2010 to 2019

-0.800 to -0.062	-0.062 to 0.007	0.007 to 0.052	0.052 to 0.093	0.093 to 0.132	0.132 to 0.174	0.174 to 0.223	0.223 to 0.287	0.287 to 0.372	0.372 to 1.738

	1	2	3	4	5	6	7	8	9	10
Cheshire West and Chester	Smoking	High blood pressure	High fasting plasma glucose	High body-mass index	High LDL	Low temperature	Alcohol use	Occupational carcinogens	Low whole grains	Kidney dysfunction
Cumbria	Smoking	High blood pressure	High fasting plasma glucose	High body-mass index	High LDL	Low temperature	Kidney dysfunction	Low whole grains	Occupational carcinogens	Alcohol use
Halton	Smoking	High blood pressure	High fasting plasma glucose	High body-mass index	High LDL	Low temperature	Alcohol use	Occupational carcinogens	Low whole grains	Kidney dysfunction
Knowsley	Smoking	High blood pressure	High fasting plasma glucose	High body-mass index	High LDL	Low temperature	Occupational carcinogens	Alcohol use	Low whole grains	Kidney dysfunction
Lancashire	Smoking	High blood pressure	High fasting plasma glucose	High body-mass index	High LDL	Low temperature	Alcohol use	Kidney dysfunction	Low whole grains	Occupational carcinogens
Liverpool	Smoking	High blood pressure	High fasting plasma glucose	High body-mass index	High LDL	Low temperature	Alcohol use	Occupational carcinogens	Low whole grains	Kidney dysfunction
Manchester	Smoking	High blood pressure	High fasting plasma glucose	High body-mass index	High LDL	Alcohol use	Low temperature	Low whole grains	Kidney dysfunction	Particulate matter
Oldham	Smoking	High blood pressure	High fasting plasma glucose	High body-mass index	High LDL	Low temperature	Low whole grains	Kidney dysfunction	Alcohol use	Occupational carcinogens
Rochdale	Smoking	High blood pressure	High fasting plasma glucose	High body-mass index	High LDL	Low temperature	Kidney dysfunction	Low whole grains	Alcohol use	Occupational carcinogens
Salford	Smoking	High blood pressure	High fasting plasma glucose	High body-mass index	High LDL	Low temperature	Alcohol use	Occupational carcinogens	Low whole grains	Kidney dysfunction
Sefton	Smoking	High blood pressure	High fasting plasma glucose	High body-mass index	High LDL	Low temperature	Kidney dysfunction	Alcohol use	Occupational carcinogens	Low whole grains
St Helens	Smoking	High blood pressure	High fasting plasma glucose	High body-mass index	High LDL	Low temperature	Alcohol use	Low whole grains	Occupational carcinogens	Kidney dysfunction
Stockport	Smoking	High blood pressure	High fasting plasma glucose	High body-mass index	High LDL	Low temperature	Alcohol use	Low whole grains	Kidney dysfunction	Occupational carcinogens
Tameside	Smoking	High blood pressure	High fasting plasma glucose	High LDL	High body-mass index	Low temperature	Low whole grains	Occupational carcinogens	Kidney dysfunction	Alcohol use
Trafford	Smoking	High blood pressure	High fasting plasma glucose	High body-mass index	High LDL	Low temperature	Alcohol use	Kidney dysfunction	Low whole grains	Occupational carcinogens
Warrington	Smoking	High blood pressure	High fasting plasma glucose	High body-mass index	High LDL	Low temperature	Alcohol use	Low whole grains	Kidney dysfunction	Occupational carcinogens
Wigan	Smoking	High blood pressure	High fasting plasma glucose	High body-mass index	High LDL	Low temperature	Occupational carcinogens	Low whole grains	Alcohol use	Kidney dysfunction
Wirral	Smoking	High blood pressure	High fasting plasma glucose	High body-mass index	High LDL	Low temperature	Occupational carcinogens	Alcohol use	Kidney dysfunction	Low whole grains
South East England	Smoking	High blood pressure	High fasting plasma glucose	High body-mass index	High LDL	Low temperature	Alcohol use	Kidney dysfunction	Low whole grains	Occupational carcinogens
Bracknell Forest	Smoking	High blood pressure	High fasting plasma glucose	High body-mass index	High LDL	Low temperature	Alcohol use	Kidney dysfunction	Low whole grains	Occupational carcinogens
Brighton and Hove	Smoking	High blood pressure	High fasting plasma glucose	High body-mass index	High LDL	Alcohol use	Low temperature	Kidney dysfunction	Low whole grains	Occupational carcinogens
Buckinghamshire	Smoking	High blood pressure	High fasting plasma glucose	High body-mass index	High LDL	Low temperature	Alcohol use	Kidney dysfunction	Low whole grains	Occupational carcinogens
East Sussex	Smoking	High blood pressure	High fasting plasma glucose	High body-mass index	High LDL	Low temperature	Alcohol use	Low whole grains	Kidney dysfunction	Occupational carcinogens
Hampshire	Smoking	High blood pressure	High fasting plasma glucose	High body-mass index	High LDL	Low temperature	Alcohol use	Kidney dysfunction	Low whole grains	Occupational carcinogens
Isle of Wight	Smoking	High blood pressure	High fasting plasma glucose	High body-mass index	High LDL	Low temperature	Kidney dysfunction	Low whole grains	Alcohol use	Occupational carcinogens
Kent	Smoking	High blood pressure	High fasting plasma glucose	High body-mass index	High LDL	Low temperature	Alcohol use	Kidney dysfunction	Occupational carcinogens	Low whole grains
Medway	Smoking	High blood pressure	High fasting plasma glucose	High body-mass index	High LDL	Low temperature	Occupational carcinogens	Alcohol use	Kidney dysfunction	Low whole grains
Milton Keynes	Smoking	High blood pressure	High fasting plasma glucose	High body-mass index	High LDL	Low temperature	Alcohol use	Occupational carcinogens	Kidney dysfunction	Low whole grains
Oxfordshire	Smoking	High blood pressure	High fasting plasma glucose	High body-mass index	High LDL	Low temperature	Alcohol use	Kidney dysfunction	Low whole grains	Occupational carcinogens
Portsmouth	Smoking	High blood pressure	High fasting plasma glucose	High body-mass index	High LDL	Low temperature	Alcohol use	Occupational carcinogens	Kidney dysfunction	Low whole grains
Reading	Smoking	High blood pressure	High fasting plasma glucose	High body-mass index	High LDL	Alcohol use	Low temperature	Kidney dysfunction	Low whole grains	Occupational carcinogens
Slough	Smoking	High blood pressure	High fasting plasma glucose	High body-mass index	High LDL	Low temperature	Alcohol use	Low whole grains	Kidney dysfunction	Particulate matter

Figure S2. Leading 10 level 3 risk factors for deaths in 2019 by location. Each risk is colored by the percentage change in all-age deaths from 2010 to 2019.

Percentage change in all-age deaths from 2010 to 2019

-0.800 to -0.062	-0.062 to 0.007	0.007 to 0.052	0.052 to 0.093	0.093 to 0.132	0.132 to 0.174	0.174 to 0.223	0.223 to 0.287	0.287 to 0.372	0.372 to 1.738

	1	2	3	4	5	6	7	8	9	10
Southampton	Smoking	High blood pressure	High fasting plasma glucose	High body-mass index	High LDL	Low temperature	Alcohol use	Occupational carcinogens	Kidney dysfunction	Low whole grains
Surrey	Smoking	High blood pressure	High fasting plasma glucose	High body-mass index	High LDL	Low temperature	Alcohol use	Kidney dysfunction	Low whole grains	Occupational carcinogens
West Berkshire	Smoking	High blood pressure	High fasting plasma glucose	High body-mass index	High LDL	Low temperature	Alcohol use	Occupational carcinogens	Kidney dysfunction	Low whole grains
West Sussex	Smoking	High blood pressure	High fasting plasma glucose	High body-mass index	High LDL	Low temperature	Alcohol use	Kidney dysfunction	Low whole grains	Occupational carcinogens
Windsor and Maidenhead	Smoking	High blood pressure	High fasting plasma glucose	High body-mass index	High LDL	Low temperature	Alcohol use	Kidney dysfunction	Low whole grains	Occupational carcinogens
Wokingham	Smoking	High blood pressure	High fasting plasma glucose	High body-mass index	High LDL	Low temperature	Alcohol use	Kidney dysfunction	Low whole grains	Occupational carcinogens
South West England	Smoking	High blood pressure	High fasting plasma glucose	High body-mass index	High LDL	Low temperature	Alcohol use	Kidney dysfunction	Low whole grains	Occupational carcinogens
Bath and North East Somerset	Smoking	High blood pressure	High fasting plasma glucose	High body-mass index	High LDL	Low temperature	Alcohol use	Kidney dysfunction	Low whole grains	Occupational carcinogens
Bournemouth	Smoking	High blood pressure	High fasting plasma glucose	High body-mass index	High LDL	Low temperature	Alcohol use	Kidney dysfunction	Low whole grains	Occupational carcinogens
Bristol, City of	Smoking	High blood pressure	High fasting plasma glucose	High body-mass index	High LDL	Low temperature	Alcohol use	Kidney dysfunction	Occupational carcinogens	Low whole grains
Cornwall	Smoking	High blood pressure	High fasting plasma glucose	High body-mass index	High LDL	Low temperature	Alcohol use	Kidney dysfunction	Low whole grains	Occupational carcinogens
Devon	Smoking	High blood pressure	High fasting plasma glucose	High body-mass index	High LDL	Low temperature	Alcohol use	Kidney dysfunction	Low whole grains	Occupational carcinogens
Dorset	Smoking	High blood pressure	High fasting plasma glucose	High body-mass index	High LDL	Low temperature	Kidney dysfunction	Alcohol use	Low whole grains	Occupational carcinogens
Gloucestershire	Smoking	High blood pressure	High fasting plasma glucose	High body-mass index	High LDL	Low temperature	Alcohol use	Kidney dysfunction	Low whole grains	Occupational carcinogens
North Somerset	Smoking	High blood pressure	High fasting plasma glucose	High body-mass index	High LDL	Low temperature	Alcohol use	Kidney dysfunction	Low whole grains	Occupational carcinogens
Plymouth	Smoking	High blood pressure	High fasting plasma glucose	High body-mass index	High LDL	Low temperature	Alcohol use	Occupational carcinogens	Kidney dysfunction	Low whole grains
Poole	Smoking	High blood pressure	High fasting plasma glucose	High body-mass index	High LDL	Low temperature	Alcohol use	Kidney dysfunction	Low whole grains	Occupational carcinogens
Somerset	Smoking	High fasting plasma glucose	High blood pressure	High body-mass index	High LDL	Low temperature	Kidney dysfunction	Alcohol use	Low whole grains	Occupational carcinogens
South Gloucestershire	Smoking	High blood pressure	High fasting plasma glucose	High body-mass index	High LDL	Low temperature	Alcohol use	Kidney dysfunction	Low whole grains	Occupational carcinogens
Swindon	Smoking	High blood pressure	High fasting plasma glucose	High body-mass index	High LDL	Low temperature	Alcohol use	Kidney dysfunction	Low whole grains	Occupational carcinogens
Torbay	Smoking	High blood pressure	High fasting plasma glucose	High LDL	High body-mass index	Low temperature	Kidney dysfunction	Alcohol use	Low whole grains	Occupational carcinogens
Wiltshire	Smoking	High blood pressure	High fasting plasma glucose	High body-mass index	High LDL	Low temperature	Alcohol use	Kidney dysfunction	Low whole grains	Occupational carcinogens
West Midlands	Smoking	High blood pressure	High fasting plasma glucose	High body-mass index	High LDL	Low temperature	Alcohol use	Kidney dysfunction	Low whole grains	Occupational carcinogens
Birmingham	Smoking	High blood pressure	High fasting plasma glucose	High body-mass index	High LDL	Low temperature	Alcohol use	Kidney dysfunction	Low whole grains	Particulate matter
Coventry	Smoking	High blood pressure	High fasting plasma glucose	High body-mass index	High LDL	Low temperature	Alcohol use	Kidney dysfunction	Low whole grains	Occupational carcinogens
Dudley	Smoking	High blood pressure	High fasting plasma glucose	High body-mass index	High LDL	Low temperature	Kidney dysfunction	Alcohol use	Low whole grains	Occupational carcinogens
Herefordshire, County of	Smoking	High blood pressure	High fasting plasma glucose	High body-mass index	High LDL	Low temperature	Kidney dysfunction	Low whole grains	Alcohol use	Occupational carcinogens
Sandwell	Smoking	High blood pressure	High fasting plasma glucose	High body-mass index	High LDL	Low temperature	Alcohol use	Kidney dysfunction	Low whole grains	Particulate matter
Shropshire	Smoking	High blood pressure	High fasting plasma glucose	High body-mass index	High LDL	Low temperature	Kidney dysfunction	Alcohol use	Low whole grains	Low physical activity
Solihull	Smoking	High blood pressure	High fasting plasma glucose	High body-mass index	High LDL	Low temperature	Alcohol use	Kidney dysfunction	Low whole grains	Occupational carcinogens
Staffordshire	Smoking	High blood pressure	High fasting plasma glucose	High body-mass index	High LDL	Low temperature	Kidney dysfunction	Alcohol use	Low whole grains	Occupational carcinogens
Stoke-on-Trent	Smoking	High blood pressure	High fasting plasma glucose	High body-mass index	High LDL	Low temperature	Alcohol use	Occupational carcinogens	Low whole grains	Particulate matter

Figure S2. Leading 10 level 3 risk factors for deaths in 2019 by location. Each risk is colored by the percentage change in all-age deaths from 2010 to 2019.

	Percentage change in all-age deaths from 2010 to 2019									
	-0.800 to -0.062	-0.062 to 0.007	0.007 to 0.052	0.052 to 0.093	0.093 to 0.132	0.132 to 0.174	0.174 to 0.223	0.223 to 0.287	0.287 to 0.372	0.372 to 1.738
	1	2	3	4	5	6	7	8	9	10
Telford and Wrekin	Smoking	High blood pressure	High fasting plasma glucose	High body-mass index	High LDL	Low temperature	Alcohol use	Kidney dysfunction	Low whole grains	Occupational carcinogens
Walsall	Smoking	High blood pressure	High fasting plasma glucose	High LDL	High body-mass index	Low temperature	Kidney dysfunction	Low whole grains	Alcohol use	Occupational carcinogens
Warwickshire	Smoking	High blood pressure	High fasting plasma glucose	High body-mass index	High LDL	Low temperature	Alcohol use	Kidney dysfunction	Low whole grains	Occupational carcinogens
Wolverhampton	Smoking	High blood pressure	High fasting plasma glucose	High body-mass index	High LDL	Low temperature	Kidney dysfunction	Alcohol use	Low whole grains	Occupational carcinogens
Worcestershire	Smoking	High blood pressure	High fasting plasma glucose	High body-mass index	High LDL	Low temperature	Alcohol use	Kidney dysfunction	Low whole grains	Occupational carcinogens
Yorkshire and the Humber	Smoking	High blood pressure	High fasting plasma glucose	High body-mass index	High LDL	Low temperature	Kidney dysfunction	Low whole grains	Occupational carcinogens	Alcohol use
Barnsley	Smoking	High blood pressure	High fasting plasma glucose	High LDL	High body-mass index	Low temperature	Low whole grains	Kidney dysfunction	Particulate matter	Occupational carcinogens
Bradford	Smoking	High blood pressure	High fasting plasma glucose	High body-mass index	High LDL	Low temperature	Low whole grains	Kidney dysfunction	Alcohol use	Occupational carcinogens
Calderdale	Smoking	High blood pressure	High fasting plasma glucose	High body-mass index	High LDL	Low temperature	Kidney dysfunction	Occupational carcinogens	Low whole grains	Alcohol use
Doncaster	Smoking	High blood pressure	High fasting plasma glucose	High body-mass index	High LDL	Low temperature	Occupational carcinogens	Low whole grains	Kidney dysfunction	Particulate matter
East Riding of Yorkshire	Smoking	High blood pressure	High fasting plasma glucose	High body-mass index	High LDL	Low temperature	Kidney dysfunction	Low whole grains	Occupational carcinogens	Alcohol use
Kingston upon Hull, City of	Smoking	High blood pressure	High fasting plasma glucose	High body-mass index	High LDL	Low temperature	Occupational carcinogens	Low whole grains	Kidney dysfunction	Alcohol use
Kirklees	Smoking	High blood pressure	High fasting plasma glucose	High body-mass index	High LDL	Low temperature	Kidney dysfunction	Low whole grains	Occupational carcinogens	Alcohol use
Leeds	Smoking	High blood pressure	High fasting plasma glucose	High body-mass index	High LDL	Low temperature	Alcohol use	Occupational carcinogens	Low whole grains	Kidney dysfunction
North East Lincolnshire	Smoking	High blood pressure	High fasting plasma glucose	High body-mass index	High LDL	Low temperature	Kidney dysfunction	Low whole grains	Occupational carcinogens	Alcohol use
North Lincolnshire	Smoking	High blood pressure	High fasting plasma glucose	High body-mass index	High LDL	Low temperature	Kidney dysfunction	Low whole grains	Occupational carcinogens	Particulate matter
North Yorkshire	Smoking	High blood pressure	High fasting plasma glucose	High body-mass index	High LDL	Low temperature	Kidney dysfunction	Low whole grains	Alcohol use	Occupational carcinogens
Rotherham	Smoking	High blood pressure	High fasting plasma glucose	High body-mass index	High LDL	Low temperature	Low whole grains	Occupational carcinogens	Kidney dysfunction	Particulate matter
Sheffield	Smoking	High blood pressure	High fasting plasma glucose	High body-mass index	High LDL	Low temperature	Low whole grains	Alcohol use	Occupational carcinogens	Kidney dysfunction
Wakefield	Smoking	High blood pressure	High fasting plasma glucose	High body-mass index	High LDL	Low temperature	Occupational carcinogens	Low whole grains	Kidney dysfunction	Alcohol use
York	Smoking	High blood pressure	High fasting plasma glucose	High body-mass index	High LDL	Low temperature	Alcohol use	Kidney dysfunction	Low whole grains	Occupational carcinogens
Northern Ireland	Smoking	High blood pressure	High fasting plasma glucose	High body-mass index	High LDL	Low temperature	Alcohol use	Kidney dysfunction	Low whole grains	Low physical activity
Scotland	Smoking	High blood pressure	High fasting plasma glucose	High body-mass index	High LDL	Low temperature	Alcohol use	Kidney dysfunction	Low whole grains	Occupational carcinogens
Wales	Smoking	High blood pressure	High fasting plasma glucose	High body-mass index	High LDL	Low temperature	Low physical activity	Alcohol use	Kidney dysfunction	Low whole grains
**Latin America and Caribbean**	High blood pressure	High fasting plasma glucose	High body-mass index	Smoking	Kidney dysfunction	High LDL	Particulate matter	Alcohol use	Low whole grains	High sodium
**Andean Latin America**	High blood pressure	High body-mass index	High fasting plasma glucose	Kidney dysfunction	Particulate matter	High LDL	Alcohol use	Smoking	Low birth weight & short gestation	Low temperature
Bolivia	High fasting plasma glucose	High blood pressure	High body-mass index	Particulate matter	Kidney dysfunction	Smoking	High LDL	Alcohol use	Low birth weight & short gestation	Low temperature
Ecuador	High body-mass index	High fasting plasma glucose	High blood pressure	Kidney dysfunction	High LDL	Smoking	Particulate matter	Alcohol use	Low whole grains	Unsafe sex
Peru	High blood pressure	High body-mass index	High fasting plasma glucose	Particulate matter	Kidney dysfunction	High LDL	Alcohol use	Smoking	Low temperature	Unsafe sex
**Caribbean**	High blood pressure	High fasting plasma glucose	High body-mass index	Smoking	Particulate matter	High LDL	Kidney dysfunction	Alcohol use	Low whole grains	Low physical activity
Antigua and Barbuda	High fasting plasma glucose	High blood pressure	High body-mass index	Kidney dysfunction	Smoking	High LDL	Particulate matter	Alcohol use	Low whole grains	Low physical activity
The Bahamas	High blood pressure	High body-mass index	High fasting plasma glucose	Kidney dysfunction	High LDL	Smoking	Alcohol use	Unsafe sex	Particulate matter	Low whole grains

Figure S2. Leading 10 level 3 risk factors for deaths in 2019 by location. Each risk is colored by the percentage change in all-age deaths from 2010 to 2019.

Percentage change in all-age deaths from 2010 to 2019

-0.800 to -0.062	-0.062 to 0.007	0.007 to 0.052	0.052 to 0.093	0.093 to 0.132	0.132 to 0.174	0.174 to 0.223	0.223 to 0.287	0.287 to 0.372	0.372 to 1.738

	1	2	3	4	5	6	7	8	9	10
Barbados	High fasting plasma glucose	High blood pressure	High body-mass index	Kidney dysfunction	High LDL	Particulate matter	Smoking	Alcohol use	Low physical activity	Low whole grains
Belize	High blood pressure	High fasting plasma glucose	High body-mass index	Kidney dysfunction	Smoking	Particulate matter	Alcohol use	High LDL	Unsafe sex	Low birth weight & short gestation
Bermuda	High blood pressure	High fasting plasma glucose	High body-mass index	Smoking	High LDL	Kidney dysfunction	Low whole grains	Alcohol use	Low physical activity	Low legumes
Cuba	Smoking	High blood pressure	High fasting plasma glucose	High body-mass index	High LDL	Kidney dysfunction	Particulate matter	Alcohol use	Low whole grains	Low physical activity
Dominica	High blood pressure	High fasting plasma glucose	High body-mass index	Kidney dysfunction	Smoking	Particulate matter	High LDL	Alcohol use	Low whole grains	Low physical activity
Dominican Republic	High blood pressure	High fasting plasma glucose	High body-mass index	Smoking	High LDL	Kidney dysfunction	Particulate matter	Alcohol use	Low birth weight & short gestation	Low whole grains
Grenada	High fasting plasma glucose	High blood pressure	High body-mass index	Kidney dysfunction	Smoking	Particulate matter	High LDL	Alcohol use	Low whole grains	Low physical activity
Guyana	High blood pressure	High fasting plasma glucose	High body-mass index	High LDL	Kidney dysfunction	Alcohol use	Particulate matter	Smoking	Low whole grains	Unsafe sex
Haiti	High blood pressure	Particulate matter	High fasting plasma glucose	Low birth weight & short gestation	High body-mass index	High LDL	Unsafe sex	Child growth failure	Kidney dysfunction	Alcohol use
Jamaica	High fasting plasma glucose	High blood pressure	High body-mass index	Smoking	Kidney dysfunction	Particulate matter	High LDL	Low physical activity	Low whole grains	Unsafe sex
Puerto Rico	High fasting plasma glucose	High blood pressure	High body-mass index	Kidney dysfunction	Smoking	High LDL	Alcohol use	Low whole grains	Low physical activity	Low legumes
Saint Kitts and Nevis	High blood pressure	High fasting plasma glucose	High body-mass index	Kidney dysfunction	Unsafe sex	High LDL	Alcohol use	Smoking	Low whole grains	Low fibre
Saint Lucia	High fasting plasma glucose	High blood pressure	High body-mass index	Kidney dysfunction	Smoking	Alcohol use	Particulate matter	High LDL	Low whole grains	Low physical activity
Saint Vincent and the Grenadines	High fasting plasma glucose	High blood pressure	High body-mass index	Kidney dysfunction	Particulate matter	High LDL	Smoking	Alcohol use	Low whole grains	Low physical activity
Suriname	High fasting plasma glucose	High blood pressure	High body-mass index	Smoking	Kidney dysfunction	High LDL	Particulate matter	Alcohol use	Unsafe sex	Low fibre
Trinidad and Tobago	High fasting plasma glucose	High blood pressure	High body-mass index	High LDL	Kidney dysfunction	Smoking	Particulate matter	Low whole grains	Low physical activity	Alcohol use
Virgin Islands	High blood pressure	High fasting plasma glucose	High body-mass index	High LDL	Kidney dysfunction	Smoking	Low whole grains	Alcohol use	Low legumes	Particulate matter
**Central Latin America**	High fasting plasma glucose	High blood pressure	High body-mass index	Kidney dysfunction	High LDL	Smoking	Particulate matter	Alcohol use	High sodium	Low whole grains
Colombia	High blood pressure	High fasting plasma glucose	High body-mass index	High LDL	Kidney dysfunction	Smoking	Particulate matter	High sodium	Alcohol use	Low whole grains
Costa Rica	High blood pressure	High fasting plasma glucose	High body-mass index	Kidney dysfunction	Smoking	High LDL	Alcohol use	Particulate matter	Low whole grains	High sodium
El Salvador	High blood pressure	High fasting plasma glucose	Kidney dysfunction	High body-mass index	High LDL	Particulate matter	Alcohol use	Smoking	High sodium	Low whole grains
Guatemala	High fasting plasma glucose	High blood pressure	Particulate matter	Kidney dysfunction	High body-mass index	Alcohol use	High LDL	Child growth failure	Smoking	Low birth weight & short gestation
Honduras	High blood pressure	High fasting plasma glucose	High body-mass index	Particulate matter	Kidney dysfunction	Smoking	High LDL	Alcohol use	Lead	Low birth weight & short gestation
Mexico	High fasting plasma glucose	High blood pressure	High body-mass index	Kidney dysfunction	High LDL	Alcohol use	Smoking	Particulate matter	Low temperature	Low whole grains
Aguascalientes	High fasting plasma glucose	High blood pressure	High body-mass index	Kidney dysfunction	Smoking	Alcohol use	High LDL	Particulate matter	Low temperature	Low whole grains
Baja California	High fasting plasma glucose	High body-mass index	High blood pressure	Kidney dysfunction	High LDL	Alcohol use	Smoking	Particulate matter	Low temperature	Low whole grains
Baja California Sur	High fasting plasma glucose	High body-mass index	High blood pressure	Kidney dysfunction	High LDL	Smoking	Alcohol use	Particulate matter	Low whole grains	High red meat
Campeche	High fasting plasma glucose	High body-mass index	High blood pressure	Kidney dysfunction	High LDL	Alcohol use	Particulate matter	Smoking	Unsafe sex	Low whole grains
Chiapas	High fasting plasma glucose	High blood pressure	High body-mass index	Kidney dysfunction	Particulate matter	Alcohol use	High LDL	Low birth weight & short gestation	Smoking	Child growth failure
Chihuahua	High fasting plasma glucose	High blood pressure	High body-mass index	Kidney dysfunction	Smoking	High LDL	Alcohol use	Particulate matter	Low temperature	Low whole grains
Coahuila	High fasting plasma glucose	High blood pressure	High body-mass index	Kidney dysfunction	Smoking	High LDL	Particulate matter	Alcohol use	Low temperature	High red meat
Colima	High fasting plasma glucose	High body-mass index	High blood pressure	Kidney dysfunction	Alcohol use	High LDL	Smoking	Particulate matter	Low whole grains	High red meat

Figure S2. Leading 10 level 3 risk factors for deaths in 2019 by location. Each risk is colored by the percentage change in all-age deaths from 2010 to 2019.

	Percentage change in all-age deaths from 2010 to 2019									
	-0.800 to -0.062	-0.062 to 0.007	0.007 to 0.052	0.052 to 0.093	0.093 to 0.132	0.132 to 0.174	0.174 to 0.223	0.223 to 0.287	0.287 to 0.372	0.372 to 1.738
	**1**	**2**	**3**	**4**	**5**	**6**	**7**	**8**	**9**	**10**
Durango	High fasting plasma glucose	High blood pressure	High body-mass index	Kidney dysfunction	High LDL	Smoking	Particulate matter	Alcohol use	Low temperature	Low whole grains
Guanajuato	High fasting plasma glucose	High body-mass index	High blood pressure	Kidney dysfunction	Smoking	High LDL	Alcohol use	Particulate matter	Low temperature	Low whole grains
Guerrero	High fasting plasma glucose	High body-mass index	High blood pressure	Kidney dysfunction	Particulate matter	Alcohol use	High LDL	Smoking	Low whole grains	Lead
Hidalgo	High fasting plasma glucose	High blood pressure	High body-mass index	Kidney dysfunction	Alcohol use	High LDL	Particulate matter	Smoking	Low temperature	Low whole grains
Jalisco	High fasting plasma glucose	High body-mass index	High blood pressure	Kidney dysfunction	Smoking	High LDL	Alcohol use	Particulate matter	Low temperature	Lead
México	High fasting plasma glucose	High body-mass index	High blood pressure	Kidney dysfunction	Alcohol use	Particulate matter	Smoking	High LDL	Low temperature	Low birth weight & short gestation
Mexico City	High fasting plasma glucose	High body-mass index	High blood pressure	Kidney dysfunction	Smoking	High LDL	Particulate matter	Alcohol use	Low temperature	Low physical activity
Michoacán de Ocampo	High fasting plasma glucose	High blood pressure	High body-mass index	Kidney dysfunction	Smoking	Particulate matter	High LDL	Alcohol use	Low temperature	Low whole grains
Morelos	High fasting plasma glucose	High body-mass index	High blood pressure	Kidney dysfunction	Alcohol use	High LDL	Particulate matter	Smoking	Low whole grains	Low temperature
Nayarit	High fasting plasma glucose	High blood pressure	High body-mass index	Kidney dysfunction	Smoking	High LDL	Alcohol use	Particulate matter	Low whole grains	High sodium
Nuevo León	High fasting plasma glucose	High blood pressure	High body-mass index	Kidney dysfunction	Smoking	High LDL	Particulate matter	Alcohol use	Low temperature	Low physical activity
Oaxaca	High fasting plasma glucose	High blood pressure	High body-mass index	Kidney dysfunction	Alcohol use	Particulate matter	High LDL	Smoking	Lead	Child growth failure
Puebla	High fasting plasma glucose	High blood pressure	High body-mass index	Kidney dysfunction	Alcohol use	Particulate matter	High LDL	Smoking	Low temperature	Lead
Querétaro	High fasting plasma glucose	High blood pressure	High body-mass index	Kidney dysfunction	Alcohol use	High LDL	Smoking	Particulate matter	Low temperature	Low whole grains
Quintana Roo	High fasting plasma glucose	High body-mass index	High blood pressure	Kidney dysfunction	Alcohol use	High LDL	Smoking	Particulate matter	Unsafe sex	Low birth weight & short gestation
San Luis Potosí	High fasting plasma glucose	High blood pressure	High body-mass index	Kidney dysfunction	High LDL	Particulate matter	Smoking	Alcohol use	Low temperature	Low whole grains
Sinaloa	High fasting plasma glucose	High blood pressure	High body-mass index	Kidney dysfunction	High LDL	Smoking	Alcohol use	Particulate matter	Low whole grains	High red meat
Sonora	High fasting plasma glucose	High blood pressure	High body-mass index	Kidney dysfunction	High LDL	Smoking	Alcohol use	Particulate matter	Low whole grains	Low temperature
Tabasco	High fasting plasma glucose	High body-mass index	High blood pressure	Kidney dysfunction	Particulate matter	High LDL	Alcohol use	Smoking	Unsafe sex	Low whole grains
Tamaulipas	High fasting plasma glucose	High blood pressure	High body-mass index	Kidney dysfunction	High LDL	Smoking	Particulate matter	Alcohol use	Low whole grains	High red meat
Tlaxcala	High fasting plasma glucose	High body-mass index	High blood pressure	Kidney dysfunction	Alcohol use	Particulate matter	Low temperature	High LDL	Smoking	Low whole grains
Veracruz de Ignacio de la Llave	High fasting plasma glucose	High blood pressure	High body-mass index	Kidney dysfunction	Alcohol use	Particulate matter	High LDL	Smoking	Low whole grains	Unsafe sex
Yucatán	High fasting plasma glucose	High blood pressure	High body-mass index	Kidney dysfunction	High LDL	Alcohol use	Particulate matter	Smoking	Low whole grains	Unsafe sex
Zacatecas	High fasting plasma glucose	High blood pressure	High body-mass index	Kidney dysfunction	Smoking	High LDL	Alcohol use	Particulate matter	Low temperature	Low whole grains
Nicaragua	High blood pressure	High fasting plasma glucose	Kidney dysfunction	High body-mass index	Particulate matter	High LDL	Alcohol use	Smoking	High sodium	Low whole grains
Panama	High blood pressure	High fasting plasma glucose	High body-mass index	Kidney dysfunction	High LDL	Smoking	Alcohol use	Particulate matter	Unsafe sex	High sodium
Venezuela	High blood pressure	High fasting plasma glucose	High body-mass index	Kidney dysfunction	Smoking	High LDL	Particulate matter	Alcohol use	High sodium	Low whole grains
**Tropical Latin America**	High blood pressure	High body-mass index	High fasting plasma glucose	Smoking	High LDL	Alcohol use	Kidney dysfunction	Particulate matter	Low physical activity	High red meat
Brazil	High blood pressure	High body-mass index	High fasting plasma glucose	Smoking	High LDL	Alcohol use	Kidney dysfunction	Particulate matter	Low physical activity	High red meat
Acre	High blood pressure	Smoking	High body-mass index	High fasting plasma glucose	Alcohol use	Kidney dysfunction	High LDL	Low birth weight & short gestation	Particulate matter	Low physical activity
Alagoas	High blood pressure	High fasting plasma glucose	High body-mass index	Smoking	High LDL	Alcohol use	Kidney dysfunction	Particulate matter	Low physical activity	High red meat
Amapá	High blood pressure	High body-mass index	High fasting plasma glucose	Smoking	Alcohol use	Low birth weight & short gestation	High LDL	Kidney dysfunction	Particulate matter	Unsafe sex

Figure S2. Leading 10 level 3 risk factors for deaths in 2019 by location. Each risk is colored by the percentage change in all-age deaths from 2010 to 2019.

Percentage change in all-age deaths from 2010 to 2019

-0.800 to -0.062	-0.062 to 0.007	0.007 to 0.052	0.052 to 0.093	0.093 to 0.132	0.132 to 0.174	0.174 to 0.223	0.223 to 0.287	0.287 to 0.372	0.372 to 1.738

	1	2	3	4	5	6	7	8	9	10
Amazonas	High blood pressure	High body-mass index	High fasting plasma glucose	Smoking	Alcohol use	Kidney dysfunction	High LDL	Unsafe sex	Low birth weight & short gestation	Particulate matter
Bahia	High blood pressure	High fasting plasma glucose	High body-mass index	Smoking	High LDL	Alcohol use	Kidney dysfunction	Particulate matter	Low physical activity	High red meat
Ceará	High blood pressure	High fasting plasma glucose	High body-mass index	Smoking	High LDL	Alcohol use	Kidney dysfunction	Particulate matter	Low physical activity	Low birth weight & short gestation
Distrito Federal	High blood pressure	High body-mass index	High fasting plasma glucose	Smoking	Alcohol use	High LDL	Kidney dysfunction	Low physical activity	High red meat	Particulate matter
Espírito Santo	High blood pressure	High body-mass index	High fasting plasma glucose	Smoking	High LDL	Alcohol use	Kidney dysfunction	Particulate matter	Low physical activity	High red meat
Goiás	High blood pressure	Smoking	High body-mass index	High fasting plasma glucose	High LDL	Alcohol use	Kidney dysfunction	Particulate matter	Low physical activity	High red meat
Maranhão	High blood pressure	High fasting plasma glucose	High body-mass index	Smoking	High LDL	Particulate matter	Kidney dysfunction	Alcohol use	Low birth weight & short gestation	Low physical activity
Mato Grosso	High blood pressure	High body-mass index	High fasting plasma glucose	Smoking	High LDL	Alcohol use	Kidney dysfunction	Particulate matter	Low physical activity	High red meat
Mato Grosso do Sul	High blood pressure	High body-mass index	High fasting plasma glucose	Smoking	High LDL	Alcohol use	Kidney dysfunction	Particulate matter	Low physical activity	High red meat
Minas Gerais	High blood pressure	Smoking	High body-mass index	High fasting plasma glucose	High LDL	Alcohol use	Kidney dysfunction	Particulate matter	Low physical activity	High red meat
Pará	High blood pressure	High fasting plasma glucose	High body-mass index	Smoking	High LDL	Alcohol use	Kidney dysfunction	Particulate matter	Low birth weight & short gestation	Low physical activity
Paraíba	High blood pressure	High fasting plasma glucose	High body-mass index	Smoking	High LDL	Kidney dysfunction	Particulate matter	Alcohol use	Low physical activity	High red meat
Paraná	High blood pressure	Smoking	High body-mass index	High fasting plasma glucose	High LDL	Alcohol use	Kidney dysfunction	Particulate matter	Low physical activity	High red meat
Pernambuco	High blood pressure	High fasting plasma glucose	High body-mass index	Smoking	High LDL	Alcohol use	Kidney dysfunction	Particulate matter	Low physical activity	High red meat
Piauí	High blood pressure	High fasting plasma glucose	High body-mass index	Smoking	High LDL	Particulate matter	Kidney dysfunction	Alcohol use	Low physical activity	Low birth weight & short gestation
Rio de Janeiro	High blood pressure	High body-mass index	High fasting plasma glucose	Smoking	High LDL	Kidney dysfunction	Alcohol use	Particulate matter	Low physical activity	High red meat
Rio Grande do Norte	High blood pressure	High fasting plasma glucose	High body-mass index	Smoking	High LDL	Alcohol use	Kidney dysfunction	Particulate matter	Low physical activity	Low whole grains
Rio Grande do Sul	High blood pressure	Smoking	High body-mass index	High fasting plasma glucose	High LDL	Alcohol use	Kidney dysfunction	Particulate matter	Low physical activity	Low temperature
Rondônia	High blood pressure	High body-mass index	High fasting plasma glucose	Smoking	High LDL	Kidney dysfunction	Alcohol use	Particulate matter	Low physical activity	High red meat
Roraima	High blood pressure	High body-mass index	High fasting plasma glucose	Smoking	Low birth weight & short gestation	Alcohol use	High LDL	Kidney dysfunction	Particulate matter	Low physical activity
Santa Catarina	High blood pressure	Smoking	High body-mass index	High fasting plasma glucose	High LDL	Alcohol use	Kidney dysfunction	Particulate matter	Low physical activity	Low temperature
São Paulo	High blood pressure	Smoking	High body-mass index	High fasting plasma glucose	High LDL	Alcohol use	Kidney dysfunction	Particulate matter	High red meat	Low physical activity
Sergipe	High blood pressure	High fasting plasma glucose	High body-mass index	Smoking	Alcohol use	High LDL	Kidney dysfunction	Particulate matter	Low physical activity	High red meat
Tocantins	High blood pressure	High body-mass index	High fasting plasma glucose	Smoking	High LDL	Kidney dysfunction	Alcohol use	Particulate matter	Low physical activity	High red meat
Paraguay	High blood pressure	High fasting plasma glucose	High body-mass index	Smoking	Kidney dysfunction	High LDL	Particulate matter	Alcohol use	High red meat	Low whole grains
**North Africa and Middle East**	High blood pressure	High body-mass index	High fasting plasma glucose	High LDL	Particulate matter	Smoking	Kidney dysfunction	Low whole grains	Low physical activity	Low temperature
Afghanistan	Particulate matter	High blood pressure	High fasting plasma glucose	Low birth weight & short gestation	High body-mass index	High LDL	Child growth failure	Kidney dysfunction	Low whole grains	Smoking
Algeria	High blood pressure	High fasting plasma glucose	High body-mass index	High LDL	Kidney dysfunction	Particulate matter	Smoking	Low whole grains	Low physical activity	Second-hand smoke
Bahrain	High fasting plasma glucose	High body-mass index	High blood pressure	Particulate matter	Smoking	High LDL	Kidney dysfunction	Low whole grains	Low physical activity	Second-hand smoke
Egypt	High blood pressure	High body-mass index	High fasting plasma glucose	Particulate matter	High LDL	Smoking	Kidney dysfunction	Low whole grains	Low physical activity	High trans fat
Iran	High blood pressure	High fasting plasma glucose	High body-mass index	High LDL	Particulate matter	Smoking	Kidney dysfunction	Low whole grains	Low temperature	Lead
Iraq	High blood pressure	High fasting plasma glucose	High body-mass index	Particulate matter	Smoking	High LDL	Kidney dysfunction	Low whole grains	Low physical activity	Low birth weight & short gestation

Figure S2. Leading 10 level 3 risk factors for deaths in 2019 by location. Each risk is colored by the percentage change in all-age deaths from 2010 to 2019.

Percentage change in all-age deaths from 2010 to 2019

-0.800 to -0.062	-0.062 to 0.007	0.007 to 0.052	0.052 to 0.093	0.093 to 0.132	0.132 to 0.174	0.174 to 0.223	0.223 to 0.287	0.287 to 0.372	0.372 to 1.738

	1	2	3	4	5	6	7	8	9	10
Jordan	High blood pressure	High body-mass index	High fasting plasma glucose	Smoking	High LDL	Kidney dysfunction	Particulate matter	Low whole grains	Low birth weight & short gestation	Low temperature
Kuwait	High blood pressure	High body-mass index	High fasting plasma glucose	High LDL	Smoking	Particulate matter	Kidney dysfunction	Low whole grains	Low physical activity	Low legumes
Lebanon	High blood pressure	Smoking	High fasting plasma glucose	High body-mass index	High LDL	Kidney dysfunction	Particulate matter	Low whole grains	Low physical activity	Low temperature
Libya	High blood pressure	High body-mass index	High fasting plasma glucose	High LDL	Smoking	Particulate matter	Kidney dysfunction	Low whole grains	Low physical activity	Low legumes
Morocco	High blood pressure	High fasting plasma glucose	High body-mass index	High LDL	Particulate matter	Kidney dysfunction	Smoking	Low whole grains	Low temperature	Low physical activity
Oman	High blood pressure	High body-mass index	High fasting plasma glucose	High LDL	Particulate matter	Kidney dysfunction	Smoking	Low whole grains	High temperature	Low physical activity
Palestine	High fasting plasma glucose	High blood pressure	High body-mass index	Smoking	High LDL	Particulate matter	Kidney dysfunction	Low whole grains	Low physical activity	Low birth weight & short gestation
Qatar	High body-mass index	High fasting plasma glucose	High blood pressure	Particulate matter	High LDL	Smoking	Kidney dysfunction	Low whole grains	High temperature	Low physical activity
Saudi Arabia	High blood pressure	High body-mass index	High fasting plasma glucose	High LDL	Particulate matter	Kidney dysfunction	Smoking	Low whole grains	Low physical activity	Low legumes
Sudan	High blood pressure	Particulate matter	High fasting plasma glucose	High body-mass index	High LDL	Low birth weight & short gestation	Smoking	Kidney dysfunction	Low whole grains	Low physical activity
Syria	High blood pressure	High fasting plasma glucose	High LDL	High body-mass index	Smoking	Particulate matter	Kidney dysfunction	Low whole grains	Low temperature	Low legumes
Tunisia	High blood pressure	High fasting plasma glucose	High body-mass index	Smoking	High LDL	Kidney dysfunction	Particulate matter	Low whole grains	Low temperature	Second-hand smoke
Turkey	High blood pressure	Smoking	High body-mass index	High fasting plasma glucose	High LDL	Kidney dysfunction	Particulate matter	Low whole grains	Low temperature	Low physical activity
United Arab Emirates	High body-mass index	High blood pressure	High fasting plasma glucose	High LDL	Smoking	Particulate matter	Kidney dysfunction	Low whole grains	High temperature	Low physical activity
Yemen	High blood pressure	Particulate matter	Low birth weight & short gestation	Smoking	High LDL	High fasting plasma glucose	High body-mass index	Low whole grains	Kidney dysfunction	Child growth failure
**South Asia**	Particulate matter	High blood pressure	High fasting plasma glucose	Smoking	High LDL	High body-mass index	Low birth weight & short gestation	Unsafe water	Kidney dysfunction	Alcohol use
Bangladesh	High blood pressure	Particulate matter	Smoking	High fasting plasma glucose	High LDL	High body-mass index	Low birth weight & short gestation	Low fibre	Kidney dysfunction	Lead
Bhutan	High blood pressure	Particulate matter	High fasting plasma glucose	High LDL	Smoking	High body-mass index	Kidney dysfunction	Low temperature	Low birth weight & short gestation	Low whole grains
India	Particulate matter	High blood pressure	High fasting plasma glucose	Smoking	High LDL	High body-mass index	Unsafe water	Kidney dysfunction	Low birth weight & short gestation	Alcohol use
Andhra Pradesh	High blood pressure	Particulate matter	High fasting plasma glucose	Smoking	High LDL	High body-mass index	Unsafe water	Kidney dysfunction	Low whole grains	Lead
Arunachal Pradesh	Smoking	High blood pressure	Particulate matter	High fasting plasma glucose	Alcohol use	Low birth weight & short gestation	High body-mass index	Unsafe water	Kidney dysfunction	Low temperature
Assam	Particulate matter	High blood pressure	Smoking	High fasting plasma glucose	Alcohol use	Low birth weight & short gestation	High body-mass index	Unsafe water	Kidney dysfunction	High LDL
Bihar	Particulate matter	High blood pressure	Unsafe water	High fasting plasma glucose	Smoking	Low birth weight & short gestation	Unsafe sanitation	High LDL	Handwashing	Kidney dysfunction
Chhattisgarh	Particulate matter	High blood pressure	High fasting plasma glucose	Smoking	Unsafe water	Low birth weight & short gestation	High LDL	Alcohol use	High body-mass index	Kidney dysfunction
Delhi	Particulate matter	High blood pressure	High fasting plasma glucose	Smoking	High body-mass index	High LDL	Kidney dysfunction	Alcohol use	Low birth weight & short gestation	Low whole grains
Goa	High blood pressure	High fasting plasma glucose	Particulate matter	High body-mass index	High LDL	Kidney dysfunction	Alcohol use	Smoking	Low whole grains	Low fruit
Gujarat	Particulate matter	High blood pressure	High fasting plasma glucose	Smoking	High LDL	High body-mass index	Kidney dysfunction	Low birth weight & short gestation	Low whole grains	Alcohol use
Haryana	Particulate matter	High blood pressure	Smoking	High fasting plasma glucose	High LDL	High body-mass index	Kidney dysfunction	Low birth weight & short gestation	Alcohol use	Low whole grains
Himachal Pradesh	Smoking	High blood pressure	Particulate matter	High fasting plasma glucose	High LDL	High body-mass index	Low temperature	Kidney dysfunction	Alcohol use	Ozone
Jammu & Kashmir and Ladakh	Particulate matter	Smoking	High blood pressure	High fasting plasma glucose	High LDL	Low temperature	High body-mass index	Kidney dysfunction	Second-hand smoke	Low birth weight & short gestation
Jharkhand	Particulate matter	High blood pressure	Unsafe water	High fasting plasma glucose	Low birth weight & short gestation	Unsafe sanitation	Smoking	Kidney dysfunction	High body-mass index	Handwashing
Karnataka	High blood pressure	High fasting plasma glucose	Particulate matter	Smoking	High LDL	High body-mass index	Kidney dysfunction	Alcohol use	Unsafe water	Low fruit

Figure S2. Leading 10 level 3 risk factors for deaths in 2019 by location. Each risk is colored by the percentage change in all-age deaths from 2010 to 2019.

Percentage change in all-age deaths from 2010 to 2019

-0.800 to -0.062	-0.062 to 0.007	0.007 to 0.052	0.052 to 0.093	0.093 to 0.132	0.132 to 0.174	0.174 to 0.223	0.223 to 0.287	0.287 to 0.372	0.372 to 1.738

	1	2	3	4	5	6	7	8	9	10
Kerala	High blood pressure	High fasting plasma glucose	Particulate matter	High LDL	Smoking	High body-mass index	Kidney dysfunction	Low legumes	Low fruit	Alcohol use
Madhya Pradesh	Particulate matter	High blood pressure	Smoking	High fasting plasma glucose	Low birth weight & short gestation	Unsafe water	High LDL	High body-mass index	Kidney dysfunction	Unsafe sanitation
Maharashtra	High blood pressure	Particulate matter	High fasting plasma glucose	High LDL	High body-mass index	Smoking	Kidney dysfunction	Alcohol use	Low whole grains	Low fruit
Manipur	High blood pressure	Smoking	High fasting plasma glucose	Particulate matter	Alcohol use	High body-mass index	Kidney dysfunction	High LDL	Unsafe water	Low birth weight & short gestation
Meghalaya	Smoking	High blood pressure	Particulate matter	High fasting plasma glucose	Alcohol use	Low birth weight & short gestation	Unsafe water	High body-mass index	Kidney dysfunction	High LDL
Mizoram	Smoking	Particulate matter	High blood pressure	High fasting plasma glucose	Alcohol use	Low birth weight & short gestation	Unsafe sex	High body-mass index	Kidney dysfunction	Second-hand smoke
Nagaland	High blood pressure	Particulate matter	High fasting plasma glucose	Smoking	Alcohol use	Kidney dysfunction	High LDL	High body-mass index	Low birth weight & short gestation	Unsafe sex
Odisha	High blood pressure	Particulate matter	High fasting plasma glucose	Unsafe water	Unsafe sanitation	Smoking	Low birth weight & short gestation	Kidney dysfunction	High body-mass index	Alcohol use
Punjab	High blood pressure	High fasting plasma glucose	Particulate matter	High body-mass index	High LDL	Kidney dysfunction	Low whole grains	Smoking	Low fruit	Low legumes
Rajasthan	Particulate matter	Smoking	High blood pressure	Low birth weight & short gestation	High fasting plasma glucose	High LDL	Unsafe water	High body-mass index	Second-hand smoke	Occupational particulates
Sikkim	High blood pressure	Particulate matter	Smoking	High fasting plasma glucose	High body-mass index	Alcohol use	Low temperature	High LDL	Kidney dysfunction	Second-hand smoke
Tamil Nadu	High fasting plasma glucose	High blood pressure	Particulate matter	High body-mass index	High LDL	Kidney dysfunction	Smoking	Low whole grains	Lead	Low fruit
Telangana	High blood pressure	Particulate matter	High fasting plasma glucose	Smoking	High LDL	High body-mass index	Unsafe water	Kidney dysfunction	Low whole grains	Lead
Tripura	High blood pressure	Particulate matter	Smoking	High fasting plasma glucose	Kidney dysfunction	High LDL	High body-mass index	Alcohol use	Unsafe water	Low birth weight & short gestation
Other Union Territories	High fasting plasma glucose	High blood pressure	Particulate matter	High body-mass index	High LDL	Smoking	Kidney dysfunction	Alcohol use	Low whole grains	Low fruit
Uttar Pradesh	Particulate matter	Smoking	High blood pressure	High fasting plasma glucose	Low birth weight & short gestation	Unsafe water	Unsafe sanitation	High LDL	Alcohol use	High body-mass index
Uttarakhand	Smoking	Particulate matter	High blood pressure	High fasting plasma glucose	High body-mass index	High LDL	Low temperature	Alcohol use	Kidney dysfunction	Second-hand smoke
West Bengal	High blood pressure	Particulate matter	Smoking	High fasting plasma glucose	High LDL	Kidney dysfunction	High body-mass index	Unsafe water	Second-hand smoke	Low fruit
Nepal	Particulate matter	Smoking	High blood pressure	High fasting plasma glucose	Low temperature	High LDL	High body-mass index	Kidney dysfunction	Low birth weight & short gestation	Occupational particulates
Pakistan	Particulate matter	High blood pressure	Low birth weight & short gestation	High fasting plasma glucose	Smoking	High body-mass index	High LDL	Kidney dysfunction	Unsafe water	Child growth failure
**Southeast Asia, east Asia, and Oceania**	High blood pressure	Smoking	Particulate matter	High fasting plasma glucose	High LDL	High body-mass index	High sodium	Kidney dysfunction	Alcohol use	Low temperature
**East Asia**	High blood pressure	Smoking	Particulate matter	High fasting plasma glucose	High LDL	High sodium	High body-mass index	Low temperature	Kidney dysfunction	Alcohol use
China	High blood pressure	Smoking	Particulate matter	High fasting plasma glucose	High LDL	High sodium	High body-mass index	Low temperature	Kidney dysfunction	Alcohol use
North Korea	Particulate matter	High blood pressure	Smoking	High fasting plasma glucose	High LDL	High sodium	Low temperature	Kidney dysfunction	Second-hand smoke	Alcohol use
Taiwan (province of China)	Smoking	High fasting plasma glucose	High blood pressure	High body-mass index	Kidney dysfunction	Particulate matter	Alcohol use	High LDL	Low whole grains	Second-hand smoke
**Oceania**	High fasting plasma glucose	Particulate matter	High blood pressure	Smoking	High body-mass index	High LDL	Child growth failure	Low birth weight & short gestation	Unsafe sex	Kidney dysfunction
American Samoa	High fasting plasma glucose	High body-mass index	High blood pressure	Smoking	Kidney dysfunction	High LDL	Low whole grains	Second-hand smoke	Particulate matter	Low physical activity
Cook Islands	High fasting plasma glucose	High body-mass index	High blood pressure	Smoking	Kidney dysfunction	High LDL	Second-hand smoke	Alcohol use	High sodium	Low whole grains
Fiji	High fasting plasma glucose	High body-mass index	High blood pressure	Smoking	High LDL	Particulate matter	Kidney dysfunction	Low whole grains	Low fruit	Second-hand smoke
Guam	High blood pressure	High body-mass index	High fasting plasma glucose	Smoking	High LDL	Kidney dysfunction	Low whole grains	High sodium	Second-hand smoke	Particulate matter
Kiribati	High fasting plasma glucose	Smoking	High body-mass index	High blood pressure	Particulate matter	High LDL	Kidney dysfunction	Second-hand smoke	Low whole grains	Low birth weight & short gestation
Marshall Islands	High fasting plasma glucose	High blood pressure	High body-mass index	Smoking	High LDL	Particulate matter	Kidney dysfunction	Low whole grains	Second-hand smoke	High sodium

Figure S2. Leading 10 level 3 risk factors for deaths in 2019 by location. Each risk is colored by the percentage change in all-age deaths from 2010 to 2019.

	Percentage change in all-age deaths from 2010 to 2019									
	-0.800 to -0.062	-0.062 to 0.007	0.007 to 0.052	0.052 to 0.093	0.093 to 0.132	0.132 to 0.174	0.174 to 0.223	0.223 to 0.287	0.287 to 0.372	0.372 to 1.738
	1	2	3	4	5	6	7	8	9	10
Federated States of Micronesia	High fasting plasma glucose	High body-mass index	High blood pressure	Smoking	High LDL	Kidney dysfunction	Particulate matter	Unsafe sex	Low whole grains	Second-hand smoke
Nauru	High blood pressure	High body-mass index	High fasting plasma glucose	Smoking	High LDL	Kidney dysfunction	Low whole grains	Second-hand smoke	Alcohol use	Low birth weight & short gestation
Niue	High fasting plasma glucose	High blood pressure	High body-mass index	Smoking	Kidney dysfunction	High LDL	Second-hand smoke	Low whole grains	High sodium	Low physical activity
Northern Mariana Islands	High body-mass index	High fasting plasma glucose	High blood pressure	Smoking	Kidney dysfunction	High LDL	Particulate matter	Low whole grains	High sodium	Second-hand smoke
Palau	High fasting plasma glucose	High body-mass index	High blood pressure	Smoking	High LDL	Kidney dysfunction	Second-hand smoke	Low whole grains	High sodium	Low legumes
Papua New Guinea	Particulate matter	High fasting plasma glucose	Smoking	High blood pressure	High body-mass index	Child growth failure	Low birth weight & short gestation	High LDL	Unsafe sex	Unsafe water
Samoa	High fasting plasma glucose	High blood pressure	High body-mass index	Smoking	Particulate matter	Kidney dysfunction	High LDL	Low whole grains	Second-hand smoke	Low vegetables
Solomon Islands	Particulate matter	High fasting plasma glucose	Smoking	High body-mass index	High blood pressure	High LDL	Kidney dysfunction	Low whole grains	Second-hand smoke	Low vegetables
Tokelau	High fasting plasma glucose	High blood pressure	High body-mass index	Smoking	High LDL	Kidney dysfunction	Second-hand smoke	Low whole grains	High sodium	Low physical activity
Tonga	High fasting plasma glucose	High blood pressure	High body-mass index	Smoking	Kidney dysfunction	High LDL	Particulate matter	Second-hand smoke	Low whole grains	High sodium
Tuvalu	High fasting plasma glucose	High blood pressure	High body-mass index	Smoking	High LDL	Kidney dysfunction	Low whole grains	Second-hand smoke	High sodium	Particulate matter
Vanuatu	High blood pressure	High fasting plasma glucose	Particulate matter	High body-mass index	Smoking	High LDL	Kidney dysfunction	Low whole grains	High sodium	Low legumes
**Southeast Asia**	High blood pressure	High fasting plasma glucose	Smoking	Particulate matter	High body-mass index	Kidney dysfunction	High LDL	High sodium	Alcohol use	Second-hand smoke
Cambodia	Particulate matter	Smoking	High blood pressure	High fasting plasma glucose	Alcohol use	High LDL	High body-mass index	Kidney dysfunction	Low birth weight & short gestation	High sodium
Indonesia	High blood pressure	High fasting plasma glucose	Smoking	High body-mass index	Particulate matter	High LDL	Kidney dysfunction	High sodium	Second-hand smoke	Low fruit
Aceh	High blood pressure	High fasting plasma glucose	Smoking	High body-mass index	Particulate matter	Kidney dysfunction	High LDL	High sodium	Second-hand smoke	Unsafe water
Bali	High blood pressure	High fasting plasma glucose	Smoking	High body-mass index	Particulate matter	Kidney dysfunction	High LDL	High sodium	Second-hand smoke	Low fruit
Bangka-Belitung Islands	High blood pressure	High fasting plasma glucose	High body-mass index	Smoking	Particulate matter	Kidney dysfunction	High LDL	High sodium	Second-hand smoke	Low fruit
Banten	High blood pressure	High fasting plasma glucose	Smoking	Particulate matter	High body-mass index	High LDL	Kidney dysfunction	High sodium	Low birth weight & short gestation	Second-hand smoke
Bengkulu	High blood pressure	High fasting plasma glucose	Smoking	High body-mass index	Particulate matter	Kidney dysfunction	High LDL	High sodium	Second-hand smoke	Low fruit
Gorontalo	High blood pressure	High fasting plasma glucose	Particulate matter	Smoking	High body-mass index	Kidney dysfunction	Child growth failure	High sodium	High LDL	Second-hand smoke
Jakarta	High blood pressure	High body-mass index	High fasting plasma glucose	Smoking	Particulate matter	High LDL	Kidney dysfunction	High sodium	Second-hand smoke	Low legumes
Jambi	High blood pressure	Smoking	High fasting plasma glucose	High body-mass index	Particulate matter	Kidney dysfunction	High LDL	High sodium	Second-hand smoke	Low fruit
West Java	High blood pressure	High fasting plasma glucose	Smoking	Particulate matter	High body-mass index	High LDL	Kidney dysfunction	High sodium	Second-hand smoke	Low legumes
Central Java	High blood pressure	High fasting plasma glucose	Smoking	Particulate matter	High body-mass index	Kidney dysfunction	High sodium	High LDL	Low fruit	Low fibre
East Java	High blood pressure	Smoking	High fasting plasma glucose	Particulate matter	High body-mass index	High LDL	Kidney dysfunction	High sodium	Unsafe water	Second-hand smoke
West Kalimantan	High blood pressure	High fasting plasma glucose	Smoking	Particulate matter	High body-mass index	Kidney dysfunction	High LDL	High sodium	Second-hand smoke	Low fruit
South Kalimantan	High blood pressure	High fasting plasma glucose	Smoking	High body-mass index	Particulate matter	High LDL	Kidney dysfunction	High sodium	Second-hand smoke	Low legumes
Central Kalimantan	High blood pressure	High fasting plasma glucose	Smoking	High body-mass index	Particulate matter	High LDL	Kidney dysfunction	High sodium	Second-hand smoke	Low legumes
East Kalimantan	High blood pressure	High body-mass index	Smoking	High fasting plasma glucose	Particulate matter	Kidney dysfunction	High LDL	High sodium	Second-hand smoke	Low birth weight & short gestation
North Kalimantan	High fasting plasma glucose	High body-mass index	High blood pressure	Smoking	Particulate matter	Kidney dysfunction	Low birth weight & short gestation	High LDL	High sodium	Second-hand smoke
Riau Islands	High blood pressure	High body-mass index	High fasting plasma glucose	Smoking	Particulate matter	High LDL	Kidney dysfunction	High sodium	Low birth weight & short gestation	Second-hand smoke

Figure S2. Leading 10 level 3 risk factors for deaths in 2019 by location. Each risk is colored by the percentage change in all-age deaths from 2010 to 2019.

Percentage change in all-age deaths from 2010 to 2019

	-0.800 to -0.062	-0.062 to 0.007	0.007 to 0.052	0.052 to 0.093	0.093 to 0.132	0.132 to 0.174	0.174 to 0.223	0.223 to 0.287	0.287 to 0.372	0.372 to 1.738
	1	2	3	4	5	6	7	8	9	10
Lampung	High blood pressure	Smoking	High fasting plasma glucose	Particulate matter	High body-mass index	Kidney dysfunction	High sodium	High LDL	Second-hand smoke	Low fruit
Maluku	High blood pressure	High fasting plasma glucose	Smoking	Particulate matter	High body-mass index	High LDL	Kidney dysfunction	Unsafe water	High sodium	Low birth weight & short gestation
North Maluku	High blood pressure	High fasting plasma glucose	Particulate matter	Smoking	High body-mass index	High LDL	Kidney dysfunction	High sodium	Second-hand smoke	Low birth weight & short gestation
West Nusa Tenggara	High blood pressure	High fasting plasma glucose	Smoking	Particulate matter	High body-mass index	Kidney dysfunction	High LDL	High sodium	Unsafe water	Second-hand smoke
East Nusa Tenggara	High blood pressure	Particulate matter	High fasting plasma glucose	Smoking	High body-mass index	Kidney dysfunction	High sodium	High LDL	Unsafe water	Low birth weight & short gestation
Papua	High blood pressure	High body-mass index	Smoking	High fasting plasma glucose	Particulate matter	Low birth weight & short gestation	Kidney dysfunction	Child growth failure	High LDL	Unsafe water
West Papua	High blood pressure	High fasting plasma glucose	High body-mass index	Smoking	Particulate matter	Kidney dysfunction	High LDL	High sodium	Low birth weight & short gestation	Second-hand smoke
Riau	High blood pressure	High body-mass index	Smoking	High fasting plasma glucose	Particulate matter	High LDL	Kidney dysfunction	High sodium	Second-hand smoke	Low birth weight & short gestation
West Sulawesi	High blood pressure	High fasting plasma glucose	Particulate matter	Smoking	High body-mass index	Kidney dysfunction	High LDL	High sodium	Low birth weight & short gestation	Second-hand smoke
South Sulawesi	High blood pressure	High fasting plasma glucose	Smoking	High body-mass index	Particulate matter	Kidney dysfunction	High LDL	High sodium	Second-hand smoke	Low fruit
Central Sulawesi	High blood pressure	High fasting plasma glucose	Smoking	High body-mass index	Particulate matter	Kidney dysfunction	High LDL	High sodium	Second-hand smoke	Low birth weight & short gestation
Southeast Sulawesi	High blood pressure	High fasting plasma glucose	Smoking	High body-mass index	Particulate matter	High LDL	Kidney dysfunction	High sodium	Low birth weight & short gestation	Second-hand smoke
North Sulawesi	High blood pressure	High fasting plasma glucose	Smoking	High body-mass index	Particulate matter	Kidney dysfunction	High sodium	High LDL	Second-hand smoke	Low fruit
West Sumatra	High blood pressure	Smoking	High fasting plasma glucose	High body-mass index	Particulate matter	Kidney dysfunction	High LDL	High sodium	Second-hand smoke	Unsafe water
South Sumatra	High blood pressure	Smoking	High fasting plasma glucose	High body-mass index	Particulate matter	High LDL	Kidney dysfunction	High sodium	Second-hand smoke	Low birth weight & short gestation
North Sumatra	High blood pressure	High fasting plasma glucose	Smoking	High body-mass index	Particulate matter	Kidney dysfunction	High LDL	High sodium	Second-hand smoke	Low birth weight & short gestation
Yogyakarta	High blood pressure	High fasting plasma glucose	Smoking	High body-mass index	Particulate matter	Kidney dysfunction	High LDL	High sodium	Low fruit	Low fibre
Laos	High blood pressure	Particulate matter	High fasting plasma glucose	Smoking	High body-mass index	Kidney dysfunction	High LDL	Low birth weight & short gestation	Alcohol use	High sodium
Malaysia	High blood pressure	High fasting plasma glucose	Smoking	High LDL	High body-mass index	Kidney dysfunction	Particulate matter	High sodium	Low whole grains	Second-hand smoke
Maldives	High blood pressure	High fasting plasma glucose	Smoking	High LDL	Kidney dysfunction	High body-mass index	Particulate matter	High sodium	Low whole grains	Low birth weight & short gestation
Mauritius	High fasting plasma glucose	High blood pressure	High body-mass index	Kidney dysfunction	Smoking	High LDL	Particulate matter	High sodium	Low whole grains	Second-hand smoke
Myanmar	High blood pressure	Particulate matter	High fasting plasma glucose	Smoking	High body-mass index	Kidney dysfunction	High LDL	Alcohol use	High sodium	Low birth weight & short gestation
Philippines	High blood pressure	Smoking	High fasting plasma glucose	Particulate matter	High body-mass index	Kidney dysfunction	High LDL	Alcohol use	High sodium	Second-hand smoke
Seychelles	High blood pressure	High fasting plasma glucose	Smoking	High body-mass index	Kidney dysfunction	High LDL	Alcohol use	Particulate matter	High sodium	Low whole grains
Sri Lanka	High fasting plasma glucose	High blood pressure	High body-mass index	Particulate matter	High LDL	Smoking	Kidney dysfunction	Alcohol use	High sodium	Low whole grains
Thailand	High blood pressure	High fasting plasma glucose	Smoking	High body-mass index	Kidney dysfunction	Particulate matter	Alcohol use	High LDL	High sodium	Unsafe sex
Timor-Leste	High blood pressure	Particulate matter	High fasting plasma glucose	Smoking	Low birth weight & short gestation	Kidney dysfunction	High LDL	High sodium	Child growth failure	Alcohol use
Vietnam	High blood pressure	High fasting plasma glucose	Smoking	Particulate matter	Alcohol use	Kidney dysfunction	High LDL	High body-mass index	High sodium	Low fibre
**Sub-Saharan Africa**	Particulate matter	Low birth weight & short gestation	High blood pressure	Child growth failure	Unsafe sex	Unsafe water	High fasting plasma glucose	Unsafe sanitation	Handwashing	High body-mass index
**Central sub-Saharan Africa**	Particulate matter	High blood pressure	Low birth weight & short gestation	High fasting plasma glucose	Child growth failure	Unsafe water	Unsafe sex	Unsafe sanitation	High body-mass index	Handwashing
Angola	High blood pressure	Low birth weight & short gestation	Unsafe sex	Particulate matter	Alcohol use	Child growth failure	Unsafe water	High fasting plasma glucose	Smoking	Handwashing
Central African Republic	Particulate matter	Child growth failure	Unsafe water	Low birth weight & short gestation	Unsafe sanitation	Unsafe sex	High blood pressure	Handwashing	High fasting plasma glucose	Alcohol use

Figure S2. Leading 10 level 3 risk factors for deaths in 2019 by location. Each risk is colored by the percentage change in all-age deaths from 2010 to 2019.

	Percentage change in all-age deaths from 2010 to 2019									
	-0.800 to -0.062	-0.062 to 0.007	0.007 to 0.052	0.052 to 0.093	0.093 to 0.132	0.132 to 0.174	0.174 to 0.223	0.223 to 0.287	0.287 to 0.372	0.372 to 1.738
	1	2	3	4	5	6	7	8	9	10
Congo (Brazzaville)	High blood pressure	Unsafe sex	Particulate matter	High fasting plasma glucose	High body-mass index	Low birth weight & short gestation	Alcohol use	Unsafe water	Smoking	High LDL
DR Congo	Particulate matter	High blood pressure	Low birth weight & short gestation	High fasting plasma glucose	Child growth failure	Unsafe water	Handwashing	High body-mass index	Unsafe sanitation	Smoking
Equatorial Guinea	Unsafe sex	High blood pressure	High body-mass index	High fasting plasma glucose	Particulate matter	Low birth weight & short gestation	Alcohol use	Intimate partner violence	Kidney dysfunction	Smoking
Gabon	High blood pressure	High fasting plasma glucose	High body-mass index	Unsafe sex	Alcohol use	Particulate matter	Kidney dysfunction	Smoking	Low birth weight & short gestation	High LDL
**Eastern sub-Saharan Africa**	Particulate matter	Low birth weight & short gestation	Unsafe sex	High blood pressure	Child growth failure	Unsafe water	High fasting plasma glucose	Unsafe sanitation	Handwashing	Alcohol use
Burundi	Particulate matter	Child growth failure	Unsafe water	Low birth weight & short gestation	High blood pressure	Unsafe sanitation	Alcohol use	Handwashing	High fasting plasma glucose	Smoking
Comoros	High blood pressure	Particulate matter	High fasting plasma glucose	Low birth weight & short gestation	High body-mass index	Smoking	Unsafe water	Child growth failure	High LDL	Kidney dysfunction
Djibouti	Unsafe sex	High blood pressure	Particulate matter	Low birth weight & short gestation	Smoking	Child growth failure	High fasting plasma glucose	High body-mass index	Unsafe water	Handwashing
Eritrea	Particulate matter	High blood pressure	Unsafe water	Child growth failure	Low birth weight & short gestation	Unsafe sanitation	High fasting plasma glucose	Handwashing	Unsafe sex	High body-mass index
Ethiopia	Particulate matter	Low birth weight & short gestation	Unsafe water	Child growth failure	High blood pressure	Unsafe sanitation	Handwashing	High fasting plasma glucose	Unsafe sex	Alcohol use
Kenya	Unsafe sex	Particulate matter	High blood pressure	Low birth weight & short gestation	Alcohol use	Unsafe water	High body-mass index	High fasting plasma glucose	Child growth failure	Unsafe sanitation
Baringo	Particulate matter	Low birth weight & short gestation	High blood pressure	Unsafe water	Child growth failure	Unsafe sanitation	Alcohol use	Handwashing	High body-mass index	High fasting plasma glucose
Bomet	Unsafe sex	Particulate matter	High blood pressure	Low birth weight & short gestation	Alcohol use	Unsafe water	Drug use	High body-mass index	Unsafe sanitation	High fasting plasma glucose
Bungoma	Particulate matter	High blood pressure	Unsafe water	Low birth weight & short gestation	Child growth failure	Unsafe sanitation	High fasting plasma glucose	Handwashing	Alcohol use	High body-mass index
Busia	Particulate matter	Unsafe sex	Low birth weight & short gestation	High blood pressure	Unsafe water	Child growth failure	Unsafe sanitation	Handwashing	Alcohol use	High fasting plasma glucose
Elgeyo Marakwet	High blood pressure	Particulate matter	High fasting plasma glucose	Alcohol use	High body-mass index	Unsafe sex	Unsafe water	Unsafe sanitation	Handwashing	Smoking
Embu	High blood pressure	Unsafe sex	Particulate matter	High body-mass index	Alcohol use	High fasting plasma glucose	Unsafe water	Low birth weight & short gestation	Smoking	Unsafe sanitation
Garissa	Particulate matter	Low birth weight & short gestation	High blood pressure	Unsafe sex	Unsafe water	Child growth failure	Smoking	Unsafe sanitation	Alcohol use	Handwashing
Homa Bay	Unsafe sex	Particulate matter	Child growth failure	Unsafe water	High blood pressure	Intimate partner violence	Low birth weight & short gestation	Unsafe sanitation	Drug use	Handwashing
Isiolo	Low birth weight & short gestation	Particulate matter	Unsafe sex	Child growth failure	High blood pressure	Unsafe water	Handwashing	Unsafe sanitation	Alcohol use	Smoking
Kajiado	Unsafe sex	High blood pressure	High body-mass index	Particulate matter	Alcohol use	High fasting plasma glucose	Low birth weight & short gestation	Smoking	Unsafe water	Kidney dysfunction
Kakamega	Unsafe sex	Particulate matter	High blood pressure	Low birth weight & short gestation	Child growth failure	Unsafe water	Alcohol use	High body-mass index	Unsafe sanitation	Handwashing
Kericho	High blood pressure	Particulate matter	Unsafe sex	Low birth weight & short gestation	High body-mass index	High fasting plasma glucose	Unsafe water	Alcohol use	Unsafe sanitation	Handwashing
Kiambu	High blood pressure	Unsafe sex	High body-mass index	Alcohol use	High fasting plasma glucose	Particulate matter	Smoking	Low birth weight & short gestation	Kidney dysfunction	Unsafe water
Kilifi	Unsafe sex	Particulate matter	High blood pressure	Low birth weight & short gestation	Unsafe water	Child growth failure	Alcohol use	High fasting plasma glucose	Handwashing	High body-mass index
Kirinyaga	High blood pressure	Unsafe sex	Particulate matter	High body-mass index	Alcohol use	High fasting plasma glucose	Smoking	Low birth weight & short gestation	Unsafe water	Kidney dysfunction
Kisii	High blood pressure	Particulate matter	Unsafe sex	High body-mass index	Alcohol use	High fasting plasma glucose	Unsafe water	Low birth weight & short gestation	Unsafe sanitation	Handwashing
Kisumu	Unsafe sex	High blood pressure	Particulate matter	High body-mass index	High fasting plasma glucose	Alcohol use	Child growth failure	Smoking	Intimate partner violence	Low birth weight & short gestation
Kitui	High blood pressure	Particulate matter	Unsafe sex	Unsafe water	Low birth weight & short gestation	Alcohol use	High body-mass index	High fasting plasma glucose	Unsafe sanitation	Handwashing
Kwale	Particulate matter	Unsafe sex	Low birth weight & short gestation	High blood pressure	Alcohol use	High fasting plasma glucose	High body-mass index	Unsafe water	Smoking	Handwashing
Laikipia	Unsafe sex	High blood pressure	Particulate matter	High body-mass index	Smoking	High fasting plasma glucose	Alcohol use	Unsafe water	Kidney dysfunction	Intimate partner violence
Lamu	Particulate matter	Low birth weight & short gestation	Unsafe sex	High blood pressure	Alcohol use	High body-mass index	High fasting plasma glucose	Child growth failure	Smoking	Unsafe water

Figure S2. Leading 10 level 3 risk factors for deaths in 2019 by location. Each risk is colored by the percentage change in all-age deaths from 2010 to 2019.

Percentage change in all-age deaths from 2010 to 2019

-0.800 to -0.062	-0.062 to 0.007	0.007 to 0.052	0.052 to 0.093	0.093 to 0.132	0.132 to 0.174	0.174 to 0.223	0.223 to 0.287	0.287 to 0.372	0.372 to 1.738

	1	2	3	4	5	6	7	8	9	10
Machakos	High blood pressure	Unsafe sex	Particulate matter	High body-mass index	High fasting plasma glucose	Alcohol use	Unsafe water	Smoking	Handwashing	Unsafe sanitation
Makueni	Unsafe sex	High blood pressure	Particulate matter	Unsafe water	Alcohol use	Intimate partner violence	High fasting plasma glucose	High body-mass index	Unsafe sanitation	Low birth weight & short gestation
Mandera	Particulate matter	Child growth failure	Low birth weight & short gestation	Unsafe sex	Unsafe water	Unsafe sanitation	Handwashing	High blood pressure	Alcohol use	High fasting plasma glucose
Marsabit	Low birth weight & short gestation	Particulate matter	Unsafe sex	Unsafe water	Child growth failure	Unsafe sanitation	High blood pressure	Handwashing	Alcohol use	High fasting plasma glucose
Meru	Unsafe sex	High blood pressure	Particulate matter	Alcohol use	High body-mass index	High fasting plasma glucose	Low birth weight & short gestation	Unsafe water	Smoking	Unsafe sanitation
Migori	Unsafe sex	Child growth failure	Particulate matter	Low birth weight & short gestation	Unsafe water	Unsafe sanitation	High blood pressure	Handwashing	Intimate partner violence	Alcohol use
Mombasa	Unsafe sex	High blood pressure	Low birth weight & short gestation	Alcohol use	Particulate matter	High body-mass index	High fasting plasma glucose	Drug use	Smoking	Intimate partner violence
Murang'a	High blood pressure	Unsafe sex	Particulate matter	High body-mass index	High fasting plasma glucose	Alcohol use	Smoking	Unsafe water	Kidney dysfunction	Handwashing
Nairobi	Unsafe sex	High blood pressure	High body-mass index	Alcohol use	High fasting plasma glucose	Low birth weight & short gestation	Smoking	Particulate matter	Kidney dysfunction	High LDL
Nakuru	Unsafe sex	High blood pressure	Particulate matter	Alcohol use	Low birth weight & short gestation	High body-mass index	High fasting plasma glucose	Smoking	Unsafe water	Child growth failure
Nandi	Unsafe sex	High blood pressure	Particulate matter	Alcohol use	High fasting plasma glucose	High body-mass index	Smoking	Unsafe water	Low birth weight & short gestation	Handwashing
Narok	Unsafe sex	Particulate matter	High blood pressure	Unsafe water	Low birth weight & short gestation	Alcohol use	Unsafe sanitation	Child growth failure	High body-mass index	Handwashing
Nyamira	Unsafe sex	High blood pressure	Particulate matter	Alcohol use	High body-mass index	High fasting plasma glucose	Smoking	Low birth weight & short gestation	Unsafe water	Handwashing
Nyandarua	High blood pressure	Unsafe sex	Particulate matter	High body-mass index	Alcohol use	High fasting plasma glucose	Smoking	Low birth weight & short gestation	Unsafe water	Low temperature
Nyeri	Unsafe sex	High blood pressure	Particulate matter	High body-mass index	High fasting plasma glucose	Alcohol use	Smoking	Low temperature	Kidney dysfunction	Unsafe water
Samburu	Unsafe sex	Low birth weight & short gestation	Particulate matter	Unsafe water	Child growth failure	Unsafe sanitation	Handwashing	High blood pressure	Alcohol use	Smoking
Siaya	Unsafe sex	Particulate matter	High blood pressure	Low birth weight & short gestation	Unsafe water	Child growth failure	High fasting plasma glucose	Intimate partner violence	Unsafe sanitation	Handwashing
Taita Taveta	Unsafe sex	High blood pressure	Particulate matter	High body-mass index	High fasting plasma glucose	Alcohol use	Smoking	Kidney dysfunction	Unsafe water	Handwashing
Tana River	Particulate matter	Low birth weight & short gestation	Unsafe sex	High blood pressure	Alcohol use	Unsafe water	Child growth failure	High fasting plasma glucose	Handwashing	High body-mass index
Tharaka Nithi	High blood pressure	Particulate matter	Alcohol use	High body-mass index	High fasting plasma glucose	Unsafe water	Unsafe sex	Smoking	Unsafe sanitation	Handwashing
Trans Nzoia	Unsafe sex	High blood pressure	Particulate matter	Alcohol use	Unsafe water	High body-mass index	High fasting plasma glucose	Unsafe sanitation	Low birth weight & short gestation	Handwashing
Turkana	Unsafe sex	Unsafe water	Particulate matter	Low birth weight & short gestation	Child growth failure	Unsafe sanitation	Handwashing	High blood pressure	Alcohol use	Drug use
Uasin Gishu	Unsafe sex	High blood pressure	Particulate matter	High body-mass index	Alcohol use	Low birth weight & short gestation	Unsafe water	High fasting plasma glucose	Unsafe sanitation	Smoking
Vihiga	Particulate matter	High blood pressure	Unsafe sex	Smoking	High fasting plasma glucose	Low birth weight & short gestation	High body-mass index	Alcohol use	Unsafe water	Handwashing
Wajir	Low birth weight & short gestation	Particulate matter	Unsafe water	Child growth failure	Unsafe sanitation	Handwashing	High blood pressure	Alcohol use	Suboptimal breastfeeding	High fasting plasma glucose
West Pokot	Low birth weight & short gestation	Unsafe water	Particulate matter	Child growth failure	Unsafe sanitation	Handwashing	High blood pressure	Unsafe sex	Alcohol use	High body-mass index
Madagascar	Particulate matter	High blood pressure	Child growth failure	Unsafe water	Low birth weight & short gestation	Unsafe sanitation	Handwashing	High body-mass index	High fasting plasma glucose	Smoking
Malawi	Unsafe sex	Particulate matter	High blood pressure	Low birth weight & short gestation	High fasting plasma glucose	Unsafe water	Child growth failure	Unsafe sanitation	Smoking	Handwashing
Mozambique	Unsafe sex	Particulate matter	High blood pressure	Low birth weight & short gestation	High fasting plasma glucose	Child growth failure	Smoking	High body-mass index	Unsafe water	Handwashing
Rwanda	Particulate matter	High blood pressure	Alcohol use	Smoking	Low birth weight & short gestation	High fasting plasma glucose	Unsafe sex	Child growth failure	High body-mass index	Unsafe water
Somalia	Child growth failure	Particulate matter	Low birth weight & short gestation	Unsafe water	High blood pressure	Unsafe sanitation	Handwashing	High fasting plasma glucose	Smoking	Unsafe sex
South Sudan	Particulate matter	Child growth failure	Low birth weight & short gestation	Unsafe water	High blood pressure	Unsafe sanitation	Handwashing	Unsafe sex	High fasting plasma glucose	High body-mass index

Figure S2. Leading 10 level 3 risk factors for deaths in 2019 by location. Each risk is colored by the percentage change in all-age deaths from 2010 to 2019.

	Percentage change in all-age deaths from 2010 to 2019									
	-0.800 to -0.062	-0.062 to 0.007	0.007 to 0.052	0.052 to 0.093	0.093 to 0.132	0.132 to 0.174	0.174 to 0.223	0.223 to 0.287	0.287 to 0.372	0.372 to 1.738
	1	2	3	4	5	6	7	8	9	10
Uganda	Particulate matter	Low birth weight & short gestation	Unsafe sex	High blood pressure	Alcohol use	Child growth failure	High fasting plasma glucose	Unsafe water	High body-mass index	Handwashing
Tanzania	Particulate matter	High blood pressure	Low birth weight & short gestation	Unsafe sex	Child growth failure	Alcohol use	High fasting plasma glucose	Smoking	High body-mass index	Unsafe water
Zambia	Unsafe sex	Particulate matter	High blood pressure	Low birth weight & short gestation	Child growth failure	Alcohol use	High fasting plasma glucose	High body-mass index	Unsafe water	Smoking
**Southern sub-Saharan Africa**	Unsafe sex	High blood pressure	High fasting plasma glucose	High body-mass index	Particulate matter	Smoking	Alcohol use	Low birth weight & short gestation	Kidney dysfunction	Intimate partner violence
Botswana	Unsafe sex	High blood pressure	High fasting plasma glucose	High body-mass index	Smoking	Particulate matter	Alcohol use	Low birth weight & short gestation	Kidney dysfunction	Intimate partner violence
eSwatini	Unsafe sex	High body-mass index	High fasting plasma glucose	High blood pressure	Particulate matter	Alcohol use	Low birth weight & short gestation	Unsafe water	Kidney dysfunction	Smoking
Lesotho	Unsafe sex	High blood pressure	High fasting plasma glucose	Particulate matter	High body-mass index	Smoking	Alcohol use	Unsafe water	Intimate partner violence	Low birth weight & short gestation
Namibia	Unsafe sex	High blood pressure	High fasting plasma glucose	Particulate matter	High body-mass index	Smoking	Alcohol use	Low birth weight & short gestation	Kidney dysfunction	High LDL
South Africa	Unsafe sex	High blood pressure	High body-mass index	High fasting plasma glucose	Smoking	Particulate matter	Alcohol use	Kidney dysfunction	Low birth weight & short gestation	Intimate partner violence
Zimbabwe	Unsafe sex	Particulate matter	High blood pressure	High fasting plasma glucose	Smoking	Low birth weight & short gestation	High body-mass index	Child growth failure	Alcohol use	Handwashing
**Western sub-Saharan Africa**	Particulate matter	Child growth failure	Low birth weight & short gestation	Unsafe water	High blood pressure	Unsafe sanitation	Handwashing	Unsafe sex	High fasting plasma glucose	High body-mass index
Benin	Particulate matter	Low birth weight & short gestation	Child growth failure	High blood pressure	High fasting plasma glucose	Unsafe water	High body-mass index	Handwashing	Unsafe sanitation	Kidney dysfunction
Burkina Faso	Particulate matter	Child growth failure	Low birth weight & short gestation	High blood pressure	Unsafe water	Handwashing	Unsafe sanitation	High fasting plasma glucose	Alcohol use	High body-mass index
Cape Verde	High blood pressure	High fasting plasma glucose	Particulate matter	High body-mass index	High LDL	Alcohol use	Kidney dysfunction	Smoking	Low whole grains	Low birth weight & short gestation
Cameroon	Unsafe sex	Particulate matter	High blood pressure	Child growth failure	Low birth weight & short gestation	Unsafe water	High body-mass index	High fasting plasma glucose	Unsafe sanitation	Handwashing
Chad	Child growth failure	Unsafe water	Particulate matter	Unsafe sanitation	Low birth weight & short gestation	Handwashing	High blood pressure	Suboptimal breastfeeding	High fasting plasma glucose	Unsafe sex
Côte d'Ivoire	Low birth weight & short gestation	Particulate matter	High blood pressure	Unsafe sex	High fasting plasma glucose	Child growth failure	High body-mass index	Alcohol use	Smoking	Unsafe water
The Gambia	Particulate matter	High blood pressure	Low birth weight & short gestation	Unsafe sex	High fasting plasma glucose	High body-mass index	Smoking	Alcohol use	High LDL	Kidney dysfunction
Ghana	High blood pressure	Particulate matter	High body-mass index	High fasting plasma glucose	Unsafe sex	Low birth weight & short gestation	Alcohol use	High LDL	Kidney dysfunction	Child growth failure
Guinea	Particulate matter	Low birth weight & short gestation	Child growth failure	High blood pressure	Unsafe water	High fasting plasma glucose	Handwashing	Unsafe sex	Smoking	High body-mass index
Guinea-Bissau	Particulate matter	High blood pressure	Low birth weight & short gestation	Child growth failure	Unsafe water	Unsafe sex	High fasting plasma glucose	Unsafe sanitation	Handwashing	High body-mass index
Liberia	Particulate matter	High blood pressure	Low birth weight & short gestation	Unsafe water	High fasting plasma glucose	Unsafe sex	Child growth failure	High body-mass index	Unsafe sanitation	Handwashing
Mali	Child growth failure	Low birth weight & short gestation	Particulate matter	Unsafe water	High blood pressure	Unsafe sanitation	Handwashing	High fasting plasma glucose	High body-mass index	Unsafe sex
Mauritania	High blood pressure	Particulate matter	Low birth weight & short gestation	High body-mass index	High fasting plasma glucose	Unsafe water	Child growth failure	Unsafe sanitation	Kidney dysfunction	Handwashing
Niger	Child growth failure	Particulate matter	Unsafe water	Low birth weight & short gestation	Unsafe sanitation	Handwashing	High blood pressure	High fasting plasma glucose	Suboptimal breastfeeding	High temperature
Nigeria	Child growth failure	Low birth weight & short gestation	Particulate matter	Unsafe water	High blood pressure	Unsafe sanitation	Handwashing	Unsafe sex	High fasting plasma glucose	High body-mass index
São Tomé and PrÍncipe	High blood pressure	Particulate matter	High body-mass index	High fasting plasma glucose	Kidney dysfunction	Alcohol use	High LDL	Smoking	Low birth weight & short gestation	Low whole grains
Senegal	Particulate matter	High blood pressure	Low birth weight & short gestation	High fasting plasma glucose	Unsafe water	High body-mass index	Child growth failure	Unsafe sanitation	Kidney dysfunction	Handwashing
Sierra Leone	Particulate matter	High blood pressure	Low birth weight & short gestation	Child growth failure	Unsafe water	Unsafe sex	Handwashing	High fasting plasma glucose	Unsafe sanitation	Smoking
Togo	Particulate matter	High blood pressure	Unsafe water	Low birth weight & short gestation	Child growth failure	Unsafe sanitation	Unsafe sex	Handwashing	High body-mass index	High fasting plasma glucose

Appendix Figure 3. Leading 10 level 3 risk factors for DALYs in 2019 by location. Each risk is colored by the percentage change in all-age DALYs from 2010 to 2019. DALYs=disability-adjusted life-years.

Percentage change in all-age DALYs from 2010 to 2019

-0.828 to -0.164	-0.164 to -0.066	-0.066 to -0.010	-0.010 to 0.029	0.029 to 0.073	0.073 to 0.120	0.120 to 0.173	0.173 to 0.235	0.235 to 0.325	0.325 to 1.699

	1	2	3	4	5	6	7	8	9	10
**Global**	High blood pressure	Particulate matter	Smoking	High fasting plasma glucose	Low birth weight & short gestation	High body-mass index	High LDL	Alcohol use	Child growth failure	Kidney dysfunction
**Low SDI**	Low birth weight & short gestation	Child growth failure	Particulate matter	Unsafe water	Unsafe sanitation	Handwashing	High blood pressure	High fasting plasma glucose	Unsafe sex	Smoking
**Low-middle SDI**	Particulate matter	Low birth weight & short gestation	High blood pressure	High fasting plasma glucose	Smoking	High body-mass index	Child growth failure	Unsafe water	High LDL	Alcohol use
**Middle SDI**	High blood pressure	Smoking	High fasting plasma glucose	Particulate matter	High body-mass index	High LDL	Kidney dysfunction	Low birth weight & short gestation	Alcohol use	High sodium
**High-middle SDI**	High blood pressure	Smoking	High body-mass index	High fasting plasma glucose	High LDL	Particulate matter	Alcohol use	Kidney dysfunction	High sodium	Low whole grains
**High SDI**	Smoking	High body-mass index	High fasting plasma glucose	High blood pressure	Alcohol use	High LDL	Drug use	Kidney dysfunction	Particulate matter	Low whole grains
**Central Europe, eastern Europe, and central Asia**	High blood pressure	Smoking	High body-mass index	High LDL	Alcohol use	High fasting plasma glucose	Particulate matter	Low whole grains	Kidney dysfunction	Low legumes
**Central Asia**	High blood pressure	High body-mass index	High fasting plasma glucose	Smoking	Particulate matter	High LDL	Alcohol use	Low whole grains	Low birth weight & short gestation	Kidney dysfunction
Armenia	High blood pressure	Smoking	High body-mass index	High fasting plasma glucose	Particulate matter	High LDL	Low whole grains	Kidney dysfunction	Alcohol use	Low legumes
Azerbaijan	High blood pressure	High body-mass index	Smoking	High fasting plasma glucose	High LDL	Particulate matter	Alcohol use	Low birth weight & short gestation	Low whole grains	Kidney dysfunction
Georgia	High blood pressure	High fasting plasma glucose	High body-mass index	Smoking	High LDL	Particulate matter	Alcohol use	Low whole grains	Kidney dysfunction	Low temperature
Kazakhstan	High blood pressure	High body-mass index	Smoking	High fasting plasma glucose	Alcohol use	High LDL	Particulate matter	Low whole grains	Kidney dysfunction	High red meat
Kyrgyzstan	High blood pressure	Smoking	High body-mass index	Alcohol use	Particulate matter	High LDL	Low birth weight & short gestation	High fasting plasma glucose	Low whole grains	Kidney dysfunction
Mongolia	High blood pressure	Alcohol use	High body-mass index	Smoking	Particulate matter	High LDL	High red meat	Low birth weight & short gestation	Kidney dysfunction	High fasting plasma glucose
Tajikistan	High blood pressure	Particulate matter	High fasting plasma glucose	Low birth weight & short gestation	Child growth failure	High body-mass index	High LDL	Smoking	Alcohol use	Low whole grains
Turkmenistan	High blood pressure	High body-mass index	High LDL	Smoking	High fasting plasma glucose	Particulate matter	Alcohol use	Low birth weight & short gestation	Kidney dysfunction	Low whole grains
Uzbekistan	High blood pressure	High body-mass index	High fasting plasma glucose	High LDL	Particulate matter	Smoking	Low whole grains	Kidney dysfunction	Alcohol use	Low birth weight & short gestation
**Central Europe**	High blood pressure	Smoking	High body-mass index	High fasting plasma glucose	High LDL	Alcohol use	Particulate matter	High sodium	Kidney dysfunction	Low whole grains
Albania	High blood pressure	Smoking	High body-mass index	High LDL	High fasting plasma glucose	Particulate matter	High sodium	Low whole grains	Kidney dysfunction	Alcohol use
Bosnia and Herzegovina	Smoking	High fasting plasma glucose	High blood pressure	High body-mass index	Particulate matter	High LDL	High sodium	Alcohol use	Kidney dysfunction	Low temperature
Bulgaria	High blood pressure	Smoking	High body-mass index	High fasting plasma glucose	High LDL	High sodium	Alcohol use	Particulate matter	Kidney dysfunction	Low whole grains
Croatia	Smoking	High blood pressure	High body-mass index	High fasting plasma glucose	High LDL	Alcohol use	High sodium	Particulate matter	Kidney dysfunction	Low whole grains
Czech Republic	Smoking	High fasting plasma glucose	High body-mass index	High blood pressure	High LDL	Alcohol use	High sodium	Particulate matter	Kidney dysfunction	Low whole grains
Hungary	Smoking	High blood pressure	High body-mass index	High fasting plasma glucose	High LDL	Alcohol use	High sodium	Particulate matter	Low whole grains	Kidney dysfunction
Montenegro	Smoking	High blood pressure	High body-mass index	High fasting plasma glucose	Particulate matter	High LDL	Alcohol use	High sodium	Kidney dysfunction	Low whole grains
North Macedonia	High blood pressure	Smoking	High fasting plasma glucose	High body-mass index	Particulate matter	High LDL	High sodium	Kidney dysfunction	Alcohol use	Low whole grains
Poland	Smoking	High blood pressure	High body-mass index	High fasting plasma glucose	Alcohol use	High LDL	Particulate matter	High sodium	Low whole grains	Kidney dysfunction
Romania	High blood pressure	Smoking	High body-mass index	High LDL	Alcohol use	High fasting plasma glucose	High sodium	Particulate matter	Kidney dysfunction	Low whole grains
Serbia	High blood pressure	Smoking	High fasting plasma glucose	High body-mass index	High LDL	Particulate matter	High sodium	Alcohol use	Kidney dysfunction	Low whole grains
Slovakia	High blood pressure	High body-mass index	Smoking	High fasting plasma glucose	High LDL	Alcohol use	High sodium	Particulate matter	Kidney dysfunction	Low whole grains
Slovenia	Smoking	High blood pressure	High body-mass index	High fasting plasma glucose	Alcohol use	High LDL	High sodium	Particulate matter	Low bone mineral density	Kidney dysfunction
**Eastern Europe**	High blood pressure	Smoking	High body-mass index	High LDL	Alcohol use	High fasting plasma glucose	Low whole grains	Kidney dysfunction	Particulate matter	Low legumes

Appendix Figure 3. Leading 10 level 3 risk factors for DALYs in 2019 by location. Each risk is colored by the percentage change in all-age DALYs from 2010 to 2019. DALYs=disability-adjusted life-years.

Percentage change in all-age DALYs from 2010 to 2019

	-0.828 to -0.164	-0.164 to -0.066	-0.066 to -0.010	-0.010 to 0.029	0.029 to 0.073	0.073 to 0.120	0.120 to 0.173	0.173 to 0.235	0.235 to 0.325	0.325 to 1.699
	1	2	3	4	5	6	7	8	9	10
Belarus	High blood pressure	Smoking	High body-mass index	High LDL	Alcohol use	High fasting plasma glucose	Low whole grains	Low legumes	Particulate matter	Kidney dysfunction
Estonia	High blood pressure	High body-mass index	Smoking	Alcohol use	High LDL	High fasting plasma glucose	Kidney dysfunction	Drug use	Low whole grains	Low legumes
Latvia	High blood pressure	High body-mass index	Smoking	High LDL	Alcohol use	High fasting plasma glucose	Low whole grains	Kidney dysfunction	Particulate matter	Low legumes
Lithuania	High blood pressure	High body-mass index	Smoking	High LDL	Alcohol use	High fasting plasma glucose	Low whole grains	Kidney dysfunction	Low legumes	Particulate matter
Moldova	High blood pressure	High body-mass index	Smoking	Alcohol use	High LDL	High fasting plasma glucose	Particulate matter	Low whole grains	Kidney dysfunction	Low legumes
Russia	High blood pressure	Smoking	High body-mass index	Alcohol use	High LDL	High fasting plasma glucose	Kidney dysfunction	Low whole grains	Particulate matter	Low legumes
Ukraine	High blood pressure	Smoking	High LDL	High body-mass index	Alcohol use	High fasting plasma glucose	Low whole grains	Particulate matter	Low legumes	Kidney dysfunction
**High income**	Smoking	High fasting plasma glucose	High body-mass index	High blood pressure	Alcohol use	High LDL	Drug use	Kidney dysfunction	Low whole grains	Particulate matter
**Australasia**	Smoking	High body-mass index	High blood pressure	High fasting plasma glucose	Alcohol use	High LDL	Drug use	Kidney dysfunction	High red meat	Low bone mineral density
Australia	High body-mass index	Smoking	High blood pressure	High fasting plasma glucose	Alcohol use	High LDL	Drug use	Kidney dysfunction	High red meat	Low bone mineral density
New Zealand	Smoking	High body-mass index	High blood pressure	High fasting plasma glucose	Alcohol use	High LDL	Kidney dysfunction	High red meat	Low whole grains	Low bone mineral density
**High-income Asia Pacific**	Smoking	High blood pressure	High fasting plasma glucose	Alcohol use	High body-mass index	Kidney dysfunction	High LDL	Particulate matter	High sodium	Low temperature
Brunei	High fasting plasma glucose	High blood pressure	Smoking	High body-mass index	High LDL	Kidney dysfunction	Low birth weight & short gestation	Low whole grains	High sodium	Occupational injury
Japan	Smoking	High blood pressure	High fasting plasma glucose	High body-mass index	Alcohol use	Kidney dysfunction	High LDL	Particulate matter	High sodium	Low temperature
Aichi	Smoking	High blood pressure	High fasting plasma glucose	High body-mass index	Alcohol use	Kidney dysfunction	High LDL	Particulate matter	High sodium	Low temperature
Akita	Smoking	High blood pressure	High fasting plasma glucose	Kidney dysfunction	High body-mass index	Alcohol use	High LDL	Low temperature	High sodium	Particulate matter
Aomori	Smoking	High blood pressure	High fasting plasma glucose	Kidney dysfunction	High body-mass index	Alcohol use	High LDL	Low temperature	High sodium	Particulate matter
Chiba	Smoking	High blood pressure	High fasting plasma glucose	High body-mass index	Alcohol use	Kidney dysfunction	High LDL	Particulate matter	High sodium	Low temperature
Ehime	Smoking	High blood pressure	High fasting plasma glucose	Kidney dysfunction	High body-mass index	Alcohol use	High LDL	Particulate matter	High sodium	Low temperature
Fukui	Smoking	High blood pressure	High fasting plasma glucose	High body-mass index	Kidney dysfunction	High LDL	Alcohol use	Particulate matter	Low temperature	High sodium
Fukuoka	Smoking	High blood pressure	High fasting plasma glucose	Alcohol use	High body-mass index	Kidney dysfunction	High LDL	Particulate matter	High sodium	Low whole grains
Fukushima	Smoking	High blood pressure	High fasting plasma glucose	High body-mass index	Kidney dysfunction	High LDL	Alcohol use	Low temperature	High sodium	Particulate matter
Gifu	Smoking	High blood pressure	High fasting plasma glucose	Kidney dysfunction	High body-mass index	High LDL	Alcohol use	Particulate matter	Low temperature	High sodium
Gunma	Smoking	High blood pressure	High fasting plasma glucose	High body-mass index	Kidney dysfunction	Alcohol use	High LDL	Particulate matter	Low temperature	High sodium
Hiroshima	Smoking	High blood pressure	High fasting plasma glucose	High body-mass index	Alcohol use	Kidney dysfunction	High LDL	Particulate matter	Low temperature	High sodium
Hokkaidō	Smoking	High blood pressure	High fasting plasma glucose	High body-mass index	Kidney dysfunction	Alcohol use	High LDL	High sodium	Particulate matter	Low temperature
Hyōgo	Smoking	High blood pressure	High fasting plasma glucose	High body-mass index	Alcohol use	Kidney dysfunction	High LDL	Particulate matter	High sodium	Low temperature
Ibaraki	Smoking	High blood pressure	High fasting plasma glucose	High body-mass index	Kidney dysfunction	Alcohol use	High LDL	Particulate matter	High sodium	Low temperature
Ishikawa	Smoking	High blood pressure	High fasting plasma glucose	High body-mass index	Kidney dysfunction	Alcohol use	High LDL	Particulate matter	Low temperature	High sodium
Iwate	Smoking	High blood pressure	High fasting plasma glucose	High body-mass index	Kidney dysfunction	Alcohol use	High LDL	Low temperature	High sodium	Particulate matter
Kagawa	Smoking	High blood pressure	High fasting plasma glucose	Kidney dysfunction	High body-mass index	Alcohol use	High LDL	Particulate matter	High sodium	Low temperature
Kagoshima	Smoking	High blood pressure	High fasting plasma glucose	High body-mass index	Kidney dysfunction	Alcohol use	High LDL	Particulate matter	High sodium	Low whole grains

Appendix Figure 3. Leading 10 level 3 risk factors for DALYs in 2019 by location. Each risk is colored by the percentage change in all-age DALYs from 2010 to 2019. DALYs=disability-adjusted life-years.

	Percentage change in all-age DALYs from 2010 to 2019									
	-0.828 to -0.164	-0.164 to -0.066	-0.066 to -0.010	-0.010 to 0.029	0.029 to 0.073	0.073 to 0.120	0.120 to 0.173	0.173 to 0.235	0.235 to 0.325	0.325 to 1.699
	1	2	3	4	5	6	7	8	9	10
Kanagawa	Smoking	High blood pressure	High fasting plasma glucose	Alcohol use	High body-mass index	Kidney dysfunction	High LDL	Particulate matter	High sodium	Low whole grains
Kōchi	Smoking	High blood pressure	High fasting plasma glucose	Kidney dysfunction	High body-mass index	Alcohol use	High LDL	Particulate matter	High sodium	Low temperature
Kumamoto	Smoking	High blood pressure	High fasting plasma glucose	Kidney dysfunction	High body-mass index	Alcohol use	High LDL	Particulate matter	High sodium	Low temperature
Kyōto	Smoking	High blood pressure	High fasting plasma glucose	High body-mass index	Kidney dysfunction	Alcohol use	High LDL	Particulate matter	High sodium	Low temperature
Mie	Smoking	High blood pressure	High fasting plasma glucose	High body-mass index	Kidney dysfunction	High LDL	Alcohol use	Particulate matter	High sodium	Low temperature
Miyagi	Smoking	High blood pressure	High fasting plasma glucose	High body-mass index	Kidney dysfunction	Alcohol use	High LDL	Low temperature	High sodium	Particulate matter
Miyazaki	Smoking	High blood pressure	High fasting plasma glucose	Kidney dysfunction	High body-mass index	Alcohol use	High LDL	Particulate matter	High sodium	Low temperature
Nagano	Smoking	High blood pressure	High fasting plasma glucose	High body-mass index	Kidney dysfunction	High LDL	Alcohol use	High sodium	Low temperature	Particulate matter
Nagasaki	Smoking	High blood pressure	High fasting plasma glucose	Kidney dysfunction	High body-mass index	Alcohol use	High LDL	Particulate matter	High sodium	Low whole grains
Nara	Smoking	High blood pressure	High fasting plasma glucose	High body-mass index	Kidney dysfunction	Alcohol use	High LDL	Particulate matter	Low temperature	High sodium
Niigata	Smoking	High blood pressure	High fasting plasma glucose	High body-mass index	Kidney dysfunction	High LDL	Alcohol use	Low temperature	High sodium	Particulate matter
Ōita	Smoking	High blood pressure	High fasting plasma glucose	High body-mass index	Kidney dysfunction	Alcohol use	High LDL	Particulate matter	High sodium	Low temperature
Okayama	Smoking	High blood pressure	High fasting plasma glucose	High body-mass index	Kidney dysfunction	Alcohol use	High LDL	Particulate matter	Low temperature	High sodium
Okinawa	Smoking	High blood pressure	High fasting plasma glucose	Alcohol use	High body-mass index	Kidney dysfunction	High LDL	Particulate matter	Low whole grains	High sodium
Ōsaka	Smoking	High blood pressure	High fasting plasma glucose	Alcohol use	High body-mass index	Kidney dysfunction	High LDL	Particulate matter	High sodium	Low whole grains
Saga	Smoking	High blood pressure	High fasting plasma glucose	High body-mass index	Kidney dysfunction	Alcohol use	High LDL	Particulate matter	High sodium	Low temperature
Saitama	Smoking	High blood pressure	High fasting plasma glucose	High LDL	Kidney dysfunction	High body-mass index	Alcohol use	Particulate matter	High sodium	Low temperature
Shiga	Smoking	High blood pressure	High fasting plasma glucose	High body-mass index	Kidney dysfunction	Alcohol use	High LDL	Particulate matter	High sodium	Low temperature
Shimane	Smoking	High blood pressure	High fasting plasma glucose	High body-mass index	Kidney dysfunction	Alcohol use	High LDL	Particulate matter	Low temperature	High sodium
Shizuoka	Smoking	High blood pressure	High fasting plasma glucose	High body-mass index	Kidney dysfunction	Alcohol use	High LDL	Particulate matter	High sodium	Low temperature
Tochigi	Smoking	High blood pressure	High fasting plasma glucose	High body-mass index	Kidney dysfunction	High LDL	Alcohol use	Particulate matter	Low temperature	High sodium
Tokushima	Smoking	High blood pressure	High fasting plasma glucose	Kidney dysfunction	High body-mass index	Alcohol use	High LDL	Particulate matter	Low temperature	High sodium
Tōkyō	Smoking	High blood pressure	High fasting plasma glucose	Alcohol use	High body-mass index	Kidney dysfunction	High LDL	Particulate matter	High sodium	Low temperature
Tottori	Smoking	High blood pressure	High fasting plasma glucose	High body-mass index	Kidney dysfunction	High LDL	Alcohol use	Particulate matter	High sodium	Low temperature
Toyama	Smoking	High blood pressure	High fasting plasma glucose	High body-mass index	Kidney dysfunction	Alcohol use	High LDL	Particulate matter	Low temperature	High sodium
Wakayama	Smoking	High blood pressure	High fasting plasma glucose	High body-mass index	Kidney dysfunction	High LDL	Alcohol use	Particulate matter	High sodium	Low temperature
Yamagata	Smoking	High blood pressure	High fasting plasma glucose	High body-mass index	Kidney dysfunction	High LDL	Alcohol use	Low temperature	High sodium	Particulate matter
Yamaguchi	Smoking	High blood pressure	High fasting plasma glucose	High body-mass index	Kidney dysfunction	Alcohol use	High LDL	Particulate matter	High sodium	Low temperature
Yamanashi	Smoking	High blood pressure	High fasting plasma glucose	High body-mass index	Kidney dysfunction	Alcohol use	High LDL	Low temperature	Particulate matter	High sodium
South Korea	Smoking	High fasting plasma glucose	Alcohol use	High blood pressure	High body-mass index	Particulate matter	High LDL	Kidney dysfunction	High sodium	Low bone mineral density
Singapore	High fasting plasma glucose	Smoking	High body-mass index	High blood pressure	High LDL	Particulate matter	Kidney dysfunction	Occupational injury	High sodium	Low whole grains
**High-income North America**	Smoking	High body-mass index	High fasting plasma glucose	High blood pressure	Drug use	Alcohol use	High LDL	Kidney dysfunction	Low whole grains	High red meat

Appendix Figure 3. Leading 10 level 3 risk factors for DALYs in 2019 by location. Each risk is colored by the percentage change in all-age DALYs from 2010 to 2019. DALYs=disability-adjusted life-years.

Percentage change in all-age DALYs from 2010 to 2019

-0.828 to -0.164	-0.164 to -0.066	-0.066 to -0.010	-0.010 to 0.029	0.029 to 0.073	0.073 to 0.120	0.120 to 0.173	0.173 to 0.235	0.235 to 0.325	0.325 to 1.699

	1	2	3	4	5	6	7	8	9	10
Canada	Smoking	High body-mass index	High blood pressure	High fasting plasma glucose	Alcohol use	High LDL	Drug use	Kidney dysfunction	Low whole grains	Low bone mineral density
Greenland	Smoking	High body-mass index	Alcohol use	High blood pressure	High fasting plasma glucose	High LDL	Drug use	Kidney dysfunction	Low birth weight & short gestation	High red meat
USA	Smoking	High body-mass index	High fasting plasma glucose	High blood pressure	Drug use	Alcohol use	High LDL	Kidney dysfunction	Low whole grains	High red meat
Alabama	Smoking	High body-mass index	High fasting plasma glucose	High blood pressure	Drug use	High LDL	Alcohol use	Kidney dysfunction	Low whole grains	High red meat
Alaska	Smoking	High body-mass index	Drug use	High fasting plasma glucose	Alcohol use	High blood pressure	High LDL	Kidney dysfunction	Low whole grains	High red meat
Arizona	Smoking	High body-mass index	High fasting plasma glucose	Drug use	High blood pressure	Alcohol use	High LDL	Kidney dysfunction	Low whole grains	Low temperature
Arkansas	Smoking	High body-mass index	High fasting plasma glucose	High blood pressure	Drug use	High LDL	Kidney dysfunction	Alcohol use	Low whole grains	Low temperature
California	High body-mass index	Smoking	High fasting plasma glucose	High blood pressure	Drug use	Alcohol use	High LDL	Kidney dysfunction	Low whole grains	High red meat
Colorado	Smoking	High body-mass index	High fasting plasma glucose	Drug use	High blood pressure	Alcohol use	High LDL	Kidney dysfunction	Low whole grains	High red meat
Connecticut	Smoking	High body-mass index	High fasting plasma glucose	High blood pressure	Drug use	Alcohol use	High LDL	Kidney dysfunction	Low temperature	Low whole grains
Delaware	Smoking	High body-mass index	High fasting plasma glucose	High blood pressure	Drug use	Alcohol use	High LDL	Kidney dysfunction	Low whole grains	Low temperature
Washington, DC	High body-mass index	Smoking	High blood pressure	High fasting plasma glucose	Drug use	Alcohol use	Kidney dysfunction	High LDL	Low birth weight & short gestation	Unsafe sex
Florida	Smoking	High body-mass index	High fasting plasma glucose	High blood pressure	Drug use	Alcohol use	High LDL	Kidney dysfunction	Low whole grains	High red meat
Georgia	Smoking	High body-mass index	High fasting plasma glucose	High blood pressure	Drug use	Alcohol use	Kidney dysfunction	High LDL	Low whole grains	High red meat
Hawaii	Smoking	High body-mass index	High fasting plasma glucose	High blood pressure	Alcohol use	Drug use	Kidney dysfunction	High LDL	Low whole grains	High red meat
Idaho	Smoking	High body-mass index	High fasting plasma glucose	High blood pressure	Drug use	Alcohol use	High LDL	Kidney dysfunction	Low whole grains	High red meat
Illinois	Smoking	High body-mass index	High fasting plasma glucose	High blood pressure	Drug use	Alcohol use	High LDL	Kidney dysfunction	Low temperature	Low whole grains
Indiana	Smoking	High body-mass index	High fasting plasma glucose	High blood pressure	Drug use	Alcohol use	High LDL	Kidney dysfunction	Low temperature	Low whole grains
Iowa	Smoking	High body-mass index	High fasting plasma glucose	High blood pressure	Alcohol use	Drug use	High LDL	Kidney dysfunction	Low whole grains	High red meat
Kansas	Smoking	High body-mass index	High fasting plasma glucose	High blood pressure	Drug use	Alcohol use	High LDL	Kidney dysfunction	Low temperature	Low whole grains
Kentucky	Smoking	High body-mass index	High fasting plasma glucose	Drug use	High blood pressure	High LDL	Alcohol use	Kidney dysfunction	Low temperature	Low whole grains
Louisiana	Smoking	High body-mass index	High fasting plasma glucose	High blood pressure	Drug use	Alcohol use	Kidney dysfunction	High LDL	Low whole grains	High red meat
Maine	Smoking	High body-mass index	High fasting plasma glucose	High blood pressure	Drug use	Alcohol use	High LDL	Kidney dysfunction	Low whole grains	Low temperature
Maryland	High body-mass index	Smoking	High fasting plasma glucose	High blood pressure	Alcohol use	Drug use	High LDL	Kidney dysfunction	Low whole grains	Low temperature
Massachusetts	Smoking	High body-mass index	High fasting plasma glucose	Drug use	High blood pressure	Alcohol use	Kidney dysfunction	High LDL	Low temperature	Low whole grains
Michigan	Smoking	High body-mass index	High fasting plasma glucose	High blood pressure	Drug use	Alcohol use	High LDL	Kidney dysfunction	Low whole grains	Low temperature
Minnesota	Smoking	High body-mass index	High fasting plasma glucose	High blood pressure	Alcohol use	Drug use	Kidney dysfunction	High LDL	Low bone mineral density	Low whole grains
Mississippi	Smoking	High body-mass index	High fasting plasma glucose	High blood pressure	Drug use	High LDL	Kidney dysfunction	Alcohol use	Low whole grains	High red meat
Missouri	Smoking	High body-mass index	High fasting plasma glucose	High blood pressure	Drug use	High LDL	Alcohol use	Kidney dysfunction	Low temperature	Low whole grains
Montana	Smoking	High body-mass index	High fasting plasma glucose	High blood pressure	Drug use	Alcohol use	High LDL	Kidney dysfunction	Low whole grains	High red meat
Nebraska	Smoking	High body-mass index	High fasting plasma glucose	High blood pressure	Alcohol use	Drug use	Kidney dysfunction	High LDL	Low temperature	High red meat
Nevada	Smoking	High body-mass index	High fasting plasma glucose	High blood pressure	Drug use	Alcohol use	High LDL	Kidney dysfunction	Low temperature	Low whole grains

Appendix Figure 3. Leading 10 level 3 risk factors for DALYs in 2019 by location. Each risk is colored by the percentage change in all-age DALYs from 2010 to 2019. DALYs=disability-adjusted life-years.

Percentage change in all-age DALYs from 2010 to 2019

-0.828 to -0.164	-0.164 to -0.066	-0.066 to -0.010	-0.010 to 0.029	0.029 to 0.073	0.073 to 0.120	0.120 to 0.173	0.173 to 0.235	0.235 to 0.325	0.325 to 1.699

	1	2	3	4	5	6	7	8	9	10
New Hampshire	Smoking	High body-mass index	High fasting plasma glucose	Drug use	High blood pressure	Alcohol use	High LDL	Kidney dysfunction	Low whole grains	Low temperature
New Jersey	High body-mass index	Smoking	High fasting plasma glucose	High blood pressure	Drug use	Alcohol use	High LDL	Kidney dysfunction	Low whole grains	Low temperature
New Mexico	Smoking	High body-mass index	High fasting plasma glucose	Drug use	High blood pressure	Alcohol use	High LDL	Kidney dysfunction	Low whole grains	Low temperature
New York	Smoking	High body-mass index	High fasting plasma glucose	High blood pressure	Drug use	Alcohol use	High LDL	Kidney dysfunction	Low whole grains	Low temperature
North Carolina	Smoking	High body-mass index	High fasting plasma glucose	High blood pressure	Drug use	Alcohol use	High LDL	Kidney dysfunction	Low whole grains	High red meat
North Dakota	Smoking	High body-mass index	High fasting plasma glucose	High blood pressure	Alcohol use	Drug use	High LDL	Kidney dysfunction	High red meat	Low whole grains
Ohio	Smoking	High body-mass index	High fasting plasma glucose	High blood pressure	Drug use	Alcohol use	High LDL	Kidney dysfunction	Low temperature	Low whole grains
Oklahoma	Smoking	High body-mass index	High fasting plasma glucose	High blood pressure	Drug use	High LDL	Alcohol use	Kidney dysfunction	Low whole grains	Low temperature
Oregon	Smoking	High body-mass index	High fasting plasma glucose	High blood pressure	Drug use	Alcohol use	Kidney dysfunction	High LDL	Low temperature	Low whole grains
Pennsylvania	Smoking	High body-mass index	High fasting plasma glucose	Drug use	High blood pressure	Alcohol use	High LDL	Kidney dysfunction	Low whole grains	Low temperature
Rhode Island	Smoking	High body-mass index	High fasting plasma glucose	Drug use	High blood pressure	Alcohol use	High LDL	Kidney dysfunction	Low temperature	Low whole grains
South Carolina	Smoking	High body-mass index	High fasting plasma glucose	High blood pressure	Drug use	Alcohol use	High LDL	Kidney dysfunction	Low whole grains	High red meat
South Dakota	Smoking	High body-mass index	High fasting plasma glucose	High blood pressure	Alcohol use	High LDL	Drug use	Kidney dysfunction	Low whole grains	Low temperature
Tennessee	Smoking	High body-mass index	High fasting plasma glucose	High blood pressure	Drug use	High LDL	Alcohol use	Kidney dysfunction	Low whole grains	Low temperature
Texas	High body-mass index	Smoking	High fasting plasma glucose	High blood pressure	Drug use	Alcohol use	High LDL	Kidney dysfunction	Low whole grains	High red meat
Utah	High body-mass index	Drug use	High fasting plasma glucose	Smoking	High blood pressure	Alcohol use	Kidney dysfunction	High LDL	Low birth weight & short gestation	Low whole grains
Vermont	Smoking	High body-mass index	High fasting plasma glucose	High blood pressure	Drug use	Alcohol use	High LDL	Kidney dysfunction	Low whole grains	Low bone mineral density
Virginia	Smoking	High body-mass index	High fasting plasma glucose	High blood pressure	Drug use	Alcohol use	Kidney dysfunction	High LDL	Low whole grains	Low temperature
Washington	Smoking	High body-mass index	High fasting plasma glucose	Drug use	High blood pressure	Alcohol use	High LDL	Kidney dysfunction	Low whole grains	Low temperature
West Virginia	Smoking	High body-mass index	High fasting plasma glucose	Drug use	High blood pressure	High LDL	Kidney dysfunction	Alcohol use	Low temperature	Low whole grains
Wisconsin	Smoking	High body-mass index	High fasting plasma glucose	High blood pressure	Drug use	Alcohol use	Kidney dysfunction	High LDL	Low whole grains	Low temperature
Wyoming	Smoking	High body-mass index	High fasting plasma glucose	High blood pressure	Drug use	Alcohol use	High LDL	Kidney dysfunction	Low whole grains	High red meat
**Southern Latin America**	Smoking	High blood pressure	High body-mass index	High fasting plasma glucose	Alcohol use	Kidney dysfunction	High LDL	Particulate matter	Low birth weight & short gestation	High red meat
Argentina	Smoking	High blood pressure	High body-mass index	High fasting plasma glucose	Alcohol use	Kidney dysfunction	High LDL	Particulate matter	Low birth weight & short gestation	High red meat
Chile	High body-mass index	High blood pressure	High fasting plasma glucose	Smoking	Alcohol use	Particulate matter	Kidney dysfunction	High LDL	Drug use	Low birth weight & short gestation
Uruguay	Smoking	High blood pressure	High body-mass index	High fasting plasma glucose	Alcohol use	High LDL	Kidney dysfunction	Low whole grains	High red meat	Particulate matter
**Western Europe**	Smoking	High blood pressure	High fasting plasma glucose	High body-mass index	Alcohol use	High LDL	Kidney dysfunction	Low whole grains	Particulate matter	Low temperature
Andorra	Smoking	High body-mass index	High blood pressure	Alcohol use	High fasting plasma glucose	High LDL	Kidney dysfunction	Occupational carcinogens	Low bone mineral density	Low whole grains
Austria	Smoking	High blood pressure	High fasting plasma glucose	High body-mass index	Alcohol use	High LDL	Kidney dysfunction	Particulate matter	Low whole grains	High red meat
Belgium	Smoking	High blood pressure	High fasting plasma glucose	High body-mass index	Alcohol use	High LDL	Kidney dysfunction	Particulate matter	Low temperature	Low bone mineral density
Cyprus	Smoking	High fasting plasma glucose	High blood pressure	High body-mass index	High LDL	Alcohol use	Kidney dysfunction	Particulate matter	Low whole grains	Low legumes
Denmark	Smoking	High blood pressure	Alcohol use	High fasting plasma glucose	High body-mass index	High LDL	Kidney dysfunction	Low temperature	Low whole grains	Drug use

Appendix Figure 3. Leading 10 level 3 risk factors for DALYs in 2019 by location. Each risk is colored by the percentage change in all-age DALYs from 2010 to 2019. DALYs=disability-adjusted life-years.

Percentage change in all-age DALYs from 2010 to 2019

	-0.828 to -0.164	-0.164 to -0.066	-0.066 to -0.010	-0.010 to 0.029	0.029 to 0.073	0.073 to 0.120	0.120 to 0.173	0.173 to 0.235	0.235 to 0.325	0.325 to 1.699
	1	2	3	4	5	6	7	8	9	10
Finland	High blood pressure	High fasting plasma glucose	Smoking	High body-mass index	Alcohol use	High LDL	Low whole grains	Kidney dysfunction	Drug use	Low legumes
France	Smoking	High blood pressure	Alcohol use	High body-mass index	High fasting plasma glucose	High LDL	Kidney dysfunction	Low bone mineral density	Occupational carcinogens	Low whole grains
Germany	Smoking	High blood pressure	High fasting plasma glucose	High body-mass index	Alcohol use	High LDL	Kidney dysfunction	Particulate matter	Low whole grains	Low temperature
Greece	Smoking	High blood pressure	High fasting plasma glucose	High body-mass index	High LDL	Kidney dysfunction	Low whole grains	Alcohol use	Particulate matter	Low temperature
Iceland	Smoking	High body-mass index	High blood pressure	High fasting plasma glucose	High LDL	Alcohol use	Drug use	Low whole grains	Kidney dysfunction	High red meat
Ireland	Smoking	High blood pressure	High body-mass index	High fasting plasma glucose	Alcohol use	High LDL	Kidney dysfunction	Drug use	High red meat	Low temperature
Israel	Smoking	High fasting plasma glucose	High body-mass index	High blood pressure	Kidney dysfunction	Particulate matter	High LDL	Low whole grains	Alcohol use	Low birth weight & short gestation
Italy	Smoking	High fasting plasma glucose	High blood pressure	High body-mass index	Alcohol use	High LDL	Low whole grains	Particulate matter	Kidney dysfunction	Low temperature
Luxembourg	Smoking	High fasting plasma glucose	High blood pressure	High body-mass index	Alcohol use	High LDL	Kidney dysfunction	Drug use	High red meat	Low whole grains
Malta	High fasting plasma glucose	Smoking	High blood pressure	High body-mass index	High LDL	Low whole grains	Kidney dysfunction	Alcohol use	Particulate matter	Low physical activity
Monaco	Smoking	High blood pressure	High body-mass index	High fasting plasma glucose	High LDL	Alcohol use	Occupational carcinogens	Kidney dysfunction	Particulate matter	Low whole grains
Netherlands	Smoking	High blood pressure	High body-mass index	High fasting plasma glucose	Alcohol use	High LDL	Kidney dysfunction	Occupational carcinogens	Particulate matter	Low temperature
Norway	Smoking	High blood pressure	High fasting plasma glucose	High body-mass index	High LDL	Alcohol use	Low whole grains	Drug use	Kidney dysfunction	Low bone mineral density
Portugal	High fasting plasma glucose	Smoking	High blood pressure	High body-mass index	Alcohol use	High LDL	Kidney dysfunction	Low temperature	High red meat	Low whole grains
San Marino	Smoking	High blood pressure	High body-mass index	High fasting plasma glucose	Alcohol use	High LDL	Low whole grains	Kidney dysfunction	High red meat	Occupational carcinogens
Spain	Smoking	High fasting plasma glucose	High body-mass index	High blood pressure	Alcohol use	High LDL	Kidney dysfunction	Low temperature	Low whole grains	Particulate matter
Sweden	Smoking	High blood pressure	High fasting plasma glucose	High body-mass index	Alcohol use	High LDL	Kidney dysfunction	Low whole grains	Drug use	Low bone mineral density
Stockholm	Smoking	High blood pressure	High fasting plasma glucose	High body-mass index	Alcohol use	High LDL	Low whole grains	Drug use	Kidney dysfunction	Low bone mineral density
Sweden except Stockholm	Smoking	High blood pressure	High fasting plasma glucose	High body-mass index	High LDL	Alcohol use	Kidney dysfunction	Low whole grains	Low temperature	Low bone mineral density
Switzerland	Smoking	High blood pressure	High fasting plasma glucose	High body-mass index	Alcohol use	High LDL	Kidney dysfunction	Low bone mineral density	Low whole grains	Drug use
UK	Smoking	High fasting plasma glucose	High body-mass index	High blood pressure	Alcohol use	High LDL	Low temperature	Drug use	Low whole grains	Kidney dysfunction
England	Smoking	High fasting plasma glucose	High body-mass index	High blood pressure	Alcohol use	High LDL	Low temperature	Drug use	Low whole grains	Kidney dysfunction
East Midlands	Smoking	High fasting plasma glucose	High body-mass index	High blood pressure	Alcohol use	High LDL	Low temperature	Low whole grains	Kidney dysfunction	Particulate matter
Derby	Smoking	High fasting plasma glucose	High body-mass index	High blood pressure	Alcohol use	High LDL	Low temperature	Drug use	Particulate matter	Low whole grains
Derbyshire	Smoking	High fasting plasma glucose	High body-mass index	High blood pressure	Alcohol use	High LDL	Low temperature	Low whole grains	Kidney dysfunction	Particulate matter
Leicester	Smoking	High fasting plasma glucose	High body-mass index	High blood pressure	Alcohol use	High LDL	Drug use	Particulate matter	Low temperature	Low whole grains
Leicestershire	Smoking	High body-mass index	High fasting plasma glucose	High blood pressure	Alcohol use	High LDL	Low temperature	Low whole grains	Kidney dysfunction	Particulate matter
Lincolnshire	Smoking	High fasting plasma glucose	High body-mass index	High blood pressure	High LDL	Alcohol use	Low temperature	Low whole grains	Kidney dysfunction	Particulate matter
Northamptonshire	Smoking	High fasting plasma glucose	High body-mass index	High blood pressure	Alcohol use	High LDL	Low temperature	Low whole grains	Drug use	Kidney dysfunction
Nottingham	Smoking	High body-mass index	High fasting plasma glucose	High blood pressure	Alcohol use	High LDL	Drug use	Low temperature	Particulate matter	Low whole grains
Nottinghamshire	Smoking	High fasting plasma glucose	High body-mass index	High blood pressure	Alcohol use	High LDL	Low temperature	Low whole grains	Kidney dysfunction	Particulate matter
Rutland	Smoking	High body-mass index	High fasting plasma glucose	High blood pressure	Alcohol use	High LDL	Low temperature	Kidney dysfunction	Low whole grains	Particulate matter

Appendix Figure 3. Leading 10 level 3 risk factors for DALYs in 2019 by location. Each risk is colored by the percentage change in all-age DALYs from 2010 to 2019. DALYs=disability-adjusted life-years.

Percentage change in all-age DALYs from 2010 to 2019

-0.828 to -0.164	-0.164 to -0.066	-0.066 to -0.010	-0.010 to 0.029	0.029 to 0.073	0.073 to 0.120	0.120 to 0.173	0.173 to 0.235	0.235 to 0.325	0.325 to 1.699

	1	2	3	4	5	6	7	8	9	10
East of England	Smoking	High fasting plasma glucose	High body-mass index	High blood pressure	Alcohol use	High LDL	Low temperature	Low whole grains	Particulate matter	Kidney dysfunction
Bedford	Smoking	High fasting plasma glucose	High body-mass index	High blood pressure	Alcohol use	High LDL	Low temperature	Low whole grains	Drug use	Kidney dysfunction
Cambridgeshire	Smoking	High body-mass index	High fasting plasma glucose	High blood pressure	Alcohol use	High LDL	Low temperature	Drug use	Low whole grains	Particulate matter
Central Bedfordshire	Smoking	High fasting plasma glucose	High body-mass index	High blood pressure	Alcohol use	High LDL	Low temperature	Low whole grains	Kidney dysfunction	Drug use
Essex	Smoking	High fasting plasma glucose	High body-mass index	High blood pressure	Alcohol use	High LDL	Low temperature	Particulate matter	Occupational carcinogens	Low whole grains
Hertfordshire	Smoking	High body-mass index	High fasting plasma glucose	High blood pressure	Alcohol use	High LDL	Low temperature	Particulate matter	Drug use	Low whole grains
Luton	Smoking	High fasting plasma glucose	High body-mass index	High blood pressure	Alcohol use	High LDL	Drug use	Low temperature	Low birth weight & short gestation	Low whole grains
Norfolk	Smoking	High fasting plasma glucose	High body-mass index	High blood pressure	Alcohol use	High LDL	Low temperature	Low whole grains	Kidney dysfunction	Drug use
Peterborough	Smoking	High fasting plasma glucose	High body-mass index	High blood pressure	Alcohol use	High LDL	Drug use	Low temperature	Particulate matter	Low whole grains
Southend-on-Sea	Smoking	High fasting plasma glucose	High body-mass index	High blood pressure	Alcohol use	High LDL	Low temperature	Drug use	Particulate matter	Low whole grains
Suffolk	Smoking	High body-mass index	High fasting plasma glucose	High blood pressure	Alcohol use	High LDL	Low temperature	Low whole grains	Kidney dysfunction	Occupational carcinogens
Thurrock	Smoking	High fasting plasma glucose	High body-mass index	High blood pressure	Alcohol use	High LDL	Low temperature	Particulate matter	Occupational carcinogens	Low whole grains
Greater London	Smoking	High body-mass index	High fasting plasma glucose	High blood pressure	Alcohol use	High LDL	Drug use	Particulate matter	Low temperature	Low whole grains
Barking and Dagenham	Smoking	High fasting plasma glucose	High body-mass index	High blood pressure	Alcohol use	High LDL	Drug use	Particulate matter	Low temperature	Occupational carcinogens
Barnet	Smoking	High body-mass index	High fasting plasma glucose	High blood pressure	Alcohol use	High LDL	Drug use	Particulate matter	Low temperature	Low whole grains
Bexley	Smoking	High fasting plasma glucose	High body-mass index	High blood pressure	Alcohol use	High LDL	Low temperature	Particulate matter	Drug use	Occupational carcinogens
Brent	Smoking	High fasting plasma glucose	High body-mass index	High blood pressure	Alcohol use	High LDL	Drug use	Particulate matter	Low temperature	Low whole grains
Bromley	Smoking	High fasting plasma glucose	High body-mass index	High blood pressure	Alcohol use	High LDL	Particulate matter	Low temperature	Low whole grains	Drug use
Camden	Smoking	High body-mass index	High fasting plasma glucose	Alcohol use	High blood pressure	Drug use	High LDL	Particulate matter	Low temperature	Kidney dysfunction
Croydon	Smoking	High fasting plasma glucose	High body-mass index	High blood pressure	Alcohol use	High LDL	Low temperature	Particulate matter	Drug use	Low whole grains
Ealing	Smoking	High body-mass index	High fasting plasma glucose	High blood pressure	Alcohol use	High LDL	Drug use	Particulate matter	Low temperature	Low whole grains
Enfield	Smoking	High fasting plasma glucose	High body-mass index	High blood pressure	Alcohol use	High LDL	Particulate matter	Drug use	Low temperature	Low whole grains
Greenwich	Smoking	High fasting plasma glucose	High body-mass index	High blood pressure	Alcohol use	High LDL	Drug use	Particulate matter	Low birth weight & short gestation	Low temperature
Hackney	Smoking	High body-mass index	High fasting plasma glucose	Alcohol use	High blood pressure	Drug use	High LDL	Particulate matter	Low birth weight & short gestation	Low temperature
Hammersmith and Fulham	Smoking	High body-mass index	High fasting plasma glucose	Alcohol use	High blood pressure	Drug use	High LDL	Particulate matter	Low temperature	Kidney dysfunction
Haringey	Smoking	High body-mass index	High fasting plasma glucose	Alcohol use	High blood pressure	High LDL	Drug use	Particulate matter	Low temperature	Low whole grains
Harrow	Smoking	High fasting plasma glucose	High body-mass index	High blood pressure	Alcohol use	High LDL	Drug use	Particulate matter	Low temperature	Low birth weight & short gestation
Havering	Smoking	High fasting plasma glucose	High body-mass index	High blood pressure	Alcohol use	High LDL	Low temperature	Particulate matter	Occupational carcinogens	Low whole grains
Hillingdon	Smoking	High body-mass index	High fasting plasma glucose	High blood pressure	Alcohol use	High LDL	Drug use	Particulate matter	Low temperature	Low whole grains
Hounslow	Smoking	High body-mass index	High fasting plasma glucose	Alcohol use	High blood pressure	High LDL	Drug use	Particulate matter	Low birth weight & short gestation	Low temperature
Islington	Smoking	High body-mass index	High fasting plasma glucose	Alcohol use	High blood pressure	Drug use	High LDL	Particulate matter	Low temperature	Low whole grains
Kensington and Chelsea	Smoking	High body-mass index	High fasting plasma glucose	Alcohol use	High blood pressure	Drug use	High LDL	Particulate matter	Low temperature	Kidney dysfunction

Appendix Figure 3. Leading 10 level 3 risk factors for DALYs in 2019 by location. Each risk is colored by the percentage change in all-age DALYs from 2010 to 2019. DALYs=disability-adjusted life-years.

Percentage change in all-age DALYs from 2010 to 2019

-0.828 to -0.164	-0.164 to -0.066	-0.066 to -0.010	-0.010 to 0.029	0.029 to 0.073	0.073 to 0.120	0.120 to 0.173	0.173 to 0.235	0.235 to 0.325	0.325 to 1.699

	1	2	3	4	5	6	7	8	9	10
Kingston upon Thames	Smoking	High body-mass index	High fasting plasma glucose	High blood pressure	Alcohol use	High LDL	Drug use	Low temperature	Particulate matter	Kidney dysfunction
Lambeth	Smoking	High body-mass index	High fasting plasma glucose	Alcohol use	High blood pressure	Drug use	High LDL	Low birth weight & short gestation	Particulate matter	Low temperature
Lewisham	Smoking	High fasting plasma glucose	High body-mass index	Alcohol use	High blood pressure	High LDL	Drug use	Particulate matter	Low temperature	Low birth weight & short gestation
Merton	Smoking	High body-mass index	High fasting plasma glucose	High blood pressure	Alcohol use	High LDL	Drug use	Low temperature	Particulate matter	Low whole grains
Newham	Smoking	High body-mass index	High fasting plasma glucose	Alcohol use	High blood pressure	High LDL	Drug use	Particulate matter	Low birth weight & short gestation	Low temperature
Redbridge	Smoking	High fasting plasma glucose	High body-mass index	High blood pressure	Alcohol use	High LDL	Drug use	Particulate matter	Low temperature	Kidney dysfunction
Richmond upon Thames	Smoking	High body-mass index	High fasting plasma glucose	Alcohol use	High blood pressure	High LDL	Drug use	Low temperature	Particulate matter	Low whole grains
Southwark	Smoking	High body-mass index	High fasting plasma glucose	Alcohol use	High blood pressure	Drug use	High LDL	Particulate matter	Low birth weight & short gestation	Low temperature
Sutton	Smoking	High fasting plasma glucose	High body-mass index	High blood pressure	Alcohol use	High LDL	Low temperature	Drug use	Particulate matter	Low whole grains
Tower Hamlets	Smoking	High body-mass index	High fasting plasma glucose	Alcohol use	High blood pressure	Drug use	High LDL	Low birth weight & short gestation	Particulate matter	Low temperature
Waltham Forest	Smoking	High fasting plasma glucose	High body-mass index	High blood pressure	Alcohol use	High LDL	Drug use	Particulate matter	Low temperature	Low whole grains
Wandsworth	Smoking	High body-mass index	High fasting plasma glucose	Alcohol use	High blood pressure	Drug use	High LDL	Low temperature	Low birth weight & short gestation	Particulate matter
Westminster	Smoking	High body-mass index	High fasting plasma glucose	Alcohol use	High blood pressure	Drug use	High LDL	Particulate matter	Low temperature	Kidney dysfunction
North East England	Smoking	High fasting plasma glucose	High body-mass index	High blood pressure	Alcohol use	High LDL	Low temperature	Occupational carcinogens	Low whole grains	Drug use
County Durham	Smoking	High fasting plasma glucose	High body-mass index	High blood pressure	Alcohol use	High LDL	Low temperature	Low whole grains	Occupational carcinogens	Kidney dysfunction
Darlington	Smoking	High fasting plasma glucose	High body-mass index	High blood pressure	Alcohol use	High LDL	Low temperature	Low whole grains	Occupational carcinogens	Drug use
Gateshead	Smoking	High fasting plasma glucose	High body-mass index	High blood pressure	Alcohol use	High LDL	Low temperature	Occupational carcinogens	Drug use	Low whole grains
Hartlepool	Smoking	High fasting plasma glucose	High body-mass index	High blood pressure	Alcohol use	High LDL	Occupational carcinogens	Low temperature	Low whole grains	Drug use
Middlesbrough	Smoking	High fasting plasma glucose	High body-mass index	High blood pressure	Alcohol use	High LDL	Low temperature	Occupational carcinogens	Low whole grains	Drug use
Newcastle upon Tyne	Smoking	High fasting plasma glucose	High body-mass index	High blood pressure	Alcohol use	High LDL	Drug use	Occupational carcinogens	Low temperature	Low whole grains
North Tyneside	Smoking	High fasting plasma glucose	High body-mass index	High blood pressure	Alcohol use	High LDL	Occupational carcinogens	Low temperature	Drug use	Low whole grains
Northumberland	Smoking	High fasting plasma glucose	High body-mass index	High blood pressure	Alcohol use	High LDL	Low temperature	Low whole grains	Occupational carcinogens	Kidney dysfunction
Redcar and Cleveland	Smoking	High fasting plasma glucose	High body-mass index	High blood pressure	High LDL	Alcohol use	Low temperature	Occupational carcinogens	Low whole grains	Kidney dysfunction
South Tyneside	Smoking	High fasting plasma glucose	High body-mass index	High blood pressure	Alcohol use	High LDL	Occupational carcinogens	Low temperature	Low whole grains	Drug use
Stockton-on-Tees	Smoking	High fasting plasma glucose	High body-mass index	High blood pressure	Alcohol use	High LDL	Occupational carcinogens	Low temperature	Low whole grains	Drug use
Sunderland	Smoking	High fasting plasma glucose	High body-mass index	High blood pressure	Alcohol use	High LDL	Occupational carcinogens	Low temperature	Low whole grains	Kidney dysfunction
North West England	Smoking	High fasting plasma glucose	High body-mass index	High blood pressure	Alcohol use	High LDL	Low temperature	Drug use	Low whole grains	Occupational carcinogens
Blackburn with Darwen	Smoking	High fasting plasma glucose	High body-mass index	High blood pressure	Alcohol use	High LDL	Drug use	Low temperature	Low whole grains	Kidney dysfunction
Blackpool	Smoking	High fasting plasma glucose	High blood pressure	High body-mass index	Alcohol use	High LDL	Low temperature	Drug use	Low whole grains	Occupational carcinogens
Bolton	Smoking	High fasting plasma glucose	High body-mass index	High blood pressure	Alcohol use	High LDL	Low temperature	Drug use	Low whole grains	Particulate matter
Bury	Smoking	High fasting plasma glucose	High body-mass index	High blood pressure	Alcohol use	High LDL	Low temperature	Low whole grains	Drug use	Kidney dysfunction
Cheshire East	Smoking	High fasting plasma glucose	High body-mass index	High blood pressure	Alcohol use	High LDL	Low temperature	Low whole grains	Kidney dysfunction	Occupational carcinogens

Appendix Figure 3. Leading 10 level 3 risk factors for DALYs in 2019 by location. Each risk is colored by the percentage change in all-age DALYs from 2010 to 2019. DALYs=disability-adjusted life-years.

Percentage change in all-age DALYs from 2010 to 2019

-0.828 to -0.164	-0.164 to -0.066	-0.066 to -0.010	-0.010 to 0.029	0.029 to 0.073	0.073 to 0.120	0.120 to 0.173	0.173 to 0.235	0.235 to 0.325	0.325 to 1.699

	1	2	3	4	5	6	7	8	9	10
Cheshire West and Chester	Smoking	High fasting plasma glucose	High body-mass index	High blood pressure	Alcohol use	High LDL	Low temperature	Low whole grains	Occupational carcinogens	Drug use
Cumbria	Smoking	High fasting plasma glucose	High body-mass index	High blood pressure	High LDL	Alcohol use	Low temperature	Low whole grains	Occupational carcinogens	Kidney dysfunction
Halton	Smoking	High fasting plasma glucose	High body-mass index	High blood pressure	Alcohol use	High LDL	Low temperature	Occupational carcinogens	Low whole grains	Drug use
Knowsley	Smoking	High fasting plasma glucose	High body-mass index	High blood pressure	Alcohol use	High LDL	Low temperature	Occupational carcinogens	Low whole grains	Drug use
Lancashire	Smoking	High body-mass index	High fasting plasma glucose	High blood pressure	Alcohol use	High LDL	Low temperature	Low whole grains	Drug use	Kidney dysfunction
Liverpool	Smoking	High fasting plasma glucose	High body-mass index	High blood pressure	Alcohol use	High LDL	Drug use	Low temperature	Occupational carcinogens	Low whole grains
Manchester	Smoking	High body-mass index	High fasting plasma glucose	Alcohol use	High blood pressure	High LDL	Drug use	Low birth weight & short gestation	Low temperature	Particulate matter
Oldham	Smoking	High fasting plasma glucose	High body-mass index	High blood pressure	Alcohol use	High LDL	Low temperature	Drug use	Low whole grains	Particulate matter
Rochdale	Smoking	High fasting plasma glucose	High body-mass index	High blood pressure	High LDL	Alcohol use	Drug use	Low temperature	Low whole grains	Particulate matter
Salford	Smoking	High body-mass index	High fasting plasma glucose	High blood pressure	Alcohol use	High LDL	Drug use	Low temperature	Low whole grains	Particulate matter
Sefton	Smoking	High fasting plasma glucose	High body-mass index	High blood pressure	Alcohol use	High LDL	Low temperature	Low whole grains	Occupational carcinogens	Kidney dysfunction
St Helens	Smoking	High fasting plasma glucose	High body-mass index	High blood pressure	Alcohol use	High LDL	Low temperature	Low whole grains	Particulate matter	Occupational carcinogens
Stockport	Smoking	High body-mass index	High fasting plasma glucose	High blood pressure	Alcohol use	High LDL	Low temperature	Low whole grains	Drug use	Particulate matter
Tameside	Smoking	High fasting plasma glucose	High body-mass index	High blood pressure	High LDL	Alcohol use	Low temperature	Low whole grains	Drug use	Particulate matter
Trafford	Smoking	High body-mass index	High fasting plasma glucose	High blood pressure	Alcohol use	High LDL	Low temperature	Drug use	Particulate matter	Low whole grains
Warrington	Smoking	High fasting plasma glucose	High body-mass index	High blood pressure	Alcohol use	High LDL	Low temperature	Drug use	Low whole grains	Particulate matter
Wigan	Smoking	High fasting plasma glucose	High blood pressure	High body-mass index	Alcohol use	High LDL	Low temperature	Low whole grains	Occupational carcinogens	Drug use
Wirral	Smoking	High fasting plasma glucose	High body-mass index	High blood pressure	Alcohol use	High LDL	Low temperature	Occupational carcinogens	Low whole grains	Drug use
South East England	Smoking	High fasting plasma glucose	High body-mass index	High blood pressure	Alcohol use	High LDL	Low temperature	Drug use	Low whole grains	Kidney dysfunction
Bracknell Forest	Smoking	High fasting plasma glucose	High body-mass index	High blood pressure	Alcohol use	High LDL	Low temperature	Drug use	Low whole grains	Kidney dysfunction
Brighton and Hove	Smoking	High body-mass index	High fasting plasma glucose	Alcohol use	High blood pressure	Drug use	High LDL	Low temperature	Low whole grains	Occupational carcinogens
Buckinghamshire	Smoking	High body-mass index	High fasting plasma glucose	High blood pressure	Alcohol use	High LDL	Low temperature	Low whole grains	Kidney dysfunction	Drug use
East Sussex	Smoking	High fasting plasma glucose	High body-mass index	High blood pressure	Alcohol use	High LDL	Low temperature	Low whole grains	Occupational carcinogens	Particulate matter
Hampshire	Smoking	High fasting plasma glucose	High body-mass index	High blood pressure	Alcohol use	High LDL	Low temperature	Low whole grains	Kidney dysfunction	Occupational carcinogens
Isle of Wight	Smoking	High fasting plasma glucose	High body-mass index	High blood pressure	High LDL	Alcohol use	Low temperature	Low whole grains	Kidney dysfunction	Occupational carcinogens
Kent	Smoking	High fasting plasma glucose	High body-mass index	High blood pressure	Alcohol use	High LDL	Low temperature	Particulate matter	Low whole grains	Drug use
Medway	Smoking	High fasting plasma glucose	High body-mass index	High blood pressure	Alcohol use	High LDL	Occupational carcinogens	Low temperature	Particulate matter	Drug use
Milton Keynes	Smoking	High fasting plasma glucose	High body-mass index	High blood pressure	Alcohol use	High LDL	Drug use	Low temperature	Low whole grains	Particulate matter
Oxfordshire	Smoking	High body-mass index	High fasting plasma glucose	High blood pressure	Alcohol use	High LDL	Low temperature	Drug use	Low whole grains	Kidney dysfunction
Portsmouth	Smoking	High fasting plasma glucose	High body-mass index	High blood pressure	Alcohol use	High LDL	Drug use	Low temperature	Particulate matter	Occupational carcinogens
Reading	Smoking	High fasting plasma glucose	High body-mass index	Alcohol use	High blood pressure	High LDL	Drug use	Low temperature	Low whole grains	Kidney dysfunction
Slough	Smoking	High body-mass index	High fasting plasma glucose	High blood pressure	Alcohol use	High LDL	Drug use	Low birth weight & short gestation	Low temperature	Low whole grains

Appendix Figure 3. Leading 10 level 3 risk factors for DALYs in 2019 by location. Each risk is colored by the percentage change in all-age DALYs from 2010 to 2019. DALYs=disability-adjusted life-years.

Percentage change in all-age DALYs from 2010 to 2019: -0.828 to -0.164 | -0.164 to -0.066 | -0.066 to -0.010 | -0.010 to 0.029 | 0.029 to 0.073 | 0.073 to 0.120 | 0.120 to 0.173 | 0.173 to 0.235 | 0.235 to 0.325 | 0.325 to 1.699

	1	2	3	4	5	6	7	8	9	10
Southampton	Smoking	High fasting plasma glucose	High body-mass index	High blood pressure	Alcohol use	High LDL	Drug use	Low temperature	Particulate matter	Occupational carcinogens
Surrey	Smoking	High body-mass index	High fasting plasma glucose	High blood pressure	Alcohol use	High LDL	Low temperature	Low whole grains	Kidney dysfunction	Drug use
West Berkshire	Smoking	High fasting plasma glucose	High body-mass index	High blood pressure	Alcohol use	High LDL	Low temperature	Low whole grains	Occupational carcinogens	Drug use
West Sussex	Smoking	High fasting plasma glucose	High body-mass index	High blood pressure	Alcohol use	High LDL	Low temperature	Low whole grains	Kidney dysfunction	Particulate matter
Windsor and Maidenhead	Smoking	High fasting plasma glucose	High body-mass index	High blood pressure	Alcohol use	High LDL	Low temperature	Drug use	Low whole grains	Kidney dysfunction
Wokingham	Smoking	High fasting plasma glucose	High body-mass index	High blood pressure	Alcohol use	High LDL	Low temperature	Low whole grains	Kidney dysfunction	Drug use
South West England	Smoking	High fasting plasma glucose	High body-mass index	High blood pressure	Alcohol use	High LDL	Low temperature	Low whole grains	Kidney dysfunction	Drug use
Bath and North East Somerset	Smoking	High body-mass index	High fasting plasma glucose	High blood pressure	Alcohol use	High LDL	Drug use	Low temperature	Low whole grains	Kidney dysfunction
Bournemouth	Smoking	High fasting plasma glucose	High body-mass index	High blood pressure	Alcohol use	High LDL	Drug use	Low temperature	Low whole grains	Kidney dysfunction
Bristol, City of	Smoking	High body-mass index	High fasting plasma glucose	High blood pressure	Alcohol use	High LDL	Drug use	Low temperature	Low whole grains	Particulate matter
Cornwall	Smoking	High fasting plasma glucose	High body-mass index	High blood pressure	Alcohol use	High LDL	Low temperature	Low whole grains	Kidney dysfunction	Occupational carcinogens
Devon	Smoking	High fasting plasma glucose	High body-mass index	High blood pressure	Alcohol use	High LDL	Low temperature	Low whole grains	Kidney dysfunction	Occupational carcinogens
Dorset	Smoking	High fasting plasma glucose	High body-mass index	High blood pressure	Alcohol use	High LDL	Low temperature	Kidney dysfunction	Low whole grains	Occupational carcinogens
Gloucestershire	Smoking	High fasting plasma glucose	High body-mass index	High blood pressure	Alcohol use	High LDL	Low temperature	Low whole grains	Kidney dysfunction	Drug use
North Somerset	Smoking	High fasting plasma glucose	High body-mass index	High blood pressure	Alcohol use	High LDL	Low temperature	Low whole grains	Kidney dysfunction	Drug use
Plymouth	Smoking	High fasting plasma glucose	High body-mass index	High blood pressure	Alcohol use	High LDL	Drug use	Low temperature	Occupational carcinogens	Low whole grains
Poole	Smoking	High fasting plasma glucose	High body-mass index	High blood pressure	Alcohol use	High LDL	Low temperature	Low whole grains	Kidney dysfunction	Drug use
Somerset	Smoking	High fasting plasma glucose	High body-mass index	High blood pressure	Alcohol use	High LDL	Low temperature	Low whole grains	Kidney dysfunction	Occupational carcinogens
South Gloucestershire	Smoking	High body-mass index	High fasting plasma glucose	High blood pressure	Alcohol use	High LDL	Low temperature	Low whole grains	Kidney dysfunction	Drug use
Swindon	Smoking	High body-mass index	High fasting plasma glucose	High blood pressure	Alcohol use	High LDL	Low temperature	Drug use	Low whole grains	Occupational carcinogens
Torbay	Smoking	High fasting plasma glucose	High blood pressure	High body-mass index	High LDL	Alcohol use	Low temperature	Low whole grains	Kidney dysfunction	Occupational carcinogens
Wiltshire	Smoking	High body-mass index	High fasting plasma glucose	High blood pressure	Alcohol use	High LDL	Low temperature	Low whole grains	Kidney dysfunction	Occupational carcinogens
West Midlands	Smoking	High body-mass index	High fasting plasma glucose	High blood pressure	Alcohol use	High LDL	Low temperature	Low whole grains	Kidney dysfunction	Drug use
Birmingham	Smoking	High body-mass index	High fasting plasma glucose	High blood pressure	Alcohol use	High LDL	Low birth weight & short gestation	Drug use	Low temperature	Low whole grains
Coventry	Smoking	High fasting plasma glucose	High body-mass index	High blood pressure	Alcohol use	High LDL	Drug use	Low temperature	Kidney dysfunction	Low whole grains
Dudley	Smoking	High fasting plasma glucose	High body-mass index	High blood pressure	Alcohol use	High LDL	Low temperature	Low whole grains	Kidney dysfunction	Particulate matter
Herefordshire, County of	Smoking	High body-mass index	High fasting plasma glucose	High blood pressure	Alcohol use	High LDL	Low temperature	Kidney dysfunction	Low whole grains	Drug use
Sandwell	Smoking	High fasting plasma glucose	High body-mass index	High blood pressure	Alcohol use	High LDL	Low temperature	Low whole grains	Particulate matter	Low birth weight & short gestation
Shropshire	Smoking	High body-mass index	High fasting plasma glucose	High blood pressure	Alcohol use	High LDL	Low temperature	Low whole grains	Kidney dysfunction	Drug use
Solihull	Smoking	High body-mass index	High fasting plasma glucose	High blood pressure	Alcohol use	High LDL	Low temperature	Kidney dysfunction	Low whole grains	Drug use
Staffordshire	Smoking	High body-mass index	High fasting plasma glucose	High blood pressure	Alcohol use	High LDL	Low temperature	Low whole grains	Kidney dysfunction	Particulate matter
Stoke-on-Trent	Smoking	High fasting plasma glucose	High body-mass index	High blood pressure	Alcohol use	High LDL	Drug use	Low temperature	Particulate matter	Occupational carcinogens

Appendix Figure 3. Leading 10 level 3 risk factors for DALYs in 2019 by location. Each risk is colored by the percentage change in all-age DALYs from 2010 to 2019. DALYs=disability-adjusted life-years.

Percentage change in all-age DALYs from 2010 to 2019

	-0.828 to -0.164	-0.164 to -0.066	-0.066 to -0.010	-0.010 to 0.029	0.029 to 0.073	0.073 to 0.120	0.120 to 0.173	0.173 to 0.235	0.235 to 0.325	0.325 to 1.699
	1	2	3	4	5	6	7	8	9	10
Telford and Wrekin	Smoking	High body-mass index	High fasting plasma glucose	High blood pressure	Alcohol use	High LDL	Low temperature	Drug use	Low whole grains	Kidney dysfunction
Walsall	Smoking	High fasting plasma glucose	High body-mass index	High blood pressure	Alcohol use	High LDL	Low temperature	Low whole grains	Kidney dysfunction	Particulate matter
Warwickshire	Smoking	High body-mass index	High fasting plasma glucose	High blood pressure	Alcohol use	High LDL	Low temperature	Kidney dysfunction	Low whole grains	Drug use
Wolverhampton	Smoking	High fasting plasma glucose	High body-mass index	High blood pressure	Alcohol use	High LDL	Low temperature	Kidney dysfunction	Low whole grains	Particulate matter
Worcestershire	Smoking	High body-mass index	High fasting plasma glucose	High blood pressure	Alcohol use	High LDL	Low temperature	Kidney dysfunction	Low whole grains	Drug use
Yorkshire and the Humber	Smoking	High body-mass index	High fasting plasma glucose	High blood pressure	High LDL	Alcohol use	Low temperature	Low whole grains	Drug use	Kidney dysfunction
Barnsley	Smoking	High fasting plasma glucose	High body-mass index	High blood pressure	High LDL	Alcohol use	Low temperature	Low whole grains	Particulate matter	Drug use
Bradford	Smoking	High body-mass index	High fasting plasma glucose	High blood pressure	High LDL	Alcohol use	Low temperature	Drug use	Low whole grains	Kidney dysfunction
Calderdale	Smoking	High body-mass index	High fasting plasma glucose	High blood pressure	Alcohol use	High LDL	Low temperature	Low whole grains	Drug use	Occupational carcinogens
Doncaster	Smoking	High fasting plasma glucose	High body-mass index	High blood pressure	High LDL	Alcohol use	Low temperature	Occupational carcinogens	Low whole grains	Particulate matter
East Riding of Yorkshire	Smoking	High body-mass index	High fasting plasma glucose	High blood pressure	High LDL	Alcohol use	Low temperature	Low whole grains	Kidney dysfunction	Occupational carcinogens
Kingston upon Hull, City of	Smoking	High fasting plasma glucose	High body-mass index	High blood pressure	Alcohol use	High LDL	Low temperature	Drug use	Occupational carcinogens	Low whole grains
Kirklees	Smoking	High fasting plasma glucose	High body-mass index	High blood pressure	Alcohol use	High LDL	Low temperature	Low whole grains	Kidney dysfunction	Drug use
Leeds	Smoking	High body-mass index	High fasting plasma glucose	High blood pressure	Alcohol use	High LDL	Drug use	Low temperature	Low whole grains	Occupational carcinogens
North East Lincolnshire	Smoking	High fasting plasma glucose	High body-mass index	High blood pressure	High LDL	Alcohol use	Low temperature	Low whole grains	Kidney dysfunction	Drug use
North Lincolnshire	Smoking	High body-mass index	High fasting plasma glucose	High blood pressure	High LDL	Alcohol use	Low temperature	Kidney dysfunction	Low whole grains	Particulate matter
North Yorkshire	Smoking	High body-mass index	High fasting plasma glucose	High blood pressure	High LDL	Alcohol use	Low temperature	Low whole grains	Kidney dysfunction	Occupational carcinogens
Rotherham	Smoking	High fasting plasma glucose	High body-mass index	High blood pressure	High LDL	Alcohol use	Low temperature	Low whole grains	Particulate matter	Occupational carcinogens
Sheffield	Smoking	High body-mass index	High fasting plasma glucose	High blood pressure	Alcohol use	High LDL	Drug use	Low temperature	Low whole grains	Particulate matter
Wakefield	Smoking	High fasting plasma glucose	High body-mass index	High blood pressure	High LDL	Alcohol use	Low temperature	Low whole grains	Occupational carcinogens	Particulate matter
York	Smoking	High body-mass index	High fasting plasma glucose	High blood pressure	Alcohol use	High LDL	Low temperature	Drug use	Low whole grains	Kidney dysfunction
Northern Ireland	Smoking	High blood pressure	High body-mass index	High fasting plasma glucose	Alcohol use	High LDL	Low temperature	Low whole grains	Kidney dysfunction	Drug use
Scotland	Smoking	High body-mass index	High fasting plasma glucose	High blood pressure	Alcohol use	High LDL	Drug use	Low temperature	Low whole grains	Kidney dysfunction
Wales	Smoking	High blood pressure	High body-mass index	High fasting plasma glucose	Alcohol use	High LDL	Low temperature	Drug use	Low whole grains	Low physical activity
**Latin America and Caribbean**	High body-mass index	High fasting plasma glucose	High blood pressure	Alcohol use	Smoking	Kidney dysfunction	Low birth weight & short gestation	Particulate matter	High LDL	Unsafe sex
**Andean Latin America**	High body-mass index	High fasting plasma glucose	High blood pressure	Low birth weight & short gestation	Particulate matter	Kidney dysfunction	Alcohol use	High LDL	Smoking	Unsafe sex
Bolivia	High body-mass index	Low birth weight & short gestation	High fasting plasma glucose	Particulate matter	High blood pressure	Kidney dysfunction	Alcohol use	Child growth failure	Smoking	High LDL
Ecuador	High body-mass index	High fasting plasma glucose	High blood pressure	Kidney dysfunction	Low birth weight & short gestation	Alcohol use	Particulate matter	Smoking	High LDL	Unsafe sex
Peru	High body-mass index	High fasting plasma glucose	High blood pressure	Low birth weight & short gestation	Particulate matter	Alcohol use	Kidney dysfunction	High LDL	Unsafe sex	Smoking
**Caribbean**	High fasting plasma glucose	High blood pressure	High body-mass index	Smoking	Low birth weight & short gestation	Particulate matter	Alcohol use	High LDL	Kidney dysfunction	Child growth failure
Antigua and Barbuda	High fasting plasma glucose	High body-mass index	High blood pressure	Kidney dysfunction	Alcohol use	Smoking	Particulate matter	High LDL	Low whole grains	Unsafe sex
The Bahamas	High body-mass index	High blood pressure	High fasting plasma glucose	Alcohol use	Kidney dysfunction	Unsafe sex	Smoking	High LDL	Particulate matter	Low whole grains

Appendix Figure 3. Leading 10 level 3 risk factors for DALYs in 2019 by location. Each risk is colored by the percentage change in all-age DALYs from 2010 to 2019. DALYs=disability-adjusted life-years.

Percentage change in all-age DALYs from 2010 to 2019: -0.828 to -0.164 | -0.164 to -0.066 | -0.066 to -0.010 | -0.010 to 0.029 | 0.029 to 0.073 | 0.073 to 0.120 | 0.120 to 0.173 | 0.173 to 0.235 | 0.235 to 0.325 | 0.325 to 1.699

	1	2	3	4	5	6	7	8	9	10
Barbados	High fasting plasma glucose	High body-mass index	High blood pressure	Kidney dysfunction	Alcohol use	Particulate matter	Smoking	High LDL	Low physical activity	Low whole grains
Belize	High body-mass index	High fasting plasma glucose	High blood pressure	Alcohol use	Low birth weight & short gestation	Kidney dysfunction	Unsafe sex	Smoking	Particulate matter	High LDL
Bermuda	High body-mass index	High fasting plasma glucose	High blood pressure	Smoking	Kidney dysfunction	High LDL	Alcohol use	Low whole grains	Low physical activity	High red meat
Cuba	Smoking	High fasting plasma glucose	High body-mass index	High blood pressure	High LDL	Alcohol use	Kidney dysfunction	Particulate matter	Occupational injury	Low whole grains
Dominica	High fasting plasma glucose	High body-mass index	High blood pressure	Kidney dysfunction	Alcohol use	Smoking	Particulate matter	Low birth weight & short gestation	High LDL	Low whole grains
Dominican Republic	High blood pressure	High body-mass index	Low birth weight & short gestation	High fasting plasma glucose	Smoking	High LDL	Alcohol use	Particulate matter	Kidney dysfunction	Unsafe sex
Grenada	High fasting plasma glucose	High body-mass index	High blood pressure	Kidney dysfunction	Alcohol use	Smoking	Particulate matter	High LDL	Low birth weight & short gestation	Low whole grains
Guyana	High fasting plasma glucose	High body-mass index	High blood pressure	Alcohol use	Low birth weight & short gestation	Kidney dysfunction	Particulate matter	High LDL	Smoking	Unsafe sex
Haiti	Particulate matter	Low birth weight & short gestation	High blood pressure	Child growth failure	High fasting plasma glucose	Unsafe water	Unsafe sex	High body-mass index	Unsafe sanitation	Alcohol use
Jamaica	High fasting plasma glucose	High body-mass index	High blood pressure	Smoking	Kidney dysfunction	Low birth weight & short gestation	Particulate matter	Unsafe sex	High LDL	Alcohol use
Puerto Rico	High fasting plasma glucose	High body-mass index	High blood pressure	Smoking	Kidney dysfunction	Alcohol use	High LDL	Low physical activity	Low whole grains	Low birth weight & short gestation
Saint Kitts and Nevis	High fasting plasma glucose	High body-mass index	High blood pressure	Kidney dysfunction	Unsafe sex	Alcohol use	Smoking	High LDL	Low birth weight & short gestation	Low whole grains
Saint Lucia	High fasting plasma glucose	High body-mass index	High blood pressure	Alcohol use	Smoking	Kidney dysfunction	Particulate matter	Low birth weight & short gestation	High LDL	Low whole grains
Saint Vincent and the Grenadines	High fasting plasma glucose	High body-mass index	High blood pressure	Alcohol use	Kidney dysfunction	Particulate matter	Smoking	High LDL	Unsafe sex	Low birth weight & short gestation
Suriname	High fasting plasma glucose	High body-mass index	High blood pressure	Smoking	Low birth weight & short gestation	Kidney dysfunction	Particulate matter	Alcohol use	High LDL	Unsafe sex
Trinidad and Tobago	High fasting plasma glucose	High body-mass index	High blood pressure	Smoking	Kidney dysfunction	High LDL	Particulate matter	Alcohol use	Low whole grains	Low physical activity
Virgin Islands	High body-mass index	High fasting plasma glucose	High blood pressure	Smoking	High LDL	Kidney dysfunction	Alcohol use	Low whole grains	Particulate matter	Low legumes
**Central Latin America**	High fasting plasma glucose	High body-mass index	High blood pressure	Kidney dysfunction	Alcohol use	Particulate matter	Smoking	Low birth weight & short gestation	High LDL	Unsafe sex
Colombia	High fasting plasma glucose	High body-mass index	High blood pressure	Smoking	Alcohol use	Particulate matter	Kidney dysfunction	High LDL	Low birth weight & short gestation	Unsafe sex
Costa Rica	High blood pressure	High body-mass index	High fasting plasma glucose	Kidney dysfunction	Smoking	Alcohol use	High LDL	Particulate matter	Low birth weight & short gestation	Low whole grains
El Salvador	High fasting plasma glucose	High body-mass index	High blood pressure	Kidney dysfunction	Alcohol use	Particulate matter	High LDL	Smoking	Low birth weight & short gestation	Unsafe sex
Guatemala	High fasting plasma glucose	Alcohol use	High body-mass index	Particulate matter	High blood pressure	Low birth weight & short gestation	Child growth failure	Kidney dysfunction	Unsafe water	Smoking
Honduras	High blood pressure	High fasting plasma glucose	High body-mass index	Particulate matter	Low birth weight & short gestation	Kidney dysfunction	Alcohol use	Smoking	High LDL	Unsafe water
Mexico	High fasting plasma glucose	High body-mass index	High blood pressure	Kidney dysfunction	Alcohol use	Particulate matter	Smoking	High LDL	Low birth weight & short gestation	Unsafe sex
Aguascalientes	High body-mass index	High fasting plasma glucose	High blood pressure	Kidney dysfunction	Alcohol use	Smoking	Low birth weight & short gestation	Particulate matter	High LDL	Low temperature
Baja California	High body-mass index	High fasting plasma glucose	High blood pressure	Kidney dysfunction	Alcohol use	Smoking	High LDL	Particulate matter	Unsafe sex	Low birth weight & short gestation
Baja California Sur	High body-mass index	High fasting plasma glucose	High blood pressure	Kidney dysfunction	Alcohol use	High LDL	Smoking	Particulate matter	Low birth weight & short gestation	Unsafe sex
Campeche	High body-mass index	High fasting plasma glucose	High blood pressure	Kidney dysfunction	Alcohol use	Particulate matter	Low birth weight & short gestation	High LDL	Smoking	Unsafe sex
Chiapas	High fasting plasma glucose	High body-mass index	Kidney dysfunction	High blood pressure	Alcohol use	Particulate matter	Low birth weight & short gestation	High LDL	Child growth failure	Unsafe sex
Chihuahua	High fasting plasma glucose	High body-mass index	High blood pressure	Alcohol use	Kidney dysfunction	Smoking	High LDL	Particulate matter	Low birth weight & short gestation	Low temperature
Coahuila	High fasting plasma glucose	High body-mass index	High blood pressure	Kidney dysfunction	Smoking	Alcohol use	High LDL	Low birth weight & short gestation	Particulate matter	High red meat
Colima	High body-mass index	High fasting plasma glucose	High blood pressure	Kidney dysfunction	Alcohol use	Smoking	High LDL	Particulate matter	Low birth weight & short gestation	Unsafe sex

Appendix Figure 3. Leading 10 level 3 risk factors for DALYs in 2019 by location. Each risk is colored by the percentage change in all-age DALYs from 2010 to 2019. DALYs=disability-adjusted life-years.

Percentage change in all-age DALYs from 2010 to 2019: -0.828 to -0.164 | -0.164 to -0.066 | -0.066 to -0.010 | -0.010 to 0.029 | 0.029 to 0.073 | 0.073 to 0.120 | 0.120 to 0.173 | 0.173 to 0.235 | 0.235 to 0.325 | 0.325 to 1.699

	1	2	3	4	5	6	7	8	9	10
Durango	High fasting plasma glucose	High body-mass index	High blood pressure	Kidney dysfunction	Alcohol use	High LDL	Low birth weight & short gestation	Smoking	Particulate matter	Low temperature
Guanajuato	High fasting plasma glucose	High body-mass index	High blood pressure	Kidney dysfunction	Alcohol use	Smoking	Particulate matter	High LDL	Low birth weight & short gestation	High red meat
Guerrero	High fasting plasma glucose	High body-mass index	Kidney dysfunction	High blood pressure	Alcohol use	Particulate matter	Low birth weight & short gestation	High LDL	Smoking	Child growth failure
Hidalgo	High fasting plasma glucose	High body-mass index	High blood pressure	Kidney dysfunction	Alcohol use	Particulate matter	High LDL	Smoking	Low birth weight & short gestation	Low temperature
Jalisco	High fasting plasma glucose	High body-mass index	High blood pressure	Kidney dysfunction	Alcohol use	Smoking	Particulate matter	High LDL	Low birth weight & short gestation	High red meat
México	High fasting plasma glucose	High body-mass index	Kidney dysfunction	High blood pressure	Alcohol use	Particulate matter	Low birth weight & short gestation	Smoking	High LDL	Low temperature
Mexico City	High fasting plasma glucose	High body-mass index	High blood pressure	Kidney dysfunction	Alcohol use	Smoking	Particulate matter	High LDL	Low temperature	Low birth weight & short gestation
Michoacán de Ocampo	High fasting plasma glucose	High body-mass index	High blood pressure	Kidney dysfunction	Alcohol use	Particulate matter	Smoking	High LDL	Low birth weight & short gestation	Child growth failure
Morelos	High fasting plasma glucose	High body-mass index	Kidney dysfunction	High blood pressure	Alcohol use	Particulate matter	Smoking	High LDL	Low birth weight & short gestation	Unsafe sex
Nayarit	High fasting plasma glucose	High body-mass index	High blood pressure	Kidney dysfunction	Alcohol use	Smoking	High LDL	Particulate matter	Low birth weight & short gestation	Unsafe sex
Nuevo León	High body-mass index	High fasting plasma glucose	High blood pressure	Kidney dysfunction	Smoking	Alcohol use	High LDL	Particulate matter	Low birth weight & short gestation	High red meat
Oaxaca	High fasting plasma glucose	High body-mass index	High blood pressure	Kidney dysfunction	Alcohol use	Particulate matter	High LDL	Low birth weight & short gestation	Child growth failure	Smoking
Puebla	High fasting plasma glucose	High body-mass index	Kidney dysfunction	High blood pressure	Alcohol use	Particulate matter	Low birth weight & short gestation	High LDL	Smoking	Child growth failure
Querétaro	High body-mass index	High fasting plasma glucose	High blood pressure	Kidney dysfunction	Alcohol use	Smoking	Particulate matter	High LDL	Low birth weight & short gestation	Low temperature
Quintana Roo	High body-mass index	High fasting plasma glucose	Alcohol use	High blood pressure	Kidney dysfunction	Low birth weight & short gestation	Unsafe sex	Particulate matter	High LDL	Smoking
San Luis Potosí	High fasting plasma glucose	High body-mass index	High blood pressure	Kidney dysfunction	Alcohol use	Particulate matter	Smoking	High LDL	Low birth weight & short gestation	High red meat
Sinaloa	High fasting plasma glucose	High body-mass index	High blood pressure	Kidney dysfunction	Alcohol use	High LDL	Smoking	Particulate matter	Low birth weight & short gestation	Low whole grains
Sonora	High body-mass index	High fasting plasma glucose	High blood pressure	Kidney dysfunction	Alcohol use	Smoking	High LDL	Particulate matter	Low birth weight & short gestation	Low whole grains
Tabasco	High fasting plasma glucose	High body-mass index	High blood pressure	Kidney dysfunction	Alcohol use	Particulate matter	Low birth weight & short gestation	High LDL	Unsafe sex	Smoking
Tamaulipas	High fasting plasma glucose	High body-mass index	High blood pressure	Kidney dysfunction	Alcohol use	High LDL	Smoking	Particulate matter	Low birth weight & short gestation	High red meat
Tlaxcala	High fasting plasma glucose	High body-mass index	Kidney dysfunction	High blood pressure	Alcohol use	Particulate matter	Low birth weight & short gestation	Smoking	High LDL	Low temperature
Veracruz de Ignacio de la Llave	High fasting plasma glucose	High body-mass index	High blood pressure	Kidney dysfunction	Alcohol use	Particulate matter	High LDL	Low birth weight & short gestation	Smoking	Unsafe sex
Yucatán	High body-mass index	High fasting plasma glucose	High blood pressure	Kidney dysfunction	Alcohol use	Particulate matter	High LDL	Low birth weight & short gestation	Smoking	Unsafe sex
Zacatecas	High fasting plasma glucose	High body-mass index	High blood pressure	Kidney dysfunction	Alcohol use	Smoking	High LDL	Particulate matter	Low birth weight & short gestation	Low temperature
Nicaragua	High fasting plasma glucose	High blood pressure	High body-mass index	Kidney dysfunction	Particulate matter	Alcohol use	Low birth weight & short gestation	High LDL	Smoking	Unsafe sex
Panama	High fasting plasma glucose	High body-mass index	High blood pressure	Kidney dysfunction	Alcohol use	Unsafe sex	Low birth weight & short gestation	Smoking	High LDL	Particulate matter
Venezuela	High fasting plasma glucose	High blood pressure	High body-mass index	Kidney dysfunction	Smoking	Alcohol use	High LDL	Particulate matter	Low birth weight & short gestation	Occupational injury
**Tropical Latin America**	High body-mass index	High blood pressure	High fasting plasma glucose	Smoking	Alcohol use	High LDL	Low birth weight & short gestation	Kidney dysfunction	Particulate matter	High red meat
Brazil	High body-mass index	High blood pressure	High fasting plasma glucose	Smoking	Alcohol use	High LDL	Low birth weight & short gestation	Kidney dysfunction	Particulate matter	High red meat
Acre	Low birth weight & short gestation	High body-mass index	Smoking	Alcohol use	High blood pressure	High fasting plasma glucose	Child growth failure	Kidney dysfunction	High LDL	Particulate matter
Alagoas	High body-mass index	High fasting plasma glucose	High blood pressure	Alcohol use	Low birth weight & short gestation	Smoking	High LDL	Particulate matter	Kidney dysfunction	Child growth failure
Amapá	Low birth weight & short gestation	High body-mass index	Alcohol use	High fasting plasma glucose	High blood pressure	Smoking	Kidney dysfunction	High LDL	Unsafe sex	Particulate matter

Appendix Figure 3. Leading 10 level 3 risk factors for DALYs in 2019 by location. Each risk is colored by the percentage change in all-age DALYs from 2010 to 2019. DALYs=disability-adjusted life-years.

Percentage change in all-age DALYs from 2010 to 2019: -0.828 to -0.164 | -0.164 to -0.066 | -0.066 to -0.010 | -0.010 to 0.029 | 0.029 to 0.073 | 0.073 to 0.120 | 0.120 to 0.173 | 0.173 to 0.235 | 0.235 to 0.325 | 0.325 to 1.699

	1	2	3	4	5	6	7	8	9	10
Amazonas	High body-mass index	High fasting plasma glucose	Low birth weight & short gestation	Alcohol use	High blood pressure	Smoking	Unsafe sex	Kidney dysfunction	High LDL	Drug use
Bahia	High body-mass index	High blood pressure	High fasting plasma glucose	Alcohol use	Low birth weight & short gestation	Smoking	High LDL	Kidney dysfunction	Particulate matter	High red meat
Ceará	High body-mass index	High blood pressure	High fasting plasma glucose	Alcohol use	Smoking	Low birth weight & short gestation	High LDL	Kidney dysfunction	Particulate matter	Low physical activity
Distrito Federal	High body-mass index	Alcohol use	High fasting plasma glucose	High blood pressure	Smoking	Low birth weight & short gestation	High LDL	Kidney dysfunction	High red meat	Particulate matter
Espírito Santo	High body-mass index	High blood pressure	High fasting plasma glucose	Alcohol use	Smoking	High LDL	Low birth weight & short gestation	Kidney dysfunction	Particulate matter	High red meat
Goiás	High body-mass index	High blood pressure	Smoking	High fasting plasma glucose	Alcohol use	High LDL	Low birth weight & short gestation	Kidney dysfunction	Particulate matter	High red meat
Maranhão	High fasting plasma glucose	High body-mass index	High blood pressure	Low birth weight & short gestation	Alcohol use	Smoking	Particulate matter	High LDL	Kidney dysfunction	Child growth failure
Mato Grosso	High body-mass index	High fasting plasma glucose	High blood pressure	Alcohol use	Smoking	High LDL	Low birth weight & short gestation	Kidney dysfunction	Particulate matter	High red meat
Mato Grosso do Sul	High body-mass index	High blood pressure	High fasting plasma glucose	Smoking	Alcohol use	High LDL	Low birth weight & short gestation	Kidney dysfunction	Particulate matter	High red meat
Minas Gerais	High body-mass index	High blood pressure	Smoking	High fasting plasma glucose	Alcohol use	High LDL	Low birth weight & short gestation	Kidney dysfunction	Particulate matter	High red meat
Pará	High body-mass index	High fasting plasma glucose	High blood pressure	Low birth weight & short gestation	Alcohol use	Smoking	High LDL	Kidney dysfunction	Particulate matter	Unsafe sex
Paraíba	High body-mass index	High fasting plasma glucose	High blood pressure	Smoking	Alcohol use	High LDL	Low birth weight & short gestation	Particulate matter	Kidney dysfunction	Low physical activity
Paraná	High body-mass index	High blood pressure	Smoking	High fasting plasma glucose	Alcohol use	High LDL	Kidney dysfunction	Low birth weight & short gestation	Particulate matter	High red meat
Pernambuco	High body-mass index	High blood pressure	High fasting plasma glucose	Smoking	Alcohol use	Low birth weight & short gestation	High LDL	Kidney dysfunction	Particulate matter	High red meat
Piauí	High body-mass index	High blood pressure	High fasting plasma glucose	Low birth weight & short gestation	Alcohol use	Smoking	Particulate matter	High LDL	Kidney dysfunction	High red meat
Rio de Janeiro	High body-mass index	High blood pressure	High fasting plasma glucose	Smoking	Alcohol use	High LDL	Kidney dysfunction	Particulate matter	Low physical activity	High red meat
Rio Grande do Norte	High body-mass index	High fasting plasma glucose	High blood pressure	Alcohol use	Smoking	High LDL	Low birth weight & short gestation	Kidney dysfunction	Particulate matter	Low physical activity
Rio Grande do Sul	Smoking	High body-mass index	High blood pressure	High fasting plasma glucose	Alcohol use	High LDL	Kidney dysfunction	Particulate matter	Low birth weight & short gestation	Unsafe sex
Rondônia	High body-mass index	High blood pressure	High fasting plasma glucose	Smoking	Alcohol use	Low birth weight & short gestation	High LDL	Kidney dysfunction	Particulate matter	Low physical activity
Roraima	Low birth weight & short gestation	High body-mass index	High fasting plasma glucose	Alcohol use	High blood pressure	Smoking	Kidney dysfunction	High LDL	Particulate matter	Unsafe sex
Santa Catarina	High body-mass index	High blood pressure	Smoking	High fasting plasma glucose	Alcohol use	High LDL	Low birth weight & short gestation	Kidney dysfunction	Particulate matter	Unsafe sex
São Paulo	High body-mass index	High blood pressure	Smoking	High fasting plasma glucose	Alcohol use	High LDL	Kidney dysfunction	Particulate matter	Low birth weight & short gestation	High red meat
Sergipe	High body-mass index	High fasting plasma glucose	High blood pressure	Alcohol use	Low birth weight & short gestation	Smoking	High LDL	Kidney dysfunction	Particulate matter	High red meat
Tocantins	High body-mass index	High fasting plasma glucose	High blood pressure	Alcohol use	Smoking	Low birth weight & short gestation	High LDL	Kidney dysfunction	Particulate matter	High red meat
Paraguay	High body-mass index	High fasting plasma glucose	High blood pressure	Smoking	Alcohol use	Kidney dysfunction	Particulate matter	High LDL	Low birth weight & short gestation	High red meat
**North Africa and Middle East**	High blood pressure	High body-mass index	High fasting plasma glucose	Particulate matter	Smoking	High LDL	Low birth weight & short gestation	Kidney dysfunction	Low whole grains	Child growth failure
Afghanistan	Low birth weight & short gestation	Particulate matter	Child growth failure	High blood pressure	High body-mass index	High fasting plasma glucose	High LDL	Unsafe water	Kidney dysfunction	Smoking
Algeria	High blood pressure	High body-mass index	High fasting plasma glucose	Low birth weight & short gestation	Particulate matter	High LDL	Smoking	Kidney dysfunction	Low whole grains	Second-hand smoke
Bahrain	High body-mass index	High fasting plasma glucose	High blood pressure	Particulate matter	Smoking	High LDL	Kidney dysfunction	Low whole grains	Low physical activity	Second-hand smoke
Egypt	High blood pressure	High body-mass index	Particulate matter	High fasting plasma glucose	High LDL	Smoking	Kidney dysfunction	Low whole grains	Child growth failure	Low birth weight & short gestation
Iran	High blood pressure	High body-mass index	High fasting plasma glucose	Smoking	High LDL	Particulate matter	Kidney dysfunction	Low birth weight & short gestation	Low whole grains	Drug use
Iraq	High blood pressure	High body-mass index	High fasting plasma glucose	Particulate matter	Smoking	Low birth weight & short gestation	High LDL	Kidney dysfunction	Low whole grains	Second-hand smoke

Appendix Figure 3. Leading 10 level 3 risk factors for DALYs in 2019 by location. Each risk is colored by the percentage change in all-age DALYs from 2010 to 2019. DALYs=disability-adjusted life-years.

Percentage change in all-age DALYs from 2010 to 2019: -0.828 to -0.164 | -0.164 to -0.066 | -0.066 to -0.010 | -0.010 to 0.029 | 0.029 to 0.073 | 0.073 to 0.120 | 0.120 to 0.173 | 0.173 to 0.235 | 0.235 to 0.325 | 0.325 to 1.699

	1	2	3	4	5	6	7	8	9	10
Jordan	High body-mass index	High blood pressure	High fasting plasma glucose	Smoking	Low birth weight & short gestation	Particulate matter	High LDL	Kidney dysfunction	Low whole grains	Second-hand smoke
Kuwait	High body-mass index	High fasting plasma glucose	High blood pressure	Smoking	Particulate matter	High LDL	Low birth weight & short gestation	Kidney dysfunction	Low whole grains	Low physical activity
Lebanon	High blood pressure	Smoking	High body-mass index	High fasting plasma glucose	High LDL	Particulate matter	Kidney dysfunction	Low whole grains	Low birth weight & short gestation	Low physical activity
Libya	High body-mass index	High blood pressure	High fasting plasma glucose	Smoking	High LDL	Particulate matter	Kidney dysfunction	Low whole grains	Second-hand smoke	Low birth weight & short gestation
Morocco	High blood pressure	High body-mass index	High fasting plasma glucose	High LDL	Particulate matter	Smoking	Kidney dysfunction	Low birth weight & short gestation	Low whole grains	Low physical activity
Oman	High body-mass index	High blood pressure	High fasting plasma glucose	High LDL	Particulate matter	Low birth weight & short gestation	Smoking	Kidney dysfunction	High temperature	Low whole grains
Palestine	High fasting plasma glucose	High body-mass index	High blood pressure	Smoking	Low birth weight & short gestation	Particulate matter	High LDL	Kidney dysfunction	Low whole grains	Second-hand smoke
Qatar	High body-mass index	High fasting plasma glucose	Particulate matter	High blood pressure	Smoking	High LDL	Low birth weight & short gestation	Kidney dysfunction	Occupational injury	Low whole grains
Saudi Arabia	High body-mass index	High blood pressure	High fasting plasma glucose	Particulate matter	High LDL	Smoking	Kidney dysfunction	Low whole grains	Low physical activity	Second-hand smoke
Sudan	Low birth weight & short gestation	High blood pressure	Particulate matter	High body-mass index	High fasting plasma glucose	Child growth failure	High LDL	Unsafe water	Smoking	Unsafe sanitation
Syria	High blood pressure	High body-mass index	High fasting plasma glucose	High LDL	Smoking	Particulate matter	Kidney dysfunction	Low whole grains	Low legumes	Low birth weight & short gestation
Tunisia	High blood pressure	High body-mass index	High fasting plasma glucose	Smoking	High LDL	Particulate matter	Kidney dysfunction	Low whole grains	Low birth weight & short gestation	Second-hand smoke
Turkey	Smoking	High body-mass index	High blood pressure	High fasting plasma glucose	Particulate matter	High LDL	Kidney dysfunction	Low birth weight & short gestation	Low whole grains	Low temperature
United Arab Emirates	High body-mass index	High blood pressure	High fasting plasma glucose	Smoking	High LDL	Particulate matter	Kidney dysfunction	Drug use	Low whole grains	High temperature
Yemen	Low birth weight & short gestation	Particulate matter	High blood pressure	Child growth failure	Smoking	Unsafe water	High LDL	High body-mass index	High fasting plasma glucose	Iron deficiency
**South Asia**	Particulate matter	Low birth weight & short gestation	High blood pressure	High fasting plasma glucose	Smoking	High body-mass index	High LDL	Unsafe water	Child growth failure	Kidney dysfunction
Bangladesh	Particulate matter	High blood pressure	Low birth weight & short gestation	Smoking	High fasting plasma glucose	High body-mass index	High LDL	Kidney dysfunction	Unsafe water	Low fibre
Bhutan	Low birth weight & short gestation	Particulate matter	High blood pressure	High fasting plasma glucose	High body-mass index	High LDL	Smoking	Iron deficiency	Kidney dysfunction	Alcohol use
India	Particulate matter	Low birth weight & short gestation	High blood pressure	High fasting plasma glucose	Smoking	High body-mass index	High LDL	Unsafe water	Alcohol use	Kidney dysfunction
Andhra Pradesh	High blood pressure	Particulate matter	High fasting plasma glucose	Low birth weight & short gestation	Smoking	High body-mass index	High LDL	Unsafe water	Kidney dysfunction	Alcohol use
Arunachal Pradesh	Low birth weight & short gestation	Particulate matter	Smoking	Alcohol use	High blood pressure	High fasting plasma glucose	Child growth failure	High body-mass index	Unsafe water	Iron deficiency
Assam	Low birth weight & short gestation	Particulate matter	High blood pressure	Smoking	High fasting plasma glucose	Alcohol use	High body-mass index	Unsafe water	Child growth failure	Kidney dysfunction
Bihar	Low birth weight & short gestation	Particulate matter	Unsafe water	High blood pressure	High fasting plasma glucose	Child growth failure	Unsafe sanitation	Smoking	Iron deficiency	Handwashing
Chhattisgarh	Particulate matter	Low birth weight & short gestation	High blood pressure	High fasting plasma glucose	Alcohol use	Smoking	Unsafe water	High body-mass index	High LDL	Kidney dysfunction
Delhi	Particulate matter	High fasting plasma glucose	High blood pressure	High body-mass index	Low birth weight & short gestation	Smoking	High LDL	Alcohol use	Kidney dysfunction	Iron deficiency
Goa	High fasting plasma glucose	High blood pressure	High body-mass index	Particulate matter	High LDL	Alcohol use	Kidney dysfunction	Low birth weight & short gestation	Smoking	Low whole grains
Gujarat	Particulate matter	High blood pressure	Low birth weight & short gestation	High fasting plasma glucose	High body-mass index	Smoking	High LDL	Kidney dysfunction	Iron deficiency	Alcohol use
Haryana	Particulate matter	High blood pressure	Low birth weight & short gestation	Smoking	High fasting plasma glucose	High body-mass index	High LDL	Alcohol use	Iron deficiency	Kidney dysfunction
Himachal Pradesh	Particulate matter	Smoking	High blood pressure	High fasting plasma glucose	Low birth weight & short gestation	High body-mass index	High LDL	Alcohol use	Kidney dysfunction	Low temperature
Jammu & Kashmir and Ladakh	Particulate matter	Smoking	High blood pressure	Low birth weight & short gestation	High fasting plasma glucose	High body-mass index	High LDL	Kidney dysfunction	Low temperature	Iron deficiency
Jharkhand	Low birth weight & short gestation	Particulate matter	High blood pressure	High fasting plasma glucose	Unsafe water	Iron deficiency	Unsafe sanitation	Child growth failure	High body-mass index	Alcohol use
Karnataka	High blood pressure	Particulate matter	High fasting plasma glucose	Smoking	High body-mass index	Low birth weight & short gestation	High LDL	Alcohol use	Kidney dysfunction	Unsafe water

Appendix Figure 3. Leading 10 level 3 risk factors for DALYs in 2019 by location. Each risk is colored by the percentage change in all-age DALYs from 2010 to 2019. DALYs=disability-adjusted life-years.

Percentage change in all-age DALYs from 2010 to 2019

-0.828 to -0.164	-0.164 to -0.066	-0.066 to -0.010	-0.010 to 0.029	0.029 to 0.073	0.073 to 0.120	0.120 to 0.173	0.173 to 0.235	0.235 to 0.325	0.325 to 1.699

	1	2	3	4	5	6	7	8	9	10
Kerala	High blood pressure	High fasting plasma glucose	Particulate matter	High body-mass index	High LDL	Smoking	Kidney dysfunction	Alcohol use	Low birth weight & short gestation	Low fruit
Madhya Pradesh	Particulate matter	Low birth weight & short gestation	High blood pressure	Smoking	High fasting plasma glucose	Child growth failure	Unsafe water	Iron deficiency	High body-mass index	High LDL
Maharashtra	Particulate matter	High blood pressure	High fasting plasma glucose	Low birth weight & short gestation	High body-mass index	High LDL	Alcohol use	Smoking	Kidney dysfunction	Low fruit
Manipur	Particulate matter	High fasting plasma glucose	High blood pressure	Smoking	Low birth weight & short gestation	Alcohol use	High body-mass index	Kidney dysfunction	Unsafe sex	High LDL
Meghalaya	Low birth weight & short gestation	Particulate matter	Smoking	Alcohol use	High blood pressure	High fasting plasma glucose	Child growth failure	Iron deficiency	High body-mass index	Unsafe water
Mizoram	Smoking	Low birth weight & short gestation	Particulate matter	High fasting plasma glucose	Unsafe sex	Alcohol use	High blood pressure	High body-mass index	Child growth failure	Second-hand smoke
Nagaland	Low birth weight & short gestation	Particulate matter	High blood pressure	High fasting plasma glucose	Smoking	Alcohol use	Unsafe sex	Child growth failure	High body-mass index	Kidney dysfunction
Odisha	Particulate matter	Low birth weight & short gestation	High blood pressure	High fasting plasma glucose	Unsafe water	Alcohol use	High body-mass index	Unsafe sanitation	Smoking	Kidney dysfunction
Punjab	High blood pressure	High fasting plasma glucose	Particulate matter	High body-mass index	High LDL	Low birth weight & short gestation	Kidney dysfunction	Low whole grains	Alcohol use	Smoking
Rajasthan	Particulate matter	Low birth weight & short gestation	Smoking	High blood pressure	High fasting plasma glucose	Child growth failure	High LDL	Iron deficiency	High body-mass index	Unsafe water
Sikkim	High blood pressure	Particulate matter	High fasting plasma glucose	Smoking	High body-mass index	Alcohol use	Low birth weight & short gestation	Kidney dysfunction	High LDL	Low temperature
Tamil Nadu	High fasting plasma glucose	High blood pressure	High body-mass index	Particulate matter	High LDL	Kidney dysfunction	Smoking	Low birth weight & short gestation	Alcohol use	Low fruit
Telangana	Particulate matter	High blood pressure	High fasting plasma glucose	Low birth weight & short gestation	High body-mass index	Smoking	High LDL	Alcohol use	Iron deficiency	Unsafe water
Tripura	Particulate matter	High blood pressure	Smoking	Low birth weight & short gestation	High fasting plasma glucose	Alcohol use	High body-mass index	Kidney dysfunction	High LDL	Iron deficiency
Other Union Territories	High fasting plasma glucose	High blood pressure	Particulate matter	High body-mass index	Low birth weight & short gestation	Alcohol use	High LDL	Smoking	Kidney dysfunction	Iron deficiency
Uttar Pradesh	Low birth weight & short gestation	Particulate matter	Smoking	High fasting plasma glucose	Unsafe water	High blood pressure	Child growth failure	Unsafe sanitation	Alcohol use	High body-mass index
Uttarakhand	Particulate matter	Smoking	High blood pressure	High fasting plasma glucose	Low birth weight & short gestation	High body-mass index	Alcohol use	High LDL	Kidney dysfunction	Iron deficiency
West Bengal	Particulate matter	High blood pressure	Smoking	High fasting plasma glucose	Low birth weight & short gestation	High body-mass index	High LDL	Kidney dysfunction	Alcohol use	Iron deficiency
Nepal	Particulate matter	Low birth weight & short gestation	Smoking	High blood pressure	High fasting plasma glucose	High body-mass index	Alcohol use	Kidney dysfunction	Child growth failure	High LDL
Pakistan	Low birth weight & short gestation	Particulate matter	High blood pressure	Child growth failure	High fasting plasma glucose	Smoking	Unsafe water	High body-mass index	High LDL	Kidney dysfunction
**Southeast Asia, east Asia, and Oceania**	High blood pressure	Smoking	Particulate matter	High fasting plasma glucose	High body-mass index	High LDL	High sodium	Alcohol use	Kidney dysfunction	Second-hand smoke
**East Asia**	Smoking	High blood pressure	Particulate matter	High fasting plasma glucose	High body-mass index	High sodium	High LDL	Alcohol use	Kidney dysfunction	Second-hand smoke
China	Smoking	High blood pressure	Particulate matter	High fasting plasma glucose	High body-mass index	High sodium	High LDL	Alcohol use	Kidney dysfunction	Second-hand smoke
North Korea	Particulate matter	Smoking	High blood pressure	High fasting plasma glucose	High LDL	High sodium	Kidney dysfunction	Alcohol use	Low temperature	High body-mass index
Taiwan (province of China)	High fasting plasma glucose	Smoking	High blood pressure	High body-mass index	Alcohol use	Kidney dysfunction	Particulate matter	High LDL	High red meat	Drug use
**Oceania**	Particulate matter	High fasting plasma glucose	Child growth failure	Low birth weight & short gestation	High body-mass index	High blood pressure	Smoking	High LDL	Unsafe sex	Unsafe water
American Samoa	High body-mass index	High fasting plasma glucose	High blood pressure	Smoking	Kidney dysfunction	High LDL	Second-hand smoke	Low whole grains	Particulate matter	Low birth weight & short gestation
Cook Islands	High body-mass index	High fasting plasma glucose	High blood pressure	Smoking	Kidney dysfunction	High LDL	Alcohol use	Second-hand smoke	Low whole grains	High sodium
Fiji	High fasting plasma glucose	High body-mass index	High blood pressure	Smoking	High LDL	Particulate matter	Kidney dysfunction	Low whole grains	Low birth weight & short gestation	Low fruit
Guam	High body-mass index	High blood pressure	High fasting plasma glucose	Smoking	High LDL	Kidney dysfunction	Low birth weight & short gestation	Low whole grains	Second-hand smoke	Particulate matter
Kiribati	High fasting plasma glucose	High body-mass index	Smoking	High blood pressure	Particulate matter	High LDL	Low birth weight & short gestation	Kidney dysfunction	Second-hand smoke	Child growth failure
Marshall Islands	High fasting plasma glucose	High body-mass index	High blood pressure	Smoking	High LDL	Particulate matter	Kidney dysfunction	Low birth weight & short gestation	Low whole grains	Second-hand smoke

Appendix Figure 3. Leading 10 level 3 risk factors for DALYs in 2019 by location. Each risk is colored by the percentage change in all-age DALYs from 2010 to 2019. DALYs=disability-adjusted life-years.

Percentage change in all-age DALYs from 2010 to 2019

-0.828 to -0.164	-0.164 to -0.066	-0.066 to -0.010	-0.010 to 0.029	0.029 to 0.073	0.073 to 0.120	0.120 to 0.173	0.173 to 0.235	0.235 to 0.325	0.325 to 1.699

	1	2	3	4	5	6	7	8	9	10
Federated States of Micronesia	High body-mass index	High fasting plasma glucose	Smoking	High blood pressure	High LDL	Kidney dysfunction	Particulate matter	Unsafe sex	Low whole grains	Second-hand smoke
Nauru	High body-mass index	High blood pressure	High fasting plasma glucose	Smoking	High LDL	Kidney dysfunction	Low birth weight & short gestation	Alcohol use	Low whole grains	Second-hand smoke
Niue	High fasting plasma glucose	High body-mass index	High blood pressure	Smoking	Kidney dysfunction	High LDL	Second-hand smoke	Low whole grains	High sodium	Particulate matter
Northern Mariana Islands	High body-mass index	High fasting plasma glucose	High blood pressure	Smoking	Kidney dysfunction	High LDL	Particulate matter	Low whole grains	Second-hand smoke	Alcohol use
Palau	High fasting plasma glucose	High body-mass index	High blood pressure	Smoking	High LDL	Kidney dysfunction	Second-hand smoke	Low whole grains	Unsafe sex	High red meat
Papua New Guinea	Particulate matter	Child growth failure	Low birth weight & short gestation	High fasting plasma glucose	Smoking	High blood pressure	High body-mass index	Unsafe sex	Unsafe water	Unsafe sanitation
Samoa	High body-mass index	High fasting plasma glucose	High blood pressure	Smoking	Particulate matter	Kidney dysfunction	High LDL	Second-hand smoke	Low whole grains	Low birth weight & short gestation
Solomon Islands	Particulate matter	High fasting plasma glucose	High body-mass index	Smoking	High blood pressure	High LDL	Kidney dysfunction	Low birth weight & short gestation	Second-hand smoke	Low whole grains
Tokelau	High fasting plasma glucose	High body-mass index	High blood pressure	Smoking	High LDL	Kidney dysfunction	Second-hand smoke	Low whole grains	High sodium	Low fruit
Tonga	High fasting plasma glucose	High body-mass index	High blood pressure	Smoking	Particulate matter	Kidney dysfunction	High LDL	Low birth weight & short gestation	Second-hand smoke	Low whole grains
Tuvalu	High fasting plasma glucose	High body-mass index	High blood pressure	Smoking	High LDL	Kidney dysfunction	Second-hand smoke	Low whole grains	Particulate matter	Low fruit
Vanuatu	High blood pressure	Particulate matter	High fasting plasma glucose	High body-mass index	Smoking	High LDL	Low birth weight & short gestation	Kidney dysfunction	Low whole grains	Child growth failure
**Southeast Asia**	High blood pressure	High fasting plasma glucose	Smoking	Particulate matter	High body-mass index	Kidney dysfunction	Low birth weight & short gestation	High LDL	Alcohol use	High sodium
Cambodia	Particulate matter	Smoking	Low birth weight & short gestation	High fasting plasma glucose	Alcohol use	High blood pressure	Child growth failure	High body-mass index	Kidney dysfunction	High LDL
Indonesia	High blood pressure	High fasting plasma glucose	Smoking	High body-mass index	Particulate matter	Kidney dysfunction	High LDL	Low birth weight & short gestation	High sodium	Child growth failure
Aceh	High blood pressure	High body-mass index	High fasting plasma glucose	Smoking	Particulate matter	Low birth weight & short gestation	Kidney dysfunction	High LDL	Child growth failure	High sodium
Bali	High blood pressure	High fasting plasma glucose	High body-mass index	Smoking	Particulate matter	Kidney dysfunction	High LDL	High sodium	Occupational injury	Second-hand smoke
Bangka-Belitung Islands	High blood pressure	High body-mass index	High fasting plasma glucose	Smoking	Particulate matter	Kidney dysfunction	High LDL	Low birth weight & short gestation	High sodium	Second-hand smoke
Banten	High blood pressure	Smoking	High fasting plasma glucose	High body-mass index	Particulate matter	Low birth weight & short gestation	Kidney dysfunction	High LDL	Child growth failure	High sodium
Bengkulu	High blood pressure	High fasting plasma glucose	High body-mass index	Smoking	Particulate matter	Low birth weight & short gestation	Kidney dysfunction	High LDL	High sodium	Child growth failure
Gorontalo	High blood pressure	High fasting plasma glucose	Particulate matter	High body-mass index	Smoking	Low birth weight & short gestation	Child growth failure	Kidney dysfunction	Unsafe water	Second-hand smoke
Jakarta	High blood pressure	High body-mass index	High fasting plasma glucose	Smoking	Particulate matter	High LDL	Kidney dysfunction	Low birth weight & short gestation	High sodium	Second-hand smoke
Jambi	High blood pressure	High body-mass index	Smoking	High fasting plasma glucose	Particulate matter	Kidney dysfunction	Low birth weight & short gestation	High LDL	High sodium	Child growth failure
West Java	High blood pressure	High fasting plasma glucose	Smoking	High body-mass index	Particulate matter	High LDL	Low birth weight & short gestation	Kidney dysfunction	High sodium	Child growth failure
Central Java	High blood pressure	Smoking	High fasting plasma glucose	Particulate matter	High body-mass index	Kidney dysfunction	High LDL	High sodium	Low birth weight & short gestation	Low fruit
East Java	High blood pressure	Smoking	High fasting plasma glucose	High body-mass index	Particulate matter	Kidney dysfunction	High LDL	High sodium	Low birth weight & short gestation	Second-hand smoke
West Kalimantan	High blood pressure	High fasting plasma glucose	Smoking	High body-mass index	Particulate matter	Kidney dysfunction	Low birth weight & short gestation	High LDL	High sodium	Second-hand smoke
South Kalimantan	High blood pressure	High fasting plasma glucose	High body-mass index	Smoking	Particulate matter	High LDL	Kidney dysfunction	Low birth weight & short gestation	High sodium	Child growth failure
Central Kalimantan	High blood pressure	High body-mass index	High fasting plasma glucose	Smoking	Particulate matter	High LDL	Low birth weight & short gestation	Kidney dysfunction	High sodium	Second-hand smoke
East Kalimantan	High body-mass index	High blood pressure	Smoking	High fasting plasma glucose	Particulate matter	Kidney dysfunction	High LDL	Low birth weight & short gestation	High sodium	Second-hand smoke
North Kalimantan	High body-mass index	High fasting plasma glucose	Smoking	Low birth weight & short gestation	High blood pressure	Particulate matter	Kidney dysfunction	Child growth failure	Occupational injury	Unsafe sex
Riau Islands	High body-mass index	High blood pressure	High fasting plasma glucose	Smoking	Low birth weight & short gestation	Particulate matter	Kidney dysfunction	High LDL	Child growth failure	Unsafe water

Appendix Figure 3. Leading 10 level 3 risk factors for DALYs in 2019 by location. Each risk is colored by the percentage change in all-age DALYs from 2010 to 2019. DALYs=disability-adjusted life-years.

	Percentage change in all-age DALYs from 2010 to 2019									
	-0.828 to -0.164	-0.164 to -0.066	-0.066 to -0.010	-0.010 to 0.029	0.029 to 0.073	0.073 to 0.120	0.120 to 0.173	0.173 to 0.235	0.235 to 0.325	0.325 to 1.699
	**1**	**2**	**3**	**4**	**5**	**6**	**7**	**8**	**9**	**10**
Lampung	High blood pressure	Smoking	High fasting plasma glucose	Particulate matter	High body-mass index	Low birth weight & short gestation	Kidney dysfunction	High LDL	Child growth failure	High sodium
Maluku	High blood pressure	High fasting plasma glucose	Particulate matter	Smoking	High body-mass index	Low birth weight & short gestation	Child growth failure	Unsafe water	Kidney dysfunction	High LDL
North Maluku	High blood pressure	High fasting plasma glucose	Particulate matter	High body-mass index	Smoking	Low birth weight & short gestation	Child growth failure	Kidney dysfunction	High LDL	Second-hand smoke
West Nusa Tenggara	High blood pressure	High fasting plasma glucose	Smoking	Particulate matter	High body-mass index	Low birth weight & short gestation	Kidney dysfunction	High LDL	High sodium	Unsafe water
East Nusa Tenggara	High blood pressure	Particulate matter	High fasting plasma glucose	Smoking	Low birth weight & short gestation	High body-mass index	Kidney dysfunction	Child growth failure	Unsafe water	High LDL
Papua	Low birth weight & short gestation	High body-mass index	High blood pressure	Particulate matter	High fasting plasma glucose	Smoking	Child growth failure	Unsafe water	Kidney dysfunction	High LDL
West Papua	High blood pressure	High body-mass index	High fasting plasma glucose	Low birth weight & short gestation	Smoking	Particulate matter	Kidney dysfunction	High LDL	Child growth failure	Second-hand smoke
Riau	High body-mass index	High blood pressure	Smoking	High fasting plasma glucose	Low birth weight & short gestation	Particulate matter	Kidney dysfunction	High LDL	Child growth failure	Unsafe water
West Sulawesi	High blood pressure	Particulate matter	High fasting plasma glucose	High body-mass index	Smoking	Low birth weight & short gestation	Child growth failure	Kidney dysfunction	High LDL	Unsafe water
South Sulawesi	High blood pressure	High fasting plasma glucose	Smoking	High body-mass index	Particulate matter	Kidney dysfunction	Low birth weight & short gestation	High LDL	High sodium	Second-hand smoke
Central Sulawesi	High blood pressure	High body-mass index	High fasting plasma glucose	Smoking	Particulate matter	Low birth weight & short gestation	Kidney dysfunction	High LDL	Child growth failure	Second-hand smoke
Southeast Sulawesi	High blood pressure	High fasting plasma glucose	High body-mass index	Particulate matter	Smoking	Low birth weight & short gestation	Child growth failure	High LDL	Kidney dysfunction	Unsafe water
North Sulawesi	High blood pressure	High fasting plasma glucose	High body-mass index	Smoking	Particulate matter	Kidney dysfunction	High LDL	High sodium	Low birth weight & short gestation	Second-hand smoke
West Sumatra	High blood pressure	Smoking	High fasting plasma glucose	High body-mass index	Particulate matter	Kidney dysfunction	High LDL	High sodium	Low birth weight & short gestation	Second-hand smoke
South Sumatra	High blood pressure	High body-mass index	Smoking	High fasting plasma glucose	Particulate matter	Low birth weight & short gestation	Kidney dysfunction	High LDL	Child growth failure	High sodium
North Sumatra	High blood pressure	High body-mass index	High fasting plasma glucose	Smoking	Particulate matter	Low birth weight & short gestation	Kidney dysfunction	High LDL	Child growth failure	High sodium
Yogyakarta	High blood pressure	High fasting plasma glucose	Smoking	High body-mass index	Particulate matter	Kidney dysfunction	High LDL	High sodium	Second-hand smoke	Low fruit
Laos	Particulate matter	Low birth weight & short gestation	High blood pressure	High fasting plasma glucose	Smoking	Child growth failure	High body-mass index	Alcohol use	Kidney dysfunction	High LDL
Malaysia	High blood pressure	High body-mass index	High fasting plasma glucose	Smoking	High LDL	Kidney dysfunction	Particulate matter	High sodium	Low whole grains	Second-hand smoke
Maldives	High blood pressure	High fasting plasma glucose	Smoking	Low birth weight & short gestation	High body-mass index	Kidney dysfunction	High LDL	Particulate matter	Occupational injury	Low whole grains
Mauritius	High fasting plasma glucose	High body-mass index	High blood pressure	Kidney dysfunction	Smoking	High LDL	Particulate matter	Alcohol use	High sodium	Second-hand smoke
Myanmar	Particulate matter	High blood pressure	High fasting plasma glucose	Smoking	Low birth weight & short gestation	High body-mass index	Alcohol use	Kidney dysfunction	High LDL	Child growth failure
Philippines	High blood pressure	Smoking	Particulate matter	High fasting plasma glucose	High body-mass index	Low birth weight & short gestation	Kidney dysfunction	Alcohol use	High LDL	Child growth failure
Seychelles	High blood pressure	High fasting plasma glucose	High body-mass index	Smoking	Alcohol use	Kidney dysfunction	High LDL	Particulate matter	Low birth weight & short gestation	High sodium
Sri Lanka	High fasting plasma glucose	High blood pressure	High body-mass index	Particulate matter	Smoking	High LDL	Kidney dysfunction	Alcohol use	High sodium	Low fruit
Thailand	High fasting plasma glucose	Smoking	High body-mass index	High blood pressure	Alcohol use	Particulate matter	Kidney dysfunction	High LDL	Unsafe sex	Drug use
Timor-Leste	Low birth weight & short gestation	Particulate matter	High blood pressure	High fasting plasma glucose	Smoking	Child growth failure	Kidney dysfunction	High LDL	Alcohol use	Unsafe water
Vietnam	High blood pressure	Smoking	High fasting plasma glucose	Particulate matter	Alcohol use	High body-mass index	Kidney dysfunction	High LDL	High sodium	Low fibre
**Sub-Saharan Africa**	Low birth weight & short gestation	Child growth failure	Particulate matter	Unsafe water	Unsafe sex	Unsafe sanitation	Handwashing	High blood pressure	High fasting plasma glucose	High body-mass index
**Central sub-Saharan Africa**	Low birth weight & short gestation	Particulate matter	Child growth failure	Unsafe water	Unsafe sanitation	High blood pressure	Unsafe sex	Handwashing	High fasting plasma glucose	Alcohol use
Angola	Low birth weight & short gestation	Child growth failure	Unsafe sex	Unsafe water	Particulate matter	Alcohol use	Unsafe sanitation	High blood pressure	Handwashing	High fasting plasma glucose
Central African Republic	Child growth failure	Unsafe water	Low birth weight & short gestation	Particulate matter	Unsafe sanitation	Handwashing	Unsafe sex	High blood pressure	Suboptimal breastfeeding	High fasting plasma glucose

Appendix Figure 3. Leading 10 level 3 risk factors for DALYs in 2019 by location. Each risk is colored by the percentage change in all-age DALYs from 2010 to 2019. DALYs=disability-adjusted life-years.

Percentage change in all-age DALYs from 2010 to 2019

-0.828 to -0.164	-0.164 to -0.066	-0.066 to -0.010	-0.010 to 0.029	0.029 to 0.073	0.073 to 0.120	0.120 to 0.173	0.173 to 0.235	0.235 to 0.325	0.325 to 1.699

	1	2	3	4	5	6	7	8	9	10
Congo (Brazzaville)	Unsafe sex	Low birth weight & short gestation	Particulate matter	High blood pressure	High body-mass index	Unsafe water	High fasting plasma glucose	Child growth failure	Alcohol use	Unsafe sanitation
DR Congo	Low birth weight & short gestation	Particulate matter	Child growth failure	Unsafe water	High blood pressure	Unsafe sanitation	Handwashing	High fasting plasma glucose	High body-mass index	Alcohol use
Equatorial Guinea	Unsafe sex	Low birth weight & short gestation	Particulate matter	High body-mass index	High blood pressure	High fasting plasma glucose	Intimate partner violence	Alcohol use	Child growth failure	Unsafe water
Gabon	Unsafe sex	Low birth weight & short gestation	High blood pressure	High body-mass index	High fasting plasma glucose	Alcohol use	Particulate matter	Smoking	Kidney dysfunction	Iron deficiency
**Eastern sub-Saharan Africa**	Low birth weight & short gestation	Particulate matter	Child growth failure	Unsafe sex	Unsafe water	Unsafe sanitation	Handwashing	High blood pressure	Alcohol use	High fasting plasma glucose
Burundi	Low birth weight & short gestation	Child growth failure	Unsafe water	Particulate matter	Unsafe sanitation	Handwashing	Alcohol use	High blood pressure	Unsafe sex	High fasting plasma glucose
Comoros	Low birth weight & short gestation	Particulate matter	High blood pressure	Child growth failure	Unsafe water	High body-mass index	High fasting plasma glucose	Unsafe sanitation	Handwashing	Smoking
Djibouti	Low birth weight & short gestation	Unsafe sex	Child growth failure	Particulate matter	High blood pressure	Unsafe water	Smoking	High fasting plasma glucose	High body-mass index	Handwashing
Eritrea	Child growth failure	Low birth weight & short gestation	Particulate matter	Unsafe water	Unsafe sanitation	Handwashing	High blood pressure	Unsafe sex	High fasting plasma glucose	Alcohol use
Ethiopia	Low birth weight & short gestation	Particulate matter	Child growth failure	Unsafe water	Unsafe sanitation	Handwashing	Unsafe sex	High blood pressure	Alcohol use	High fasting plasma glucose
Kenya	Unsafe sex	Low birth weight & short gestation	Particulate matter	Child growth failure	Unsafe water	Unsafe sanitation	High blood pressure	Alcohol use	Handwashing	High body-mass index
Baringo	Low birth weight & short gestation	Unsafe water	Child growth failure	Particulate matter	Unsafe sanitation	Handwashing	High blood pressure	Alcohol use	Unsafe sex	High body-mass index
Bomet	Unsafe sex	Low birth weight & short gestation	Particulate matter	Unsafe water	Drug use	High blood pressure	Unsafe sanitation	Alcohol use	Intimate partner violence	Handwashing
Bungoma	Low birth weight & short gestation	Particulate matter	Child growth failure	Unsafe water	Unsafe sanitation	High blood pressure	Handwashing	Unsafe sex	Alcohol use	High body-mass index
Busia	Low birth weight & short gestation	Particulate matter	Unsafe sex	Child growth failure	Unsafe water	Unsafe sanitation	Handwashing	High blood pressure	Alcohol use	High body-mass index
Elgeyo Marakwet	Particulate matter	Low birth weight & short gestation	High blood pressure	Unsafe sex	Unsafe water	Alcohol use	High body-mass index	Unsafe sanitation	High fasting plasma glucose	Child growth failure
Embu	Unsafe sex	Low birth weight & short gestation	Particulate matter	High blood pressure	Unsafe water	High body-mass index	Alcohol use	Child growth failure	Unsafe sanitation	High fasting plasma glucose
Garissa	Low birth weight & short gestation	Particulate matter	Unsafe water	Child growth failure	Unsafe sanitation	Unsafe sex	Iron deficiency	Handwashing	High blood pressure	Alcohol use
Homa Bay	Unsafe sex	Child growth failure	Low birth weight & short gestation	Unsafe water	Particulate matter	Unsafe sanitation	Intimate partner violence	Handwashing	Drug use	High blood pressure
Isiolo	Low birth weight & short gestation	Child growth failure	Particulate matter	Unsafe sex	Unsafe water	Handwashing	Unsafe sanitation	High blood pressure	Alcohol use	High body-mass index
Kajiado	Unsafe sex	Low birth weight & short gestation	High blood pressure	High body-mass index	Particulate matter	Alcohol use	Unsafe water	High fasting plasma glucose	Child growth failure	Unsafe sanitation
Kakamega	Unsafe sex	Low birth weight & short gestation	Child growth failure	Particulate matter	Unsafe water	Unsafe sanitation	Handwashing	High blood pressure	Alcohol use	High body-mass index
Kericho	Low birth weight & short gestation	Unsafe sex	Particulate matter	Unsafe water	High blood pressure	Unsafe sanitation	Child growth failure	Alcohol use	High body-mass index	Handwashing
Kiambu	Unsafe sex	Low birth weight & short gestation	High blood pressure	High body-mass index	Alcohol use	Particulate matter	High fasting plasma glucose	Smoking	Child growth failure	Unsafe water
Kilifi	Low birth weight & short gestation	Unsafe sex	Particulate matter	Child growth failure	Unsafe water	Unsafe sanitation	Handwashing	High blood pressure	Alcohol use	High body-mass index
Kirinyaga	Unsafe sex	Low birth weight & short gestation	Particulate matter	High blood pressure	High body-mass index	Alcohol use	High fasting plasma glucose	Unsafe water	Child growth failure	Smoking
Kisii	Unsafe sex	Particulate matter	Low birth weight & short gestation	High blood pressure	Unsafe water	High body-mass index	Alcohol use	Unsafe sanitation	High fasting plasma glucose	Handwashing
Kisumu	Unsafe sex	Particulate matter	High blood pressure	Child growth failure	Low birth weight & short gestation	High body-mass index	Alcohol use	High fasting plasma glucose	Intimate partner violence	Smoking
Kitui	Low birth weight & short gestation	Particulate matter	Unsafe sex	Unsafe water	High blood pressure	Unsafe sanitation	Child growth failure	Alcohol use	Handwashing	High body-mass index
Kwale	Low birth weight & short gestation	Particulate matter	Unsafe sex	Child growth failure	Unsafe water	High blood pressure	Alcohol use	Handwashing	Unsafe sanitation	High body-mass index
Laikipia	Unsafe sex	Particulate matter	High blood pressure	Low birth weight & short gestation	High body-mass index	Alcohol use	Smoking	High fasting plasma glucose	Unsafe water	Intimate partner violence
Lamu	Low birth weight & short gestation	Particulate matter	Unsafe sex	Child growth failure	High blood pressure	Alcohol use	Unsafe water	Handwashing	High body-mass index	High fasting plasma glucose

Appendix Figure 3. Leading 10 level 3 risk factors for DALYs in 2019 by location. Each risk is colored by the percentage change in all-age DALYs from 2010 to 2019. DALYs=disability-adjusted life-years.

Percentage change in all-age DALYs from 2010 to 2019

	1	2	3	4	5	6	7	8	9	10
	-0.828 to -0.164	-0.164 to -0.066	-0.066 to -0.010	-0.010 to 0.029	0.029 to 0.073	0.073 to 0.120	0.120 to 0.173	0.173 to 0.235	0.235 to 0.325	0.325 to 1.699
Machakos	Unsafe sex	Low birth weight & short gestation	Particulate matter	High blood pressure	Unsafe water	High body-mass index	Alcohol use	High fasting plasma glucose	Unsafe sanitation	Handwashing
Makueni	Unsafe sex	Low birth weight & short gestation	Particulate matter	Unsafe water	High blood pressure	Intimate partner violence	Unsafe sanitation	Drug use	Alcohol use	Handwashing
Mandera	Child growth failure	Low birth weight & short gestation	Particulate matter	Unsafe water	Unsafe sex	Unsafe sanitation	Handwashing	Iron deficiency	High blood pressure	Alcohol use
Marsabit	Low birth weight & short gestation	Particulate matter	Child growth failure	Unsafe sex	Unsafe water	Unsafe sanitation	Handwashing	High blood pressure	Alcohol use	High fasting plasma glucose
Meru	Unsafe sex	Low birth weight & short gestation	Particulate matter	Unsafe water	High blood pressure	Child growth failure	Alcohol use	Unsafe sanitation	High body-mass index	Handwashing
Migori	Unsafe sex	Child growth failure	Low birth weight & short gestation	Unsafe water	Particulate matter	Unsafe sanitation	Handwashing	Intimate partner violence	Suboptimal breastfeeding	High blood pressure
Mombasa	Unsafe sex	Low birth weight & short gestation	Particulate matter	Alcohol use	High blood pressure	High body-mass index	Child growth failure	Iron deficiency	Drug use	Unsafe water
Murang'a	Unsafe sex	Particulate matter	High blood pressure	Low birth weight & short gestation	High body-mass index	Alcohol use	High fasting plasma glucose	Unsafe water	Smoking	Handwashing
Nairobi	Unsafe sex	Low birth weight & short gestation	High body-mass index	Alcohol use	High blood pressure	High fasting plasma glucose	Smoking	Particulate matter	Child growth failure	Kidney dysfunction
Nakuru	Unsafe sex	Low birth weight & short gestation	Particulate matter	High blood pressure	Child growth failure	Unsafe water	Alcohol use	High body-mass index	Unsafe sanitation	Handwashing
Nandi	Unsafe sex	Particulate matter	Low birth weight & short gestation	High blood pressure	Alcohol use	Unsafe water	High body-mass index	High fasting plasma glucose	Unsafe sanitation	Handwashing
Narok	Low birth weight & short gestation	Unsafe sex	Particulate matter	Unsafe water	Child growth failure	Unsafe sanitation	Handwashing	Alcohol use	High blood pressure	High body-mass index
Nyamira	Unsafe sex	Particulate matter	Low birth weight & short gestation	High blood pressure	Alcohol use	High body-mass index	High fasting plasma glucose	Unsafe water	Unsafe sanitation	Intimate partner violence
Nyandarua	Unsafe sex	Low birth weight & short gestation	Particulate matter	High blood pressure	Alcohol use	High body-mass index	Child growth failure	High fasting plasma glucose	Unsafe water	Smoking
Nyeri	Unsafe sex	High blood pressure	Particulate matter	High body-mass index	Low birth weight & short gestation	Alcohol use	High fasting plasma glucose	Smoking	Unsafe water	Child growth failure
Samburu	Low birth weight & short gestation	Unsafe sex	Child growth failure	Particulate matter	Unsafe water	Unsafe sanitation	Handwashing	High blood pressure	Alcohol use	Intimate partner violence
Siaya	Unsafe sex	Low birth weight & short gestation	Particulate matter	Child growth failure	Unsafe water	Unsafe sanitation	Intimate partner violence	Handwashing	High blood pressure	Drug use
Taita Taveta	Unsafe sex	High blood pressure	Particulate matter	High body-mass index	Alcohol use	High fasting plasma glucose	Low birth weight & short gestation	Smoking	Unsafe water	Drug use
Tana River	Low birth weight & short gestation	Particulate matter	Unsafe sex	Child growth failure	Unsafe water	Handwashing	High blood pressure	Alcohol use	Unsafe sanitation	High fasting plasma glucose
Tharaka Nithi	Particulate matter	Low birth weight & short gestation	High blood pressure	Alcohol use	Unsafe water	High body-mass index	Child growth failure	Unsafe sex	Unsafe sanitation	High fasting plasma glucose
Trans Nzoia	Unsafe sex	Low birth weight & short gestation	Particulate matter	Unsafe water	High blood pressure	Unsafe sanitation	Alcohol use	Child growth failure	High body-mass index	Handwashing
Turkana	Unsafe sex	Low birth weight & short gestation	Child growth failure	Unsafe water	Particulate matter	Unsafe sanitation	Handwashing	Alcohol use	Drug use	High blood pressure
Uasin Gishu	Unsafe sex	Low birth weight & short gestation	Particulate matter	Unsafe water	High blood pressure	Child growth failure	Alcohol use	Unsafe sanitation	High body-mass index	Handwashing
Vihiga	Unsafe sex	Particulate matter	Low birth weight & short gestation	High blood pressure	Unsafe water	Child growth failure	Unsafe sanitation	Alcohol use	Handwashing	High body-mass index
Wajir	Low birth weight & short gestation	Unsafe water	Particulate matter	Child growth failure	Unsafe sanitation	Handwashing	Suboptimal breastfeeding	Iron deficiency	High blood pressure	Alcohol use
West Pokot	Low birth weight & short gestation	Child growth failure	Unsafe water	Unsafe sanitation	Particulate matter	Handwashing	Suboptimal breastfeeding	Unsafe sex	High blood pressure	Alcohol use
Madagascar	Child growth failure	Low birth weight & short gestation	Unsafe water	Particulate matter	Unsafe sanitation	High blood pressure	Handwashing	High body-mass index	High fasting plasma glucose	Unsafe sex
Malawi	Low birth weight & short gestation	Unsafe sex	Particulate matter	Child growth failure	Unsafe water	Unsafe sanitation	High blood pressure	Handwashing	High fasting plasma glucose	Alcohol use
Mozambique	Unsafe sex	Low birth weight & short gestation	Particulate matter	Child growth failure	High blood pressure	Unsafe water	Handwashing	Unsafe sanitation	High fasting plasma glucose	High body-mass index
Rwanda	Low birth weight & short gestation	Particulate matter	Child growth failure	Alcohol use	Unsafe water	Unsafe sex	High blood pressure	Handwashing	Unsafe sanitation	Smoking
Somalia	Child growth failure	Particulate matter	Low birth weight & short gestation	Unsafe water	Unsafe sanitation	Handwashing	Suboptimal breastfeeding	High blood pressure	High fasting plasma glucose	Vitamin A deficiency
South Sudan	Child growth failure	Low birth weight & short gestation	Particulate matter	Unsafe water	Unsafe sanitation	Handwashing	Unsafe sex	High blood pressure	Suboptimal breastfeeding	High body-mass index

Appendix Figure 3. Leading 10 level 3 risk factors for DALYs in 2019 by location. Each risk is colored by the percentage change in all-age DALYs from 2010 to 2019. DALYs=disability-adjusted life-years.

Percentage change in all-age DALYs from 2010 to 2019: -0.828 to -0.164 | -0.164 to -0.066 | -0.066 to -0.010 | -0.010 to 0.029 | 0.029 to 0.073 | 0.073 to 0.120 | 0.120 to 0.173 | 0.173 to 0.235 | 0.235 to 0.325 | 0.325 to 1.699

	1	2	3	4	5	6	7	8	9	10
Uganda	Low birth weight & short gestation	Particulate matter	Unsafe sex	Child growth failure	Unsafe water	Alcohol use	High blood pressure	Unsafe sanitation	Handwashing	High fasting plasma glucose
Tanzania	Low birth weight & short gestation	Particulate matter	Child growth failure	Unsafe sex	High blood pressure	Alcohol use	Handwashing	Unsafe water	High body-mass index	Smoking
Zambia	Unsafe sex	Low birth weight & short gestation	Particulate matter	Child growth failure	Unsafe water	Alcohol use	Unsafe sanitation	High blood pressure	Handwashing	High body-mass index
**Southern sub-Saharan Africa**	Unsafe sex	Low birth weight & short gestation	High body-mass index	High blood pressure	High fasting plasma glucose	Particulate matter	Alcohol use	Smoking	Child growth failure	Intimate partner violence
Botswana	Unsafe sex	Low birth weight & short gestation	High body-mass index	High blood pressure	High fasting plasma glucose	Particulate matter	Smoking	Alcohol use	Child growth failure	Intimate partner violence
eSwatini	Unsafe sex	High body-mass index	Low birth weight & short gestation	High fasting plasma glucose	Particulate matter	High blood pressure	Alcohol use	Child growth failure	Unsafe water	Unsafe sanitation
Lesotho	Unsafe sex	Low birth weight & short gestation	Particulate matter	Alcohol use	Smoking	High fasting plasma glucose	High body-mass index	High blood pressure	Child growth failure	Unsafe water
Namibia	Unsafe sex	Low birth weight & short gestation	Particulate matter	High blood pressure	Alcohol use	High body-mass index	High fasting plasma glucose	Child growth failure	Unsafe water	Smoking
South Africa	Unsafe sex	High body-mass index	Low birth weight & short gestation	High fasting plasma glucose	High blood pressure	Alcohol use	Smoking	Particulate matter	Intimate partner violence	Child growth failure
Zimbabwe	Unsafe sex	Low birth weight & short gestation	Particulate matter	Child growth failure	High blood pressure	High fasting plasma glucose	Smoking	Alcohol use	High body-mass index	Unsafe water
**Western sub-Saharan Africa**	Child growth failure	Low birth weight & short gestation	Particulate matter	Unsafe water	Unsafe sanitation	Handwashing	Unsafe sex	High blood pressure	Suboptimal breastfeeding	High body-mass index
Benin	Low birth weight & short gestation	Child growth failure	Particulate matter	Unsafe water	Handwashing	Unsafe sanitation	High blood pressure	High body-mass index	High fasting plasma glucose	Unsafe sex
Burkina Faso	Child growth failure	Low birth weight & short gestation	Particulate matter	Unsafe water	Handwashing	Unsafe sanitation	High blood pressure	Alcohol use	Iron deficiency	High fasting plasma glucose
Cape Verde	High blood pressure	Particulate matter	High body-mass index	High fasting plasma glucose	Low birth weight & short gestation	Alcohol use	Smoking	High LDL	Kidney dysfunction	Unsafe sex
Cameroon	Child growth failure	Low birth weight & short gestation	Unsafe water	Unsafe sex	Particulate matter	Unsafe sanitation	Handwashing	High blood pressure	High body-mass index	High fasting plasma glucose
Chad	Child growth failure	Unsafe water	Unsafe sanitation	Low birth weight & short gestation	Particulate matter	Handwashing	Suboptimal breastfeeding	Unsafe sex	High blood pressure	Iron deficiency
Côte d'Ivoire	Low birth weight & short gestation	Particulate matter	Child growth failure	Unsafe sex	High blood pressure	Unsafe water	Handwashing	High body-mass index	Unsafe sanitation	Alcohol use
The Gambia	Low birth weight & short gestation	Particulate matter	Unsafe sex	High blood pressure	Child growth failure	High body-mass index	Unsafe water	Iron deficiency	High fasting plasma glucose	Handwashing
Ghana	Low birth weight & short gestation	Particulate matter	Unsafe sex	High blood pressure	High body-mass index	Child growth failure	High fasting plasma glucose	Alcohol use	Unsafe water	Handwashing
Guinea	Low birth weight & short gestation	Particulate matter	Child growth failure	Unsafe water	Handwashing	Unsafe sanitation	High blood pressure	Unsafe sex	High fasting plasma glucose	High body-mass index
Guinea-Bissau	Low birth weight & short gestation	Particulate matter	Child growth failure	Unsafe water	Unsafe sanitation	Unsafe sex	High blood pressure	Handwashing	High fasting plasma glucose	Alcohol use
Liberia	Low birth weight & short gestation	Particulate matter	Child growth failure	Unsafe water	Unsafe sanitation	Unsafe sex	High blood pressure	Handwashing	High body-mass index	High fasting plasma glucose
Mali	Low birth weight & short gestation	Child growth failure	Particulate matter	Unsafe water	Unsafe sanitation	Handwashing	Iron deficiency	High blood pressure	Unsafe sex	High fasting plasma glucose
Mauritania	Low birth weight & short gestation	Particulate matter	Child growth failure	Unsafe water	Unsafe sanitation	High blood pressure	High body-mass index	Handwashing	High fasting plasma glucose	Iron deficiency
Niger	Child growth failure	Low birth weight & short gestation	Unsafe water	Particulate matter	Unsafe sanitation	Handwashing	Suboptimal breastfeeding	Vitamin A deficiency	High blood pressure	Iron deficiency
Nigeria	Child growth failure	Low birth weight & short gestation	Particulate matter	Unsafe water	Unsafe sanitation	Handwashing	Unsafe sex	Suboptimal breastfeeding	High blood pressure	Iron deficiency
São Tomé and PrÍncipe	High blood pressure	Particulate matter	Low birth weight & short gestation	High body-mass index	High fasting plasma glucose	Alcohol use	Kidney dysfunction	Child growth failure	High LDL	Smoking
Senegal	Low birth weight & short gestation	Particulate matter	Child growth failure	Unsafe water	High blood pressure	Unsafe sanitation	High fasting plasma glucose	High body-mass index	Handwashing	Iron deficiency
Sierra Leone	Low birth weight & short gestation	Particulate matter	Child growth failure	Unsafe water	Handwashing	High blood pressure	Unsafe sex	Unsafe sanitation	Iron deficiency	Alcohol use
Togo	Low birth weight & short gestation	Unsafe water	Child growth failure	Particulate matter	Unsafe sanitation	Handwashing	Unsafe sex	High blood pressure	High body-mass index	High fasting plasma glucose

Supplementary Results Table 1. Alabama age-standardised Summary Exposure Values (SEVs) for most-detailed risk factors and percentage change, by sex, 1990, 2010, and 2019. Data in parentheses are 95% uncertainty intervals.

	Male					Female				
	1990	2010	2019	Percentage change 1990-2010	Percentage change 2010-2019	1990	2010	2019	Percentage change 1990-2010	Percentage change 2010-2019
Unsafe sanitation	8·97 (4·65–15·8)	4·64 (2·04–8·86)	3·95 (1·72–7·38)	-48·2 (-78·4–19)	-15 (-54·5–67·2)	--	--	--	--	--
Ambient particulate matter pollution	14·1 (5·47–27·3)	8·88 (6·06–12·2)	5·15 (2·61–8·01)	-37·2 (-67·6–45·4)	-42 (-58–33·2)	14·1 (5·45–27·3)	8·88 (6·05–12·1)	5·15 (2·61–8·01)	-37·1 (-67·6–44·7)	-42 (-58–33·1)
Household air pollution from solid fuels	0·0473 (0·0121–0·123)	0·024 (0·00434–0·0757)	0·0162 (0·00264–0·0523)	-49·2 (-80·7–20·7)	-32·6 (-66·7–25·9)	0·0624 (0·0169–0·167)	0·0318 (0·00608–0·0977)	0·0215 (0·00377–0·0701)	-49 (-80·6–21·5)	-32·4 (-66·6–26·1)
Ambient ozone pollution	75·6 (34–100)	55·8 (23·6–94·2)	26·4 (9·95–48)	-26·3 (-35·9–5·75)	-52·6 (-65·1–42·6)	75·6 (34–100)	55·8 (23·6–94·2)	26·4 (9·95–48)	-26·3 (-35·9–5·75)	-52·6 (-65·1–42·6)
Residential radon	17·2 (2·77–54·6)	17·2 (2·77–54·6)	17·2 (2·77–54·6)	0 (0–0)	0 (0–0)	17·2 (2·77–54·6)	17·2 (2·77–54·6)	17·2 (2·77–54·6)	0 (0–0)	0 (0–0)
Lead exposure	52·4 (24·4–75·7)	31·8 (11·4–54·2)	22·7 (6·65–42·3)	-39·3 (-56·4–26·7)	-28·6 (-43·8–20·4)	29·7 (2·73–58·3)	19·1 (1·23–43·8)	13·8 (0·534–34·6)	-35·7 (-67·8–22·6)	-27·4 (-58·7–19·1)
Child underweight	0·985 (0·558–1·5)	0·981 (0·535–1·5)	0·961 (0·544–1·47)	-0·348 (-17·3–21)	-2·08 (-14·8–11·2)	0·832 (0·461–1·26)	0·865 (0·465–1·33)	0·887 (0·493–1·39)	4·03 (-17·1–30·5)	2·48 (-11·2–18·1)
Iron deficiency	--	--	--	--	--	12·6 (10·7–14·8)	13·2 (11·1–15·4)	13·4 (11·2–16)	4·25 (-8·19–18·1)	2·05 (-7·3–13)
Vitamin A deficiency	1·11 (0·285–2·89)	1·43 (0·359–3·92)	1·06 (0·274–3·12)	28·5 (-67·4–500)	-26·1 (-66·9–74·9)	2·06 (0·783–4·24)	2·07 (0·795–4·83)	1·85 (0·717–4·35)	0·664 (-63·7–207)	-11 (-50·6–63·8)
Zinc deficiency	2·58 (0–8·16)	1·31 (0–4·24)	1·38 (0–4·42)	-49·1 (-100–NA)	5·26 (-14·6–NA)	2·66 (0–8·25)	1·32 (0–4·46)	1·35 (0–4·24)	-50·3 (-100–NA)	2·34 (-14·6–NA)
Smoking	28 (25·3–31)	21·9 (19·8–24)	20·2 (18·3–22·3)	-22 (-27·4–16·3)	-7·6 (-11·2–4·01)	18·3 (15·8–21)	14·9 (13·3–16·5)	13·9 (12·4–15·6)	-18·7 (-27·4–8·24)	-6·52 (-11–1·69)
Secondhand smoke	38 (35·5–40·7)	27·6 (26–29·3)	27·2 (24·3–30·2)	-27·5 (-33·7–20·7)	-1·26 (-10·1–8·53)	36·6 (34·6–38·7)	26·7 (25·4–28)	26·4 (24·3–28·5)	-26·9 (-32·8–21·1)	-1·42 (-8·15–5·6)
Alcohol use	12·7 (7·94–17·7)	13·5 (8·14–18·9)	14·3 (8·76–20·7)	6·5 (-23·1–42·3)	5·67 (-16·5–34·7)	7·89 (4·67–11·4)	8·93 (5·4–12·6)	8·98 (5·31–13·6)	13·2 (-18–57·6)	0·537 (-21·5–25·8)
Drug use	0·504 (0·286–0·888)	0·995 (0·71–1·4)	1·52 (1·14–2·01)	97·5 (42–183)	52·5 (31·1–78·7)	0·369 (0·23–0·58)	0·749 (0·558–1·03)	1·25 (0·964–1·62)	103 (57·9–163)	67 (41·5–96)
High fasting plasma glucose	14·1 (11·7–17)	20·2 (17·8–23)	21·3 (18·5–24·2)	43·1 (17·8–72)	5·4 (-7·83–20·5)	12·2 (9·95–15·1)	17 (15·1–19·3)	18·9 (16·5–22·1)	38·8 (14·2–69·4)	11·5 (-1·56–28·6)
High systolic blood pressure	26 (20·7–31·5)	17·4 (15·4–19·6)	20·8 (16–25·9)	-32·9 (-45·3–17·6)	19·3 (-5·16–48·4)	24·4 (18·1–31·3)	14·7 (13–16·3)	19·7 (14·4–25·9)	-39·8 (-53·8–18)	34·1 (-1·2–77·4)
High body-mass index	24·7 (17·1–34·3)	41·5 (30·3–53)	41·6 (30·9–52·7)	68·3 (47·8–101)	0·19 (-6·23–7)	30·8 (22·6–39·4)	46·5 (35·8–55·8)	46·4 (36·1–55·9)	51·1 (32·6–75·6)	-0·227 (-6·38–7·26)
Low bone mineral density	12·4 (7·34–19·6)	13·4 (8·04–20·8)	14·2 (8·5–21·1)	8·5 (-17·5–41·8)	5·67 (-18·6–35·7)	15·6 (9·88–22·6)	16·4 (10·6–23·6)	18·9 (12·8–26·1)	5·19 (-16·6–31·6)	15·2 (-7·47–50·9)
Diet low in fruits	55·8 (44·4–68·4)	56·9 (46–69·2)	57·6 (45·5–70·6)	1·86 (-9·37–14)	1·21 (-7·56–10·4)	56·9 (45·1–69·6)	56·3 (45–69·6)	56·8 (44·5–69·8)	-1·09 (-10·6–11·3)	0·909 (-7·68–9·81)
Diet low in vegetables	48·3 (28·6–69·7)	76·2 (60·1–90·1)	74·8 (58·3–89)	57·7 (22·5–128)	-1·92 (-7·1–1·73)	47·8 (28·5–68·5)	76·5 (60·1–89·5)	74·8 (57·7–88·7)	59·9 (27–131)	-2·1 (-7·33–1·47)
Diet low in whole grains	80 (70·6–88·8)	81·9 (73·3–89·8)	82·1 (72·8–89·9)	2·38 (-1·12–7·02)	0·148 (-2·34–2·67)	77·5 (67·1–86·7)	80·1 (71–88·4)	80 (70·4–88·8)	3·44 (-0·485–9·53)	-0·125 (-3·12–2·65)
Diet low in nuts and seeds	31 (9·55–58)	26·5 (8·75–56·6)	22·6 (7·44–54·7)	-14·5 (-45·2–46·9)	-14·7 (-40·7–12·8)	26·5 (6·89–55·7)	23·1 (6·8–54·9)	21 (6·24–54·5)	-12·5 (-45·7–53·3)	-9·3 (-36·5–20·8)
Diet low in milk	51·3 (27·6–72)	46·1 (22·5–66·3)	45·7 (22·5–66·7)	-10·2 (-28·4–1·62)	-0·758 (-11·5–11·4)	51 (28–71·9)	45·9 (21·6–65·4)	46 (22·8–65·7)	-9·9 (-28·5–1·51)	0·184 (-10·8–11·5)
Diet high in red meat	75·7 (61·5–87·1)	73·9 (59·3–85·4)	73·3 (59·1–85·8)	-2·34 (-18·1–14·2)	-0·788 (-12·8–12·1)	76·9 (63·1–88·2)	73·1 (58·3–84·3)	72·6 (57·8–84·9)	-4·91 (-19·5–12·5)	-0·697 (-12·4–12·3)
Diet high in processed meat	77·6 (53·2–97·9)	85·8 (66·3–99·6)	85·7 (66·8–99·6)	10·6 (1·01–29·8)	-0·171 (-4·61–4·85)	78·7 (54·8–97·7)	88·4 (73–99·7)	87 (69·2–99·6)	12·4 (1·5–37·1)	-1·62 (-6·8–2·1)
Diet high in sugar-sweetened beverages	42·7 (25·2–62·9)	58·9 (39·7–76·6)	58·8 (39·8–76·7)	37·9 (-4·33–119)	-0·29 (-22·1–26·8)	46·8 (28·4–67·2)	64·8 (45·9–80·2)	63·1 (44·4–80·1)	38·4 (-1·09–114)	-2·65 (-22·3–22·7)
Diet low in fiber	46·4 (30·7–62·7)	32·4 (20·3–46·2)	30·3 (17·7–44·4)	-30·2 (-45·4–14·9)	-6·55 (-24–12·4)	46·5 (30·2–63·3)	37·7 (23·2–52·1)	33·6 (19·8–49·3)	-18·9 (-33·7–2·9)	-11 (-26·2–4·22)
Diet low in seafood omega-3 fatty acids	85·5 (69–99·9)	81·6 (63·1–99·6)	82·1 (64·5–98·8)	-4·53 (-19·4–8·32)	0·586 (-12·3–18·2)	87·9 (73·6–99·7)	85·3 (69·4–99·6)	85 (70·1–99·8)	-2·96 (-13·6–6·55)	-0·347 (-10·3–10·5)
Diet low in polyunsaturated fatty acids	15·9 (4·61–43·6)	8·06 (2·4–28·7)	7·81 (2·28–27)	-49·3 (-75·4–9·38)	-3·07 (-23·9–19·4)	17·6 (4·97–45·7)	8·27 (2·39–28·1)	7·96 (2·32–27·3)	-53·1 (-76·3–10·1)	-3·79 (-25·5–19·2)
Diet high in trans fatty acids	89·5 (81·5–100)	87·8 (79–100)	87·4 (78·4–100)	-1·86 (-9·95–5·7)	-0·532 (-9·11–7·9)	88·3 (80·9–100)	86·7 (78·3–100)	86·2 (77–100)	-1·83 (-9·54–6·64)	-0·58 (-8·7–7·96)
Diet high in sodium	18·2 (4·51–41·3)	39·8 (11·7–63·6)	38·8 (11·7–64·6)	118 (38·7–422)	-2·33 (-29·1–28·6)	20·9 (4·86–45·5)	21·2 (4·91–44·4)	21·8 (4·94–45·8)	1·34 (-27–47·9)	2·9 (-17·1–28·2)
Low physical activity	2·34 (0·849–4·96)	1·94 (0·713–5·06)	2·13 (0·78–4·59)	-16·8 (-58·5–32·2)	9·49 (-25·9–99·2)	--	--	--	--	--
Occupational asthmagens	23·7 (19·8–28·6)	20·9 (17·5–24·8)	21·7 (17·9–26·1)	-11·9 (-22·6–0·182)	4·11 (-5·14–13·3)	11 (9–13·3)	10·4 (8·63–12·4)	10·7 (8·69–12·9)	-5·39 (-18·7–10·3)	2·9 (-8·94–15)
Occupational particulate matter, gases, and fumes	9·96 (7·1–14·4)	9·34 (6·69–13·5)	8·98 (6·47–13)	-6·17 (-11·4–0·155)	-3·95 (-7·52–0·738)	5·28 (3·67–7·86)	5·12 (3·63–7·34)	4·93 (3·5–7·2)	-2·95 (-12·3–7·54)	-3·79 (-9·29–1·67)
Occupational noise	8·66 (7·84–9·52)	8·29 (7·7–9)	8·01 (7·47–8·67)	-4·3 (-7·86–0·0333)	-3·44 (-5·56–1·28)	5·67 (4·92–6·45)	5·91 (5·36–6·51)	5·88 (5·46–6·43)	4·26 (-2·56–12·9)	-0·457 (-3·63–3·15)
Occupational injuries	--	--	--	--	--	--	--	--	--	--
Occupational ergonomic factors	12·9 (10·5–15·6)	10·8 (9·06–12·9)	11·2 (9·22–13·3)	-16·1 (-27·5–3·53)	3·79 (-4·65–13·8)	7·25 (5·75–9·03)	6·81 (5·53–8·26)	7·17 (5·7–8·97)	-6·02 (-21·8–12·2)	5·29 (-7·09–18·7)
Childhood sexual abuse	7·4 (5·05–10·5)	6·97 (4·6–9·93)	6·85 (4·54–9·67)	-5·83 (-12·2–5·83)	-1·68 (-8·51–5·53)	12·3 (8·94–17·3)	17·4 (12·9–24)	17·8 (13·1–24·7)	41·9 (30·7–54·8)	2·17 (-5–11)
Intimate partner violence	--	--	--	--	--	27·8 (15·2–36·7)	26·2 (14·6–35·2)	27·3 (15–36·2)	-5·75 (-13·8–8·64)	4·3 (-5·2–21·6)
Non-exclusive breastfeeding	26·1 (21·7–30·8)	24·8 (21·3–28·7)	24·8 (20·6–29·9)	-5·11 (-19·3–13·1)	0·247 (-14·2–15·4)	26·1 (21·7–30·8)	24·8 (21·3–28·7)	24·8 (20·6–29·9)	-5·1 (-19·3–13·1)	0·239 (-14·2–15·4)
Discontinued breastfeeding	27·3 (26·4–28·2)	25·3 (24–26·6)	26 (24·8–27·3)	-7·3 (-13–1·56)	2·76 (-3·13–9)	27·4 (26·4–28·3)	25·3 (23·9–26·6)	26 (24·8–27·2)	-7·58 (-13·3–1·85)	2·78 (-3·12–9·03)
Diet low in calcium	18 (8·55–33·5)	15·1 (7·3–27·9)	14·9 (7·04–27·7)	-16·3 (-26·5–4·44)	-1·41 (-10·1–6·44)	13·6 (5·71–26·4)	12·2 (5·4–23·3)	12·4 (5·32–23·5)	-10·4 (-24·2–6·62)	1·32 (-8·02–10·4)
Occupational exposure to asbestos	7·59 (6·81–8·52)	6·69 (6·1–7·29)	5·99 (4·3–7·98)	-11·9 (-23·8–0·294)	-10·5 (-35–20·6)	--	--	--	--	--
Occupational exposure to arsenic	0·549 (0–1·29)	0·532 (0–1·28)	0·56 (0–1·3)	-2·97 (-16·5–NA)	5·23 (-6·58–NA)	0·481 (0–1·12)	0·468 (0–1·12)	0·485 (0–1·17)	-2·67 (-19·2–NA)	3·72 (-9·82–NA)
Occupational exposure to benzene	0·792 (0·0469–2·31)	0·753 (0·0436–2·16)	0·786 (0·0469–2·27)	-4·89 (-12·6–7·86)	4·41 (-3·14–14·9)	1·07 (0·0627–2·96)	1·15 (0·0672–3·26)	1·22 (0·076–3·59)	7·54 (-4·59–31·3)	6·17 (-3·69–19·8)
Occupational exposure to beryllium	0·0239 (0·0224–0·0253)	0·023 (0·0224–0·0235)	0·0243 (0·0228–0·0258)	-3·72 (-9·77–3·3)	5·78 (-0·751–12·2)	0·0248 (0·0224–0·0271)	0·0251 (0·0245–0·0258)	0·0264 (0·0245–0·0284)	1·37 (-7·65–12·5)	5·13 (-2·67–12·5)
Occupational exposure to cadmium	0·0635 (0·0571–0·0703)	0·0619 (0·0563–0·068)	0·0652 (0·0573–0·0732)	-2·53 (-14·8–11·4)	5·3 (-5·63–16·3)	0·0568 (0·0497–0·0644)	0·0558 (0·0495–0·0629)	0·0579 (0·0497–0·0674)	-1·83 (-16·7–17·8)	3·75 (-8·78–17·5)
Occupational exposure to chromium	0·151 (0·137–0·165)	0·15 (0·139–0·162)	0·157 (0·141–0·175)	-0·637 (-11·7–11·3)	5·29 (-4·31–14·9)	0·14 (0·124–0·157)	0·14 (0·128–0·154)	0·146 (0·128–0·166)	0·355 (-13·2–17·3)	3·83 (-7·64–15·4)
Occupational exposure to diesel engine exhaust	0·724 (0·668–0·796)	0·757 (0·709–0·808)	0·801 (0·728–0·878)	4·47 (-5·96–16·7)	5·9 (-2·52–15·8)	0·615 (0·544–0·692)	0·646 (0·585–0·711)	0·665 (0·586–0·753)	4·92 (-9·45–22·1)	2·91 (-7·81–14·4)
Occupational exposure to formaldehyde	0·317 (0·284–0·353)	0·301 (0·266–0·334)	0·314 (0·273–0·354)	-5·16 (-17·8–9·04)	4·45 (-6·81–16·2)	--	--	--	--	--
Occupational exposure to nickel	0·479 (0–2·09)	0·441 (0–1·89)	0·463 (0–1·96)	-7·98 (-20·4–NA)	5·05 (-6·24–NA)	0·385 (0–1·66)	0·362 (0–1·56)	0·377 (0–1·68)	-5·84 (-22·6–NA)	4·2 (-10·7–NA)
Occupational exposure to polycyclic aromatic hydrocarbons	0·296 (0·27–0·323)	0·293 (0·272–0·316)	0·308 (0·277–0·34)	-1·1 (-11·6–10·8)	5·13 (-4·4–14·5)	0·293 (0·26–0·326)	0·302 (0·28–0·327)	0·314 (0·282–0·351)	3·21 (-9·64–18·8)	3·98 (-6·2–14·2)
Occupational exposure to silica	4·59 (0·647–14·9)	4·05 (0·571–13)	4·31 (0·618–13·6)	-11·7 (-23·5–4·14)	6·23 (-5·3–19·4)	3·29 (0·452–10·8)	3 (0·399–9·65)	3·09 (0·424–9·77)	-8·82 (-26·1–13·7)	3·13 (-11·4–22·4)
Occupational exposure to sulfuric acid	0·94 (0·198–3·04)	0·995 (0·21–3·16)	1·03 (0·209–3·36)	5·83 (-6·91–21·5)	3·89 (-6·24–14·5)	0·854 (0·178–2·7)	1 (0·213–3·32)	1·01 (0·215–3·16)	17·7 (-3·44–44)	0·155 (-14–16·8)
Unsafe sex	--	--	--	--	--	--	--	--	--	--
Occupational exposure to trichloroethylene	0·0862 (0·0799–0·0931)	0·088 (0·0842–0·0926)	0·0928 (0·0863–0·1)	2·09 (-6·34–11·4)	5·45 (-1·79–13)	0·0798 (0·0715–0·0887)	0·0813 (0·0761–0·0871)	0·0843 (0·0757–0·0934)	1·92 (-9·68–16·5)	3·65 (-6·15–13·5)
No access to handwashing facility	3·46 (3·2–3·75)	3·15 (2·91–3·4)	3·12 (2·88–3·38)	-8·89 (-16·9–0·0244)	-0·937 (-6·92–4·95)	3·46 (3·2–3·75)	3·15 (2·91–3·4)	3·12 (2·88–3·38)	-8·89 (-16·9–0·0244)	-0·937 (-6·92–4·95)
Child wasting	0·753 (0·497–1·04)	0·731 (0·48–0·995)	0·731 (0·476–0·995)	-2·92 (-11·5–6·81)	0·0704 (-6·78–7·35)	--	--	--	--	--
Child stunting	2·54 (1·39–3·64)	2·15 (1·12–3·24)	2·1 (1·09–3·09)	-15·1 (-33–9·3)	-2·47 (-17·8–14·5)	2·01 (0·996–3)	1·84 (0·94–2·79)	1·83 (0·906–2·81)	-8·56 (-28·4–16·6)	-0·54 (-16·3–18·8)
Chewing tobacco	9·61 (7·96–11·4)	7·77 (5·96–9·97)	7·11 (5·31–9·24)	-19·1 (-39·5–7·81)	-8·46 (-26·2–14·9)	0·488 (0·313–0·731)	0·458 (0·289–0·718)	0·445 (0·273–0·7)	-6·09 (-46·2–62·1)	-2·91 (-33·3–39·6)
Diet low in legumes	74·9 (40·2–99·5)	77·8 (47·5–99·8)	78 (47·1–99·7)	3·93 (-0·111–23·5)	0·266 (-4·08–6·11)	73·3 (36·9–99·4)	76·2 (43·3–99·7)	76·7 (45·9–99·5)	3·88 (-0·461–18·6)	0·685 (-4·1–8·66)
Short gestation	38·1 (31·5–46·1)	37·7 (31·4–44·6)	33·1 (26·8–39·8)	-1·03 (-21·9–22·8)	-12·4 (-30–9·43)	34·2 (28·4–40·2)	36·8 (31·2–43·1)	32·7 (27·1–38·8)	7·61 (-12·2–36·1)	-11·3 (-27·7–8·74)
Low birth weight	15·4 (12·9–18·4)	16·3 (13·9–19·1)	15·2 (12·5–18·4)	5·97 (-16·4–34·1)	-6·81 (-25·5–16·4)	15·9 (13·5–18·9)	18·9 (16·3–21·8)	18·1 (15·4–21·6)	18·8 (-5·06–49·2)	-4·41 (-21·3–14·9)
High temperature	25·1 (17–33·9)	27·6 (19·3–36·4)	23·4 (15·4–32·1)	9·87 (5·05–15·4)	-15·4 (-21·8–9·71)	25·1 (17–33·9)	27·6 (19·3–36·4)	23·4 (15·4–32·1)	9·87 (5·05–15·4)	-15·4 (-21·8–9·71)
Low temperature	36 (27·4–46·3)	40·6 (33·3–49·7)	37·4 (29·1–47·2)	12·9 (6·33–22·1)	-7·82 (-13·3–3·55)	36 (27·4–46·3)	40·6 (33·3–49·7)	37·4 (29·1–47·2)	12·9 (6·33–22·1)	-7·82 (-13·3–3·55)
Kidney dysfunction	20·8 (14·9–27·9)	21·6 (15·4–29)	21·7 (15·7–29)	3·72 (0·257–8·13)	0·654 (-3–4·2)	--	--	--	--	--
Bullying victimization	16·5 (6·83–32·1)	18·4 (8·74–34·2)	18·5 (7·69–34·1)	11·4 (-9·65–62·1)	0·518 (-18·1–13·3)	16·4 (6·85–32·1)	17·9 (8·34–33·5)	18·2 (7·75–33·5)	9·19 (-13·4–54·8)	1·93 (-16·3–16·1)
High LDL cholesterol	66·4 (61·9–71·1)	34·5 (31·2–38·1)	30·6 (27–34·5)	-48 (-53·3–43)	-11·4 (-16·5–6·28)	64·6 (61–68·2)	34·8 (31·6–38·2)	31·9 (28·3–35·4)	-46·1 (-50·8–41·4)	-8·48 (-13–4·31)

Supplementary Results Table 1. Alaska age-standardised Summary Exposure Values (SEVs) for most-detailed risk factors and percentage change, by sex, 1990, 2010, and 2019. Data in parentheses are 95% uncertainty intervals.

	Male					Female				
	1990	2010	2019	Percentage change 1990-2010	Percentage change 2010-2019	1990	2010	2019	Percentage change 1990-2010	Percentage change 2010-2019
Unsafe sanitation	7·96 (3·91–13·4)	4·07 (1·8–7·66)	3·04 (1·27–5·81)	-48·9 (-78·8–21)	-25·1 (-62·8–48·2)	--	--	--	--	--
Ambient particulate matter pollution	6·84 (1·64–15·6)	4·49 (2·25–7·21)	5·45 (3·01–8·34)	-34·4 (-71·9–105)	21·5 (11·5–46·2)	6·85 (1·69–15·6)	4·5 (2·29–7·21)	5·46 (3·05–8·32)	-34·2 (-71·6–102)	21·2 (11·2–45·3)
Household air pollution from solid fuels	0·129 (0·0264–0·371)	0·0704 (0·0112–0·241)	0·0577 (0·00939–0·197)	-45·6 (-80·2–27·8)	-18 (-55·5–65)	0·171 (0·0374–0·478)	0·0936 (0·0151–0·321)	0·0765 (0·013–0·252)	-45·2 (-80–26·7)	-18·3 (-55·7–64·2)
Ambient ozone pollution	16·4 (4·89–34·3)	11·5 (3·46–24·4)	7·03 (1·75–17·5)	-29·8 (-61·5–27·2)	-39·1 (-71·9–33·2)	--	--	--	--	--
Residential radon	30·3 (4·23–82)	30·3 (4·23–82)	30·3 (4·23–82)	0 (0–0)	0 (0–0)	30·3 (4·23–82)	30·3 (4·23–82)	30·3 (4·23–82)	0 (0–0)	0 (0–0)
Lead exposure	46·4 (18·2–71·1)	27·8 (8·45–49·6)	19·6 (4·55–39)	-40 (-59·7—26·7)	-29·7 (-48·5—20·8)	25·2 (1·03–53·6)	16·1 (0·424–40)	11·6 (0·181–31·8)	-36 (-72·6—21·1)	-27·8 (-66·3—19·4)
Child underweight	0·975 (0·56–1·49)	0·962 (0·519–1·46)	0·934 (0·515–1·43)	-1·28 (-19·3–19)	-2·96 (-16·7–12·1)	0·814 (0·435–1·21)	0·845 (0·467–1·3)	0·861 (0·473–1·33)	3·85 (-16·4–28·5)	1·9 (-12–18·5)
Iron deficiency	--	--	--	--	--	12·4 (10·3–14·6)	12·2 (10·2–14·2)	12·4 (10·2–14·7)	-1·53 (-13·9–12·6)	1·78 (-7·7–12·9)
Vitamin A deficiency	0·969 (0·251–2·53)	1·26 (0·349–3·46)	0·836 (0·224–2·36)	30·3 (-66·3–481)	-33·8 (-71·6–76·6)	1·87 (0·681–3·99)	1·76 (0·665–3·83)	1·45 (0·528–3·24)	-6·01 (-63·9–177)	-17·4 (-54·6–55)
Zinc deficiency	1·71 (0–5·57)	1·03 (0–3·19)	1·05 (0–3·34)	-39·7 (-100–NA)	1·76 (-19·1–NA)	1·76 (0–5·78)	1·03 (0–3·36)	1·01 (0–3·33)	-41·1 (-100–NA)	-2·38 (-26·2–NA)
Smoking	29·1 (26·2–32·2)	20·6 (18·6–22·9)	17·9 (16–19·9)	-29 (-34·1—23·5)	-13·4 (-16·9—9·71)	20·8 (18–23·9)	16·3 (14·6–18·3)	14·3 (12·6–16)	-21·3 (-30·4—9·44)	-12·4 (-16·8—7·52)
Secondhand smoke	42·5 (39·9–45·5)	26·7 (25–28·5)	26·2 (23·4–29)	-37·1 (-43—30·8)	-1·98 (-10·9–6·18)	40·6 (38·3–42·9)	25·5 (24·2–26·8)	25 (22·9–27·3)	-37·1 (-42·4—31·5)	-2·06 (-9·39–5·29)
Alcohol use	13·7 (7·9–20·2)	13 (7·49–19·4)	14·4 (8·64–20·7)	-5·4 (-33·5–36·4)	10·8 (-13·8–43·2)	9·29 (5·13–14·2)	9·98 (5·95–14·8)	10·5 (6·27–15·5)	7·45 (-23·5–63·3)	4·91 (-19·6–36·3)
Drug use	0·623 (0·34–1·13)	0·964 (0·658–1·4)	1·41 (1·03–1·91)	54·7 (19·1–109)	46·4 (26·2–73)	0·418 (0·26–0·681)	0·813 (0·593–1·12)	1·16 (0·884–1·5)	94·5 (54·5–150)	42·8 (23·4–65·5)
High fasting plasma glucose	12·4 (10·3–15)	15·8 (13·5–18·8)	17·7 (15–20·7)	27·4 (5·03–54·6)	11·6 (-3·23–29·1)	10·7 (8·78–13·2)	14 (11·8–17)	16 (13·3–19·1)	31·4 (7·67–63)	14 (-3·87–36)
High systolic blood pressure	25·7 (20·9–31·4)	16·2 (14·2–18·4)	20·2 (15·5–25·1)	-37·1 (-49·1—21)	24·6 (-1·09–54·4)	23·7 (17·4–30·8)	13·5 (11·9–15·2)	18·3 (13·1–24·5)	-43 (-56·8—23·5)	35·4 (-0·711–82)
High body-mass index	27·3 (18·9–38)	39·8 (29·2–51·5)	42·2 (31–53·9)	45·8 (29·5–67·8)	6·09 (0·0237–13·5)	36·6 (26·8–45·5)	45·4 (35·3–54·6)	46·6 (36·2–55·8)	24·1 (12·6–42·5)	2·72 (-3·69–9·25)
Low bone mineral density	12·5 (7·25–19·6)	9·61 (5·18–15·8)	12·1 (6·99–18·8)	-22·9 (-42·3–0·379)	26·4 (-1·1–65·5)	16·5 (10·7–23·1)	17·1 (11·3–24·5)	19·6 (13·3–27·3)	3·48 (-17·7–29·4)	15·1 (-7·05–46·1)
Diet low in fruits	50·1 (37·6–63·5)	49·5 (38–62·2)	49·2 (36·6–62·4)	-1·16 (-11·8–10·7)	-0·749 (-11·4–10·1)	49·9 (37·6–63·6)	49·4 (37·5–62·1)	49·2 (37·3–62·3)	-1·11 (-10·8–11)	-0·404 (-12·3–11·4)
Diet low in vegetables	23·9 (11·1–43·1)	45·4 (26–67·5)	41·9 (23·9–63·2)	90·4 (35·2–212)	-7·79 (-21–2·73)	20·7 (8·64–39·8)	39·4 (22·2–61·4)	36·6 (20·2–57·3)	90·7 (33–225)	-7·11 (-22–5·78)
Diet low in whole grains	78·6 (68·4–87·8)	81·1 (72·6–89·3)	81·2 (72·1–89·5)	3·19 (-0·634–8·53)	0·0691 (-2·59–2·91)	75·8 (64·5–86·1)	79·1 (69·8–88·1)	79 (69·4–88·1)	4·35 (-0·09–10·7)	-0·235 (-3·23–2·9)
Diet low in nuts and seeds	22·6 (6·54–53·7)	24·7 (7·74–55·1)	20·7 (6·51–53·6)	9·7 (-31·1–103)	-16·2 (-42·4–11)	18·9 (4·56–52·6)	20·9 (6·22–54)	18·4 (5–53)	10·6 (-32–122)	-11·9 (-39·6–19·9)
Diet low in milk	42·4 (20·5–63·3)	39·5 (16·9–59·6)	38·9 (16·5–58·7)	-6·8 (-25·9–11·4)	-1·72 (-15·7–13·4)	--	--	--	--	--
Diet high in red meat	84·4 (74·3–92·3)	82·7 (70·7–91·7)	81·9 (69·2–92·2)	-2·06 (-14·3–10·9)	-1·01 (-11·2–8·52)	81·6 (70–90·4)	77·9 (65·1–88·4)	77·4 (64·2–88·3)	-4·56 (-18·2–10·4)	-0·661 (-10·3–11·1)
Diet high in processed meat	73·7 (49·1–95·6)	82·3 (61–99·2)	82·1 (61–99·1)	11·7 (2·33–32)	-0·211 (-4·84–5·48)	73·8 (48·4–95·7)	83·7 (65·5–99·5)	82·4 (63·2–99·1)	13·4 (2·58–37·3)	-1·56 (-7·03–2·82)
Diet high in sugar-sweetened beverages	51·9 (31·7–71·3)	66·3 (47·4–83)	66·2 (47·6–82·6)	27·8 (-7·53–90·3)	-0·199 (-18·4–22·4)	56·5 (36·4–76·2)	72·1 (53·8–87·3)	70·4 (51·3–85·7)	27·7 (-6·69–88)	-2·4 (-19·1–18·2)
Diet low in fiber	41·3 (25·2–57·6)	30·9 (18·2–45·2)	28·5 (15·7–43)	-25·1 (-42·3—5·43)	-7·95 (-24·5–11·1)	43·5 (27·1–60)	39·4 (25·6–54·3)	34·6 (20·8–50·1)	-9·26 (-24·7–12·6)	-12·3 (-28·5–1·61)
Diet low in seafood omega-3 fatty acids	78·7 (61·3–99·4)	76·3 (57·7–98·3)	76·5 (57·7–99·1)	-3·1 (-18·1–14·1)	0·302 (-15·5–19·4)	82·3 (65·1–99·4)	81·1 (64·1–99·5)	80·6 (63·2–99·5)	-1·48 (-14–12·1)	-0·654 (-13·5–13)
Diet low in polyunsaturated fatty acids	10·9 (3·32–34·3)	6·6 (1·84–24·4)	6·45 (1·8–24·5)	-39·4 (-71·7—5·93)	-2·23 (-22·2–23·2)	11·2 (3–36·9)	6·82 (1·88–24·7)	6·49 (1·77–24)	-39·3 (-71·7—3·05)	-4·87 (-29·1–18·7)
Diet high in trans fatty acids	89·5 (81·5–100)	87·9 (79·2–100)	87·5 (78·1–100)	-1·77 (-10–5·95)	-0·452 (-8·56–8·82)	88·4 (81·1–100)	86·7 (78·1–100)	86·3 (77·6–100)	-1·94 (-9·93–6·22)	-0·431 (-7·98–8·34)
Diet high in sodium	18·2 (4·58–42)	39·7 (11·9–64)	38·8 (10·4–63·6)	117 (38·6–451)	-2·14 (-29·9–30·3)	20·9 (4·78–45)	21·2 (4·91–45·4)	21·7 (5·15–45·6)	1·78 (-30·1–53·1)	2·23 (-15·9–30)
Low physical activity	1·64 (0·495–4·92)	1·42 (0·488–4·75)	1·42 (0·394–4·82)	-13·8 (-46·8–23·2)	0·193 (-31·5–31·5)	--	--	--	--	--
Occupational asthmagens	19 (16–22·5)	18·7 (15·9–22·2)	18·8 (15·8–22·6)	-1·41 (-13·9–12)	0·725 (-8·31–10·9)	10·3 (8·44–12·4)	10·6 (8·79–12·7)	10·5 (8·59–12·9)	2·99 (-13·3–20·7)	-0·477 (-11·6–12)
Occupational particulate matter, gases, and fumes	7·17 (5·05–10·4)	7·41 (5·28–10·6)	7·36 (5·27–10·6)	3·27 (-2·69–10·8)	-0·56 (-4·44–3·33)	4·22 (2·92–6·16)	4·29 (3·02–6·2)	4·21 (2·98–6)	1·76 (-7·69–14)	-2·08 (-7·11–3·15)
Occupational noise	7·23 (6·58–7·96)	7·58 (7·06–8·22)	7·55 (7·05–8·18)	4·81 (0·103–10·3)	-0·413 (-2·87–2·44)	5·23 (4·55–5·95)	5·72 (5·21–6·34)	5·8 (5·39–6·36)	9·49 (2·61–18·5)	1·37 (-1·77–4·8)
Occupational injuries	--	--	--	--	--	--	--	--	--	--
Occupational ergonomic factors	14·3 (11·7–17·5)	12·5 (10·5–15)	12·9 (10·8–15·6)	-12·5 (-25–1·86)	2·93 (-6·92–13·8)	8·09 (6·28–10·2)	7·84 (6·37–9·65)	8·22 (6·54–10·5)	-2·97 (-20–18·9)	4·77 (-8·65–20·9)
Childhood sexual abuse	7·4 (5·05–10·5)	6·97 (4·6–9·93)	6·85 (4·54–9·67)	-5·83 (-12·2–5·83)	-1·68 (-8·51–5·53)	12·3 (8·94–17·3)	17·4 (12·9–24)	17·8 (13·1–24·7)	41·9 (30·7–54·8)	2·17 (-5–11)
Intimate partner violence	--	--	--	--	--	35·9 (20·2–45·5)	34·1 (19·7–43·9)	35 (20·6–45·4)	-4·93 (-11·9–6·97)	2·66 (-4·7–21·4)
Non-exclusive breastfeeding	26·1 (21·3–31)	25 (21·2–29)	24·9 (20·4–29·4)	-4 (-19·6–14·1)	-0·558 (-13·3–14)	26·1 (21·3–31)	25 (21·2–29)	24·9 (20·4–29·4)	-4 (-19·6–14·1)	-0·568 (-13·3–14)
Discontinued breastfeeding	27·5 (26·4–28·4)	26·1 (24·8–27·3)	25·6 (24·2–26·8)	-5·04 (-10·3–0·356)	-1·81 (-7·06–4·16)	27·5 (26·4–28·4)	26 (24·7–27·3)	25·6 (24·3–26·9)	-5·17 (-10·5–0·217)	-1·66 (-6·91–4·32)
Diet low in calcium	15·6 (6·62–29·8)	15·1 (6·76–28·1)	14·7 (6·59–27·6)	-3·45 (-15·6–13·3)	-2·08 (-10·7–6·05)	13·5 (6·05–26)	15·8 (7·84–28·8)	15·7 (7·83–28·7)	16·9 (-0·0898–46·9)	-0·528 (-8·41–8·57)
Occupational exposure to asbestos	12 (10·7–13·2)	10·3 (9·37–11·2)	9·52 (7·35–11·7)	-14·2 (-24·5—1·78)	-7·43 (-27·5–14·6)	--	--	--	--	--
Occupational exposure to arsenic	0·36 (0–0·854)	0·395 (0–0·941)	0·415 (0–0·975)	9·62 (-4·68–NA)	5·21 (-6·64–NA)	0·382 (0–0·886)	0·414 (0–1·01)	0·424 (0–1·04)	8·42 (-7·77–NA)	2·44 (-8·43–NA)
Occupational exposure to benzene	0·765 (0·0446–2·27)	0·817 (0·048–2·44)	0·841 (0·0508–2·49)	6·78 (-3·77–23·8)	2·89 (-5·56–13)	1·11 (0·0656–3·18)	1·33 (0·0816–3·6)	1·38 (0·0826–3·72)	19·6 (5·22–45·4)	3·71 (-5·56–18·1)
Occupational exposure to beryllium	0·0237 (0·0224–0·0251)	0·0244 (0·0236–0·0252)	0·0254 (0·024–0·0268)	2·85 (-3·57–9·49)	4·08 (-1·38–9·97)	0·0266 (0·0243–0·0291)	0·0285 (0·0274–0·0296)	0·0294 (0·0275–0·0315)	7·33 (-2·23–17·5)	3·2 (-3·57–10·2)
Occupational exposure to cadmium	0·044 (0·04–0·0486)	0·0483 (0·0442–0·0532)	0·0507 (0·0456–0·0567)	9·76 (-3·11–23·9)	4·97 (-5·64–15·7)	0·0471 (0·0421–0·0534)	0·0514 (0·0468–0·0565)	0·0526 (0·0468–0·0592)	9·07 (-5·7–25·9)	2·38 (-7·09–13·3)
Occupational exposure to chromium	0·105 (0·0961–0·115)	0·12 (0·111–0·13)	0·126 (0·114–0·139)	14·3 (1·79–27·5)	4·73 (-4·55–14·7)	0·118 (0·106–0·132)	0·133 (0·123–0·144)	0·136 (0·122–0·15)	12·7 (-1·28–27·9)	2·02 (-6·63–11·9)
Occupational exposure to diesel engine exhaust	0·714 (0·654–0·781)	0·833 (0·771–0·897)	0·858 (0·778–0·941)	16·7 (4·29–31·2)	2·99 (-4·8–11·8)	0·672 (0·597–0·753)	0·741 (0·67–0·823)	0·737 (0·652–0·834)	10·3 (-4·72–27·5)	-0·601 (-10·6–10·7)
Occupational exposure to formaldehyde	0·201 (0·18–0·226)	0·223 (0·199–0·248)	0·232 (0·204–0·264)	10·6 (-4·26–28·1)	3·98 (-7·45–16·2)	--	--	--	--	--
Occupational exposure to nickel	0·352 (0–1·52)	0·351 (0–1·53)	0·36 (0–1·55)	-0·29 (-13·4–NA)	2·54 (-8·11–NA)	0·311 (0–1·38)	0·318 (0–1·39)	0·326 (0–1·41)	2·53 (-14·6–NA)	2·5 (-8·74–NA)
Occupational exposure to polycyclic aromatic hydrocarbons	0·213 (0·194–0·232)	0·242 (0·224–0·262)	0·252 (0·229–0·279)	13·9 (1·35–27·7)	4·18 (-4·98–13·4)	0·255 (0·229–0·285)	0·298 (0·278–0·319)	0·304 (0·276–0·335)	16·9 (3·18–31·4)	2·24 (-5·97–11)
Occupational exposure to silica	3·66 (0·522–11·1)	3·51 (0·512–11)	3·57 (0·515–11·4)	-4·06 (-20·4–17·5)	1·61 (-10·1–16·4)	2·76 (0·4–8·99)	2·64 (0·367–8·26)	2·67 (0·383–8·66)	-4·26 (-19·6–17·2)	1·03 (-10·6–16·9)
Occupational exposure to sulfuric acid	0·723 (0·154–2·35)	0·898 (0·186–2·8)	0·921 (0·192–2·99)	24·2 (4·05–47·8)	2·62 (-9·2–16·1)	0·804 (0·161–2·71)	1·06 (0·212–3·36)	1·06 (0·217–3·39)	32·2 (5·06–65·9)	-0·665 (-17·2–16·7)
Unsafe sex	--	--	--	--	--	--	--	--	--	--
Occupational exposure to trichloroethylene	0·0682 (0·0635–0·0734)	0·0793 (0·0749–0·0843)	0·083 (0·0769–0·0902)	16·3 (5·94–27·6)	4·69 (-2·73–12·9)	0·0742 (0·0673–0·0826)	0·0828 (0·0771–0·0885)	0·084 (0·076–0·0922)	11·6 (-0·593–25·9)	1·4 (-6·49–10·6)
No access to handwashing facility	3·42 (3·14–3·69)	3·13 (2·91–3·39)	3·09 (2·85–3·35)	-8·37 (-16·1–0·551)	-1·45 (-7·26–4·41)	3·42 (3·14–3·69)	3·13 (2·91–3·39)	3·09 (2·85–3·35)	-8·37 (-16·1–0·551)	-1·45 (-7·26–4·41)
Child wasting	0·748 (0·499–1·03)	0·727 (0·48–0·986)	0·725 (0·474–0·983)	-2·74 (-12·2–7·54)	-0·267 (-7·8–7·96)	1·05 (0·703–1·38)	0·699 (0·373–0·971)	0·695 (0·363–0·968)	-33·4 (-59·3—0·566)	-0·682 (-8·08–7·18)
Child stunting	2·49 (1·27–3·68)	2·08 (0·996–3·07)	2·01 (1·01–2·98)	-16·4 (-35·8–6·37)	-3·18 (-17·1–13·4)	1·98 (0·98–2·98)	1·8 (0·88–2·72)	1·76 (0·874–2·67)	-9·27 (-28·9–14·2)	-2·1 (-17·6–16·5)
Chewing tobacco	4·36 (3·37–5·6)	4·36 (3·11–5·95)	4·08 (2·78–5·8)	-0·0225 (-33·1–48·9)	-6·44 (-26·9–19·9)	0·699 (0·443–1·08)	0·721 (0·456–1·12)	0·681 (0·416–1·08)	3·15 (-39·4–82·8)	-5·64 (-35·5–34·7)
Diet low in legumes	72·1 (34·7–98·8)	76 (42·4–99·6)	76 (42·3–99·6)	5·38 (0·00604–29·6)	0·0944 (-5·3–6·31)	70·4 (31·5–98)	74·4 (39·4–99·3)	74·7 (41·5–99·4)	5·57 (0·0537–32)	0·476 (-4·69–7·74)
Short gestation	18·7 (15·2–22·9)	18·8 (15·5–22·7)	18·9 (15·1–23·5)	0·359 (-19·5–26·7)	0·468 (-20·2–25·8)	17·8 (14·5–21·3)	18·3 (15·2–22·1)	18·5 (15·5–22)	3·12 (-19–35)	1·08 (-17·5–25·2)
Low birth weight	6·94 (5·67–8·55)	7·63 (6·33–9·2)	7·58 (6·03–9·26)	9·96 (-12·6–40·1)	-0·594 (-22·2–26·6)	7·25 (5·97–8·63)	8·16 (6·83–9·58)	8·78 (7·38–10·4)	12·6 (-12·3–48·5)	7·59 (-13·4–33·1)
High temperature	19·4 (7·42–34·2)	17·9 (5·72–32·7)	20·2 (7·03–35·1)	-7·54 (-39·1–19)	12·7 (-2·1–35·7)	19·4 (7·42–34·2)	17·9 (5·72–32·7)	20·2 (7·03–35·1)	-7·54 (-39·1–19)	12·7 (-2·1–35·7)
Low temperature	42 (34·3–49·8)	43·3 (37·1–49·3)	39·1 (33·1–45)	3·05 (-3·41–10·8)	-9·76 (-15—4·61)	42 (34·3–49·8)	43·3 (37·1–49·3)	39·1 (33·1–45)	3·05 (-3·41–10·8)	-9·76 (-15—4·61)
Kidney dysfunction	18·5 (13–25·5)	19·3 (13·4–26·5)	19·4 (13·7–26·5)	4·13 (-0·0392–9·44)	0·584 (-3·35–4·5)	--	--	--	--	--
Bullying victimization	5·85 (2·42–12)	9·07 (3·62–18)	8·75 (3·33–18·1)	55·1 (35·1–78)	-3·61 (-13·4–2·61)	4·29 (1·72–9·02)	8 (3·18–15·8)	7·83 (2·92–16·1)	86·4 (61·8–116)	-2·1 (-12·6–4·8)
High LDL cholesterol	66·7 (62·3–71·2)	34·6 (31·3–38·2)	30·9 (27·3–34·7)	-48·1 (-53·3—42·8)	-10·7 (-15·7—5·79)	64·8 (61·2–68·7)	34·2 (30·9–37·4)	31·4 (27·8–35)	-47·3 (-52·2—42·8)	-8·12 (-12·3—3·95)

Supplementary Results Table 1. Arizona age-standardised Summary Exposure Values (SEVs) for most-detailed risk factors and percentage change, by sex, 1990, 2010, and 2019. Data in parentheses are 95% uncertainty intervals.

	Male					Female				
	1990	2010	2019	Percentage change 1990-2010	Percentage change 2010-2019	1990	2010	2019	Percentage change 1990-2010	Percentage change 2010-2019
Unsafe sanitation	8·48 (4·06–15)	4·25 (1·91–8·11)	3·32 (1·56–6·33)	-49·9 (-79·3–16·5)	-21·9 (-57·6–49·5)	--	--	--	--	--
Ambient particulate matter pollution	12·5 (4·65–25)	4·65 (2·35–7·35)	3·81 (1·64–6·45)	-62·9 (-83·4—7·93)	-18·1 (-32·8—11·9)	12·5 (4·68–24·9)	4·66 (2·37–7·34)	3·82 (1·65–6·43)	-62·8 (-83·2—7·8)	-18·1 (-32·5—11·8)
Household air pollution from solid fuels	0·104 (0·0264–0·276)	0·0436 (0·00678–0·154)	0·0297 (0·00411–0·1)	-57·9 (-85·3—2·94)	-31·9 (-65·8–25·4)	0·136 (0·0365–0·362)	0·0579 (0·00946–0·21)	0·0395 (0·0056–0·13)	-57·4 (-85·1—2·05)	-31·8 (-66–25·3)
Ambient ozone pollution	80·2 (37·3–100)	83·2 (38·9–100)	79 (36–100)	3·73 (-2·27–14·9)	-5·1 (-14·3–0)	--	--	--	--	--
Residential radon	15·9 (2·39–44·9)	15·9 (2·39–44·9)	15·9 (2·39–44·9)	0 (0–0)	0 (0–0)	15·9 (2·39–44·9)	15·9 (2·39–44·9)	15·9 (2·39–44·9)	0 (0–0)	0 (0–0)
Lead exposure	71·5 (47–88)	43 (22·5–62·6)	30·4 (13·6–48·3)	-39·9 (-53·1—26·8)	-29·4 (-39·9—22·3)	45·3 (13·2–72·2)	25·8 (4·42–50·2)	17·6 (1·81–37·8)	-42·9 (-69·2—27·7)	-31·8 (-58·8—23·5)
Child underweight	0·978 (0·548–1·49)	0·969 (0·538–1·48)	0·946 (0·52–1·45)	-0·883 (-17·2–19·7)	-2·4 (-16·2–12·8)	0·815 (0·451–1·24)	0·848 (0·46–1·26)	0·869 (0·471–1·32)	3·96 (-16–29·2)	2·54 (-11·7–18·1)
Iron deficiency	--	--	--	--	--	11·7 (9·71–13·6)	12 (10·1–13·9)	12·2 (10·2–14·4)	2·25 (-9·45–16)	1·67 (-7·91–12·6)
Vitamin A deficiency	1·04 (0·262–2·69)	1·32 (0·333–3·25)	0·942 (0·251–2·63)	27 (-67·2–529)	-28·7 (-70·1–69·4)	1·94 (0·748–4·02)	1·89 (0·72–3·87)	1·6 (0·592–3·47)	-2·83 (-64·1–182)	-15·1 (-53–63)
Zinc deficiency	2·3 (0–7·74)	1·24 (0–3·94)	1·36 (0–4·34)	-45·8 (-100–NA)	8·97 (-9·22–NA)	2·36 (0–7·67)	1·26 (0–4·16)	1·33 (0–4·26)	-46·7 (-100–NA)	5·46 (-14·6–NA)
Smoking	25·5 (23–28·3)	18·3 (16·5–20·2)	15·8 (14·2–17·6)	-28·4 (-32·8—23·2)	-13·7 (-17·4—9·9)	19·5 (17·2–22·1)	13·7 (12·3–15·3)	11·5 (10·2–12·8)	-29·5 (-35·9—22·4)	-16·6 (-20·9—12·1)
Secondhand smoke	38·8 (36·3–41·5)	24·1 (22·8–25·7)	24·3 (21·9–27)	-37·8 (-43·4—31·9)	0·703 (-8·24–9·91)	35·6 (33·6–37·6)	23 (21·9–24·1)	23·2 (21·2–25·2)	-35·3 (-40—30·1)	0·822 (-6·09–8·36)
Alcohol use	13·5 (7·97–19)	14·1 (8·18–20·6)	15·4 (9·02–22·5)	4·9 (-26·4–48·4)	9·36 (-13·3–40·3)	9·2 (5·19–13·4)	10·2 (5·89–14·9)	10·2 (5·82–14·9)	10·5 (-23·7–58·7)	0·631 (-20·8–28·7)
Drug use	0·763 (0·46–1·27)	1·22 (0·88–1·68)	1·51 (1·12–2·01)	60·2 (20·6–113)	23·7 (8·34–42·6)	0·576 (0·324–1)	0·957 (0·683–1·33)	1·31 (0·994–1·7)	66·2 (25–123)	37 (20·6–59)
High fasting plasma glucose	12·5 (10·4–15·2)	17·9 (15·5–20·6)	19·5 (16·9–22·4)	42·9 (17·9–73·7)	8·92 (-5·22–25·3)	9·22 (7·91–11)	13·1 (11·3–15·1)	14·4 (12·3–16·9)	41·8 (19·9–66·6)	10·4 (-4·81–28)
High systolic blood pressure	25·2 (20·2–31·1)	15·9 (13·8–18·2)	19·6 (14·9–24·5)	-36·9 (-49·8—20)	23 (-2·57–53·5)	22·6 (17–29·4)	12·9 (11·4–14·6)	17·4 (12·6–23·6)	-42·7 (-57·3—24·2)	34·4 (-1·3–83)
High body-mass index	22·6 (15·8–31)	36·6 (26·7–48·5)	37·3 (27·1–49·1)	61·9 (42·7–91)	1·81 (-5·05–10)	26·9 (19·6–36·1)	39·9 (31·5–48·8)	40·8 (31·8–49·7)	48·1 (28·3–75·4)	2·18 (-4·13–9·53)
Low bone mineral density	12·2 (7·36–18·8)	9·26 (4·95–15·4)	11·6 (6·85–18·5)	-23·9 (-43·9—0·298)	25·2 (-3·19–69)	16 (10·8–23)	16·4 (10·8–23·6)	18·8 (12·8–26·1)	2·65 (-19·4–29·4)	14·6 (-11·2–44·8)
Diet low in fruits	52·8 (41·4–65·9)	42 (30·3–54·6)	43·4 (31·2–56·6)	-20·5 (-32·7—11·1)	3·5 (-9·12–19·2)	52·9 (40·9–66)	43·8 (32·1–57·2)	47·1 (34·1–61·6)	-17·2 (-28·8—7·29)	7·55 (-4·61–21·4)
Diet low in vegetables	15·7 (6·49–31·2)	15·5 (8·61–26·6)	15·5 (8·54–27·4)	-1·11 (-32·7–81·7)	0·0495 (-20–21·9)	20·5 (8·63–39·6)	30·1 (17·7–46·5)	29·7 (17·2–46·1)	47 (3·1–160)	-1·31 (-16·1–14·3)
Diet low in whole grains	79·7 (70·2–88·4)	81·9 (73·3–89·5)	82 (73·3–90)	2·69 (-0·841–7·6)	0·152 (-2·25–2·78)	77 (66·6–86·8)	79·9 (70·9–89)	79·9 (70·4–88·7)	3·89 (-0·118–9·64)	-0·0406 (-2·67–2·67)
Diet low in nuts and seeds	28·6 (8·2–57·2)	26 (7·97–55·5)	22·1 (5·76–54·3)	-9·25 (-40·4–60·8)	-14·9 (-42·8–11·7)	24·4 (5·77–54·6)	22·8 (7·37–54·1)	20·5 (5·79–53·7)	-6·64 (-41–69·7)	-10 (-37·8–19·6)
Diet low in milk	49·3 (25·4–70·1)	44·9 (20·7–64·5)	45 (20·9–66·3)	-8·92 (-27–4)	0·315 (-11–14·9)	--	--	--	--	--
Diet high in red meat	78·8 (66·4–88·7)	78·5 (65·8–89·3)	77·5 (63·1–88·7)	-0·347 (-14·5–15·8)	-1·23 (-12·8–9·85)	75·5 (62·9–85·8)	72·7 (58·9–84·2)	72·1 (57·4–84·7)	-3·75 (-19·6–12·2)	-0·824 (-13·1–11·4)
Diet high in processed meat	75·6 (51·2–96·9)	83 (63·7–99·3)	82·8 (62·3–99·1)	9·71 (1·16–26·8)	-0·282 (-5·02–4·6)	75·1 (49·7–96·2)	84·2 (65·7–98·9)	82·7 (63·2–98·8)	12·1 (2·07–34·7)	-1·82 (-7·98–2·26)
Diet high in sugar-sweetened beverages	45·5 (28–64·3)	60·5 (42·1–77·8)	59·7 (40–78·8)	33 (-5·34–109)	-1·37 (-22·3–23·9)	49·4 (30·3–69·5)	66·4 (47·4–82·7)	64·3 (44·2–81·5)	34·2 (-2·97–108)	-3·16 (-22·9–18·8)
Diet low in fiber	44·1 (28·8–59·1)	31·6 (18·3–45·6)	29·9 (16·6–44)	-28·3 (-45·5—11·6)	-5·42 (-23·8–12·6)	44·4 (27·7–60·2)	36·9 (22·2–51·7)	33·1 (18·7–48·3)	-16·8 (-33·2–0·49)	-10·4 (-25·9–5·16)
Diet low in seafood omega-3 fatty acids	83·6 (65·3–99·9)	81·1 (63·3–99·8)	81·4 (64·2–99·3)	-2·99 (-17·3–12·5)	0·326 (-13·2–17·2)	86·5 (71·2–99·8)	84·7 (70·4–99·1)	85 (69·6–99·9)	-2·04 (-11·7–9·37)	0·309 (-9·98–12·5)
Diet low in polyunsaturated fatty acids	14·3 (4·17–40·4)	7·73 (2·22–26·3)	7·67 (2·26–26·4)	-45·9 (-73·1—7·06)	-0·872 (-21·9–26·3)	16 (4·54–46·5)	7·88 (2·35–27·6)	7·7 (2·3–26·7)	-50·6 (-75·8—7·83)	-2·33 (-23·2–20)
Diet high in trans fatty acids	89·5 (81·3–100)	88 (78·8–100)	87·3 (78–100)	-1·71 (-9·94–5·6)	-0·767 (-8·99–7·5)	88·4 (80·8–100)	86·7 (78·5–100)	86·3 (77·8–100)	-1·91 (-9·44–5·55)	-0·448 (-8·31–8·36)
Diet high in sodium	18 (4·53–40·6)	39·4 (10·9–63·7)	38·8 (10·6–64·5)	118 (42·4–417)	-1·38 (-28·2–30·7)	20·9 (4·81–45·5)	21·1 (5·03–44·8)	21·6 (5·13–46)	0·822 (-30–41·8)	2·29 (-18·4–28)
Low physical activity	2·01 (0·634–5·16)	1·88 (0·68–5·08)	1·85 (0·549–4·97)	-6·7 (-38·4–40·2)	-1·49 (-33·6–40·7)	--	--	--	--	--
Occupational asthmagens	21 (17·6–25·2)	18·6 (15·7–22)	19·4 (16·3–23·3)	-11·3 (-21·7—0·45)	4·13 (-4·78–13·2)	10·5 (8·52–12·7)	9·93 (8·24–11·9)	10·1 (8·17–12·4)	-5·08 (-19–12)	2·01 (-10·7–15·4)
Occupational particulate matter, gases, and fumes	8·83 (6·25–12·7)	8·32 (5·96–11·9)	8·04 (5·79–11·5)	-5·77 (-11·5–0·0613)	-3·36 (-7·04–0·171)	4·78 (3·33–6·95)	4·63 (3·32–6·71)	4·43 (3·2–6·45)	-3·15 (-12·5–6·98)	-4·2 (-9·4–1·31)
Occupational noise	8·39 (7·64–9·18)	8·01 (7·44–8·68)	7·73 (7·23–8·38)	-4·5 (-7·92—0·143)	-3·5 (-5·55—1·25)	5·62 (4·89–6·47)	5·87 (5·35–6·48)	5·84 (5·42–6·39)	4·36 (-2·04–12·5)	-0·496 (-3·58–3)
Occupational injuries	--	--	--	--	--	--	--	--	--	--
Occupational ergonomic factors	11·9 (9·88–14·4)	10·2 (8·65–12·4)	10·5 (8·75–12·7)	-14·3 (-23·5—4·66)	2·9 (-4·86–11·3)	6·8 (5·47–8·53)	6·46 (5·3–7·91)	6·75 (5·42–8·51)	-5·01 (-19–12·8)	4·51 (-7·44–18·8)
Childhood sexual abuse	7·4 (5·05–10·5)	6·97 (4·6–9·93)	6·85 (4·54–9·67)	-5·83 (-12·2–5·83)	-1·68 (-8·51–5·53)	12·3 (8·94–17·3)	17·4 (12·9–24)	17·8 (13·1–24·7)	41·9 (30·7–54·8)	2·17 (-5–11)
Intimate partner violence	--	--	--	--	--	20·7 (11·5–29·4)	19·8 (11·2–28·1)	22·2 (12·8–31·8)	-4·41 (-14·2–12·5)	12·2 (-2·28–33·3)
Non-exclusive breastfeeding	26·3 (21·6–31·6)	24·9 (21·2–28·7)	24·9 (20·7–29·6)	-5·42 (-20·3–12·3)	0·319 (-13·2–14·5)	26·3 (21·6–31·6)	24·9 (21·2–28·7)	24·9 (20·7–29·6)	-5·4 (-20·2–12·3)	0·313 (-13·2–14·5)
Discontinued breastfeeding	27·4 (26·4–28·3)	25·3 (24–26·6)	25·7 (24·4–27)	-7·59 (-13·3—2·37)	1·58 (-3·79–7·97)	27·4 (26·4–28·3)	25·3 (24–26·6)	25·7 (24·4–26·9)	-7·66 (-13·4—2·44)	1·51 (-3·87–7·89)
Diet low in calcium	16·7 (7·9–30·7)	14·5 (6·85–26·5)	14·5 (6·68–27)	-13·3 (-24·7—2·13)	-0·135 (-9·63–8·13)	12·1 (5·11–23·1)	11·5 (4·96–21·6)	11·9 (5·13–22·5)	-4·6 (-19–14·6)	3·24 (-6·88–13·7)
Occupational exposure to asbestos	7·4 (6·52–8·29)	6·78 (6·16–7·42)	6·54 (4·69–8·73)	-8·35 (-19·9–4·33)	-3·53 (-31·1–29)	1·41 (1·08–1·97)	1·33 (1·01–1·83)	1·35 (0·888–2·07)	-5·14 (-19·1–11·1)	1·19 (-27·6–38·2)
Occupational exposure to arsenic	0·444 (0–1·05)	0·447 (0–1·07)	0·476 (0–1·12)	0·584 (-14·7–NA)	6·59 (-4·67–NA)	0·423 (0–1)	0·422 (0–1·01)	0·437 (0–1·04)	-0·245 (-17·6–NA)	3·35 (-9·59–NA)
Occupational exposure to benzene	0·831 (0·0476–2·4)	0·798 (0·0469–2·26)	0·826 (0·0505–2·34)	-4·06 (-12·6–8·5)	3·54 (-4·39–13·4)	1·14 (0·065–3·13)	1·25 (0·0764–3·48)	1·31 (0·0806–3·8)	9·6 (-3·04–28·7)	5·06 (-3·71–18)
Occupational exposure to beryllium	0·0231 (0·0217–0·0245)	0·0223 (0·0219–0·0228)	0·0236 (0·0223–0·025)	-3·21 (-8·9–3·26)	5·85 (0·326–12·3)	0·0253 (0·0231–0·0276)	0·0258 (0·0252–0·0263)	0·0268 (0·0247–0·0287)	1·7 (-7·09–12)	4·09 (-3·89–11·2)
Occupational exposure to cadmium	0·0529 (0·0475–0·0587)	0·0532 (0·0483–0·0586)	0·0566 (0·0505–0·0634)	0·64 (-12·8–15·3)	6·36 (-3·73–16·9)	0·0513 (0·0449–0·0584)	0·0515 (0·0466–0·0571)	0·0531 (0·0465–0·0609)	0·388 (-15·5–17·7)	3·17 (-8·47–15·6)
Occupational exposure to chromium	0·129 (0·118–0·142)	0·133 (0·123–0·143)	0·141 (0·127–0·155)	2·48 (-9·04–15·2)	6·15 (-2·57–15·5)	0·13 (0·115–0·145)	0·133 (0·123–0·144)	0·137 (0·123–0·153)	2·41 (-11·3–18·2)	3·05 (-7·31–13·7)
Occupational exposure to diesel engine exhaust	0·746 (0·686–0·804)	0·77 (0·724–0·818)	0·817 (0·746–0·884)	3·3 (-6·76–14·1)	6·02 (-2·09–15·1)	0·66 (0·583–0·74)	0·67 (0·608–0·739)	0·673 (0·593–0·764)	1·45 (-12·4–17·3)	0·562 (-9·98–12·4)
Occupational exposure to formaldehyde	0·258 (0·207–0·292)	0·258 (0·232–0·287)	0·267 (0·212–0·304)	0·0178 (-14·7–22·6)	3·44 (-14·5–15·1)	--	--	--	--	--
Occupational exposure to nickel	0·39 (0–1·72)	0·359 (0–1·57)	0·382 (0–1·61)	-8 (-20·8–NA)	6·55 (-4·96–NA)	0·334 (0–1·47)	0·321 (0–1·48)	0·331 (0–1·46)	-3·83 (-22–NA)	3·01 (-10·3–NA)
Occupational exposure to polycyclic aromatic hydrocarbons	0·26 (0·238–0·285)	0·265 (0·247–0·283)	0·279 (0·254–0·307)	1·71 (-9·57–14·1)	5·54 (-2·89–14·1)	0·278 (0·247–0·308)	0·294 (0·276–0·313)	0·304 (0·275–0·336)	5·83 (-7·24–20·2)	3·14 (-6·33–12·4)
Occupational exposure to silica	3·82 (0·536–12·4)	3·28 (0·458–10·1)	3·5 (0·471–10·8)	-14·1 (-27·1–1·07)	6·81 (-4·13–20·9)	2·88 (0·401–8·74)	2·61 (0·355–8·24)	2·68 (0·367–8·43)	-9·32 (-26·8–13)	2·47 (-11·8–20·7)
Occupational exposure to sulfuric acid	0·887 (0·19–2·72)	0·978 (0·204–3·08)	1·01 (0·202–3·2)	10·2 (-5·87–28·3)	3·13 (-8–14·8)	0·848 (0·174–2·72)	1·01 (0·204–3·3)	0·998 (0·21–3·14)	19·6 (-3·56–49)	-1·65 (-17–15·1)
Unsafe sex	--	--	--	--	--	--	--	--	--	--
Occupational exposure to trichloroethylene	0·0789 (0·0733–0·0853)	0·0824 (0·0789–0·0865)	0·0875 (0·0812–0·0942)	4·53 (-4·06–14·4)	6·13 (-0·976–13·8)	0·0778 (0·0697–0·0861)	0·0796 (0·0749–0·0846)	0·0815 (0·0737–0·0896)	2·29 (-8·82–15·8)	2·4 (-6·91–12)
No access to handwashing facility	3·43 (3·16–3·73)	3·14 (2·91–3·4)	3·1 (2·88–3·34)	-8·63 (-16·6–0·0342)	-1·23 (-6·88–4·48)	3·43 (3·16–3·73)	3·14 (2·91–3·4)	3·1 (2·88–3·34)	-8·63 (-16·6–0·0342)	-1·23 (-6·88–4·48)
Child wasting	0·75 (0·493–1·02)	0·728 (0·47–0·983)	0·726 (0·475–0·992)	-3·05 (-12·7–6·71)	-0·151 (-6·81–7·36)	--	--	--	--	--
Child stunting	2·48 (1·35–3·6)	2·09 (1·1–3·13)	2·04 (1·04–3·04)	-15·7 (-34·2–6·82)	-2·26 (-17·3–13·6)	1·99 (0·992–3·03)	1·79 (0·886–2·71)	1·78 (0·89–2·71)	-9·62 (-29·8–13·8)	-0·904 (-16·5–17·7)
Chewing tobacco	2·88 (2·18–3·75)	2·64 (1·87–3·62)	2·58 (1·81–3·72)	-8·28 (-38·7–36·3)	-2·33 (-24·6–29·1)	0·258 (0·166–0·399)	0·292 (0·183–0·454)	0·307 (0·191–0·476)	13·5 (-35·1–97·7)	4·85 (-26–48·5)
Diet low in legumes	74·2 (39·2–99·3)	77·6 (45·9–99·7)	77·9 (47–99·7)	4·66 (0·0239–21·9)	0·378 (-4·44–6·08)	72·6 (36·6–99·1)	75·9 (42·7–99·6)	76·5 (44·9–99·6)	4·52 (-0·0727–23·1)	0·84 (-3·99–9·58)
Short gestation	21·9 (17·8–26·5)	20·9 (17·5–24·7)	20 (16·2–24·2)	-4·53 (-22·7–18·4)	-4·31 (-23·3–17·3)	20·1 (16·7–24)	19·4 (16·6–22·4)	18·8 (15·9–22·2)	-3·73 (-23·1–19·4)	-2·63 (-18·5–16·3)
Low birth weight	8·94 (7·41–10·7)	8·49 (7·19–10)	8·52 (7–10·3)	-5·04 (-24·5–20·8)	0·399 (-19·9–25·1)	9·56 (7·95–11·2)	8·88 (7·63–10·4)	9·64 (8·21–11·3)	-7·1 (-26·7–16·9)	8·54 (-10·3–31·1)
High temperature	39 (24–53·9)	42·9 (26·8–59·1)	43·8 (26·8–60·9)	10·1 (5·53–15·7)	1·98 (-0·895–4·31)	39 (24–53·9)	42·9 (26·8–59·1)	43·8 (26·8–60·9)	10·1 (5·53–15·7)	1·98 (-0·895–4·31)
Low temperature	38·6 (30·1–48·2)	37·6 (27·8–48·1)	37·4 (27·9–47·8)	-2·69 (-10–3·36)	-0·428 (-3·26–2·55)	38·6 (30·1–48·2)	37·6 (27·8–48·1)	37·4 (27·9–47·8)	-2·69 (-10–3·36)	-0·428 (-3·26–2·55)
Kidney dysfunction	19·3 (13·7–26·3)	20 (14·2–26·9)	20·1 (14·5–27·3)	3·46 (-0·212–7·53)	0·53 (-3·17–4·35)	--	--	--	--	--
Bullying victimization	5·87 (2·42–12·1)	8·95 (3·54–17·7)	8·74 (3·36–18)	52·4 (32–75·7)	-2·28 (-11·8–3·69)	4·26 (1·74–8·94)	7·86 (3·14–15·7)	7·82 (2·93–16·1)	84·6 (58·8–114)	-0·598 (-11·1–6·36)
High LDL cholesterol	67·1 (62·8–71·5)	34·5 (30·9–38·2)	30·7 (27·3–34·5)	-48·6 (-54—43)	-11·1 (-15·8—5·83)	65·5 (62·1–69·4)	34·5 (31·2–37·9)	31·8 (28·3–35·4)	-47·4 (-52·3—42·8)	-7·68 (-11·9—3·55)

Supplementary Results Table 1. Arkansas age-standardised Summary Exposure Values (SEVs) for most-detailed risk factors and percentage change, by sex, 1990, 2010, and 2019. Data in parentheses are 95% uncertainty intervals.										
	Male					Female				
	1990	2010	2019	Percentage change 1990-2010	Percentage change 2010-2019	1990	2010	2019	Percentage change 1990-2010	Percentage change 2010-2019
Unsafe sanitation	10·8 (5·37–19·5)	5·57 (2·53–10·5)	4·42 (1·92–8·06)	-48·6 (-79·6–21·6)	-20·6 (-59·3–57·8)	--	--	--	--	--
Ambient particulate matter pollution	11·8 (3·89–24)	8·45 (5·58–11·8)	5·66 (3·02–8·67)	-28·2 (-64·2–87·4)	-33 (-46·4—25·6)	11·8 (3·92–24)	8·44 (5·59–11·8)	5·66 (3·02–8·67)	-28·1 (-64·2–86·7)	-32·9 (-46·2—25·5)
Household air pollution from solid fuels	0·0507 (0·013–0·135)	0·026 (0·00512–0·0789)	0·0177 (0·0034–0·0544)	-48·8 (-81·2–27·5)	-32 (-66·2–33·1)	0·0668 (0·0188–0·173)	0·0343 (0·00718–0·102)	0·0233 (0·00475–0·0715)	-48·7 (-81·2–27·5)	-31·9 (-66–33·1)
Ambient ozone pollution	67·3 (29·1–100)	59·5 (25·6–100)	34·3 (14·1–60·5)	-11·5 (-19·7–0)	-42·4 (-53·8—33)	67·3 (29·1–100)	59·5 (25·6–100)	34·3 (14·1–60·5)	-11·5 (-19·7–0)	-42·4 (-53·8—33)
Residential radon	18·4 (2·78–53·6)	18·4 (2·78–53·6)	18·4 (2·78–53·6)	0 (0–0)	0 (0–0)	18·4 (2·78–53·6)	18·4 (2·78–53·6)	18·4 (2·78–53·6)	0 (0–0)	0 (0–0)
Lead exposure	48·8 (19·8–73·1)	31 (10·6–53·7)	22·5 (6·18–42·5)	-36·3 (-53·5—24·3)	-27·5 (-44·3—19)	27·9 (1·75–56·3)	19·2 (1·2–43·6)	14·2 (0·639–35·4)	-31·2 (-58·1—4·94)	-25·7 (-57·5—17·4)
Child underweight	0·995 (0·565–1·51)	0·992 (0·548–1·5)	0·972 (0·541–1·48)	-0·297 (-17·5–21·1)	-2·02 (-15·4–12·8)	0·828 (0·46–1·26)	0·868 (0·484–1·35)	0·892 (0·483–1·35)	4·92 (-14·9–31·3)	2·67 (-12–18)
Iron deficiency	--	--	--	--	--	12·4 (10·4–14·7)	13 (11–15·1)	13·2 (11–15·5)	4·77 (-7·27–19·7)	1·96 (-7·84–12·5)
Vitamin A deficiency	1·31 (0·362–3·35)	1·63 (0·432–4·72)	1·12 (0·289–3·02)	24·5 (-69·6–434)	-31·5 (-71·3–70·1)	2·35 (0·918–4·88)	2·28 (0·835–4·91)	1·93 (0·695–4·18)	-2·92 (-63·3–182)	-15·5 (-54–68·1)
Zinc deficiency	2·71 (0–8·75)	1·36 (0–4·38)	1·36 (0–4·53)	-49·9 (-100–NA)	0·168 (-19·1–NA)	2·79 (0–8·57)	1·37 (0–4·56)	1·34 (0–4·32)	-50·9 (-100–NA)	-1·98 (-21·5–NA)
Smoking	30·9 (27·8–34·3)	22·8 (20·7–25)	20·8 (18·7–23)	-26·3 (-31·4—20·8)	-8·55 (-12·1—4·59)	21·1 (17·8–24·4)	17·2 (15·5–19)	16 (14·3–17·9)	-18·7 (-27·8—7·27)	-6·66 (-11·3—1·57)
Secondhand smoke	40·7 (38·2–43·1)	29·6 (27·8–31·4)	28·7 (25·8–31·4)	-27·3 (-33·4—20·8)	-3·09 (-11·4–5·14)	39·5 (37·6–41·5)	28·1 (26·8–29·3)	27·1 (25–29·4)	-28·8 (-33·5—23·9)	-3·44 (-9·95–3·7)
Alcohol use	12·9 (8·1–18·2)	13·2 (8·08–19·1)	13·3 (8·21–19·1)	2·94 (-27·8–41)	0·815 (-19·8–26·9)	7·67 (4·47–11·3)	9·07 (5·67–12·9)	8·98 (5·3–13)	18·2 (-16·3–66·7)	-0·93 (-22·3–25)
Drug use	0·61 (0·314–1·16)	0·908 (0·618–1·34)	1·25 (0·91–1·69)	49 (8·54–117)	37·4 (18·5–62·4)	0·483 (0·286–0·813)	0·728 (0·508–1·04)	1·11 (0·838–1·42)	50·9 (19·7–97·3)	51·9 (29·7–78·5)
High fasting plasma glucose	12·7 (10·5–15·4)	18·4 (15·8–21·4)	20·2 (17·5–23·3)	44·3 (18·9–74·2)	10·1 (-4·29–26·1)	--	--	--	--	--
High systolic blood pressure	25·8 (20·5–31·6)	16·7 (14·6–18·8)	20·3 (15·7–25·5)	-35·3 (-47·3—19·5)	21·5 (-2·88–53·4)	23·3 (17·4–30·9)	14·1 (12·4–15·9)	19·2 (14·1–25·7)	-39·3 (-54·2—16·8)	35·8 (-0·273–84·6)
High body-mass index	24·4 (16·9–34)	40·7 (29·8–52·3)	41·8 (30·7–53·3)	67 (46–100)	2·74 (-3·75–9·99)	31·1 (22·6–40·5)	45·1 (34·8–54·2)	45·9 (35·6–55·6)	45·2 (27·9–69)	1·67 (-4·83–8·86)
Low bone mineral density	12·2 (7·11–19)	9·34 (5·02–15·3)	11·9 (6·8–19·2)	-23·5 (-43·3—0·926)	27·8 (-0·48–71·2)	16·2 (10·8–23·1)	16·8 (11·3–23·8)	19·2 (13·2–26·8)	3·45 (-16·7–28·6)	14·6 (-7·58–44·7)
Diet low in fruits	55·8 (44·2–69)	53·7 (42·1–66·7)	52·5 (39·2–66)	-3·78 (-13·7–5·84)	-2·35 (-13·1–6·84)	57·6 (46·4–70·2)	54·8 (43·6–67·9)	53·9 (41·1–67·1)	-4·82 (-15–4·58)	-1·67 (-10·9–7·76)
Diet low in vegetables	33·5 (17·5–54·8)	54·3 (33–75·9)	51 (30·6–72·4)	62·2 (22·6–146)	-6·09 (-16·6–1·88)	38·8 (21·7–60·3)	55·5 (34–76·7)	52·1 (31·1–72·6)	43 (14·4–96)	-6·22 (-15·7–1·18)
Diet low in whole grains	80·1 (71·3–88·7)	82 (73·8–89·6)	82 (73·8–89·7)	2·41 (-1·19–6·81)	-0·0389 (-2·51–2·4)	77·6 (66·8–87·2)	80·2 (70·9–88·7)	79·9 (70·3–88·4)	3·39 (-0·692–9)	-0·349 (-3·08–2·23)
Diet low in nuts and seeds	31·6 (10·3–58·7)	26·5 (8·32–56·3)	22·3 (6·94–54·9)	-16·4 (-47–42·8)	-15·6 (-41·7–9·68)	27·4 (7·32–56·6)	23·5 (6·7–55·6)	20·8 (6·03–53·7)	-14·1 (-46·6–46)	-11·7 (-39–15·9)
Diet low in milk	52·1 (28·5–73·1)	47 (24·2–67·1)	45·5 (23·7–66·4)	-9·82 (-26·4–1·17)	-2·99 (-14·8–8·57)	--	--	--	--	--
Diet high in red meat	75·3 (62·5–86·4)	73·4 (58·9–85·3)	73·6 (59·3–86)	-2·53 (-18·6–14·9)	0·17 (-10·8–12·7)	76·3 (63·5–87·8)	72·6 (58·5–84·1)	73·1 (57·7–85)	-4·75 (-20·2–12)	0·627 (-11·4–14·7)
Diet high in processed meat	77·5 (53·3–97·7)	85·6 (66·5–99·6)	85·4 (66·5–99·7)	10·4 (1·14–30·5)	-0·162 (-4·83–4·73)	78·3 (54·4–97·8)	88·1 (71·1–99·7)	86·9 (68·8–99·6)	12·5 (1·32–36·4)	-1·41 (-6·85–2·47)
Diet high in sugar-sweetened beverages	42·1 (24·6–60·6)	57·9 (37·3–75·4)	58·8 (37·6–77·4)	37·6 (-7·43–129)	1·64 (-21·6–30)	46·1 (27–65·4)	64·1 (44·4–81)	63·3 (42·3–81·4)	39·1 (-3·94–121)	-1·27 (-21·1–22·6)
Diet low in fiber	47 (30·1–62·2)	33·4 (19·8–47·2)	30·4 (16·8–43·7)	-29 (-45·2—12·4)	-9·09 (-25·6–7·31)	47·5 (30·5–63·8)	38·8 (24·5–53·6)	33·5 (19·8–48·8)	-18·5 (-33·4—2·99)	-13·5 (-28·7–1·47)
Diet low in seafood omega-3 fatty acids	84·1 (66·7–99·5)	79·6 (60·7–99·7)	79·3 (61·4–99·5)	-5·3 (-19·9–7·16)	-0·414 (-13·4–16·6)	86·4 (70·3–99·9)	83·7 (68·2–99·1)	83·2 (66·6–99·3)	-3·15 (-14·4–5·67)	-0·673 (-11·4–10·5)
Diet low in polyunsaturated fatty acids	16·5 (4·89–43)	8·33 (2·36–28·6)	7·79 (2·25–27·4)	-49·5 (-75—8·55)	-6·44 (-30·6–16·1)	18·3 (5·4–47·9)	8·52 (2·54–28·3)	7·86 (2·35–26·7)	-53·5 (-77—9·48)	-7·76 (-29·9–10·4)
Diet high in trans fatty acids	89·4 (81·9–100)	87·8 (78·6–100)	87·3 (78·1–100)	-1·79 (-9·79–5·73)	-0·496 (-8·29–7·95)	88·3 (80·9–100)	86·7 (77·8–100)	86·3 (76·7–100)	-1·82 (-9·67–5·68)	-0·539 (-8·64–7·93)
Diet high in sodium	18·3 (4·64–41·3)	40 (12·7–64·6)	39·3 (11·5–64·6)	119 (41·3–435)	-1·83 (-28·4–30·2)	21 (4·94–45·8)	21·2 (4·98–44·9)	21·7 (5·1–45·9)	0·996 (-29·5–48)	2·36 (-16·8–29)
Low physical activity	1·82 (0·558–5·05)	1·61 (0·547–4·78)	1·68 (0·499–4·89)	-11·6 (-41·4–28·8)	4·04 (-23–40·3)	--	--	--	--	--
Occupational asthmagens	23·5 (19·6–28·7)	21·3 (18·1–25·3)	22·2 (18·3–26·8)	-9·28 (-19·9–1·75)	4·1 (-5·03–15)	11·7 (9·57–14·2)	11·3 (9·41–13·6)	11·5 (9·33–14·1)	-3·46 (-17–13·3)	1·25 (-10·6–13·6)
Occupational particulate matter, gases, and fumes	10·2 (7·3–14·4)	9·71 (6·98–13·8)	9·36 (6·7–13·3)	-5·06 (-10·3–0·764)	-3·65 (-7·22—0·293)	5·83 (4·09–8·51)	5·67 (4·08–8·1)	5·44 (3·92–7·67)	-2·77 (-11·7–7·6)	-3·95 (-9·21–1·61)
Occupational noise	8·93 (8·16–9·85)	8·58 (7·99–9·32)	8·3 (7·77–9)	-3·95 (-7·35–0·0774)	-3·26 (-5·46—0·994)	6·05 (5·27–6·92)	6·34 (5·77–6·97)	6·31 (5·84–6·9)	4·7 (-1·45–12·4)	-0·376 (-3·35–3·1)
Occupational injuries	--	--	--	--	--	--	--	--	--	--
Occupational ergonomic factors	13·6 (11·2–16·5)	11·3 (9·59–13·4)	11·7 (9·65–14·1)	-16·9 (-28·2—4·38)	3·77 (-5·26–14·6)	7·84 (6·14–9·85)	7·45 (6·07–9·19)	7·74 (6·18–9·64)	-5·04 (-21·8–13·3)	3·99 (-7·69–17·5)
Childhood sexual abuse	5·98 (3·56–9·11)	5·65 (3·71–7·74)	5·53 (3·74–7·61)	-5·44 (-22·9–18·3)	-2·17 (-8·19–4·43)	18·1 (13–25·7)	16·1 (12·9–21·4)	16·2 (12·7–21·6)	-11 (-23–4·9)	0·514 (-5·6–8·23)
Intimate partner violence	--	--	--	--	--	32 (18·1–41·6)	30·4 (17·2–39·9)	31·3 (18–40·7)	-5 (-13·5–9·14)	2·99 (-6·08–21·8)
Non-exclusive breastfeeding	25·9 (21·4–30·9)	24·9 (21·3–28·8)	24·8 (20·3–29·8)	-4·23 (-17·7–13·4)	-0·0912 (-13–14·7)	25·9 (21·4–30·9)	24·8 (21·3–28·8)	24·8 (20·3–29·8)	-4·22 (-17·7–13·4)	-0·098 (-13–14·7)
Discontinued breastfeeding	27·1 (26·2–28·1)	25·2 (23·9–26·4)	25·7 (24·5–26·9)	-7·1 (-12·6—1·92)	2·04 (-3·48–8·1)	27·2 (26·3–28·1)	25·3 (24–26·5)	25·8 (24·5–26·9)	-7·19 (-12·6—2·02)	1·89 (-3·62–7·93)
Diet low in calcium	18·6 (8·8–34·8)	15·6 (7·51–28·8)	14·8 (6·99–27·7)	-16·3 (-26·6—5·34)	-5·11 (-13·5–2·46)	14·2 (6·08–27·3)	12·7 (5·61–23·8)	12·2 (5·28–23)	-10·4 (-23·9–5·09)	-4 (-13·5–4·88)
Occupational exposure to asbestos	5·56 (4·98–6·15)	4·94 (4·47–5·44)	4·92 (3·53–6·57)	-11·1 (-22·8–3·43)	-0·346 (-28·9–33·8)	0·841 (0·577–1·97)	0·879 (0·627–1·96)	0·915 (0·523–2·22)	4·58 (-10·3–24·9)	4·07 (-27·2–42·6)
Occupational exposure to arsenic	0·541 (0–1·26)	0·537 (0–1·3)	0·566 (0–1·36)	-0·678 (-14–NA)	5·42 (-6·71–NA)	0·529 (0–1·27)	0·519 (0–1·25)	0·53 (0–1·29)	-1·94 (-18·8–NA)	2·25 (-11·3–NA)
Occupational exposure to benzene	0·784 (0·0459–2·2)	0·756 (0·044–2·22)	0·788 (0·0433–2·38)	-3·59 (-11·6–7·19)	4·28 (-2·89–14·4)	1·11 (0·0617–3·1)	1·22 (0·0731–3·4)	1·29 (0·0747–3·69)	10·4 (-1·66–30·9)	5·12 (-4·57–18·3)
Occupational exposure to beryllium	0·0239 (0·0224–0·0253)	0·0231 (0·0225–0·0238)	0·0244 (0·023–0·0259)	-3·1 (-9·32–3·3)	5·64 (-0·615–11·9)	0·0261 (0·0237–0·0285)	0·0271 (0·0264–0·0278)	0·0282 (0·0261–0·0304)	3·89 (-5·11–15·1)	3·99 (-3·29–11·2)
Occupational exposure to cadmium	0·0628 (0·0567–0·0695)	0·0625 (0·057–0·0689)	0·0658 (0·0578–0·0743)	-0·431 (-12·5–14·3)	5·25 (-5·55–15·8)	0·0621 (0·0543–0·0704)	0·0616 (0·0552–0·0684)	0·0631 (0·0546–0·0733)	-0·846 (-16·2–17·7)	2·34 (-9·73–15·5)
Occupational exposure to chromium	0·149 (0·136–0·163)	0·151 (0·14–0·164)	0·159 (0·143–0·176)	1·43 (-9·58–14·1)	5·23 (-4·03–14·7)	0·152 (0·135–0·17)	0·154 (0·141–0·168)	0·158 (0·14–0·18)	1·55 (-11·9–18·1)	2·4 (-8·28–14·1)
Occupational exposure to diesel engine exhaust	0·719 (0·661–0·78)	0·758 (0·71–0·808)	0·801 (0·726–0·878)	5·45 (-4·57–17·2)	5·69 (-2·73–14·1)	0·649 (0·567–0·73)	0·697 (0·632–0·767)	0·705 (0·629–0·794)	7·34 (-6·22–24·6)	1·21 (-8·42–11·9)
Occupational exposure to formaldehyde	0·307 (0·246–0·347)	0·3 (0·24–0·338)	0·312 (0·251–0·358)	-2·5 (-14·6–12·3)	4·01 (-6·98–14·9)	--	--	--	--	--
Occupational exposure to nickel	0·464 (0–2·03)	0·441 (0–1·94)	0·466 (0–1·96)	-4·9 (-17·4–NA)	5·64 (-6·16–NA)	0·423 (0–1·83)	0·403 (0–1·76)	0·413 (0–1·82)	-4·6 (-22·5–NA)	2·39 (-12·3–NA)
Occupational exposure to polycyclic aromatic hydrocarbons	0·292 (0·267–0·319)	0·295 (0·274–0·318)	0·31 (0·28–0·342)	0·927 (-9·77–12·5)	5·14 (-4·06–14·2)	0·316 (0·284–0·352)	0·33 (0·307–0·355)	0·339 (0·304–0·378)	4·49 (-7·7–19·8)	2·76 (-7·12–13·3)
Occupational exposure to silica	4·36 (0·619–13·9)	3·97 (0·553–12·7)	4·2 (0·587–12·9)	-8·8 (-20·9–5·39)	5·81 (-4·87–19·5)	3·63 (0·511–11·8)	3·3 (0·447–10·4)	3·37 (0·444–10·9)	-9·04 (-25·5–16·3)	1·99 (-12·5–21·8)
Occupational exposure to sulfuric acid	0·946 (0·201–3·03)	1·01 (0·201–3·15)	1·05 (0·214–3·37)	6·56 (-6·13–21·7)	4·07 (-6·69–14·9)	0·944 (0·199–2·96)	1·11 (0·234–3·58)	1·11 (0·237–3·47)	17·6 (-2·3–42·1)	-0·224 (-13·6–15·2)
Unsafe sex	--	--	--	--	--	--	--	--	--	--
Occupational exposure to trichloroethylene	0·086 (0·0801–0·0928)	0·089 (0·085–0·0935)	0·0937 (0·0869–0·101)	3·49 (-4·96–12·8)	5·35 (-2·23–12·8)	0·0857 (0·0767–0·0952)	0·0888 (0·0829–0·0949)	0·0907 (0·0822–0·0999)	3·54 (-7·77–18·3)	2·15 (-6·34–12·1)
No access to handwashing facility	3·52 (3·24–3·8)	3·17 (2·92–3·43)	3·13 (2·89–3·38)	-9·8 (-18·2—1·05)	-1·19 (-6·79–4·32)	3·52 (3·24–3·8)	3·17 (2·92–3·43)	3·13 (2·89–3·38)	-9·8 (-18·2—1·05)	-1·19 (-6·79–4·32)
Child wasting	0·755 (0·499–1·03)	0·732 (0·486–0·997)	0·731 (0·484–0·993)	-3·05 (-13–8·02)	-0·127 (-7·16–7·68)	0·778 (0·516–1·07)	0·769 (0·514–1·05)	0·765 (0·514–1·05)	-1·24 (-12–9·95)	-0·428 (-8·09–8·22)
Child stunting	2·56 (1·41–3·74)	2·18 (1·15–3·3)	2·12 (1·13–3·24)	-15 (-33·4–9·07)	-2·62 (-17·7–14)	2·02 (1·01–3·01)	1·86 (0·965–2·84)	1·84 (0·922–2·78)	-8·14 (-27·9–14·5)	-0·782 (-16–18·6)
Chewing tobacco	7·18 (5·46–9·31)	7·64 (5·68–10·1)	7·47 (5·43–10·2)	6·43 (-25·6–54·7)	-2·15 (-22·6–23·7)	0·417 (0·271–0·635)	0·461 (0·287–0·708)	0·481 (0·298–0·703)	10·7 (-34·3–87·3)	4·27 (-26·8–44·5)
Diet low in legumes	75·2 (40·2–99·5)	78·2 (48·6–99·8)	78 (47·2–99·7)	3·9 (-0·298–21·9)	-0·237 (-6·26–4·85)	73·6 (37·4–99·3)	76·5 (44·1–99·6)	76·6 (46·1–99·6)	3·92 (-0·249–20·8)	0·212 (-5·47–7·23)
Short gestation	27·4 (22·9–32·7)	23·7 (20·1–28·2)	22·6 (18·5–27·6)	-13·3 (-28·6–6·21)	-4·84 (-22·9–15·8)	22·8 (19·2–26·9)	22·3 (19·1–26·1)	21 (17·7–24·6)	-2·17 (-20·2–19·9)	-5·95 (-21·4–13·9)
Low birth weight	11·5 (9·71–13·5)	10·6 (8·88–12·5)	10·7 (8·99–12·7)	-7·95 (-26–13·3)	1·02 (-18–24·5)	11·8 (10·1–13·7)	12·1 (10·4–14)	11·7 (9·96–13·8)	2·37 (-17·1–25·5)	-2·84 (-18·9–17·6)
High temperature	29·1 (20·4–38·5)	32·8 (23·8–42)	24·7 (16·3–34)	12·8 (7·6–19·8)	-24·6 (-32·5—17)	29·1 (20·4–38·5)	32·8 (23·8–42)	24·7 (16·3–34)	12·8 (7·6–19·8)	-24·6 (-32·5—17)
Low temperature	35·9 (30·2–43)	38·2 (33·6–44·5)	37·5 (32·5–44)	6·54 (2·19–12·4)	-1·7 (-4·89–0·91)	35·9 (30·2–43)	38·2 (33·6–44·5)	37·5 (32·5–44)	6·54 (2·19–12·4)	-1·7 (-4·89–0·91)
Kidney dysfunction	20·1 (14·4–27)	20·9 (15–28)	21·1 (15·2–28·4)	4·02 (0·277–8·59)	0·742 (-2·83–4·61)	22·5 (16–30·2)	23·8 (17·1–31·8)	24·8 (17·9–32·6)	5·92 (1·9–11·5)	4·1 (0·126–8·86)
Bullying victimization	5·91 (2·42–12·1)	8·91 (3·55–17·5)	8·69 (3·36–17·9)	50·8 (31–74·2)	-2·43 (-12·2–3·73)	4·27 (1·74–8·99)	7·8 (3·13–15·5)	7·74 (2·91–16)	82·5 (58·2–111)	-0·796 (-11·2–6·1)
High LDL cholesterol	65·1 (60·6–69·3)	34·4 (31–38)	30·4 (26·9–34·2)	-47·1 (-52·6—41·3)	-11·6 (-16·8—6·82)	64·5 (60·8–68·3)	34·7 (31·4–38·3)	31·8 (28·4–35·4)	-46·1 (-50·8—40·9)	-8·33 (-12·4—4·35)

Supplementary Results Table 1. California age-standardised Summary Exposure Values (SEVs) for most-detailed risk factors and percentage change, by sex, 1990, 2010, and 2019. Data in parentheses are 95% uncertainty intervals.

	Male					Female				
	1990	2010	2019	Percentage change 1990-2010	Percentage change 2010-2019	1990	2010	2019	Percentage change 1990-2010	Percentage change 2010-2019
Unsafe sanitation	7·25 (3·59–12·6)	3·27 (1·5–6·33)	2·52 (1·12–4·77)	-54·9 (-82·3–9·98)	-22·9 (-59·3–46·4)	--	--	--	--	--
Ambient particulate matter pollution	14·4 (5·69–27·5)	8·08 (5·5–11·1)	6·95 (4·43–9·87)	-43·7 (-70·8–29·4)	-14 (-20·4–10·4)	14·4 (5·7–27·5)	8·08 (5·5–11·1)	6·95 (4·44–9·85)	-43·7 (-70·8–29)	-14 (-20·4–10·3)
Household air pollution from solid fuels	0·0108 (0·00278–0·0293)	0·00643 (0·00108–0·022)	0·00475 (0·000812–0·0162)	-40·3 (-77·5–29·8)	-26 (-60·8–37·4)	0·0142 (0·0038–0·0379)	0·0085 (0·00159–0·0272)	0·00629 (0·00114–0·0218)	-40 (-77·3–29·7)	-26·1 (-60·7–37·5)
Ambient ozone pollution	90·1 (44·9–100)	72·2 (32–100)	75·8 (33·4–100)	-19·9 (-34·4–0)	5·07 (0–11·1)	--	--	--	--	--
Residential radon	17·6 (2·51–52·9)	17·6 (2·51–52·9)	17·6 (2·51–52·9)	0 (0–0)	0 (0–0)	17·6 (2·51–52·9)	17·6 (2·51–52·9)	17·6 (2·51–52·9)	0 (0–0)	0 (0–0)
Lead exposure	79 (55·1–92·7)	46·9 (25·8–65·4)	32·9 (15·6–49·6)	-40·6 (-53·7–27·6)	-30 (-39·6–24)	54·2 (21·5–78·8)	29·7 (6·41–53·8)	19·9 (2·64–39·4)	-45·2 (-70·2–29·3)	-33·1 (-56·9–25·6)
Child underweight	0·963 (0·548–1·48)	0·944 (0·521–1·42)	0·918 (0·506–1·4)	-1·98 (-19·1–17·2)	-2·68 (-14·9–10·4)	0·804 (0·423–1·21)	0·833 (0·447–1·29)	0·847 (0·445–1·3)	3·64 (-16·3–29·9)	1·68 (-12·3–17·1)
Iron deficiency	--	--	--	--	--	11·7 (9·74–13·7)	11·6 (9·72–13·6)	11·6 (9·62–13·9)	-0·922 (-10·7–9·81)	0·169 (-8·25–9·45)
Vitamin A deficiency	0·853 (0·258–2·14)	1·01 (0·297–2·54)	0·689 (0·202–1·74)	18·7 (-70·1–391)	-32 (-69·4–51)	1·76 (0·734–3·53)	1·59 (0·632–3·39)	1·32 (0·517–2·89)	-9·65 (-63·9–156)	-17·4 (-53·3–48·1)
Zinc deficiency	1·94 (0–6·53)	1·05 (0–3·33)	1·06 (0–3·32)	-45·9 (-100–NA)	0·885 (-23·8–NA)	1·99 (0–6·67)	1·06 (0–3·41)	1·05 (0–3·31)	-46·8 (-100–NA)	-1·09 (-19·4–NA)
Smoking	24·7 (22·1–27·4)	16·1 (14·4–17·9)	13·2 (11·7–14·8)	-34·8 (-39–30)	-17·9 (-21·3–14·7)	18·5 (16·3–20·8)	10·7 (9·52–12·1)	7·97 (6·97–8·99)	-41·9 (-47–36·4)	-25·7 (-29·9–21·3)
Secondhand smoke	37·3 (34·9–40)	21·1 (19·6–22·7)	21·7 (19·3–24·2)	-43·4 (-48·8–37·5)	2·62 (-6·31–11·9)	33·9 (31·9–35·7)	19·8 (18·8–20·9)	20·4 (18·6–22·3)	-41·4 (-46·3–36·4)	2·9 (-4·77–11·3)
Alcohol use	14·4 (9–20·5)	15·8 (10–22·8)	17·6 (10·9–24·9)	10·2 (-19–57·7)	11·3 (-8·88–36·4)	9·76 (5·76–14·8)	10·6 (6·58–15·3)	11·2 (6·67–16·3)	8·42 (-21·2–61·4)	5·53 (-15·2–31·5)
Drug use	0·642 (0·374–1·14)	0·768 (0·534–1·11)	0·949 (0·683–1·32)	19·6 (-5·09–54·4)	23·5 (8·46–42·4)	0·407 (0·27–0·621)	0·607 (0·445–0·821)	0·807 (0·627–1·03)	49·3 (24·5–81·8)	32·9 (15·8–53·7)
High fasting plasma glucose	11·1 (9·26–13·4)	16·2 (14–18·7)	17·7 (15·2–20·5)	45·2 (22·1–75·3)	9·29 (-4·39–26·9)	8·61 (7·26–10·4)	12·4 (10·4–15·1)	13·9 (11·5–17)	44·3 (20–73)	12 (-5·21–33·7)
High systolic blood pressure	24·4 (20·1–29·6)	15·4 (13·5–17·4)	19·1 (14·9–24·3)	-36·7 (-48·2–23·7)	24·2 (-0·279–53·5)	21·6 (16·1–27·8)	12·7 (11·2–14·4)	16·7 (12·3–21·9)	-41 (-55–21·5)	31·1 (-3·01–72·3)
High body-mass index	22 (15·2–30·7)	37·4 (27·2–49·2)	39 (28·6–50·6)	69·6 (47·2–101)	4·25 (-1·89–11·2)	28·2 (20·4–37·6)	42 (32·3–51)	43·1 (33·7–52·3)	48·8 (30–73·5)	2·66 (-3·32–10)
Low bone mineral density	12·3 (7·34–19·4)	13·4 (7·86–20·6)	14·2 (8·77–21·9)	8·86 (-16–46·2)	6·2 (-17·1–38·3)	15·6 (10·3–22·4)	15·8 (10·1–22·7)	18·2 (12·4–25·6)	1·52 (-19·4–28·6)	15 (-7·64–46)
Diet low in fruits	48·8 (37·3–62·5)	40·3 (29·1–53)	39·7 (28·1–53·2)	-17·6 (-29·1–7·14)	-1·36 (-15·6–12·9)	51·6 (39·8–65)	42·2 (30·6–54·4)	41 (28·9–54·2)	-18·3 (-30–8·67)	-2·7 (-15·7–10·6)
Diet low in vegetables	11·5 (4·43–24·9)	8·69 (4·21–18·5)	7·79 (3·62–17·1)	-24·2 (-49·1–21·7)	-10·4 (-36·8–20)	15·2 (5·79–31·7)	17·8 (8·16–33·3)	15·9 (6·66–30·9)	17 (-13·3–85·4)	-10·6 (-36·4–17·8)
Diet low in whole grains	79·3 (70–88·1)	81·3 (72·7–89·4)	81·2 (72·4–89·2)	2·59 (-0·678–7·29)	-0·0822 (-2·34–2·26)	76·5 (66–86·1)	79·4 (69·8–88·3)	79·1 (69·5–88·2)	3·76 (-0·196–9·13)	-0·377 (-3·11–2·38)
Diet low in nuts and seeds	24·9 (6·56–55·1)	24 (7·11–55·2)	19 (5·07–53·7)	-3·53 (-40–76·6)	-20·9 (-46·9–3·47)	20·8 (4·64–53·2)	20·4 (6·51–53·6)	17·2 (5·29–52·4)	-1·98 (-38·8–87·4)	-15·9 (-41·8–9·81)
Diet low in milk	46 (22·9–66·6)	40·7 (18·7–60·3)	39·1 (17·5–59·2)	-11·7 (-30–1·84)	-3·84 (-16·9–8·98)	--	--	--	--	--
Diet high in red meat	80 (68·8–89·6)	77·6 (65·1–88·7)	78 (65·9–88·8)	-3 (-15·7–10·7)	0·51 (-9·82–11·8)	80·8 (69·3–90·2)	77·1 (64–87·4)	77·2 (63·9–88·2)	-4·56 (-16·6–9·38)	0·199 (-10·5–12·6)
Diet high in processed meat	78·6 (54·2–98·4)	86·4 (67·6–99·8)	86·4 (67·9–99·7)	9·86 (0·968–28·8)	-0·0321 (-4·7–4·54)	79·9 (57–98·4)	89·4 (74·4–99·7)	88·1 (71·8–99·7)	11·9 (1·15–35·9)	-1·47 (-6·94–2·39)
Diet high in sugar-sweetened beverages	48·2 (29·7–66·9)	65·9 (47·7–82)	66·8 (47·4–83·4)	36·7 (-2·19–106)	1·32 (-18·4–23·7)	52·5 (33·4–71·3)	71·7 (54·8–86·9)	70·7 (52·5–86·1)	36·6 (3·29–108)	-1·35 (-18·4–20)
Diet low in fiber	40·7 (25·3–56·5)	28 (15·7–41·1)	24·8 (13·4–38·4)	-31·3 (-48–14·8)	-11·4 (-29·1–6·16)	41·1 (25·2–56·4)	33·3 (20·4–47·1)	27·9 (15·4–41·4)	-19 (-33·9–2·43)	-16·2 (-32·6–2·02)
Diet low in seafood omega-3 fatty acids	81·4 (64–99·9)	76·8 (57·8–98·4)	75·7 (56·7–98·8)	-5·67 (-20·6–10·3)	-1·39 (-17·6–15·6)	84·2 (67·2–99·7)	81·3 (63·5–99·1)	80·2 (61·9–99·2)	-3·48 (-17–9·76)	-1·31 (-14·1–12·2)
Diet low in polyunsaturated fatty acids	12·2 (3·52–37·5)	6·6 (1·91–24·4)	6·27 (1·79–24·5)	-46·1 (-74·9–6·24)	-5·02 (-26·9–11·9)	13·7 (3·93–39·3)	6·78 (1·96–24·6)	6·35 (1·82–24·6)	-50·5 (-77·3–9·36)	-6·38 (-30·1–9·3)
Diet high in trans fatty acids	89·5 (82–100)	88 (79·4–100)	87·4 (78·5–100)	-1·6 (-9·28–5·45)	-0·67 (-8·25–7·18)	--	--	--	--	--
Diet high in sodium	18 (4·71–39·9)	39·2 (11·7–63·9)	38·4 (10·7–64)	118 (43·7–410)	-2 (-25·7–26)	20·8 (5·01–45·2)	21 (4·98–45·2)	21·6 (4·98–46)	1·08 (-28·3–48)	2·62 (-17·3–25·9)
Low physical activity	1·94 (0·69–4·34)	1·47 (0·546–3·87)	1·84 (0·599–4·11)	-24 (-52·6–7·51)	24·7 (-6·39–85)	4·25 (2·02–8·35)	3·72 (1·85–6·98)	4·17 (2·04–8·24)	-12·5 (-34·2–17·4)	12 (-5·34–31·2)
Occupational asthmagens	21·5 (18·1–25·7)	19·2 (16·2–22·7)	19·9 (16·7–23·9)	-10·7 (-21·1–0·361)	3·79 (-4·29–12·1)	10·8 (8·8–13·3)	10·2 (8·5–12·3)	10·5 (8·6–12·8)	-5·6 (-19·2–10·4)	2·6 (-9·21–14·5)
Occupational particulate matter, gases, and fumes	8·84 (6·27–12·6)	8·25 (5·92–11·8)	7·97 (5·68–11·3)	-6·63 (-12–0·868)	-3·49 (-7·02–0·0366)	4·81 (3·34–7·1)	4·61 (3·26–6·85)	4·4 (3·15–6·39)	-4·21 (-12·4–6)	-4·57 (-9·62–0·713)
Occupational noise	8·17 (7·49–8·96)	7·92 (7·38–8·58)	7·69 (7·2–8·34)	-3·1 (-6·49–0·699)	-2·95 (-4·83–0·918)	5·66 (4·99–6·37)	5·87 (5·41–6·48)	5·82 (5·43–6·41)	3·7 (-2·08–10·6)	-0·806 (-3·68–2·28)
Occupational injuries	--	--	--	--	--	--	--	--	--	--
Occupational ergonomic factors	11·5 (9·5–14)	10·1 (8·46–12·1)	10·4 (8·6–12·6)	-11·9 (-20·8–2·09)	3·08 (-3·99–11·1)	6·34 (5·03–8·04)	6·16 (5·03–7·62)	6·46 (5·17–7·98)	-2·93 (-16·7–13·5)	4·93 (-6·17–17·7)
Childhood sexual abuse	7·4 (5·05–10·5)	6·97 (4·6–9·93)	6·85 (4·54–9·67)	-5·83 (-12·2–5·83)	-1·68 (-8·51–5·53)	12·3 (8·94–17·3)	17·4 (12·9–24)	17·8 (13·1–24·7)	41·9 (30·7–54·8)	2·17 (-5–11)
Intimate partner violence	--	--	--	--	--	15 (7·57–24·1)	14 (7·45–22·1)	16·5 (7·51–26·5)	-6·6 (-18·9–16·4)	17·4 (-5·22–51·9)
Non-exclusive breastfeeding	26·5 (21·9–31·4)	25 (21·5–28·5)	25 (20·5–29·6)	-5·55 (-19·6–10)	-0·12 (-13·7–13·2)	26·5 (21·9–31·4)	25 (21·5–28·5)	25 (20·5–29·6)	-5·54 (-19·6–10)	-0·128 (-13·7–13·2)
Discontinued breastfeeding	27·8 (26·9–28·7)	25·4 (24·2–26·6)	25·5 (24·2–26·6)	-8·65 (-14–3·81)	0·0985 (-5·07–5·69)	27·8 (26·9–28·7)	25·4 (24·2–26·6)	25·5 (24·2–26·6)	-8·64 (-14–3·8)	0·068 (-5·1–5·65)
Diet low in calcium	14·6 (7–26·9)	12·3 (6·02–22·6)	11·3 (5·49–20·7)	-15·7 (-26·5–3·15)	-8·12 (-18·1–0·0606)	9·89 (4·3–19·2)	9·23 (4·06–17·1)	8·6 (3·67–16)	-6·71 (-22·1–12·5)	-6·74 (-18·5–3·12)
Occupational exposure to asbestos	8·02 (7·21–8·92)	6·61 (6·13–7·08)	5·75 (4·21–7·62)	-17·6 (-27·5–6·96)	-13·1 (-36·5–13·9)	1·58 (1·16–2·12)	1·41 (0·976–1·79)	1·34 (0·849–1·92)	-10·7 (-28·7–7·15)	-5·01 (-30·8–29)
Occupational exposure to arsenic	0·495 (0–1·18)	0·49 (0–1·17)	0·524 (0–1·25)	-0·975 (-14·4–NA)	6·95 (-4·12–NA)	0·452 (0–1·05)	0·445 (0–1·06)	0·457 (0–1·12)	-1·53 (-19–NA)	2·6 (-10·1–NA)
Occupational exposure to benzene	0·882 (0·0526–2·46)	0·858 (0·0502–2·45)	0·887 (0·0543–2·6)	-2·65 (-9·96–8·25)	3·34 (-4·43–13)	1·18 (0·0733–3·28)	1·27 (0·0772–3·68)	1·34 (0·081–3·84)	7·79 (-2·98–26·2)	5·48 (-3·75–17·2)
Occupational exposure to beryllium	0·0235 (0·0223–0·0248)	0·0232 (0·0227–0·0236)	0·0245 (0·0233–0·0258)	-1·32 (-6·65–3·97)	5·72 (0·517–11·1)	0·0254 (0·0232–0·0276)	0·0256 (0·0251–0·0261)	0·0266 (0·0249–0·0283)	0·746 (-7·38–10·6)	4·02 (-3·26–11·3)
Occupational exposure to cadmium	0·0581 (0·0528–0·0642)	0·0577 (0·0528–0·0629)	0·0615 (0·0546–0·0684)	-0·591 (-12·5–12·7)	6·63 (-3·16–17·1)	0·0542 (0·0482–0·061)	0·0535 (0·0482–0·0596)	0·055 (0·0477–0·0633)	-1·18 (-16·4–15·8)	2·76 (-8·81–15·8)
Occupational exposure to chromium	0·14 (0·129–0·153)	0·142 (0·132–0·152)	0·15 (0·135–0·165)	1·44 (-9·01–13)	6·04 (-2·27–14·8)	0·136 (0·123–0·152)	0·137 (0·126–0·149)	0·141 (0·126–0·158)	0·6 (-12·6–14·8)	2·71 (-7·22–13·4)
Occupational exposure to diesel engine exhaust	0·678 (0·629–0·732)	0·737 (0·692–0·786)	0·768 (0·703–0·836)	8·68 (-1·29–19·3)	4·2 (-3·56–12·3)	0·631 (0·561–0·703)	0·63 (0·58–0·693)	0·631 (0·561–0·703)	-0·265 (-12·8–14·9)	0·266 (-9·39–10·7)
Occupational exposure to formaldehyde	0·294 (0·266–0·326)	0·284 (0·257–0·311)	0·299 (0·264–0·335)	-3·18 (-15·3–10·6)	5·23 (-4·69–16·1)	--	--	--	--	--
Occupational exposure to nickel	0·412 (0–1·83)	0·391 (0–1·66)	0·42 (0–1·76)	-5·13 (-17·7–NA)	7·43 (-3·95–NA)	0·355 (0–1·56)	0·337 (0–1·48)	0·349 (0–1·6)	-5·04 (-22·8–NA)	3·6 (-11·5–NA)
Occupational exposure to polycyclic aromatic hydrocarbons	0·281 (0·259–0·306)	0·284 (0·265–0·303)	0·3 (0·272–0·328)	1·16 (-8·53–12·5)	5·73 (-2·18–13·9)	0·291 (0·262–0·321)	0·302 (0·283–0·322)	0·311 (0·282–0·343)	3·78 (-8·14–17·2)	3·05 (-6·01–12·5)
Occupational exposure to silica	3·69 (0·516–11·6)	3·4 (0·466–10·7)	3·67 (0·496–11·5)	-7·7 (-20–7·37)	7·77 (-3·39–21·8)	2·98 (0·429–9·08)	2·72 (0·365–8·86)	2·77 (0·381–8·77)	-8·57 (-26·7–13·9)	1·83 (-12·6–21·9)
Occupational exposure to sulfuric acid	0·922 (0·197–2·87)	1 (0·208–3·15)	1·04 (0·219–3·4)	8·62 (-4·77–24·8)	4·25 (-5·57–15·2)	0·835 (0·171–2·69)	0·966 (0·198–3·14)	0·956 (0·2–2·99)	15·7 (-5·58–39·9)	-1·07 (-14·3–13·4)
Unsafe sex	--	--	--	--	--	--	--	--	--	--
Occupational exposure to trichloroethylene	0·0818 (0·0768–0·0878)	0·0853 (0·0818–0·0894)	0·0899 (0·0837–0·0964)	4·27 (-3·47–12·9)	5·45 (-0·91–12·4)	0·0792 (0·0716–0·0877)	0·0795 (0·0751–0·0848)	0·0812 (0·0737–0·0894)	0·398 (-10·2–12·9)	2·1 (-6·48–11·5)
No access to handwashing facility	3·38 (3·12–3·62)	3·1 (2·87–3·32)	3·07 (2·85–3·29)	-8·15 (-15·9–0·47)	-1·12 (-6·44–4·67)	3·38 (3·12–3·62)	3·1 (2·87–3·32)	3·07 (2·85–3·29)	-8·15 (-15·9–0·47)	-1·12 (-6·44–4·67)
Child wasting	0·748 (0·496–1·04)	0·725 (0·469–0·989)	0·723 (0·478–0·99)	-3·04 (-10·8–7·71)	-0·284 (-7·81–7·04)	0·773 (0·506–1·06)	1·03 (0·702–1·34)	0·755 (0·508–1·04)	33·6 (2·61–58·8)	-26·9 (-37·4–5·88)
Child stunting	2·41 (1·25–3·52)	2·03 (1·05–3·02)	1·97 (1·01–2·94)	-15·9 (-32·1–5·64)	-2·94 (-16·2–12·5)	1·94 (0·968–2·9)	1·75 (0·828–2·65)	1·72 (0·849–2·61)	-9·67 (-27·8–11·9)	-1·71 (-15·7–14·9)
Chewing tobacco	2·29 (1·71–3·02)	2·42 (1·76–3·29)	2·38 (1·68–3·26)	5·7 (-27·9–53·9)	-1·54 (-23·2–24·8)	0·263 (0·175–0·388)	0·281 (0·181–0·426)	0·287 (0·182–0·449)	6·86 (-37·2–70)	2·37 (-26·3–40·9)
Diet low in legumes	73·1 (37–99·4)	76·3 (44·7–99·8)	76·1 (43·8–99·6)	4·39 (0–26·1)	-0·266 (-5·4–4·76)	71·4 (32·9–98·8)	74·5 (39·4–99·4)	74·6 (41·4–99·4)	4·29 (0·0422–23·6)	0·181 (-5–6·53)
Short gestation	24·7 (20·6–29·3)	16·8 (14·2–19·7)	17·6 (14·6–21)	-32·1 (-45·1–17·4)	4·66 (-13·2–28·8)	21·8 (18–26·3)	16·2 (13·7–19·1)	17 (14·3–20)	-25·6 (-39·9–6·36)	4·98 (-13·4–28·6)
Low birth weight	10·3 (8·57–12·3)	7·62 (6·51–8·82)	7·9 (6·63–9·34)	-26·1 (-41–8·75)	3·69 (-15·5–28·8)	10·1 (8·4–12·1)	8·39 (7·12–9·79)	8·85 (7·53–10·5)	-17 (-34–6·08)	5·48 (-12·9–30·3)
High temperature	31 (23·2–39·8)	29·7 (21·9–38·1)	34·6 (26·1–44·2)	-4·15 (-9·18–1·5)	16·6 (9·14–24·3)	31 (23·2–39·8)	29·7 (21·9–38·1)	34·6 (26·1–44·2)	-4·15 (-9·18–1·5)	16·6 (9·14–24·3)
Low temperature	32·4 (26·6–40·5)	33·7 (27·7–41·7)	31·8 (25·8–39·9)	3·88 (1·52–6·42)	-5·53 (-7·45–3·46)	32·4 (26·6–40·5)	33·7 (27·7–41·7)	31·8 (25·8–39·9)	3·88 (1·52–6·42)	-5·53 (-7·45–3·46)
Kidney dysfunction	16·3 (11·6–22·8)	16·4 (11·9–22·7)	16·7 (12–23)	0·729 (-4·89–6·47)	1·91 (-3·24–7·69)	21·2 (15·2–28·6)	22 (15·7–29·4)	22 (15·9–29·4)	3·51 (-1–8·51)	0·263 (-4·44–5·42)
Bullying victimization	5·78 (2·42–11·8)	8·96 (3·59–17·8)	8·69 (3·35–17·9)	54·9 (34–78·5)	-3 (-12·7–3·15)	4·22 (1·73–8·76)	7·88 (3·15–15·7)	7·77 (2·93–16)	86·9 (60·8–117)	-1·47 (-11·8–5·45)
High LDL cholesterol	77·9 (73·4–82·3)	34·2 (30·7–37·9)	30·5 (27–34·2)	-56·1 (-61·2–51·3)	-10·9 (-15·8–5·88)	66·2 (62·9–69·3)	34 (30·7–37·4)	31·3 (27·8–34·8)	-48·6 (-52·9–44·3)	-7·93 (-12–4·12)

Supplementary Results Table 1. Colorado age-standardised Summary Exposure Values (SEVs) for most-detailed risk factors and percentage change, by sex, 1990, 2010, and 2019. Data in parentheses are 95% uncertainty intervals.

	Male					Female				
	1990	2010	2019	Percentage change 1990-2010	Percentage change 2010-2019	1990	2010	2019	Percentage change 1990-2010	Percentage change 2010-2019
Unsafe sanitation	5·43 (2·51–9·81)	3·01 (1·27–5·73)	2·34 (1·05–4·74)	-44·7 (-77·2–41·2)	-22·2 (-59·4–53)	5·43 (2·51–9·81)	3·01 (1·27–5·73)	2·34 (1·05–4·74)	-44·7 (-77·2–41·2)	-22·2 (-59·4–53)
Ambient particulate matter pollution	6·63 (1·07–15·8)	3·64 (1·35–6·35)	2·94 (0·807–5·59)	-45·1 (-81·2–134)	-19·3 (-41·6–-11·7)	6·63 (1·09–15·8)	3·64 (1·35–6·36)	2·94 (0·808–5·59)	-45·1 (-80·9–135)	-19·3 (-41·7–-11·7)
Household air pollution from solid fuels	0·0192 (0·00295–0·058)	0·0109 (0·00147–0·0377)	0·0078 (0·000867–0·0284)	-43·1 (-81·5–67·5)	-28·5 (-64·4–29·9)	0·0254 (0·00412–0·0733)	0·0145 (0·00206–0·0483)	0·0104 (0·00121–0·0396)	-42·7 (-81·3–68·2)	-28·5 (-64·4–30·2)
Ambient ozone pollution	69·4 (30·8–100)	84·1 (39·8–100)	79·9 (36·3–100)	21·2 (0–46)	-5·02 (-15·2–0)	--	--	--	--	--
Residential radon	23·7 (4·2–65·2)	23·7 (4·2–65·2)	23·7 (4·2–65·2)	0 (0–0)	0 (0–0)	23·7 (4·2–65·2)	23·7 (4·2–65·2)	23·7 (4·2–65·2)	0 (0–0)	0 (0–0)
Lead exposure	64·5 (38·6–84)	36·4 (16·1–57·6)	25 (9·02–43·4)	-43·5 (-58·9–-30·2)	-31·4 (-44·8–-24)	38·8 (8·21–66·9)	21·1 (2·03–45·8)	14·1 (0·681–34·3)	-45·7 (-77·6–-29·3)	-32·8 (-68·3–-24·5)
Child underweight	0·941 (0·524–1·44)	0·943 (0·52–1·42)	0·924 (0·506–1·41)	0·238 (-16·7–21·6)	-2·02 (-15–12·4)	0·792 (0·429–1·21)	0·835 (0·454–1·28)	0·852 (0·456–1·29)	5·36 (-16·8–36·4)	2·09 (-12·3–19·4)
Iron deficiency	--	--	--	--	--	11·4 (9·46–13·4)	11·6 (9·66–13·5)	11·8 (9·73–14)	2·41 (-9·82–14·9)	1·46 (-8·6–11·5)
Vitamin A deficiency	0·747 (0·197–2·01)	1 (0·247–2·69)	0·677 (0·183–1·89)	33·9 (-66·3–492)	-32·3 (-70·5–65·8)	1·5 (0·591–2·97)	1·51 (0·571–3·24)	1·26 (0·478–2·89)	0·769 (-60·7–215)	-16·7 (-53·6–62·3)
Zinc deficiency	2·08 (0–6·71)	1·11 (0–3·53)	1·12 (0–3·56)	-46·5 (-100–NA)	0·316 (-20·2–NA)	2·14 (0–6·83)	1·13 (0–3·62)	1·09 (0–3·44)	-47·2 (-100–NA)	-2·95 (-24·1–NA)
Smoking	26·8 (23·8–29·7)	18·1 (16·1–20)	15·3 (13·6–17·1)	-32·7 (-37·5–-27·1)	-15·1 (-18·8–-11·1)	19·4 (16·8–22·5)	14 (12·5–15·6)	11·7 (10·3–13·1)	-27·8 (-36·3–-18·3)	-16·7 (-20·7–-12·4)
Secondhand smoke	38·7 (36·3–41·4)	23·2 (21·6–24·8)	23·3 (20·6–26·1)	-40·1 (-45·7–-34·4)	0·402 (-9·17–10·3)	35·7 (33·6–37·7)	21·7 (20·6–22·8)	21·9 (19·9–23·8)	-39·2 (-43·9–-33·9)	0·773 (-7·03–8·64)
Alcohol use	15·4 (9·79–21·8)	15·7 (9·07–23·2)	16·9 (9·91–24·9)	2·03 (-27·6–43·1)	7·84 (-15·1–39·7)	10·6 (6·33–15·6)	12·1 (7·08–18)	12·1 (6·94–18)	14·2 (-19·8–66·3)	0·299 (-21·8–26·3)
Drug use	0·565 (0·28–1·13)	0·883 (0·579–1·36)	1·17 (0·812–1·7)	56·2 (17·2–123)	32·5 (14·7–54·4)	0·401 (0·23–0·706)	0·704 (0·5–1)	1·03 (0·77–1·38)	75·7 (36·1–132)	46·5 (24·8–70·7)
High fasting plasma glucose	11·2 (9·32–13·6)	14·3 (12·2–17·2)	16·2 (13·8–19·2)	28·4 (5·96–57·3)	13 (-3·56–32·7)	8·65 (7·32–10·3)	10·5 (8·91–12·8)	11·5 (9·73–13·7)	21·1 (0·671–46·5)	9·93 (-6·22–29·1)
High systolic blood pressure	24·5 (19·7–30·2)	15·2 (13·2–17·3)	19·3 (14·7–24·6)	-38·2 (-50·1–-23·2)	26·9 (-0·203–57·9)	22 (15·7–28·4)	12·3 (10·8–13·7)	17·1 (12·4–22·9)	-44·4 (-57·2–-23·7)	39·4 (-0·127–84·1)
High body-mass index	22·7 (15·9–31·6)	34·8 (25·4–46·7)	37·5 (27·7–49·3)	53 (34·5–78·6)	7·84 (0·709–16·6)	28·1 (20–37·9)	39·4 (31·7–47·7)	41·4 (33–50·3)	39·9 (20·7–66·9)	4·99 (-1·65–11·6)
Low bone mineral density	12·4 (7·35–19·5)	9·51 (5·17–15·7)	11·8 (6·89–18·9)	-23·4 (-42·2–-1·65)	24·2 (-2·68–61)	16·5 (10·8–23·4)	17 (11·2–24·2)	19·3 (13·1–26·6)	3·36 (-19·2–32·7)	13·5 (-9·48–46·1)
Diet low in fruits	52·4 (40–65·5)	46·7 (34·6–59·8)	45·5 (33·1–59·3)	-10·9 (-22·4–0·299)	-2·5 (-14·4–9·13)	52·7 (40·5–65·2)	46·4 (34·8–59·2)	44·9 (32·3–58·9)	-12 (-23·9–-0·963)	-3·25 (-15–8·64)
Diet low in vegetables	24·6 (11·2–45·3)	39·7 (21·9–62·8)	36·5 (19·5–57·7)	61·6 (21·7–162)	-8·15 (-23–4·17)	22·8 (9·76–42·2)	37·8 (20·4–59·6)	35·2 (18·9–55·8)	65·9 (21·8–182)	-6·92 (-22·1–7)
Diet low in whole grains	79·3 (69·5–88·3)	81·4 (72·5–89·3)	81·3 (72·4–89·1)	2·61 (-0·911–7·31)	-0·17 (-2·99–2·39)	76·6 (65·9–86·9)	79·5 (70–88·2)	79·1 (69·4–87·9)	3·71 (-0·85–9·41)	-0·419 (-3·4–2·44)
Diet low in nuts and seeds	26·1 (7·47–55·1)	25·6 (8·26–55·9)	19·9 (6·02–53·4)	-1·91 (-41·2–91·2)	-22·3 (-49·1–1·6)	22·3 (5·74–53·5)	21·9 (6·76–54)	18·1 (5·15–52·9)	-1·52 (-38·8–94·8)	-17·2 (-45·5–11·9)
Diet low in milk	46·9 (24·1–66·7)	41·5 (18·5–61·9)	39·7 (17·7–59·8)	-11·5 (-30·4–2·51)	-4·36 (-18·6–7·42)	--	--	--	--	--
Diet high in red meat	86·8 (75·8–94·6)	84·2 (72–93·2)	83·8 (71–93·3)	-2·92 (-14·9–9·2)	-0·528 (-9·17–8·5)	85 (73·8–93·5)	80·7 (67·9–90·8)	80·5 (68·1–91)	-5·05 (-19·1–8·84)	-0·232 (-9·8–10·5)
Diet high in processed meat	78·2 (54·2–98·2)	85·9 (67·1–99·8)	85·9 (66·6–99·8)	9·8 (0·821–29·1)	0·00491 (-4·54–5·14)	78·1 (54·1–97·5)	87·5 (71·1–99·6)	86·4 (68·9–99·6)	12·1 (1·5–36·9)	-1·23 (-6·21–2·59)
Diet high in sugar-sweetened beverages	47·5 (29·1–67·1)	64·3 (44·9–80·6)	65·4 (45·3–82)	35·2 (-4·82–109)	1·82 (-17·1–25·3)	51·3 (30·6–70·7)	69·6 (50·4–85·4)	69·1 (49·5–85·8)	35·8 (-2·48–106)	-0·733 (-18·1–22·4)
Diet low in fiber	41·7 (26·3–57·3)	29 (17·1–43·5)	25·6 (14–39·8)	-30·5 (-47·7–-12)	-11·5 (-30·2–7·58)	42·3 (25·9–58·3)	34·1 (20·7–48·2)	28·6 (15·2–42·8)	-19·4 (-35·1–-0·501)	-16·1 (-33·1–0·749)
Diet low in seafood omega-3 fatty acids	79·2 (60·3–99·4)	75·2 (55·9–98·2)	73·8 (55–97·8)	-5·11 (-21·2–10·3)	-1·89 (-16·4–15·7)	82·7 (65·2–99·5)	78·9 (60·5–98·5)	78·8 (61·5–98·4)	-4·56 (-18·5–8·88)	-0·0948 (-12·2–14·1)
Diet low in polyunsaturated fatty acids	12·9 (3·83–39·3)	6·92 (1·95–26·3)	6·48 (1·83–24·4)	-46·4 (-75·4–-6·78)	-6·31 (-30·7–12·4)	14·4 (4·08–41·7)	7·05 (2·04–24·6)	6·55 (1·89–24·6)	-51 (-78·2–-7·83)	-7·04 (-32·7–10·3)
Diet high in trans fatty acids	89·4 (81·3–100)	87·8 (78·8–100)	87·3 (77·7–100)	-1·77 (-10–6·23)	-0·594 (-8·74–7·75)	--	--	--	--	--
Diet high in sodium	18·5 (4·45–41·9)	40·1 (10·4–64·3)	39·5 (10·3–64·2)	116 (36·9–417)	-1·48 (-28·6–33·4)	--	--	--	--	--
Low physical activity	1·88 (0·567–5·04)	1·61 (0·552–4·89)	1·58 (0·451–4·84)	-14·7 (-47–20·8)	-1·67 (-31·7–36·2)	--	--	--	--	--
Occupational asthmagens	21·8 (18·3–26)	20·2 (17–23·8)	20·8 (17·3–25·4)	-7·36 (-18·8–4·94)	2·82 (-6·41–13·4)	11·3 (9·32–13·7)	11·4 (9·5–13·6)	11·5 (9·34–14)	0·571 (-13·8–17·2)	0·605 (-10·1–12·9)
Occupational particulate matter, gases, and fumes	9·01 (6·5–13·1)	8·61 (6·23–12·5)	8·39 (6·08–12)	-4·46 (-10·2–1·54)	-2·53 (-6·45–1·01)	5·04 (3·61–7·38)	4·96 (3·58–7·18)	4·8 (3·49–6·9)	-1·57 (-9·58–8·23)	-3·12 (-8·37–2·15)
Occupational noise	8·62 (7·92–9·44)	8·37 (7·83–9·08)	8·16 (7·66–8·8)	-2·85 (-6·67–1·4)	-2·49 (-4·59–-0·197)	6·06 (5·31–6·84)	6·42 (5·87–7·04)	6·44 (6–7)	6·08 (0·128–13·3)	0·321 (-2·75–3·45)
Occupational injuries	--	--	--	--	--	--	--	--	--	--
Occupational ergonomic factors	12·3 (10·1–14·9)	11·1 (9·36–13·3)	11·5 (9·57–13·9)	-9·89 (-20–1·55)	3·5 (-4·92–12·6)	7·06 (5·54–9·02)	7·16 (5·86–8·87)	7·46 (5·92–9·51)	1·44 (-14·9–20·9)	4·16 (-7·91–17·9)
Childhood sexual abuse	7·4 (5·05–10·5)	6·97 (4·6–9·93)	6·85 (4·54–9·67)	-5·83 (-12·2–5·83)	-1·68 (-8·51–5·53)	12·3 (8·94–17·3)	17·4 (12·9–24)	17·8 (13·1–24·7)	41·9 (30·7–54·8)	2·17 (-5–11)
Intimate partner violence	--	--	--	--	--	29 (16–37·8)	27·4 (15·2–36·1)	28·4 (15·3–37·5)	-5·5 (-13·8–5·96)	3·56 (-5·66–21·2)
Non-exclusive breastfeeding	26·2 (21·4–30·9)	25 (21·4–29·1)	25 (20·6–29·6)	-4·54 (-18·9–14·2)	-0·152 (-13·7–15)	26·2 (21·4–30·9)	25 (21·4–29·1)	25 (20·6–29·6)	-4·53 (-18·9–14·2)	-0·158 (-13·7–15)
Discontinued breastfeeding	26·9 (26–27·9)	25·2 (24–26·6)	25·2 (23·9–26·4)	-6·19 (-12–-0·677)	-0·219 (-6·19–5·46)	26·9 (26–27·9)	25·2 (23·9–26·6)	25·2 (23·9–26·4)	-6·39 (-12·2–-0·886)	-0·134 (-6·11–5·55)
Diet low in calcium	15·2 (7·18–27·6)	13 (6·36–23·5)	11·8 (5·48–21·7)	-14·6 (-25–-2·02)	-9·52 (-20·2–-0·989)	10·5 (4·48–19·8)	9·8 (4·3–18·2)	9 (3·91–16·2)	-6·64 (-20·7–13·1)	-8·13 (-19·5–1·88)
Occupational exposure to asbestos	7·32 (6·46–8·22)	5·7 (5·04–6·34)	5·81 (4·12–7·72)	-22·1 (-34·2–-8·42)	1·91 (-26·9–37·8)	1·25 (1·01–2·01)	1·19 (0·948–1·87)	1·14 (0·753–1·92)	-4·78 (-19–12·7)	-4·43 (-31·3–31·1)
Occupational exposure to arsenic	0·462 (0–1·07)	0·484 (0–1·16)	0·51 (0–1·21)	4·79 (-9·98–NA)	5·46 (-5·65–NA)	0·445 (0–1·07)	0·467 (0–1·1)	0·475 (0–1·13)	4·77 (-11·8–NA)	1·79 (-9·45–NA)
Occupational exposure to benzene	0·888 (0·0521–2·53)	0·899 (0·0538–2·52)	0·918 (0·055–2·7)	1·28 (-7·64–14·1)	2·07 (-4·98–11·7)	1·25 (0·0709–3·51)	1·42 (0·0826–3·9)	1·47 (0·0823–4·2)	13·5 (1·57–34·9)	3·72 (-4·47–17·8)
Occupational exposure to beryllium	0·0241 (0·0228–0·0256)	0·0246 (0·0241–0·0251)	0·0257 (0·0244–0·0271)	1·79 (-4·39–7·77)	4·63 (-0·716–10·3)	0·0272 (0·0249–0·0296)	0·0288 (0·0282–0·0295)	0·0296 (0·0276–0·0315)	5·84 (-2·75–15·6)	2·78 (-3·95–8·87)
Occupational exposure to cadmium	0·0551 (0·0496–0·061)	0·0578 (0·053–0·0628)	0·0609 (0·054–0·0688)	4·91 (-7·97–19·6)	5·33 (-4·72–16·2)	0·0541 (0·048–0·0612)	0·057 (0·0515–0·0634)	0·058 (0·0512–0·0655)	5·37 (-9·34–23·4)	1·76 (-8·37–13·5)
Occupational exposure to chromium	0·136 (0·124–0·148)	0·145 (0·135–0·155)	0·152 (0·137–0·168)	6·85 (-4·65–19·8)	4·69 (-4·05–14·1)	0·138 (0·124–0·153)	0·148 (0·138–0·161)	0·15 (0·136–0·166)	7·39 (-5·98–22·5)	1·52 (-7·41–11·7)
Occupational exposure to diesel engine exhaust	0·783 (0·72–0·85)	0·851 (0·801–0·901)	0·876 (0·808–0·95)	8·72 (-1·49–19·8)	2·96 (-4·06–10·9)	0·715 (0·64–0·797)	0·754 (0·688–0·828)	0·745 (0·666–0·834)	5·46 (-7·42–20·9)	-1·17 (-10·1–9·52)
Occupational exposure to formaldehyde	0·272 (0·24–0·304)	0·277 (0·221–0·308)	0·293 (0·258–0·331)	1·64 (-13·9–17·9)	5·66 (-5·78–29·3)	--	--	--	--	--
Occupational exposure to nickel	0·396 (0–1·76)	0·386 (0–1·72)	0·409 (0–1·73)	-2·52 (-16·3–NA)	5·94 (-5·54–NA)	0·351 (0–1·48)	0·353 (0–1·6)	0·36 (0–1·56)	0·608 (-16·6–NA)	1·89 (-10·6–NA)
Occupational exposure to polycyclic aromatic hydrocarbons	0·272 (0·249–0·297)	0·289 (0·271–0·309)	0·302 (0·275–0·332)	6·3 (-4·76–18·1)	4·39 (-3·92–13·1)	0·298 (0·269–0·329)	0·329 (0·31–0·352)	0·335 (0·307–0·367)	10·6 (-1·34–25·1)	1·82 (-6·11–10·3)
Occupational exposure to silica	3·8 (0·539–12·3)	3·48 (0·515–11·1)	3·7 (0·548–11·7)	-8·56 (-22–8·28)	6·43 (-4·81–19·9)	3·05 (0·43–9·52)	2·87 (0·394–9·1)	2·89 (0·391–9·2)	-5·86 (-22·1–17·8)	0·709 (-12–17·5)
Occupational exposure to sulfuric acid	0·922 (0·195–2·95)	1·05 (0·216–3·4)	1·08 (0·221–3·56)	14·2 (-3·98–34·2)	2·81 (-8·24–14·9)	0·911 (0·192–2·93)	1·13 (0·236–3·51)	1·11 (0·231–3·56)	23·7 (-0·0875–54)	-1·41 (-15·6–14·2)
Unsafe sex	--	--	--	--	--	--	--	--	--	--
Occupational exposure to trichloroethylene	0·0832 (0·0778–0·0897)	0·0906 (0·0869–0·095)	0·0944 (0·0876–0·101)	8·94 (0·41–18·1)	4·14 (-2·67–11·1)	0·083 (0·0755–0·0916)	0·0889 (0·084–0·0948)	0·0896 (0·0816–0·0976)	7·05 (-3·64–20·5)	0·817 (-6·69–9·82)
No access to handwashing facility	3·31 (3·06–3·58)	3·09 (2·85–3·35)	3·05 (2·82–3·31)	-6·63 (-14·8–2·34)	-1·32 (-6·83–4·32)	3·31 (3·06–3·58)	3·09 (2·85–3·35)	3·05 (2·82–3·31)	-6·63 (-14·8–2·34)	-1·32 (-6·83–4·32)
Child wasting	0·748 (0·491–1·02)	0·726 (0·465–0·985)	0·723 (0·464–0·992)	-2·93 (-12·1–8·18)	-0·437 (-7·31–7·25)	--	--	--	--	--
Child stunting	2·33 (1·22–3·42)	2·02 (1·05–3·05)	1·98 (1·03–3·02)	-13·4 (-31·2–10·4)	-2·19 (-16·7–13·3)	1·9 (0·938–2·91)	1·75 (0·869–2·66)	1·73 (0·855–2·67)	-8·29 (-28·3–17·5)	-1·1 (-16·3–17·8)
Chewing tobacco	4·11 (3·11–5·33)	3·92 (2·9–5·33)	3·81 (2·7–5·3)	-4·68 (-36–40·5)	-2·84 (-25·5–27·5)	0·271 (0·171–0·433)	0·309 (0·189–0·49)	0·319 (0·197–0·514)	14·1 (-34·8–103)	3·19 (-26·9–45·5)
Diet low in legumes	73·5 (36·6–99·1)	76·5 (44–99·7)	76·2 (43·8–99·5)	4·13 (-0·0909–22·4)	-0·353 (-6·59–5·76)	71·8 (33·9–98·6)	74·9 (40·7–99·4)	74·8 (43·2–99·3)	4·32 (-0·0839–24·7)	-0·0418 (-5·81–7·68)
Short gestation	22·8 (18·5–27·7)	24 (20·1–28·6)	22·2 (18·2–27)	5·27 (-16·9–32·4)	-7·31 (-23·8–13·9)	18·6 (15·9–21·7)	20·7 (17·8–24·3)	19·2 (16·3–22·5)	11·2 (-8·2–33·4)	-7·27 (-22·1–11·3)
Low birth weight	10·8 (9·02–12·9)	11·7 (10–13·8)	11·3 (9·55–13·3)	8·04 (-14·7–36·2)	-3·73 (-21·3–18·5)	11·2 (9·62–12·9)	12·4 (10·9–14·3)	11·9 (10·3–13·9)	10·7 (-8·35–32·7)	-4·13 (-19·9–14·2)
High temperature	27 (17·9–37·6)	25·2 (16·1–36·4)	25·9 (16·6–37)	-6·82 (-23·2–10·7)	2·78 (-14·6–21·6)	27 (17·9–37·6)	25·2 (16·1–36·4)	25·9 (16·6–37)	-6·82 (-23·2–10·7)	2·78 (-14·6–21·6)
Low temperature	36 (32–41)	35·5 (31·9–40)	36·5 (33–40·9)	-1·28 (-4·06–1·1)	2·89 (1·92–3·79)	36 (32–41)	35·5 (31·9–40)	36·5 (33–40·9)	-1·28 (-4·06–1·1)	2·89 (1·92–3·79)
Kidney dysfunction	18·7 (13·2–25·6)	19·2 (13·6–26·2)	19·3 (13·7–26·1)	2·45 (-1·39–6·81)	0·52 (-3·22–4·4)	21·1 (14·9–28·6)	21·8 (15·3–29·4)	22·6 (16·1–30·4)	2·95 (-1·78–8·18)	3·62 (-1·03–9·01)
Bullying victimization	5·86 (2·44–12·1)	8·95 (3·58–17·8)	8·76 (3·38–18·1)	52·8 (32·3–75·4)	-2·11 (-11·7–4·05)	4·26 (1·71–8·95)	7·87 (3·14–15·7)	7·84 (2·93–16·1)	84·7 (58·8–113)	-0·394 (-10·7–6·36)
High LDL cholesterol	67·1 (62·8–71·4)	34·4 (31–37·9)	30·9 (27·5–34·7)	-48·7 (-53·6–-43·4)	-10·2 (-15–-5·22)	65·9 (62·2–69·8)	34·1 (30·9–37·6)	31·7 (28·2–35·3)	-48·2 (-53–-43·7)	-7·06 (-11·3–-2·66)

Supplementary Results Table 1. Connecticut age-standardised Summary Exposure Values (SEVs) for most-detailed risk factors and percentage change, by sex, 1990, 2010, and 2019. Data in parentheses are 95% uncertainty intervals.

	Male					Female				
	1990	2010	2019	Percentage change 1990-2010	Percentage change 2010-2019	1990	2010	2019	Percentage change 1990-2010	Percentage change 2010-2019
Unsafe sanitation	3·77 (1·79–6·8)	2·16 (0·939–4·13)	1·78 (0·808–3·48)	-42·8 (-77·6–36·8)	-17·3 (-57–75·3)	--	--	--	--	--
Ambient particulate matter pollution	9·12 (2·18–20·1)	5·35 (2·68–8·43)	3·26 (0·88–6·09)	-41·3 (-74·6–92·3)	-39·1 (-67·2—27·4)	9·12 (2·19–20·1)	5·35 (2·68–8·43)	3·26 (0·887–6·09)	-41·3 (-74·6–90·6)	-39·1 (-66·9—27·4)
Household air pollution from solid fuels	0·00657 (0·00114–0·0208)	0·00392 (0·000579–0·0131)	0·0025 (0·000251–0·00923)	-40·4 (-78·6–54·4)	-36·2 (-73·4–27·1)	0·0087 (0·00154–0·0278)	0·00521 (0·000814–0·0174)	0·00333 (0·000332–0·0125)	-40·1 (-78·5–54·1)	-36·2 (-73·2–28·3)
Ambient ozone pollution	82·3 (37·7–100)	53·8 (22·3–90·7)	58·4 (24·5–98·4)	-34·5 (-49—9·32)	8·52 (-4·24–26·8)	--	--	--	--	--
Residential radon	21·8 (3·61–64·3)	21·8 (3·61–64·3)	21·8 (3·61–64·3)	0 (0–0)	0 (0–0)	21·8 (3·61–64·3)	21·8 (3·61–64·3)	21·8 (3·61–64·3)	0 (0–0)	0 (0–0)
Lead exposure	55 (27·2–77)	29·2 (9·77–50·8)	19·5 (4·81–37·6)	-46·9 (-65·6—32·4)	-33·3 (-51·7—24·6)	30·9 (3·36–58·7)	16·3 (0·47–40)	10·9 (0·112–29·8)	-47·3 (-86·9—31)	-32·9 (-77·5—24·9)
Child underweight	0·911 (0·529–1·38)	0·919 (0·501–1·39)	0·901 (0·491–1·37)	0·913 (-16·2–24·9)	-2·03 (-16·1–11·7)	0·774 (0·402–1·18)	0·819 (0·439–1·25)	0·838 (0·458–1·28)	5·76 (-16·1–44·9)	2·43 (-12·6–19·4)
Iron deficiency	--	--	--	--	--	11·5 (9·59–13·8)	11·8 (9·85–13·8)	11·9 (9·88–14·1)	2·57 (-9·26–16)	1·03 (-8·09–11·1)
Vitamin A deficiency	0·539 (0·13–1·44)	0·763 (0·198–2·06)	0·55 (0·142–1·42)	41·4 (-65·5–486)	-27·9 (-69·1–74·4)	1·17 (0·45–2·33)	1·22 (0·476–2·68)	1·07 (0·392–2·3)	4·28 (-63–217)	-12·5 (-52·4–69·8)
Zinc deficiency	1·72 (0–5·69)	0·914 (0–2·71)	0·949 (0–2·9)	-46·8 (-98·9–NA)	3·76 (-24·5–NA)	1·76 (0–5·81)	0·922 (0–2·8)	0·929 (0–2·9)	-47·7 (-100–NA)	0·683 (-27·3–NA)
Smoking	25·8 (23·3–28·6)	18·2 (16·4–20·2)	15·2 (13·6–16·9)	-29·5 (-34·6—24)	-16·5 (-20·2—12·6)	20·4 (17·8–23)	15 (13·4–16·8)	12 (10·7–13·6)	-26·5 (-33·2—18·7)	-19·8 (-23·7—15·5)
Secondhand smoke	40 (37·4–42·4)	23·7 (22·2–25·4)	23·7 (21·2–26·5)	-40·8 (-46—35)	0·257 (-9–9·95)	35·6 (33·6–37·5)	21·7 (20·5–22·8)	21·9 (19·8–23·9)	-39·1 (-43·8—34·2)	1·08 (-7·07–9·01)
Alcohol use	17·1 (11·2–23·4)	16·1 (10·2–22·3)	16·7 (10·9–22·6)	-5·91 (-36–35·8)	3·87 (-18·6–34·3)	--	--	--	--	--
Drug use	0·716 (0·384–1·32)	1·24 (0·865–1·78)	1·58 (1·16–2·2)	72·9 (28·4–138)	27·9 (12·5–48·2)	0·475 (0·283–0·807)	0·774 (0·565–1·09)	1·09 (0·831–1·41)	62·9 (28·5–114)	41·1 (20·4–64·8)
High fasting plasma glucose	11·8 (9·91–14·3)	17·5 (15·2–20·3)	18·4 (15·9–21·5)	48·1 (22·1–78·4)	5·18 (-7·44–21·1)	8·78 (7·48–10·4)	13·5 (11·4–15·9)	14·5 (12·2–17·3)	53·6 (28·4–84·3)	7·82 (-8·17–28·5)
High systolic blood pressure	25·5 (20·7–31)	15·8 (13·8–17·9)	19·7 (15·2–25)	-38 (-50—23·3)	24·7 (-1·25–55·8)	22·4 (16·5–29·2)	12·7 (11·1–14·3)	17·5 (12·4–23·5)	-43·4 (-56·9—23·4)	37·7 (-2·59–83·6)
High body-mass index	26·1 (18·6–36·6)	39·7 (29·3–51·6)	41·8 (30·9–54)	52·5 (34·5–78·4)	5·18 (-1·37–13)	29·7 (21·3–38·7)	42·2 (33·8–50·4)	44·1 (34·9–53·3)	42·1 (22·9–68·9)	4·48 (-2·57–11·7)
Low bone mineral density	12·1 (6·98–19)	9·17 (4·96–15·6)	11·4 (6·36–18·1)	-24·1 (-43·3—2·82)	24·6 (-3·78–66·5)	16 (10·3–22·6)	16·4 (10·9–23·3)	18·6 (12·6–25·7)	2·63 (-19·9–30·3)	13·2 (-9·75–43·6)
Diet low in fruits	46·2 (34·5–59·4)	41·6 (29·9–54·2)	42·3 (30–55·7)	-9·98 (-23·3–2·57)	1·84 (-12·3–17)	49·7 (37·7–63·2)	43 (31·7–56·6)	42·6 (30·2–55·6)	-13·5 (-25—2·78)	-0·939 (-13·7–11·7)
Diet low in vegetables	22·3 (9·6–43·5)	37·5 (20·1–58·4)	35·8 (19·2–56·4)	68·3 (22·8–176)	-4·71 (-19·9–8·81)	25·2 (11·4–45)	34·7 (18–56·3)	32·7 (15·9–53·5)	37·7 (8·6–100)	-5·72 (-22·1–9·27)
Diet low in whole grains	78·6 (68·6–87·9)	80·3 (70·6–89·1)	80·5 (71–89)	2·17 (-1·6–6·91)	0·265 (-2·35–2·91)	75·8 (64·8–86)	78·3 (68·4–87·6)	78·2 (68·6–87·6)	3·35 (-1·1–9·14)	-0·0938 (-3·34–2·88)
Diet low in nuts and seeds	22·8 (6–54·2)	23·6 (6·19–54·8)	19·3 (5·24–52·9)	3·64 (-40·5–105)	-18·2 (-47·3–10·3)	18·9 (4·8–52·5)	19·4 (6·35–53·3)	17 (4·78–52·6)	2·58 (-43·4–109)	-12·5 (-41·3–18·4)
Diet low in milk	42·5 (19·8–62)	34 (13·9–53·4)	34·2 (14·5–53·7)	-20·1 (-42·6—2·73)	0·576 (-15·4–21·1)	--	--	--	--	--
Diet high in red meat	81·1 (69·8–90·7)	81·1 (68·3–91·2)	80·2 (66·9–91)	-0·075 (-13·7–14·4)	-1·03 (-11–10·2)	82 (69·8–91·8)	79·9 (65·9–90·4)	78·9 (65·3–90)	-2·51 (-16–11·7)	-1·24 (-12·8–10·3)
Diet high in processed meat	78·9 (55·5–98·4)	87·2 (68·8–99·8)	87 (69·5–99·7)	10·6 (0·826–30·7)	-0·252 (-5·15–4·84)	80·6 (58·3–98·5)	90·3 (76·6–99·7)	88·8 (73·1–99·7)	12·1 (1·12–35·5)	-1·73 (-7·37–1·85)
Diet high in sugar-sweetened beverages	52 (32·7–71·1)	72·3 (53·5–87·1)	71 (50·9–86·9)	39 (2·93–109)	-1·88 (-19·4–16·5)	56·1 (34·7–74·1)	77·3 (60·8–90·7)	74·7 (56·2–89·2)	37·7 (3·76–106)	-3·39 (-19·9–12·4)
Diet low in fiber	38·1 (23·3–52·7)	23·7 (12·9–36·2)	22 (11·7–35)	-37·9 (-54·7—19·7)	-6·9 (-28–16·2)	38·3 (23·2–54·7)	28·3 (16·6–42·6)	24·5 (13·4–38·8)	-26·1 (-43·2—5·98)	-13·4 (-32·4–4·66)
Diet low in seafood omega-3 fatty acids	79·2 (61·2–99·6)	72·9 (54·6–97·2)	73·7 (54·5–97·7)	-7·98 (-23·7–10·8)	1·11 (-15·5–21·2)	82·3 (64·7–99)	77·3 (58·8–99)	77·4 (58·5–98·6)	-6·08 (-21·6–8·46)	0·155 (-14·7–17·6)
Diet low in polyunsaturated fatty acids	11·1 (3·21–34·2)	5·92 (1·61–24·3)	5·89 (1·59–24·4)	-46·6 (-79—5·9)	-0·422 (-20·5–25·8)	12·3 (3·58–35·6)	6 (1·63–24·4)	5·95 (1·64–24·5)	-51·2 (-81·1—7·3)	-0·927 (-18–22·4)
Diet high in trans fatty acids	89·5 (81·7–100)	87·9 (79·4–100)	87·6 (78·4–100)	-1·77 (-9·71–6·02)	-0·351 (-7·89–8·53)	--	--	--	--	--
Diet high in sodium	18·1 (4·3–41·4)	39·8 (10·6–64·2)	39·3 (9·51–64)	119 (39·4–431)	-1·09 (-28·4–32·7)	--	--	--	--	--
Low physical activity	2·37 (0·867–5·11)	1·89 (0·658–5·05)	2·06 (0·639–5·16)	-20·1 (-60·9–28·4)	8·65 (-21·3–54·4)	--	--	--	--	--
Occupational asthmagens	25·5 (21–31·4)	21·7 (18·4–26·1)	22·7 (18·7–28·1)	-15·1 (-27·8—1·34)	4·66 (-5·67–17·1)	12·1 (9·9–14·9)	12·2 (10·2–14·5)	12·3 (10–15·1)	0·216 (-15·5–18·6)	1·22 (-10·4–15)
Occupational particulate matter, gases, and fumes	10·1 (7·14–14·6)	9·16 (6·53–13·1)	8·78 (6·35–12·5)	-9·45 (-14·7—3·94)	-4·09 (-7·63—0·662)	5·56 (3·93–8·15)	5·12 (3·65–7·31)	4·87 (3·45–7·03)	-8·05 (-16·3–2·16)	-4·8 (-10·1–0·946)
Occupational noise	8·76 (8·03–9·6)	8·43 (7·84–9·25)	8·2 (7·66–8·95)	-3·82 (-7·34–0·227)	-2·7 (-4·83—0·536)	6·23 (5·43–7·05)	6·54 (6·01–7·16)	6·55 (6·13–7·12)	5·03 (-0·787–12·4)	0·181 (-2·65–3·59)
Occupational injuries	--	--	--	--	--	--	--	--	--	--
Occupational ergonomic factors	12 (9·88–14·6)	10·9 (9·27–13·1)	11·3 (9·39–13·7)	-9·16 (-18·8–1·74)	3·77 (-3·66–12·7)	7 (5·43–8·8)	7·26 (5·92–8·97)	7·6 (6·05–9·61)	3·69 (-12·9–23·1)	4·72 (-7·04–19·8)
Childhood sexual abuse	7·4 (5·05–10·5)	6·97 (4·6–9·93)	6·85 (4·54–9·67)	-5·83 (-12·2–5·83)	-1·68 (-8·51–5·53)	12·3 (8·94–17·3)	17·4 (12·9–24)	17·8 (13·1–24·7)	41·9 (30·7–54·8)	2·17 (-5–11)
Intimate partner violence	--	--	--	--	--	29·2 (16·3–38·7)	27·5 (15·9–36·9)	28·6 (15·4–38)	-5·85 (-14·6–8·9)	4·02 (-4·49–19·7)
Non-exclusive breastfeeding	26·4 (21·7–31·4)	25·1 (21·4–29)	25 (20·6–29·9)	-5·06 (-19·9–13·2)	-0·106 (-13·6–14·9)	26·4 (21·7–31·4)	25·1 (21·4–29)	25 (20·6–29·9)	-5·05 (-19·9–13·2)	-0·112 (-13·6–14·9)
Discontinued breastfeeding	27·6 (26·5–28·5)	24·9 (23·4–26·1)	25·6 (24·2–26·8)	-9·82 (-15·4—4·63)	3·13 (-2·68–9·63)	27·6 (26·5–28·5)	24·8 (23·4–26)	25·6 (24·2–26·8)	-9·92 (-15·5—4·73)	3·18 (-2·64–9·69)
Diet low in calcium	13·2 (6·48–23·8)	10·3 (5·24–18·5)	10·1 (5·1–18·2)	-22·5 (-33·1—9·52)	-1·36 (-10·6–7·95)	8·45 (3·54–15·7)	6·9 (3·01–12·6)	7·09 (3·05–13·2)	-18·4 (-33·5–3·26)	2·79 (-9·98–15·5)
Occupational exposure to asbestos	9·08 (8·1–10·2)	7·5 (6·79–8·21)	7·19 (5·1–9·4)	-17·4 (-28·9—4·65)	-4·08 (-31·4–27·6)	1·65 (1·06–2·03)	1·45 (0·888–1·76)	1·42 (0·787–2)	-12·1 (-27–5·09)	-2·64 (-30–33·6)
Occupational exposure to arsenic	0·599 (0–1·42)	0·572 (0–1·36)	0·603 (0–1·44)	-4·6 (-17·7–NA)	5·46 (-6·3–NA)	0·522 (0–1·21)	0·512 (0–1·23)	0·521 (0–1·24)	-1·91 (-20·4–NA)	1·72 (-11·5–NA)
Occupational exposure to benzene	0·912 (0·055–2·65)	0·936 (0·0547–2·74)	0·953 (0·0587–2·79)	2·67 (-5·92–15·9)	1·75 (-5·57–12·6)	1·31 (0·0759–3·66)	1·52 (0·0905–4·19)	1·57 (0·0962–4·66)	15·7 (4·11–37·7)	3·23 (-4·95–15·8)
Occupational exposure to beryllium	0·0251 (0·0234–0·0266)	0·0253 (0·0247–0·0259)	0·0265 (0·025–0·028)	1·03 (-5·43–7·96)	4·59 (-1·32–10·9)	0·028 (0·0254–0·0304)	0·0298 (0·0291–0·0306)	0·0306 (0·0286–0·0327)	6·66 (-2·13–17·9)	2·69 (-3·56–9·63)
Occupational exposure to cadmium	0·0691 (0·0621–0·0759)	0·0667 (0·0605–0·0736)	0·0701 (0·0622–0·0786)	-3·55 (-15·7–10·6)	5·13 (-5·49–15·8)	0·062 (0·0538–0·0706)	0·0617 (0·0552–0·0689)	0·0628 (0·0547–0·0723)	-0·458 (-16·8–18·7)	1·71 (-10–14·7)
Occupational exposure to chromium	0·164 (0·149–0·178)	0·162 (0·15–0·176)	0·17 (0·154–0·187)	-1·23 (-12·1–10·9)	4·74 (-4·57–14·3)	0·155 (0·136–0·174)	0·159 (0·146–0·173)	0·162 (0·144–0·181)	2·87 (-11–18·8)	1·63 (-8·08–12·5)
Occupational exposure to diesel engine exhaust	0·693 (0·631–0·751)	0·768 (0·722–0·819)	0·799 (0·728–0·873)	10·9 (0·477–23·6)	4·01 (-3·66–13·2)	0·666 (0·587–0·743)	0·725 (0·66–0·8)	0·726 (0·639–0·828)	8·9 (-5·91–26·4)	0·143 (-9·5–11·5)
Occupational exposure to formaldehyde	0·353 (0·316–0·389)	0·331 (0·299–0·365)	0·343 (0·302–0·386)	-6·22 (-18·6–7·7)	3·64 (-6·92–14·5)	--	--	--	--	--
Occupational exposure to nickel	0·495 (0–2·17)	0·458 (0–2·07)	0·483 (0–2·11)	-7·5 (-20–NA)	5·42 (-6·09–NA)	0·41 (0–1·76)	0·386 (0–1·69)	0·394 (0–1·74)	-5·8 (-24·4–NA)	1·99 (-12·3–NA)
Occupational exposure to polycyclic aromatic hydrocarbons	0·324 (0·297–0·352)	0·322 (0·3–0·347)	0·337 (0·306–0·369)	-0·628 (-11·1–11)	4·43 (-4·03–13·7)	0·329 (0·291–0·366)	0·35 (0·328–0·375)	0·357 (0·324–0·394)	6·53 (-6·05–21·5)	1·86 (-6·54–11·5)
Occupational exposure to silica	4·33 (0·582–13·9)	3·97 (0·569–12·4)	4·22 (0·586–12·8)	-8·28 (-20·8–6·93)	6·46 (-4·84–20·5)	--	--	--	--	--
Occupational exposure to sulfuric acid	1·02 (0·224–3·25)	1·08 (0·219–3·43)	1·12 (0·235–3·55)	5·53 (-8·26–21·3)	3·54 (-6·96–15·2)	0·906 (0·189–2·97)	1·04 (0·204–3·23)	1·03 (0·218–3·28)	14·4 (-6·59–40·1)	-0·916 (-14·8–15·5)
Unsafe sex	--	--	--	--	--	--	--	--	--	--
Occupational exposure to trichloroethylene	0·0919 (0·0853–0·099)	0·0944 (0·0901–0·0998)	0·0986 (0·0916–0·107)	2·72 (-5·6–11·8)	4·47 (-2·58–12·3)	0·0881 (0·0789–0·0983)	0·0923 (0·0866–0·099)	0·0934 (0·0853–0·103)	4·73 (-6·97–19·5)	1·28 (-7·25–11)
No access to handwashing facility	3·23 (2·97–3·47)	3·06 (2·83–3·3)	3·03 (2·8–3·27)	-5·21 (-13·2–4·45)	-0·956 (-6·6–4·87)	3·23 (2·97–3·47)	3·06 (2·83–3·3)	3·03 (2·8–3·27)	-5·21 (-13·2–4·45)	-0·956 (-6·6–4·87)
Child wasting	0·742 (0·486–1·01)	0·72 (0·462–0·99)	0·719 (0·458–0·978)	-3·01 (-12·1–8·46)	-0·0953 (-7·04–7·34)	0·767 (0·508–1·05)	0·692 (0·372–0·956)	0·752 (0·5–1·05)	-9·8 (-48·3–41·6)	8·61 (-29·1–84·8)
Child stunting	2·24 (1·12–3·29)	1·96 (1·01–2·89)	1·92 (1–2·88)	-12·1 (-30·5–10·7)	-2·17 (-17·5–14·9)	1·84 (0·933–2·77)	1·71 (0·839–2·67)	1·69 (0·812–2·65)	-6·94 (-26·9–20·9)	-1·19 (-16·5–17·1)
Chewing tobacco	2·18 (1·6–2·96)	2·23 (1·55–3·14)	2·25 (1·51–3·22)	2·26 (-33·7–58·9)	0·633 (-24·7–34·8)	0·291 (0·189–0·44)	0·315 (0·197–0·499)	0·328 (0·2–0·502)	8·05 (-37·2–88·2)	4·18 (-28·9–47·5)
Diet low in legumes	72·1 (34–98·8)	74·5 (39·1–99·3)	74·7 (40·1–99·5)	3·33 (-0·889–19·9)	0·274 (-5·16–7·3)	70·4 (30·7–98)	72·8 (34·5–99)	73·4 (38·3–99·1)	3·32 (-1·21–19·6)	0·87 (-4·28–8·63)
Short gestation	21·7 (17·7–26·2)	24·8 (20·5–30)	25·4 (20·6–30·7)	14·3 (-9·14–47·3)	2·36 (-18·5–28·9)	19·4 (16–23·2)	22·5 (19·2–26·3)	22·2 (18·7–26)	16 (-6·74–42·3)	-1·29 (-18·5–18·3)
Low birth weight	9·33 (7·63–11·1)	10·6 (8·79–12·5)	11·4 (9·4–13·6)	13·4 (-10·3–46·4)	7·88 (-13·8–36·4)	9·46 (7·95–11·2)	11·7 (10·2–13·7)	11·9 (10·3–13·9)	23·5 (-0·661–53)	2·31 (-14–22·4)
High temperature	25·7 (13·6–39)	28·2 (15·7–41·4)	26·6 (14·1–39·9)	9·8 (4·06–23·8)	-5·61 (-15·5—2·21)	25·7 (13·6–39)	28·2 (15·7–41·4)	26·6 (14·1–39·9)	9·8 (4·06–23·8)	-5·61 (-15·5—2·21)
Low temperature	33·4 (28·7–38·5)	34·5 (30–39·3)	35·4 (31·1–40·2)	3·31 (1·85–4·88)	2·81 (1·76–4·11)	33·4 (28·7–38·5)	34·5 (30–39·3)	35·4 (31·1–40·2)	3·31 (1·85–4·88)	2·81 (1·76–4·11)
Kidney dysfunction	19·5 (13·9–26·5)	20 (14·3–27·1)	19·9 (14·3–27·1)	2·72 (-1·34–7·35)	-0·318 (-4·98–4·04)	21·5 (15·1–29·3)	22·2 (15·7–29·9)	22·9 (16·3–30·8)	3·17 (-1·22–8·41)	3·09 (-1·11–8·43)
Bullying victimization	5·9 (2·46–12)	9·12 (3·61–18·1)	8·82 (3·36–18·2)	54·6 (34–79·4)	-3·33 (-13·1–2·9)	4·28 (1·74–8·88)	8·01 (3·17–15·9)	7·86 (2·96–16·2)	87·2 (60·1–119)	-1·75 (-12·4–5·34)
High LDL cholesterol	68·1 (63·7–72·7)	34·3 (30·8–38)	30·8 (27·2–34·6)	-49·6 (-54·7—44·1)	-10·2 (-15·1—5·44)	65·9 (62–69·7)	34·1 (30·9–37·6)	31·8 (28·3–35·4)	-48·2 (-53—43·6)	-6·8 (-11·1—3·18)

Supplementary Results Table 1. Delaware age-standardised Summary Exposure Values (SEVs) for most-detailed risk factors and percentage change, by sex, 1990, 2010, and 2019. Data in parentheses are 95% uncertainty intervals.

	Male					Female				
	1990	2010	2019	Percentage change 1990-2010	Percentage change 2010-2019	1990	2010	2019	Percentage change 1990-2010	Percentage change 2010-2019
Unsafe sanitation	5·54 (2·62–10·2)	3·07 (1·35–5·68)	2·63 (1·13–4·87)	-44·6 (-78·7–29·6)	-14·5 (-55·9–70·6)	--	--	--	--	--
Ambient particulate matter pollution	13·8 (5·13–26·8)	8·21 (5·39–11·5)	4·78 (2·21–7·67)	-40·5 (-69·6–43·6)	-41·8 (-59·4–-32·4)	13·8 (5·12–26·8)	8·2 (5·39–11·5)	4·78 (2·21–7·67)	-40·5 (-69·5–43·8)	-41·7 (-59·5–-32·4)
Household air pollution from solid fuels	0·0222 (0·00516–0·0622)	0·0139 (0·00253–0·0453)	0·00959 (0·00135–0·0332)	-37·3 (-76·8–40·8)	-31·2 (-66·5–24·5)	0·0293 (0·00692–0·0823)	0·0185 (0·0034–0·0577)	0·0127 (0·00185–0·0439)	-36·9 (-76·5–40·7)	-31·1 (-66·4–24·9)
Ambient ozone pollution	86·4 (42–100)	66·3 (28·8–100)	52·3 (21·7–90)	-23·3 (-41·6–0)	-21·1 (-37·7–-5·33)	86·4 (42–100)	66·3 (28·8–100)	52·3 (21·7–90)	-23·3 (-41·6–0)	-21·1 (-37·7–-5·33)
Residential radon	20·6 (2·93–60)	20·6 (2·93–60)	20·6 (2·93–60)	0 (0–0)	0 (0–0)	20·6 (2·93–60)	20·6 (2·93–60)	20·6 (2·93–60)	0 (0–0)	0 (0–0)
Lead exposure	59·6 (31·8–81)	33·5 (13·4–55)	23·1 (7·31–41·6)	-43·7 (-60·1–-29·9)	-31·1 (-46·2–-23·2)	34·3 (5·44–61·9)	18·9 (1·32–43)	12·9 (0·428–32·1)	-44·9 (-79·6–-28·8)	-31·7 (-68·8–-23·6)
Child underweight	0·951 (0·534–1·45)	0·948 (0·52–1·44)	0·926 (0·508–1·43)	-0·263 (-17–21·3)	-2·31 (-15·4–11·9)	0·803 (0·435–1·23)	0·838 (0·474–1·28)	0·858 (0·475–1·3)	4·4 (-16·1–32·6)	2·32 (-12·6–19·6)
Iron deficiency	--	--	--	--	--	12·5 (10·4–14·9)	12·5 (10·6–14·4)	12·7 (10·6–14·9)	-0·0952 (-12·7–12·9)	1·52 (-8·26–12·6)
Vitamin A deficiency	0·726 (0·17–1·93)	1·04 (0·293–2·75)	0·757 (0·202–2·08)	43·8 (-61·6–520)	-27·4 (-68·2–68)	1·5 (0·562–3·38)	1·53 (0·616–3·23)	1·37 (0·51–2·76)	2·01 (-62·2–218)	-10·3 (-50–70·7)
Zinc deficiency	2·03 (0–6·63)	1·07 (0–3·37)	1·17 (0–3·82)	-47·4 (-100–NA)	9·47 (-12·8–NA)	2·07 (0–6·81)	1·08 (0–3·55)	1·15 (0–3·73)	-47·9 (-100–NA)	6·15 (-11·8–NA)
Smoking	28·2 (25·2–31·3)	20·9 (18·8–23·1)	17·8 (16–19·7)	-26·1 (-31·5–-19·6)	-14·6 (-18·3–-11·1)	20·8 (18·1–23·8)	16·2 (14·5–18)	13·6 (12–15·2)	-22·3 (-31·1–-11·1)	-16·2 (-20·3–-11·8)
Secondhand smoke	41·9 (39–44·6)	26 (24·3–27·7)	26·2 (23·3–29·1)	-37·8 (-44–-31·5)	0·718 (-8·25–10·2)	39·9 (37·8–42)	25·1 (23·8–26·4)	25·4 (23·1–27·6)	-37·1 (-41·6–-32)	1·05 (-6·37–8·6)
Alcohol use	15·6 (9·7–22)	15·4 (9·29–21·9)	16·9 (10–24·5)	-1·68 (-29·2–35·3)	10·1 (-14·6–41·3)	10·2 (6·04–15)	11·9 (7·21–17·1)	11·5 (6·63–17)	16·5 (-16·5–65·7)	-3·07 (-24·1–23·2)
Drug use	0·715 (0·394–1·28)	1·2 (0·83–1·7)	1·6 (1·18–2·17)	67·5 (26·5–128)	33·8 (16·7–55·7)	0·513 (0·324–0·815)	0·95 (0·69–1·27)	1·45 (1·1–1·87)	85·2 (47–136)	52·5 (31·5–79·8)
High fasting plasma glucose	13·9 (11·5–16·9)	19·8 (17·3–22·7)	20·7 (18–23·7)	42·5 (18·6–72·2)	4·45 (-9·14–19·6)	9·74 (8·69–10·8)	12·6 (11·9–13·3)	13·4 (12·2–14·6)	29·4 (18·6–43·1)	6·1 (-1·12–13·5)
High systolic blood pressure	26 (20·8–31·5)	17 (14·9–19)	20·6 (15·9–26)	-34·6 (-46·7–-19·5)	21·1 (-5·8–52·3)	23·4 (17–30·8)	12·9 (11·2–14·8)	17·5 (12·3–23·5)	-44·7 (-58·7–-25·7)	35·2 (-3·02–84)
High body-mass index	25·9 (18·1–36·4)	40·1 (29·8–52·1)	40·8 (30–52·4)	54·8 (35·5–81)	1·71 (-5·15–8·83)	33·2 (24·6–42)	44·5 (35–53·4)	45 (35·3–54·6)	34·2 (20·3–53)	0·964 (-4·78–7·91)
Low bone mineral density	11·9 (6·96–19)	9·14 (4·71–15·3)	11·6 (6·49–18·5)	-23·3 (-44·8–-0·0177)	26·5 (-3–69·1)	15·8 (10·3–22·5)	16·4 (10·7–23·4)	18·7 (12·9–26·2)	3·86 (-17·1–32·7)	14 (-12·5–46·4)
Diet low in fruits	53·1 (41·4–65·9)	51·9 (39·9–64·9)	53·4 (40·6–67)	-2·44 (-12·7–10·5)	2·9 (-6·77–13·3)	54·2 (43·1–68·1)	53·1 (41·5–66·2)	54 (40·8–67·9)	-1·89 (-12·2–9·57)	1·62 (-8·82–10·9)
Diet low in vegetables	42 (23·5–64)	72·7 (54·8–88·2)	71·6 (52·7–87)	73·3 (29·4–163)	-1·61 (-7–2·6)	42·5 (24·1–63·3)	73 (55·4–88)	71·8 (54·1–87·4)	71·5 (29–157)	-1·59 (-6·63–2·52)
Diet low in whole grains	79·2 (69·5–88·1)	81·2 (72·4–89·5)	81·5 (72·3–89·6)	2·58 (-0·948–7·26)	0·295 (-2·41–2·84)	76·5 (65·4–86·5)	79·3 (69·5–88·1)	79·3 (69·6–88·1)	3·73 (-0·691–9·71)	0·0245 (-3·18–2·92)
Diet low in nuts and seeds	25·8 (7·72–55)	24·9 (8·28–55·2)	21·2 (6·76–53·9)	-3·4 (-39·8–79·4)	-14·9 (-42·8–15·1)	21·7 (4·93–53·7)	21 (6·35–54·1)	19·2 (5·54–53·2)	-3·51 (-40·2–82·8)	-8·62 (-37·5–26·9)
Diet low in milk	46·1 (22·8–66)	40·7 (17·7–60·9)	41·5 (19·1–61·7)	-11·7 (-31·9–2·79)	1·94 (-10·4–20·4)	45·8 (22·7–66)	40·7 (17·5–61·6)	41·9 (18·7–62·9)	-11·2 (-29·8–5·85)	2·91 (-10·8–18)
Diet high in red meat	79·4 (66·6–89·2)	77·5 (64–88·8)	76·3 (61·9–88·5)	-2·39 (-15·9–12·8)	-1·53 (-13–9·83)	80·2 (68–90·4)	76·5 (63·4–87·9)	75·2 (59·9–87·3)	-4·59 (-18·1–12·2)	-1·71 (-13·5–10·5)
Diet high in processed meat	78·4 (54·3–98)	86·5 (69·3–99·7)	86·1 (67·6–99·5)	10·3 (1–29)	-0·456 (-5·61–4·43)	79·7 (57–98)	89·3 (72·1–99·7)	87·6 (70·7–99·6)	12 (1·36–35·7)	-1·9 (-7·1–2·02)
Diet high in sugar-sweetened beverages	48·4 (28·9–68·3)	65 (44·8–81·4)	63·6 (43·2–81·9)	34·1 (-5·54–113)	-2·19 (-22·5–20·6)	52·4 (32–72·1)	70·6 (51·3–86·2)	67·6 (48–84·3)	34·8 (-4·36–108)	-4·29 (-23·6–16·3)
Diet low in fiber	41·1 (26·1–57·1)	28·4 (16·9–41·3)	27·1 (15·1–40·9)	-31 (-47·6–-13·4)	-4·45 (-23·5–15·1)	41·6 (24·2–57·3)	33·5 (19·9–47·5)	30·1 (17·3–44·5)	-19·5 (-36·9–-0·893)	-10·2 (-26·9–5·46)
Diet low in seafood omega-3 fatty acids	81·9 (64–99·6)	77·3 (57·1–99·4)	78·8 (60·9–99·1)	-5·69 (-22·2–10·9)	2·03 (-12·4–20·4)	84·6 (67·8–100)	82 (64·8–99·1)	82·7 (66·1–99·4)	-3·06 (-15·3–9·56)	0·81 (-10·4–15·9)
Diet low in polyunsaturated fatty acids	12·7 (3·74–39)	6·81 (1·97–25·7)	6·9 (1·95–26)	-46·3 (-75–-6·76)	1·3 (-18·7–34·5)	14·2 (4·13–42·9)	6·92 (2·02–25·9)	6·94 (2·06–25·7)	-51·3 (-78·9–-7·27)	0·28 (-18·4–25·6)
Diet high in trans fatty acids	89·4 (81·2–100)	87·8 (78·9–100)	87·3 (77·8–100)	-1·83 (-9·53–6·18)	-0·511 (-9·17–8·23)	--	--	--	--	--
Diet high in sodium	18·6 (4·28–41·7)	40·3 (11·4–64·4)	39·6 (11·4–65·9)	117 (38·6–433)	-1·81 (-27·5–32·6)	--	--	--	--	--
Low physical activity	2·88 (1·11–6·06)	2·55 (0·955–5·81)	2·49 (0·924–5·26)	-11·7 (-54·6–41·2)	-2·16 (-33·3–78·4)	--	--	--	--	--
Occupational asthmagens	23 (19·3–27·9)	20·1 (16·8–24)	21·2 (17·4–25·8)	-12·5 (-24·5–-0·312)	5·04 (-4·78–16·8)	11·3 (9·14–13·7)	11·5 (9·47–13·8)	11·7 (9·46–14·2)	1·78 (-13·8–19·7)	1·63 (-9·4–15·4)
Occupational particulate matter, gases, and fumes	9·56 (6·83–13·9)	8·78 (6·28–12·6)	8·43 (6·09–12·1)	-8·23 (-13·8–-2·38)	-3·9 (-7·45–-0·0166)	5·25 (3·71–7·68)	4·97 (3·57–7·15)	4·78 (3·44–6·87)	-5·37 (-14–3·97)	-3·72 (-8·4–1·27)
Occupational noise	8·42 (7·7–9·24)	8·07 (7·49–8·82)	7·82 (7·3–8·52)	-4·14 (-7·97–0·268)	-3·07 (-5·21–-0·585)	6 (5·24–6·8)	6·24 (5·7–6·85)	6·25 (5·81–6·82)	4·12 (-1·57–11·3)	0·0263 (-2·93–3·05)
Occupational injuries	--	--	--	--	--	--	--	--	--	--
Occupational ergonomic factors	12 (9·9–14·6)	10·7 (8·95–12·7)	11·1 (9·16–13·4)	-11 (-21·4–0·573)	4·07 (-4·13–13·1)	7·1 (5·62–9)	7·28 (5·89–9·19)	7·69 (6·12–9·82)	2·54 (-14·5–22·7)	5·51 (-8·11–20·8)
Childhood sexual abuse	7·4 (5·05–10·5)	6·97 (4·6–9·93)	6·85 (4·54–9·67)	-5·83 (-12·2–5·83)	-1·68 (-8·51–5·53)	12·3 (8·94–17·3)	17·4 (12·9–24)	17·8 (13·1–24·7)	41·9 (30·7–54·8)	2·17 (-5–11)
Intimate partner violence	--	--	--	--	--	30·5 (16·5–39·7)	28·8 (15·9–37·6)	29·8 (16·6–39·5)	-5·65 (-14–7·29)	3·7 (-4·7–19·2)
Non-exclusive breastfeeding	26·3 (21·5–31)	25 (21·4–29)	25 (20·7–29·9)	-5·03 (-19·1–12·6)	-0·00678 (-13·2–14·9)	26·3 (21·5–31)	25 (21·4–29)	25 (20·7–29·9)	-5·01 (-19–12·6)	-0·0249 (-13·2–14·8)
Discontinued breastfeeding	27·6 (26·6–28·5)	25·5 (24·1–26·8)	25·9 (24·6–27·2)	-7·4 (-13·2–-1·83)	1·58 (-4·01–7·71)	27·5 (26·5–28·4)	25·3 (23·9–26·6)	25·8 (24·5–27)	-7·96 (-13·7–-2·42)	1·89 (-3·74–8·09)
Diet low in calcium	14·9 (7·3–27·5)	12·5 (6·23–22·8)	12·8 (6·25–23·6)	-16·3 (-28·2–-4·09)	2·19 (-7·83–11·7)	10·2 (4·51–19·3)	9·41 (4·25–17·5)	10·1 (4·43–19·2)	-7·68 (-23·5–11·9)	7·23 (-4·17–19·9)
Occupational exposure to asbestos	12 (10·6–13·4)	9·66 (8·82–10·5)	9·06 (7·09–11·4)	-19·2 (-30·4–-7·28)	-6·17 (-26·9–20·2)	1·64 (1·13–2·18)	1·47 (1·04–1·85)	1·39 (0·919–1·93)	-10·3 (-26·6–8·26)	-5·21 (-28·4–22·8)
Occupational exposure to arsenic	0·536 (0–1·29)	0·511 (0–1·21)	0·541 (0–1·27)	-4·73 (-18·4–NA)	5·89 (-6·56–NA)	0·474 (0–1·1)	0·477 (0–1·14)	0·49 (0–1·15)	0·737 (-15–NA)	2·69 (-9·76–NA)
Occupational exposure to benzene	0·855 (0·052–2·52)	0·838 (0·0488–2·41)	0·862 (0·0506–2·51)	-1·96 (-10·7–10·6)	2·9 (-4·55–12·3)	1·22 (0·0677–3·43)	1·38 (0·0824–4·02)	1·43 (0·0865–4·08)	12·8 (0·388–33)	3·43 (-5·5–15·6)
Occupational exposure to beryllium	0·024 (0·0226–0·0255)	0·0236 (0·0229–0·0243)	0·0249 (0·0235–0·0264)	-1·68 (-7·6–5·24)	5·41 (-0·499–11·5)	0·027 (0·0247–0·0293)	0·0287 (0·0277–0·0297)	0·0296 (0·0276–0·0316)	6·1 (-2·54–16·1)	3·05 (-3·99–9·8)
Occupational exposure to cadmium	0·0624 (0·0563–0·0695)	0·0601 (0·0541–0·0659)	0·0635 (0·0557–0·0716)	-3·66 (-16·2–11·3)	5·61 (-5·29–17·9)	0·0569 (0·0499–0·0645)	0·0581 (0·0526–0·0645)	0·0595 (0·0524–0·068)	2 (-12·2–20)	2·53 (-8·57–15·8)
Occupational exposure to chromium	0·15 (0·136–0·164)	0·147 (0·135–0·159)	0·155 (0·139–0·173)	-1·44 (-12·8–11·8)	5·47 (-3·75–15·7)	0·143 (0·127–0·16)	0·15 (0·139–0·163)	0·153 (0·137–0·171)	4·64 (-7·79–20·5)	2·32 (-7·25–12·9)
Occupational exposure to diesel engine exhaust	0·712 (0·656–0·773)	0·772 (0·72–0·825)	0·818 (0·747–0·893)	8·42 (-2·32–20·4)	5·85 (-2·3–14·7)	0·692 (0·616–0·778)	0·756 (0·686–0·828)	0·759 (0·675–0·849)	9·17 (-5·93–25·9)	0·434 (-9·14–11·8)
Occupational exposure to formaldehyde	0·313 (0·278–0·351)	0·289 (0·231–0·324)	0·304 (0·262–0·349)	-7·63 (-23·6–8·73)	5·32 (-6·7–21·6)	--	--	--	--	--
Occupational exposure to nickel	0·442 (0–2)	0·407 (0–1·71)	0·432 (0–1·83)	-7·8 (-21·4–NA)	5·92 (-5·83–NA)	0·37 (0–1·62)	0·359 (0–1·57)	0·37 (0–1·62)	-3·09 (-18·6–NA)	3·1 (-10·9–NA)
Occupational exposure to polycyclic aromatic hydrocarbons	0·295 (0·27–0·323)	0·292 (0·269–0·313)	0·306 (0·276–0·338)	-1·31 (-12·3–11·4)	5·04 (-3·71–14·6)	0·304 (0·272–0·336)	0·328 (0·307–0·352)	0·336 (0·304–0·371)	7·68 (-3·88–22)	2·44 (-6·41–11·3)
Occupational exposure to silica	3·96 (0·578–13)	3·58 (0·508–11·6)	3·81 (0·548–11·8)	-9·56 (-22·3–6·92)	6·5 (-4·68–21·8)	3·09 (0·435–9·44)	2·89 (0·418–9·41)	2·96 (0·394–9·69)	-6·46 (-23·2–16·8)	2·38 (-11·7–23·1)
Occupational exposure to sulfuric acid	0·965 (0·2–3·01)	1·03 (0·216–3·26)	1·06 (0·224–3·47)	6·4 (-8·04–22·9)	3·56 (-7·44–14·6)	0·888 (0·188–2·97)	1·06 (0·233–3·4)	1·06 (0·221–3·32)	19·6 (-3·27–47)	-0·403 (-14·6–16·3)
Unsafe sex	--	--	--	--	--	--	--	--	--	--
Occupational exposure to trichloroethylene	0·087 (0·0807–0·0938)	0·0889 (0·0846–0·0938)	0·0938 (0·0872–0·102)	2·16 (-5·89–11·5)	5·54 (-1·8–13·7)	0·0848 (0·0759–0·0938)	0·0901 (0·0846–0·0969)	0·0917 (0·0834–0·101)	6·34 (-4·57–19·3)	1·76 (-6·72–11·5)
No access to handwashing facility	3·32 (3·06–3·59)	3·1 (2·87–3·35)	3·07 (2·83–3·31)	-6·53 (-14·5–2·6)	-0·84 (-6·12–5·06)	3·32 (3·06–3·59)	3·1 (2·87–3·35)	3·07 (2·83–3·31)	-6·53 (-14·5–2·6)	-0·84 (-6·12–5·06)
Child wasting	0·749 (0·499–1·01)	0·725 (0·475–0·987)	0·725 (0·482–0·975)	-3·12 (-12·4–6·98)	-0·0284 (-7·22–7·48)	--	--	--	--	--
Child stunting	2·39 (1·24–3·53)	2·04 (1·03–3·02)	1·98 (1·05–3)	-14·8 (-33·5–10·4)	-2·68 (-16·9–13·8)	1·94 (0·982–2·9)	1·76 (0·879–2·65)	1·74 (0·885–2·69)	-8·96 (-28·3–16·9)	-0·994 (-15·5–17·5)
Chewing tobacco	2·3 (1·71–3·02)	2·19 (1·53–3·08)	2·17 (1·49–3·06)	-4·62 (-35·9–43·5)	-1·03 (-24·5–28·8)	0·257 (0·162–0·4)	0·267 (0·165–0·416)	0·284 (0·176–0·438)	3·68 (-38·8–74·1)	6·43 (-28·3–48·4)
Diet low in legumes	73·2 (37–99·1)	76·4 (43·1–99·6)	76·8 (43·8–99·6)	4·32 (-0·15–23)	0·557 (-4·24–6·86)	71·6 (33·9–98·5)	74·7 (39·7–99·5)	75·5 (42·5–99·5)	4·31 (-0·564–25·4)	1·13 (-2·9–9·61)
Short gestation	40·4 (33–48·5)	37·9 (31·7–45·3)	35·2 (28·3–43·9)	-6·17 (-23·2–15·3)	-7·05 (-24·3–14·4)	33 (27·9–39·8)	34·4 (28·8–40·8)	31 (25·9–37·2)	4·21 (-18–28·4)	-9·8 (-25·9–11·6)
Low birth weight	15·9 (13·3–19·2)	16·1 (13·9–19·3)	16 (13·1–19·9)	1·25 (-17·1–24·8)	-0·908 (-19·2–23·9)	14·6 (12·3–17·3)	18 (15·3–21·2)	16·9 (14·3–20)	23 (-4·21–53·5)	-5·67 (-22·7–16·7)
High temperature	23·3 (12·3–36·5)	28·3 (16·9–41·2)	26·1 (15·1–38·5)	21·4 (9·3–42·4)	-7·97 (-13·5–-4·65)	23·3 (12·3–36·5)	28·3 (16·9–41·2)	26·1 (15·1–38·5)	21·4 (9·3–42·4)	-7·97 (-13·5–-4·65)
Low temperature	34·4 (28·9–40·7)	36·8 (32–42·5)	36·2 (31·2–42·1)	7·03 (4·03–10·8)	-1·71 (-3·19–-0·254)	34·4 (28·9–40·7)	36·8 (32–42·5)	36·2 (31·2–42·1)	7·03 (4·03–10·8)	-1·71 (-3·19–-0·254)
Kidney dysfunction	20·2 (14·5–27·3)	20·9 (15–28·3)	21·1 (15·2–28·4)	3·53 (-1·04–8·11)	0·788 (-2·94–4·91)	22·3 (15·8–29·9)	23·4 (16·6–31·2)	24·1 (17·3–31·8)	5·15 (0·721–10·2)	3·18 (-1·08–7·99)
Bullying victimization	5·85 (2·44–12)	8·96 (3·6–17·8)	8·7 (3·35–17·9)	53 (32·3–77)	-2·82 (-12·4–3·41)	4·22 (1·74–8·81)	7·82 (3·16–15·6)	7·74 (2·95–16)	85·3 (59–115)	-0·946 (-11·5–5·9)
High LDL cholesterol	67·8 (63·2–72·6)	34·5 (31–38)	30·8 (27·4–34·5)	-49·1 (-54·3–-44·1)	-10·7 (-16·1–-5·8)	65·2 (61·4–69·3)	34 (30·7–37·7)	31·6 (28·1–35·2)	-47·8 (-52·8–-43·2)	-7·19 (-11·4–-2·88)

Supplementary Results Table 1. Washington, DC age-standardised Summary Exposure Values (SEVs) for most-detailed risk factors and percentage change, by sex, 1990, 2010, and 2019. Data in parentheses are 95% uncertainty intervals.

	Male					Female				
	1990	2010	2019	Percentage change 1990-2010	Percentage change 2010-2019	1990	2010	2019	Percentage change 1990-2010	Percentage change 2010-2019
Unsafe sanitation	5·63 (2·71–10·3)	2·41 (1·07–4·55)	2·3 (1·01–4·27)	-57·2 (-82·2–2·42)	-4·75 (-51·4–85·8)	--	--	--	--	--
Ambient particulate matter pollution	15·6 (6·31–30)	9·73 (6·48–13·3)	5·72 (2·87–8·79)	-37·7 (-66·6–39·9)	-41·2 (-57·1–32·9)	15·6 (6·31–30)	9·73 (6·47–13·3)	5·72 (2·85–8·8)	-37·7 (-66·6–39·9)	-41·2 (-57–32·9)
Household air pollution from solid fuels	0·00597 (0·00142–0·018)	0·00274 (0·000443–0·00847)	0·00184 (0·000281–0·00597)	-54 (-82·7–2·44)	-32·8 (-66·6–28·3)	0·00784 (0·00193–0·0222)	0·00362 (0·000629–0·0111)	0·00244 (0·000379–0·00737)	-53·8 (-82·5–2·29)	-32·7 (-66·5–28·7)
Ambient ozone pollution	87·3 (42·2–100)	69·3 (28·3–100)	53·5 (20·4–99·6)	-20·6 (-48·6–0)	-22·8 (-52·8–12·7)	--	--	--	--	--
Residential radon	19·7 (3·12–56·7)	19·7 (3·12–56·7)	19·7 (3·12–56·7)	0 (0–0)	0 (0–0)	19·7 (3·12–56·7)	19·7 (3·12–56·7)	19·7 (3·12–56·7)	0 (0–0)	0 (0–0)
Lead exposure	98·1 (90·6–100)	63·6 (46·3–77·9)	45·3 (31·3–58)	-35·2 (-48·9–22)	-28·7 (-33·3–24·8)	87·2 (62·6–98·9)	51·8 (27–72·3)	35·6 (15·7–52·6)	-40·6 (-56·9–25·8)	-31·3 (-41·8–26·8)
Child underweight	1·03 (0·599–1·55)	0·96 (0·527–1·46)	0·934 (0·509–1·44)	-6·54 (-23·6–13·2)	-2·7 (-15·3–11·5)	0·863 (0·479–1·29)	0·849 (0·48–1·28)	0·866 (0·471–1·35)	-1·62 (-19·5–22·1)	1·91 (-12–19)
Iron deficiency	--	--	--	--	--	15·2 (12·9–17·7)	13·7 (11·6–15·9)	13·6 (11·4–16·2)	-9·98 (-20·7–1·72)	-0·366 (-10–9·56)
Vitamin A deficiency	0·739 (0·196–1·93)	0·837 (0·208–2·33)	0·656 (0·165–1·81)	13·3 (-72·9–428)	-21·7 (-67·3–97·6)	1·5 (0·554–3·13)	1·31 (0·488–2·77)	1·21 (0·482–2·59)	-12·5 (-67·3–144)	-7·68 (-49·2–80·4)
Zinc deficiency	1·6 (0–5·5)	0·766 (0–2·49)	0·752 (0–2·5)	-52·2 (-100–NA)	-1·81 (-29·5–NA)	1·63 (0–5·58)	0·77 (0–2·56)	0·743 (0–2·37)	-52·8 (-100–NA)	-3·6 (-33–NA)
Smoking	22·7 (20·4–25·1)	16·2 (14·4–18)	14·7 (13–16·3)	-28·5 (-33·8–22·3)	-9·6 (-13·4–5·49)	18·2 (15·8–20·9)	12·5 (11·1–14·1)	10·9 (9·6–12·4)	-31·2 (-38–23·6)	-12·9 (-17·8–7·99)
Secondhand smoke	36·4 (33·6–39·3)	20·9 (19·5–22·6)	21·2 (18·8–23·7)	-42·5 (-48·5–35·8)	1·57 (-7·98–12·2)	31·6 (29·5–33·6)	18·8 (17·6–19·9)	19·2 (17·4–21·1)	-40·5 (-45·8–34·9)	2·09 (-6·13–10·3)
Alcohol use	14·4 (9·38–19·6)	14·4 (8·71–20·4)	14·4 (8·94–20·4)	-0·223 (-33·7–48·4)	0·35 (-25·5–34·7)	--	--	--	--	--
Drug use	0·975 (0·434–2·01)	1·03 (0·568–1·86)	1·16 (0·695–1·95)	5·33 (-13·2–36·6)	13·1 (0·0579–31·3)	0·585 (0·282–1·16)	0·789 (0·477–1·33)	0·953 (0·636–1·48)	34·9 (10·2–81)	20·8 (4·97–42·7)
High fasting plasma glucose	15·1 (12·4–18·3)	13·8 (11·7–16·6)	16·5 (14–19·4)	-8·61 (-26·2–12·1)	19·6 (2·28–40·8)	12·4 (10·2–15)	12·9 (10·7–15·7)	15·3 (12·4–18·3)	3·97 (-16·1–31·4)	18·2 (-2·28–40·7)
High systolic blood pressure	24·4 (19·4–29·7)	15 (13–17·1)	19·1 (14·6–23·9)	-38·5 (-50·1–23·6)	27·1 (0·483–58·1)	25·5 (19·1–32·6)	13·5 (11·9–15·1)	18·3 (13·4–24·2)	-46·9 (-58·8–30·1)	35·2 (-1·08–80)
High body-mass index	21·9 (15·3–30·9)	35·5 (26·1–46·8)	36·1 (26·2–47·2)	62·2 (41·1–95·9)	1·81 (-5·23–10·1)	32·1 (23·3–41·5)	45·1 (36·6–53·5)	45·1 (35·8–53·5)	40·3 (24–65·4)	-0·0117 (-6·55–6·88)
Low bone mineral density	11·5 (6·61–18·1)	9·06 (4·68–15·5)	11·3 (6·42–18·1)	-21·1 (-42·7–1·46)	24·8 (-1·97–70·5)	15·2 (9·99–22·2)	16·4 (10·8–23·6)	18·5 (12·6–25·9)	7·6 (-14–37·8)	12·7 (-10·6–43·6)
Diet low in fruits	37·9 (27·1–49·8)	41·6 (29·8–54·7)	42·3 (29·2–55·9)	9·97 (-7–31·9)	1·5 (-12·9–15·9)	42·8 (30·9–55)	43 (31·9–55·9)	43·5 (30·9–57)	0·507 (-13·3–15·6)	1·08 (-13·1–14·1)
Diet low in vegetables	28·2 (13·5–49·7)	65·4 (45·4–83)	63 (42–81·4)	132 (53·6–319)	-3·75 (-11·1–1·87)	28·7 (13·7–49·8)	63·9 (43·9–82·4)	61·4 (40·3–80·5)	123 (44·9–293)	-3·96 (-12·3–1·41)
Diet low in whole grains	78·7 (68·5–87·8)	80·3 (70·9–88·7)	80 (70·1–88·7)	2·03 (-1·68–7·06)	-0·279 (-3·06–2·37)	75·8 (65·2–86)	78·2 (68·1–87·4)	77·8 (67·6–87·2)	3·16 (-1·37–8·84)	-0·567 (-3·68–2·56)
Diet low in nuts and seeds	22·8 (6·55–54·3)	25·5 (8·2–55·1)	18·3 (5·72–52·5)	11·6 (-37·1–132)	-28·4 (-55·4–2·11)	19 (4·21–52·7)	20·7 (5·85–53·6)	15·9 (4·56–52·3)	9·05 (-35·7–117)	-22·9 (-52·5–3·98)
Diet low in milk	42·9 (20–62·7)	33·8 (13·7–53·3)	31·3 (12·5–50·3)	-21·1 (-43·7–3·7)	-7·43 (-26·9–9·19)	--	--	--	--	--
Diet high in red meat	82·1 (70·7–92·2)	83·8 (72·4–93·1)	84 (71·9–93·6)	2·11 (-9·94–16·6)	0·236 (-9·42–10·5)	76·3 (63·2–86·6)	77 (64·3–87·4)	77·7 (64–88·8)	1·04 (-13·7–19·6)	0·849 (-10·8–13·8)
Diet high in processed meat	79·2 (56·1–98·3)	87·1 (69·7–99·8)	87·1 (69·1–99·8)	9·95 (0·859–30·2)	-0·0536 (-4·52–4·94)	81·3 (60·2–98·5)	90·6 (76·2–99·6)	89·4 (74–99·6)	11·3 (0·936–33)	-1·32 (-6·81–2·3)
Diet high in sugar-sweetened beverages	51·6 (33·1–70·9)	72·5 (53·7–87·2)	73·7 (55·6–88·3)	40·5 (2·89–115)	1·67 (-17·6–22·2)	56 (34·6–74·7)	77·5 (60·2–90·8)	77 (60·5–90·8)	38·5 (3·85–114)	-0·655 (-15·6–15·8)
Diet low in fiber	38·1 (23·3–53·2)	23·4 (13·4–36)	20 (10·3–32·5)	-38·5 (-54·7–20·6)	-14·8 (-35·5–6)	38·6 (22·9–54·4)	28·3 (16–41·1)	22·5 (11·4–35·2)	-26·6 (-43·6–6·08)	-20·5 (-39·3–3·47)
Diet low in seafood omega-3 fatty acids	79 (60·1–98·8)	72·9 (55–97·6)	70·6 (52·9–97·2)	-7·73 (-23·6–11·9)	-3·19 (-19·7–16·9)	82·3 (64·2–99·5)	77·3 (59·7–98·2)	75 (56·1–98·5)	-6·02 (-21·3–8·73)	-3·03 (-17·4–13·4)
Diet low in polyunsaturated fatty acids	11·1 (3·23–35)	5·91 (1·55–24·4)	5·61 (1·47–22·3)	-46·9 (-78·7–6·41)	-5·03 (-27·2–7·41)	12·5 (3·65–37·8)	5·99 (1·61–24·5)	5·65 (1·5–24·5)	-51·9 (-81·4–7·23)	-5·63 (-29–6·5)
Diet high in trans fatty acids	89·5 (81·3–100)	87·8 (78·5–100)	87·4 (77·8–100)	-1·82 (-10·3–6·22)	-0·485 (-8·31–8·49)	--	--	--	--	--
Diet high in sodium	18·6 (4·42–42·1)	40·3 (11·3–64·3)	39·5 (9·84–65·4)	116 (38·1–423)	-1·93 (-30·2–27·1)	--	--	--	--	--
Low physical activity	2·19 (0·677–5·39)	2·2 (0·934–4·52)	2·43 (0·889–5·06)	0·142 (-35·1–109)	10·8 (-18·2–53·4)	--	--	--	--	--
Occupational asthmagens	15·7 (12·9–18·9)	14·2 (11·8–17·1)	14·6 (12–18)	-9·85 (-21·8–4·85)	3·31 (-7·6–15·4)	9·08 (7·38–11)	9·89 (7·99–12·3)	10 (7·98–12·6)	8·93 (-9·28–31·7)	1·55 (-13·2–17·2)
Occupational particulate matter, gases, and fumes	5·38 (3·73–7·92)	5·08 (3·61–7·42)	4·94 (3·5–7·28)	-5·57 (-13–2·08)	-2·79 (-6·84–1·85)	2·87 (1·97–4·22)	2·99 (2·09–4·37)	2·96 (2·07–4·32)	4·2 (-6·68–17·2)	-0·806 (-6·56–5·19)
Occupational noise	6·24 (5·61–6·91)	6·25 (5·78–6·86)	6·14 (5·73–6·75)	0·121 (-4·26–5·2)	-1·77 (-4·16–0·836)	4·52 (3·9–5·19)	5·1 (4·67–5·61)	5·25 (4·87–5·76)	12·9 (5·39–21·7)	2·96 (-0·311–6·49)
Occupational injuries	--	--	--	--	--	--	--	--	--	--
Occupational ergonomic factors	10·7 (8·77–13·3)	9·41 (7·83–11·3)	9·85 (8·06–12·1)	-12·5 (-22·1–2·38)	4·66 (-3·37–13·6)	6·1 (4·75–7·76)	6·32 (5·02–7·91)	6·71 (5·28–8·6)	3·74 (-12·8–25)	6·14 (-7·35–21·1)
Childhood sexual abuse	6·89 (4·45–10·1)	6·54 (4·76–8·66)	6·37 (4·7–8·34)	-5·04 (-21·5–15·9)	-2·63 (-8·37–3·01)	16·3 (11·4–22·9)	14·7 (11·2–19·1)	14·8 (11·3–19·5)	-9·83 (-23·1–9·24)	0·799 (-3·95–7·95)
Intimate partner violence	--	--	--	--	--	21·6 (11·7–29·8)	24·2 (14·2–32·8)	27·4 (15·2–36·4)	12·1 (2·12–26·9)	13·3 (1·85–32·7)
Non-exclusive breastfeeding	26·4 (22–31·4)	25·3 (21·4–29·5)	24·8 (20·3–29·5)	-4·36 (-19·2–13·2)	-1·83 (-14·6–12·1)	26·4 (22–31·4)	25·3 (21·4–29·5)	24·8 (20·3–29·5)	-4·3 (-19·2–13·2)	-1·83 (-14·6–12·1)
Discontinued breastfeeding	28·4 (27·3–29·3)	26·6 (25·4–27·9)	26·3 (24·8–27·5)	-6·12 (-11·7–0·572)	-1·43 (-7·25–4·32)	28·3 (27·3–29·3)	26·9 (25·6–28·1)	26·4 (24·9–27·7)	-5·13 (-10·7–0·447)	-1·78 (-7·55–3·92)
Diet low in calcium	13·1 (6·25–23·7)	10·7 (5·65–18·5)	9·03 (4·48–16·1)	-18·3 (-29·9–0·564)	-15·7 (-26·8–6·03)	8·45 (3·67–15·7)	7·14 (3·37–12·9)	6·02 (2·8–11·1)	-15·6 (-31·1–7·36)	-15·7 (-27·6–4·29)
Occupational exposure to asbestos	3·87 (3·36–4·44)	3·22 (2·85–3·62)	2·93 (2·06–4·02)	-16·8 (-31·1–0·94)	-9·15 (-35·7–24·2)	0·952 (0·598–2·55)	0·977 (0·651–2·39)	0·817 (0·46–2·1)	2·66 (-19·6–30·3)	-16·4 (-39·8–13·4)
Occupational exposure to arsenic	0·335 (0–0·798)	0·346 (0–0·816)	0·372 (0–0·849)	3 (-11·7–NA)	7·56 (-3·95–NA)	0·338 (0–0·809)	0·374 (0–0·902)	0·387 (0–0·912)	10·4 (-4–NA)	3·47 (-7·04–NA)
Occupational exposure to benzene	0·96 (0·0567–2·78)	0·967 (0·0559–2·8)	1·01 (0·0568–2·96)	0·722 (-9·93–16·2)	4·08 (-4·9–16·7)	1·22 (0·0644–3·26)	1·48 (0·093–4·14)	1·55 (0·0994–4·41)	21·5 (7·3–44·9)	4·45 (-5·3–18·8)
Occupational exposure to beryllium	0·0217 (0·0201–0·0232)	0·0214 (0·0206–0·0222)	0·0226 (0·021–0·0241)	-1·23 (-8·12–6·6)	5·87 (-0·903–12·6)	0·0253 (0·0231–0·0275)	0·0274 (0·0264–0·0284)	0·0284 (0·0264–0·0303)	8·16 (-1·05–18·8)	3·85 (-2·77–10·5)
Occupational exposure to cadmium	0·0409 (0·0367–0·0455)	0·0423 (0·0385–0·0465)	0·0453 (0·04–0·0514)	3·27 (-9·99–17·8)	7·18 (-3·18–19·7)	0·0421 (0·0377–0·047)	0·0465 (0·0429–0·0512)	0·0482 (0·043–0·0545)	10·6 (-2·22–27·3)	3·53 (-6·06–14·5)
Occupational exposure to chromium	0·101 (0·0915–0·111)	0·107 (0·0979–0·116)	0·113 (0·101–0·127)	5·37 (-7·12–18·6)	6·1 (-3·17–16·8)	0·105 (0·095–0·117)	0·121 (0·113–0·131)	0·126 (0·113–0·14)	15·2 (2·64–30·6)	3·45 (-5–13)
Occupational exposure to diesel engine exhaust	0·546 (0·5–0·596)	0·58 (0·536–0·625)	0·591 (0·533–0·651)	6·27 (-4·8–18·5)	1·92 (-6·66–11·7)	0·493 (0·438–0·549)	0·553 (0·503–0·609)	0·562 (0·495–0·632)	12·3 (-2·72–30·3)	1·51 (-8·61–13·3)
Occupational exposure to formaldehyde	0·204 (0·162–0·231)	0·208 (0·166–0·234)	0·22 (0·173–0·256)	2·21 (-11·6–17)	5·76 (-4·62–18·1)	--	--	--	--	--
Occupational exposure to nickel	0·265 (0–1·14)	0·265 (0–1·13)	0·288 (0–1·28)	-0·275 (-15·5–NA)	8·69 (-3·6–NA)	0·259 (0–1·15)	0·276 (0–1·21)	0·288 (0–1·25)	6·57 (-9·42–NA)	4·07 (-7·78–NA)
Occupational exposure to polycyclic aromatic hydrocarbons	0·214 (0·194–0·234)	0·228 (0·211–0·246)	0·242 (0·217–0·268)	6·48 (-5·51–19·2)	5·98 (-3·15–15·7)	0·236 (0·213–0·262)	0·284 (0·265–0·303)	0·294 (0·266–0·321)	20·2 (7·51–34·6)	3·47 (-4·65–12·1)
Occupational exposure to silica	2·27 (0·319–7·22)	2·17 (0·301–6·71)	2·36 (0·33–7·26)	-4·26 (-20·1–15·5)	8·83 (-3·95–26·7)	2 (0·264–6·34)	2·05 (0·281–6·33)	2·12 (0·311–6·81)	2·45 (-15·1–29·3)	3·55 (-9·72–22·7)
Occupational exposure to sulfuric acid	0·687 (0·148–2·13)	0·82 (0·163–2·75)	0·854 (0·175–2·79)	19·4 (-1·85–44·3)	4·15 (-9·93–19·7)	0·619 (0·133–1·99)	0·851 (0·176–2·76)	0·845 (0·17–2·69)	37·4 (7·77–72·7)	-0·613 (-16·9–19·1)
Unsafe sex	--	--	--	--	--	--	--	--	--	--
Occupational exposure to trichloroethylene	0·063 (0·0579–0·0684)	0·0664 (0·0623–0·0708)	0·0695 (0·0634–0·0765)	5·38 (-5·2–16·9)	4·76 (-3·17–14·1)	0·0635 (0·0576–0·0702)	0·0716 (0·0671–0·0768)	0·0736 (0·0667–0·081)	12·6 (1·19–26·4)	2·91 (-5·04–12·4)
No access to handwashing facility	3·32 (3·07–3·58)	3·07 (2·84–3·33)	3·05 (2·82–3·31)	-7·55 (-15·6–1·95)	-0·434 (-5·99–5·38)	3·32 (3·07–3·58)	3·07 (2·84–3·33)	3·05 (2·82–3·31)	-7·55 (-15·6–1·95)	-0·434 (-5·99–5·38)
Child wasting	0·754 (0·497–1·04)	0·725 (0·479–0·983)	0·724 (0·478–0·983)	-3·9 (-12·9–6·46)	-0·133 (-7·18–7·11)	0·778 (0·523–1·06)	0·761 (0·51–1·05)	0·755 (0·497–1·04)	-2·16 (-12·2–8·58)	-0·693 (-8·02–7·64)
Child stunting	2·78 (1·46–4·02)	2·11 (1·11–3·2)	2·03 (1·05–3·01)	-24·1 (-42–1·97)	-3·77 (-19·1–12·6)	2·13 (1·05–3·22)	1·81 (0·899–2·78)	1·77 (0·84–2·75)	-15·4 (-34·2–7·44)	-1·83 (-17·1–16·4)
Chewing tobacco	1·33 (1·01–1·72)	1·3 (0·954–1·75)	1·23 (0·896–1·69)	-2·26 (-32·7–41)	-5·07 (-25·3–21·3)	0·302 (0·188–0·463)	0·313 (0·198–0·501)	0·315 (0·192–0·483)	3·42 (-38·8–76·6)	0·802 (-29·3–45·2)
Diet low in legumes	72·2 (34·7–98·9)	74·4 (40–99·1)	73·9 (37·8–99·1)	2·97 (-1·33–19·2)	-0·642 (-8·4–4·78)	70·5 (30·9–98·6)	72·8 (36·2–98·8)	72·6 (35·8–99·1)	3·23 (-1·45–21·5)	-0·215 (-6·47–6·05)
Short gestation	64·7 (54·2–77·8)	42·8 (35·2–51·7)	37·6 (30·2–46·7)	-33·9 (-46·7–16·8)	-12·1 (-30·3–9·04)	53·6 (46·4–61·5)	40·1 (34·3–46·7)	32·6 (27·3–39)	-25·2 (-34·9–13·4)	-18·7 (-31·8–2·13)
Low birth weight	27·5 (23·2–33·3)	17·9 (14·9–21·5)	16·8 (13·6–20·8)	-34·7 (-47·7–17·6)	-6·21 (-25·7–17·3)	26·3 (23·2–29·6)	19·4 (17–22·1)	17·1 (14·7–19·9)	-26·2 (-35·3–14·6)	-12 (-26·5–3·57)
High temperature	24·5 (9·89–40)	31·4 (15·6–48)	26·1 (11–42·3)	28·2 (14·5–62·3)	-16·8 (-32·3–9·22)	24·5 (9·89–40)	31·4 (15·6–48)	26·1 (11–42·3)	28·2 (14·5–62·3)	-16·8 (-32·3–9·22)
Low temperature	33·7 (26·6–41·9)	37·5 (31·4–44·5)	36·8 (30·2–44·6)	11·4 (5·64–18·6)	-1·84 (-5·76–2·17)	33·7 (26·6–41·9)	37·5 (31·4–44·5)	36·8 (30·2–44·6)	11·4 (5·64–18·6)	-1·84 (-5·76–2·17)
Kidney dysfunction	20·6 (14·9–27·8)	21·3 (15·2–28·7)	21 (15·1–28·2)	3·09 (-0·417–7·56)	-1·07 (-4·83–2·48)	22·5 (15·9–30)	23·5 (16·8–31·5)	24·4 (17·6–32·1)	4·73 (0·816–9·39)	3·57 (0·315–7·76)
Bullying victimization	5·78 (2·45–11·7)	8·69 (3·55–17)	8·29 (3·24–16·8)	50·4 (29·8–74·3)	-4·53 (-14·1–1·6)	4·17 (1·72–8·54)	7·58 (3·12–14·9)	7·35 (2·86–14·9)	81·8 (56·4–113)	-2·93 (-13·7–4·01)
High LDL cholesterol	66·5 (62–71·2)	34·3 (30·8–37·9)	30·7 (27·3–34·6)	-48·4 (-53·8–43·2)	-10·6 (-15·6–5·54)	66·1 (62·4–70)	34·3 (31·1–37·7)	31·6 (28–35·2)	-48·1 (-52·9–43·2)	-7·84 (-12·4–3·84)

Supplementary Results Table 1. Florida age-standardised Summary Exposure Values (SEVs) for most-detailed risk factors and percentage change, by sex, 1990, 2010, and 2019. Data in parentheses are 95% uncertainty intervals.

	Male					Female				
	1990	2010	2019	Percentage change 1990-2010	Percentage change 2010-2019	1990	2010	2019	Percentage change 1990-2010	Percentage change 2010-2019
Unsafe sanitation	7·09 (3·44–12·5)	3·46 (1·53–6·58)	2·87 (1·28–5·38)	-51·1 (-80·3–18·8)	-17·3 (-58–59·8)	--	--	--	--	--
Ambient particulate matter pollution	9·32 (2·37–19·9)	5·31 (2·79–8·23)	2·79 (0·683–5·47)	-43·1 (-75·1–83·2)	-47·4 (-75·4—33·5)	9·32 (2·37–19·9)	5·31 (2·79–8·21)	2·79 (0·684–5·47)	-43 (-75·3–83)	-47·4 (-75·4—33·7)
Household air pollution from solid fuels	0·0082 (0·0016–0·0231)	0·00402 (0·00069–0·0121)	0·00232 (0·000246–0·00896)	-51 (-82·3–24·5)	-42·4 (-79·7–13·8)	0·0108 (0·00233–0·0318)	0·00534 (0·000949–0·0167)	0·00308 (0·000334–0·0118)	-50·7 (-82·3–24·1)	-42·4 (-79·9–13·6)
Ambient ozone pollution	47·4 (20–80·8)	45·9 (18·8–79·1)	16·4 (5·54–32·6)	-3·24 (-12·1–5·49)	-64·3 (-76·5—54·6)	--	--	--	--	--
Residential radon	14·6 (2–43·3)	14·6 (2–43·3)	14·6 (2–43·3)	0 (0–0)	0 (0–0)	14·6 (2–43·3)	14·6 (2–43·3)	14·6 (2–43·3)	0 (0–0)	0 (0–0)
Lead exposure	75·7 (51·5–90·7)	44·2 (24·5–63·3)	30·9 (14·5–48)	-41·5 (-54·1—28·7)	-30·1 (-39·7—23·5)	50·1 (18–74·9)	27·4 (5·72–50·8)	18·4 (2·34–37·3)	-45·4 (-70·6—29·8)	-32·8 (-58—24·7)
Child underweight	0·958 (0·539–1·46)	0·949 (0·516–1·42)	0·926 (0·504–1·39)	-0·889 (-17·9–19·3)	-2·44 (-15·1–12·8)	0·806 (0·437–1·2)	0·837 (0·449–1·28)	0·855 (0·463–1·32)	3·89 (-16·4–32·3)	2·22 (-11·6–17)
Iron deficiency	--	--	--	--	--	11·7 (9·82–13·9)	12·1 (10·2–14·1)	12·3 (10·2–14·6)	3 (-8·87–16·9)	1·94 (-7·45–12·2)
Vitamin A deficiency	0·897 (0·24–2·16)	1·13 (0·288–2·81)	0·823 (0·213–2·24)	26·6 (-70·1–457)	-27·5 (-67·8–64·3)	1·74 (0·694–3·56)	1·65 (0·655–3·36)	1·44 (0·536–3·07)	-5·33 (-63·7–156)	-12·7 (-51·4–65·5)
Zinc deficiency	2·15 (0–6·88)	1·14 (0–3·7)	1·23 (0–3·95)	-46·8 (-100–NA)	7·35 (-14·8–NA)	2·21 (0–7·26)	1·14 (0–3·73)	1·2 (0–3·78)	-48·4 (-100–NA)	5·08 (-14·3–NA)
Smoking	28·1 (25·4–31·1)	20·6 (18·6–22·7)	17·4 (15·6–19·3)	-27 (-31·6—21·8)	-15·4 (-18·9—12)	21·7 (19·2–24·4)	15·6 (14–17·4)	12·9 (11·4–14·5)	-28 (-34—21)	-17·5 (-21·5—13·2)
Secondhand smoke	40·7 (38·3–43·3)	25·1 (23·6–26·6)	25 (22·4–27·9)	-38·5 (-44·2—32·7)	-0·218 (-8·51–10·4)	38·3 (36·3–40·5)	24·1 (23–25·3)	24·2 (22·2–26·1)	-37 (-41·7—31·8)	0·103 (-6·72–7·44)
Alcohol use	14·6 (8·95–20·9)	16·7 (10·2–24·4)	17·1 (10·3–25·4)	14·8 (-17·2–64·8)	2·24 (-18·8–29)	10·1 (6·07–14·9)	11·2 (6·96–16)	11·3 (6·65–16·9)	10·6 (-19·2–56·2)	0·397 (-21·3–26·9)
Drug use	0·675 (0·333–1·29)	1·2 (0·815–1·78)	1·46 (1·05–2·05)	77·8 (28·4–164)	21·4 (7·25–39·6)	0·396 (0·247–0·647)	0·799 (0·579–1·08)	1·09 (0·832–1·42)	102 (56·6–163)	36·8 (17·7–57·8)
High fasting plasma glucose	12·6 (10·5–15·2)	18·9 (16·5–21·5)	20 (17·2–23·3)	49·9 (25·6–79)	5·96 (-7·33–21·5)	--	--	--	--	--
High systolic blood pressure	25·2 (20·5–30·3)	15·9 (13·9–17·9)	19·8 (15·3–24·7)	-36·9 (-48·1—22·7)	24·5 (-1·6–53·5)	22 (16·3–28·4)	13·2 (11·6–14·9)	17·8 (12·8–23·7)	-39·9 (-54·5—20·1)	34·5 (-2·02–81·8)
High body-mass index	23·1 (16·1–32)	37·6 (27·4–49·3)	38·4 (28·1–50)	63 (42·7–94·6)	2·02 (-4·02–9·17)	29·1 (20·8–38·5)	42·1 (33–50·8)	42·7 (33·9–51·6)	44·6 (26–69·6)	1·47 (-4·65–8·16)
Low bone mineral density	12·1 (7·04–18·6)	9·14 (4·8–15·4)	11·5 (6·78–18·4)	-24·2 (-45·5—0·212)	25·6 (-1·38–66·5)	15·9 (10·3–22·8)	16·5 (11–24)	18·8 (12·6–26)	3·89 (-18–32·3)	13·3 (-8·86–41·9)
Diet low in fruits	49·6 (37·3–62·5)	44·6 (33·3–57·3)	45·2 (33–59)	-10 (-22·1–0·984)	1·28 (-10·7–14·5)	32·9 (10·4–73·9)	29·1 (7·77–69·4)	31·3 (8·36–70·8)	-11·5 (-47·8–20·6)	7·3 (-14–63·2)
Diet low in vegetables	33·6 (17·1–55·9)	49·8 (33·2–68·6)	48·5 (32·2–66·4)	48·4 (16·7–121)	-2·56 (-10·9–5·81)	36·3 (18·8–58·2)	45 (27·5–65·1)	43·6 (25·7–64·1)	23·9 (5·43–61·1)	-3·18 (-15·5–6·98)
Diet low in whole grains	79·4 (69·6–88·2)	81·5 (73–89·6)	81·7 (73–89·8)	2·66 (-0·808–7·26)	0·239 (-2·26–2·88)	76·7 (66–86·6)	79·5 (70·4–88·3)	79·5 (69·8–88·2)	3·7 (-0·703–9·46)	-0·0534 (-2·96–2·58)
Diet low in nuts and seeds	27 (7·67–55·4)	25 (8·33–55·1)	20·8 (6·72–53·7)	-7·33 (-41·4–66·4)	-16·8 (-44·8–10·4)	22·7 (5·26–54·2)	21·5 (6·28–54·5)	19·1 (5·72–53·2)	-5·23 (-39·6–63·4)	-11·4 (-40·3–16)
Diet low in milk	47·9 (25·3–67·6)	42·8 (18·8–63·1)	42·8 (19·7–63·8)	-10·6 (-30·1–2·16)	-0·104 (-11·6–13·5)	--	--	--	--	--
Diet high in red meat	78·8 (66·4–88·7)	76·5 (63·3–87·2)	75·7 (61·9–87·4)	-2·81 (-16·8–12·1)	-1·06 (-12·2–11·3)	79·4 (67–89·4)	75·8 (62·3–86·7)	75 (61·6–86·8)	-4·56 (-17·5–9·75)	-0·974 (-11·9–11·1)
Diet high in processed meat	78·2 (53·5–98·4)	86·2 (68·3–99·8)	85·9 (68–99·8)	10·2 (0·943–30·8)	-0·279 (-5·17–3·56)	79·5 (56·7–98·3)	89 (72·4–99·7)	87·4 (69·7–99·7)	12 (1·26–35·2)	-1·81 (-7·86–1·54)
Diet high in sugar-sweetened beverages	46·9 (27·3–65·4)	63·3 (42·7–80·3)	62·3 (41·9–80·5)	35 (-4·23–108)	-1·49 (-22·2–22·4)	50·6 (30·5–68·9)	69·1 (48·4–85·8)	66·7 (48·1–84·3)	36·7 (0·32–115)	-3·49 (-22·4–19·9)
Diet low in fiber	42·4 (26·8–57·7)	29·6 (17–43)	27·9 (15·3–42·1)	-30·1 (-46·6—13·1)	-5·91 (-24·3–12·1)	43 (26·6–58·9)	35 (20·7–49·5)	30·9 (17·6–45·8)	-18·6 (-33·9—0·944)	-11·6 (-28·4–4·02)
Diet low in seafood omega-3 fatty acids	82·5 (63·6–99·6)	78·8 (59·4–98·9)	79·3 (60·4–99·7)	-4·4 (-20·7–12·3)	0·568 (-14·2–19·2)	85·8 (69·3–99·9)	82·8 (66–99·7)	83 (66·3–99·7)	-3·43 (-15·7–8·41)	0·192 (-12–14·9)
Diet low in polyunsaturated fatty acids	13·3 (3·93–37·9)	7·03 (1·99–25·4)	6·92 (2–25·5)	-47·2 (-75·5—7·72)	-1·61 (-21·8–25·7)	14·9 (4·44–42·9)	7·29 (2·11–26·4)	7·09 (2·1–24·6)	-51·2 (-78·3—8·16)	-2·66 (-24·2–21·5)
Diet high in trans fatty acids	89·5 (81·9–100)	87·8 (78·7–100)	87·4 (78·4–100)	-1·82 (-9·4–5·57)	-0·518 (-8·45–8·03)	--	--	--	--	--
Diet high in sodium	18·6 (4·53–41·1)	40·1 (9·95–64)	39·4 (10·3–64·4)	116 (39·3–388)	-1·96 (-27·3–32)	--	--	--	--	--
Low physical activity	1·99 (0·598–5·15)	1·8 (0·63–4·98)	1·77 (0·519–4·87)	-9·36 (-39·8–32·7)	-1·76 (-30·5–33·9)	--	--	--	--	--
Occupational asthmagens	19·7 (16·6–23·3)	17·6 (14·9–20·7)	18·4 (15·3–22·1)	-10·8 (-20·8—0·259)	4·72 (-4·13–14·7)	10·6 (8·63–12·8)	10·3 (8·54–12·4)	10·5 (8·46–12·7)	-2·88 (-17·4–11·8)	1·97 (-9·79–14·2)
Occupational particulate matter, gases, and fumes	8·4 (5·93–12)	7·81 (5·56–11·1)	7·53 (5·39–10·8)	-7 (-12·2—1·08)	-3·58 (-6·93—0·128)	4·73 (3·31–7·06)	4·55 (3·23–6·58)	4·37 (3·13–6·26)	-3·85 (-12·7–6·07)	-3·87 (-8·83–1·54)
Occupational noise	7·92 (7·18–8·81)	7·61 (7·03–8·32)	7·36 (6·87–8·04)	-3·96 (-7·53—0·0742)	-3·22 (-5·21—0·894)	5·66 (4·91–6·48)	5·93 (5·41–6·55)	5·93 (5·51–6·48)	4·73 (-1·29–12·1)	-0·0553 (-2·97–3·36)
Occupational injuries	--	--	--	--	--	--	--	--	--	--
Occupational ergonomic factors	11·5 (9·59–14)	9·91 (8·39–11·9)	10·3 (8·61–12·4)	-13·6 (-22·6—3·34)	3·57 (-3·69–11·3)	6·62 (5·15–8·55)	6·54 (5·29–8·11)	6·84 (5·47–8·68)	-1·12 (-17·6–18·2)	4·58 (-6·82–17·8)
Childhood sexual abuse	7·4 (5·05–10·5)	6·97 (4·6–9·93)	6·85 (4·54–9·67)	-5·83 (-12·2–5·83)	-1·68 (-8·51–5·53)	12·3 (8·94–17·3)	17·4 (12·9–24)	17·8 (13·1–24·7)	41·9 (30·7–54·8)	2·17 (-5–11)
Intimate partner violence	--	--	--	--	--	30 (17·7–39·2)	28·4 (16–37·6)	29·4 (15·7–38·9)	-5·51 (-14·8–6·78)	3·42 (-4·9–20·9)
Non-exclusive breastfeeding	26·3 (21·9–30·9)	24·9 (20·9–28·6)	24·9 (20·6–29·7)	-5·47 (-19·4–11·2)	0·0149 (-13·1–14·5)	26·3 (21·9–30·9)	24·9 (20·9–28·6)	24·9 (20·6–29·7)	-5·46 (-19·4–11·2)	0·00704 (-13·1–14·5)
Discontinued breastfeeding	27·5 (26·6–28·4)	25·4 (24–26·5)	25·9 (24·6–27·1)	-7·81 (-13·7—2·65)	2·29 (-3·1–8·47)	27·5 (26·5–28·3)	25·3 (23·9–26·5)	25·9 (24·5–27)	-7·94 (-13·8—2·79)	2·34 (-3·05–8·55)
Diet low in calcium	15·8 (7·65–28·8)	13·4 (6·53–24·3)	13·2 (6·13–24·2)	-15·4 (-27·1—3·75)	-1·45 (-11·3–7·21)	11·1 (4·6–20·9)	10·3 (4·51–19·4)	10·5 (4·47–20)	-6·92 (-20·5–12·8)	2·05 (-9·58–12·6)
Occupational exposure to asbestos	7·63 (6·82–8·58)	6·21 (5·7–6·69)	5·51 (3·98–7·4)	-18·6 (-28·5—7·44)	-11·4 (-35·2–17·9)	1·22 (0·962–1·8)	1·11 (0·89–1·69)	1·13 (0·746–1·82)	-9·11 (-22·1–4·28)	1·81 (-27·7–38·9)
Occupational exposure to arsenic	0·424 (0–1·01)	0·419 (0–0·999)	0·45 (0–1·1)	-1·23 (-15·2–NA)	7·44 (-3·98–NA)	0·412 (0–0·964)	0·415 (0–0·991)	0·429 (0–1·03)	0·648 (-16·3–NA)	3·39 (-7·98–NA)
Occupational exposure to benzene	0·846 (0·0522–2·45)	0·806 (0·0464–2·35)	0·838 (0·0521–2·43)	-4·66 (-12·7–6·4)	3·86 (-4·2–14·1)	1·19 (0·0701–3·33)	1·3 (0·0786–3·6)	1·37 (0·0788–3·89)	10·1 (-1·89–29·7)	4·96 (-4·12–18·4)
Occupational exposure to beryllium	0·0227 (0·0214–0·0242)	0·0219 (0·0215–0·0224)	0·0233 (0·0221–0·0246)	-3·28 (-9·15–2·69)	6·34 (0·382–12·4)	0·0254 (0·0233–0·0276)	0·0262 (0·0257–0·0267)	0·0273 (0·0255–0·0289)	3·33 (-5·12–12·8)	4·14 (-2·57–10·7)
Occupational exposure to cadmium	0·0508 (0·0459–0·0564)	0·0503 (0·0459–0·0551)	0·0539 (0·0482–0·0607)	-1·04 (-13·6–12·8)	7·15 (-2·89–17·6)	0·0502 (0·0444–0·0572)	0·0509 (0·0466–0·0564)	0·0526 (0·0463–0·0596)	1·38 (-14·3–18·1)	3·46 (-7·19–15·3)
Occupational exposure to chromium	0·126 (0·115–0·138)	0·127 (0·118–0·137)	0·136 (0·123–0·15)	0·799 (-10·3–12·9)	6·78 (-2·05–15·6)	0·129 (0·115–0·144)	0·133 (0·124–0·144)	0·138 (0·124–0·152)	3·41 (-10·5–17·1)	3·32 (-5·66–12·8)
Occupational exposure to diesel engine exhaust	0·737 (0·683–0·797)	0·771 (0·725–0·817)	0·818 (0·753–0·887)	4·67 (-4·82–14·9)	6·1 (-1·29–14·3)	0·674 (0·593–0·753)	0·695 (0·632–0·755)	0·701 (0·623–0·787)	3·13 (-9·8–20·4)	0·927 (-9·13–10·8)
Occupational exposure to formaldehyde	0·253 (0·226–0·281)	0·244 (0·22–0·269)	0·259 (0·229–0·292)	-3·43 (-16·2–10·8)	5·82 (-4·79–17)	0·244 (0·195–0·28)	0·249 (0·201–0·277)	0·259 (0·205–0·294)	2·37 (-12·1–17·8)	3·73 (-6·24–14·3)
Occupational exposure to nickel	0·35 (0–1·53)	0·33 (0–1·45)	0·356 (0–1·55)	-5·79 (-19·1–NA)	7·82 (-3·68–NA)	0·318 (0–1·43)	0·308 (0–1·33)	0·321 (0–1·38)	-3·08 (-20–NA)	4·05 (-9·36–NA)
Occupational exposure to polycyclic aromatic hydrocarbons	0·253 (0·232–0·276)	0·254 (0·237–0·271)	0·27 (0·247–0·296)	0·534 (-10·1–11·4)	6·18 (-2·19–14·6)	0·277 (0·249–0·307)	0·296 (0·279–0·315)	0·306 (0·279–0·333)	6·94 (-5·24–20·3)	3·38 (-5·04–12·1)
Occupational exposure to silica	3·26 (0·475–10·8)	2·95 (0·395–9·62)	3·2 (0·421–10·1)	-9·43 (-22–6·52)	8·26 (-3·12–22·7)	2·68 (0·39–8·2)	2·5 (0·342–7·9)	2·57 (0·348–8·25)	-6·81 (-23·9–15·8)	2·72 (-10–20·7)
Occupational exposure to sulfuric acid	0·873 (0·183–2·74)	0·948 (0·197–3·04)	0·984 (0·208–3·17)	8·68 (-7·38–27·9)	3·79 (-7·3–16·6)	0·818 (0·164–2·67)	0·991 (0·197–3·14)	0·974 (0·205–3·04)	21·2 (-2·29–49·5)	-1·73 (-15·8–14·7)
Unsafe sex	--	--	--	--	--	--	--	--	--	--
Occupational exposure to trichloroethylene	0·0786 (0·073–0·085)	0·0807 (0·0775–0·0846)	0·086 (0·0805–0·0927)	2·72 (-5·59–12·1)	6·51 (-0·618–14·1)	0·0782 (0·0711–0·0864)	0·0808 (0·0765–0·086)	0·0829 (0·0755–0·0902)	3·35 (-8·06–16·4)	2·63 (-5·62–11·5)
No access to handwashing facility	3·38 (3·13–3·64)	3·11 (2·89–3·37)	3·08 (2·85–3·32)	-7·95 (-15·9–0·8)	-1·03 (-6·56–4·51)	3·38 (3·13–3·64)	3·11 (2·89–3·37)	3·08 (2·85–3·32)	-7·95 (-15·9–0·8)	-1·03 (-6·56–4·51)
Child wasting	0·749 (0·501–1·04)	0·726 (0·468–0·994)	0·726 (0·478–0·982)	-3·1 (-12·3–7·16)	-0·00535 (-6·38–7·58)	--	--	--	--	--
Child stunting	2·39 (1·29–3·59)	2·05 (1·06–3·12)	1·99 (1–2·99)	-14·3 (-34·2–9·13)	-2·94 (-17·9–12·1)	1·93 (0·988–2·9)	1·76 (0·896–2·67)	1·74 (0·87–2·7)	-9·07 (-28·6–14·4)	-1·21 (-15·5–16·8)
Chewing tobacco	3·98 (3·11–5·01)	3·64 (2·64–4·87)	3·57 (2·48–4·95)	-8·57 (-35·3–30·2)	-1·82 (-24·3–26·2)	0·366 (0·237–0·561)	0·379 (0·24–0·585)	0·395 (0·249–0·642)	3·58 (-40·3–75·5)	4·04 (-27·2–45)
Diet low in legumes	73·7 (39·2–99·4)	76·8 (44·8–99·6)	77·1 (44·7–99·6)	4·27 (0–22·1)	0·294 (-4·68–5·18)	72·1 (35·4–98·7)	75·2 (41·5–99·5)	75·8 (43·4–99·6)	4·32 (-0·015–21·9)	0·824 (-3·5–9·57)
Short gestation	31 (26–36·8)	24·8 (21·2–28·8)	22·5 (18·7–26·6)	-20 (-34·2—1·91)	-9·31 (-24·5–8·95)	28·9 (24·4–34·1)	24·1 (20·6–28)	22·7 (19·3–26·9)	-16·5 (-31·6–1·35)	-5·81 (-20·1–11·9)
Low birth weight	12·1 (10·2–14·3)	10·5 (9·11–12·2)	10 (8·42–11·8)	-13·3 (-29·9–7·47)	-4·88 (-22·8–16·9)	13 (11–15·5)	12·3 (10·6–14)	12·4 (10·6–14·5)	-5·74 (-23·9–16·3)	0·842 (-14·9–22·6)
High temperature	0 (0–0)	26·3 (17·5–36·6)	25·1 (16·5–35·2)	--	-4·56 (-6·83—2·42)	0 (0–0)	26·3 (17·5–36·6)	25·1 (16·5–35·2)	--	-4·56 (-6·83—2·42)
Low temperature	31·5 (21–43·6)	37·1 (28–47·1)	34·2 (23·5–46·4)	17·6 (4·33–42·7)	-7·8 (-17·4—0·535)	31·5 (21–43·6)	37·1 (28–47·1)	34·2 (23·5–46·4)	17·6 (4·33–42·7)	-7·8 (-17·4—0·535)
Kidney dysfunction	19·5 (14–26·5)	20·3 (14·5–27·5)	20·5 (14·6–27·6)	4·12 (-0·154–8·31)	0·675 (-2·78–4·26)	21·6 (15·2–29·1)	22·7 (16–30·5)	23·6 (17·1–31·3)	5·38 (1·26–10·9)	3·79 (-0·4–9·08)
Bullying victimization	5·88 (2·45–12·1)	8·99 (3·61–17·8)	8·72 (3·35–17·9)	53 (32·9–76·5)	-3·08 (-12·4–3·1)	4·26 (1·73–8·89)	7·89 (3·17–15·7)	7·78 (2·95–16)	85·1 (59·8–116)	-1·38 (-11·9–5·47)
High LDL cholesterol	66 (61·7–70·4)	35 (31·5–38·8)	31·3 (27·7–35)	-46·9 (-52·2—41·4)	-10·7 (-15·6—5·94)	65·2 (61·6–69·2)	34·6 (31·4–38)	32·1 (28·6–35·7)	-46·9 (-51·3—42·3)	-7·21 (-11·4—2·94)

Supplementary Results Table 1. Azerbaijan age-standardised Summary Exposure Values (SEVs) for most-detailed risk factors and percentage change, by sex, 1990, 2010, and 2019. Data in parentheses are 95% uncertainty intervals.										
	Male					Female				
	1990	2010	2019	Percentage change 1990-2010	Percentage change 2010-2019	1990	2010	2019	Percentage change 1990-2010	Percentage change 2010-2019
Unsafe sanitation	58·6 (49–67·6)	43·4 (39·9–46·9)	37·3 (28·5–46)	-25·9 (-35·2—12·1)	-14·1 (-33·4–4·6)	58·6 (49–67·6)	43·4 (39·9–46·9)	37·3 (28·5–46)	-25·9 (-35·2—12·1)	-14·1 (-33·4–4·6)
Ambient particulate matter pollution	17 (7·3–33·2)	23·3 (12·6–37)	24·2 (13·6–38·3)	37·2 (-23·7–159)	4·05 (-18·4–32·7)	15·3 (6·54–30·8)	22·6 (12·3–36·4)	23·8 (13·4–37·5)	47·7 (-18·5–182)	5·51 (-17–34·6)
Household air pollution from solid fuels	10·6 (4·94–19·3)	1·91 (0·736–4·19)	0·985 (0·333–2·19)	-81·9 (-89·3—71·5)	-48·3 (-65·8—25·8)	12·4 (6·42–20·8)	2·42 (0·994–4·86)	1·26 (0·448–2·77)	-80·6 (-88·5—69·5)	-47·7 (-65·4—25·4)
Ambient ozone pollution	58·8 (25·4–98·6)	44·4 (18·1–75·5)	67·2 (29–100)	-24·5 (-36·2—12·3)	51·3 (24·7–89·1)	58·8 (25·4–98·6)	44·4 (18·1–75·5)	67·2 (29–100)	-24·5 (-36·2—12·3)	51·3 (24·7–89·1)
Residential radon	27·7 (4·04–100)	27·7 (4·04–100)	27·7 (4·04–100)	0 (0–0)	0 (0–0)	--	--	--	--	--
Lead exposure	47·4 (25·1–68·4)	43·6 (23·4–63·5)	37·2 (18·2–57·3)	-7·98 (-19·6–8·07)	-14·8 (-23·4—9·19)	23·5 (0·224–50·4)	24·6 (2·94–49·2)	21·6 (2·76–45·4)	4·55 (-8·51–1380)	-12·1 (-24·7–1·3)
Child underweight	4·79 (3·43–6·14)	2·94 (1·93–3·99)	2·32 (1·4–3·26)	-38·6 (-51·3—27)	-21·1 (-33·2—11·2)	5·4 (3·94–6·61)	3·43 (2·32–4·51)	2·75 (1·79–3·75)	-36·4 (-47·3—27·1)	-19·8 (-30·1—11·9)
Iron deficiency	--	--	--	--	--	22·4 (20·2–24·6)	19·9 (17·8–22)	18·8 (16·4–21)	-11·2 (-19—2·78)	-5·86 (-11·4—0·369)
Vitamin A deficiency	7·81 (3·26–15·2)	5·4 (2·29–10·4)	3·8 (1·64–7·48)	-30·9 (-70·5–71·6)	-29·6 (-59·8–27·6)	4·64 (2·38–8·18)	4·96 (2·43–8·32)	3·76 (1·89–6·32)	6·77 (-44·6–122)	-24 (-49·6–20·7)
Zinc deficiency	21·7 (6·59–36)	8·54 (0·829–21·7)	6·07 (0·17–17)	-60·6 (-89·3—40·4)	-28·9 (-68·8—15·8)	20·9 (6·35–35·7)	8·21 (0·676–20·3)	5·91 (0·0756–16·6)	-60·7 (-89·3—40·5)	-28 (-70·9—14·8)
Smoking	28·8 (24·8–32·9)	28·3 (25·2–31·6)	28·5 (25·6–31·5)	-1·71 (-8·89–6·99)	0·803 (-2·94–4·97)	0·67 (0·463–0·945)	0·761 (0·563–0·996)	0·884 (0·66–1·15)	13·6 (-12·9–49·3)	16·2 (-1·5–35·1)
Secondhand smoke	33·2 (31·4–34·9)	34·6 (32·9–36·2)	34·6 (32·1–37)	4·31 (-3–12·6)	0·0436 (-5·1–5·56)	54·4 (52·6–56)	58·1 (56·6–59·4)	57·8 (55·4–59·9)	6·8 (2·78–11·2)	-0·472 (-3·29–2·34)
Alcohol use	11·5 (8·11–15·2)	10·4 (6·99–14·2)	11·6 (7·68–15·7)	-9 (-24·2–5·35)	11·6 (-2·74–28·6)	3·56 (1·97–5·54)	3·09 (1·65–4·97)	3·51 (1·78–5·5)	-13·1 (-36·2–15·7)	13·6 (-11·6–44·6)
Drug use	0·135 (0·0763–0·252)	0·142 (0·0825–0·261)	0·147 (0·0872–0·263)	4·76 (-4·91–17·8)	3·81 (-3·52–12·7)	0·076 (0·0418–0·144)	0·076 (0·0414–0·147)	0·0787 (0·0435–0·146)	-0·0505 (-7·25–8·2)	3·48 (-4·34–12·2)
High fasting plasma glucose	5·64 (4·9–6·48)	9·72 (7·8–11·9)	12·5 (10·1–15·1)	72·4 (43·7–108)	28·4 (7·03–61·5)	5·66 (5·01–6·4)	11·6 (9·87–13·8)	12·8 (10·7–15·1)	106 (77·6–141)	9·65 (-6·61–27·7)
High systolic blood pressure	32·9 (26–40·5)	30·7 (25·6–35·8)	32·8 (26–39·5)	-6·73 (-28·8–20·7)	6·74 (-10·4–26)	31·4 (22·3–41·6)	24·2 (18·2–31·2)	26·3 (18·7–35·3)	-22·9 (-47·7–16·7)	8·61 (-16·2–41·1)
High body-mass index	15·3 (10·4–21·2)	23·1 (16·8–30·7)	29·4 (21·8–38·4)	51·1 (32·6–79·9)	27·2 (17·7–39·8)	22·5 (16·8–30·2)	29·7 (22·4–39·3)	35·4 (27–46·1)	31·9 (19·1–48·3)	19·1 (12·2–27·9)
Low bone mineral density	8·67 (4·6–14·3)	8·43 (4·62–13·8)	8·04 (4·2–13·3)	-2·79 (-25·4–25·7)	-4·69 (-27·8–23·4)	16·1 (10·7–23·1)	15·9 (10·1–23·2)	15·2 (9·53–22·3)	-1·5 (-23·3–25·4)	-4·31 (-26·5–24)
Diet low in fruits	48·9 (37·2–62·3)	40·6 (28·7–53·4)	36·9 (25·6–49·4)	-17·1 (-26·4—10·7)	-9·06 (-18·7—0·594)	50·4 (38·7–63·8)	42·1 (30·4–55·5)	38·9 (27·5–51·8)	-16·4 (-24·8—10·4)	-7·79 (-17·5–0·866)
Diet low in vegetables	22·9 (9·65–44·6)	5·49 (2·28–13·9)	3·55 (1·69–9·94)	-76·1 (-86·6—55·6)	-35·4 (-63·5—5·45)	--	--	--	--	--
Diet low in whole grains	99·9 (100–100)	99·9 (100–100)	99·9 (100–100)	0 (0–0)	0 (0–0)	99·9 (100–100)	99·9 (100–100)	99·9 (100–100)	0 (0–0)	0 (0–0)
Diet low in nuts and seeds	69·5 (34·1–84)	69·9 (33·6–83·5)	65·7 (30·4–80)	0·646 (-2·61–7·45)	-6·07 (-14·2–1·54)	65·3 (32–81·6)	65·4 (31·4–80·7)	60·1 (27·9–76·9)	0·0177 (-3·78–5·64)	-7·96 (-17·9—1·78)
Diet low in milk	78·1 (53·8–97·7)	75·1 (48·8–95·8)	73·4 (46·5–94·6)	-3·87 (-11—0·608)	-2·24 (-6·52—0·252)	77·7 (53·4–97·6)	74·7 (48–95·8)	73 (46·1–94·7)	-3·9 (-10·7—0·595)	-2·32 (-6·26—0·23)
Diet high in red meat	25·3 (15·8–34·4)	30·6 (20·3–40·7)	36·1 (25·2–47·5)	21·2 (14·5–32·8)	18·1 (7·24–35·5)	25·2 (15·5–34·5)	30·5 (20–40·3)	36 (24·6–47·7)	21·2 (14·5–32·1)	17·9 (7·6–35·5)
Diet high in processed meat	40·9 (22·2–64·8)	45·1 (25·4–69·1)	47 (26·3–70·7)	10·4 (-12·1–44·2)	4·17 (-10·1–22)	47·2 (26·4–71)	52·9 (30·7–76·3)	55·2 (32·7–78)	12·1 (-8·1–43·9)	4·31 (-6·23–19·3)
Diet high in sugar-sweetened beverages	24·8 (15·3–41·5)	26·2 (14·9–42·3)	29 (16·7–45·2)	5·65 (-34·3–63·6)	10·8 (-14·9–50·3)	22 (12·7–37·2)	26·5 (13·7–44)	30·1 (15·7–47·6)	20·4 (-27·7–98·8)	13·8 (-12·1–57·6)
Diet low in fiber	39·3 (23·8–55·2)	22·3 (12·6–33·5)	18·8 (9·99–29·5)	-43·3 (-51·6—36·4)	-15·7 (-30·1—1·31)	39·5 (23·6–56·1)	21·7 (11·7–33·2)	18·2 (9·41–29·3)	-44·9 (-53·8—37·2)	-16·1 (-30·7—0·533)
Diet low in seafood omega-3 fatty acids	97·8 (91·4–100)	97·2 (91·2–100)	95·1 (86·7–100)	-0·609 (-5·87–4·16)	-2·21 (-9·04–1·43)	98·5 (93·2–100)	98·3 (94·2–100)	96·6 (90·6–100)	-0·183 (-3·77–4·35)	-1·74 (-6·98–0·507)
Diet low in polyunsaturated fatty acids	83·7 (50–100)	79·3 (43·4–100)	77·2 (39·3–100)	-5·31 (-17·2–0)	-2·65 (-9·33–0)	84·8 (53–100)	80·5 (45·2–100)	78·4 (39·9–100)	-5·06 (-16·8–0)	-2·57 (-9·21–0)
Diet high in trans fatty acids	85 (75·9–100)	75·4 (66·3–100)	72·8 (63·2–99·9)	-11·3 (-18·8–0)	-3·43 (-11·1–4·16)	83·4 (74·7–100)	74·4 (65·3–100)	72·2 (63·2–100)	-10·8 (-17·6–0)	-3·01 (-10·8–3·76)
Diet high in sodium	63·2 (38·5–81·3)	43·3 (14·3–66·7)	42·7 (13·9–67·2)	-31·4 (-66·6—10·1)	-1·5 (-23–21)	30·9 (11·2–53·9)	18·6 (4·4–41·8)	18·2 (4·33–40·9)	-39·8 (-70·6—18·3)	-2·14 (-19·2–14·5)
Low physical activity	1·9 (0·92–4·12)	2·08 (1·02–4·25)	2·25 (1·11–4·52)	9·56 (-0·766–23·2)	8·27 (-1·71–22·1)	1·56 (0·847–3·16)	1·69 (0·903–3·41)	1·82 (0·979–3·53)	8·33 (-0·462–21·5)	7·43 (-1·45–19·4)
Occupational asthmagens	20·3 (16·1–25·7)	21·1 (17·1–25·7)	22·1 (17·7–27·2)	3·74 (-9·29–19·5)	4·61 (-3·88–14·2)	11·2 (8·2–15·4)	12·9 (10·1–16·4)	14 (10·5–18·3)	14·9 (-9·18–51·1)	8·88 (-6·36–25·2)
Occupational particulate matter, gases, and fumes	10·8 (8·36–14·4)	9·91 (7·67–13·3)	9·8 (7·65–13·1)	-8·06 (-12·7—3·11)	-1·12 (-3·41–1·68)	7·24 (5·38–10·1)	6·7 (5·09–9·21)	6·82 (5·19–9·4)	-7·48 (-14·1—1·01)	1·84 (-2·12–5·99)
Occupational noise	11·7 (10·6–12·8)	11·4 (10·7–12·3)	11·6 (11–12·4)	-2·27 (-6·4–2·38)	1·87 (0·0377–3·98)	8·22 (7·09–9·35)	8·33 (7·65–9·1)	8·92 (8·4–9·65)	1·34 (-4·94–9·93)	7·13 (3·88–10·7)
Occupational injuries	--	--	--	--	--	--	--	--	--	--
Occupational ergonomic factors	21·8 (18·3–25·5)	22·5 (20·6–24·6)	23·1 (20·3–26·2)	3·48 (-12·3–22·8)	2·32 (-8·21–14·6)	14·7 (11·2–18·8)	17·6 (15·5–19·7)	18·8 (15·6–22·3)	20 (-6·48–59·1)	6·63 (-9·92–25·7)
Childhood sexual abuse	2·52 (1·65–3·56)	2·52 (1·65–3·56)	2·52 (1·65–3·56)	0 (0–0)	0 (0–0)	3·14 (2·26–4·3)	3·14 (2·26–4·3)	3·14 (2·26–4·3)	0 (0–0)	0 (0–0)
Intimate partner violence	--	--	--	--	--	15·1 (8·61–21·9)	13·2 (8·12–17·7)	15·1 (8·61–21·9)	-12·8 (-22·9–4·13)	14·7 (0·738–36·4)
Non-exclusive breastfeeding	28·7 (19·2–41·5)	28·4 (20–40)	27·5 (18·9–39·2)	-1·1 (-7·1–7·34)	-3·07 (-8·88–2·63)	28·7 (19·2–41·5)	28·4 (20·1–40·1)	27·5 (18·9–39·3)	-0·96 (-6·97–7·48)	-3·12 (-8·93–2·57)
Discontinued breastfeeding	18·4 (16·7–20·1)	18·9 (17·3–20·5)	17·1 (15·6–18·7)	2·6 (-8·7–16·8)	-9·61 (-19·9–2·4)	18·4 (16·7–20)	18·9 (17·3–20·5)	17·1 (15·6–18·7)	2·46 (-8·83–16·6)	-9·48 (-19·8–2·55)
Diet low in calcium	56·5 (42·7–76·8)	39·2 (25·4–61·8)	33·1 (19·8–55·8)	-30·5 (-43—15·8)	-15·6 (-24·5—7·97)	47·3 (32·9–68·8)	30·6 (17·3–54·2)	25·2 (12·6–47·4)	-35·3 (-50·9—19·3)	-17·5 (-30—5·49)
Occupational exposure to asbestos	0·399 (0·109–0·681)	0·651 (0·232–0·894)	0·551 (0·187–0·85)	63·2 (3·18–147)	-15·3 (-41·8–22)	0·397 (0·216–0·675)	0·673 (0·388–1·05)	0·666 (0·357–1·11)	69·7 (-2·42–254)	-1·03 (-44·8–68·9)
Occupational exposure to arsenic	0·366 (0·143–0·618)	0·305 (0·125–0·511)	0·31 (0·126–0·529)	-16·7 (-23·7—8·16)	1·75 (-4·29–8·43)	0·371 (0·152–0·616)	0·353 (0·141–0·601)	0·37 (0·146–0·631)	-4·96 (-15·9–8·51)	5·09 (-1·11–11·9)
Occupational exposure to benzene	0·738 (0·45–1·34)	0·964 (0·586–1·73)	0·987 (0·609–1·78)	30·7 (21·2–44·2)	2·38 (-3·27–8·83)	0·797 (0·49–1·38)	1·22 (0·74–2·2)	1·34 (0·811–2·42)	53·7 (36·5–81·8)	9·58 (2·77–18)
Occupational exposure to beryllium	0·0941 (0·0878–0·1)	0·0979 (0·0965–0·0994)	0·0978 (0·095–0·101)	4·12 (-2·36–11·7)	-0·14 (-3·31–2·79)	0·0962 (0·0858–0·106)	0·119 (0·116–0·122)	0·125 (0·12–0·13)	23·7 (11·5–38·9)	4·87 (0·711–9·09)
Occupational exposure to cadmium	0·195 (0·18–0·211)	0·168 (0·161–0·176)	0·171 (0·162–0·181)	-13·6 (-20·6—5·47)	1·63 (-3·66–7·46)	0·198 (0·174–0·22)	0·197 (0·189–0·205)	0·207 (0·195–0·219)	-0·553 (-11·8–13·4)	5·26 (-0·577–11·4)
Occupational exposure to chromium	0·437 (0·404–0·472)	0·399 (0·383–0·415)	0·41 (0·39–0·433)	-8·65 (-16—0·493)	2·8 (-2·33–8·27)	0·441 (0·389–0·491)	0·463 (0·444–0·483)	0·493 (0·465–0·524)	4·93 (-6·65–19·4)	6·54 (0·178–13·1)
Occupational exposure to diesel engine exhaust	2·51 (2·31–2·69)	2·89 (2·79–2·98)	2·97 (2·81–3·12)	15·2 (6·11–25·2)	3·01 (-1·94–8·4)	1·95 (1·71–2·18)	2·49 (2·38–2·6)	2·64 (2·45–2·84)	27·7 (13·3–46·3)	6·24 (-0·558–13·5)
Occupational exposure to formaldehyde	0·91 (0·834–0·991)	0·808 (0·765–0·852)	0·833 (0·78–0·894)	-11·1 (-19·1—1·99)	3·02 (-3·1–9·57)	0·858 (0·748–0·96)	0·876 (0·831–0·922)	0·943 (0·876–1·01)	2·14 (-9·51–17·1)	7·64 (0·801–15)
Occupational exposure to nickel	0·399 (0·128–1·04)	0·369 (0·118–0·979)	0·373 (0·123–0·973)	-7·4 (-14·9–1·56)	1·1 (-4·6–6·95)	0·355 (0·115–0·918)	0·33 (0·107–0·875)	0·345 (0·112–0·898)	-7·11 (-17·9–6·57)	4·67 (-1·55–11·8)
Occupational exposure to polycyclic aromatic hydrocarbons	0·922 (0·856–0·995)	0·888 (0·858–0·919)	0·912 (0·871–0·958)	-3·73 (-11–4·32)	2·67 (-1·89–7·45)	0·913 (0·804–1·02)	1 (0·963–1·04)	1·07 (1·01–1·14)	9·59 (-2·41–24·6)	7·07 (1·14–13·3)
Occupational exposure to silica	4·1 (2·11–9)	4·53 (2·37–9·92)	4·63 (2·43–10·3)	10·6 (0·701–22·8)	2·23 (-5·25–10·6)	2·92 (1·49–6·5)	2·77 (1·42–6·08)	2·91 (1·45–6·28)	-5·05 (-15·8–8·37)	4·93 (-1·44–12·6)
Occupational exposure to sulfuric acid	1·17 (0·83–2·05)	0·889 (0·642–1·55)	0·886 (0·644–1·54)	-23·8 (-30·5—16·4)	-0·243 (-5·84–5·85)	0·984 (0·696–1·79)	0·91 (0·666–1·6)	0·939 (0·678–1·65)	-7·51 (-18·2–5·35)	3·18 (-2·41–9·07)
Unsafe sex	--	--	--	--	--	--	--	--	--	--
Occupational exposure to trichloroethylene	0·256 (0·239–0·276)	0·245 (0·238–0·253)	0·251 (0·24–0·263)	-4·57 (-11·3–3·52)	2·55 (-1·65–7·27)	0·256 (0·226–0·286)	0·29 (0·28–0·302)	0·308 (0·291–0·326)	13·4 (1·43–29·5)	6·04 (0·274–12·2)
No access to handwashing facility	22·9 (21·9–23·9)	13·7 (13·1–14·4)	11·3 (10·7–11·9)	-40·2 (-43·2—36·8)	-17·7 (-20·6—14·6)	--	--	--	--	--
Child wasting	2·62 (2·02–3·23)	2·48 (1·88–3·04)	2·48 (1·9–3·04)	-5·6 (-16·5–7·3)	0·316 (-8·05–9·72)	2·56 (1·97–3·13)	2·38 (1·83–2·92)	2·61 (2·03–3·18)	-6·97 (-17·6–4·74)	9·53 (0·433–19·3)
Child stunting	15·1 (10·4–18)	10·4 (7·19–12·8)	8·5 (5·46–11)	-30·8 (-43—19·9)	-18·6 (-30·7—7·68)	15·3 (10·8–18·2)	10·9 (7·32–13·2)	8·61 (5·57–11)	-29 (-42—17·5)	-21 (-33—10·2)
Chewing tobacco	0·237 (0·184–0·309)	0·227 (0·173–0·29)	0·233 (0·18–0·297)	-4·12 (-29·2–30·3)	2·37 (-16–24·2)	--	--	--	--	--
Diet low in legumes	100 (100–100)	99·7 (98·8–100)	99·4 (98·2–100)	-0·313 (-1·15–0)	-0·257 (-1·07–0·138)	100 (99·9–100)	99·1 (97·5–100)	98·7 (96·3–100)	-0·849 (-2·51–0)	-0·444 (-1·65–0·198)
Short gestation	11 (7·24–17·1)	10·3 (6·9–15·6)	11·2 (7·56–16·5)	-6·77 (-44·6–69·4)	8·75 (-30·4–68·7)	12 (7·68–19·6)	12·2 (7·72–19·2)	12·1 (7·73–19·5)	1·67 (-46·8–90·9)	-0·862 (-39·5–62·3)
Low birth weight	6·51 (4·62–9·58)	5·44 (3·83–7·84)	5·58 (4·07–7·83)	-16·4 (-48·4–38·6)	2·69 (-32·8–54·4)	7·35 (4·93–11·5)	6·53 (4·29–9·96)	6·37 (4·22–9·67)	-11·1 (-51·2–55·2)	-2·49 (-40·1–57·7)
High temperature	22·3 (15·1–31·3)	29·5 (21·5–38·4)	27·3 (19·7–35·9)	32·2 (14·9–52·6)	-7·5 (-11—2·89)	22·3 (15·1–31·3)	29·5 (21·5–38·4)	27·3 (19·7–35·9)	32·2 (14·9–52·6)	-7·5 (-11—2·89)
Low temperature	34·5 (30–39·9)	35·2 (30·7–40·6)	35·2 (30·5–40·6)	2·06 (0·314–3·91)	-0·134 (-1·56–1·32)	34·5 (30–39·9)	35·2 (30·7–40·6)	35·2 (30·5–40·6)	2·06 (0·314–3·91)	-0·134 (-1·56–1·32)
Kidney dysfunction	20·3 (13·4–28·2)	22·6 (15·5–30·7)	24·6 (17·2–32·7)	11·7 (7·37–18·6)	8·57 (4·76–14·5)	20·6 (13·9–28·3)	22·7 (15·6–30·9)	24·6 (17·4–32·8)	10·3 (6·23–16·1)	8·51 (4·83–14·2)
Bullying victimization	2·4 (0·999–5)	2·56 (1·06–5·22)	2·54 (1·05–5·2)	6·61 (0·56–14·4)	-0·687 (-3·53–1·96)	1·39 (0·59–2·85)	1·51 (0·621–3·1)	1·5 (0·624–3·08)	8·62 (0·907–18·6)	-0·374 (-3·41–2·95)
High LDL cholesterol	31·4 (27·9–34·9)	30·1 (26·5–33·6)	29·9 (26·6–33·4)	-4 (-9·55–2·02)	-0·722 (-4·77–3·29)	33·1 (29·7–36·5)	32 (28·7–35·5)	32 (28·7–35·5)	-3·3 (-8·23–1·26)	-0·0652 (-3·49–3·54)

Supplementary Results Table 1. Georgia age-standardised Summary Exposure Values (SEVs) for most-detailed risk factors and percentage change, by sex, 1990, 2010, and 2019. Data in parentheses are 95% uncertainty intervals.

	Male					Female				
	1990	2010	2019	Percentage change 1990-2010	Percentage change 2010-2019	1990	2010	2019	Percentage change 1990-2010	Percentage change 2010-2019
Unsafe sanitation	8·37 (3·98–15·1)	4·19 (1·94–8·11)	3·31 (1·49–6·14)	-50 (-81–21·8)	-21 (-58·8–53·1)	--	--	--	--	--
Ambient particulate matter pollution	14·9 (6·04–28·7)	9·3 (6·5–12·7)	6·02 (3·49–9·16)	-37·7 (-67·3–43·5)	-35·3 (-47·7—27·8)	14·9 (6·03–28·7)	9·3 (6·49–12·7)	6·02 (3·5–9·15)	-37·7 (-67·3–43·3)	-35·3 (-47·5—27·8)
Household air pollution from solid fuels	0·0318 (0·00786–0·0809)	0·0156 (0·00296–0·047)	0·0106 (0·00184–0·0346)	-51 (-81·8–8·93)	-31·9 (-65·5–28·6)	0·0419 (0·0107–0·105)	0·0206 (0·00413–0·06)	0·0141 (0·00252–0·0447)	-50·8 (-81·7–9·16)	-31·7 (-65·4–28·6)
Ambient ozone pollution	83·6 (39–100)	64 (28·4–100)	32·9 (12·8–58·2)	-23·5 (-34·6–0)	-48·6 (-58·9—40·1)	83·6 (39–100)	64 (28·4–100)	32·9 (12·8–58·2)	-23·5 (-34·6–0)	-48·6 (-58·9—40·1)
Residential radon	17·9 (2·46–51·4)	17·9 (2·46–51·4)	17·9 (2·46–51·4)	0 (0–0)	0 (0–0)	17·9 (2·46–51·4)	17·9 (2·46–51·4)	17·9 (2·46–51·4)	0 (0–0)	0 (0–0)
Lead exposure	62·2 (35·2–82·6)	36·8 (16·3–58·1)	25·9 (9·63–44·9)	-40·9 (-55·8—28·2)	-29·6 (-42·2—21·9)	36·7 (6·9–65)	21·5 (2·31–46·2)	14·9 (0·923–35·7)	-41·5 (-71·7—27·1)	-30·6 (-62·5—22·3)
Child underweight	0·977 (0·551–1·48)	0·967 (0·543–1·46)	0·944 (0·52–1·43)	-1·02 (-18·4–19·9)	-2·42 (-15·1–13)	0·824 (0·439–1·23)	0·856 (0·47–1·31)	0·873 (0·476–1·32)	3·94 (-16·4–29·1)	2·03 (-11·9–17·5)
Iron deficiency	--	--	--	--	--	12·7 (10·7–14·8)	12·7 (10·7–14·6)	12·9 (10·7–15·1)	-0·143 (-11·3–12·3)	1·68 (-8·13–12·2)
Vitamin A deficiency	1·04 (0·274–2·73)	1·28 (0·343–3·24)	0·889 (0·233–2·34)	22·3 (-64·8–388)	-30·3 (-69·6–81·3)	1·92 (0·762–3·97)	1·86 (0·738–4·01)	1·57 (0·584–3·41)	-3·42 (-63·4–172)	-15·7 (-54·2–68·1)
Zinc deficiency	2·34 (0–7·46)	1·23 (0–3·84)	1·33 (0–4·17)	-47·5 (-100–NA)	8·69 (-9·53–NA)	2·4 (0–7·64)	1·24 (0–4·11)	1·3 (0–4·19)	-48·3 (-100–NA)	4·7 (-14–NA)
Smoking	27·5 (24·8–30·5)	19·3 (17·5–21·2)	17·2 (15·5–19)	-29·8 (-34·3—24·9)	-10·7 (-14·4—7·28)	18·2 (16–20·6)	13·8 (12·3–15·3)	11·9 (10·6–13·3)	-24·5 (-31·6—16·3)	-13·4 (-17·6—9·1)
Secondhand smoke	38·1 (35·6–40·9)	25·3 (23·8–27)	24·4 (21·7–27·1)	-33·6 (-39·1—27·3)	-3·77 (-12·2–5·03)	36·3 (34·2–38·3)	24·2 (23–25·4)	23·3 (21·4–25·2)	-33·3 (-38·2—28)	-3·72 (-11·2–3·39)
Alcohol use	12·9 (7·82–18·6)	13·2 (7·81–19·3)	14·4 (8·56–20·7)	2·78 (-28·6–45·6)	8·69 (-16·2–39·5)	8·29 (4·8–12·5)	9·71 (5·7–14·3)	9·9 (5·8–14·6)	17·2 (-19·2–69·7)	1·94 (-22·5–31·6)
Drug use	0·504 (0·258–0·956)	0·814 (0·56–1·19)	1·12 (0·81–1·54)	61·4 (16·9–132)	37·6 (16·7–63·8)	0·418 (0·26–0·657)	0·729 (0·531–0·991)	1·05 (0·81–1·35)	74·3 (39·2–121)	43·7 (24–67)
High fasting plasma glucose	13·2 (10·9–15·8)	19·3 (17–22·2)	20·5 (17·9–23·5)	46·2 (21–75·4)	6·33 (-7·44–21·5)	10·9 (8·98–13·5)	16·9 (14·6–19·7)	18·2 (15·7–21·3)	54·5 (26·4–89·4)	8·06 (-8·7–27·5)
High systolic blood pressure	25·6 (20·5–31·3)	16·5 (14·4–18·6)	20 (15·3–24·8)	-35·5 (-47·5—20·5)	21 (-3·89–51·4)	23·6 (17·7–30·2)	14 (12·4–15·7)	18·9 (13·5–25)	-40·4 (-54·5—21·1)	34·3 (-2·77–80·5)
High body-mass index	23·8 (16·7–33·2)	38·7 (28·5–50·2)	39·5 (29·3–50·8)	62·8 (42–94·3)	2·17 (-4·76–10·4)	30·3 (22·1–39·6)	44·1 (33·8–53·3)	44·8 (35·1–53·8)	45·6 (28–72)	1·41 (-4·98–9·06)
Low bone mineral density	11 (6·45–17·2)	8·41 (4·4–14·3)	10·6 (6·08–16·9)	-23·5 (-45·5—1·64)	26·2 (-2·83–70·3)	14·7 (9·37–21·4)	15·1 (9·49–21·7)	17·5 (11·5–24·2)	2·86 (-20·1–29·4)	15·8 (-9·18–52·7)
Diet low in fruits	51·1 (38·3–64·2)	49 (37·9–61·8)	49·8 (37·9–63)	-4·11 (-15·4–9·03)	1·75 (-9·59–14)	--	--	--	--	--
Diet low in vegetables	39·9 (21·8–61·4)	51·2 (36·1–65·9)	50·6 (34·4–65·7)	28·4 (1·23–83·8)	-1·1 (-9·09–5·82)	24·9 (12–42·9)	20·5 (12·7–32·1)	21·5 (12·8–33·6)	-17·5 (-36·7–23)	4·83 (-12·5–25)
Diet low in whole grains	79·8 (70·5–88·5)	81·7 (73–89·7)	81·9 (73·4–89·9)	2·5 (-1–7)	0·188 (-2·36–2·74)	77·1 (66·2–86·5)	79·9 (70·8–88·4)	79·9 (71–88·8)	3·73 (-0·182–9·2)	-0·0287 (-2·9–2·77)
Diet low in nuts and seeds	28·8 (8·25–57)	25·7 (8·11–55·5)	22 (6·65–53·9)	-10·8 (-44·5–62·9)	-14·5 (-41·7–12·6)	24·7 (6·29–54·9)	22·6 (6·65–54·6)	20·2 (5·31–54)	-8·28 (-42·2–63·6)	-10·5 (-38·7–20·1)
Diet low in milk	49·5 (24·8–69·9)	44·5 (19·7–63·7)	44·4 (20·4–65·1)	-10·1 (-28·8–1·83)	-0·195 (-12·2–11·8)	--	--	--	--	--
Diet high in red meat	77·3 (64·2–87·6)	75·1 (61·6–86·4)	74·4 (59·9–86·3)	-2·89 (-16·8–13·6)	-0·86 (-12·8–10·9)	77·9 (64·5–88·5)	74·4 (60·7–85·5)	73·7 (58·8–86·1)	-4·44 (-19·7–12·2)	-0·944 (-13·6–12·2)
Diet high in processed meat	78 (54·5–98·1)	86·2 (67·8–99·7)	85·9 (67·4–99·6)	10·5 (0·949–31·5)	-0·37 (-5·24–3·89)	79·2 (55·5–97·8)	88·7 (72·1–99·7)	87·1 (68·1–99·6)	12 (1·35–34·9)	-1·76 (-7·52–1·97)
Diet high in sugar-sweetened beverages	44·5 (24·9–64·1)	60·4 (39·5–76·9)	60·2 (39·3–77·9)	35·9 (-5·75–120)	-0·318 (-23·5–26)	48·9 (30·6–68·1)	66·6 (47·4–83·3)	64·7 (44–82·6)	36·4 (-2·29–104)	-2·97 (-22·2–19·7)
Diet low in fiber	44·6 (27·9–60)	31·3 (18·5–45·9)	29·4 (16·5–43·9)	-29·8 (-46·1—13·8)	-6·11 (-23·8–12)	44·8 (27·9–61·4)	36·4 (21·9–51·4)	32·4 (18·1–48·3)	-18·6 (-34·6—2·03)	-11 (-26·7–3·63)
Diet low in seafood omega-3 fatty acids	84·1 (66·9–99·9)	80·6 (62·4–99·5)	80·8 (63–99·6)	-4·19 (-18·5–9·9)	0·316 (-12·7–16·8)	86·9 (71·4–100)	84·2 (68·3–99·6)	84·6 (69·7–99·8)	-3·09 (-13·7–7·53)	0·48 (-9·45–13)
Diet low in polyunsaturated fatty acids	14·6 (4·14–40·7)	7·59 (2·16–26·3)	7·45 (2·15–26·2)	-48·1 (-74·9—7·79)	-1·85 (-23·8–23·5)	--	--	--	--	--
Diet high in trans fatty acids	89·5 (81·4–100)	87·9 (79–100)	87·4 (77·5–100)	-1·81 (-9·92–6·17)	-0·551 (-8·85–8·31)	--	--	--	--	--
Diet high in sodium	17·9 (4·53–40·5)	39·4 (11·9–65)	38·8 (11·3–65·6)	120 (40·9–431)	-1·69 (-26·6–27·2)	--	--	--	--	--
Low physical activity	2·02 (0·62–5·23)	1·85 (0·645–5·02)	1·64 (0·476–4·89)	-8·79 (-39·9–37·6)	-11·2 (-38·5–10·8)	--	--	--	--	--
Occupational asthmagens	22·1 (18·4–26·4)	19·5 (16·6–23·2)	20·3 (17–24·6)	-11·6 (-22—0·605)	4·03 (-5·23–13·9)	11 (8·89–13·1)	10·3 (8·54–12·4)	10·5 (8·52–12·8)	-5·75 (-19–9·58)	1·7 (-10·3–14)
Occupational particulate matter, gases, and fumes	9·3 (6·66–13·4)	8·62 (6·13–12·3)	8·28 (5·89–11·7)	-7·32 (-12·9—1·67)	-3·89 (-7·29—0·163)	5·29 (3·67–7·78)	4·99 (3·54–7·25)	4·76 (3·38–6·88)	-5·6 (-14·6–3·9)	-4·69 (-10–1·03)
Occupational noise	8·19 (7·43–9·05)	7·84 (7·28–8·58)	7·59 (7·11–8·26)	-4·22 (-7·89—0·0547)	-3·24 (-5·44—0·861)	5·68 (4·97–6·51)	5·89 (5·37–6·5)	5·85 (5·44–6·39)	3·62 (-2·6–11·2)	-0·585 (-3·74–2·73)
Occupational injuries	--	--	--	--	--	--	--	--	--	--
Occupational ergonomic factors	12·3 (10·2–14·8)	10·4 (8·71–12·5)	10·8 (8·97–13·1)	-15·5 (-25·4—4·16)	3·43 (-4·36–12·2)	6·97 (5·52–8·8)	6·65 (5·35–8·18)	6·96 (5·54–8·81)	-4·66 (-19·6–12·2)	4·76 (-7·32–19)
Childhood sexual abuse	7·4 (5·05–10·5)	6·97 (4·6–9·93)	6·85 (4·54–9·67)	-5·83 (-12·2–5·83)	-1·68 (-8·51–5·53)	12·3 (8·94–17·3)	17·4 (12·9–24)	17·8 (13·1–24·7)	41·9 (30·7–54·8)	2·17 (-5–11)
Intimate partner violence	--	--	--	--	--	30·6 (17·6–39·5)	29 (16·6–37·9)	29·9 (16·8–39·4)	-5·29 (-13·6–8·77)	3·23 (-4·93–17·3)
Non-exclusive breastfeeding	26·3 (21·6–31·3)	24·8 (21·2–28·6)	24·8 (20·6–29·6)	-5·66 (-19·9–11·5)	0·308 (-13·4–14·7)	26·3 (21·6–31·3)	24·8 (21·2–28·6)	24·8 (20·6–29·6)	-5·65 (-19·9–11·6)	0·304 (-13·4–14·7)
Discontinued breastfeeding	27·6 (26·6–28·4)	25·2 (23·8–26·4)	25·8 (24·5–27)	-8·6 (-14—3·3)	2·36 (-3·54–8·6)	27·6 (26·6–28·5)	25·2 (23·8–26·5)	25·8 (24·5–27)	-8·85 (-14·2—3·57)	2·3 (-3·58–8·54)
Diet low in calcium	16·9 (8·25–31·7)	14·2 (6·9–26·2)	14·1 (6·74–26·1)	-15·8 (-26·6—4·98)	-0·557 (-9·94–7·7)	12·3 (5·25–23·3)	11·3 (4·81–21·3)	11·6 (4·99–21·9)	-8·37 (-23·2–8·13)	2·54 (-6·93–12·7)
Occupational exposure to asbestos	6·24 (5·56–6·93)	4·88 (4·44–5·33)	4·41 (3·18–5·93)	-21·9 (-31·2—10·1)	-9·49 (-34·4–21·8)	1·11 (0·865–2·09)	1·03 (0·805–1·9)	1·01 (0·651–1·95)	-7·58 (-22·9–7·37)	-1·16 (-28·9–34·5)
Occupational exposure to arsenic	0·512 (0–1·22)	0·492 (0–1·15)	0·52 (0–1·23)	-3·91 (-16·3–NA)	5·8 (-5·92–NA)	0·485 (0–1·15)	0·464 (0–1·12)	0·479 (0–1·16)	-4·41 (-20·2–NA)	3·26 (-10–NA)
Occupational exposure to benzene	0·803 (0·0476–2·28)	0·776 (0·0472–2·24)	0·801 (0·0498–2·33)	-3·38 (-11·6–9·71)	3·26 (-3·8–14·3)	1·1 (0·0626–3·15)	1·21 (0·0751–3·35)	1·28 (0·0755–3·63)	10·3 (-1·8–29·8)	5·88 (-3·67–19·7)
Occupational exposure to beryllium	0·0235 (0·022–0·0249)	0·0227 (0·0222–0·0232)	0·024 (0·0227–0·0254)	-3·43 (-9·06–2·61)	5·79 (-0·312–11·9)	0·0253 (0·023–0·0274)	0·0259 (0·0253–0·0264)	0·027 (0·025–0·0289)	2·3 (-6·22–12·2)	4·48 (-3·11–11·7)
Occupational exposure to cadmium	0·0598 (0·0537–0·0661)	0·0579 (0·0527–0·0638)	0·0612 (0·0541–0·0688)	-3·09 (-14·3–10·2)	5·67 (-5·02–16·5)	0·0575 (0·0503–0·0657)	0·0557 (0·0501–0·0621)	0·0575 (0·0495–0·0667)	-3·04 (-17·7–14·6)	3·23 (-8·78–16·4)
Occupational exposure to chromium	0·143 (0·13–0·157)	0·142 (0·131–0·153)	0·15 (0·135–0·166)	-0·815 (-10·8–11)	5·54 (-3·64–15·2)	0·142 (0·125–0·16)	0·141 (0·13–0·154)	0·146 (0·128–0·164)	-0·266 (-13·1–15)	3·16 (-7·2–14·4)
Occupational exposure to diesel engine exhaust	0·713 (0·655–0·772)	0·759 (0·711–0·806)	0·804 (0·737–0·871)	6·41 (-3·71–17·5)	5·94 (-2–14·1)	0·626 (0·551–0·698)	0·664 (0·608–0·737)	0·67 (0·59–0·761)	6·05 (-7·76–22·9)	1·04 (-9·21–12·4)
Occupational exposure to formaldehyde	0·292 (0·237–0·33)	0·277 (0·223–0·311)	0·289 (0·233–0·331)	-5·32 (-17·1–8·87)	4·33 (-6·48–15·8)	--	--	--	--	--
Occupational exposure to nickel	0·43 (0–1·9)	0·394 (0–1·73)	0·415 (0–1·8)	-8·23 (-20·2–NA)	5·2 (-6·11–NA)	0·387 (0–1·7)	0·356 (0–1·63)	0·367 (0–1·7)	-8·02 (-24·3–NA)	3·11 (-10·7–NA)
Occupational exposure to polycyclic aromatic hydrocarbons	0·281 (0·256–0·308)	0·279 (0·259–0·3)	0·293 (0·265–0·323)	-0·998 (-10·9–10·4)	5·2 (-3·79–14·8)	0·297 (0·265–0·332)	0·306 (0·287–0·328)	0·317 (0·283–0·351)	3·17 (-9·21–17·1)	3·4 (-6·02–13·1)
Occupational exposure to silica	3·93 (0·554–13)	3·51 (0·496–11·2)	3·75 (0·52–11·8)	-10·7 (-23·2–3·54)	6·66 (-5·21–20·4)	3·24 (0·469–10·5)	2·89 (0·401–9·27)	2·96 (0·395–9·83)	-10·8 (-27·7–13)	2·51 (-11·6–21·6)
Occupational exposure to sulfuric acid	0·921 (0·191–2·97)	0·976 (0·191–3·12)	1·01 (0·208–3·28)	5·92 (-7·88–21·8)	3·82 (-6·92–15·5)	0·875 (0·178–2·86)	1·02 (0·218–3·27)	1·02 (0·221–3·29)	17·2 (-4·43–40·9)	-0·737 (-14·6–14·8)
Unsafe sex	--	--	--	--	--	--	--	--	--	--
Occupational exposure to trichloroethylene	0·0843 (0·0783–0·0905)	0·086 (0·0825–0·0903)	0·0909 (0·0847–0·0981)	2·09 (-5·58–10·6)	5·67 (-1·22–13)	0·0812 (0·0728–0·0901)	0·0826 (0·0777–0·0882)	0·0848 (0·0761–0·0939)	1·77 (-9·5–14·9)	2·66 (-6·04–12·4)
No access to handwashing facility	3·42 (3·15–3·68)	3·13 (2·9–3·37)	3·1 (2·86–3·34)	-8·24 (-16–0·742)	-1·21 (-6·62–4·66)	3·42 (3·15–3·68)	3·13 (2·9–3·37)	3·1 (2·86–3·34)	-8·24 (-16–0·742)	-1·21 (-6·62–4·66)
Child wasting	0·752 (0·491–1·03)	0·729 (0·48–0·987)	0·729 (0·476–0·987)	-3·03 (-12·3–6·76)	-0·0472 (-6·69–7·44)	0·776 (0·518–1·06)	0·766 (0·511–1·05)	0·763 (0·504–1·05)	-1·29 (-11·5–9·44)	-0·324 (-8·15–7·94)
Child stunting	2·5 (1·3–3·67)	2·12 (1·12–3·14)	2·06 (1·07–3·06)	-15·2 (-34·2–9·9)	-2·83 (-17·4–13·7)	1·99 (0·987–3)	1·82 (0·933–2·77)	1·79 (0·888–2·73)	-8·57 (-28·9–15·1)	-1·33 (-15·7–16)
Chewing tobacco	7·74 (6·44–9·34)	5·97 (4·49–7·8)	5·48 (4·03–7·09)	-22·8 (-43·9–5·15)	-8·25 (-27·2–14·4)	1·04 (0·728–1·49)	0·614 (0·404–0·905)	0·553 (0·35–0·843)	-41·2 (-62·5—4·18)	-9·83 (-35·4–25·8)
Diet low in legumes	74·3 (38·7–99)	77·4 (47·2–99·7)	77·6 (46·8–99·7)	4·25 (-0·000585–22·8)	0·256 (-5·07–6·08)	72·7 (35·7–99)	75·7 (42·5–99·6)	76·3 (44·2–99·7)	4·2 (-0·0488–23·1)	0·709 (-4·18–7·8)
Short gestation	40·2 (32·8–48·6)	30·3 (25·5–35·6)	29 (24·1–34·6)	-24·7 (-39·1—6·88)	-4·44 (-22·3–16·3)	37·7 (31·2–45·9)	29·6 (25·1–34·8)	28·6 (24–34)	-21·6 (-37—0·239)	-3·42 (-20·2–15·6)
Low birth weight	15·8 (13·2–19)	13·6 (11·8–15·9)	13·5 (11·3–16·2)	-13·9 (-31·5–6·52)	-1·23 (-20·2–21·5)	18·2 (15·2–21·4)	15·7 (13·5–18·1)	15·6 (13·3–18·4)	-13·8 (-30·8–7·86)	-0·169 (-17·6–21·8)
High temperature	24·3 (16·5–33·1)	25·7 (17·5–34·2)	23·5 (15·3–32·2)	5·72 (-4·57–18·1)	-8·54 (-20·6–4·15)	24·3 (16·5–33·1)	25·7 (17·5–34·2)	23·5 (15·3–32·2)	5·72 (-4·57–18·1)	-8·54 (-20·6–4·15)
Low temperature	35·1 (27·5–44)	39·5 (33·1–47·7)	35·3 (28·3–44)	12·3 (6·1–20·8)	-10·5 (-15·9—5·75)	35·1 (27·5–44)	39·5 (33·1–47·7)	35·3 (28·3–44)	12·3 (6·1–20·8)	-10·5 (-15·9—5·75)
Kidney dysfunction	20·6 (14·8–27·6)	21·4 (15·5–28·8)	21·5 (15·6–29)	4·02 (-0·0813–8·96)	0·281 (-3·9–4·84)	23·2 (16·6–30·9)	24·4 (17·6–32·5)	25·3 (18·5–33·4)	5·22 (0·582–10·9)	3·63 (-1·26–9·04)
Bullying victimization	5·87 (2·45–12·1)	8·94 (3·58–17·7)	8·75 (3·38–18)	52·2 (31·8–76)	-2·13 (-11·7–3·95)	--	--	--	--	--
High LDL cholesterol	66·1 (61·7–70·6)	34·6 (31·2–38·2)	31 (27·5–34·9)	-47·6 (-53·1—42·2)	-10·4 (-15·1—5·39)	65 (61·3–68·6)	34·5 (31·1–37·9)	31·8 (28·2–35·4)	-47 (-51·9—42)	-7·89 (-12—3·99)

Supplementary Results Table 1. Hawaii age-standardised Summary Exposure Values (SEVs) for most-detailed risk factors and percentage change, by sex, 1990, 2010, and 2019. Data in parentheses are 95% uncertainty intervals.

	Male					Female				
	1990	2010	2019	Percentage change 1990-2010	Percentage change 2010-2019	1990	2010	2019	Percentage change 1990-2010	Percentage change 2010-2019
Unsafe sanitation	6·63 (3·12–12·4)	3·4 (1·44–6·51)	2·7 (1·18–5·02)	-48·8 (-80·5–27)	-20·5 (-58·9–59·6)	--	--	--	--	--
Ambient particulate matter pollution	1·74 (0·0254–7·03)	1·6 (0·126–3·96)	0·981 (0·0719–2·96)	-7·96 (-89·4–3850)	-38·8 (-71·1–-23·9)	1·74 (0·03–7·02)	1·6 (0·133–3·96)	0·983 (0·0742–2·96)	-7·94 (-88·9–4160)	-38·7 (-71·6–-23·8)
Household air pollution from solid fuels	0·00305 (3·26e-05–0·0144)	0·00226 (8·62e-05–0·0103)	0·00135 (3·64e-05–0·00659)	-25·8 (-91·3–1970)	-40·5 (-80·4–11·7)	0·00403 (4·74e-05–0·0181)	0·00302 (0·000111–0·0131)	0·00179 (5·38e-05–0·00882)	-25·1 (-91·1–1850)	-40·6 (-79·7–11·7)
Ambient ozone pollution	15·3 (1·33–44·7)	18 (1·35–49·5)	16·3 (0·348–50·9)	17·6 (-86·9–865)	-9·33 (-96·3–884)	15·3 (1·33–44·7)	18 (1·35–49·5)	16·3 (0·348–50·9)	17·6 (-86·9–865)	-9·33 (-96·3–884)
Residential radon	14·4 (1·96–48·9)	14·4 (1·96–48·9)	14·4 (1·96–48·9)	0 (0–0)	0 (0–0)	14·4 (1·96–48·9)	14·4 (1·96–48·9)	14·4 (1·96–48·9)	0 (0–0)	0 (0–0)
Lead exposure	55·2 (27·6–77·2)	31·7 (11·7–53·4)	21·9 (6·28–40·8)	-42·6 (-60·2–-29)	-30·8 (-47·4–-22·6)	30·6 (2·46–59·4)	17·7 (0·745–41·9)	12·4 (0·285–32·2)	-42·1 (-78·1–-27·2)	-30·4 (-67·9–-22·6)
Child underweight	0·937 (0·532–1·38)	0·943 (0·515–1·43)	0·922 (0·507–1·4)	0·596 (-16·9–22·4)	-2·23 (-15·2–12·1)	0·792 (0·415–1·21)	0·835 (0·455–1·28)	0·856 (0·465–1·32)	5·47 (-16·4–40·3)	2·52 (-12·4–18·8)
Iron deficiency	--	--	--	--	--	11·5 (9·56–13·5)	11·8 (9·89–13·8)	11·9 (9·82–14·2)	2·27 (-9·6–15·4)	1·17 (-8·28–11·6)
Vitamin A deficiency	0·828 (0·218–2·16)	1·07 (0·298–2·59)	0·768 (0·199–2·13)	29·1 (-63·3–462)	-28·2 (-69·8–78·4)	1·64 (0·656–3·63)	1·62 (0·617–3·41)	1·36 (0·543–2·87)	-1·02 (-62·4–198)	-16·1 (-53·7–63)
Zinc deficiency	1·96 (0–6·41)	1·09 (0–3·52)	1·12 (0–3·66)	-44·7 (-100–NA)	3·35 (-15–NA)	2·01 (0–6·75)	1·1 (0–3·67)	1·1 (0–3·59)	-45·4 (-100–NA)	-0·341 (-20·3–NA)
Smoking	25·6 (23·1–28·3)	17·9 (16·1–19·9)	15·4 (13·7–17·2)	-30·1 (-35·2–-25)	-13·8 (-17·5–-10·1)	17·2 (14·9–19·9)	12·4 (11–13·8)	10·8 (9·42–12·1)	-28·1 (-35·5–-19·5)	-13·1 (-17·6–-8·27)
Secondhand smoke	40·3 (38–42·8)	26·5 (24·9–28·3)	26·4 (23·8–29·2)	-34·3 (-39·9–-28)	-0·383 (-8·89–7·83)	39·2 (37·1–41·3)	26 (24·6–27·2)	25·8 (23·6–28)	-33·9 (-39–-28·7)	-0·628 (-7·78–6·62)
Alcohol use	15·1 (9·86–20·9)	14·5 (9·31–19·5)	14·9 (9·51–20·6)	-4·32 (-34·2–38·1)	3·35 (-20·3–32·3)	--	--	--	--	--
Drug use	0·522 (0·259–1·01)	0·717 (0·466–1·13)	0·86 (0·587–1·29)	37·2 (6·14–94·6)	19·9 (5·69–39·4)	0·392 (0·234–0·66)	0·522 (0·367–0·747)	0·654 (0·485–0·88)	33·2 (9·41–67·5)	25·4 (9·22–46)
High fasting plasma glucose	10·3 (8·85–12·3)	15·8 (13·9–17·9)	17·2 (14·9–19·5)	53·7 (30·5–78·4)	8·71 (-3·78–25)	9·35 (7·73–11·6)	12·8 (10·8–15·4)	10·5 (9·62–11·5)	36·9 (10·6–67·8)	-17·9 (-31·7–-1·7)
High systolic blood pressure	27·2 (22·6–31·7)	15·5 (13·4–17·5)	18·5 (14·4–23·3)	-43·2 (-52–31·6)	19·8 (-5·96–49·4)	27·9 (22·1–33·8)	12·9 (11·3–14·6)	17·6 (12·6–23·5)	-53·8 (-62·6–-41·4)	36·3 (-2–85·5)
High body-mass index	23·9 (17·3–32·5)	38·5 (28·5–49·5)	40·4 (29·9–51·3)	61·1 (40·3–90·7)	4·8 (-1·78–13·3)	26·2 (19·1–36·1)	40·1 (32·9–48·2)	41·7 (33·1–50·5)	53 (26·7–85·9)	3·9 (-3·48–10·5)
Low bone mineral density	12·4 (7·3–19·2)	9·61 (5·23–15·7)	11·9 (6·86–18·6)	-22·5 (-41·7–-1·72)	24·3 (-2·79–60·8)	16·4 (10·7–23·4)	17·1 (11·4–24·1)	19·5 (13·3–26·5)	3·78 (-17·7–33·7)	14·2 (-9·45–43·6)
Diet low in fruits	51·5 (39·7–64·7)	46·9 (34·8–59·8)	49·1 (36·4–62·8)	-8·91 (-20·9–3·2)	4·68 (-7·06–17·7)	53·4 (41·4–66·4)	46·9 (34·9–60·8)	48·9 (36·3–63·2)	-12·3 (-23·3–-1·87)	4·31 (-6·21–16·9)
Diet low in vegetables	26·9 (12·3–47·6)	64·6 (44·4–83)	63·5 (42·9–82)	140 (52·6–345)	-1·78 (-8·27–3·69)	21·1 (9–40·7)	62 (42·3–81·3)	60·8 (41·3–80·1)	194 (75·7–503)	-1·98 (-8·97–4·06)
Diet low in whole grains	79·1 (68·8–88)	81·3 (72·5–89·5)	81·4 (72·5–89·6)	2·87 (-0·891–7·79)	0·0839 (-2·37–2·67)	76·5 (65·7–86·6)	79·5 (70·2–87·8)	79·3 (69·6–88·1)	4 (-0·295–10·6)	-0·221 (-3·39–2·79)
Diet low in nuts and seeds	25·5 (7·63–55·3)	25·2 (8·1–55·1)	20·6 (6·06–54)	-1·15 (-39·2–74·6)	-18·3 (-44·3–6·78)	21·5 (5·34–53·1)	21·7 (6·49–53·9)	19 (5·79–53·3)	0·746 (-38–80·3)	-12·6 (-40·4–17·6)
Diet low in milk	46 (23·1–66·9)	42 (19·4–62·5)	41·2 (18·9–62·2)	-8·69 (-27·8–4·68)	-1·93 (-16·2–12·2)	45·7 (23·2–66·1)	41·9 (19·5–61·5)	41·4 (19·4–60·8)	-8·42 (-27·8–7·04)	-1·07 (-13·3–12)
Diet high in red meat	89·1 (80·4–95·2)	86·8 (76·3–94·2)	85·9 (75·8–93·9)	-2·57 (-12·8–7·28)	-1·01 (-9·37–7·25)	88·9 (81–94·6)	84·6 (74·4–92·7)	83·6 (71–93)	-4·86 (-15–4·09)	-1·16 (-10·3–7·64)
Diet high in processed meat	72·9 (47·5–95·2)	82·1 (60·5–99·3)	81·9 (61·3–99·3)	12·6 (2·3–33·6)	-0·145 (-4·81–4·56)	74·8 (51·4–95·4)	84·7 (66·3–98·6)	83·4 (65·3–98·6)	13·3 (2·52–35·5)	-1·6 (-6·88–2·48)
Diet high in sugar-sweetened beverages	48·5 (30·1–67·1)	63·8 (44·1–80·8)	64·1 (43·7–81·3)	31·5 (-8·93–96·1)	0·517 (-20·2–24·7)	52·5 (31·6–73·2)	69·6 (50·2–85·2)	67·8 (48·7–85·9)	32·4 (-4·85–101)	-2·52 (-19·9–19·2)
Diet low in fiber	41 (26–56·6)	29·1 (16·2–42·9)	26·6 (13·7–40·4)	-29·1 (-46·9–-12·1)	-8·53 (-29·3–10)	41·6 (25·4–58·1)	34·3 (20·6–48·6)	29·7 (16·6–45·4)	-17·5 (-33·4–1·73)	-13·5 (-31–3·63)
Diet low in seafood omega-3 fatty acids	82·1 (63·9–99·6)	78·5 (60·1–99·3)	78·5 (61–99·2)	-4·48 (-18·8–11·3)	0·045 (-14·7–17·9)	84·6 (69·1–99·5)	82·4 (65·2–99·4)	82·4 (65·8–99·3)	-2·56 (-15·1–10·3)	-0·0873 (-13·1–13·9)
Diet low in polyunsaturated fatty acids	12·5 (3·55–37·7)	7·03 (2·02–24·5)	6·81 (1·98–24·6)	-43·8 (-73·8–-6·34)	-3·12 (-23·4–18·5)	--	--	--	--	--
Diet high in trans fatty acids	89·5 (82·1–100)	87·9 (79–100)	87·3 (78·5–100)	-1·74 (-9·78–6·17)	-0·686 (-8·55–8·01)	--	--	--	--	--
Diet high in sodium	20 (4·4–44·8)	39·5 (10·5–63·8)	38·9 (9·85–64·3)	97·8 (29·3–394)	-1·48 (-32·2–34·9)	--	--	--	--	--
Low physical activity	1·91 (0·58–5·09)	1·67 (0·607–4·82)	1·45 (0·412–4·94)	-12·3 (-44·8–34·8)	-13·4 (-44·4–10·9)	--	--	--	--	--
Occupational asthmagens	16·8 (14–20·1)	16·7 (14–20·3)	17·3 (14·4–21)	-0·3 (-13·2–13·1)	3·28 (-6·18–12·8)	10·5 (8·49–12·8)	11 (9·08–13·3)	11·1 (8·92–13·6)	5·35 (-9·32–21·5)	0·863 (-10·1–13·2)
Occupational particulate matter, gases, and fumes	7·09 (5–10·4)	7·08 (5·03–10·4)	7·03 (5·02–10·3)	-0·152 (-6·65–7·47)	-0·714 (-4·87–2·93)	4·89 (3·42–7·11)	4·87 (3·49–7)	4·73 (3·39–6·78)	-0·502 (-9·58–10·4)	-2·81 (-7·81–2·33)
Occupational noise	6·84 (6·18–7·58)	7·1 (6·55–7·75)	7·09 (6·58–7·75)	3·75 (-1–9·33)	-0·0405 (-2·56–2·52)	5·69 (4·98–6·49)	6·18 (5·67–6·78)	6·24 (5·82–6·8)	8·59 (2·15–16·3)	1·03 (-1·94–4·06)
Occupational injuries	--	--	--	--	--	--	--	--	--	--
Occupational ergonomic factors	12·6 (10·2–15·2)	11·5 (9·56–13·8)	11·9 (9·94–14·3)	-8·82 (-19·7–2·8)	3·47 (-5·07–13·3)	7·32 (5·81–9·25)	7·52 (6·09–9·38)	7·9 (6·24–10)	2·73 (-15–22·2)	5·11 (-7·36–20·6)
Childhood sexual abuse	7·74 (5·25–10·9)	7·06 (5·05–9·15)	7·1 (5·03–9·4)	-8·73 (-20·9–7·23)	0·591 (-4·7–7·56)	17·5 (12·6–24·7)	15 (11·4–19·5)	15·1 (11·7–19·7)	-14·6 (-26·6–2·39)	1·11 (-6·05–8·7)
Intimate partner violence	--	--	--	--	--	19·4 (11·5–27·8)	18·8 (10·5–26·5)	20·7 (11·2–30·5)	-3·03 (-13–11·3)	10·2 (-5·04–35·3)
Non-exclusive breastfeeding	26·1 (21·8–31·3)	25 (21·2–28·9)	24·9 (20·2–29·5)	-4·48 (-18·3–14·2)	-0·326 (-14·1–14·3)	26·1 (21·8–31·3)	25 (21·2–28·9)	24·9 (20·2–29·5)	-4·49 (-18·3–14·2)	-0·337 (-14·1–14·3)
Discontinued breastfeeding	27·6 (26·7–28·6)	25·9 (24·5–27·1)	25·7 (24·3–26·9)	-6·4 (-11·9–-1·14)	-0·816 (-6·33–4·98)	27·6 (26·6–28·5)	26·1 (24·7–27·3)	25·8 (24·4–27)	-5·53 (-11·1–0·214)	-1·07 (-6·57–4·7)
Diet low in calcium	14·8 (7·11–27·3)	13 (6·31–23·4)	12·4 (5·87–22·8)	-12·3 (-23·7–0·531)	-4·28 (-14·3–4·37)	9·97 (3·99–18·9)	9·74 (4·07–18)	9·56 (4·02–18·1)	-2·3 (-19·6–24)	-1·85 (-13·2–9·18)
Occupational exposure to asbestos	5·74 (5·06–6·44)	5·34 (4·68–5·97)	4·7 (3·36–6·6)	-7 (-22–9·45)	-11·9 (-37·7–23·3)	0·73 (0·48–1·72)	0·654 (0·445–1·62)	0·658 (0·379–1·71)	-10·4 (-28·2–10·5)	0·522 (-27·1–31·8)
Occupational exposure to arsenic	0·382 (0–0·903)	0·412 (0–0·976)	0·435 (0–1·05)	7·92 (-7·62–NA)	5·46 (-6·04–NA)	0·423 (0–1·01)	0·451 (0–1·09)	0·46 (0–1·09)	6·69 (-9·53–NA)	1·99 (-8·76–NA)
Occupational exposure to benzene	0·795 (0·0466–2·33)	0·849 (0·0505–2·49)	0·868 (0·0541–2·53)	6·72 (-4·76–23·7)	2·33 (-5·98–13·6)	1·18 (0·0621–3·31)	1·39 (0·0831–3·84)	1·44 (0·0861–4·17)	17·6 (4·55–40)	3·63 (-5·61–15·9)
Occupational exposure to beryllium	0·0235 (0·022–0·025)	0·0244 (0·0237–0·025)	0·0254 (0·0239–0·0267)	3·8 (-2·57–11)	4·27 (-1·24–9·8)	0·0273 (0·0253–0·0295)	0·0296 (0·0288–0·0304)	0·0303 (0·0284–0·0322)	8·35 (0·0983–17·6)	2·57 (-3·89–9·17)
Occupational exposure to cadmium	0·0464 (0·042–0·0515)	0·0502 (0·0456–0·0556)	0·0528 (0·0467–0·0593)	8·12 (-5·47–23·6)	5·14 (-5·1–16·3)	0·052 (0·0463–0·0585)	0·0558 (0·0509–0·061)	0·0568 (0·0504–0·0642)	7·15 (-7·1–22·6)	1·92 (-7·86–14·4)
Occupational exposure to chromium	0·11 (0·0998–0·121)	0·123 (0·113–0·135)	0·13 (0·116–0·144)	11·8 (-1·21–26·6)	5·01 (-4·58–14·7)	0·132 (0·119–0·146)	0·144 (0·134–0·155)	0·147 (0·133–0·162)	9·75 (-3·31–23·6)	1·71 (-7·56–12·4)
Occupational exposure to diesel engine exhaust	0·645 (0·588–0·702)	0·761 (0·702–0·815)	0·795 (0·719–0·869)	18·1 (5·6–31·2)	4·46 (-3·74–12·8)	0·721 (0·642–0·813)	0·789 (0·716–0·867)	0·783 (0·69–0·879)	9·38 (-5·43–26·8)	-0·79 (-10·4–10·2)
Occupational exposure to formaldehyde	0·216 (0·192–0·244)	0·232 (0·206–0·261)	0·242 (0·21–0·277)	7·55 (-8·07–25·3)	4·27 (-7·23–16·3)	0·246 (0·217–0·277)	0·268 (0·245–0·294)	0·274 (0·243–0·308)	9·15 (-6–25·1)	2·22 (-7·76–13·9)
Occupational exposure to nickel	0·313 (0–1·32)	0·325 (0–1·44)	0·343 (0–1·47)	3·91 (-10·7–NA)	5·67 (-5·79–NA)	0·325 (0–1·37)	0·336 (0–1·52)	0·345 (0–1·53)	3·41 (-13·5–NA)	2·69 (-9·49–NA)
Occupational exposure to polycyclic aromatic hydrocarbons	0·225 (0·204–0·247)	0·254 (0·234–0·276)	0·265 (0·237–0·292)	12·9 (-0·24–27·4)	4·14 (-5·03–13·4)	0·284 (0·258–0·314)	0·324 (0·305–0·345)	0·33 (0·3–0·36)	14·2 (2·18–26·6)	1·61 (-6·48–10·8)
Occupational exposure to silica	2·77 (0·378–8·84)	2·84 (0·417–9·3)	3·04 (0·424–9·45)	2·84 (-12·6–22·5)	6·78 (-5·57–22·1)	2·71 (0·382–8·69)	2·69 (0·368–8·46)	2·73 (0·372–8·82)	-0·605 (-17·5–20·3)	1·33 (-11·4–22·1)
Occupational exposure to sulfuric acid	0·911 (0·19–2·82)	1·12 (0·232–3·53)	1·12 (0·238–3·51)	23·3 (1·84–53·3)	-0·382 (-14·1–14·4)	0·999 (0·211–3·31)	1·29 (0·262–4·19)	1·24 (0·249–3·97)	28·9 (1·39–63·7)	-3·55 (-17·8–14·7)
Unsafe sex	--	--	--	--	--	--	--	--	--	--
Occupational exposure to trichloroethylene	0·0709 (0·0654–0·0766)	0·0802 (0·0756–0·0851)	0·084 (0·0774–0·0913)	13·2 (3·61–24·6)	4·74 (-2·44–12·6)	0·0819 (0·0745–0·0902)	0·0894 (0·0842–0·0958)	0·0903 (0·0831–0·0988)	9·12 (-2·09–22)	1·03 (-7·36–10·5)
No access to handwashing facility	3·36 (3·11–3·62)	3·11 (2·85–3·37)	3·08 (2·84–3·33)	-7·3 (-15·4–1·78)	-1·07 (-6·55–4·82)	3·36 (3·11–3·62)	3·11 (2·85–3·37)	3·08 (2·84–3·33)	-7·3 (-15·4–1·78)	-1·07 (-6·55–4·82)
Child wasting	0·747 (0·496–1·03)	0·724 (0·468–0·983)	0·723 (0·465–0·978)	-3·09 (-12·7–7·02)	-0·159 (-7·37–7·75)	--	--	--	--	--
Child stunting	2·34 (1·2–3·48)	2·02 (1·04–3·09)	1·97 (1·03–2·98)	-13·7 (-33–11)	-2·62 (-18–13·7)	1·91 (0·966–2·94)	1·74 (0·853–2·66)	1·72 (0·843–2·65)	-8·64 (-30·2–16·6)	-1·4 (-16·9–15·8)
Chewing tobacco	1·88 (1·41–2·53)	2·2 (1·52–3·02)	2·24 (1·53–3·16)	16·9 (-23·2–77·1)	1·86 (-23·9–34·1)	0·307 (0·193–0·476)	0·345 (0·213–0·541)	0·35 (0·213–0·531)	12·3 (-34·6–92·1)	1·42 (-30·3–46·4)
Diet low in legumes	73·1 (36–99·2)	76·6 (43·5–99·6)	76·7 (45·1–99·7)	4·9 (0–28·2)	0·0374 (-4·77–6·47)	71·5 (33·3–98·7)	75 (41·1–99·6)	75·3 (43·5–99·4)	4·8 (-3·79e-05–25)	0·451 (-4·5–7·83)
Short gestation	26·1 (21·1–32·1)	23·4 (19·4–28·1)	22·3 (18·1–27)	-10·6 (-30·5–14·3)	-4·66 (-24–17·6)	19·3 (16·4–22·6)	22·5 (18·8–26·8)	20·5 (17·2–24·1)	16·3 (-6·13–42·6)	-8·91 (-24·9–10)
Low birth weight	11·2 (9·13–13·7)	10·4 (8·59–12·3)	10·3 (8·64–12·4)	-7·44 (-28·6–19·7)	-0·756 (-20–23)	9·81 (8·45–11·5)	11·7 (10–13·7)	11·5 (9·94–13·4)	19·3 (-3·42–47·3)	-1·74 (-19·3–19)
High temperature	21·2 (7·52–36·8)	0 (0–0)	21·5 (8·2–36·7)	-100 (-100–-100)	--	21·2 (7·52–36·8)	0 (0–0)	21·5 (8·2–36·7)	-100 (-100–-100)	--
Low temperature	29·1 (19–41)	28·9 (19·1–40·5)	28·4 (18·5–40·7)	-0·7 (-2·33–1·47)	-1·63 (-7·83–3·86)	29·1 (19–41)	28·9 (19·1–40·5)	28·4 (18·5–40·7)	-0·7 (-2·33–1·47)	-1·63 (-7·83–3·86)
Kidney dysfunction	19·3 (13·7–26·4)	20 (14·3–27·2)	20·1 (14·4–27·4)	3·72 (0·153–8·13)	0·634 (-3·72–5·33)	21·6 (15·3–29)	22·5 (16–30·4)	23·4 (16·9–31·3)	4·25 (-0·186–9·42)	3·86 (-0·358–8·51)
Bullying victimization	14·2 (5·96–28·8)	19·3 (8·52–35)	19 (7·96–36·1)	36·3 (14·6–63·3)	-1·61 (-10·3–4·02)	13·1 (5·39–26·3)	17·5 (7·44–33·2)	17·1 (6·87–34)	33·6 (15·8–57·1)	-2·49 (-11–2·99)
High LDL cholesterol	66·1 (61·9–70·8)	34·2 (30·7–37·6)	30·7 (27·1–34·4)	-48·3 (-53·6–-42·9)	-10·1 (-15–-4·96)	63·6 (59·9–67·4)	34 (30·7–37·3)	31·6 (28·1–35)	-46·6 (-51·2–-41·9)	-7·04 (-11·2–-2·96)

Supplementary Results Table 1. Idaho age-standardised Summary Exposure Values (SEVs) for most-detailed risk factors and percentage change, by sex, 1990, 2010, and 2019. Data in parentheses are 95% uncertainty intervals.

	Male					Female				
	1990	2010	2019	Percentage change 1990-2010	Percentage change 2010-2019	1990	2010	2019	Percentage change 1990-2010	Percentage change 2010-2019
Unsafe sanitation	8·1 (4–14·6)	4·39 (2·01–8·37)	3·57 (1·57–6·59)	-45·9 (-77·7–36·8)	-18·6 (-57–55·9)	--	--	--	--	--
Ambient particulate matter pollution	4·58 (0·413–12·1)	3·1 (0·928–5·76)	3·61 (1·28–6·37)	-32·3 (-79·8–362)	16·3 (8·67–43·1)	4·59 (0·43–12·1)	3·11 (0·941–5·77)	3·61 (1·29–6·38)	-32·3 (-79·9–335)	16·2 (8·57–43·8)
Household air pollution from solid fuels	0·0374 (0·00394–0·128)	0·0187 (0·00239–0·0701)	0·0161 (0·00232–0·0586)	-50·1 (-85·5–124)	-13·6 (-53·3–71·8)	0·0496 (0·00496–0·162)	0·0249 (0·00312–0·089)	0·0214 (0·00332–0·0766)	-49·9 (-85·4–116)	-13·8 (-53·2–70·2)
Ambient ozone pollution	52·4 (22·1–90·6)	59·2 (25·1–98·6)	51·6 (21·3–88)	12·9 (-3·56–35·2)	-12·8 (-28·4–4·26)	52·4 (22·1–90·6)	59·2 (25·1–98·6)	51·6 (21·3–88)	12·9 (-3·56–35·2)	-12·8 (-28·4–4·26)
Residential radon	23·3 (3·12–67·3)	23·3 (3·12–67·3)	23·3 (3·12–67·3)	0 (0–0)	0 (0–0)	23·3 (3·12–67·3)	23·3 (3·12–67·3)	23·3 (3·12–67·3)	0 (0–0)	0 (0–0)
Lead exposure	47·2 (18·7–71·7)	28·7 (8·45–51·1)	20·4 (4·62–40·3)	-39·2 (-58·3–26·1)	-28·8 (-47·6–20·1)	26 (1·13–54·6)	16·6 (0·552–40·5)	12 (0·217–31·8)	-36·1 (-74·1–19·7)	-27·8 (-66·3–19·7)
Child underweight	0·978 (0·567–1·47)	0·968 (0·539–1·46)	0·943 (0·524–1·42)	-1·02 (-18·8–19·8)	-2·63 (-14·8–10·8)	0·819 (0·457–1·24)	0·851 (0·468–1·31)	0·872 (0·482–1·32)	3·92 (-17·1–27)	2·44 (-11·1–19·5)
Iron deficiency	--	--	--	--	--	11·5 (9·61–13·5)	11·9 (10·1–13·8)	12·1 (10·1–14·4)	3·5 (-8·09–15·6)	1·91 (-7·42–12·9)
Vitamin A deficiency	1·06 (0·29–2·71)	1·37 (0·355–3·79)	0·976 (0·265–2·74)	29 (-67·7–476)	-28·6 (-68–71·9)	1·95 (0·719–4·2)	1·95 (0·697–4·24)	1·67 (0·601–3·7)	0·331 (-65·2–197)	-14·7 (-53·7–65·2)
Zinc deficiency	2·65 (0–8·36)	1·33 (0–4·2)	1·4 (0–4·51)	-49·7 (-100–NA)	4·68 (-13·4–NA)	2·71 (0–8·6)	1·34 (0–4·3)	1·36 (0–4·32)	-50·4 (-100–NA)	0·868 (-18·6–NA)
Smoking	25·1 (22·6–27·9)	17·8 (16·1–19·7)	15·3 (13·8–17)	-29 (-34–23·8)	-14 (-17·9–10·4)	17·5 (15·4–19·6)	13·8 (12·3–15·4)	11·6 (10·3–13·1)	-21·1 (-29·2–12·5)	-15·8 (-19·8–11·4)
Secondhand smoke	36·6 (34·2–39·1)	24·8 (23·1–26·4)	24·7 (22·1–27·2)	-32·3 (-38·2–25·6)	-0·478 (-9·57–8·66)	33·5 (31·7–35·3)	23 (21·8–24·1)	22·8 (20·9–24·9)	-31·4 (-36·5–26)	-0·525 (-7·45–7·1)
Alcohol use	13 (7·86–18·4)	13 (8·09–18·9)	13·4 (8·09–19·4)	0·112 (-28·9–43·3)	3·14 (-17·6–32·1)	9·13 (5·33–13·4)	9·72 (6·09–14·1)	9·43 (5·52–13·9)	6·37 (-23·6–57·4)	-2·94 (-25·6–23·7)
Drug use	0·46 (0·242–0·849)	0·679 (0·455–1·01)	1·1 (0·789–1·49)	47·7 (6·66–106)	61·9 (35·8–96·8)	0·503 (0·334–0·747)	0·896 (0·666–1·17)	1·33 (1·03–1·68)	78·1 (45·8–117)	47·9 (28·2–72·3)
High fasting plasma glucose	12·2 (10·2–14·9)	16·3 (14·1–18·8)	17·7 (15·3–20·6)	34 (10·6–61)	8·9 (-4·93–25·8)	9·37 (7·91–11·4)	12·8 (11–14·8)	14·4 (12·2–16·8)	36·2 (14·7–62·8)	12·7 (-2·91–31·9)
High systolic blood pressure	24·9 (20–30·1)	15·6 (13·6–17·7)	19 (14·8–23·8)	-37·3 (-48·8–21·6)	22·1 (-3·65–53·4)	22 (16–29·5)	12·8 (11·1–14·4)	17·2 (12·2–22·7)	-42·1 (-56·3–20·9)	34·6 (-3·76–80·8)
High body-mass index	23·2 (16–32·6)	37·5 (27·2–49)	38·4 (27·5–49·7)	61·7 (41·5–92)	2·62 (-3·99–10·4)	29 (20·8–38·6)	42·6 (33·8–51·1)	43·2 (34·2–51·9)	47·1 (27·3–73·3)	1·36 (-5·2–7·9)
Low bone mineral density	12·1 (7·23–18·8)	9·32 (4·94–15·7)	11·7 (6·7–18·5)	-22·8 (-43·8–0·267)	25·9 (-2·13–70·2)	15·8 (10·2–22·7)	16·5 (10·9–23·9)	19·1 (12·8–26·2)	4·7 (-18·3–31·7)	15·7 (-8·43–48·6)
Diet low in fruits	57 (45·4–69·8)	50·8 (38·8–64·4)	52·5 (39·8–66·1)	-11 (-22·3–2·25)	3·45 (-6·62–15·1)	60·5 (45·4–79·5)	53·8 (38·9–74·1)	54 (40·2–69·2)	-11 (-21–1·8)	0·305 (-21·7–32·4)
Diet low in vegetables	33·6 (18·4–55·6)	68·5 (49·1–85·7)	67·4 (48·1–84·8)	104 (39·1–241)	-1·53 (-7·2–3·12)	28·5 (13·1–49·8)	64 (44·4–81·1)	62·6 (42·9–80·5)	124 (44·9–303)	-2·18 (-8·88–3·83)
Diet low in whole grains	80 (70·3–88·9)	82 (73·4–89·9)	82 (73·5–90)	2·47 (-1·09–6·71)	2·8e-05 (-2·55–2·5)	77·3 (66·7–87)	80·1 (70·7–88·7)	80 (70·1–88·7)	3·66 (-0·522–9·76)	-0·181 (-3·2–2·61)
Diet low in nuts and seeds	30·5 (9·49–58·4)	26·5 (9·04–55·9)	22·1 (7·14–54·4)	-13 (-45·8–55·3)	-16·6 (-43·1–8·63)	26·3 (7·17–55·8)	23·2 (7·27–55·1)	20·5 (5·89–53·6)	-11·9 (-45·1–48·5)	-11·5 (-39·5–15·3)
Diet low in milk	51 (27·3–72·1)	46·2 (22·6–66·8)	45·5 (22·1–65·8)	-9·44 (-27·9–2·19)	-1·51 (-12·4–10)	50·8 (27·3–70·8)	46·1 (22–66·2)	45·7 (22·2–65·9)	-9·23 (-26·5–3·01)	-0·921 (-11·2–11)
Diet high in red meat	85·1 (74·4–92·9)	84·5 (72·4–93·7)	83·6 (70·8–92·6)	-0·692 (-13·4–12·1)	-1·09 (-10·7–8·17)	82·4 (71·4–91·5)	79·7 (67·3–90·5)	78·9 (65·1–89·9)	-3·26 (-17·2–12·1)	-1·05 (-12·2–10)
Diet high in processed meat	77·4 (52·8–98)	84·9 (66–99·5)	84·8 (65·3–99·5)	9·71 (0·937–29·4)	-0·131 (-4·6–4·55)	77·6 (52·9–97·9)	86·1 (68·7–99·3)	84·7 (66·1–99·3)	11 (0·887–32·2)	-1·59 (-6·86–2·53)
Diet high in sugar-sweetened beverages	43·2 (25·6–62·2)	58·3 (39·2–75·6)	58·7 (39–77·3)	34·9 (-9·09–111)	0·661 (-21·7–25·2)	46·7 (28·1–66·4)	64·3 (45·1–81·9)	62·9 (40·9–80·8)	37·6 (-5·72–117)	-2·19 (-22·6–20·7)
Diet low in fiber	46 (29·8–61·9)	32·8 (19·2–48·5)	30·1 (17·1–45·5)	-28·8 (-45·6–13)	-8·15 (-26–8·58)	46·3 (29·8–62·8)	38 (23·5–52·9)	33·4 (18·7–49·3)	-17·8 (-34·4–0·645)	-12·3 (-28·2–2·36)
Diet low in seafood omega-3 fatty acids	83·1 (65·1–99·8)	79·5 (61·2–99·2)	78·9 (60·8–99·6)	-4·33 (-17·3–8·36)	-0·676 (-14·4–15)	85·8 (70·7–100)	83 (66·9–99·8)	83·2 (66·5–99·7)	-3·27 (-14·7–6·22)	0·208 (-9·71–11·7)
Diet low in polyunsaturated fatty acids	15·6 (4·61–43)	8·07 (2·28–27·4)	7·71 (2·21–26·6)	-48·3 (-73·9–7·09)	-4·45 (-25·4–18·5)	--	--	--	--	--
Diet high in trans fatty acids	89·4 (81·8–100)	87·9 (79·6–100)	87·2 (77·9–100)	-1·66 (-9·35–6·18)	-0·809 (-8·39–7·68)	--	--	--	--	--
Diet high in sodium	18·4 (4·72–41·6)	39·9 (11·1–63·2)	38·9 (10·3–63·3)	117 (37·7–433)	-2·51 (-30·1–30)	56·7 (0–100)	58·2 (0–100)	58·4 (0–100)	2·63 (-17·8–NA)	0·307 (-12·5–NA)
Low physical activity	1·81 (0·55–5·01)	1·48 (0·468–4·9)	1·52 (0·419–4·75)	-18·1 (-49·8–11·2)	2·85 (-26·4–46·9)	--	--	--	--	--
Occupational asthmagens	20·7 (17·2–25)	20 (17–23·9)	20·8 (17·4–25·1)	-3·37 (-14·7–8·57)	4·02 (-4·99–14)	11 (8·9–13·5)	11 (9·13–13·2)	11·1 (8·99–13·5)	0·116 (-14·6–18·2)	0·85 (-11·1–13·5)
Occupational particulate matter, gases, and fumes	9·58 (6·85–13·6)	9·16 (6·63–13·1)	8·9 (6·46–12·6)	-4·3 (-9·94–1·52)	-2·93 (-6·6–0·527)	5·44 (3·78–8·05)	5·39 (3·86–7·75)	5·24 (3·77–7·53)	-0·819 (-9·39–9·61)	-2·89 (-8·28–2·63)
Occupational noise	8·84 (8·05–9·66)	8·53 (7·92–9·23)	8·28 (7·77–8·92)	-3·6 (-7·15–0·532)	-2·91 (-5·05–0·657)	6·04 (5·27–6·83)	6·4 (5·82–7)	6·42 (5·94–6·99)	6·07 (-0·232–14·4)	0·297 (-2·68–3·72)
Occupational injuries	--	--	--	--	--	--	--	--	--	--
Occupational ergonomic factors	13·3 (10·9–16·4)	11·3 (9·54–13·5)	11·7 (9·82–14·1)	-14·8 (-25·8–2·7)	3·66 (-4·21–13·5)	7·73 (6·07–9·84)	7·42 (6·09–9·25)	7·7 (6·13–9·71)	-4·08 (-19·4–14·9)	3·85 (-8·91–18·5)
Childhood sexual abuse	7·4 (5·05–10·5)	6·97 (4·6–9·93)	6·85 (4·54–9·67)	-5·83 (-12·2–5·83)	-1·68 (-8·51–5·53)	12·3 (8·94–17·3)	17·4 (12·9–24)	17·8 (13·1–24·7)	41·9 (30·7–54·8)	2·17 (-5–11)
Intimate partner violence	--	--	--	--	--	26·6 (14·2–35·8)	25 (13·5–33·9)	26·2 (13·9–35·5)	-5·93 (-14·9–9·71)	4·86 (-4·11–18·9)
Non-exclusive breastfeeding	26·3 (21·6–31·3)	24·8 (20·9–29)	24·8 (20·1–29·6)	-5·73 (-19·6–12)	0·191 (-12·6–15·9)	26·3 (21·6–31·3)	24·8 (20·9–29)	24·8 (20·1–29·6)	-5·73 (-19·6–12)	0·185 (-12·6–15·9)
Discontinued breastfeeding	26·7 (25·8–27·6)	25·3 (24–26·6)	25·7 (24·4–26·9)	-5·34 (-10·8–0·00273)	1·52 (-4·02–7·41)	26·9 (25·9–27·8)	25·4 (24·1–26·7)	25·7 (24·4–27)	-5·46 (-10·9–0·12)	1·21 (-4·31–7·05)
Diet low in calcium	17·9 (8·51–33·6)	15·2 (7·51–28)	14·7 (6·95–27·7)	-15·1 (-26·2–3·5)	-3·76 (-13·8–4·04)	13·4 (5·74–25·8)	12·3 (5·45–23·3)	12·2 (5·3–22·9)	-8·35 (-21·8–7·95)	-1·11 (-10·9–7·56)
Occupational exposure to asbestos	8·49 (7·59–9·39)	7·85 (7·13–8·64)	7·22 (5·26–9·31)	-7·45 (-19·9–7·3)	-8·07 (-32·9–23·1)	1·63 (1·05–2)	1·66 (1·06–1·97)	1·67 (1·02–2·3)	1·36 (-12·7–20)	0·845 (-23·8–32·7)
Occupational exposure to arsenic	0·459 (0–1·08)	0·49 (0–1·16)	0·517 (0–1·22)	6·82 (-9·41–NA)	5·5 (-5·8–NA)	0·452 (0–1·07)	0·476 (0–1·16)	0·489 (0–1·18)	5·24 (-11·5–NA)	2·65 (-10–NA)
Occupational exposure to benzene	0·792 (0·0469–2·25)	0·802 (0·0471–2·32)	0·831 (0·0494–2·35)	1·23 (-7·72–14·1)	3·57 (-4·04–14·2)	1·13 (0·0644–3·21)	1·27 (0·0767–3·44)	1·33 (0·0786–3·83)	12·7 (0·126–34·1)	4·38 (-5·34–16·9)
Occupational exposure to beryllium	0·0235 (0·0221–0·0249)	0·0236 (0·023–0·0242)	0·0248 (0·0235–0·0262)	0·157 (-5·66–6·78)	5·11 (-0·532–11·4)	0·0264 (0·0242–0·0287)	0·0276 (0·0268–0·0284)	0·0285 (0·0264–0·0306)	4·48 (-4·79–14·8)	3·33 (-4·02–10·7)
Occupational exposure to cadmium	0·0543 (0·049–0·06)	0·0578 (0·0523–0·0641)	0·0611 (0·054–0·07)	6·49 (-7·55–22·8)	5·58 (-5·01–17)	0·0546 (0·0478–0·0611)	0·0575 (0·0516–0·0647)	0·0589 (0·0511–0·0682)	5·31 (-9·87–24·8)	2·45 (-8·74–14·8)
Occupational exposure to chromium	0·13 (0·119–0·143)	0·142 (0·13–0·154)	0·15 (0·135–0·168)	8·84 (-3·46–22)	5·51 (-3·55–15·2)	0·136 (0·121–0·151)	0·146 (0·134–0·16)	0·15 (0·132–0·169)	7·23 (-6·59–23·5)	2·39 (-7·66–13·1)
Occupational exposure to diesel engine exhaust	0·719 (0·662–0·779)	0·788 (0·738–0·838)	0·83 (0·759–0·904)	9·7 (-0·43–21)	5·27 (-2·76–13·6)	0·687 (0·607–0·772)	0·72 (0·653–0·786)	0·724 (0·634–0·819)	4·75 (-9·09–21·6)	0·6 (-10·2–11·8)
Occupational exposure to formaldehyde	0·265 (0·237–0·294)	0·279 (0·25–0·312)	0·292 (0·256–0·334)	5·43 (-8·87–22·2)	4·5 (-6·09–16·3)	0·262 (0·229–0·293)	0·279 (0·252–0·31)	0·286 (0·249–0·328)	6·75 (-8·22–25·7)	2·58 (-8·06–14·3)
Occupational exposure to nickel	0·396 (0–1·75)	0·396 (0–1·74)	0·419 (0–1·79)	0·106 (-14·4–NA)	5·8 (-5·63–NA)	0·36 (0–1·53)	0·365 (0–1·62)	0·375 (0–1·62)	1·27 (-15·7–NA)	2·88 (-11·1–NA)
Occupational exposure to polycyclic aromatic hydrocarbons	0·259 (0·237–0·282)	0·28 (0·259–0·303)	0·295 (0·266–0·328)	8·11 (-3·56–21·3)	5·25 (-3·43–14·5)	0·29 (0·259–0·32)	0·32 (0·299–0·345)	0·328 (0·295–0·365)	10·4 (-2·43–25·6)	2·62 (-6·4–12)
Occupational exposure to silica	3·77 (0·553–12·4)	3·58 (0·503–11·4)	3·8 (0·54–11·9)	-5·14 (-19–11·3)	6·33 (-5·43–20·2)	3·08 (0·421–10)	2·97 (0·398–9·79)	3·03 (0·424–9·33)	-3·65 (-20·3–21·5)	2·01 (-11·9–20·4)
Occupational exposure to sulfuric acid	0·879 (0·19–2·87)	1·01 (0·208–3·21)	1·04 (0·214–3·35)	14·3 (-0·375–32)	3·6 (-6·92–16·3)	0·928 (0·189–3)	1·14 (0·24–3·49)	1·14 (0·242–3·56)	22·8 (-1·21–49·7)	-0·254 (-15·1–16·8)
Unsafe sex	--	--	--	--	--	--	--	--	--	--
Occupational exposure to trichloroethylene	0·0791 (0·0737–0·0851)	0·087 (0·0827–0·092)	0·0916 (0·0852–0·0993)	9·88 (1·22–19·7)	5·38 (-1·44–13·1)	0·0816 (0·0734–0·0902)	0·0867 (0·0816–0·0933)	0·0884 (0·0793–0·0976)	6·32 (-5·08–20·5)	1·91 (-6·99–11·3)
No access to handwashing facility	3·42 (3·17–3·69)	3·14 (2·9–3·37)	3·11 (2·86–3·36)	-8·28 (-16·1–0·247)	-0·842 (-6·65–5·01)	3·42 (3·17–3·69)	3·14 (2·9–3·37)	3·11 (2·86–3·36)	-8·28 (-16·1–0·247)	-0·842 (-6·65–5·01)
Child wasting	0·75 (0·487–1·02)	0·727 (0·479–0·994)	0·726 (0·476–0·983)	-3·08 (-12·6–7)	-0·126 (-6·87–8·01)	0·775 (0·517–1·06)	0·765 (0·511–1·05)	0·761 (0·502–1·03)	-1·31 (-11·7–9·83)	-0·515 (-7·7–6·97)
Child stunting	2·47 (1·26–3·64)	2·1 (1·09–3·17)	2·03 (1·06–3)	-14·9 (-34·5–9·74)	-3·05 (-17·5–14)	1·98 (0·992–3)	1·8 (0·873–2·7)	1·77 (0·87–2·69)	-9·02 (-28·4–18·5)	-1·5 (-17·2–16·8)
Chewing tobacco	5·46 (4·22–6·9)	5·83 (4·24–7·99)	5·75 (4·01–7·93)	6·77 (-27·9–55)	-1·28 (-22·8–27·6)	0·295 (0·182–0·474)	0·345 (0·209–0·551)	0·36 (0·22–0·558)	17 (-33·3–106)	4·44 (-27·9–50·1)
Diet low in legumes	74·8 (40–99·4)	77·9 (46·7–99·7)	77·9 (48·4–99·7)	4·12 (-0·0574–18·6)	-0·0201 (-5·05–5·16)	73·2 (37·2–99)	76·2 (44·1–99·7)	76·6 (45·4–99·6)	4·14 (-0·244–22·8)	0·476 (-3·76–6·69)
Short gestation	18·2 (14·8–22·3)	16·7 (13·7–20)	17·3 (14–21·2)	-8·61 (-28·1–15·1)	3·79 (-17·3–28·2)	14·5 (12·1–17·1)	15·9 (13·6–18·5)	17·2 (14·5–20·1)	9·94 (-8·12–32·1)	7·9 (-9·74–26·7)
Low birth weight	7·49 (6·11–9·01)	7·23 (6·04–8·58)	7·4 (6·14–8·98)	-3·47 (-25·1–24·3)	2·39 (-19–29·6)	6·98 (5·92–8·22)	8·14 (7·05–9·38)	9·01 (7·65–10·5)	16·7 (-2·94–41·8)	10·7 (-8·02–31)
High temperature	28·2 (17·7–39·6)	28·1 (16·7–40·2)	33 (21·2–45·1)	-0·358 (-10·9–7·17)	17·6 (7·27–37·2)	28·2 (17·7–39·6)	28·1 (16·7–40·2)	33 (21·2–45·1)	-0·358 (-10·9–7·17)	17·6 (7·27–37·2)
Low temperature	36·3 (33·1–40·7)	36 (32·8–40)	36·5 (33·3–40·4)	-0·815 (-3·96–1·82)	1·37 (0·212–2·45)	36·3 (33·1–40·7)	36 (32·8–40)	36·5 (33·3–40·4)	-0·815 (-3·96–1·82)	1·37 (0·212–2·45)
Kidney dysfunction	18·8 (13·3–25·7)	19·4 (13·8–26·6)	19·5 (14–26·5)	3·56 (0·0137–7·83)	0·553 (-3·09–4·43)	21·2 (14·9–28·6)	22·2 (15·8–29·9)	23 (16·5–30·6)	5·05 (0·958–9·76)	3·5 (-0·317–8·3)
Bullying victimization	5·96 (2·47–12·2)	8·96 (3·59–17·8)	8·78 (3·39–18·2)	50·4 (31–73·2)	-2·08 (-11·5–4·02)	--	--	--	--	--
High LDL cholesterol	66 (61·7–70·8)	34·6 (30·9–38·2)	30·8 (27·2–34·7)	-47·6 (-53·2–42·3)	-10·9 (-15·9–5·68)	64 (60·5–67·9)	34·3 (31–37·8)	31·4 (28–34·9)	-46·5 (-51·1–41·6)	-8·28 (-12·4–4·26)

Supplementary Results Table 1. Illinois age-standardised Summary Exposure Values (SEVs) for most-detailed risk factors and percentage change, by sex, 1990, 2010, and 2019. Data in parentheses are 95% uncertainty intervals.

	Male					Female				
	1990	2010	2019	Percentage change 1990-2010	Percentage change 2010-2019	1990	2010	2019	Percentage change 1990-2010	Percentage change 2010-2019
Unsafe sanitation	6·13 (2·96–11·2)	2·97 (1·32–5·75)	2·4 (1·05–4·66)	-51·5 (-80–16·8)	-19·1 (-60·6–58·3)	--	--	--	--	--
Ambient particulate matter pollution	13·9 (5·26–26·9)	8·72 (5·93–11·8)	6·31 (3·7–9·27)	-37·4 (-67·7–50·1)	-27·7 (-38·7–21·6)	13·9 (5·26–26·9)	8·72 (5·92–11·8)	6·3 (3·68–9·27)	-37·4 (-67·7–50·5)	-27·7 (-38·6–21·6)
Household air pollution from solid fuels	0·00787 (0·00186–0·0212)	0·00438 (0·000774–0·0134)	0·00313 (5e-04–0·0104)	-44·4 (-78·9–24·5)	-28·6 (-64–33·4)	0·0104 (0·00274–0·0279)	0·0058 (0·00112–0·0174)	0·00414 (0·00069–0·0138)	-44·2 (-78·5–25·6)	-28·7 (-63·9–33·4)
Ambient ozone pollution	51·2 (21·6–87·5)	46·3 (19·3–78·9)	50·3 (21·3–87)	-9·59 (-19·2–1·45)	8·7 (-0·0383–20)	--	--	--	--	--
Residential radon	22·5 (3·26–66·4)	22·5 (3·26–66·4)	22·5 (3·26–66·4)	0 (0–0)	0 (0–0)	22·5 (3·26–66·4)	22·5 (3·26–66·4)	22·5 (3·26–66·4)	0 (0–0)	0 (0–0)
Lead exposure	75·6 (51·6–90·8)	43·9 (24–64)	30·5 (14·3–47·9)	-41·9 (-54·8–29·1)	-30·5 (-40·2–23·8)	50·3 (17·1–75·7)	27·3 (5·11–51·6)	18·2 (2·12–38)	-45·8 (-71·8–29·6)	-33·1 (-59·9–25·2)
Child underweight	0·961 (0·54–1·46)	0·947 (0·503–1·44)	0·925 (0·509–1·4)	-1·44 (-19–20·3)	-2·31 (-15·6–12·4)	0·809 (0·437–1·22)	0·839 (0·466–1·3)	0·856 (0·467–1·31)	3·71 (-16·2–31·2)	1·99 (-12·2–17·4)
Iron deficiency	--	--	--	--	--	12 (9·96–14·1)	12·1 (10·2–14·1)	12·2 (10·1–14·5)	0·546 (-10·9–13·6)	1·55 (-7·48–12)
Vitamin A deficiency	0·817 (0·192–2·18)	1·02 (0·279–2·72)	0·703 (0·195–1·93)	24·6 (-65·8–434)	-31 (-71·1–76·4)	1·6 (0·539–3·28)	1·52 (0·612–3·14)	1·28 (0·507–2·86)	-5·03 (-66·7–182)	-15·8 (-51·6–64·5)
Zinc deficiency	2·02 (0–6·52)	1·1 (0–3·42)	1·13 (0–3·55)	-45·7 (-100–NA)	2·47 (-18·5–NA)	2·08 (0–6·63)	1·11 (0–3·61)	1·1 (0–3·48)	-46·6 (-100–NA)	-0·408 (-21·6–NA)
Smoking	27·1 (24·5–30)	19·9 (18–22)	17 (15·2–18·9)	-26·6 (-31·3–21·5)	-14·5 (-18–11)	21·1 (18·4–23·8)	14·4 (12·8–16·1)	12 (10·7–13·4)	-31·6 (-37·7–24·8)	-16·7 (-20·6–12·4)
Secondhand smoke	41·2 (38·6–43·8)	25·3 (23·6–26·9)	25·5 (22·9–28·3)	-38·5 (-43·8–32·6)	0·665 (-8·6–10·1)	38·7 (36·7–40·8)	24·4 (23·3–25·6)	24·6 (22·7–26·7)	-36·8 (-41·5–32)	0·853 (-6·66–8·01)
Alcohol use	14·9 (9·65–21·1)	14·4 (9·15–19·8)	15·1 (9·84–21)	-3·33 (-36·4–44·4)	5·14 (-18·6–39·6)	--	--	--	--	--
Drug use	0·603 (0·332–1·07)	0·941 (0·658–1·34)	1·18 (0·853–1·61)	56·2 (19·4–111)	25·7 (10·6–45·1)	0·401 (0·249–0·65)	0·676 (0·494–0·911)	0·942 (0·719–1·23)	68·7 (32·5–118)	39·3 (20·6–63·4)
High fasting plasma glucose	13·1 (11–15·7)	17·3 (15·1–19·9)	18·8 (16·3–21·6)	32·3 (11·4–58·3)	8·82 (-5·5–26·2)	9·94 (8·43–11·8)	13·3 (11·6–15·5)	14·9 (12·7–17·2)	34·1 (13·4–58·5)	11·4 (-4·08–29·6)
High systolic blood pressure	25·8 (20·7–30·9)	16·1 (14·1–18·2)	19·7 (15·3–24·4)	-37·5 (-48·9–22·4)	22·4 (-2·55–50·8)	24·5 (18·3–31·5)	13·3 (11·6–15)	17·5 (12·4–23·1)	-45·9 (-57·7–28·8)	32·1 (-3·85–75·3)
High body-mass index	24·2 (16·9–34)	38·8 (28·6–50·3)	39·9 (29·4–51·4)	60·5 (40·9–88·3)	2·79 (-3·07–9·64)	30·9 (22·8–40·1)	43·7 (34·3–52·8)	44·6 (35·4–53·9)	41·7 (25·7–63·5)	1·98 (-3·73–8·01)
Low bone mineral density	12·3 (7·32–19·2)	9·39 (5·12–15·8)	11·6 (6·66–18·4)	-23·5 (-44·1–0·482)	24 (-3·18–68)	16·2 (10·4–23·1)	16·7 (11·2–23·8)	19 (13·2–26·2)	3·22 (-19·4–32·5)	14 (-12·1–46·7)
Diet low in fruits	58·1 (47·1–71)	52·5 (41·1–65·3)	54·9 (42·2–68·2)	-9·66 (-19·8–0·312)	4·53 (-5·06–15·3)	--	--	--	--	--
Diet low in vegetables	45·9 (26·6–67·3)	70·7 (56–82·5)	69·3 (54·8–81·3)	54 (18·3–127)	-1·88 (-6·79–2·21)	43 (23·7–65·5)	70·5 (53–86·3)	69·7 (51·1–85·6)	64·1 (25·9–146)	-1·13 (-6·63–3·31)
Diet low in whole grains	79·2 (70–88·3)	81·2 (72·4–89·5)	81·3 (72·1–89·4)	2·59 (-0·841–7·33)	0·102 (-2·39–2·76)	76·6 (66·1–86·3)	79·4 (70·1–88·1)	79·2 (69·5–88·2)	3·72 (-0·665–9·18)	-0·238 (-3·06–2·72)
Diet low in nuts and seeds	25·9 (7·62–55·7)	25 (7·95–55·3)	20·3 (6·62–53·7)	-3·5 (-37·9–77·5)	-18·6 (-45·3–8·47)	21·8 (5·55–53)	21·5 (6·29–54·1)	18·6 (4·81–53·1)	-1·57 (-39·5–83·6)	-13·3 (-42·7–16·7)
Diet low in milk	46·5 (21·5–67)	41·1 (18·4–60·9)	40·5 (18·3–59·6)	-11·6 (-31–1·77)	-1·44 (-14·6–12·8)	--	--	--	--	--
Diet high in red meat	86·8 (77·9–93·5)	85·8 (75·1–93·6)	84·8 (72·8–93·3)	-1·15 (-12·4–10·3)	-1·22 (-10·2–7·54)	81·5 (70·6–90·2)	78·8 (65·8–88·7)	78·3 (64·3–88·8)	-3·27 (-16·1–11·2)	-0·708 (-11·5–9·82)
Diet high in processed meat	78·3 (53·8–98)	85·6 (67·1–99·6)	85·4 (65·6–99·7)	9·33 (1·06–28·6)	-0·289 (-4·93–4·28)	78·7 (54·5–98·4)	87·1 (69·5–99·6)	85·7 (66·9–99·6)	10·8 (1·01–34·4)	-1·59 (-7·23–2·85)
Diet high in sugar-sweetened beverages	48 (29·4–67·9)	64·9 (46·9–81·1)	64·6 (44·6–81·2)	35·2 (-3·9–114)	-0·525 (-19·9–22·4)	51·9 (30·3–70·4)	70·5 (51–85·8)	68·9 (48·5–85·7)	36 (-0·739–111)	-2·34 (-20·4–17·9)
Diet low in fiber	41·3 (24·6–56·8)	28·7 (16·1–42·2)	26·5 (13·9–40·8)	-30·6 (-47·2–13·8)	-7·76 (-26·6–10·8)	41·8 (25·5–57·8)	33·6 (19·7–48·1)	29 (16·4–43·4)	-19·6 (-35·6–1·4)	-13·5 (-30·6–0·755)
Diet low in seafood omega-3 fatty acids	82·3 (64·8–99·6)	77·4 (57·4–98·2)	77·8 (59·8–98·3)	-5·9 (-20·3–10·1)	0·494 (-14·3–20·6)	84·9 (68·7–99·8)	82·4 (65·5–99·5)	81·9 (64·7–99·5)	-2·94 (-15·6–8·56)	-0·692 (-12·6–12·2)
Diet low in polyunsaturated fatty acids	12·8 (3·77–38·8)	6·8 (1·97–24·5)	6·61 (1·93–24·6)	-46·7 (-74·8–6·89)	-2·83 (-23·6–19·5)	--	--	--	--	--
Diet high in trans fatty acids	89·6 (81·8–100)	88 (78·8–100)	87·4 (78·2–100)	-1·83 (-10·1–5·37)	-0·639 (-8·58–7·47)	--	--	--	--	--
Diet high in sodium	20·3 (4·55–45·7)	40 (10·4–64·3)	39·4 (9·94–64·9)	97·1 (29·1–375)	-1·56 (-25·8–34·8)	51·4 (0–100)	52·1 (0–100)	52·2 (0–100)	1·33 (-27·4–NA)	0·292 (-14·9–NA)
Low physical activity	1·57 (0·548–3·89)	1·47 (0·586–3·79)	1·74 (0·609–3·96)	-5·92 (-36·2–43·3)	17·8 (-10·7–62·1)	--	--	--	--	--
Occupational asthmagens	24·5 (20·4–30·1)	21 (17·8–25·1)	22 (18–27·1)	-14·3 (-26·2–2·77)	4·77 (-5·06–17·9)	11·6 (9·55–14·2)	11·4 (9·43–13·7)	11·6 (9·45–14·2)	-1·76 (-16·3–14·1)	1·66 (-9·37–15·3)
Occupational particulate matter, gases, and fumes	9·87 (7·11–14·2)	9·1 (6·55–13·1)	8·75 (6·26–12·6)	-7·77 (-13·2–2·28)	-3·91 (-7·3–0·483)	5·36 (3·73–8·03)	5·1 (3·63–7·38)	4·88 (3·48–7·01)	-4·96 (-13·4–4·53)	-4·31 (-9·6–1·26)
Occupational noise	8·67 (7·94–9·49)	8·31 (7·76–9·06)	8·06 (7·55–8·77)	-4·1 (-7·7–0·137)	-3·07 (-5·11–0·898)	5·98 (5·26–6·8)	6·3 (5·8–6·95)	6·31 (5·89–6·89)	5·39 (-0·777–12·9)	0·172 (-2·82–3·3)
Occupational injuries	--	--	--	--	--	--	--	--	--	--
Occupational ergonomic factors	11·9 (9·8–14·4)	10·5 (8·92–12·6)	10·8 (9·1–13·2)	-11·8 (-21·5–0·974)	3·28 (-4·12–12·2)	6·75 (5·29–8·46)	6·8 (5·52–8·42)	7·13 (5·68–8·86)	0·802 (-15·4–18·6)	4·74 (-7·91–19·1)
Childhood sexual abuse	7·4 (5·05–10·5)	6·97 (4·6–9·93)	6·85 (4·54–9·67)	-5·83 (-12·2–5·83)	-1·68 (-8·51–5·53)	12·3 (8·94–17·3)	17·4 (12·9–24)	17·8 (13·1–24·7)	41·9 (30·7–54·8)	2·17 (-5–11)
Intimate partner violence	--	--	--	--	--	32·4 (17·3–42·3)	30·6 (17·1–39·4)	31·6 (18–41·2)	-5·54 (-14·5–8·69)	3·47 (-5·76–19·8)
Non-exclusive breastfeeding	26·3 (21·9–31·1)	24·9 (21·5–28·9)	24·9 (20·5–29·5)	-5·45 (-19·8–12·6)	0·037 (-13–14·4)	26·3 (21·9–31·1)	24·9 (21·5–28·9)	24·9 (20·5–29·5)	-5·44 (-19·8–12·6)	0·0304 (-13–14·4)
Discontinued breastfeeding	27·5 (26·6–28·4)	25·2 (23·9–26·4)	25·7 (24·5–26·9)	-8·54 (-13·8–3·39)	2·14 (-3·35–8·02)	27·6 (26·6–28·5)	25·2 (23·9–26·4)	25·7 (24·4–26·9)	-8·71 (-14–3·57)	2·08 (-3·4–7·96)
Diet low in calcium	15 (7·18–27·5)	12·7 (6·2–22·9)	12·3 (5·93–22·2)	-15·1 (-26·4–2·56)	-3·84 (-13·8–4·81)	10·3 (4·42–19·7)	9·59 (4·22–18)	9·5 (4·08–17·6)	-7·3 (-22·3–12·5)	-0·899 (-12–9·42)
Occupational exposure to asbestos	10·2 (9·07–11·3)	8·29 (7·68–8·86)	7·29 (5·27–9·41)	-18·7 (-28·3–6·5)	-12·1 (-36·3–15·3)	1·77 (1·19–2·23)	1·62 (1·07–1·95)	1·57 (0·994–2·18)	-8·48 (-22·9–7·16)	-3·11 (-28·6–32·1)
Occupational exposure to arsenic	0·572 (0–1·36)	0·548 (0–1·3)	0·578 (0–1·38)	-4·26 (-16·8–NA)	5·44 (-5·51–NA)	0·499 (0–1·17)	0·492 (0–1·19)	0·502 (0–1·23)	-1·47 (-17·6–NA)	2·12 (-9·92–NA)
Occupational exposure to benzene	0·87 (0·0525–2·49)	0·858 (0·0511–2·43)	0·882 (0·0519–2·48)	-1·46 (-9·8–9·81)	2·83 (-4·6–13·1)	1·21 (0·0716–3·46)	1·39 (0·083–3·88)	1·45 (0·0903–4·08)	14·9 (2·57–33·2)	4·35 (-3·69–17·5)
Occupational exposure to beryllium	0·0244 (0·0231–0·0258)	0·0241 (0·0236–0·0246)	0·0253 (0·0239–0·0267)	-1·37 (-7–4·65)	4·94 (-0·724–10·9)	0·0268 (0·0245–0·0291)	0·028 (0·0274–0·0286)	0·029 (0·027–0·0311)	4·57 (-4·52–14·4)	3·53 (-3·44–11)
Occupational exposure to cadmium	0·0661 (0·0601–0·0725)	0·064 (0·0585–0·0703)	0·0673 (0·0597–0·0759)	-3·27 (-14·6–9·9)	5·24 (-4·65–16)	0·0595 (0·0528–0·0672)	0·0591 (0·0533–0·0657)	0·0604 (0·0527–0·0691)	-0·729 (-15·4–15·3)	2·23 (-8·55–15·1)
Occupational exposure to chromium	0·158 (0·145–0·172)	0·156 (0·144–0·168)	0·163 (0·148–0·18)	-1·16 (-11·1–10·2)	4·92 (-3·65–14·3)	0·149 (0·134–0·166)	0·152 (0·14–0·164)	0·155 (0·138–0·174)	1·95 (-10·8–16)	2·23 (-7·03–13·3)
Occupational exposure to diesel engine exhaust	0·71 (0·657–0·764)	0·766 (0·717–0·816)	0·799 (0·728–0·87)	7·89 (-2·39–19·1)	4·24 (-3·38–11·8)	0·675 (0·599–0·758)	0·703 (0·642–0·77)	0·705 (0·627–0·796)	4·09 (-9·01–19·9)	0·419 (-9·75–10·7)
Occupational exposure to formaldehyde	0·335 (0·301–0·37)	0·315 (0·286–0·347)	0·328 (0·289–0·371)	-5·88 (-17·7–7·49)	3·9 (-6·2–14·8)	0·293 (0·26–0·329)	0·295 (0·268–0·326)	0·302 (0·267–0·342)	0·806 (-13–16·6)	2·42 (-7·7–14·2)
Occupational exposure to nickel	0·477 (0–2·09)	0·44 (0–1·93)	0·463 (0–1·96)	-7·84 (-20·4–NA)	5·24 (-5·43–NA)	0·391 (0–1·67)	0·371 (0–1·62)	0·38 (0–1·68)	-5·14 (-21·5–NA)	2·49 (-11–NA)
Occupational exposure to polycyclic aromatic hydrocarbons	0·312 (0·287–0·337)	0·308 (0·287–0·331)	0·323 (0·292–0·354)	-1·15 (-10·7–9·53)	4·69 (-3·42–13·6)	0·314 (0·285–0·347)	0·332 (0·312–0·356)	0·341 (0·308–0·378)	5·77 (-6·7–18·8)	2·59 (-5·83–12)
Occupational exposure to silica	4·28 (0·616–14)	3·84 (0·523–12·3)	4·09 (0·568–12·9)	-10·3 (-21·7–3·76)	6·42 (-4·91–20·4)	3·27 (0·471–10·2)	2·98 (0·427–9·46)	3·04 (0·427–9·87)	-8·64 (-25·4–14)	1·71 (-12–20·1)
Occupational exposure to sulfuric acid	1·01 (0·211–3·17)	1·07 (0·212–3·53)	1·1 (0·22–3·52)	5·33 (-7·75–21·3)	3·32 (-6·51–14·8)	0·91 (0·188–2·97)	1·05 (0·218–3·35)	1·05 (0·218–3·45)	15·7 (-6·07–39·2)	-0·617 (-15·3–15·1)
Unsafe sex	--	--	--	--	--	--	--	--	--	--
Occupational exposure to trichloroethylene	0·0896 (0·0842–0·0962)	0·0917 (0·088–0·0962)	0·096 (0·0897–0·103)	2·33 (-5·05–11·1)	4·67 (-2·17–12)	0·0859 (0·0777–0·0954)	0·0882 (0·0827–0·0946)	0·0898 (0·0813–0·0992)	2·7 (-8·66–14·7)	1·78 (-6·53–11·6)
No access to handwashing facility	3·34 (3·08–3·6)	3·09 (2·86–3·35)	3·06 (2·83–3·31)	-7·4 (-15·3–1·22)	-0·992 (-6·56–4·78)	3·34 (3·08–3·6)	3·09 (2·86–3·35)	3·06 (2·83–3·31)	-7·4 (-15·3–1·22)	-0·992 (-6·56–4·78)
Child wasting	0·747 (0·485–1·03)	0·723 (0·477–0·978)	0·722 (0·463–0·973)	-3·31 (-12–6·75)	-0·0296 (-6·59–7·57)	0·772 (0·516–1·05)	0·76 (0·505–1·04)	0·757 (0·501–1·03)	-1·47 (-12·2–10·4)	-0·481 (-7·96–8·02)
Child stunting	2·41 (1·25–3·51)	2·03 (1·06–3·05)	1·98 (1·04–2·96)	-15·5 (-34·2–7·21)	-2·73 (-16·3–14·2)	1·94 (0·945–2·96)	1·76 (0·892–2·7)	1·74 (0·854–2·66)	-9·35 (-28·6–13·9)	-1·25 (-16·6–16·9)
Chewing tobacco	3·67 (2·86–4·77)	3·47 (2·62–4·71)	3·41 (2·43–4·57)	-5·23 (-33·4–36·1)	-1·97 (-24·2–26·4)	--	--	--	--	--
Diet low in legumes	73·2 (37·4–99·1)	76·4 (44·2–99·7)	76·5 (43·8–99·6)	4·4 (-0·143–24·5)	0·132 (-5·68–6·29)	71·7 (34·8–98·8)	74·7 (40·8–99·5)	75·1 (42–99·7)	4·18 (-0·197–23·4)	0·588 (-4·86–8·7)
Short gestation	37·9 (31·6–45·6)	28·9 (24·7–34)	27·7 (22·8–33·5)	-23·8 (-37·3–7·84)	-4·25 (-22·1–15·9)	33·2 (28·3–39·1)	29·5 (25–34·3)	27·5 (23·2–32·4)	-10·9 (-27·2–7·07)	-7·02 (-23·7–11·9)
Low birth weight	14·4 (12·1–17·1)	11·9 (10·3–13·7)	12 (10–14·4)	-17·1 (-32·4–1·76)	0·909 (-18·1–24·9)	15·6 (13·2–18·2)	14·5 (12·4–16·8)	14·5 (12·3–16·9)	-7·05 (-24·3–13·3)	0·118 (-18·7–20·9)
High temperature	27·1 (17·2–39·2)	26·8 (16·7–38·9)	25·8 (16·4–37·5)	-1·35 (-4·14–1·5)	-3·47 (-7·28–1·65)	27·1 (17·2–39·2)	26·8 (16·7–38·9)	25·8 (16·4–37·5)	-1·35 (-4·14–1·5)	-3·47 (-7·28–1·65)
Low temperature	35·3 (31·5–39·5)	35·8 (32·1–39·8)	37·3 (33·6–41·5)	1·36 (-1·27–3·89)	4·39 (2·85–6·24)	35·3 (31·5–39·5)	35·8 (32·1–39·8)	37·3 (33·6–41·5)	1·36 (-1·27–3·89)	4·39 (2·85–6·24)
Kidney dysfunction	20·2 (14·5–27·3)	20·8 (14·9–28)	20·7 (15–27·9)	2·97 (-1·24–7·76)	-0·118 (-4·18–4·24)	22·3 (16–29·9)	23·2 (16·8–31·1)	24 (17·5–32)	4·16 (-0·684–9·51)	3·33 (-1·73–9·31)
Bullying victimization	5·88 (2·47–12·1)	9 (3·6–17·9)	8·74 (3·35–18)	53 (32·3–76·1)	-2·87 (-12·6–3·4)	4·26 (1·73–8·87)	7·91 (3·14–15·7)	7·81 (2·94–16·1)	85·5 (59·6–115)	-1·25 (-12–5·61)
High LDL cholesterol	67 (62·7–71·2)	34·9 (31·7–38·5)	31·4 (27·9–35·5)	-47·9 (-52·9–42·6)	-10·1 (-14·9–4·97)	65 (61·3–68·5)	34·6 (31·4–38)	32·1 (28·7–35·5)	-46·8 (-51·4–42·4)	-7·14 (-11·3–3·06)

Supplementary Results Table 1. Indiana age-standardised Summary Exposure Values (SEVs) for most-detailed risk factors and percentage change, by sex, 1990, 2010, and 2019. Data in parentheses are 95% uncertainty intervals.

	Male					Female				
	1990	2010	2019	Percentage change 1990-2010	Percentage change 2010-2019	1990	2010	2019	Percentage change 1990-2010	Percentage change 2010-2019
Unsafe sanitation	7·21 (3·33–13·3)	4·13 (1·93–7·84)	3·3 (1·47–6·22)	-42·7 (-75·3—44·1)	-20·1 (-58·8—60)	--	--	--	--	--
Ambient particulate matter pollution	14 (5·32–27·5)	8·99 (6·2–12·2)	6·43 (3·83–9·43)	-35·7 (-66·6—51·6)	-28·5 (-38·9—22·3)	14 (5·33–27·4)	8·99 (6·21–12·2)	6·43 (3·82–9·43)	-35·7 (-66·5—52·2)	-28·5 (-38·9—22·4)
Household air pollution from solid fuels	0·0189 (0·00489–0·0528)	0·0115 (0·0021–0·0363)	0·00812 (0·00142–0·0252)	-39·4 (-77·8—37·7)	-29·2 (-66—33·1)	0·025 (0·00672–0·0696)	0·0152 (0·0031–0·0485)	0·0108 (0·002–0·0328)	-39·1 (-77·4—38·2)	-29·3 (-66—33·7)
Ambient ozone pollution	75·8 (34·3–100)	58·7 (24·7–98·9)	42 (16·9–72·7)	-22·6 (-32·2—1·06)	-28·5 (-38·8—20·4)	75·8 (34·3–100)	58·7 (24·7–98·9)	42 (16·9–72·7)	-22·6 (-32·2—1·06)	-28·5 (-38·8—20·4)
Residential radon	20·9 (3·4–64·3)	20·9 (3·4–64·3)	20·9 (3·4–64·3)	0 (0–0)	0 (0–0)	20·9 (3·4–64·3)	20·9 (3·4–64·3)	20·9 (3·4–64·3)	0 (0–0)	0 (0–0)
Lead exposure	57·1 (29·7–78·6)	33·5 (13·1–55)	23·6 (7·48–42·8)	-41·4 (-58·1—28·1)	-29·6 (-44·3—21·5)	32·5 (3·94–60·6)	19·2 (1·18–43·4)	13·4 (0·458–33·5)	-41 (-74·3—26·1)	-29·8 (-63·4—21·8)
Child underweight	0·966 (0·557–1·49)	0·966 (0·532–1·47)	0·943 (0·521–1·43)	0·0803 (-18–19·3)	-2·39 (-15·3–12·7)	0·813 (0·441–1·24)	0·854 (0·464–1·28)	0·877 (0·473–1·35)	4·98 (-16·4–32·7)	2·71 (-11·2–18·9)
Iron deficiency	--	--	--	--	--	11·8 (9·8–13·9)	12·3 (10·5–14·4)	12·6 (10·5–14·8)	4·57 (-6·79–18·2)	1·85 (-7·49–12·4)
Vitamin A deficiency	0·949 (0·25–2·69)	1·31 (0·346–3·35)	0·901 (0·223–2·44)	38·1 (-64·5–562)	-31·2 (-72–80·1)	1·78 (0·7–3·69)	1·89 (0·741–4·03)	1·59 (0·608–3·5)	5·9 (-60·6–220)	-15·8 (-52·8–61·7)
Zinc deficiency	2·39 (0–7·34)	1·26 (0–4·06)	1·28 (0–4·12)	-47·1 (-100–NA)	1·61 (-16·8–NA)	2·45 (0–7·84)	1·27 (0–4·17)	1·26 (0–4·07)	-48·2 (-100–NA)	-0·615 (-19·2–NA)
Smoking	29 (26·2–31·9)	23 (20·8–25·3)	20·5 (18·5–22·6)	-20·7 (-25·8—14·9)	-11·1 (-14·7—7·67)	19·9 (17·5–22·6)	17·1 (15·4–18·9)	15·4 (13·8–17·2)	-14·2 (-22·9—5·48)	-9·87 (-14—5·81)
Secondhand smoke	40·6 (38·1–43)	28·8 (27·1–30·6)	28·3 (25·5–31·1)	-29 (-35·1—22·6)	-1·88 (-9·98–6·47)	38·2 (36·2–40·2)	27·9 (26·7–29·1)	27·4 (25·3–29·6)	-26·9 (-31·7—21·6)	-1·97 (-8·83–4·84)
Alcohol use	14 (9·04–19·4)	12·9 (7·81–18)	13·4 (8·49–18·7)	-7·98 (-40·2–32)	4·21 (-22·9–40·2)	--	--	--	--	--
Drug use	0·436 (0·243–0·784)	0·913 (0·663–1·25)	1·36 (1·01–1·78)	109 (47·8–204)	49 (28–73·5)	0·401 (0·27–0·597)	0·801 (0·604–1·06)	1·29 (0·999–1·65)	99·7 (62·9–148)	60·9 (38·5–87·2)
High fasting plasma glucose	13·6 (11·3–16·4)	19·1 (16·8–21·9)	20·7 (17·9–23·8)	40·2 (16·4–70·7)	8·46 (-5·75–25·1)	11·1 (9·28–13·4)	15·8 (14–18·1)	17·1 (15–19·7)	42·5 (17·2–70·3)	8·24 (-4·82–23·1)
High systolic blood pressure	25·6 (20·6–31·3)	16·9 (14·8–19)	20·4 (15·8–25·2)	-34·2 (-46·9—18·1)	20·8 (-4·27–49·9)	23·2 (17·3–30)	14 (12·3–15·6)	18·3 (13–24·8)	-39·8 (-54·3—19·2)	31·2 (-3·59–76·3)
High body-mass index	25·2 (17·7–34·9)	39·4 (28·8–51·1)	40·5 (30·1–52·3)	56·3 (37·4–82·5)	2·92 (-3·52–10·2)	32 (23·9–40·3)	45·4 (36·4–54·4)	46 (36·2–55·3)	42·1 (25·8–61·9)	1·26 (-4·87–8·02)
Low bone mineral density	12·1 (6·97–18·8)	9·45 (5·16–15·1)	12 (6·97–18·8)	-21·7 (-42·1–0·321)	26·6 (-1·19–69·3)	15·9 (10·5–22·9)	16·9 (11·2–24·1)	19·3 (13·2–27·1)	5·81 (-16·4–34·9)	14·5 (-7·09–42·5)
Diet low in fruits	57·6 (45·1–71·5)	51·7 (37·4–67)	52·4 (38·3–67·3)	-10·4 (-21·8—1·67)	1·46 (-9·11–12)	--	--	--	--	--
Diet low in vegetables	43·4 (24·1–64·7)	56·5 (39·4–73·5)	53·2 (36·3–70·2)	30·3 (8·21–76·2)	-5·89 (-13·4–1·41)	39·6 (21·3–60·4)	53·8 (35·6–72·5)	51·2 (33·1–70·9)	35·9 (13·8–83·8)	-4·81 (-13–2·62)
Diet low in whole grains	79·7 (69·9–88·3)	81·7 (73–89·8)	81·7 (73–89·8)	2·5 (-1·18–7·07)	0·026 (-2·5–2·74)	77·1 (66·7–87·1)	80 (71·1–88·8)	79·8 (70·6–88·7)	3·77 (-0·44–10·4)	-0·297 (-3·23–2·34)
Diet low in nuts and seeds	29 (8·16–56·9)	25·6 (7·55–56)	21·3 (5·88–54·6)	-11·6 (-42·8–52·7)	-16·7 (-43–8·03)	24·8 (5·85–54·6)	22·6 (7·43–54·2)	19·8 (6·15–53·1)	-9·22 (-44–60·6)	-12·1 (-39·2–13·7)
Diet low in milk	49·5 (26·2–70)	45 (22·4–65)	44 (22–64·1)	-9·16 (-26·3–3·2)	-2·13 (-14·1–9·96)	--	--	--	--	--
Diet high in red meat	76·9 (63·6–87·9)	74·8 (60·5–86·2)	74·7 (61–86·4)	-2·75 (-17·1–13·9)	-0·201 (-12·3–12·3)	77·9 (64·3–88·3)	74 (60·3–85·7)	73·8 (59·2–85·7)	-4·92 (-19·9–10·4)	-0·276 (-12·9–13·4)
Diet high in processed meat	76·9 (52·7–97·3)	85·2 (66·4–99·6)	85 (66–99·6)	10·8 (1·53–33·3)	-0·222 (-5·18–4·04)	77·9 (53·7–97·5)	87·8 (70·5–99·7)	86·5 (67·9–99·7)	12·7 (1·47–37·7)	-1·52 (-6·41–2·22)
Diet high in sugar-sweetened beverages	45·1 (27·2–64)	60·2 (39·9–78·1)	60·7 (40·5–79·2)	33·7 (-8·59–112)	0·782 (-20·6–29·2)	48·4 (28–68·8)	66·1 (46·7–82·8)	64·9 (46·3–83·5)	36·4 (-4·17–114)	-1·8 (-22·5–21·7)
Diet low in fiber	44·7 (28·4–60·3)	31·5 (18·4–46·3)	28·8 (16·5–43·9)	-29·4 (-46·8—12·3)	-8·58 (-26·4–9·27)	45 (29·1–61·1)	36·9 (22·8–51·3)	32·1 (17·9–47·5)	-18·1 (-33·4—1·06)	-12·9 (-28·3–1·37)
Diet low in seafood omega-3 fatty acids	84·4 (67·4–100)	80·9 (62·9–99·5)	80·9 (63–99·6)	-4·13 (-18·5–10·7)	-0·062 (-13·1–18·2)	86·7 (70·5–100)	84·4 (68·4–99·9)	84·3 (68·6–99·6)	-2·69 (-15·9–8·3)	-0·152 (-10·8–12·2)
Diet low in polyunsaturated fatty acids	14·4 (4·05–42·7)	7·6 (2·21–27)	7·27 (2·1–26·3)	-47·4 (-74·4—6·63)	-4·37 (-28·4–20·4)	--	--	--	--	--
Diet high in trans fatty acids	89·4 (81·4–100)	88 (79·1–100)	87·4 (78·5–100)	-1·65 (-9·4–6·62)	-0·689 (-8·75–7·7)	--	--	--	--	--
Diet high in sodium	18·1 (4·53–41)	39·3 (10·9–64)	38·7 (10·3–64·5)	118 (40·6–419)	-1·56 (-29·2–27·7)	--	--	--	--	--
Low physical activity	2 (0·625–5·22)	1·8 (0·622–4·99)	2·17 (0·8–4·64)	-9·71 (-40–31·4)	20·2 (-22·7–134)	--	--	--	--	--
Occupational asthmagens	27·4 (22·2–33·8)	23·1 (19·5–27·4)	24·2 (20·1–29·9)	-15·7 (-27·9—2·42)	4·92 (-4·31–18·3)	11·8 (9·67–14·3)	11·8 (9·77–14·1)	11·9 (9·65–14·4)	-0·509 (-15·4–15·6)	0·835 (-9·91–13)
Occupational particulate matter, gases, and fumes	11 (7·78–15·4)	10·3 (7·34–14·6)	9·87 (7·05–13·9)	-6·54 (-12—0·933)	-4·13 (-7·6—0·753)	5·82 (4·12–8·62)	5·71 (4·14–8·13)	5·51 (3·97–7·96)	-1·91 (-10·7–8·13)	-3·56 (-8·52–2·34)
Occupational noise	9·18 (8·39–10·1)	8·83 (8·22–9·62)	8·54 (8–9·26)	-3·84 (-7·57—0·279)	-3·21 (-5·23—1)	6·15 (5·4–7)	6·5 (5·96–7·17)	6·51 (6·07–7·09)	5·7 (-0·666–13·1)	0·125 (-2·78–3·45)
Occupational injuries	--	--	--	--	--	--	--	--	--	--
Occupational ergonomic factors	12·6 (10·4–15·2)	10·9 (9·2–13)	11·2 (9·37–13·5)	-13·4 (-24·2—1·64)	3·14 (-5·15–11·9)	7·28 (5·74–9·22)	7·24 (5·9–8·99)	7·56 (6·02–9·53)	-0·588 (-16·1–17)	4·41 (-7·73–17·8)
Childhood sexual abuse	7·4 (5·05–10·5)	6·97 (4·6–9·93)	6·85 (4·54–9·67)	-5·83 (-12·2–5·83)	-1·68 (-8·51–5·53)	12·3 (8·94–17·3)	17·4 (12·9–24)	17·8 (13·1–24·7)	41·9 (30·7–54·8)	2·17 (-5–11)
Intimate partner violence	--	--	--	--	--	33·9 (19·6–44)	32·1 (17·8–41·8)	33 (18·3–42·6)	-5·36 (-14–5·65)	2·79 (-5·81–15·5)
Non-exclusive breastfeeding	26·2 (21·6–31)	24·9 (21·3–28·9)	24·9 (20·4–29·8)	-5·05 (-20–11)	0·023 (-13·8–13)	26·2 (21·6–31)	24·9 (21·3–28·9)	24·9 (20·4–29·8)	-5·04 (-20–11·1)	0·0212 (-13·8–13)
Discontinued breastfeeding	27·4 (26·4–28·3)	25·3 (23·8–26·5)	25·6 (24·3–26·8)	-7·71 (-13—2·26)	1·48 (-3·89–7·38)	27·4 (26·4–28·3)	25·3 (23·9–26·5)	25·7 (24·3–26·8)	-7·57 (-12·9—2·11)	1·35 (-4·01–7·24)
Diet low in calcium	17 (8·33–31·5)	14·4 (7·04–26·8)	13·8 (6·46–25·6)	-15·2 (-25·9—3·82)	-4·33 (-13·7–3·76)	12·4 (5·29–23·6)	11·5 (5·14–21·3)	11·2 (4·7–21·1)	-7·46 (-22·2–8·93)	-2·4 (-12·3–6·83)
Occupational exposure to asbestos	8·67 (7·75–9·72)	7·93 (7·21–8·6)	7·57 (5·41–9·87)	-8·45 (-20·3–4·79)	-4·61 (-31·3–26·1)	1·51 (1·15–2·14)	1·53 (1·13–2·09)	1·51 (1·01–2·21)	0·959 (-12·8–18·2)	-1·15 (-29·5–30·8)
Occupational exposure to arsenic	0·656 (0–1·56)	0·62 (0–1·47)	0·647 (0–1·53)	-5·61 (-18·1–NA)	4·36 (-5·77–NA)	0·541 (0–1·28)	0·541 (0–1·28)	0·547 (0–1·34)	-0·00613 (-17·4–NA)	1·13 (-12·3–NA)
Occupational exposure to benzene	0·825 (0·0466–2·38)	0·804 (0·048–2·31)	0·832 (0·0508–2·37)	-2·51 (-10·3–8·58)	3·42 (-3·16–14·5)	1·16 (0·0693–3·25)	1·3 (0·0755–3·64)	1·36 (0·0793–3·83)	12·4 (-0·122–32·1)	4·94 (-3·73–16·7)
Occupational exposure to beryllium	0·0249 (0·0235–0·0264)	0·0243 (0·0237–0·0248)	0·0255 (0·024–0·0271)	-2·58 (-8·41–3·55)	5·04 (-0·677–11·5)	0·0269 (0·0247–0·0291)	0·0282 (0·0275–0·0289)	0·0292 (0·0272–0·0313)	4·83 (-3·8–14·8)	3·48 (-3·18–10·8)
Occupational exposure to cadmium	0·0747 (0·0676–0·0818)	0·0711 (0·0646–0·0776)	0·0742 (0·0662–0·0833)	-4·82 (-16·4–8·78)	4·42 (-4·93–15·3)	0·0636 (0·0554–0·0719)	0·0642 (0·0577–0·0719)	0·0651 (0·0568–0·0757)	1·07 (-15–21·8)	1·42 (-10·7–14·5)
Occupational exposure to chromium	0·174 (0·159–0·189)	0·169 (0·157–0·182)	0·177 (0·16–0·196)	-2·79 (-13·1–9·15)	4·43 (-3·87–13·7)	0·156 (0·138–0·174)	0·161 (0·149–0·177)	0·164 (0·147–0·186)	3·43 (-10·9–21·2)	1·62 (-8·64–12·8)
Occupational exposure to diesel engine exhaust	0·688 (0·634–0·745)	0·744 (0·695–0·794)	0·784 (0·72–0·853)	8·22 (-1·55–20·1)	5·3 (-2·81–13·8)	0·671 (0·597–0·752)	0·721 (0·657–0·793)	0·728 (0·646–0·82)	7·51 (-6·81–24)	0·89 (-9·13–11·3)
Occupational exposure to formaldehyde	0·379 (0·342–0·416)	0·351 (0·318–0·384)	0·362 (0·321–0·408)	-7·41 (-19·1–6·36)	3·17 (-6·22–14·4)	0·309 (0·271–0·349)	0·314 (0·283–0·349)	0·319 (0·28–0·366)	1·74 (-13·9–21·6)	1·62 (-10·2–14·2)
Occupational exposure to nickel	0·553 (0–2·44)	0·507 (0–2·28)	0·527 (0–2·31)	-8·32 (-20·2–NA)	4·07 (-5·68–NA)	0·431 (0–1·97)	0·419 (0–1·87)	0·424 (0–1·9)	-2·76 (-20·8–NA)	1·33 (-13·3–NA)
Occupational exposure to polycyclic aromatic hydrocarbons	0·342 (0·313–0·371)	0·332 (0·309–0·355)	0·346 (0·314–0·382)	-2·77 (-12·8–8·64)	4·3 (-3·85–13·4)	0·327 (0·292–0·364)	0·348 (0·325–0·374)	0·355 (0·321–0·396)	6·42 (-5·75–22·4)	2·07 (-7·12–12·4)
Occupational exposure to silica	4·9 (0·722–15·5)	4·4 (0·644–14)	4·62 (0·717–14·6)	-10·1 (-21·5–3·72)	5·04 (-4·58–18·3)	3·62 (0·502–11·2)	3·41 (0·482–11)	3·43 (0·485–10·9)	-5·9 (-23·9–21·5)	0·506 (-13·7–19·6)
Occupational exposure to sulfuric acid	1·11 (0·24–3·51)	1·15 (0·233–3·63)	1·18 (0·242–3·71)	3·28 (-8·94–17·5)	3·38 (-6·16–13·5)	1 (0·215–3·23)	1·17 (0·243–3·75)	1·16 (0·247–3·72)	17·1 (-3·96–41·7)	-0·916 (-14·9–15·2)
Unsafe sex	--	--	--	--	--	--	--	--	--	--
Occupational exposure to trichloroethylene	0·095 (0·089–0·102)	0·0957 (0·0917–0·1)	0·1 (0·0937–0·108)	0·79 (-7·24–9·77)	4·68 (-1·97–12·6)	--	--	--	--	--
No access to handwashing facility	3·38 (3·13–3·64)	3·14 (2·92–3·38)	3·1 (2·88–3·36)	-7·25 (-15·7–1·38)	-1·18 (-6·96–4·97)	3·38 (3·13–3·64)	3·14 (2·92–3·38)	3·1 (2·88–3·36)	-7·25 (-15·7–1·38)	-1·18 (-6·96–4·97)
Child wasting	0·75 (0·493–1·02)	0·728 (0·482–0·987)	0·727 (0·47–0·992)	-2·96 (-12·2–7·56)	-0·106 (-6·99–8·3)	0·774 (0·516–1·05)	0·764 (0·513–1·05)	0·761 (0·498–1·04)	-1·29 (-10·7–10·1)	-0·438 (-7·92–7·21)
Child stunting	2·42 (1·3–3·59)	2·09 (1·12–3·14)	2·04 (1·04–3·05)	-13·8 (-32·2–9·32)	-2·19 (-17–13·6)	1·95 (0·984–2·89)	1·8 (0·906–2·73)	1·79 (0·885–2·73)	-7·76 (-27·4–18·4)	-0·774 (-15·7–18·2)
Chewing tobacco	5·24 (4·24–6·43)	4·3 (3·18–5·66)	4·02 (2·9–5·4)	-18 (-42·4–12·8)	-6·49 (-25·9–18·3)	0·286 (0·183–0·431)	0·286 (0·18–0·434)	0·292 (0·185–0·443)	-0·00967 (-41·8–69·6)	2·12 (-29·3–43)
Diet low in legumes	74·3 (41·2–99·3)	77·5 (46·5–99·7)	77·5 (46·7–99·8)	4·32 (-0·0251–22·2)	-0·0421 (-5·18–5·3)	72·7 (36–99·2)	75·9 (42·7–99·6)	76·3 (44·8–99·6)	4·28 (-0·0803–21·7)	0·52 (-4·5–8·83)
Short gestation	26·6 (21·9–32·6)	28·7 (24·2–33·5)	27·5 (22·5–33·6)	7·6 (-15·4–32·9)	-4·13 (-22·2–18·5)	22·9 (19·2–27·5)	25·4 (21·2–30)	23·9 (19·9–28·3)	10·8 (-11·9–37·3)	-5·89 (-22·8–13·7)
Low birth weight	10·7 (8·83–12·9)	11·7 (9·91–13·8)	11·8 (9·6–14·2)	9·72 (-14·5–37·5)	0·5 (-20·4–24·4)	10·4 (8·7–12·3)	12·6 (10·9–14·8)	12·2 (10·3–14·2)	22 (-2·43–55·4)	-3·9 (-22·7–17·2)
High temperature	25·9 (17·3–36·6)	26·7 (18·1–37·2)	25·7 (17·1–36·2)	3·2 (-1·62–7·85)	-3·71 (-7·47—0·933)	25·9 (17·3–36·6)	26·7 (18·1–37·2)	25·7 (17·1–36·2)	3·2 (-1·62–7·85)	-3·71 (-7·47—0·933)
Low temperature	34·3 (30·9–38·8)	36·2 (32·7–40·3)	36·9 (33·5–41·2)	5·35 (2·44–8·43)	2 (0·196–3·93)	34·3 (30·9–38·8)	36·2 (32·7–40·3)	36·9 (33·5–41·2)	5·35 (2·44–8·43)	2 (0·196–3·93)
Kidney dysfunction	20·2 (14·5–27·3)	20·9 (14·9–28·1)	21 (15·2–28·2)	3·35 (-0·23–7·38)	0·758 (-2·91–4·28)	22·6 (16·2–30·1)	23·7 (16·9–31·6)	24·5 (17·6–32·6)	4·78 (0·687–9·96)	3·58 (-0·483–8·57)

Bullying victimization	3·51 (1·33–7·56)	5·12 (1·97–10·5)	5·14 (1·93–10·9)	45·8 (22·1–76·8)	0·275 (-10·3–7·46)	--	--	--	--	--
High LDL cholesterol	67·5 (63–72·1)	34·7 (31·4–38·1)	31·1 (27·6–34·9)	-48·6 (-54·1–-43·1)	-10·4 (-15·5–-5·56)	66·1 (62·6–70·1)	34·6 (31·5–37·8)	31·9 (28·4–35·5)	-47·6 (-52·1–-43)	-7·77 (-12–-3·52)

Supplementary Results Table 1. Iowa age-standardised Summary Exposure Values (SEVs) for most-detailed risk factors and percentage change, by sex, 1990, 2010, and 2019. Data in parentheses are 95% uncertainty intervals.										
	Male					Female				
	1990	2010	2019	Percentage change 1990-2010	Percentage change 2010-2019	1990	2010	2019	Percentage change 1990-2010	Percentage change 2010-2019
Unsafe sanitation	5·86 (2·74–10·7)	3·41 (1·53–6·36)	2·64 (1·13–4·99)	-41·7 (-75·7–38·2)	-22·6 (-60·5–56·6)	--	--	--	--	--
Ambient particulate matter pollution	10·3 (2·8–22·1)	6·77 (4–9·84)	4·99 (2·4–7·86)	-34·5 (-69·1–87·2)	-26·3 (-40·9—19·2)	10·3 (2·81–22)	6·77 (4–9·83)	4·99 (2·4–7·86)	-34·5 (-69·1–85·9)	-26·3 (-41·1—19·4)
Household air pollution from solid fuels	0·0135 (0·00276–0·04)	0·00768 (0·00136–0·0249)	0·0051 (0·000751–0·0173)	-43 (-79–37·8)	-33·6 (-66·8–21·8)	0·0178 (0·00389–0·0492)	0·0102 (0·00192–0·0314)	0·00677 (0·00105–0·0212)	-42·8 (-79–38·9)	-33·5 (-66·8–21·9)
Ambient ozone pollution	30·9 (12·1–54·8)	49·3 (20·4–85·5)	43·2 (17·8–75·1)	59·8 (35·7–96·2)	-12·3 (-25·6–0·441)	30·9 (12·1–54·8)	49·3 (20·4–85·5)	43·2 (17·8–75·1)	59·8 (35·7–96·2)	-12·3 (-25·6–0·441)
Residential radon	22·7 (3·45–62·8)	22·7 (3·45–62·8)	22·7 (3·45–62·8)	0 (0–0)	0 (0–0)	22·7 (3·45–62·8)	22·7 (3·45–62·8)	22·7 (3·45–62·8)	0 (0–0)	0 (0–0)
Lead exposure	33·6 (5·22–60·9)	20·3 (2·78–43)	14·3 (1·3–34·4)	-39·7 (-63·2—26·2)	-29·4 (-55·7—19·8)	18·6 (0·143–46·1)	12·1 (0·051–35·1)	8·89 (0·0167–28·2)	-35 (-82·9—16·9)	-26·6 (-80·1—18·3)
Child underweight	0·937 (0·528–1·4)	0·95 (0·514–1·44)	0·924 (0·498–1·37)	1·4 (-16·6–22·3)	-2·68 (-15·2–11·6)	0·789 (0·407–1·21)	0·839 (0·441–1·28)	0·858 (0·469–1·31)	6·28 (-14·1–36·3)	2·35 (-11·9–19·1)
Iron deficiency	--	--	--	--	--	11·3 (9·46–13·3)	11·9 (9·98–13·9)	12·1 (10–14·3)	5·02 (-7·76–18·7)	1·78 (-7·25–12·6)
Vitamin A deficiency	0·8 (0·209–2·26)	1·16 (0·288–3·08)	0·775 (0·197–2·08)	44·5 (-68·4–555)	-32·9 (-70·1–71·2)	1·56 (0·602–3·22)	1·68 (0·65–3·59)	1·4 (0·541–3·14)	7·69 (-60·2–222)	-16·8 (-54·5–64·3)
Zinc deficiency	2·39 (0–7·72)	1·21 (0–3·86)	1·22 (0–3·82)	-49·3 (-100–NA)	1·05 (-19·5–NA)	2·46 (0–7·79)	1·21 (0–3·93)	1·19 (0–3·94)	-50·5 (-100–NA)	-1·83 (-23·3–NA)
Smoking	26·1 (23·5–28·8)	20·2 (18·3–22·2)	17·4 (15·6–19·2)	-22·5 (-27·9—16·5)	-13·9 (-17·1—10·5)	19·4 (16·8–22·3)	15·2 (13·5–16·9)	13·1 (11·7–14·6)	-22 (-30·6—12·1)	-13·3 (-17·4—8·98)
Secondhand smoke	38·8 (36·3–41·3)	25·3 (23·6–26·9)	25·3 (22·5–28·1)	-34·9 (-40·6—28·7)	0·122 (-8·59–9·65)	35·6 (33·6–37·5)	24·1 (22·9–25·3)	24·1 (22–26·1)	-32·3 (-37·6—26·7)	0·0272 (-7·39–7·16)
Alcohol use	15·4 (9·95–21·4)	14·1 (8·95–19·9)	14·9 (9·4–20·6)	-8·39 (-39–37·9)	5·4 (-20·5–39·9)	--	--	--	--	--
Drug use	0·382 (0·194–0·699)	0·574 (0·378–0·869)	0·901 (0·644–1·26)	50·4 (12·6–117)	56·9 (32·1–89·9)	0·306 (0·185–0·509)	0·471 (0·327–0·673)	0·705 (0·521–0·935)	53·8 (23·9–93·7)	49·9 (27·3–78·5)
High fasting plasma glucose	11·7 (9·69–14·2)	16·7 (14·3–19·6)	18·3 (15·5–21·2)	42·4 (17·2–72·2)	9·37 (-4·14–27·2)	8·64 (7·32–10·3)	12 (10·5–14·1)	13·8 (11·7–16·3)	39·3 (17·3–67·5)	14·8 (-1·82–33·8)
High systolic blood pressure	25·6 (20·5–31·5)	16·1 (14–18·2)	19·9 (15·6–24·4)	-37·4 (-50·1—22·2)	23·7 (-2·16–54·6)	24·3 (17·9–31·4)	13·5 (11·9–15·1)	18·5 (13·6–24·4)	-44·5 (-57·3—24·6)	37·4 (1·64–86·2)
High body-mass index	27·5 (19·1–38)	41·2 (30·4–52·9)	43·2 (32·3–54·5)	50·1 (32–72·7)	4·67 (-1·68–11·8)	32·5 (23·6–41·5)	44·1 (34·4–53·5)	45·9 (35·1–55·8)	35·4 (21–54·6)	4·18 (-2·29–11·5)
Low bone mineral density	11·8 (6·74–18·7)	9·15 (4·85–15·3)	11·5 (6·61–18·4)	-22·2 (-42–0·0674)	25·8 (-5·9–67·2)	15·6 (10·2–22·6)	16·4 (10·7–23·6)	18·7 (12·5–26·1)	5·08 (-15·6–31·8)	14·2 (-8·49–46·1)
Diet low in fruits	57·5 (45·6–70·1)	47·6 (36–59·4)	47·1 (34·7–60·2)	-17·2 (-28·7—7·92)	-1·15 (-13·2–11·4)	57·8 (46·3–70·8)	49·3 (37·8–62·5)	48·3 (36·1–62·1)	-14·8 (-26·3—5·68)	-1·88 (-13·2–8·53)
Diet low in vegetables	38·1 (20·8–59·4)	42·4 (26·2–61·5)	39·5 (24·3–58·5)	11·2 (-4·77–44·3)	-6·75 (-18·1–4·24)	34·8 (18·5–56)	47·3 (31·6–64·6)	45 (29·1–62·1)	35·7 (6·96–99·1)	-4·81 (-14·1–3·49)
Diet low in whole grains	79·6 (69·9–88·4)	81·6 (72·8–89·7)	81·5 (72·2–89·4)	2·43 (-1·18–7·32)	-0·0529 (-2·81–2·45)	77 (66·7–86·7)	79·7 (70·1–88·4)	79·4 (69·6–88·5)	3·55 (-0·608–9·17)	-0·374 (-3·4–2·31)
Diet low in nuts and seeds	28·4 (7·6–56·4)	25·5 (7·73–55·4)	21·1 (6·18–54)	-10·1 (-45·5–72·2)	-17·3 (-43·7–11·3)	24·3 (6·25–55)	22·2 (7·16–53·7)	19·5 (6·07–52·8)	-8·83 (-43·1–70·2)	-12·1 (-39·9–14·1)
Diet low in milk	49·2 (25·2–69·7)	43·7 (20·4–63·1)	42·5 (20·4–63·1)	-11·3 (-29·3–1·68)	-2·78 (-15·4–9·68)	--	--	--	--	--
Diet high in red meat	92·6 (84·9–97·7)	91·1 (82–97·1)	89·8 (79·1–97·1)	-1·61 (-11·3–7·16)	-1·37 (-8·68–5·55)	90·1 (81·7–96·1)	86·5 (75·5–94·4)	85·4 (73·1–94·3)	-3·97 (-15·3–6·48)	-1·29 (-10·7–8·39)
Diet high in processed meat	79·2 (55·5–98·9)	86·2 (66·8–99·8)	86 (66·5–99·8)	8·83 (0·542–26·5)	-0·281 (-4·72–4·14)	79·7 (55·8–98·8)	87·8 (70·8–99·9)	86·4 (68·8–99·7)	10·2 (0·816–31·5)	-1·62 (-7·1–1·9)
Diet high in sugar-sweetened beverages	45·3 (28–64·6)	62·1 (43·2–78·2)	62·6 (43·2–79·8)	37·3 (-1·57–119)	0·75 (-19·3–23·6)	49·4 (30·5–69)	67·7 (49–84·4)	66·6 (46·2–84·1)	37·1 (-2·69–117)	-1·59 (-21·2–19·4)
Diet low in fiber	44·2 (27·7–59·5)	30·6 (18·1–44·6)	27·8 (15·4–41·3)	-30·9 (-47·8—13·7)	-8·98 (-27·1–9·25)	44·6 (28·1–60·3)	35·9 (21·8–49·4)	30·8 (16·9–45·6)	-19·3 (-35·9—3·42)	-14·4 (-32·2–0·0404)
Diet low in seafood omega-3 fatty acids	81·7 (64·1–99·9)	76·7 (57·6–99·3)	76·1 (57·6–98·9)	-6·18 (-21·5–9·14)	-0·78 (-16·3–18)	84·6 (67·9–99·7)	81·6 (64·9–99·6)	80·7 (63·8–99·2)	-3·59 (-16·4–8·96)	-1·06 (-13·8–12·7)
Diet low in polyunsaturated fatty acids	14·4 (4·19–41·7)	7·39 (2·08–26·6)	7·03 (1·96–26·5)	-48·6 (-75·2—8·19)	-4·85 (-28·9–18)	--	--	--	--	--
Diet high in trans fatty acids	89·5 (81·4–100)	87·9 (78·6–100)	87·4 (77·5–100)	-1·77 (-9·82–5·83)	-0·512 (-8·73–8·24)	--	--	--	--	--
Diet high in sodium	18·2 (4·62–40·8)	39·7 (11·3–63·8)	38·7 (10·6–63·4)	117 (38·2–434)	-2·49 (-27·2–28·8)	56·6 (0–100)	58·1 (0–100)	58·4 (0–100)	2·62 (-17–NA)	0·482 (-13·5–NA)
Low physical activity	2·27 (0·717–5·42)	2·21 (0·791–5·32)	2·59 (0·978–5·66)	-2·65 (-31·9–48·7)	17 (-24–111)	--	--	--	--	--
Occupational asthmagens	24·4 (20·1–30)	24·1 (20·4–28·6)	25·4 (20·8–31·2)	-1·28 (-15·1–14·6)	5·44 (-6·38–20·1)	12·3 (10·1–14·8)	13·3 (11–16)	13·2 (10·8–16·1)	7·59 (-7·69–24·9)	-0·575 (-11·6–13·8)
Occupational particulate matter, gases, and fumes	10·8 (7·69–15·1)	10·5 (7·61–14·7)	10·2 (7·42–14·5)	-2·96 (-8·18–2·77)	-2·45 (-5·67–0·859)	5·8 (4·1–8·49)	5·9 (4·29–8·56)	5·78 (4·2–8·33)	1·63 (-7·25–12·4)	-2·02 (-7·36–3·46)
Occupational noise	9·43 (8·62–10·3)	9·3 (8·68–10)	9·12 (8·6–9·86)	-1·45 (-5–2·61)	-1·91 (-3·89–0·0812)	6·55 (5·8–7·38)	7·12 (6·6–7·71)	7·22 (6·79–7·78)	8·67 (2·71–16·3)	1·42 (-1·28–4·45)
Occupational injuries	--	--	--	--	--	--	--	--	--	--
Occupational ergonomic factors	14·1 (11·7–17·2)	12·7 (10·8–15·1)	13·1 (10·9–15·9)	-9·58 (-21–4·2)	2·89 (-6·15–13·5)	8·53 (6·64–11)	8·77 (7·08–11)	9·41 (7·29–12·2)	2·74 (-15·1–26)	7·31 (-8·27–26·1)
Childhood sexual abuse	5·96 (3·55–9·11)	5·27 (3·66–7·04)	5·26 (3·74–6·98)	-11·7 (-29·4–12·6)	-0·131 (-6·98–7·07)	16·9 (12·5–23·6)	15 (11·8–19·9)	15·1 (11·8–20)	-11·5 (-21·7—1·79)	1·05 (-5·91–7·54)
Intimate partner violence	--	--	--	--	--	17 (9·03–25·9)	18 (10·1–26·6)	18·8 (9·9–28·2)	6·01 (-6·57–23·9)	4·18 (-11·1–30·5)
Non-exclusive breastfeeding	26·2 (21·6–31·2)	25 (21·5–29)	24·9 (20·5–29·6)	-4·77 (-18·9–12·4)	-0·314 (-13·4–14·4)	26·2 (21·6–31·2)	25 (21·5–29)	24·9 (20·5–29·6)	-4·76 (-18·9–12·5)	-0·328 (-13·4–14·4)
Discontinued breastfeeding	26·9 (25·9–27·8)	25·3 (23·9–26·5)	25·5 (24·2–26·7)	-6·25 (-11·6—0·676)	1 (-5·01–7·19)	27 (25·9–27·9)	25·3 (23·9–26·6)	25·5 (24·2–26·7)	-6·26 (-11·6—0·683)	0·92 (-5·09–7·1)
Diet low in calcium	16·7 (8·01–31)	13·9 (6·94–25·5)	13·2 (6·41–24·6)	-16·8 (-27·8—4·72)	-5·2 (-14·9–2·59)	12·1 (4·98–23·1)	10·8 (4·49–20·7)	10·5 (4·39–19·8)	-10·7 (-24·8–7·01)	-3·33 (-13·1–6)
Occupational exposure to asbestos	6·7 (5·91–7·5)	6·19 (5·62–6·81)	5·82 (4·2–7·8)	-7·66 (-20·2–6·42)	-5·96 (-32·1–25·3)	1·42 (0·969–1·76)	1·49 (0·954–1·78)	1·48 (0·87–2·06)	4·81 (-9·71–23·8)	-0·947 (-29·2–31·9)
Occupational exposure to arsenic	0·571 (0–1·38)	0·612 (0–1·45)	0·635 (0–1·49)	7·15 (-8·12–NA)	3·85 (-6·22–NA)	0·522 (0–1·22)	0·574 (0–1·38)	0·575 (0–1·4)	10·1 (-7·71–NA)	0·122 (-11·9–NA)
Occupational exposure to benzene	0·848 (0·0492–2·52)	0·895 (0·0532–2·64)	0·918 (0·0567–2·65)	5·54 (-2·96–18·7)	2·49 (-4·38–11·7)	1·3 (0·074–3·72)	1·54 (0·0919–4·34)	1·59 (0·0957–4·37)	18·1 (6·56–39)	3·23 (-4·88–14)
Occupational exposure to beryllium	0·0255 (0·0241–0·0269)	0·0265 (0·0259–0·0271)	0·0275 (0·0262–0·0289)	4·08 (-1·43–10·6)	3·89 (-0·928–9·26)	0·0292 (0·0269–0·0315)	0·0323 (0·0316–0·0331)	0·0329 (0·031–0·0348)	10·7 (2·35–20·5)	1·92 (-3·54–7·96)
Occupational exposure to cadmium	0·0664 (0·0598–0·073)	0·0713 (0·0649–0·078)	0·0741 (0·0661–0·0824)	7·33 (-6·41–23·3)	3·89 (-5·15–13·9)	0·0625 (0·0555–0·0702)	0·069 (0·0617–0·0775)	0·0692 (0·0603–0·0799)	10·4 (-5·14–30·2)	0·238 (-10–12·5)
Occupational exposure to chromium	0·157 (0·143–0·171)	0·172 (0·16–0·186)	0·179 (0·163–0·196)	9·47 (-2·62–23·4)	3·85 (-4·34–12·5)	0·156 (0·141–0·173)	0·176 (0·162–0·192)	0·177 (0·157–0·198)	12·6 (-0·478–29·1)	0·332 (-8·1–10·3)
Occupational exposure to diesel engine exhaust	0·744 (0·685–0·803)	0·847 (0·793–0·905)	0·88 (0·805–0·952)	13·8 (3·67–24·9)	3·95 (-3·15–11·3)	0·741 (0·662–0·827)	0·834 (0·763–0·911)	0·826 (0·739–0·929)	12·6 (-1·82–29·5)	-1·03 (-10·5–9·74)
Occupational exposure to formaldehyde	0·33 (0·296–0·364)	0·349 (0·314–0·383)	0·358 (0·317–0·402)	5·67 (-8·57–22·7)	2·75 (-6·9–13)	0·305 (0·272–0·342)	0·34 (0·307–0·377)	0·342 (0·3–0·389)	11·5 (-3·51–30·8)	0·45 (-9·44–12)
Occupational exposure to nickel	0·474 (0–2·09)	0·495 (0–2·13)	0·514 (0–2·25)	4·32 (-11·3–NA)	3·99 (-6·47–NA)	0·409 (0–1·79)	0·438 (0–1·93)	0·439 (0–1·96)	7·11 (-11·6–NA)	0·3 (-13·3–NA)
Occupational exposure to polycyclic aromatic hydrocarbons	0·309 (0·282–0·336)	0·338 (0·314–0·363)	0·35 (0·32–0·382)	9·36 (-2·1–22·9)	3·69 (-4·28–11·9)	0·332 (0·301–0·365)	0·383 (0·359–0·41)	0·386 (0·351–0·426)	15·4 (3·45–30·6)	0·767 (-6·79–9·71)
Occupational exposure to silica	4·28 (0·588–13·4)	4·32 (0·646–14)	4·52 (0·632–13·9)	0·887 (-12·8–19·3)	4·74 (-5·44–16·6)	3·47 (0·489–10·7)	3·55 (0·487–11·5)	3·53 (0·492–11·6)	2·56 (-15–28·7)	-0·629 (-14·3–19·1)
Occupational exposure to sulfuric acid	1·02 (0·224–3·19)	1·16 (0·241–3·7)	1·19 (0·247–3·75)	14 (-0·0674–30·8)	2·28 (-6·8–12·9)	1 (0·209–3·31)	1·27 (0·262–3·94)	1·24 (0·257–3·91)	26·6 (2·94–53·9)	-2·07 (-15·5–12·8)
Unsafe sex	--	--	--	--	--	--	--	--	--	--
Occupational exposure to trichloroethylene	0·0914 (0·0853–0·0979)	0·101 (0·0972–0·106)	0·105 (0·0991–0·113)	11 (2·66–20·9)	3·84 (-2·26–10·6)	--	--	--	--	--
No access to handwashing facility	3·32 (3·06–3·59)	3·11 (2·86–3·36)	3·07 (2·84–3·32)	-6·39 (-15·1–2·51)	-1·25 (-6·69–4·75)	3·32 (3·06–3·59)	3·11 (2·86–3·36)	3·07 (2·84–3·32)	-6·39 (-15·1–2·51)	-1·25 (-6·69–4·75)
Child wasting	0·747 (0·497–1·02)	0·728 (0·482–0·994)	0·725 (0·467–0·987)	-2·52 (-12·1–7·41)	-0·441 (-8·35–7·18)	0·771 (0·518–1·05)	0·761 (0·511–1·04)	0·757 (0·506–1·04)	-1·23 (-11·8–9·22)	-0·586 (-8·14–8·2)
Child stunting	2·31 (1·19–3·31)	2·04 (1·07–3·05)	1·98 (1·02–3·02)	-11·5 (-32–13·5)	-2·81 (-17·6–14·9)	1·89 (0·916–2·9)	1·76 (0·867–2·69)	1·74 (0·858–2·66)	-7·06 (-28·6–19·3)	-1·38 (-16·6–17·2)
Chewing tobacco	5·37 (4·25–6·79)	5·61 (4·11–7·47)	5·46 (3·82–7·43)	4·39 (-26·5–49·1)	-2·66 (-24·8–28·5)	0·263 (0·165–0·404)	0·257 (0·161–0·4)	0·262 (0·162–0·41)	-2·17 (-43·5–68·1)	1·62 (-29·2–44·3)
Diet low in legumes	74·2 (37·7–99·2)	77·2 (45·6–99·7)	77·1 (46·1–99·6)	4·04 (-0·357–22·5)	-0·0846 (-5·24–4·93)	72·6 (36·2–98·7)	75·5 (42·2–99·6)	75·7 (42–99·6)	3·94 (-0·509–20·4)	0·264 (-4·61–7·16)
Short gestation	19·5 (16–23·9)	20·7 (17–24·9)	20·7 (16·6–25·5)	5·72 (-17·1–34·2)	0·0976 (-21·1–27·4)	16·3 (13·5–20)	16·8 (13·9–19·9)	18·1 (15–21·7)	3·03 (-21·2–30·9)	7·72 (-12·3–32·8)
Low birth weight	7·72 (6·31–9·48)	8·37 (6·86–9·88)	8·48 (6·89–10·4)	8·53 (-16·4–40·8)	1·3 (-20·7–31·3)	7·55 (6·21–9·19)	8·18 (6·94–9·72)	8·92 (7·53–10·6)	8·44 (-17·1–40)	8·99 (-12·4–36·2)
High temperature	28·8 (18·2–41·1)	26 (15·2–38·7)	26·7 (15·7–39·5)	-9·62 (-18·5—3·85)	2·84 (-0·821–6·78)	28·8 (18·2–41·1)	26 (15·2–38·7)	26·7 (15·7–39·5)	-9·62 (-18·5—3·85)	2·84 (-0·821–6·78)
Low temperature	35·8 (32·2–39·6)	35·4 (31·5–39·9)	37·3 (33·5–41·6)	-1·12 (-4·26–2·26)	5·61 (4·03–7·41)	35·8 (32·2–39·6)	35·4 (31·5–39·9)	37·3 (33·5–41·6)	-1·12 (-4·26–2·26)	5·61 (4·03–7·41)
Kidney dysfunction	18·7 (13·2–25·7)	19·3 (13·6–26·4)	19·5 (14–26·4)	3·47 (0·0109–7·78)	0·772 (-2·79–4·35)	20·9 (14·7–28·4)	21·9 (15·3–29·6)	22·8 (16·1–30·5)	4·52 (0·605–9·66)	4·15 (0·803–8·7)
Bullying victimization	5·87 (2·45–12)	8·92 (3·57–17·8)	8·69 (3·35–17·9)	51·9 (31·8–75·7)	-2·54 (-12–3·7)	4·25 (1·74–8·89)	7·81 (3·18–15·5)	7·75 (2·93–15·9)	83·8 (59·2–112)	-0·783 (-11·5–6·19)
High LDL cholesterol	67 (62·5–71·4)	34·2 (30·7–37·7)	30·6 (27·1–34·2)	-48·9 (-54·2—43·6)	-10·5 (-15·4—5·41)	65·2 (61·6–68·9)	34·6 (31·2–37·9)	32 (28·6–35·5)	-47 (-52·1—42·2)	-7·29 (-11·2—3·34)

Supplementary Results Table 1. Kansas age-standardised Summary Exposure Values (SEVs) for most-detailed risk factors and percentage change, by sex, 1990, 2010, and 2019. Data in parentheses are 95% uncertainty intervals.

	Male					Female				
	1990	2010	2019	Percentage change 1990-2010	Percentage change 2010-2019	1990	2010	2019	Percentage change 1990-2010	Percentage change 2010-2019
Unsafe sanitation	6·4 (2·99–11·6)	3·82 (1·68–7·23)	2·84 (1·25–5·19)	-40·3 (-76–41·9)	-25·9 (-61·8–47·4)	--	--	--	--	--
Ambient particulate matter pollution	9·95 (2·74–20·8)	6·5 (3·75–9·59)	4·34 (1·87–7·2)	-34·7 (-69·3–94)	-33·2 (-52·1—24·5)	9·95 (2·74–20·8)	6·5 (3·75–9·59)	4·34 (1·88–7·19)	-34·7 (-69·3–93·4)	-33·2 (-52·1—24·4)
Household air pollution from solid fuels	0·00921 (0·00192–0·025)	0·00584 (0·00105–0·0186)	0·00386 (0·000561–0·0133)	-36·6 (-76·1–63)	-33·9 (-69·6–22·5)	0·0122 (0·00279–0·0327)	0·00773 (0·00144–0·0239)	0·00512 (0·000774–0·0173)	-36·5 (-75·7–63)	-33·8 (-69·6–22·9)
Ambient ozone pollution	48 (19·4–82·8)	65·6 (28·5–100)	52·5 (22·1–91·3)	36·7 (18·6–61·9)	-19·9 (-31·5—6·67)	--	--	--	--	--
Residential radon	19·9 (2·74–59·2)	19·9 (2·74–59·2)	19·9 (2·74–59·2)	0 (0–0)	0 (0–0)	19·9 (2·74–59·2)	19·9 (2·74–59·2)	19·9 (2·74–59·2)	0 (0–0)	0 (0–0)
Lead exposure	51·9 (23·1–75·8)	30·2 (9·48–51·6)	21 (4·92–40·1)	-41·9 (-61·4—28·2)	-30·5 (-48·3—21·9)	28·1 (1·8–57·2)	16·6 (0·536–40·8)	11·6 (0·19–31·4)	-40·8 (-79·8—24·7)	-30 (-69·9—21·8)
Child underweight	0·946 (0·544–1·42)	0·961 (0·539–1·47)	0·933 (0·512–1·4)	1·5 (-16·2–23·8)	-2·91 (-15·8–10·1)	0·794 (0·427–1·2)	0·846 (0·471–1·28)	0·864 (0·476–1·31)	6·49 (-14·1–34·1)	2·17 (-13·2–17·7)
Iron deficiency	--	--	--	--	--	12·6 (10·5–14·8)	13·2 (11·1–15·5)	13·4 (11·2–15·9)	4·78 (-7·44–18·7)	1·81 (-7·78–13·4)
Vitamin A deficiency	0·843 (0·223–2·3)	1·21 (0·318–3·45)	0·796 (0·215–2·1)	43·3 (-67·7–513)	-34·1 (-72·6–73·5)	1·66 (0·622–3·46)	1·82 (0·725–3·66)	1·47 (0·541–3·13)	9·3 (-58·7–250)	-19·3 (-54·4–56·3)
Zinc deficiency	2·24 (0–7·1)	1·17 (0–3·8)	1·19 (0–3·87)	-47·6 (-100–NA)	1·2 (-18·1–NA)	2·31 (0–7·58)	1·18 (0–3·97)	1·16 (0–3·66)	-48·8 (-100–NA)	-2·31 (-20·4–NA)
Smoking	27·9 (25–30·9)	19·1 (17·4–21)	16·6 (14·9–18·3)	-31·4 (-36·5—25·8)	-13·4 (-17·1—9·6)	19·4 (16·7–22·7)	13·8 (12·4–15·3)	12·7 (11·3–14·2)	-29 (-37·7—18·5)	-8·15 (-12·6—3·53)
Secondhand smoke	37·9 (35·4–40·2)	24·9 (23·4–26·5)	25·3 (22·7–28·1)	-34·2 (-40·2—27·7)	1·52 (-7–11)	35·8 (33·8–37·7)	23·5 (22·4–24·7)	23·9 (21·9–26)	-34·3 (-39·1—28·8)	1·75 (-5·55–9·32)
Alcohol use	13·8 (8·81–19·4)	14·1 (8·4–20·5)	15·4 (9·2–22·1)	2·71 (-27·9–38·9)	8·67 (-14·4–41·6)	11·7 (7·27–17·1)	12·7 (7·09–19·1)	12·8 (6·71–20·6)	8·86 (-28·3–56·1)	1·15 (-25·2–30·8)
Drug use	0·412 (0·211–0·771)	0·667 (0·448–0·987)	0·962 (0·684–1·32)	61·9 (14·5–139)	44·4 (21·9–71·2)	0·392 (0·259–0·599)	0·682 (0·512–0·91)	1·03 (0·8–1·33)	74·2 (40·2–115)	51·5 (29·3–74·7)
High fasting plasma glucose	12·5 (10·4–15·2)	17·4 (15–20·3)	19 (16·5–22)	39 (16–68·9)	9·15 (-5·35–26·3)	9·65 (8·24–11·5)	14·3 (12·4–16·4)	15·5 (13·4–18·1)	47·8 (25·8–73·1)	8·84 (-5·01–25·4)
High systolic blood pressure	25·4 (20·5–30·7)	16·1 (14–18·1)	20 (15·6–25)	-36·7 (-48·8—21)	24·4 (-2·59–55·8)	22·2 (16·4–29)	12·7 (11·1–14·4)	17·6 (12·8–23·6)	-42·6 (-56·9—21·4)	38·3 (0·988–82·6)
High body-mass index	25·6 (17·9–35·1)	39·4 (28·7–51)	41·6 (30·5–53·2)	53·7 (35·8–78·5)	5·77 (-0·665–13·2)	31·5 (22·9–40·8)	44·2 (34·1–53)	45·9 (36·1–55·2)	40·3 (23·9–65·1)	3·88 (-2·34–10·8)
Low bone mineral density	12·2 (7·2–19·3)	9·23 (4·97–15·7)	11·5 (6·53–18·3)	-24·2 (-43·2—3·85)	24·7 (-5·16–65·4)	16·2 (10·7–23·2)	16·5 (10·8–23·4)	18·9 (12·6–26·3)	1·75 (-18·1–29·3)	14·5 (-8·97–45·1)
Diet low in fruits	56·2 (43·4–70·6)	52·8 (39·4–67·3)	52·2 (38–67·3)	-6·12 (-16·2–2·71)	-1·11 (-11·6–8·37)	--	--	--	--	--
Diet low in vegetables	28·9 (13·8–49·9)	45·1 (26·4–65·8)	42·5 (24·9–62·2)	56·2 (20·2–136)	-5·79 (-17·7–4·15)	25·8 (11·1–45·5)	42·7 (25·5–62·8)	40·6 (23·6–60·5)	65·7 (21·7–168)	-4·96 (-16·7–6·17)
Diet low in whole grains	79·6 (70·7–88·3)	81·5 (72·7–89·6)	81·5 (72·3–89·5)	2·42 (-1·14–7·23)	-0·0099 (-2·66–2·69)	76·9 (66·5–86·5)	79·7 (70·4–88·2)	79·4 (69·6–88·2)	3·67 (-0·364–9·35)	-0·287 (-3·36–2·4)
Diet low in nuts and seeds	27·6 (7·94–56·2)	25·3 (7·87–55·8)	20·8 (6·26–54)	-8·4 (-42·8–66·4)	-17·7 (-44–9·7)	23·8 (5·55–55·3)	22·3 (6·48–54·4)	19·3 (5·48–53·3)	-6·35 (-40·1–73·6)	-13·1 (-41·2–16·6)
Diet low in milk	48·2 (24·5–68·8)	42·9 (20–62·8)	41·8 (20·2–61·8)	-11·1 (-29·2–1·65)	-2·54 (-15·4–12)	--	--	--	--	--
Diet high in red meat	87·1 (77–94·4)	85·1 (74·1–93·6)	84·3 (72·4–93·2)	-2·2 (-13·4–8·8)	-0·991 (-10·9–8·58)	86·9 (76·7–94·4)	83·1 (70·4–91·8)	82 (68·7–91·6)	-4·4 (-16·1–7·86)	-1·34 (-11·7–8·68)
Diet high in processed meat	77·9 (52·6–98·1)	85·6 (66·4–99·6)	85·5 (66·5–99·5)	9·96 (1·08–29·9)	-0·103 (-4·46–4·33)	79·3 (56·2–98)	88·1 (70·6–99·8)	86·7 (67·9–99·7)	11·1 (1·28–33·4)	-1·54 (-6·79–1·99)
Diet high in sugar-sweetened beverages	46·5 (28·5–66)	62·7 (42·4–80·8)	63·2 (43·2–82·1)	34·9 (-4·84–115)	0·74 (-20·1–25·7)	50 (29·9–70·3)	68·4 (49·2–84·1)	67·1 (47·9–84·3)	36·6 (0·0703–107)	-1·79 (-19·1–21·1)
Diet low in fiber	40·9 (22·8–58·2)	27·6 (13·8–42·6)	24·8 (11·7–39·4)	-32·5 (-51·6—13·4)	-10 (-31·3–10·4)	--	--	--	--	--
Diet low in seafood omega-3 fatty acids	80·5 (63·2–100)	76·1 (58·2–98·2)	76·3 (57·5–98·5)	-5·42 (-21·5–11·8)	0·147 (-15–20·1)	83·8 (66–99·4)	81·4 (64·9–99·1)	80·4 (62·7–99·3)	-2·92 (-15·3–10·5)	-1·14 (-13·8–12·2)
Diet low in polyunsaturated fatty acids	13·8 (3·93–41·1)	7·28 (2·05–25·1)	6·97 (1·98–24·5)	-47·1 (-74·8—6)	-4·34 (-27·7–16·7)	--	--	--	--	--
Diet high in trans fatty acids	89·4 (81·6–100)	87·8 (79–100)	87·4 (77·8–100)	-1·82 (-10·3–5·57)	-0·385 (-8·43–8·7)	--	--	--	--	--
Diet high in sodium	18·2 (4·51–40·4)	39·7 (12·3–64·1)	39·1 (11·2–64·2)	118 (38·3–395)	-1·62 (-29–31·8)	20·8 (5·02–44·9)	21·4 (5·09–44·6)	21·7 (5·08–45·2)	2·56 (-26·4–50·2)	1·63 (-16·9–27·1)
Low physical activity	2·74 (1·04–5·58)	2·73 (1·2–5·31)	3·05 (1·2–6·48)	-0·186 (-31·7–60·7)	11·6 (-14·4–44·5)	5·96 (2·89–11·2)	6·54 (3·67–11·8)	6·5 (3·45–12·1)	9·78 (-17·9–55·4)	-0·619 (-13·1–11·3)
Occupational asthmagens	23·1 (19·2–27·6)	22·2 (18·7–26·1)	22·9 (18·8–28·2)	-3·98 (-16–9·14)	3·39 (-5·55–15)	11·9 (9·8–14·4)	12·4 (10·5–14·9)	12·3 (10–15)	3·95 (-10·8–21·1)	-1·03 (-11·4–10·3)
Occupational particulate matter, gases, and fumes	9·96 (7·12–14·3)	9·59 (6·93–13·6)	9·33 (6·69–13·1)	-3·78 (-8·99–2·38)	-2·65 (-6·17–0·744)	5·46 (3·89–7·94)	5·46 (3·97–7·75)	5·32 (3·84–7·65)	0·00195 (-8·55–10·4)	-2·59 (-7·47–2·97)
Occupational noise	8·99 (8·2–9·82)	8·77 (8·15–9·5)	8·56 (8·01–9·29)	-2·4 (-5·99–1·59)	-2·35 (-4·48—0·0558)	6·34 (5·55–7·09)	6·84 (6·29–7·42)	6·91 (6·46–7·46)	7·91 (1·96–15·7)	0·995 (-1·73–4·01)
Occupational injuries	--	--	--	--	--	--	--	--	--	--
Occupational ergonomic factors	13·1 (10·8–16)	11·7 (9·89–14·1)	12·1 (10–14·6)	-10·3 (-20·8–1·32)	3·04 (-5·25–13·2)	7·85 (6·17–10·1)	7·97 (6·53–9·87)	8·27 (6·54–10·3)	1·6 (-15·6–22·4)	3·71 (-8·71–17·9)
Childhood sexual abuse	7·4 (5·05–10·5)	6·97 (4·6–9·93)	6·85 (4·54–9·67)	-5·83 (-12·2–5·83)	-1·68 (-8·51–5·53)	12·3 (8·94–17·3)	17·4 (12·9–24)	17·8 (13·1–24·7)	41·9 (30·7–54·8)	2·17 (-5–11)
Intimate partner violence	--	--	--	--	--	26·3 (14·1–35·1)	24·7 (13·6–33·6)	25·9 (14·1–35·2)	-5·86 (-14·5–6·3)	4·81 (-4·48–19·7)
Non-exclusive breastfeeding	26·1 (21·5–30·8)	24·9 (21·2–28·9)	24·9 (20·2–29·7)	-4·67 (-19·7–14·2)	-0·0194 (-13·4–15·6)	--	--	--	--	--
Discontinued breastfeeding	27 (26–27·9)	25·6 (24·2–26·9)	25·3 (23·9–26·6)	-5·2 (-11–0·816)	-0·889 (-6·75–4·87)	27 (26–27·8)	25·5 (24·1–26·8)	25·3 (23·9–26·5)	-5·5 (-11·3–0·503)	-0·77 (-6·64–5·01)
Diet low in calcium	16·1 (7·65–29·9)	13·5 (6·53–24·8)	12·8 (6·15–23·8)	-15·9 (-26·5—4·11)	-4·91 (-15·2–3·4)	11·5 (4·93–21·6)	10·5 (4·64–19·5)	10·2 (4·39–19·3)	-8·31 (-22·7–8·73)	-2·99 (-13·3–7·99)
Occupational exposure to asbestos	7·21 (6·34–8·21)	6·01 (5·41–6·65)	5·44 (3·86–7·43)	-16·7 (-28·4—2·31)	-9·4 (-35·8–20·7)	1·13 (0·876–1·74)	1·21 (0·968–1·87)	1·2 (0·792–1·98)	6·72 (-8·62–26·4)	-0·486 (-29–35·9)
Occupational exposure to arsenic	0·536 (0–1·28)	0·57 (0–1·35)	0·596 (0–1·43)	6·33 (-9·07–NA)	4·7 (-6·86–NA)	0·496 (0–1·15)	0·534 (0–1·3)	0·536 (0–1·29)	7·61 (-10·3–NA)	0·464 (-10·4–NA)
Occupational exposure to benzene	0·846 (0·0473–2·51)	0·876 (0·0525–2·53)	0·895 (0·0556–2·59)	3·55 (-4·85–15)	2·1 (-5·2–12·9)	1·3 (0·0713–3·64)	1·51 (0·0923–4·27)	1·56 (0·0957–4·41)	16·2 (3·93–36·2)	3·13 (-5·29–14·7)
Occupational exposure to beryllium	0·0249 (0·0235–0·0264)	0·0257 (0·0251–0·0263)	0·0267 (0·0253–0·0282)	3·1 (-3·43–9·32)	4·14 (-1·27–9·85)	0·0285 (0·0262–0·0307)	0·0311 (0·0304–0·0319)	0·0317 (0·0299–0·0337)	9·14 (1·21–19)	1·95 (-4·14–8·08)
Occupational exposure to cadmium	0·0625 (0·0564–0·0691)	0·0666 (0·0609–0·0732)	0·0696 (0·0618–0·0785)	6·57 (-7·78–23·4)	4·41 (-5·73–15·4)	0·0596 (0·0526–0·0674)	0·0644 (0·0582–0·0724)	0·0649 (0·057–0·0744)	8·13 (-8·14–26·9)	0·657 (-9·26–12)
Occupational exposure to chromium	0·149 (0·135–0·163)	0·162 (0·15–0·175)	0·168 (0·152–0·187)	8·46 (-4·64–22·7)	4·19 (-4·6–13·6)	0·15 (0·134–0·167)	0·165 (0·153–0·181)	0·166 (0·149–0·185)	10·3 (-3·63–26·8)	0·581 (-7·97–10·3)
Occupational exposure to diesel engine exhaust	0·743 (0·686–0·798)	0·821 (0·771–0·875)	0·851 (0·787–0·926)	10·5 (0·31–21·8)	3·69 (-3·58–11·8)	0·717 (0·636–0·806)	0·79 (0·722–0·867)	0·777 (0·691–0·882)	10·2 (-4·95–28)	-1·64 (-10·8–8·45)
Occupational exposure to formaldehyde	0·31 (0·276–0·346)	0·325 (0·293–0·361)	0·335 (0·295–0·381)	4·64 (-9·77–21·6)	3·14 (-7·23–14·7)	0·293 (0·259–0·329)	0·319 (0·288–0·354)	0·322 (0·286–0·363)	9·16 (-6·19–27·1)	0·848 (-8·59–11·2)
Occupational exposure to nickel	0·462 (0–2)	0·467 (0–2·11)	0·487 (0–2·11)	1·2 (-12·8–NA)	4·15 (-7·09–NA)	0·391 (0–1·73)	0·407 (0–1·78)	0·41 (0–1·79)	3·93 (-14·7–NA)	0·856 (-11·1–NA)
Occupational exposure to polycyclic aromatic hydrocarbons	0·295 (0·269–0·322)	0·319 (0·297–0·342)	0·331 (0·3–0·366)	8·25 (-3·84–21·9)	3·9 (-4·68–12·7)	0·32 (0·289–0·354)	0·363 (0·341–0·391)	0·367 (0·335–0·402)	13·4 (0·85–27·6)	0·921 (-6·49–8·91)
Occupational exposure to silica	4·32 (0·613–14)	4·14 (0·586–13·1)	4·35 (0·59–14·3)	-3·98 (-17·7–12·5)	4·96 (-6·04–17·6)	3·32 (0·467–10·7)	3·29 (0·445–10·3)	3·28 (0·46–10·2)	-0·736 (-18·9–23·9)	-0·458 (-12·7–17·1)
Occupational exposure to sulfuric acid	0·973 (0·204–3·1)	1·12 (0·226–3·53)	1·15 (0·241–3·61)	15·2 (0·415–34·7)	2·35 (-7·4–13·7)	0·945 (0·201–3·02)	1·19 (0·246–3·68)	1·16 (0·251–3·69)	26·2 (1·91–54·5)	-2·53 (-15·8–14)
Unsafe sex	--	--	--	--	--	--	--	--	--	--
Occupational exposure to trichloroethylene	0·0871 (0·081–0·0938)	0·0959 (0·0916–0·101)	0·0998 (0·0931–0·107)	10 (0·401–20·1)	4·1 (-2·37–11·2)	--	--	--	--	--
No access to handwashing facility	3·35 (3·08–3·63)	3·13 (2·89–3·38)	3·09 (2·87–3·32)	-6·67 (-14·8–2·23)	-1·37 (-7·11–4·37)	3·35 (3·08–3·63)	3·13 (2·89–3·38)	3·09 (2·87–3·32)	-6·67 (-14·8–2·23)	-1·37 (-7·11–4·37)
Child wasting	0·748 (0·488–1·02)	0·729 (0·481–0·997)	0·727 (0·476–0·987)	-2·57 (-11·8–8·62)	-0·273 (-7·3–7·15)	0·772 (0·518–1·06)	0·762 (0·502–1·04)	0·758 (0·5–1·04)	-1·25 (-11·3–9·14)	-0·569 (-7·85–7·53)
Child stunting	2·34 (1·27–3·38)	2·08 (1·06–3·08)	2·01 (1·04–3·03)	-11·2 (-30·8–12·9)	-3·62 (-18·5–12·5)	1·91 (0·932–2·88)	1·79 (0·864–2·76)	1·76 (0·855–2·7)	-6·23 (-26·5–21·2)	-1·8 (-16·9–15·8)
Chewing tobacco	5·8 (4·52–7·27)	5·95 (4·32–8)	5·7 (3·97–7·93)	2·46 (-28·1–50·5)	-4·21 (-26·8–23·3)	--	--	--	--	--
Diet low in legumes	73·9 (37·2–99·3)	76·9 (46·1–99·6)	76·9 (47–99·5)	4·1 (-0·0985–23·1)	-0·0435 (-5·33–5·81)	72·2 (33·8–98·8)	75·2 (41·1–99·5)	75·6 (43·2–99·4)	4·16 (-0·262–22·9)	0·454 (-4·58–7·41)
Short gestation	22·6 (18·3–27·4)	23·4 (19·4–28·2)	23·6 (19·2–28·9)	3·62 (-18·2–30·6)	0·856 (-18·6–25)	20·9 (17·1–25·4)	24 (20·1–28·7)	23·2 (19·3–27·4)	15·1 (-10·2–50·2)	-3·47 (-21·2–16·7)
Low birth weight	8·55 (6·87–10·4)	9·26 (7·69–11·1)	9·71 (7·92–11·9)	8·27 (-16·4–39·7)	4·89 (-16·3–31)	9·55 (7·79–11·4)	12 (10·2–14·3)	11·9 (10–13·9)	26·1 (-1·67–64·7)	-1·32 (-19·6–21·6)
High temperature	33·6 (22·4–45·8)	32 (20·5–44·3)	27·5 (16·6–39·9)	-4·92 (-10·9—0·793)	-14 (-22·4—8·25)	33·6 (22·4–45·8)	32 (20·5–44·3)	27·5 (16·6–39·9)	-4·92 (-10·9—0·793)	-14 (-22·4—8·25)
Low temperature	36·9 (32·4–42·1)	37·9 (33·7–43·1)	38·5 (34·3–43·4)	2·83 (0·599–5·31)	1·53 (0·444–2·6)	36·9 (32·4–42·1)	37·9 (33·7–43·1)	38·5 (34·3–43·4)	2·83 (0·599–5·31)	1·53 (0·444–2·6)
Kidney dysfunction	19·7 (14·1–26·6)	20·3 (14·5–27·4)	20·4 (14·7–27·6)	2·94 (-0·636–6·84)	0·838 (-3·22–5·29)	22·1 (15·8–29·7)	23·2 (16·5–31)	24 (17·4–32)	4·63 (0·424–9·78)	3·73 (-0·428–8·66)
Bullying victimization	5·83 (2·44–11·9)	8·95 (3·57–17·8)	8·73 (3·32–18·1)	53·4 (32·9–76·6)	-2·46 (-11·9–3·61)	--	--	--	--	--
High LDL cholesterol	65·6 (61·5–70·2)	34·1 (30·7–37·8)	30·8 (27·4–34·7)	-48 (-53·3—42·4)	-9·87 (-15—4·69)	64·7 (61–68·2)	34·5 (31·3–37·8)	32 (28·6–35·6)	-46·7 (-51·4—42·1)	-7·05 (-11·3—3·03)

Supplementary Results Table 1. Kentucky age-standardised Summary Exposure Values (SEVs) for most-detailed risk factors and percentage change, by sex, 1990, 2010, and 2019. Data in parentheses are 95% uncertainty intervals.

	Male					Female				
	1990	2010	2019	Percentage change 1990-2010	Percentage change 2010-2019	1990	2010	2019	Percentage change 1990-2010	Percentage change 2010-2019
Unsafe sanitation	9·2 (4·38–16·8)	5·13 (2·45–9·69)	4·15 (1·86–7·8)	-44·3 (-77·3–-39·2)	-19·1 (-57·3–-53·4)	--	--	--	--	--
Ambient particulate matter pollution	14·6 (5·81–28·3)	8·52 (5·76–11·7)	5·26 (2·81–8·08)	-41·8 (-69·8–-33·7)	-38·3 (-52·2–-30)	14·6 (5·79–28·1)	8·52 (5·76–11·7)	5·26 (2·82–8·07)	-41·7 (-69·8–-33·7)	-38·2 (-51·9–-30)
Household air pollution from solid fuels	0·0531 (0·0148–0·136)	0·026 (0·0045–0·0745)	0·0167 (0·00286–0·0556)	-51 (-80·6–11·1)	-35·6 (-67·5–19·1)	0·07 (0·0192–0·174)	0·0344 (0·00665–0·0984)	0·0222 (0·00407–0·0725)	-50·8 (-80·6–11·2)	-35·4 (-67·5–19·7)
Ambient ozone pollution	79·9 (36·5–100)	70·6 (31·4–100)	44·5 (18·5–76·1)	-11·7 (-21·2–0)	-37 (-47·7–-23·9)	79·9 (36·5–100)	70·6 (31·4–100)	44·5 (18·5–76·1)	-11·7 (-21·2–0)	-37 (-47·7–-23·9)
Residential radon	19·7 (3·09–56·3)	19·7 (3·09–56·3)	19·7 (3·09–56·3)	0 (0–0)	0 (0–0)	19·7 (3·09–56·3)	19·7 (3·09–56·3)	19·7 (3·09–56·3)	0 (0–0)	0 (0–0)
Lead exposure	52·9 (23·5–76·5)	32·4 (10·8–54·9)	23·3 (6·48–43·2)	-38·8 (-55·8–-25·9)	-28·2 (-43·9–-20·1)	29·8 (2·55–58)	19·2 (1·2–43·6)	14 (0·55–34·4)	-35·7 (-67·6–-21·3)	-27·3 (-59·9–-19·4)
Child underweight	0·977 (0·541–1·5)	0·98 (0·538–1·48)	0·959 (0·533–1·43)	0·267 (-17·6–22·9)	-2·05 (-14·9–12·5)	0·82 (0·441–1·24)	0·862 (0·461–1·3)	0·886 (0·481–1·33)	5·13 (-15·1–35·1)	2·83 (-11·8–19·9)
Iron deficiency	--	--	--	--	--	11·9 (9·87–14·1)	12·6 (10·5–14·6)	12·9 (10·6–15·1)	5·63 (-6·08–18·7)	2·24 (-6·56–13·4)
Vitamin A deficiency	1·13 (0·297–3·12)	1·5 (0·377–4·14)	1·06 (0·269–3·02)	33 (-68·9–512)	-29·5 (-69·7–70·6)	2·08 (0·788–3·92)	2·1 (0·797–4·47)	1·8 (0·692–3·89)	0·928 (-58·2–193)	-14·1 (-51·2–69·7)
Zinc deficiency	2·63 (0–8·29)	1·32 (0–4·3)	1·36 (0–4·51)	-49·8 (-100–NA)	3·13 (-14·3–NA)	2·71 (0–8·48)	1·33 (0–4·43)	1·34 (0–4·34)	-50·8 (-100–NA)	0·368 (-18·4–NA)
Smoking	32·2 (29·1–35·3)	25·6 (23·4–28·1)	22·7 (20·6–25·1)	-20·2 (-25–-15)	-11·4 (-14·6–-7·98)	22·7 (20–25·5)	19·7 (17·8–21·7)	18·2 (16·3–20·2)	-13·2 (-20·7–-4·54)	-7·46 (-11·3–-3·22)
Secondhand smoke	41·4 (38·8–43·9)	32 (30·3–33·7)	30·6 (27·8–33·6)	-22·8 (-29–-16·4)	-4·33 (-12·3–3·37)	40·4 (38·4–42·4)	30·9 (29·6–32·2)	29·6 (27·3–32)	-23·5 (-28·8–-18)	-4·34 (-10·4–1·89)
Alcohol use	13·1 (8·72–17·5)	11·9 (7·59–16·2)	12·1 (7·86–16·5)	-8·78 (-36·7–29·9)	1·46 (-22·1–30·7)	--	--	--	--	--
Drug use	0·53 (0·337–0·848)	1·41 (1·05–1·86)	2·1 (1·6–2·68)	166 (97·7–252)	48·7 (29·2–73·8)	0·447 (0·319–0·631)	1·18 (0·905–1·53)	1·98 (1·53–2·5)	164 (116–222)	67·6 (46·1–94·2)
High fasting plasma glucose	14 (11·5–17·2)	20 (17·5–23)	21·4 (18·7–24·3)	43·3 (18·6–74·2)	6·75 (-6·61–23·5)	11 (9·25–13·3)	16·4 (14·4–18·7)	18 (15·6–20·5)	48·8 (26·2–75·4)	9·44 (-3·95–25·5)
High systolic blood pressure	26·4 (21·3–31·9)	17·3 (15·3–19·5)	21·2 (16·6–26·1)	-34·3 (-45·9–-19)	22·3 (-1·65–49·1)	24·3 (18·4–31·8)	14·3 (12·6–16·1)	18·9 (13·6–24·7)	-41·1 (-55·4–-20·7)	32 (-4·18–76·4)
High body-mass index	24·6 (17·4–34·3)	40 (29·7–51·6)	40·6 (30–52·1)	62·6 (41·4–93·8)	1·69 (-4·52–8·1)	31·9 (23·4–41·5)	45·3 (35·2–54·6)	45·6 (35·4–54·9)	41·8 (25·7–63·1)	0·63 (-5·45–7·87)
Low bone mineral density	12·2 (7·12–19)	9·53 (5·1–15·9)	12·1 (6·98–19·3)	-21·8 (-41–0·967)	26·7 (-0·483–68·6)	16·2 (10·7–22·9)	17 (11·1–24·1)	19·6 (13·6–26·7)	5·4 (-16·3–33)	14·8 (-8·7–49·1)
Diet low in fruits	63·1 (52·5–75)	56·3 (45·5–68·4)	55·4 (43·7–68·3)	-10·8 (-20–-3·55)	-1·6 (-10·8–7·33)	61·1 (49·8–73·4)	57·6 (46·3–70·5)	56·4 (44·5–69·3)	-5·69 (-13·5–2·25)	-2·03 (-11–6·49)
Diet low in vegetables	48·7 (29·4–70·8)	57·9 (42·7–72·4)	55·9 (39·8–70·8)	18·9 (0·0829–56·1)	-3·53 (-10·3–3·09)	47·5 (27·1–70·3)	55 (35·6–75·7)	52·3 (33·2–72·4)	15·7 (3·92–38·1)	-4·95 (-13·8–2·95)
Diet low in whole grains	80·1 (70·3–88·7)	82 (73·5–89·8)	82·1 (73·3–89·6)	2·36 (-0·95–6·77)	0·12 (-2·43–2·57)	77·5 (67·1–87·4)	80·1 (70·9–88·8)	80 (70·6–88·5)	3·33 (-0·846–9·14)	-0·144 (-3·06–2·52)
Diet low in nuts and seeds	31·1 (9·22–57·9)	26·3 (8·93–55·9)	22·7 (6·72–54·3)	-15·3 (-44·2–48·5)	-13·6 (-41·1–10·7)	26·8 (6·9–55·9)	23·1 (6·41–54·7)	21 (6·59–53·8)	-13·8 (-47–52·9)	-9 (-37·6–20·4)
Diet low in milk	51·7 (27·7–72)	46·4 (22·9–66·6)	45·8 (23·3–66·8)	-10·2 (-28·5–0·752)	-1·26 (-12·4–10·3)	--	--	--	--	--
Diet high in red meat	82·5 (71·3–91·7)	80·9 (68·5–90·8)	80·1 (67·2–90·1)	-1·96 (-14·9–12·1)	-0·959 (-11·9–10·3)	83·3 (73·3–91·7)	79·3 (67·3–89·2)	78·4 (65·6–89·2)	-4·83 (-17–8·03)	-1·15 (-11·7–10·4)
Diet high in processed meat	81·6 (59·5–99·2)	88·2 (70·8–99·7)	87·9 (69·8–99·8)	8·14 (0·277–25)	-0·414 (-5·44–4·74)	82·3 (61·2–98·7)	90·3 (76–99·6)	88·7 (72·5–99·6)	9·72 (0·545–29·4)	-1·76 (-7·51–1·7)
Diet high in sugar-sweetened beverages	42·8 (25·1–62·5)	58·8 (38·2–75·5)	58·7 (38·3–76·7)	37·3 (-6·8–117)	-0·019 (-22·3–24·7)	46·8 (27·5–68·1)	64·3 (45·2–81·1)	62·7 (43·9–80·5)	37·4 (-4·47–132)	-2·44 (-23·6–21·8)
Diet low in fiber	46·6 (30·8–62·1)	32·7 (20·1–46·5)	30·5 (17·3–44·6)	-29·7 (-46–-14·7)	-6·73 (-24·2–12·2)	46·9 (30·1–62·9)	38·1 (23·6–52·8)	33·7 (19·1–48·6)	-18·7 (-34·3–-2·13)	-11·5 (-27·6–3·36)
Diet low in seafood omega-3 fatty acids	85·6 (68·5–100)	82·4 (64·1–100)	82·1 (64·6–99·4)	-3·72 (-17·9–9·54)	-0·303 (-12·9–14·1)	88 (73·8–100)	85·3 (70·8–99·9)	85·2 (69·9–99·6)	-3·06 (-14–6·38)	-0·098 (-10·4–10·1)
Diet low in polyunsaturated fatty acids	15·9 (4·65–43·9)	8·09 (2·42–28·2)	7·79 (2·32–26·8)	-49 (-73·8–-8·69)	-3·67 (-25·5–20·9)	--	--	--	--	--
Diet high in trans fatty acids	89·5 (81·5–100)	87·9 (79·2–100)	87·4 (78·3–100)	-1·79 (-9·35–6·33)	-0·545 (-8·48–7·99)	--	--	--	--	--
Diet high in sodium	18·1 (4·57–41)	39·5 (11·4–63·6)	38·8 (10·3–63)	118 (41·8–384)	-1·87 (-28·8–30)	--	--	--	--	--
Low physical activity	2·49 (0·896–5·12)	2·34 (0·993–4·72)	2·09 (0·743–4·55)	-6·11 (-37·9–45·5)	-10·7 (-34·7–13·2)	--	--	--	--	--
Occupational asthmagens	24·3 (20·7–29·1)	21·2 (18–25·1)	21·9 (18·3–26·6)	-13 (-23·4–-1·22)	3·45 (-5·3–13·9)	11·1 (8·98–13·6)	10·8 (8·92–12·8)	11 (9·04–13·2)	-3·29 (-17·6–13·2)	1·92 (-9·34–14·7)
Occupational particulate matter, gases, and fumes	10·3 (7·28–14·7)	9·67 (6·95–14)	9·28 (6·67–13·2)	-5·85 (-11·1–-0·353)	-3·99 (-7·67–-0·847)	5·45 (3·8–8·03)	5·32 (3·81–7·59)	5·13 (3·69–7·35)	-2·33 (-11·1–8·7)	-3·55 (-8·69–2·02)
Occupational noise	9·34 (8·53–10·2)	8·78 (8·17–9·52)	8·42 (7·9–9·1)	-6·04 (-9·79–-2·03)	-4·07 (-6·18–-1·86)	5·83 (5·03–6·69)	6·15 (5·61–6·79)	6·16 (5·74–6·76)	5·56 (-1·23–13·7)	0·0285 (-3·1–3·6)
Occupational injuries	--	--	--	--	--	--	--	--	--	--
Occupational ergonomic factors	12·9 (10·7–15·8)	10·9 (9·12–13)	11·2 (9·36–13·5)	-16·1 (-26·7–-5·39)	3·33 (-4·84–12·9)	7·38 (5·82–9·29)	7·04 (5·76–8·67)	7·39 (5·96–9·48)	-4·49 (-20·6–14·6)	4·94 (-7·5–17·7)
Childhood sexual abuse	7·4 (5·05–10·5)	6·97 (4·6–9·93)	6·85 (4·54–9·67)	-5·83 (-12·2–5·83)	-1·68 (-8·51–5·53)	12·3 (8·94–17·3)	17·4 (12·9–24)	17·8 (13·1–24·7)	41·9 (30·7–54·8)	2·17 (-5–11)
Intimate partner violence	--	--	--	--	--	32·2 (17·2–41·9)	30·5 (17·2–40·1)	31·4 (17·8–41·1)	-5·42 (-14·4–7·1)	2·99 (-5·59–17·4)
Non-exclusive breastfeeding	26·2 (21·7–30·9)	24·8 (21·3–28·9)	24·8 (20·2–30·1)	-5·56 (-19·2–11·3)	0·0967 (-13·9–14·3)	26·2 (21·7–30·9)	24·8 (21·3–28·9)	24·8 (20·2–30·1)	-5·56 (-19·2–11·3)	0·091 (-13·9–14·3)
Discontinued breastfeeding	27·3 (26·2–28·2)	25·2 (23·9–26·5)	25·8 (24·5–27)	-7·45 (-12·9–-1·77)	2·16 (-3·53–8·11)	27·3 (26·3–28·2)	25·3 (23·9–26·6)	25·8 (24·5–27)	-7·24 (-12·7–-1·56)	1·93 (-3·74–7·86)
Diet low in calcium	18·3 (8·8–34·2)	15·1 (7·27–27·9)	14·9 (7·11–27·6)	-17·2 (-28·6–-6·1)	-1·61 (-11·2–6·06)	13·8 (5·83–26·3)	12·3 (5·25–22·7)	12·3 (5·29–23·2)	-11·2 (-24·8–3·36)	0·753 (-8·08–9·32)
Occupational exposure to asbestos	6·88 (6·17–7·72)	5·98 (5·4–6·55)	5·72 (4·2–7·45)	-13·1 (-25–0·436)	-4·43 (-29·6–26·5)	1·24 (0·982–2·04)	1·28 (1·04–2·12)	1·32 (0·865–2·36)	3·4 (-11·3–22·4)	3·2 (-26–39·8)
Occupational exposure to arsenic	0·523 (0–1·25)	0·514 (0–1·22)	0·542 (0–1·28)	-1·58 (-15·8–NA)	5·38 (-6·46–NA)	0·482 (0–1·17)	0·475 (0–1·1)	0·491 (0–1·2)	-1·5 (-19–NA)	3·3 (-9·98–NA)
Occupational exposure to benzene	0·769 (0·0464–2·21)	0·739 (0·0432–2·15)	0·773 (0·0446–2·24)	-3·84 (-11·7–7·82)	4·62 (-2·92–16·2)	1·08 (0·0665–3·11)	1·2 (0·0716–3·33)	1·27 (0·0778–3·65)	10·5 (-2·02–30·7)	5·97 (-2·79–19·6)
Occupational exposure to beryllium	0·0237 (0·0223–0·0251)	0·0225 (0·0219–0·023)	0·0238 (0·0224–0·0253)	-5·31 (-11–0·902)	6·07 (0·0586–12·7)	0·0252 (0·0227–0·0273)	0·0258 (0·0252–0·0265)	0·0271 (0·025–0·0291)	2·68 (-5·82–13·9)	4·92 (-2·92–12·6)
Occupational exposure to cadmium	0·0606 (0·0547–0·0676)	0·0599 (0·0543–0·0658)	0·0631 (0·0559–0·0707)	-1·28 (-14·5–12·9)	5·47 (-4·92–17)	0·057 (0·0498–0·0645)	0·0568 (0·0508–0·0634)	0·0587 (0·0507–0·0679)	-0·496 (-16·7–18·6)	3·4 (-8·6–17·4)
Occupational exposure to chromium	0·144 (0·131–0·159)	0·145 (0·134–0·157)	0·153 (0·138–0·168)	0·433 (-11–12·4)	5·53 (-3·75–15·6)	0·141 (0·124–0·158)	0·143 (0·132–0·157)	0·148 (0·132–0·167)	1·89 (-12·3–18·4)	3·47 (-6·79–15·5)
Occupational exposure to diesel engine exhaust	0·755 (0·693–0·822)	0·743 (0·693–0·796)	0·789 (0·721–0·858)	-1·6 (-10·7–9·21)	6·19 (-1·67–14·9)	0·633 (0·561–0·705)	0·667 (0·606–0·732)	0·681 (0·602–0·769)	5·36 (-8·68–24)	2·21 (-8·16–14)
Occupational exposure to formaldehyde	0·301 (0·269–0·337)	0·292 (0·263–0·324)	0·304 (0·267–0·343)	-3·01 (-16·3–11·4)	4·21 (-6·72–16·1)	0·276 (0·241–0·313)	0·277 (0·25–0·308)	0·287 (0·251–0·328)	0·184 (-15·4–18·3)	3·55 (-7·65–16·7)
Occupational exposure to nickel	0·489 (0–2·09)	0·436 (0–1·98)	0·457 (0–2·01)	-10·8 (-23·2–NA)	4·75 (-6·3–NA)	0·387 (0–1·72)	0·368 (0–1·63)	0·38 (0–1·75)	-5 (-22·2–NA)	3·44 (-10·7–NA)
Occupational exposure to polycyclic aromatic hydrocarbons	0·286 (0·262–0·314)	0·285 (0·264–0·306)	0·3 (0·271–0·329)	-0·377 (-11·7–11·1)	5·32 (-3·43–15·1)	0·296 (0·264–0·33)	0·311 (0·29–0·334)	0·322 (0·29–0·359)	5·04 (-8·52–20·3)	3·73 (-5·63–14·3)
Occupational exposure to silica	4·92 (0·681–16)	4·05 (0·558–12·9)	4·27 (0·632–13·4)	-17·7 (-29·4–-4·53)	5·23 (-5·62–18·2)	3·3 (0·452–10·5)	3·02 (0·401–9·52)	3·1 (0·416–10·5)	-8·62 (-24·8–15·1)	2·81 (-11·9–22·3)
Occupational exposure to sulfuric acid	0·921 (0·194–2·91)	0·981 (0·202–3·12)	1·02 (0·211–3·25)	6·57 (-8·17–21·7)	4·11 (-6·42–15·3)	0·901 (0·184–2·98)	1·07 (0·228–3·56)	1·07 (0·222–3·3)	18·2 (-3·62–46·9)	0·0989 (-14·3–18·3)
Unsafe sex	--	--	--	--	--	--	--	--	--	--
Occupational exposure to trichloroethylene	0·083 (0·0774–0·0898)	0·085 (0·0814–0·0897)	0·0901 (0·0833–0·0973)	2·43 (-5·54–12)	5·92 (-0·886–13·8)	--	--	--	--	--
No access to handwashing facility	3·46 (3·2–3·73)	3·16 (2·9–3·42)	3·12 (2·87–3·39)	-8·65 (-17·1–0·217)	-1·1 (-6·81–5)	3·46 (3·2–3·73)	3·16 (2·9–3·42)	3·12 (2·87–3·39)	-8·65 (-17·1–0·217)	-1·1 (-6·81–5)
Child wasting	0·751 (0·49–1·03)	0·729 (0·486–1)	0·73 (0·478–1·01)	-2·83 (-12·9–8·58)	0·0406 (-7·28–7·56)	0·776 (0·514–1·06)	0·766 (0·512–1·07)	0·762 (0·507–1·05)	-1·23 (-11·5–9·63)	-0·583 (-7·93–8·06)
Child stunting	2·48 (1·34–3·6)	2·15 (1·09–3·23)	2·09 (1·07–3·12)	-13·3 (-31·9–12·2)	-2·66 (-17·4–14·5)	1·98 (0·967–3·03)	1·83 (0·909–2·86)	1·82 (0·925–2·8)	-7·58 (-26·8–17·8)	-0·719 (-16·3–17·4)
Chewing tobacco	9·73 (8·04–11·7)	8·8 (6·86–11·1)	8·45 (6·3–11)	-9·53 (-34·1–21)	-3·97 (-22·2–18·6)	--	--	--	--	--
Diet low in legumes	75·1 (41–99·4)	77·9 (46·5–99·8)	78 (47–99·8)	3·7 (-0·357–17·8)	0·106 (-4·91–6·74)	73·5 (36–98·9)	76·3 (45·4–99·6)	76·7 (45–99·6)	3·79 (-0·519–19·8)	0·6 (-4·68–8·21)
Short gestation	25·7 (21·1–31·6)	23·8 (20·1–28·3)	23·1 (19·1–28)	-7·21 (-26–15·8)	-3·12 (-20·3–18·2)	22·1 (18·3–26·5)	22·7 (19·2–26·8)	21·6 (18·2–25·6)	2·52 (-18·8–28·2)	-4·83 (-20·6–14·1)
Low birth weight	10·6 (8·7–12·7)	10·1 (8·57–12)	10·5 (8·75–12·5)	-4·76 (-25·2–19)	3·48 (-16·4–27·1)	10·5 (8·75–12·7)	11·7 (10·1–13·6)	11·7 (10–13·5)	11·4 (-12·7–40·5)	-0·258 (-17–19·5)
High temperature	24·5 (14·9–35·1)	26·1 (16–37·1)	23·5 (14–34·4)	6·69 (1·74–11·7)	-10 (-15·7–-5·01)	24·5 (14·9–35·1)	26·1 (16–37·1)	23·5 (14–34·4)	6·69 (1·74–11·7)	-10 (-15·7–-5·01)
Low temperature	33·8 (28·9–39·7)	37·1 (32·9–42·3)	36·2 (31·3–41·9)	9·86 (5·75–14·7)	-2·56 (-5·73–0·263)	33·8 (28·9–39·7)	37·1 (32·9–42·3)	36·2 (31·3–41·9)	9·86 (5·75–14·7)	-2·56 (-5·73–0·263)
Kidney dysfunction	20·5 (14·7–27·7)	21·2 (15·2–28·5)	21·4 (15·4–28·6)	3·22 (-0·62–7·73)	0·717 (-3·11–4·74)	22·8 (16·5–30·4)	24·1 (17·3–31·9)	25 (18·2–33)	5·55 (1·65–10·9)	3·78 (-0·435–8·68)

Bullying victimization	5·87<br>(2·44–12)	8·92<br>(3·58–17·8)	8·69<br>(3·35–17·9)	51·9<br>(31·4–74·4)	-2·55<br>(-12·2–3·59)	--	--	--	--	--
High LDL cholesterol	66·5<br>(62–71·1)	34·5<br>(31·2–38)	30·6<br>(27·1–34·3)	-48·1<br>(-53·4–-42·8)	-11·3<br>(-16·3–-6·04)	65·8<br>(62·3–69·2)	35<br>(31·7–38·4)	32·1<br>(28·6–35·7)	-46·8<br>(-51·3–-42·2)	-8·2<br>(-12·4–-4·08)

Supplementary Results Table 1. Louisiana age-standardised Summary Exposure Values (SEVs) for most-detailed risk factors and percentage change, by sex, 1990, 2010, and 2019. Data in parentheses are 95% uncertainty intervals.

	Male					Female				
	1990	2010	2019	Percentage change 1990-2010	Percentage change 2010-2019	1990	2010	2019	Percentage change 1990-2010	Percentage change 2010-2019
Unsafe sanitation	9·82 (4·6–17·5)	4·89 (2·28–8·97)	3·87 (1·8–7·54)	-50·2 (-78·7–23·7)	-20·8 (-58·7–47·4)	9·82 (4·6–17·5)	4·89 (2·28–8·97)	3·87 (1·8–7·54)	-50·2 (-78·7–23·7)	-20·8 (-58·7–47·4)
Ambient particulate matter pollution	11·5 (3·87–23·5)	7·64 (4·79–10·9)	5·04 (2·41–7·98)	-33·6 (-67·3–76·2)	-34·1 (-49·9–-25·7)	11·5 (3·92–23·5)	7·64 (4·8–10·9)	5·04 (2·43–7·99)	-33·6 (-67·3–75·7)	-34 (-50–-25·8)
Household air pollution from solid fuels	0·0163 (0·00414–0·0408)	0·00866 (0·00165–0·0252)	0·00583 (0·000999–0·0185)	-46·8 (-79·6–29·7)	-32·7 (-67·6–27·1)	0·0215 (0·00532–0·0537)	0·0115 (0·00229–0·033)	0·00775 (0·00137–0·0243)	-46·6 (-79·5–29·8)	-32·3 (-67·6–27·6)
Ambient ozone pollution	57·4 (24·1–99·1)	51·1 (21·6–87·3)	23·5 (8·58–44·3)	-11 (-21·4–0)	-53·9 (-68·1–-42·8)	--	--	--	--	--
Residential radon	15·9 (1·91–47·8)	15·9 (1·91–47·8)	15·9 (1·91–47·8)	0 (0–0)	0 (0–0)	15·9 (1·91–47·8)	15·9 (1·91–47·8)	15·9 (1·91–47·8)	0 (0–0)	0 (0–0)
Lead exposure	67·4 (42·9–86·2)	41·1 (21·4–62·3)	29·4 (13–48·4)	-39 (-52–-26·1)	-28·4 (-39·7–-21·1)	41·2 (9·23–67·9)	24·9 (3·85–48·7)	17·6 (1·66–37·8)	-39·7 (-66·4–-26)	-29·4 (-56–-21·2)
Child underweight	0·999 (0·566–1·49)	0·98 (0·549–1·46)	0·959 (0·527–1·44)	-1·91 (-19·3–19)	-2·12 (-15–12·5)	0·838 (0·456–1·27)	0·864 (0·486–1·34)	0·885 (0·489–1·35)	3·18 (-17–26·9)	2·43 (-11·8–18·9)
Iron deficiency	--	--	--	--	--	12·7 (10·5–15)	13 (10·9–15·2)	13·3 (11·1–15·9)	2·47 (-9·82–16·7)	1·92 (-8·19–12·7)
Vitamin A deficiency	1·16 (0·308–3·04)	1·45 (0·387–3·76)	1·01 (0·256–2·87)	25·2 (-64·7–447)	-30·3 (-69–65·2)	2·13 (0·766–4·35)	2·08 (0·768–4·41)	1·75 (0·638–3·69)	-2·27 (-62·9–166)	-16 (-54–59)
Zinc deficiency	2·55 (0–8·28)	1·27 (0–4·07)	1·25 (0–4·12)	-50·4 (-100–NA)	-1·26 (-22·2–NA)	2·6 (0–8·43)	1·28 (0–4·31)	1·23 (0–4·02)	-50·9 (-100–NA)	-3·36 (-25·3–NA)
Smoking	28·4 (25·5–31·5)	21·1 (19·1–23·3)	19·9 (17·9–22)	-25·7 (-30·9–-20·2)	-5·87 (-9·69–-1·96)	18·9 (16·1–21·8)	14·7 (13·1–16·3)	13·7 (12·1–15·3)	-22·2 (-31–-11·3)	-6·98 (-11·2–-2·41)
Secondhand smoke	39·9 (37·2–42·5)	27·4 (25·8–29·2)	27·2 (24·4–30)	-31·2 (-37·5–-24·7)	-0·79 (-9·45–8·04)	38·4 (36·4–40·7)	26·9 (25·7–28·3)	26·8 (24·6–28·8)	-30 (-35·4–-24·4)	-0·507 (-7·71–6·27)
Alcohol use	13·5 (8·17–19·1)	14·2 (8·73–20·5)	14·1 (8·48–20·4)	5·14 (-26·2–49·5)	-0·388 (-21·7–24·5)	8·46 (4·79–12·7)	9·7 (5·92–14·1)	9·53 (5·49–14·3)	14·7 (-20·1–69·6)	-1·73 (-24·2–24·1)
Drug use	0·617 (0·337–1·1)	1·2 (0·854–1·68)	1·6 (1·2–2·12)	94·6 (42·8–175)	33 (16·4–54·2)	0·449 (0·281–0·727)	0·887 (0·646–1·19)	1·34 (1·03–1·72)	97·5 (53·7–153)	50·9 (30·2–75·2)
High fasting plasma glucose	15·1 (12·5–18)	20 (17·6–22·5)	21·1 (18·4–24)	32 (10·1–60·3)	5·48 (-6·53–20·1)	13·5 (11·1–15·9)	17·4 (15·4–19·8)	18·3 (16–20·7)	29·2 (8–56·4)	4·99 (-7·59–19·2)
High systolic blood pressure	26 (20·9–32·1)	17·6 (15·6–19·8)	21·2 (16·4–26·9)	-32·2 (-46·3–-15·6)	20·4 (-3·47–50·4)	24·8 (18·7–32·7)	14·6 (12·9–16·3)	20·1 (14·7–26·8)	-41·1 (-54·6–-20·8)	38·1 (2·64–85)
High body-mass index	24 (16·7–33·3)	40·2 (29·3–51·7)	41·5 (30·5–52·7)	67·3 (45·9–98·7)	3·31 (-2·99–10·9)	32·9 (24·3–41·7)	46·3 (35·8–55·5)	47·3 (36·8–56·9)	40·8 (25·6–62·9)	2·13 (-4·07–9·27)
Low bone mineral density	10 (5·5–16·2)	9·21 (4·73–15·2)	8·8 (4·64–14·6)	-8·27 (-32·2–19·8)	-4·44 (-29·6–23·7)	15·9 (10·4–22·9)	14·9 (9·81–21·7)	15·2 (9·68–22·1)	-6·36 (-26·4–18·3)	1·58 (-21·4–33·5)
Diet low in fruits	58 (44·7–72·6)	51·9 (38·4–66·9)	51·7 (38·3–66·1)	-10·7 (-22–-1·62)	-0·338 (-10·2–10·8)	--	--	--	--	--
Diet low in vegetables	45 (25·6–66·9)	61·1 (42·8–78·4)	58·5 (40·7–75·6)	36 (13·6–81·1)	-4·31 (-12·1–1·93)	46·1 (26–68·5)	55·5 (35·9–75·5)	53·3 (33·4–73·4)	20·5 (5·45–49·5)	-4·11 (-12·8–3·12)
Diet low in whole grains	80 (70·5–88·8)	81·8 (73–89·9)	81·8 (73·1–89·6)	2·3 (-1·17–6·9)	-0·067 (-2·58–2·52)	77·4 (66·9–87)	80 (70·8–88·6)	79·7 (70·9–88·7)	3·32 (-0·776–9·23)	-0·359 (-3·36–2·35)
Diet low in nuts and seeds	30·3 (8·46–58·5)	26·2 (8·65–56·3)	21·6 (6·55–54·5)	-13·4 (-46–54·1)	-17·6 (-43·9–6·88)	26·1 (6·77–55·5)	23 (7·17–54·8)	20 (5·82–53·9)	-11·8 (-44·3–51·2)	-13·2 (-41·4–16·9)
Diet low in milk	51·1 (27·3–71·7)	45 (22·5–65·9)	45 (21·5–64·6)	-11·9 (-31·1–-0·12)	-3·2 (-17·1–9·02)	--	--	--	--	--
Diet high in red meat	87·6 (77·3–95·3)	84·6 (73–93·4)	83·9 (71·9–93·3)	-3·43 (-14·2–7·66)	-0·781 (-10·4–8·96)	89·1 (80·4–95·6)	84·1 (72·5–93·1)	83·4 (71·1–93·1)	-5·6 (-17·4–4·89)	-0·908 (-10·4–8·95)
Diet high in processed meat	76·7 (52·7–97·5)	84·8 (65·1–99·6)	84·8 (65·5–99·6)	10·6 (1·42–29·9)	-0·0322 (-4·57–5·4)	80·2 (57–98·8)	88·9 (73·4–99·7)	87·6 (70·5–99·7)	10·8 (0·642–33·5)	-1·4 (-6·88–2·43)
Diet high in sugar-sweetened beverages	43·4 (25·8–62·7)	59·9 (40·2–77·2)	61·1 (42·2–79·1)	38·2 (-4·37–123)	1·99 (-20·1–27·8)	47·3 (27·5–67·3)	66 (47·1–83)	65·3 (44·9–81·7)	39·5 (0·445–119)	-1·07 (-22·7–23·1)
Diet low in fiber	46·1 (30–61·2)	31·9 (19·2–45·5)	28·7 (15·9–42·6)	-30·9 (-46·3–-15·6)	-9·85 (-27·7–8·43)	46·4 (29·4–62·1)	37·1 (22·4–51·6)	31·7 (17·9–46·8)	-20·1 (-35·2–-2·66)	-14·4 (-31–0·909)
Diet low in seafood omega-3 fatty acids	85·7 (68·5–100)	81·2 (62·9–99·7)	80·1 (62·2–99·3)	-5·27 (-18·2–7·54)	-1·34 (-15·2–15·8)	87·7 (74·6–99·7)	84·6 (68·8–99·8)	83·9 (67·9–99·4)	-3·52 (-15·1–5·69)	-0·878 (-11·9–10·3)
Diet low in polyunsaturated fatty acids	15·8 (4·52–44·6)	7·77 (2·23–27·3)	7·28 (2·06–24·8)	-50·7 (-76·9–-8·63)	-6·39 (-30·2–13·9)	--	--	--	--	--
Diet high in trans fatty acids	89·4 (81·7–100)	87·9 (78·9–100)	87·5 (78·4–100)	-1·7 (-9·19–6·16)	-0·52 (-8·47–8·09)	--	--	--	--	--
Diet high in sodium	18·1 (4·49–41·7)	39·6 (12·1–64·3)	38·9 (11·8–64·1)	118 (40·7–421)	-1·68 (-28·2–31·9)	--	--	--	--	--
Low physical activity	2·49 (0·898–5·03)	2·32 (1·06–4·6)	2·34 (0·864–4·89)	-6·58 (-38·3–49·4)	0·873 (-26–29·8)	--	--	--	--	--
Occupational asthmagens	23·8 (20·3–28·4)	20·4 (17·5–24·1)	21 (17·7–25·1)	-14·3 (-24·8–-2·41)	2·74 (-6·05–12·4)	10·9 (8·91–13·2)	10·6 (8·85–12·6)	10·7 (8·76–13)	-2·84 (-15·7–14·7)	1·47 (-9·91–14·3)
Occupational particulate matter, gases, and fumes	9·6 (6·86–13·8)	8·92 (6·47–12·8)	8·54 (6·15–12·1)	-7·05 (-12·4–-1·09)	-4·24 (-7·98–-0·62)	4·65 (3·26–6·83)	4·61 (3·3–6·65)	4·48 (3·18–6·49)	-0·768 (-9·63–9·69)	-2·79 (-8·12–2·95)
Occupational noise	9·12 (8·31–10)	8·63 (8·05–9·34)	8·27 (7·72–8·94)	-5·32 (-9·29–-0·972)	-4·24 (-6·43–-1·88)	5·69 (4·96–6·45)	6·03 (5·51–6·64)	6·05 (5·64–6·61)	6·09 (-0·586–14)	0·298 (-2·69–3·86)
Occupational injuries	--	--	--	--	--	--	--	--	--	--
Occupational ergonomic factors	12·6 (10·3–15·3)	10·7 (9·05–12·8)	11·1 (9·21–13·3)	-14·9 (-25·5–-3·9)	3·53 (-4·4–13·5)	7·34 (5·75–9·25)	7·15 (5·88–8·9)	7·53 (5·98–9·46)	-2·49 (-17·6–17·4)	5·2 (-6·87–20·4)
Childhood sexual abuse	5·8 (3·47–9·07)	5·49 (3·94–7·2)	5·42 (3·9–7·23)	-5·39 (-25·6–25·8)	-1·15 (-6·48–6·02)	16 (11·6–22·5)	14·6 (11·3–19·4)	14·6 (11·3–19·5)	-9·15 (-21·8–4·89)	0·115 (-5·82–6·87)
Intimate partner violence	--	--	--	--	--	29·5 (16·4–38·6)	27·7 (15·7–37)	28·8 (15·7–38·1)	-6·01 (-14–5·61)	3·91 (-4·13–17·3)
Non-exclusive breastfeeding	26 (21·4–30·8)	24·8 (21·1–28·9)	24·7 (20·3–29·3)	-4·71 (-18·8–11·9)	-0·469 (-12·9–15·2)	--	--	--	--	--
Discontinued breastfeeding	27 (26–28)	25·6 (24·3–26·8)	25·4 (24·1–26·5)	-5·29 (-11–0·173)	-0·769 (-6·45–5·05)	27 (26–28)	25·5 (24·2–26·8)	25·3 (24·1–26·5)	-5·44 (-11·1–0·00759)	-0·793 (-6·48–5·03)
Diet low in calcium	17·7 (8·17–33·6)	13·9 (6·22–25·8)	13·1 (5·89–24·5)	-21·5 (-34·5–-11·3)	-5·79 (-15·1–2·76)	14·7 (6·07–28·2)	12·1 (4·95–22·7)	11·5 (4·77–21·8)	-18 (-32·6–-4·21)	-4·45 (-14·9–5·05)
Occupational exposure to asbestos	10·4 (9·46–11·6)	9·27 (8·57–9·98)	9·09 (6·69–11·6)	-11·3 (-21·1–0·0358)	-1·97 (-28·1–25·1)	2·1 (1·11–2·5)	2 (1·05–2·36)	2·03 (1·01–2·88)	-4·5 (-17·1–11·2)	1·55 (-25·9–36·7)
Occupational exposure to arsenic	0·464 (0–1·1)	0·465 (0–1·1)	0·496 (0–1·18)	0·251 (-15·1–NA)	6·57 (-5·16–NA)	0·398 (0–0·937)	0·419 (0–1·02)	0·435 (0–1·06)	5·43 (-11·5–NA)	3·74 (-8·13–NA)
Occupational exposure to benzene	0·808 (0·0453–2·3)	0·788 (0·046–2·27)	0·819 (0·05–2·41)	-2·4 (-10·8–10·7)	3·85 (-4·35–13·6)	1·18 (0·0661–3·29)	1·31 (0·0757–3·68)	1·37 (0·0813–3·95)	11·1 (-1·31–31)	4·65 (-3·89–19·7)
Occupational exposure to beryllium	0·0239 (0·0223–0·0253)	0·0231 (0·0226–0·0236)	0·0243 (0·0229–0·0258)	-3·31 (-9·17–3·72)	5·58 (-0·397–12)	0·0253 (0·023–0·0274)	0·0266 (0·026–0·0272)	0·0277 (0·0258–0·0297)	5·12 (-3·39–16·3)	4·23 (-3·21–11·4)
Occupational exposure to cadmium	0·0549 (0·0492–0·0607)	0·0552 (0·0502–0·0602)	0·0587 (0·0522–0·0668)	0·427 (-13·1–14·7)	6·42 (-4·08–18·5)	0·0488 (0·0432–0·0547)	0·0515 (0·0469–0·0571)	0·0533 (0·0468–0·0607)	5·46 (-9·4–25)	3·63 (-7·58–16·2)
Occupational exposure to chromium	0·134 (0·122–0·147)	0·137 (0·127–0·147)	0·145 (0·131–0·162)	1·71 (-10·2–14·1)	6·05 (-3·16–16·1)	0·126 (0·112–0·14)	0·134 (0·125–0·145)	0·139 (0·125–0·155)	6·71 (-6·42–23·1)	3·4 (-6·18–14·1)
Occupational exposure to diesel engine exhaust	0·844 (0·776–0·917)	0·823 (0·771–0·875)	0·854 (0·779–0·931)	-2·55 (-12·5–8·16)	3·84 (-3·8–12·6)	0·693 (0·611–0·779)	0·714 (0·652–0·785)	0·72 (0·636–0·811)	3·13 (-11·2–18·7)	0·712 (-10·4–11·5)
Occupational exposure to formaldehyde	0·271 (0·241–0·303)	0·267 (0·241–0·294)	0·281 (0·246–0·322)	-1·64 (-15·6–13·5)	5·17 (-5·77–17·5)	0·24 (0·212–0·27)	0·254 (0·233–0·279)	0·264 (0·235–0·298)	5·87 (-8·77–23·8)	3·81 (-6·55–15·7)
Occupational exposure to nickel	0·464 (0–1·96)	0·413 (0–1·89)	0·431 (0–1·91)	-10·9 (-23·5–NA)	4·29 (-5·47–NA)	0·322 (0–1·4)	0·32 (0–1·44)	0·332 (0–1·43)	-0·698 (-16·8–NA)	3·89 (-8·92–NA)
Occupational exposure to polycyclic aromatic hydrocarbons	0·27 (0·246–0·292)	0·273 (0·254–0·291)	0·288 (0·262–0·319)	1·09 (-9·85–12·8)	5·6 (-3·2–15·2)	0·272 (0·244–0·301)	0·299 (0·282–0·318)	0·309 (0·281–0·34)	9·92 (-2·32–24·5)	3·42 (-5·24–13)
Occupational exposure to silica	5·01 (0·724–15·9)	4·09 (0·595–13·1)	4·23 (0·609–12·9)	-18·5 (-30·9–-2·01)	3·43 (-7·2–17·9)	2·88 (0·386–9·21)	2·69 (0·373–8·57)	2·76 (0·381–9·03)	-6·58 (-23·1–14·9)	2·55 (-10·6–21·3)
Occupational exposure to sulfuric acid	0·871 (0·183–2·67)	0·966 (0·19–3·04)	1·01 (0·205–3·25)	10·9 (-3·58–29·3)	4·1 (-6·25–16·5)	0·793 (0·167–2·54)	1·02 (0·213–3·49)	1·01 (0·209–3·27)	29 (4·6–58·4)	-1·44 (-16·4–15·2)
Unsafe sex	--	--	--	--	--	--	--	--	--	--
Occupational exposure to trichloroethylene	0·0809 (0·0746–0·0869)	0·0837 (0·0798–0·0882)	0·0886 (0·0824–0·0961)	3·53 (-5·22–13·9)	5·86 (-1·56–14·3)	0·0767 (0·0688–0·0854)	0·0814 (0·0768–0·087)	0·0836 (0·0756–0·092)	6·12 (-5·59–21·1)	2·69 (-6·54–11·7)
No access to handwashing facility	3·47 (3·2–3·73)	3·16 (2·91–3·42)	3·12 (2·88–3·36)	-9·11 (-17·3–-0·642)	-1·28 (-7·27–4·49)	3·47 (3·2–3·73)	3·16 (2·91–3·42)	3·12 (2·88–3·36)	-9·11 (-17·3–-0·642)	-1·28 (-7·27–4·49)
Child wasting	0·754 (0·496–1·04)	0·73 (0·488–0·999)	0·73 (0·483–0·994)	-3·2 (-12·3–7·83)	-0·0374 (-6·83–7·63)	0·778 (0·52–1·08)	0·765 (0·5–1·05)	0·763 (0·506–1·05)	-1·69 (-11·6–9·71)	-0·293 (-7·68–7·87)
Child stunting	2·58 (1·35–3·79)	2·14 (1·11–3·13)	2·09 (1·06–3·15)	-17 (-35·5–6·79)	-2·38 (-18·1–13·7)	2·03 (1·02–3·08)	1·84 (0·906–2·8)	1·82 (0·904–2·73)	-9·67 (-29·3–15·4)	-0·951 (-15·4–16·4)
Chewing tobacco	4·39 (3·41–5·51)	4·47 (3·32–5·87)	4·25 (3·05–5·76)	1·86 (-26·4–42·6)	-4·86 (-24·1–21·2)	--	--	--	--	--
Diet low in legumes	74·8 (39·8–99·5)	77·6 (46·2–99·8)	77·5 (46·5–99·7)	3·76 (-0·528–21·6)	-0·245 (-5·58–5·15)	73·2 (37·4–99·3)	75·9 (43–99·6)	76·1 (43·5–99·5)	3·66 (-0·831–22·5)	0·199 (-5·35–6·79)
Short gestation	38·2 (31·3–45·6)	41·9 (34·9–50)	36·8 (30–44·8)	9·62 (-11·2–36·2)	-12·1 (-28·8–8·87)	35·1 (29–41·6)	41·3 (35–48)	35·9 (29·6–43)	17·8 (-3·79–43·6)	-13 (-28·4–4·67)
Low birth weight	15·2 (12·6–18·3)	18·1 (15·2–21·4)	17·4 (14·4–21)	18·9 (-4·83–50)	-3·63 (-21·5–19·9)	16·9 (14·2–19·7)	21·1 (18·2–24·3)	19·5 (16·6–22·8)	24·9 (2·89–52·3)	-7·62 (-22·7–11·8)
High temperature	27·1 (16·8–38)	30·2 (19·1–42)	27·9 (17·1–39·4)	11·7 (4·16–22·3)	-7·8 (-12·9–-3·73)	27·1 (16·8–38)	30·2 (19·1–42)	27·9 (17·1–39·4)	11·7 (4·16–22·3)	-7·8 (-12·9–-3·73)
Low temperature	37·6 (24–50·9)	41·1 (28·6–53·1)	38·7 (25·2–52)	9·28 (2·97–20·7)	-5·84 (-13–-1·21)	37·6 (24–50·9)	41·1 (28·6–53·1)	38·7 (25·2–52)	9·28 (2·97–20·7)	-5·84 (-13–-1·21)
Kidney dysfunction	21·2 (15·3–28·3)	22 (15·9–29·4)	22·1 (16·1–29·6)	4·11 (0·257–8·59)	0·376 (-3·34–4·63)	23·9 (17·3–31·5)	25·2 (18·3–33·2)	26·2 (19·2–34·2)	5·7 (1·2–11·2)	3·79 (-1·05–9·28)
Bullying victimization	5·87 (2·42–12)	8·93 (3·57–17·9)	8·67 (3·33–17·9)	52·2 (32·2–74·9)	-2·97 (-12·2–3·17)	--	--	--	--	--
High LDL cholesterol	65·6 (61·2–70·1)	34·8 (31·2–38·3)	30·9 (27·4–34·6)	-47 (-52·2–-41·4)	-11 (-15·9–-6·15)	65·4 (61·7–69·4)	34·7 (31·4–38·1)	32 (28·4–35·7)	-46·9 (-51·8–-42·2)	-7·89 (-12·3–-3·78)

Supplementary Results Table 1. Maine age-standardised Summary Exposure Values (SEVs) for most-detailed risk factors and percentage change, by sex, 1990, 2010, and 2019. Data in parentheses are 95% uncertainty intervals.

	Male					Female				
	1990	2010	2019	Percentage change 1990-2010	Percentage change 2010-2019	1990	2010	2019	Percentage change 1990-2010	Percentage change 2010-2019
Unsafe sanitation	6·15 (2·96–11)	3·27 (1·4–6·15)	2·72 (1·17–5·41)	-46·8 (-78·4–27·3)	-16·9 (-57·8–67·5)	--	--	--	--	--
Ambient particulate matter pollution	4·98 (0·454–12·7)	2·44 (0·507–5)	1·18 (0·133–3·22)	-50·9 (-89·1–179)	-51·9 (-80·8–35·2)	4·99 (0·502–12·7)	2·46 (0·531–5·02)	1·2 (0·151–3·23)	-50·6 (-88·5–169)	-51·5 (-79·4–35·1)
Household air pollution from solid fuels	0·0752 (0·00713–0·26)	0·0312 (0·00249–0·128)	0·0154 (0·000611–0·0678)	-58·5 (-91·3–64·5)	-50·5 (-87–-5·52)	0·0995 (0·0106–0·321)	0·0417 (0·00358–0·164)	0·0208 (0·000872–0·0851)	-58·1 (-91·1–58·4)	-50 (-86·3–-3·52)
Ambient ozone pollution	52·2 (22·1–87·8)	33·1 (12·9–59·3)	14 (4·29–28·4)	-36·5 (-50·7–-24·8)	-57·7 (-73·2–-44·1)	--	--	--	--	--
Residential radon	24·8 (4·04–71)	24·8 (4·04–71)	24·8 (4·04–71)	0 (0–0)	0 (0–0)	24·8 (4·04–71)	24·8 (4·04–71)	24·8 (4·04–71)	0 (0–0)	0 (0–0)
Lead exposure	26·5 (1·49–54·2)	16·3 (0·922–39·3)	11·6 (0·462–31·3)	-38·6 (-64–-23·8)	-28·6 (-58·4–-19·2)	16·3 (0·0323–43·3)	11·1 (0·0123–33·5)	8·38 (2·44e-05–27·7)	-31·7 (-84·9–34·6)	-24·5 (-81·8–-15·7)
Child underweight	0·934 (0·516–1·41)	0·947 (0·522–1·4)	0·924 (0·518–1·4)	1·37 (-16·2–23·2)	-2·45 (-15·5–12)	0·789 (0·42–1·21)	0·84 (0·458–1·32)	0·858 (0·475–1·32)	6·46 (-14·1–37·8)	2·14 (-12·7–17·5)
Iron deficiency	--	--	--	--	--	11·3 (9·36–13·3)	11·8 (9·95–13·8)	12·2 (10·1–14·5)	4·5 (-7·58–18·6)	2·79 (-6·48–13·3)
Vitamin A deficiency	0·832 (0·215–2·26)	1·09 (0·304–3·05)	0·788 (0·211–2·21)	31·4 (-68·3–490)	-28 (-69·9–64·4)	1·61 (0·626–3·26)	1·65 (0·621–3·41)	1·42 (0·538–3·1)	2·33 (-62·8–187)	-13·7 (-54·3–62·4)
Zinc deficiency	2·44 (0–8·12)	1·25 (0–4·04)	1·28 (0–4·17)	-48·7 (-100–NA)	2·22 (-17·9–NA)	2·51 (0–8·14)	1·26 (0–4·09)	1·26 (0–4)	-49·9 (-100–NA)	0·137 (-17·3–NA)
Smoking	29·1 (26·3–32·1)	22·1 (20–24·3)	20·1 (18·2–22·3)	-23·9 (-29·1–-18·6)	-9·08 (-12·5–-5·5)	22·6 (19·8–25·8)	17·3 (15·6–19·2)	15·5 (13·8–17·3)	-23·4 (-31·3–-15·1)	-10·2 (-14·2–-5·45)
Secondhand smoke	40·5 (38·1–43)	27·3 (25·6–29)	27·2 (24·2–30·1)	-32·6 (-38·5–-26·3)	-0·168 (-8·97–8·79)	38·1 (36·2–40·1)	26·2 (25–27·4)	26·3 (24·1–28·5)	-31·2 (-36·1–-26)	0·24 (-6·77–7·75)
Alcohol use	14·8 (9·49–20·8)	14 (8·43–19·8)	14·6 (8·83–20·5)	-5·19 (-38·3–45·1)	4·28 (-21·4–40·7)	--	--	--	--	--
Drug use	0·498 (0·288–0·869)	1 (0·728–1·34)	1·56 (1·17–2)	101 (44·1–176)	55·6 (33·7–81·9)	0·406 (0·272–0·619)	0·776 (0·588–1·01)	1·18 (0·931–1·48)	91·1 (50·3–140)	52·3 (32·5–76·9)
High fasting plasma glucose	13·1 (10·7–15·9)	18·1 (15·9–20·9)	19·4 (16·9–21·9)	38·6 (11·8–69·4)	6·72 (-6·32–22·4)	9·58 (8·2–11·4)	13·5 (11·9–15·5)	14·8 (12·8–17)	41·4 (19·6–66·8)	9·61 (-3·78–25·8)
High systolic blood pressure	26 (20·9–32·2)	16·1 (14–18·3)	19·7 (15·2–25)	-38·2 (-49·5–-22·1)	22·8 (-2·97–54·3)	23·7 (17·4–30·4)	13·2 (11·5–14·8)	17·9 (12·9–24)	-44·4 (-58–-25·4)	35·9 (-2·72–81·5)
High body-mass index	26·7 (18·6–36·8)	41·1 (30·2–52·8)	42·9 (32·1–54·7)	53·9 (34·8–80)	4·43 (-1·96–11·3)	32·3 (23·7–41·8)	44·6 (35·2–53·7)	46 (36·1–55·5)	38·3 (21·5–62·5)	3·02 (-3·12–10·2)
Low bone mineral density	12·2 (7·25–19)	9·45 (5·07–15·5)	11·9 (6·86–18·9)	-22·3 (-43·2–-0·32)	25·4 (-2·22–68·6)	16 (10·6–22·9)	16·8 (11·1–23·9)	19·2 (13·1–26·7)	4·65 (-17·5–32·8)	14·6 (-10·8–44·5)
Diet low in fruits	51·7 (38·1–65·3)	46·2 (33·1–59·8)	46·1 (33·8–59·1)	-10·8 (-22·5–0·588)	-0·0228 (-13·2–16·6)	--	--	--	--	--
Diet low in vegetables	26·4 (12·4–46·9)	40·5 (25–58)	39·8 (24·7–57·5)	53·2 (12·8–140)	-1·7 (-12·5–9·03)	28·1 (13·1–48·1)	33·6 (19·7–50·2)	32·7 (19–48·8)	19·6 (-7·17–84·8)	-2·81 (-16·2–10·5)
Diet low in whole grains	79·7 (70–88·6)	81·6 (73·2–89·7)	81·8 (73–89·8)	2·42 (-1·35–7·09)	0·158 (-2·2–2·73)	77·1 (66·7–86·5)	79·8 (70·6–88·6)	79·7 (70·4–88·5)	3·43 (-0·377–8·97)	-0·139 (-2·83–2·63)
Diet low in nuts and seeds	29·2 (9·09–57·2)	25·6 (8·92–55·5)	21·6 (7·11–54)	-12·3 (-45·8–53·4)	-15·7 (-43·4–10·8)	25·2 (6·59–55·3)	22·4 (7·18–54·2)	19·9 (6·27–53·5)	-10·9 (-43·9–66·8)	-11·1 (-38·7–18·6)
Diet low in milk	50 (27·3–70·8)	44·2 (20·5–65·2)	43·8 (21–65·2)	-11·5 (-29–1·62)	-1·04 (-13·7–13)	--	--	--	--	--
Diet high in red meat	76·8 (63·2–88)	75·7 (61·6–87·3)	75·4 (61·3–87·5)	-1·46 (-16·3–15·6)	-0·36 (-11·4–10·4)	78 (64·7–88·8)	74·8 (60·7–85·8)	74·2 (59·9–86·4)	-4·16 (-19·3–12·1)	-0·821 (-12·5–12·5)
Diet high in processed meat	77·9 (54–97·6)	85·9 (66·6–99·7)	85·7 (65·9–99·6)	10·3 (1·22–29·6)	-0·275 (-5·46–4·56)	79·1 (56–97·9)	88·9 (73·3–99·6)	87·5 (69·8–99·7)	12·4 (1·32–37·2)	-1·64 (-7·6–2·14)
Diet high in sugar-sweetened beverages	44·6 (27·4–64·3)	61·4 (40·9–79·1)	61·4 (40·8–78·8)	37·6 (-3·88–111)	0·0451 (-20·3–26·5)	48·4 (28·2–67·5)	66·8 (46·8–83·2)	65·4 (46·1–82·3)	38 (-0·117–114)	-2·1 (-21·6–20·9)
Diet low in fiber	44·8 (27·8–60·3)	30·9 (18·4–45·7)	28·8 (15·9–43·3)	-31 (-47·2–-15)	-6·97 (-26·2–12·5)	45·3 (28·7–62·1)	36·2 (21·7–50·2)	31·6 (18·1–46·9)	-20·2 (-34·8–-3·28)	-12·6 (-29·5–1·27)
Diet low in seafood omega-3 fatty acids	84·9 (67·3–100)	79·9 (61·9–99·4)	80·8 (62·9–99·6)	-5·85 (-19·8–8·96)	1·09 (-13·1–19·5)	87·4 (72·7–99·9)	83·9 (67·9–99·8)	84 (67·5–99·6)	-3·98 (-15·7–6·31)	0·0697 (-12·1–11·4)
Diet low in polyunsaturated fatty acids	14·8 (4·38–41)	7·4 (2·16–26·5)	7·27 (2·1–24·8)	-50·1 (-76·4–-8·41)	-1·76 (-23·9–25·3)	--	--	--	--	--
Diet high in trans fatty acids	89·5 (82–100)	87·9 (79·1–100)	87·3 (78–100)	-1·83 (-9·8–5·73)	-0·592 (-8·72–8·59)	--	--	--	--	--
Diet high in sodium	18·2 (4·65–41·7)	39·7 (11·6–64·5)	39 (10·8–64·6)	118 (38–428)	-1·8 (-29·2–35)	21 (4·93–45·5)	21·1 (5–45·4)	21·7 (4·97–45·9)	0·907 (-28·1–49·5)	2·69 (-18·5–33·6)
Low physical activity	1·71 (0·519–4·82)	1·4 (0·543–3·75)	1·46 (0·414–4·89)	-18·4 (-44·4–42·7)	4·3 (-39·9–37·5)	--	--	--	--	--
Occupational asthmagens	23·7 (19·7–29·3)	21·5 (18–25·5)	22·4 (18·7–26·9)	-9·15 (-21·2–3·19)	3·97 (-4·82–14·3)	12·3 (10·1–14·9)	12·4 (10·4–14·9)	12·5 (10·2–15·2)	1·1 (-12·9–17·5)	0·209 (-10·3–11·7)
Occupational particulate matter, gases, and fumes	10·1 (7·26–14·4)	9·56 (6·91–13·8)	9·27 (6·75–13·3)	-5·46 (-10·5–0·933)	-3·07 (-6·25–0·532)	5·94 (4·19–8·77)	5·65 (4·08–8·1)	5·46 (3·93–7·8)	-4·87 (-13·2–4·76)	-3·44 (-8·57–1·79)
Occupational noise	8·81 (8·04–9·63)	8·59 (7·99–9·31)	8·38 (7·85–9·03)	-2·51 (-5·94–1·61)	-2·39 (-4·38–-0·0539)	6·37 (5·59–7·26)	6·78 (6·22–7·39)	6·83 (6·36–7·43)	6·41 (0·13–14·1)	0·722 (-2·15–3·98)
Occupational injuries	--	--	--	--	--	--	--	--	--	--
Occupational ergonomic factors	13·8 (11·3–16·8)	11·8 (9·92–14·2)	12·3 (10·1–15)	-14·4 (-26·6–-1·08)	3·92 (-5·46–14)	8·54 (6·63–10·8)	8·51 (6·93–10·7)	9·14 (7·26–11·6)	-0·353 (-18–19·3)	7·4 (-7·38–25·2)
Childhood sexual abuse	7·4 (5·05–10·5)	6·97 (4·6–9·93)	6·85 (4·54–9·67)	-5·83 (-12·2–5·83)	-1·68 (-8·51–5·53)	12·3 (8·94–17·3)	17·4 (12·9–24)	17·8 (13·1–24·7)	41·9 (30·7–54·8)	2·17 (-5–11)
Intimate partner violence	--	--	--	--	--	31·6 (17·8–41·1)	29·9 (16·4–39·2)	30·9 (16·8–40·4)	-5·29 (-13·6–6·15)	3·35 (-6·21–17·4)
Non-exclusive breastfeeding	26·1 (21·6–30·9)	24·9 (21·2–29)	24·9 (20·2–30)	-4·42 (-19·6–14·5)	-0·0758 (-13·4–14·7)	--	--	--	--	--
Discontinued breastfeeding	26·9 (25·9–27·8)	24·8 (23·4–26·1)	25·4 (24·1–26·6)	-7·63 (-13·3–-2·16)	2·31 (-3·34–8·52)	26·9 (25·9–27·8)	24·9 (23·5–26·1)	25·4 (24·1–26·6)	-7·4 (-13·1–-1·93)	2·17 (-3·47–8·35)
Diet low in calcium	17·2 (8·47–31·5)	14 (6·77–25·5)	13·7 (6·48–25·3)	-18·4 (-29·2–-6·85)	-2·43 (-12·2–6·45)	12·7 (5·29–24·6)	11·1 (4·86–20·6)	11·1 (4·74–20·8)	-12·6 (-26·6–4·18)	-0·109 (-9·82–10·7)
Occupational exposure to asbestos	12·7 (11·5–14)	10·9 (10–11·8)	10 (7·67–12·5)	-13·9 (-24·7–-1·96)	-8·54 (-30·2–14·5)	2·76 (1·04–3·53)	2·65 (1·02–3·28)	2·78 (0·991–4·03)	-4·24 (-18·6–12·5)	5·13 (-20·3–37·2)
Occupational exposure to arsenic	0·544 (0–1·31)	0·535 (0–1·27)	0·563 (0–1·34)	-1·61 (-16·8–NA)	5·18 (-6·31–NA)	0·528 (0–1·22)	0·524 (0–1·26)	0·534 (0–1·27)	-0·633 (-17·2–NA)	1·87 (-9·97–NA)
Occupational exposure to benzene	0·837 (0·0494–2·42)	0·826 (0·0474–2·41)	0·849 (0·0506–2·42)	-1·31 (-9·24–11·9)	2·84 (-4·12–12·7)	1·24 (0·0737–3·54)	1·45 (0·0856–3·9)	1·5 (0·086–4·24)	16·6 (4·09–38·1)	3·42 (-5·48–15·4)
Occupational exposure to beryllium	0·0243 (0·0228–0·0257)	0·0241 (0·0234–0·0248)	0·0253 (0·0239–0·0267)	-0·744 (-7–6·3)	5·07 (-0·204–11·2)	0·0282 (0·0257–0·0305)	0·0303 (0·0295–0·0312)	0·0312 (0·0291–0·0332)	7·73 (-0·839–18·1)	2·78 (-3·6–9·08)
Occupational exposure to cadmium	0·0632 (0·0567–0·0699)	0·0625 (0·0564–0·0692)	0·0657 (0·0579–0·0741)	-1·09 (-14·4–13·4)	5·07 (-5·2–16·3)	0·0627 (0·0552–0·0703)	0·0633 (0·0571–0·0702)	0·0645 (0·0562–0·0741)	0·993 (-14–19·6)	1·92 (-8·78–14·4)
Occupational exposure to chromium	0·15 (0·136–0·164)	0·152 (0·139–0·166)	0·16 (0·143–0·177)	1·41 (-10·8–14·3)	4·97 (-4·15–14·6)	0·155 (0·138–0·172)	0·162 (0·149–0·176)	0·165 (0·147–0·184)	4·56 (-8·12–22·2)	1·71 (-7·54–11·6)
Occupational exposure to diesel engine exhaust	0·694 (0·639–0·752)	0·776 (0·728–0·83)	0·818 (0·751–0·888)	11·9 (1·34–24)	5·35 (-2·88–13·7)	0·685 (0·608–0·767)	0·781 (0·713–0·86)	0·782 (0·697–0·88)	14 (-1·53–32·8)	0·0824 (-10·1–10·3)
Occupational exposure to formaldehyde	0·316 (0·281–0·353)	0·305 (0·271–0·341)	0·316 (0·276–0·36)	-3·49 (-17·3–11·5)	3·83 (-6·85–15·6)	0·305 (0·269–0·345)	0·311 (0·282–0·344)	0·317 (0·278–0·36)	1·98 (-12·4–19·6)	1·82 (-8·18–13·1)
Occupational exposure to nickel	0·452 (0–1·95)	0·429 (0–1·86)	0·452 (0–1·95)	-5·05 (-19·6–NA)	5·27 (-6·12–NA)	0·419 (0–1·77)	0·4 (0–1·7)	0·409 (0–1·8)	-4·5 (-21·5–NA)	2·23 (-10·8–NA)
Occupational exposure to polycyclic aromatic hydrocarbons	0·296 (0·27–0·323)	0·299 (0·277–0·324)	0·313 (0·283–0·346)	1·28 (-10·3–13·6)	4·67 (-4·1–13·8)	0·327 (0·295–0·362)	0·354 (0·332–0·381)	0·361 (0·328–0·397)	8·2 (-3·47–22·4)	1·89 (-6·58–11·2)
Occupational exposure to silica	4·02 (0·565–13·2)	3·74 (0·507–12·1)	3·96 (0·537–12·4)	-6·92 (-20·4–9·17)	5·75 (-5·44–20·4)	3·49 (0·488–11·3)	3·22 (0·447–10·4)	3·28 (0·464–10·6)	-7·73 (-24·2–16)	1·85 (-12·1–19·8)
Occupational exposure to sulfuric acid	0·979 (0·211–3·24)	1·06 (0·216–3·47)	1·1 (0·233–3·56)	8·4 (-5·76–25·7)	3·65 (-7·57–14·9)	0·983 (0·209–3·2)	1·19 (0·248–3·86)	1·19 (0·253–3·94)	21·3 (-1·35–49·5)	-0·246 (-15·8–15·8)
Unsafe sex	--	--	--	--	--	--	--	--	--	--
Occupational exposure to trichloroethylene	0·0865 (0·0804–0·0931)	0·0908 (0·0862–0·0962)	0·0954 (0·0882–0·103)	4·89 (-3·89–15·4)	5·06 (-1·64–12·7)	0·0888 (0·08–0·0981)	0·0954 (0·0897–0·102)	0·0967 (0·0881–0·106)	7·48 (-4·19–23)	1·32 (-6·51–10·1)
No access to handwashing facility	3·34 (3·09–3·61)	3·09 (2·86–3·34)	3·06 (2·82–3·32)	-7·24 (-16·1–1·55)	-0·968 (-6·5–5·16)	3·34 (3·09–3·61)	3·09 (2·86–3·34)	3·06 (2·82–3·32)	-7·24 (-16·1–1·55)	-0·968 (-6·5–5·16)
Child wasting	0·745 (0·481–1·02)	0·726 (0·479–0·989)	0·725 (0·469–0·984)	-2·54 (-12–8·01)	-0·167 (-6·83–7·76)	0·771 (0·513–1·04)	0·763 (0·502–1·04)	0·758 (0·5–1·04)	-1·03 (-11·7–10·3)	-0·642 (-7·94–7·19)
Child stunting	2·3 (1·2–3·4)	2·03 (1·07–3·09)	1·98 (1·01–3)	-11·7 (-30–12·8)	-2·58 (-17·5–14·9)	1·88 (0·946–2·85)	1·75 (0·861–2·62)	1·73 (0·864–2·61)	-7 (-27·5–16·4)	-0·885 (-15·9–18·1)
Chewing tobacco	2·33 (1·77–3·01)	2·38 (1·68–3·23)	2·36 (1·68–3·35)	2·31 (-29·7–46)	-0·794 (-23·9–29)	--	--	--	--	--
Diet low in legumes	74·3 (40·3–99·3)	77·2 (46·8–99·7)	77·3 (46·2–99·7)	3·79 (-0·34–19·6)	0·233 (-5–6)	72·8 (35·8–98·8)	75·6 (43·5–99·6)	76 (44·1–99·6)	3·78 (-0·345–22·2)	0·555 (-4·43–8·02)
Short gestation	20·8 (16·9–25·4)	21·1 (17·3–25·5)	21·6 (17·2–27·2)	1·47 (-19·9–28)	2·41 (-17·9–28·4)	16·6 (14–19·7)	20·7 (17·5–24·3)	20·9 (17·4–24·8)	24·6 (2·29–49·6)	0·705 (-17·2–22·1)
Low birth weight	8·08 (6·69–9·79)	9·08 (7·56–10·9)	9·37 (7·57–11·6)	12·4 (-11·3–42·8)	3·18 (-18·3–32·7)	8·07 (6·93–9·28)	9·78 (8·21–11·5)	10·4 (8·72–12·2)	21·2 (-1·62–47·7)	6·06 (-13·9–28·5)
High temperature	22 (12–33·7)	24 (13·8–35·2)	25·5 (14·9–37)	9·1 (-1·17–22·7)	6·45 (0·659–12·9)	22 (12–33·7)	24 (13·8–35·2)	25·5 (14·9–37)	9·1 (-1·17–22·7)	6·45 (0·659–12·9)
Low temperature	34·1 (30·5–38·5)	33·2 (29·7–37·6)	35·6 (32·4–39·7)	-2·68 (-3·57–-1·87)	7·3 (4·45–10·4)	34·1 (30·5–38·5)	33·2 (29·7–37·6)	35·6 (32·4–39·7)	-2·68 (-3·57–-1·87)	7·3 (4·45–10·4)
Kidney dysfunction	19·5 (13·9–26·4)	19·9 (14·2–27·1)	19·9 (14·3–27·1)	1·92 (-2·36–6·72)	0·0415 (-4·76–5·1)	21·9 (15·5–29·3)	22·7 (16·3–30·4)	23·4 (16·9–31·2)	3·62 (-0·805–8·66)	3·16 (-1·42–8·27)

Bullying victimization	5·9<br>(2·42–12·1)	9·05<br>(3·64–17·9)	8·77<br>(3·37–18·1)	53·4<br>(32·5–76·8)	-3·16<br>(-12·6–2·92)	--	--	--	--	--
High LDL cholesterol	67·6<br>(63·1–72·1)	34·3<br>(30·8–37·9)	30·6<br>(27–34·4)	-49·2<br>(-54·3–-44·1)	-10·8<br>(-16·1–-5·81)	64·5<br>(60·7–68)	34·1<br>(30·7–37·5)	31·4<br>(27·9–35)	-47·1<br>(-51·8–-42·3)	-7·83<br>(-11·9–-3·74)

Supplementary Results Table 1. Georgia age-standardised Summary Exposure Values (SEVs) for most-detailed risk factors and percentage change, by sex, 1990, 2010, and 2019. Data in parentheses are 95% uncertainty intervals.

	Male					Female				
	1990	2010	2019	Percentage change 1990-2010	Percentage change 2010-2019	1990	2010	2019	Percentage change 1990-2010	Percentage change 2010-2019
Unsafe sanitation	36·9 (27·4–47·3)	43·3 (39·9–46·7)	28·5 (20·9–37·4)	17·2 (-8·31–56·5)	-34·1 (-50·9–-15·2)	36·9 (27·4–47·3)	43·3 (39·9–46·7)	28·5 (20·9–37·4)	17·2 (-8·31–56·5)	-34·1 (-50·9–-15·2)
Ambient particulate matter pollution	15 (7·27–27)	14·9 (10·5–20·6)	15·9 (11·1–21·8)	-0·94 (-43·5–82·9)	6·79 (-16·7–39·7)	13·7 (6·5–25)	13·8 (9·47–19·3)	15·1 (10·6–21·2)	0·359 (-42·9–85·8)	9·62 (-14·6–44)
Household air pollution from solid fuels	10 (4·15–19·3)	7·47 (3·13–14·9)	4·55 (1·64–9·84)	-25·4 (-43·4–4·4)	-39·1 (-57·2–-20·2)	12·1 (5·66–21·7)	9·14 (4·34–16·6)	5·7 (2·17–11·8)	-24·5 (-42·5–4·24)	-37·6 (-56·2–18·5)
Ambient ozone pollution	63·6 (26·7–100)	51·9 (21·6–89·5)	69·9 (29·7–100)	-18·3 (-33·4–-1·75)	34·6 (7·13–72·9)	63·6 (26·7–100)	51·9 (21·6–89·5)	69·9 (29·7–100)	-18·3 (-33·4–-1·75)	34·6 (7·13–72·9)
Residential radon	30·9 (3·91–100)	30·9 (3·91–100)	30·9 (3·91–100)	0 (0–0)	0 (0–0)	--	--	--	--	--
Lead exposure	39·3 (11·7–62·7)	39·5 (17·9–60·5)	34·2 (14·6–54·8)	0·456 (-12·5–61·6)	-13·4 (-23·7–-7·76)	18 (0·0222–44·6)	20·2 (0·565–45·4)	18·3 (0·913–42·6)	12 (-1·99–3600)	-9·25 (-18·1–50·6)
Child underweight	1·46 (0·948–2·04)	1·11 (0·695–1·59)	1·01 (0·612–1·48)	-23·6 (-34–-12·5)	-8·97 (-16·9–-0·621)	1·44 (0·916–2)	1·14 (0·707–1·61)	1·04 (0·629–1·49)	-21 (-30·2–-11·7)	-8·44 (-16·1–-0·684)
Iron deficiency	--	--	--	--	--	20 (17·6–22·3)	20 (18·1–21·9)	19·7 (17·6–22)	0·029 (-8·6–10·1)	-1·23 (-6·78–5·09)
Vitamin A deficiency	3·81 (1·55–7·7)	5 (2·03–9·78)	3·37 (1·36–7·09)	31·3 (-49·4–263)	-32·5 (-61·2–23·6)	2·67 (1·28–5)	4·56 (2·4–8·1)	3·29 (1·69–5·74)	70·8 (-15–253)	-28 (-51–12·9)
Zinc deficiency	7·14 (0·547–18·4)	8·08 (0·653–20·9)	5·71 (0·0982–16·3)	13·1 (-3·42–58·8)	-29·3 (-72·2–-16·3)	6·98 (0·379–18·3)	7·73 (0·544–19·7)	5·49 (0–15·6)	10·8 (-6·43–48·2)	-28·9 (-68·9–15·5)
Smoking	28·8 (25·4–32·4)	27·2 (24·2–30·3)	29 (25·8–32·5)	-5·56 (-12–0·977)	6·81 (2·71–11·2)	2·75 (2·08–3·53)	2·64 (2·16–3·21)	2·85 (2·32–3·43)	-3·84 (-22·4–22·8)	7·86 (-4·52–22·3)
Secondhand smoke	34·1 (31·7–36·6)	36·9 (35·3–38·3)	38 (35·5–40·4)	8·11 (-0·613–17·9)	2·96 (-1·98–8·11)	51·6 (49·2–53·8)	58·5 (57–59·8)	60 (57·7–62·2)	13·3 (7·77–19·7)	2·65 (-0·273–5·6)
Alcohol use	10·5 (6·88–15)	14·7 (10·3–20·4)	16·7 (11·7–22·7)	40·6 (10·8–83·5)	13·4 (-3·68–34·3)	1·3 (0·614–2·46)	2·01 (1·03–3·52)	2·49 (1·24–4·32)	54·8 (4·27–144)	23·6 (-8·79–69·6)
Drug use	0·167 (0·104–0·285)	0·176 (0·113–0·292)	0·19 (0·127–0·309)	5·43 (-3·88–17·3)	7·95 (-0·594–18·7)	0·101 (0·0634–0·17)	0·101 (0·0641–0·167)	0·106 (0·0673–0·176)	-0·491 (-8·65–8·2)	4·74 (-2·97–13·6)
High fasting plasma glucose	6·33 (5·52–7·26)	15·1 (12·7–17·8)	17·6 (15·2–20)	139 (104–182)	16·1 (1·51–35·4)	6·02 (5·28–6·88)	12·8 (10·8–15·3)	13·8 (11·7–16·2)	113 (80·8–149)	7·66 (-9·58–27·5)
High systolic blood pressure	54·3 (47–62·3)	39·3 (34·3–44·3)	43·2 (35·3–52)	-27·5 (-41–-12)	9·79 (-7·21–29·1)	46·4 (36·7–56·4)	27·6 (21·9–33·5)	31·1 (22·7–41)	-40·6 (-54·9–-20·2)	12·8 (-12·9–43·4)
High body-mass index	24·3 (18·2–32·1)	26·5 (20·2–34·3)	31 (24–39·7)	8·82 (-0·467–22·1)	17 (8·11–28)	25·6 (20–33)	27·8 (21·8–35·6)	31·4 (25·2–39·3)	8·7 (-3·83–23·7)	12·8 (2·78–24·5)
Low bone mineral density	8·38 (4·55–14·2)	8·48 (4·42–14·2)	8·46 (4·49–13·9)	1·1 (-25·5–32·8)	-0·218 (-23–31·7)	15·7 (10·2–22·7)	16 (10·6–22·6)	15·8 (10·3–23·5)	1·81 (-19·4–34·1)	-1·34 (-21·4–22·6)
Diet low in fruits	49·9 (38·4–63·4)	63·8 (53·7–76)	60·3 (49·1–73·1)	28 (15·7–46·1)	-5·45 (-11·7–-0·804)	51·7 (40–64·8)	65·1 (55–76·9)	61·7 (50·5–74·3)	26 (14·7–43)	-5·27 (-10·9–-1·05)
Diet low in vegetables	24·5 (10·7–45·2)	45·9 (25·5–69·3)	38 (20·8–60)	87·7 (28·5–239)	-17·3 (-33·5–-5·8)	--	--	--	--	--
Diet low in whole grains	99·9 (100–100)	99·9 (100–100)	99·9 (100–100)	0 (0–0)	0 (0–0)	99·9 (100–100)	99·9 (100–100)	99·9 (100–100)	0 (0–0)	0 (0–0)
Diet low in nuts and seeds	53·3 (22·9–72·1)	71·1 (35·4–85)	66·5 (33–81·5)	33·3 (14·8–68·8)	-6·4 (-14·2–-1·48)	47·8 (20–68·8)	67·2 (32·2–83)	62 (28·9–78·9)	40·5 (16·9–90·1)	-7·78 (-17–-1·84)
Diet low in milk	47·3 (21·4–68·5)	41·8 (16·6–61·9)	54·9 (29·9–76·5)	-11·4 (-25·7–-1·97)	31·2 (6·24–103)	46·3 (20·5–66·5)	40·6 (15·4–60·9)	54 (27·7–75·6)	-12·2 (-28·3–-2·45)	33·1 (6·49–106)
Diet high in red meat	35·1 (24·2–46·1)	27·7 (17·8–37·4)	30·4 (19·5–40·9)	-21·1 (-30·1–-14·8)	9·83 (0·422–22·3)	34·9 (24·1–45·7)	27·6 (17·6–37·3)	30·4 (19·8–41·3)	-21·1 (-30·2–-14·9)	10·2 (1·19–22·9)
Diet high in processed meat	43·1 (23·7–66·6)	43 (22·8–66)	44·7 (24·2–67·9)	-0·243 (-21·8–31·7)	3·98 (-10·1–22·5)	50·2 (28·3–76·5)	50·3 (27·8–75·6)	52·5 (30·2–77·5)	0·307 (-19·1–25·3)	4·36 (-7·21–20·4)
Diet high in sugar-sweetened beverages	23·6 (13·8–39·4)	23·2 (12·7–38·3)	25·3 (14·3–41·6)	-1·67 (-33–48·2)	8·78 (-16·9–47·9)	22·1 (12·2–37·4)	23·1 (12·5–38·4)	26·1 (13·2–42·7)	4·37 (-31·7–61·7)	13 (-14·3–56·5)
Diet low in fiber	20·4 (10·8–31·5)	31·4 (18·3–45·5)	26·4 (14·5–40·5)	54·1 (37–82)	-15·8 (-28·5–-4·28)	19·9 (10·3–31)	31·2 (17·6–45·9)	26 (14·1–39·3)	56·5 (38·3–90·5)	-16·4 (-29·1–-3·92)
Diet low in seafood omega-3 fatty acids	92·3 (78·9–100)	96·1 (88·6–100)	93·6 (85·6–100)	4·08 (-1·42–19·3)	-2·65 (-8·52–1·73)	94 (83·2–100)	97·5 (92·9–100)	95·1 (87·7–100)	3·72 (-0·149–15·1)	-2·46 (-8·32–0·33)
Diet low in polyunsaturated fatty acids	79·7 (44·1–100)	73·7 (34·9–100)	69·9 (30·8–100)	-7·61 (-22·1–0)	-5·15 (-15·2–0)	80·9 (45·9–100)	75·2 (37·2–100)	71·5 (33–100)	-7·14 (-21·1–0)	-4·84 (-14·3–0)
Diet high in trans fatty acids	55·2 (45·3–98·4)	43·2 (32–100)	42·8 (31·5–99·9)	-21·7 (-37·1–3·15)	-1·01 (-9·33–6·49)	55·4 (45·5–99·1)	45·6 (33·3–100)	45·3 (32·7–100)	-17·8 (-34·6–9·37)	-0·561 (-8·47–5·03)
Diet high in sodium	64·6 (40·3–83·8)	42·3 (14·8–65·8)	42·3 (14·3–66·9)	-34·5 (-69·3–-12·9)	-0·0617 (-22·5–24·6)	31·9 (11–55·4)	18·5 (4·38–41·2)	18·3 (4·25–41·7)	-41·9 (-70·6–-19·3)	-1·35 (-18·5–14)
Low physical activity	1·9 (0·909–4·06)	1·58 (0·759–3·48)	2·11 (1·02–4·35)	-17 (-35·6–1·76)	34·1 (7·75–80·3)	1·61 (0·87–3·31)	1·53 (0·813–3·08)	1·75 (0·938–3·43)	-4·79 (-22·3–13·5)	14·6 (-3·96–43·3)
Occupational asthmagens	25·9 (20·2–32·7)	20·5 (16·1–25·7)	17·5 (14·1–21·9)	-20·9 (-31·7–-8·65)	-14·3 (-24–-4·11)	16·6 (12·3–22·3)	13·5 (10·2–18)	10·7 (7·92–14·7)	-18·8 (-34·5–0·142)	-20·8 (-34·6–-5·51)
Occupational particulate matter, gases, and fumes	11·2 (8·39–15·5)	10·9 (8·22–14·8)	10·6 (8·11–14·4)	-2·74 (-7·72–2·72)	-2·55 (-4·58–-0·528)	6·81 (4·98–9·57)	6·66 (5·01–9·32)	6·35 (4·73–8·81)	-2·28 (-9·72–6·43)	-4·63 (-7·63–-1·31)
Occupational noise	12·6 (11·5–13·7)	12·3 (11·7–13·1)	12·1 (11·5–12·8)	-1·94 (-5·97–2·88)	-1·89 (-3·34–-0·276)	8·42 (7·36–9·6)	8·49 (7·91–9·31)	8·34 (7·85–9·02)	0·918 (-5·3–8·57)	-1·81 (-3·89–0·725)
Occupational injuries	--	--	--	--	--	--	--	--	--	--
Occupational ergonomic factors	31·5 (27·5–35·4)	25·9 (22·9–28·7)	19·3 (16·1–22·7)	-17·8 (-30·1–-3·94)	-25·5 (-35·1–-15·7)	24·9 (20·9–28·8)	20·7 (17·8–23·4)	15·3 (11·7–19·5)	-16·7 (-33·4–3·2)	-26·2 (-39·4–-11·6)
Childhood sexual abuse	2·52 (1·65–3·56)	2·52 (1·65–3·56)	2·52 (1·65–3·56)	0 (0–0)	0 (0–0)	3·14 (2·26–4·3)	3·14 (2·26–4·3)	3·14 (2·26–4·3)	0 (0–0)	0 (0–0)
Intimate partner violence	--	--	--	--	--	3·39 (2·01–4·92)	3·14 (1·81–4·23)	3·39 (2·01–4·92)	-7·38 (-18·7–9·05)	7·97 (-3·73–28·7)
Non-exclusive breastfeeding	27·5 (21·4–35·4)	24·4 (19·1–31·4)	24·6 (19–32)	-11·5 (-19·6–-2·5)	1 (-6·78–10·3)	27·5 (21·4–35·3)	24·4 (19·1–31·4)	24·6 (19–32)	-11·2 (-19·4–-2·2)	0·886 (-6·89–10·2)
Discontinued breastfeeding	17·8 (16·2–19·4)	18·9 (18–19·9)	17·5 (15·9–19·1)	6·44 (-3·96–18·1)	-7·49 (-15·7–1·01)	17·7 (16·1–19·3)	19 (18·1–20)	17·5 (15·9–19·1)	7·34 (-3·14–19·1)	-7·83 (-16–0·631)
Diet low in calcium	31·2 (17·6–54·8)	32·8 (19·3–55·5)	27·6 (15·2–49·4)	5·18 (0·78–12·7)	-15·7 (-26·2–-6·78)	--	--	--	--	--
Occupational exposure to asbestos	0·2 (0·125–0·276)	1·39 (1·16–1·61)	4·66 (3·58–6·05)	592 (387–971)	236 (158–334)	0·195 (0·121–0·367)	0·281 (0·143–0·365)	0·444 (0·125–0·623)	44·2 (-22·9–127)	58·1 (-25·2–125)
Occupational exposure to arsenic	0·366 (0·151–0·627)	0·325 (0·13–0·549)	0·334 (0·135–0·567)	-11·4 (-19·4–-2·35)	2·81 (-3·63–9·47)	0·341 (0·135–0·571)	0·309 (0·123–0·518)	0·317 (0·129–0·529)	-9·51 (-18·2–1·28)	2·76 (-3·62–9·96)
Occupational exposure to benzene	0·913 (0·566–1·59)	0·829 (0·515–1·48)	0·91 (0·556–1·64)	-9·17 (-16·7–2·08)	9·69 (2·64–18·6)	1·09 (0·67–2·03)	1·1 (0·676–1·95)	1·29 (0·793–2·31)	0·848 (-9·93–14·3)	17·8 (8·84–30)
Occupational exposure to beryllium	0·109 (0·101–0·115)	0·103 (0·101–0·105)	0·104 (0·1–0·107)	-5·12 (-11–1·9)	0·512 (-3·28–4·22)	0·115 (0·105–0·125)	0·111 (0·108–0·113)	0·113 (0·108–0·119)	-3·92 (-11·9–5·38)	2·33 (-3·06–7·77)
Occupational exposure to cadmium	0·199 (0·183–0·215)	0·178 (0·171–0·185)	0·183 (0·172–0·195)	-10·6 (-17·9–-2·36)	2·88 (-2·99–8·77)	0·188 (0·17–0·206)	0·172 (0·166–0·178)	0·178 (0·167–0·189)	-8·6 (-17·1–1·83)	3·37 (-2·82–9·91)
Occupational exposure to chromium	0·442 (0·409–0·478)	0·394 (0·379–0·41)	0·417 (0·392–0·445)	-11 (-18·4–-2·89)	5·82 (-0·18–12)	0·415 (0·373–0·457)	0·382 (0·368–0·396)	0·414 (0·388–0·444)	-7·95 (-17–2·51)	8·28 (1·41–15·4)
Occupational exposure to diesel engine exhaust	2·53 (2·33–2·72)	2·47 (2·38–2·57)	2·64 (2·48–2·8)	-2·18 (-10·6–6·85)	6·77 (0·859–12·6)	1·86 (1·68–2·06)	1·75 (1·67–1·83)	1·88 (1·73–2·03)	-6·02 (-15·6–5·26)	7·2 (-0·37–15·2)
Occupational exposure to formaldehyde	0·904 (0·825–0·988)	0·781 (0·74–0·822)	0·834 (0·767–0·904)	-13·6 (-21·9–-4·6)	6·84 (-0·413–14·1)	0·796 (0·71–0·882)	0·735 (0·701–0·77)	0·818 (0·754–0·885)	-7·65 (-17·3–4·05)	11·2 (3·34–19·4)
Occupational exposure to nickel	0·379 (0·123–1·01)	0·349 (0·114–0·902)	0·354 (0·113–0·931)	-8·01 (-15·9–1·08)	1·61 (-5–8·09)	0·32 (0·104–0·869)	0·288 (0·0935–0·759)	0·285 (0·0929–0·746)	-10·1 (-18·7–1·01)	-1·19 (-7·27–6·41)
Occupational exposure to polycyclic aromatic hydrocarbons	0·943 (0·874–1·02)	0·845 (0·814–0·875)	0·899 (0·848–0·954)	-10·4 (-17·5–-2·53)	6·41 (0·456–12·1)	0·908 (0·817–0·998)	0·848 (0·82–0·879)	0·932 (0·875–0·996)	-6·58 (-15·7–3·73)	9·82 (2·85–17·2)
Occupational exposure to silica	3·68 (1·9–8·07)	3·62 (1·85–8·03)	3·72 (1·93–8·23)	-1·74 (-11·1–9·99)	2·99 (-4·69–11·7)	2·48 (1·28–5·5)	2·22 (1·14–4·88)	2·11 (1·08–4·65)	-10·5 (-19·5–0·344)	-5·15 (-11·1–2·37)
Occupational exposure to sulfuric acid	1·19 (0·858–2·1)	1·07 (0·77–1·87)	1·09 (0·785–1·92)	-10·2 (-18·2–-0·0878)	2·16 (-4·09–8·79)	0·962 (0·686–1·71)	0·871 (0·63–1·54)	0·866 (0·626–1·52)	-9·47 (-17·5–0·738)	-0·608 (-6·76–6·16)
Unsafe sex	--	--	--	--	--	--	--	--	--	--
Occupational exposure to trichloroethylene	0·271 (0·252–0·291)	0·247 (0·24–0·256)	0·26 (0·247–0·274)	-8·74 (-15·7–-1·09)	5·33 (0·0695–10·9)	0·251 (0·226–0·277)	0·233 (0·226–0·242)	0·25 (0·235–0·267)	-6·89 (-15·8–3·8)	7·21 (0·506–14·2)
No access to handwashing facility	15·6 (14·8–16·4)	13·8 (13·1–14·5)	10·6 (10·1–11·2)	-11·5 (-16·6–-6·02)	-23 (-25·6–-19·9)	15·6 (14·8–16·4)	13·8 (13·1–14·5)	10·6 (10·1–11·2)	-11·5 (-16·6–-6·02)	-23 (-25·6–-19·9)
Child wasting	1·87 (1·5–2·24)	1·77 (1·42–2·14)	1·72 (1·37–2·08)	-5·09 (-19·2–9·64)	-2·68 (-12·8–8·03)	1·23 (0·948–1·53)	1·17 (0·901–1·45)	1·15 (0·873–1·42)	-4·77 (-14·5–6·01)	-2·15 (-10·1–6·26)
Child stunting	9·33 (6·19–11·7)	7·52 (4·79–9·57)	6·72 (4·24–8·73)	-19·3 (-34·2–-2·9)	-10·6 (-23·2–1·95)	8·75 (5·98–11·2)	7·54 (4·9–9·64)	6·63 (4·11–8·62)	-13·9 (-28·2–3·29)	-12 (-24·8–1·23)
Chewing tobacco	0·346 (0·264–0·443)	0·342 (0·261–0·444)	0·349 (0·266–0·452)	-1·21 (-27·1–31·5)	1·99 (-15·8–23·5)	--	--	--	--	--
Diet low in legumes	100 (100–100)	99·9 (99·3–100)	99·7 (98·8–100)	-0·146 (-0·686–0)	-0·166 (-0·816–0·139)	100 (99·8–100)	99·5 (98·3–100)	99·1 (97·3–100)	-0·493 (-1·56–0)	-0·341 (-1·45–0·147)
Short gestation	6·73 (5·22–8·63)	7·82 (6·47–9·34)	10·2 (8·41–12·2)	16·2 (-12·3–54·4)	30·6 (6·23–60·6)	4·98 (4·11–6·51)	5·05 (4·35–5·88)	6·54 (5·46–7·72)	1·37 (-23·5–24·9)	29·5 (8·11–56·2)
Low birth weight	3·21 (2·56–3·99)	3·53 (2·92–4·27)	4·29 (3·61–5·08)	10·1 (-15·8–43·8)	21·3 (-4·84–54)	2·55 (2·12–3·26)	2·71 (2·32–3·16)	3·24 (2·66–3·85)	6·22 (-18·3–33·1)	19·6 (-4–47)

High temperature	24·6<br>(14·7–36·1)	28·5<br>(19·7–39·4)	27·9<br>(19·3–38·5)	16<br>(0·936–45·9)	-2·15<br>(-5·08–1·21)	24·6<br>(14·7–36·1)	28·5<br>(19·7–39·4)	27·9<br>(19·3–38·5)	16<br>(0·936–45·9)	-2·15<br>(-5·08–1·21)
Low temperature	34·2<br>(31·4–37·9)	33<br>(29·8–37)	33·9<br>(30·9–37·9)	-3·62<br>(-5·97–-1·23)	2·88<br>(1·4–4·31)	34·2<br>(31·4–37·9)	33<br>(29·8–37)	33·9<br>(30·9–37·9)	-3·62<br>(-5·97–-1·23)	2·88<br>(1·4–4·31)
Kidney dysfunction	20·6<br>(13·7–28·6)	22·5<br>(15·4–30·6)	24·2<br>(16·9–32·4)	9·28<br>(5·55–15·2)	7·73<br>(3·63–13·3)	20·3<br>(13·6–28)	21·7<br>(14·6–29·7)	23·3<br>(16·3–31·4)	6·95<br>(3·77–11·4)	7·44<br>(3·63–13)
Bullying victimization	2·48<br>(1·03–5·07)	2·53<br>(1·06–5·2)	2·61<br>(1·1–5·35)	1·95<br>(-2·45–8·26)	3·24<br>(1·42–5·13)	1·42<br>(0·581–2·93)	1·5<br>(0·627–3·03)	1·55<br>(0·644–3·15)	5·87<br>(-0·302–14·6)	3·35<br>(1·11–5·52)
High LDL cholesterol	24·4<br>(21·2–27·8)	25·4<br>(22·1–29·1)	26·2<br>(22·8–29·9)	3·74<br>(-2·53–10·2)	3·49<br>(-0·973–7·41)	27·7<br>(24·5–31)	28·5<br>(25·3–32·1)	29·4<br>(26·2–33·1)	3·12<br>(-1·56–8·25)	2·84<br>(-0·735–6·45)

Supplementary Results Table 1. Maryland age-standardised Summary Exposure Values (SEVs) for most-detailed risk factors and percentage change, by sex, 1990, 2010, and 2019. Data in parentheses are 95% uncertainty intervals.

	Male					Female				
	1990	2010	2019	Percentage change 1990-2010	Percentage change 2010-2019	1990	2010	2019	Percentage change 1990-2010	Percentage change 2010-2019
Unsafe sanitation	4·84 (2·21–8·6)	2·47 (1·04–4·56)	2·08 (0·902–3·82)	-48·9 (-79·8–25·8)	-15·8 (-55·7–72·3)	--	--	--	--	--
Ambient particulate matter pollution	14·2 (5·29–27·4)	8·48 (5·56–11·7)	4·92 (2·38–7·76)	-40·2 (-69·2–41·7)	-41·9 (-58·7—32·8)	14·2 (5·33–27·4)	8·48 (5·56–11·7)	4·93 (2·38–7·76)	-40·2 (-69·2–41·9)	-41·9 (-58·7—32·8)
Household air pollution from solid fuels	0·0123 (0·00268–0·0351)	0·00697 (0·00121–0·0217)	0·0048 (0·000658–0·0168)	-43·4 (-78·2–30·5)	-31·1 (-65·4–39·5)	0·0162 (0·00386–0·043)	0·00922 (0·00155–0·0292)	0·00635 (0·000914–0·0222)	-43·1 (-78·1–30·7)	-31·1 (-65·4–39·6)
Ambient ozone pollution	89·4 (44·4–100)	70·3 (30·8–100)	56·2 (23·5–94·4)	-21·4 (-37·2–0)	-20·1 (-31·5—5·12)	--	--	--	--	--
Residential radon	20·4 (3·42–58·8)	20·4 (3·42–58·8)	20·4 (3·42–58·8)	0 (0–0)	0 (0–0)	20·4 (3·42–58·8)	20·4 (3·42–58·8)	20·4 (3·42–58·8)	0 (0–0)	0 (0–0)
Lead exposure	62·8 (36·5–83·4)	34·6 (14·8–55·6)	23·4 (7·84–41·8)	-45 (-61—31)	-32·3 (-47·1—24·6)	37·2 (6·68–65·7)	19·6 (1·51–44)	13·1 (0·477–32·4)	-47·2 (-79·4—30·6)	-33·5 (-70·5—25·2)
Child underweight	0·941 (0·538–1·43)	0·938 (0·524–1·4)	0·916 (0·509–1·38)	-0·318 (-18·3–22·6)	-2·35 (-15·6–12·6)	0·793 (0·426–1·21)	0·829 (0·451–1·28)	0·853 (0·465–1·34)	4·54 (-16·9–37·3)	2·86 (-10·9–19·6)
Iron deficiency	--	--	--	--	--	12·2 (10·2–14·4)	12·3 (10·4–14·4)	12·6 (10·4–14·8)	0·856 (-11·1–14·3)	2·36 (-6·89–13)
Vitamin A deficiency	0·679 (0·17–1·81)	0·873 (0·227–2·43)	0·628 (0·169–1·69)	28·6 (-68–517)	-28 (-69·3–81·9)	1·39 (0·484–2·88)	1·33 (0·519–2·75)	1·17 (0·393–2·61)	-4·43 (-64·9–194)	-11·7 (-54·5–72·6)
Zinc deficiency	1·91 (0–6·49)	0·987 (0–3·04)	1·03 (0–3·27)	-48·2 (-100–NA)	4·41 (-13·5–NA)	1·96 (0–6·43)	0·993 (0–3·27)	1·02 (0–3·27)	-49·3 (-100–NA)	2·87 (-15·9–NA)
Smoking	24·8 (22·1–27·5)	17·6 (15·8–19·3)	14·8 (13·2–16·4)	-29·2 (-34·3—23·5)	-15·6 (-19·3—11·9)	19·6 (17·1–22·5)	13·6 (12·2–15·1)	11·2 (9·92–12·5)	-30·8 (-37·9—23)	-17·7 (-21·9—13)
Secondhand smoke	39·7 (37–42·3)	24 (22·4–25·7)	23·9 (21·1–26·4)	-39·4 (-44·9—32·7)	-0·756 (-9·66–8·31)	36·5 (34·3–38·7)	22·2 (21·1–23·4)	22·2 (20·2–24·2)	-39 (-44·5—33·7)	-0·138 (-7·85–7·07)
Alcohol use	14·8 (9·45–20·9)	16·3 (10·1–22·9)	17·9 (11–25·6)	10·1 (-19·1–50·9)	9·4 (-10·8–37·1)	--	--	--	--	--
Drug use	0·505 (0·273–0·934)	0·709 (0·482–1·06)	0·893 (0·636–1·26)	40·4 (6·68–93)	26 (10·4–45·9)	0·416 (0·265–0·659)	0·477 (0·327–0·68)	0·62 (0·463–0·834)	14·7 (-1·05–37·9)	30·2 (12·6–52·9)
High fasting plasma glucose	13·7 (11·3–16·5)	18·4 (16·1–21·1)	19·4 (17–22·5)	35 (12–64·1)	5·4 (-6·82–21)	11 (9·26–13·1)	14·7 (12·9–16·8)	15·7 (13·7–18·2)	34·2 (11·2–60·6)	6·88 (-7·32–22·6)
High systolic blood pressure	25·3 (20·6–31·2)	15·4 (13·4–17·6)	19·3 (15·1–24·1)	-39 (-51·2—24·8)	25·1 (-1·56–58·4)	22·9 (16·6–30·4)	13·1 (11·5–14·8)	17·5 (12·7–23·1)	-42·9 (-57·6—21·9)	33·4 (-2·73–76·2)
High body-mass index	25·8 (18·1–36·1)	40·2 (29·7–51·8)	41·6 (30·6–53)	56·2 (37·7–82·4)	3·25 (-3·18–10·7)	32 (23·7–40·7)	44·7 (35·2–53·7)	45·9 (35·6–55·4)	39·8 (24·6–60·8)	2·63 (-3·76–9·56)
Low bone mineral density	11·9 (6·91–18·8)	8·94 (4·88–14·8)	11·3 (6·45–18·5)	-25 (-44—4·53)	26·8 (-1·87–74·1)	15·8 (10·3–22·9)	16·1 (10·5–22·9)	18·4 (12·4–25·7)	2 (-18·8–27·6)	14·4 (-7·81–50·4)
Diet low in fruits	51·5 (39·8–64·6)	45·1 (33·5–57·8)	46·6 (34·3–60·1)	-12·5 (-23·3—2·72)	3·48 (-8·75–16·6)	35·9 (12·2–79·4)	23·7 (4·84–61·2)	24·9 (5·19–61·7)	-33·9 (-70·7—12·2)	5·11 (-20·9–74·5)
Diet low in vegetables	24·4 (11·7–42·8)	14·4 (9·66–21·9)	14·2 (9·35–21·8)	-40·7 (-60·8–4·69)	-1·34 (-14·6–10·8)	25·4 (12–46)	8·64 (5·01–16·8)	8·64 (4·76–17·1)	-66 (-76·3—41·6)	-0·016 (-29·4–30·7)
Diet low in whole grains	79·1 (69·2–88·1)	80·9 (72·3–89·5)	81 (71·7–89·3)	2·32 (-1·52–7·37)	0·0528 (-2·52–2·58)	76·2 (64·9–86·1)	78·9 (69·6–87·7)	78·8 (69·5–87·9)	3·56 (-0·711–9·1)	-0·16 (-3·09–2·88)
Diet low in nuts and seeds	24·8 (6·82–54·5)	24·5 (7·93–55·1)	19·6 (5·7–53·7)	-1·14 (-39·5–90·6)	-19·8 (-48·3–8·01)	20·7 (4·96–53·9)	20·8 (5·84–54·1)	17·8 (5·39–53·3)	0·699 (-39·3–102)	-14·7 (-44·8–17·5)
Diet low in milk	45·2 (22·2–64·3)	38·1 (15·6–57·6)	37·6 (15·9–57·4)	-15·7 (-36·6—0·984)	-1·46 (-15·5–14·1)	--	--	--	--	--
Diet high in red meat	75·6 (63·8–85·7)	78·2 (65–88·5)	77·9 (64·5–89·1)	3·33 (-10·2–18·8)	-0·365 (-10·4–10·8)	67·8 (54·7–79·9)	69·8 (56·1–81·7)	70·3 (55–83·3)	2·96 (-13·1–23·3)	0·716 (-12–15·2)
Diet high in processed meat	74·5 (50·3–95·2)	83·6 (63·6–99)	83·4 (64·5–99·1)	12·2 (2·79–33·7)	-0·178 (-4·54–4·7)	74·7 (48·7–95·9)	84·8 (66·1–98·9)	83·4 (63·4–98·7)	13·6 (2·25–38·2)	-1·6 (-7·39–2·35)
Diet high in sugar-sweetened beverages	49·6 (30·8–68)	68·2 (49·8–84)	67·8 (47·9–84·5)	37·6 (-1·71–107)	-0·541 (-19·7–20·8)	54 (32·9–73·5)	73·6 (54·8–88)	71·8 (51·7–87·4)	36·2 (-0·269–113)	-2·35 (-18·7–18)
Diet low in fiber	40·2 (24·9–55·4)	26·1 (14·3–39·2)	24 (12·8–37·7)	-34·9 (-51·7—17·5)	-8·12 (-26·5–12·2)	40·5 (24·6–56·5)	31·4 (18·7–45·4)	27 (14·9–41·3)	-22·5 (-38·7—3·95)	-14·1 (-31·5–2·99)
Diet low in seafood omega-3 fatty acids	80·7 (61·9–99·7)	75·7 (57·2–98·7)	76 (58·1–98·4)	-6·16 (-22·7–12·2)	0·354 (-15·1–17·5)	83·9 (67–100)	80 (61·5–99·1)	80 (62·2–99·4)	-4·64 (-18·5–7·51)	-0·0327 (-13·2–14·2)
Diet low in polyunsaturated fatty acids	12 (3·62–36·7)	6·36 (1·78–24·4)	6·24 (1·74–24·5)	-47·2 (-77·6—6·77)	-1·79 (-23·1–20·4)	--	--	--	--	--
Diet high in trans fatty acids	89·5 (81·4–100)	87·9 (79·1–100)	87·5 (77·4–100)	-1·8 (-9·9–6·09)	-0·437 (-8·71–8·28)	88·4 (81·1–100)	86·7 (77·5–100)	86·3 (77·5–100)	-1·87 (-9·6–5·87)	-0·55 (-8·58–8·42)
Diet high in sodium	18·2 (4·6–41·5)	39·6 (11–64·5)	39 (11·3–63·8)	118 (36·7–434)	-1·68 (-27·4–30·8)	42·3 (2·61–67·7)	22 (4·27–45·5)	58·2 (0–100)	-48 (-80·6–71·7)	164 (-100–700)
Low physical activity	2·48 (0·914–5·24)	2·04 (0·757–5·12)	2·41 (0·892–5·03)	-17·8 (-59·1–34)	18 (-23·4–119)	--	--	--	--	--
Occupational asthmagens	19·9 (16·5–24·1)	18·7 (15·7–22·1)	19·3 (16·1–23)	-5·59 (-16·8–6·19)	2·74 (-6·14–12·4)	10·6 (8·69–12·7)	11·2 (9·28–13·4)	11·2 (9·1–13·8)	5·61 (-9·79–23·1)	0·577 (-11·1–13·7)
Occupational particulate matter, gases, and fumes	7·96 (5·6–11·6)	7·58 (5·39–10·9)	7·4 (5·31–10·8)	-4·74 (-10·5–1·8)	-2·37 (-6·14–1·34)	4·33 (2·94–6·48)	4·26 (3·03–6·06)	4·14 (2·95–6·02)	-1·75 (-10·9–8·34)	-2·68 (-7·74–2·64)
Occupational noise	7·57 (6·93–8·36)	7·54 (7–8·19)	7·43 (6·94–8·04)	-0·365 (-4·08–4·12)	-1·57 (-3·84–0·931)	5·48 (4·8–6·2)	5·99 (5·5–6·57)	6·08 (5·66–6·62)	9·23 (2·45–17·4)	1·62 (-1·32–5·05)
Occupational injuries	--	--	--	--	--	--	--	--	--	--
Occupational ergonomic factors	12·3 (10·1–14·9)	11 (9·32–13·2)	11·5 (9·45–13·8)	-10·1 (-20·2–0·987)	3·66 (-4·07–12·6)	7·16 (5·6–9·18)	7·36 (5·98–9·17)	7·79 (6·12–9·81)	2·83 (-14·1–23·3)	5·86 (-7·74–22)
Childhood sexual abuse	7·4 (5·05–10·5)	6·97 (4·6–9·93)	6·85 (4·54–9·67)	-5·83 (-12·2–5·83)	-1·68 (-8·51–5·53)	12·3 (8·94–17·3)	17·4 (12·9–24)	17·8 (13·1–24·7)	41·9 (30·7–54·8)	2·17 (-5–11)
Intimate partner violence	--	--	--	--	--	34·8 (20·1–44·2)	33·1 (19·2–43)	34 (19·5–44)	-4·84 (-13·3–8·32)	2·57 (-5·19–15·7)
Non-exclusive breastfeeding	26·3 (22–31·2)	25·1 (21·3–29·1)	25 (20·7–29·9)	-4·73 (-18·9–12·6)	-0·428 (-13·6–15)	--	--	--	--	--
Discontinued breastfeeding	27·6 (26·6–28·6)	25·3 (23·9–26·7)	25·9 (24·6–27)	-8·35 (-14·1—2·61)	2·13 (-3·57–8·24)	27·6 (26·5–28·5)	25·4 (24–26·7)	25·9 (24·6–27)	-7·99 (-13·8—2·24)	2·03 (-3·67–8·13)
Diet low in calcium	14·4 (7·04–26·1)	11·6 (5·61–20·8)	11·1 (5·37–20·2)	-19·3 (-30·6—6·47)	-4·39 (-13·9–4·7)	9·61 (3·97–18·1)	8·32 (3·56–15·3)	8·18 (3·45–15·2)	-13·4 (-29·2–5·65)	-1·63 (-13·5–9·86)
Occupational exposure to asbestos	9·72 (8·55–10·9)	7·39 (6·73–8·05)	6·79 (4·85–9·03)	-24 (-34·6—11·9)	-8·12 (-33·2–21·6)	1·61 (1·14–2·14)	1·39 (1·02–1·81)	1·37 (0·918–1·95)	-13·4 (-27·9–3·95)	-1·28 (-30–32·7)
Occupational exposure to arsenic	0·448 (0–1·06)	0·465 (0–1·1)	0·49 (0–1·17)	3·73 (-12–NA)	5·41 (-5·4–NA)	0·419 (0–0·987)	0·448 (0–1·09)	0·456 (0–1·09)	7·05 (-8·62–NA)	1·82 (-8·63–NA)
Occupational exposure to benzene	0·895 (0·0515–2·61)	0·925 (0·0543–2·65)	0·941 (0·0594–2·71)	3·4 (-5·81–17)	1·77 (-6·77–13·6)	1·26 (0·0714–3·57)	1·5 (0·0899–4·29)	1·55 (0·0903–4·47)	19·8 (7·16–41·8)	2·92 (-5·48–16)
Occupational exposure to beryllium	0·024 (0·0227–0·0255)	0·0246 (0·0241–0·0251)	0·0257 (0·0243–0·0271)	2·6 (-3·43–8·5)	4·32 (-1·16–9·99)	0·0273 (0·0251–0·0295)	0·0298 (0·0292–0·0304)	0·0306 (0·0287–0·0325)	9·05 (0·759–18·9)	2·7 (-3·44–9·39)
Occupational exposure to cadmium	0·0534 (0·048–0·0598)	0·0557 (0·0505–0·0611)	0·0585 (0·052–0·0659)	4·18 (-9·73–19)	5·1 (-4·44–16·6)	0·0511 (0·0455–0·0571)	0·0551 (0·0503–0·0609)	0·0562 (0·0503–0·0633)	7·84 (-5·95–25·3)	2·01 (-7·69–13·2)
Occupational exposure to chromium	0·13 (0·118–0·143)	0·138 (0·128–0·149)	0·145 (0·131–0·161)	6·6 (-6·27–19·7)	4·57 (-3·8–14·6)	0·129 (0·115–0·142)	0·143 (0·134–0·154)	0·146 (0·132–0·16)	11·3 (-1·11–26·5)	1·79 (-6·83–10·7)
Occupational exposure to diesel engine exhaust	0·7 (0·647–0·755)	0·784 (0·736–0·836)	0·811 (0·742–0·883)	11·9 (1·54–23·8)	3·54 (-4·36–12·2)	0·632 (0·564–0·701)	0·704 (0·642–0·772)	0·703 (0·624–0·787)	11·5 (-2·24–28·2)	-0·224 (-10·2–10·8)
Occupational exposure to formaldehyde	0·265 (0·236–0·297)	0·271 (0·244–0·3)	0·281 (0·247–0·32)	2·36 (-12·5–17·8)	3·64 (-6·42–15·3)	0·249 (0·22–0·278)	0·276 (0·254–0·302)	0·281 (0·253–0·313)	10·8 (-3·27–27·7)	2 (-7·32–12·2)
Occupational exposure to nickel	0·366 (0–1·59)	0·365 (0–1·55)	0·387 (0–1·61)	-0·0768 (-15·1–NA)	5·99 (-4·95–NA)	0·322 (0–1·35)	0·332 (0–1·47)	0·341 (0–1·53)	3·09 (-13·2–NA)	2·61 (-9·59–NA)
Occupational exposure to polycyclic aromatic hydrocarbons	0·26 (0·236–0·285)	0·278 (0·258–0·299)	0·289 (0·263–0·321)	6·96 (-5·11–19·3)	4·17 (-3·79–13·9)	0·278 (0·25–0·305)	0·321 (0·302–0·342)	0·327 (0·299–0·358)	15·5 (3·04–29·9)	1·89 (-6·06–10·3)
Occupational exposure to silica	3·29 (0·474–10·3)	3·2 (0·443–9·8)	3·41 (0·465–10·5)	-3 (-17·5–13·9)	6·61 (-4·14–22)	2·61 (0·35–8·48)	2·59 (0·359–8·24)	2·63 (0·377–8·46)	-0·792 (-17·8–24)	1·51 (-11·3–19·3)
Occupational exposure to sulfuric acid	0·84 (0·181–2·63)	0·967 (0·197–3·05)	0·993 (0·207–3·12)	15·2 (-1·6–35·6)	2·68 (-8·83–16·4)	0·765 (0·157–2·49)	0·982 (0·208–3·17)	0·964 (0·2–3·1)	28·4 (4–58·7)	-1·8 (-15·5–15)
Unsafe sex	--	--	--	--	--	--	--	--	--	--
Occupational exposure to trichloroethylene	0·0796 (0·074–0·0862)	0·0864 (0·082–0·0909)	0·09 (0·0835–0·0976)	8·55 (-0·597–18·8)	4·18 (-2·6–12·7)	0·0776 (0·0706–0·0848)	0·0859 (0·0813–0·0914)	0·087 (0·0796–0·0951)	10·7 (-0·0629–25·1)	1·29 (-6·32–9·75)
No access to handwashing facility	3·28 (3·03–3·55)	3·07 (2·85–3·33)	3·04 (2·81–3·29)	-6·44 (-15–2·5)	-0·868 (-6·87–4·8)	3·28 (3·03–3·55)	3·07 (2·85–3·33)	3·04 (2·81–3·29)	-6·44 (-15–2·5)	-0·868 (-6·87–4·8)
Child wasting	0·746 (0·488–1·03)	0·725 (0·479–0·991)	0·723 (0·464–0·974)	-2·8 (-12·3–8·39)	-0·312 (-7·31–7·33)	0·771 (0·508–1·05)	0·757 (0·497–1·05)	0·754 (0·499–1·04)	-1·8 (-11·5–8·59)	-0·358 (-8·12–7·87)
Child stunting	2·34 (1·19–3·44)	2·02 (1·03–3·04)	1·97 (1·03–3·01)	-13·8 (-33·2–8·26)	-2·54 (-16·9–14·4)	1·91 (0·962–2·86)	1·74 (0·85–2·65)	1·73 (0·855–2·65)	-8·56 (-28·9–14·9)	-0·87 (-16–18·3)
Chewing tobacco	1·7 (1·28–2·26)	2·03 (1·43–2·73)	2·08 (1·46–2·91)	19·7 (-18·8–74·8)	2·37 (-21·7–32·3)	--	--	--	--	--
Diet low in legumes	72·9 (35·5–99)	75·6 (42·9–99·6)	75·7 (42·8–99·6)	3·74 (-0·461–20·7)	0·0497 (-5·46–6·6)	71·3 (31·8–98·9)	74 (38–99·2)	74·4 (41–99·4)	3·84 (-0·634–22·1)	0·569 (-4·46–8·14)
Short gestation	34·5 (27·7–41·9)	34·8 (29·1–40·9)	31·4 (25·3–38·4)	0·819 (-17·2–22·8)	-9·82 (-26·6–10·3)	28·7 (23·7–34)	32·6 (27·3–38·1)	30·7 (25·7–36·5)	13·5 (-6·55–40·6)	-5·91 (-21·9–14·4)
Low birth weight	14·2 (11·6–17·2)	14·8 (12·7–17·1)	13·9 (11·4–16·7)	4·11 (-16·1–27·9)	-6·28 (-23·4–15·5)	13·7 (11·5–16·1)	16·1 (13·9–18·6)	16 (13·7–18·6)	17·4 (-4·65–44·5)	-0·189 (-16·5–21·5)
High temperature	24·3 (14·3–36)	29·4 (18·6–41·6)	25·3 (15·1–37·1)	21 (9·65–36·5)	-14·2 (-22·6—8·3)	24·3 (14·3–36)	29·4 (18·6–41·6)	25·3 (15·1–37·1)	21 (9·65–36·5)	-14·2 (-22·6—8·3)
Low temperature	34·3 (29·2–40·3)	37·3 (32·7–42·5)	36·3 (31·3–42·1)	8·86 (5·59–12·7)	-2·88 (-5·1—0·857)	34·3 (29·2–40·3)	37·3 (32·7–42·5)	36·3 (31·3–42·1)	8·86 (5·59–12·7)	-2·88 (-5·1—0·857)
Kidney dysfunction	20·3 (14·4–27·4)	20·8 (15·1–28·3)	20·7 (14·9–27·8)	2·77 (-1·99–8·11)	-0·734 (-5·4–3·88)	22·4 (16·1–30)	23·2 (16·6–31·4)	23·8 (17·2–31·6)	3·8 (-1·17–10·1)	2·66 (-2–8·34)
Bullying victimization	14·1 (5·9–29·3)	20·7 (10·1–35·7)	20·1 (8·97–36·4)	47·3 (11·7–134)	-2·93 (-11·9–4·06)	12·9 (5·35–25·8)	17·9 (8·44–33)	17·3 (7·63–32·8)	39·2 (4·99–109)	-3·8 (-11·6–2·79)
High LDL cholesterol	68·3 (64·2–72·8)	34·6 (31·1–38·3)	30·7 (27·1–34·6)	-49·4 (-54·6—44·3)	-11 (-16·2—6·09)	66·2 (62·8–69·9)	33·9 (30·6–37·3)	31·3 (27·7–34·8)	-48·7 (-53·3—44·2)	-7·78 (-11·9—3·58)

Supplementary Results Table 1. Massachusetts age-standardised Summary Exposure Values (SEVs) for most-detailed risk factors and percentage change, by sex, 1990, 2010, and 2019. Data in parentheses are 95% uncertainty intervals.

	Male					Female				
	1990	2010	2019	Percentage change 1990-2010	Percentage change 2010-2019	1990	2010	2019	Percentage change 1990-2010	Percentage change 2010-2019
Unsafe sanitation	3·71 (1·7–6·94)	2·02 (0·883–3·81)	1·72 (0·764–3·3)	-45·5 (-79·3–35·1)	-15 (-57·2–66·5)	--	--	--	--	--
Ambient particulate matter pollution	7·5 (1·14–17·3)	4·46 (1·88–7·3)	2·37 (0·299–4·94)	-40·5 (-76·9–173)	-46·9 (-84·3–31·6)	7·5 (1·13–17·3)	4·46 (1·89–7·3)	2·37 (0·298–4·94)	-40·5 (-77–175)	-46·9 (-84·2–31·6)
Household air pollution from solid fuels	0·00671 (0·000936–0·0223)	0·00362 (0·00045–0·0134)	0·00211 (0·000119–0·00851)	-46·1 (-82·6–69·9)	-41·8 (-85·6–11·2)	0·00887 (0·00122–0·0282)	0·0048 (0·000601–0·0171)	0·00279 (0·000152–0·0115)	-45·9 (-82·6–69·1)	-41·9 (-85·5–10·9)
Ambient ozone pollution	64·1 (26·7–100)	45·1 (18·4–76·9)	33·3 (12·5–60·9)	-29·6 (-42·8–16·7)	-26·1 (-43·4–10·9)	64·1 (26·7–100)	45·1 (18·4–76·9)	33·3 (12·5–60·9)	-29·6 (-42·8–16·7)	-26·1 (-43·4–10·9)
Residential radon	22·1 (3·85–62·3)	22·1 (3·85–62·3)	22·1 (3·85–62·3)	0 (0–0)	0 (0–0)	22·1 (3·85–62·3)	22·1 (3·85–62·3)	22·1 (3·85–62·3)	0 (0–0)	0 (0–0)
Lead exposure	60·9 (32·9–81·5)	32·4 (12·2–53·8)	21·6 (6·36–40·1)	-46·7 (-63·4–32·5)	-33·4 (-49·5–25·4)	35·7 (5·35–64)	18·3 (0·947–42·8)	12 (0·231–31)	-48·8 (-84·5–31·7)	-34·1 (-76·1–26)
Child underweight	0·91 (0·5–1·39)	0·919 (0·506–1·41)	0·896 (0·499–1·36)	0·965 (-17·1–24·9)	-2·45 (-14·7–11·9)	0·771 (0·403–1·16)	0·816 (0·451–1·24)	0·834 (0·458–1·29)	5·79 (-16·9–54·3)	2·3 (-12·9–19·6)
Iron deficiency	--	--	--	--	--	11·6 (9·62–13·6)	11·9 (10–13·9)	12 (10–14·3)	2·56 (-8·58–16·4)	1·21 (-8·15–11·1)
Vitamin A deficiency	0·537 (0·143–1·38)	0·753 (0·185–2·07)	0·545 (0·133–1·5)	40·1 (-63·8–500)	-27·5 (-69–83·7)	1·17 (0·435–2·37)	1·19 (0·454–2·5)	1·05 (0·394–2·22)	1·6 (-62·3–224)	-11·6 (-52·1–67·8)
Zinc deficiency	1·87 (0–6·3)	0·975 (0–2·97)	0·955 (0–2·94)	-48 (-100–NA)	-2·04 (-50–NA)	1·91 (0–6·38)	0·981 (0–3·14)	0·935 (0–2·93)	-48·7 (-100–NA)	-4·66 (-30·9–NA)
Smoking	26·7 (23·9–29·3)	18·3 (16·5–20·2)	15·4 (13·8–17·1)	-31·3 (-36·1–26)	-15·6 (-19·1–12·2)	22·4 (19·9–25·3)	15·7 (14·1–17·5)	12·6 (11·1–14)	-30·2 (-36·2–23·3)	-19·6 (-23·5–15·9)
Secondhand smoke	40·8 (38·3–43·4)	23·6 (21·9–25·2)	24 (21·3–26·6)	-42·2 (-47·9–36·4)	1·63 (-8·04–11·6)	36·6 (34·6–38·5)	21·7 (20·7–22·9)	22·3 (20·3–24·3)	-40·6 (-44·9–35·6)	2·38 (-5–10·5)
Alcohol use	17 (11·2–23·2)	16·3 (10·4–21·8)	17 (11·3–22·7)	-4·1 (-33·9–39·5)	3·98 (-19–36·3)	--	--	--	--	--
Drug use	0·546 (0·299–0·973)	1·23 (0·889–1·71)	1·75 (1·33–2·29)	126 (60·2–228)	41·9 (24–64·7)	0·483 (0·283–0·833)	0·793 (0·579–1·11)	1·17 (0·886–1·53)	64·1 (29–117)	46·9 (27·1–72·6)
High fasting plasma glucose	12·6 (10·3–15·4)	16·9 (14·5–19·7)	17·9 (15·3–20·8)	34·5 (9·76–63·5)	5·68 (-7·98–22·9)	8·73 (7·53–10·3)	11·5 (9·98–13·3)	12·6 (10·8–14·9)	31·7 (12–55·2)	9·62 (-5·21–28·5)
High systolic blood pressure	25 (19·9–30·2)	15·6 (13·7–17·7)	19·6 (15·1–24·3)	-37·4 (-49·8–22·1)	25·2 (-0·212–54·2)	23·2 (17·2–30·3)	12·7 (11–14·3)	17·2 (12·6–22·9)	-45·3 (-59·1–26·2)	35·6 (-0·414–81·4)
High body-mass index	26·1 (18·3–35·9)	39·3 (29·1–51·6)	41·3 (30·4–53·4)	50·5 (32·6–74·3)	5·02 (-1·52–12·7)	27·6 (19·6–37·4)	41·1 (32·8–49·1)	43·2 (34·3–52·4)	48·7 (26·2–78·2)	5·14 (-1·7–11·7)
Low bone mineral density	6·83 (3·36–11·9)	6·72 (3·31–12)	6·68 (3·23–11·7)	-1·62 (-25·4–31·7)	-0·653 (-27·8–33·5)	18 (12·2–25·2)	15·9 (10·3–22·9)	16·9 (11·4–24·1)	-11·7 (-30–10·5)	6·19 (-15·1–34·1)
Diet low in fruits	46·8 (35·1–59·8)	41·7 (30·4–54·1)	42 (29·4–55·5)	-10·9 (-23·5–1·52)	0·736 (-14–16·1)	--	--	--	--	--
Diet low in vegetables	30·9 (15·4–51·8)	36·8 (20·8–57·5)	33·8 (17·8–55·8)	19 (-0·572–57·7)	-8·11 (-24·4–6·22)	31·7 (16–52·3)	33·6 (18·5–53·4)	31·2 (16·4–50·9)	6·09 (-10·8–33·8)	-7·19 (-24–7·94)
Diet low in whole grains	78·9 (68·8–87·8)	80·8 (71·8–88·9)	80·6 (71·1–89)	2·33 (-1·25–7·25)	-0·178 (-2·99–2·39)	76·2 (65·5–85·9)	78·9 (69·2–87·6)	78·5 (68·9–87·6)	3·53 (-0·662–9·69)	-0·506 (-3·63–2·67)
Diet low in nuts and seeds	24·5 (6·81–55·2)	24·4 (6·8–54·7)	18·7 (5·05–53·2)	-0·483 (-41·6–93·7)	-23·4 (-49·9–4·23)	20·6 (4·76–53·3)	20·7 (6·19–53·5)	16·9 (4·85–52·6)	0·428 (-41·3–92·6)	-18·2 (-46·3–8·91)
Diet low in milk	45 (22·7–65·2)	37·3 (15·7–57·2)	35·6 (14·7–55·6)	-16·9 (-38·7–1·37)	-4·7 (-20·9–11·3)	--	--	--	--	--
Diet high in red meat	75·5 (62·1–86·1)	77·8 (64·7–88·5)	78·2 (65·2–89·6)	3·15 (-11·6–21·3)	0·427 (-10·4–11·6)	69·5 (55·9–80·9)	71·7 (57·5–83·7)	72·7 (59·7–84·3)	3·11 (-13·9–21·5)	1·4 (-11–16·2)
Diet high in processed meat	74·3 (48·8–96·3)	83·7 (64·7–99·4)	83·9 (64·5–99·4)	12·8 (2·14–35·1)	0·141 (-4·69–5·66)	74·1 (49·4–96)	85·2 (66·5–99·4)	84·1 (65·6–99·4)	15 (2·54–41·8)	-1·37 (-6·66–2·51)
Diet high in sugar-sweetened beverages	49·5 (30·1–69·4)	68·8 (50·1–84·5)	68·9 (50·6–85·8)	38·9 (1·63–115)	1·44 (-17·1–24·4)	54·2 (33·9–72·9)	74·2 (56·5–88·8)	73·7 (56·5–88·6)	36·9 (2·02–111)	-0·771 (-18·1–17·5)
Diet low in fiber	40·7 (26–56·1)	26·5 (14·8–40·9)	23·4 (11·7–37·7)	-34·7 (-53·1–16)	-11·7 (-30·8–9·34)	41·2 (25·3–57·3)	32 (19·1–47·1)	26·6 (14·6–41·1)	-22·3 (-38·8–4·77)	-17 (-34·5–0·652)
Diet low in seafood omega-3 fatty acids	96·5 (87·5–100)	96·7 (86·9–100)	95·3 (83·6–100)	0·166 (-8·46–10·3)	-1·39 (-9·88–6·27)	98·8 (92·5–100)	98·8 (92·5–100)	98·7 (92·9–100)	0·0448 (-6·61–7·52)	-0·144 (-5·23–5·55)
Diet low in polyunsaturated fatty acids	17·9 (6·08–47·2)	13·6 (5·01–37·7)	12·6 (4·64–33·9)	-24 (-42·9–2·38)	-7·9 (-24·2–8·19)	--	--	--	--	--
Diet high in trans fatty acids	89·4 (81·8–100)	87·9 (78·8–100)	87·4 (77·7–100)	-1·76 (-9·62–6·25)	-0·564 (-8·41–7·85)	88·4 (81·2–100)	86·7 (78·2–100)	86·1 (77·1–100)	-1·92 (-9·62–5·76)	-0·631 (-8·95–8·16)
Diet high in sodium	18·5 (4·34–41·2)	39·4 (11·2–63·9)	38·8 (10·6–63·9)	113 (38–406)	-1·59 (-28·9–32·9)	--	--	--	--	--
Low physical activity	2·14 (0·761–4·6)	1·8 (0·73–4·1)	1·77 (0·613–4·13)	-16 (-46·1–26·5)	-1·63 (-28·5–32·1)	4·78 (2·3–9·42)	4·5 (2·35–8·38)	4·62 (2·32–9·06)	-5·75 (-29·9–29·2)	2·71 (-10·9–16·9)
Occupational asthmagens	23·4 (19·5–28·5)	20·7 (17·4–24·5)	21·5 (17·7–26·3)	-11·9 (-24·4–1·45)	4·05 (-5·9–17)	11·9 (9·65–14·6)	12·1 (9·97–14·6)	12·3 (10–15·1)	2·17 (-13·1–21·3)	1·27 (-9·98–15·7)
Occupational particulate matter, gases, and fumes	9·33 (6·57–13·3)	8·56 (6·13–12·2)	8·24 (5·87–11·7)	-8·3 (-13·5–2·29)	-3·7 (-7·39–0·0472)	5·32 (3·71–7·88)	4·98 (3·53–7·21)	4·76 (3·38–6·86)	-6·42 (-14·8–2·62)	-4·38 (-9·34–1·1)
Occupational noise	8·45 (7·75–9·29)	8·19 (7·62–8·95)	7·98 (7·47–8·71)	-3·14 (-6·65–1·15)	-2·57 (-4·66–0·252)	6·17 (5·46–6·94)	6·55 (6·01–7·16)	6·59 (6·15–7·19)	6·14 (0·243–12·7)	0·578 (-2·12–3·61)
Occupational injuries	--	--	--	--	--	--	--	--	--	--
Occupational ergonomic factors	11·8 (9·69–14·5)	10·6 (9·02–12·8)	11 (9·07–13·4)	-9·76 (-19·5–1·51)	3·78 (-4·08–12·6)	6·86 (5·33–8·78)	7·14 (5·79–8·86)	7·51 (5·91–9·57)	3·96 (-14·2–24·1)	5·21 (-7·34–21)
Childhood sexual abuse	7·4 (5·05–10·5)	6·97 (4·6–9·93)	6·85 (4·54–9·67)	-5·83 (-12·2–5·83)	-1·68 (-8·51–5·53)	12·3 (8·94–17·3)	17·4 (12·9–24)	17·8 (13·1–24·7)	41·9 (30·7–54·8)	2·17 (-5–11)
Intimate partner violence	--	--	--	--	--	28·4 (15·1–37·5)	26·7 (15·2–35·7)	27·8 (15·6–36·8)	-5·92 (-13·5–5·92)	4·12 (-4·85–17·9)
Non-exclusive breastfeeding	26·4 (22·1–31·1)	25·1 (21·3–28·8)	25 (20·3–29·7)	-5·13 (-20–11·3)	-0·383 (-14·1–13·6)	--	--	--	--	--
Discontinued breastfeeding	27·6 (26·5–28·5)	25·3 (23·9–26·6)	25·7 (24·3–27)	-8·22 (-13·5–2·39)	1·5 (-4·09–8·02)	27·7 (26·6–28·6)	25·3 (23·9–26·5)	25·7 (24·3–26·9)	-8·83 (-14·1–3·04)	1·63 (-3·97–8·18)
Diet low in calcium	13·1 (5·11–24·9)	8·84 (3·6–16·5)	7·86 (3·18–14·8)	-32·4 (-46·1–20)	-11·1 (-23·5–0·599)	9 (3·44–17·1)	6·64 (2·62–12·3)	5·99 (2·27–11·3)	-26·2 (-44·2–4·72)	-9·74 (-25·8–5·6)
Occupational exposure to asbestos	11·8 (10·6–13)	9·36 (8·71–9·97)	8·62 (6·47–11)	-20·8 (-29·7–11·3)	-7·97 (-31·5–18·9)	1·8 (1·07–2·21)	1·64 (0·915–1·96)	1·61 (0·88–2·23)	-9·12 (-25·8–8·87)	-1·92 (-28·9–30·4)
Occupational exposure to arsenic	0·549 (0–1·3)	0·534 (0–1·25)	0·569 (0–1·35)	-2·58 (-16·6–NA)	6·48 (-5·53–NA)	0·502 (0–1·18)	0·499 (0–1·21)	0·508 (0–1·2)	-0·609 (-16·3–NA)	1·81 (-9·37–NA)
Occupational exposure to benzene	0·936 (0·0562–2·65)	0·951 (0·0568–2·81)	0·976 (0·0592–2·86)	1·63 (-6·99–15·5)	2·61 (-5·2–13·1)	1·32 (0·0743–3·77)	1·55 (0·0904–4·3)	1·61 (0·0953–4·56)	17 (5·43–34·6)	3·82 (-4·83–15·8)
Occupational exposure to beryllium	0·0245 (0·023–0·026)	0·0247 (0·0242–0·0253)	0·0259 (0·0245–0·0274)	0·894 (-5·28–7·91)	4·87 (-0·941–11)	0·0278 (0·0254–0·0301)	0·0298 (0·0292–0·0304)	0·0307 (0·0289–0·0326)	7·17 (-1·06–17·2)	2·97 (-3·38–8·89)
Occupational exposure to cadmium	0·0637 (0·0574–0·0706)	0·0628 (0·0568–0·0692)	0·0666 (0·0591–0·0757)	-1·45 (-14·2–13·4)	6·08 (-4·85–17·5)	0·0599 (0·0525–0·0676)	0·0604 (0·0547–0·0669)	0·0616 (0·0544–0·0698)	0·791 (-13·2–18·7)	2·06 (-8·25–14·5)
Occupational exposure to chromium	0·153 (0·139–0·168)	0·154 (0·142–0·167)	0·162 (0·146–0·181)	0·757 (-10·3–13·7)	5·3 (-4·09–15·2)	0·15 (0·134–0·167)	0·157 (0·146–0·169)	0·16 (0·144–0·176)	4·26 (-8·21–19·9)	1·93 (-6·75–11·8)
Occupational exposure to diesel engine exhaust	0·691 (0·638–0·749)	0·761 (0·712–0·81)	0·779 (0·71–0·849)	10·1 (0·349–21·5)	2·45 (-5·16–10·8)	0·657 (0·581–0·734)	0·72 (0·658–0·788)	0·719 (0·64–0·807)	9·49 (-5·44–25·9)	-0·114 (-9·5–10·5)
Occupational exposure to formaldehyde	0·326 (0·292–0·363)	0·313 (0·282–0·345)	0·327 (0·289–0·373)	-3·93 (-16·6–11·1)	4·68 (-6·27–15·9)	0·299 (0·263–0·335)	0·307 (0·282–0·337)	0·314 (0·28–0·353)	2·72 (-10·2–19·3)	2·13 (-7·31–12·9)
Occupational exposure to nickel	0·449 (0–1·98)	0·425 (0–1·86)	0·455 (0–1·91)	-5·47 (-19·4–NA)	7·05 (-5·12–NA)	0·392 (0–1·74)	0·373 (0–1·67)	0·383 (0–1·74)	-4·66 (-20·4–NA)	2·55 (-10·2–NA)
Occupational exposure to polycyclic aromatic hydrocarbons	0·306 (0·279–0·334)	0·309 (0·288–0·332)	0·325 (0·296–0·36)	1·19 (-9·15–13·5)	5·11 (-3·64–14·5)	0·322 (0·288–0·353)	0·349 (0·329–0·371)	0·356 (0·326–0·388)	8·23 (-3·14–22·7)	2·25 (-5·94–10·7)
Occupational exposure to silica	3·93 (0·563–12·9)	3·65 (0·497–11·9)	3·92 (0·53–12·3)	-7·09 (-21·2–9·83)	7·33 (-4·34–22·7)	3·22 (0·442–9·93)	2·96 (0·396–9·68)	3·02 (0·405–10)	-8·19 (-24·3–15·8)	1·91 (-11·5–20·8)
Occupational exposure to sulfuric acid	0·98 (0·204–3·09)	1·06 (0·224–3·35)	1·1 (0·226–3·54)	7·98 (-6·24–25·5)	4·08 (-7·59–15·9)	0·895 (0·183–2·81)	1·06 (0·228–3·33)	1·05 (0·221–3·36)	18 (-3·28–45·4)	-0·556 (-14·1–15·5)
Unsafe sex	--	--	--	--	--	--	--	--	--	--
Occupational exposure to trichloroethylene	0·0876 (0·0812–0·0947)	0·0911 (0·0869–0·0959)	0·0951 (0·0882–0·104)	3·98 (-4·63–13·7)	4·36 (-2·8–12·1)	0·086 (0·0774–0·0949)	0·091 (0·086–0·097)	0·0923 (0·0844–0·101)	5·76 (-5·29–19·5)	1·41 (-6·22–9·84)
No access to handwashing facility	3·22 (3–3·48)	3·04 (2·82–3·28)	3·02 (2·8–3·27)	-5·67 (-12·9–3·24)	-0·676 (-6·57–5·33)	3·22 (3–3·48)	3·04 (2·82–3·28)	3·02 (2·8–3·27)	-5·67 (-12·9–3·24)	-0·676 (-6·57–5·33)
Child wasting	0·743 (0·49–1·02)	0·721 (0·473–0·989)	0·718 (0·472–0·972)	-2·99 (-12·4–7·98)	-0·436 (-7·76–7·44)	0·765 (0·503–1·04)	0·757 (0·501–1·04)	0·752 (0·491–1·03)	-1·06 (-10·7–9·53)	-0·596 (-7·83–6·65)
Child stunting	2·21 (1·18–3·24)	1·95 (1·02–2·91)	1·9 (0·964–2·87)	-11·9 (-30·7–12·4)	-2·51 (-16·9–14·3)	1·83 (0·9–2·77)	1·69 (0·843–2·61)	1·67 (0·83–2·62)	-7·56 (-27·6–18·5)	-1·12 (-17–18·6)
Chewing tobacco	1·61 (1·22–2·13)	2·06 (1·47–2·76)	2·14 (1·54–2·98)	28·2 (-13·1–86·3)	4·01 (-20·1–34)	0·284 (0·176–0·451)	0·341 (0·21–0·522)	0·36 (0·214–0·579)	20·1 (-30–108)	5·5 (-27·2–51·6)
Diet low in legumes	72·8 (36–99)	75·4 (41–99·6)	75 (41·4–99·5)	3·46 (-0·792–21·8)	-0·42 (-6·83–5·12)	71·2 (32·5–98·5)	73·7 (37·4–99·2)	73·8 (39·7–99·1)	3·61 (-0·678–23·5)	0·0867 (-6·14–7·83)
Short gestation	28 (22·5–34·2)	26·4 (21·9–32·2)	26·2 (21·1–32·5)	-5·43 (-26–18·1)	-0·93 (-22–23·6)	22·1 (18·6–26)	23·9 (20·4–27·8)	24·9 (20·7–29·4)	8·42 (-9·36–29·9)	4·03 (-12·8–24·6)
Low birth weight	11·3 (9·22–13·9)	11·3 (9·52–13·7)	11·5 (9·46–14)	0·519 (-22–28·4)	1·78 (-21–30·3)	10·3 (8·74–12)	12·3 (10·7–14·1)	13·4 (11·3–15·6)	19·8 (-0·89–44·7)	8·67 (-8·64–31·5)
High temperature	25·7 (14·3–38·7)	27 (15·4–39·9)	27·4 (15·8–40·4)	5·01 (0·904–10·7)	1·61 (-1·59–4·34)	25·7 (14·3–38·7)	27 (15·4–39·9)	27·4 (15·8–40·4)	5·01 (0·904–10·7)	1·61 (-1·59–4·34)
Low temperature	33·5 (29·4–37·8)	34·4 (30·3–38·6)	35·6 (31·6–39·7)	2·6 (1·38–4)	3·59 (2·39–4·92)	33·5 (29·4–37·8)	34·4 (30·3–38·6)	35·6 (31·6–39·7)	2·6 (1·38–4)	3·59 (2·39–4·92)
Kidney dysfunction	22·5 (15·8–30·3)	22·4 (16–30·1)	23·2 (16·7–30·7)	-0·421 (-4·53–3·52)	3·55 (-0·363–8)	25·4 (17·9–33·3)	25·1 (17·9–33·5)	25·8 (18·5–34·1)	-1·46 (-5·83–3·46)	2·99 (-1·19–7·9)
Bullying victimization	5·87 (2·44–11·9)	9·02 (3·6–17·7)	8·73 (3·35–17·9)	53·7 (32·7–77·4)	-3·23 (-12·8–3·08)	--	--	--	--	--
High LDL cholesterol	67·6 (62·9–72·1)	34·2 (30·8–37·8)	30·8 (27·3–34·6)	-49·4 (-54·3–44·2)	-9·92 (-14·9–5·22)	66·3 (62·5–70)	34·3 (31–37·7)	32 (28·5–35·8)	-48·2 (-53–43·6)	-6·8 (-11·1–2·81)

Supplementary Results Table 1. Michigan age-standardised Summary Exposure Values (SEVs) for most-detailed risk factors and percentage change, by sex, 1990, 2010, and 2019. Data in parentheses are 95% uncertainty intervals.

	Male					Female				
	1990	2010	2019	Percentage change 1990-2010	Percentage change 2010-2019	1990	2010	2019	Percentage change 1990-2010	Percentage change 2010-2019
Unsafe sanitation	6·16 (2·98–11·1)	3·35 (1·5–6·52)	2·69 (1·13–4·96)	-45·6 (-77·6–32·8)	-19·8 (-58·6–58·1)	6·16 (2·98–11·1)	3·35 (1·5–6·52)	2·69 (1·13–4·96)	-45·6 (-77·6–32·8)	-19·8 (-58·6–58·1)
Ambient particulate matter pollution	10·4 (3·09–21·9)	6·04 (3·4–8·97)	5·06 (2·53–7·92)	-41·8 (-73·2–62·7)	-16·1 (-26·7–-11·1)	10·4 (3·03–21·9)	6·04 (3·41–8·98)	5·07 (2·53–7·91)	-41·8 (-73·2–62·8)	-16·1 (-26·6–-11·1)
Household air pollution from solid fuels	0·015 (0·00317–0·0426)	0·00862 (0·00147–0·0267)	0·00627 (0·000917–0·0224)	-42·7 (-80·2–37·2)	-27·2 (-62·7–33·3)	0·0198 (0·00443–0·0535)	0·0114 (0·00194–0·0357)	0·00833 (0·00124–0·0283)	-42·3 (-80·1–38·3)	-27·2 (-62·7–33·4)
Ambient ozone pollution	63·5 (27–100)	47·8 (19·7–83·1)	51 (21·8–86·3)	-24·7 (-34·4–-15·7)	6·68 (-1·75–16·9)	63·5 (27–100)	47·8 (19·7–83·1)	51 (21·8–86·3)	-24·7 (-34·4–-15·7)	6·68 (-1·75–16·9)
Residential radon	22·8 (3·32–68·3)	22·8 (3·32–68·3)	22·8 (3·32–68·3)	0 (0–0)	0 (0–0)	22·8 (3·32–68·3)	22·8 (3·32–68·3)	22·8 (3·32–68·3)	0 (0–0)	0 (0–0)
Lead exposure	65·8 (40·5–84·5)	37·6 (17·8–58·3)	26·1 (10·2–44·2)	-42·9 (-57·5–-29·4)	-30·6 (-43·1–-23·1)	39·9 (9·1–68·3)	22 (2·61–46·6)	15 (0·971–34·7)	-45 (-74·8–-29·9)	-31·9 (-62·8–-23·9)
Child underweight	0·96 (0·56–1·44)	0·963 (0·533–1·46)	0·942 (0·512–1·43)	0·307 (-18·7–20·7)	-2·23 (-14·6–11·1)	0·808 (0·417–1·23)	0·848 (0·459–1·29)	0·866 (0·473–1·32)	5·01 (-15·7–32·4)	2·17 (-12·7–17·7)
Iron deficiency	--	--	--	--	--	12·1 (10·1–14·3)	12·5 (10·5–14·4)	12·7 (10·6–14·9)	3·42 (-9·31–16·8)	1·46 (-8·35–11·4)
Vitamin A deficiency	0·801 (0·222–2·04)	1·11 (0·286–2·92)	0·79 (0·192–2·16)	38·5 (-67–550)	-28·8 (-69·4–67·8)	1·63 (0·629–3·45)	1·65 (0·63–3·46)	1·39 (0·494–2·96)	1·13 (-60·7–204)	-15·7 (-53·8–61·2)
Zinc deficiency	2·23 (0–7·24)	1·24 (0–4·04)	1·24 (0–4·03)	-44·2 (-100–NA)	-0·00799 (-21·7–NA)	2·28 (0–7·48)	1·25 (0–4·14)	1·21 (0–3·85)	-45 (-100–NA)	-2·93 (-24·6–NA)
Smoking	28·4 (25·7–31·4)	21·6 (19·6–23·7)	19·3 (17·4–21·4)	-23·8 (-28·8–-18·5)	-10·6 (-13·8–-7·12)	21 (18·4–23·9)	16·6 (14·8–18·4)	14·7 (13·1–16·4)	-21·2 (-28·7–-12·1)	-11·3 (-15·6–-7·28)
Secondhand smoke	42·2 (39·6–44·8)	27·5 (25·9–29·2)	27·1 (24·3–30)	-34·8 (-40·4–-28·8)	-1·74 (-10·7–6·7)	39·2 (37·2–41·3)	26·6 (25·3–27·8)	26·1 (24–28·2)	-32·2 (-37·4–-26·7)	-1·61 (-8·45–5·49)
Alcohol use	14·9 (9·59–20·7)	13·7 (8·01–19·1)	14·6 (8·56–20·3)	-7·84 (-39·4–39)	6·3 (-18·9–42)	--	--	--	--	--
Drug use	0·521 (0·269–0·992)	0·986 (0·678–1·46)	1·41 (1·03–1·97)	89·3 (36·2–174)	43·4 (23·8–67·5)	0·434 (0·274–0·699)	0·849 (0·618–1·14)	1·29 (1–1·66)	95·7 (56·6–147)	51·7 (30·6–76·3)
High fasting plasma glucose	13·9 (11·2–16·8)	18·7 (16·4–21·4)	20·1 (17·5–23·1)	34·7 (11·5–62·4)	7·52 (-5·62–23·2)	10·7 (8·88–12·9)	15·3 (13·5–17·3)	16·6 (14·5–19)	43·2 (17·8–70·6)	8·46 (-4·14–24)
High systolic blood pressure	26 (21·2–31·2)	16·5 (14·4–18·5)	20·2 (15·7–24·7)	-36·6 (-47·9–-23·2)	22·4 (-2·65–50·5)	24 (17·5–30·9)	13·8 (12·2–15·5)	18·2 (13·4–24·2)	-42·4 (-56·1–-22)	31·9 (-3·13–75·5)
High body-mass index	26 (18·5–36·1)	40·4 (29·7–51·7)	41·4 (30·7–53)	55·4 (35·9–81·5)	2·45 (-3·91–9·91)	33·1 (24·7–41·9)	45·6 (35–54·8)	46·3 (36·2–55·4)	37·6 (23·2–56)	1·53 (-3·99–8·51)
Low bone mineral density	12 (7·04–19·1)	9·24 (4·86–15·4)	11·6 (6·58–18·5)	-23·2 (-43–-1·62)	25·9 (-3·89–72·9)	15·9 (10·6–23)	16·5 (10·8–23·3)	19·1 (12·9–26·7)	3·6 (-17·4–29·9)	15·8 (-8·06–44·4)
Diet low in fruits	48·7 (34·7–63·1)	47·7 (34·4–62·3)	47·9 (34·5–62·8)	-1·92 (-13·7–11·6)	0·387 (-11·5–12·9)	--	--	--	--	--
Diet low in vegetables	27·7 (13·5–47·5)	46·2 (29·3–65·5)	44·8 (28·3–62·8)	66·5 (24·8–159)	-3 (-13–7·17)	30·1 (14–51)	45·4 (28·1–65)	44·1 (27·4–63·8)	50·9 (17·7–128)	-2·87 (-13·3–6·82)
Diet low in whole grains	79·6 (70–88·3)	81·7 (72·9–90)	81·7 (73–89·6)	2·69 (-0·963–7·44)	-0·00308 (-2·5–2·54)	76·9 (66·3–87)	79·9 (70·6–88·7)	79·6 (69·7–88·4)	3·88 (-0·399–9·69)	-0·304 (-3·13–2·36)
Diet low in nuts and seeds	27·3 (7·83–55·6)	25 (7·67–55·1)	20·8 (6·81–53·5)	-8·57 (-40·7–50·7)	-16·9 (-43·7–8·77)	23·4 (5·51–54·7)	22·1 (7·09–53·9)	19·5 (5·54–53·1)	-5·52 (-41·9–74·6)	-12·1 (-42·1–15·8)
Diet low in milk	48·1 (24·5–68·7)	44·2 (21·5–64·1)	43·1 (20·2–63)	-8·24 (-25–4·92)	-2·27 (-15·1–10·2)	--	--	--	--	--
Diet high in red meat	78·2 (64·7–88·8)	75·5 (62–86·8)	75·2 (61·5–87·9)	-3·49 (-17·1–12·3)	-0·347 (-11·5–12·6)	79 (67·2–88·9)	74·6 (60·6–86)	74·3 (59·9–86·6)	-5·6 (-20·3–10·2)	-0·453 (-12·1–11·2)
Diet high in processed meat	78 (53·8–97·7)	86 (66·4–99·6)	85·8 (66·6–99·4)	10·2 (1·31–30·3)	-0·156 (-4·9–4·63)	79·3 (56·5–98)	88·9 (74·2–99·7)	87·4 (71·2–99·7)	12·1 (1·22–37·2)	-1·59 (-7·19–1·78)
Diet high in sugar-sweetened beverages	46·2 (27·9–66)	61·1 (41·3–79·2)	61·5 (41·2–80·2)	32·3 (-6·61–104)	0·746 (-20·7–25·1)	50·1 (31·1–71·2)	67·3 (49·1–83·6)	66·2 (48·4–82·7)	34·1 (-4·2–103)	-1·51 (-20·9–22·3)
Diet low in fiber	43·1 (26·9–58·6)	31·1 (18·2–44·9)	28·4 (15·4–42·9)	-27·9 (-45·1–-11·2)	-8·69 (-26·1–8·03)	43·2 (26·7–59·2)	36·1 (22–51·2)	31·4 (17·9–47·1)	-16·4 (-33·2–1·79)	-13 (-28·8–2·27)
Diet low in seafood omega-3 fatty acids	83·1 (65·9–99·2)	79·9 (60·8–98·9)	80·3 (63·2–99)	-3·85 (-18·6–12·8)	0·49 (-13·9–19)	86·1 (69·5–99·9)	84·1 (68·5–99·6)	83·9 (68·3–98·9)	-2·26 (-13·7–9·64)	-0·214 (-9·55–12·3)
Diet low in polyunsaturated fatty acids	13·7 (4–39·7)	7·46 (2·17–26·4)	7·13 (2·09–25·6)	-45·6 (-74–-7·47)	-4·44 (-27·4–17·7)	--	--	--	--	--
Diet high in trans fatty acids	89·6 (81·5–100)	87·9 (78·9–100)	87·4 (78–100)	-1·9 (-10·5–6·15)	-0·532 (-8·69–7·93)	88·3 (80·9–100)	86·7 (78·2–100)	86·2 (77·6–100)	-1·83 (-9·43–5·46)	-0·548 (-8·26–8·21)
Diet high in sodium	18·5 (4·29–41·6)	40·1 (11·2–63·8)	39·4 (10·7–65·1)	116 (40·2–434)	-1·69 (-26·4–29·3)	--	--	--	--	--
Low physical activity	2·25 (0·832–4·78)	1·89 (0·791–4·15)	1·74 (0·628–4·02)	-16 (-44·4–27·4)	-7·84 (-34·5–15·9)	5·29 (2·59–10·4)	5·12 (2·75–9·37)	4·98 (2·52–9·77)	-3·19 (-26·5–34·2)	-2·81 (-16·3–10·5)
Occupational asthmagens	26·1 (21·4–32·2)	21·4 (18·1–25·3)	22·5 (18·5–27·1)	-18 (-28·8–-7·34)	4·96 (-4·89–15·4)	11·3 (9·19–13·5)	11·2 (9·34–13·2)	11·3 (9·2–13·6)	-0·974 (-15·7–16·1)	1·29 (-10–14·2)
Occupational particulate matter, gases, and fumes	10·7 (7·66–15·3)	9·81 (7·06–14)	9·34 (6·68–13·2)	-8·43 (-13·4–-3·46)	-4·78 (-8·06–-1·58)	5·33 (3·76–7·87)	5·26 (3·77–7·59)	5·08 (3·69–7·35)	-1·36 (-9·94–8·45)	-3·29 (-8·71–1·52)
Occupational noise	8·96 (8·17–9·84)	8·47 (7·88–9·23)	8·15 (7·63–8·85)	-5·48 (-8·92–-1·68)	-3·86 (-5·87–-1·66)	5·96 (5·2–6·69)	6·28 (5·77–6·86)	6·28 (5·88–6·84)	5·52 (-0·707–13)	-0·0366 (-2·99–3·13)
Occupational injuries	--	--	--	--	--	--	--	--	--	--
Occupational ergonomic factors	11·6 (9·53–14)	9·91 (8·29–11·9)	10·3 (8·58–12·4)	-14·6 (-24·4–-3·93)	3·72 (-4·37–12·5)	6·86 (5·49–8·75)	6·78 (5·56–8·41)	7·1 (5·62–9)	-1·09 (-15·8–17·9)	4·69 (-7·95–18·8)
Childhood sexual abuse	7·4 (5·05–10·5)	6·97 (4·6–9·93)	6·85 (4·54–9·67)	-5·83 (-12·2–5·83)	-1·68 (-8·51–5·53)	12·3 (8·94–17·3)	17·4 (12·9–24)	17·8 (13·1–24·7)	41·9 (30·7–54·8)	2·17 (-5–11)
Intimate partner violence	--	--	--	--	--	34·6 (19·8–44·6)	33·1 (18·8–43·2)	33·9 (19·7–43·6)	-4·27 (-13·2–9·49)	2·48 (-5·92–16·2)
Non-exclusive breastfeeding	26·3 (21·9–31·1)	24·9 (21·3–28·8)	24·8 (20·6–29·5)	-5·23 (-19·5–11·3)	-0·16 (-13·7–13·9)	--	--	--	--	--
Discontinued breastfeeding	27·4 (26·5–28·4)	25 (23·7–26·3)	25·5 (24·1–26·6)	-8·71 (-14·1–-3·04)	1·84 (-3·97–7·85)	27·4 (26·5–28·4)	25·1 (23·7–26·3)	25·5 (24·1–26·7)	-8·65 (-14·1–-2·98)	1·71 (-4·09–7·71)
Diet low in calcium	16 (7·91–29·8)	13·9 (6·66–26)	13·3 (6·33–24·8)	-13 (-24·8–-2·13)	-4·54 (-14·6–3·79)	11·4 (4·72–21·8)	11 (4·8–20·4)	10·7 (4·6–20·1)	-3·29 (-19·1–15·1)	-2·46 (-13·2–6·54)
Occupational exposure to asbestos	8·76 (7·87–9·71)	7·95 (7·32–8·56)	7·44 (5·49–9·64)	-9·28 (-19·4–2·97)	-6·41 (-30·7–21·5)	1·64 (1·1–2·05)	1·56 (1·03–1·91)	1·5 (0·965–2·14)	-5·04 (-18·8–12)	-3·74 (-30·6–28)
Occupational exposure to arsenic	0·633 (0–1·52)	0·567 (0–1·36)	0·6 (0–1·42)	-10·5 (-22·4–NA)	5·76 (-5·25–NA)	0·494 (0–1·16)	0·491 (0–1·19)	0·498 (0–1·18)	-0·592 (-17·8–NA)	1·39 (-11·3–NA)
Occupational exposure to benzene	0·813 (0·0489–2·33)	0·75 (0·0449–2·2)	0·786 (0·0476–2·29)	-7·68 (-14·9–3·02)	4·82 (-2·98–15·7)	1·17 (0·0678–3·28)	1·27 (0·0745–3·56)	1·33 (0·0818–3·68)	8·57 (-3·16–26·6)	4·84 (-3·76–17)
Occupational exposure to beryllium	0·0238 (0·0225–0·0253)	0·0222 (0·0217–0·0226)	0·0236 (0·0221–0·0249)	-7·05 (-12·6–-1·28)	6·36 (0·254–12·6)	0·026 (0·0236–0·0282)	0·0266 (0·026–0·0272)	0·0276 (0·0257–0·0295)	2·32 (-5·85–12·7)	3·84 (-3·62–10·9)
Occupational exposure to cadmium	0·0721 (0·0658–0·0791)	0·065 (0·0594–0·0707)	0·0687 (0·0607–0·0767)	-9·79 (-20·6–2·47)	5·7 (-4·43–16·1)	0·0585 (0·0514–0·0664)	0·0586 (0·0524–0·0656)	0·0597 (0·0519–0·0687)	0·13 (-15·6–17·9)	1·91 (-9·83–14·9)
Occupational exposure to chromium	0·168 (0·155–0·183)	0·155 (0·143–0·166)	0·164 (0·147–0·18)	-7·77 (-17·5–2·93)	5·69 (-3·3–14·8)	0·145 (0·129–0·162)	0·148 (0·136–0·162)	0·151 (0·135–0·169)	2·15 (-11–18)	2·03 (-8·19–13)
Occupational exposure to diesel engine exhaust	0·635 (0·585–0·685)	0·666 (0·622–0·711)	0·71 (0·644–0·774)	4·93 (-5·57–17·1)	6·58 (-1·53–15·4)	0·643 (0·566–0·722)	0·671 (0·613–0·743)	0·679 (0·598–0·767)	4·44 (-9·77–21·2)	1·11 (-9·32–12·3)
Occupational exposure to formaldehyde	0·367 (0·334–0·404)	0·322 (0·292–0·351)	0·336 (0·295–0·376)	-12·4 (-23·1–-0·59)	4·46 (-5·64–14·9)	0·287 (0·252–0·323)	0·289 (0·261–0·319)	0·295 (0·258–0·334)	0·69 (-13·8–18·6)	2·08 (-8·94–14·3)
Occupational exposure to nickel	0·531 (0–2·34)	0·461 (0–2·02)	0·487 (0–2·13)	-13·2 (-24·6–NA)	5·71 (-5·15–NA)	0·389 (0–1·67)	0·376 (0–1·71)	0·383 (0–1·77)	-3·22 (-20·7–NA)	1·83 (-11·7–NA)
Occupational exposure to polycyclic aromatic hydrocarbons	0·33 (0·306–0·359)	0·304 (0·284–0·324)	0·321 (0·29–0·352)	-7·83 (-17–2·05)	5·55 (-3·03–14·3)	0·308 (0·276–0·342)	0·323 (0·302–0·347)	0·331 (0·301–0·364)	4·93 (-7·05–19·4)	2·47 (-6·66–12·1)
Occupational exposure to silica	4·65 (0·666–15·2)	3·98 (0·583–12·9)	4·24 (0·632–13·7)	-14·3 (-25·4–-1·31)	6·52 (-4·02–19·2)	3·27 (0·441–10·6)	3·06 (0·423–9·76)	3·09 (0·414–10·3)	-6·64 (-24·3–17)	1·12 (-13–20·3)
Occupational exposure to sulfuric acid	1·07 (0·228–3·45)	1·05 (0·216–3·39)	1·1 (0·223–3·59)	-2·09 (-14–11·5)	4·37 (-5·85–14·5)	0·93 (0·194–2·96)	1·09 (0·22–3·56)	1·08 (0·223–3·46)	16·7 (-5·03–43·9)	-0·138 (-13·4–16·1)
Unsafe sex	--	--	--	--	--	--	--	--	--	--
Occupational exposure to trichloroethylene	0·0906 (0·0849–0·0979)	0·087 (0·0836–0·0911)	0·0922 (0·0854–0·0994)	-3·89 (-11·4–4·16)	5·93 (-1·45–13·4)	0·0831 (0·0749–0·0922)	0·0854 (0·08–0·0912)	0·0871 (0·0788–0·096)	2·84 (-8·74–16·7)	1·89 (-7–11·3)
No access to handwashing facility	3·34 (3·1–3·63)	3·11 (2·88–3·36)	3·07 (2·84–3·33)	-7·13 (-15·2–2·09)	-1·13 (-7·24–4·94)	3·34 (3·1–3·63)	3·11 (2·88–3·36)	3·07 (2·84–3·33)	-7·13 (-15·2–2·09)	-1·13 (-7·24–4·94)
Child wasting	0·749 (0·499–1·03)	0·728 (0·482–0·986)	0·727 (0·477–0·984)	-2·81 (-12·2–7·43)	-0·149 (-6·78–8·18)	0·772 (0·517–1·05)	0·763 (0·498–1·06)	0·76 (0·501–1·04)	-1·16 (-10·8–10)	-0·366 (-8·03–7·75)
Child stunting	2·43 (1·31–3·54)	2·11 (1·09–3·18)	2·04 (1·07–3·04)	-13·2 (-32·9–11·2)	-3 (-16·9–14·7)	1·94 (0·944–2·96)	1·79 (0·903–2·72)	1·78 (0·889–2·7)	-7·77 (-26·4–16·2)	-0·741 (-16–16·4)
Chewing tobacco	3·7 (2·77–4·9)	3·87 (2·8–5·24)	3·89 (2·75–5·41)	4·74 (-28·7–54·6)	0·415 (-22·1–28·9)	--	--	--	--	--
Diet low in legumes	74 (37·3–99·2)	77·3 (45·5–99·7)	77·2 (46·2–99·7)	4·48 (0–25·2)	-0·0675 (-5·72–5·74)	72·2 (34·3–98·7)	75·6 (43·3–99·6)	75·9 (42·8–99·6)	4·73 (0–27·5)	0·426 (-4·65–7·06)
Short gestation	38·6 (31·6–46·1)	30·8 (26–35·9)	30·1 (24·4–36·6)	-20·3 (-34·9–-2·2)	-2·17 (-20·6–19·7)	34·7 (29·1–40·9)	28 (23·4–33·2)	26·9 (22·3–32·1)	-19·2 (-34–0·336)	-3·91 (-21·5–16·1)
Low birth weight	15 (12·5–18)	12·9 (10·8–15·2)	13·3 (10·9–16·3)	-13·9 (-30·1–7·59)	3·17 (-17·5–26·9)	16·2 (13·8–18·9)	14·3 (12·4–16·5)	14·1 (11·8–16·7)	-11·9 (-27·6–8·67)	-1·56 (-20·3–19·9)
High temperature	24·3 (13–38)	25·1 (13·6–38·7)	26·5 (14·6–40·4)	3·31 (-0·473–8·06)	5·67 (0·661–12·2)	24·3 (13–38)	25·1 (13·6–38·7)	26·5 (14·6–40·4)	3·31 (-0·473–8·06)	5·67 (0·661–12·2)
Low temperature	34·4 (30·5–38·4)	35·4 (31·7–39·5)	36·9 (33·1–40·9)	2·91 (1·12–4·88)	4·2 (2·36–6·37)	34·4 (30·5–38·4)	35·4 (31·7–39·5)	36·9 (33·1–40·9)	2·91 (1·12–4·88)	4·2 (2·36–6·37)
Kidney dysfunction	19·8 (14·1–26·7)	20·4 (14·4–27·6)	20·4 (14·5–27·6)	2·91 (-0·795–7·08)	0·195 (-3·86–4·23)	22·2 (15·7–29·6)	23·1 (16·5–31)	23·9 (17·2–32)	4·01 (-0·663–9·7)	3·38 (-1·48–8·79)
Bullying victimization	5·91 (2·47–12·1)	9·03 (3·6–17·9)	8·73 (3·34–17·9)	52·9 (31·9–76·4)	-3·35 (-12·9–2·77)	4·27 (1·72–8·93)	7·91 (3·18–15·7)	7·78 (2·93–15·9)	85·4 (59·8–115)	-1·62 (-12·3–5·31)
High LDL cholesterol	65·7 (61·6–70)	34·6 (31·1–38·2)	30·9 (27·3–34·7)	-47·4 (-52·6–-41·7)	-10·6 (-15·3–-5·9)	65·4 (61·9–69)	34·5 (31·2–37·9)	31·9 (28·4–35·5)	-47·2 (-51·9–-42·5)	-7·54 (-11·7–-3·45)

Supplementary Results Table 1. Minnesota age-standardised Summary Exposure Values (SEVs) for most-detailed risk factors and percentage change, by sex, 1990, 2010, and 2019. Data in parentheses are 95% uncertainty intervals.

	Male					Female				
	1990	2010	2019	Percentage change 1990-2010	Percentage change 2010-2019	1990	2010	2019	Percentage change 1990-2010	Percentage change 2010-2019
Unsafe sanitation	4·67 (2·22–8·81)	2·67 (1·13–5·45)	2·12 (0·968–4·11)	-42·9 (-79·1–39·3)	-20·6 (-59·9–60·9)	4·67 (2·22–8·81)	2·67 (1·13–5·45)	2·12 (0·968–4·11)	-42·9 (-79·1–39·3)	-20·6 (-59·9–60·9)
Ambient particulate matter pollution	7·65 (1·49–17·3)	4·61 (2·18–7·42)	3·53 (1·24–6·2)	-39·7 (-75·4–123)	-23·5 (-44—15·5)	7·65 (1·49–17·4)	4·62 (2·2–7·41)	3·53 (1·23–6·2)	-39·7 (-75·4–121)	-23·5 (-44·3—15·5)
Household air pollution from solid fuels	0·0124 (0·00235–0·038)	0·00682 (0·00108–0·0241)	0·00484 (0·000616–0·0182)	-45·2 (-81·3–64·9)	-29 (-67–30·2)	0·0164 (0·0031–0·0515)	0·00908 (0·00141–0·0314)	0·00646 (0·000836–0·024)	-44·7 (-81·3–67·2)	-28·8 (-67·2–30·8)
Ambient ozone pollution	29·1 (11·5–52·3)	41·5 (17·3–72·8)	26·6 (9·62–48·5)	42·4 (19·6–80·2)	-35·9 (-49·9—22·6)	--	--	--	--	--
Residential radon	24 (3·47–67·6)	24 (3·47–67·6)	24 (3·47–67·6)	0 (0–0)	0 (0–0)	24 (3·47–67·6)	24 (3·47–67·6)	24 (3·47–67·6)	0 (0–0)	0 (0–0)
Lead exposure	51·5 (23–74·9)	28·3 (8·79–50·4)	19·2 (4·35–38·1)	-45 (-64·9—30·5)	-32·2 (-51·4—23·4)	28·2 (1·88–57·4)	15·8 (0·361–39·5)	10·8 (0·127–29·6)	-44·1 (-85—28·6)	-31·3 (-75—23·3)
Child underweight	0·916 (0·516–1·39)	0·928 (0·494–1·4)	0·911 (0·504–1·38)	1·25 (-16·4–22·8)	-1·78 (-15·6–12·9)	0·775 (0·403–1·17)	0·824 (0·439–1·27)	0·845 (0·459–1·29)	6·3 (-14·2–46·6)	2·51 (-12·5–20·3)
Iron deficiency	--	--	--	--	--	10·8 (9·02–12·8)	11·3 (9·53–13·3)	11·6 (9·59–13·7)	4·63 (-6·69–19)	2·03 (-7·12–12·6)
Vitamin A deficiency	0·673 (0·182–1·91)	0·929 (0·247–2·44)	0·658 (0·17–1·68)	38 (-68·4–493)	-29·2 (-66·4–63·9)	1·36 (0·479–2·75)	1·44 (0·548–3·03)	1·22 (0·457–2·6)	6·26 (-60·5–213)	-15·8 (-53·2–59·5)
Zinc deficiency	2·2 (0–7·03)	1·09 (0–3·5)	1·12 (0–3·67)	-50·3 (-100–NA)	2·47 (-15·8–NA)	2·25 (0–7·22)	1·11 (0–3·5)	1·1 (0–3·52)	-50·7 (-100–NA)	-1 (-20·3–NA)
Smoking	27·2 (24·5–30)	18·4 (16·6–20·3)	16·6 (14·9–18·4)	-32·5 (-37—27·8)	-9·8 (-13·6—5·92)	20·7 (18·2–23·5)	14 (12·5–15·6)	12·6 (11·2–14·2)	-32·7 (-38·5—26·5)	-9·48 (-13·9—5)
Secondhand smoke	38·6 (36·1–41·3)	24·2 (22·6–25·9)	24·3 (21·8–27·2)	-37·2 (-42·9—30·9)	0·141 (-8·68–9·75)	35 (33·1–37)	22·8 (21·6–23·9)	22·9 (21·1–25·1)	-34·8 (-40—29·3)	0·626 (-6·74–9·2)
Alcohol use	15·1 (9·54–21·3)	15·3 (9·26–21·8)	16·8 (11–24·1)	1·59 (-25·5–42·7)	9·75 (-11·8–39·4)	11·5 (6·08–17·8)	7·15 (2·39–13·5)	6·95 (2·24–13·3)	-37·8 (-75·8–17)	-2·72 (-38·9–55·9)
Drug use	0·384 (0·21–0·724)	0·616 (0·428–0·873)	0·844 (0·611–1·13)	60·3 (13·4–133)	37 (18·4–59·7)	0·297 (0·184–0·489)	0·448 (0·327–0·63)	0·654 (0·49–0·863)	51 (22·6–89)	45·9 (23·5–71·4)
High fasting plasma glucose	12·1 (9·98–14·9)	16·3 (14–19)	17·4 (14·9–20·6)	34·4 (10·8–62·9)	7·22 (-8·58–25·8)	8·69 (7·33–10·4)	11·6 (10–13·6)	13 (11–15·2)	33·8 (12·4–59·3)	11·7 (-3·58–29·4)
High systolic blood pressure	25·1 (20·2–30·3)	15·1 (13–17·2)	18·9 (14·6–23·6)	-39·8 (-50·6—24·9)	25·4 (-0·189–53·9)	22·2 (16–28·5)	11·8 (10·2–13·4)	16·5 (11·6–22·3)	-46·9 (-59·4—24·8)	40·4 (0·468–88·2)
High body-mass index	25·8 (18–35·9)	39·7 (29–51·2)	41·6 (31–52·9)	53·9 (35·1–80·4)	4·88 (-1·42–13·1)	28·9 (20·9–38·6)	42·9 (34–51·9)	44·5 (35·5–54·1)	48·5 (27·8–76·9)	3·72 (-2·62–11)
Low bone mineral density	12·5 (7·52–19·6)	13·3 (7·99–20·6)	14 (8·45–21·2)	5·77 (-17·3–35·2)	5·25 (-18·1–38·5)	20·5 (14–27·8)	20·1 (13·7–27·7)	21 (14·4–28·8)	-2·08 (-21·1–23·9)	4·41 (-17·7–33)
Diet low in fruits	51·8 (38·3–66·1)	48·1 (35·2–62·6)	47·6 (34·4–62·1)	-7·09 (-18·9–3·68)	-1·1 (-12·6–10·9)	--	--	--	--	--
Diet low in vegetables	26·5 (12·1–47·2)	45·7 (26·2–66·2)	43·2 (24·7–63·2)	72·4 (24·7–185)	-5·6 (-17·4–5·53)	27·4 (12·9–48·6)	42·8 (24·5–63·8)	41·7 (23·6–61·9)	56·3 (18–147)	-2·5 (-14·3–9·82)
Diet low in whole grains	79·3 (69·4–88)	81·2 (72·2–89·2)	81·2 (72–89·2)	2·42 (-1·2–7·07)	-0·0211 (-2·7–2·54)	76·6 (66·1–86·4)	79·4 (69·9–88·2)	79·2 (69·6–88·4)	3·68 (-0·559–9·81)	-0·312 (-3·35–2·57)
Diet low in nuts and seeds	26·8 (7·32–56)	25·2 (7·07–55)	20·3 (6·26–53·6)	-6·17 (-41·9–72·9)	-19·5 (-46·7–5·76)	22·7 (6·3–54·1)	21·6 (6·89–54·4)	18·5 (5·55–53·2)	-4·91 (-41–69·8)	-14·5 (-43·9–14·7)
Diet low in milk	47·5 (24·7–68·7)	41·1 (17·9–61·5)	40·1 (18·1–60·6)	-13·5 (-35·5–0·65)	-2·5 (-15·8–12)	47·3 (23·9–68·1)	41 (18·7–60·5)	40·3 (18·2–60·1)	-13·4 (-34·5–0·338)	-1·65 (-14·1–12·2)
Diet high in red meat	78·5 (65·2–89)	77·1 (64–88·2)	77 (64–88·7)	-1·83 (-15·2–13·8)	-0·158 (-11·7–12·2)	79·1 (65·8–89·5)	76 (61·5–87·6)	75·8 (61·7–87·4)	-3·97 (-18·9–12·9)	-0·192 (-11·1–11·5)
Diet high in processed meat	75·6 (50·6–96·5)	84·5 (65·2–99·5)	84·4 (65·8–99·4)	11·7 (1·95–32·1)	-0·00879 (-4·62–4·99)	76·4 (51·5–97·1)	87 (70·4–99·6)	85·7 (66·7–99·5)	13·8 (1·83–39)	-1·43 (-7·08–2·49)
Diet high in sugar-sweetened beverages	47·1 (28·6–66·4)	64·9 (47·3–80·8)	65·1 (46·6–81·6)	37·8 (-1·34–113)	0·274 (-18·9–24·7)	51 (31·9–70·6)	70·5 (51–86)	69 (50·7–84)	38·3 (-2·1–115)	-2·03 (-20·3–19·1)
Diet low in fiber	42·8 (27·5–58·1)	28·6 (16·4–42·5)	26 (13·8–39·5)	-33 (-50·1—15·1)	-9·27 (-26·3–9·46)	42·8 (26·2–58·7)	33·7 (21·2–47·6)	28·8 (15·9–43)	-21·3 (-37·4–2·85)	-14·4 (-31·8–1·49)
Diet low in seafood omega-3 fatty acids	83 (65–99·7)	78 (59·2–98·9)	77·8 (59·2–99·3)	-6·05 (-22·7–8·25)	-0·227 (-14·9–18·2)	85·7 (69–99·5)	82·1 (65·5–99·6)	81·7 (64·7–99·4)	-4·2 (-17·1–8·49)	-0·4 (-13–13·9)
Diet low in polyunsaturated fatty acids	11·8 (3·49–35)	6·48 (1·8–24·3)	6·22 (1·73–24·3)	-45 (-75—6·38)	-4·08 (-25·5–15·9)	--	--	--	--	--
Diet high in trans fatty acids	91·5 (85–100)	84·6 (76·9–100)	83·9 (76–100)	-7·45 (-14·6–0)	-0·853 (-8·15–6·87)	91·1 (84·6–100)	84·5 (76·7–100)	83·8 (75·6–100)	-7·2 (-14·4–0)	-0·885 (-8·44–6·61)
Diet high in sodium	18·4 (4·37–41·5)	39·9 (11·5–64·3)	39·3 (10·6–64·8)	117 (38·7–430)	-1·53 (-29·2–28)	--	--	--	--	--
Low physical activity	2·82 (1·07–6·01)	2·92 (1·25–5·85)	2·89 (1·13–6·08)	3·51 (-30·1–65·6)	-1·05 (-22·2–22·4)	5·84 (2·84–11·3)	6·47 (3·54–11·8)	6·38 (3·34–12)	10·9 (-16·9–59·2)	-1·43 (-13–9·66)
Occupational asthmagens	24·4 (19·9–29·9)	23 (19·3–27·4)	24·4 (19·8–30·4)	-5·9 (-20·2–10·1)	6·04 (-5·95–21·3)	12·5 (10·2–15·1)	13·2 (11–15·8)	13·3 (10·6–16·6)	5·98 (-10·5–23·8)	0·459 (-10·8–17·7)
Occupational particulate matter, gases, and fumes	10·1 (7·24–14·5)	9·71 (7·04–13·9)	9·46 (6·8–13·4)	-3·98 (-9·41–1·72)	-2·59 (-5·89–0·794)	5·76 (4·07–8·45)	5·73 (4·14–8·19)	5·59 (4·06–8·03)	-0·455 (-8·99–9·57)	-2·5 (-7·57–3·25)
Occupational noise	9·09 (8·33–10)	8·89 (8·29–9·67)	8·7 (8·15–9·44)	-2·19 (-5·58–1·59)	-2·14 (-3·98—0·0642)	6·59 (5·82–7·36)	7·13 (6·61–7·74)	7·22 (6·79–7·82)	8·15 (2·25–15·1)	1·32 (-1·11–4·34)
Occupational injuries	--	--	--	--	--	--	--	--	--	--
Occupational ergonomic factors	13 (10·6–15·8)	11·7 (9·85–14·1)	12·1 (10·1–14·8)	-9·62 (-19·7–2·57)	3·6 (-4·8–13·2)	7·92 (6·23–9·94)	8·23 (6·77–10·2)	8·84 (6·89–11·2)	3·86 (-14·7–25·8)	7·42 (-7·32–25·4)
Childhood sexual abuse	5·75 (3·45–8·97)	5·07 (3·67–6·86)	5·05 (3·65–6·73)	-11·9 (-30·7–18·4)	-0·363 (-6·3–6·29)	15·2 (11–21·4)	13·6 (10·5–18·1)	13·7 (10·6–17·9)	-10·7 (-21·7–3·42)	0·689 (-5·5–8·34)
Intimate partner violence	--	--	--	--	--	29·7 (16·6–39·3)	28 (15·8–37)	29·1 (16·6–37·9)	-5·85 (-13·6–5·83)	3·8 (-5·23–19)
Non-exclusive breastfeeding	26·2 (21·6–31)	25 (21·5–29)	25 (20·6–29·9)	-4·48 (-18·8–13)	-0·0593 (-13·4–14·7)	--	--	--	--	--
Discontinued breastfeeding	27 (26–27·9)	25·2 (23·9–26·4)	25·6 (24·3–26·8)	-6·43 (-11·9—1·45)	1·36 (-4·21–7·78)	27 (26–27·9)	25·2 (23·8–26·4)	25·5 (24·3–26·7)	-6·59 (-12·1—1·61)	1·38 (-4·2–7·79)
Diet low in calcium	15·7 (7·54–28·6)	12·7 (6·23–23·3)	12 (5·85–22·2)	-18·9 (-29·2—6·99)	-5·51 (-15·9–3·06)	10·9 (4·7–20·5)	9·59 (4·19–18·2)	9·29 (4·01–17·3)	-12·4 (-27·6–5·06)	-3·11 (-14·6–7·55)
Occupational exposure to asbestos	11·2 (10–12·4)	9·6 (8·95–10·3)	8·97 (6·64–11·3)	-14·2 (-24·1—2·5)	-6·52 (-30·5–18·4)	1·97 (1·01–2·42)	1·96 (0·911–2·36)	1·88 (0·815–2·64)	-0·517 (-17·9–17·9)	-4·17 (-31·6–27·8)
Occupational exposure to arsenic	0·551 (0–1·3)	0·579 (0–1·35)	0·604 (0–1·43)	5·05 (-10·1–NA)	4·32 (-6·42–NA)	0·522 (0–1·24)	0·558 (0–1·36)	0·562 (0–1·37)	6·9 (-10·3–NA)	0·694 (-10·6–NA)
Occupational exposure to benzene	0·886 (0·0514–2·5)	0·906 (0·0542–2·57)	0·927 (0·0565–2·63)	2·28 (-5·32–13·7)	2·29 (-4·43–11·3)	1·34 (0·0721–3·69)	1·57 (0·0928–4·47)	1·61 (0·0975–4·74)	17·6 (5·82–37·9)	2·76 (-4·4–15)
Occupational exposure to beryllium	0·0249 (0·0236–0·0263)	0·0255 (0·025–0·0261)	0·0266 (0·0252–0·0279)	2·31 (-3·47–8·44)	4·16 (-0·945–9·3)	0·0292 (0·0269–0·0313)	0·032 (0·0313–0·0327)	0·0326 (0·0308–0·0346)	9·58 (2·03–18·9)	2·03 (-3·27–7·74)
Occupational exposure to cadmium	0·0643 (0·0583–0·071)	0·0676 (0·061–0·0738)	0·0705 (0·0624–0·0793)	5·14 (-8·32–20·9)	4·32 (-5·31–14·8)	0·0625 (0·0558–0·0704)	0·0671 (0·0606–0·0749)	0·0677 (0·0591–0·0773)	7·41 (-8·01–25·9)	0·782 (-9·39–12·1)
Occupational exposure to chromium	0·154 (0·142–0·168)	0·165 (0·152–0·177)	0·172 (0·155–0·19)	6·83 (-4·81–20·6)	4·1 (-4·37–13)	0·157 (0·142–0·174)	0·172 (0·159–0·188)	0·174 (0·155–0·194)	9·81 (-3·53–25·3)	0·751 (-8·08–10·6)
Occupational exposure to diesel engine exhaust	0·75 (0·694–0·813)	0·821 (0·771–0·872)	0·851 (0·782–0·924)	9·44 (-1·17–20·3)	3·69 (-3·79–12·2)	0·73 (0·652–0·811)	0·808 (0·74–0·892)	0·801 (0·715–0·9)	10·6 (-3·59–28·2)	-0·86 (-9·33–8·66)
Occupational exposure to formaldehyde	0·323 (0·291–0·359)	0·333 (0·298–0·367)	0·343 (0·303–0·388)	3·14 (-10·2–19·5)	3·08 (-6·79–13·5)	0·308 (0·275–0·346)	0·335 (0·304–0·369)	0·337 (0·297–0·381)	8·73 (-6·11–26·2)	0·882 (-8·76–11·8)
Occupational exposure to nickel	0·463 (0–2·06)	0·466 (0–2·06)	0·486 (0–2·15)	0·727 (-13·1–NA)	4·34 (-6·36–NA)	0·409 (0–1·73)	0·423 (0–1·92)	0·426 (0–1·87)	3·36 (-14·7–NA)	0·655 (-11·5–NA)
Occupational exposure to polycyclic aromatic hydrocarbons	0·305 (0·281–0·331)	0·326 (0·301–0·349)	0·338 (0·308–0·37)	6·66 (-4·35–19·6)	3·92 (-4·05–12·4)	0·335 (0·304–0·367)	0·378 (0·355–0·405)	0·382 (0·35–0·418)	13 (0·602–27·3)	1·07 (-6·43–9·26)
Occupational exposure to silica	4·19 (0·617–13·7)	4·07 (0·584–12·8)	4·29 (0·601–13·9)	-2·83 (-16·5–14·7)	5·3 (-5·29–19)	3·4 (0·465–11)	3·4 (0·463–11·3)	3·4 (0·493–11·2)	-0·23 (-18·4–24·9)	0·177 (-12·4–19·3)
Occupational exposure to sulfuric acid	1 (0·213–3·14)	1·12 (0·233–3·65)	1·15 (0·241–3·74)	12 (-3·05–30·1)	2·7 (-6·91–14)	0·986 (0·211–3·15)	1·22 (0·25–3·87)	1·2 (0·246–3·94)	23·7 (-0·203–51·8)	-1·8 (-14–13·6)
Unsafe sex	--	--	--	--	--	--	--	--	--	--
Occupational exposure to trichloroethylene	0·0899 (0·0843–0·0965)	0·0973 (0·0928–0·102)	0·101 (0·0941–0·108)	8·21 (-0·119–17·4)	3·95 (-2·34–11)	0·0915 (0·0833–0·101)	0·101 (0·0947–0·108)	0·101 (0·0923–0·111)	10 (-0·829–23·6)	0·379 (-7·02–8·84)
No access to handwashing facility	3·28 (3·02–3·53)	3·08 (2·85–3·35)	3·04 (2·81–3·29)	-6·14 (-14·7–3·27)	-1·11 (-6·95–4·96)	3·28 (3·02–3·53)	3·08 (2·85–3·35)	3·04 (2·81–3·29)	-6·14 (-14·7–3·27)	-1·11 (-6·95–4·96)
Child wasting	0·744 (0·495–1·02)	0·724 (0·471–0·984)	0·722 (0·475–0·986)	-2·63 (-12–7·43)	-0·228 (-7·76–8·01)	0·768 (0·514–1·05)	0·758 (0·501–1·03)	0·756 (0·5–1·03)	-1·35 (-11·3–10·3)	-0·324 (-7·83–7·72)
Child stunting	2·23 (1·18–3·32)	1·98 (1·04–2·92)	1·93 (1–2·9)	-11·5 (-31·6–13·3)	-2·23 (-16·7–13·9)	1·84 (0·92–2·8)	1·71 (0·84–2·61)	1·7 (0·838–2·63)	-7·16 (-27·2–19)	-0·894 (-16·3–16·7)
Chewing tobacco	3·93 (3·07–4·99)	3·95 (2·94–5·37)	3·92 (2·87–5·33)	0·523 (-29·8–45·5)	-0·839 (-22·9–26)	--	--	--	--	--
Diet low in legumes	73·6 (38·1–99·2)	76·5 (44·6–99·6)	76·4 (44·6–99·5)	3·93 (-0·435–22·2)	-0·139 (-5·21–5·2)	72 (33·6–98·9)	74·7 (41·2–99·5)	75 (41·8–99·4)	3·81 (-0·36–23·6)	0·34 (-4·54–8·03)
Short gestation	23·4 (18·9–28·7)	23·6 (19·6–28·3)	23·6 (18·9–29·3)	1·13 (-21·4–30·2)	-0·255 (-20·9–27·3)	18·8 (15·7–22·5)	19·8 (16·5–23·5)	21·3 (17·9–25·3)	4·98 (-16·7–31·3)	7·79 (-11·9–30·1)
Low birth weight	8·9 (7·3–10·8)	9·99 (8·3–12)	9·9 (8·15–12·2)	12·2 (-13·6–45·3)	-0·849 (-21·6–26·4)	8·18 (6·8–9·77)	9·32 (7·82–10·9)	10·6 (8·97–12·6)	13·9 (-8·74–43·5)	13·8 (-8·72–40·1)
High temperature	24·5 (12–37·6)	24·6 (12–37·9)	24·1 (11·4–37·3)	0·74 (-4·98–5·71)	-2·09 (-4·78—0·508)	24·5 (12–37·6)	24·6 (12–37·9)	24·1 (11·4–37·3)	0·74 (-4·98–5·71)	-2·09 (-4·78—0·508)
Low temperature	36·3 (32·4–41·2)	34·3 (30·3–39·6)	37·5 (33·4–42·7)	-5·38 (-8·98—1·86)	9·14 (5·21–13·4)	36·3 (32·4–41·2)	34·3 (30·3–39·6)	37·5 (33·4–42·7)	-5·38 (-8·98—1·86)	9·14 (5·21–13·4)
Kidney dysfunction	18·3 (12·9–24·9)	18·9 (13·3–25·9)	19 (13·6–25·7)	3·52 (-0·318–7·95)	0·782 (-3·56–5·36)	20·9 (14·8–28·1)	21·8 (15·1–29·5)	22·4 (16·1–30·2)	4·26 (-0·0343–9·44)	2·97 (-1·78–8·5)
Bullying victimization	4·31 (1·56–9·29)	3·73 (1·41–8·07)	4·71 (1·88–9·59)	-13·4 (-34·8–3·57)	26·1 (14·1–44·3)	--	--	--	--	--
High LDL cholesterol	66·2 (62·1–70·7)	34·2 (30·9–37·9)	30·7 (27·1–34·4)	-48·4 (-53·5—43·1)	-10·2 (-15·3—5)	64·6 (61·1–68·2)	34·1 (30·9–37·4)	31·6 (28·1–35·2)	-47·3 (-52—42·6)	-7·14 (-10·8—3·03)

Supplementary Results Table 1. Mississippi age-standardised Summary Exposure Values (SEVs) for most-detailed risk factors and percentage change, by sex, 1990, 2010, and 2019. Data in parentheses are 95% uncertainty intervals.										
	Male					Female				
	1990	2010	2019	Percentage change 1990-2010	Percentage change 2010-2019	1990	2010	2019	Percentage change 1990-2010	Percentage change 2010-2019
Unsafe sanitation	11·3<br>(5·48–19·5)	6·04<br>(2·67–11·2)	4·65<br>(2·09–8·69)	-46·3<br>(-76·7–27)	-23·1<br>(-58·5–48·4)	--	--	--	--	--
Ambient particulate matter pollution	12·7<br>(4·61–25·3)	7·96<br>(5·11–11·3)	4·83<br>(2·31–7·8)	-37·4<br>(-68·8–55·6)	-39·3<br>(-55·8–-30·2)	12·7<br>(4·59–25·3)	7·95<br>(5·12–11·3)	4·84<br>(2·33–7·81)	-37·4<br>(-68·8–55·5)	-39·2<br>(-55·8–-30·2)
Household air pollution from solid fuels	0·0531<br>(0·015–0·137)	0·0279<br>(0·00553–0·0893)	0·0175<br>(0·00293–0·0538)	-47·5<br>(-80·7–33·8)	-37·4<br>(-69·4–18·5)	0·0701<br>(0·0201–0·183)	0·0369<br>(0·0077–0·112)	0·0232<br>(0·00421–0·0749)	-47·3<br>(-80·6–33·6)	-37·3<br>(-69·4–18·7)
Ambient ozone pollution	65·1<br>(28·4–100)	52·3<br>(21·7–89)	24·3<br>(8·66–46)	-19·7<br>(-29·4–-9·9)	-53·6<br>(-66·6–-43·3)	65·1<br>(28·4–100)	52·3<br>(21·7–89)	24·3<br>(8·66–46)	-19·7<br>(-29·4–-9·9)	-53·6<br>(-66·6–-43·3)
Residential radon	16·5<br>(2·46–52·6)	16·5<br>(2·46–52·6)	16·5<br>(2·46–52·6)	0<br>(0–0)	0<br>(0–0)	16·5<br>(2·46–52·6)	16·5<br>(2·46–52·6)	16·5<br>(2·46–52·6)	0<br>(0–0)	0<br>(0–0)
Lead exposure	54·2<br>(26·2–77)	34·1<br>(13·7–56·4)	24·8<br>(8·29–44·9)	-37<br>(-52·8–-24·7)	-27·4<br>(-41·2–-19·1)	31·7<br>(3·43–60)	21·6<br>(2·15–45·9)	16<br>(1·19–37·3)	-32<br>(-57·8–-18·1)	-25·9<br>(-52·1–-17·9)
Child underweight	1<br>(0·577–1·5)	0·999<br>(0·565–1·49)	0·974<br>(0·556–1·48)	-0·162<br>(-16·4–20)	-2·51<br>(-15·2–10·7)	0·844<br>(0·472–1·27)	0·877<br>(0·479–1·34)	0·899<br>(0·492–1·35)	3·93<br>(-16·6–27·8)	2·43<br>(-11·5–18·4)
Iron deficiency	--	--	--	--	--	12·9<br>(10·8–15·1)	13·6<br>(11·6–15·8)	13·8<br>(11·5–16·2)	5·14<br>(-7·57–18·6)	1·58<br>(-8·13–12·1)
Vitamin A deficiency	1·33<br>(0·357–3·2)	1·68<br>(0·433–4·06)	1·15<br>(0·315–2·97)	26·4<br>(-66·8–401)	-31·5<br>(-71·2–69·7)	2·39<br>(0·836–5·18)	2·36<br>(0·896–4·99)	1·97<br>(0·733–4·28)	-1·31<br>(-62·2–199)	-16·6<br>(-51·5–55·9)
Zinc deficiency	2·91<br>(0–9·02)	1·4<br>(0–4·49)	1·49<br>(0–4·82)	-52<br>(-100–NA)	6·33<br>(-14–NA)	2·99<br>(0–9·34)	1·41<br>(0–4·65)	1·45<br>(0–4·7)	-52·8<br>(-100–NA)	2·93<br>(-16–NA)
Smoking	29·7<br>(26·8–32·7)	22·1<br>(20–24·3)	20·5<br>(18·4–22·6)	-25·8<br>(-30·9–-20·2)	-7·3<br>(-11·2–-3·54)	17·8<br>(15·3–20·6)	14·5<br>(13–16·1)	13·8<br>(12·3–15·3)	-18·5<br>(-27·7–-6·52)	-4·88<br>(-9·4–-0·152)
Secondhand smoke	38·7<br>(36·1–41·1)	27·9<br>(26·3–29·6)	27·6<br>(24·8–30·7)	-27·8<br>(-34·2–-20·8)	-1·01<br>(-9·69–7·22)	38·6<br>(36·6–40·8)	27·6<br>(26·4–28·8)	27·2<br>(25·2–29·3)	-28·5<br>(-33·4–-23)	-1·55<br>(-8·28–5·51)
Alcohol use	12·1<br>(7·41–16·8)	12·8<br>(7·75–18·3)	13·2<br>(8·25–19·4)	5·33<br>(-24–44·1)	3·23<br>(-16·8–31)	--	--	--	--	--
Drug use	0·526<br>(0·268–0·999)	0·889<br>(0·602–1·3)	1·43<br>(1·06–1·92)	69<br>(23·4–141)	61·3<br>(35·3–97·5)	0·419<br>(0·26–0·677)	0·756<br>(0·552–1·03)	1·14<br>(0·864–1·48)	80·3<br>(42·7–131)	51·1<br>(30–78)
High fasting plasma glucose	12·7<br>(10·6–15·1)	18·9<br>(16·4–21·5)	20·9<br>(18·1–23·8)	48·9<br>(26·1–76·5)	10·8<br>(-3·42–28)	11·1<br>(9·38–13·1)	16·2<br>(14·1–18·5)	18·2<br>(15·9–20·8)	46<br>(22·6–73·2)	12<br>(-2·3–28·3)
High systolic blood pressure	26·4<br>(21·3–32·2)	17·4<br>(15·3–19·7)	20·8<br>(16·2–26)	-34·2<br>(-46–-18·3)	19·8<br>(-4·3–51·7)	25<br>(18·6–32·7)	15<br>(13·2–16·9)	20·1<br>(15–26·3)	-40<br>(-54·8–-18·7)	33·7<br>(0·5–75·7)
High body-mass index	24·3<br>(17·2–33·5)	40·8<br>(30·6–52·5)	41·6<br>(31·2–53·5)	67·8<br>(46·7–101)	2·09<br>(-4·79–9·67)	33·2<br>(25·3–41·2)	47·9<br>(36·6–57·4)	47·9<br>(37·1–57·4)	44·5<br>(29·6–64·5)	-0·0969<br>(-6·26–7·13)
Low bone mineral density	11·8<br>(6·91–18·1)	9·04<br>(4·79–15·4)	11·5<br>(6·42–18·1)	-23·2<br>(-42·4–-2·35)	27·4<br>(-2·63–71·8)	15·5<br>(10·2–22·3)	16·1<br>(10·5–23·3)	18·8<br>(12·8–26·5)	3·91<br>(-19·8–31·8)	16·5<br>(-7·74–50)
Diet low in fruits	57·3<br>(44·1–71·2)	55·7<br>(42·3–70·3)	56·3<br>(42–70·7)	-2·8<br>(-13·6–7·14)	1·08<br>(-7·91–10·6)	--	--	--	--	--
Diet low in vegetables	57·5<br>(36·3–78)	74·8<br>(59·6–87·4)	72·4<br>(57·1–85·6)	30<br>(10·2–68·9)	-3·13<br>(-8·23–0·763)	50·2<br>(29–72)	55<br>(36·4–73·3)	53·9<br>(35·3–73)	9·65<br>(-1·83–32·2)	-1·95<br>(-10·4–5·71)
Diet low in whole grains	80·4<br>(70·9–89·3)	82·2<br>(73·8–89·7)	82·2<br>(73·5–89·9)	2·26<br>(-1·19–6·83)	0·0486<br>(-2·26–2·47)	77·8<br>(67·6–87·3)	80·4<br>(71·4–88·7)	80·2<br>(71·1–89·1)	3·39<br>(-0·766–8·96)	-0·214<br>(-3·27–2·41)
Diet low in nuts and seeds	33·5<br>(10·3–59·6)	27<br>(8·84–56)	23·2<br>(7·16–54·1)	-19·4<br>(-50–32·3)	-13·8<br>(-40·4–11·7)	29·1<br>(7·62–56·9)	23·8<br>(7·02–55)	21·4<br>(6·39–54·2)	-18·3<br>(-48·2–34·4)	-10<br>(-36·6–20·3)
Diet low in milk	53·4<br>(29–73·5)	47·7<br>(24·3–67·6)	47·1<br>(24·4–68)	-10·6<br>(-28·9–0·14)	-1·19<br>(-11·8–9·22)	53·2<br>(29·8–74·4)	47·8<br>(23·7–69)	47·4<br>(23·9–67·8)	-10·2<br>(-27·9–1·22)	-0·809<br>(-10·7–10·6)
Diet high in red meat	84·9<br>(75·1–92·1)	82·3<br>(70·4–90·9)	81·4<br>(69·3–90·8)	-3·01<br>(-14·8–8·59)	-1·15<br>(-11·3–8·37)	86·3<br>(76·2–94·2)	81·1<br>(68·5–90·6)	80·1<br>(66·2–90·3)	-6·1<br>(-18·6–5·67)	-1·24<br>(-12·1–8·61)
Diet high in processed meat	79·6<br>(56·4–98·3)	86·8<br>(69·6–99·7)	86·6<br>(69–99·5)	9·09<br>(0·783–28·4)	-0·304<br>(-5·63–4·49)	82·1<br>(59·6–99·3)	90·3<br>(77·1–99·8)	88·7<br>(72–99·8)	9·95<br>(0·405–32·4)	-1·77<br>(-7·68–1·62)
Diet high in sugar-sweetened beverages	40·6<br>(23·9–60·6)	56·7<br>(37–73·5)	57·1<br>(36·1–76·5)	39·6<br>(-4·88–120)	0·59<br>(-22·6–30·3)	44·4<br>(25·9–64·6)	62·5<br>(43·5–80)	61·1<br>(42·6–79)	40·7<br>(-1·38–130)	-2·23<br>(-26–24)
Diet low in fiber	48·5<br>(32–64·2)	33·7<br>(20·3–48·5)	31·3<br>(17·9–46·3)	-30·5<br>(-46·7–-14·9)	-7·12<br>(-25·2–10·2)	49·1<br>(31·8–64·3)	39·6<br>(24·4–54)	34·9<br>(20–50·1)	-19·5<br>(-35·6–-3·75)	-11·9<br>(-26·3–2·38)
Diet low in seafood omega-3 fatty acids	87·5<br>(72·9–99·7)	83·2<br>(65·7–99·4)	83·3<br>(66·6–99·5)	-4·9<br>(-19·5–6·25)	0·138<br>(-11·9–15·5)	88·9<br>(76–99·9)	86·4<br>(72–99·8)	86<br>(71·5–99·7)	-2·81<br>(-12·8–4·82)	-0·501<br>(-10·5–9·95)
Diet low in polyunsaturated fatty acids	17·6<br>(5·14–46)	8·67<br>(2·47–28·2)	8·35<br>(2·39–26·6)	-50·7<br>(-75·3–-9·33)	-3·74<br>(-26·6–21·1)	--	--	--	--	--
Diet high in trans fatty acids	89·4<br>(81·4–100)	87·9<br>(79–100)	87·5<br>(77·8–100)	-1·7<br>(-9·64–5·71)	-0·516<br>(-8·26–7·48)	--	--	--	--	--
Diet high in sodium	18·6<br>(4·36–42·2)	40·2<br>(10·9–65·1)	39·3<br>(10·1–65)	116<br>(36·3–418)	-2·04<br>(-27·6–29·5)	--	--	--	--	--
Low physical activity	2·46<br>(0·889–5·26)	2·26<br>(1–4·62)	2·38<br>(0·898–5)	-8·05<br>(-38·8–40·7)	4·99<br>(-19·7–37)	6·15<br>(3·02–11·4)	6·84<br>(3·8–12·3)	6·89<br>(3·64–12·7)	11·3<br>(-16–53·6)	0·758<br>(-11·7–12·6)
Occupational asthmagens	22·8<br>(18·9–27·3)	19·8<br>(16·8–23·4)	20·7<br>(17·2–25)	-12·8<br>(-22·6–-2·02)	4·3<br>(-4·65–14·2)	11·2<br>(9·13–13·6)	10·5<br>(8·75–12·7)	10·7<br>(8·69–13)	-5·79<br>(-20·2–11·4)	1·52<br>(-10·2–14·4)
Occupational particulate matter, gases, and fumes	9·68<br>(6·87–13·7)	8·94<br>(6·42–12·7)	8·52<br>(6·11–12)	-7·66<br>(-13·1–-2·05)	-4·68<br>(-8·14–-1·25)	5·35<br>(3·68–7·9)	5·15<br>(3·65–7·47)	4·93<br>(3·52–7·06)	-3·64<br>(-12·6–6·77)	-4·33<br>(-9·89–1·4)
Occupational noise	8·63<br>(7·86–9·47)	8·09<br>(7·47–8·78)	7·74<br>(7·23–8·38)	-6·25<br>(-9·77–-2·15)	-4·4<br>(-6·63–-1·9)	5·67<br>(4·93–6·5)	5·86<br>(5·33–6·51)	5·8<br>(5·39–6·4)	3·23<br>(-3·03–10·8)	-0·973<br>(-4·41–2·78)
Occupational injuries	--	--	--	--	--	--	--	--	--	--
Occupational ergonomic factors	13<br>(10·7–15·8)	10·6<br>(8·94–12·6)	11<br>(9·16–13·1)	-18·5<br>(-29·2–-6·52)	3·81<br>(-5·38–14·8)	7·47<br>(5·89–9·51)	6·93<br>(5·67–8·48)	7·25<br>(5·82–9·15)	-7·24<br>(-24·2–11·3)	4·65<br>(-7·73–19·1)
Childhood sexual abuse	7·4<br>(5·05–10·5)	6·97<br>(4·6–9·93)	6·85<br>(4·54–9·67)	-5·83<br>(-12·2–5·83)	-1·68<br>(-8·51–5·53)	12·3<br>(8·94–17·3)	17·4<br>(12·9–24)	17·8<br>(13·1–24·7)	41·9<br>(30·7–54·8)	2·17<br>(-5–11)
Intimate partner violence	--	--	--	--	--	33·7<br>(18·4–42·8)	32·1<br>(17·9–41·7)	32·8<br>(18·6–42·4)	-4·66<br>(-13·1–7·37)	2·28<br>(-6·37–16·1)
Non-exclusive breastfeeding	26<br>(21·7–31·3)	24·6<br>(20·9–28·7)	24·6<br>(20·2–29·7)	-5·68<br>(-20–11·5)	0·257<br>(-13·5–15·2)	--	--	--	--	--
Discontinued breastfeeding	27·2<br>(26·2–28·1)	25·3<br>(24–26·5)	25·8<br>(24·5–27)	-6·92<br>(-12·5–-1·47)	2·06<br>(-3·44–8·29)	27·3<br>(26·3–28·2)	25·3<br>(24·1–26·6)	25·8<br>(24·5–27)	-7·16<br>(-12·7–-1·73)	1·87<br>(-3·62–8·08)
Diet low in calcium	19·6<br>(9·12–36·6)	16·1<br>(7·62–29·8)	15·7<br>(7·56–29·3)	-18<br>(-29·8–-7·68)	-2·07<br>(-11·5–5·42)	15·2<br>(6·38–29·9)	13·3<br>(5·73–25·4)	13·3<br>(5·57–25·3)	-12·6<br>(-26·7–1·56)	0·332<br>(-8·67–8·73)
Occupational exposure to asbestos	6·73<br>(5·98–7·56)	5·87<br>(5·28–6·46)	5·88<br>(4·27–7·79)	-12·8<br>(-24·9–2·08)	0·0855<br>(-27·2–34·4)	1·11<br>(0·85–2·05)	1·19<br>(0·953–2·15)	1·19<br>(0·757–2·22)	6·88<br>(-8·17–30·5)	-0·0944<br>(-29·7–34·7)
Occupational exposure to arsenic	0·509<br>(0–1·21)	0·494<br>(0–1·17)	0·524<br>(0–1·23)	-2·93<br>(-17–NA)	6·08<br>(-6·33–NA)	0·499<br>(0–1·17)	0·486<br>(0–1·16)	0·497<br>(0–1·19)	-2·63<br>(-20·4–NA)	2·27<br>(-11·3–NA)
Occupational exposure to benzene	0·758<br>(0·045–2·2)	0·715<br>(0·0423–2·09)	0·749<br>(0·0458–2·15)	-5·68<br>(-13·5–5·39)	4·74<br>(-3·39–16·3)	1·07<br>(0·0577–2·97)	1·14<br>(0·0679–3·17)	1·21<br>(0·075–3·43)	6·52<br>(-5·59–25·2)	6·17<br>(-4·39–20·3)
Occupational exposure to beryllium	0·0232<br>(0·0218–0·0247)	0·0219<br>(0·0213–0·0224)	0·0232<br>(0·0218–0·0248)	-5·9<br>(-11·7–0·241)	6·29<br>(-0·184–13·5)	0·0248<br>(0·0224–0·0273)	0·0251<br>(0·0244–0·0258)	0·0263<br>(0·024–0·0284)	0·933<br>(-8·42–12·4)	4·71<br>(-3·48–12·7)
Occupational exposure to cadmium	0·0592<br>(0·0534–0·0659)	0·0576<br>(0·0523–0·063)	0·061<br>(0·0538–0·0685)	-2·7<br>(-15·5–11·2)	5·81<br>(-5·23–17·4)	0·0586<br>(0·0513–0·0667)	0·0575<br>(0·051–0·0647)	0·0589<br>(0·0501–0·0684)	-1·93<br>(-17·6–17·6)	2·54<br>(-10·2–17·8)
Occupational exposure to chromium	0·141<br>(0·128–0·155)	0·14<br>(0·129–0·15)	0·148<br>(0·132–0·164)	-0·957<br>(-12·3–11·6)	5·87<br>(-3·84–16)	--	--	--	--	--
Occupational exposure to diesel engine exhaust	0·72<br>(0·666–0·78)	0·725<br>(0·678–0·777)	0·774<br>(0·704–0·852)	0·715<br>(-9·3–12·4)	6·72<br>(-2·18–15·9)	0·603<br>(0·533–0·684)	0·628<br>(0·575–0·691)	0·642<br>(0·565–0·729)	4·19<br>(-10·6–21·5)	2·28<br>(-8·52–14·3)
Occupational exposure to formaldehyde	0·294<br>(0·263–0·329)	0·28<br>(0·251–0·309)	0·293<br>(0·256–0·331)	-4·67<br>(-18·1–10·2)	4·53<br>(-6·55–16·7)	0·285<br>(0·249–0·324)	0·279<br>(0·248–0·312)	0·287<br>(0·246–0·331)	-1·91<br>(-17·1–16·2)	2·69<br>(-9·59–17)
Occupational exposure to nickel	0·454<br>(0–1·94)	0·415<br>(0–1·79)	0·438<br>(0–1·81)	-8·58<br>(-21·2–NA)	5·45<br>(-5·84–NA)	0·401<br>(0–1·66)	0·378<br>(0–1·68)	0·388<br>(0–1·68)	-5·74<br>(-23·8–NA)	2·63<br>(-11·9–NA)
Occupational exposure to polycyclic aromatic hydrocarbons	0·277<br>(0·253–0·305)	0·273<br>(0·253–0·293)	0·289<br>(0·259–0·32)	-1·48<br>(-12·4–10)	5·63<br>(-3·81–15·3)	0·299<br>(0·266–0·335)	0·307<br>(0·282–0·333)	0·316<br>(0·279–0·354)	2·67<br>(-9·91–17·7)	3·12<br>(-7·6–14·4)
Occupational exposure to silica	4·44<br>(0·633–14·5)	3·83<br>(0·552–12·1)	4·06<br>(0·556–12·8)	-13·7<br>(-25·1–-0·147)	5·98<br>(-5·22–20·2)	3·43<br>(0·466–11·2)	3·12<br>(0·444–10·2)	3·18<br>(0·428–10·3)	-9·14<br>(-26·9–15·4)	2<br>(-12·8–23·3)
Occupational exposure to sulfuric acid	0·881<br>(0·183–2·76)	0·932<br>(0·187–3·04)	0·975<br>(0·195–3·17)	5·8<br>(-7·57–20·4)	4·54<br>(-6·8–16·6)	0·868<br>(0·182–2·71)	1·01<br>(0·217–3·18)	1·01<br>(0·213–3·13)	16·5<br>(-4·24–42·9)	-0·0497<br>(-15–16)
Unsafe sex	--	--	--	--	--	--	--	--	--	--
Occupational exposure to trichloroethylene	0·0819<br>(0·0763–0·0884)	0·0827<br>(0·0789–0·0868)	0·0878<br>(0·0812–0·0951)	0·962<br>(-7·41–9·94)	6·22<br>(-1·76–14·5)	0·0804<br>(0·0719–0·0903)	0·0816<br>(0·0761–0·0876)	0·0839<br>(0·0743–0·0925)	1·48<br>(-10·4–15·8)	2·75<br>(-7·66–13·5)
No access to handwashing facility	3·52<br>(3·27–3·81)	3·18<br>(2·92–3·45)	3·14<br>(2·89–3·4)	-9·83<br>(-17·9–-1·04)	-1·28<br>(-7·04–4·41)	3·52<br>(3·27–3·81)	3·18<br>(2·92–3·45)	3·14<br>(2·89–3·4)	-9·83<br>(-17·9–-1·04)	-1·28<br>(-7·04–4·41)
Child wasting	0·756<br>(0·496–1·03)	0·732<br>(0·479–0·994)	0·731<br>(0·468–0·995)	-3·23<br>(-12·8–6·71)	-0·103<br>(-7·04–8·42)	0·779<br>(0·522–1·05)	0·77<br>(0·51–1·07)	0·765<br>(0·51–1·05)	-1·23<br>(-11·7–9·98)	-0·572<br>(-8·34–8·11)
Child stunting	2·61<br>(1·37–3·75)	2·2<br>(1·13–3·34)	2·13<br>(1·13–3·23)	-15·6<br>(-35·5–8·79)	-3·17<br>(-18·3–15·5)	2·05<br>(1·06–3·08)	1·89<br>(0·942–2·87)	1·86<br>(0·926–2·86)	-7·98<br>(-25·8–16·7)	-1·4<br>(-15·9–16·4)
Chewing tobacco	8·03<br>(6·3–10)	7·44<br>(5·62–9·51)	7·18<br>(5·38–9·48)	-7·33<br>(-33·6–28·6)	-3·56<br>(-22–19·3)	--	--	--	--	--
Diet low in legumes	75·7<br>(41·7–99·6)	78·4<br>(48·1–99·8)	78·4<br>(47·5–99·8)	3·51<br>(-0·38–18·2)	0·0729<br>(-4·9–5·22)	74<br>(37·7–99·2)	76·7<br>(45·3–99·7)	77·1<br>(46·2–99·7)	3·64<br>(-0·51–20·1)	0·565<br>(-4·22–7·38)
Short gestation	35·8<br>(29·8–42·5)	36<br>(30·3–42·4)	30·4<br>(25·1–36·6)	0·581<br>(-19–24)	-15·5<br>(-29·9–3·31)	31·8<br>(26·8–37·5)	35·3<br>(29·9–41·2)	30<br>(25·1–35·7)	10·9<br>(-9·58–37·4)	-14·9<br>(-29·9–2·01)
Low birth weight	15<br>(12·5–17·7)	15·9<br>(13·6–18·5)	14·7<br>(12·2–17·4)	6·08<br>(-14·7–32·8)	-7·97<br>(-24·1–12·3)	15·7<br>(13·5–18·4)	18·7<br>(16·1–21·6)	17·3<br>(14·7–20·2)	19·1<br>(-4·19–46·5)	-7·96<br>(-23·3–11·3)
High temperature	26·6<br>(16·5–37·2)	30<br>(19·6–40·9)	24·9<br>(15·2–35·5)	12·5<br>(7·04–20·7)	-16·8<br>(-24·3–-11)	26·6<br>(16·5–37·2)	30<br>(19·6–40·9)	24·9<br>(15·2–35·5)	12·5<br>(7·04–20·7)	-16·8<br>(-24·3–-11)
Low temperature	37·1<br>(27·4–49·3)	41·1<br>(32·5–51·9)	38·5<br>(28·9–50·2)	10·7<br>(4·47–19·3)	-6·31<br>(-11·4–-2·37)	37·1<br>(27·4–49·3)	41·1<br>(32·5–51·9)	38·5<br>(28·9–50·2)	10·7<br>(4·47–19·3)	-6·31<br>(-11·4–-2·37)
Kidney dysfunction	20·9<br>(14·9–28)	21·8<br>(15·7–28·9)	22<br>(16–29·4)	4·45<br>(0·423–9·31)	0·902<br>(-2·77–4·63)	23·4<br>(16·9–31·2)	24·9<br>(17·9–32·8)	25·9<br>(18·9–33·7)	6·28<br>(1·92–11·7)	3·8<br>(0·546–7·88)

Bullying victimization	7·25 (3·21–14·4)	9·88 (4·07–19·6)	10·7 (4·19–21·7)	36·2 (19–55·6)	8·6 (-2·24–19·8)	--	--	--	--	--
High LDL cholesterol	66 (61·8–70·6)	34·8 (31·3–38·5)	31 (27·2–34·9)	-47·3 (-52·9–-42·1)	-11 (-16·2–-6·04)	65·3 (61·6–69·3)	34·8 (31·6–38·2)	31·9 (28·4–35·6)	-46·7 (-51·4–-42·1)	-8·34 (-12·8–-4·27)

Supplementary Results Table 1. Missouri age-standardised Summary Exposure Values (SEVs) for most-detailed risk factors and percentage change, by sex, 1990, 2010, and 2019. Data in parentheses are 95% uncertainty intervals.

	Male					Female				
	1990	2010	2019	Percentage change 1990-2010	Percentage change 2010-2019	1990	2010	2019	Percentage change 1990-2010	Percentage change 2010-2019
Unsafe sanitation	7·15 (3·37–12·6)	4·01 (1·87–7·61)	3·19 (1·46–6·05)	-43·9 (-76·2–31·9)	-20·4 (-59·2–52·2)	--	--	--	--	--
Ambient particulate matter pollution	12·1 (4·02–24·7)	8·13 (5·04–11·3)	5·33 (2·57–8·15)	-32·8 (-65·9–76·3)	-34·5 (-50·1—26·7)	12·1 (4·04–24·7)	8·13 (5·05–11·3)	5·33 (2·57–8·13)	-32·8 (-65·9–74·3)	-34·5 (-50—26·6)
Household air pollution from solid fuels	0·0214 (0·00479–0·0562)	0·0125 (0·00251–0·0378)	0·00847 (0·00152–0·027)	-41·5 (-77·3–34·5)	-32·4 (-66–25·6)	0·0283 (0·0069–0·0747)	0·0166 (0·00341–0·0518)	0·0112 (0·002–0·0363)	-41·5 (-77·3–35)	-32·3 (-65·9–26·3)
Ambient ozone pollution	56·8 (23·8–93·7)	53·6 (22·6–90·4)	48·9 (19·9–84·7)	-5·71 (-14·3–3·12)	-8·7 (-18—0·184)	--	--	--	--	--
Residential radon	19·3 (3·02–56·5)	19·3 (3·02–56·5)	19·3 (3·02–56·5)	0 (0–0)	0 (0–0)	19·3 (3·02–56·5)	19·3 (3·02–56·5)	19·3 (3·02–56·5)	0 (0–0)	0 (0–0)
Lead exposure	60·2 (32·4–81·1)	35·3 (14·2–57)	24·8 (8·27–44)	-41·3 (-58—28)	-29·7 (-43·7—21·9)	34·9 (5·17–63·3)	20·4 (1·69–45·3)	14·2 (0·662–34·5)	-41·6 (-74·1—26·5)	-30·2 (-63·5—22·1)
Child underweight	0·963 (0·548–1·46)	0·965 (0·534–1·44)	0·94 (0·515–1·44)	0·208 (-16–20·9)	-2·52 (-16·2–11·1)	0·809 (0·454–1·22)	0·847 (0·463–1·3)	0·868 (0·47–1·32)	4·65 (-13·9–29·6)	2·48 (-11·9–18·4)
Iron deficiency	--	--	--	--	--	11·8 (9·81–14)	12·2 (10·3–14·2)	12·5 (10·4–14·9)	3·48 (-8·41–17·2)	2·33 (-6·66–12·5)
Vitamin A deficiency	0·894 (0·236–2·3)	1·24 (0·359–3·19)	0·876 (0·239–2·45)	39 (-63–479)	-29·5 (-69·3–63·7)	1·76 (0·696–3·53)	1·82 (0·693–3·77)	1·56 (0·563–3·59)	2·93 (-59·5–220)	-14·1 (-54·3–65·5)
Zinc deficiency	2·31 (0–7·54)	1·21 (0–3·81)	1·28 (0–4·09)	-47·5 (-100–NA)	5·38 (-14–NA)	2·36 (0–7·58)	1·23 (0–4·17)	1·25 (0–4·1)	-48·2 (-100–NA)	2·12 (-15·3–NA)
Smoking	28·8 (26–31·8)	22·9 (20·8–25·2)	20·3 (18·3–22·5)	-20·5 (-26·1—14·8)	-11·5 (-14·9—7·87)	20·2 (17·8–22·8)	17·4 (15·7–19·3)	15·8 (14·1–17·6)	-14 (-21·9—4·07)	-8·8 (-13·2—4·21)
Secondhand smoke	39·7 (37·1–42·2)	29·3 (27·7–31·1)	28·7 (25·7–31·7)	-26·2 (-32·2—19·2)	-2·29 (-10·6–6·47)	37·7 (35·8–39·7)	28·5 (27·2–29·8)	27·8 (25·5–30·1)	-24·6 (-30·1—19·1)	-2·48 (-9·36–4·42)
Alcohol use	14·3 (9·02–20·2)	14·6 (8·87–20·6)	15·4 (9·16–21·9)	1·97 (-27–43·9)	4·99 (-18·2–32·1)	--	--	--	--	--
Drug use	0·414 (0·255–0·694)	0·907 (0·648–1·23)	1·3 (0·966–1·73)	119 (57·1–192)	43·8 (24·7–69·2)	0·341 (0·236–0·488)	0·782 (0·598–1·01)	1·21 (0·94–1·53)	129 (85·5–184)	54·1 (32·7–76·2)
High fasting plasma glucose	13 (10·6–15·8)	18·6 (16·3–21·1)	19·5 (16·9–22·5)	43·2 (17·7–72·6)	4·97 (-7·52–20·6)	9·72 (8·3–11·4)	15·1 (13·2–17·1)	16·1 (13·9–18·6)	55·4 (31·1–83)	6·77 (-7·57–23·3)
High systolic blood pressure	25·6 (20·6–31)	16·2 (14·2–18·4)	19·9 (15·4–24·9)	-36·5 (-48·6—21)	22·8 (-3·65–52·1)	23·9 (17·6–30·7)	13·8 (12·2–15·5)	18·7 (13·7–25)	-42·3 (-55·6—22·2)	35·5 (-0·599–84·9)
High body-mass index	24·9 (17·4–34·1)	40·1 (29·4–51·7)	41 (30·5–52·7)	61·3 (41·3–89·7)	2·24 (-4·38–10)	30·9 (22·8–39·8)	44·9 (34·6–54·2)	45·5 (35·5–55·2)	45·4 (28·3–69·6)	1·39 (-4·45–8·16)
Low bone mineral density	12·2 (7·17–19)	9·47 (4·93–15·7)	12 (6·98–18·8)	-22·4 (-43·1—0·826)	27·2 (1·32–69·5)	16·1 (10·5–22·7)	16·9 (11–24·2)	19·6 (13·2–27)	5·29 (-15·9–31·9)	15·5 (-10·5–48·2)
Diet low in fruits	55·5 (43·4–68·7)	48·7 (37·6–61)	49·2 (37·3–62)	-12·2 (-22·3—2·39)	1·08 (-10·1–12·4)	--	--	--	--	--
Diet low in vegetables	38·2 (21·1–59·4)	46·2 (30·1–64·9)	44·4 (28·5–62·3)	21·1 (3–59·9)	-3·97 (-14·4–5·63)	41·4 (22·8–63·8)	57·9 (39·1–76·3)	56·1 (37·6–74·9)	39·9 (14·6–91·4)	-3·04 (-10·4–3·45)
Diet low in whole grains	79·6 (69·9–88·7)	81·7 (72·9–89·7)	81·8 (72·9–89·6)	2·62 (-0·975–7·14)	0·17 (-2·49–2·77)	77 (66·9–86·6)	79·8 (70·6–88·3)	79·7 (69·9–88·4)	3·62 (-0·992–9·34)	-0·102 (-2·89–2·8)
Diet low in nuts and seeds	28 (8–56·9)	25·4 (8·69–56·1)	21·9 (7·25–54·7)	-9·09 (-43·5–62·6)	-13·9 (-41·5–16·3)	24·1 (6·24–54·4)	22·1 (6·46–54·2)	20·1 (6·03–53·1)	-8·26 (-41·3–62·5)	-9·11 (-36·4–23·9)
Diet low in milk	48·9 (25·5–69·5)	44 (19·7–64·4)	43·9 (20·1–64·8)	-10 (-28·6–3·21)	-0·0697 (-11·6–12·5)	48·7 (25·7–69·1)	44 (21·4–64)	44·2 (21·3–63·9)	-9·71 (-26·9–3·49)	0·579 (-10–13·5)
Diet high in red meat	90 (81·4–96·2)	86·4 (75·8–94)	85·1 (73·1–93·9)	-3·96 (-14·3–5·68)	-1·56 (-10·5–7)	90 (82–95·9)	85 (73·5–93·7)	83·5 (71·3–93·1)	-5·61 (-16·6–4·46)	-1·72 (-11–7·33)
Diet high in processed meat	80·8 (56·6–99·2)	87·6 (69·7–99·6)	87·4 (68·9–99·8)	8·43 (0·215–25·2)	-0·336 (-5·21–4·41)	82·6 (61·8–99·3)	90·4 (77–99·9)	88·7 (73·3–99·8)	9·4 (0·358–29·4)	-1·83 (-7·85–1·69)
Diet high in sugar-sweetened beverages	45·8 (27·5–64·8)	62 (42·4–79·3)	61·2 (41·9–78·7)	35·6 (-4–116)	-1·32 (-21·8–23·7)	49·5 (28·7–69·7)	67·2 (47·4–83·1)	64·9 (44·5–83)	35·8 (-2·92–117)	-3·49 (-24·8–16·4)
Diet low in fiber	43·7 (27·9–59·3)	30·7 (17·7–45)	28·9 (15·6–43·3)	-29·8 (-46·6—12·9)	-5·76 (-25·2–14·1)	44·3 (27·1–60·9)	36·1 (22–50·2)	31·9 (17·5–46·3)	-18·5 (-34·4—0·859)	-11·7 (-27·3–2·71)
Diet low in seafood omega-3 fatty acids	80·8 (61·7–99·7)	77·1 (58·5–99·2)	77·3 (59·1–98·8)	-4·66 (-20·7–11)	0·361 (-14·1–19·1)	84·3 (67–99·6)	81·7 (65–99·7)	81·4 (63·7–99·2)	-3 (-15·4–8·54)	-0·369 (-12·3–13·2)
Diet low in polyunsaturated fatty acids	14·2 (4·01–40·1)	7·47 (2·13–27·2)	7·32 (2·13–26·8)	-47·2 (-74·9—5·85)	-2·05 (-23·1–24·4)	--	--	--	--	--
Diet high in trans fatty acids	89·4 (81·9–100)	88 (79–100)	87·5 (78·5–100)	-1·64 (-10·1–6·07)	-0·519 (-8·54–7·74)	88·3 (80·8–100)	86·6 (78·6–100)	86·3 (77·3–100)	-1·93 (-9·72–5·94)	-0·407 (-8·98–7·85)
Diet high in sodium	18·6 (4·5–41·8)	40 (11·2–64·1)	39·3 (10·3–64·3)	116 (37·4–403)	-1·79 (-26·7–28·4)	--	--	--	--	--
Low physical activity	2·29 (0·853–4·87)	2·08 (0·908–4·36)	1·88 (0·678–4·2)	-9·54 (-40·3–44·3)	-9·47 (-36·2–16·5)	5·56 (2·69–10·6)	5·8 (3·18–10·6)	5·66 (2·85–10·8)	4·39 (-22·3–43·8)	-2·37 (-15·7–10·9)
Occupational asthmagens	23·2 (19·3–28·2)	21 (17·8–24·8)	21·9 (18·2–26·6)	-9·64 (-21·4–2·49)	4·29 (-5·09–15·8)	11·6 (9·81–14)	11·9 (9·86–14·2)	12 (9·76–14·6)	2·05 (-13·5–18·3)	0·81 (-9·8–14·1)
Occupational particulate matter, gases, and fumes	9·79 (6·94–14·2)	9·27 (6·68–13·5)	8·97 (6·49–12·9)	-5·3 (-10·9–1·22)	-3·21 (-6·75–0·168)	5·5 (3·88–8·09)	5·4 (3·87–7·78)	5·24 (3·77–7·56)	-1·89 (-11–8·23)	-2·91 (-7·68–2·81)
Occupational noise	8·67 (7·95–9·57)	8·42 (7·85–9·18)	8·19 (7·69–8·91)	-2·9 (-6·43–1·63)	-2·66 (-4·75—0·537)	6·12 (5·34–6·94)	6·53 (6·01–7·13)	6·58 (6·14–7·16)	6·76 (0·543–14·6)	0·732 (-2–4·08)
Occupational injuries	--	--	--	--	--	--	--	--	--	--
Occupational ergonomic factors	12·6 (10·4–15·5)	11 (9·29–13·2)	11·4 (9·38–13·8)	-12·9 (-23·8—1·44)	3·71 (-4·57–12·7)	7·51 (5·94–9·37)	7·64 (6·3–9·51)	8·02 (6·36–10·2)	1·74 (-14·6–20·4)	4·97 (-8·2–20)
Childhood sexual abuse	7·4 (5·05–10·5)	6·97 (4·6–9·93)	6·85 (4·54–9·67)	-5·83 (-12·2–5·83)	-1·68 (-8·51–5·53)	12·3 (8·94–17·3)	17·4 (12·9–24)	17·8 (13·1–24·7)	41·9 (30·7–54·8)	2·17 (-5–11)
Intimate partner violence	--	--	--	--	--	22·4 (12·5–30·8)	22·6 (12·4–30·7)	23·6 (13·3–32·6)	0·805 (-8·38–14·4)	4·53 (-7·26–20·8)
Non-exclusive breastfeeding	26·2 (21·4–30·9)	24·9 (21·3–28·8)	24·9 (20·3–29·6)	-4·63 (-18·7–12·3)	-0·284 (-13·3–14)	--	--	--	--	--
Discontinued breastfeeding	27·2 (26·3–28·2)	25·4 (24·1–26·7)	25·6 (24·1–26·7)	-6·69 (-11·9—1·38)	0·586 (-5·02–6·22)	27·2 (26·3–28·2)	25·4 (24·1–26·7)	25·5 (24·1–26·7)	-6·85 (-12·1—1·55)	0·599 (-5·01–6·23)
Diet low in calcium	16·5 (7·84–30·5)	13·9 (6·74–25·4)	13·9 (6·59–25·5)	-15·6 (-27·2—3·45)	-0·233 (-9·1–7·89)	11·8 (4·9–22·4)	10·9 (4·6–20·2)	11·2 (4·63–21·3)	-7·9 (-21·8–9·39)	2·55 (-7·23–13·1)
Occupational exposure to asbestos	7·57 (6·76–8·48)	6·49 (5·92–7·04)	5·94 (4·34–7·85)	-14·3 (-25·3—0·571)	-8·44 (-33·5–21·5)	1·29 (1·04–1·96)	1·31 (1·03–1·93)	1·29 (0·857–2·08)	1·98 (-11·1–19·9)	-2·05 (-30·7–31·9)
Occupational exposure to arsenic	0·535 (0–1·26)	0·527 (0–1·24)	0·556 (0–1·33)	-1·46 (-15·6–NA)	5·49 (-5·73–NA)	0·499 (0–1·17)	0·51 (0–1·22)	0·519 (0–1·25)	2·01 (-14·9–NA)	1·9 (-9·94–NA)
Occupational exposure to benzene	0·842 (0·0478–2·41)	0·823 (0·0484–2·4)	0·851 (0·0506–2·44)	-2·24 (-10·3–9·05)	3·38 (-4·68–14·9)	1·21 (0·0671–3·52)	1·38 (0·0796–3·85)	1·44 (0·086–4·13)	14·1 (1·7–35·7)	4·04 (-4·27–15·8)
Occupational exposure to beryllium	0·0241 (0·0227–0·0256)	0·0237 (0·0232–0·0242)	0·025 (0·0236–0·0265)	-1·59 (-7·42–4·38)	5·36 (-0·437–11·5)	0·0272 (0·0249–0·0295)	0·029 (0·0284–0·0297)	0·03 (0·0281–0·0318)	6·79 (-1·84–17·5)	3·2 (-2·77–9·71)
Occupational exposure to cadmium	0·0624 (0·0565–0·0689)	0·0617 (0·0561–0·0676)	0·0649 (0·0575–0·0728)	-1·14 (-14·1–14·1)	5·25 (-4·85–16·7)	0·0596 (0·0527–0·0668)	0·0614 (0·0553–0·0688)	0·0626 (0·0549–0·0722)	3·02 (-12·5–22·4)	2·04 (-8·55–14·7)
Occupational exposure to chromium	0·149 (0·137–0·163)	0·151 (0·14–0·162)	0·158 (0·143–0·175)	0·805 (-10·4–13·6)	5·17 (-3·75–14·9)	--	--	--	--	--
Occupational exposure to diesel engine exhaust	0·724 (0·669–0·786)	0·778 (0·727–0·828)	0·822 (0·751–0·894)	7·39 (-2·92–18·2)	5·66 (-1·94–14)	0·68 (0·599–0·758)	0·746 (0·678–0·819)	0·749 (0·661–0·836)	9·73 (-4·23–26·7)	0·336 (-9·18–10·2)
Occupational exposure to formaldehyde	0·313 (0·281–0·348)	0·302 (0·272–0·331)	0·314 (0·277–0·354)	-3·57 (-16·6–12)	3·94 (-6·55–15·8)	0·291 (0·258–0·325)	0·302 (0·273–0·335)	0·308 (0·272–0·351)	3·79 (-11–21·9)	2·16 (-7·92–14·3)
Occupational exposure to nickel	0·448 (0–1·95)	0·424 (0–1·9)	0·446 (0–1·85)	-5·42 (-18·9–NA)	5·29 (-5·2–NA)	0·394 (0–1·7)	0·388 (0–1·76)	0·397 (0–1·8)	-1·5 (-19·1–NA)	2·28 (-11·2–NA)
Occupational exposure to polycyclic aromatic hydrocarbons	0·295 (0·272–0·322)	0·298 (0·277–0·318)	0·312 (0·283–0·343)	0·743 (-10–12·7)	4·82 (-3·64–14·1)	0·314 (0·283–0·346)	0·342 (0·32–0·368)	0·349 (0·317–0·383)	8·84 (-3·78–23·9)	2·2 (-5·6–11·1)
Occupational exposure to silica	4·07 (0·578–13·3)	3·76 (0·543–11·8)	3·99 (0·561–12·5)	-7·76 (-20·3–8·21)	6·08 (-4·52–19·4)	3·32 (0·461–10·7)	3·15 (0·444–10·1)	3·2 (0·435–10·1)	-5·26 (-22·5–20·2)	1·61 (-12·4–20·4)
Occupational exposure to sulfuric acid	0·98 (0·203–3·08)	1·06 (0·217–3·41)	1·09 (0·222–3·49)	8·04 (-6·2–24·8)	3·22 (-7·3–14·8)	0·94 (0·195–3·08)	1·14 (0·229–3·72)	1·12 (0·24–3·58)	21·6 (-1·92–49·7)	-1·59 (-15·7–13·8)
Unsafe sex	--	--	--	--	--	--	--	--	--	--
Occupational exposure to trichloroethylene	0·0871 (0·0812–0·0938)	0·09 (0·0862–0·0944)	0·0948 (0·088–0·102)	3·37 (-4·95–12·8)	5·32 (-1·5–12·7)	0·086 (0·0775–0·0945)	0·092 (0·0862–0·0984)	0·0935 (0·0849–0·103)	6·98 (-4·71–21·1)	1·6 (-5·92–10·5)
No access to handwashing facility	3·39 (3·11–3·65)	3·13 (2·9–3·37)	3·1 (2·85–3·33)	-7·62 (-15·6–1·43)	-1·01 (-6·34–4·97)	3·39 (3·11–3·65)	3·13 (2·9–3·37)	3·1 (2·85–3·33)	-7·62 (-15·6–1·43)	-1·01 (-6·34–4·97)
Child wasting	0·75 (0·497–1·02)	0·728 (0·485–0·991)	0·727 (0·48–0·983)	-2·95 (-12·7–7·77)	-0·142 (-7·39–7·99)	0·774 (0·526–1·05)	0·765 (0·512–1·05)	0·762 (0·506–1·04)	-1·19 (-11·5–9·9)	-0·356 (-7·6–7·53)
Child stunting	2·4 (1·23–3·47)	2·08 (1·03–3·11)	2·02 (1·05–3·03)	-13·5 (-32·5–11·1)	-3·06 (-17·4–13·5)	1·94 (1·01–3·02)	1·79 (0·886–2·74)	1·78 (0·898–2·74)	-7·62 (-26·9–16·9)	-0·935 (-16·5–17·4)
Chewing tobacco	5·16 (4·08–6·51)	5·53 (4·01–7·38)	5·43 (3·9–7·35)	7·23 (-24·3–51·3)	-1·78 (-24–25·6)	--	--	--	--	--
Diet low in legumes	74·1 (40·3–99·1)	77·2 (45·1–99·7)	77·4 (46·3–99·8)	4·22 (-0·0223–21·9)	0·305 (-4·17–6·63)	72·5 (34·9–98·7)	75·5 (43–99·6)	76·1 (45·7–99·5)	4·19 (-0·153–24·3)	0·741 (-4·24–9·75)
Short gestation	35·8 (29·3–43·6)	30·5 (25·5–35·7)	28·1 (23–34·6)	-14·8 (-32·5–5·97)	-8·09 (-24·8–13·3)	31·8 (26·9–37·4)	30·6 (25·9–36)	27·4 (23–32·4)	-3·77 (-20·9–16·3)	-10·5 (-25·8–8·56)
Low birth weight	14·8 (12·4–17·9)	12·7 (10·7–14·8)	12·7 (10·5–15·6)	-14·2 (-31·9–6·33)	0·262 (-18·4–25·6)	14·6 (12·5–17·2)	15·2 (13–17·6)	14·4 (12·3–16·8)	3·73 (-16·9–26·5)	-5·29 (-21·8–14·7)
High temperature	28·2 (18–39·8)	29 (18·5–40·6)	25·8 (15·9–37·4)	2·74 (0·413–5·27)	-11·1 (-17·7—5·79)	28·2 (18–39·8)	29 (18·5–40·6)	25·8 (15·9–37·4)	2·74 (0·413–5·27)	-11·1 (-17·7—5·79)
Low temperature	35·7 (31·3–41)	37·5 (33·3–42·4)	38·1 (33·9–43·2)	4·84 (1·93–8·39)	1·73 (-0·451–3·71)	35·7 (31·3–41)	37·5 (33·3–42·4)	38·1 (33·9–43·2)	4·84 (1·93–8·39)	1·73 (-0·451–3·71)
Kidney dysfunction	19·9 (14·3–27)	20·7 (14·9–27·9)	20·8 (15–28·1)	3·89 (0·472–8·65)	0·48 (-3·52–4·37)	22·2 (15·9–29·6)	23·4 (16·8–31·2)	24·2 (17·5–31·9)	5·28 (0·634–10·2)	3·36 (-1·54–9·12)

Bullying victimization	5·88 (2·45–12)	8·98 (3·59–17·8)	8·72 (3·35–17·9)	52·8 (32·6–76·2)	-2·83 (-12·5–3·09)	--	--	--	--	--
High LDL cholesterol	66·1 (61·8–70·6)	34·5 (31·1–38·1)	30·9 (27·3–34·7)	-47·8 (-52·8–-42·5)	-10·7 (-15·8–-5·59)	65·2 (61·9–68·8)	34·8 (31·5–38·1)	32·1 (28·5–35·8)	-46·7 (-51·5–-42·1)	-7·56 (-11·4–-3·46)

Supplementary Results Table 1. Montana age-standardised Summary Exposure Values (SEVs) for most-detailed risk factors and percentage change, by sex, 1990, 2010, and 2019. Data in parentheses are 95% uncertainty intervals.

	Male					Female				
	1990	2010	2019	Percentage change 1990-2010	Percentage change 2010-2019	1990	2010	2019	Percentage change 1990-2010	Percentage change 2010-2019
Unsafe sanitation	6·95 (3·16–12·7)	3·82 (1·66–7·45)	2·87 (1·27–5·57)	-45·1 (-77–32·5)	-24·9 (-61·1–44·1)	--	--	--	--	--
Ambient particulate matter pollution	6·32 (1·27–15·2)	2·71 (0·88–5·18)	3·03 (1·02–5·57)	-57·1 (-86·9–53·9)	11·7 (5·82–29·1)	6·32 (1·29–15·2)	2·72 (0·897–5·17)	3·03 (1·03–5·59)	-57 (-86·7–54·1)	11·5 (5·72–28·5)
Household air pollution from solid fuels	0·0467 (0·00809–0·148)	0·0201 (0·00223–0·0725)	0·0161 (0·00202–0·0596)	-57 (-88–14·5)	-19·8 (-56·8–60·5)	0·0618 (0·0106–0·192)	0·0268 (0·00298–0·091)	0·0214 (0·00258–0·0769)	-56·6 (-87·8–14·1)	-20 (-57–60·3)
Ambient ozone pollution	39·8 (16·1–70)	47·9 (19·2–84·6)	40·6 (15·5–69·8)	20·3 (-1·85–48·6)	-15·2 (-34·6–6·92)	--	--	--	--	--
Residential radon	25 (3·69–71·9)	25 (3·69–71·9)	25 (3·69–71·9)	0 (0–0)	0 (0–0)	25 (3·69–71·9)	25 (3·69–71·9)	25 (3·69–71·9)	0 (0–0)	0 (0–0)
Lead exposure	31·8 (4·46–59·3)	19·8 (2·32–42·3)	14·1 (1·09–33·6)	-37·8 (-60·5–-23·3)	-28·6 (-55·6–-19·4)	18·1 (0·105–45·9)	12·3 (0·0435–35·2)	9·21 (0·0169–28·7)	-32 (-78·6–20·9)	-25·2 (-74·5–-16·8)
Child underweight	0·958 (0·541–1·48)	0·968 (0·529–1·47)	0·939 (0·53–1·41)	1·04 (-17·1–22·9)	-3·02 (-15·6–11·5)	0·811 (0·452–1·24)	0·854 (0·473–1·31)	0·87 (0·487–1·34)	5·25 (-17·1–34·5)	1·85 (-12·1–17·4)
Iron deficiency	--	--	--	--	--	11·5 (9·62–13·6)	12 (10–13·9)	12·3 (10·3–14·6)	4·02 (-7·97–17·6)	2·94 (-6·76–14·1)
Vitamin A deficiency	0·901 (0·242–2·26)	1·19 (0·311–3·09)	0·806 (0·227–2·19)	31·8 (-63·2–406)	-32·2 (-70·1–66)	1·74 (0·618–3·65)	1·77 (0·657–3·59)	1·47 (0·564–3·23)	1·94 (-61·4–185)	-17·1 (-54·4–57·5)
Zinc deficiency	2·56 (0–7·88)	1·29 (0–4·08)	1·28 (0–4·07)	-49·5 (-100–NA)	-0·525 (-21·2–NA)	2·63 (0–8·3)	1·3 (0–4·4)	1·24 (0–3·92)	-50·6 (-100–NA)	-4·83 (-24·7–NA)
Smoking	25·5 (22·8–28·3)	17·9 (16·2–19·8)	16·7 (15–18·6)	-29·5 (-34·2–-24·1)	-6·78 (-10·6–-2·72)	21·1 (18·7–23·7)	15·9 (14·3–17·7)	14·3 (12·7–16)	-24·7 (-30·9–-17·5)	-9·83 (-14·3–-5·25)
Secondhand smoke	37·9 (35·3–40·5)	25·4 (23·8–27·1)	25·1 (22·6–27·8)	-32·9 (-38·8–-25·8)	-1·1 (-10·3–8·35)	33·5 (31·6–35·6)	23·8 (22·6–24·9)	23·8 (21·6–25·8)	-29·2 (-34·7–-23·6)	-0·0238 (-7·81–7·01)
Alcohol use	14·7 (8·99–21·2)	13·9 (8·3–20·6)	15·2 (9·16–22·3)	-5·02 (-35·2–35·1)	9·18 (-14·7–40·8)	10·3 (6·02–15·5)	11·5 (6·98–17·1)	11·7 (6·96–17·3)	11·4 (-21·7–61·7)	1·77 (-20·8–28·7)
Drug use	0·454 (0·243–0·851)	0·728 (0·494–1·07)	1·24 (0·909–1·66)	60·3 (19·1–124)	70 (42·8–105)	0·42 (0·272–0·649)	0·777 (0·58–1·03)	1·22 (0·932–1·58)	85·1 (49·1–130)	56·7 (33·6–81·9)
High fasting plasma glucose	11·8 (9·81–14·3)	15 (12·8–17·7)	17·1 (14·7–19·9)	26·9 (4·06–52·3)	14 (-2·34–31·9)	9·29 (7·92–11·1)	10·9 (9·52–12·6)	12·9 (10·9–15·3)	17·4 (-0·52–39·2)	18·4 (3·38–38·4)
High systolic blood pressure	25·5 (20·7–30·8)	15·8 (13·7–17·9)	19·9 (15·3–25·3)	-38 (-50·3–-23·5)	26·1 (-1·07–60·6)	23·5 (17·3–30)	12·5 (10·9–14·1)	17·4 (12–23·6)	-46·5 (-59·2–-27·7)	38·4 (-1·53–87·7)
High body-mass index	24·6 (17·3–34·1)	38·4 (28–49·9)	41·4 (30·9–53)	56·1 (35·9–84·1)	7·86 (1·29–17·2)	28·6 (20·5–38·5)	41·3 (32·6–50·2)	43·6 (33·9–53·2)	44·2 (23·1–71·6)	5·52 (-1·24–12·8)
Low bone mineral density	12·2 (7·14–19)	9·47 (5·13–15·7)	11·9 (6·81–18·8)	-22·2 (-42·6–-1·89)	26 (-1·8–68·3)	15·9 (10·6–22·5)	17·1 (11·6–24·1)	19·4 (13·2–26·8)	7 (-13·7–34·9)	13·8 (-8·63–43·8)
Diet low in fruits	53·7 (41·8–67·1)	50·1 (38·6–62·9)	50·6 (37–63·8)	-6·79 (-18·2–4·78)	0·961 (-9·67–12·3)	54·3 (42·5–67·7)	48·9 (37–62)	48·9 (36·8–62·6)	-10·1 (-20·8–-0·0525)	0·133 (-11·4–12·1)
Diet low in vegetables	30·2 (15·4–51·1)	56·3 (37·1–76)	53·5 (34·3–72·6)	86·5 (32–196)	-4·84 (-13·1–2·45)	25·4 (11·4–46·3)	49·9 (31·2–69·7)	48·1 (30·1–67·5)	96·4 (34·3–254)	-3·72 (-13·9–5·47)
Diet low in whole grains	79·9 (70·4–88·3)	81·8 (73·4–89·6)	81·7 (72·8–89·6)	2·46 (-1·42–7·17)	-0·161 (-2·93–2·24)	77·3 (67·1–86·7)	80 (71·2–88·4)	79·6 (70·1–88·2)	3·45 (-0·666–8·69)	-0·459 (-3·64–2·51)
Diet low in nuts and seeds	30·2 (9·55–58·1)	25·9 (7·96–55·7)	21·1 (5·96–53·9)	-14·2 (-44·7–51·9)	-18·5 (-46·9–6·41)	25·9 (6·99–55·1)	22·8 (6·64–54·3)	19·6 (5·36–53·3)	-11·9 (-43·1–53·1)	-14·1 (-40·9–10·9)
Diet low in milk	50·7 (27·8–71·6)	45·4 (22·1–65·3)	43·4 (20·5–63·9)	-10·5 (-29·2–1·21)	-4·3 (-17·6–5·86)	50·5 (26·6–71)	45·5 (22·2–66·6)	43·8 (21·2–64·2)	-9·85 (-27·1–2·88)	-3·7 (-15·7–9·3)
Diet high in red meat	87·7 (77·9–95·2)	84·1 (71·7–93·2)	83·6 (69·7–93·6)	-4·03 (-16·3–7·9)	-0·603 (-10·4–8·81)	89·5 (80·1–96·4)	83·9 (71·7–93·1)	83·4 (71·1–93·4)	-6·26 (-18·3–5·59)	-0·575 (-10·4–10·1)
Diet high in processed meat	78·2 (54·3–97·8)	85·9 (67·1–99·7)	85·8 (66·9–99·5)	9·8 (1·03–28·4)	-0·0571 (-4·55–5·13)	78·7 (54·7–98·1)	87·8 (70·4–99·6)	86·5 (67·8–99·6)	11·5 (1·05–35·3)	-1·45 (-6·93–2·41)
Diet high in sugar-sweetened beverages	43·3 (25·2–63·2)	59·1 (39·2–77·6)	61·1 (41–79·1)	36·6 (-6·07–119)	3·28 (-17·2–28·5)	47·4 (27·2–66·8)	65·3 (45–81·4)	65·5 (45·2–82·2)	37·9 (-2·27–123)	0·196 (-19–24·8)
Diet low in fiber	45·8 (29·5–62·5)	32·1 (18·4–47·6)	28·6 (15·7–44·1)	-29·8 (-46·4–-13·4)	-11·1 (-27·8–7·04)	46·2 (28·3–62·4)	37·4 (23–53·5)	31·7 (18·1–47·8)	-19·1 (-35·3–-1·17)	-15 (-31·9–-1·24)
Diet low in seafood omega-3 fatty acids	85·4 (69·1–100)	81·3 (63·8–99·1)	80·6 (63·4–99)	-4·8 (-19·5–8·17)	-0·885 (-15·4–15·9)	87·5 (72·5–100)	84·9 (69·6–99·4)	83·9 (67·5–99·4)	-2·94 (-13·5–5·88)	-1·2 (-13·3–9·35)
Diet low in polyunsaturated fatty acids	15·4 (4·48–42·1)	7·78 (2·25–26·8)	7·21 (2·11–24·5)	-49·5 (-74·6–-8·89)	-7·38 (-32·6–12·9)	--	--	--	--	--
Diet high in trans fatty acids	89·5 (81·7–100)	87·8 (78·8–100)	87·3 (78·2–100)	-1·87 (-10·2–6·05)	-0·513 (-8·74–8·45)	88·3 (80·8–100)	86·7 (77·9–100)	86·2 (77·3–100)	-1·85 (-10·2–6·21)	-0·538 (-8·71–7·92)
Diet high in sodium	18·7 (4·5–41·8)	40·1 (11·4–63·5)	39·4 (10–64)	115 (37·2–397)	-1·76 (-27–30·7)	--	--	--	--	--
Low physical activity	1·81 (0·627–4·27)	1·48 (0·619–3·77)	1·35 (0·473–3·87)	-18·3 (-49·9–23·3)	-9·06 (-35·6–13·4)	4·67 (2·28–9·28)	4·29 (2·21–7·93)	4·35 (2·15–8·56)	-8·26 (-31·2–26)	1·61 (-12·5–17·6)
Occupational asthmagens	20·1 (16·8–23·7)	19·1 (16·1–22·4)	19·7 (16·4–23·5)	-5 (-15·7–8·32)	3·16 (-5·18–12·4)	11·1 (9·02–13·5)	11·7 (9·64–14)	11·6 (9·4–14·1)	5·21 (-10·1–22·8)	-0·62 (-10·9–11·5)
Occupational particulate matter, gases, and fumes	9·27 (6·61–13·7)	8·81 (6·38–12·8)	8·52 (6·13–12·3)	-4·91 (-10·5–1·26)	-3·27 (-6·87–0·227)	5·14 (3·58–7·8)	5·23 (3·82–7·49)	5·13 (3·71–7·56)	1·64 (-7·2–12·6)	-1·9 (-6·8–3·03)
Occupational noise	8·93 (8·16–9·73)	8·56 (7·96–9·28)	8·29 (7·78–9·01)	-4·11 (-7·82–0·317)	-3·23 (-5·42–-0·98)	6·18 (5·41–6·98)	6·69 (6·16–7·3)	6·76 (6·32–7·33)	8·26 (2·08–16)	1·12 (-1·75–4·23)
Occupational injuries	--	--	--	--	--	--	--	--	--	--
Occupational ergonomic factors	13·8 (11·2–16·8)	12 (9·96–14·4)	12·3 (10·2–14·7)	-13·2 (-24·6–1·97)	2·97 (-6·3–13·6)	8·6 (6·62–11)	8·47 (6·93–10·5)	9·02 (7·11–11·6)	-1·57 (-19·8–19·5)	6·54 (-7·98–23·4)
Childhood sexual abuse	7·46 (4·99–10·6)	7·34 (5·46–9·26)	7·31 (5·44–9·22)	-1·67 (-16·3–20·2)	-0·398 (-5·7–6·64)	21·1 (15·2–29·6)	18·7 (14·7–24·6)	18·7 (14·8–24·7)	-11·6 (-23–4·65)	0·266 (-5·71–6·35)
Intimate partner violence	--	--	--	--	--	33·2 (18·2–42·9)	31·6 (18·4–41·1)	32·4 (18·6–41·8)	-4·67 (-12·8–9·52)	2·39 (-6·41–20·2)
Non-exclusive breastfeeding	26·1 (21·6–30·9)	24·9 (21·5–29)	24·8 (20·4–29·6)	-4·45 (-19·3–13·6)	-0·425 (-13·6–15·3)	--	--	--	--	--
Discontinued breastfeeding	26·4 (25·5–27·3)	25·4 (24–26·7)	25·3 (23·8–26·5)	-4·02 (-9·62–1·62)	-0·509 (-6·22–5·19)	26·5 (25·6–27·4)	25·5 (24·1–26·8)	25·3 (23·9–26·5)	-3·92 (-9·51–1·71)	-0·719 (-6·41–4·98)
Diet low in calcium	17·6 (8·42–32·8)	14·7 (7·03–27)	13·5 (6·31–25·1)	-16·5 (-27·7–-4·95)	-8·17 (-17·9–-0·238)	13·1 (5·37–25)	11·8 (5·17–21·7)	11 (4·87–20·6)	-9·87 (-23–6·55)	-7·36 (-18·2–2·34)
Occupational exposure to asbestos	9·35 (8·33–10·5)	8·02 (7·19–8·8)	7·96 (5·78–10·5)	-14·2 (-26·6–0·199)	-0·78 (-27·7–30·1)	2·17 (1·13–2·63)	2·13 (1·09–2·53)	2·17 (1·06–2·99)	-1·84 (-15·4–15)	1·56 (-22·1–32·7)
Occupational exposure to arsenic	0·404 (0–0·962)	0·433 (0–1·01)	0·46 (0–1·08)	7·32 (-7·01–NA)	6·22 (-4·42–NA)	0·417 (0–0·985)	0·461 (0–1·1)	0·469 (0–1·1)	10·7 (-6·32–NA)	1·78 (-9·04–NA)
Occupational exposure to benzene	0·795 (0·0476–2·35)	0·812 (0·0485–2·38)	0·838 (0·0482–2·42)	2·09 (-6·58–15·1)	3·15 (-3·97–13·7)	1·22 (0·0705–3·42)	1·42 (0·0792–3·96)	1·47 (0·0795–4·14)	16·1 (3·63–36·3)	3·33 (-5·49–16)
Occupational exposure to beryllium	0·0236 (0·0222–0·0249)	0·0237 (0·023–0·0244)	0·0249 (0·0235–0·0264)	0·67 (-5·21–7·4)	4·9 (-0·502–10·8)	0·0278 (0·0255–0·03)	0·0301 (0·0292–0·0309)	0·0308 (0·0288–0·0327)	8·24 (-0·0356–18·1)	2·45 (-3·67–8·77)
Occupational exposure to cadmium	0·0487 (0·0441–0·0538)	0·0523 (0·0477–0·0577)	0·0554 (0·0491–0·0627)	7·32 (-5·52–23)	5·95 (-3·96–17·5)	0·0514 (0·0463–0·0574)	0·0568 (0·0519–0·0625)	0·0578 (0·0512–0·065)	10·7 (-4·01–27·5)	1·74 (-7·9–13·4)
Occupational exposure to chromium	0·119 (0·108–0·13)	0·13 (0·121–0·142)	0·138 (0·124–0·153)	9·79 (-1·3–24)	5·69 (-3·13–15·5)	--	--	--	--	--
Occupational exposure to diesel engine exhaust	0·769 (0·709–0·829)	0·83 (0·781–0·882)	0·866 (0·795–0·945)	7·95 (-2·6–18·9)	4·31 (-3·43–12·2)	0·728 (0·64–0·81)	0·794 (0·719–0·866)	0·785 (0·697–0·877)	9·15 (-5·2–26·5)	-1·12 (-10·5–8·93)
Occupational exposure to formaldehyde	0·232 (0·207–0·259)	0·248 (0·224–0·278)	0·26 (0·228–0·299)	6·58 (-7·64–23·9)	4·96 (-5·22–17·2)	0·244 (0·218–0·274)	0·275 (0·249–0·303)	0·28 (0·248–0·313)	12·3 (-1·98–28·8)	1·99 (-6·98–12·7)
Occupational exposure to nickel	0·37 (0–1·63)	0·361 (0–1·6)	0·381 (0–1·63)	-2·38 (-15·1–NA)	5·54 (-5·6–NA)	0·328 (0–1·42)	0·349 (0–1·56)	0·357 (0–1·57)	6·32 (-10·6–NA)	2·28 (-9·71–NA)
Occupational exposure to polycyclic aromatic hydrocarbons	0·239 (0·219–0·261)	0·261 (0·242–0·283)	0·275 (0·249–0·304)	9·18 (-1·52–21·9)	5·31 (-3·14–14·5)	0·285 (0·259–0·312)	0·331 (0·311–0·353)	0·336 (0·307–0·366)	16·1 (4·41–28·8)	1·68 (-5·59–10)
Occupational exposure to silica	3·82 (0·561–12·7)	3·44 (0·512–10·9)	3·63 (0·543–11·6)	-9·95 (-22·6–6·53)	5·62 (-4·65–20)	2·86 (0·378–9·5)	2·88 (0·381–9·27)	2·9 (0·399–9·77)	0·503 (-15·7–22·5)	0·766 (-11·5–17·3)
Occupational exposure to sulfuric acid	0·845 (0·179–2·62)	0·999 (0·203–3·07)	1·03 (0·21–3·25)	18·2 (1·02–37·4)	3·23 (-8·38–15·3)	0·968 (0·209–3·13)	1·28 (0·257–4·18)	1·25 (0·265–4·04)	32 (6·31–67·2)	-1·94 (-18·2–15·5)
Unsafe sex	--	--	--	--	--	--	--	--	--	--
Occupational exposure to trichloroethylene	0·0754 (0·0702–0·0815)	0·0836 (0·0793–0·0884)	0·088 (0·0815–0·0951)	10·8 (2·2–21·1)	5·29 (-1·33–13·3)	0·0811 (0·0731–0·0889)	0·0902 (0·0848–0·0965)	0·0909 (0·083–0·0995)	11·3 (0·0831–24·5)	0·806 (-6·61–9·34)
No access to handwashing facility	3·37 (3·13–3·63)	3·12 (2·88–3·38)	3·08 (2·84–3·34)	-7·45 (-15·9–2·17)	-1·37 (-7·12–4·74)	3·37 (3·13–3·63)	3·12 (2·88–3·38)	3·08 (2·84–3·34)	-7·45 (-15·9–2·17)	-1·37 (-7·12–4·74)
Child wasting	0·751 (0·502–1·02)	0·729 (0·479–0·98)	0·727 (0·478–0·982)	-2·91 (-12·5–7·39)	-0·297 (-7·6–8·35)	0·775 (0·517–1·06)	0·765 (0·509–1·04)	0·761 (0·504–1·04)	-1·24 (-11·6–9·94)	-0·63 (-8·39–7·22)
Child stunting	2·41 (1·23–3·55)	2·09 (1·07–3·1)	2·03 (1·02–3·08)	-13 (-32–10·6)	-2·91 (-17·1–13·1)	1·95 (0·96–2·96)	1·8 (0·872–2·75)	1·77 (0·866–2·75)	-7·66 (-27·6–16·5)	-1·46 (-16·2–17·3)
Chewing tobacco	9·33 (7·57–11·4)	9·13 (6·88–12·2)	8·4 (6·12–11·5)	-2·14 (-30·2–37·1)	-7·94 (-28–17·9)	--	--	--	--	--
Diet low in legumes	74·7 (40·9–99·5)	77·7 (47·9–99·7)	77·4 (46–99·8)	3·94 (-0·0445–20·5)	-0·355 (-6·05–4·71)	73·1 (35·4–98·9)	76 (42·2–99·6)	76 (43·2–99·7)	4·02 (-0·343–21·4)	-0·0267 (-5·42–5·46)
Short gestation	16·8 (13·7–20·6)	21·3 (17·6–25·5)	19·9 (16·2–24·7)	26·4 (0·489–56·9)	-6·32 (-25·5–16·9)	14·7 (12·1–18)	18·6 (15·5–22·1)	18·8 (15·6–22·3)	26·6 (-0·599–57·5)	0·98 (-17·5–23·5)
Low birth weight	7·07 (5·8–8·5)	9·47 (7·88–11·4)	8·7 (7·19–10·5)	34 (5·77–69)	-8·17 (-26·3–14·8)	7·21 (5·99–8·64)	9·69 (8·22–11·3)	9·99 (8·44–11·7)	34·4 (4·83–68·3)	3·16 (-15·3–26·7)
High temperature	26·3 (15·6–38·3)	25·1 (14·2–38)	26·2 (15·8–38·1)	-4·24 (-19·8–6·94)	4·32 (-7·04–26·5)	26·3 (15·6–38·3)	25·1 (14·2–38)	26·2 (15·8–38·1)	-4·24 (-19·8–6·94)	4·32 (-7·04–26·5)
Low temperature	37·4 (32·8–43)	36·7 (32·4–41·8)	38·1 (33·8–43·3)	-2·08 (-6·99–1·22)	4·01 (1·79–7·06)	37·4 (32·8–43)	36·7 (32·4–41·8)	38·1 (33·8–43·3)	-2·08 (-6·99–1·22)	4·01 (1·79–7·06)
Kidney dysfunction	18·8 (13·3–25·8)	19·2 (13·5–26·2)	19·2 (13·7–26·3)	2·15 (-1·75–6·82)	0·00255 (-3·73–3·89)	21·2 (15–28·5)	22·1 (15·6–29·6)	22·8 (16·3–30·6)	4·16 (-0·000404–8·8)	3·47 (-0·335–8·14)

Bullying victimization	5·93 (2·45–12·2)	8·98 (3·59–17·9)	8·71 (3·37–18)	51·4 (31·6–73·5)	-3·06 (-12·5–3·17)	--	--	--	--	--
High LDL cholesterol	66·5 (62·2–71)	35 (31·5–38·5)	31·4 (27·8–35)	-47·4 (-52·9–-42)	-10·4 (-15·2–-5·26)	64·6 (60·7–68·5)	34·2 (30·9–37·7)	31·7 (28·2–35·3)	-47 (-51·7–-42·2)	-7·18 (-11·2–-2·86)

Supplementary Results Table 1. Nebraska age-standardised Summary Exposure Values (SEVs) for most-detailed risk factors and percentage change, by sex, 1990, 2010, and 2019. Data in parentheses are 95% uncertainty intervals.

	Male					Female				
	1990	2010	2019	Percentage change 1990-2010	Percentage change 2010-2019	1990	2010	2019	Percentage change 1990-2010	Percentage change 2010-2019
Unsafe sanitation	5·78 (2·71–10·6)	3·47 (1·46–6·72)	2·67 (1·17–5·23)	-39·9 (-76·9–56·8)	-23·1 (-60·6–55·2)	5·78 (2·71–10·6)	3·47 (1·46–6·72)	2·67 (1·17–5·23)	-39·9 (-76·9–56·8)	-23·1 (-60·6–55·2)
Ambient particulate matter pollution	8·88 (2·19–19·2)	6·3 (3·48–9·45)	4·33 (1·84–7·22)	-29 (-67·7–136)	-31·3 (-49·1–-23)	8·88 (2·21–19·2)	6·3 (3·47–9·43)	4·33 (1·83–7·22)	-29 (-67·6–135)	-31·3 (-49·2–-23)
Household air pollution from solid fuels	0·0111 (0·00219–0·0321)	0·00674 (0·00109–0·0217)	0·00439 (0·000648–0·015)	-39·4 (-78·6–59·1)	-34·9 (-66·9–26·5)	0·0147 (0·00294–0·0422)	0·00895 (0·00143–0·0278)	0·00582 (0·000856–0·0199)	-39 (-78·6–58·8)	-35 (-66·8–27·2)
Ambient ozone pollution	38·4 (15·4–68·6)	55·4 (23·2–93·2)	49·6 (21–84·6)	44·3 (16·5–91·3)	-10·6 (-29·1–8·83)	38·4 (15·4–68·6)	55·4 (23·2–93·2)	49·6 (21–84·6)	44·3 (16·5–91·3)	-10·6 (-29·1–8·83)
Residential radon	21·7 (3·4–62·6)	21·7 (3·4–62·6)	21·7 (3·4–62·6)	0 (0–0)	0 (0–0)	21·7 (3·4–62·6)	21·7 (3·4–62·6)	21·7 (3·4–62·6)	0 (0–0)	0 (0–0)
Lead exposure	45·5 (16·4–71·2)	26·2 (6·85–49·2)	18·1 (3·51–37·7)	-42·4 (-63·6–-29·2)	-30·8 (-51·5–-21·9)	24·5 (1·02–52·4)	14·6 (0·227–37·7)	10·3 (0·0634–29·6)	-40·4 (-83·4–-25·3)	-29·5 (-74·8–-21·4)
Child underweight	0·938 (0·527–1·42)	0·942 (0·524–1·41)	0·919 (0·505–1·38)	0·505 (-16·6–21·9)	-2·51 (-15·5–11·4)	0·791 (0·411–1·2)	0·838 (0·452–1·29)	0·856 (0·465–1·34)	5·88 (-15·3–42·4)	2·2 (-11·9–18·8)
Iron deficiency	--	--	--	--	--	11·3 (9·55–13·4)	11·8 (9·94–13·7)	12·1 (9·93–14·3)	4·22 (-8·39–17·9)	2·36 (-7·16–13·3)
Vitamin A deficiency	0·777 (0·21–2·04)	1·11 (0·296–3·09)	0·743 (0·189–2·05)	42·7 (-62–514)	-33 (-71–70·3)	1·54 (0·574–3·2)	1·66 (0·692–3·55)	1·37 (0·509–2·96)	7·88 (-59·6–254)	-17·7 (-55·5–56·9)
Zinc deficiency	2·29 (0–7·12)	1·15 (0–3·61)	1·15 (0–3·57)	-49·6 (-100–NA)	-0·0527 (-18·5–NA)	2·35 (0–7·62)	1·17 (0–3·89)	1·12 (0–3·48)	-50·1 (-100–NA)	-4·34 (-25·1–NA)
Smoking	26 (23·4–28·9)	19·6 (17·8–21·5)	16·7 (15–18·6)	-24·8 (-30–-18·7)	-14·6 (-18·5–-10·8)	18·8 (16·2–21·4)	14·4 (12·9–16)	13·1 (11·5–14·7)	-23·3 (-31·5–-13·5)	-9·37 (-14·3–-4·05)
Secondhand smoke	38 (35·6–40·6)	24·7 (23–26·3)	25·3 (22·6–28·1)	-35·1 (-41·3–-28·1)	2·59 (-6·8–12·1)	35·1 (33·1–37·1)	23·5 (22·3–24·6)	23·9 (22–26)	-33·2 (-38·5–-27·8)	2·03 (-5·26–9·7)
Alcohol use	14·6 (8·95–21·1)	14·5 (8·59–21·3)	16·1 (9·63–23·4)	-0·892 (-29·4–41)	11 (-14·5–43·1)	9·59 (4·81–14·6)	13·1 (7·18–19·6)	6·27 (1·47–12·4)	36·2 (-25–159)	-52 (-85·6–-6·48)
Drug use	0·36 (0·176–0·716)	0·463 (0·304–0·699)	0·652 (0·455–0·903)	28·8 (-5·76–91·7)	40·9 (17·8–71·1)	0·368 (0·238–0·566)	0·553 (0·415–0·749)	0·793 (0·614–1·02)	50·2 (23·3–87)	43·5 (23·5–68·5)
High fasting plasma glucose	11·9 (9·91–14·2)	16·8 (14·4–19·7)	18·4 (15·7–21·5)	41·4 (16–70)	9·87 (-5·7–27·9)	8·81 (7·6–10·5)	13·1 (11·5–15·3)	14·9 (12·8–17·3)	48·9 (27·6–74·3)	13·9 (-0·488–31·3)
High systolic blood pressure	24·7 (19·9–30·2)	15·6 (13·6–17·6)	19·6 (15–24·5)	-36·7 (-49–-21·5)	25·8 (-0·257–54·4)	22·9 (17–29·8)	13 (11·4–14·6)	18 (12·9–23·8)	-43·2 (-56·8–-22·9)	38·3 (0·674–83·5)
High body-mass index	26·6 (18·5–36·6)	40·2 (29·6–52)	42·3 (31·3–54)	50·9 (32·8–75·8)	5·31 (-1·5–12·7)	31·1 (22·5–40·1)	43·1 (33·3–52·2)	45·1 (35–54·5)	38·6 (21·9–58·2)	4·67 (-2·28–11·9)
Low bone mineral density	10·9 (6·3–17·5)	8·37 (4·38–14·2)	10·6 (6·02–17·1)	-23·6 (-43·2–-1·63)	26·4 (-3·66–70·6)	14·4 (9·14–20·9)	15·1 (9·8–21·7)	17·4 (11·3–24·8)	4·7 (-19·5–34·7)	15·4 (-8·9–47·6)
Diet low in fruits	55·6 (44·4–68·1)	47·3 (36·1–60·2)	47·1 (34·7–60·1)	-14·9 (-26·6–-5·42)	-0·397 (-12·4–12·5)	54·5 (35–80·1)	29·4 (7·5–70·5)	29·6 (7·41–69·8)	-46·1 (-84–0·588)	0·864 (-24·5–47·4)
Diet low in vegetables	34·1 (17·8–55·1)	51·3 (31·8–72·4)	48·6 (30–68·9)	50·4 (16·8–115)	-5·3 (-15·1–2·95)	30·7 (14·2–52·4)	45·7 (27·5–66·3)	42·8 (25·1–62·2)	48·9 (15·6–119)	-6·31 (-17·3–3·24)
Diet low in whole grains	78·1 (67·5–88·3)	80·6 (71·2–89·6)	80 (70–89·4)	3·11 (-1·25–9·1)	-0·679 (-3·79–2·12)	--	--	--	--	--
Diet low in nuts and seeds	27·7 (7·98–56·3)	25·6 (8·37–55·2)	20·6 (6·56–53·7)	-7·51 (-40·4–75·3)	-19·8 (-47·6–6·08)	23·7 (5·95–54)	22 (7·18–53·8)	18·6 (5·36–52·9)	-6·95 (-43·3–72)	-15·5 (-45·2–14·6)
Diet low in milk	50·5 (25·7–71·3)	43·8 (19·5–64)	41·9 (18·6–62·2)	-13·3 (-33·9–-0·234)	-4·29 (-18·5–7·92)	50 (25–71·6)	44 (19·7–65·3)	42·4 (17–63·2)	-12·1 (-32·5–0·685)	-3·67 (-17·4–8·93)
Diet high in red meat	92·9 (85·4–98·2)	89 (79·3–96)	88·2 (77·4–96)	-4·2 (-14–3·88)	-0·958 (-9·03–6·65)	92·8 (84·7–97·8)	87·2 (75·9–95·3)	86·4 (73·4–94·9)	-6·01 (-16·4–2·99)	-0·954 (-9·88–8·23)
Diet high in processed meat	80·1 (55·7–99)	86·9 (69–99·8)	86·9 (68–99·8)	8·49 (0·395–26·6)	-0·00929 (-4·28–4·57)	80·5 (56·8–98·7)	88·8 (73–99·8)	87·5 (69–99·8)	10·3 (0·784–29·2)	-1·47 (-6·64–2·2)
Diet high in sugar-sweetened beverages	46·4 (27·2–66·7)	63·2 (43·7–79·5)	64·3 (43·8–81·6)	36·4 (-4·34–109)	1·72 (-18·1–29·5)	49·9 (28·4–70·2)	68·6 (49·9–85·9)	68·1 (48·1–84·2)	37·5 (-1·04–124)	-0·626 (-19·2–22·3)
Diet low in fiber	43·5 (28·4–58·4)	30 (17·5–43·7)	26·8 (14·5–40)	-31 (-47·1–-14·5)	-11 (-30·6–7·59)	43·5 (27·8–59·6)	34·9 (21·5–49·6)	29·4 (16·9–44·2)	-19·8 (-35·7–-2·15)	-15·7 (-31·8–-0·566)
Diet low in seafood omega-3 fatty acids	80·7 (61·8–99·2)	75·9 (57·2–99)	74·6 (55·4–98·7)	-5·86 (-21·6–10·4)	-1·78 (-17·7–15·6)	84 (67·4–99·8)	80·6 (62·8–99·5)	79·9 (62·9–99)	-4·01 (-16·9–8·57)	-0·929 (-13·3–12·4)
Diet low in polyunsaturated fatty acids	14 (4·04–42·3)	7·18 (2·05–24·4)	6·74 (1·89–24·5)	-48·6 (-76·2–-8·28)	-6·13 (-29·9–14)	--	--	--	--	--
Diet high in trans fatty acids	89·5 (81·4–100)	87·9 (79·3–100)	87·3 (78·2–100)	-1·79 (-9·84–6·01)	-0·665 (-8·57–8·2)	--	--	--	--	--
Diet high in sodium	18·6 (4·45–42·2)	40·3 (11·3–64·7)	39·3 (11–64·3)	117 (38·6–404)	-2·6 (-28·8–26)	--	--	--	--	--
Low physical activity	2·77 (1·06–5·92)	2·82 (1·22–5·6)	2·85 (1·12–5·99)	1·75 (-30·2–56·8)	1·04 (-20·1–28·9)	5·83 (2·83–11·1)	6·49 (3·54–11·5)	6·01 (3·07–11·4)	11·2 (-17–57·3)	-7·38 (-19·9–4·16)
Occupational asthmagens	20·9 (17·5–24·9)	21·6 (18·3–25·8)	22·5 (18·7–27)	3·12 (-9·23–17·5)	4·26 (-6·61–16·7)	12·1 (9·9–14·6)	13 (10·8–15·8)	13 (10·5–16)	7·59 (-8·24–25·9)	-0·523 (-11·6–13·6)
Occupational particulate matter, gases, and fumes	9·77 (6·99–13·9)	9·53 (6·87–13·4)	9·31 (6·7–13·2)	-2·47 (-7·67–3·37)	-2·32 (-5·8–1·21)	5·52 (3·84–7·96)	5·67 (4·08–8·29)	5·58 (4·02–8·1)	2·74 (-6·04–12·8)	-1·59 (-7–3·71)
Occupational noise	8·91 (8·16–9·81)	8·81 (8·24–9·54)	8·64 (8·12–9·33)	-1·11 (-4·92–3·14)	-1·96 (-4·18–0·216)	6·5 (5·78–7·27)	7·11 (6·58–7·73)	7·22 (6·78–7·82)	9·29 (3·3–16·6)	1·6 (-1·01–4·7)
Occupational injuries	--	--	--	--	--	--	--	--	--	--
Occupational ergonomic factors	13·6 (11·3–16·6)	12·4 (10·5–14·9)	12·7 (10·5–15·4)	-8·69 (-20·6–3·73)	2·56 (-6·45–12·3)	8·33 (6·52–10·7)	8·66 (7·02–10·8)	9·21 (7·17–11·9)	3·94 (-15·8–27)	6·39 (-8·51–26·1)
Childhood sexual abuse	7·4 (5·05–10·5)	6·97 (4·6–9·93)	6·85 (4·54–9·67)	-5·83 (-12·2–5·83)	-1·68 (-8·51–5·53)	12·3 (8·94–17·3)	17·4 (12·9–24)	17·8 (13·1–24·7)	41·9 (30·7–54·8)	2·17 (-5–11)
Intimate partner violence	--	--	--	--	--	32·7 (18·5–42·5)	31·2 (17·8–40·7)	32 (18·3–41·6)	-4·63 (-13·1–6·53)	2·57 (-5·49–20)
Non-exclusive breastfeeding	26·1 (21·4–31)	25 (21·3–29)	24·9 (20·8–29·7)	-4·39 (-19·2–13·5)	-0·391 (-13·5–14·2)	--	--	--	--	--
Discontinued breastfeeding	26·9 (26–27·8)	25·5 (24·2–26·7)	25·6 (24·2–26·8)	-5·36 (-10·9–0·143)	0·388 (-5·59–6·12)	26·8 (25·9–27·7)	25·5 (24·2–26·7)	25·6 (24·2–26·8)	-5·1 (-10·6–0·422)	0·454 (-5·53–6·19)
Diet low in calcium	16·2 (7·66–30·3)	13·5 (6·8–24·3)	12·4 (6·09–22·4)	-16·5 (-27·5–-3·67)	-7·96 (-17·1–0·316)	--	--	--	--	--
Occupational exposure to asbestos	8·22 (7·24–9·28)	7·25 (6·58–7·97)	6·44 (4·7–8·74)	-11·8 (-24·2–2·7)	-11·2 (-35·9–21·3)	1·69 (1·01–2)	1·7 (0·973–2·02)	1·7 (0·921–2·3)	0·764 (-14·4–18·9)	0·0688 (-25·5–30·5)
Occupational exposure to arsenic	0·488 (0–1·13)	0·55 (0–1·29)	0·577 (0–1·36)	12·9 (-3·41–NA)	4·77 (-7·31–NA)	0·497 (0–1·16)	0·555 (0–1·34)	0·557 (0–1·36)	11·7 (-7·44–NA)	0·47 (-11–NA)
Occupational exposure to benzene	0·852 (0·0484–2·41)	0·913 (0·0532–2·64)	0·935 (0·0564–2·7)	7·12 (-1·64–19·3)	2·41 (-4·48–12·1)	1·33 (0·0794–3·61)	1·58 (0·0879–4·29)	1·62 (0·0948–4·49)	18·2 (6·28–37·8)	3·06 (-4·79–14·5)
Occupational exposure to beryllium	0·025 (0·0235–0·0265)	0·0265 (0·0259–0·0271)	0·0275 (0·0259–0·029)	5·85 (-0·523–13·1)	3·77 (-1·91–9·33)	0·0296 (0·0275–0·0318)	0·033 (0·0321–0·0338)	0·0335 (0·0315–0·0357)	11·5 (3·01–21·1)	1·68 (-4·23–7·94)
Occupational exposure to cadmium	0·0578 (0·0519–0·0641)	0·0651 (0·0589–0·0718)	0·068 (0·06–0·0768)	12·5 (-1·49–29·5)	4·4 (-6–15·8)	0·0601 (0·0537–0·0675)	0·0674 (0·0607–0·0753)	0·0677 (0·0595–0·0777)	12·2 (-5·27–31·9)	0·447 (-9·65–12·4)
Occupational exposure to chromium	0·139 (0·127–0·153)	0·16 (0·147–0·174)	0·167 (0·15–0·185)	14·7 (2·38–30·1)	4·15 (-4·9–14)	--	--	--	--	--
Occupational exposure to diesel engine exhaust	0·771 (0·712–0·837)	0·883 (0·828–0·942)	0·911 (0·836–0·991)	14·6 (3·7–26·6)	3·18 (-4·67–11)	0·754 (0·68–0·844)	0·853 (0·779–0·936)	0·837 (0·75–0·94)	13·1 (-1·86–30·1)	-1·85 (-12–7·49)
Occupational exposure to formaldehyde	0·282 (0·25–0·316)	0·315 (0·281–0·351)	0·325 (0·285–0·371)	11·5 (-3·47–29·9)	3·32 (-7·62–15·4)	0·293 (0·262–0·329)	0·331 (0·3–0·368)	0·334 (0·295–0·379)	13·1 (-3·37–32·7)	0·758 (-8·79–11·9)
Occupational exposure to nickel	0·409 (0–1·82)	0·444 (0–1·97)	0·465 (0–2·04)	8·45 (-6·99–NA)	4·74 (-6·94–NA)	0·39 (0–1·66)	0·424 (0–1·94)	0·425 (0–1·93)	8·81 (-10·7–NA)	0·34 (-12–NA)
Occupational exposure to polycyclic aromatic hydrocarbons	0·276 (0·252–0·302)	0·315 (0·292–0·341)	0·328 (0·297–0·363)	14·2 (2·36–28·2)	4·03 (-4·76–13·3)	0·326 (0·297–0·358)	0·381 (0·357–0·409)	0·384 (0·35–0·422)	16·9 (3·6–31·9)	0·721 (-7·06–9·3)
Occupational exposure to silica	3·89 (0·534–12·3)	3·98 (0·595–12·5)	4·2 (0·637–13·3)	2·33 (-10·6–21·7)	5·54 (-5·47–19·9)	3·34 (0·458–10·2)	3·44 (0·476–11·2)	3·43 (0·462–11·5)	2·98 (-17·1–28·2)	-0·13 (-13·2–16·6)
Occupational exposure to sulfuric acid	0·924 (0·198–2·86)	1·11 (0·235–3·45)	1·13 (0·237–3·63)	19·7 (3·11–38·6)	2·52 (-7·6–13·7)	0·992 (0·21–3·2)	1·3 (0·264–4·23)	1·26 (0·259–3·96)	31·2 (4·23–62·3)	-3·26 (-16·9–13·6)
Unsafe sex	--	--	--	--	--	--	--	--	--	--
Occupational exposure to trichloroethylene	0·0854 (0·0792–0·0919)	0·0981 (0·0935–0·103)	0·102 (0·0946–0·11)	14·9 (5·82–25·5)	3·75 (-3·12–10·9)	0·0904 (0·0827–0·0993)	0·103 (0·0963–0·109)	0·103 (0·0932–0·113)	13·8 (1·49–28)	-0·169 (-8·27–8·72)
No access to handwashing facility	3·32 (3·08–3·59)	3·11 (2·86–3·37)	3·08 (2·83–3·34)	-6·38 (-14·5–2·94)	-1·15 (-6·7–4·68)	3·32 (3·08–3·59)	3·11 (2·86–3·37)	3·08 (2·83–3·34)	-6·38 (-14·5–2·94)	-1·15 (-6·7–4·68)
Child wasting	0·748 (0·492–1·03)	0·725 (0·47–0·988)	0·723 (0·476–0·984)	-3·12 (-12·2–7·6)	-0·201 (-7·7–8·11)	0·771 (0·51–1·05)	0·76 (0·515–1·05)	0·756 (0·503–1·04)	-1·43 (-11·6–9·38)	-0·587 (-8·04–7·68)
Child stunting	2·32 (1·21–3·41)	2·03 (1·05–3·07)	1·97 (1·04–2·99)	-12·7 (-31·2–10·6)	-2·8 (-16·8–13·2)	1·9 (0·962–2·88)	1·75 (0·883–2·65)	1·73 (0·887–2·65)	-7·92 (-28·1–19)	-1·1 (-17–18·4)
Chewing tobacco	5·34 (4·22–6·8)	5·05 (3·59–6·91)	4·63 (3·13–6·44)	-5·3 (-35·3–40·2)	-8·39 (-29·2–21·7)	0·277 (0·174–0·419)	0·276 (0·17–0·444)	0·28 (0·17–0·445)	-0·0193 (-42·4–73·3)	1·18 (-30–46·2)
Diet low in legumes	73·9 (37·7–99·2)	76·9 (44·7–99·7)	76·7 (44·3–99·6)	4·08 (-0·358–22·2)	-0·342 (-6·46–5·45)	72·3 (33·8–98·8)	75·2 (41·1–99·6)	75·4 (43·1–99·5)	4·05 (-0·374–21·1)	0·14 (-5·18–7·94)
Short gestation	22·8 (18·6–27·7)	23·4 (19·4–28)	23·3 (19–28·6)	2·48 (-18·1–30·2)	-0·287 (-19·3–23·1)	18·3 (15·6–21·3)	19·2 (16·4–22·3)	19·5 (16·4–23·1)	5·17 (-11–23·9)	1·56 (-15·5–21·7)
Low birth weight	8·92 (7·35–11)	9·52 (8–11·4)	9·75 (8·04–11·8)	6·72 (-16·2–33·6)	2·41 (-17·9–28)	8·58 (7·28–10)	9·59 (8·24–11·2)	10·1 (8·57–11·9)	11·7 (-5·75–33·9)	5·7 (-11·4–27·7)
High temperature	34·7 (21·2–48·4)	29 (15·5–43·3)	28·4 (14·7–43)	-16·3 (-32·4–-8)	-2·36 (-6·84–0·346)	34·7 (21·2–48·4)	29 (15·5–43·3)	28·4 (14·7–43)	-16·3 (-32·4–-8)	-2·36 (-6·84–0·346)
Low temperature	36 (31·6–40·9)	36·3 (31·9–41·3)	37·9 (33·5–42·8)	0·833 (-1·36–3·05)	4·36 (3·33–5·48)	36 (31·6–40·9)	36·3 (31·9–41·3)	37·9 (33·5–42·8)	0·833 (-1·36–3·05)	4·36 (3·33–5·48)
Kidney dysfunction	19·4 (13·9–26·4)	20 (14·3–27·2)	20 (14·5–27·1)	2·96 (-0·573–7·17)	0·134 (-3·88–4)	21·8 (15·5–29·4)	22·8 (16·2–30·5)	23·7 (17·1–31·4)	4·77 (0·327–9·89)	3·98 (0·203–8·6)
Bullying victimization	5·88 (2·42–12·1)	8·92 (3·57–17·6)	8·71 (3·32–17·9)	51·6 (31·3–74·7)	-2·41 (-11·8–3·81)	--	--	--	--	--
High LDL cholesterol	66·9 (62·8–71·4)	34·2 (30·7–37·8)	30·8 (27·2–34·6)	-48·8 (-53·9–-44)	-10·1 (-14·8–-4·92)	65·4 (61·8–69)	34·4 (31–37·9)	31·9 (28·2–35·7)	-47·4 (-52·2–-42·8)	-7·18 (-11·4–-3·15)

Supplementary Results Table 1. Nevada age-standardised Summary Exposure Values (SEVs) for most-detailed risk factors and percentage change, by sex, 1990, 2010, and 2019. Data in parentheses are 95% uncertainty intervals.										
	Male					Female				
	1990	2010	2019	Percentage change 1990-2010	Percentage change 2010-2019	1990	2010	2019	Percentage change 1990-2010	Percentage change 2010-2019
Unsafe sanitation	7·81 (3·51–14·7)	3·79 (1·61–7·09)	3·42 (1·57–6·53)	-51·4 (-82–18·9)	-9·88 (-52·7–72)	7·81 (3·51–14·7)	3·79 (1·61–7·09)	3·42 (1·57–6·53)	-51·4 (-82–18·9)	-9·88 (-52·7–72)
Ambient particulate matter pollution	8·96 (2·46–19·1)	4·2 (1·68–7·24)	4·07 (1·55–7·16)	-53·1 (-82·3–39·7)	-3·17 (-10·1–0·357)	8·96 (2·45–19·1)	4·2 (1·69–7·26)	4·07 (1·54–7·17)	-53·1 (-82·3–38·2)	-3·19 (-10·2–0·363)
Household air pollution from solid fuels	0·0145 (0·00353–0·0418)	0·00706 (0·00105–0·0245)	0·00595 (0·000848–0·0192)	-51·4 (-85–16·7)	-15·7 (-55·5–66·6)	0·0192 (0·00463–0·0527)	0·00936 (0·00139–0·0302)	0·00789 (0·0012–0·026)	-51·2 (-84·9–17·4)	-15·6 (-55·6–66·8)
Ambient ozone pollution	74·1 (33–100)	88·6 (43·1–100)	80·1 (35·6–100)	19·5 (0–51·4)	-9·58 (-23·3–0)	--	--	--	--	--
Residential radon	18·9 (3·06–56·9)	18·9 (3·06–56·9)	18·9 (3·06–56·9)	0 (0–0)	0 (0–0)	18·9 (3·06–56·9)	18·9 (3·06–56·9)	18·9 (3·06–56·9)	0 (0–0)	0 (0–0)
Lead exposure	68·4 (42–86·7)	40 (19·2–60·6)	28 (11·5–45·8)	-41·5 (-55·6—28·1)	-30·1 (-41·9—22·8)	42·5 (10·3–69·9)	23·4 (3·06–48·2)	15·8 (1·23–35·9)	-44·9 (-74·4—29)	-32·6 (-62·2—24·1)
Child underweight	0·989 (0·566–1·47)	0·971 (0·543–1·44)	0·95 (0·529–1·43)	-1·86 (-19·2–19·3)	-2·13 (-15–12)	0·826 (0·447–1·26)	0·851 (0·45–1·3)	0·873 (0·483–1·33)	3·11 (-16·2–27·4)	2·57 (-11·3–18)
Iron deficiency	--	--	--	--	--	12·2 (10·2–14·2)	12·4 (10·4–14·4)	12·5 (10·4–14·9)	1·95 (-10·2–14·5)	0·995 (-8·4–11)
Vitamin A deficiency	0·971 (0·248–2·61)	1·18 (0·321–2·86)	0·922 (0·232–2·53)	21·3 (-70·8–427)	-21·7 (-66·6–94·5)	1·87 (0·709–3·77)	1·76 (0·663–3·56)	1·62 (0·606–3·43)	-5·74 (-63·8–166)	-8·17 (-50–79·7)
Zinc deficiency	2·13 (0–6·92)	1·11 (0–3·59)	1·31 (0–4·28)	-47·6 (-100–NA)	17·5 (-5·36–NA)	2·17 (0–7·03)	1·13 (0–3·71)	1·27 (0–4·1)	-48·1 (-100–NA)	12·6 (-11·1–NA)
Smoking	30·3 (27·3–33·6)	21·5 (19·5–23·6)	17·8 (16–19·6)	-28·9 (-34·1—23·4)	-17·5 (-20·9—13·9)	22·8 (19·8–26)	17·3 (15·5–19·2)	13·3 (11·9–14·8)	-24·2 (-32·5—13·6)	-22·9 (-26·7—18·7)
Secondhand smoke	43·4 (40·8–45·9)	27·4 (25·6–29·1)	26·7 (24·1–29·6)	-36·9 (-42·6—30·9)	-2·26 (-10·7–6·1)	40·5 (38·4–42·6)	25·9 (24·7–27·2)	25·5 (23·3–27·6)	-36 (-41—31)	-1·81 (-9·23–5·01)
Alcohol use	12·3 (7·49–17·1)	12·9 (7·94–18·7)	13·7 (7·97–20·2)	4·93 (-30·3–61·6)	6·75 (-17·8–37·6)	--	--	--	--	--
Drug use	0·649 (0·363–1·15)	1·09 (0·769–1·52)	1·39 (1·02–1·89)	67·8 (26·5–131)	27·2 (10·9–47)	0·505 (0·33–0·744)	1·07 (0·803–1·38)	1·5 (1·18–1·91)	111 (68·7–167)	40·8 (22·8–60·3)
High fasting plasma glucose	12 (9·91–14·4)	18·2 (15·8–21·1)	18·6 (15·9–21·5)	51 (26·8–80·2)	2·12 (-11·6–18·3)	9·37 (7·89–11·2)	13·8 (12·1–15·7)	14·8 (12·7–16·9)	46·9 (22·5–72·9)	7·8 (-6·53–23·3)
High systolic blood pressure	25·5 (20·4–31·4)	16·2 (14·3–18·3)	19·6 (15·4–24·8)	-36·5 (-49·1—20·1)	21·2 (-4·6–50)	22·8 (16·8–29·3)	13 (11·4–14·7)	17·2 (12·2–23·1)	-42·8 (-56·2—23·7)	32·2 (-4·49–75·6)
High body-mass index	23·8 (16·7–32·9)	36·8 (26·7–49)	35·9 (25·9–47·8)	54·5 (35·1–81·6)	-2·25 (-8·72–4·37)	29·2 (21·4–39)	40·5 (32–49·1)	40·2 (31·2–49)	38·8 (20·1–62·1)	-0·806 (-7·49–6·15)
Low bone mineral density	12·4 (7·19–19·2)	9·4 (5·03–15·7)	11·8 (6·8–18·9)	-24·4 (-44·6—1·58)	25·5 (-5·06–69·1)	16·4 (10·8–23·4)	16·9 (11–24·1)	19·2 (13–26·5)	2·72 (-20·4–33·5)	14 (-8·79–48·2)
Diet low in fruits	49·8 (37·4–63·7)	47·2 (35·6–58·9)	49·4 (37·6–62·3)	-5·35 (-16·7–7·89)	4·75 (-6·38–17)	51·7 (38·1–66·5)	49·5 (36·1–64·4)	51·6 (38·2–65·3)	-4·37 (-16·2–8·52)	4·32 (-5·96–16·3)
Diet low in vegetables	28·6 (13·9–48·5)	56·4 (39–72·7)	57 (39·7–72·9)	97·1 (35·4–225)	0·972 (-5·82–8·03)	24·3 (11·2–43·3)	50·7 (32·3–69·9)	52·8 (34·7–72·2)	109 (41·7–266)	4·28 (-2·97–13·9)
Diet low in whole grains	77·8 (66·5–88·4)	80 (69·4–89·6)	80·4 (70–90)	2·78 (-0·889–8·07)	0·497 (-2·1–3·13)	--	--	--	--	--
Diet low in nuts and seeds	26·1 (7·61–56)	25·3 (7·59–55·6)	22 (6·38–54·2)	-3·12 (-39·5–77·8)	-13·2 (-39·4–16·2)	22·4 (5·57–54·1)	21·8 (6·97–54·3)	20·1 (6·02–53·5)	-2·67 (-38·9–77·5)	-7·65 (-37·2–26·2)
Diet low in milk	46·8 (23·6–66·6)	42·2 (18·5–61·9)	43·9 (21–64·3)	-9·88 (-30·7–3·17)	4·14 (-7·49–20·9)	46·4 (23·7–66)	42 (18·7–61·1)	44 (21–64·4)	-9·48 (-28·7–5·1)	4·73 (-6·29–21·6)
Diet high in red meat	78·9 (66·3–90)	76·4 (62·7–87·6)	74·7 (59·9–86·2)	-3·13 (-17·2–12·4)	-2·27 (-14·1–8·47)	79·6 (67·9–90)	75·6 (62–86·7)	74·2 (60·2–86·7)	-4·93 (-18·5–10)	-1·92 (-13·5–11)
Diet high in processed meat	77·1 (52·9–97·5)	85·6 (66–99·7)	85·3 (65·3–99·6)	11·1 (1·4–31·4)	-0·367 (-5·02–4·32)	78·2 (55–97·6)	88·4 (71·6–99·6)	86·7 (68·9–99·5)	13·1 (1·57–36·7)	-1·87 (-7·45–1·62)
Diet high in sugar-sweetened beverages	47·5 (29·2–65)	63·7 (43·6–79·7)	60·8 (41·3–78·3)	34·2 (-3·57–108)	-4·57 (-23·8–18·7)	52 (32·1–71·8)	69·3 (50·8–86·3)	64·9 (44·2–82·3)	33·2 (-3·12–107)	-6·3 (-25·8–13·9)
Diet low in fiber	41·7 (26·4–57·3)	29·3 (16·6–43·1)	28·9 (16·4–43·2)	-29·8 (-46·8—12·4)	-1·39 (-20·5–20·1)	42 (26·3–57·8)	34·4 (20·7–49·5)	31·8 (17·9–47·2)	-18·1 (-34·3—1·64)	-7·73 (-25·1–8·2)
Diet low in seafood omega-3 fatty acids	82·1 (64·7–99·8)	78·7 (60·1–99)	80·5 (62·7–98·7)	-4·14 (-20·6–12)	2·31 (-11·7–22·4)	85·2 (69·3–100)	83 (66·8–99·5)	84·1 (67·5–99·4)	-2·62 (-14·4–9·27)	1·26 (-8·75–14·1)
Diet low in polyunsaturated fatty acids	12·9 (3·81–38)	7·06 (2·04–25·2)	7·35 (2·13–26·4)	-45·5 (-74·9–7·77)	4·11 (-13·8–40·6)	--	--	--	--	--
Diet high in trans fatty acids	89·5 (81·5–100)	87·9 (78·8–100)	87·5 (78–100)	-1·78 (-10·8–6·16)	-0·448 (-7·9–8·16)	--	--	--	--	--
Diet high in sodium	18·5 (4·35–41·9)	39·9 (11·3–64·1)	39 (10·9–64·3)	116 (34·4–405)	-2·26 (-32·7–27·4)	--	--	--	--	--
Low physical activity	2·12 (0·779–4·6)	1·91 (0·857–4·11)	1·79 (0·669–4·13)	-9·52 (-41·6–42·5)	-6·59 (-32·6–22·6)	4·96 (2·42–9·81)	4·78 (2·54–8·67)	4·89 (2·47–9·39)	-3·52 (-26·5–33·5)	2·16 (-11·2–15·4)
Occupational asthmagens	19·6 (16·4–23·2)	18 (15·2–21·3)	18·9 (15·8–22·6)	-8·3 (-19·1–3·83)	5·02 (-3·68–15·3)	10·3 (8·32–12·6)	9·89 (8·22–11·9)	10·2 (8·16–12·3)	-3·62 (-17·6–13·4)	2·67 (-9·27–15·7)
Occupational particulate matter, gases, and fumes	8·33 (5·92–11·9)	7·96 (5·72–11·4)	7·77 (5·57–11·2)	-4·38 (-10·2–1·66)	-2·49 (-6·09–1·07)	4·79 (3·36–6·92)	4·68 (3·29–6·74)	4·52 (3·23–6·51)	-2·26 (-11·4–8·49)	-3·56 (-8·58–1·98)
Occupational noise	8·27 (7·53–9·1)	8·09 (7·53–8·74)	7·88 (7·38–8·51)	-2·11 (-5·94–2·23)	-2·68 (-4·74—0·474)	5·74 (5·01–6·53)	6·02 (5·53–6·57)	6·01 (5·62–6·51)	5 (-1·85–12·8)	-0·265 (-3·33–3·33)
Occupational injuries	--	--	--	--	--	--	--	--	--	--
Occupational ergonomic factors	12 (9·82–14·6)	10·3 (8·71–12·4)	10·7 (8·91–12·9)	-13·5 (-22·9—3·2)	3·85 (-3·82–12·7)	6·77 (5·28–8·52)	6·45 (5·22–8·07)	6·76 (5·37–8·56)	-4·72 (-19·9–15·7)	4·79 (-8·04–18·9)
Childhood sexual abuse	9·6 (6·44–13·6)	8·06 (5·83–10·7)	8·14 (5·87–10·9)	-16·1 (-27·6—3·26)	1·02 (-4·37–7·22)	22·6 (16·4–32·1)	19·9 (15·7–26)	20 (15·8–26·1)	-12·3 (-24·1–0·767)	0·514 (-5·13–7·8)
Intimate partner violence	--	--	--	--	--	29·5 (16·4–38·9)	29·2 (16·7–38·1)	30·5 (17·2–39·8)	-1·2 (-10·6–12·4)	4·72 (-5·67–20·8)
Non-exclusive breastfeeding	26·5 (21·8–31·4)	25 (21·2–28·8)	24·9 (20·1–29·4)	-5·79 (-20–11·6)	-0·0718 (-13·3–13·9)	26·5 (21·8–31·4)	25 (21·2–28·8)	24·9 (20·1–29·4)	-5·77 (-20–11·6)	-0·0779 (-13·3–13·9)
Discontinued breastfeeding	27·6 (26·6–28·5)	25·4 (24·1–26·7)	26·2 (24·8–27·4)	-7·96 (-13·5—2·41)	3·07 (-2·66–9·25)	27·6 (26·6–28·5)	25·5 (24·1–26·7)	26·2 (24·8–27·4)	-7·85 (-13·4—2·3)	2·85 (-2·87–9·01)
Diet low in calcium	15·3 (7·54–28·1)	13·2 (6·35–24·1)	13·9 (6·58–25·5)	-13·8 (-25·4—0·869)	5·32 (-4·4–14·4)	10·5 (4·57–20·1)	10·1 (4·42–18·7)	11·2 (4·61–21·1)	-3·47 (-19·7–17·6)	11·2 (-0·329–24)
Occupational exposure to asbestos	8·05 (7·24–8·87)	6·46 (5·83–7·12)	5·72 (4·14–7·6)	-19·7 (-30·4—7·7)	-11·5 (-35·2–18·2)	1·86 (1·27–2·47)	1·68 (1·19–2·21)	1·61 (1·1–2·26)	-10·1 (-22·9–4·24)	-3·85 (-27·5–24·4)
Occupational exposure to arsenic	0·404 (0–0·971)	0·415 (0–0·979)	0·443 (0–1·07)	2·63 (-11·6–NA)	6·97 (-4·86–NA)	--	--	--	--	--
Occupational exposure to benzene	0·868 (0·0524–2·55)	0·85 (0·0496–2·47)	0·877 (0·0524–2·53)	-2·16 (-11–10·3)	3·24 (-4·48–12·5)	1·15 (0·0661–3·21)	1·27 (0·0703–3·52)	1·32 (0·072–3·81)	10·7 (-1·33–30)	3·79 (-4·78–17·2)
Occupational exposure to beryllium	0·023 (0·0216–0·0244)	0·0226 (0·0221–0·0231)	0·0239 (0·0226–0·0253)	-1·74 (-7·48–4·86)	5·8 (-0·413–11·9)	0·0257 (0·0234–0·0279)	0·0262 (0·0255–0·0269)	0·0271 (0·0252–0·0291)	2·01 (-6·34–12·1)	3·63 (-3·71–11·4)
Occupational exposure to cadmium	0·0488 (0·0438–0·0537)	0·0502 (0·0457–0·0547)	0·0535 (0·0474–0·0601)	2·89 (-9·66–16·9)	6·65 (-3·96–18·3)	0·0501 (0·044–0·0566)	0·0508 (0·0463–0·0564)	0·0526 (0·0463–0·0606)	1·27 (-12·7–18·3)	3·56 (-7·88–16·2)
Occupational exposure to chromium	0·12 (0·109–0·131)	0·127 (0·117–0·136)	0·135 (0·122–0·149)	5·19 (-5·87–17·5)	6·68 (-2·1–16·7)	--	--	--	--	--
Occupational exposure to diesel engine exhaust	0·744 (0·689–0·803)	0·791 (0·742–0·837)	0·846 (0·775–0·921)	6·31 (-3·6–16·3)	6·95 (-1·64–15·4)	0·686 (0·605–0·777)	0·687 (0·624–0·759)	0·698 (0·615–0·784)	0·127 (-13·6–15·9)	1·64 (-8·17–12·9)
Occupational exposure to formaldehyde	0·237 (0·21–0·264)	0·242 (0·218–0·267)	0·255 (0·225–0·291)	1·75 (-11·5–16·5)	5·57 (-4·93–17·4)	0·24 (0·212–0·272)	0·247 (0·225–0·272)	0·256 (0·227–0·291)	2·81 (-11·2–20)	3·94 (-7·75–16)
Occupational exposure to nickel	0·35 (0–1·55)	0·335 (0–1·45)	0·356 (0–1·54)	-4·28 (-17·6–NA)	6·43 (-5·54–NA)	0·32 (0–1·42)	0·313 (0–1·38)	0·326 (0–1·47)	-2·32 (-18·9–NA)	4·2 (-9·78–NA)
Occupational exposure to polycyclic aromatic hydrocarbons	0·255 (0·233–0·276)	0·263 (0·246–0·281)	0·277 (0·253–0·304)	3·08 (-6·78–13·9)	5·38 (-3·02–14·4)	0·282 (0·253–0·313)	0·3 (0·283–0·321)	0·309 (0·282–0·342)	6·71 (-5·29–20)	2·94 (-6·48–12·9)
Occupational exposure to silica	3·43 (0·476–11·3)	3·1 (0·426–9·81)	3·31 (0·464–10·3)	-9·63 (-22–6·62)	6·69 (-4·71–21·2)	2·79 (0·391–8·68)	2·56 (0·356–7·95)	2·65 (0·356–8·49)	-8·04 (-23·9–15·1)	3·36 (-10·6–21·9)
Occupational exposure to sulfuric acid	1·14 (0·242–3·53)	1·15 (0·237–3·66)	1·15 (0·245–3·72)	0·99 (-16·5–22·7)	0·705 (-11·6–15·1)	1·08 (0·228–3·41)	1·25 (0·255–3·95)	1·22 (0·249–3·9)	16·6 (-9·91–51)	-2·61 (-19·8–15·5)
Unsafe sex	--	--	--	--	--	--	--	--	--	--
Occupational exposure to trichloroethylene	0·0754 (0·0698–0·0812)	0·0806 (0·0769–0·0847)	0·0861 (0·0801–0·0929)	6·82 (-1·61–16·8)	6·87 (-0·451–14·9)	0·0777 (0·0695–0·0861)	0·0793 (0·0748–0·0847)	0·0817 (0·0741–0·0905)	2·1 (-9·05–15·1)	3·02 (-6·05–13·1)
No access to handwashing facility	3·4 (3·14–3·66)	3·13 (2·89–3·4)	3·1 (2·86–3·37)	-8·05 (-16·2–1·27)	-0·72 (-6·34–5·28)	3·4 (3·14–3·66)	3·13 (2·89–3·4)	3·1 (2·86–3·37)	-8·05 (-16·2–1·27)	-0·72 (-6·34–5·28)
Child wasting	0·751 (0·499–1·03)	0·729 (0·474–0·984)	0·728 (0·48–0·987)	-2·97 (-12·3–7·58)	-0·15 (-7·55–7·48)	0·774 (0·514–1·05)	0·764 (0·504–1·05)	0·762 (0·501–1·05)	-1·37 (-10·5–10·1)	-0·25 (-7·68–8·39)
Child stunting	2·53 (1·34–3·72)	2·11 (1·11–3·15)	2·06 (1·06–3·09)	-16·6 (-35·2–7·41)	-2·21 (-15·7–15)	2 (1·02–3·03)	1·81 (0·911–2·79)	1·79 (0·845–2·75)	-9·46 (-28·7–14·9)	-1·09 (-16–17·1)
Chewing tobacco	3·4 (2·49–4·63)	3·26 (2·3–4·47)	3·13 (2·17–4·45)	-4·04 (-36·3–43·8)	-4·13 (-25·6–24·4)	--	--	--	--	--
Diet low in legumes	73·4 (35·9–99·3)	76·8 (44·2–99·6)	77·5 (46·9–99·8)	4·58 (0–28)	0·973 (-3–8·72)	71·8 (32·6–98·6)	75·1 (40–99·6)	76·1 (44·5–99·4)	4·58 (-0·0285–24·3)	1·42 (-2·75–11)
Short gestation	25·4 (20·7–31·1)	20·7 (17·3–24·6)	20·6 (17–24·8)	-18·3 (-34·9—2·84)	-0·65 (-19·1–22·4)	20·5 (17·2–24·1)	20·3 (17·2–23·7)	18·5 (15·6–21·8)	-0·728 (-17·9–21·2)	-8·95 (-22·9–7·67)
Low birth weight	10·4 (8·55–12·7)	9·79 (8·37–11·5)	9·64 (7·98–11·6)	-5·57 (-24·9–18·8)	-1·62 (-20·9–22)	10·5 (8·93–12·2)	10·6 (9·09–12·2)	9·93 (8·5–11·5)	0·89 (-18·1–23·6)	-5·86 (-22·6–12·2)
High temperature	40 (27·9–52·2)	43·7 (30·7–56·7)	42·5 (29·6–55·3)	9·17 (5·66–13·4)	-2·73 (-4·2—0·977)	40 (27·9–52·2)	43·7 (30·7–56·7)	42·5 (29·6–55·3)	9·17 (5·66–13·4)	-2·73 (-4·2—0·977)
Low temperature	39·8 (32·3–49·1)	42·2 (33·8–52·4)	42·1 (33·7–52·4)	5·91 (1·72–10·7)	-0·212 (-1·07–0·558)	39·8 (32·3–49·1)	42·2 (33·8–52·4)	42·1 (33·7–52·4)	5·91 (1·72–10·7)	-0·212 (-1·07–0·558)
Kidney dysfunction	20·3 (14·6–27·3)	20·8 (14·9–28·1)	20·8 (15·1–27·9)	2·12 (-2·34–6·98)	0·0236 (-3·85–4·28)	22·4 (16·1–30)	23·2 (16·6–31·2)	23·9 (17·3–31·7)	3·41 (-2·05–9·42)	3·33 (-0·691–8·85)
Bullying victimization	5·88 (2·44–12)	9·02 (3·59–17·8)	8·76 (3·34–18·1)	53·4 (32·7–77·5)	-2·89 (-12·6–3·3)	--	--	--	--	--
High LDL cholesterol	66·1 (61·6–70·2)	34·9 (31·5–38·5)	30·8 (27–34·7)	-47·2 (-52·7—41·7)	-11·8 (-17—7·19)	65·6 (62–69·3)	34·7 (31·4–38·1)	31·9 (28·1–35·5)	-47·1 (-51·6—42·4)	-8·14 (-12·3—4·2)

Supplementary Results Table 1. New Hampshire age-standardised Summary Exposure Values (SEVs) for most-detailed risk factors and percentage change, by sex, 1990, 2010, and 2019. Data in parentheses are 95% uncertainty intervals.

	Male					Female				
	1990	2010	2019	Percentage change 1990-2010	Percentage change 2010-2019	1990	2010	2019	Percentage change 1990-2010	Percentage change 2010-2019
Unsafe sanitation	4·27 (2·02–8·5)	2·35 (1·01–4·54)	1·88 (0·835–3·7)	-45 (-79·3–38·8)	-20 (-60–57·2)	--	--	--	--	--
Ambient particulate matter pollution	5·78 (0·512–14·2)	3·34 (0·992–6·04)	1·74 (0·173–4·11)	-42·3 (-82·1–265)	-47·8 (-84·8—31·6)	5·79 (0·534–14·2)	3·35 (1·01–6·05)	1·75 (0·181–4·12)	-42·2 (-82–257)	-47·7 (-84·3—31·7)
Household air pollution from solid fuels	0·0397 (0·00392–0·136)	0·02 (0·00207–0·0734)	0·0105 (0·000394–0·0473)	-49·7 (-86·1–80·1)	-47·5 (-88·3–8·77)	0·0526 (0·00572–0·172)	0·0265 (0·00296–0·0954)	0·0139 (0·000546–0·064)	-49·6 (-85·9–78·9)	-47·5 (-87·9–9·35)
Ambient ozone pollution	59·5 (25–99)	45 (19–78·2)	23·2 (8·3–43·8)	-24·4 (-38·2—10)	-48·4 (-64·9—34·4)	--	--	--	--	--
Residential radon	24·3 (3·69–70·1)	24·3 (3·69–70·1)	24·3 (3·69–70·1)	0 (0–0)	0 (0–0)	24·3 (3·69–70·1)	24·3 (3·69–70·1)	24·3 (3·69–70·1)	0 (0–0)	0 (0–0)
Lead exposure	26·9 (2·03–54·7)	15·5 (0·927–37·4)	10·7 (0·338–28·9)	-42·4 (-71·4—28·4)	-30·7 (-65·5—21·1)	15·7 (0·0241–42·8)	9·83 (6·99e-12–31·5)	7·15 (3·62e-12–25·1)	-37·3 (-100—20·7)	-27·3 (-100—9·29)
Child underweight	0·921 (0·523–1·4)	0·927 (0·486–1·41)	0·905 (0·484–1·38)	0·597 (-18–23·7)	-2·31 (-15·3–12·8)	0·779 (0·402–1·2)	0·823 (0·457–1·26)	0·843 (0·46–1·27)	5·61 (-16·9–47·4)	2·54 (-11·3–21·2)
Iron deficiency	--	--	--	--	--	11·3 (9·46–13·5)	11·6 (9·68–13·7)	11·9 (9·87–14·2)	2·73 (-9·18–15·9)	2·03 (-7·43–12·8)
Vitamin A deficiency	0·624 (0·148–1·75)	0·816 (0·208–2·07)	0·57 (0·144–1·47)	30·9 (-67·3–590)	-30·2 (-69·5–78·9)	1·3 (0·504–2·73)	1·29 (0·493–2·79)	1·09 (0·411–2·4)	-0·964 (-61·1–195)	-15·5 (-54–64·2)
Zinc deficiency	2·06 (0–6·86)	1·07 (0–3·36)	1·09 (0–3·48)	-48·1 (-100–NA)	1·85 (-21·5–NA)	2·1 (0–6·84)	1·09 (0–3·47)	1·07 (0–3·43)	-48·3 (-100–NA)	-1·09 (-22·2–NA)
Smoking	27·6 (24·9–30·5)	20·6 (18·7–22·7)	17·5 (15·7–19·4)	-25·3 (-30·6—19·7)	-15·1 (-18·8—11·6)	21·9 (19·3–24·8)	17·2 (15·3–19·2)	14·4 (12·8–16·1)	-21·5 (-29—13·3)	-16·4 (-20·4—12·2)
Secondhand smoke	39·7 (37·2–42·3)	25·6 (24·1–27·3)	25·5 (22·9–28·3)	-35·6 (-41·3—29·5)	-0·14 (-8·89–9·14)	36·7 (34·8–38·8)	24·3 (23·2–25·6)	24·3 (22·2–26·5)	-33·8 (-38·7—28·1)	-0·229 (-7·71–7·71)
Alcohol use	16 (9·65–22·5)	17 (10·6–24·1)	19·4 (12–28·3)	6·29 (-22·3–51·6)	14·5 (-8·98–47·5)	12·5 (7·26–18·2)	15·1 (9·42–21·4)	14·4 (8·99–20·8)	20·6 (-12·3–75·5)	-4·49 (-24·5–21·4)
Drug use	0·648 (0·353–1·21)	1·24 (0·868–1·73)	1·89 (1·43–2·5)	91·5 (40·8–172)	52·3 (30·2–81·5)	0·497 (0·304–0·812)	0·897 (0·655–1·2)	1·41 (1·08–1·79)	80·6 (41·6–132)	56·7 (35·2–82·8)
High fasting plasma glucose	12·9 (10·6–15·7)	18 (15·7–20·7)	19·3 (16·8–22·3)	40 (15·7–68·7)	7·1 (-7·02–22·2)	9·24 (7·77–11·1)	12·7 (11–14·8)	14·1 (12–16·5)	37·8 (14·6–64·2)	10·9 (-2·9–26·6)
High systolic blood pressure	25·4 (20·7–30·8)	15·8 (13·9–18)	19·5 (15·1–24·6)	-37·7 (-49·2—22·8)	23·2 (-3·01–53·3)	22·3 (16·1–29·8)	12·6 (11–14·2)	17·2 (12·4–23·3)	-43·6 (-58·6—21·8)	36·6 (-1·58–86·1)
High body-mass index	27·2 (19·4–37·9)	42·1 (31·5–53·9)	44·5 (33·4–56)	54·6 (35·9–80·6)	5·86 (-0·0785–13·5)	30·6 (22·1–40·1)	43·3 (34·4–52)	45·7 (36·7–54·6)	41·6 (23·3–66·4)	5·52 (-1·06–13·1)
Low bone mineral density	12·3 (7·08–19·2)	9·43 (5·04–15·9)	11·7 (6·72–18·7)	-23·6 (-42·6—3)	24·3 (-4·26–65·7)	16·2 (10·7–23·1)	16·8 (11·3–24·2)	19·1 (13–26·4)	3·71 (-18·7–30·3)	13·4 (-10·6–48·6)
Diet low in fruits	49·1 (37–62)	44·5 (33·1–57·4)	45·3 (33·1–58·9)	-9·25 (-22·6–4·52)	1·6 (-10·9–14·1)	52·1 (38–66·2)	47·4 (33·4–62·1)	48·6 (33·8–66·7)	-8·91 (-22·4–5·93)	2·46 (-21·5–24·6)
Diet low in vegetables	30·5 (15·3–51·3)	42·9 (28·1–59·7)	41·9 (27–57·9)	40·6 (7·92–117)	-2·38 (-12·5–8·39)	32·4 (15·9–52·8)	40·1 (23·5–60·1)	38·4 (22–58·2)	23·5 (2·55–69·3)	-4·22 (-16·2–9·16)
Diet low in whole grains	79·2 (69·6–88)	79·6 (69·2–89·2)	81·1 (71·3–89·3)	0·512 (-6·75–8·8)	1·78 (-5·09–8·61)	--	--	--	--	--
Diet low in nuts and seeds	26·1 (7·17–56·6)	24·2 (7·72–54·9)	20 (6·38–53·7)	-7·39 (-44·9–78·3)	-17·4 (-43·6–8·79)	22·2 (5·65–54·4)	20·5 (5·77–53·6)	18·1 (5·15–52·8)	-7·7 (-43·5–69·6)	-11·7 (-40·2–23·7)
Diet low in milk	46·5 (23·6–67·2)	39·3 (17–59·3)	38·8 (17·5–59·6)	-15·5 (-36·2—1·89)	-1·43 (-14·2–13·9)	46·3 (22·6–67·1)	39·4 (17·3–58·8)	39·1 (17·8–58·8)	-15 (-34·9—0·315)	-0·638 (-14·4–14·8)
Diet high in red meat	74·3 (61·7–85·3)	77·1 (63·7–88·3)	76·8 (63·1–87·7)	3·78 (-11–20·8)	-0·401 (-10·5–11·6)	67·7 (53·1–79·3)	69·8 (57·5–81·8)	69·9 (56·1–82·4)	3·19 (-12·6–23·8)	0·145 (-12·6–14·2)
Diet high in processed meat	75·4 (50·5–97·1)	84·1 (64·2–99·5)	83·9 (63·8–99·2)	11·6 (1·39–31·7)	-0·208 (-4·92–4·65)	73·5 (48·4–94·9)	84·2 (64·2–99·3)	82·9 (62·1–99·1)	14·6 (3·31–41·4)	-1·6 (-7·27–2·49)
Diet high in sugar-sweetened beverages	48·4 (31·2–67·5)	66·9 (47·8–83·2)	66·8 (47·4–83·8)	38·1 (-0·322–108)	-0·128 (-18·6–21·6)	52·3 (31·9–71·9)	72·4 (54·5–87·2)	70·5 (51·3–86·1)	38·3 (1·9–113)	-2·63 (-20·5–18·9)
Diet low in fiber	41·4 (26–56·8)	27·2 (15·5–40·3)	25 (13·7–37·9)	-34·3 (-51—15·9)	-7·99 (-27·1–11·2)	42 (24·8–57·8)	32·2 (18·9–46·9)	27·9 (15·1–41·7)	-23·3 (-40·1—4·16)	-13·6 (-30·6–2·37)
Diet low in seafood omega-3 fatty acids	82·5 (64·4–99·4)	76·6 (57·1–99·1)	76·6 (58·7–98·4)	-7·22 (-24·2–7·94)	0·0254 (-15·6–17·9)	85 (68·8–99·5)	80·7 (62·8–98·6)	80·6 (62·5–98·9)	-5·04 (-19–7·78)	-0·146 (-13·3–14·8)
Diet low in polyunsaturated fatty acids	12·8 (3·83–38·5)	6·53 (1·82–24·5)	6·42 (1·81–25·6)	-49·1 (-79·1—7·91)	-1·63 (-21·6–21·2)	--	--	--	--	--
Diet high in trans fatty acids	89·4 (81·7–100)	87·9 (79–100)	87·5 (78·3–100)	-1·77 (-9·27–6·58)	-0·422 (-8·21–8·08)	--	--	--	--	--
Diet high in sodium	18·6 (4·31–41·8)	40·2 (11·9–63·8)	39·5 (10·1–64·5)	116 (38·7–431)	-1·72 (-28–30·2)	--	--	--	--	--
Low physical activity	2·09 (0·772–4·55)	1·7 (0·683–4·02)	1·98 (0·69–4·31)	-18·6 (-47·6–17·4)	16·5 (-14·9–64·5)	4·96 (2·37–9·85)	4·78 (2·5–8·87)	5·09 (2·56–9·94)	-3·63 (-27·8–34·6)	6·42 (-7–20·6)
Occupational asthmagens	27·6 (22·3–33·9)	24·2 (20·1–29)	25·5 (20·6–31·8)	-12·4 (-26–4·35)	5·54 (-6·99–23)	13·1 (10·7–15·9)	13·3 (11–16)	13·3 (10·8–16·2)	1·65 (-14·1–19·4)	0·0251 (-11·2–14·5)
Occupational particulate matter, gases, and fumes	10·5 (7·49–14·9)	9·96 (7·16–14·1)	9·66 (6·99–13·8)	-5·47 (-10·5–0·202)	-2·98 (-6·67–0·546)	6·52 (4·5–9·38)	6·14 (4·44–8·73)	5·89 (4·28–8·49)	-5·73 (-13·5–3·26)	-4·13 (-9·32–0·834)
Occupational noise	9·04 (8·27–9·95)	8·85 (8·24–9·62)	8·67 (8·13–9·44)	-2·08 (-5·64–2·03)	-2·05 (-4·07–0·154)	6·73 (6–7·58)	7·14 (6·56–7·78)	7·18 (6·72–7·8)	6·02 (0·232–13)	0·507 (-2·28–3·56)
Occupational injuries	--	--	--	--	--	--	--	--	--	--
Occupational ergonomic factors	13·5 (11·2–16·5)	12·1 (10·1–14·5)	12·6 (10·4–15·2)	-10·5 (-21·6–1·81)	3·93 (-5·79–14·6)	8·31 (6·6–10·9)	8·55 (6·87–10·8)	9·17 (7·1–11·9)	2·87 (-16·8–25)	7·22 (-8·34–25·8)
Childhood sexual abuse	7·4 (5·05–10·5)	6·97 (4·6–9·93)	6·85 (4·54–9·67)	-5·83 (-12·2–5·83)	-1·68 (-8·51–5·53)	12·3 (8·94–17·3)	17·4 (12·9–24)	17·8 (13·1–24·7)	41·9 (30·7–54·8)	2·17 (-5–11)
Intimate partner violence	--	--	--	--	--	33·9 (19·6–44)	32·1 (17·8–41·8)	33 (18·3–42·6)	-5·36 (-14–5·65)	2·79 (-5·81–15·5)
Non-exclusive breastfeeding	26·2 (21·5–31·1)	25 (21·3–29·1)	25 (20·5–29·9)	-4·39 (-18·6–13·1)	-0·0142 (-13·7–15·1)	--	--	--	--	--
Discontinued breastfeeding	27·2 (26·2–28·1)	24·8 (23·4–26·1)	25·4 (24–26·6)	-8·8 (-14·3—3·1)	2·38 (-3·19–8·73)	27·2 (26·2–28·1)	24·8 (23·3–26)	25·4 (24–26·6)	-8·97 (-14·5—3·28)	2·37 (-3·2–8·73)
Diet low in calcium	15·2 (7·41–27·7)	11·8 (5·96–21·7)	11·5 (5·59–21·2)	-22·2 (-34·2—9·32)	-2·88 (-12·8–5·83)	10·4 (4·37–19·7)	8·7 (3·8–16)	8·67 (3·8–16·1)	-16·2 (-31·2—1·99)	-0·432 (-11·5–11·8)
Occupational exposure to asbestos	11·1 (9·99–12·4)	9·07 (8·24–9·89)	8·67 (6·39–10·9)	-18·6 (-30—5·96)	-4·4 (-29·4–21·9)	1·49 (1·08–1·96)	1·36 (0·946–1·77)	1·35 (0·885–1·92)	-8·36 (-24·2–9·77)	-0·665 (-24·6–33·3)
Occupational exposure to arsenic	0·622 (0–1·45)	0·62 (0–1·46)	0·643 (0–1·52)	-0·251 (-14·1–NA)	3·64 (-7·82–NA)	0·593 (0–1·41)	0·576 (0–1·39)	0·58 (0–1·38)	-2·77 (-20·1–NA)	0·55 (-11·3–NA)
Occupational exposure to benzene	0·893 (0·0539–2·53)	0·912 (0·0542–2·65)	0·932 (0·0573–2·65)	2·07 (-6·24–14·2)	2·29 (-4·85–11·1)	1·29 (0·0672–3·61)	1·5 (0·0897–4·24)	1·54 (0·0887–4·5)	16·5 (5·69–39·5)	2·83 (-5·17–15·2)
Occupational exposure to beryllium	0·0256 (0·0241–0·0271)	0·0263 (0·0255–0·027)	0·0273 (0·0258–0·0288)	2·77 (-3·5–9·48)	3·83 (-1·71–9·57)	0·0294 (0·0271–0·0317)	0·0316 (0·0308–0·0326)	0·0323 (0·0304–0·0343)	7·64 (-0·673–17·2)	2·2 (-4·06–8·18)
Occupational exposure to cadmium	0·0715 (0·0648–0·0787)	0·072 (0·0653–0·0785)	0·0746 (0·0657–0·0839)	0·734 (-11·8–14·9)	3·51 (-6·95–15·2)	0·0697 (0·0613–0·0786)	0·069 (0·0616–0·0777)	0·0695 (0·0605–0·0796)	-0·983 (-17–17·1)	0·745 (-9·98–13·6)
Occupational exposure to chromium	0·169 (0·155–0·185)	0·174 (0·161–0·188)	0·18 (0·162–0·199)	2·85 (-7·89–15·5)	3·37 (-5·64–13)	--	--	--	--	--
Occupational exposure to diesel engine exhaust	0·73 (0·674–0·789)	0·83 (0·779–0·885)	0·86 (0·792–0·931)	13·7 (2·59–25·6)	3·64 (-4·34–12)	0·701 (0·628–0·784)	0·799 (0·723–0·885)	0·796 (0·708–0·895)	14 (-1·25–32·3)	-0·467 (-9·48–10)
Occupational exposure to formaldehyde	0·363 (0·327–0·401)	0·355 (0·321–0·39)	0·363 (0·318–0·41)	-2·12 (-14·4–12·5)	2·27 (-8·05–13·9)	0·34 (0·3–0·385)	0·34 (0·305–0·38)	0·343 (0·302–0·39)	0·0196 (-15·2–17)	0·798 (-9·32–13·3)
Occupational exposure to nickel	0·512 (0–2·25)	0·498 (0–2·25)	0·517 (0–2·26)	-2·66 (-16·6–NA)	3·84 (-7·81–NA)	0·472 (0–2)	0·443 (0–2·02)	0·448 (0–1·95)	-6·1 (-24·1–NA)	1·07 (-12·4–NA)
Occupational exposure to polycyclic aromatic hydrocarbons	0·334 (0·306–0·363)	0·344 (0·319–0·368)	0·354 (0·32–0·391)	2·92 (-7·74–14·9)	3·17 (-5·52–12·2)	0·358 (0·322–0·395)	0·38 (0·353–0·41)	0·384 (0·347–0·422)	6·15 (-6·54–20·4)	1·12 (-7·01–10·3)
Occupational exposure to silica	4·51 (0·651–15·1)	4·29 (0·594–13·5)	4·47 (0·636–13·8)	-4·78 (-17·6–11·9)	4·25 (-6·98–18·7)	3·89 (0·576–12·9)	3·53 (0·482–11·2)	3·54 (0·508–11·4)	-9·07 (-27–14·3)	0·114 (-13·3–20·3)
Occupational exposure to sulfuric acid	1·09 (0·231–3·44)	1·2 (0·24–3·92)	1·22 (0·256–4)	10·2 (-3·61–25·5)	2·09 (-7·57–13·1)	1·06 (0·22–3·39)	1·23 (0·251–3·72)	1·21 (0·254–3·93)	15·4 (-5·67–40)	-0·905 (-15–14·7)
Unsafe sex	--	--	--	--	--	--	--	--	--	--
Occupational exposure to trichloroethylene	0·0953 (0·0886–0·103)	0·101 (0·0969–0·107)	0·105 (0·0977–0·113)	6·46 (-2·16–16·1)	3·45 (-3·36–10·7)	0·0956 (0·0867–0·105)	0·101 (0·0948–0·109)	0·102 (0·0927–0·111)	6·05 (-5·33–19·7)	0·489 (-7·28–9·46)
No access to handwashing facility	3·25 (2·99–3·5)	3·06 (2·82–3·32)	3·03 (2·78–3·29)	-5·83 (-14·7–3·49)	-0·984 (-6·6–4·68)	3·25 (2·99–3·5)	3·06 (2·82–3·32)	3·03 (2·78–3·29)	-5·83 (-14·7–3·49)	-0·984 (-6·6–4·68)
Child wasting	0·744 (0·483–1·03)	0·723 (0·471–0·98)	0·721 (0·473–0·985)	-2·89 (-12·2–8·02)	-0·208 (-7·48–7·26)	--	--	--	--	--
Child stunting	2·26 (1·16–3·38)	1·98 (1·01–2·99)	1·93 (0·994–2·92)	-12·2 (-31·3–13·5)	-2·58 (-17·3–14)	1·86 (0·901–2·88)	1·71 (0·841–2·6)	1·7 (0·855–2·66)	-7·95 (-28–17·7)	-0·792 (-17·5–18)
Chewing tobacco	1·37 (1·01–1·86)	1·76 (1·18–2·6)	1·79 (1·19–2·63)	28·7 (-15·9–96·6)	1·5 (-24·5–36·6)	0·243 (0·151–0·379)	0·272 (0·168–0·429)	0·282 (0·176–0·448)	11·7 (-38·2–92·2)	3·58 (-28·1–46·4)
Diet low in legumes	73·2 (37–99·3)	75·9 (42·2–99·6)	76 (43·5–99·6)	3·65 (-0·416–20·7)	0·0888 (-5·76–5·79)	71·7 (34–98·8)	74·1 (38·5–99·5)	74·6 (41–99·4)	3·48 (-0·528–20·6)	0·668 (-4·68–9)
Short gestation	20·7 (16·7–25·4)	22·1 (18·1–27·1)	20·7 (16·6–25·6)	7·1 (-18·5–40·9)	-6·38 (-26·9–20·4)	14·9 (12·7–17·3)	18·9 (16–22·5)	19·5 (16·3–22·8)	27·1 (5·27–54·3)	2·93 (-15·5–23·1)
Low birth weight	8·07 (6·53–9·77)	9·55 (7·79–11·6)	9·05 (7·43–11·2)	18·4 (-10·8–54·5)	-5·24 (-27·2–21·2)	7·02 (6·01–8·14)	9·31 (7·89–10·9)	9·95 (8·39–11·6)	32·6 (7·72–65·5)	6·79 (-12·5–29·9)
High temperature	26 (15·7–38·7)	27·8 (17·1–40·2)	29 (18·2–41·4)	6·7 (1·57–17·3)	4·43 (-0·293–9·62)	26 (15·7–38·7)	27·8 (17·1–40·2)	29 (18·2–41·4)	6·7 (1·57–17·3)	4·43 (-0·293–9·62)
Low temperature	34 (30·7–37·7)	34·5 (31·3–38·1)	35·9 (32·9–39·3)	1·36 (0·449–2·4)	4·28 (3·06–5·59)	34 (30·7–37·7)	34·5 (31·3–38·1)	35·9 (32·9–39·3)	1·36 (0·449–2·4)	4·28 (3·06–5·59)
Kidney dysfunction	19·2 (13·7–26·1)	19·6 (13·7–26·5)	19·5 (14–26·5)	2·11 (-1·75–6·37)	-0·223 (-4·51–4·37)	21·3 (14·9–28·8)	22·1 (15·5–29·8)	22·8 (16·3–30·5)	3·53 (-0·523–8·2)	3·49 (-1·07–8·73)
Bullying victimization	5·88 (2·44–12·1)	9·12 (3·62–18)	8·79 (3·37–18·1)	55·2 (33·6–79·1)	-3·55 (-13–2·69)	4·24 (1·73–8·82)	7·97 (3·19–15·9)	7·83 (2·96–16·2)	87·9 (61·7–117)	-1·76 (-12·2–5·18)
High LDL cholesterol	67·4 (62·9–71·7)	34·4 (30·8–38)	30·9 (27·3–34·7)	-48·9 (-54·3—43·5)	-10·2 (-14·9—5·31)	65·1 (61·5–68·9)	34·2 (30·8–37·7)	31·7 (28·2–35·4)	-47·5 (-52·1—42·6)	-7·21 (-11·1—3·02)

Supplementary Results Table 1. Kazakhstan age-standardised Summary Exposure Values (SEVs) for most-detailed risk factors and percentage change, by sex, 1990, 2010, and 2019. Data in parentheses are 95% uncertainty intervals.

	Male					Female				
	1990	2010	2019	Percentage change 1990-2010	Percentage change 2010-2019	1990	2010	2019	Percentage change 1990-2010	Percentage change 2010-2019
Unsafe sanitation	63·2 (56·4–69·7)	53·7 (51–55·9)	32·2 (27·4–37·6)	-15 (-22·8—5·54)	-40 (-49·4—29·4)	63·2 (56·4–69·7)	53·7 (51–55·9)	32·2 (27·4–37·6)	-15 (-22·8—5·54)	-40 (-49·4—29·4)
Ambient particulate matter pollution	16·4 (7·65–31·2)	17·2 (11·2–24·1)	18·9 (12·1–28·1)	4·74 (-41·3–102)	9·54 (-15·7–42·3)	15 (6·89–29·1)	16·6 (10·9–23·6)	18·5 (11·5–27·6)	10·9 (-38·6–116)	11·2 (-14·4–43·8)
Household air pollution from solid fuels	8·27 (3·66–15·3)	2·47 (0·956–5·15)	1·35 (0·438–3·14)	-70·2 (-82·9—49·4)	-45·3 (-64—25·2)	9·86 (4·78–17·2)	3·13 (1·32–6·46)	1·73 (0·613–3·95)	-68·3 (-81·8—47·4)	-44·6 (-63·7—24·4)
Ambient ozone pollution	64·6 (27·3–100)	50·7 (21·6–85·5)	49·1 (20·5–83·4)	-21·6 (-29·1—13·9)	-3·02 (-10·7–4·84)	64·6 (27·3–100)	50·7 (21·6–85·5)	49·1 (20·5–83·4)	-21·6 (-29·1—13·9)	-3·02 (-10·7–4·84)
Residential radon	37·6 (6·48–100)	37·6 (6·48–100)	37·6 (6·48–100)	0 (0–0)	0 (0–0)	--	--	--	--	--
Lead exposure	46 (22·7–67·4)	38·9 (19·6–59·1)	31·7 (14·1–52·1)	-15·3 (-29·1—4·3)	-18·5 (-29·9—11·6)	22·7 (0·167–50)	21·7 (1·6–46·3)	18·3 (1·29–41·1)	-4·4 (-15·9–1100)	-16 (-33·6—8·37)
Child underweight	2·25 (1·47–3·11)	1·65 (1·07–2·31)	1·33 (0·843–1·9)	-26·8 (-38·9—12·2)	-19·4 (-29·3—8·86)	2·41 (1·62–3·25)	1·61 (1·03–2·22)	1·29 (0·821–1·83)	-33 (-43·4—23·2)	-20 (-30—10·4)
Iron deficiency	--	--	--	--	--	22·2 (20·3–24·2)	21 (18·7–23·4)	19·1 (16·8–21·6)	-5·54 (-13·8–3·65)	-8·8 (-14·2—3·24)
Vitamin A deficiency	33·1 (18–49·1)	19·6 (11·2–29·8)	13·9 (6·7–24·5)	-40·6 (-69·3–26·2)	-29·3 (-55·9–11·9)	17·9 (10·2–26·5)	16·2 (9·6–24·1)	11·2 (5·78–18·2)	-9·55 (-48·3–76)	-31 (-52—1·29)
Zinc deficiency	4·05 (0–12·4)	2·87 (0–9·4)	2·37 (0–7·66)	-29·1 (-100–NA)	-17·4 (-53·1–NA)	3·95 (0–12·1)	2·8 (0–8·88)	2·31 (0–7·51)	-29 (-100–NA)	-17·6 (-51·7–NA)
Smoking	27·7 (23·7–31·7)	24·3 (21–27·8)	23·8 (20·6–27·4)	-12·3 (-19·6—4·5)	-1·85 (-6·09–2·44)	2·72 (1·98–3·79)	2·61 (2·04–3·22)	2·82 (2·22–3·47)	-4·17 (-23·7–24)	8·1 (-3·95–20·6)
Secondhand smoke	35·3 (33·3–37·3)	35·4 (33·7–37)	33·7 (31·3–36)	0·361 (-6·52–8·58)	-4·82 (-9·76–0·306)	57·5 (55·9–59·2)	55·4 (54·3–56·6)	53 (51–54·9)	-3·63 (-7·05—0·256)	-4·44 (-7·38—1·44)
Alcohol use	13·9 (9·19–19·5)	13·1 (9·18–17·9)	12·8 (8·67–17·7)	-6 (-23–18)	-2·39 (-15·2–10·7)	6·18 (3·53–9·84)	5·04 (3·08–7·68)	4·88 (2·8–7·63)	-18·6 (-41·9–14·2)	-2·98 (-20·4–17·9)
Drug use	0·245 (0·17–0·378)	0·391 (0·284–0·566)	0·35 (0·259–0·509)	59·9 (36·3–95·1)	-10·5 (-20·8–2·18)	0·146 (0·102–0·218)	0·182 (0·134–0·257)	0·173 (0·125–0·248)	24·4 (10·1–42·2)	-5·04 (-14·6–4·77)
High fasting plasma glucose	6·14 (5·32–7·02)	10·5 (8·76–12·4)	12·9 (10·8–15·2)	71·6 (46–105)	22·4 (4·35–47·9)	8·33 (7·06–10·1)	14·9 (12·6–17·3)	16 (13·8–18·5)	78·5 (49·9–115)	7·53 (-7·63–26·1)
High systolic blood pressure	45 (37–54·1)	41·8 (37–47)	44·5 (37·6–51·6)	-7·08 (-23·6–14·6)	6·5 (-10·1–26·8)	49·4 (38–62·7)	40·7 (35–47·1)	42 (33·3–52·5)	-17·6 (-37–7·34)	3·33 (-19·2–31·1)
High body-mass index	17·3 (12·4–23·6)	25·4 (19–33·8)	31·7 (23·7–41·8)	46·8 (30·4–71·8)	24·4 (16–35·1)	25·2 (18·3–34·1)	31·6 (24–41·1)	36·5 (27·8–46·1)	25·4 (12·4–42·2)	15·4 (8·01–25·8)
Low bone mineral density	8·09 (4·19–13·5)	7·63 (3·95–12·9)	7·42 (3·79–12·8)	-5·65 (-29·1–22·8)	-2·88 (-26–28·5)	15·2 (9·64–22)	14·6 (9·07–21·9)	14 (8·78–21·2)	-4 (-27·8–21·2)	-4·01 (-25·4–24·8)
Diet low in fruits	80·8 (74·2–88)	61·4 (50·7–74·3)	56 (44·4–69·9)	-23·9 (-33·3—15·1)	-8·88 (-15·7—3·27)	81·7 (75·5–88·6)	62·8 (52·4–75·2)	57·4 (45·9–70·4)	-23·1 (-31·7—14·3)	-8·7 (-15·6—3·6)
Diet low in vegetables	36·3 (19·4–58·1)	7·62 (3·04–19·2)	4·86 (2·18–13)	-79 (-89·4—61·4)	-36·2 (-61·9—6·57)	--	--	--	--	--
Diet low in whole grains	99·9 (100–100)	99·9 (100–100)	99·9 (100–100)	0 (0–0)	0 (0–0)	99·9 (100–100)	99·9 (100–100)	99·9 (100–100)	0 (0–0)	0 (0–0)
Diet low in nuts and seeds	88·1 (45·8–96)	76·2 (38·4–88·6)	69·8 (34·4–84·7)	-13·5 (-33·6–0·569)	-8·4 (-17·1—3·05)	89·2 (45·7–96·5)	73·1 (37·4–87)	65·8 (32·2–81·6)	-18·1 (-37·8—5·13)	-9·89 (-20—4·31)
Diet low in milk	31·8 (11·3–51·3)	9·52 (2·15–26·6)	16·8 (4·38–36·2)	-70·1 (-90·8—39)	76·5 (26·4–278)	30·6 (10·8–48·6)	8·75 (2·03–24·8)	15·7 (3·78–34·7)	-71·4 (-91·2—41·4)	79·4 (28·9–260)
Diet high in red meat	87 (81·4–91·5)	80·1 (72·5–86·6)	85·8 (77·7–92)	-7·85 (-12—4·75)	7·07 (-0·255–15·2)	87 (81·4–91·3)	80·1 (72·8–86·6)	85·8 (77·3–92·2)	-7·91 (-11·8—4·71)	7·12 (-0·184–15·7)
Diet high in processed meat	49·8 (28·3–73·9)	48·6 (27–72·8)	50·5 (29·5–74·9)	-2·48 (-21–19·4)	4·01 (-7·84–19·5)	58·6 (35·5–83·2)	57·4 (33·7–82·7)	59·7 (34·9–84·1)	-1·93 (-18·8–16·5)	3·86 (-5·77–17·2)
Diet high in sugar-sweetened beverages	30·7 (16·1–47·4)	32 (17·1–49·2)	36·9 (20·3–54·2)	4·07 (-31·7–69·4)	15·3 (-10·2–58·2)	33·5 (16·6–51·8)	34·3 (18·6–53·2)	39·8 (21·7–58·3)	2·36 (-33·4–63·7)	16·1 (-12·1–60·7)
Diet low in fiber	37 (22·4–51·6)	33·3 (20–47·6)	27·2 (15·3–40·7)	-10·1 (-15·7—6·21)	-18·3 (-30·7—7·2)	36·9 (21·9–52·2)	33 (19·2–47·4)	26·8 (15·1–40·3)	-10·5 (-15·8—6·31)	-18·8 (-30·7—8·11)
Diet low in seafood omega-3 fatty acids	95·7 (86·5–100)	91·5 (78·3–100)	87·3 (70·4–100)	-4·37 (-14·9–0·972)	-4·62 (-18·3–3·92)	96 (87–100)	92·8 (81·7–100)	89·2 (75·3–100)	-3·24 (-11·2–1·14)	-3·94 (-14·2–1·87)
Diet low in polyunsaturated fatty acids	65·3 (24·7–100)	54·5 (16·6–95·2)	49·5 (14·2–91·9)	-16·7 (-36·7—0·946)	-9·11 (-20·8—1·25)	67·2 (26·1–100)	56·7 (17·7–96·6)	52 (15·5–93·7)	-15·5 (-36·2—0·856)	-8·34 (-20·1—1·02)
Diet high in trans fatty acids	30·2 (21·7–83·2)	25 (15·7–93·4)	24·9 (15·5–91·2)	-17·5 (-40·1–14·1)	-0·358 (-9·09–8·56)	31 (21·9–89·4)	26·8 (16·3–98·3)	26·8 (16·2–97·4)	-13·6 (-36·2–18·5)	-0·0556 (-8·81–7·06)
Diet high in sodium	62·7 (38·7–80·8)	43·1 (13·9–67·3)	42·4 (13·1–67)	-31·3 (-68·3—8·86)	-1·61 (-22–20·9)	30·9 (10·8–54·2)	18·5 (4·25–40·9)	18 (4·15–40·4)	-40·3 (-70·9—16·9)	-2·68 (-19·5–12·9)
Low physical activity	2·43 (1·19–4·88)	2·43 (1·25–4·65)	2·83 (1·4–5·38)	-0·116 (-20·7–24·2)	16·5 (-5·15–50·1)	2·44 (1·36–4·37)	2·69 (1·57–4·63)	2·82 (1·56–4·88)	10·5 (-6·26–32·8)	4·71 (-11·6–24)
Occupational asthmagens	21·6 (17·6–26·5)	19·9 (16·8–23·5)	19·4 (16·3–23·1)	-8·24 (-19·8–6·59)	-2·26 (-9·64–5·4)	13 (9·73–17·5)	12·3 (10·2–15)	12 (9·81–14·7)	-5·54 (-23·1–18·1)	-2·61 (-13·7–9·33)
Occupational particulate matter, gases, and fumes	12·9 (9·91–16·9)	12·4 (9·65–16·5)	12·2 (9·5–16·1)	-3·52 (-7·1–0·823)	-2·17 (-3·94—0·454)	7·81 (5·8–11)	7·55 (5·75–10·5)	7·31 (5·63–10)	-3·32 (-9·07–3·8)	-3·13 (-5·87—0·23)
Occupational noise	14·4 (13·4–15·5)	14·1 (13·5–14·9)	14 (13·4–14·8)	-2·06 (-5·06–1·48)	-0·876 (-2·18–0·416)	9·8 (8·76–10·9)	10·1 (9·44–10·9)	10·3 (9·72–10·9)	3·31 (-1·75–9·53)	1·34 (-0·735–3·68)
Occupational injuries	--	--	--	--	--	--	--	--	--	--
Occupational ergonomic factors	22·8 (19·2–26·7)	19 (16·7–21·4)	17·1 (14·5–19·8)	-16·9 (-29·7—1·57)	-9·89 (-18·5—0·546)	--	--	--	--	--
Childhood sexual abuse	2·52 (1·65–3·56)	2·27 (1·58–3·06)	2·52 (1·65–3·56)	-10 (-21–1·14)	11·1 (-0·371–27·7)	3·14 (2·26–4·3)	2·85 (2·11–3·75)	3·14 (2·26–4·3)	-9·26 (-18·3–1·9)	10·2 (-1·36–25·5)
Intimate partner violence	--	--	--	--	--	11·6 (6·88–16·7)	10·7 (6·64–14·2)	11·6 (6·88–16·7)	-7·84 (-20–15·5)	8·51 (-6·24–36)
Non-exclusive breastfeeding	24 (15·4–36·2)	21·7 (13·8–32·3)	20·8 (13·2–30·9)	-9·31 (-15·9—1·5)	-4·28 (-12·6–5·06)	23·9 (15·4–36·1)	21·7 (13·8–32·3)	20·8 (13·2–30·9)	-9·23 (-15·8—1·42)	-4·26 (-12·6–5·09)
Discontinued breastfeeding	14·2 (12·8–15·7)	15·2 (14·5–16)	13·2 (11·8–14·7)	7·56 (-3·9–21)	-13·2 (-23·6—2·24)	14·1 (12·7–15·6)	15·2 (14·5–16)	13·2 (11·8–14·7)	7·92 (-3·6–21·4)	-13·2 (-23·6—2·27)
Diet low in calcium	22·3 (10·6–43·4)	14·9 (7·31–27·2)	12 (5·87–21·5)	-33·3 (-43·3—21·1)	-19·4 (-26·6—14·2)	--	--	--	--	--
Occupational exposure to asbestos	2·19 (1·81–2·72)	2·81 (2·33–3·36)	2·04 (1·64–2·54)	28 (-1·64–62)	-27·2 (-44·4—4·22)	3·13 (0·567–10·5)	2·3 (0·659–5·48)	1·9 (0·561–4·57)	-26·4 (-83·3–197)	-17·5 (-73·4–152)
Occupational exposure to arsenic	0·365 (0·148–0·616)	0·37 (0·147–0·625)	0·366 (0·148–0·619)	1·31 (-6·84–10·1)	-0·822 (-5·99–4·68)	0·38 (0·153–0·649)	0·394 (0·161–0·666)	0·399 (0·166–0·673)	3·65 (-6·79–15·9)	1·2 (-4·43–7·49)
Occupational exposure to benzene	0·844 (0·51–1·52)	0·928 (0·573–1·65)	0·965 (0·599–1·74)	10·1 (2·79–20·6)	3·98 (-0·867–9·74)	1·11 (0·672–2)	1·39 (0·873–2·5)	1·51 (0·945–2·75)	25·8 (12·7–43·3)	8·51 (2·42–16·5)
Occupational exposure to beryllium	0·109 (0·103–0·115)	0·108 (0·106–0·11)	0·106 (0·103–0·109)	-1·37 (-6·79–4·41)	-1·8 (-4·39–0·82)	0·118 (0·109–0·127)	0·127 (0·124–0·13)	0·128 (0·124–0·132)	8 (-0·206–16·8)	0·756 (-2·84–4·17)
Occupational exposure to cadmium	0·197 (0·184–0·213)	0·201 (0·197–0·205)	0·199 (0·191–0·208)	1·76 (-5·9–9·6)	-0·678 (-5·26–4·07)	0·208 (0·19–0·229)	0·219 (0·213–0·226)	0·222 (0·211–0·234)	5·24 (-4·74–16·4)	1·51 (-3·78–7·06)
Occupational exposure to chromium	0·447 (0·415–0·48)	0·471 (0·462–0·481)	0·476 (0·456–0·496)	5·57 (-2·29–13·8)	0·95 (-3·3–5·34)	0·485 (0·443–0·534)	0·535 (0·518–0·552)	0·555 (0·528–0·585)	10·2 (-0·603–21·8)	3·78 (-1·38–9·23)
Occupational exposure to diesel engine exhaust	3·29 (3·04–3·56)	3·47 (3·38–3·56)	3·52 (3·35–3·68)	5·22 (-2·94–14·5)	1·56 (-3·25–6·3)	2·76 (2·51–3·04)	3·11 (2·99–3·24)	3·19 (3·01–3·38)	12·8 (1·15–25·1)	2·64 (-3·11–8·52)
Occupational exposure to formaldehyde	0·878 (0·708–0·971)	0·931 (0·769–0·973)	0·945 (0·763–1·01)	6·03 (-3·07–15·8)	1·53 (-4·02–7·03)	--	--	--	--	--
Occupational exposure to nickel	0·486 (0·155–1·27)	0·462 (0·152–1·22)	0·457 (0·148–1·21)	-4·81 (-13·1–4·68)	-1·09 (-6·17–4·72)	0·394 (0·131–1·04)	0·387 (0·128–1·03)	0·381 (0·125–0·983)	-1·65 (-11·5–9·44)	-1·66 (-7·44–4·74)
Occupational exposure to polycyclic aromatic hydrocarbons	0·974 (0·912–1·04)	1·02 (1·01–1·05)	1·04 (0·999–1·08)	5·27 (-2·12–13·2)	1·32 (-2·85–5·51)	1·05 (0·957–1·15)	1·17 (1·13–1·21)	1·22 (1·16–1·28)	11·6 (1·13–23)	4·17 (-0·856–9·43)
Occupational exposure to silica	5·5 (2·9–12·2)	5·48 (2·9–11·9)	5·54 (2·88–12·3)	-0·234 (-10·5–12·6)	1 (-5·76–8·72)	3·62 (1·83–7·86)	3·57 (1·85–8)	3·46 (1·79–7·4)	-1·33 (-11·9–11·7)	-3·06 (-9·56–4·35)
Occupational exposure to sulfuric acid	1·21 (0·873–2·14)	1·15 (0·837–2·03)	1·11 (0·797–1·93)	-4·86 (-13·5–4·52)	-3·54 (-9·17–2·4)	1·08 (0·762–1·9)	1·06 (0·77–1·86)	1·03 (0·746–1·82)	-1·85 (-11·6–8·03)	-2·75 (-8·53–3·21)
Unsafe sex	--	--	--	--	--	--	--	--	--	--
Occupational exposure to trichloroethylene	0·269 (0·253–0·288)	0·286 (0·282–0·293)	0·288 (0·279–0·299)	6·44 (-0·609–13·6)	0·629 (-3·14–4·52)	0·296 (0·273–0·324)	0·331 (0·322–0·343)	0·343 (0·328–0·362)	11·8 (1·35–23·1)	3·52 (-1·41–8·86)
No access to handwashing facility	22·9 (21·9–23·9)	13·9 (13·1–14·6)	11·1 (10·6–11·7)	-39·6 (-42·9—36)	-19·7 (-22·7—16·8)	--	--	--	--	--
Child wasting	2·07 (1·6–2·5)	2 (1·54–2·46)	1·92 (1·45–2·41)	-3·05 (-15·8–11·3)	-4·21 (-14·9–5·95)	2·5 (1·96–2·98)	2·41 (1·89–2·91)	2·29 (1·79–2·78)	-3·44 (-14·9–9·69)	-5·16 (-13·5–3·31)
Child stunting	9·92 (6·75–12·2)	8·01 (5·33–10)	5·98 (3·69–7·86)	-19·3 (-32·8—5·07)	-25·4 (-35·9—14·8)	10·8 (7·34–13·1)	8·2 (5·43–10·2)	6·15 (3·89–7·97)	-23·9 (-37·5—9·73)	-25·1 (-36·5—14·3)
Chewing tobacco	0·793 (0·619–0·986)	0·787 (0·626–0·976)	0·892 (0·687–1·12)	-0·794 (-25·8–33·6)	13·4 (-5·69–35·5)	--	--	--	--	--
Diet low in legumes	100 (99·9–100)	97 (92·5–100)	96·8 (91·7–100)	-2·95 (-7·51–0)	-0·257 (-1·72–0·691)	99·8 (99·2–100)	95·7 (89·4–100)	95·4 (88·6–100)	-4·11 (-10–0)	-0·25 (-1·68–0·75)
Short gestation	16·8 (12–22·7)	16·3 (12·9–20·8)	17·1 (13·8–20·8)	-2·99 (-31·2–35·1)	5·05 (-21·5–40·5)	13 (9–18)	11·2 (8·84–13·8)	10·4 (8·37–12·6)	-14·1 (-40·8–27·5)	-6·99 (-30·1–23·2)
Low birth weight	8·65 (6·5–11·5)	7·54 (6·16–9·37)	7·65 (6·36–9·34)	-12·8 (-37·1–20·9)	1·46 (-23·6–34·3)	7 (5·07–9·61)	5·85 (4·75–7·18)	5·46 (4·53–6·58)	-16·4 (-41·2–18·2)	-6·52 (-29–23·6)

High temperature	30<br>(22·3–39·3)	37·5<br>(28·1–47·3)	37·3<br>(28·7–46·6)	24·9<br>(12·3–40·4)	-0·433<br>(-6·66–7·23)	30<br>(22·3–39·3)	37·5<br>(28·1–47·3)	37·3<br>(28·7–46·6)	24·9<br>(12·3–40·4)	-0·433<br>(-6·66–7·23)
Low temperature	37·7<br>(33·5–43·1)	39·2<br>(33·8–45·4)	38<br>(33·5–43·3)	3·88<br>(-2·37–12·2)	-3<br>(-9·41–2·3)	37·7<br>(33·5–43·1)	39·2<br>(33·8–45·4)	38<br>(33·5–43·3)	3·88<br>(-2·37–12·2)	-3<br>(-9·41–2·3)
Kidney dysfunction	20·9<br>(13·9–29)	22·7<br>(15·6–30·9)	24·6<br>(17·3–32·9)	8·74<br>(5·01–14·4)	8·3<br>(4·61–13·3)	20·6<br>(13·7–28·4)	21·8<br>(14·8–29·8)	23·4<br>(16·2–31·6)	6·04<br>(2·57–10·4)	7·24<br>(4·01–12·1)
Bullying victimization	2·75<br>(1·13–5·71)	2·79<br>(1·16–5·74)	2·74<br>(1·13–5·58)	1·45<br>(-0·165–3·87)	-2·04<br>(-3·46–-0·782)	1·69<br>(0·705–3·48)	1·7<br>(0·701–3·49)	1·66<br>(0·687–3·42)	0·774<br>(-0·607–2·55)	-2·19<br>(-3·42–-0·948)
High LDL cholesterol	31·4<br>(27·9–35)	31·5<br>(28·1–34·7)	31·9<br>(28·4–35·3)	0·333<br>(-5·19–6·09)	1·2<br>(-2·87–5·48)	30·6<br>(27·3–34)	30<br>(26·7–33·4)	30·3<br>(27–33·5)	-2<br>(-6·75–2·89)	0·835<br>(-2·53–4·53)

Supplementary Results Table 1. New Jersey age-standardised Summary Exposure Values (SEVs) for most-detailed risk factors and percentage change, by sex, 1990, 2010, and 2019. Data in parentheses are 95% uncertainty intervals.

	Male					Female				
	1990	2010	2019	Percentage change 1990-2010	Percentage change 2010-2019	1990	2010	2019	Percentage change 1990-2010	Percentage change 2010-2019
Unsafe sanitation	4·15 (1·96–7·46)	2·33 (1·04–4·58)	1·97 (0·844–3·68)	-43·8 (-78–34·1)	-15·6 (-56·6–64)	--	--	--	--	--
Ambient particulate matter pollution	13·4 (4·95–26·5)	7·2 (4·53–10·3)	4·81 (2·32–7·67)	-46·3 (-72·9–31·6)	-33·2 (-49·7–-25)	13·4 (4·95–26·5)	7·2 (4·52–10·3)	4·81 (2·32–7·66)	-46·3 (-72·9–32·1)	-33·2 (-49·8–-25·1)
Household air pollution from solid fuels	0·00517 (0·00112–0·0152)	0·00309 (0·000515–0·0107)	0·0022 (0·000312–0·00745)	-40·2 (-78–39·6)	-28·7 (-67–41·7)	0·00681 (0·00148–0·0203)	0·00411 (0·000701–0·0148)	0·00293 (0·000423–0·00991)	-39·7 (-77·9–40·2)	-28·7 (-66·9–41·9)
Ambient ozone pollution	75·9 (33·5–100)	59·6 (25·1–100)	46·2 (18·8–80·3)	-21·4 (-33·3–0)	-22·5 (-36·7–-9·87)	--	--	--	--	--
Residential radon	21·3 (3·41–61·9)	21·3 (3·41–61·9)	21·3 (3·41–61·9)	0 (0–0)	0 (0–0)	21·3 (3·41–61·9)	21·3 (3·41–61·9)	21·3 (3·41–61·9)	0 (0–0)	0 (0–0)
Lead exposure	75·9 (52·4–91·7)	42·8 (22·6–62·3)	29·3 (13·4–46·1)	-43·6 (-56·9–-30·3)	-31·6 (-42·8–-25·2)	51·1 (18·9–76·6)	26·6 (5·03–51·3)	17·4 (1·99–37·1)	-48 (-74·4–-31·4)	-34·3 (-62·4–-26·5)
Child underweight	0·932 (0·541–1·4)	0·931 (0·506–1·4)	0·908 (0·49–1·37)	-0·168 (-18·4–21·8)	-2·43 (-15·6–13·1)	0·789 (0·432–1·2)	0·828 (0·444–1·23)	0·845 (0·466–1·28)	4·9 (-16–36·2)	2·12 (-12·6–20·1)
Iron deficiency	--	--	--	--	--	11·9 (9·9–14)	12·1 (10·2–14·1)	12·1 (10·1–14·4)	1·13 (-10·5–15)	0·642 (-8·3–11·3)
Vitamin A deficiency	0·589 (0·159–1·5)	0·823 (0·218–2·2)	0·602 (0·152–1·74)	39·8 (-65·5–521)	-26·9 (-67·5–83·9)	1·26 (0·466–2·59)	1·29 (0·503–2·67)	1·12 (0·438–2·37)	2·2 (-59·6–216)	-12·9 (-52·5–71·3)
Zinc deficiency	1·79 (0–6·03)	0·973 (0–3·07)	1·01 (0–3·23)	-45·6 (-100–NA)	3·38 (-23·9–NA)	1·84 (0–6·11)	0·975 (0–3·06)	0·997 (0–3·15)	-46·9 (-100–NA)	2·19 (-20·1–NA)
Smoking	25 (22·4–27·9)	17·5 (15·8–19·3)	14·1 (12·6–15·8)	-29·9 (-35·1–-24)	-19·2 (-22·8–-15·3)	18·7 (16·1–21·6)	14·3 (12·8–15·9)	11·1 (9·77–12·4)	-23·9 (-32·4–-13·8)	-22·2 (-26·4–-18)
Secondhand smoke	40·8 (38·1–43·4)	23·8 (22·1–25·5)	24·1 (21·6–26·8)	-41·7 (-47·8–-35·8)	1·56 (-7·57–11·3)	37·4 (35·4–39·4)	22·3 (21·2–23·4)	22·8 (20·9–24·7)	-40·2 (-44·8–-35·4)	2·22 (-4·95–9·7)
Alcohol use	16 (10·1–22·6)	17·9 (11·5–24·8)	18·5 (11·4–26·4)	12 (-19–51·9)	3·2 (-15·6–27·4)	11·5 (6·71–17)	13·1 (8·56–18·3)	12·8 (7·87–18·1)	14·4 (-15·3–59·4)	-2·59 (-22·2–20·8)
Drug use	0·7 (0·396–1·21)	1·07 (0·748–1·52)	1·36 (0·983–1·85)	53·1 (16·7–108)	27·3 (11·6–47·8)	0·426 (0·263–0·7)	0·707 (0·522–0·967)	0·956 (0·734–1·24)	65·9 (31·6–113)	35·1 (17·9–57)
High fasting plasma glucose	14 (11·6–16·9)	18·1 (15·8–20·7)	19·2 (16·9–22·1)	30 (7·39–55·6)	5·86 (-6·61–20·9)	10·1 (8·62–12)	13·3 (11·5–15·4)	14·6 (12·4–17·1)	31·6 (10·1–56·1)	10·2 (-4·59–26·7)
High systolic blood pressure	25·7 (20·7–31·3)	15·8 (13·6–17·7)	19·7 (15·3–24·5)	-38·7 (-50–-24·4)	25 (-0·893–57)	23·5 (17·4–30·2)	13·2 (11·6–14·7)	18 (13–23·8)	-43·9 (-57·1–-24·8)	36·7 (1·32–81·6)
High body-mass index	25·4 (17·5–35·6)	38·7 (28·5–50·2)	40·1 (29·9–51·7)	52·4 (34·5–77·4)	3·7 (-2·69–11·2)	30 (21·7–39·8)	42·2 (33·4–50·8)	43·5 (33·9–52·1)	40·6 (23–65·3)	3·05 (-3·11–9·68)
Low bone mineral density	11·8 (6·89–18·7)	8·94 (4·76–15·3)	11·1 (6·18–17·8)	-24·5 (-43·7–-3·24)	24·5 (-2·36–67·6)	15·7 (10·1–22·5)	16 (10·6–22·9)	18·3 (12·4–25·3)	1·99 (-18·9–29·5)	14·6 (-7·84–50·4)
Diet low in fruits	48·7 (35·1–63·9)	45·2 (32–59·2)	44·7 (32·1–58·1)	-7·36 (-18·4–2·47)	-1·04 (-14·8–15·9)	--	--	--	--	--
Diet low in vegetables	28·4 (13·5–50)	39·4 (21·4–61·6)	38·8 (21·3–60·2)	38·8 (12·7–94·8)	-1·37 (-13·7–11·6)	26·1 (12·2–48·2)	34·5 (19–54)	33·8 (18·4–54·8)	32·3 (7·52–87·3)	-1·92 (-16·6–13·1)
Diet low in whole grains	78·7 (68·5–87·7)	80·7 (71·9–89·1)	80·7 (71·8–89)	2·48 (-1·24–7·25)	0·0788 (-2·51–2·85)	--	--	--	--	--
Diet low in nuts and seeds	23·5 (6·64–54·2)	24·6 (7·86–55·1)	19·7 (5·96–53·6)	4·26 (-37·8–109)	-19·7 (-46·7–5·64)	19·7 (4·68–53·1)	20·6 (5·76–53·8)	17·5 (4·66–52·7)	4·96 (-36·8–112)	-15·1 (-46·3–17·9)
Diet low in milk	43·7 (20·9–64·1)	37·3 (16–57·1)	37 (16·5–56·1)	-14·7 (-36·7–1·36)	-0·839 (-15·6–18·3)	43·3 (20·5–63·2)	37·3 (16·1–56·6)	37·2 (16·9–56·2)	-14 (-35·2–1·23)	-0·0739 (-13·7–15·9)
Diet high in red meat	68·8 (55·4–80·1)	72·8 (59·5–84·4)	72·9 (59·6–85·2)	5·85 (-11·2–24·9)	0·17 (-11·7–13·6)	68·2 (54–79·3)	69·4 (54·9–81·7)	69·5 (53·5–81·6)	1·78 (-14·6–21·4)	0·146 (-11·9–13·7)
Diet high in processed meat	72·3 (47·8–95)	81·7 (60·6–99·2)	81·5 (59·5–99·1)	13·1 (2·75–34·3)	-0·209 (-5·2–5·04)	73·7 (48·7–95·5)	84 (65·4–99)	82·7 (63·1–98·7)	14 (2·64–37·9)	-1·56 (-7·02–2·82)
Diet high in sugar-sweetened beverages	50·8 (30·2–70·2)	69·1 (50·5–84·2)	68·5 (49·3–85·2)	36·1 (-1·47–102)	-0·872 (-20·4–19·5)	55 (35·2–73·5)	74·8 (55·7–89·2)	72·5 (53·5–88·4)	35·9 (-0·223–97)	-3·06 (-19·8–15·5)
Diet low in fiber	39 (24·7–55·3)	25·8 (14·4–39)	23·7 (12·7–36·4)	-34 (-51·4–-16·8)	-8·08 (-27·5–13)	39·2 (23·8–55·6)	30·8 (18·1–45·3)	26·7 (14·6–40·7)	-21·3 (-37·8–-0·926)	-13·5 (-31·4–3·52)
Diet low in seafood omega-3 fatty acids	79·7 (61·1–99·5)	74·9 (56·3–97·9)	74·8 (57·6–98·5)	-6·04 (-22·2–12·4)	-0·0613 (-15·4–19·6)	82·8 (66·5–99·7)	78·9 (60·1–99·1)	78·6 (59·6–99)	-4·8 (-20·6–9·47)	-0·324 (-14·9–16·2)
Diet low in polyunsaturated fatty acids	11·5 (3·41–36·5)	6·2 (1·71–24·3)	6·1 (1·71–24·4)	-46 (-76·3–-5·93)	-1·74 (-21·5–18·1)	--	--	--	--	--
Diet high in trans fatty acids	89·5 (82·1–100)	87·9 (79·2–100)	87·4 (78·1–100)	-1·87 (-9·82–6·07)	-0·471 (-8·34–7·84)	--	--	--	--	--
Diet high in sodium	18·3 (4·49–41·1)	39·7 (11·5–64·3)	39·3 (11–64·6)	116 (39·1–418)	-1·1 (-25·8–27·2)	--	--	--	--	--
Low physical activity	2·27 (0·848–4·89)	1·94 (0·798–4·15)	1·79 (0·61–4·09)	-14·5 (-44·6–28·3)	-7·62 (-32·5–17·9)	5·09 (2·46–9·69)	4·94 (2·59–9·04)	4·68 (2·34–9·21)	-3 (-26·1–29·8)	-5·3 (-18·9–7·86)
Occupational asthmagens	23·1 (19–28·5)	20·1 (16·8–23·9)	21·1 (17·3–25·8)	-13 (-26·3–1·04)	4·84 (-4·93–18·6)	11·6 (9·44–14·3)	11·6 (9·65–13·8)	11·8 (9·59–14·5)	-0·0709 (-14·8–17·7)	1·91 (-10–15·6)
Occupational particulate matter, gases, and fumes	9·15 (6·48–13·3)	8·31 (5·93–11·9)	8 (5·69–11·4)	-9·19 (-15–-3·37)	-3·76 (-7·19–0·00999)	5·25 (3·66–7·92)	4·87 (3·47–7·05)	4·63 (3·3–6·87)	-7·27 (-15·9–2·28)	-4·91 (-10·2–0·342)
Occupational noise	8·24 (7·53–9·13)	7·92 (7·35–8·64)	7·7 (7·19–8·44)	-3·89 (-7·58–0·375)	-2·69 (-4·74–-0·492)	5·92 (5·19–6·72)	6·21 (5·7–6·79)	6·22 (5·79–6·79)	4·95 (-1–12·4)	0·0264 (-2·92–3·27)
Occupational injuries	--	--	--	--	--	--	--	--	--	--
Occupational ergonomic factors	11·6 (9·51–14·2)	10·5 (8·86–12·5)	10·9 (9·05–13·2)	-9·83 (-19·1–0·693)	3·87 (-3·57–13·4)	6·47 (5·1–8·23)	6·62 (5·37–8·18)	6·95 (5·51–8·71)	2·36 (-13·2–21·4)	4·88 (-7·21–19)
Childhood sexual abuse	7·4 (5·05–10·5)	6·97 (4·6–9·93)	6·85 (4·54–9·67)	-5·83 (-12·2–5·83)	-1·68 (-8·51–5·53)	12·3 (8·94–17·3)	17·4 (12·9–24)	17·8 (13·1–24·7)	41·9 (30·7–54·8)	2·17 (-5–11)
Intimate partner violence	--	--	--	--	--	24 (13·1–33)	22·8 (12·7–31·1)	24·1 (13·6–32·9)	-5·18 (-12·7–7·47)	5·85 (-3·63–24·5)
Non-exclusive breastfeeding	26·5 (22–31·4)	25 (21·3–29)	25 (20·5–29·5)	-5·57 (-19·4–11·7)	0·0204 (-12·9–15·3)	--	--	--	--	--
Discontinued breastfeeding	27·7 (26·7–28·6)	25·2 (23·8–26·4)	25·8 (24·4–26·9)	-9·24 (-14·7–-3·87)	2·46 (-3·21–8·64)	27·8 (26·8–28·7)	25·1 (23·8–26·4)	25·8 (24·4–26·9)	-9·43 (-14·9–-4·08)	2·42 (-3·25–8·6)
Diet low in calcium	13·7 (6·55–25·2)	11·4 (5·66–20·7)	10·9 (5·35–19·8)	-16·8 (-28·2–-3·1)	-4·13 (-14·1–4·61)	8·93 (3·73–16·8)	8·05 (3·71–14·9)	7·96 (3·57–15·1)	-9·9 (-26·6–11·6)	-1·11 (-13–11·1)
Occupational exposure to asbestos	12 (10·9–13·1)	9·56 (8·97–10·2)	7·98 (5·98–10·2)	-20·4 (-28·5–-11·4)	-16·5 (-37·2–6·52)	1·91 (1·11–2·34)	1·68 (0·935–1·98)	1·63 (0·9–2·29)	-12·1 (-28–7·45)	-2·93 (-29·8–28·3)
Occupational exposure to arsenic	0·53 (0–1·26)	0·515 (0–1·24)	0·546 (0–1·31)	-2·87 (-16·6–NA)	6·03 (-5·24–NA)	0·494 (0–1·18)	0·483 (0–1·17)	0·495 (0–1·22)	-2·27 (-19·9–NA)	2·44 (-9·91–NA)
Occupational exposure to benzene	0·917 (0·0533–2·6)	0·925 (0·0547–2·7)	0·942 (0·0581–2·69)	0·933 (-7·71–14·7)	1·77 (-5·02–13·8)	1·25 (0·0717–3·57)	1·44 (0·0868–4·02)	1·5 (0·0884–4·39)	14·8 (3·65–34·4)	4·22 (-4·42–16·1)
Occupational exposure to beryllium	0·0243 (0·0229–0·0258)	0·0245 (0·024–0·025)	0·0256 (0·0242–0·027)	0·554 (-5·43–7·06)	4·72 (-0·853–10·3)	0·0269 (0·0245–0·0291)	0·0282 (0·0276–0·0288)	0·0292 (0·0272–0·0313)	4·96 (-3·44–15·7)	3·44 (-3·62–10·6)
Occupational exposure to cadmium	0·0621 (0·0558–0·0684)	0·0609 (0·0552–0·0666)	0·0643 (0·0568–0·0724)	-1·96 (-14·2–12·9)	5·69 (-4·47–17·2)	0·059 (0·052–0·0663)	0·0584 (0·0522–0·0654)	0·0597 (0·0519–0·0685)	-0·949 (-16·6–16)	2·24 (-8·73–15·7)
Occupational exposure to chromium	0·15 (0·137–0·164)	0·151 (0·139–0·162)	0·158 (0·142–0·175)	0·37 (-10·3–13·4)	5·09 (-3·7–14·9)	--	--	--	--	--
Occupational exposure to diesel engine exhaust	0·725 (0·67–0·785)	0·794 (0·743–0·846)	0·821 (0·757–0·893)	9·51 (-0·865–21·1)	3·39 (-4·23–11·5)	0·655 (0·581–0·736)	0·698 (0·64–0·767)	0·701 (0·622–0·786)	6·49 (-7·45–22·9)	0·449 (-9·27–11·4)
Occupational exposure to formaldehyde	0·315 (0·281–0·349)	0·301 (0·271–0·331)	0·314 (0·278–0·355)	-4·45 (-17–10·4)	4·3 (-5·65–16·3)	0·293 (0·259–0·329)	0·296 (0·268–0·327)	0·302 (0·268–0·344)	0·975 (-13·7–16·3)	2·36 (-7·43–14·5)
Occupational exposure to nickel	0·434 (0–1·91)	0·405 (0–1·87)	0·431 (0–1·85)	-6·75 (-19·9–NA)	6·59 (-5·18–NA)	0·385 (0–1·64)	0·366 (0–1·7)	0·374 (0–1·67)	-5·01 (-22·3–NA)	2·2 (-10·8–NA)
Occupational exposure to polycyclic aromatic hydrocarbons	0·298 (0·273–0·324)	0·3 (0·279–0·322)	0·315 (0·285–0·345)	0·694 (-9·58–12·9)	4·81 (-2·93–14·2)	0·313 (0·281–0·346)	0·332 (0·311–0·355)	0·34 (0·309–0·375)	5·97 (-6·22–18·9)	2·57 (-5·75–12·1)
Occupational exposure to silica	3·87 (0·533–11·9)	3·55 (0·514–11·4)	3·8 (0·564–12·1)	-8·38 (-21·3–9·21)	7·11 (-4·77–22·9)	3·2 (0·433–10·3)	2·89 (0·42–9·3)	2·93 (0·424–9·04)	-9·7 (-26·7–12·8)	1·48 (-12·2–23·1)
Occupational exposure to sulfuric acid	0·953 (0·198–3·01)	1·02 (0·209–3·39)	1·06 (0·218–3·47)	7·38 (-6·7–24·1)	3·45 (-6·61–15·1)	0·865 (0·182–2·84)	0·983 (0·198–3·26)	0·979 (0·209–3·22)	13·7 (-6·04–35·6)	-0·39 (-14·5–15·4)
Unsafe sex	--	--	--	--	--	--	--	--	--	--
Occupational exposure to trichloroethylene	0·0879 (0·0819–0·0947)	0·0912 (0·0873–0·0957)	0·0953 (0·0889–0·103)	3·74 (-4·41–13)	4·47 (-2·25–11·9)	0·085 (0·077–0·094)	0·0879 (0·0824–0·094)	0·0895 (0·0812–0·0983)	3·5 (-7·39–16·7)	1·76 (-6·97–11·1)
No access to handwashing facility	3·25 (3–3·5)	3·07 (2·85–3·31)	3·04 (2·81–3·28)	-5·71 (-13·6–3·07)	-0·89 (-6·65–4·6)	3·25 (3–3·5)	3·07 (2·85–3·31)	3·04 (2·81–3·28)	-5·71 (-13·6–3·07)	-0·89 (-6·65–4·6)
Child wasting	0·746 (0·485–1·03)	0·722 (0·471–0·987)	0·722 (0·474–0·978)	-3·12 (-13·5–7·91)	-0·0194 (-7·27–8·03)	0·77 (0·509–1·05)	0·758 (0·502–1·03)	0·755 (0·503–1·04)	-1·44 (-11·2–9·61)	-0·393 (-7·71–7·89)
Child stunting	2·31 (1·13–3·35)	2·01 (1·03–3·04)	1·94 (1–2·95)	-13·2 (-31·4–9·43)	-3·12 (-16·8–12·5)	1·88 (0·925–2·85)	1·73 (0·879–2·63)	1·7 (0·857–2·62)	-7·83 (-28·5–18)	-1·62 (-17·5–18·6)
Chewing tobacco	1·9 (1·45–2·55)	1·89 (1·32–2·59)	1·93 (1·35–2·71)	-0·45 (-32·5–44·9)	2·11 (-20·4–32·2)	0·274 (0·174–0·411)	0·304 (0·189–0·466)	0·322 (0·196–0·494)	10·8 (-36·4–86·4)	5·8 (-29·2–53·4)
Diet low in legumes	72·5 (33·7–98·5)	75·4 (42·1–99·4)	75·5 (41·3–99·4)	4 (-0·3–21·9)	0·176 (-5·63–7·76)	70·8 (32·6–98·5)	73·6 (36·9–99·4)	74·2 (40·7–99·5)	4 (-0·301–24)	0·685 (-4·07–10·3)
Short gestation	32·4 (26·9–38·4)	28·3 (23·7–33·2)	26·2 (21·2–31·8)	-12·6 (-27·5–7·47)	-7·47 (-25·9–13·7)	30·2 (25·4–35·1)	25 (21·6–29)	23·2 (19·5–27·6)	-17·2 (-30·5–-1·3)	-7·27 (-22·7–9·96)
Low birth weight	12·6 (10·7–15)	11·8 (10·1–13·8)	11·8 (9·84–14·2)	-6·52 (-22·9–16·4)	-0·03 (-20·7–23·2)	13·7 (11·7–15·8)	12·8 (11·2–14·6)	12·2 (10·4–14·3)	-6·55 (-22–12·5)	-4·68 (-20·3–13·5)
High temperature	23·9 (13·7–35·4)	29·5 (19·1–41·2)	26·8 (16·8–38·7)	23·5 (10–45·2)	-9·22 (-16·8–-4·9)	23·9 (13·7–35·4)	29·5 (19·1–41·2)	26·8 (16·8–38·7)	23·5 (10–45·2)	-9·22 (-16·8–-4·9)
Low temperature	33·9 (29·6–39·2)	36·1 (32·3–41·1)	35·8 (31·9–40·9)	6·64 (4·17–9·44)	-0·755 (-1·83–0·26)	33·9 (29·6–39·2)	36·1 (32·3–41·1)	35·8 (31·9–40·9)	6·64 (4·17–9·44)	-0·755 (-1·83–0·26)
Kidney dysfunction	20·3 (14·6–27·2)	20·8 (14·9–28)	20·7 (15–28·1)	2·45 (-1·29–6·32)	-0·121 (-3·8–4·01)	22·2 (15·8–29·9)	22·9 (16·5–30·8)	23·6 (17·2–31·6)	3·18 (-1·8–9·3)	3·05 (-1·34–8·29)
Bullying victimization	5·93 (2·47–12·2)	9·1 (3·6–17·8)	8·8 (3·37–18·1)	53·4 (32·8–78·1)	-3·28 (-13·1–2·92)	4·3 (1·74–8·97)	8 (3·14–15·9)	7·87 (2·94–16·2)	85·9 (59·2–115)	-1·7 (-12·6–5·39)
High LDL cholesterol	68·2 (64·1–73)	34·4 (30·8–38·1)	30·9 (27·4–34·6)	-49·6 (-55·1–-44·5)	-10·1 (-15·1–-5·54)	65·6 (62–69·2)	34·2 (31–37·8)	31·7 (28·2–35·4)	-47·8 (-52·6–-43·3)	-7·26 (-11·6–-3·19)

Supplementary Results Table 1. New Mexico age-standardised Summary Exposure Values (SEVs) for most-detailed risk factors and percentage change, by sex, 1990, 2010, and 2019. Data in parentheses are 95% uncertainty intervals.

	Male					Female				
	1990	2010	2019	Percentage change 1990-2010	Percentage change 2010-2019	1990	2010	2019	Percentage change 1990-2010	Percentage change 2010-2019
Unsafe sanitation	9·85 (4·82–16·8)	4·99 (2·19–9·83)	3·89 (1·73–7·27)	-49·3 (-79·5–-22·7)	-22 (-60–52·3)	--	--	--	--	--
Ambient particulate matter pollution	5·07 (1·04–12·3)	2·32 (0·79–4·53)	1·87 (0·494–4·02)	-54·2 (-85·8–62·4)	-19·2 (-41·4–-10)	5·08 (1·1–12·2)	2·36 (0·842–4·54)	1·91 (0·528–4·02)	-53·5 (-85–57·1)	-18·9 (-41·5–-9·68)
Household air pollution from solid fuels	0·23 (0·0414–0·676)	0·0875 (0·0104–0·312)	0·0574 (0·0055–0·226)	-62 (-90·1–7·83)	-34·4 (-72·2–20·5)	0·305 (0·0551–0·875)	0·118 (0·0153–0·404)	0·0778 (0·00743–0·3)	-61·2 (-90–8·71)	-34·3 (-72·1–21·8)
Ambient ozone pollution	73·2 (32·6–100)	78·5 (35·4–100)	77·7 (35·1–100)	7·25 (-1·28–21·2)	-0·98 (-11·5–9·48)	--	--	--	--	--
Residential radon	20·4 (3·18–60·6)	20·4 (3·18–60·6)	20·4 (3·18–60·6)	0 (0–0)	0 (0–0)	20·4 (3·18–60·6)	20·4 (3·18–60·6)	20·4 (3·18–60·6)	0 (0–0)	0 (0–0)
Lead exposure	55·8 (27·4–78·6)	34·4 (13·6–56·8)	24·6 (7·95–44·7)	-38·4 (-55·1–-25·8)	-28·5 (-42·8–-20·6)	31·6 (3·48–60·2)	19·9 (1·51–44·2)	14·2 (0·64–34·8)	-36·8 (-68–-23·3)	-28·7 (-61·8–-20·5)
Child underweight	1 (0·579–1·52)	0·981 (0·547–1·47)	0·96 (0·513–1·45)	-2·23 (-19·6–18·6)	-2·2 (-14·9–12·6)	0·834 (0·464–1·26)	0·861 (0·488–1·32)	0·884 (0·493–1·36)	3·23 (-16·8–27·9)	2·7 (-11·8–19·7)
Iron deficiency	--	--	--	--	--	12·3 (10·3–14·4)	12·6 (10·6–14·7)	12·8 (10·6–15·2)	2·27 (-9·48–16·3)	2·01 (-8·02–13·3)
Vitamin A deficiency	1·25 (0·321–3·15)	1·5 (0·419–3·79)	1·02 (0·269–2·71)	19·8 (-67·7–421)	-32·2 (-69·7–61·6)	2·19 (0·798–4·64)	2·13 (0·787–4·67)	1·79 (0·699–3·91)	-2·75 (-62·4–207)	-16·3 (-53·5–65)
Zinc deficiency	2·56 (0–8·26)	1·33 (0–4·29)	1·42 (0–4·63)	-47·9 (-100–NA)	6·26 (-11·5–NA)	2·63 (0–8·47)	1·36 (0–4·48)	1·38 (0–4·4)	-48·4 (-100–NA)	1·99 (-16·1–NA)
Smoking	25·5 (22·8–28·3)	19 (17·1–20·9)	17·1 (15·3–18·9)	-25·4 (-30·8–-19·8)	-10 (-13·9–-6·33)	18·3 (15·9–21)	14 (12·5–15·6)	11·9 (10·6–13·4)	-23·8 (-31·5–-14·6)	-14·5 (-18·8–-10·2)
Secondhand smoke	38·2 (35·8–41·1)	24 (22·4–25·7)	24·2 (21·6–26·9)	-37·3 (-43·7–-30·7)	1·1 (-8·44–10·4)	35 (32·8–37·1)	23 (21·8–24·1)	23 (21·3–25·3)	-34·4 (-39·5–-28·9)	1·08 (-6·16–8·4)
Alcohol use	13·4 (7·92–19·3)	14·5 (8·1–21·3)	14·5 (8·79–22)	7·83 (-26·1–54·9)	0·408 (-21·7–28·4)	4·38 (0·9–9·4)	5·3 (1·6–10·8)	5·06 (1·44–10·3)	21 (-41·2–226)	-4·45 (-46·8–61·3)
Drug use	0·834 (0·49–1·4)	1·36 (0·964–1·9)	1·67 (1·24–2·26)	62·9 (26·1–113)	23·1 (7·96–42·5)	0·477 (0·305–0·751)	0·981 (0·729–1·29)	1·34 (1·03–1·72)	106 (59·4–160)	36·6 (18·5–55·3)
High fasting plasma glucose	14·2 (11·9–17)	18·8 (16·4–21·6)	20·3 (17·8–23·4)	32·3 (9·2–60·7)	7·78 (-5·65–23·1)	11·7 (9·65–14·3)	15·6 (13·6–17·7)	16·7 (14·6–18·9)	33·2 (9·56–61)	7·25 (-4·77–21·6)
High systolic blood pressure	24·3 (18·8–29·8)	15·9 (13·8–18)	19·7 (15·3–24·5)	-34·5 (-46·9–-17·5)	23·5 (-0·31–54)	21·7 (15·9–28·3)	12·7 (11·2–14·2)	17 (12·3–22·8)	-41·3 (-56–-19·6)	33·6 (-2·27–76·7)
High body-mass index	22 (15·3–30·8)	36·6 (27·1–48·2)	38·1 (28·1–49·8)	66·4 (44·6–97·6)	3·96 (-2·75–11·3)	27·3 (19·1–37·4)	41·1 (31·4–50·5)	42·2 (32·8–51·7)	50·6 (29–78·3)	2·71 (-3·62–10·6)
Low bone mineral density	12·2 (7·06–19·3)	9·33 (5·12–15·5)	11·8 (6·83–18·6)	-23·7 (-44·5–2·86)	26·1 (-2·45–69·8)	16·1 (10·7–23·2)	16·5 (10·9–23·8)	19·1 (12·9–26·3)	3 (-18·6–32·1)	15·4 (-7·47–48·7)
Diet low in fruits	53·6 (39·8–68·2)	44 (31·2–59)	43·7 (31·5–58·5)	-17·9 (-30·6–-6·8)	-0·606 (-13·9–13·7)	--	--	--	--	--
Diet low in vegetables	32 (16·5–54)	44·1 (26·5–64·3)	43·1 (25·5–63·2)	37·9 (10·9–98)	-2·37 (-14·2–8·81)	29·2 (13·7–49·2)	46·4 (27·3–67·5)	46·4 (27·3–67·2)	59·3 (20·8–148)	-0·0231 (-9·89–11·5)
Diet low in whole grains	78·6 (68·3–89)	80·6 (71·1–89·7)	80·7 (71–89·8)	2·55 (-1·12–8·14)	0·161 (-2·42–2·73)	--	--	--	--	--
Diet low in nuts and seeds	30·5 (8·69–57·7)	26·3 (8·38–56·1)	23·1 (6·64–54·9)	-13·8 (-44·7–47·2)	-12·1 (-39·9–15·3)	26·2 (7·24–55·9)	23·2 (7·27–54·9)	21·4 (6·57–54·3)	-11·6 (-43–48·8)	-7·51 (-32·9–20·2)
Diet low in milk	51·3 (27·1–71)	46·4 (23·2–66·8)	46·4 (23·6–67·2)	-9·53 (-27·4–2·7)	0·0351 (-10·5–13·1)	51 (26·9–71·7)	46·3 (22·2–66·4)	46·6 (22·6–67·1)	-9·17 (-25·5–3·4)	0·642 (-10·3–13·1)
Diet high in red meat	76·1 (62·7–86·9)	73·8 (60·4–85·6)	73·2 (59·2–84·9)	-3·03 (-18·5–14·5)	-0·74 (-13·1–11·7)	77 (63·7–87·6)	73·1 (58·3–84·6)	72·5 (57·7–84·5)	-5·04 (-20·6–11·8)	-0·876 (-12·6–11·5)
Diet high in processed meat	77·7 (53·5–98)	85·8 (66·3–99·7)	85·4 (66·4–99·7)	10·3 (1·14–29·2)	-0·362 (-5·63–3·91)	78·9 (55·6–97·6)	88·4 (71·5–99·6)	86·9 (68·4–99·5)	12·1 (1·29–34·1)	-1·68 (-8·24–2·2)
Diet high in sugar-sweetened beverages	43·5 (25·7–63·4)	58·6 (40·2–76·7)	58·2 (38·9–75·6)	34·7 (-4·38–108)	-0·7 (-23·5–23·7)	47·3 (27·2–67·7)	64·2 (44·4–82·7)	62·4 (41·8–80)	35·8 (-5·94–114)	-2·71 (-23–21·2)
Diet low in fiber	46·2 (29·9–61·8)	32·9 (19·7–47·2)	31·1 (17·8–45·4)	-28·8 (-45·1–-12·2)	-5·57 (-22·4–12·4)	46·5 (29·2–62·9)	38·2 (24·2–52·5)	34·3 (20·3–49·6)	-17·8 (-33·5–-1·35)	-10·2 (-25·3–4·9)
Diet low in seafood omega-3 fatty acids	83·1 (66·5–99·8)	79·3 (60·4–98·7)	79·4 (61·7–98·4)	-4·64 (-20·3–10)	0·141 (-13·9–17·2)	86 (71–99·9)	83·5 (67·3–99·2)	83·6 (67·2–99·4)	-2·93 (-13·5–6·54)	0·151 (-11·2–11·3)
Diet low in polyunsaturated fatty acids	15·7 (4·64–45·1)	8·17 (2·34–28·7)	8·01 (2·33–28·9)	-48 (-74·3–-8·34)	-1·94 (-24·1–24·6)	--	--	--	--	--
Diet high in trans fatty acids	89·5 (81·6–100)	87·9 (79·4–100)	87·3 (77·6–100)	-1·71 (-9·86–6·43)	-0·727 (-8·42–7·86)	88·4 (80·8–100)	86·6 (78–100)	86·2 (77·5–100)	-2·02 (-9·94–5·73)	-0·556 (-8·43–7·85)
Diet high in sodium	18·8 (4·32–42)	39·8 (10·6–64·9)	39·3 (10·1–65·4)	112 (37·7–377)	-1·23 (-27·5–30·3)	--	--	--	--	--
Low physical activity	2·11 (0·779–4·56)	1·78 (0·733–4·09)	1·74 (0·603–4·04)	-15·4 (-46·1–25·6)	-2·34 (-28·6–28·1)	4·89 (2·41–9·66)	4·66 (2·44–8·64)	4·67 (2·33–9·21)	-4·68 (-28·5–32·7)	0·0863 (-12·6–13·8)
Occupational asthmagens	20·9 (17·7–24·7)	18·1 (15·4–21·3)	18·7 (15·6–22·6)	-13·6 (-23·9–-3·4)	3·15 (-5·56–12·5)	10·3 (8·48–12·5)	9·83 (8·12–11·9)	9·91 (8·01–12·1)	-4·7 (-18·7–10·6)	0·891 (-10·6–13·8)
Occupational particulate matter, gases, and fumes	8·35 (5·86–12)	7·85 (5·63–11·3)	7·58 (5·45–10·8)	-6·03 (-11·7–0·317)	-3·42 (-7·24–0·0633)	4·57 (3·16–6·82)	4·47 (3·16–6·36)	4·31 (3·08–6·1)	-2·21 (-11·2–8·8)	-3·51 (-8·98–2·21)
Occupational noise	8·63 (7·86–9·47)	8·09 (7·49–8·75)	7·75 (7·23–8·38)	-6·26 (-10·1–-1·61)	-4·24 (-6·54–-1·95)	5·54 (4·82–6·27)	5·81 (5·3–6·42)	5·8 (5·39–6·35)	4·99 (-1·61–12·7)	-0·249 (-3·46–3·21)
Occupational injuries	--	--	--	--	--	--	--	--	--	--
Occupational ergonomic factors	12·8 (10·5–15·6)	10·7 (9·07–12·8)	11·1 (9·22–13·5)	-15·8 (-27–-4·37)	3·62 (-5·39–12·8)	7·4 (5·8–9·27)	6·92 (5·64–8·48)	7·22 (5·81–9·09)	-6·55 (-21·2–11·6)	4·32 (-7·77–17·2)
Childhood sexual abuse	7·4 (5·05–10·5)	6·97 (4·6–9·93)	6·85 (4·54–9·67)	-5·83 (-12·2–5·83)	-1·68 (-8·51–5·53)	12·3 (8·94–17·3)	17·4 (12·9–24)	17·8 (13·1–24·7)	41·9 (30·7–54·8)	2·17 (-5–11)
Intimate partner violence	--	--	--	--	--	30·1 (16·8–39·4)	28·4 (16·5–37·4)	29·4 (15·7–38·5)	-5·57 (-13·1–9·18)	3·37 (-6·31–18·3)
Non-exclusive breastfeeding	26·1 (21·5–30·5)	24·8 (21·2–28·6)	24·8 (20·4–29·2)	-5·1 (-19·3–11·9)	0·155 (-14–16·7)	--	--	--	--	--
Discontinued breastfeeding	26·9 (25·9–27·8)	25·3 (24–26·6)	25·7 (24·4–26·9)	-5·65 (-11·3–-0·117)	1·61 (-4·16–7·42)	27 (26–27·9)	25·4 (24–26·6)	25·8 (24·5–27)	-5·9 (-11·5–-0·393)	1·53 (-4·23–7·33)
Diet low in calcium	18 (8·38–33·5)	15·3 (7·39–28·1)	15·4 (7·46–28·6)	-14·9 (-25·7–-3·78)	0·434 (-8·63–8·94)	13·5 (5·67–25·6)	12·3 (5·18–23·3)	12·8 (5·36–24·5)	-8·6 (-22·3–8·39)	3·75 (-4·57–13·1)
Occupational exposure to asbestos	6·6 (5·89–7·32)	5·51 (4·93–6·09)	5·52 (3·91–7·52)	-16·6 (-28·3–-3·93)	0·193 (-29·2–37·5)	1·32 (1·06–2·03)	1·23 (0·977–1·85)	1·27 (0·878–1·95)	-6·27 (-20·4–11·4)	2·84 (-22·6–37·3)
Occupational exposure to arsenic	0·38 (0–0·898)	0·399 (0–0·945)	0·427 (0–1·02)	5·08 (-10·6–NA)	6·84 (-4·79–NA)	0·391 (0–0·923)	0·404 (0–0·958)	0·417 (0–0·98)	3·19 (-14–NA)	3·22 (-9·61–NA)
Occupational exposure to benzene	0·825 (0·0473–2·42)	0·799 (0·0471–2·35)	0·828 (0·048–2·38)	-3·16 (-12·2–9·28)	3·61 (-4·93–15·9)	1·13 (0·0643–3·23)	1·24 (0·0701–3·42)	1·13 (0·0692–3·76)	9·89 (-1·93–28·6)	5·02 (-4·32–18·7)
Occupational exposure to beryllium	0·0229 (0·0215–0·0244)	0·0223 (0·0218–0·0228)	0·0236 (0·0222–0·0251)	-2·85 (-8·98–3·48)	5·81 (-0·123–12·4)	--	--	--	--	--
Occupational exposure to cadmium	0·046 (0·0417–0·0511)	0·0483 (0·044–0·053)	0·0515 (0·0456–0·0579)	4·89 (-8·82–19·3)	6·62 (-3·79–17·7)	0·0479 (0·0424–0·0543)	0·0495 (0·0449–0·0545)	0·0511 (0·045–0·0581)	3·37 (-11·8–19·8)	3·25 (-8·63–15·4)
Occupational exposure to chromium	0·114 (0·104–0·125)	0·121 (0·112–0·131)	0·129 (0·116–0·143)	6·53 (-5·62–19)	6·44 (-2·88–16·2)	--	--	--	--	--
Occupational exposure to diesel engine exhaust	0·785 (0·717–0·856)	0·785 (0·731–0·837)	0·831 (0·762–0·902)	0·0228 (-10·1–10·7)	5·78 (-2·11–14·4)	0·656 (0·581–0·742)	0·667 (0·607–0·738)	0·671 (0·594–0·755)	1·67 (-11·7–18·2)	0·7 (-10·2–11·7)
Occupational exposure to formaldehyde	0·225 (0·202–0·251)	0·232 (0·21–0·256)	0·244 (0·214–0·279)	3·19 (-11·5–18·1)	5·29 (-5·39–16·9)	0·231 (0·205–0·262)	0·242 (0·22–0·265)	0·251 (0·22–0·283)	4·87 (-9·68–21·7)	3·41 (-7·75–15·2)
Occupational exposure to nickel	0·386 (0–1·68)	0·342 (0–1·51)	0·36 (0–1·55)	-11·2 (-24–NA)	5·2 (-6·19–NA)	0·314 (0–1·39)	0·307 (0–1·37)	0·319 (0–1·43)	-2·18 (-19·1–NA)	3·82 (-10·2–NA)
Occupational exposure to polycyclic aromatic hydrocarbons	0·234 (0·216–0·255)	0·245 (0·229–0·264)	0·259 (0·235–0·287)	4·8 (-6·59–16·2)	5·7 (-3·27–15·1)	0·263 (0·238–0·293)	0·286 (0·268–0·304)	0·295 (0·267–0·325)	8·54 (-3·65–22·5)	3·15 (-5·88–12·4)
Occupational exposure to silica	4·25 (0·615–13·6)	3·35 (0·486–10·6)	3·51 (0·473–10·9)	-21·1 (-33·8–-6·69)	4·53 (-6·73–18·5)	2·78 (0·374–8·91)	2·55 (0·335–7·94)	2·6 (0·354–8·27)	-8·58 (-25·2–13·7)	2·16 (-11·7–19·1)
Occupational exposure to sulfuric acid	0·773 (0·167–2·48)	0·902 (0·185–2·91)	0·938 (0·195–3·03)	16·7 (-0·504–39·2)	3·9 (-8·54–16·8)	0·829 (0·179–2·77)	1·02 (0·217–3·29)	1·01 (0·219–3·19)	23·1 (-0·751–53·2)	-0·859 (-16·9–17·6)
Unsafe sex	--	--	--	--	--	--	--	--	--	--
Occupational exposure to trichloroethylene	0·0715 (0·0663–0·0775)	0·0775 (0·0738–0·0817)	0·0826 (0·0765–0·0895)	8·34 (-1·15–18·4)	6·61 (-0·982–15·2)	0·0742 (0·067–0·0827)	0·0774 (0·0729–0·0825)	0·0793 (0·0719–0·0872)	4·39 (-7–17·8)	2·45 (-6·9–11·7)
No access to handwashing facility	3·49 (3·23–3·78)	3·16 (2·91–3·41)	3·12 (2·86–3·38)	-9·62 (-17·5–-0·738)	-1·27 (-6·92–4·47)	3·49 (3·23–3·78)	3·16 (2·91–3·41)	3·12 (2·86–3·38)	-9·62 (-17·5–-0·738)	-1·27 (-6·92–4·47)
Child wasting	0·754 (0·509–1·04)	0·732 (0·48–0·995)	0·731 (0·471–0·985)	-3·02 (-12·3–7·04)	-0·0751 (-7·53–7·34)	--	--	--	--	--
Child stunting	2·59 (1·4–3·74)	2·14 (1·1–3·2)	2·09 (1·06–3·1)	-17·5 (-35·8–6·75)	-2·35 (-16·9–14·4)	2·05 (1·02–3·14)	1·84 (0·879–2·76)	1·82 (0·897–2·75)	-10·2 (-29·1–12)	-1·15 (-15·9–15·4)
Chewing tobacco	4·58 (3·57–5·81)	4·34 (3·08–5·92)	4·19 (2·91–5·77)	-5·1 (-34·8–35·7)	-3·48 (-25·4–26·4)	--	--	--	--	--
Diet low in legumes	74·9 (41·3–99·4)	78 (47·9–99·8)	78·2 (48·8–99·8)	4·06 (-0·0788–21·8)	0·375 (-4·74–7·16)	73·3 (36·6–99·3)	76·4 (43·7–99·7)	76·9 (46·6–99·7)	4·19 (-0·228–22·7)	0·771 (-4·16–8·25)
Short gestation	29·6 (24·4–35·8)	27 (22·2–32·6)	26·5 (21·1–33·2)	-8·81 (-26·6–14·3)	-1·92 (-21·1–27·3)	21·5 (17·8–26)	22·1 (18·4–26·4)	21·9 (18·2–25·6)	2·87 (-20·1–33·5)	-1·03 (-20·6–21·4)
Low birth weight	12·7 (10·5–15·3)	12 (9·92–14·4)	12·6 (10·2–15·7)	-5·75 (-25–19)	5·07 (-17·5–37·6)	11·3 (9·48–13·4)	11·8 (9·81–13·8)	12 (10·1–14)	3·84 (-20·2–34·2)	1·79 (-18·8–24·3)
High temperature	29·5 (21·9–38·5)	28 (19·9–38)	28·2 (20·3–38)	-5·31 (-11·2–1·16)	1·01 (-1·26–3·7)	29·5 (21·9–38·5)	28 (19·9–38)	28·2 (20·3–38)	-5·31 (-11·2–1·16)	1·01 (-1·26–3·7)
Low temperature	36 (32·2–41·4)	36·5 (32·5–42·2)	36·7 (32·8–42·1)	1·46 (0·0178–2·83)	0·514 (-0·275–1·42)	36 (32·2–41·4)	36·5 (32·5–42·2)	36·7 (32·8–42·1)	1·46 (0·0178–2·83)	0·514 (-0·275–1·42)
Kidney dysfunction	19·2 (13·5–26·2)	20 (14·3–27·1)	20·1 (14·4–27·4)	4·59 (0·863–9·12)	0·395 (-3·68–4·39)	21·4 (15·2–28·9)	22·8 (16·2–30·7)	23·6 (17–31·5)	6·49 (2·16–11·9)	3·57 (-0·483–8·32)

Bullying victimization	5·91 (2·44–12·2)	8·97 (3·6–17·8)	8·75 (3·38–18)	51·7 (32·1–74·2)	-2·48 (-11·9–3·77)	--	--	--	--	--
High LDL cholesterol	66·1 (61·7–70·6)	34·2 (30·7–37·8)	30·5 (26·9–34·3)	-48·2 (-53·6–-43)	-10·9 (-15·9–-6·13)	63·6 (59·9–67·3)	34·4 (31·1–37·7)	31·6 (27·9–35·2)	-46 (-50·9–-41·4)	-8·14 (-12·2–-4·05)

Supplementary Results Table 1. New York age-standardised Summary Exposure Values (SEVs) for most-detailed risk factors and percentage change, by sex, 1990, 2010, and 2019. Data in parentheses are 95% uncertainty intervals.

	Male					Female				
	1990	2010	2019	Percentage change 1990-2010	Percentage change 2010-2019	1990	2010	2019	Percentage change 1990-2010	Percentage change 2010-2019
Unsafe sanitation	4·79 (2·2–8·59)	2·58 (1·18–4·9)	2·14 (0·941–4·23)	-46 (-79·1–38·8)	-17·4 (-56·6–55·9)	4·79 (2·2–8·59)	2·58 (1·18–4·9)	2·14 (0·941–4·23)	-46 (-79·1–38·8)	-17·4 (-56·6–55·9)
Ambient particulate matter pollution	10·9 (3·47–22·6)	5·94 (3·41–8·93)	4·06 (1·75–6·86)	-45·6 (-74·8–47·1)	-31·6 (-49·3—22·8)	10·9 (3·46–22·6)	5·94 (3·4–8·92)	4·06 (1·74–6·86)	-45·5 (-74·8–46)	-31·6 (-49·2—22·9)
Household air pollution from solid fuels	0·0113 (0·00234–0·0326)	0·00627 (0·000968–0·0209)	0·00416 (0·000552–0·0152)	-44·6 (-79·2–35·7)	-33·7 (-67·9–26·8)	0·0149 (0·00326–0·0428)	0·00831 (0·00128–0·0278)	0·00551 (0·000764–0·0206)	-44·4 (-79–36·3)	-33·7 (-67·7–27)
Ambient ozone pollution	63·5 (27·9–100)	49 (20·6–83·2)	44·1 (18–76·6)	-22·8 (-35·3—11·8)	-10·1 (-25·4–6·44)	--	--	--	--	--
Residential radon	22·1 (3·36–61·9)	22·1 (3·36–61·9)	22·1 (3·36–61·9)	0 (0–0)	0 (0–0)	22·1 (3·36–61·9)	22·1 (3·36–61·9)	22·1 (3·36–61·9)	0 (0–0)	0 (0–0)
Lead exposure	72·5 (47·1–88·8)	40·9 (20·5–60·9)	28 (11·7–45·3)	-43·6 (-57·5—30·1)	-31·5 (-42·9—24·7)	46·8 (13·9–73·2)	24·5 (3·5–48·9)	16·2 (1·29–35·4)	-47·6 (-75·8—31·1)	-34 (-64—26)
Child underweight	0·947 (0·543–1·43)	0·935 (0·513–1·42)	0·909 (0·506–1·38)	-1·3 (-17·3–18·9)	-2·74 (-15·2–12)	0·798 (0·422–1·22)	0·831 (0·454–1·27)	0·846 (0·467–1·29)	4·08 (-15·5–34·7)	1·88 (-11·8–17·8)
Iron deficiency	--	--	--	--	--	12·1 (10·1–14·3)	12·1 (10·3–14)	12·2 (10·2–14·4)	-0·549 (-11·8–12)	0·954 (-8·11–10·5)
Vitamin A deficiency	0·661 (0·196–1·76)	0·893 (0·245–2·26)	0·63 (0·171–1·71)	35 (-64·7–487)	-29·4 (-69·7–74·3)	1·36 (0·527–2·87)	1·37 (0·535–2·82)	1·18 (0·475–2·55)	0·781 (-63·2–166)	-13·7 (-52–59·4)
Zinc deficiency	1·87 (0–6·13)	0·999 (0–3·15)	0·991 (0–3·16)	-46·5 (-100–NA)	-0·833 (-22·8–NA)	1·91 (0–6·43)	1·01 (0–3·24)	0·971 (0–3·13)	-47 (-100–NA)	-4·17 (-25·6–NA)
Smoking	25·8 (23·2–28·6)	18·8 (16·9–20·8)	15·9 (14·2–17·8)	-27·2 (-31·9—21·9)	-15·5 (-18·8—11·8)	21·8 (19·2–24·7)	15 (13·3–16·7)	12 (10·5–13·5)	-31·4 (-37·6—24·7)	-20 (-23·9—15·9)
Secondhand smoke	41·6 (39–44·2)	24·5 (22·9–26·2)	24·9 (22·3–27·7)	-41·2 (-46·7—35·1)	1·74 (-6·79–11·1)	38 (36·1–40·2)	23 (21·8–24·2)	23·5 (21·5–25·5)	-39·6 (-44·5—34·3)	2·15 (-5·34–9·79)
Alcohol use	15·5 (10·3–21·2)	16·4 (10·4–23·3)	17·8 (11·2–24·9)	5·92 (-20·7–38·9)	8·66 (-11·8–35·6)	10·7 (6·73–15)	12·2 (7·82–17·4)	12·5 (7·78–18·3)	14·3 (-14·4–52·9)	2·61 (-18·8–27·1)
Drug use	0·803 (0·407–1·51)	0·984 (0·649–1·53)	1·24 (0·86–1·79)	22·6 (-6·25–67)	25·9 (9·48–46)	0·458 (0·264–0·799)	0·623 (0·435–0·912)	0·838 (0·618–1·15)	36·1 (9·9–76·4)	34·6 (14–57·8)
High fasting plasma glucose	12·6 (10·6–15·3)	18·9 (16·5–21·8)	19·6 (16·9–22·4)	50·1 (24·2–79)	3·7 (-9·46–20·2)	9·18 (7·87–10·8)	14·8 (13–16·8)	15·5 (13·5–17·7)	61·3 (38·3–87·1)	4·66 (-7·03–20·7)
High systolic blood pressure	25·4 (20·7–30·6)	15·8 (13·8–17·9)	19·4 (15·1–24)	-37·6 (-48·7—23·6)	22·5 (-0·384–50·6)	23·1 (17·4–29·4)	13 (11·5–14·7)	17·5 (12·9–23·2)	-43·5 (-56·2—25·8)	34·2 (-0·786–78·9)
High body-mass index	24·4 (17–33·8)	38·3 (28·5–50·2)	40·1 (29·8–51·7)	57·1 (38·3–85·3)	4·83 (-0·833–12·1)	29·1 (20·9–38·9)	42·6 (33·2–51·5)	43·7 (34·3–52·9)	46·4 (27·1–71·3)	2·49 (-3·32–9·67)
Low bone mineral density	11·9 (6·97–18·7)	9 (4·73–15·1)	11·3 (6·46–18·2)	-24·3 (-43·4—3·49)	25·2 (-2·98–69·7)	15·7 (10·1–22·8)	16·2 (10·6–23)	18·3 (12·4–25·3)	2·96 (-18·6–32)	13·5 (-11·2–46·8)
Diet low in fruits	50 (36·2–65·2)	45·5 (32·4–60·1)	44·7 (31·2–59·3)	-8·94 (-18·8–0·0628)	-1·77 (-14·7–10·8)	--	--	--	--	--
Diet low in vegetables	30·4 (15–52·4)	37·5 (21·4–57·5)	34·9 (19·9–55·7)	23·3 (2·72–67·3)	-6·82 (-19·8–6·54)	29·5 (13·7–50·1)	33·6 (18·4–53·5)	31·1 (16·8–50·2)	13·8 (-4·27–50)	-7·34 (-22·1–6·71)
Diet low in whole grains	79·1 (69·4–87·9)	81 (71·7–89·2)	80·9 (71·5–89·2)	2·43 (-1·2–6·96)	-0·128 (-2·66–2·55)	--	--	--	--	--
Diet low in nuts and seeds	24·5 (6–54·8)	24·8 (7·76–55·4)	19·5 (4·8–53·4)	1·44 (-37·6–95·2)	-21·5 (-48·5–3·55)	20·5 (5·45–53·5)	21 (6·56–53·8)	17·4 (4·73–52·4)	2·3 (-36·5–110)	-17·1 (-44·7–10·5)
Diet low in milk	45 (22·5–65·2)	39·1 (17·1–58·6)	37·4 (16·6–57·7)	-13·2 (-33·8–0·0634)	-4·29 (-19·4–11)	44·7 (20·2–64·5)	39·1 (17·3–58)	37·7 (16·3–56·5)	-12·3 (-32·2–2·63)	-3·69 (-17·7–10·8)
Diet high in red meat	77·2 (64·6–87·7)	78·5 (65·6–88·6)	78·7 (65·4–88·8)	1·69 (-12·3–16)	0·366 (-10·3–12·9)	74·9 (62·6–85·2)	74·5 (61·5–86)	74·9 (60·2–86·6)	-0·608 (-15·9–17·3)	0·618 (-11–12·9)
Diet high in processed meat	74·9 (49·7–96·8)	83·2 (63·8–99·2)	83·2 (62·9–99·1)	11·1 (1·59–28·5)	-0·0131 (-4·39–4·82)	76·1 (50·5–97·3)	85·3 (66·8–99·7)	84·2 (65·9–99·6)	12 (1·76–37)	-1·34 (-6·56–2·43)
Diet high in sugar-sweetened beverages	49·5 (31·3–67·3)	66·9 (48·7–82·7)	67·7 (48·4–83·5)	35·1 (-1·12–103)	1·21 (-17·4–20·7)	53·6 (33·5–72·7)	72·9 (54·4–87·9)	72·1 (51·3–87·7)	36 (1·69–100)	-1·05 (-19·1–19·3)
Diet low in fiber	40 (24·2–55·4)	27 (15·5–40·2)	23·8 (12·7–37)	-32·5 (-48·5—14·7)	-11·7 (-31·2–7·31)	40·3 (23·6–57·1)	32·2 (18·4–46·7)	26·7 (14·1–41·3)	-20·2 (-36·8—2·68)	-17 (-34·3—0·719)
Diet low in seafood omega-3 fatty acids	80·7 (62·6–99·2)	75·8 (57·1–98·9)	75·2 (56·2–99·3)	-6·11 (-22–11·3)	-0·776 (-18·7–17·4)	83·9 (67·5–99·9)	80·2 (62·2–99·1)	79·1 (61·5–99·3)	-4·44 (-18·5–10)	-1·35 (-14·3–14)
Diet low in polyunsaturated fatty acids	11·9 (3·5–35·9)	6·42 (1·87–24·3)	6·12 (1·74–24·4)	-45·9 (-75·6—6·12)	-4·72 (-27·1–11·2)	--	--	--	--	--
Diet high in trans fatty acids	89·5 (81·7–100)	88 (79·1–100)	87·5 (78·2–100)	-1·62 (-9·46–5·84)	-0·585 (-8·59–8·1)	88·4 (80·9–100)	86·7 (78·1–100)	86·2 (77·3–100)	-1·95 (-9·69–5·99)	-0·486 (-8·94–8·14)
Diet high in sodium	18·5 (4·27–42)	39·9 (11–65·1)	39·3 (9·95–64·4)	115 (41·5–410)	-1·54 (-30–29·8)	--	--	--	--	--
Low physical activity	2·33 (0·87–4·89)	2·09 (0·897–4·38)	2·15 (0·796–4·67)	-10·3 (-40·7–38·8)	2·97 (-23·2–38·7)	5·27 (2·59–10·1)	5·36 (2·89–10·1)	5·64 (2·95–10·8)	1·7 (-23·2–37·3)	5·3 (-8·16–18·4)
Occupational asthmagens	21·1 (17·6–25·6)	18·7 (15·6–22·2)	19·4 (16–23·6)	-11·5 (-23·6—0·377)	3·88 (-4·69–14·1)	11 (8·84–13·3)	10·8 (8·97–13)	11 (8·92–13·4)	-1·4 (-15·6–15·8)	1·37 (-10–13·9)
Occupational particulate matter, gases, and fumes	8·49 (6·03–12·4)	7·73 (5·54–11·2)	7·44 (5·38–10·6)	-8·93 (-14·5—2·55)	-3·8 (-7·58—0·0744)	4·7 (3·31–6·92)	4·37 (3·09–6·41)	4·17 (2·94–6·1)	-7·08 (-16·6–2·64)	-4·61 (-9·87–1·38)
Occupational noise	8·02 (7·33–8·78)	7·68 (7·11–8·37)	7·45 (6·96–8·12)	-4·33 (-8·05—0·339)	-3·01 (-5·11—0·934)	5·68 (4·96–6·44)	5·95 (5·46–6·52)	5·94 (5·55–6·5)	4·72 (-1·64–12·1)	-0·0744 (-2·81–3·06)
Occupational injuries	--	--	--	--	--	--	--	--	--	--
Occupational ergonomic factors	11·5 (9·47–14·1)	10·2 (8·56–12·2)	10·6 (8·74–12·7)	-11·4 (-20·7—1·45)	3·75 (-3·67–11·9)	6·45 (5·12–8·13)	6·52 (5·27–8·14)	6·85 (5·5–8·57)	1·14 (-14·4–19·4)	4·95 (-7·03–18)
Childhood sexual abuse	7·4 (5·05–10·5)	6·97 (4·6–9·93)	6·85 (4·54–9·67)	-5·83 (-12·2–5·83)	-1·68 (-8·51–5·53)	12·3 (8·94–17·3)	17·4 (12·9–24)	17·8 (13·1–24·7)	41·9 (30·7–54·8)	2·17 (-5–11)
Intimate partner violence	--	--	--	--	--	28·8 (15·6–38)	27·2 (14·9–36·1)	28·1 (15·4–37)	-5·6 (-15·4–8·95)	3·43 (-5·81–19·6)
Non-exclusive breastfeeding	26·5 (22–31·2)	25·1 (21·4–28·9)	25 (20·3–29·8)	-5·09 (-18·8–11·6)	-0·53 (-13·9–13·8)	--	--	--	--	--
Discontinued breastfeeding	27·7 (26·7–28·6)	25·7 (24·4–26·9)	25·8 (24·5–26·9)	-7·18 (-12·6—1·8)	0·264 (-5·09–5·99)	27·6 (26·7–28·5)	25·7 (24·3–26·8)	25·7 (24·5–26·9)	-7·03 (-12·5—1·65)	0·257 (-5·1–5·99)
Diet low in calcium	14·3 (6·66–26·3)	12·1 (6·06–22·1)	11 (5·34–20·3)	-15·3 (-26·6—0·103)	-8·86 (-17·9—0·0315)	9·54 (4·07–18·1)	8·75 (3·96–16·1)	8·05 (3·5–15)	-8·3 (-24·1–12·2)	-8·04 (-19·2–2·28)
Occupational exposure to asbestos	7·5 (6·68–8·38)	6·24 (5·73–6·74)	5·3 (3·83–7·19)	-16·8 (-27—5·55)	-15·1 (-39·3–14·7)	1·33 (1·01–2)	1·12 (0·894–1·7)	1·07 (0·705–1·76)	-15·8 (-32·8–5·45)	-4·8 (-32·6–28·8)
Occupational exposure to arsenic	0·484 (0–1·14)	0·469 (0–1·11)	0·504 (0–1·21)	-2·98 (-17·4–NA)	7·38 (-3·73–NA)	0·449 (0–1·06)	0·443 (0–1·04)	0·457 (0–1·13)	-1·32 (-16·5–NA)	3·22 (-8·75–NA)
Occupational exposure to benzene	0·923 (0·054–2·73)	0·9 (0·0545–2·67)	0·927 (0·0545–2·65)	-2·49 (-9·78–9·8)	2·97 (-4·57–13·9)	1·26 (0·0736–3·49)	1·42 (0·0844–3·84)	1·47 (0·0932–4·25)	12·6 (0·73–34·4)	4·21 (-3·84–15·8)
Occupational exposure to beryllium	0·0235 (0·0221–0·0248)	0·0232 (0·0227–0·0236)	0·0244 (0·0232–0·026)	-1·24 (-7·04–4·74)	5·54 (-0·03–11·7)	--	--	--	--	--
Occupational exposure to cadmium	0·057 (0·0517–0·0631)	0·0557 (0·0507–0·0613)	0·0595 (0·0528–0·0674)	-2·27 (-15·1–11·2)	6·68 (-3·24–19·6)	0·054 (0·0481–0·0608)	0·0538 (0·0487–0·0594)	0·0555 (0·0486–0·0632)	-0·325 (-13·8–16·7)	3·12 (-7·88–15)
Occupational exposure to chromium	0·139 (0·127–0·152)	0·138 (0·129–0·149)	0·147 (0·132–0·163)	-0·254 (-11·3—11·7)	5·92 (-2·62–16·5)	--	--	--	--	--
Occupational exposure to diesel engine exhaust	0·683 (0·634–0·737)	0·736 (0·693–0·781)	0·763 (0·703–0·828)	7·75 (-1·99–19·1)	3·56 (-3·94–11·5)	0·619 (0·554–0·687)	0·65 (0·596–0·711)	0·654 (0·585–0·732)	5·09 (-8·21–21·6)	0·595 (-8·96–11·2)
Occupational exposure to formaldehyde	0·29 (0·262–0·322)	0·276 (0·25–0·303)	0·29 (0·255–0·33)	-4·99 (-17·5–8·54)	5·24 (-4·56–18)	0·271 (0·243–0·303)	0·275 (0·251–0·302)	0·283 (0·253–0·318)	1·34 (-10·9–17)	3·1 (-6·85–14·2)
Occupational exposure to nickel	0·398 (0–1·73)	0·371 (0–1·62)	0·399 (0–1·72)	-6·77 (-19·8–NA)	7·59 (-3·27–NA)	0·348 (0–1·54)	0·332 (0–1·47)	0·343 (0–1·53)	-4·47 (-20·8–NA)	3·31 (-9·85–NA)
Occupational exposure to polycyclic aromatic hydrocarbons	0·28 (0·257–0·305)	0·279 (0·262–0·299)	0·295 (0·269–0·325)	-0·153 (-10·1–10·5)	5·64 (-2·29–15·4)	0·294 (0·267–0·324)	0·312 (0·294–0·331)	0·322 (0·293–0·351)	5·95 (-4·85–19·4)	3·17 (-5·44–11·5)
Occupational exposure to silica	3·56 (0·532–11·7)	3·24 (0·466–10·4)	3·5 (0·478–11·1)	-8·96 (-22·3–6·69)	8·16 (-3·22–24·9)	2·88 (0·387–9·63)	2·62 (0·372–8·42)	2·69 (0·388–8·88)	-9·11 (-25–13·3)	2·87 (-11·2–21·6)
Occupational exposure to sulfuric acid	0·913 (0·188–2·9)	0·986 (0·201–3·21)	1·03 (0·216–3·39)	7·98 (-5·91–24·8)	4·5 (-6·54–17·5)	0·792 (0·171–2·69)	0·915 (0·183–2·82)	0·918 (0·192–2·74)	15·5 (-5·08–39·6)	0·242 (-12·7–16·3)
Unsafe sex	--	--	--	--	--	--	--	--	--	--
Occupational exposure to trichloroethylene	0·0818 (0·0763–0·088)	0·0841 (0·0806–0·0882)	0·0884 (0·0823–0·0954)	2·81 (-5·06–12)	5·1 (-1·56–13·1)	0·0792 (0·072–0·0875)	0·0817 (0·0773–0·0875)	0·0836 (0·0762–0·0918)	3·24 (-7·33–17·7)	2·31 (-6·03–11·6)
No access to handwashing facility	3·28 (3·04–3·52)	3·08 (2·85–3·32)	3·05 (2·81–3·29)	-6·16 (-14·1–3·43)	-0·928 (-6·48–4·77)	3·28 (3·04–3·52)	3·08 (2·85–3·32)	3·05 (2·81–3·29)	-6·16 (-14·1–3·43)	-0·928 (-6·48–4·77)
Child wasting	0·748 (0·499–1·03)	0·723 (0·476–0·985)	0·722 (0·465–0·987)	-3·29 (-12–7·31)	-0·22 (-6·69–7·13)	0·77 (0·516–1·06)	0·759 (0·506–1·05)	0·754 (0·502–1·03)	-1·42 (-11·1–10·7)	-0·706 (-7·79–7·24)
Child stunting	2·38 (1·25–3·52)	2 (1·06–2·98)	1·94 (1·01–2·88)	-15·7 (-35·1–7·84)	-3·29 (-17·2–13)	1·93 (0·938–2·91)	1·75 (0·866–2·69)	1·71 (0·826–2·65)	-9·54 (-28·3–14·8)	-1·8 (-16·3–16·9)
Chewing tobacco	1·59 (1·19–2·08)	2·05 (1·45–2·79)	2·13 (1·46–3·04)	29 (-13·5–93·2)	3·98 (-18·1–34·1)	0·29 (0·185–0·441)	0·339 (0·214–0·519)	0·354 (0·219–0·541)	17 (-29·8–99·4)	4·34 (-25–47·1)
Diet low in legumes	72·8 (35·6–98·8)	75·9 (42·2–99·6)	75·6 (42·8–99·6)	4·19 (0–22·8)	-0·323 (-6·54–5·27)	71·1 (33·8–98·8)	74·2 (39·6–99·4)	74·2 (40–99·4)	4·3 (-0·0164–23·6)	0·0673 (-5·85–6·73)
Short gestation	27·2 (22·7–32·2)	23·7 (20·2–27·6)	23 (18·6–28·2)	-12·9 (-28–4·38)	-2·97 (-21·4–20·1)	25 (21·2–29·3)	23 (19·6–26·7)	21·8 (18·2–25·8)	-8·29 (-25–11)	-5·11 (-21·6–15)
Low birth weight	11·4 (9·73–13·5)	10·6 (9·07–12·3)	10·3 (8·59–12·4)	-6·85 (-23·8–13·7)	-2·73 (-22·1–20·7)	12·2 (10·4–14·5)	11·9 (10·3–13·7)	11·6 (9·78–13·5)	-2·52 (-20·2–18·6)	-2·76 (-20·3–18·1)
High temperature	23·3 (12·4–35·8)	28·4 (17·3–40·6)	26·5 (15·5–38·3)	21·7 (9·1—44·5)	-6·59 (-12·9—2·11)	23·3 (12·4–35·8)	28·4 (17·3–40·6)	26·5 (15·5–38·3)	21·7 (9·1—44·5)	-6·59 (-12·9—2·11)
Low temperature	33·6 (30·1–38·3)	34·9 (31·6–39)	35·8 (32·3–40·1)	3·93 (2·1–5·77)	2·51 (1·43–3·63)	33·6 (30·1–38·3)	34·9 (31·6–39)	35·8 (32·3–40·1)	3·93 (2·1–5·77)	2·51 (1·43–3·63)
Kidney dysfunction	19·6 (14·1–26·6)	20·3 (14·4–27·5)	20·2 (14·5–27·3)	3·36 (-0·756–7·92)	-0·335 (-4·58–3·38)	21·5 (15·1–29·1)	22·5 (15·8–30·1)	23·2 (16·7–31)	4·57 (-0·136–10·2)	3·22 (-1·15–8·66)
Bullying victimization	2·77 (0·973–6·09)	3·38 (1·25–7·14)	3·8 (1·33–8·31)	21·9 (-2·45–54·5)	12·3 (0·388–22·7)	--	--	--	--	--
High LDL cholesterol	68·1 (63·5–72·6)	34·3 (30·8–37·7)	30·7 (27–34·4)	-49·7 (-54·9—44·6)	-10·5 (-15·5—5·83)	66·2 (62·5–69·7)	34·2 (30·9–37·6)	31·4 (27·8–35)	-48·3 (-53—43·9)	-8·11 (-12·5—4·17)

Supplementary Results Table 1. North Carolina age-standardised Summary Exposure Values (SEVs) for most-detailed risk factors and percentage change, by sex, 1990, 2010, and 2019. Data in parentheses are 95% uncertainty intervals.

	Male					Female				
	1990	2010	2019	Percentage change 1990-2010	Percentage change 2010-2019	1990	2010	2019	Percentage change 1990-2010	Percentage change 2010-2019
Unsafe sanitation	7·99 (3·75–15·1)	4·25 (2·08–8·16)	3·35 (1·52–6·33)	-46·8 (-78·2–29·6)	-21·3 (-59·5–49·7)	--	--	--	--	--
Ambient particulate matter pollution	14·8 (5·85–28·4)	7·19 (4·58–10·3)	4·31 (1·92–7·11)	-51·5 (-75·3–12·5)	-40 (-59·2–-30)	14·8 (5·86–28·3)	7·19 (4·58–10·3)	4·32 (1·92–7·11)	-51·5 (-75·3–12·4)	-39·9 (-58·9–-29·9)
Household air pollution from solid fuels	0·0598 (0·0154–0·157)	0·026 (0·00488–0·0792)	0·0164 (0·0022–0·0573)	-56·6 (-84·3–4·16)	-36·9 (-70·5–15·7)	0·0786 (0·0215–0·201)	0·0345 (0·00674–0·11)	0·0218 (0·00324–0·0758)	-56·2 (-84·2–6·44)	-36·8 (-70·3–16)
Ambient ozone pollution	83·7 (39·3–100)	68·7 (30·1–100)	44·1 (18·4–76·1)	-17·9 (-28·4–0)	-35·8 (-45·1–-23·9)	--	--	--	--	--
Residential radon	18·9 (2·71–53·3)	18·9 (2·71–53·3)	18·9 (2·71–53·3)	0 (0–0)	0 (0–0)	18·9 (2·71–53·3)	18·9 (2·71–53·3)	18·9 (2·71–53·3)	0 (0–0)	0 (0–0)
Lead exposure	41·3 (11·8–66·4)	25·2 (5·84–47·8)	17·8 (3·06–37·4)	-39·1 (-59·8–-25·9)	-29·2 (-50·3–-20)	23 (0·551–51·4)	14·9 (0·25–38·8)	10·8 (0·0977–30·7)	-35·5 (-74·6–-10·9)	-27·6 (-68·6–-19·3)
Child underweight	0·971 (0·542–1·48)	0·963 (0·522–1·45)	0·934 (0·509–1·39)	-0·829 (-17·7–23)	-3 (-15·1–11)	0·82 (0·457–1·24)	0·853 (0·457–1·31)	0·873 (0·477–1·32)	4 (-16·7–28)	2·34 (-11·5–18)
Iron deficiency	--	--	--	--	--	12·4 (10·4–14·4)	12·4 (10·5–14·5)	12·6 (10·5–15)	0·282 (-12·1–13·1)	1·68 (-8·13–13)
Vitamin A deficiency	0·984 (0·274–2·57)	1·27 (0·36–3·53)	0·889 (0·243–2·46)	29·2 (-69–457)	-30·1 (-69–65·1)	1·88 (0·674–3·86)	1·85 (0·701–3·72)	1·56 (0·578–3·37)	-1·72 (-63·2–197)	-15·5 (-54·6–59·9)
Zinc deficiency	2·39 (0–7·64)	1·24 (0–4·06)	1·33 (0–4·32)	-48·1 (-100–NA)	7·14 (-11·4–NA)	2·44 (0–7·67)	1·24 (0–4·08)	1·29 (0–4·26)	-49 (-100–NA)	3·71 (-13·8–NA)
Smoking	29 (26·3–32)	22·4 (20·2–24·6)	19·2 (17·2–21·2)	-23 (-27·6–-18·1)	-14·2 (-17·3–-11)	19·8 (17·5–22·3)	15·7 (14·1–17·5)	13·5 (12–15·2)	-20·6 (-27·3–-12·5)	-14 (-18·2–-9·66)
Secondhand smoke	39·8 (37·3–42·3)	25·8 (24·2–27·5)	25·6 (22·8–28·4)	-35·1 (-41–-28·8)	-0·916 (-9·73–8·65)	38·8 (36·7–40·8)	24·9 (23·8–26·1)	24·7 (22·7–26·7)	-35·7 (-40·4–-30·9)	-1·01 (-8·14–6·19)
Alcohol use	11·7 (7·68–15·9)	13·7 (8·16–19·9)	14·1 (8·45–20·2)	16·9 (-20·6–61)	3·06 (-16·6–29·1)	--	--	--	--	--
Drug use	0·562 (0·295–1·04)	1·02 (0·719–1·49)	1·36 (0·991–1·85)	81·4 (32·7–162)	33·4 (16·1–54·4)	0·383 (0·222–0·672)	0·692 (0·496–0·966)	1·05 (0·782–1·39)	80·5 (40·6–143)	51·7 (29·1–79·7)
High fasting plasma glucose	13·8 (11·6–16·5)	19·4 (17·1–22)	20·3 (17·6–23·3)	40·5 (17·2–70·4)	4·7 (-8·89–19·7)	11 (9·26–13)	16·2 (14·3–18·3)	17 (14·9–19·5)	46·6 (22·8–73·7)	5·41 (-7·2–20·4)
High systolic blood pressure	25·4 (20·5–30·7)	16·2 (14·2–18·3)	19·8 (15·5–24·5)	-36·1 (-48·1–-21·3)	21·9 (-3·44–51·7)	23·6 (17·6–30·2)	13·4 (11·7–15)	17·7 (12·9–23·3)	-43·4 (-56·9–-24·7)	32·2 (-4·33–76·6)
High body-mass index	25·1 (17·8–34·6)	40·2 (30–51·9)	41 (30·4–52·4)	59·8 (39·9–88·4)	2·04 (-4·27–9·72)	32·2 (23·8–40·9)	46·1 (35·6–55·5)	46·3 (35·9–55·7)	43·3 (27·4–65·5)	0·443 (-5·69–7·8)
Low bone mineral density	11·9 (6·87–18·5)	9·06 (4·79–14·7)	11·4 (6·49–18·4)	-23·9 (-42·9–4·15)	26·2 (-3·96–69·7)	15·9 (10·3–22·8)	16·4 (10·8–23·6)	18·7 (12·7–26·1)	2·81 (-18·8–30·2)	14·3 (-9·14–45·6)
Diet low in fruits	57·9 (45·1–71·7)	51·4 (37·3–66·8)	51·6 (37·2–66·6)	-11·2 (-22·4–-2·13)	0·395 (-9·6–10)	--	--	--	--	--
Diet low in vegetables	35·4 (19·3–56·3)	43·7 (27·1–62·1)	46 (28·3–66·2)	23·4 (5·9–61·8)	5·34 (-5·62–17·5)	36·3 (19·4–57)	46·4 (28·2–66·1)	48·1 (29·3–69·2)	27·9 (7·94–70·2)	3·68 (-6·91–14·3)
Diet low in whole grains	79·8 (70·4–88·2)	81·7 (72·9–89·9)	81·9 (73·5–89·9)	2·46 (-1·11–6·56)	0·192 (-2·24–2·67)	--	--	--	--	--
Diet low in nuts and seeds	29·1 (8·8–57·2)	25·6 (8·86–55·5)	21·9 (7·23–54·1)	-11·8 (-45·1–63·3)	-14·8 (-43·1–12·9)	24·9 (6·54–55)	22·3 (6·48–54·5)	20 (5·37–53·6)	-10·3 (-43·4–57·3)	-10·3 (-39·6–18·6)
Diet low in milk	49·8 (25·9–70·1)	44·3 (19·8–64·7)	44·3 (20·5–64·9)	-11 (-28–0·203)	-0·104 (-10·8–12·6)	49·5 (24·8–69·7)	44·3 (20·2–63·9)	44·5 (20·7–64·7)	-10·6 (-29·5–1·88)	0·645 (-11·2–14·8)
Diet high in red meat	79·3 (67–89·4)	78·2 (65·4–88·4)	77·2 (63·9–88·4)	-1·45 (-16·2–13·1)	-1·3 (-12·7–11)	80·9 (68·9–91·1)	77·4 (64·6–88·3)	76·5 (62·1–87·5)	-4·23 (-17·5–10·9)	-1·17 (-12·2–10·3)
Diet high in processed meat	75·5 (50·9–97·1)	84·5 (65·3–99·6)	84·3 (64·6–99·5)	11·9 (1·86–33·7)	-0·136 (-4·51–4·49)	76·4 (52·3–96·7)	87 (70·6–99·6)	85·8 (66·9–99·5)	13·9 (1·97–39·7)	-1·41 (-6·61–2·39)
Diet high in sugar-sweetened beverages	44·7 (26·9–64·4)	61 (38·9–79·4)	60·3 (40–78·7)	36·7 (-6·12–117)	-1·14 (-21·6–24·8)	48·3 (28·2–68·4)	66·7 (48·8–82·8)	64·9 (45·2–80·9)	38 (-2·26–115)	-2·66 (-21·5–18·4)
Diet low in fiber	47·3 (30·4–64)	32 (18·8–46·9)	30·1 (17·1–44·8)	-32·4 (-49·3–-17·6)	-5·82 (-23·8–13·3)	48·2 (31–64·7)	38·1 (24·3–53·4)	33·9 (19·9–50·4)	-20·8 (-36·6–-4·65)	-11·1 (-28·1–2·72)
Diet low in seafood omega-3 fatty acids	84·7 (67·3–99·9)	80·9 (62·8–99·7)	81·1 (63·2–99·2)	-4·46 (-19–9·42)	0·238 (-13·4–16·3)	86·9 (72·3–99·8)	84·6 (68·4–99·9)	84·4 (68·5–99·8)	-2·68 (-13·6–6·81)	-0·249 (-11·6–12)
Diet low in polyunsaturated fatty acids	14·8 (4·23–42·6)	7·61 (2·16–25·7)	7·46 (2·15–24·6)	-48·7 (-75·7–-8·12)	-1·94 (-23–21·6)	--	--	--	--	--
Diet high in trans fatty acids	84·4 (76·2–100)	85·9 (77·6–100)	85·1 (76·2–100)	1·71 (-5·76–10·8)	-0·942 (-8·76–7·39)	81·2 (72·1–100)	85 (76–100)	84·5 (76·1–100)	4·68 (-3·07–15)	-0·563 (-8·25–8·67)
Diet high in sodium	18·5 (4·37–41·5)	40 (11–64·7)	39 (11·2–64·9)	116 (40·6–426)	-2·33 (-29·2–28·1)	--	--	--	--	--
Low physical activity	2·94 (1·12–6·09)	3·02 (1·37–5·81)	2·7 (1·02–5·83)	2·58 (-29·4–64)	-10·4 (-33·9–12·2)	6·38 (3·16–12)	7·56 (4·22–13·2)	6·59 (3·42–12·2)	18·5 (-10·1–65·3)	-12·8 (-25·2–-1·88)
Occupational asthmagens	23·7 (19·6–28·7)	21 (17·6–24·9)	21·8 (18·2–26·4)	-11·2 (-22·8–1·29)	3·55 (-5·69–14)	12·1 (9·83–14·6)	11·2 (9·32–13·4)	11·3 (9·21–13·6)	-7·59 (-21·3–8·21)	0·795 (-9·98–13·5)
Occupational particulate matter, gases, and fumes	10·1 (7·15–14·5)	9·41 (6·79–13·5)	9·05 (6·48–12·8)	-6·73 (-12·4–-0·923)	-3·87 (-7·33–-0·561)	6·31 (4·43–9·15)	5·83 (4·17–8·28)	5·5 (3·96–7·94)	-7·7 (-15·9–1·69)	-5·53 (-10·5–-0·265)
Occupational noise	8·59 (7·86–9·47)	8·27 (7·71–8·99)	8·02 (7·51–8·73)	-3·75 (-7·77–0·516)	-3·05 (-5·27–-0·885)	6·21 (5·38–7·06)	6·39 (5·82–7·06)	6·33 (5·88–6·92)	2·89 (-3·6–11)	-0·894 (-3·89–2·3)
Occupational injuries	--	--	--	--	--	--	--	--	--	--
Occupational ergonomic factors	13 (10·7–15·7)	10·9 (9·13–13·1)	11·3 (9·32–13·5)	-16·2 (-26·6–-3·62)	3·14 (-5·3–12·2)	7·52 (5·89–9·44)	7·03 (5·75–8·69)	7·33 (5·84–9·23)	-6·45 (-22·4–11·7)	4·32 (-8·28–17)
Childhood sexual abuse	6·12 (3·68–9·41)	5·7 (4·06–7·58)	5·66 (4·05–7·49)	-6·91 (-26·3–21·3)	-0·609 (-6·87–5·47)	16·5 (11·8–23·1)	15·4 (11·9–20·5)	15·5 (12·1–20·6)	-6·75 (-17·8–8·6)	0·656 (-5·52–8·68)
Intimate partner violence	--	--	--	--	--	35·7 (20·5–45·8)	34·1 (19·6–44·1)	35·1 (20–44·8)	-4·49 (-13–9·54)	2·79 (-4·14–18·5)
Non-exclusive breastfeeding	26·2 (21·6–31·1)	24·9 (21·3–28·9)	24·9 (20·6–29·9)	-5·13 (-18·9–12·5)	0·108 (-12·2–13·8)	--	--	--	--	--
Discontinued breastfeeding	27·7 (26·8–28·6)	25·3 (24–26·5)	25·8 (24·5–26·9)	-8·58 (-14·1–-3·42)	1·62 (-4·08–7·54)	27·8 (26·9–28·6)	25·3 (24–26·5)	25·7 (24·5–26·9)	-8·77 (-14·3–-3·62)	1·61 (-4·09–7·54)
Diet low in calcium	16·9 (7·09–32·5)	13·4 (5·63–25·4)	13·5 (5·67–25·6)	-20·8 (-32·3–-9·48)	1·25 (-7·98–9·75)	12·8 (5·07–24·7)	11 (4·6–20·5)	11·4 (4·65–21·9)	-14 (-28·3–-2·81)	3·44 (-6·4–14·4)
Occupational exposure to asbestos	7·26 (6·44–8·15)	6·24 (5·68–6·78)	5·39 (3·96–7·23)	-14·1 (-24·8–-1·86)	-13·7 (-36–17·4)	1·08 (0·854–1·94)	1·05 (0·823–1·86)	1·05 (0·671–1·93)	-3·02 (-17·2–14·5)	0·189 (-27·9–35·5)
Occupational exposure to arsenic	0·565 (0–1·33)	0·542 (0–1·29)	0·569 (0–1·34)	-4·08 (-17·2–NA)	4·99 (-7·53–NA)	0·58 (0–1·38)	0·535 (0–1·3)	0·544 (0–1·33)	-7·74 (-24·1–NA)	1·66 (-11·2–NA)
Occupational exposure to benzene	0·793 (0·0468–2·28)	0·775 (0·0472–2·25)	0·805 (0·049–2·31)	-2·24 (-9·46–10·4)	3·86 (-3·57–13·3)	1·1 (0·0565–2·93)	1·23 (0·0742–3·5)	1·3 (0·0815–3·75)	12·7 (0·123–32·3)	5·45 (-4–17·4)
Occupational exposure to beryllium	0·0241 (0·0226–0·0254)	0·0234 (0·0229–0·024)	0·0247 (0·0233–0·0261)	-2·76 (-8·23–3·44)	5·27 (-0·733–11·7)	0·0264 (0·024–0·0287)	0·0269 (0·0263–0·0276)	0·028 (0·026–0·0299)	1·91 (-6·86–12·5)	4 (-3·29–11·2)
Occupational exposure to cadmium	0·0653 (0·0591–0·0718)	0·0631 (0·0573–0·0692)	0·0662 (0·0582–0·0743)	-3·3 (-15·8–11·7)	4·88 (-6·52–15·4)	0·0674 (0·0586–0·0758)	0·0631 (0·0562–0·0707)	0·0643 (0·0552–0·0741)	-6·4 (-21·1–11·9)	1·99 (-9·8–15·5)
Occupational exposure to chromium	0·154 (0·14–0·167)	0·152 (0·14–0·164)	0·16 (0·143–0·177)	-0·861 (-11·8–12·1)	4·86 (-4·96–14)	--	--	--	--	--
Occupational exposure to diesel engine exhaust	0·686 (0·633–0·742)	0·746 (0·7–0·795)	0·788 (0·72–0·865)	8·74 (-1·81–21·1)	5·67 (-2·45–15)	0·613 (0·543–0·691)	0·66 (0·604–0·726)	0·67 (0·593–0·753)	7·7 (-7·36–24·9)	1·56 (-9·06–12·4)
Occupational exposure to formaldehyde	0·326 (0·292–0·36)	0·308 (0·277–0·339)	0·319 (0·28–0·361)	-5·48 (-18·2–9·88)	3·61 (-8·23–14·5)	0·327 (0·283–0·367)	0·308 (0·276–0·342)	0·315 (0·27–0·357)	-5·61 (-19·8–12·3)	2·15 (-8·8–14·4)
Occupational exposure to nickel	0·475 (0–2·1)	0·44 (0–1·93)	0·461 (0–1·99)	-7·38 (-20·3–NA)	4·88 (-7·58–NA)	0·47 (0–2·07)	0·416 (0–1·8)	0·424 (0–1·86)	-11·6 (-27·2–NA)	1·87 (-11·5–NA)
Occupational exposure to polycyclic aromatic hydrocarbons	0·3 (0·275–0·327)	0·298 (0·276–0·319)	0·311 (0·28–0·343)	-0·935 (-11·7–11·7)	4·6 (-4·8–13·4)	0·335 (0·297–0·37)	0·336 (0·312–0·363)	0·345 (0·307–0·383)	0·437 (-11·4–15·5)	2·53 (-6·51–12·2)
Occupational exposure to silica	4·26 (0·593–13·8)	3·84 (0·551–12·1)	4·05 (0·603–13)	-9·96 (-22·3–5·97)	5·62 (-6·28–19·2)	3·9 (0·548–12·4)	3·37 (0·48–10·6)	3·4 (0·486–10·9)	-13·8 (-30·2–8·82)	1·09 (-12·9–20·2)
Occupational exposure to sulfuric acid	0·978 (0·207–3·14)	1·03 (0·213–3·24)	1·06 (0·22–3·37)	5·08 (-8·32–20·2)	3·27 (-7·29–14·2)	0·988 (0·209–3·19)	1·11 (0·229–3·57)	1·1 (0·221–3·48)	11·9 (-7·85–38·6)	-0·716 (-13·9–13·6)
Unsafe sex	--	--	--	--	--	--	--	--	--	--
Occupational exposure to trichloroethylene	0·0874 (0·0811–0·0938)	0·0895 (0·0855–0·0943)	0·0941 (0·0873–0·102)	2·46 (-5·69–11·9)	5·09 (-2·2–13·1)	0·0883 (0·079–0·0978)	0·0883 (0·0828–0·0944)	0·0902 (0·0816–0·0996)	-0·0167 (-11·1–14·7)	2·14 (-6·65–11·4)
No access to handwashing facility	3·4 (3·15–3·68)	3·13 (2·88–3·37)	3·1 (2·86–3·35)	-8·03 (-16–0·512)	-1·05 (-6·66–5·09)	3·4 (3·15–3·68)	3·13 (2·88–3·37)	3·1 (2·86–3·35)	-8·03 (-16–0·512)	-1·05 (-6·66–5·09)
Child wasting	0·751 (0·487–1·03)	0·728 (0·474–0·985)	0·727 (0·482–0·987)	-3·09 (-12–7·02)	-0·139 (-7·29–7·05)	0·774 (0·509–1·06)	0·764 (0·512–1·05)	0·759 (0·505–1·05)	-1·25 (-10·9–10·6)	-0·627 (-7·93–7·11)
Child stunting	2·45 (1·25–3·59)	2·08 (1·08–3·1)	2·02 (1·07–3·01)	-15·2 (-33·2–9·28)	-2·97 (-17·4–14·1)	1·97 (0·999–2·98)	1·8 (0·872–2·68)	1·77 (0·866–2·67)	-8·72 (-29–15·9)	-1·28 (-15·8–18·1)
Chewing tobacco	9·49 (7·86–11·4)	6·53 (5·07–8·31)	5·97 (4·39–7·97)	-31·2 (-49·5–-6·34)	-8·56 (-26·9–14·5)	--	--	--	--	--
Diet low in legumes	74·5 (37·7–99·1)	77·4 (45·6–99·7)	77·6 (46·8–99·6)	3·96 (-0·249–20·9)	0·287 (-4·12–7·22)	72·8 (34·4–99)	75·7 (42·3–99·7)	76·4 (45·1–99·7)	3·94 (-0·219–23)	0·923 (-3·68–10·1)
Short gestation	39·2 (32·2–47·7)	32·9 (28–38·8)	29·8 (24·1–36·7)	-16·1 (-32·2–5·01)	-9·43 (-26·7–10·5)	33·9 (28·5–40·4)	29·1 (24·8–33·8)	26·7 (22·3–31·4)	-14·3 (-29·8–5·23)	-8·26 (-23–10·6)
Low birth weight	15·5 (12·7–18·8)	14·2 (12·2–16·6)	13·2 (11–15·8)	-8·66 (-27·3–17·1)	-6·52 (-24·5–14·7)	15·5 (13·2–18·3)	14·3 (12·4–16·4)	14·2 (12·1–16·5)	-7·78 (-25·4–14·2)	-0·777 (-18·5–19·5)
High temperature	23 (14·5–32·1)	25·2 (16·3–34·6)	21·1 (12·4–30·7)	9·71 (3·65–17·1)	-16·3 (-25–-10·1)	23 (14·5–32·1)	25·2 (16·3–34·6)	21·1 (12·4–30·7)	9·71 (3·65–17·1)	-16·3 (-25–-10·1)
Low temperature	32·5 (27·7–38·9)	36·9 (32·6–43)	34·3 (29·5–40·7)	13·6 (9·28–18·6)	-7·14 (-11–-3·99)	32·5 (27·7–38·9)	36·9 (32·6–43)	34·3 (29·5–40·7)	13·6 (9·28–18·6)	-7·14 (-11–-3·99)
Kidney dysfunction	20·2 (14·5–27·3)	21·1 (15·2–28·3)	21·1 (15·3–28·4)	4·13 (0·208–8·66)	0·109 (-3·89–3·77)	22·6 (16·2–30·2)	23·9 (17·2–31·9)	24·6 (17·7–32·5)	5·77 (1·09–11·6)	2·74 (-2·13–8·92)

Bullying victimization	5·83 (2·43–11·9)	8·91 (3·58–17·7)	8·71 (3·34–17·9)	52·7 (31·8–75·9)	-2·18 (-12·2–3·93)	4·25 (1·75–8·83)	7·83 (3·16–15·6)	7·78 (2·94–16·1)	84·2 (58·7–113)	-0·545 (-11·2–6·28)
High LDL cholesterol	66·9 (62–71·5)	34·3 (30·9–37·9)	30·5 (26·9–34·4)	-48·7 (-53·9–-43·5)	-11·2 (-15·9–-6·14)	65·6 (61·8–69·4)	34·4 (31·1–37·9)	31·6 (28·1–35·2)	-47·5 (-52·3–-42·9)	-8·03 (-12·2–-4·22)

Supplementary Results Table 1. North Dakota age-standardised Summary Exposure Values (SEVs) for most-detailed risk factors and percentage change, by sex, 1990, 2010, and 2019. Data in parentheses are 95% uncertainty intervals.										
	Male					Female				
	1990	2010	2019	Percentage change 1990-2010	Percentage change 2010-2019	1990	2010	2019	Percentage change 1990-2010	Percentage change 2010-2019
Unsafe sanitation	5·95 (2·78–11·4)	3·46 (1·6–6·65)	2·39 (1·03–4·71)	-42 (-76·3–44·1)	-31 (-64·3–35·7)	5·95 (2·78–11·4)	3·46 (1·6–6·65)	2·39 (1·03–4·71)	-42 (-76·3–44·1)	-31 (-64·3–35·7)
Ambient particulate matter pollution	5·19 (0·343–13·7)	3·31 (0·929–6·2)	2·78 (0·587–5·58)	-36·3 (-79·4–400)	-15·8 (-40·5—9·16)	5·19 (0·355–13·7)	3·31 (0·904–6·2)	2·79 (0·592–5·56)	-36·3 (-79·4–414)	-15·8 (-39·8—9·2)
Household air pollution from solid fuels	0·00778 (0·000635–0·0266)	0·00418 (0·000418–0·0162)	0·00265 (0·000225–0·00985)	-46·3 (-84·9–137)	-36·7 (-73·3–18·7)	0·0103 (0·00089–0·0366)	0·00555 (0·000653–0·021)	0·00352 (0·000301–0·0134)	-45·9 (-84·9–120)	-36·7 (-73·2–18·3)
Ambient ozone pollution	27·2 (10·3–48·9)	35·4 (13·9–64·1)	30·1 (11·3–55·9)	30·3 (-4·92–85·1)	-15 (-39·8–17·6)	--	--	--	--	--
Residential radon	25·6 (4·07–74·3)	25·6 (4·07–74·3)	25·6 (4·07–74·3)	0 (0–0)	0 (0–0)	25·6 (4·07–74·3)	25·6 (4·07–74·3)	25·6 (4·07–74·3)	0 (0–0)	0 (0–0)
Lead exposure	37 (7·85–64)	21·9 (3·38–45)	15·3 (1·65–35)	-40·8 (-64·4—27·6)	-30·2 (-55·8—20·6)	20·3 (0·288–48·1)	12·9 (0·122–35·9)	9·38 (0·0346–28·3)	-36·2 (-80·1—16·8)	-27·5 (-75·6—18·7)
Child underweight	0·946 (0·545–1·44)	0·948 (0·51–1·45)	0·923 (0·505–1·4)	0·313 (-16·8–23·1)	-2·69 (-16·2–11·8)	0·793 (0·43–1·22)	0·839 (0·465–1·3)	0·856 (0·472–1·31)	5·73 (-16·2–43·7)	2·03 (-11·9–18·6)
Iron deficiency	--	--	--	--	--	11·3 (9·39–13·3)	11·9 (9·98–13·9)	12·2 (10·1–14·4)	5·07 (-6·62–17·8)	2·76 (-6·68–12·9)
Vitamin A deficiency	0·811 (0·211–2·2)	1·14 (0·315–3·16)	0·689 (0·186–1·89)	40·7 (-59·2–516)	-39·6 (-73·4–46)	1·57 (0·598–3·28)	1·64 (0·678–3·45)	1·26 (0·494–2·75)	4·29 (-57·7–196)	-22·8 (-57·9–50·6)
Zinc deficiency	2·46 (0–7·92)	1·14 (0–3·72)	1·04 (0–3·24)	-53·7 (-100–NA)	-8·9 (-39·1–NA)	2·52 (0–7·86)	1·14 (0–3·79)	0·991 (0–3·14)	-54·8 (-100–NA)	-13·2 (-46·8–NA)
Smoking	27·4 (24·7–30·2)	19·7 (17·9–21·8)	17·3 (15·7–19·3)	-27·9 (-32·6—22·4)	-12·3 (-15·9—8·54)	19·5 (17–22·3)	14·9 (13·3–16·7)	13 (11·5–14·6)	-23·9 (-31·5—15·2)	-12·6 (-17·6—7·35)
Secondhand smoke	37·5 (35–40·1)	25·1 (23·4–26·8)	25·5 (22·7–28·7)	-33·1 (-39·4—26·2)	1·82 (-7·47–11·4)	34·6 (32·8–36·6)	24·1 (22·9–25·3)	24·7 (22·6–26·8)	-30·4 (-35·8—24·9)	2·47 (-5·03–10·3)
Alcohol use	14·8 (9·23–21·5)	15·8 (9·3–23·2)	17·1 (9·98–24·8)	6·83 (-26–51·1)	8·16 (-15·6–38·4)	9·98 (5·86–15·1)	11·6 (7·03–17)	12·9 (7·42–19·2)	16·6 (-17·2–65·7)	10·9 (-14·5–42·3)
Drug use	0·38 (0·179–0·742)	0·473 (0·297–0·766)	0·771 (0·537–1·08)	24·4 (-6·72–80·6)	63·2 (29·4–106)	0·367 (0·23–0·6)	0·481 (0·341–0·699)	0·691 (0·511–0·915)	31·1 (11–59·2)	43·6 (21–68·6)
High fasting plasma glucose	12·6 (10·4–15·4)	16·9 (14·5–19·6)	18·9 (16·3–21·7)	33·6 (10·6–63·1)	11·7 (-2·11–30·1)	9·45 (7·97–11·1)	12·5 (10·8–14·5)	14·4 (12·2–16·9)	32·2 (10·5–57·8)	15·6 (-0·955–35·3)
High systolic blood pressure	25·6 (20·7–31)	16·1 (14·2–18·2)	20·6 (15·9–25·8)	-37·1 (-48·4—22·9)	28·1 (1·57–59·2)	24 (17·8–31·1)	13 (11·4–14·6)	17·8 (12·6–23·9)	-45·7 (-58·6—26·6)	36·3 (-2·32–81·6)
High body-mass index	27·1 (19·2–37·4)	41·9 (30·7–54·1)	46 (34·1–58·2)	54·4 (36·3–81·8)	9·79 (3·15–17·7)	31·2 (22·8–40)	42·6 (32·8–51·8)	46·1 (35·3–56·2)	36·6 (22·3–57·3)	8·23 (1·47–16·2)
Low bone mineral density	11·8 (6·87–18·7)	9·3 (4·97–15·6)	11·5 (6·47–18·3)	-21 (-41–3·65)	23·6 (-2·29–64·9)	15·6 (10·3–22·3)	16·5 (10·7–23·7)	18·8 (12·8–26)	5·97 (-16·3–34·8)	14·1 (-9·27–44·9)
Diet low in fruits	56·3 (44·5–69·1)	49·5 (36·1–64·1)	48 (33·9–62·8)	-12·1 (-27·4—0·627)	-3·02 (-13·4–7·13)	--	--	--	--	--
Diet low in vegetables	29·8 (14·9–50·4)	40·6 (24·9–57·4)	35·4 (20·8–52·9)	36·5 (4·12–102)	-12·8 (-25·6—2·51)	25·5 (11·5–46·1)	20·5 (12·4–32·8)	17·7 (10·2–30·4)	-19·4 (-39·9–28·1)	-13·7 (-31·2–2·14)
Diet low in whole grains	79·8 (70·2–88·3)	81·5 (72·8–89·3)	80·9 (72–89·1)	2·08 (-1·51–6·78)	-0·675 (-3·44–1·8)	--	--	--	--	--
Diet low in nuts and seeds	29·4 (8·65–57·4)	25 (8·18–55·1)	19·4 (5·61–53·8)	-14·9 (-46·6–54·1)	-22·5 (-49·1–1·98)	25·3 (6·49–55·9)	21·8 (7·11–54·6)	17·8 (5·21–53)	-13·6 (-47·8–55·7)	-18·7 (-46·1–6·86)
Diet low in milk	50·2 (25·4–69·7)	42·9 (19·7–63·1)	38 (16·9–58·2)	-14·5 (-33·8—1·7)	-11·5 (-28·8—0·258)	49·9 (25·8–70·9)	42·7 (20·7–62·9)	38·1 (16·6–58·3)	-14·3 (-33·9—1·34)	-10·7 (-26·9–1·5)
Diet high in red meat	87·5 (76·7–95·3)	85·4 (73·9–93·9)	86·3 (74·8–95)	-2·4 (-14·4–9·18)	1·01 (-7·54–11·2)	89 (79·9–95·8)	84·9 (73·3–93·2)	85·5 (72·6–94·7)	-4·62 (-16·9–6·59)	0·789 (-7·67–10·3)
Diet high in processed meat	78·8 (53·7–98·2)	86·3 (66·6–99·7)	86·6 (67·6–99·7)	9·59 (0·957–29·3)	0·312 (-4–5·34)	79·8 (57·1–98·3)	88·8 (73·3–99·7)	87·9 (71·5–99·5)	11·3 (1·1–34·4)	-0·949 (-6·18–3·24)
Diet high in sugar-sweetened beverages	44·3 (26·4–63·5)	63·2 (43·7–79·2)	67·8 (46·9–84·1)	42·7 (-0·79–129)	7·19 (-12·5–34·6)	48·5 (29·3–68·6)	68·5 (48·6–84·5)	71·1 (51·6–87·9)	41·3 (-2·04–125)	3·73 (-15·3–27·6)
Diet low in fiber	45·2 (28·3–61)	29·7 (17–43)	24 (12·4–36·8)	-34·2 (-51·2—18·6)	-19·4 (-36·7—2·02)	45·4 (28·5–61·8)	34·9 (21·1–49·4)	26·9 (14·3–40·8)	-23·3 (-39·8—6·19)	-22·8 (-39·2—7·36)
Diet low in seafood omega-3 fatty acids	84·7 (67·9–99·9)	79·3 (60·2–99·4)	75·2 (55·7–99)	-6·46 (-22·5—7·57)	-5·17 (-21·4–12·2)	87·2 (72–99·9)	83·3 (66·8–99·4)	80·1 (62·4–99·1)	-4·45 (-16·5–5·59)	-3·81 (-16·8–7·37)
Diet low in polyunsaturated fatty acids	14·9 (4·31–43·4)	7·19 (2·09–24·5)	6·25 (1·76–24·4)	-51·8 (-78·4—9·04)	-13·1 (-40·8–1·65)	--	--	--	--	--
Diet high in trans fatty acids	89·5 (82–100)	87·8 (78·6–100)	87·4 (77·8–100)	-1·85 (-10·2–5·7)	-0·463 (-8·77–8·1)	88·4 (80·7–100)	86·7 (77·9–100)	86·2 (77·4–100)	-1·97 (-9·76–5·43)	-0·595 (-8·41–7·58)
Diet high in sodium	18·5 (4·36–41·3)	39·9 (10·8–64·9)	39·5 (10·1–64·4)	115 (38·9–404)	-1·07 (-26·2–25·8)	--	--	--	--	--
Low physical activity	2·47 (0·906–5·21)	2·34 (1·04–4·64)	2·81 (1·09–5·88)	-5·37 (-37·3–50·6)	20·1 (-10·7–65)	5·56 (2·73–10·5)	5·79 (3·09–10·4)	6·14 (3·17–11·8)	4·15 (-23·2–45·6)	6·03 (-7·16–20·5)
Occupational asthmagens	19·6 (16·5–23·5)	20·8 (17·7–24·9)	21 (17·5–25·5)	6·24 (-5·92–19·3)	1·04 (-7·27–11)	11·6 (9·52–14·1)	12·9 (10·8–15·5)	12·6 (10·2–15·2)	11 (-5·35–31·2)	-2·9 (-13·5–9·36)
Occupational particulate matter, gases, and fumes	9·81 (6·97–13·9)	9·61 (6·93–13·8)	9·36 (6·66–13·3)	-2·1 (-7·42–3·63)	-2·51 (-5·9–0·512)	5·23 (3·71–7·5)	5·33 (3·8–7·56)	5·22 (3·75–7·52)	1·89 (-6·94–11·7)	-1·95 (-6·78–3·11)
Occupational noise	9·3 (8·57–10·1)	9·17 (8·62–9·9)	8·98 (8·45–9·71)	-1·33 (-4·88–2·77)	-2·07 (-4·19–0·0306)	6·48 (5·77–7·24)	7·13 (6·59–7·73)	7·27 (6·82–7·82)	9·99 (4·06–17·1)	1·9 (-0·671–4·94)
Occupational injuries	--	--	--	--	--	--	--	--	--	--
Occupational ergonomic factors	14·8 (12·2–17·8)	13·8 (11·6–16·6)	13·9 (11·4–17)	-6·71 (-17·4–5·61)	0·924 (-8·22–10·4)	9·03 (6·95–11·5)	9·82 (7·64–12·4)	10·4 (7·94–13·5)	8·71 (-13·9–35·5)	6·09 (-11–26)
Childhood sexual abuse	7·4 (5·05–10·5)	6·97 (4·6–9·93)	6·85 (4·54–9·67)	-5·83 (-12·2–5·83)	-1·68 (-8·51–5·53)	12·3 (8·94–17·3)	17·4 (12·9–24)	17·8 (13·1–24·7)	41·9 (30·7–54·8)	2·17 (-5–11)
Intimate partner violence	--	--	--	--	--	23·4 (13–32)	22·2 (13·7–31)	23·6 (13·6–32·3)	-4·85 (-13·7–9·27)	6 (-3·97–21·1)
Non-exclusive breastfeeding	25·9 (21·4–30·8)	25·1 (21·4–29·6)	24·8 (20·4–29·5)	-3·09 (-17·6–14·8)	-1·18 (-14·3–12·7)	--	--	--	--	--
Discontinued breastfeeding	26·6 (25·6–27·5)	26·2 (24·9–27·5)	25·3 (23·9–26·6)	-1·37 (-7·11–4·21)	-3·43 (-8·87–2·24)	26·8 (25·8–27·7)	26·3 (24·9–27·5)	25·3 (23·9–26·6)	-1·81 (-7·51–3·75)	-3·62 (-9·04–2·03)
Diet low in calcium	17·3 (8·2–32·1)	13·4 (6·45–24·4)	11 (5·29–20·2)	-22·7 (-32·9—10·9)	-17·5 (-26·1—9·21)	12·8 (5·54–24·3)	10·3 (4·48–19·3)	8·18 (3·52–15·2)	-19·3 (-32—4·82)	-20·9 (-31·2—11·6)
Occupational exposure to asbestos	10 (8·85–11·3)	8·78 (7·97–9·64)	8·42 (6·3–10·8)	-12·5 (-25·1–2·1)	-4·14 (-29·1–23·6)	1·42 (0·959–1·77)	1·41 (0·904–1·72)	1·45 (0·905–1·95)	-1·02 (-15·6–18)	3·16 (-21·8–32·9)
Occupational exposure to arsenic	0·415 (0–0·989)	0·486 (0–1·15)	0·512 (0–1·2)	17·1 (1·03–NA)	5·35 (-6·38–NA)	0·436 (0–1·04)	0·506 (0–1·22)	0·507 (0–1·19)	15·9 (0·356–NA)	0·302 (-9·76–NA)
Occupational exposure to benzene	0·801 (0·05–2·29)	0·887 (0·0539–2·62)	0·911 (0·0543–2·57)	10·7 (1·32–24·3)	2·76 (-3·98–12·1)	1·36 (0·0842–3·8)	1·67 (0·0996–4·72)	1·73 (0·105–5·02)	22·4 (9·61–42·7)	3·42 (-5·03–14·6)
Occupational exposure to beryllium	0·0248 (0·0234–0·0262)	0·0264 (0·0257–0·0272)	0·0273 (0·026–0·0287)	6·36 (0·144–12·6)	3·42 (-1·52–8·54)	0·0295 (0·0273–0·0316)	0·0337 (0·0326–0·0346)	0·0342 (0·0323–0·0361)	14·1 (6·09–23·2)	1·61 (-3·91–7·3)
Occupational exposure to cadmium	0·0501 (0·0453–0·0552)	0·0585 (0·0533–0·0644)	0·0613 (0·0549–0·0691)	16·7 (2·25–33·4)	4·92 (-5·64–15·8)	0·0539 (0·0484–0·0597)	0·0626 (0·057–0·0686)	0·0629 (0·0563–0·0699)	16·2 (2·19–33·2)	0·387 (-8·5–10·7)
Occupational exposure to chromium	0·121 (0·11–0·132)	0·145 (0·133–0·157)	0·151 (0·136–0·167)	19·3 (5·76–34·7)	4·18 (-5–13·3)	--	--	--	--	--
Occupational exposure to diesel engine exhaust	0·791 (0·732–0·85)	0·901 (0·844–0·96)	0·904 (0·831–0·984)	13·9 (4·24–24·6)	0·302 (-6·95–7·87)	0·766 (0·68–0·853)	0·877 (0·794–0·966)	0·846 (0·754–0·947)	14·4 (0·718–31·6)	-3·56 (-12·7–6·35)
Occupational exposure to formaldehyde	0·235 (0·21–0·262)	0·277 (0·249–0·309)	0·288 (0·255–0·328)	17·6 (1·65–36·4)	4·05 (-7·45–15·5)	0·263 (0·235–0·291)	0·311 (0·285–0·34)	0·313 (0·281–0·346)	18·3 (4·37–34·7)	0·731 (-7·81–10·2)
Occupational exposure to nickel	0·375 (0–1·65)	0·408 (0–1·8)	0·428 (0–1·77)	8·78 (-5·54–NA)	5·1 (-6·24–NA)	0·339 (0–1·49)	0·38 (0–1·72)	0·383 (0–1·73)	12·1 (-4·9–NA)	0·836 (-10·7–NA)
Occupational exposure to polycyclic aromatic hydrocarbons	0·242 (0·221–0·263)	0·288 (0·267–0·31)	0·3 (0·274–0·331)	19·1 (6·18–33·7)	4·36 (-4·69–12·8)	0·304 (0·278–0·331)	0·371 (0·351–0·394)	0·373 (0·346–0·4)	21·9 (10·5–35·3)	0·667 (-5·93–7·94)
Occupational exposure to silica	3·87 (0·531–12·7)	3·94 (0·593–12·5)	4·14 (0·598–13·2)	1·94 (-12·1–20·5)	4·91 (-6·08–17·8)	2·94 (0·396–9·45)	3·11 (0·425–9·73)	3·08 (0·453–10)	5·49 (-11–28·9)	-0·868 (-12·9–15·2)
Occupational exposure to sulfuric acid	0·834 (0·175–2·63)	1·04 (0·22–3·31)	1·07 (0·22–3·43)	25·3 (8·61–45·1)	2·77 (-7·99–15·5)	0·949 (0·19–3·08)	1·29 (0·268–4·03)	1·25 (0·269–3·79)	36 (8·82–70·5)	-2·96 (-18–14·7)
Unsafe sex	--	--	--	--	--	--	--	--	--	--
Occupational exposure to trichloroethylene	0·0779 (0·0724–0·0838)	0·0919 (0·0873–0·097)	0·0945 (0·0879–0·102)	18 (7·95–28·6)	2·91 (-3·94–9·74)	0·0858 (0·0778–0·0935)	0·1 (0·0947–0·107)	0·0993 (0·0917–0·108)	16·8 (6·09–30·8)	-0·906 (-7·86–7·48)
No access to handwashing facility	3·33 (3·07–3·6)	3·11 (2·87–3·37)	3·06 (2·83–3·3)	-6·52 (-14·5–2·36)	-1·57 (-7·06–3·98)	3·33 (3·07–3·6)	3·11 (2·87–3·37)	3·06 (2·83–3·3)	-6·52 (-14·5–2·36)	-1·57 (-7·06–3·98)
Child wasting	0·748 (0·487–1·03)	0·726 (0·464–0·99)	0·723 (0·472–0·983)	-2·86 (-12·6–7·9)	-0·462 (-7·99–7·49)	0·772 (0·514–1·06)	0·763 (0·505–1·05)	0·757 (0·495–1·03)	-1·17 (-11·2–10·1)	-0·758 (-8·47–6·89)
Child stunting	2·34 (1·25–3·46)	2·04 (1·04–3·07)	1·98 (1·01–3·02)	-12·8 (-31·2–10·7)	-2·92 (-16·9–14·1)	1·91 (0·919–2·89)	1·76 (0·865–2·71)	1·74 (0·855–2·65)	-7·55 (-28·6–21·3)	-1·48 (-17·2–16·2)
Chewing tobacco	5·72 (4·47–7·24)	4·91 (3·61–6·53)	4·72 (3·33–6·5)	-14 (-39·4–21·7)	-4·01 (-25·7–22·9)	0·258 (0·162–0·398)	0·307 (0·195–0·489)	0·321 (0·196–0·509)	19·1 (-32·4–109)	4·45 (-27·6–51)
Diet low in legumes	74·5 (40·7–99·4)	76·9 (46–99·7)	75·8 (41·2–99·5)	3·22 (-0·79–17·4)	-1·55 (-9·39–2·23)	72·9 (36·3–99)	75·2 (42·2–99·6)	74·4 (40·4–99·2)	3·15 (-1·09–18·5)	-1·07 (-8·79–3·69)
Short gestation	23·6 (18·8–29·3)	23·8 (19·7–28·9)	22·8 (18·7–27·6)	0·718 (-21·1–29·1)	-4·16 (-23·6–18·7)	20 (16·7–23·6)	21·4 (18–25·5)	20·8 (17·4–24·8)	7·02 (-15–34·9)	-2·91 (-19·9–18·4)
Low birth weight	9·29 (7·59–11·3)	9·43 (7·62–11·5)	9·88 (8·17–12)	1·56 (-22·5–32·8)	4·71 (-17·6–33·1)	8·8 (7·42–10·4)	10·3 (8·72–12·1)	10·2 (8·67–12·2)	16·9 (-6·84–47·2)	-0·32 (-18·2–22·1)
High temperature	22·5 (9·62–37·5)	23·4 (10·1–38·1)	24·6 (10·7–39·7)	3·9 (-1·13–11·4)	5·03 (0·0539–10·6)	22·5 (9·62–37·5)	23·4 (10·1–38·1)	24·6 (10·7–39·7)	3·9 (-1·13–11·4)	5·03 (0·0539–10·6)
Low temperature	38·3 (32·2–45)	35·5 (29·6–42·4)	38·9 (32·6–45·9)	-7·34 (-14·2—0·951)	9·75 (4·4–17·1)	38·3 (32·2–45)	35·5 (29·6–42·4)	38·9 (32·6–45·9)	-7·34 (-14·2—0·951)	9·75 (4·4–17·1)
Kidney dysfunction	19·1 (13·6–26)	19·8 (14–26·9)	19·8 (14·2–26·9)	3·51 (-0·368–7·79)	0·357 (-3·49–4·73)	21·3 (15–28·9)	22·4 (15·9–30·3)	23·2 (16·6–30·9)	5·3 (1·07–10·5)	3·42 (-0·0635–7·65)
Bullying victimization	5·84 (2·4–12)	8·84 (3·58–17·5)	8·55 (3·3–17·6)	51·5 (32–74·6)	-3·34 (-13–2·55)	4·24 (1·71–8·87)	7·76 (3·17–15·4)	7·63 (2·9–15·6)	83·1 (58·4–111)	-1·69 (-12·3–5·19)
High LDL cholesterol	66·2 (61·5–70·7)	34 (30·6–37·4)	30·5 (27–34·2)	-48·7 (-54—43·2)	-10·1 (-14·8—5·31)	65·8 (62–69·9)	33·8 (30·5–37·1)	31·1 (27·6–34·6)	-48·7 (-53·1—43·8)	-7·98 (-11·9—4·25)

Supplementary Results Table 1. Ohio age-standardised Summary Exposure Values (SEVs) for most-detailed risk factors and percentage change, by sex, 1990, 2010, and 2019. Data in parentheses are 95% uncertainty intervals.

	Male					Female				
	1990	2010	2019	Percentage change 1990-2010	Percentage change 2010-2019	1990	2010	2019	Percentage change 1990-2010	Percentage change 2010-2019
Unsafe sanitation	6·7 (3·22–11·8)	3·76 (1·71–6·9)	3·09 (1·39–5·93)	-43·9 (-77·3–39·2)	-17·9 (-57·8–58·7)	--	--	--	--	--
Ambient particulate matter pollution	14·1 (5·45–27·4)	8·3 (5·64–11·5)	6·24 (3·68–9·21)	-41·1 (-69·7–39·3)	-24·9 (-35·2—19·1)	14·1 (5·47–27·4)	8·3 (5·62–11·5)	6·24 (3·68–9·22)	-41·1 (-69·7–39·6)	-24·8 (-35·2—19·1)
Household air pollution from solid fuels	0·0133 (0·00299–0·0367)	0·00793 (0·00139–0·0257)	0·00567 (0·000957–0·0183)	-40·5 (-77·9–38·8)	-28·6 (-62·7–34·9)	0·0176 (0·0042–0·0473)	0·0105 (0·00188–0·0353)	0·00753 (0·00127–0·0252)	-40 (-77·8–39·2)	-28·5 (-62·4–35·3)
Ambient ozone pollution	83 (39·1–100)	63·5 (26·9–100)	50 (21·3–85)	-23·5 (-35·2–0)	-21·3 (-30—13·7)	--	--	--	--	--
Residential radon	22·4 (3·43–60·2)	22·4 (3·43–60·2)	22·4 (3·43–60·2)	0 (0–0)	0 (0–0)	22·4 (3·43–60·2)	22·4 (3·43–60·2)	22·4 (3·43–60·2)	0 (0–0)	0 (0–0)
Lead exposure	62·9 (35·3–83·5)	36·4 (15·7–58·3)	25·4 (8·87–44·7)	-42·2 (-57·8—29)	-30·2 (-44·1—22·5)	37·1 (6·25–66)	20·9 (1·97–45·3)	14·4 (0·775–34·4)	-43·7 (-75—28·7)	-31·2 (-64·3—23·1)
Child underweight	0·961 (0·554–1·47)	0·963 (0·518–1·45)	0·942 (0·513–1·41)	0·218 (-17·7–20·9)	-2·21 (-14·1–12·1)	0·804 (0·439–1·21)	0·853 (0·473–1·3)	0·873 (0·482–1·33)	6 (-13·9–33·8)	2·42 (-12·8–18·8)
Iron deficiency	--	--	--	--	--	11·9 (9·82–13·8)	12·4 (10·4–14·4)	12·7 (10·6–15)	4·79 (-6·53–18·3)	2·2 (-7·43–13·4)
Vitamin A deficiency	0·839 (0·228–2·1)	1·21 (0·314–3·2)	0·857 (0·218–2·23)	44·2 (-62·2–487)	-29·2 (-67·7–76·3)	1·69 (0·632–3·4)	1·75 (0·672–3·66)	1·5 (0·581–3·11)	3·96 (-62·6–176)	-14·3 (-53·2–60·7)
Zinc deficiency	2·27 (0–7·24)	1·23 (0–3·97)	1·24 (0–4·01)	-46 (-100–NA)	0·791 (-17·7–NA)	2·33 (0–7·53)	1·24 (0–3·99)	1·21 (0–3·95)	-46·8 (-100–NA)	-2·08 (-23·1–NA)
Smoking	27·7 (25–30·5)	21·8 (19·6–23·8)	19·4 (17·4–21·3)	-21·4 (-26·3—15·9)	-11 (-14·3—7·6)	21·4 (18·9–24·2)	17·6 (15·8–19·5)	15·9 (14·2–17·7)	-18 (-25·4—9·56)	-9·52 (-13·8—5·01)
Secondhand smoke	41·4 (38·9–44·1)	29·3 (27·7–30·9)	28·1 (25·2–31)	-29·3 (-35·8—22·7)	-4 (-11·9–4·56)	38·3 (36·5–40·2)	27·5 (26·2–28·7)	26·4 (24·3–28·6)	-28·3 (-33·2—23·1)	-3·7 (-10·3–2·68)
Alcohol use	13·9 (8·63–19·5)	15·1 (8·98–21·6)	15·5 (9·5–22·7)	8·41 (-23·3–49·7)	2·96 (-18·8–29·7)	--	--	--	--	--
Drug use	0·574 (0·304–1·06)	1·23 (0·875–1·71)	1·82 (1·35–2·44)	114 (56·4–212)	48·5 (27·9–73·2)	0·537 (0·321–0·909)	1·08 (0·776–1·47)	1·72 (1·3–2·22)	100 (52–162)	59·6 (38·5–86·8)
High fasting plasma glucose	14·9 (12·2–17·7)	19·6 (17·1–22·3)	20·9 (18·2–23·7)	31·5 (8·33–59·6)	6·83 (-6·61–22·8)	11·6 (9·75–13·8)	16·3 (14·4–18·3)	17·3 (15·3–19·5)	40·5 (17·2–66·4)	6·15 (-5·73–20·2)
High systolic blood pressure	25·6 (20·7–30·6)	16·5 (14·5–18·5)	20·4 (16·2–25·2)	-35·6 (-47·4—19·1)	24 (0·154–50·9)	24 (18·3–30·8)	13·7 (12–15·4)	18 (13·2–23·8)	-43 (-56·4—24·4)	31·3 (-3·97–73·4)
High body-mass index	25 (17·6–34·8)	39·9 (29·5–51·3)	41 (30·3–52·2)	59·7 (40·3–87·5)	2·74 (-3·97–10·4)	31·6 (23·2–40·7)	44·3 (34·5–53·3)	45·2 (35·3–54·1)	40·1 (24–61·8)	2·03 (-4·19–8·36)
Low bone mineral density	12·1 (6·89–19·2)	9·33 (5·05–15·3)	11·7 (6·68–18·8)	-22·6 (-41·1—0·65)	25·9 (-2·43–71·9)	15·9 (10·3–22·6)	16·7 (10·9–24·1)	19·1 (12·9–27)	5·17 (-16·7–32·2)	14·3 (-9·32–46·3)
Diet low in fruits	55·9 (44·2–69)	49·6 (37·6–62·4)	49·3 (37·1–62·4)	-11·3 (-22·5—1·93)	-0·577 (-10·9–11·5)	56·7 (44·3–69·4)	51·2 (39·3–64·2)	50·5 (38·9–64·3)	-9·76 (-19—1·01)	-1·45 (-13·2–8·77)
Diet low in vegetables	18·9 (7·74–36·6)	15·3 (7·76–28·1)	14·2 (6·5–26)	-19 (-41·1–24·5)	-7·4 (-32·6–20)	16·2 (6·91–32·2)	7·38 (3·23–17·3)	7·26 (3–17·8)	-54·3 (-72·3—21·6)	-1·64 (-33·8–47)
Diet low in whole grains	79·6 (69·8–88·4)	81·6 (73·3–89·7)	81·7 (72·8–89·8)	2·61 (-0·806–7·3)	0·00864 (-2·68–2·35)	--	--	--	--	--
Diet low in nuts and seeds	27·8 (8·48–56)	25·1 (8·43–55·2)	20·9 (5·78–53·8)	-9·74 (-44·4–60·4)	-16·9 (-45·8–9·24)	23·6 (5·29–54·5)	22·2 (6·51–54·7)	19·4 (5·82–53·6)	-6 (-39·8–75·1)	-12·6 (-40·7–15·9)
Diet low in milk	48·6 (25·7–68·8)	44·1 (20·9–64·2)	43 (19·7–64·7)	-9·23 (-27·8–2·81)	-2·54 (-14·1–9·66)	48·4 (24·3–69)	43·8 (19·6–64)	43·1 (19·5–63·5)	-9·36 (-29·1–3·65)	-1·67 (-13·5–10)
Diet high in red meat	79·9 (67·5–90)	78·9 (66·1–89·9)	78·6 (65·9–90·1)	-1·21 (-15·1–13·1)	-0·475 (-10·5–10·2)	83·3 (71·7–92·2)	78·9 (66·2–89·3)	78·7 (65·4–89·7)	-5·18 (-18·4–9·25)	-0·282 (-11·1–10·9)
Diet high in processed meat	80·8 (57·8–99·2)	87·7 (69·9–99·9)	87·5 (69–99·9)	8·5 (0·425–27)	-0·258 (-5·2–3·69)	82·1 (61–98·9)	90·2 (77–99·8)	88·8 (74–99·7)	9·82 (0·571–29·2)	-1·6 (-7·12–2·22)
Diet high in sugar-sweetened beverages	46 (28·3–64·4)	61·5 (41·9–79·3)	61·8 (42·5–79·9)	33·7 (-6·26–108)	0·453 (-19·5–24·6)	49·9 (30·9–68·7)	67·3 (47·9–83·5)	66·1 (46·1–83)	34·9 (-2·74–109)	-1·77 (-21·6–22·3)
Diet low in fiber	43·7 (27·7–59·9)	30·7 (18–44·9)	28 (15·4–41·7)	-29·7 (-46·4—13·1)	-8·81 (-26·6–9·54)	43·9 (27·3–59·9)	36·2 (22·1–50·3)	31·1 (17·6–46·2)	-17·7 (-33·8—0·931)	-13·9 (-30·4–0·84)
Diet low in seafood omega-3 fatty acids	83·4 (65·1–100)	80·4 (61·8–99·2)	80·2 (62·5–99·4)	-3·67 (-18·2–11·8)	-0·227 (-14–15·2)	86·1 (69·9–99·9)	84 (68·1–99·7)	83·6 (66·9–99·7)	-2·43 (-14·1–9·94)	-0·397 (-10·7–11·8)
Diet low in polyunsaturated fatty acids	13·9 (3·99–39·1)	7·41 (2·13–26·8)	7·1 (2·05–26·2)	-46·8 (-74·4—6·48)	-4·26 (-26·7–16·2)	15·5 (4·4–44·4)	7·64 (2·24–26·8)	7·22 (2·12–25·4)	-50·8 (-76·9—7·72)	-5·51 (-27·2–18·2)
Diet high in trans fatty acids	89·4 (81·6–100)	87·9 (79·4–100)	87·4 (77·3–100)	-1·63 (-9·33–5·84)	-0·635 (-8·63–8·44)	88·4 (81·4–100)	86·6 (78–100)	86·3 (77·7–100)	-2·03 (-9·89–5·76)	-0·411 (-8·69–8·6)
Diet high in sodium	18·6 (4·38–41·7)	40·2 (11·5–63·9)	39·5 (10·7–64·3)	117 (38·4–424)	-1·73 (-27–27·6)	--	--	--	--	--
Low physical activity	2·29 (0·85–4·97)	2·07 (0·894–4·32)	2 (0·739–4·33)	-9·83 (-40·6–39·2)	-3·32 (-31·3–28·7)	5·37 (2·54–10·4)	5·38 (2·86–9·59)	5·28 (2·66–10·1)	0·098 (-24·7–41·2)	-1·89 (-15·6–11·4)
Occupational asthmagens	26·4 (21·6–32·7)	22 (18·8–25·9)	23 (19·3–27·7)	-16·4 (-28·5—5·24)	4·59 (-4·95–16·6)	11·7 (9·61–14·2)	11·8 (9·82–14)	12 (9·86–14·4)	0·798 (-14·1–17·4)	1·28 (-9·72–13·1)
Occupational particulate matter, gases, and fumes	10·7 (7·62–15·4)	9·81 (7·06–14)	9·38 (6·75–13·4)	-8·16 (-13·6—2·99)	-4·42 (-8·04—0·896)	5·6 (3·99–8·37)	5·52 (3·99–8·07)	5·35 (3·84–7·72)	-1·42 (-10·2–7·5)	-3·09 (-7·94–2·31)
Occupational noise	9·02 (8·28–9·88)	8·59 (8–9·28)	8·29 (7·77–9)	-4·83 (-8·39—1·1)	-3·44 (-5·42—1·3)	6·15 (5·42–6·96)	6·52 (5·99–7·16)	6·55 (6·1–7·2)	6·05 (-0·00738–13·2)	0·456 (-2·48–3·77)
Occupational injuries	--	--	--	--	--	--	--	--	--	--
Occupational ergonomic factors	12·1 (10·1–14·7)	10·6 (9·01–12·7)	11 (9·15–13·2)	-12·4 (-22·1—1·82)	3·52 (-4·08–12·1)	7·14 (5·67–8·97)	7·26 (5·9–8·94)	7·6 (6·09–9·57)	1·66 (-14·5–20·9)	4·8 (-7·52–18·3)
Childhood sexual abuse	7·4 (5·05–10·5)	6·97 (4·6–9·93)	6·85 (4·54–9·67)	-5·83 (-12·2–5·83)	-1·68 (-8·51–5·53)	12·3 (8·94–17·3)	17·4 (12·9–24)	17·8 (13·1–24·7)	41·9 (30·7–54·8)	2·17 (-5–11)
Intimate partner violence	--	--	--	--	--	25·6 (13·9–34·3)	25·3 (14·5–33·5)	26·8 (15·1–35·7)	-1·14 (-9·89–14·8)	6·06 (-4·47–22·9)
Non-exclusive breastfeeding	26·3 (21·6–31)	24·9 (21·2–29)	24·8 (20–30)	-5·37 (-19·2–11·1)	-0·222 (-14–14·3)	--	--	--	--	--
Discontinued breastfeeding	27·3 (26·4–28·1)	25·2 (23·8–26·5)	25·6 (24·3–26·7)	-7·59 (-13·1—2·4)	1·39 (-4·01–7·34)	27·3 (26·4–28·2)	25·2 (23·8–26·5)	25·6 (24·3–26·7)	-7·64 (-13·1—2·46)	1·29 (-4·11–7·23)
Diet low in calcium	16·3 (8·01–30·2)	14 (6·87–25·8)	13·3 (6·34–24·5)	-14·3 (-24·4—1·89)	-4·79 (-14·4–2·94)	11·7 (4·94–22·3)	11 (4·86–20·4)	10·7 (4·58–20·2)	-5·68 (-20·2–13·3)	-2·95 (-13·5–6·57)
Occupational exposure to asbestos	9·65 (8·64–10·6)	8·45 (7·75–9·1)	8·06 (6–10·3)	-12·4 (-22—0·522)	-4·57 (-28·6–21·4)	1·66 (1·18–2·21)	1·57 (1·14–2·11)	1·55 (0·995–2·23)	-5·07 (-18·4–9·34)	-1·66 (-28·4–32·6)
Occupational exposure to arsenic	0·631 (0–1·5)	0·584 (0–1·39)	0·614 (0–1·48)	-7·55 (-19·5–NA)	5·21 (-6–NA)	0·516 (0–1·23)	0·523 (0–1·26)	0·532 (0–1·32)	1·27 (-14·7–NA)	1·64 (-10·9–NA)
Occupational exposure to benzene	0·839 (0·0485–2·4)	0·812 (0·0473–2·33)	0·843 (0·0519–2·41)	-3·17 (-10·8–7·77)	3·77 (-3·41–13·4)	1·19 (0·0695–3·44)	1·34 (0·0755–3·62)	1·41 (0·0878–4·13)	12·4 (0·534–32·3)	4·76 (-3·74–16·6)
Occupational exposure to beryllium	0·0246 (0·0233–0·026)	0·0238 (0·0232–0·0243)	0·0251 (0·0236–0·0265)	-3·33 (-8·96–2·6)	5·46 (-0·551–11·4)	0·0269 (0·0247–0·0293)	0·0285 (0·0279–0·0291)	0·0295 (0·0276–0·0313)	5·63 (-2·75–15·7)	3·54 (-2·99–10·2)
Occupational exposure to cadmium	0·072 (0·0657–0·0792)	0·0673 (0·0612–0·0735)	0·0708 (0·0627–0·0785)	-6·53 (-17·9–5·88)	5·12 (-4·9–15·8)	0·0612 (0·0541–0·0689)	0·0625 (0·0561–0·0695)	0·0637 (0·0556–0·0729)	2·13 (-12·6–20·3)	2 (-9·39–15)
Occupational exposure to chromium	0·169 (0·156–0·184)	0·161 (0·149–0·173)	0·17 (0·153–0·185)	-4·39 (-14·4–6·48)	5·01 (-4–14·6)	--	--	--	--	--
Occupational exposure to diesel engine exhaust	0·681 (0·628–0·735)	0·732 (0·687–0·782)	0·77 (0·707–0·842)	7·48 (-2·73–18·2)	5·31 (-2·36–13·7)	0·678 (0·606–0·758)	0·73 (0·669–0·8)	0·735 (0·652–0·827)	7·59 (-6·11–24·3)	0·74 (-9·16–11·2)
Occupational exposure to formaldehyde	0·366 (0·333–0·404)	0·332 (0·301–0·365)	0·345 (0·305–0·386)	-9·22 (-20·5–3·37)	3·86 (-6·58–14·7)	0·299 (0·265–0·336)	0·307 (0·277–0·339)	0·314 (0·276–0·356)	2·61 (-11·9–19·3)	2·27 (-8·42–14·8)
Occupational exposure to nickel	0·533 (0–2·38)	0·476 (0–2·13)	0·5 (0–2·16)	-10·7 (-22·7–NA)	5·09 (-5·74–NA)	0·408 (0–1·76)	0·399 (0–1·78)	0·408 (0–1·86)	-2·03 (-18·5–NA)	2·09 (-11·6–NA)
Occupational exposure to polycyclic aromatic hydrocarbons	0·332 (0·307–0·361)	0·318 (0·296–0·341)	0·333 (0·302–0·363)	-4·33 (-13·9–5·81)	4·85 (-4·17–14·1)	0·32 (0·289–0·355)	0·344 (0·322–0·368)	0·352 (0·321–0·389)	7·44 (-4·35–21·6)	2·4 (-5·76–11·8)
Occupational exposure to silica	4·71 (0·662–15·1)	4·13 (0·574–13·2)	4·37 (0·611–13·9)	-12·3 (-24–0·875)	5·77 (-5·05–19·6)	3·44 (0·483–10·7)	3·25 (0·459–10·2)	3·29 (0·435–10·2)	-5·55 (-21·7–18·2)	1·35 (-12·4–19·9)
Occupational exposure to sulfuric acid	1·08 (0·231–3·48)	1·1 (0·228–3·49)	1·14 (0·235–3·74)	2·09 (-9·95–16·3)	3·61 (-6·28–14·7)	0·972 (0·206–3·02)	1·17 (0·237–3·76)	1·15 (0·241–3·62)	20 (-1·56–46·5)	-1·2 (-15·5–13·9)
Unsafe sex	--	--	--	--	--	--	--	--	--	--
Occupational exposure to trichloroethylene	0·0926 (0·0871–0·0997)	0·0923 (0·0881–0·0967)	0·097 (0·0898–0·104)	-0·344 (-7·79–8·21)	5·08 (-2·13–12·5)	0·087 (0·0791–0·0958)	0·0917 (0·0862–0·0981)	0·0934 (0·0852–0·102)	5·41 (-5·55–18·9)	1·79 (-6·35–11·2)
No access to handwashing facility	3·36 (3·1–3·64)	3·11 (2·87–3·38)	3·08 (2·86–3·34)	-7·4 (-15·2–1·69)	-0·978 (-6·51–4·8)	3·36 (3·1–3·64)	3·11 (2·87–3·38)	3·08 (2·86–3·34)	-7·4 (-15·2–1·69)	-0·978 (-6·51–4·8)
Child wasting	0·749 (0·494–1·02)	0·728 (0·478–0·999)	0·725 (0·468–0·982)	-2·84 (-11·6–8·17)	-0·343 (-7·54–7·73)	0·773 (0·507–1·05)	0·764 (0·504–1·05)	0·76 (0·5–1·04)	-1·13 (-11–9·44)	-0·546 (-8·23–7·41)
Child stunting	2·4 (1·29–3·52)	2·08 (1·08–3·14)	2·03 (1·04–3·09)	-13·3 (-32·1–10·5)	-2·37 (-17·8–13·7)	1·95 (0·974–2·93)	1·79 (0·903–2·76)	1·77 (0·873–2·69)	-7·85 (-27·9–17·3)	-1·09 (-16·4–16·9)
Chewing tobacco	4·96 (3·93–6·08)	3·88 (2·89–5·11)	3·61 (2·66–4·82)	-21·8 (-43·3–9·35)	-6·97 (-26·2–18·4)	--	--	--	--	--
Diet low in legumes	74 (37·9–99·3)	77·3 (46–99·8)	77·2 (46·1–99·7)	4·36 (0–23·2)	-0·099 (-5·32–5·13)	72·4 (35·7–98·8)	75·6 (42·6–99·6)	75·9 (44·3–99·6)	4·46 (0–24·7)	0·436 (-5–7·94)
Short gestation	32·8 (27–39·3)	27·9 (23·8–33)	26·2 (21·8–31·9)	-14·9 (-30·4–5·49)	-6·19 (-23·8–16·4)	31·3 (26·3–36·8)	27·7 (23·6–32·6)	24·9 (21·1–29·2)	-11·4 (-28·8–9·91)	-9·93 (-24·6–8·23)
Low birth weight	13·2 (11–15·7)	12·1 (10·3–14·2)	11·8 (9·78–14·3)	-7·94 (-26–14·8)	-2·73 (-21·8–21·8)	14·6 (12·2–17·4)	14·2 (12·1–16·6)	13·3 (11·3–15·5)	-2·75 (-23·8–20·2)	-6·01 (-22·3–14)
High temperature	25·2 (16·1–37)	25·6 (16·2–37·2)	25·7 (16·3–37·4)	1·38 (-2·38–4·09)	0·504 (-1·59–2·5)	25·2 (16·1–37)	25·6 (16·2–37·2)	25·7 (16·3–37·4)	1·38 (-2·38–4·09)	0·504 (-1·59–2·5)
Low temperature	33·1 (29·4–37·6)	35·6 (32–39·9)	35·5 (31·9–40)	7·61 (4·74–10·7)	-0·117 (-1·56–1·29)	33·1 (29·4–37·6)	35·6 (32–39·9)	35·5 (31·9–40)	7·61 (4·74–10·7)	-0·117 (-1·56–1·29)
Kidney dysfunction	20·1 (14·3–27·3)	20·7 (14·8–27·7)	20·8 (15–28)	2·92 (-0·894–7·21)	0·307 (-3·25–4·46)	22·5 (16–30)	23·5 (16·8–31·5)	24·4 (17·7–32·3)	4·78 (0·382–10)	3·66 (-0·62–8·63)
Bullying victimization	15·8 (6·61–31)	16 (6·65–31·1)	18·7 (8·13–32·9)	1·14 (-25·6–27)	17·2 (4·65–37·8)	--	--	--	--	--
High LDL cholesterol	66·9 (62·6–71·3)	34·1 (30·5–37·5)	30·2 (26·6–33·9)	-49 (-54·1—43·7)	-11·3 (-16·3—6·58)	66·2 (62·5–70)	34 (30·7–37·2)	31 (27·5–34·6)	-48·7 (-53·6—44·3)	-8·65 (-12·9—4·61)

Supplementary Results Table 1. Oklahoma age-standardised Summary Exposure Values (SEVs) for most-detailed risk factors and percentage change, by sex, 1990, 2010, and 2019. Data in parentheses are 95% uncertainty intervals.

	Male					Female				
	1990	2010	2019	Percentage change 1990-2010	Percentage change 2010-2019	1990	2010	2019	Percentage change 1990-2010	Percentage change 2010-2019
Unsafe sanitation	8·59 (4·16–15·1)	5 (2·31–8·82)	3·86 (1·75–7·36)	-41·8 (-76·9–34·9)	-22·8 (-61·7–53·6)	8·59 (4·16–15·1)	5 (2·31–8·82)	3·86 (1·75–7·36)	-41·8 (-76·9–34·9)	-22·8 (-61·7–53·6)
Ambient particulate matter pollution	11·8 (3·83–23·6)	7·98 (5·02–11·2)	5·64 (2·91–8·67)	-32·2 (-66·2–73·6)	-29·3 (-43–-22·4)	11·8 (3·83–23·6)	7·98 (5–11·2)	5·64 (2·91–8·66)	-32·2 (-66·2–74·4)	-29·3 (-43·1–-22·4)
Household air pollution from solid fuels	0·0191 (0·00464–0·0511)	0·0125 (0·0024–0·0369)	0·00861 (0·0014–0·0261)	-34·3 (-76–50·1)	-31·2 (-66·2–35·1)	0·0251 (0·00618–0·0665)	0·0166 (0·00315–0·0495)	0·0114 (0·00194–0·0354)	-34 (-76–49·9)	-31·2 (-66·2–35·3)
Ambient ozone pollution	71·9 (31–100)	74·1 (32·9–100)	48·5 (19·7–83·6)	3·17 (-3·73–12·3)	-34·6 (-46·4–-16·4)	--	--	--	--	--
Residential radon	18·3 (2·8–57·7)	18·3 (2·8–57·7)	18·3 (2·8–57·7)	0 (0–0)	0 (0–0)	18·3 (2·8–57·7)	18·3 (2·8–57·7)	18·3 (2·8–57·7)	0 (0–0)	0 (0–0)
Lead exposure	52·1 (23·7–75·9)	32·1 (11·5–54·4)	23 (6·77–43·2)	-38·5 (-56·1–-25·7)	-28·4 (-44·7–-20·2)	28·8 (2·13–57·7)	18·5 (0·88–42·9)	13·4 (0·36–33·9)	-35·9 (-69·4–-21·8)	-27·7 (-62·9–-19·7)
Child underweight	0·966 (0·534–1·47)	0·982 (0·545–1·5)	0·959 (0·532–1·45)	1·59 (-15·5–23·2)	-2·27 (-15·6–12·4)	0·812 (0·441–1·25)	0·859 (0·462–1·3)	0·88 (0·478–1·36)	5·72 (-14·6–31·5)	2·43 (-12·5–19)
Iron deficiency	--	--	--	--	--	11·9 (10–14·1)	12·9 (10·9–14·9)	13·1 (10·9–15·4)	8·11 (-4·5–23·2)	1·4 (-7·8–11·8)
Vitamin A deficiency	1·06 (0·292–2·74)	1·46 (0·405–3·85)	0·984 (0·257–2·51)	37·3 (-67·4–519)	-32·4 (-71·3–64·9)	1·99 (0·74–4·17)	2·08 (0·773–4·56)	1·72 (0·635–3·61)	4·56 (-60·6–210)	-17·5 (-51·6–59·7)
Zinc deficiency	2·43 (0–7·92)	1·25 (0–4·05)	1·24 (0–3·98)	-48·5 (-100–NA)	-0·536 (-22·4–NA)	2·5 (0–8·12)	1·26 (0–4·11)	1·23 (0–3·89)	-49·7 (-100–NA)	-2·39 (-22·9–NA)
Smoking	28 (25·2–30·9)	21·5 (19·5–23·7)	20 (18·1–22·1)	-23 (-28·6–-17·4)	-7·15 (-10·6–-3·32)	20·7 (17·9–23·6)	16·9 (15·1–18·7)	15·6 (13·9–17·3)	-18·2 (-27·1–-8·24)	-7·96 (-12–-3·47)
Secondhand smoke	40 (37·4–42·8)	28·7 (26·9–30·4)	27·9 (25–30·8)	-28·4 (-34·8–-20·9)	-2·65 (-11·8–5·98)	36·9 (34·9–38·9)	27·1 (25·8–28·4)	26·4 (24·2–28·7)	-26·6 (-32·1–-20·7)	-2·47 (-9·74–5·13)
Alcohol use	12·8 (8·27–17·6)	13·1 (8·3–18·8)	13·5 (8·58–19·2)	2·19 (-25–40·8)	3·08 (-17·5–29)	8·22 (5·14–11·7)	9·27 (5·91–13·3)	9·06 (5·46–13·2)	12·8 (-19–58·3)	-2·25 (-24·6–22·1)
Drug use	0·53 (0·309–0·917)	1·13 (0·822–1·53)	1·62 (1·22–2·12)	113 (52·1–211)	43·9 (24·1–66·6)	0·431 (0·284–0·674)	0·953 (0·703–1·27)	1·51 (1·18–1·91)	121 (73·8–176)	58·5 (35·6–83·5)
High fasting plasma glucose	12·6 (10·4–15·4)	19·4 (17·1–22·1)	20·9 (18·1–24·2)	53·3 (27·5–86·7)	8·03 (-5·78–24·9)	9·95 (8·47–12)	15·9 (14–18)	17 (14·9–19·7)	59·5 (34·5–87·6)	7·41 (-6·05–23·1)
High systolic blood pressure	27 (22–32·9)	17·1 (15·1–19·3)	21 (16·6–25·3)	-36·6 (-48·6–22·5)	23 (-1·45–50·7)	24 (17·4–30·8)	14·7 (13–16·3)	19·7 (14·4–26·2)	-39 (-53–-16·6)	34·1 (-1·6–80·2)
High body-mass index	24·5 (17–33·9)	40·1 (29·4–51·6)	42 (31·2–53·6)	64 (42·7–93·7)	4·79 (-1·75–12·8)	30·5 (22·8–39·6)	44·8 (34·7–54)	46·3 (36·3–55·9)	46·7 (30·3–68·6)	3·24 (-3·16–11)
Low bone mineral density	12·1 (7·14–18·5)	9·32 (5·06–15·6)	11·7 (6·68–19)	-22·9 (-42·5–-1·06)	26·1 (-0·343–69·3)	16·1 (10·2–23·3)	16·6 (11–23·6)	19·1 (12·9–26·5)	3·28 (-18·7–32·1)	15·3 (-8·74–48·5)
Diet low in fruits	56·9 (45·4–69·9)	51·9 (40·4–65·5)	50·9 (37·9–64·7)	-8·67 (-18·9–0·0359)	-1·97 (-12·7–8·58)	57·5 (45·8–70·8)	53·8 (42·2–66·6)	52·8 (40·5–65·6)	-6·33 (-16·1–3·08)	-1·89 (-11·9–7·42)
Diet low in vegetables	36·3 (19·4–57·6)	46·6 (29·3–66)	44·2 (27·2–64·6)	28·5 (7·55–73·1)	-5·11 (-15·9–4·91)	37·5 (20·9–59·8)	47·4 (28·1–69·1)	44·9 (26·4–66·6)	26·3 (6·72–61·8)	-5·39 (-17·5–4·09)
Diet low in whole grains	79·8 (70·1–88·6)	81·8 (73·5–89·5)	81·7 (72·8–89·7)	2·47 (-1·08–6·69)	-0·082 (-2·71–2·5)	77·2 (67–87)	79·9 (70·9–88·9)	79·6 (70·1–88·7)	3·58 (-0·505–9·71)	-0·396 (-3·37–2·23)
Diet low in nuts and seeds	29·4 (8·02–56·9)	25·7 (7·9–55·8)	21·6 (6·42–54·3)	-12·7 (-44·6–54·5)	-16·2 (-42·2–10·6)	25 (7·01–55·4)	22·4 (7·1–54·2)	19·8 (6·22–53·6)	-10·3 (-45·5–57)	-11·6 (-41–16·6)
Diet low in milk	50 (26·4–71)	44·6 (21·9–64·6)	43·3 (20·1–64)	-10·7 (-30·5–1·24)	-2·9 (-14·7–8·96)	49·8 (25·9–71·2)	44·8 (21·7–65)	43·8 (20·7–63·7)	-10 (-26·3–1·97)	-2·33 (-14·1–9·25)
Diet high in red meat	85·9 (75·7–93·1)	84·9 (73·8–93·4)	84·2 (72·1–93·7)	-1·09 (-13·1–10·5)	-0·831 (-10·5–8·28)	84·8 (75·4–92·1)	81·4 (68·9–90·7)	81 (68·2–91)	-3·97 (-17·1–8·37)	-0·486 (-9·62–9·8)
Diet high in processed meat	80·4 (57·1–99·2)	87·4 (69·8–99·8)	87·3 (69·2–99·7)	8·73 (0·448–27·1)	-0·2 (-4·81–4·32)	82·6 (62·2–99·4)	90·5 (76·6–99·9)	89·1 (73·9–99·9)	9·53 (0·306–29·9)	-1·55 (-6·66–1·59)
Diet high in sugar-sweetened beverages	44·1 (26·2–64·8)	60·7 (41·9–77·9)	61·8 (42·9–79·5)	37·5 (-2·34–115)	1·89 (-18·9–28·7)	48·8 (29·3–67·7)	66·9 (47–82·8)	66·2 (46·7–82·9)	37·2 (-2·58–121)	-1·09 (-18·6–24·2)
Diet low in fiber	44·9 (28–61·7)	31·4 (18·3–46·8)	28·4 (15·2–42·6)	-30·1 (-45·6–12·8)	-9·33 (-27·6–7·98)	45·4 (28·4–61·6)	36·8 (22·5–51·7)	31·5 (17·7–46·4)	-19 (-34·4–-2·16)	-14·3 (-31·7–0·57)
Diet low in seafood omega-3 fatty acids	81·7 (63·5–99·6)	77·7 (59–98·9)	77 (59·3–98·9)	-4·91 (-20·4–12)	-0·836 (-16·3–16·4)	84·9 (69·6–99·9)	82·4 (65·5–99·6)	81·2 (63·9–99·1)	-2·95 (-15·1–8·27)	-1·48 (-13·4–11)
Diet low in polyunsaturated fatty acids	14·7 (4·22–44·1)	7·66 (2·23–27·9)	7·25 (2·17–24·7)	-48 (-74·5–-7·37)	-5·3 (-28·5–17·6)	16·5 (5·05–46·1)	7·89 (2·23–28·3)	7·34 (2·13–26·5)	-52·2 (-77·9–-9·33)	-7·03 (-30–10·9)
Diet high in trans fatty acids	89·4 (81·3–100)	87·9 (79·1–100)	87·3 (78·9–100)	-1·65 (-9·33–5·94)	-0·669 (-8·33–8·46)	88·5 (80·9–100)	86·7 (77·9–100)	86·2 (77·5–100)	-1·99 (-9·65–5·83)	-0·519 (-8·23–7·78)
Diet high in sodium	18·2 (4·58–40·9)	39·7 (11·6–64)	39 (11–64·3)	118 (38·8–426)	-1·68 (-28·4–28·9)	20·8 (4·85–45·1)	21 (4·76–44·8)	21·7 (4·99–45·6)	1·24 (-30–50·6)	3·01 (-19·7–32·7)
Low physical activity	2·86 (1·13–6·11)	2·97 (1·3–5·8)	3·06 (1·2–6·5)	3·91 (-28–68·8)	3·16 (-20·9–33)	6·07 (2·99–11·4)	7·12 (3·97–12·5)	6·76 (3·54–12·5)	17·1 (-11·8–63·7)	-5·02 (-16·6–5·74)
Occupational asthmagens	23·5 (19·9–27·7)	21·2 (18·1–25)	21·7 (18·2–26·1)	-9·67 (-19·8–1·32)	2·46 (-6·03–11·6)	11·5 (9·39–13·8)	11·3 (9·41–13·3)	11·3 (9·15–13·6)	-1·94 (-16·4–14·9)	0·174 (-10·9–13)
Occupational particulate matter, gases, and fumes	9·7 (6·84–13·9)	9·2 (6·58–13·3)	8·91 (6·36–12·8)	-5·08 (-10·3–0·871)	-3·19 (-6·63–0·512)	5·22 (3·59–7·57)	5·14 (3·7–7·42)	4·98 (3·57–7·28)	-1·49 (-10·3–8·71)	-3·05 (-7·96–2·14)
Occupational noise	9·14 (8·39–10)	8·7 (8·12–9·42)	8·41 (7·92–9·09)	-4·8 (-8·44–-0·826)	-3·38 (-5·38–-1·06)	6·06 (5·29–6·89)	6·37 (5·84–7·01)	6·37 (5·95–6·93)	5·2 (-0·799–12·7)	-0·0358 (-3·1–3·54)
Occupational injuries	--	--	--	--	--	--	--	--	--	--
Occupational ergonomic factors	13·2 (10·9–16·1)	11·5 (9·76–13·8)	11·9 (9·95–14·3)	-12·6 (-23·9–-0·486)	3·14 (-5·39–12·2)	7·61 (6·05–9·38)	7·5 (6·09–9·13)	7·77 (6·28–9·7)	-1·57 (-17·8–16·5)	3·73 (-9·15–17·6)
Childhood sexual abuse	8 (5·46–11·3)	7·17 (5·24–9·24)	7·18 (5·22–9·3)	-10·3 (-23–4·62)	0·144 (-5·43–7·04)	18·6 (13·5–26·1)	17 (13·4–22·7)	17·2 (13·4–22·7)	-8·43 (-20·7–5·53)	1·02 (-5·23–8·23)
Intimate partner violence	--	--	--	--	--	23·5 (13·4–32·2)	23·5 (13·3–31·8)	25 (14·4–33·6)	0·0988 (-9·23–15·6)	6·37 (-4·46–25·4)
Non-exclusive breastfeeding	26·1 (21·3–30·9)	24·8 (21·2–28·9)	24·8 (20·1–29·7)	-4·88 (-19·2–13·6)	-0·172 (-13·5–14·1)	26·1 (21·3–30·9)	24·8 (21·2–28·9)	24·8 (20·1–29·7)	-4·89 (-19·2–13·6)	-0·174 (-13·5–14·1)
Discontinued breastfeeding	27 (26–27·8)	25·5 (24·2–26·7)	25·5 (24·2–26·7)	-5·4 (-11–0·549)	0·0872 (-5·21–5·78)	27 (26·1–27·9)	25·5 (24·3–26·7)	25·5 (24·2–26·7)	-5·63 (-11·2–0·312)	-0·0494 (-5·33–5·64)
Diet low in calcium	17·2 (8·17–32·1)	14·3 (6·9–26·1)	13·5 (6·52–24·9)	-16·9 (-28·6–-5·92)	-5·37 (-14·4–2·86)	12·7 (5·33–24·4)	11·4 (4·93–21·8)	10·9 (4·75–21)	-10·3 (-24·7–7·16)	-4·09 (-14·9–5·47)
Occupational exposure to asbestos	5·81 (5·21–6·43)	5·19 (4·68–5·72)	4·99 (3·65–6·65)	-10·6 (-23–3·37)	-3·82 (-29·6–26·8)	1·25 (0·987–2·01)	1·34 (1·08–2·15)	1·34 (0·865–2·23)	6·51 (-9·19–26·4)	0·472 (-28·5–34·8)
Occupational exposure to arsenic	0·487 (0–1·15)	0·506 (0–1·22)	0·534 (0–1·26)	4·02 (-11·2–NA)	5·53 (-6·58–NA)	0·445 (0–1·03)	0·462 (0–1·11)	0·474 (0–1·14)	3·78 (-14·6–NA)	2·66 (-9·54–NA)
Occupational exposure to benzene	0·816 (0·0465–2·29)	0·821 (0·0487–2·38)	0·848 (0·052–2·47)	0·53 (-7·97–14·3)	3·34 (-4·25–12·4)	1·17 (0·0674–3·33)	1·3 (0·0779–3·62)	1·36 (0·0816–3·92)	11·4 (-1·12–30·3)	4·22 (-5·3–16·6)
Occupational exposure to beryllium	0·0242 (0·0228–0·0257)	0·0242 (0·0236–0·0248)	0·0254 (0·024–0·0269)	-0·184 (-6·34–6·17)	4·96 (-1–10·8)	0·0263 (0·0241–0·0287)	0·0275 (0·0269–0·0281)	0·0285 (0·0264–0·0306)	4·49 (-4·48–14·7)	3·48 (-4·05–10·8)
Occupational exposure to cadmium	0·0572 (0·0521–0·0634)	0·0596 (0·0538–0·0657)	0·0629 (0·0553–0·0711)	4·2 (-9·06–18·8)	5·42 (-5·85–16·9)	0·0537 (0·0475–0·0609)	0·056 (0·0504–0·0621)	0·0575 (0·0503–0·0658)	4·33 (-11·7–21·9)	2·66 (-8·26–16·1)
Occupational exposure to chromium	0·138 (0·126–0·151)	0·146 (0·134–0·158)	0·153 (0·138–0·171)	5·86 (-5·85–19·1)	5·13 (-4·76–15·1)	--	--	--	--	--
Occupational exposure to diesel engine exhaust	0·789 (0·726–0·853)	0·815 (0·761–0·87)	0·846 (0·774–0·919)	3·23 (-6·49–14·6)	3·89 (-3·81–12·3)	0·701 (0·625–0·792)	0·729 (0·667–0·8)	0·727 (0·64–0·82)	4·03 (-10–20·4)	-0·362 (-10·2–11·2)
Occupational exposure to formaldehyde	0·282 (0·255–0·314)	0·289 (0·259–0·322)	0·301 (0·261–0·342)	2·23 (-11·4–18·1)	4·11 (-7·44–15·9)	0·261 (0·232–0·295)	0·274 (0·248–0·302)	0·282 (0·248–0·32)	4·97 (-10·7–21·6)	2·73 (-8·43–15·8)
Occupational exposure to nickel	0·458 (0–2·01)	0·43 (0–1·85)	0·449 (0–1·95)	-6·21 (-18·6–NA)	4·54 (-6·75–NA)	0·365 (0–1·67)	0·358 (0–1·63)	0·367 (0–1·62)	-2·1 (-20·6–NA)	2·7 (-10·7–NA)
Occupational exposure to polycyclic aromatic hydrocarbons	0·275 (0·253–0·299)	0·289 (0·268–0·312)	0·303 (0·274–0·335)	5·15 (-6·05–17·8)	4·8 (-4·29–14·2)	0·29 (0·261–0·321)	0·315 (0·295–0·337)	0·323 (0·291–0·357)	8·81 (-4·65–22·4)	2·63 (-6·17–12·1)
Occupational exposure to silica	4·67 (0·683–15)	4 (0·597–12·4)	4·18 (0·633–13·2)	-14·3 (-26·1–1·88)	4·49 (-6·52–18)	3·27 (0·464–10·3)	3 (0·401–9·74)	3·04 (0·425–9·86)	-8·1 (-25·5–12·3)	1·29 (-11·8–20·6)
Occupational exposure to sulfuric acid	0·891 (0·191–2·85)	1·02 (0·214–3·28)	1·05 (0·226–3·49)	14·2 (-0·775–31·9)	3·51 (-7·65–15·5)	0·86 (0·178–2·8)	1·06 (0·215–3·46)	1·05 (0·22–3·41)	23·4 (-0·858–51·4)	-0·597 (-15·3–15·7)
Unsafe sex	--	--	--	--	--	--	--	--	--	--
Occupational exposure to trichloroethylene	0·0821 (0·0765–0·0885)	0·0885 (0·0843–0·0935)	0·0929 (0·0862–0·1)	7·8 (-0·721–18)	4·99 (-2·17–12·6)	0·0808 (0·0733–0·0897)	0·0858 (0·0805–0·0917)	0·0873 (0·0794–0·0965)	6·23 (-5·96–19·3)	1·77 (-6·7–12)
No access to handwashing facility	3·44 (3·17–3·72)	3·16 (2·91–3·42)	3·12 (2·88–3·37)	-7·94 (-15·5–0·691)	-1·45 (-6·89–4·59)	3·44 (3·17–3·72)	3·16 (2·91–3·42)	3·12 (2·88–3·37)	-7·94 (-15·5–0·691)	-1·45 (-6·89–4·59)
Child wasting	0·75 (0·495–1·03)	0·73 (0·484–0·999)	0·728 (0·474–0·986)	-2·79 (-12·2–7·16)	-0·215 (-7·33–7·6)	0·775 (0·516–1·05)	0·767 (0·511–1·05)	0·762 (0·509–1·04)	-1·06 (-11·4–9·3)	-0·618 (-8·47–7·65)
Child stunting	2·44 (1·29–3·6)	2·15 (1·11–3·21)	2·08 (1·08–3·07)	-12 (-30·3–16·1)	-3·17 (-17·8–13)	1·97 (0·98–2·99)	1·84 (0·896–2·81)	1·81 (0·91–2·79)	-6·55 (-26·3–20·4)	-1·18 (-16·5–18·4)
Chewing tobacco	5·38 (4·33–6·6)	5·13 (3·9–6·65)	4·79 (3·47–6·44)	-4·57 (-30·5–32·5)	-6·74 (-25·4–17·2)	--	--	--	--	--
Diet low in legumes	74·5 (39·8–99·4)	77·5 (46·9–99·6)	77·4 (46·2–99·7)	4·06 (-0·152–22·7)	-0·196 (-5·75–4·68)	72·9 (35·7–99·2)	75·8 (42·3–99·6)	75·9 (44·5–99·6)	4·04 (-0·22–21·9)	0·19 (-5·6–7·32)
Short gestation	20·7 (16·7–25·8)	25·5 (21·1–30·4)	24·6 (19·9–29·7)	23·3 (-4·76–59)	-3·74 (-22·5–21)	18·7 (15·4–22·4)	25·2 (21·7–29·3)	23·9 (20–28·5)	34·8 (9·73–66·4)	-4·88 (-20·4–13·9)
Low birth weight	8·89 (7·13–10·9)	10·6 (8·84–12·6)	10·8 (8·87–13·1)	19·6 (-8·44–55·9)	1·98 (-18·8–27·8)	9·09 (7·58–10·9)	12·9 (11·2–14·7)	12·7 (10·8–14·9)	41·8 (13·3–75·3)	-1·07 (-16·8–18·3)
High temperature	33·1 (21·8–43·9)	35·7 (24·6–46·4)	29·4 (18·5–40·1)	8·09 (4·65–13·5)	-17·8 (-26–-12·5)	33·1 (21·8–43·9)	35·7 (24·6–46·4)	29·4 (18·5–40·1)	8·09 (4·65–13·5)	-17·8 (-26–-12·5)
Low temperature	36·6 (31·3–43·3)	38·9 (34·1–45·3)	37·9 (32·9–44·6)	6·12 (3·32–9·86)	-2·43 (-4·16–-0·884)	36·6 (31·3–43·3)	38·9 (34·1–45·3)	37·9 (32·9–44·6)	6·12 (3·32–9·86)	-2·43 (-4·16–-0·884)
Kidney dysfunction	20 (14·2–27·1)	20·6 (14·7–27·8)	20·8 (15–28·1)	2·86 (-0·778–7·03)	1·14 (-2·31–5·1)	22·5 (16·1–30·2)	23·5 (16·7–31·2)	24·5 (17·7–32·3)	4·71 (0·885–10)	4·39 (0·436–9·04)

Bullying victimization	5·86 (2·43–12)	8·87 (3·57–17·6)	8·67 (3·34–17·8)	51·3 (31·2–73·6)	-2·26 (-11·8–3·56)	--	--	--	--	--
High LDL cholesterol	65·6 (61·2–70)	34·4 (30·9–37·9)	30·7 (27·2–34·5)	-47·5 (-52·7–-42·1)	-10·8 (-15·9–-5·56)	64·9 (61·2–68·6)	34·6 (31·2–38)	31·8 (28·2–35·3)	-46·6 (-51·3–-42)	-8·25 (-12·1–-4·16)

Supplementary Results Table 1. Oregon age-standardised Summary Exposure Values (SEVs) for most-detailed risk factors and percentage change, by sex, 1990, 2010, and 2019. Data in parentheses are 95% uncertainty intervals.

	Male					Female				
	1990	2010	2019	Percentage change 1990-2010	Percentage change 2010-2019	1990	2010	2019	Percentage change 1990-2010	Percentage change 2010-2019
Unsafe sanitation	6·32 (2·9–11·4)	3·31 (1·35–6·33)	2·59 (1·14–5·23)	-47·5 (-79·6–31·4)	-21·7 (-59·8–62·7)	--	--	--	--	--
Ambient particulate matter pollution	5·4 (0·594–13·5)	3·54 (1·33–6·22)	3·55 (1·35–6·24)	-34·5 (-78·1–277)	0·301 (-4·99–5·57)	5·41 (0·604–13·5)	3·54 (1·33–6·2)	3·55 (1·34–6·22)	-34·5 (-78·2–266)	0·287 (-5·13–5·38)
Household air pollution from solid fuels	0·0243 (0·00294–0·0753)	0·0136 (0·00178–0·0478)	0·0106 (0·00147–0·0375)	-44 (-82·4–129)	-22·5 (-58·1–51·1)	0·0321 (0·00388–0·101)	0·018 (0·0026–0·0604)	0·014 (0·00201–0·0465)	-43·8 (-82·3–109)	-22·4 (-58·1–50·2)
Ambient ozone pollution	26·2 (10–47·4)	25·3 (9·36–47·1)	41·1 (16·1–71·1)	-3·41 (-28·6–25)	62·1 (29·1–116)	--	--	--	--	--
Residential radon	21·8 (3·41–60·9)	21·8 (3·41–60·9)	21·8 (3·41–60·9)	0 (0–0)	0 (0–0)	21·8 (3·41–60·9)	21·8 (3·41–60·9)	21·8 (3·41–60·9)	0 (0–0)	0 (0–0)
Lead exposure	59·3 (32–80·6)	33·9 (13·4–55·8)	23·4 (7·36–42·2)	-42·9 (-60—28·8)	-30·9 (-46·5—22·8)	34·1 (4·91–62·2)	19·1 (1·34–43·3)	13·1 (0·456–32·8)	-43·9 (-78·3—28·5)	-31·6 (-68·7—23·5)
Child underweight	0·946 (0·527–1·41)	0·952 (0·526–1·43)	0·933 (0·519–1·41)	0·555 (-16·6–23·7)	-1·98 (-15·1–12·7)	0·798 (0·416–1·21)	0·837 (0·456–1·29)	0·859 (0·471–1·31)	4·88 (-15·1–32)	2·6 (-12·3–18·5)
Iron deficiency	--	--	--	--	--	11·2 (9·3–13·2)	11·6 (9·64–13·5)	11·8 (9·89–14·1)	3·53 (-8·4–17·1)	1·68 (-7·75–12·3)
Vitamin A deficiency	0·815 (0·231–2·06)	1·1 (0·279–2·83)	0·753 (0·201–2·1)	34·8 (-65·7–430)	-31·5 (-71·6–80·8)	1·63 (0·602–3·17)	1·65 (0·658–3·43)	1·38 (0·531–2·92)	1·26 (-59–181)	-16·2 (-53·9–63·8)
Zinc deficiency	2·38 (0–7·75)	1·22 (0–4·02)	1·23 (0–3·87)	-48·9 (-100–NA)	1·14 (-18·4–NA)	2·43 (0–7·72)	1·23 (0–4·07)	1·21 (0–3·93)	-49·4 (-100–NA)	-1·4 (-21·2–NA)
Smoking	26·5 (23·7–29·4)	18·6 (16·7–20·6)	16·4 (14·6–18·4)	-29·9 (-35–24·2)	-11·9 (-15·8—7·87)	19·5 (16·9–22·1)	14·9 (13·3–16·7)	13 (11·5–14·6)	-23·6 (-31·3—14·9)	-12·7 (-16·8—7·87)
Secondhand smoke	38·1 (35·7–40·6)	24·5 (23–26·2)	24·9 (22·1–27·7)	-35·7 (-41·4—29·2)	1·52 (-8·32–11)	35 (33–37·2)	23 (21·8–24·2)	23·4 (21·3–25·4)	-34·2 (-39·6—28·3)	1·61 (-6·16–9·2)
Alcohol use	15 (9·28–21·7)	15·7 (9·32–23·1)	16·8 (10·1–24·7)	4·89 (-28·5–49·3)	6·73 (-16·9–37)	--	--	--	--	--
Drug use	0·686 (0·41–1·16)	1·06 (0·765–1·43)	1·42 (1·07–1·85)	54·7 (17·2–105)	33·5 (16–54·2)	0·463 (0·297–0·731)	0·796 (0·597–1·05)	1·19 (0·929–1·53)	72 (38·7–116)	49·9 (29·5–76·7)
High fasting plasma glucose	12·3 (10·3–14·9)	16·5 (14·2–19·3)	18 (15·4–20·9)	33·9 (9·93–61·4)	9·26 (-4·4–27·3)	9·32 (7·91–11·3)	11·9 (10·2–13·9)	13·6 (11·6–15·9)	28 (5·65–53·6)	14·3 (-1·95–33·9)
High systolic blood pressure	25·5 (20·5–31)	15·8 (13·8–17·9)	19·8 (15·2–24·7)	-38·1 (-50—23·6)	25 (-0·726–55·7)	23·3 (17·1–30·1)	13·2 (11·6–14·8)	18 (13·3–23·6)	-43·4 (-57·1—23·7)	36·8 (2·11–81·2)
High body-mass index	24·1 (16·6–33·5)	38·5 (28·4–49·9)	40·2 (29·9–51·8)	59·9 (39·3–87·9)	4·58 (-2·07–12·3)	32 (23·4–41)	43 (33·8–51·4)	44·3 (34·6–53)	34·6 (19·5–55)	2·91 (-2·92–10·2)
Low bone mineral density	12·4 (7·32–19·4)	13·4 (7·93–20·6)	14·2 (8·9–21·4)	8·1 (-17·9–42·7)	5·77 (-17·1–37)	15·9 (10·5–22·7)	16·5 (11–23·8)	19 (13·2–26·1)	3·89 (-19·9–30·5)	15·2 (-6·51–48·7)
Diet low in fruits	53·6 (42·1–66·8)	46·8 (35–59·9)	47·2 (34·7–60·4)	-12·8 (-25·6—1·56)	0·959 (-10·9–13·2)	55·1 (42·9–68·1)	45·1 (33·2–58)	44·7 (32–58·3)	-18·2 (-31·1—7·64)	-0·923 (-14·3–11·9)
Diet low in vegetables	28·5 (14·2–48·5)	43·3 (28·4–59·8)	41·4 (26·2–58·9)	51·8 (11·5–142)	-4·39 (-14·9–4·92)	28·5 (13·2–49·4)	41·5 (23·8–62·2)	39·7 (22·5–60·4)	45·8 (14·3–117)	-4·31 (-17·5–7·36)
Diet low in whole grains	78·3 (67·5–89)	80·2 (70·5–90)	80·2 (69·9–89·6)	2·43 (-1·16–7·34)	-0·0338 (-2·82–2·78)	--	--	--	--	--
Diet low in nuts and seeds	28·5 (7·78–56·4)	25·4 (8·04–55·4)	20·7 (5·95–53·6)	-11 (-42·9–54·8)	-18·3 (-45·4–5·76)	24·2 (6·01–55·3)	21·8 (7·18–54·6)	18·8 (5·38–53·3)	-10·1 (-43·4–61·9)	-13·6 (-43·8–12·9)
Diet low in milk	49 (24·6–69·7)	43·7 (20·6–62·8)	42·4 (19·3–61·9)	-10·7 (-28·3–2·65)	-3·01 (-16·3–10·1)	48·7 (25·2–70·3)	43·9 (21·1–65·3)	42·9 (20·7–63·8)	-9·89 (-28–3·33)	-2·2 (-14·6–10·2)
Diet high in red meat	77·2 (64–88·1)	75·4 (62·5–87·1)	75·7 (61·7–88·1)	-2·33 (-17·6–13·4)	0·381 (-10·7–13)	78·3 (66–88·5)	74·8 (61·8–85·8)	74·9 (60·6–85·9)	-4·47 (-20–11)	0·134 (-11·1–13·9)
Diet high in processed meat	78 (54·6–98·3)	86·1 (67·5–99·7)	86 (67·4–99·6)	10·4 (0·839–29·3)	-0·146 (-4·51–4·06)	79·3 (55·2–98)	88·9 (73·8–99·7)	87·6 (70–99·6)	12·1 (1·45–35·8)	-1·42 (-7·01–2·5)
Diet high in sugar-sweetened beverages	45·6 (26·3–64·4)	61·6 (43·3–78·8)	62·2 (42·6–79·8)	35·2 (-4·82–116)	0·979 (-20·3–27·4)	49·5 (29·6–69·5)	67·4 (48·8–83·5)	66·4 (47·2–83)	36·2 (-2·65–115)	-1·51 (-21·9–21)
Diet low in fiber	44 (28·4–59·6)	30·9 (18·4–44·3)	27·9 (15·2–41·2)	-29·7 (-47—12·6)	-9·56 (-26·8–7·68)	44·4 (27·1–60·4)	35·9 (22–50·6)	30·8 (17·5–45·9)	-19·3 (-35·3—2·29)	-14 (-30–1·69)
Diet low in seafood omega-3 fatty acids	84·4 (67·8–99·6)	80·2 (60·7–99·4)	79·7 (62–99·2)	-4·9 (-20·9–10·3)	-0·681 (-14·6–16·6)	87·1 (71·5–100)	84 (68·3–99·4)	83·4 (66·9–99·3)	-3·53 (-14·8–6·21)	-0·683 (-11·9–11)
Diet low in polyunsaturated fatty acids	14·4 (4·18–39·9)	7·52 (2·08–26·9)	7·07 (1·99–25·4)	-47·8 (-74·7—8)	-5·98 (-29·9–16·6)	16 (4·75–44)	7·64 (2·19–26·8)	7·09 (2·01–26)	-52·3 (-78—9)	-7·11 (-29·5–13·6)
Diet high in trans fatty acids	89·4 (81·4–100)	87·9 (78·8–100)	87·3 (78·2–100)	-1·73 (-9·85–6·07)	-0·614 (-9·34–9·13)	88·4 (81–100)	86·7 (78·2–100)	86·2 (77·2–100)	-1·94 (-9·78–5·48)	-0·509 (-8·67–8·14)
Diet high in sodium	18·2 (4·52–41)	39·6 (11–64·5)	38·8 (11·1–63·8)	117 (37·7–426)	-1·95 (-26·6–28·2)	--	--	--	--	--
Low physical activity	2·11 (0·785–4·53)	1·69 (0·675–4·06)	1·82 (0·627–4·16)	-19·8 (-49·8–13·9)	7·77 (-19·6–44·8)	4·72 (2·33–9·19)	4·34 (2·22–8·1)	4·58 (2·25–9·08)	-8·13 (-31·8–26·1)	5·58 (-8·8–21·9)
Occupational asthmagens	21·8 (18·3–26·6)	19·6 (16·6–23·4)	20·5 (17·2–24·8)	-10·1 (-20·5–1·87)	4·58 (-4·43–14·6)	10·9 (8·95–13·3)	10·9 (9·04–13·1)	11 (8·8–13·4)	-0·518 (-15·7–15·7)	1·42 (-10·7–14·1)
Occupational particulate matter, gases, and fumes	9·56 (6·85–13·6)	8·95 (6·48–12·8)	8·61 (6·26–12·3)	-6·44 (-11·9—0·891)	-3·73 (-7·3—0·156)	5·18 (3·67–7·56)	5·04 (3·68–7·15)	4·86 (3·53–6·95)	-2·81 (-11·6–7·46)	-3·52 (-8·33–1·61)
Occupational noise	8·58 (7·83–9·43)	8·22 (7·63–8·93)	7·94 (7·43–8·63)	-4·22 (-7·88—0·167)	-3·4 (-5·65—0·956)	5·97 (5·18–6·77)	6·28 (5·71–6·85)	6·28 (5·83–6·82)	5·15 (-1·13–13·3)	0·0126 (-2·79–3·38)
Occupational injuries	--	--	--	--	--	--	--	--	--	--
Occupational ergonomic factors	11·8 (9·79–14·3)	10·2 (8·59–12·3)	10·5 (8·74–12·7)	-13·8 (-24—2·47)	3·4 (-4·83–11·7)	7 (5·48–8·83)	6·91 (5·64–8·63)	7·22 (5·72–9·13)	-1·26 (-16·4–17·9)	4·48 (-7·74–18)
Childhood sexual abuse	7·4 (5·05–10·5)	6·97 (4·6–9·93)	6·85 (4·54–9·67)	-5·83 (-12·2–5·83)	-1·68 (-8·51–5·53)	12·3 (8·94–17·3)	17·4 (12·9–24)	17·8 (13·1–24·7)	41·9 (30·7–54·8)	2·17 (-5–11)
Intimate partner violence	--	--	--	--	--	32 (18·1–41·6)	30·4 (17·2–39·9)	31·3 (18–40·7)	-5 (-13·5–9·14)	2·99 (-6·08–21·8)
Non-exclusive breastfeeding	26·2 (21·6–31·1)	24·8 (20·9–28·7)	24·8 (20·2–29·5)	-5 (-19·5–12·5)	-0·203 (-13·5–15·8)	26·2 (21·6–31·1)	24·8 (20·9–28·7)	24·8 (20·2–29·5)	-4·98 (-19·4–12·5)	-0·216 (-13·5–15·7)
Discontinued breastfeeding	27·1 (26·1–28·1)	25·4 (24·2–26·6)	25·3 (24–26·5)	-6·19 (-11·9—0·55)	-0·457 (-6·24–5·89)	27 (26–28)	25·4 (24·1–26·6)	25·3 (24–26·5)	-6·14 (-11·9—0·486)	-0·337 (-6·13–6·03)
Diet low in calcium	16·6 (7·93–30·9)	13·9 (6·7–25·4)	13 (6·02–23·9)	-16·5 (-27·2—4·84)	-6·21 (-16·3–2·4)	12·1 (5·08–23·1)	11 (4·69–20·8)	10·5 (4·52–20)	-9·09 (-24·3–8·09)	-4·77 (-14·9–4·91)
Occupational exposure to asbestos	9·96 (9–10·9)	8·62 (7·93–9·29)	7·74 (5·73–9·89)	-13·4 (-23·4—2·64)	-10·2 (-33·5–16)	2·02 (1·13–2·42)	2·03 (1·1–2·42)	1·93 (0·979–2·7)	0·611 (-12·9–17·8)	-5·16 (-30·5–25·9)
Occupational exposure to arsenic	0·503 (0–1·18)	0·495 (0–1·17)	0·528 (0–1·23)	-1·5 (-16·2–NA)	6·57 (-4·65–NA)	0·45 (0–1·06)	0·458 (0–1·09)	0·47 (0–1·14)	1·75 (-15·5–NA)	2·68 (-9·62–NA)
Occupational exposure to benzene	0·828 (0·0474–2·42)	0·788 (0·0464–2·26)	0·823 (0·05–2·32)	-4·81 (-13·1–7)	4·39 (-3·81–15)	1·19 (0·0631–3·32)	1·32 (0·0765–3·67)	1·38 (0·0832–3·94)	11·5 (-1·38–31·4)	4·48 (-4·43–16·2)
Occupational exposure to beryllium	0·0233 (0·0219–0·0247)	0·0225 (0·022–0·023)	0·0238 (0·0224–0·0253)	-3·31 (-9·34–3·37)	6·03 (-0·0666–12·1)	0·0263 (0·024–0·0286)	0·0272 (0·0266–0·0278)	0·0282 (0·0261–0·0301)	3·56 (-5·26–14)	3·61 (-3·85–10·3)
Occupational exposure to cadmium	0·0588 (0·0528–0·0651)	0·058 (0·0525–0·0634)	0·0617 (0·0544–0·0693)	-1·35 (-14·5–14·2)	6·41 (-3·73–17·3)	0·0543 (0·0479–0·0613)	0·0555 (0·0502–0·0618)	0·057 (0·05–0·0657)	2·3 (-13·2–18)	2·74 (-8·39–16·1)
Occupational exposure to chromium	0·141 (0·128–0·155)	0·142 (0·131–0·152)	0·151 (0·135–0·166)	0·577 (-10·6–14·3)	6·15 (-2·61–15·2)	--	--	--	--	--
Occupational exposure to diesel engine exhaust	0·692 (0·64–0·748)	0·738 (0·693–0·785)	0·779 (0·713–0·845)	6·69 (-3·13–17·4)	5·5 (-3·4–14)	0·673 (0·592–0·753)	0·703 (0·637–0·772)	0·704 (0·622–0·792)	4·44 (-9·77–22·9)	0·118 (-10·4–10·9)
Occupational exposure to formaldehyde	0·295 (0·264–0·328)	0·284 (0·256–0·312)	0·299 (0·263–0·337)	-3·61 (-16·8–12·4)	5·18 (-4·95–16·2)	0·265 (0·233–0·298)	0·274 (0·248–0·302)	0·282 (0·249–0·323)	3·65 (-11·3–18·8)	2·9 (-7·29–14·9)
Occupational exposure to nickel	0·417 (0–1·77)	0·395 (0–1·75)	0·422 (0–1·81)	-5·32 (-19·4–NA)	6·91 (-4·7–NA)	0·353 (0–1·6)	0·348 (0–1·54)	0·358 (0–1·57)	-1·64 (-19·5–NA)	2·89 (-10·5–NA)
Occupational exposure to polycyclic aromatic hydrocarbons	0·28 (0·256–0·306)	0·281 (0·26–0·3)	0·297 (0·269–0·327)	0·142 (-10·8–12·6)	5·92 (-2·48–14·7)	0·293 (0·263–0·326)	0·316 (0·296–0·337)	0·325 (0·295–0·358)	7·63 (-5·05–20·5)	2·85 (-5·55–12·1)
Occupational exposure to silica	3·76 (0·533–12·1)	3·47 (0·511–10·9)	3·73 (0·551–11·9)	-7·72 (-21·9–10·2)	7·34 (-3·47–21·2)	2·99 (0·403–9·78)	2·81 (0·377–8·92)	2·87 (0·387–9·44)	-6·09 (-23·4–15·1)	2·17 (-11·9–20·7)
Occupational exposure to sulfuric acid	0·936 (0·207–2·98)	0·996 (0·211–3·18)	1·04 (0·209–3·24)	6·43 (-7·91–23·3)	4·33 (-6·31–16)	0·914 (0·188–2·92)	1·09 (0·218–3·52)	1·08 (0·222–3·39)	18·9 (-5·44–46·8)	-0·256 (-15–16·4)
Unsafe sex	--	--	--	--	--	--	--	--	--	--
Occupational exposure to trichloroethylene	0·0828 (0·0768–0·0893)	0·0852 (0·0815–0·0894)	0·0902 (0·0837–0·0975)	2·87 (-5·47–12·8)	5·92 (-1·16–13·3)	0·0811 (0·0734–0·09)	0·0847 (0·0801–0·0905)	0·0863 (0·0783–0·0951)	4·44 (-7·18–17·3)	1·91 (-6·52–11·5)
No access to handwashing facility	3·35 (3·09–3·61)	3·11 (2·88–3·34)	3·07 (2·82–3·32)	-7·18 (-15–1·69)	-1·15 (-7·09–4·71)	3·35 (3·09–3·61)	3·11 (2·88–3·34)	3·07 (2·82–3·32)	-7·18 (-15–1·69)	-1·15 (-7·09–4·71)
Child wasting	0·749 (0·497–1·03)	0·726 (0·472–0·989)	0·725 (0·465–0·984)	-3·05 (-12·3–6·53)	-0·211 (-7·39–8·11)	0·769 (0·508–1·05)	0·761 (0·51–1·04)	0·758 (0·504–1·04)	-1·08 (-11·4–9·21)	-0·359 (-8·35–7·91)
Child stunting	2·35 (1·25–3·46)	2·03 (1·04–3·01)	1·99 (1·03–2·96)	-13·7 (-32·3–7·93)	-2·11 (-16·9–13·6)	1·92 (0·935–2·93)	1·77 (0·875–2·74)	1·74 (0·862–2·73)	-7·84 (-28·1–18)	-1·31 (-16·3–16·2)
Chewing tobacco	4·54 (3·43–5·95)	4·57 (3·3–6·27)	4·56 (3·2–6·41)	0·614 (-31·9–49·5)	-0·0905 (-24–30·3)	--	--	--	--	--
Diet low in legumes	74·2 (39·1–99·3)	77·3 (45·5–99·7)	77·2 (46–99·7)	4·23 (-0·00193–25)	-0·138 (-5·77–5·34)	72·5 (34·9–99)	75·6 (42·7–99·6)	75·8 (45·2–99·6)	4·2 (0–23·5)	0·347 (-4·81–7·86)
Short gestation	19 (15·4–23·2)	19·8 (16·4–23·9)	20·4 (16·3–25·1)	3·98 (-16·8–29·8)	3·11 (-18·8–26·4)	15·6 (13·1–18·4)	16·1 (13·5–19·1)	16·6 (13·9–19·5)	3·2 (-15·5–25·1)	3·09 (-15–23·6)
Low birth weight	7·44 (6·12–9·02)	8·27 (6·84–9·86)	8·79 (7·18–10·6)	11·2 (-12·4–41·6)	6·23 (-15·7–32·9)	7·24 (6·12–8·42)	7·95 (6·75–9·27)	8·32 (7·09–9·7)	9·78 (-10·3–35·8)	4·7 (-14·3–26·7)
High temperature	26·5 (17–38·6)	25·3 (15·6–37·1)	26·4 (16·3–38·1)	-4·8 (-10·3—1·19)	4·6 (-2·4–11·4)	26·5 (17–38·6)	25·3 (15·6–37·1)	26·4 (16·3–38·1)	-4·8 (-10·3—1·19)	4·6 (-2·4–11·4)
Low temperature	33·1 (29–38·4)	32·7 (28·5–38·1)	32·5 (28·5–37·9)	-1·1 (-2·55–0·115)	-0·625 (-2·03–0·87)	33·1 (29–38·4)	32·7 (28·5–38·1)	32·5 (28·5–37·9)	-1·1 (-2·55–0·115)	-0·625 (-2·03–0·87)
Kidney dysfunction	18·9 (13·4–25·7)	19·5 (13·8–26·5)	19·6 (14–26·7)	3·07 (-0·822–7·18)	0·682 (-3·26–4·44)	21·3 (15·1–28·7)	22·2 (15·7–29·8)	23 (16·4–30·6)	4·03 (-0·243–8·79)	3·78 (-0·459–8·61)
Bullying victimization	5·92 (2·43–12·1)	8·98 (3·58–17·9)	8·73 (3·37–18)	51·6 (31·8–74·5)	-2·78 (-12–3·23)	4·28 (1·74–8·94)	7·87 (3·14–15·6)	7·78 (2·94–16)	83·8 (58·5–112)	-1·07 (-11·7–5·9)
High LDL cholesterol	66·8 (62·4–71·2)	34·7 (31·4–38·2)	31·1 (27·6–34·8)	-48·1 (-53·5—42·4)	-10·5 (-15·5—5·45)	65·3 (61·6–69·3)	34 (30·7–37·4)	31·4 (27·9–35)	-47·9 (-52·8—43·1)	-7·64 (-11·8—3·74)

Supplementary Results Table 1. Pennsylvania age-standardised Summary Exposure Values (SEVs) for most-detailed risk factors and percentage change, by sex, 1990, 2010, and 2019. Data in parentheses are 95% uncertainty intervals.										
	Male					Female				
	1990	2010	2019	Percentage change 1990-2010	Percentage change 2010-2019	1990	2010	2019	Percentage change 1990-2010	Percentage change 2010-2019
Unsafe sanitation	5·52 (2·73–10·3)	3·01 (1·36–5·76)	2·45 (1·06–4·65)	-45·5 (-78·3–38·9)	-18·7 (-57·6–51·4)	--	--	--	--	--
Ambient particulate matter pollution	15·2 (6·21–29·1)	8·69 (5·97–12)	6·05 (3·52–9·03)	-42·7 (-70–31)	-30·4 (-41·7–-23·7)	15·2 (6·2–29·1)	8·69 (5·97–12)	6·05 (3·52–9·02)	-42·7 (-70–31·5)	-30·4 (-41·7–-23·7)
Household air pollution from solid fuels	0·026 (0·00608–0·0721)	0·0143 (0·00239–0·0447)	0·00969 (0·0016–0·031)	-45·1 (-79·4–20·5)	-32·1 (-65·5–23·4)	0·0341 (0·00849–0·098)	0·0188 (0·00327–0·0567)	0·0128 (0·00218–0·0404)	-44·8 (-79·2–20·8)	-31·8 (-65·2–23·8)
Ambient ozone pollution	81·6 (37·3–100)	62·1 (26·8–100)	48·9 (20·5–83·8)	-23·9 (-34·4–0)	-21·3 (-29·8–-14)	--	--	--	--	--
Residential radon	21·1 (2·98–62·2)	21·1 (2·98–62·2)	21·1 (2·98–62·2)	0 (0–0)	0 (0–0)	21·1 (2·98–62·2)	21·1 (2·98–62·2)	21·1 (2·98–62·2)	0 (0–0)	0 (0–0)
Lead exposure	59 (31·4–79·7)	33·1 (13–54·5)	22·7 (6·97–41·1)	-43·9 (-60·6–-30·1)	-31·4 (-46·7–-23·3)	33·5 (3·87–61·6)	18·5 (1·02–42·2)	12·7 (0·368–32·1)	-44·7 (-78·9–-29)	-31·7 (-68–-23·6)
Child underweight	0·95 (0·538–1·47)	0·948 (0·525–1·45)	0·923 (0·503–1·4)	-0·261 (-18–21·2)	-2·58 (-15·7–13·3)	0·8 (0·424–1·22)	0·838 (0·472–1·3)	0·855 (0·469–1·3)	4·74 (-14·8–36)	2·13 (-13·3–17·4)
Iron deficiency	--	--	--	--	--	12·2 (10·2–14·3)	12·6 (10·6–14·8)	12·8 (10·7–15·1)	3·06 (-8·4–16)	1·73 (-7·4–12)
Vitamin A deficiency	0·762 (0·221–1·99)	1·01 (0·28–2·61)	0·719 (0·192–1·94)	33 (-69·2–495)	-29 (-69·3–67·6)	1·51 (0·577–2·91)	1·53 (0·595–3·13)	1·31 (0·503–2·83)	1·66 (-60·4–187)	-14·9 (-51·9–64·4)
Zinc deficiency	2·2 (0–7·3)	1·13 (0–3·71)	1·14 (0–3·8)	-48·8 (-100–NA)	1·28 (-17·8–NA)	2·24 (0–7·33)	1·14 (0–3·77)	1·12 (0–3·62)	-49·2 (-100–NA)	-1·98 (-23·1–NA)
Smoking	28 (25·2–30·9)	20·3 (18·3–22·4)	18·3 (16·5–20·2)	-27·5 (-32·4–-22·1)	-9·87 (-13·3–-6·53)	19·9 (17·1–22·7)	16·6 (14·7–18·5)	14·5 (12·9–16·3)	-16·9 (-26·2–-6·49)	-12·6 (-16·4–-8·56)
Secondhand smoke	41·9 (39·3–44·5)	27·4 (25·7–29)	27·7 (24·8–30·6)	-34·7 (-40·6–-28·3)	1·29 (-7·71–10·6)	39·1 (37–41·1)	25·7 (24·5–26·9)	26·1 (24–28·3)	-34·2 (-38·7–-29)	1·62 (-5·19–9·04)
Alcohol use	15·5 (9·61–21·8)	16·9 (10·3–24·4)	17·5 (11·3–25·3)	9·29 (-21·2–50·6)	3·58 (-14·9–29·3)	--	--	--	--	--
Drug use	0·762 (0·42–1·36)	1·37 (0·973–1·94)	1·82 (1·34–2·46)	79·9 (37·5–146)	32·7 (16·2–53·8)	0·46 (0·302–0·691)	0·982 (0·739–1·29)	1·48 (1·14–1·87)	113 (66·2–169)	50·2 (30·1–72·5)
High fasting plasma glucose	15·6 (12·8–18·7)	18·4 (16·1–21·1)	19·2 (16·5–22·3)	18 (-3·68–44)	4·13 (-9·22–20·6)	12·3 (10·5–14·8)	14·2 (12·2–16·3)	14·6 (12·2–17)	15·1 (-2·84–36·8)	2·52 (-12·3–17·6)
High systolic blood pressure	25·7 (20·5–31·2)	16·3 (14·3–18·4)	19·9 (15·2–24·6)	-36·6 (-47·2–-21·8)	22·4 (-3·02–50·8)	23·9 (17·9–31)	13·4 (11·8–15)	17·4 (12·7–23·2)	-44 (-56·8–-25·6)	29·9 (-5·11–73·3)
High body-mass index	26·1 (18·3–35·6)	40·4 (29·7–52·2)	41·9 (31·2–53·4)	54·5 (35·6–79·9)	3·86 (-2·28–11·5)	32·2 (23·8–41·8)	44·6 (35–53·4)	45·7 (36·1–55·2)	38·2 (23·2–58·3)	2·52 (-4·12–9·68)
Low bone mineral density	10·1 (5·64–16·3)	9·96 (5·51–16·4)	10·2 (5·7–16·6)	-1·01 (-25·2–29·5)	2·08 (-24·1–34·1)	13·7 (8·81–20·1)	16 (10·3–23)	14 (8·87–20·4)	16·8 (-7·62–49·4)	-12·1 (-33·4–12·3)
Diet low in fruits	51·9 (40·3–64·7)	45·2 (33·4–58)	44·7 (32·2–58·1)	-12·9 (-23·8–-3·49)	-1·18 (-14·6–12·1)	53·9 (40·9–68·9)	47·5 (33·8–62·5)	47·5 (33·8–62·3)	-11·9 (-23·1–-3·35)	0·03 (-10·9–11·9)
Diet low in vegetables	38·2 (20·8–59·7)	45·4 (26·9–67·2)	41·8 (24·6–62·5)	19 (3·25–49·9)	-7·87 (-19·1–2·02)	35·9 (19·3–57·3)	40·9 (23·6–61·9)	36·9 (20·2–57·7)	13·9 (-0·155–38·9)	-9·68 (-23·8–1·68)
Diet low in whole grains	78 (66·6–88·5)	80 (70·7–89·7)	80 (70·2–89·5)	2·58 (-1·15–7·69)	-0·0185 (-2·63–2·6)	--	--	--	--	--
Diet low in nuts and seeds	27·3 (7·26–56·3)	25 (8·65–54·9)	20·5 (6·64–53·7)	-8·48 (-44·4–69·9)	-18 (-45·4–10·6)	23·2 (5·72–53·8)	21·6 (6·6–53·9)	18·6 (5·4–52·7)	-6·65 (-42·6–66·6)	-14 (-42–15·4)
Diet low in milk	47·9 (25·4–68·6)	41·9 (19·4–62·6)	40·7 (19–61·6)	-12·6 (-31·1–0·317)	-2·71 (-16·5–10·1)	47·6 (24·1–68·2)	41·9 (19·6–60·7)	41·1 (19–60·6)	-11·9 (-31·1–1·69)	-1·95 (-14·3–10·8)
Diet high in red meat	79·3 (66·6–89)	79·3 (67·7–89·5)	79 (65·1–90)	0·0138 (-14–15)	-0·434 (-10·9–10·1)	76·1 (63·4–86·3)	74·8 (61·9–86)	74·7 (60·7–86·8)	-1·67 (-16·6–14·1)	-0·118 (-12–12·9)
Diet high in processed meat	77·8 (52·7–97·8)	85·7 (66·3–99·5)	85·5 (66·3–99·5)	10·2 (1·25–31·6)	-0·189 (-4·71–4·15)	77 (52·8–97·4)	86·8 (68·8–99·5)	85·5 (66·6–99·6)	12·7 (1·55–36·8)	-1·48 (-6·19–2·67)
Diet high in sugar-sweetened beverages	46·3 (27·5–65·8)	64·1 (46·2–80·8)	64·5 (45·1–80·9)	38·6 (-1·97–117)	0·549 (-20·5–22·5)	50·2 (30·5–70·1)	69·6 (50·1–86·5)	68·2 (48·5–85·5)	38·8 (-2·59–114)	-1·96 (-20·3–22·4)
Diet low in fiber	43 (26·9–58·2)	29·2 (17·1–42·9)	26·4 (14·1–41)	-32·2 (-48·7–-14·9)	-9·55 (-29·1–9·26)	43·3 (27–59·5)	34·4 (20·6–48·1)	29·5 (16·4–43·9)	-20·6 (-37·7–-3·77)	-14·3 (-31·5–0·0918)
Diet low in seafood omega-3 fatty acids	83·1 (66–99·5)	78·5 (60·2–98·9)	78·1 (59·5–99·1)	-5·58 (-21–10·4)	-0·438 (-16·4–15·7)	86 (70·2–100)	82·4 (65·4–99·3)	82 (65·1–99·5)	-4·09 (-16·3–7·65)	-0·532 (-13·5–13·2)
Diet low in polyunsaturated fatty acids	13·5 (3·94–39·8)	7·01 (1·93–25·1)	6·7 (1·86–24·6)	-48·3 (-76·4–-7·85)	-4·34 (-27·4–14·5)	15·1 (4·52–41·1)	7·15 (2·08–26·5)	6·78 (1·94–24·6)	-52·7 (-78·7–-9·22)	-5·21 (-28·6–14)
Diet high in trans fatty acids	89·4 (81·3–100)	87·9 (79–100)	87·5 (78·2–100)	-1·66 (-9·91–5·81)	-0·493 (-8·09–7·42)	88·4 (81·2–100)	86·7 (78·5–100)	86·2 (77·3–100)	-1·92 (-8·97–5·62)	-0·62 (-8·58–7·12)
Diet high in sodium	18·1 (4·56–40·8)	39·6 (11–64·3)	38·8 (10·7–64·2)	119 (43–415)	-2·1 (-27·4–29·6)	--	--	--	--	--
Low physical activity	2·32 (0·871–5·08)	2·06 (0·84–4·32)	2·19 (0·8–4·75)	-11·4 (-41·5–37·9)	6·53 (-20·5–43·1)	5·36 (2·64–10·5)	5·45 (2·89–10·1)	5·67 (2·91–10·8)	1·62 (-23·2–42·8)	4·14 (-8·97–18·4)
Occupational asthmagens	25·1 (21–30·7)	21·5 (18·2–25·5)	22·4 (18·4–27·5)	-14·5 (-26·7–-2·4)	4·23 (-4·85–16·1)	11·7 (9·64–14·1)	11·7 (9·72–14·1)	11·8 (9·64–14·5)	-0·0396 (-14·3–16)	0·881 (-9·97–13·3)
Occupational particulate matter, gases, and fumes	10·2 (7·27–14·7)	9·36 (6·72–13·4)	8·96 (6·44–12·9)	-8·48 (-13·7–-2·76)	-4·3 (-7·91–-0·707)	5·62 (4–8·2)	5·32 (3·81–7·61)	5·1 (3·62–7·23)	-5·36 (-13–4·58)	-4·2 (-9·32–0·807)
Occupational noise	8·92 (8·17–9·77)	8·5 (7·9–9·24)	8·22 (7·68–8·92)	-4·72 (-8·4–-0·952)	-3·3 (-5·44–-1·15)	6·12 (5·39–6·91)	6·47 (5·96–7·12)	6·48 (6·06–7·07)	5·62 (-0·0273–13)	0·262 (-2·62–3·14)
Occupational injuries	--	--	--	--	--	--	--	--	--	--
Occupational ergonomic factors	12·2 (10–14·9)	10·7 (9·03–12·8)	11·1 (9·21–13·2)	-12·2 (-22·1–-0·898)	3·34 (-4·62–12)	7·11 (5·67–9·01)	7·22 (5·87–8·84)	7·56 (6·08–9·37)	1·52 (-13·9–21·7)	4·67 (-7·87–19·1)
Childhood sexual abuse	7·4 (5·05–10·5)	6·97 (4·6–9·93)	6·85 (4·54–9·67)	-5·83 (-12·2–5·83)	-1·68 (-8·51–5·53)	12·3 (8·94–17·3)	17·4 (12·9–24)	17·8 (13·1–24·7)	41·9 (30·7–54·8)	2·17 (-5–11)
Intimate partner violence	--	--	--	--	--	32·4 (17·3–42·3)	30·6 (17·1–39·4)	31·6 (18–41·2)	-5·54 (-14·5–8·69)	3·47 (-5·76–19·8)
Non-exclusive breastfeeding	26·3 (21·8–31·1)	25 (21·4–28·8)	24·9 (20·2–29·9)	-4·92 (-18·8–12)	-0·631 (-14·4–13·8)	26·3 (21·8–31·1)	25 (21·4–28·8)	24·9 (20·2–29·9)	-4·93 (-18·8–12)	-0·637 (-14·4–13·8)
Discontinued breastfeeding	27·3 (26·3–28·2)	25·3 (23·9–26·5)	25·6 (24·2–26·8)	-7·44 (-13–-2·06)	1·19 (-4·57–7·14)	27·3 (26·4–28·3)	25·3 (23·9–26·5)	25·6 (24·2–26·8)	-7·56 (-13·2–-2·18)	1·15 (-4·61–7·1)
Diet low in calcium	15·9 (7·71–29·4)	13 (6·27–23·9)	12·3 (5·81–22·8)	-18·4 (-29·3–-6·86)	-5·47 (-15·5–2·62)	11·2 (4·86–21·3)	9·89 (4·48–18·2)	9·59 (4·11–18)	-11·9 (-25·7–6·16)	-3·03 (-14·3–7·64)
Occupational exposure to asbestos	11·3 (10·3–12·6)	9·58 (9·02–10·1)	8·4 (6·39–10·6)	-15·6 (-24·5–-5·63)	-12·3 (-33·7–10·6)	1·87 (1·18–2·28)	1·85 (1·09–2·15)	1·78 (1·02–2·49)	-1·01 (-18·8–19·3)	-3·76 (-29·7–27·4)
Occupational exposure to arsenic	0·585 (0–1·38)	0·554 (0–1·31)	0·585 (0–1·38)	-5·36 (-18·9–NA)	5·63 (-5·87–NA)	0·508 (0–1·2)	0·497 (0–1·21)	0·506 (0–1·24)	-2·24 (-18–NA)	1·86 (-10·5–NA)
Occupational exposure to benzene	0·864 (0·0505–2·49)	0·855 (0·0506–2·44)	0·882 (0·0522–2·52)	-0·969 (-9·08–12·4)	3·12 (-3·99–12·9)	1·21 (0·0698–3·46)	1·39 (0·0795–3·87)	1·45 (0·0859–4·15)	15 (2·56–34·7)	4·53 (-3·8–16·3)
Occupational exposure to beryllium	0·0245 (0·0232–0·0259)	0·024 (0·0235–0·0246)	0·0253 (0·0239–0·0267)	-1·89 (-7·67–4·21)	5·23 (-0·45–11·6)	0·027 (0·0246–0·0291)	0·0284 (0·0279–0·0291)	0·0294 (0·0275–0·0313)	5·41 (-2·59–15·6)	3·38 (-3·33–10)
Occupational exposure to cadmium	0·0675 (0·0613–0·074)	0·0645 (0·0585–0·0706)	0·068 (0·06–0·0767)	-4·39 (-16·9–10·5)	5·41 (-5–16·8)	0·0603 (0·0534–0·0677)	0·0599 (0·0537–0·0666)	0·0611 (0·0537–0·0696)	-0·786 (-15·2–17)	2·03 (-9·23–15·1)
Occupational exposure to chromium	0·16 (0·147–0·174)	0·157 (0·145–0·168)	0·165 (0·147–0·183)	-2·21 (-13·2–10·8)	5·13 (-4–14·9)	--	--	--	--	--
Occupational exposure to diesel engine exhaust	0·711 (0·658–0·769)	0·759 (0·711–0·807)	0·794 (0·727–0·864)	6·88 (-3·55–18)	4·57 (-3·21–13·4)	0·674 (0·6–0·752)	0·729 (0·664–0·799)	0·731 (0·653–0·82)	8·13 (-5·73–24·7)	0·276 (-9·78–10·7)
Occupational exposure to formaldehyde	0·342 (0·31–0·376)	0·319 (0·287–0·35)	0·332 (0·291–0·377)	-6·88 (-19·3–8·26)	4·1 (-6·47–15·4)	0·296 (0·263–0·332)	0·298 (0·269–0·328)	0·304 (0·269–0·342)	0·51 (-12·9–17·8)	2·17 (-8·33–13·3)
Occupational exposure to nickel	0·495 (0–2·24)	0·45 (0–1·98)	0·476 (0–2·1)	-9·07 (-22·3–NA)	5·75 (-5·43–NA)	0·4 (0–1·77)	0·379 (0–1·65)	0·386 (0–1·71)	-5·35 (-22·2–NA)	1·89 (-11·3–NA)
Occupational exposure to polycyclic aromatic hydrocarbons	0·317 (0·292–0·343)	0·31 (0·288–0·332)	0·325 (0·293–0·358)	-2·06 (-12·4–10·1)	4·92 (-3·85–13·7)	0·317 (0·285–0·348)	0·336 (0·315–0·359)	0·344 (0·311–0·377)	6·09 (-5·23–20·3)	2·38 (-6·15–11·1)
Occupational exposure to silica	4·52 (0·682–14·3)	3·97 (0·56–12·7)	4·22 (0·622–13)	-12 (-24·6–3·05)	6·3 (-4·12–20·1)	3·35 (0·468–10·4)	3·06 (0·417–9·86)	3·1 (0·429–9·88)	-8·88 (-25·8–13·5)	1·47 (-12·6–20·4)
Occupational exposure to sulfuric acid	1·02 (0·211–3·34)	1·06 (0·226–3·45)	1·1 (0·23–3·7)	4·26 (-8·91–20)	3·99 (-7·25–15)	0·926 (0·197–2·96)	1·08 (0·222–3·41)	1·08 (0·224–3·3)	17·1 (-3·04–41·8)	-0·619 (-13·9–14·6)
Unsafe sex	--	--	--	--	--	--	--	--	--	--
Occupational exposure to trichloroethylene	0·0901 (0·0843–0·0967)	0·0914 (0·0875–0·0959)	0·0959 (0·0892–0·103)	1·42 (-6·43–10·4)	4·93 (-2·07–12·3)	0·0864 (0·0778–0·0947)	0·09 (0·0848–0·0959)	0·0914 (0·084–0·1)	4·18 (-6·06–18)	1·58 (-6·65–11·3)
No access to handwashing facility	3·31 (3·08–3·57)	3·1 (2·86–3·35)	3·06 (2·84–3·32)	-6·59 (-14·8–3·23)	-1 (-6·73–4·82)	3·31 (3·08–3·57)	3·1 (2·86–3·35)	3·06 (2·84–3·32)	-6·59 (-14·8–3·23)	-1 (-6·73–4·82)
Child wasting	0·748 (0·494–1·02)	0·724 (0·48–0·989)	0·724 (0·483–0·981)	-3·2 (-12·8–6·71)	-0·0925 (-6·7–7·86)	0·771 (0·51–1·06)	0·762 (0·511–1·03)	0·758 (0·5–1·03)	-1·19 (-11·1–9·12)	-0·488 (-8·58–7·68)
Child stunting	2·36 (1·21–3·42)	2·05 (1·03–3·12)	1·99 (1·03–2·94)	-13·4 (-32·9–10·2)	-2·84 (-17·9–13·7)	1·92 (0·95–2·97)	1·76 (0·853–2·75)	1·74 (0·868–2·72)	-8·11 (-28·4–17·2)	-1·31 (-17·3–18·3)
Chewing tobacco	3·74 (2·89–4·82)	3·68 (2·72–4·88)	3·69 (2·63–4·98)	-1·46 (-31·6–41·1)	0·159 (-21·6–28·7)	0·299 (0·186–0·466)	0·332 (0·205–0·512)	0·342 (0·21–0·539)	11·1 (-35·4–94·1)	2·78 (-28–49·5)
Diet low in legumes	73·8 (38·1–99·1)	76·7 (44·8–99·7)	76·6 (43·7–99·6)	3·97 (-0·0384–20·6)	-0·0517 (-5·73–5·55)	72·2 (35–98·8)	75 (42–99·6)	75·2 (42·8–99·6)	3·83 (-0·33–20·9)	0·343 (-4·98–7·6)
Short gestation	31·2 (25·4–37·7)	27·2 (22·6–32·1)	25·6 (21–31·2)	-12·9 (-29·9–8·26)	-5·82 (-24·9–17·4)	28·2 (23·3–33·6)	24·5 (20·7–28·8)	24·6 (20·4–28·9)	-13·3 (-28·8–7·51)	0·599 (-16·5–21·3)
Low birth weight	12·4 (10·3–14·8)	11·8 (9·97–13·8)	11·5 (9·58–13·9)	-4·75 (-23·4–20·5)	-2·39 (-21·8–23·6)	13·1 (11–15·5)	12·9 (11·1–14·9)	13 (10·9–15)	-1·65 (-20·7–22·1)	0·658 (-16·4–22·1)
High temperature	24 (14·8–35·7)	28·1 (18·5–40·1)	26·1 (16·9–37·8)	16·8 (5·28–32·7)	-7·18 (-12·2–-3·9)	24 (14·8–35·7)	28·1 (18·5–40·1)	26·1 (16·9–37·8)	16·8 (5·28–32·7)	-7·18 (-12·2–-3·9)
Low temperature	33 (29·6–37·2)	35·3 (32·1–39·2)	34·9 (31·7–39)	6·93 (4·73–9·31)	-1·14 (-2·48–0·247)	33 (29·6–37·2)	35·3 (32·1–39·2)	34·9 (31·7–39)	6·93 (4·73–9·31)	-1·14 (-2·48–0·247)
Kidney dysfunction	20·6 (14·8–27·7)	20·6 (14·7–27·8)	20·7 (15–27·8)	-0·00716 (-3·78–3·69)	0·254 (-3·77–4·63)	22·8 (16·3–30·5)	23·1 (16·4–31·2)	23·9 (17·4–31·8)	1·54 (-2·93–6·24)	3·34 (-1·46–8·75)

Bullying victimization	5·88 (2·45–12)	9 (3·57–17·9)	8·71 (3·34–17·9)	53·1 (32·3–76)	-3·23 (-12·7–2·9)	--	--	--	--	--
High LDL cholesterol	67·6 (63·3–72·1)	34·3 (30·9–37·9)	30·5 (27·1–34·2)	-49·2 (-54·6–-44)	-11·1 (-15·9–-6·04)	66·4 (62·7–70·3)	34·2 (31–37·7)	31·3 (27·7–34·9)	-48·4 (-52·9–-43·8)	-8·62 (-12·6–-4·55)

Supplementary Results Table 1. Rhode Island age-standardised Summary Exposure Values (SEVs) for most-detailed risk factors and percentage change, by sex, 1990, 2010, and 2019. Data in parentheses are 95% uncertainty intervals.

	Male					Female				
	1990	2010	2019	Percentage change 1990-2010	Percentage change 2010-2019	1990	2010	2019	Percentage change 1990-2010	Percentage change 2010-2019
Unsafe sanitation	4·87 (2·33–8·99)	2·69 (1·18–5·31)	2·18 (0·947–4·42)	-44·7 (-77·1–39·7)	-18·9 (-58·5–53·4)	4·87 (2·33–8·99)	2·69 (1·18–5·31)	2·18 (0·947–4·42)	-44·7 (-77·1–39·7)	-18·9 (-58·5–53·4)
Ambient particulate matter pollution	8·26 (1·46–18·3)	5·12 (2·43–8·01)	2·76 (0·525–5·43)	-38 (-74·3–141)	-46 (-79·1—32)	8·26 (1·46–18·3)	5·12 (2·43–8·01)	2·76 (0·525–5·43)	-38 (-74·3–140)	-46 (-79·1—32·1)
Household air pollution from solid fuels	0·00888 (0·0015–0·0269)	0·00503 (0·000738–0·0167)	0·00289 (0·00025–0·0114)	-43·4 (-80·7–55)	-42·5 (-82–16·8)	0·0117 (0·00204–0·0358)	0·00667 (0·00102–0·0215)	0·00383 (0·000344–0·015)	-43·2 (-80·8–55·5)	-42·5 (-81·9–16·8)
Ambient ozone pollution	80·7 (36–100)	55·5 (22·9–95·3)	42·9 (16·9–75·5)	-31·2 (-51·9—3·72)	-22·8 (-43·8–1·45)	80·7 (36–100)	55·5 (22·9–95·3)	42·9 (16·9–75·5)	-31·2 (-51·9—3·72)	-22·8 (-43·8–1·45)
Residential radon	22·3 (3·81–65·2)	22·3 (3·81–65·2)	22·3 (3·81–65·2)	0 (0–0)	0 (0–0)	22·3 (3·81–65·2)	22·3 (3·81–65·2)	22·3 (3·81–65·2)	0 (0–0)	0 (0–0)
Lead exposure	72·8 (48·4–89·6)	41·2 (21·6–61·3)	28·3 (12·4–45·9)	-43·4 (-56·8—29·9)	-31·3 (-43·3—24·8)	46·9 (13·4–73·5)	24·8 (3·64–48·7)	16·4 (1·4–35·8)	-47·2 (-75·5—31·1)	-33·6 (-62·9—25·8)
Child underweight	0·927 (0·537–1·39)	0·934 (0·509–1·41)	0·914 (0·5–1·38)	0·817 (-18·9–22·4)	-2·18 (-15·7–13·7)	0·785 (0·413–1·19)	0·828 (0·453–1·24)	0·849 (0·461–1·29)	5·37 (-17–44)	2·59 (-11·9–20·2)
Iron deficiency	--	--	--	--	--	11·7 (9·86–13·9)	11·9 (10·1–13·9)	12·1 (10–14·5)	1·78 (-10·2–14·5)	1·48 (-8·88–12)
Vitamin A deficiency	0·686 (0·191–1·94)	0·924 (0·248–2·38)	0·668 (0·178–1·91)	34·7 (-65·2–445)	-27·7 (-69·7–84·5)	1·39 (0·523–2·94)	1·41 (0·549–3·01)	1·23 (0·5–2·82)	0·898 (-62·2–205)	-12·8 (-54·9–70·5)
Zinc deficiency	2·11 (0–6·98)	1·12 (0–3·54)	1·14 (0–3·67)	-47·2 (-100–NA)	2·09 (-15·8–NA)	2·16 (0–7·36)	1·12 (0–3·74)	1·1 (0–3·61)	-48·1 (-100–NA)	-1·61 (-27·5–NA)
Smoking	27·3 (24·5–30·2)	19·8 (17·9–21·9)	16·8 (15–18·6)	-27·6 (-32·3—22·4)	-15·2 (-18·8—11·6)	23·1 (20·4–26·1)	16·6 (14·9–18·5)	13·6 (12–15·2)	-28·2 (-34·3—21·4)	-18·2 (-21·7—14·2)
Secondhand smoke	41·1 (38·6–43·5)	25·3 (23·7–27)	25·5 (22·8–28·5)	-38·5 (-43·5—32·1)	0·732 (-8·17–10·1)	36·9 (35–38·8)	23·6 (22·5–24·8)	23·9 (22–26·1)	-35·9 (-40·7—30·8)	1·21 (-6·37–8·69)
Alcohol use	16·1 (10–22·8)	16·8 (9·99–24·2)	17·5 (10·6–24·7)	4·04 (-26·6–46·7)	4·3 (-17·3–34)	11·7 (6·9–17·2)	13·2 (7·79–18·6)	13·5 (7·68–19·4)	12·9 (-19–65·3)	2·5 (-20·7–33·5)
Drug use	0·792 (0·411–1·5)	1·33 (0·873–2)	1·91 (1·36–2·73)	68·3 (28·7–136)	43·5 (21·8–68·4)	0·625 (0·347–1·13)	0·972 (0·673–1·44)	1·4 (1·04–1·85)	55·4 (21·1–107)	43·7 (22–68·6)
High fasting plasma glucose	12·7 (10·6–15·5)	17·7 (15·4–20·4)	18·8 (16·2–21·8)	39·8 (14–71·2)	5·98 (-7·8–21·6)	8·77 (7·46–10·5)	12·1 (10·4–14·1)	13·7 (11·7–16·1)	37·4 (15·9–64·4)	13·9 (-2·04–31·9)
High systolic blood pressure	25·7 (20·4–31·4)	16 (13·9–18)	20 (15·6–24·8)	-37·7 (-49·5—21·1)	25·1 (-1·35–54·7)	23·7 (17·5–30·6)	13·2 (11·7–14·9)	17·7 (12·9–23·4)	-44·2 (-57·3—24·1)	34·5 (-3·16–79·5)
High body-mass index	24·3 (16·9–33·5)	38·2 (27·8–49·8)	40 (29·3–51·6)	57·2 (38·7–84·5)	4·69 (-2–12·5)	27·3 (19·6–36·6)	42 (33·7–50·3)	43·5 (34·3–52·3)	54·1 (31·8–82·9)	3·56 (-3·09–10·2)
Low bone mineral density	12·2 (7·01–18·1)	9·21 (4·99–15·4)	11·5 (6·6–18·2)	-24·3 (-45·2—2·26)	24·4 (-3·48–68·7)	16 (10·1–22·8)	16·6 (11–23·2)	18·7 (12·7–25·6)	3·35 (-16·8–34·7)	12·7 (-8·61–39·9)
Diet low in fruits	48·6 (36·4–62·1)	49·6 (37·7–63·1)	49·7 (37·1–63·8)	2·17 (-8·7–14·5)	0·177 (-11–10·6)	52·9 (41·7–65·8)	52·2 (40·4–64·8)	51 (38·7–64·2)	-1·23 (-11·1–10·1)	-2·38 (-12·5–6·79)
Diet low in vegetables	34·1 (17·4–55·8)	50·5 (31·1–70·5)	49·1 (30·3–69·6)	48·3 (17·1–112)	-2·89 (-12·7–6)	36·6 (19·1–57·9)	47·7 (28·1–69)	46·6 (27–67·2)	30·5 (8·96–75·5)	-2·27 (-12·8–7·98)
Diet low in whole grains	77·9 (67·4–88·6)	79·9 (69·7–89·5)	79·9 (68·8–89·6)	2·52 (-1·33–7·79)	0·0535 (-2·68–3·01)	--	--	--	--	--
Diet low in nuts and seeds	26·9 (7·58–55·8)	25·2 (8·49–55·3)	20·6 (6·16–53·9)	-6·28 (-41·9–73·2)	-18·5 (-45·5–9·91)	22·9 (5·84–54·2)	21·5 (5·84–54·3)	18·8 (5·23–53·5)	-5·84 (-42·8–70·9)	-12·9 (-43·6–21·9)
Diet low in milk	47·6 (23·2–68·3)	41·7 (19·2–62·4)	40·7 (18·9–61·1)	-12·5 (-31·7–1·93)	-2·34 (-15·8–11·8)	47·3 (23·9–69)	41·4 (19·1–62)	40·8 (18·9–61·7)	-12·5 (-32·1–1·78)	-1·3 (-15·1–12·1)
Diet high in red meat	78·3 (65·5–88·6)	77 (63·8–88·5)	76·7 (63·9–87·8)	-1·71 (-15·2–13·6)	-0·358 (-12–11·4)	79 (65·6–89)	76·1 (61·8–86·8)	75·9 (60·8–87·6)	-3·64 (-18·4–12·3)	-0·197 (-11·7–13·4)
Diet high in processed meat	78·2 (53·5–97·9)	86·4 (67·1–99·6)	86·2 (66·6–99·7)	10·5 (1·14–29·4)	-0·22 (-4·89–4·57)	79·6 (56·5–98·1)	89·2 (73·1–99·7)	87·9 (70·6–99·6)	12·1 (1·3–35·7)	-1·44 (-6·68–2·14)
Diet high in sugar-sweetened beverages	47 (29·4–67)	64·1 (46·5–79·9)	64·5 (44·1–81·3)	36·6 (-3·69–105)	0·5 (-21·2–23)	51·2 (30·3–70·9)	70·1 (51·1–86·5)	68·9 (47·6–85·8)	37 (-0·98–109)	-1·71 (-19·8–20·4)
Diet low in fiber	42·4 (26·7–58·9)	28·9 (16·4–42·6)	26·4 (14·4–40·4)	-31·9 (-48·5—14·5)	-8·79 (-28·1–9·6)	42·8 (26·6–60·3)	34·1 (20·7–49)	29·4 (16·2–44)	-20·3 (-36·5—2·73)	-13·7 (-30·7–1·88)
Diet low in seafood omega-3 fatty acids	82·6 (64·2–99·8)	78·4 (59·1–99·4)	78·3 (59·5–99·2)	-5·04 (-20·4–10·6)	-0·207 (-14–16·8)	85·5 (69·2–99·7)	82·6 (65·7–98·9)	82 (64·2–99·8)	-3·4 (-16·1–9·46)	-0·734 (-14·1–12·7)
Diet low in polyunsaturated fatty acids	13·4 (3·91–40·4)	6·93 (1·98–26)	6·69 (1·83–24·4)	-48·2 (-76·7—7·69)	-3·44 (-25·3–19·6)	14·9 (4·31–41·3)	7·1 (2·08–26·2)	6·76 (2–24·8)	-52·2 (-78·8—9·17)	-4·78 (-27–14·9)
Diet high in trans fatty acids	89·5 (81·1–100)	87·8 (78·9–100)	87·5 (77·5–100)	-1·81 (-9·79–5·84)	-0·397 (-8·74–8·35)	88·4 (81·1–100)	86·6 (77·2–100)	86·2 (77·5–100)	-2·07 (-9·98–5·53)	-0·468 (-7·98–7·76)
Diet high in sodium	18·6 (4·47–41·8)	39·7 (12·6–63·3)	39·1 (11·5–63·4)	114 (33·4–425)	-1·6 (-27·6–31)	--	--	--	--	--
Low physical activity	2·36 (0·883–5·07)	2·14 (0·937–4·47)	2·35 (0·876–5)	-9·34 (-40·6–39)	9·78 (-19·6–50·1)	5·48 (2·69–10·7)	5·65 (2·99–10·2)	6·36 (3·29–12·2)	3·11 (-22·1–39·2)	12·6 (-2·44–27·4)
Occupational asthmagens	23·9 (19·8–29·3)	20·5 (17·3–24·3)	21·3 (17·7–25·8)	-14·1 (-26·2—1·4)	4·07 (-5·33–14·7)	12·5 (10·1–15·4)	12·3 (10·3–14·8)	12·5 (10–15·3)	-1·58 (-17·8–16·9)	1·64 (-10·4–15·4)
Occupational particulate matter, gases, and fumes	9·81 (6·97–14·1)	8·99 (6·38–13)	8·61 (6·17–12·4)	-8·39 (-14—2·28)	-4·19 (-7·6—0·392)	6·27 (4·38–9·11)	5·75 (4·1–8·28)	5·45 (3·88–7·86)	-8·33 (-16·1—1·3)	-5·17 (-10·2—0·66)
Occupational noise	8·54 (7·8–9·39)	8·2 (7·59–8·93)	7·95 (7·42–8·63)	-3·99 (-7·87—0·0534)	-3·12 (-5·27—0·593)	6·45 (5·66–7·38)	6·78 (6·2–7·45)	6·79 (6·33–7·4)	5·14 (-0·791–12·7)	0·0807 (-2·93–3·38)
Occupational injuries	--	--	--	--	--	--	--	--	--	--
Occupational ergonomic factors	11·5 (9·45–14)	10·3 (8·57–12·3)	10·6 (8·75–12·8)	-11·1 (-21·2—0·401)	3·53 (-4–12)	6·93 (5·36–8·8)	7·16 (5·77–8·85)	7·51 (5·96–9·54)	3·23 (-15·4–24·5)	4·91 (-7·85–20·1)
Childhood sexual abuse	7·4 (5·05–10·5)	6·97 (4·6–9·93)	6·85 (4·54–9·67)	-5·83 (-12·2–5·83)	-1·68 (-8·51–5·53)	12·3 (8·94–17·3)	17·4 (12·9–24)	17·8 (13·1–24·7)	41·9 (30·7–54·8)	2·17 (-5–11)
Intimate partner violence	--	--	--	--	--	20·8 (11·4–29·2)	20·4 (10·8–28·5)	22·6 (12·3–31·8)	-1·99 (-10·3–10·4)	11 (-3·94–35·3)
Non-exclusive breastfeeding	26·3 (21·8–31·5)	25 (21·5–28·7)	24·9 (20·4–29·4)	-5·08 (-19·6–12·6)	-0·272 (-13·8–14·4)	26·3 (21·8–31·5)	25 (21·5–28·7)	24·9 (20·4–29·4)	-5·07 (-19·6–12·6)	-0·276 (-13·8–14·4)
Discontinued breastfeeding	27·5 (26·5–28·4)	25·3 (23·9–26·5)	25·8 (24·4–27)	-8·16 (-13·8—2·68)	1·93 (-4·06–7·88)	27·7 (26·8–28·7)	25·2 (23·8–26·4)	25·7 (24·4–27)	-9·24 (-14·8—3·83)	2·21 (-3·81–8·2)
Diet low in calcium	15·7 (7·44–28·9)	12·9 (6·28–23·5)	12·3 (5·93–22·4)	-17·8 (-28·3—4·51)	-4·49 (-14·3–4·18)	10·9 (4·7–20·8)	9·77 (4·33–18·3)	9·55 (4·18–17·9)	-10·6 (-26·4–8·58)	-2·25 (-12·8–8·68)
Occupational exposure to asbestos	10·5 (9·23–11·8)	9·04 (8·21–9·88)	8·11 (5·83–10·6)	-13·8 (-25·4—0·0774)	-10·4 (-35·5–17·2)	1·42 (1·07–1·94)	1·3 (0·957–1·77)	1·35 (0·898–1·97)	-8·83 (-25·2–10·5)	4·3 (-22·5–38·3)
Occupational exposure to arsenic	0·575 (0–1·36)	0·539 (0–1·29)	0·572 (0–1·38)	-6·28 (-19·3–NA)	6·14 (-5·67–NA)	0·579 (0–1·36)	0·537 (0–1·29)	0·548 (0–1·36)	-7·27 (-23·5–NA)	1·99 (-11·3–NA)
Occupational exposure to benzene	0·872 (0·0496–2·58)	0·852 (0·0494–2·44)	0·88 (0·0528–2·55)	-2·19 (-10·5–10·1)	3·27 (-4·41–14·5)	1·24 (0·0686–3·57)	1·44 (0·0839–3·92)	1·49 (0·0857–4·29)	16 (3·48–38·5)	3·76 (-5·18–15·4)
Occupational exposure to beryllium	0·0242 (0·0227–0·0256)	0·0236 (0·0229–0·0244)	0·0249 (0·0234–0·0264)	-2·24 (-8·59–4·49)	5·59 (-0·895–11·9)	0·0279 (0·0256–0·0304)	0·0293 (0·0284–0·0303)	0·0302 (0·0282–0·0323)	5 (-3·95–14·4)	3·02 (-3·59–9·44)
Occupational exposure to cadmium	0·0663 (0·06–0·0731)	0·0628 (0·0571–0·0692)	0·0665 (0·0588–0·0748)	-5·27 (-17·3–8·37)	5·86 (-4·78–16·8)	0·0677 (0·0594–0·0765)	0·0642 (0·0571–0·0723)	0·0653 (0·057–0·0758)	-5·25 (-19·9–12·6)	1·83 (-9·87–14·9)
Occupational exposure to chromium	0·157 (0·143–0·171)	0·152 (0·14–0·165)	0·16 (0·144–0·178)	-3·05 (-13·9–9·07)	5·51 (-3·5–15·1)	--	--	--	--	--
Occupational exposure to diesel engine exhaust	0·667 (0·611–0·727)	0·725 (0·678–0·779)	0·759 (0·693–0·832)	8·63 (-1·91–20·1)	4·78 (-3·64–13·4)	0·638 (0·567–0·717)	0·706 (0·639–0·782)	0·707 (0·63–0·796)	10·7 (-3·75–28·3)	0·114 (-10·2–10·3)
Occupational exposure to formaldehyde	0·337 (0·304–0·372)	0·31 (0·28–0·343)	0·324 (0·285–0·365)	-7·97 (-20·3–6·02)	4·5 (-6·06–15·7)	0·333 (0·294–0·376)	0·32 (0·286–0·358)	0·326 (0·287–0·373)	-3·88 (-17·9–13·2)	1·87 (-9·04–13·6)
Occupational exposure to nickel	0·473 (0–2·09)	0·431 (0–1·84)	0·457 (0–1·94)	-8·87 (-22–NA)	6·16 (-5·66–NA)	0·461 (0–1·99)	0·411 (0–1·85)	0·421 (0–1·93)	-10·8 (-27–NA)	2·35 (-12·3–NA)
Occupational exposure to polycyclic aromatic hydrocarbons	0·311 (0·286–0·337)	0·303 (0·282–0·327)	0·319 (0·288–0·351)	-2·66 (-13–8·83)	5·22 (-3·57–14·4)	0·346 (0·311–0·385)	0·356 (0·332–0·385)	0·364 (0·328–0·404)	2·85 (-9·08–16·7)	2·09 (-6·68–11·4)
Occupational exposure to silica	4·14 (0·545–13·3)	3·72 (0·507–12)	3·98 (0·586–12·5)	-10·1 (-23·1–5·58)	6·95 (-4·57–20·6)	3·78 (0·534–12·8)	3·27 (0·452–10·5)	3·3 (0·463–10·5)	-13·6 (-29·9–11·4)	1·19 (-13·1–22·2)
Occupational exposure to sulfuric acid	1·03 (0·231–3·21)	1·08 (0·218–3·54)	1·12 (0·227–3·66)	4·75 (-8·38–21·5)	3·81 (-6·85–15·9)	1·01 (0·206–3·37)	1·14 (0·232–3·72)	1·13 (0·234–3·54)	13·1 (-7·15–37·8)	-0·746 (-14·4–15·6)
Unsafe sex	--	--	--	--	--	--	--	--	--	--
Occupational exposure to trichloroethylene	0·0881 (0·0818–0·0948)	0·0887 (0·0843–0·094)	0·0934 (0·086–0·101)	0·685 (-7·88–10·3)	5·28 (-2·54–13)	0·0909 (0·0825–0·101)	0·0927 (0·0864–0·1)	0·094 (0·0856–0·104)	2 (-10·1–15·4)	1·41 (-7·08–10·4)
No access to handwashing facility	3·29 (3·04–3·55)	3·08 (2·83–3·33)	3·05 (2·82–3·29)	-6·31 (-14·9–2·43)	-0·886 (-6·46–5·16)	3·29 (3·04–3·55)	3·08 (2·83–3·33)	3·05 (2·82–3·29)	-6·31 (-14·9–2·43)	-0·886 (-6·46–5·16)
Child wasting	0·746 (0·494–1·03)	0·724 (0·481–0·99)	0·722 (0·47–0·969)	-2·92 (-12·5–7·49)	-0·343 (-7·59–7·35)	0·769 (0·503–1·04)	0·758 (0·502–1·05)	0·754 (0·51–1·03)	-1·45 (-11·4–8·97)	-0·489 (-7·78–7·72)
Child stunting	2·28 (1·13–3·39)	1·99 (1·04–2·92)	1·94 (1–2·93)	-12·7 (-31·6–11)	-2·64 (-16·4–12·7)	1·87 (0·926–2·84)	1·73 (0·83–2·64)	1·71 (0·841–2·66)	-7·69 (-28·9–17·7)	-1·27 (-18·1–17·8)
Chewing tobacco	1·43 (1·06–1·91)	1·6 (1·13–2·23)	1·65 (1·12–2·37)	12 (-24·1–70·2)	2·78 (-24·2–35·2)	0·283 (0·178–0·431)	0·325 (0·207–0·492)	0·333 (0·209–0·53)	14·9 (-33·6–102)	2·39 (-30·8–46·7)
Diet low in legumes	73·6 (36·9–99·1)	76·6 (46·1–99·7)	76·6 (45–99·6)	4·02 (-0·328–22·6)	0·0206 (-5·05–5·51)	72 (34·8–98·8)	74·9 (41·2–99·6)	75·2 (42·3–99·6)	4·04 (-0·305–24·6)	0·426 (-4·56–6·68)
Short gestation	22·6 (18·4–27·5)	27·7 (23·1–32·9)	25·3 (20·6–31·1)	22·8 (-0·824–52·2)	-8·77 (-25·1–11·6)	18·5 (15·2–22·5)	24 (20·2–28·1)	21·9 (18·5–25·6)	29·6 (1·87–65·5)	-8·75 (-24·2–8·46)
Low birth weight	9·02 (7·51–10·9)	11·4 (9·68–13·2)	11·1 (9·26–13·3)	26·3 (1·29–55·2)	-2·73 (-20·7–20·8)	8·22 (6·84–9·87)	12·1 (10·3–14)	11·6 (9·87–13·4)	47·1 (15·3–88)	-4·14 (-21–14·7)
High temperature	24·4 (11·1–39·1)	25·3 (11·4–40·5)	25·4 (11·6–40·7)	3·46 (-1·43–7·12)	0·473 (-0·456–1·36)	24·4 (11·1–39·1)	25·3 (11·4–40·5)	25·4 (11·6–40·7)	3·46 (-1·43–7·12)	0·473 (-0·456–1·36)
Low temperature	33·1 (27·7–39·1)	34·2 (29–40)	35·3 (30·3–40·8)	3·21 (1·68–5·02)	3·29 (2·05–4·8)	33·1 (27·7–39·1)	34·2 (29–40)	35·3 (30·3–40·8)	3·21 (1·68–5·02)	3·29 (2·05–4·8)
Kidney dysfunction	19·3 (13·8–26·4)	19·8 (14–27)	20 (14·3–27·3)	2·64 (-0·98–7·13)	0·817 (-3·03–4·94)	21·1 (14·7–28·6)	22·1 (15·5–29·7)	22·9 (16·2–30·7)	4·46 (0·397–9·5)	3·73 (0·167–8·21)
Bullying victimization	5·82 (2·43–11·9)	8·93 (3·6–17·5)	8·66 (3·38–17·6)	53·4 (32·2–76·6)	-3·04 (-12·6–2·96)	--	--	--	--	--
High LDL cholesterol	67·1 (62·8–71·7)	34·4 (31–37·9)	30·9 (27·6–34·5)	-48·8 (-53·8—43·3)	-10·1 (-14·9—5·11)	65·7 (61·8–69·6)	34·2 (31·1–37·6)	31·7 (28·2–35·3)	-47·9 (-52·7—43·3)	-7·4 (-11·4—3·34)

Supplementary Results Table 1. Kyrgyzstan age-standardised Summary Exposure Values (SEVs) for most-detailed risk factors and percentage change, by sex, 1990, 2010, and 2019. Data in parentheses are 95% uncertainty intervals.

	Male					Female				
	1990	2010	2019	Percentage change 1990-2010	Percentage change 2010-2019	1990	2010	2019	Percentage change 1990-2010	Percentage change 2010-2019
Unsafe sanitation	64·7 (57·3–72·3)	75·3 (70·5–79·3)	72·1 (65·7–77·9)	16·5 (6·8–28·7)	-4·31 (-12–2·67)	64·7 (57·3–72·3)	75·3 (70·5–79·3)	72·1 (65·7–77·9)	16·5 (6·8–28·7)	-4·31 (-12–2·67)
Ambient particulate matter pollution	13·5 (5·81–26·7)	17·3 (10·5–26·5)	19·7 (11·8–30·7)	27·4 (-28·7–139)	14·4 (-10·6–46·9)	11·8 (4·93–23·7)	15·5 (9·24–24·4)	18·3 (10·7–28·9)	31·6 (-28·1–151)	18 (-8·44–52·1)
Household air pollution from solid fuels	18·4 (9·1–31·6)	9·9 (5·02–17)	5·9 (2·75–10·8)	-46·1 (-59·9–-25)	-40·4 (-56·6–-22)	20·4 (11·2–33)	11·6 (6·4–18·5)	7·15 (3·64–12·3)	-43·3 (-56·9–-22·9)	-38·2 (-54·8–-20·4)
Ambient ozone pollution	87·5 (41·9–100)	75·9 (33·9–100)	86·2 (41·1–100)	-13·3 (-26·9–0)	13·5 (0–33·1)	87·5 (41·9–100)	75·9 (33·9–100)	86·2 (41·1–100)	-13·3 (-26·9–0)	13·5 (0–33·1)
Residential radon	35·5 (5·32–100)	35·5 (5·32–100)	35·5 (5·32–100)	0 (0–0)	0 (0–0)	--	--	--	--	--
Lead exposure	54·6 (32·5–73·6)	52·3 (32·9–70·6)	48 (29·2–66·5)	-4·23 (-13·8–9·93)	-8·22 (-14·5–-3·73)	29·9 (1·97–56·4)	33 (8·59–56·8)	31·6 (9·18–54·7)	10·6 (-4·68–395)	-4·38 (-11·9–13·6)
Child underweight	3·09 (2·08–4·03)	1·93 (1·25–2·69)	1·71 (1·07–2·46)	-37·6 (-50·4–-25·8)	-11·4 (-23·4–0·747)	3·4 (2·42–4·41)	2·21 (1·42–3·02)	1·94 (1·23–2·7)	-35·1 (-46·7–-25)	-12·1 (-21·8–-2·51)
Iron deficiency	--	--	--	--	--	23·9 (21·5–26·4)	22·1 (19·9–24·3)	20·6 (18·5–22·9)	-7·7 (-15·8–1·74)	-6·53 (-12·1–-0·961)
Vitamin A deficiency	11·9 (4·87–21·9)	8·86 (3·79–16·8)	6·69 (2·91–12·8)	-25·4 (-67·7–81·1)	-24·5 (-56·3–34·9)	6·9 (3·55–11·5)	7·99 (4·05–13·3)	6·24 (3·13–10·9)	15·8 (-39·7–136)	-21·8 (-46·8–16·2)
Zinc deficiency	5·94 (0·221–16·3)	5·37 (0·0337–15·3)	4·73 (0–14)	-9·65 (-34·9–9·86)	-12 (-48·1–NA)	5·77 (0·154–16)	5·24 (0–15)	4·6 (0–13·6)	-9·15 (-36·9–18·4)	-12·3 (-45·8–NA)
Smoking	31·4 (28·3–34·8)	24·2 (21·5–27)	26·1 (23·2–29·3)	-22·9 (-27·8–-18)	8·05 (3·85–12·9)	1·84 (1·39–2·41)	1·73 (1·42–2·1)	2·43 (1·97–2·94)	-6·33 (-27·1–21·9)	40·4 (23·2–59·2)
Secondhand smoke	32·6 (30·7–34·8)	35·9 (34·9–36·9)	36·4 (34·5–38·1)	9·92 (2·51–17·5)	1·43 (-3·58–6·18)	52·1 (50·1–54)	55·5 (54·5–56·5)	58·4 (56·6–60)	6·58 (2·15–10·8)	5·23 (2·12–8·45)
Alcohol use	9·35 (6·72–12·6)	12 (8·9–15·6)	10·7 (7·65–14·3)	28·2 (12·1–48·3)	-11 (-23–-0·052)	1·7 (1·02–2·68)	2·91 (1·92–4·26)	2·48 (1·49–3·81)	70·6 (32·4–125)	-14·6 (-34–6·12)
Drug use	0·175 (0·113–0·294)	0·22 (0·153–0·347)	0·195 (0·13–0·311)	25·3 (9·87–47·8)	-11·5 (-21·5–-1·68)	0·0947 (0·0587–0·164)	0·109 (0·0719–0·179)	0·102 (0·0655–0·169)	15 (3·76–28·8)	-6·08 (-14·6–1·6)
High fasting plasma glucose	4·79 (4·09–5·53)	6·11 (5·28–6·94)	6·85 (5·9–7·85)	27·6 (14·5–40·9)	12 (1·32–24·3)	4·6 (3·98–5·24)	6·14 (5·38–7·11)	6·94 (5·98–8·04)	33·6 (20·7–50·1)	13·1 (-0·715–29·3)
High systolic blood pressure	23·5 (18·5–29·5)	18·1 (14·2–22·3)	19 (14·4–23·7)	-23·2 (-42–1·04)	5·22 (-14·8–33·3)	22·9 (16·1–32)	14·9 (10·6–20)	15·4 (10·3–21·2)	-35 (-57·3–2·27)	3·37 (-28–45·9)
High body-mass index	15·1 (10·8–20·8)	17 (12·5–23)	20·1 (15·1–26·6)	12·4 (0·794–28·2)	18·1 (9·6–31·3)	19·7 (14·5–26·5)	21·1 (15·4–28·5)	23·4 (17·3–31·6)	6·96 (-4·39–17·9)	10·8 (3·09–19·6)
Low bone mineral density	9·03 (5–14·6)	9·08 (4·92–14·9)	8·96 (4·96–14·7)	0·462 (-22·7–30·4)	-1·31 (-23·4–29·8)	16·8 (11–24·2)	17 (11·2–24·1)	16·6 (10·7–24·2)	1·05 (-22·5–29·5)	-2·24 (-23–23·5)
Diet low in fruits	77·6 (70·5–85·9)	68·1 (58·7–79·2)	66·5 (56·1–77·8)	-12·2 (-17·7–-7·11)	-2·33 (-7·15–1·42)	78·6 (71·8–86·4)	69·4 (60·3–80·2)	67·7 (57·9–78·4)	-11·7 (-16·9–-6·83)	-2·33 (-7·13–1·33)
Diet low in vegetables	22 (8·98–43·6)	11·8 (4·47–26·2)	9·87 (3·71–23·1)	-46·5 (-61·8–-30·5)	-16·3 (-42·5–12·2)	--	--	--	--	--
Diet low in whole grains	99·9 (100–100)	99·9 (100–100)	99·9 (100–100)	0 (0–0)	0 (0–0)	99·9 (100–100)	99·9 (100–100)	99·9 (100–100)	0 (0–0)	0 (0–0)
Diet low in nuts and seeds	85·3 (43·9–93·4)	83·8 (43·6–92·8)	82 (42·3–92·3)	-1·71 (-10·8–7·8)	-2·17 (-10·1–7·69)	82·3 (42·7–92·3)	81·5 (42·3–91·6)	79 (41·4–90·3)	-1·02 (-9·44–8·07)	-2·99 (-11–5·82)
Diet low in milk	26·8 (8·72–45·4)	17·8 (5·3–37·2)	18·9 (5·6–37·7)	-33·6 (-59·3–-10·4)	5·94 (-14·2–42·4)	25·5 (8·16–44)	16·7 (4·22–36·6)	17·7 (4·87–36·5)	-34·6 (-63·6–-11)	6·41 (-13·4–51·1)
Diet high in red meat	74·2 (65·4–82)	52·7 (41·4–63·2)	54·7 (43–66)	-28·9 (-38·2–-21·2)	3·76 (-6·09–15·4)	74·1 (65·8–82·1)	52·5 (41·4–63·1)	54·6 (42·7–66·1)	-29·1 (-39·1–-21·2)	3·9 (-6·52–15·9)
Diet high in processed meat	45·9 (24·8–71·4)	44·5 (23·6–68·6)	45·6 (24·5–69·7)	-3·14 (-23·5–21·3)	2·46 (-10·5–20)	53·5 (31–78·6)	52·4 (30·3–77)	53·7 (31·1–77·8)	-2·02 (-21·3–20·7)	2·44 (-9·93–17·2)
Diet high in sugar-sweetened beverages	22·5 (12–37·8)	21·7 (10·9–36·8)	23 (11·1–39·1)	-3·72 (-40·8–51)	6·27 (-20·7–44·5)	23·4 (11·7–39·3)	23·6 (10·7–40·1)	25·2 (11·4–43·3)	0·731 (-40·1–67·2)	6·92 (-20·6–43·7)
Diet low in fiber	35 (20·5–50)	24·4 (13·8–36·3)	23·3 (12·8–34·7)	-30·2 (-37·2–-24·3)	-4·84 (-19·4–9·48)	35 (20·4–50·5)	23·9 (13·3–36·2)	22·8 (12·1–34·8)	-31·7 (-39·3–-25·2)	-4·8 (-19·8–11·9)
Diet low in seafood omega-3 fatty acids	97·8 (92·3–100)	98·8 (95·2–100)	97·8 (93·5–100)	1·05 (-1·38–6·31)	-1·06 (-4·62–1·33)	98·8 (95·5–100)	99·4 (98·1–100)	98·5 (94·7–100)	0·681 (-1·68e-05–3·4)	-0·935 (-4·14–0·0673)
Diet low in polyunsaturated fatty acids	76·9 (39·5–100)	83·1 (48·7–100)	81 (46·4–100)	8·06 (0–29·4)	-2·45 (-8·22–0)	78·2 (41·8–100)	84·1 (52·2–100)	82·2 (48·2–100)	7·56 (0–26)	-2·34 (-7·49–0)
Diet high in trans fatty acids	41·9 (31–99)	38·4 (28·1–97·8)	37·1 (27·2–97·8)	-8·19 (-18–0·724)	-3·32 (-14·8–6·52)	43·6 (32·5–100)	40 (29·3–100)	39 (28–99·8)	-8·14 (-17·7–0)	-2·62 (-11·2–5·65)
Diet high in sodium	64 (40·1–82·4)	43·7 (14·2–66·7)	42·5 (13·6–66·1)	-31·8 (-67–-11·4)	-2·69 (-23·3–18·5)	31·1 (11·5–54·2)	18·9 (4·64–41·4)	18·3 (4·49–41)	-39·3 (-69·7–-18·2)	-3·04 (-20·3–10·5)
Low physical activity	1·85 (0·883–4·02)	1·92 (0·922–4·11)	2·01 (0·974–4·17)	3·29 (-6·14–15·9)	4·7 (-4·97–16·7)	1·54 (0·831–3·13)	1·59 (0·852–3·2)	1·65 (0·892–3·26)	3·35 (-4·98–15·1)	3·67 (-4·78–14·4)
Occupational asthmagens	25·7 (20·3–32·5)	23·1 (18·5–28·6)	21·6 (17·3–27)	-10·4 (-21·8–3·89)	-6·16 (-15·6–3·03)	13·5 (9·77–18·3)	13·4 (10·5–16·9)	11·9 (9·09–15·5)	-0·837 (-18·1–22·6)	-10·8 (-23–3·05)
Occupational particulate matter, gases, and fumes	13·5 (10·6–17·9)	12·9 (10·1–17·1)	12·7 (9·95–16·8)	-4·99 (-9·52–-0·0245)	-1·37 (-3·34–0·684)	6·97 (5·14–9·71)	6·94 (5·27–9·49)	6·69 (5·12–9·16)	-0·437 (-8·06–8·61)	-3·63 (-6·69–-0·706)
Occupational noise	14·4 (13·2–15·7)	14·1 (13·3–15)	14·1 (13·4–14·9)	-2·15 (-5·84–2·29)	-0·138 (-1·66–1·52)	8·31 (7·23–9·45)	8·64 (8·03–9·34)	8·58 (8·1–9·21)	3·89 (-3·16–12·6)	-0·636 (-3·06–1·88)
Occupational injuries	--	--	--	--	--	--	--	--	--	--
Occupational ergonomic factors	31·4 (27·3–35·6)	28·5 (26·1–31)	25·3 (22·1–28·6)	-9·42 (-20·8–5·44)	-11·2 (-20·7–-1·46)	--	--	--	--	--
Childhood sexual abuse	2·52 (1·65–3·56)	2·52 (1·65–3·56)	2·52 (1·65–3·56)	0 (0–0)	0 (0–0)	3·14 (2·26–4·3)	3·14 (2·26–4·3)	3·14 (2·26–4·3)	0 (0–0)	0 (0–0)
Intimate partner violence	--	--	--	--	--	18·8 (11·1–26)	18·1 (11·1–24·1)	18·8 (11·1–26)	-3·46 (-14·8–17·1)	3·58 (-8·86–27·9)
Non-exclusive breastfeeding	20·1 (12·7–29·7)	16·3 (10·7–23·5)	16·3 (10·3–23·7)	-18·8 (-25·9–-10·2)	-0·346 (-9·66–9·16)	20·1 (12·6–29·7)	16·3 (10·7–23·4)	16·3 (10·3–23·7)	-18·7 (-25·8–-10·1)	-0·296 (-9·61–9·22)
Discontinued breastfeeding	10·9 (9·38–12·5)	11·7 (10·7–12·9)	11·5 (9·98–12·9)	7·86 (-9·31–28)	-2·1 (-17·4–13·1)	10·8 (9·33–12·4)	11·7 (10·6–12·9)	11·5 (9·97–12·9)	8·24 (-8·99–28·5)	-2·03 (-17·3–13·2)
Diet low in calcium	22·3 (10·5–43)	20·8 (10·3–39·2)	19·1 (9·1–36)	-6·59 (-12·4–1·45)	-8·09 (-15·4–-3·53)	--	--	--	--	--
Occupational exposure to asbestos	0·377 (0·291–0·47)	0·884 (0·766–1)	0·951 (0·763–1·15)	135 (87·4–195)	7·53 (-12·7–30·3)	0·173 (0·102–0·393)	0·347 (0·188–0·436)	0·309 (0·141–0·429)	100 (-7·91–227)	-11 (-35·6–16·9)
Occupational exposure to arsenic	0·417 (0·167–0·699)	0·361 (0·147–0·608)	0·361 (0·147–0·612)	-13·4 (-20·7–-4·45)	-0·253 (-5·97–6·28)	0·365 (0·141–0·617)	0·361 (0·144–0·608)	0·343 (0·142–0·577)	-1·21 (-11·3–11·7)	-4·83 (-12·7–3·2)
Occupational exposure to benzene	0·87 (0·536–1·54)	0·911 (0·563–1·6)	0·919 (0·564–1·63)	4·66 (-2·83–16)	0·953 (-3·74–6·52)	0·985 (0·606–1·81)	1·06 (0·645–1·92)	1·09 (0·664–1·93)	7·45 (-4·24–23·8)	3 (-4·41–12·6)
Occupational exposure to beryllium	0·109 (0·102–0·116)	0·113 (0·112–0·115)	0·111 (0·109–0·114)	3·85 (-2·54–11·4)	-1·55 (-4·17–1·35)	0·098 (0·0886–0·107)	0·106 (0·104–0·108)	0·0999 (0·0954–0·104)	8·29 (-1–20·5)	-5·82 (-10·2–-1·13)
Occupational exposure to cadmium	0·221 (0·204–0·237)	0·198 (0·191–0·207)	0·198 (0·187–0·21)	-10·3 (-17·3–-2·12)	-0·101 (-5·23–5·64)	0·194 (0·175–0·214)	0·197 (0·188–0·206)	0·187 (0·174–0·202)	1·26 (-8·61–13·9)	-4·68 (-11·5–2·66)
Occupational exposure to chromium	0·491 (0·454–0·53)	0·457 (0·44–0·476)	0·465 (0·441–0·491)	-6·91 (-14–1·68)	1·79 (-3·33–7·38)	0·439 (0·395–0·484)	0·459 (0·439–0·481)	0·449 (0·417–0·483)	4·58 (-6·2–18·2)	-2·33 (-9·18–5·36)
Occupational exposure to diesel engine exhaust	2·93 (2·71–3·16)	3·61 (3·45–3·78)	3·79 (3·59–4·01)	23·3 (13·5–34·6)	4·97 (-0·445–11)	1·76 (1·58–1·95)	2·27 (2·16–2·38)	2·19 (2·03–2·38)	28·9 (15·5–45)	-3·15 (-10·4–4·53)
Occupational exposure to formaldehyde	1·03 (0·822–1·13)	0·881 (0·719–0·946)	0·895 (0·705–0·978)	-14·3 (-21·8–-5·23)	1·55 (-4·72–8·79)	--	--	--	--	--
Occupational exposure to nickel	0·501 (0·16–1·35)	0·462 (0·153–1·23)	0·464 (0·15–1·22)	-7·75 (-14·7–1·13)	0·526 (-4·73–7·37)	0·358 (0·114–0·925)	0·336 (0·11–0·874)	0·312 (0·105–0·829)	-6·1 (-15·7–6·43)	-7·11 (-14·6–1·65)
Occupational exposure to polycyclic aromatic hydrocarbons	1·06 (0·982–1·14)	1·01 (0·975–1·05)	1·03 (0·979–1·08)	-4·47 (-11·4–3·99)	1·47 (-3·04–6·47)	0·952 (0·857–1·05)	0·977 (0·936–1·02)	0·958 (0·894–1·02)	2·62 (-7·56–15·5)	-1·94 (-8·42–5·34)
Occupational exposure to silica	5·76 (3·03–12·9)	6·11 (3·19–13·3)	6·31 (3·21–14·1)	6·06 (-4·77–19·4)	3·23 (-4·74–13)	3·1 (1·57–6·87)	2·81 (1·43–6·01)	2·54 (1·28–5·57)	-9·35 (-18·7–2·63)	-9·49 (-16·2–-1·61)
Occupational exposure to sulfuric acid	1·14 (0·821–2·01)	1·13 (0·824–1·97)	1·09 (0·78–1·93)	-0·938 (-8·97–7·64)	-3·45 (-8·64–1·86)	0·895 (0·631–1·52)	0·93 (0·668–1·61)	0·849 (0·604–1·49)	3·91 (-6·12–16·6)	-8·65 (-14·4–-2·32)
Unsafe sex	--	--	--	--	--	--	--	--	--	--
Occupational exposure to trichloroethylene	0·276 (0·257–0·298)	0·283 (0·275–0·293)	0·289 (0·278–0·303)	2·48 (-4·8–11·6)	2·21 (-2·02–7·09)	0·241 (0·218–0·267)	0·276 (0·267–0·288)	0·269 (0·252–0·288)	14·5 (3·44–28·8)	-2·73 (-8·74–4·64)
No access to handwashing facility	21·3 (20·3–22·3)	15·1 (14·4–15·9)	13 (12·4–13·6)	-29 (-33–-25)	-13·9 (-17·1–-10·6)	--	--	--	--	--
Child wasting	1·05 (0·761–1·36)	1·01 (0·737–1·31)	1 (0·729–1·28)	-3·03 (-11·7–5·86)	-1·35 (-7·8–5·46)	1·61 (1·26–2)	1·53 (1·17–1·9)	1·5 (1·16–1·85)	-5·13 (-15·3–6·33)	-1·62 (-9·62–7·96)
Child stunting	14·3 (9·98–17·4)	10·7 (7·26–13)	9·05 (5·78–11·7)	-25·2 (-35·8–-13·9)	-15·3 (-27·3–-4·47)	15·9 (11·2–18·9)	11·3 (7·8–13·6)	9·5 (6·2–12·1)	-29·3 (-39·3–-19·5)	-15·6 (-26·6–-4·76)
Chewing tobacco	5·83 (4·65–7·33)	6·33 (5·36–7·4)	6·15 (4·97–7·51)	8·48 (-13·3–41·1)	-2·79 (-18·6–14·9)	--	--	--	--	--
Diet low in legumes	97·8 (94·4–100)	89·3 (75–100)	90·2 (76·3–100)	-8·62 (-20·6–0)	1 (-0·348–3·5)	96·5 (91·1–100)	87 (69·7–100)	88·1 (71·8–100)	-9·81 (-23·8–0)	1·21 (-0·254–4·98)
Short gestation	8·8 (6·6–11·8)	9·46 (8·1–11)	9·72 (7·84–12)	7·54 (-23·4–48·1)	2·76 (-18·2–29·3)	7·56 (5·99–9·73)	7·63 (6·5–8·88)	7·29 (6·14–8·6)	0·977 (-25·2–29·2)	-4·46 (-21·2–14·5)
Low birth weight	4·11 (3·18–5·39)	4·8 (4·18–5·57)	4·89 (4·04–5·9)	17 (-14·2–57·1)	1·76 (-19·5–28·1)	3·66 (2·97–4·66)	3·73 (3·21–4·32)	3·75 (3·22–4·37)	1·8 (-22·8–29·5)	0·625 (-17·7–23)
High temperature	26·7 (18·1–36·7)	24·3 (15·6–34·8)	28·4 (19·7–38·3)	-9·05 (-19·1–-0·76)	17·3 (1·94–36·8)	26·7 (18·1–36·7)	24·3 (15·6–34·8)	28·4 (19·7–38·3)	-9·05 (-19·1–-0·76)	17·3 (1·94–36·8)
Low temperature	37·3 (33·7–42)	36·3 (32·5–41·3)	37·4 (33·8–42·2)	-2·7 (-4·55–-0·695)	2·93 (1·57–4·34)	37·3 (33·7–42)	36·3 (32·5–41·3)	37·4 (33·8–42·2)	-2·7 (-4·55–-0·695)	2·93 (1·57–4·34)
Kidney dysfunction	20·7 (13·8–28·9)	21·7 (14·8–29·8)	22·9 (15·6–31·1)	4·79 (1·34–9·67)	5·31 (2·37–9·57)	20·6 (13·7–28·5)	21·2 (14·2–29·2)	22·2 (15·3–30·2)	2·94 (-0·675–7·12)	4·96 (1·81–9·3)

Bullying victimization	2·51 (1·02–5·17)	2·64 (1·1–5·39)	2·59 (1·08–5·29)	4·93 (0·836–10·1)	-1·7 (-3·75–0·635)	1·49 (0·626–3·05)	1·6 (0·667–3·28)	1·58 (0·658–3·23)	6·99 (1·93–14·4)	-1·38 (-3·46–0·842)
High LDL cholesterol	30·5 (26·9–34·1)	29·1 (25·7–32·4)	28·9 (25·4–32·3)	-4·48 (-9·89–1·29)	-0·852 (-5·05–3·22)	32 (28·6–35·4)	30·7 (27·4–34·2)	30·5 (27·1–33·9)	-4·11 (-8·97–0·61)	-0·665 (-4·02–2·68)

Supplementary Results Table 1. South Carolina age-standardised Summary Exposure Values (SEVs) for most-detailed risk factors and percentage change, by sex, 1990, 2010, and 2019. Data in parentheses are 95% uncertainty intervals.

	Male					Female				
	1990	2010	2019	Percentage change 1990-2010	Percentage change 2010-2019	1990	2010	2019	Percentage change 1990-2010	Percentage change 2010-2019
Unsafe sanitation	8·86 (4·09–15·9)	4·4 (1·92–8·53)	3·5 (1·56–6·75)	-50·3 (-80–25·5)	-20·6 (-58·5–51)	--	--	--	--	--
Ambient particulate matter pollution	14·2 (5·55–27·4)	7·57 (4·73–10·7)	4·67 (2·08–7·53)	-46·8 (-73–24·4)	-38·4 (-57·3–-29·3)	14·2 (5·54–27·4)	7·57 (4·75–10·7)	4·67 (2·06–7·52)	-46·7 (-73–24·4)	-38·4 (-57·2–-29·2)
Household air pollution from solid fuels	0·0442 (0·0115–0·117)	0·019 (0·00338–0·0588)	0·012 (0·00187–0·0402)	-57 (-84·4–-0·649)	-36·7 (-70·7–22·7)	0·0582 (0·0156–0·155)	0·0252 (0·00457–0·083)	0·016 (0·00265–0·0554)	-56·7 (-84·2–0·545)	-36·5 (-70·6–23·2)
Ambient ozone pollution	77·8 (35·7–100)	60·4 (26·4–100)	36·3 (14·3–63·7)	-22·4 (-32·4–0)	-39·9 (-49·8–-30·7)	--	--	--	--	--
Residential radon	17·7 (2·36–55)	17·7 (2·36–55)	17·7 (2·36–55)	0 (0–0)	0 (0–0)	17·7 (2·36–55)	17·7 (2·36–55)	17·7 (2·36–55)	0 (0–0)	0 (0–0)
Lead exposure	42 (12·3–67·3)	25·7 (6·39–48·3)	18·3 (3·5–38·3)	-38·7 (-59–-26·2)	-28·9 (-49·2–-19·7)	23·7 (0·659–52·3)	15·6 (0·336–39·6)	11·4 (0·149–31·9)	-34·2 (-72·7–-14·2)	-27 (-65·8–-18·6)
Child underweight	0·988 (0·556–1·51)	0·978 (0·54–1·46)	0·954 (0·515–1·46)	-1·05 (-19·3–19·5)	-2·48 (-14·6–12·3)	0·834 (0·475–1·25)	0·862 (0·473–1·32)	0·882 (0·479–1·32)	3·35 (-13·9–27)	2·27 (-12·8–18·9)
Iron deficiency	--	--	--	--	--	12·8 (10·7–14·9)	12·7 (10·7–14·8)	13 (10·8–15·2)	-0·376 (-11·5–12·2)	1·97 (-7·9–12·2)
Vitamin A deficiency	1·11 (0·301–2·88)	1·41 (0·383–3·69)	0·946 (0·217–2·5)	26·7 (-69·1–417)	-32·9 (-70–61·2)	2·03 (0·782–4·23)	1·97 (0·755–3·94)	1·65 (0·641–3·52)	-2·96 (-60·7–204)	-15·9 (-52·6–57·3)
Zinc deficiency	2·59 (0–8·52)	1·31 (0–4·33)	1·38 (0–4·52)	-49·2 (-100–NA)	4·93 (-12·6–NA)	2·65 (0–8·47)	1·33 (0–4·41)	1·36 (0–4·39)	-49·8 (-100–NA)	2·3 (-15·9–NA)
Smoking	28·3 (25·6–31·1)	21·3 (19·3–23·4)	19·2 (17·4–21·3)	-24·9 (-29·5–-19·9)	-9·73 (-13·3–-5·87)	18·7 (16·3–21·2)	14·9 (13·3–16·5)	13·4 (11·9–15·1)	-20·7 (-28·5–-12)	-9·66 (-14·1–-4·94)
Secondhand smoke	39·2 (36·7–41·7)	26·3 (24·8–28)	26·1 (23·4–28·7)	-32·9 (-38·7–-26·2)	-0·918 (-9·48–7·81)	38·4 (36·3–40·3)	25·9 (24·7–27·1)	25·7 (23·5–27·8)	-32·5 (-37·1–-27·2)	-0·742 (-7·87–6·47)
Alcohol use	13·6 (8·93–19·3)	14·9 (9·13–21·8)	15·9 (9·59–23·4)	9·73 (-20·5–48)	6·71 (-15·5–34·3)	8·01 (5·14–11·8)	10 (6·22–14·7)	10·1 (5·89–14·8)	24·8 (-9·57–72·7)	0·83 (-22·1–28)
Drug use	0·609 (0·31–1·14)	1·02 (0·694–1·53)	1·41 (1·02–1·97)	67·9 (26·5–140)	37·7 (18·4–62·4)	0·491 (0·302–0·806)	0·917 (0·664–1·23)	1·37 (1·04–1·77)	86·9 (46·8–141)	49·7 (30·1–72·3)
High fasting plasma glucose	13·8 (11·4–16·6)	20·1 (17·5–22·7)	20·9 (18·1–24·1)	46 (22–77·1)	4·04 (-8·78–19·1)	11·7 (9·86–13·8)	16·8 (14·8–19·2)	17·6 (15·5–20·2)	44 (20·3–70)	5·07 (-7·77–19·7)
High systolic blood pressure	25·8 (20·6–31·2)	16·7 (14·6–18·7)	20·3 (15·9–25·2)	-35·3 (-47·2–-19·6)	22·2 (-1·8–49·6)	23·9 (17·3–30·7)	14·1 (12·5–15·7)	19·8 (14·7–26)	-40·9 (-54·6–-19·9)	39·6 (4·8–85·7)
High body-mass index	24·9 (17·6–33·8)	40·6 (30·5–51·9)	41·6 (30·9–52·8)	62·9 (41·8–93)	2·66 (-3·34–9·89)	31·4 (23·4–40·8)	45·9 (35·2–55·3)	46·6 (36·3–56·4)	46·3 (29–67·6)	1·5 (-5·23–8·68)
Low bone mineral density	12 (6·99–18·7)	9·28 (4·94–15·3)	11·7 (6·85–18·8)	-22·9 (-43·3–-1·09)	26·5 (-2·94–72·7)	15·9 (10·5–22·9)	16·7 (10·9–23·7)	19·1 (13·1–26·6)	4·51 (-16·5–29·9)	14·7 (-6·86–45·9)
Diet low in fruits	58·9 (45·7–73)	50·2 (36·5–64·8)	50·2 (36·2–64·5)	-14·8 (-25·4–-6·57)	-0·0965 (-10·2–10·3)	--	--	--	--	--
Diet low in vegetables	45·1 (25·6–66·6)	54·9 (35–76·3)	54·2 (34–73·7)	21·9 (6·72–54)	-1·41 (-9·49–6·44)	44 (24·8–64·6)	49·7 (31–70·9)	48·5 (29·1–68·6)	13·1 (1·67–33)	-2·51 (-12·1–7·13)
Diet low in whole grains	78·7 (68·2–88·7)	82 (73·4–89·6)	82 (73·6–89·5)	4·23 (-3·35–12·7)	-0·0242 (-2·6–2·49)	--	--	--	--	--
Diet low in nuts and seeds	31 (9·18–57·4)	26·3 (8·57–55·8)	21·9 (7·16–54·1)	-15 (-45·7–46·7)	-16·8 (-42·5–7·22)	26·6 (7·23–55·6)	23·4 (7·38–55·4)	20·5 (5·89–54)	-12 (-43·8–47·7)	-12·2 (-40·7–13·8)
Diet low in milk	51·2 (27·8–72·4)	46·2 (23·6–66·1)	45·4 (23·2–65·4)	-9·77 (-26·5–1·67)	-1·83 (-13·1–10·2)	51·1 (27·2–72·2)	46·4 (23·8–66·6)	45·7 (23·6–66·2)	-9·36 (-26·9–2·79)	-1·46 (-12·8–10·9)
Diet high in red meat	85·2 (75·7–92·1)	84·4 (73·7–92·6)	83·6 (71·8–92·9)	-0·847 (-12·3–11)	-0·959 (-9·92–7·58)	83 (73–90·2)	80 (68·8–88·9)	79·3 (67·3–89·3)	-3·51 (-15·7–9·37)	-0·99 (-10·5–9·84)
Diet high in processed meat	78·9 (55·5–98·8)	86·1 (66·5–99·8)	85·9 (66·6–99·7)	9·1 (0·639–27·5)	-0·304 (-5·35–4·74)	80·7 (58·8–98·6)	89 (73·6–99·5)	87·6 (69·7–99·5)	10·3 (0·694–31·6)	-1·58 (-6·92–2·26)
Diet high in sugar-sweetened beverages	43 (25·4–62·1)	58·5 (39·3–76·9)	59 (37·9–78·3)	36·2 (-6·79–129)	0·891 (-20·8–30·2)	47·2 (28–65·4)	64·6 (44·3–81·1)	63·5 (41·8–79·8)	36·7 (-3·47–110)	-1·63 (-21·4–24·1)
Diet low in fiber	46·7 (30·8–61·1)	33 (19·9–47·1)	30·2 (17·4–44·5)	-29·4 (-44·9–-12·7)	-8·54 (-25·3–8·3)	46·6 (30·2–62·6)	38 (24·1–52·7)	33·1 (18·3–48·1)	-18·4 (-34·1–-2·76)	-12·9 (-29·2–1·64)
Diet low in seafood omega-3 fatty acids	85·7 (70·1–100)	82·1 (63·7–99·7)	82·1 (64·6–99·6)	-4·27 (-18·7–8·34)	-0·0277 (-13·5–15·1)	87·8 (73·3–99·8)	85·5 (70·8–99·7)	85 (69·8–99·8)	-2·62 (-13·4–7·01)	-0·639 (-10·4–8·84)
Diet low in polyunsaturated fatty acids	15·8 (4·54–44·1)	8·09 (2·31–27·5)	7·71 (2·21–26·3)	-48·6 (-74·7–-8·58)	-4·72 (-26·4–18·4)	17·6 (5·18–47·9)	8·32 (2·35–28·1)	7·8 (2·27–27·5)	-52·8 (-76·5–-9·61)	-6·26 (-27·8–13·5)
Diet high in trans fatty acids	89·6 (81·8–100)	87·8 (79·1–100)	87·5 (78·6–100)	-1·92 (-9·99–5·63)	-0·401 (-8·45–9·28)	88·4 (80·7–100)	86·7 (78–100)	86·2 (77–100)	-1·91 (-9·99–6·27)	-0·654 (-8·15–8·41)
Diet high in sodium	18·6 (4·33–41·2)	40·2 (11·4–63·6)	39·4 (10·8–63·9)	116 (39·2–426)	-1·92 (-29·1–32·1)	--	--	--	--	--
Low physical activity	2·15 (0·788–4·62)	1·86 (0·795–4·14)	1·99 (0·695–4·4)	-13·4 (-41·9–30·8)	6·88 (-24·7–47·8)	5·47 (2·64–10·4)	5·85 (3·15–10·7)	6·41 (3·27–12)	6·98 (-19·6–49·6)	9·58 (-4·89–25·8)
Occupational asthmagens	23·8 (19·9–28·7)	20·6 (17·4–24·5)	21·4 (17·8–25·8)	-13·4 (-23·7–-2·2)	3·7 (-5·62–13·9)	11·8 (9·66–14·4)	10·9 (9·16–13)	11 (8·92–13·4)	-7·73 (-21·3–8·21)	1·16 (-10–13·4)
Occupational particulate matter, gases, and fumes	10·1 (7·17–14·4)	9·43 (6·79–13·5)	9·05 (6·53–12·7)	-6·46 (-11·8–-0·393)	-4·04 (-7·86–-0·383)	6·07 (4·22–8·87)	5·7 (4·1–8·25)	5·41 (3·91–7·76)	-6·15 (-14·6–3·36)	-5·01 (-10·5–0·566)
Occupational noise	8·54 (7·74–9·49)	8·23 (7·63–9·01)	7·96 (7·43–8·71)	-3·67 (-7·42–0·738)	-3·21 (-5·3–-0·812)	6·05 (5·27–6·92)	6·25 (5·71–6·91)	6·21 (5·78–6·79)	3·41 (-2·82–11·3)	-0·64 (-3·97–2·85)
Occupational injuries	--	--	--	--	--	--	--	--	--	--
Occupational ergonomic factors	13 (10·8–15·7)	10·7 (8·99–12·7)	11 (9·08–13·2)	-17·8 (-28·4–-6·24)	3·3 (-5·56–12·4)	7·48 (5·93–9·42)	7·04 (5·71–8·61)	7·36 (5·82–9·23)	-5·9 (-19·9–13·2)	4·57 (-7·29–17·1)
Childhood sexual abuse	7·4 (5·05–10·5)	6·97 (4·6–9·93)	6·85 (4·54–9·67)	-5·83 (-12·2–5·83)	-1·68 (-8·51–5·53)	12·3 (8·94–17·3)	17·4 (12·9–24)	17·8 (13·1–24·7)	41·9 (30·7–54·8)	2·17 (-5–11)
Intimate partner violence	--	--	--	--	--	34·5 (19·6–43·9)	32·8 (19·3–42·4)	33·6 (19·6–43·2)	-4·75 (-13·8–8·18)	2·43 (-4·88–17)
Non-exclusive breastfeeding	26·1 (21·4–31)	24·9 (21·1–28·9)	25 (20·5–29·8)	-4·83 (-19·8–12·2)	0·378 (-12·7–15)	26·1 (21·4–31)	24·9 (21·1–28·9)	25 (20·5–29·8)	-4·83 (-19·8–12·2)	0·371 (-12·8–15)
Discontinued breastfeeding	27·7 (26·7–28·6)	25·3 (23·8–26·6)	25·9 (24·4–27·2)	-8·75 (-14·4–-3·42)	2·52 (-3·29–9·28)	27·6 (26·7–28·5)	25·3 (23·9–26·6)	25·9 (24·4–27·2)	-8·29 (-14–-2·95)	2·23 (-3·56–8·96)
Diet low in calcium	18·2 (8·62–34·2)	15·2 (7·4–28·2)	14·5 (6·92–27·3)	-16·3 (-27·6–-5·63)	-4·47 (-14·9–3·05)	13·7 (5·62–26·1)	12·3 (5·4–23·3)	12·1 (5·17–22·6)	-10·1 (-22·5–5·11)	-2·1 (-11·6–6·78)
Occupational exposure to asbestos	7·95 (7·09–8·94)	6·94 (6·33–7·66)	6·53 (4·81–8·7)	-12·6 (-24·9–0·671)	-5·86 (-30·8–26·8)	1·27 (1·03–2·06)	1·23 (1–1·94)	1·22 (0·801–2)	-3·48 (-18·8–13)	-1·14 (-28·6–32·8)
Occupational exposure to arsenic	0·564 (0–1·34)	0·529 (0–1·28)	0·558 (0–1·34)	-6·23 (-19·9–NA)	5·44 (-6·33–NA)	0·551 (0–1·29)	0·514 (0–1·21)	0·524 (0–1·23)	-6·76 (-24–NA)	2 (-11·8–NA)
Occupational exposure to benzene	0·778 (0·0462–2·2)	0·74 (0·044–2·1)	0·769 (0·0463–2·26)	-4·95 (-12·8–6·73)	3·98 (-3·14–14·5)	1·07 (0·0613–2·9)	1·19 (0·0683–3·3)	1·26 (0·0708–3·65)	11·2 (-2·22–29·7)	6·45 (-2·84–18·9)
Occupational exposure to beryllium	0·0238 (0·0225–0·0253)	0·0226 (0·0221–0·0231)	0·024 (0·0226–0·0254)	-4·88 (-10·5–1·03)	5·96 (-0·019–12·8)	0·0258 (0·0234–0·0284)	0·0264 (0·0257–0·027)	0·0275 (0·0256–0·0295)	2·15 (-7·8–12·6)	4·3 (-2·74–11·7)
Occupational exposure to cadmium	0·0651 (0·0587–0·072)	0·0615 (0·056–0·0674)	0·0648 (0·0576–0·0733)	-5·59 (-17·8–7·96)	5·46 (-5·06–16·9)	0·0645 (0·0563–0·0739)	0·0607 (0·0542–0·0684)	0·0621 (0·0528–0·0719)	-5·81 (-21·9–13·9)	2·22 (-10·5–15·9)
Occupational exposure to chromium	0·153 (0·14–0·167)	0·148 (0·137–0·16)	0·156 (0·141–0·174)	-3·3 (-14·2–8·39)	5·41 (-3·76–15·3)	--	--	--	--	--
Occupational exposure to diesel engine exhaust	0·682 (0·63–0·746)	0·728 (0·681–0·778)	0·771 (0·704–0·845)	6·65 (-4·16–18·8)	5·91 (-2·34–14·6)	0·616 (0·541–0·691)	0·662 (0·606–0·725)	0·671 (0·591–0·755)	7·47 (-7·56–25·9)	1·45 (-8·7–12·8)
Occupational exposure to formaldehyde	0·325 (0·292–0·363)	0·299 (0·271–0·331)	0·312 (0·274–0·357)	-7·94 (-20·6–6·01)	4·2 (-6·31–15·8)	0·312 (0·272–0·356)	0·294 (0·265–0·328)	0·302 (0·257–0·347)	-5·62 (-20·6–12·6)	2·51 (-9·12–15·2)
Occupational exposure to nickel	0·479 (0–2·07)	0·433 (0–1·93)	0·456 (0–1·94)	-9·64 (-22·3–NA)	5·39 (-6·34–NA)	0·448 (0–1·9)	0·402 (0–1·78)	0·41 (0–1·76)	-10·3 (-27·7–NA)	1·98 (-12·8–NA)
Occupational exposure to polycyclic aromatic hydrocarbons	0·3 (0·274–0·327)	0·29 (0·27–0·312)	0·305 (0·276–0·339)	-3·27 (-13·7–7·89)	5·19 (-3·82–15)	0·322 (0·288–0·362)	0·325 (0·303–0·351)	0·334 (0·299–0·372)	0·747 (-12·3–16)	2·8 (-6·73–12·9)
Occupational exposure to silica	4·37 (0·617–13·9)	3·88 (0·584–12·4)	4·13 (0·579–12·8)	-11·3 (-23·4–2·59)	6·41 (-4·93–19·7)	3·78 (0·545–12·1)	3·31 (0·433–10·9)	3·34 (0·451–11·4)	-12·5 (-29·8–10)	1·14 (-13·8–20·2)
Occupational exposure to sulfuric acid	0·977 (0·203–3·11)	1·02 (0·205–3·3)	1·06 (0·218–3·42)	4·53 (-8·56–19·5)	3·63 (-6·4–15·9)	0·966 (0·197–3·13)	1·12 (0·226–3·64)	1·11 (0·224–3·43)	16 (-6·64–42·5)	-0·998 (-15·9–14·9)
Unsafe sex	--	--	--	--	--	--	--	--	--	--
Occupational exposure to trichloroethylene	0·0866 (0·0806–0·0936)	0·0867 (0·0827–0·091)	0·0915 (0·0849–0·099)	0·117 (-7·71–8·81)	5·56 (-1·54–13·7)	0·0858 (0·0766–0·0963)	0·086 (0·0807–0·0922)	0·088 (0·0792–0·0982)	0·221 (-12·4–14·5)	2·29 (-6·54–11·9)
No access to handwashing facility	3·44 (3·18–3·74)	3·14 (2·91–3·4)	3·11 (2·87–3·35)	-8·73 (-16·8–0·0757)	-1·17 (-7·03–4·71)	3·44 (3·18–3·74)	3·14 (2·91–3·4)	3·11 (2·87–3·35)	-8·73 (-16·8–0·0757)	-1·17 (-7·03–4·71)
Child wasting	0·754 (0·5–1·03)	0·731 (0·477–0·99)	0·729 (0·477–0·993)	-3·01 (-12·7–6·54)	-0·264 (-7·65–7·35)	0·779 (0·514–1·07)	0·768 (0·51–1·06)	0·762 (0·504–1·05)	-1·42 (-11·8–8·72)	-0·731 (-9–7·2)
Child stunting	2·55 (1·41–3·78)	2·13 (1·11–3·2)	2·07 (1·06–3·06)	-16·5 (-34·7–8)	-3·05 (-18·2–13·6)	2·02 (0·997–3·05)	1·82 (0·917–2·78)	1·8 (0·906–2·75)	-9·94 (-30·1–15·3)	-0·825 (-15·7–17·1)
Chewing tobacco	7·24 (5·86–8·78)	5·25 (3·88–7·07)	4·99 (3·55–6·85)	-27·4 (-48·3–1·85)	-4·95 (-25·2–19·7)	0·754 (0·505–1·08)	0·55 (0·359–0·835)	0·512 (0·33–0·808)	-27·1 (-56–19)	-6·89 (-34–32·5)
Diet low in legumes	75 (41·7–99·3)	78 (47·5–99·7)	77·9 (46·4–99·8)	3·95 (-0·0298–21·4)	-0·0683 (-5·48–5·68)	73·4 (38·1–99·4)	76·3 (44·8–99·6)	76·6 (46·1–99·6)	4·01 (-0·179–24·1)	0·345 (-5·51–6·35)
Short gestation	37·2 (30·5–45)	31 (26·3–35·9)	28·9 (23·9–35·1)	-16·7 (-33·1–3·37)	-6·74 (-22·9–13·1)	29·3 (24·3–34·9)	31·4 (26·5–36·9)	27·1 (23–31·9)	7·28 (-14·9–33·2)	-13·8 (-27·9–2·72)
Low birth weight	15·7 (13·1–19·1)	13·7 (11·6–15·9)	13·9 (11·6–16·6)	-12·6 (-30–8·57)	1·24 (-16·3–24·7)	14·7 (12·4–17·2)	16·1 (13·9–18·7)	15·2 (13·1–17·6)	9·83 (-12·9–36·8)	-6·07 (-21·7–12)
High temperature	25·5 (16·2–35·1)	27·4 (18·4–37·1)	23 (14–32·6)	7·66 (1·9–15·8)	-16 (-25–-9·07)	25·5 (16·2–35·1)	27·4 (18·4–37·1)	23 (14–32·6)	7·66 (1·9–15·8)	-16 (-25–-9·07)
Low temperature	34·7 (26·8–44·7)	39·5 (32·4–48·5)	36·9 (29–46·6)	13·7 (7·44–22·3)	-6·73 (-11–-3·03)	34·7 (26·8–44·7)	39·5 (32·4–48·5)	36·9 (29–46·6)	13·7 (7·44–22·3)	-6·73 (-11–-3·03)
Kidney dysfunction	20·3 (14·6–27·4)	21·3 (15·2–28·5)	21·4 (15·4–28·6)	4·57 (0·562–8·94)	0·486 (-3·1–4)	22·7 (16·2–30·4)	24·2 (17·3–32·2)	25·1 (18·3–33·2)	6·25 (1·73–11·8)	3·9 (-0·417–9·3)

Bullying victimization	5·84 (2·45–11·9)	8·9 (3·59–17·7)	8·67 (3·35–17·9)	52·5 (31·7–75·6)	-2·52 (-12–3·58)	4·24 (1·73–8·8)	7·8 (3·15–15·5)	7·74 (2·95–15·8)	83·8 (58·9–114)	-0·758 (-11·5–6·09)
High LDL cholesterol	66·8 (62·6–71·1)	34·5 (30·8–38·1)	30·7 (27–34·4)	-48·4 (-53·9–-43·5)	-11 (-15·8–-6·08)	66·1 (62·3–70)	34·6 (31·5–38·2)	31·9 (28·5–35·4)	-47·6 (-52–-42·7)	-7·99 (-12–-4·02)

Supplementary Results Table 1. South Dakota age-standardised Summary Exposure Values (SEVs) for most-detailed risk factors and percentage change, by sex, 1990, 2010, and 2019. Data in parentheses are 95% uncertainty intervals.

	Male				
	1990	2010	2019	Percentage change 1990-2010	Percentage change 2010-2019
Unsafe sanitation	7·34 (3·46–13·1)	4·04 (1·76–7·36)	3·01 (1·4–5·9)	-44·9 (-77·3–40·7)	-25·6 (-61·6–39·5)
Ambient particulate matter pollution	6·27 (1·02–14·6)	3·95 (1·59–6·78)	3·03 (0·895–5·72)	-37 (-76·2–181)	-23·2 (-44·9—14·9)
Household air pollution from solid fuels	0·0302 (0·00456–0·09)	0·0148 (0·00208–0·0487)	0·00932 (0·00108–0·034)	-51·1 (-84·8–60·1)	-36·9 (-70·7–18·1)
Ambient ozone pollution	30·8 (12·2–56·3)	45·5 (18·7–79·2)	44·1 (17·8–77)	47·7 (16·3–98·8)	-3·1 (-23·5–20)
Residential radon	24·1 (3·64–65·7)	24·1 (3·64–65·7)	24·1 (3·64–65·7)	0 (0–0)	0 (0–0)
Lead exposure	32·9 (4·68–60·3)	20·6 (2·64–43·2)	14·7 (1·36–34·5)	-37·3 (-61·7—23·3)	-28·4 (-54·4—19·3)
Child underweight	0·958 (0·545–1·48)	0·957 (0·526–1·42)	0·932 (0·505–1·41)	-0·0886 (-17·4–22·4)	-2·6 (-14·9–12·2)
Iron deficiency	--	--	--	--	--
Vitamin A deficiency	0·98 (0·254–2·88)	1·26 (0·342–3·34)	0·842 (0·197–2·2)	28·8 (-68·5–497)	-33·3 (-69·6–68)
Zinc deficiency	2·54 (0–7·92)	1·15 (0–3·7)	1·17 (0–3·78)	-54·6 (-100–NA)	1·53 (-23·7–NA)
Smoking	27·2 (24·3–29·9)	19·8 (17·9–21·8)	17·2 (15·5–19)	-27·2 (-32·6—21·7)	-12·9 (-16·3—9·24)
Secondhand smoke	37·7 (35·4–40·3)	25·5 (23·8–27·1)	25·5 (22·8–28·3)	-32·5 (-38·9—25·8)	0·0428 (-8·84–8·91)
Alcohol use	14·1 (8·43–20·2)	13·7 (7·81–20·1)	15·4 (9·13–22·4)	-2·65 (-32·3–39·8)	12·2 (-11·4–45·3)
Drug use	0·385 (0·183–0·753)	0·49 (0·302–0·803)	0·738 (0·514–1·05)	27·2 (-5·23–86·5)	50·8 (22·2–87·5)
High fasting plasma glucose	12·1 (10·1–14·5)	16·5 (14·2–19·2)	18·7 (16–21·8)	36·2 (14·5–63·2)	13·4 (-1·58–33)
High systolic blood pressure	25·2 (20·2–31·2)	16 (14–18·1)	20 (15·6–24·9)	-36·5 (-49—20·2)	24·4 (-0·623–55·2)
High body-mass index	25·7 (18–35·5)	40·6 (29·7–52·6)	43 (31·9–54·9)	58·1 (38·9–85·2)	5·93 (-0·38–14)
Low bone mineral density	11·8 (6·45–18·2)	9·15 (4·93–15)	11·5 (6·52–18·1)	-22·2 (-42·6–1·06)	25·7 (-3·77–66·4)
Diet low in fruits	55·2 (43·4–68·6)	51·1 (38·9–64·5)	51·1 (38·3–64·9)	-7·45 (-18·8–3·68)	0·0245 (-10·8–9·66)
Diet low in vegetables	13·2 (5·63–28·5)	6·15 (3·66–13·2)	6·16 (3·42–13·4)	-53·2 (-72·4—13·9)	0·0878 (-25·6–40·9)
Diet low in whole grains	79·9 (69·8–88·8)	81·5 (72–89·7)	80 (69·7–89·5)	2·01 (-1·82–6·63)	-1·78 (-7·87–5·27)
Diet low in nuts and seeds	29·9 (9–57·6)	25·4 (8·9–55·4)	20·5 (6·86–53·5)	-15·1 (-49·5–50·8)	-19·1 (-46·1–5·02)
Diet low in milk	50·5 (26·4–70·9)	42·9 (19·2–63·1)	41·4 (18·7–61·3)	-15·1 (-34·2—2·16)	-3·39 (-17·5–10·7)
Diet high in red meat	76·3 (63·2–87·4)	76·3 (62·3–87·6)	76·4 (63·3–87·8)	0·0533 (-15–16·5)	0·0605 (-10·8–13·1)
Diet high in processed meat	74·3 (48·6–95·8)	83·7 (64·4–99·4)	83·8 (64–99·4)	12·6 (2·03–35·3)	0·101 (-4·58–5·31)
Diet high in sugar-sweetened beverages	43·9 (26·5–64·6)	62·6 (44·3–79·6)	63·5 (42·8–81)	42·5 (-2·69–123)	1·41 (-18·8–26·4)
Diet low in fiber	45·5 (29·6–60·7)	29·9 (17·2–43·5)	27 (13·9–40·7)	-34·2 (-50·1—18·8)	-9·97 (-28·4–9·43)
Diet low in seafood omega-3 fatty acids	85·1 (68·6–100)	79·4 (60·7–98·7)	78·5 (59·6–99·4)	-6·7 (-21·6–6·11)	-1·1 (-17–16·8)
Diet low in polyunsaturated fatty acids	15·3 (4·4–42·7)	7·22 (2·07–26·6)	6·86 (1·95–24·5)	-52·8 (-78·7—9·95)	-5·02 (-27·5–15·3)
Diet high in trans fatty acids	89·5 (81·1–100)	87·8 (78·9–100)	87·4 (78·3–100)	-1·85 (-9·32–5·79)	-0·506 (-8·3–8·48)
Diet high in sodium	18·3 (4·53–42)	39·6 (12·2–63·9)	38·8 (11·4–64·3)	116 (37·8–401)	-1·79 (-28·4–30·1)
Low physical activity	2·82 (1·07–5·89)	2·91 (1·32–5·42)	2·95 (1·14–6·27)	3·23 (-28·1–61·7)	1·17 (-23·8–31·5)
Occupational asthmagens	20·1 (16·9–24·1)	21 (17·7–24·7)	21·6 (18·1–26)	4·52 (-7·58–18·4)	3·01 (-5·95–13·2)
Occupational particulate matter, gases, and fumes	9·89 (7·2–14)	9·64 (6·99–13·7)	9·4 (6·81–13·2)	-2·51 (-7·7–3·35)	-2·57 (-6·24–0·756)
Occupational noise	9·21 (8·47–9·96)	9·04 (8·46–9·73)	8·82 (8·28–9·56)	-1·94 (-5·81–2·17)	-2·34 (-4·39—0·103)
Occupational injuries	--	--	--	--	--
Occupational ergonomic factors	14·5 (12·1–17·4)	12·9 (10·9–15·5)	13·2 (11–15·9)	-11·2 (-23·4–1·52)	2·27 (-7·01–12·6)
Childhood sexual abuse	7·4 (5·05–10·5)	6·97 (4·6–9·93)	6·85 (4·54–9·67)	-5·83 (-12·2–5·83)	-1·68 (-8·51–5·53)
Intimate partner violence	--	--	--	--	--
Non-exclusive breastfeeding	26 (21·6–31·2)	25 (21·3–29·1)	24·8 (20·4–29·6)	-4 (-19·1–14·9)	-0·708 (-13·8–13·3)
Discontinued breastfeeding	26·9 (25·9–27·8)	25·7 (24·2–26·9)	25·5 (24·2–26·8)	-4·46 (-10·2–0·818)	-0·658 (-6·08–5·48)
Diet low in calcium	17·6 (8·54–32·9)	13·5 (6·5–24·7)	12·5 (5·97–23·3)	-23·5 (-34·4—12·1)	-6·84 (-16·3–1·78)
Occupational exposure to asbestos	7·63 (6·7–8·62)	6·64 (5·93–7·36)	6·69 (4·9–8·88)	-13 (-26·6–2·94)	0·801 (-26·8–33)
Occupational exposure to arsenic	0·445 (0–1·07)	0·517 (0–1·21)	0·543 (0–1·29)	16·3 (-0·965–NA)	4·94 (-6·9–NA)
Occupational exposure to benzene	0·814 (0·0504–2·34)	0·884 (0·0525–2·52)	0·908 (0·0536–2·64)	8·62 (-0·521–21·6)	2·69 (-4·92–13·1)
Occupational exposure to beryllium	0·0248 (0·0233–0·0262)	0·0262 (0·0254–0·027)	0·0272 (0·0257–0·0287)	5·81 (-0·473–12·6)	3·73 (-1·72–9·35)
Occupational exposure to cadmium	0·0532 (0·048–0·0587)	0·0615 (0·055–0·068)	0·0644 (0·0567–0·0723)	15·7 (0·68–32·1)	4·75 (-5·61–15·7)
Occupational exposure to chromium	0·128 (0·116–0·14)	0·151 (0·138–0·163)	0·158 (0·142–0·174)	18·3 (4·89–33·2)	4·6 (-4·51–14·3)
Occupational exposure to diesel engine exhaust	0·75 (0·691–0·808)	0·866 (0·807–0·928)	0·897 (0·82–0·978)	15·5 (4·36–27·9)	3·49 (-4·54–11·8)
Occupational exposure to formaldehyde	0·254 (0·226–0·284)	0·294 (0·261–0·328)	0·305 (0·267–0·346)	15·9 (-0·663–34·1)	3·83 (-7·13–15·6)
Occupational exposure to nickel	0·382 (0–1·65)	0·421 (0–1·86)	0·443 (0–1·95)	10·2 (-6·16–NA)	5·05 (-6·94–NA)
Occupational exposure to polycyclic aromatic hydrocarbons	0·254 (0·232–0·277)	0·299 (0·275–0·323)	0·312 (0·281–0·343)	17·8 (4·96–31·9)	4·36 (-4·49–13·2)
Occupational exposure to silica	3·68 (0·52–12)	3·83 (0·558–12·5)	4·02 (0·563–12·7)	4·03 (-10·9–22·2)	5·05 (-5·95–19·3)
Occupational exposure to sulfuric acid	0·881 (0·187–2·81)	1·09 (0·218–3·39)	1·11 (0·228–3·49)	23·1 (6–41·6)	2·62 (-7·95–14·3)
Unsafe sex	--	--	--	--	--
Occupational exposure to trichloroethylene	0·0798 (0·0742–0·0859)	0·0938 (0·0889–0·0994)	0·0978 (0·091–0·106)	17·6 (7·59–29·3)	4·18 (-2·75–11·3)
No access to handwashing facility	3·38 (3·13–3·65)	3·13 (2·89–3·4)	3·09 (2·86–3·35)	-7·41 (-15·6–2·31)	-1·39 (-7·19–4·63)
Child wasting	0·75 (0·495–1·03)	0·727 (0·474–0·996)	0·725 (0·466–0·984)	-3·11 (-12·8–7·63)	-0·22 (-7·5–7·7)
Child stunting	2·4 (1·26–3·49)	2·07 (1·05–3·03)	2·01 (1·03–2·99)	-14 (-32·7–10·8)	-2·6 (-16·7–15·1)
Chewing tobacco	6·34 (4·83–8·06)	6·94 (4·96–9·39)	6·6 (4·55–9·13)	9·48 (-25·7–58·3)	-4·8 (-26·8–23·6)
Diet low in legumes	74·7 (41·2–99·1)	76·9 (45·3–99·6)	76·8 (44·9–99·6)	2·99 (-1·1–14·9)	-0·159 (-5·34–5·6)
Short gestation	20·1 (16·5–24·4)	22·9 (18·8–27·3)	22·3 (17·9–27·6)	13·8 (-9·81–45·1)	-2·41 (-20·7–20·6)
Low birth weight	7·65 (6·22–9·41)	9·39 (7·81–11·2)	9·67 (7·99–11·7)	22·8 (-3·69–54·8)	3·01 (-17·3–28·8)
High temperature	28·6 (18–39·5)	26 (15·7–37)	27·7 (17·2–38·9)	-9·08 (-17·7—4·15)	6·34 (3·01–11·3)
Low temperature	36·5 (32·9–40·9)	35·3 (32–39·5)	37·7 (34·1–42·2)	-3·41 (-6·24—0·411)	6·71 (4·54–8·94)
Kidney dysfunction	18·8 (13·4–25·8)	19·5 (13·8–26·4)	19·5 (13·9–26·5)	3·32 (-0·466–7·49)	0·0573 (-3·42–3·82)
Bullying victimization	5·86 (2·44–12)	8·92 (3·56–17·6)	8·7 (3·37–17·9)	52·2 (32·5–75·4)	-2·53 (-12·3–3·72)
High LDL cholesterol	67·7 (63·1–72)	33·7 (30·4–37·2)	30 (26·5–33·6)	-50·2 (-55·3—44·7)	-11·1 (-15·8—6·31)

	Female				
	1990	2010	2019	Percentage change 1990-2010	Percentage change 2010-2019
Unsafe sanitation	--	--	--	--	--
Ambient particulate matter pollution	6·27 (1·04–14·6)	3·95 (1·6–6·78)	3·03 (0·91–5·73)	-37 (-76·1–177)	-23·2 (-44·9—14·9)
Household air pollution from solid fuels	0·04 (0·00621–0·116)	0·0196 (0·00291–0·0622)	0·0123 (0·00143–0·043)	-51 (-84·8–57·7)	-37·1 (-71–18·9)
Ambient ozone pollution	--	--	--	--	--
Residential radon	24·1 (3·64–65·7)	24·1 (3·64–65·7)	24·1 (3·64–65·7)	0 (0–0)	0 (0–0)
Lead exposure	18·7 (0·145–46)	12·7 (0·079–35·9)	9·44 (0·0291–29)	-32·2 (-76·5–18·3)	-25·6 (-74·2—17)
Child underweight	0·805 (0·442–1·23)	0·845 (0·466–1·3)	0·866 (0·476–1·33)	4·99 (-16·7–33·1)	2·42 (-12·5–18·4)
Iron deficiency	11·5 (9·57–13·4)	12 (10·2–14)	12·3 (10·3–14·6)	4·55 (-7·5–17·5)	3·09 (-6·16–12·9)
Vitamin A deficiency	1·8 (0·655–3·64)	1·8 (0·69–3·93)	1·45 (0·552–3·09)	-0·0209 (-60·8–178)	-19·2 (-57·2–51)
Zinc deficiency	2·6 (0–8·15)	1·17 (0–3·81)	1·13 (0–3·64)	-55·2 (-100–NA)	-2·81 (-23·8–NA)
Smoking	18·8 (16·4–21·6)	14·7 (13·2–16·5)	13·4 (11·8–15·1)	-21·7 (-30·1—12·4)	-9·11 (-13·5—4·69)
Secondhand smoke	34·7 (32·8–36·7)	24 (22·8–25·2)	24 (21·9–26·2)	-31 (-36·2—25)	0·137 (-7·47–7·52)
Alcohol use	9·67 (5·63–14·5)	10·5 (6·19–15·4)	10·4 (6·05–15·9)	8·94 (-24–61)	-1·04 (-22·7–25·3)
Drug use	0·356 (0·221–0·576)	0·525 (0·371–0·743)	0·73 (0·548–0·98)	47·5 (22·4–82)	39 (19–62·8)
High fasting plasma glucose	8·86 (7·61–10·3)	11·7 (10·1–13·6)	13·7 (11·6–16·3)	32·2 (11·3–56·1)	16·9 (1·08–35·3)
High systolic blood pressure	23 (17·1–30·7)	13·2 (11·7–14·8)	17·9 (13–24·4)	-42·6 (-57—23·7)	34·9 (-1·61–81·7)
High body-mass index	29·9 (21·3–39·8)	43 (33·3–52·4)	45 (34·7–54·5)	43·8 (25·7–69·7)	4·68 (-1·64–12·3)
Low bone mineral density	15·6 (10·2–22·6)	16·4 (10·6–23·9)	18·8 (12·6–26·3)	5·15 (-17·3–33·6)	14·8 (-9·76–45)
Diet low in fruits	57 (45·4–70·3)	49·5 (38·8–61·6)	48·3 (36·8–60·8)	-13·1 (-23·2—3·21)	-2·51 (-13·4–7·9)
Diet low in vegetables	11·1 (4·33–24·7)	3·57 (1·81–10·4)	4·04 (1·89–10·7)	-67·8 (-84·9—29·3)	13·1 (-24·2–88)
Diet low in whole grains	--	--	--	--	--
Diet low in nuts and seeds	25·8 (6·38–55·9)	21·8 (7·43–54·3)	18·7 (5·96–53·2)	-15·4 (-46·8–53·4)	-14·3 (-42·8–14·2)
Diet low in milk	50·3 (26·9–71)	43·1 (20·1–63·3)	41·9 (20·8–62)	-14·3 (-32·1—1·28)	-2·82 (-15·7–10·2)
Diet high in red meat	77·2 (64–87·9)	75·2 (61·3–86·4)	75·4 (61·8–87·1)	-2·58 (-17·1–14·5)	0·368 (-10·9–12·8)
Diet high in processed meat	75 (51·1–96·3)	86·1 (67·3–99·5)	85·1 (66·6–99·4)	14·9 (2·76–39·5)	-1·19 (-6·47–2·86)
Diet high in sugar-sweetened beverages	47·7 (28·1–67·5)	68·2 (49·3–84·2)	67·5 (48·5–84·1)	42·8 (0·424–126)	-0·946 (-19·5–21·1)
Diet low in fiber	45·7 (29·4–61·7)	35 (21·1–49·9)	29·7 (16·3–45)	-23·5 (-38·7—7·56)	-15·2 (-30·9–0·0177)
Diet low in seafood omega-3 fatty acids	87·4 (72·2–99·7)	83·5 (66·9–99·9)	82·8 (65·8–99·2)	-4·49 (-17·5–4·99)	-0·801 (-13·2–12·3)
Diet low in polyunsaturated fatty acids	17 (5·16–46·8)	7·42 (2·18–25·5)	6·95 (2·04–24·6)	-56·3 (-80·5—9·17)	-6·3 (-31·1–13·4)
Diet high in trans fatty acids	88·4 (80·8–100)	86·6 (78·3–100)	86·3 (77·5–100)	-1·97 (-9·46–5·63)	-0·429 (-8·42–8·81)
Diet high in sodium	--	--	--	--	--
Low physical activity	5·8 (2·81–10·9)	6·21 (3·44–11·3)	5·97 (3·07–11·4)	7·07 (-19·9–50·7)	-3·93 (-17·5–8·16)
Occupational asthmagens	11·8 (9·62–14·2)	12·6 (10·4–15·1)	12·4 (10·1–15·3)	6·82 (-7·57–24·1)	-0·97 (-11·9–11·5)
Occupational particulate matter, gases, and fumes	5·41 (3·82–8·11)	5·52 (3·99–7·9)	5·42 (3·88–7·9)	1·98 (-7·59–13·1)	-1·78 (-7·15–3·88)
Occupational noise	6·36 (5·62–7·12)	6·87 (6·36–7·47)	6·96 (6·5–7·53)	8·05 (2·04–15·3)	1·28 (-1·57–4·35)
Occupational injuries	--	--	--	--	--
Occupational ergonomic factors	8·85 (6·9–11·3)	9·04 (7·3–11·3)	9·64 (7·48–12·5)	2·12 (-18·2–27)	6·68 (-9·82–25·7)
Childhood sexual abuse	12·3 (8·94–17·3)	17·4 (12·9–24)	17·8 (13·1–24·7)	41·9 (30·7–54·8)	2·17 (-5–11)
Intimate partner violence	29·7 (16·6–39·3)	28 (15·8–37)	29·1 (16·6–37·9)	-5·85 (-13·6–5·83)	3·8 (-5·23–19)
Non-exclusive breastfeeding	26 (21·6–31·1)	25 (21·2–29·1)	24·8 (20·4–29·6)	-3·98 (-19·1–14·9)	-0·727 (-13·8–13·3)
Discontinued breastfeeding	27 (26–27·9)	25·6 (24·2–26·9)	25·5 (24·2–26·7)	-4·97 (-10·7–0·273)	-0·595 (-6·02–5·56)
Diet low in calcium	13·1 (5·72–25·3)	10·5 (4·46–19·5)	9·9 (4·15–18·5)	-20·1 (-32·7—4·87)	-5·45 (-16·2–4·13)
Occupational exposure to asbestos	0·783 (0·53–1·85)	0·8 (0·561–1·81)	0·82 (0·483–1·96)	2·17 (-13·3–25·5)	2·47 (-25–36·7)
Occupational exposure to arsenic	0·475 (0–1·11)	0·536 (0–1·28)	0·54 (0–1·31)	12·9 (-5·06–NA)	0·796 (-10·7–NA)
Occupational exposure to benzene	1·28 (0·0715–3·55)	1·5 (0·0902–4·27)	1·55 (0·0958–4·43)	17·4 (5·71–36·2)	3·47 (-4·5–16)
Occupational exposure to beryllium	0·0288 (0·0265–0·0309)	0·0319 (0·0309–0·0329)	0·0325 (0·0307–0·0345)	10·7 (2·51–20·7)	2·07 (-3·16–7·57)
Occupational exposure to cadmium	0·0576 (0·0513–0·0645)	0·065 (0·0584–0·0726)	0·0656 (0·0575–0·0754)	12·9 (-3·47–32·7)	0·867 (-9·33–11·7)
Occupational exposure to chromium	--	--	--	--	--
Occupational exposure to diesel engine exhaust	0·727 (0·645–0·813)	0·825 (0·75–0·91)	0·813 (0·727–0·906)	13·4 (-1·15–31·5)	-1·43 (-10·1–8·5)
Occupational exposure to formaldehyde	0·278 (0·249–0·311)	0·317 (0·287–0·354)	0·32 (0·281–0·366)	14 (-1·76–32·8)	1·11 (-8·55–11·5)
Occupational exposure to nickel	0·374 (0–1·55)	0·407 (0–1·75)	0·411 (0–1·8)	8·82 (-10·4–NA)	1·04 (-12–NA)
Occupational exposure to polycyclic aromatic hydrocarbons	0·311 (0·283–0·34)	0·365 (0·341–0·392)	0·369 (0·337–0·407)	17·4 (4·7–32·9)	1·16 (-6·05–9·01)
Occupational exposure to silica	3·17 (0·443–10·1)	3·31 (0·449–10·5)	3·31 (0·445–11)	4·19 (-14·7–31·3)	0·0528 (-13·1–16·6)
Occupational exposure to sulfuric acid	0·979 (0·206–3·1)	1·27 (0·259–4·11)	1·24 (0·255–3·9)	29·5 (4·71–59·8)	-2·22 (-15·3–13)
Unsafe sex	--	--	--	--	--
Occupational exposure to trichloroethylene	0·0867 (0·0786–0·0951)	0·0992 (0·0932–0·107)	0·0994 (0·0916–0·109)	14·4 (2·25–29·2)	0·214 (-7·19–8·32)
No access to handwashing facility	3·38 (3·13–3·65)	3·13 (2·89–3·4)	3·09 (2·86–3·35)	-7·41 (-15·6–2·31)	-1·39 (-7·19–4·63)
Child wasting	0·774 (0·515–1·05)	0·762 (0·503–1·04)	0·759 (0·504–1·04)	-1·51 (-11·4–9·42)	-0·452 (-7·92–7·67)
Child stunting	1·93 (0·936–2·99)	1·77 (0·898–2·73)	1·76 (0·878–2·64)	-8·35 (-28·1–18·5)	-0·945 (-16·5–18)
Chewing tobacco	0·276 (0·172–0·455)	0·31 (0·187–0·519)	0·325 (0·195–0·548)	12·3 (-36·1–98·8)	4·85 (-30–53·2)
Diet low in legumes	73·1 (37·1–99·3)	75·3 (43·1–99·6)	75·4 (42·4–99·5)	2·93 (-1·27–18·2)	0·242 (-5·15–7·49)
Short gestation	19·5 (16·1–23·5)	21 (17·8–24·8)	21 (17·6–25)	7·34 (-15·2–37·4)	0·166 (-17–20·5)
Low birth weight	9·06 (7·44–11)	10·8 (9·17–12·4)	11·1 (9·42–13·1)	18·8 (-7·75–51·4)	3·02 (-14·3–26·2)
High temperature	28·6 (18–39·5)	26 (15·7–37)	27·7 (17·2–38·9)	-9·08 (-17·7—4·15)	6·34 (3·01–11·3)
Low temperature	36·5 (32·9–40·9)	35·3 (32–39·5)	37·7 (34·1–42·2)	-3·41 (-6·24—0·411)	6·71 (4·54–8·94)
Kidney dysfunction	21·1 (14·9–28·6)	22·2 (15·8–29·7)	22·9 (16·3–30·7)	4·99 (0·946–10·4)	3·36 (-0·338–7·74)
Bullying victimization	--	--	--	--	--
High LDL cholesterol	66·1 (62·3–69·8)	33·7 (30·3–36·9)	30·9 (27·4–34·5)	-49·1 (-53·7—44·4)	-8·07 (-12·3—4·16)

Supplementary Results Table 1. Tennessee age-standardised Summary Exposure Values (SEVs) for most-detailed risk factors and percentage change, by sex, 1990, 2010, and 2019. Data in parentheses are 95% uncertainty intervals.

	Male					Female				
	1990	2010	2019	Percentage change 1990-2010	Percentage change 2010-2019	1990	2010	2019	Percentage change 1990-2010	Percentage change 2010-2019
Unsafe sanitation	8·94 (4·08–16·2)	4·83 (2·26–9·48)	3·64 (1·61–6·77)	-46 (-78·8–29·9)	-24·5 (-60·1–41·5)	8·94 (4·08–16·2)	4·83 (2·26–9·48)	3·64 (1·61–6·77)	-46 (-78·8–29·9)	-24·5 (-60·1–41·5)
Ambient particulate matter pollution	13·1 (4·75–26·1)	7·65 (4·86–10·8)	4·61 (2·07–7·48)	-41·5 (-70·5–43·4)	-39·7 (-57·4—30·4)	13·1 (4·73–26·1)	7·65 (4·86–10·8)	4·61 (2·08–7·48)	-41·4 (-70·5–43·9)	-39·7 (-57·3—30·2)
Household air pollution from solid fuels	0·0411 (0·0108–0·108)	0·0209 (0·00381–0·0632)	0·0124 (0·00183–0·0413)	-49·1 (-81·5–16)	-40·7 (-71·4–10·1)	0·0541 (0·0143–0·138)	0·0276 (0·00511–0·0839)	0·0164 (0·00263–0·0534)	-48·9 (-81·4–17)	-40·6 (-71·4–10·1)
Ambient ozone pollution	80·2 (36·5–100)	66·1 (29–100)	40·3 (16·5–70·3)	-17·5 (-27·4–0)	-39·1 (-49·7—29·4)	--	--	--	--	--
Residential radon	19·2 (3·17–54·7)	19·2 (3·17–54·7)	19·2 (3·17–54·7)	0 (0–0)	0 (0–0)	19·2 (3·17–54·7)	19·2 (3·17–54·7)	19·2 (3·17–54·7)	0 (0–0)	0 (0–0)
Lead exposure	51·7 (22·4–74·8)	31·4 (10·7–53·6)	22·4 (6·13–42·1)	-39·2 (-56·8—26·9)	-28·7 (-44·4—20·3)	28·7 (2·07–57·7)	18·2 (0·814–42·6)	13·1 (0·393–33·7)	-36·7 (-70·4—22·6)	-27·9 (-61·7—19·9)
Child underweight	0·98 (0·542–1·47)	0·987 (0·523–1·51)	0·946 (0·511–1·45)	0·786 (-18·2–21·1)	-4·14 (-16·4–8·74)	0·824 (0·446–1·25)	0·867 (0·484–1·35)	0·88 (0·493–1·38)	5·22 (-14·2–31·7)	1·4 (-12·9–17·6)
Iron deficiency	--	--	--	--	--	12·2 (10·2–14·5)	12·6 (10·5–14·6)	12·8 (10·8–15·2)	3·22 (-8·29–16·9)	1·61 (-7·27–12·4)
Vitamin A deficiency	1·09 (0·279–2·79)	1·44 (0·375–3·72)	0·951 (0·263–2·57)	32·2 (-67·2–445)	-33·8 (-70·9–57·3)	2·04 (0·74–4·16)	2·08 (0·813–4·36)	1·68 (0·643–3·64)	1·64 (-59·3–205)	-18·9 (-55·4–51·2)
Zinc deficiency	2·47 (0–7·99)	1·25 (0–3·98)	1·28 (0–4·16)	-49·3 (-100–NA)	1·92 (-17·2–NA)	2·53 (0–8)	1·26 (0–4·23)	1·25 (0–4·17)	-50·2 (-100–NA)	-1·01 (-19·5–NA)
Smoking	28·9 (26–31·7)	22·1 (20·1–24·3)	20 (18–22)	-23·3 (-28·1—18·3)	-9·8 (-13·2—5·94)	19·8 (17·5–22·4)	16·9 (15·1–18·7)	15·2 (13·6–16·9)	-14·7 (-21·9—5·83)	-9·83 (-13·9—5·38)
Secondhand smoke	41 (38·5–43·5)	29·6 (27·9–31·2)	28·4 (25·7–31·3)	-27·9 (-33·9—21·4)	-3·88 (-12·2–3·81)	39·1 (37·1–40·9)	28·6 (27·4–29·7)	27·4 (25·2–29·5)	-26·9 (-31·7—21·9)	-4·07 (-10·4–2·63)
Alcohol use	12·2 (7·98–16·7)	13 (8·23–18·3)	13·2 (8·32–18·6)	6·9 (-21·3–41·6)	0·945 (-19·2–24·7)	4·73 (1·85–8·79)	5·32 (2·21–9·49)	8·88 (4·89–13)	12·4 (-36·9–100)	67 (1·22–250)
Drug use	0·591 (0·325–1·09)	1·16 (0·819–1·61)	1·7 (1·27–2·23)	96·3 (41·4–177)	46·3 (24·7–71·1)	0·465 (0·301–0·733)	1 (0·758–1·32)	1·59 (1·24–2·03)	116 (67·5–178)	58 (36·7–83·1)
High fasting plasma glucose	13·5 (11·3–16·5)	19·7 (17·2–22·5)	21 (18–24·1)	45·9 (19·7–75·3)	6·58 (-7·41–21·8)	11·1 (9·17–13·5)	17·1 (14·7–20)	18·8 (15·9–22·1)	54·1 (27·4–87·4)	10·4 (-6·23–29·5)
High systolic blood pressure	26 (20·8–31·6)	16·8 (14·8–19)	20·7 (16·1–25·7)	-35·3 (-47·7—19·9)	23·1 (-2·51–53·3)	23·2 (17·5–30·1)	13·7 (12–15·5)	18·6 (13·8–24·5)	-41 (-55·7—21·5)	35·4 (-1·22–80·1)
High body-mass index	24 (16·7–33·3)	39·9 (29·6–51·8)	41·3 (30·6–53)	66·2 (44·7–95·2)	3·62 (-2·9–11·4)	30·4 (22·8–39)	45·5 (35·5–55)	46·5 (36·1–55·9)	50 (31·9–75)	2·06 (-4·28–9·34)
Low bone mineral density	8·42 (4·59–14)	8·11 (4·32–13·8)	7·85 (3·99–13·6)	-3·67 (-27·3–25·6)	-3·19 (-27·4–26)	13·7 (8·66–20·3)	16 (10·4–22·9)	14 (8·94–20·8)	16·9 (-5·7–48·5)	-12·5 (-31·8–10·8)
Diet low in fruits	58·5 (47·3–71·6)	46·8 (35·5–60·2)	48·8 (36·8–62·1)	-19·9 (-31·1—9·91)	4·35 (-7·51–17·4)	59·5 (47·8–72·6)	49·4 (37·4–62·4)	49·8 (37·2–63·8)	-17 (-27·8—8·19)	0·753 (-10·7–12·1)
Diet low in vegetables	45·2 (25·4–68·4)	58·5 (40–76·9)	57·9 (39·8–75·8)	29·5 (9·64–68)	-1·07 (-8·37–5·09)	42·6 (23·9–64·7)	44·4 (25·8–64·9)	41·3 (23·9–62·1)	4·19 (-6·54–19·9)	-6·89 (-18·9–3·41)
Diet low in whole grains	79·8 (70·5–88·6)	81·8 (72·9–89·7)	81·8 (72·9–89·4)	2·45 (-1·42–7·3)	0·0157 (-2·65–2·65)	77·3 (66·8–87·1)	80 (70·8–88·7)	79·8 (70·6–88·6)	3·51 (-0·745–9·36)	-0·249 (-2·96–2·38)
Diet low in nuts and seeds	29·5 (8·45–57·4)	25·6 (8·24–56·3)	21·5 (6·56–54·5)	-13·4 (-46·9–54·1)	-16·1 (-43·6–9·39)	25·8 (6·68–54·7)	22·8 (7·16–54·6)	20 (6·05–53·4)	-11·6 (-43·4–53·7)	-12·5 (-40·3–17·2)
Diet low in milk	50·4 (26·7–70·7)	44·8 (19·9–65·6)	43·8 (21–64·1)	-10·9 (-31·1–1·76)	-2·3 (-13·5–9·9)	50·2 (26·5–71·4)	44·9 (21·4–65·5)	44·3 (21·2–64·1)	-10·7 (-30·5–0·874)	-1·34 (-13·5–10·7)
Diet high in red meat	74·7 (62·5–85·8)	76·6 (63·7–88·2)	76·3 (62·4–88·1)	2·54 (-13·5–19·7)	-0·363 (-11·8–11)	80·1 (69·1–89·4)	77·6 (65·2–88·8)	77·2 (63·3–88·6)	-3·07 (-17·7–11·9)	-0·497 (-11·7–11·3)
Diet high in processed meat	76·7 (52·1–98)	84·2 (65–99·8)	84·1 (64·4–99·6)	9·78 (0·825–29·2)	-0·141 (-5·08–4·54)	82·4 (61–99·4)	90·5 (76·9–99·9)	88·9 (72·9–99·8)	9·78 (0·274–29·4)	-1·7 (-7·27–1·86)
Diet high in sugar-sweetened beverages	43·8 (26–62·9)	59·9 (40·5–77·2)	60·5 (40·8–78·5)	36·8 (-2·49–119)	0·978 (-20·9–27·5)	48 (29·1–68·5)	66·5 (46·9–84)	65·3 (45·4–83·8)	38·7 (-1·78–117)	-1·8 (-22·3–22)
Diet low in fiber	45·2 (28·9–60·5)	31·6 (18·9–45·9)	28·9 (16·4–43·2)	-30·1 (-46–14·2)	-8·69 (-26·8–8)	45·7 (28·9–62·6)	36·9 (22·9–52·1)	31·9 (18·3–48)	-19·3 (-34·5—4·06)	-13·4 (-30·2–1·7)
Diet low in seafood omega-3 fatty acids	85·1 (67·6–99·8)	80·9 (62·6–99·5)	80·3 (61·9–99·5)	-4·94 (-19·4–9·88)	-0·737 (-15·4–16·4)	87·7 (74–99·8)	84·8 (69·6–99·9)	84·2 (68·3–99·7)	-3·21 (-14·7–5·5)	-0·819 (-11·3–10·2)
Diet low in polyunsaturated fatty acids	15·2 (4·43–43·3)	7·67 (2·21–26·5)	7·31 (2·13–25·4)	-49·7 (-76·2—8·93)	-4·62 (-26·5–16·8)	16·9 (4·98–45·2)	7·88 (2·31–26·9)	7·4 (2·19–25·2)	-53·4 (-77·9—8·51)	-6·09 (-28·6–13·3)
Diet high in trans fatty acids	89·4 (81·5–100)	87·8 (78·6–100)	87·5 (77·9–100)	-1·79 (-9·91–6·19)	-0·406 (-8·26–8·2)	88·5 (80·7–100)	86·7 (77·6–100)	86·2 (77·6–100)	-2 (-10·7–5·91)	-0·544 (-8·31–7·36)
Diet high in sodium	18·2 (4·67–40·9)	39·7 (11·4–64·5)	39·1 (10·8–64·4)	118 (38·5–416)	-1·62 (-27·5–32·6)	21 (4·87–45·6)	21·2 (5·09–45·1)	21·8 (5·2–46·1)	0·797 (-30·5–47·3)	2·88 (-16·9–32·5)
Low physical activity	3·07 (1·15–6·32)	3·23 (1·48–6·15)	3·6 (1·51–7·59)	5·23 (-28·8–67)	11·3 (-13·6–42·7)	6·46 (3·08–12·2)	8 (4·43–13·9)	8·1 (4·42–14·2)	23·7 (-10·2–81·1)	1·27 (-10·3–12·3)
Occupational asthmagens	23·9 (20–29·2)	21·1 (17·9–25)	21·9 (18·3–26·6)	-11·7 (-23·1–0·0794)	3·83 (-4·89–13·9)	11·6 (9·45–14·1)	11·1 (9·19–13·3)	11·1 (9–13·6)	-4·24 (-18·4–11·7)	0·599 (-10·6–13)
Occupational particulate matter, gases, and fumes	10 (7·22–14·5)	9·4 (6·78–13·6)	9·04 (6·56–13)	-6·41 (-12·1—0·838)	-3·77 (-7·29—0·118)	5·76 (4·01–8·73)	5·52 (3·97–8·04)	5·28 (3·81–7·64)	-4·16 (-13·1–5·97)	-4·3 (-9·56–1·37)
Occupational noise	8·75 (8–9·61)	8·38 (7·8–9·07)	8·11 (7·58–8·81)	-4·23 (-7·96—0·291)	-3·23 (-5·26—0·959)	5·92 (5·13–6·73)	6·19 (5·63–6·79)	6·17 (5·75–6·72)	4·48 (-1·95–12·3)	-0·342 (-3·49–3·24)
Occupational injuries	--	--	--	--	--	--	--	--	--	--
Occupational ergonomic factors	12·9 (10·6–15·3)	10·8 (9·18–12·9)	11·2 (9·37–13·5)	-15·6 (-26·1—4·13)	3·34 (-5·59–12·4)	7·31 (5·8–9·28)	7·02 (5·74–8·67)	7·29 (5·86–9·15)	-4 (-20·2–13·4)	3·9 (-8·98–17·3)
Childhood sexual abuse	6·37 (3·95–9·41)	6·09 (4·27–8·11)	6·04 (4·27–8·02)	-4·38 (-20·3–20·3)	-0·855 (-7·05–5·57)	16·5 (11·9–23·2)	15·1 (11·7–20·1)	15·3 (11·8–20·5)	-8·42 (-20·5–7·74)	1·13 (-5·6–8·59)
Intimate partner violence	--	--	--	--	--	33·6 (17·9–43·3)	32 (17·5–41·6)	32·8 (18·7–42·8)	-4·91 (-13·4–7·68)	2·69 (-6·55–15·9)
Non-exclusive breastfeeding	26·2 (21·5–31)	25 (21·4–29·2)	24·9 (20·5–29·6)	-4·44 (-19–14·4)	-0·476 (-14·1–13·3)	26·2 (21·5–31)	25 (21·4–29·2)	24·9 (20·5–29·6)	-4·43 (-19–14·4)	-0·481 (-14·1–13·3)
Discontinued breastfeeding	27·5 (26·6–28·4)	25·4 (24–26·7)	25·6 (24·3–26·8)	-7·57 (-12·7—2·1)	0·713 (-5·09–6·92)	27·5 (26·5–28·4)	25·4 (24–26·7)	25·6 (24·3–26·8)	-7·44 (-12·6—1·97)	0·618 (-5·18–6·82)
Diet low in calcium	17·5 (8·49–32·2)	14·4 (7·04–26·5)	13·8 (6·5–25·5)	-17·3 (-28·2—6·23)	-4·4 (-14·2–3·62)	12·9 (5·5–24·5)	11·5 (5–21·8)	11·2 (4·58–21·5)	-11·2 (-24·1—2·96)	-2·56 (-11·8–6·3)
Occupational exposure to asbestos	7·09 (6·3–7·88)	6·55 (5·95–7·13)	5·98 (4·26–7·95)	-7·64 (-20–5·87)	-8·64 (-34·7–20·9)	1·18 (0·922–2)	1·24 (1·02–1·99)	1·25 (0·83–2·16)	5·35 (-9·29–26·1)	0·661 (-28·8–36·8)
Occupational exposure to arsenic	0·555 (0–1·31)	0·538 (0–1·29)	0·567 (0–1·33)	-3·12 (-16·3–NA)	5·48 (-5·92–NA)	0·531 (0–1·28)	0·513 (0–1·23)	0·522 (0–1·31)	-3·47 (-21·1–NA)	1·81 (-11·8–NA)
Occupational exposure to benzene	0·817 (0·0492–2·38)	0·784 (0·0455–2·29)	0·814 (0·0469–2·37)	-4·02 (-11·2–7·42)	3·78 (-3·81–14·4)	1·09 (0·0599–2·9)	1·21 (0·0724–3·36)	1·29 (0·0789–3·65)	11·6 (-0·899–31·8)	6·07 (-3·87–19·3)
Occupational exposure to beryllium	0·0239 (0·0226–0·0253)	0·0231 (0·0226–0·0236)	0·0244 (0·0231–0·0259)	-3·6 (-9·4–2·69)	5·89 (-0·11–12·4)	0·0257 (0·0232–0·0282)	0·0266 (0·0259–0·0273)	0·0277 (0·0255–0·0297)	3·25 (-5·72–14·3)	4·16 (-3·83–11·5)
Occupational exposure to cadmium	0·0643 (0·0576–0·0709)	0·0626 (0·0569–0·0683)	0·066 (0·0587–0·0742)	-2·68 (-14·4–12·2)	5·41 (-4·94–17·1)	0·0623 (0·054–0·071)	0·0608 (0·054–0·0681)	0·0621 (0·0536–0·0716)	-2·37 (-18·3–17·2)	2·14 (-10·3–15·9)
Occupational exposure to chromium	0·153 (0·139–0·167)	0·152 (0·141–0·163)	0·16 (0·145–0·177)	-0·91 (-11·5–11·8)	5·35 (-3·37–15·6)	--	--	--	--	--
Occupational exposure to diesel engine exhaust	0·722 (0·664–0·781)	0·748 (0·7–0·796)	0·794 (0·728–0·867)	3·72 (-6·03–14)	6·04 (-2·24–14·7)	0·633 (0·56–0·716)	0·681 (0·617–0·745)	0·686 (0·602–0·775)	7·47 (-6·24–25·1)	0·821 (-9·86–12·7)
Occupational exposure to formaldehyde	0·323 (0·289–0·358)	0·307 (0·276–0·336)	0·319 (0·282–0·361)	-5·04 (-17·1–9·75)	4·1 (-6·3–16·2)	0·302 (0·264–0·344)	0·296 (0·266–0·329)	0·303 (0·264–0·347)	-1·9 (-16·9–16·1)	2·39 (-10·2–15·5)
Occupational exposure to nickel	0·476 (0–2·14)	0·438 (0–1·91)	0·462 (0–2·03)	-8·04 (-19·8–NA)	5·45 (-5·77–NA)	0·427 (0–1·86)	0·396 (0–1·78)	0·405 (0–1·74)	-7·25 (-24·8–NA)	2·2 (-12·6–NA)
Occupational exposure to polycyclic aromatic hydrocarbons	0·302 (0·276–0·328)	0·298 (0·278–0·319)	0·314 (0·286–0·344)	-1·18 (-11–11)	5·12 (-3·67–14·9)	0·316 (0·28–0·352)	0·327 (0·304–0·352)	0·336 (0·301–0·374)	3·54 (-8·71–19)	2·72 (-7·38–13)
Occupational exposure to silica	4·45 (0·634–14·5)	3·91 (0·532–12·3)	4·15 (0·57–13)	-12·2 (-23·5–1·02)	6·17 (-4·45–20·9)	3·63 (0·502–11·8)	3·26 (0·445–11)	3·3 (0·45–11·5)	-10·3 (-27·5–12·4)	1·37 (-13·4–20·4)
Occupational exposure to sulfuric acid	0·979 (0·207–3·02)	1·04 (0·21–3·31)	1·08 (0·224–3·55)	6·24 (-6·76–22·8)	3·82 (-6·74–14·4)	0·945 (0·196–3·1)	1·1 (0·221–3·45)	1·09 (0·221–3·45)	16·4 (-4·49–43·7)	-0·912 (-14·5–15)
Unsafe sex	--	--	--	--	--	--	--	--	--	--
Occupational exposure to trichloroethylene	0·0875 (0·0815–0·094)	0·0889 (0·0852–0·0932)	0·0938 (0·0878–0·101)	1·6 (-6·3–11·3)	5·57 (-1·39–13·6)	0·0851 (0·0757–0·0946)	0·0873 (0·0813–0·0936)	0·0891 (0·0801–0·0983)	2·67 (-9·43–17·2)	1·96 (-7·43–11·9)
No access to handwashing facility	3·45 (3·2–3·71)	3·15 (2·91–3·41)	3·11 (2·85–3·38)	-8·53 (-16·5—0·0778)	-1·42 (-6·93–4·12)	3·45 (3·2–3·71)	3·15 (2·91–3·41)	3·11 (2·85–3·38)	-8·53 (-16·5—0·0778)	-1·42 (-6·93–4·12)
Child wasting	0·753 (0·504–1·04)	0·73 (0·478–0·989)	0·728 (0·473–0·994)	-3·05 (-12·7–6·73)	-0·363 (-7·51–8·09)	0·776 (0·522–1·06)	0·767 (0·512–1·06)	0·762 (0·506–1·04)	-1·04 (-10·9–9·74)	-0·741 (-8·46–6·7)
Child stunting	2·5 (1·31–3·63)	2·16 (1·15–3·17)	2·06 (1·08–3·11)	-13·5 (-32·7–10·3)	-4·66 (-19–12·5)	1·99 (0·976–3·04)	1·84 (0·916–2·77)	1·79 (0·894–2·72)	-7·86 (-27·2–16·4)	-2·48 (-17·8–16·4)
Chewing tobacco	8·82 (7·07–10·9)	7·97 (6·06–10·4)	7·76 (5·69–10·2)	-9·6 (-34·4–22·1)	-2·66 (-22·1–22·8)	0·655 (0·419–1·01)	0·561 (0·362–0·875)	0·568 (0·362–0·866)	-14·4 (-49·9–47·1)	1·23 (-27·6–43)
Diet low in legumes	74·6 (39·4–99·2)	77·5 (46–99·7)	77·5 (47·6–99·7)	3·91 (0–19·5)	0·0225 (-4·8–5·38)	73 (37–98·6)	75·9 (43·5–99·4)	76·2 (44·2–99·4)	3·95 (-0·158–24·5)	0·391 (-4·69–7·08)
Short gestation	31·9 (26·4–38·1)	30·6 (26–35·8)	27·1 (22·6–32·4)	-4·15 (-21·7–17·8)	-11·3 (-27·1–8·25)	32·3 (27·2–38)	29·2 (25·2–33·4)	25·5 (21·3–30·2)	-9·76 (-23·9–8·7)	-12·5 (-26·8–5·02)
Low birth weight	13·4 (11·3–15·9)	13·5 (11·6–15·7)	12·8 (10·6–15·1)	0·53 (-18·8–25·8)	-5·24 (-22·4–16·4)	15·7 (13·5–18·3)	15·3 (13·7–17·4)	14·3 (12·2–16·8)	-2·54 (-18·5–18·5)	-6·74 (-20·5–11·6)
High temperature	24·4 (16·4–33·9)	26·5 (18·1–35·9)	21·3 (13–31·1)	8·63 (3·18–15·4)	-19·6 (-28·9—12·1)	24·4 (16·4–33·9)	26·5 (18·1–35·9)	21·3 (13–31·1)	8·63 (3·18–15·4)	-19·6 (-28·9—12·1)
Low temperature	33·8 (29·4–39·8)	37·4 (33·5–42·9)	35·3 (30·6–41·5)	10·5 (6·12–15·8)	-5·48 (-9·57—1·93)	33·8 (29·4–39·8)	37·4 (33·5–42·9)	35·3 (30·6–41·5)	10·5 (6·12–15·8)	-5·48 (-9·57—1·93)
Kidney dysfunction	19·9 (14·3–27·1)	20·6 (14·7–27·7)	20·8 (14·8–28·1)	3·38 (-0·646–8·12)	0·912 (-2·97–4·65)	22·3 (15·9–29·9)	23·5 (16·7–31·3)	24·4 (17·6–32·4)	5·2 (0·952–10·1)	4·05 (-0·185–9·03)
Bullying victimization	5·87 (2·42–11·9)	8·94 (3·57–17·7)	8·72 (3·32–17·8)	52·2 (31·8–75·9)	-2·49 (-12–3·69)	4·26 (1·72–8·83)	7·83 (3·13–15·5)	7·77 (2·92–16)	83·9 (58·3–113)	-0·749 (-11·4–6)
High LDL cholesterol	66·7 (62·2–71·3)	34·3 (30·9–38)	30·7 (27·3–34·5)	-48·5 (-53·5—42·9)	-10·6 (-15·3—6·02)	65·3 (61·8–69)	34·6 (31·4–38)	31·7 (28·1–35·3)	-47·1 (-51·5—42·3)	-8·26 (-12·4—4·16)

Supplementary Results Table 1. Texas age-standardised Summary Exposure Values (SEVs) for most-detailed risk factors and percentage change, by sex, 1990, 2010, and 2019. Data in parentheses are 95% uncertainty intervals.

	Male					Female				
	1990	2010	2019	Percentage change 1990-2010	Percentage change 2010-2019	1990	2010	2019	Percentage change 1990-2010	Percentage change 2010-2019
Unsafe sanitation	8·96 (4·48–15·5)	4·93 (2·27–9·24)	3·63 (1·7–7)	-44·9 (-76·8–29)	-26·4 (-61–40·1)	8·96 (4·48–15·5)	4·93 (2·27–9·24)	3·63 (1·7–7)	-44·9 (-76·8–29)	-26·4 (-61–40·1)
Ambient particulate matter pollution	12·6 (4·56–25)	8·65 (5·7–12)	6·32 (3·62–9·38)	-31·4 (-64·5–68·9)	-27 (-37·7–-21)	12·6 (4·56–25)	8·65 (5·7–12)	6·32 (3·59–9·37)	-31·4 (-64·6–69)	-27 (-37·8–-21·1)
Household air pollution from solid fuels	0·0137 (0·00348–0·0358)	0·00813 (0·0015–0·0251)	0·00568 (0·000885–0·0189)	-40·5 (-78·6–43·8)	-30·2 (-64·7–31·9)	0·018 (0·00489–0·045)	0·0107 (0·00202–0·0326)	0·00749 (0·00134–0·0238)	-40·4 (-78·5–45)	-30·3 (-64·5–32·3)
Ambient ozone pollution	56·4 (24·1–94·4)	53·8 (22·5–91·5)	32·5 (12·2–57·8)	-4·62 (-10·3–0·731)	-39·7 (-48·5–-32·5)	56·4 (24·1–94·4)	53·8 (22·5–91·5)	32·5 (12·2–57·8)	-4·62 (-10·3–0·731)	-39·7 (-48·5–-32·5)
Residential radon	16·2 (2·26–46·6)	16·2 (2·26–46·6)	16·2 (2·26–46·6)	0 (0–0)	0 (0–0)	16·2 (2·26–46·6)	16·2 (2·26–46·6)	16·2 (2·26–46·6)	0 (0–0)	0 (0–0)
Lead exposure	70·6 (45·5–87·9)	42·8 (22·1–62·6)	30·4 (13·4–48·6)	-39·4 (-52·6–-26·6)	-28·9 (-39·3–-21·7)	44 (12–71·2)	25·5 (4·1–50·2)	17·6 (1·83–38·2)	-42 (-69·4–-27·3)	-31·2 (-58–-23·2)
Child underweight	0·981 (0·564–1·45)	0·965 (0·539–1·45)	0·94 (0·515–1·41)	-1·67 (-17·4–18·7)	-2·62 (-14·9–11·5)	0·821 (0·447–1·23)	0·853 (0·464–1·29)	0·871 (0·47–1·33)	3·89 (-16·7–29·2)	2·11 (-11·5–18)
Iron deficiency	--	--	--	--	--	11·3 (9·33–13·4)	11·7 (9·86–13·7)	11·8 (9·82–14·1)	2·98 (-8·01–16·2)	0·888 (-8·64–10·7)
Vitamin A deficiency	1·06 (0·29–2·45)	1·42 (0·393–3·55)	0·934 (0·253–2·46)	34 (-63·7–499)	-34·2 (-70·5–57·7)	1·98 (0·772–3·98)	2·02 (0·886–3·92)	1·63 (0·649–3·48)	2·09 (-58·9–193)	-19·2 (-55·1–47·5)
Zinc deficiency	2·26 (0–7·29)	1·19 (0–3·81)	1·2 (0–3·8)	-47·5 (-100–NA)	1·41 (-15·2–NA)	2·33 (0–7·7)	1·2 (0–3·84)	1·18 (0–3·74)	-48·5 (-100–NA)	-1·89 (-22·5–NA)
Smoking	26 (23·3–28·9)	18·5 (16·6–20·4)	15·6 (13·9–17·4)	-29·1 (-34·2–-23·9)	-15·5 (-19–-11·9)	18·5 (16·2–21·1)	12·6 (11·3–14·1)	10·2 (9·06–11·4)	-31·8 (-38·6–-23·9)	-19·1 (-23·5–-14·5)
Secondhand smoke	38·7 (36·1–41·5)	22·8 (21·2–24·4)	23·3 (20·9–26·1)	-41·1 (-47·1–-34·3)	2·53 (-6·66–11·6)	36·1 (34–38·2)	21·9 (20·7–23·1)	22·4 (20·5–24·4)	-39·4 (-44·3–-33·8)	2·22 (-5·33–9·91)
Alcohol use	11·9 (7·22–16·8)	12·8 (7·81–18·2)	12·7 (7·6–18·5)	7·89 (-25·8–57·1)	-0·81 (-26·8–30·8)	--	--	--	--	--
Drug use	0·501 (0·261–0·945)	0·762 (0·517–1·12)	0·968 (0·677–1·36)	52·2 (12·6–120)	27 (9·55–48·3)	0·346 (0·226–0·528)	0·629 (0·473–0·836)	0·87 (0·667–1·12)	81·8 (48·5–128)	38·4 (20·4–58·4)
High fasting plasma glucose	13·7 (11·4–16·6)	19·2 (17–22)	19·9 (17·2–22·7)	39·7 (16·2–68·9)	3·41 (-9·79–17·3)	11·3 (9·34–13·9)	16·7 (14·3–19·3)	17·3 (14·8–20·2)	47·1 (21·2–78·7)	3·65 (-11–19·4)
High systolic blood pressure	25·4 (20·6–30·8)	16·4 (14·3–18·5)	20·1 (15·6–25·2)	-35·5 (-47·8–-19·9)	22·5 (-1·74–51·7)	22·8 (17·1–29·1)	13·4 (11·7–15·1)	17·7 (13·1–23·5)	-41·3 (-54·5–-21·3)	32·2 (-1·66–78·5)
High body-mass index	23·9 (16·4–33·4)	38·6 (28·2–50·1)	40·2 (29·7–51·8)	61·7 (41·4–93·4)	4·15 (-2·51–11·6)	30·7 (22·4–40)	44 (33·7–53·4)	45·2 (35·1–54·6)	43·5 (27·6–65)	2·67 (-3·59–10·2)
Low bone mineral density	11·9 (7·07–18·7)	8·88 (4·76–14·8)	11·1 (6·2–17·7)	-25·2 (-44–-1·96)	25 (-3·03–65·8)	15·7 (10·5–22·3)	15·8 (10·5–22·9)	18·2 (12·4–25·6)	1·06 (-20·1–28·5)	15·2 (-7·09–49·5)
Diet low in fruits	53·6 (42–67·1)	46·1 (34·3–59·4)	45·8 (33·4–59·5)	-14 (-25·6–-3·97)	-0·687 (-13·3–12·2)	55·5 (44·3–67·6)	46·6 (34·3–59·9)	45·6 (32·6–59·8)	-16 (-27·8–-6·79)	-2·06 (-13·7–9·53)
Diet low in vegetables	30 (14·5–51·6)	46 (26·7–67·2)	43·7 (25·4–65·2)	53·3 (18·3–128)	-4·96 (-16·7–5·65)	30·7 (15·3–52·9)	38·1 (22·2–57·9)	35·4 (19·8–55·4)	23·8 (1·68–66·3)	-6·97 (-19·7–5·26)
Diet low in whole grains	79·6 (70·7–88·3)	81·6 (72·7–89·5)	81·5 (72·4–89·4)	2·42 (-1·07–6·65)	-0·0978 (-2·7–2·38)	77·1 (66·3–86·5)	79·8 (70·4–88·5)	79·6 (69·8–88·3)	3·59 (-0·125–8·99)	-0·328 (-3·11–2·3)
Diet low in nuts and seeds	27·9 (7·87–56)	24·9 (8·27–55·4)	20·4 (6·71–53·9)	-10·6 (-43·6–58·6)	-17·9 (-44–6·6)	24 (6·39–54·1)	21·8 (6·6–54)	18·9 (5·54–53·1)	-9·2 (-44·3–67·3)	-13·4 (-40·1–16·1)
Diet low in milk	48·8 (25·4–69·7)	43·7 (21–64)	42·2 (19·7–63·8)	-10·6 (-28·9–1·59)	-3·38 (-17·1–8·78)	48·5 (23·4–68·6)	43·5 (19·1–63)	42·4 (19·1–62·2)	-10·3 (-28·8–2·29)	-2·64 (-15·6–9·66)
Diet high in red meat	89·1 (80·1–95·4)	87·4 (76·9–94·6)	86·7 (76–94·8)	-1·89 (-11·4–7·52)	-0·784 (-8·62–7·22)	88·6 (79·2–95)	85·7 (75·4–93·3)	84·7 (73·1–93·3)	-3·33 (-14·3–7·74)	-1·14 (-10·1–7·01)
Diet high in processed meat	78·3 (54·1–98·2)	85·9 (66·7–99·8)	85·9 (66·5–99·7)	9·79 (1·08–29·2)	-0·0823 (-4·83–4·37)	79·4 (56·3–98·3)	88·3 (71·7–99·7)	87·1 (69–99·6)	11·3 (1·12–33·1)	-1·42 (-6·58–2·25)
Diet high in sugar-sweetened beverages	45·4 (27·7–65·3)	62 (42·5–79·2)	63 (41·6–80·9)	36·5 (-3·34–110)	1·63 (-18·8–25·7)	49·6 (29·7–68·4)	68·2 (48·5–84·1)	67·2 (47·2–83·2)	37·4 (-0·589–113)	-1·47 (-19·5–19·3)
Diet low in fiber	43·9 (28·4–58·9)	30·4 (18·4–43·1)	27·4 (15·2–40·3)	-30·7 (-46·2–-14·4)	-9·96 (-27·2–6·99)	44·2 (28–60·7)	35·7 (22·3–50)	30·2 (17·6–45·5)	-19·2 (-33·8–-2·39)	-15·4 (-30·9–0·842)
Diet low in seafood omega-3 fatty acids	83·7 (66·6–99·5)	79·9 (60·8–99)	79·3 (60·2–98·9)	-4·5 (-20·2–9·45)	-0·775 (-15·8–16·5)	86·4 (71·7–99·8)	83·7 (67–99·4)	82·7 (66·3–99·6)	-3·11 (-14·1–7·32)	-1·14 (-11·7–10·3)
Diet low in polyunsaturated fatty acids	14 (4·13–40·7)	7·3 (2·09–27)	6·89 (1·99–24·4)	-47·9 (-75–6·86)	-5·5 (-27·4–15·9)	15·7 (4·56–44·4)	7·47 (2·19–26·8)	6·98 (2·04–25·6)	-52·3 (-78·2–7·37)	-6·62 (-28·9–12)
Diet high in trans fatty acids	89·5 (81·8–100)	88 (79·2–100)	87·5 (78·2–100)	-1·71 (-9·78–6·55)	-0·486 (-8·23–7·73)	88·5 (81·1–100)	86·8 (78–100)	86·3 (77·6–100)	-1·93 (-9·04–5·35)	-0·541 (-8·35–7·27)
Diet high in sodium	18·1 (4·46–41·4)	39·2 (10·7–63·5)	38·5 (9·83–63·1)	117 (40·7–387)	-1·65 (-28·6–30·3)	--	--	--	--	--
Low physical activity	2·32 (0·867–4·95)	2·15 (0·892–4·45)	2·48 (0·891–5·38)	-7·53 (-38·5–42·2)	15·3 (-11·1–55·6)	5·43 (2·63–10·2)	5·7 (3·06–10·4)	6·05 (3·09–11·5)	5 (-19·8–46·9)	6·08 (-6·88–19·9)
Occupational asthmagens	23·6 (19·8–28)	21·1 (17·9–24·7)	21·7 (18·2–26·1)	-10·7 (-21·4–0·444)	2·76 (-5·45–13·2)	11·1 (9·17–13·3)	10·5 (8·73–12·5)	10·5 (8·62–12·6)	-5·8 (-18·6–9·98)	0·63 (-10·3–12·6)
Occupational particulate matter, gases, and fumes	9·64 (6·91–13·9)	9·07 (6·56–13·1)	8·77 (6·34–12·4)	-5·87 (-11·1–-0·654)	-3·32 (-6·67–-0·0598)	4·97 (3·52–7·21)	4·79 (3·42–6·91)	4·59 (3·32–6·5)	-3·62 (-12·4–6·46)	-4·1 (-8·87–1·42)
Occupational noise	8·91 (8·17–9·75)	8·56 (7·99–9·31)	8·29 (7·79–9)	-3·95 (-7·38–-0·227)	-3·14 (-5·08–-1·17)	5·83 (5·11–6·63)	6·08 (5·56–6·7)	6·06 (5·64–6·63)	4·39 (-1·67–12·2)	-0·416 (-3·28–2·93)
Occupational injuries	--	--	--	--	--	--	--	--	--	--
Occupational ergonomic factors	12·7 (10·5–15·3)	11·1 (9·37–13·3)	11·5 (9·51–13·8)	-12·7 (-22·9–-2·17)	3·07 (-5·3–11·5)	6·99 (5·54–8·85)	6·75 (5·47–8·36)	7·01 (5·68–8·75)	-3·41 (-18·8–15)	3·87 (-6·83–16·5)
Childhood sexual abuse	7·4 (5·05–10·5)	6·97 (4·6–9·93)	6·85 (4·54–9·67)	-5·83 (-12·2–5·83)	-1·68 (-8·51–5·53)	12·3 (8·94–17·3)	17·4 (12·9–24)	17·8 (13·1–24·7)	41·9 (30·7–54·8)	2·17 (-5–11)
Intimate partner violence	--	--	--	--	--	30·3 (16·7–39·3)	28·6 (16·2–37·8)	29·5 (16·5–38·5)	-5·47 (-14·4–10·2)	3·11 (-6·32–18·6)
Non-exclusive breastfeeding	26·3 (21·8–31·2)	24·9 (21·4–28·8)	24·9 (20·8–29·4)	-5·4 (-18·5–11·9)	-0·0309 (-13–15·1)	26·3 (21·8–31·2)	24·9 (21·4–28·8)	24·9 (20·8–29·4)	-5·39 (-18·5–11·9)	-0·0386 (-13·1–15·1)
Discontinued breastfeeding	27·3 (26·4–28·2)	25·4 (24·1–26·6)	25·3 (24–26·4)	-7·01 (-12·3–-1·81)	-0·434 (-6·12–5·51)	27·3 (26·4–28·2)	25·4 (24·1–26·6)	25·3 (24–26·4)	-6·98 (-12·3–-1·78)	-0·503 (-6·19–5·43)
Diet low in calcium	16·4 (7·73–30·5)	13·7 (6·64–25·1)	12·9 (6·18–23·8)	-16·2 (-26·6–-5·11)	-5·97 (-14·9–1·45)	11·9 (5·12–22·5)	10·8 (4·64–20)	10·3 (4·25–19)	-9·23 (-22·4–7·6)	-4·65 (-14·4–4·4)
Occupational exposure to asbestos	7·62 (6·86–8·43)	6·15 (5·64–6·64)	5·62 (4·09–7·55)	-19·3 (-28·9–-8·17)	-8·65 (-34–21·7)	1·2 (0·974–1·93)	1·15 (0·932–1·87)	1·13 (0·751–1·89)	-4·34 (-17·4–10·4)	-2·09 (-29·8–32·7)
Occupational exposure to arsenic	0·499 (0–1·17)	0·513 (0–1·21)	0·542 (0–1·29)	2·89 (-10·5–NA)	5·67 (-5·52–NA)	0·432 (0–0·995)	0·443 (0–1·05)	0·455 (0–1·1)	2·52 (-14·2–NA)	2·78 (-8·51–NA)
Occupational exposure to benzene	0·85 (0·0497–2·44)	0·858 (0·0517–2·53)	0·882 (0·0548–2·53)	0·912 (-7·31–13·2)	2·79 (-4·69–12·5)	1·17 (0·0669–3·39)	1·3 (0·0772–3·6)	1·36 (0·0808–3·8)	10·8 (-0·922–30·5)	4·88 (-3·6–18·7)
Occupational exposure to beryllium	0·0244 (0·0231–0·0258)	0·0245 (0·0241–0·025)	0·0257 (0·0244–0·0271)	0·592 (-4·75–6·53)	4·79 (-0·777–10·3)	0·0256 (0·0233–0·0279)	0·0266 (0·0261–0·0271)	0·0276 (0·0258–0·0293)	3·71 (-5·17–13·8)	3·72 (-2·99–10·5)
Occupational exposure to cadmium	0·0587 (0·0531–0·0648)	0·0606 (0·0552–0·0663)	0·0638 (0·0566–0·0719)	3·21 (-9·1–17·8)	5·34 (-4·65–15·9)	0·0524 (0·0464–0·0591)	0·0538 (0·0489–0·0596)	0·0553 (0·0488–0·0632)	2·77 (-12–19·8)	2·81 (-7·27–15)
Occupational exposure to chromium	0·143 (0·131–0·156)	0·15 (0·14–0·161)	0·158 (0·143–0·174)	5·01 (-5·69–16·9)	4·92 (-3·79–13·9)	--	--	--	--	--
Occupational exposure to diesel engine exhaust	0·809 (0·747–0·87)	0·853 (0·803–0·906)	0·882 (0·817–0·954)	5·49 (-4·41–16·1)	3·4 (-4·05–10·6)	0·69 (0·614–0·777)	0·701 (0·644–0·768)	0·698 (0·624–0·789)	1·65 (-12·7–16·4)	-0·471 (-10·2–9·73)
Occupational exposure to formaldehyde	0·293 (0·264–0·325)	0·296 (0·267–0·326)	0·307 (0·27–0·348)	0·92 (-12–15·8)	4·01 (-6·26–15)	0·258 (0·23–0·291)	0·267 (0·245–0·294)	0·275 (0·245–0·312)	3·54 (-10·4–19)	3·05 (-6·63–14·5)
Occupational exposure to nickel	0·449 (0–2·04)	0·425 (0–1·94)	0·446 (0–1·97)	-5·36 (-18·4–NA)	5·05 (-6·41–NA)	0·351 (0–1·53)	0·339 (0–1·51)	0·35 (0–1·58)	-3·54 (-18·6–NA)	3·23 (-9·42–NA)
Occupational exposure to polycyclic aromatic hydrocarbons	0·284 (0·261–0·308)	0·297 (0·277–0·318)	0·311 (0·282–0·341)	4·43 (-5·83–15·6)	4·62 (-3·97–13·2)	0·285 (0·258–0·315)	0·307 (0·29–0·327)	0·316 (0·289–0·349)	7·63 (-4·01–21)	2·9 (-4·93–12·1)
Occupational exposure to silica	4·47 (0·649–14·3)	3·92 (0·595–12·4)	4·11 (0·589–12·7)	-12·2 (-24·4–1·06)	4·64 (-5·64–17·7)	3·14 (0·455–9·96)	2·81 (0·362–8·59)	2·85 (0·399–9·03)	-10·4 (-25·4–10·1)	1·49 (-11·2–18·9)
Occupational exposure to sulfuric acid	0·925 (0·197–3)	1·04 (0·214–3·33)	1·07 (0·219–3·5)	12·4 (-1·91–30·9)	3·25 (-6·93–14·7)	0·825 (0·167–2·76)	1·01 (0·211–3·09)	1 (0·21–3·16)	22·8 (0·96–49·5)	-1·02 (-14·3–14·2)
Unsafe sex	--	--	--	--	--	--	--	--	--	--
Occupational exposure to trichloroethylene	0·0856 (0·0803–0·092)	0·0919 (0·0883–0·0964)	0·0962 (0·0895–0·103)	7·36 (-0·812–16·6)	4·62 (-1·84–11·9)	0·0795 (0·0718–0·088)	0·0829 (0·0784–0·0885)	0·0844 (0·0774–0·0927)	4·26 (-6·84–17·1)	1·83 (-6·11–10·8)
No access to handwashing facility	3·45 (3·2–3·7)	3·16 (2·93–3·41)	3·11 (2·88–3·35)	-8·57 (-15·7–-0·312)	-1·47 (-6·88–3·78)	3·45 (3·2–3·7)	3·16 (2·93–3·41)	3·11 (2·88–3·35)	-8·57 (-15·7–-0·312)	-1·47 (-6·88–3·78)
Child wasting	0·753 (0·501–1·03)	0·728 (0·476–0·984)	0·727 (0·476–0·996)	-3·23 (-12·7–6·7)	-0·175 (-7·04–7·02)	0·774 (0·52–1·06)	0·763 (0·505–1·05)	0·761 (0·501–1·04)	-1·43 (-11·1–9·17)	-0·346 (-7·91–7·64)
Child stunting	2·48 (1·31–3·59)	2·1 (1·07–3·13)	2·04 (1·03–3·09)	-15·5 (-33·2–6·61)	-3·02 (-17–13·7)	1·99 (0·982–3·02)	1·81 (0·904–2·8)	1·78 (0·889–2·75)	-9·06 (-28·2–13·7)	-1·61 (-16·1–14·9)
Chewing tobacco	3·3 (2·6–4·24)	3·46 (2·56–4·58)	3·28 (2·39–4·42)	4·75 (-26·9–48·9)	-5·12 (-23·9–19·2)	--	--	--	--	--
Diet low in legumes	74·1 (40·8–99·4)	77·1 (46·6–99·7)	76·9 (45·8–99·6)	4·02 (-0–20·5)	-0·213 (-5·09–4·73)	72·5 (35·9–98·4)	75·4 (42·6–99·7)	75·7 (43·3–99·5)	4·08 (-0·07–22·1)	0·278 (-5·19–7·71)
Short gestation	31·3 (25·7–38·1)	25·7 (21·6–30·2)	24·8 (20·4–30·2)	-18 (-34·6–0·709)	-3·55 (-22·6–18·9)	25·9 (21·8–31·1)	24·5 (20·9–28·8)	23·1 (19·4–27·3)	-5·42 (-25·4–16·7)	-5·88 (-21·8–13·1)
Low birth weight	12·7 (10·4–15·3)	10·8 (9·2–12·7)	10·5 (8·61–12·6)	-14·8 (-33·2–7·4)	-3·25 (-22·5–20·1)	11·9 (10–14·3)	12·1 (10·3–13·9)	11·9 (10·2–13·8)	1·17 (-20·1–25·8)	-1·4 (-19·9–20)
High temperature	30·7 (21·6–40·4)	33·5 (23·6–43·5)	33·2 (23·4–43·3)	9·08 (4·79–13·6)	-0·914 (-3·87–2·34)	30·7 (21·6–40·4)	33·5 (23·6–43·5)	33·2 (23·4–43·3)	9·08 (4·79–13·6)	-0·914 (-3·87–2·34)
Low temperature	38·9 (27·6–50·8)	41 (30·5–52·2)	40·3 (29·1–52)	5·41 (1·48–11·3)	-1·58 (-4·51–0·181)	38·9 (27·6–50·8)	41 (30·5–52·2)	40·3 (29·1–52)	5·41 (1·48–11·3)	-1·58 (-4·51–0·181)
Kidney dysfunction	14·7 (10·6–20·9)	15·5 (11·1–21·6)	15·8 (11·5–22)	5·03 (0·129–10·9)	2·31 (-2·7–7·45)	16·1 (11·6–22·4)	17·1 (12·2–23·7)	18·1 (13·2–24·7)	5·96 (0·209–13)	6·1 (-0·238–13·6)
Bullying victimization	5·85 (2·41–12)	8·91 (3·55–17·8)	8·72 (3·31–18·1)	52·5 (32·3–75·9)	-2·17 (-12–3·96)	--	--	--	--	--
High LDL cholesterol	67·4 (63·1–71·8)	34·5 (31·1–38·3)	30·7 (27·1–34·5)	-48·8 (-54·2–-43·4)	-11 (-15·7–-6·29)	65·2 (61·9–68·9)	34·4 (31·2–37·9)	31·5 (27·9–35·1)	-47·2 (-51·8–-43)	-8·61 (-12·7–-4·49)

Supplementary Results Table 1. Utah age-standardised Summary Exposure Values (SEVs) for most-detailed risk factors and percentage change, by sex, 1990, 2010, and 2019. Data in parentheses are 95% uncertainty intervals.

	Male					Female				
	1990	2010	2019	Percentage change 1990-2010	Percentage change 2010-2019	1990	2010	2019	Percentage change 1990-2010	Percentage change 2010-2019
Unsafe sanitation	7·72 (3·67–13·7)	3·98 (1·93–7·38)	3 (1·47–5·78)	-48·4 (-78·1–24·7)	-24·7 (-61·4–50·2)	--	--	--	--	--
Ambient particulate matter pollution	8·17 (1·88–18·3)	5·37 (2·91–8·26)	5·23 (2·77–8·08)	-34·3 (-70·8–123)	-2·66 (-6·96–0·245)	8·17 (1·87–18·3)	5·37 (2·92–8·25)	5·23 (2·77–8·09)	-34·3 (-70·7–121)	-2·69 (-6·96–0·264)
Household air pollution from solid fuels	0·0189 (0·00434–0·0547)	0·0106 (0·00193–0·033)	0·00825 (0·00138–0·0266)	-43·9 (-79·3–51·2)	-22·2 (-60–48·3)	0·0251 (0·00593–0·0701)	0·0141 (0·00253–0·045)	0·0109 (0·00195–0·0356)	-43·8 (-79·3–51·8)	-22·5 (-60·2–48·1)
Ambient ozone pollution	77·9 (35·7–100)	79·9 (37–100)	80·7 (37·3–100)	2·55 (-7·04–15·7)	0·938 (-8·62–12·5)	--	--	--	--	--
Residential radon	22·8 (4·15–63·8)	22·8 (4·15–63·8)	22·8 (4·15–63·8)	0 (0–0)	0 (0–0)	22·8 (4·15–63·8)	22·8 (4·15–63·8)	22·8 (4·15–63·8)	0 (0–0)	0 (0–0)
Lead exposure	76·3 (52·4–91·7)	46·3 (25·1–66·4)	32·8 (15·2–50·6)	-39·3 (-52·9—26·1)	-29·1 (-38·7—22·8)	49·6 (17·1–75·1)	28·1 (5·23–52·4)	19·2 (2·31–39·3)	-43·3 (-69·3—27·4)	-31·8 (-58·7—24·3)
Child underweight	0·978 (0·551–1·47)	0·961 (0·521–1·45)	0·943 (0·526–1·44)	-1·74 (-18·7–19·4)	-1·8 (-15·3–12·6)	0·821 (0·452–1·23)	0·845 (0·46–1·31)	0·864 (0·466–1·33)	2·98 (-17·1–27·9)	2·28 (-11·2–18·4)
Iron deficiency	--	--	--	--	--	11·8 (9·81–13·7)	11·9 (9·96–14)	12·1 (9·85–14·4)	1·28 (-10·4–14·4)	1·88 (-7·4–12·4)
Vitamin A deficiency	0·992 (0·274–2·6)	1·27 (0·354–3·56)	0·859 (0·225–2·3)	28·1 (-64·2–473)	-32·4 (-70·9–60·8)	1·83 (0·7–3·94)	1·81 (0·735–3·76)	1·51 (0·598–3·19)	-1·04 (-59·8–184)	-16·7 (-53·1–55·7)
Zinc deficiency	2·68 (0–8·39)	1·32 (0–4·3)	1·34 (0–4·3)	-50·5 (-100–NA)	1·25 (-19·1–NA)	2·74 (0–8·71)	1·34 (0–4·5)	1·31 (0–4·25)	-51·2 (-100–NA)	-2·34 (-20·6–NA)
Smoking	20·5 (18·3–22·8)	13 (11·7–14·5)	11 (9·8–12·2)	-36·3 (-41·3—31·1)	-16 (-19·8—12·2)	12 (10·4–13·8)	8·48 (7·52–9·53)	6·81 (5·98–7·68)	-29·4 (-37·1—20·1)	-19·7 (-24·1—14·6)
Secondhand smoke	32·9 (30·6–35·6)	19·3 (17·9–20·8)	20·1 (17·7–22·5)	-41·4 (-47·6—34·4)	3·99 (-5·93–14·5)	30·2 (28·4–32·1)	17·6 (16·6–18·5)	18·4 (16·5–20·2)	-41·7 (-46·4—36·7)	4·69 (-4–13·5)
Alcohol use	11·2 (7·57–15·4)	10·7 (7·25–14·5)	11·6 (7·83–15·8)	-4·71 (-25·8–22·6)	8·2 (-9·64–28·5)	7·03 (4·53–9·92)	7·15 (4·85–9·85)	7·82 (5·23–10·9)	1·64 (-21·9–32·7)	9·31 (-10·4–31·2)
Drug use	0·52 (0·284–0·948)	0·894 (0·63–1·27)	1·27 (0·937–1·68)	71·7 (24·6–145)	41·7 (22·4–64·7)	0·469 (0·305–0·707)	0·824 (0·623–1·09)	1·31 (1·03–1·67)	75·8 (40·9–120)	59·5 (37·2–84·1)
High fasting plasma glucose	14 (11·6–16·7)	17·3 (15–20)	19 (16·4–21·9)	23·4 (1·69–51)	9·51 (-4·25–27·2)	12·1 (9·66–14·9)	15 (12·8–17·5)	16·8 (14·1–19·6)	24·3 (2·08–54)	11·8 (-4·76–30·5)
High systolic blood pressure	24·4 (19·6–29·8)	15·2 (13·2–17·3)	18·8 (14·4–23·5)	-37·7 (-49·5—22·8)	23·8 (-1·13–52·9)	21·6 (15·7–28·6)	12·5 (10·9–14)	16·8 (12–22·7)	-42·4 (-57·7—20·5)	34·8 (-4·94–80·5)
High body-mass index	21·1 (14·4–29·3)	35·2 (25·4–46·9)	36·9 (26·6–48·4)	66·6 (44·8–99·9)	4·79 (-2·51–12·4)	30·4 (20·1–43·7)	45·1 (33·1–57)	46·5 (34·5–58·8)	48·4 (27·1–81·1)	3·06 (-3·49–10·7)
Low bone mineral density	11·5 (6·62–17·8)	8·78 (4·62–14·8)	11·1 (6·15–17·7)	-23·5 (-43·7–0·0352)	26·1 (-4·32–69·7)	15·3 (10–22·2)	15·7 (10·4–22·6)	18·1 (12·1–25·4)	2·04 (-19·7–29·7)	15·6 (-9·09–46·4)
Diet low in fruits	52·6 (41·1–66·4)	46·4 (34·8–59·4)	47·7 (34·8–60·4)	-11·9 (-23·4—1·71)	2·81 (-9·18–16·1)	53·4 (40·9–65·7)	47·3 (36–61)	48·4 (36–61·9)	-11·5 (-22·5—0·669)	2·27 (-9·64–13·9)
Diet low in vegetables	29·4 (14–50·7)	41·7 (24–61·4)	40·2 (23·6–60·3)	41·9 (12·2–111)	-3·52 (-16·3–8·82)	29·3 (13·7–50·8)	43·5 (25·2–63·8)	43·3 (24·6–64·7)	48·6 (15·4–122)	-0·401 (-12·2–12·4)
Diet low in whole grains	80·1 (70·1–88·6)	82 (73·3–89·6)	81·9 (72·9–89·6)	2·32 (-1·26–6·92)	-0·057 (-2·62–2·19)	76 (63·9–87)	78·8 (68–89)	78·5 (68·1–89·3)	3·59 (-1·08–10·1)	-0·328 (-3·23–2·69)
Diet low in nuts and seeds	31·2 (10·1–58·3)	26·8 (9·6–56·1)	22 (7·26–53·9)	-14·1 (-46–47·5)	-17·7 (-43·5–8·63)	27·1 (7·15–55·8)	23·7 (7·44–55·3)	20·5 (6·28–53·9)	-12·5 (-44–51·8)	-13·5 (-42·1–14·1)
Diet low in milk	51·8 (27·9–72·4)	46·5 (22·3–67)	45·1 (21·1–65)	-10·2 (-27·8–0·895)	-3·04 (-14·4–8·8)	51·5 (27·8–72·7)	46·5 (23·1–66·9)	45·4 (23·6–66·4)	-9·65 (-26·9–2·59)	-2·52 (-13·4–10·1)
Diet high in red meat	75·7 (62·8–86·7)	75·8 (62·7–87·1)	75·8 (62·6–88)	0·208 (-14·2–18·2)	0·0302 (-11·4–12·7)	75·7 (61·7–87·3)	73·7 (59–85·4)	74·1 (59·3–86·4)	-2·67 (-18·6–14·4)	0·509 (-12·8–14·5)
Diet high in processed meat	75·2 (51–96·6)	83·7 (64·1–99·4)	83·6 (63·6–99·4)	11·3 (1·78–31·2)	-0·0753 (-4·57–4·62)	75·4 (49·9–95·8)	85·4 (66·3–99·2)	84·2 (65–99·1)	13·2 (2·48–37·7)	-1·46 (-7·08–2·47)
Diet high in sugar-sweetened beverages	43·1 (26·1–62·5)	59·2 (40·2–76·2)	60·1 (40–78·4)	37·4 (-7·21–120)	1·43 (-21·1–27·6)	46·4 (27·2–66·1)	64·3 (44·9–81·3)	63·8 (43·3–81·7)	38·6 (-3·82–115)	-0·857 (-21·6–21·7)
Diet low in fiber	46·6 (29·4–61·9)	32·6 (19·3–47)	29·5 (16·7–43·8)	-30 (-45·8—15·1)	-9·6 (-27·5–8·26)	47·2 (30·5–63·5)	38·6 (24·3–52·3)	33·3 (19·2–48·2)	-18·2 (-34—0·134)	-13·8 (-28·9—0·836)
Diet low in seafood omega-3 fatty acids	85·8 (69·6–100)	82 (64·4–99·5)	81·4 (63·4–99)	-4·41 (-17·8–9·29)	-0·719 (-13·9–15·3)	87·7 (73·2–99·9)	85·5 (71·5–99·5)	84·7 (69·4–99·4)	-2·57 (-12·2–6·36)	-0·925 (-11·6–9·04)
Diet low in polyunsaturated fatty acids	16 (4·76–45·2)	8·15 (2·38–28)	7·59 (2·25–26·8)	-49 (-74·4—8·79)	-6·77 (-29·4–14·7)	17·8 (5·13–46·7)	8·38 (2·41–28)	7·69 (2·24–26·4)	-52·8 (-76·8—9·31)	-8·27 (-32·1–11)
Diet high in trans fatty acids	89·5 (81·6–100)	87·9 (78·1–100)	87·4 (77·8–100)	-1·88 (-10·3–5·77)	-0·488 (-9·23–7·61)	88·4 (81·3–100)	86·5 (77·7–100)	86·2 (77·1–100)	-2·08 (-10·1–5·97)	-0·39 (-8·05–8·89)
Diet high in sodium	18·3 (4·8–41)	39·8 (12·3–63·8)	38·7 (11·2–64·3)	117 (42–402)	-2·54 (-30–30·9)	21 (5–45·9)	21·4 (4·85–46)	21·8 (5·18–46·7)	1·73 (-27·2–50)	2·06 (-18·4–30·6)
Low physical activity	2·31 (0·857–4·95)	1·97 (0·779–4·25)	2·07 (0·71–4·59)	-14·9 (-45·8–27·9)	5·35 (-22·2–42·6)	5·08 (2·41–9·89)	5·09 (2·63–9·46)	5·34 (2·74–10·2)	0·122 (-24·1–36·5)	5·05 (-7·46–19)
Occupational asthmagens	23·2 (19·4–28·2)	22 (18·7–25·9)	22·7 (18·8–27·6)	-5·08 (-17·5–7·2)	3·16 (-6·19–15·2)	10·8 (8·81–13·1)	10·8 (9·11–13)	11 (8·97–13·3)	0·231 (-14·1–16·2)	0·972 (-10·5–14·2)
Occupational particulate matter, gases, and fumes	9·28 (6·69–13·5)	9·1 (6·58–13·1)	8·95 (6·45–13)	-1·98 (-7·22–4·08)	-1·67 (-5·42–1·81)	5·18 (3·67–7·71)	5·06 (3·67–7·23)	4·88 (3·55–7·07)	-2·38 (-11·5–7·47)	-3·61 (-8·7–1·63)
Occupational noise	8·86 (8·1–9·68)	8·7 (8·13–9·43)	8·53 (8·03–9·23)	-1·73 (-5·37–2·26)	-1·93 (-4·01–0·247)	5·89 (5·16–6·67)	6·25 (5·73–6·85)	6·26 (5·85–6·8)	6·15 (-0·249–13·9)	0·173 (-2·83–3·61)
Occupational injuries	--	--	--	--	--	--	--	--	--	--
Occupational ergonomic factors	12·8 (10·6–15·6)	11·7 (9·67–14)	12 (10–14·4)	-8·86 (-19·6–2·18)	3·22 (-5·1–12·1)	6·85 (5·44–8·6)	6·78 (5·53–8·35)	7·08 (5·67–8·89)	-0·978 (-16·9–18)	4·37 (-7·69–17·7)
Childhood sexual abuse	7·4 (5·05–10·5)	6·97 (4·6–9·93)	6·85 (4·54–9·67)	-5·83 (-12·2–5·83)	-1·68 (-8·51–5·53)	12·3 (8·94–17·3)	17·4 (12·9–24)	17·8 (13·1–24·7)	41·9 (30·7–54·8)	2·17 (-5–11)
Intimate partner violence	--	--	--	--	--	31·8 (17·5–41·3)	30·1 (16·9–39)	31 (17·2–40·3)	-5·37 (-13·9–6·24)	3·06 (-5·48–15·7)
Non-exclusive breastfeeding	26·1 (21·5–31·3)	24·9 (21·1–28·6)	24·9 (20·6–29·5)	-4·66 (-19·1–11·4)	-0·186 (-13·1–14·5)	26·1 (21·5–31·3)	24·9 (21·1–28·6)	24·9 (20·6–29·5)	-4·65 (-19·1–11·4)	-0·194 (-13·1–14·5)
Discontinued breastfeeding	27·2 (26·1–28·1)	25·7 (24·4–26·9)	25·5 (24·2–26·7)	-5·57 (-11·1—0·262)	-0·614 (-6·17–4·91)	27·2 (26·1–28·1)	25·8 (24·6–27·1)	25·6 (24·2–26·8)	-5·01 (-10·5–0·327)	-0·869 (-6·4–4·65)
Diet low in calcium	17·7 (7·61–34)	14·8 (6·85–27·5)	13·9 (6·16–26·3)	-16·4 (-27·1—4·23)	-5·95 (-15·4–1·4)	13·5 (5·48–25·6)	12·2 (5·37–22·9)	11·6 (4·96–22)	-9·47 (-22·2–10·6)	-5·12 (-15·3–3·58)
Occupational exposure to asbestos	8·3 (7·41–9·32)	7·31 (6·57–8·05)	6·65 (4·77–8·85)	-11·9 (-23·9–1·49)	-9·09 (-34–21·1)	1·31 (1·03–1·91)	1·28 (1·03–1·84)	1·28 (0·89–2)	-1·99 (-16–15·2)	0·23 (-25·2–33·3)
Occupational exposure to arsenic	0·492 (0–1·19)	0·533 (0–1·27)	0·558 (0–1·34)	8·37 (-6·29–NA)	4·6 (-6·19–NA)	0·452 (0–1·1)	0·458 (0–1·09)	0·468 (0–1·12)	1·42 (-15·2–NA)	2·16 (-10·1–NA)
Occupational exposure to benzene	0·894 (0·0497–2·55)	0·925 (0·0527–2·68)	0·939 (0·056–2·69)	3·52 (-5·19–15·7)	1·54 (-5·27–11·5)	1·18 (0·0691–3·22)	1·31 (0·0775–3·59)	1·37 (0·0814–3·84)	11·7 (0·219–33·2)	4·42 (-4·66–16·9)
Occupational exposure to beryllium	0·0249 (0·0236–0·0262)	0·0258 (0·0252–0·0264)	0·0268 (0·0255–0·0282)	3·84 (-2·05–9·88)	4·03 (-0·808–9·31)	0·0264 (0·0241–0·0287)	0·0273 (0·0266–0·0281)	0·0283 (0·0263–0·0304)	3·44 (-5·18–13·3)	3·66 (-3·79–10·8)
Occupational exposure to cadmium	0·0583 (0·053–0·0643)	0·0632 (0·0572–0·0694)	0·066 (0·0582–0·0739)	8·49 (-4·8–22)	4·41 (-5·01–15·5)	0·0545 (0·0482–0·0619)	0·0556 (0·0503–0·0618)	0·057 (0·0502–0·0655)	2·04 (-13·2–19·6)	2·52 (-8·99–15·8)
Occupational exposure to chromium	0·143 (0·131–0·156)	0·157 (0·145–0·169)	0·163 (0·147–0·18)	10·2 (-1·52–22·2)	4·01 (-4·25–13·4)	0·137 (0·123–0·154)	0·143 (0·132–0·156)	0·147 (0·131–0·164)	4·42 (-8·7–19·8)	2·28 (-7·49–13·7)
Occupational exposure to diesel engine exhaust	0·809 (0·752–0·878)	0·887 (0·831–0·946)	0·915 (0·837–0·993)	9·59 (-0·305–20·4)	3·13 (-4·54–11·4)	0·686 (0·607–0·77)	0·716 (0·651–0·783)	0·712 (0·63–0·808)	4·34 (-10–20·9)	-0·463 (-11·1–10·8)
Occupational exposure to formaldehyde	0·291 (0·262–0·324)	0·31 (0·278–0·34)	0·319 (0·28–0·359)	6·42 (-7·76–20·7)	2·9 (-6·93–14·5)	0·265 (0·235–0·299)	0·274 (0·248–0·304)	0·281 (0·247–0·32)	3·54 (-10·8–20)	2·57 (-7·78–15·2)
Occupational exposure to nickel	0·431 (0–1·85)	0·431 (0–1·88)	0·451 (0–1·92)	0·142 (-13·4–NA)	4·54 (-6·21–NA)	0·356 (0–1·52)	0·346 (0–1·53)	0·357 (0–1·64)	-2·67 (-19·6–NA)	3·14 (-11·2–NA)
Occupational exposure to polycyclic aromatic hydrocarbons	0·285 (0·262–0·31)	0·312 (0·289–0·334)	0·323 (0·292–0·355)	9·55 (-1·79–21·2)	3·66 (-4·13–12·8)	0·293 (0·262–0·326)	0·316 (0·296–0·338)	0·324 (0·294–0·358)	7·87 (-4·39–21·4)	2·58 (-6·26–12)
Occupational exposure to silica	4·21 (0·608–13·6)	3·9 (0·585–12·3)	4·08 (0·597–12·7)	-7·4 (-20–8·13)	4·61 (-5·44–18·1)	3·02 (0·413–9·67)	2·81 (0·388–8·94)	2·85 (0·39–9·36)	-6·74 (-24–15·3)	1·42 (-12·3–20·3)
Occupational exposure to sulfuric acid	0·929 (0·2–2·91)	1·1 (0·225–3·49)	1·13 (0·23–3·58)	18·3 (1·81–36·4)	2·61 (-8·68–15·1)	0·903 (0·188–2·8)	1·09 (0·217–3·46)	1·08 (0·21–3·35)	20·5 (-1·91–50)	-1·06 (-16·4–16·5)
Unsafe sex	--	--	--	--	--	--	--	--	--	--
Occupational exposure to trichloroethylene	0·0863 (0·0811–0·0928)	0·0965 (0·0922–0·101)	0·1 (0·0936–0·108)	11·8 (3·1–21)	3·84 (-2·7–11·2)	0·0819 (0·0742–0·0907)	0·0855 (0·0803–0·0911)	0·0868 (0·0789–0·0958)	4·36 (-7·33–18·2)	1·6 (-7·5–11·2)
No access to handwashing facility	3·4 (3·13–3·66)	3·12 (2·88–3·4)	3·09 (2·83–3·35)	-8·04 (-16·2–1·22)	-1·18 (-6·68–4·89)	3·4 (3·13–3·66)	3·12 (2·88–3·4)	3·09 (2·83–3·35)	-8·04 (-16·2–1·22)	-1·18 (-6·68–4·89)
Child wasting	0·752 (0·5–1·02)	0·726 (0·487–0·99)	0·726 (0·485–0·997)	-3·48 (-12·5–6·87)	-0·0718 (-7·64–8·26)	0·776 (0·52–1·06)	0·764 (0·517–1·04)	0·76 (0·505–1·05)	-1·5 (-11–9·47)	-0·623 (-8·37–7·54)
Child stunting	2·49 (1·28–3·65)	2·06 (1·07–3·08)	2·02 (1·07–3·06)	-17·1 (-36·6–5·71)	-2·04 (-16·5–14)	1·98 (0·982–3·03)	1·79 (0·91–2·72)	1·78 (0·887–2·71)	-9·8 (-29·4–16·4)	-0·695 (-15·6–18)
Chewing tobacco	2·6 (1·96–3·34)	2·92 (2·05–4·13)	2·95 (1·99–4·23)	12·4 (-24·8–65·3)	1·29 (-22·8–32·4)	--	--	--	--	--
Diet low in legumes	75·2 (41·1–99·5)	78·1 (48·6–99·8)	77·9 (47·2–99·7)	3·82 (-0·06–20·1)	-0·316 (-5·8–4·17)	73·5 (37·6–99·4)	76·3 (44·9–99·6)	76·5 (45·1–99·6)	3·83 (-0·457–20·9)	0·213 (-4·8–6·78)
Short gestation	18·9 (15·3–23)	19·7 (16·3–23·6)	19·9 (16·3–24·5)	3·91 (-18·6–31·4)	1·3 (-19·8–27·7)	16 (13·4–18·7)	17·1 (14·3–20·1)	17·5 (14·7–20·8)	6·75 (-15·1–32·6)	2·87 (-15·1–24·1)
Low birth weight	7·84 (6·47–9·5)	8·46 (7·12–10·2)	8·73 (7·22–10·5)	7·97 (-15·7–38·7)	3·24 (-18·4–31·4)	8·05 (6·76–9·53)	8·65 (7·36–10·1)	9·18 (7·76–10·7)	7·46 (-14·2–33·8)	6·08 (-12·8–29·2)
High temperature	27·5 (16·9–39·5)	30·3 (19·9–41·9)	33 (22·5–44·7)	10·1 (3·33–22·4)	8·74 (3·51–15·8)	27·5 (16·9–39·5)	30·3 (19·9–41·9)	33 (22·5–44·7)	10·1 (3·33–22·4)	8·74 (3·51–15·8)
Low temperature	37 (33·4–40·9)	37·9 (34·8–41·5)	38·6 (35·5–42·2)	2·46 (-0·336–4·91)	1·86 (1·27–2·51)	37 (33·4–40·9)	37·9 (34·8–41·5)	38·6 (35·5–42·2)	2·46 (-0·336–4·91)	1·86 (1·27–2·51)
Kidney dysfunction	19 (13·4–26·1)	19·9 (14·2–27)	20·1 (14·4–27·1)	4·8 (1·2–9·8)	0·843 (-3·1–4·82)	21·7 (15·4–29·2)	22·9 (16·2–30·7)	23·6 (17·2–31·4)	5·43 (1·5–10·8)	3·45 (-0·736–8·11)

Bullying victimization	5·94 (2·44–12·2)	8·86 (3·54–17·7)	8·74 (3·39–18·1)	49·2 (29·8–71·3)	-1·39 (-11·3–4·38)	--	--	--	--	--
High LDL cholesterol	66·2 (61·5–70·8)	33·7 (30–37·1)	30 (26·5–33·7)	-49·1 (-54·2–-43·9)	-10·8 (-15·9–-5·99)	64·4 (60·9–68·3)	33·9 (30·6–37·3)	31·2 (27·6–34·9)	-47·4 (-52·1–-42·8)	-7·86 (-12·3–-3·72)

Supplementary Results Table 1. Vermont age-standardised Summary Exposure Values (SEVs) for most-detailed risk factors and percentage change, by sex, 1990, 2010, and 2019. Data in parentheses are 95% uncertainty intervals.

	Male					Female				
	1990	2010	2019	Percentage change 1990-2010	Percentage change 2010-2019	1990	2010	2019	Percentage change 1990-2010	Percentage change 2010-2019
Unsafe sanitation	4·79 (2·23–8·5)	2·61 (1·14–5·07)	2·06 (0·924–3·9)	-45·5 (-79·2–33·2)	-21·2 (-61·1–51·4)	4·79 (2·23–8·5)	2·61 (1·14–5·07)	2·06 (0·924–3·9)	-45·5 (-79·2–33·2)	-21·2 (-61·1–51·4)
Ambient particulate matter pollution	4·99 (0·379–12·4)	2·86 (0·667–5·65)	1·42 (0·128–3·77)	-42·6 (-84·8–288)	-50·3 (-83·8—33·2)	5 (0·43–12·3)	2·88 (0·69–5·67)	1·44 (0·15–3·78)	-42·4 (-84·5–254)	-49·9 (-82·7—33·1)
Household air pollution from solid fuels	0·0579 (0·00416–0·208)	0·0293 (0·00246–0·118)	0·0151 (0·000491–0·0724)	-49·4 (-88–109)	-48·3 (-88·3—0·645)	0·0769 (0·00657–0·277)	0·0391 (0·00339–0·151)	0·0203 (0·000741–0·0948)	-49·2 (-87·8–101)	-48 (-87·3–1·32)
Ambient ozone pollution	54·5 (22·7–92·9)	37·3 (14·3–66·3)	21·9 (7·48–40·4)	-31·6 (-46·6—18·6)	-41·4 (-58·3—23·9)	--	--	--	--	--
Residential radon	24·4 (3·99–72·7)	24·4 (3·99–72·7)	24·4 (3·99–72·7)	0 (0–0)	0 (0–0)	24·4 (3·99–72·7)	24·4 (3·99–72·7)	24·4 (3·99–72·7)	0 (0–0)	0 (0–0)
Lead exposure	19·4 (0·187–46·9)	11·9 (0·125–33·4)	8·53 (0·045–26·8)	-38·5 (-70–0·586)	-28·5 (-71—19·3)	12·7 (6·05e-12–37·9)	8·73 (4·4e-12–29·4)	6·64 (3·03e-12–24·3)	-31·4 (-100–60)	-24 (-100–21·3)
Child underweight	0·941 (0·532–1·4)	0·94 (0·514–1·4)	0·915 (0·494–1·38)	-0·132 (-17·3–20·6)	-2·6 (-15·4–11·9)	0·798 (0·436–1·21)	0·829 (0·438–1·26)	0·847 (0·451–1·29)	3·93 (-17·6–32·2)	2·1 (-12·3–18·5)
Iron deficiency	--	--	--	--	--	11·8 (9·75–13·9)	11·7 (9·81–13·7)	11·8 (9·92–14·1)	-0·848 (-12·2–11·4)	1·41 (-8·12–11·5)
Vitamin A deficiency	0·68 (0·186–1·85)	0·909 (0·234–2·55)	0·645 (0·169–1·9)	33·8 (-65·5–508)	-29·1 (-69·2–75·8)	1·42 (0·533–2·99)	1·43 (0·561–2·84)	1·2 (0·439–2·55)	0·202 (-62·5–177)	-15·8 (-53·9–60·7)
Zinc deficiency	2·37 (0–7·57)	1·18 (0–3·73)	1·15 (0–3·73)	-50·1 (-100–NA)	-2·5 (-27·5–NA)	2·44 (0–7·91)	1·19 (0–3·78)	1·14 (0–3·7)	-51·3 (-100–NA)	-4·39 (-31·3–NA)
Smoking	27·6 (24·7–30·8)	20·3 (18·3–22·3)	17·7 (15·9–19·6)	-26·6 (-32—20·9)	-12·7 (-15·8—9·06)	19·3 (16·5–22·3)	16·5 (14·7–18·4)	13·7 (12·2–15·4)	-14·6 (-24—3·63)	-16·6 (-20·4—12·3)
Secondhand smoke	38·5 (36·1–41)	23·8 (22·2–25·5)	23·8 (21·3–26·6)	-38·1 (-43·6—32)	-0·0418 (-9·26–9·41)	36 (34–38)	22·7 (21·5–23·8)	22·7 (20·7–25)	-37 (-41·7—31·8)	0·385 (-7·38–8·95)
Alcohol use	15·9 (9·81–22·7)	16·1 (9·44–23·5)	19·1 (12·1–27·4)	1·71 (-29·5–45)	18·5 (-5·87–55·8)	11·1 (6·62–16·5)	13·6 (8·27–19·4)	13·8 (8·65–20)	22 (-14·8–82)	1·68 (-19·3–30·7)
Drug use	0·53 (0·309–0·919)	0·91 (0·643–1·27)	1·33 (0·991–1·77)	71·7 (27·2–133)	46·6 (26·5–71·4)	0·448 (0·295–0·664)	0·712 (0·523–0·955)	0·975 (0·749–1·26)	59 (33·2–95·9)	37 (17·7–57·2)
High fasting plasma glucose	12·4 (10·3–15·2)	15·9 (13·7–18·7)	17·4 (14·9–20·4)	28·4 (6·61–56·8)	9·47 (-5·08–26·8)	9·54 (7·94–11·6)	11·7 (9·88–13·9)	13·4 (11–16·1)	22·9 (0·384–50·8)	14 (-2·44–34·7)
High systolic blood pressure	24·9 (20–30·5)	15·7 (13·6–17·8)	19·5 (14·8–24·9)	-37 (-49·5—21)	24·1 (-2·5–55)	23·3 (17·7–29·8)	12·6 (11–14·1)	17·4 (12·7–23·1)	-45·9 (-58·7—28·7)	38·4 (1·86–85·2)
High body-mass index	27 (19·1–36·9)	41 (31·1–52·6)	44·3 (33–55·9)	52·1 (34·2–77·4)	7·86 (1·7–15·2)	32·1 (23·7–41·8)	42·9 (34·9–51·6)	45·6 (36·2–54·8)	33·6 (17·3–55·9)	6·33 (-0·604–13·5)
Low bone mineral density	12·2 (7·25–19)	9·31 (4·93–15·5)	11·7 (6·78–18·4)	-23·8 (-44·8—2·53)	25·5 (-2·17–72·8)	16·1 (10·4–23·2)	16·7 (11–23·8)	19 (13·1–26·5)	3·88 (-18·4–29·3)	13·9 (-8·91–44·1)
Diet low in fruits	52·5 (40·8–65·5)	48·7 (37·3–62·1)	49·3 (36·7–62·5)	-7·25 (-18·8–2·74)	1·08 (-10·1–12·9)	53·2 (40·9–66·8)	50·1 (38·7–62·9)	50·3 (37·8–63·3)	-5·89 (-16·9–5·51)	0·453 (-10·1–11)
Diet low in vegetables	34·3 (17·4–57·1)	48·4 (28·9–70·2)	45·9 (27–66·3)	41·3 (13·4–103)	-5·09 (-17·5–4·27)	35·8 (18·8–57·5)	48·4 (28·2–70·1)	46·6 (27·4–68·2)	35·4 (10·7–84·3)	-3·85 (-14·3–4·82)
Diet low in whole grains	78·2 (67·6–88·6)	80·1 (70·5–89·5)	80 (69·7–89·4)	2·39 (-1·53–7·58)	-0·0772 (-2·79–2·66)	--	--	--	--	--
Diet low in nuts and seeds	28·6 (8·36–56·7)	25·1 (8·73–55·3)	20·4 (6·48–53·7)	-12·2 (-45·5–57·9)	-18·9 (-45·3–8·69)	24·6 (6·33–55·3)	22 (6·52–54)	19 (5·58–53·4)	-10·6 (-45·5–67·5)	-13·7 (-41·3–16·7)
Diet low in milk	49·4 (25·8–70)	42·5 (18·5–63·6)	41·2 (18·4–62·4)	-13·9 (-34—1·62)	-3·04 (-15·8–12·3)	49·1 (25·2–70·2)	42·5 (19·8–62·5)	41·5 (19·1–61·7)	-13·3 (-32·6–0·179)	-2·41 (-14·5–10·3)
Diet high in red meat	77·1 (62·5–88·3)	76·6 (62·3–87·7)	76·5 (63–87·9)	-0·7 (-14·5–18·4)	-0·00986 (-10·8–12·9)	78 (64·6–88·4)	75·2 (61·7–86·2)	75·3 (60·8–87·4)	-3·69 (-18·6–13·4)	0·176 (-11–13)
Diet high in processed meat	78 (53·5–97·7)	86·3 (67·2–99·7)	86·2 (67–99·6)	10·6 (1·17–31·6)	-0·15 (-4·54–4·94)	79·1 (56–97·9)	89·2 (74·2–99·8)	87·9 (71·6–99·7)	12·7 (1·56–34·1)	-1·47 (-6·7–2·18)
Diet high in sugar-sweetened beverages	45·7 (26·5–63·8)	63·4 (43·9–79·7)	63·9 (42·9–82·1)	38·6 (-2·92–122)	0·845 (-19·4–24·4)	49·4 (29·4–68·1)	68·8 (49–84·1)	67·9 (48·9–84·4)	39·4 (-0·255–124)	-1·29 (-21·1–21·7)
Diet low in fiber	44 (28–60·3)	29·4 (17–43·2)	26·4 (14·8–40·1)	-33·2 (-50—16·4)	-10 (-28–8·97)	44·8 (28·7–60·4)	34·7 (21·6–49·4)	29·6 (16·7–44·4)	-22·4 (-38·3—5·69)	-14·8 (-32·1–1·05)
Diet low in seafood omega-3 fatty acids	84·4 (67·1–100)	79·2 (60·6–99·1)	78·6 (59·7–99·2)	-6·23 (-21·2–8·53)	-0·727 (-16·1–16·5)	86·9 (71·9–100)	83·1 (66·8–99·7)	82·2 (65·1–99·8)	-4·36 (-17·2–5·69)	-1·16 (-13·5–11·3)
Diet low in polyunsaturated fatty acids	14·5 (4·23–41·5)	7·12 (2·08–25·7)	6·81 (1·89–26·2)	-50·8 (-77·4—7·27)	-4·35 (-26·9–15·9)	16·3 (4·6–44·1)	7·31 (2·12–26·9)	6·86 (1·96–24·6)	-55·1 (-80·1—8·25)	-6·19 (-28·7–11·7)
Diet high in trans fatty acids	89·4 (81·2–100)	87·8 (79·2–100)	87·4 (78·1–100)	-1·79 (-10·1–6·25)	-0·506 (-9·26–8·57)	88·4 (80·9–100)	86·6 (77·8–100)	86·2 (77·4–100)	-1·99 (-9·83–6·03)	-0·486 (-8·44–7·69)
Diet high in sodium	18·2 (4·45–41·9)	39·6 (12·4–63·9)	38·9 (10·9–63·4)	117 (38·6–432)	-1·77 (-25·3–27·3)	21·1 (4·94–44·9)	21·2 (4·96–44·8)	21·7 (5·13–45·5)	0·463 (-31·4–41·7)	2·33 (-17·6–32·1)
Low physical activity	1·9 (0·67–4·36)	1·41 (0·557–3·78)	1·4 (0·466–3·88)	-25·6 (-54·7–7·37)	-1·03 (-27·6–26·7)	4·88 (2·33–9·39)	4·54 (2·34–8·49)	4·64 (2·3–9·08)	-7·11 (-31·1–28·1)	2·32 (-11·1–17·4)
Occupational asthmagens	24·9 (20·8–30·3)	23·2 (19·5–27·8)	24 (19·7–29·3)	-7·15 (-19–6·33)	3·67 (-5·93–16·1)	12·2 (9·96–14·7)	12·6 (10·4–15·1)	12·6 (10·3–15·3)	2·81 (-12–18·8)	0·0833 (-10·4–11·8)
Occupational particulate matter, gases, and fumes	10·5 (7·41–15)	10·1 (7·28–14·4)	9·79 (7·13–13·7)	-4·12 (-9·43–1·88)	-2·69 (-6·05–0·66)	5·68 (4–8·32)	5·54 (4·04–7·98)	5·38 (3·91–7·7)	-2·4 (-11·3–7·01)	-2·91 (-8·25–2·82)
Occupational noise	9·26 (8·47–10·2)	9·1 (8·47–9·86)	8·92 (8·37–9·66)	-1·72 (-5·53–2·61)	-2·06 (-4·09–0·0467)	6·46 (5·75–7·3)	6·91 (6·39–7·55)	6·97 (6·51–7·54)	7·05 (1·13–14)	0·746 (-2·12–3·82)
Occupational injuries	--	--	--	--	--	--	--	--	--	--
Occupational ergonomic factors	14·2 (11·6–17)	12·6 (10·6–15)	13·1 (10·8–15·6)	-10·9 (-23·1–2·91)	3·59 (-6·02–14·5)	8·63 (6·79–11·1)	8·66 (7·06–10·6)	9·26 (7·28–11·7)	0·287 (-16·9–19·9)	6·93 (-7·39–26·1)
Childhood sexual abuse	6·89 (4·53–10·1)	6·5 (5·17–7·93)	6·41 (5·11–7·82)	-5·75 (-26·3–22)	-1·28 (-7·07–6·37)	17·2 (12·2–24·2)	14·4 (11·8–18·2)	14·4 (11·8–17·9)	-16·1 (-30·9–2·27)	0·12 (-5·46–6·62)
Intimate partner violence	--	--	--	--	--	20·2 (11–28·9)	20·5 (11·2–28·4)	21·9 (12·1–30·9)	1·7 (-8·71–16·4)	6·74 (-7·03–28·2)
Non-exclusive breastfeeding	26·2 (21·8–30·8)	24·9 (21·2–28·9)	24·9 (20·5–29·8)	-4·7 (-19·6–13·1)	0·0561 (-13·7–15)	26·2 (21·8–30·8)	24·9 (21·2–28·9)	24·9 (20·5–29·8)	-4·69 (-19·6–13·2)	0·0347 (-13·7–15)
Discontinued breastfeeding	27 (26–27·9)	25 (23·6–26·2)	25·6 (24·2–26·9)	-7·34 (-13—1·56)	2·4 (-3·68–8·71)	27·1 (26·1–28)	24·8 (23·4–26)	25·4 (24·1–26·7)	-8·54 (-14·2—2·84)	2·76 (-3·37–9·12)
Diet low in calcium	16·8 (8·23–30·7)	13·2 (6·46–24·2)	12·4 (6–23)	-21·2 (-32·4—9·14)	-6·11 (-15·8–1·96)	12·2 (5·32–23·4)	10·2 (4·49–18·9)	9·73 (4·25–18·2)	-16·4 (-30·3—1·24)	-4·66 (-15·1–5·43)
Occupational exposure to asbestos	9 (7·84–10·3)	7·38 (6·57–8·23)	6·97 (5·34–8·8)	-17·9 (-30·9—2·35)	-5·63 (-28·3–21·6)	1·48 (1·07–1·93)	1·29 (0·914–1·75)	1·36 (0·882–1·94)	-12·7 (-30·6–7·4)	5·32 (-21·1–35·6)
Occupational exposure to arsenic	0·568 (0–1·35)	0·59 (0–1·4)	0·614 (0–1·43)	3·78 (-12–NA)	4·09 (-7·01–NA)	0·512 (0–1·22)	0·531 (0–1·29)	0·538 (0–1·29)	3·79 (-14·3–NA)	1·34 (-10·8–NA)
Occupational exposure to benzene	0·893 (0·0507–2·56)	0·907 (0·0543–2·57)	0·931 (0·0572–2·61)	1·62 (-7·03–13·4)	2·61 (-5·37–13·2)	1·31 (0·079–3·62)	1·51 (0·0927–4·38)	1·55 (0·0911–4·27)	15 (3·3–34·4)	2·86 (-5·44–14·3)
Occupational exposure to beryllium	0·0254 (0·0238–0·0271)	0·0263 (0·0255–0·0272)	0·0274 (0·0259–0·0291)	3·74 (-2·71–10·8)	4·01 (-1·83–10·3)	0·0286 (0·0264–0·031)	0·031 (0·0299–0·0319)	0·0317 (0·0297–0·0338)	8·26 (-0·673–18·3)	2·47 (-3·72–8·51)
Occupational exposure to cadmium	0·0661 (0·0596–0·073)	0·0689 (0·062–0·0761)	0·0716 (0·063–0·0806)	4·3 (-9·66–19·4)	3·91 (-6·54–15·4)	0·0613 (0·0543–0·0696)	0·064 (0·058–0·0714)	0·0649 (0·0566–0·0745)	4·42 (-11·6–22·4)	1·39 (-9·43–13·6)
Occupational exposure to chromium	0·157 (0·143–0·172)	0·167 (0·153–0·182)	0·173 (0·156–0·192)	6·13 (-6·06–19·2)	3·75 (-5·23–13·8)	--	--	--	--	--
Occupational exposure to diesel engine exhaust	0·74 (0·684–0·802)	0·841 (0·788–0·904)	0·873 (0·801–0·954)	13·6 (3·04–26·4)	3·75 (-3·78–12·1)	0·68 (0·603–0·757)	0·762 (0·695–0·841)	0·761 (0·679–0·854)	12·2 (-2·81–28·8)	-0·162 (-9·86–10·6)
Occupational exposure to formaldehyde	0·331 (0·296–0·367)	0·336 (0·297–0·373)	0·345 (0·301–0·39)	1·5 (-12·9–17·3)	2·71 (-7·86–14·4)	0·301 (0·268–0·339)	0·317 (0·287–0·351)	0·321 (0·282–0·365)	5·26 (-10·5–22·9)	1·3 (-8·8–12·4)
Occupational exposure to nickel	0·474 (0–2·08)	0·475 (0–2·03)	0·495 (0–2·04)	0·18 (-14·4–NA)	4·15 (-7·08–NA)	0·405 (0–1·84)	0·402 (0–1·77)	0·409 (0–1·81)	-0·734 (-18·1–NA)	1·65 (-11–NA)
Occupational exposure to polycyclic aromatic hydrocarbons	0·313 (0·287–0·341)	0·332 (0·307–0·36)	0·344 (0·311–0·38)	6·02 (-5·23–18·3)	3·54 (-5·15–13·3)	0·328 (0·297–0·363)	0·362 (0·339–0·388)	0·367 (0·333–0·403)	10·3 (-2·65–24·3)	1·42 (-6·72–10·1)
Occupational exposure to silica	4·25 (0·602–13·3)	4·19 (0·585–13·7)	4·4 (0·626–13·7)	-1·51 (-15·8–15·6)	5·13 (-6·06–19)	3·36 (0·46–10·6)	3·26 (0·449–11·1)	3·29 (0·449–10·6)	-3·03 (-21–21·9)	0·97 (-13–18·7)
Occupational exposure to sulfuric acid	1·07 (0·227–3·36)	1·22 (0·257–3·79)	1·25 (0·266–4·05)	13·8 (-1·23–30·5)	2·15 (-8·44–13·6)	0·993 (0·211–3·18)	1·22 (0·257–3·87)	1·21 (0·254–3·73)	23·2 (-0·231–50·4)	-1 (-14·2–15·1)
Unsafe sex	--	--	--	--	--	--	--	--	--	--
Occupational exposure to trichloroethylene	0·0909 (0·0848–0·0979)	0·0989 (0·0939–0·105)	0·103 (0·0951–0·111)	8·82 (-0·12–18·9)	3·72 (-3·4–11·6)	0·0877 (0·0794–0·0967)	0·0953 (0·0893–0·102)	0·0962 (0·0871–0·106)	8·59 (-3·7–22·3)	0·966 (-7·01–9·82)
No access to handwashing facility	3·29 (3·04–3·54)	3·08 (2·85–3·34)	3·04 (2·81–3·31)	-6·38 (-14·3–2·64)	-1·05 (-6·9–4·64)	3·29 (3·04–3·54)	3·08 (2·85–3·34)	3·04 (2·81–3·31)	-6·38 (-14·3–2·64)	-1·05 (-6·9–4·64)
Child wasting	0·747 (0·494–1·03)	0·724 (0·472–1)	0·723 (0·468–0·991)	-3·07 (-12·5–7·29)	-0·147 (-7·17–8·08)	0·772 (0·512–1·06)	0·759 (0·505–1·03)	0·755 (0·495–1·04)	-1·65 (-11·9–9·77)	-0·576 (-8·59–8·1)
Child stunting	2·34 (1·22–3·46)	2 (1·05–3·08)	1·95 (1·03–2·95)	-14·4 (-33–10·5)	-2·45 (-16·7–14·3)	1·9 (0·932–2·89)	1·74 (0·856–2·67)	1·71 (0·874–2·65)	-8·63 (-27·2–14·9)	-1·61 (-17·1–16·5)
Chewing tobacco	3·36 (2·49–4·63)	3·61 (2·52–5·03)	3·64 (2·51–5·11)	7·48 (-29·1–64·3)	0·938 (-23·3–32·1)	--	--	--	--	--
Diet low in legumes	74·2 (39·1–99·5)	76·8 (44·7–99·6)	76·7 (46·2–99·6)	3·47 (-0·329–19·1)	-0·0848 (-5·23–5·36)	72·6 (34·4–98·8)	75·1 (40·7–99·5)	75·3 (42·2–99·6)	3·42 (-1·45–19·9)	0·243 (-5·24–6·86)
Short gestation	22·9 (18·2–28·7)	20·6 (16·9–25·4)	20·8 (16·7–25·6)	-9·78 (-30·2–16·9)	0·655 (-19·7–26·1)	15·4 (12·8–18·4)	18·6 (15·3–22·5)	18·1 (15·1–21·6)	20·7 (-5·64–56·3)	-2·8 (-21–21·5)
Low birth weight	9·37 (7·6–11·6)	9·43 (7·75–11·4)	9·54 (7·75–11·7)	0·595 (-23·3–30·2)	1·22 (-21–29·6)	8·02 (6·8–9·5)	9·49 (7·93–11·3)	9·39 (7·92–11·1)	18·4 (-7·21–53·4)	-1·04 (-20·9–23·3)
High temperature	21 (10·7–32·5)	25·3 (14·8–36·9)	27 (16–38·8)	20·7 (1·5–57·5)	6·58 (0·951–12·8)	21 (10·7–32·5)	25·3 (14·8–36·9)	27 (16–38·8)	20·7 (1·5–57·5)	6·58 (0·951–12·8)
Low temperature	34·2 (30·3–39·1)	34·3 (30·6–39·2)	36·2 (32·6–41)	0·435 (-0·574–1·65)	5·41 (3·32–7·84)	34·2 (30·3–39·1)	34·3 (30·6–39·2)	36·2 (32·6–41)	0·435 (-0·574–1·65)	5·41 (3·32–7·84)
Kidney dysfunction	18·5 (13·1–25·3)	19 (13·4–25·9)	19 (13·6–25·9)	2·41 (-1·47–7·1)	0·177 (-3·96–4·58)	20·9 (14·7–28·3)	21·5 (15·1–29·3)	22·3 (16–30·1)	3·25 (-1·79–9·1)	3·62 (-1·06–9·14)
Bullying victimization	5·85 (2·44–11·9)	9·02 (3·64–17·9)	8·71 (3·39–17·9)	54·1 (33·5–77·6)	-3·41 (-13·3–2·75)	--	--	--	--	--
High LDL cholesterol	68·1 (63·9–72·5)	34·5 (31–38·2)	30·5 (26·9–34·4)	-49·3 (-54·4—44·1)	-11·4 (-16·3—6·4)	66 (62·3–69·7)	33·9 (30·7–37·5)	31·3 (27·9–34·8)	-48·6 (-53·4—43·8)	-7·67 (-11·8—3·68)

Supplementary Results Table 1. Virginia age-standardised Summary Exposure Values (SEVs) for most-detailed risk factors and percentage change, by sex, 1990, 2010, and 2019. Data in parentheses are 95% uncertainty intervals.										
	Male					Female				
	1990	2010	2019	Percentage change 1990-2010	Percentage change 2010-2019	1990	2010	2019	Percentage change 1990-2010	Percentage change 2010-2019
Unsafe sanitation	5·49 (2·59–10·1)	2·78 (1·25–5·17)	2·3 (1·05–4·28)	-49·4 (-80·2–20)	-17·2 (-57·7–62·1)	--	--	--	--	--
Ambient particulate matter pollution	12·9 (4·78–25·7)	6·8 (4·07–9·88)	3·77 (1·39–6·52)	-47·3 (-73·7–28·4)	-44·5 (-66·9—33)	12·9 (4·79–25·6)	6·8 (4·08–9·88)	3·78 (1·41–6·51)	-47·3 (-73·6–28·5)	-44·4 (-66·5—33)
Household air pollution from solid fuels	0·0839 (0·0195–0·235)	0·0378 (0·00631–0·118)	0·0237 (0·00294–0·0832)	-54·9 (-82·4—1·36)	-37·4 (-72·3–15·9)	0·11 (0·0259–0·297)	0·0502 (0·00845–0·161)	0·0315 (0·00394–0·114)	-54·5 (-82·3—1·77)	-37·3 (-72·1–16·2)
Ambient ozone pollution	85·9 (41–100)	68·6 (29·6–100)	43·6 (18–75·7)	-20·1 (-33–0)	-36·5 (-49·2—24·3)	--	--	--	--	--
Residential radon	19·4 (3·04–56·4)	19·4 (3·04–56·4)	19·4 (3·04–56·4)	0 (0–0)	0 (0–0)	19·4 (3·04–56·4)	19·4 (3·04–56·4)	19·4 (3·04–56·4)	0 (0–0)	0 (0–0)
Lead exposure	58·5 (31·5–80·7)	32·7 (13·3–54·8)	22·3 (7·19–41·3)	-44·1 (-61·3—30·4)	-31·8 (-47·1—23·4)	33·2 (4·08–61·6)	18·3 (1·08–42·8)	12·4 (0·341–32·1)	-44·8 (-79·5—28·8)	-32·1 (-68·8—23·8)
Child underweight	0·953 (0·535–1·44)	0·939 (0·504–1·4)	0·92 (0·513–1·41)	-1·48 (-17·6–18)	-2·04 (-14·8–12·5)	0·799 (0·427–1·2)	0·831 (0·458–1·29)	0·852 (0·475–1·29)	3·98 (-16·5–32·8)	2·59 (-12·9–19)
Iron deficiency	--	--	--	--	--	11·9 (9·9–14)	12·1 (10·1–14·2)	12·3 (10·1–14·7)	1·2 (-11–15·5)	1·76 (-7·77–12·5)
Vitamin A deficiency	0·728 (0·189–1·87)	0·949 (0·254–2·64)	0·68 (0·17–1·84)	30·3 (-67·1–527)	-28·3 (-70·1–86·6)	1·48 (0·573–3·02)	1·44 (0·554–2·94)	1·25 (0·476–2·73)	-2·97 (-60·2–190)	-13 (-52·9–60·4)
Zinc deficiency	2·04 (0–6·63)	1·05 (0–3·35)	1·1 (0–3·54)	-48·6 (-100–NA)	4·63 (-15·2–NA)	2·09 (0–6·78)	1·06 (0–3·55)	1·08 (0–3·5)	-49 (-100–NA)	1·45 (-21·2–NA)
Smoking	26·9 (24·2–29·9)	19 (17·1–20·9)	16·7 (15–18·5)	-29·4 (-34·5—24·2)	-11·8 (-15·3—8·23)	18·5 (16–21)	14·7 (13·2–16·3)	12·5 (11·2–14)	-20·5 (-29·3—10·8)	-14·7 (-18·5—10·5)
Secondhand smoke	39·5 (36·9–42·2)	25·8 (24·4–27·4)	25·3 (22·6–28·2)	-34·7 (-40·5—28·1)	-2·16 (-11–6·81)	37·3 (35·2–39·3)	24·4 (23·2–25·6)	23·9 (22–25·9)	-34·6 (-39·6—29·7)	-1·96 (-8·86–5·28)
Alcohol use	14·8 (9·43–20·8)	16·3 (10·3–23·1)	17·2 (10·9–24·5)	10·3 (-18·9–52·4)	5·79 (-15·3–30·1)	10·3 (6·23–15)	12·4 (8·01–17·3)	12·2 (7·66–17·4)	20·5 (-11·8–75·1)	-1·65 (-21–19·9)
Drug use	0·486 (0·251–0·911)	0·759 (0·515–1·12)	1·04 (0·735–1·44)	56·2 (16·6–127)	37·1 (18·6–62·3)	0·41 (0·261–0·661)	0·682 (0·506–0·91)	0·95 (0·729–1·21)	66·3 (32·5–111)	39·3 (21·1–62·9)
High fasting plasma glucose	12·5 (10·4–15·1)	18·4 (16–21·2)	19·4 (16·7–22·3)	46·7 (20·8–76·2)	5·35 (-8·62–21·4)	10·4 (8·66–12·6)	16·6 (14·1–19·3)	17·1 (14·5–20)	59·3 (31·4–95·1)	3·2 (-11·1–22·4)
High systolic blood pressure	25·8 (20·8–30·9)	15·9 (13·9–18)	19·8 (15·6–24·5)	-38·3 (-50·2—23·2)	24 (-0·71–53·5)	23·3 (17·4–30·4)	13·5 (11·9–15·1)	18·3 (13·2–24·3)	-42 (-56·4—22·5)	35·3 (0·864–81·7)
High body-mass index	25·1 (17·6–34·8)	39·6 (29·7–51)	41·1 (30·5–52·5)	58 (37·5–85·9)	3·77 (-2·73–11·1)	31 (23–40·2)	44 (34·5–52·6)	45·2 (35·2–54·5)	41·6 (24·4–63·7)	2·82 (-3·41–10·1)
Low bone mineral density	12·2 (7·21–19·3)	9·42 (5·04–15·4)	11·8 (6·95–18·9)	-22·5 (-42·4—1·87)	24·8 (-2·96–63·9)	16·1 (10·7–22·7)	16·6 (11·2–23·4)	19 (13–26·4)	3·38 (-19·7–31·1)	14·2 (-8·99–44·1)
Diet low in fruits	52·6 (40·7–66·3)	45·6 (34·4–58)	46·8 (34·5–60·6)	-13·4 (-25—2·74)	2·8 (-9·35–17·1)	54·4 (42·6–67·2)	48 (35·9–61·6)	48 (35·7–62·1)	-11·7 (-22·7—1·66)	0·0124 (-11·7–13)
Diet low in vegetables	30·1 (14·8–51·4)	34·8 (20·4–52·7)	34·7 (20–52·9)	15·5 (-5·83–65)	-0·338 (-13·9–14)	28·6 (13·5–49·6)	26·3 (14–42·2)	26·8 (13·9–43)	-8·2 (-26·4–26·2)	1·99 (-14·7–21·1)
Diet low in whole grains	79·2 (69·5–88)	81·1 (72·2–89·1)	81·2 (71·7–89·2)	2·33 (-1·36–6·89)	0·127 (-2·47–2·76)	75·1 (62·8–86·4)	77·8 (67·3–88·4)	82·1 (65·8–99·7)	3·71 (-0·806–10·1)	5·42 (-11·8–26·1)
Diet low in nuts and seeds	25·7 (6·92–55·5)	24·7 (8·61–54·8)	20·4 (6·3–53·5)	-3·71 (-41·3–76)	-17·7 (-45·1–9·25)	21·8 (5·59–54·3)	21·1 (6·67–53·7)	18·6 (5·69–53)	-3·16 (-41–90·9)	-12·2 (-41·8–18·9)
Diet low in milk	46·7 (24·4–67·4)	40·3 (18·3–59·4)	40 (18–60·2)	-13·7 (-34–0·303)	-0·723 (-13·7–15·3)	46·1 (23·3–66·3)	40·2 (18–59·6)	40·3 (18·5–60·1)	-12·7 (-31·8–1·02)	0·261 (-13·2–16·7)
Diet high in red meat	81·4 (71·5–89·3)	83·1 (72·3–91·3)	82 (69·7–91·7)	2·08 (-9·68–15·5)	-1·4 (-10·3–7·69)	76·7 (65–86)	75·4 (62·4–86)	74·7 (61·2–86·5)	-1·67 (-15·8–14·3)	-0·962 (-12·7–11·3)
Diet high in processed meat	78·3 (54–98)	85·8 (66·4–99·7)	85·6 (65·8–99·5)	9·52 (1–28·5)	-0·245 (-5·18–4·23)	79 (56·1–98·1)	87·5 (70·3–99·5)	86·1 (67·6–99·5)	10·8 (1·14–33)	-1·66 (-7–1·92)
Diet high in sugar-sweetened beverages	47·9 (30·3–67·4)	66 (45·8–82·2)	65·4 (45–82·7)	37·8 (-2·66–113)	-0·967 (-19·9–21·4)	52·5 (32·6–71·4)	71·9 (51·8–87·2)	69·7 (50–86·7)	37 (-0·899–112)	-3·13 (-19·8–18·6)
Diet low in fiber	41·5 (26·1–56·6)	27·8 (16·5–40·7)	25·9 (13·9–39·2)	-33 (-49·5—15·9)	-7·01 (-26·1–11·9)	41·8 (25·1–58·3)	32·8 (19·3–47)	28·6 (16–42·9)	-21·4 (-37·8—4·6)	-12·8 (-31·1–2·4)
Diet low in seafood omega-3 fatty acids	82·3 (65·2–99·5)	77 (58·3–99·3)	77·2 (58·7–99·1)	-6·39 (-21·1–7·54)	0·309 (-15·7–18·8)	84·9 (69·2–99·8)	81·2 (63–99·2)	81·5 (64–99·4)	-4·28 (-17·9–7·79)	0·304 (-13·5–15·8)
Diet low in polyunsaturated fatty acids	12·9 (3·78–38·8)	6·66 (1·89–26)	6·57 (1·83–25·9)	-48·3 (-76·5—6·84)	-1·36 (-22–26·7)	14·4 (4·35–42·1)	6·81 (1·96–26)	6·64 (1·9–24·6)	-52·6 (-79·2—8·14)	-2·53 (-25·5–20·8)
Diet high in trans fatty acids	89·4 (81·2–100)	87·9 (79–100)	87·4 (78·5–100)	-1·77 (-9·76–6·11)	-0·545 (-8·8–8·11)	88·3 (80·5–100)	86·7 (78·1–100)	86·2 (77·1–100)	-1·82 (-9·42–6·19)	-0·658 (-8·69–7·37)
Diet high in sodium	18·2 (4·66–40·6)	39·6 (11·5–63·9)	38·9 (10·6–64·8)	118 (38·3–406)	-1·82 (-27·6–29·2)	20·9 (4·83–45·3)	21·1 (4·93–45·3)	21·8 (5·06–46·3)	1·23 (-29·3–48·7)	3·29 (-15·3–30·8)
Low physical activity	2·03 (0·726–4·54)	1·71 (0·701–4·01)	1·73 (0·595–4·06)	-15·6 (-46·3–28·8)	1·38 (-27·4–36·2)	4·99 (2·45–9·7)	4·71 (2·52–8·73)	4·71 (2·33–9·16)	-5·56 (-30·4–27·5)	-0·105 (-14·4–14·8)
Occupational asthmagens	20·7 (17·4–24·6)	19·6 (16·5–23·1)	20·2 (16·6–24·1)	-5·5 (-15·6–6·52)	3·2 (-6·21–13·1)	11 (9–13·4)	11·2 (9·24–13·4)	11·2 (9·15–13·7)	1·21 (-13·8–19)	0·558 (-10·9–12·1)
Occupational particulate matter, gases, and fumes	8·47 (6·02–12·3)	8·12 (5·85–11·8)	7·95 (5·74–11·4)	-4·08 (-10–1·8)	-2·21 (-6·17–1·49)	4·91 (3·43–7·18)	4·8 (3·42–6·88)	4·65 (3·27–6·79)	-2·21 (-10·8–7·47)	-3·29 (-8·49–2·16)
Occupational noise	7·82 (7·15–8·63)	7·74 (7·2–8·39)	7·61 (7·1–8·23)	-1·04 (-5·19–3·37)	-1·69 (-3·88–0·656)	5·77 (5·1–6·47)	6·17 (5·71–6·72)	6·21 (5·83–6·76)	6·85 (0·303–14·4)	0·698 (-2·23–3·86)
Occupational injuries	--	--	--	--	--	--	--	--	--	--
Occupational ergonomic factors	12·7 (10·3–15·4)	11·1 (9·46–13·4)	11·6 (9·6–14)	-12 (-21·6—1·69)	3·91 (-4·18–13·2)	7·22 (5·71–9·12)	7·26 (5·84–8·98)	7·63 (6·01–9·72)	0·587 (-16–19·3)	5 (-7·3–19·9)
Childhood sexual abuse	7·4 (5·05–10·5)	6·97 (4·6–9·93)	6·85 (4·54–9·67)	-5·83 (-12·2–5·83)	-1·68 (-8·51–5·53)	12·3 (8·94–17·3)	17·4 (12·9–24)	17·8 (13·1–24·7)	41·9 (30·7–54·8)	2·17 (-5–11)
Intimate partner violence	--	--	--	--	--	21·8 (11·6–29·9)	22·2 (12·1–30·9)	23·2 (13·3–32·3)	1·67 (-6·57–13·1)	4·71 (-7·13–23·8)
Non-exclusive breastfeeding	26·2 (21·6–31·1)	25 (21·4–29·1)	25 (20·2–29·6)	-4·62 (-19·4–12·2)	-0·0702 (-14·4–14·8)	26·2 (21·6–31·1)	25 (21·4–29·1)	25 (20·2–29·6)	-4·61 (-19·4–12·2)	-0·0793 (-14·4–14·8)
Discontinued breastfeeding	27·6 (26·7–28·5)	25·3 (24–26·5)	25·8 (24·3–27)	-8·23 (-13·5—3·1)	1·7 (-4·14–7·41)	27·6 (26·7–28·5)	25·3 (24–26·5)	25·8 (24·3–27)	-8·26 (-13·5—3·12)	1·62 (-4·22–7·32)
Diet low in calcium	15·1 (7·25–28·1)	12·3 (6·09–22·7)	12·1 (5·86–22·2)	-18·1 (-29—5·18)	-2·03 (-12·1–7·12)	10·3 (4·47–19·4)	9·11 (4·15–16·6)	9·21 (3·96–17·3)	-11·5 (-26·5–6·04)	1·18 (-9·51–12·2)
Occupational exposure to asbestos	11·3 (10·2–12·5)	9·22 (8·54–9·84)	7·94 (5·64–10·1)	-18·7 (-28·1—7·82)	-13·9 (-37·7–11·6)	1·62 (1·09–2·02)	1·49 (1·02–1·84)	1·46 (0·925–2·08)	-7·81 (-23·3–9·36)	-2·35 (-29·5–31·1)
Occupational exposure to arsenic	0·474 (0–1·13)	0·494 (0–1·18)	0·521 (0–1·25)	4·32 (-11–NA)	5·49 (-6·53–NA)	0·459 (0–1·09)	0·474 (0–1·13)	0·483 (0–1·16)	3·35 (-13·9–NA)	1·82 (-9·83–NA)
Occupational exposure to benzene	0·829 (0·0512–2·38)	0·856 (0·0496–2·44)	0·877 (0·0566–2·52)	3·3 (-6·08–16·8)	2·34 (-5·34–12·8)	1·22 (0·0694–3·39)	1·39 (0·0804–3·91)	1·44 (0·087–4·13)	14·4 (2·94–34·7)	3·66 (-4·53–16·7)
Occupational exposure to beryllium	0·0241 (0·0227–0·0256)	0·0245 (0·024–0·025)	0·0256 (0·0242–0·027)	1·62 (-4·68–8·07)	4·59 (-1·02–10·2)	0·0269 (0·0246–0·029)	0·0287 (0·0282–0·0294)	0·0296 (0·0277–0·0315)	6·74 (-0·921–17·1)	3·06 (-3·26–9·88)
Occupational exposure to cadmium	0·0559 (0·0503–0·0621)	0·0584 (0·0531–0·0642)	0·0615 (0·0539–0·0691)	4·62 (-9·11–20)	5·19 (-5·61–16·6)	0·0552 (0·0488–0·062)	0·0575 (0·0521–0·0647)	0·0586 (0·0519–0·067)	4·24 (-10·9–24)	1·82 (-8·57–14·1)
Occupational exposure to chromium	0·133 (0·121–0·147)	0·142 (0·131–0·154)	0·149 (0·133–0·166)	6·97 (-5·62–20·6)	4·88 (-4·83–15)	--	--	--	--	--
Occupational exposure to diesel engine exhaust	0·689 (0·635–0·745)	0·762 (0·708–0·814)	0·794 (0·726–0·868)	10·6 (-0·641–21·8)	4·24 (-3·76–12·7)	0·639 (0·57–0·714)	0·701 (0·64–0·77)	0·701 (0·621–0·789)	9·66 (-5·2–26)	0·088 (-9·49–10·7)
Occupational exposure to formaldehyde	0·274 (0·244–0·307)	0·282 (0·252–0·312)	0·293 (0·253–0·332)	3·03 (-11·2–20·2)	3·91 (-6·92–15·1)	0·269 (0·239–0·301)	0·284 (0·26–0·317)	0·289 (0·257–0·328)	5·63 (-8·81–24·8)	1·91 (-8·23–13·7)
Occupational exposure to nickel	0·4 (0–1·79)	0·397 (0–1·8)	0·419 (0–1·79)	-0·812 (-15·5–NA)	5·5 (-5·58–NA)	0·359 (0–1·55)	0·36 (0–1·54)	0·367 (0–1·57)	0·311 (-16·9–NA)	2·09 (-11·3–NA)
Occupational exposure to polycyclic aromatic hydrocarbons	0·264 (0·24–0·289)	0·283 (0·262–0·305)	0·295 (0·265–0·326)	7·18 (-5·08–20·7)	4·53 (-4·97–13·9)	0·294 (0·263–0·323)	0·324 (0·306–0·349)	0·331 (0·3–0·364)	10·5 (-1·58–26·2)	1·98 (-6·4–11·1)
Occupational exposure to silica	3·63 (0·511–11·6)	3·49 (0·483–11·1)	3·71 (0·518–11·8)	-3·96 (-18·2–12·9)	6·5 (-5·01–21·3)	2·97 (0·41–9·2)	2·84 (0·409–9·11)	2·87 (0·398–9·09)	-4·52 (-21·9–20·6)	1·06 (-12·5–19·3)
Occupational exposure to sulfuric acid	0·858 (0·183–2·72)	0·988 (0·214–3·18)	1·02 (0·212–3·23)	15·1 (-1·41–34·6)	3·02 (-8·38–14·8)	0·843 (0·17–2·76)	1·04 (0·221–3·37)	1·03 (0·215–3·22)	23·7 (0·966–52·1)	-1·67 (-15·1–14·1)
Unsafe sex	--	--	--	--	--	--	--	--	--	--
Occupational exposure to trichloroethylene	0·08 (0·0742–0·0868)	0·0869 (0·0826–0·0919)	0·0909 (0·0839–0·0986)	8·64 (-0·535–19)	4·63 (-3·01–12·5)	0·0807 (0·0728–0·0892)	0·0869 (0·0816–0·0934)	0·0881 (0·0805–0·0969)	7·72 (-3·36–22)	1·39 (-6·76–10·3)
No access to handwashing facility	3·31 (3·06–3·57)	3·08 (2·86–3·33)	3·06 (2·81–3·32)	-6·79 (-15·4–2·33)	-0·875 (-6·24–5·32)	3·31 (3·06–3·57)	3·08 (2·86–3·33)	3·06 (2·81–3·32)	-6·79 (-15·4–2·33)	-0·875 (-6·24–5·32)
Child wasting	0·747 (0·493–1·03)	0·724 (0·474–0·977)	0·723 (0·472–0·992)	-3·15 (-12–7·71)	-0·161 (-7·21–8·08)	0·77 (0·511–1·06)	0·761 (0·509–1·03)	0·756 (0·502–1·03)	-1·24 (-10·7–10·2)	-0·592 (-7·92–7·39)
Child stunting	2·37 (1·26–3·49)	2·02 (1·03–2·99)	1·97 (0·998–2·93)	-15 (-32·6–7·99)	-2·29 (-15·8–14·1)	1·93 (0·947–2·94)	1·74 (0·864–2·63)	1·73 (0·861–2·66)	-9·58 (-29·2–16·3)	-0·643 (-15·7–17·5)
Chewing tobacco	4·29 (3·35–5·38)	4·28 (3·19–5·49)	4·08 (2·95–5·46)	-0·17 (-29·3–40·4)	-4·65 (-24·6–19·6)	0·297 (0·191–0·47)	0·336 (0·219–0·52)	0·349 (0·223–0·538)	13·1 (-32·7–90·1)	3·71 (-28·6–44·7)
Diet low in legumes	73·3 (35·5–98·8)	76·1 (42·6–99·7)	76·3 (43·6–99·6)	3·79 (-0·47–23)	0·251 (-4·6–6·44)	71·7 (33·6–98·5)	74·4 (39·2–99·3)	74·9 (41·2–99·4)	3·83 (-0·475–23·6)	0·667 (-4·5–8·71)
Short gestation	32·7 (26·7–40)	30·2 (25·1–36·1)	29 (23·8–35·3)	-7·65 (-27·4–16·5)	-4·08 (-23·5–19·2)	27·6 (22·8–32·8)	28·1 (23·8–32·9)	25 (20·9–29·7)	1·88 (-17·7–28·9)	-11·2 (-26·9–6·87)
Low birth weight	12·6 (10·4–15·4)	12·9 (10·8–15·4)	12·8 (10·5–15·6)	2·25 (-20·7–32·3)	-0·259 (-21–24·6)	12·8 (10·7–15·2)	14·2 (12·3–16·5)	13·1 (11·1–15·5)	10·9 (-11·1–38·9)	-7·84 (-23·7–11·7)
High temperature	23·3 (15·3–33·1)	28·2 (19·4–38·3)	23·6 (15·2–33·7)	21·3 (11–34·6)	-16·4 (-24—10·1)	23·3 (15·3–33·1)	28·2 (19·4–38·3)	23·6 (15·2–33·7)	21·3 (11–34·6)	-16·4 (-24—10·1)
Low temperature	33·1 (28·8–38·5)	36·9 (33–41·7)	35·1 (30·8–40·5)	11·5 (8·02–15·5)	-4·71 (-7·05—2·66)	33·1 (28·8–38·5)	36·9 (33–41·7)	35·1 (30·8–40·5)	11·5 (8·02–15·5)	-4·71 (-7·05—2·66)
Kidney dysfunction	20·3 (14·5–27·4)	21 (15·2–28·1)	21·1 (15·4–28·3)	3·52 (-0·61–8·69)	0·167 (-4·5–4·61)	22·7 (16·3–30·4)	23·7 (17·1–31·7)	24·5 (17·9–32·7)	4·29 (-0·685–10·3)	3·4 (-1·62–9)
Bullying victimization	5·48 (2·25–11·2)	6·61 (2·95–13·1)	6·72 (2·89–13·2)	20·7 (-3·42–61·8)	1·7 (-5·32–7·93)	--	--	--	--	--
High LDL cholesterol	67·1 (62·6–71·4)	34·2 (30·6–37·7)	30·4 (26·9–34·1)	-49 (-54·3—43·6)	-11·2 (-16·3—6·29)	66·1 (62·6–69·7)	34 (30·8–37·3)	31·2 (27·8–34·6)	-48·5 (-53·2—44·1)	-8·2 (-12·3—4·15)

Supplementary Results Table 1. Washington age-standardised Summary Exposure Values (SEVs) for most-detailed risk factors and percentage change, by sex, 1990, 2010, and 2019. Data in parentheses are 95% uncertainty intervals.

	Male					Female				
	1990	2010	2019	Percentage change 1990-2010	Percentage change 2010-2019	1990	2010	2019	Percentage change 1990-2010	Percentage change 2010-2019
Unsafe sanitation	5·81 (2·63–10·6)	2·9 (1·32–5·49)	2·33 (1·05–4·41)	-50·1 (-80·2–35·2)	-19·8 (-58·1–52·9)	--	--	--	--	--
Ambient particulate matter pollution	5·43 (0·641–13·3)	3·11 (1·04–5·62)	3·05 (1·05–5·54)	-42·8 (-81·7–191)	-1·71 (-7·32–4·53)	5·43 (0·666–13·3)	3·11 (1·05–5·62)	3·06 (1·07–5·55)	-42·7 (-81·3–188)	-1·73 (-7·15–4·66)
Household air pollution from solid fuels	0·0205 (0·00258–0·0631)	0·0111 (0·00141–0·0418)	0·00889 (0·00106–0·0312)	-45·7 (-84·6–99·8)	-20·1 (-58·2–45·6)	0·0271 (0·00345–0·0867)	0·0148 (0·0019–0·0532)	0·0118 (0·0015–0·0407)	-45·6 (-84·4–97·2)	-20·2 (-58·4–47·3)
Ambient ozone pollution	18·1 (6·19–35)	17·9 (6·38–34·1)	29·6 (11·6–54·1)	-0·986 (-24·3–29·4)	65·4 (32·1–121)	--	--	--	--	--
Residential radon	22·2 (3·28–60·4)	22·2 (3·28–60·4)	22·2 (3·28–60·4)	0 (0–0)	0 (0–0)	22·2 (3·28–60·4)	22·2 (3·28–60·4)	22·2 (3·28–60·4)	0 (0–0)	0 (0–0)
Lead exposure	60·7 (33·8–81·7)	34 (13·8–55·8)	23·3 (7·48–42·1)	-44 (-60·9–-29·8)	-31·6 (-46·8–-23·9)	35 (5·55–63·4)	19 (1·08–42·9)	12·8 (0·388–32·3)	-45·7 (-81·2–-29·2)	-32·6 (-71·2–-24·3)
Child underweight	0·941 (0·526–1·43)	0·942 (0·522–1·44)	0·92 (0·501–1·39)	0·145 (-17·3–22·6)	-2·35 (-16·3–13·1)	0·79 (0·434–1·22)	0·829 (0·45–1·26)	0·848 (0·47–1·3)	4·96 (-16·3–33·2)	2·31 (-12–19·2)
Iron deficiency	--	--	--	--	--	11·2 (9·26–13·3)	11·5 (9·7–13·5)	11·7 (9·64–13·8)	2·83 (-9·28–16·9)	1·39 (-7·56–12·7)
Vitamin A deficiency	0·774 (0·214–2·12)	0·957 (0·261–2·42)	0·661 (0·192–1·73)	23·6 (-68·5–425)	-30·9 (-69·2–61·6)	1·51 (0·565–3·21)	1·49 (0·585–2·95)	1·25 (0·519–2·7)	-1·9 (-62·9–183)	-15·8 (-52·8–63·8)
Zinc deficiency	2·18 (0–7·2)	1·09 (0–3·59)	1·08 (0–3·52)	-49·9 (-100–NA)	-0·685 (-25·2–NA)	2·23 (0–7·24)	1·1 (0–3·63)	1·07 (0–3·42)	-50·4 (-100–NA)	-3·08 (-22·6–NA)
Smoking	26·5 (23·9–29·4)	18·4 (16·5–20·5)	14·9 (13·3–16·7)	-30·6 (-35·6–-24·6)	-18·9 (-22·6–-15·3)	20 (17·3–22·9)	14·9 (13·3–16·6)	12·3 (10·7–13·8)	-25·8 (-33–-16·7)	-17·5 (-22·2–-12·6)
Secondhand smoke	38·6 (36–41·2)	23·2 (21·5–24·8)	23·6 (21·1–26·4)	-39·9 (-45·6–-33·6)	1·67 (-8·13–11·2)	35·1 (33·2–37·2)	21·4 (20·3–22·5)	21·9 (20–23·9)	-38·9 (-43·6–-33·9)	2·05 (-5·64–9·69)
Alcohol use	14·7 (9·4–21·3)	15·9 (9·58–23·4)	16·8 (10·4–24·5)	8·25 (-25·5–55·2)	5·64 (-15·8–34·7)	11 (6·77–16·3)	12·2 (7·4–17·3)	12·2 (7·5–17·7)	10·5 (-21·4–58)	0·161 (-20·7–25·6)
Drug use	0·705 (0·366–1·35)	1·05 (0·707–1·56)	1·34 (0·957–1·88)	48·4 (11·7–110)	28·5 (11·5–49·2)	0·532 (0·3–0·991)	0·88 (0·622–1·24)	1·23 (0·918–1·62)	65·2 (23·4–125)	39·6 (20·7–61·8)
High fasting plasma glucose	12·4 (10·4–15·1)	16·5 (14·2–19·3)	18·2 (15·7–21·3)	32·9 (10·2–60·8)	10 (-5·28–27·9)	9·6 (8·01–11·8)	12·8 (10·7–15·5)	14·5 (12–17·5)	33·5 (9·36–65·3)	13·4 (-4·77–35·9)
High systolic blood pressure	25 (20·1–30·7)	15·7 (13·7–17·9)	19·8 (15·3–24·6)	-37·1 (-49·1–-21·4)	25·6 (-1·08–55·5)	22·7 (16·8–29·6)	12·8 (11·2–14·5)	17·1 (12·2–22·6)	-43·7 (-57·3–-23·9)	34·3 (-3·93–80·4)
High body-mass index	24 (16·6–33·2)	38·9 (28·8–50)	40·9 (30·6–52·7)	62·3 (41·9–93·4)	5·28 (-1·09–13·3)	30·1 (22–39·3)	43·7 (34·7–52·3)	45·3 (35·9–54·5)	45 (26·8–69)	3·82 (-2·08–10·6)
Low bone mineral density	12·1 (7–19·2)	9·18 (4·88–15·4)	11·3 (6·5–18·3)	-24 (-44·1–2·76)	23 (-5·5–63·4)	15·9 (10·4–22·8)	16·3 (10·7–23·1)	18·5 (12·6–25·3)	2·68 (-17–27·8)	13·5 (-11·6–43·1)
Diet low in fruits	53·7 (41·5–66·5)	49·3 (37·5–62·8)	48·7 (36·3–63)	-8·1 (-19·7–2·7)	-1·19 (-11·8–10·2)	55·6 (43·7–68·7)	50·6 (39·4–63·6)	50 (38·2–63·9)	-8·97 (-19·5–1·38)	-1·2 (-12–8·82)
Diet low in vegetables	32·5 (16–54·8)	45·7 (25·9–66·6)	42·2 (23·2–63·2)	40·7 (12·3–97·7)	-7·64 (-19·9–2·18)	32·6 (16·6–54·1)	47·1 (28·2–69·4)	44·2 (25·1–66·2)	44·5 (15·5–101)	-6·06 (-17·9–3·15)
Diet low in whole grains	77·9 (67–88·7)	79·9 (70·1–89·6)	79·8 (69·8–89·8)	2·54 (-1·28–7·84)	-0·148 (-2·75–2·47)	--	--	--	--	--
Diet low in nuts and seeds	26·8 (7·58–55·6)	25·1 (8·12–55·8)	19·4 (5·9–53·7)	-6·53 (-45·2–80·6)	-22·4 (-50·3–1·07)	22·5 (5·72–53·5)	21·5 (6·12–53·8)	17·8 (5·08–52·6)	-4·64 (-42·7–70·8)	-17·2 (-45·9–10·6)
Diet low in milk	47·4 (24·3–66·9)	41 (17·7–60·7)	39·3 (17·6–59·1)	-13·5 (-35–0·833)	-4·19 (-20–10·1)	47 (24·4–67·4)	40·8 (18–60·5)	39·4 (17·1–58·5)	-13·3 (-35·5–1·69)	-3·28 (-17·7–10·5)
Diet high in red meat	78·5 (66·4–89·3)	77·2 (64·2–87·9)	77·5 (64·1–88·8)	-1·67 (-15·3–12·7)	0·492 (-10·2–12·5)	79·3 (66·8–89·3)	76·4 (63·4–87·1)	76·9 (63·3–87·9)	-3·65 (-18·3–11·6)	0·643 (-9·97–13·2)
Diet high in processed meat	78·2 (54·3–97·8)	86·4 (68–99·8)	86·3 (67·7–99·7)	10·5 (1·29–30·4)	-0·107 (-4·72–4·47)	79·7 (56·5–98·2)	89·5 (74·1–99·6)	88·2 (71–99·7)	12·2 (1·28–35·8)	-1·43 (-6·92–2·1)
Diet high in sugar-sweetened beverages	47 (29·1–66·4)	64·7 (46·4–81·9)	65·6 (46·6–82·4)	37·8 (-3·97–112)	1·36 (-17·9–23·9)	51·1 (31·9–70·5)	70·7 (51·8–86·2)	70 (50·9–86·1)	38·4 (0·127–110)	-0·99 (-19·3–19·7)
Diet low in fiber	42·4 (25·8–58·7)	28·6 (16·5–42·4)	25·4 (14·1–38·5)	-32·4 (-48–-15·5)	-11·4 (-29·4–7·85)	42·7 (26·3–59·2)	33·6 (21·1–48·2)	28·1 (16·1–42·5)	-21·1 (-36·7–-2·46)	-16·4 (-31·9–0·0491)
Diet low in seafood omega-3 fatty acids	82·9 (65·8–99·8)	78·1 (60·1–99)	76·8 (57·8–98·5)	-5·86 (-20·2–9·77)	-1·63 (-17·1–15·8)	85·6 (70–99·9)	82·3 (66·3–99·8)	81·4 (64·1–99·4)	-3·78 (-15·4–9·08)	-1·1 (-12·3–11·3)
Diet low in polyunsaturated fatty acids	13·2 (3·84–38·9)	6·86 (1·98–24·4)	6·44 (1·8–24·4)	-48·1 (-77·1–-7·39)	-6·15 (-30·1–11·9)	14·8 (4·24–42·2)	6·98 (2·01–24·6)	6·48 (1·85–24·6)	-52·8 (-79·6–-9·18)	-7·05 (-31·4–10·3)
Diet high in trans fatty acids	89·5 (81·8–100)	87·9 (79·1–100)	87·4 (78·3–100)	-1·78 (-10–5·93)	-0·6 (-8·16–8·07)	88·4 (80·7–100)	86·6 (78·1–100)	86·2 (77·2–100)	-2·12 (-10·1–5·94)	-0·442 (-8·35–8·26)
Diet high in sodium	18·1 (4·59–40·9)	39·7 (12–64·1)	38·9 (12·2–63·2)	119 (41·7–439)	-1·93 (-27–30·4)	20·9 (5·06–45·1)	21·3 (5·03–44·8)	21·7 (5·15–45·8)	1·69 (-29–48·1)	2·06 (-18·4–31)
Low physical activity	2·33 (0·861–4·97)	1·98 (0·781–4·25)	2·3 (0·834–4·93)	-14·9 (-43·8–28·2)	16·1 (-13·6–60·9)	5·02 (2·41–9·89)	4·98 (2·59–9·38)	5·42 (2·77–10·3)	-0·83 (-25·8–36·9)	8·92 (-3·99–22·9)
Occupational asthmagens	21·7 (17·9–26·2)	19·9 (16·8–23·6)	20·6 (17–24·7)	-8·13 (-19·2–3·73)	3·29 (-5·28–13)	10·9 (8·89–13·3)	10·8 (9·02–13·1)	11 (8·87–13·4)	-0·937 (-15·6–15·9)	1·15 (-10·4–13·8)
Occupational particulate matter, gases, and fumes	9·28 (6·63–13·4)	8·7 (6·29–12·5)	8·41 (6·02–11·9)	-6·29 (-11·6–-0·831)	-3·37 (-7·01–-0·0832)	5·01 (3·43–7·44)	4·87 (3·44–7)	4·69 (3·33–6·74)	-2·72 (-12·1–7·63)	-3·67 (-9·05–1·66)
Occupational noise	8·38 (7·65–9·29)	8·1 (7·52–8·84)	7·87 (7·36–8·59)	-3·35 (-7·07–0·544)	-2·82 (-4·93–-0·613)	5·91 (5·16–6·76)	6·2 (5·66–6·83)	6·19 (5·74–6·79)	4·88 (-1·42–12·3)	-0·248 (-3·19–3·18)
Occupational injuries	--	--	--	--	--	--	--	--	--	--
Occupational ergonomic factors	12 (9·9–14·7)	10·6 (8·96–12·8)	10·9 (9·1–13·2)	-12 (-22·2–1·27)	3·44 (-3·9–12·2)	6·89 (5·46–8·66)	6·79 (5·54–8·26)	7·06 (5·62–8·84)	-1·43 (-17·7–17·2)	3·96 (-8·75–17·3)
Childhood sexual abuse	7·86 (5·28–11·1)	7·54 (5·55–9·71)	7·5 (5·65–9·53)	-4·11 (-17·5–15·7)	-0·559 (-6·45–5·43)	26·8 (19·3–37·2)	23·2 (18·3–30·5)	23·3 (18·3–30·4)	-13·2 (-24·9–1·38)	0·207 (-4·77–6·52)
Intimate partner violence	--	--	--	--	--	35 (20·2–44·8)	33·4 (19·5–43)	34·2 (20·1–43·9)	-4·69 (-13·2–14·2)	2·62 (-5·7–20·9)
Non-exclusive breastfeeding	26·3 (21·7–31·1)	25·1 (21·2–29·1)	25 (20·4–29·9)	-4·61 (-18·8–12·7)	-0·415 (-13·6–14·9)	26·3 (21·7–31·1)	25·1 (21·2–29·1)	25 (20·4–29·9)	-4·6 (-18·8–12·8)	-0·424 (-13·6–14·9)
Discontinued breastfeeding	27·2 (26·2–28·1)	25·5 (24·2–26·7)	25·3 (23·9–26·5)	-6·19 (-11·8–-0·721)	-0·811 (-6·4–4·77)	27·2 (26·2–28·1)	25·6 (24·2–26·8)	25·4 (23·9–26·5)	-5·86 (-11·5–-0·371)	-0·967 (-6·54–4·61)
Diet low in calcium	15·6 (7·6–28·7)	12·7 (6·3–23·2)	11·5 (5·56–21·3)	-18·7 (-29·6–-6·23)	-9·23 (-20–-0·774)	10·8 (4·58–20·6)	9·54 (4·31–17·9)	8·82 (3·73–16·5)	-12 (-26·3–5·49)	-7·56 (-19·5–2·83)
Occupational exposure to asbestos	11·9 (10·8–12·9)	10·1 (9·39–10·7)	8·81 (6·73–10·9)	-15·2 (-24·1–-6·21)	-12·4 (-33·9–9·03)	2·22 (1·15–2·69)	2·13 (1·04–2·54)	2·01 (0·963–2·89)	-4·04 (-18·2–12·5)	-5·77 (-32–27·5)
Occupational exposure to arsenic	0·503 (0–1·19)	0·505 (0–1·22)	0·534 (0–1·28)	0·375 (-12·9–NA)	5·84 (-4·98–NA)	0·439 (0–1·05)	0·448 (0–1·09)	0·459 (0–1·11)	2·06 (-14–NA)	2·5 (-9·61–NA)
Occupational exposure to benzene	0·845 (0·0509–2·47)	0·83 (0·0493–2·39)	0·856 (0·0538–2·51)	-1·86 (-10·9–10·8)	3·24 (-3·86–12·8)	1·21 (0·0693–3·29)	1·33 (0·0779–3·79)	1·38 (0·0801–3·95)	10·1 (-1·7–29·1)	4·02 (-4·61–15·8)
Occupational exposure to beryllium	0·0237 (0·0224–0·0251)	0·0235 (0·023–0·024)	0·0247 (0·0234–0·0261)	-0·928 (-6·84–5·27)	5·18 (-0·586–10·8)	0·0263 (0·0241–0·0286)	0·0271 (0·0265–0·0277)	0·028 (0·0261–0·03)	2·96 (-5·77–13·1)	3·29 (-3·58–10·7)
Occupational exposure to cadmium	0·0588 (0·053–0·0648)	0·0594 (0·0537–0·0652)	0·0628 (0·0552–0·0703)	0·986 (-11·7–15·8)	5·66 (-4·03–16·8)	0·0532 (0·0472–0·0601)	0·0545 (0·0494–0·0603)	0·0559 (0·049–0·0643)	2·44 (-12·1–18·6)	2·48 (-8·48–15·6)
Occupational exposure to chromium	0·141 (0·129–0·154)	0·145 (0·134–0·156)	0·152 (0·136–0·168)	2·8 (-8·63–16)	5·15 (-3·68–14·8)	--	--	--	--	--
Occupational exposure to diesel engine exhaust	0·691 (0·639–0·747)	0·744 (0·701–0·789)	0·77 (0·712–0·835)	7·73 (-1·92–18·6)	3·51 (-4·03–11·1)	0·68 (0·598–0·765)	0·7 (0·64–0·774)	0·696 (0·613–0·781)	2·95 (-11–20·3)	-0·655 (-11·2–9·18)
Occupational exposure to formaldehyde	0·294 (0·262–0·326)	0·29 (0·261–0·322)	0·303 (0·265–0·342)	-1·25 (-14·3–14·2)	4·34 (-5·59–15·5)	0·261 (0·231–0·293)	0·27 (0·244–0·298)	0·277 (0·245–0·315)	3·57 (-10·7–19·5)	2·61 (-8·13–14·5)
Occupational exposure to nickel	0·415 (0–1·81)	0·404 (0–1·75)	0·429 (0–1·85)	-2·77 (-16·3–NA)	6·24 (-5·01–NA)	0·342 (0–1·54)	0·338 (0–1·49)	0·349 (0–1·57)	-1·13 (-18·4–NA)	3·24 (-10·6–NA)
Occupational exposure to polycyclic aromatic hydrocarbons	0·28 (0·256–0·304)	0·287 (0·266–0·308)	0·301 (0·271–0·331)	2·56 (-8·18–14·6)	5·02 (-3·37–13·8)	0·29 (0·261–0·32)	0·311 (0·292–0·331)	0·319 (0·29–0·352)	7·16 (-4·4–20·4)	2·56 (-6·29–12·3)
Occupational exposure to silica	3·73 (0·541–11·9)	3·52 (0·494–11)	3·75 (0·543–11·6)	-5·66 (-18·7–11)	6·65 (-4·35–21)	2·89 (0·434–9·11)	2·74 (0·377–8·5)	2·79 (0·399–8·67)	-5·4 (-22·8–15·8)	1·83 (-11·4–21·6)
Occupational exposure to sulfuric acid	0·924 (0·202–2·91)	1 (0·207–3·22)	1·04 (0·219–3·31)	8·71 (-5·94–25·2)	3·72 (-6·49–15·2)	0·878 (0·176–3·01)	1·04 (0·212–3·39)	1·04 (0·208–3·3)	18·2 (-3·02–44·5)	-0·0725 (-14·4–16·9)
Unsafe sex	--	--	--	--	--	--	--	--	--	--
Occupational exposure to trichloroethylene	0·083 (0·0775–0·0893)	0·0869 (0·0828–0·0914)	0·0909 (0·0843–0·0977)	4·73 (-3·7–14·2)	4·63 (-2·54–11·7)	0·0809 (0·0728–0·0891)	0·084 (0·0791–0·0894)	0·0851 (0·0773–0·0935)	3·82 (-7·63–17·2)	1·4 (-7·35–11·1)
No access to handwashing facility	3·32 (3·06–3·59)	3·09 (2·86–3·36)	3·06 (2·81–3·31)	-6·85 (-15–2·22)	-1·15 (-6·74–4·91)	3·32 (3·06–3·59)	3·09 (2·86–3·36)	3·06 (2·81–3·31)	-6·85 (-15–2·22)	-1·15 (-6·74–4·91)
Child wasting	0·747 (0·495–1·03)	0·726 (0·482–0·977)	0·723 (0·479–0·98)	-2·82 (-12–8·15)	-0·351 (-7·32–7·07)	0·77 (0·512–1·07)	0·76 (0·501–1·05)	0·756 (0·498–1·03)	-1·34 (-11·4–9·72)	-0·583 (-7·53–8·03)
Child stunting	2·32 (1·2–3·43)	2·02 (1·02–3·02)	1·97 (1·01–3)	-12·9 (-32·3–11·5)	-2·65 (-16·7–14·9)	1·89 (0·942–2·85)	1·74 (0·875–2·62)	1·72 (0·878–2·65)	-7·9 (-28·5–19·1)	-1·33 (-16·7–16)
Chewing tobacco	3·19 (2·44–4·25)	3·19 (2·24–4·5)	3·04 (2·08–4·32)	0·0381 (-34·8–50·5)	-4·91 (-27·8–27·4)	--	--	--	--	--
Diet low in legumes	73·6 (38–99·1)	76·4 (44·5–99·7)	76·2 (43·4–99·6)	3·81 (-0·383–21)	-0·299 (-5·97–5·15)	72 (32·9–98·6)	74·7 (40·3–99·5)	74·8 (40·8–99·4)	3·78 (-0·53–20·5)	0·0691 (-5·49–7·88)
Short gestation	20·5 (16·8–25)	20·2 (16·6–24·3)	21 (17–25·9)	-1·34 (-22·3–24·4)	3·68 (-16·5–31·3)	18·1 (15–21·7)	17·9 (15–21·2)	18·4 (15·4–21·8)	-0·676 (-21·7–28·2)	2·58 (-17–27·5)
Low birth weight	8·37 (6·87–10·2)	8·38 (7·05–10)	8·73 (7·27–10·6)	0·083 (-24–28·1)	4·22 (-16–32·4)	8·38 (6·87–10·1)	8·67 (7·29–10·2)	8·95 (7·48–10·5)	3·48 (-19·2–34·6)	3·23 (-16·4–27·9)
High temperature	29·6 (20·4–40·5)	27·1 (17·9–38·8)	33·1 (23·3–43·9)	-8·46 (-21·5–2·19)	22·5 (10·1–39·6)	29·6 (20·4–40·5)	27·1 (17·9–38·8)	33·1 (23·3–43·9)	-8·46 (-21·5–2·19)	22·5 (10·1–39·6)
Low temperature	32·9 (29·3–37·1)	32·6 (28·8–36·9)	32·2 (28·6–36·3)	-0·88 (-2·27–0·287)	-1·28 (-2·62–0·222)	32·9 (29·3–37·1)	32·6 (28·8–36·9)	32·2 (28·6–36·3)	-0·88 (-2·27–0·287)	-1·28 (-2·62–0·222)
Kidney dysfunction	18·6 (13·1–25·6)	19·2 (13·5–26·3)	19·4 (13·9–26·3)	3·53 (-0·255–7·89)	0·91 (-2·8–4·62)	21 (14·8–28·7)	21·9 (15·3–29·6)	22·8 (16·4–30·7)	4·46 (0·198–9·57)	3·99 (0·244–8·47)

Bullying victimization	5·86<br>(2·43–11·9)	9<br>(3·62–17·9)	8·72<br>(3·35–18·1)	53·4<br>(33·5–76·5)	-3·09<br>(-12·5–2·98)	--	--	--	--	--
High LDL cholesterol	66·4<br>(62·2–70·9)	34·2<br>(30·6–37·8)	30·6<br>(27–34·5)	-48·5<br>(-53·6–-43·3)	-10·7<br>(-15·4–-5·92)	65·1<br>(61·5–68·9)	34·1<br>(30·8–37·5)	31·6<br>(28·2–35·3)	-47·6<br>(-52·6–-42·9)	-7·45<br>(-11·7–-3·67)

Supplementary Results Table 1. West Virginia age-standardised Summary Exposure Values (SEVs) for most-detailed risk factors and percentage change, by sex, 1990, 2010, and 2019. Data in parentheses are 95% uncertainty intervals.

	Male					Female				
	1990	2010	2019	Percentage change 1990-2010	Percentage change 2010-2019	1990	2010	2019	Percentage change 1990-2010	Percentage change 2010-2019
Unsafe sanitation	8·84 (4·35–15·7)	5·46 (2·5–9·6)	4·27 (2·04–8·11)	-38·2 (-74·1–45·4)	-21·8 (-59·3–48·9)	--	--	--	--	--
Ambient particulate matter pollution	14·2 (5·41–27·6)	6·82 (4·12–9·93)	3·17 (1·23–5·76)	-51·8 (-76·1–12·4)	-53·5 (-71·7–-41·5)	14·1 (5·41–27·5)	6·82 (4·13–9·92)	3·18 (1·25–5·77)	-51·7 (-76–12·3)	-53·3 (-71·2–-41·3)
Household air pollution from solid fuels	0·105 (0·0265–0·281)	0·0548 (0·00968–0·16)	0·0291 (0·00438–0·108)	-47·9 (-80–18·1)	-46·8 (-77·4–-1·85)	0·138 (0·0372–0·372)	0·0725 (0·0142–0·211)	0·0387 (0·00605–0·136)	-47·5 (-79·9–18·7)	-46·5 (-77·2–-1·54)
Ambient ozone pollution	79 (36·4–100)	62·7 (26·9–100)	44·7 (18·1–76)	-20·6 (-31·2–0)	-28·6 (-39–-19·5)	79 (36·4–100)	62·7 (26·9–100)	44·7 (18·1–76)	-20·6 (-31·2–0)	-28·6 (-39–-19·5)
Residential radon	21·5 (3·2–64·4)	21·5 (3·2–64·4)	21·5 (3·2–64·4)	0 (0–0)	0 (0–0)	21·5 (3·2–64·4)	21·5 (3·2–64·4)	21·5 (3·2–64·4)	0 (0–0)	0 (0–0)
Lead exposure	41·2 (11·2–67·2)	26 (6·21–48·4)	18·9 (3·61–38·9)	-37 (-57–-24·3)	-27·2 (-46·4–-18·6)	23·1 (0·681–51·5)	16·1 (0·482–40·1)	12·2 (0·235–32·7)	-30·4 (-66·1–23·4)	-24 (-56·9–-16·4)
Child underweight	0·984 (0·55–1·49)	0·99 (0·554–1·51)	0·964 (0·544–1·44)	0·655 (-16·8–22)	-2·62 (-15·3–10·3)	0·821 (0·44–1·24)	0·869 (0·482–1·32)	0·892 (0·477–1·36)	5·86 (-15·1–34·6)	2·61 (-11·7–19·2)
Iron deficiency	--	--	--	--	--	12·3 (10·3–14·6)	12·9 (11–15·1)	13·2 (11–15·6)	4·92 (-8·11–18·3)	1·88 (-7·3–12·3)
Vitamin A deficiency	1·14 (0·317–3·09)	1·65 (0·443–4·36)	1·12 (0·294–3·07)	44·7 (-62·2–548)	-32 (-71·4–78·8)	2 (0·802–4·03)	2·21 (0·892–4·55)	1·87 (0·743–4·23)	10·6 (-57·2–229)	-15·6 (-54·3–61·7)
Zinc deficiency	2·77 (0–8·5)	1·38 (0–4·42)	1·42 (0–4·71)	-50·2 (-100–NA)	2·76 (-17·6–NA)	2·83 (0–8·85)	1·4 (0–4·62)	1·38 (0–4·58)	-50·5 (-100–NA)	-1·46 (-21·5–NA)
Smoking	29·3 (26·5–32·3)	23·5 (21·4–26)	22·3 (20·2–24·8)	-19·7 (-24·8–-14·3)	-5·12 (-8·56–-1·18)	21 (18·6–23·6)	19·1 (17·2–21·1)	18·4 (16·5–20·4)	-8·91 (-18–0·0197)	-3·7 (-8·34–1·45)
Secondhand smoke	41·1 (38·4–43·7)	33·4 (31·5–35·3)	32·1 (29–35·2)	-18·7 (-24·9–-11·2)	-3·93 (-11·6–3·63)	38·1 (36–40)	31·9 (30·6–33·3)	30·6 (28·3–33)	-16·1 (-21·6–-9·72)	-4·24 (-10·5–2·2)
Alcohol use	13·6 (9·16–18·6)	13·5 (8·68–18·9)	14·3 (9·17–20·2)	-0·979 (-28·2–30·9)	6·22 (-13·9–31·6)	7·54 (4·91–10·7)	9·08 (5·95–12·9)	8·37 (5·37–12)	20·4 (-12·4–66·4)	-7·8 (-26·4–14·9)
Drug use	0·632 (0·36–1·12)	1·53 (1·12–2·05)	2·54 (1·97–3·29)	142 (73·9–242)	66·2 (41·3–94·8)	0·509 (0·335–0·757)	1·32 (0·993–1·72)	2·23 (1·72–2·81)	159 (102–230)	69·2 (47·4–95·4)
High fasting plasma glucose	14·6 (12·2–17·4)	21 (18·4–23·8)	22·2 (19·8–25·2)	43·4 (20·3–72)	6·05 (-6·16–22·1)	13·1 (10·7–16·3)	19·3 (16·9–22·1)	20·5 (17·5–23·5)	47 (20·8–80·1)	5·96 (-8·62–23·2)
High systolic blood pressure	26·7 (21·3–32·3)	16·7 (14·6–19)	20·2 (15·6–25·2)	-37·2 (-48·7–-21·7)	20·5 (-4·72–50·7)	24 (17·9–30·8)	13·9 (12·2–15·6)	18·9 (14–25·2)	-41·9 (-56–-21·3)	35·6 (-0·773–82·4)
High body-mass index	25·9 (18·2–35·7)	41·1 (30·3–52·3)	42·4 (31·3–53·7)	58·9 (38·9–88·5)	3·12 (-3·3–10·3)	32·3 (24·1–41)	45·6 (35·3–54·8)	46·4 (36·3–55·9)	41·4 (27·5–61·7)	1·7 (-4·69–8·91)
Low bone mineral density	11·8 (6·85–18·6)	9·31 (5·09–15·6)	11·9 (6·89–18·9)	-21 (-40·3–1·06)	27·9 (0·105–68·2)	15·5 (10·2–22·6)	16·6 (10·9–23·5)	19·3 (13·2–26·5)	7·6 (-13·5–35·7)	16·3 (-9·02–45·9)
Diet low in fruits	54·5 (42·8–67·8)	47·7 (36·1–60·6)	49 (37–62·6)	-12·5 (-24·3–-2·27)	2·84 (-9·09–15·2)	54·9 (43·2–68)	46·3 (34·9–59·3)	47·3 (35·3–60·8)	-15·8 (-27·3–-5·76)	2·27 (-9·85–14·7)
Diet low in vegetables	38·1 (20·9–60·6)	53·4 (34·8–71·3)	51·3 (33–69·3)	40·1 (11–105)	-3·81 (-12·6–4·27)	33·9 (17·3–55·1)	41 (24·1–61)	39·7 (22·9–59·7)	21·1 (3–58·5)	-3·09 (-14·9–8·99)
Diet low in whole grains	80·2 (70·2–88·7)	82·1 (72·9–89·8)	82·1 (73·7–89·8)	2·36 (-1·11–6·82)	0·0917 (-2·31–2·48)	76·1 (63·5–87·1)	78·8 (68·2–88·7)	78·7 (68·3–88·8)	3·57 (-0·584–9·76)	-0·143 (-3·32–2·67)
Diet low in nuts and seeds	32 (9·15–58·9)	26·1 (8·54–56·5)	22·9 (7·14–55·2)	-18·4 (-47·2–33·6)	-12·2 (-38·6–12·7)	27·9 (7·74–55·9)	23·4 (6·77–54·6)	21·5 (6·57–54·3)	-16 (-49·3–40·9)	-8·41 (-36·3–21·3)
Diet low in milk	52·1 (27·5–73·1)	47 (23·3–67·4)	46·4 (23·4–66·7)	-9·89 (-27·8–1·75)	-1·27 (-12·8–10·1)	52·1 (28·1–72·9)	47 (23·3–67)	46·7 (23·5–66·7)	-9·72 (-27·2–2·43)	-0·657 (-11·2–10)
Diet high in red meat	80·2 (68·3–90)	78·8 (65·7–89·3)	78·2 (64·4–89·3)	-1·72 (-15·3–13·6)	-0·818 (-11·5–11·1)	77·4 (64·8–87·4)	74·8 (60·6–86)	74·3 (60·7–86)	-3·39 (-18·2–12·4)	-0·668 (-12·9–11·2)
Diet high in processed meat	81·1 (58·7–99·1)	87·8 (70·7–99·8)	87·5 (69·7–99·8)	8·34 (0·437–25·7)	-0·347 (-4·86–3·95)	81·7 (59·4–98·3)	89·9 (75·6–99·7)	88·4 (72·8–99·6)	10·1 (0·867–31·4)	-1·73 (-7·01–2·07)
Diet high in sugar-sweetened beverages	41·9 (23·3–61·7)	57·5 (37·2–75·3)	58 (37·4–76·2)	37·4 (-6·75–124)	0·829 (-22·1–28·4)	45·7 (27·6–64·4)	63·6 (44·7–79·7)	62·2 (42·1–80·1)	39·1 (-1·73–122)	-2·18 (-23·5–20·7)
Diet low in fiber	47·5 (30·8–63·1)	33·6 (20·5–47·8)	31·4 (18·6–45·4)	-29·3 (-44·6–-13·8)	-6·58 (-23·5–9·68)	47·7 (30·2–64·1)	38·8 (24–53·9)	34·2 (19·8–49·6)	-18·6 (-33·3–-2·65)	-11·9 (-27·2–2·74)
Diet low in seafood omega-3 fatty acids	84·3 (67·3–99·6)	79·7 (61·5–99·2)	79·8 (61·2–99·4)	-5·38 (-19·7–8·15)	0·111 (-13·9–16·5)	86·6 (72·1–99·9)	84·2 (69·8–99·6)	83·8 (68·6–99·4)	-2·79 (-12·5–6·51)	-0·476 (-10·9–9·64)
Diet low in polyunsaturated fatty acids	16·6 (4·83–44·4)	8·44 (2·41–27·8)	8·12 (2·37–26·4)	-49·2 (-74·1–-8·79)	-3·76 (-27·5–22·3)	18·4 (5·34–47·1)	8·64 (2·49–28·2)	8·19 (2·45–27·8)	-53·1 (-76·7–-8·92)	-5·21 (-25·9–17·3)
Diet high in trans fatty acids	89·5 (81·6–100)	87·8 (79·1–100)	87·4 (78·1–100)	-1·84 (-9·52–5·7)	-0·525 (-8·77–8·42)	88·4 (81·1–100)	86·6 (78·1–100)	86·2 (77·3–100)	-2·03 (-9·68–5·66)	-0·399 (-8·12–8·55)
Diet high in sodium	18·2 (4·61–40·5)	39·7 (11·3–64·5)	39 (10·4–65·1)	118 (41·1–420)	-1·64 (-27·9–31·2)	20·9 (4·86–45·4)	21·2 (5·01–45·1)	21·7 (5·02–45·6)	1·43 (-29–47·5)	2·2 (-16·9–27·6)
Low physical activity	2·5 (0·925–5·13)	2·28 (1·02–4·64)	2·33 (0·876–5·02)	-8·86 (-38·7–38·5)	2·27 (-24·4–33·7)	6·12 (2·98–11·4)	6·61 (3·66–12)	6·74 (3·53–12·3)	8 (-17–50·5)	2·05 (-11·2–15·6)
Occupational asthmagens	26 (22–30·8)	20·6 (17·6–24)	21·1 (17·8–25·5)	-20·8 (-30·8–-10·6)	2·79 (-6·53–12)	10·4 (8·5–12·5)	10·2 (8·43–12·2)	10·4 (8·57–12·6)	-2·3 (-17·2–15·1)	2·11 (-9·48–14·4)
Occupational particulate matter, gases, and fumes	10·5 (7·42–15·1)	9·26 (6·63–13·3)	8·73 (6·24–12·4)	-11·5 (-16·3–-6·39)	-5·73 (-9–-2·29)	4·78 (3·28–7·11)	4·63 (3·26–6·59)	4·49 (3·16–6·44)	-3·21 (-12·3–7·77)	-3·04 (-8·71–2·74)
Occupational noise	10·2 (9·32–11·1)	9·02 (8·36–9·79)	8·45 (7·88–9·16)	-11·6 (-14·9–-7·84)	-6·3 (-8·34–-4)	5·53 (4·78–6·32)	5·77 (5·24–6·37)	5·77 (5·34–6·29)	4·39 (-2·15–12·8)	-0·12 (-3·67–3·44)
Occupational injuries	--	--	--	--	--	--	--	--	--	--
Occupational ergonomic factors	13 (10·5–15·8)	10·8 (8·91–12·9)	11·2 (9·22–13·4)	-17·2 (-29–-4·1)	4·04 (-5·61–14·4)	7·44 (5·85–9·27)	7·1 (5·78–8·67)	7·44 (5·96–9·35)	-4·68 (-21·4–15·1)	4·85 (-7·66–19·1)
Childhood sexual abuse	7·4 (5·05–10·5)	6·97 (4·6–9·93)	6·85 (4·54–9·67)	-5·83 (-12·2–5·83)	-1·68 (-8·51–5·53)	12·3 (8·94–17·3)	17·4 (12·9–24)	17·8 (13·1–24·7)	41·9 (30·7–54·8)	2·17 (-5–11)
Intimate partner violence	--	--	--	--	--	29·7 (15·9–38·6)	28 (16–36·6)	29 (15·7–38)	-5·82 (-15–13·7)	3·6 (-5·34–19·3)
Non-exclusive breastfeeding	26·1 (21·8–31)	24·8 (21–28·7)	24·7 (20·2–29·6)	-5·06 (-20·4–12·7)	-0·413 (-14–14·5)	26·1 (21·8–30·9)	24·8 (21–28·7)	24·7 (20·2–29·6)	-5·06 (-20·4–12·7)	-0·416 (-14–14·5)
Discontinued breastfeeding	26·9 (25·9–27·8)	25·3 (24–26·6)	25·3 (24–26·6)	-5·76 (-11–-0·685)	0·0338 (-5·24–5·79)	26·9 (26–27·8)	25·5 (24·2–26·7)	25·4 (24·1–26·7)	-5·3 (-10·6–-0·237)	-0·228 (-5·49–5·48)
Diet low in calcium	18·7 (9·07–34·8)	15·6 (7·72–28·9)	15·4 (7·59–28·7)	-16·8 (-27·5–-5·39)	-1·43 (-9·4–6·51)	14·3 (6·09–27·6)	12·8 (5·55–23·9)	12·9 (5·5–24·2)	-10·8 (-24·1–4·28)	0·705 (-8·04–9·96)
Occupational exposure to asbestos	11·4 (10·3–12·5)	11 (10·2–11·9)	10·3 (7·75–13·1)	-3·47 (-14·7–9·15)	-6·38 (-28·9–18·6)	1·36 (1·07–2·01)	1·45 (1·08–2·1)	1·52 (1·01–2·35)	6·38 (-8·48–25·3)	4·72 (-24·8–39·4)
Occupational exposure to arsenic	0·474 (0–1·13)	0·445 (0–1·05)	0·478 (0–1·13)	-5·96 (-19·9–NA)	7·2 (-5·31–NA)	0·403 (0–0·953)	0·4 (0–0·967)	0·418 (0–1·01)	-0·604 (-17·2–NA)	4·32 (-8·16–NA)
Occupational exposure to benzene	0·754 (0·0443–2·14)	0·718 (0·0432–2·09)	0·751 (0·0477–2·19)	-4·83 (-12·4–7·02)	4·67 (-2·18–16·2)	1·08 (0·0565–3·1)	1·16 (0·0663–3·21)	1·22 (0·0715–3·45)	7·23 (-4·32–29·9)	5·72 (-3·84–19·5)
Occupational exposure to beryllium	0·0233 (0·0217–0·0248)	0·0213 (0·0207–0·0219)	0·0227 (0·0213–0·024)	-8·45 (-14·4–-2·13)	6·39 (0·227–12·9)	0·0241 (0·0217–0·0263)	0·0244 (0·0237–0·0252)	0·0257 (0·0238–0·0277)	1·37 (-7·55–12·3)	5·28 (-2·73–13·3)
Occupational exposure to cadmium	0·0555 (0·0497–0·0619)	0·0524 (0·0476–0·0579)	0·0561 (0·0497–0·0636)	-5·56 (-18·2–10)	7·03 (-4·18–19·5)	0·0487 (0·0424–0·0551)	0·0488 (0·0443–0·0542)	0·051 (0·0446–0·0585)	0·199 (-15·4–18·1)	4·39 (-6·95–17·9)
Occupational exposure to chromium	0·134 (0·122–0·148)	0·129 (0·119–0·14)	0·138 (0·124–0·153)	-3·9 (-15·3–9·16)	6·89 (-2·61–17·3)	--	--	--	--	--
Occupational exposure to diesel engine exhaust	0·853 (0·779–0·925)	0·768 (0·719–0·819)	0·807 (0·74–0·882)	-9·96 (-18·7–-0·163)	5·19 (-2·98–13·7)	0·641 (0·56–0·724)	0·664 (0·6–0·73)	0·678 (0·596–0·773)	3·58 (-10·2–21·5)	2·14 (-8·89–14·5)
Occupational exposure to formaldehyde	0·275 (0·245–0·309)	0·255 (0·23–0·284)	0·27 (0·237–0·308)	-7·39 (-20·4–8·38)	5·78 (-5·57–18·7)	0·237 (0·207–0·268)	0·239 (0·217–0·265)	0·25 (0·22–0·286)	0·829 (-14·8–18·8)	4·49 (-6·32–17)
Occupational exposure to nickel	0·519 (0–2·36)	0·411 (0–1·8)	0·427 (0–1·83)	-20·6 (-31·2–NA)	3·86 (-6·63–NA)	0·321 (0–1·33)	0·306 (0–1·34)	0·322 (0–1·44)	-4·69 (-22·4–NA)	5·17 (-8·73–NA)
Occupational exposure to polycyclic aromatic hydrocarbons	0·272 (0·248–0·297)	0·258 (0·24–0·278)	0·274 (0·247–0·304)	-5·2 (-15·9–6·57)	6·34 (-2·65–15·9)	0·264 (0·234–0·293)	0·278 (0·261–0·298)	0·29 (0·262–0·324)	5·48 (-7·25–20·6)	4·36 (-5·05–15·2)
Occupational exposure to silica	5·99 (0·866–19·7)	4·24 (0·609–13·7)	4·37 (0·622–13·7)	-29·2 (-39·9–-16·5)	2·94 (-8·55–16·1)	2·9 (0·409–9·14)	2·61 (0·349–8·26)	2·7 (0·364–8·92)	-10·1 (-27·2–11·3)	3·47 (-9·98–22·8)
Occupational exposure to sulfuric acid	0·851 (0·177–2·67)	0·883 (0·18–2·8)	0·931 (0·195–3·05)	3·76 (-9·47–21·4)	5·44 (-5·97–17·5)	0·799 (0·166–2·58)	0·978 (0·193–3·27)	0·988 (0·199–3·12)	22·4 (-1·19–53·9)	0·942 (-14·5–18·2)
Unsafe sex	--	--	--	--	--	--	--	--	--	--
Occupational exposure to trichloroethylene	0·0779 (0·0721–0·0844)	0·0775 (0·0739–0·082)	0·0831 (0·0772–0·0901)	-0·523 (-9·09–9·37)	7·19 (-0·718–15)	0·0739 (0·0656–0·0822)	0·0762 (0·0716–0·0814)	0·079 (0·0714–0·0876)	3·12 (-8·95–19·1)	3·67 (-5·58–14·2)
No access to handwashing facility	3·45 (3·2–3·73)	3·17 (2·92–3·42)	3·13 (2·88–3·39)	-8·03 (-15·9–1·33)	-1·33 (-7·05–4·46)	3·45 (3·2–3·73)	3·17 (2·92–3·42)	3·13 (2·88–3·39)	-8·03 (-15·9–1·33)	-1·33 (-7·05–4·46)
Child wasting	0·752 (0·502–1·03)	0·731 (0·473–1)	0·731 (0·478–0·993)	-2·83 (-11·9–6·94)	0·0116 (-7·32–8·45)	0·778 (0·524–1·06)	0·769 (0·51–1·05)	0·765 (0·507–1·05)	-1·23 (-10·9–10·1)	-0·485 (-7·48–7·65)
Child stunting	2·51 (1·37–3·66)	2·18 (1·15–3·25)	2·11 (1·12–3·14)	-13·4 (-31·8–11)	-3·17 (-17·7–13·2)	2 (1·01–2·98)	1·86 (0·937–2·83)	1·84 (0·913–2·85)	-7·18 (-27·2–17·2)	-1·02 (-15·8–15·9)
Chewing tobacco	13·5 (11·4–15·9)	9·06 (6·99–11·4)	8·39 (6·17–11)	-33 (-49·1–-10·9)	-7·41 (-25·4–14)	--	--	--	--	--
Diet low in legumes	75·4 (40·8–99·6)	78·1 (47·2–99·8)	78·2 (47·4–99·8)	3·64 (-0·309–18·9)	0·148 (-4·84–5·63)	73·6 (36·8–98·8)	76·5 (44·8–99·7)	77 (46·3–99·6)	4·01 (-0·284–21·4)	0·596 (-3·95–7·63)
Short gestation	28·5 (23·3–34·7)	29 (23·8–35·3)	27·1 (21·9–33·2)	1·77 (-21·5–29·8)	-6·71 (-24·9–16·5)	19·8 (17–23·1)	21·7 (18·5–25·3)	21 (17·6–24·9)	9·6 (-7·09–29·3)	-3·51 (-19·3–15·4)
Low birth weight	12 (9·83–14·6)	13·4 (11·1–16·1)	13·1 (10·8–15·9)	11·7 (-13·4–42·4)	-2·03 (-21·7–22·6)	9·9 (8·57–11·4)	12·1 (10·5–13·8)	12·1 (10·3–14·1)	21·8 (1·52–43·4)	0·368 (-16·6–20·1)
High temperature	23·3 (12·3–35·4)	24·9 (14·1–37·1)	23·5 (12·8–35·5)	7 (1·37–13·5)	-5·7 (-9·73–-2·49)	23·3 (12·3–35·4)	24·9 (14·1–37·1)	23·5 (12·8–35·5)	7 (1·37–13·5)	-5·7 (-9·73–-2·49)
Low temperature	31·7 (27·8–36·5)	34·7 (31·1–38·9)	33·6 (29·7–38·1)	9·49 (5·9–13·3)	-3·06 (-4·97–-1·25)	31·7 (27·8–36·5)	34·7 (31·1–38·9)	33·6 (29·7–38·1)	9·49 (5·9–13·3)	-3·06 (-4·97–-1·25)
Kidney dysfunction	20·4 (14·5–27·5)	21·2 (15·1–28·4)	21·4 (15·5–28·7)	3·93 (0·0428–8·05)	1·17 (-2·33–5·06)	22·8 (16·2–30·4)	24 (17·2–31·9)	25·1 (18·3–33·2)	5·68 (1·52–11)	4·54 (-0·329–9·61)

Bullying victimization	5·95 (2·46–12·1)	8·89 (3·55–17·5)	8·65 (3·36–17·7)	49·3 (30·1–71·6)	-2·65 (-12–3·36)	4·31 (1·76–8·98)	7·79 (3·14–15·5)	7·73 (2·93–15·8)	81·1 (57·1–109)	-0·872 (-11·5–5·98)
High LDL cholesterol	66·5 (62·2–71·1)	34·2 (30·8–37·8)	30·3 (26·9–34)	-48·5 (-54–-43·3)	-11·4 (-16·3–-6·78)	66·5 (62·8–70·4)	34·7 (31·4–38·2)	31·6 (28·1–35·3)	-47·8 (-52·7–-42·8)	-8·95 (-13·6–-5·01)

Supplementary Results Table 1. Wisconsin age-standardised Summary Exposure Values (SEVs) for most-detailed risk factors and percentage change, by sex, 1990, 2010, and 2019. Data in parentheses are 95% uncertainty intervals.

	Male					Female				
	1990	2010	2019	Percentage change 1990-2010	Percentage change 2010-2019	1990	2010	2019	Percentage change 1990-2010	Percentage change 2010-2019
Unsafe sanitation	5·46 (2·44–10·3)	3·1 (1·44–5·79)	2·44 (1·08–4·69)	-43·2 (-76·5–38·8)	-21·2 (-60·1–47·8)	--	--	--	--	--
Ambient particulate matter pollution	10·5 (3·15–22)	6·08 (3·4–9·23)	4·97 (2·41–7·96)	-42 (-73–63·2)	-18·3 (-30·6—12·8)	10·5 (3·15–22)	6·08 (3·39–9·22)	4·97 (2·41–7·97)	-42 (-73·2–62·3)	-18·3 (-30·7—12·7)
Household air pollution from solid fuels	0·0197 (0·00439–0·056)	0·0111 (0·00188–0·0369)	0·00807 (0·00115–0·0264)	-43·8 (-80·6–32·6)	-27·2 (-64·8–34·9)	0·0261 (0·00604–0·0728)	0·0147 (0·00258–0·0482)	0·0107 (0·0016–0·0349)	-43·6 (-80·5–33·3)	-27·2 (-64·8–35·3)
Ambient ozone pollution	54 (22·5–91·6)	44·2 (18·3–77·2)	38·6 (15·6–67·1)	-18·1 (-30·2—6·83)	-12·7 (-25·4—2·28)	54 (22·5–91·6)	44·2 (18·3–77·2)	38·6 (15·6–67·1)	-18·1 (-30·2—6·83)	-12·7 (-25·4—2·28)
Residential radon	24·2 (3·54–74·4)	24·2 (3·54–74·4)	24·2 (3·54–74·4)	0 (0–0)	0 (0–0)	24·2 (3·54–74·4)	24·2 (3·54–74·4)	24·2 (3·54–74·4)	0 (0–0)	0 (0–0)
Lead exposure	44·8 (15·7–70·1)	25·5 (5·9–47·6)	17·6 (2·83–36·7)	-43·2 (-64·1—29·3)	-31·1 (-52·3—22)	24 (0·819–52·8)	14·2 (0·184–37·8)	10 (0·0469–29·5)	-40·7 (-84·1—25·5)	-29·4 (-76·5—21·1)
Child underweight	0·935 (0·517–1·45)	0·943 (0·509–1·43)	0·918 (0·513–1·39)	0·878 (-17·2–22·1)	-2·63 (-16–11·7)	0·783 (0·394–1·19)	0·836 (0·451–1·29)	0·857 (0·472–1·32)	6·72 (-15·1–40)	2·51 (-12·6–19·2)
Iron deficiency	--	--	--	--	--	11·2 (9·28–13·1)	11·7 (9·92–13·7)	11·9 (9·9–14)	4·91 (-7·37–17·7)	1·42 (-7·91–11·1)
Vitamin A deficiency	0·753 (0·206–1·93)	1·05 (0·295–2·82)	0·733 (0·21–1·87)	39·8 (-65·1–498)	-30·4 (-67·6–75·7)	1·49 (0·595–3·08)	1·57 (0·634–3·33)	1·34 (0·52–3·08)	5·53 (-60·7–211)	-14·4 (-51·6–66·4)
Zinc deficiency	2·32 (0–7·44)	1·19 (0–3·74)	1·21 (0–4·02)	-48·9 (-100–NA)	2·46 (-18·7–NA)	2·38 (0–7·42)	1·2 (0–4·04)	1·2 (0–3·9)	-49·4 (-100–NA)	-0·287 (-20·7–NA)
Smoking	26·9 (24·2–29·7)	20·5 (18·4–22·6)	17·6 (15·7–19·5)	-24 (-29—18·5)	-14·1 (-17·5—10·8)	19·8 (17·4–22·5)	16 (14·2–17·8)	13·7 (12·1–15·4)	-19·5 (-26·6—10·6)	-14·3 (-18·3—10)
Secondhand smoke	37·3 (34·5–40·2)	25·8 (24·2–27·5)	25·4 (22·8–28·3)	-30·9 (-37·6—23·2)	-1·45 (-10·3–7·14)	34·5 (32·3–36·9)	24·2 (23·1–25·4)	23·9 (21·8–26·1)	-29·9 (-35·6—23·7)	-1·26 (-8·66–6·33)
Alcohol use	15·4 (9·41–21·9)	15·6 (9·3–22·6)	16·4 (9·57–23·7)	1·3 (-29–45·9)	4·91 (-16·8–32·4)	11·4 (6·67–16·7)	13·1 (7·93–18·9)	12·8 (7·39–18·9)	15 (-18–66·9)	-1·96 (-23–23·9)
Drug use	0·551 (0·276–1·09)	0·892 (0·609–1·34)	1·24 (0·889–1·73)	61·9 (17·9–141)	39·3 (20·8–64·3)	0·485 (0·298–0·787)	0·814 (0·594–1·12)	1·14 (0·87–1·47)	67·8 (32·7–120)	40·3 (20·6–64·9)
High fasting plasma glucose	13·8 (11·5–16·4)	16·8 (14·5–19·4)	17·6 (15·2–20·5)	22 (1·78–46·7)	4·84 (-10·2–21)	11·5 (9·42–14·3)	13·9 (11·4–16·5)	14·6 (11·8–18)	21·1 (-3·63–51·1)	5·27 (-12·6–26·6)
High systolic blood pressure	25·7 (20·7–31·1)	16·1 (14·1–18·2)	19·9 (15·6–25·1)	-37·5 (-49·2—22·7)	23·8 (-1·64–54·1)	23·3 (17·2–30·2)	13·4 (11·8–15)	17·7 (13–23·4)	-42·6 (-56·4—22·6)	32 (-2·78–76·8)
High body-mass index	26·5 (18·3–36·5)	40·4 (29·8–52·1)	42 (31·3–53·6)	52·6 (34·2–76·8)	3·92 (-2·62–10·7)	31·8 (23·7–41)	43 (33·9–52·2)	44·6 (34·7–54·4)	35·3 (20·3–55·2)	3·55 (-2·56–10·5)
Low bone mineral density	12·1 (7·1–18·7)	9·37 (4·91–15·7)	11·7 (6·61–18·7)	-22·5 (-43·1–0·425)	24·7 (-3·73–69·3)	15·9 (10·3–22·8)	16·8 (11·2–24·1)	19 (13·1–26·1)	6·09 (-16·6–32·7)	12·8 (-9·73–43)
Diet low in fruits	53 (41·2–65·8)	48·4 (37·1–60·9)	49·3 (36·9–62·4)	-8·73 (-20·6–3·17)	1·87 (-9·07–14·3)	53·5 (41·7–66·6)	49·3 (37·3–62·3)	49·4 (36·9–63·4)	-7·8 (-19·5–4·06)	0·218 (-10·4–10·6)
Diet low in vegetables	32·3 (16·6–54·7)	51·8 (34·8–70·1)	50·6 (33·4–69·1)	60·3 (21·3–142)	-2·38 (-11·4–5·26)	30·3 (14·6–50·9)	37·8 (21·1–58)	36·4 (20·5–55·5)	24·5 (2·89–77·4)	-3·53 (-16·4–10·3)
Diet low in whole grains	78·2 (67·7–88·6)	80·1 (70·1–89·3)	80·2 (70·3–89·4)	2·48 (-1·19–7·22)	0·0516 (-2·53–2·62)	--	--	--	--	--
Diet low in nuts and seeds	28 (7·96–56·8)	25·7 (8·2–56)	21·3 (6·51–54·6)	-8·31 (-42·8–68·2)	-17·2 (-43·4–8·19)	23·9 (6·01–54·8)	22 (7·16–54)	19·4 (5·85–52·9)	-8·01 (-41·9–66·1)	-11·7 (-39·9–16·9)
Diet low in milk	48·5 (25–69·2)	43·1 (19·8–64·3)	42·5 (19·6–63·2)	-11·1 (-30·7–1·64)	-1·44 (-13·9–11·7)	48·5 (25·7–68·4)	43·2 (19·1–62·9)	42·8 (18·7–63·1)	-11 (-31·1–2·48)	-0·971 (-12·3–12·4)
Diet high in red meat	77·5 (64·8–87·9)	75·8 (63·2–87·4)	75·6 (61·9–87·4)	-2·14 (-16·9–14·9)	-0·368 (-11–12·1)	79 (65·8–89·1)	75·3 (61·8–86·7)	75 (60·3–87·5)	-4·63 (-19·2–11)	-0·498 (-11·9–12·1)
Diet high in processed meat	77·9 (54·7–97·6)	86·1 (67·6–99·7)	85·9 (67·4–99·8)	10·5 (1·31–29·5)	-0·274 (-4·93–4·51)	79·2 (54·1–98·2)	89 (72·6–99·7)	87·5 (69·1–99·6)	12·3 (1·22–36·9)	-1·61 (-7·51–2·07)
Diet high in sugar-sweetened beverages	46 (27·7–66·4)	63 (43·9–79·9)	62·7 (43·6–81)	36·8 (-4·69–116)	-0·434 (-20·5–24·4)	50·1 (31·5–68·6)	68·4 (49·8–85)	66·7 (46·9–84·8)	36·4 (-1·19–109)	-2·36 (-20·7–19·8)
Diet low in fiber	43·5 (28·5–59·2)	30·2 (17·3–44·8)	27·7 (14·8–41·6)	-30·7 (-48·1—13·4)	-8·19 (-26·5–11·5)	43·9 (27·6–59·3)	35·4 (21·7–49·4)	30·9 (17·7–46·2)	-19·4 (-35·4—2·22)	-12·8 (-29·7–3·62)
Diet low in seafood omega-3 fatty acids	83·5 (65·4–99·3)	78·9 (60·9–99)	79·3 (59·9–99·1)	-5·46 (-19·7–8·88)	0·524 (-15–18·4)	86·3 (70·4–100)	83·7 (67·2–99·4)	83·2 (66·7–99·7)	-3 (-14·9–8·15)	-0·654 (-10·7–11·6)
Diet low in polyunsaturated fatty acids	13·9 (3·97–40·9)	7·22 (2·04–26·9)	6·99 (2·03–26·8)	-48·2 (-75·9—7·11)	-3·08 (-25·4–17·5)	15·6 (4·35–42·9)	7·4 (2·11–27·2)	7·08 (2·08–26·6)	-52·7 (-78·2—8·97)	-4·3 (-27·2–18·5)
Diet high in trans fatty acids	89·5 (81·5–100)	87·9 (79·5–100)	87·5 (77·9–100)	-1·72 (-9·86–6·07)	-0·531 (-8·18–7·82)	88·4 (80·9–100)	86·6 (77·7–100)	86·2 (77·3–100)	-2·04 (-10·1–5·79)	-0·421 (-8·35–8·47)
Diet high in sodium	18·3 (4·72–40·9)	39·7 (12·4–63·6)	39 (11·3–64·5)	117 (42·3–412)	-1·94 (-28·2–29·2)	20·9 (4·85–45·9)	21·3 (5–45)	21·8 (5·04–45·9)	1·71 (-29·2–47·2)	2·35 (-18·1–31·7)
Low physical activity	2·05 (0·741–4·53)	1·65 (0·666–3·99)	1·8 (0·597–4·1)	-19·4 (-48·5–20·9)	9·23 (-20·3–55)	4·86 (2·3–9·62)	4·59 (2·36–8·71)	4·9 (2·46–9·62)	-5·46 (-29·8–27·8)	6·68 (-8·11–22·8)
Occupational asthmagens	26·9 (21·8–33·4)	24·1 (20·3–28·6)	25·4 (20·8–31·5)	-10·3 (-23·2–3·35)	5·43 (-5·29–19·8)	12·7 (10·5–15·5)	13·3 (11·1–15·9)	13·3 (10·6–16·4)	4·62 (-11·1–21·5)	0·233 (-10·6–14·6)
Occupational particulate matter, gases, and fumes	11·1 (7·89–15·8)	10·7 (7·67–15·1)	10·3 (7·4–14·6)	-4·22 (-9·47–1·66)	-3·07 (-6·41–0·232)	6·25 (4·36–8·95)	6·27 (4·48–8·88)	6·11 (4·4–8·89)	0·428 (-8·11–9·88)	-2·65 (-7·94–2·37)
Occupational noise	9·38 (8·58–10·3)	9·17 (8·55–9·91)	8·96 (8·39–9·65)	-2·2 (-5·87–1·85)	-2·3 (-4·24—0·116)	6·68 (5·91–7·55)	7·2 (6·63–7·89)	7·27 (6·81–7·92)	7·78 (2–15·1)	1·03 (-1·67–4·08)
Occupational injuries	--	--	--	--	--	--	--	--	--	--
Occupational ergonomic factors	13 (10·7–15·8)	11·6 (9·72–13·8)	12 (10–14·6)	-11·2 (-22·1–0·987)	3·75 (-5·01–13·3)	7·98 (6·3–10)	8·14 (6·69–9·94)	8·73 (6·92–11·3)	2·06 (-14·5–21·8)	7·21 (-7·63–25·2)
Childhood sexual abuse	6·67 (4·29–9·79)	5·61 (4·6–6·64)	5·56 (4·59–6·58)	-16 (-36·1–16)	-0·799 (-6·61–5·2)	17·9 (12·9–25·1)	14·7 (12·3–17·7)	14·8 (12·5–17·7)	-17·6 (-36·4–6·28)	0·675 (-4·85–7·49)
Intimate partner violence	--	--	--	--	--	28·8 (15·7–38·1)	27·2 (15·1–36·3)	28·2 (15·1–37)	-5·62 (-14·5–9·49)	3·48 (-4·82–19·8)
Non-exclusive breastfeeding	26·2 (21·4–31·1)	24·9 (21·3–28·6)	24·9 (20·6–29·4)	-5·11 (-19·1–12·3)	0·0162 (-14–15·2)	26·2 (21·4–31·1)	24·9 (21·3–28·6)	24·9 (20·6–29·4)	-5·1 (-19·1–12·3)	0·0092 (-14–15·1)
Discontinued breastfeeding	26·8 (25·9–27·7)	25·1 (23·8–26·4)	25·5 (24·1–26·7)	-6·32 (-11·8—0·721)	1·36 (-4·41–7·43)	26·9 (26–27·8)	25·2 (23·8–26·4)	25·5 (24·1–26·7)	-6·33 (-11·8—0·731)	1·26 (-4·49–7·32)
Diet low in calcium	16·4 (7·91–30·3)	13·6 (6·56–24·7)	13·1 (6·17–24)	-17 (-28—6·02)	-3·42 (-12·9–4·6)	11·8 (5·1–22·4)	10·6 (4·58–19·9)	10·5 (4·45–19·7)	-9·91 (-24·1–7·53)	-1·12 (-10·8–9·09)
Occupational exposure to asbestos	10·1 (8·97–11·4)	9·01 (8·33–9·73)	8·3 (6·22–10·5)	-11·1 (-21·4—0·83)	-7·89 (-32–17·2)	1·97 (1·04–2·39)	1·98 (0·944–2·38)	2 (0·915–2·88)	0·765 (-15·8–17·9)	0·897 (-27·6–37·9)
Occupational exposure to arsenic	0·647 (0–1·51)	0·641 (0–1·51)	0·664 (0–1·57)	-1·04 (-13·7–NA)	3·62 (-6·45–NA)	0·578 (0–1·38)	0·603 (0–1·48)	0·601 (0–1·47)	4·49 (-13·2–NA)	-0·447 (-11·8–NA)
Occupational exposure to benzene	0·847 (0·0475–2·47)	0·852 (0·0496–2·46)	0·876 (0·0525–2·57)	0·558 (-7·95–11·7)	2·86 (-3·98–11·9)	1·26 (0·072–3·58)	1·47 (0·084–4·11)	1·52 (0·0916–4·37)	16·2 (4·79–34·9)	3·61 (-4·52–15·7)
Occupational exposure to beryllium	0·0253 (0·0239–0·0268)	0·0255 (0·0249–0·0261)	0·0266 (0·0252–0·028)	0·562 (-5·12–6·48)	4·4 (-0·94–9·74)	0·0292 (0·0268–0·0314)	0·0316 (0·0309–0·0324)	0·0322 (0·0304–0·0343)	8·41 (0·616–17·9)	2·01 (-3·97–7·87)
Occupational exposure to cadmium	0·0739 (0·067–0·0806)	0·0736 (0·0674–0·0803)	0·0764 (0·0677–0·0852)	-0·344 (-12·2–13·4)	3·78 (-5·67–14·1)	0·0681 (0·0602–0·0768)	0·0716 (0·0642–0·0802)	0·0715 (0·0622–0·0821)	5·21 (-10·5–23·8)	-0·13 (-10·4–11·5)
Occupational exposure to chromium	0·172 (0·158–0·186)	0·175 (0·163–0·189)	0·182 (0·164–0·2)	1·69 (-8·89–14)	3·81 (-4·36–12·6)	--	--	--	--	--
Occupational exposure to diesel engine exhaust	0·685 (0·634–0·742)	0·766 (0·716–0·817)	0·802 (0·73–0·873)	11·8 (1·96–23·2)	4·79 (-3·19–12·8)	0·708 (0·633–0·787)	0·788 (0·719–0·861)	0·786 (0·702–0·878)	11·4 (-2·89–29·5)	-0·363 (-9·72–10)
Occupational exposure to formaldehyde	0·373 (0·336–0·408)	0·363 (0·33–0·397)	0·372 (0·328–0·415)	-2·84 (-14·8–11·1)	2·56 (-6·69–13)	0·331 (0·293–0·372)	0·351 (0·316–0·388)	0·351 (0·308–0·397)	6·09 (-8·46–23·9)	0·0758 (-9·79–11·4)
Occupational exposure to nickel	0·539 (0–2·37)	0·52 (0–2·27)	0·539 (0–2·32)	-3·59 (-16·1–NA)	3·72 (-6·65–NA)	0·458 (0–2)	0·465 (0–2·05)	0·465 (0–1·98)	1·47 (-16·7–NA)	0·13 (-13·5–NA)
Occupational exposure to polycyclic aromatic hydrocarbons	0·339 (0·31–0·365)	0·344 (0·321–0·367)	0·357 (0·323–0·39)	1·59 (-8·39–13·7)	3·68 (-4·21–12)	0·351 (0·316–0·387)	0·388 (0·363–0·417)	0·39 (0·356–0·427)	10·6 (-1·4–24·4)	0·611 (-7·39–9·26)
Occupational exposure to silica	4·72 (0·669–15·7)	4·45 (0·62–14·1)	4·66 (0·651–14·5)	-5·59 (-17·6–9·73)	4·65 (-5·6–17·7)	3·83 (0·527–12·3)	3·75 (0·539–11·6)	3·72 (0·571–12·3)	-2·11 (-19·5–23·4)	-0·922 (-14–15·7)
Occupational exposure to sulfuric acid	1·11 (0·239–3·45)	1·19 (0·244–3·82)	1·22 (0·247–3·91)	7 (-6·17–21·5)	2·64 (-6·51–13·2)	1·08 (0·23–3·39)	1·3 (0·28–4·21)	1·27 (0·263–4·08)	20·7 (-1·33–46·8)	-2·1 (-15–12·6)
Unsafe sex	--	--	--	--	--	--	--	--	--	--
Occupational exposure to trichloroethylene	0·0947 (0·0887–0·102)	0·0993 (0·0953–0·104)	0·103 (0·0964–0·111)	4·86 (-2·87–14·4)	4·07 (-2·3–10·7)	0·0943 (0·0861–0·103)	0·103 (0·0965–0·11)	0·103 (0·0939–0·112)	8·74 (-2·89–22·4)	0·0907 (-7·66–8·08)
No access to handwashing facility	3·31 (3·07–3·57)	3·1 (2·86–3·35)	3·06 (2·85–3·3)	-6·52 (-14·8–2·7)	-1·07 (-7–4·99)	3·31 (3·07–3·57)	3·1 (2·86–3·35)	3·06 (2·85–3·3)	-6·52 (-14·8–2·7)	-1·07 (-7–4·99)
Child wasting	0·746 (0·493–1·02)	0·725 (0·481–0·983)	0·724 (0·48–0·977)	-2·78 (-12·2–7·75)	-0·176 (-7·15–8·04)	0·77 (0·517–1·05)	0·761 (0·497–1·05)	0·756 (0·494–1·03)	-1·18 (-11·5–9·67)	-0·637 (-8·24–7·63)
Child stunting	2·29 (1·2–3·42)	2·02 (1·04–2·98)	1·97 (1·02–2·91)	-11·6 (-30·2–12)	-2·57 (-16·7–14·3)	1·88 (0·961–2·81)	1·74 (0·883–2·63)	1·72 (0·862–2·62)	-7·08 (-26·7–16·5)	-1·18 (-16·1–17·1)
Chewing tobacco	3·39 (2·62–4·42)	3·7 (2·65–5·12)	3·7 (2·55–5·23)	9·28 (-25·2–59·2)	-0·0995 (-22·9–29·4)	--	--	--	--	--
Diet low in legumes	74·1 (39·5–99·1)	77 (45·1–99·7)	77 (45·6–99·7)	3·93 (0–19)	0·0668 (-5·03–5·59)	72·4 (35·3–99·2)	75·4 (40·3–99·6)	75·7 (43·2–99·6)	4·11 (-0·267–23·5)	0·469 (-4·99–6·9)
Short gestation	25·7 (20·9–31·2)	26·3 (21·7–31·9)	26·7 (21·6–33·4)	2·43 (-19·5–30·7)	1·42 (-18·5–29·6)	22·1 (18·5–26·3)	25·3 (21·1–30·3)	24·5 (20–29)	14·5 (-9·67–47·4)	-2·89 (-21·6–20)
Low birth weight	9·91 (8·18–12)	10·6 (8·73–12·8)	11·2 (9·24–13·7)	7·18 (-16·5–37·8)	5·49 (-17·7–36·8)	10 (8·44–11·8)	11·8 (9·83–14·1)	11·8 (9·84–14·1)	17·8 (-6·91–54·7)	-0·138 (-20·7–24·1)
High temperature	24·8 (13·7–37·4)	24·1 (12·7–36·7)	23·6 (12·7–35·9)	-2·85 (-9·47–1·03)	-2·25 (-7·91–4·46)	24·8 (13·7–37·4)	24·1 (12·7–36·7)	23·6 (12·7–35·9)	-2·85 (-9·47–1·03)	-2·25 (-7·91–4·46)
Low temperature	35·1 (31·9–39·3)	34·3 (31·2–38·6)	36·6 (33·3–40·8)	-2·28 (-4·91–0·158)	6·54 (3·45–10)	35·1 (31·9–39·3)	34·3 (31·2–38·6)	36·6 (33·3–40·8)	-2·28 (-4·91–0·158)	6·54 (3·45–10)
Kidney dysfunction	20·3 (14·5–27·4)	21·1 (15·3–28·3)	20·8 (15·2–28)	3·9 (-0·122–8·34)	-1·03 (-5·79–3·85)	21·4 (14·5–29·5)	23·6 (16·9–31·3)	24·2 (17·3–32·3)	10·2 (-0·986–27·3)	2·24 (-2·75–7·31)

Bullying victimization	5·89<br>(2·43–12)	9<br>(3·61–17·9)	8·75<br>(3·36–18)	52·9<br>(32·3–76·6)	-2·79<br>(-12·1–3·33)	4·25<br>(1·74–8·85)	7·88<br>(3·17–15·7)	7·8<br>(2·94–16·1)	85·5<br>(60·8–115)	-1·06<br>(-11·9–5·73)
High LDL cholesterol	66·7<br>(62·7–71·2)	34·3<br>(30·8–37·8)	30·7<br>(27·3–34·5)	-48·6<br>(-54·1–-43·2)	-10·4<br>(-15·9–-5·64)	65·9<br>(62·3–69·5)	34<br>(30·7–37·4)	31·3<br>(27·8–35·1)	-48·3<br>(-52·9–-43·6)	-8·16<br>(-12·1–-4·19)

Supplementary Results Table 1. Mongolia age-standardised Summary Exposure Values (SEVs) for most-detailed risk factors and percentage change, by sex, 1990, 2010, and 2019. Data in parentheses are 95% uncertainty intervals.

	Male					Female				
	1990	2010	2019	Percentage change 1990-2010	Percentage change 2010-2019	1990	2010	2019	Percentage change 1990-2010	Percentage change 2010-2019
Unsafe sanitation	82·8 (77·3–87·6)	71·9 (68·3–75·4)	65·6 (58·1–72·5)	-13·1 (-17·9–-7·18)	-8·68 (-18·2–0·561)	82·8 (77·3–87·6)	71·9 (68·3–75·4)	65·6 (58·1–72·5)	-13·1 (-17·9–-7·18)	-8·68 (-18·2–0·561)
Ambient particulate matter pollution	12·7 (5·44–24·2)	20·9 (14·5–28·3)	29 (20·6–38·7)	63·6 (-8·04–226)	38·8 (12·1–74·5)	10·9 (4·67–21·3)	18·8 (12·8–26)	27 (18·5–36·8)	72·1 (-4·87–250)	43·9 (15·1–81·2)
Household air pollution from solid fuels	28·3 (15·6–46·1)	12·5 (6·04–22·5)	6·73 (2·96–13·7)	-55·9 (-69·6–-40)	-45·9 (-60·4–-31·9)	30·7 (18·6–48)	14·7 (7·74–25·2)	8·21 (3·79–15·3)	-52·1 (-65·8–-37·1)	-44·1 (-58·9–-29·9)
Ambient ozone pollution	64·5 (28·2–100)	69·8 (30·3–100)	46·4 (18·9–79·2)	8·28 (0–18·7)	-33·6 (-46·5–-19·8)	64·5 (28·2–100)	69·8 (30·3–100)	46·4 (18·9–79·2)	8·28 (0–18·7)	-33·6 (-46·5–-19·8)
Residential radon	43·9 (6·68–100)	43·9 (6·68–100)	43·9 (6·68–100)	0 (0–0)	0 (0–0)	--	--	--	--	--
Lead exposure	68·2 (48·9–83·1)	56·6 (38·3–73·5)	48 (31·2–66)	-17 (-25·1–-10·1)	-15·2 (-21·3–-9·72)	46·9 (19·8–69·9)	39·7 (17·8–62·2)	33·6 (14·1–55·6)	-15·4 (-28·2–0·939)	-15·3 (-25·2–-9·25)
Child underweight	5·1 (3·71–6·47)	2·78 (1·88–3·67)	2·15 (1·39–2·95)	-45·5 (-55·3–-34·6)	-22·5 (-33·2–-12·5)	5·15 (3·73–6·43)	2·8 (1·87–3·67)	2·25 (1·48–3·04)	-45·7 (-55–-36·9)	-19·6 (-29·4–-11·3)
Iron deficiency	--	--	--	--	--	29·8 (27·2–32·4)	24·2 (22–26·4)	22·6 (20·2–25·2)	-18·8 (-28·6–-8·79)	-6·39 (-12·1–-0·563)
Vitamin A deficiency	16·8 (8·03–27·3)	3·7 (1·81–6·38)	2·08 (0·812–4·12)	-78 (-91–-45·1)	-43·7 (-67·6–-3·2)	7·78 (4·23–12·2)	2·96 (1·68–4·57)	1·89 (0·984–3·32)	-61·9 (-80·1–-20·3)	-36·2 (-56·1–-2·45)
Zinc deficiency	4·87 (0–14·3)	3·88 (0–11·8)	2·73 (0–9·01)	-20·4 (-100–NA)	-29·5 (-100–NA)	4·76 (0–13·9)	3·8 (0–11·9)	2·67 (0–8·7)	-20·2 (-100–NA)	-29·8 (-100–NA)
Smoking	20·2 (17·3–23·5)	19·8 (17–22·9)	21·1 (18·1–24·5)	-1·97 (-7·77–4·45)	6·6 (2·61–10·9)	1·76 (1·31–2·27)	1·88 (1·53–2·3)	2·22 (1·78–2·74)	6·59 (-14·9–33·1)	18·3 (3·92–32·8)
Secondhand smoke	32·2 (30–34·3)	33·4 (32·2–34·4)	35·6 (33·6–37·6)	3·76 (-3·91–12·5)	6·7 (1·27–12·6)	53·8 (51·5–56·1)	57·2 (56–58·2)	61·7 (59·5–63·7)	6·17 (1·33–11·5)	7·86 (4·46–11·2)
Alcohol use	7·81 (5·19–11·4)	12 (8·43–16·2)	14·2 (10–19·3)	53·3 (29–87·7)	18·4 (1·25–40·6)	1·48 (0·755–2·67)	2·85 (1·57–4·69)	3·58 (2·01–5·92)	92·3 (45·4–178)	25·6 (-6·06–66·3)
Drug use	0·145 (0·085–0·263)	0·18 (0·118–0·3)	0·18 (0·115–0·304)	23·8 (9·39–47)	0·151 (-8·48–9·4)	0·0803 (0·0456–0·149)	0·0903 (0·0551–0·158)	0·0919 (0·056–0·161)	12·5 (1·98–26·6)	1·73 (-5·93–9·53)
High fasting plasma glucose	3·45 (2·89–4·15)	4·81 (4·14–5·54)	5·19 (4·45–5·98)	39·3 (24·7–54·3)	7·98 (0·137–17·6)	3·1 (2·65–3·61)	4 (3·49–4·58)	4·56 (3·97–5·23)	28·9 (16·9–40·7)	14·1 (5·63–22·8)
High systolic blood pressure	36·6 (30·6–43·4)	39·4 (33·8–44·7)	40·6 (33·4–48·5)	7·62 (-14–35·1)	2·95 (-12·5–20·4)	34·3 (26–43·4)	32·5 (26–39·2)	33·4 (25·1–43·2)	-5·22 (-31·3–31·7)	3·04 (-20·1–31)
High body-mass index	13·1 (8·83–18·4)	18·9 (14·4–24·5)	23·3 (18·1–30·4)	43·7 (25·2–73·6)	23·3 (14·5–37·4)	18 (13–24·7)	22·1 (16·6–29)	25·6 (19·3–33·3)	22·6 (10·1–41·3)	16 (7·47–28·7)
Low bone mineral density	8·72 (4·78–14·3)	8·6 (4·79–14·3)	8·53 (4·68–14·2)	-1·41 (-24·9–29·1)	-0·763 (-24·5–30·3)	16·2 (10·6–23·5)	16·2 (10·4–23·1)	16 (10·5–23·1)	0·106 (-22·7–26·3)	-1·35 (-21·3–25·8)
Diet low in fruits	99·7 (99·4–100)	96·3 (94·6–98·2)	94·6 (91·9–97·2)	-3·33 (-4·92–-1·69)	-1·84 (-3·15–-0·746)	99·7 (99·5–100)	96·7 (95–98·4)	95·2 (92·7–97·7)	-3·09 (-4·57–-1·49)	-1·47 (-2·71–-0·514)
Diet low in vegetables	97·2 (94·8–99·1)	77·2 (61·4–90·6)	70·1 (50·2–86·6)	-20·6 (-35·5–-8·5)	-9·11 (-18·3–-3·4)	--	--	--	--	--
Diet low in whole grains	99·9 (100–100)	99·9 (100–100)	99·9 (100–100)	0·0013 (0–0)	-0·00117 (0–0)	99·9 (100–100)	99·9 (100–100)	99·9 (100–100)	0 (0–0)	0 (0–0)
Diet low in nuts and seeds	95·3 (49·9–100)	94·2 (47·9–98·9)	93·9 (47·7–98·6)	-1·13 (-8·15–7·78)	-0·401 (-4·49–5·44)	92·4 (49–99·4)	92·9 (47·2–97·7)	91·5 (46·9–97·2)	0·557 (-6·5–13·3)	-1·51 (-6·1–2·14)
Diet low in milk	64·6 (36·3–86·6)	48·8 (23·3–70·1)	42·9 (18·2–63·4)	-24·5 (-52·8–-5·53)	-12 (-26·6–-2·72)	64 (35·7–86·4)	47·9 (22·4–69·4)	42 (17·8–62·3)	-25·2 (-53·5–-5·77)	-12·3 (-27·4–-2·99)
Diet high in red meat	99·9 (99·6–100)	99·8 (99·4–100)	99·8 (99·4–100)	-0·109 (-0·486–0·189)	0·0379 (-0·322–0·343)	99·9 (99·5–100)	99·8 (99·4–100)	99·8 (99·4–100)	-0·109 (-0·479–0·205)	0·0442 (-0·3–0·385)
Diet high in processed meat	43·1 (22·9–67·6)	41·1 (22·2–64·3)	43·8 (24·1–67·6)	-4·63 (-27–20·2)	6·69 (-5·83–25·6)	49·9 (27·4–73·4)	47·7 (26·6–71·7)	51·2 (28·8–75·1)	-4·44 (-25·7–19·6)	7·37 (-4·4–25·9)
Diet high in sugar-sweetened beverages	21·5 (11·9–37·2)	23·8 (14·4–41·2)	25·2 (15·4–41·5)	10·7 (-21·5–65·8)	5·86 (-19·1–41·5)	20·7 (10·7–35·3)	22·1 (12–37·2)	24·8 (13·9–40·8)	6·68 (-30·6–64)	12·3 (-17·9–55·4)
Diet low in fiber	87·6 (79·2–94·5)	74·8 (61–85·9)	67·3 (51·2–80)	-14·6 (-23·2–-8·61)	-10·1 (-17·2–-5·51)	87·9 (79·3–94·7)	75·2 (61·1–86·7)	67·6 (51·8–81·1)	-14·5 (-23·3–-8·21)	-10 (-17·2–-5)
Diet low in seafood omega-3 fatty acids	97·7 (91·9–100)	98·1 (93·7–100)	95·4 (88·3–100)	0·418 (-3·01–5·87)	-2·82 (-8·69–0)	98·6 (94·6–100)	98·7 (94·5–100)	96·5 (90·9–100)	0·147 (-3·63–3·58)	-2·26 (-6·83–0·00202)
Diet low in polyunsaturated fatty acids	92·9 (73·5–100)	73·5 (34·8–100)	70·5 (30·9–100)	-20·9 (-53–0)	-3·99 (-13–0)	93·6 (75·8–100)	74·9 (37–100)	72·1 (33·3–100)	-19·9 (-50·5–0)	-3·83 (-12·1–0)
Diet high in trans fatty acids	41·9 (31·9–99·1)	38·5 (28·6–97·9)	37·2 (27·1–97·8)	-8·31 (-18·4–0·728)	-3·22 (-13·3–6·14)	43·6 (32·5–100)	40 (29·3–99·5)	38·9 (28–100)	-8·15 (-18–0)	-2·83 (-12·4–5·19)
Diet high in sodium	62·3 (38·2–80·3)	43·7 (14·3–67)	43 (13·9–67·4)	-29·8 (-66·9–-7·21)	-1·75 (-24·2–19·7)	30·6 (11·2–53·5)	18·8 (4·46–41·8)	18·4 (4·3–41·5)	-38·5 (-68·8–-15·3)	-2·09 (-19·6–12·6)
Low physical activity	1·89 (0·888–4·09)	1·91 (0·922–4·05)	1·99 (0·976–4·16)	0·823 (-10·7–13·8)	4·43 (-4·92–16·4)	1·5 (0·802–3·05)	1·51 (0·817–3·09)	1·54 (0·83–3·18)	0·566 (-9·54–11·9)	1·97 (-6·16–12·4)
Occupational asthmagens	23·6 (18·6–29·9)	23·3 (19·4–28·2)	21·8 (18–26·6)	-1·31 (-13·1–13·4)	-6·49 (-14·1–1·95)	16·4 (12·2–22·1)	15·1 (12·2–18·8)	13·6 (11–17·2)	-7·87 (-22·3–11)	-9·52 (-19·6–2·07)
Occupational particulate matter, gases, and fumes	11 (8·38–14·8)	10·6 (8·21–14·2)	10·3 (8·07–13·8)	-3·57 (-8·61–2·7)	-2·37 (-4·62–0·0929)	7·93 (5·95–11)	7·7 (5·9–10·4)	7·11 (5·5–9·59)	-2·96 (-9·99–4·71)	-7·62 (-10·3–-4·82)
Occupational noise	12·1 (10·9–13·4)	12 (11·3–12·8)	12 (11·4–12·7)	-1·17 (-6·06–4·65)	-0·183 (-2·01–2·04)	9·04 (7·99–10·2)	9·37 (8·72–10·2)	9·17 (8·66–9·87)	3·63 (-2·97–11·2)	-2·15 (-4·3–0·12)
Occupational injuries	--	--	--	--	--	--	--	--	--	--
Occupational ergonomic factors	28·2 (24·7–31·4)	22·7 (20·8–24·6)	19·2 (16·6–22)	-19·6 (-29–-6·55)	-15 (-24·7–-4·18)	--	--	--	--	--
Childhood sexual abuse	2·52 (1·65–3·56)	2·52 (1·65–3·56)	2·52 (1·65–3·56)	0 (0–0)	0 (0–0)	3·14 (2·26–4·3)	3·14 (2·26–4·3)	3·14 (2·26–4·3)	0 (0–0)	0 (0–0)
Intimate partner violence	--	--	--	--	--	11·3 (6·65–16·1)	11·3 (6·65–16·1)	11·3 (6·65–16·1)	0 (0–0)	0 (0–0)
Non-exclusive breastfeeding	15·4 (10·2–22·5)	14·1 (9·45–20·2)	14·1 (9·48–20·4)	-8·72 (-19·2–4·36)	-0·111 (-10·2–12·5)	15·4 (10·2–22·5)	14·1 (9·45–20·2)	14·1 (9·49–20·4)	-8·48 (-19–4·64)	-0·122 (-10·2–12·4)
Discontinued breastfeeding	6·83 (5·67–8·29)	6·61 (6·02–7·27)	6·56 (5·38–7·93)	-3·31 (-22·1–19·6)	-0·675 (-19·9–22·8)	6·82 (5·66–8·28)	6·59 (6·01–7·26)	6·55 (5·37–7·92)	-3·38 (-22·2–19·5)	-0·692 (-19·9–22·7)
Diet low in calcium	56·9 (43·2–77·3)	43 (29·1–64·7)	35·1 (21·6–57·5)	-24·5 (-34·9–-12·5)	-18·4 (-27·9–-9·83)	--	--	--	--	--
Occupational exposure to asbestos	1·23 (0·848–1·7)	1·77 (1·14–2·49)	1·32 (0·856–1·89)	43·1 (-15·1–139)	-25 (-50·2–12·2)	0·637 (0·285–1·13)	0·622 (0·249–1·05)	0·579 (0·219–1·05)	-2·48 (-49·4–110)	-6·84 (-49–66·7)
Occupational exposure to arsenic	0·292 (0·118–0·494)	0·314 (0·126–0·532)	0·314 (0·125–0·535)	7·58 (-3·74–20·8)	-0·192 (-6·65–7·12)	0·352 (0·134–0·613)	0·372 (0·147–0·623)	0·361 (0·141–0·603)	5·71 (-7·03–20·6)	-2·95 (-10·1–5·36)
Occupational exposure to benzene	0·687 (0·419–1·18)	0·835 (0·515–1·5)	0·867 (0·536–1·52)	21·6 (10·3–37·5)	3·82 (-1·85–11·5)	0·839 (0·495–1·45)	1·11 (0·688–1·98)	1·19 (0·738–2·13)	32·7 (18·9–52·3)	6·76 (0·55–13·9)
Occupational exposure to beryllium	0·0925 (0·0848–0·1)	0·0961 (0·0946–0·0976)	0·0936 (0·0905–0·0965)	3·84 (-4·63–13·5)	-2·63 (-6·07–0·579)	0·107 (0·0963–0·117)	0·112 (0·111–0·114)	0·108 (0·104–0·112)	5·34 (-4·13–16·2)	-3·47 (-7·33–0·349)
Occupational exposure to cadmium	0·158 (0·144–0·175)	0·172 (0·165–0·178)	0·171 (0·161–0·182)	8·4 (-2·23–20·6)	-0·134 (-5·96–6·31)	0·19 (0·169–0·213)	0·205 (0·196–0·214)	0·2 (0·187–0·215)	7·47 (-4·55–21·7)	-2·42 (-8·77–4·6)
Occupational exposure to chromium	0·347 (0·315–0·384)	0·404 (0·389–0·419)	0·413 (0·39–0·435)	16·5 (5·45–29·2)	2·01 (-3·52–8·02)	0·421 (0·371–0·472)	0·491 (0·472–0·512)	0·495 (0·466–0·53)	16·5 (3·07–32·4)	0·882 (-5·3–7·8)
Occupational exposure to diesel engine exhaust	2·58 (2·29–2·88)	3·17 (3·02–3·32)	3·21 (3·02–3·4)	22·9 (9·21–38·6)	1·29 (-5·01–7·79)	2·42 (2·15–2·72)	2·91 (2·78–3·04)	2·95 (2·76–3·15)	20 (6·57–36·3)	1·4 (-5·03–8·04)
Occupational exposure to formaldehyde	0·684 (0·54–0·776)	0·794 (0·645–0·847)	0·813 (0·658–0·886)	16 (3·3–30·9)	2·44 (-4·04–9·73)	--	--	--	--	--
Occupational exposure to nickel	0·413 (0·134–1·05)	0·428 (0·142–1·14)	0·415 (0·136–1·13)	3·78 (-8·4–18·6)	-3·16 (-10·6–4·02)	0·4 (0·127–1·04)	0·383 (0·122–1·02)	0·357 (0·117–0·934)	-4·37 (-15·3–8·89)	-6·79 (-13·6–2·13)
Occupational exposure to polycyclic aromatic hydrocarbons	0·769 (0·696–0·847)	0·886 (0·855–0·917)	0·905 (0·861–0·951)	15·1 (4·45–27·7)	2·19 (-2·66–7·23)	0·906 (0·804–1·01)	1·04 (1·01–1·09)	1·06 (0·998–1·12)	15·3 (2·9–30·2)	1·11 (-4·93–7·51)
Occupational exposure to silica	4·67 (2·42–10·1)	5·13 (2·7–11·3)	5·07 (2·64–11)	9·72 (-4·52–28·6)	-1·13 (-10–7·61)	3·95 (2–8·68)	3·6 (1·86–7·99)	3·31 (1·71–7·17)	-8·89 (-20·1–4·16)	-8·12 (-15–0·669)
Occupational exposure to sulfuric acid	0·974 (0·699–1·77)	0·94 (0·684–1·67)	0·927 (0·669–1·63)	-3·47 (-12·7–8·3)	-1·32 (-7·66–5·39)	1·02 (0·718–1·78)	0·964 (0·698–1·68)	0·898 (0·644–1·56)	-5·31 (-15·9–6·21)	-6·81 (-12·6–-0·294)
Unsafe sex	--	--	--	--	--	--	--	--	--	--
Occupational exposure to trichloroethylene	0·207 (0·189–0·227)	0·246 (0·239–0·254)	0·251 (0·239–0·263)	19 (7·81–31·6)	1·99 (-2·62–7·17)	0·251 (0·224–0·279)	0·302 (0·294–0·314)	0·306 (0·291–0·325)	20·4 (8·51–36)	1·38 (-4·2–7·4)
No access to handwashing facility	36·4 (35–37·7)	34·8 (33·6–36)	29·3 (28·2–30·5)	-4·24 (-8·17–-0·0789)	-15·7 (-18·2–-13·3)	36·4 (35–37·7)	34·8 (33·6–36)	29·3 (28·2–30·5)	-4·24 (-8·17–-0·0789)	-15·7 (-18·2–-13·3)
Child wasting	1·25 (0·943–1·56)	1·14 (0·863–1·45)	1·06 (0·792–1·34)	-8·72 (-17·3–0·565)	-6·95 (-13·5–-0·374)	1·8 (1·38–2·23)	1·63 (1·23–2·01)	1·53 (1·15–1·92)	-9·46 (-20·9–3·67)	-6·26 (-18–4·72)
Child stunting	19 (13–22·2)	10·8 (7·1–13·5)	8·71 (5·17–11·3)	-43·4 (-55·1–-33·6)	-19·2 (-32·3–-8·47)	19·8 (13·9–23·2)	11·7 (7·64–14·5)	9·54 (6·08–12·4)	-40·9 (-53–-30·5)	-18·5 (-31·4–-7·6)
Chewing tobacco	1·06 (0·803–1·38)	0·976 (0·737–1·27)	1·03 (0·767–1·36)	-7·99 (-32·1–26·3)	5·32 (-14·2–28·9)	--	--	--	--	--
Diet low in legumes	100 (100–100)	100 (100–100)	100 (100–100)	0 (0–0)	0 (0–0)	100 (100–100)	100 (100–100)	100 (100–100)	0 (0–0)	-0·000738 (0–0)
Short gestation	7·2 (4·83–10·9)	7·94 (5·8–11·1)	9·69 (7·13–13·4)	10·3 (-29·4–70·1)	22·1 (-18·4–77·1)	6·16 (4·2–9·09)	5·97 (4·44–8·07)	6·09 (4·24–8·27)	-3·04 (-39·7–51·3)	1·98 (-28·6–46·3)
Low birth weight	3·57 (2·6–5·07)	3·94 (2·99–5·3)	4·44 (3·32–6·07)	10·4 (-23·9–59·7)	12·7 (-22·2–64)	3·24 (2·39–4·56)	3·2 (2·48–4·19)	3·24 (2·4–4·24)	-1·39 (-34·7–46)	1·34 (-25·7–41·1)
High temperature	22·4 (9·16–37)	29·9 (15–45·7)	24·2 (9·95–39)	33·7 (9·97–78·1)	-19·3 (-35·2–-8·08)	22·4 (9·16–37)	29·9 (15–45·7)	24·2 (9·95–39)	33·7 (9·97–78·1)	-19·3 (-35·2–-8·08)
Low temperature	41·2 (33·6–48·8)	43 (34·2–52)	41·7 (33·4–50·1)	4·41 (-2·38–11·7)	-2·96 (-7–0·953)	41·2 (33·6–48·8)	43 (34·2–52)	41·7 (33·4–50·1)	4·41 (-2·38–11·7)	-2·96 (-7–0·953)
Kidney dysfunction	21·2 (14·2–29·4)	23·7 (16·4–31·9)	24·3 (17·2–32·6)	11·6 (7·26–18·3)	2·72 (-0·997–7·24)	21·9 (14·9–29·8)	23·3 (16·2–31·3)	24 (16·8–32·4)	6·09 (2·94–10·3)	3·03 (-0·215–7·47)
Bullying victimization	5·71 (2·32–11·9)	6·38 (2·98–12·4)	6·62 (3–12·7)	11·7 (-11·5–49·5)	3·8 (-5·4–13)	4·36 (1·78–8·97)	4·83 (2·21–9·59)	4·91 (2·24–9·94)	10·8 (-8·79–47·7)	1·73 (-5·87–10·6)
High LDL cholesterol	35·3 (31·5–39·1)	38·8 (35·4–42·4)	39·1 (35·5–42·7)	10·1 (3·67–17·6)	0·619 (-3·44–5·22)	35·3 (31·9–38·8)	38·5 (35·1–41·8)	38·7 (35·3–42·2)	9·15 (4·06–15)	0·447 (-3–4·07)

Supplementary Results Table 1. Wyoming age-standardised Summary Exposure Values (SEVs) for most-detailed risk factors and percentage change, by sex, 1990, 2010, and 2019. Data in parentheses are 95% uncertainty intervals.

	Male					Female				
	1990	2010	2019	Percentage change 1990-2010	Percentage change 2010-2019	1990	2010	2019	Percentage change 1990-2010	Percentage change 2010-2019
Unsafe sanitation	7·27 (3·39–13·2)	3·51 (1·62–6·85)	2·86 (1·22–5·46)	-51·7 (-79·9–17·9)	-18·4 (-59–60·9)	7·27 (3·39–13·2)	3·51 (1·62–6·85)	2·86 (1·22–5·46)	-51·7 (-79·9–17·9)	-18·4 (-59–60·9)
Ambient particulate matter pollution	3·06 (0·088–9·5)	1·05 (0·124–2·91)	1·05 (0·123–2·9)	-65·7 (-95·5–265)	0·295 (-14·6–19·9)	3·07 (0·101–9·51)	1·06 (0·132–2·9)	1·06 (0·134–2·9)	-65·5 (-95·3–237)	0·0961 (-16·5–17·6)
Household air pollution from solid fuels	0·0179 (0·000502–0·0679)	0·00602 (0·000251–0·0291)	0·0048 (0·000213–0·0213)	-66·4 (-96–123)	-20·3 (-61·1–61·5)	0·0237 (0·000805–0·0901)	0·00804 (0·000376–0·0379)	0·00638 (0·000304–0·0274)	-66·1 (-95·9–110)	-20·7 (-61·6–60·6)
Ambient ozone pollution	59·7 (25·3–100)	73·8 (32·8–100)	65·6 (28·2–100)	23·6 (0–52)	-11 (-23–0)	59·7 (25·3–100)	73·8 (32·8–100)	65·6 (28·2–100)	23·6 (0–52)	-11 (-23–0)
Residential radon	26 (3·83–71·8)	26 (3·83–71·8)	26 (3·83–71·8)	0 (0–0)	0 (0–0)	26 (3·83–71·8)	26 (3·83–71·8)	26 (3·83–71·8)	0 (0–0)	0 (0–0)
Lead exposure	26·4 (1·43–54·5)	17·3 (0·988–40·8)	12·5 (0·488–32·9)	-34·4 (-59·2–5·83)	-27·7 (-57·7–18·3)	16·1 (0·0385–43·1)	11·3 (0·0171–33·7)	8·53 (0·000121–28)	-29·6 (-81·8–76·6)	-24·7 (-84·3–15·7)
Child underweight	0·958 (0·542–1·47)	0·959 (0·545–1·45)	0·936 (0·519–1·4)	0·116 (-17·8–20·6)	-2·47 (-15·9–12·6)	0·803 (0·438–1·23)	0·846 (0·466–1·29)	0·865 (0·477–1·33)	5·39 (-16–33·7)	2·29 (-12–18·2)
Iron deficiency	--	--	--	--	--	11·6 (9·71–13·8)	12·2 (10·4–14·2)	12·3 (10·2–14·7)	4·69 (-6·97–18·9)	1·26 (-7·94–11·8)
Vitamin A deficiency	0·921 (0·257–2·41)	1·14 (0·29–3·01)	0·78 (0·207–2·09)	23·6 (-72·1–406)	-31·5 (-69·8–66·7)	1·78 (0·654–3·88)	1·68 (0·628–3·62)	1·42 (0·53–2·99)	-5·54 (-63·7–163)	-15·4 (-55·4–57·3)
Zinc deficiency	2·29 (0–7·29)	1·07 (0–3·39)	1·08 (0–3·44)	-53·1 (-100–NA)	0·328 (-21–NA)	2·33 (0–7·35)	1·08 (0–3·59)	1·08 (0–3·43)	-53·8 (-100–NA)	-0·0203 (-22·3–NA)
Smoking	29·2 (26·4–32·7)	19·1 (17·2–21)	17·2 (15·4–19·1)	-34·8 (-39·6–29·5)	-9·83 (-13·9–5·68)	21·1 (18·1–24·3)	16·6 (15–18·5)	14·4 (12·9–16·1)	-21 (-30·5–9·49)	-13·4 (-17·9–8·94)
Secondhand smoke	37·7 (34·6–40·9)	27 (25·4–28·7)	25·7 (23–28·6)	-28·4 (-35·2–20·5)	-4·78 (-13·6–4·35)	35·5 (32·9–38)	25·9 (24·7–27·1)	24·7 (22·6–26·8)	-27·1 (-32·8–20·5)	-4·61 (-11·3–2·34)
Alcohol use	14 (8·57–20·4)	15 (9·09–21·8)	15·7 (9·6–23·2)	7·48 (-23·1–56)	4·35 (-18·9–31·8)	9·51 (5·57–14·6)	11 (6·71–15·6)	10·7 (6·47–15·8)	15·8 (-18–71·2)	-2·48 (-24–22)
Drug use	0·45 (0·236–0·837)	0·728 (0·485–1·08)	1·29 (0·926–1·76)	61·7 (18·3–128)	77·8 (44·2–117)	0·359 (0·223–0·575)	0·702 (0·512–0·971)	1·08 (0·818–1·41)	95·5 (56·5–149)	53·4 (30·3–78·2)
High fasting plasma glucose	11·7 (9·65–14·2)	14·4 (12·3–17·1)	16·5 (13·9–19·5)	23·6 (2·42–50·6)	14·2 (-1·67–35·1)	9·43 (7·89–11·5)	11·6 (9·68–14·1)	13·5 (11·3–16·5)	23·3 (1·61–48)	16·5 (-1·27–36·6)
High systolic blood pressure	25·4 (20·2–31)	16 (13·9–18·1)	20 (15·3–25·3)	-36·9 (-48·6–20·5)	24·7 (-1·43–54·1)	22·3 (16·5–28·9)	13·1 (11·6–14·8)	17·6 (12·5–23·8)	-41·2 (-55·2–20·4)	34·3 (-3·51–77·7)
High body-mass index	25·9 (18·2–36·3)	40 (29·2–51·8)	42·5 (31·6–54)	54·7 (36·3–79·9)	6·11 (-0·175–13·9)	36 (23·5–48·6)	47·6 (34·3–59·3)	49·9 (36–62·3)	32·4 (15·7–61·4)	4·77 (-2·7–13)
Low bone mineral density	12·5 (7·45–19·3)	9·56 (5·31–15·9)	12 (7·13–19·5)	-23·4 (-42·4–0·238)	26 (-3·04–66·5)	16·5 (10·9–23·4)	17·1 (11·1–24·5)	19·5 (13·3–27·2)	3·37 (-19·5–33·5)	14·2 (-9·91–47·1)
Diet low in fruits	49 (37–62·5)	46·5 (34·1–60·2)	48·8 (36·5–63)	-5·17 (-17·8–8·39)	4·99 (-6·17–18·4)	52·2 (40·2–65)	47·2 (35·2–60·4)	48·7 (36·2–62)	-9·52 (-21·6–1·78)	3·21 (-8·3–15·4)
Diet low in vegetables	32·5 (17·2–53·2)	54·9 (34·1–75·6)	52·6 (32·5–73)	69·1 (25–156)	-4·18 (-13·1–2·9)	32·4 (16·6–54)	53 (32·8–74·3)	51·6 (31–72·6)	63·5 (23·7–146)	-2·75 (-12–5·63)
Diet low in whole grains	78·1 (67·4–88·3)	79·8 (69·9–89·5)	79·7 (69·6–89·5)	2·22 (-1·66–7·43)	-0·141 (-2·93–2·54)	--	--	--	--	--
Diet low in nuts and seeds	27·7 (7·47–56·6)	25·4 (7·88–55)	20·2 (6·1–53·9)	-8·21 (-44·9–75·1)	-20·3 (-46·8–5·42)	23·6 (5·72–54·1)	21·9 (6·54–54)	18·3 (5·61–53·1)	-7·4 (-43·7–70·3)	-16·4 (-43–12·7)
Diet low in milk	48·4 (25·4–70)	40·5 (18·3–60)	38·9 (18·2–58·9)	-16·3 (-37·9–2·6)	-4·12 (-18·3–9·99)	48·1 (23·6–68·4)	40·5 (17·6–60·4)	39·2 (17·1–59·3)	-15·9 (-36·6–1·5)	-3·27 (-16·5–9·74)
Diet high in red meat	77·6 (65·1–88·6)	77·3 (64·5–87·5)	77·6 (65–88·7)	-0·39 (-14·9–16·3)	0·369 (-10·2–11·6)	78·6 (66·8–89·2)	76·3 (62·9–87·5)	76·5 (63·3–87·5)	-2·93 (-18–12·8)	0·216 (-10·1–12·3)
Diet high in processed meat	78·1 (54·9–97·9)	86·5 (68·1–99·7)	86·6 (68·8–99·6)	10·8 (1·38–30·8)	0·027 (-4·6–5·15)	79·3 (56–98·2)	89·4 (74·6–99·7)	88·2 (72·5–99·7)	12·8 (1·12–41·1)	-1·43 (-7·02–2·25)
Diet high in sugar-sweetened beverages	45·8 (26·2–65)	65·5 (46·2–81·2)	66·4 (45·8–83·1)	42·8 (-1·58–129)	1·42 (-17·5–25)	50 (30·6–69·9)	71·3 (52·3–87)	70·7 (49·6–87·1)	42·5 (2–123)	-0·811 (-18·4–22·6)
Diet low in fiber	43·3 (27·5–58·8)	28·1 (16·8–41·5)	25·1 (13·8–38·2)	-35 (-50·7–19·6)	-10·8 (-29·8–8·98)	43·7 (27·4–59)	33·3 (20·3–47·2)	27·7 (15·2–40·9)	-24 (-39·4–6·19)	-16·6 (-33·9–0·315)
Diet low in seafood omega-3 fatty acids	80·5 (62·3–99·1)	73·8 (55·1–98·2)	73·7 (54·5–99·1)	-8·34 (-24·1–8·44)	-0·0559 (-16·6–18·3)	84·2 (68·9–99·7)	78·7 (60·8–99·4)	77·7 (59·5–98·4)	-6·55 (-21·1–4·81)	-1·3 (-14·4–13·2)
Diet low in polyunsaturated fatty acids	13·8 (3·99–40·3)	6·74 (1·9–24·5)	6·42 (1·82–24·5)	-51·2 (-79·3–7·62)	-4·73 (-29·9–13·2)	15·4 (4·32–44·4)	6·91 (1·99–24·6)	6·51 (1·89–24·6)	-55·2 (-80·4–8·53)	-5·83 (-29·7–11·4)
Diet high in trans fatty acids	89·4 (81·5–100)	87·9 (78·6–100)	87·4 (77·9–100)	-1·72 (-10·1–6·29)	-0·588 (-8·97–7·96)	88·3 (80·7–100)	86·7 (78·2–100)	86·2 (77–100)	-1·82 (-9·45–5·87)	-0·603 (-8·58–7·82)
Diet high in sodium	18·2 (4·46–41·1)	39·7 (11·7–64·2)	38·8 (11·2–64·5)	118 (36·3–406)	-2·05 (-27·2–31)	20·9 (4·93–45)	21·3 (4·92–44·7)	21·8 (4·98–46·2)	1·66 (-31–55·3)	2·23 (-17·6–31·2)
Low physical activity	1·88 (0·654–4·34)	1·54 (0·632–3·87)	1·47 (0·505–3·88)	-17·9 (-47·4–27·5)	-4·46 (-31·1–22·8)	4·67 (2·3–9·32)	4·26 (2·22–8·07)	4·54 (2·27–8·85)	-8·86 (-32·4–25·2)	6·71 (-7·98–22·9)
Occupational asthmagens	27 (23·1–31·5)	22·5 (19·3–26·2)	22·2 (18·7–26·6)	-16·5 (-27·8–3·68)	-1·3 (-10·3–8·35)	11·6 (9·57–13·9)	11·1 (9·28–13·3)	11·2 (9·15–13·5)	-3·53 (-17·3–13·3)	0·243 (-11·2–12·6)
Occupational particulate matter, gases, and fumes	10·6 (7·63–15)	9·88 (7·19–14)	9·44 (6·9–13·4)	-6·45 (-11·4–0·716)	-4·49 (-7·75–1·39)	5·28 (3·74–7·86)	5·09 (3·69–7·46)	4·89 (3·57–7·14)	-3·66 (-11·8–5·77)	-3·92 (-8·79–1·32)
Occupational noise	11·2 (10·2–12·2)	10·3 (9·61–11·1)	9·74 (9·12–10·6)	-7·59 (-11·7–3·23)	-5·51 (-7·77–3·03)	6·3 (5·54–7·17)	6·55 (6·04–7·2)	6·52 (6·09–7·12)	4·07 (-1·79–11·5)	-0·457 (-3·48–2·84)
Occupational injuries	--	--	--	--	--	--	--	--	--	--
Occupational ergonomic factors	14·6 (12–17·6)	13·1 (11–15·9)	13·5 (11·2–16·2)	-10·1 (-21·8–3·42)	2·98 (-6·16–13·6)	8·26 (6·49–10·5)	7·98 (6·49–9·88)	8·43 (6·62–10·8)	-3·33 (-19·8–15·7)	5·53 (-8·23–21·6)
Childhood sexual abuse	7·4 (5·05–10·5)	6·97 (4·6–9·93)	6·85 (4·54–9·67)	-5·83 (-12·2–5·83)	-1·68 (-8·51–5·53)	12·3 (8·94–17·3)	17·4 (12·9–24)	17·8 (13·1–24·7)	41·9 (30·7–54·8)	2·17 (-5–11)
Intimate partner violence	--	--	--	--	--	31·2 (17·4–40·3)	29·4 (16·6–38·5)	30·4 (16·8–39·5)	-5·64 (-14·1–6·44)	3·44 (-4·88–19·1)
Non-exclusive breastfeeding	25·9 (21·2–30·7)	24·8 (21·1–28·5)	24·9 (20·3–29·5)	-4·04 (-18·6–13·7)	0·255 (-13·1–15·6)	25·9 (21·2–30·6)	24·8 (21·1–28·5)	24·9 (20·3–29·5)	-4·03 (-18·6–13·8)	0·238 (-13·1–15·6)
Discontinued breastfeeding	26·7 (25·7–27·6)	25·4 (24–26·7)	25·6 (24·3–26·8)	-4·99 (-10·5–0·675)	0·802 (-4·7–6·74)	26·1 (25–27)	25·5 (24·1–26·8)	25·6 (24·3–26·8)	-2·17 (-7·77–3·78)	0·308 (-5·18–6·21)
Diet low in calcium	16·1 (7·46–30·1)	12·6 (6·24–22·7)	11·6 (5·76–21·2)	-21·7 (-32·7–8·22)	-7·86 (-17·2–0·0916)	11·6 (4·9–22)	9·39 (4·23–17·5)	8·74 (3·86–16·1)	-19 (-31·6–1·39)	-6·95 (-17·7–3·77)
Occupational exposure to asbestos	11·5 (10·4–12·7)	10 (9·19–11)	9·18 (7·27–11·2)	-12·8 (-23·3–0·644)	-8·51 (-27·8–13·7)	1·98 (1·07–2·36)	1·96 (1·04–2·34)	1·93 (1–2·61)	-1·12 (-15·4–16)	-1·49 (-24·5–27·9)
Occupational exposure to arsenic	0·409 (0–0·986)	0·453 (0–1·07)	0·477 (0–1·13)	10·6 (-3·72–NA)	5·39 (-6·21–NA)	0·409 (0–0·972)	0·434 (0–1·06)	0·447 (0–1·1)	6·09 (-8·55–NA)	2·93 (-7·86–NA)
Occupational exposure to benzene	0·783 (0·0463–2·24)	0·85 (0·0498–2·47)	0·877 (0·0543–2·58)	8·52 (-1·77–22·1)	3·23 (-4·17–13·8)	1·19 (0·0675–3·43)	1·36 (0·0772–3·82)	1·41 (0·0823–4·11)	14·7 (1·92–34·8)	3·76 (-4·7–17·1)
Occupational exposure to beryllium	0·0253 (0·0239–0·0267)	0·0259 (0·0251–0·0267)	0·0268 (0·0253–0·0284)	2·18 (-3·45–8·09)	3·53 (-1·95–9·6)	0·0272 (0·0246–0·0294)	0·0286 (0·0276–0·0296)	0·0295 (0·0276–0·0316)	5·25 (-2·97–16·2)	3·15 (-3·66–9·78)
Occupational exposure to cadmium	0·0494 (0·0451–0·0544)	0·0547 (0·0498–0·0607)	0·0575 (0·0511–0·0652)	10·7 (-2·23–25·6)	5·09 (-5·03–15·9)	0·0503 (0·0445–0·0565)	0·0536 (0·0487–0·0593)	0·055 (0·049–0·0623)	6·52 (-6·52–23·9)	2·69 (-6·87–13·5)
Occupational exposure to chromium	0·122 (0·112–0·133)	0·136 (0·126–0·149)	0·143 (0·129–0·159)	11·9 (0·417–25·3)	4·66 (-4·46–14·7)	--	--	--	--	--
Occupational exposure to diesel engine exhaust	1·01 (0·93–1·1)	0·967 (0·901–1·04)	0·969 (0·881–1·05)	-4·53 (-14·1–6·52)	0·291 (-7·22–8·65)	0·759 (0·669–0·847)	0·755 (0·685–0·835)	0·753 (0·667–0·852)	-0·45 (-13·8–14·9)	-0·287 (-10·1–10·8)
Occupational exposure to formaldehyde	0·234 (0·212–0·26)	0·257 (0·23–0·288)	0·267 (0·236–0·308)	9·56 (-4·65–26·5)	4·18 (-7·01–16·1)	0·24 (0·213–0·268)	0·259 (0·236–0·287)	0·267 (0·236–0·3)	8·18 (-5·84–25·1)	2·8 (-7·04–13·5)
Occupational exposure to nickel	0·537 (0–2·32)	0·452 (0–1·96)	0·452 (0–1·89)	-15·9 (-28·1–NA)	0·103 (-9·96–NA)	0·349 (0–1·51)	0·339 (0–1·53)	0·348 (0–1·58)	-2·85 (-17–NA)	2·56 (-9·15–NA)
Occupational exposure to polycyclic aromatic hydrocarbons	0·254 (0·234–0·275)	0·278 (0·258–0·301)	0·289 (0·262–0·321)	9·46 (-0·951–21·4)	4·03 (-4·72–13·4)	--	--	--	--	--
Occupational exposure to silica	6·8 (1·03–22·4)	5·01 (0·734–15·8)	4·88 (0·722–15·3)	-26·4 (-40·5–9)	-2·57 (-14–12·3)	3·31 (0·455–10·2)	2·88 (0·396–9·28)	2·92 (0·43–9·59)	-12·9 (-26·8–7·29)	1·17 (-10·6–15·4)
Occupational exposure to sulfuric acid	0·834 (0·178–2·59)	1·03 (0·215–3·34)	1·06 (0·227–3·39)	24 (6·36–43·9)	2·55 (-9·47–16·9)	0·967 (0·205–3·13)	1·24 (0·247–4·14)	1·22 (0·246–4)	28 (1·86–61·2)	-1·41 (-17–18·8)
Unsafe sex	--	--	--	--	--	--	--	--	--	--
Occupational exposure to trichloroethylene	0·0754 (0·0702–0·0811)	0·0865 (0·082–0·0917)	0·0904 (0·0836–0·0986)	14·6 (5·34–25)	4·48 (-3·07–12·8)	0·0795 (0·0712–0·0878)	0·0846 (0·0795–0·0906)	0·0861 (0·0786–0·0946)	6·45 (-4·85–21·2)	1·82 (-6·35–10·2)
No access to handwashing facility	3·38 (3·13–3·64)	3·11 (2·88–3·37)	3·08 (2·84–3·33)	-7·94 (-15·9–0·548)	-1·09 (-6·81–4·59)	3·38 (3·13–3·64)	3·11 (2·88–3·37)	3·08 (2·84–3·33)	-7·94 (-15·9–0·548)	-1·09 (-6·81–4·59)
Child wasting	0·749 (0·495–1·02)	0·727 (0·476–0·993)	0·724 (0·471–0·987)	-2·98 (-11·8–8·01)	-0·381 (-9·3–7·28)	0·771 (0·512–1·07)	0·762 (0·504–1·04)	0·757 (0·492–1·04)	-1·23 (-11·1–9·45)	-0·589 (-8·59–7·59)
Child stunting	2·4 (1·27–3·5)	2·07 (1·03–3·12)	2·01 (1·02–3·06)	-13·6 (-32·7–8·68)	-2·8 (-18·7–14·1)	1·94 (0·993–2·91)	1·78 (0·896–2·73)	1·76 (0·878–2·72)	-7·91 (-28·5–16·5)	-1·19 (-16·5–18·1)
Chewing tobacco	8·38 (6·34–10·9)	8·53 (6·31–11·4)	8·19 (5·79–11·4)	1·89 (-30·9–52)	-3·99 (-25·1–23·5)	--	--	--	--	--
Diet low in legumes	73·9 (37·7–99·1)	76·2 (42·7–99·6)	76 (43·8–99·6)	3·12 (-1·39–17·3)	-0·279 (-6·23–5·27)	72·2 (34·3–99)	74·5 (39·3–99·5)	74·7 (40·3–99·4)	3·19 (-1·05–20·9)	0·188 (-4·97–7·64)
Short gestation	14·9 (12·2–18·4)	16·2 (13·2–19·5)	16·8 (13·6–20·6)	8·85 (-15·3–39·7)	4·03 (-18·2–30·6)	13·9 (11·7–16·5)	14·6 (12·2–17·2)	15·5 (12·9–18·2)	5·09 (-15·9–32·8)	6·08 (-12·9–26·5)
Low birth weight	6·46 (5·28–7·88)	7·58 (6·22–9·21)	7·91 (6·52–9·56)	17·3 (-10·1–51·3)	4·4 (-18·9–30·8)	7·8 (6·57–9·19)	8·63 (7·36–10·1)	9·13 (7·82–10·6)	10·7 (-11·6–41)	5·8 (-12·7–27·5)
High temperature	26 (15·5–37·2)	24·4 (14–35·3)	25·4 (14·7–36·5)	-6·3 (-13·9–0·0584)	4·35 (-2·58–11·1)	26 (15·5–37·2)	24·4 (14–35·3)	25·4 (14·7–36·5)	-6·3 (-13·9–0·0584)	4·35 (-2·58–11·1)
Low temperature	37·2 (33–42·2)	37 (33·2–41·7)	37·8 (34·2–42·4)	-0·474 (-4·32–2·22)	2·24 (1·1–3·6)	37·2 (33–42·2)	37 (33·2–41·7)	37·8 (34·2–42·4)	-0·474 (-4·32–2·22)	2·24 (1·1–3·6)
Kidney dysfunction	18·7 (13·2–25·7)	19·1 (13·4–26·1)	19·2 (13·6–26·2)	1·86 (-2·17–6·3)	0·351 (-3·34–4·53)	21·1 (15–28·4)	21·9 (15·4–29·7)	22·7 (16·3–30·4)	3·68 (-0·404–8·79)	3·86 (-0·199–8·5)
Bullying victimization	5·94 (2·48–12·2)	8·92 (3·57–17·6)	8·73 (3·34–18)	50·1 (30·9–71·8)	-2·12 (-11·7–3·88)	4·31 (1·74–8·9)	7·82 (3·15–15·6)	7·77 (2·93–16·1)	81·5 (57·9–109)	-0·593 (-10·9–6·07)
High LDL cholesterol	65·7 (61·7–70·5)	34·2 (30·8–37·8)	30·4 (26·9–34·2)	-47·9 (-53·2–42·7)	-11·1 (-16–6·15)	64·7 (61·1–68·4)	34·4 (31·1–37·8)	31·6 (28–35·4)	-46·8 (-51·5–42·1)	-8·01 (-12·3–3·63)

Supplementary Results Table 1. Southern Latin America age-standardised Summary Exposure Values (SEVs) for most-detailed risk factors and percentage change, by sex, 1990, 2010, and 2019. Data in parentheses are 95% uncertainty intervals.

	Male					Female				
	1990	2010	2019	Percentage change 1990-2010	Percentage change 2010-2019	1990	2010	2019	Percentage change 1990-2010	Percentage change 2010-2019
Unsafe sanitation	35·6 (30·5–40·5)	8·16 (5·85–11·3)	5·56 (3·88–7·8)	-77·1 (-83·9–-67·7)	-31·9 (-50·4–-7·77)	37·9 (31·4–44·9)	7·61 (4·75–11·7)	5·25 (3·1–8·22)	-79·9 (-87·7–-68·5)	-31 (-56·7–1·82)
Ambient particulate matter pollution	14·3 (5·65–26·2)	14·2 (10·2–18·5)	14·6 (10–19·8)	-0·728 (-43·6–124)	2·92 (-17·3–27·3)	13·5 (5·3–25·1)	14 (10–18·1)	14·4 (9·91–19·6)	3·41 (-41·8–134)	3·57 (-17·1–28·9)
Household air pollution from solid fuels	3·26 (1·46–6·31)	0·774 (0·287–1·71)	0·432 (0·139–1·02)	-76·3 (-84·4–-66·8)	-44·2 (-59·9–-26·6)	4·01 (2·04–6·94)	1 (0·4–2·11)	0·561 (0·199–1·27)	-75 (-83·6–-64·8)	-43·8 (-59·7–-26·2)
Ambient ozone pollution	18·8 (6·93–35·2)	17·1 (6·25–31·5)	20·3 (7·65–37·7)	-9·03 (-26·5–8·52)	18·8 (0·223–46·3)	18·8 (6·94–35·2)	17 (6·24–31·5)	20·3 (7·66–37·7)	-9·2 (-26·6–8·28)	19 (0·247–46·5)
Residential radon	12·7 (3·25–34)	12·7 (3·27–33·8)	12·7 (3·23–34)	0·128 (-4·08–3·64)	-0·0918 (-1·98–0·725)	12·7 (3·26–34)	12·7 (3·25–33·9)	12·7 (3·23–34·2)	0·0614 (-3·95–2·64)	-0·104 (-1·94–0·801)
Lead exposure	39·6 (5·66–65·3)	33·7 (9·55–57·1)	26·7 (6·84–49·5)	-15 (-25·8–65·8)	-20·8 (-34·8–-13)	18·9 (1·53–44·9)	16·5 (2·25–40·2)	13·3 (1·61–35·1)	-13·1 (-23·4–45·8)	-19·2 (-30·7–-12·2)
Child underweight	1·63 (1·03–2·28)	1·28 (0·769–1·84)	1·2 (0·718–1·74)	-21·5 (-30·4–-12·9)	-6·09 (-12·5–0·664)	1·59 (0·998–2·22)	1·27 (0·775–1·81)	1·2 (0·724–1·73)	-19·8 (-28·5–-11·6)	-5·69 (-11·8–0·624)
Iron deficiency	--	--	--	--	--	14·4 (12·7–16·2)	12·7 (11–14·4)	12·3 (10·5–13·9)	-12 (-18–-5·92)	-3·2 (-8·1–1·77)
Vitamin A deficiency	23·8 (15·5–31·7)	14·9 (8·06–23·7)	11·2 (5·77–19)	-37·5 (-64·5–15·4)	-25 (-49·7–13·1)	16 (10·6–21·5)	12·3 (7·6–17·7)	9·6 (5·3–14·3)	-23 (-51·8–21·7)	-21·9 (-42·5–7·27)
Zinc deficiency	3·49 (0·0452–10·3)	2·72 (0–8·79)	2·29 (0–7·36)	-22·1 (-100–21)	-16 (-90·7–NA)	3·39 (0·0551–10)	2·64 (0–8·36)	2·23 (0–7·05)	-22·3 (-100–30·6)	-15·5 (-73·4–NA)
Smoking	22·4 (19·6–25·5)	18·6 (16·1–21·2)	17·1 (14·7–19·5)	-17 (-21·1–-12·5)	-8·16 (-11·2–-4·89)	15·2 (12·6–18·3)	13·5 (11·2–16)	12·2 (10·3–14·3)	-11·4 (-18·6–3·34)	-9·84 (-14·5–-4·87)
Secondhand smoke	44·6 (42·5–46·8)	38·2 (36–40·3)	37·2 (35–39·3)	-14·4 (-19·5–-9·22)	-2·59 (-6·45–1·63)	50·7 (48·8–52·5)	42·3 (40·5–43·9)	41·1 (39·4–42·8)	-16·6 (-20·4–-12·9)	-2·73 (-5·48–0·187)
Alcohol use	18·3 (13·5–24)	16·8 (12–23)	18 (12·5–24·7)	-7·95 (-14·6–0·813)	6·62 (-3·61–17·2)	10·3 (7·05–14·6)	8·67 (5·71–12·8)	9·22 (6–13·5)	-15·6 (-24–-8·21)	6·44 (-4·79–19·2)
Drug use	0·24 (0·0993–0·523)	0·261 (0·1–0·573)	0·254 (0·103–0·547)	8·74 (-6·1–26·7)	-2·45 (-14·4–10·2)	0·137 (0·064–0·278)	0·162 (0·0752–0·322)	0·151 (0·0754–0·299)	17·7 (2·85–37·1)	-6·39 (-18·4–5·75)
High fasting plasma glucose	6·48 (5·66–7·39)	9·41 (8·19–10·8)	12·8 (10·9–14·8)	45·1 (32·4–60)	36·4 (21·6–53·7)	6·98 (5·89–8·19)	9·99 (8·68–11·5)	11·6 (9·99–13·5)	43·2 (30·6–58·1)	16·1 (6·33–29·3)
High systolic blood pressure	19·3 (16·3–22·5)	26·2 (23–29·5)	26·2 (22·3–30·7)	35·9 (15·6–57·9)	0·0894 (-12·5–13·9)	16·1 (13–19·6)	24·2 (21·3–27·2)	22·7 (18·3–28)	50·3 (23·1–87·9)	-5·94 (-22·1–11·9)
High body-mass index	13·4 (8·55–19·4)	21·9 (15·7–29·9)	24·6 (17·7–33·1)	63·8 (43·3–99·5)	12·2 (7·39–18·7)	17·4 (10·4–27)	29·3 (20·3–42·6)	31·9 (22·2–45·6)	68·3 (41·9–118)	8·57 (2·4–16·7)
Low bone mineral density	10·7 (6·09–17)	9·96 (5·66–16·1)	9·62 (5·39–15·7)	-6·8 (-23·4–12·8)	-3·41 (-21–16·2)	19·6 (13·8–26·9)	17·8 (12·4–24·6)	17·2 (11·8–24·1)	-9·38 (-24·6–7·93)	-3·4 (-18–14)
Diet low in fruits	49·4 (38·2–62)	35·8 (26–47·3)	34·1 (24·4–44·8)	-27·5 (-35·6–-20·5)	-4·7 (-13·1–3·45)	51 (40–63·4)	37·8 (27·8–49·1)	36·2 (26·6–47·1)	-26 (-34·4–-18·9)	-4·14 (-13–4·07)
Diet low in vegetables	58·5 (38–78·6)	44·5 (26·1–65)	42·4 (25·1–63·1)	-23·8 (-37·2–-11·4)	-4·73 (-11·3–-0·0725)	56·4 (36–77)	42·3 (24·3–62·8)	40·3 (23·3–60·6)	-25 (-38·6–-12·5)	-4·78 (-11·6–0·921)
Diet low in whole grains	99·6 (99·3–99·9)	91·3 (86·6–95·8)	90·9 (86·1–95·7)	-8·33 (-12·7–-4·14)	-0·35 (-0·949–0·121)	98·5 (97·7–99·5)	91 (86·1–95·9)	88·9 (83–94·6)	-7·62 (-12–-3·52)	-2·39 (-3·89–-1·14)
Diet low in nuts and seeds	82·8 (43·5–93·1)	80 (41·1–90·1)	79 (39·6–90)	-3·36 (-15·9–12·4)	-1·21 (-13·6–12·5)	81·9 (42·3–92·4)	78·1 (41–89·1)	77·5 (39·5–88·8)	-4·62 (-18·1–13·1)	-0·748 (-13·6–14·1)
Diet low in milk	76·6 (54·8–93·9)	78·3 (56·9–95·4)	77 (55·6–94·3)	2·31 (1·03–4·68)	-1·7 (-4·09–-0·568)	76·2 (54·4–93·7)	77·9 (56·2–95·2)	76·5 (54·9–94)	2·21 (0·972–4·4)	-1·79 (-4·14–-0·678)
Diet high in red meat	85·9 (82·6–88·8)	90·5 (87·5–93·4)	91·2 (87·1–94·4)	5·38 (3·07–7·84)	0·724 (-1·63–3·01)	85·7 (82·5–88·8)	90·7 (87·6–93·5)	91·4 (87·4–94·5)	5·72 (3·48–8·3)	0·776 (-1·42–3·09)
Diet high in processed meat	43·8 (26·3–64·4)	51·2 (32·3–71·2)	56 (34·9–78·1)	17 (6·66–32·9)	9·27 (2·48–18·9)	49·4 (30·5–71·4)	57·3 (36·4–78·9)	62·7 (40·3–85·6)	16 (6·07–31·9)	9·44 (2·85–19·7)
Diet high in sugar-sweetened beverages	47·3 (32·8–62·2)	61·4 (47·4–74·4)	66·6 (52·2–78·6)	29·9 (9·99–61·5)	8·39 (-2·59–22·3)	53·7 (38·2–68·5)	69·4 (56·8–80·5)	73·9 (61·1–84·7)	29·2 (10·5–59)	6·41 (-1·75–17·8)
Diet low in fiber	48·5 (32·5–64)	43·9 (28·7–58·8)	41·8 (26·4–56·6)	-9·4 (-13·1–-6·72)	-4·91 (-12·1–1·14)	48·6 (31·7–64·5)	44 (28–59·2)	41·8 (26·6–56·8)	-9·52 (-13·4–-6·66)	-5·04 (-11·7–0·948)
Diet low in seafood omega-3 fatty acids	85·6 (70·5–99·8)	80·7 (64·1–99·2)	77·4 (61·7–98·3)	-5·79 (-18·2–4·33)	-4·1 (-15·7–8·7)	88·1 (75·1–100)	82·9 (66·4–99·9)	80·3 (64·6–99·6)	-5·96 (-18·5–3·19)	-3·14 (-14·7–8·92)
Diet low in polyunsaturated fatty acids	54·9 (17·5–95)	47·9 (14–89·4)	45·1 (12·9–87·4)	-12·7 (-24·7–-2·36)	-5·99 (-13–-1·19)	57·2 (18·9–96·5)	50·4 (15–91·4)	47·6 (13·7–88·7)	-11·8 (-23·9–-2·07)	-5·53 (-12·3–-1·13)
Diet high in trans fatty acids	36·7 (27·3–93·9)	33·8 (24·8–92·1)	33·4 (24·3–91·8)	-7·83 (-16·8–2·01e-14)	-1·12 (-8·82–6)	37·9 (28·2–98·6)	35·2 (25·5–96·5)	34·9 (24·6–97·9)	-7·21 (-15·6–0)	-0·949 (-8·47–5·39)
Diet high in sodium	40·4 (11·4–64·7)	39·1 (9·69–63·6)	40·5 (10·3–65·3)	-3·17 (-30·4–19·8)	3·59 (-11·8–24·1)	23·7 (5·33–47·4)	22·6 (4·45–47·7)	22·2 (4·34–47·5)	-4·71 (-33·1–9·79)	-1·43 (-13·6–7·93)
Low physical activity	1·09 (0·388–3·37)	1·15 (0·444–3·28)	1·27 (0·497–3·43)	5·67 (-21·6–46)	10·1 (0·17–27·5)	1·77 (0·782–3·61)	2·15 (1·1–4·02)	2·33 (1·2–4·29)	21·7 (-4·97–69·1)	8·25 (1·73–15·1)
Occupational asthmagens	22 (18·9–25·9)	20·6 (17·9–24·2)	20·3 (17·5–23·7)	-6·05 (-11·5–0·064)	-1·77 (-5·76–2·7)	8·84 (7·27–10·6)	10·9 (9·3–12·9)	11·9 (10–14)	23·5 (11·9–37·9)	8·94 (2·12–15·7)
Occupational particulate matter, gases, and fumes	11·5 (9·22–14·9)	10·9 (8·84–14)	10·8 (8·82–13·9)	-5·48 (-8·73–-2·07)	-0·451 (-2·13–1·29)	4·04 (3·17–5·32)	3·83 (3·03–5·13)	3·8 (3–5·06)	-5·34 (-12·2–2·98)	-0·806 (-3·97–2·62)
Occupational noise	12·8 (11·9–13·7)	12·6 (11·9–13·4)	12·8 (12·2–13·6)	-1·11 (-3·72–1·55)	1·36 (0·249–2·53)	6·13 (5·41–6·98)	6·61 (6·13–7·26)	7·03 (6·64–7·65)	7·83 (1·98–15·1)	6·25 (3·94–8·7)
Occupational injuries	--	--	--	--	--	--	--	--	--	--
Occupational ergonomic factors	14·1 (12·3–16·5)	14·2 (12·4–16·4)	13·9 (12–16·1)	1·27 (-5·87–8·77)	-2·75 (-7·29–1·77)	5·89 (4·88–7·16)	7·29 (6·18–8·72)	7·56 (6·33–9·06)	23·8 (11·4–37·8)	3·61 (-1·7–9·03)
Childhood sexual abuse	6·21 (4·15–8·84)	6·02 (4·21–8·14)	6·21 (4·15–8·82)	-3·12 (-11·3–6·64)	3·17 (-5·51–13·5)	12·1 (9–17)	11·1 (8·79–14·4)	12·2 (9·03–17·1)	-8·14 (-19·5–4·58)	9·11 (-2·9–26·3)
Intimate partner violence	--	--	--	--	--	15·4 (8·37–20·8)	15·5 (8·61–20·8)	15·4 (8·53–20·9)	0·767 (-1·23–3·75)	-0·29 (-1·67–1·62)
Non-exclusive breastfeeding	20·9 (15·2–28·6)	20·6 (15–27·8)	20·3 (14·7–27·3)	-1·28 (-8·81–7·99)	-1·49 (-8·43–6·15)	20·9 (15·2–28·6)	20·6 (15–27·8)	20·3 (14·7–27·3)	-1·27 (-8·8–8)	-1·5 (-8·45–6·13)
Discontinued breastfeeding	15·8 (14·6–17·1)	15·4 (14·8–16·1)	14·5 (13·4–15·5)	-2·55 (-9·21–5·9)	-6·33 (-14·2–2·26)	15·8 (14·6–17·1)	15·4 (14·8–16·1)	14·5 (13·4–15·5)	-2·33 (-9·02–6·13)	-6·32 (-14·2–2·28)
Diet low in calcium	42·4 (28·9–63·4)	38·7 (25·3–60)	35·9 (22·9–57·3)	-8·81 (-13·5–-4·72)	-7·08 (-11·6–-3·65)	34 (20·9–55·9)	30·5 (17·7–52·1)	28 (15·7–49·1)	-10·2 (-16·1–-5·44)	-8·17 (-14·1–-4·24)
Occupational exposure to asbestos	2·78 (2·55–3·06)	3·66 (3·44–3·87)	3·53 (2·73–4·48)	31·7 (19–44·2)	-3·68 (-25·4–22·1)	1·02 (0·785–1·77)	1·4 (0·852–1·59)	1·46 (0·824–1·97)	36·5 (-24–81·4)	4·6 (-20·6–34·9)
Occupational exposure to arsenic	0·555 (0·222–0·949)	0·514 (0·206–0·87)	0·517 (0·208–0·877)	-7·47 (-14·2–0·222)	0·57 (-4·61–6·39)	0·373 (0·154–0·637)	0·392 (0·162–0·661)	0·397 (0·163–0·674)	5·28 (-6·3–18)	1·26 (-4·34–7·63)
Occupational exposure to benzene	1·01 (0·609–1·86)	1·13 (0·699–2·02)	1·16 (0·712–2·06)	12·1 (6·55–18·8)	1·89 (-1·18–5·92)	1·19 (0·733–2·17)	1·56 (0·951–2·77)	1·71 (1·05–3·01)	30·5 (19·2–45·3)	9·68 (5·09–15·5)
Occupational exposure to beryllium	0·119 (0·114–0·124)	0·126 (0·125–0·127)	0·127 (0·124–0·129)	5·79 (1·16–10·3)	0·906 (-1·31–3·08)	0·0881 (0·0805–0·0953)	0·108 (0·106–0·109)	0·115 (0·111–0·118)	22·1 (12·3–33·5)	6·51 (3·32–9·78)
Occupational exposure to cadmium	0·294 (0·276–0·312)	0·276 (0·268–0·284)	0·277 (0·264–0·292)	-6·12 (-12·4–0·591)	0·621 (-4·01–5·64)	0·199 (0·179–0·22)	0·214 (0·208–0·221)	0·219 (0·208–0·232)	7·82 (-3·28–20)	2·38 (-2·69–7·85)
Occupational exposure to chromium	0·703 (0·663–0·745)	0·675 (0·659–0·693)	0·681 (0·654–0·711)	-3·99 (-9·84–2·24)	0·906 (-3·1–5·08)	0·504 (0·475–0·535)	0·65 (0·629–0·672)	0·673 (0·631–0·72)	29·1 (21·1–38·2)	3·56 (-2·39–9·77)
Occupational exposure to diesel engine exhaust	3·94 (3·75–4·15)	4·64 (4·53–4·75)	4·7 (4·53–4·87)	17·6 (11·1–24·4)	1·42 (-1·92–4·99)	1·9 (1·73–2·07)	2·39 (2·32–2·46)	2·61 (2·47–2·75)	25·6 (14·6–38·3)	9·04 (3·7–14·8)
Occupational exposure to formaldehyde	1·45 (1·36–1·55)	1·35 (1·29–1·4)	1·35 (1·28–1·43)	-7·12 (-13·7–0·235)	0·248 (-4·57–5·68)	1·01 (0·913–1·11)	1·12 (1·08–1·15)	1·16 (1·1–1·22)	10·8 (0·18–22·3)	3·52 (-1·16–8·64)
Occupational exposure to nickel	0·544 (0·178–1·45)	0·578 (0·193–1·51)	0·585 (0·192–1·52)	6·31 (-0·73–14·5)	1·16 (-3·39–6·16)	0·314 (0·101–0·822)	0·326 (0·108–0·856)	0·324 (0·106–0·864)	3·62 (-8·35–17·6)	-0·553 (-6·91–6·79)
Occupational exposure to polycyclic aromatic hydrocarbons	1·38 (1·3–1·46)	1·39 (1·37–1·43)	1·41 (1·36–1·47)	1·12 (-4·67–7·55)	1·32 (-2·18–5·16)	1·07 (0·976–1·17)	1·22 (1·2–1·24)	1·28 (1·23–1·33)	13·6 (3·41–25·2)	4·89 (0·946–9·09)
Occupational exposure to silica	4·97 (2·56–11·1)	6·82 (3·58–15)	7·02 (3·69–15·6)	37·2 (27–50·1)	2·89 (-2·72–9·43)	2·21 (1·12–4·9)	2·32 (1·17–5·06)	2·27 (1·16–4·9)	4·53 (-7·6–18·7)	-1·94 (-8·11–6·06)
Occupational exposure to sulfuric acid	1·32 (0·959–2·31)	1·21 (0·885–2·1)	1·2 (0·868–2·05)	-8·29 (-13·9–-1·88)	-1·45 (-5·8–3·21)	0·824 (0·581–1·44)	0·825 (0·596–1·45)	0·802 (0·58–1·43)	0·0582 (-11–12·1)	-2·8 (-8·45–3·26)
Unsafe sex	--	--	--	--	--	--	--	--	--	--
Occupational exposure to trichloroethylene	0·419 (0·4–0·443)	0·406 (0·4–0·416)	0·409 (0·398–0·424)	-2·94 (-7·84–2·35)	0·691 (-2·36–3·96)	0·274 (0·249–0·299)	0·317 (0·311–0·325)	0·336 (0·323–0·351)	15·6 (5·32–28)	6·08 (1·8–10·3)
No access to handwashing facility	22·1 (21·2–22·9)	8·64 (8·3–8·98)	7·53 (7·23–7·85)	-60·9 (-62·5–-59)	-12·8 (-15·3–-10·1)	22·1 (21·2–22·9)	8·64 (8·3–8·99)	7·53 (7·23–7·85)	-60·8 (-62·5–-59)	-12·8 (-15·3–-10·1)
Child wasting	1·31 (0·938–1·69)	1·28 (0·925–1·66)	1·24 (0·891–1·62)	-2·37 (-9·14–6·57)	-3·51 (-9·93–1·55)	1·29 (0·939–1·68)	1·24 (0·893–1·61)	1·21 (0·864–1·57)	-4·05 (-13·6–6·63)	-2·23 (-11·1–6·98)
Child stunting	6·43 (4·07–8·19)	4·83 (2·89–6·44)	4·34 (2·53–5·83)	-24·8 (-37·4–-13·9)	-10·2 (-19·3–-1·2)	6·37 (4·04–8·13)	4·37 (2·63–5·93)	4·07 (2·37–5·58)	-31·4 (-44·1–-19·9)	-6·82 (-18·7–3·66)
Chewing tobacco	0·174 (0·147–0·204)	0·161 (0·137–0·19)	0·157 (0·132–0·184)	-7·35 (-23·7–13)	-2·41 (-13·9–10·1)	0·189 (0·137–0·257)	0·186 (0·135–0·255)	0·179 (0·13–0·244)	-1·35 (-31·4–43)	-4·1 (-24·1–20·8)
Diet low in legumes	87·9 (72·2–99·9)	80·7 (56–98·7)	81·7 (58·3–99)	-8·23 (-22·8–-1·11)	1·26 (-0·134–4·55)	85·6 (66·8–99·7)	77·9 (50·7–97·6)	79 (53·5–98·2)	-9·05 (-24·1–-1·78)	1·46 (-0·281–5·29)
Short gestation	12·8 (10·7–15·4)	16 (13·9–18·2)	16·3 (13·6–19·2)	24·7 (5·1–48·1)	1·66 (-13·8–21·3)	13·5 (11·5–15·9)	16·2 (14·3–18·4)	15·9 (13·6–18·5)	20·7 (3·27–42·6)	-2·1 (-16·6–14·7)
Low birth weight	6·64 (5·65–7·82)	7·93 (7·06–8·97)	8·46 (7·24–9·87)	19·4 (-0·81–42·8)	6·68 (-10·9–27·2)	7·6 (6·6–8·77)	9·17 (8·17–10·2)	8·83 (7·62–10·1)	20·6 (3·02–41·9)	-3·66 (-17·8–12·7)

High temperature	25·4 (18–33·5)	23·4 (17·5–30·3)	25·3 (19·2–32)	-7·99 (-26·2–18·5)	8·1 (4·1–13·4)	25·4 (18–33·5)	23·4 (17·5–30·3)	25·3 (19·2–32)	-7·99 (-26·2–18·5)	8·1 (4·1–13·4)
Low temperature	32·6 (27·4–38·9)	33·1 (28–39·3)	31·3 (26·2–37·4)	1·51 (0·626–2·53)	-5·44 (-6·74–-4·12)	32·6 (27·4–38·9)	33·1 (28–39·3)	31·3 (26·2–37·4)	1·51 (0·626–2·53)	-5·44 (-6·74–-4·12)
Kidney dysfunction	16·6 (11·4–23·2)	18·3 (13–25·1)	18·6 (13·3–25·7)	10·8 (6·44–17·1)	1·44 (-1·86–4·65)	17·7 (12–24·7)	19·8 (14·1–27)	20·1 (14·2–27·2)	11·9 (7·62–18·5)	1·48 (-1·37–4·93)
Bullying victimization	7·24 (2·96–14·4)	7·83 (3·59–15)	7·3 (3·31–14)	8·18 (-11·9–49·4)	-6·77 (-13·8–-3·01)	6·15 (2·5–12·4)	6·61 (3·05–12·7)	6·15 (2·7–12)	7·42 (-15·8–50·6)	-6·94 (-15·3–-2·87)
High LDL cholesterol	36·5 (33·2–39·9)	40·1 (37–43·3)	40·8 (37·6–44·3)	9·96 (5·48–14·3)	1·87 (-1·05–5·02)	37·6 (34·4–41)	40·8 (37·7–44·1)	41·6 (38·4–44·7)	8·37 (4·61–12·6)	1·97 (-0·501–4·51)

Supplementary Results Table 1. Argentina age-standardised Summary Exposure Values (SEVs) for most-detailed risk factors and percentage change, by sex, 1990, 2010, and 2019. Data in parentheses are 95% uncertainty intervals.

	Male					Female				
	1990	2010	2019	Percentage change 1990-2010	Percentage change 2010-2019	1990	2010	2019	Percentage change 1990-2010	Percentage change 2010-2019
Unsafe sanitation	37·9 (31·4–44·9)	7·61 (4·75–11·7)	5·25 (3·1–8·22)	-79·9 (-87·7–-68·5)	-31 (-56·7–1·82)	37·9 (31·4–44·9)	7·61 (4·75–11·7)	5·25 (3·1–8·22)	-79·9 (-87·7–-68·5)	-31 (-56·7–1·82)
Ambient particulate matter pollution	13·5 (4·81–25·7)	12·3 (7·95–17·2)	12·1 (7·13–17·7)	-9·13 (-49·2–113)	-1·66 (-22·9–25)	12·8 (4·55–24·8)	12·1 (7·88–16·9)	12 (7·09–17·6)	-5·58 (-47·7–117)	-1·04 (-22·8–25)
Household air pollution from solid fuels	2·58 (1·11–5·05)	0·63 (0·225–1·43)	0·365 (0·116–0·924)	-75·6 (-85–-62·5)	-42 (-63–-14·4)	3·2 (1·51–5·65)	0·817 (0·325–1·74)	0·477 (0·156–1·15)	-74·5 (-84·3–-60·7)	-41·6 (-62·7–-14·1)
Ambient ozone pollution	16·8 (6·03–32·2)	16·6 (6·02–31·3)	21·5 (8·4–39·8)	-1·36 (-23·3–25·6)	29·5 (4·73–66·7)	16·8 (6·03–32·2)	16·6 (6·02–31·3)	21·5 (8·4–39·8)	-1·36 (-23·3–25·6)	29·5 (4·73–66·7)
Residential radon	12 (1·74–41·3)	12 (1·74–41·3)	12 (1·74–41·3)	0 (0–0)	0 (0–0)	12 (1·74–41·3)	12 (1·74–41·3)	12 (1·74–41·3)	0 (0–0)	0 (0–0)
Lead exposure	44·6 (1·99–72·6)	40·3 (9·33–65·4)	32·2 (6·93–56·8)	-9·7 (-21·6–482)	-20 (-34·7–-12·3)	18·2 (1·78e-12–46·6)	16·5 (2·47e-12–42·7)	13·6 (2·84e-12–37·5)	-9·47 (-46·5–2·09e+13)	-17·6 (-40·9–1·05e+11)
Child underweight	1·81 (1·13–2·54)	1·39 (0·847–2)	1·31 (0·783–1·9)	-23 (-33·9–-12)	-5·57 (-13·3–3·16)	1·75 (1·1–2·45)	1·41 (0·859–2·02)	1·33 (0·798–1·93)	-19·5 (-29·7–-9·29)	-5·48 (-13–2·48)
Iron deficiency	--	--	--	--	--	14·7 (12·9–16·7)	13·1 (11·3–15)	12·7 (10·7–14·6)	-10·9 (-19–-2·63)	-3·26 (-9·82–3·44)
Vitamin A deficiency	25·9 (15·1–36·8)	16·2 (7·5–28·5)	12·2 (5·09–23·4)	-37·3 (-69·6–33·3)	-24·5 (-54·7–26·6)	17·5 (10·7–25·1)	13·6 (7·45–20·8)	10·7 (5·47–17)	-22·6 (-56·7–38·5)	-21·4 (-46·8–18·2)
Zinc deficiency	2·51 (0–8)	2·37 (0–7·56)	2·04 (0–6·67)	-5·5 (-28·8–NA)	-14·1 (-51·9–NA)	2·46 (0–7·56)	2·3 (0–7·36)	1·99 (0–6·45)	-6·55 (-32·6–NA)	-13·4 (-41·8–NA)
Smoking	22·8 (20–26·1)	18·4 (16–21)	17 (14·8–19·4)	-19·3 (-24·8–-13·3)	-7·62 (-12–-3·02)	15·1 (12·3–18·4)	13 (10·8–15·5)	11·7 (9·88–13·7)	-13·4 (-23·6–-1·55)	-10·7 (-17–-3·58)
Secondhand smoke	42·6 (39·6–45·8)	36 (33·1–39)	34·9 (32–37·8)	-15·5 (-22·5–-7·99)	-3·12 (-8·67–2·58)	49·5 (47–52·1)	40·8 (38·3–43·1)	39·4 (37·2–41·7)	-17·6 (-22·8–-12·5)	-3·38 (-7·31–0·749)
Alcohol use	19·5 (14·4–25·5)	17·1 (12·1–23·4)	17·9 (12·4–25)	-12·5 (-20·2–-4·94)	4·98 (-8·5–19)	11·2 (7·73–15·7)	8·91 (5·86–13·3)	9·34 (5·95–14)	-20·2 (-30·1–-11·1)	4·84 (-11·7–22·3)
Drug use	0·227 (0·0828–0·518)	0·255 (0·0942–0·573)	0·254 (0·0982–0·54)	12·3 (-5·01–35·1)	-0·221 (-14·9–17·3)	0·129 (0·0584–0·269)	0·153 (0·0691–0·31)	0·144 (0·0702–0·296)	18·2 (0·696–42)	-5·78 (-21·2–10·4)
High fasting plasma glucose	6·56 (5·7–7·54)	8·74 (7·58–10·2)	12·2 (10·1–14·6)	33·1 (20·5–48·8)	40·2 (20·1–63·9)	7·02 (5·93–8·29)	9·04 (7·76–10·5)	10·8 (9·1–13)	28·7 (14·1–43·5)	19·4 (6·14–37·2)
High systolic blood pressure	15·2 (11·7–19·2)	24·7 (20·7–29·1)	23·9 (18·7–29·6)	62·9 (26·2–114)	-3·32 (-20·6–16·5)	11·7 (8·03–15·8)	25·4 (21·6–29·5)	22·5 (15·9–29·9)	116 (54·1–216)	-11·4 (-33·5–12·8)
High body-mass index	12·1 (7·61–17·7)	19·1 (13·5–26·2)	21·7 (15·4–29·4)	57·8 (36·4–96·2)	13·9 (6·56–24·3)	15·2 (8·53–24·1)	26·1 (17·8–38·7)	28·8 (19·6–41·7)	71·7 (39·3–140)	10·1 (0·0965–22·9)
Low bone mineral density	10·9 (6·2–17·5)	10·3 (5·72–16·8)	9·93 (5·53–16·3)	-5·93 (-29·2–23)	-3·36 (-26·5–27·2)	20 (13·7–27·4)	18·3 (12·3–25·7)	17·6 (12–24·7)	-8·58 (-28·8–15·8)	-3·79 (-23·7–23)
Diet low in fruits	44·6 (32·6–58)	27·7 (18·1–37·9)	26·9 (17·4–37·4)	-37·9 (-49·4–-26·9)	-2·95 (-17·8–13·4)	46·3 (34·6–59·5)	30·1 (20·5–41·1)	29·4 (19·6–40·1)	-35 (-47·2–-24)	-2·38 (-18·4–13)
Diet low in vegetables	60·8 (39·4–81·3)	47·7 (27·2–70·3)	46·4 (26·8–69·6)	-21·6 (-36·3–-9·09)	-2·7 (-10·7–3·19)	58·9 (37·3–79·5)	45·5 (25·8–67·7)	44·3 (25–66·9)	-22·8 (-37·8–-9·7)	-2·66 (-10·9–4·45)
Diet low in whole grains	99·9 (99·7–100)	90·9 (85·8–96)	90·8 (85·6–96)	-8·97 (-14·1–-4·02)	-0·117 (-0·864–0·537)	--	--	--	--	--
Diet low in nuts and seeds	85·6 (47·5–96·7)	85·9 (46·9–96·8)	86·3 (45·5–96·3)	0·395 (-15·9–18·3)	0·392 (-17·4–21·7)	82·9 (47·2–95·4)	83·4 (44·6–94·9)	84·6 (46·3–95·7)	0·55 (-16·5–23·5)	1·49 (-16·1–22·2)
Diet low in milk	75 (49·4–95·3)	75·6 (49·9–96·2)	74 (47·5–95·2)	0·869 (-0·333–2·43)	-2·06 (-6·5–0·235)	74·6 (48·8–95)	75·2 (49·3–95·9)	73·6 (46·9–94·8)	0·865 (-0·264–2·43)	-2·12 (-6·11–0·223)
Diet high in red meat	99·3 (98·7–99·7)	99·1 (98·4–99·6)	98·8 (97·2–99·7)	-0·25 (-0·84–0·269)	-0·279 (-1·67–0·567)	99·3 (98·7–99·7)	99·1 (98·4–99·6)	98·8 (97·3–99·7)	-0·232 (-0·791–0·288)	-0·248 (-1·62–0·6)
Diet high in processed meat	34·2 (18·2–55·6)	39·5 (21·8–62·5)	46 (26–69·3)	15·4 (0·0982–39·5)	16·5 (3·26–39·6)	40·3 (21·5–63·3)	47·4 (27·1–71)	54·7 (32·2–79·7)	17·8 (1·64–46)	15·4 (3·53–37·2)
Diet high in sugar-sweetened beverages	53·5 (37·3–69·5)	66·4 (52·6–78·3)	69·5 (53·5–83)	24·1 (2·32–60)	4·76 (-9·52–20·3)	60·8 (43·2–76·9)	74·6 (62·1–85)	77·1 (62·7–88)	22·7 (2·97–56·3)	3·4 (-7·51–15·7)
Diet low in fiber	47·4 (31·6–62·8)	47·1 (31·4–61·9)	45·7 (29·7–61·5)	-0·681 (-3·74–3·08)	-2·86 (-11·8–5·34)	47·5 (30·6–63·5)	47·1 (30·5–62·5)	45·7 (29·8–60·8)	-0·7 (-3·93–3·4)	-3·12 (-11·3–4·5)
Diet low in seafood omega-3 fatty acids	82 (62·2–100)	78·5 (58·8–99·3)	74·7 (57·3–97·9)	-4·33 (-20–13·1)	-4·85 (-21·1–13·4)	84·9 (67·5–100)	80·3 (60·4–100)	77·3 (59·4–99·4)	-5·44 (-23–9·91)	-3·64 (-20·6–15·4)
Diet low in polyunsaturated fatty acids	53·2 (15·7–94·4)	48·7 (13·9–90·5)	45·6 (12·8–88·6)	-8·41 (-17·7–-1·14)	-6·38 (-15·5–-0·773)	55·6 (17·5–95·8)	51·3 (14·8–92·5)	48·3 (13·8–89·9)	-7·75 (-16·5–-0·951)	-5·8 (-13·9–-0·547)
Diet high in trans fatty acids	34·6 (25·2–92·8)	34 (25–91·1)	33·9 (24·5–90·9)	-1·55 (-12·2–9·56)	-0·521 (-11·7–9·24)	35·8 (25·7–97·9)	35·3 (25·5–95·7)	35·2 (25–98)	-1·22 (-10·7–9·58)	-0·299 (-11–9·2)
Diet high in sodium	39·5 (10·1–65·3)	38·2 (8·92–63·5)	39·8 (9·6–66)	-3·35 (-37·5–33·4)	4·05 (-16·7–31·8)	22·3 (5·02–46·2)	22·2 (4·28–47·9)	21·9 (4·21–47·8)	-0·203 (-32·6–22)	-1·5 (-18·1–14·6)
Low physical activity	0·719 (0·253–3·04)	0·707 (0·253–3)	0·765 (0·265–3·12)	-1·68 (-26·8–17·8)	8·19 (-1·79–25·4)	0·864 (0·358–2·56)	0·877 (0·368–2·51)	1·02 (0·418–2·72)	1·52 (-22·1–29·4)	16·5 (5·21–35·3)
Occupational asthmagens	22·1 (18·9–26·4)	20·6 (17·7–24·2)	20·3 (17·3–24)	-7·17 (-14·4–1·52)	-1·26 (-6·72–5·26)	9·97 (7·97–12·2)	11·5 (9·76–13·6)	12·3 (10·2–14·7)	15·8 (1·16–34)	6·28 (-2·65–15·3)
Occupational particulate matter, gases, and fumes	12 (9·5–15·6)	10·8 (8·76–13·9)	10·7 (8·73–13·7)	-10·2 (-14·3–-5·75)	-0·62 (-3·02–2·1)	4·6 (3·48–6·13)	4·02 (3·15–5·37)	3·82 (3–5·11)	-12·4 (-19·8–-3·51)	-4·99 (-8·81–-0·536)
Occupational noise	13·2 (12·1–14·4)	12·6 (11·8–13·5)	12·7 (12–13·6)	-4·45 (-7·91–-0·944)	1·04 (-0·553–2·79)	6·99 (5·98–8·16)	7·17 (6·52–7·94)	7·35 (6·86–8·07)	2·68 (-3·97–11·4)	2·51 (-0·213–5·65)
Occupational injuries	--	--	--	--	--	--	--	--	--	--
Occupational ergonomic factors	13·2 (11·1–15·9)	13·9 (12–16·2)	13·7 (11·7–16·2)	5·8 (-4·13–17·1)	-1·51 (-7·93–4·83)	6·47 (5·2–7·98)	7·24 (6·09–8·69)	7·35 (6·06–8·85)	12 (-2·83–30·3)	1·46 (-5·9–8·66)
Childhood sexual abuse	6·2 (4·16–8·83)	5·92 (4·23–7·89)	6·2 (4·16–8·83)	-4·51 (-16–9·58)	4·72 (-7·66–19·9)	12·9 (9·48–18·1)	11·4 (9·04–14·5)	12·9 (9·48–18·1)	-11·1 (-26·9–6·46)	12·5 (-4·23–38·4)
Intimate partner violence	--	--	--	--	--	9·71 (5·14–14·2)	9·71 (5·14–14·2)	9·71 (5·14–14·2)	0 (0–0)	0 (0–0)
Non-exclusive breastfeeding	22·3 (15·8–30·8)	22·1 (16–29·9)	21·7 (15·5–29·5)	-0·701 (-9·98–10·7)	-1·81 (-10·7–7·57)	22·3 (15·8–30·8)	22·1 (16–29·9)	21·7 (15·5–29·5)	-0·685 (-9·97–10·8)	-1·82 (-10·7–7·57)
Discontinued breastfeeding	14·8 (13·2–16·5)	15·3 (14·6–16)	14·4 (12·9–15·8)	3·06 (-7·83–16·6)	-5·7 (-16·3–5·99)	14·8 (13·2–16·5)	15·2 (14·6–16)	14·4 (12·9–15·8)	3·29 (-7·62–16·9)	-5·72 (-16·3–5·98)
Diet low in calcium	36·2 (22·1–59)	32·7 (19·2–55·8)	30·6 (17·4–53·1)	-9·44 (-16·3–-4·18)	-6·51 (-12·4–-2·18)	27·8 (15–51)	25·1 (12·7–47·2)	23·3 (11·4–45·1)	-9·93 (-18·6–-2·55)	-6·97 (-15·6–-1·48)
Occupational exposure to asbestos	2·41 (2·15–2·71)	4·03 (3·76–4·3)	3·9 (3·03–4·99)	67·5 (46·6–88·9)	-3·39 (-25·4–23·1)	1·02 (0·782–1·78)	1·62 (0·93–1·86)	1·74 (0·91–2·35)	59 (-18·8–116)	7·06 (-18·4–37·2)
Occupational exposure to arsenic	0·574 (0·228–0·981)	0·525 (0·213–0·895)	0·529 (0·21–0·908)	-8·49 (-17·8–1·54)	0·822 (-6·23–9·08)	0·406 (0·167–0·704)	0·393 (0·16–0·662)	0·384 (0·161–0·648)	-3·21 (-17·5–13·2)	-2·28 (-10–6·87)
Occupational exposure to benzene	1·04 (0·618–1·92)	1·16 (0·716–2·08)	1·17 (0·72–2·1)	11·8 (3·84–22·5)	1·03 (-3·21–6·38)	1·37 (0·849–2·51)	1·64 (1·01–2·93)	1·75 (1·07–3·08)	19·6 (6·32–38·9)	6·7 (0·753–14·5)
Occupational exposure to beryllium	0·12 (0·112–0·128)	0·126 (0·125–0·128)	0·127 (0·123–0·13)	5·43 (-1·09–12·1)	0·637 (-2·35–3·7)	0·096 (0·0851–0·107)	0·108 (0·106–0·109)	0·111 (0·107–0·116)	12·2 (0·206–26·8)	3·34 (-0·758–7·74)
Occupational exposure to cadmium	0·304 (0·278–0·331)	0·281 (0·271–0·292)	0·283 (0·265–0·304)	-7·43 (-15·6–1·88)	0·744 (-5·54–7·93)	0·217 (0·188–0·247)	0·214 (0·205–0·224)	0·212 (0·196–0·23)	-1·04 (-14·8–14·1)	-1·12 (-8·13–6·63)
Occupational exposure to chromium	0·737 (0·679–0·798)	0·694 (0·673–0·716)	0·699 (0·659–0·741)	-5·9 (-13·5–2·68)	0·739 (-4·66–6·66)	--	--	--	--	--
Occupational exposure to diesel engine exhaust	4·13 (3·86–4·44)	4·76 (4·6–4·92)	4·78 (4·54–5·03)	15·2 (6·77–24·4)	0·426 (-4·21–5·22)	1·99 (1·74–2·25)	2·23 (2·14–2·32)	2·36 (2·18–2·55)	11·7 (-1·64–28·1)	5·94 (-1·65–14·1)
Occupational exposure to formaldehyde	1·52 (1·38–1·66)	1·39 (1·32–1·45)	1·39 (1·29–1·5)	-8·66 (-17·4–1·32)	0·211 (-6·51–7·47)	1·13 (0·98–1·28)	1·14 (1·1–1·19)	1·15 (1·07–1·23)	1·44 (-11·9–16·1)	0·365 (-5·73–6·97)
Occupational exposure to nickel	0·534 (0·175–1·4)	0·583 (0·195–1·54)	0·592 (0·193–1·51)	9·24 (-1·09–21·4)	1·55 (-4·52–8·39)	0·339 (0·108–0·881)	0·324 (0·108–0·851)	0·311 (0·102–0·814)	-4·34 (-19·2–13·4)	-4·06 (-13·6–6·26)
Occupational exposure to polycyclic aromatic hydrocarbons	1·43 (1·32–1·55)	1·43 (1·4–1·47)	1·45 (1·38–1·53)	0·361 (-7·69–9·27)	1·11 (-3·74–6·19)	1·19 (1·04–1·33)	1·24 (1·21–1·28)	1·27 (1·2–1·34)	4·69 (-6·94–18·7)	1·63 (-3·56–7·26)
Occupational exposure to silica	4·71 (2·4–10·6)	7·01 (3·65–15·3)	7·19 (3·75–16)	49·1 (33·8–70·7)	2·56 (-5·12–11·7)	2·34 (1·18–5·05)	2·25 (1·15–4·96)	2·12 (1·08–4·6)	-3·75 (-18·6–15)	-5·82 (-14·9–5·77)
Occupational exposure to sulfuric acid	1·35 (0·97–2·35)	1·2 (0·875–2·07)	1·19 (0·859–2·05)	-10·8 (-18·2–-2·13)	-0·991 (-7·15–5·62)	0·913 (0·634–1·59)	0·818 (0·59–1·44)	0·755 (0·536–1·34)	-10·4 (-22·9–4·73)	-7·69 (-15·3–0·884)
Unsafe sex	--	--	--	--	--	--	--	--	--	--
Occupational exposure to trichloroethylene	0·445 (0·416–0·479)	0·417 (0·407–0·43)	0·418 (0·402–0·438)	-6·29 (-12·8–0·816)	0·192 (-3·94–4·57)	0·296 (0·259–0·334)	0·31 (0·302–0·321)	0·319 (0·301–0·339)	4·71 (-7·39–20·8)	2·71 (-2·85–9·12)
No access to handwashing facility	27·2 (26·1–28·5)	9·2 (8·73–9·67)	7·96 (7·53–8·4)	-66·2 (-68–-64·2)	-13·4 (-16·7–-9·99)	27·2 (26·1–28·5)	9·2 (8·73–9·67)	7·96 (7·53–8·4)	-66·2 (-68–-64·2)	-13·4 (-16·7–-9·99)
Child wasting	1·5 (1·1–1·94)	1·41 (1·02–1·83)	1·38 (1–1·79)	-6·42 (-13·9–1·26)	-2·2 (-7·7–3·74)	1·51 (1·11–1·93)	1·41 (1·02–1·81)	1·37 (0·98–1·79)	-6·55 (-14·8–1·47)	-2·32 (-7·93–3·3)
Child stunting	7·27 (4·66–9·24)	5·11 (3·04–6·9)	4·73 (2·76–6·4)	-29·6 (-44·1–-16·6)	-7·52 (-18·7–4·39)	7·29 (4·73–9·3)	5·04 (3·06–6·85)	4·66 (2·71–6·41)	-30·9 (-45·6–-16·4)	-7·57 (-21·8–5·42)
Chewing tobacco	0·139 (0·109–0·177)	0·136 (0·108–0·173)	0·136 (0·105–0·169)	-2·17 (-26·6–29·1)	-0·194 (-17–19·2)	--	--	--	--	--
Diet low in legumes	92·8 (82·6–100)	86·7 (68·9–100)	87·7 (71·4–100)	-6·59 (-17·3–0)	1·17 (-0·214–4·43)	90·9 (78·6–100)	84·2 (63–100)	85·4 (65·4–100)	-7·37 (-20·6–0)	1·35 (-0·352–5·81)
Short gestation	11·3 (8·73–14·4)	14·8 (12·5–17·1)	15·2 (12·3–18·8)	30·5 (1·45–70·5)	3·22 (-17·2–29·9)	12 (9·63–15)	15·4 (13·2–17·9)	15·1 (12·3–18·5)	28·3 (0·876–66·1)	-1·97 (-21·1–22·2)
Low birth weight	6 (4·78–7·54)	7·46 (6·37–8·74)	8·21 (6·74–9·96)	24·2 (-4·99–61·9)	10 (-13·1–40·3)	7·05 (5·77–8·59)	9·14 (7·96–10·5)	8·79 (7·25–10·4)	29·7 (1·97–64·8)	-3·84 (-22·6–18·5)

High temperature	27<br>(20·3–34·8)	25·7<br>(19–33·7)	27·5<br>(20·7–35·5)	-4·89<br>(-10·8–0·696)	7·19<br>(3·82–10·8)	27<br>(20·3–34·8)	25·7<br>(19–33·7)	27·5<br>(20·7–35·5)	-4·89<br>(-10·8–0·696)	7·19<br>(3·82–10·8)
Low temperature	33·1<br>(27·2–41)	33·5<br>(27·6–41·4)	31·8<br>(26–39·3)	1·13<br>(-0·0187–2·42)	-5·04<br>(-6·56–-3·43)	33·1<br>(27·2–41)	33·5<br>(27·6–41·4)	31·8<br>(26–39·3)	1·13<br>(-0·0187–2·42)	-5·04<br>(-6·56–-3·43)
Kidney dysfunction	16·7<br>(11·4–23·5)	18·3<br>(13–25)	18·4<br>(13–25·4)	9·38<br>(4·46–16·5)	0·39<br>(-3·96–4·63)	17·7<br>(12·1–24·7)	19·6<br>(13·8–26·8)	19·8<br>(14–27·1)	11·1<br>(5·94–18·8)	0·737<br>(-3·41–5·23)
Bullying victimization	6·16<br>(2·47–12·3)	6·83<br>(3·16–13·1)	6·78<br>(3·17–12·9)	10·8<br>(-14–50·2)	-0·724<br>(-7·12–4·66)	5·36<br>(2·19–10·8)	5·8<br>(2·7–11·4)	5·74<br>(2·63–11)	8·16<br>(-17·5–48·9)	-0·98<br>(-7·92–4·47)
High LDL cholesterol	37<br>(33·6–40·6)	40·3<br>(37·2–43·7)	41·2<br>(37·7–44·6)	8·95<br>(3·2–15)	2·03<br>(-1·84–6·28)	37·4<br>(34·1–41·1)	40·8<br>(37·6–44·1)	41·7<br>(38·5–45·1)	9·08<br>(3·83–14·8)	2·09<br>(-1·22–5·83)

Supplementary Results Table 1. Chile age-standardised Summary Exposure Values (SEVs) for most-detailed risk factors and percentage change, by sex, 1990, 2010, and 2019. Data in parentheses are 95% uncertainty intervals.

	Male					Female				
	1990	2010	2019	Percentage change 1990-2010	Percentage change 2010-2019	1990	2010	2019	Percentage change 1990-2010	Percentage change 2010-2019
Unsafe sanitation	33·5 (26·7–40·6)	10·4 (6·62–15·6)	7 (4·09–10·8)	-68·8 (-81–-51·9)	-33 (-56·4–0·232)	--	--	--	--	--
Ambient particulate matter pollution	17·7 (7·95–31)	20·2 (16·6–24·7)	22·1 (17–27·9)	14·3 (-33·4–138)	9·42 (-9·1–31·1)	16·4 (7·24–29·3)	19·9 (16·2–24·4)	21·9 (16·8–27·5)	20·8 (-31–155)	10·4 (-8·31–32)
Household air pollution from solid fuels	5·25 (2·49–9·72)	1·17 (0·403–2·68)	0·619 (0·179–1·51)	-77·7 (-87·5–-65)	-47·2 (-65·5–-24·6)	6·38 (3·22–11)	1·51 (0·559–3·33)	0·8 (0·252–1·97)	-76·4 (-86·7–-63)	-46·8 (-65·1–-24·5)
Ambient ozone pollution	26·5 (10·1–48·6)	19·7 (7·03–36·8)	19 (6·54–36·2)	-25·6 (-46·6–4·28)	-3·52 (-27·4–29·8)	26·5 (10·1–48·6)	19·7 (7·03–36·8)	19 (6·54–36·2)	-25·6 (-46·6–4·28)	-3·52 (-27·4–29·8)
Residential radon	14·2 (2·12–50·4)	14·2 (2·12–50·4)	14·2 (2·12–50·4)	0 (0–0)	0 (0–0)	14·2 (2·12–50·4)	14·2 (2·12–50·4)	14·2 (2·12–50·4)	0 (0–0)	0 (0–0)
Lead exposure	13·5 (1·94e-12–40·4)	11·4 (1·89e-12–35·7)	9·04 (2·4e-12–30·9)	-15·4 (-56·9–17000)	-20·8 (-61·4–165)	7·94 (1·49e-12–30·9)	6·67 (1·46e-12–27·3)	5·37 (1·56e-12–23·6)	-15·9 (-61·8–306)	-19·5 (-75·2–145)
Child underweight	1·08 (0·634–1·57)	0·834 (0·48–1·25)	0·764 (0·444–1·14)	-23 (-30·3–-16·5)	-8·44 (-14·9–-0·0233)	1·06 (0·627–1·52)	0·782 (0·451–1·15)	0·731 (0·411–1·08)	-26 (-32·9–-19·4)	-6·51 (-14·3–1·77)
Iron deficiency	--	--	--	--	--	13·8 (11·8–15·8)	11·6 (9·87–13·3)	11·2 (9·4–12·9)	-16·2 (-24·7–-6·8)	-3·77 (-9·45–1·98)
Vitamin A deficiency	19·1 (8·7–33·7)	10·9 (4·5–20·3)	7·97 (3·41–15·4)	-42·7 (-73·5–29·3)	-27·2 (-56–30·8)	12·5 (6·77–21·3)	8·77 (4·42–14·7)	6·62 (3·13–10·9)	-29·9 (-62·6–33)	-24·5 (-48·9–14·5)
Zinc deficiency	5·78 (0·161–16)	3·63 (0–11)	2·99 (0–9·42)	-37·2 (-100–-23)	-17·5 (-68·5–NA)	5·6 (0·198–15·5)	3·52 (0–10·9)	2·91 (0–9·12)	-37·1 (-100–-13·6)	-17·4 (-58·6–NA)
Smoking	20·5 (17·2–24·1)	18·1 (15·1–21·3)	16·6 (13·8–19·5)	-11·8 (-18·1–-5·27)	-7·94 (-12·1–-3·71)	16·3 (13–20)	14·3 (11·6–17·5)	13·3 (10·9–16·4)	-11·8 (-21·1–-0·577)	-7·02 (-13·3–-0·288)
Secondhand smoke	50·6 (48·9–52·5)	43·4 (41–45·8)	42·8 (40·1–45·5)	-14·2 (-20·1–-8·19)	-1·36 (-5·95–3·13)	54·1 (52·4–55·9)	45·8 (44–47·7)	45·3 (43·1–47·7)	-15·4 (-19·5–-11·1)	-1·03 (-4·38–2·27)
Alcohol use	16·2 (11·7–21·8)	16·6 (11·5–22·4)	18·3 (12·8–24·9)	2·79 (-7·23–13·7)	9·71 (-2·68–24·6)	8·76 (5·88–12·7)	8·26 (5·37–12·2)	9·04 (5·8–13·3)	-5·68 (-17·6–6·8)	9·38 (-6·52–27·4)
Drug use	0·242 (0·117–0·496)	0·25 (0·1–0·523)	0·235 (0·1–0·498)	3·42 (-18·3–21·3)	-6·07 (-17·6–6·62)	0·128 (0·0599–0·262)	0·158 (0·0754–0·307)	0·15 (0·0763–0·285)	23 (7·92–44·1)	-5·21 (-15·7–6·71)
High fasting plasma glucose	7·42 (6·4–8·7)	12·2 (10·1–14·1)	14·5 (12·3–17·1)	63·8 (37·6–92·5)	19·7 (3·54–44·5)	8·13 (6·53–10·2)	13·7 (11·6–16)	14·3 (11·9–16·8)	67·9 (36·5–106)	4·85 (-8·71–21·9)
High systolic blood pressure	29·2 (24·5–34)	29·7 (26·6–32·8)	30·9 (25·7–37·1)	1·6 (-15·3–22·3)	3·99 (-12–22·6)	26·6 (22–32·1)	22 (19·6–24·4)	23 (17·6–29·4)	-17·3 (-32–1·09)	4·84 (-19·9–33·8)
High body-mass index	17·2 (11·1–24·3)	29·6 (21·1–39·7)	32·4 (23·4–43·1)	72·2 (50·2–106)	9·49 (4·02–16·2)	23·4 (15–36·2)	37·4 (25·6–53·2)	39·4 (27·5–55·1)	59·5 (37·6–93·4)	5·42 (-1·44–15·7)
Low bone mineral density	10·2 (5·68–16·7)	9·29 (5·19–15·5)	8·99 (4·82–14·9)	-8·57 (-31·2–18·3)	-3·27 (-26·2–26·2)	18·8 (12·7–26·2)	16·6 (11·7–23·1)	16·3 (10·7–23)	-11·6 (-31·7–13·2)	-1·77 (-23·3–26·3)
Diet low in fruits	60·4 (49·5–73·2)	51·7 (40·1–65·1)	48·4 (36·5–61·5)	-14·5 (-21·7–-8·33)	-6·22 (-13·9–-0·301)	61·8 (51·3–74·2)	52·9 (41·5–65·8)	49·9 (38·4–63·1)	-14·4 (-21·8–-8·69)	-5·59 (-13·3–1·09)
Diet low in vegetables	46·3 (26–68·4)	32·3 (16·7–53·9)	29 (14·1–49·9)	-30·1 (-49·1–-11·4)	-10·3 (-25·3–0·728)	43·8 (24·3–65·8)	29·7 (14·1–51·7)	26·5 (12–47·6)	-32·1 (-54·1–-12·8)	-11·1 (-26·8–2·05)
Diet low in whole grains	99·5 (99–100)	90·6 (85·8–95·2)	89·9 (84·7–94·7)	-8·94 (-13·4–-4·5)	-0·76 (-1·73–-0·0435)	99·7 (99·3–100)	89·9 (84·2–95·2)	87·4 (80·7–94)	-9·85 (-15·4–-4·54)	-2·71 (-4·45–-1·28)
Diet low in nuts and seeds	77·2 (41·2–89·6)	67 (34·1–82·8)	63·4 (31·2–80·9)	-13·2 (-41–15·8)	-5·42 (-15·4–-0·812)	79·8 (41·7–91·7)	66 (33·2–82·1)	62 (30·3–79·8)	-17·3 (-44·6–5·95)	-6·07 (-15·9–-0·802)
Diet low in milk	89·1 (73·4–100)	90·2 (75·4–100)	89·6 (74·3–100)	1·23 (-0·0119–3·81)	-0·601 (-1·99–0·0219)	88·9 (73·5–100)	90 (75·4–100)	89·4 (74·1–100)	1·21 (-0·0184–3·81)	-0·64 (-2·2–0)
Diet high in red meat	46·8 (34·5–57·9)	70·9 (61·9–79·4)	73·8 (61·7–83·5)	51·6 (32·1–86·9)	4·05 (-5·98–13·4)	46·6 (35–57·7)	71 (62–79·7)	74 (62·5–83·6)	52·2 (32·4–85·5)	4·31 (-5·33–14·7)
Diet high in processed meat	66 (40·3–89·9)	76·4 (50·3–98·8)	77·4 (52·2–99)	15·7 (5·23–35·5)	1·3 (-1·37–5·31)	70·2 (44·8–94·4)	78·5 (53·6–99·7)	79·9 (56·9–99·7)	11·9 (3·61–26·9)	1·8 (-0·322–6·89)
Diet high in sugar-sweetened beverages	33·1 (19–50·5)	50·8 (35–66·9)	60·1 (43·7–75)	53·4 (10·8–122)	18·3 (-1·29–48·3)	37·9 (20·6–55·6)	59 (44–72·5)	67·1 (51·8–80·5)	55·8 (16·9–144)	13·8 (-2·14–36·2)
Diet low in fiber	49·6 (33·4–65·2)	38·4 (23·8–53·3)	34·5 (20·6–48·6)	-22·6 (-30·9–-16·5)	-10 (-21–-0·881)	49·7 (32·6–65·9)	38·3 (23·4–53·7)	34·5 (20·2–50)	-22·9 (-31·2–-16·6)	-9·89 (-20·5–-0·0568)
Diet low in seafood omega-3 fatty acids	94·6 (86·2–100)	84·4 (66·7–100)	82·3 (63·1–100)	-10·7 (-27·3–0)	-2·52 (-17·6–11·8)	96·1 (89·1–100)	87·5 (72·5–100)	85·7 (69·5–100)	-8·91 (-22·5–0)	-2·03 (-13·5–8·11)
Diet low in polyunsaturated fatty acids	56·7 (19·1–95·9)	49·3 (15·1–90·5)	46·9 (14–88·8)	-13 (-26–-2·16)	-5·02 (-13·8–-0·666)	59 (21–97·1)	51·8 (15·9–92·8)	49·3 (14·8–91)	-12·2 (-25·8–-1·88)	-4·75 (-12·3–-0·542)
Diet high in trans fatty acids	43 (33–95·1)	34·1 (24·5–94·9)	33·2 (23·5–95·4)	-20·7 (-33·9–0)	-2·58 (-11·8–7·48)	44·3 (33·8–100)	35·8 (25·2–98·9)	34·9 (24·2–98·3)	-19·3 (-32·1–0)	-2·5 (-11·5–6·27)
Diet high in sodium	42·1 (11·4–66·6)	40·7 (9·73–66·3)	41·8 (10·6–68·3)	-3·3 (-41·1–38·1)	2·72 (-16·8–32·5)	26·6 (5·72–51·1)	23·1 (4·54–47·9)	22·9 (4·47–48)	-13·1 (-43·3–5·41)	-1·19 (-17–15·3)
Low physical activity	1·32 (0·397–3·87)	1·54 (0·527–3·89)	1·64 (0·575–4·02)	16·6 (-27·2–111)	6·23 (-6·63–26)	4·15 (1·87–8·05)	5·08 (2·66–9·3)	5·25 (2·83–9·47)	22·5 (-8·58–82·7)	3·29 (-3·96–11·4)
Occupational asthmagens	21·9 (19–25·7)	20·6 (17·9–24·2)	20 (17·4–23·2)	-5·82 (-13·1–1·33)	-3·11 (-8·87–3·33)	6·24 (5·27–7·32)	9·32 (7·94–11)	10·9 (9·2–12·9)	49·4 (37·3–61·8)	16·9 (7·83–26·3)
Occupational particulate matter, gases, and fumes	10·4 (8·27–13·9)	11·1 (8·93–14·7)	11·1 (8·92–14·4)	6·85 (2·98–11·4)	-0·548 (-2·33–1·01)	2·5 (1·93–3·41)	3·28 (2·56–4·38)	3·65 (2·85–4·9)	31·3 (21·1–42·8)	11·3 (7·38–15·1)
Occupational noise	11·6 (10·9–12·4)	12·6 (12·1–13·3)	12·8 (12·3–13·6)	8·83 (6·06–12)	1·79 (0·873–2·67)	3·82 (3·51–4·27)	5·22 (4·94–5·63)	6·15 (5·86–6·61)	36·5 (29·2–43·9)	17·8 (16·1–19·7)
Occupational injuries	--	--	--	--	--	--	--	--	--	--
Occupational ergonomic factors	16·7 (14·7–18·9)	14·7 (12·7–16·9)	13·9 (11·9–16·2)	-12 (-20·7–2·32)	-5·3 (-12·7–2·39)	4·6 (3·9–5·47)	7·1 (6·01–8·42)	7·75 (6·53–9·27)	54·5 (45–64·6)	9·04 (1·15–17)
Childhood sexual abuse	6·22 (4·11–8·87)	6·22 (4·11–8·87)	6·22 (4·11–8·87)	0 (0–0)	0 (0–0)	11 (8·15–15·4)	11 (8·15–15·4)	11 (8·15–15·4)	0 (0–0)	0 (0–0)
Intimate partner violence	--	--	--	--	--	29·5 (16·9–38·9)	29·5 (16·9–38·9)	29·5 (16·9–38·9)	0 (0–0)	0 (0–0)
Non-exclusive breastfeeding	18 (13·2–24·8)	16·7 (12·3–22·2)	16·5 (12·2–22)	-7·41 (-17·4–4·31)	-0·965 (-8·38–7·34)	18 (13·2–24·8)	16·7 (12·3–22·2)	16·5 (12·2–22)	-7·37 (-17·3–4·36)	-0·975 (-8·39–7·33)
Discontinued breastfeeding	18 (16·4–19·6)	15·9 (14·2–17·6)	14·6 (12·9–16·1)	-11·2 (-21–1·2)	-8·72 (-17–0·215)	17·9 (16·3–19·6)	15·9 (14·2–17·7)	14·6 (12·9–16·1)	-11·1 (-20·9–1·31)	-8·67 (-16·9–0·268)
Diet low in calcium	61·2 (47·6–80·9)	55·2 (41·1–75·7)	50·8 (36·4–71·7)	-9·85 (-15·2–-4·56)	-7·99 (-12·9–-3·43)	52·2 (38·3–73·2)	45·9 (31·2–67·5)	41·5 (27·1–64)	-12·1 (-19–-5·47)	-9·6 (-15·8–-4·55)
Occupational exposure to asbestos	4·11 (3·58–4·72)	3·14 (2·85–3·42)	2·86 (2·14–3·72)	-23·5 (-34·8–-11·5)	-8·88 (-30·6–17·7)	1·18 (0·806–1·9)	0·975 (0·741–1·41)	0·966 (0·621–1·42)	-17 (-38·6–10·5)	-0·941 (-30·5–35)
Occupational exposure to arsenic	0·522 (0·206–0·873)	0·488 (0·191–0·821)	0·486 (0·186–0·817)	-6·52 (-13·8–1·7)	-0·399 (-8·22–7·31)	0·279 (0·113–0·471)	0·376 (0·154–0·643)	0·413 (0·166–0·706)	35 (22·5–49·2)	9·7 (1·09–19·4)
Occupational exposure to benzene	0·955 (0·587–1·69)	1·06 (0·655–1·89)	1·1 (0·685–1·94)	11·4 (6·4–17)	3·82 (-0·259–9·13)	0·811 (0·493–1·45)	1·29 (0·79–2·26)	1·55 (0·957–2·76)	58·6 (48·5–71·7)	20·3 (13·3–29·1)
Occupational exposure to beryllium	0·119 (0·117–0·122)	0·124 (0·123–0·126)	0·126 (0·123–0·13)	4·22 (1·44–6·92)	1·56 (-1·5–4·67)	0·0678 (0·0652–0·0701)	0·103 (0·101–0·105)	0·118 (0·114–0·123)	52·2 (46–59·3)	14·9 (9·73–20·1)
Occupational exposure to cadmium	0·274 (0·26–0·29)	0·262 (0·249–0·277)	0·262 (0·247–0·278)	-4·46 (-11·2–2·87)	0·019 (-6·75–6·72)	0·15 (0·14–0·16)	0·207 (0·196–0·218)	0·229 (0·214–0·245)	38·1 (26·8–50·6)	10·8 (2·83–19)
Occupational exposure to chromium	0·633 (0·603–0·664)	0·634 (0·606–0·664)	0·64 (0·608–0·674)	0·0429 (-6·21–6·81)	0·948 (-4·85–6·86)	--	--	--	--	--
Occupational exposure to diesel engine exhaust	3·54 (3·4–3·68)	4·45 (4·29–4·61)	4·61 (4·43–4·81)	25·7 (18·9–32·5)	3·64 (-1·06–8·53)	1·67 (1·57–1·77)	2·71 (2·57–2·85)	3·12 (2·93–3·33)	62·4 (50·4–75·9)	15·1 (7·33–23·6)
Occupational exposure to formaldehyde	1·32 (1·24–1·4)	1·25 (1·18–1·33)	1·25 (1·16–1·33)	-5·28 (-12·5–3·02)	-0·121 (-7·28–7·24)	0·733 (0·685–0·783)	1·01 (0·964–1·07)	1·14 (1·06–1·22)	38·4 (27·5–49·6)	12·6 (4·73–20·5)
Occupational exposure to nickel	0·573 (0·185–1·53)	0·574 (0·189–1·52)	0·574 (0·189–1·5)	0·227 (-6·76–8·1)	-0·0577 (-6·4–7·01)	0·241 (0·0799–0·628)	0·318 (0·106–0·826)	0·342 (0·116–0·899)	32 (19·3–48·2)	7·51 (-1·52–17·9)
Occupational exposure to polycyclic aromatic hydrocarbons	1·28 (1·23–1·34)	1·31 (1·26–1·37)	1·33 (1·27–1·4)	2·49 (-3·5–8·54)	1·45 (-3·7–6·67)	0·775 (0·734–0·815)	1·12 (1·08–1·16)	1·27 (1·21–1·34)	44·3 (35·3–53·9)	13·9 (7·14–20·3)
Occupational exposure to silica	5·57 (2·91–12)	6·59 (3·47–14·6)	6·79 (3·55–15·2)	18·3 (9·06–28·9)	3·1 (-3·81–11·2)	1·8 (0·91–3·88)	2·4 (1·21–5·25)	2·55 (1·3–5·63)	33·5 (20·6–49·3)	6·09 (-2·74–16·6)
Occupational exposure to sulfuric acid	1·28 (0·931–2·22)	1·21 (0·877–2·13)	1·19 (0·846–2·06)	-5·16 (-11·5–1·75)	-2·27 (-8·23–3·61)	0·591 (0·424–1·05)	0·812 (0·582–1·41)	0·876 (0·632–1·55)	37·4 (25·1–50·6)	7·85 (-0·695–17)
Unsafe sex	--	--	--	--	--	--	--	--	--	--
Occupational exposure to trichloroethylene	0·364 (0·353–0·379)	0·383 (0·372–0·397)	0·389 (0·376–0·407)	5·06 (0·603–9·84)	1·68 (-2·77–6·5)	0·215 (0·205–0·226)	0·321 (0·31–0·334)	0·366 (0·348–0·386)	49·5 (41–59·1)	13·8 (7·13–20·6)
No access to handwashing facility	11·7 (11·1–12·2)	7·29 (6·91–7·67)	6·49 (6·16–6·88)	-37·4 (-41·1–-33·2)	-10·9 (-14·2–-7·35)	11·7 (11·1–12·2)	7·29 (6·91–7·67)	6·49 (6·16–6·88)	-37·4 (-41·1–-33·2)	-10·9 (-14·2–-7·35)
Child wasting	0·854 (0·495–1·15)	0·907 (0·642–1·21)	0·808 (0·45–1·11)	6·16 (-3·42–75·1)	-10·9 (-48·1–-1·86)	0·769 (0·422–1·1)	0·73 (0·383–1·08)	0·707 (0·366–1·04)	-5·14 (-45·7–65·3)	-3·11 (-46·7–68·8)
Child stunting	3·81 (2·25–5·26)	3·37 (1·94–4·61)	2·56 (1·38–3·71)	-11·4 (-23–1·26)	-24 (-35·2–-15·3)	3·75 (2·2–5·2)	2·18 (1·16–3·18)	2·12 (1·07–3·11)	-41·8 (-51·9–-32·4)	-3·01 (-11·5–6·97)
Chewing tobacco	0·27 (0·215–0·341)	0·215 (0·169–0·273)	0·204 (0·16–0·261)	-20·5 (-39·4–6·15)	-4·82 (-20·3–14)	--	--	--	--	--
Diet low in legumes	73·7 (38–99·5)	65·1 (21·3–95·9)	66·7 (22·8–96·6)	-11·6 (-47·1–-0·797)	2·43 (-0·598–18)	70·5 (30–98·9)	61·2 (16·6–92·9)	63 (18·1–94·5)	-13·2 (-50·6–-1·23)	2·83 (-1·34–21)
Short gestation	16·5 (13·7–20·3)	20·2 (17·2–23·5)	19·9 (15·8–24·7)	22·4 (-1·63–52·2)	-1·91 (-23·1–24·1)	16·7 (13·8–20·1)	19 (16·2–22·3)	18·4 (15·2–22·5)	13·9 (-9·83–44·2)	-3·42 (-23·4–21·3)
Low birth weight	8·2 (6·82–9·89)	9·45 (8·12–11)	9·4 (7·64–11·6)	15·2 (-8·58–44)	-0·563 (-21·7–27·5)	8·58 (7·18–10·5)	9·22 (7·94–10·6)	8·77 (7·27–10·6)	7·48 (-17–34·2)	-4·83 (-24·6–19·5)
High temperature	21·1 (7·66–35·6)	18 (5·81–31·7)	20 (8·46–32·7)	-15 (-74·4–182)	11·2 (-1·08–56·8)	21·1 (7·66–35·6)	18 (5·81–31·7)	20 (8·46–32·7)	-15 (-74·4–182)	11·2 (-1·08–56·8)
Low temperature	31·4 (27·2–36·4)	32·3 (28·2–37·3)	30·3 (26·2–35·3)	2·88 (1·89–3·82)	-6·37 (-7·94–-4·54)	31·4 (27·2–36·4)	32·3 (28·2–37·3)	30·3 (26·2–35·3)	2·88 (1·89–3·82)	-6·37 (-7·94–-4·54)
Kidney dysfunction	16·2 (11·1–22·8)	18·8 (13·3–25·6)	19·4 (13·9–26·5)	15·9 (9·95–24·4)	3·16 (-1·63–8·8)	18·1 (12·5–25)	20·8 (15–28·1)	21·3 (15·4–28·6)	14·9 (9·51–22·8)	2·08 (-2·2–6·86)

Bullying victimization	10·3<br>(4·14–20·7)	11·1<br>(5·05–21·2)	9·24<br>(3·81–18·3)	7·25<br>(-12–55·8)	-16·7<br>(-28·4–-9·8)	8·41<br>(3·37–17·2)	9·21<br>(4·17–17·5)	7·63<br>(3·14–15·4)	9·4<br>(-14–59·2)	-17·1<br>(-28·6–-10·4)
High LDL cholesterol	33·5<br>(30–36·9)	38·4<br>(35–41·7)	39·1<br>(35·6–42·8)	14·5<br>(8·57–20·9)	1·85<br>(-1·83–5·96)	36·9<br>(33·5–40·3)	39·5<br>(36·3–42·9)	40·3<br>(37·1–43·8)	7·11<br>(2·03–12·5)	1·97<br>(-1·42–5·45)

Supplementary Results Table 1. Uruguay age-standardised Summary Exposure Values (SEVs) for most-detailed risk factors and percentage change, by sex, 1990, 2010, and 2019. Data in parentheses are 95% uncertainty intervals.

	Male					Female				
	1990	2010	2019	Percentage change 1990-2010	Percentage change 2010-2019	1990	2010	2019	Percentage change 1990-2010	Percentage change 2010-2019
Unsafe sanitation	20·2 (14·7–26·7)	3·57 (2·16–5·47)	1·87 (1·08–2·99)	-82·3 (-89·6–-70·5)	-47·6 (-66·7–-18·6)	--	--	--	--	--
Ambient particulate matter pollution	8·63 (2·6–18·6)	7·31 (3·83–11·7)	7·4 (3·58–12·3)	-15·3 (-56·9–125)	1·23 (-26·6–37·7)	8·21 (2·59–17·8)	7·24 (3·86–11·5)	7·37 (3·63–12·2)	-11·9 (-55·2–129)	1·87 (-25·2–37·4)
Household air pollution from solid fuels	2·22 (0·821–4·39)	0·537 (0·18–1·22)	0·299 (0·0826–0·761)	-75·9 (-85·7–-62)	-44·3 (-65·8–-18·2)	2·75 (1·14–4·99)	0·698 (0·249–1·48)	0·393 (0·121–0·957)	-74·6 (-85·2–-59·6)	-43·7 (-65·5–-17·5)
Ambient ozone pollution	9·44 (2·82–20·6)	9·67 (2·4–22·3)	12·6 (3·42–26·9)	2·51 (-54·8–119)	30·2 (-46·4–228)	9·44 (2·82–20·6)	9·67 (2·4–22·3)	12·6 (3·42–26·9)	2·51 (-54·8–119)	30·2 (-46·4–228)
Residential radon	13·6 (1·94–49)	13·6 (1·94–49)	13·6 (1·94–49)	0 (0–0)	0 (0–0)	13·6 (1·94–49)	13·6 (1·94–49)	13·6 (1·94–49)	0 (0–0)	0 (0–0)
Lead exposure	81·7 (58·4–93·9)	69·1 (49·2–83·7)	57·1 (36·7–74·6)	-15·4 (-26·2–-6·98)	-17·4 (-25·5–-10·4)	63·6 (20·2–86·4)	60·4 (33·8–79·8)	49·5 (24·9–71)	-4·97 (-19–62·6)	-18·1 (-27·8–-10·6)
Child underweight	2·32 (1·54–3·15)	1·86 (1·16–2·6)	1·7 (1·03–2·43)	-19·7 (-32·8–-6·51)	-8·34 (-19·2–2·52)	2·3 (1·52–3·14)	1·7 (1·06–2·42)	1·56 (0·965–2·25)	-25·8 (-36–-16)	-8·44 (-15·6–-0·272)
Iron deficiency	--	--	--	--	--	13·8 (11·8–16)	12·5 (10·5–14·4)	12·2 (10·3–14·1)	-9·64 (-19·1–0·302)	-2·08 (-7·99–3·63)
Vitamin A deficiency	22·9 (10·8–39)	15 (6·23–27·8)	11·1 (4·26–19·6)	-34·4 (-69·2–43·6)	-26·5 (-55·8–24·9)	14·5 (7·66–23·7)	11·3 (5·64–18·8)	8·67 (4·3–14·7)	-21·8 (-56·8–48)	-23·4 (-48·5–13·9)
Zinc deficiency	3·71 (0–11·3)	3·28 (0–10)	2·42 (0–7·83)	-11·4 (-49·2–NA)	-26·3 (-100–NA)	3·61 (0–11·2)	3·19 (0–10)	2·35 (0–7·65)	-11·8 (-45–NA)	-26·1 (-90·8–NA)
Smoking	22·8 (19·6–26)	22·1 (19·3–25)	20·1 (17·5–22·8)	-3·14 (-9·76–3·88)	-8·89 (-12·9–-4·42)	12·5 (10·2–15·1)	13·7 (11·5–16·3)	12·3 (10·4–14·6)	9·23 (-3·63–24·9)	-9·8 (-16·3–-2·81)
Secondhand smoke	41·7 (38·7–44·5)	38·7 (35·9–41·6)	37·5 (34·8–40·5)	-7·15 (-15·1–1·01)	-2·96 (-8·17–2·68)	49·1 (46·9–51·4)	43·9 (41·2–46·4)	42·3 (39·9–44·7)	-10·7 (-16·3–-4·93)	-3·59 (-7·61–0·509)
Alcohol use	14·7 (10·2–20·1)	16 (11·1–21·7)	17·7 (12·3–24·4)	8·45 (-1·97–20·8)	10·5 (-4·03–27·1)	7·59 (4·94–11·3)	7·91 (5·16–11·8)	8·63 (5·48–12·7)	4·24 (-8·17–18·7)	9·06 (-8·91–28·8)
Drug use	0·359 (0·143–0·778)	0·4 (0·159–0·872)	0·374 (0·152–0·826)	11·3 (-0·635–25·4)	-6·41 (-16–5·04)	0·259 (0·116–0·562)	0·299 (0·129–0·635)	0·267 (0·121–0·567)	15·3 (3·14–28·4)	-10·8 (-19·4–-0·438)
High fasting plasma glucose	3·25 (2·64–4·06)	4·9 (4·17–5·7)	10·2 (8·5–12·1)	51·1 (31·5–71·9)	109 (78·5–145)	3·86 (3·09–4·84)	5·61 (4·5–6·89)	7·47 (6·22–8·98)	45·1 (26·1–65·3)	33·3 (19·4–51·1)
High systolic blood pressure	26·1 (20·6–33·1)	27·4 (22·5–33)	28·6 (22·7–35·3)	5·13 (-20·4–38·1)	4·48 (-13·3–26·1)	24·2 (16·6–33·5)	24·1 (18·9–30·2)	24·5 (18–32·9)	-0·425 (-32·4–47·2)	1·71 (-22·9–33·3)
High body-mass index	11·8 (7·43–17·2)	19·2 (13·5–26·7)	22·4 (15·9–30·5)	62·8 (41·3–105)	16·8 (9·13–27)	16·1 (9·53–25)	26·3 (17·9–38·5)	29·9 (21·3–42·8)	63·5 (36·5–119)	13·9 (3·65–30·1)
Low bone mineral density	10·2 (5·44–16·4)	9·83 (5·51–15·9)	9·51 (5·22–15·4)	-3·31 (-25·1–22·4)	-3·25 (-25–25·8)	18·7 (12·8–26·2)	17·5 (11·7–24·9)	16·9 (11·2–24)	-6·35 (-26·9–15)	-3·18 (-22·7–20·5)
Diet low in fruits	54·9 (43·4–68·1)	48·3 (36·7–62)	44·8 (32·7–58·2)	-12 (-18·7–-7·13)	-7·39 (-16·1–-0·394)	56·5 (45·3–69·9)	50 (38·6–63·4)	46·2 (34·9–59·1)	-11·5 (-17·3–-6·97)	-7·59 (-15·8–-1)
Diet low in vegetables	80 (65·6–92)	69·3 (50·2–85·6)	66·6 (46·7–84·3)	-13·4 (-24·2–-4·93)	-3·93 (-9·75–-0·481)	78·7 (63·8–91·4)	67·7 (47·9–85)	64·9 (44·5–83·4)	-14 (-25·3–-5·21)	-4·07 (-9·72–-0·708)
Diet low in whole grains	96·5 (94·5–98·9)	98·3 (97·1–99·8)	98·2 (96·9–99·8)	1·79 (0·67–3·11)	-0·0779 (-0·595–0·365)	94·6 (91·2–98)	96·7 (94·5–99·3)	96·6 (94·3–99·2)	2·29 (0·801–4·1)	-0·095 (-0·757–0·568)
Diet low in nuts and seeds	77·3 (41·4–93·2)	77·1 (40·1–89·4)	75·4 (39–89·3)	-0·248 (-23·5–32·4)	-2·23 (-13·1–11·3)	79·9 (42·8–93·2)	76·9 (40·1–90)	73·2 (37·6–87·6)	-3·75 (-27·9–21·5)	-4·84 (-13·9–3·43)
Diet low in milk	46·6 (21·4–67·4)	50·5 (24·9–71·7)	43·9 (18·8–64·5)	8·32 (1·8–23·1)	-13 (-29·9–-3·9)	45·5 (20·3–66·2)	49·5 (24·2–70·9)	42·9 (18·9–63)	8·8 (2·04–24·8)	-13·4 (-27·2–-3·72)
Diet high in red meat	98·9 (98·2–99·5)	91·3 (86·7–94·8)	92·7 (86·3–96·8)	-7·62 (-11·9–-4·51)	1·5 (-3·35–6·35)	98·9 (98·2–99·5)	91·4 (86·7–94·8)	92·6 (86·1–96·8)	-7·59 (-11·8–-4·38)	1·33 (-3·6–5·69)
Diet high in processed meat	54·5 (32·5–78·4)	59·9 (36·5–83·6)	61·6 (37·4–84·9)	9·86 (-6·98–37·5)	2·81 (-5·69–14·3)	60·3 (36·8–84)	66·1 (40·7–89·7)	67·8 (42·1–91·6)	9·57 (-4·78–30·8)	2·5 (-4·71–12·6)
Diet high in sugar-sweetened beverages	43·4 (24·5–63)	57·5 (38·5–75·6)	64 (45·5–81·7)	32·5 (-4·52–100)	11·4 (-7·41–37·3)	47·9 (28·7–67·2)	63 (44·4–80·6)	69·2 (50·3–85·5)	31·5 (-3·27–99·9)	9·92 (-5·86–33·2)
Diet low in fiber	55·1 (38·8–70)	35·8 (21·7–50·3)	31·9 (18·8–45·9)	-35 (-45·3–-26·8)	-10·9 (-21·2–-0·564)	55·3 (37·9–70·5)	35·6 (20·9–50·9)	31·7 (18·4–46·1)	-35·6 (-46·4–-26·5)	-11 (-23–-0·947)
Diet low in seafood omega-3 fatty acids	87·2 (70·7–100)	88·1 (72·9–100)	83·5 (66·1–99·9)	1·11 (-9–14·8)	-5·26 (-19·1–4·44)	89·7 (76·3–100)	90 (78–100)	86·8 (71–100)	0·289 (-6·82–9·85)	-3·5 (-15·1–3·15)
Diet low in polyunsaturated fatty acids	64·5 (24·2–99·8)	31·7 (8·84–70·7)	27·9 (7·99–64·3)	-50·9 (-74·9–-14)	-12 (-22·4–-0·501)	66·3 (26·2–100)	34·5 (9·96–75·6)	30·7 (8·96–69·1)	-48 (-73·5–-12·8)	-11·1 (-21–-0·585)
Diet high in trans fatty acids	33·7 (23·5–97·8)	29·5 (20·2–94·5)	28·9 (19·8–90·9)	-12·4 (-23·7–0)	-2·12 (-10·7–7·01)	35·5 (24·4–99·8)	31·2 (20·3–99·6)	30·5 (19·9–98·5)	-12·2 (-23·1–0)	-1·97 (-10·9–7·08)
Diet high in sodium	42·7 (11·8–67·6)	40·9 (10·7–66)	41·7 (11·2–67·3)	-4·06 (-38–33·2)	1·77 (-19·2–32·3)	26·4 (5·91–51·5)	23·3 (4·37–48·8)	22·9 (4·38–48·3)	-11·5 (-44–8·13)	-1·59 (-19·3–15·4)
Low physical activity	3·81 (1·46–8·51)	4·45 (1·99–9·04)	5·42 (2·22–11·3)	16·7 (-15·5–88·8)	21·9 (-12·2–69·5)	1·91 (0·741–4·11)	2·42 (1·05–4·85)	2·83 (1·19–5·72)	26·7 (-10·1–97·9)	16·6 (-19–69·4)
Occupational asthmagens	20·9 (18·2–24·4)	21·7 (18·5–25·6)	21·2 (18–25·3)	4·22 (-2·91–12·2)	-2·27 (-8·33–4·11)	8·28 (6·99–9·78)	12·1 (10·2–14·3)	12·8 (10·5–15·3)	46·6 (33·7–60·1)	5·04 (-3·18–14·4)
Occupational particulate matter, gases, and fumes	10·6 (8·56–14)	10·7 (8·64–14·1)	11 (8·86–14·4)	0·952 (-3·6–6·42)	2·22 (-0·142–4·63)	4·09 (3·22–5·44)	4·14 (3·28–5·46)	4·34 (3·4–5·79)	1·12 (-7·38–9·75)	4·78 (0·517–9·31)
Occupational noise	12·4 (11·6–13·4)	12·6 (12–13·4)	13 (12·4–13·8)	1·37 (-1·71–4·9)	3·38 (2·23–4·58)	5·9 (5·36–6·55)	6·98 (6·6–7·53)	7·91 (7·53–8·48)	18·3 (11·8–24·9)	13·3 (11·6–15·1)
Occupational injuries	--	--	--	--	--	--	--	--	--	--
Occupational ergonomic factors	12·8 (11–14·8)	16 (14·1–18·2)	15·1 (13–17·8)	25·3 (15·1–36·3)	-5·64 (-12·5–1·93)	5·48 (4·59–6·58)	9·1 (7·72–10·8)	9·36 (7·79–11·2)	66·2 (54·7–79·1)	2·86 (-4·65–11·3)
Childhood sexual abuse	6·27 (4·15–8·89)	5·94 (4·27–7·76)	6·27 (4·15–8·89)	-5·25 (-17·6–9·39)	5·54 (-7·28–23)	9·4 (6·9–13·1)	8·39 (6·79–10·6)	9·4 (6·9–13·1)	-10·7 (-24·1–6·09)	12 (-4·5–34)
Intimate partner violence	--	--	--	--	--	13·1 (6·88–18·7)	11·7 (7·65–14·9)	13·1 (6·88–18·7)	-11 (-26·5–18·1)	12·3 (-7·78–50)
Non-exclusive breastfeeding	18·8 (13·6–25·7)	17·4 (12·4–23·3)	17·4 (12·7–23·6)	-7·75 (-17·9–3·77)	0·168 (-8·05–9·4)	18·8 (13·6–25·7)	17·4 (12·4–23·3)	17·4 (12·7–23·6)	-7·77 (-17·9–3·75)	0·193 (-8·02–9·42)
Discontinued breastfeeding	17·5 (16–19·1)	15·5 (13·9–17·1)	15·1 (13·5–16·7)	-11·5 (-23–0·131)	-2·98 (-11·7–7·1)	17·6 (16–19·2)	15·5 (13·9–17·1)	15·1 (13·5–16·7)	-11·6 (-23–0·214)	-2·92 (-11·6–7·16)
Diet low in calcium	33·5 (19·8–56·7)	23·6 (12–44·1)	20·3 (9·8–38·3)	-29·7 (-45·2–-13·4)	-13·7 (-23·4–-6·81)	25·6 (13·2–48·2)	17·5 (7·23–34·5)	14·6 (5·93–28·1)	-31·6 (-55·8–-11·5)	-16·5 (-29·4–-7·04)
Occupational exposure to asbestos	2·38 (2·12–2·63)	2·19 (1·97–2·45)	2·93 (2·21–3·78)	-7·98 (-19·6–6·25)	33·5 (1·63–70·8)	0·631 (0·456–1·17)	0·809 (0·522–1·01)	0·859 (0·547–1·2)	28·1 (-26–79·4)	6·14 (-20·9–38·6)
Occupational exposure to arsenic	0·497 (0·202–0·836)	0·517 (0·204–0·865)	0·528 (0·207–0·904)	3·95 (-4·05–13·1)	2·24 (-5·01–10·1)	0·391 (0·161–0·664)	0·458 (0·185–0·768)	0·468 (0·185–0·798)	17·2 (7·35–28·7)	2·21 (-5·93–11)
Occupational exposure to benzene	0·971 (0·593–1·72)	1·15 (0·715–2·06)	1·19 (0·73–2·12)	18·9 (12·2–27·2)	3·37 (-1·21–8·9)	1·08 (0·663–1·92)	1·94 (1·2–3·48)	2 (1·23–3·56)	78·9 (68·7–91·7)	3 (-2·15–9·69)
Occupational exposure to beryllium	0·109 (0·106–0·111)	0·127 (0·125–0·129)	0·128 (0·125–0·131)	16·6 (13·5–19·8)	0·905 (-1·83–3·83)	0·0867 (0·0838–0·0898)	0·127 (0·125–0·129)	0·133 (0·128–0·137)	46·9 (41·6–52·5)	4·19 (0·288–7·99)
Occupational exposure to cadmium	0·262 (0·247–0·277)	0·277 (0·265–0·292)	0·283 (0·262–0·304)	5·87 (-1·28–13·8)	2·03 (-4·17–8·88)	0·206 (0·193–0·221)	0·25 (0·24–0·261)	0·257 (0·237–0·28)	21·3 (12·2–31·8)	2·75 (-4·34–10·5)
Occupational exposure to chromium	0·622 (0·592–0·653)	0·668 (0·643–0·699)	0·684 (0·643–0·729)	7·35 (0·862–14·4)	2·32 (-3·08–8·24)	0·504 (0·475–0·535)	0·65 (0·629–0·672)	0·673 (0·631–0·72)	29·1 (21·1–38·2)	3·56 (-2·39–9·77)
Occupational exposure to diesel engine exhaust	3·55 (3·38–3·71)	4·15 (3·99–4·32)	4·19 (3·99–4·39)	16·9 (10·2–24)	1·06 (-3·55–5·99)	1·87 (1·77–1·98)	2·59 (2·47–2·71)	2·84 (2·64–3·03)	38·2 (28–48·9)	9·61 (2·57–17·3)
Occupational exposure to formaldehyde	1·31 (1·23–1·39)	1·34 (1·27–1·42)	1·37 (1·28–1·48)	2·69 (-4·82–11·3)	2·18 (-4·31–9·5)	1·03 (0·963–1·1)	1·34 (1·28–1·38)	1·36 (1·27–1·46)	29·5 (20·8–39·3)	2 (-4·08–8·59)
Occupational exposure to nickel	0·533 (0·175–1·4)	0·536 (0·173–1·43)	0·55 (0·175–1·47)	0·524 (-7–9·2)	2·62 (-3·74–10·3)	0·344 (0·115–0·872)	0·376 (0·123–0·973)	0·38 (0·127–0·995)	9·52 (-0·0646–21·9)	0·833 (-8·13–11·1)
Occupational exposure to polycyclic aromatic hydrocarbons	1·26 (1·21–1·32)	1·37 (1·33–1·43)	1·41 (1·33–1·49)	8·51 (2·47–15·1)	2·79 (-2·12–8·12)	1·06 (1·01–1·12)	1·46 (1·42–1·49)	1·5 (1·42–1·58)	36·9 (29·4–45·1)	2·88 (-2·32–8·3)
Occupational exposure to silica	5·32 (2·76–11·4)	5·68 (2·94–12·5)	5·95 (3·06–13)	6·87 (-3·12–18·8)	4·6 (-2·86–13·3)	2·53 (1·31–5·42)	2·58 (1·32–5·6)	2·59 (1·3–5·62)	1·74 (-7·46–13·5)	0·447 (-9·39–11)
Occupational exposure to sulfuric acid	1·22 (0·883–2·13)	1·3 (0·934–2·32)	1·28 (0·91–2·24)	7·04 (-0·206–15·3)	-1·56 (-7·51–5·07)	0·825 (0·601–1·44)	0·957 (0·691–1·71)	0·965 (0·683–1·76)	16 (6·24–27·4)	0·763 (-7·25–9·62)
Unsafe sex	--	--	--	--	--	--	--	--	--	--
Occupational exposure to trichloroethylene	0·358 (0·346–0·373)	0·402 (0·391–0·417)	0·408 (0·391–0·428)	12·2 (7·04–18·1)	1·4 (-2·61–5·78)	0·273 (0·26–0·287)	0·362 (0·352–0·376)	0·383 (0·363–0·407)	32·8 (25·5–41)	5·85 (0·517–11·8)
No access to handwashing facility	11·5 (10·9–12·1)	8·47 (8·06–8·93)	7·27 (6·88–7·63)	-26·2 (-31–-21·4)	-14·2 (-17·6–-10·9)	11·5 (10·9–12·1)	8·47 (8·06–8·93)	7·27 (6·88–7·63)	-26·2 (-31–-21·4)	-14·2 (-17·6–-10·9)
Child wasting	1·32 (0·961–1·72)	1·27 (0·905–1·67)	1·25 (0·884–1·63)	-4·06 (-10·7–3·48)	-1·5 (-7·56–4·21)	1·35 (0·982–1·74)	1·32 (0·955–1·7)	1·3 (0·949–1·68)	-2·29 (-9·28–4·93)	-1·43 (-7·02–4·25)
Child stunting	9·83 (6·52–12·1)	8·02 (5·18–10·3)	7·41 (4·65–9·57)	-18·4 (-32·5–-3·49)	-7·59 (-19·9–6·48)	8·48 (5·51–10·8)	5·44 (3·23–7·23)	5·07 (2·95–6·81)	-35·8 (-49·5–-23·5)	-6·93 (-20·3–5·8)
Chewing tobacco	0·172 (0·135–0·219)	0·169 (0·132–0·213)	0·157 (0·123–0·2)	-1·85 (-26·7–29·6)	-6·98 (-21·9–11·5)	0·189 (0·137–0·257)	0·186 (0·135–0·255)	0·179 (0·13–0·244)	-1·35 (-31·4–43)	-4·1 (-24·1–20·8)
Diet low in legumes	92·6 (82·5–100)	89·7 (76·2–100)	88·7 (73·8–100)	-3·16 (-8·35–0)	-1·13 (-4·21–0·216)	90·7 (78–100)	87·4 (70·8–100)	86·3 (67·9–100)	-3·56 (-9·68–0)	-1·26 (-4·93–0·248)
Short gestation	12 (9·59–15·2)	13·6 (11·7–16)	14·3 (11·7–17·4)	13·3 (-11·6–45·5)	5·33 (-15·4–30·2)	13·7 (11·2–16·6)	14·2 (12–16·6)	15·1 (12·3–18·3)	3·44 (-16·8–31·6)	6·27 (-13·7–32·1)
Low birth weight	6·13 (4·98–7·65)	7·33 (6·35–8·52)	7·61 (6·32–9·18)	19·5 (-6·2–53·6)	3·77 (-16·5–29·8)	9·34 (7·77–11·1)	9·36 (8·09–11)	9·81 (8·19–11·8)	0·12 (-20·5–25·5)	4·86 (-14·5–28·4)
High temperature	26·6 (17·6–36·4)	22 (13·2–31·7)	23·6 (15·1–32·9)	-17·5 (-29·5–-7·03)	7·37 (-1·44–18·8)	26·6 (17·6–36·4)	22 (13·2–31·7)	23·6 (15·1–32·9)	-17·5 (-29·5–-7·03)	7·37 (-1·44–18·8)
Low temperature	32·9 (25·3–43·1)	32·9 (25·3–43)	30·9 (23·3–40·9)	0·101 (-1·22–1·61)	-6·18 (-8·68–-3·78)	32·9 (25·3–43·1)	32·9 (25·3–43)	30·9 (23·3–40·9)	0·101 (-1·22–1·61)	-6·18 (-8·68–-3·78)
Kidney dysfunction	16 (10·9–22·8)	17·1 (11·9–23·8)	17·4 (12·2–24·3)	6·73 (1·89–13·2)	1·82 (-2·5–6·39)	16·7 (11·2–23·4)	17·6 (11·9–24·5)	18 (12·4–24·9)	5·49 (1·65–10·9)	2·32 (-1·44–6·67)

Bullying victimization	4·19 (1·69–8·36)	4·4 (2·02–8·52)	4·2 (1·89–8·21)	5·05 (-13·6–46·5)	-4·46 (-11·8–1·46)	4 (1·61–8·14)	4·15 (1·87–7·98)	4·04 (1·79–7·82)	3·72 (-17·8–44·5)	-2·59 (-7·96–2·03)
High LDL cholesterol	42·2 (38·7–45·8)	45·3 (42–48·6)	46·7 (43·1–50·3)	7·37 (1·41–13·9)	3·08 (-1·25–7·55)	42·7 (39·2–46·1)	45·9 (42·5–49·2)	47·1 (43·8–50·5)	7·37 (2·25–13·2)	2·73 (-0·832–6·44)

Supplementary Results Table 1. Western Europe age-standardised Summary Exposure Values (SEVs) for most-detailed risk factors and percentage change, by sex, 1990, 2010, and 2019. Data in parentheses are 95% uncertainty intervals.

	Male					Female				
	1990	2010	2019	Percentage change 1990-2010	Percentage change 2010-2019	1990	2010	2019	Percentage change 1990-2010	Percentage change 2010-2019
Unsafe sanitation	3·12 (2·64–3·67)	1·2 (0·994–1·44)	0·915 (0·765–1·1)	-61·7 (-69·3—51·7)	-23·5 (-34·6—10·6)	3·39 (2·84–4·06)	1·32 (1·08–1·62)	1 (0·816–1·21)	-61 (-69·2—49·9)	-24·4 (-36·9—10·2)
Ambient particulate matter pollution	18 (7·29–31·9)	13·8 (10·8–17·4)	9·85 (7·11–13·1)	-23·4 (-57·5–74·8)	-28·7 (-34·5—24·4)	18 (7·3–31·8)	13·8 (10·8–17·4)	9·86 (7·12–13·1)	-23·2 (-57·4–74·7)	-28·7 (-34·4—24·3)
Household air pollution from solid fuels	0·179 (0·0656–0·418)	0·0392 (0·0116–0·0938)	0·0223 (0·00602–0·0595)	-78·1 (-86·9—66·2)	-43 (-56·5—28·1)	0·231 (0·0894–0·509)	0·0517 (0·0163–0·121)	0·0299 (0·00858–0·0787)	-77·6 (-86·4—65·7)	-42·2 (-56—27·1)
Ambient ozone pollution	43·4 (19·3–65·6)	43·2 (18·1–69·8)	44·5 (19·4–69)	-0·45 (-7·21–6·81)	3·15 (-1·73–8·03)	43·5 (19·4–65·7)	43·2 (18·1–69·9)	44·7 (19·5–69·2)	-0·636 (-7·59–6·7)	3·43 (-1·57–8·4)
Residential radon	26 (16·2–38·8)	26 (16·3–38·6)	25·9 (16·1–38·5)	-0·00591 (-1·06–1·22)	-0·383 (-1·15–0·313)	25·9 (16·4–38·5)	25·9 (16·4–38·3)	25·8 (16·3–38·2)	-0·203 (-1·29–1·01)	-0·341 (-0·911–0·293)
Lead exposure	48·6 (26·4–68·7)	35·3 (17–56)	25·8 (10·8–45·9)	-27·2 (-36—18)	-26·9 (-36·5—17·9)	30·7 (10·4–53·8)	22·6 (7·12–44)	16·2 (4·07–35·9)	-26·6 (-37—17·6)	-28·3 (-42·7—18·3)
Child underweight	0·947 (0·548–1·37)	0·773 (0·442–1·15)	0·77 (0·428–1·14)	-18·3 (-24·4—13·4)	-0·497 (-5·05–4·12)	1·08 (0·636–1·55)	0·919 (0·525–1·35)	0·907 (0·518–1·33)	-15 (-20·1—10·3)	-1·38 (-4·3–1·69)
Iron deficiency	--	--	--	--	--	10·3 (8·91–11·9)	9·24 (7·9–10·7)	9·09 (7·76–10·6)	-10·6 (-13·8—7·49)	-1·52 (-3·59–0·44)
Vitamin A deficiency	3·7 (2·77–4·75)	2 (1·39–2·66)	1·76 (1·26–2·38)	-45·9 (-60·3—22·9)	-11·9 (-29·1–12)	2·85 (2·27–3·45)	2·1 (1·62–2·57)	1·86 (1·41–2·32)	-26·4 (-42·2—5·84)	-11·5 (-23·8–5·33)
Zinc deficiency	3·13 (0·00738–9·7)	2·58 (0·00126–8·22)	2·47 (0·00132–7·85)	-17·6 (-91·3—11·4)	-4·4 (-20–125)	3·07 (0·00499–9·51)	2·51 (0·00104–7·94)	2·4 (0·0012–7·59)	-18·3 (-91·5—11·4)	-4·18 (-17·2–228)
Smoking	27·5 (24·7–30·4)	21·8 (19·5–24·3)	19·2 (17·1–21·6)	-20·8 (-23—18·6)	-11·6 (-13—10·3)	16·6 (14·7–18·9)	14·4 (12·5–16·4)	13·2 (11·5–15·2)	-13·8 (-16·9—10·9)	-8·07 (-9·76—6·49)
Secondhand smoke	42·9 (41·7–44·1)	35·8 (34·9–36·7)	34·4 (33·4–35·3)	-16·6 (-19·9—13·1)	-3·94 (-5·75—2·07)	45·4 (44·4–46·5)	36·4 (35·7–37·1)	34·9 (34–35·7)	-19·7 (-22·4—17·1)	-4·13 (-5·58—2·7)
Alcohol use	20·5 (15·3–26·4)	21·4 (15·2–28·7)	21·2 (15–28·7)	4·01 (-4·72–15·5)	-0·871 (-4·49–2·39)	11·3 (7·98–15·6)	10·5 (7·2–14·7)	10·5 (7·09–14·8)	-6·4 (-12·4–1·85)	-0·83 (-6·83–5·4)
Drug use	0·251 (0·141–0·466)	0·342 (0·211–0·597)	0·324 (0·194–0·567)	36·3 (23·9–53·3)	-5·28 (-9·74—0·863)	0·147 (0·0852–0·271)	0·212 (0·143–0·348)	0·208 (0·132–0·342)	44·1 (23·7–72·9)	-1·98 (-9·9–5·43)
High fasting plasma glucose	8·48 (7·43–9·65)	12·5 (11·3–13·9)	14·4 (12·8–16·2)	47·5 (39·2–57·4)	15·2 (9·45–22·4)	7·81 (6·84–8·93)	10·2 (9·12–11·4)	12 (10·5–13·6)	30·4 (22·9–39·2)	17·9 (11·5–25·1)
High systolic blood pressure	37·7 (35·8–39·4)	31·2 (29·1–33·3)	32·8 (30·2–35·5)	-17·3 (-20·6—13·8)	5·25 (0·103–10·8)	33·2 (31·7–34·8)	22·3 (20·7–24)	24·5 (21·9–27·1)	-32·9 (-36·5—29·2)	9·81 (1·15–18·8)
High body-mass index	18·2 (13·2–24·5)	24·4 (18·3–31·9)	25·6 (19·1–33·4)	34 (25·6–47·4)	4·7 (2·68–7·17)	18·7 (14–25)	26·2 (21·1–33·5)	27·5 (22·3–35·2)	40·1 (30–54·3)	5·01 (2·41–8·21)
Low bone mineral density	9·07 (5·25–14·9)	8·37 (4·66–14)	8·35 (4·49–14)	-7·69 (-15·8—0·49)	-0·349 (-8·44–7·52)	14·6 (10·2–20·7)	13·9 (9·4–19·9)	13·6 (8·95–19·8)	-5·21 (-11·9–0·538)	-2·17 (-9·87–5·08)
Diet low in fruits	44 (34·7–54·9)	41·9 (32·7–52·4)	42·5 (33·2–53·3)	-4·62 (-6·57—3·01)	1·49 (-1·33–4·46)	45·6 (36·1–56·4)	43·8 (34·6–54·6)	44·3 (34·8–55·3)	-3·85 (-5·63—2·21)	1·22 (-1·52–4·21)
Diet low in vegetables	37·5 (22·7–54·7)	37·4 (21·7–56·6)	37·4 (21·9–56·1)	-0·255 (-7·87–6·03)	-0·201 (-3·42–2·97)	35·2 (20·5–52)	35·1 (20·1–53·5)	34·9 (19·9–53·3)	-0·235 (-7·85–6·43)	-0·484 (-4·16–2·65)
Diet low in whole grains	86·4 (80·3–92·6)	88·3 (82·9–93·9)	88·9 (83·8–94·1)	2·2 (1·13–3·42)	0·678 (0·235–1·24)	91·9 (88·4–95·4)	91·8 (88·2–95·4)	91·7 (88·1–95·5)	-0·119 (-0·653–0·432)	-0·081 (-0·621–0·433)
Diet low in nuts and seeds	53·6 (23·2–71·8)	45·8 (18·4–66·9)	45·9 (19·3–66·8)	-14·6 (-22·9—6·36)	0·094 (-2·96–5·02)	49·3 (20·5–69·1)	41·5 (15·8–63·9)	41·4 (15·9–64)	-15·8 (-24·6—5·95)	-0·211 (-3·58–4·65)
Diet low in milk	57 (33·5–75·9)	49·6 (27·1–68·9)	50·5 (28·7–69·5)	-13 (-29—4·27)	1·77 (-0·146–5·22)	57 (33·6–75·7)	49·7 (27·8–68·7)	50·6 (29–69·9)	-12·9 (-28·1—4·04)	1·87 (-0·0156–5·22)
Diet high in red meat	82·5 (77·6–87)	79·1 (73·1–84·4)	78·8 (73–84·1)	-4·2 (-6·09—2·87)	-0·354 (-2·75–2·09)	82·5 (77·6–87·1)	79 (73·3–84·3)	78·7 (72·6–84·1)	-4·32 (-6—2·96)	-0·319 (-2·7–2·12)
Diet high in processed meat	72·8 (49·2–92·5)	76·9 (53·9–95·8)	76·5 (53·9–95·6)	5·67 (2·88–10·5)	-0·514 (-1·6–0·366)	76·3 (53·5–95·2)	80·2 (59–97·6)	79·8 (58·5–97·2)	5·04 (2·18–9·68)	-0·452 (-1·56–0·404)
Diet high in sugar-sweetened beverages	38·3 (26·1–53·1)	43·5 (29·4–57·8)	46·4 (33·3–59·9)	13·6 (0·397–26·2)	6·61 (0·249–17·6)	42·3 (30·3–55·7)	48·4 (35·4–62·2)	50·9 (38·4–64·4)	14·2 (3·26–27·5)	5·22 (-1·31–14)
Diet low in fiber	34·1 (21·5–47·2)	33 (20·4–46·1)	32·7 (20·2–45·7)	-3·26 (-7·91–0·975)	-1·02 (-4·99–3·53)	34 (20·8–47·8)	33·2 (19·7–46·7)	32·8 (19·8–46·5)	-2·43 (-6·68–1·43)	-1·12 (-5·12–3·12)
Diet low in seafood omega-3 fatty acids	89·9 (80·2–99·9)	88·6 (78·8–99·7)	88·2 (78·1–99·4)	-1·46 (-4·49–0·842)	-0·462 (-3·24–1·61)	91·6 (83·5–99·9)	90·5 (82·1–99·8)	90·1 (81·4–99·8)	-1·29 (-4·02–0·402)	-0·435 (-2·7–1·39)
Diet low in polyunsaturated fatty acids	49·1 (15·4–88·3)	42·5 (12·7–82·3)	41·5 (12·5–81·1)	-13·4 (-25·2—2·92)	-2·3 (-5·14—0·222)	51·4 (16·7–90·1)	45 (13·4–85·1)	44 (13·4–84·4)	-12·5 (-24·7—2·66)	-2·2 (-4·79—0·203)
Diet high in trans fatty acids	49·4 (41–94·6)	42·4 (34·1–93·9)	42·2 (33·8–93·4)	-14·2 (-20—1·01)	-0·31 (-3·86–3·68)	49·8 (41–96·6)	42·8 (33·4–96·7)	42·5 (33·4–96·2)	-14·1 (-20·1—2·01e-14)	-0·717 (-3·99–2·68)
Diet high in sodium	31·1 (9·72–53)	33·5 (10·8–55·3)	36 (12·3–57·9)	7·54 (-5·61–28·6)	7·48 (-0·413–22·9)	22·5 (3·67–43)	23 (3·62–43·7)	23·8 (3·67–45·2)	2·34 (-7·8–14·5)	3·14 (-3·41–11·6)
Low physical activity	3·97 (1·76–7·87)	4·19 (1·97–8·37)	4·5 (2·03–8·91)	5·58 (-9·3–29·6)	7·37 (-9·94–24·6)	6·36 (3·42–11·7)	6·59 (3·68–11·7)	7·04 (3·84–12·6)	3·7 (-6·59–18·9)	6·78 (-4·6–17·6)
Occupational asthmagens	24·3 (20·9–28·6)	21·1 (18·1–24·8)	21 (18–24·7)	-13·4 (-16·3—10·5)	-0·524 (-3·43–2·74)	11·6 (9·73–13·8)	11 (9·33–13)	11·7 (9·77–13·9)	-4·86 (-12·8–4·01)	6·25 (-1–13·6)
Occupational particulate matter, gases, and fumes	9·13 (6·53–13·1)	8·26 (5·88–11·8)	7·81 (5·56–11·2)	-9·47 (-11·7—7·22)	-5·53 (-6·64—4·36)	3·9 (2·82–5·64)	3·67 (2·64–5·29)	3·51 (2·53–5·05)	-5·8 (-9·86—2·32)	-4·41 (-6·39—2·19)
Occupational noise	8·36 (7·96–9·02)	7·86 (7·49–8·51)	7·6 (7·22–8·26)	-6·02 (-6·68—5·29)	-3·35 (-3·68—2·99)	4·41 (4·19–4·75)	4·76 (4·54–5·11)	4·93 (4·7–5·29)	8·02 (6·78–9·11)	3·6 (3·15–4·04)
Occupational injuries	--	--	--	--	--	--	--	--	--	--
Occupational ergonomic factors	12·3 (10·8–14·1)	10·7 (9·28–12·4)	10·6 (9·19–12·3)	-12·9 (-14·8—11·1)	-0·929 (-3·12–1·42)	6·29 (5·42–7·37)	7·27 (6·19–8·67)	7·68 (6·51–9·12)	15·5 (11·1–19·5)	5·69 (2·7–9·14)
Childhood sexual abuse	6·68 (4·46–9·16)	6·65 (4·49–9·17)	6·74 (4·41–9·48)	-0·399 (-3·18–2·25)	1·31 (-2·56–5·16)	12 (9·22–16)	11·9 (9·2–15·8)	11·9 (9·17–16)	-1·38 (-4·14–1·7)	0·55 (-1·52–2·9)
Intimate partner violence	--	--	--	--	--	19·3 (10·9–26·2)	17·9 (10·4–23·6)	19 (10·7–26)	-7·23 (-13·1–3·33)	6·28 (-1·03–17)
Non-exclusive breastfeeding	25·2 (21·5–30)	24·2 (20·6–28·7)	24 (20·6–28·6)	-4·13 (-8·28–0·228)	-0·767 (-3·98–2·8)	25·9 (21·9–30·8)	24·7 (21–29·3)	24·4 (20·8–29·2)	-4·58 (-9·61–0·768)	-1·19 (-5·46–3·39)
Discontinued breastfeeding	25·6 (25·4–26)	25·1 (24·9–25·5)	24·8 (24·5–25·2)	-1·94 (-3·47—0·227)	-1·31 (-2·71–0·0104)	25·6 (25·4–26)	25·1 (24·9–25·5)	24·8 (24·5–25·2)	-1·9 (-3·43—0·198)	-1·32 (-2·73—0·000226)
Diet low in calcium	17·9 (8·81–33·1)	15·8 (7·65–29·1)	15 (7·18–28)	-11·8 (-17—7·74)	-4·75 (-8·01—2·8)	12·4 (5·37–23·3)	10·1 (4·48–18·6)	9·55 (4·05–17·6)	-18·4 (-25·1—13)	-5·5 (-9·68—2·68)
Occupational exposure to asbestos	12·3 (11·7–12·8)	12·1 (11·8–12·4)	11·6 (10·4–12·8)	-0·903 (-5·4–3·62)	-4·34 (-14·2–5·61)	3·01 (1·95–4)	2·56 (1·64–2·95)	2·48 (1·6–3·04)	-15·2 (-35·4–3)	-2·99 (-15·4–10·5)
Occupational exposure to arsenic	0·535 (0–1·26)	0·486 (0–1·15)	0·495 (0–1·16)	-9·1 (-11·5–NA)	1·75 (-0·815–NA)	0·412 (0–0·968)	0·416 (0–0·974)	0·424 (0–0·997)	0·893 (-2·56–NA)	1·93 (-0·82–NA)
Occupational exposure to benzene	0·761 (0·0449–2·17)	0·81 (0·0478–2·31)	0·839 (0·0489–2·4)	6·49 (4·83–8·77)	3·49 (2·07–5·4)	0·889 (0·0533–2·55)	1·25 (0·0742–3·57)	1·35 (0·081–3·95)	40·3 (38–44·2)	8·55 (6·8–11·1)
Occupational exposure to beryllium	0·0228 (0·0227–0·0229)	0·0229 (0·0229–0·023)	0·0236 (0·0234–0·0238)	0·589 (-0·0226–1·15)	2·77 (1·83–3·66)	0·0208 (0·0207–0·0209)	0·0252 (0·0251–0·0253)	0·0267 (0·0264–0·0269)	21·1 (20·2–22)	5·94 (4·87–6·99)
Occupational exposure to cadmium	0·0613 (0·0601–0·0626)	0·0565 (0·0557–0·0574)	0·0576 (0·0563–0·059)	-7·83 (-10·1—5·77)	1·91 (-0·376–4·33)	0·0485 (0·0473–0·0497)	0·0503 (0·0494–0·0511)	0·0516 (0·0504–0·0528)	3·69 (0·701–6·58)	2·68 (0·33–5·24)
Occupational exposure to chromium	0·143 (0·14–0·145)	0·134 (0·133–0·136)	0·137 (0·135–0·14)	-5·74 (-7·74—3·84)	2·27 (0·295–4·39)	0·114 (0·11–0·117)	0·125 (0·122–0·127)	0·128 (0·125–0·131)	9·62 (5·72–13·5)	2·84 (0·527–5·3)
Occupational exposure to diesel engine exhaust	0·649 (0·64–0·659)	0·69 (0·683–0·698)	0·712 (0·701–0·725)	6·25 (4·35–8·11)	3·25 (1·56–4·86)	0·502 (0·493–0·512)	0·607 (0·597–0·617)	0·643 (0·629–0·657)	20·9 (17·9–23·7)	5·92 (3·81–8·18)
Occupational exposure to formaldehyde	0·306 (0·293–0·313)	0·274 (0·264–0·279)	0·278 (0·268–0·285)	-10·5 (-12·8—8·34)	1·52 (-0·773–4·01)	0·232 (0·222–0·238)	0·25 (0·24–0·254)	0·259 (0·248–0·265)	7·56 (4·73–10·4)	3·66 (1·48–6)
Occupational exposure to nickel	0·502 (0–2·19)	0·465 (0–2·03)	0·473 (0–2·07)	-7·45 (-9·78–NA)	1·75 (-0·4–NA)	0·33 (0–1·46)	0·322 (0–1·42)	0·326 (0–1·45)	-2·42 (-5·88–NA)	1·27 (-1·78–NA)
Occupational exposure to polycyclic aromatic hydrocarbons	0·289 (0·285–0·294)	0·277 (0·274–0·28)	0·284 (0·279–0·289)	-4·06 (-5·85—2·37)	2·35 (0·613–4·28)	0·249 (0·244–0·254)	0·279 (0·275–0·282)	0·292 (0·287–0·298)	12·1 (9·5–14·6)	4·88 (2·71–7·13)
Occupational exposure to silica	5·57 (0·823–17·9)	5·57 (0·814–18)	5·7 (0·844–18)	-0·0678 (-2·73–2·9)	2·34 (0·0298–5·04)	2·81 (0·408–8·82)	2·76 (0·396–8·75)	2·8 (0·402–8·89)	-1·65 (-5·35–2·01)	1·23 (-1·87–4·9)
Occupational exposure to sulfuric acid	0·87 (0·182–2·69)	0·774 (0·162–2·46)	0·774 (0·161–2·44)	-11·1 (-13·3—8·99)	0·0368 (-2·25–2·22)	0·611 (0·124–1·94)	0·6 (0·125–1·89)	0·604 (0·124–1·88)	-1·85 (-4·95–1·39)	0·709 (-1·88–3·61)
Unsafe sex	--	--	--	--	--	--	--	--	--	--
Occupational exposure to trichloroethylene	0·0775 (0·0769–0·0788)	0·0751 (0·0747–0·0761)	0·0771 (0·0762–0·0785)	-3·12 (-4·51—1·67)	2·59 (1·12–4·18)	0·0676 (0·0669–0·0688)	0·0761 (0·0756–0·0773)	0·0796 (0·0786–0·0812)	12·6 (10·6–14·5)	4·57 (2·95–6·31)
No access to handwashing facility	3·37 (3·31–3·44)	3·2 (3·14–3·26)	3·16 (3·1–3·23)	-5·03 (-7·13—3·05)	-1·21 (-2·66–0·126)	3·37 (3·31–3·44)	3·2 (3·14–3·27)	3·16 (3·11–3·23)	-5·06 (-7·15—3·06)	-1·19 (-2·64–0·13)
Child wasting	1·19 (0·83–1·57)	1·16 (0·817–1·54)	1·14 (0·803–1·52)	-1·98 (-4·7–0·771)	-1·52 (-3·36–0·474)	0·99 (0·698–1·29)	0·986 (0·693–1·3)	0·955 (0·674–1·26)	-0·375 (-4·74–3·74)	-3·19 (-5·82—0·452)
Child stunting	1·57 (0·808–2·31)	1·18 (0·569–1·77)	1·16 (0·547–1·74)	-24·8 (-32·8—18·6)	-1·59 (-10·5–5·55)	1·9 (0·998–2·73)	1·55 (0·783–2·28)	1·53 (0·782–2·24)	-18·3 (-25—12·7)	-1·64 (-5·62–2·69)
Chewing tobacco	0·214 (0·196–0·234)	0·222 (0·206–0·24)	0·24 (0·222–0·261)	3·78 (-5·88–14·5)	8·14 (1·39–15)	0·149 (0·131–0·172)	0·152 (0·133–0·173)	0·157 (0·138–0·178)	1·79 (-12·6–19·8)	3·32 (-7·08–14·3)
Diet low in legumes	79·3 (51·6–99)	75·6 (46·4–95·9)	77·3 (48·8–97·7)	-4·67 (-10·7—1·71)	2·27 (0·276–6·24)	76·5 (45·5–98)	71·9 (41·5–92·9)	73·9 (42·1–95·6)	-6·08 (-11·8—2·62)	2·79 (0·267–6·62)
Short gestation	20·2 (18–23)	20·8 (18·7–22·9)	19·8 (17·8–21·9)	2·81 (-5·46–10·5)	-4·72 (-9·74–0·616)	20·3 (18·2–22·5)	19·5 (17·8–21·4)	18·3 (16·5–20·2)	-3·69 (-10·4–3·54)	-6·47 (-11·5—1·1)
Low birth weight	9·79 (9·02–10·7)	10·2 (9·52–10·9)	9·74 (9·01–10·5)	3·82 (-4·52–11·5)	-4·16 (-9·24–1·19)	10·1 (9·28–10·9)	9·95 (9·38–10·6)	9·19 (8·56–9·92)	-1·16 (-8·16–6·05)	-7·69 (-12·6—2·44)
High temperature	21·5 (15·3–28·8)	20·6 (15·7–26·6)	22·2 (16·9–28·8)	-4·23 (-10·9–5·1)	8·13 (3·75–12·3)	21·5 (15·3–28·8)	20·6 (15·7–26·6)	22·2 (16·9–28·8)	-4·23 (-10·9–5·1)	8·13 (3·75–12·3)
Low temperature	32·4 (29·5–35·5)	34·5 (31·9–37·4)	32·3 (29·4–35·4)	6·53 (4·7–8·56)	-6·48 (-8·17—4·7)	32·4 (29·5–35·5)	34·5 (31·9–37·4)	32·3 (29·4–35·4)	6·53 (4·7–8·56)	-6·48 (-8·17—4·7)
Kidney dysfunction	14·8 (10·1–21·3)	15 (10·4–21·4)	15·1 (10·4–21·5)	1·38 (-0·348–3·52)	0·285 (-1·13–1·73)	15·6 (10·6–22·1)	15 (10·8–20·7)	16 (11·1–22·6)	-3·75 (-7·42–1·59)	6·69 (2·01–10·2)
Bullying victimization	6·38 (2·6–12·8)	7·14 (3·29–13·7)	7·25 (3·29–13·8)	11·9 (-2·43–45·9)	1·51 (-1·99–4·34)	5·26 (2·17–10·6)	5·83 (2·67–11)	5·94 (2·7–11·2)	10·9 (-5·2–44·8)	1·82 (-1·42–4·12)
High LDL cholesterol	55·2 (52·9–57·8)	44·9 (42·2–47·9)	45·2 (42·3–48·1)	-18·7 (-20·8—16·7)	0·623 (-0·763–1·97)	53·2 (50·7–55·8)	45·2 (42·4–48·2)	45·9 (43–48·9)	-15·1 (-16·9—13·1)	1·52 (0·357–2·73)

Supplementary Results Table 1. Andorra age-standardised Summary Exposure Values (SEVs) for most-detailed risk factors and percentage change, by sex, 1990, 2010, and 2019. Data in parentheses are 95% uncertainty intervals.

	Male					Female				
	1990	2010	2019	Percentage change 1990-2010	Percentage change 2010-2019	1990	2010	2019	Percentage change 1990-2010	Percentage change 2010-2019
Unsafe sanitation	1·44 (0·801–2·36)	0·671 (0·376–1·09)	0·578 (0·326–0·929)	-53·2 (-76·2–-5·86)	-13·9 (-46·5–43·4)	--	--	--	--	--
Ambient particulate matter pollution	13 (3·53–25·3)	9·52 (6·1–13·4)	6·72 (3·62–10·3)	-26·7 (-63·7–126)	-29·4 (-41·8–-23·1)	13 (3·6–25·3)	9·52 (6·1–13·4)	6·72 (3·63–10·3)	-26·7 (-63·7–126)	-29·4 (-41·5–-23·1)
Household air pollution from solid fuels	0·0488 (0·0103–0·152)	0·0122 (0·00229–0·038)	0·00871 (0·00142–0·0291)	-75 (-88·7–-48·7)	-28·6 (-55·5–10·3)	0·0642 (0·014–0·189)	0·0161 (0·0029–0·0501)	0·0116 (0·00193–0·0391)	-74·9 (-88·5–-48·4)	-28·2 (-55·6–10·5)
Ambient ozone pollution	51 (17·9–98)	74·4 (30·9–100)	64·3 (23·5–100)	45·9 (-1·64–178)	-13·6 (-44·6–22·3)	51 (17·9–98)	74·4 (30·9–100)	64·3 (23·5–100)	45·9 (-1·64–178)	-13·6 (-44·6–22·3)
Residential radon	25·2 (4·16–75·1)	25·2 (4·16–75·1)	25·2 (4·16–75·1)	0 (0–0)	0 (0–0)	25·2 (4·16–75·1)	25·2 (4·16–75·1)	25·2 (4·16–75·1)	0 (0–0)	0 (0–0)
Lead exposure	33 (4·14–59·9)	29·5 (7·23–52·9)	20·8 (3·42–42·2)	-10·5 (-23·2–95·5)	-29·6 (-51·4–-18·7)	19·8 (0·0398–47·3)	17·5 (0·199–41·8)	12·2 (0·036–33·5)	-11·3 (-23·1–831)	-30·2 (-77·5–-18·8)
Child underweight	0·857 (0·478–1·27)	0·734 (0·406–1·11)	0·734 (0·413–1·12)	-14·3 (-25·4–-2·26)	-0·0822 (-9·39–10·6)	0·993 (0·579–1·47)	0·878 (0·512–1·34)	0·878 (0·506–1·32)	-11·5 (-22·8–0·652)	-0·0842 (-8·66–9·6)
Iron deficiency	--	--	--	--	--	9·63 (7·98–11·5)	8·85 (7·39–10·6)	8·81 (7·35–10·6)	-8·11 (-17·1–1·92)	-0·372 (-6·03–6·04)
Vitamin A deficiency	2·27 (0·921–4·69)	1·23 (0·477–2·55)	1·12 (0·44–2·34)	-45·9 (-75·8–43·6)	-8·76 (-49·1–78·1)	2·01 (0·954–3·43)	1·48 (0·749–2·69)	1·37 (0·688–2·34)	-26·5 (-63·5–63·7)	-7·77 (-39·8–42·9)
Zinc deficiency	1·9 (0–6·16)	1·73 (0–5·41)	1·8 (0–5·73)	-9·24 (-38·5–NA)	4·26 (-9·21–NA)	1·87 (0–5·87)	1·69 (0–5·38)	1·76 (0–5·49)	-9·94 (-41·2–NA)	4·52 (-8·1–NA)
Smoking	25·9 (21·5–30·3)	21·9 (18·4–25·9)	19·8 (16·5–23·6)	-15·3 (-24–-5·26)	-9·78 (-15–-3·74)	16·1 (12·5–19·6)	13·6 (10·9–16·9)	12·7 (10–15·9)	-15·1 (-30–2·06)	-6·76 (-16·6–3·98)
Secondhand smoke	42·4 (39·3–45·5)	37·5 (34·7–40·6)	35·3 (32·4–38·2)	-11·4 (-18·8–-3·38)	-6·05 (-12–0·632)	44·9 (42·6–47·4)	39 (36·5–41·2)	36·7 (34·5–39)	-13·3 (-19·1–-7·03)	-5·86 (-10·1–-1·34)
Alcohol use	22·5 (16·1–30·7)	23 (16·2–30·9)	22·7 (15·5–31·5)	2·46 (-13·3–24·8)	-1·21 (-11·3–10·9)	11·3 (7·15–16·6)	11 (7·31–15·9)	10·8 (6·67–15·9)	-2·76 (-25–29)	-1·99 (-17·4–14·4)
Drug use	0·207 (0·106–0·406)	0·278 (0·167–0·482)	0·296 (0·183–0·516)	34·4 (16·1–68·5)	6·74 (-2·53–19·2)	0·101 (0·0489–0·206)	0·137 (0·0801–0·245)	0·15 (0·0902–0·258)	35·2 (15·3–72·6)	9·23 (0·72–21·1)
High fasting plasma glucose	6·13 (5·26–7·09)	9·07 (7·56–11)	11·1 (9·25–13·3)	47·9 (31·7–71·3)	22·2 (6·5–42·5)	5·14 (4·31–6·07)	7·19 (6·14–8·45)	9·06 (7·56–10·8)	39·7 (25·5–57·4)	26·1 (13·2–44·3)
High systolic blood pressure	38·6 (31·2–47·3)	38·6 (31·1–47·1)	38·6 (31·5–46·4)	0·131 (-23–28·3)	-0·00973 (-16·1–20·4)	30·9 (22·8–41)	29·5 (20·7–39·8)	29·1 (21·1–39)	-4·38 (-35·2–46·7)	-1·43 (-26·9–33·1)
High body-mass index	25·1 (18·5–32·8)	27·3 (20·5–35·5)	28·5 (21·4–36·8)	8·69 (0·114–20·7)	4·61 (-1·68–11·6)	25·1 (16·9–37·1)	28·6 (20·1–40·3)	30·3 (21·9–42·5)	13·9 (0·363–35·4)	6·1 (-4·79–18·8)
Low bone mineral density	9·35 (4·93–15·4)	8·76 (4·64–15)	8·58 (4·43–14·4)	-6·31 (-28·5–20·4)	-2·06 (-24·8–28·5)	15 (9·72–21·6)	14·2 (9·06–20·9)	13·9 (8·48–20·4)	-5·03 (-25·5–20)	-2·21 (-23–24·3)
Diet low in fruits	36·5 (24·3–49·7)	34·4 (22·4–48·5)	35·1 (22·7–49·1)	-5·81 (-27–22·1)	2·01 (-13·2–19·8)	38·7 (26·2–52·2)	36·6 (24·2–49·9)	37 (25·7–51·4)	-5·42 (-26·2–21·3)	1·05 (-12·2–17·4)
Diet low in vegetables	27·5 (12·8–47·3)	26 (12–45·7)	27·3 (12·6–48·1)	-5·5 (-31·9–26·5)	4·88 (-11·1–28·3)	25·3 (11·4–46·4)	23·5 (10·5–42·1)	24·8 (11·5–44·3)	-7·14 (-34·5–29·3)	5·52 (-11·7–31·6)
Diet low in whole grains	86·8 (80–93·6)	87·2 (80·8–93·8)	87·7 (81·6–93·9)	0·524 (-0·168–1·48)	0·528 (-0·347–1·68)	--	--	--	--	--
Diet low in nuts and seeds	37·4 (13·9–62·1)	36 (13·6–61·7)	36 (14·2–61·9)	-3·65 (-27·1–28·3)	-0·0402 (-16·5–17·9)	32·3 (10·5–59·7)	31 (9·76–59)	31·1 (10·7–58·4)	-4·11 (-31·3–33)	0·481 (-16–19·3)
Diet low in milk	45·6 (22·6–65·9)	45 (21·3–64·8)	46·1 (21·9–66·1)	-1·31 (-15·1–14·1)	2·29 (-5·69–12·7)	44·8 (22·2–64·5)	44·2 (19·8–64·1)	45·2 (21·1–65·3)	-1·36 (-15·5–13)	2·31 (-6·69–11·8)
Diet high in red meat	84·1 (73·6–92·9)	84·1 (72·9–93)	83·6 (72–92·1)	0·0787 (-12·1–12·9)	-0·696 (-8·07–7·3)	83·9 (72·1–92·6)	83·5 (72·5–92·4)	82·8 (71·2–91·7)	-0·476 (-12·6–14·3)	-0·875 (-8·15–6·94)
Diet high in processed meat	75·3 (50·2–96·9)	75·9 (50·2–96·9)	75·7 (50·3–97·4)	0·767 (-6·16–8·24)	-0·176 (-4·51–4·33)	79 (55·3–98·6)	79·2 (54·8–98·8)	79·1 (55·5–98·8)	0·309 (-5·28–6·88)	-0·181 (-3·92–3·76)
Diet high in sugar-sweetened beverages	55·3 (36·2–73·2)	57·1 (39·4–74·7)	55·8 (37·6–73·9)	3·22 (-24·4–43·1)	-2·32 (-20·7–20·3)	60·5 (40·2–78·9)	61·9 (43·6–79·4)	60·4 (41·1–78·7)	2·34 (-23·2–39·4)	-2·4 (-19·3–18·5)
Diet low in fiber	23·5 (12·3–36·8)	22·7 (12·1–35·7)	23·6 (12·4–37·3)	-3·37 (-28·9–31·4)	4·14 (-13·9–24·8)	23·3 (11·8–36·1)	22·4 (11·3–36)	23·3 (11·8–36·9)	-4·13 (-32·5–31·8)	4·29 (-13·9–26·4)
Diet low in seafood omega-3 fatty acids	80·3 (62·2–98·9)	76·9 (59·4–98·8)	77·1 (57·6–99·4)	-4·22 (-21·9–14·1)	0·183 (-15·9–20·1)	83·1 (65·3–100)	80·5 (62·2–100)	80·9 (62·5–99·6)	-3·13 (-18–13·4)	0·418 (-12·5–18·2)
Diet low in polyunsaturated fatty acids	35·8 (9·18–79)	33·2 (8·56–77·2)	34·1 (8·78–78·2)	-7·18 (-23·3–5·36)	2·81 (-5·09–14·5)	38·6 (9·96–82·3)	36·1 (9·28–79·8)	37 (9·52–79·9)	-6·47 (-21·8–4·58)	2·48 (-4·77–12·7)
Diet high in trans fatty acids	48 (38–100)	41·3 (31·6–96·8)	40·3 (30·4–96·4)	-14 (-24·2–-1·87)	-2·34 (-13–7·4)	49·2 (38·6–100)	42·6 (32·1–99·1)	41·5 (31·3–99·3)	-13·4 (-23·5–0)	-2·49 (-11·5–6·2)
Diet high in sodium	23·7 (4·62–48·9)	25·2 (4·99–50·1)	26·5 (5·38–51·6)	6·7 (-25·6–73·9)	4·98 (-14·9–40·8)	13 (3·56–33·2)	13·4 (3·53–34·8)	13·8 (3·56–35·2)	2·95 (-27·2–35·9)	3·17 (-13–27)
Low physical activity	4·04 (1·69–8·23)	4·92 (2·16–10)	4·95 (2·31–10·4)	21·7 (2·04–49·6)	0·625 (-13–15·9)	6·86 (3·59–12·6)	7·78 (4·14–14·1)	7·85 (4·21–14)	13·5 (2·6–29)	0·869 (-8·05–10·1)
Occupational asthmagens	22·5 (18·1–28)	21 (16·8–25·8)	20·8 (16·9–25·7)	-6·92 (-21·5–10)	-0·881 (-11·7–11)	10·8 (8·73–13·3)	10·9 (8·84–13·4)	11·2 (9·13–13·8)	1·11 (-14·2–17·2)	2·98 (-6·65–14·5)
Occupational particulate matter, gases, and fumes	8·61 (6·04–12·5)	8·07 (5·63–11·7)	7·69 (5·35–11·2)	-6·28 (-13·1–0·146)	-4·65 (-8·33–-1·25)	3·77 (2·62–5·66)	3·5 (2·44–5·17)	3·34 (2·31–4·9)	-7·32 (-17·2–4·05)	-4·45 (-9·44–0·718)
Occupational noise	8·12 (7·39–8·93)	7·87 (7·23–8·63)	7·67 (7·07–8·35)	-3·07 (-7·5–1·89)	-2·53 (-4·87–-0·226)	4·79 (4·18–5·52)	5·05 (4·57–5·7)	5·17 (4·74–5·75)	5·49 (-1·65–14·8)	2·4 (-1·02–5·86)
Occupational injuries	--	--	--	--	--	--	--	--	--	--
Occupational ergonomic factors	12·5 (10·6–15·1)	10·8 (8·9–12·8)	10·8 (9·03–12·9)	-13·9 (-25·9–-1·31)	-0·208 (-8·26–8·84)	7·2 (5·6–9·08)	7·59 (5·88–9·68)	8 (6·22–10·1)	5·48 (-14·2–28·2)	5·31 (-8·12–20·8)
Childhood sexual abuse	7·16 (4·65–10·1)	7·16 (4·65–10·1)	7·16 (4·65–10·1)	0 (0–0)	0 (0–0)	10·6 (7·84–14·3)	10·6 (7·84–14·3)	10·6 (7·84–14·3)	0 (0–0)	0 (0–0)
Intimate partner violence	--	--	--	--	--	15 (8·02–22·3)	15 (8·02–22·3)	15 (8·02–22·3)	0 (0–0)	0 (0–0)
Non-exclusive breastfeeding	21·2 (17–26·4)	19·9 (15·9–24·5)	19·7 (15·6–24·6)	-6·1 (-17·8–7·58)	-1·05 (-9·68–8·51)	21·2 (17–26·4)	19·9 (15·9–24·5)	19·7 (15·6–24·6)	-6·09 (-17·8–7·59)	-1·06 (-9·69–8·5)
Discontinued breastfeeding	23·6 (22·4–24·8)	20·1 (18·7–21·4)	20·4 (19·2–21·7)	-15 (-21·6–-8·27)	1·85 (-3·67–7·72)	24·1 (22·8–25·3)	21·6 (20·3–23)	20·5 (19·2–21·8)	-10·1 (-16·8–-3·25)	-5·47 (-10·6–-0·207)
Diet low in calcium	9·11 (4·04–16·7)	8·64 (3·75–16)	8·79 (3·78–16·6)	-5·14 (-16·7–10·3)	1·75 (-8·26–10·4)	4·03 (1·7–7·43)	3·86 (1·58–6·98)	3·95 (1·6–7·32)	-4·37 (-24·5–20)	2·57 (-11·7–18)
Occupational exposure to asbestos	17·3 (11·1–22·9)	16·9 (11·1–21·5)	16·2 (10·6–21·1)	-2·4 (-26·5–31·1)	-3·74 (-22·5–16·7)	3·06 (1·01–5·42)	2·23 (0·871–4)	2·36 (0·856–4·29)	-27 (-61·2–38·6)	5·73 (-37·9–80·5)
Occupational exposure to arsenic	0·518 (0–1·22)	0·485 (0–1·15)	0·485 (0–1·15)	-6·34 (-19·1–NA)	-0·0388 (-9·29–NA)	0·416 (0–0·99)	0·407 (0–0·992)	0·411 (0–0·965)	-2·04 (-17·6–NA)	0·839 (-8·16–NA)
Occupational exposure to benzene	0·811 (0·0499–2·27)	0·862 (0·0507–2·41)	0·878 (0·0516–2·55)	6·21 (-2·42–19·6)	1·94 (-3·83–11·8)	1·08 (0·0634–3·02)	1·36 (0·0876–4·03)	1·44 (0·0899–4·17)	26·3 (12·3–53·6)	5·65 (-1·72–15·8)
Occupational exposure to beryllium	0·0236 (0·0221–0·0251)	0·0237 (0·0222–0·025)	0·0241 (0·0226–0·0256)	0·18 (-6·89–7·24)	2·04 (-2·93–7·27)	0·0235 (0·021–0·026)	0·0264 (0·0243–0·0285)	0·0275 (0·0255–0·0297)	12·4 (0·573–25·1)	4·22 (-1·85–10·5)
Occupational exposure to cadmium	0·0601 (0·0538–0·0667)	0·0568 (0·0511–0·0628)	0·0568 (0·0508–0·0627)	-5·6 (-17·4–6·99)	0·138 (-8·31–9·27)	0·0498 (0·0433–0·0575)	0·0501 (0·0446–0·0559)	0·0508 (0·0458–0·0568)	0·563 (-14·4–16·3)	1·41 (-6·56–10·4)
Occupational exposure to chromium	0·141 (0·127–0·155)	0·136 (0·124–0·148)	0·137 (0·124–0·149)	-3·7 (-14·6–7·92)	0·621 (-6·95–8·58)	--	--	--	--	--
Occupational exposure to diesel engine exhaust	0·689 (0·635–0·745)	0·715 (0·66–0·769)	0·734 (0·679–0·789)	3·64 (-5·29–13·6)	2·79 (-3·35–9·82)	0·58 (0·507–0·656)	0·636 (0·563–0·71)	0·663 (0·588–0·741)	9·55 (-5·99–25·7)	4·25 (-4·7–13·9)
Occupational exposure to formaldehyde	0·295 (0·235–0·334)	0·272 (0·22–0·306)	0·269 (0·215–0·303)	-7·53 (-19·6–5·49)	-1·18 (-9·67–7·94)	--	--	--	--	--
Occupational exposure to nickel	0·488 (0–2·16)	0·466 (0–2·12)	0·467 (0–2·12)	-4·37 (-16·4–NA)	0·199 (-8·26–NA)	0·325 (0–1·37)	0·309 (0–1·29)	0·309 (0–1·33)	-4·88 (-21·3–NA)	-0·0108 (-9·75–NA)
Occupational exposure to polycyclic aromatic hydrocarbons	0·287 (0·262–0·314)	0·281 (0·258–0·305)	0·284 (0·26–0·308)	-2·03 (-12·2–8·58)	0·844 (-6·46–8·01)	0·262 (0·231–0·296)	0·286 (0·259–0·313)	0·294 (0·269–0·321)	9·11 (-4·25–24)	3·07 (-3·88–10·7)
Occupational exposure to silica	5·67 (0·777–18·6)	5·69 (0·844–18·2)	5·72 (0·836–18·3)	0·32 (-11·6–14·3)	0·629 (-8·22–10·7)	2·81 (0·4–8·6)	2·61 (0·366–8·37)	2·6 (0·36–8·56)	-7·11 (-22·7–12·7)	-0·362 (-10·1–11·5)
Occupational exposure to sulfuric acid	0·835 (0·179–2·69)	0·763 (0·156–2·36)	0·758 (0·157–2·34)	-8·62 (-19–3·27)	-0·629 (-8·95–8·71)	0·599 (0·123–1·93)	0·579 (0·119–1·82)	0·579 (0·121–1·86)	-3·27 (-18·6–13·1)	0·0578 (-8·59–9·83)
Unsafe sex	--	--	--	--	--	--	--	--	--	--
Occupational exposure to trichloroethylene	0·0784 (0·0727–0·0848)	0·0765 (0·0708–0·0826)	0·0775 (0·0715–0·084)	-2·45 (-11·1–6·84)	1·35 (-4·77–8·32)	0·0739 (0·0654–0·0834)	0·0785 (0·0711–0·0866)	0·0808 (0·0732–0·0885)	6·23 (-6·6–20·2)	3·01 (-3·82–11·1)
No access to handwashing facility	3·21 (3·03–3·39)	3·13 (2·96–3·3)	3·11 (2·93–3·29)	-2·43 (-8·62–4·23)	-0·69 (-4·52–3·49)	3·21 (3·03–3·39)	3·13 (2·96–3·3)	3·11 (2·93–3·29)	-2·43 (-8·62–4·23)	-0·69 (-4·52–3·49)
Child wasting	1·22 (0·866–1·61)	1·18 (0·841–1·56)	1·18 (0·839–1·56)	-3·17 (-10·7–5·11)	0·128 (-5·98–6·4)	1·03 (0·712–1·36)	0·989 (0·674–1·33)	0·99 (0·682–1·32)	-3·81 (-10·9–3·14)	0·0983 (-4·91–5·85)
Child stunting	1·55 (0·793–2·34)	1·15 (0·538–1·76)	1·16 (0·54–1·77)	-25·7 (-38–-11·4)	0·107 (-11·8–14)	1·82 (0·93–2·7)	1·49 (0·752–2·26)	1·49 (0·728–2·25)	-17·8 (-31·3–-2·61)	-0·268 (-11·1–13·4)
Chewing tobacco	0·206 (0·165–0·257)	0·218 (0·171–0·273)	0·238 (0·186–0·299)	5·73 (-19·5–38·3)	9·19 (-7·73–29·5)	0·15 (0·109–0·203)	0·156 (0·111–0·212)	0·164 (0·119–0·225)	3·49 (-26·6–50·1)	5·52 (-17·3–36·2)
Diet low in legumes	79·2 (51·5–99·9)	78·4 (49·5–99·9)	78·7 (49·4–99·9)	-0·97 (-7·84–3·22)	0·369 (-2·82–4·93)	76·3 (42·7–99·7)	75·5 (42·4–99·5)	75·9 (44·1–99·6)	-1·05 (-9·11–3·23)	0·495 (-3·14–5·81)
Short gestation	18·8 (12·3–27·5)	20·9 (13·6–30·9)	18·7 (12–28·4)	11·2 (-35·5–95·6)	-10·4 (-41·9–38·1)	19·6 (13·3–28·7)	20·2 (13·7–28·7)	18·1 (12·4–25·4)	2·89 (-37·7–79·8)	-10·3 (-40·3–32·6)
Low birth weight	9·36 (6·3–13·5)	10·2 (6·79–15·2)	9·18 (6·09–13·9)	9 (-36·3–86·3)	-10·1 (-41·8–36·7)	10·3 (6·96–14·8)	10·2 (7·09–14·3)	9·14 (6·39–13)	-0·52 (-37·9–67·4)	-10·5 (-40·8–31·5)
High temperature	0 (0–0)	0 (0–0)	0 (0–0)	--	--	0 (0–0)	0 (0–0)	0 (0–0)	--	--
Low temperature	36·3 (30·5–42·3)	38 (32·7–43·5)	36·1 (30·2–42·1)	4·57 (2·5–7·73)	-4·94 (-9·07–-1·88)	36·3 (30·5–42·3)	38 (32·7–43·5)	36·1 (30·2–42·1)	4·57 (2·5–7·73)	-4·94 (-9·07–-1·88)
Kidney dysfunction	14·4 (9·68–20·8)	14·6 (10–21·1)	14·5 (9·77–21·1)	1·89 (-1·76–6·2)	-0·916 (-4·75–2·95)	15·7 (10·7–22·3)	16·2 (11·3–22·7)	16 (10·9–22·5)	2·81 (-1·13–7·53)	-0·982 (-4·98–2·98)

Bullying victimization	6·47<br>(2·46–13·4)	6·45<br>(2·45–13·4)	6·57<br>(2·47–13·7)	-0·263<br>(-1·83–1·5)	1·84<br>(0·876–2·7)	5·1<br>(1·97–10·7)	5·06<br>(1·97–10·6)	5·14<br>(2–10·8)	-0·95<br>(-2·32–0·445)	1·61<br>(0·818–2·29)
High LDL cholesterol	53·2<br>(49·7–57)	50·2<br>(46·7–53·8)	50·9<br>(47·4–54·6)	-5·6<br>(-11·8–0·954)	1·26<br>(-3·79–6·58)	51<br>(47·6–54·5)	48·5<br>(45·3–51·8)	49<br>(45·8–52·2)	-4·86<br>(-9·83–0·27)	1·03<br>(-2·7–5·21)

Supplementary Results Table 1. Austria age-standardised Summary Exposure Values (SEVs) for most-detailed risk factors and percentage change, by sex, 1990, 2010, and 2019. Data in parentheses are 95% uncertainty intervals.										
	Male					Female				
	1990	2010	2019	Percentage change 1990-2010	Percentage change 2010-2019	1990	2010	2019	Percentage change 1990-2010	Percentage change 2010-2019
Unsafe sanitation	5·78 (3·69–8·59)	3·66 (2·17–5·66)	2·94 (1·71–4·72)	-36·6 (-65·5–15·5)	-19·8 (-49·4–26)	5·78 (3·69–8·59)	3·66 (2·17–5·66)	2·94 (1·71–4·72)	-36·6 (-65·5–15·5)	-19·8 (-49·4–26)
Ambient particulate matter pollution	20·6 (9·09–35·5)	14·9 (11·6–18·5)	10·6 (7·74–13·8)	-28 (-58·8–55·1)	-28·4 (-34–-24·2)	20·6 (9·11–35·5)	14·8 (11·6–18·5)	10·6 (7·75–13·8)	-27·9 (-58·8–55·1)	-28·4 (-34–-24·2)
Household air pollution from solid fuels	0·0972 (0·0259–0·239)	0·031 (0·00714–0·0868)	0·0211 (0·0046–0·0619)	-68·1 (-85–-38·7)	-31·9 (-58·4–3·43)	0·127 (0·0354–0·325)	0·0408 (0·00979–0·114)	0·0279 (0·00626–0·0808)	-68 (-85–-38·3)	-31·5 (-58·3–4·02)
Ambient ozone pollution	57·9 (24·6–97·4)	57·2 (24·6–96·8)	62·8 (26·8–100)	-1·18 (-6·78–4·43)	9·72 (2·15–17·7)	57·9 (24·6–97·4)	57·2 (24·6–96·8)	62·8 (26·8–100)	-1·18 (-6·78–4·43)	9·72 (2·15–17·7)
Residential radon	44 (21·8–72)	44 (21·8–72)	44 (21·8–72)	0 (0–0)	0 (0–0)	44 (21·8–72)	44 (21·8–72)	44 (21·8–72)	0 (0–0)	0 (0–0)
Lead exposure	14·2 (2·41e-12–40·9)	11·1 (2·71e-12–34·2)	8·2 (2·25e-12–28·1)	-21·7 (-56·6–67300000)	-26·4 (-83·1–106)	32·9 (3·46–59·5)	23·5 (3·37–47·1)	16·3 (1·42–37·8)	-28·6 (-42·6–8·1)	-30·6 (-60·2–-19·2)
Child underweight	0·944 (0·552–1·41)	0·777 (0·421–1·16)	0·755 (0·412–1·12)	-17·6 (-28·3–-5·09)	-2·95 (-11·5–6·45)	1·1 (0·649–1·6)	0·926 (0·529–1·36)	0·901 (0·513–1·33)	-15·6 (-25–-4·32)	-2·74 (-11·3–5·61)
Iron deficiency	--	--	--	--	--	9·99 (8·4–11·8)	9·16 (7·62–10·9)	9 (7·48–10·6)	-8·31 (-17–1·06)	-1·81 (-7·56–4·04)
Vitamin A deficiency	4·54 (1·84–8·97)	2 (0·803–4·1)	1·72 (0·661–3·7)	-55·8 (-82·3–20·6)	-14·3 (-49·8–68·2)	3·43 (1·68–5·65)	2·09 (1·08–3·44)	1·84 (0·899–3·17)	-38·9 (-68·9–27·9)	-12 (-42·1–43·4)
Zinc deficiency	2·55 (0–8·08)	2·17 (0–6·99)	1·96 (0–6·24)	-15 (-54–NA)	-9·57 (-33·9–NA)	2·5 (0–8·04)	2·11 (0–6·67)	1·93 (0–6·18)	-15·6 (-61–NA)	-8·78 (-27·6–NA)
Smoking	23·4 (20·9–26)	24·1 (21·4–27·1)	23·5 (20·9–26·4)	3·19 (-2·15–8·3)	-2·64 (-6·24–1·04)	13·2 (11·3–15·3)	15·8 (13·6–18·2)	15·3 (13·3–17·6)	19·4 (10·1–28·5)	-2·94 (-8·29–2·22)
Secondhand smoke	36·9 (35·1–38·9)	40·7 (39–42·3)	40·6 (38·1–42·9)	10·3 (2·61–18·4)	-0·215 (-5·1–4·77)	38 (36·2–39·7)	41·7 (40–43·1)	41·3 (39·1–43·3)	9·7 (2·89–16·7)	-0·978 (-5·09–2·69)
Alcohol use	22 (16·5–28·1)	24·4 (17·1–32·8)	24·1 (16·7–32·9)	10·9 (-1·45–28·1)	-1·18 (-8·38–5·29)	11·4 (7·78–16)	11·2 (7·45–15·7)	11·1 (7·27–15·8)	-1·96 (-11·7–8·99)	-1·24 (-14·1–12·6)
Drug use	0·284 (0·165–0·513)	0·377 (0·253–0·607)	0·333 (0·197–0·57)	32·5 (15·2–56·9)	-11·5 (-25·7–-0·135)	0·144 (0·091–0·243)	0·208 (0·152–0·305)	0·237 (0·167–0·342)	44·3 (19·7–82·6)	13·9 (-4·37–38·6)
High fasting plasma glucose	4·95 (4·3–5·67)	9·77 (7·97–12)	10·8 (8·75–12·9)	97·4 (66·3–136)	10·9 (-9·57–35·5)	4·54 (3·91–5·31)	7·56 (6·16–9·33)	8·75 (7·17–10·8)	66·5 (43·4–101)	15·7 (-2·06–40·3)
High systolic blood pressure	33·2 (29·6–36·9)	36·1 (29·2–43·5)	37 (30·3–45·7)	8·75 (-13·6–31·7)	2·4 (-14·6–23·1)	29·9 (25·4–34·5)	30·1 (21–40·7)	30·5 (22·1–40·4)	0·606 (-29·7–38·6)	1·49 (-25·1–37·4)
High body-mass index	16·5 (11·7–22·6)	21·9 (16·4–28·9)	24·1 (17·7–31·6)	32·5 (17·8–53·7)	9·87 (2·96–18·1)	16·4 (10·6–23·6)	22·3 (15·6–31·2)	25·7 (18·5–35·5)	36·2 (18·3–69·7)	15·1 (2·16–33·6)
Low bone mineral density	9·5 (5·18–15·9)	9·79 (5·22–16)	9·47 (5·04–15·7)	3·04 (-24·5–34·4)	-3·26 (-26·6–25·2)	15·3 (9·74–22·1)	15·6 (9·97–22·9)	15·2 (9·73–22·2)	2·05 (-18·5–27·3)	-2·33 (-21·5–25·5)
Diet low in fruits	39·9 (28·7–52·4)	32·2 (22·4–43·9)	30·7 (20·3–42·7)	-19·3 (-27·1–-12·8)	-4·67 (-17·7–7·81)	41·9 (30·7–54)	34·2 (23·9–46·3)	32·8 (22·4–44·8)	-18·4 (-25·9–-12)	-4·13 (-15–7·78)
Diet low in vegetables	57·1 (35·7–79)	35 (18·4–57)	36·7 (19·1–58·8)	-38·8 (-59·1–-17·8)	5·12 (-3·76–18·3)	55·2 (33·4–77·3)	32·4 (16·3–54·6)	34·3 (17·9–56·4)	-41·3 (-62·8–-18·4)	6·06 (-4·35–23)
Diet low in whole grains	74·1 (63·2–84·6)	77·1 (67·5–86·5)	78·2 (68·7–87·2)	4·09 (2·04–6·75)	1·42 (-0·0257–3·37)	69·1 (55·8–83)	72·4 (60·1–84·9)	73·6 (61·4–85·7)	4·76 (2·06–8·6)	1·68 (-0·0241–3·91)
Diet low in nuts and seeds	44·5 (17·3–66·2)	34·9 (12·3–60·1)	34 (12–59·5)	-21·5 (-38·1–-7·51)	-2·78 (-16·8–12·3)	39 (13·8–62·8)	29·5 (9·26–57·1)	28·7 (8·82–57·1)	-24·3 (-42·7–-7·35)	-2·75 (-18·7–17·5)
Diet low in milk	54·6 (29·7–75·6)	44·5 (20·1–65·1)	46·5 (22·8–66·6)	-18·6 (-40·5–-5·4)	4·55 (-3·09–17·8)	53·9 (30·3–74)	43·3 (19·6–63·9)	45·5 (21·9–65·8)	-19·7 (-42·9–-5·38)	5·12 (-2·64–19·1)
Diet high in red meat	95·7 (93·7–97·4)	82·7 (75·8–88·3)	83·7 (74·9–90·9)	-13·6 (-19·4–-8·75)	1·16 (-6·17–8·5)	95·8 (93·6–97·5)	82·8 (75·7–88·7)	83·6 (74·9–90·7)	-13·6 (-19·8–-8·66)	1·03 (-6·16–8·55)
Diet high in processed meat	59·5 (35·8–84·8)	74·5 (47·6–97·3)	70·5 (44·4–94·7)	25·2 (9·45–55·1)	-5·26 (-12–-1·14)	66 (40·4–90·5)	77·1 (51·2–99)	74·8 (48·2–97·4)	16·8 (5·44–35·5)	-3·02 (-8–-0·404)
Diet high in sugar-sweetened beverages	30·3 (16·8–47·7)	42·4 (26·7–59·2)	43·6 (27·4–60·4)	39·7 (6·98–92·3)	2·94 (-14·7–26·1)	34·6 (19·8–51·4)	49·7 (33–65·7)	50·6 (32·9–66·4)	43·5 (10·5–106)	1·78 (-15·7–24·7)
Diet low in fiber	31·9 (19·3–45·7)	22·8 (12·7–34·3)	22·5 (11·9–34·5)	-28·5 (-38·7–-20·3)	-1·24 (-16·2–14·8)	31·7 (18·7–46)	22·3 (12·1–34·4)	21·9 (11·5–33·8)	-29·8 (-40–-21·3)	-1·54 (-16·9–14·6)
Diet low in seafood omega-3 fatty acids	89·8 (76–100)	88·4 (75–100)	87·1 (71·9–100)	-1·59 (-10·3–6·08)	-1·52 (-10·7–7)	91·6 (80·2–100)	90·4 (78·8–100)	89 (76–100)	-1·4 (-8·36–5·06)	-1·51 (-9·66–5·33)
Diet low in polyunsaturated fatty acids	66·7 (25·9–100)	61·7 (20·8–99·5)	60 (19·8–98·9)	-7·55 (-22·2–-0·132)	-2·64 (-9·73–0)	68·5 (28·5–100)	63·6 (23·1–99·9)	62 (21·8–99·2)	-7·09 (-21·9–-0·00403)	-2·46 (-8·78–0)
Diet high in trans fatty acids	36·8 (27·6–91·4)	30·5 (21·7–89·3)	30·5 (21·6–86·4)	-17·2 (-28·9–-1·65)	-0·0715 (-9·75–11·1)	38 (28·3–97·6)	31·6 (22–94·7)	31·5 (22·1–93·1)	-16·8 (-29·7–-0·818)	-0·278 (-8·96–11·8)
Diet high in sodium	30·2 (6·23–55·8)	32·1 (7·07–58·2)	33·1 (7·16–59·3)	6·28 (-21·1–56·7)	3·26 (-15·3–32·2)	16·7 (4·08–39·4)	17·4 (4·1–40·9)	17·6 (4·09–41·1)	3·73 (-19·8–34·4)	1·46 (-13–18·6)
Low physical activity	2·04 (0·701–4·48)	1·95 (0·782–4·31)	2·56 (0·974–5·45)	-4·58 (-31·8–38·4)	31·5 (-2·73–94·1)	6·72 (3·49–12·4)	6·06 (3·38–11·1)	7·7 (4·14–13·9)	-9·76 (-27·6–13·9)	27 (2·98–57·8)
Occupational asthmagens	22·8 (19·5–26·9)	24·8 (20·7–29·7)	23·2 (19·6–28·2)	8·68 (-2·52–18·9)	-6·08 (-14·3–2·53)	10·4 (8·68–12·3)	13·8 (11·6–16·2)	13·7 (11·3–16·3)	32·6 (22·1–44·1)	-0·477 (-7·65–7·58)
Occupational particulate matter, gases, and fumes	9·87 (7·07–14·1)	9·35 (6·69–13·4)	9·1 (6·5–13·1)	-5·22 (-8·03–-2·35)	-2·73 (-4·6–-0·992)	4·66 (3·36–6·7)	4·73 (3·42–6·76)	4·62 (3·29–6·55)	1·53 (-3·81–6·77)	-2·53 (-5·92–0·699)
Occupational noise	8·4 (7·98–9·08)	8·3 (7·87–8·9)	8·28 (7·86–8·88)	-1·23 (-3·01–0·598)	-0·239 (-1·2–0·661)	4·58 (4·32–4·94)	5·27 (5·01–5·63)	5·55 (5·26–5·95)	14·9 (11·8–18·3)	5·26 (4·01–6·59)
Occupational injuries	--	--	--	--	--	--	--	--	--	--
Occupational ergonomic factors	12·4 (10·7–14·2)	12·5 (10·9–14·4)	12·1 (10·4–14·2)	1·02 (-5·27–7·26)	-2·88 (-9·02–4·41)	7·47 (6·43–8·73)	10·1 (8·79–11·7)	10·5 (8·72–12·5)	35 (22·1–48·5)	3·75 (-6·77–15·8)
Childhood sexual abuse	7·16 (4·65–10·1)	7·16 (4·65–10·1)	7·16 (4·65–10·1)	0 (0–0)	0 (0–0)	10·6 (7·84–14·3)	10·6 (7·84–14·3)	10·6 (7·84–14·3)	0 (0–0)	0 (0–0)
Intimate partner violence	--	--	--	--	--	9·39 (5·04–13·7)	8·83 (5·06–11·7)	9·39 (5·04–13·7)	-5·96 (-18·2–12·9)	6·33 (-6·59–32·9)
Non-exclusive breastfeeding	21·1 (17–26·3)	20·3 (16·1–25·5)	20 (16–25·2)	-4·01 (-15·7–8·48)	-1·39 (-9·13–8·23)	21·1 (17–26·3)	20·3 (16·1–25·5)	20 (16–25·2)	-4·03 (-15·7–8·46)	-1·34 (-9·08–8·28)
Discontinued breastfeeding	28·1 (27·7–28·6)	27·9 (27·4–28·4)	28·1 (27·6–28·7)	-0·835 (-3·41–1·76)	0·768 (-1·26–2·91)	28·1 (27·7–28·6)	27·8 (27·3–28·4)	28·1 (27·6–28·7)	-1·07 (-3·65–1·52)	1·06 (-0·976–3·21)
Diet low in calcium	17·9 (8·47–33·7)	16·8 (7·55–32·2)	15·9 (7·1–30·3)	-6·46 (-18·3–0·189)	-5·23 (-11·1–-0·754)	12·1 (4·93–22·5)	11·4 (4·14–21·1)	10·5 (3·88–19·1)	-5·34 (-18·2–3·05)	-8·16 (-17·4–-1·42)
Occupational exposure to asbestos	6·41 (5·7–7·07)	7·99 (7·39–8·58)	6·86 (5·52–8·6)	24·5 (10·1–40)	-14·1 (-32–8·83)	2·21 (1·36–3·15)	1·86 (1·26–2·24)	1·53 (1·08–2·14)	-16 (-37·4–16·6)	-17·5 (-38·8–8·11)
Occupational exposure to arsenic	0·52 (0–1·21)	0·537 (0–1·28)	0·545 (0–1·29)	3·16 (-7·09–NA)	1·54 (-5·74–NA)	0·388 (0–0·91)	0·463 (0–1·1)	0·472 (0–1·12)	19·3 (6·67–NA)	1·82 (-5·74–NA)
Occupational exposure to benzene	0·663 (0·0388–1·89)	0·811 (0·048–2·35)	0·83 (0·0491–2·36)	22·3 (17·4–30)	2·29 (-1·69–9·48)	0·743 (0·0422–2·16)	1·23 (0·0732–3·59)	1·31 (0·0797–3·8)	65·5 (56·3–80·7)	6·82 (1·53–13·8)
Occupational exposure to beryllium	0·0219 (0·0216–0·0222)	0·0244 (0·0241–0·0248)	0·0249 (0·0243–0·0255)	11·7 (9·53–13·9)	1·8 (-0·615–4·34)	0·0197 (0·0193–0·0201)	0·0277 (0·0274–0·0281)	0·029 (0·0283–0·0298)	40·8 (37·3–44·4)	4·6 (1·84–7·69)
Occupational exposure to cadmium	0·0598 (0·0568–0·0629)	0·0623 (0·0575–0·067)	0·0632 (0·0583–0·0687)	4·13 (-5·16–13·2)	1·48 (-5·19–8·92)	0·046 (0·0432–0·0491)	0·0564 (0·0525–0·061)	0·0576 (0·0533–0·0629)	22·5 (11·6–34·8)	2·13 (-4·45–9·74)
Occupational exposure to chromium	0·139 (0·133–0·146)	0·148 (0·138–0·157)	0·15 (0·14–0·161)	6·06 (-1·95–14·3)	1·37 (-4·43–7·76)	--	--	--	--	--
Occupational exposure to diesel engine exhaust	0·657 (0·632–0·683)	0·762 (0·73–0·794)	0·764 (0·726–0·803)	16·1 (9·69–22·7)	0·261 (-4·42–4·7)	0·545 (0·517–0·571)	0·762 (0·72–0·802)	0·776 (0·723–0·831)	39·7 (30·8–50·2)	1·84 (-4·29–8·22)
Occupational exposure to formaldehyde	0·294 (0·276–0·311)	0·3 (0·275–0·325)	0·302 (0·277–0·329)	2·06 (-7·67–11·7)	0·508 (-6·27–8·38)	0·215 (0·201–0·23)	0·27 (0·252–0·291)	0·276 (0·256–0·3)	25·6 (14·9–37·8)	2·25 (-4·32–9·18)
Occupational exposure to nickel	0·468 (0–2·12)	0·503 (0–2·22)	0·517 (0–2·23)	7·47 (-1·72–NA)	2·66 (-3·63–NA)	0·31 (0–1·36)	0·361 (0–1·55)	0·367 (0–1·62)	16·4 (3·15–NA)	1·65 (-6·62–NA)
Occupational exposure to polycyclic aromatic hydrocarbons	0·275 (0·263–0·286)	0·3 (0·283–0·317)	0·306 (0·288–0·326)	9·13 (1·87–16·7)	1·94 (-3·18–7·83)	0·225 (0·214–0·237)	0·301 (0·287–0·318)	0·311 (0·295–0·33)	33·5 (25·1–43·5)	3·41 (-1·78–8·71)
Occupational exposure to silica	4·92 (0·679–15·8)	5·87 (0·82–18·9)	6·1 (0·867–19·3)	19·2 (10·3–31)	3·92 (-2·53–12·2)	2·68 (0·382–8·48)	3·18 (0·472–10·1)	3·27 (0·439–10·7)	18·4 (6·15–36)	2·97 (-6·08–13·6)
Occupational exposure to sulfuric acid	0·867 (0·177–2·73)	0·875 (0·177–2·79)	0·87 (0·181–2·79)	0·946 (-7·13–9·58)	-0·602 (-7·06–6·02)	0·596 (0·12–1·9)	0·695 (0·143–2·23)	0·695 (0·14–2·25)	16·6 (5·29–28·9)	0·00258 (-7·19–8·25)
Unsafe sex	--	--	--	--	--	--	--	--	--	--
Occupational exposure to trichloroethylene	0·0782 (0·076–0·0809)	0·0834 (0·08–0·0872)	0·0839 (0·0802–0·0883)	6·64 (1·51–12·1)	0·514 (-3·71–5·01)	0·0671 (0·0646–0·0699)	0·0887 (0·0854–0·0926)	0·0907 (0·0865–0·0957)	32·1 (24·9–39·6)	2·34 (-2·12–7·46)
No access to handwashing facility	3·52 (3·33–3·72)	3·17 (3–3·36)	3·14 (2·97–3·33)	-9·9 (-15·5–-4·28)	-0·928 (-5·05–3·42)	3·52 (3·33–3·72)	3·17 (3–3·36)	3·14 (2·97–3·33)	-9·9 (-15·5–-4·28)	-0·928 (-5·05–3·42)
Child wasting	1·26 (0·895–1·67)	1·21 (0·862–1·58)	1·19 (0·849–1·58)	-4·58 (-12·4–3·67)	-1·02 (-7·01–4·88)	1·07 (0·75–1·39)	1·01 (0·71–1·35)	0·999 (0·693–1·33)	-5·83 (-14·5–3·12)	-0·786 (-6·62–5·19)
Child stunting	1·7 (0·861–2·53)	1·21 (0·578–1·89)	1·19 (0·557–1·86)	-28·5 (-41·4–-12·9)	-2·26 (-14·3–12)	2 (1·02–2·93)	1·59 (0·808–2·38)	1·54 (0·78–2·28)	-20·8 (-34·4–-4·71)	-2·84 (-13·3–9·39)
Chewing tobacco	0·234 (0·185–0·302)	0·261 (0·206–0·321)	0·288 (0·234–0·356)	11·8 (-16·1–50·3)	10·1 (-7·32–30·9)	--	--	--	--	--
Diet low in legumes	93 (83–100)	90·7 (78–100)	89·2 (74·9–100)	-2·5 (-6·74–0)	-1·61 (-5·32–0)	91·1 (79·1–100)	88·5 (73·5–100)	87 (69·9–100)	-2·84 (-7·88–0)	-1·74 (-5·47–0)
Short gestation	16·3 (13·4–19·7)	20·7 (16·5–25·4)	16·9 (13·3–21·2)	27·1 (-3·99–65·2)	-18 (-37·1–4·77)	18·7 (15·7–21·9)	22·5 (18·5–26·9)	16·7 (14–19·8)	19·9 (-3·37–47·6)	-25·5 (-38·9–-7·59)
Low birth weight	8·41 (6·95–10·1)	10·2 (8·28–12·4)	7·8 (6·1–9·77)	21·2 (-8·25–57·8)	-23·5 (-41·6–0·952)	9·51 (8·19–11)	10·9 (9·03–13·2)	8·3 (7·04–9·77)	14·9 (-7·71–42·8)	-24 (-39·4–-6·01)
High temperature	23·8 (11·8–36·7)	24·8 (14–37·5)	24·8 (13·5–37·7)	4·33 (-16·8–44·3)	0·0228 (-7·44–4·95)	23·8 (11·8–36·7)	24·8 (14–37·5)	24·8 (13·5–37·7)	4·33 (-16·8–44·3)	0·0228 (-7·44–4·95)
Low temperature	34·6 (31–38·6)	35·3 (31·9–39·1)	32·9 (29·3–37·2)	1·93 (0·158–3·98)	-6·76 (-9·29–-4·35)	34·6 (31–38·6)	35·3 (31·9–39·1)	32·9 (29·3–37·2)	1·93 (0·158–3·98)	-6·76 (-9·29–-4·35)
Kidney dysfunction	14·5 (9·85–21)	15·7 (10·9–22·3)	16 (11·1–22·7)	8·27 (4·52–13·6)	1·89 (-2·12–6·4)	15·5 (10·4–22)	16·7 (11·5–23·3)	17 (11·8–23·6)	7·65 (3·79–12·9)	1·83 (-1·96–6·11)

Bullying victimization	8·02 (3·18–16·6)	9·72 (4·48–18·5)	11 (5·19–20·5)	21·3 (-3·98–113)	13 (3·81–25·3)	5·3 (2·1–10·8)	6·6 (3·01–12·5)	7·31 (3·48–13·6)	24·4 (-2·89–130)	10·7 (1·07–22·6)
High LDL cholesterol	57·4 (53·6–60·8)	55 (51·2–58·8)	55·5 (52·2–59·4)	-4·16 (-10·7–1·99)	1·05 (-3·34–5·57)	54·3 (51–57·5)	52·5 (49·3–55·9)	53·1 (49·7–56·4)	-3·18 (-8·2–1·98)	1·04 (-2·46–4·88)

Supplementary Results Table 1. Belgium age-standardised Summary Exposure Values (SEVs) for most-detailed risk factors and percentage change, by sex, 1990, 2010, and 2019. Data in parentheses are 95% uncertainty intervals.

	Male					Female				
	1990	2010	2019	Percentage change 1990-2010	Percentage change 2010-2019	1990	2010	2019	Percentage change 1990-2010	Percentage change 2010-2019
Unsafe sanitation	3·29 (1·87–5·4)	1·15 (0·68–1·88)	0·879 (0·487–1·46)	-65 (-82·4—31·4)	-23·7 (-53·1–20·7)	3·29 (1·87–5·4)	1·15 (0·68–1·88)	0·879 (0·487–1·46)	-65 (-82·4—31·4)	-23·7 (-53·1–20·7)
Ambient particulate matter pollution	20·5 (8·43–35·4)	16·3 (12·9–20·2)	11·3 (8·33–14·7)	-20·5 (-55–76·7)	-30·6 (-36·3—26·4)	20·4 (8·41–35·4)	16·2 (12·9–20·2)	11·3 (8·35–14·7)	-20·5 (-55–76·7)	-30·6 (-36·2—26·4)
Household air pollution from solid fuels	0·0648 (0·0174–0·16)	0·0162 (0·00354–0·0446)	0·0101 (0·00207–0·0313)	-74·9 (-87·9—51·3)	-38 (-62·2—4·81)	0·085 (0·0241–0·216)	0·0214 (0·00483–0·0593)	0·0133 (0·00277–0·0411)	-74·8 (-87·8—49·8)	-37·7 (-61·9—4·6)
Ambient ozone pollution	24·5 (9·07–44·7)	27·8 (10·1–51·2)	31·8 (12·2–57·6)	13·2 (-2·17–29·5)	14·6 (2·57–34·9)	24·5 (9·07–44·7)	27·8 (10·1–51·2)	31·8 (12·2–57·6)	13·2 (-2·17–29·5)	14·6 (2·57–34·9)
Residential radon	24·9 (8·07–56·1)	24·9 (8·07–56·1)	24·9 (8·07–56·1)	0 (0–0)	0 (0–0)	24·9 (8·07–56·1)	24·9 (8·07–56·1)	24·9 (8·07–56·1)	0 (0–0)	0 (0–0)
Lead exposure	87·9 (71·8–96·3)	67·8 (50·2–82·4)	54·3 (36·8–71·6)	-22·9 (-33·5—12·9)	-19·9 (-27·3—12·6)	78·1 (57·5–90·6)	58·9 (39·3–76)	46 (27·6–64·9)	-24·5 (-35·7—14·4)	-21·9 (-30·5—14)
Child underweight	0·917 (0·519–1·35)	0·757 (0·423–1·14)	0·745 (0·415–1·12)	-17·4 (-28·1—4·84)	-1·61 (-10·1–7·85)	1·06 (0·625–1·54)	0·907 (0·534–1·35)	0·892 (0·51–1·31)	-14·8 (-24—3·7)	-1·65 (-9·96–7·62)
Iron deficiency	--	--	--	--	--	9·76 (8·2–11·5)	8·94 (7·4–10·5)	8·73 (7·12–10·3)	-8·47 (-17·5—0·831)	-2·28 (-8·06–3·28)
Vitamin A deficiency	4·37 (1·75–8·71)	1·76 (0·718–3·48)	1·44 (0·604–2·99)	-59·7 (-83·2–5·83)	-18·1 (-53·9–55·6)	3·33 (1·61–5·64)	2·05 (1·05–3·44)	1·74 (0·824–3·01)	-38·3 (-67·1—31·4)	-15·4 (-45·8–32·3)
Zinc deficiency	3·78 (0–11·4)	3·16 (0–9·74)	2·87 (0–9·26)	-16·2 (-68·6–NA)	-9·34 (-26·7–NA)	3·67 (0–11·4)	3·05 (0–9·49)	2·78 (0–8·72)	-16·9 (-63·1–NA)	-8·87 (-26·8–NA)
Smoking	26·5 (23·7–29·4)	22·4 (20·2–24·8)	19·4 (17·5–21·7)	-15·3 (-19·4—10·3)	-13·3 (-16—10·6)	17·3 (14·9–20·1)	15·2 (13·3–17·4)	14·1 (12·4–16)	-12·4 (-20·1—3·42)	-7·28 (-11·6—2·73)
Secondhand smoke	38·9 (36·4–41·5)	34·1 (32–36·1)	32·7 (30·1–35·4)	-12·4 (-20·3—3·69)	-3·89 (-9·76–1·53)	40·3 (38·3–42·5)	33·6 (32–35·3)	32·2 (30·1–34·3)	-16·5 (-22·5—10·1)	-4·28 (-8·44–0·287)
Alcohol use	21 (15·5–27)	22·4 (16–29·8)	21·8 (15·2–29·2)	6·63 (-2·64–18)	-2·45 (-13·1–7·07)	11·6 (8·05–16·3)	12 (8·12–16·7)	11·4 (7·44–16·4)	3·24 (-8·24–16·3)	-4·65 (-17·4–8·38)
Drug use	0·211 (0·12–0·382)	0·257 (0·16–0·429)	0·283 (0·181–0·462)	21·6 (8·13–42·7)	10·1 (1·43–23·1)	0·142 (0·0882–0·238)	0·182 (0·122–0·278)	0·205 (0·141–0·303)	28·6 (13·9–48·9)	12·2 (3·62–23·6)
High fasting plasma glucose	6·3 (5·38–7·29)	9 (7·66–10·8)	10·9 (9·1–13·2)	43 (26·2–65·6)	21·6 (3·02–43·5)	5·93 (5·04–7·05)	7·89 (6·49–9·72)	9·61 (7·97–11·7)	33·2 (16·2–59·8)	21·7 (3·37–46·9)
High systolic blood pressure	25·9 (23·1–28·8)	32 (29·2–34·9)	33·8 (28·3–39·2)	23·5 (10·4–38·5)	5·55 (-10·4–23·6)	18·1 (14·7–22)	29·2 (26·3–32·2)	30·4 (23·3–38·4)	61·6 (32·1–99·1)	4·21 (-19·4–32·1)
High body-mass index	16·1 (11·5–21·7)	21·4 (15·8–28·3)	23 (17–30·5)	32·8 (19·3–55·1)	7·93 (1·08–16·5)	17·3 (11·5–23·9)	25 (18·2–33·2)	26·7 (19·8–35)	44 (23·5–82·3)	6·8 (-5·5–23·3)
Low bone mineral density	10·3 (5·63–16·9)	9·47 (5·02–15·4)	9·19 (4·9–15·5)	-7·96 (-30·7–19·5)	-2·99 (-25·5–26·5)	16·3 (10·9–23·3)	15·2 (9·83–21·8)	15·1 (9·47–22·1)	-7·07 (-25·2–18·5)	-0·738 (-22·4–25·2)
Diet low in fruits	56·5 (45·2–69·8)	44·2 (32·4–57·8)	44·4 (32·7–57·5)	-21·9 (-32·4—13·7)	0·481 (-7·9–9·24)	58·1 (47·2–71)	46 (34·1–59·1)	46·3 (34·3–59·3)	-20·8 (-30·6—12·4)	0·699 (-7·41–8·63)
Diet low in vegetables	39·4 (21·5–60·6)	21·5 (8·82–42·1)	21 (8·65–39·8)	-45·4 (-68·6—22·2)	-2·27 (-17·5–21·5)	36·8 (19·9–59·1)	18·8 (7·28–38·3)	18·4 (7·34–36·8)	-48·9 (-71·8—26·6)	-2·15 (-20·3–21·1)
Diet low in whole grains	83·3 (75·2–91·5)	84·9 (77·7–92·3)	86·7 (80–93·3)	1·93 (0·887–3·52)	2·1 (0·826–3·69)	--	--	--	--	--
Diet low in nuts and seeds	46·9 (18·8–68·2)	35 (11·1–59·9)	35·2 (11·6–60·3)	-25·5 (-47·1—9·39)	0·73 (-12·1–18·3)	41·6 (14·9–64·7)	29·7 (8·13–56·6)	30 (8·06–56·9)	-28·6 (-52—9·3)	1·03 (-13·2–25·6)
Diet low in milk	67·7 (40·6–88·8)	51·3 (26·3–71·8)	53·7 (28·7–74·7)	-24·2 (-50·7—6·31)	4·66 (-0·623–16·6)	67·3 (39·5–88·7)	50·3 (25·3–71·1)	52·6 (27·1–73·5)	-25·2 (-51·9—6·44)	4·62 (-0·642–17·4)
Diet high in red meat	65·6 (55·8–74·7)	90·9 (86·5–94·5)	87·7 (80–93·5)	38·7 (25–57·2)	-3·62 (-9·79–1·66)	65·4 (55·3–74·9)	91 (86·5–94·5)	87·7 (80·2–93·3)	39·2 (24·8–60·5)	-3·69 (-9·58–1·34)
Diet high in processed meat	74 (47·3–96·5)	78·4 (53·6–99·3)	78·9 (54·7–99·2)	6·02 (1·47–14·5)	0·535 (-1·3–3·43)	77 (51·3–98·4)	80·8 (58–99·8)	81·3 (59·1–99·8)	4·85 (0·738–13·6)	0·639 (-1·07–3·57)
Diet high in sugar-sweetened beverages	40·2 (24·3–58)	54·8 (39–69·4)	55·5 (38·7–71·6)	36·4 (7·9–84)	1·37 (-16–20·3)	47·2 (29·9–64·7)	63·9 (49·1–77·3)	63·5 (47·6–77·8)	35·3 (8·21–85·9)	-0·552 (-14·9–14·6)
Diet low in fiber	37·9 (23·3–52·9)	35·5 (21·3–50·4)	31·2 (18·2–45·6)	-6·37 (-11·5—2·39)	-12·1 (-23—2·31)	37·7 (22·6–52·8)	35·6 (21–50·9)	32·2 (18·6–47·2)	-5·6 (-10·6—1·35)	-9·7 (-20·1–0·875)
Diet low in seafood omega-3 fatty acids	88·8 (73·9–100)	85·7 (70·2–100)	85·3 (69–99·7)	-3·57 (-14·7–5·57)	-0·41 (-11·5–11)	90·7 (78·7–100)	87·8 (74·7–99·9)	88 (75·2–99·9)	-3·22 (-11·8–3·6)	0·17 (-6·82–8·37)
Diet low in polyunsaturated fatty acids	31 (8·8–69·3)	21·7 (6·15–54·5)	23·3 (6·67–57·7)	-30 (-45·5—5·12)	7·22 (-1·68–20·8)	33·8 (9·46–74·1)	24·3 (6·71–59·4)	25·8 (7·11–63·1)	-28·2 (-43·7—5·33)	6·43 (-1·64–20·3)
Diet high in trans fatty acids	62·4 (52·7–98·3)	53·3 (43·7–96·7)	50·9 (41·2–96·9)	-14·7 (-24·2—2·01)	-4·36 (-14·2–4·99)	62·2 (52–100)	53·4 (43·5–99·5)	51·4 (41·3–100)	-14·1 (-23·2—0·52)	-3·72 (-12·7–4·11)
Diet high in sodium	32·8 (6·89–57·1)	31·3 (6·44–58)	31·8 (6·43–58·8)	-4·43 (-38–28·9)	1·36 (-20·3–27·6)	15·7 (3·95–37·6)	15·5 (3·62–38·6)	15·6 (3·61–38·3)	-1·59 (-31·7–25·5)	1·2 (-14·5–20·3)
Low physical activity	4·08 (1·72–8·31)	4·22 (1·95–8·62)	5 (2·24–10·1)	3·55 (-22·7–51·2)	18·4 (-12·6–60·3)	6·84 (3·58–12·7)	6·81 (3·67–12·3)	7·85 (4·27–13·8)	-0·454 (-18·6–24·8)	15·4 (-5·6–40·1)
Occupational asthmagens	23·6 (19·4–28·7)	22·3 (18·8–26·6)	19·8 (16·5–23·9)	-5·48 (-15·6–7·15)	-11·3 (-19·1—2·05)	--	--	--	--	--
Occupational particulate matter, gases, and fumes	8·02 (5·68–11·6)	7·35 (5·18–10·6)	6·98 (4·93–10·1)	-8·37 (-12·3—4·67)	-5·06 (-7·31—2·94)	2·99 (2·05–4·48)	2·84 (1·99–4·25)	2·75 (1·93–4·06)	-4·8 (-12–3·05)	-3·25 (-7·12–0·705)
Occupational noise	7·49 (7·06–8·17)	7·07 (6·7–7·74)	6·86 (6·51–7·52)	-5·54 (-7·41—3·54)	-2·94 (-3·81—2)	3·63 (3·39–3·96)	4·12 (3·91–4·4)	4·38 (4·17–4·67)	13·3 (9·99–17·6)	6·33 (4·99–7·73)
Occupational injuries	--	--	--	--	--	--	--	--	--	--
Occupational ergonomic factors	9·55 (8·21–11·3)	8·84 (7·56–10·4)	8·59 (7·31–10·2)	-7·43 (-12·1—2·34)	-2·8 (-7·69–2·53)	4·53 (3·79–5·48)	6·11 (5·09–7·41)	6·63 (5·47–8·09)	34·7 (24·5–44·7)	8·55 (1·29–16·2)
Childhood sexual abuse	7·16 (4·65–10·1)	7·16 (4·65–10·1)	7·16 (4·65–10·1)	0 (0–0)	0 (0–0)	10·6 (7·84–14·3)	10·6 (7·84–14·3)	10·6 (7·84–14·3)	0 (0–0)	0 (0–0)
Intimate partner violence	--	--	--	--	--	17·9 (9·81–25·7)	16·6 (9·74–22·5)	17·9 (9·81–25·7)	-7·6 (-18·6–11)	8·23 (-4·6–29·8)
Non-exclusive breastfeeding	20·9 (16·7–25·9)	20·1 (16–25·1)	19·8 (15·9–24·8)	-3·61 (-15·7–9·12)	-1·58 (-10–8·04)	20·9 (16·7–25·9)	20·1 (16–25·1)	19·8 (15·9–24·8)	-3·62 (-15·7–9·12)	-1·59 (-10·1–8·03)
Discontinued breastfeeding	26·3 (25·4–27·1)	25·9 (25–26·8)	25·3 (24·4–26·2)	-1·32 (-6·05–3·94)	-2·34 (-6·96–2·68)	26·3 (25·4–27·1)	25·9 (25–26·8)	25·3 (24·3–26·2)	-1·45 (-6·18–3·81)	-2·23 (-6·86–2·79)
Diet low in calcium	16·8 (7·85–31·4)	13·9 (6·39–26)	13·6 (6·23–25·3)	-17·4 (-24·6—12·3)	-1·76 (-7·05–3·7)	10·9 (4·27–20·4)	7·97 (3·08–14·5)	7·79 (3·08–14·5)	-27·2 (-36·7—20·1)	-2·23 (-10·4–6·24)
Occupational exposure to asbestos	13·6 (12·4–14·7)	15·4 (14·7–16·2)	14·2 (11·8–16·7)	13·2 (3·4–25·2)	-8·15 (-24·6–8·96)	2·32 (1·79–3·74)	2·59 (1·75–3·17)	2·41 (1·58–3·32)	11·5 (-25·8–43·7)	-6·84 (-29·7–21·2)
Occupational exposure to arsenic	0·482 (0–1·13)	0·478 (0–1·13)	0·465 (0–1·09)	-0·815 (-8·71–NA)	-2·83 (-10·7–NA)	0·291 (0–0·695)	0·357 (0–0·842)	0·362 (0–0·865)	22·8 (10·3–NA)	1·23 (-5·87–NA)
Occupational exposure to benzene	0·709 (0·0419–2)	0·792 (0·0472–2·27)	0·794 (0·0473–2·29)	11·8 (7·65–17·1)	0·179 (-4·21–6·71)	0·72 (0·0421–2·04)	1·25 (0·0742–3·63)	1·32 (0·0791–3·84)	74 (66·6–86·8)	5·62 (1·01–11·5)
Occupational exposure to beryllium	0·0203 (0·02–0·0206)	0·0212 (0·0211–0·0214)	0·0213 (0·0208–0·0218)	4·52 (2·75–6·31)	0·187 (-2·24–2·69)	0·0158 (0·0155–0·0161)	0·0231 (0·0229–0·0233)	0·0241 (0·0235–0·0247)	46·3 (43·4–49·1)	4·45 (1·83–7·29)
Occupational exposure to cadmium	0·0554 (0·0511–0·0599)	0·0552 (0·054–0·0564)	0·0539 (0·0503–0·0574)	-0·266 (-7·48–7·8)	-2·41 (-9·64–4·32)	0·0347 (0·0318–0·0383)	0·0438 (0·0429–0·0447)	0·0446 (0·0422–0·0473)	25·9 (14·6–37·5)	1·81 (-4·28–9·09)
Occupational exposure to chromium	0·13 (0·121–0·139)	0·131 (0·129–0·134)	0·129 (0·122–0·136)	1·21 (-5·18–8·05)	-1·88 (-8·04–3·87)	--	--	--	--	--
Occupational exposure to diesel engine exhaust	0·583 (0·553–0·612)	0·611 (0·597–0·624)	0·622 (0·595–0·649)	4·67 (-0·292–10·3)	1·92 (-2·58–6·64)	0·385 (0·364–0·409)	0·515 (0·496–0·535)	0·538 (0·504–0·57)	33·6 (25·2–42·3)	4·48 (-1·4–10·9)
Occupational exposure to formaldehyde	0·28 (0·257–0·304)	0·274 (0·265–0·281)	0·264 (0·246–0·282)	-2·11 (-9·43–6·01)	-3·73 (-10·6–3·03)	0·17 (0·156–0·187)	0·227 (0·219–0·231)	0·231 (0·219–0·244)	33·3 (22·1–45·2)	2·08 (-3·17–8·11)
Occupational exposure to nickel	0·44 (0–1·89)	0·439 (0–1·93)	0·43 (0–1·94)	-0·0663 (-7·67–NA)	-2·22 (-9·33–NA)	0·23 (0–1)	0·276 (0–1·2)	0·277 (0–1·23)	19·9 (6·9–NA)	0·2 (-7·44–NA)
Occupational exposure to polycyclic aromatic hydrocarbons	0·261 (0·246–0·277)	0·269 (0·264–0·274)	0·265 (0·252–0·278)	3·1 (-2·81–9·48)	-1·45 (-6·87–3·49)	0·181 (0·17–0·193)	0·255 (0·251–0·259)	0·263 (0·254–0·274)	41 (32–50·2)	3·24 (-0·938–7·83)
Occupational exposure to silica	4·76 (0·688–15)	4·94 (0·727–15·5)	4·95 (0·706–15·6)	3·82 (-2·77–12·5)	0·184 (-6·84–7·86)	2·02 (0·295–6·23)	2·4 (0·348–7·48)	2·37 (0·346–7·51)	18·7 (6·14–34·3)	-1·23 (-9·22–10·3)
Occupational exposure to sulfuric acid	0·752 (0·154–2·3)	0·728 (0·152–2·26)	0·707 (0·142–2·21)	-3·24 (-10·4–4·84)	-2·88 (-9·8–4·18)	0·416 (0·0914–1·36)	0·496 (0·103–1·54)	0·499 (0·102–1·53)	19·3 (8·18–31·3)	0·61 (-6·74–9·25)
Unsafe sex	--	--	--	--	--	--	--	--	--	--
Occupational exposure to trichloroethylene	0·071 (0·0682–0·0743)	0·072 (0·0711–0·0734)	0·0713 (0·0685–0·0746)	1·37 (-2·61–5·96)	-0·985 (-5·36–3·47)	0·0503 (0·0479–0·0532)	0·0663 (0·0653–0·0677)	0·0686 (0·0661–0·0717)	31·7 (24·9–38·7)	3·48 (-0·861–8·45)
No access to handwashing facility	3·34 (3·15–3·55)	3·19 (3–3·38)	3·15 (2·97–3·33)	-4·62 (-10·7–2·01)	-1·1 (-5·12–2·96)	3·34 (3·15–3·55)	3·19 (3–3·38)	3·15 (2·97–3·33)	-4·62 (-10·7–2·01)	-1·1 (-5·12–2·96)
Child wasting	1·25 (0·875–1·65)	1·19 (0·831–1·59)	1·19 (0·827–1·59)	-4·55 (-12·5–3·9)	-0·653 (-6·64–5·64)	1·06 (0·732–1·42)	0·999 (0·693–1·34)	0·995 (0·688–1·34)	-5·35 (-13·5–2·68)	-0·456 (-6·03–5·25)
Child stunting	1·65 (0·837–2·42)	1·18 (0·561–1·79)	1·17 (0·552–1·79)	-28·1 (-41·3—13·9)	-1·18 (-13·4–12·9)	1·94 (0·986–2·85)	1·55 (0·773–2·37)	1·52 (0·764–2·29)	-20·3 (-33·4—5·25)	-1·83 (-12–10·9)
Chewing tobacco	0·148 (0·117–0·188)	0·149 (0·118–0·19)	0·152 (0·121–0·192)	0·458 (-22·5–31·5)	1·62 (-14·5–21·1)	0·137 (0·0985–0·184)	0·137 (0·0985–0·189)	0·137 (0·101–0·181)	0·199 (-30·3–43·6)	0·0758 (-21·4–26·5)
Diet low in legumes	78·4 (48·7–100)	75·3 (42–99·7)	75·5 (43·1–99·7)	-3·93 (-15—0·0772)	0·339 (-2·62–4·54)	75·4 (42·3–99·7)	72·2 (33·4–99·4)	72·5 (34·1–99·3)	-4·22 (-18·4—0·124)	0·413 (-2·62–5·38)
Short gestation	15·3 (12·9–17·9)	10·3 (8·58–12·1)	10·8 (9·26–12·6)	-32·8 (-44·6—17·3)	4·88 (-13·3–25·9)	14·9 (12·2–18·3)	11·2 (9·44–13·2)	11·1 (9·19–13·2)	-24·8 (-43—0·00261)	-1·19 (-19·6–21)
Low birth weight	9·51 (8·13–11)	6·32 (5·36–7·36)	6·57 (5·68–7·57)	-33·5 (-46—17·8)	3·99 (-14–26·1)	8·55 (6·99–10·5)	6·44 (5·44–7·51)	6·43 (5·42–7·61)	-24·6 (-41·9—1·2)	-0·15 (-20·1–23·7)
High temperature	24 (12–36·8)	25·1 (13·5–38)	25·9 (14·2–38·6)	4·82 (0·742–11·2)	3·2 (1·09–6·91)	24 (12–36·8)	25·1 (13·5–38)	25·9 (14·2–38·6)	4·82 (0·742–11·2)	3·2 (1·09–6·91)
Low temperature	31·8 (27–36·9)	34·6 (30·5–39)	31·2 (26·5–36·4)	8·84 (5·46–12·9)	-9·71 (-13·2—6·49)	31·8 (27–36·9)	34·6 (30·5–39)	31·2 (26·5–36·4)	8·84 (5·46–12·9)	-9·71 (-13·2—6·49)
Kidney dysfunction	14·9 (10·1–21·4)	15·6 (10·7–22·1)	15·4 (10·6–22)	4·7 (0·109–10·9)	-1·08 (-5·71–3·64)	16·3 (11·2–22·9)	17 (12–23·7)	16·8 (11·7–23·5)	4·26 (0·376–9·55)	-1·06 (-6·28–3·84)
Bullying victimization	9·15 (3·43–19)	10·5 (4·87–19·7)	11·3 (5·24–21)	15 (-13·4–85·5)	7·35 (-1·38–15·4)	6·54 (2·63–13·6)	7·11 (3·31–13·3)	7·63 (3·54–14·2)	8·7 (-17·5–62·9)	7·33 (0·616–16·7)
High LDL cholesterol	58·9 (55·6–62·2)	37·1 (33·6–40·7)	37·1 (33·6–40·4)	-36·9 (-41·6—32·3)	-0·249 (-4·21–3·98)	54·4 (51–57·6)	43·2 (39·9–46·6)	43·9 (40·6–47·3)	-20·5 (-24·9—16·2)	1·45 (-1·88–5·15)

Supplementary Results Table 1. Cyprus age-standardised Summary Exposure Values (SEVs) for most-detailed risk factors and percentage change, by sex, 1990, 2010, and 2019. Data in parentheses are 95% uncertainty intervals.

	Male					Female				
	1990	2010	2019	Percentage change 1990-2010	Percentage change 2010-2019	1990	2010	2019	Percentage change 1990-2010	Percentage change 2010-2019
Unsafe sanitation	6·48 (3·84–10·1)	1·13 (0·641–1·84)	0·929 (0·501–1·54)	-82·6 (-90·8–-65·9)	-17·8 (-48–32·8)	6·48 (3·84–10·1)	1·13 (0·641–1·84)	0·929 (0·501–1·54)	-82·6 (-90·8–-65·9)	-17·8 (-48–32·8)
Ambient particulate matter pollution	16·6 (6·04–30·4)	18·4 (14·2–23·3)	14·7 (10·9–19·1)	11 (-38·8–177)	-19·9 (-23·8–-17·1)	16·5 (6·06–30·3)	18·4 (14·2–23·3)	14·7 (10·8–19·1)	11·4 (-38·6–178)	-19·9 (-23·8–-17·2)
Household air pollution from solid fuels	0·215 (0·0696–0·514)	0·0238 (0·0051–0·0738)	0·0153 (0·00306–0·0475)	-88·9 (-95·5–-76·7)	-35·8 (-59·1–0·0312)	0·281 (0·095–0·662)	0·0313 (0·00732–0·0923)	0·0201 (0·00433–0·0591)	-88·9 (-95·5–-76·4)	-35·6 (-59–0·375)
Ambient ozone pollution	79·6 (35·5–100)	74 (33·9–100)	78·1 (35·5–100)	-7·12 (-26·8–10·7)	5·59 (-9·23–27·1)	79·6 (35·5–100)	74 (33·9–100)	78·1 (35·5–100)	-7·12 (-26·8–10·7)	5·59 (-9·23–27·1)
Residential radon	2·72 (1–5·75)	2·72 (1–5·75)	2·72 (1–5·75)	0 (0–0)	0 (0–0)	2·72 (1–5·75)	2·72 (1–5·75)	2·72 (1–5·75)	0 (0–0)	0 (0–0)
Lead exposure	66·9 (45·4–83·2)	49·3 (29·8–68·7)	36·1 (19–56·4)	-26·3 (-36·9–-16·8)	-26·7 (-37·3–-17·9)	43·5 (14·1–67·9)	32·6 (11·3–55·6)	22·8 (5·77–44·6)	-25·2 (-39·8–-12·8)	-30 (-48·8–-19·1)
Child underweight	1·02 (0·591–1·5)	0·815 (0·459–1·23)	0·803 (0·451–1·2)	-20·1 (-30·7–-7·91)	-1·43 (-10·9–8·36)	1·22 (0·738–1·74)	1·03 (0·599–1·5)	1·01 (0·587–1·46)	-15·8 (-25·3–-3·75)	-1·92 (-9·59–6·03)
Iron deficiency	--	--	--	--	--	11·5 (9·61–13·4)	9·44 (7·83–11·2)	9·19 (7·58–10·9)	-17·8 (-26·4–-9·1)	-2·69 (-8·5–3·7)
Vitamin A deficiency	8·17 (3·6–16·3)	2·2 (0·91–4·31)	1·94 (0·759–4·1)	-73·1 (-88·5–-26·2)	-11·8 (-51·5–55·5)	5·53 (2·63–9·55)	2·3 (1·15–4·02)	2·07 (1·05–3·61)	-58·5 (-79·3–-17·6)	-9·91 (-40·3–41·5)
Zinc deficiency	6·65 (0·281–17·9)	6·51 (0·382–17·9)	6·44 (0·314–17·5)	-2·14 (-17·3–23·4)	-1·14 (-19·4–31·2)	6·41 (0·352–17·4)	6·34 (0·366–17·3)	6·25 (0·321–17·2)	-1·03 (-17·6–30·4)	-1·54 (-21·8–38·6)
Smoking	27·4 (24·2–30·9)	28 (24·8–31·3)	27·4 (24·2–30·9)	2·23 (-4·09–9·88)	-2·03 (-6·34–2·36)	12·7 (10·2–15·8)	11·8 (10·1–13·9)	11·4 (9·72–13·2)	-7·15 (-20·7–10·3)	-4 (-13·7–6·55)
Secondhand smoke	38·8 (36·5–41·3)	39·7 (37·4–42)	38·6 (36–41·2)	2·15 (-6·33–11·7)	-2·79 (-7·66–2·59)	48·8 (46·5–50·9)	49 (46·8–51·1)	47·1 (44·5–49·6)	0·382 (-5·48–6·66)	-3·89 (-7·23–-0·469)
Alcohol use	18·1 (13·3–23·6)	25 (17·5–34·2)	23 (16·2–31·6)	38·6 (17·1–72·4)	-8·13 (-21·3–2·93)	5·99 (3·65–9·45)	7·16 (4·51–10·7)	6·72 (4–10·6)	19·7 (1·04–44·6)	-6·23 (-26·5–16)
Drug use	0·168 (0·0754–0·344)	0·197 (0·102–0·375)	0·22 (0·121–0·4)	17 (2·22–43·5)	11·9 (-1·71–33)	0·08 (0·0415–0·151)	0·0915 (0·0554–0·163)	0·108 (0·0671–0·182)	14·4 (-0·0884–39·7)	18·2 (1·61–46·8)
High fasting plasma glucose	13·3 (11·2–15·2)	15·1 (13·1–17·3)	15·7 (13·7–18)	13·3 (-1·07–32·5)	4·19 (-7·57–18·4)	11·1 (9·64–12·9)	13·3 (11·2–15·4)	13·8 (11·6–16·1)	19·5 (4·68–37·3)	4·06 (-8·77–20·8)
High systolic blood pressure	34·4 (29–40·2)	36·5 (29–44·4)	36·7 (29·5–44·8)	6·21 (-16·8–35·3)	0·452 (-17·2–23·1)	27·9 (22–34·7)	29·1 (20·5–39)	28·5 (20·2–38·6)	4·15 (-28·9–44·9)	-2·11 (-27·1–31·1)
High body-mass index	13·3 (9·16–18·8)	20·6 (15·5–27·1)	20·6 (15·2–26·9)	55 (35–87·4)	-0·427 (-6·67–6·2)	12·4 (7·18–18·5)	18·9 (12·8–26·4)	19·5 (13·5–27)	52·2 (24·9–113)	3·04 (-8·45–19)
Low bone mineral density	10·5 (5·98–17·1)	10 (5·51–16·4)	9·91 (5·45–16·2)	-4·59 (-29–26·4)	-0·874 (-24·2–29·6)	16·6 (10·9–23·9)	16 (10·4–23)	15·9 (10·2–22·9)	-3·73 (-25·2–22·3)	-0·83 (-21·8–28·2)
Diet low in fruits	29·8 (20·1–40·7)	38·1 (26·8–51·2)	40·3 (28·6–53·5)	27·9 (16·1–44·6)	5·86 (-3·8–17·7)	31·8 (21·8–43·3)	39·7 (28·6–52·8)	42 (30·5–54·7)	24·8 (14·7–39·6)	5·8 (-3·89–17·2)
Diet low in vegetables	45 (25·4–67·9)	39·9 (22·3–61·6)	45·8 (26–67·6)	-11·4 (-21·7–-3·63)	14·8 (4·09–34·8)	42·8 (23·8–65)	37·4 (20·2–59·1)	43·6 (24·1–64·8)	-12·7 (-25·3–-3·88)	16·5 (4·16–38)
Diet low in whole grains	97·6 (95·9–99·8)	96·9 (94·7–99·6)	96·9 (94·6–99·5)	-0·775 (-1·65–-0·131)	-0·00758 (-0·839–0·779)	--	--	--	--	--
Diet low in nuts and seeds	17·5 (4·11–51·8)	20·3 (4·55–52·4)	22·7 (5·32–53·6)	15·9 (0·522–42·9)	11·8 (-5·27–54·1)	13·9 (2·46–51·1)	16·2 (3·19–51·4)	18·3 (4·54–52·1)	16·6 (-0·0364–51·6)	12·9 (-5·47–56·9)
Diet low in milk	67·3 (39·4–90)	55 (28·1–76·2)	58·2 (31·4–79·5)	-18·2 (-42·5–-2·96)	5·8 (0·491–19·5)	66·9 (38·9–89·1)	54·2 (29·7–75·5)	57·6 (31·6–79·1)	-18·9 (-43·9–-3·09)	6·26 (0·532–21·4)
Diet high in red meat	68·5 (58·6–77·2)	63·2 (53·3–72·8)	56 (44·1–67·2)	-7·73 (-12·9–-3·88)	-11·4 (-21–-2·91)	68·3 (58·6–77·3)	63·1 (53–72·8)	56 (44·7–67·8)	-7·59 (-12·4–-3·68)	-11·2 (-21·2–-1·6)
Diet high in processed meat	58·7 (34·4–83·4)	52·2 (29·9–76·1)	56·4 (32·7–81)	-11·1 (-30·5–6·47)	8·02 (-2·74–23·3)	65·1 (40·9–88·8)	60·3 (36–85·1)	63 (38·2–87·5)	-7·33 (-21·6–6·86)	4·37 (-3·96–14·8)
Diet high in sugar-sweetened beverages	41·5 (23·7–59·3)	40·3 (24·2–56·8)	37·5 (21·1–55·4)	-2·87 (-35·1–49·4)	-6·92 (-29–17·2)	47·2 (27·1–65·2)	46·8 (29·6–62·7)	42·8 (24·8–61)	-0·904 (-31·6–50·9)	-8·61 (-29·8–13·7)
Diet low in fiber	47·6 (31·3–62·8)	53 (36·9–68·3)	52·4 (36·1–67·6)	11·5 (7·5–18)	-1·2 (-7·66–6·06)	47·7 (31·1–64)	53·2 (36·1–69)	52·7 (35·4–68·8)	11·6 (7·43–18·3)	-1·01 (-8·05–5·71)
Diet low in seafood omega-3 fatty acids	91·1 (77·9–100)	89·7 (76·5–100)	90·5 (78·3–100)	-1·6 (-10·3–6·38)	0·934 (-5·48–10·3)	92·6 (81·4–100)	91·5 (81–100)	92·5 (83–100)	-1·19 (-8·17–5·76)	1·01 (-4·45–8·97)
Diet low in polyunsaturated fatty acids	19·6 (7·03–48·9)	30·2 (10·4–65·3)	30·8 (10·1–66·6)	53·9 (12–94·2)	1·83 (-6·62–9·24)	21·6 (7·41–52·2)	32·6 (11·1–68·3)	33·3 (11–69·4)	51·2 (12–91·2)	2·08 (-6·3–9·64)
Diet high in trans fatty acids	47·9 (37·8–99·2)	41·2 (31·4–97·4)	40·3 (30·4–97)	-14 (-25·2–-0·19)	-2·19 (-12–7·61)	49·1 (38·7–100)	42·6 (32·2–99·7)	41·6 (31·1–99)	-13·3 (-23·4–-0·302)	-2·43 (-10·6–6·5)
Diet high in sodium	30·3 (5·9–56·2)	33·6 (7·52–59·1)	35·4 (8·13–61)	10·9 (-25·5–74·9)	5·17 (-17·4–43·2)	15·1 (3·82–37)	16 (3·92–39)	16·7 (3·94–40·3)	5·88 (-19–50·7)	4·42 (-11–28·9)
Low physical activity	2·86 (1·1–6·05)	3·23 (1·41–6·43)	3·43 (1·36–7·25)	13 (-19·7–76·2)	6·14 (-28·3–48·5)	3·97 (1·82–7·86)	4·66 (2·32–9·07)	4·67 (2·24–9·32)	17·5 (-8·83–62·1)	0·207 (-24–27·8)
Occupational asthmagens	20·9 (17·7–24·8)	21·2 (18·2–24·9)	18·8 (15·8–22·5)	1·17 (-7·42–10·5)	-11 (-17–-4·52)	--	--	--	--	--
Occupational particulate matter, gases, and fumes	7·8 (5·62–11·3)	7·35 (5·27–10·6)	6·7 (4·79–9·68)	-5·79 (-10·3–-1·14)	-8·83 (-11·3–-6·55)	3·86 (2·71–5·69)	3·66 (2·59–5·31)	3·32 (2·34–4·86)	-5·11 (-12·1–2·04)	-9·33 (-13·4–-5·67)
Occupational noise	7·65 (7·11–8·38)	7·61 (7·18–8·24)	7·22 (6·85–7·86)	-0·555 (-3·98–2·77)	-5·03 (-6·24–-3·8)	3·68 (3·32–4·13)	4·27 (4·01–4·63)	4·37 (4·13–4·71)	16·1 (9·7–23·5)	2·4 (0·67–4·19)
Occupational injuries	--	--	--	--	--	--	--	--	--	--
Occupational ergonomic factors	15 (12·9–17·4)	12·3 (10·7–14·3)	10·9 (9·35–12·7)	-18 (-26·7–-9·14)	-11·4 (-17·1–-4·64)	6·23 (5·11–7·55)	7·52 (6·35–9·03)	7·55 (6·28–9·1)	20·7 (4·2–36·9)	0·407 (-7–8·37)
Childhood sexual abuse	7·16 (4·65–10·1)	7·16 (4·65–10·1)	7·16 (4·65–10·1)	0 (0–0)	0 (0–0)	10·6 (7·84–14·3)	10·6 (7·84–14·3)	10·6 (7·84–14·3)	0 (0–0)	0 (0–0)
Intimate partner violence	--	--	--	--	--	9·5 (5·19–13·6)	9·03 (5·23–12·6)	9·5 (5·19–13·6)	-5·01 (-15·7–13·9)	5·27 (-6·06–28·6)
Non-exclusive breastfeeding	21·2 (16·7–26·9)	20·1 (16–25·1)	19·8 (15·9–24·7)	-5·05 (-16·9–8·64)	-1·88 (-10·4–7·85)	21·2 (16·7–26·9)	20·1 (16–25·1)	19·8 (15·9–24·7)	-5·05 (-16·9–8·63)	-1·89 (-10·4–7·83)
Discontinued breastfeeding	23·4 (22·1–24·5)	22·8 (21·5–24)	21·8 (20·6–23·1)	-2·45 (-9·15–4·72)	-4·28 (-9·2–0·895)	23·4 (22·2–24·6)	22·8 (21·4–24)	21·8 (20·6–23·2)	-2·56 (-9·25–4·6)	-4·12 (-9·04–1·06)
Diet low in calcium	29 (16·2–51·2)	27·3 (15–49)	26·8 (14·7–48·5)	-5·95 (-12·4–-1·62)	-1·48 (-6·23–2·25)	21·9 (10·2–42·6)	20·5 (9·07–40·4)	20·2 (8·92–39·8)	-6·28 (-15·5–-1·31)	-1·47 (-6·75–2·55)
Occupational exposure to asbestos	11·8 (9·59–14·6)	10·3 (8·57–11·5)	7·56 (5·98–9·26)	-12·7 (-31·1–6·86)	-26·4 (-40·7–-9·66)	2·51 (1·14–3·7)	1·58 (0·861–1·91)	1·6 (0·808–2·08)	-37·2 (-56·3–-5·66)	1·56 (-20·5–25·3)
Occupational exposure to arsenic	0·461 (0–1·09)	0·393 (0–0·932)	0·364 (0–0·859)	-14·7 (-22·1–NA)	-7·27 (-14·3–NA)	0·41 (0–0·965)	0·364 (0–0·854)	0·35 (0–0·828)	-11·2 (-20·3–NA)	-3·73 (-9·83–NA)
Occupational exposure to benzene	0·78 (0·0449–2·25)	0·97 (0·0563–2·78)	0·849 (0·0499–2·37)	24·3 (18·3–32·6)	-12·5 (-17·8–-5·05)	0·67 (0·0398–1·91)	1·19 (0·0694–3·37)	1·16 (0·0666–3·28)	77·5 (67·5–91·2)	-2·5 (-7·7–3·69)
Occupational exposure to beryllium	0·0238 (0·0232–0·0245)	0·0242 (0·0239–0·0245)	0·0222 (0·0215–0·0229)	1·51 (-1·41–4·32)	-8·41 (-11·5–-4·97)	0·02 (0·0194–0·0206)	0·0255 (0·0252–0·0258)	0·0248 (0·024–0·0256)	27·6 (23·5–32)	-2·71 (-6·02–0·879)
Occupational exposure to cadmium	0·0542 (0·0499–0·0586)	0·0477 (0·0462–0·0491)	0·0442 (0·0413–0·0472)	-12 (-18·6–-4·8)	-7·35 (-13·5–-0·716)	0·048 (0·0434–0·0528)	0·0456 (0·0444–0·0467)	0·044 (0·0417–0·0464)	-5·02 (-13·8–4·76)	-3·51 (-8·94–2·78)
Occupational exposure to chromium	0·128 (0·119–0·136)	0·12 (0·117–0·123)	0·111 (0·104–0·117)	-6·26 (-12·5–0·689)	-7·69 (-13·1–-2·04)	--	--	--	--	--
Occupational exposure to diesel engine exhaust	0·741 (0·703–0·781)	0·811 (0·787–0·837)	0·752 (0·711–0·791)	9·39 (3·55–16·2)	-7·22 (-12·2–-2·44)	0·501 (0·469–0·532)	0·702 (0·674–0·731)	0·682 (0·638–0·73)	40·1 (30·6–51·1)	-2·86 (-9·43–3·95)
Occupational exposure to formaldehyde	0·262 (0·239–0·286)	0·234 (0·225–0·242)	0·212 (0·197–0·227)	-10·9 (-18·4–-2·61)	-9·4 (-15·7–-2·82)	0·216 (0·194–0·239)	0·22 (0·213–0·226)	0·212 (0·201–0·224)	1·86 (-7·84–13·3)	-3·87 (-9·05–2·24)
Occupational exposure to nickel	0·461 (0–2)	0·398 (0–1·77)	0·369 (0–1·62)	-13·7 (-20·1–NA)	-7·15 (-13·4–NA)	0·34 (0–1·47)	0·281 (0–1·26)	0·268 (0–1·21)	-17·3 (-25·8–NA)	-4·69 (-11·3–NA)
Occupational exposure to polycyclic aromatic hydrocarbons	0·261 (0·245–0·278)	0·256 (0·25–0·262)	0·235 (0·223–0·247)	-1·86 (-8·1–4·7)	-8·36 (-13·1–-3·53)	0·224 (0·207–0·243)	0·255 (0·25–0·26)	0·247 (0·237–0·258)	13·8 (4·96–23·7)	-3·13 (-7·37–1·96)
Occupational exposure to silica	5·69 (0·825–18·2)	5·53 (0·812–17·8)	5·14 (0·756–16·5)	-2·73 (-11–7·6)	-7·09 (-15·5–1·76)	3·08 (0·421–9·66)	2·55 (0·354–7·92)	2·42 (0·329–7·54)	-17·2 (-26·1–-7·41)	-5·21 (-12·8–3·5)
Occupational exposure to sulfuric acid	0·748 (0·155–2·37)	0·639 (0·133–2·04)	0·591 (0·125–1·85)	-14·5 (-21–-7·22)	-7·53 (-13·8–-0·919)	0·602 (0·122–1·92)	0·531 (0·11–1·67)	0·511 (0·106–1·59)	-11·8 (-20·5–-2·51)	-3·79 (-9·82–3·37)
Unsafe sex	--	--	--	--	--	--	--	--	--	--
Occupational exposure to trichloroethylene	0·0745 (0·0714–0·0782)	0·073 (0·0714–0·075)	0·0675 (0·0647–0·0709)	-2·02 (-6·95–3·18)	-7·45 (-11·9–-2·68)	0·0658 (0·062–0·0699)	0·077 (0·0753–0·0794)	0·0747 (0·0714–0·0787)	16·9 (9·96–24·6)	-2·94 (-7·5–2·27)
No access to handwashing facility	3·47 (3·29–3·66)	3·19 (3·02–3·37)	3·16 (3–3·33)	-8·05 (-13·7–-2·1)	-0·863 (-4·87–3·61)	3·47 (3·29–3·66)	3·19 (3·02–3·37)	3·16 (3–3·33)	-8·05 (-13·7–-2·1)	-0·863 (-4·87–3·61)
Child wasting	1·32 (0·929–1·73)	1·27 (0·896–1·67)	1·26 (0·883–1·64)	-3·63 (-11·9–5·71)	-0·53 (-6·74–5·69)	1·12 (0·782–1·51)	1·06 (0·738–1·42)	1·05 (0·735–1·4)	-5·29 (-14·3–5·9)	-0·592 (-8·22–7·79)
Child stunting	1·82 (0·919–2·69)	1·24 (0·569–1·9)	1·23 (0·566–1·88)	-31·7 (-43·6–-17·7)	-1·04 (-13·1–13·2)	2·2 (1·11–3·18)	1·64 (0·811–2·44)	1·62 (0·814–2·45)	-25·3 (-38·3–-11·2)	-1·63 (-13·7–10·5)
Chewing tobacco	0·163 (0·129–0·205)	0·163 (0·13–0·208)	0·168 (0·132–0·212)	0·0893 (-23·9–30·8)	2·82 (-13·5–22·5)	0·136 (0·101–0·183)	0·137 (0·1–0·186)	0·139 (0·102–0·185)	0·536 (-30·1–45·7)	1·59 (-19·7–28·6)
Diet low in legumes	74·2 (39·7–99·6)	78·8 (49·6–100)	79·5 (51·2–100)	6·11 (0·251–29·5)	0·87 (-1·46–6·07)	71·1 (31·9–98·9)	75·8 (43·1–99·7)	76·5 (43·6–99·7)	6·61 (0·318–33·5)	0·948 (-1·66–6·28)
Short gestation	9·01 (6·53–14·9)	11·1 (9·2–13·4)	11 (8·92–13·7)	23·2 (-27·5–74·8)	-1·25 (-20·4–23·6)	18·6 (13·2–27·4)	16·9 (13·1–21·2)	13·4 (10·5–17·1)	-9·4 (-43·7–31·8)	-20·7 (-40·3–4·66)
Low birth weight	9·63 (7·73–14·1)	11·1 (9·68–12·8)	11 (9·4–13)	15·6 (-22·4–49)	-1·24 (-18–19·5)	17·3 (12·9–24·5)	15·5 (12·8–19)	12·6 (10·5–15·4)	-9·91 (-40·1–24·2)	-18·7 (-36·5–2·41)
High temperature	29 (18–39·8)	34·8 (23·3–46·5)	33·9 (22·3–45·7)	19·9 (12·9–31·8)	-2·48 (-5·24–-0·261)	29 (18–39·8)	34·8 (23·3–46·5)	33·9 (22·3–45·7)	19·9 (12·9–31·8)	-2·48 (-5·24–-0·261)
Low temperature	39·3 (26·3–52·7)	35·6 (22·5–49·5)	38·1 (24·9–51·4)	-9·5 (-17·9–-3·52)	7·06 (2·62–13·8)	39·3 (26·3–52·7)	35·6 (22·5–49·5)	38·1 (24·9–51·4)	-9·5 (-17·9–-3·52)	7·06 (2·62–13·8)
Kidney dysfunction	16·7 (11·7–23·2)	17·7 (12·6–24·4)	17·3 (12·1–23·9)	6·15 (1·77–12)	-2·21 (-6·5–1·91)	17·3 (12–23·9)	18 (12·8–24·7)	17·5 (12·2–24·3)	4·57 (1·22–8·57)	-2·76 (-6·52–0·478)

Bullying victimization	5·28<br>(2·04–10·8)	6·3<br>(2·42–13)	6·29<br>(2·4–13·1)	19·3<br>(15·2–23·5)	-0·067<br>(-1·87–1·51)	4·12<br>(1·63–8·52)	4·94<br>(1·97–10·3)	4·94<br>(1·95–10·3)	19·9<br>(15·8–23·8)	-0·167<br>(-1·99–1·47)
High LDL cholesterol	48·9<br>(45·3–52·6)	49·6<br>(46·1–53)	50·2<br>(46·6–53·9)	1·4<br>(-4·55–8·26)	1·24<br>(-3·34–6·13)	48·1<br>(44·5–51·3)	48·3<br>(45–51·9)	48·9<br>(45·6–52·4)	0·506<br>(-4·85–5·83)	1·15<br>(-2·47–4·82)

Supplementary Results Table 1. Tajikistan age-standardised Summary Exposure Values (SEVs) for most-detailed risk factors and percentage change, by sex, 1990, 2010, and 2019. Data in parentheses are 95% uncertainty intervals.

	Male					Female				
	1990	2010	2019	Percentage change 1990-2010	Percentage change 2010-2019	1990	2010	2019	Percentage change 1990-2010	Percentage change 2010-2019
Unsafe sanitation	64·2 (55·8–73·2)	61·7 (54–70·5)	59·8 (51·3–69·2)	-3·95 (-10·8–5·7)	-3·15 (-9·73–2·91)	64·2 (55·8–73·2)	61·7 (54–70·5)	59·8 (51·3–69·2)	-3·95 (-10·8–5·7)	-3·15 (-9·73–2·91)
Ambient particulate matter pollution	14·4 (4·95–32·1)	21·4 (10·1–39)	27·9 (13·4–48·2)	48·4 (-13·1–183)	30·5 (4·17–72·2)	12·3 (4·35–28·1)	18·9 (8·6–34)	25·5 (12·1–45)	53·2 (-12·3–197)	35·2 (6·61–79·7)
Household air pollution from solid fuels	28·9 (15·6–46·5)	13·4 (6·78–23·3)	7·74 (3·43–15·1)	-53·6 (-64·7–-39·6)	-42·2 (-58·8–-23·6)	31·1 (17·9–48·1)	15·3 (8·6–24·7)	9·21 (4·51–16·6)	-50·9 (-61·6–-38·4)	-39·7 (-56–-21·8)
Ambient ozone pollution	93·5 (50·2–100)	81·9 (37·7–100)	81·4 (37·4–100)	-12·4 (-30·4–0)	-0·602 (-8·86–6·85)	93·5 (50·2–100)	81·9 (37·7–100)	81·4 (37·4–100)	-12·4 (-30·4–0)	-0·602 (-8·86–6·85)
Residential radon	27 (4·25–96·2)	27 (4·25–96·2)	27 (4·25–96·2)	0 (0–0)	0 (0–0)	--	--	--	--	--
Lead exposure	84·3 (67·9–93·9)	75·2 (60·6–86·9)	68·7 (53·3–82·3)	-10·8 (-17·9–-4·82)	-8·66 (-14·4–-4·38)	66·9 (44–83·7)	60·6 (41·4–77·1)	55·6 (36·7–73)	-9·39 (-17·1–0·305)	-8·3 (-14·8–-3·69)
Child underweight	8·41 (6·43–10·2)	5·85 (4·17–7·41)	4·64 (3·15–6·11)	-30·4 (-43·1–-18)	-20·7 (-31·7–-10·6)	8·86 (6·92–10·6)	7·16 (5·31–8·78)	5·87 (4·11–7·36)	-19·2 (-30·7–-8·05)	-18 (-27·7–-10)
Iron deficiency	--	--	--	--	--	23·1 (20·6–25·5)	21·3 (19·4–23·1)	20·7 (18·8–22·8)	-8·05 (-16·5–1·39)	-2·58 (-7·68–2·38)
Vitamin A deficiency	25·4 (13·3–38·3)	20·5 (11·5–31·1)	13·8 (6·45–24·7)	-19·1 (-57·9–68)	-32·6 (-57·9–4·09)	16·5 (9·41–24·4)	18·6 (11·7–25·6)	13·7 (7·52–21)	13·1 (-33·2–108)	-26·6 (-48·3–2·6)
Zinc deficiency	39·1 (26·2–48·2)	55·3 (46·7–65·5)	39·1 (25·8–48·5)	41·4 (9·53–129)	-29·3 (-55·6–-11·2)	38 (24·1–47·8)	53·5 (44·3–62·6)	37·4 (22·7–47·1)	40·8 (8·91–132)	-30 (-56·6–-11·2)
Smoking	26·9 (23·6–30·1)	17·8 (15·9–20)	15·2 (13·4–17·3)	-33·8 (-40–-26)	-14·7 (-19·9–-9·54)	1·49 (1·08–1·98)	1·13 (0·889–1·43)	1·08 (0·832–1·37)	-24·3 (-42·9–1·53)	-4·6 (-17·8–11·3)
Secondhand smoke	28·9 (26·2–31·7)	23·8 (21·3–26·2)	23·6 (21·3–26)	-17·7 (-26·7–-6·66)	-0·964 (-8·56–6·33)	40·5 (38–43)	31·6 (29·5–33·9)	31·4 (29·3–33·6)	-21·8 (-27·7–-15·4)	-0·753 (-5·71–4·63)
Alcohol use	9·69 (6·71–13)	5·99 (3·94–8·63)	7·08 (4·68–10·1)	-38·1 (-49·9–-26)	18·1 (-2·18–40·8)	2·05 (1·14–3·36)	1·06 (0·578–1·87)	1·31 (0·699–2·29)	-48 (-64–-27·4)	22·9 (-7·2–63)
Drug use	0·127 (0·0672–0·242)	0·127 (0·0669–0·248)	0·129 (0·0699–0·25)	-0·11 (-7·86–7·99)	1·77 (-5·82–10·4)	0·0708 (0·0372–0·14)	0·0702 (0·0369–0·138)	0·0712 (0·0374–0·138)	-0·92 (-8·52–7·67)	1·4 (-5·73–9·3)
High fasting plasma glucose	5·43 (4·67–6·24)	9·13 (7·54–11·4)	13·6 (11·3–16·2)	68·2 (43·2–106)	48·8 (21·9–83·4)	5·18 (4·58–5·83)	10·9 (9·09–12·9)	13·6 (11·6–15·7)	111 (79·1–150)	24·3 (9·4–45·1)
High systolic blood pressure	29·7 (22·9–37·6)	28·4 (22·1–35·3)	29·9 (23·3–37·1)	-4·4 (-28·5–30·9)	5·55 (-14·6–29·1)	--	--	--	--	--
High body-mass index	6·72 (3·85–10·5)	7·4 (4·43–11·2)	10·3 (6·81–14·7)	10·2 (-2·45–30·9)	39 (24–64·1)	9·89 (6·36–14·1)	10·5 (6·86–14·7)	14·1 (10·1–19·2)	5·8 (-8·24–23·9)	34·4 (19·8–60·5)
Low bone mineral density	9·78 (5·3–16·1)	8·92 (4·93–14·6)	8·8 (4·8–14·8)	-8·82 (-29·7–18·9)	-1·3 (-24·9–27·4)	18 (12·2–25·8)	16·7 (11·1–24·4)	16·3 (10·6–23·3)	-7·06 (-25·9–18·1)	-2·57 (-23·1–22·2)
Diet low in fruits	70·1 (61·2–80·8)	73·6 (65·5–83·1)	68·9 (59·3–79·8)	4·99 (2·76–7·72)	-6·43 (-11·3–-2·9)	71·3 (62·7–81·6)	74·7 (66·9–83·9)	70 (60·4–80·9)	4·74 (2·59–7·35)	-6·29 (-11·4–-2·6)
Diet low in vegetables	17·3 (6·66–35·8)	19·1 (7·52–38·4)	10·5 (4·01–24·5)	10·8 (-0·504–29·9)	-44·9 (-64·9–-21·8)	14·5 (5·28–31·3)	16·5 (6·4–34)	8·61 (3·24–20·4)	13·8 (0·181–38·2)	-47·8 (-66·6–-24·2)
Diet low in whole grains	99·9 (100–100)	99·9 (100–100)	99·9 (100–100)	0·000184 (0–0)	0 (0–0)	99·9 (100–100)	99·9 (100–100)	99·9 (100–100)	0·000852 (0–0)	-0·00282 (0–0)
Diet low in nuts and seeds	86·8 (43·8–94·4)	88·6 (46·4–95·7)	85·6 (44·9–94·5)	2·06 (-5·62–10·8)	-3·39 (-12·2–5·65)	83·9 (43·5–93·3)	85·6 (44·5–94·3)	83 (42·5–93)	1·94 (-9·3–13·3)	-3·01 (-13·7–8·75)
Diet low in milk	78·4 (53·8–98·1)	83 (61·5–99·8)	81·7 (59·4–99·5)	5·82 (0·412–18·1)	-1·53 (-5·03–-0·0692)	78·1 (53·6–97·9)	82·7 (61·6–99·7)	81·5 (59·2–99·3)	5·96 (0·432–18·4)	-1·55 (-4·66–-0·0679)
Diet high in red meat	24·2 (14·9–33·1)	18·8 (10·9–26·3)	21·4 (12·8–30·2)	-22·3 (-28·8–-16·3)	14 (4·45–26·5)	24·1 (15–33·1)	18·7 (11·1–26·4)	21·4 (13–29·8)	-22·4 (-29–-16)	14·2 (4·76–27·4)
Diet high in processed meat	34·8 (17·3–57·8)	33 (16·9–55·6)	34·3 (17·5–56·9)	-5·19 (-28·3–23·5)	3·92 (-10·3–23)	39 (21·1–61·1)	36·9 (20·1–58·6)	38·6 (20·5–60·8)	-5·48 (-26·1–21·5)	4·66 (-8·78–23·3)
Diet high in sugar-sweetened beverages	32·1 (20·8–53·6)	29·4 (18·2–50)	27·9 (17·1–49)	-8·26 (-32–19·3)	-4·99 (-22–16·4)	26·9 (16·2–45·6)	24·8 (14·8–43·6)	23·6 (14–41·2)	-7·73 (-34·9–27·6)	-4·97 (-25·3–18)
Diet low in fiber	55·4 (39–70·6)	54·9 (38·7–69·9)	49·7 (33·2–65·7)	-0·83 (-3·31–2·48)	-9·41 (-17·4–-2·89)	55·7 (38–71·6)	55·1 (38·1–70·7)	49·9 (32·3–65·9)	-1·1 (-3·77–2·58)	-9·38 (-17·8–-2·94)
Diet low in seafood omega-3 fatty acids	100 (99·8–100)	100 (99·9–100)	99·8 (99·1–100)	0·0108 (-0·0234–0·11)	-0·192 (-0·78–0)	100 (100–100)	100 (100–100)	99·9 (99·6–100)	-0·00175 (-0·00198–0·01)	-0·0837 (-0·414–0)
Diet low in polyunsaturated fatty acids	80·3 (45–100)	77·4 (42·7–100)	75·2 (38·6–100)	-3·62 (-10–1·68)	-2·76 (-8·61–0)	81·5 (46·4–100)	78·7 (44·1–100)	76·7 (41–100)	-3·36 (-9·6–1·47)	-2·56 (-8·25–0)
Diet high in trans fatty acids	41·9 (31·8–99·4)	38·4 (28·6–98·1)	37·2 (27·2–97·2)	-8·34 (-18·1–0·0504)	-3·05 (-13·9–5·78)	43·6 (32·4–100)	40 (29·1–100)	39 (27·7–99·4)	-8·14 (-17·7–0)	-2·64 (-12·3–5·37)
Diet high in sodium	62·7 (40·1–80·9)	43·4 (15·3–67·5)	42·7 (14·5–66·6)	-30·7 (-67·3–-8·22)	-1·66 (-22·7–20·4)	30·5 (11–52·7)	18·9 (4·43–42·2)	18·3 (4·31–41·2)	-37·9 (-68·9–-14·5)	-3·3 (-22·8–12·6)
Low physical activity	1·68 (0·791–3·75)	1·7 (0·799–3·78)	1·77 (0·834–3·88)	1·39 (-8·4–12·4)	3·92 (-5·86–16·3)	1·43 (0·755–2·95)	1·44 (0·765–3·03)	1·48 (0·78–3·03)	0·736 (-8·09–10·6)	2·71 (-5·31–12·4)
Occupational asthmagens	13·3 (9·94–17·4)	13·8 (10·9–17·7)	13·8 (10·1–17·6)	3·97 (-15·1–30·1)	-0·0461 (-12·1–13)	10·4 (7·11–15)	10·1 (7·21–13·8)	9·37 (6·64–12·9)	-2·77 (-24·7–26·9)	-7·37 (-23·4–9·91)
Occupational particulate matter, gases, and fumes	8·01 (5·92–11·4)	7·45 (5·71–10·3)	7·59 (5·86–10·3)	-6·97 (-13·6–0·866)	1·89 (-2·05–6·37)	5·04 (3·56–7·24)	5·16 (3·78–7·2)	4·91 (3·6–6·68)	2·32 (-7·63–16·3)	-4·85 (-10·6–1·03)
Occupational noise	8·78 (7·7–9·99)	8·44 (7·82–9·25)	8·75 (8·22–9·45)	-3·93 (-9·79–2·93)	3·69 (0·363–7·33)	5·47 (4·49–6·58)	5·84 (5·2–6·63)	5·77 (5·2–6·51)	6·75 (-3·08–19·5)	-1·25 (-6·51–4·45)
Occupational injuries	--	--	--	--	--	--	--	--	--	--
Occupational ergonomic factors	17·7 (14·3–21·1)	16·8 (14·4–19·3)	16·2 (13·4–19·3)	-5·05 (-23·1–18)	-3·42 (-16·8–9·77)	--	--	--	--	--
Childhood sexual abuse	2·52 (1·65–3·56)	2·52 (1·65–3·56)	2·52 (1·65–3·56)	0 (0–0)	0 (0–0)	3·14 (2·26–4·3)	3·14 (2·26–4·3)	3·14 (2·26–4·3)	0 (0–0)	0 (0–0)
Intimate partner violence	--	--	--	--	--	16·1 (9·37–23)	15·4 (9·08–20·3)	16·1 (9·37–23)	-4·51 (-17·7–14·5)	4·72 (-7·57–29·6)
Non-exclusive breastfeeding	22·2 (12·9–35·1)	20·2 (12·1–31·2)	19·8 (12·2–30·5)	-9·14 (-16–0·358)	-1·85 (-8·18–5·31)	22·2 (12·8–35·1)	20·2 (12·1–31·2)	19·8 (12·2–30·4)	-9·07 (-16–0·439)	-1·85 (-8·18–5·32)
Discontinued breastfeeding	10·1 (8·49–11·6)	11 (10·3–11·7)	9·99 (9·25–10·8)	9·09 (-6·45–30·7)	-8·92 (-17·5–0·389)	10 (8·46–11·6)	11 (10·3–11·6)	9·99 (9·25–10·8)	9·48 (-6·11–31·2)	-8·9 (-17·4–0·412)
Diet low in calcium	70·5 (58·2–87·8)	78·4 (67·5–92·9)	73·6 (62–90·1)	11·3 (5·13–17·2)	-6·08 (-9·4–-2·83)	--	--	--	--	--
Occupational exposure to asbestos	0·507 (0·305–0·93)	0·956 (0·686–1·28)	0·935 (0·636–1·29)	88·5 (-5·25–208)	-2·18 (-33–40)	0·893 (0·498–1·63)	0·904 (0·536–1·46)	0·943 (0·55–1·57)	1·25 (-48·1–91·8)	4·24 (-43–83·6)
Occupational exposure to arsenic	0·232 (0·0936–0·389)	0·233 (0·0934–0·395)	0·247 (0·0983–0·425)	0·253 (-12·3–14)	6·03 (-2·58–15·3)	0·213 (0·0835–0·367)	0·198 (0·0796–0·337)	0·201 (0·0798–0·343)	-6·71 (-22·5–13·1)	1·38 (-9·33–14)
Occupational exposure to benzene	0·555 (0·343–0·967)	0·706 (0·444–1·25)	0·731 (0·444–1·3)	27·2 (12·8–47·6)	3·61 (-4·17–13·8)	0·473 (0·279–0·853)	0·61 (0·366–1·09)	0·677 (0·403–1·18)	29·2 (6·29–62·1)	11 (-2·06–28·4)
Occupational exposure to beryllium	0·0756 (0·0675–0·0836)	0·0797 (0·0777–0·0818)	0·0824 (0·0763–0·089)	5·44 (-5·21–17·5)	3·38 (-3·4–10·3)	0·071 (0·061–0·082)	0·0716 (0·0646–0·0789)	0·0714 (0·0619–0·0818)	0·801 (-15·4–21·3)	-0·206 (-10·1–10·6)
Occupational exposure to cadmium	0·127 (0·111–0·142)	0·129 (0·123–0·135)	0·136 (0·124–0·149)	1·9 (-10·2–14·9)	5·86 (-2·17–14·4)	0·115 (0·0982–0·135)	0·11 (0·0984–0·122)	0·111 (0·0959–0·128)	-4·97 (-21–14·3)	1·63 (-8·92–13·7)
Occupational exposure to chromium	0·278 (0·244–0·312)	0·301 (0·288–0·315)	0·322 (0·294–0·353)	8·43 (-4·5–22·4)	7·07 (-0·743–15·7)	0·233 (0·197–0·275)	0·232 (0·208–0·258)	0·244 (0·21–0·281)	-0·459 (-17·5–20·7)	5·3 (-5·46–18·3)
Occupational exposure to diesel engine exhaust	2·14 (1·89–2·4)	2·58 (2·44–2·72)	2·79 (2·53–3·05)	20·6 (6·76–38·8)	7·82 (-0·623–16·5)	1·11 (0·943–1·29)	1·09 (0·976–1·21)	1·13 (0·976–1·31)	-2·06 (-18·6–19·1)	4·17 (-6·9–17·3)
Occupational exposure to formaldehyde	0·539 (0·424–0·623)	0·581 (0·471–0·625)	0·62 (0·499–0·69)	7·84 (-6·69–23·5)	6·7 (-2·29–16·3)	--	--	--	--	--
Occupational exposure to nickel	0·31 (0·0994–0·789)	0·324 (0·108–0·856)	0·34 (0·113–0·864)	4·42 (-8·29–19·4)	5·1 (-3·56–14·8)	0·227 (0·0753–0·604)	0·202 (0·0657–0·526)	0·198 (0·0624–0·515)	-11·1 (-26·8–8·29)	-1·82 (-12·2–10·1)
Occupational exposure to polycyclic aromatic hydrocarbons	0·62 (0·547–0·693)	0·699 (0·669–0·727)	0·744 (0·683–0·812)	12·6 (-0·252–27·1)	6·5 (-1·19–14·7)	0·497 (0·421–0·584)	0·513 (0·462–0·569)	0·543 (0·466–0·626)	3·1 (-14·7–24·3)	5·82 (-5·15–18·7)
Occupational exposure to silica	4·04 (2·09–8·67)	4·72 (2·47–10·3)	5 (2·55–11)	16·8 (1·52–38·6)	5·88 (-4·91–18·5)	2·08 (1·06–4·57)	1·76 (0·9–3·86)	1·68 (0·865–3·8)	-15·2 (-30·1–4·43)	-4·66 (-14·8–7·18)
Occupational exposure to sulfuric acid	0·726 (0·505–1·28)	0·727 (0·525–1·27)	0·774 (0·553–1·35)	0·114 (-11·8–14·1)	6·57 (-2–15·6)	0·606 (0·419–1·05)	0·555 (0·387–0·972)	0·548 (0·376–0·989)	-8·35 (-23·9–9·37)	-1·32 (-11·7–10)
Unsafe sex	--	--	--	--	--	--	--	--	--	--
Occupational exposure to trichloroethylene	0·169 (0·15–0·189)	0·185 (0·179–0·193)	0·199 (0·183–0·217)	9·53 (-2·43–23·3)	7·32 (-0·215–15·6)	0·14 (0·119–0·165)	0·14 (0·126–0·156)	0·147 (0·126–0·17)	0·192 (-16·9–21·9)	4·71 (-5·79–18·2)
No access to handwashing facility	34·3 (32·9–35·6)	29·6 (28·4–30·8)	23·8 (22·8–24·9)	-13·6 (-17·4–-9·57)	-19·6 (-22·1–-17·1)	34·3 (32·9–35·6)	29·6 (28·4–30·8)	23·8 (22·8–24·9)	-13·6 (-17·4–-9·57)	-19·6 (-22·1–-17·1)
Child wasting	4·42 (3·47–5·26)	4·3 (3·4–5·11)	3·9 (3·05–4·67)	-2·78 (-14·1–9·94)	-9·23 (-16·5–-1·9)	5·05 (4·04–5·92)	4·96 (3·98–5·82)	4·54 (3·59–5·37)	-1·74 (-12·7–11·2)	-8·51 (-16·7–-0·437)
Child stunting	21·1 (14·8–24·6)	19 (13·3–22·2)	15·8 (10·4–19·6)	-10 (-20·7–1·87)	-16·8 (-29·3–-7·49)	20·8 (14·3–24·5)	19 (13·1–22·3)	15·5 (10·4–19)	-8·49 (-18·5–2·86)	-18·7 (-30·7–-9·18)
Chewing tobacco	5·43 (4·19–6·82)	5·63 (4·45–7·11)	6·35 (5–8·08)	3·68 (-20·4–38·3)	12·9 (-5·14–35·4)	--	--	--	--	--
Diet low in legumes	100 (100–100)	99·2 (97·4–100)	99·2 (97·6–100)	-0·825 (-2·56–0)	0·0198 (-0·812–0·862)	100 (100–100)	97·9 (94·7–100)	98·1 (94·9–100)	-2·12 (-5·32–0)	0·219 (-0·694–1·35)
Short gestation	8·26 (6·23–11·3)	7·96 (6·15–10·5)	8·02 (6·26–10·6)	-3·61 (-34·8–39·6)	0·739 (-25·8–37·4)	5·36 (4·06–7·34)	5·34 (4·02–7·32)	5·19 (3·97–6·85)	-0·416 (-31·1–46·7)	-2·69 (-29·7–33·6)
Low birth weight	6·25 (5·12–8)	5·98 (4·95–7·55)	5·44 (4·46–6·85)	-4·38 (-31·1–29·7)	-9·04 (-31·1–16·8)	3·58 (2·82–4·6)	3·6 (2·84–4·67)	3·44 (2·72–4·31)	0·458 (-27·2–39·3)	-4·46 (-27·8–25)
High temperature	34 (26·4–42·2)	32·7 (25·2–41)	40 (32·5–48·2)	-3·8 (-8·93–-1·41)	22·3 (13·2–33·1)	34 (26·4–42·2)	32·7 (25·2–41)	40 (32·5–48·2)	-3·8 (-8·93–-1·41)	22·3 (13·2–33·1)
Low temperature	38·3 (34·9–43·4)	36·2 (32·6–41·3)	37·6 (33·9–42·8)	-5·46 (-6·98–-3·82)	3·91 (2·27–5·55)	38·3 (34·9–43·4)	36·2 (32·6–41·3)	37·6 (33·9–42·8)	-5·46 (-6·98–-3·82)	3·91 (2·27–5·55)
Kidney dysfunction	18·7 (12·1–26·4)	19·9 (13·2–27·6)	21·6 (14·6–29·7)	6·04 (2·72–10·9)	8·69 (4·76–14·2)	19·1 (12·5–26·9)	20 (13·3–27·6)	21·5 (14·7–29·5)	4·76 (1·9–8·6)	7·66 (3·86–13)

Bullying victimization	1·05 (0·369–2·22)	1·24 (0·563–2·43)	1·22 (0·528–2·42)	18·6 (-3·68–83·4)	-2·18 (-13·8–3·33)	0·684 (0·24–1·45)	0·827 (0·362–1·7)	0·838 (0·345–1·75)	20·9 (-4·59–63·8)	1·23 (-8·42–8·41)
High LDL cholesterol	28·4 (25·1–31·9)	28 (24·7–31·5)	28 (24·6–31·5)	-1·38 (-7·09–4·56)	-0·0099 (-3·98–4·31)	29·3 (26·1–32·8)	28·8 (25·6–32·1)	28·7 (25·4–32)	-1·73 (-6·2–3·31)	-0·484 (-3·9–3·09)

Supplementary Results Table 1. Denmark age-standardised Summary Exposure Values (SEVs) for most-detailed risk factors and percentage change, by sex, 1990, 2010, and 2019. Data in parentheses are 95% uncertainty intervals.

	Male					Female				
	1990	2010	2019	Percentage change 1990-2010	Percentage change 2010-2019	1990	2010	2019	Percentage change 1990-2010	Percentage change 2010-2019
Unsafe sanitation	1·86 (1·03–3·01)	0·704 (0·4–1·17)	0·585 (0·325–0·956)	-62·2 (-81–-24·8)	-16·9 (-47·1–29·8)	--	--	--	--	--
Ambient particulate matter pollution	16·4 (5·91–30·1)	12 (8·67–16)	7·65 (4·68–11·1)	-26·7 (-60·8–89·5)	-36·4 (-46·3–-30·1)	16·4 (5·93–30·1)	12 (8·68–16)	7·65 (4·68–11·1)	-26·7 (-60·9–90)	-36·4 (-46·2–-30·1)
Household air pollution from solid fuels	0·0317 (0·00718–0·0896)	0·0104 (0·00208–0·0322)	0·00664 (0·00118–0·0223)	-67·3 (-85·2–-32·6)	-35·9 (-61·2–1·33)	0·0416 (0·0102–0·114)	0·0137 (0·00288–0·0412)	0·00882 (0·00169–0·0302)	-67·1 (-85·1–-32·6)	-35·6 (-61·1–1·5)
Ambient ozone pollution	22·7 (8·39–42·6)	28·9 (10·8–53)	27·6 (10·2–49·8)	27·1 (4·68–55·8)	-4·53 (-19·3–13·7)	22·7 (8·39–42·6)	28·9 (10·8–53)	27·6 (10·2–49·8)	27·1 (4·68–55·8)	-4·53 (-19·3–13·7)
Residential radon	26·4 (10·9–50)	26·4 (10·9–50)	26·4 (10·9–50)	0 (0–0)	0 (0–0)	26·4 (10·9–50)	26·4 (10·9–50)	26·4 (10·9–50)	0 (0–0)	0 (0–0)
Lead exposure	23·3 (3·43e-12–52·2)	18·1 (0·00168–42·9)	13·2 (0·00268–35·1)	-22·3 (-34·9–2·12e+13)	-27·3 (-61·6–-3·46)	22·7 (2·85e-12–51·7)	18·6 (0·0141–44)	13·9 (0·0138–36·5)	-17·9 (-28·9–1·73e+13)	-25·5 (-59·7–9·05)
Child underweight	0·925 (0·545–1·35)	0·787 (0·44–1·17)	0·77 (0·421–1·16)	-15 (-25·7–-1·59)	-2·11 (-9·92–7·55)	1·11 (0·645–1·61)	0·959 (0·559–1·42)	0·939 (0·535–1·39)	-13·3 (-24·3–-2·23)	-2·13 (-10·2–6·46)
Iron deficiency	--	--	--	--	--	9·16 (7·6–10·9)	8·21 (6·8–9·75)	8·06 (6·65–9·66)	-10·4 (-18·7–-1·97)	-1·79 (-7·66–4·32)
Vitamin A deficiency	2·97 (1·2–5·82)	1·39 (0·569–2·93)	1·2 (0·453–2·38)	-53·1 (-81·4–21·3)	-13·7 (-50·2–69·7)	2·45 (1·25–4·11)	1·62 (0·793–2·8)	1·42 (0·708–2·49)	-33·8 (-66·2–33·2)	-12·4 (-43·3–35·1)
Zinc deficiency	2·88 (0–9·12)	2·18 (0–7·07)	2·33 (0–7·61)	-24·1 (-76·5–NA)	6·92 (-4·61–NA)	2·82 (0–8·98)	2·14 (0–6·96)	2·28 (0–7·4)	-24 (-66·6–NA)	6·4 (-6·2–NA)
Smoking	28 (24·9–31·2)	21·6 (19·3–24·1)	18·2 (16·2–20·4)	-22·7 (-27–-17·9)	-15·9 (-19·2–-12·1)	23·5 (20·7–26·8)	18 (15·7–20·6)	14·6 (12·8–16·7)	-23·6 (-29·3–-17·5)	-18·7 (-22·6–-14·2)
Secondhand smoke	45·2 (42·5–47·7)	34·4 (32·5–36·2)	32·5 (29·8–35·1)	-23·8 (-29·8–-16·6)	-5·64 (-11·3–-0·0101)	41·1 (39–43·1)	33·1 (31·5–34·7)	31·2 (29·1–33·3)	-19·4 (-25·1–-13·1)	-5·97 (-10·6–-1·53)
Alcohol use	19·4 (14–25·7)	20·1 (13·7–27·9)	18·8 (12·6–26·2)	3·94 (-7·51–20·1)	-6·37 (-18·4–7·01)	12 (8·04–17·3)	11·2 (7·41–16·2)	10·8 (6·77–16·3)	-6·54 (-16–3·21)	-3·16 (-17·9–11·8)
Drug use	0·285 (0·148–0·556)	0·336 (0·196–0·608)	0·341 (0·206–0·607)	17·7 (2·12–39·9)	1·62 (-9·34–14·5)	0·131 (0·0704–0·245)	0·151 (0·0922–0·263)	0·17 (0·106–0·284)	15·7 (1·71–36)	12·5 (2–24·4)
High fasting plasma glucose	4·9 (4·21–5·65)	8·15 (6·91–9·7)	9·27 (7·88–11)	66·4 (46·3–94·2)	13·8 (-0·844–35·1)	4·31 (3·68–5·09)	6·78 (5·68–8·17)	8·43 (6·98–10·2)	57·1 (38·7–84·5)	24·4 (7·08–47·4)
High systolic blood pressure	35 (32·1–38)	38·8 (32·8–46)	38·8 (31·3–46·9)	11 (-7·07–32·4)	-0·0265 (-16–17·5)	33·5 (30·7–36·3)	31·3 (24·1–39·2)	30·3 (22·5–39·9)	-6·38 (-27·8–16·2)	-3·21 (-25·9–24·3)
High body-mass index	16·4 (11·7–21·8)	22·7 (17–29·5)	23·5 (17·7–30·8)	38·7 (22·7–61)	3·79 (-2·68–11·3)	14·6 (9·19–21·1)	25·6 (19·3–34·2)	26·9 (20·6–35·3)	75·2 (43·6–136)	4·95 (-6·03–20·2)
Low bone mineral density	9·23 (4·75–15·5)	8·68 (4·53–14·7)	8·63 (4·54–14·7)	-5·98 (-30·3–24)	-0·533 (-25·9–32·8)	12·6 (7·81–18·7)	11·6 (7·05–17·7)	11·4 (6·78–17·3)	-7·95 (-30·7–17·9)	-1·21 (-24·1–28·3)
Diet low in fruits	59·1 (48·3–72·5)	29·9 (20·3–40·5)	33 (22·4–44·7)	-49·5 (-62·6–-37·4)	10·6 (-3·53–27·2)	60·1 (49·5–72·6)	32·2 (22·7–43·6)	35·3 (24·9–48)	-46·4 (-58·7–-34·2)	9·75 (-3·22–24·7)
Diet low in vegetables	53·4 (32·5–75·4)	31·5 (16·1–53·4)	31·4 (15·7–53·2)	-40·9 (-62–-18·5)	-0·544 (-11·6–13·5)	51·3 (31–73·6)	28·9 (13·6–51)	29 (13·5–49·9)	-43·6 (-65·6–-18·9)	0·229 (-12·7–15·9)
Diet low in whole grains	71·5 (60–83·3)	87·3 (80·9–93·7)	89·2 (83·4–94·7)	22 (11–35·9)	2·21 (0·73–4·3)	--	--	--	--	--
Diet low in nuts and seeds	64·4 (32–80·9)	48·9 (19·9–69·5)	47·4 (21·1–69·1)	-24 (-41·9–-11·7)	-3·07 (-14·3–5·85)	60·1 (29·2–78·1)	43·6 (16·5–66)	42 (15·3–65·1)	-27·4 (-47·2–-13)	-3·72 (-17·6–7·57)
Diet low in milk	56·2 (31·1–77·3)	40·3 (18·1–59·7)	43·9 (20·9–64·9)	-28·3 (-52·2–-10·2)	8·96 (0·394–26·2)	55·5 (30·9–75·7)	39 (16·7–58·9)	42·9 (19·7–63·5)	-29·7 (-54·8–-11·2)	9·96 (0·56–28·7)
Diet high in red meat	89·1 (84·1–93)	83·8 (77·2–89·2)	83·5 (75–90·2)	-5·91 (-9·21–-3·55)	-0·387 (-7·59–6·31)	89·2 (84·3–93)	83·8 (77·2–89·4)	83·3 (75–90·1)	-6·01 (-9·39–-3·43)	-0·58 (-7·57–6·13)
Diet high in processed meat	74·8 (48·2–97·4)	78·3 (53·2–99·3)	78·2 (53–99·1)	4·63 (0·941–12·1)	-0·11 (-2·15–1·97)	77·6 (52–98·7)	80·6 (57·7–99·7)	80·7 (57·8–99·6)	3·89 (0·491–11·4)	0·113 (-1·84–2·62)
Diet high in sugar-sweetened beverages	36·1 (20·9–52·4)	41·4 (26·1–57·4)	44 (27·1–61·2)	14·6 (-16·4–64·1)	6·32 (-15–37·1)	40·5 (24·1–59·2)	48 (31·7–64·3)	50·7 (33·7–67·8)	18·7 (-9·35–65·7)	5·59 (-15·2–32·9)
Diet low in fiber	33·7 (19·8–48·3)	27·8 (15·9–40·7)	28·5 (16·1–42)	-17·3 (-25·2–-10·8)	2·41 (-11·1–16·1)	33·5 (19·3–47·9)	28 (15·5–41·8)	28·1 (15·6–42·2)	-16·5 (-25·3–-8·97)	0·674 (-13–15·6)
Diet low in seafood omega-3 fatty acids	88 (72·3–100)	78·3 (58·7–99·8)	78·5 (60·5–99·9)	-11 (-27·1–0·917)	0·271 (-17·2–19·5)	89·8 (76·6–100)	82·2 (64–100)	82·6 (64·1–99·9)	-8·4 (-23·3–1·6)	0·482 (-14·1–17·8)
Diet low in polyunsaturated fatty acids	43·8 (11·8–87·1)	53·4 (15·5–95·2)	51·8 (14·5–94·3)	21·9 (2·45–54·6)	-2·99 (-9·72–0·201)	46·5 (12·9–89·5)	55·7 (16·7–96·3)	54·2 (16·1–95·4)	19·8 (2·05–49·2)	-2·72 (-9·35–0·00793)
Diet high in trans fatty acids	56·9 (46·9–98)	49·4 (39·8–95·9)	48·5 (38·7–95)	-13·2 (-22·8–-0·817)	-1·84 (-12–8·39)	57·3 (47·3–100)	49·8 (40·4–99·6)	49 (39·1–98·7)	-13 (-22·2–0)	-1·72 (-11·3–6·93)
Diet high in sodium	31·1 (6·16–57)	32·9 (6·66–59·7)	33·3 (6·2–59·8)	5·89 (-30·9–61·9)	1·26 (-20·9–31·5)	14·4 (3·24–36·6)	14·5 (3·35–36·8)	14·6 (3·32–36·9)	1·02 (-24·7–37·9)	0·796 (-16·1–21·3)
Low physical activity	3·01 (1·18–6·2)	3·14 (1·28–6·44)	3·63 (1·51–7·45)	4·59 (-21·5–55·1)	15·5 (-16·4–55·4)	5·4 (2·73–10·2)	4·78 (2·47–8·86)	6 (3·1–11·1)	-11·4 (-28·4–10·9)	25·4 (-0·218–57·1)
Occupational asthmagens	22·9 (19·4–27·5)	20·9 (17·8–24·6)	21·1 (17·8–25·4)	-8·88 (-17·5–-0·0753)	1·27 (-7·69–12·4)	--	--	--	--	--
Occupational particulate matter, gases, and fumes	9·26 (6·56–13·2)	8·74 (6·26–12·6)	8·27 (5·92–11·9)	-5·7 (-9·24–-1·82)	-5·32 (-7·93–-3·13)	4·97 (3·53–7·27)	4·57 (3·28–6·67)	4·28 (3·09–6·22)	-8·04 (-14·3–-1·11)	-6·37 (-9·49–-3·17)
Occupational noise	8·51 (8·04–9·19)	8·42 (8·01–9·06)	8·22 (7·82–8·88)	-1·15 (-3·32–1·15)	-2·32 (-3·34–-1·24)	6·08 (5·66–6·63)	6·28 (5·92–6·78)	6·27 (5·93–6·81)	3·2 (0·522–6·27)	-0·0834 (-1·3–1·13)
Occupational injuries	--	--	--	--	--	--	--	--	--	--
Occupational ergonomic factors	12·4 (10·7–14·3)	11·3 (9·83–13·2)	11·1 (9·54–13·1)	-8·64 (-14·1–-2·92)	-1·54 (-7·42–4·63)	8·32 (7·02–9·97)	8·96 (7·53–10·8)	8·69 (7·22–10·5)	7·71 (0·956–14·8)	-2·94 (-10·8–5·9)
Childhood sexual abuse	5·77 (3·78–8·2)	6 (3·99–8·37)	5·77 (3·78–8·2)	4 (-2·78–13·7)	-3·84 (-11·7–3·42)	6·68 (5–8·88)	6·81 (5·02–9·29)	6·68 (5–8·88)	1·97 (-4·8–11)	-1·93 (-9·19–5·54)
Intimate partner violence	--	--	--	--	--	21·5 (12–29·6)	21·2 (12·5–27·6)	21·5 (12–29·6)	-1·65 (-15·2–23·9)	1·68 (-13·1–28·9)
Non-exclusive breastfeeding	24·5 (19·8–30)	21·6 (17·8–26·3)	21·8 (18–26·5)	-12 (-22·9–0·89)	1·01 (-9·89–14·2)	24·5 (19·8–30)	21·6 (17·8–26·3)	21·8 (18–26·5)	-12 (-22·9–0·919)	0·994 (-9·9–14·2)
Discontinued breastfeeding	22·6 (21·3–23·9)	19 (17·5–20·6)	19·3 (17·8–20·9)	-16 (-23·8–-7·59)	1·69 (-6·99–11·1)	22·5 (21·3–23·8)	18·9 (17·4–20·5)	19·3 (17·8–20·8)	-16·1 (-23·9–-7·72)	2·03 (-6·68–11·5)
Diet low in calcium	17·6 (8·25–33·3)	10·8 (5·07–19·9)	10·7 (5·07–19·9)	-38·5 (-47·6–-30·7)	-1·34 (-7·84–4·65)	11·9 (4·71–22·2)	5·13 (2·09–9·16)	5·07 (2·04–9·28)	-56·9 (-67·1–-47·8)	-0·993 (-11·9–10·1)
Occupational exposure to asbestos	11·6 (10·6–12·7)	13·8 (12·9–14·7)	13·4 (10·7–16·3)	19·1 (6·94–32·7)	-2·67 (-23·2–19·7)	2·26 (1·79–4·06)	2·26 (1·8–3·26)	2·21 (1·51–3·12)	0·18 (-27·7–23·7)	-2·03 (-27·3–30·5)
Occupational exposure to arsenic	0·543 (0–1·28)	0·48 (0–1·14)	0·503 (0–1·19)	-11·4 (-19·2–NA)	4·75 (-3·61–NA)	0·53 (0–1·25)	0·475 (0–1·12)	0·471 (0–1·13)	-10·2 (-20–NA)	-0·953 (-8·62–NA)
Occupational exposure to benzene	0·835 (0·0492–2·4)	0·929 (0·0556–2·71)	0·962 (0·057–2·78)	11·3 (6·87–17·8)	3·52 (-0·76–9·58)	1·32 (0·0787–3·79)	1·53 (0·0924–4·37)	1·6 (0·0991–4·65)	15·9 (9·96–25·2)	4·54 (-0·716–11·8)
Occupational exposure to beryllium	0·0244 (0·024–0·0248)	0·0251 (0·0248–0·0254)	0·026 (0·0253–0·0267)	2·77 (0·703–4·9)	3·74 (0·899–6·83)	0·0289 (0·0283–0·0295)	0·031 (0·0306–0·0313)	0·0312 (0·0302–0·0321)	7·16 (4·87–9·56)	0·654 (-2·57–3·98)
Occupational exposure to cadmium	0·0626 (0·0575–0·0682)	0·0568 (0·0547–0·0587)	0·0594 (0·0555–0·0635)	-9·31 (-16·5–-0·504)	4·58 (-2·9–12·2)	0·0631 (0·0576–0·0695)	0·0585 (0·0561–0·0611)	0·0581 (0·0544–0·0626)	-7·3 (-16·4–1·91)	-0·707 (-7·39–6·33)
Occupational exposure to chromium	0·147 (0·136–0·158)	0·138 (0·133–0·142)	0·144 (0·136–0·152)	-6·32 (-12·8–1·41)	4·62 (-1·67–11·1)	--	--	--	--	--
Occupational exposure to diesel engine exhaust	0·705 (0·672–0·738)	0·77 (0·745–0·796)	0·795 (0·758–0·834)	9·27 (3·66–15·4)	3·29 (-1·4–8·03)	0·7 (0·658–0·745)	0·774 (0·738–0·808)	0·755 (0·711–0·804)	10·6 (3·25–18·8)	-2·36 (-8·21–4·29)
Occupational exposure to formaldehyde	0·312 (0·283–0·341)	0·276 (0·265–0·287)	0·286 (0·266–0·306)	-11·5 (-19·1–-2·45)	3·55 (-4·22–11·3)	0·309 (0·283–0·34)	0·291 (0·279–0·303)	0·293 (0·274–0·313)	-5·97 (-14·7–2·94)	0·629 (-5·64–7·27)
Occupational exposure to nickel	0·499 (0–2·22)	0·45 (0–1·98)	0·471 (0–2·1)	-9·81 (-17·1–NA)	4·79 (-3·2–NA)	0·423 (0–1·81)	0·367 (0–1·59)	0·361 (0–1·58)	-13·1 (-23·3–NA)	-1·65 (-10·2–NA)
Occupational exposure to polycyclic aromatic hydrocarbons	0·296 (0·277–0·317)	0·284 (0·276–0·292)	0·298 (0·283–0·313)	-4·1 (-10·2–2·92)	4·73 (-1·14–10·7)	0·33 (0·31–0·352)	0·329 (0·32–0·339)	0·334 (0·318–0·351)	-0·101 (-7·13–6·57)	1·33 (-3·65–6·44)
Occupational exposure to silica	5·5 (0·823–17·9)	5·36 (0·78–17·5)	5·61 (0·81–17·5)	-2·37 (-10·7–7·53)	4·47 (-3·47–13)	3·66 (0·527–11·9)	3·21 (0·458–10·3)	3·09 (0·42–9·39)	-12·3 (-22·8–-0·865)	-3·85 (-12·1–6·95)
Occupational exposure to sulfuric acid	0·853 (0·168–2·71)	0·761 (0·157–2·38)	0·786 (0·167–2·51)	-10·8 (-17·4–-2·49)	3·38 (-3·9–10·9)	0·753 (0·156–2·4)	0·687 (0·144–2·19)	0·669 (0·142–2·17)	-8·78 (-18·6–1·36)	-2·61 (-9·94–5·78)
Unsafe sex	--	--	--	--	--	--	--	--	--	--
Occupational exposure to trichloroethylene	0·0822 (0·0787–0·0864)	0·0805 (0·0786–0·0829)	0·0837 (0·08–0·0879)	-2·13 (-6·72–3·13)	4·03 (-0·716–8·95)	0·0911 (0·0866–0·0964)	0·0924 (0·0897–0·0953)	0·0917 (0·0877–0·0966)	1·47 (-4·15–7·25)	-0·747 (-5·51–4·41)
No access to handwashing facility	3·24 (3·08–3·42)	3·14 (2·97–3·31)	3·11 (2·94–3·3)	-3·22 (-8·89–3·64)	-0·784 (-5·02–3·45)	3·24 (3·08–3·42)	3·14 (2·97–3·31)	3·11 (2·94–3·3)	-3·22 (-8·89–3·64)	-0·784 (-5·02–3·45)
Child wasting	1·28 (0·908–1·68)	1·23 (0·873–1·62)	1·22 (0·872–1·6)	-3·85 (-11·5–4·54)	-0·78 (-6·75–6·03)	1·08 (0·751–1·44)	1·02 (0·705–1·36)	1·02 (0·703–1·37)	-5·32 (-14·1–4·08)	-0·57 (-7·5–6·15)
Child stunting	1·66 (0·817–2·47)	1·22 (0·573–1·83)	1·19 (0·562–1·84)	-26·8 (-39·1–-11·6)	-1·91 (-13·9–11)	1·96 (1·03–2·86)	1·6 (0·797–2·38)	1·56 (0·771–2·34)	-18·4 (-31·4–-3·63)	-2·5 (-13·6–9·54)
Chewing tobacco	0·267 (0·211–0·328)	0·283 (0·225–0·35)	0·309 (0·244–0·386)	6·1 (-17·9–36·7)	9·21 (-8·61–30·2)	--	--	--	--	--
Diet low in legumes	88·5 (73–100)	93·5 (84·6–100)	92·5 (82·1–100)	5·68 (0–16·5)	-1·12 (-3·57–0)	86·2 (68·1–100)	91·7 (80·5–100)	90·5 (77·8–100)	6·41 (0–19·2)	-1·27 (-4·28–0)
Short gestation	20·4 (15·8–26·2)	31·4 (24·9–39)	30·1 (23·2–39·1)	54·2 (12·4–108)	-4·42 (-26·1–25·1)	18·4 (14·9–22·6)	23·7 (19·6–28·3)	23·2 (18·7–28·5)	29 (0·371–65·2)	-2·07 (-21·3–20)
Low birth weight	6·11 (4·71–7·96)	9·34 (7·3–11·7)	8·78 (6·68–11·5)	52·7 (9·06–112)	-5·94 (-31–28·5)	5·56 (4·5–6·8)	6·97 (5·78–8·44)	6·96 (5·61–8·56)	25·2 (-3·72–62·6)	-0·0582 (-22·1–26·8)
High temperature	20·8 (6·79–36)	20·9 (7·34–35·9)	21·3 (8·09–35·9)	0·332 (-2·08–8·36)	2·08 (-1·92–11·6)	20·8 (6·79–36)	20·9 (7·34–35·9)	21·3 (8·09–35·9)	0·332 (-2·08–8·36)	2·08 (-1·92–11·6)
Low temperature	31 (26·8–36)	35·6 (32·3–39·7)	30·8 (26·9–35·8)	14·8 (9·41–20·8)	-13·4 (-17·1–-9·27)	31 (26·8–36)	35·6 (32·3–39·7)	30·8 (26·9–35·8)	14·8 (9·41–20·8)	-13·4 (-17·1–-9·27)
Kidney dysfunction	14·8 (9·96–21·4)	15·9 (10·9–22·5)	15·9 (11·1–22·5)	7·7 (3·53–13·7)	-0·119 (-4·63–4·49)	15·6 (10·5–22·1)	16·6 (11·4–23·3)	16·7 (11·4–23·3)	6·42 (2·66–11·4)	0·247 (-3·35–4·23)
Bullying victimization	5·43 (2·52–10·4)	5·57 (2·58–10·6)	5·17 (2·32–9·88)	2·54 (-8·75–14·3)	-7·14 (-15·3–1·8)	4·65 (2·15–8·88)	5·04 (2·35–9·43)	4·76 (2·18–9·04)	8·33 (-7·02–23·8)	-5·51 (-13·3–2·42)
High LDL cholesterol	58·7 (55·6–62·1)	45·6 (42·3–49·3)	45·5 (41·9–49·2)	-22·3 (-27–-17·4)	-0·217 (-4·33–4·26)	56·8 (53·8–59·9)	47·1 (43·8–50·7)	47·3 (43·7–50·7)	-17 (-21·3–-12·5)	0·403 (-3·24–3·94)

Supplementary Results Table 1. Finland age-standardised Summary Exposure Values (SEVs) for most-detailed risk factors and percentage change, by sex, 1990, 2010, and 2019. Data in parentheses are 95% uncertainty intervals.

	Male					Female				
	1990	2010	2019	Percentage change 1990-2010	Percentage change 2010-2019	1990	2010	2019	Percentage change 1990-2010	Percentage change 2010-2019
Unsafe sanitation	2·95 (1·6–4·72)	1·06 (0·58–1·71)	0·832 (0·47–1·36)	-64·1 (-81·6—28·7)	-21·4 (-50·2–25·5)	2·95 (1·6–4·72)	1·06 (0·58–1·71)	0·832 (0·47–1·36)	-64·1 (-81·6—28·7)	-21·4 (-50·2–25·5)
Ambient particulate matter pollution	6·38 (0·719–14·7)	4·31 (1·99–7·04)	2·21 (0·445–4·61)	-32·5 (-75·4–304)	-48·6 (-78·1—33·6)	6·38 (0·777–14·6)	4·31 (2·01–7·04)	2·22 (0·453–4·62)	-32·5 (-75·6–297)	-48·5 (-78—33·7)
Household air pollution from solid fuels	0·0668 (0·00956–0·196)	0·0163 (0·00278–0·0518)	0·00795 (0·000688–0·0321)	-75·5 (-91·4—19·5)	-51·3 (-82·8—15·7)	0·0885 (0·014–0·259)	0·0217 (0·00382–0·0703)	0·0106 (0·000971–0·0417)	-75·4 (-91·4—21·4)	-51·2 (-82·6—15·5)
Ambient ozone pollution	13 (4·47–24·7)	23·8 (8·75–44·1)	12·2 (3·88–24·6)	82·6 (62·4–115)	-48·9 (-61·4—39)	13 (4·47–24·7)	23·8 (8·75–44·1)	12·2 (3·88–24·6)	82·6 (62·4–115)	-48·9 (-61·4—39)
Residential radon	35·4 (14·1–67·2)	35·4 (14·1–67·2)	35·4 (14·1–67·2)	0 (0–0)	0 (0–0)	35·4 (14·1–67·2)	35·4 (14·1–67·2)	35·4 (14·1–67·2)	0 (0–0)	0 (0–0)
Lead exposure	6·74 (1·34e-12–27·8)	5·37 (1·27e-12–23·8)	4·08 (1·2e-12–20·4)	-20·3 (-76·4–156)	-24 (-81·6–195)	4·06 (1·05e-12–21·1)	2·96 (1·06e-12–17·6)	2·2 (8·25e-13–14·8)	-27 (-100–174)	-25·6 (-100–215)
Child underweight	0·92 (0·541–1·4)	0·78 (0·431–1·21)	0·778 (0·437–1·17)	-15·2 (-26·1—3·69)	-0·302 (-9·14–9·98)	1·1 (0·651–1·61)	0·962 (0·562–1·41)	0·953 (0·555–1·42)	-12·7 (-23·9—0·87)	-0·937 (-8·7–7·88)
Iron deficiency	--	--	--	--	--	12·1 (10·4–13·9)	10·5 (8·92–12·2)	10·2 (8·69–12)	-13·5 (-22·7—4·3)	-2·31 (-7·77–3·41)
Vitamin A deficiency	3·83 (1·56–7·72)	1·79 (0·712–3·72)	1·5 (0·62–3·02)	-53·3 (-81·2–19·5)	-15·9 (-51·8–56·5)	3·17 (1·64–5·38)	2·04 (1·07–3·48)	1·76 (0·891–3·02)	-35·7 (-66·8–26·6)	-13·6 (-43·2–40·5)
Zinc deficiency	3·04 (0–9·78)	2·57 (0–8·1)	2·46 (0–7·85)	-15·5 (-55·5–NA)	-4·2 (-18·6–NA)	3·04 (0–9·35)	2·5 (0–8·04)	2·41 (0–7·75)	-17·7 (-66·3–NA)	-3·63 (-19·5–NA)
Smoking	21·9 (19·2–24·8)	17·2 (15–19·7)	15·9 (13·9–18·3)	-21·7 (-26·5—16·5)	-7·38 (-10·6—4·12)	14·2 (12–16·8)	10·5 (8·98–12·2)	10·1 (8·71–11·9)	-26·1 (-33·3—18)	-3·72 (-9·09–1·5)
Secondhand smoke	32·7 (30·3–35·2)	29·7 (27·9–31·4)	29·2 (26·7–31·8)	-9·21 (-17·4–0·287)	-1·74 (-7·54–4·5)	33·8 (31·7–35·7)	28·7 (27·2–30·1)	28·1 (26·1–30)	-15·2 (-21·6—7·94)	-2·07 (-6·83–2·93)
Alcohol use	18 (13·1–23·9)	22 (15·9–29·3)	20·5 (14·8–27·7)	22·2 (10·3–39)	-7·16 (-16·6–2·25)	9·27 (5·88–13·9)	10·5 (6·92–15·2)	9·72 (6·19–14·6)	13·3 (0·557–30)	-7·46 (-21·1–7·42)
Drug use	0·233 (0·113–0·489)	0·328 (0·193–0·597)	0·379 (0·237–0·663)	40·6 (15·8–82·1)	15·5 (1·34–34·5)	0·13 (0·0601–0·278)	0·176 (0·101–0·331)	0·196 (0·118–0·352)	34·9 (12·1–74)	11·5 (-3·52–29·5)
High fasting plasma glucose	9·17 (8·05–10·4)	12·9 (11·1–15·2)	15 (12·8–17·4)	41·1 (24·3–62·3)	15·9 (1·08–34·7)	8·89 (7·67–10·4)	12·4 (10·7–14·5)	14·1 (12–16·4)	39·6 (23·3–57·9)	13·3 (-0·461–30·1)
High systolic blood pressure	54·2 (51·6–56·8)	39·5 (36·5–42·3)	40·6 (34·6–47·4)	-27 (-32·4—21·9)	2·64 (-11–18·6)	46·6 (44–48·9)	31·2 (28·3–34·3)	32·2 (25·2–40·2)	-33·1 (-39·3—26·5)	3·42 (-19·2–28·6)
High body-mass index	18·6 (13·3–24·9)	23·7 (17·4–31·4)	25·1 (18·4–33·1)	27·6 (15–45·4)	5·79 (-0·706–12·8)	18·8 (13·8–25·3)	26·4 (20·9–34·2)	27·5 (21·8–35·7)	40·6 (23·1–65)	4·17 (-3·63–13·8)
Low bone mineral density	9·78 (5·6–15·6)	8·82 (4·92–14·7)	9·02 (4·86–15·1)	-9·77 (-27·7–13·3)	2·22 (-22·1–33·8)	16·1 (11·6–22·5)	14 (9·66–19·9)	14·8 (9·57–21·9)	-12·8 (-27·4–3·35)	5·68 (-16·9–36·1)
Diet low in fruits	59·8 (49·1–72·6)	54·2 (42·8–67·4)	56·5 (44·8–69·3)	-9·35 (-14·7—5·25)	4·23 (-1·1–11·4)	61·7 (51·3–73·9)	55·8 (44·9–68·2)	58·4 (47·4–71·1)	-9·51 (-14·9—5·69)	4·65 (-0·534–11·3)
Diet low in vegetables	63·7 (42·8–82·4)	48·8 (28·1–71·1)	47·4 (26·9–70)	-23·4 (-38·9—10·5)	-2·89 (-10·3–2·89)	61·8 (40·8–81·7)	46·2 (26·1–68·7)	45·3 (25·8–66·3)	-25·3 (-41·2—11·2)	-1·83 (-10·4–5·37)
Diet low in whole grains	83·8 (76–91·9)	83 (74·7–91·3)	83·7 (75·8–91·7)	-0·987 (-1·95—0·253)	0·808 (-0·209–2·13)	--	--	--	--	--
Diet low in nuts and seeds	77·7 (41·1–89·9)	64 (31·6–81·5)	60·6 (29·3–79·1)	-17·6 (-33·6—7·29)	-5·37 (-15·7—0·611)	75·4 (38·8–89·1)	59·9 (28·5–78·6)	56·3 (24·7–76·4)	-20·5 (-38·9—9·74)	-6·05 (-17·8—0·26)
Diet low in milk	17·3 (4·81–35·7)	8·02 (1·42–23)	9·13 (1·63–24·6)	-53·7 (-81·6—29·8)	13·9 (-11·3–83·9)	16·1 (4·69–34·8)	7·35 (1·25–22·4)	8·34 (1·49–23·2)	-54·5 (-82·5—32·4)	13·6 (-14–95·6)
Diet high in red meat	80·3 (73–86·5)	69·8 (60·7–78·4)	74 (63·2–83·8)	-13·1 (-18·7—8·26)	5·96 (-3·48–16·1)	80·3 (72·9–86·9)	69·6 (60·3–78·4)	73·8 (63–82·9)	-13·3 (-19—8·54)	5·96 (-2·9–16·2)
Diet high in processed meat	63·1 (38·3–87·6)	77·7 (52·6–99)	70·5 (43·3–94·2)	23·2 (7·68–51·3)	-9·31 (-19·3—3·15)	69·1 (42·3–93·2)	79·9 (56–99·4)	74·7 (47·6–97·7)	15·7 (4·62–38)	-6·5 (-15·9—1·11)
Diet high in sugar-sweetened beverages	30·6 (16·9–46·6)	36·8 (22·5–53·2)	40·6 (25·7–57·6)	20·1 (-4·87–58·6)	10·4 (-8·01–39·3)	34·7 (19·6–52·1)	42·8 (27·1–59·2)	46·8 (29·3–63·9)	23·3 (-6·79–68·9)	9·32 (-9·98–37·3)
Diet low in fiber	40·4 (25·1–55·3)	30·6 (17·8–43·9)	25·9 (14·6–38·3)	-24·2 (-32·3—18·1)	-15·5 (-27·7—4·09)	40·4 (25–56·2)	30·8 (17·4–45)	26·6 (14·7–40·4)	-23·7 (-31·7—17·4)	-13·7 (-25·5—2·31)
Diet low in seafood omega-3 fatty acids	89·4 (75·2–100)	87·1 (70·8–100)	87·1 (71·8–100)	-2·59 (-14·4–5·5)	-0·0222 (-10·6–12)	91·5 (80·3–100)	89·3 (77·1–100)	89·1 (75·9–100)	-2·35 (-10·5–3·95)	-0·19 (-8·63–8·77)
Diet low in polyunsaturated fatty acids	82 (47·4–100)	78·6 (41·5–100)	76·6 (37·5–100)	-4·18 (-13·6–0)	-2·47 (-8·51–0)	83·1 (48·5–100)	79·8 (44–100)	78 (40·9–100)	-3·97 (-12·7–0)	-2·32 (-7·95–0)
Diet high in trans fatty acids	51·5 (41·1–99·9)	40·7 (30·9–98·9)	40 (30–97)	-21 (-32·3—0·981)	-1·69 (-11·1–9·35)	52·8 (42·2–100)	42·4 (31·3–100)	41·5 (30·8–100)	-19·8 (-30·1–0)	-1·98 (-9·24–6·71)
Diet high in sodium	36·7 (6·62–65·5)	34·3 (7·27–58·9)	36·9 (8·56–62·3)	-6·37 (-35·7–50)	7·37 (-12–49·7)	15·6 (3·49–39·2)	15·1 (3·6–36·9)	16 (3·71–38·9)	-3·1 (-25·8–31·2)	5·89 (-9·49–35·5)
Low physical activity	3·29 (1·34–6·73)	3·41 (1·57–6·65)	4 (1·68–8·11)	3·49 (-22·5–46·5)	17·4 (-13·9–55·7)	4·99 (2·5–9·57)	4·94 (2·5–9·29)	5·64 (2·91–10·8)	-1·07 (-20·7–25·1)	14·3 (-6·1–42·8)
Occupational asthmagens	21·4 (18·3–25·6)	20·5 (17·5–24·3)	19·9 (16·9–23·7)	-4·44 (-9·36–0·835)	-2·68 (-8·49–3·55)	--	--	--	--	--
Occupational particulate matter, gases, and fumes	9·69 (6·96–14)	8·68 (6·19–12·4)	8·25 (5·92–11·7)	-10·4 (-12·6—8·05)	-4·98 (-6·6—3·51)	5·91 (4·29–8·48)	4·49 (3·26–6·43)	4·01 (2·9–5·85)	-24 (-28·4—19·6)	-10·6 (-13·1—8·25)
Occupational noise	8·71 (8·27–9·36)	8·07 (7·7–8·72)	7·85 (7·47–8·48)	-7·39 (-8·6—5·93)	-2·76 (-3·48—2·09)	6·59 (6·24–7·18)	6·06 (5·76–6·62)	5·94 (5·63–6·49)	-8·1 (-9·46—6·68)	-1·99 (-2·77—1·17)
Occupational injuries	--	--	--	--	--	--	--	--	--	--
Occupational ergonomic factors	12·9 (11·2–14·9)	11·6 (10·2–13·4)	11·2 (9·57–13·1)	-10·2 (-17·5—2·72)	-3·07 (-9·66–4·56)	9·09 (7·66–10·7)	10 (8·64–11·8)	9·73 (8·18–11·7)	10·3 (-1·36–22·6)	-2·93 (-11·9–6·61)
Childhood sexual abuse	6·54 (4·31–9·15)	6·45 (4·2–9·05)	6·45 (4·2–9·05)	-1·3 (-6·84–5·33)	0 (0–0)	5·89 (4·41–8·02)	5·93 (4·37–8·18)	5·93 (4·37–8·18)	0·695 (-6·33–8·65)	0 (0–0)
Intimate partner violence	--	--	--	--	--	24·1 (13·7–32·6)	22 (13·2–28·3)	24·1 (13·7–32·6)	-8·53 (-20·4–15·7)	9·33 (-6·39–30·4)
Non-exclusive breastfeeding	23·4 (18·7–28·9)	22·8 (18·4–28·3)	22·7 (18·7–27·8)	-2·69 (-15·1–12·1)	-0·264 (-10·6–11·3)	--	--	--	--	--
Discontinued breastfeeding	21·8 (20·4–23·1)	21·3 (20–22·8)	20·3 (18·9–21·8)	-2·24 (-10·4–7·42)	-4·72 (-11·2–3·09)	21·8 (20·4–23·1)	21·4 (20–22·8)	20·3 (18·9–21·8)	-2·26 (-10·5–7·39)	-4·7 (-11·2–3·1)
Diet low in calcium	11·3 (5·38–20·9)	8·63 (4·17–15·6)	8·15 (4·05–14·8)	-23·8 (-28·9—18·9)	-5·49 (-11·8–2·66)	5·56 (2·2–9·94)	3·73 (1·62–6·61)	3·58 (1·59–6·45)	-32·8 (-39·8—23·9)	-3·99 (-14–7·68)
Occupational exposure to asbestos	10·9 (9·82–12·1)	11·7 (10·8–12·6)	10·4 (8·14–13·3)	7·59 (-4·14–21·3)	-11·2 (-31·6–14·3)	2·89 (1·83–3·87)	2·2 (1·54–2·8)	2·07 (1·35–2·86)	-24 (-41·7–4·97)	-5·61 (-29–24·1)
Occupational exposure to arsenic	0·486 (0–1·15)	0·502 (0–1·19)	0·495 (0–1·16)	3·29 (-0·931–NA)	-1·35 (-7·68–NA)	--	--	--	--	--
Occupational exposure to benzene	0·701 (0·041–1·99)	0·796 (0·0469–2·3)	0·812 (0·0471–2·3)	13·5 (9·79–19)	1·98 (-1·79–8·9)	1·24 (0·0731–3·52)	1·52 (0·0894–4·4)	1·56 (0·0912–4·52)	22·3 (17·1–30·6)	2·36 (-2·34–9·02)
Occupational exposure to beryllium	0·0215 (0·0212–0·0218)	0·0232 (0·023–0·0235)	0·0236 (0·0229–0·0242)	7·93 (5·89–9·95)	1·52 (-1·51–4·46)	0·0277 (0·0273–0·0281)	0·0286 (0·0283–0·029)	0·0292 (0·0284–0·03)	3·51 (1·74–5·51)	2·01 (-0·811–5·03)
Occupational exposure to cadmium	0·0558 (0·0544–0·0573)	0·0581 (0·0565–0·0596)	0·0576 (0·0543–0·0613)	4·09 (0·161–8·07)	-0·869 (-6·76–5·49)	0·0605 (0·0583–0·0629)	0·0547 (0·0527–0·057)	0·0546 (0·0513–0·0582)	-9·55 (-14·5—4·49)	-0·314 (-6·07–6·1)
Occupational exposure to chromium	0·129 (0·126–0·132)	0·137 (0·134–0·141)	0·137 (0·13–0·145)	6·42 (2·83–10·1)	-0·304 (-5·74–5·41)	--	--	--	--	--
Occupational exposure to diesel engine exhaust	0·611 (0·596–0·627)	0·698 (0·682–0·714)	0·716 (0·685–0·746)	14·1 (10·1–18·1)	2·57 (-1·86–7·21)	0·633 (0·612–0·655)	0·661 (0·639–0·686)	0·679 (0·638–0·724)	4·42 (-0·634–9·83)	2·73 (-3·05–9·38)
Occupational exposure to formaldehyde	0·276 (0·266–0·284)	0·282 (0·271–0·291)	0·276 (0·259–0·294)	2·2 (-1·71–6·13)	-2·13 (-8·38–4·43)	0·295 (0·283–0·306)	0·282 (0·27–0·292)	0·28 (0·264–0·297)	-4·43 (-8·98–0·221)	-0·492 (-5·55–4·82)
Occupational exposure to nickel	0·468 (0–2·05)	0·481 (0–2·12)	0·477 (0–2·14)	2·74 (-0·873–NA)	-0·746 (-6·52–NA)	0·409 (0–1·84)	0·341 (0–1·5)	0·336 (0–1·43)	-16·6 (-22·2–NA)	-1·46 (-8·68–NA)
Occupational exposure to polycyclic aromatic hydrocarbons	0·264 (0·258–0·27)	0·282 (0·276–0·288)	0·282 (0·269–0·296)	6·8 (3·62–9·96)	0·0451 (-4·9–5·21)	0·314 (0·306–0·322)	0·316 (0·308–0·324)	0·318 (0·305–0·332)	0·635 (-3·1–4·32)	0·723 (-3·4–5·07)
Occupational exposure to silica	5·42 (0·801–17·1)	5·77 (0·841–18·6)	5·79 (0·843–18·3)	6·54 (1·13–12·7)	0·275 (-6·05–7·55)	3·52 (0·509–11·2)	2·87 (0·414–8·78)	2·83 (0·382–8·73)	-18·4 (-24·8—12)	-1·57 (-9·53–8·06)
Occupational exposure to sulfuric acid	0·81 (0·167–2·52)	0·786 (0·16–2·48)	0·774 (0·159–2·42)	-2·87 (-6·93–1·44)	-1·6 (-7·38–4·74)	0·748 (0·152–2·34)	0·635 (0·13–2·01)	0·626 (0·131–2·01)	-15·1 (-20·3—9·02)	-1·42 (-8·3–5·7)
Unsafe sex	--	--	--	--	--	--	--	--	--	--
Occupational exposure to trichloroethylene	0·0703 (0·0691–0·0722)	0·0763 (0·0751–0·078)	0·0767 (0·0738–0·0803)	8·61 (5·5–11·8)	0·561 (-3·65–5·17)	0·0851 (0·0831–0·0876)	0·0839 (0·0821–0·0864)	0·085 (0·0814–0·0893)	-1·44 (-4·81–2·2)	1·26 (-2·96–6·16)
No access to handwashing facility	3·31 (3·13–3·51)	3·18 (3–3·36)	3·15 (2·96–3·33)	-4 (-10·4–2·4)	-1·03 (-5·08–3·73)	3·31 (3·13–3·51)	3·18 (3–3·36)	3·15 (2·96–3·33)	-4 (-10·4–2·4)	-1·03 (-5·08–3·73)
Child wasting	1·28 (0·905–1·69)	1·24 (0·874–1·63)	1·23 (0·867–1·63)	-3·34 (-11·3–4·89)	-0·26 (-6·48–6·1)	1·08 (0·738–1·44)	1·03 (0·703–1·37)	1·02 (0·7–1·36)	-4·99 (-14·3–4·74)	-0·379 (-6·9–6·78)
Child stunting	1·65 (0·819–2·44)	1·21 (0·557–1·85)	1·2 (0·562–1·85)	-26·8 (-39·3—11·5)	-0·49 (-12·4–12·4)	1·95 (1–2·88)	1·59 (0·808–2·39)	1·59 (0·792–2·38)	-18·5 (-32·3—3·73)	-0·123 (-11·5–12·8)
Chewing tobacco	0·249 (0·192–0·318)	0·294 (0·225–0·377)	0·351 (0·27–0·451)	17·8 (-13·3–57·8)	19·6 (-1·03–47·2)	--	--	--	--	--
Diet low in legumes	96·9 (92·3–100)	97·3 (93·1–100)	96 (89·8–100)	0·5 (0–1·48)	-1·37 (-3·8–0)	95·4 (88·7–100)	95·9 (89·9–100)	94·4 (86·7–100)	0·498 (0–1·52)	-1·48 (-4·11–0)
Short gestation	13·5 (10·4–17·4)	12·8 (10·4–15·5)	10·5 (8·44–12·9)	-5·19 (-30·4–26)	-18·4 (-35·8–3·81)	13·7 (11·4–16·5)	12·4 (10·4–14·7)	10·5 (8·94–12·4)	-10·2 (-27·1–11·8)	-15 (-30·1–4)
Low birth weight	6·03 (4·81–7·44)	6·06 (4·96–7·26)	5·18 (4·19–6·27)	0·544 (-26·1–33·2)	-14·5 (-34·2–8·89)	6·16 (5·13–7·33)	5·85 (4·93–6·92)	5 (4·22–5·9)	-5 (-25·4–20·3)	-14·6 (-31·1–4·82)
High temperature	17·5 (5·17–31·7)	21·9 (8·2–37·2)	19·8 (6·83–34·6)	25·2 (-4·23–79)	-9·57 (-23·3–2·29)	17·5 (5·17–31·7)	21·9 (8·2–37·2)	19·8 (6·83–34·6)	25·2 (-4·23–79)	-9·57 (-23·3–2·29)
Low temperature	37·1 (31·4–43)	38·8 (33·3–44·7)	36·5 (31–42·6)	4·57 (-3·01–14·6)	-5·9 (-12·9–0·312)	37·1 (31·4–43)	38·8 (33·3–44·7)	36·5 (31–42·6)	4·57 (-3·01–14·6)	-5·9 (-12·9–0·312)
Kidney dysfunction	13·3 (8·56–19·9)	13·7 (9·06–20·1)	13·7 (8·96–20·1)	3·02 (-0·189–7·47)	-0·563 (-4·25–2·83)	14·6 (9·47–21·1)	15·4 (10·4–22·1)	15·8 (10·6–22·4)	5·65 (1·88–11·3)	2·75 (-0·669–7·07)
Bullying victimization	5·17 (2·32–9·91)	6·21 (2·94–11·6)	7·05 (3·38–13·1)	20·3 (3·44–38·7)	13·5 (6·06–29·7)	3·31 (1·38–6·68)	4·72 (2·2–8·89)	5·77 (2·82–10·6)	42·5 (17–84·8)	22·2 (11·3–45·5)
High LDL cholesterol	57·3 (54·5–60·2)	43·3 (40–46·8)	43·5 (40–47)	-24·4 (-28·6—20·1)	0·3 (-3·72–4·62)	54·3 (51·2–57·7)	39·9 (36·5–43·4)	39·9 (36·7–43·3)	-26·6 (-30·9—22·4)	0·0855 (-3·26–3·9)

Supplementary Results Table 1. France age-standardised Summary Exposure Values (SEVs) for most-detailed risk factors and percentage change, by sex, 1990, 2010, and 2019. Data in parentheses are 95% uncertainty intervals.

	Male					Female				
	1990	2010	2019	Percentage change 1990-2010	Percentage change 2010-2019	1990	2010	2019	Percentage change 1990-2010	Percentage change 2010-2019
Unsafe sanitation	3·37 (1·94–5·65)	1·33 (0·752–2·2)	1·04 (0·57–1·73)	-60·4 (-79·8—22·4)	-22·1 (-51·3–23·6)	3·37 (1·94–5·65)	1·33 (0·752–2·2)	1·04 (0·57–1·73)	-60·4 (-79·8—22·4)	-22·1 (-51·3–23·6)
Ambient particulate matter pollution	17·7 (7·03–31·9)	14·1 (11–17·8)	9·69 (6·9–12·9)	-20·4 (-56·5–87·2)	-31·3 (-37·8—26·6)	17·7 (7–31·8)	14·1 (11–17·7)	9·69 (6·91–12·9)	-20·4 (-56·3–87·5)	-31·3 (-37·7—26·6)
Household air pollution from solid fuels	0·084 (0·0233–0·213)	0·0229 (0·00561–0·0637)	0·0147 (0·00306–0·0415)	-72·8 (-87—44·1)	-35·8 (-60·4—1·54)	0·11 (0·0331–0·271)	0·0301 (0·008–0·0842)	0·0194 (0·0042–0·0564)	-72·5 (-87—44)	-35·5 (-60·2—1·18)
Ambient ozone pollution	53·1 (22·8–88·4)	46·8 (19·5–80·1)	44·3 (17·8–75·8)	-11·9 (-19—5·57)	-5·25 (-9·82—1·1)	53·1 (22·8–88·4)	46·8 (19·5–80·1)	44·3 (17·8–75·8)	-11·9 (-19—5·57)	-5·25 (-9·82—1·1)
Residential radon	22·4 (10–41)	22·4 (10–41)	22·4 (10–41)	0 (0–0)	0 (0–0)	22·4 (10–41)	22·4 (10–41)	22·4 (10–41)	0 (0–0)	0 (0–0)
Lead exposure	66·7 (44·2–83·2)	45·3 (26·1–64·6)	32·7 (16·3–52)	-32·1 (-43·8—21·1)	-27·7 (-38·2—18·9)	47·8 (20·3–70·6)	32·9 (12–55)	23·1 (6·45–43·9)	-31 (-46·1—20·3)	-30 (-47·9—19·8)
Child underweight	0·926 (0·533–1·38)	0·771 (0·436–1·18)	0·758 (0·428–1·14)	-16·7 (-28—3·83)	-1·68 (-10·6–8·55)	1·07 (0·624–1·56)	0·921 (0·522–1·34)	0·903 (0·508–1·36)	-13·8 (-23·8—2·16)	-1·94 (-10·8–7·45)
Iron deficiency	--	--	--	--	--	9·47 (7·94–11·1)	8·47 (6·91–10·1)	8·31 (6·81–9·95)	-10·6 (-18·2—2·46)	-1·85 (-7·17–3·59)
Vitamin A deficiency	0·646 (0·253–1·44)	0·512 (0·178–1·13)	0·429 (0·165–0·929)	-20·7 (-68·3–122)	-16·2 (-54·7–50·7)	0·606 (0·304–1·04)	0·607 (0·309–1·08)	0·515 (0·259–0·898)	0·12 (-49·4–103)	-15 (-44·7–32·5)
Zinc deficiency	1·89 (0–6·13)	2·14 (0–6·99)	2·05 (0–6·64)	12·9 (-1·75–NA)	-3·83 (-23·3–NA)	1·85 (0–6·07)	2·07 (0–6·61)	1·99 (0–6·38)	11·9 (-2·13–NA)	-3·99 (-23·2–NA)
Smoking	25·3 (22·3–28·7)	19·5 (16·9–22·3)	18·2 (15·7–21)	-23·1 (-28·3—17·7)	-6·65 (-10·5—2·58)	12·6 (10·5–14·9)	12·5 (10·5–14·7)	12·7 (10·7–14·9)	-0·467 (-10·5–9·64)	1·19 (-4·48–6·79)
Secondhand smoke	42·3 (40·9–43·7)	36 (34·4–37·5)	34·3 (31·8–36·7)	-15 (-19·7—10·2)	-4·75 (-9·69–0·434)	46·8 (45·5–48)	36·1 (34·8–37·2)	34·2 (32·2–36·1)	-22·8 (-26·5—19·3)	-5·18 (-8·94—1·29)
Alcohol use	22·9 (17·2–29·6)	22·8 (16·1–30·7)	22·5 (15·8–30·3)	-0·397 (-11·1–11·5)	-1·31 (-12·6–9·03)	12·1 (8·39–16·9)	11 (7·4–15·5)	10·9 (6·86–16·1)	-8·74 (-19·4–3·4)	-1·3 (-17·4–16·4)
Drug use	0·141 (0·0884–0·235)	0·19 (0·131–0·278)	0·198 (0·133–0·298)	35·1 (14·9–67·1)	4·06 (-7·72–15·1)	0·096 (0·0628–0·148)	0·23 (0·157–0·287)	0·176 (0·128–0·236)	140 (68·1–241)	-23·4 (-40·6–2)
High fasting plasma glucose	4·63 (3·94–5·5)	5·6 (4·93–6·39)	6·53 (5·67–7·46)	20·8 (9·83–32·9)	16·7 (7·46–27·7)	3·66 (3·05–4·41)	4·98 (4·3–5·76)	5·74 (4·94–6·66)	36 (23·5–51·4)	15·2 (5·6–26·1)
High systolic blood pressure	35·4 (31·5–39·4)	33 (28·7–37·3)	35·1 (28·6–42·4)	-6·7 (-19·8–8·26)	6·3 (-9·36–22·9)	28·9 (25·1–33·1)	20 (16·3–24·4)	22·8 (16·4–30·4)	-30·9 (-45·6—14)	14·1 (-12·8–46·6)
High body-mass index	16 (11·6–21·8)	21 (15·6–27·3)	23 (17·3–30·2)	30·8 (17·7–50·7)	9·8 (3·2–19·1)	15·2 (9·65–21·6)	25·4 (19·5–32·9)	27·8 (21·4–36)	67·7 (37·1–128)	9·23 (-3·08–24·7)
Low bone mineral density	6·85 (3·54–12·1)	6·35 (2·97–11·1)	6·15 (2·92–10·9)	-7·31 (-30·8–23·3)	-3·2 (-27·7–35)	12·8 (8·12–19·1)	11·3 (6·79–17·7)	11·2 (6·67–17·2)	-11·6 (-35·7–16·5)	-0·983 (-24·1–25·5)
Diet low in fruits	53·5 (42·1–67·1)	57·1 (46·3–69·9)	56·1 (44·5–69·4)	6·59 (3·3–11·4)	-1·71 (-8·33–3·56)	54·6 (43·2–67·5)	59 (48·3–71·7)	57·7 (46·7–70·5)	8·13 (4·3–14·1)	-2·22 (-8·08–2·79)
Diet low in vegetables	41·2 (22·7–62·8)	50·7 (29·8–73)	50·1 (29·4–72·7)	23 (7·85–48·1)	-1·13 (-7·49–4·9)	38·5 (20·7–60·4)	48·5 (28·1–71·1)	47·8 (26·8–70·3)	25·9 (8·3–53·8)	-1·51 (-8·68–5·42)
Diet low in whole grains	94·2 (90·7–97·8)	97·4 (95·6–99·6)	97·4 (95·4–99·6)	3·48 (1·32–6·21)	-0·0206 (-0·701–0·681)	--	--	--	--	--
Diet low in nuts and seeds	53 (23·5–73)	53 (23·4–72·4)	50·1 (21·8–70·6)	-0·14 (-3·87–4·77)	-5·32 (-16·1—2·11)	48 (19·7–68·9)	47·9 (19·5–68·9)	45·1 (18·1–67·2)	-0·111 (-5·2–5·92)	-5·99 (-18·8—2·96)
Diet low in milk	60·7 (34·1–81·9)	56·4 (30·9–77·5)	56·7 (31·9–78)	-7·07 (-19·2—0·941)	0·476 (-4·22–6·76)	60·1 (33·6–81·2)	55·8 (30·9–76·4)	56·2 (30·9–76·9)	-7·13 (-20·4—1)	0·652 (-3·79–6·81)
Diet high in red meat	98·6 (97·7–99·2)	94·4 (91·4–96·6)	93·4 (88·1–96·9)	-4·26 (-6·74—2·45)	-1·05 (-5·38–2·29)	98·6 (97·7–99·2)	94·4 (91·7–96·5)	93·2 (87·8–96·9)	-4·22 (-6·65—2·35)	-1·23 (-5·7–2·1)
Diet high in processed meat	75 (49·7–97·2)	78·7 (53·9–99·5)	78·6 (53·6–99·3)	4·82 (0·979–12·1)	-0·0683 (-2·05–2·3)	78 (52·9–98·9)	81 (58·5–99·9)	81·1 (58·6–99·7)	3·9 (0·476–11·8)	0·0997 (-2–2·68)
Diet high in sugar-sweetened beverages	34·2 (20·4–49·9)	35·2 (21·7–51·5)	39·9 (24·3–56·6)	2·97 (-22·2–32·8)	13·6 (-7·8–46·7)	37·9 (22·1–55·3)	41 (25·9–58)	46·2 (28·3–63·6)	8·27 (-17·3–45·5)	12·7 (-9·41–40·8)
Diet low in fiber	41·1 (26·1–56·5)	42·2 (26·8–57·5)	40·8 (25·4–56)	2·62 (-1·56–7·44)	-3·31 (-12·6–5·58)	41 (25·4–56·5)	42·2 (26·3–58·1)	40·8 (24·5–57·3)	3·01 (-1·39–8·08)	-3·35 (-13·1–5·89)
Diet low in seafood omega-3 fatty acids	85·8 (68·8–100)	86·8 (71·1–99·9)	86·7 (72·6–100)	1·18 (-8·73–15·7)	-0·149 (-9·57–10·7)	88 (74·1–100)	88·9 (76·4–100)	88·7 (76·5–100)	1·05 (-6·21–12)	-0·221 (-8·57–8·29)
Diet low in polyunsaturated fatty acids	60·1 (19·8–98·7)	41·3 (10·5–85·1)	39·1 (10·5–83·1)	-31·3 (-55·9—4·63)	-5·36 (-14–0·318)	62·1 (22·1–99·7)	44·1 (11·9–87·5)	41·9 (11·2–85·7)	-29 (-54·8—4·5)	-4·9 (-12·9–0·383)
Diet high in trans fatty acids	51·6 (41·8–97·6)	45·3 (36·3–92·4)	44·8 (35·1–92)	-12·2 (-22·8–0)	-1·16 (-11·2–9·61)	52·1 (42–99·6)	45·9 (36·3–97·2)	45·3 (35·4–97·5)	-11·9 (-21·2—0·996)	-1·26 (-10·1–8·53)
Diet high in sodium	19 (4·06–43)	24·4 (5·03–49·3)	26·7 (5·53–52·1)	28·6 (-3·37–144)	9·6 (-16–49·7)	11·6 (3–31·6)	13·1 (3·34–33·4)	13·9 (3·49–34·9)	12·4 (-11·8–62·1)	6·58 (-6·85–34·1)
Low physical activity	5·56 (2·54–11·2)	5·87 (2·83–11·8)	6·47 (2·98–12·6)	5·49 (-19·6–48·6)	10·2 (-17·4–45·1)	8·55 (4·76–14·9)	9·15 (5·3–15·6)	9·35 (5·28–15·8)	7 (-11·7–32·9)	2·15 (-15·8–23·5)
Occupational asthmagens	24·4 (20–29·5)	20·5 (17·2–24·6)	19·4 (16·2–23·2)	-15·9 (-24·6—6·11)	-5·65 (-13–2·59)	--	--	--	--	--
Occupational particulate matter, gases, and fumes	7·75 (5·5–11·3)	7·25 (5·16–10·4)	6·97 (4·98–10·1)	-6·48 (-10·5—2·83)	-3·88 (-5·82—1·82)	3·2 (2·26–4·68)	3·06 (2·14–4·51)	2·95 (2·07–4·29)	-4·46 (-10·6–1·6)	-3·46 (-6·59—0·167)
Occupational noise	7·18 (6·78–7·75)	7·03 (6·66–7·64)	6·92 (6·56–7·53)	-2·06 (-3·83—0·208)	-1·63 (-2·44—0·849)	3·92 (3·67–4·25)	4·38 (4·15–4·71)	4·59 (4·35–4·92)	11·7 (9–14·7)	4·71 (3·69–5·86)
Occupational injuries	--	--	--	--	--	--	--	--	--	--
Occupational ergonomic factors	12 (10·5–13·8)	10·7 (9·44–12·3)	10·4 (8·87–12·2)	-10·3 (-16·3—3·57)	-3·09 (-10·4–5·05)	7·03 (5·99–8·26)	7·34 (6·28–8·71)	7·63 (6·47–9·2)	4·41 (-4·77–13·3)	3·98 (-3·76–12·4)
Childhood sexual abuse	4·2 (2·67–5·88)	4·17 (2·7–5·93)	4·17 (2·7–5·93)	-0·623 (-7·86–7·19)	0 (0–0)	4·63 (3·45–6·37)	4·96 (3·69–6·84)	4·96 (3·69–6·84)	7·09 (-2·88–21·3)	0 (0–0)
Intimate partner violence	--	--	--	--	--	16·5 (8·93–23·9)	16·5 (9·6–21·6)	16·5 (8·93–23·9)	0·0281 (-15·5–23·9)	-0·0281 (-14·9–25·7)
Non-exclusive breastfeeding	20·9 (16·7–25·7)	20·1 (16·1–25·1)	19·8 (15·9–24·7)	-3·78 (-15–8·93)	-1·47 (-9·89–8·64)	--	--	--	--	--
Discontinued breastfeeding	27·4 (26·8–28)	27·5 (26·9–28·1)	27 (26·4–27·6)	0·395 (-2·63–3·44)	-1·94 (-4·35–0·515)	27·4 (26·8–28)	27·5 (26·9–28·1)	27 (26·4–27·6)	0·479 (-2·55–3·52)	-2·04 (-4·45–0·414)
Diet low in calcium	12·7 (5·83–23·7)	15·7 (7·18–29·8)	14·9 (6·69–27·8)	24 (16–33·5)	-5 (-11·5—0·536)	6·83 (2·64–12·4)	9·76 (3·71–17·9)	9·1 (3·35–16·7)	42·9 (28·7–61·8)	-6·78 (-15·6—0·706)
Occupational exposure to asbestos	13 (11·9–14·2)	13·9 (13·1–14·6)	15·6 (12·3–19·3)	7·17 (-2·8–18·8)	12·2 (-10·6–38·9)	2·39 (1·74–3·25)	2·54 (1·51–2·89)	2·37 (1·32–3·25)	6·31 (-29·2–35·4)	-6·9 (-30·1–21·1)
Occupational exposure to arsenic	0·474 (0–1·13)	0·442 (0–1·05)	0·441 (0–1·04)	-6·75 (-13·4–NA)	-0·28 (-8·13–NA)	0·376 (0–0·892)	0·383 (0–0·897)	0·382 (0–0·894)	1·87 (-5·52–NA)	-0·257 (-7·49–NA)
Occupational exposure to benzene	0·744 (0·0449–2·1)	0·781 (0·0465–2·24)	0·789 (0·0458–2·26)	4·99 (0·423–11·3)	1·09 (-3·7–7·11)	0·963 (0·0556–2·74)	1·3 (0·0749–3·77)	1·35 (0·0789–3·92)	35 (28·3–45·4)	3·76 (-1·21–10·6)
Occupational exposure to beryllium	0·0211 (0·0208–0·0214)	0·0213 (0·0211–0·0215)	0·0215 (0·021–0·0219)	1·06 (-0·382–2·59)	0·783 (-1·71–3·13)	0·0212 (0·0209–0·0215)	0·0247 (0·0244–0·0249)	0·0252 (0·0245–0·0258)	16·2 (14·3–18·3)	2·09 (-0·816–5·01)
Occupational exposure to cadmium	0·055 (0·0518–0·0582)	0·0516 (0·0498–0·0536)	0·0515 (0·0484–0·0547)	-6·12 (-12·1—0·0163)	-0·23 (-7·1–6·91)	0·0454 (0·0427–0·0484)	0·0469 (0·0454–0·0485)	0·047 (0·0443–0·0498)	3·4 (-3·14–10·6)	0·0501 (-6·19–7·03)
Occupational exposure to chromium	0·13 (0·124–0·137)	0·124 (0·12–0·128)	0·124 (0·118–0·131)	-4·94 (-9·93–0·396)	0·103 (-5·89–6·23)	--	--	--	--	--
Occupational exposure to diesel engine exhaust	0·638 (0·61–0·664)	0·656 (0·635–0·679)	0·662 (0·633–0·695)	2·85 (-1·94–8·05)	0·96 (-3·4–5·81)	0·564 (0·532–0·596)	0·577 (0·546–0·61)	0·583 (0·545–0·618)	2·28 (-4·64–9·68)	0·994 (-4·87–7·64)
Occupational exposure to formaldehyde	0·276 (0·259–0·293)	0·253 (0·241–0·264)	0·25 (0·234–0·265)	-8·45 (-14·2—2·44)	-1·31 (-8·29–6)	0·221 (0·208–0·235)	0·24 (0·232–0·247)	0·241 (0·228–0·255)	8·63 (1·96–15·5)	0·538 (-4·95–6·74)
Occupational exposure to nickel	0·431 (0–1·88)	0·421 (0–1·83)	0·419 (0–1·81)	-2·33 (-8·65–NA)	-0·527 (-6·86–NA)	0·29 (0–1·34)	0·294 (0–1·27)	0·291 (0–1·31)	1·37 (-6·98–NA)	-1·04 (-8·31–NA)
Occupational exposure to polycyclic aromatic hydrocarbons	0·263 (0·252–0·275)	0·256 (0·25–0·263)	0·257 (0·246–0·269)	-2·55 (-6·91–2·12)	0·342 (-4·83–5·54)	0·239 (0·229–0·25)	0·27 (0·265–0·276)	0·274 (0·263–0·286)	13·2 (8·25–18·3)	1·49 (-2·77–6·23)
Occupational exposure to silica	4·81 (0·714–15·5)	5·11 (0·756–16)	5·11 (0·753–16·3)	6·07 (-2·31–16·5)	0·12 (-6·84–8·31)	2·48 (0·36–7·76)	2·55 (0·369–8)	2·51 (0·351–7·7)	3·03 (-6·52–14·6)	-1·91 (-9·86–7·37)
Occupational exposure to sulfuric acid	0·769 (0·162–2·37)	0·7 (0·145–2·22)	0·693 (0·147–2·25)	-9·06 (-14·7—2·89)	-0·952 (-7·92–6·1)	0·562 (0·116–1·78)	0·546 (0·115–1·73)	0·539 (0·11–1·68)	-2·94 (-10·4–5·08)	-1·31 (-8·3–7·07)
Unsafe sex	--	--	--	--	--	--	--	--	--	--
Occupational exposure to trichloroethylene	0·0735 (0·0713–0·0762)	0·0701 (0·0685–0·0721)	0·0703 (0·0676–0·0735)	-4·73 (-8·38—0·77)	0·348 (-3·98–4·75)	0·0695 (0·067–0·0725)	0·0723 (0·0704–0·0748)	0·0729 (0·0699–0·0765)	4 (-0·563–9·03)	0·879 (-3·88–5·93)
No access to handwashing facility	3·43 (3·24–3·63)	3·26 (3·07–3·45)	3·21 (3·03–3·4)	-5·03 (-11·6–1·36)	-1·42 (-5·23–2·73)	3·43 (3·24–3·63)	3·26 (3·07–3·45)	3·21 (3·03–3·4)	-5·03 (-11·6–1·36)	-1·42 (-5·23–2·73)
Child wasting	1·25 (0·879–1·65)	1·2 (0·85–1·6)	1·19 (0·833–1·58)	-3·56 (-11·2–5·14)	-0·851 (-7·19–5·12)	1·05 (0·728–1·41)	1 (0·691–1·34)	0·999 (0·691–1·34)	-4·7 (-13·2–4·16)	-0·434 (-5·65–5·3)
Child stunting	1·67 (0·85–2·47)	1·21 (0·576–1·84)	1·19 (0·558–1·82)	-27·8 (-40—12·6)	-1·57 (-13·4–12)	1·96 (1·01–2·86)	1·58 (0·797–2·37)	1·54 (0·766–2·31)	-19·4 (-33·5—5·2)	-2·2 (-13·1–9·82)
Chewing tobacco	0·148 (0·117–0·185)	0·147 (0·117–0·184)	0·15 (0·118–0·19)	-0·56 (-24·1–31·8)	2·43 (-14·7–21·5)	--	--	--	--	--
Diet low in legumes	71·1 (30·6–98·9)	76·6 (44·9–99·8)	76·9 (45·6–99·8)	7·77 (0·28–44·3)	0·356 (-2·53–5·61)	67·9 (25·9–97·7)	73·6 (37·8–99·6)	73·8 (38–99·5)	8·41 (0·291–49·8)	0·263 (-4·06–5·12)
Short gestation	11·7 (9·79–13·9)	7·02 (5·92–8·17)	7·48 (6·09–9·17)	-40·2 (-50·8—28·4)	6·52 (-13·2–31·9)	11·2 (9·4–13·3)	6·81 (5·75–8·01)	7·15 (5·94–8·46)	-39·2 (-49·7—25·3)	5·09 (-13·6–30·8)
Low birth weight	5·11 (4·29–6·08)	3·19 (2·74–3·7)	3·27 (2·69–3·95)	-37·6 (-49·7—23·4)	2·67 (-17·6–29·4)	4·98 (4·23–5·85)	3·06 (2·58–3·61)	3·1 (2·55–3·72)	-38·5 (-51·8—23)	1·31 (-19·4–27·8)
High temperature	25·3 (17·3–35·3)	23·4 (15·9–33·8)	26·9 (18·6–37·5)	-7·77 (-14·8–0·33)	15·3 (4·14–27·8)	25·3 (17·3–35·3)	23·4 (15·9–33·8)	26·9 (18·6–37·5)	-7·77 (-14·8–0·33)	15·3 (4·14–27·8)
Low temperature	31·6 (28–36)	33·9 (30·5–37·7)	31·6 (27·8–36·1)	7·25 (4·87–9·82)	-6·77 (-8·99—4·68)	31·6 (28–36)	33·9 (30·5–37·7)	31·6 (27·8–36·1)	7·25 (4·87–9·82)	-6·77 (-8·99—4·68)
Kidney dysfunction	13·9 (9·18–20·4)	14 (9·52–20·3)	14·5 (9·76–21·1)	1·24 (-4·09–6·87)	3·43 (-1·1–9·07)	14·5 (9·67–20·9)	9·02 (7·53–10·9)	15·6 (10·7–22·2)	-37·8 (-59—4·38)	72·8 (14·2–156)

Bullying victimization	7·38<br>(2·84–15·2)	8·69<br>(3·98–16·3)	9·22<br>(4·25–17·3)	17·6<br>(-5·17–74·9)	6·16<br>(-0·217–15·7)	6·94<br>(2·74–14·3)	8·07<br>(3·82–15·1)	8·47<br>(3·97–15·8)	16·3<br>(-10·2–87·4)	4·96<br>(-0·636–11·5)
High LDL cholesterol	57·4<br>(54·3–60·9)	49·9<br>(46·7–53·4)	49·9<br>(46·6–53·3)	-13·1<br>(-17·8–-8·29)	0·0525<br>(-3·76–4·24)	54<br>(50·7–57·4)	47·3<br>(44·2–50·6)	47·9<br>(44·5–51·4)	-12·3<br>(-16·7–-7·8)	1·15<br>(-2·22–4·73)

Supplementary Results Table 1. Germany age-standardised Summary Exposure Values (SEVs) for most-detailed risk factors and percentage change, by sex, 1990, 2010, and 2019. Data in parentheses are 95% uncertainty intervals.										
	Male					Female				
	1990	2010	2019	Percentage change 1990-2010	Percentage change 2010-2019	1990	2010	2019	Percentage change 1990-2010	Percentage change 2010-2019
Unsafe sanitation	1·64 (0·948–2·69)	0·661 (0·382–1·07)	0·546 (0·304–0·937)	-59·7 (-79—20·9)	-17·3 (-48·9–31·9)	--	--	--	--	--
Ambient particulate matter pollution	19·9 (8·18–35)	15·3 (12·2–19·1)	10·2 (7·4–13·5)	-23·2 (-56·8–71·5)	-33·4 (-39·7—28·7)	19·9 (8·19–35)	15·3 (12·2–19·1)	10·2 (7·41–13·5)	-23·2 (-56·8–71·2)	-33·4 (-39·7—28·7)
Household air pollution from solid fuels	0·0193 (0·00447–0·0543)	0·00845 (0·00165–0·024)	0·00605 (0·00113–0·0173)	-56·3 (-78·7—12·6)	-28·3 (-56·9–12·6)	0·0253 (0·00628–0·0731)	0·0112 (0·00239–0·0334)	0·00803 (0·00155–0·0245)	-56 (-78·7—12·5)	-28 (-56·7–13·1)
Ambient ozone pollution	31·3 (12·4–55·3)	39·2 (15·4–68·7)	40·4 (16·3–70·1)	25·5 (20·4–32·1)	2·92 (-0·327–7·29)	31·3 (12·4–55·3)	39·2 (15·4–68·7)	40·4 (16·3–70·1)	25·5 (20·4–32·1)	2·92 (-0·327–7·29)
Residential radon	23·6 (11–46·1)	23·6 (11–46·1)	23·6 (11–46·1)	0 (0–0)	0 (0–0)	23·6 (11–46·1)	23·6 (11–46·1)	23·6 (11–46·1)	0 (0–0)	0 (0–0)
Lead exposure	30·4 (0·743–58)	24·5 (2·81–49·4)	17·8 (1·33–40·8)	-19·5 (-31·2–377)	-27·1 (-53·8—16·9)	13 (2·36e-12–38·7)	11·7 (2·43e-12–35)	8·95 (2·42e-12–29·6)	-10·6 (-50·3–7·89e+10)	-23·2 (-71·9–107)
Child underweight	1·05 (0·619–1·53)	0·907 (0·51–1·35)	0·896 (0·519–1·33)	-13·6 (-24·4—1·51)	-1·18 (-9·68–8·13)	1·11 (0·629–1·59)	0·912 (0·511–1·35)	0·901 (0·512–1·33)	-17·6 (-27·6—5·95)	-1·18 (-9·7–8·79)
Iron deficiency	--	--	--	--	--	10·9 (9·09–12·7)	9·8 (8·2–11·5)	9·65 (8·03–11·4)	-9·79 (-18·7—0·0663)	-1·49 (-7·01–4·18)
Vitamin A deficiency	0·944 (0·373–1·96)	0·856 (0·345–1·75)	0·728 (0·282–1·47)	-9·32 (-62·8–137)	-15 (-52·5–57·3)	0·782 (0·385–1·34)	0·932 (0·461–1·61)	0·814 (0·368–1·4)	19·3 (-41·9–155)	-12·6 (-43·7–40·7)
Zinc deficiency	3·2 (0–10)	2·86 (0–9·13)	2·47 (0–8·02)	-10·7 (-33·6–NA)	-13·5 (-52·8–NA)	3·12 (0–9·77)	2·77 (0–8·68)	2·4 (0–7·87)	-11·3 (-36·3–NA)	-13·2 (-40·8–NA)
Smoking	26·3 (23·4–29·7)	22·2 (19·7–25)	20 (17·6–22·6)	-15·6 (-21·7—8·92)	-10·1 (-13·6—6·59)	17 (14·5–20)	14·2 (12·2–16·5)	12·9 (10·9–15)	-16·5 (-24·8—7·6)	-9·33 (-13·6—4·79)
Secondhand smoke	40 (37·1–42·7)	35 (33–37·2)	33·8 (31·4–36·3)	-12·5 (-20·3—3·66)	-3·5 (-9·06–1·83)	40·2 (37·8–42·4)	33·6 (32–35·1)	32·6 (30·6–34·8)	-16·4 (-22·3—9·91)	-2·97 (-7·11–1·45)
Alcohol use	23 (17·1–29·3)	24·5 (17·4–33·1)	24·9 (17·6–33·6)	6·52 (-5·3–22·8)	1·47 (-6·13–9·01)	13·9 (9·76–18·9)	13·2 (9·11–18·6)	13·6 (9·04–19·3)	-4·61 (-15·1–7·65)	2·95 (-9·2–18·5)
Drug use	0·226 (0·116–0·432)	0·306 (0·201–0·514)	0·273 (0·159–0·484)	35·4 (10·8–75·9)	-10·9 (-24·5–2·25)	0·12 (0·0669–0·227)	0·165 (0·117–0·262)	0·171 (0·109–0·275)	36·9 (7·74–83·5)	3·67 (-14·6–24)
High fasting plasma glucose	10·8 (8·78–13)	13·1 (11·3–15·3)	17·1 (14·9–19·6)	21·1 (4·79–43·8)	30·3 (14·9–52·9)	10·9 (8·9–13·1)	11·9 (9·92–14·1)	16·1 (13·6–18·9)	9·1 (-6·97–29·9)	35·6 (16·9–60)
High systolic blood pressure	42·5 (39·7–45·1)	28·2 (25·4–31·3)	32·6 (26·6–38·9)	-33·7 (-40·5—26·9)	15·6 (-1·6–34·3)	37·1 (34·4–39·6)	20·9 (18·6–23·4)	27 (20·6–34·7)	-43·7 (-50·1—36·6)	29·4 (-0·181–62·6)
High body-mass index	19·4 (13·9–26·1)	26 (19·4–34·2)	27·7 (20·8–36·5)	33·9 (21·2–53·8)	6·54 (0·666–13·2)	20 (13·3–29·1)	29·6 (22·5–39·8)	31·4 (23·9–42)	47·8 (27·8–85·2)	6·19 (-3·14–18·3)
Low bone mineral density	8·33 (4·55–14·1)	7·91 (4·26–13·6)	7·46 (3·64–13·2)	-4·99 (-26·9–21·1)	-5·72 (-32–24·7)	13 (8·48–19·1)	12·3 (7·96–18)	11·7 (7·09–18)	-5·58 (-25·9–14·7)	-4·35 (-27·4–25·2)
Diet low in fruits	41·9 (29·9–55·1)	48·9 (37·1–62·1)	49 (36·9–61·7)	16·7 (9·34–27·8)	0·21 (-6·65–6·96)	43·6 (32·1–56·5)	50·5 (38·8–63·9)	50·6 (38·9–64)	15·7 (9·39–26·2)	0·295 (-5·92–7·36)
Diet low in vegetables	50·8 (29·6–73)	46·2 (25·7–68·7)	44·2 (24·4–65·8)	-9·08 (-17—3·22)	-4·28 (-12·1—1·74)	48·6 (28–71·4)	43·9 (24·1–65·7)	41·9 (23–62·9)	-9·69 (-18·3—3·39)	-4·49 (-13·8—2·9)
Diet low in whole grains	69·8 (56·6–83)	78 (67·5–88·4)	79·2 (69·2–89)	11·7 (5·11–20·1)	1·55 (-0·0384–3·7)	--	--	--	--	--
Diet low in nuts and seeds	44·1 (17–66·5)	31·9 (9·22–58·1)	32·3 (11–58·5)	-27·7 (-47·6—9·54)	1·41 (-13·6–22)	38·8 (12·7–62·8)	26·7 (7·01–55·1)	27·3 (7·55–55·6)	-31·1 (-54·2—8·74)	2·25 (-13·2–29·7)
Diet low in milk	63·8 (36·4–85·4)	48·8 (23·5–69·7)	48 (23·3–68·1)	-23·5 (-50·3—6·26)	-1·56 (-8·83–4·88)	63·2 (35·8–84·6)	47·8 (22·5–68·9)	47 (22·8–68)	-24·4 (-51·2—6·58)	-1·69 (-8·74–5·38)
Diet high in red meat	91·1 (87·1–94·2)	80·8 (73·5–87·1)	83·2 (73·7–90)	-11·3 (-16·4—7·3)	2·9 (-4·91–10·6)	91·2 (87·3–94·6)	80·9 (73·8–87)	83·2 (74·2–90·4)	-11·4 (-16·7—7·31)	2·85 (-4·83–10·4)
Diet high in processed meat	78·9 (54·2–99·4)	83·7 (64·6–99·9)	82 (61·2–99·9)	6·18 (0·396–18·4)	-2·1 (-6·78–5·74e-07)	81·3 (58·9–99·8)	86·6 (69·8–100)	84·6 (64·6–100)	6·54 (0·189–19·9)	-2·38 (-7·41–0·0129)
Diet high in sugar-sweetened beverages	43·5 (26·7–61·5)	49·1 (33–64·7)	51·5 (35·3–66·7)	13 (-9·99–46·8)	4·85 (-12·7–28·5)	49·9 (30·9–67·4)	57·5 (41·7–71·7)	59·5 (42·7–74·8)	15·3 (-9·36–54·8)	3·41 (-12·8–23·2)
Diet low in fiber	30·8 (18·1–44·1)	31·3 (18·4–45·3)	31·7 (18·5–46·4)	1·67 (-7·47–10·4)	1·44 (-10·7–15·5)	30·9 (17·8–44·8)	32·1 (18·4–46·5)	32·3 (18–47·4)	3·82 (-3·96–12·1)	0·877 (-11·6–13·2)
Diet low in seafood omega-3 fatty acids	90·3 (78·4–100)	90·8 (79·8–100)	90·2 (77·5–100)	0·535 (-5·93–9·32)	-0·676 (-9·38–6·44)	92·2 (81·8–100)	92·6 (83·2–100)	91·9 (80·8–100)	0·447 (-5·74–8·53)	-0·72 (-8·32–5·27)
Diet low in polyunsaturated fatty acids	44·8 (12·1–88·1)	41·1 (11–84·8)	40 (10·8–82·8)	-8·08 (-16·9—0·762)	-2·72 (-9·59–3)	47·4 (12·8–90)	43·9 (12–87·1)	42·8 (11·7–86·6)	-7·35 (-16—0·599)	-2·58 (-9·83–2·64)
Diet high in trans fatty acids	64·8 (54·8–100)	55·8 (46·3–98·6)	54·3 (44·8–98·7)	-13·9 (-22·4—0·979)	-2·72 (-11·8–6·5)	64·9 (55·4–100)	56·1 (46·3–99·7)	54·5 (45–100)	-13·6 (-22·2—0·333)	-2·72 (-11·8–6·42)
Diet high in sodium	37·9 (8·34–64)	37·4 (9·37–61·5)	40·8 (11·7–64·3)	-1·18 (-31·3–55·8)	9·08 (-11·6–56·9)	15·9 (3·74–39·4)	15·9 (3·76–38)	17·1 (3·92–40·4)	0·189 (-23–41·6)	7·59 (-6·51–35·5)
Low physical activity	2·19 (0·803–4·88)	2·37 (0·877–5·12)	2·79 (1·06–6·08)	8·01 (-22·3–65·6)	17·8 (-15·5–68·3)	3·95 (1·83–8·01)	3·66 (1·85–7·04)	4·68 (2·2–9·08)	-7·18 (-27·8–24·6)	27·6 (-0·517–65·6)
Occupational asthmagens	27·6 (23·7–32·6)	24 (20·5–28·4)	25·4 (21·3–30·6)	-13·2 (-19·4—6·21)	5·75 (-3·45–17·1)	--	--	--	--	--
Occupational particulate matter, gases, and fumes	10·2 (7·33–14·5)	9·13 (6·51–13)	8·81 (6·3–12·6)	-10·7 (-13·9—7·51)	-3·53 (-5·16—1·94)	4·56 (3·22–6·81)	4·21 (2·99–6·09)	4·09 (2·91–5·93)	-7·7 (-14·2—1·32)	-2·73 (-5·98–0·709)
Occupational noise	9·2 (8·72–9·9)	8·51 (8·08–9·18)	8·33 (7·92–8·99)	-7·51 (-8·93—5·99)	-2·1 (-2·81—1·33)	5·08 (4·79–5·49)	5·3 (5·04–5·67)	5·52 (5·26–5·89)	4·23 (1·72–6·71)	4·1 (3·11–5·09)
Occupational injuries	--	--	--	--	--	--	--	--	--	--
Occupational ergonomic factors	11·5 (9·86–13·4)	10·3 (8·92–12·1)	10·8 (9·28–12·7)	-9·99 (-13·1—6·82)	4·82 (-0·8–11·3)	6·35 (5·4–7·62)	7·36 (6·26–8·79)	8 (6·73–9·69)	15·9 (9·89–22·3)	8·71 (0·716–18)
Childhood sexual abuse	5·17 (3·37–7·25)	5·1 (3·33–7·24)	5·1 (3·33–7·24)	-1·42 (-8·75–5·45)	0 (0–0)	19·7 (14·9–26·2)	19·5 (14·5–26·8)	19·5 (14·5–26·8)	-1·06 (-7·38–6·07)	0 (0–0)
Intimate partner violence	--	--	--	--	--	22·3 (13·2–30·5)	20·7 (12·3–26·7)	22·3 (13·2–30·5)	-7·17 (-18·8–13·9)	7·72 (-7·95–38·8)
Non-exclusive breastfeeding	27·4 (23·3–32·2)	27·6 (24–32·4)	27·5 (23·4–32·4)	0·717 (-10·8–14·1)	-0·369 (-9·62–10·4)	--	--	--	--	--
Discontinued breastfeeding	25·9 (25–26·8)	24 (22·9–25)	24·6 (23·5–25·7)	-7·53 (-12·2—2·16)	2·5 (-2·33–7·76)	26 (25–26·8)	24 (22·9–25·1)	24·6 (23·5–25·8)	-7·58 (-12·2—2·21)	2·57 (-2·26–7·83)
Diet low in calcium	19·1 (9·11–35·9)	13·7 (6·35–25·7)	12·8 (5·91–23·9)	-28·4 (-39·1—20·2)	-6·43 (-12·1—1·11)	13·3 (5·43–25·4)	7·97 (3·15–14·4)	7 (2·78–12·5)	-40·2 (-52·2—30·9)	-12·1 (-21·4—3·93)
Occupational exposure to asbestos	9·92 (9–10·9)	10·3 (9·72–10·9)	9·67 (7·56–12·1)	4·31 (-5·96–15·5)	-6·5 (-26·9–18)	2·54 (1·76–3·74)	1·94 (1·46–2·58)	1·91 (1·32–2·68)	-23·7 (-40·1—2·94)	-1·56 (-24·6–25·3)
Occupational exposure to arsenic	0·616 (0–1·46)	0·578 (0–1·38)	0·612 (0–1·44)	-6·15 (-9·96–NA)	5·75 (-0·839–NA)	0·467 (0–1·1)	0·479 (0–1·12)	0·514 (0–1·22)	2·44 (-3·81–NA)	7·42 (0·296–NA)
Occupational exposure to benzene	0·803 (0·0459–2·31)	0·866 (0·0506–2·48)	0·924 (0·055–2·64)	7·75 (3·89–13·4)	6·71 (2·81–13)	0·951 (0·0574–2·74)	1·35 (0·0797–3·88)	1·5 (0·0892–4·44)	42 (36·4–50·6)	11·3 (6·78–17·9)
Occupational exposure to beryllium	0·0237 (0·0235–0·0239)	0·0243 (0·0241–0·0245)	0·0261 (0·0255–0·0267)	2·57 (1·23–3·88)	7·3 (4·86–9·93)	0·0226 (0·0223–0·0229)	0·0277 (0·0274–0·0279)	0·0305 (0·0298–0·0312)	22·6 (20·6–24·6)	10·2 (7·59–13)
Occupational exposure to cadmium	0·0697 (0·0674–0·0717)	0·0662 (0·0645–0·0679)	0·0702 (0·0665–0·074)	-4·93 (-8·49—0·984)	5·94 (0·067–12·1)	0·0548 (0·0525–0·0573)	0·0577 (0·0557–0·0597)	0·0622 (0·0585–0·0664)	5·24 (-0·478–11·1)	7·87 (1·52–15·4)
Occupational exposure to chromium	0·16 (0·155–0·164)	0·155 (0·152–0·159)	0·165 (0·157–0·173)	-3·07 (-6·3–0·479)	6·32 (1·16–11·6)	--	--	--	--	--
Occupational exposure to diesel engine exhaust	0·633 (0·615–0·652)	0·673 (0·657–0·69)	0·731 (0·7–0·761)	6·23 (2·29–10·5)	8·6 (4·04–13·2)	0·547 (0·529–0·567)	0·664 (0·643–0·686)	0·732 (0·695–0·772)	21·3 (15·8–27·2)	10·2 (4·73–16·1)
Occupational exposure to formaldehyde	0·354 (0·341–0·366)	0·327 (0·317–0·336)	0·342 (0·322–0·361)	-7·58 (-11·1—3·8)	4·53 (-1·31–10·6)	0·264 (0·252–0·276)	0·286 (0·275–0·296)	0·309 (0·29–0·328)	8·57 (2·98–14·2)	8·04 (2·22–14·5)
Occupational exposure to nickel	0·583 (0–2·54)	0·537 (0–2·37)	0·567 (0–2·55)	-7·91 (-11·6–NA)	5·6 (-0·17–NA)	0·379 (0–1·66)	0·376 (0–1·67)	0·402 (0–1·8)	-0·803 (-7·5–NA)	6·94 (-1·23–NA)
Occupational exposure to polycyclic aromatic hydrocarbons	0·325 (0·317–0·332)	0·317 (0·311–0·323)	0·337 (0·323–0·352)	-2·33 (-5·31–0·656)	6·43 (1·77–11·2)	0·274 (0·265–0·283)	0·315 (0·308–0·322)	0·343 (0·329–0·358)	14·9 (10·3–19·5)	9·12 (4·47–14)
Occupational exposure to silica	6·33 (0·944–19·8)	5·97 (0·869–19)	6·35 (0·933–20·1)	-5·75 (-11·4–0·473)	6·4 (0·263–13·4)	3·33 (0·509–10·4)	3·27 (0·473–10·3)	3·46 (0·511–11·1)	-1·74 (-8·46–5·61)	5·83 (-1·99–16·4)
Occupational exposure to sulfuric acid	0·946 (0·199–2·92)	0·883 (0·182–2·81)	0·932 (0·197–2·98)	-6·61 (-10·5—2·54)	5·48 (-0·609–11·5)	0·68 (0·141–2·16)	0·687 (0·144–2·14)	0·735 (0·149–2·35)	0·999 (-5·11–7·21)	7·04 (-0·246–15·1)
Unsafe sex	--	--	--	--	--	--	--	--	--	--
Occupational exposure to trichloroethylene	0·0827 (0·0811–0·0847)	0·0829 (0·0816–0·0848)	0·0888 (0·0858–0·0924)	0·357 (-2·33–3·47)	7·01 (2·98–11·2)	0·075 (0·0733–0·0774)	0·0853 (0·0837–0·0876)	0·0932 (0·0898–0·0974)	13·7 (9·74–17·7)	9·26 (5·19–14·1)
No access to handwashing facility	3·2 (3·03–3·4)	3·11 (2·93–3·28)	3·08 (2·91–3·25)	-2·93 (-9·02–3·17)	-0·84 (-5·06–3·42)	3·2 (3·03–3·4)	3·11 (2·93–3·28)	3·08 (2·91–3·25)	-2·93 (-9·02–3·17)	-0·84 (-5·06–3·42)
Child wasting	0·877 (0·606–1·18)	0·847 (0·568–1·15)	0·844 (0·567–1·14)	-3·44 (-9·83–3·76)	-0·332 (-5·11–4·82)	0·731 (0·487–0·999)	0·71 (0·473–0·967)	0·708 (0·469–0·969)	-2·97 (-10–5·48)	-0·278 (-5·55–5·67)
Child stunting	1·11 (0·533–1·72)	1·04 (0·382–1·69)	1·02 (0·369–1·64)	-6·98 (-60·7–16·7)	-1·81 (-58·5–123)	1·62 (0·818–2·41)	1·47 (0·72–2·2)	1·45 (0·696–2·2)	-9·53 (-23·5–9·02)	-1·33 (-12·7–10·8)
Chewing tobacco	0·176 (0·14–0·226)	0·196 (0·155–0·25)	0·227 (0·178–0·287)	10·9 (-16·1–46)	15·9 (-2·87–37·9)	--	--	--	--	--
Diet low in legumes	95·8 (89·5–100)	94·7 (87–100)	93·6 (84·6–100)	-1·14 (-2·93–0)	-1·17 (-3·58–0)	94·3 (85·8–100)	93 (83·2–100)	91·8 (80·4–100)	-1·34 (-3·65–0)	-1·27 (-3·99–0)
Short gestation	32·1 (25·4–40·5)	25·2 (21·4–29·5)	22·6 (18–27·9)	-21·5 (-38·3–1·07)	-10·6 (-28·5–10·5)	30·4 (24·3–37·5)	22·4 (19·2–25·9)	20·3 (16·6–24·9)	-26·3 (-40·8—6·76)	-9·31 (-25·6–11·5)
Low birth weight	14·3 (11·4–17·9)	10·9 (9·27–12·8)	9·87 (7·89–12·1)	-23·5 (-41·1—1·29)	-9·88 (-29–14·2)	13·5 (10·8–16·9)	10 (8·78–11·6)	8·83 (7·26–10·7)	-25·7 (-42·2—4·78)	-12 (-30–11·4)
High temperature	24·3 (12·9–37·4)	26·3 (15·3–39)	27 (15·9–39·6)	8·26 (-8·24–33·9)	2·68 (-0·378–5·79)	24·3 (12·9–37·4)	26·3 (15·3–39)	27 (15·9–39·6)	8·26 (-8·24–33·9)	2·68 (-0·378–5·79)
Low temperature	32·1 (28·3–36)	34·9 (31·7–38·3)	31·6 (27·9–35·6)	8·73 (5·68–12·3)	-9·45 (-12·3—6·6)	32·1 (28·3–36)	34·9 (31·7–38·3)	31·6 (27·9–35·6)	8·73 (5·68–12·3)	-9·45 (-12·3—6·6)
Kidney dysfunction	15 (10·3–21·5)	15 (10·4–21·3)	15·5 (10·9–22·1)	0·0426 (-4·15–4·69)	3·66 (-0·376–8·24)	15·8 (10·9–22·4)	15·9 (11·1–22·3)	16·6 (11·6–23·1)	0·454 (-3·83–4·77)	4·08 (0·0879–9·28)
Bullying victimization	7·28 (3·06–14·4)	8·65 (3·95–16·2)	8·64 (3·91–16·5)	18·9 (-2·91–61·7)	-0·11 (-7·79–5·66)	6·18 (2·67–12·3)	7·25 (3·49–13·4)	7·47 (3·44–14)	17·2 (-6·3–62·1)	3·01 (-5·47–9·29)
High LDL cholesterol	61 (58·4–63·7)	45·1 (42–48·5)	46·8 (43·3–50·3)	-26 (-30—22·2)	3·69 (-0·658–7·95)	57·2 (54·5–60)	47·2 (43·9–50·6)	48·9 (45·5–52·4)	-17·4 (-21·5—13·4)	3·54 (0·173–7·39)

Supplementary Results Table 1. Greece age-standardised Summary Exposure Values (SEVs) for most-detailed risk factors and percentage change, by sex, 1990, 2010, and 2019. Data in parentheses are 95% uncertainty intervals.

	Male					Female				
	1990	2010	2019	Percentage change 1990-2010	Percentage change 2010-2019	1990	2010	2019	Percentage change 1990-2010	Percentage change 2010-2019
Unsafe sanitation	7·71 (5·21–10·9)	1·82 (1·04–2·93)	1·59 (0·897–2·62)	-76·4 (-87·9–-59·2)	-12·2 (-45–41·5)	--	--	--	--	--
Ambient particulate matter pollution	19·5 (8·32–34)	18·6 (15–23·1)	13·1 (9·94–17)	-4·6 (-46·1–111)	-29·4 (-34·3–-25·7)	19·4 (8·22–33·9)	18·6 (15–23)	13·1 (9·93–17)	-3·96 (-45·6–112)	-29·4 (-34·2–-25·6)
Household air pollution from solid fuels	0·436 (0·131–1·02)	0·0767 (0·0228–0·178)	0·0501 (0·0128–0·13)	-82·4 (-90·7–-65·1)	-34·6 (-59·5–1·49)	0·567 (0·186–1·33)	0·1 (0·0319–0·237)	0·066 (0·0178–0·174)	-82·3 (-90·6–-64·8)	-34·3 (-59·3–0·896)
Ambient ozone pollution	73 (30·8–100)	73·1 (31·5–100)	60 (25–100)	0·173 (-11·1–13·7)	-18 (-30·4–0)	73 (30·8–100)	73·1 (31·5–100)	60 (25–100)	0·173 (-11·1–13·7)	-18 (-30·4–0)
Residential radon	35·4 (15·4–63·9)	35·4 (15·4–63·9)	35·4 (15·4–63·9)	0 (0–0)	0 (0–0)	35·4 (15·4–63·9)	35·4 (15·4–63·9)	35·4 (15·4–63·9)	0 (0–0)	0 (0–0)
Lead exposure	66·9 (45·4–83·3)	53·6 (34–71·9)	41·2 (23·4–60·8)	-20 (-29·9–-11·9)	-23·1 (-31·9–-15·3)	25·3 (0·0351–53·4)	19 (0·302–44)	13·7 (0·107–36·3)	-24·7 (-40·5–1070)	-28·2 (-67·7–-16·9)
Child underweight	0·926 (0·525–1·39)	0·75 (0·416–1·12)	0·754 (0·421–1·13)	-19·1 (-29·6–-6·95)	0·628 (-8·85–10·9)	1·05 (0·618–1·53)	0·893 (0·5–1·32)	0·9 (0·517–1·34)	-14·6 (-25·3–-3·23)	0·747 (-7·5–10·3)
Iron deficiency	--	--	--	--	--	9·93 (8·26–11·7)	9·24 (7·67–11·1)	9·3 (7·72–11·1)	-6·98 (-16·4–3·58)	0·652 (-5·42–7)
Vitamin A deficiency	6·44 (2·63–12·6)	2·52 (1·07–5·28)	2·37 (0·973–4·68)	-60·9 (-83–-0·19)	-6·04 (-44·9–75·4)	4·89 (2·49–8·35)	2·72 (1·33–4·73)	2·55 (1·27–4·46)	-44·4 (-70·3–-20·2)	-5·97 (-36·4–47·8)
Zinc deficiency	2·56 (0–7·84)	2·13 (0–7·01)	2·67 (0–8·34)	-16·8 (-93·3–NA)	25·3 (7·99–NA)	2·5 (0–7·95)	2·09 (0–6·8)	2·61 (0–8·02)	-16·3 (-70·3–NA)	24·5 (9·14–NA)
Smoking	35·8 (32·2–39·5)	32·1 (28·6–35·7)	29·2 (25·8–32·5)	-10·3 (-16–-3·79)	-9·19 (-12·6–-5·81)	14·8 (12·7–17·1)	18·2 (15·6–21)	18·4 (15·8–21·3)	23·3 (10·6–37·4)	0·704 (-4·22–6·01)
Secondhand smoke	49·2 (45·9–52·4)	44·2 (42·1–46·3)	40·9 (38·2–43·4)	-10·3 (-17·2–-2·31)	-7·43 (-11·6–-3·57)	58·9 (56·2–61·7)	48·1 (46·3–49·8)	45 (42·8–47·2)	-18·4 (-23·4–-13·1)	-6·35 (-9·28–-3·24)
Alcohol use	20·1 (14·9–26)	22·1 (15·4–30·5)	20·4 (13·9–28·1)	9·85 (-2·94–27·4)	-7·6 (-19·8–2·91)	8·11 (5·19–12·2)	7·2 (4·46–11·3)	6·72 (3·81–10·7)	-11·2 (-24·7–3·18)	-6·69 (-26·2–14·2)
Drug use	0·15 (0·107–0·227)	0·244 (0·192–0·318)	0·249 (0·193–0·329)	63 (35·8–104)	1·81 (-14·8–17·1)	0·0716 (0·0468–0·115)	0·0999 (0·0761–0·141)	0·125 (0·0915–0·17)	39·6 (19–72·5)	25·1 (7·86–49·2)
High fasting plasma glucose	6·27 (5·5–7·21)	10·1 (8·35–12·4)	12·4 (10–14·9)	60·2 (34·8–92·4)	23·2 (3·5–49·7)	5·39 (4·69–6·19)	7·39 (6·42–8·53)	8·63 (7·31–10·1)	37 (24·2–53·5)	16·9 (2·52–33·8)
High systolic blood pressure	25·3 (21·9–28·9)	26·4 (23–30)	23·6 (19·5–28·5)	4·1 (-11·5–20)	-10·5 (-24·9–7·88)	33·5 (28·8–38·5)	21·6 (18·1–25·8)	21·8 (16·6–27·3)	-35·3 (-47·8–-18·5)	0·795 (-24·2–30)
High body-mass index	20·9 (15–27·9)	28·8 (21·5–37·6)	27·3 (20·2–35·8)	37·7 (24·6–56·3)	-5·18 (-10·4–-0·144)	20·8 (14·1–29·8)	27·7 (20·1–38·6)	27·1 (19·6–37·3)	32·9 (16·2–62·1)	-2·16 (-12·9–9·3)
Low bone mineral density	14·8 (9·51–21·7)	12·3 (7·21–19)	12·3 (7·04–19·3)	-16·5 (-36·7–6·19)	0·0653 (-22·3–28·9)	21 (15·8–27·5)	18·7 (12·6–25·7)	18·2 (12·3–25·2)	-11·2 (-31·5–13·4)	-2·52 (-22·9–21·2)
Diet low in fruits	13·2 (7·93–20)	13·7 (8·39–20·4)	17·7 (10·4–26·6)	4·19 (-6·94–17·6)	29·1 (-0·934–67·5)	15·6 (9·66–23)	16·2 (9·97–23·8)	20·3 (12·3–29·3)	4·12 (-5·6–14·7)	25·1 (-1·52–57·3)
Diet low in vegetables	2·58 (1·28–8·21)	2·73 (1·4–8·26)	4·05 (1·82–11·1)	5·53 (-0·682–28·3)	48·5 (3·21–218)	2·57 (1·26–8·22)	2·64 (1·31–8·32)	3·44 (1·58–10·4)	2·81 (-2·26–18·3)	30·1 (1·25–142)
Diet low in whole grains	100 (100–100)	100 (100–100)	100 (100–100)	6·35e-08 (0–0)	-0·000285 (0–0)	--	--	--	--	--
Diet low in nuts and seeds	34·9 (12·1–60)	22·6 (6·27–53·8)	26·4 (7·1–55·2)	-35·2 (-57·5–-9·47)	16·7 (-0·313–52)	29·2 (8·91–57·3)	18·2 (4·92–52·3)	21·6 (5·51–53·7)	-37·8 (-60·8–-7·52)	18·9 (-0·00335–60·8)
Diet low in milk	76 (50·9–95·9)	63·2 (35·8–84·9)	66 (37·8–87·3)	-16·8 (-37·1–-3·39)	4·39 (0·566–12·5)	75·7 (50·8–95·7)	62·6 (35·2–84)	65·6 (37·8–87)	-17·3 (-37·5–-3·53)	4·66 (0·553–13·6)
Diet high in red meat	79 (71·4–85·8)	93·7 (90·6–96·1)	88·2 (81·2–93·7)	18·6 (11·5–28)	-5·92 (-11·7–-1·24)	79 (71·1–86)	93·8 (90·6–96·2)	88·1 (80·5–93·9)	18·7 (11·6–28·7)	-6·04 (-12·8–-1·22)
Diet high in processed meat	50 (28·2–73·4)	60·9 (36·3–85·3)	59·7 (35·5–84·1)	21·7 (4·69–52·2)	-1·97 (-12·4–7·26)	58·6 (34·6–83·6)	68·6 (42·1–92·5)	66·9 (41·3–90·3)	17·1 (4·52–40·3)	-2·49 (-10·1–3·04)
Diet high in sugar-sweetened beverages	33·4 (18·3–49·6)	36·1 (22–52·8)	34·9 (20–51·7)	7·96 (-19·7–42·5)	-3·18 (-21·2–17·8)	38·3 (21·3–56·5)	42 (26·4–59·1)	40·8 (24·5–57·7)	9·61 (-16·3–51·3)	-2·91 (-22·3–20·3)
Diet low in fiber	16 (8·65–25)	18·6 (10–28·7)	22·6 (11·8–34·9)	16·1 (7·9–26·9)	21·4 (4·87–43·2)	15·4 (7·95–24·3)	18 (9·49–28·3)	22·1 (11·7–34·2)	17·5 (8·3–28·9)	22·4 (6·28–46·5)
Diet low in seafood omega-3 fatty acids	92·4 (81·3–100)	90·9 (79·6–100)	92·4 (82·1–100)	-1·58 (-9·06–5·24)	1·62 (-3·91–10·5)	94 (85·1–100)	92·7 (83–100)	93·7 (84·4–100)	-1·41 (-8–4·4)	1·08 (-4·63–8·71)
Diet low in polyunsaturated fatty acids	70·2 (29·9–100)	56·1 (16·8–96·4)	58·1 (18·7–97·4)	-20·1 (-48·2–-0·885)	3·49 (0·113–13·6)	71·8 (31·7–100)	58·3 (18·2–97·8)	60·2 (21·1–98·3)	-18·8 (-46·8–-0·701)	3·27 (0·0275–12·9)
Diet high in trans fatty acids	52·4 (42·5–99·8)	40·4 (30·7–96·6)	39·8 (30·1–97·9)	-22·8 (-34·2–-0·953)	-1·47 (-10·1–9·02)	53·4 (43·2–100)	42 (31·3–100)	41·4 (30·6–99·8)	-21·2 (-33·3–0)	-1·48 (-9·28–7·81)
Diet high in sodium	30·1 (6·63–55·8)	34·1 (8·08–60·9)	36·3 (8·75–62·5)	13·4 (-20·5–75·7)	6·33 (-15·1–44·3)	15·5 (3·96–38·1)	15·9 (3·88–38·4)	16·7 (3·99–40)	2·69 (-21·7–41·9)	4·9 (-12·2–29·4)
Low physical activity	1·81 (0·646–4·35)	1·77 (0·67–4·06)	2·18 (0·797–4·78)	-2·21 (-28·8–36·1)	23·1 (-6·52–76·1)	3·17 (1·43–6·24)	3·1 (1·5–5·96)	3·77 (1·78–7·7)	-2·12 (-25·7–35·3)	21·4 (-5·69–58·6)
Occupational asthmagens	22·8 (19·2–27·4)	19·7 (16·7–23·8)	17·4 (14·6–21·2)	-13·6 (-19·9–-7·87)	-11·9 (-17·9–-5·03)	--	--	--	--	--
Occupational particulate matter, gases, and fumes	9·55 (6·89–13·6)	8·15 (5·9–11·6)	7 (5·06–10·2)	-14·7 (-18·5–-11·1)	-14 (-17·1–-11·4)	4·23 (3·1–5·96)	3·66 (2·64–5·3)	3·21 (2·28–4·67)	-13·4 (-19·4–-8·22)	-12·4 (-16·2–-8·79)
Occupational noise	8·75 (8·3–9·39)	7·83 (7·44–8·46)	6·97 (6·6–7·59)	-10·5 (-12·1–-8·83)	-11 (-11·8–-10·1)	4·07 (3·81–4·45)	4·06 (3·85–4·4)	3·84 (3·64–4·15)	-0·25 (-3·7–2·96)	-5·52 (-6·7–-4·32)
Occupational injuries	--	--	--	--	--	--	--	--	--	--
Occupational ergonomic factors	19·3 (17·7–21·3)	14·6 (13·3–16·2)	12·9 (11·3–14·7)	-24·2 (-28·6–-19·7)	-11·8 (-20–-2·01)	10·7 (9·79–11·7)	9·98 (9·1–11·1)	9·31 (7·88–10·8)	-6·51 (-14·8–1·52)	-6·76 (-19–7·46)
Childhood sexual abuse	7·16 (4·65–10·1)	7·16 (4·65–10·1)	7·16 (4·65–10·1)	0 (0–0)	0 (0–0)	10·6 (7·84–14·3)	10·6 (7·84–14·3)	10·6 (7·84–14·3)	0 (0–0)	0 (0–0)
Intimate partner violence	--	--	--	--	--	14·5 (7·87–21·4)	13·5 (7·9–18·1)	14·5 (7·87–21·4)	-6·95 (-19·6–15·4)	7·47 (-5·74–30·6)
Non-exclusive breastfeeding	28·6 (24·4–32·9)	28·1 (24·3–32·3)	27·9 (24·1–32·1)	-1·73 (-14·2–12·6)	-0·615 (-12·1–10·5)	--	--	--	--	--
Discontinued breastfeeding	23·5 (22·5–24·6)	24·9 (23·6–26)	23·8 (22·6–24·9)	5·8 (-0·923–12·9)	-4·41 (-9·19–0·933)	23·5 (22·5–24·6)	24·9 (23·6–26)	23·7 (22·5–24·9)	5·75 (-0·965–12·8)	-4·53 (-9·3–0·815)
Diet low in calcium	15·6 (7·09–29·6)	11·9 (5·6–21·9)	13·9 (6·25–26)	-23·5 (-30·2–-15·8)	16·9 (10·3–24·3)	10·2 (3·8–18·8)	5·89 (2·46–10·7)	7·7 (3·08–13·9)	-42·2 (-51·8–-33·2)	30·8 (17·5–46·9)
Occupational exposure to asbestos	3·03 (2·58–3·63)	3·01 (2·58–3·53)	3·1 (2·33–4·08)	-0·425 (-19·3–21)	2·7 (-23·2–38·1)	1·95 (0·38–7·06)	1·12 (0·347–2·98)	1·03 (0·314–2·83)	-42·8 (-87·4–174)	-7·35 (-76·4–315)
Occupational exposure to arsenic	0·443 (0–1·05)	0·37 (0–0·873)	0·321 (0–0·754)	-16·7 (-22·1–NA)	-13·3 (-19·1–NA)	0·338 (0–0·798)	0·305 (0–0·706)	0·281 (0–0·661)	-9·9 (-17·8–NA)	-7·77 (-15·1–NA)
Occupational exposure to benzene	0·679 (0·0413–1·92)	0·722 (0·0426–2·05)	0·633 (0·0365–1·8)	6·3 (1·34–12·9)	-12·2 (-16·6–-5·77)	0·585 (0·0346–1·66)	0·837 (0·049–2·44)	0·801 (0·0476–2·28)	43·1 (34·7–54·6)	-4·34 (-9·54–2·74)
Occupational exposure to beryllium	0·0231 (0·0228–0·0233)	0·0209 (0·0206–0·0211)	0·0183 (0·0177–0·0189)	-9·55 (-11–-8·07)	-12·3 (-15·3–-9·56)	0·0165 (0·0162–0·0167)	0·0191 (0·0188–0·0194)	0·0183 (0·0176–0·019)	16·1 (13·5–18·8)	-4·27 (-8·05–-0·434)
Occupational exposure to cadmium	0·0523 (0·0509–0·054)	0·0443 (0·0422–0·0463)	0·0386 (0·0362–0·041)	-15·4 (-20·2–-10·7)	-13 (-18·4–-6·9)	0·0393 (0·0373–0·0414)	0·0373 (0·0352–0·0397)	0·0347 (0·0324–0·0373)	-5·18 (-12·3–3·62)	-6·97 (-13·4–0·114)
Occupational exposure to chromium	0·123 (0·12–0·126)	0·108 (0·104–0·113)	0·0949 (0·0897–0·1)	-11·6 (-16–-7·22)	-12·5 (-17·1–-7·22)	0·0906 (0·0861–0·0952)	0·0943 (0·09–0·0995)	0·0889 (0·0836–0·0945)	4·17 (-2·81–12·4)	-5·75 (-11·5–0·066)
Occupational exposure to diesel engine exhaust	0·718 (0·692–0·743)	0·696 (0·668–0·726)	0·615 (0·584–0·648)	-3·03 (-7·58–1·56)	-11·6 (-15·9–-7·09)	0·327 (0·31–0·347)	0·482 (0·45–0·512)	0·481 (0·447–0·52)	47·3 (35·9–59·1)	-0·0363 (-6·98–8·13)
Occupational exposure to formaldehyde	0·246 (0·236–0·255)	0·209 (0·198–0·22)	0·18 (0·167–0·193)	-15 (-20·2–-9·72)	-13·9 (-19·5–-7·86)	0·179 (0·168–0·19)	0·176 (0·165–0·187)	0·162 (0·152–0·174)	-1·81 (-9·46–7·27)	-7·61 (-13·8–-1·02)
Occupational exposure to nickel	0·42 (0–1·85)	0·353 (0–1·55)	0·305 (0–1·36)	-16 (-21·1–NA)	-13·7 (-19·3–NA)	0·28 (0–1·23)	0·236 (0–1·01)	0·215 (0–0·945)	-15·8 (-23·7–NA)	-8·99 (-16·4–NA)
Occupational exposure to polycyclic aromatic hydrocarbons	0·246 (0·24–0·252)	0·223 (0·216–0·231)	0·195 (0·185–0·205)	-9·27 (-13·2–-5·42)	-12·6 (-17–-7·8)	0·185 (0·176–0·193)	0·198 (0·189–0·207)	0·186 (0·176–0·196)	7·09 (0·387–14·6)	-6·01 (-11·3–-0·681)
Occupational exposure to silica	4·81 (0·672–14·9)	4·42 (0·602–13·8)	3·84 (0·538–12·1)	-8·13 (-16·1–0·979)	-13 (-19·9–-5·49)	2·39 (0·324–7·28)	2·01 (0·276–6·2)	1·84 (0·254–5·77)	-16·2 (-24–-6·06)	-8·4 (-16·1–0·909)
Occupational exposure to sulfuric acid	0·821 (0·17–2·55)	0·653 (0·135–2·03)	0·551 (0·114–1·74)	-20·5 (-25·7–-15)	-15·6 (-20·8–-9·92)	0·532 (0·109–1·68)	0·479 (0·0997–1·47)	0·437 (0·0905–1·42)	-10·1 (-16·9–-1·57)	-8·8 (-15·3–-1·1)
Unsafe sex	--	--	--	--	--	--	--	--	--	--
Occupational exposure to trichloroethylene	0·0738 (0·0725–0·0759)	0·0666 (0·0648–0·0689)	0·0586 (0·0562–0·0615)	-9·72 (-13·2–-6·22)	-12 (-16–-7·81)	0·0498 (0·0481–0·0522)	0·0579 (0·0558–0·0606)	0·0558 (0·0527–0·0593)	16·2 (9·75–23·5)	-3·56 (-8·95–1·99)
No access to handwashing facility	3·31 (3·12–3·5)	3·2 (3·03–3·39)	3·18 (3·01–3·35)	-3·25 (-9·43–3·55)	-0·673 (-4·91–3·33)	3·31 (3·12–3·5)	3·2 (3·03–3·39)	3·18 (3·01–3·35)	-3·25 (-9·43–3·55)	-0·673 (-4·91–3·33)
Child wasting	1·23 (0·865–1·62)	1·18 (0·827–1·56)	1·19 (0·831–1·56)	-3·62 (-11·4–5·36)	0·566 (-5·18–6·81)	1·03 (0·725–1·39)	0·99 (0·68–1·32)	0·994 (0·69–1·32)	-4·29 (-12–3·21)	0·388 (-5·08–5·69)
Child stunting	1·66 (0·849–2·46)	1·17 (0·56–1·81)	1·19 (0·578–1·8)	-29·2 (-41·5–-15·1)	0·946 (-10·8–15·6)	1·95 (1·03–2·89)	1·54 (0·762–2·35)	1·55 (0·769–2·34)	-21·1 (-34·7–-5·58)	0·915 (-10·6–14·4)
Chewing tobacco	0·161 (0·124–0·203)	0·162 (0·129–0·203)	0·164 (0·128–0·207)	0·777 (-24·3–33·5)	1·38 (-16–20·5)	--	--	--	--	--
Diet low in legumes	69·5 (27·8–98·3)	63·8 (18·7–95)	67·7 (25·7–96·7)	-8·12 (-35·5–-0·632)	6 (0·303–42·4)	66·2 (23·1–96·7)	59·7 (14·6–91·5)	64·1 (18·9–94·6)	-9·81 (-42–-0·911)	7·29 (0·326–48·6)
Short gestation	23·3 (19·2–28·2)	46·8 (37–59·9)	38·3 (29·9–49·8)	101 (55·3–172)	-18·2 (-38–6·57)	29·5 (25·6–34)	50·1 (40·2–61)	37·9 (30·6–46·7)	70·1 (34·9–113)	-24·4 (-41·1–-4·67)
Low birth weight	13·9 (11·5–16·9)	23·3 (18·2–29·6)	20·4 (16·4–26·3)	68 (25·7–128)	-12·4 (-35·5–17·1)	17·5 (15·1–20·1)	25·6 (20·8–30·6)	20·4 (16·6–24·6)	46 (16·5–81·5)	-20·2 (-38·5–0·953)
High temperature	21·7 (13·4–31·2)	28 (19·3–37·9)	22·7 (14·4–32·3)	29·3 (15·4–51·5)	-19·1 (-27·8–-11·9)	21·7 (13·4–31·2)	28 (19·3–37·9)	22·7 (14·4–32·3)	29·3 (15·4–51·5)	-19·1 (-27·8–-11·9)
Low temperature	34·3 (28·2–42·7)	32·9 (26·9–41·2)	35·6 (29·4–44·2)	-3·93 (-5·41–-2·6)	8·09 (5·54–10·6)	34·3 (28·2–42·7)	32·9 (26·9–41·2)	35·6 (29·4–44·2)	-3·93 (-5·41–-2·6)	8·09 (5·54–10·6)
Kidney dysfunction	16 (11–22·5)	16·4 (11·4–22·8)	15·8 (11–22·4)	2·55 (-1·09–7·07)	-3·62 (-7·97–0·045)	17·5 (12·3–24·1)	17·7 (12·6–24·3)	17·1 (12–23·8)	1·25 (-2·31–5·31)	-3·63 (-8·04–0·00506)

Bullying victimization	4·91<br>(1·93–10·3)	6·13<br>(2·8–11·8)	6·26<br>(2·69–12·3)	24·8<br>(2·64–64·2)	2·09<br>(-7·03–7·91)	3·95<br>(1·57–7·93)	5·05<br>(2·3–9·7)	5·22<br>(2·3–10·2)	27·9<br>(7·65–70·7)	3·39<br>(-6·24–9·34)
High LDL cholesterol	40·8<br>(37·2–44·2)	40·4<br>(37·1–43·9)	40·8<br>(37·5–44·2)	-0·967<br>(-6·77–5·01)	0·936<br>(-3·44–5·38)	40·6<br>(37·4–44)	40·6<br>(37·3–44·1)	41·2<br>(37·8–44·5)	0·0574<br>(-5·02–5·54)	1·34<br>(-2·09–4·86)

Supplementary Results Table 1. Iceland age-standardised Summary Exposure Values (SEVs) for most-detailed risk factors and percentage change, by sex, 1990, 2010, and 2019. Data in parentheses are 95% uncertainty intervals.

	Male					Female				
	1990	2010	2019	Percentage change 1990-2010	Percentage change 2010-2019	1990	2010	2019	Percentage change 1990-2010	Percentage change 2010-2019
Unsafe sanitation	2·75 (1·59–4·55)	0·895 (0·501–1·47)	0·674 (0·369–1·09)	-67·4 (-83·7—38·1)	-24·7 (-53·1–21·2)	2·75 (1·59–4·55)	0·895 (0·501–1·47)	0·674 (0·369–1·09)	-67·4 (-83·7—38·1)	-24·7 (-53·1–21·2)
Ambient particulate matter pollution	3·92 (0·333–10·2)	3·05 (0·887–5·42)	2·06 (0·433–4·24)	-22·2 (-76–514)	-32·5 (-59·6—21·3)	--	--	--	--	--
Household air pollution from solid fuels	0·0505 (0·00533–0·161)	0·0115 (0·00146–0·0377)	0·00631 (0·000554–0·0235)	-77·3 (-92·4—5·86)	-45 (-74·7—11)	0·0671 (0·00836–0·212)	0·0153 (0·00215–0·0494)	0·00839 (0·000733–0·0307)	-77·2 (-92·3—15·6)	-45·1 (-74·7—11·1)
Ambient ozone pollution	12·8 (3·96–26·8)	25·8 (9·29–49)	21·5 (7·43–43·1)	101 (26·2–259)	-16·6 (-52·1–37·8)	12·8 (3·96–26·8)	25·8 (9·29–49)	21·5 (7·43–43·1)	101 (26·2–259)	-16·6 (-52·1–37·8)
Residential radon	4·83 (1·86–9·97)	4·83 (1·86–9·97)	4·83 (1·86–9·97)	0 (0–0)	0 (0–0)	4·83 (1·86–9·97)	4·83 (1·86–9·97)	4·83 (1·86–9·97)	0 (0–0)	0 (0–0)
Lead exposure	34·7 (5·4–60·7)	22·7 (3·92–45·7)	15·1 (1·56–35·6)	-34·6 (-50·4—18·2)	-33·4 (-60·2—21·3)	24 (0·179–51·4)	16·2 (0·224–38·9)	10·9 (0·0658–30·2)	-32·6 (-50·9–73·6)	-32·4 (-73·5—21·1)
Child underweight	0·854 (0·485–1·28)	0·765 (0·425–1·16)	0·759 (0·42–1·13)	-10·3 (-22·9–2·81)	-0·875 (-10·8–8·84)	1 (0·581–1·47)	0·925 (0·53–1·39)	0·915 (0·515–1·36)	-7·56 (-19·2–5·23)	-1·14 (-9·99–8·77)
Iron deficiency	--	--	--	--	--	9·01 (7·5–10·7)	8·34 (6·95–9·98)	8·35 (6·95–9·94)	-7·5 (-16·2–2·16)	0·0919 (-5·7–6·32)
Vitamin A deficiency	3·84 (1·44–8·01)	1·59 (0·62–3·2)	1·31 (0·543–2·69)	-58·6 (-83–10·9)	-17·5 (-53·1–51·2)	3·02 (1·45–5·06)	1·82 (0·907–3·03)	1·56 (0·785–2·74)	-39·7 (-69·3–27·7)	-14 (-43·5–37·1)
Zinc deficiency	3·27 (0–10·1)	2·5 (0–7·94)	2·43 (0–7·64)	-23·6 (-86·5–NA)	-3·02 (-16·7–NA)	3·18 (0–10)	2·45 (0–7·91)	2·34 (0–7·4)	-22·9 (-80·6–NA)	-4·69 (-21·3–NA)
Smoking	26 (23·4–28·9)	18·7 (16·7–20·9)	15·6 (13·9–17·6)	-27·9 (-33·5—21·9)	-16·6 (-20·6—12·3)	17·4 (14·8–20·3)	13·7 (11·9–15·6)	11 (9·5–12·7)	-21·2 (-31·7—9·25)	-19·6 (-26·1—12·2)
Secondhand smoke	45·2 (41·8–48·2)	31·4 (28·6–34·4)	32 (29·2–34·9)	-30·4 (-36·7—23·5)	2·01 (-4·93–9·11)	43·1 (40·6–45·4)	30·6 (28·3–32·9)	31·3 (29–33·8)	-28·9 (-34—23·3)	2·33 (-2·97–7·84)
Alcohol use	11·4 (7·46–17)	14·9 (10·2–20·9)	17·1 (11·7–23·5)	31·5 (17·9–52·1)	14·6 (1·54–30·7)	5·6 (3·2–9·75)	7·47 (4·63–11·9)	8·39 (5·19–13)	33·5 (15·6–59·6)	12·3 (-2·83–32·9)
Drug use	0·253 (0·146–0·457)	0·294 (0·172–0·527)	0·34 (0·205–0·593)	15·9 (6·26–28·1)	15·8 (4·08–32·3)	0·178 (0·118–0·281)	0·217 (0·146–0·334)	0·228 (0·156–0·341)	22·2 (10·2–35·7)	4·93 (-4·37–15·3)
High fasting plasma glucose	5·68 (4·84–6·62)	9·03 (7·66–11·1)	11·4 (9·42–13·3)	59 (40·1–87·7)	25·9 (8·9–47·1)	4·85 (4·12–5·69)	7·05 (6·09–8·26)	8·7 (7·44–10·2)	45·4 (30·7–63·5)	23·5 (11·6–38·5)
High systolic blood pressure	21·6 (18·5–24·8)	28 (22·7–33·7)	28·8 (23–34·8)	29·5 (3·61–61·7)	2·86 (-14–22·3)	16·8 (13·6–20·5)	23·1 (16·8–30·1)	22·7 (15·9–30·9)	37·2 (-6·36–93·7)	-1·63 (-28·4–30·9)
High body-mass index	22·8 (16·3–30·8)	27·4 (20·1–36·5)	29·7 (22–39·3)	20·4 (9·27–34·5)	8·24 (2·19–15·1)	26·3 (18–38·1)	33·7 (25·2–45·3)	34·9 (26·6–47·6)	28·2 (12·6–53·8)	3·75 (-5·69–15·2)
Low bone mineral density	9·7 (5·3–16)	8·72 (4·44–14·9)	8·69 (4·51–14·7)	-10·1 (-33·9–15·8)	-0·313 (-26·1–33·3)	15·6 (10·1–22·7)	14·2 (9·07–21·1)	14 (8·96–20·9)	-9·03 (-29·2–14·4)	-1·1 (-22·8–24·6)
Diet low in fruits	64·4 (54·4–76·3)	53·3 (41·9–66·3)	51·5 (39·6–64·4)	-17·3 (-25—10·9)	-3·41 (-10·2–2·02)	65·7 (56·2–77·4)	55·1 (44·2–67·8)	53·4 (41·6–66)	-16·2 (-23·3—10·3)	-3·03 (-9·14–2·33)
Diet low in vegetables	75·3 (58·2–89·6)	55·2 (34·1–76·6)	52·9 (31·7–74·7)	-26·7 (-43·4—11·2)	-4·11 (-11·5–0·885)	73·8 (56·5–88·8)	53·2 (32·1–75·1)	50·9 (30·5–72·4)	-27·9 (-46·2—11·9)	-4·42 (-12·2–0·932)
Diet low in whole grains	97·4 (95·6–99·4)	88·3 (82·4–93·8)	88 (82·3–93·7)	-9·39 (-14·3—4·92)	-0·286 (-1·29–0·679)	96·4 (94·1–98·7)	86·6 (80·4–92·7)	86·3 (79·7–92·5)	-10·2 (-15·2—5·51)	-0·346 (-1·52–0·77)
Diet low in nuts and seeds	69·3 (35·9–85·6)	50·2 (21–71·2)	47·2 (19·7–69·4)	-27·7 (-50·4—13·8)	-5·83 (-18·6–3·48)	65·8 (33·5–83·6)	45·2 (16·9–67·8)	42·3 (14·7–65·9)	-31·4 (-57·2—15)	-6·38 (-20·4–4·09)
Diet low in milk	17 (4·87–35·8)	42·3 (18·8–62·2)	39·5 (17·3–59·9)	149 (37·8–500)	-6·8 (-19–0·776)	15·9 (4·47–35)	41·3 (17·5–61)	38·2 (15·5–57·4)	160 (38·2–576)	-7·44 (-21–1·22)
Diet high in red meat	98·2 (97·2–99)	95·4 (93·1–97·2)	93·3 (87·7–97·1)	-2·89 (-4·53—1·54)	-2·17 (-6·64–1·07)	98·2 (97·2–99)	95·4 (93–97·2)	93·2 (88·2–97)	-2·89 (-4·82—1·6)	-2·29 (-6·69–0·936)
Diet high in processed meat	73·4 (47·9–95·7)	72·5 (47·3–95·2)	73·1 (48·2–95·8)	-1·19 (-10·3–7·87)	0·921 (-4·32–7·2)	77·1 (52·6–97·6)	76 (51·1–97·1)	76·7 (51·9–97·5)	-1·39 (-8·79–4·92)	0·863 (-3·17–6·55)
Diet high in sugar-sweetened beverages	43·8 (25·5–62·3)	39 (23·3–56·1)	41·3 (24·1–59·2)	-11 (-40·5–30·5)	5·94 (-15·7–36)	48·9 (28·9–68·7)	42·4 (24·1–61·5)	45·5 (25·6–64·2)	-13·4 (-44·5–29·8)	7·47 (-14·9–39·9)
Diet low in fiber	57·7 (41·1–72·1)	54·3 (38·2–69·1)	50·6 (33·4–66·5)	-5·89 (-10·7—2·58)	-6·92 (-14·7—0·247)	58 (40·5–73)	54·8 (37·4–70·2)	51·2 (33·9–67)	-5·51 (-10·4—2·03)	-6·48 (-14·6—0·411)
Diet low in seafood omega-3 fatty acids	89·5 (75·5–100)	80·9 (62–100)	80·8 (60·5–99·7)	-9·54 (-25·2–1·51)	-0·173 (-16·9–18·5)	91·8 (80·9–100)	84·1 (65·5–99·9)	84·5 (67·4–100)	-8·37 (-23·8–0·54)	0·442 (-13·1–16·5)
Diet low in polyunsaturated fatty acids	51·7 (14·4–93·4)	44·5 (12·4–87·6)	43·7 (11·9–87·1)	-13·8 (-27—1·99)	-1·85 (-7·95–3·06)	54·1 (16·2–95)	47·2 (13–89·7)	46·4 (12·7–89·7)	-12·7 (-26·7—1·87)	-1·71 (-7·94–3·26)
Diet high in trans fatty acids	48·1 (37·5–99·2)	44 (34·3–95)	42·5 (33–94·6)	-8·55 (-18–2)	-3·56 (-14·7–6·96)	49·3 (38·3–100)	44·6 (34·7–98·9)	43·2 (33·2–98·6)	-9·39 (-18·2—0·00322)	-3·15 (-12·7–6·44)
Diet high in sodium	33·6 (6·82–58·8)	35·4 (7·48–61·8)	35·5 (6·45–62)	5·43 (-27·4–64·4)	0·329 (-24·6–28·5)	14·2 (3·39–35·7)	14·5 (3·35–37·1)	14·7 (3·42–37·5)	2·37 (-23·9–35·6)	1·49 (-14·7–23·4)
Low physical activity	4·16 (1·79–8·56)	4·9 (2·2–9·92)	5·08 (2·25–9·99)	17·6 (-0·221–44·7)	3·77 (-9·5–21·6)	6·99 (3·72–12·6)	7·78 (4·17–14·1)	7·95 (4·35–14)	11·2 (1·42–25·5)	2·13 (-6·45–13·2)
Occupational asthmagens	25·7 (21·9–30·4)	22·4 (19·3–26·2)	24·9 (21·3–29·7)	-13 (-19·8—6·44)	11·4 (4·46–20·8)	--	--	--	--	--
Occupational particulate matter, gases, and fumes	9·79 (7·08–13·8)	9·47 (6·93–13·4)	9·16 (6·67–13)	-3·2 (-7·28–1·33)	-3·34 (-4·86—1·78)	5·43 (3·81–7·84)	4·86 (3·52–7)	4·6 (3·35–6·71)	-10·5 (-16·6—3·24)	-5·48 (-8·22—2·69)
Occupational noise	9·5 (8·84–10·3)	9·34 (8·85–10·1)	9·15 (8·68–9·87)	-1·7 (-4·34–1·36)	-1·98 (-2·98—1·02)	6·91 (6·34–7·62)	7·03 (6·64–7·67)	7·03 (6·67–7·67)	1·73 (-2·01–6·11)	-0·0207 (-1·18–1·25)
Occupational injuries	--	--	--	--	--	--	--	--	--	--
Occupational ergonomic factors	20·9 (18·5–23·6)	15·4 (13·7–17·5)	16·4 (14·2–19·2)	-26·3 (-32·4—19·4)	6·73 (-1·3–16·4)	12·6 (10·7–14·7)	10·8 (9·26–12·8)	11·8 (9·89–14·2)	-13·8 (-21·9—4·81)	8·88 (-1·58–19·5)
Childhood sexual abuse	4·3 (2·79–6·04)	4·46 (3·12–6·13)	4·23 (2·93–5·99)	3·87 (-8·74–17·8)	-5·28 (-11·5–3·04)	9·47 (7·07–12·9)	9·17 (6·9–12·7)	8·76 (6·41–12·2)	-3·1 (-20·7–14·4)	-4·52 (-12–2·5)
Intimate partner violence	--	--	--	--	--	6·02 (3·3–8·68)	5·67 (3·08–7·97)	6·02 (3·3–8·68)	-5·76 (-14·6–7·4)	6·12 (-3·7–26·5)
Non-exclusive breastfeeding	22·7 (18·5–28)	22·1 (17·9–27)	22·2 (18·3–27·1)	-2·63 (-14·6–10·9)	0·246 (-10·5–11·9)	22·7 (18·5–28)	22·1 (17·9–27)	22·2 (18·3–27·1)	-2·65 (-14·7–10·9)	0·227 (-10·5–11·9)
Discontinued breastfeeding	23·3 (22–24·6)	24·8 (23·5–26)	23·6 (22·4–24·7)	6·64 (-0·611–15·1)	-4·81 (-9·54–0·539)	22·9 (21·6–24·2)	24·9 (23·6–26·1)	23·5 (22·3–24·6)	8·88 (1·48–17·5)	-5·53 (-10·2—0·218)
Diet low in calcium	9·98 (4·52–18·6)	8·94 (4·34–16·2)	7·65 (3·64–14·2)	-10·5 (-18·4–2·59)	-14·4 (-22·6—7·65)	4·39 (1·86–7·77)	3·72 (1·64–6·51)	2·87 (1·3–5)	-15·1 (-25·9–1·29)	-23 (-34—12·5)
Occupational exposure to asbestos	8·13 (7·22–9·02)	9·79 (8·71–10·9)	10·5 (9–12·2)	20·5 (3·49–40·4)	7·49 (-9·68–27)	1·97 (1·34–3·02)	1·35 (0·981–1·78)	1·29 (0·882–1·74)	-31·1 (-50—7·71)	-4·66 (-24·2–20·2)
Occupational exposure to arsenic	0·587 (0–1·38)	0·527 (0–1·25)	0·589 (0–1·38)	-10·2 (-14·8–NA)	11·7 (4·66–NA)	0·641 (0–1·53)	0·521 (0–1·22)	0·55 (0–1·3)	-18·7 (-24·1–NA)	5·57 (-0·779–NA)
Occupational exposure to benzene	1·01 (0·0604–2·91)	1·01 (0·0603–2·91)	1·12 (0·0667–3·23)	0·272 (-3·23–5·47)	10·5 (6·53–16·1)	1·7 (0·103–4·98)	1·81 (0·107–5·29)	1·95 (0·119–5·61)	6·52 (2·07–12·7)	7·36 (2·62–14·1)
Occupational exposure to beryllium	0·0297 (0·0292–0·0302)	0·0286 (0·0283–0·0288)	0·0315 (0·0309–0·0323)	-3·76 (-5·49—1·95)	10·4 (7·72–13·2)	0·0351 (0·0343–0·0358)	0·0352 (0·0348–0·0356)	0·038 (0·0371–0·0389)	0·345 (-2·08–2·83)	7·92 (5·11–10·9)
Occupational exposure to cadmium	0·0687 (0·0659–0·0717)	0·0624 (0·0608–0·0643)	0·0698 (0·0658–0·074)	-9·12 (-13·2—4·46)	11·7 (5·35–18·8)	0·0764 (0·0726–0·0804)	0·0646 (0·0622–0·0669)	0·0685 (0·065–0·0726)	-15·5 (-20·5—9·47)	6·14 (0·701–12·7)
Occupational exposure to chromium	0·161 (0·155–0·168)	0·15 (0·146–0·154)	0·168 (0·16–0·177)	-7·01 (-11·1—2·4)	12·1 (6·21–18·4)	0·192 (0·184–0·2)	0·17 (0·165–0·175)	0·182 (0·174–0·191)	-11·3 (-15·4—6·11)	7·02 (2·44–12·3)
Occupational exposure to diesel engine exhaust	0·876 (0·844–0·91)	0·879 (0·858–0·9)	0·972 (0·934–1·01)	0·307 (-4·21–5·09)	10·6 (6·22–14·9)	0·793 (0·759–0·831)	0·84 (0·816–0·866)	0·933 (0·882–0·991)	5·97 (-0·0332–11·8)	11 (4·67–18·1)
Occupational exposure to formaldehyde	0·337 (0·32–0·354)	0·299 (0·288–0·309)	0·331 (0·31–0·353)	-11·3 (-15·9—5·89)	10·8 (3·85–18·4)	0·384 (0·364–0·402)	0·328 (0·315–0·339)	0·346 (0·327–0·366)	-14·5 (-19—9·25)	5·56 (0·614–11·4)
Occupational exposure to nickel	0·572 (0–2·58)	0·512 (0–2·26)	0·566 (0–2·5)	-10·4 (-14·3–NA)	10·4 (4·1–NA)	0·502 (0–2·25)	0·392 (0–1·74)	0·409 (0–1·79)	-22 (-27·7–NA)	4·32 (-2·51–NA)
Occupational exposure to polycyclic aromatic hydrocarbons	0·334 (0·322–0·346)	0·313 (0·306–0·319)	0·349 (0·335–0·366)	-6·32 (-10·2—2·24)	11·8 (6·76–17·4)	0·408 (0·393–0·422)	0·372 (0·363–0·38)	0·397 (0·382–0·414)	-8·8 (-12·6—4·39)	6·8 (2·79–11·4)
Occupational exposure to silica	6·99 (1·05–22·8)	6·48 (0·961–20·5)	7·01 (1·03–22)	-7·26 (-14·4—0·983)	8·17 (1·41–16·6)	4·07 (0·585–13·1)	3·18 (0·445–9·83)	3·31 (0·467–10·5)	-21·8 (-28·4—13·8)	3·94 (-3·6–13·9)
Occupational exposure to sulfuric acid	0·981 (0·204–3·04)	0·86 (0·176–2·7)	0·951 (0·201–3·01)	-12·3 (-16·9—7·31)	10·5 (4·36–17·9)	--	--	--	--	--
Unsafe sex	--	--	--	--	--	--	--	--	--	--
Occupational exposure to trichloroethylene	0·0921 (0·0897–0·0952)	0·088 (0·0866–0·09)	0·0986 (0·0953–0·103)	-4·52 (-7·77—0·802)	12·1 (7·74–16·9)	0·109 (0·105–0·113)	0·103 (0·101–0·105)	0·111 (0·107–0·116)	-5·73 (-9·41—1·32)	8·54 (4·23–13·4)
No access to handwashing facility	3·3 (3·12–3·48)	3·16 (2·99–3·33)	3·12 (2·95–3·31)	-4·32 (-10·4–2·02)	-1·14 (-5·22–3)	3·3 (3·12–3·48)	3·16 (2·99–3·33)	3·12 (2·95–3·31)	-4·32 (-10·4–2·02)	-1·14 (-5·22–3)
Child wasting	1·23 (0·877–1·62)	1·21 (0·849–1·61)	1·21 (0·849–1·59)	-1·64 (-10–6·65)	-0·499 (-6·54–5·59)	1·04 (0·718–1·38)	1·01 (0·699–1·36)	1·01 (0·688–1·35)	-2·51 (-11·3–6·97)	-0·364 (-6·37–6·41)
Child stunting	1·53 (0·777–2·27)	1·17 (0·578–1·78)	1·18 (0·549–1·77)	-23·5 (-35·9—8·08)	0·277 (-12·6–15·2)	1·81 (0·903–2·66)	1·54 (0·79–2·32)	1·53 (0·781–2·27)	-14·9 (-28·2–0·97)	-0·301 (-12–12·7)
Chewing tobacco	0·523 (0·41–0·663)	0·728 (0·558–0·925)	1·03 (0·795–1·29)	39·2 (3·91–86)	41·2 (17·9–69·6)	--	--	--	--	--
Diet low in legumes	81·6 (56·5–100)	83·1 (60·7–100)	82·2 (58·1–100)	1·86 (-1·54–10·4)	-1·1 (-5·58–1·19)	78·9 (50·7–100)	80·5 (53·9–100)	79·6 (51·7–100)	2 (-1·58–12·6)	-1·08 (-6·73–1·74)
Short gestation	28·4 (22·4–35·7)	20·2 (16·2–24·8)	19·7 (15·8–24·7)	-28·8 (-47·5—4·56)	-2·35 (-24·5–26·1)	24·1 (19·6–29·3)	17·7 (14·3–21·6)	17·2 (14·6–20·4)	-26·4 (-44·5—0·675)	-2·6 (-20·7–21·3)
Low birth weight	14·3 (11·4–17·8)	10·3 (8·32–12·7)	9·73 (7·73–12)	-27·7 (-45·1—4·33)	-5·7 (-28·2–21·6)	12·2 (9·98–14·6)	9 (7·47–10·8)	8·93 (7·58–10·5)	-26·1 (-43·9–0·674)	-0·862 (-19·8–23)
High temperature	0 (0–0)	0 (0–0)	0 (0–0)	--	--	0 (0–0)	0 (0–0)	0 (0–0)	--	--
Low temperature	42·7 (37·1–47·7)	38·9 (32·7–44·7)	39·9 (33·4–45·8)	-9·03 (-12·5—5·84)	2·6 (0·391–4·63)	42·7 (37·1–47·7)	38·9 (32·7–44·7)	39·9 (33·4–45·8)	-9·03 (-12·5—5·84)	2·6 (0·391–4·63)
Kidney dysfunction	13·3 (8·87–19·4)	13·6 (9·12–19·9)	13·6 (9·15–20·1)	2·15 (-1·79–6·75)	0·0417 (-3·84–4·48)	14·5 (9·67–21·1)	15·2 (10·2–21·9)	15·1 (10·1–21·6)	4·8 (0·711–10·5)	-0·814 (-4·89–3·03)
Bullying victimization	3·7 (1·42–7·53)	4·08 (1·88–7·63)	4·24 (1·95–8)	10·1 (-15·1–76·1)	4·07 (-2·26–11·3)	2·77 (1·03–5·64)	2·95 (1·35–5·61)	3·08 (1·45–5·83)	6·63 (-14·6–71·2)	4·37 (-0·918–12·8)
High LDL cholesterol	59·1 (55·8–62·3)	56·3 (52·5–60)	57·3 (53·4–61)	-4·75 (-10·8–0·855)	1·75 (-3·22–6·75)	55·9 (52·9–59)	55·3 (52·1–58·6)	55·8 (52·7–59·2)	-1·03 (-6·04–3·88)	0·971 (-2·97–4·67)

Supplementary Results Table 1. Ireland age-standardised Summary Exposure Values (SEVs) for most-detailed risk factors and percentage change, by sex, 1990, 2010, and 2019. Data in parentheses are 95% uncertainty intervals.

	Male					Female				
	1990	2010	2019	Percentage change 1990-2010	Percentage change 2010-2019	1990	2010	2019	Percentage change 1990-2010	Percentage change 2010-2019
Unsafe sanitation	9·34 (6·02–14·2)	2·55 (1·45–4·11)	1·85 (1·07–3·08)	-72·7 (-85·5—50·4)	-27·6 (-55·7–18·8)	9·34 (6·02–14·2)	2·55 (1·45–4·11)	1·85 (1·07–3·08)	-72·7 (-85·5—50·4)	-27·6 (-55·7–18·8)
Ambient particulate matter pollution	11·2 (2·93–22·1)	7·81 (5·05–11)	5·13 (2·56–7·98)	-30·1 (-66·8–127)	-34·3 (-49·9—26·2)	11·2 (2·96–22)	7·81 (5·06–11)	5·13 (2·56–7·97)	-30 (-66·6–126)	-34·3 (-49·7—26·1)
Household air pollution from solid fuels	0·1 (0·0279–0·255)	0·0198 (0·0042–0·0584)	0·0107 (0·00197–0·0377)	-80·2 (-92·1—57·3)	-45·8 (-70·2—15·4)	0·132 (0·0397–0·337)	0·0262 (0·00596–0·073)	0·0142 (0·00255–0·0473)	-80·1 (-92—57)	-45·7 (-70·1—15·2)
Ambient ozone pollution	22·6 (8·29–42·1)	14 (4·7–28·1)	4·63 (1·26–10·3)	-37·7 (-55·1—23)	-67 (-79·2—54·4)	22·6 (8·29–42·1)	14 (4·7–28·1)	4·63 (1·26–10·3)	-37·7 (-55·1—23)	-67 (-79·2—54·4)
Residential radon	33·5 (14·6–60·7)	33·5 (14·6–60·7)	33·5 (14·6–60·7)	0 (0–0)	0 (0–0)	--	--	--	--	--
Lead exposure	59·6 (35·8–78)	40·7 (20·9–60·8)	29·3 (12·5–49·2)	-31·7 (-45—21·2)	-28 (-41·1—18·7)	34·2 (5·14–61·1)	24 (3·89–47·8)	16·8 (1·78–38·1)	-29·8 (-46·2—5·39)	-29·9 (-57·1—18·8)
Child underweight	0·948 (0·545–1·4)	0·757 (0·426–1·13)	0·738 (0·411–1·1)	-20·2 (-30·1—7·93)	-2·44 (-11·9–7·28)	1·08 (0·63–1·56)	0·903 (0·503–1·34)	0·879 (0·503–1·31)	-16·2 (-26·2—5·11)	-2·67 (-11·6–7·03)
Iron deficiency	--	--	--	--	--	10·5 (8·7–12·4)	9·09 (7·56–10·8)	8·84 (7·35–10·6)	-13·3 (-21·6—3·4)	-2·74 (-8·58–2·77)
Vitamin A deficiency	3·77 (1·58–7·48)	1·58 (0·626–3·41)	1·2 (0·515–2·61)	-58·2 (-82·8–8·8)	-23·7 (-55·7–45)	3·33 (1·7–5·75)	1·88 (0·897–3·3)	1·49 (0·707–2·54)	-43·6 (-72·1–18·3)	-20·4 (-47·6–23·1)
Zinc deficiency	2·11 (0–6·62)	1·83 (0–5·97)	1·53 (0–5·04)	-13·2 (-100–NA)	-16·3 (-41·6–NA)	2·06 (0–6·46)	1·8 (0–5·94)	1·51 (0–5)	-12·8 (-100–NA)	-16 (-37·7–NA)
Smoking	24·6 (22–27·6)	19 (16·8–21·4)	15·3 (13·4–17·5)	-22·8 (-27·6—18)	-19·5 (-22·9—15·9)	19·2 (16·8–21·9)	14·4 (12·4–16·6)	13·1 (11·3–15·2)	-24·8 (-30·2—18·8)	-8·89 (-13·7—4·12)
Secondhand smoke	48·4 (46·9–50·1)	34·8 (33·6–36)	32·1 (30–34)	-28·2 (-31·9—24·5)	-7·75 (-12·7—3·11)	46·7 (45·3–48·1)	32·7 (31·8–33·6)	30·4 (28·7–32)	-30 (-33·1—26·8)	-7·14 (-11·3—2·84)
Alcohol use	17·7 (13·1–23·1)	20 (14·6–26·1)	19·2 (13·8–25·3)	12·6 (3·61–26)	-3·6 (-13·2–5·79)	11·3 (7·82–15·6)	12·1 (8·47–16·6)	11·9 (8·13–16·7)	6·98 (-3·74–19·1)	-1·28 (-14·9–11·8)
Drug use	0·205 (0·12–0·356)	0·364 (0·26–0·535)	0·384 (0·273–0·561)	78 (43·6–136)	5·36 (-7·61–21·6)	0·13 (0·076–0·228)	0·212 (0·147–0·31)	0·23 (0·162–0·334)	62·4 (30·7–109)	8·79 (-1·36–20·5)
High fasting plasma glucose	4·57 (3·83–5·45)	8·08 (6·98–9·5)	11·9 (10–14)	76·6 (54·1–106)	46·8 (26·2–69·6)	3·67 (3·09–4·38)	5·86 (5·1–6·7)	8·56 (7·29–9·95)	59·5 (42·7–76·8)	46·1 (30·9–66·9)
High systolic blood pressure	39·3 (32·1–46·7)	37·6 (32–44)	38·8 (31·5–47·1)	-4·31 (-24·5–21·5)	3·15 (-14·6–24·3)	33·5 (25·4–43·3)	24·3 (19·7–29·8)	25·7 (18·6–33·8)	-27·4 (-47·7–3·53)	5·74 (-19·4–40·4)
High body-mass index	21 (14·9–28·3)	27·7 (20·6–36·6)	29·6 (22·3–38·7)	32 (18·7–51·7)	6·9 (1·1–13·6)	20·3 (13·2–29·3)	30·1 (22·4–41·2)	32·1 (23·9–43·7)	48·1 (28·7–85·6)	6·65 (-2·92–19·8)
Low bone mineral density	9·26 (5·11–15·2)	8·38 (4·51–14·2)	8·11 (4·21–14·1)	-9·46 (-33·5–20·4)	-3·24 (-26·3–29)	14·4 (10·1–20·5)	12·9 (8·18–19·4)	12·6 (7·76–18·7)	-9·85 (-31·5–15·2)	-2·57 (-24·4–26·3)
Diet low in fruits	67·1 (57·5–78·6)	48·2 (36·7–61·5)	45·8 (33·9–58·7)	-28·2 (-39·1—18·5)	-4·82 (-12·4–2·01)	69·1 (59·9–79·9)	49·5 (38–62·7)	47·6 (35·8–60·7)	-28·3 (-38·7—18·4)	-3·87 (-11·9–2·78)
Diet low in vegetables	54·2 (32·7–76·5)	38·6 (20·9–60·9)	30·2 (15·1–52·1)	-28·8 (-46·7—11·9)	-21·7 (-41·6—7·46)	52·1 (30·7–74·1)	36·1 (19·2–58·8)	27·9 (12·9–50·9)	-30·7 (-49·7—12·4)	-22·8 (-42·8—8·05)
Diet low in whole grains	75 (64·7–85)	68·1 (56–80·6)	70·4 (58·2–82·1)	-9·19 (-14·3—4·68)	3·31 (0·745–6·62)	71·9 (60·5–83·1)	64·2 (51·2–78·2)	66·7 (54·2–79·7)	-10·7 (-16·6—5·62)	3·91 (0·814–8·16)
Diet low in nuts and seeds	75·3 (38·3–89)	53·8 (22·8–74·3)	44·3 (16–67·2)	-28·5 (-52·5—14·5)	-17·6 (-39·4—6·72)	72·2 (37·7–87·1)	48·9 (19·7–70·9)	39·2 (13·1–63)	-32·3 (-57·1—16·4)	-19·7 (-41·3—6·82)
Diet low in milk	11·9 (2·45–29·4)	28·2 (9·8–46·7)	24·1 (7·94–42·8)	137 (38·3–596)	-14·5 (-34·5—1·1)	10·9 (2·2–27·3)	26·9 (9·45–44·8)	22·9 (7·2–41·5)	147 (43·2–692)	-14·8 (-36·2—0·915)
Diet high in red meat	94·8 (91·7–97·2)	99 (98·2–99·6)	98·6 (96·9–99·7)	4·41 (2·12–7·72)	-0·392 (-1·85–0·473)	94·8 (91·7–97·2)	99 (98·3–99·6)	98·7 (97–99·7)	4·4 (2·05–7·87)	-0·333 (-1·78–0·569)
Diet high in processed meat	59·6 (34·8–84·3)	74·5 (48·1–97·3)	70·7 (43·9–94·1)	25·1 (8·83–53·5)	-5·2 (-12·4—1·04)	66·5 (40·3–90·9)	77·2 (51·2–98·7)	75 (48·8–97·3)	16 (4·52–39·2)	-2·85 (-7·67—0·321)
Diet high in sugar-sweetened beverages	42·9 (25–60·9)	52·5 (37–67·6)	63·9 (45·4–78·3)	22·4 (-3·85–68·8)	21·7 (2·9–49·2)	49·7 (30·5–67·4)	61·4 (46·9–75·1)	71·5 (55·1–84·4)	23·6 (-2·24–72·8)	16·4 (0·974–36·6)
Diet low in fiber	24·5 (13·7–36·7)	25·2 (13·9–37·8)	21·3 (11–34)	2·82 (-8·63–14·9)	-15·3 (-30·4—0·501)	24 (13·1–36·3)	26·2 (14·1–39·3)	22·2 (12·1–34·8)	9·47 (1·29–18·5)	-15·4 (-30·2—1·07)
Diet low in seafood omega-3 fatty acids	85·7 (68·2–100)	82·9 (66–100)	80·7 (63·6–99·1)	-3·21 (-16·7–10·2)	-2·71 (-14·6–12·1)	88·5 (73·1–100)	86·2 (72·4–100)	84 (69·3–99·1)	-2·65 (-11·4–7·06)	-2·45 (-12·6–6·77)
Diet low in polyunsaturated fatty acids	61·6 (21·1–99·5)	59·3 (19·2–98·3)	54 (15·6–95·1)	-3·73 (-10·8–0)	-8·96 (-23—0·546)	63·6 (23·2–99·9)	61·3 (20·8–99·2)	56·2 (16·9–96·3)	-3·52 (-10·2–0)	-8·35 (-22·3—0·385)
Diet high in trans fatty acids	41·9 (32·6–93·2)	33·4 (24·5–89·7)	33·2 (24·2–88·9)	-20·3 (-32·4—1·75)	-0·453 (-10·6–12)	42·7 (32·9–98)	34·3 (24·6–94·6)	34·1 (24·5–94·8)	-19·7 (-31·7–0)	-0·539 (-9·61–10·4)
Diet high in sodium	23·6 (4·43–49·4)	24·4 (4·76–49·7)	25·7 (4·94–51·9)	3·05 (-29–49·1)	5·48 (-16·9–37·5)	11·3 (3·11–29·9)	10·9 (3·01–28·5)	11·3 (3·1–29·8)	-3·3 (-29·7–29)	3·66 (-14·5–26·7)
Low physical activity	5·24 (2·31–10·8)	5·76 (2·81–11·1)	6·69 (3·14–13·5)	10·1 (-15·7–57·1)	16 (-11·9–49·2)	11·3 (6·8–18·1)	11·4 (7·14–17·3)	12·2 (7·51–18·7)	0·909 (-12·4–20·4)	6·72 (-8·04–23)
Occupational asthmagens	21·6 (18·4–25·7)	18·3 (15·7–21·8)	19·3 (16·4–23)	-15 (-20·8—8·93)	5·14 (-1·77–12)	--	--	--	--	--
Occupational particulate matter, gases, and fumes	9·36 (6·73–13·4)	8·41 (6·07–12)	7·71 (5·53–11)	-10·1 (-12·7—7·41)	-8·35 (-10·5—6·33)	3·34 (2·4–4·92)	3·46 (2·46–5·14)	3·32 (2·35–4·89)	3·33 (-5·32–11·8)	-3·83 (-8·13–0·977)
Occupational noise	8·46 (8–9·09)	7·91 (7·51–8·56)	7·46 (7·07–8·11)	-6·61 (-8·24—4·81)	-5·66 (-6·57—4·72)	3·53 (3·32–3·8)	4·33 (4·12–4·58)	4·56 (4·35–4·83)	22·5 (18·5–26·8)	5·42 (4·07–6·74)
Occupational injuries	--	--	--	--	--	--	--	--	--	--
Occupational ergonomic factors	15·8 (14·2–17·7)	11·9 (10·5–13·7)	12·1 (10·4–14)	-24·6 (-31·3—17)	1·1 (-7·63–11·2)	4·44 (3·72–5·42)	6·37 (5·32–7·69)	7·04 (5·79–8·56)	43·5 (32–55·5)	10·6 (2·98–19)
Childhood sexual abuse	11·8 (7·6–16·5)	10·4 (9·21–11·7)	11·8 (7·6–16·5)	-12 (-33·4–25·6)	13·6 (-18·9–54·3)	6·05 (4·51–8·12)	5·14 (4·52–5·92)	6·05 (4·51–8·12)	-15 (-34–9·97)	17·6 (-7·86–55·1)
Intimate partner violence	--	--	--	--	--	15 (8·34–20·3)	11·6 (6·57–15·5)	13·2 (7·25–19)	-22·5 (-29·8—12·3)	13·7 (-1·27–40·4)
Non-exclusive breastfeeding	22·3 (17·5–28·1)	21·4 (16·8–27)	21·1 (16·7–26·6)	-3·96 (-14·9–8·91)	-1·6 (-10·1–7·53)	--	--	--	--	--
Discontinued breastfeeding	26·7 (26·2–27·2)	28·7 (28·1–29·2)	27·2 (26·6–27·8)	7·43 (4·66–10·1)	-5·1 (-7·06—2·88)	26·7 (26·2–27·2)	28·6 (28·1–29·2)	27·2 (26·6–27·8)	7·41 (4·63–10)	-5·1 (-7·06—2·87)
Diet low in calcium	13·2 (6·22–24·6)	11·1 (5·05–20·4)	7·86 (3·32–14·3)	-15·7 (-24·4—9·63)	-29·2 (-40—20·7)	7·2 (2·82–13·1)	5·69 (2·34–10·2)	3·09 (1·29–5·6)	-21 (-31·3—12·1)	-45·7 (-57·2—33·7)
Occupational exposure to asbestos	9·01 (7·27–11·2)	7·83 (6·95–8·75)	6·97 (5·32–9·2)	-13·1 (-32·8–12·3)	-11 (-33·1–17·2)	2·71 (0·528–9)	0·965 (0·463–1·65)	1·16 (0·365–3·13)	-64·4 (-90·4–39·9)	19·9 (-62·7–226)
Occupational exposure to arsenic	0·475 (0–1·12)	0·403 (0–0·946)	0·446 (0–1·03)	-15·1 (-20·6–NA)	10·6 (3·08–NA)	0·297 (0–0·711)	0·366 (0–0·851)	0·39 (0–0·92)	23·2 (14·5–NA)	6·58 (-1·38–NA)
Occupational exposure to benzene	0·666 (0·0393–1·9)	0·724 (0·042–2·02)	0·793 (0·0456–2·24)	8·7 (4·51–14·8)	9·52 (4·48–17·1)	0·64 (0·0383–1·82)	1·16 (0·0696–3·26)	1·28 (0·0779–3·71)	81·7 (72·8–95)	9·92 (3·96–19·1)
Occupational exposure to beryllium	0·022 (0·0216–0·0223)	0·0212 (0·0209–0·0215)	0·0234 (0·0227–0·0242)	-3·41 (-5·38—1·65)	10·4 (6·66–14·1)	0·0148 (0·0144–0·0151)	0·0235 (0·0232–0·0239)	0·0259 (0·0251–0·0269)	59·4 (54·9–64·3)	10·2 (6·34–14·3)
Occupational exposure to cadmium	0·0549 (0·0528–0·0573)	0·0477 (0·0457–0·0499)	0·0527 (0·0491–0·0567)	-13·1 (-18·2—7·88)	10·5 (3·6–17·8)	0·0351 (0·0334–0·0368)	0·0452 (0·0432–0·0472)	0·0483 (0·0451–0·0519)	28·7 (20·7–37·5)	7·05 (0·0169–14·5)
Occupational exposure to chromium	0·127 (0·122–0·132)	0·115 (0·111–0·12)	0·127 (0·12–0·135)	-9·17 (-14—4·33)	10·2 (3·96–16·6)	0·0869 (0·0834–0·0907)	0·119 (0·115–0·123)	0·128 (0·121–0·135)	37 (30–44·6)	7·21 (1·24–13·4)
Occupational exposure to diesel engine exhaust	0·632 (0·609–0·657)	0·688 (0·663–0·715)	0·749 (0·711–0·79)	8·86 (3·66–14·6)	8·85 (3·44–14·6)	0·377 (0·357–0·4)	0·619 (0·586–0·653)	0·669 (0·616–0·727)	64 (52·3–76·1)	8·16 (0·103–16·7)
Occupational exposure to formaldehyde	0·266 (0·251–0·279)	0·227 (0·216–0·239)	0·248 (0·229–0·267)	-14·4 (-19·9—8·85)	8·97 (1·74–16·7)	0·17 (0·161–0·179)	0·225 (0·215–0·234)	0·239 (0·223–0·256)	31·8 (24·2–40·3)	6·33 (-0·00127–13·6)
Occupational exposure to nickel	0·443 (0–1·97)	0·385 (0–1·7)	0·429 (0–1·86)	-13·2 (-18·5–NA)	11·4 (3·98–NA)	0·234 (0–1·03)	0·275 (0–1·24)	0·293 (0–1·32)	17·4 (8·14–NA)	6·43 (-2·41–NA)
Occupational exposure to polycyclic aromatic hydrocarbons	0·254 (0·246–0·263)	0·236 (0·229–0·244)	0·261 (0·247–0·276)	-6·97 (-11·3—2·54)	10·4 (4·8–16·4)	0·178 (0·171–0·185)	0·254 (0·247–0·262)	0·273 (0·259–0·288)	42·7 (36·1–50)	7·59 (2·48–13·3)
Occupational exposure to silica	4·87 (0·699–15·6)	4·74 (0·664–15)	5·29 (0·755–16·9)	-2·8 (-10·8–7·26)	11·7 (4·1–22·5)	1·98 (0·269–6·19)	2·28 (0·316–7·14)	2·41 (0·332–7·49)	15·3 (5·78–28·3)	6·01 (-3·01–17·7)
Occupational exposure to sulfuric acid	0·799 (0·169–2·52)	0·677 (0·143–2·16)	0·729 (0·154–2·27)	-15·3 (-20·4—10·2)	7·71 (1·06–15·2)	0·447 (0·0918–1·47)	0·55 (0·111–1·73)	0·571 (0·114–1·77)	23·1 (14·1–33·1)	3·75 (-3·95–12)
Unsafe sex	--	--	--	--	--	--	--	--	--	--
Occupational exposure to trichloroethylene	0·0722 (0·0703–0·0745)	0·0686 (0·0668–0·071)	0·0751 (0·0719–0·0792)	-4·95 (-8·72—0·819)	9·5 (4·66–14·9)	0·0499 (0·0481–0·052)	0·0729 (0·0707–0·0756)	0·0787 (0·0748–0·0835)	46·2 (39–53·8)	7·98 (2·24–14·1)
No access to handwashing facility	3·36 (3·18–3·56)	3·18 (3·02–3·37)	3·14 (2·96–3·32)	-5·27 (-11–1·19)	-1·46 (-5·4–2·74)	3·36 (3·18–3·56)	3·18 (3·02–3·37)	3·14 (2·96–3·32)	-5·27 (-11–1·19)	-1·46 (-5·4–2·74)
Child wasting	1·25 (0·877–1·64)	1·19 (0·834–1·57)	1·18 (0·814–1·56)	-4·45 (-12–3·99)	-1·07 (-6·79–4·82)	1·05 (0·737–1·4)	0·997 (0·699–1·33)	0·988 (0·694–1·32)	-5·09 (-13·4–3·25)	-0·912 (-6·36–4·37)
Child stunting	1·7 (0·874–2·54)	1·18 (0·564–1·85)	1·16 (0·553–1·76)	-30·6 (-43·4—16·4)	-1·55 (-13·5–12·1)	2·01 (1·05–2·93)	1·54 (0·767–2·33)	1·5 (0·764–2·26)	-23·3 (-36·4—8·36)	-2·44 (-13·4–10·8)
Chewing tobacco	0·145 (0·114–0·183)	0·146 (0·115–0·185)	0·149 (0·117–0·186)	0·695 (-22·7–33)	1·91 (-15–20·8)	--	--	--	--	--
Diet low in legumes	86·1 (67·2–100)	82·4 (59–100)	81·2 (56·7–100)	-4·25 (-12·7–0)	-1·51 (-6·47–0·182)	83·5 (61–100)	79·6 (51·9–100)	78·3 (48·3–99·9)	-4·65 (-15·8–0)	-1·65 (-8·08–0·304)
Short gestation	21 (16·7–26·3)	16·8 (13·5–20·8)	16·6 (12·7–21·6)	-20·2 (-39·6–3·69)	-1·27 (-25·1–30·4)	20 (16·7–23·7)	14·4 (11·9–17·3)	15·1 (12·3–18·5)	-27·9 (-42·4—11·4)	4·39 (-17–30·4)
Low birth weight	9·21 (7·34–11·5)	7·22 (5·88–8·88)	7·91 (6·18–10)	-21·7 (-41–3·24)	9·62 (-17·4–46·7)	9·29 (7·86–10·8)	6·61 (5·53–7·88)	7·35 (5·99–8·92)	-28·8 (-43·5—10·7)	11·1 (-14·8–41·6)
High temperature	0 (0–0)	0 (0–0)	0 (0–0)	--	--	0 (0–0)	0 (0–0)	0 (0–0)	--	--
Low temperature	31·3 (26·4–36·6)	34·3 (29·9–39·1)	30·8 (25·8–36·3)	9·47 (6·59–13·1)	-10 (-13·6—6·95)	31·3 (26·4–36·6)	34·3 (29·9–39·1)	30·8 (25·8–36·3)	9·47 (6·59–13·1)	-10 (-13·6—6·95)
Kidney dysfunction	17·1 (11·7–23·8)	17·4 (12·4–24)	17·9 (12·6–24·7)	1·71 (-6·79–9·43)	3·24 (-0·927–8·53)	19·2 (13·3–26·4)	17·7 (12·5–24·4)	18·2 (12·8–24·9)	-7·81 (-15·6–0·816)	2·89 (-0·639–7·77)
Bullying victimization	5·03 (1·9–10·4)	6·02 (2·84–11·2)	6·53 (3·01–12·2)	19·8 (-8·38–95·9)	8·41 (0·296–16·6)	3·5 (1·32–7·38)	4·22 (1·98–7·8)	4·53 (2·11–8·46)	20·6 (-3·18–104)	7·43 (-0·409–14·3)
High LDL cholesterol	42 (38·7–45·6)	38·6 (35·2–42·5)	39·3 (35·8–43)	-8·07 (-13·6—2)	1·81 (-2·33–6·53)	43·8 (40·5–47·4)	40·5 (37–44·1)	41·2 (37·9–44·7)	-7·58 (-12·3—2·54)	1·81 (-1·9–5·62)

Supplementary Results Table 1. Israel age-standardised Summary Exposure Values (SEVs) for most-detailed risk factors and percentage change, by sex, 1990, 2010, and 2019. Data in parentheses are 95% uncertainty intervals.										
	Male					Female				
	1990	2010	2019	Percentage change 1990-2010	Percentage change 2010-2019	1990	2010	2019	Percentage change 1990-2010	Percentage change 2010-2019
Unsafe sanitation	3·72 (2·1–5·95)	1·59 (0·897–2·51)	1·27 (0·736–2·18)	-57·2 (-78·6–-15·1)	-20·4 (-50·8–23·2)	3·72 (2·1–5·95)	1·59 (0·897–2·51)	1·27 (0·736–2·18)	-57·2 (-78·6–-15·1)	-20·4 (-50·8–23·2)
Ambient particulate matter pollution	21·9 (10·3–36·7)	24·8 (20·6–29·7)	19·3 (15·5–23·7)	13·3 (-32·3–126)	-22·1 (-25·2–-19·7)	21·8 (10·4–36·7)	24·8 (20·6–29·7)	19·3 (15·5–23·7)	13·4 (-32·3–126)	-22·1 (-25·1–-19·7)
Household air pollution from solid fuels	0·0738 (0·0217–0·183)	0·022 (0·00552–0·0572)	0·0161 (0·00354–0·0446)	-70·2 (-86·3–-42·6)	-26·7 (-54·2–14·2)	0·0964 (0·0306–0·243)	0·0287 (0·00758–0·0756)	0·0211 (0·00541–0·0568)	-70·3 (-86·3–-42·6)	-26·3 (-53·9–14·8)
Ambient ozone pollution	88·6 (44·2–100)	87·6 (43–100)	80·2 (35·4–100)	-1·15 (-10·8–7·31)	-8·47 (-23·1–0)	88·6 (44·2–100)	87·6 (43–100)	80·2 (35·4–100)	-1·15 (-10·8–7·31)	-8·47 (-23·1–0)
Residential radon	18·7 (7·66–37·8)	18·7 (7·66–37·8)	18·7 (7·66–37·8)	0 (0–0)	0 (0–0)	18·7 (7·66–37·8)	18·7 (7·66–37·8)	18·7 (7·66–37·8)	0 (0–0)	0 (0–0)
Lead exposure	39·6 (11·7–63·3)	27·7 (8·22–50)	19·9 (4·07–41·7)	-30 (-46·2–-18·3)	-28·1 (-50·5–-16·8)	24·3 (0·353–49·1)	15·4 (0·866–36·4)	10·5 (0·302–29·6)	-36·4 (-53·3–122)	-31·8 (-67·1–19)
Child underweight	0·906 (0·514–1·37)	0·76 (0·434–1·13)	0·752 (0·419–1·11)	-16·1 (-27–-3·63)	-1·04 (-9·98–8·7)	1·06 (0·611–1·55)	0·903 (0·515–1·34)	0·892 (0·507–1·34)	-14·6 (-25·3–-3·75)	-1·2 (-10·1–7·71)
Iron deficiency	--	--	--	--	--	11·2 (9·39–13·1)	9·99 (8·28–11·8)	9·77 (8·12–11·5)	-11·1 (-20·8–-1·25)	-2·12 (-8·11–3·91)
Vitamin A deficiency	24·5 (15·9–33·7)	13·7 (6·8–24·3)	11·9 (5·36–22·2)	-43·8 (-73·2–18·6)	-13·4 (-48·1–46·9)	17·7 (10·9–25·2)	13·1 (6·78–20·6)	11·1 (5·98–18·3)	-26·4 (-57·7–29·4)	-14·8 (-41·6–25·4)
Zinc deficiency	2·39 (0–7·7)	1·52 (0–5·06)	1·44 (0–4·77)	-36·4 (-100–NA)	-5·22 (-18·4–NA)	2·32 (0–7·62)	1·48 (0–5·02)	1·41 (0–4·72)	-36·1 (-100–NA)	-4·6 (-21·3–NA)
Smoking	26·3 (23·1–29·7)	20·3 (17·9–22·9)	17·6 (15·6–19·8)	-22·8 (-28·2–-17)	-13·3 (-16·9–-9·67)	14·9 (12·1–17·8)	10·5 (8·98–12·3)	8·65 (7·4–10·2)	-29·9 (-39·4–-18·2)	-17·3 (-23–-11·3)
Secondhand smoke	41·7 (40–43·6)	35 (32·5–37·8)	32·9 (30·2–35·6)	-16·1 (-23·1–-8·76)	-5·94 (-11·4–0·164)	45·8 (44·1–47·5)	39 (36·8–41·4)	36·6 (34·1–38·9)	-14·9 (-20·5–-9·22)	-6·2 (-10·8–-1·85)
Alcohol use	5·44 (2·96–9·27)	6·87 (4·22–11)	8·07 (4·64–12·7)	26·5 (3·78–63·4)	17·4 (-7·35–47·9)	2·12 (1·03–3·96)	2·71 (1·49–4·73)	3·17 (1·67–5·53)	27·7 (-0·783–74·6)	17·2 (-10·7–52)
Drug use	0·17 (0·0848–0·339)	0·196 (0·114–0·356)	0·212 (0·125–0·371)	15·3 (0·314–40)	8·12 (0·427–18·8)	0·123 (0·0669–0·231)	0·153 (0·0941–0·253)	0·164 (0·105–0·264)	24·4 (5·51–54·8)	7·67 (-0·376–18·3)
High fasting plasma glucose	6·45 (5·61–7·49)	11·3 (10·3–12·5)	11·8 (9·85–14·1)	75 (52·6–100)	4·59 (-10·9–21·2)	6·64 (5·63–7·89)	10·9 (9·9–12·1)	10·9 (9·28–12·6)	64 (40·4–89·9)	-0·139 (-13·3–12·9)
High systolic blood pressure	31·4 (27·4–35·7)	32·4 (27·6–37·5)	34·7 (27·8–42·2)	2·96 (-15·5–25·5)	7·15 (-8·91–25)	29·6 (24·8–34·9)	27·7 (21·6–34·5)	29·1 (21·3–38·8)	-6·35 (-30·8–24·4)	5·2 (-18·8–33·9)
High body-mass index	19·2 (13·8–25·9)	24·5 (18·1–32·2)	25·8 (19–33·8)	27·2 (14·5–43·7)	5·71 (-0·531–13·2)	21·1 (14·4–29·8)	27·2 (19·5–38·6)	28·9 (20·9–40·7)	28·9 (12·4–53·6)	6·06 (-3·73–18·6)
Low bone mineral density	9·33 (5·11–15·8)	8·63 (4·62–14·4)	8·33 (4·28–14·2)	-7·52 (-31·5–21·5)	-3·48 (-28·7–26·5)	15 (9·69–21·7)	14 (9·03–20·5)	13·6 (8·58–19·9)	-6·6 (-27·4–21·2)	-2·54 (-23·5–24·9)
Diet low in fruits	15 (9·2–22·1)	20·8 (13·2–29·4)	19·9 (12·1–29·2)	38·8 (25–60·1)	-4·1 (-24·1–15·7)	17·5 (11·1–25·4)	23·5 (15·2–32·7)	22·2 (14–31·4)	34·5 (22·8–51·8)	-5·24 (-23·4–11·9)
Diet low in vegetables	5·25 (2·21–14)	2·69 (1·34–8·49)	2·91 (1·45–8·74)	-48·9 (-77·7–-12·1)	8·27 (-3·72–55·8)	4·03 (1·86–11·3)	2·62 (1·32–8·38)	2·73 (1·37–8·49)	-34·9 (-68·7–-5·52)	4·18 (-3·27–30·4)
Diet low in whole grains	99·9 (100–100)	99·8 (99·7–100)	99·7 (99·4–100)	-0·101 (-0·322–0·014)	-0·0736 (-0·289–0·0542)	99·9 (99·9–100)	99·5 (99–100)	99·3 (98·6–100)	-0·421 (-0·888–0·0031)	-0·168 (-0·589–0·0792)
Diet low in nuts and seeds	11·9 (1·91–50·8)	8·83 (1·13–50·5)	9·11 (1·44–50·5)	-25·9 (-55·9–-0·561)	3·19 (-15·6–40·8)	9·69 (1·29–50·5)	7·83 (0·845–50·4)	8·08 (0·855–50·4)	-19·2 (-53·9–-0·0485)	3·2 (-9·94–38·4)
Diet low in milk	76·4 (52–96·1)	67 (38·8–89·1)	66·2 (37·2–88)	-12·3 (-28·2–-2·41)	-1·27 (-5·1–1·11)	76·1 (51·2–96·2)	66·5 (38·1–88·1)	65·7 (37·6–87·7)	-12·6 (-28·9–-2·32)	-1·24 (-5·54–1·23)
Diet high in red meat	35·8 (24·9–46·6)	48·9 (37·6–59·7)	51·3 (39·1–62·5)	36·5 (22·2–60·9)	4·96 (-4·28–16)	35·7 (24·7–46·6)	48·7 (37·5–59·2)	51·2 (39·5–62·8)	36·3 (21·8–61·8)	5·04 (-3·57–16·5)
Diet high in processed meat	41 (22·3–64)	45·8 (25·9–69·7)	50·6 (29–75·2)	11·7 (-3·41–33·9)	10·6 (0·329–28·9)	48·4 (27·4–72·3)	55·2 (32·4–80·4)	59·7 (34·9–84·3)	14·1 (-0·201–37)	8·3 (-1·23–22·7)
Diet high in sugar-sweetened beverages	43 (25·7–60·3)	54·2 (38·6–69·2)	58·5 (41·5–74·3)	26 (1·02–75·1)	7·85 (-8·23–28·3)	50 (31·5–66·8)	63·4 (48·6–76·8)	67 (50·1–80·9)	26·8 (4·01–69·7)	5·76 (-8·76–21·3)
Diet low in fiber	12·1 (6·41–19·3)	11·8 (6·2–18·7)	11·8 (5·96–19·5)	-2·86 (-11·5–5·41)	0·117 (-20·1–23·9)	11·4 (5·71–18·7)	11·1 (5·58–18·1)	11·1 (5·46–18·7)	-2·91 (-11·9–6·92)	0·351 (-21–25·4)
Diet low in seafood omega-3 fatty acids	83·3 (65·5–99·9)	72·3 (54·5–98·4)	71·7 (53·1–96·8)	-13·2 (-29·1–3·04)	-0·918 (-18·3–20·1)	86·7 (71·7–100)	75·1 (55·4–99)	74·7 (56·7–98·9)	-13·5 (-29·7–0·414)	-0·5 (-17·2–19·5)
Diet low in polyunsaturated fatty acids	20·4 (6·06–50·9)	17·8 (5·08–47·7)	17·4 (4·91–47·8)	-13 (-24·2–-1·79)	-2·03 (-13·4–9·29)	22·9 (6·63–55·1)	20·1 (5·62–50·8)	19·7 (5·64–49·5)	-12·3 (-23·7–-1·47)	-1·88 (-12·5–9·41)
Diet high in trans fatty acids	34·1 (24·8–91)	29·4 (20·7–86·9)	29·4 (20·9–84)	-13·8 (-27·2–-0·0131)	0·129 (-9·53–12·5)	35·2 (25·6–97·9)	30·5 (21·1–94·7)	30·5 (21·2–90·8)	-13·4 (-25·6–0)	-0·0803 (-9·15–12·1)
Diet high in sodium	30 (6·23–56·1)	34 (7·84–59·9)	35·7 (8·82–61·9)	13·5 (-18·5–90·9)	4·91 (-15·2–38·2)	15·1 (3·84–37)	15·9 (3·85–38·5)	16·7 (3·96–40·6)	4·9 (-18·2–43)	5·37 (-9·11–29·3)
Low physical activity	4·04 (1·69–8·25)	4·71 (2·05–9·62)	4·78 (2·11–9·49)	16·4 (-0·946–40)	1·52 (-11·6–15·9)	6·85 (3·62–12·6)	7·6 (4·14–13·6)	7·66 (4·18–13·6)	11 (1·29–24·8)	0·757 (-8·09–11·3)
Occupational asthmagens	18·6 (15·8–21·9)	19·7 (17–23·2)	21 (17·9–24·7)	6·14 (-1·56–14·8)	6·6 (-0·559–15·2)	--	--	--	--	--
Occupational particulate matter, gases, and fumes	7·16 (5·11–10·4)	6·76 (4·86–9·79)	6·82 (4·9–9·96)	-5·62 (-9·62–-0·779)	0·881 (-1·21–3·48)	2·69 (1·89–3·9)	2·77 (1·96–4·03)	2·9 (2·07–4·21)	2·86 (-4·52–11·4)	4·65 (1·23–8·55)
Occupational noise	6·74 (6·29–7·36)	6·51 (6·14–7·11)	6·7 (6·34–7·3)	-3·43 (-6·02–-0·591)	2·97 (1·72–4·25)	3·33 (3·07–3·67)	3·96 (3·74–4·3)	4·54 (4·3–4·9)	18·8 (14–23·5)	14·7 (12·9–16·3)
Occupational injuries	--	--	--	--	--	--	--	--	--	--
Occupational ergonomic factors	9·28 (8·01–11)	10 (8·61–11·8)	10·5 (8·88–12·3)	7·92 (-0·0582–15·9)	4·85 (-1·75–11·5)	4·05 (3·38–4·96)	6·46 (5·39–7·9)	7·23 (5·99–8·86)	59·3 (47·2–72·6)	12 (3·6–21)
Childhood sexual abuse	11·9 (7·82–16·8)	12·7 (8·47–17·9)	11·9 (7·82–16·8)	7·32 (-1·07–17·7)	-6·82 (-14·2–1·89)	8·18 (6·18–11·2)	8·13 (6·16–11)	8·18 (6·18–11·2)	-0·647 (-6·92–6·56)	0·652 (-5·73–8·63)
Intimate partner violence	--	--	--	--	--	24·4 (13·7–33·1)	24·5 (14·1–32·7)	24·4 (13·7–33·1)	0·434 (-6·45–11·9)	-0·432 (-7·43–15)
Non-exclusive breastfeeding	21 (16·6–26·5)	20·4 (16·1–26)	20·1 (16–25·6)	-3·1 (-14·6–9·87)	-1·23 (-9·31–7·13)	--	--	--	--	--
Discontinued breastfeeding	22·6 (21·3–23·9)	22·8 (21·4–24·1)	22·4 (21–23·8)	0·818 (-6·25–8·57)	-1·71 (-6·39–3·14)	22·6 (21·3–23·9)	22·8 (21·4–24·1)	22·4 (21–23·8)	0·902 (-6·17–8·66)	-1·82 (-6·49–3·02)
Diet low in calcium	19·3 (9·13–36·3)	15·5 (7·06–29·2)	14·1 (6·56–26·4)	-19·4 (-27·7–-13·1)	-8·97 (-15–-4·12)	13·6 (5·49–25·8)	9·62 (3·83–17·5)	8·15 (3·12–14·7)	-29·1 (-40·7–-20·5)	-15·3 (-24·6–-7·69)
Occupational exposure to asbestos	4·46 (3·97–4·96)	4·7 (4·27–5·15)	3·64 (2·73–4·75)	5·44 (-8·15–21·4)	-22·5 (-41·9–2·28)	1·32 (0·961–2·27)	1·07 (0·842–1·57)	1·01 (0·689–1·55)	-18·7 (-41·1–7·36)	-5·69 (-29·3–22·5)
Occupational exposure to arsenic	0·477 (0–1·12)	0·487 (0–1·14)	0·519 (0–1·24)	2·15 (-6·78–NA)	6·55 (-2·67–NA)	--	--	--	--	--
Occupational exposure to benzene	0·708 (0·0417–2·06)	0·861 (0·0502–2·54)	0·94 (0·0539–2·7)	21·7 (16·2–30·3)	9·1 (3·62–16·3)	0·779 (0·0448–2·23)	1·38 (0·0801–3·96)	1·56 (0·0892–4·55)	76·8 (67–92·8)	12·9 (7·13–20·8)
Occupational exposure to beryllium	0·0207 (0·0202–0·0213)	0·0239 (0·0235–0·0244)	0·0259 (0·025–0·0268)	15·3 (11·5–19)	8·33 (4·08–12·5)	0·0174 (0·0169–0·018)	0·0263 (0·0258–0·0268)	0·0291 (0·0281–0·0301)	51 (45·8–57)	10·8 (6·41–15)
Occupational exposure to cadmium	0·0548 (0·0502–0·0593)	0·0572 (0·0549–0·0596)	0·061 (0·0578–0·0644)	4·25 (-4·17–13·8)	6·72 (-1·57–15)	0·0379 (0·0346–0·0416)	0·0491 (0·0471–0·051)	0·053 (0·0502–0·0559)	29·5 (17·7–42·6)	8·11 (0·848–16·7)
Occupational exposure to chromium	0·128 (0·119–0·137)	0·138 (0·133–0·143)	0·148 (0·141–0·155)	7·7 (-0·0493–16·2)	7·23 (-0·0374–14·4)	0·0936 (0·0866–0·101)	0·128 (0·124–0·132)	0·14 (0·133–0·146)	36·8 (26·5–47·7)	9·04 (2·52–16·3)
Occupational exposure to diesel engine exhaust	0·581 (0·55–0·615)	0·747 (0·721–0·774)	0·811 (0·773–0·845)	28·4 (21·1–36·6)	8·63 (2·45–14·9)	0·406 (0·381–0·431)	0·595 (0·565–0·626)	0·648 (0·61–0·689)	46·7 (36·8–57·9)	8·89 (1·35–16·5)
Occupational exposure to formaldehyde	0·274 (0·249–0·298)	0·278 (0·265–0·291)	0·294 (0·277–0·312)	1·5 (-7·12–11)	5·61 (-2·82–14·3)	0·184 (0·168–0·202)	0·251 (0·24–0·261)	0·273 (0·258–0·287)	36·1 (24·3–48·7)	8·88 (2·27–16·8)
Occupational exposure to nickel	0·438 (0–1·93)	0·459 (0–2·03)	0·487 (0–2·13)	4·73 (-3·86–NA)	6·29 (-1·52–NA)	0·257 (0–1·12)	0·315 (0–1·39)	0·334 (0–1·51)	22·5 (9·08–NA)	6·1 (-2·09–NA)
Occupational exposure to polycyclic aromatic hydrocarbons	0·258 (0·24–0·275)	0·282 (0·273–0·292)	0·303 (0·29–0·317)	9·57 (2·19–17·4)	7·55 (0·799–14)	0·196 (0·183–0·209)	0·282 (0·275–0·291)	0·311 (0·298–0·324)	44·1 (34·1–54·4)	10 (4·31–16·4)
Occupational exposure to silica	4·74 (0·718–15·2)	5·44 (0·81–17·1)	5·81 (0·829–18·2)	14·9 (6·31–25·9)	6·77 (-1·25–16·1)	2·29 (0·313–7·4)	2·73 (0·375–8·69)	2·85 (0·379–9·01)	19·2 (6·92–36·8)	4·49 (-4·14–16·4)
Occupational exposure to sulfuric acid	0·75 (0·157–2·38)	0·766 (0·158–2·37)	0·811 (0·171–2·48)	2·19 (-5·49–11·2)	5·79 (-2·13–13·9)	0·451 (0·0917–1·4)	0·551 (0·117–1·68)	0·588 (0·123–1·93)	22·2 (10·3–36·6)	6·76 (-1·87–16·5)
Unsafe sex	--	--	--	--	--	--	--	--	--	--
Occupational exposure to trichloroethylene	0·0704 (0·0671–0·0742)	0·0792 (0·0773–0·0818)	0·0853 (0·0822–0·0892)	12·5 (6·72–19)	7·72 (1·97–13·5)	0·0536 (0·0507–0·057)	0·0747 (0·0724–0·0774)	0·0816 (0·0783–0·0856)	39·2 (31·2–48·7)	9·21 (3·31–15·8)
No access to handwashing facility	3·39 (3·2–3·59)	3·24 (3·06–3·42)	3·2 (3·03–3·37)	-4·46 (-9·74–2)	-1·19 (-5·04–2·95)	3·39 (3·2–3·59)	3·24 (3·06–3·42)	3·2 (3·03–3·37)	-4·46 (-9·74–2)	-1·19 (-5·04–2·95)
Child wasting	1·23 (0·867–1·63)	1·18 (0·825–1·56)	1·18 (0·821–1·55)	-4·12 (-12·2–4·57)	-0·519 (-6·54–5·58)	1·04 (0·72–1·37)	0·99 (0·688–1·32)	0·987 (0·679–1·31)	-4·65 (-13·2–3·6)	-0·268 (-5·26–5·12)
Child stunting	1·66 (0·819–2·43)	1·2 (0·553–1·81)	1·18 (0·553–1·83)	-27·8 (-40·5–-14·3)	-1·08 (-13–12·9)	1·95 (1·01–2·83)	1·57 (0·776–2·36)	1·54 (0·774–2·31)	-19·8 (-33·7–-3·86)	-1·78 (-12·4–10·1)
Chewing tobacco	0·207 (0·164–0·256)	0·216 (0·169–0·275)	0·237 (0·184–0·3)	4·61 (-21·4–37·9)	9·55 (-7·68–31·2)	0·151 (0·109–0·204)	0·156 (0·112–0·216)	0·163 (0·118–0·221)	3·16 (-29·7–50·6)	4·79 (-19·2–34·8)
Diet low in legumes	72·4 (33·9–98·8)	50 (10·1–81·8)	57·5 (13·8–89·5)	-30·9 (-77·1–-8·08)	15 (1·56–77·8)	69·2 (27·6–97·9)	40·3 (6·35–70·7)	50·9 (11·3–82·2)	-41·7 (-83·8–-11·8)	26·3 (3·3–102)
Short gestation	26·8 (20·1–35·1)	16·7 (14·1–19·5)	15·1 (12·3–18·4)	-37·8 (-53·4–-14·5)	-9·58 (-28·4–12·7)	27·2 (22–33·3)	18·7 (16–22·3)	15·5 (12·8–18·4)	-31·1 (-45·3–-13·6)	-17·3 (-32·4–1·31)
Low birth weight	11·9 (9·04–15·2)	7·7 (6·55–8·96)	7·19 (5·87–8·56)	-35·2 (-51·5–-10·8)	-6·63 (-26·2–15·4)	12·7 (10·4–15·3)	9·11 (7·73–10·7)	7·42 (6·11–8·75)	-28·2 (-43–-8·64)	-18·5 (-34·9–1·25)
High temperature	23·3 (11·4–37·2)	27·7 (14·5–43·1)	25·8 (12·9–41)	18·7 (6·24–35·4)	-6·67 (-12·5–-3·23)	23·3 (11·4–37·2)	27·7 (14·5–43·1)	25·8 (12·9–41)	18·7 (6·24–35·4)	-6·67 (-12·5–-3·23)
Low temperature	35 (19·9–50·4)	32 (16·8–48·2)	35·3 (20·4–50·8)	-8·44 (-18·6–-2·6)	10·2 (2·91–23·7)	35 (19·9–50·4)	32 (16·8–48·2)	35·3 (20·4–50·8)	-8·44 (-18·6–-2·6)	10·2 (2·91–23·7)
Kidney dysfunction	16·7 (11·6–23·3)	18·2 (12·9–24·9)	17·8 (12·7–24·6)	8·98 (2·51–16·5)	-1·76 (-6·84–3·15)	17·7 (12·3–24·3)	18·9 (13·5–25·8)	18·6 (13·3–25·3)	6·94 (1·9–13·2)	-1·95 (-6·77–3·12)

Bullying victimization	8·01<br>(3·24–16·6)	9·76<br>(4·45–18·5)	9·4<br>(4·26–17·8)	21·8<br>(3·36–60·4)	-3·7<br>(-11·8–3·06)	4·88<br>(2·05–9·9)	5·51<br>(2·52–10·4)	5·26<br>(2·37–10·2)	12·9<br>(-6·01–52·1)	-4·66<br>(-12–2·85)
High LDL cholesterol	47·9<br>(44·1–51·4)	40·4<br>(36·7–44)	40·9<br>(37·3–44·5)	-15·6<br>(-21·1–-9·62)	1·27<br>(-3·13–6·04)	48<br>(44·6–51·5)	41·9<br>(38·5–45·5)	42·4<br>(39·1–45·9)	-12·6<br>(-17·5–-7·81)	1·01<br>(-2·64–4·92)

Supplementary Results Table 1. Italy age-standardised Summary Exposure Values (SEVs) for most-detailed risk factors and percentage change, by sex, 1990, 2010, and 2019. Data in parentheses are 95% uncertainty intervals.

	Male					Female				
	1990	2010	2019	Percentage change 1990-2010	Percentage change 2010-2019	1990	2010	2019	Percentage change 1990-2010	Percentage change 2010-2019
Unsafe sanitation	3·63 (2·09–5·77)	1·75 (0·989–2·81)	1·42 (0·826–2·44)	-51·8 (-75·3—1·43)	-19 (-50·1–28·1)	3·64 (2·1–5·78)	1·76 (0·992–2·82)	1·42 (0·826–2·44)	-51·8 (-75·3—1·39)	-19·1 (-50·3–27·9)
Ambient particulate matter pollution	24·8 (11·7–41·2)	19·5 (16–23·7)	14·9 (11·7–18·6)	-21·6 (-53·8–57·5)	-23·5 (-27—20·6)	24·7 (11·7–41)	19·4 (16–23·6)	14·9 (11·7–18·6)	-21·5 (-53·7–57·6)	-23·3 (-26·8—20·5)
Household air pollution from solid fuels	0·251 (0·0746–0·59)	0·0575 (0·0154–0·155)	0·0367 (0·00952–0·102)	-77·1 (-89·3—54·7)	-36·2 (-60·8–2·73)	0·323 (0·105–0·759)	0·0753 (0·0218–0·204)	0·0482 (0·0134–0·134)	-76·7 (-89·1—54·6)	-35·9 (-60·8–2·92)
Ambient ozone pollution	89·3 (44·9–100)	65·8 (29·5–92·5)	83 (40·5–99·9)	-26·2 (-37·8—7·46)	26·1 (7·94–40·6)	89·3 (44·9–100)	65·6 (29·4–92·4)	83 (40·5–99·9)	-26·5 (-38·1—7·59)	26·5 (8·08–41·1)
Residential radon	29·8 (16·6–47)	30 (16·5–47·3)	30·1 (16·6–47·7)	0·447 (-1·04–1·55)	0·433 (-0·462–1·35)	29·9 (16·6–47·1)	30 (16·6–47·3)	30·1 (16·6–47·6)	0·307 (-0·948–1·33)	0·416 (-0·677–1·45)
Lead exposure	59 (36·7–77·2)	38·6 (20–58·9)	26·8 (11·4–46·7)	-34·6 (-47·4—22·5)	-30·5 (-43·5—19·8)	39·7 (11·6–64)	25·5 (7·41–48·1)	16·8 (3·15–37·3)	-35·6 (-51·9—22·7)	-34·1 (-56·7—21·4)
Child underweight	0·954 (0·541–1·4)	0·75 (0·426–1·12)	0·747 (0·414–1·12)	-21·4 (-31·8—8·79)	-0·326 (-9·29–9·13)	1·08 (0·62–1·57)	0·889 (0·502–1·31)	0·886 (0·503–1·32)	-17·7 (-28—6·01)	-0·419 (-8·72–8·94)
Iron deficiency	--	--	--	--	--	9·99 (8·35–11·8)	8·9 (7·33–10·6)	8·81 (7·23–10·5)	-10·9 (-19·5—1·13)	-1·06 (-6·65–4·65)
Vitamin A deficiency	5·96 (2·41–11·6)	2·88 (1·1–5·9)	2·5 (1·01–5·18)	-51·8 (-80·1—30)	-13·2 (-48·3–56)	4·34 (2·3–7·35)	2·88 (1·43–5·03)	2·56 (1·29–4·5)	-33·7 (-65·7–29·9)	-11·2 (-41·8–35·5)
Zinc deficiency	2·87 (0–8·99)	2·29 (0–7·28)	2·33 (0–7·29)	-20·1 (-98·5–NA)	1·64 (-14·7–NA)	2·8 (0–8·53)	2·22 (0–7·07)	2·27 (0–7·21)	-20·6 (-76·9–NA)	2·2 (-14–NA)
Smoking	27·1 (23·9–30·5)	20·1 (17·7–22·7)	17·7 (15·6–20·1)	-25·6 (-30·6—20·5)	-12 (-15·3—8·39)	14·1 (11·9–16·4)	12·3 (10·5–14·2)	11·7 (9·96–13·5)	-12·9 (-20·3—3·89)	-4·82 (-9·3–0·0356)
Secondhand smoke	44·7 (41·8–47·6)	38·8 (36·7–40·8)	37·1 (34·4–40)	-13·4 (-20·5—4·89)	-4·17 (-9·34–1·15)	48·3 (46·1–50·5)	41·2 (39·6–42·8)	39·2 (37–41·4)	-14·7 (-19·8—9·25)	-4·9 (-8·68—1·21)
Alcohol use	21·2 (15·7–27·5)	20·1 (14–27·7)	20·3 (13·6–28·7)	-5·31 (-17·6–9·34)	1·23 (-9·41–12·1)	10·8 (7·48–14·9)	8·46 (5·67–12)	8·6 (5·38–12·4)	-21·4 (-33·8—7·89)	1·65 (-16·3–20·8)
Drug use	0·301 (0·17–0·557)	0·358 (0·197–0·681)	0·227 (0·114–0·461)	18·7 (10·2–26·2)	-36·5 (-45·3—29·6)	0·164 (0·0865–0·319)	0·23 (0·129–0·419)	0·16 (0·0898–0·3)	40·2 (29·1–58·3)	-30·4 (-36·4—24·1)
High fasting plasma glucose	9·5 (8·22–10·9)	16 (14·4–17·6)	14·9 (13–17)	68·4 (53·7–86·4)	-7·04 (-13·2—0·623)	8·45 (7·42–9·56)	11·2 (10·1–12·5)	11·5 (10–13·1)	32·7 (23·3–44·2)	2·24 (-5–10·1)
High systolic blood pressure	39·8 (37·3–42·4)	29·5 (26·7–32·2)	28·5 (23·5–34)	-25·8 (-32·2—18·9)	-3·44 (-18·8–14)	35·9 (33·4–38·4)	25·1 (22·8–27·6)	23·6 (18·1–29·9)	-29·9 (-36·7—22·8)	-6·14 (-26–16·9)
High body-mass index	18·2 (13–24·4)	23·3 (17·4–30·5)	23·8 (17·8–31·3)	28 (16·3–43·5)	2·19 (-3·28–7·98)	17·3 (12·7–23·4)	21·4 (16·4–28·4)	22·4 (17·3–29·6)	23·6 (10·6–41·5)	4·74 (-2·55–13)
Low bone mineral density	9·26 (5·23–15·1)	8·65 (4·75–14·2)	8·5 (4·69–14·2)	-6·59 (-14–1·05)	-1·77 (-9·55–5·7)	14·7 (9·76–21·2)	15 (10–21·4)	14·8 (9·9–20·9)	2·38 (-3·75–9·9)	-1·66 (-8·43–4·75)
Diet low in fruits	26·8 (19·1–35·9)	23 (16·1–31·4)	24·6 (17·1–33·6)	-14·2 (-19·6—8·97)	7·31 (-7·63–23·5)	28·8 (20·6–38·1)	25·1 (17·8–33·6)	26·8 (18·4–36·1)	-12·7 (-18·1—7·98)	6·65 (-7·55–22·3)
Diet low in vegetables	11·7 (5·36–24·2)	24·1 (11·9–42·7)	26·9 (14·1–45·3)	106 (57·1–167)	11·7 (-1·78–32·6)	9·96 (4·46–22·1)	21·4 (10·4–39·1)	24·2 (11·6–43·7)	114 (59·1–180)	13·3 (-2·28–34)
Diet low in whole grains	99·3 (98·9–99·6)	99·8 (99·8–100)	99·7 (99·6–99·9)	0·544 (0·299–0·871)	-0·0886 (-0·182—0·0105)	99·3 (99–99·6)	99·6 (99·5–99·9)	99·5 (99·4–99·8)	0·371 (0·188–0·611)	-0·0983 (-0·206—0·0155)
Diet low in nuts and seeds	56·8 (25·6–73·6)	49·6 (21·4–69·4)	51·1 (23·6–70·5)	-12·6 (-23·4—4·84)	3·1 (-4·7–13·9)	51·6 (22–70·4)	44·6 (17·9–66)	46·1 (18·8–67)	-13·6 (-25·6—4·92)	3·37 (-5·77–16·1)
Diet low in milk	67·8 (41·5–88·6)	55·2 (31·6–74·8)	57·7 (33·1–77·9)	-18·5 (-37·1—6·29)	4·44 (0·0897–12·3)	67·4 (41·5–87·8)	54·4 (31·7–73·9)	56·9 (33·2–77·3)	-19·2 (-39·4—6·86)	4·58 (0·181–13)
Diet high in red meat	80 (73·8–85·4)	77·4 (70·9–83·3)	75·1 (66·9–82·8)	-3·25 (-6—0·478)	-2·97 (-9·19–3·14)	80 (74·2–85·5)	77·2 (70·5–83·2)	75 (66·8–82·6)	-3·44 (-6·46—0·809)	-2·91 (-9·51–2·68)
Diet high in processed meat	67·2 (42·9–88·9)	73 (48·3–94)	72·5 (48–93·7)	8·53 (2·78–18·1)	-0·715 (-4·5–2·85)	71 (46·6–92·8)	76·5 (52·5–96·5)	76·2 (52·6–96·1)	7·77 (2·38–17·3)	-0·497 (-3·58–2·5)
Diet high in sugar-sweetened beverages	33 (22·9–47)	34·8 (24·3–48·6)	36·1 (24·9–50·5)	5·5 (-11·5–26·3)	3·74 (-9·29–20·5)	34·7 (23·4–49)	37·8 (27·1–51·3)	39 (26·9–53·7)	9·02 (-10·2–32·7)	3 (-11·1–19·9)
Diet low in fiber	28·2 (17·3–40)	26·1 (15·8–37·4)	29 (17·6–41·8)	-7·47 (-12·6—2·4)	10·8 (-1·69–24·9)	27·9 (16·7–40·2)	26·1 (15·7–37·5)	28·7 (16·6–41·2)	-6·63 (-12·1—1·2)	9·97 (-2·6–25)
Diet low in seafood omega-3 fatty acids	90·3 (81·3–99·8)	90·8 (82·4–99·7)	90·6 (81·8–99·7)	0·597 (-1·31–3·29)	-0·181 (-2·76–2·11)	91·7 (83·7–99·8)	92·2 (84·7–99·9)	92 (84·1–100)	0·528 (-1·3–3·16)	-0·15 (-2·41–1·87)
Diet low in polyunsaturated fatty acids	41·3 (11·5–84·2)	34·4 (9·68–75·4)	33·9 (9·46–73·5)	-16·7 (-28·8—2·55)	-1·44 (-8·06–5·63)	44·1 (12·5–86·5)	37·2 (10·4–79·1)	36·7 (10·2–78·7)	-15·5 (-27·8—2·4)	-1·42 (-7·96–5·5)
Diet high in trans fatty acids	26·6 (18·7–84·1)	25·1 (17·1–86·2)	25·5 (17·5–83·2)	-5·7 (-18·7–4·86)	1·58 (-7·55–16)	27·6 (19–88·3)	26·4 (17·5–88·5)	26·7 (17·8–88·1)	-4·63 (-17–5·07)	1·17 (-6·86–11·3)
Diet high in sodium	50·6 (22·7–72·2)	49·3 (21–70·6)	51 (23·3–72·1)	-2·46 (-26·5–31·7)	3·51 (-11·3–24·9)	67·2 (0·201–97·2)	66·5 (0·197–97·1)	67·5 (0·208–97·1)	-1·03 (-19·7–25)	1·57 (-11·8–19·4)
Low physical activity	4·81 (2·1–9·65)	4·52 (1·94–9·38)	4·24 (1·81–8·72)	-6·01 (-16·6–2·05)	-6·11 (-17·4–6·61)	8·96 (4·98–15·5)	8·85 (4·94–15·1)	8·73 (4·83–15·1)	-1·19 (-6·41–3·17)	-1·29 (-6·38–3·44)
Occupational asthmagens	23·5 (19·8–27·9)	18·7 (16–22·3)	17·3 (14·7–20·4)	-20·3 (-26·5—13·9)	-7·52 (-13·5—1·09)	--	--	--	--	--
Occupational particulate matter, gases, and fumes	8·79 (6·28–12·6)	8 (5·66–11·4)	7·34 (5·21–10·6)	-9·01 (-12·9—5·15)	-8·25 (-10·9—5·31)	3·59 (2·52–5·22)	3·38 (2·39–5·02)	3·15 (2·24–4·66)	-5·73 (-12·7—1·93)	-6·81 (-11—2·54)
Occupational noise	8·1 (7·66–8·72)	7·49 (7·11–8·11)	7·02 (6·66–7·65)	-7·5 (-9·23—5·82)	-6·29 (-7·15—5·42)	3·64 (3·41–3·94)	3·83 (3·64–4·11)	3·84 (3·64–4·11)	5·41 (2·55–8·46)	0·175 (-0·981–1·43)
Occupational injuries	--	--	--	--	--	--	--	--	--	--
Occupational ergonomic factors	13·4 (11·7–15·4)	9·69 (8·33–11·4)	9·27 (7·85–11)	-27·7 (-31·6—23·7)	-4·41 (-10·4–2·7)	5·85 (4·96–6·85)	5·29 (4·4–6·36)	5·54 (4·56–6·76)	-9·6 (-18·6—0·0813)	4·69 (-3·94–14·2)
Childhood sexual abuse	7·82 (5·11–11·2)	7·82 (5·11–11·2)	7·82 (5·11–11·2)	-2·2e-14 (-4·05e-14–2·14e-14)	2·2e-14 (-2·19e-14–3·97e-14)	12·2 (8·99–16·8)	12·2 (8·99–16·8)	12·2 (8·99–16·8)	0 (-3·55e-14–2·67e-14)	0 (-3·34e-14–2·63e-14)
Intimate partner violence	--	--	--	--	--	28·4 (16·3–37·2)	27·9 (15·2–36·8)	28·4 (16·3–37·2)	-1·67 (-8·44–8·92)	1·7 (-6·35–12·8)
Non-exclusive breastfeeding	25·7 (21·5–30·4)	25·1 (21–29·6)	25 (21·2–29·5)	-2·26 (-15·1–12·2)	-0·519 (-11·1–11·7)	25·7 (21·6–30·5)	25·1 (21–29·6)	25 (21·2–29·4)	-2·23 (-15·9–12·1)	-0·548 (-11·1–12)
Discontinued breastfeeding	24·1 (23–25·2)	23·4 (22·2–24·6)	22·7 (21·4–23·8)	-3·13 (-9·8–3·57)	-3·05 (-8·57–2·81)	24·1 (22·9–25·2)	23·4 (22·3–24·7)	22·7 (21·4–23·9)	-2·76 (-9·46–3·96)	-3·27 (-8·78–2·58)
Diet low in calcium	20·1 (10·2–37)	16·4 (7·99–30·3)	16·8 (8·37–31·4)	-18·3 (-26·5—12·3)	2·46 (-1·76–7·22)	13·5 (5·85–25·4)	9·95 (4·14–18·4)	10·4 (4·34–19·2)	-26·3 (-35·6—18·7)	4·49 (-2·15–12·1)
Occupational exposure to asbestos	13·3 (12·4–14·4)	12·6 (12·2–13)	11·7 (9·55–14)	-4·91 (-12·8–2·74)	-7·08 (-23·4–10·7)	4·54 (2·36–5·95)	3·66 (2·12–4·09)	3·49 (1·83–4·61)	-19·3 (-37·4–3·69)	-4·79 (-25·1–19·9)
Occupational exposure to arsenic	0·538 (0–1·28)	0·465 (0–1·09)	0·451 (0–1·05)	-13·5 (-20·3–NA)	-2·87 (-10·3–NA)	0·369 (0–0·852)	0·373 (0–0·874)	0·371 (0–0·874)	1·02 (-9·4–NA)	-0·523 (-8·06–NA)
Occupational exposure to benzene	0·742 (0·0447–2·11)	0·702 (0·0418–2)	0·701 (0·0402–2·04)	-5·41 (-9·58—0·48)	-0·187 (-4·44–6·6)	0·633 (0·0397–1·85)	0·865 (0·0514–2·46)	0·934 (0·0553–2·67)	36·5 (29·7–47·8)	8·03 (2·19–15·5)
Occupational exposure to beryllium	0·0226 (0·0223–0·023)	0·0213 (0·0211–0·0215)	0·0212 (0·0206–0·0218)	-5·87 (-7·54—4·26)	-0·597 (-3·72–2·25)	0·0161 (0·0158–0·0165)	0·0193 (0·0191–0·0195)	0·0203 (0·0197–0·021)	19·6 (16·6–22·7)	5·29 (1·73–9·13)
Occupational exposure to cadmium	0·0616 (0·0576–0·0658)	0·0538 (0·0526–0·055)	0·0524 (0·0489–0·0556)	-12·6 (-18·9—5·94)	-2·59 (-9·1–3·59)	0·0416 (0·0383–0·045)	0·0429 (0·0416–0·0442)	0·0431 (0·0402–0·0462)	2·96 (-5·79–13·2)	0·501 (-6·61–8·16)
Occupational exposure to chromium	0·143 (0·135–0·152)	0·127 (0·125–0·129)	0·124 (0·117–0·131)	-11·3 (-16·9—5·38)	-2·21 (-7·92–3·08)	0·0993 (0·0924–0·106)	0·107 (0·105–0·11)	0·109 (0·103–0·116)	8·29 (0·644–17·1)	1·67 (-4·5–8·12)
Occupational exposure to diesel engine exhaust	0·654 (0·622–0·684)	0·666 (0·648–0·683)	0·665 (0·632–0·697)	1·89 (-3·32–7·38)	-0·155 (-5·16–4·87)	0·364 (0·344–0·387)	0·472 (0·448–0·499)	0·501 (0·466–0·542)	29·7 (20–40·2)	6·23 (-0·638–13·8)
Occupational exposure to formaldehyde	0·307 (0·285–0·33)	0·259 (0·25–0·266)	0·249 (0·232–0·266)	-15·7 (-22—8·69)	-3·9 (-10·7–2·48)	0·196 (0·179–0·211)	0·206 (0·199–0·212)	0·208 (0·194–0·222)	5·3 (-2·84–15·7)	0·788 (-5·82–8·16)
Occupational exposure to nickel	0·501 (0–2·15)	0·453 (0–2)	0·441 (0–1·92)	-9·66 (-15·7–NA)	-2·57 (-8·33–NA)	0·291 (0–1·24)	0·282 (0–1·27)	0·279 (0–1·25)	-3·17 (-12·9–NA)	-1·14 (-8·73–NA)
Occupational exposure to polycyclic aromatic hydrocarbons	0·289 (0·274–0·304)	0·261 (0·256–0·265)	0·255 (0·242–0·268)	-9·72 (-14·8—4·44)	-1·98 (-7·07–2·74)	0·201 (0·189–0·213)	0·224 (0·219–0·229)	0·23 (0·218–0·242)	11·2 (4·28–19·5)	2·58 (-2·92–8·38)
Occupational exposure to silica	5·5 (0·755–17·9)	5·58 (0·82–17·7)	5·53 (0·813–17·7)	1·58 (-5·57–10·1)	-0·949 (-7·37–6·3)	2·46 (0·342–7·38)	2·35 (0·321–7·33)	2·38 (0·343–7·48)	-4·18 (-14·1–8·27)	0·931 (-7·61–11·1)
Occupational exposure to sulfuric acid	0·879 (0·18–2·81)	0·74 (0·157–2·35)	0·702 (0·143–2·25)	-15·8 (-21·6—9·17)	-5·15 (-11·4–1·12)	0·505 (0·107–1·58)	0·506 (0·107–1·58)	0·503 (0·104–1·56)	0·341 (-8·88–10·7)	-0·7 (-8·52–7·76)
Unsafe sex	--	--	--	--	--	--	--	--	--	--
Occupational exposure to trichloroethylene	0·0781 (0·0756–0·0814)	0·0707 (0·0697–0·0722)	0·0696 (0·0668–0·0729)	-9·51 (-13·5—5·4)	-1·58 (-6·07–2·63)	0·0543 (0·0518–0·0572)	0·062 (0·0605–0·064)	0·064 (0·0607–0·0676)	14·2 (7·88–21·1)	3·22 (-1·93–8·51)
No access to handwashing facility	3·39 (3·21–3·6)	3·24 (3·07–3·43)	3·2 (3·04–3·4)	-4·52 (-10·7–1·7)	-1·1 (-5·31–3·02)	3·39 (3·21–3·6)	3·24 (3·07–3·43)	3·2 (3·04–3·4)	-4·52 (-10·7–1·69)	-1·11 (-5·32–3·02)
Child wasting	1·25 (0·887–1·65)	1·18 (0·83–1·55)	1·18 (0·839–1·55)	-5·04 (-12·7–3·1)	-0·0487 (-5·24–5·88)	0·991 (0·691–1·27)	1·22 (0·858–1·55)	0·93 (0·624–1·2)	22·7 (-3·51–53·2)	-23·5 (-41·4—2·77)
Child stunting	1·7 (0·877–2·53)	1·18 (0·553–1·8)	1·17 (0·549–1·79)	-30·9 (-44·3—17)	-0·323 (-14–15·7)	2·01 (1·05–2·91)	1·53 (0·75–2·29)	1·53 (0·718–2·32)	-23·6 (-35·9—9·71)	-0·349 (-11·5–12·5)
Chewing tobacco	0·17 (0·135–0·214)	0·181 (0·144–0·227)	0·185 (0·147–0·237)	6·5 (-17·6–35·2)	1·98 (-14·6–22)	0·136 (0·0954–0·189)	0·136 (0·0965–0·187)	0·138 (0·0984–0·187)	0·0765 (-33·1–45·1)	0·955 (-20·8–30·8)
Diet low in legumes	71·4 (32·2–98·6)	56·9 (15·6–87·7)	63·1 (21·2–92)	-20·3 (-56·7—5·91)	11 (2·2–54·1)	68·1 (26·5–96·8)	50·2 (11·6–79·6)	58·2 (17–88·1)	-26·3 (-63·8—9·41)	15·8 (3·81–67·9)
Short gestation	18·8 (16·9–20·9)	13·4 (12–14·8)	12·7 (11·4–14·1)	-28·7 (-33·9—23·5)	-4·96 (-11·2–0·929)	19·8 (18–21·8)	13·5 (12·3–14·8)	12·4 (11·2–13·8)	-32·2 (-36·6—27·5)	-7·61 (-14·2—1·57)
Low birth weight	10·8 (10–11·6)	7·82 (7·26–8·46)	7·43 (6·85–8)	-27·3 (-32·7—21·9)	-5·01 (-11·1–1·56)	11·3 (10·6–12·1)	7·82 (7·24–8·41)	7·15 (6·61–7·72)	-31 (-35·5—26·1)	-8·52 (-14·5—2·2)
High temperature	20·6 (13·9–28·3)	23·1 (16–31·2)	23·8 (16·8–31·7)	12·5 (5·2–21)	3·01 (-0·249–6·88)	20·6 (13·9–28·3)	23·1 (16–31·2)	23·8 (16·8–31·7)	12·5 (5·2–21)	3·01 (-0·249–6·88)
Low temperature	32·5 (28·8–36·6)	33·9 (30·3–38)	33·2 (29·4–37·5)	4·25 (3·39–5·24)	-1·87 (-3·02—0·754)	32·5 (28·8–36·6)	33·9 (30·3–38)	33·2 (29·4–37·5)	4·25 (3·39–5·24)	-1·87 (-3·02—0·754)
Kidney dysfunction	15·5 (10·6–22)	15·2 (10·4–21·8)	15·1 (10·2–21·6)	-1·81 (-3·44—0·429)	-0·899 (-2·38–0·458)	16·3 (11–23·1)	16 (10·8–22·6)	16·2 (11–23·1)	-1·96 (-3·7—0·547)	1·32 (-0·0939–2·79)
Bullying victimization	5·23 (2·11–10·6)	5·38 (2·2–10·8)	5·32 (2·09–10·8)	2·75 (-6·52–12·2)	-1·1 (-7·42–3·22)	4·49 (1·88–9·07)	4·41 (1·82–8·81)	4·37 (1·71–8·86)	-1·6 (-9·5–8·03)	-0·951 (-7·79–3·51)
High LDL cholesterol	49·1 (46·1–52·2)	43·6 (40·4–46·7)	43·3 (39·7–46·7)	-11·2 (-15·1—7·3)	-0·769 (-4·77–3·68)	46·8 (43·8–49·8)	44·3 (41·2–47·6)	44·6 (41·3–48)	-5·42 (-9·03—1·57)	0·69 (-2·7–4·25)

Supplementary Results Table 1. Luxembourg age-standardised Summary Exposure Values (SEVs) for most-detailed risk factors and percentage change, by sex, 1990, 2010, and 2019. Data in parentheses are 95% uncertainty intervals.

	Male					Female				
	1990	2010	2019	Percentage change 1990-2010	Percentage change 2010-2019	1990	2010	2019	Percentage change 1990-2010	Percentage change 2010-2019
Unsafe sanitation	1·59 (0·912–2·67)	0·691 (0·395–1·14)	0·592 (0·342–0·935)	-56·4 (-78·6–-14·9)	-14·4 (-47·7–37·2)	1·59 (0·912–2·67)	0·691 (0·395–1·14)	0·592 (0·342–0·935)	-56·4 (-78·6–-14·9)	-14·4 (-47·7–37·2)
Ambient particulate matter pollution	15·2 (5·21–28·4)	11·9 (8·86–15·5)	8·03 (5·25–11·2)	-21·8 (-58·5–108)	-32·6 (-41·1–-27)	15·2 (5·23–28·4)	11·9 (8·85–15·5)	8·03 (5·25–11·3)	-21·8 (-58·5–107)	-32·6 (-41–-27·1)
Household air pollution from solid fuels	0·0464 (0·0113–0·124)	0·0168 (0·0035–0·0507)	0·0101 (0·00193–0·0314)	-63·9 (-82·8–-25·8)	-39·5 (-63·4–-2·68)	0·061 (0·0151–0·16)	0·0222 (0·00467–0·0653)	0·0134 (0·00254–0·0424)	-63·6 (-82·6–-25·4)	-39·4 (-63·1–-2·35)
Ambient ozone pollution	32·9 (12·1–61·3)	42·1 (16·9–73·9)	42·5 (16·8–73·6)	27·9 (-4·63–86·7)	1·13 (-22·9–30·8)	32·9 (12·1–61·3)	42·1 (16·9–73·9)	42·5 (16·8–73·6)	27·9 (-4·63–86·7)	1·13 (-22·9–30·8)
Residential radon	40·7 (17·7–72·9)	40·7 (17·7–72·9)	40·7 (17·7–72·9)	0 (0–0)	0 (0–0)	40·7 (17·7–72·9)	40·7 (17·7–72·9)	40·7 (17·7–72·9)	0 (0–0)	0 (0–0)
Lead exposure	37·1 (6·58–63·2)	25·4 (5·09–48·3)	17·3 (2·42–37·7)	-31·7 (-47·2–-18·1)	-31·8 (-55·7–-20·4)	20·7 (0·0306–48·2)	14·5 (0·0997–37·3)	9·98 (0·0192–29·8)	-30·1 (-47·6–223)	-31·2 (-77·6–-20)
Child underweight	0·927 (0·539–1·38)	0·781 (0·431–1·16)	0·769 (0·437–1·14)	-15·7 (-26·4–-3·93)	-1·54 (-10·4–8·24)	1·11 (0·661–1·62)	0·973 (0·56–1·43)	0·95 (0·548–1·42)	-12·6 (-22·9–0·553)	-2·3 (-10·4–6·18)
Iron deficiency	--	--	--	--	--	10·4 (8·55–12·3)	9·05 (7·48–10·7)	8·95 (7·35–10·7)	-12·9 (-21·4–-3·96)	-1·05 (-6·84–4·93)
Vitamin A deficiency	2·67 (1·03–5·54)	1·29 (0·542–2·55)	1·15 (0·492–2·22)	-51·6 (-79·9–36·8)	-11·3 (-50·9–69·7)	2·24 (1·1–4·01)	1·52 (0·761–2·72)	1·36 (0·697–2·43)	-32·1 (-67·1–36)	-10·6 (-40·7–39)
Zinc deficiency	2·63 (0–8·55)	2·02 (0–6·48)	1·94 (0–6·31)	-23·3 (-64·5–NA)	-4·09 (-28·7–NA)	2·57 (0–8·31)	1·98 (0–6·59)	1·9 (0–6·27)	-23 (-67·9–NA)	-3·9 (-20·2–NA)
Smoking	24·5 (20·5–28·7)	20·6 (17·4–24·1)	18·3 (15·3–21·5)	-15·9 (-25·2–-5·37)	-11·4 (-16·7–-5·92)	16·8 (13·7–20·2)	14·6 (12–17·6)	12·4 (10·1–15)	-13·2 (-25·1–1·54)	-15·2 (-21·5–-7·83)
Secondhand smoke	40·4 (37·5–43·1)	35·3 (33·2–37·4)	33·8 (31·1–36·5)	-12·6 (-20·6–-4·24)	-4·38 (-9·71–1·04)	42·2 (39·8–44·4)	36·9 (35·1–38·6)	35 (32·8–37·2)	-12·5 (-18·4–-6·36)	-5·03 (-9·29–-0·966)
Alcohol use	24·8 (18·4–31·7)	21·2 (15·4–27·6)	23·4 (16·6–31·9)	-14·3 (-21·9–-7·21)	10·5 (-1·72–24·5)	11 (7·39–15·7)	9·93 (6·45–14·6)	10·3 (6·56–15·3)	-9·32 (-21·9–3·13)	3·76 (-12·2–21·9)
Drug use	0·262 (0·17–0·442)	0·38 (0·24–0·594)	0·324 (0·214–0·531)	45 (17·6–82·9)	-14·6 (-30·7–3·62)	0·147 (0·0932–0·242)	0·248 (0·189–0·357)	0·235 (0·164–0·341)	68·4 (41·5–112)	-5·18 (-19·8–11·1)
High fasting plasma glucose	4·85 (4·12–5·68)	12·7 (10·7–14·7)	17·2 (15–19·7)	161 (120–209)	35·5 (19–55·9)	4·31 (3·7–5·09)	8·2 (7·02–9·72)	13·2 (11·4–15·3)	90·1 (64·5–122)	61·5 (39–87·6)
High systolic blood pressure	38·3 (30·7–46·4)	39·6 (34·6–45)	40·1 (33·4–48·2)	3·27 (-17·3–32·5)	1·38 (-13·7–20·1)	33·8 (24·2–44·7)	26·6 (21·3–31·6)	27·4 (19·7–35·9)	-21·3 (-43·4–13·3)	2·93 (-20·6–30·9)
High body-mass index	18·2 (13·4–24·1)	20·9 (15·5–27·7)	22·5 (16·8–29·7)	14·6 (3·59–28·8)	7·77 (0·887–15·7)	18·2 (13·5–24·4)	20·9 (15·9–28)	23 (17·8–30·2)	15·2 (1·22–34·9)	10·1 (-0·531–24·1)
Low bone mineral density	9·71 (5·31–16)	9·27 (5·03–15·6)	9·02 (4·76–14·9)	-4·52 (-28·3–28·6)	-2·72 (-26·1–28·5)	15·6 (9·86–22·8)	14·9 (9·69–21·7)	14·6 (9·2–21·4)	-4·18 (-24·6–26·1)	-1·87 (-23·7–28·5)
Diet low in fruits	48·4 (37·3–61)	48·1 (37·3–60·5)	48·8 (37·4–61·7)	-0·494 (-3·04–2·56)	1·39 (-5·71–8·64)	50·6 (39·8–63)	49·9 (39·3–61·8)	50·4 (39·5–63)	-1·43 (-3·93–1·2)	1·13 (-5·65–8·81)
Diet low in vegetables	49·4 (28·6–71·8)	36·9 (19·9–59·3)	34·1 (17·4–55·5)	-25·3 (-42·6–-9·75)	-7·67 (-21–1·52)	47·3 (26·7–70)	34·3 (18·1–55·9)	31·5 (15·6–53·2)	-27·4 (-45·7–-9·7)	-8·23 (-21·9–1·96)
Diet low in whole grains	83·3 (75·8–90·6)	80·8 (72·4–88·9)	81·9 (73·6–89·5)	-3·02 (-4·87–-1·46)	1·34 (0·121–2·98)	81 (72·5–89·1)	78·2 (68·8–87·2)	79·4 (70·3–88)	-3·45 (-5·79–-1·65)	1·58 (0·211–3·54)
Diet low in nuts and seeds	78·9 (37·6–88·4)	82·7 (39·2–89·7)	81 (39·4–88·9)	4·74 (0·22–14·8)	-2·04 (-7·34–2·06)	74·1 (35·9–85·6)	77·5 (36–86·4)	75·7 (35·2–85·3)	4·67 (-0·185–19)	-2·36 (-9·02–2·65)
Diet low in milk	36·2 (14·7–56·3)	35·4 (13·8–55·4)	33·8 (13·1–53·5)	-2·16 (-22·1–18·8)	-4·52 (-18·8–7·45)	35 (13·1–54·5)	34 (12–53·5)	32·4 (12·2–51·7)	-2·85 (-24·5–17·4)	-4·52 (-18·3–8·37)
Diet high in red meat	91·6 (87·2–94·8)	96·2 (94·1–97·7)	93·5 (88–97·3)	5·02 (2·66–8·48)	-2·83 (-7·41–0·304)	91·6 (87·3–94·8)	96·2 (94–97·8)	93·5 (88·3–97·1)	4·95 (2·42–8·41)	-2·76 (-7·23–0·209)
Diet high in processed meat	83·8 (64·4–99·6)	88·4 (73·8–99·8)	85·5 (66·6–99·7)	5·42 (0·0405–17·2)	-3·22 (-9·91–0·0198)	86·6 (68·1–99·8)	91·8 (80·9–99·9)	88·5 (72·5–99·8)	6·05 (-0·0132–19·7)	-3·62 (-10·4–-0·0114)
Diet high in sugar-sweetened beverages	51·8 (32·6–69)	61·8 (47·4–76·1)	57·6 (39·6–74·6)	19·2 (-10·6–73·6)	-6·67 (-25·3–9·19)	58·2 (40–75)	70·5 (57·4–82·7)	64·5 (48·1–80·1)	21·1 (-6·95–70·4)	-8·51 (-25·2–4·23)
Diet low in fiber	39 (24·1–54·1)	35·5 (21·2–50·2)	31·8 (18·4–45·9)	-8·96 (-14·5–-4·86)	-10·3 (-21·8–-0·353)	38·9 (23·7–54·5)	35·3 (20·3–50·5)	31·7 (17·6–47·2)	-9·21 (-14·9–-4·7)	-10·3 (-21·6–-0·263)
Diet low in seafood omega-3 fatty acids	88·6 (74·2–100)	83·3 (65·2–99·9)	82·4 (63·3–100)	-6 (-20·9–3·24)	-1·11 (-16·4–15·5)	90·9 (80–100)	86·5 (71·4–100)	85·5 (68·7–100)	-4·87 (-17·2–2·05)	-1·09 (-12·6–12·6)
Diet low in polyunsaturated fatty acids	54·3 (15·4–95·5)	55·2 (16·9–95·8)	53·6 (15·5–94·6)	1·72 (-1·47–7·5)	-2·98 (-10·1–0·146)	56·6 (17·4–96·6)	57·4 (18·1–97)	55·9 (17·3–96·5)	1·54 (-1·41–7·02)	-2·67 (-9·41–0·0414)
Diet high in trans fatty acids	47·9 (38·1–98·8)	41·2 (31–97·3)	40·3 (30·7–96)	-14 (-24·8–-0·491)	-2·34 (-12·1–8·76)	49·2 (38·5–100)	42·5 (32·2–100)	41·6 (30·6–99·8)	-13·6 (-23·2–0)	-2·3 (-10·9–7·05)
Diet high in sodium	31·1 (5·82–56·5)	34·1 (7·35–59·1)	36 (7·67–60·9)	9·59 (-23·3–77·2)	5·57 (-14·8–40·4)	--	--	--	--	--
Low physical activity	3·37 (1·36–6·91)	3·25 (1·4–6·6)	3·83 (1·63–8·02)	-3·55 (-26·1–32·7)	17·9 (-8·41–52·5)	5·22 (2·61–9·93)	4·95 (2·53–9·47)	5·86 (3·08–11)	-5·17 (-23·3–19·7)	18·4 (-4·62–48·7)
Occupational asthmagens	24·2 (20·3–29)	19·5 (16–24)	18·5 (14·9–23·2)	-19·3 (-29·7–-7·38)	-5·06 (-17–8·02)	--	--	--	--	--
Occupational particulate matter, gases, and fumes	9·32 (6·61–13·4)	7·23 (5·08–10·5)	6·35 (4·47–9·24)	-22·4 (-29–-16·5)	-12·2 (-15·3–-9·63)	3·28 (2·3–4·87)	2·57 (1·79–3·8)	2·39 (1·67–3·52)	-21·6 (-31·1–-13·7)	-6·87 (-10·8–-3·48)
Occupational noise	8·53 (8·07–9·16)	7·47 (7·09–8·11)	6·94 (6·58–7·56)	-12·4 (-14·1–-10·6)	-7·04 (-8–-6·06)	3·82 (3·58–4·12)	3·91 (3·71–4·17)	4·13 (3·93–4·37)	2·36 (-0·77–5·65)	5·52 (4·15–6·88)
Occupational injuries	--	--	--	--	--	--	--	--	--	--
Occupational ergonomic factors	11·3 (9·62–13·3)	8·57 (7·37–10·1)	8·46 (7·13–10·2)	-24·4 (-29·5–-19)	-1·34 (-7·7–5·8)	--	--	--	--	--
Childhood sexual abuse	7·16 (4·65–10·1)	7·16 (4·65–10·1)	7·16 (4·65–10·1)	0 (0–0)	0 (0–0)	10·6 (7·84–14·3)	10·6 (7·84–14·3)	10·6 (7·84–14·3)	0 (0–0)	0 (0–0)
Intimate partner violence	--	--	--	--	--	13·6 (8·13–19·8)	13·7 (7·98–20)	13·6 (8·13–19·8)	0·741 (-4·34–12·5)	-0·735 (-7·18–7·6)
Non-exclusive breastfeeding	20·6 (16·5–25·4)	19·7 (15·8–24·4)	19·5 (15·5–24·2)	-4·55 (-16·9–8·5)	-1·12 (-9·43–8·17)	--	--	--	--	--
Discontinued breastfeeding	23·7 (22·4–24·9)	22·9 (21·6–24·1)	22·7 (21·5–24)	-3·53 (-10·3–3·94)	-0·83 (-6·98–5·5)	23·9 (22·6–25·1)	23 (21·7–24·2)	22·7 (21·5–24)	-3·95 (-10·7–3·46)	-1·14 (-7·26–5·15)
Diet low in calcium	12·1 (5·4–22·9)	9·62 (4·67–17·4)	8·47 (4·15–15·2)	-20·3 (-27–-8·76)	-12 (-18·5–-5·66)	6·19 (2·46–11)	4·08 (1·74–7·26)	3·33 (1·47–6·01)	-34 (-42·5–-20·8)	-18·4 (-28·5–-8·09)
Occupational exposure to asbestos	12·1 (10·9–13·4)	12·2 (11–13·3)	10·9 (8·77–13·3)	0·521 (-12·8–14·9)	-10·7 (-28·4–10·8)	1·35 (0·96–2·04)	1·16 (0·821–1·5)	0·969 (0·638–1·43)	-14·6 (-36·7–9·63)	-16·1 (-38·4–13·7)
Occupational exposure to arsenic	0·49 (0–1·16)	0·338 (0–0·803)	0·344 (0–0·811)	-31 (-36·7–NA)	1·81 (-5·42–NA)	0·26 (0–0·61)	0·281 (0–0·666)	0·306 (0–0·723)	8·05 (0·738–NA)	8·93 (2·97–NA)
Occupational exposure to benzene	0·767 (0·0444–2·18)	0·858 (0·0506–2·48)	0·843 (0·0509–2·4)	11·8 (6·97–18)	-1·72 (-6·94–5·33)	0·725 (0·0434–2·1)	1·18 (0·0718–3·39)	1·28 (0·0763–3·64)	63·2 (56·1–73·1)	8·19 (3·24–14·8)
Occupational exposure to beryllium	0·0222 (0·022–0·0225)	0·0205 (0·0203–0·0208)	0·0207 (0·0203–0·0212)	-7·55 (-8·96–-6·13)	0·856 (-1·38–3·19)	0·0168 (0·0165–0·0171)	0·0219 (0·0217–0·0222)	0·0239 (0·0232–0·0245)	30·2 (27·5–32·9)	8·98 (5·83–12)
Occupational exposure to cadmium	0·0566 (0·0541–0·0592)	0·0406 (0·0382–0·043)	0·0412 (0·0384–0·0446)	-28·3 (-33·4–-22·9)	1·64 (-4·54–8·7)	0·032 (0·0305–0·0335)	0·0355 (0·0339–0·0371)	0·0386 (0·0366–0·0408)	10·8 (4·37–18)	8·95 (3·57–14·5)
Occupational exposure to chromium	0·133 (0·128–0·138)	0·0997 (0·0947–0·105)	0·101 (0·095–0·108)	-24·8 (-29·4–-20·1)	1·41 (-3·83–7·4)	0·0816 (0·0785–0·0847)	0·0948 (0·0917–0·0983)	0·103 (0·0983–0·108)	16·2 (10·6–22·2)	8·82 (3·88–13·6)
Occupational exposure to diesel engine exhaust	0·648 (0·626–0·67)	0·579 (0·559–0·6)	0·585 (0·557–0·616)	-10·6 (-14·8–-6·17)	1·09 (-3·67–6·18)	0·452 (0·434–0·471)	0·477 (0·455–0·501)	0·516 (0·483–0·552)	5·42 (-0·575–12·2)	8·13 (1·76–15·6)
Occupational exposure to formaldehyde	0·283 (0·268–0·297)	0·201 (0·188–0·214)	0·201 (0·185–0·218)	-28·8 (-34·1–-23·1)	-0·263 (-6·56–6·96)	0·151 (0·144–0·159)	0·183 (0·175–0·191)	0·198 (0·188–0·209)	21·1 (14·2–29·1)	8·21 (2·81–13·5)
Occupational exposure to nickel	0·465 (0–2·08)	0·324 (0–1·42)	0·328 (0–1·42)	-30·3 (-35·4–NA)	1·12 (-5·4–NA)	0·203 (0–0·907)	0·212 (0–0·931)	0·231 (0–1·02)	4·39 (-3·14–NA)	8·75 (1·97–NA)
Occupational exposure to polycyclic aromatic hydrocarbons	0·27 (0·26–0·279)	0·214 (0·205–0·223)	0·216 (0·204–0·229)	-20·7 (-25–-16·3)	0·965 (-3·76–6·27)	0·169 (0·163–0·175)	0·214 (0·208–0·221)	0·233 (0·223–0·243)	26·9 (21·2–32·9)	8·79 (4·1–13·2)
Occupational exposure to silica	5·39 (0·792–17·5)	4·07 (0·567–12·8)	4·12 (0·582–13·5)	-24·4 (-31·1–-17·3)	1·08 (-7·33–9·82)	1·83 (0·253–5·56)	1·8 (0·258–5·6)	1·93 (0·284–5·89)	-1·57 (-9·74–8·68)	7·25 (-0·216–17·6)
Occupational exposure to sulfuric acid	0·756 (0·158–2·33)	0·518 (0·107–1·6)	0·525 (0·109–1·63)	-31·5 (-36·4–-26·3)	1·43 (-5·1–8·81)	0·381 (0·0785–1·18)	0·375 (0·0779–1·16)	0·407 (0·0836–1·26)	-1·82 (-8·6–6·43)	8·54 (1·84–15·5)
Unsafe sex	--	--	--	--	--	--	--	--	--	--
Occupational exposure to trichloroethylene	0·0734 (0·0717–0·0758)	0·0586 (0·0569–0·0607)	0·0595 (0·057–0·0628)	-20·1 (-23·6–-16·7)	1·55 (-2·53–6·06)	0·0516 (0·05–0·0532)	0·0576 (0·0561–0·0597)	0·0627 (0·0601–0·0657)	11·8 (7·29–16·7)	8·77 (4·04–13·8)
No access to handwashing facility	3·22 (3·06–3·4)	3·14 (2·95–3·33)	3·11 (2·94–3·29)	-2·6 (-9·21–3·7)	-0·845 (-4·67–3·2)	3·22 (3·06–3·4)	3·14 (2·95–3·33)	3·11 (2·94–3·29)	-2·6 (-9·21–3·7)	-0·845 (-4·67–3·2)
Child wasting	1·28 (0·903–1·69)	1·24 (0·877–1·63)	1·23 (0·875–1·62)	-3·19 (-11·4–5·9)	-1·05 (-6·9–5·16)	--	--	--	--	--
Child stunting	1·66 (0·839–2·43)	1·19 (0·576–1·82)	1·19 (0·56–1·79)	-28·1 (-40–-14·7)	-0·793 (-13·3–13·1)	1·95 (0·975–2·86)	1·56 (0·789–2·35)	1·55 (0·775–2·3)	-19·8 (-32·6–-4·55)	-1·25 (-12·1–11·5)
Chewing tobacco	0·147 (0·116–0·182)	0·148 (0·114–0·188)	0·151 (0·119–0·189)	1·19 (-23·9–35·4)	1·82 (-14·7–21·7)	0·137 (0·0977–0·187)	0·137 (0·0996–0·189)	0·139 (0·1–0·19)	0·465 (-30–47·3)	1·6 (-19·5–28·9)
Diet low in legumes	92·5 (82·2–100)	95 (87·9–100)	93·6 (84·5–100)	2·72 (0–7·07)	-1·51 (-4·28–0)	90·6 (77·9–100)	93·4 (84·1–100)	91·8 (80·3–100)	3·1 (0–8·61)	-1·68 (-5·27–0)
Short gestation	11·9 (9·62–14·8)	10·2 (8·47–12·2)	10 (8·24–12·1)	-14·1 (-32·6–10)	-2·04 (-20–21·7)	11·2 (9·6–13·2)	9·28 (7·65–11)	9·03 (7·6–10·8)	-17·2 (-32·6–0·684)	-2·72 (-20·3–20)
Low birth weight	6·26 (5·09–7·67)	5·56 (4·69–6·64)	5·2 (4·32–6·34)	-11·1 (-30·6–15·4)	-6·5 (-26·5–18·8)	6·13 (5·21–7·18)	5·02 (4·23–6·01)	4·92 (4·17–5·82)	-18·1 (-34–3·08)	-2·13 (-21·5–21·1)
High temperature	22·1 (11–35)	22·8 (11·2–36·7)	25·1 (13·5–38·6)	3·6 (-11·3–17·5)	9·7 (-11·2–44·8)	22·1 (11–35)	22·8 (11·2–36·7)	25·1 (13·5–38·6)	3·6 (-11·3–17·5)	9·7 (-11·2–44·8)
Low temperature	32·5 (28·6–36·7)	34·3 (30·9–38·1)	31·6 (27·7–36)	5·39 (2·92–8·12)	-7·77 (-10·4–-5·26)	32·5 (28·6–36·7)	34·3 (30·9–38·1)	31·6 (27·7–36)	5·39 (2·92–8·12)	-7·77 (-10·4–-5·26)
Kidney dysfunction	15·4 (10·5–22)	16·7 (11·6–23·1)	16 (11·1–22·6)	8·5 (3·65–15·8)	-4·12 (-9·1–0·846)	17·1 (11·7–23·8)	18·4 (12·9–25·2)	17·8 (12·4–24·6)	7·82 (2·49–15·2)	-3·12 (-8·06–1·66)

Bullying victimization	7·17<br>(2·81–14·7)	8·03<br>(3·75–15)	8·28<br>(3·81–15·7)	12·1<br>(-15·4–72·7)	3·14<br>(-4·01–9·69)	6·84<br>(2·67–14·1)	7·48<br>(3·51–14)	7·54<br>(3·48–14·2)	9·38<br>(-18–66)	0·705<br>(-5·91–6·47)
High LDL cholesterol	52·6<br>(48·9–56·5)	45·9<br>(42·3–49·4)	46<br>(42·8–49·8)	-12·9<br>(-18·2–-7·04)	0·404<br>(-3·75–4·92)	51·2<br>(47·7–54·6)	45·4<br>(41·7–48·8)	45·4<br>(42–48·9)	-11·3<br>(-16–-6·13)	0·0125<br>(-3·52–3·71)

Supplementary Results Table 1. Turkmenistan age-standardised Summary Exposure Values (SEVs) for most-detailed risk factors and percentage change, by sex, 1990, 2010, and 2019. Data in parentheses are 95% uncertainty intervals.										
	Male					Female				
	1990	2010	2019	Percentage change 1990-2010	Percentage change 2010-2019	1990	2010	2019	Percentage change 1990-2010	Percentage change 2010-2019
Unsafe sanitation	63·9 (54·7–73)	49·6 (42·2–57·1)	40·8 (33·7–48·4)	-22·5 (-30·9—11·6)	-17·7 (-29·4—5·74)	63·9 (54·7–73)	49·6 (42·2–57·1)	40·8 (33·7–48·4)	-22·5 (-30·9—11·6)	-17·7 (-29·4—5·74)
Ambient particulate matter pollution	23·3 (8·1–48·8)	25·2 (13·7–39·7)	25·3 (14·2–40·5)	8·24 (-38–126)	0·293 (-21·4–28·6)	23·1 (8–48·5)	25·2 (13·7–39·7)	25·3 (14·2–40·5)	8·77 (-37·9–127)	0·346 (-21·4–28·6)
Household air pollution from solid fuels	0·233 (0·0873–0·545)	0·0522 (0·0173–0·126)	0·0172 (0·00505–0·0431)	-77·6 (-89·8—53·2)	-67·1 (-80·3—47·8)	0·301 (0·117–0·639)	0·0679 (0·024–0·152)	0·0224 (0·00684–0·0548)	-77·5 (-89·8—53)	-67 (-80·5—47·8)
Ambient ozone pollution	77·4 (35–100)	54·7 (23·2–93·2)	61·3 (26·1–100)	-29·4 (-39·3—6·85)	12·1 (0·428–25·2)	77·4 (35–100)	54·7 (23·2–93·2)	61·3 (26·1–100)	-29·4 (-39·3—6·85)	12·1 (0·428–25·2)
Residential radon	24·1 (3·21–93·6)	24·1 (3·21–93·6)	24·1 (3·21–93·6)	0 (0–0)	0 (0–0)	--	--	--	--	--
Lead exposure	46·5 (22·6–67·6)	41·9 (20·9–61·8)	34·8 (15·9–55)	-9·93 (-22·6–6·74)	-17 (-26·2—10·7)	26 (0·585–52·9)	26·4 (3·89–50·5)	22·5 (3·14–45·9)	1·61 (-10·2–745)	-14·8 (-29·1—8·32)
Child underweight	6·29 (4·87–7·58)	3·88 (2·69–4·95)	3·17 (2·2–4·17)	-38·2 (-50—27·1)	-18·2 (-28·2—9·57)	5·44 (4·05–6·69)	3·3 (2·31–4·29)	2·28 (1·51–3·09)	-39·4 (-50—30·5)	-31 (-39·5—24·2)
Iron deficiency	--	--	--	--	--	22·3 (19·7–24·8)	20·1 (17·8–22·5)	18·8 (16·5–21·2)	-9·68 (-19·1—0·549)	-6·48 (-12·2—0·695)
Vitamin A deficiency	10·4 (4·35–19·2)	5·55 (2·29–10·5)	3·14 (1·28–6·19)	-46·6 (-76·5—25·9)	-43·5 (-68·2—3·57)	6·25 (3·17–10·6)	5·02 (2·54–8·74)	3·12 (1·62–5·32)	-19·7 (-58–74·3)	-37·9 (-59·3—2·51)
Zinc deficiency	11·9 (1·65–26·2)	6·89 (0·407–18·2)	3·92 (0–12·2)	-42·2 (-75·1—27·6)	-43·1 (-100—29·9)	11·5 (1·5–25·6)	6·67 (0·43–17·7)	3·84 (0–11·6)	-41·9 (-74·2—28·9)	-42·5 (-100—30·1)
Smoking	31·2 (28·1–34·4)	23 (20·6–25·6)	19·5 (17·3–21·8)	-26·4 (-31·2—21·1)	-14·9 (-19·2—10·3)	2·7 (2–3·6)	2·23 (1·66–2·88)	2·09 (1·6–2·65)	-17·5 (-35·7–9·79)	-6·32 (-19·6–9·17)
Secondhand smoke	36·9 (34·3–39·4)	36·2 (34·5–37·8)	38·2 (35·7–40·8)	-1·98 (-9·7–6·58)	5·63 (-0·184–11·9)	54·9 (52·5–57·4)	53·2 (51·5–54·7)	56·1 (53·5–58·4)	-3·1 (-7·97–1·77)	5·39 (1·52–9·46)
Alcohol use	6·22 (3·72–9·62)	10·6 (7·53–14·5)	11·6 (8·01–15·9)	70·9 (34·1–135)	9·58 (-4·42–25·7)	0·983 (0·475–1·85)	2·08 (1·15–3·5)	2·35 (1·24–3·99)	111 (49·8–227)	13·3 (-11·4–43·8)
Drug use	0·141 (0·0817–0·261)	0·178 (0·114–0·301)	0·182 (0·118–0·304)	25·9 (9·52–52)	2·48 (-6·4–13·6)	0·0821 (0·0473–0·152)	0·0956 (0·059–0·165)	0·1 (0·0632–0·17)	16·5 (4·35–34·2)	4·85 (-4·07–16·1)
High fasting plasma glucose	5·37 (4·58–6·25)	8·08 (6·95–9·75)	10·5 (8·41–13·3)	50·4 (31·6–77·1)	30·3 (7·64–63·3)	5·34 (4·66–6·11)	9·06 (7·48–10·9)	10·5 (8·83–12·6)	69·5 (43·8–101)	16·3 (-1·2–42·9)
High systolic blood pressure	34·5 (27·3–42·5)	35·2 (28·4–43·2)	38·2 (30·8–46·4)	1·99 (-21–33·3)	8·4 (-10·3–28·8)	33·3 (24·1–43·8)	31·8 (22·5–41·4)	33·5 (23·9–44·1)	-4·7 (-34·4–43·8)	5·57 (-19·9–38)
High body-mass index	17·1 (12·5–23·1)	27·3 (20·9–35·1)	36 (28·2–45·9)	59·5 (39·1–91)	32·2 (22·3–44·6)	21·9 (16·5–28·8)	30·5 (24–39·3)	37 (29·1–47·2)	39·3 (24·3–59)	21·4 (10·9–32·8)
Low bone mineral density	9·23 (5·11–15·4)	8·25 (4·31–13·6)	7·9 (4·07–13·5)	-10·6 (-32·8–14·8)	-4·25 (-28·1–25·4)	17·2 (11·5–24·5)	15·6 (10·1–23)	14·9 (9·43–21·7)	-9·36 (-29·7–15·4)	-3·99 (-26·1–22·6)
Diet low in fruits	58·4 (46·9–71·7)	47·4 (35·7–61)	42·1 (30·2–55·5)	-18·8 (-28—11·7)	-11·2 (-21·1—3·85)	59·8 (48·8–72·9)	49·1 (37·2–62·3)	43·8 (31·8–56·8)	-17·9 (-26·5—10·9)	-10·8 (-20·3—3·83)
Diet low in vegetables	22·8 (9·66–44)	7·11 (2·7–18)	3·13 (1·52–9·51)	-68·7 (-81·1—47·5)	-56 (-78·1—18·5)	20·1 (8·02–40·4)	5·28 (2·2–13·9)	2·86 (1·41–9·17)	-73·7 (-85—49)	-45·8 (-74·6—10·5)
Diet low in whole grains	99·9 (100–100)	99·9 (100–100)	99·9 (100–100)	0 (0–0)	0 (0–0)	99·9 (100–100)	99·9 (100–100)	99·9 (100–100)	0 (0–0)	0 (0–0)
Diet low in nuts and seeds	96 (50–100)	97·3 (50–100)	97·6 (50–100)	1·37 (-5·1–10·2)	0·281 (-5·45–6·71)	93·6 (49·4–99·9)	96·9 (50–100)	96·7 (49·9–100)	3·51 (-4·27–15·5)	-0·172 (-7·06–6·48)
Diet low in milk	52·7 (27·4–74·2)	41·5 (16·4–62·3)	35·1 (13·8–54·9)	-21·2 (-44·1—6·07)	-15·3 (-32·3—3·91)	51·8 (25·7–73·4)	40·4 (15·5–60·5)	33·9 (12·3–53·6)	-22·1 (-44·5—6·46)	-15·9 (-32·9—3·16)
Diet high in red meat	63·4 (53·4–72·9)	83·3 (76·7–88·9)	91·4 (84·8–95·7)	31·5 (20·3–46·8)	9·65 (3·5–16·9)	63·2 (52·8–73·4)	83·3 (76·9–88·9)	91·3 (85·1–95·7)	31·9 (20·5–48·4)	9·5 (3·61–17·2)
Diet high in processed meat	46·4 (24·7–71·3)	48·5 (26·8–73·3)	51·7 (30·1–77·4)	4·44 (-15·6–33·2)	6·71 (-5·63–24·7)	54·1 (31·9–77·4)	57·3 (34·4–81·7)	60·8 (36·9–83·8)	5·84 (-11·3–29·9)	6·23 (-3·28–21·1)
Diet high in sugar-sweetened beverages	24·3 (12·9–39·6)	30·4 (16–47·8)	38·9 (20·2–58)	25·4 (-22·4–105)	27·8 (-1·4–83·4)	25·3 (12·8–41·9)	33·3 (17·3–51·2)	42·7 (23–61·3)	31·5 (-20·8–119)	28·4 (-1·86–81)
Diet low in fiber	54·1 (37·1–69·5)	43·8 (28·2–59·2)	35·3 (21·2–50·5)	-19·1 (-25·8—13·8)	-19·4 (-30·5—10·7)	54·4 (37–70·2)	43·9 (27·9–59·8)	35·2 (20·8–51)	-19·3 (-27—13·6)	-19·7 (-30·9—11)
Diet low in seafood omega-3 fatty acids	97·6 (92·5–100)	95·7 (87·4–100)	90·5 (78·9–100)	-1·88 (-9·05–0·774)	-5·44 (-14–0)	98·4 (94·1–100)	96·8 (90·2–100)	92·5 (83·2–100)	-1·59 (-7·58–1·25)	-4·5 (-12·3–0)
Diet low in polyunsaturated fatty acids	94 (76·8–100)	92 (70·9–100)	89·1 (62·5–100)	-2·06 (-7·86–0)	-3·19 (-11·4–0)	94·6 (79·3–100)	92·7 (73·3–100)	89·9 (64·7–100)	-1·96 (-7·42–0)	-3·03 (-11–0)
Diet high in trans fatty acids	42 (32–98·9)	38·5 (28·4–98·1)	37·2 (27·4–98·3)	-8·41 (-19·5–1·68)	-3·24 (-13·8–6·69)	43·5 (32·6–100)	40·1 (29·1–100)	38·9 (27·8–100)	-7·9 (-17·3–0)	-2·89 (-13·1–5·83)
Diet high in sodium	61·9 (38·7–79·9)	43·5 (15–67·8)	42·9 (14·1–68)	-29·6 (-66·6—6·12)	-1·38 (-23·4–21)	30·5 (10·6–53·2)	18·9 (4·3–42·3)	18·4 (4·13–41·1)	-38·2 (-69·8—15·7)	-2·77 (-19·9–13·8)
Low physical activity	1·9 (0·91–4·1)	2·18 (1·07–4·47)	2·46 (1·24–4·83)	14·7 (2·91–30·5)	12·9 (2·46–28·7)	1·57 (0·844–3·19)	1·76 (0·928–3·48)	1·98 (1·07–3·68)	12·1 (2–27·5)	12·7 (3–27·3)
Occupational asthmagens	20·3 (15·7–26·1)	19·6 (15·5–24·9)	19·4 (15·5–24·5)	-3·21 (-18·9–16·5)	-0·877 (-12–11)	8·15 (5·72–11·4)	8·53 (6·09–11·7)	8·36 (6·12–11·2)	4·62 (-25·5–43·4)	-2·04 (-20·1–19·8)
Occupational particulate matter, gases, and fumes	10·7 (8·07–14·6)	10·2 (7·8–13·6)	10·2 (7·79–13·6)	-4·15 (-10·3–3·48)	-0·67 (-3·94–2·72)	4·15 (2·97–5·91)	4·14 (3·11–5·77)	4·16 (3·07–5·82)	-0·251 (-10·8–14·1)	0·457 (-5·49–6·76)
Occupational noise	11·6 (10·5–12·8)	11·4 (10·7–12·2)	11·6 (10·9–12·4)	-1·73 (-7·21–4·66)	1·42 (-1·47–4·43)	4·82 (4·01–5·62)	5·08 (4·65–5·58)	5·41 (4·92–5·97)	5·37 (-4·5–19·6)	6·5 (0·643–13)
Occupational injuries	--	--	--	--	--	--	--	--	--	--
Occupational ergonomic factors	25·4 (21·7–29·1)	22·3 (18·5–26·3)	20 (16·2–24·2)	-12 (-27·4–7·17)	-10·3 (-22·2–1·94)	--	--	--	--	--
Childhood sexual abuse	2·52 (1·65–3·56)	2·52 (1·65–3·56)	2·52 (1·65–3·56)	0 (0–0)	0 (0–0)	3·14 (2·26–4·3)	3·14 (2·26–4·3)	3·14 (2·26–4·3)	0 (0–0)	0 (0–0)
Intimate partner violence	--	--	--	--	--	11·3 (6·65–16·1)	11·3 (6·65–16·1)	11·3 (6·65–16·1)	0 (0–0)	0 (0–0)
Non-exclusive breastfeeding	23·9 (13·9–37·5)	20 (11·8–31·4)	16 (9·46–24·6)	-16·2 (-22·8—8·8)	-20·2 (-27·2—12·2)	23·9 (13·8–37·4)	20 (11·8–31·4)	16 (9·45–24·6)	-16·1 (-22·7—8·68)	-20·2 (-27·1—12·1)
Discontinued breastfeeding	11·6 (10–13·3)	11·7 (10·7–12·9)	11·7 (10·5–12·8)	1·52 (-14·1–21·3)	-0·715 (-13·3–12·7)	11·5 (10–13·3)	11·8 (10·7–12·9)	11·7 (10·5–12·8)	1·78 (-13·8–21·6)	-0·667 (-13·3–12·8)
Diet low in calcium	39·6 (25·5–62·3)	33·8 (20·1–56·9)	25·5 (13·3–47·1)	-14·7 (-23—7·31)	-24·5 (-38—10·6)	--	--		--	--
Occupational exposure to asbestos	0·366 (0·235–0·574)	0·701 (0·496–1)	0·776 (0·492–1·17)	91·3 (25·6–205)	10·7 (-28·8–66·3)	1·51 (0·316–7·29)	2·04 (0·376–9·17)	2·22 (0·372–10·4)	35·2 (-82·2–1100)	8·87 (-80·9–504)
Occupational exposure to arsenic	0·299 (0·116–0·509)	0·316 (0·127–0·526)	0·328 (0·134–0·561)	5·85 (-7·11–20·7)	3·73 (-4·75–12·5)	0·199 (0·0772–0·341)	0·237 (0·0942–0·411)	0·252 (0·098–0·443)	19·4 (-5·94–51·3)	6·27 (-5·82–20·9)
Occupational exposure to benzene	0·67 (0·41–1·19)	0·823 (0·509–1·46)	0·925 (0·569–1·66)	22·8 (9·01–42)	12·5 (3·93–23·3)	0·488 (0·293–0·919)	0·737 (0·447–1·31)	0·926 (0·546–1·67)	51 (22–95·8)	25·6 (10·5–43·1)
Occupational exposure to beryllium	0·0907 (0·084–0·0971)	0·0972 (0·0903–0·104)	0·0991 (0·0913–0·107)	7·18 (-3·32–19·5)	1·93 (-4·12–7·72)	0·0593 (0·0513–0·0671)	0·0739 (0·0652–0·0828)	0·0789 (0·0679–0·0902)	24·7 (1·56–54·3)	6·74 (-3·98–17·8)
Occupational exposure to cadmium	0·161 (0·147–0·177)	0·173 (0·158–0·189)	0·18 (0·163–0·199)	7·03 (-5·01–21·1)	3·94 (-4·11–11·9)	0·107 (0·0918–0·124)	0·13 (0·112–0·149)	0·139 (0·117–0·161)	21·3 (-3·04–52·8)	7·01 (-4·92–20·3)
Occupational exposure to chromium	0·353 (0·322–0·387)	0·397 (0·363–0·432)	0·425 (0·385–0·469)	12·3 (-0·388–27·6)	7·07 (-0·701–15·4)	0·234 (0·2–0·272)	0·301 (0·26–0·347)	0·338 (0·287–0·39)	28·5 (3·49–61·4)	12·4 (0·062–26·3)
Occupational exposure to diesel engine exhaust	2·38 (2·16–2·6)	2·83 (2·59–3·08)	3·06 (2·77–3·36)	19 (4·8–36·7)	8·22 (0·412–16·7)	1·12 (0·954–1·3)	1·5 (1·29–1·73)	1·69 (1·43–1·96)	34·4 (8·73–68)	12·6 (-0·186–26·8)
Occupational exposure to formaldehyde	0·699 (0·558–0·793)	0·778 (0·615–0·876)	0·84 (0·659–0·96)	11·2 (-3·27–27·6)	7·99 (-1·07–17·5)	--	--	--	--	--
Occupational exposure to nickel	0·372 (0·121–0·981)	0·382 (0·123–1·01)	0·393 (0·127–1·04)	2·74 (-9·73–18)	2·9 (-5·21–11·7)	0·202 (0·0674–0·529)	0·224 (0·0735–0·599)	0·229 (0·0761–0·606)	11·2 (-10·8–42·4)	2·3 (-9·34–17·1)
Occupational exposure to polycyclic aromatic hydrocarbons	0·766 (0·7–0·835)	0·868 (0·797–0·943)	0·938 (0·855–1·03)	13·4 (0·885–28·2)	8·03 (0·279–15·6)	0·498 (0·428–0·576)	0·648 (0·561–0·742)	0·74 (0·631–0·85)	30·1 (4·47–62·5)	14·3 (1·58–28·4)
Occupational exposure to silica	4·19 (2·12–9·08)	4·54 (2·38–10·1)	4·82 (2·48–10·8)	8·33 (-6·69–28·5)	6·17 (-3·39–18·1)	1·79 (0·873–3·78)	1·89 (0·959–4·08)	1·89 (0·929–4·02)	5·61 (-14·4–36·8)	-0·193 (-11·8–14·2)
Occupational exposure to sulfuric acid	0·95 (0·673–1·72)	1·01 (0·721–1·79)	1·02 (0·73–1·79)	6·08 (-6·46–21·8)	1·69 (-6·96–10·7)	0·532 (0·365–0·944)	0·616 (0·426–1·07)	0·623 (0·431–1·09)	15·9 (-5·79–45·4)	1·23 (-10·2–13·7)
Unsafe sex	--	--	--	--	--	--	--	--	--	--
Occupational exposure to trichloroethylene	0·212 (0·195–0·231)	0·244 (0·224–0·265)	0·259 (0·238–0·284)	14·9 (2·23–30·2)	6·5 (-0·764–13·9)	0·138 (0·119–0·16)	0·182 (0·157–0·208)	0·204 (0·173–0·235)	31·9 (6·47–65·7)	11·8 (-0·65–25·1)
No access to handwashing facility	30·1 (28·9–31·4)	19·9 (18·9–20·8)	15 (14·3–15·8)	-34·1 (-37·8—30·7)	-24·4 (-27—21·5)	--	--	--	--	--
Child wasting	3·63 (2·86–4·34)	3·3 (2·59–3·96)	3·09 (2·43–3·75)	-9·16 (-19·8–1·49)	-6·33 (-13·3–1·3)	3·57 (2·8–4·25)	3·21 (2·51–3·86)	3 (2·31–3·64)	-9·9 (-19·4–2·32)	-6·49 (-14·1–1·86)
Child stunting	15·1 (10·4–17·8)	9·57 (6·29–11·8)	7·23 (4·65–9·3)	-36·5 (-48·3—24·8)	-24·4 (-36·2—14·1)	15·6 (10·5–18·7)	9·95 (6·8–12·4)	7·42 (4·64–9·54)	-36·1 (-48·2—23·9)	-25·5 (-35·8—15)
Chewing tobacco	0·377 (0·293–0·473)	0·359 (0·279–0·455)	0·338 (0·264–0·435)	-4·81 (-29·6–29)	-5·84 (-23·3–14·4)	--	--	--	--	--
Diet low in legumes	100 (99·8–100)	99·1 (97·1–100)	98·2 (94·9–100)	-0·875 (-2·7–0)	-0·918 (-2·79–0)	99·9 (99·3–100)	98·2 (95–100)	97·1 (92·5–100)	-1·64 (-4·54–0)	-1·15 (-3·32–0)
Short gestation	7·19 (5·08–10·2)	9·46 (6·52–13·4)	9·93 (7·2–14)	31·5 (-1·05–94·8)	4·94 (-19·2–34·6)	7·37 (5·44–11·5)	8·29 (5·93–12·3)	7·71 (5·64–11·5)	12·6 (-9·01–40·6)	-6·98 (-24·2–14·3)
Low birth weight	3·05 (2·24–4·24)	3·47 (2·48–4·93)	3·64 (2·68–4·99)	13·9 (-14·6–67·4)	4·64 (-19·6–35·9)	3·02 (2·26–4·67)	3·11 (2·23–4·64)	2·77 (2·05–4·19)	2·81 (-17·2–27·8)	-10·7 (-26·2–9·84)
High temperature	44·6 (35·4–53·8)	48·8 (39·2–57·9)	49·7 (40·2–58·8)	9·32 (3·55–13·1)	1·95 (0·29–3·33)	44·6 (35·4–53·8)	48·8 (39·2–57·9)	49·7 (40·2–58·8)	9·32 (3·55–13·1)	1·95 (0·29–3·33)
Low temperature	42 (37·3–49·3)	39·5 (34·4–47·1)	41·1 (35·6–49·1)	-5·99 (-8·16—3·77)	4·1 (1·3–6·75)	42 (37·3–49·3)	39·5 (34·4–47·1)	41·1 (35·6–49·1)	-5·99 (-8·16—3·77)	4·1 (1·3–6·75)
Kidney dysfunction	21·1 (14·1–29·2)	23·4 (16·4–31·8)	25·1 (17·7–33·7)	11·1 (6·06–19·3)	7·37 (2·89–13·3)	21·1 (14·2–28·8)	22·7 (15·6–31)	24·4 (17·1–32·6)	7·74 (3·87–13·4)	7·26 (3·45–12·4)
Bullying victimization	2·75 (1·12–5·67)	2·83 (1·17–5·85)	2·77 (1·14–5·66)	2·96 (1·02–5·42)	-2·38 (-4·08—0·66)	1·61 (0·662–3·3)	1·7 (0·703–3·48)	1·67 (0·686–3·45)	5·09 (2·24–8·54)	-1·8 (-3·65—0·0301)
High LDL cholesterol	31 (27·8–34·4)	31·5 (28·3–35)	32·4 (28·9–36)	1·65 (-4·13–7·91)	2·64 (-1·56–7·02)	--	--	--	--	--

Supplementary Results Table 1. Malta age-standardised Summary Exposure Values (SEVs) for most-detailed risk factors and percentage change, by sex, 1990, 2010, and 2019. Data in parentheses are 95% uncertainty intervals.

	Male					Female				
	1990	2010	2019	Percentage change 1990-2010	Percentage change 2010-2019	1990	2010	2019	Percentage change 1990-2010	Percentage change 2010-2019
Unsafe sanitation	6·9 (4·12–11)	1·98 (1·13–3·22)	1·37 (0·782–2·18)	-71·4 (-84·8—45·1)	-30·7 (-57·1–16·5)	6·9 (4·12–11)	1·98 (1·13–3·22)	1·37 (0·782–2·18)	-71·4 (-84·8—45·1)	-30·7 (-57·1–16·5)
Ambient particulate matter pollution	15·5 (5·64–28·8)	17 (13·3–21·3)	11·7 (8·46–15·4)	9·63 (-41·4–192)	-31·4 (-37·3—27·2)	15·5 (5·59–28·7)	17 (13·3–21·3)	11·7 (8·47–15·3)	10 (-41·3–192)	-31·3 (-37·2—27·2)
Household air pollution from solid fuels	0·189 (0·0612–0·451)	0·0319 (0·00823–0·0812)	0·0173 (0·00398–0·0459)	-83·1 (-92·4—64·3)	-45·7 (-66·3—17·5)	0·247 (0·0828–0·576)	0·0419 (0·0114–0·106)	0·0229 (0·00566–0·0622)	-83 (-92·4—64·5)	-45·4 (-66·2—17)
Ambient ozone pollution	93·3 (50·2–100)	66·7 (27·2–100)	84·6 (38–100)	-28·6 (-55·1–0)	26·9 (-0·702–87·2)	93·3 (50·2–100)	66·7 (27·2–100)	84·6 (38–100)	-28·6 (-55·1–0)	26·9 (-0·702–87·2)
Residential radon	16·3 (2·76–50·1)	16·3 (2·76–50·1)	16·3 (2·76–50·1)	0 (0–0)	0 (0–0)	16·3 (2·76–50·1)	16·3 (2·76–50·1)	16·3 (2·76–50·1)	0 (0–0)	0 (0–0)
Lead exposure	100 (100–100)	92·2 (82·4–97·4)	78 (65·1–88·5)	-7·79 (-17·6—2·57)	-15·4 (-22·2—8·9)	53·5 (25·9–75·1)	40·1 (18·3–61·6)	28·9 (10·7–50·6)	-25 (-37·8—15)	-27·9 (-42·5—17·9)
Child underweight	0·998 (0·582–1·48)	0·824 (0·465–1·23)	0·782 (0·432–1·16)	-17·5 (-28·2—5·12)	-5·12 (-13·6–4·87)	1·15 (0·703–1·69)	0·997 (0·587–1·47)	0·946 (0·543–1·42)	-13·6 (-23·7—3·28)	-5·07 (-12·5–3·88)
Iron deficiency	--	--	--	--	--	10·7 (8·99–12·5)	9·57 (7·98–11·4)	9·38 (7·75–11·2)	-10·3 (-19·1—0·455)	-1·95 (-7·69–3·91)
Vitamin A deficiency	8·46 (3·31–17·1)	3·13 (1·21–6·27)	2·35 (0·913–4·81)	-62·9 (-84·9–3·83)	-25·1 (-57·2–38·2)	5·6 (2·9–9·51)	3·14 (1·58–5·24)	2·48 (1·21–4·34)	-43·9 (-70·2–15·8)	-21 (-47·6–27·1)
Zinc deficiency	3·66 (0–11·2)	3·02 (0–9·5)	2·56 (0–8·58)	-17·5 (-51·9–NA)	-15·3 (-48·8–NA)	3·58 (0–11)	3·05 (0–9·45)	2·49 (0–7·93)	-14·8 (-34·2–NA)	-18·5 (-72·7–NA)
Smoking	27·1 (24·1–30·4)	21 (18·9–23·3)	19·1 (17–21·3)	-22·6 (-28·1—16·5)	-9·08 (-14—4·16)	14·2 (11·9–16·7)	11·7 (10·3–13·4)	12·7 (10·9–14·5)	-17·6 (-28·3—6·84)	7·89 (-1·02–17·1)
Secondhand smoke	38·5 (35·5–41·4)	33·6 (30·8–36·6)	32 (29·1–35·1)	-12·7 (-20·7—3·46)	-4·82 (-11–1·34)	45 (42·5–47·4)	35·7 (33·3–37·9)	33·8 (31·7–35·9)	-20·6 (-26—15·2)	-5·25 (-9·89—0·332)
Alcohol use	14·1 (9·75–19·6)	17·4 (11·8–24·3)	19 (12·9–26·7)	23·3 (8·48–45·3)	9·19 (-5·15–25·7)	5·6 (3·36–9·09)	6·3 (3·81–9·83)	7·07 (4·24–11·1)	12·5 (-3·83–34·5)	12·2 (-9·08–37·2)
Drug use	0·199 (0·122–0·345)	0·405 (0·316–0·583)	0·279 (0·183–0·461)	103 (60·8–171)	-31·2 (-45—18·5)	0·132 (0·082–0·216)	0·235 (0·189–0·326)	0·183 (0·124–0·273)	78·6 (42·5–145)	-22·3 (-38·9—5·56)
High fasting plasma glucose	10·2 (8·41–12·4)	14·1 (11·9–16·4)	14·5 (12·3–16·7)	37·5 (17·2–61·1)	3·02 (-10·2–17·4)	9·87 (8·35–11·8)	12·6 (10·8–14·8)	14·4 (12·4–16·7)	28·1 (11·2–50)	14 (1·33–29·6)
High systolic blood pressure	37·8 (32·4–43·6)	38·9 (31·6–46·9)	39·9 (32·1–48)	2·94 (-17·2–27·7)	2·75 (-15·1–24·7)	37·7 (30·8–44·6)	33·3 (24·2–43·3)	34·2 (24·8–45·5)	-11·6 (-36·7–20·3)	2·59 (-22–34·7)
High body-mass index	13·7 (9·13–19)	21·3 (15·7–28·3)	21·7 (16·2–28·8)	55·5 (35·6–88·4)	2·26 (-3·56–8·52)	14·8 (10·4–20·5)	24·1 (18·5–32·3)	24·6 (18·8–32·8)	62·5 (39·3–100)	2·13 (-6·14–12)
Low bone mineral density	9·7 (5·13–15·8)	8·64 (4·47–14·5)	8·56 (4·53–14·7)	-10·9 (-32·5–17·5)	-0·893 (-25·5–32·2)	15·5 (9·91–22·8)	14 (8·86–20·5)	13·9 (8·92–20·8)	-9·63 (-29·8–13·8)	-0·602 (-20·7–27)
Diet low in fruits	48·9 (37·2–62·4)	46·7 (34·8–60)	47·7 (35·4–61·7)	-4·33 (-7·99—1·55)	1·99 (-5·21–10·4)	50·4 (38·8–63·9)	48·5 (37–62)	49 (37·3–62·3)	-3·81 (-7·42—1·35)	1·07 (-5·39–8·26)
Diet low in vegetables	33·2 (16·8–54·9)	12·6 (4·7–27·5)	9·93 (3·84–23·1)	-61·9 (-79·1—39·6)	-21·4 (-45·3–3·76)	30·5 (15·1–52·1)	10 (3·83–23·5)	7·97 (3·01–19·9)	-67·1 (-81·7—46·3)	-20·6 (-46·1–8·2)
Diet low in whole grains	99·9 (100–100)	99·9 (100–100)	99·9 (100–100)	0·00258 (-0·000712–0·0228)	-0·00126 (-0·0144–0·00032)	99·9 (99·9–100)	99·9 (100–100)	99·9 (99·9–100)	0·0113 (-0·00615–0·0568)	-0·00976 (-0·0815–0·0055)
Diet low in nuts and seeds	47·5 (18·7–69·1)	37·4 (12–61·5)	34·4 (10·8–59·7)	-21·3 (-40·2—7·78)	-7·99 (-24–6·51)	42·4 (15–65·3)	32·2 (9·27–57·9)	29·3 (8·91–56·7)	-24·2 (-45·4—7·76)	-8·91 (-26·6–6·7)
Diet low in milk	73·6 (47·4–94·6)	71·1 (43·3–92·9)	68·8 (40·9–91·1)	-3·43 (-10·2—0·317)	-3·26 (-8·8—0·263)	73·2 (46·6–94·3)	70·6 (42·5–92·5)	68·2 (40·1–90·4)	-3·53 (-10·1—0·311)	-3·38 (-9·19—0·285)
Diet high in red meat	65·8 (56–74·9)	63·1 (52·9–72·6)	63 (51–73·7)	-3·98 (-7·76—0·871)	-0·218 (-10·1–9·71)	65·5 (55·8–75·1)	62·9 (53·3–72·8)	62·6 (51·6–73)	-3·94 (-7·97—0·612)	-0·578 (-10·2–9·39)
Diet high in processed meat	81·8 (60·5–99·3)	84·8 (66·5–99·7)	83·3 (63·3–99·6)	3·65 (-0·279–13·6)	-1·84 (-6·89–0·607)	84·7 (66–99·7)	87·9 (72·5–99·9)	86·2 (66·7–99·8)	3·74 (-0·375–13·6)	-1·94 (-6·79–0·415)
Diet high in sugar-sweetened beverages	58·9 (39·2–75·5)	70·6 (55·8–83)	66·3 (48·9–81·6)	19·9 (-9·06–70·5)	-6·14 (-21·4–7·62)	62·2 (41·1–80·8)	73 (57·6–85·9)	69·1 (49·5–85·2)	17·4 (-11·1–68·3)	-5·4 (-22–9·69)
Diet low in fiber	34 (20–48·4)	22·7 (12·4–34·2)	21·6 (11·1–33·8)	-33·2 (-42·3—25·8)	-5·1 (-20·3–10·6)	33·8 (19·4–48·7)	22·3 (12–34·4)	21·2 (10·9–33·8)	-34 (-44·3—25·9)	-4·84 (-21·5–11·5)
Diet low in seafood omega-3 fatty acids	92·3 (81·3–100)	87·4 (71·1–100)	85·2 (68·9–99·6)	-5·3 (-18·4–1·2)	-2·52 (-13·7–9·6)	93·8 (84·1–100)	89·6 (76·7–100)	87·4 (74·4–99·7)	-4·4 (-14·6–0·52)	-2·54 (-10·8–5·75)
Diet low in polyunsaturated fatty acids	21·8 (6·74–53·1)	36 (10·4–78·7)	31·5 (8·73–72)	64·8 (8·36–119)	-12·4 (-25·6—2·44)	24·2 (7·21–56·4)	38·8 (10·9–80·7)	34·3 (9·51–75)	60·1 (7·91–114)	-11·5 (-24—2·28)
Diet high in trans fatty acids	47·9 (38·1–99·5)	41·3 (31·6–96·7)	40·2 (30·8–94·9)	-13·8 (-24—0·549)	-2·54 (-11·8–7·8)	49·1 (38·5–100)	42·7 (32–99·9)	41·6 (31·1–99)	-13·1 (-23·8–0)	-2·55 (-11·7–6·63)
Diet high in sodium	42·3 (13–66·6)	45·8 (15·2–70·1)	48·1 (16–72·7)	8·49 (-22·7–70·5)	4·95 (-16·3–41·4)	--	--	--	--	--
Low physical activity	8·82 (4·42–16·5)	11·5 (6·2–19·6)	10·3 (5·31–18·4)	29·9 (7·69–68·2)	-10·5 (-26·1–4·33)	12·9 (8·24–19·3)	14·1 (9·29–20)	13·9 (9·03–20·1)	9·02 (-1·32–25·2)	-1·4 (-10·3–7·97)
Occupational asthmagens	24·7 (20·6–29·7)	22·2 (19·1–26·2)	23·9 (20–28·7)	-10·2 (-19·5—0·465)	7·47 (-1·25–18·4)	--	--	--	--	--
Occupational particulate matter, gases, and fumes	9·26 (6·6–13·3)	8·42 (5·98–12·2)	7·89 (5·6–11·3)	-8·99 (-13·6—4·73)	-6·4 (-8·43—4·32)	3·95 (2·67–6·06)	3·44 (2·4–5·02)	3·29 (2·29–4·8)	-12·7 (-21—2·65)	-4·53 (-9·93–1·05)
Occupational noise	8·54 (7·82–9·33)	8·15 (7·65–8·8)	7·88 (7·46–8·51)	-4·51 (-7·56—1·17)	-3·31 (-4·48—2·06)	3·97 (3·37–4·59)	3·87 (3·54–4·23)	4·13 (3·86–4·41)	-2·45 (-8·99–6·08)	6·53 (3·43–9·87)
Occupational injuries	--	--	--	--	--	--	--	--	--	--
Occupational ergonomic factors	12·3 (10·2–14·7)	11·2 (9·59–13·2)	11·4 (9·7–13·6)	-9·02 (-18·6–0·57)	2·23 (-3·88–8·87)	4·32 (3·37–5·51)	4·97 (4·11–6·13)	6·48 (5·26–7·96)	15·2 (-2·47–36·9)	30·3 (19·1–43·2)
Childhood sexual abuse	7·16 (4·65–10·1)	7·16 (4·65–10·1)	7·16 (4·65–10·1)	0 (0–0)	0 (0–0)	10·6 (7·84–14·3)	10·6 (7·84–14·3)	10·6 (7·84–14·3)	0 (0–0)	0 (0–0)
Intimate partner violence	--	--	--	--	--	10·8 (5·83–16·1)	10·2 (5·85–13·8)	10·8 (5·83–16·1)	-5·27 (-17·6–14)	5·56 (-7·97–28·6)
Non-exclusive breastfeeding	21·3 (16·8–26·4)	20·4 (16–25·6)	19·9 (15·6–24·7)	-4·12 (-14·9–8·38)	-2·18 (-11·5–6·79)	21·3 (16·8–26·4)	20·4 (16–25·6)	19·9 (15·6–24·7)	-4·05 (-14·9–8·45)	-2·22 (-11·5–6·75)
Discontinued breastfeeding	23·2 (22–24·4)	22·6 (21·3–23·9)	22 (20·7–23·3)	-2·91 (-9·34–4·35)	-2·53 (-7·9–2·74)	23·4 (22·1–24·5)	22·9 (21·6–24·3)	22·2 (20·8–23·5)	-2·05 (-8·52–5·26)	-3·21 (-8·55–1·98)
Diet low in calcium	22·8 (11·3–42·9)	19 (8·89–36·4)	16·1 (7·32–30·8)	-16·6 (-28·4—8·38)	-15·2 (-24·9—8·3)	17 (7·03–33·2)	13·5 (5·3–25·8)	10·6 (4·03–20·2)	-20·6 (-35·7—9·59)	-21·1 (-33·5—12·4)
Occupational exposure to asbestos	10·7 (9·37–12·1)	12·9 (11·7–14·1)	12 (10·1–13·9)	20·6 (3·11–41·1)	-7·37 (-23·2–11·3)	1·62 (1·03–2·37)	1·26 (0·832–1·56)	1·22 (0·816–1·64)	-22·4 (-43–3·27)	-3·22 (-23·5–21·1)
Occupational exposure to arsenic	0·538 (0–1·28)	0·462 (0–1·09)	0·48 (0–1·12)	-14·2 (-21–NA)	3·84 (-2·25–NA)	0·334 (0–0·798)	0·297 (0–0·702)	0·366 (0–0·863)	-11·2 (-23·4–NA)	23·6 (14·4–NA)
Occupational exposure to benzene	0·815 (0·0479–2·3)	0·879 (0·0527–2·51)	0·948 (0·0555–2·72)	7·85 (2·32–15·1)	7·85 (3·93–13·4)	0·601 (0·0351–1·72)	0·828 (0·0494–2·41)	1·13 (0·0693–3·23)	37·7 (22·2–62·5)	36·7 (29·4–46·6)
Occupational exposure to beryllium	0·0236 (0·0227–0·0245)	0·0235 (0·0234–0·0237)	0·025 (0·0245–0·0255)	-0·34 (-3·91–3·61)	6·35 (4·01–8·71)	0·015 (0·0134–0·0166)	0·0175 (0·0172–0·0177)	0·0231 (0·0224–0·0238)	16·4 (5·19–30·8)	32·3 (27·5–36·8)
Occupational exposure to cadmium	0·0622 (0·0576–0·0668)	0·0547 (0·0535–0·0559)	0·057 (0·0541–0·0599)	-12 (-18·4—4·96)	4·23 (-1·28–9·93)	0·0388 (0·0334–0·044)	0·0359 (0·0344–0·0377)	0·0449 (0·0418–0·0484)	-7·27 (-19·5–7·86)	24·9 (16·8–34·1)
Occupational exposure to chromium	0·147 (0·138–0·157)	0·135 (0·132–0·137)	0·141 (0·135–0·147)	-8·75 (-14·7—2·41)	4·66 (0·0425–9·44)	0·0939 (0·0816–0·106)	0·0924 (0·0893–0·0959)	0·117 (0·11–0·124)	-1·55 (-13·5–13·5)	26·6 (19·6–33·9)
Occupational exposure to diesel engine exhaust	0·742 (0·698–0·786)	0·786 (0·771–0·8)	0·822 (0·792–0·853)	5·94 (-0·456–13·2)	4·56 (0·779–8·72)	0·377 (0·334–0·425)	0·446 (0·429–0·463)	0·576 (0·542–0·613)	18·4 (5·33–34·8)	29·2 (21·5–37·9)
Occupational exposure to formaldehyde	0·312 (0·287–0·337)	0·268 (0·258–0·275)	0·276 (0·262–0·292)	-14·3 (-21·1—7·34)	3·29 (-2·52–9·07)	0·185 (0·16–0·21)	0·176 (0·169–0·184)	0·222 (0·207–0·237)	-4·73 (-17·1–10·6)	25·8 (17·9–34·4)
Occupational exposure to nickel	0·507 (0–2·18)	0·432 (0–1·89)	0·445 (0–1·98)	-14·8 (-20·9–NA)	3·06 (-2·62–NA)	0·264 (0–1·17)	0·226 (0–0·995)	0·277 (0–1·21)	-14·5 (-27·1–NA)	22·5 (12·8–NA)
Occupational exposure to polycyclic aromatic hydrocarbons	0·299 (0·282–0·317)	0·278 (0·273–0·282)	0·291 (0·279–0·302)	-7·13 (-12·7—1·31)	4·76 (0·501–9·02)	0·189 (0·165–0·212)	0·194 (0·189–0·201)	0·249 (0·237–0·263)	2·79 (-9·24–18·4)	28·5 (21·9–35·4)
Occupational exposure to silica	5·73 (0·846–18·5)	5·2 (0·758–16·5)	5·34 (0·758–16·9)	-9·2 (-16·8—0·0992)	2·6 (-3·87–10)	2·3 (0·308–6·97)	1·9 (0·262–6·11)	2·3 (0·322–7·32)	-17·2 (-29·3—1·2)	20·7 (11–34·5)
Occupational exposure to sulfuric acid	0·897 (0·188–2·76)	0·795 (0·168–2·5)	0·796 (0·168–2·49)	-11·4 (-18·3—4·16)	0·127 (-5·29–5·9)	0·484 (0·0972–1·55)	0·426 (0·0891–1·34)	0·519 (0·109–1·64)	-11·8 (-23·5–3·65)	21·8 (13·3–31·8)
Unsafe sex	--	--	--	--	--	--	--	--	--	--
Occupational exposure to trichloroethylene	0·0825 (0·0788–0·087)	0·0795 (0·0787–0·0809)	0·0836 (0·0811–0·0864)	-3·58 (-8·51–1·53)	5·08 (1·68–8·66)	0·0526 (0·0464–0·0586)	0·0552 (0·0538–0·057)	0·0706 (0·0675–0·0744)	5·07 (-5·75–20)	27·8 (21·9–34·1)
No access to handwashing facility	3·48 (3·29–3·68)	3·25 (3·09–3·43)	3·2 (3·04–3·38)	-6·45 (-12·3—0·408)	-1·62 (-5·68–2·43)	3·48 (3·29–3·68)	3·25 (3·09–3·43)	3·2 (3·04–3·38)	-6·45 (-12·3—0·408)	-1·62 (-5·68–2·43)
Child wasting	1·29 (0·913–1·69)	1·25 (0·881–1·66)	1·22 (0·863–1·64)	-3·43 (-11·9–4·56)	-1·82 (-7·54–4·32)	--	--	--	--	--
Child stunting	1·77 (0·904–2·59)	1·26 (0·626–1·92)	1·21 (0·582–1·83)	-28·6 (-41·5—14·8)	-4·22 (-15–8·07)	2·11 (1·11–3·01)	1·67 (0·822–2·47)	1·59 (0·784–2·38)	-20·6 (-33·4—5·5)	-5·01 (-15·8–6·61)
Chewing tobacco	0·159 (0·125–0·2)	0·158 (0·125–0·198)	0·165 (0·128–0·205)	-0·654 (-24·4–28·4)	4·14 (-13·5–25)	0·137 (0·1–0·187)	0·138 (0·1–0·19)	0·14 (0·1–0·194)	0·46 (-32·2–47·1)	1·24 (-22–29·2)
Diet low in legumes	75·5 (42·2–99·6)	69·7 (29·1–98·2)	70·6 (30·9–98·7)	-7·72 (-31·1—0·477)	1·27 (-1·57–10·2)	72·4 (34·6–99·3)	66·4 (23·6–97)	67·3 (24·9–97·1)	-8·31 (-35·4—0·504)	1·37 (-1·93–11·7)
Short gestation	43·3 (34·2–55·3)	32·2 (25·6–40)	31·1 (24·3–39·7)	-25·6 (-44·6–0·364)	-3·41 (-26·3–28·4)	39·3 (32·6–46·7)	28·5 (23·2–34)	27·1 (21·5–33·2)	-27·4 (-43·9—8·64)	-5 (-23·8–17·1)
Low birth weight	21·3 (16·8–26·9)	16·3 (13·3–20·1)	15·9 (12·4–20)	-23·4 (-42·9–5·52)	-2·7 (-25·9–28·5)	19·6 (16·5–23·1)	14·4 (11·9–17·1)	13·7 (11–16·6)	-26·8 (-43·4—5·55)	-4·49 (-24–18·7)
High temperature	22·4 (7·08–38)	23 (7·36–39·1)	22·8 (7·08–38·9)	2·51 (-0·246–5·91)	-1·01 (-3·59–0·766)	22·4 (7·08–38)	23 (7·36–39·1)	22·8 (7·08–38·9)	2·51 (-0·246–5·91)	-1·01 (-3·59–0·766)
Low temperature	30·8 (15·8–47)	29·8 (15–46)	29·6 (14·7–45·8)	-3·28 (-8·91–0·0743)	-0·516 (-3·36–1·76)	30·8 (15·8–47)	29·8 (15–46)	29·6 (14·7–45·8)	-3·28 (-8·91–0·0743)	-0·516 (-3·36–1·76)
Kidney dysfunction	15·8 (10·8–22·6)	16·5 (11·4–23·2)	16·3 (11·4–22·9)	4·64 (-0·0919–10·4)	-1·42 (-6·32–3·66)	17 (11·8–23·7)	17·8 (12·7–24·4)	17·7 (12·5–24·6)	4·63 (0·163–11·5)	-0·654 (-5·62–4·03)
Bullying victimization	4·95 (1·97–9·97)	5·61 (2·63–10·6)	5·39 (2·38–10·3)	13·1 (-13·4–75·2)	-3·92 (-19·1–8·39)	2·31 (0·911–4·63)	2·65 (1·2–5·01)	2·56 (1·09–4·95)	14·6 (-9·2–73·4)	-3·48 (-17·3–8·24)
High LDL cholesterol	54·9 (51·4–58·5)	51·1 (47·3–54·8)	51·4 (47·5–55)	-7 (-13—0·993)	0·642 (-3·58–5·8)	55·8 (52·4–59·2)	51·1 (47·9–54·7)	51·1 (47·9–54·5)	-8·45 (-13·3—3·26)	-0·00352 (-3·75–4·02)

Supplementary Results Table 1. Monaco age-standardised Summary Exposure Values (SEVs) for most-detailed risk factors and percentage change, by sex, 1990, 2010, and 2019. Data in parentheses are 95% uncertainty intervals.

	Male					Female				
	1990	2010	2019	Percentage change 1990-2010	Percentage change 2010-2019	1990	2010	2019	Percentage change 1990-2010	Percentage change 2010-2019
Unsafe sanitation	1·37 (0·773–2·19)	0·603 (0·343–1·02)	0·517 (0·296–0·849)	-56 (-77·2–-11·8)	-14·2 (-46·7–38·7)	1·37 (0·773–2·19)	0·603 (0·343–1·02)	0·517 (0·296–0·849)	-56 (-77·2–-11·8)	-14·2 (-46·7–38·7)
Ambient particulate matter pollution	8·07 (0·685–18·3)	13·3 (8·29–18·4)	10 (5·75–14·6)	64·6 (-23·6–1420)	-24·3 (-32·5–-20)	8·07 (0·687–18·3)	13·3 (8·29–18·4)	10 (5·76–14·6)	64·6 (-23·2–1380)	-24·3 (-32·5–-20)
Household air pollution from solid fuels	0·0182 (0·00191–0·0545)	0·0072 (0·00134–0·0227)	0·00512 (0·000926–0·0161)	-60·4 (-82·7–69·2)	-28·8 (-56·9–9·43)	0·0241 (0·00304–0·0764)	0·00948 (0·00193–0·0292)	0·00676 (0·00133–0·0214)	-60·6 (-82·7–64·5)	-28·6 (-56·8–9·89)
Ambient ozone pollution	75·7 (20·6–100)	86·8 (40·6–100)	67 (26·5–100)	14·7 (-28·5–271)	-22·8 (-56·3–4·69)	75·7 (20·6–100)	86·8 (40·6–100)	67 (26·5–100)	14·7 (-28·5–271)	-22·8 (-56·3–4·69)
Residential radon	20·7 (3·67–58·8)	20·7 (3·67–58·8)	20·7 (3·67–58·8)	0 (0–0)	0 (0–0)	20·7 (3·67–58·8)	20·7 (3·67–58·8)	20·7 (3·67–58·8)	0 (0–0)	0 (0–0)
Lead exposure	41·3 (11·2–65·8)	30·5 (9·41–52·7)	21·3 (4·8–41·9)	-26·3 (-40·8–-9·43)	-30 (-49·9–-19·3)	22·7 (0·104–50·1)	17·2 (0·401–40·8)	12 (0·139–33)	-24·3 (-38·3–328)	-30·3 (-70·1–-19·3)
Child underweight	0·795 (0·445–1·16)	0·722 (0·397–1·09)	0·713 (0·394–1·05)	-9·18 (-21·2–3·83)	-1·33 (-10·7–8·79)	0·918 (0·533–1·34)	0·849 (0·476–1·26)	0·839 (0·471–1·26)	-7·52 (-18·5–5·13)	-1·18 (-10·5–8·3)
Iron deficiency	--	--	--	--	--	9·4 (7·8–11·2)	8·88 (7·37–10·6)	8·78 (7·3–10·5)	-5·48 (-14·5–4·49)	-1·13 (-7·17–5·21)
Vitamin A deficiency	1·86 (0·721–3·76)	0·942 (0·349–1·85)	0·823 (0·327–1·7)	-49·3 (-79·7–38·4)	-12·7 (-52·3–58·7)	1·85 (0·867–3·18)	1·19 (0·584–2·12)	1·06 (0·486–1·82)	-35·8 (-67·2–34·1)	-11·1 (-41·5–41·6)
Zinc deficiency	0·781 (0–2·38)	0·697 (0–2·05)	0·655 (0–1·85)	-10·8 (-68·7–NA)	-6·05 (-57·2–NA)	0·774 (0–2·37)	0·684 (0–2·03)	0·651 (0–1·82)	-11·6 (-69·9–NA)	-4·85 (-42·9–NA)
Smoking	25·5 (21·1–29·9)	21·5 (17·9–25·6)	19·5 (16·3–23)	-15·7 (-25·1–-5·18)	-9·17 (-14·8–3·19)	16·8 (13·4–20·8)	14·5 (11·5–18·3)	13·3 (10·5–16·8)	-13·5 (-28·7–5·62)	-8·04 (-18·2–3·72)
Secondhand smoke	40·7 (37·8–43·6)	36·6 (33·5–39·4)	34·3 (31·5–37·3)	-10·1 (-18·4–-1·88)	-6·15 (-11·9–-0·167)	42·3 (40–44·8)	37·1 (34·7–39·4)	34·9 (32·6–37·4)	-12·5 (-18·7–-6·15)	-5·77 (-10·1–-1·41)
Alcohol use	14·8 (1·07–28·8)	15 (1·07–28·3)	15·1 (0·739–29·8)	1·35 (-93·8–1220)	1·09 (-94·5–1510)	7·25 (0·0246–16)	6·97 (0·049–15)	7·11 (0–15·8)	-3·9 (-99·3–23200)	2·04 (-100–20100)
Drug use	0·251 (0·149–0·439)	0·288 (0·179–0·488)	0·298 (0·188–0·496)	14·6 (2·37–30·5)	3·49 (-6·38–15·4)	0·135 (0·0788–0·234)	0·171 (0·109–0·277)	0·184 (0·12–0·291)	26·9 (12·8–46·2)	7·46 (-1·01–17·9)
High fasting plasma glucose	5·63 (4·81–6·57)	8·62 (7·33–10·2)	10·8 (8·92–13)	53 (36·5–75·5)	24·9 (9·97–45·3)	5·08 (4·27–5·99)	7·19 (6·16–8·38)	8·82 (7·63–10·2)	41·4 (27·7–56·4)	22·7 (12·8–35·6)
High systolic blood pressure	40 (32·6–48·2)	39·4 (32·2–47·7)	39·7 (31·7–48·5)	-1·41 (-25–27·9)	0·836 (-15·3–20·4)	33·7 (24·2–45)	30·5 (21·5–40·8)	30·3 (21·4–40·8)	-9·43 (-38·9–35·8)	-0·658 (-24·9–30·6)
High body-mass index	28·5 (20·8–37·8)	33·4 (24·9–43·7)	35 (26·2–45·5)	17·3 (7·94–28·9)	4·63 (-0·34–11)	31·1 (24·2–40·8)	36·2 (29·1–46·2)	37·8 (30·1–47·9)	16·6 (3·8–30·8)	4·39 (-2·65–13)
Low bone mineral density	8·79 (4·57–14·7)	8·22 (4·4–14·3)	8·07 (4·15–13·7)	-6·48 (-28·5–22·8)	-1·86 (-25·2–28)	14·2 (8·9–20·7)	13·5 (8·29–20·3)	13·2 (8·25–19·7)	-5·13 (-25·9–23·5)	-1·94 (-24·4–23·8)
Diet low in fruits	21·7 (11·6–33·6)	18·7 (9·61–30·3)	16·1 (8·02–27·4)	-13·8 (-46·1–31·5)	-13·9 (-39·9–14·3)	23·1 (13·4–34·9)	20 (11–30·8)	17·3 (8·75–28·5)	-13·4 (-45–29·2)	-13·7 (-37·8–11·9)
Diet low in vegetables	7·33 (2·68–20·5)	5·9 (2·12–16·9)	4·34 (1·7–12·8)	-19·4 (-61·4–55·8)	-26·5 (-58·7–10·7)	6·23 (2·17–16·9)	4·95 (1·86–14)	3·78 (1·55–10·9)	-20·5 (-63·3–58·1)	-23·7 (-57·8–17·1)
Diet low in whole grains	89·4 (84–94·6)	89·3 (83·5–94·4)	89 (83·3–94·3)	-0·111 (-2·32–2·12)	-0·391 (-1·66–0·93)	87·8 (81·1–93·6)	87·6 (81·3–93·5)	87·2 (80·7–93·2)	-0·163 (-2·58–2·33)	-0·451 (-1·93–0·833)
Diet low in nuts and seeds	13·8 (2·79–50·9)	11·8 (2·44–50·7)	10 (1·72–50·6)	-14·1 (-52·7–42·8)	-15·1 (-49·6–10·4)	11·3 (2·16–50·6)	10·1 (1·1–50·5)	8·82 (1·27–50·5)	-10·7 (-52·9–43·2)	-12·3 (-47·8–10·4)
Diet low in milk	22·2 (6·98–41·4)	20 (6·01–39)	16 (4·32–33·8)	-9·73 (-40·7–29·8)	-20·3 (-47·8–-3·23)	21·4 (6·87–39·9)	19·3 (5·63–37·1)	15·2 (4·05–32·6)	-9·93 (-40·9–31·4)	-21·2 (-50·3–-2·71)
Diet high in red meat	93·3 (85·4–98·1)	93·9 (87·2–98·2)	95 (88·7–98·9)	0·626 (-6·42–8·35)	1·19 (-3·03–5·96)	93 (84·3–98)	93·5 (85·7–98·4)	94·7 (87·4–98·8)	0·501 (-6·2–9·18)	1·31 (-2·91–7)
Diet high in processed meat	78·4 (53·8–98·2)	79 (55·2–98·7)	79·7 (57·5–98·9)	0·857 (-4·56–7·55)	0·814 (-2·41–5·48)	82·4 (61·9–99·6)	82·9 (62·7–99·7)	83·5 (62·8–99·6)	0·684 (-5·08–7·29)	0·729 (-2·65–5·12)
Diet high in sugar-sweetened beverages	79·6 (62·2–92·9)	82·1 (66·3–93·9)	85·8 (71·2–95·7)	3·21 (-13·9–25·8)	4·51 (-6·09–18·6)	83·5 (68·3–95·4)	85·7 (72–96)	88·9 (76·9–97·7)	2·65 (-13·4–22·3)	3·69 (-5·76–16)
Diet low in fiber	8·76 (3·8–16·4)	7·76 (3·36–14·5)	6·1 (2·61–11·9)	-11·4 (-48·1–49·9)	-21·4 (-45·7–9·91)	8·32 (3·77–15·4)	7·49 (3·15–14·3)	5·89 (2·58–11·5)	-10·1 (-49·6–63·8)	-21·3 (-46·3–12·3)
Diet low in seafood omega-3 fatty acids	64·6 (47·6–96·1)	59·3 (42·1–97·8)	54·8 (38·9–95·6)	-8·16 (-19·2–3·54)	-7·54 (-18·7–3·98)	69·6 (53·3–96·9)	64·3 (47·1–96·5)	60·6 (44·3–96·1)	-7·66 (-19·4–5·23)	-5·68 (-14·9–5·28)
Diet low in polyunsaturated fatty acids	17·2 (4·03–50·4)	14·6 (3·43–43·7)	11·9 (2·89–38·4)	-14·9 (-41–11)	-18·5 (-40·2–-0·816)	19·8 (4·8–54·1)	16·9 (3·89–49·3)	13·8 (3·22–42·7)	-14·6 (-39·1–4·85)	-18·3 (-37·4–-1·09)
Diet high in trans fatty acids	49·8 (38·9–100)	43·3 (32·7–99·9)	42·6 (31·5–100)	-13·1 (-22·9–0)	-1·59 (-9·7–7·77)	50·2 (38·8–100)	44 (32·6–100)	43·3 (32–100)	-12·2 (-22·4–0)	-1·57 (-9·74–7)
Diet high in sodium	29·3 (4·89–55·6)	32·4 (5·47–59·1)	34·3 (6·34–61·1)	10·6 (-24·5–84·4)	6·06 (-15·8–52·5)	--	--	--	--	--
Low physical activity	5·16 (2·3–10·7)	5·9 (2·74–11·7)	6·09 (2·83–12·1)	14·4 (-1·14–34·7)	3·06 (-8·17–19·5)	7·97 (4·28–14·3)	8·66 (4·85–15·1)	8·81 (4·95–15·3)	8·71 (-0·0313–21·3)	1·77 (-5·39–11·2)
Occupational asthmagens	21·9 (17·8–27·7)	19·8 (16·4–24·3)	19·4 (16·2–23·3)	-9·91 (-23·2–5·43)	-1·88 (-11·6–8·38)	--	--	--	--	--
Occupational particulate matter, gases, and fumes	8·25 (5·82–11·9)	7·56 (5·3–11·2)	7·11 (4·97–10·5)	-8·41 (-14·5–-1·92)	-5·92 (-9·38–-2·7)	3·59 (2·45–5·34)	3·18 (2·2–4·74)	2·92 (2·01–4·23)	-11·3 (-19·8–-1·27)	-8·31 (-12·7–-3·59)
Occupational noise	7·79 (7·08–8·59)	7·49 (6·89–8·19)	7·25 (6·73–7·91)	-3·83 (-8·06–0·783)	-3·21 (-5·05–-1·31)	4·58 (4–5·34)	4·73 (4·27–5·33)	4·68 (4·3–5·19)	3·21 (-3·54–11)	-1·09 (-3·69–1·91)
Occupational injuries	--	--	--	--	--	--	--	--	--	--
Occupational ergonomic factors	10·5 (8·67–12·6)	9·7 (8·18–11·7)	9·47 (7·96–11·2)	-7·39 (-17·1–3·52)	-2·38 (-9·34–5·75)	--	--	--	--	--
Childhood sexual abuse	7·16 (4·65–10·1)	7·16 (4·65–10·1)	7·16 (4·65–10·1)	0 (0–0)	0 (0–0)	10·6 (7·84–14·3)	10·6 (7·84–14·3)	10·6 (7·84–14·3)	0 (0–0)	0 (0–0)
Intimate partner violence	--	--	--	--	--	15 (8·02–22·3)	15 (8·02–22·3)	15 (8·02–22·3)	0 (0–0)	0 (0–0)
Non-exclusive breastfeeding	20·9 (16·9–25·9)	20·1 (16·3–25·1)	19·8 (15·9–24·6)	-3·91 (-15·1–8·92)	-1·34 (-10·4–7·97)	20·9 (16·9–25·9)	20·1 (16·3–25·1)	19·8 (15·9–24·6)	-3·89 (-15·1–8·95)	-1·34 (-10·4–7·97)
Discontinued breastfeeding	24 (22·8–25·3)	22·7 (21·4–24·1)	22 (20·7–23·3)	-5·22 (-12–2·05)	-3·21 (-8·29–2·18)	23·9 (22·7–25·2)	22 (20·6–23·3)	21·2 (19·9–22·5)	-7·93 (-14·6–-0·781)	-3·59 (-8·82–1·9)
Diet low in calcium	0·597 (0·436–0·817)	0·576 (0·429–0·777)	0·549 (0·412–0·735)	-3·44 (-15·8–5·25)	-4·74 (-13·1–-0·484)	0·538 (0·404–0·722)	0·538 (0·403–0·722)	0·538 (0·403–0·722)	-0·0114 (-0·168–0·107)	-0·0194 (-0·144–0·0643)
Occupational exposure to asbestos	13·8 (10·6–17·5)	13·7 (11·2–16·3)	12·5 (9·73–15·4)	-0·503 (-25·4–35·4)	-9·01 (-28·2–13·5)	0·967 (0·538–1·56)	0·829 (0·492–1·23)	0·794 (0·432–1·26)	-14·2 (-52·7–58·6)	-4·19 (-41·8–63·9)
Occupational exposure to arsenic	0·534 (0–1·26)	0·489 (0–1·19)	0·489 (0–1·18)	-8·42 (-19·9–NA)	0·00461 (-9–NA)	0·406 (0–0·971)	0·366 (0–0·873)	0·345 (0–0·831)	-9·85 (-22·2–NA)	-5·67 (-15·1–NA)
Occupational exposure to benzene	0·795 (0·0472–2·27)	0·856 (0·0522–2·49)	0·871 (0·0506–2·5)	7·65 (-1·35–21·1)	1·74 (-4·47–11·3)	1·06 (0·062–2·99)	1·25 (0·0715–3·51)	1·24 (0·0725–3·58)	18·4 (5·87–40·8)	-1·23 (-8·68–10·3)
Occupational exposure to beryllium	0·0225 (0·0211–0·0239)	0·0229 (0·022–0·0238)	0·0233 (0·0225–0·0241)	1·8 (-4·12–8·73)	1·66 (-3·35–6·51)	0·0226 (0·0204–0·025)	0·0239 (0·0225–0·0252)	0·0232 (0·0221–0·0242)	5·35 (-4·83–17)	-2·82 (-9·49–4·29)
Occupational exposure to cadmium	0·0608 (0·0548–0·0672)	0·0565 (0·051–0·0622)	0·0567 (0·0516–0·0624)	-7·02 (-18·1–5·11)	0·362 (-7·93–9·96)	0·0486 (0·0421–0·0553)	0·045 (0·0409–0·0495)	0·0427 (0·0393–0·0465)	-7·4 (-19·3–8·47)	-5·17 (-13·9–3·66)
Occupational exposure to chromium	0·14 (0·127–0·153)	0·133 (0·121–0·144)	0·133 (0·123–0·145)	-5·19 (-15·4–6·02)	0·61 (-6·86–9·34)	0·122 (0·107–0·136)	0·118 (0·109–0·128)	0·113 (0·105–0·12)	-3·11 (-14·3–11·7)	-4·46 (-12·4–3·42)
Occupational exposure to diesel engine exhaust	0·583 (0·538–0·628)	0·619 (0·579–0·665)	0·627 (0·588–0·668)	6·21 (-2·64–16·4)	1·26 (-5·44–7·8)	0·531 (0·469–0·598)	0·549 (0·498–0·605)	0·528 (0·483–0·58)	3·42 (-10·4–19)	-3·95 (-12·8–6·48)
Occupational exposure to formaldehyde	0·307 (0·275–0·34)	0·278 (0·25–0·307)	0·276 (0·251–0·303)	-9·42 (-20·3–2·84)	-0·829 (-9·09–8·95)	0·239 (0·207–0·271)	0·229 (0·21–0·251)	0·218 (0·201–0·236)	-4·15 (-15·8–11·5)	-4·82 (-12·9–3·45)
Occupational exposure to nickel	0·504 (0–2·22)	0·475 (0–2·15)	0·474 (0–2·1)	-5·75 (-15·8–NA)	-0·132 (-7·43–NA)	0·319 (0–1·43)	0·279 (0–1·18)	0·261 (0–1·14)	-12·6 (-25·2–NA)	-6·31 (-16·1–NA)
Occupational exposure to polycyclic aromatic hydrocarbons	0·288 (0·263–0·313)	0·279 (0·258–0·301)	0·281 (0·262–0·302)	-3·14 (-11·9–7·01)	0·914 (-5·87–8·78)	0·256 (0·226–0·285)	0·258 (0·241–0·278)	0·248 (0·233–0·264)	0·87 (-10·2–15·5)	-3·71 (-10·9–4·05)
Occupational exposure to silica	5·76 (0·816–18)	5·79 (0·835–19)	5·83 (0·843–18·9)	0·405 (-9·64–14)	0·692 (-7·21–10·1)	2·71 (0·374–8·39)	2·33 (0·321–7·37)	2·18 (0·323–6·89)	-14 (-27·4–4·13)	-6·6 (-17·3–5·23)
Occupational exposure to sulfuric acid	0·825 (0·168–2·63)	0·747 (0·155–2·33)	0·745 (0·156–2·36)	-9·4 (-20–1·99)	-0·39 (-9·14–9·03)	0·579 (0·125–1·89)	0·514 (0·11–1·66)	0·481 (0·103–1·52)	-11·2 (-24·5–4·07)	-6·4 (-15·8–3·38)
Unsafe sex	--	--	--	--	--	--	--	--	--	--
Occupational exposure to trichloroethylene	0·0737 (0·068–0·0799)	0·0714 (0·0669–0·0761)	0·072 (0·0676–0·0768)	-3·1 (-11·4–6·4)	0·827 (-5·41–7·84)	0·0705 (0·0629–0·0787)	0·0696 (0·0648–0·075)	0·0667 (0·0626–0·0711)	-1·25 (-12·1–12·9)	-4·24 (-11·8–3·6)
No access to handwashing facility	3·2 (3·04–3·39)	3·12 (2·96–3·29)	3·09 (2·93–3·27)	-2·69 (-8·97–3·48)	-0·816 (-4·71–3·13)	3·2 (3·04–3·39)	3·12 (2·96–3·29)	3·09 (2·93–3·27)	-2·69 (-8·97–3·48)	-0·816 (-4·71–3·13)
Child wasting	1·18 (0·823–1·56)	1·15 (0·813–1·53)	1·15 (0·807–1·53)	-2·17 (-9·56–5·29)	-0·722 (-5·97–5·2)	--	--	--	--	--
Child stunting	1·45 (0·734–2·26)	1·14 (0·532–1·76)	1·13 (0·514–1·73)	-21·6 (-34·3–-6·08)	-1·02 (-13·6–13·7)	1·68 (0·847–2·51)	1·46 (0·744–2·21)	1·45 (0·741–2·2)	-13 (-25·9–2·96)	-1·13 (-13–11·7)
Chewing tobacco	0·207 (0·163–0·258)	0·217 (0·171–0·274)	0·237 (0·186–0·297)	4·81 (-19·9–39·7)	9·59 (-7·89–30·1)	0·151 (0·109–0·207)	0·157 (0·115–0·209)	0·165 (0·119–0·223)	4·06 (-28·4–52·3)	5·51 (-17–32·9)
Diet low in legumes	72·8 (36·2–98·9)	71·6 (33·3–98·4)	70·2 (30·6–98·3)	-1·67 (-11·4–3·74)	-1·96 (-12·4–0·777)	69·8 (29·8–98·2)	68·4 (27·2–97·6)	67 (23·4–96·6)	-1·87 (-14·1–4·67)	-2·15 (-12·4–1·21)
Short gestation	9·71 (6·92–13·9)	10·1 (7·65–13·9)	9·96 (7·53–13·3)	4·39 (-31·2–60·2)	-1·7 (-30·2–38)	9·31 (6·79–12·5)	9·35 (7·31–12·1)	9·22 (7·05–12·2)	0·514 (-32·1–44·8)	-1·45 (-28–34·8)
Low birth weight	5·01 (3·64–6·92)	5·2 (3·99–6·94)	5·05 (3·87–6·6)	3·86 (-31·1–59·8)	-2·96 (-31–34·9)	5·11 (3·89–6·85)	4·94 (3·95–6·27)	5·05 (3·95–6·64)	-3·33 (-33·2–33·9)	2·24 (-26·1–39·6)
High temperature	0 (0–0)	0 (0–0)	18·8 (6·15–32·6)	--	--	0 (0–0)	0 (0–0)	18·8 (6·15–32·6)	--	--
Low temperature	31·4 (25·3–38·4)	34 (27·9–40·9)	30·3 (23·8–37·8)	8·18 (5·32–11·5)	-10·8 (-15·5–-6·36)	31·4 (25·3–38·4)	34 (27·9–40·9)	30·3 (23·8–37·8)	8·18 (5·32–11·5)	-10·8 (-15·5–-6·36)
Kidney dysfunction	14·5 (9·73–21·2)	15 (10·2–21·7)	15 (10·3–21·5)	3·46 (-0·485–8·35)	0·0336 (-3·75–4·25)	15·6 (10·5–22·1)	16·3 (11·2–23)	16·4 (11·3–23·1)	4·55 (0·691–9·71)	0·982 (-2·71–4·9)
Bullying victimization	6·37 (2·43–13·3)	6·51 (2·45–13·6)	6·5 (2·45–13·5)	2·21 (0·3–4·4)	-0·115 (-0·96–0·558)	4·98 (1·97–10·4)	5·11 (1·98–10·7)	5·1 (1·97–10·7)	2·79 (0·0276–4·85)	-0·346 (-1·38–0·294)
High LDL cholesterol	57·3 (53·6–61·1)	53·9 (50·3–57·9)	54·5 (50·9–58·3)	-5·86 (-12–0·494)	1·09 (-3·65–6·12)	55·8 (52·4–59)	53·3 (50–56·6)	53·8 (50·4–57·2)	-4·43 (-9·84–0·646)	0·861 (-2·85–5·05)

Supplementary Results Table 1. Netherlands age-standardised Summary Exposure Values (SEVs) for most-detailed risk factors and percentage change, by sex, 1990, 2010, and 2019. Data in parentheses are 95% uncertainty intervals.

	Male					Female				
	1990	2010	2019	Percentage change 1990-2010	Percentage change 2010-2019	1990	2010	2019	Percentage change 1990-2010	Percentage change 2010-2019
Unsafe sanitation	2·01 (1·15–3·28)	0·763 (0·434–1·28)	0·627 (0·342–1·02)	-62·1 (-80·6—27·4)	-17·8 (-48·6–35·5)	2·01 (1·15–3·28)	0·763 (0·434–1·28)	0·627 (0·342–1·02)	-62·1 (-80·6—27·4)	-17·8 (-48·6–35·5)
Ambient particulate matter pollution	19·3 (7·73–34)	14·9 (11·7–18·7)	10·5 (7·54–13·9)	-22·8 (-56·8–74·9)	-29·6 (-35·5—25·2)	19·3 (7·72–33·9)	14·9 (11·7–18·7)	10·5 (7·56–13·9)	-22·8 (-56·8–74·6)	-29·6 (-35·5—25·3)
Household air pollution from solid fuels	0·0478 (0·0116–0·138)	0·0111 (0·00217–0·0333)	0·00705 (0·00133–0·0221)	-76·9 (-89·5—52·7)	-36·3 (-61·1—1·5)	0·0626 (0·0149–0·178)	0·0146 (0·00303–0·043)	0·00931 (0·00185–0·0274)	-76·7 (-89·4—52·4)	-36·1 (-60·7—1·49)
Ambient ozone pollution	26·1 (9·97–48)	21·3 (7·33–41·1)	28·7 (10·8–52·2)	-18·4 (-34·1—5·94)	34·7 (19·9–62·6)	26·1 (9·97–48)	21·3 (7·33–41·1)	28·7 (10·8–52·2)	-18·4 (-34·1—5·94)	34·7 (19·9–62·6)
Residential radon	8·78 (2·93–19·8)	8·78 (2·93–19·8)	8·78 (2·93–19·8)	0 (0–0)	0 (0–0)	8·78 (2·93–19·8)	8·78 (2·93–19·8)	8·78 (2·93–19·8)	0 (0–0)	0 (0–0)
Lead exposure	44·5 (16·2–68·1)	29·5 (10·1–51·7)	20·5 (4·92–41·6)	-33·8 (-49·9—22·1)	-30·6 (-50·3—19·6)	23·6 (0·133–51·1)	16·1 (0·382–39·4)	11·2 (0·145–31·5)	-31·6 (-50·2–129)	-30·6 (-69·7—19·4)
Child underweight	0·866 (0·489–1·3)	0·756 (0·424–1·13)	0·751 (0·421–1·13)	-12·7 (-24·6—0·24)	-0·62 (-9·63–9·64)	1·02 (0·603–1·51)	0·92 (0·522–1·4)	0·909 (0·509–1·36)	-10·1 (-20·7–1·77)	-1·26 (-9·64–7·26)
Iron deficiency	--	--	--	--	--	9·73 (8·14–11·6)	8·98 (7·39–10·7)	8·83 (7·31–10·5)	-7·7 (-16·6–2·21)	-1·7 (-7·43–4·5)
Vitamin A deficiency	3·2 (1·36–6·19)	1·68 (0·637–3·8)	1·41 (0·501–3·03)	-47·6 (-78·9–32)	-15·7 (-52·2–62)	2·55 (1·25–4·31)	1·75 (0·888–2·97)	1·54 (0·791–2·5)	-31·4 (-64·7–37·5)	-11·9 (-42·2–35·8)
Zinc deficiency	2·91 (0–9·17)	2·29 (0–7·33)	2·25 (0–7·23)	-21·3 (-62·7–NA)	-1·8 (-15·4–NA)	2·84 (0–9·09)	2·22 (0–7)	2·2 (0–7·14)	-21·8 (-61·2–NA)	-0·99 (-16–NA)
Smoking	33·7 (29·9–37·8)	20·7 (18·1–23·4)	17·6 (15·3–20·1)	-38·6 (-42·8—34·4)	-15 (-18·1—11·8)	25·2 (21·9–28·7)	15·9 (13·6–18·3)	13·6 (11·6–15·7)	-36·9 (-42·1—30·9)	-14·4 (-18·2—10·6)
Secondhand smoke	44·4 (41·2–47·4)	34·1 (31·2–37·2)	33·5 (30·5–36·7)	-23·2 (-29·9—15·7)	-1·7 (-8·12–4·92)	43·6 (41–46·1)	33·6 (31·2–36·1)	32·9 (30·6–35·2)	-23 (-28·8—16·9)	-2 (-6·68–2·97)
Alcohol use	17·9 (13–23·8)	18·4 (12·9–25·3)	19 (13·2–26·5)	3 (-5·76–14·7)	3·08 (-8·27–16·1)	9·93 (6·64–14·3)	9·86 (6·6–14·4)	9·86 (6·45–14·5)	-0·707 (-10·7–10·8)	0·0302 (-13–13·9)
Drug use	0·22 (0·0924–0·461)	0·244 (0·112–0·505)	0·278 (0·142–0·535)	10·7 (-2·91–29·3)	13·8 (-0·729–35·9)	0·127 (0·0568–0·258)	0·131 (0·0672–0·252)	0·171 (0·0972–0·311)	3·21 (-11·3–27·8)	31·1 (13·8–60·3)
High fasting plasma glucose	6·41 (5·52–7·48)	8·25 (6·72–9·88)	9·36 (7·87–11)	28·8 (10·8–53·7)	13·4 (-4·05–35·6)	5·46 (4·67–6·41)	6·33 (5·41–7·46)	7·24 (6·18–8·49)	16 (-0·28–34·2)	14·3 (1·53–32·3)
High systolic blood pressure	27·1 (24·1–30·2)	35·4 (29·4–41·8)	36·2 (29·7–44·1)	30·7 (7·93–58·5)	2·31 (-14·5–22)	22·4 (19·9–25·2)	22·6 (16·9–28·9)	24·4 (17·1–31·7)	0·942 (-23·5–28·5)	7·98 (-16·9–40·3)
High body-mass index	14·5 (9·64–20·2)	18·5 (13·1–25·1)	20 (14·1–27)	27·7 (15·5–46·6)	8·41 (2·21–16·4)	16·6 (12·1–22·3)	22·7 (17·5–29·9)	25·6 (20–33·8)	36·7 (21·5–60·2)	13·1 (3·62–24·9)
Low bone mineral density	12·6 (7·84–19·3)	11·7 (6·81–18·6)	11·5 (6·5–18·1)	-7·28 (-29·6–19·3)	-1·4 (-23·9–29)	14·3 (9·76–21)	13·4 (8·46–20)	13·2 (8·16–19·8)	-6·82 (-29–17·3)	-1·54 (-24·8–29·4)
Diet low in fruits	49 (36·9–62·4)	44·3 (32·3–57·5)	43 (30·6–56·8)	-9·64 (-19·4—2·75)	-2·84 (-11·1–6·05)	51·3 (40–64·1)	46·3 (34·6–59·6)	44·9 (32·7–58·4)	-9·69 (-17·3—3·61)	-2·96 (-11·2–5·29)
Diet low in vegetables	54·5 (33·3–76·6)	52·1 (31·1–74·2)	50·5 (29·8–72·4)	-4·52 (-10·8—0·741)	-3·07 (-10·1–2·77)	52·6 (31·3–75)	50·2 (29·4–72·5)	48·2 (28·4–70·3)	-4·61 (-10·6—0·854)	-3·81 (-11·5–2)
Diet low in whole grains	80·4 (71·1–89·9)	75·9 (65·1–86·8)	81·9 (73·3–90·7)	-5·57 (-9·09—2·54)	7·78 (3·57–13·3)	--	--	--	--	--
Diet low in nuts and seeds	44·4 (17·1–66·6)	36·4 (13·1–61·1)	34·7 (12·1–59·9)	-18·1 (-29·9—4·97)	-4·71 (-20·1–10·9)	39·1 (13·2–63)	31·1 (9·39–57·9)	29·4 (9·33–57·5)	-20·6 (-34·7—4·51)	-5·34 (-21·7–12·4)
Diet low in milk	41·5 (18·3–61·2)	55·9 (30·7–76·8)	52·8 (28·4–74·3)	34·5 (10·9–97·5)	-5·47 (-14·6—0·59)	40·3 (17·4–60·1)	55·1 (30·1–76·2)	51·8 (27·2–72·3)	36·9 (10·7–96·4)	-5·92 (-17·3—0·735)
Diet high in red meat	80·7 (73·5–87·1)	86 (80·3–90·9)	85·2 (76·7–92)	6·51 (2·46–11·9)	-0·96 (-7·93–5·53)	81 (73·2–87·2)	85·9 (80–90·9)	85·7 (77·1–92)	6·05 (1·46–12)	-0·201 (-7·03–6·12)
Diet high in processed meat	65·1 (40·2–88·5)	71·5 (44·7–94·8)	72·1 (45·7–95·2)	9·83 (1·52–25·8)	0·865 (-3·64–5·63)	70·6 (44·8–94·1)	75·2 (48·6–98)	75·8 (50·1–97·9)	6·52 (1·11–16·4)	0·761 (-1·92–4·47)
Diet high in sugar-sweetened beverages	40·5 (24·3–57·6)	49·8 (33·9–65·1)	54·7 (37·9–70·7)	23 (-2·26–67·1)	9·97 (-8·84–36·4)	46·9 (28·4–63·9)	58·2 (43–72·9)	62·5 (45–77·4)	23·9 (-0·0435–71·6)	7·39 (-8·54–27·1)
Diet low in fiber	46·8 (31·2–61·9)	37 (22·5–51·8)	33 (19·2–47·6)	-20·9 (-29·3—14·5)	-10·8 (-23—0·75)	47·3 (31·5–63)	37·5 (22·3–53)	33·1 (19·1–48·1)	-20·7 (-29·9—13·9)	-11·8 (-23·1—2·36)
Diet low in seafood omega-3 fatty acids	91·1 (78·4–100)	90 (77·7–100)	89·2 (76·2–100)	-1·17 (-9·01–6·83)	-0·927 (-9·89–6·83)	92·5 (81·9–100)	91·8 (81·5–100)	90·9 (79·8–100)	-0·7 (-6·82–6·69)	-0·984 (-8·66–5·24)
Diet low in polyunsaturated fatty acids	26·4 (7·76–61·9)	29·2 (8·8–64·9)	29·9 (9·2–66·1)	10·4 (1·88–26·9)	2·45 (-5·47–10·9)	29·1 (8·46–67·1)	31·8 (9·77–69·6)	32·5 (9·67–71·5)	9·29 (1·54–24·9)	2·23 (-4·6–10·7)
Diet high in trans fatty acids	63·5 (53·1–100)	61·2 (51·6–100)	59·1 (49·3–99·6)	-3·61 (-13·5–7·14)	-3·41 (-11·9–4·7)	65 (54·3–100)	61·4 (51·8–100)	59·6 (49·9–100)	-5·5 (-15·7–5·61)	-3·03 (-10·9–4·86)
Diet high in sodium	28·2 (5·76–52·3)	32·7 (7·28–58)	35·1 (7·47–61·6)	16·2 (-26·9–91·9)	7·1 (-19·2–47·1)	--	--	--	--	--
Low physical activity	1·61 (0·564–3·97)	1·45 (0·506–3·89)	1·9 (0·681–4·29)	-10·1 (-35·8–21·3)	30·8 (-1·2–96·3)	2 (0·838–4·09)	1·85 (0·802–3·75)	2·33 (1–4·75)	-7·69 (-29·4–19·7)	26·2 (0·679–65·6)
Occupational asthmagens	23·4 (19·5–28·4)	29 (24·3–35)	25·7 (21·2–31·8)	24·1 (8·21–40·7)	-11·4 (-21·4—1·16)	--	--	--	--	--
Occupational particulate matter, gases, and fumes	7·47 (5·36–10·9)	7·8 (5·57–11·4)	7·8 (5·59–11·4)	4·39 (-0·28–10·3)	0·0563 (-2·63–2·48)	2·55 (1·81–3·86)	3·24 (2·29–4·83)	3·39 (2·39–5·08)	27 (17–38·3)	4·51 (0·0667–9·15)
Occupational noise	7·15 (6·74–7·76)	7·77 (7·37–8·34)	7·92 (7·52–8·49)	8·63 (6·28–11)	2 (0·85–3·13)	3·32 (3·12–3·58)	4·98 (4·76–5·25)	5·58 (5·33–5·9)	49·8 (44·3–55·1)	12·1 (10·7–13·4)
Occupational injuries	--	--	--	--	--	--	--	--	--	--
Occupational ergonomic factors	9·5 (8·13–11·2)	10·4 (8·95–12·3)	10·5 (8·97–12·4)	9·9 (3·82–15·9)	0·393 (-4·54–6·28)	5·28 (4·4–6·38)	7·94 (6·67–9·62)	8·6 (7·13–10·5)	50·2 (40·9–60·8)	8·42 (0·562–16·5)
Childhood sexual abuse	4·13 (2·66–5·93)	3·54 (3·18–4·03)	3·54 (3·18–4·03)	-14·3 (-36·9–24·2)	0 (0–0)	23·9 (18·1–31·9)	19·2 (17·9–20·6)	19·2 (17·9–20·6)	-19·6 (-39·8–7·12)	0 (0–0)
Intimate partner violence	--	--	--	--	--	19·2 (11·3–25·4)	17·7 (10·2–23·8)	19·6 (10·5–27·7)	-7·95 (-14·7–4·61)	10·7 (-3·69–32·9)
Non-exclusive breastfeeding	33·3 (29·6–37·2)	32·8 (29·4–36·4)	32·9 (29·4–36·5)	-1·41 (-12·1–9·75)	0·299 (-8·27–9·77)	33·3 (29·6–37·2)	32·8 (29·4–36·4)	32·9 (29·4–36·5)	-1·37 (-12·1–9·79)	0·295 (-8·27–9·76)
Discontinued breastfeeding	24·6 (23·5–25·7)	23·9 (22·7–25)	23·8 (22·7–25)	-3·12 (-9·27–3·33)	-0·128 (-5·24–4·98)	24·6 (23·5–25·7)	23·9 (22·7–25)	23·8 (22·7–25)	-3·07 (-9·23–3·38)	-0·111 (-5·23–5)
Diet low in calcium	8·25 (4·04–15·2)	8·97 (4·26–16·4)	7·57 (3·56–14)	8·81 (-1·02–18·3)	-15·6 (-23·4—8·69)	--	--	--	--	--
Occupational exposure to asbestos	21·9 (20·5–23·3)	19·2 (18·5–20)	17·3 (14·9–20)	-12·2 (-18·3—5·35)	-9·89 (-22·6–4·28)	2·95 (2·28–4·19)	2·88 (2·03–3·61)	2·93 (1·97–3·97)	-2·1 (-22·4–14·3)	1·78 (-22·1–29·7)
Occupational exposure to arsenic	0·465 (0–1·08)	0·516 (0–1·23)	0·526 (0–1·26)	10·9 (-0·517–NA)	1·97 (-5·78–NA)	0·297 (0–0·711)	0·433 (0–1·02)	0·433 (0–1·02)	45·9 (30·4–NA)	0·0399 (-6·14–NA)
Occupational exposure to benzene	0·784 (0·0467–2·26)	1 (0·0585–2·84)	1 (0·0582–2·89)	27·8 (21·6–35·9)	-0·107 (-4·02–6·07)	0·866 (0·0486–2·51)	1·6 (0·0934–4·57)	1·64 (0·0946–4·74)	84·9 (75·6–103)	2·2 (-2·12–8·8)
Occupational exposure to beryllium	0·0212 (0·0209–0·0215)	0·0258 (0·0255–0·0262)	0·0265 (0·0259–0·0271)	21·8 (19·1–24·4)	2·51 (-0·0755–5·04)	0·0178 (0·0174–0·0182)	0·0295 (0·0291–0·0299)	0·0303 (0·0295–0·0311)	65·7 (61·8–69·9)	2·81 (-0·309–5·52)
Occupational exposure to cadmium	0·0538 (0·0505–0·0573)	0·0606 (0·0567–0·0647)	0·0619 (0·0569–0·0675)	12·6 (1·64–24·6)	2·14 (-4·93–9·65)	0·0358 (0·0335–0·0384)	0·0535 (0·0505–0·0571)	0·0539 (0·0506–0·0581)	49·3 (35·7–65·1)	0·798 (-4·67–6·99)
Occupational exposure to chromium	0·127 (0·12–0·134)	0·147 (0·139–0·155)	0·15 (0·139–0·161)	15·5 (5·73–25·8)	2·25 (-3·65–8·53)	0·0909 (0·086–0·0961)	0·142 (0·136–0·149)	0·145 (0·138–0·153)	56·5 (45·6–69·5)	1·57 (-2·98–6·5)
Occupational exposure to diesel engine exhaust	0·585 (0·56–0·609)	0·755 (0·726–0·787)	0·785 (0·747–0·823)	29·2 (21·6–37·1)	3·92 (-0·808–8·9)	0·414 (0·393–0·436)	0·674 (0·638–0·711)	0·713 (0·666–0·761)	62·9 (51–75·3)	5·74 (-0·408–12·1)
Occupational exposure to formaldehyde	0·272 (0·254–0·291)	0·299 (0·278–0·32)	0·301 (0·276–0·329)	9·86 (-1·22–22·1)	0·489 (-6·48–8)	0·179 (0·167–0·191)	0·278 (0·263–0·293)	0·278 (0·262–0·296)	55·3 (43·7–69·8)	0·235 (-4·57–5·82)
Occupational exposure to nickel	0·421 (0–1·82)	0·47 (0–2·1)	0·482 (0–2·17)	11·7 (1·45–NA)	2·5 (-4·38–NA)	0·234 (0–1·05)	0·326 (0–1·44)	0·324 (0–1·46)	39·1 (23·5–NA)	-0·659 (-7·89–NA)
Occupational exposure to polycyclic aromatic hydrocarbons	0·258 (0·246–0·271)	0·302 (0·288–0·317)	0·308 (0·289–0·328)	17·2 (8·41–26·4)	1·89 (-3·17–7·41)	--	--	--	--	--
Occupational exposure to silica	4·49 (0·63–14·5)	5·32 (0·775–16·9)	5·46 (0·812–17·1)	18·5 (7·9–32·7)	2·68 (-4·65–11·9)	2·04 (0·293–6·36)	2·72 (0·385–8·63)	2·69 (0·36–8·52)	33·3 (17–57·1)	-1·06 (-8·71–8·91)
Occupational exposure to sulfuric acid	0·734 (0·152–2·34)	0·803 (0·164–2·51)	0·808 (0·172–2·62)	9·49 (-0·929–20·4)	0·61 (-6·3–7·7)	0·422 (0·0882–1·37)	0·608 (0·127–1·9)	0·606 (0·127–1·9)	44·1 (28·7–62·1)	-0·269 (-6·94–7·3)
Unsafe sex	--	--	--	--	--	--	--	--	--	--
Occupational exposure to trichloroethylene	0·0706 (0·0683–0·0737)	0·084 (0·0812–0·0877)	0·0864 (0·0827–0·0912)	18·9 (12·5–25·6)	2·87 (-1·33–7·48)	0·053 (0·0509–0·0554)	0·084 (0·0812–0·0877)	0·0868 (0·0831–0·0912)	58·4 (50·2–69·1)	3·25 (-1·1–7·38)
No access to handwashing facility	3·26 (3·08–3·47)	3·15 (2·98–3·33)	3·12 (2·94–3·28)	-3·5 (-9·51–2·88)	-0·963 (-5·28–3·38)	3·26 (3·08–3·47)	3·15 (2·98–3·33)	3·12 (2·94–3·28)	-3·5 (-9·51–2·88)	-0·963 (-5·28–3·38)
Child wasting	1·24 (0·867–1·64)	1·21 (0·852–1·6)	1·2 (0·852–1·59)	-2·6 (-11·2–6·15)	-0·631 (-6·53–5·47)	--	--	--	--	--
Child stunting	1·56 (0·819–2·33)	1·18 (0·557–1·81)	1·18 (0·566–1·81)	-24·2 (-38·3—8·43)	-0·0412 (-12·2–14·6)	1·83 (0·928–2·67)	1·54 (0·766–2·33)	1·53 (0·765–2·27)	-15·7 (-29·6—0·601)	-1·18 (-12·5–11·1)
Chewing tobacco	0·149 (0·116–0·187)	0·148 (0·116–0·187)	0·15 (0·119–0·192)	-1·2 (-25–31·4)	1·85 (-15–22·5)	0·135 (0·0978–0·188)	0·137 (0·101–0·184)	0·138 (0·0994–0·188)	0·89 (-29·7–49·4)	1·21 (-19·6–27·9)
Diet low in legumes	79·5 (51·8–100)	84·4 (63·7–100)	83·4 (60·7–100)	6·12 (0–24·5)	-1·11 (-5·42–0·733)	76·6 (44·3–99·9)	81·7 (57·6–100)	80·8 (55·5–100)	6·69 (0·106–29·5)	-1·14 (-5·28–0·864)
Short gestation	14·7 (12·3–17·9)	12·1 (10·1–14·4)	13·4 (10·8–16·3)	-17·6 (-33·2–2·15)	10·7 (-10·9–38·6)	14·5 (12·2–17)	11·6 (9·87–13·6)	12·3 (10·2–14·9)	-19·5 (-33·5—2·88)	5·79 (-14·5–30·1)
Low birth weight	7·86 (6·65–9·37)	6·7 (5·69–7·88)	7·15 (5·89–8·76)	-14·8 (-33·3–6·77)	6·72 (-13·9–32·9)	7·87 (6·65–9·22)	6·58 (5·64–7·73)	6·6 (5·4–7·89)	-16·4 (-32–1·76)	0·25 (-20·4–24·7)
High temperature	23·4 (11·2–36·6)	26·1 (13·6–40)	27·7 (15–41·5)	11·6 (-3·75–31)	5·89 (2·78–11·6)	23·4 (11·2–36·6)	26·1 (13·6–40)	27·7 (15–41·5)	11·6 (-3·75–31)	5·89 (2·78–11·6)
Low temperature	30·8 (26·3–35·8)	34·4 (30·5–38·8)	30·4 (25·9–35·3)	11·6 (7·78–16·7)	-11·8 (-15·4—8·49)	30·8 (26·3–35·8)	34·4 (30·5–38·8)	30·4 (25·9–35·3)	11·6 (7·78–16·7)	-11·8 (-15·4—8·49)
Kidney dysfunction	15·9 (10·7–22·8)	16·4 (11·1–23)	16·6 (11·5–23·4)	2·92 (-0·465–7·17)	1·77 (-3·21–6·69)	16·3 (10·9–23·1)	17 (11·5–23·7)	17·4 (11·9–24·2)	4·18 (0·584–9·23)	2·01 (-2·02–6·87)
Bullying victimization	7·01 (3·12–14)	7·19 (3·2–14)	7·21 (3·18–13·9)	2·49 (-21·4–24·2)	0·262 (-8·16–7·8)	6·25 (2·7–12·7)	5·83 (2·54–11·4)	5·72 (2·48–11·2)	-6·65 (-30·9–18·1)	-1·83 (-9·78–5·62)
High LDL cholesterol	60 (56·3–63·6)	44·4 (40·7–48)	44·4 (40·6–48·2)	-26 (-31·2—21·4)	0·0581 (-4·11–4·96)	57·5 (54·2–60·8)	52·1 (48·7–55·7)	53·3 (49·9–56·7)	-9·33 (-14·2—3·84)	2·14 (-1·57–6·3)

Supplementary Results Table 1. Norway age-standardised Summary Exposure Values (SEVs) for most-detailed risk factors and percentage change, by sex, 1990, 2010, and 2019. Data in parentheses are 95% uncertainty intervals.

	Male					Female				
	1990	2010	2019	Percentage change 1990-2010	Percentage change 2010-2019	1990	2010	2019	Percentage change 1990-2010	Percentage change 2010-2019
Unsafe sanitation	1·84 (1·03–2·95)	0·605 (0·342–1·04)	0·489 (0·28–0·803)	-67·1 (-82·5–-32·4)	-19·1 (-51·5–33·3)	1·83 (1·02–2·94)	0·603 (0·34–1·03)	0·488 (0·28–0·801)	-67 (-82·5–-32·2)	-19·1 (-51·5–33·4)
Ambient particulate matter pollution	9·64 (2·19–19·6)	5·34 (2·87–8·3)	3·5 (1·36–6·19)	-44·6 (-77·1–91·8)	-34·5 (-54·3–-24·7)	9·72 (2·26–19·7)	5·38 (2·9–8·34)	3·52 (1·38–6·23)	-44·7 (-77–88·2)	-34·4 (-54·1–-24·7)
Household air pollution from solid fuels	0·0336 (0·00671–0·0922)	0·00857 (0·00144–0·0257)	0·00495 (0·000641–0·0179)	-74·5 (-89·7–-39·6)	-42·2 (-70·3–-9·19)	0·044 (0·00967–0·123)	0·0114 (0·00199–0·0365)	0·0066 (0·000837–0·0248)	-74·1 (-89·5–-39·7)	-42·2 (-70–-8·88)
Ambient ozone pollution	27·8 (10·9–50)	23·8 (8·9–44)	15·3 (5·34–29·5)	-14·2 (-26·1–-2·9)	-35·7 (-46·5–-25·8)	27·9 (10·9–50·2)	23·8 (8·88–43·9)	15·3 (5·31–29·4)	-14·8 (-27–-3·32)	-35·8 (-46·7–-25·9)
Residential radon	29·3 (15·2–48)	29·2 (15–47·8)	29·1 (14·9–47·7)	-0·447 (-3·97–3·26)	-0·128 (-1·63–1·41)	29·3 (15·2–48·3)	29·2 (15–47·7)	29·1 (14·9–47·8)	-0·384 (-3·42–2·7)	0·148 (-1·8–1·55)
Lead exposure	41·7 (15·8–64·7)	31·6 (11·7–53·2)	22·7 (6·73–43·4)	-24·4 (-36·1–-15·6)	-28 (-43·1–-18·1)	16·7 (0·699–41·9)	13·3 (0·486–36·1)	9·72 (0·237–30·1)	-20·7 (-43·5–8·59)	-26·7 (-58·1–-16·4)
Child underweight	0·89 (0·512–1·31)	0·761 (0·434–1·16)	0·752 (0·425–1·15)	-14·5 (-25·5–-1·91)	-1·27 (-10–7·96)	1·05 (0·623–1·53)	0·926 (0·521–1·36)	0·913 (0·516–1·34)	-12 (-22·4–-0·381)	-1·4 (-10·3–7·55)
Iron deficiency	--	--	--	--	--	10·2 (8·69–11·8)	9·04 (7·53–10·7)	8·95 (7·37–10·7)	-11·2 (-20·1–-2·71)	-1·03 (-6·35–4·46)
Vitamin A deficiency	3·1 (1·33–6·3)	1·31 (0·538–2·68)	1·1 (0·408–2·27)	-57·7 (-83·4–8·14)	-16·1 (-53–53·6)	2·53 (1·25–4·23)	1·47 (0·694–2·57)	1·28 (0·649–2·16)	-41·9 (-68·4–22)	-13·2 (-44·3–36·5)
Zinc deficiency	4·92 (0·0757–14·1)	2·97 (0–9·37)	2·9 (0–9·23)	-39·6 (-100–25·4)	-2·38 (-17·4–NA)	4·76 (0·0578–13·8)	2·9 (0–9)	2·82 (0–9)	-39 (-100–23·9)	-2·91 (-22·6–NA)
Smoking	21·3 (18·4–24·4)	17·5 (15·1–20·1)	14 (12·1–16)	-18 (-23·7–-12)	-19·9 (-23·5–-16·4)	17·9 (14·9–21·1)	13·2 (11·1–15·5)	9·58 (8·13–11·2)	-26·4 (-34·2–-17·5)	-27·2 (-32·5–-21·6)
Secondhand smoke	43·9 (40·8–46·9)	33·5 (30·6–36·5)	32·7 (29·9–35·9)	-23·8 (-30·5–-16·6)	-2·13 (-8·47–4·71)	41·7 (39·2–44·2)	32·6 (30·4–34·9)	31·9 (29·6–34·2)	-21·8 (-28–-15·6)	-2·09 (-6·85–3·22)
Alcohol use	13·4 (8·77–19·6)	17·1 (11·8–24·4)	17·4 (11·3–25·2)	27·9 (15–43)	1·73 (-10·5–14·6)	6·89 (4·02–11·2)	8·59 (5·29–13·3)	8·45 (5·09–13·2)	24·8 (9·5–44·1)	-1·63 (-16·7–14·8)
Drug use	0·254 (0·176–0·386)	0·356 (0·24–0·552)	0·35 (0·245–0·527)	40·1 (32·5–50·7)	-1·76 (-6·47–3·03)	0·14 (0·1–0·203)	0·188 (0·136–0·271)	0·193 (0·141–0·275)	34·4 (28·3–40·5)	2·65 (-0·74–6·82)
High fasting plasma glucose	9·4 (8·14–10·8)	11 (9·45–12·7)	12·9 (11·2–14·8)	16·8 (9·18–25·8)	17·8 (10·6–26·3)	7·88 (6·83–9·1)	8·8 (7·63–10·1)	11·1 (9·55–12·7)	11·6 (3·34–20·6)	25·7 (18·1–33·8)
High systolic blood pressure	47·1 (42·9–51·7)	36·4 (32·4–40·6)	39·7 (33·2–47)	-22·8 (-33–-10·9)	9·13 (-5·82–25·9)	38·9 (34·1–44·2)	31·4 (26·3–36·8)	32·4 (24·3–40·6)	-19·2 (-34·7–-1·87)	3·07 (-17·2–26·6)
High body-mass index	16·5 (12·1–21·7)	20·2 (15·2–26·5)	22·2 (16·6–29·1)	22 (11·6–37·8)	9·74 (2·73–17·7)	16·7 (12·5–21·7)	21·6 (16·7–27·6)	23·2 (18·3–29·4)	28·7 (14·2–47·8)	7·65 (0·317–16·1)
Low bone mineral density	9·4 (5·23–15·3)	8·7 (4·86–14·4)	8·43 (4·67–14·3)	-7·43 (-14·6–-0·495)	-3·05 (-10·5–4·07)	14·7 (9·85–20·9)	13·3 (9·02–19·4)	13·2 (8·57–19·3)	-9·68 (-16·6–-2·87)	-0·866 (-7·48–6·22)
Diet low in fruits	51·4 (40·6–63·3)	39 (29·2–50·6)	38·6 (28·8–50·1)	-24 (-32–-17·6)	-1·13 (-11·2–8·6)	53·2 (42·9–65)	40·4 (30·2–52·1)	40·2 (29·6–51·9)	-23·9 (-31·5–-17·3)	-0·494 (-9·63–9·59)
Diet low in vegetables	64·3 (44·3–82·3)	56·7 (36–77·6)	53·4 (33·3–74·3)	-11·9 (-20·1–-5·28)	-5·84 (-13·4–-0·785)	62·5 (42–81·7)	54·4 (34·2–75·2)	51·7 (32·5–72·6)	-13 (-21·7–-6·19)	-4·82 (-11·3–0·493)
Diet low in whole grains	96·5 (94·2–99)	88·8 (83·1–94·6)	92·4 (87·9–96·5)	-7·96 (-12·2–-3·63)	3·98 (1·66–6·68)	--	--	--	--	--
Diet low in nuts and seeds	64·3 (30·4–80·3)	54·7 (23·9–72·8)	51·9 (24·2–71·8)	-15·1 (-26·5–-7·45)	-5·13 (-15·7–2·22)	60·1 (27·3–77·4)	50·4 (21·1–70)	47·2 (19·4–68·4)	-16·2 (-27·5–-7·63)	-6·24 (-17·1–1·09)
Diet low in milk	40·7 (19–59·5)	33·3 (13·6–51·4)	33·3 (13·8–51·7)	-18·1 (-35·5–-5·77)	0·0729 (-11·3–12·2)	39·7 (18·3–58·5)	33·7 (13·5–51·5)	33·9 (15–51·9)	-15·2 (-31·1–-4·3)	0·711 (-10·2–13·3)
Diet high in red meat	64·1 (55·6–72·4)	76·4 (69·9–82·3)	76·8 (68·3–84)	19·1 (13–27·5)	0·597 (-6·16–6·95)	64 (55·4–72·2)	74·3 (67·1–80·7)	74·9 (66·2–82·3)	16·1 (11·1–22·8)	0·797 (-5·91–7·4)
Diet high in processed meat	88·4 (72·6–99·8)	92·2 (81·8–99·9)	91·7 (81·3–99·9)	4·24 (0·0577–13)	-0·517 (-3·02–1·68)	90·9 (78·4–99·9)	94·1 (86·5–100)	93·5 (84·9–100)	3·59 (0·0143–11·9)	-0·715 (-3·17–1·08)
Diet high in sugar-sweetened beverages	34·7 (24·5–48·9)	43·8 (33·3–56·8)	44·4 (32·9–58·3)	26·3 (6·47–55·2)	1·48 (-10·5–16·8)	36·2 (24·6–50)	46·3 (34·9–58·8)	46·8 (34·7–60·7)	27·9 (6·8–60·8)	1·14 (-12·4–15·4)
Diet low in fiber	43·8 (29·1–58·5)	30·8 (19·3–42·6)	25·8 (15·8–37·5)	-29·6 (-35·6–-25)	-16·3 (-27·4–-5·71)	43·8 (28·5–58·8)	31·9 (19·6–44·9)	27·2 (16·3–39·3)	-27·2 (-33·1–-22)	-14·8 (-26–-4·75)
Diet low in seafood omega-3 fatty acids	91·7 (83·5–99·9)	88 (76·7–99·6)	87·2 (76–99·4)	-4·07 (-9·46–-0·0177)	-0·825 (-4·29–2·41)	93 (86·1–100)	89·9 (80·5–99·8)	89·1 (78·8–99·8)	-3·38 (-7·93–0·068)	-0·877 (-4·28–1·36)
Diet low in polyunsaturated fatty acids	16·1 (5·78–41·3)	14·5 (4·53–40·2)	15 (4·63–42·1)	-10·3 (-28·2–2·21)	3·68 (-6·87–17·5)	17·8 (6·54–43·5)	16 (4·93–42·5)	16·5 (5·06–43·6)	-9·82 (-28·2–2·18)	2·78 (-7·65–16·6)
Diet high in trans fatty acids	47·4 (37·9–94·9)	36·1 (25·9–97·8)	39·2 (29·8–95·3)	-23·9 (-38·4–2·01e-14)	8·72 (-4·48–26·1)	48·2 (38·2–98·5)	38·4 (27·6–99·3)	40·9 (30·9–96·8)	-20·5 (-34·5–0)	6·61 (-5·28–23·1)
Diet high in sodium	31·5 (7·88–56·4)	30·4 (7·93–53·5)	31·4 (8·21–55·1)	-3·28 (-31·3–29·5)	3·27 (-14·2–28·1)	23·3 (2·84–45·5)	23·4 (2·55–45·4)	24·5 (2·43–46·8)	0·564 (-26·6–21·6)	4·4 (-14·5–27·6)
Low physical activity	4·05 (1·72–8·31)	4·16 (1·81–8·4)	4·4 (1·88–8·93)	2·8 (-3·13–9·92)	5·73 (0·0467–13·1)	6·45 (3·31–12·2)	6·74 (3·54–12·5)	7·17 (3·78–13·2)	4·59 (0·206–10·9)	6·32 (2·19–12·2)
Occupational asthmagens	24·4 (20·4–29·1)	20·7 (17·9–24·2)	20·3 (17·3–24)	-15 (-24–-5·45)	-2·17 (-10·2–6·95)	--	--	--	--	--
Occupational particulate matter, gases, and fumes	9·67 (7–13·8)	8·52 (6·15–12·4)	7·98 (5·75–11·6)	-11·9 (-15·1–-8·43)	-6·27 (-8·27–-4·36)	4·46 (3·17–6·43)	3·84 (2·75–5·59)	3·5 (2·51–5·12)	-13·9 (-19·7–-8·36)	-8·83 (-11·8–-6·1)
Occupational noise	9·25 (8·75–9·92)	8·6 (8·15–9·23)	8·34 (7·91–8·97)	-7·03 (-8·9–-5·18)	-3·09 (-4·01–-2·12)	5·66 (5·3–6·2)	5·95 (5·63–6·45)	6·08 (5·78–6·59)	5·25 (2·72–7·88)	2·1 (1·06–3·23)
Occupational injuries	--	--	--	--	--	--	--	--	--	--
Occupational ergonomic factors	13·6 (11·9–15·8)	11·8 (10·3–13·7)	10·9 (9·43–12·9)	-13·2 (-19·9–-6·39)	-7·3 (-12·9–-1·08)	9·13 (7·58–11)	11·6 (9·72–14)	10 (8·26–12·4)	26·7 (13·5–40·5)	-13·2 (-21·5–-3·63)
Childhood sexual abuse	7·19 (4·63–10·3)	7·56 (4·94–10·8)	7·56 (4·94–10·8)	5·2 (-1·84–12·4)	0 (-3·19e-14–2·1e-14)	9·88 (7·47–13·2)	13·3 (9·71–18·3)	13·3 (9·71–18·3)	35 (24·9–47·8)	0 (-2·42e-14–3·34e-14)
Intimate partner violence	--	--	--	--	--	16·6 (9·01–24·4)	15·6 (8·55–23·2)	15 (8·2–22·4)	-6·22 (-13·6–5·66)	-3·62 (-9·35–6·5)
Non-exclusive breastfeeding	16·2 (12–21·5)	15·9 (11·9–21·1)	15·8 (11·8–20·8)	-1·99 (-13–10·4)	-0·419 (-9·23–9·21)	16·2 (12–21·5)	15·9 (11·9–21·1)	15·8 (11·8–20·9)	-1·95 (-13–11·1)	-0·486 (-9·11–9·31)
Discontinued breastfeeding	19·7 (18·1–21·2)	19 (17·4–20·6)	18·3 (16·8–19·8)	-3·85 (-13·5–7·61)	-3·53 (-11·8–6·47)	19·7 (18·1–21·2)	18·9 (17·4–20·6)	18·3 (16·8–19·8)	-4·27 (-13·9–7·12)	-3·19 (-11·4–6·84)
Diet low in calcium	13·1 (6·16–24·4)	13·4 (6·68–24·3)	13·1 (6·7–23·7)	2·29 (-6·57–18·5)	-1·99 (-7·34–5·63)	7·44 (2·87–13·6)	7·92 (3·26–14·5)	7·56 (3·15–14)	6·4 (-7·36–25·4)	-4·49 (-12·2–5·84)
Occupational exposure to asbestos	7·21 (6·78–7·64)	11·1 (10·6–11·7)	9·97 (8·15–12)	54·7 (45·1–65)	-10·6 (-26·7–6·74)	13·1 (4·78–16·2)	4·7 (3·86–7·23)	3·69 (2·47–7·12)	-64·2 (-73·7–-1·31)	-21·5 (-45·8–7·3)
Occupational exposure to arsenic	0·508 (0–1·21)	0·439 (0–1·03)	0·446 (0–1·05)	-13·7 (-21·3–NA)	1·66 (-5·52–NA)	0·47 (0–1·14)	0·428 (0–1·01)	0·426 (0–0·994)	-9 (-17·6–NA)	-0·45 (-5·73–NA)
Occupational exposure to benzene	0·835 (0·049–2·42)	0·955 (0·0558–2·74)	0·954 (0·0554–2·69)	14·3 (9·55–21·3)	-0·109 (-4·26–5·82)	1·3 (0·0766–3·78)	1·8 (0·107–5·32)	1·82 (0·109–5·37)	38·4 (32·2–48·8)	1·09 (-3·6–6·98)
Occupational exposure to beryllium	0·0248 (0·0244–0·0252)	0·0255 (0·0253–0·0258)	0·0255 (0·0249–0·0262)	2·99 (1·18–4·67)	-0·0944 (-2·72–2·58)	0·0287 (0·0281–0·0293)	0·0318 (0·0314–0·0322)	0·0318 (0·031–0·0327)	10·7 (7·99–13·4)	0·0441 (-2·89–3·21)
Occupational exposure to cadmium	0·0594 (0·0547–0·0643)	0·0526 (0·0513–0·0541)	0·0533 (0·0499–0·0567)	-11·4 (-18·3–-4·04)	1·36 (-5·21–8·34)	0·0573 (0·0527–0·0622)	0·0541 (0·0528–0·0553)	0·0538 (0·0515–0·0565)	-5·65 (-13·5–2·52)	-0·463 (-5·03–4·57)
Occupational exposure to chromium	0·14 (0·131–0·151)	0·13 (0·127–0·133)	0·132 (0·124–0·139)	-7·4 (-13·7–-0·657)	1·19 (-4·4–6·98)	0·146 (0·137–0·156)	0·149 (0·146–0·151)	0·148 (0·143–0·154)	1·49 (-5·2–8·2)	-0·227 (-4·04–3·91)
Occupational exposure to diesel engine exhaust	0·768 (0·731–0·806)	0·871 (0·849–0·893)	0·853 (0·818–0·89)	13·5 (7·52–19·5)	-2·09 (-6·41–2·13)	0·767 (0·721–0·812)	0·781 (0·753–0·81)	0·766 (0·719–0·813)	1·84 (-4·6–8·61)	-2·02 (-7·63–4·26)
Occupational exposure to formaldehyde	0·293 (0·268–0·32)	0·255 (0·245–0·263)	0·256 (0·238–0·274)	-12·9 (-20·2–-4·84)	0·474 (-6·13–7·66)	0·278 (0·257–0·301)	0·287 (0·277–0·294)	0·287 (0·274–0·3)	3·2 (-4·65–11·6)	-0·169 (-4·17–4·28)
Occupational exposure to nickel	0·499 (0–2·26)	0·46 (0–1·99)	0·46 (0–2)	-7·87 (-14·2–NA)	0·00998 (-5·98–NA)	0·375 (0–1·72)	0·323 (0–1·44)	0·32 (0–1·46)	-13·9 (-22·4–NA)	-0·984 (-6·81–NA)
Occupational exposure to polycyclic aromatic hydrocarbons	0·286 (0·269–0·303)	0·275 (0·27–0·281)	0·278 (0·265–0·292)	-3·66 (-9·42–2·46)	1·13 (-3·7–6·1)	--	--	--	--	--
Occupational exposure to silica	5·82 (0·86–18·8)	6·25 (0·918–20·3)	6·15 (0·915–20)	7·3 (-0·536–17·2)	-1·52 (-7·79–6·28)	3·38 (0·466–10·6)	2·8 (0·391–8·85)	2·74 (0·382–8·55)	-17·1 (-25·6–-7·16)	-2·06 (-9·06–7·21)
Occupational exposure to sulfuric acid	0·837 (0·176–2·67)	0·716 (0·147–2·26)	0·719 (0·147–2·27)	-14·4 (-20·9–-7·02)	0·405 (-6·4–7·31)	0·699 (0·145–2·21)	0·612 (0·126–1·9)	0·601 (0·129–1·89)	-12·4 (-20·4–-4·16)	-1·78 (-7·43–5·15)
Unsafe sex	--	--	--	--	--	--	--	--	--	--
Occupational exposure to trichloroethylene	0·0804 (0·077–0·0847)	0·0779 (0·0766–0·0798)	0·0782 (0·0753–0·0816)	-3·13 (-7·62–1·82)	0·378 (-3·89–4·77)	0·0894 (0·0855–0·094)	0·0905 (0·0888–0·0928)	0·0897 (0·0865–0·0939)	1·23 (-3·64–6·08)	-0·861 (-4·79–3·72)
No access to handwashing facility	3·24 (3·07–3·43)	3·12 (2·95–3·3)	3·09 (2·92–3·26)	-3·83 (-9·66–2·45)	-0·988 (-4·71–3·05)	3·24 (3·06–3·43)	3·12 (2·95–3·3)	3·09 (2·92–3·26)	-3·82 (-9·64–2·49)	-0·987 (-4·71–3·07)
Child wasting	1·25 (0·881–1·65)	1·21 (0·859–1·59)	1·21 (0·851–1·59)	-3·14 (-10·7–4·87)	-0·64 (-6·51–5·14)	1·06 (0·74–1·42)	1·02 (0·695–1·35)	1·01 (0·683–1·35)	-3·9 (-13·6–7·53)	-0·765 (-7·81–6·77)
Child stunting	1·6 (0·791–2·38)	1·19 (0·567–1·82)	1·18 (0·576–1·83)	-25·8 (-38·9–-10·8)	-1·14 (-13·1–12·3)	1·88 (0·951–2·71)	1·55 (0·748–2·3)	1·53 (0·772–2·31)	-17·6 (-30·9–-1·9)	-1·25 (-12–12·1)
Chewing tobacco	0·617 (0·488–0·772)	0·934 (0·731–1·16)	1·37 (1·08–1·7)	51·5 (11·6–101)	46·2 (21·9–74·1)	0·314 (0·228–0·421)	0·414 (0·308–0·561)	0·534 (0·388–0·723)	31·6 (-7·66–93)	29·1 (1·49–67·2)
Diet low in legumes	97·3 (93·5–100)	88·9 (74–100)	88·3 (72·7–100)	-8·66 (-21·3–2·01e-14)	-0·689 (-3·29–0·669)	96 (90·1–100)	86·6 (68·8–100)	85·9 (66·6–100)	-9·78 (-24–0)	-0·836 (-4·2–0·769)
Short gestation	10·2 (9·16–11·2)	9·83 (8·85–10·9)	9·79 (8·74–10·8)	-3·37 (-9·29–2·85)	-0·401 (-6·68–5·47)	9·59 (8·66–10·6)	9·17 (8·33–10·1)	8·49 (7·66–9·4)	-4·37 (-9·92–1·33)	-7·37 (-12·6–-1·89)
Low birth weight	4·47 (4·17–4·82)	4·69 (4·37–5·05)	4·55 (4·22–4·92)	5·08 (-1·41–12)	-3·09 (-9·27–3·27)	4·62 (4·32–4·97)	4·73 (4·4–5·08)	4·28 (3·98–4·62)	2·36 (-4·12–9·03)	-9·41 (-15–-3·41)
High temperature	0·314 (0·0988–0·562)	0 (0–0)	17·7 (8·06–28·9)	-100 (-100–-100)	--	0·314 (0·0988–0·562)	0 (0–0)	17·7 (8·06–28·9)	-100 (-100–-100)	--
Low temperature	35·2 (30·1–40·5)	38·1 (33·9–42·8)	34·9 (30·3–40·1)	8·22 (2·64–15·5)	-8·3 (-13·2–-4·17)	35·2 (30·1–40·5)	38·1 (33·9–42·8)	34·9 (30·3–40·1)	8·22 (2·64–15·5)	-8·3 (-13·2–-4·17)
Kidney dysfunction	14·2 (9·38–20·6)	14·7 (9·82–21·3)	14·7 (9·87–21·3)	3·53 (1·09–6·47)	0·329 (-1·2–1·85)	13·7 (8·85–20·2)	14·6 (9·67–21·1)	14·9 (10–21·3)	6·49 (3·72–9·58)	1·66 (-0·0455–3·96)
Bullying victimization	5·03 (2·04–10·2)	5·54 (2·22–11·2)	5·33 (2·11–10·9)	10·1 (2·61–19)	-3·74 (-8·86–0·00226)	4·03 (1·62–8·07)	4·53 (1·85–9·1)	4·38 (1·77–8·89)	12·3 (5·2–21·4)	-3·21 (-8·37–1·2)
High LDL cholesterol	53·9 (50·4–57·4)	51·3 (48–54·9)	52·4 (48·6–56)	-4·86 (-10·5–1·18)	2·07 (-2·15–6·62)	54·2 (50·9–57·4)	52·1 (49–55·6)	52·7 (49·3–56·4)	-3·93 (-8·81–1·67)	1·16 (-2·6–4·76)

Supplementary Results Table 1. Portugal age-standardised Summary Exposure Values (SEVs) for most-detailed risk factors and percentage change, by sex, 1990, 2010, and 2019. Data in parentheses are 95% uncertainty intervals.

	Male					Female				
	1990	2010	2019	Percentage change 1990-2010	Percentage change 2010-2019	1990	2010	2019	Percentage change 1990-2010	Percentage change 2010-2019
Unsafe sanitation	19·9 (15·7–24·6)	7·13 (4·4–11·2)	5·31 (3·11–8·22)	-64·2 (-78·6–-41·4)	-25·5 (-51·9–15)	19·9 (15·7–24·6)	7·13 (4·4–11·2)	5·31 (3·11–8·22)	-64·2 (-78·6–-41·4)	-25·5 (-51·9–15)
Ambient particulate matter pollution	9·76 (2·93–19·3)	7·79 (5·23–10·8)	5·68 (3·33–8·55)	-20·2 (-62·6–136)	-27 (-37·8–-20·6)	9·57 (2·9–19·1)	7·77 (5·24–10·8)	5·68 (3·32–8·53)	-18·8 (-62–132)	-26·9 (-37·7–-20·3)
Household air pollution from solid fuels	0·806 (0·249–1·89)	0·116 (0·0311–0·307)	0·0519 (0·0116–0·142)	-85·6 (-93·3–-67·9)	-55·4 (-74–-30·3)	1·03 (0·345–2·39)	0·154 (0·0425–0·382)	0·0689 (0·0161–0·183)	-85·1 (-93·2–-68·1)	-55·2 (-74–-30·2)
Ambient ozone pollution	25·1 (10–45·1)	45·1 (18·7–77·6)	33·2 (12·8–60·2)	79·7 (54·7–115)	-26·4 (-36·1–-17·8)	25·1 (10–45·1)	45·1 (18·7–77·6)	33·2 (12·8–60·2)	79·7 (54·7–115)	-26·4 (-36·1–-17·8)
Residential radon	28·2 (12–50)	28·2 (12–50)	28·2 (12–50)	0 (0–0)	0 (0–0)	28·2 (12–50)	28·2 (12–50)	28·2 (12–50)	0 (0–0)	0 (0–0)
Lead exposure	88·9 (74·4–96·7)	66·8 (50·9–80·8)	52·4 (36·7–69)	-24·8 (-34·5–-15)	-21·6 (-28·2–-14·5)	67·6 (44·6–84·4)	48·3 (27·9–68·2)	36·1 (18·1–56·3)	-28·6 (-39·8–-18)	-25·2 (-35·6–-16·7)
Child underweight	1·02 (0·595–1·5)	0·804 (0·461–1·2)	0·785 (0·44–1·17)	-21·5 (-32·3–-10)	-2·41 (-11·2–7·29)	1·21 (0·712–1·78)	0·978 (0·584–1·44)	0·953 (0·561–1·39)	-19·3 (-29·4–-7·69)	-2·59 (-10·6–5·46)
Iron deficiency	--	--	--	--	--	11·4 (9·57–13·4)	9·66 (7·91–11·4)	9·35 (7·7–11)	-15·6 (-24·5–-6·39)	-3·15 (-8·55–2·77)
Vitamin A deficiency	11·3 (4·52–21·7)	4·08 (1·66–8·42)	3·31 (1·31–6·96)	-64 (-84·7–-9·06)	-19 (-54·8–49·7)	7·46 (3·78–12·7)	4·06 (2·02–7·11)	3·34 (1·72–5·83)	-45·6 (-72·1–6·76)	-17·7 (-45·6–28·5)
Zinc deficiency	4·72 (0–13·7)	2·81 (0–8·85)	2·91 (0–8·9)	-40·5 (-100–NA)	3·47 (-10–NA)	4·57 (0–13)	2·75 (0–8·87)	2·84 (0–9·07)	-39·8 (-100–NA)	3·38 (-8·94–NA)
Smoking	24·1 (21·1–27·2)	20·9 (18·3–23·6)	18·5 (16·1–21·1)	-13·5 (-20·1–-6·79)	-11·2 (-14·8–-7·26)	7·49 (6·12–8·94)	7·23 (6·02–8·59)	7·98 (6·58–9·48)	-3·48 (-17·3–10·8)	10·3 (2·43–18·6)
Secondhand smoke	40·1 (38·4–41·7)	32·8 (31·4–34·2)	31·5 (29·5–33·6)	-18·1 (-23·2–-13)	-4·02 (-9·49–1·63)	51·7 (49·9–53·4)	39·4 (37·9–40·9)	37·6 (35·5–39·4)	-23·8 (-27·8–-19·9)	-4·68 (-8·59–-1·01)
Alcohol use	20·6 (15·6–26·2)	22·3 (16·2–29·7)	23·2 (16·5–31·9)	8·3 (-3·04–24·3)	4·3 (-5·69–17·5)	8·03 (5·52–11·3)	7·59 (4·99–10·9)	7·87 (5·02–11·6)	-5·49 (-19·9–13)	3·72 (-11·9–21·3)
Drug use	0·151 (0·0728–0·31)	0·156 (0·0959–0·269)	0·194 (0·11–0·352)	3·46 (-19·5–41·8)	24·8 (7·64–44·3)	0·101 (0·0503–0·204)	0·12 (0·0743–0·198)	0·151 (0·0923–0·251)	18·6 (-8·19–61·1)	25·7 (13·5–39·9)
High fasting plasma glucose	9·13 (7·66–10·9)	15·9 (13·9–17·9)	15·8 (13·5–18)	74·1 (51·6–103)	-0·739 (-13·6–12·3)	9·08 (7·48–11·2)	12·7 (11·3–14·2)	15·1 (12·6–18·1)	40·4 (15·6–67·7)	18·5 (1·13–39·9)
High systolic blood pressure	35·3 (30·6–40·9)	30·3 (26·9–34·2)	33·4 (27·7–40)	-14 (-27·4–2·75)	10·2 (-8·03–31·4)	36·1 (29·9–42·4)	19·3 (16·7–22·2)	22·5 (16·3–29·3)	-46·5 (-56·9–-33·6)	16·2 (-13·6–52·5)
High body-mass index	13·8 (9·01–19·8)	22·1 (15·8–29·9)	22·3 (15·8–30)	59·5 (40·2–90·6)	0·763 (-4·72–7·03)	15·8 (11·1–22·1)	23·6 (18–31·9)	23·7 (17·8–31·8)	49·5 (31·3–78·7)	0·293 (-6·8–8·46)
Low bone mineral density	9·77 (5·34–15·9)	8·81 (4·75–14·6)	8·59 (4·69–14·5)	-9·89 (-30·6–16·3)	-2·46 (-27·6–34·4)	16·2 (10·9–23·5)	15 (9·67–21·5)	14·5 (9·17–21·1)	-7·61 (-26·2–14·9)	-3·37 (-25·2–22·2)
Diet low in fruits	41·9 (30·1–55)	38·2 (27·3–50·9)	37·8 (26·7–51·1)	-8·85 (-13·4–-5·1)	-1·16 (-9·95–8·34)	43·7 (32·1–56·7)	39·7 (28·2–52·5)	39·3 (28·4–52·1)	-9·14 (-13·9–-5·4)	-1·19 (-10–7·99)
Diet low in vegetables	15·8 (6·22–32·9)	10·9 (3·96–24·6)	11·1 (4·19–24·8)	-31·2 (-46·3–-16·5)	1·61 (-24·5–37·8)	13·2 (4·9–28·6)	8·48 (3·13–21·4)	8·97 (3·27–22·4)	-35·6 (-51·9–-16·8)	5·82 (-24·9–45·6)
Diet low in whole grains	85·9 (78·9–93·1)	87·5 (81·2–93·9)	87·9 (81·6–93·9)	1·78 (0·822–3·09)	0·456 (-0·275–1·31)	--	--	--	--	--
Diet low in nuts and seeds	63·9 (30·7–80·4)	52·3 (21·8–72·4)	54·1 (23·1–74·2)	-18·1 (-34·4–-8·52)	3·44 (-4·11–15·6)	59·5 (27·5–77·8)	47·2 (18·5–68·8)	49·1 (20·7–70·1)	-20·7 (-39·4–-9·45)	4·05 (-4·76–18·4)
Diet low in milk	64·3 (36·2–86·3)	38·1 (15–57·8)	40·6 (17·5–60·2)	-40·7 (-70·1–-15·7)	6·49 (-3·07–23·4)	63·8 (35·6–84·9)	36·9 (14·4–56·3)	39·5 (16·6–59·6)	-42·2 (-72–-16·2)	6·99 (-2·39–23·9)
Diet high in red meat	61·1 (50·7–71·4)	97·7 (96·5–98·7)	95·2 (91·1–98)	59·9 (37·6–91·2)	-2·55 (-6·5–-0·0943)	60·9 (50·2–70·9)	97·8 (96·6–98·8)	95·3 (91·3–98)	60·5 (38·8–93·5)	-2·47 (-6·02–-0·0474)
Diet high in processed meat	34 (17·6–56·4)	44 (25–67·9)	42·6 (23·4–66·5)	29·3 (8·33–66·1)	-3·06 (-15·1–9·9)	40·1 (22–63)	53 (31·2–77·3)	51·2 (29·4–75)	32·3 (11·2–72·3)	-3·44 (-14·8–8·09)
Diet high in sugar-sweetened beverages	28·5 (10·8–50·9)	34·2 (12·8–58·4)	37·2 (14·3–60·4)	19·9 (-16·2–62·5)	8·84 (-10·5–47·2)	--	--	--	--	--
Diet low in fiber	23·4 (13·3–35)	27·8 (15·8–40·7)	26 (14·4–39)	18·6 (10·8–29·1)	-6·24 (-19·1–7·45)	23 (12·7–34·9)	27·7 (15·5–41·2)	26·7 (14·7–40·2)	20·6 (13–31·8)	-3·62 (-16·9–9·91)
Diet low in seafood omega-3 fatty acids	93·5 (85·1–100)	88·3 (73·7–100)	88·3 (74·5–100)	-5·59 (-17·7–0·617)	0·065 (-7·93–9·26)	95·1 (88·2–100)	90·3 (79·5–100)	90·3 (79·3–100)	-5·02 (-14·4–0)	-0·00765 (-7·17–7·79)
Diet low in polyunsaturated fatty acids	45·2 (12·1–89)	50·3 (13·9–92·9)	48·3 (13·1–91·9)	11·3 (1·45–28·9)	-3·97 (-11·5–0)	47·9 (12·9–90·8)	52·7 (14·9–94·2)	50·8 (14·3–93·3)	10·1 (1·36–26·8)	-3·7 (-11·4–0)
Diet high in trans fatty acids	51·9 (41·7–100)	41·8 (31·6–99·2)	40·9 (30·7–98·6)	-19·4 (-29·5–0·458)	-2·23 (-11·4–7·14)	53·4 (42·6–100)	43·5 (32·3–100)	42·6 (31·7–100)	-18·7 (-29·2–0)	-1·93 (-9·71–6·39)
Diet high in sodium	33·5 (8·37–57·5)	42·7 (13·5–65·5)	46·9 (16·3–70·8)	27·6 (-9·31–133)	9·87 (-11·7–43·7)	17·9 (4·13–42)	20·3 (4·54–43·5)	21·8 (4·49–45·8)	13·7 (-13·7–80·1)	7·28 (-11–33·6)
Low physical activity	5·69 (2·54–11·6)	6·34 (3·11–12·3)	6·84 (3·26–13·6)	11·4 (-14–55·9)	7·94 (-18·6–38)	6·86 (3·55–12·4)	7·54 (4·04–13·4)	7·92 (4·27–13·7)	9·85 (-9·82–43)	5·07 (-15·6–28·2)
Occupational asthmagens	23·7 (20·2–28)	20 (17–23·7)	19·8 (16·6–23·8)	-15·5 (-21·6–-9·15)	-1·31 (-7·41–5·18)	--	--	--	--	--
Occupational particulate matter, gases, and fumes	11·3 (8·13–15·8)	9·48 (6·79–13·6)	8·46 (6·06–12·1)	-15·7 (-20·4–-11·6)	-10·8 (-13·5–-8·13)	6·47 (4·7–9·31)	5·6 (4·02–8·02)	5·03 (3·55–7·23)	-13·4 (-20·4–-7·21)	-10·3 (-14·1–-6·75)
Occupational noise	9·98 (9·48–10·7)	8·74 (8·3–9·4)	7·98 (7·56–8·63)	-12·4 (-13·8–-10·9)	-8·68 (-9·56–-7·77)	6·03 (5·73–6·52)	6·01 (5·71–6·5)	5·81 (5·51–6·31)	-0·41 (-2·11–1·39)	-3·22 (-4·18–-2·12)
Occupational injuries	--	--	--	--	--	--	--	--	--	--
Occupational ergonomic factors	17 (15·1–19·1)	14 (12·5–15·8)	13·1 (11·4–15·2)	-17·5 (-22·9–-11·4)	-6·29 (-14·1–2·75)	--	--	--	--	--
Childhood sexual abuse	7·16 (4·65–10·1)	7·16 (4·65–10·1)	7·16 (4·65–10·1)	0 (0–0)	0 (0–0)	10·6 (7·84–14·3)	10·6 (7·84–14·3)	10·6 (7·84–14·3)	0 (0–0)	0 (0–0)
Intimate partner violence	--	--	--	--	--	9·06 (4·86–13·2)	9·34 (5·17–13·3)	9·06 (4·86–13·2)	3·12 (-4·61–13·8)	-3·02 (-9·88–7·8)
Non-exclusive breastfeeding	29·6 (25·9–33·7)	29·3 (25·4–33)	29 (25·2–32·7)	-0·869 (-12–10·8)	-1·18 (-12·7–10·8)	29·6 (25·9–33·7)	29·3 (25·5–33·1)	29 (25·2–32·7)	-0·814 (-11·9–10·8)	-1·24 (-12·7–10·7)
Discontinued breastfeeding	25 (24·1–25·8)	24·8 (23·8–25·8)	24·8 (23·8–25·9)	-0·505 (-5·82–4·95)	-0·254 (-5·94–5·93)	25 (24·2–25·9)	24·9 (23·8–25·9)	24·8 (23·8–25·9)	-0·458 (-5·76–4·99)	-0·455 (-6·13–5·7)
Diet low in calcium	30·4 (17·1–53·5)	19·6 (9·41–36·9)	19·4 (9·26–36·7)	-35·7 (-53–-19·4)	-0·991 (-6·47–3·41)	23·1 (10·9–44·9)	13·9 (5·74–26·4)	13·8 (5·6–26·3)	-39·8 (-63·4–-19)	-0·258 (-6·15–4·54)
Occupational exposure to asbestos	1·92 (1·64–2·17)	3·12 (2·84–3·4)	3·35 (2·54–4·36)	62·3 (40·5–88)	7·4 (-19·2–40·6)	0·562 (0·346–1·14)	0·539 (0·395–0·766)	0·606 (0·342–0·862)	-4·05 (-45·2–52·7)	12·4 (-24·7–59·4)
Occupational exposure to arsenic	0·582 (0–1·37)	0·483 (0–1·13)	0·477 (0–1·11)	-17 (-23·1–NA)	-1·29 (-8·93–NA)	0·631 (0–1·48)	0·532 (0–1·24)	0·516 (0–1·24)	-15·7 (-23·9–NA)	-2·93 (-11·1–NA)
Occupational exposure to benzene	0·74 (0·0439–2·12)	0·729 (0·0421–2·08)	0·73 (0·0428–2·06)	-1·5 (-6·15–5·5)	0·0828 (-4·6–7·44)	0·926 (0·0567–2·59)	1·22 (0·0698–3·46)	1·29 (0·0718–3·67)	31·9 (25·2–42·7)	5·57 (0·332–12·5)
Occupational exposure to beryllium	0·026 (0·0257–0·0263)	0·0234 (0·0231–0·0237)	0·0231 (0·0224–0·0238)	-10·1 (-11·7–-8·52)	-1·1 (-4–2·2)	0·025 (0·0246–0·0254)	0·0266 (0·0261–0·027)	0·0275 (0·0267–0·0284)	6·15 (3·63–8·75)	3·74 (0·32–6·97)
Occupational exposure to cadmium	0·0668 (0·065–0·0688)	0·0562 (0·0526–0·0601)	0·0556 (0·051–0·0599)	-15·9 (-21·5–-9·84)	-1·15 (-7·94–5·21)	0·0714 (0·0686–0·0743)	0·0623 (0·0576–0·0677)	0·0613 (0·0558–0·0675)	-12·7 (-20–-4·62)	-1·74 (-9·27–6·68)
Occupational exposure to chromium	0·152 (0·147–0·156)	0·132 (0·125–0·14)	0·131 (0·122–0·14)	-12·9 (-18·3–-7·24)	-0·486 (-6·54–5·37)	0·163 (0·158–0·169)	0·154 (0·144–0·164)	0·153 (0·142–0·166)	-6·01 (-12·6–1·07)	-0·192 (-6·53–6·58)
Occupational exposure to diesel engine exhaust	0·726 (0·694–0·758)	0·747 (0·71–0·784)	0·75 (0·707–0·794)	2·91 (-3·65–9·1)	0·375 (-4·71–6·03)	0·449 (0·429–0·47)	0·586 (0·558–0·617)	0·635 (0·589–0·682)	30·5 (22–40·2)	8·3 (0·844–15·6)
Occupational exposure to formaldehyde	0·321 (0·307–0·332)	0·266 (0·248–0·285)	0·261 (0·238–0·283)	-17·1 (-23·4–-10·5)	-1·92 (-9·17–4·92)	0·337 (0·323–0·351)	0·305 (0·284–0·329)	0·299 (0·274–0·328)	-9·59 (-16·8–-1·97)	-1·91 (-8·77–5·78)
Occupational exposure to nickel	0·567 (0–2·51)	0·491 (0–2·17)	0·483 (0–2·16)	-13·5 (-19·1–NA)	-1·71 (-7·62–NA)	0·524 (0–2·34)	0·423 (0–1·85)	0·406 (0–1·84)	-19·4 (-27·2–NA)	-3·93 (-12·7–NA)
Occupational exposure to polycyclic aromatic hydrocarbons	0·306 (0·298–0·314)	0·273 (0·26–0·286)	0·271 (0·255–0·288)	-11 (-15·7–-6·06)	-0·513 (-5·82–4·71)	--	--	--	--	--
Occupational exposure to silica	6·53 (0·939–20·8)	6·38 (0·923–20·3)	6·32 (0·895–20·3)	-2·27 (-11·5–7·81)	-0·917 (-8·17–8·2)	4·33 (0·611–14·1)	3·54 (0·476–11)	3·42 (0·492–11·1)	-18·4 (-27·1–-8·5)	-3·31 (-12·1–7·96)
Occupational exposure to sulfuric acid	0·984 (0·207–3·07)	0·786 (0·162–2·44)	0·758 (0·16–2·38)	-20·1 (-25·4–-14·4)	-3·65 (-9·76–2·77)	0·901 (0·187–2·85)	0·755 (0·16–2·33)	0·735 (0·153–2·3)	-16·2 (-24–-7·98)	-2·64 (-10·5–6·55)
Unsafe sex	--	--	--	--	--	--	--	--	--	--
Occupational exposure to trichloroethylene	0·0832 (0·0814–0·0857)	0·0745 (0·0716–0·0778)	0·0744 (0·0708–0·0786)	-10·4 (-14·5–-6)	-0·179 (-4·45–4·63)	0·0829 (0·0806–0·0857)	0·0845 (0·081–0·0889)	0·0867 (0·0818–0·0921)	2·02 (-3·27–7·94)	2·53 (-2·67–8·54)
No access to handwashing facility	4·86 (4·6–5·11)	3·45 (3·28–3·64)	3·37 (3·19–3·57)	-28·9 (-33·4–-24·5)	-2·51 (-6·55–1·35)	4·86 (4·6–5·11)	3·45 (3·28–3·64)	3·37 (3·19–3·57)	-28·9 (-33·4–-24·5)	-2·51 (-6·55–1·35)
Child wasting	1·3 (0·915–1·72)	1·24 (0·884–1·62)	1·23 (0·876–1·61)	-4·92 (-12·9–3·52)	-0·832 (-7·04–5·39)	1·1 (0·774–1·45)	0·926 (0·537–1·24)	1·02 (0·711–1·36)	-15·7 (-50·6–23·7)	10·2 (-25·5–83·1)
Child stunting	1·84 (0·958–2·73)	1·24 (0·584–1·89)	1·22 (0·564–1·86)	-32·6 (-45–-19·5)	-1·81 (-14–11·4)	2·24 (1·19–3·21)	1·66 (0·837–2·44)	1·62 (0·819–2·42)	-25·5 (-39–-11·4)	-2·67 (-13·1–9·36)
Chewing tobacco	0·144 (0·114–0·185)	0·146 (0·115–0·182)	0·148 (0·117–0·184)	1·06 (-24·2–32·7)	1·14 (-14·9–19·8)	0·136 (0·0997–0·189)	0·136 (0·0991–0·181)	0·137 (0·0976–0·179)	0·0809 (-30·8–45·4)	0·68 (-19·7–25·7)
Diet low in legumes	71·2 (31·6–99)	72·5 (34·3–99·4)	73·1 (35·7–99·4)	1·75 (0·0309–9·37)	0·841 (-1·9–6·72)	68 (24·5–97·7)	69·3 (26·9–98·3)	69·9 (28·4–98·2)	1·96 (0·0423–10·9)	0·817 (-2·38–7·92)
Short gestation	13·5 (11·1–16·2)	10 (8·28–11·9)	10·4 (8·64–12·6)	-25·9 (-40·7–-8·15)	3·62 (-16·2–29·6)	15·1 (12·8–17·9)	9·76 (8·29–11·5)	9·5 (7·94–11·2)	-35·5 (-47–-20·5)	-2·72 (-20·9–18·3)
Low birth weight	6·45 (5·42–7·77)	5·85 (4·92–6·92)	7·49 (6·32–8·95)	-9·43 (-28·1–13·6)	28·1 (3·66–57·6)	7·54 (6·31–8·85)	5·99 (5·18–7·02)	6·83 (5·77–7·91)	-20·5 (-36·4–-1·07)	13·9 (-6·92–38)
High temperature	27·7 (20·4–36·5)	26·4 (18·9–35·2)	29·3 (21·4–37·8)	-4·75 (-11–0·802)	10·9 (4·15–16·7)	27·7 (20·4–36·5)	26·4 (18·9–35·2)	29·3 (21·4–37·8)	-4·75 (-11–0·802)	10·9 (4·15–16·7)
Low temperature	30·5 (26·2–36·5)	31·4 (27–37·4)	30·4 (26–36·4)	2·93 (2·09–3·85)	-3·21 (-4·11–-2·4)	30·5 (26·2–36·5)	31·4 (27–37·4)	30·4 (26–36·4)	2·93 (2·09–3·85)	-3·21 (-4·11–-2·4)
Kidney dysfunction	14·8 (9·87–21·4)	16·3 (11·1–23)	15·2 (10·3–21·8)	9·98 (4·96–17·5)	-6·99 (-12·5–-2·82)	15·7 (10·5–22·2)	18 (12·5–24·8)	16·5 (11·4–23·2)	14·9 (8·36–24·8)	-8·59 (-14·4–-3·96)

Bullying victimization	7·17 (2·96–14·7)	8·32 (3·67–16·1)	7·77 (3·28–15·4)	15·9 (-3·75–60·7)	-6·64 (-13·6–3·67)	4·88 (1·91–9·9)	5·8 (2·52–11·3)	5·52 (2·39–11·1)	18·9 (-5·23–138)	-4·89 (-12·1–0·0573)
High LDL cholesterol	50·6 (47–54·1)	50·1 (46·5–54)	50·8 (46·9–54·8)	-0·929 (-6·9–5·7)	1·51 (-3·08–6·17)	48·1 (44·6–51·4)	48·5 (45–51·9)	49·1 (45·7–52·9)	0·814 (-4·31–6·33)	1·16 (-2·42–5·05)

Supplementary Results Table 1. San Marino age-standardised Summary Exposure Values (SEVs) for most-detailed risk factors and percentage change, by sex, 1990, 2010, and 2019. Data in parentheses are 95% uncertainty intervals.										
	Male					Female				
	1990	2010	2019	Percentage change 1990-2010	Percentage change 2010-2019	1990	2010	2019	Percentage change 1990-2010	Percentage change 2010-2019
Unsafe sanitation	1·76 (1·02–2·94)	0·713 (0·393–1·16)	0·641 (0·362–1·06)	-59·5 (-79·6–-19·9)	-10·1 (-43·3–48·8)	1·76 (1·02–2·94)	0·713 (0·393–1·16)	0·641 (0·362–1·06)	-59·5 (-79·6–-19·9)	-10·1 (-43·3–48·8)
Ambient particulate matter pollution	11 (2·22–24·6)	6·66 (2·56–11·4)	7·81 (3·2–13·1)	-39·3 (-75·7–100)	17·3 (-20·3–88·1)	11 (2·26–24·6)	6·66 (2·58–11·4)	7·81 (3·2–13·1)	-39·3 (-75·3–99·2)	17·3 (-20·2–87·1)
Household air pollution from solid fuels	0·0595 (0·0119–0·177)	0·0161 (0·0026–0·0543)	0·0124 (0·00204–0·0413)	-72·9 (-88·7–-39·6)	-23·3 (-52–32·2)	0·0784 (0·0169–0·232)	0·0214 (0·00367–0·07)	0·0165 (0·0029–0·0509)	-72·7 (-88·6–-39·7)	-23·2 (-52·1–32·1)
Ambient ozone pollution	88·3 (38·8–100)	70·8 (27·3–100)	77·6 (34·4–100)	-19·9 (-55·2–22·9)	9·61 (-27·6–79·8)	88·3 (38·8–100)	70·8 (27·3–100)	77·6 (34·4–100)	-19·9 (-55·2–22·9)	9·61 (-27·6–79·8)
Residential radon	21·9 (3·38–64·4)	21·9 (3·38–64·4)	21·9 (3·38–64·4)	0 (0–0)	0 (0–0)	21·9 (3·38–64·4)	21·9 (3·38–64·4)	21·9 (3·38–64·4)	0 (0–0)	0 (0–0)
Lead exposure	44·9 (15–68·9)	32·9 (12·1–55·2)	23·3 (6·45–44·3)	-26·7 (-40·7–-14·4)	-29·2 (-48·1–-18·8)	24·3 (0·18–52·2)	18·6 (0·727–42·6)	13·1 (0·261–34·3)	-23·5 (-38–320)	-29·8 (-65·2–-18·8)
Child underweight	0·839 (0·475–1·26)	0·764 (0·41–1·13)	0·779 (0·423–1·15)	-8·86 (-19·9–4·63)	1·95 (-7·31–12·1)	0·98 (0·574–1·47)	0·905 (0·525–1·35)	0·923 (0·537–1·37)	-7·66 (-18·5–4·32)	1·98 (-6·84–11·5)
Iron deficiency	--	--	--	--	--	9·6 (7·89–11·4)	8·93 (7·41–10·5)	8·85 (7·28–10·5)	-6·96 (-15·5–3·14)	-0·898 (-6·53–4·82)
Vitamin A deficiency	2·71 (1·06–5·78)	1·23 (0·496–2·6)	1·21 (0·491–2·46)	-54·4 (-82·2–21)	-1·9 (-46–84·6)	2·29 (1·17–3·98)	1·52 (0·747–2·63)	1·47 (0·754–2·47)	-33·9 (-65·7–29·7)	-3·05 (-35·9–51·1)
Zinc deficiency	1·98 (0–6·44)	1·44 (0–4·84)	1·9 (0–6·27)	-27·5 (-100–NA)	32·2 (14·9–NA)	1·93 (0–6·1)	1·41 (0–4·7)	1·86 (0–5·99)	-27·2 (-100–NA)	32 (13·7–NA)
Smoking	24·8 (20·7–29·4)	19·6 (16·4–23·1)	17 (14·1–20)	-21·1 (-30·3–-10·6)	-13·3 (-19·1–-6·87)	16·3 (12·8–20·6)	13·1 (10·4–16·5)	11·5 (9·19–14·3)	-19·5 (-33·3–-2·83)	-12·8 (-22·1–-2·28)
Secondhand smoke	39·6 (36·7–42·4)	36·5 (33·5–39·3)	35·5 (32·5–38·5)	-7·77 (-15·8–0·976)	-2·85 (-8·55–3·31)	41·1 (38·8–43·5)	37·1 (34·5–39·3)	36·1 (33·7–38·3)	-9·92 (-16–-3·34)	-2·59 (-7·19–2·05)
Alcohol use	17·3 (1·77–25·7)	18·9 (2·39–28·1)	19·2 (1·7–29)	9·02 (-84·8–834)	1·86 (-88·3–458)	9·02 (0·546–15·4)	9·68 (0·877–16)	9·92 (0·567–16·4)	7·31 (-88·8–1390)	2·49 (-92·5–637)
Drug use	0·253 (0·155–0·432)	0·288 (0·181–0·483)	0·298 (0·192–0·499)	13·7 (1·41–29·5)	3·51 (-6–14·6)	0·134 (0·0787–0·23)	0·171 (0·108–0·278)	0·184 (0·123–0·286)	27·2 (13·8–47·9)	7·75 (-0·248–18·2)
High fasting plasma glucose	6·28 (5·4–7·3)	9·55 (8·03–11·6)	11·8 (9·95–13·9)	52·1 (34·3–76·8)	23·5 (8·02–44)	5·7 (4·77–6·74)	8·24 (6·91–9·85)	10·2 (8·42–12·3)	44·6 (29–66·7)	24·1 (9·48–43·8)
High systolic blood pressure	38·2 (30·5–46·6)	38·1 (30·7–46·2)	38·1 (30·6–46·2)	-0·366 (-23·7–29·9)	-0·0831 (-16·3–21·9)	32 (23·3–42·5)	29 (20·8–38·4)	28·9 (20·4–39·6)	-9·43 (-39–32·1)	-0·122 (-25·5–35·5)
High body-mass index	25·3 (18·6–33·8)	31·6 (23·8–41·2)	31 (23·3–40·7)	25 (13·4–40·3)	-1·82 (-7·36–3·93)	26·2 (20·2–35)	32·9 (25·9–42·6)	32·4 (26–41·7)	25·3 (11·8–43·8)	-1·39 (-8·67–7·77)
Low bone mineral density	9·26 (4·96–15·2)	8·62 (4·39–14·7)	8·5 (4·55–14·5)	-6·97 (-30·3–23·8)	-1·32 (-27·1–33·4)	14·9 (9·41–21·7)	13·9 (8·53–20·2)	13·8 (8·77–19·9)	-6·25 (-27·1–19·7)	-1·04 (-21·2–27·1)
Diet low in fruits	39·1 (26·3–52·8)	33 (21–47)	37·3 (25·7–51·7)	-15·7 (-36·5–6·13)	13·1 (-1·1–35·9)	40·5 (28·2–54)	34·7 (22·8–48·2)	38·9 (26·4–52)	-14·4 (-33·4–5·74)	12·3 (-0·682–32·5)
Diet low in vegetables	31·5 (16·1–52·1)	24·2 (10·7–44·8)	30·9 (14·7–52·1)	-23·1 (-47·5–-1·65)	27·6 (7·06–67·6)	28·9 (13·6–48·6)	21·7 (9·22–41·9)	28·3 (14–48·5)	-24·8 (-49–0·791)	30·2 (8·73–71·8)
Diet low in whole grains	90·6 (85·1–95·6)	90·2 (84·7–95·4)	90·8 (85·2–95·7)	-0·421 (-2·58–1·57)	0·676 (-0·506–2·19)	--	--	--	--	--
Diet low in nuts and seeds	37·5 (12·3–62·9)	29·4 (8·1–57·8)	36·6 (11·6–62·4)	-21·5 (-50–0·649)	24·2 (4·28–72·8)	33 (9·58–58·8)	25·1 (6·36–54·5)	31·8 (9·19–59·3)	-23·8 (-54·3–1·41)	26·5 (3·7–79·4)
Diet low in milk	49 (24·8–69·5)	43·3 (19·3–64·1)	49·1 (25·6–70·4)	-11·6 (-29·9–0·54)	13·3 (3·12–35·2)	48·1 (24–68·9)	42·7 (19·9–62·6)	48·5 (25·1–68·5)	-11·4 (-28·9–0·693)	13·6 (3·23–35·7)
Diet high in red meat	82·4 (70–91·9)	84·8 (73·3–93·8)	81·4 (69·3–91·3)	2·99 (-9·56–17·9)	-4·05 (-12–2·87)	82·1 (69·9–91·5)	84·6 (73·2–93·1)	81·2 (67–91·5)	3·05 (-10–17·1)	-4·07 (-12·4–3·54)
Diet high in processed meat	74·6 (50·3–95·7)	75·9 (51·4–96·8)	75 (50·4–95·9)	1·75 (-5·08–11·2)	-1·21 (-5·87–2·88)	78·4 (54·5–98·6)	79·5 (56·2–99·1)	78·5 (55·1–98·5)	1·37 (-3·95–8·39)	-1·25 (-5·81–2·19)
Diet high in sugar-sweetened beverages	51·4 (32·2–71·2)	59 (39·4–76·1)	52·2 (33·9–69·5)	14·7 (-17–66·2)	-11·4 (-29·2–6·78)	57·1 (37·9–74·8)	64·9 (45·3–80·9)	57·7 (38·6–75·1)	13·5 (-15·5–63)	-11 (-28·2–6·64)
Diet low in fiber	26·5 (13·9–40·9)	21·3 (10·7–34·9)	26·5 (14·3–41)	-19·4 (-42·8–9·02)	24·2 (5·13–55·7)	26·1 (13·9–40·1)	21·2 (10·5–34)	26·5 (14·1–40·6)	-18·7 (-42·8–9·6)	24·9 (3·69–56·2)
Diet low in seafood omega-3 fatty acids	83·4 (67·1–99·9)	78·3 (59·7–99·3)	81·5 (65·2–99·4)	-6·16 (-18·1–4·41)	4·18 (-6·41–20·3)	85·3 (68·6–99·2)	80·5 (62·3–99·7)	84·1 (69·3–99·3)	-5·58 (-16·3–3·54)	4·48 (-5·63–21·2)
Diet low in polyunsaturated fatty acids	39·7 (10·2–83·6)	32·5 (8·07–77·1)	38·2 (10·1–82·6)	-18·1 (-37·7–-2·39)	17·5 (2·78–44·3)	42·5 (11·2–86·5)	35·4 (8·66–78·8)	41·1 (10·4–84·8)	-16·6 (-36·9–-2·12)	16 (2·36–42·2)
Diet high in trans fatty acids	49·8 (39·3–100)	43·3 (32·1–100)	42·6 (31·8–100)	-13·1 (-23·3–0)	-1·63 (-10·5–7·22)	50·2 (38·8–100)	44 (32·8–100)	43·3 (32–100)	-12·3 (-22·2–0)	-1·57 (-10·2–6·96)
Diet high in sodium	29·6 (4·9–56·4)	32·6 (6·01–59·1)	34·4 (6·47–61)	10 (-26·7–97·6)	5·48 (-17·5–50·8)	14·4 (3·02–37·6)	15·2 (3·23–37·9)	16 (3·5–39·5)	5·33 (-19·5–53·4)	5·17 (-9·05–38·5)
Low physical activity	4·41 (1·87–8·64)	5·11 (2·28–10·3)	5·02 (2·28–10·2)	15·8 (0·314–39·7)	-1·66 (-14·8–11·9)	7·25 (3·82–13·2)	8·01 (4·35–14·4)	7·92 (4·28–13·9)	10·5 (0·973–25)	-1·11 (-9·87–8·26)
Occupational asthmagens	25 (20·1–31)	23·7 (18·9–30·1)	25·4 (20–32·4)	-5·11 (-23–16·4)	6·85 (-7·78–23)	--	--	--	--	--
Occupational particulate matter, gases, and fumes	8·82 (6·32–12·6)	8·2 (5·85–11·9)	7·8 (5·52–11·3)	-6·99 (-13·1–-1·73)	-4·87 (-7·65–-2·22)	3·82 (2·65–5·8)	3·42 (2·39–5·03)	3·23 (2·25–4·73)	-10·7 (-19·2–-0·666)	-5·5 (-9·74–-1·25)
Occupational noise	8·22 (7·57–8·95)	7·99 (7·53–8·62)	7·79 (7·38–8·42)	-2·8 (-6·49–1·23)	-2·45 (-3·83–-0·975)	4·63 (4·07–5·23)	4·7 (4·33–5·14)	4·77 (4·44–5·21)	1·45 (-4·81–8·74)	1·48 (-0·855–4·06)
Occupational injuries	--	--	--	--	--	--	--	--	--	--
Occupational ergonomic factors	13·8 (11·6–16·5)	12·8 (10·9–15·2)	13·1 (11–15·6)	-6·93 (-19·1–6·1)	1·83 (-7·07–11·3)	--	--	--	--	--
Childhood sexual abuse	7·16 (4·65–10·1)	7·16 (4·65–10·1)	7·16 (4·65–10·1)	0 (0–0)	0 (0–0)	10·6 (7·84–14·3)	10·6 (7·84–14·3)	10·6 (7·84–14·3)	0 (0–0)	0 (0–0)
Intimate partner violence	--	--	--	--	--	15 (8·02–22·3)	15 (8·02–22·3)	15 (8·02–22·3)	0 (0–0)	0 (0–0)
Non-exclusive breastfeeding	21·1 (17·1–26·1)	20·1 (15·9–24·7)	19·9 (15·7–24·8)	-4·82 (-16·5–8·1)	-1·06 (-10·1–8·93)	21·1 (17·1–26·1)	20·1 (15·9–24·7)	19·9 (15·7–24·8)	-4·79 (-16·4–8·14)	-1·06 (-10·1–8·92)
Discontinued breastfeeding	22·5 (21·2–23·7)	21·7 (20·4–23·1)	21·6 (20·3–22·9)	-3·73 (-10·6–3·62)	-0·521 (-6·03–4·8)	22·5 (21·2–23·6)	21·7 (20·4–23)	21·6 (20·2–22·9)	-3·52 (-10·4–3·85)	-0·481 (-5·99–4·85)
Diet low in calcium	9·44 (3·64–17·2)	5·72 (2·24–10·5)	9·49 (3·99–17·5)	-39·4 (-53·5–-26·2)	65·9 (39·8–112)	3·96 (1·5–7·43)	1·47 (0·702–2·88)	3·99 (1·6–7·53)	-62·9 (-75·3–-41·4)	172 (87·9–279)
Occupational exposure to asbestos	8·38 (6·25–13·2)	9·61 (6·19–15)	9·65 (6·42–14·6)	14·7 (-24·6–67·6)	0·46 (-21·1–28·9)	3·07 (1·04–5·17)	2·44 (0·91–4·37)	2·4 (0·89–4·35)	-20·5 (-55·3–39·7)	-1·92 (-41·7–70·9)
Occupational exposure to arsenic	0·526 (0–1·26)	0·484 (0–1·13)	0·481 (0–1·12)	-8 (-18·6–NA)	-0·561 (-8·31–NA)	0·411 (0–0·965)	0·39 (0–0·911)	0·411 (0–0·957)	-4·99 (-18·3–NA)	5·27 (-3·98–NA)
Occupational exposure to benzene	0·801 (0·0448–2·28)	0·858 (0·0496–2·41)	0·866 (0·0508–2·49)	7·06 (-0·0111–17)	0·937 (-3·72–7·21)	1 (0·0593–2·76)	1·24 (0·0732–3·51)	1·36 (0·0826–3·92)	24·2 (12·9–42·3)	9·66 (2·79–18·7)
Occupational exposure to beryllium	0·0239 (0·0231–0·0247)	0·024 (0·0234–0·0246)	0·0244 (0·0239–0·0249)	0·429 (-3·8–4·76)	1·48 (-1·34–4·46)	0·0229 (0·0214–0·0243)	0·0252 (0·0241–0·0263)	0·0273 (0·0262–0·0283)	10·3 (1·77–20·2)	8·14 (3·08–13·7)
Occupational exposure to cadmium	0·0608 (0·0555–0·0666)	0·0566 (0·0517–0·0616)	0·0565 (0·0516–0·0618)	-6·97 (-17·3–4·13)	-0·0568 (-7·26–7·2)	0·0493 (0·0446–0·0548)	0·0481 (0·0439–0·0527)	0·0508 (0·0466–0·0555)	-2·42 (-15·1–11·8)	5·72 (-2·47–14·5)
Occupational exposure to chromium	0·142 (0·131–0·154)	0·134 (0·124–0·145)	0·136 (0·126–0·146)	-5·4 (-14·8–4·58)	0·827 (-5·25–7·29)	0·123 (0·112–0·135)	0·125 (0·116–0·135)	0·134 (0·125–0·143)	2 (-9·29–14·6)	6·63 (-0·352–14·3)
Occupational exposure to diesel engine exhaust	0·7 (0·66–0·742)	0·717 (0·678–0·76)	0·75 (0·709–0·793)	2·51 (-4·39–10·6)	4·57 (-0·462–9·98)	0·575 (0·523–0·63)	0·628 (0·577–0·682)	0·68 (0·628–0·743)	9·26 (-2·76–22·8)	8·32 (0·382–17·4)
Occupational exposure to formaldehyde	0·301 (0·272–0·332)	0·274 (0·249–0·299)	0·27 (0·246–0·296)	-9·07 (-19·7–2·65)	-1·31 (-8·43–6·22)	0·237 (0·214–0·264)	0·239 (0·22–0·259)	0·253 (0·234–0·275)	0·745 (-12–14·6)	5·96 (-1·56–14·6)
Occupational exposure to nickel	0·498 (0–2·23)	0·471 (0–2·1)	0·467 (0–2·16)	-5·33 (-15·1–NA)	-0·95 (-7·76–NA)	0·325 (0–1·42)	0·299 (0–1·33)	0·313 (0–1·39)	-7·99 (-22·3–NA)	4·54 (-5·7–NA)
Occupational exposure to polycyclic aromatic hydrocarbons	0·289 (0·268–0·311)	0·28 (0·262–0·298)	0·282 (0·264–0·301)	-3·1 (-11·4–5·93)	0·561 (-4·8–6·37)	--	--	--	--	--
Occupational exposure to silica	5·75 (0·807–18)	5·87 (0·86–18·9)	5·82 (0·812–19·3)	2·03 (-8·01–14·4)	-0·768 (-8·19–7·58)	2·86 (0·374–8·87)	2·6 (0·326–8·44)	2·7 (0·358–8·92)	-9·11 (-23·4–8·65)	4·08 (-5·89–16·3)
Occupational exposure to sulfuric acid	0·846 (0·17–2·69)	0·764 (0·153–2·47)	0·761 (0·155–2·45)	-9·72 (-19·4–0·517)	-0·306 (-7·19–6·58)	0·598 (0·122–1·97)	0·559 (0·115–1·75)	0·586 (0·121–1·93)	-6·54 (-19·5–8·26)	4·78 (-4·22–14·7)
Unsafe sex	--	--	--	--	--	--	--	--	--	--
Occupational exposure to trichloroethylene	0·0793 (0·0752–0·0844)	0·0758 (0·0719–0·0802)	0·0776 (0·0739–0·0818)	-4·35 (-11·6–2·57)	2·31 (-2·39–7·6)	0·0727 (0·0672–0·0787)	0·0758 (0·0711–0·081)	0·0812 (0·0765–0·0864)	4·24 (-6·07–16·1)	7·2 (0·806–14·2)
No access to handwashing facility	3·24 (3·05–3·43)	3·14 (2·96–3·31)	3·11 (2·95–3·29)	-3·2 (-9·45–3·34)	-0·685 (-4·92–3·67)	3·24 (3·05–3·43)	3·14 (2·96–3·31)	3·11 (2·95–3·29)	-3·2 (-9·45–3·34)	-0·685 (-4·92–3·67)
Child wasting	1·22 (0·847–1·61)	1·19 (0·826–1·58)	1·21 (0·843–1·58)	-1·91 (-9·6–6·7)	1·16 (-4·17–7·83)	--	--	--	--	--
Child stunting	1·51 (0·739–2·25)	1·19 (0·575–1·84)	1·21 (0·588–1·86)	-21·4 (-34·9–-5·75)	1·52 (-10·8–14)	1·77 (0·898–2·58)	1·55 (0·765–2·3)	1·57 (0·773–2·35)	-12·2 (-25·9–3·69)	1·38 (-10·2–14·1)
Chewing tobacco	0·206 (0·163–0·259)	0·216 (0·172–0·276)	0·236 (0·188–0·3)	4·88 (-20·3–38·8)	9·04 (-7·33–30)	0·15 (0·11–0·203)	0·154 (0·112–0·21)	0·161 (0·115–0·22)	2·48 (-30·8–47·8)	4·44 (-18·5–32·1)
Diet low in legumes	80·2 (53·2–99·9)	78 (48·8–99·8)	79·7 (52–99·9)	-2·7 (-11·9–0·743)	2·19 (-0·426–10·5)	77·2 (46·4–99·7)	75 (41·2–99·4)	76·8 (45·5–99·7)	-2·83 (-14·9–0·908)	2·41 (-0·148–13·6)
Short gestation	7·53 (5·57–10·3)	8·32 (6·26–11·2)	8·39 (6·34–11·1)	10·5 (-21·2–59·7)	0·744 (-24–35·5)	8·36 (6·28–11·2)	9·32 (7·09–12·6)	9·24 (6·91–12·3)	11·4 (-26·3–66·1)	-0·819 (-28–36·9)
Low birth weight	4·32 (3·35–5·71)	4·86 (3·83–6·28)	4·79 (3·78–6·21)	12·7 (-18–59·8)	-1·52 (-23·1–28·7)	4·86 (3·7–6·32)	5·07 (4·01–6·64)	5·12 (3·95–6·74)	4·34 (-28·1–49·1)	0·856 (-26·6–35·8)
High temperature	20·6 (7·3–34·9)	22·1 (8·32–36·8)	19 (6·56–32·9)	7·05 (3·28–17·6)	-13·8 (-28–-4·16)	20·6 (7·3–34·9)	22·1 (8·32–36·8)	19 (6·56–32·9)	7·05 (3·28–17·6)	-13·8 (-28–-4·16)
Low temperature	32·7 (26·4–39·8)	34·4 (28·3–41·4)	32·6 (26·1–40)	5·33 (3·26–7·8)	-5·24 (-8·32–-2·28)	32·7 (26·4–39·8)	34·4 (28·3–41·4)	32·6 (26·1–40)	5·33 (3·26–7·8)	-5·24 (-8·32–-2·28)
Kidney dysfunction	13·8 (8·96–20·2)	14·1 (9·34–20·7)	14·1 (9·37–20·7)	2·4 (-1·22–6·55)	0·0858 (-3·83–4·13)	14·9 (9·82–21·4)	15·4 (10·4–22)	15·6 (10·5–22·1)	3·63 (-0·249–8·44)	1·07 (-2·7–5·1)

Bullying victimization	6·43<br>(2·43–13·3)	6·46<br>(2·44–13·4)	6·53<br>(2·46–13·5)	0·499<br>(-0·881–2·22)	0·941<br>(0·332–1·6)	5·06<br>(1·99–10·7)	5·06<br>(1·98–10·7)	5·1<br>(1·99–10·7)	-0·0371<br>(-1·09–1·38)	0·788<br>(0·263–1·24)
High LDL cholesterol	55·1<br>(51·5–58·8)	52<br>(48·3–55·4)	52<br>(48·4–55·6)	-5·56<br>(-11·8–0·697)	-0·169<br>(-5–4·54)	53·5<br>(50·1–56·9)	51·3<br>(48–54·6)	51·1<br>(47·7–54·5)	-4·07<br>(-9·51–1·54)	-0·284<br>(-4·03–3·78)

Supplementary Results Table 1. Spain age-standardised Summary Exposure Values (SEVs) for most-detailed risk factors and percentage change, by sex, 1990, 2010, and 2019. Data in parentheses are 95% uncertainty intervals.

	Male					Female				
	1990	2010	2019	Percentage change 1990-2010	Percentage change 2010-2019	1990	2010	2019	Percentage change 1990-2010	Percentage change 2010-2019
Unsafe sanitation	3·02 (1·79–4·73)	0·821 (0·471–1·32)	0·481 (0·269–0·784)	-72·8 (-86·1—47·1)	-41·4 (-64·1—8·49)	3·02 (1·79–4·73)	0·821 (0·471–1·32)	0·481 (0·269–0·784)	-72·8 (-86·1—47·1)	-41·4 (-64·1—8·49)
Ambient particulate matter pollution	12·5 (4·35–23·6)	9·81 (7·06–13·1)	7·6 (4·98–10·6)	-21·8 (-60·3–104)	-22·5 (-29·9—17·9)	12·4 (4·33–23·1)	9·79 (7·07–13)	7·59 (4·98–10·6)	-20·7 (-59–103)	-22·4 (-29·8—17·6)
Household air pollution from solid fuels	0·796 (0·267–1·89)	0·15 (0·0417–0·367)	0·0865 (0·0217–0·238)	-81·1 (-91·4—59·9)	-42·5 (-64·2—13·7)	1·03 (0·378–2·36)	0·198 (0·0587–0·477)	0·114 (0·0302–0·309)	-80·7 (-91·3—59·7)	-42·2 (-64—13·2)
Ambient ozone pollution	37 (14·8–63·8)	57·6 (24·4–97·2)	65 (28·8–100)	55·6 (39·9–79·5)	12·7 (2·85–21·1)	37 (14·8–63·8)	57·6 (24·4–97·2)	65 (28·8–100)	55·6 (39·9–79·5)	12·7 (2·85–21·1)
Residential radon	30·9 (14·2–55·2)	30·9 (14·2–55·2)	30·9 (14·2–55·2)	0 (0–0)	0 (0–0)	30·9 (14·2–55·2)	30·9 (14·2–55·2)	30·9 (14·2–55·2)	0 (0–0)	0 (0–0)
Lead exposure	70·8 (49·6–85·7)	53·9 (34·8–71·9)	41·1 (23·9–60)	-23·8 (-34·2—14·5)	-23·7 (-32·1—15·7)	49·3 (22·4–71·5)	37·8 (16·9–58·8)	27·6 (9·87–48·2)	-23·4 (-35·7—12·3)	-27·1 (-41·1—17·4)
Child underweight	0·892 (0·51–1·31)	0·628 (0·264–0·981)	0·615 (0·228–0·982)	-29·6 (-71·2—15·7)	-2·18 (-59·2–131)	1·08 (0·646–1·57)	0·919 (0·536–1·35)	0·905 (0·533–1·35)	-14·7 (-23·8—4·06)	-1·43 (-10·4–7·24)
Iron deficiency	--	--	--	--	--	10·9 (9·14–12·7)	9·41 (7·78–11·1)	9·25 (7·7–10·9)	-13·7 (-22·2—4·94)	-1·75 (-7·24–4·07)
Vitamin A deficiency	2·77 (1·08–5·78)	1·32 (0·526–2·68)	1·13 (0·467–2·44)	-52·2 (-81·4–29·2)	-14·6 (-51·3–55·5)	2·74 (1·45–4·72)	1·47 (0·745–2·6)	1·26 (0·653–2·22)	-46·4 (-71·7–15·1)	-14·4 (-41·8–36·4)
Zinc deficiency	4·22 (0–12·5)	3·69 (0–11·2)	3·57 (0–11)	-12·6 (-72·7–NA)	-3·36 (-16·7–NA)	4·1 (0–12·4)	3·58 (0–11)	3·46 (0–10·5)	-12·5 (-71·7–NA)	-3·35 (-16·5–NA)
Smoking	34·4 (31–38·4)	27·5 (24·6–30·7)	23·8 (21·1–27)	-20·2 (-25·3—14·9)	-13·5 (-16·8—10·2)	15·6 (13·2–18·4)	15·4 (13·1–18)	15·5 (13·2–18·2)	-1·29 (-11·6–11)	0·641 (-4·28–5·77)
Secondhand smoke	46·2 (42·7–49·7)	38·7 (35·9–41·4)	37·2 (34·2–40·1)	-16·3 (-23·8—8·16)	-3·79 (-9·21–2·02)	50·8 (48·1–53·8)	40·4 (37·9–42·8)	39 (36·6–41·3)	-20·4 (-26·2—14·4)	-3·6 (-7·62–0·583)
Alcohol use	20·8 (15·8–26·5)	22·3 (15·8–30·2)	21·9 (15–30·3)	6·98 (-6·34–25·4)	-1·8 (-13·6–8·23)	--	--	--	--	--
Drug use	0·335 (0·169–0·64)	0·332 (0·162–0·67)	0·365 (0·188–0·682)	-0·898 (-16·7–18·1)	9·96 (-9·11–34·9)	0·192 (0·0934–0·379)	0·196 (0·101–0·376)	0·253 (0·147–0·431)	2·42 (-13·8–27·4)	29 (3·76–69·5)
High fasting plasma glucose	9·97 (8·6–11·6)	14·6 (12·7–16·3)	15·1 (12·9–17·6)	46·1 (27·7–67·8)	3·53 (-9·39–18·9)	9·55 (7·89–11·4)	12·5 (10·9–14·2)	12·8 (10·5–15·5)	30·4 (11·1–54·3)	2·67 (-13·4–19)
High systolic blood pressure	26·5 (23·8–29·2)	35·4 (32·3–38·5)	36·6 (30·3–43·8)	33·9 (20·7–48·7)	3·27 (-11·8–20·7)	24·4 (21·7–27·2)	20·2 (17·8–22·7)	24 (18·1–31·5)	-17·3 (-28·6—5·13)	19 (-8·55–52·9)
High body-mass index	19·4 (13·9–26·2)	27·8 (21·1–36·4)	27·1 (20·3–35·4)	43·3 (27·7–68·2)	-2·74 (-8·76–2·97)	19·6 (14·1–27)	28 (21·8–37·2)	28·2 (21·8–36·9)	43·2 (26·9–66·8)	0·722 (-7·04–9·06)
Low bone mineral density	9·99 (6·08–16)	9·2 (4·96–15·4)	8·96 (4·69–15)	-7·86 (-33–18·5)	-2·65 (-26·1–26·4)	17·2 (12·4–23·5)	16 (10·9–22·6)	15·6 (10·2–22·5)	-6·54 (-23·9–12·3)	-2·92 (-23·4–23·2)
Diet low in fruits	24·7 (16·2–34·3)	23·5 (15·3–33)	24·9 (15·8–35·1)	-5·14 (-20·9–11·5)	6·34 (-12·5–28·3)	26·4 (17·5–36·5)	25·8 (17–36·2)	27·4 (17·9–38·5)	-2·48 (-17·3–14·4)	6·46 (-10·9–25·8)
Diet low in vegetables	8·32 (3·14–20·8)	24·5 (10·8–45·2)	24·5 (10·9–45·5)	194 (82·3–417)	0·161 (-16·8–19·9)	6·35 (2·52–16·6)	21·5 (8·94–42·1)	22 (9·24–42·9)	239 (95·1–497)	2·05 (-15·9–26·4)
Diet low in whole grains	89 (83·5–94·7)	83·4 (75·4–91·5)	83 (74·9–91·4)	-6·32 (-9·9—3·14)	-0·41 (-1·65–0·741)	--	--	--	--	--
Diet low in nuts and seeds	39·3 (14·1–63·2)	35·9 (11·8–60·8)	35 (11·8–60·5)	-8·79 (-26—0·858)	-2·49 (-16·9–12·1)	33·7 (11·3–59·3)	30·6 (8·57–57·5)	29·9 (8·5–56·9)	-9·21 (-27·5—0·896)	-2·4 (-19·2–15·3)
Diet low in milk	34·3 (12·6–54·8)	33·7 (13·1–52·7)	37·1 (15·9–56·8)	-1·76 (-16·8–17)	9·9 (-1·26–31·6)	33·1 (12·2–52·2)	32·5 (12·8–51·1)	36 (14·9–54·4)	-1·71 (-19·3–21·5)	10·8 (-0·00269–33·9)
Diet high in red meat	83·4 (76·6–88·7)	79·8 (72·4–86·2)	79·2 (69·3–87·5)	-4·3 (-7·83—1·67)	-0·72 (-8·55–6·42)	83·4 (76·5–89·1)	79·8 (72–86·4)	79·1 (69·4–87·5)	-4·33 (-8·18—1·34)	-0·858 (-9·19–6·69)
Diet high in processed meat	82 (60·4–99·6)	77 (51·4–98·8)	77·5 (52·3–98·6)	-6·08 (-15·4—0·592)	0·597 (-1·64–3·8)	84·8 (66·4–99·8)	79·4 (54·9–99·4)	80 (56·5–99·5)	-6·38 (-16·7—0·245)	0·863 (-1·02–4·77)
Diet high in sugar-sweetened beverages	38·9 (15–61·6)	49·8 (22·2–71·8)	54·1 (26·3–75·2)	27·8 (-3·02–94·6)	8·74 (-10·5–44)	--	--	--	--	--
Diet low in fiber	27·6 (16–39·9)	37·7 (22·8–53)	36·5 (21·6–51·4)	36·8 (22·5–59·8)	-3·22 (-14·1–7·55)	27·9 (15·7–40·9)	37·8 (23–52·4)	36·6 (21·8–51·9)	35·4 (23·1–55·3)	-3·15 (-14·1–7·52)
Diet low in seafood omega-3 fatty acids	89·3 (75–100)	86·9 (71·4–100)	87 (72·3–99·9)	-2·74 (-13·3–5·96)	0·163 (-8·42–10·9)	91·2 (79·5–100)	88·6 (75·9–100)	88·8 (76·3–100)	-2·81 (-10·9–3·46)	0·119 (-7·1–8·74)
Diet low in polyunsaturated fatty acids	51·3 (14·3–93·3)	43 (11·8–85)	42·2 (11·8–84·6)	-16·2 (-29·3—1·46)	-1·84 (-8·2–3·7)	53·7 (15·5–94·8)	45·7 (13·1–87·4)	44·9 (12·9–87)	-15 (-28·9—1·82)	-1·77 (-7·99–3·03)
Diet high in trans fatty acids	28 (19·7–86·4)	25·5 (17·2–89·8)	25·9 (17·5–86·2)	-8·92 (-23·9–5·06)	1·77 (-6·15–14·2)	29 (19·9–88·6)	26·9 (17·5–93·6)	27·3 (17·9–92·7)	-7·32 (-21·5–6·45)	1·4 (-6·02–12·2)
Diet high in sodium	22·1 (5·15–44·6)	33·4 (8·42–57·8)	39·5 (11·1–64·9)	51·5 (9·39–191)	18·3 (-2·22–74)	11·5 (3·33–29)	16·1 (4·15–37·3)	18·5 (4·33–41·2)	39·9 (-11–155)	15·2 (-6·5–55·7)
Low physical activity	5·14 (2·35–10·4)	5·4 (2·62–10·4)	5·92 (2·73–11·5)	5·03 (-23·7–52·6)	9·7 (-21·5–49·3)	5·18 (2·56–9·8)	5·28 (2·68–9·71)	5·85 (3·02–11·1)	2·06 (-21·1–37)	10·8 (-16·2–41·2)
Occupational asthmagens	20·6 (17·5–24·2)	17·6 (14·9–20·8)	16·7 (14·2–20)	-14·6 (-20·6—8·52)	-4·64 (-10·8–1·58)	--	--	--	--	--
Occupational particulate matter, gases, and fumes	9·49 (6·76–13·5)	8·08 (5·78–11·6)	7·08 (5·08–10·3)	-14·9 (-18·3—11·3)	-12·3 (-15·5—9·75)	2·97 (2·09–4·26)	2·92 (2·07–4·3)	2·82 (1·99–4·15)	-1·95 (-9·75–5·69)	-3·25 (-8·21–0·986)
Occupational noise	8·52 (8·06–9·16)	7·53 (7·12–8·16)	6·84 (6·47–7·44)	-11·6 (-13·2—9·91)	-9·18 (-10·1—8·24)	3·01 (2·82–3·27)	3·5 (3·33–3·73)	3·71 (3·53–3·93)	16·3 (12·3–20·3)	5·95 (4·51–7·43)
Occupational injuries	--	--	--	--	--	--	--	--	--	--
Occupational ergonomic factors	13·7 (12·1–15·5)	10·6 (9·16–12·3)	9·93 (8·48–11·6)	-22·7 (-27·4—17·7)	-5·91 (-12·3–1·07)	5·27 (4·55–6·13)	7·03 (5·95–8·43)	7·21 (5·98–8·64)	33·6 (22·4–44·6)	2·52 (-4·94–11·6)
Childhood sexual abuse	5·82 (4·45–7·21)	4·65 (3·45–6·07)	5·77 (3·74–8·19)	-20·1 (-28·1—9·84)	24·2 (4·69–50·7)	4·52 (3·66–5·72)	4·77 (3·76–6·11)	5·19 (3·85–7·08)	5·58 (-4·49–16·6)	8·91 (-5·47–29·5)
Intimate partner violence	--	--	--	--	--	6·75 (3·94–9·96)	6·13 (3·68–8·37)	6·75 (3·94–9·96)	-9·18 (-21·3–7·9)	10·1 (-3·67–41·3)
Non-exclusive breastfeeding	27·9 (24–32·2)	27·7 (23·8–31·9)	27·4 (23·7–31·7)	-0·665 (-12·7–13·8)	-0·893 (-10·6–9·95)	27·9 (24–32·2)	27·7 (23·8–31·9)	27·4 (23·7–31·7)	-0·661 (-12·7–13·8)	-0·919 (-10·6–9·92)
Discontinued breastfeeding	23 (21·9–24·2)	23·5 (22·3–24·7)	22·3 (21–23·6)	2·24 (-5·31–10·3)	-5·27 (-10·9–0·367)	23 (21·9–24·2)	23·5 (22·2–24·7)	22·3 (21–23·5)	2·22 (-5·34–10·2)	-5·06 (-10·6–0·602)
Diet low in calcium	25·3 (13·4–46·3)	27·7 (14·8–49·7)	26·1 (13·5–47·7)	9·63 (3·05–21·5)	-5·87 (-12·3—1·71)	19 (8·24–37·6)	20·8 (9·45–40·7)	19·7 (8·7–38·7)	9·56 (2·01–25·8)	-5·4 (-14·5—1·11)
Occupational exposure to asbestos	5·31 (4·69–5·95)	6·28 (5·85–6·66)	5·77 (4·45–7·4)	18·3 (4·54–36·3)	-8·22 (-29·5–18)	1·71 (1·24–2·9)	1·48 (1·12–2·05)	1·45 (0·978–2·1)	-13·2 (-40·7–16·3)	-2·26 (-26·5–27·9)
Occupational exposure to arsenic	0·488 (0–1·15)	0·398 (0–0·948)	0·386 (0–0·925)	-18·5 (-25·2–NA)	-3·05 (-10·3–NA)	0·269 (0–0·638)	0·337 (0–0·787)	0·337 (0–0·793)	25·3 (12·7–NA)	0·0883 (-7·88–NA)
Occupational exposure to benzene	0·606 (0·0353–1·7)	0·693 (0·0408–1·96)	0·673 (0·0392–1·93)	14·4 (9·85–21)	-2·85 (-7·72–3·14)	0·515 (0·0303–1·46)	1·01 (0·0616–2·84)	1·05 (0·0643–2·91)	95·5 (85·1–110)	4·71 (-0·247–12·7)
Occupational exposure to beryllium	0·0212 (0·0209–0·0215)	0·0203 (0·02–0·0206)	0·0197 (0·0191–0·0203)	-4·27 (-6·09—2·28)	-3·12 (-5·95–0·0375)	0·0131 (0·0128–0·0134)	0·0213 (0·021–0·0216)	0·022 (0·0213–0·0228)	62·8 (58·4–67·3)	3·42 (-0·19–6·9)
Occupational exposure to cadmium	0·0559 (0·0532–0·0587)	0·0468 (0·0442–0·0496)	0·0454 (0·0421–0·0491)	-16·3 (-22·6—9·35)	-2·99 (-9·5–4·26)	0·0315 (0·0295–0·0335)	0·0414 (0·0389–0·0441)	0·0417 (0·0387–0·0448)	31·4 (19·7–44·2)	0·745 (-6·28–7·51)
Occupational exposure to chromium	0·127 (0·122–0·133)	0·112 (0·107–0·118)	0·109 (0·103–0·117)	-11·8 (-17·7—5·37)	-2·38 (-8·09–3·85)	0·0757 (0·0716–0·0799)	0·108 (0·103–0·113)	0·11 (0·103–0·116)	42·4 (32·2–53·3)	1·75 (-4·08–7·19)
Occupational exposure to diesel engine exhaust	0·6 (0·574–0·624)	0·653 (0·625–0·68)	0·642 (0·608–0·678)	8·93 (2·92–15·5)	-1·72 (-6·92–3·69)	0·31 (0·294–0·328)	0·573 (0·541–0·609)	0·597 (0·554–0·646)	85 (71·3–99·8)	4·18 (-3·28–12·5)
Occupational exposure to formaldehyde	0·27 (0·254–0·285)	0·224 (0·21–0·239)	0·215 (0·199–0·234)	-16·9 (-23·7—9·14)	-3·89 (-10·7–3·72)	0·148 (0·139–0·158)	0·202 (0·191–0·215)	0·203 (0·189–0·218)	36·2 (25–48·9)	0·654 (-5·74–7·14)
Occupational exposure to nickel	0·471 (0–2·07)	0·392 (0–1·72)	0·38 (0–1·67)	-16·6 (-22·4–NA)	-3·27 (-9·43–NA)	0·217 (0–0·973)	0·261 (0–1·17)	0·259 (0–1·16)	20·5 (7·62–NA)	-0·681 (-9·34–NA)
Occupational exposure to polycyclic aromatic hydrocarbons	0·256 (0·246–0·267)	0·232 (0·223–0·243)	0·226 (0·213–0·24)	-9·47 (-14·7—3·9)	-2·69 (-7·7–2·78)	--	--	--	--	--
Occupational exposure to silica	5·32 (0·759–17·1)	4·99 (0·692–15·4)	4·84 (0·711–15)	-6·27 (-14·5–3·23)	-3 (-10·4–4·72)	1·87 (0·246–5·67)	2·36 (0·335–7·37)	2·34 (0·35–7·17)	26·2 (13·3–44·1)	-0·63 (-9·31–9·38)
Occupational exposure to sulfuric acid	0·848 (0·175–2·62)	0·659 (0·141–2·04)	0·615 (0·127–1·94)	-22·3 (-27·9—15·8)	-6·69 (-13–0·108)	0·395 (0·0832–1·22)	0·493 (0·0994–1·55)	0·489 (0·102–1·6)	24·7 (13·6–38·4)	-0·639 (-8·09–6·82)
Unsafe sex	--	--	--	--	--	--	--	--	--	--
Occupational exposure to trichloroethylene	0·0696 (0·0678–0·0722)	0·065 (0·0629–0·0675)	0·0636 (0·0608–0·067)	-6·68 (-11—2·18)	-2·06 (-6·48–2·82)	0·0429 (0·0412–0·0448)	0·0661 (0·0638–0·0688)	0·0679 (0·0642–0·0717)	54 (45·7–62·9)	2·77 (-2·43–8·07)
No access to handwashing facility	3·4 (3·21–3·58)	3·28 (3·11–3·47)	3·24 (3·07–3·41)	-3·52 (-9·69–3·44)	-1·2 (-5·12–2·79)	3·4 (3·21–3·58)	3·28 (3·11–3·47)	3·24 (3·07–3·41)	-3·52 (-9·69–3·44)	-1·2 (-5·12–2·79)
Child wasting	1·31 (0·919–1·72)	1·31 (0·917–1·73)	1·3 (0·912–1·7)	-0·102 (-7·78–8·33)	-0·837 (-6·12–4·56)	--	--	--	--	--
Child stunting	1·8 (0·911–2·64)	1·24 (0·605–1·86)	1·22 (0·6–1·88)	-31·4 (-44·1—18)	-1 (-12·8–11·6)	2·01 (1·06–2·92)	1·54 (0·773–2·31)	1·51 (0·763–2·28)	-23·6 (-37·4—10·1)	-1·74 (-11·5–10·9)
Chewing tobacco	0·143 (0·112–0·18)	0·146 (0·114–0·183)	0·148 (0·117–0·186)	1·65 (-20·9–34·2)	1·53 (-15·5–21·7)	0·137 (0·101–0·187)	0·137 (0·0994–0·185)	0·138 (0·1–0·185)	0·284 (-32·5–42·8)	0·383 (-21·8–27·2)
Diet low in legumes	66·5 (24·4–96·4)	60·6 (15·6–92·2)	63·7 (19·7–94·3)	-8·82 (-38—0·941)	5·05 (0–32·6)	63·1 (17·5–94)	55·5 (11·8–86·9)	59·4 (15–90·6)	-12 (-44·3—1·54)	6·98 (-0·0103–39·9)
Short gestation	19·4 (16·6–22·6)	11·5 (9·77–13·5)	10·2 (8·25–12·6)	-40·4 (-50·2—28·9)	-11·9 (-28·3–8·65)	17·7 (15·1–20·7)	10·7 (9·25–12·3)	9·53 (7·89–11·3)	-39·7 (-49·7—27·5)	-10·8 (-26·1–7·6)
Low birth weight	8·68 (7·51–10)	7·37 (6·4–8·38)	6·85 (5·81–8·31)	-15·1 (-30·1–2·26)	-7·05 (-24·2–15·5)	8·91 (7·62–10·5)	8·64 (7·6–9·77)	7·66 (6·49–8·92)	-3·07 (-19–18·7)	-11·3 (-25·7–5·99)
High temperature	26·5 (18·7–35·1)	26·8 (18·7–35·3)	27·1 (19·5–35·2)	0·987 (-2·13–3·32)	1·48 (-4·08–7·02)	26·5 (18·7–35·1)	26·8 (18·7–35·3)	27·1 (19·5–35·2)	0·987 (-2·13–3·32)	1·48 (-4·08–7·02)
Low temperature	34·5 (29·5–41·7)	35·8 (30·7–43·1)	34·5 (29·3–41·8)	3·81 (2·45–5·05)	-3·71 (-4·95—2·25)	34·5 (29·5–41·7)	35·8 (30·7–43·1)	34·5 (29·3–41·8)	3·81 (2·45–5·05)	-3·71 (-4·95—2·25)
Kidney dysfunction	14·3 (9·72–20·6)	14·1 (9·58–20·5)	13·5 (9·01–19·7)	-1·23 (-5·99–2·93)	-4·44 (-9·5–0·0246)	15·6 (10·7–22·1)	15·6 (10·8–22·3)	14·8 (10–21·4)	-0·0549 (-4·18–4·36)	-4·96 (-10·3—0·881)
Bullying victimization	4·92 (1·96–9·93)	4·85 (2·15–9·43)	4·89 (2·17–9·47)	-1·51 (-21·6–46·3)	0·927 (-4·47–7·1)	3·82 (1·49–7·65)	3·71 (1·66–7·14)	3·74 (1·67–7·2)	-2·75 (-24·3–41·7)	0·65 (-5·58–6·8)
High LDL cholesterol	48·2 (45–51·6)	40·9 (37·9–44·1)	39·6 (36·3–43·1)	-15·2 (-19·5—10·7)	-3·35 (-7·04–0·22)	47·8 (44·5–51·1)	40·5 (37·5–43·7)	40·6 (37·3–44·1)	-15·3 (-19·5—11·1)	0·415 (-2·87–3·78)

Supplementary Results Table 1. Sweden age-standardised Summary Exposure Values (SEVs) for most-detailed risk factors and percentage change, by sex, 1990, 2010, and 2019. Data in parentheses are 95% uncertainty intervals.										
	Male					Female				
	1990	2010	2019	Percentage change 1990-2010	Percentage change 2010-2019	1990	2010	2019	Percentage change 1990-2010	Percentage change 2010-2019
Unsafe sanitation	2·61 (1·51–4·31)	0·862 (0·476–1·42)	0·695 (0·398–1·11)	-67 (-83·6—32·2)	-19·4 (-51·7—37·2)	2·61 (1·51–4·3)	0·862 (0·476–1·42)	0·695 (0·398–1·11)	-67 (-83·6—32·5)	-19·4 (-51·7—37·1)
Ambient particulate matter pollution	7·53 (1·21–16·6)	4·54 (2·17–7·31)	2·32 (0·576–4·77)	-39·7 (-76·8–202)	-48·9 (-73·6—34·5)	7·53 (1·21–16·6)	4·54 (2·19–7·29)	2·32 (0·58–4·76)	-39·7 (-76·7–198)	-48·9 (-73·8—34·4)
Household air pollution from solid fuels	0·048 (0·00836–0·145)	0·012 (0·00199–0·0395)	0·00557 (0·000534–0·0205)	-74·9 (-90·3—32·7)	-53·8 (-80·4—22·5)	0·063 (0·0124–0·193)	0·0159 (0·00267–0·049)	0·00738 (0·000747–0·0267)	-74·7 (-90·3—32·1)	-53·7 (-80·5—22·2)
Ambient ozone pollution	23·3 (8·76–42·7)	27·8 (10·3–50·5)	18·5 (6·35–35·3)	19·5 (5·53–39)	-33·4 (-47·6—20·3)	23·3 (8·7–42·7)	27·8 (10·3–50·6)	18·5 (6·34–35·2)	19·6 (5·29–39·5)	-33·5 (-47·8—20·3)
Residential radon	28·8 (8·87–69·3)	29·5 (9·1–68·7)	29·8 (9·28–67·9)	2·1 (-2–10·3)	1·22 (-1·21–5·55)	29 (8·94–68·9)	29·6 (9·14–68·4)	29·9 (9·31–67·7)	1·88 (-1·81–9·02)	1·14 (-1·14–5·11)
Lead exposure	19·5 (0·409–47·2)	15·3 (0·504–39)	11·1 (0·189–31·6)	-21·3 (-34·4–47·3)	-27·5 (-64·5—18·4)	9·14 (3·61e-12–32·2)	6·95 (7·7e-12–26·3)	5·03 (4·56e-12–21·3)	-23·9 (-45·9–5·87e+11)	-27·7 (-89·5—17·4)
Child underweight	0·883 (0·508–1·29)	0·761 (0·435–1·13)	0·753 (0·418–1·12)	-13·9 (-25·1—0·422)	-0·998 (-9·93–8·27)	1·05 (0·602–1·55)	0·925 (0·529–1·37)	0·912 (0·518–1·36)	-12·3 (-22·5–0·0558)	-1·34 (-9·27–7·61)
Iron deficiency	--	--	--	--	--	9·6 (8–11·4)	9 (7·49–10·6)	8·9 (7·31–10·5)	-6·28 (-14·9–3·2)	-1·07 (-6·6–4·42)
Vitamin A deficiency	4·05 (1·73–8·16)	1·59 (0·649–3·25)	1·37 (0·58–2·89)	-60·8 (-83·9—2·55)	-13·6 (-49·7–55·6)	3·15 (1·56–5·43)	1·87 (0·94–3·23)	1·6 (0·807–2·77)	-40·6 (-70—22·1)	-14·7 (-45·5–35·7)
Zinc deficiency	3·4 (0–10·4)	2·26 (0–7·23)	2·18 (0–7·09)	-33·6 (-100–NA)	-3·29 (-18·6–NA)	3·32 (0–10·4)	2·21 (0–7·11)	2·12 (0–6·92)	-33·6 (-100–NA)	-3·73 (-18·7–NA)
Smoking	16·7 (14·8–18·6)	12·5 (11·1–14·1)	11·7 (10·3–13·2)	-24·9 (-30·4—19·5)	-6·84 (-11·6—1·77)	17 (14·6–19·7)	14·1 (12·2–16·2)	12 (10·5–13·7)	-17·2 (-24·4—9·32)	-14·6 (-19·8—9·04)
Secondhand smoke	30·3 (27·8–32·9)	26 (24·1–28)	25·8 (23·4–28·3)	-14·3 (-23·2—3·79)	-0·818 (-7·14–5·5)	25·5 (23·7–27·4)	22·5 (21·1–23·7)	22·6 (20·8–24·3)	-11·8 (-20—2·66)	0·371 (-5·3–5·96)
Alcohol use	16·7 (11·6–23)	17·7 (11·8–25·4)	18 (12–25·2)	6 (-6·41–20·4)	1·66 (-11·2–17)	--	--	--	--	--
Drug use	0·12 (0·0625–0·239)	0·253 (0·162–0·419)	0·302 (0·199–0·492)	110 (68·4–187)	19·6 (11·4–29·6)	0·0942 (0·046–0·193)	0·131 (0·0753–0·24)	0·148 (0·0861–0·266)	39·1 (20·5–77·5)	12·8 (6·29–20·5)
High fasting plasma glucose	7·25 (6·28–8·38)	10·9 (9·13–12·9)	11·6 (9·83–13·6)	50·6 (30·9–74·3)	6·31 (-7·34–22·4)	6·24 (5·27–7·32)	8·92 (7·48–10·6)	9·29 (7·82–10·9)	42·8 (23·6–68·3)	4·12 (-9·49–21·2)
High systolic blood pressure	33·1 (29·9–36·2)	33 (28·3–38)	34·9 (28·2–42·5)	-0·223 (-15·3–17·6)	5·95 (-11·7–24·7)	29 (25·4–32·7)	28·1 (22·8–33·6)	29·3 (21·8–38·5)	-2·88 (-22·2–20·7)	4·13 (-19·6–31·1)
High body-mass index	16·5 (11·6–23·1)	22·5 (16·1–29·7)	25 (18·3–33)	35·9 (23·7–55)	11·1 (5·05–19·2)	17 (12–23·5)	25·7 (20·2–33·9)	27·9 (21·8–37·1)	51·1 (31·4–79·7)	8·87 (0·4–18·8)
Low bone mineral density	9·95 (5·68–15·9)	9·87 (5·54–16)	9·71 (5·4–16)	-0·821 (-19·5–24)	-1·69 (-23·8–21)	18·8 (12·9–25·7)	19·2 (13·4–26·4)	19·1 (13·1–26·7)	2·54 (-15·6–25·3)	-0·585 (-17·8–19·3)
Diet low in fruits	55·6 (44·7–67·9)	46·5 (35·6–59·2)	45 (33·6–57·7)	-16·3 (-24—10·4)	-3·3 (-11·2–4·08)	57·2 (46·6–69·3)	47·5 (36·6–60·3)	46·4 (34·7–59)	-16·9 (-24·5—10·8)	-2·33 (-11·6–5·46)
Diet low in vegetables	63·3 (43·1–81·5)	51·4 (31·6–72·4)	48 (28·9–69·1)	-18·8 (-30·8—9·3)	-6·62 (-14·6—0·855)	61·3 (40·8–80·7)	48·7 (28·8–70·3)	45 (26·2–66·2)	-20·5 (-33·1—9·33)	-7·62 (-16·1—1)
Diet low in whole grains	89·7 (84·6–94·7)	88·7 (83–94·3)	90·4 (85·5–95·3)	-1·09 (-2·8–0·0512)	1·92 (0·796–3·37)	--	--	--	--	--
Diet low in nuts and seeds	58·3 (26·5–76·7)	43·4 (16·5–65·8)	38·4 (13·5–62·1)	-25·6 (-43·6—11·9)	-11·5 (-27·3—0·832)	53·8 (24·5–73·5)	39·4 (13·6–62·9)	34·6 (11–59·5)	-26·8 (-47·2—11·6)	-12·1 (-28·8—0·97)
Diet low in milk	28 (13·4–45·1)	26·7 (12·9–44·3)	28·2 (13·3–45·5)	-4·55 (-19·2–12·1)	5·35 (-6·82–19·9)	26·9 (11·5–44·9)	25·7 (11·4–43·3)	27·3 (12·4–44·5)	-4·3 (-19·6–14·7)	6·14 (-6·22–23·1)
Diet high in red meat	68·8 (60·4–76·8)	77·2 (69·9–83·7)	79 (70·2–86·7)	12·3 (7·21–19·4)	2·3 (-4·62–9·83)	68·4 (59·4–77·2)	74·6 (66·6–81·9)	76·2 (66·7–84·3)	9·05 (4·45–15·4)	2·15 (-6·13–10·8)
Diet high in processed meat	79·6 (57·2–98·4)	84 (65·5–99·7)	84·8 (66·6–99·5)	5·57 (0·731–14·9)	0·922 (-1·45–5·02)	82·4 (61·5–99·5)	86·8 (70·2–99·9)	87·6 (72·6–99·9)	5·35 (0·308–15·9)	0·937 (-1·49–5·01)
Diet high in sugar-sweetened beverages	30·9 (18·5–47·4)	39 (24·7–54·4)	41·1 (25·9–57)	26·4 (-0·887–62·1)	5·41 (-11·4–26·7)	32·6 (13–55·7)	41·4 (17·1–64·9)	42·3 (17·9–64·3)	26·9 (-8·7–84·7)	2·34 (-22–40·3)
Diet low in fiber	45·7 (29·6–60·9)	32·4 (19·7–45·2)	29·2 (17·4–42·1)	-29·1 (-39·5—20·5)	-9·74 (-22·5–2·07)	45·7 (29·7–61·2)	34·2 (20·5–48·1)	30·5 (18–44·4)	-25·1 (-33·2—18·5)	-10·9 (-23·9–0·153)
Diet low in seafood omega-3 fatty acids	90·2 (78·5–99·9)	82·8 (66·5–99·1)	81·5 (64·1–99·1)	-8·21 (-20·1–0·434)	-1·63 (-13·3–11)	91·9 (82·1–100)	85·6 (70·4–99·7)	84·9 (70·5–99·5)	-6·89 (-18·6–0·261)	-0·78 (-9·99–10)
Diet low in polyunsaturated fatty acids	58·2 (19·6–96·4)	62·2 (22·4–98·5)	60·5 (21·6–97·2)	6·96 (0·688–19·4)	-2·86 (-7·85–0)	60·5 (22·4–96·2)	63·8 (24·2–97·9)	62·1 (24–96·3)	5·46 (0·527–15·8)	-2·6 (-6·36–1·08)
Diet high in trans fatty acids	35·9 (25·8–93·2)	30 (20·7–93·5)	29·9 (20·9–90·9)	-16·5 (-29·7–0)	-0·0787 (-8·85–11)	37·4 (26·8–99·4)	31·7 (21·1–97·5)	31·5 (21·1–95·8)	-15·2 (-28·8–0)	-0·524 (-7·96–9·24)
Diet high in sodium	33·2 (7·59–58·4)	31·8 (7·06–56·2)	33·2 (7·87–58·4)	-4·05 (-36·1–25·8)	4·29 (-11·9–33·9)	15·6 (4·08–36·9)	15·6 (4·05–37·7)	15·9 (4·01–38·2)	-0·603 (-24·6–21·4)	2·21 (-10·6–20·9)
Low physical activity	3·1 (1·19–6·46)	2·68 (0·914–5·84)	2·79 (0·991–6·16)	-13·4 (-33·5–3·34)	3·83 (-16·1–30·5)	4·72 (2·31–9·21)	5·11 (2·53–9·8)	4·8 (2·34–9·17)	8·2 (-4·91–24·5)	-6·13 (-17·1–6·03)
Occupational asthmagens	25 (20·6–30·5)	20·7 (17·8–24·2)	21 (17·9–24·9)	-17·3 (-26—7·82)	1·7 (-6·18–11·2)	--	--	--	--	--
Occupational particulate matter, gases, and fumes	10·2 (7·3–14·5)	8·45 (6·05–12·2)	7·89 (5·65–11·3)	-17 (-21·7—12·4)	-6·62 (-8·54—4·44)	5·29 (3·75–7·76)	4·19 (2·95–6·2)	3·79 (2·69–5·67)	-20·9 (-27·8—14·4)	-9·37 (-12·7—6·07)
Occupational noise	9·3 (8·79–9·99)	8·2 (7·78–8·86)	7·91 (7·49–8·56)	-11·7 (-13·3—10·1)	-3·63 (-4·45—2·76)	7·02 (6·6–7·62)	6·53 (6·18–7·13)	6·43 (6·09–7·04)	-7·06 (-9·13—5·01)	-1·47 (-2·43—0·453)
Occupational injuries	--	--	--	--	--	--	--	--	--	--
Occupational ergonomic factors	12 (10·5–13·9)	11 (9·47–12·8)	10·9 (9·28–12·7)	-8·6 (-13·9—3·04)	-1·08 (-6·32–4·45)	--	--	--	--	--
Childhood sexual abuse	6·07 (3·87–8·57)	6·12 (4·06–8·58)	5·32 (3·5–7·58)	0·884 (-6·13–9·01)	-13·2 (-19·1—6·38)	12·4 (9·29–16·7)	16·8 (12·6–22·8)	16·6 (12·3–22·9)	35·7 (27·3–45·5)	-1·29 (-7·49–5·29)
Intimate partner violence	--	--	--	--	--	18·4 (10·4–25·8)	21·1 (12·1–29·1)	18·4 (10·4–25·8)	14·6 (5·2–29·3)	-12·7 (-18·7—1·57)
Non-exclusive breastfeeding	21·4 (17·7–26·2)	18·9 (15–23·8)	18·7 (14·8–23·9)	-11·7 (-23—0·548)	-0·99 (-12·2–13·6)	21·4 (17·7–26·2)	18·9 (15–23·8)	18·7 (14·8–23·9)	-11·7 (-23—0·541)	-1·01 (-12·2–13·6)
Discontinued breastfeeding	24·1 (22·8–25·4)	23·3 (22·1–24·5)	22·5 (21·2–23·8)	-3·28 (-10·2–4·94)	-3·28 (-10·2–4·18)	24·1 (22·8–25·4)	23·3 (22·1–24·5)	22·5 (21·2–23·8)	-3·24 (-10·2–4·98)	-3·25 (-10·2–4·2)
Diet low in calcium	10·2 (5·03–18·4)	8·67 (4·11–15·8)	8·2 (4–14·8)	-15·2 (-23·4—9·23)	-5·41 (-12·1—2·83)	4·73 (2·07–8·38)	3·67 (1·54–6·55)	3·44 (1·48–6·22)	-22·5 (-35·8—10·4)	-6·26 (-17·7–6·49)
Occupational exposure to asbestos	10·8 (9·83–11·8)	9·87 (9·22–10·6)	8·9 (7·15–10·9)	-8·41 (-17·9–2·68)	-9·85 (-28·1–11)	1·75 (1·31–2·66)	1·61 (1·21–2·1)	1·63 (1·1–2·18)	-7·93 (-29·8–14·4)	1·65 (-20·3–27·6)
Occupational exposure to arsenic	0·582 (0–1·37)	0·493 (0–1·16)	0·507 (0–1·19)	-15·3 (-22·4–NA)	2·88 (-4·75–NA)	0·563 (0–1·33)	0·459 (0–1·09)	0·468 (0–1·11)	-18·5 (-26·8–NA)	1·88 (-4·6–NA)
Occupational exposure to benzene	0·867 (0·0526–2·53)	0·942 (0·0569–2·68)	0·97 (0·0571–2·73)	8·55 (4·41–14·5)	2·96 (-0·98–8·79)	1·62 (0·0964–4·73)	1·75 (0·106–4·98)	1·86 (0·111–5·4)	7·55 (2·26–15·6)	6·72 (2·21–12·2)
Occupational exposure to beryllium	0·0252 (0·0248–0·0257)	0·0252 (0·025–0·0254)	0·0259 (0·0253–0·0265)	-0·245 (-2·02–1·52)	2·88 (0·424–5·38)	0·0322 (0·0316–0·0328)	0·031 (0·0308–0·0313)	0·0325 (0·0318–0·0333)	-3·68 (-5·54—1·85)	4·86 (2·29–7·58)
Occupational exposure to cadmium	0·0669 (0·0616–0·0722)	0·0579 (0·0567–0·0592)	0·0596 (0·0558–0·0634)	-13·4 (-20·1—6·43)	2·84 (-3·8–10·4)	0·0676 (0·0617–0·074)	0·0566 (0·0553–0·058)	0·0581 (0·0551–0·0613)	-16·3 (-23·7—7·83)	2·52 (-3·23–9·02)
Occupational exposure to chromium	0·156 (0·146–0·167)	0·14 (0·137–0·143)	0·144 (0·137–0·152)	-10·4 (-16·5—4·32)	3·01 (-2·9–9·31)	0·172 (0·161–0·185)	0·151 (0·148–0·153)	0·156 (0·15–0·163)	-12·6 (-18·8—5·89)	3·53 (-0·872–8·46)
Occupational exposure to diesel engine exhaust	0·726 (0·689–0·763)	0·773 (0·757–0·79)	0·792 (0·757–0·825)	6·59 (1·44–11·9)	2·39 (-2·2–7·07)	0·749 (0·703–0·793)	0·683 (0·658–0·709)	0·71 (0·67–0·755)	-8·92 (-14·5—2·55)	3·99 (-1·93–10·1)
Occupational exposure to formaldehyde	0·337 (0·308–0·366)	0·285 (0·275–0·292)	0·289 (0·27–0·309)	-15·5 (-22·3—8·37)	1·68 (-5·27–9·14)	0·344 (0·317–0·375)	0·3 (0·289–0·307)	0·31 (0·295–0·325)	-12·9 (-19·7—5·14)	3·15 (-1·65–8·25)
Occupational exposure to nickel	0·544 (0–2·41)	0·471 (0–2·08)	0·484 (0–2·16)	-13·5 (-19·4–NA)	2·85 (-4·02–NA)	0·441 (0–2)	0·35 (0–1·54)	0·353 (0–1·58)	-20·7 (-29·4–NA)	0·901 (-6·38–NA)
Occupational exposure to polycyclic aromatic hydrocarbons	0·317 (0·299–0·336)	0·291 (0·286–0·296)	0·3 (0·286–0·315)	-8·26 (-13·8—2·9)	3·21 (-1·96–8·72)	--	--	--	--	--
Occupational exposure to silica	6·12 (0·908–19·6)	5·78 (0·834–19)	5·92 (0·884–19)	-5·53 (-12–2·46)	2·46 (-4·61–10·3)	3·76 (0·533–11·8)	2·95 (0·425–9·49)	2·96 (0·412–9·48)	-21·6 (-30·5—10·5)	0·224 (-7·96–9·89)
Occupational exposure to sulfuric acid	0·915 (0·187–2·8)	0·769 (0·16–2·41)	0·785 (0·164–2·44)	-16 (-22·4—9·36)	2·06 (-4·66–9·63)	0·802 (0·163–2·44)	0·637 (0·13–1·99)	0·645 (0·134–2·07)	-20·6 (-28·5—11·5)	1·16 (-5·92–9·35)
Unsafe sex	--	--	--	--	--	--	--	--	--	--
Occupational exposure to trichloroethylene	0·0855 (0·082–0·0897)	0·0804 (0·0793–0·082)	0·0826 (0·0797–0·0863)	-6 (-10·3—1·47)	2·79 (-1·5–7·27)	0·0986 (0·0938–0·104)	0·0872 (0·0858–0·0893)	0·0905 (0·0872–0·0947)	-11·5 (-16·2—6·37)	3·73 (-0·512–8·39)
No access to handwashing facility	3·3 (3·11–3·48)	3·16 (2·99–3·33)	3·13 (2·97–3·3)	-4·16 (-9·87–2·24)	-1·04 (-4·83–3·04)	3·3 (3·11–3·48)	3·16 (2·99–3·33)	3·13 (2·97–3·3)	-4·16 (-9·89–2·23)	-1·04 (-4·83–3·05)
Child wasting	1·26 (0·882–1·68)	1·21 (0·857–1·62)	1·2 (0·853–1·59)	-3·71 (-11·7–5·24)	-0·83 (-6·43–5·2)	1·44 (0·998–1·86)	1·02 (0·695–1·37)	1·36 (0·946–1·76)	-28·9 (-42·1—8·4)	33·4 (4·19–59·4)
Child stunting	1·58 (0·798–2·36)	1·19 (0·563–1·81)	1·18 (0·544–1·84)	-24·9 (-38·6—10·1)	-0·659 (-13·9–12·2)	1·87 (0·985–2·71)	1·55 (0·768–2·34)	1·53 (0·745–2·3)	-17 (-30·3—0·337)	-1·34 (-11·5–10·9)
Chewing tobacco	1·91 (1·46–2·44)	1·97 (1·54–2·48)	1·93 (1·49–2·44)	3·18 (-23·1–38·4)	-2·2 (-19·2–17·2)	0·355 (0·247–0·518)	0·392 (0·271–0·559)	0·392 (0·273–0·557)	10·4 (-28·9–70·2)	-0·0259 (-24·7–34)
Diet low in legumes	90·4 (77·6–100)	82·4 (58·8–100)	82·9 (59·9–100)	-8·83 (-24·9–0)	0·562 (-1·65–4·31)	88·2 (72–100)	79·7 (52·2–100)	80·2 (53·6–99·9)	-9·72 (-29·4—0·0425)	0·638 (-2·03–4·86)
Short gestation	12·5 (10·3–14·8)	11 (9·2–13·2)	10·4 (8·71–12·5)	-11·6 (-28·1–11·1)	-5·67 (-22·6–17)	11·2 (9·79–12·9)	9·64 (8·16–11·2)	10·3 (8·56–12·5)	-14·1 (-28·6–2·22)	6·91 (-11·9–32·5)
Low birth weight	5·18 (4·35–6·12)	4·43 (3·76–5·29)	4·22 (3·55–5·01)	-14·4 (-31·4–9·27)	-4·91 (-23–18·4)	4·92 (4·31–5·61)	3·84 (3·24–4·53)	4·09 (3·39–4·96)	-21·8 (-36—4·06)	6·42 (-15·4–33·1)
High temperature	16·7 (7·69–27·1)	21 (11–32)	21 (10·3–32·6)	25·8 (-24·3–126)	-0·28 (-11·2–8·49)	16·7 (7·69–27·1)	21 (11–32)	21 (10·3–32·6)	25·8 (-24·3–126)	-0·28 (-11·2–8·49)
Low temperature	33·5 (28·7–38·9)	37·2 (33·4–41·7)	33·8 (29·4–38·9)	11 (4·87–19·2)	-9·16 (-14—5·05)	33·5 (28·7–38·9)	37·2 (33·4–41·7)	33·8 (29·4–38·9)	11 (4·87–19·2)	-9·16 (-14—5·05)
Kidney dysfunction	19·1 (12·9–26·4)	19·6 (13·5–26·9)	19·4 (13·3–26·7)	2·52 (-0·51–6·8)	-1·17 (-3·83–1·43)	14·9 (9·77–21·5)	14·7 (9·64–21·3)	14·6 (9·55–21·2)	-1·22 (-4·8–2·11)	-0·668 (-3·71–2·11)
Bullying victimization	6·39 (2·54–12·9)	5·3 (2·19–10·8)	5·49 (2·26–11)	-17·1 (-27·3—7·66)	3·55 (-1·57–13·1)	5·18 (2·09–10·5)	4·38 (1·78–9)	4·46 (1·84–8·96)	-15·4 (-25·4—5·61)	1·81 (-2·77–9·73)
High LDL cholesterol	60·2 (57·1–63·4)	44·8 (41·3–48·3)	44 (40·3–47·6)	-25·6 (-30·4—21·1)	-1·88 (-6·38–2·67)	57·4 (54·6–60·5)	45·6 (42·4–48·9)	46·9 (43·5–50·2)	-20·5 (-24·9—16·5)	2·76 (-0·842–6·84)

Supplementary Results Table 1. Stockholm age-standardised Summary Exposure Values (SEVs) for most-detailed risk factors and percentage change, by sex, 1990, 2010, and 2019. Data in parentheses are 95% uncertainty intervals.

	Male					Female				
	1990	2010	2019	Percentage change 1990-2010	Percentage change 2010-2019	1990	2010	2019	Percentage change 1990-2010	Percentage change 2010-2019
Unsafe sanitation	2·62 (1·03–5·33)	0·912 (0·374–1·82)	0·745 (0·326–1·41)	-65·3 (-88—0·682)	-18·2 (-58·5–69·3)	2·62 (1·03–5·33)	0·912 (0·374–1·82)	0·745 (0·326–1·41)	-65·3 (-88—0·682)	-18·2 (-58·5–69·3)
Ambient particulate matter pollution	6·87 (0·511–15·9)	4·24 (1·46–7·48)	2·04 (0·0739–4·91)	-38·3 (-78·7–392)	-51·9 (-95·1—34·2)	6·87 (0·523–15·9)	4·24 (1·48–7·49)	2·04 (0·0719–4·91)	-38·3 (-78·5–356)	-51·9 (-95·2—34·2)
Household air pollution from solid fuels	0·0246 (0·00228–0·0802)	0·00683 (0·000807–0·0257)	0·00303 (6·22e-05–0·0124)	-72·2 (-92·9–64·9)	-55·7 (-96·5—7·74)	0·0325 (0·00303–0·115)	0·00908 (0·00107–0·0332)	0·00402 (7·11e-05–0·0168)	-72 (-92·9–62·3)	-55·8 (-96·3—7·68)
Ambient ozone pollution	19 (6–40·2)	25·6 (7·75–51·9)	11·7 (2·38–27·8)	35 (-34·9–174)	-54·4 (-85—0·819)	19 (6–40·2)	25·6 (7·75–51·9)	11·7 (2·38–27·8)	35 (-34·9–174)	-54·4 (-85—0·819)
Residential radon	46·1 (12·2–100)	46·1 (12·2–100)	46·1 (12·2–100)	0 (0–0)	0 (0–0)	46·1 (12·2–100)	46·1 (12·2–100)	46·1 (12·2–100)	0 (0–0)	0 (0–0)
Lead exposure	29·4 (2·92–54·3)	20·5 (3·37–41·5)	12·9 (1·25–30·8)	-30·2 (-41·6–18·2)	-37·3 (-64·6—25·6)	18·3 (3·51e-12–43·6)	12·4 (8·82e-12–32·4)	7·71 (5·65e-12–23·4)	-32 (-44·8–3·43e+12)	-38 (-88·8—25·9)
Child underweight	0·901 (0·517–1·33)	0·769 (0·432–1·2)	0·763 (0·416–1·18)	-14·6 (-31·5–5·44)	-0·878 (-13·3–12·8)	1·08 (0·628–1·61)	0·939 (0·531–1·43)	0·928 (0·534–1·4)	-12·8 (-28·3–5·89)	-1·18 (-12·8–12·4)
Iron deficiency	--	--	--	--	--	9·79 (7·81–12)	9·14 (7·27–11)	9·09 (7·18–11)	-6·72 (-19·6–8·12)	-0·553 (-8·58–8·48)
Vitamin A deficiency	4·56 (1·19–10·9)	1·78 (0·473–4·39)	1·56 (0·406–4·3)	-61 (-88·3–66·5)	-12·6 (-60·2–120)	3·31 (1·12–6·71)	1·98 (0·731–4·23)	1·71 (0·59–3·58)	-40 (-78·1–80·5)	-13·7 (-56·1–70·1)
Zinc deficiency	3·67 (0–11·1)	2·46 (0–8·06)	2·38 (0–7·55)	-32·9 (-100–NA)	-3·51 (-26·9–NA)	3·61 (0–11·1)	2·4 (0–7·7)	2·3 (0–7·57)	-33·3 (-100–NA)	-4·17 (-22·6–NA)
Smoking	19·2 (16·8–22)	13·2 (11·6–15)	12 (10·5–13·6)	-31·4 (-38·2—24)	-9·19 (-15·7—2·34)	19·3 (16–23·2)	14·2 (12–16·6)	11·8 (10·1–13·8)	-26·3 (-36·5—14·9)	-17·1 (-24·9—8·95)
Secondhand smoke	33·6 (29·6–38)	27 (23·5–30·6)	25·9 (22·6–29·5)	-19·8 (-32—5·36)	-3·88 (-13·3–5·94)	27·7 (24·8–30·9)	23·5 (21·1–26)	22·8 (20·2–25·5)	-15·3 (-26·7—2·56)	-3·13 (-10·8–5·58)
Alcohol use	17·6 (9·74–26)	17·4 (8·81–26)	18·3 (9·64–27·2)	-1·26 (-40–53·4)	5·18 (-22·2–41)	--	--	--	--	--
Drug use	0·139 (0·0784–0·264)	0·277 (0·178–0·445)	0·311 (0·2–0·508)	99·3 (61·9–161)	12·3 (0·601–24·8)	0·103 (0·0527–0·201)	0·156 (0·094–0·273)	0·178 (0·11–0·303)	51·7 (27·1–102)	13·8 (4·55–24)
High fasting plasma glucose	7 (5·97–8·29)	9·89 (8·17–12·2)	11 (8·93–13·3)	41·2 (19·8–69·6)	10·9 (-7·05–34·8)	5·18 (4·38–6·16)	7·62 (6·23–9·33)	8·79 (7·13–11·1)	47·2 (24·6–74·8)	15·3 (-1·35–37·8)
High systolic blood pressure	23·5 (17·6–29·9)	25·8 (19·1–33·7)	27·5 (19·5–36·8)	9·72 (-24·1–55·2)	6·91 (-20·5–40·8)	23·7 (15·1–33·8)	20·7 (12·5–30)	21·9 (12·8–34·5)	-13 (-51·3–53·9)	5·97 (-32·5–69·4)
High body-mass index	13·9 (9·25–19·9)	18·7 (13·1–25·4)	20·8 (14·9–28·2)	34·8 (17·3–60·8)	11·1 (2·37–21·9)	14·5 (9·58–21)	21·6 (15·8–29·4)	23·9 (17·8–32·8)	49·5 (23–97·2)	10·6 (-1·63–25·5)
Low bone mineral density	9·95 (5·29–16·2)	9·34 (5·13–15·8)	9·09 (4·91–15·1)	-6·14 (-27·7–25·9)	-2·65 (-26·7–27·8)	19 (12·7–26·1)	18 (12·1–24·9)	17·9 (12·1–25)	-5·11 (-25·4–18·7)	-0·651 (-21·8–25·7)
Diet low in fruits	68·1 (58·3–78·5)	54·9 (43·9–67·5)	51·8 (39–64·7)	-19·4 (-30·6—9·29)	-5·73 (-17·2–3·99)	70·4 (60·8–80)	57·6 (46·1–69·9)	55 (43–67·7)	-18·2 (-28·7—9·34)	-4·52 (-14–4·84)
Diet low in vegetables	47·8 (28·9–68·6)	35·8 (19–57)	30·2 (14·7–50·8)	-25·2 (-44·4—9·71)	-15·7 (-34·4—1·1)	50·6 (31·2–72·1)	36·7 (20·3–58·1)	32·4 (16·4–53·4)	-27·4 (-45·9—10·6)	-11·8 (-28·9–1·43)
Diet low in whole grains	99·6 (98·8–100)	97·7 (95·9–99·3)	98·7 (97·3–99·9)	-1·94 (-3·48—0·566)	1·06 (0·18–2·26)	--	--	--	--	--
Diet low in nuts and seeds	67 (32–81·8)	55·5 (25·8–75·3)	49·4 (21·6–70·6)	-17·2 (-36·1—4·37)	-11 (-27·7–0·844)	45·9 (19·9–67·5)	30·2 (10·2–57·7)	26·1 (8·24–55·6)	-34·1 (-59—11·2)	-13·6 (-38·9–6·72)
Diet low in milk	53·9 (31·1–74)	57 (33·6–78·2)	56·4 (32·3–77·9)	5·93 (-4·83–21·8)	-1·09 (-8·92–6·02)	48·2 (25–67·7)	51·6 (27·3–71·1)	51·4 (27·1–71·8)	7·07 (-6·3–28·4)	-0·266 (-9·5–9·88)
Diet high in red meat	55 (41·5–67·3)	64·4 (51–76·4)	66·8 (52–80·6)	17·2 (-4·49–49·6)	3·67 (-10·1–20)	57·6 (45·1–69·2)	58·5 (45·1–71)	59·5 (44·5–72·7)	1·43 (-17·9–23·6)	1·74 (-12·7–17·8)
Diet high in processed meat	73·1 (48·7–95·5)	78·9 (54·4–99·1)	79·2 (56·2–98·5)	7·89 (-0·591–23·2)	0·471 (-4·57–6·43)	79 (55·6–98·5)	85 (66·3–99·8)	85·8 (66·6–99·7)	7·63 (0·413–24·2)	0·87 (-2·95–6·69)
Diet high in sugar-sweetened beverages	35·2 (19–57·7)	36·2 (16·6–58)	43·3 (21·9–64·9)	2·79 (-40·2–61·1)	19·6 (-9·36–79·7)	--	--	--	--	--
Diet low in fiber	49·6 (34·4–64·3)	29·1 (17·2–41·6)	24·8 (13·7–37·4)	-41·4 (-57·1—27·2)	-14·6 (-32·9–4·44)	42·1 (26·5–57·6)	23·5 (13–35·3)	19·3 (9·76–30·3)	-44·1 (-59—30)	-18·1 (-39·4–2·11)
Diet low in seafood omega-3 fatty acids	87·6 (73–100)	79·5 (61·3–98·9)	76·8 (57·9–98·6)	-9·23 (-24–2·56)	-3·48 (-20·7–15·7)	89·6 (76·7–100)	82 (63·2–99·6)	80·5 (63–99·6)	-8·43 (-23·7–2·17)	-1·84 (-16·9–15·2)
Diet low in polyunsaturated fatty acids	44·8 (11·8–88·4)	53·7 (16·4–94·8)	47·6 (12·6–91·5)	19·7 (2·32–59·7)	-11·4 (-27·4—1·4)	42·8 (10·8–86·4)	50·4 (13·6–93)	43·4 (10·6–86·2)	17·7 (1·79–53·3)	-13·9 (-31·2—1·74)
Diet high in trans fatty acids	33·4 (22·5–94·3)	28·1 (19·3–90)	28 (19·2–89·9)	-15·8 (-28·7–0)	-0·65 (-12·5–14·3)	36·4 (25·2–98·8)	31·9 (22–94·3)	31·1 (21·5–92·7)	-12·3 (-25·4–5·14)	-2·44 (-15·7–12·4)
Diet high in sodium	28·4 (6·13–54·6)	28·8 (6·35–54·4)	30·2 (6·8–56·5)	1·43 (-38·9–61·2)	4·66 (-21·8–56)	15·3 (4·09–36·3)	15·5 (4·14–37·6)	15·9 (4·13–38·4)	1·11 (-31·4–43·5)	2·74 (-17–33·8)
Low physical activity	3·3 (1·3–6·79)	3·01 (1·03–6·23)	3·06 (1·11–6·34)	-8·78 (-28·8–12·9)	1·77 (-17·3–26·9)	4·97 (2·46–9·42)	5·49 (2·71–10·5)	5·15 (2·54–9·88)	10·3 (-4·83–30·1)	-6·2 (-17·9–6·3)
Occupational asthmagens	29 (22·7–36·9)	22·5 (18·2–27·5)	23 (18·4–28·6)	-22·5 (-37·3—3·16)	2·47 (-12·5–22)	--	--	--	--	--
Occupational particulate matter, gases, and fumes	10·5 (7·5–15·2)	8·84 (6·19–12·9)	8·25 (5·83–12)	-16·1 (-22·5—9·97)	-6·6 (-9·53—3·51)	5·61 (3·77–8·52)	4·44 (3·09–6·63)	3·98 (2·79–5·86)	-21 (-29·9—11·2)	-10·3 (-15—5·21)
Occupational noise	9·52 (8·71–10·5)	8·5 (7·92–9·29)	8·18 (7·66–8·91)	-10·7 (-14—7·3)	-3·68 (-5·39—1·91)	7·52 (6·81–8·31)	7·01 (6·52–7·69)	6·83 (6·41–7·45)	-6·82 (-10·7—2·53)	-2·57 (-4·3—0·728)
Occupational injuries	--	--	--	--	--	--	--	--	--	--
Occupational ergonomic factors	11·6 (9·56–13·9)	10·5 (8·82–12·6)	10·5 (8·72–12·7)	-8·89 (-19·1–2·46)	-0·573 (-8·29–8·14)	--	--	--	--	--
Childhood sexual abuse	6·07 (3·87–8·57)	6·12 (4·06–8·58)	5·32 (3·5–7·58)	0·884 (-6·13–9·01)	-13·2 (-19·1—6·38)	12·4 (9·29–16·7)	16·8 (12·6–22·8)	16·6 (12·3–22·9)	35·7 (27·3–45·5)	-1·29 (-7·49–5·29)
Intimate partner violence	--	--	--	--	--	18·4 (10·4–25·8)	21·1 (12·1–29·1)	18·4 (10·4–25·8)	14·6 (5·2–29·3)	-12·7 (-18·7—1·57)
Non-exclusive breastfeeding	21·5 (17·3–26·8)	19 (14·7–24·6)	18·8 (14·3–24·1)	-11·5 (-27·6–6·86)	-1·23 (-14·9–16·8)	21·5 (17·3–26·8)	19 (14·7–24·6)	18·8 (14·3–24·1)	-11·5 (-27·6–6·87)	-1·25 (-14·9–16·8)
Discontinued breastfeeding	24·5 (22·4–26·5)	23·4 (21·4–25·2)	22·6 (20·6–24·5)	-4·82 (-14·5–6·55)	-3·33 (-11·9–6·08)	24·5 (22·4–26·5)	23·4 (21·4–25·2)	22·6 (20·6–24·5)	-4·7 (-14·4–6·69)	-3·21 (-11·8–6·21)
Diet low in calcium	14·6 (8·49–24)	10·5 (5·52–18·6)	9·52 (5·02–17)	-28·1 (-41·4—17·5)	-9·37 (-18·4—0·249)	6·63 (3·28–12)	4·23 (1·85–7·6)	3·75 (1·63–6·77)	-36·2 (-52·3—20·7)	-11·3 (-24·5–4·46)
Occupational exposure to asbestos	11 (9·72–12·4)	9·43 (8·45–10·4)	8·43 (6·32–10·8)	-13·9 (-26·1—0·757)	-10·6 (-32·6–16·8)	2·2 (1·33–2·85)	1·9 (1·09–2·26)	1·85 (0·992–2·54)	-13·8 (-37–12)	-2·63 (-28–25·4)
Occupational exposure to arsenic	0·633 (0–1·52)	0·529 (0–1·24)	0·537 (0–1·25)	-16·5 (-28·2–NA)	1·52 (-9·65–NA)	0·616 (0–1·47)	0·488 (0–1·2)	0·485 (0–1·16)	-20·8 (-33·8–NA)	-0·543 (-9·87–NA)
Occupational exposure to benzene	0·928 (0·0554–2·73)	0·984 (0·0569–2·8)	1 (0·0574–2·79)	5·98 (-2·73–19·2)	1·95 (-3·88–11·7)	1·79 (0·109–5·02)	1·86 (0·107–5·31)	1·93 (0·112–5·5)	3·99 (-4·81–17·8)	4·14 (-2·5–12·8)
Occupational exposure to beryllium	0·0266 (0·0252–0·028)	0·0261 (0·0256–0·0266)	0·0266 (0·0257–0·0276)	-1·8 (-6·97–3·54)	1·97 (-2–6·07)	0·0347 (0·0331–0·0365)	0·0325 (0·032–0·0331)	0·0333 (0·0322–0·0345)	-6·39 (-11—1·27)	2·37 (-1·46–6·1)
Occupational exposure to cadmium	0·0727 (0·0641–0·0819)	0·0621 (0·0565–0·0683)	0·0632 (0·0565–0·0702)	-14·6 (-25·9—1·62)	1·66 (-8·42–13·2)	0·0736 (0·0635–0·0863)	0·06 (0·0552–0·0656)	0·0601 (0·0546–0·0665)	-18·5 (-30·6—4·94)	0·163 (-8·08–9·68)
Occupational exposure to chromium	0·171 (0·153–0·19)	0·151 (0·139–0·163)	0·153 (0·14–0·168)	-11·8 (-21·9—0·715)	1·81 (-6·94–11·3)	0·188 (0·168–0·214)	0·16 (0·15–0·171)	0·162 (0·15–0·175)	-15·1 (-25·1—4·75)	1·08 (-5·51–8·58)
Occupational exposure to diesel engine exhaust	0·778 (0·708–0·846)	0·814 (0·766–0·86)	0·826 (0·763–0·888)	4·63 (-4·52–15·5)	1·47 (-5·06–8·4)	0·797 (0·717–0·883)	0·704 (0·647–0·763)	0·714 (0·65–0·789)	-11·7 (-21·6—0·154)	1·39 (-7·2–10·4)
Occupational exposure to formaldehyde	0·369 (0·325–0·417)	0·307 (0·278–0·337)	0·308 (0·276–0·344)	-16·8 (-28—4·04)	0·437 (-9·89–11·9)	0·378 (0·333–0·434)	0·32 (0·297–0·343)	0·322 (0·297–0·35)	-15·5 (-26·3—4·05)	0·76 (-6·71–8·41)
Occupational exposure to nickel	0·585 (0–2·61)	0·499 (0–2·28)	0·507 (0–2·22)	-14·6 (-25–NA)	1·56 (-8·64–NA)	0·478 (0–2·19)	0·37 (0–1·59)	0·364 (0–1·62)	-22·7 (-36·6–NA)	-1·54 (-12·2–NA)
Occupational exposure to polycyclic aromatic hydrocarbons	0·345 (0·314–0·378)	0·311 (0·291–0·333)	0·317 (0·293–0·343)	-9·87 (-18·9—0·0446)	1·99 (-5·89–10·5)	--	--	--	--	--
Occupational exposure to silica	6·47 (0·92–20·6)	5·97 (0·819–19·5)	6·05 (0·911–19·9)	-7·69 (-19·3–8·74)	1·33 (-8·88–14·7)	4 (0·577–13·3)	3·06 (0·42–9·45)	3 (0·411–10)	-23·5 (-37·9—4·96)	-1·93 (-13·7–12·7)
Occupational exposure to sulfuric acid	0·986 (0·203–3·09)	0·823 (0·169–2·61)	0·83 (0·166–2·57)	-16·5 (-27·1—4·88)	0·876 (-8·63–11·9)	0·873 (0·176–2·76)	0·678 (0·14–2·17)	0·67 (0·145–2·15)	-22·4 (-35·4—7·67)	-1·12 (-10·9–10·7)
Unsafe sex	--	--	--	--	--	--	--	--	--	--
Occupational exposure to trichloroethylene	0·093 (0·0862–0·101)	0·0861 (0·082–0·0911)	0·0876 (0·082–0·0936)	-7·46 (-15–0·564)	1·71 (-4·37–8·7)	0·107 (0·0976–0·118)	0·0916 (0·0871–0·0972)	0·0927 (0·087–0·1)	-14·2 (-22·2—5·14)	1·22 (-4·56–8·32)
No access to handwashing facility	3·29 (3·03–3·56)	3·17 (2·93–3·42)	3·13 (2·89–3·4)	-3·78 (-12·1–5·55)	-1 (-6·82–5·51)	3·29 (3·03–3·56)	3·17 (2·93–3·42)	3·13 (2·89–3·4)	-3·78 (-12·1–5·55)	-1 (-6·82–5·51)
Child wasting	1·27 (0·884–1·7)	1·22 (0·859–1·62)	1·21 (0·84–1·6)	-3·57 (-14·9–9·18)	-0·882 (-8·96–7·82)	1·44 (0·998–1·86)	1·02 (0·695–1·37)	1·36 (0·946–1·76)	-28·9 (-42·1—8·4)	33·4 (4·19–59·4)
Child stunting	1·6 (0·787–2·43)	1·19 (0·554–1·89)	1·19 (0·513–1·9)	-25·3 (-42·3—3)	-0·373 (-19·1–22·5)	1·9 (0·939–2·81)	1·57 (0·733–2·4)	1·54 (0·734–2·38)	-17·6 (-36·5–4·68)	-1·39 (-17·4–16·5)
Chewing tobacco	2·06 (1·39–2·97)	2·12 (1·47–2·98)	2·09 (1·44–2·85)	3·14 (-32–61·8)	-1·44 (-24·5–28)	0·382 (0·21–0·66)	0·419 (0·239–0·704)	0·407 (0·237–0·643)	9·52 (-39·5–103)	-2·93 (-35·2–42·1)
Diet low in legumes	91·2 (78·2–100)	83·7 (60·5–100)	84·1 (62·7–100)	-8·26 (-24·2–0)	0·507 (-3·24–4·55)	89·2 (73·6–100)	81 (55·2–100)	81·5 (57·2–99·9)	-9·17 (-26·4—0·0316)	0·65 (-3·43–6·23)
Short gestation	11·2 (9·02–13·8)	9·94 (8·11–12·2)	9·47 (7·61–11·8)	-11·3 (-31·6–13·7)	-4·72 (-26·6–25·5)	11·1 (8·94–13·4)	9·39 (7·77–11·3)	10·1 (8–12·6)	-15·1 (-34·4–11·8)	7·26 (-14·3–36·7)
Low birth weight	4·74 (3·82–5·88)	4·11 (3·3–5·06)	3·83 (3·08–4·73)	-13·3 (-34–14·2)	-6·94 (-30·4–24·7)	4·79 (3·94–5·76)	3·74 (3·06–4·6)	4·07 (3·25–5·08)	-21·8 (-41·8–5·27)	8·77 (-15·5–40·7)
High temperature	0 (0–0)	19·5 (6·81–33·5)	19·4 (6·53–33·4)	--	-0·421 (-2·56–2·9)	0 (0–0)	19·5 (6·81–33·5)	19·4 (6·53–33·4)	--	-0·421 (-2·56–2·9)
Low temperature	33 (28–38·8)	36·8 (33·1–41·6)	33·8 (29·1–39·5)	11·5 (4·36–20·7)	-8·23 (-13·5—3·06)	33 (28–38·8)	36·8 (33·1–41·6)	33·8 (29·1–39·5)	11·5 (4·36–20·7)	-8·23 (-13·5—3·06)
Kidney dysfunction	15·1 (10·2–21·7)	15·2 (10·3–21·7)	15 (10–21·5)	0·614 (-3·97–5·52)	-1·27 (-4·88–2·05)	14·3 (9·3–20·8)	14·2 (9·34–20·7)	14·2 (9·26–20·6)	-0·447 (-5–3·91)	-0·162 (-3·29–3·23)
Bullying victimization	4·83 (1·92–10·1)	4·36 (1·77–8·96)	4·46 (1·82–9·09)	-9·83 (-19—1·12)	2·24 (-2·78–8·94)	3·9 (1·58–7·84)	3·59 (1·46–7·31)	3·62 (1·48–7·36)	-7·92 (-18·7–4·19)	0·827 (-3·83–6·77)
High LDL cholesterol	50·9 (46·5–55·2)	49·3 (44·9–53·7)	48·2 (43·5–52·6)	-3·17 (-11·5–5·94)	-2·31 (-8·16–4·21)	57·9 (54·1–61·9)	58·5 (54·3–62·7)	59·4 (55·1–63·7)	0·958 (-6·56–8·94)	1·63 (-3·8–7·69)

Supplementary Results Table 1. Sweden except Stockholm age-standardised Summary Exposure Values (SEVs) for most-detailed risk factors and percentage change, by sex, 1990, 2010, and 2019. Data in parentheses are 95% uncertainty intervals.

	Male					Female				
	1990	2010	2019	Percentage change 1990-2010	Percentage change 2010-2019	1990	2010	2019	Percentage change 1990-2010	Percentage change 2010-2019
Unsafe sanitation	2·61 (1·49–4·24)	0·848 (0·455–1·47)	0·68 (0·372–1·14)	-67·5 (-84·1–-30·4)	-19·8 (-51·5–38·7)	2·61 (1·49–4·24)	0·848 (0·455–1·47)	0·68 (0·372–1·14)	-67·5 (-84·1–-30·4)	-19·8 (-51·5–38·7)
Ambient particulate matter pollution	7·68 (1·31–16·8)	4·63 (2·28–7·4)	2·4 (0·693–4·79)	-39·8 (-76·7–183)	-48 (-70·2–-34·4)	7·69 (1·38–16·7)	4·63 (2·3–7·4)	2·41 (0·699–4·79)	-39·8 (-76·6–179)	-48 (-70·1–-34·3)
Household air pollution from solid fuels	0·0533 (0·00976–0·16)	0·0135 (0·0022–0·0443)	0·00633 (0·000626–0·0235)	-74·7 (-90·4–-30·6)	-53·1 (-79·3–-21·5)	0·0704 (0·0138–0·214)	0·0179 (0·00295–0·0561)	0·00842 (0·000851–0·0307)	-74·5 (-90·3–-30·4)	-53 (-79·5–-21)
Ambient ozone pollution	24·3 (9·02–44·5)	28·5 (10·7–51·2)	20·6 (7·22–39·3)	17·1 (5·92–32·2)	-27·6 (-40·1–-16·2)	24·3 (9·02–44·5)	28·5 (10·7–51·2)	20·6 (7·22–39·3)	17·1 (5·92–32·2)	-27·6 (-40·1–-16·2)
Residential radon	25 (4·34–74·4)	25 (4·34–74·4)	25 (4·34–74·4)	0 (0–0)	0 (0–0)	25 (4·34–74·4)	25 (4·34–74·4)	25 (4·34–74·4)	0 (0–0)	0 (0–0)
Lead exposure	17·6 (1·6e-12–45·5)	14·4 (2·69e-12–38·6)	10·9 (3·12e-12–31·9)	-18·4 (-39·1–1·36e+13)	-24·3 (-50·5–7·81e+10)	7·16 (1·32e-12–29·5)	5·77 (1·02e-12–25·1)	4·46 (1·18e-12–21·2)	-19·4 (-75·1–381)	-22·7 (-67·3–332)
Child underweight	0·879 (0·501–1·29)	0·758 (0·435–1·14)	0·75 (0·422–1·12)	-13·8 (-25·1–0·58)	-1·03 (-10·6–8·49)	1·05 (0·594–1·53)	0·92 (0·529–1·34)	0·907 (0·513–1·34)	-12·3 (-23–0·282)	-1·38 (-9·6–7·94)
Iron deficiency	--	--	--	--	--	9·54 (7·96–11·4)	8·94 (7·39–10·7)	8·84 (7·34–10·5)	-6·27 (-15·3–3·66)	-1·21 (-7·07–4·69)
Vitamin A deficiency	3·93 (1·64–8·17)	1·52 (0·538–3·09)	1·31 (0·477–2·7)	-61·2 (-84·8–4·59)	-13·8 (-53·2–79)	3·11 (1·5–5·52)	1·83 (0·902–3·17)	1·56 (0·727–2·82)	-41·1 (-71·3–23·6)	-14·9 (-47·7–44)
Zinc deficiency	3·34 (0–10·4)	2·19 (0–7·16)	2·12 (0–6·84)	-34·3 (-100–NA)	-3·14 (-15·9–NA)	3·26 (0–10·2)	2·14 (0–7·17)	2·07 (0–6·68)	-34·2 (-100–NA)	-3·49 (-18·8–NA)
Smoking	16·1 (14·3–18)	12·4 (11–13·9)	11·6 (10·2–13)	-23·4 (-29·5–-17·3)	-6·29 (-11–-1·32)	16·5 (14·1–19·2)	14 (12·2–16·1)	12·1 (10·6–13·8)	-14·8 (-22·6–-6·07)	-13·8 (-19·3–-7·74)
Secondhand smoke	29·6 (26·9–32·2)	25·7 (23·5–27·9)	25·7 (23·1–28·4)	-13·1 (-22·9–-1·72)	0·057 (-6·69–7·04)	25 (23·1–26·8)	22·2 (20·7–23·5)	22·5 (20·7–24·5)	-11·1 (-19·2–-2·14)	1·41 (-4·52–7·46)
Alcohol use	16·5 (11·2–22·8)	17·8 (11·8–25·8)	17·9 (11·6–25·5)	7·85 (-9·28–27·7)	0·567 (-14·4–18)	--	--	--	--	--
Drug use	0·115 (0·0582–0·235)	0·246 (0·157–0·412)	0·3 (0·199–0·487)	113 (68–195)	21·9 (11·3–35·6)	0·0919 (0·044–0·19)	0·123 (0·0687–0·229)	0·138 (0·0776–0·253)	34·4 (16·8–69)	12 (3·92–21)
High fasting plasma glucose	7·29 (6·28–8·51)	11·1 (9·25–13·3)	11·8 (9·84–14)	52·8 (30·8–79·9)	5·53 (-9·88–23·9)	6·48 (5·45–7·66)	9·21 (7·62–11·1)	9·4 (7·93–11·1)	42·1 (20·3–69·6)	2·07 (-13·6–22·4)
High systolic blood pressure	35·1 (31·5–38·7)	34·9 (29·5–40·9)	37·2 (29·6–45·9)	-0·447 (-16·2–20)	6·47 (-11·6–26·6)	30·1 (25·7–34·6)	30 (24–36·5)	31·4 (23·1–42·1)	-0·232 (-21·4–26·6)	4·59 (-21·1–33·2)
High body-mass index	17·1 (11·9–23·9)	23·5 (16·8–31·4)	26·2 (19·2–34·9)	37·3 (23·5–57·7)	11·6 (4·83–20·3)	17·6 (12·4–24·5)	26·9 (21–35·6)	29·2 (22·8–38·4)	52·9 (32·7–85·3)	8·69 (-0·814–19·4)
Low bone mineral density	9·96 (5·57–16·1)	10 (5·61–16·2)	9·87 (5·28–16·3)	0·443 (-22·4–30·6)	-1·29 (-27·6–28·4)	18·7 (12·6–25·7)	19·5 (13·5–26·9)	19·5 (13–27·2)	4·37 (-17·6–33)	-0·382 (-21·2–25·7)
Diet low in fruits	52·6 (41–65·5)	44 (32·1–57·2)	42·8 (30·5–55·9)	-16·2 (-25·3–-9·15)	-2·76 (-11·5–5·99)	53·9 (42·5–66·8)	44·6 (33·2–58·1)	43·7 (31·5–56·3)	-17·3 (-26–-10·3)	-2 (-12·3–7·19)
Diet low in vegetables	66·9 (46·3–84·6)	55·7 (35·2–77·5)	53·4 (32·5–75·3)	-16·7 (-29·5–-6·77)	-4·13 (-12·1–1·46)	64 (43·3–82·9)	52·2 (31·2–74·2)	48·9 (28·5–71·5)	-18·4 (-32–-7·6)	-6·21 (-14·8–0·652)
Diet low in whole grains	87·3 (81·3–93·5)	86·1 (79·2–93·2)	87·8 (81·6–94·2)	-1·38 (-3·59–0·117)	1·97 (0·676–3·63)	--	--	--	--	--
Diet low in nuts and seeds	56·2 (25·1–75·9)	40 (13·8–63·7)	35·1 (10·8–60)	-28·7 (-50·1–-12·9)	-12·3 (-30·4–0·287)	55·7 (25·8–75·7)	42 (14·6–65·1)	37·3 (12·3–61·3)	-24·5 (-46·3–-10·4)	-11·2 (-28·5–0·504)
Diet low in milk	22 (7·4–40·6)	18·3 (5·23–37·6)	19·7 (5·82–39·1)	-16·6 (-47·7–3)	7·65 (-12·7–43·5)	21·6 (7·27–40·2)	18·1 (4·96–37·3)	19·6 (5·75–38·5)	-16·3 (-45·8–3·01)	8·63 (-10·4–46·6)
Diet high in red meat	72·1 (63·6–80)	81 (73·4–87·3)	82·8 (73·9–90)	12·3 (5·36–21·5)	2·18 (-4·97–10·2)	70·8 (61·4–80·4)	79 (70·5–86·4)	81·3 (70·8–89·1)	11·6 (4·42–21·5)	2·86 (-5·96–12)
Diet high in processed meat	81·1 (59·4–99·6)	85·6 (67·7–99·9)	86·6 (69·3–99·9)	5·45 (0·223–15·7)	1·17 (-1·05–5·41)	83·3 (63·2–99·9)	87·3 (71·6–100)	88·2 (72·5–100)	4·89 (0·0732–15)	0·976 (-1·58–5·35)
Diet high in sugar-sweetened beverages	30·2 (17·4–47)	40·1 (26–55·4)	40·6 (25–57·2)	32·7 (2·6–79·6)	1·27 (-17·5–22·6)	32·6 (13–55·7)	41·4 (17·1–64·9)	42·3 (17·9–64·3)	26·9 (-8·7–84·7)	2·34 (-22–40·3)
Diet low in fiber	44·8 (28·1–60·4)	33·3 (20–46·7)	30·6 (18·2–44·1)	-25·6 (-36·9–-15·8)	-8 (-21·2–4·51)	46·5 (30·3–62·4)	37·3 (22·6–51·9)	34·1 (20·2–49·6)	-19·8 (-28·6–-12·4)	-8·79 (-22·5–2·37)
Diet low in seafood omega-3 fatty acids	90·9 (78·8–100)	83·8 (65·6–100)	82·9 (64·2–99·8)	-7·8 (-22·4–1·46)	-0·994 (-16·1–14·7)	92·5 (81·9–100)	86·6 (70·1–100)	86·3 (70·3–99·8)	-6·35 (-20–1·28)	-0·354 (-11·7–13·2)
Diet low in polyunsaturated fatty acids	61·1 (21·6–98·9)	64·3 (23·8–99·5)	64·1 (23·9–99·3)	5·35 (0·0713–17·6)	-0·373 (-4·15–3·03)	64·7 (24·9–99·7)	67·4 (27–100)	67·7 (26·8–99·9)	4·27 (0–15·6)	0·458 (-2·41–4·44)
Diet high in trans fatty acids	36·5 (26·5–92·4)	30·5 (21–94)	30·6 (21·2–91·6)	-16·3 (-30·7–0)	0·206 (-8·98–12·6)	37·7 (27·1–100)	31·7 (20·5–98·6)	31·7 (20·9–98·2)	-15·8 (-31·6–0)	0·0537 (-8·48–11·7)
Diet high in sodium	34·4 (7·98–59·7)	32·8 (7·21–57·1)	34·2 (8·01–60·1)	-4·59 (-38·8–28·1)	4·31 (-12·3–38·7)	15·7 (4·05–37·3)	15·6 (4·02–37·5)	15·9 (3·94–38·5)	-0·927 (-27·4–21·5)	2·11 (-11·5–21·8)
Low physical activity	3·05 (1·16–6·32)	2·6 (0·861–5·73)	2·71 (0·955–6·05)	-15 (-36·6–5·2)	4·24 (-18·3–36·1)	4·66 (2·27–9·19)	5 (2·42–9·68)	4·69 (2·24–8·97)	7·27 (-7·88–25·2)	-6·22 (-19·5–7·72)
Occupational asthmagens	24 (20–29·6)	20·1 (17·3–23·7)	20·3 (17·3–24·1)	-16·2 (-25·2–-7·12)	1·11 (-6·15–10·8)	--	--	--	--	--
Occupational particulate matter, gases, and fumes	10·1 (7·19–14·4)	8·34 (5·97–12)	7·78 (5·57–11·2)	-17·4 (-22·2–-12·6)	-6·72 (-8·78–-4·38)	5·22 (3·71–7·66)	4·12 (2·91–6·03)	3·74 (2·66–5·49)	-21·1 (-28·2–-14·3)	-9·17 (-12·7–-5·47)
Occupational noise	9·25 (8·73–9·95)	8·12 (7·7–8·76)	7·82 (7·42–8·49)	-12·2 (-13·9–-10·4)	-3·69 (-4·63–-2·72)	6·9 (6·48–7·49)	6·39 (6·04–6·98)	6·31 (5·98–6·93)	-7·43 (-9·65–-5·2)	-1·23 (-2·36–-0·134)
Occupational injuries	--	--	--	--	--	--	--	--	--	--
Occupational ergonomic factors	12·1 (10·5–14)	11·1 (9·6–12·9)	11 (9·38–12·8)	-8·38 (-14·4–-2·08)	-1·16 (-6·6–4·95)	--	--	--	--	--
Childhood sexual abuse	6·07 (3·87–8·57)	6·12 (4·06–8·58)	5·32 (3·5–7·58)	0·884 (-6·13–9·01)	-13·2 (-19·1–-6·38)	12·4 (9·29–16·7)	16·8 (12·6–22·8)	16·6 (12·3–22·9)	35·7 (27·3–45·5)	-1·29 (-7·49–5·29)
Intimate partner violence	--	--	--	--	--	18·4 (10·4–25·8)	21·1 (12·1–29·1)	18·4 (10·4–25·8)	14·6 (5·2–29·3)	-12·7 (-18·7–1·57)
Non-exclusive breastfeeding	21·4 (17·6–26·1)	18·9 (14·9–23·8)	18·7 (14·8–23·9)	-11·8 (-23–-0·52)	-0·906 (-12·3–14·5)	21·4 (17·6–26·1)	18·9 (14·9–23·9)	18·7 (14·8–23·9)	-11·8 (-23–-0·512)	-0·923 (-12·4–14·5)
Discontinued breastfeeding	24 (22·6–25·3)	23·3 (22–24·6)	22·5 (21·1–23·8)	-2·94 (-10·2–5·61)	-3·26 (-10·2–4·41)	24 (22·6–25·3)	23·3 (22–24·6)	22·5 (21·1–23·8)	-2·92 (-10·2–5·64)	-3·26 (-10·2–4·41)
Diet low in calcium	9·19 (4·15–17·1)	8·16 (3·66–15·1)	7·83 (3·56–14·6)	-11·2 (-19·4–-3·58)	-4·04 (-11·5–5·46)	4·26 (1·74–7·6)	3·49 (1·44–6·27)	3·33 (1·38–6·09)	-18·1 (-33·2–-2·94)	-4·59 (-17·9–10·5)
Occupational exposure to asbestos	10·7 (9·72–11·9)	9·96 (9·21–10·8)	9 (7·15–11)	-7·27 (-17·5–5·1)	-9·64 (-28·6–11·7)	1·65 (1·26–2·63)	1·54 (1·23–2·14)	1·59 (1·11–2·2)	-6·34 (-28·2–18·6)	2·69 (-19·6–31·9)
Occupational exposure to arsenic	0·569 (0–1·33)	0·482 (0–1·14)	0·497 (0–1·18)	-15·2 (-23·1–NA)	3·12 (-5·59–NA)	0·55 (0–1·3)	0·451 (0–1·05)	0·462 (0–1·1)	-18·1 (-27·2–NA)	2·53 (-4·5–NA)
Occupational exposure to benzene	0·852 (0·0518–2·47)	0·929 (0·0568–2·65)	0·959 (0·057–2·71)	9·01 (4·43–15·2)	3·21 (-1·13–9·5)	1·58 (0·093–4·65)	1·71 (0·104–4·89)	1·84 (0·11–5·36)	8·26 (2·39–17·2)	7·53 (2·68–13·4)
Occupational exposure to beryllium	0·0249 (0·0244–0·0254)	0·0249 (0·0246–0·0251)	0·0256 (0·025–0·0263)	-0·0236 (-2·2–2·25)	3·07 (0·478–5·68)	0·0316 (0·031–0·0322)	0·0306 (0·0303–0·0309)	0·0323 (0·0315–0·0331)	-3·16 (-5·18–-1)	5·55 (2·82–8·49)
Occupational exposure to cadmium	0·0655 (0·0603–0·0712)	0·0568 (0·0546–0·0587)	0·0585 (0·0543–0·0629)	-13·4 (-20·6–-5·67)	3·02 (-4·52–10·6)	0·0661 (0·0602–0·0727)	0·0557 (0·0538–0·0575)	0·0574 (0·054–0·0609)	-15·9 (-23·9–-6·86)	3·16 (-3–10·3)
Occupational exposure to chromium	0·153 (0·142–0·165)	0·137 (0·133–0·141)	0·141 (0·133–0·151)	-10·4 (-17–-3·57)	3·19 (-3·17–9·83)	0·169 (0·157–0·181)	0·148 (0·144–0·152)	0·154 (0·147–0·162)	-12·1 (-18·9–-5·32)	4·2 (-0·709–9·62)
Occupational exposure to diesel engine exhaust	0·713 (0·677–0·752)	0·762 (0·741–0·783)	0·781 (0·748–0·816)	6·85 (1·15–12·8)	2·54 (-2·16–7·52)	0·738 (0·69–0·784)	0·676 (0·65–0·705)	0·708 (0·666–0·753)	-8·32 (-14·3–-1·52)	4·75 (-1·54–11·7)
Occupational exposure to formaldehyde	0·329 (0·3–0·359)	0·278 (0·265–0·289)	0·283 (0·263–0·307)	-15·5 (-22·8–-7·58)	1·95 (-5·62–9·89)	0·335 (0·307–0·366)	0·294 (0·283–0·303)	0·305 (0·289–0·322)	-12·4 (-19·9–-4·83)	3·9 (-1·15–9·66)
Occupational exposure to nickel	0·535 (0–2·39)	0·463 (0–2)	0·477 (0–2·1)	-13·5 (-20·1–NA)	3·1 (-4·05–NA)	0·432 (0–1·97)	0·344 (0–1·54)	0·35 (0–1·57)	-20·4 (-29·9–NA)	1·57 (-6·4–NA)
Occupational exposure to polycyclic aromatic hydrocarbons	0·31 (0·292–0·331)	0·285 (0·277–0·293)	0·295 (0·279–0·311)	-8·12 (-14·1–-1·95)	3·42 (-2·19–9·05)	--	--	--	--	--
Occupational exposure to silica	6·03 (0·906–19·1)	5·72 (0·826–18·4)	5·88 (0·866–19)	-5·13 (-12·2–3·29)	2·73 (-4·71–11·3)	3·71 (0·526–11·8)	2·92 (0·422–9·23)	2·94 (0·419–9·32)	-21·2 (-31·3–-9·63)	0·835 (-7·8–11·6)
Occupational exposure to sulfuric acid	0·898 (0·185–2·75)	0·754 (0·156–2·35)	0·771 (0·163–2·41)	-16·1 (-23·1–-8·54)	2·25 (-5·2–10·4)	0·785 (0·163–2·42)	0·626 (0·128–1·96)	0·637 (0·13–2·04)	-20·3 (-28·9–-10·8)	1·76 (-5·8–10·4)
Unsafe sex	--	--	--	--	--	--	--	--	--	--
Occupational exposure to trichloroethylene	0·0837 (0·0798–0·0883)	0·0788 (0·0772–0·0808)	0·0811 (0·0778–0·0852)	-5·92 (-10·6–-0·969)	2·94 (-1·78–7·81)	0·0965 (0·0917–0·103)	0·0859 (0·0841–0·0883)	0·0897 (0·0862–0·0941)	-11 (-15·9–-5·52)	4·43 (0·108–9·58)
No access to handwashing facility	3·3 (3·1–3·49)	3·16 (2·98–3·34)	3·12 (2·96–3·3)	-4·26 (-10·4–2·51)	-1·06 (-4·86–3·12)	3·3 (3·1–3·49)	3·16 (2·98–3·34)	3·12 (2·96–3·3)	-4·26 (-10·4–2·51)	-1·06 (-4·86–3·12)
Child wasting	1·26 (0·878–1·67)	1·21 (0·858–1·62)	1·2 (0·845–1·58)	-3·83 (-12·3–5·53)	-0·803 (-7·33–6·07)	--	--	--	--	--
Child stunting	1·57 (0·795–2·35)	1·18 (0·563–1·82)	1·17 (0·553–1·83)	-24·9 (-38·8–-8·85)	-0·744 (-13·7–12·9)	1·86 (0·959–2·7)	1·54 (0·766–2·33)	1·52 (0·755–2·3)	-16·9 (-31·1–0·934)	-1·31 (-12·5–11·5)
Chewing tobacco	1·88 (1·41–2·41)	1·93 (1·48–2·47)	1·88 (1·41–2·41)	2·77 (-25·8–40·4)	-2·74 (-20·5–17·3)	0·348 (0·236–0·508)	0·384 (0·258–0·559)	0·387 (0·265–0·547)	10·2 (-31·5–73·3)	0·848 (-25·9–37·7)
Diet low in legumes	90·2 (77·4–100)	82·1 (58·3–100)	82·5 (59·5–100)	-9·03 (-25·4–0)	0·54 (-1·92–4·74)	88 (71·8–100)	79·3 (50·9–100)	79·8 (52·6–99·9)	-9·93 (-30·6–-0·0313)	0·596 (-2·34–4·99)
Short gestation	12·8 (10·3–15·5)	11·4 (9·04–14·1)	10·7 (8·64–13·3)	-11 (-31·4–16·4)	-6·06 (-27·3–22·3)	11·3 (9·66–13·2)	9·73 (7·96–11·7)	10·4 (8·35–13·2)	-13·6 (-31·9–8·14)	6·76 (-17·7–39·5)
Low birth weight	5·28 (4·29–6·48)	4·54 (3·69–5·65)	4·34 (3·5–5·36)	-14·1 (-34·3–16·2)	-4·43 (-26·5–25·8)	4·95 (4·23–5·78)	3·88 (3·12–4·73)	4·1 (3·26–5·19)	-21·7 (-39·1–2·3)	5·66 (-20·7–40·5)
High temperature	20·5 (9·44–33·3)	21·4 (11·8–32·7)	21·4 (11·1–33·1)	4·45 (-31·4–80·8)	-0·0808 (-12·8–11·1)	20·5 (9·44–33·3)	21·4 (11·8–32·7)	21·4 (11·1–33·1)	4·45 (-31·4–80·8)	-0·0808 (-12·8–11·1)
Low temperature	33·7 (28·8–39·1)	37·3 (33·5–42·2)	33·8 (29·4–39·2)	10·9 (4·64–19)	-9·4 (-14·2–-5·36)	33·7 (28·8–39·1)	37·3 (33·5–42·2)	33·8 (29·4–39·2)	10·9 (4·64–19)	-9·4 (-14·2–-5·36)
Kidney dysfunction	20 (13·5–27·7)	20·7 (14·2–28·4)	20·6 (14·1–28·2)	3·73 (0·335–8·48)	-0·604 (-3·76–2·57)	15 (9·9–21·6)	14·8 (9·71–21·4)	14·7 (9·62–21·4)	-1·23 (-5·15–2·5)	-0·738 (-4·34–2·66)
Bullying victimization	6·73 (2·68–13·5)	5·56 (2·3–11·2)	5·8 (2·38–11·6)	-17·5 (-28·4–-7·32)	4·34 (-1·58–15·5)	5·48 (2·2–11·2)	4·61 (1·86–9·43)	4·72 (1·95–9·48)	-15·9 (-26·5–-5·26)	2·49 (-3·12–11·7)
High LDL cholesterol	62·4 (59–65·5)	43·6 (40·1–47·2)	42·7 (39·1–46·5)	-30·1 (-35·2–-25·1)	-1·95 (-7·13–2·67)	57·3 (54·4–60·3)	41·9 (38·4–45·2)	43 (39·3–46·4)	-26·9 (-31·2–-22·6)	2·55 (-1·24–6·63)

Supplementary Results Table 1. Uzbekistan age-standardised Summary Exposure Values (SEVs) for most-detailed risk factors and percentage change, by sex, 1990, 2010, and 2019. Data in parentheses are 95% uncertainty intervals.

	Male					Female				
	1990	2010	2019	Percentage change 1990-2010	Percentage change 2010-2019	1990	2010	2019	Percentage change 1990-2010	Percentage change 2010-2019
Unsafe sanitation	68·4 (61·4–75·1)	69·1 (62·5–75·9)	56·5 (47·3–66·8)	0·995 (-7·18–10·9)	-18·2 (-29·2–-8·22)	68·4 (61·4–75·1)	69·1 (62·5–75·9)	56·5 (47·3–66·8)	0·995 (-7·18–10·9)	-18·2 (-29·2–-8·22)
Ambient particulate matter pollution	23·1 (8·76–45·4)	27·6 (15·6–42·3)	31·3 (17·7–47·6)	19·8 (-30·3–132)	13·3 (-8·85–42·9)	20·6 (7·66–42·6)	26·2 (14·4–41·2)	30·5 (17–47)	26·8 (-28·3–150)	16·4 (-6·7–47·4)
Household air pollution from solid fuels	10·7 (5·1–19·7)	3·5 (1·49–7·08)	1·69 (0·624–3·78)	-67·4 (-81·6–-42·3)	-51·7 (-69·4–-29·9)	12·3 (6·32–21·1)	4·33 (1·99–8·15)	2·14 (0·82–4·58)	-64·9 (-79·5–-40·4)	-50·7 (-68·7–-28·6)
Ambient ozone pollution	89·4 (44·3–100)	72·2 (31·6–100)	76·9 (35·1–100)	-19·2 (-33·5–0)	6·44 (0–15·5)	89·4 (44·3–100)	72·2 (31·6–100)	76·9 (35·1–100)	-19·2 (-33·5–0)	6·44 (0–15·5)
Residential radon	27·3 (3·56–99)	27·3 (3·56–99)	27·3 (3·56–99)	0 (0–0)	0 (0–0)	--	--	--	--	--
Lead exposure	58·9 (33·2–79)	49·9 (27·6–70·1)	41 (20·9–61·9)	-15·2 (-24·7–-6·23)	-17·8 (-26·1–-11·1)	43 (13·1–68·1)	38·9 (15–62·6)	32·6 (11·1–55·9)	-9·54 (-21·4–27·9)	-16 (-27·2–-9·6)
Child underweight	3·77 (2·58–4·83)	2·6 (1·71–3·47)	2·14 (1·37–2·96)	-31 (-44·7–-18·4)	-17·7 (-29·1–-6·31)	4·65 (3·55–5·76)	3·12 (2·22–4·05)	2·56 (1·77–3·47)	-32·8 (-41·9–-24·4)	-17·9 (-27·1–-9·25)
Iron deficiency	--	--	--	--	--	23·8 (21·3–26·2)	21·4 (19–23·8)	20·4 (18–23)	-10·2 (-18·4–-1·44)	-4·51 (-9·45–0·892)
Vitamin A deficiency	20·8 (9·15–36·8)	7·28 (2·82–14·7)	4·52 (1·84–9·26)	-65·1 (-84·1–-18·6)	-37·9 (-63·9–10·3)	10·9 (5·4–17·8)	6·54 (3·51–11)	4·72 (2·37–8·46)	-39·8 (-68–22·2)	-27·8 (-51·9–10·9)
Zinc deficiency	9·78 (1·13–22·9)	9·48 (1·09–22·7)	6·2 (0·298–17)	-3 (-21–15·2)	-34·7 (-71·6–-21·9)	9·44 (1·06–22·7)	9·18 (0·853–22·1)	6·02 (0·206–16·7)	-2·81 (-19·4–15·5)	-34·5 (-72·9–-22·4)
Smoking	13·9 (11·6–16·3)	14·1 (12·4–16·1)	14·9 (13–17)	1·42 (-8·77–12·5)	5·3 (0·404–10·5)	0·855 (0·624–1·14)	0·882 (0·675–1·13)	0·972 (0·745–1·23)	3·05 (-18·5–30·1)	10·2 (-3·1–25·5)
Secondhand smoke	26·4 (24·4–28·5)	28 (26·3–29·6)	26·4 (24·2–28·7)	6·03 (-3·65–16·7)	-5·63 (-11·4–0·757)	37·6 (35·7–39·4)	38·4 (36·6–40)	36 (33·9–38·2)	2 (-5·08–9·41)	-6·07 (-10·1–-1·93)
Alcohol use	7·5 (4·69–11)	8·48 (5·85–11·8)	9·27 (6·13–13)	13 (-9·77–49·5)	9·31 (-7·53–26·6)	1·13 (0·553–2·08)	1·44 (0·786–2·57)	1·65 (0·876–2·97)	28 (-10·7–87·5)	14·2 (-11·3–45·5)
Drug use	0·118 (0·0597–0·233)	0·146 (0·0875–0·267)	0·15 (0·0891–0·269)	23·2 (8·7–48·7)	2·83 (-5·48–10·6)	0·0667 (0·0328–0·135)	0·078 (0·0423–0·147)	0·081 (0·0458–0·153)	17·1 (3·68–37·2)	3·85 (-3·87–12·4)
High fasting plasma glucose	5·17 (4·41–5·99)	11·5 (9·23–13·9)	14·2 (11·9–16·6)	122 (83·8–167)	23·5 (6·22–49·9)	4·28 (3·71–4·91)	9·93 (8·19–11·9)	11·9 (9·95–14·2)	132 (91·4–181)	20·1 (2·45–46·4)
High systolic blood pressure	22·2 (18·2–26·9)	26·9 (22·7–31·6)	32·5 (27·2–38·1)	21·2 (-3·39–55·1)	20·6 (0·946–45·3)	21·1 (15·8–26·6)	24·7 (19·7–30·2)	28·5 (21·9–35·7)	17·1 (-15·1–65·3)	15·6 (-10·7–51·6)
High body-mass index	16·5 (11·7–22·8)	21·2 (16–27·9)	27·9 (21·8–36·1)	28·1 (13·5–47·7)	31·6 (22·2–45·4)	21·1 (15·5–29·1)	26·6 (20·6–35·4)	33·5 (26·6–43·6)	26 (11·7–44·7)	26 (16–38·6)
Low bone mineral density	8·02 (4·15–13·6)	7·66 (3·89–13·4)	7·31 (3·8–12·4)	-4·5 (-28·7–25·3)	-4·66 (-26·8–25·7)	15·1 (9·77–22·3)	14·6 (9·44–21·1)	13·8 (8·54–20·4)	-3·53 (-24·4–23·1)	-5·61 (-27·1–20·6)
Diet low in fruits	66·7 (56·9–78·4)	53·1 (41·4–66·6)	47 (34·8–61·1)	-20·4 (-29·3–-12·4)	-11·5 (-20·3–-4·88)	68 (58·6–79·1)	54·6 (42·9–67·7)	48·4 (36·6–61·7)	-19·8 (-28·7–-12·1)	-11·2 (-19·4–-4·43)
Diet low in vegetables	11·1 (4·08–25·4)	2·81 (1·44–8·46)	2·64 (1·32–8·31)	-74·7 (-89·2–-32·8)	-5·8 (-20–0·0688)	8·61 (3·15–21·9)	2·73 (1·38–8·67)	2·62 (1·29–8·49)	-68·3 (-87·7–-24·3)	-3·87 (-14·3–0·226)
Diet low in whole grains	99·9 (100–100)	99·9 (100–100)	99·9 (100–100)	0 (0–0)	0 (0–0)	99·9 (100–100)	99·9 (100–100)	99·9 (100–100)	-1·24e-05 (0–0)	1·24e-05 (0–0)
Diet low in nuts and seeds	93·8 (48·6–99·3)	93·5 (47·6–98·2)	92·7 (47·4–97·8)	-0·326 (-4·45–8·13)	-0·924 (-4·5–1·94)	93·5 (48–98·4)	91 (45·8–96·6)	90 (45·1–96·2)	-2·62 (-7·39–3·76)	-1·08 (-6·37–3·65)
Diet low in milk	44·9 (19·1–65·9)	52·6 (26·4–73·8)	59·3 (33·2–80·8)	17·1 (4·49–45·9)	12·8 (1·46–40·6)	43·8 (18·8–64·1)	51·7 (25·3–72·9)	58·7 (31·9–80·2)	18 (4·77–50·7)	13·7 (1·55–44·5)
Diet high in red meat	46·1 (34·9–57)	47·7 (36·3–58·7)	56·3 (43·9–67·2)	3·43 (0·73–7·22)	18 (6·83–34·9)	46 (34·7–56·9)	47·6 (36·4–58·5)	56·2 (45–66·7)	3·37 (0·487–7·18)	18·1 (6·26–36·9)
Diet high in processed meat	44·4 (24·3–69·8)	42·3 (22·8–65·7)	44·8 (24·8–69·8)	-4·78 (-26·8–22·4)	5·87 (-7·8–25·5)	--	--	--	--	--
Diet high in sugar-sweetened beverages	21·1 (11·7–34·8)	21·4 (11·8–35·5)	23·4 (12·8–38·7)	1·09 (-31·6–51)	9·51 (-20·7–50·8)	21·1 (10·6–35·8)	20·9 (10·9–34·5)	24·4 (12·5–39·4)	-0·798 (-37–57·1)	16·4 (-16·6–65·6)
Diet low in fiber	47·2 (31·4–62·6)	34·3 (20·5–48·5)	26 (14·6–38·9)	-27·3 (-36–-20·8)	-24·2 (-36·1–-13·3)	47·3 (30·9–63·5)	34·1 (19·9–48·9)	25·5 (13·9–39·3)	-28 (-37·2–-20·6)	-25·1 (-37·8–-14·3)
Diet low in seafood omega-3 fatty acids	98·4 (93·9–100)	98·4 (93·9–100)	95·3 (87·2–100)	-0·0654 (-3·43–3·75)	-3·13 (-10·1–0)	99·2 (96·5–100)	99·2 (96·6–100)	96·6 (90·2–100)	-0·0144 (-2·18–2·16)	-2·55 (-8·6–0)
Diet low in polyunsaturated fatty acids	85·3 (53·1–100)	82·7 (48·8–100)	79·4 (43·3–100)	-3·07 (-9·85–0)	-3·99 (-13–0)	86·3 (56–100)	83·8 (50·5–100)	80·6 (45·6–100)	-2·9 (-9·35–0)	-3·74 (-11·7–0)
Diet high in trans fatty acids	28·7 (19·5–89·3)	26·5 (17–94·2)	26·6 (16·6–93·3)	-7·84 (-24·8–7·07)	0·344 (-7·59–8·18)	30·4 (19·8–97·4)	28·7 (17·4–100)	28·8 (17·2–100)	-5·3 (-21·7–11)	0·0226 (-7·69–7·49)
Diet high in sodium	61·5 (39·2–78·6)	44·1 (14·9–67·8)	44·3 (14·3–70·8)	-28·3 (-65·8–5·25)	0·521 (-20·3–21·7)	30·1 (11·2–52·3)	19 (4·59–42·2)	18·7 (4·4–42·7)	-37·1 (-69·7–14·5)	-1·49 (-20·8–11·8)
Low physical activity	1·84 (0·886–3·99)	1·85 (0·885–3·97)	2·18 (1·05–4·41)	0·698 (-10·8–13·9)	17·3 (4·31–36·9)	1·37 (0·717–2·93)	1·33 (0·704–2·89)	1·54 (0·836–3·14)	-2·97 (-16·6–10·6)	16·2 (2·71–39)
Occupational asthmagens	40·9 (33·6–49·1)	30·9 (25–38·1)	28·1 (22·7–34·6)	-24·5 (-36·6–-11·7)	-9 (-18·1–2·04)	13·5 (10–18·3)	14·7 (10·9–19·8)	14·4 (10·6–19·4)	8·71 (-14·4–35·6)	-1·74 (-16·9–15·6)
Occupational particulate matter, gases, and fumes	16·9 (13·2–21·8)	15·8 (12·4–20·4)	15 (11·8–19·5)	-6·83 (-10·9–-2·8)	-4·94 (-6·88–-3·01)	5·93 (4·35–8·26)	5·66 (4·29–7·78)	5·96 (4·48–8·24)	-4·48 (-12·2–5·86)	5·29 (1·06–10·4)
Occupational noise	19·7 (18·2–21·2)	18·6 (17·6–19·6)	17·8 (17–18·8)	-5·48 (-9–-1·75)	-4·04 (-5·52–-2·51)	7·27 (6·23–8·39)	7·22 (6·64–7·86)	7·92 (7·48–8·54)	-0·739 (-7·81–8·3)	9·7 (6·18–13·8)
Occupational injuries	--	--	--	--	--	--	--	--	--	--
Occupational ergonomic factors	40·7 (35·7–45·6)	33·2 (28·5–38·5)	29·8 (25·2–34·7)	-18·4 (-31·1–-4·49)	-10·4 (-21·1–1·39)	--	--	--	--	--
Childhood sexual abuse	2·52 (1·65–3·56)	2·52 (1·65–3·56)	2·52 (1·65–3·56)	0 (0–0)	0 (0–0)	3·14 (2·26–4·3)	3·14 (2·26–4·3)	3·14 (2·26–4·3)	0 (0–0)	0 (0–0)
Intimate partner violence	--	--	--	--	--	11·3 (6·65–16·1)	11·3 (6·65–16·1)	11·3 (6·65–16·1)	0 (0–0)	0 (0–0)
Non-exclusive breastfeeding	26·6 (16–40·5)	23·6 (14·5–35·8)	24 (14·5–36·5)	-11·1 (-16·1–-5·13)	1·64 (-4·26–8·07)	26·5 (16–40·4)	23·6 (14·5–35·8)	24 (14·5–36·5)	-11 (-16–-5·05)	1·69 (-4·22–8·12)
Discontinued breastfeeding	10·6 (9·2–12·2)	10 (8·52–11·5)	9·61 (8·25–11·2)	-5·45 (-23·5–16·5)	-4·25 (-21·5–18)	10·6 (9·2–12·2)	10 (8·5–11·5)	9·61 (8·24–11·2)	-5·67 (-23·7–16·3)	-4·07 (-21·4–18·2)
Diet low in calcium	37·1 (23·1–60)	36·1 (22·3–59)	28·8 (15·9–50·9)	-2·91 (-6·47–-0·615)	-20·2 (-31·6–-9·38)	--	--	--	--	--
Occupational exposure to asbestos	0·651 (0·545–0·773)	0·411 (0·331–0·5)	0·745 (0·559–0·949)	-36·9 (-47·2–-24·4)	81·4 (40·4–135)	0·23 (0·0835–0·579)	0·354 (0·248–0·749)	0·551 (0·334–0·765)	54·1 (-3·85–244)	55·4 (-19·5–138)
Occupational exposure to arsenic	0·363 (0·147–0·607)	0·351 (0·142–0·598)	0·353 (0·143–0·598)	-3·24 (-9·43–4·03)	0·617 (-2·95–4·42)	0·201 (0·082–0·335)	0·284 (0·115–0·479)	0·31 (0·125–0·522)	41·3 (25·7–59·8)	9·19 (3·67–15·6)
Occupational exposure to benzene	0·947 (0·581–1·64)	1·05 (0·639–1·87)	1·12 (0·681–2·01)	10·5 (2·46–22·5)	7·07 (1·59–14·5)	0·605 (0·367–1·12)	1·02 (0·621–1·8)	1·26 (0·774–2·22)	68·7 (44·9–101)	23·8 (15·2–35·2)
Occupational exposure to beryllium	0·15 (0·141–0·159)	0·137 (0·134–0·139)	0·134 (0·131–0·138)	-8·95 (-13·9–-2·61)	-1·78 (-4·29–0·917)	0·0812 (0·0723–0·091)	0·11 (0·108–0·113)	0·119 (0·114–0·125)	35·7 (20·9–53·8)	7·99 (3·04–13)
Occupational exposure to cadmium	0·202 (0·19–0·213)	0·198 (0·193–0·203)	0·2 (0·193–0·208)	-1·91 (-7·68–5·03)	1·09 (-2·19–4·58)	0·113 (0·1–0·127)	0·161 (0·156–0·167)	0·177 (0·168–0·187)	42·6 (27·2–61·1)	9·85 (4·32–15·7)
Occupational exposure to chromium	0·456 (0·428–0·483)	0·461 (0·446–0·476)	0·477 (0·458–0·497)	1·05 (-5·65–8·7)	3·62 (0·0365–7·51)	0·249 (0·221–0·279)	0·373 (0·357–0·39)	0·428 (0·401–0·454)	49·9 (32·4–70·5)	14·7 (8·22–21·8)
Occupational exposure to diesel engine exhaust	6·16 (5·57–6·79)	4·91 (4·55–5·32)	4·8 (4·48–5·16)	-20·3 (-29·8–-8·72)	-2·15 (-8·6–4·46)	2·14 (1·86–2·43)	2·57 (2·39–2·78)	2·81 (2·57–3·06)	20·2 (2·58–41·3)	9·22 (0·856–18·1)
Occupational exposure to formaldehyde	0·806 (0·641–0·871)	0·829 (0·679–0·881)	0·865 (0·706–0·93)	2·76 (-4·79–11·1)	4·38 (0·0939–9·16)	--	--	--	--	--
Occupational exposure to nickel	0·928 (0·298–2·38)	0·63 (0·211–1·59)	0·568 (0·185–1·45)	-32·1 (-41·4–-19·5)	-9·77 (-17·1–0·76)	0·32 (0·105–0·839)	0·332 (0·109–0·85)	0·335 (0·11–0·884)	3·83 (-10·9–22·7)	0·998 (-5·63–8·6)
Occupational exposure to polycyclic aromatic hydrocarbons	1·1 (1·03–1·17)	1·07 (1·03–1·11)	1·1 (1·05–1·15)	-3·12 (-10·1–4·89)	2·89 (-0·949–6·98)	0·563 (0·499–0·631)	0·828 (0·789–0·867)	0·957 (0·896–1·01)	47 (29·7–67·5)	15·6 (9·04–23·1)
Occupational exposure to silica	12·2 (6·33–27·8)	8·29 (4·28–18·7)	7·6 (3·92–17·5)	-32 (-42·7–-18·5)	-8·33 (-17·2–2·73)	3·66 (1·83–7·99)	3·34 (1·7–7·11)	3·25 (1·69–7·05)	-8·67 (-23·5–10·5)	-2·72 (-10·7–6·59)
Occupational exposure to sulfuric acid	1·21 (0·872–2·15)	1·25 (0·902–2·21)	1·27 (0·907–2·23)	3·34 (-6·79–14·1)	1·35 (-5·63–8·66)	0·579 (0·408–1·04)	0·802 (0·584–1·4)	0·844 (0·614–1·5)	38·4 (22·2–57·7)	5·31 (-1·21–12·5)
Unsafe sex	--	--	--	--	--	--	--	--	--	--
Occupational exposure to trichloroethylene	0·272 (0·257–0·289)	0·295 (0·288–0·306)	0·31 (0·299–0·324)	8·46 (1·72–16·4)	5·02 (1·43–8·97)	0·157 (0·139–0·177)	0·242 (0·231–0·256)	0·275 (0·257–0·294)	53·7 (36·1–75·4)	13·9 (7·23–21·4)
No access to handwashing facility	29·2 (27·9–30·4)	19·9 (19–20·8)	14·8 (14·1–15·6)	-31·7 (-35·2–-28·3)	-25·4 (-27·9–-22·9)	--	--	--	--	--
Child wasting	4·62 (3·83–5·34)	4·15 (3·38–4·91)	3·95 (3·22–4·67)	-10·2 (-21·1–3)	-4·63 (-13·8–4·88)	4·95 (4·08–5·69)	4·76 (3·92–5·52)	4·54 (3·79–5·23)	-3·99 (-15·4–7·82)	-4·48 (-13·5–4·75)
Child stunting	21·2 (14·7–24·5)	13·9 (9·58–16·9)	12 (8·23–14·8)	-34·2 (-45·5–-22·9)	-14·1 (-25·3–-2·54)	23·2 (16–26·3)	12·9 (8·64–15·9)	11 (7·2–14)	-44·3 (-54·4–-34·5)	-14·6 (-27·3–-2·76)
Chewing tobacco	6·29 (4·82–8·11)	6·46 (5–8·26)	6·9 (5·35–8·81)	2·8 (-25·3–41·8)	6·71 (-10·5–29·1)	--	--	--	--	--
Diet low in legumes	100 (100–100)	100 (100–100)	100 (100–100)	0 (0–0)	0 (0–0)	100 (100–100)	100 (100–100)	100 (100–100)	0 (0–0)	0 (0–0)
Short gestation	4·9 (3·79–6·03)	5·61 (4·75–6·73)	9·34 (7·66–11·6)	14·5 (-12·1–50·6)	66·5 (30·2–111)	4·15 (3·28–5·32)	4·69 (4·11–5·32)	6·94 (5·91–8·1)	13 (-12·1–42·6)	48 (25·9–73)
Low birth weight	2·28 (1·77–2·79)	2·52 (2·13–3·02)	3·8 (3·15–4·73)	10·7 (-14·6–48·6)	50·8 (15·5–95·6)	2·26 (1·81–2·81)	2·61 (2·29–3·01)	3·55 (3·02–4·19)	15·5 (-10·2–48·1)	35·8 (11·9–63·3)

High temperature	36·6 (28·4–44·7)	37·3 (29·3–45·8)	43·4 (34·8–51·4)	2·16 (-2·05–4·95)	16·3 (11·2–21·9)	36·6 (28·4–44·7)	37·3 (29·3–45·8)	43·4 (34·8–51·4)	2·16 (-2·05–4·95)	16·3 (11·2–21·9)
Low temperature	39 (35·7–43·7)	36·8 (33·4–41·6)	37·9 (34·2–43·3)	-5·75 (-7·15–-4·3)	3·12 (0·377–5·49)	39 (35·7–43·7)	36·8 (33·4–41·6)	37·9 (34·2–43·3)	-5·75 (-7·15–-4·3)	3·12 (0·377–5·49)
Kidney dysfunction	21·4 (14·5–29·3)	24·1 (16·9–32·2)	25·8 (18·2–33·8)	12·6 (8·12–19·1)	7·07 (3·96–11·4)	22·3 (15·4–30·4)	24·1 (16·9–32·6)	25·6 (18·2–34)	7·98 (4·56–13·1)	6·11 (2·72–10·4)
Bullying victimization	2·55 (1·05–5·22)	2·64 (1·1–5·4)	2·62 (1·09–5·34)	3·64 (-0·127–8·63)	-0·632 (-2·7–1·51)	1·47 (0·609–2·96)	1·57 (0·654–3·18)	1·57 (0·654–3·2)	6·92 (1·74–14·3)	-0·0258 (-2·38–2·42)
High LDL cholesterol	31·3 (27·8–34·9)	32·7 (29·2–36·2)	34·1 (30·8–37·7)	4·69 (-1·25–10·7)	4·22 (0·0937–8·78)	37·1 (33·7–40·7)	38·8 (35·5–42·3)	40·8 (37·6–44·4)	4·7 (-0·367–10·1)	4·97 (1·32–8·95)

Supplementary Results Table 1. Switzerland age-standardised Summary Exposure Values (SEVs) for most-detailed risk factors and percentage change, by sex, 1990, 2010, and 2019. Data in parentheses are 95% uncertainty intervals.

	Male					Female				
	1990	2010	2019	Percentage change 1990-2010	Percentage change 2010-2019	1990	2010	2019	Percentage change 1990-2010	Percentage change 2010-2019
Unsafe sanitation	1·03 (0·585–1·69)	0·473 (0·254–0·782)	0·404 (0·235–0·655)	-54·1 (-76·6–-8·59)	-14·7 (-49·6–42)	1·03 (0·585–1·69)	0·473 (0·254–0·782)	0·404 (0·235–0·655)	-54·1 (-76·6–-8·59)	-14·7 (-49·6–42)
Ambient particulate matter pollution	17·1 (6·61–30·8)	11·8 (8·87–15·2)	7·79 (5·13–10·9)	-30·9 (-62·6–65·9)	-34·2 (-42·8–-28·2)	17·1 (6·64–30·8)	11·8 (8·87–15·2)	7·79 (5·13–10·9)	-30·9 (-62·6–65·5)	-34·2 (-42·8–-28·2)
Household air pollution from solid fuels	0·0142 (0·00277–0·045)	0·0063 (0·00112–0·0193)	0·00428 (0·000643–0·0139)	-55·6 (-78·1–-13·8)	-32·2 (-59·1–8·79)	0·0186 (0·00378–0·0587)	0·00834 (0·00151–0·0243)	0·00567 (0·000914–0·0183)	-55·3 (-78–-13·4)	-32 (-59·1–8·94)
Ambient ozone pollution	69·3 (30·5–100)	62·9 (26·5–100)	65·8 (28·4–100)	-9·26 (-17·3–0)	4·53 (-2·13–13·1)	69·3 (30·5–100)	62·9 (26·5–100)	65·8 (28·4–100)	-9·26 (-17·3–0)	4·53 (-2·13–13·1)
Residential radon	31 (13·1–62·3)	31 (13·1–62·3)	31 (13·1–62·3)	0 (0–0)	0 (0–0)	31 (13·1–62·3)	31 (13·1–62·3)	31 (13·1–62·3)	0 (0–0)	0 (0–0)
Lead exposure	34·2 (3·04–61·9)	30·1 (6·26–55·3)	22·9 (3·52–47·1)	-11·8 (-23·2–115)	-24·1 (-46·1–-14·2)	18 (3·47e-12–44·9)	14·9 (0·00183–39·2)	11 (0·00301–32·5)	-17 (-30·2–3·23e+12)	-26·1 (-65·4–27·2)
Child underweight	0·863 (0·479–1·29)	0·75 (0·42–1·13)	0·744 (0·417–1·13)	-13 (-24·2–-1·22)	-0·868 (-10·3–10·1)	1·01 (0·591–1·49)	0·91 (0·505–1·33)	0·901 (0·504–1·34)	-10·1 (-21·1–2·17)	-0·926 (-9·31–7·95)
Iron deficiency	--	--	--	--	--	9·71 (8·09–11·6)	8·94 (7·41–10·6)	8·9 (7·36–10·5)	-7·89 (-16·8–1·12)	-0·507 (-6·34–5·95)
Vitamin A deficiency	1·87 (0·731–3·99)	1 (0·378–2·08)	0·882 (0·349–1·75)	-46·4 (-77·6–51·9)	-12 (-50·7–75·6)	1·68 (0·833–2·89)	1·27 (0·617–2·15)	1·14 (0·56–1·91)	-24·3 (-61·4–55·3)	-10·5 (-41–38·9)
Zinc deficiency	2·71 (0–8·7)	3·28 (0–10·1)	2·92 (0–8·9)	20·9 (6·56–NA)	-11 (-40·1–NA)	2·64 (0–8·44)	3·17 (0–9·95)	2·83 (0–8·78)	20·3 (5·95–NA)	-10·8 (-38·3–NA)
Smoking	24·7 (21·8–27·4)	20·3 (17·9–22·9)	17·5 (15·2–19·9)	-17·5 (-23·5–-11·4)	-13·9 (-18·1–-9·63)	14·6 (12·7–16·7)	13·8 (12–15·9)	12·1 (10·3–14·1)	-5·18 (-14–4·66)	-12·6 (-18·6–-6·31)
Secondhand smoke	45·2 (41·7–48·7)	34·1 (31·1–36·9)	33·1 (30·3–35·8)	-24·6 (-31·5–-17·4)	-2·84 (-8·55–3·3)	44·9 (42·4–47·6)	34·5 (32·2–36·9)	33·6 (31·2–36)	-23·2 (-28·8–-17)	-2·58 (-7·55–2·31)
Alcohol use	20·6 (15·2–27)	19·6 (13·6–26·7)	19·9 (13·2–28·6)	-4·92 (-14·4–7·09)	1·6 (-12·3–15·1)	7·59 (3·23–14·1)	5·79 (2·08–11·4)	5·49 (1·57–10·9)	-23·8 (-42·9–-10·8)	-5·22 (-35·7–23·6)
Drug use	0·382 (0·275–0·56)	0·28 (0·175–0·478)	0·289 (0·183–0·48)	-26·8 (-43·8–-9·97)	3·17 (-6·49–14·3)	0·24 (0·166–0·365)	0·198 (0·125–0·328)	0·203 (0·131–0·321)	-17·5 (-32·3–-4·38)	2·39 (-5·6–12·7)
High fasting plasma glucose	7·4 (6·49–8·56)	9·28 (8·11–10·5)	10·9 (9·3–13·2)	25·4 (12–40·4)	17·2 (2·6–36·1)	6·59 (5·6–7·77)	8·52 (7·31–9·83)	9·2 (7·83–10·9)	29·4 (13·2–48·8)	7·94 (-3·7–22·5)
High systolic blood pressure	29·2 (26·2–32·5)	25·1 (21·9–28·7)	26·8 (21·6–32·4)	-14·2 (-25·3–-1·86)	6·67 (-10·3–26·4)	20·4 (17·2–23·8)	12·3 (10·2–14·5)	12·4 (8·8–16·8)	-39·8 (-51·9–-24·5)	0·799 (-26–33·9)
High body-mass index	19·5 (14·3–25·9)	21·7 (16·5–28·4)	23·7 (17·8–30·9)	11·6 (2·14–23·3)	9·06 (1·84–17·1)	17·7 (13–23·3)	20·1 (15·2–26·1)	23·1 (18–29·6)	13·9 (0·336–30·6)	14·8 (4·15–29·5)
Low bone mineral density	9·94 (5·46–16·1)	9·11 (4·93–15)	8·8 (4·49–14·8)	-8·37 (-32·3–21·9)	-3·37 (-27–24·6)	15·2 (10·7–21·9)	14 (9·26–20·4)	13·7 (8·74–20·2)	-8·27 (-26·5–11·7)	-1·96 (-24·8–23·7)
Diet low in fruits	44·4 (32·7–57·1)	46·1 (34·5–59·5)	44·8 (33·1–58·6)	3·9 (0·715–8·41)	-2·88 (-10·5–4·11)	46·8 (35·3–59·8)	47·8 (36·4–61)	46·4 (34·9–59·6)	2·03 (-1·25–5·94)	-2·97 (-10·2–3·97)
Diet low in vegetables	49·7 (29·2–72·3)	48·8 (27·8–71·6)	45·2 (25·2–67·6)	-1·82 (-5·73–0·703)	-7·46 (-18·2–-1·13)	47·6 (27·1–70·3)	46·6 (25·8–69·5)	42·7 (24–64·6)	-2·03 (-6·25–1)	-8·34 (-18·3–-1·1)
Diet low in whole grains	89·6 (84·1–95)	95·3 (92·2–98·8)	94·9 (91·6–98·3)	6·39 (2·52–11·2)	-0·411 (-1·52–0·54)	--	--	--	--	--
Diet low in nuts and seeds	25·8 (7·85–55·4)	30·4 (9·89–57·6)	30·2 (9·39–58·2)	17·6 (3·28–44·7)	-0·806 (-16·3–17·8)	20·9 (5·58–53·1)	25·1 (7·52–54·9)	25 (7·75–55·1)	20 (2·92–50·2)	-0·277 (-17·7–21·4)
Diet low in milk	46·2 (22·5–65·8)	50·2 (25·1–71)	48·1 (23·2–68·7)	8·66 (2·33–19·1)	-4·22 (-14·1–0·768)	45·2 (21·6–64·5)	49·3 (24·4–70·1)	47·2 (22·3–68·5)	8·98 (2·43–19·2)	-4·12 (-12·6–1·1)
Diet high in red meat	95·7 (93·7–97·3)	91 (86·7–94·3)	90·6 (84·2–95·5)	-4·96 (-7·93–-2·8)	-0·365 (-5·72–4·04)	95·8 (93·7–97·4)	91·1 (87–94·4)	90·7 (84·1–95·2)	-4·92 (-7·66–-2·7)	-0·358 (-5·41–3·81)
Diet high in processed meat	68·9 (43·1–92·6)	73·7 (47–96·9)	74·3 (47·3–96·8)	6·94 (1·47–17·3)	0·758 (-2·04–4·31)	73·6 (48–95·9)	76·6 (50·4–98·7)	77·3 (52–98·6)	4·21 (0·506–11·3)	0·903 (-1·05–4·18)
Diet high in sugar-sweetened beverages	32·6 (18·3–48·9)	34·1 (20·6–50·7)	38·1 (22·6–55·1)	4·81 (-19·5–34·7)	11·5 (-6·92–39·3)	37·2 (21·8–54·8)	39·6 (24·4–56·5)	44·2 (26·3–60·9)	6·43 (-18·7–41·3)	11·5 (-8·03–40·1)
Diet low in fiber	47·9 (31·9–63·4)	53·5 (36·9–68·4)	50·2 (33·6–65·6)	11·8 (7·12–19)	-6·22 (-14–0·0733)	48 (31·2–63·9)	53·7 (36·9–69·5)	50·3 (33·3–65·9)	12 (7·27–19·3)	-6·29 (-14·3–0·0524)
Diet low in seafood omega-3 fatty acids	93 (82·1–100)	93·1 (83·2–100)	92 (81·7–100)	0·154 (-5·73–7·86)	-1·19 (-8·28–4·95)	94·6 (86·7–100)	94·7 (86·9–100)	93·7 (84·9–100)	0·163 (-4·9–6·29)	-1·07 (-7·64–3·48)
Diet low in polyunsaturated fatty acids	71·5 (32·1–100)	66·1 (25·8–100)	64·5 (24·5–99·7)	-7·64 (-22·9–0)	-2·29 (-8·31–0)	73·1 (33·3–100)	67·8 (28·2–100)	66·4 (26·5–100)	-7·16 (-21·4–0)	-2·15 (-7·54–0)
Diet high in trans fatty acids	61·8 (52–100)	51·4 (41·4–100)	49·6 (39·5–99·8)	-16·9 (-25·8–0)	-3·45 (-11·5–4·94)	63 (52·8–100)	52·7 (41·9–100)	50·9 (40·1–100)	-16·3 (-24·3–0)	-3·53 (-11·6–3·91)
Diet high in sodium	30·4 (6·44–55·8)	33·5 (7·88–58·5)	35·2 (7·98–60·6)	10·5 (-19·9–82·4)	4·95 (-18–40·7)	15·6 (3·93–38)	16 (4·07–38·5)	16·7 (4·05–39·7)	2·77 (-21·2–43·6)	4·39 (-9·55–29·4)
Low physical activity	3·95 (1·68–8·18)	4·12 (1·73–8·3)	4·37 (1·9–8·77)	4·39 (-9·66–22·3)	6·21 (-7·27–25·1)	6·76 (3·53–12·5)	7·06 (3·71–12·8)	7·27 (3·87–13)	4·34 (-4·81–16)	3·1 (-6·15–14·3)
Occupational asthmagens	39·8 (34–46·8)	29·3 (24·4–35·4)	27·6 (22·5–33·7)	-26·3 (-35·1–-16·6)	-5·9 (-16·7–6·4)	--	--	--	--	--
Occupational particulate matter, gases, and fumes	11·4 (8·19–15·9)	10·2 (7·37–14·5)	9·61 (6·97–13·7)	-10·5 (-13·1–-7·67)	-5·85 (-7·75–-3·93)	5·38 (3·88–7·82)	4·99 (3·59–7·21)	4·73 (3·4–6·9)	-7·2 (-14·2–-1·3)	-5·21 (-8·35–-1·81)
Occupational noise	9·83 (9·3–10·6)	9·32 (8·84–10)	9·03 (8·57–9·72)	-5·2 (-6·58–-3·55)	-3·13 (-4·06–-2·22)	5·75 (5·32–6·23)	6·2 (5·88–6·66)	6·34 (6·02–6·81)	7·94 (4·13–11·8)	2·26 (0·88–3·6)
Occupational injuries	--	--	--	--	--	--	--	--	--	--
Occupational ergonomic factors	14·4 (12·7–16·6)	12·4 (10·9–14·4)	12·4 (10·8–14·5)	-13·7 (-19·2–-7·78)	-0·00663 (-7·22–8·49)	--	--	--	--	--
Childhood sexual abuse	5·1 (3·45–7·14)	4·98 (3·53–6·84)	4·98 (3·53–6·84)	-2·36 (-9·37–7·3)	0 (0–0)	13·8 (10–19·2)	13·4 (10·6–17·6)	14·6 (11–19·6)	-2·99 (-14·2–10·4)	8·98 (-0·244–19·3)
Intimate partner violence	--	--	--	--	--	8·07 (4·77–11·6)	8·07 (4·77–11·6)	8·07 (4·77–11·6)	0 (0–0)	0 (0–0)
Non-exclusive breastfeeding	23·8 (20·1–27·9)	24·4 (20·5–28·9)	24 (20–28·6)	2·41 (-10·6–17·1)	-1·64 (-12·2–10·5)	23·8 (20·1–27·9)	24·4 (20·5–28·9)	24 (20–28·6)	2·43 (-10·6–17·1)	-1·64 (-12·2–10·5)
Discontinued breastfeeding	24·7 (23·7–25·6)	24·5 (23·5–25·7)	24·1 (22·9–25·2)	-0·854 (-6·79–5·24)	-1·85 (-6·5–3·42)	24·7 (23·6–25·6)	24·5 (23·4–25·7)	24·1 (22·9–25·2)	-0·778 (-6·72–5·32)	-1·73 (-6·4–3·54)
Diet low in calcium	9·42 (4·18–17·6)	11·6 (5·17–21·7)	10·9 (4·76–20·6)	23·2 (15·7–33·8)	-6·47 (-13·9–-0·787)	3·89 (1·61–6·92)	5·8 (2·28–10·5)	5·12 (2·04–9·29)	49·1 (30·7–71·7)	-11·7 (-22·8–-1·14)
Occupational exposure to asbestos	13·8 (12·5–15·1)	15·9 (15–16·7)	13·9 (11·2–16·8)	15·8 (5·37–28·2)	-12·5 (-30·6–5·52)	2·22 (1·55–3·1)	2·07 (1·38–2·5)	1·98 (1·23–2·69)	-7·06 (-29·1–14·3)	-3·94 (-27·7–23·8)
Occupational exposure to arsenic	0·698 (0–1·63)	0·594 (0–1·36)	0·591 (0–1·38)	-14·9 (-22·2–NA)	-0·395 (-7·22–NA)	0·516 (0–1·21)	0·498 (0–1·18)	0·511 (0–1·21)	-3·58 (-13·8–NA)	2·79 (-4·89–NA)
Occupational exposure to benzene	0·94 (0·0557–2·69)	0·958 (0·0572–2·71)	0·98 (0·0574–2·78)	1·94 (-2·64–8·26)	2·28 (-1·75–8·13)	1·21 (0·073–3·44)	1·51 (0·0915–4·32)	1·59 (0·1–4·55)	24·9 (17·2–35·5)	5·6 (0·293–13·3)
Occupational exposure to beryllium	0·0282 (0·0279–0·0286)	0·0273 (0·027–0·0277)	0·0281 (0·0275–0·0287)	-3·16 (-4·85–-1·41)	2·7 (0·479–5·07)	0·0279 (0·0271–0·0287)	0·0311 (0·0306–0·0317)	0·0326 (0·0317–0·0336)	11·6 (8·16–15·1)	4·76 (1·41–8·26)
Occupational exposure to cadmium	0·0798 (0·0772–0·0825)	0·0689 (0·064–0·0737)	0·0689 (0·0631–0·0745)	-13·7 (-20·3–-6·67)	0·0347 (-6·22–6·91)	0·0621 (0·0581–0·0665)	0·0609 (0·0564–0·0662)	0·0627 (0·0579–0·0683)	-1·93 (-11·1–8·12)	2·92 (-3·84–10·7)
Occupational exposure to chromium	0·186 (0·181–0·192)	0·163 (0·153–0·173)	0·163 (0·152–0·174)	-12·5 (-18·1–-6·29)	0·152 (-5·25–5·9)	0·16 (0·152–0·168)	0·159 (0·151–0·169)	0·164 (0·154–0·175)	-0·242 (-7·37–7·66)	2·65 (-2·69–9·07)
Occupational exposure to diesel engine exhaust	0·802 (0·777–0·827)	0·788 (0·754–0·824)	0·804 (0·764–0·847)	-1·76 (-6·73–3·2)	2·04 (-2·55–6·5)	0·847 (0·805–0·89)	0·819 (0·773–0·867)	0·82 (0·762–0·879)	-3·27 (-10·1–3·84)	0·111 (-6·29–6·61)
Occupational exposure to formaldehyde	0·401 (0·383–0·416)	0·335 (0·31–0·361)	0·33 (0·301–0·358)	-16·5 (-22·9–-9·42)	-1·52 (-7·87–5·51)	0·301 (0·282–0·322)	0·302 (0·281–0·324)	0·31 (0·287–0·335)	0·182 (-7·98–9·77)	2·7 (-3·56–9·76)
Occupational exposure to nickel	0·616 (0–2·7)	0·541 (0–2·4)	0·544 (0–2·46)	-12·3 (-18·9–NA)	0·561 (-5·55–NA)	0·396 (0–1·73)	0·378 (0–1·67)	0·389 (0–1·68)	-4·4 (-15·3–NA)	2·91 (-5·49–NA)
Occupational exposure to polycyclic aromatic hydrocarbons	0·371 (0·362–0·381)	0·331 (0·314–0·35)	0·333 (0·312–0·353)	-10·7 (-15·7–-5·29)	0·433 (-4·37–5·72)	--	--	--	--	--
Occupational exposure to silica	6·29 (0·926–20·1)	6·01 (0·867–19)	6·14 (0·904–19·8)	-4·44 (-12·6–4·34)	2·05 (-4·06–10·9)	3·37 (0·474–10·4)	3·23 (0·422–10·5)	3·32 (0·476–10·7)	-4·21 (-16·1–10)	2·68 (-6·01–14·2)
Occupational exposure to sulfuric acid	1·16 (0·244–3·52)	0·947 (0·196–3)	0·919 (0·188–2·91)	-18·2 (-24·1–-11·7)	-3·01 (-8·55–3·45)	0·785 (0·164–2·48)	0·719 (0·152–2·21)	0·727 (0·155–2·27)	-8·48 (-17·4–2·53)	1·13 (-5·94–9·89)
Unsafe sex	--	--	--	--	--	--	--	--	--	--
Occupational exposure to trichloroethylene	0·102 (0·0999–0·105)	0·0915 (0·0881–0·0957)	0·092 (0·0877–0·097)	-10·3 (-14·3–-6·01)	0·607 (-3·15–4·77)	0·0978 (0·0943–0·102)	0·0969 (0·0929–0·102)	0·0988 (0·0939–0·105)	-0·893 (-6·17–5·04)	2 (-2·82–7·24)
No access to handwashing facility	3·16 (2·99–3·34)	3·09 (2·92–3·26)	3·06 (2·9–3·23)	-2·38 (-8·56–4·42)	-0·842 (-4·92–3·42)	3·16 (2·99–3·34)	3·09 (2·92–3·26)	3·06 (2·9–3·23)	-2·38 (-8·56–4·42)	-0·842 (-4·92–3·42)
Child wasting	1·24 (0·878–1·63)	1·2 (0·849–1·58)	1·2 (0·844–1·58)	-2·95 (-11·3–5·75)	-0·326 (-6–6·06)	1·04 (0·731–1·4)	1 (0·697–1·34)	1·34 (0·942–1·71)	-3·78 (-12·4–4·07)	33·9 (5–56)
Child stunting	1·55 (0·761–2·28)	1·17 (0·554–1·79)	1·16 (0·546–1·81)	-24·6 (-37·9–-9·98)	-0·609 (-12·2–13)	1·81 (0·916–2·67)	1·52 (0·723–2·26)	1·51 (0·739–2·27)	-15·9 (-29·3–-0·68)	-0·691 (-12·4–10·5)
Chewing tobacco	0·207 (0·164–0·258)	0·22 (0·173–0·281)	0·241 (0·187–0·302)	5·88 (-20·1–41)	9·69 (-8·1–31·3)	0·151 (0·111–0·207)	0·156 (0·114–0·211)	0·164 (0·118–0·223)	2·88 (-29·2–49·8)	5·03 (-17·2–32·5)
Diet low in legumes	91·5 (79·8–100)	91·5 (80·1–100)	90·6 (77·9–100)	-0·00692 (-0·699–0·63)	-1 (-3·64–0·0998)	89·5 (75·5–100)	89·5 (75·3–100)	88·5 (73·2–100)	-0·0144 (-0·717–0·712)	-1·06 (-4·08–0·221)
Short gestation	18 (14·1–23·2)	14·4 (11·9–17·3)	14·1 (11·4–17·3)	-19·9 (-40·4–6·98)	-2·15 (-21·8–21·7)	19·8 (15·9–24·3)	16·4 (13·8–19·6)	15·7 (12·8–18·5)	-17·4 (-34·7–6·51)	-4·36 (-22·3–17·8)
Low birth weight	9·31 (7·33–11·8)	8·03 (6·67–9·38)	7·79 (6·48–9·35)	-13·8 (-34·7–14·4)	-2·99 (-22·1–20·9)	10·4 (8·47–12·6)	8·56 (7·32–10)	8·17 (6·7–9·68)	-17·7 (-35·1–5·69)	-4·56 (-23·9–19·4)
High temperature	0 (0–0)	21·8 (11–35·4)	24·3 (13·7–37·2)	--	11·5 (-4·11–38·3)	0 (0–0)	21·8 (11–35·4)	24·3 (13·7–37·2)	--	11·5 (-4·11–38·3)
Low temperature	33·9 (30·3–37·9)	35·2 (31·9–39·1)	33·4 (29·7–37·6)	3·81 (2·07–5·71)	-5·18 (-7·46–-3·12)	33·9 (30·3–37·9)	35·2 (31·9–39·1)	33·4 (29·7–37·6)	3·81 (2·07–5·71)	-5·18 (-7·46–-3·12)
Kidney dysfunction	15·2 (10·3–21·8)	15·2 (10·4–21·7)	15·4 (10·5–22)	-0·224 (-4·27–3·55)	1·55 (-2·73–6·24)	17·6 (12·1–24·3)	17·8 (12·4–24·7)	17·6 (12·3–24·5)	1·26 (-2·49–5·22)	-0·923 (-5·09–2·89)
Bullying victimization	6·44 (2·61–13·3)	8·53 (4·02–16)	8·79 (4·16–16·4)	32·5 (5·61–118)	2·99 (-5·42–10·7)	5·27 (2·12–10·7)	6·53 (3·07–12)	6·84 (3·26–12·6)	24 (-1·05–90·1)	4·66 (-2·18–13·1)
High LDL cholesterol	65·2 (62–68·5)	49·2 (45·9–52·6)	49·1 (45·6–52·5)	-24·7 (-29·8–-19·3)	-0·116 (-4·23–4·48)	--	--	--	--	--

Supplementary Results Table 1. UK age-standardised Summary Exposure Values (SEVs) for most-detailed risk factors and percentage change, by sex, 1990, 2010, and 2019. Data in parentheses are 95% uncertainty intervals.

	Male					Female				
	1990	2010	2019	Percentage change 1990-2010	Percentage change 2010-2019	1990	2010	2019	Percentage change 1990-2010	Percentage change 2010-2019
Unsafe sanitation	0·561 (0·317–0·906)	0·315 (0·171–0·526)	0·246 (0·133–0·406)	-43·8 (-70·5–16·2)	-22 (-53–25·9)	0·564 (0·317–0·904)	0·319 (0·173–0·537)	0·249 (0·134–0·41)	-43·4 (-70·3–16·7)	-22·1 (-52·9–26·5)
Ambient particulate matter pollution	16·5 (6·06–29·9)	11 (8·08–14·4)	8·07 (5·42–11·2)	-33·4 (-64·3–63·4)	-26·6 (-33·9—21·7)	16·5 (6·04–29·9)	11 (8·07–14·4)	8·05 (5·41–11·2)	-33·4 (-64·4–63·3)	-26·6 (-33·9—21·7)
Household air pollution from solid fuels	0·0156 (0·00417–0·0402)	0·00529 (0·00121–0·0142)	0·00322 (0·000642–0·0101)	-66 (-84·1—27·5)	-39·1 (-64·2–0·386)	0·0204 (0·00584–0·0536)	0·00701 (0·00163–0·0196)	0·00427 (0·000924–0·0128)	-65·7 (-83·9—26·8)	-39·1 (-64·2–0·394)
Ambient ozone pollution	16·2 (5·64–30·7)	11·4 (3·48–23·1)	8·86 (2·63–18·8)	-29·6 (-45·6—19·2)	-22·2 (-35·2—13·5)	16·3 (5·65–30·8)	11·5 (3·5–23·3)	8·81 (2·63–18·8)	-29·7 (-45·7—19·2)	-23·1 (-36·2—13·6)
Residential radon	23·9 (14·9–36·2)	23·9 (14·9–36·2)	23·8 (14·9–36)	-0·0918 (-1·05–1·02)	-0·151 (-0·667–0·495)	24·1 (14·9–35·3)	24·1 (15·1–35·2)	24 (15·1–35·2)	-0·113 (-0·95–0·896)	-0·15 (-0·672–0·456)
Lead exposure	34·3 (11·9–57·3)	22·5 (6·55–43·8)	15·8 (3·6–35·3)	-34·3 (-46·3—22·8)	-29·7 (-45·8—19)	16·8 (1·62–39·3)	10·9 (1·14–29·9)	7·63 (0·562–24·2)	-35·1 (-48·1—23)	-30·1 (-50·8—18·8)
Child underweight	0·92 (0·523–1·36)	0·78 (0·427–1·16)	0·768 (0·418–1·16)	-15·2 (-26·4—1·89)	-1·51 (-10·5–8·62)	1·08 (0·621–1·58)	0·938 (0·54–1·38)	0·923 (0·528–1·36)	-13·1 (-23·5—1·38)	-1·58 (-9·73–6·59)
Iron deficiency	--	--	--	--	--	10·7 (9·23–12·3)	9·59 (8·17–11·1)	9·42 (7·93–11)	-10·7 (-18·5—2·79)	-1·75 (-7·18–3·55)
Vitamin A deficiency	4·57 (1·81–8·86)	2 (0·832–4·01)	1·69 (0·673–3·39)	-56·2 (-81·1—10·8)	-15·6 (-51·6–54·8)	3·49 (1·71–6·02)	2·2 (1·15–3·75)	1·89 (0·96–3·21)	-36·8 (-66·7–36)	-14·2 (-43–35·6)
Zinc deficiency	3·8 (0·0155–11·5)	2·53 (0–7·89)	2·55 (0·000103–8·03)	-33·3 (-97·7—17·3)	0·622 (-16·4–NA)	3·79 (0·0238–11·3)	2·48 (0–7·95)	2·5 (0–7·93)	-34·7 (-98·7—17·6)	0·896 (-17·2–NA)
Smoking	28·5 (25·6–31·8)	21·3 (18·9–24)	18·2 (16–20·6)	-25·2 (-29—21·6)	-14·7 (-17·3—12)	23·3 (20·6–26·4)	18·2 (15·9–20·8)	14·7 (12·8–17)	-22·2 (-26·5—17·9)	-19·1 (-22—15·9)
Secondhand smoke	45·5 (41·7–49·1)	33·9 (31–37)	33·1 (30·3–36)	-25·6 (-33·4—16·5)	-2·29 (-7·97–3·91)	47·4 (44·5–50·7)	34·3 (31·7–36·6)	33·5 (31·1–35·7)	-27·7 (-34·5—21)	-2·35 (-6·93–2·48)
Alcohol use	17·6 (12·9–23·1)	20 (14·4–26·4)	19·3 (13·6–26·2)	13·5 (2·96–26·4)	-3·48 (-14·1–7·12)	10·5 (7·26–14·8)	11·1 (7·64–15·3)	10·6 (6·84–15·2)	5·08 (-6·23–18·3)	-4·58 (-19·8–9·93)
Drug use	0·348 (0·183–0·694)	0·652 (0·386–1·14)	0·611 (0·364–1·08)	87·6 (53·4–133)	-6·35 (-10·3—2·65)	0·221 (0·126–0·406)	0·344 (0·213–0·578)	0·339 (0·205–0·58)	55·8 (25–90·2)	-1·65 (-6·66–3·54)
High fasting plasma glucose	9·5 (8·26–10·9)	17 (15·2–19·1)	21·8 (19·1–25·3)	78·8 (64·1–96)	28·2 (20·9–37·7)	7·72 (6·7–8·92)	12·6 (11·2–14·2)	15·9 (14–18·2)	63·4 (50·3–77)	26·4 (18·2–34·8)
High systolic blood pressure	46·1 (44·1–48·2)	28·8 (26·9–30·7)	29·7 (27·5–32)	-37·6 (-40·8—34·4)	3·29 (-1·97–8·62)	40 (37·4–42·4)	20·5 (18·7–22·3)	20·9 (18·7–23·3)	-48·7 (-52·9—44·1)	2·03 (-7·06–12)
High body-mass index	20·2 (14·3–27·4)	27·9 (20·2–37·2)	29·1 (21·2–38·8)	38·4 (28·6–52·7)	4·4 (2·67–6·39)	22·5 (17·1–30·4)	30·2 (23·7–39·6)	31·3 (24·6–40·7)	34·2 (26·2–44·6)	3·46 (1·51–5·6)
Low bone mineral density	8·75 (4·87–14·4)	7·59 (4·02–13)	8·85 (4·94–14·5)	-13·3 (-18·6—9·46)	16·6 (11·3–24·7)	14·8 (10–21·2)	14 (9·32–20·2)	13·7 (9·06–19·8)	-5·64 (-9·89—2·53)	-2·27 (-5·67–1·78)
Diet low in fruits	65·7 (56·9–75·7)	58·7 (49·5–69·3)	57·8 (48·2–68·5)	-10·7 (-15·2—7·2)	-1·43 (-6·47–4·27)	66·9 (58·3–76·9)	60 (50·6–70·7)	59·3 (49·4–69·8)	-10·4 (-14·7—6·91)	-1·18 (-6·28–3·61)
Diet low in vegetables	51·1 (32·3–70·8)	45·2 (28–64·6)	44·2 (26·4–63)	-11·7 (-19·9—5·15)	-2·07 (-9·68–5·25)	47·8 (30·1–67·5)	43 (25·7–62·4)	41·8 (25·4–61·8)	-9·93 (-17·6—4)	-2·76 (-11·2–4·78)
Diet low in whole grains	89·5 (84·3–94·3)	89·1 (83·9–94)	89·2 (84·1–94·2)	-0·416 (-1·8–0·918)	0·158 (-0·927–1·26)	88 (82·2–93·5)	87·6 (81·7–93·2)	87·8 (81·8–93·3)	-0·443 (-1·77–0·773)	0·199 (-0·924–1·42)
Diet low in nuts and seeds	81·6 (40·6–90·8)	74·9 (37·1–85·6)	76·8 (38·1–86·8)	-8·24 (-15·6—3·41)	2·52 (-0·849–7·18)	81·6 (41·5–91·1)	74 (36·3–85·2)	75·1 (38·1–86·4)	-9·36 (-17·5—4·14)	1·6 (-2·46–6·65)
Diet low in milk	57·4 (33·8–77·1)	58·1 (34·2–77·1)	58·2 (34·3–77·1)	1·16 (-4·41–7·98)	0·209 (-3·32–4·31)	60·4 (35·4–80·6)	62·9 (37·1–82·8)	62·8 (37·3–82·8)	4·11 (-0·239–12·8)	-0·0713 (-2·93–3)
Diet high in red meat	66·1 (58·2–73·3)	53·5 (44·6–62)	55·4 (45·2–64·9)	-19 (-25·5—13·7)	3·56 (-5·03–13)	66·4 (58·7–73·6)	53·5 (44·6–62·1)	55·6 (44·9–64·8)	-19·4 (-26—14·1)	3·93 (-4·47–13)
Diet high in processed meat	77·5 (55·2–95·8)	82 (62·4–98·2)	81·9 (62·4–98·1)	5·78 (1·33–15·1)	-0·105 (-2·72–2·92)	80·8 (61·1–97·4)	85·4 (67·8–99·2)	85·5 (68·5–99)	5·73 (1·22–14)	0·0895 (-2·28–3·06)
Diet high in sugar-sweetened beverages	43·6 (33·3–58·2)	47·9 (38·1–61·6)	49·8 (39·7–63·3)	9·87 (-4·82–27·8)	3·9 (-5·78–15·4)	44·7 (34·4–57·8)	50·3 (40·6–62·4)	51·6 (41·6–64·5)	12·6 (-1·65–31·7)	2·61 (-7·34–14·2)
Diet low in fiber	40·5 (27·4–54·5)	33·7 (20·3–47·3)	33·7 (21–47·8)	-16·8 (-34·5–0·641)	-0·212 (-12·6–13·2)	40·6 (25·7–55·2)	33·7 (20·1–48·4)	33·6 (20·4–47·7)	-17·1 (-34·2–0·217)	-0·246 (-12·5–14·1)
Diet low in seafood omega-3 fatty acids	92·6 (85·2–99·7)	90 (81·1–99·2)	89·4 (80–99·1)	-2·79 (-6·72—0·0731)	-0·652 (-2·88–0·83)	93·8 (87·4–99·8)	91·6 (83·5–99·6)	91·2 (82·7–99·6)	-2·34 (-6·31—0·00212)	-0·433 (-2·57–1·15)
Diet low in polyunsaturated fatty acids	52·4 (15·5–93)	47·5 (13·4–89)	46·5 (12·9–88·4)	-9·28 (-19·5—1·45)	-2·19 (-7·36–1·35)	54·4 (17–94·2)	49·7 (13·9–91·1)	48·6 (13·5–90·5)	-8·65 (-19·2—1·31)	-2·1 (-7·54–1·56)
Diet high in trans fatty acids	57·3 (47·6–99·8)	50·5 (40·7–98·3)	50·4 (40·7–98·1)	-11·9 (-20—0·349)	-0·168 (-7·61–8·56)	56·1 (46–99·9)	48·1 (37·3–99·7)	48 (37·4–99·6)	-14·2 (-23·9–0)	-0·281 (-6·68–7·69)
Diet high in sodium	19·2 (5·77–39·7)	20·6 (6·19–40·1)	22·9 (6·89–43·7)	7·28 (-21·5–66·1)	11·2 (-8·41–42·5)	13·2 (4·21–31·1)	13·1 (4·13–30·5)	13·9 (4·13–32·6)	-0·751 (-21·5–42·5)	6·55 (-10·4–36·7)
Low physical activity	4·58 (2·03–9·18)	5·22 (2·46–10·4)	5·21 (2·44–10·4)	14·1 (4·29–26·2)	-0·203 (-2·7–2·22)	7·42 (4·05–13·3)	8·42 (4·72–14·6)	8·48 (4·74–14·7)	13·4 (7·57–22·3)	0·689 (-1·05–2·52)
Occupational asthmagens	22·9 (19·3–26·8)	19·9 (16·9–23·5)	21·5 (18–25·9)	-13·1 (-21·2—4·39)	8·16 (-0·822–17·8)	11·7 (9·86–14·1)	10·7 (8·97–12·5)	11·4 (9·52–13·6)	-9·28 (-17·9—0·0686)	7·43 (-1·2–16)
Occupational particulate matter, gases, and fumes	8·74 (6·23–12·7)	8·21 (5·88–11·9)	7·87 (5·6–11·4)	-6·08 (-10·3—1·88)	-4·06 (-6·43—1·85)	3·88 (2·73–5·83)	3·85 (2·7–5·63)	3·63 (2·58–5·28)	-0·649 (-8·41–8·63)	-5·7 (-9·74—1·69)
Occupational noise	8·29 (7·8–8·94)	8·1 (7·7–8·76)	7·94 (7·54–8·63)	-2·24 (-4·55–0·1)	-2·01 (-3·02—0·904)	4·91 (4·57–5·37)	5·48 (5·19–5·93)	5·58 (5·29–6·04)	11·6 (8·1–15·4)	1·87 (0·651–3·26)
Occupational injuries	--	--	--	--	--	--	--	--	--	--
Occupational ergonomic factors	10·5 (8·95–12·5)	10·3 (8·77–12·2)	10·7 (9·03–12·7)	-2·62 (-6·97–1·39)	3·95 (-0·769–9·21)	5·98 (4·99–7·32)	7·59 (6·3–9·19)	8·37 (6·94–10·3)	26·9 (17·4–37·1)	10·3 (-0·0306–20·9)
Childhood sexual abuse	10·8 (7·14–14·7)	11·1 (7·38–15·5)	10·9 (7·06–15·4)	2·97 (-2·7–9·49)	-1·95 (-10·3–6·42)	13·5 (10·2–18·1)	14·1 (10·5–19·1)	14·1 (10·4–19·2)	4·01 (-2·9–10·7)	-0·0373 (-7·5–9·74)
Intimate partner violence	--	--	--	--	--	22·2 (12·2–30·5)	19·4 (11·8–26·5)	22·4 (12·3–30·6)	-12·9 (-19·3—1·86)	15·7 (6·3–28·2)
Non-exclusive breastfeeding	25·8 (19·7–33·1)	23·2 (18·1–29·6)	23·3 (18·1–29·8)	-10·2 (-19·6–1·24)	0·351 (-8·36–9·09)	25·8 (19·7–33·1)	23·2 (18·1–29·6)	23·3 (18·1–29·8)	-10·2 (-19·6–1·26)	0·354 (-8·36–9·09)
Discontinued breastfeeding	27·9 (27·3–28·6)	27·8 (27·2–28·4)	27·6 (26·9–28·2)	-0·601 (-3·34–2·61)	-0·719 (-3·31–1·72)	27·9 (27·3–28·6)	27·8 (27·2–28·4)	27·6 (26·9–28·2)	-0·62 (-3·36–2·59)	-0·736 (-3·33–1·71)
Diet low in calcium	18·9 (8·84–35·5)	13·5 (5·72–25·3)	13·4 (5·87–25·1)	-28·4 (-45—17)	-0·754 (-6·62–6)	13·3 (5·44–25·8)	8·05 (3·01–14·4)	7·97 (3·05–14·5)	-39·6 (-56·8—24·4)	-0·972 (-10·7–9·15)
Occupational exposure to asbestos	20·8 (20–21·8)	18·9 (18·7–19·2)	17·3 (15·2–19·4)	-9·2 (-13·7—4·96)	-8·53 (-19·5–2·31)	4·17 (2·8–5·85)	4·15 (2·54–4·59)	4·23 (2·44–5·46)	-0·464 (-32·2–24·4)	2·02 (-18·5–25·7)
Occupational exposure to arsenic	0·516 (0–1·19)	0·496 (0–1·18)	0·52 (0–1·22)	-3·89 (-13·4–NA)	4·78 (-3·09–NA)	0·482 (0–1·13)	0·445 (0–1·06)	0·448 (0–1·06)	-7·51 (-18·9–NA)	0·638 (-6·99–NA)
Occupational exposure to benzene	0·826 (0·0485–2·35)	0·886 (0·0521–2·53)	0·936 (0·0535–2·7)	7·35 (2·93–13·5)	5·59 (1·32–11·4)	1·11 (0·0666–3·2)	1·41 (0·0864–4·03)	1·55 (0·0957–4·51)	27·6 (20·9–38·4)	9·61 (4·53–16·6)
Occupational exposure to beryllium	0·0233 (0·023–0·0237)	0·0245 (0·0242–0·0248)	0·0259 (0·0252–0·0265)	5·17 (3·17–7·17)	5·62 (2·99–8·15)	0·025 (0·0246–0·0256)	0·0278 (0·0274–0·0283)	0·0298 (0·029–0·0306)	11·1 (8·45–13·7)	7·24 (4·32–10·2)
Occupational exposure to cadmium	0·0595 (0·0554–0·064)	0·0578 (0·0541–0·0614)	0·0608 (0·0558–0·0656)	-2·76 (-11·5–6·84)	5·07 (-2·11–12·6)	0·057 (0·0525–0·0622)	0·0543 (0·0507–0·0582)	0·0552 (0·0511–0·0597)	-4·86 (-15–5·57)	1·73 (-5·08–8·75)
Occupational exposure to chromium	0·139 (0·131–0·148)	0·137 (0·129–0·144)	0·145 (0·134–0·155)	-1·55 (-9·31–6·65)	5·56 (-0·726–12·2)	0·14 (0·131–0·151)	0·14 (0·133–0·148)	0·145 (0·137–0·154)	0·0692 (-8·61–8·63)	3·25 (-2·44–9·02)
Occupational exposure to diesel engine exhaust	0·666 (0·636–0·698)	0·715 (0·689–0·74)	0·764 (0·725–0·809)	7·28 (1·1–13·8)	6·89 (1·95–12·1)	0·597 (0·567–0·632)	0·651 (0·619–0·684)	0·7 (0·65–0·751)	8·97 (0·745–17·2)	7·46 (0·951–14·3)
Occupational exposure to formaldehyde	0·298 (0·275–0·323)	0·279 (0·26–0·298)	0·29 (0·265–0·315)	-6·47 (-15·3–3·15)	4·15 (-3·31–11·8)	0·274 (0·249–0·299)	0·271 (0·25–0·29)	0·277 (0·255–0·297)	-1·37 (-10·9–8·23)	2·42 (-3·72–9·02)
Occupational exposure to nickel	0·497 (0–2·19)	0·488 (0–2·17)	0·512 (0–2·24)	-1·94 (-10–NA)	5·05 (-1·28–NA)	0·389 (0–1·75)	0·347 (0–1·54)	0·346 (0–1·54)	-10·7 (-22·3–NA)	-0·424 (-8·74–NA)
Occupational exposure to polycyclic aromatic hydrocarbons	0·288 (0·272–0·304)	0·288 (0·274–0·301)	0·303 (0·285–0·322)	0·0279 (-6·84–7·13)	5·4 (-0·0906–10·9)	0·288 (0·272–0·305)	0·303 (0·29–0·316)	0·316 (0·3–0·333)	5·06 (-2·19–12·7)	4·41 (-0·332–9·42)
Occupational exposure to silica	5·74 (0·842–18·2)	6·03 (0·875–19)	6·36 (0·922–19·4)	4·99 (-3·07–15·6)	5·48 (-1·51–14·3)	3·14 (0·452–10·2)	2·92 (0·415–9·05)	2·94 (0·401–9·21)	-6·98 (-19·6–8·07)	0·449 (-8·83–11·1)
Occupational exposure to sulfuric acid	0·873 (0·185–2·81)	0·816 (0·172–2·51)	0·832 (0·181–2·61)	-6·56 (-15·3–2·26)	1·96 (-5·45–9·11)	0·787 (0·16–2·52)	0·686 (0·141–2·13)	0·662 (0·138–2·06)	-12·8 (-22·6—1·56)	-3·6 (-11·2–4·2)
Unsafe sex	--	--	--	--	--	--	--	--	--	--
Occupational exposure to trichloroethylene	0·0759 (0·0729–0·0795)	0·0762 (0·0733–0·0793)	0·0807 (0·0767–0·0849)	0·36 (-4·91–6·12)	5·96 (1·2–11)	0·0794 (0·0758–0·0834)	0·082 (0·0792–0·0859)	0·0861 (0·0821–0·0909)	3·35 (-2·95–10·1)	5·04 (0·287–10·2)
No access to handwashing facility	3·34 (3·16–3·53)	3·19 (3·01–3·37)	3·15 (2·99–3·33)	-4·39 (-10·6–1·96)	-1·22 (-5–2·96)	3·34 (3·15–3·52)	3·19 (3·01–3·37)	3·15 (2·99–3·33)	-4·37 (-10·5–2·02)	-1·21 (-5·09–2·94)
Child wasting	1·26 (0·889–1·67)	1·22 (0·857–1·62)	1·21 (0·852–1·62)	-3·65 (-11–4·13)	-0·47 (-5·72–5·23)	1·08 (0·764–1·44)	1·03 (0·726–1·37)	1·02 (0·708–1·35)	-4·78 (-12·7–3·8)	-0·888 (-6·84–5·2)
Child stunting	1·65 (0·849–2·44)	1·22 (0·592–1·83)	1·21 (0·564–1·82)	-26·1 (-39—11·7)	-1·35 (-13·9–13·3)	1·95 (1·01–2·83)	1·6 (0·773–2·41)	1·57 (0·781–2·35)	-18·1 (-30·7—3·58)	-1·69 (-12·7–10·2)
Chewing tobacco	0·173 (0·134–0·222)	0·166 (0·128–0·209)	0·169 (0·131–0·212)	-4·31 (-27·6–27·5)	2·02 (-15–21·5)	0·136 (0·0988–0·182)	0·135 (0·0988–0·183)	0·137 (0·0991–0·183)	-0·382 (-30·8–45·5)	0·925 (-20·7–26·1)
Diet low in legumes	73·5 (38·3–98·6)	72·6 (36·3–98·5)	73·5 (38·2–98·1)	-1·32 (-6·36–0·6)	1·32 (-1·17–8·15)	70·3 (30·8–97·4)	69·2 (30·4–96·3)	70·3 (31·9–96·7)	-1·5 (-7·06–0·82)	1·51 (-1·58–10)
Short gestation	21·4 (19·2–23·8)	46·4 (41·5–51)	43·1 (38·6–47·6)	117 (106–129)	-7·13 (-10·8—3·15)	23·3 (21·2–25·5)	42·1 (38·2–46·1)	38·5 (34·6–42·5)	80·7 (73·5–87·9)	-8·59 (-12·8—4·38)
Low birth weight	11·2 (10·4–12·1)	22·3 (20·9–23·8)	21·1 (19·5–22·6)	98·8 (88·9–109)	-5·33 (-9·23—1·38)	12·4 (11·7–13·3)	20·7 (19·5–22)	19·3 (18–20·7)	66·7 (59·9–73·4)	-7·02 (-11·6—2·59)
High temperature	15·3 (7·23–24·1)	0 (0–0)	4·86 (2·26–7·67)	-100 (-100—100)	--	15·3 (7·23–24·1)	0 (0–0)	4·86 (2·26–7·67)	-100 (-100—100)	--
Low temperature	31·1 (27·1–35·5)	34 (30·4–38)	30·5 (26·5–34·9)	9·34 (6·49–12·8)	-10·5 (-13·3—7·97)	31·1 (27·1–35·5)	34 (30·4–38)	30·5 (26·5–34·9)	9·34 (6·49–12·8)	-10·5 (-13·3—7·97)
Kidney dysfunction	13·9 (9·36–20·4)	14·4 (9·85–20·9)	14·2 (9·67–20·7)	3·34 (0·918–6·72)	-1·33 (-3·16–0·228)	14·9 (10–21·5)	15·2 (10·3–21·7)	15·2 (10·3–21·8)	2·35 (0·471–4·93)	0·203 (-1·69–2·17)
Bullying victimization	5·91 (2·43–11·6)	5·98 (2·45–11·8)	5·65 (2·32–11·4)	1·09 (-7·48–10·5)	-5·57 (-10·4—1·43)	4·57 (1·87–9·36)	4·89 (1·95–9·89)	4·64 (1·83–9·51)	6·9 (-4·61–18·2)	-5·07 (-11·8—0·37)
High LDL cholesterol	55·1 (52·7–57·7)	43·9 (41–47)	44 (41·2–47)	-20·3 (-22·9—18)	0·357 (-0·931–1·57)	57·4 (55·1–59·9)	42·9 (40–45·9)	42·9 (40–46)	-25·3 (-27·9—22·8)	0·0102 (-1·27–1·34)

Supplementary Results Table 1. East Midlands age-standardised Summary Exposure Values (SEVs) for most-detailed risk factors and percentage change, by sex, 1990, 2010, and 2019. Data in parentheses are 95% uncertainty intervals.

	Male					Female				
	1990	2010	2019	Percentage change 1990-2010	Percentage change 2010-2019	1990	2010	2019	Percentage change 1990-2010	Percentage change 2010-2019
Unsafe sanitation	0·647 (0·281–1·28)	0·37 (0·166–0·708)	0·28 (0·12–0·525)	-42·8 (-77·6–47·8)	-24·2 (-62·7–50·7)	0·647 (0·281–1·28)	0·37 (0·166–0·71)	0·281 (0·12–0·527)	-42·7 (-77·6–47·8)	-24·2 (-62·6–50·5)
Ambient particulate matter pollution	18·3 (6·9–33·1)	11·2 (7·9–15)	8·5 (5·45–11·9)	-38·5 (-67·4–45·7)	-24·4 (-31·8—19·9)	18·3 (6·89–33·1)	11·2 (7·89–15)	8·49 (5·45–11·9)	-38·5 (-67·5–45·6)	-24·4 (-31·9—19·9)
Household air pollution from solid fuels	0·0198 (0·00476–0·0558)	0·00691 (0·00139–0·0212)	0·00429 (0·000739–0·014)	-65 (-87·7—8·52)	-38 (-70·8–19·8)	0·026 (0·00637–0·0741)	0·00921 (0·00188–0·0276)	0·00572 (0·00106–0·0182)	-64·6 (-87·7—7·26)	-37·8 (-70·5–20·2)
Ambient ozone pollution	14·2 (4·62–28·3)	8·62 (2·3–18·5)	8·66 (2·23–18·8)	-39·2 (-62·5—18·5)	0·495 (-30·7–46·7)	14·2 (4·62–28·3)	8·65 (2·31–18·6)	8·74 (2·25–18·9)	-38·9 (-62·3—18)	0·962 (-30–46·9)
Residential radon	29·4 (15·1–49·6)	29·4 (15·2–49·6)	29·3 (15·3–49·3)	-0·0315 (-2·4–2·63)	-0·292 (-1·47–1·61)	29·4 (15·1–49·5)	29·4 (15·2–49·6)	29·4 (15·2–49·5)	0·111 (-2·16–2·47)	-0·0954 (-1·26–1·46)
Lead exposure	30·8 (6·29–55)	19·9 (3·24–42)	13·7 (1·28–34)	-35·5 (-54·1—23·3)	-30·9 (-60·8—19·1)	12·4 (0·0355–34·5)	7·45 (0·0123–25·6)	5·12 (7·27e-12–20·8)	-40 (-74·4—25·3)	-31·3 (-100—18·8)
Child underweight	0·922 (0·535–1·4)	0·785 (0·426–1·21)	0·773 (0·417–1·19)	-14·9 (-30·8–3·5)	-1·55 (-14·1–13·3)	1·08 (0·629–1·61)	0·946 (0·539–1·38)	0·93 (0·528–1·38)	-12·8 (-26·1–3·26)	-1·61 (-12·4–10·2)
Iron deficiency	--	--	--	--	--	11·1 (9·1–13·1)	10 (8·28–11·8)	9·79 (8·06–11·6)	-9·51 (-21·5–2·65)	-2·11 (-9·83–6·38)
Vitamin A deficiency	4·73 (1·27–11·7)	2·22 (0·643–5·98)	1·87 (0·495–5·05)	-53·1 (-86·1–72·5)	-15·5 (-60·9–98·4)	3·59 (1·31–7·22)	2·35 (0·871–4·8)	2·02 (0·744–4·01)	-34·6 (-74·7–74·8)	-14 (-51·9–78·8)
Zinc deficiency	4·36 (0–12·9)	2·79 (0–8·53)	2·8 (0–8·59)	-36 (-100–NA)	0·305 (-24–NA)	4·2 (0–12·4)	2·67 (0–8·52)	2·67 (0–8·47)	-36·4 (-100–NA)	-0·0428 (-22·4–NA)
Smoking	28·2 (24·9–31·7)	21·7 (19·2–24·5)	18·3 (16–20·7)	-22·8 (-27·8—17·7)	-15·8 (-19·5—12·1)	23·2 (20·2–26·4)	18·4 (16·1–21·1)	14·5 (12·5–16·9)	-20·5 (-26·8—13·1)	-21·2 (-25·5—16·5)
Secondhand smoke	45·2 (40·6–50·3)	33·2 (29·3–37·4)	32·8 (29–36·6)	-26·4 (-36·6—14·9)	-1·37 (-9·85–7·16)	47·3 (43·2–51·6)	33·7 (30·6–36·9)	33·4 (30·2–36·5)	-28·6 (-36·9—19·8)	-1·1 (-7·17–5·93)
Alcohol use	16·8 (10·2–23·8)	19·6 (12·2–27·8)	18·5 (11·2–26·7)	16·6 (-16·1–60·6)	-5·54 (-25·6–15·2)	5·56 (0·632–13·9)	5·68 (0·463–13·7)	5·15 (0·348–13·3)	2·11 (-80·4–324)	-9·34 (-76·4–167)
Drug use	0·312 (0·155–0·645)	0·586 (0·34–1·04)	0·561 (0·317–1·01)	87·7 (48·7–145)	-4·37 (-10·3–0·243)	0·195 (0·105–0·377)	0·309 (0·182–0·527)	0·301 (0·171–0·54)	58·8 (21·8–105)	-2·56 (-10·9–4·16)
High fasting plasma glucose	9·52 (8·1–11·2)	17·8 (15·6–20·5)	22·7 (19·5–26·9)	87·2 (66–112)	27·5 (17·4–42·1)	7·87 (6·57–9·53)	13·4 (11·4–15·5)	17 (14·5–19·8)	69·8 (47·3–95)	27·1 (15·5–42·7)
High systolic blood pressure	35·2 (28·6–43)	32·3 (29·5–35·3)	32·3 (27·1–38)	-8·19 (-25·3–15)	-0·1 (-15·5–17·2)	33·5 (22–47·4)	21·2 (14·2–29·2)	19·5 (12·1–28·4)	-36·8 (-60·8–4·2)	-7·88 (-40·8–38·2)
High body-mass index	21·3 (14·9–29·1)	27 (18·8–36·4)	28·1 (19·7–37·9)	27 (16·1–41·7)	4·22 (-0·742–9·74)	24·1 (18·1–32·7)	31·2 (24·4–41·1)	32 (25·1–42·4)	29·2 (15·6–44·3)	2·57 (-3·24–8·82)
Low bone mineral density	8·73 (4·79–14·4)	7·6 (3·97–13·1)	9·26 (5·16–15·1)	-12·9 (-23·7—3)	21·8 (9·8–39·2)	14·6 (9·65–20·9)	14 (9·4–20·3)	13·8 (9·09–20)	-3·93 (-13·3–5·54)	-1·82 (-11·3–8·63)
Diet low in fruits	67·1 (56·2–78·3)	60·1 (49·2–71·7)	59 (47·2–70·9)	-10·3 (-20·4—1·5)	-1·93 (-10·1–6·46)	68·2 (58·1–78·3)	60·6 (49·1–71·5)	59·8 (48·6–71·4)	-11·1 (-20·4—1·94)	-1·26 (-8·19–6·84)
Diet low in vegetables	53 (32·4–73·8)	48·3 (28·8–69)	47·3 (28·6–67·1)	-8·91 (-23·6–4·15)	-2·12 (-12·7–8·91)	50·4 (30·8–71·8)	45·2 (26·6–65·4)	43·9 (25·6–65·2)	-10·4 (-25·8–2·4)	-2·92 (-15·6–8·39)
Diet low in whole grains	89·3 (83·3–94·7)	88·9 (83·2–94·2)	89 (83·2–94·5)	-0·377 (-2·94–2·14)	0·133 (-1·44–1·77)	--	--	--	--	--
Diet low in nuts and seeds	83·1 (42·5–92·4)	75·4 (39·7–87·1)	77·4 (39·5–89)	-9·28 (-20·9—1·62)	2·67 (-3·49–11·2)	83·1 (43·3–92·6)	75·4 (39–87·7)	76·4 (39·3–88·4)	-9·27 (-20·6—1·21)	1·33 (-5·58–9·71)
Diet low in milk	59·7 (34·9–80·6)	60·8 (35·9–81·4)	60·7 (36·3–81·5)	1·9 (-6·55–13·7)	-0·0826 (-5·74–6·06)	62·1 (37–82·7)	64·4 (39·5–84·9)	64·3 (39·6–85·5)	3·76 (-2·75–17)	-0·188 (-4·96–4·64)
Diet high in red meat	65·2 (53–76·5)	51 (39–63·4)	53·2 (39·9–66·1)	-21·7 (-35·4—6·1)	4·18 (-9·94–21·1)	64·8 (52·1–76·7)	52·2 (38·9–64·6)	54·6 (40·2–68·2)	-19·5 (-34·3—3·58)	4·67 (-9·5–20·7)
Diet high in processed meat	76·5 (52·6–95·3)	81·2 (60·6–98)	81·3 (60·3–98·1)	6·18 (-0·862–19)	0·109 (-4·35–5·16)	79·5 (58·5–97·2)	84·7 (66·2–99)	84·9 (66·3–99)	6·56 (0·388–18·9)	0·248 (-3·97–4·8)
Diet high in sugar-sweetened beverages	44·8 (30·6–65·8)	48·6 (33·2–68·5)	50·6 (35–69·5)	8·62 (-17–44·5)	4·09 (-12·7–25·2)	45 (28·3–67·1)	48·2 (30–68·8)	50·4 (33·6–71·3)	7·19 (-27·5–56)	4·46 (-18·8–38·6)
Diet low in fiber	43·3 (28·5–58·4)	36 (21·1–51·6)	35·8 (21·5–51·5)	-16·9 (-38·3–6·21)	-0·292 (-16·4–18·8)	43·6 (27·2–59·6)	36·2 (20·8–52·3)	35·9 (21·6–52·1)	-17·1 (-39·9–9·52)	-0·773 (-16·7–19·6)
Diet low in seafood omega-3 fatty acids	94·4 (87·2–100)	92 (83·2–99·8)	91·5 (82·4–99·7)	-2·49 (-7·96–0·868)	-0·543 (-4·44–2·74)	95·3 (89·2–100)	93·2 (85·2–99·9)	92·8 (84·3–99·8)	-2·24 (-6·98–0·44)	-0·431 (-3·74–2·57)
Diet low in polyunsaturated fatty acids	55·2 (17·1–95)	50·1 (14·9–91·8)	49·1 (14·3–91·3)	-9·2 (-23·7—0·665)	-2·15 (-10·2–4·42)	57·5 (18·9–96·5)	52·4 (16·3–93·9)	51·4 (16–93)	-8·8 (-23·5—0·619)	-1·97 (-9·55–4·12)
Diet high in trans fatty acids	57·8 (47·3–99·9)	50·8 (40–98·9)	50·9 (40·1–97·5)	-12·1 (-25·5–0)	0·184 (-11·5–13·4)	55·8 (45–99·7)	47·7 (36·3–99·5)	47·7 (36·6–99·4)	-14·5 (-26·8—2·01e-14)	-0·0247 (-10·4–12·9)
Diet high in sodium	18·4 (5·17–40·2)	22·3 (5·57–44·9)	24·5 (6·38–48·7)	21·1 (-32·5–131)	9·86 (-23·3–70·6)	11·2 (3·26–28·7)	11·7 (3·5–28·9)	12·5 (3·61–31·1)	4·07 (-29·8–75·1)	7·39 (-21·4–51)
Low physical activity	4·48 (1·93–9·23)	4·86 (2·14–9·8)	4·91 (2·2–9·97)	8·63 (1·05–19·3)	1·05 (-4·35–6·82)	6·99 (3·63–13)	7·76 (4·17–14)	7·84 (4·25–14·2)	11·1 (5·24–20·3)	1·01 (-2·77–5·21)
Occupational asthmagens	22·3 (18·8–26·5)	21 (17·7–25)	22 (18·2–26·7)	-5·98 (-17·4–6·5)	4·84 (-4·92–15·8)	11·7 (9·4–14·5)	11 (8·97–13·4)	11·6 (9·16–14·3)	-6·32 (-22·2–11·5)	5·67 (-6·44–18)
Occupational particulate matter, gases, and fumes	9·14 (6·46–13·1)	8·72 (6·28–12·7)	8·52 (6·09–12·3)	-4·57 (-10·8–2·51)	-2·33 (-5·66–0·958)	4·35 (2·97–6·8)	4·31 (3·04–6·4)	4·1 (2·9–6·09)	-0·945 (-12·3–12·8)	-5·03 (-10·1–0·347)
Occupational noise	8·75 (8–9·69)	8·55 (8·02–9·28)	8·42 (7·93–9·11)	-2·25 (-6·46–2·73)	-1·55 (-3·41–0·4)	5·12 (4·44–5·83)	5·7 (5·27–6·19)	5·84 (5·44–6·31)	11·3 (4–20·8)	2·37 (-0·027–5·04)
Occupational injuries	--	--	--	--	--	--	--	--	--	--
Occupational ergonomic factors	11·2 (9·43–13·2)	10·8 (9·14–12·9)	11·3 (9·45–13·5)	-2·72 (-9·88–4·99)	3·99 (-3·3–11·6)	6·43 (5·13–8·07)	8·07 (6·47–9·96)	8·82 (6·99–11·2)	25·4 (5·1–49·4)	9·35 (-5·71–27·9)
Childhood sexual abuse	10·6 (6·74–14·7)	11 (7·04–15·8)	10·7 (6·96–15·2)	4·38 (-2·5–12·8)	-2·79 (-11–6·54)	13·3 (9·72–18·3)	14·1 (10·3–19·6)	13·7 (10·1–18·8)	6·28 (-2·16–15·1)	-2·58 (-10·4–8·27)
Intimate partner violence	--	--	--	--	--	22·8 (12·5–31·3)	20 (11·3–28·2)	22·8 (12·5–31·3)	-12·2 (-19·2–0·696)	13·9 (2·98–28·7)
Non-exclusive breastfeeding	25·9 (19·7–33·8)	23·2 (17·7–30·5)	23·3 (17·7–30·4)	-10·3 (-22·8–4·73)	0·256 (-11·3–13·3)	25·9 (19·6–33·8)	23·2 (17·7–30·5)	23·3 (17·7–30·4)	-10·3 (-22·8–4·75)	0·258 (-11·3–13·4)
Discontinued breastfeeding	28 (27·1–28·7)	27·7 (26·9–28·5)	27·6 (26·7–28·4)	-0·883 (-4·81–3·42)	-0·36 (-3·54–3·02)	27·9 (27·1–28·7)	27·7 (26·9–28·5)	27·6 (26·6–28·4)	-0·913 (-4·83–3·38)	-0·444 (-3·62–2·93)
Diet low in calcium	20·7 (9·77–39·2)	14·9 (6·52–28·4)	14·7 (6·4–28)	-28 (-47·5—14)	-1·58 (-9·25–6·95)	15·1 (6·23–29·5)	9·48 (3·5–17·4)	9·29 (3·37–17·2)	-37·4 (-58·3—19·3)	-1·94 (-15–11)
Occupational exposure to asbestos	19·1 (18–20·3)	17·8 (17·3–18·3)	16 (13·5–18·5)	-7·2 (-13—1·13)	-9·66 (-23·5–4·27)	3·43 (2·68–5·57)	3·44 (2·46–4·38)	3·62 (2·39–4·81)	0·155 (-31·1–28·3)	5·18 (-20·1–34·8)
Occupational exposure to arsenic	0·492 (0–1·15)	0·538 (0–1·26)	0·562 (0–1·34)	9·24 (-7·34–NA)	4·45 (-6·96–NA)	0·466 (0–1·12)	0·464 (0–1·07)	0·469 (0–1·11)	-0·313 (-16·8–NA)	1·16 (-10·1–NA)
Occupational exposure to benzene	0·829 (0·0489–2·36)	0·882 (0·0514–2·55)	0·924 (0·0528–2·67)	6·35 (-1·17–15·8)	4·76 (-1·79–13·3)	1·11 (0·0684–3·24)	1·42 (0·0862–4·04)	1·55 (0·0971–4·47)	27·7 (16·1–45·4)	9·07 (1·77–19·6)
Occupational exposure to beryllium	0·024 (0·023–0·025)	0·0252 (0·0246–0·0258)	0·0264 (0·0253–0·0275)	5·01 (0·499–10·2)	5 (0·728–9·27)	0·0256 (0·024–0·0271)	0·0281 (0·0273–0·0289)	0·0301 (0·0287–0·0315)	9·68 (2·58–17·5)	7·1 (1·74–12·3)
Occupational exposure to cadmium	0·0573 (0·0509–0·0642)	0·0624 (0·0565–0·0681)	0·0653 (0·0576–0·0736)	8·87 (-6·27–24·9)	4·74 (-5·77–15·5)	0·0555 (0·0488–0·0635)	0·0563 (0·0507–0·0624)	0·0575 (0·0515–0·0646)	1·41 (-13·7–20·9)	2·19 (-8·15–13·1)
Occupational exposure to chromium	0·135 (0·122–0·15)	0·147 (0·135–0·159)	0·155 (0·139–0·171)	8·69 (-4·36–22·5)	5·04 (-4·12–14·6)	0·137 (0·122–0·154)	0·145 (0·134–0·157)	0·15 (0·137–0·165)	6·14 (-7·17–22·2)	3·44 (-5·3–12·1)
Occupational exposure to diesel engine exhaust	0·757 (0·701–0·816)	0·762 (0·717–0·81)	0·801 (0·741–0·863)	0·656 (-8·68–11·1)	5·13 (-1·96–12·7)	0·625 (0·568–0·687)	0·676 (0·624–0·733)	0·723 (0·65–0·804)	8·14 (-4·58–23·4)	6·94 (-3·26–17·2)
Occupational exposure to formaldehyde	0·285 (0·251–0·323)	0·304 (0·274–0·333)	0·315 (0·277–0·356)	6·57 (-8·76–23·2)	3·5 (-7·34–14·5)	0·266 (0·234–0·305)	0·282 (0·256–0·308)	0·289 (0·261–0·32)	5·98 (-8·96–24·3)	2·66 (-6·95–12·3)
Occupational exposure to nickel	0·505 (0–2·22)	0·54 (0–2·36)	0·562 (0–2·43)	6·94 (-6·73–NA)	4·05 (-5·35–NA)	0·384 (0–1·72)	0·37 (0–1·6)	0·371 (0–1·62)	-3·63 (-20·5–NA)	0·278 (-11·9–NA)
Occupational exposure to polycyclic aromatic hydrocarbons	0·283 (0·259–0·31)	0·308 (0·287–0·329)	0·323 (0·295–0·354)	8·84 (-2·41–20·3)	4·85 (-2·95–13·3)	--	--	--	--	--
Occupational exposure to silica	6·41 (0·94–21·4)	6·74 (0·955–22·3)	6·99 (1–22·7)	5·16 (-7·13–22·5)	3·72 (-5·8–14·9)	3·55 (0·495–11·4)	3·38 (0·476–10·6)	3·4 (0·475–11·1)	-4·77 (-21·1–18·6)	0·609 (-11·6–16·3)
Occupational exposure to sulfuric acid	0·838 (0·177–2·77)	0·876 (0·182–2·73)	0·898 (0·189–2·81)	4·47 (-9·54–19·4)	2·55 (-7·11–12·6)	0·81 (0·17–2·51)	0·772 (0·159–2·51)	0·755 (0·157–2·33)	-4·71 (-21·9–16·5)	-2·13 (-13·4–10·3)
Unsafe sex	--	--	--	--	--	--	--	--	--	--
Occupational exposure to trichloroethylene	0·0757 (0·0707–0·0818)	0·0806 (0·0761–0·0854)	0·0849 (0·0782–0·0918)	6·5 (-2·6–16)	5·33 (-1·53–12·1)	0·0789 (0·0722–0·0861)	0·084 (0·0794–0·0897)	0·088 (0·0815–0·0953)	6·54 (-4·23–19·9)	4·75 (-2·66–12·7)
No access to handwashing facility	3·37 (3·11–3·63)	3·21 (2·95–3·48)	3·17 (2·92–3·43)	-4·65 (-12·9–4·35)	-1·41 (-6·76–4·22)	3·37 (3·11–3·63)	3·21 (2·95–3·48)	3·17 (2·92–3·43)	-4·65 (-12·9–4·34)	-1·41 (-6·76–4·2)
Child wasting	1·27 (0·878–1·68)	1·22 (0·861–1·63)	1·22 (0·858–1·62)	-3·63 (-14·3–8·89)	-0·374 (-7·81–8·47)	1·07 (0·759–1·44)	0·967 (0·668–1·24)	1·02 (0·7–1·37)	-9·94 (-29·7–16)	5·07 (-17·1–32·8)
Child stunting	1·65 (0·816–2·47)	1·22 (0·587–1·89)	1·21 (0·561–1·89)	-26 (-43—4·62)	-0·938 (-18·9–17·9)	1·95 (0·982–2·92)	1·6 (0·77–2·42)	1·57 (0·773–2·4)	-17·8 (-34·7–3·78)	-1·76 (-17·4–16·6)
Chewing tobacco	0·174 (0·122–0·247)	0·166 (0·115–0·231)	0·17 (0·118–0·236)	-4·7 (-37–46·6)	2·64 (-21·1–32·6)	0·131 (0·0819–0·199)	0·131 (0·0823–0·199)	0·133 (0·0825–0·207)	-0·423 (-42–70·1)	1·91 (-28–44)
Diet low in legumes	74·3 (37·9–99·1)	73·2 (37·7–98·8)	74·1 (37·9–98·8)	-1·44 (-10·1–4·68)	1·17 (-3·22–9·97)	70·7 (31·6–97·8)	69·5 (29·7–97·3)	70·3 (31·3–97·2)	-1·65 (-12·8–4·95)	1·12 (-4·21–11)
Short gestation	23·1 (20·4–26·2)	50·9 (44·6–57)	47·1 (41·1–53·1)	120 (98·6–143)	-7·44 (-16–1·51)	25·7 (23–28·5)	48·6 (43·4–54)	44·6 (39·5–49·7)	89 (71·5–107)	-8·18 (-15·9—0·6)
Low birth weight	11·9 (10·9–13·1)	24·9 (22·5–27·2)	23·1 (20·6–25·7)	109 (85·6–132)	-6·97 (-15·8–2·04)	13·4 (12·2–14·6)	23·9 (22–26·1)	22·6 (20·6–24·8)	78·3 (62·2–95·8)	-5·59 (-13·7–2·42)
High temperature	22·6 (8·8–38)	0 (0–0)	0 (0–0)	-100 (-100—100)	--	22·6 (8·8–38)	0 (0–0)	0 (0–0)	-100 (-100—100)	--
Low temperature	31·2 (26·2–36·6)	34 (29·6–38·8)	30·4 (25·6–35·7)	9·22 (5·83–13·6)	-10·6 (-14·1—7·59)	31·2 (26·2–36·6)	34 (29·6–38·8)	30·4 (25·6–35·7)	9·22 (5·83–13·6)	-10·6 (-14·1—7·59)
Kidney dysfunction	13·5 (9·02–19·8)	14·3 (9·79–20·7)	14·1 (9·65–20·4)	5·81 (2·82–10)	-1·01 (-3·51–1·26)	14·4 (9·69–20·9)	15·1 (10·3–21·6)	15·2 (10·2–21·8)	4·81 (2·06–9·05)	0·461 (-2·06–3·04)
Bullying victimization	6·09 (2·44–12·1)	6·05 (2·4–12·2)	5·6 (2·25–11·5)	-0·714 (-9·94–9)	-7·44 (-12·8—2·81)	4·67 (1·91–9·54)	4·98 (1·99–10·1)	4·71 (1·85–9·65)	6·58 (-4·91–18)	-5·44 (-12—0·584)
High LDL cholesterol	49·6 (46·1–53·2)	42·8 (39·5–46·4)	43·7 (40·3–47·3)	-13·6 (-19·2—8·34)	1·92 (-2·18–5·96)	--	--	--	--	--

Supplementary Results Table 1. Derby age-standardised Summary Exposure Values (SEVs) for most-detailed risk factors and percentage change, by sex, 1990, 2010, and 2019. Data in parentheses are 95% uncertainty intervals.

	Male					Female				
	1990	2010	2019	Percentage change 1990-2010	Percentage change 2010-2019	1990	2010	2019	Percentage change 1990-2010	Percentage change 2010-2019
Unsafe sanitation	0·606 (0·215–1·38)	0·326 (0·124–0·732)	0·233 (0·0796–0·509)	-46·3 (-83·9–67)	-28·4 (-71·3–70·7)	0·606 (0·215–1·38)	0·326 (0·124–0·732)	0·233 (0·0796–0·509)	-46·3 (-83·9–67)	-28·4 (-71·3–70·7)
Ambient particulate matter pollution	20·7 (7·7–37·5)	12·6 (8·57–17·4)	9·54 (5·86–13·7)	-39 (-66·3–38·3)	-24·5 (-32·1—20·1)	20·7 (7·72–37·5)	12·6 (8·6–17·4)	9·54 (5·86–13·7)	-39 (-66·4–38·4)	-24·4 (-32—20·2)
Household air pollution from solid fuels	0·0107 (0·00202–0·0329)	0·00362 (0·000677–0·012)	0·00226 (0·000321–0·0076)	-66·1 (-90·8–19·2)	-37·4 (-73·1–34·2)	0·0141 (0·00292–0·0444)	0·00479 (0·000923–0·017)	0·003 (0·000464–0·0101)	-66 (-90·7–20·6)	-37·4 (-73–34·2)
Ambient ozone pollution	9·18 (0–30)	4·12 (0–17·1)	4·77 (0–20·6)	-55·1 (-100–NA)	15·6 (-100–NA)	9·18 (0–30)	4·12 (0–17·1)	4·77 (0–20·6)	-55·1 (-100–NA)	15·6 (-100–NA)
Residential radon	25 (3·99–79·4)	25 (3·99–79·4)	25 (3·99–79·4)	0 (0–0)	0 (0–0)	25 (3·99–79·4)	25 (3·99–79·4)	25 (3·99–79·4)	0 (0–0)	0 (0–0)
Lead exposure	30·8 (6·39–55·9)	19·4 (3·09–41·7)	13·2 (1·12–33·3)	-37 (-60·3–23·6)	-32 (-63—19·6)	12·4 (0·0173–34·5)	7·2 (1e-11–25·4)	4·85 (3·56e-12–20·4)	-41·7 (-98·7–23·9)	-32·7 (-100—18·8)
Child underweight	0·928 (0·526–1·41)	0·786 (0·429–1·21)	0·767 (0·391–1·21)	-15·3 (-33·8–7·66)	-2·39 (-16·8–14·6)	1·09 (0·609–1·64)	0·945 (0·529–1·4)	0·923 (0·525–1·4)	-13·3 (-29·8–7·83)	-2·25 (-16·1–13·7)
Iron deficiency	--	--	--	--	--	11·5 (9·1–13·9)	10·3 (8·34–12·3)	10 (7·93–12·1)	-10·2 (-25·3–6·59)	-2·39 (-12·4–8·12)
Vitamin A deficiency	4·33 (0·833–11·7)	2·06 (0·394–6·2)	1·66 (0·301–4·89)	-52·4 (-90·2–145)	-19·4 (-70·4–135)	3·4 (0·97–7·98)	2·15 (0·584–5·09)	1·8 (0·509–4·48)	-36·7 (-79·4–120)	-16·1 (-60·5–93·9)
Zinc deficiency	3·58 (0–10·8)	2·34 (0–7·42)	2·33 (0–7·26)	-34·6 (-100–NA)	-0·699 (-27·9–NA)	3·46 (0–11)	2·23 (0–7·33)	2·19 (0–7·11)	-35·5 (-100–NA)	-1·96 (-27–NA)
Smoking	27·8 (24·2–32)	22 (19·2–25·2)	18·6 (16·2–21·7)	-20·8 (-28·2—12·6)	-15·4 (-20·4—9·79)	23 (19·2–27·4)	18·6 (15·5–22·4)	14·8 (12·2–17·8)	-19·1 (-30·4—5·51)	-20·6 (-28·6—11·8)
Secondhand smoke	47·1 (40·8–53·6)	33·4 (28·7–38·9)	31·4 (26·8–36·2)	-29·1 (-41·5—15·2)	-5·87 (-15·5–4·37)	49·2 (44·1–54·6)	33·8 (30·3–37·7)	32 (28·2–36)	-31·3 (-40·7—21·2)	-5·44 (-13·3–2·7)
Alcohol use	16·8 (8·82–24·8)	19·3 (10·9–28·5)	17·3 (9·29–25·7)	14·9 (-28·6–81·7)	-10·6 (-36·7–18·3)	5·56 (0·632–13·9)	5·68 (0·463–13·7)	5·15 (0·348–13·3)	2·11 (-80·4–324)	-9·34 (-76·4–167)
Drug use	0·334 (0·167–0·685)	0·663 (0·401–1·13)	0·64 (0·375–1·12)	98·6 (53·2–168)	-3·49 (-13·6–6·95)	0·198 (0·106–0·383)	0·314 (0·183–0·539)	0·302 (0·17–0·543)	58·8 (21–108)	-3·83 (-15·7–6·84)
High fasting plasma glucose	9·1 (7·57–11·2)	17·2 (14·6–20·3)	21·4 (18·1–25·9)	89·4 (57·2–125)	24·4 (7·5–48·6)	7·52 (6·06–9·49)	12·9 (10·6–16)	16·3 (13·4–20)	71·5 (39·6–113)	26·7 (6·27–53·6)
High systolic blood pressure	37·3 (27·3–48·3)	34·5 (27·3–43·2)	34·7 (26·1–45·2)	-7·28 (-34·4–34)	0·514 (-21·6–29·8)	33·5 (22–47·4)	21·2 (14·2–29·2)	19·5 (12·1–28·4)	-36·8 (-60·8–4·2)	-7·88 (-40·8–38·2)
High body-mass index	19·2 (13·1–26·5)	24·8 (16·9–33·7)	26·6 (18·4–36·3)	29 (14·5–50·9)	7·09 (-0·759–15·1)	22·8 (16·2–31·7)	30·1 (22·8–40·1)	31·5 (24·3–41·8)	31·8 (13·6–57·5)	4·85 (-4·64–16·7)
Low bone mineral density	8·72 (4·59–14·6)	7·63 (3·75–13·3)	9·27 (5–15·3)	-12·4 (-35·7–14·9)	21·5 (-7·28–69·3)	14·5 (9·11–21·1)	14 (8·92–20·5)	13·7 (8·5–20·6)	-3·33 (-25·8–24·2)	-2·19 (-23·6–24·5)
Diet low in fruits	64·9 (51·9–77·3)	57·8 (44·3–70·4)	56·4 (42·6–69·5)	-11 (-24·8–2·36)	-2·51 (-13·7–9·68)	65·9 (54–78)	58 (44·6–70·8)	57 (43·6–70·5)	-11·9 (-26·8–1·25)	-1·72 (-11·6–9·17)
Diet low in vegetables	49·6 (29·6–70·1)	44·6 (25–66)	43·3 (24·6–64·6)	-10·1 (-29·5–9·4)	-3·03 (-17·4–13·4)	46·7 (27·5–69·1)	41·2 (22·8–62·8)	39·6 (21·6–59·7)	-11·9 (-32·9–8·51)	-3·87 (-21–13·3)
Diet low in whole grains	89·7 (83·8–94·8)	89·4 (83·6–94·4)	89·5 (83·6–94·6)	-0·298 (-3·66–3·19)	0·0918 (-1·82–2·33)	--	--	--	--	--
Diet low in nuts and seeds	81·8 (42·2–92·7)	73·7 (38·3–87·3)	75·9 (40·1–88·8)	-9·87 (-23·6–1·89)	2·98 (-5·91–14·9)	81·9 (42·2–92·7)	73·6 (39–87·6)	74·6 (38·9–88·4)	-10·2 (-24·1–1·72)	1·41 (-8·38–12·5)
Diet low in milk	57·1 (32·8–77·9)	58·2 (34·2–78·8)	57·9 (33·7–79·2)	1·91 (-10·5–19·4)	-0·5 (-8·78–9·03)	59·9 (34·5–80·7)	62·1 (37·3–83·1)	61·8 (37·8–83·2)	3·66 (-7·51–19·9)	-0·491 (-7·22–6·2)
Diet high in red meat	67 (52·1–80·8)	53·2 (37·4–69·6)	55·4 (39·5–71·6)	-20·6 (-40·5—0·169)	4·24 (-13–26·6)	66·6 (49·9–81·8)	54·6 (38·9–70)	57·4 (40·9–72·4)	-18 (-37·9–3·38)	5·15 (-11·8–26·3)
Diet high in processed meat	77·2 (53·9–96·2)	81·9 (61·2–98·9)	81·9 (61·8–98·7)	6·08 (-4·03–21·9)	0·0608 (-5·86–6·97)	80·2 (58·7–98)	85·3 (65·5–99·3)	85·7 (66·5–99·4)	6·39 (-2·01–20·8)	0·378 (-4·93–7·06)
Diet high in sugar-sweetened beverages	43·9 (28·5–64)	50 (33·1–68·2)	52 (34·2–71·3)	13·8 (-22·4–74·7)	4·06 (-20·3–39·2)	45·4 (28·6–65·1)	50·9 (32·7–68·5)	52·2 (33·3–71·5)	12·2 (-22·9–64·9)	2·57 (-21·9–35·4)
Diet low in fiber	40·3 (25·2–56·9)	33·1 (18·5–49·3)	32·7 (17·9–48·5)	-17·8 (-45·7–14·3)	-1·33 (-22·3–25·3)	40·4 (23·3–57·9)	33 (18·7–49·5)	32·4 (18·4–48·9)	-18·4 (-44·1–18·1)	-1·74 (-23·7–26·1)
Diet low in seafood omega-3 fatty acids	93 (82–100)	90·5 (75·8–100)	90·2 (75·7–100)	-2·74 (-15·4–6·33)	-0·318 (-11·9–12)	94 (84·8–100)	91·7 (80·3–100)	91·5 (79·3–100)	-2·39 (-11·9–4·07)	-0·218 (-10·2–10·2)
Diet low in polyunsaturated fatty acids	52·1 (15·4–93·4)	46·5 (13–88·6)	45·2 (12·1–88)	-10·7 (-28·5–0)	-2·94 (-13·2–6·41)	54·6 (16·7–95·2)	49 (13·7–92·9)	47·7 (13·4–91·1)	-10·1 (-28–0)	-2·73 (-13·7–6·15)
Diet high in trans fatty acids	57·9 (47–100)	50·9 (39·4–98·3)	51·1 (39·9–98·7)	-12 (-26·4–1·56)	0·34 (-14·8–17·8)	55·9 (44·5–100)	47·9 (35·9–99·8)	47·8 (36·1–99·3)	-14·3 (-28·7–0)	-0·187 (-13·2–15)
Diet high in sodium	18·7 (4·4–40·9)	22·6 (4·9–46·3)	24·8 (5·26–51)	20·7 (-46·9–161)	9·74 (-36–96·4)	11·3 (2·78–28·8)	11·8 (3·24–29·5)	12·7 (3·42–31·8)	4·86 (-41·3–113)	7·24 (-30·5–72·4)
Low physical activity	4·6 (1·97–9·31)	5·08 (2·18–10·2)	5·19 (2·38–10·7)	10·5 (-4·79–30·5)	2·08 (-10·7–16·9)	7·09 (3·71–13·2)	7·96 (4·31–14·3)	8·09 (4·4–14·5)	12·2 (2·41–26·7)	1·66 (-6·4–10·9)
Occupational asthmagens	22·5 (18·2–28)	21·5 (17·4–26·6)	22·6 (17·6–28·6)	-4·13 (-21–16·7)	4·77 (-9·22–22·8)	--	--	--	--	--
Occupational particulate matter, gases, and fumes	9·14 (6·44–13·7)	8·71 (6·16–12·9)	8·51 (6·05–12·5)	-4·61 (-13·2–5·69)	-2·37 (-7·09–2·52)	4·34 (2·8–6·86)	4·21 (2·9–6·3)	3·98 (2·72–6·13)	-3·01 (-18·7–16·4)	-5·56 (-12·2–2·31)
Occupational noise	8·53 (7·5–9·69)	8·41 (7·64–9·3)	8·3 (7·59–9·12)	-1·45 (-7·69–5·63)	-1·24 (-4·03–1·82)	5·11 (4·16–6·13)	5·7 (5·08–6·45)	5·85 (5·27–6·49)	11·6 (0·753–26·2)	2·52 (-1·52–7·12)
Occupational injuries	--	--	--	--	--	--	--	--	--	--
Occupational ergonomic factors	10·3 (8·48–12·5)	10·1 (8·31–12·4)	10·5 (8·67–13)	-1·57 (-13·2–11·1)	3·91 (-4·97–13·3)	--	--	--	--	--
Childhood sexual abuse	10·6 (6·74–14·7)	11 (7·04–15·8)	10·7 (6·96–15·2)	4·38 (-2·5–12·8)	-2·79 (-11–6·54)	13·3 (9·72–18·3)	14·1 (10·3–19·6)	13·7 (10·1–18·8)	6·28 (-2·16–15·1)	-2·58 (-10·4–8·27)
Intimate partner violence	--	--	--	--	--	22·8 (12·5–31·3)	20 (11·3–28·2)	22·8 (12·5–31·3)	-12·2 (-19·2–0·696)	13·9 (2·98–28·7)
Non-exclusive breastfeeding	26·1 (19·2–34·1)	23·3 (17·4–30·9)	23·3 (17·2–31·1)	-10·5 (-25·8–8·43)	0·0722 (-13·5–15·9)	26 (19·2–34·1)	23·3 (17·4–30·9)	23·3 (17·2–31·1)	-10·5 (-25·7–8·47)	0·0788 (-13·5–15·9)
Discontinued breastfeeding	28·3 (27·2–29·2)	28 (26·9–29)	27·4 (26·2–28·3)	-0·868 (-5·84–4·7)	-2·33 (-6·11–1·55)	28·3 (27·2–29·3)	28·2 (27·1–29·1)	27·4 (26·3–28·4)	-0·51 (-5·49–5·07)	-2·71 (-6·46–1·15)
Diet low in calcium	18·4 (8·46–34·7)	12·5 (5·2–23·8)	12 (5·1–22·6)	-32·1 (-50·2—17·7)	-4·17 (-15·5–7·82)	12·9 (5·2–24·9)	6·98 (2·56–12·9)	6·66 (2·4–12·5)	-46 (-66·8—25·1)	-4·66 (-24·4–20·3)
Occupational exposure to asbestos	24·5 (22·7–26·3)	22·9 (21·6–24·3)	19·7 (16·8–22·6)	-6·56 (-14·5–2·13)	-13·9 (-26·9—0·255)	3·46 (2·46–6·18)	3·56 (2·63–4·68)	3·51 (2·41–4·95)	2·75 (-30·1–44·5)	-1·22 (-28·2–34·9)
Occupational exposure to arsenic	0·516 (0–1·22)	0·557 (0–1·31)	0·578 (0–1·36)	7·95 (-11·4–NA)	3·78 (-9·63–NA)	0·471 (0–1·12)	0·464 (0–1·1)	0·467 (0–1·13)	-1·44 (-21·9–NA)	0·526 (-13·7–NA)
Occupational exposure to benzene	0·842 (0·0483–2·35)	0·9 (0·0553–2·52)	0·94 (0·0575–2·82)	6·88 (-5·48–26·5)	4·5 (-4·33–17·4)	1·14 (0·0667–3·33)	1·46 (0·0857–4·16)	1·59 (0·1–4·62)	28 (10·5–60·7)	9·44 (-0·756–25·4)
Occupational exposure to beryllium	0·024 (0·0222–0·0257)	0·0252 (0·0235–0·0271)	0·0265 (0·0246–0·0284)	5·12 (-3·64–15·2)	4·94 (-1·29–11·3)	0·0255 (0·0227–0·0283)	0·0279 (0·0257–0·0303)	0·0299 (0·0273–0·0324)	9·53 (-2·69–24·3)	7·1 (-0·64–15·2)
Occupational exposure to cadmium	0·0598 (0·0511–0·0693)	0·0643 (0·0555–0·0737)	0·067 (0·0576–0·0782)	7·62 (-10·5–29·5)	4·13 (-8·72–18·5)	0·0559 (0·0459–0·0673)	0·0562 (0·0485–0·0657)	0·0571 (0·0487–0·0668)	0·561 (-18·9–27)	1·5 (-11·3–15·8)
Occupational exposure to chromium	0·141 (0·123–0·162)	0·152 (0·134–0·172)	0·159 (0·138–0·182)	7·59 (-8·63–26·9)	4·41 (-6·69–16·9)	0·138 (0·116–0·162)	0·145 (0·128–0·165)	0·149 (0·13–0·17)	5·39 (-12·5–29·3)	2·96 (-8·04–15·2)
Occupational exposure to diesel engine exhaust	0·742 (0·66–0·83)	0·753 (0·676–0·835)	0·792 (0·708–0·885)	1·43 (-11·4–17)	5·17 (-4·37–15·6)	0·594 (0·506–0·681)	0·639 (0·552–0·737)	0·681 (0·584–0·794)	7·58 (-11·4–31·1)	6·68 (-7·19–21·2)
Occupational exposure to formaldehyde	0·3 (0·255–0·351)	0·315 (0·27–0·363)	0·324 (0·278–0·38)	5·08 (-12·8–27)	2·79 (-9·91–16·9)	0·271 (0·224–0·324)	0·285 (0·248–0·33)	0·292 (0·252–0·335)	5·38 (-14–29·9)	2·21 (-9·8–14·6)
Occupational exposure to nickel	0·506 (0–2·18)	0·542 (0–2·33)	0·562 (0–2·44)	7·08 (-9·41–NA)	3·71 (-8·58–NA)	0·383 (0–1·76)	0·364 (0–1·59)	0·361 (0–1·62)	-4·89 (-25·5–NA)	-0·877 (-15·7–NA)
Occupational exposure to polycyclic aromatic hydrocarbons	0·293 (0·258–0·331)	0·316 (0·282–0·354)	0·33 (0·292–0·372)	8·03 (-6·68–25·7)	4·29 (-5·94–15·6)	--	--	--	--	--
Occupational exposure to silica	6·2 (0·868–18·7)	6·58 (0·858–21·6)	6·81 (0·954–22·3)	6·13 (-10·5–31·3)	3·62 (-8·47–19·4)	3·38 (0·501–10·9)	3·19 (0·43–10·1)	3·17 (0·425–10·3)	-5·71 (-27·1–25·9)	-0·713 (-16·3–20·2)
Occupational exposure to sulfuric acid	0·86 (0·179–2·72)	0·891 (0·197–2·86)	0·91 (0·193–2·8)	3·55 (-14·2–24)	2·09 (-9·77–15·1)	0·831 (0·172–2·71)	0·785 (0·164–2·55)	0·759 (0·159–2·3)	-5·54 (-27·1–23·6)	-3·35 (-16·1–12·4)
Unsafe sex	--	--	--	--	--	--	--	--	--	--
Occupational exposure to trichloroethylene	0·0785 (0·0708–0·0873)	0·0829 (0·0751–0·0921)	0·0869 (0·0774–0·0969)	5·66 (-6·33–21·7)	4·84 (-4·02–14·8)	0·0783 (0·0674–0·0907)	0·0828 (0·0743–0·0926)	0·0863 (0·0759–0·0972)	5·71 (-10·4–27·2)	4·31 (-5·65–15·2)
No access to handwashing facility	3·36 (3·03–3·69)	3·2 (2·88–3·53)	3·15 (2·84–3·45)	-4·65 (-15·2–7·55)	-1·65 (-8·41–5·65)	3·36 (3·03–3·69)	3·2 (2·88–3·53)	3·15 (2·84–3·45)	-4·65 (-15·2–7·55)	-1·65 (-8·41–5·65)
Child wasting	1·27 (0·88–1·7)	1·22 (0·868–1·65)	1·21 (0·843–1·64)	-3·64 (-16·4–11·4)	-0·765 (-10·4–9·38)	--	--	--	--	--
Child stunting	1·67 (0·818–2·55)	1·23 (0·59–1·97)	1·22 (0·526–1·94)	-26·1 (-46·6–1·69)	-1·1 (-22·3–25·3)	1·96 (0·982–3·02)	1·6 (0·771–2·48)	1·57 (0·786–2·4)	-18·7 (-37·9–8·63)	-1·88 (-20·9–20·9)
Chewing tobacco	0·176 (0·11–0·258)	0·168 (0·106–0·257)	0·172 (0·11–0·261)	-4·62 (-42·6–69·6)	2·26 (-26·7–41·5)	0·134 (0·075–0·228)	0·133 (0·0756–0·232)	0·134 (0·0741–0·23)	-0·693 (-49·2–91·8)	1·3 (-35–57·4)
Diet low in legumes	73·1 (34·6–98·9)	72 (34·4–97·9)	72·8 (34·9–98·3)	-1·51 (-15·2–7·73)	1·11 (-5·48–12·6)	69·5 (29·3–97·1)	68·2 (26·9–96·4)	68·9 (28·4–96·6)	-1·83 (-14·8–8·76)	0·961 (-7·19–14·5)
Short gestation	20·7 (16·4–25·7)	47·6 (37–61)	52·4 (38·2–72·2)	131 (67·4–213)	10·1 (-22–57·4)	25·6 (20·6–31·7)	47·5 (37·8–59·2)	48·7 (37·9–64)	85·2 (29·3–160)	2·69 (-25·7–47·8)
Low birth weight	10·8 (8·57–13·3)	23·6 (18·4–30)	25·4 (18·5–35·6)	118 (56·1–197)	7·63 (-24·8–53·8)	13·9 (11·3–16·9)	24 (19·2–30·4)	24·7 (19·3–32)	73·5 (19·2–147)	2·62 (-25·4–45·5)
High temperature	22·5 (6·73–39·6)	0 (0–0)	0 (0–0)	-100 (-100—100)	--	22·5 (6·73–39·6)	0 (0–0)	0 (0–0)	-100 (-100—100)	--
Low temperature	31·5 (26·1–37·4)	34·3 (29·5–39·7)	30·9 (25·8–36·8)	9·06 (5·43–13·5)	-9·94 (-13·2—6·88)	31·5 (26·1–37·4)	34·3 (29·5–39·7)	30·9 (25·8–36·8)	9·06 (5·43–13·5)	-9·94 (-13·2—6·88)
Kidney dysfunction	13·3 (8·81–19·6)	14 (9·53–20·5)	13·9 (9·41–20·4)	5·53 (0·492–12·3)	-0·463 (-4·99–4·44)	14·2 (9·52–20·7)	14·9 (10·1–21·4)	15 (10·1–21·6)	5·02 (0·765–10·4)	0·57 (-3·48–4·8)
Bullying victimization	6·08 (2·43–12·2)	5·97 (2·37–12)	5·54 (2·23–11·4)	-1·93 (-11–8)	-7·09 (-12·3—2·42)	4·63 (1·9–9·39)	4·89 (1·96–9·91)	4·65 (1·84–9·53)	5·75 (-5·78–17·1)	-5 (-11·7—0·184)
High LDL cholesterol	49·9 (45·7–54·5)	43·2 (39·4–47)	44·1 (40·5–48·3)	-13·4 (-21·4—5·42)	2·22 (-3·48–8·3)	--	--	--	--	--

Supplementary Results Table 1. Derbyshire age-standardised Summary Exposure Values (SEVs) for most-detailed risk factors and percentage change, by sex, 1990, 2010, and 2019. Data in parentheses are 95% uncertainty intervals.

	Male					Female				
	1990	2010	2019	Percentage change 1990-2010	Percentage change 2010-2019	1990	2010	2019	Percentage change 1990-2010	Percentage change 2010-2019
Unsafe sanitation	0·722 (0·265–1·61)	0·42 (0·159–0·857)	0·314 (0·117–0·651)	-41·8 (-81·6–89)	-25·4 (-67·9–68·1)	0·722 (0·265–1·61)	0·42 (0·159–0·857)	0·314 (0·117–0·651)	-41·8 (-81·6–89)	-25·4 (-67·9–68·1)
Ambient particulate matter pollution	18·7 (6·9–33·7)	11·3 (7·83–15·3)	8·44 (5·26–12)	-39·3 (-67·6–42·9)	-25·6 (-33·7—20·9)	18·7 (6·89–33·7)	11·3 (7·84–15·3)	8·44 (5·27–12)	-39·2 (-67·5–42·9)	-25·6 (-33·7—20·9)
Household air pollution from solid fuels	0·0238 (0·00511–0·0758)	0·00809 (0·00141–0·0265)	0·0049 (0·00084–0·0164)	-66 (-89·3—1·58)	-39·5 (-74·2–28·2)	0·0312 (0·00683–0·0982)	0·0107 (0·0019–0·0336)	0·00648 (0·00111–0·0214)	-65·7 (-89·2—1·24)	-39·4 (-74·1–28·4)
Ambient ozone pollution	15·7 (4·59–30·6)	6·47 (0·993–17·6)	5·92 (0·814–16·2)	-58·7 (-88·8—18·3)	-8·51 (-74·1–205)	15·7 (4·59–30·6)	6·47 (0·993–17·6)	5·92 (0·814–16·2)	-58·7 (-88·8—18·3)	-8·51 (-74·1–205)
Residential radon	36·4 (8·95–83·5)	36·4 (8·95–83·5)	36·4 (8·95–83·5)	0 (0–0)	0 (0–0)	36·4 (8·95–83·5)	36·4 (8·95–83·5)	36·4 (8·95–83·5)	0 (0–0)	0 (0–0)
Lead exposure	32·4 (7·02–57·5)	21·1 (3·59–44)	14·7 (1·46–35·3)	-34·8 (-58·9—22)	-30·5 (-61·2—18·8)	13 (0·0302–35·4)	7·96 (0·0127–26·6)	5·51 (5·38e-12–21·8)	-38·8 (-82·7—21·7)	-30·8 (-100—18)
Child underweight	0·924 (0·533–1·4)	0·787 (0·416–1·19)	0·775 (0·419–1·2)	-14·8 (-32·4–6·48)	-1·44 (-15·4–15·3)	1·09 (0·63–1·63)	0·949 (0·547–1·38)	0·937 (0·534–1·4)	-12·9 (-29·2–7·69)	-1·26 (-14·5–14·8)
Iron deficiency	--	--	--	--	--	11 (8·9–13·3)	9·89 (8·01–11·9)	9·76 (7·85–11·9)	-10·4 (-24·2–5·19)	-1·3 (-11·1–8·94)
Vitamin A deficiency	5·16 (1·09–14·9)	2·47 (0·531–7·82)	2·05 (0·393–6·14)	-52·1 (-89–125)	-17·1 (-67·7–141)	3·89 (1·25–9·09)	2·59 (0·827–5·81)	2·16 (0·699–4·88)	-33·4 (-77·9–118)	-16·6 (-57·9–87·9)
Zinc deficiency	4·97 (0–14)	3·2 (0–9·87)	3·15 (0–9·58)	-35·6 (-100–NA)	-1·46 (-28·1–NA)	4·78 (0–13·9)	3·04 (0–9·6)	2·99 (0–9·5)	-36·2 (-100–NA)	-1·83 (-27·6–NA)
Smoking	28·3 (24·7–32·1)	21·5 (18·8–24·4)	18 (15·5–20·6)	-24·1 (-31·2—16·4)	-16·3 (-21·1—11·1)	23·2 (19·4–27·1)	18·2 (15·3–21·6)	14·3 (11·9–17·2)	-21·5 (-32·5—7·8)	-21·7 (-29·3—13·6)
Secondhand smoke	43·4 (38·1–48·8)	33·4 (28·7–38·5)	33·6 (28·8–38·4)	-23·1 (-35·7—9·76)	0·501 (-9·48–11·4)	45·5 (41·1–49·8)	34 (30·3–37·7)	34·3 (30·5–38)	-25·2 (-35—14·8)	0·746 (-7·42–9·53)
Alcohol use	16·6 (8·96–24·9)	18·4 (10·2–27·6)	19·3 (10·4–29·8)	10·8 (-30·4–69·4)	4·76 (-25·2–43·2)	--	--	--	--	--
Drug use	0·309 (0·149–0·633)	0·577 (0·323–1·02)	0·571 (0·319–1·06)	86·7 (44·5–148)	-0·95 (-9·94–9·06)	0·192 (0·1–0·382)	0·309 (0·18–0·543)	0·309 (0·177–0·547)	60·6 (21·5–111)	0·259 (-10·6–9·74)
High fasting plasma glucose	9·63 (7·84–12)	17·1 (14·5–20·2)	21·2 (17·6–26·2)	77·6 (48·9–115)	24·1 (8·34–47·3)	7·72 (6·21–9·76)	12·6 (10·4–15·1)	15·8 (13–19)	63·3 (32·8–103)	25 (5·45–51·1)
High systolic blood pressure	34·3 (25·6–44·9)	31·1 (24·6–38·5)	31·2 (23·9–40·5)	-9·32 (-33·4–27)	0·251 (-21·2–28·4)	--	--	--	--	--
High body-mass index	20·9 (14·4–28·8)	26·1 (18·3–35·4)	27·4 (19·3–37·3)	25·1 (11·9–44·7)	4·97 (-1·85–13·3)	23·2 (16·9–31·9)	29·8 (22·8–39·8)	30·8 (23·4–40·8)	28·5 (9·82–49·6)	3·29 (-5·52–13·6)
Low bone mineral density	8·46 (4·38–14·4)	7·33 (3·66–12·9)	8·99 (4·68–15·2)	-13·3 (-35·2–14)	22·7 (-6·82–65·8)	14·2 (8·92–21)	13·6 (8·59–20·3)	13·3 (8·33–19·8)	-4·01 (-26–21·1)	-2·45 (-23·6–27·8)
Diet low in fruits	68·5 (56·5–80·1)	61·6 (48·4–74·2)	60·3 (47·1–72·9)	-10·1 (-23·8–2·13)	-2·18 (-10·8–7·77)	69·7 (58·2–79·9)	62·1 (50·1–73·4)	61·2 (49–73·1)	-10·8 (-22·5–0·389)	-1·49 (-9·86–7·69)
Diet low in vegetables	55·3 (34·3–76·6)	50·8 (30·6–72·3)	49·5 (29·4–70·6)	-8·13 (-23·7–7·79)	-2·69 (-15·4–9·79)	52·7 (32·4–73·7)	47·8 (27·5–68·9)	46·3 (27·2–67·5)	-9·26 (-28·3–6·63)	-3·31 (-17·1–9·63)
Diet low in whole grains	89·1 (82·5–95·1)	88·8 (82–94·7)	88·9 (82·4–94·7)	-0·335 (-3·41–2·9)	0·0884 (-1·96–2·14)	--	--	--	--	--
Diet low in nuts and seeds	83·8 (43·1–93·7)	76·6 (40·5–89)	78·5 (41·8–90·5)	-8·59 (-21·7–1·93)	2·45 (-5·56–12·6)	84 (44·7–93·7)	76·6 (40–89·5)	77·6 (40·3–90·4)	-8·81 (-23·4–1·3)	1·34 (-7·23–12)
Diet low in milk	61·3 (37·2–82·8)	62·4 (36·8–83·4)	62·1 (37·1–83)	1·73 (-8·26–15·1)	-0·415 (-6·72–6·54)	63·6 (37·9–84·8)	65·9 (40·6–86·8)	65·6 (40·4–86·7)	3·61 (-4·25–19)	-0·479 (-6·08–5·13)
Diet high in red meat	63·9 (48·6–78·4)	49·6 (34·4–65·1)	51·8 (36·5–68)	-22·4 (-39·9—1·49)	4·54 (-12·3–28·1)	63·3 (47–77·8)	50·6 (35·3–64·7)	53·3 (36·9–68·7)	-20·1 (-40–2·49)	5·3 (-12·9–26·5)
Diet high in processed meat	76·2 (53–96·5)	81 (59·8–98·4)	81·2 (59·9–98·5)	6·24 (-2·95–21·3)	0·287 (-5·2–6·81)	79·1 (58·1–97·5)	84·4 (64·4–99·5)	84·6 (64·9–99·4)	6·64 (-1·53–21·6)	0·29 (-5·3–6·75)
Diet high in sugar-sweetened beverages	40 (25·2–59·8)	44·2 (28·9–61·7)	46·6 (30·8–64·1)	10·5 (-23·4–65·4)	5·52 (-20·6–39·6)	44·9 (25·6–70·5)	47·2 (26–71)	49·6 (30·5–72·8)	5·27 (-34·8–63·6)	5 (-22–50·5)
Diet low in fiber	45·6 (29·1–62·1)	38·3 (21·7–55)	38·1 (21–54·4)	-15·9 (-41·4–11·9)	-0·643 (-18–22·1)	45·6 (27–63)	38·2 (21·2–56)	37·7 (21·7–55·3)	-16·2 (-41·8–11·1)	-1·27 (-19·3–24·5)
Diet low in seafood omega-3 fatty acids	95·1 (87·1–100)	92·8 (80·8–100)	92·5 (81·5–100)	-2·46 (-13·6–3·8)	-0·272 (-10·1–10·1)	96 (88·2–100)	93·5 (83·2–100)	93·4 (82·9–100)	-2·54 (-11·2–1·98)	-0·197 (-8·43–8·01)
Diet low in polyunsaturated fatty acids	57·3 (18·5–96·5)	52·4 (16·2–93·5)	51·1 (14·8–93·1)	-8·57 (-23·2–0)	-2·57 (-11·7–4·12)	59·6 (20·8–97·5)	54·7 (17·3–95·1)	53·3 (16·8–94·1)	-8·28 (-26·4–0)	-2·44 (-11·3–4·47)
Diet high in trans fatty acids	57·7 (46·5–100)	50·8 (39·3–98·5)	50·8 (39·7–97·9)	-11·9 (-26·6–1·54)	-0·0189 (-13·9–16·2)	55·8 (44·8–100)	47·8 (36–99·4)	47·6 (36·1–100)	-14·4 (-28·6–0)	-0·228 (-13·7–14·9)
Diet high in sodium	18·3 (4·24–39·9)	22·2 (4·77–46·3)	24·5 (5·22–49·8)	21·3 (-42·1–161)	9·98 (-32·7–90·5)	11·2 (2·8–28·8)	11·6 (3·09–29·4)	12·5 (3·25–31·1)	3·95 (-41·9–102)	7·67 (-33·2–77·4)
Low physical activity	4·37 (1·82–9·08)	4·69 (2·03–9·59)	4·74 (2·07–9·87)	7·39 (-6·23–27·6)	1·03 (-12·2–16·7)	6·86 (3·55–12·6)	7·6 (3·98–13·6)	7·68 (4·14–13·7)	10·8 (1·31–26·9)	1·13 (-7·88–11·3)
Occupational asthmagens	22·3 (18·2–27)	20·9 (17·4–25·2)	21·9 (17·8–27·3)	-6·17 (-20·2–11·2)	4·78 (-7·16–18·3)	--	--	--	--	--
Occupational particulate matter, gases, and fumes	9·14 (6·47–13·1)	8·73 (6·2–12·7)	8·54 (6·06–12·4)	-4·42 (-12·8–4·69)	-2·22 (-6·46–2·11)	4·35 (2·89–6·7)	4·34 (2·99–6·49)	4·13 (2·84–6·21)	-0·239 (-15–18·2)	-4·87 (-11·2–2·36)
Occupational noise	8·77 (7·79–9·82)	8·57 (7·87–9·4)	8·44 (7·8–9·25)	-2·27 (-8·15–4·41)	-1·56 (-4·14–1·24)	5·11 (4·31–6)	5·69 (5·07–6·35)	5·82 (5·26–6·52)	11·3 (0·91–24·8)	2·38 (-1·27–6·68)
Occupational injuries	--	--	--	--	--	--	--	--	--	--
Occupational ergonomic factors	11·5 (9·47–13·9)	11·1 (9·32–13·3)	11·5 (9·53–13·9)	-3·23 (-14·7–8·9)	3·8 (-6·34–14·7)	--	--	--	--	--
Childhood sexual abuse	10·6 (6·74–14·7)	11 (7·04–15·8)	10·7 (6·96–15·2)	4·38 (-2·5–12·8)	-2·79 (-11–6·54)	13·3 (9·72–18·3)	14·1 (10·3–19·6)	13·7 (10·1–18·8)	6·28 (-2·16–15·1)	-2·58 (-10·4–8·27)
Intimate partner violence	--	--	--	--	--	22·8 (12·5–31·3)	20 (11·3–28·2)	22·8 (12·5–31·3)	-12·2 (-19·2–0·696)	13·9 (2·98–28·7)
Non-exclusive breastfeeding	26 (19·2–34·6)	23·2 (17·4–30·7)	23·3 (17·2–30·8)	-10·7 (-25·9–8·65)	0·487 (-13–16)	26 (19·2–34·6)	23·2 (17·4–30·7)	23·3 (17·2–30·8)	-10·6 (-25·9–8·67)	0·487 (-13–16)
Discontinued breastfeeding	28 (26·9–28·9)	27·4 (26·3–28·4)	27·5 (26·3–28·4)	-2·1 (-6·69–2·64)	0·247 (-3·44–4·39)	28 (26·9–28·9)	27·4 (26·3–28·3)	27·5 (26·3–28·4)	-2·09 (-6·69–2·65)	0·288 (-3·4–4·44)
Diet low in calcium	22·3 (10·6–42·2)	16·6 (7·23–31·9)	16·2 (7·05–31·4)	-25·5 (-46·7—11·1)	-2·39 (-11–7·77)	16·6 (6·75–32)	11·2 (3·96–21·2)	10·8 (3·84–20·1)	-32·4 (-55·4—11·9)	-3·79 (-18·4–11·2)
Occupational exposure to asbestos	19·3 (17·3–21·1)	17·4 (16·3–18·5)	15·7 (13·1–18·6)	-9·66 (-19·1–2·32)	-9·86 (-25·8–6·15)	3·43 (2·51–5·56)	3·28 (2·39–4·25)	3·52 (2·37–4·75)	-4·13 (-33·8–29·9)	7·12 (-19·5–40·7)
Occupational exposure to arsenic	0·49 (0–1·18)	0·54 (0–1·31)	0·563 (0–1·36)	10·2 (-10·7–NA)	4·26 (-9·19–NA)	0·467 (0–1·12)	0·468 (0–1·12)	0·473 (0–1·14)	0·195 (-20·5–NA)	1·03 (-12·8–NA)
Occupational exposure to benzene	0·828 (0·0488–2·37)	0·878 (0·0519–2·39)	0·922 (0·0537–2·68)	6·05 (-4·84–20·8)	4·91 (-3·44–16·4)	1·1 (0·0706–3·17)	1·41 (0·0882–3·95)	1·53 (0·0962–4·51)	27·5 (9·05–55·6)	9·02 (-1·64–23·9)
Occupational exposure to beryllium	0·024 (0·0223–0·0254)	0·0252 (0·0237–0·0267)	0·0264 (0·0246–0·0281)	5·09 (-2·03–13·4)	4·82 (-0·943–11·1)	0·0256 (0·0229–0·0282)	0·0281 (0·0258–0·0304)	0·0301 (0·0277–0·0325)	9·56 (-2·48–23·9)	7·11 (-0·634–15·5)
Occupational exposure to cadmium	0·057 (0·0488–0·0657)	0·0625 (0·0543–0·0707)	0·0654 (0·0562–0·076)	9·65 (-9·53–32·6)	4·7 (-7·92–18·2)	0·0557 (0·0466–0·0659)	0·0566 (0·0484–0·0668)	0·0578 (0·0496–0·0674)	1·66 (-17·5–27·1)	2·19 (-10·6–17·7)
Occupational exposure to chromium	0·135 (0·117–0·152)	0·147 (0·13–0·164)	0·155 (0·135–0·177)	9·4 (-6·78–28·4)	4·99 (-6·22–17)	0·137 (0·117–0·159)	0·146 (0·128–0·167)	0·151 (0·132–0·171)	6·27 (-11·5–28·8)	3·41 (-8·06–16·7)
Occupational exposure to diesel engine exhaust	0·755 (0·679–0·831)	0·76 (0·694–0·838)	0·797 (0·711–0·886)	0·651 (-10·6–15·4)	4·97 (-4·65–14·8)	0·632 (0·544–0·728)	0·681 (0·596–0·77)	0·729 (0·635–0·836)	7·67 (-9·56–28)	7·05 (-5·77–21·4)
Occupational exposure to formaldehyde	0·283 (0·24–0·329)	0·304 (0·263–0·345)	0·315 (0·27–0·368)	7·29 (-12·3–31·1)	3·6 (-9·27–17·8)	0·266 (0·222–0·314)	0·283 (0·245–0·329)	0·29 (0·251–0·334)	6·21 (-12·9–31·6)	2·63 (-9·62–16·9)
Occupational exposure to nickel	0·504 (0–2·19)	0·543 (0–2·46)	0·563 (0–2·54)	7·81 (-9·91–NA)	3·7 (-7·83–NA)	0·385 (0–1·72)	0·373 (0–1·62)	0·375 (0–1·63)	-2·99 (-24·5–NA)	0·502 (-13·6–NA)
Occupational exposure to polycyclic aromatic hydrocarbons	0·282 (0·25–0·314)	0·308 (0·277–0·34)	0·323 (0·286–0·363)	9·43 (-5·62–26·4)	4·78 (-5·23–15·8)	--	--	--	--	--
Occupational exposure to silica	6·4 (0·932–20·9)	6·75 (0·97–21·5)	7 (1·01–23)	5·46 (-10·8–29·6)	3·74 (-8·45–18·4)	3·6 (0·488–11·1)	3·44 (0·471–10·9)	3·45 (0·488–11·3)	-4·47 (-25·2–24·8)	0·511 (-14·5–21·6)
Occupational exposure to sulfuric acid	0·837 (0·183–2·77)	0·881 (0·186–2·79)	0·901 (0·192–2·89)	5·2 (-12·4–25·5)	2·32 (-8·9–15·3)	0·805 (0·168–2·53)	0·773 (0·158–2·59)	0·758 (0·157–2·39)	-3·97 (-24·2–22·2)	-1·93 (-15·2–14·7)
Unsafe sex	--	--	--	--	--	--	--	--	--	--
Occupational exposure to trichloroethylene	0·0752 (0·0679–0·0828)	0·0805 (0·0732–0·0881)	0·0847 (0·0761–0·0938)	7·02 (-4·49–20·5)	5·25 (-3·38–14·7)	0·0792 (0·0686–0·0901)	0·0844 (0·0751–0·0951)	0·0884 (0·0788–0·0983)	6·52 (-8·62–25·6)	4·79 (-4·95–17·1)
No access to handwashing facility	3·39 (3·07–3·72)	3·23 (2·93–3·55)	3·18 (2·9–3·49)	-4·66 (-14·6–5·61)	-1·49 (-8·03–5·25)	3·39 (3·07–3·72)	3·23 (2·93–3·55)	3·18 (2·9–3·49)	-4·66 (-14·6–5·61)	-1·49 (-8·03–5·25)
Child wasting	1·27 (0·895–1·69)	1·22 (0·864–1·63)	1·22 (0·864–1·63)	-3·75 (-16·5–9·44)	-0·295 (-9·49–10·1)	--	--	--	--	--
Child stunting	1·64 (0·789–2·54)	1·22 (0·591–1·92)	1·21 (0·536–1·95)	-25·7 (-45·1–1·32)	-0·586 (-22·1–25)	1·95 (0·989–2·97)	1·61 (0·785–2·52)	1·58 (0·766–2·48)	-17·6 (-37·4–10)	-1·61 (-20·1–21)
Chewing tobacco	0·174 (0·113–0·257)	0·166 (0·106–0·248)	0·17 (0·11–0·245)	-4·45 (-41·9–55·1)	2·16 (-25·1–39·8)	0·129 (0·0736–0·208)	0·129 (0·0736–0·22)	0·132 (0·0753–0·225)	-0·0619 (-47·2–87·8)	2·53 (-32·6–55·9)
Diet low in legumes	75 (40·1–99·4)	74 (39·8–98·9)	74·7 (40·1–99·1)	-1·37 (-11·8–5·7)	0·975 (-5·5–9·85)	71·5 (33·6–98·3)	70·4 (31·3–97·5)	71 (31·3–98)	-1·55 (-13·2–6·49)	0·948 (-5·87–11·7)
Short gestation	22 (17·6–27·8)	45·7 (36·7–55·9)	41·8 (32·6–52·3)	108 (53·7–177)	-8·56 (-29·5–15·9)	25·3 (21·7–29·9)	45·9 (38·3–55)	41·1 (33·6–49·7)	81·4 (47–124)	-10·4 (-26·9–10·2)
Low birth weight	10·9 (8·69–13·5)	22·4 (18–27·4)	21·3 (16·7–26·8)	105 (52·2–175)	-5·1 (-27·6–19·8)	13·1 (11·2–15·3)	22·8 (19·1–26·7)	21·5 (17·8–25·8)	74·5 (38·3–117)	-5·53 (-24·4–16·8)
High temperature	22·1 (11·3–35·2)	0 (0–0)	0 (0–0)	-100 (-100—100)	--	22·1 (11·3–35·2)	0 (0–0)	0 (0–0)	-100 (-100—100)	--
Low temperature	31·6 (27·5–35·7)	34·3 (30·9–38)	30·6 (26·7–34·7)	8·72 (6·01–12)	-10·8 (-13·5—8·21)	31·6 (27·5–35·7)	34·3 (30·9–38)	30·6 (26·7–34·7)	8·72 (6·01–12)	-10·8 (-13·5—8·21)
Kidney dysfunction	13·4 (8·89–19·7)	14·1 (9·65–20·4)	14 (9·52–20·2)	5·74 (1·01–12·2)	-1·05 (-5·85–3·66)	14·2 (9·49–20·6)	14·9 (10·1–21·3)	15 (10–21·7)	4·76 (-0·0228–11·2)	0·394 (-3·96–4·99)
Bullying victimization	6·13 (2·45–12·1)	6·13 (2·42–12·4)	5·66 (2·26–11·7)	0·036 (-9·39–9·84)	-7·65 (-13·3—2·94)	4·69 (1·92–9·56)	5·05 (2·01–10·2)	4·77 (1·87–9·82)	7·75 (-4–19·3)	-5·62 (-12·4—0·714)
High LDL cholesterol	49·3 (45·1–53·3)	42·4 (38·6–46·4)	43·3 (39·7–47·1)	-13·9 (-20·7—6·84)	2·08 (-3·39–8·16)	--	--	--	--	--

Supplementary Results Table 1. Leicester age-standardised Summary Exposure Values (SEVs) for most-detailed risk factors and percentage change, by sex, 1990, 2010, and 2019. Data in parentheses are 95% uncertainty intervals.

	Male					Female				
	1990	2010	2019	Percentage change 1990-2010	Percentage change 2010-2019	1990	2010	2019	Percentage change 1990-2010	Percentage change 2010-2019
Unsafe sanitation	0·693 (0·247–1·52)	0·359 (0·138–0·816)	0·282 (0·0995–0·62)	-48·2 (-82·9–68)	-21·6 (-68·8–84·2)	0·693 (0·247–1·52)	0·359 (0·138–0·816)	0·282 (0·0995–0·62)	-48·2 (-82·9–68)	-21·6 (-68·8–84·2)
Ambient particulate matter pollution	21·1 (8·55–36·9)	13·7 (10·1–17·7)	10·4 (7·11–14)	-35 (-63·4–46·2)	-24·4 (-30·5—20·4)	21·1 (8·56–36·9)	13·7 (10·1–17·7)	10·4 (7·12–14)	-35 (-63·3–46·2)	-24·4 (-30·5—20·4)
Household air pollution from solid fuels	0·0145 (0·00284–0·0445)	0·00467 (0·000785–0·0137)	0·00305 (0·00046–0·0107)	-67·9 (-91·7–9·01)	-34·6 (-71·8–42·9)	0·0191 (0·00414–0·0586)	0·00617 (0·00112–0·0185)	0·00403 (0·000665–0·0135)	-67·7 (-91·6–9·58)	-34·7 (-71·7–43·5)
Ambient ozone pollution	14·1 (0–47·5)	4·68 (0–21)	6·21 (0–24·6)	-66·8 (-100–NA)	32·7 (-100–NA)	14·1 (0–47·5)	4·68 (0–21)	6·21 (0–24·6)	-66·8 (-100–NA)	32·7 (-100–NA)
Residential radon	24·9 (3·66–75·4)	24·9 (3·66–75·4)	24·9 (3·66–75·4)	0 (0–0)	0 (0–0)	24·9 (3·66–75·4)	24·9 (3·66–75·4)	24·9 (3·66–75·4)	0 (0–0)	0 (0–0)
Lead exposure	34 (8·75–57·9)	21·3 (4·24–43·2)	14·4 (1·7–34·5)	-37·5 (-60·6—23·4)	-32 (-62·1—19·8)	13·5 (0·0472–35·8)	7·83 (0·0128–26·3)	5·26 (5·43e-12–20·9)	-41·8 (-86·7—24·9)	-32·8 (-100—19·2)
Child underweight	0·94 (0·535–1·42)	0·793 (0·414–1·2)	0·776 (0·403–1·19)	-15·6 (-33·1–6·8)	-2·11 (-18·3–16·9)	1·11 (0·637–1·65)	0·958 (0·549–1·43)	0·934 (0·532–1·39)	-13·3 (-29·8–5·68)	-2·51 (-15·8–12·5)
Iron deficiency	--	--	--	--	--	11·6 (9·39–14·1)	10·6 (8·52–12·8)	10·1 (8·09–12·3)	-8·69 (-23·9–7·52)	-4·69 (-14·2–6·44)
Vitamin A deficiency	4·95 (1·02–15·4)	2·14 (0·466–7·51)	1·85 (0·38–5·81)	-56·8 (-90·6–122)	-13·2 (-68–144)	3·78 (1·18–8·28)	2·31 (0·735–5·54)	2·01 (0·644–4·63)	-38·8 (-81–114)	-13·1 (-57·1–88·7)
Zinc deficiency	3·81 (0–11·6)	2·57 (0–8·2)	2·65 (0–8·44)	-32·6 (-100–NA)	3·36 (-22·8–NA)	3·68 (0–11·4)	2·46 (0–8·13)	2·55 (0–8·26)	-33 (-100–NA)	3·5 (-20·4–NA)
Smoking	27·9 (24·4–32)	22·8 (19·9–26·3)	19·5 (16·9–22·5)	-18·4 (-26·2—10·3)	-14·2 (-19·5—8·55)	23·2 (19·3–27·3)	19·2 (15·9–22·6)	15·4 (12·8–18·7)	-17 (-28·7—2·2)	-19·8 (-27·3—11·6)
Secondhand smoke	47·4 (41·6–54·4)	33·4 (28·9–38·2)	33 (28·4–37·6)	-29·5 (-41·2—16)	-1·36 (-11·4–8·85)	49·7 (44·6–55)	33·9 (30·1–37·9)	33·6 (29·8–37·4)	-31·7 (-41·4—20·9)	-1·11 (-8·8–7·14)
Alcohol use	16·5 (8·7–24·5)	17·5 (9·64–25·8)	16·6 (8·23–25·1)	6·35 (-32·1–68·8)	-5·08 (-34–26·9)	--	--	--	--	--
Drug use	0·317 (0·158–0·65)	0·611 (0·358–1·06)	0·562 (0·316–1·01)	92·8 (50·2–158)	-8·03 (-17·9–1·23)	0·194 (0·102–0·387)	0·318 (0·188–0·549)	0·299 (0·17–0·547)	64·2 (24·7–119)	-6·22 (-17·7–4·18)
High fasting plasma glucose	10·8 (8·9–13·3)	18·6 (15·8–21·8)	23·3 (19·5–28·3)	72·2 (43·4–111)	25·4 (7·7–52·2)	8·09 (6·48–10·3)	13·3 (11·2–16·3)	16·4 (13·5–19·8)	64·6 (30·8–107)	23·4 (5·14–49·8)
High systolic blood pressure	35·5 (25·9–47)	33 (25·9–41·1)	33·6 (25·6–42·8)	-7·08 (-34·5–39·4)	1·8 (-21·2–31·3)	--	--	--	--	--
High body-mass index	18·3 (12·5–25·4)	24 (16·5–32·7)	24·9 (17·3–34·3)	31 (15·4–52·9)	3·49 (-3·52–12)	21·6 (15·7–29·9)	29 (22·2–38·9)	29·6 (22·3–39·9)	34·2 (16·7–58·5)	1·75 (-8·2–12·2)
Low bone mineral density	8·84 (4·7–14·7)	7·86 (3·95–13·4)	9·57 (5·07–16·1)	-11·1 (-33·5–16·4)	21·8 (-7·34–65·1)	14·8 (9·59–21·6)	14·3 (8·97–21)	14·2 (8·91–20·6)	-3·54 (-26·3–23·5)	-0·687 (-22·7–27·7)
Diet low in fruits	66 (53·6–78·1)	59·5 (47–72·1)	58·7 (45·4–72·9)	-9·88 (-23·9–3·85)	-1·22 (-11·8–10·3)	67·1 (55·1–79·5)	59·7 (45·8–72·1)	59·4 (45·9–72·2)	-10·9 (-24·1–2·74)	-0·57 (-10·1–10·9)
Diet low in vegetables	51·4 (30·6–72·9)	47·2 (27·8–68)	46·9 (26·8–68·1)	-8·26 (-26·4–10·4)	-0·455 (-13·5–14·6)	48·7 (29·2–70·1)	43·8 (24·4–64·3)	43·3 (24·5–63·8)	-10·1 (-29·7–9·7)	-1·1 (-16·1–16·4)
Diet low in whole grains	88·7 (82·1–94·8)	88·4 (81·7–94·6)	88·6 (81·9–94·8)	-0·312 (-4·01–3·39)	0·224 (-1·93–2·45)	--	--	--	--	--
Diet low in nuts and seeds	82·5 (42·5–93·6)	74·7 (38·2–87·9)	77·2 (39·8–89·7)	-9·49 (-23·8–1·7)	3·38 (-4·65–15)	82·4 (42·6–93·3)	74·5 (39·8–88·2)	76·1 (39·7–89·4)	-9·5 (-23·9–2)	2·09 (-6·45–13·5)
Diet low in milk	58·3 (33·6–79·4)	59·9 (34·6–80)	60·4 (35·1–80·9)	2·72 (-7·97–20)	0·837 (-5·83–9·3)	60·9 (35·2–82·7)	63·7 (38·6–85·1)	64·1 (39·8–85)	4·61 (-4·57–22·1)	0·682 (-4·46–7·74)
Diet high in red meat	66·1 (50·6–80·8)	51·7 (37·1–67·6)	53·5 (38·7–67·7)	-21·7 (-41·1—1·82)	3·36 (-13·4–26·1)	65·9 (49·4–81·5)	53·3 (37–69·4)	55 (38·6–71·3)	-19·2 (-40·9–4·67)	3·27 (-14·8–23·8)
Diet high in processed meat	77 (52·8–96·3)	81·6 (60·9–98·2)	81·5 (60·1–98·4)	5·99 (-2·93–22·2)	-0·0968 (-6·6–6·46)	80·1 (58·3–97·5)	85·1 (65·5–99·3)	85·2 (66·1–99·4)	6·32 (-2·32–21·4)	0·051 (-5·63–5·98)
Diet high in sugar-sweetened beverages	47·2 (28·7–71·9)	50·7 (31·5–75·3)	52·1 (33·2–76·6)	7·21 (-30–60·7)	2·88 (-21·4–42·3)	--	--	--	--	--
Diet low in fiber	39·6 (21·3–57·5)	32·9 (16·4–50·5)	33·4 (16·8–52·3)	-17 (-47–18·2)	1·67 (-21·3–31·8)	--	--	--	--	--
Diet low in seafood omega-3 fatty acids	93·6 (83·5–100)	91·4 (78·9–100)	91·2 (78–100)	-2·32 (-13·2–5·63)	-0·21 (-9·65–11·4)	94·7 (85·3–100)	92·4 (80·8–100)	92·5 (81·6–100)	-2·38 (-12–3·1)	0·0698 (-6·21–8·87)
Diet low in polyunsaturated fatty acids	53·6 (15·9–94·1)	49·1 (14·2–92)	48·7 (13·9–91·7)	-8·39 (-24·8–1·38)	-0·886 (-10–9·61)	56 (17·6–96·8)	51·4 (16·1–92·6)	51 (15·8–92·5)	-8·12 (-23·6–0·553)	-0·921 (-10–8·28)
Diet high in trans fatty acids	57·8 (46·4–100)	51 (39·8–98·4)	51 (39·8–97·7)	-11·9 (-26·1–1·49)	0·184 (-13·4–16·5)	55·9 (44·2–100)	47·8 (36·2–100)	47·8 (35·9–100)	-14·6 (-29–0)	0·141 (-12·6–17)
Diet high in sodium	18·5 (4·27–41·5)	22·4 (4·67–45·4)	24·7 (4·87–49·5)	20·8 (-45·7–184)	10·2 (-32·2–107)	11·2 (2·85–29·8)	11·8 (2·95–30·1)	12·7 (3·11–31·8)	5·11 (-44·3–111)	7·55 (-34·3–85·7)
Low physical activity	4·48 (1·94–9·31)	4·94 (2·17–9·93)	4·93 (2·14–9·9)	10·1 (-3·9–31·8)	-0·178 (-13·4–13·6)	6·98 (3·75–12·9)	7·83 (4·21–14)	7·88 (4·22–14)	12·2 (2·7–28·3)	0·563 (-7·76–9·26)
Occupational asthmagens	22·7 (18·1–28·2)	21·5 (17·9–26·6)	22·6 (18–28·7)	-5·02 (-21·7–15·9)	4·93 (-8·63–21·9)	--	--	--	--	--
Occupational particulate matter, gases, and fumes	9·22 (6·36–13·2)	8·78 (6·21–12·6)	8·58 (6·11–12·5)	-4·71 (-13·6–5·17)	-2·33 (-6·88–2·64)	4·5 (2·9–6·83)	4·36 (2·96–6·49)	4·11 (2·84–6·2)	-2·91 (-16·9–16)	-5·85 (-12·4–2·11)
Occupational noise	8·62 (7·65–9·71)	8·45 (7·75–9·24)	8·34 (7·69–9·19)	-1·87 (-7·94–4·81)	-1·31 (-4·07–1·64)	5·1 (4·1–6·07)	5·68 (4·96–6·4)	5·83 (5·21–6·5)	11·5 (0·883–26)	2·48 (-1·38–7·38)
Occupational injuries	--	--	--	--	--	--	--	--	--	--
Occupational ergonomic factors	10·4 (8·61–12·7)	10·2 (8·4–12·3)	10·7 (8·72–13)	-1·99 (-12·9–10·9)	4·3 (-4·54–13·9)	--	--	--	--	--
Childhood sexual abuse	10·6 (6·74–14·7)	11 (7·04–15·8)	10·7 (6·96–15·2)	4·38 (-2·5–12·8)	-2·79 (-11–6·54)	13·3 (9·72–18·3)	14·1 (10·3–19·6)	13·7 (10·1–18·8)	6·28 (-2·16–15·1)	-2·58 (-10·4–8·27)
Intimate partner violence	--	--	--	--	--	22·8 (12·5–31·3)	20 (11·3–28·2)	22·8 (12·5–31·3)	-12·2 (-19·2–0·696)	13·9 (2·98–28·7)
Non-exclusive breastfeeding	26 (19·1–34·5)	23·4 (17·5–30·6)	23·5 (17·3–31·3)	-9·9 (-25·9–10·8)	0·165 (-14·2–16·4)	26 (19·1–34·5)	23·4 (17·5–30·6)	23·5 (17·3–31·3)	-9·88 (-25·9–10·8)	0·162 (-14·2–16·4)
Discontinued breastfeeding	28·2 (27·2–29·1)	28·3 (27·1–29·3)	28·1 (26·9–29·1)	0·418 (-4·47–5·34)	-0·716 (-4·38–2·99)	28·2 (27·2–29)	28·3 (27·1–29·3)	28 (26·8–29)	0·445 (-4·45–5·38)	-0·955 (-4·63–2·75)
Diet low in calcium	19·5 (9·04–37·5)	14·1 (5·93–26·7)	14·4 (6·1–26·9)	-27·6 (-46·6—13·8)	2·05 (-7·75–13·5)	14·1 (5·62–27·1)	8·59 (3·29–16·2)	8·98 (3·3–16·9)	-38·9 (-60·5—18·4)	4·57 (-13·8–26·6)
Occupational exposure to asbestos	16·5 (14·9–18·3)	15·9 (14·6–17·1)	13·9 (11·1–16·3)	-3·86 (-14·7–9·84)	-12·3 (-29–5·12)	2·76 (1·96–6·04)	2·78 (2·2–4·77)	2·69 (1·82–4·71)	0·763 (-26·6–33·2)	-3·07 (-28·7–33·6)
Occupational exposure to arsenic	0·523 (0–1·23)	0·563 (0–1·3)	0·587 (0–1·41)	7·79 (-12·2–NA)	4·23 (-9·54–NA)	0·477 (0–1·15)	0·467 (0–1·09)	0·472 (0–1·14)	-2·02 (-23·3–NA)	1·01 (-14–NA)
Occupational exposure to benzene	0·842 (0·0487–2·39)	0·894 (0·051–2·56)	0·934 (0·0512–2·75)	6·13 (-4·92–26)	4·56 (-3·67–15·6)	1·09 (0·066–3·1)	1·41 (0·085–3·97)	1·56 (0·0935–4·41)	29·5 (11·2–59·6)	10 (-0·748–25·7)
Occupational exposure to beryllium	0·0242 (0·0224–0·026)	0·0254 (0·0238–0·0272)	0·0267 (0·0248–0·0287)	5·02 (-3·23–14·4)	5·09 (-1·11–12)	0·025 (0·0219–0·0278)	0·0275 (0·0251–0·0299)	0·0296 (0·027–0·0321)	9·99 (-3·02–24·9)	7·58 (-0·539–16·9)
Occupational exposure to cadmium	0·0605 (0·051–0·0709)	0·0651 (0·0569–0·0737)	0·068 (0·0584–0·0793)	7·68 (-10·6–30·4)	4·47 (-8·15–18·7)	0·0563 (0·0461–0·068)	0·0563 (0·0479–0·0662)	0·0575 (0·049–0·0676)	0·0472 (-20·3–26·1)	2·1 (-11·5–17·4)
Occupational exposure to chromium	0·143 (0·123–0·164)	0·154 (0·136–0·172)	0·161 (0·141–0·184)	7·64 (-9·18–27·3)	4·74 (-6·57–17·2)	0·138 (0·115–0·162)	0·145 (0·127–0·166)	0·15 (0·131–0·171)	4·94 (-13·8–27·9)	3·53 (-8·69–16·6)
Occupational exposure to diesel engine exhaust	0·749 (0·67–0·83)	0·758 (0·68–0·84)	0·799 (0·712–0·892)	1·29 (-12–15·6)	5·32 (-4·43–15·1)	0·587 (0·505–0·687)	0·632 (0·551–0·718)	0·678 (0·582–0·792)	7·59 (-10·6–29·7)	7·34 (-5·58–22·3)
Occupational exposure to formaldehyde	0·303 (0·254–0·356)	0·319 (0·278–0·363)	0·329 (0·282–0·384)	5·1 (-13·6–28·7)	3·14 (-9·48–17·4)	0·271 (0·222–0·327)	0·284 (0·244–0·33)	0·292 (0·252–0·338)	4·75 (-15·7–29·9)	2·8 (-10·3–16·8)
Occupational exposure to nickel	0·513 (0–2·28)	0·55 (0–2·35)	0·574 (0–2·46)	7·23 (-10·6–NA)	4·38 (-7·37–NA)	0·39 (0–1·72)	0·368 (0–1·6)	0·37 (0–1·64)	-5·49 (-27·2–NA)	0·394 (-15·5–NA)
Occupational exposure to polycyclic aromatic hydrocarbons	0·295 (0·259–0·335)	0·319 (0·285–0·352)	0·334 (0·297–0·376)	8·07 (-6·84–25·6)	4·62 (-5·27–15·9)	--	--	--	--	--
Occupational exposure to silica	6·28 (0·858–20)	6·66 (0·884–21·3)	6·92 (0·994–22·4)	5·97 (-10·8–30·7)	3·86 (-8·55–20·2)	3·47 (0·501–11·4)	3·24 (0·458–10·3)	3·25 (0·461–10·4)	-6·47 (-28·3–25·1)	0·301 (-16·2–21·9)
Occupational exposure to sulfuric acid	0·874 (0·186–2·84)	0·906 (0·196–2·85)	0·928 (0·195–2·93)	3·69 (-13·9–25·4)	2·46 (-9·2–16·3)	0·837 (0·176–2·6)	0·787 (0·163–2·57)	0·767 (0·158–2·33)	-5·95 (-26·8–23·7)	-2·53 (-16·6–14·2)
Unsafe sex	--	--	--	--	--	--	--	--	--	--
Occupational exposure to trichloroethylene	0·0792 (0·071–0·0882)	0·0837 (0·076–0·0919)	0·0879 (0·0794–0·0984)	5·69 (-7·45–20·8)	5·09 (-3·47–14·9)	0·078 (0·0668–0·0897)	0·0823 (0·0736–0·0923)	0·0862 (0·0758–0·0972)	5·48 (-9·91–25·5)	4·84 (-5·77–16·5)
No access to handwashing facility	3·38 (3·07–3·7)	3·21 (2·9–3·53)	3·17 (2·89–3·49)	-5·08 (-15·1–6·51)	-1·33 (-7·95–5·95)	3·38 (3·07–3·7)	3·21 (2·9–3·53)	3·17 (2·89–3·49)	-5·08 (-15·1–6·51)	-1·33 (-7·95–5·95)
Child wasting	1·27 (0·878–1·71)	1·23 (0·866–1·65)	1·22 (0·856–1·63)	-3·64 (-16·2–11·4)	-0·592 (-9·83–10·1)	1·08 (0·757–1·44)	0·927 (0·542–1·25)	1·02 (0·7–1·38)	-13·8 (-48·2–29·1)	10·1 (-25·8–75·5)
Child stunting	1·69 (0·778–2·59)	1·24 (0·577–1·99)	1·22 (0·546–1·94)	-26·7 (-46·4—0·204)	-1·45 (-20·9–23·6)	2 (1·03–3·07)	1·62 (0·796–2·48)	1·58 (0·75–2·45)	-18·9 (-38·8–7·91)	-2·82 (-19·4–19·4)
Chewing tobacco	0·176 (0·11–0·261)	0·168 (0·105–0·257)	0·172 (0·107–0·261)	-4·97 (-43·6–61·8)	2·65 (-27·9–46·2)	0·135 (0·0749–0·229)	0·134 (0·072–0·231)	0·136 (0·0751–0·243)	-0·846 (-49·6–96·9)	2·08 (-34·6–57·1)
Diet low in legumes	73·6 (36·5–98·5)	72·8 (38·3–98·5)	73·9 (39·5–98·8)	-1·16 (-11·5–8·26)	1·52 (-4·14–13·5)	70 (29·3–97·8)	69·1 (29·8–96·8)	70·1 (31·2–97·8)	-1·38 (-13·7–9·71)	1·48 (-4·68–15·4)
Short gestation	24·4 (19·5–31·2)	60·8 (48·3–76·1)	52·1 (41·1–66·3)	150 (86–226)	-14·3 (-34·3–10·6)	27·1 (21·7–33·2)	57·5 (46·6–71·4)	49·5 (40·8–59·1)	112 (50·8–196)	-14 (-31·9–7·47)
Low birth weight	11·9 (9·57–15·1)	27·1 (21·8–33·5)	23·8 (18·7–30·5)	127 (67·2–201)	-12·1 (-32·8–15·9)	13·8 (11·2–16·6)	26·3 (21·1–32·7)	23·8 (20–28·1)	91·5 (35·7–170)	-9·47 (-28·8–15·5)
High temperature	22·5 (6·73–39·6)	0 (0–0)	0 (0–0)	-100 (-100—100)	--	22·5 (6·73–39·6)	0 (0–0)	0 (0–0)	-100 (-100—100)	--
Low temperature	31·4 (26–37·3)	34·2 (29·4–39·6)	30·9 (25·8–36·8)	8·92 (5·32–13·3)	-9·69 (-12·9—6·6)	31·4 (26–37·3)	34·2 (29·4–39·6)	30·9 (25·8–36·8)	8·92 (5·32–13·3)	-9·69 (-12·9—6·6)
Kidney dysfunction	13·6 (9·05–20·1)	14·4 (9·79–20·8)	14·4 (9·73–20·7)	5·43 (0·404–11·8)	-0·051 (-4·97–4·92)	14·5 (9·76–21)	15·2 (10·3–21·7)	15·3 (10·2–22)	4·35 (-0·0642–10)	1·1 (-3·15–5·91)
Bullying victimization	5·96 (2·38–11·8)	5·92 (2·35–11·9)	5·53 (2·24–11·4)	-0·675 (-10·1–9·07)	-6·55 (-11·8—1·79)	4·54 (1·86–9·2)	4·83 (1·94–9·75)	4·61 (1·84–9·41)	6·37 (-5·3–18)	-4·73 (-11·4–0·156)
High LDL cholesterol	49·5 (44·9–54)	43·3 (39·5–47·5)	43·9 (40–48)	-12·5 (-20·2—4·62)	1·22 (-4·47–7·19)	--	--	--	--	--

Supplementary Results Table 1. Leicestershire age-standardised Summary Exposure Values (SEVs) for most-detailed risk factors and percentage change, by sex, 1990, 2010, and 2019. Data in parentheses are 95% uncertainty intervals.

	Male					Female				
	1990	2010	2019	Percentage change 1990-2010	Percentage change 2010-2019	1990	2010	2019	Percentage change 1990-2010	Percentage change 2010-2019
Unsafe sanitation	0·532 (0·201–1·17)	0·283 (0·108–0·581)	0·225 (0·0795–0·484)	-46·8 (-82·8–61·3)	-20·5 (-66·9–87·5)	0·532 (0·201–1·17)	0·283 (0·108–0·581)	0·225 (0·0795–0·484)	-46·8 (-82·8–61·3)	-20·5 (-66·9–87·5)
Ambient particulate matter pollution	17·9 (6·49–32·9)	11·3 (7·68–15·5)	8·33 (5·01–12)	-36·5 (-66·3–56·5)	-26·5 (-35·1—21·7)	17·9 (6·52–32·9)	11·3 (7·67–15·5)	8·33 (5·01–12)	-36·5 (-66·3–56·9)	-26·5 (-35—21·7)
Household air pollution from solid fuels	0·0153 (0·00322–0·0494)	0·00527 (0·000907–0·0178)	0·00344 (0·000496–0·0119)	-65·6 (-90·8–10·1)	-34·8 (-72·5–31·8)	0·0201 (0·00435–0·0627)	0·00699 (0·00126–0·0235)	0·00456 (0·000698–0·0159)	-65·3 (-90·8–10·4)	-34·8 (-72·4–32·3)
Ambient ozone pollution	12·7 (2·87–28·9)	6·05 (0·939–16·4)	7·17 (1·02–18·5)	-52·3 (-86·8–14)	18·5 (-60·3–258)	12·7 (2·87–28·9)	6·05 (0·939–16·4)	7·17 (1·02–18·5)	-52·3 (-86·8–14)	18·5 (-60·3–258)
Residential radon	26 (6·13–69·1)	26 (6·13–69·1)	26 (6·13–69·1)	0 (0–0)	0 (0–0)	26 (6·13–69·1)	26 (6·13–69·1)	26 (6·13–69·1)	0 (0–0)	0 (0–0)
Lead exposure	26·2 (1·75–51·3)	16·8 (1·06–38·6)	11·5 (0·398–30·8)	-36·1 (-62·1—21·4)	-31·3 (-68·9—19·3)	10·9 (7·16e-12–32·8)	6·44 (4·29e-12–24·1)	4·4 (2·79e-12–19·3)	-40·9 (-100—0·471)	-31·7 (-100–27·9)
Child underweight	0·894 (0·5–1·35)	0·768 (0·406–1·22)	0·76 (0·408–1·2)	-14 (-31·7–8·62)	-1·02 (-16·8–16·9)	1·05 (0·595–1·57)	0·924 (0·534–1·38)	0·916 (0·513–1·38)	-12 (-28·5–8·26)	-0·911 (-14·2–14·7)
Iron deficiency	--	--	--	--	--	10·5 (8·48–12·8)	9·62 (7·78–11·7)	9·48 (7·54–11·7)	-8·7 (-22·8–7·3)	-1·49 (-10·7–8·66)
Vitamin A deficiency	4·15 (0·753–12·1)	1·85 (0·379–5·35)	1·62 (0·322–4·79)	-55·4 (-89·8–121)	-12·7 (-68–138)	3·21 (1·01–7·66)	2·04 (0·615–4·79)	1·79 (0·505–4·1)	-36·4 (-79·2–121)	-12·2 (-54·8–112)
Zinc deficiency	4·21 (0–12·5)	2·72 (0–8·24)	2·68 (0–8·29)	-35·5 (-100–NA)	-1·37 (-24·9–NA)	4·08 (0–11·9)	2·61 (0–8·34)	2·56 (0–8·23)	-36·1 (-100–NA)	-1·68 (-27·8–NA)
Smoking	28·3 (24·8–32·3)	21·7 (18·9–24·7)	18 (15·5–20·7)	-23·3 (-30·1—15·8)	-17 (-22·3—11·7)	23·2 (19·5–27·5)	18·4 (15·5–21·6)	14·4 (11·8–17·3)	-20·5 (-31·9—7·39)	-21·9 (-29·2—13·6)
Secondhand smoke	44·9 (39·4–50·7)	32·8 (28·5–37·7)	32·9 (28·5–37·5)	-26·9 (-37·9—13·8)	0·104 (-9·58–11·3)	46·9 (42·2–51·8)	33·2 (29·7–36·8)	33·4 (29·7–36·8)	-29·1 (-38·3—18·9)	0·34 (-7·28–9·32)
Alcohol use	17 (9·56–24·9)	20·7 (12·2–29·2)	20 (11·4–29·2)	21·5 (-19·5–88·1)	-3·15 (-27·9–25·2)	--	--	--	--	--
Drug use	0·305 (0·152–0·635)	0·541 (0·306–0·972)	0·528 (0·293–0·951)	77·4 (39·1–132)	-2·33 (-12·2–6·99)	0·197 (0·105–0·373)	0·309 (0·179–0·536)	0·294 (0·165–0·531)	56·7 (17·8–106)	-4·62 (-16·2–5·37)
High fasting plasma glucose	7·79 (6·54–9·3)	16·1 (13·7–19·1)	19·6 (16·2–23·7)	106 (71·5–144)	22·1 (6·79–44·4)	6·9 (5·53–8·73)	12·3 (10–15·3)	14·9 (12·4–18·1)	78·1 (42·2–125)	21·2 (1·12–46·1)
High systolic blood pressure	35·3 (26·2–46·8)	32·6 (25·7–40·1)	32·2 (24·7–42·3)	-7·82 (-34·1–32·8)	-1·04 (-23·3–25·3)	--	--	--	--	--
High body-mass index	23·3 (16·3–31·5)	29·9 (21·2–40·5)	31 (22·2–41·5)	28·3 (13–47·5)	3·46 (-3·65–10·9)	26 (19·4–35·3)	33·6 (25·4–44·6)	34·2 (26·3–45·1)	29·2 (11·4–50·5)	1·85 (-6·56–11·7)
Low bone mineral density	8·74 (4·57–14·6)	7·54 (3·82–13·1)	9·21 (4·84–15·1)	-13·7 (-37·4–15·5)	22·2 (-7·2–67·9)	14·6 (9·08–21·3)	13·9 (8·85–20·5)	13·7 (8·46–20·2)	-4·72 (-25·2–21·3)	-1·11 (-23·2–28·3)
Diet low in fruits	66·6 (53·9–78·8)	59·5 (47–72·2)	58·1 (44·7–70·7)	-10·7 (-24·3–2·02)	-2·31 (-13·3–8·33)	67·6 (55·6–78·9)	59·8 (47·2–72·3)	58·9 (45·5–71·7)	-11·5 (-23·5–1·64)	-1·5 (-11·6–8·8)
Diet low in vegetables	52 (31·4–73·4)	47·3 (27·7–68·1)	46 (26·6–67·3)	-9·07 (-28·5–9·82)	-2·69 (-16·3–10·7)	49·6 (29·4–71·4)	44·2 (25·8–65·4)	42·6 (23·8–64·6)	-11 (-29–7·28)	-3·57 (-19·4–11·1)
Diet low in whole grains	88·8 (82–94·8)	88·4 (81·8–94·6)	88·5 (81·7–94·9)	-0·393 (-3·6–3)	0·1 (-2·15–2·18)	--	--	--	--	--
Diet low in nuts and seeds	82·9 (42·6–93·3)	74·8 (37·9–87·8)	76·8 (39·7–88·9)	-9·72 (-23·8–0·715)	2·64 (-5·36–14)	83 (43·5–93·4)	75·1 (38·8–88·2)	76 (39·2–89·4)	-9·48 (-22·8–1·75)	1·1 (-8·13–12·2)
Diet low in milk	59 (34·4–80·3)	60·2 (35·4–80·7)	59·9 (35·2–80·7)	1·96 (-8·95–16·3)	-0·359 (-7·63–7·52)	61·6 (36·4–82·7)	63·8 (38·5–84·6)	63·6 (38·6–84·4)	3·66 (-5·59–19·8)	-0·391 (-5·8–6·07)
Diet high in red meat	65·8 (50·9–79·6)	51·6 (36·7–66·5)	53·9 (38·3–68·9)	-21·5 (-40·1—1·2)	4·42 (-12·3–26·4)	65·4 (49–79·8)	52·7 (37·2–67·8)	55·2 (38·5–70·4)	-19·3 (-38·8–2·04)	4·76 (-12·8–25·9)
Diet high in processed meat	76·7 (53·5–96·4)	81·4 (60·3–98·7)	81·5 (60·3–98·5)	6·17 (-2·24–21·4)	0·0936 (-5·96–6·36)	79·9 (59·1–97·9)	85 (65·8–99·3)	85·2 (65·9–99·2)	6·4 (-1·39–23·4)	0·256 (-4·9–6·51)
Diet high in sugar-sweetened beverages	46·3 (28·3–73·5)	49·8 (30·8–73·6)	51·7 (31·9–75·4)	7·64 (-27·9–64·1)	3·73 (-21–39)	--	--	--	--	--
Diet low in fiber	42·4 (26·2–59·5)	35 (19·2–52·2)	34·7 (19–51·1)	-17·3 (-42–11·5)	-0·972 (-20·4–23·5)	42·7 (25·9–61·2)	35 (19·3–52·6)	34·5 (19·1–51·3)	-17·9 (-43·8–14·9)	-1·4 (-21·1–25)
Diet low in seafood omega-3 fatty acids	93·9 (83·4–100)	91·7 (79·1–100)	90·8 (77·3–100)	-2·39 (-12·4–5·87)	-0·971 (-11·3–8·97)	94·9 (86–100)	92·6 (81·2–100)	91·9 (80·2–100)	-2·43 (-11·9–4·39)	-0·776 (-9·93–9·45)
Diet low in polyunsaturated fatty acids	54·3 (15·9–94·9)	49·1 (13·8–91·6)	47·8 (13·5–91·4)	-9·59 (-26·5–0)	-2·64 (-13·6–06)	56·7 (18·9–96)	51·4 (15·8–94·3)	50·2 (15–92·6)	-9·32 (-27–0)	-2·31 (-12·2–6·18)
Diet high in trans fatty acids	57·8 (47·1–100)	50·7 (39·6–99·5)	50·9 (39·2–97·5)	-12·2 (-28–0·45)	0·241 (-14–16·5)	55·8 (44·1–100)	47·7 (36·2–99·8)	47·7 (36·2–99·7)	-14·4 (-28·7–0)	-0·0889 (-12·9–16·1)
Diet high in sodium	18·3 (4·31–41·1)	22·3 (4·86–45·9)	24·6 (5·43–49·3)	22·2 (-46·1–183)	9·91 (-31·6–92·2)	11·3 (2·84–29·3)	11·7 (2·98–29·4)	12·6 (3·16–31·2)	3·5 (-44·2–116)	7·82 (-34·3–70·7)
Low physical activity	4·57 (1·94–9·55)	5·04 (2·29–10·1)	5·11 (2·27–10·5)	10·3 (-3·98–31·6)	1·41 (-12·2–17·1)	7·11 (3·69–13·2)	7·95 (4·21–14·2)	8·01 (4·39–14·1)	11·8 (2·13–25·9)	0·785 (-7·53–11)
Occupational asthmagens	21·9 (17·7–27·1)	20·6 (17·1–25·1)	21·6 (17·4–26·9)	-5·61 (-20·8–12·5)	4·86 (-7·11–20)	--	--	--	--	--
Occupational particulate matter, gases, and fumes	8·98 (6·26–13·1)	8·61 (6·09–12·3)	8·42 (5·93–12·2)	-4·12 (-12·5–6·16)	-2·24 (-6·71–2·13)	4·29 (2·84–6·68)	4·25 (2·97–6·43)	4·04 (2·81–6·05)	-0·857 (-14·3–16·8)	-5·06 (-11·8–1·91)
Occupational noise	8·6 (7·62–9·65)	8·45 (7·74–9·26)	8·34 (7·71–9·07)	-1·71 (-7·5–4·73)	-1·32 (-4·14–1·59)	5·12 (4·23–6·06)	5·7 (5·04–6·36)	5·83 (5·22–6·44)	11·3 (0·833–25·5)	2·41 (-1·54–6·91)
Occupational injuries	--	--	--	--	--	--	--	--	--	--
Occupational ergonomic factors	11 (9·11–13·2)	10·7 (8·87–13)	11·2 (9·22–13·6)	-2·92 (-14·8–9·75)	4·32 (-4·73–15)	--	--	--	--	--
Childhood sexual abuse	10·6 (6·74–14·7)	11 (7·04–15·8)	10·7 (6·96–15·2)	4·38 (-2·5–12·8)	-2·79 (-11–6·54)	13·3 (9·72–18·3)	14·1 (10·3–19·6)	13·7 (10·1–18·8)	6·28 (-2·16–15·1)	-2·58 (-10·4–8·27)
Intimate partner violence	--	--	--	--	--	22·8 (12·5–31·3)	20 (11·3–28·2)	22·8 (12·5–31·3)	-12·2 (-19·2–0·696)	13·9 (2·98–28·7)
Non-exclusive breastfeeding	25·8 (19·3–33·9)	23·1 (17·3–30·7)	23·2 (17·3–30·7)	-10·3 (-26·4–8·64)	0·424 (-14·2–15·8)	25·8 (19·3–33·9)	23·1 (17·3–30·7)	23·2 (17·3–30·7)	-10·3 (-26·4–8·65)	0·424 (-14·2–15·8)
Discontinued breastfeeding	27·7 (26·7–28·6)	27·1 (26–28·1)	27·5 (26·4–28·6)	-2·21 (-6·84–3·1)	1·6 (-2·25–5·83)	27·7 (26·6–28·6)	27·1 (26–28·2)	27·5 (26·3–28·5)	-2·04 (-6·68–3·28)	1·17 (-2·65–5·39)
Diet low in calcium	20 (9·16–38·2)	14·3 (6·01–27·2)	13·9 (5·89–26·1)	-28·8 (-48·5—14·5)	-2·8 (-12·7–7·48)	14·6 (5·94–27·6)	8·67 (3·2–16·2)	8·31 (3·08–15·7)	-40·4 (-63·1—19·7)	-4·17 (-21·2–13·1)
Occupational exposure to asbestos	18·4 (16·5–20·2)	17·6 (16·6–18·8)	15·4 (12·8–18·1)	-4·11 (-13·8–7·94)	-12·5 (-27·5–3·19)	3·23 (2·43–4·9)	3·22 (2·23–4·09)	3·42 (2·12–4·76)	-0·357 (-33·3–34·3)	6·31 (-22·6–42·1)
Occupational exposure to arsenic	0·484 (0–1·12)	0·525 (0–1·24)	0·548 (0–1·31)	8·4 (-11·6–NA)	4·4 (-9·85–NA)	0·461 (0–1·1)	0·455 (0–1·06)	0·461 (0–1·11)	-1·39 (-21·8–NA)	1·34 (-12·2–NA)
Occupational exposure to benzene	0·828 (0·0471–2·36)	0·877 (0·0486–2·55)	0·917 (0·0509–2·56)	5·83 (-5·53–21·4)	4·63 (-3·3–17·9)	1·13 (0·066–3·21)	1·43 (0·0828–4·14)	1·55 (0·0942–4·67)	26·9 (9·56–56·4)	8·68 (-0·774–25·5)
Occupational exposure to beryllium	0·0238 (0·0221–0·0255)	0·0249 (0·0234–0·0265)	0·0262 (0·0244–0·0279)	4·77 (-3·55–14)	5·02 (-0·751–11·9)	0·0258 (0·0231–0·0284)	0·0283 (0·0261–0·0306)	0·0303 (0·0277–0·0328)	9·48 (-2·21–22·5)	6·94 (-0·412–14·8)
Occupational exposure to cadmium	0·0565 (0·0483–0·0657)	0·0611 (0·0532–0·0697)	0·0639 (0·0544–0·0742)	8·2 (-10·2–30·1)	4·62 (-8·65–18·3)	0·0551 (0·046–0·0661)	0·0555 (0·0482–0·0648)	0·0568 (0·049–0·0671)	0·824 (-18·2–25·4)	2·29 (-9·73–16)
Occupational exposure to chromium	0·134 (0·117–0·153)	0·145 (0·128–0·162)	0·152 (0·132–0·174)	8·09 (-8·05–27·2)	4·89 (-6·47–17·3)	0·136 (0·116–0·159)	0·144 (0·128–0·163)	0·149 (0·131–0·171)	5·79 (-11·5–26·9)	3·45 (-6·7–14·8)
Occupational exposure to diesel engine exhaust	0·754 (0·679–0·832)	0·762 (0·69–0·837)	0·801 (0·716–0·885)	1·06 (-10·7–15·6)	5·16 (-4·46–14·9)	0·634 (0·542–0·732)	0·69 (0·606–0·786)	0·735 (0·637–0·856)	8·82 (-9·34–33)	6·5 (-6·08–19·4)
Occupational exposure to formaldehyde	0·28 (0·238–0·332)	0·297 (0·257–0·339)	0·307 (0·261–0·358)	5·86 (-12·8–28·7)	3·47 (-9·37–17·7)	0·264 (0·221–0·316)	0·278 (0·243–0·317)	0·286 (0·249–0·331)	5·54 (-13·4–29)	2·8 (-8·24–15·7)
Occupational exposure to nickel	0·493 (0–2·21)	0·527 (0–2·27)	0·55 (0–2·4)	6·88 (-9·48–NA)	4·36 (-7·56–NA)	0·379 (0–1·72)	0·361 (0–1·55)	0·363 (0–1·61)	-4·76 (-25·4–NA)	0·544 (-13·5–NA)
Occupational exposure to polycyclic aromatic hydrocarbons	0·28 (0·248–0·316)	0·303 (0·272–0·335)	0·317 (0·28–0·359)	8·34 (-6·09–25·9)	4·75 (-5·12–15·9)	--	--	--	--	--
Occupational exposure to silica	6·3 (0·921–20·9)	6·65 (0·948–21·8)	6·91 (1–21·9)	5·42 (-10·7–31·1)	4·02 (-7·43–19·7)	3·51 (0·503–11·4)	3·31 (0·451–10·7)	3·35 (0·438–11·3)	-5·44 (-26·7–26·1)	0·985 (-13·3–20·7)
Occupational exposure to sulfuric acid	0·829 (0·177–2·6)	0·858 (0·176–2·73)	0·88 (0·185–2·74)	3·53 (-15·2–24·2)	2·54 (-10·5–16)	0·796 (0·163–2·55)	0·757 (0·159–2·4)	0·742 (0·156–2·32)	-4·91 (-24·5–19·7)	-1·95 (-15·4–12·6)
Unsafe sex	--	--	--	--	--	--	--	--	--	--
Occupational exposure to trichloroethylene	0·0752 (0·0681–0·0838)	0·0796 (0·0725–0·0872)	0·0837 (0·0754–0·0932)	5·94 (-5·93–20·3)	5·14 (-3·9–15·5)	0·0791 (0·0689–0·0901)	0·0842 (0·0763–0·0934)	0·0881 (0·0784–0·0991)	6·52 (-8·57–25·4)	4·61 (-5·09–14·8)
No access to handwashing facility	3·34 (3·04–3·67)	3·18 (2·88–3·5)	3·14 (2·86–3·46)	-4·59 (-14·1–6·73)	-1·27 (-7·87–5·69)	3·34 (3·04–3·67)	3·18 (2·88–3·5)	3·14 (2·86–3·46)	-4·59 (-14·1–6·73)	-1·27 (-7·87–5·69)
Child wasting	1·26 (0·875–1·66)	1·21 (0·85–1·62)	1·21 (0·837–1·61)	-3·45 (-15·4–11·1)	-0·274 (-9·89–10·1)	--	--	--	--	--
Child stunting	1·6 (0·773–2·43)	1·2 (0·556–1·89)	1·2 (0·533–1·88)	-24·9 (-44·8–0·73)	-0·502 (-20·7–23·4)	1·88 (0·935–2·93)	1·56 (0·738–2·39)	1·54 (0·744–2·36)	-16·8 (-38·3–9·38)	-1·05 (-18·8–21·3)
Chewing tobacco	0·174 (0·109–0·258)	0·166 (0·105–0·245)	0·171 (0·105–0·253)	-4·81 (-42·6–60·8)	3·14 (-24·9–42·9)	0·131 (0·0741–0·214)	0·13 (0·076–0·214)	0·132 (0·0763–0·226)	-0·176 (-48·3–91·2)	1·64 (-34·9–53·8)
Diet low in legumes	74 (38·5–99)	72·9 (36·5–99)	73·7 (37·8–99)	-1·49 (-12·1–6·36)	1·1 (-4·77–10·8)	70·4 (31–97·6)	69·2 (29–97·1)	69·8 (31·5–97·3)	-1·68 (-15·8–7·77)	0·95 (-6·62–12·5)
Short gestation	21·7 (17·1–27·5)	47·6 (37·3–59·6)	42·9 (32·7–54·2)	119 (63·3–191)	-10 (-31·1–18·7)	23·8 (19–28·7)	45 (35·9–55·3)	41·2 (34·4–48·8)	88·9 (38·8–161)	-8·56 (-27·7–16·6)
Low birth weight	11·9 (9·66–14·8)	23·7 (19–29·1)	22·5 (17·3–28·2)	98 (45·3–167)	-5·08 (-27·9–25·8)	13·5 (11·1–16·2)	22·8 (18·3–27·9)	21·5 (17·9–25·7)	69·3 (24·6–135)	-5·43 (-25·8–21·7)
High temperature	22·4 (6·68–39·3)	0 (0–0)	0 (0–0)	-100 (-100—100)	--	22·4 (6·68–39·3)	0 (0–0)	0 (0–0)	-100 (-100—100)	--
Low temperature	31·3 (25·9–37·2)	34·1 (29·3–39·5)	30·6 (25·1–36·6)	9·07 (5·44–13·6)	-10·4 (-14·4—6·91)	31·3 (25·9–37·2)	34·1 (29·3–39·5)	30·6 (25·1–36·6)	9·07 (5·44–13·6)	-10·4 (-14·4—6·91)
Kidney dysfunction	13·5 (8·95–19·9)	14·2 (9·59–20·5)	14 (9·63–20·4)	5·38 (1·11–11·6)	-1·11 (-5·66–3·46)	14·3 (9·49–20·8)	14·8 (9·95–21·4)	14·9 (10–21·5)	3·84 (-0·56–9·37)	0·401 (-4·29–5·04)
Bullying victimization	6·11 (2·45–12·1)	6·03 (2·39–12·2)	5·59 (2·23–11·6)	-1·3 (-10·5–8·36)	-7·16 (-12·4—2·52)	4·71 (1·91–9·69)	4·99 (1·99–10·1)	4·72 (1·86–9·73)	6·01 (-5·14–17·1)	-5·41 (-12—0·581)
High LDL cholesterol	50 (45·6–54·3)	43·5 (39·8–47·3)	44·2 (40·2–48·3)	-13 (-20·5—5·05)	1·71 (-4·04–7·64)	--	--	--	--	--

Supplementary Results Table 1. Lincolnshire age-standardised Summary Exposure Values (SEVs) for most-detailed risk factors and percentage change, by sex, 1990, 2010, and 2019. Data in parentheses are 95% uncertainty intervals.

	Male					Female				
	1990	2010	2019	Percentage change 1990-2010	Percentage change 2010-2019	1990	2010	2019	Percentage change 1990-2010	Percentage change 2010-2019
Unsafe sanitation	0·709 (0·26–1·63)	0·434 (0·161–0·91)	0·315 (0·118–0·664)	-38·7 (-81·2–90·1)	-27·5 (-68·9–59·3)	0·709 (0·26–1·63)	0·434 (0·161–0·91)	0·315 (0·118–0·664)	-38·7 (-81·2–90·1)	-27·5 (-68·9–59·3)
Ambient particulate matter pollution	17·4 (6·26–33)	10·3 (6·62–14·7)	8·27 (4·77–12·3)	-40·6 (-68·9–47·3)	-20 (-28·2–-15·8)	17·4 (6·26–33)	10·3 (6·62–14·7)	8·27 (4·78–12·3)	-40·6 (-68·9–47·1)	-20 (-28–-15·8)
Household air pollution from solid fuels	0·0329 (0·00679–0·0994)	0·0119 (0·00227–0·0361)	0·00724 (0·00114–0·0243)	-63·8 (-88·9–5·61)	-39·1 (-73·2–30·3)	0·0433 (0·00907–0·135)	0·0157 (0·00306–0·0496)	0·00959 (0·00161–0·0318)	-63·6 (-88·9–6·26)	-39 (-73·1–30·8)
Ambient ozone pollution	16·3 (5·23–32·9)	13·6 (3·87–27·9)	13·6 (3·82–27·5)	-16·6 (-56·8–47·6)	0·274 (-44·7–82·5)	16·3 (5·23–32·9)	13·6 (3·87–27·9)	13·6 (3·82–27·5)	-16·6 (-56·8–47·6)	0·274 (-44·7–82·5)
Residential radon	34·2 (8·02–88·6)	34·2 (8·02–88·6)	34·2 (8·02–88·6)	0 (0–0)	0 (0–0)	34·2 (8·02–88·6)	34·2 (8·02–88·6)	34·2 (8·02–88·6)	0 (0–0)	0 (0–0)
Lead exposure	31·8 (6–57·3)	20·9 (3·12–43·6)	14·6 (1·28–35·3)	-34·4 (-57·1–-21·8)	-30·2 (-61·4–-18·4)	12·8 (0·0206–35)	7·79 (0·005–26·3)	5·4 (5·07e-12–21·5)	-38·9 (-83·2–-21·4)	-30·7 (-100–-17·6)
Child underweight	0·925 (0·524–1·46)	0·789 (0·427–1·21)	0·779 (0·42–1·2)	-14·7 (-32·7–8·42)	-1·25 (-15·4–15·9)	1·08 (0·623–1·62)	0·949 (0·539–1·4)	0·936 (0·525–1·38)	-12·5 (-28·5–6·91)	-1·33 (-14–13·6)
Iron deficiency	--	--	--	--	--	10·9 (8·71–13·1)	9·9 (7·94–12·1)	9·76 (7·73–11·8)	-9·11 (-23·3–7·77)	-1·4 (-11–8·89)
Vitamin A deficiency	5·07 (1·04–13·4)	2·5 (0·59–7·43)	2·03 (0·434–5·65)	-50·6 (-86·9–138)	-18·8 (-69–137)	3·75 (1·11–8·59)	2·59 (0·831–6·04)	2·22 (0·728–5·12)	-31·1 (-77·5–131)	-14·2 (-57·4–88)
Zinc deficiency	4·72 (0–14)	2·97 (0–8·91)	3·01 (0–9·21)	-37 (-100–NA)	1·25 (-24·1–NA)	4·54 (0–13·2)	2·86 (0–9·09)	2·86 (0–8·94)	-37 (-100–NA)	-0·0139 (-27–NA)
Smoking	28 (24·3–31·9)	21·1 (18·4–24·1)	17·7 (15·4–20·3)	-24·6 (-31·4–-16·6)	-16 (-21·3–-10·9)	23·1 (19·6–27·2)	18 (15·1–21·2)	14·2 (11·7–16·9)	-22 (-32·8–-9·35)	-21·3 (-28·5–-13·3)
Secondhand smoke	44·2 (38·7–50·3)	32·7 (28·1–37·6)	32·6 (28–37·3)	-26 (-37·5–-13·5)	-0·361 (-10·8–9·98)	46·4 (41·8–51)	33·3 (29·4–37)	33·3 (29·4–37)	-28·2 (-37·9–-18·4)	-0·0608 (-7·83–9·18)
Alcohol use	17·1 (9·4–25·3)	20 (11·1–29·4)	18·2 (9·84–27·1)	16·8 (-24·5–82·8)	-8·76 (-35·9–21·5)	--	--	--	--	--
Drug use	0·315 (0·153–0·653)	0·584 (0·337–1·05)	0·575 (0·326–1·05)	85·7 (44·3–146)	-1·55 (-11·9–9·34)	0·194 (0·103–0·382)	0·297 (0·173–0·521)	0·298 (0·168–0·541)	53·1 (17·5–102)	0·204 (-10·6–10)
High fasting plasma glucose	9·7 (7·96–12)	17·6 (15–20·8)	22·1 (18·5–26·6)	81 (50·3–118)	25·9 (8·75–48·3)	7·89 (6·4–10)	12·9 (10·7–15·5)	16·1 (13·4–19·3)	63·4 (31·5–105)	24·9 (5·6–51·5)
High systolic blood pressure	34·9 (25·6–46·2)	31·5 (24·8–39·3)	31·4 (23·9–40)	-9·79 (-35·4–26·3)	-0·257 (-23–26·5)	--	--	--	--	--
High body-mass index	23·1 (16·2–31·8)	28·6 (20·1–38·9)	30·1 (21·4–40·6)	24 (9·57–41)	5·18 (-2·07–14·1)	24·1 (15·9–36·6)	30·8 (20·7–47·7)	31·6 (21–48·8)	27·9 (-5·1–73·2)	2·55 (-14–22·6)
Low bone mineral density	8·81 (4·69–14·8)	7·71 (3·94–13·4)	9·29 (4·92–15·5)	-12·4 (-35·1–17)	20·6 (-9·83–64·3)	14·7 (9·33–21·4)	14·1 (8·94–21·2)	13·9 (8·7–20·4)	-4·3 (-25·8–23·6)	-1·17 (-22·6–27·5)
Diet low in fruits	67·6 (55·3–79·6)	60·9 (48·2–74·1)	59·9 (46·9–72·6)	-9·89 (-22·2–2·94)	-1·52 (-10·5–8·95)	68·7 (57·7–79·5)	61·4 (48·8–73·6)	60·7 (48·3–73·2)	-10·7 (-23·1–1·78)	-1·03 (-9·6–8·49)
Diet low in vegetables	54 (33·2–75·4)	49·6 (29·5–70·7)	48·7 (28·9–69·3)	-8·04 (-26·5–9·41)	-1·82 (-14·4–11·2)	51·4 (31·1–72·8)	46·4 (26·9–68)	45·3 (26·3–66·7)	-9·79 (-28·5–8·13)	-2·47 (-17·5–13)
Diet low in whole grains	89 (82–94·8)	88·7 (82·3–94·7)	88·8 (82·3–94·9)	-0·366 (-3·7–2·8)	0·131 (-1·83–2·28)	--	--	--	--	--
Diet low in nuts and seeds	83·5 (43·5–93·5)	76 (39·5–88·1)	78 (40–89·9)	-9·03 (-22·7–1·52)	2·71 (-5·14–12·3)	83·4 (42·8–93·6)	76 (37·9–88·8)	77 (38·5–90·3)	-8·85 (-23·2–3·32)	1·35 (-7–11·7)
Diet low in milk	60·5 (35·1–81·7)	61·8 (37·1–82·8)	61·8 (37·4–82·5)	2·19 (-7·74–16·9)	-0·0314 (-6·21–6·92)	62·7 (37·1–82·9)	65·2 (39·2–85·3)	65·2 (40·2–86·1)	4·02 (-4·89–19·6)	-0·0389 (-5·09–5·64)
Diet high in red meat	64·6 (49·2–78·2)	50·2 (36·2–65·9)	52·1 (36·7–67·6)	-22·2 (-41·3–-1·95)	3·84 (-14·3–28·6)	64·3 (48·4–78·4)	51·4 (35·1–66·8)	53·9 (37·6–69·9)	-20 (-38·5–2·47)	4·79 (-12·3–26·9)
Diet high in processed meat	75·7 (52·8–96·2)	80·6 (59·3–98·5)	80·6 (58·8–98·4)	6·46 (-2·78–21·8)	0·0193 (-5·99–6·34)	78·6 (56·5–97·3)	84·1 (64·6–99·3)	84·3 (65·1–99·2)	6·94 (-1·46–22·2)	0·284 (-5·21–5·58)
Diet high in sugar-sweetened beverages	45·4 (27·6–72·2)	48·3 (27·2–73·8)	50·3 (29·4–74·2)	6·43 (-33·8–64)	4·08 (-24·5–39·7)	--	--	--	--	--
Diet low in fiber	44·3 (27·6–60·7)	37·4 (21·1–54)	37·3 (22·2–55·2)	-15·6 (-40·2–13·3)	-0·121 (-19·2–23·1)	44·4 (27·4–63·1)	37·3 (21–54·9)	37·2 (21·3–54·5)	-16 (-40·9–15·1)	-0·357 (-19·1–23·7)
Diet low in seafood omega-3 fatty acids	95·8 (88·1–100)	93·7 (84·3–100)	93·4 (83–100)	-2·22 (-11·4–3·3)	-0·336 (-8·14–9·22)	96·6 (89·6–100)	94·7 (84·5–100)	94·5 (84·7–100)	-1·94 (-10–2·79)	-0·234 (-7·93–7·52)
Diet low in polyunsaturated fatty acids	56·1 (17·8–95·9)	51·5 (15·6–92·1)	50·6 (15–92)	-8·19 (-23·6–0·000979)	-1·85 (-11·7–7·11)	58·3 (19·9–97·6)	53·7 (16·5–94·7)	52·8 (16·7–93·9)	-7·92 (-24·8–0·000101)	-1·65 (-10·4–6·95)
Diet high in trans fatty acids	57·8 (46·6–99·8)	50·8 (39·5–99·3)	50·8 (39·8–97·9)	-12·2 (-26·5–1·29)	0·0938 (-13·3–16·9)	55·8 (44·5–100)	47·7 (36–99·7)	47·7 (36·1–98·6)	-14·5 (-28·3–0)	-0·0245 (-12·3–15·3)
Diet high in sodium	18·4 (4·18–40·7)	22·2 (4·54–45·9)	24·5 (5·11–48·6)	20·6 (-49–208)	10·3 (-32·9–101)	11·3 (2·72–29·2)	11·7 (2·93–29·2)	12·5 (3·12–30·8)	3·27 (-41·9–89·5)	7·27 (-32·6–71·1)
Low physical activity	4·4 (1·89–8·91)	4·71 (2·12–9·58)	4·79 (2·13–9·88)	7·09 (-5·51–27·2)	1·72 (-11·9–17·4)	6·95 (3·63–12·8)	7·61 (3·99–13·8)	7·72 (4·11–13·8)	9·53 (0·0794–23·3)	1·43 (-7·04–11·5)
Occupational asthmagens	23·1 (18·9–28)	21 (17·4–25·5)	21·9 (17·6–26·8)	-9·15 (-23·4–6·39)	4·06 (-8·44–18·1)	--	--	--	--	--
Occupational particulate matter, gases, and fumes	9·36 (6·57–13·5)	8·86 (6·31–12·8)	8·62 (6·14–12·6)	-5·37 (-13·3–3·34)	-2·69 (-7·17–1·71)	4·34 (2·84–6·75)	4·35 (2·96–6·64)	4·16 (2·89–6·27)	0·334 (-14·1–19)	-4·5 (-10·7–2·64)
Occupational noise	9·29 (8·22–10·5)	8·89 (8·13–9·79)	8·69 (7·99–9·52)	-4·29 (-10·2–2·4)	-2·26 (-5·13–0·623)	5·17 (4·29–6·15)	5·71 (5·09–6·42)	5·83 (5·26–6·47)	10·3 (0·337–23·8)	2·15 (-1·64–6·31)
Occupational injuries	--	--	--	--	--	--	--	--	--	--
Occupational ergonomic factors	12·1 (9·91–14·4)	11·8 (9·86–14·1)	12·2 (10·1–14·7)	-2·84 (-15·5–12·1)	4·02 (-6·47–17)	--	--	--	--	--
Childhood sexual abuse	10·6 (6·74–14·7)	11 (7·04–15·8)	10·7 (6·96–15·2)	4·38 (-2·5–12·8)	-2·79 (-11–6·54)	13·3 (9·72–18·3)	14·1 (10·3–19·6)	13·7 (10·1–18·8)	6·28 (-2·16–15·1)	-2·58 (-10·4–8·27)
Intimate partner violence	--	--	--	--	--	22·8 (12·5–31·3)	20 (11·3–28·2)	22·8 (12·5–31·3)	-12·2 (-19·2–0·696)	13·9 (2·98–28·7)
Non-exclusive breastfeeding	26 (19·5–34)	23·2 (17·2–30·5)	23·2 (17·4–30·2)	-10·5 (-25·4–8·34)	-0·0236 (-13·5–15·6)	26 (19·5–34)	23·2 (17·2–30·5)	23·2 (17·4–30·2)	-10·5 (-25·4–8·36)	-0·0211 (-13·5–15·6)
Discontinued breastfeeding	27·8 (26·7–28·7)	27·9 (26·9–28·8)	27·6 (26·5–28·7)	0·354 (-4·27–5·58)	-0·884 (-4·49–2·75)	27·8 (26·7–28·6)	27·8 (26·8–28·7)	27·6 (26·5–28·7)	0·147 (-4·47–5·38)	-0·638 (-4·26–3)
Diet low in calcium	21·4 (10·2–40·5)	15·8 (6·74–30·3)	15·6 (6·83–29·8)	-26·2 (-46·4–-11·6)	-0·867 (-9·94–9·56)	15·8 (6·53–30·6)	10·4 (3·84–19·3)	10·3 (3·82–19·2)	-34·3 (-56·6–-14)	-0·731 (-16·4–14·9)
Occupational exposure to asbestos	16·8 (15·1–18·7)	16·1 (15–17·3)	15·6 (13–18·3)	-3·75 (-14·9–7·94)	-3·32 (-19·1–13·5)	3·53 (2·6–5·32)	3·55 (2·37–4·3)	3·8 (2·32–5·16)	0·809 (-29·8–37·4)	6·91 (-21·4–43)
Occupational exposure to arsenic	0·469 (0–1·1)	0·516 (0–1·21)	0·538 (0–1·3)	10·2 (-10·1–NA)	4·19 (-9·53–NA)	0·454 (0–1·12)	0·46 (0–1·1)	0·466 (0–1·12)	1·29 (-20·5–NA)	1·37 (-12·2–NA)
Occupational exposure to benzene	0·809 (0·0453–2·31)	0·862 (0·0516–2·39)	0·902 (0·0524–2·54)	6·51 (-4·25–22·5)	4·65 (-2·64–17)	1·09 (0·07–3·14)	1·39 (0·0803–4·02)	1·51 (0·0887–4·47)	27·2 (9·97–58·3)	8·67 (-1·8–23)
Occupational exposure to beryllium	0·024 (0·0224–0·0257)	0·0251 (0·0236–0·0266)	0·0263 (0·0245–0·0281)	4·62 (-2·93–13·9)	4·78 (-0·432–11)	0·0255 (0·023–0·0282)	0·028 (0·0259–0·0302)	0·0301 (0·0277–0·0326)	9·74 (-1·59–23·5)	7·29 (-0·55–15·3)
Occupational exposure to cadmium	0·0547 (0·0469–0·0638)	0·0601 (0·0522–0·0685)	0·0628 (0·0536–0·0736)	9·89 (-8·97–31·1)	4·47 (-8·16–19·5)	0·0543 (0·0456–0·0651)	0·0558 (0·048–0·0654)	0·0572 (0·0494–0·0672)	2·72 (-18–26·3)	2·47 (-10·2–17·1)
Occupational exposure to chromium	0·129 (0·112–0·148)	0·142 (0·125–0·159)	0·148 (0·129–0·172)	9·63 (-6·55–28·7)	4·85 (-6·69–18·1)	0·134 (0·114–0·156)	0·144 (0·127–0·165)	0·149 (0·132–0·17)	7·19 (-11·7–28·4)	3·68 (-7·08–15·7)
Occupational exposure to diesel engine exhaust	0·785 (0·696–0·88)	0·773 (0·698–0·849)	0·811 (0·73–0·904)	-1·58 (-13·5–13·5)	5 (-3·81–15)	0·642 (0·553–0·737)	0·695 (0·612–0·784)	0·75 (0·646–0·859)	8·16 (-8·66–30·2)	7·96 (-5·6–21·7)
Occupational exposure to formaldehyde	0·269 (0·229–0·319)	0·29 (0·25–0·332)	0·3 (0·254–0·355)	7·72 (-11·3–30)	3·4 (-10–18·8)	0·259 (0·218–0·31)	0·278 (0·24–0·323)	0·285 (0·248–0·33)	7·09 (-13·1–29·9)	2·65 (-9·13–15·9)
Occupational exposure to nickel	0·517 (0–2·28)	0·54 (0–2·31)	0·56 (0–2·45)	4·43 (-11·2–NA)	3·58 (-8·05–NA)	0·381 (0–1·7)	0·37 (0–1·58)	0·372 (0–1·68)	-2·82 (-23·8–NA)	0·572 (-13·8–NA)
Occupational exposure to polycyclic aromatic hydrocarbons	0·273 (0·243–0·308)	0·298 (0·268–0·331)	0·312 (0·277–0·354)	9·28 (-5·14–25·3)	4·69 (-5·43–16)	--	--	--	--	--
Occupational exposure to silica	6·87 (0·96–22·6)	6·96 (0·964–23·2)	7·18 (1·08–23·9)	1·22 (-15·4–23·4)	3·18 (-8·61–18·3)	3·68 (0·5–11·7)	3·52 (0·513–11·2)	3·57 (0·48–11·5)	-4·24 (-24·3–25·9)	1·48 (-14·1–20·5)
Occupational exposure to sulfuric acid	0·808 (0·166–2·64)	0·853 (0·18–2·64)	0·872 (0·174–2·75)	5·45 (-11·5–25·8)	2·26 (-9·74–16·4)	--	--	--	--	--
Unsafe sex	--	--	--	--	--	--	--	--	--	--
Occupational exposure to trichloroethylene	0·0723 (0·065–0·0805)	0·0777 (0·0707–0·0852)	0·0818 (0·0733–0·0917)	7·57 (-4·29–21·9)	5·27 (-3·6–15·1)	0·078 (0·0683–0·0889)	0·0838 (0·0755–0·0937)	0·0883 (0·0789–0·0983)	7·49 (-7·38–26)	5·33 (-4·8–16·6)
No access to handwashing facility	3·38 (3·08–3·7)	3·23 (2·94–3·57)	3·18 (2·89–3·48)	-4·53 (-14·6–7·12)	-1·55 (-8·21–5·52)	3·38 (3·08–3·7)	3·23 (2·94–3·57)	3·18 (2·89–3·48)	-4·53 (-14·6–7·12)	-1·55 (-8·21–5·52)
Child wasting	1·27 (0·879–1·7)	1·23 (0·857–1·64)	1·23 (0·851–1·64)	-3·56 (-16·5–11)	-0·161 (-9·95–9·47)	--	--	--	--	--
Child stunting	1·65 (0·781–2·53)	1·23 (0·57–1·94)	1·21 (0·545–1·93)	-25·7 (-45·7–-0·492)	-1·1 (-21·3–23)	1·94 (0·956–2·98)	1·61 (0·777–2·4)	1·58 (0·757–2·48)	-17·1 (-36·3–12·1)	-1·72 (-19·6–19·7)
Chewing tobacco	0·173 (0·112–0·262)	0·167 (0·109–0·251)	0·171 (0·111–0·25)	-3·88 (-40·7–57·1)	2·76 (-24·1–41·6)	0·133 (0·0738–0·216)	0·131 (0·0746–0·218)	0·133 (0·0747–0·227)	-1·3 (-48–88·1)	1·64 (-33·7–50·9)
Diet low in legumes	74·6 (38·8–99·2)	73·6 (38–98·7)	74·6 (38·7–99)	-1·3 (-12·6–6·56)	1·26 (-4·74–11·3)	71·1 (33·1–98·2)	70·1 (30·8–97·6)	70·9 (32·1–97·8)	-1·43 (-13·8–6·88)	1·19 (-5·81–12·8)
Short gestation	24·6 (19·8–30·7)	49·6 (38·4–62·1)	44·2 (33·5–57)	102 (48·9–169)	-11 (-32·8–16·4)	26·7 (21·5–32·8)	44·5 (35·4–55·1)	38·9 (31·7–47·3)	67·1 (23·1–127)	-12·8 (-32–12·2)
Low birth weight	12·7 (10·1–15·6)	25·9 (20·4–32·2)	22·8 (17·6–29·5)	105 (50·3–173)	-12 (-34·5–18·1)	13·5 (11–16·3)	23·1 (18·7–28·1)	20·8 (16·9–25)	71·1 (23–133)	-9·72 (-28·8–17·1)
High temperature	23·1 (8·37–39·4)	0 (0–0)	0 (0–0)	-100 (-100–-100)	--	23·1 (8·37–39·4)	0 (0–0)	0 (0–0)	-100 (-100–-100)	--
Low temperature	30·6 (25·3–36·6)	33·6 (29–39)	30·1 (24·8–36·1)	9·82 (5·93–14·8)	-10·5 (-14·4–-7·04)	30·6 (25·3–36·6)	33·6 (29–39)	30·1 (24·8–36·1)	9·82 (5·93–14·8)	-10·5 (-14·4–-7·04)
Kidney dysfunction	13·7 (9·18–20)	14·5 (9·84–20·9)	14·3 (9·83–20·5)	5·51 (0·874–11·7)	-1·27 (-5·87–3·06)	14·6 (9·89–21·2)	15·3 (10·5–21·8)	15·4 (10·4–22)	4·69 (0·17–10·9)	0·212 (-3·67–4·52)
Bullying victimization	6·11 (2·43–12·2)	6·11 (2·41–12·4)	5·62 (2·25–11·6)	0·0126 (-9·41–10·1)	-8·09 (-13·6–-3·53)	4·69 (1·91–9·61)	5·04 (2–10·3)	4·74 (1·86–9·71)	7·34 (-4·62–18·6)	-5·99 (-12·7–-1·1)
High LDL cholesterol	49·2 (45–53·3)	42·3 (38·6–46·6)	43·2 (39·5–47·3)	-14 (-20·8–-6·1)	2·18 (-3·44–8·46)	--	--	--	--	--

Supplementary Results Table 1. Northamptonshire age-standardised Summary Exposure Values (SEVs) for most-detailed risk factors and percentage change, by sex, 1990, 2010, and 2019. Data in parentheses are 95% uncertainty intervals.										
	Male					Female				
	1990	2010	2019	Percentage change 1990-2010	Percentage change 2010-2019	1990	2010	2019	Percentage change 1990-2010	Percentage change 2010-2019
Unsafe sanitation	0·608 (0·222–1·36)	0·368 (0·14–0·818)	0·277 (0·0983–0·585)	-39·4 (-81·5–91·6)	-24·7 (-68·3–74·1)	0·608 (0·222–1·36)	0·368 (0·14–0·818)	0·277 (0·0983–0·585)	-39·4 (-81·5–91·6)	-24·7 (-68·3–74·1)
Ambient particulate matter pollution	14·8 (5·02–27·9)	9·31 (5·95–13·3)	6·73 (3·69–10·3)	-37·1 (-68·4–68·4)	-27·7 (-39–21·6)	14·8 (5·04–27·9)	9·31 (5·96–13·3)	6·73 (3·69–10·3)	-37·1 (-68·3–68·9)	-27·7 (-39·3–21·6)
Household air pollution from solid fuels	0·0191 (0·00405–0·0626)	0·00665 (0·00119–0·0212)	0·0041 (0·000629–0·0146)	-65·1 (-90–7·01)	-38·5 (-72·6–28·3)	0·0252 (0·0054–0·0812)	0·00882 (0·00162–0·0286)	0·00542 (0·000867–0·0189)	-65·1 (-90–6·96)	-38·6 (-72·5–28·3)
Ambient ozone pollution	14·8 (4·28–30·8)	17·3 (4·59–37)	16·1 (4·12–35)	17·1 (-39·5–114)	-6·62 (-53·8–83)	14·8 (4·28–30·8)	17·3 (4·59–37)	16·1 (4·12–35)	17·1 (-39·5–114)	-6·62 (-53·8–83)
Residential radon	31·2 (7·76–74·8)	31·2 (7·76–74·8)	31·2 (7·76–74·8)	0 (0–0)	0 (0–0)	31·2 (7·76–74·8)	31·2 (7·76–74·8)	31·2 (7·76–74·8)	0 (0–0)	0 (0–0)
Lead exposure	30 (4·38–54·7)	19·3 (2·29–41·6)	13·3 (0·795–33·8)	-35·7 (-57·9–22·7)	-31 (-63·7–19·1)	12·2 (0·0132–34·2)	7·27 (7·64e-12–25·3)	4·98 (3·73e-12–20·6)	-40·3 (-92·8–22·1)	-31·4 (-100–17·4)
Child underweight	0·925 (0·527–1·39)	0·786 (0·414–1·22)	0·771 (0·41–1·21)	-15·1 (-31·8–6·02)	-1·86 (-16–14·3)	1·08 (0·631–1·63)	0·945 (0·526–1·39)	0·925 (0·531–1·4)	-12·6 (-28·6–7·37)	-2·13 (-15–12·9)
Iron deficiency	--	--	--	--	--	11·1 (8·87–13·3)	10 (8·13–12)	9·74 (7·81–11·8)	-9·52 (-23·5–7·74)	-2·82 (-12·4–7·83)
Vitamin A deficiency	4·52 (0·897–12·5)	2·17 (0·472–6·54)	1·85 (0·373–5·68)	-52·1 (-87·9–122)	-14·7 (-66·9–142)	3·47 (1·1–7·83)	2·31 (0·778–5·19)	1·99 (0·646–4·62)	-33·3 (-78·2–117)	-14 (-57·4–85·4)
Zinc deficiency	3·95 (0–11·8)	2·52 (0–7·81)	2·59 (0–8)	-36·2 (-100–NA)	3·04 (-21·8–NA)	3·81 (0–12)	2·43 (0–7·75)	2·49 (0–8·06)	-36·1 (-100–NA)	2·3 (-22·3–NA)
Smoking	28 (24·3–32)	21·9 (19·2–25·1)	18·5 (16–21·2)	-21·7 (-28·6–14·3)	-15·7 (-20·7–10·6)	23·1 (19·4–27·1)	18·5 (15·6–22)	14·6 (12·1–17·7)	-19·9 (-31–6·05)	-21 (-28·3–12·6)
Secondhand smoke	44·3 (38·9–50)	34·4 (29·6–39·4)	32·8 (28·5–37·6)	-22·3 (-34·8–9·08)	-4·5 (-14·2–5·46)	46·2 (41·8–50·7)	34·8 (31·3–38·5)	33·4 (29·7–37)	-24·6 (-33·9–14·6)	-4·21 (-11·2–3·66)
Alcohol use	16·9 (9·37–24·7)	20·1 (11·3–30·3)	17·9 (9·74–27·1)	19·4 (-24·4–81·3)	-11 (-37·6–17·3)	--	--	--	--	--
Drug use	0·322 (0·16–0·68)	0·617 (0·361–1·07)	0·593 (0·334–1·06)	91·3 (49·5–153)	-3·85 (-13·3–5·54)	0·2 (0·11–0·384)	0·336 (0·205–0·559)	0·322 (0·188–0·579)	67·7 (26·8–121)	-4·04 (-15–5·79)
High fasting plasma glucose	10·9 (8·98–13·5)	21·4 (18·3–25)	29·6 (25–35·8)	96·4 (59·4–138)	38·6 (20·7–64·7)	8·91 (7·32–11)	16·5 (13·8–19·7)	22·7 (18·9–27·1)	85 (51–126)	37·6 (16·6–68)
High systolic blood pressure	36·1 (27·2–46·9)	33·3 (26·6–40·7)	33·3 (25·4–43·3)	-7·82 (-31·8–28·1)	-0·181 (-21–26·4)	--	--	--	--	--
High body-mass index	22·7 (15·7–31·1)	28·2 (19·3–38·2)	29·4 (20·6–39·6)	24·4 (12·3–42·7)	4·22 (-2·3–12·5)	25·4 (18·7–34·6)	32·2 (24·3–42·5)	33 (24·9–43·7)	26·7 (10·1–47·4)	2·41 (-6·15–12·7)
Low bone mineral density	8·72 (4·58–14·7)	7·63 (3·72–13)	9·27 (4·9–15·8)	-12·5 (-34·7–16·8)	21·5 (-5·57–64·5)	14·6 (9·37–21·9)	14·1 (9·13–20·7)	13·7 (8·7–20·3)	-3·14 (-24–23·7)	-2·61 (-24·2–26·4)
Diet low in fruits	65·8 (54·3–77·7)	58·9 (45·8–71·1)	57·9 (44·3–70·4)	-10·5 (-23·6–1·69)	-1·76 (-12·5–9·28)	67 (55·2–78·6)	59·4 (46·8–71·6)	58·8 (45·8–71)	-11·4 (-23·4–1·34)	-1·05 (-10·6–9·69)
Diet low in vegetables	51·2 (30–71·5)	46·4 (26·9–68·1)	45·7 (25·8–66·8)	-9·37 (-27·8–9·52)	-1·69 (-15·6–13)	48·6 (28·8–69·6)	43 (24–64·5)	42 (23·3–62·6)	-11·4 (-31·7–7·34)	-2·38 (-17·3–12·8)
Diet low in whole grains	88·7 (81·9–94·8)	88·4 (81·1–94·7)	88·5 (81·3–94·7)	-0·406 (-3·57–2·71)	0·143 (-1·88–2·23)	--	--	--	--	--
Diet low in nuts and seeds	82·6 (42·2–92·8)	74·6 (40–87·4)	76·8 (39·5–89·2)	-9·74 (-23·7–1·34)	2·9 (-5·3–13·4)	82·4 (42·8–92·8)	74·5 (38·5–88)	75·6 (40·9–88·8)	-9·64 (-24·2–1·49)	1·52 (-7·28–12·7)
Diet low in milk	58·4 (34·7–79·8)	59·5 (34·4–80·3)	59·7 (34·7–80·7)	2·03 (-8·73–17·8)	0·187 (-7·11–8·47)	61 (35·9–81·4)	63·3 (37·2–84)	63·2 (38–84·4)	3·8 (-4·96–18·7)	-0·0949 (-6·21–7·14)
Diet high in red meat	66·6 (51·5–80·4)	52·3 (37·7–66·3)	54·2 (39–68·2)	-21·4 (-40·1–0·393)	3·58 (-13·5–24·5)	66 (49·9–80·8)	53·3 (37·1–67·7)	55·6 (38·6–71·6)	-19·3 (-38·4–1·61)	4·33 (-12·4–24·9)
Diet high in processed meat	77 (53–96·7)	81·6 (60·5–98·5)	81·8 (59·3–98·8)	5·95 (-2·89–21·2)	0·167 (-5·27–6·9)	80 (58·4–97·7)	85·3 (66·4–99·5)	85·4 (66–99·2)	6·54 (-1·28–22·7)	0·166 (-5·41–6·12)
Diet high in sugar-sweetened beverages	46·3 (28·3–71)	50·2 (30·8–73·7)	52·1 (32·6–75·6)	8·47 (-30·8–67·6)	3·73 (-21·1–38·6)	--	--	--	--	--
Diet low in fiber	41·7 (25·2–58·9)	34·4 (18·6–51·2)	34·5 (19·7–51·6)	-17·5 (-42·8–11·9)	0·172 (-19·3–24)	41·6 (24·7–58·2)	34·2 (18·3–51·9)	34·4 (19·2–51·9)	-17·8 (-45·1–15·1)	0·337 (-19·6–25·8)
Diet low in seafood omega-3 fatty acids	93·6 (82·9–100)	90·9 (77·2–100)	90·3 (77·3–100)	-2·83 (-14·2–5)	-0·671 (-11·1–12·2)	94·5 (85–100)	92·3 (81–100)	91·8 (79·8–100)	-2·3 (-12·3–3·92)	-0·622 (-10–8·79)
Diet low in polyunsaturated fatty acids	53·6 (15·8–94)	48·4 (14–90·5)	47·5 (13·6–90·1)	-9·76 (-26·9–0)	-1·86 (-11·8–7·2)	55·9 (18·5–96·3)	50·6 (15·3–92·4)	49·8 (15·3–91·7)	-9·48 (-26·7–0)	-1·56 (-10·5–6·54)
Diet high in trans fatty acids	57·7 (47–99·9)	50·8 (39·3–99·1)	50·9 (39·5–98·4)	-12·1 (-27·3–1·89)	0·207 (-14·6–17·5)	55·9 (44·5–100)	47·6 (35·9–99·9)	47·6 (35·9–99·9)	-14·8 (-29·7–0)	0·0435 (-12·1–16)
Diet high in sodium	18·5 (4·33–41·4)	22·1 (4·7–46·5)	24·3 (4·84–49·9)	19·7 (-45·1–161)	9·89 (-32·3–91·9)	11·2 (2·8–29·2)	11·7 (3·08–29·5)	12·6 (3·28–31·2)	4·31 (-40·3–103)	7·63 (-34·1–67·5)
Low physical activity	4·57 (1·98–9·36)	4·93 (2·18–9·87)	4·94 (2·22–9·9)	7·93 (-5·07–26·4)	0·176 (-13·2–15·1)	7·05 (3·64–12·8)	7·82 (4·25–14·1)	7·9 (4·32–13·9)	11 (1·65–25·1)	0·975 (-7·84–10·6)
Occupational asthmagens	22 (17·9–26·7)	20·8 (17·2–25·3)	21·8 (17·6–27·2)	-5·65 (-20–10·9)	4·96 (-7·16–19·7)	12 (9·32–15·4)	11·1 (8·83–13·8)	11·8 (9·1–14·8)	-7 (-26·3–14·8)	5·81 (-8·08–21·8)
Occupational particulate matter, gases, and fumes	9·07 (6·35–13·1)	8·68 (6·13–12·6)	8·49 (5·97–12·7)	-4·32 (-12·8–5·51)	-2·2 (-6·75–2·44)	4·31 (2·85–6·84)	4·29 (2·96–6·62)	4·08 (2·83–6·06)	-0·366 (-14·5–17·6)	-4·81 (-11·6–2·29)
Occupational noise	8·67 (7·7–9·77)	8·5 (7·82–9·29)	8·38 (7·74–9·13)	-1·95 (-7·51–4·93)	-1·4 (-4·25–1·65)	5·08 (4·2–6·04)	5·67 (5·04–6·36)	5·81 (5·21–6·45)	11·5 (1·08–24·6)	2·46 (-1·45–6·93)
Occupational injuries	--	--	--	--	--	--	--	--	--	--
Occupational ergonomic factors	11·2 (9·24–13·6)	10·9 (9·06–13)	11·3 (9·32–13·7)	-2·6 (-13·7–10·1)	3·96 (-5·91–15·1)	6·51 (5·06–8·38)	8·18 (6·33–10·5)	8·97 (6·87–11·7)	25·7 (1·05–58·2)	9·62 (-7·09–30·7)
Childhood sexual abuse	10·6 (6·74–14·7)	11 (7·04–15·8)	10·7 (6·96–15·2)	4·38 (-2·5–12·8)	-2·79 (-11–6·54)	13·3 (9·72–18·3)	14·1 (10·3–19·6)	13·7 (10·1–18·8)	6·28 (-2·16–15·1)	-2·58 (-10·4–8·27)
Intimate partner violence	--	--	--	--	--	22·8 (12·5–31·3)	20 (11·3–28·2)	22·8 (12·5–31·3)	-12·2 (-19·2–0·696)	13·9 (2·98–28·7)
Non-exclusive breastfeeding	25·9 (19·1–34·3)	23·2 (17·4–30·7)	23·3 (17·6–30·7)	-10·4 (-25·1–9·68)	0·243 (-13·3–14·8)	25·9 (19·1–34·3)	23·2 (17·4–30·6)	23·3 (17·6–30·7)	-10·4 (-25·1–9·71)	0·246 (-13·3–14·8)
Discontinued breastfeeding	28 (26·9–28·9)	27·8 (26·7–28·7)	27·5 (26·4–28·5)	-0·704 (-5·68–4·16)	-0·812 (-4·51–3·17)	28 (26·9–28·9)	27·6 (26·5–28·6)	27·5 (26·3–28·4)	-1·23 (-6·19–3·62)	-0·58 (-4·3–3·43)
Diet low in calcium	19·4 (8·9–36·6)	13·6 (5·7–26)	13·5 (5·77–25·8)	-29·7 (-49·6–15·5)	-0·719 (-9·58–10·3)	13·9 (5·45–27·4)	8·02 (2·91–14·8)	8·03 (2·78–14·5)	-42·5 (-64·4–22·5)	0·0816 (-19·1–20·5)
Occupational exposure to asbestos	20·4 (18·8–22·1)	18·9 (17·7–20·2)	16·6 (14·2–19·4)	-7·33 (-16–2·38)	-12 (-24·5–4·35)	3·77 (2·73–5·47)	3·81 (2·46–4·49)	4·08 (2·41–5·51)	1·16 (-29·8–36·5)	7·11 (-21–42·3)
Occupational exposure to arsenic	0·488 (0–1·17)	0·536 (0–1·28)	0·559 (0–1·36)	9·65 (-11·7–NA)	4·33 (-10·2–NA)	0·463 (0–1·13)	0·464 (0–1·1)	0·47 (0–1·16)	0·224 (-19·8–NA)	1·26 (-13–NA)
Occupational exposure to benzene	0·826 (0·052–2·35)	0·878 (0·0517–2·54)	0·922 (0·0531–2·66)	6·35 (-4·7–21·6)	5·03 (-3·2–17·1)	1·11 (0·0659–3·28)	1·41 (0·0833–4·02)	1·54 (0·0914–4·46)	27·4 (9·97–57·1)	9·16 (-1·06–25)
Occupational exposure to beryllium	0·0239 (0·0223–0·0254)	0·0251 (0·0236–0·0267)	0·0264 (0·0245–0·0284)	5·11 (-3·08–14·1)	5·03 (-1·14–11·6)	0·0257 (0·0231–0·0283)	0·0282 (0·0259–0·0304)	0·0302 (0·0275–0·0325)	9·74 (-2·04–23·7)	7 (-0·493–15·1)
Occupational exposure to cadmium	0·0569 (0·049–0·0663)	0·0621 (0·0539–0·0705)	0·065 (0·0554–0·0761)	9·21 (-10·4–30·9)	4·74 (-8·95–18·1)	0·0553 (0·0463–0·0659)	0·0564 (0·0483–0·066)	0·0576 (0·0496–0·0671)	1·86 (-16·6–27·2)	2·17 (-10·3–17·4)
Occupational exposure to chromium	0·134 (0·118–0·154)	0·147 (0·13–0·164)	0·154 (0·133–0·177)	8·98 (-8·46–26·5)	5·04 (-7·14–17·1)	0·136 (0·116–0·159)	0·145 (0·128–0·165)	0·15 (0·132–0·171)	6·55 (-10·6–28·7)	3·38 (-7·45–16·2)
Occupational exposure to diesel engine exhaust	0·754 (0·678–0·835)	0·761 (0·691–0·829)	0·801 (0·715–0·888)	0·915 (-11·2–14·9)	5·23 (-3·8–15·1)	0·632 (0·547–0·722)	0·685 (0·602–0·774)	0·732 (0·629–0·853)	8·46 (-8·55–31·6)	6·78 (-6·22–21·9)
Occupational exposure to formaldehyde	0·283 (0·241–0·331)	0·302 (0·262–0·344)	0·313 (0·264–0·367)	6·88 (-13·4–29·1)	3·55 (-9·87–17·3)	0·265 (0·22–0·317)	0·281 (0·243–0·325)	0·289 (0·253–0·333)	6·38 (-11·9–31·2)	2·62 (-9·02–16·5)
Occupational exposure to nickel	0·499 (0–2·16)	0·537 (0–2·35)	0·559 (0–2·46)	7·66 (-9·81–NA)	4·03 (-7·37–NA)	0·381 (0–1·67)	0·373 (0–1·7)	0·373 (0–1·64)	-2·22 (-22–NA)	0·214 (-14·4–NA)
Occupational exposure to polycyclic aromatic hydrocarbons	0·281 (0·252–0·316)	0·307 (0·276–0·34)	0·322 (0·286–0·365)	9·15 (-6·4–25·3)	4·86 (-5·47–15·2)	--	--	--	--	--
Occupational exposure to silica	6·32 (0·93–20·1)	6·72 (0·955–21·8)	6·97 (0·973–22·9)	6·31 (-10·8–31·1)	3·79 (-7·93–18·5)	3·54 (0·499–11·4)	3·39 (0·447–10·7)	3·42 (0·48–11·4)	-4·25 (-22·9–26·9)	0·868 (-14·4–22·4)
Occupational exposure to sulfuric acid	0·833 (0·181–2·63)	0·87 (0·177–2·74)	0·894 (0·183–2·88)	4·46 (-13·2–24·1)	2·76 (-9·39–15)	--	--	--	--	--
Unsafe sex	--	--	--	--	--	--	--	--	--	--
Occupational exposure to trichloroethylene	0·0754 (0·0685–0·0832)	0·0803 (0·0733–0·0885)	0·0846 (0·0762–0·0942)	6·52 (-6·41–19·7)	5·35 (-3·82–14·8)	0·079 (0·0692–0·09)	0·0845 (0·0753–0·0943)	0·0884 (0·0789–0·099)	6·88 (-8·01–25·8)	4·68 (-4·86–16·6)
No access to handwashing facility	3·37 (3·06–3·68)	3·21 (2·92–3·53)	3·17 (2·89–3·45)	-4·49 (-14·7–6·09)	-1·5 (-7·91–5·28)	3·37 (3·06–3·68)	3·21 (2·92–3·53)	3·17 (2·89–3·45)	-4·49 (-14·7–6·09)	-1·5 (-7·91–5·28)
Child wasting	1·27 (0·883–1·68)	1·22 (0·859–1·64)	1·22 (0·851–1·61)	-3·6 (-14·9–10·5)	-0·45 (-9·69–9·81)	--	--	--	--	--
Child stunting	1·66 (0·809–2·55)	1·22 (0·571–1·94)	1·21 (0·528–1·9)	-26·5 (-45·8–2·66)	-1·49 (-22·3–24·3)	1·96 (0·99–3·01)	1·6 (0·785–2·49)	1·57 (0·776–2·42)	-18 (-37·6–10·7)	-2·16 (-20·2–17·5)
Chewing tobacco	0·176 (0·114–0·268)	0·166 (0·107–0·243)	0·169 (0·111–0·245)	-5·89 (-41·3–59·1)	2·32 (-25·4–41·2)	0·132 (0·0749–0·227)	0·131 (0·0756–0·222)	0·133 (0·0749–0·229)	-0·28 (-47·3–92·7)	1·61 (-35·6–58·7)
Diet low in legumes	73·7 (38·5–99·1)	72·7 (37·2–98·5)	73·6 (36·5–98·7)	-1·39 (-12·9–6·99)	1·22 (-4·83–10·8)	70 (30·4–97·5)	68·8 (27·1–97·3)	69·7 (29·9–97·2)	-1·72 (-16·1–7·94)	1·26 (-6·31–14·1)
Short gestation	24·2 (19·2–30·4)	54·3 (43·4–67·3)	49·3 (37·9–62·3)	124 (65·6–198)	-9·16 (-30·4–18·1)	26·9 (21·9–32·9)	51·1 (41·8–62·7)	46·9 (38·4–56·1)	90 (37·1–164)	-8·14 (-27·2–16·3)
Low birth weight	12·7 (10·3–15·8)	26·5 (21·2–32·9)	22·9 (17·7–28·7)	110 (53·4–179)	-13·6 (-34·9–15·4)	13·5 (11–16·4)	25·1 (20·6–30·4)	23 (19·2–27·1)	85·5 (35·2–154)	-8·41 (-27·7–14·8)
High temperature	22·3 (6·6–39·3)	0 (0–0)	0 (0–0)	-100 (-100–100)	--	22·3 (6·6–39·3)	0 (0–0)	0 (0–0)	-100 (-100–100)	--
Low temperature	30·8 (25·4–36·8)	33·7 (28·9–39·1)	30·2 (24·8–36·2)	9·09 (5·37–13·8)	-10·1 (-14·1–6·6)	30·8 (25·4–36·8)	33·7 (28·9–39·1)	30·2 (24·8–36·2)	9·09 (5·37–13·8)	-10·1 (-14·1–6·6)
Kidney dysfunction	13·5 (9·06–19·9)	14·3 (9·83–20·7)	14·1 (9·64–20·3)	5·81 (0·889–12·4)	-0·97 (-5·65–3·62)	14·5 (9·81–21)	15·3 (10·4–21·8)	15·4 (10·4–21·9)	5·28 (0·594–11·1)	0·883 (-3·18–5·17)
Bullying victimization	6·1 (2·44–12·1)	6·1 (2·4–12·3)	5·65 (2·25–11·7)	-0·102 (-9·39–10·1)	-7·37 (-12·8–2·7)	4·68 (1·91–9·55)	5 (1·99–10·1)	4·75 (1·85–9·77)	6·8 (-4·85–18)	-4·99 (-11·9–0·101)
High LDL cholesterol	49·7 (45·7–54·2)	42·7 (38·6–46·8)	43·5 (39·5–47·5)	-14·2 (-21·6–7·02)	1·87 (-3·87–8·08)	--	--	--	--	--

Supplementary Results Table 1. Central Europe age-standardised Summary Exposure Values (SEVs) for most-detailed risk factors and percentage change, by sex, 1990, 2010, and 2019. Data in parentheses are 95% uncertainty intervals.

	Male					Female				
	1990	2010	2019	Percentage change 1990-2010	Percentage change 2010-2019	1990	2010	2019	Percentage change 1990-2010	Percentage change 2010-2019
Unsafe sanitation	19·3 (16·6–22·4)	8·02 (6·36–9·79)	5·51 (4·23–7·02)	-58·4 (-67·1—48·3)	-31·2 (-43·6—17)	19·2 (16·5–22·3)	7·99 (6·36–9·77)	5·5 (4·22–7·02)	-58·3 (-67·1—48·2)	-31·1 (-43·5—16·8)
Ambient particulate matter pollution	24 (11·7–39)	22·6 (19·2–26·6)	19·2 (16·1–22·8)	-5·78 (-41·3–83·9)	-15·1 (-18·6—11·8)	22·6 (11–37·1)	22 (18·5–26·1)	18·8 (15·7–22·5)	-2·56 (-39·6–90)	-14·4 (-18—10·8)
Household air pollution from solid fuels	6·24 (2·88–11·7)	2·05 (0·763–4·27)	1·36 (0·424–3·08)	-67·1 (-78·9—53·3)	-33·8 (-46·5—21·4)	7·34 (3·62–13)	2·54 (0·974–5·27)	1·7 (0·575–3·74)	-65·4 (-78·1—50·3)	-33·1 (-46·1—20·3)
Ambient ozone pollution	60·7 (26·7–92·3)	46·4 (19·4–79)	40·4 (16·7–68·1)	-23·5 (-30·3—14·4)	-13 (-15·2—10·9)	60·4 (26·6–92·2)	46·4 (19·4–79)	40·4 (16·7–68·2)	-23·2 (-29·9—14·3)	-12·9 (-15·1—10·8)
Residential radon	25·3 (15–39·2)	25·4 (15–39)	25·5 (15·1–39·4)	0·422 (-1·29–2·24)	0·421 (-0·54–1·29)	21·1 (10·6–37)	21·1 (10·6–36·4)	21·1 (10·6–36·1)	-0·17 (-2·88–3·15)	0·0358 (-1·17–1·45)
Lead exposure	58·1 (32·5–77·5)	49·4 (28·1–69)	39·1 (20·1–59·2)	-15·1 (-20·7—9·34)	-20·8 (-28·7—13·5)	24·9 (1·86–52·2)	23·2 (2·87–48·2)	18·5 (2·15–41·7)	-6·66 (-14–69·3)	-20·5 (-30—13·4)
Child underweight	1·64 (1·11–2·22)	1·12 (0·702–1·57)	1·02 (0·645–1·42)	-32·1 (-38·2—28)	-8·71 (-12·5—5·34)	1·72 (1·19–2·31)	1·22 (0·794–1·68)	1·11 (0·716–1·53)	-29·1 (-34·3—25)	-9·31 (-12·7—6·22)
Iron deficiency	--	--	--	--	--	16·1 (14·4–17·8)	13·9 (12·3–15·7)	13·7 (12·1–15·4)	-13·3 (-17·7—9·16)	-1·99 (-4·4–0·446)
Vitamin A deficiency	20·5 (15·8–26)	11·1 (7·95–14·7)	8·63 (6·1–11·2)	-45·8 (-59·1—27·5)	-22·4 (-35·7—4·46)	18·9 (15–23·2)	12·5 (9·69–15·5)	10·1 (7·78–12·5)	-33·9 (-46·5—15·7)	-19·2 (-29·5—5·55)
Zinc deficiency	5·01 (0·287–13·5)	4·56 (0·203–12·8)	3·77 (0·104–11)	-8·87 (-35·6—5·02)	-17·4 (-46·6—12·4)	4·88 (0·235–13·5)	4·44 (0·176–12·6)	3·66 (0·0874–10·6)	-9·06 (-33·1—4·57)	-17·4 (-47·1—12·9)
Smoking	29·9 (26·9–33·2)	25·3 (22·7–28·1)	22·9 (20·5–25·6)	-15·3 (-17·6—13)	-9·66 (-11·2—8·11)	14·8 (12·8–17)	13·3 (11·6–15·3)	13 (11·4–14·9)	-10·1 (-14·3—5·68)	-2·44 (-5·09–0·228)
Secondhand smoke	45·4 (44·1–46·6)	41·6 (40·5–42·5)	39·5 (38·3–40·6)	-8·42 (-11·6—5·03)	-4·95 (-6·96—2·9)	52·8 (51·6–54)	46·6 (45·8–47·5)	44·2 (43–45·2)	-11·6 (-14·2—9·15)	-5·35 (-6·81—3·83)
Alcohol use	18·4 (13·8–23·8)	22·1 (15·8–29·7)	23·2 (16·4–31·8)	20·1 (8·54–35·5)	4·92 (0·656–10·3)	6·11 (3·96–9·3)	7·12 (4·58–10·8)	7·66 (4·94–11·6)	16·4 (4·54–33·6)	7·68 (-0·405–16·6)
Drug use	0·134 (0·0619–0·282)	0·169 (0·0892–0·33)	0·167 (0·0901–0·32)	25·5 (14–44·6)	-0·93 (-7·21–5·12)	0·085 (0·0418–0·171)	0·11 (0·0612–0·209)	0·11 (0·0623–0·198)	29·4 (18·3–45·7)	0·398 (-6·55–8·95)
High fasting plasma glucose	8·83 (7·76–10)	12·9 (11·4–14·5)	14·8 (13·1–16·7)	45·9 (37·2–56·9)	14·9 (9·52–21·2)	7·64 (6·71–8·65)	10 (8·96–11·2)	11·4 (10·2–12·7)	31·1 (24·1–39·6)	13·6 (8·31–19·3)
High systolic blood pressure	39·7 (37·4–41·9)	40·3 (37·6–43)	39·1 (36·2–42·1)	1·49 (-4·96–8·01)	-2·95 (-9·54–4·02)	38·4 (36–40·8)	32·7 (30·3–35·4)	29·6 (26·4–33)	-14·7 (-21·4—7·16)	-9·53 (-18·8—0·0163)
High body-mass index	20·3 (14·9–27·3)	27·7 (21·1–36·5)	31 (23·7–40·3)	36·7 (27·9–48·8)	11·8 (7·87–15·6)	21·2 (16·1–28·4)	26·9 (21·7–34·1)	29·5 (23·8–37·4)	27·2 (19·4–38·9)	9·63 (6·19–13·6)
Low bone mineral density	7·22 (3·68–12·2)	6·58 (3·26–11·5)	6·27 (3·02–11·1)	-8·81 (-20·4–1·49)	-4·71 (-15·5–7·45)	18·3 (12·9–25·3)	17·3 (12–24·1)	16·6 (11·3–23·2)	-5·34 (-11·2–0·454)	-4·12 (-9·98–1·57)
Diet low in fruits	56 (45·6–67·8)	53·4 (43–64·9)	50·5 (40–61·9)	-4·63 (-5·94—3·54)	-5·34 (-8·96—2·77)	57·6 (47·3–69·5)	55·2 (45·1–66·7)	52·3 (42–64·1)	-4·21 (-5·44—3·17)	-5·17 (-8·16—2·73)
Diet low in vegetables	27·9 (15·3–45·5)	19·8 (11·1–33·4)	17·4 (9·59–30·4)	-29 (-33·1—21·1)	-12 (-17·2—5·76)	25·4 (13·7–42)	18·3 (9·98–31·5)	16·1 (8·66–28·2)	-27·9 (-32·2—19·7)	-12·2 (-17·7—6·23)
Diet low in whole grains	90·7 (86·4–95·1)	85 (78·2–91·8)	84·5 (77·4–91·8)	-6·25 (-9·43—3·21)	-0·659 (-1·77–0·358)	87·7 (82–93·1)	84 (77·2–90·6)	84·7 (78·1–91)	-4·19 (-6·24—2·26)	0·889 (0·244–1·76)
Diet low in nuts and seeds	77·7 (37·6–87·5)	68·9 (33·9–83·3)	66·7 (32·1–81·5)	-11·4 (-28·2—2·6)	-3·22 (-6·89—0·98)	75·1 (36·6–86)	65·6 (32·3–81·2)	63·1 (30·8–79)	-12·6 (-30·5—3·54)	-3·77 (-8—1·49)
Diet low in milk	56·3 (33·1–75·3)	56·2 (35–73)	57·7 (35·5–75·3)	-0·304 (-10·4–20·6)	2·8 (-1·25–6·07)	59·8 (34·3–80·6)	66·1 (40·9–86·2)	64·4 (39·4–84·5)	10·5 (0·516–31·9)	-2·53 (-6·37—0·661)
Diet high in red meat	59·3 (50·4–67·5)	65·7 (57·4–73·2)	69 (61·6–76·2)	10·7 (7·93–14·5)	5·05 (1·74–9·11)	59·4 (50·7–67·6)	65·6 (57·1–73·2)	68·9 (60·6–75·9)	10·4 (7·49–14·1)	5·03 (1·63–9·26)
Diet high in processed meat	37·9 (19·6–61·1)	50·5 (29·5–72·5)	52·2 (30·8–74·1)	33·2 (12·3–62)	3·23 (-0·555–8·52)	48·2 (22·6–74·5)	55·6 (27·7–81·2)	57·5 (29·7–83·1)	15·4 (4·58–37·5)	3·32 (0·201–8·49)
Diet high in sugar-sweetened beverages	33·6 (22·4–47·4)	40·6 (27·7–55·6)	47·5 (35·3–60·6)	20·7 (6·69–37·3)	17·2 (7·53–32·9)	37·6 (25·1–51·9)	46·1 (32·5–60·7)	53·3 (40·9–66·4)	22·8 (10·2–42·4)	15·6 (6·06–30·3)
Diet low in fiber	26·6 (16·1–38·3)	27·3 (16·7–38·3)	24·3 (14·9–34·8)	2·68 (-0·581–7·3)	-11·2 (-15·9—6·47)	26·2 (15·4–37·9)	27 (16·4–38·6)	24 (14·3–34·5)	3·2 (-0·202–8·33)	-11·4 (-16·4—6·83)
Diet low in seafood omega-3 fatty acids	92·5 (84·7–99·9)	91·1 (83·1–99·8)	89 (79·9–99·6)	-1·52 (-4·04—0·00165)	-2·38 (-5·41—0·0417)	93·9 (87·6–100)	92·7 (86–100)	90·8 (83–99·7)	-1·31 (-3·51–0)	-2·08 (-4·98—0·0902)
Diet low in polyunsaturated fatty acids	69·5 (31·5–99·4)	63·2 (27–95·1)	60·8 (24·2–94·6)	-9·01 (-17·3—2·46)	-3·78 (-10·5—0·55)	71·2 (33·6–99·6)	65·1 (28·3–96·1)	62·8 (26–95·4)	-8·55 (-16·7—1·95)	-3·51 (-9·79—0·465)
Diet high in trans fatty acids	47·5 (38–97·8)	38·8 (28·8–97·7)	37·8 (27·9–96·7)	-18·4 (-27·1–0)	-2·5 (-6·2–0·913)	49·2 (38·7–99·5)	40·8 (29·5–99·5)	39·7 (28·6–99·3)	-17 (-26·1–0)	-2·71 (-6·26–0·0151)
Diet high in sodium	85·8 (74·7–91·9)	82·4 (66·1–90·9)	82·9 (66·1–91·4)	-3·96 (-14·1–2·03)	0·558 (-3·4–4·28)	58·4 (38·9–74·2)	41·5 (14·3–63·7)	39·1 (12·6–61·9)	-29 (-62·4—12·9)	-5·63 (-17·2—0·765)
Low physical activity	2·13 (0·969–4·47)	2·02 (0·93–4·16)	2·41 (1·12–4·82)	-5·06 (-13·5–3·22)	19·3 (5·75–33·9)	3·11 (1·73–5·39)	3·39 (1·93–5·81)	3·54 (1·99–6·05)	8·88 (-0·138–20·3)	4·5 (-3·86–13·3)
Occupational asthmagens	23·4 (20·2–27·8)	20·9 (17·9–24·8)	21·3 (18·2–25·3)	-11 (-14·7—7·2)	2·13 (-1·21–5·17)	12·9 (10·6–15·8)	12·6 (10·4–15·4)	12·6 (10·3–15·3)	-2·3 (-12·5–9·08)	0·22 (-8·42–8·63)
Occupational particulate matter, gases, and fumes	9·63 (6·96–13·8)	8·75 (6·27–12·4)	8·36 (5·99–11·9)	-9·17 (-12·2—6·66)	-4·45 (-5·81—3·24)	5·97 (4·32–8·55)	5·23 (3·77–7·5)	4·79 (3·43–6·92)	-12·4 (-16—9·18)	-8·26 (-10·1—6·78)
Occupational noise	8·68 (8·23–9·29)	8·05 (7·65–8·67)	7·78 (7·41–8·39)	-7·27 (-8·61—5·88)	-3·4 (-3·94—2·86)	5·56 (5·22–6·06)	5·31 (5·02–5·79)	5·16 (4·89–5·66)	-4·6 (-6·76—2·26)	-2·79 (-3·53—2)
Occupational injuries	--	--	--	--	--	--	--	--	--	--
Occupational ergonomic factors	17·3 (15·7–19·1)	14·6 (13·2–16·2)	14·2 (12·7–16·1)	-15·9 (-20·2—11·6)	-2·33 (-6·31–1·67)	14·8 (13·3–16·3)	12 (11–13·1)	11·3 (9·96–12·9)	-19·2 (-26·4—11·6)	-5·68 (-13·7–2·95)
Childhood sexual abuse	8·61 (5·66–12·2)	8·55 (5·74–12)	8·64 (5·69–12·2)	-0·698 (-2·19–1·26)	1·05 (-0·719–2·57)	6·93 (5·05–9·63)	6·64 (5·05–8·9)	6·92 (5·06–9·58)	-4·16 (-8–0·964)	4·19 (-0·624–8·97)
Intimate partner violence	--	--	--	--	--	15·8 (8·96–21·9)	14·5 (8·45–19·9)	15·6 (8·73–21·7)	-8·14 (-12·4—0·874)	7·47 (1·09–15·2)
Non-exclusive breastfeeding	22·9 (16·6–30·9)	22·7 (16·7–30·3)	22·4 (16·7–29·8)	-0·951 (-5·23–3·92)	-0·978 (-3·93–2·15)	22·9 (16·7–30·9)	22·7 (16·7–30·3)	22·4 (16·7–29·7)	-1·19 (-5·45–3·66)	-0·998 (-3·96–2·13)
Discontinued breastfeeding	20·6 (20–21·2)	21·3 (20·8–21·9)	20·8 (20·2–21·4)	3·62 (0·0945–7·65)	-2·63 (-5·47–0·34)	20·6 (20–21·1)	21·3 (20·8–21·9)	20·8 (20·2–21·4)	3·76 (0·276–7·79)	-2·56 (-5·4–0·42)
Diet low in calcium	24·6 (13·5–44·2)	22·7 (12·5–40·4)	19·6 (10·2–36)	-7·94 (-9·48—5·96)	-13·6 (-20·9—8·2)	18·2 (8·39–34·6)	16·6 (7·72–31·6)	13·9 (6·23–26·6)	-8·6 (-11—4·42)	-16 (-26·4—9·25)
Occupational exposure to asbestos	1·3 (1·18–1·43)	2·74 (2·61–2·87)	2·9 (2·46–3·37)	112 (94·1–129)	5·51 (-9·89–22)	0·938 (0·74–1·51)	0·977 (0·684–1·18)	0·975 (0·631–1·23)	4·11 (-34·4–28·6)	-0·172 (-17·7–19·2)
Occupational exposure to arsenic	0·468 (0–1·11)	0·435 (0–1·03)	0·468 (0–1·11)	-7·12 (-10·6–NA)	7·55 (4·11–NA)	0·529 (0–1·24)	0·448 (0–1·06)	0·474 (0–1·12)	-15·4 (-19·3–NA)	5·76 (2·05–NA)
Occupational exposure to benzene	0·57 (0·0339–1·64)	0·623 (0·0371–1·78)	0·677 (0·0406–1·95)	9·44 (7·29–12·5)	8·63 (6·24–11·5)	0·718 (0·0421–2·07)	0·895 (0·0533–2·57)	1·02 (0·0603–2·96)	24·8 (21·2–29·7)	14·1 (11·7–17·8)
Occupational exposure to beryllium	0·0202 (0·0199–0·0205)	0·0195 (0·0194–0·0196)	0·0209 (0·0207–0·0211)	-3·45 (-5·03—1·91)	7·33 (6·01–8·7)	0·0226 (0·0222–0·0231)	0·0222 (0·0221–0·0223)	0·0243 (0·024–0·0246)	-1·85 (-3·89–0·175)	9·5 (7·92–11)
Occupational exposure to cadmium	0·0532 (0·0516–0·055)	0·05 (0·0492–0·0508)	0·0538 (0·0522–0·0552)	-6·15 (-9·36—2·77)	7·61 (4·49–10·6)	0·0603 (0·0581–0·0627)	0·0524 (0·0513–0·0536)	0·0558 (0·054–0·0578)	-13·2 (-16·9—9·27)	6·47 (3·11–10·1)
Occupational exposure to chromium	0·119 (0·116–0·123)	0·115 (0·114–0·117)	0·125 (0·121–0·128)	-3·31 (-6·33—0·0394)	8·02 (5·15–10·7)	0·136 (0·131–0·141)	0·125 (0·123–0·128)	0·135 (0·131–0·139)	-8·28 (-12—4·37)	8·05 (5·1–11·3)
Occupational exposure to diesel engine exhaust	0·563 (0·548–0·579)	0·578 (0·57–0·586)	0·625 (0·611–0·639)	2·58 (-0·431–5·66)	8·08 (5·86–10·5)	0·459 (0·445–0·472)	0·487 (0·479–0·496)	0·547 (0·532–0·561)	6·23 (2·55–9·93)	12·2 (9·46–15)
Occupational exposure to formaldehyde	0·264 (0·242–0·276)	0·247 (0·229–0·254)	0·264 (0·24–0·274)	-6·45 (-9·89—2·71)	6·64 (3·12–9·96)	--	--	--	--	--
Occupational exposure to nickel	0·49 (0–2·2)	0·436 (0–1·92)	0·467 (0–2·06)	-11·1 (-13·9–NA)	7·16 (4·39–NA)	0·459 (0–2·02)	0·369 (0–1·63)	0·385 (0–1·74)	-19·8 (-23·4–NA)	4·5 (0·789–NA)
Occupational exposure to polycyclic aromatic hydrocarbons	0·243 (0·237–0·25)	0·238 (0·235–0·241)	0·257 (0·252–0·263)	-2·26 (-5·08–0·7)	8·22 (5·72–10·7)	0·274 (0·265–0·283)	0·259 (0·254–0·263)	0·281 (0·274–0·288)	-5·54 (-9·07—1·91)	8·67 (5·94–11·7)
Occupational exposure to silica	5·72 (0·821–18·6)	5·31 (0·769–17·1)	5·73 (0·826–18·5)	-7·08 (-10·4—3·42)	7·96 (5·24–11·6)	4·14 (0·591–13·6)	3·22 (0·457–10·3)	3·34 (0·464–10·5)	-22·1 (-25·9—18·9)	3·49 (0·0554–7·99)
Occupational exposure to sulfuric acid	0·801 (0·167–2·52)	0·744 (0·155–2·36)	0·791 (0·164–2·5)	-7·07 (-9·95—4·24)	6·27 (3·37–8·9)	0·82 (0·17–2·6)	0·69 (0·145–2·16)	0·72 (0·149–2·22)	-15·8 (-19·2—12·3)	4·4 (1·29–8·08)
Unsafe sex	--	--	--	--	--	--	--	--	--	--
Occupational exposure to trichloroethylene	0·063 (0·0617–0·0647)	0·0627 (0·0622–0·0637)	0·0677 (0·0667–0·0692)	-0·395 (-2·87–2·35)	7·95 (5·79–10·1)	0·072 (0·0703–0·0745)	0·0695 (0·0688–0·0708)	0·076 (0·0746–0·078)	-3·6 (-6·66—0·357)	9·39 (7·06–12)
No access to handwashing facility	5·73 (5·6–5·87)	3·53 (3·45–3·61)	3·23 (3·16–3·31)	-38·5 (-40·1—36·7)	-8·37 (-9·93—6·85)	5·91 (5·76–6·07)	3·56 (3·47–3·67)	3·25 (3·17–3·33)	-39·6 (-41·5—37·7)	-8·84 (-10·7—7·13)
Child wasting	1·99 (1·57–2·39)	1·92 (1·52–2·31)	1·86 (1·47–2·25)	-3·46 (-8·53–2·25)	-3·14 (-7·02–0·451)	1·58 (1·22–1·94)	1·47 (1·13–1·82)	1·43 (1·1–1·77)	-7·04 (-12·6—1·42)	-2·96 (-7·29–1·69)
Child stunting	8·19 (5·64–9·86)	5·92 (3·88–7·38)	5·27 (3·41–6·67)	-27·7 (-35·4—21·6)	-11 (-16·4—5·89)	7·59 (5·13–9·21)	5·82 (3·77–7·29)	5·19 (3·28–6·62)	-23·3 (-31·3—16·2)	-10·8 (-16·9—5·37)
Chewing tobacco	0·332 (0·301–0·366)	0·364 (0·328–0·402)	0·41 (0·369–0·456)	9·53 (-2·81–23·4)	12·7 (3·99–21·4)	0·216 (0·174–0·274)	0·23 (0·184–0·295)	0·254 (0·202–0·322)	6·15 (-19·9–39·4)	10·7 (-6·55–33)
Diet low in legumes	83·9 (62·6–99·9)	78·4 (50–99)	78·7 (50·7–99·4)	-6·56 (-19·7—0·765)	0·469 (-0·919–2·41)	81·4 (56·5–99·7)	75·3 (44–98·2)	75·8 (44·4–98·8)	-7·42 (-22·9—1·22)	0·566 (-1·32–2·73)
Short gestation	15·1 (13·2–17·4)	20·6 (18·5–22·7)	22·4 (19·9–25)	36·1 (20·3–49·7)	8·69 (0·309–16·4)	16·4 (14·7–18·6)	21·1 (19·1–23·4)	22 (19·7–24·6)	28·9 (17·1–40·2)	3·96 (-3·72–11·9)
Low birth weight	8·12 (7·38–9·11)	11·1 (10·4–12)	12 (10·9–13·1)	37·3 (23·3–49·5)	7·57 (-0·977–15·7)	9·01 (8·29–9·82)	11·6 (10·8–12·4)	11·9 (10·9–13)	28·3 (17·3–38·8)	2·97 (-4·72–11)
High temperature	21·4 (14·4–30·2)	25·1 (17·5–34·3)	23·8 (16·2–33)	17·6 (6·36–31·5)	-5·32 (-10·6—0·188)	21·4 (14·4–30·2)	25·1 (17·5–34·3)	23·8 (16·2–33)	17·6 (6·36–31·5)	-5·32 (-10·6—0·188)
Low temperature	32·3 (29·4–35·6)	33·9 (31·4–37)	32·3 (29·5–35·8)	5·2 (3·24–7·53)	-4·69 (-6·59—2·68)	32·3 (29·4–35·6)	33·9 (31·4–37)	32·3 (29·5–35·8)	5·2 (3·24–7·53)	-4·69 (-6·59—2·68)
Kidney dysfunction	17·4 (11·7–24·4)	19 (13·1–26·1)	19·6 (13·7–26·8)	9·1 (5·86–14·1)	3·14 (1·83–4·9)	18·4 (12·3–25·8)	20·3 (14·1–27·9)	21 (14·7–28·6)	10·7 (7·49–15·6)	3·41 (2·11–5·25)
Bullying victimization	5·36 (2·27–10·9)	6·12 (2·86–12)	6·17 (2·82–12·2)	14·2 (1·62–49·2)	0·802 (-2·16–3·9)	3·96 (1·65–8·06)	4·51 (2·11–8·66)	4·54 (2·11–8·78)	14 (-1·39–51·7)	0·658 (-2·72–3·47)
High LDL cholesterol	48·9 (46·2–51·8)	45·4 (42·6–48·5)	44·9 (42–47·9)	-7·12 (-9·34—4·95)	-1·12 (-2·79–0·62)	47·7 (44·9–50·7)	42 (39·1–45·1)	40·1 (37–43·3)	-12 (-14·5—9·73)	-4·54 (-6·29—2·78)

Supplementary Results Table 1. Nottingham age-standardised Summary Exposure Values (SEVs) for most-detailed risk factors and percentage change, by sex, 1990, 2010, and 2019. Data in parentheses are 95% uncertainty intervals.										
	Male					Female				
	1990	2010	2019	Percentage change 1990-2010	Percentage change 2010-2019	1990	2010	2019	Percentage change 1990-2010	Percentage change 2010-2019
Unsafe sanitation	0·524 (0·188–1·18)	0·255 (0·0859–0·566)	0·207 (0·0714–0·49)	-51·4 (-84·9–46·4)	-18·8 (-66·7–97·3)	0·524 (0·188–1·18)	0·255 (0·0859–0·566)	0·207 (0·0714–0·49)	-51·4 (-84·9–46·4)	-18·8 (-66·7–97·3)
Ambient particulate matter pollution	20·2 (8·1–36·2)	12·7 (9·44–16·5)	9·72 (6·76–13·2)	-36·9 (-64·8–46·1)	-23·7 (-29·6–19·6)	20·2 (8·06–36·2)	12·7 (9·44–16·5)	9·72 (6·76–13·2)	-36·9 (-64·9–45·6)	-23·7 (-29·6–19·7)
Household air pollution from solid fuels	0·00997 (0·00181–0·031)	0·00314 (0·000488–0·0105)	0·00216 (0·000295–0·00802)	-68·5 (-91·5–7·46)	-31·1 (-71·5–51·6)	0·0131 (0·00252–0·0419)	0·00414 (0·000673–0·0142)	0·00285 (0·000433–0·0101)	-68·4 (-91·4–8·38)	-31·2 (-71·5–52·2)
Ambient ozone pollution	11·7 (0·618–33·4)	3·11 (0–12·6)	3·63 (0–14·5)	-73·5 (-100–182)	17 (-100–NA)	11·7 (0·618–33·4)	3·11 (0–12·6)	3·63 (0–14·5)	-73·5 (-100–182)	17 (-100–NA)
Residential radon	25·4 (3·99–83·3)	25·4 (3·99–83·3)	25·4 (3·99–83·3)	0 (0–0)	0 (0–0)	25·4 (3·99–83·3)	25·4 (3·99–83·3)	25·4 (3·99–83·3)	0 (0–0)	0 (0–0)
Lead exposure	27·7 (3·37–53·6)	17·2 (1·55–39·5)	11·6 (0·483–31·3)	-38 (-63·6–24·9)	-32·6 (-69·8–20·3)	11·3 (9·72e-05–32·5)	6·46 (4·4e-12–23·4)	4·32 (2·58e-12–18·8)	-42·7 (-100–24·8)	-33·1 (-100–3·56)
Child underweight	0·931 (0·531–1·44)	0·785 (0·436–1·2)	0·77 (0·42–1·2)	-15·6 (-33·1–4·83)	-1·95 (-17·3–15·6)	1·09 (0·614–1·62)	0·943 (0·52–1·4)	0·925 (0·521–1·41)	-13·7 (-29·5–6·11)	-1·88 (-14·7–14·3)
Iron deficiency	--	--	--	--	--	11·7 (9·39–14·1)	10·6 (8·62–12·7)	10·2 (8·18–12·4)	-9·84 (-24·3–6·02)	-3·79 (-13·1–6·79)
Vitamin A deficiency	3·99 (0·79–12·8)	1·64 (0·299–5·45)	1·48 (0·275–4·71)	-58·9 (-90·8–126)	-10 (-65·5–174)	3·14 (0·935–7·44)	1·87 (0·579–4·47)	1·67 (0·519–4)	-40·4 (-81·6–129)	-10·7 (-56·9–105)
Zinc deficiency	3·12 (0–9·65)	2·13 (0–6·89)	2·15 (0–7·21)	-31·7 (-100–NA)	0·942 (-23·8–NA)	3·03 (0–9·55)	2·03 (0–6·59)	2·05 (0–6·68)	-33·1 (-100–NA)	1·37 (-22–NA)
Smoking	27·6 (24–31·3)	22·9 (19·9–26·3)	19·7 (17·2–22·7)	-17·1 (-24·9–8·57)	-14·1 (-19·1–8·88)	22·8 (18·9–27·3)	19·3 (16–23)	15·5 (12·8–18·6)	-15·6 (-28–1·11)	-19·6 (-27·6–10·9)
Secondhand smoke	47 (41–53·7)	34 (29·1–39·1)	32·7 (28·1–37·9)	-27·6 (-39·5–13·7)	-4·04 (-13·6–6·12)	49·3 (44–54·6)	34·5 (30·7–38·4)	33·1 (29·5–37·2)	-30 (-39·8–18·8)	-4·03 (-11–4·03)
Alcohol use	16·6 (8·72–24·8)	20 (11·5–29·3)	18·5 (10·1–27·5)	20·2 (-24·1–93·1)	-7·65 (-32·7–22·5)	--	--	--	--	--
Drug use	0·313 (0·152–0·663)	0·628 (0·363–1·12)	0·536 (0·295–0·995)	101 (58–166)	-14·6 (-24·7–5·33)	0·187 (0·097–0·378)	0·292 (0·164–0·514)	0·278 (0·147–0·524)	56·1 (18·4–105)	-4·83 (-17–4·66)
High fasting plasma glucose	10·3 (8·5–12·6)	18·4 (15·8–21·5)	23·1 (19·5–27·7)	77·9 (47·7–118)	25·5 (10·1–48·2)	8·5 (6·71–11)	14 (11·5–16·8)	17 (14·1–20·7)	64·3 (30–109)	22 (2·54–49)
High systolic blood pressure	36·8 (26·9–48·8)	34·6 (27·5–42·9)	33·7 (25·1–43·6)	-6·11 (-33·7–35)	-2·49 (-25·1–24·9)	--	--	--	--	--
High body-mass index	20·2 (14–27·9)	27 (18·7–36·2)	27·7 (19·4–37·2)	33·5 (16·5–55·6)	2·47 (-4·41–10·3)	23·6 (15·1–37·1)	31·3 (21·4–48·6)	31·3 (20·6–48·8)	32·9 (-5·21–83·8)	-0·145 (-16·3–21·9)
Low bone mineral density	8·75 (4·61–14·9)	7·73 (3·87–13·4)	9·43 (5–15·3)	-11·7 (-25·5–14·1)	22 (-9·16–69·4)	14·6 (9·37–21·4)	14·2 (8·91–20·7)	14 (8·82–20·6)	-3·14 (-24·7–22·8)	-1·22 (-20–28·3)
Diet low in fruits	63·5 (50·7–75·8)	56·8 (44·5–70·4)	56 (43·4–70)	-10·6 (-25·1–4·01)	-1·36 (-12·1–10·2)	64·3 (51·8–76·6)	57 (43·3–70·1)	56·5 (42·9–69·3)	-11·4 (-24·4–1·98)	-0·942 (-12–9·86)
Diet low in vegetables	47·5 (27·1–68·4)	43 (24·7–64·4)	42·5 (23·8–63·4)	-9·47 (-30–12·1)	-1·27 (-15·7–15·5)	44·7 (25·3–65·6)	39·7 (22·3–60·5)	38·9 (22·1–60)	-11·1 (-33·5–10·5)	-2·04 (-18·3–15·6)
Diet low in whole grains	89·4 (83·3–94·5)	89·2 (82·9–94·3)	89·4 (83·7–94·7)	-0·276 (-3·36–3·16)	0·247 (-1·81–2·47)	--	--	--	--	--
Diet low in nuts and seeds	81·3 (40·9–92·6)	72·7 (37·5–86·3)	75 (38·8–88·1)	-10·6 (-24·9–1·1)	3·15 (-6·45–14·5)	81·2 (41·7–92·3)	72·8 (36·2–86·4)	73·7 (38·3–88·4)	-10·4 (-25·6–3·32)	1·3 (-8·76–12·3)
Diet low in milk	55·3 (31·7–76·2)	57 (32·8–78)	57·4 (33·3–79)	2·92 (-9·47–21·4)	0·756 (-7·27–9·84)	58·1 (33·2–79·4)	61·1 (35·2–81·8)	61·4 (35·9–82)	5·27 (-5·32–25·5)	0·424 (-6·38–8·06)
Diet high in red meat	68·5 (53·4–82·2)	54·4 (38·6–70·6)	56·2 (40·2–71·5)	-20·6 (-40·1–1·01)	3·32 (-14–25·6)	68·3 (52·6–82·4)	55·7 (39·2–71·1)	57·5 (41·3–73·8)	-18·5 (-38·4–3·44)	3·37 (-13·4–24·2)
Diet high in processed meat	77·6 (54·2–96·9)	82·2 (62·1–98·7)	82·2 (62·5–98·7)	5·91 (-3·25–22)	0·00128 (-5·74–6·63)	80·8 (59·5–97·9)	85·8 (66·2–99·4)	85·9 (67·5–99·3)	6·23 (-1·38–20·9)	0·0704 (-5·39–5·8)
Diet high in sugar-sweetened beverages	49·4 (30·9–73·8)	54·1 (34·5–76·8)	55 (35·3–77·1)	9·5 (-24·8–65·1)	1·74 (-20·7–34·8)	--	--	--	--	--
Diet low in fiber	38·2 (22·9–54·4)	31·5 (16·7–48·1)	32 (17·2–48·9)	-17·4 (-46·6–17·8)	1·36 (-19·8–29·4)	38·5 (22·1–55·6)	31·3 (16–48·8)	31·6 (17·3–49·2)	-18·5 (-46·5–18·9)	0·722 (-22·4–31·7)
Diet low in seafood omega-3 fatty acids	92·3 (79·4–100)	89·5 (74·9–100)	88·7 (73·7–100)	-3·03 (-15·1–7·53)	-0·925 (-12·8–13·2)	93·1 (83·1–100)	91·1 (79–100)	90·6 (77·8–100)	-2·18 (-12·2–6·62)	-0·608 (-10·6–10·5)
Diet low in polyunsaturated fatty acids	50 (14·3–92·2)	45·1 (12·1–87·6)	44·5 (11·5–87·6)	-9·84 (-28·2–1·42)	-1·32 (-11·7–10·3)	52·5 (16·4–94·6)	47·4 (13·7–91·3)	46·9 (13·1–89·1)	-9·57 (-26·3–0·615)	-1·15 (-10·9–9·88)
Diet high in trans fatty acids	57·9 (46·7–100)	51 (39·1–99·8)	51 (39·6–98·6)	-12 (-27·2–1·57)	0·062 (-15–16·5)	55·9 (44·1–100)	47·9 (36·1–100)	47·7 (35·9–100)	-14·3 (-28·6–0)	-0·312 (-14·4–14·7)
Diet high in sodium	18·5 (4·49–40·7)	22·6 (4·68–46·3)	24·6 (4·92–49·6)	21·7 (-42·7–188)	8·88 (-34·5–87·7)	11·3 (2·97–29·6)	11·8 (2·91–29·5)	12·6 (3·24–31·7)	4·47 (-43·6–102)	7·28 (-34·1–81·6)
Low physical activity	4·74 (2·04–9·57)	5·31 (2·35–10·8)	5·29 (2·32–10·6)	12·1 (-1·97–33)	-0·396 (-13·8–14)	7·24 (3·82–13·3)	8·18 (4·46–14·4)	8·19 (4·4–14·7)	13 (3·38–27·1)	0·0895 (-8·59–9·02)
Occupational asthmagens	22·3 (18·1–28·2)	21·4 (17·5–26·3)	22·4 (18–28·4)	-3·92 (-22·4–17·2)	4·8 (-10·2–21·3)	10·9 (8·48–14·2)	10·6 (8·41–13·2)	11·2 (8·57–14·3)	-3·15 (-25·3–22·3)	6·1 (-9·79–23·8)
Occupational particulate matter, gases, and fumes	9·12 (6·28–13·2)	8·68 (6·09–12·6)	8·47 (6–12·2)	-4·8 (-13·5–6·66)	-2·38 (-7·13–2·87)	4·31 (2·77–6·9)	4·19 (2·87–6·38)	3·95 (2·68–6)	-2·88 (-17·4–16)	-5·62 (-12·3–1·76)
Occupational noise	8·49 (7·49–9·59)	8·37 (7·64–9·21)	8·27 (7·58–9·05)	-1·47 (-7·3–5·28)	-1·16 (-3·92–2·02)	5·07 (4·21–6·01)	5·66 (5–6·39)	5·81 (5·23–6·48)	11·8 (0·987–24·3)	2·56 (-1·51–6·96)
Occupational injuries	--	--	--	--	--	--	--	--	--	--
Occupational ergonomic factors	10·2 (8·37–12·4)	10 (8·21–12·3)	10·5 (8·59–12·7)	-2·16 (-13·3–9·97)	4·66 (-4·51–13·8)	--	--	--	--	--
Childhood sexual abuse	10·6 (6·74–14·7)	11 (7·04–15·8)	10·7 (6·96–15·2)	4·38 (-2·5–12·8)	-2·79 (-11–6·54)	13·3 (9·72–18·3)	14·1 (10·3–19·6)	13·7 (10·1–18·8)	6·28 (-2·16–15·1)	-2·58 (-10·4–8·27)
Intimate partner violence	--	--	--	--	--	22·8 (12·5–31·3)	20 (11·3–28·2)	22·8 (12·5–31·3)	-12·2 (-19·2–0·696)	13·9 (2·98–28·7)
Non-exclusive breastfeeding	26 (19·6–34·1)	23·4 (17·6–31·1)	23·5 (17·6–30·8)	-10·2 (-26·1–9·85)	0·481 (-14·4–15·9)	26 (19·6–34·1)	23·4 (17·6–31·1)	23·5 (17·6–30·8)	-10·1 (-26–9·9)	0·488 (-14·4–15·9)
Discontinued breastfeeding	28·3 (27·2–29·2)	28·4 (27·2–29·4)	28 (26·9–29)	0·341 (-4·61–5·25)	-1·11 (-4·86–2·71)	28·3 (27·2–29·2)	28·5 (27·4–29·6)	28·1 (27–29·1)	0·931 (-4·04–5·85)	-1·32 (-5·05–2·48)
Diet low in calcium	17 (7·73–31·8)	11·4 (4·82–21·6)	11·5 (4·74–21·9)	-32·7 (-50·4–17·5)	0·677 (-12·1–14·3)	11·3 (4·62–21·4)	5·98 (2·17–11·4)	6·13 (2·26–11·5)	-47·3 (-68–25·7)	2·52 (-20·7–28·3)
Occupational exposure to asbestos	20·8 (19·2–22·4)	18·4 (17·1–19·8)	16·6 (13·8–19·3)	-11·2 (-20–2·04)	-9·85 (-25·3–6·71)	3·95 (2·78–6·51)	4·06 (2·81–5·09)	3·85 (2·58–5·35)	2·82 (-25·6–32·5)	-5·1 (-30·4–31·7)
Occupational exposure to arsenic	0·514 (0–1·18)	0·551 (0–1·31)	0·576 (0–1·39)	7·16 (-13·2–NA)	4·59 (-9·86–NA)	0·465 (0–1·13)	0·454 (0–1·06)	0·461 (0–1·12)	-2·35 (-25·2–NA)	1·67 (-12–NA)
Occupational exposure to benzene	0·836 (0·0511–2·42)	0·896 (0·0523–2·69)	0·94 (0·0535–2·76)	7·18 (-4·56–25)	4·86 (-4·63–16·4)	1·12 (0·0677–3·26)	1·44 (0·0957–4·21)	1·58 (0·0985–4·67)	28·3 (9·53–58·5)	9·58 (-1·2–26·8)
Occupational exposure to beryllium	0·024 (0·0223–0·0256)	0·0252 (0·0235–0·0268)	0·0265 (0·0246–0·0284)	4·85 (-3·72–14·1)	5·24 (-1·16–12·5)	0·0251 (0·0223–0·028)	0·0275 (0·0251–0·0299)	0·0296 (0·0271–0·0323)	9·71 (-2·83–24·3)	7·59 (-0·276–16·2)
Occupational exposure to cadmium	0·0596 (0·0509–0·069)	0·0637 (0·055–0·0729)	0·0667 (0·0567–0·077)	6·78 (-12–29)	4·77 (-8·38–19·2)	0·0551 (0·0457–0·0671)	0·055 (0·0472–0·0644)	0·0564 (0·0484–0·0664)	-0·226 (-22·2–26)	2·67 (-9·61–17·6)
Occupational exposure to chromium	0·141 (0·123–0·16)	0·151 (0·133–0·17)	0·158 (0·137–0·179)	6·91 (-9·63–26·2)	4·98 (-6·77–17·4)	0·136 (0·114–0·16)	0·142 (0·125–0·162)	0·148 (0·13–0·17)	4·9 (-14·9–28·5)	3·98 (-6·85–16·6)
Occupational exposure to diesel engine exhaust	0·746 (0·668–0·827)	0·759 (0·679–0·838)	0·798 (0·706–0·887)	1·65 (-12·1–16·6)	5·17 (-4·12–16·5)	0·583 (0·501–0·679)	0·632 (0·552–0·724)	0·677 (0·576–0·796)	8·41 (-10·1–30)	7·04 (-6·35–22·8)
Occupational exposure to formaldehyde	0·299 (0·253–0·348)	0·312 (0·269–0·357)	0·322 (0·273–0·372)	4·29 (-14·7–26·5)	3·45 (-9·54–17·8)	0·267 (0·222–0·323)	0·279 (0·242–0·324)	0·288 (0·252–0·333)	4·64 (-16·3–30·6)	3·31 (-8·05–16·8)
Occupational exposure to nickel	0·505 (0–2·22)	0·534 (0–2·35)	0·556 (0–2·46)	5·78 (-10·9–NA)	4·12 (-7·81–NA)	0·376 (0–1·68)	0·355 (0–1·53)	0·357 (0–1·54)	-5·57 (-29·2–NA)	0·578 (-13·6–NA)
Occupational exposure to polycyclic aromatic hydrocarbons	0·292 (0·259–0·326)	0·313 (0·279–0·349)	0·329 (0·291–0·369)	7·51 (-6·97–24·6)	4·87 (-5·3–16)	--	--	--	--	--
Occupational exposure to silica	6·18 (0·865–19·8)	6·55 (0·907–21·2)	6·84 (0·976–21·6)	5·98 (-11·3–29·3)	4·38 (-8·31–19·7)	3·33 (0·487–10·8)	3·1 (0·403–9·58)	3·13 (0·414–9·98)	-6·92 (-29·7–23·8)	0·96 (-14·9–21·9)
Occupational exposure to sulfuric acid	0·859 (0·183–2·88)	0·884 (0·186–2·83)	0·908 (0·195–2·87)	2·97 (-15–24·8)	2·65 (-9·32–17·5)	--	--	--	--	--
Unsafe sex	--	--	--	--	--	--	--	--	--	--
Occupational exposure to trichloroethylene	0·0786 (0·0706–0·0867)	0·0826 (0·075–0·0913)	0·0869 (0·0778–0·0965)	5·1 (-7·19–19·3)	5·19 (-3·97–15·4)	0·0771 (0·0659–0·0896)	0·0814 (0·0726–0·0911)	0·0856 (0·0759–0·0966)	5·64 (-11–26·3)	5·07 (-5·21–16·3)
No access to handwashing facility	3·33 (3·03–3·66)	3·17 (2·85–3·52)	3·13 (2·83–3·46)	-4·94 (-15·4–5·95)	-1·14 (-7·62–5·68)	3·33 (3·03–3·66)	3·17 (2·85–3·52)	3·13 (2·83–3·46)	-4·94 (-15·4–5·95)	-1·14 (-7·62–5·68)
Child wasting	1·26 (0·883–1·68)	1·22 (0·856–1·63)	1·21 (0·853–1·64)	-3·63 (-16·2–11·1)	-0·56 (-10·3–10·5)	1·07 (0·752–1·44)	1·02 (0·698–1·39)	1·01 (0·696–1·35)	-5·1 (-18·6–10·7)	-0·585 (-9·75–9·77)
Child stunting	1·68 (0·842–2·56)	1·23 (0·576–1·97)	1·22 (0·553–1·98)	-26·8 (-45·8–0·69)	-0·837 (-20·4–23·3)	1·98 (0·988–3)	1·61 (0·77–2·45)	1·58 (0·764–2·44)	-18·4 (-37·9–8·84)	-1·99 (-20·5–19·9)
Chewing tobacco	0·177 (0·111–0·267)	0·168 (0·104–0·256)	0·172 (0·106–0·262)	-4·91 (-43·5–62·3)	2·4 (-27·1–43·9)	0·134 (0·0734–0·228)	0·133 (0·0723–0·228)	0·136 (0·0753–0·233)	-0·359 (-49·3–96·4)	1·68 (-35·5–58·5)
Diet low in legumes	72·4 (33·5–98·5)	71·4 (33·8–98·1)	72·5 (34·4–98·7)	-1·28 (-13·3–7·85)	1·49 (-4·55–13·6)	68·7 (28·8–96·5)	67·5 (25·6–96·4)	68·6 (29·1–97·1)	-1·71 (-16·8–7·17)	1·58 (-6·08–15)
Short gestation	22·7 (18–27·9)	55·2 (43·1–69·1)	53·8 (41–69·3)	143 (85·2–219)	-2·54 (-24·5–26·1)	25·3 (20·7–31·2)	54·4 (43·4–67·7)	51·2 (41·2–62·4)	116 (53·3–198)	-5·89 (-27·1–19·9)
Low birth weight	11·4 (9·25–13·9)	26·2 (20·7–32·5)	25·8 (20–33·4)	129 (71·4–200)	-1·64 (-24·5–28·5)	13·5 (11·1–16·4)	25·4 (20·4–31)	24·8 (20·2–29·7)	88·5 (33·6–161)	-2·44 (-23·8–26·9)
High temperature	22·8 (6·97–39·9)	0 (0–0)	0 (0–0)	-100 (-100–100)	--	22·8 (6·97–39·9)	0 (0–0)	0 (0–0)	-100 (-100–100)	--
Low temperature	31·2 (25·8–37·1)	34·2 (29·4–39·6)	30·8 (25·7–36·7)	9·59 (5·75–14·3)	-9·88 (-13·2–6·76)	31·2 (25·8–37·1)	34·2 (29·4–39·6)	30·8 (25·7–36·7)	9·59 (5·75–14·3)	-9·88 (-13·2–6·76)
Kidney dysfunction	13·7 (9·08–19·9)	14·4 (9·9–21)	14·3 (9·7–20·8)	5·51 (0·493–12)	-0·807 (-5·58–4·03)	14·5 (9·67–21·1)	15·2 (10·4–21·6)	15·3 (10·3–21·9)	4·7 (0·551–10·4)	0·754 (-3·43–5·34)
Bullying victimization	5·95 (2·43–11·8)	5·81 (2·33–11·5)	5·41 (2·24–11)	-2·21 (-10·8–7·75)	-6·97 (-12·2–2·25)	4·55 (1·89–9·19)	4·78 (1·95–9·55)	4·53 (1·84–9·12)	5·08 (-5·54–16·3)	-5·22 (-11·6–0·267)
High LDL cholesterol	50·3 (46·3–54·7)	44·2 (40·3–48·5)	44·9 (41–48·9)	-12·1 (-19·3–4·49)	1·46 (-4·42–7·87)	--	--	--	--	--

Supplementary Results Table 1. Nottinghamshire age-standardised Summary Exposure Values (SEVs) for most-detailed risk factors and percentage change, by sex, 1990, 2010, and 2019. Data in parentheses are 95% uncertainty intervals.

	Male					Female				
	1990	2010	2019	Percentage change 1990-2010	Percentage change 2010-2019	1990	2010	2019	Percentage change 1990-2010	Percentage change 2010-2019
Unsafe sanitation	0·696 (0·286–1·52)	0·41 (0·156–0·869)	0·319 (0·11–0·686)	-41·1 (-79·3–61·9)	-22·3 (-66·6–76·1)	0·696 (0·286–1·52)	0·41 (0·156–0·869)	0·319 (0·11–0·686)	-41·1 (-79·3–61·9)	-22·3 (-66·6–76·1)
Ambient particulate matter pollution	19·1 (7·38–35·1)	11·5 (7·71–15·7)	8·78 (5·35–12·5)	-39·8 (-67·5–40·1)	-23·8 (-31·7—19·5)	19·1 (7·38–35·1)	11·5 (7·69–15·7)	8·78 (5·35–12·5)	-39·8 (-67·5–40·1)	-23·8 (-31·6—19·4)
Household air pollution from solid fuels	0·0178 (0·00384–0·053)	0·00645 (0·00116–0·0202)	0·00411 (0·000687–0·0135)	-63·8 (-89·5–16·9)	-36·2 (-71·7–37·4)	0·0234 (0·00517–0·0728)	0·00856 (0·00155–0·0274)	0·00546 (0·000877–0·0183)	-63·4 (-89·4–18)	-36·2 (-71·5–37·5)
Ambient ozone pollution	14·1 (3·96–31·1)	5·4 (0·859–14·3)	5·5 (0·761–15·3)	-61·7 (-89—15·3)	1·93 (-69–226)	14·1 (3·96–31·1)	5·4 (0·859–14·3)	5·5 (0·761–15·3)	-61·7 (-89—15·3)	1·93 (-69–226)
Residential radon	23·9 (4·79–66·1)	23·9 (4·79–66·1)	23·9 (4·79–66·1)	0 (0–0)	0 (0–0)	23·9 (4·79–66·1)	23·9 (4·79–66·1)	23·9 (4·79–66·1)	0 (0–0)	0 (0–0)
Lead exposure	32·7 (6·25–57·3)	21·3 (3·62–43·6)	14·8 (1·47–35·6)	-34·9 (-58—21·8)	-30·4 (-60·2—18·4)	13 (0·0267–35·5)	7·86 (0·00408–26·5)	5·43 (5·35e-12–21·6)	-39·4 (-85·5—21·4)	-30·9 (-100—17·7)
Child underweight	0·924 (0·539–1·39)	0·788 (0·427–1·22)	0·778 (0·409–1·18)	-14·7 (-31·3–5·4)	-1·26 (-15·3–15·8)	1·09 (0·623–1·67)	0·953 (0·537–1·41)	0·94 (0·531–1·4)	-12·8 (-29·3–5·87)	-1·38 (-13·9–12·9)
Iron deficiency	--	--	--	--	--	11 (9·01–13·3)	9·9 (8·09–12)	9·77 (7·92–11·9)	-10·3 (-24·3–5·12)	-1·33 (-10·8–9·28)
Vitamin A deficiency	5·09 (1·01–14·1)	2·46 (0·563–7·46)	2·1 (0·471–7·07)	-51·8 (-88·1–122)	-14·6 (-66·3–137)	3·81 (1·18–9·14)	2·55 (0·778–5·85)	2·21 (0·692–5·44)	-33·1 (-78·4–108)	-13·3 (-55·4–90·2)
Zinc deficiency	5·08 (0–14·7)	3·19 (0–9·79)	3·2 (0–9·67)	-37·2 (-100–NA)	0·052 (-24·8–NA)	4·89 (0–13·7)	3·04 (0–9·43)	3·05 (0–9·64)	-37·7 (-100–NA)	0·275 (-26·3–NA)
Smoking	28·6 (25–32·2)	21·6 (18·9–24·7)	18·1 (15·7–21)	-24·3 (-31·1—16·9)	-16·2 (-21·1—11·2)	23·4 (19·4–27·9)	18·4 (15·4–21·6)	14·4 (11·9–17·4)	-21·4 (-32·2—8·33)	-21·5 (-28·6—13·7)
Secondhand smoke	46·3 (40·3–52·4)	32·5 (28·1–37)	32·7 (28·1–37·4)	-29·9 (-41·1—16·7)	0·65 (-9·15–11·9)	48·5 (43·5–53·9)	33 (29·3–36·7)	33·3 (29·9–36·8)	-32 (-41—21·6)	0·968 (-6·27–9·5)
Alcohol use	16·6 (9·16–24·3)	19·7 (11·2–29·3)	18·7 (9·9–28·5)	19·1 (-21·9–88·4)	-5·44 (-31·3–20·8)	--	--	--	--	--
Drug use	0·304 (0·146–0·62)	0·548 (0·309–0·976)	0·526 (0·285–0·962)	80·4 (43·8–138)	-4 (-13·3–5·32)	0·192 (0·102–0·385)	0·295 (0·172–0·515)	0·295 (0·164–0·54)	53·7 (15·7–101)	-0·246 (-11–10·3)
High fasting plasma glucose	9·02 (7·53–10·7)	17·1 (14·6–20·1)	21·4 (18–26)	89·2 (60·4–126)	25·3 (8·59–49·3)	7·81 (6·34–10)	12·9 (10·9–15·7)	16·3 (13·5–20)	65·7 (35–107)	26·1 (6·32–51·4)
High systolic blood pressure	34·1 (25·5–44·7)	31·2 (25·2–38·2)	31·1 (23·3–40·4)	-8·46 (-34·4–27·7)	-0·175 (-24·3–28·1)	--	--	--	--	--
High body-mass index	19·3 (13·2–26·9)	24·6 (17·1–33·8)	25·6 (17·7–35·2)	27·6 (12·7–48)	4·27 (-2·44–12·6)	20·8 (13·1–32·8)	27·4 (18·3–42·8)	27·8 (18·7–43·5)	31·3 (-4·38–80·7)	1·6 (-16–22·9)
Low bone mineral density	8·89 (4·68–15·1)	7·68 (3·97–13·6)	9·36 (4·95–15·4)	-13·5 (-36·3–11)	21·8 (-5·93–62·8)	14·8 (9·61–21·4)	14·2 (9·11–21·1)	13·9 (8·67–20·4)	-3·92 (-24–18·9)	-2·18 (-24·1–25·1)
Diet low in fruits	68·8 (57·4–79·9)	61·8 (49·3–74·1)	60·7 (47·2–73·1)	-10·2 (-22·6–0·698)	-1·77 (-11·2–7·64)	69·9 (58·9–80·5)	62·3 (49·8–74·3)	61·7 (48·8–74·4)	-10·9 (-23·7–0·00875)	-1 (-10·1–8·12)
Diet low in vegetables	55·8 (34·3–77)	50·7 (29·3–72·4)	49·9 (29·1–72)	-9 (-25·1–6·74)	-1·65 (-13·7–10·6)	53·1 (32·3–75·1)	47·8 (27·9–69·3)	46·5 (27·8–68·5)	-9·97 (-27·2–5·78)	-2·7 (-16·3–10·8)
Diet low in whole grains	90·4 (85–95·4)	90 (84·3–94·8)	90·1 (84·5–94·9)	-0·425 (-3·74–2·49)	0·132 (-1·64–2·05)	--	--	--	--	--
Diet low in nuts and seeds	84 (43·9–93·9)	76·7 (40·2–88·6)	78·6 (41·6–90·3)	-8·67 (-21·5–1·26)	2·44 (-5·34–12·4)	83·8 (44·2–93·6)	76·6 (41·3–89)	77·6 (41·7–89·7)	-8·58 (-21–2·61)	1·34 (-7·58–11·1)
Diet low in milk	61·5 (35·5–82·6)	62·3 (36·7–83·3)	62·4 (37–83·3)	1·31 (-8·9–14·2)	0·0529 (-6·18–7·07)	64 (38·5–84·9)	65·9 (41–86·8)	65·8 (41·2–87·1)	3 (-4·95–16·7)	-0·0667 (-5·55–5·72)
Diet high in red meat	63·5 (48·1–77·9)	49·4 (35·3–65)	51·7 (35·8–66·6)	-22·1 (-41–0·234)	4·61 (-13–27·6)	63·3 (46·7–78·2)	50·6 (35·2–65·8)	52·9 (36·7–69·2)	-20·1 (-40·4–4·43)	4·58 (-12·2–25·9)
Diet high in processed meat	76·2 (52·1–96)	80·9 (59·5–98·6)	80·9 (58·7–98·4)	6·22 (-3·26–21·6)	0·0578 (-5·68–6·8)	79·1 (56·2–97·5)	84·2 (63·1–99·2)	84·4 (64·5–99·2)	6·52 (-1·36–21·3)	0·263 (-5·5–6·59)
Diet high in sugar-sweetened beverages	44·4 (25·9–72)	47·8 (28·8–71·7)	49·6 (30·1–75)	7·58 (-30·6–69·5)	3·96 (-19·7–40·5)	--	--	--	--	--
Diet low in fiber	45·9 (29·1–62·6)	38·2 (22·4–55·2)	38·3 (21·8–56·2)	-16·7 (-39·9–9·21)	0·293 (-16·5–21)	46·1 (29·3–62·9)	38·4 (22·2–56·9)	38·3 (22·3–55·9)	-16·7 (-40·4–13·8)	-0·385 (-18·6–23·1)
Diet low in seafood omega-3 fatty acids	95 (85·9–100)	92·9 (81·7–100)	92·5 (80–100)	-2·27 (-11·9–3·97)	-0·416 (-9·66–9·23)	95·9 (88·3–100)	94·1 (84·7–100)	93·6 (83·6–100)	-1·88 (-10·1–3·63)	-0·485 (-7·94–6·79)
Diet low in polyunsaturated fatty acids	57·7 (18·5–96·7)	52·5 (16–93·1)	51·5 (15·5–92·9)	-9·02 (-25—0·0826)	-1·74 (-10·1–6·34)	59·7 (20·6–98·1)	54·7 (17·7–95·3)	53·7 (17–94·1)	-8·51 (-24·5–0)	-1·68 (-10·1–5·16)
Diet high in trans fatty acids	57·7 (46·2–100)	50·7 (39·7–99·1)	50·8 (39·9–97·9)	-12·1 (-25·6–2·16)	0·291 (-13·5–16·3)	55·7 (44·2–100)	47·6 (35·7–99·5)	47·7 (36·5–99·9)	-14·5 (-28·8–0)	0·204 (-12·2–15·5)
Diet high in sodium	18·4 (4·14–41)	22·3 (4·82–46·6)	24·4 (4·89–48·7)	21·3 (-43·2–183)	9·5 (-33·9–90·5)	11·2 (2·78–29)	11·6 (2·97–30)	12·4 (3·17–31·7)	4·4 (-42·1–109)	6·65 (-32·9–70·5)
Low physical activity	4·36 (1·84–9·05)	4·68 (2·03–9·49)	4·75 (2·07–9·82)	7·47 (-6·21–27·8)	1·39 (-12·7–16·7)	6·87 (3·55–12·8)	7·61 (4·02–13·6)	7·66 (4·07–13·9)	10·8 (0·816–24·8)	0·628 (-8·2–10·9)
Occupational asthmagens	22·4 (18·3–27·1)	21·1 (17·2–25·4)	22·1 (17·8–27·7)	-5·77 (-20·3–11·5)	5·02 (-7·32–20·8)	11·8 (9·18–15)	11 (8·8–13·5)	11·6 (9·04–14·4)	-6·87 (-25·1–14·6)	5·63 (-9–21·2)
Occupational particulate matter, gases, and fumes	9·15 (6·37–13·4)	8·74 (6·16–12·6)	8·54 (6·05–12·3)	-4·49 (-12·7–5·38)	-2·25 (-6·65–2·31)	4·42 (2·93–6·68)	4·37 (3·05–6·6)	4·14 (2·92–6·11)	-1·15 (-15–16·5)	-5·06 (-11–2·57)
Occupational noise	8·69 (7·73–9·7)	8·51 (7·83–9·29)	8·39 (7·75–9·19)	-2·02 (-7·67–4·56)	-1·4 (-4·14–1·5)	5·14 (4·28–6·11)	5·73 (5·13–6·39)	5·86 (5·34–6·47)	11·4 (1·56–24·4)	2·41 (-1·31–6·85)
Occupational injuries	--	--	--	--	--	--	--	--	--	--
Occupational ergonomic factors	11 (9·07–13·3)	10·8 (8·98–12·9)	11·2 (9·32–13·6)	-2·26 (-12·3–9·85)	4·24 (-4·47–14)	6·38 (5·02–8·04)	7·96 (6·31–10·1)	8·68 (6·67–11·2)	24·9 (0·945–56·7)	9 (-8·48–31·1)
Childhood sexual abuse	10·6 (6·74–14·7)	11 (7·04–15·8)	10·7 (6·96–15·2)	4·38 (-2·5–12·8)	-2·79 (-11–6·54)	13·3 (9·72–18·3)	14·1 (10·3–19·6)	13·7 (10·1–18·8)	6·28 (-2·16–15·1)	-2·58 (-10·4–8·27)
Intimate partner violence	--	--	--	--	--	22·8 (12·5–31·3)	20 (11·3–28·2)	22·8 (12·5–31·3)	-12·2 (-19·2–0·696)	13·9 (2·98–28·7)
Non-exclusive breastfeeding	25·9 (19·2–34)	23·2 (17·6–30·5)	23·2 (17·3–30·5)	-10·3 (-25·2–7·74)	0·13 (-13·5–14·7)	25·9 (19·2–34)	23·2 (17·6–30·5)	23·2 (17·3–30·5)	-10·3 (-25·2–7·75)	0·132 (-13·5–14·7)
Discontinued breastfeeding	27·9 (26·9–28·8)	27·6 (26·5–28·5)	27·4 (26·2–28·4)	-1·08 (-5·68–3·88)	-0·602 (-4·31–3·27)	27·9 (26·9–28·8)	27·6 (26·5–28·5)	27·3 (26·2–28·3)	-1·1 (-5·7–3·86)	-0·853 (-4·56–3·02)
Diet low in calcium	22·6 (11·1–42·5)	16·5 (7·12–32)	16·4 (7·03–31·5)	-26·9 (-48·3—12·4)	-0·877 (-9·35–8·04)	16·9 (6·89–32·9)	11·2 (4·02–20·4)	11·1 (4–20·5)	-33·8 (-57·5—13·8)	-1·08 (-15·6–13·3)
Occupational exposure to asbestos	19·6 (17·5–21·8)	18·1 (17–19·2)	16·6 (13·7–19·2)	-7·39 (-17·8–4·88)	-8·69 (-24·8–7·35)	3·32 (2·43–5·53)	3·39 (2·48–4·44)	3·57 (2·4–4·91)	1·97 (-30·3–40·6)	5·46 (-22·3–39·8)
Occupational exposure to arsenic	0·5 (0–1·2)	0·547 (0–1·3)	0·572 (0–1·37)	9·37 (-10·8–NA)	4·65 (-8·57–NA)	0·473 (0–1·12)	0·473 (0–1·1)	0·477 (0–1·11)	-0·143 (-20·2–NA)	0·86 (-13·1–NA)
Occupational exposure to benzene	0·836 (0·0471–2·4)	0·885 (0·0507–2·58)	0·927 (0·0519–2·71)	5·92 (-4·54–21)	4·72 (-3·26–15·2)	1·11 (0·0671–3·23)	1·43 (0·0889–4·06)	1·55 (0·0956–4·49)	28·1 (11·4–63·3)	8·73 (-0·764–22·9)
Occupational exposure to beryllium	0·024 (0·0224–0·0256)	0·0252 (0·0236–0·0269)	0·0265 (0·0246–0·0284)	5·09 (-3·04–13·9)	5·02 (-0·776–11·3)	0·0258 (0·0233–0·0281)	0·0282 (0·0262–0·0305)	0·0302 (0·0278–0·0327)	9·65 (-1·36–22·1)	6·85 (-0·5–14·8)
Occupational exposure to cadmium	0·0581 (0·0497–0·0669)	0·0633 (0·0552–0·072)	0·0664 (0·0569–0·076)	8·92 (-9·46–30·4)	4·86 (-7·24–18·7)	0·0564 (0·0473–0·0665)	0·0572 (0·0492–0·0661)	0·0582 (0·0503–0·068)	1·36 (-16·8–26·5)	1·91 (-10·8–17)
Occupational exposure to chromium	0·137 (0·12–0·155)	0·149 (0·132–0·168)	0·157 (0·137–0·177)	8·68 (-7·44–27·5)	5·11 (-5·81–17·3)	0·139 (0·119–0·16)	0·147 (0·13–0·165)	0·152 (0·135–0·172)	6·03 (-10·6–27·8)	3·17 (-7·95–15·8)
Occupational exposure to diesel engine exhaust	0·751 (0·674–0·829)	0·758 (0·683–0·833)	0·797 (0·709–0·889)	0·851 (-11·4–14·4)	5·1 (-4·08–14·7)	0·627 (0·544–0·716)	0·674 (0·589–0·762)	0·719 (0·618–0·827)	7·47 (-9·06–27·8)	6·81 (-5·52–20·1)
Occupational exposure to formaldehyde	0·29 (0·246–0·335)	0·309 (0·268–0·354)	0·32 (0·273–0·37)	6·46 (-12–27·9)	3·7 (-8·57–17·9)	0·27 (0·227–0·318)	0·286 (0·249–0·326)	0·293 (0·255–0·339)	6·08 (-12·6–30·1)	2·41 (-9·37–15·7)
Occupational exposure to nickel	0·506 (0–2·23)	0·544 (0–2·41)	0·567 (0–2·5)	7·56 (-10·3–NA)	4·27 (-6·81–NA)	0·392 (0–1·75)	0·377 (0–1·65)	0·377 (0–1·69)	-3·69 (-23·7–NA)	-0·106 (-14–NA)
Occupational exposure to polycyclic aromatic hydrocarbons	0·286 (0·254–0·319)	0·312 (0·281–0·345)	0·327 (0·29–0·365)	8·86 (-6·07–25·6)	4·92 (-4·68–16)	--	--	--	--	--
Occupational exposure to silica	6·36 (0·907–20·9)	6·73 (0·976–22·5)	6·99 (1–22·9)	5·94 (-10·5–28·9)	3·76 (-7·61–18·1)	3·57 (0·529–12)	3·42 (0·479–10·8)	3·42 (0·464–11)	-4·42 (-24·4–27·5)	0·178 (-14·4–19·9)
Occupational exposure to sulfuric acid	0·849 (0·177–2·78)	0·889 (0·176–2·89)	0·912 (0·193–2·87)	4·65 (-12·8–24·5)	2·6 (-8·24–15·6)	--	--	--	--	--
Unsafe sex	--	--	--	--	--	--	--	--	--	--
Occupational exposure to trichloroethylene	0·0766 (0·0688–0·0845)	0·0815 (0·0745–0·0894)	0·0858 (0·0772–0·0945)	6·42 (-5·76–21)	5·34 (-3·3–14·8)	0·0797 (0·0704–0·0898)	0·0847 (0·0763–0·0943)	0·0885 (0·0791–0·099)	6·22 (-7·81–24·4)	4·49 (-5·49–15·4)
No access to handwashing facility	3·38 (3·09–3·7)	3·22 (2·95–3·51)	3·18 (2·91–3·48)	-4·66 (-14·6–6·05)	-1·29 (-7·61–5·76)	3·38 (3·09–3·7)	3·22 (2·95–3·51)	3·18 (2·91–3·48)	-4·66 (-14·6–6·05)	-1·29 (-7·61–5·76)
Child wasting	1·28 (0·885–1·69)	1·23 (0·862–1·64)	1·22 (0·849–1·65)	-3·74 (-16·1–10·6)	-0·284 (-9·91–10·7)	--	--	--	--	--
Child stunting	1·66 (0·817–2·59)	1·22 (0·571–1·88)	1·22 (0·543–1·92)	-26·2 (-45·4—0·0605)	-0·669 (-20·7–22·9)	1·95 (0·994–2·92)	1·6 (0·811–2·47)	1·58 (0·77–2·46)	-17·8 (-38·8–8·19)	-1·43 (-20·8–21·3)
Chewing tobacco	0·172 (0·111–0·259)	0·165 (0·109–0·244)	0·169 (0·112–0·258)	-4·56 (-44·2–64·2)	2·97 (-23·8–40·3)	0·13 (0·0746–0·216)	0·13 (0·0745–0·22)	0·132 (0·0741–0·228)	-0·2 (-48·7–98·9)	1·95 (-33·9–59·8)
Diet low in legumes	75·2 (41–99·4)	74 (37·5–99·1)	75 (38·9–99·2)	-1·59 (-13·4–5·71)	1·24 (-4·13–12·1)	71·7 (33·3–97·9)	70·4 (31·3–97·6)	71·2 (33·1–98·2)	-1·77 (-14·6–6·75)	1·17 (-5·04–12·4)
Short gestation	23·6 (19–29·2)	49·6 (39·3–61·7)	47·5 (36·3–60·6)	111 (60·4–174)	-4·26 (-25·9–22·9)	25 (21·4–29·1)	47·4 (39·6–56·2)	45·7 (37·7–54·6)	89·3 (59·1–126)	-3·58 (-22·8–16·8)
Low birth weight	12·2 (9·95–15)	24·2 (19·1–29·2)	23·4 (17·9–29·6)	98·5 (48·8–160)	-3·13 (-26–25·5)	13·1 (11·2–15·3)	23·3 (19·6–27·6)	23·1 (19·3–27·6)	77·7 (47·4–116)	-1·22 (-21·2–21·9)
High temperature	23 (7·11–40·2)	0 (0–0)	0 (0–0)	-100 (-100—100)	--	23 (7·11–40·2)	0 (0–0)	0 (0–0)	-100 (-100—100)	--
Low temperature	31·1 (25·7–37·1)	34·1 (29·3–39·5)	30·5 (25–36·5)	9·64 (5·77–14·4)	-10·7 (-14·7—7·13)	31·1 (25·7–37·1)	34·1 (29·3–39·5)	30·5 (25–36·5)	9·64 (5·77–14·4)	-10·7 (-14·7—7·13)
Kidney dysfunction	13·4 (8·93–19·8)	14·3 (9·78–20·6)	14·1 (9·71–20·5)	6·44 (1·7–12·7)	-1·23 (-5·95–3·24)	14·5 (9·7–21·2)	15·3 (10·5–21·8)	15·3 (10·3–21·8)	5·36 (0·767–11·4)	0·114 (-3·55–4·09)
Bullying victimization	6·13 (2·45–12·2)	6·12 (2·42–12·3)	5·65 (2·26–11·6)	-0·213 (-9·66–9·87)	-7·64 (-13·2—3·07)	4·7 (1·92–9·56)	5·04 (2·01–10·2)	4·75 (1·86–9·79)	7·08 (-4·79–18·4)	-5·73 (-12·5—0·836)
High LDL cholesterol	49·4 (45·3–53·5)	42·5 (38·7–46·6)	43·3 (39·5–47·6)	-14 (-20·9—7·06)	1·92 (-3·98–7·64)	--	--	--	--	--

Supplementary Results Table 1. Rutland age-standardised Summary Exposure Values (SEVs) for most-detailed risk factors and percentage change, by sex, 1990, 2010, and 2019. Data in parentheses are 95% uncertainty intervals.

	Male					Female				
	1990	2010	2019	Percentage change 1990-2010	Percentage change 2010-2019	1990	2010	2019	Percentage change 1990-2010	Percentage change 2010-2019
Unsafe sanitation	0·474 (0·168–1·07)	0·291 (0·104–0·661)	0·227 (0·0752–0·558)	-38·7 (-80·3–115)	-21·8 (-68·5–94)	0·474 (0·168–1·07)	0·291 (0·104–0·661)	0·227 (0·0752–0·558)	-38·7 (-80·3–115)	-21·8 (-68·5–94)
Ambient particulate matter pollution	16·1 (5·3–30·8)	9·66 (5·52–14·3)	7·43 (3·64–11·6)	-40·1 (-69·1–51·4)	-23·1 (-34·5–18)	16·1 (5·29–30·8)	9·66 (5·53–14·3)	7·43 (3·64–11·6)	-40 (-69·1–51·7)	-23·1 (-34·5—17·9)
Household air pollution from solid fuels	0·018 (0·00316–0·0574)	0·00717 (0·00107–0·0265)	0·00479 (0·000649–0·0176)	-60·1 (-88–48·2)	-33·1 (-70·8–47·3)	0·0237 (0·00414–0·0762)	0·00952 (0·00149–0·0334)	0·00635 (0·000927–0·0227)	-59·9 (-88–48·8)	-33·3 (-70·7–47·6)
Ambient ozone pollution	11·9 (1·97–28)	15·7 (2·49–38·9)	16 (2·6–38·8)	31·5 (-64·2–345)	1·66 (-71·4–273)	11·9 (1·97–28)	15·7 (2·49–38·9)	16 (2·6–38·8)	31·5 (-64·2–345)	1·66 (-71·4–273)
Residential radon	42 (9·42–95·5)	42 (9·42–95·5)	42 (9·42–95·5)	0 (0–0)	0 (0–0)	42 (9·42–95·5)	42 (9·42–95·5)	42 (9·42–95·5)	0 (0–0)	0 (0–0)
Lead exposure	25·8 (1·56–51·6)	16·8 (0·952–39·3)	11·7 (0·304–31·8)	-34·9 (-59·2–19·3)	-30·5 (-68·1–18·9)	10·7 (0·003–31·6)	6·47 (6·05e-12–24·1)	4·49 (3·15e-12–19·8)	-39·5 (-100–23·4)	-30·7 (-100–16·3)
Child underweight	0·902 (0·504–1·38)	0·772 (0·399–1·19)	0·762 (0·405–1·19)	-14·4 (-32·2–11·7)	-1·29 (-17·8–18·2)	1·05 (0·608–1·6)	0·926 (0·528–1·37)	0·917 (0·501–1·39)	-12·2 (-29·4–8·42)	-0·976 (-14·5–16)
Iron deficiency	--	--	--	--	--	10·6 (8·48–12·8)	9·5 (7·73–11·5)	9·42 (7·61–11·5)	-10·2 (-24·2–6·87)	-0·805 (-10·8–10·8)
Vitamin A deficiency	3·91 (0·754–12·5)	1·91 (0·346–5·82)	1·65 (0·328–5·18)	-51·2 (-89·7–128)	-13·7 (-70·1–180)	3·09 (0·845–7·2)	2·09 (0·597–4·94)	1·85 (0·544–4·48)	-32·2 (-80·1–157)	-11·7 (-59·6–109)
Zinc deficiency	4·47 (0–12·9)	2·67 (0–8·23)	2·74 (0–8·94)	-40·3 (-100–NA)	2·74 (-23·2–NA)	4·32 (0–12·4)	2·54 (0–8·29)	2·77 (0–8·62)	-41·4 (-100–NA)	9·34 (-13·2–NA)
Smoking	28·2 (24·5–32·5)	21·3 (18·4–24·6)	17·5 (15–20·3)	-24·4 (-31·4—16·5)	-17·9 (-23—12·6)	23·4 (19·3–27·9)	18·3 (15·2–21·8)	14 (11·4–17·2)	-21·8 (-32·9—8·57)	-23·1 (-31·3—13·8)
Secondhand smoke	45·4 (39·7–51·5)	32·3 (27·5–36·8)	31·6 (27–35·9)	-28·8 (-41·4—15·2)	-2·17 (-12·4–8·9)	47·2 (42·2–52·4)	33 (29·2–36·9)	32·3 (28·6–36)	-30·1 (-39·3—19·7)	-2 (-9·79–7·29)
Alcohol use	17·5 (9·28–25·5)	21·3 (12·4–31·5)	21·4 (12·8–32)	22 (-21·7–93·7)	0·599 (-21·2–29·8)	--	--	--	--	--
Drug use	0·296 (0·143–0·607)	0·496 (0·267–0·914)	0·473 (0·247–0·893)	67·6 (33·2–117)	-4·66 (-13·5–5·05)	0·192 (0·103–0·378)	0·285 (0·164–0·493)	0·268 (0·147–0·498)	48·1 (10·8–95·8)	-5·75 (-17·6–4·94)
High fasting plasma glucose	7·87 (6·58–9·56)	15·8 (13·4–18·9)	19·1 (15·8–23·1)	100 (67·9–143)	21·2 (5·12–47·2)	6·84 (5·55–8·8)	11·8 (9·83–14·3)	14·5 (11·9–17·5)	72·7 (37·6–117)	22·8 (4·01–47·9)
High systolic blood pressure	36·5 (26–49·3)	34·1 (27–43·7)	33·4 (25·1–44·6)	-6·59 (-35·3–36·2)	-2·12 (-25·3–29)	--	--	--	--	--
High body-mass index	27·5 (19·6–37)	33·6 (24–44·7)	34·6 (25·3–46·9)	22·1 (7·56–40·1)	2·95 (-4·4–11·4)	28·1 (18–42·9)	35·4 (24·4–52·3)	35·5 (23·5–53·7)	26·2 (-5·2–73·3)	0·354 (-17·4–20·8)
Low bone mineral density	8·66 (4·54–14·5)	7·5 (3·67–12·9)	9·17 (4·92–15·4)	-13·4 (-35·6–15)	22·3 (-8·1–67·6)	14·5 (9·32–21·3)	13·9 (8·64–20·6)	13·6 (8·35–20·3)	-4·14 (-25·4–23·1)	-1·95 (-25–25·8)
Diet low in fruits	66·8 (54·3–78·8)	59·7 (46·6–72·8)	58·8 (44–71·9)	-10·5 (-24·3–2·99)	-1·49 (-12·3–9·63)	67·4 (55·8–79)	60 (46·7–72)	59·5 (46–72)	-11·1 (-25–1·34)	-0·852 (-11·1–10·5)
Diet low in vegetables	52·3 (31·3–73·8)	47·5 (27·6–68·6)	47 (26·5–66·7)	-9·23 (-27·9–8·87)	-1·11 (-14·1–14·2)	49·8 (29–70·9)	44·5 (25·1–65·6)	43·4 (24·7–65·4)	-10·8 (-31·4–8·35)	-2·3 (-17·1–12·9)
Diet low in whole grains	88·8 (82–94·9)	89·4 (83·1–94·6)	89·8 (83·6–94·9)	0·606 (-3·34–4·74)	0·49 (-1·94–2·98)	--	--	--	--	--
Diet low in nuts and seeds	82·5 (43·7–93)	74·4 (40·1–87·5)	76·6 (40·7–89)	-9·83 (-23·9–0·696)	3 (-5·29–13·5)	82·4 (43·5–93·2)	74·5 (39·2–88·2)	75·7 (40·8–89·8)	-9·65 (-24·5–1·94)	1·62 (-7·79–11·6)
Diet low in milk	59·2 (34·5–79·7)	60·3 (35·8–81·3)	60·5 (35·7–81·8)	1·87 (-9·83–18·7)	0·309 (-6·42–8·51)	61·6 (36·2–82·4)	64 (38·5–84·5)	64·1 (38·8–85)	3·87 (-5·43–19)	0·143 (-6·36–7·32)
Diet high in red meat	65·5 (49·7–79·8)	51·7 (36·8–67·1)	53·6 (37·9–68·9)	-21 (-39·9–1·15)	3·59 (-15·6–26·5)	64·8 (48·1–79·9)	52·6 (36–69·7)	54·9 (37·4–72·5)	-18·8 (-38·4–5·48)	4·29 (-13·3–26·3)
Diet high in processed meat	76·1 (51·9–96·1)	81 (59·9–98·5)	81 (59·7–98·4)	6·46 (-3·7–23·5)	0·0459 (-6·14–6·76)	79·2 (56·9–97·5)	84·6 (64·4–99·2)	84·7 (64·7–99·1)	6·86 (-1·55–24·2)	0·13 (-5·78–7·03)
Diet high in sugar-sweetened beverages	47·3 (29–72·6)	50·4 (30·5–74)	51·9 (32–76·1)	6·42 (-30·6–67·3)	3·1 (-22·2–39·9)	--	--	--	--	--
Diet low in fiber	42·9 (26·6–60)	35·6 (20·2–53·1)	35·9 (21·3–52·9)	-17·1 (-43·2–12·7)	0·899 (-19·6–26·2)	43 (24·7–61·2)	35·6 (19·7–52·6)	35·8 (20·1–53·8)	-17·1 (-44·5–18·9)	0·431 (-22·6–28·7)
Diet low in seafood omega-3 fatty acids	93·9 (83·1–100)	91·4 (78·9–100)	91·1 (78·2–100)	-2·56 (-11·9–5·7)	-0·341 (-9·47–9·53)	94·9 (85·8–100)	92·3 (81·4–100)	92·3 (81·5–100)	-2·74 (-11·6–3·14)	0·00664 (-7·59–8·39)
Diet low in polyunsaturated fatty acids	54·5 (16·5–93·8)	49·5 (14·7–90·6)	48·8 (14·7–90·4)	-9·28 (-26·7–0·0272)	-1·35 (-10·5–8·1)	56·9 (18·5–96·9)	51·8 (15·9–94·3)	51·1 (15·4–93·3)	-8·92 (-26·7–0·223)	-1·4 (-9·66–8·1)
Diet high in trans fatty acids	57·9 (46·7–100)	51 (39·4–99·3)	51·1 (39·4–98·2)	-11·9 (-27·6–2·96)	0·217 (-14·4–18·6)	56·1 (44·6–100)	48 (36–99·9)	47·9 (36·1–99·9)	-14·4 (-29·1–0·00162)	-0·146 (-13·7–15·6)
Diet high in sodium	18·6 (4·24–41·5)	22·4 (4·8–45·8)	24·7 (5·29–48·9)	20·9 (-43·5–181)	10·2 (-37·3–96·7)	11·5 (2·94–29)	12 (3·11–29·7)	12·8 (3·24–32·1)	4·46 (-45·2–110)	6·6 (-39·7–73·4)
Low physical activity	4·63 (1·97–9·31)	5·04 (2·27–10·3)	5·04 (2·23–9·97)	8·79 (-4·87–27·9)	0·0974 (-12·5–14·3)	7·18 (3·8–13·3)	7·92 (4·25–14)	8 (4·26–14·2)	10·3 (0·876–24·2)	1·09 (-7·54–10·8)
Occupational asthmagens	21·4 (17·2–26)	20·1 (16·3–24·7)	21·1 (16·7–26·6)	-5·78 (-21·2–13)	4·95 (-7·88–20·6)	12·9 (9·79–17)	11·8 (9·38–14·5)	12·5 (9·65–15·9)	-8·69 (-30·2–14·3)	5·5 (-9·38–22·1)
Occupational particulate matter, gases, and fumes	8·91 (6·25–12·9)	8·55 (6·05–12·5)	8·35 (5·93–12·2)	-4·1 (-12·7–5·81)	-2·31 (-7·39–2·54)	4·2 (2·71–6·5)	4·21 (2·91–6·3)	4·03 (2·8–6·05)	0·287 (-14–18·5)	-4·41 (-10·8–2·83)
Occupational noise	8·69 (7·66–9·8)	8·51 (7·78–9·36)	8·38 (7·71–9·21)	-2·07 (-8·22–5·71)	-1·48 (-4·41–1·7)	5·19 (4·29–6·22)	5·73 (5·04–6·54)	5·86 (5·25–6·61)	10·4 (0·361–23·7)	2·14 (-1·53–7·06)
Occupational injuries	--	--	--	--	--	--	--	--	--	--
Occupational ergonomic factors	11·8 (9·6–14·4)	11·5 (9·42–13·8)	12 (9·74–14·8)	-2·57 (-17·2–14)	4·69 (-6·38–18·8)	--	--	--	--	--
Childhood sexual abuse	10·6 (6·74–14·7)	11 (7·04–15·8)	10·7 (6·96–15·2)	4·38 (-2·5–12·8)	-2·79 (-11–6·54)	13·3 (9·72–18·3)	14·1 (10·3–19·6)	13·7 (10·1–18·8)	6·28 (-2·16–15·1)	-2·58 (-10·4–8·27)
Intimate partner violence	--	--	--	--	--	22·8 (12·5–31·3)	20 (11·3–28·2)	22·8 (12·5–31·3)	-12·2 (-19·2–0·696)	13·9 (2·98–28·7)
Non-exclusive breastfeeding	25·4 (19–33·6)	23 (17·1–30·4)	23·2 (17·2–30·3)	-9·7 (-25·8–10·2)	0·969 (-14·3–18·1)	25·4 (19–33·6)	23 (17·1–30·4)	23·2 (17·2–30·3)	-9·69 (-25·8–10·2)	0·967 (-14·3–18·1)
Discontinued breastfeeding	27·3 (26·2–28·3)	26·6 (25·3–27·6)	27·6 (26·4–28·7)	-2·9 (-7·95–2·97)	4·1 (0·132–8·54)	27·2 (26–28·2)	26·7 (25·5–27·7)	27·4 (26·2–28·4)	-1·8 (-6·91–4·12)	2·51 (-1·4–6·86)
Diet low in calcium	20·2 (9·38–38·5)	14·5 (6·17–27·7)	14·5 (6·12–27·5)	-28·3 (-50·4—12·9)	-0·0219 (-10·6–13)	14·7 (6·03–28·4)	9·02 (3·34–16·9)	9·08 (3·35–16·7)	-38·8 (-60·8—18·3)	0·671 (-17·6–19·5)
Occupational exposure to asbestos	16·6 (14·3–19)	14·9 (13·4–16·5)	13·6 (10·7–16·6)	-9·71 (-24·5–7·51)	-9·27 (-29–12·5)	3·63 (2·41–5·49)	3·3 (2·11–4·15)	3·46 (1·85–5·35)	-9·07 (-43·5–46·3)	4·75 (-30·8–59·5)
Occupational exposure to arsenic	0·455 (0–1·09)	0·499 (0–1·2)	0·522 (0–1·26)	9·55 (-11·7–NA)	4·61 (-10·2–NA)	0·446 (0–1·08)	0·452 (0–1·07)	0·458 (0–1·12)	1·43 (-20·7–NA)	1·27 (-13·2–NA)
Occupational exposure to benzene	0·809 (0·0445–2·26)	0·861 (0·0535–2·45)	0·904 (0·0552–2·66)	6·38 (-5·24–25·7)	5·01 (-3·74–19·7)	1·15 (0·0696–3·31)	1·44 (0·0873–4·12)	1·55 (0·097–4·52)	25·5 (8·04–63·5)	7·98 (-3·37–23·7)
Occupational exposure to beryllium	0·0235 (0·0216–0·0252)	0·0247 (0·023–0·0263)	0·0259 (0·024–0·0278)	5·08 (-3·78–14·9)	5·06 (-1·3–12·4)	0·0263 (0·0232–0·0292)	0·0288 (0·0262–0·0314)	0·0307 (0·0279–0·0335)	9·43 (-2·92–23·9)	6·47 (-1·13–15·2)
Occupational exposure to cadmium	0·0533 (0·0452–0·0624)	0·0582 (0·0498–0·0672)	0·061 (0·0515–0·0723)	9·31 (-10·7–31·5)	4·78 (-8·49–20·4)	0·0537 (0·044–0·0655)	0·0554 (0·0478–0·0647)	0·0566 (0·0484–0·0664)	3·17 (-17·7–28·2)	2·17 (-11–16·1)
Occupational exposure to chromium	0·126 (0·109–0·145)	0·138 (0·119–0·157)	0·145 (0·125–0·169)	9·15 (-8·21–28·1)	5·11 (-6·86–18·5)	0·134 (0·113–0·159)	0·144 (0·127–0·164)	0·149 (0·131–0·17)	7·84 (-11·7–30·7)	3·21 (-7·62–15·7)
Occupational exposure to diesel engine exhaust	0·751 (0·671–0·84)	0·757 (0·679–0·837)	0·798 (0·71–0·887)	0·902 (-12·5–16·9)	5·33 (-3·98–16·3)	0·664 (0·563–0·774)	0·726 (0·627–0·845)	0·773 (0·653–0·909)	9·4 (-10·7–32·9)	6·45 (-7·24–22·3)
Occupational exposure to formaldehyde	0·262 (0·221–0·31)	0·281 (0·237–0·326)	0·292 (0·243–0·348)	7·22 (-12·8–30·5)	3·69 (-10·1–19·4)	0·256 (0·211–0·311)	0·276 (0·24–0·318)	0·283 (0·244–0·326)	7·86 (-12·7–33·5)	2·4 (-9·36–15·2)
Occupational exposure to nickel	0·478 (0–2·18)	0·512 (0–2·26)	0·534 (0–2·37)	7·05 (-10·2–NA)	4·4 (-8·07–NA)	0·371 (0–1·64)	0·365 (0–1·61)	0·365 (0–1·69)	-1·66 (-24·2–NA)	0·0965 (-14–NA)
Occupational exposure to polycyclic aromatic hydrocarbons	0·266 (0·235–0·301)	0·291 (0·258–0·325)	0·305 (0·266–0·348)	9·26 (-6·12–25·9)	4·93 (-5·49–16·8)	--	--	--	--	--
Occupational exposure to silica	6·31 (0·924–20·2)	6·66 (0·95–21·3)	6·92 (1·01–22·1)	5·51 (-13·1–31)	3·89 (-8·82–19·7)	3·53 (0·499–10·9)	3·44 (0·484–10·9)	3·47 (0·475–11·3)	-2·53 (-25–26·3)	0·961 (-14·9–23·2)
Occupational exposure to sulfuric acid	0·793 (0·163–2·54)	0·828 (0·176–2·57)	0·849 (0·181–2·73)	4·42 (-12·8–26·3)	2·49 (-9·75–17)	--	--	--	--	--
Unsafe sex	--	--	--	--	--	--	--	--	--	--
Occupational exposure to trichloroethylene	0·0716 (0·0643–0·0799)	0·0764 (0·0691–0·085)	0·0805 (0·0718–0·091)	6·69 (-6·76–21·3)	5·4 (-4·12–16·2)	0·0791 (0·0682–0·0911)	0·0855 (0·0763–0·096)	0·0894 (0·0795–0·1)	8·07 (-8·51–28·1)	4·48 (-5·63–16·4)
No access to handwashing facility	3·32 (2·99–3·65)	3·19 (2·88–3·5)	3·15 (2·85–3·45)	-4 (-14·4–7·57)	-1·28 (-7·66–6·04)	3·32 (2·99–3·65)	3·19 (2·88–3·5)	3·15 (2·85–3·45)	-4 (-14·4–7·57)	-1·28 (-7·66–6·04)
Child wasting	1·26 (0·864–1·67)	1·22 (0·859–1·63)	1·21 (0·837–1·65)	-3·41 (-16·3–11·9)	-0·114 (-9·86–11·4)	--	--	--	--	--
Child stunting	1·62 (0·773–2·5)	1·2 (0·541–1·89)	1·19 (0·508–1·88)	-25·7 (-46·2—0·0748)	-0·531 (-24–26·5)	1·89 (0·908–2·88)	1·57 (0·743–2·43)	1·55 (0·777–2·44)	-17 (-37·3–10·4)	-1·17 (-20·1–21)
Chewing tobacco	0·177 (0·109–0·274)	0·17 (0·106–0·258)	0·175 (0·109–0·268)	-3·98 (-44·1–67·1)	2·49 (-27·3–43·7)	0·136 (0·0735–0·236)	0·135 (0·0721–0·239)	0·138 (0·0751–0·247)	-1·12 (-52–109)	2·57 (-38·2–70·1)
Diet low in legumes	74·2 (39·7–99·1)	73 (35·9–98·5)	73·9 (38·3–99·1)	-1·57 (-13·5–6·74)	1·3 (-4·98–12·2)	70·5 (32·5–97)	69·3 (30·3–97·5)	70·2 (30·6–97)	-1·67 (-13·4–8·24)	1·22 (-6·43–14·9)
Short gestation	23·3 (18·3–29·7)	40·8 (29·9–55·7)	36·3 (21·9–58·9)	75·5 (25–145)	-11·1 (-40·3–31·8)	32·2 (25·6–39·6)	46·7 (36·6–57·9)	38·8 (27·6–59·7)	45·2 (2·56–102)	-16·9 (-40·4–21)
Low birth weight	12·3 (9·74–15·6)	20·5 (15·1–27·6)	18·1 (11·4–29·1)	66·7 (18·9–134)	-11·9 (-40·3–31)	17·3 (13·8–21)	23·8 (18·7–29·3)	19·9 (14·3–30·2)	37·4 (-4·67–91·7)	-16·2 (-40·5–20)
High temperature	22·8 (6·96–39·9)	0 (0–0)	0 (0–0)	-100 (-100—100)	--	22·8 (6·96–39·9)	0 (0–0)	0 (0–0)	-100 (-100—100)	--
Low temperature	30·8 (25·3–36·8)	33·7 (29–39·2)	30·5 (25·3–36·3)	9·51 (5·63–14·4)	-9·69 (-13—6·56)	30·8 (25·3–36·8)	33·7 (29–39·2)	30·5 (25·3–36·3)	9·51 (5·63–14·4)	-9·69 (-13—6·56)
Kidney dysfunction	13·5 (9·15–19·7)	14·4 (9·97–20·7)	14·1 (9·72–20·3)	6·64 (1·4–14)	-2·02 (-7·09–3·26)	14·3 (9·56–20·8)	14·8 (10·1–21·4)	14·9 (9·95–21·5)	3·99 (-0·28–9·94)	0·237 (-4·36–4·9)
Bullying victimization	6·08 (2·51–11·8)	6·05 (2·42–12)	5·61 (2·29–11·4)	-0·643 (-10·5–9·38)	-7·17 (-12·4—2·55)	4·71 (1·95–9·59)	5·05 (2–10·2)	4·73 (1·88–9·69)	7·02 (-5·47–18·5)	-6·17 (-12·6—1·1)
High LDL cholesterol	50·8 (46·2–55·2)	43·7 (39·7–47·8)	44·5 (40·3–48·8)	-13·9 (-21·7—5·61)	1·89 (-4·55–8·67)	--	--	--	--	--

Supplementary Results Table 1. East of England age-standardised Summary Exposure Values (SEVs) for most-detailed risk factors and percentage change, by sex, 1990, 2010, and 2019. Data in parentheses are 95% uncertainty intervals.

	Male					Female				
	1990	2010	2019	Percentage change 1990-2010	Percentage change 2010-2019	1990	2010	2019	Percentage change 1990-2010	Percentage change 2010-2019
Unsafe sanitation	0·544 (0·231–1·04)	0·312 (0·133–0·626)	0·242 (0·102–0·489)	-42·6 (-78·3–61·6)	-22·5 (-61·5–55)	0·544 (0·231–1·04)	0·312 (0·133–0·626)	0·242 (0·102–0·489)	-42·7 (-78·3–61·5)	-22·5 (-61·6–55·1)
Ambient particulate matter pollution	17·9 (6·63–32·5)	11·8 (8·31–15·8)	9·13 (6–12·8)	-34·3 (-64·9–57·1)	-22·3 (-29·2—18·2)	17·9 (6·63–32·5)	11·8 (8·31–15·8)	9·13 (6–12·8)	-34·3 (-64·8–57·1)	-22·3 (-29·1—18·2)
Household air pollution from solid fuels	0·0191 (0·0046–0·0513)	0·00634 (0·00134–0·018)	0·00403 (0·000717–0·0127)	-66·8 (-88·3—9·18)	-36·4 (-69·2–27·4)	0·0251 (0·00631–0·0663)	0·00836 (0·00182–0·0226)	0·00533 (0·00101–0·0172)	-66·7 (-88·2—9·56)	-36·2 (-69·1–27·4)
Ambient ozone pollution	14·4 (4·96–27·5)	14·9 (4·78–29·9)	14·3 (4·24–29·4)	3·63 (-25·8–29·8)	-3·96 (-29·1–26)	14·4 (4·98–27·6)	14·9 (4·76–29·8)	14·3 (4·22–29·3)	3·12 (-26·5–29·2)	-3·99 (-29·1–26·7)
Residential radon	17·3 (8·13–30·8)	17·3 (8·14–30·8)	17·4 (8·18–30·9)	0·405 (-0·92–2·46)	0·336 (-0·64–1·6)	17·3 (8·11–30·8)	17·3 (8·12–30·9)	17·4 (8·15–30·9)	0·354 (-0·868–2·27)	0·296 (-0·555–1·41)
Lead exposure	23·6 (1·77–48·8)	15·2 (1·01–37)	10·6 (0·34–30·1)	-35·5 (-55—23·2)	-30·3 (-66—18·6)	10·2 (0·00357–31·3)	6·09 (0·000418–23·1)	4·24 (4·75e-12–18·9)	-40·1 (-86·9—25·3)	-30·4 (-100—17·7)
Child underweight	0·908 (0·52–1·35)	0·776 (0·427–1·16)	0·766 (0·411–1·16)	-14·5 (-29·4–4·01)	-1·34 (-14·4–12·4)	1·07 (0·608–1·56)	0·935 (0·539–1·39)	0·92 (0·522–1·37)	-12·3 (-27·1–5·11)	-1·59 (-12·9–10·5)
Iron deficiency	--	--	--	--	--	9·99 (8·2–11·9)	9·16 (7·57–10·9)	9·09 (7·48–10·9)	-8·31 (-20·6–4·45)	-0·752 (-8·45–7·91)
Vitamin A deficiency	4·3 (1·06–10·9)	1·9 (0·475–4·88)	1·57 (0·412–3·92)	-55·8 (-87·3–72·2)	-17·5 (-60·2–101)	3·23 (1·17–6·92)	2·11 (0·839–4·3)	1·8 (0·703–3·69)	-34·8 (-75–79·1)	-14·4 (-51·1–62·3)
Zinc deficiency	3·8 (0–11·5)	2·55 (0–8·04)	2·58 (0–8·13)	-32·8 (-100–NA)	1·07 (-20·3–NA)	3·68 (0–11)	2·43 (0–8·18)	2·45 (0–7·94)	-33·9 (-100–NA)	0·711 (-20·6–NA)
Smoking	27·8 (24·6–31·2)	21 (18·5–23·8)	18·7 (16·4–21·4)	-24·5 (-29·4—19·2)	-11 (-14·6—7·11)	21·3 (18·6–24·4)	17·4 (15·1–19·9)	13·8 (11·9–16)	-18·4 (-24·9—11·3)	-20·2 (-24·5—16)
Secondhand smoke	45·3 (40·1–50·5)	33·1 (29·1–37·2)	32·6 (28·5–36·7)	-26·8 (-36·1—15·6)	-1·64 (-10·1–7·23)	47·3 (43·1–51·4)	33·8 (30·6–36·9)	33·3 (30·3–36·4)	-28·5 (-36·7—20·4)	-1·65 (-8·08–5·42)
Alcohol use	18·1 (10·6–25·6)	20·8 (13–29·2)	19·8 (12·1–28·3)	14·9 (-15·3–56·8)	-4·72 (-23·4–16·7)	10·1 (5·33–15·1)	10·8 (5·72–16·2)	9·99 (5·15–15·6)	6·62 (-36·3–82)	-7·37 (-36·7–31·2)
Drug use	0·319 (0·162–0·647)	0·615 (0·363–1·06)	0·577 (0·33–1·03)	92·7 (51·9–152)	-6·19 (-12·2—0·902)	0·203 (0·112–0·384)	0·323 (0·193–0·55)	0·318 (0·184–0·559)	59·6 (22·4–106)	-1·82 (-10·5–5·74)
High fasting plasma glucose	8·89 (7·68–10·5)	17·3 (15·3–19·6)	23 (19·9–27·2)	94·9 (72·6–122)	33 (21·1–46·5)	7·52 (6·42–8·87)	12·7 (11·2–14·3)	16·8 (14·8–19·3)	68·3 (48·9–91·7)	32·8 (21·4–45·8)
High systolic blood pressure	44·2 (40·5–48·6)	27·8 (25·5–30·1)	29·1 (24·2–34·4)	-37·2 (-43·1—30·2)	4·83 (-12·1–23)	--	--	--	--	--
High body-mass index	21 (14·6–28·9)	27·7 (19·8–37·1)	29·2 (21·2–38·8)	31·8 (19·7–49·1)	5·45 (0·663–11)	21·4 (15·6–29·6)	27·4 (20·8–36·6)	29·1 (22·2–38·3)	27·9 (15·1–47·1)	6·22 (-0·616–14·9)
Low bone mineral density	8·86 (4·97–14·6)	7·86 (4·12–13·3)	8·71 (4·84–14·7)	-11·2 (-22·1—1·3)	10·8 (0·194–25·7)	14·4 (9·58–20·7)	14·1 (9·38–20·6)	13·3 (8·6–19·5)	-2·23 (-11·1–7·24)	-5·37 (-14·2–4·8)
Diet low in fruits	65·5 (54·5–76·5)	58·6 (47·1–70·5)	57·5 (45·4–69)	-10·5 (-21·5—1·45)	-1·91 (-10·1–6·82)	66·6 (56·4–77·4)	59·3 (47·6–71·2)	58·5 (46·4–70·6)	-10·9 (-20·9—1·96)	-1·38 (-9·47–7·71)
Diet low in vegetables	50·8 (31·5–71·4)	46·4 (27·9–66·7)	45·4 (26·7–66·1)	-8·73 (-23·3–4·48)	-2·19 (-12·1–7·92)	48 (29·2–68·6)	43·1 (25·5–63·6)	41·9 (24·2–62·5)	-10·2 (-25·3–3·17)	-2·81 (-15·1–10·3)
Diet low in whole grains	89·9 (84·7–94·7)	89·5 (84·1–94·6)	89·6 (83·9–94·8)	-0·402 (-2·95–1·96)	0·125 (-1·53–1·74)	88·2 (82·2–93·7)	88 (82·2–93·7)	88·2 (81·8–93·6)	-0·228 (-2·84–2·42)	0·124 (-1·68–1·98)
Diet low in nuts and seeds	82·2 (41·1–92·1)	74·6 (36·5–86·7)	76·7 (39·5–88·1)	-9·32 (-20·2—1·25)	2·91 (-3·18–10·9)	82·3 (42·7–91·9)	74·3 (35·5–87·2)	75·5 (37·2–88·1)	-9·67 (-21·9—1·18)	1·54 (-5·37–9·32)
Diet low in milk	57·9 (32·8–78·2)	59·5 (34·3–79·6)	59·5 (34·7–79·6)	2·79 (-5·55–17·2)	0·0319 (-5·74–6)	60·4 (35·8–80·9)	63·2 (37·2–83·5)	63·2 (37·3–83·4)	4·76 (-2·73–18·2)	-0·146 (-5·48–4·94)
Diet high in red meat	67 (55–78)	52·1 (40·4–63·7)	54·3 (41·2–66·7)	-22·1 (-36·9—7·81)	4·09 (-8·8–21·1)	66·1 (53·6–76·9)	53 (40·8–64·5)	55·5 (42·5–68·2)	-19·9 (-34·2—5·7)	4·77 (-8·07–21)
Diet high in processed meat	76·8 (53·7–96)	81·5 (59·9–98·3)	81·6 (60·9–98·3)	6·1 (-0·464–18·4)	0·173 (-4·35–5·07)	79·3 (57·5–97·6)	84·3 (65–99·2)	84·6 (65·7–99·2)	6·29 (-0·539–19·7)	0·321 (-3·82–5·24)
Diet high in sugar-sweetened beverages	45·8 (31·3–66·7)	49·6 (34·9–68·2)	51·3 (36·5–69·6)	8·34 (-17·1–43·4)	3·52 (-12·3–22·7)	42·2 (27·3–60·8)	47·3 (31·2–65·1)	49·2 (33·1–66·5)	12 (-25·6–65)	4·04 (-19·2–37)
Diet low in fiber	41·2 (26·1–57·2)	34·2 (19·2–50·2)	34·1 (20·4–50·4)	-16·8 (-39·1–9·27)	-0·41 (-17·3–20·6)	41·3 (25–58·3)	34·2 (19·8–49·9)	34·1 (18·9–49·6)	-17·3 (-40·1–7·27)	-0·253 (-17·5–19)
Diet low in seafood omega-3 fatty acids	93·5 (85·8–99·9)	91·1 (81·3–99·8)	90·5 (80·1–99·7)	-2·56 (-8·01–1·1)	-0·662 (-5·19–3·67)	94·6 (87·7–100)	92·5 (84·1–99·6)	92·1 (82·9–99·9)	-2·18 (-7·17–0·806)	-0·45 (-4·39–2·81)
Diet low in polyunsaturated fatty acids	53 (16·4–93·8)	48·2 (13·5–90)	47·2 (13·1–89·8)	-9 (-23·6—0·29)	-2·03 (-10·5–4·9)	55·3 (17·2–95·4)	50·8 (14·5–93)	49·8 (14·6–92·8)	-8·19 (-22·7—0·297)	-1·98 (-10·6–4·44)
Diet high in trans fatty acids	57·7 (47–99·9)	50·9 (40·6–97·6)	50·8 (39·9–98·4)	-11·8 (-23·1–0)	-0·123 (-12·8–13·5)	55·9 (45·1–100)	47·7 (36·3–99·8)	47·6 (36·2–99·4)	-14·7 (-28·2–0)	-0·134 (-10·4–13·3)
Diet high in sodium	19·1 (4·84–40·1)	21·6 (5·73–42·7)	24·5 (6·8–46·1)	13·5 (-33·4–114)	13 (-15·2–69)	11·6 (3·74–29·1)	11·9 (3·8–29)	12·9 (3·84–31·1)	2·37 (-28·6–69·8)	8·39 (-20·1–54)
Low physical activity	4·43 (1·87–9·11)	5·13 (2·32–10·3)	5·24 (2·41–10·3)	15·8 (4·08–29·5)	2·16 (-3·45–9·02)	6·94 (3·62–12·9)	8 (4·39–14·3)	8·15 (4·51–14·5)	15·2 (8·53–25·7)	1·94 (-2·23–6·75)
Occupational asthmagens	21·4 (17·7–26·1)	19·9 (16·2–24·2)	21·7 (17·3–27·7)	-7 (-21·6–9·62)	8·79 (-5·37–27·4)	11·7 (9·5–14·3)	10·3 (8·59–12·4)	11·4 (9·22–14)	-11·4 (-26–3·89)	9·77 (-1·44–23·7)
Occupational particulate matter, gases, and fumes	8·54 (5·98–12·4)	8·13 (5·79–11·8)	7·93 (5·68–11·6)	-4·73 (-11·3–2·06)	-2·47 (-5·98–0·87)	3·81 (2·59–5·72)	3·6 (2·53–5·27)	3·44 (2·41–5·02)	-5·4 (-16·9–7·23)	-4·44 (-9·29–0·78)
Occupational noise	8·34 (7·66–9·12)	8·14 (7·66–8·83)	8 (7·55–8·74)	-2·43 (-6·48–1·74)	-1·68 (-3·47–0·269)	4·9 (4·28–5·56)	5·39 (5·01–5·93)	5·53 (5·18–6·05)	9·94 (3·5–19·3)	2·61 (0·0928–5·09)
Occupational injuries	--	--	--	--	--	--	--	--	--	--
Occupational ergonomic factors	11·6 (9·85–13·7)	10·9 (9·31–12·8)	11·2 (9·48–13·4)	-5·78 (-14·8–3·65)	3·29 (-4·71–13·2)	6·06 (4·81–7·64)	7·4 (5·84–9·29)	8·15 (6·24–10·6)	22·1 (0·0936–47·2)	10·2 (-7·01–31)
Childhood sexual abuse	10·6 (6·74–14·7)	11 (7·04–15·8)	10·7 (6·96–15·2)	4·38 (-2·5–12·8)	-2·79 (-11–6·54)	13·3 (9·72–18·3)	14·1 (10·3–19·6)	13·7 (10·1–18·8)	6·28 (-2·16–15·1)	-2·58 (-10·4–8·27)
Intimate partner violence	--	--	--	--	--	22·8 (12·5–31·3)	20 (11·3–28·2)	22·8 (12·5–31·3)	-12·2 (-19·2–0·696)	13·9 (2·98–28·7)
Non-exclusive breastfeeding	25·9 (19·6–33·9)	23·3 (17·9–30·5)	23·4 (17·9–30·4)	-10 (-22·4–5·51)	0·311 (-10·7–12·5)	25·9 (19·6–33·9)	23·3 (17·9–30·5)	23·4 (17·9–30·4)	-10 (-22·4–5·52)	0·313 (-10·7–12·5)
Discontinued breastfeeding	28·1 (27·2–28·9)	27·7 (26·9–28·5)	27·6 (26·7–28·4)	-1·22 (-5·22–3·03)	-0·46 (-3·86–2·96)	28 (27·2–28·9)	27·7 (26·9–28·5)	27·6 (26·7–28·4)	-1·08 (-5·09–3·17)	-0·544 (-3·94–2·87)
Diet low in calcium	19 (8·86–36)	13·6 (5·77–25·1)	13·4 (5·77–25·1)	-28·6 (-46·3—15·9)	-1·29 (-9·55–8)	13·6 (5·43–26·1)	8·08 (3·11–15)	7·94 (2·88–14·6)	-40·4 (-60·5—22·5)	-1·67 (-15·3–13·9)
Occupational exposure to asbestos	21·6 (20·4–22·7)	19·6 (19·1–20·1)	17·9 (15·5–20·2)	-9·1 (-14·7—3·22)	-8·95 (-20·8–3)	4·61 (2·72–5·81)	4·58 (2·31–5·24)	4·75 (2·2–6·64)	-0·785 (-33–26·5)	3·76 (-21·7–34·2)
Occupational exposure to arsenic	0·469 (0–1·1)	0·501 (0–1·19)	0·518 (0–1·23)	6·96 (-9·18–NA)	3·39 (-8·23–NA)	0·403 (0–0·947)	0·419 (0–0·977)	0·442 (0–1·06)	3·86 (-12·1–NA)	5·56 (-4·54–NA)
Occupational exposure to benzene	0·896 (0·0513–2·48)	0·876 (0·0519–2·48)	0·919 (0·0548–2·6)	-2·23 (-8·52–7·71)	4·96 (-0·869–13·2)	1·18 (0·0704–3·42)	1·46 (0·0873–4·25)	1·59 (0·0982–4·59)	23·9 (12·7–41·7)	9 (1·54–19·6)
Occupational exposure to beryllium	0·0244 (0·0235–0·0253)	0·0257 (0·0251–0·0262)	0·0267 (0·0256–0·0277)	5·26 (0·785–10·1)	4·08 (-0·205–7·78)	0·0254 (0·0239–0·0269)	0·0285 (0·0278–0·0292)	0·0307 (0·0294–0·0321)	12·4 (5·6–19·7)	7·84 (2·84–13)
Occupational exposure to cadmium	0·0551 (0·0493–0·0616)	0·0588 (0·0531–0·0649)	0·061 (0·0542–0·0684)	6·73 (-8·35–24·1)	3·78 (-6·86–15·2)	0·0493 (0·0438–0·0556)	0·0518 (0·0474–0·0571)	0·0548 (0·0495–0·0615)	5·01 (-8·98–22·1)	5·9 (-3·12–16·7)
Occupational exposure to chromium	0·131 (0·119–0·145)	0·139 (0·126–0·151)	0·145 (0·13–0·16)	5·67 (-7·17–20·4)	4·41 (-5·08–14·6)	0·126 (0·114–0·14)	0·136 (0·127–0·146)	0·145 (0·133–0·159)	7·41 (-4·7–21·6)	6·62 (-1·09–15·6)
Occupational exposure to diesel engine exhaust	0·725 (0·678–0·777)	0·752 (0·71–0·793)	0·795 (0·738–0·858)	3·76 (-5·24–13·5)	5·78 (-1·07–12·9)	0·651 (0·589–0·714)	0·668 (0·617–0·722)	0·718 (0·648–0·797)	2·53 (-9·77–16)	7·52 (-2·15–17·9)
Occupational exposure to formaldehyde	0·274 (0·243–0·308)	0·28 (0·251–0·311)	0·288 (0·252–0·326)	2·26 (-13–20·3)	3·03 (-8·23–15·5)	0·241 (0·215–0·272)	0·261 (0·241–0·287)	0·277 (0·252–0·309)	8·21 (-5·27–24·8)	6·17 (-2·85–15·9)
Occupational exposure to nickel	0·453 (0–2)	0·49 (0–2·18)	0·51 (0–2·23)	8·1 (-5·88–NA)	4·1 (-5·66–NA)	0·318 (0–1·36)	0·323 (0–1·42)	0·337 (0–1·44)	1·7 (-15·3–NA)	4·35 (-6·36–NA)
Occupational exposure to polycyclic aromatic hydrocarbons	0·276 (0·253–0·3)	0·289 (0·267–0·312)	0·302 (0·275–0·33)	4·94 (-6·9–17·7)	4·53 (-3·92–14)	0·266 (0·24–0·296)	0·298 (0·277–0·32)	0·319 (0·293–0·349)	11·8 (-0·472–26·3)	7·01 (-0·9–15·8)
Occupational exposure to silica	5·57 (0·775–17·9)	6·09 (0·883–19·1)	6·41 (0·946–20·2)	9·41 (-4·17–29·3)	5·21 (-4·33–17·7)	2·83 (0·409–8·95)	2·77 (0·376–8·47)	2·89 (0·383–9·12)	-1·9 (-19·2–21·5)	4·27 (-7·58–20·8)
Occupational exposure to sulfuric acid	0·842 (0·184–2·67)	0·846 (0·176–2·55)	0·855 (0·178–2·74)	0·492 (-14·2–16·3)	1·1 (-8·81–11·8)	--	--	--	--	--
Unsafe sex	--	--	--	--	--	--	--	--	--	--
Occupational exposure to trichloroethylene	0·0752 (0·0704–0·0808)	0·0788 (0·0743–0·0839)	0·0823 (0·0762–0·0888)	4·8 (-4·73–15·3)	4·46 (-2·42–11·9)	0·0768 (0·0707–0·0836)	0·0814 (0·0771–0·0866)	0·0871 (0·081–0·0942)	5·96 (-3·86–17·9)	6·99 (0·302–15·1)
No access to handwashing facility	3·34 (3·09–3·61)	3·19 (2·95–3·46)	3·15 (2·93–3·4)	-4·41 (-12·5–3·93)	-1·21 (-6·58–5·15)	3·34 (3·09–3·61)	3·19 (2·95–3·46)	3·15 (2·93–3·4)	-4·42 (-12·5–3·92)	-1·21 (-6·58–5·14)
Child wasting	1·26 (0·881–1·66)	1·22 (0·836–1·64)	1·21 (0·837–1·62)	-3·31 (-13·9–7·7)	-0·443 (-7·62–7·95)	1·07 (0·727–1·45)	1·02 (0·702–1·38)	1·01 (0·699–1·36)	-4·72 (-17·3–10·3)	-0·807 (-11–10)
Child stunting	1·63 (0·791–2·44)	1·21 (0·541–1·87)	1·2 (0·565–1·86)	-25·4 (-42·3—3·82)	-0·679 (-19·6–19·1)	1·92 (0·998–2·89)	1·58 (0·77–2·38)	1·56 (0·775–2·36)	-17·5 (-35–4·58)	-1·51 (-17–14·8)
Chewing tobacco	0·175 (0·121–0·247)	0·165 (0·115–0·225)	0·169 (0·119–0·232)	-5·5 (-36·6–39·7)	2·5 (-20·8–30·8)	0·132 (0·0818–0·199)	0·13 (0·0827–0·193)	0·132 (0·082–0·197)	-1·4 (-42·3–73·9)	1·23 (-28·8–46)
Diet low in legumes	73·5 (36·4–99·1)	72·7 (36·4–98·9)	73·5 (38–98·6)	-1·04 (-9·81–4·23)	1·06 (-3·89–9·69)	69·8 (30·7–97·7)	68·8 (28·7–96·5)	69·6 (28·8–96·5)	-1·43 (-12·4–4·96)	1·23 (-4·62–11·9)
Short gestation	20·6 (18·3–23·3)	47·1 (42·1–52·7)	41·7 (36·9–47·5)	128 (106–154)	-11·5 (-20·3—1·96)	23·4 (20·9–25·9)	42·9 (38·6–47·7)	38·8 (34·4–43·3)	83·5 (66·7–104)	-9·46 (-17·3—1·55)
Low birth weight	10·7 (9·76–11·8)	22·5 (20·5–24·5)	20·2 (18–22·5)	109 (87–133)	-10·2 (-19—0·517)	12·3 (11·3–13·4)	20·9 (19·2–22·7)	19·3 (17·6–21)	70 (53·2–88·4)	-7·9 (-16·4–0·795)
High temperature	21 (7·1–36·8)	0 (0–0)	13·8 (4·66–24)	-100 (-100—100)	--	21 (7·1–36·8)	0 (0–0)	13·8 (4·66–24)	-100 (-100—100)	--
Low temperature	30·7 (26·1–35·7)	33·6 (29·5–38·1)	30·1 (25·6–34·9)	9·53 (6·22–13·8)	-10·5 (-14—7·52)	30·7 (26·1–35·7)	33·6 (29·5–38·1)	30·1 (25·6–34·9)	9·53 (6·22–13·8)	-10·5 (-14—7·52)
Kidney dysfunction	13·7 (9·13–20)	14·2 (9·72–20·6)	14 (9·57–20·3)	3·62 (0·566–7·8)	-1·41 (-4·06–0·963)	14·6 (9·78–21·2)	15 (10·2–21·5)	15 (10–21·7)	2·21 (-0·0815–5·29)	0·216 (-2·15–3·01)
Bullying victimization	6·11 (2·42–12·1)	6·08 (2·38–12·3)	5·64 (2·24–11·7)	-0·353 (-9·54–9·01)	-7·3 (-12·8—2·81)	4·68 (1·92–9·54)	5·01 (1·99–10·2)	4·74 (1·86–9·74)	6·97 (-5·04–18·5)	-5·44 (-12·2—0·578)
High LDL cholesterol	56·3 (53–59·7)	45·4 (42·2–48·8)	45·4 (41·9–48·8)	-19·3 (-24·2—14·7)	-0·119 (-4·13–3·81)	--	--	--	--	--

Supplementary Results Table 1. Bedford age-standardised Summary Exposure Values (SEVs) for most-detailed risk factors and percentage change, by sex, 1990, 2010, and 2019. Data in parentheses are 95% uncertainty intervals.

	Male					Female				
	1990	2010	2019	Percentage change 1990-2010	Percentage change 2010-2019	1990	2010	2019	Percentage change 1990-2010	Percentage change 2010-2019
Unsafe sanitation	0·49 (0·181–1·1)	0·292 (0·0993–0·653)	0·234 (0·0828–0·504)	-40·4 (-82·5–96·3)	-19·9 (-66·9–83·1)	0·49 (0·181–1·1)	0·292 (0·0993–0·653)	0·234 (0·0828–0·504)	-40·4 (-82·5–96·3)	-19·9 (-66·9–83·1)
Ambient particulate matter pollution	16·8 (5·94–31)	11·9 (7·96–16·5)	8·62 (5·1–12·8)	-29·4 (-63·1–73·8)	-27·5 (-36·4–-22·2)	16·8 (5·94–30·9)	11·9 (7·97–16·5)	8·62 (5·08–12·8)	-29·4 (-63–73·1)	-27·5 (-36·3–-22·3)
Household air pollution from solid fuels	0·0141 (0·00274–0·0419)	0·00504 (0·000782–0·0159)	0·00338 (0·000483–0·0114)	-64·2 (-89·9–21·3)	-32·9 (-71·2–53·4)	0·0185 (0·0038–0·0542)	0·00664 (0·00113–0·0218)	0·00447 (0·000665–0·0145)	-64·1 (-89·9–21)	-32·8 (-71–54)
Ambient ozone pollution	12 (1·96–28·3)	17·9 (4·22–40·3)	17·8 (3·55–40·7)	48·7 (-49·6–531)	-0·481 (-68·9–206)	12 (1·96–28·3)	17·9 (4·22–40·3)	17·8 (3·55–40·7)	48·7 (-49·6–531)	-0·481 (-68·9–206)
Residential radon	24 (3·86–68·4)	24 (3·86–68·4)	24 (3·86–68·4)	0 (0–0)	0 (0–0)	24 (3·86–68·4)	24 (3·86–68·4)	24 (3·86–68·4)	0 (0–0)	0 (0–0)
Lead exposure	22·8 (0·609–48·5)	14·6 (0·494–36·5)	10·2 (0·133–29·7)	-36·1 (-63·9–-16·6)	-30·3 (-73·5–-18·6)	9·88 (5·31e-12–31·1)	5·85 (3·06e-12–22·8)	4·07 (2·32e-12–18·7)	-40·8 (-100–-3·47)	-30·4 (-100–46·7)
Child underweight	0·915 (0·51–1·39)	0·777 (0·425–1·17)	0·767 (0·41–1·18)	-15·1 (-32·7–5·7)	-1·36 (-17·2–16·3)	1·08 (0·614–1·62)	0·939 (0·543–1·42)	0·923 (0·521–1·4)	-12·7 (-29·8–9·17)	-1·73 (-16–14·9)
Iron deficiency	--	--	--	--	--	10·3 (8·2–12·6)	9·42 (7·48–11·5)	9·26 (7·39–11·4)	-8·23 (-23·9–9·44)	-1·67 (-11·4–8·73)
Vitamin A deficiency	4·26 (0·778–13·4)	1·88 (0·356–5·85)	1·58 (0·278–4·75)	-55·8 (-91·2–122)	-16·1 (-66·3–142)	3·12 (0·877–7·11)	2·08 (0·612–5·17)	1·8 (0·509–4·38)	-33·3 (-78·3–134)	-13·2 (-55·7–93·8)
Zinc deficiency	3·99 (0–11·9)	2·61 (0–8·19)	2·62 (0–8·21)	-34·5 (-100–NA)	0·124 (-25·1–NA)	3·81 (0–11)	2·49 (0–8·08)	2·48 (0–7·86)	-34·6 (-100–NA)	-0·601 (-25·9–NA)
Smoking	27·9 (24·4–31·7)	21·4 (18·4–24·7)	18·9 (16·3–22·1)	-23·4 (-31–-15·9)	-11·6 (-16·7–-5·89)	21·4 (17·9–25·5)	17·7 (14·7–21·1)	14 (11·5–17·3)	-17·4 (-30·6–-3·62)	-20·5 (-28·9–-11·6)
Secondhand smoke	44·3 (38·2–50·2)	34·4 (29·5–39·3)	33 (28·3–37·5)	-22·4 (-34–-8·88)	-3·98 (-13·2–6·35)	46·2 (41·6–50·9)	35 (31·1–39)	33·7 (29·9–37·6)	-24·1 (-33·9–-13·9)	-3·73 (-11·4–4·38)
Alcohol use	17·6 (8·73–26)	20·7 (11·4–30·9)	19 (9·89–28·4)	17·8 (-25·4–94·6)	-7·99 (-32·8–23·2)	--	--	--	--	--
Drug use	0·329 (0·167–0·669)	0·65 (0·388–1·11)	0·591 (0·34–1·06)	97·8 (54·7–165)	-9·1 (-19·7–0·725)	0·201 (0·111–0·389)	0·333 (0·204–0·55)	0·323 (0·189–0·565)	65·4 (23·9–117)	-3·06 (-13·8–6·78)
High fasting plasma glucose	9·32 (7·8–11·3)	19·4 (16·6–22·8)	26·1 (22–31·1)	108 (70·3–151)	34·4 (15·5–58·6)	8·4 (6·86–10·6)	15 (12·4–17·8)	20·3 (17–24·8)	78·3 (42·2–122)	35·6 (14·5–63·2)
High systolic blood pressure	44·9 (35·4–56·1)	28·3 (22–36·2)	30·5 (22·1–39·3)	-36·9 (-53·5–-15·4)	7·8 (-17·5–44·6)	--	--	--	--	--
High body-mass index	20·4 (13·9–28·6)	27·2 (19·4–36·6)	28·3 (20–37·8)	33·4 (17·3–55·9)	3·86 (-3·55–11·7)	20·8 (12·9–34·1)	27·8 (19·2–44·6)	29 (19·3–45·2)	33·5 (-2·14–83·6)	4·42 (-13·4–26·2)
Low bone mineral density	8·77 (4·55–14·6)	7·57 (3·78–13·2)	9·17 (4·96–15·3)	-13·7 (-36·4–13·4)	21·1 (-6·63–69·9)	14·6 (9·24–21·4)	13·9 (8·49–20·8)	13·7 (8·62–19·9)	-4·41 (-25·1–21·7)	-1·55 (-22·5–25·5)
Diet low in fruits	65·8 (53·1–77·6)	58·9 (46·5–71·6)	57·6 (44·8–70·5)	-10·5 (-24·7–3·22)	-2·22 (-13·3–8·95)	67·2 (54·9–78·2)	59·7 (45·7–73·3)	58·8 (45·7–73)	-11·1 (-25·1–2·01)	-1·57 (-12–10·3)
Diet low in vegetables	51·5 (32·1–72·2)	46·7 (26·8–67·9)	45·5 (25·1–66·6)	-9·33 (-28·9–8·08)	-2·46 (-15·7–12·3)	48·8 (28·5–69·9)	43·7 (25·2–66·5)	42·3 (25·3–63·7)	-10·6 (-29·6–8·42)	-3·1 (-18·5–14·5)
Diet low in whole grains	90 (84–94·9)	89·5 (83·4–94·7)	89·6 (83·4–94·9)	-0·462 (-3·76–2·74)	0·0897 (-1·91–2·33)	--	--	--	--	--
Diet low in nuts and seeds	82·6 (42·2–93·1)	74·7 (38·3–87·4)	76·6 (41–88·8)	-9·59 (-23·4–1·01)	2·51 (-5·38–12·9)	82·5 (43·5–93·2)	74·5 (38–87·7)	75·3 (39·1–88·9)	-9·74 (-25·5–2·98)	1·07 (-7·82–11·7)
Diet low in milk	58·3 (32·6–79·1)	59·6 (34·3–79·9)	59·5 (34·3–80·2)	2·18 (-10–18·7)	-0·206 (-8·42–7·74)	60·8 (35·5–81·9)	63·4 (37·9–83·7)	63·3 (37·8–84)	4·31 (-5·56–20·7)	-0·227 (-7·19–6·19)
Diet high in red meat	66·3 (49·3–81·6)	51·9 (36·1–67·6)	54·1 (38·6–69·3)	-21·8 (-41·3–2·34)	4·35 (-12–27)	65·6 (49·7–80·3)	52·8 (38·3–68)	55·5 (39·1–70·8)	-19·6 (-39–1·11)	5·23 (-10·9–26·3)
Diet high in processed meat	76·7 (52·3–96·5)	81·6 (59·9–98·3)	81·7 (60·9–98·5)	6·34 (-2·57–23·3)	0·214 (-6·16–6·74)	79·8 (57·6–97·8)	85 (64·5–99·2)	85·2 (66–99·3)	6·41 (-2·6–22·9)	0·315 (-5·22–6·22)
Diet high in sugar-sweetened beverages	47·8 (28·5–72·8)	51·1 (32·9–74·3)	52·5 (32·7–76·1)	7·03 (-28·4–58·5)	2·76 (-24·6–38)	--	--	--	--	--
Diet low in fiber	42·1 (24·4–59·4)	34·7 (18·1–52·6)	34·3 (19·4–51·6)	-17·5 (-42·5–12·7)	-1·02 (-20·5–25·8)	42·2 (24·9–59·2)	34·7 (19·1–51·6)	34·4 (17·9–51·2)	-17·8 (-43·8–12·5)	-0·777 (-21·4–25·3)
Diet low in seafood omega-3 fatty acids	93 (82·5–100)	90·9 (77·4–100)	89·8 (76·7–100)	-2·28 (-14·4–7·56)	-1·19 (-12·1–9·61)	94·3 (84·7–100)	92 (80·9–100)	91·4 (79·2–100)	-2·41 (-12·7–4·76)	-0·608 (-9·24–9·08)
Diet low in polyunsaturated fatty acids	53·4 (16·3–93·7)	48·7 (13·7–91·5)	47·6 (12·8–90·7)	-8·82 (-25·3–0·715)	-2·35 (-12·8–6·19)	55·9 (17·8–95·6)	51·3 (15–92·9)	50·1 (14·1–92·5)	-8·14 (-25·3–0·723)	-2·27 (-12·6–6·19)
Diet high in trans fatty acids	57·8 (46·4–100)	51 (39·8–98·3)	51 (39·4–98·3)	-11·8 (-25·9–1·72)	-0·0585 (-14·8–16·2)	56·1 (44·4–100)	47·8 (35·8–100)	47·8 (36·2–99·5)	-14·7 (-29·9–0)	-0·0471 (-12·8–17·3)
Diet high in sodium	20·1 (4·36–42·9)	23·1 (4·93–46)	26·3 (5·85–51·2)	14·9 (-44·9–168)	13·8 (-30·3–101)	12·2 (3·14–31·6)	12·6 (3·34–31·6)	13·7 (3·36–34·3)	3·89 (-41·1–108)	8·64 (-33·1–68)
Low physical activity	4·45 (1·92–9·19)	5·16 (2·25–10·4)	5·23 (2·39–10·3)	16 (0·386–39·9)	1·35 (-11·1–16·6)	6·99 (3·65–12·9)	8·01 (4·35–14·2)	8·12 (4·41–14·5)	14·7 (4·62–29·8)	1·33 (-6·97–10·9)
Occupational asthmagens	21·4 (17–27)	20 (15·5–25·8)	21·8 (16·5–29·3)	-6·6 (-26·2–18·4)	8·93 (-8·89–32·9)	11·6 (8·86–15·4)	10·2 (8·13–12·7)	11·3 (8·77–14·1)	-12 (-31·6–11·6)	10·4 (-5·4–30·8)
Occupational particulate matter, gases, and fumes	8·5 (5·86–12·5)	8·11 (5·78–11·8)	7·91 (5·62–11·6)	-4·57 (-13·5–5·44)	-2·41 (-7·07–2·5)	3·8 (2·41–6·02)	3·56 (2·4–5·48)	3·4 (2·31–5·1)	-6·15 (-19·9–11·7)	-4·48 (-11·3–3·13)
Occupational noise	8·28 (7·37–9·29)	8·1 (7·45–8·91)	7·97 (7·37–8·73)	-2·18 (-8·12–4·45)	-1·58 (-4·38–1·36)	4·92 (4·01–5·83)	5·4 (4·78–6·08)	5·54 (4·93–6·18)	9·85 (0·173–23·6)	2·58 (-1·46–7·1)
Occupational injuries	--	--	--	--	--	--	--	--	--	--
Occupational ergonomic factors	11·2 (9·34–13·6)	10·7 (8·83–12·9)	11·1 (9·08–13·4)	-4·96 (-18·3–10·9)	3·98 (-7·03–16·3)	--	--	--	--	--
Childhood sexual abuse	10·6 (6·74–14·7)	11 (7·04–15·8)	10·7 (6·96–15·2)	4·38 (-2·5–12·8)	-2·79 (-11–6·54)	13·3 (9·72–18·3)	14·1 (10·3–19·6)	13·7 (10·1–18·8)	6·28 (-2·16–15·1)	-2·58 (-10·4–8·27)
Intimate partner violence	--	--	--	--	--	22·8 (12·5–31·3)	20 (11·3–28·2)	22·8 (12·5–31·3)	-12·2 (-19·2–0·696)	13·9 (2·98–28·7)
Non-exclusive breastfeeding	25·8 (19·1–33·9)	23·2 (17·4–30·9)	23·3 (17·5–30·6)	-9·86 (-25·2–8·84)	0·296 (-13·6–16·3)	25·8 (19·1–33·9)	23·2 (17·4–30·9)	23·3 (17·5–30·6)	-9·84 (-25·1–8·86)	0·302 (-13·6–16·3)
Discontinued breastfeeding	28 (26·8–28·9)	27·8 (26·6–28·8)	27·8 (26·6–28·8)	-0·493 (-5·72–4·75)	-0·124 (-4–4·02)	28 (26·9–29)	27·8 (26·6–28·8)	27·7 (26·5–28·7)	-0·679 (-5·9–4·56)	-0·337 (-4·2–3·8)
Diet low in calcium	19·6 (8·93–37)	13·9 (6–26·6)	13·6 (5·86–25·9)	-29·1 (-49·4–-14·3)	-2·18 (-12·6–9·79)	14·1 (5·35–27·6)	8·32 (3·12–16)	8·09 (2·99–15·4)	-40·9 (-64·8–-19·1)	-2·72 (-20·9–19·6)
Occupational exposure to asbestos	20·3 (18·6–22·1)	19·2 (17·9–20·5)	17·7 (15·1–20·5)	-5·24 (-15·1–5·75)	-7·71 (-21·8–8·06)	3·74 (2·71–5·49)	3·9 (2·5–4·68)	4·09 (2·42–5·71)	4·24 (-29·8–43·4)	4·77 (-24·5–42·3)
Occupational exposure to arsenic	0·47 (0–1·11)	0·503 (0–1·17)	0·521 (0–1·25)	6·82 (-14·5–NA)	3·71 (-10·7–NA)	0·404 (0–0·962)	0·418 (0–0·99)	0·443 (0–1·07)	3·6 (-18·6–NA)	5·82 (-7·04–NA)
Occupational exposure to benzene	0·899 (0·0532–2·5)	0·875 (0·0507–2·48)	0·919 (0·0547–2·59)	-2·75 (-12·8–15·8)	5·05 (-3·38–17·9)	1·2 (0·0715–3·48)	1·48 (0·0889–4·34)	1·61 (0·0947–4·62)	23 (6·33–55·2)	8·7 (-2·39–25·5)
Occupational exposure to beryllium	0·0243 (0·0226–0·026)	0·0256 (0·024–0·0274)	0·0267 (0·0247–0·0287)	5·36 (-2·68–14·4)	4·1 (-2·23–10·3)	0·0255 (0·0228–0·0283)	0·0286 (0·0263–0·031)	0·0308 (0·0282–0·0334)	12·1 (-0·0157–26·9)	7·69 (-0·276–16·3)
Occupational exposure to cadmium	0·0553 (0·0475–0·0642)	0·0589 (0·0503–0·0674)	0·0613 (0·0514–0·0718)	6·56 (-12·6–28·9)	4·01 (-9·38–19·4)	0·0495 (0·0412–0·0587)	0·0518 (0·0456–0·06)	0·0549 (0·0476–0·0634)	4·59 (-15·1–29·9)	6·06 (-5·13–19·6)
Occupational exposure to chromium	0·132 (0·115–0·15)	0·139 (0·121–0·157)	0·145 (0·124–0·166)	5·46 (-11·7–25·2)	4·6 (-7·88–18·1)	0·127 (0·109–0·148)	0·136 (0·121–0·154)	0·145 (0·128–0·165)	6·96 (-10·6–29·2)	6·68 (-3·51–18)
Occupational exposure to diesel engine exhaust	0·725 (0·654–0·799)	0·753 (0·677–0·831)	0·794 (0·709–0·888)	3·75 (-9·55–18·1)	5·51 (-4·19–15·6)	0·651 (0·557–0·748)	0·666 (0·581–0·762)	0·716 (0·617–0·842)	2·32 (-13·9–23·7)	7·41 (-5·85–24·1)
Occupational exposure to formaldehyde	0·275 (0·235–0·323)	0·28 (0·238–0·323)	0·29 (0·239–0·341)	2·01 (-17·2–24·9)	3·32 (-10·9–20·4)	0·243 (0·203–0·289)	0·262 (0·232–0·299)	0·278 (0·243–0·32)	7·75 (-11·7–31·7)	6·21 (-5·11–18·4)
Occupational exposure to nickel	0·452 (0–1·9)	0·488 (0–2·16)	0·512 (0–2·32)	7·94 (-10·3–NA)	4·9 (-8·24–NA)	0·317 (0–1·39)	0·322 (0–1·43)	0·337 (0–1·47)	1·56 (-22–NA)	4·77 (-8·24–NA)
Occupational exposure to polycyclic aromatic hydrocarbons	0·276 (0·244–0·312)	0·29 (0·255–0·323)	0·303 (0·264–0·344)	4·8 (-10·1–22·6)	4·71 (-6·08–17·4)	--	--	--	--	--
Occupational exposure to silica	5·55 (0·797–17·5)	6·09 (0·857–19·8)	6·39 (0·937–20·3)	9·85 (-9·65–38·2)	4·81 (-8·16–21·8)	2·78 (0·383–8·84)	2·73 (0·368–8·73)	2·86 (0·372–8·95)	-1·97 (-24·7–34)	4·95 (-9·2–25·6)
Occupational exposure to sulfuric acid	0·845 (0·179–2·75)	0·849 (0·174–2·6)	0·86 (0·177–2·83)	0·522 (-18·4–22)	1·31 (-12–15·9)	--	--	--	--	--
Unsafe sex	--	--	--	--	--	--	--	--	--	--
Occupational exposure to trichloroethylene	0·0755 (0·0684–0·0837)	0·079 (0·071–0·0867)	0·0826 (0·0731–0·0923)	4·58 (-8·34–19·8)	4·53 (-4·98–14·7)	0·0773 (0·0676–0·0878)	0·0815 (0·0732–0·0908)	0·0872 (0·0778–0·0985)	5·51 (-8·88–24·9)	6·98 (-2·6–18·9)
No access to handwashing facility	3·33 (3·01–3·68)	3·19 (2·9–3·5)	3·16 (2·88–3·48)	-4·17 (-14·9–6·97)	-1·04 (-7·69–6·55)	3·33 (3·01–3·68)	3·19 (2·9–3·5)	3·16 (2·88–3·48)	-4·17 (-14·9–6·97)	-1·04 (-7·69–6·55)
Child wasting	1·26 (0·86–1·7)	1·22 (0·833–1·65)	1·21 (0·832–1·63)	-3·28 (-16·6–11·8)	-0·352 (-9·95–10)	--	--	--	--	--
Child stunting	1·64 (0·776–2·52)	1·23 (0·537–1·96)	1·21 (0·57–1·92)	-25·3 (-45·2–1·04)	-1·05 (-22·3–23·7)	1·94 (0·954–2·97)	1·6 (0·734–2·48)	1·57 (0·766–2·46)	-17·8 (-40·3–10·4)	-1·42 (-19·9–21)
Chewing tobacco	0·177 (0·111–0·276)	0·167 (0·107–0·249)	0·171 (0·108–0·26)	-5·47 (-43·9–54·2)	2·1 (-26·8–43·6)	0·135 (0·07–0·232)	0·134 (0·0742–0·229)	0·135 (0·0766–0·231)	-0·699 (-50·7–98·8)	1·09 (-34·5–59·6)
Diet low in legumes	73·7 (37·9–99)	72·8 (36·4–99)	73·5 (36·4–98·8)	-1·25 (-13·2–8·14)	0·976 (-5·39–12)	70 (31·5–98·2)	68·9 (28·7–97·3)	69·7 (28·7–97·2)	-1·46 (-16·3–9·62)	1·1 (-5·93–14·5)
Short gestation	18·6 (14·9–23)	37·4 (28–49·5)	42·5 (29·9–56·4)	101 (36·8–186)	13·6 (-24·9–67·9)	19·5 (16·6–22·9)	36·4 (28·8–43·4)	38·9 (30·7–50·7)	86·6 (41·8–129)	7·01 (-19·4–60·2)
Low birth weight	10·2 (8·16–12·3)	19 (14·3–24·9)	21·3 (15·4–29·1)	87·3 (28·5–163)	12·1 (-26·2–65·3)	10·6 (9·02–12·4)	18·4 (14·9–22·1)	19·5 (15·6–24·8)	74·2 (32·1–117)	5·7 (-20·2–56·7)
High temperature	23·3 (7·41–40·6)	0 (0–0)	0 (0–0)	-100 (-100–-100)	--	23·3 (7·41–40·6)	0 (0–0)	0 (0–0)	-100 (-100–-100)	--
Low temperature	30·4 (24·9–36·5)	33·2 (28·5–38·8)	29·9 (24·5–36·1)	9·37 (5·45–14·3)	-10·1 (-14·2–-6·29)	30·4 (24·9–36·5)	33·2 (28·5–38·8)	29·9 (24·5–36·1)	9·37 (5·45–14·3)	-10·1 (-14·2–-6·29)
Kidney dysfunction	14 (9·42–20·4)	14·7 (10·1–21·1)	14·5 (9·98–20·9)	4·69 (-0·286–11·2)	-1·1 (-5·77–3·94)	14·8 (9·89–21·3)	15·2 (10·4–21·6)	15·3 (10·4–21·9)	2·75 (-1·46–8)	0·792 (-3·27–4·98)
Bullying victimization	6·13 (2·45–12·3)	6·1 (2·4–12·5)	5·64 (2·25–11·6)	-0·48 (-9·89–9·13)	-7·61 (-12·9–-3·01)	4·68 (1·93–9·55)	4·97 (1·97–10·1)	4·71 (1·85–9·7)	6·35 (-5·59–17·7)	-5·25 (-11·9–-0·317)
High LDL cholesterol	57 (52·1–61·6)	45·8 (41·8–50·2)	45·7 (41·7–49·9)	-19·5 (-26·1–-12)	-0·387 (-6·46–6·01)	--	--	--	--	--

Supplementary Results Table 1. Cambridgeshire age-standardised Summary Exposure Values (SEVs) for most-detailed risk factors and percentage change, by sex, 1990, 2010, and 2019. Data in parentheses are 95% uncertainty intervals.

	Male					Female				
	1990	2010	2019	Percentage change 1990-2010	Percentage change 2010-2019	1990	2010	2019	Percentage change 1990-2010	Percentage change 2010-2019
Unsafe sanitation	0·379 (0·133–0·886)	0·214 (0·075–0·489)	0·168 (0·0566–0·389)	-43·6 (-83·6–89·4)	-21·7 (-68–84·5)	0·379 (0·133–0·886)	0·214 (0·075–0·489)	0·168 (0·0566–0·389)	-43·6 (-83·6–89·4)	-21·7 (-68–84·5)
Ambient particulate matter pollution	17·1 (6·1–32·1)	11·4 (7·04–16·4)	8·79 (4·85–13·2)	-33·3 (-65·1–63·8)	-22·9 (-31·4–-18·5)	17·1 (6·11–32·1)	11·4 (7·05–16·4)	8·79 (4·86–13·2)	-33·3 (-65·1–63·7)	-22·9 (-31·3–-18·3)
Household air pollution from solid fuels	0·013 (0·00242–0·0397)	0·00426 (0·000697–0·0142)	0·00272 (0·000389–0·00978)	-67·4 (-90·5–8·35)	-36·2 (-72·8–40·4)	0·0172 (0·00356–0·0521)	0·00562 (0·00099–0·0185)	0·00361 (0·000537–0·0132)	-67·2 (-90·4–9·18)	-35·9 (-72·7–40·6)
Ambient ozone pollution	12·9 (4·07–27·2)	17·9 (4·94–37·7)	18·1 (5·28–39·5)	38·9 (-28–141)	1·22 (-47–102)	12·9 (4·07–27·2)	17·9 (4·94–37·7)	18·1 (5·28–39·5)	38·9 (-28–141)	1·22 (-47–102)
Residential radon	19·3 (4·11–54·3)	19·3 (4·11–54·3)	19·3 (4·11–54·3)	0 (0–0)	0 (0–0)	19·3 (4·11–54·3)	19·3 (4·11–54·3)	19·3 (4·11–54·3)	0 (0–0)	0 (0–0)
Lead exposure	18·9 (0·0797–44·3)	11·9 (0·0575–33·5)	8·24 (0·0128–26·7)	-36·9 (-68·3–-2·38)	-30·9 (-82·1–-19·4)	8·58 (3·12e-12–29·1)	4·99 (2·39e-12–21·2)	3·43 (1·99e-12–17·1)	-41·8 (-100–52·2)	-31·3 (-100–86·4)
Child underweight	0·892 (0·514–1·35)	0·766 (0·418–1·16)	0·756 (0·402–1·14)	-14·1 (-32–10·5)	-1·32 (-17·9–16·3)	1·04 (0·597–1·57)	0·92 (0·534–1·38)	0·906 (0·512–1·37)	-11·9 (-29·8–8·61)	-1·52 (-15·7–14·6)
Iron deficiency	--	--	--	--	--	9·79 (7·84–12)	8·99 (7·26–11)	8·92 (7·13–10·8)	-8·09 (-23·7–7·91)	-0·861 (-10·8–9·85)
Vitamin A deficiency	3·37 (0·611–10·5)	1·47 (0·288–4·65)	1·23 (0·205–3·88)	-56·4 (-91·7–130)	-16·3 (-67·5–175)	2·64 (0·76–6·28)	1·68 (0·539–3·73)	1·45 (0·475–3·43)	-36·4 (-79·2–109)	-13·5 (-57–92·1)
Zinc deficiency	3·53 (0–10·8)	2·3 (0–7·25)	2·26 (0–7·33)	-34·8 (-100–NA)	-1·7 (-28·8–NA)	3·42 (0–10·5)	2·21 (0–7·34)	2·14 (0–7·12)	-35·5 (-100–NA)	-2·79 (-29·5–NA)
Smoking	28·1 (24·5–32)	21·6 (18·6–24·8)	19·1 (16·5–22·2)	-23·2 (-30·1–-15·6)	-11·2 (-16·6–-5·85)	21·5 (18·1–25·6)	17·8 (14·8–21·3)	14·2 (11·7–17·2)	-17·1 (-29·2–3·34)	-20·4 (-27·8–-12·6)
Secondhand smoke	46·3 (40·2–52·5)	32·9 (28·2–37·8)	32·7 (27·9–37·4)	-28·9 (-40·3–-15·2)	-0·71 (-10·4–10·1)	48·4 (43·5–53·3)	33·7 (29·8–37·4)	33·4 (29·7–37·1)	-30·2 (-39·4–-20·4)	-0·888 (-8·7–7·3)
Alcohol use	18·6 (10·7–26·8)	22·6 (13·3–32·1)	21·3 (12·1–30·6)	21·5 (-18–88·3)	-5·74 (-29·2–22·1)	11·5 (5·5–18·8)	12·8 (6·23–20·2)	12 (5·73–20·2)	10·7 (-35·6–97·7)	-6·04 (-36·9–37·1)
Drug use	0·32 (0·163–0·624)	0·619 (0·363–1·05)	0·566 (0·315–1·02)	93·1 (48·9–156)	-8·54 (-18·8–1·19)	0·206 (0·115–0·392)	0·329 (0·199–0·56)	0·323 (0·187–0·565)	59·6 (23·1–108)	-1·82 (-13·9–8·68)
High fasting plasma glucose	9·34 (7·74–11·4)	18·6 (15·9–21·7)	25·7 (21·7–31·2)	99·2 (64·1–142)	38·2 (18·3–62·3)	7·84 (6·57–9·71)	13·6 (11·6–16)	19 (16·2–22·4)	73·6 (43·7–106)	39·8 (20·9–62·8)
High systolic blood pressure	44·7 (35·7–54·5)	28·1 (22·2–35·5)	29·7 (22·1–38·7)	-37·1 (-53–-16·2)	5·59 (-19·5–36·3)	--	--	--	--	--
High body-mass index	24·8 (17·7–34)	32·1 (23·6–42·8)	33·5 (24·6–45)	29·5 (15·3–48·3)	4·48 (-3·04–12·6)	24·8 (15·9–39·5)	32·6 (22·4–49·3)	33·8 (23·5–50·5)	31·3 (-0·761–75·3)	3·83 (-13–24·6)
Low bone mineral density	13·3 (7·86–20·5)	12·4 (7·23–19·4)	11 (6·25–18)	-6·54 (-29·5–21·6)	-11·7 (-33·7–16·9)	19·5 (13·2–26·9)	20 (12·9–27·9)	17·4 (11·4–24·4)	2·92 (-17·9–28·6)	-13·3 (-31·9–8·96)
Diet low in fruits	64·5 (52–76·6)	57·2 (44–70·8)	55·8 (42·7–68·9)	-11·2 (-27·1–2·41)	-2·48 (-13·3–9·08)	65·5 (53·8–76·8)	57·9 (44·5–71)	56·7 (43·1–70·7)	-11·6 (-25·4–2·46)	-2·14 (-12·8–10·1)
Diet low in vegetables	49·5 (30–70·7)	44·3 (25·5–66)	42·7 (23·4–64·3)	-10·5 (-31·5–9·08)	-3·61 (-17·2–11·3)	46·5 (27·1–66·8)	40·8 (21·6–61·5)	39 (20·7–60·6)	-12·2 (-33–7·97)	-4·45 (-21·4–12·2)
Diet low in whole grains	89·7 (84·1–94·9)	89·3 (83·3–94·5)	89·3 (83·3–94·5)	-0·518 (-4·04–2·71)	0·0733 (-2·03–2·27)	--	--	--	--	--
Diet low in nuts and seeds	81·7 (43·2–92)	73·4 (36·3–86·1)	75·5 (39·5–87·6)	-10·1 (-23·6–0·784)	2·94 (-6·25–13·9)	81·7 (42·7–92·4)	73·2 (35·7–87·2)	74·2 (36·6–88)	-10·4 (-25·6–1·52)	1·37 (-8·01–11·5)
Diet low in milk	56·8 (32–77·2)	58 (32·6–78·1)	57·7 (32·6–78)	2·25 (-10·3–19·3)	-0·637 (-8·59–7·05)	59·2 (34·3–80·3)	61·8 (36·3–82·6)	61·4 (36·8–82)	4·34 (-5·79–21·1)	-0·649 (-7·98–5·92)
Diet high in red meat	67·7 (52·2–81·7)	53·6 (38·8–68·1)	55·9 (39·4–71·4)	-20·7 (-39·2–0·538)	4·31 (-13·4–23·7)	66·9 (50·1–81·6)	54·2 (39·5–69·1)	56·8 (41–72·4)	-19 (-37·5–3·05)	4·88 (-12·4–26·8)
Diet high in processed meat	77·1 (52·1–96·9)	82 (59·9–98·8)	82·2 (61–98·7)	6·29 (-2·43–23·4)	0·333 (-5·42–6·65)	80·5 (58·3–98·3)	85·4 (66·3–99·5)	85·7 (66·5–99·6)	6·08 (-2·53–22·9)	0·329 (-4·96–6·31)
Diet high in sugar-sweetened beverages	47·9 (29·5–73·9)	52·1 (31·7–74·7)	54 (34–76·9)	8·69 (-30·7–70·2)	3·74 (-19·4–39·6)	--	--	--	--	--
Diet low in fiber	39·9 (23·4–56·4)	32·5 (17·3–49·1)	31·9 (17·6–48·7)	-18·4 (-44·7–14·4)	-1·9 (-21·9–25·3)	40·1 (22·3–57·8)	32·4 (17–49·4)	31·9 (16–48·9)	-19·1 (-45·3–18·5)	-1·69 (-23·5–25·1)
Diet low in seafood omega-3 fatty acids	92·1 (79·8–100)	89·3 (73·8–100)	88·3 (73·5–99·9)	-3·1 (-16·1–7·28)	-1·07 (-13·4–12·2)	93·3 (83·3–100)	91·1 (78·9–100)	90·3 (76·5–100)	-2·38 (-12·1–5·54)	-0·816 (-11·7–9·68)
Diet low in polyunsaturated fatty acids	51·4 (15–93·5)	46·2 (12·4–90·5)	44·7 (11·9–88·7)	-10·1 (-28·3–0·00296)	-3·31 (-14·3–5·5)	53·9 (16–95)	49 (14·1–91·9)	47·4 (13–91·1)	-9·17 (-27·2–0·188)	-3·2 (-13·9–5·14)
Diet high in trans fatty acids	57·7 (46·5–100)	51 (40·2–97·7)	50·9 (39·6–99)	-11·6 (-25·4–1·52)	-0·133 (-15·6–17)	55·9 (44·7–100)	47·8 (36–100)	47·7 (36·3–99·7)	-14·7 (-29·8–0)	-0·169 (-12·8–16·3)
Diet high in sodium	19·9 (4·38–43)	22·9 (5·02–46)	26·2 (6·08–50·4)	15·1 (-48·4–182)	14·4 (-24·7–101)	12 (2·96–31·2)	12·4 (3·27–31·2)	13·5 (3·39–34)	3·31 (-41·6–101)	8·53 (-30·9–82·1)
Low physical activity	4·61 (2·06–9·41)	5·47 (2·52–10·9)	5·62 (2·57–11·2)	18·6 (1·06–43·7)	2·67 (-9·75–17·3)	7·11 (3·74–13·2)	8·32 (4·55–14·6)	8·48 (4·68–14·8)	17·1 (6·29–34·1)	1·91 (-5·97–11·5)
Occupational asthmagens	20·8 (16·6–26·2)	19·4 (15·5–24·6)	21·2 (16–29)	-6·61 (-25·4–16·2)	9·5 (-9·29–33·9)	12·1 (9·22–15·5)	10·6 (8·35–13)	11·6 (9·13–14·6)	-12·3 (-30·6–9·06)	9·45 (-5·85–28·3)
Occupational particulate matter, gases, and fumes	8·36 (5·8–12·3)	7·99 (5·62–11·7)	7·8 (5·52–11·5)	-4·43 (-13·1–5·42)	-2·37 (-7·17–2·12)	3·69 (2·49–5·7)	3·51 (2·4–5·32)	3·36 (2·28–4·96)	-4·87 (-18–11·6)	-4·35 (-10·3–2·39)
Occupational noise	8·23 (7·37–9·14)	8·04 (7·43–8·8)	7·92 (7·32–8·64)	-2·27 (-8·2–4·33)	-1·59 (-4·15–1·31)	4·92 (4·11–5·8)	5·39 (4·8–6·07)	5·52 (5–6·17)	9·56 (-0·277–22·6)	2·54 (-1·06–6·71)
Occupational injuries	--	--	--	--	--	--	--	--	--	--
Occupational ergonomic factors	11·6 (9·56–14)	10·8 (8·94–12·8)	11·1 (9·01–13·3)	-7·26 (-20·4–8·88)	2·89 (-8·38–15·8)	--	--	--	--	--
Childhood sexual abuse	10·6 (6·74–14·7)	11 (7·04–15·8)	10·7 (6·96–15·2)	4·38 (-2·5–12·8)	-2·79 (-11–6·54)	13·3 (9·72–18·3)	14·1 (10·3–19·6)	13·7 (10·1–18·8)	6·28 (-2·16–15·1)	-2·58 (-10·4–8·27)
Intimate partner violence	--	--	--	--	--	22·8 (12·5–31·3)	20 (11·3–28·2)	22·8 (12·5–31·3)	-12·2 (-19·2–0·696)	13·9 (2·98–28·7)
Non-exclusive breastfeeding	25·7 (18·8–33·9)	23·2 (17·4–30·4)	23·3 (17·6–30·3)	-9·7 (-24·8–8·41)	0·355 (-12·1–14·4)	25·7 (18·8–33·8)	23·2 (17·4–30·4)	23·3 (17·6–30·3)	-9·67 (-24·7–8·43)	0·354 (-12·1–14·4)
Discontinued breastfeeding	27·9 (26·8–28·8)	27·6 (26·5–28·5)	27·4 (26·3–28·4)	-0·967 (-5·88–4·2)	-0·625 (-4·33–3·4)	27·8 (26·8–28·8)	27·7 (26·6–28·7)	27·5 (26·3–28·5)	-0·383 (-5·33–4·82)	-0·923 (-4·61–3·07)
Diet low in calcium	18 (8·09–33·8)	12·2 (5·02–22·8)	11·7 (4·88–21·7)	-32·3 (-50·1–-17·6)	-4·47 (-15·7–8·36)	12·6 (4·82–24·1)	6·6 (2·46–12·5)	6·18 (2·28–11·4)	-47·5 (-69·4–-26·6)	-6·26 (-25·1–16·9)
Occupational exposure to asbestos	20·8 (19–22·8)	18·4 (17·2–19·6)	16·9 (14·4–19·5)	-11·2 (-20·8–0·215)	-8·5 (-22·7–6·61)	4·17 (2·68–5·48)	4·01 (2·15–4·82)	4·23 (2·04–5·9)	-3·96 (-36·9–31·9)	5·6 (-22·9–42·7)
Occupational exposure to arsenic	0·451 (0–1·06)	0·482 (0–1·1)	0·499 (0–1·17)	6·91 (-13·7–NA)	3·48 (-10·8–NA)	0·395 (0–0·945)	0·411 (0–0·971)	0·432 (0–1·01)	4·04 (-17·6–NA)	5·31 (-7·86–NA)
Occupational exposure to benzene	0·881 (0·0502–2·46)	0·863 (0·0484–2·4)	0·913 (0·053–2·55)	-2·06 (-12·3–13·5)	5·76 (-3·23–17·5)	1·21 (0·0723–3·45)	1·48 (0·0892–4·25)	1·61 (0·0993–4·65)	22·1 (6·34–50·8)	9·03 (-1·04–24·7)
Occupational exposure to beryllium	0·0241 (0·0225–0·0256)	0·0254 (0·0239–0·027)	0·0264 (0·0247–0·0281)	5·08 (-2·88–13·8)	4·17 (-2·51–10·3)	0·0258 (0·0233–0·0284)	0·0289 (0·0265–0·0311)	0·0311 (0·0283–0·0335)	11·8 (0·567–25·5)	7·65 (0·166–15·8)
Occupational exposure to cadmium	0·0532 (0·0453–0·0614)	0·0568 (0·0493–0·0653)	0·059 (0·0503–0·0684)	6·78 (-11·5–27·6)	3·93 (-9·25–18·4)	0·0486 (0·0414–0·0566)	0·0511 (0·0448–0·0589)	0·054 (0·0475–0·0623)	5·09 (-13·8–27·7)	5·77 (-6·58–18·8)
Occupational exposure to chromium	0·127 (0·111–0·144)	0·134 (0·118–0·152)	0·14 (0·122–0·16)	5·74 (-10·9–24·3)	4·63 (-7·31–17·6)	0·126 (0·109–0·143)	0·135 (0·12–0·151)	0·144 (0·128–0·163)	7·39 (-8·93–27·7)	6·48 (-4·4–17·7)
Occupational exposure to diesel engine exhaust	0·729 (0·66–0·803)	0·755 (0·683–0·832)	0·798 (0·719–0·88)	3·54 (-9·88–17·7)	5·8 (-3–15·2)	0·668 (0·578–0·771)	0·685 (0·6–0·781)	0·734 (0·626–0·849)	2·54 (-13·9–22·2)	7·11 (-6·3–21·9)
Occupational exposure to formaldehyde	0·263 (0·222–0·307)	0·269 (0·23–0·313)	0·278 (0·233–0·325)	2·35 (-17–24·6)	3·34 (-10·8–18·8)	0·238 (0·203–0·277)	0·258 (0·227–0·293)	0·273 (0·241–0·312)	8·2 (-9·05–29·8)	6·09 (-5·45–18·6)
Occupational exposure to nickel	0·441 (0–1·9)	0·478 (0–2·19)	0·496 (0–2·29)	8·4 (-9·65–NA)	3·8 (-8·27–NA)	0·308 (0–1·35)	0·314 (0–1·36)	0·328 (0–1·42)	1·9 (-21·1–NA)	4·51 (-9·78–NA)
Occupational exposure to polycyclic aromatic hydrocarbons	0·268 (0·237–0·3)	0·281 (0·25–0·315)	0·294 (0·26–0·33)	4·88 (-10·5–21·5)	4·79 (-5·93–16·5)	--	--	--	--	--
Occupational exposure to silica	5·54 (0·751–18·2)	6·04 (0·817–19·6)	6·37 (0·896–20·7)	8·98 (-9·12–34·9)	5·43 (-7·55–22·6)	2·76 (0·359–8·98)	2·7 (0·364–8·44)	2·81 (0·387–9·01)	-2·3 (-23·6–29·8)	3·93 (-10·5–23·5)
Occupational exposure to sulfuric acid	0·821 (0·176–2·7)	0·819 (0·167–2·55)	0·828 (0·174–2·67)	-0·236 (-18·5–19·8)	1·14 (-12·1–14·3)	--	--	--	--	--
Unsafe sex	--	--	--	--	--	--	--	--	--	--
Occupational exposure to trichloroethylene	0·0735 (0·0666–0·0808)	0·077 (0·0697–0·0846)	0·0806 (0·0723–0·089)	4·8 (-7·84–19·1)	4·6 (-4·74–14·9)	0·0774 (0·0679–0·0873)	0·0819 (0·0736–0·0913)	0·0875 (0·0783–0·0974)	5·88 (-8·49–24)	6·77 (-3·17–17·7)
No access to handwashing facility	3·29 (2·98–3·62)	3·16 (2·87–3·45)	3·12 (2·83–3·43)	-4·07 (-14·7–6·21)	-1·07 (-7·71–5·98)	3·29 (2·98–3·62)	3·16 (2·87–3·45)	3·12 (2·83–3·43)	-4·07 (-14·7–6·21)	-1·07 (-7·71–5·98)
Child wasting	1·25 (0·851–1·67)	1·2 (0·822–1·63)	1·2 (0·825–1·61)	-3·54 (-16·7–10·7)	-0·283 (-8·98–9·93)	--	--	--	--	--
Child stunting	1·6 (0·759–2·49)	1·19 (0·518–1·92)	1·19 (0·482–1·88)	-25·4 (-47–2·03)	-0·358 (-24·5–27·8)	1·88 (0·937–2·87)	1·56 (0·753–2·4)	1·54 (0·746–2·38)	-17·1 (-37·4–9·63)	-1·48 (-20·4–19·4)
Chewing tobacco	0·175 (0·113–0·267)	0·166 (0·105–0·245)	0·17 (0·11–0·251)	-5·54 (-42·8–55·5)	2·66 (-26·7–40·6)	0·134 (0·0705–0·229)	0·133 (0·0742–0·223)	0·133 (0·0743–0·229)	-0·661 (-50·8–104)	0·222 (-34·8–53·3)
Diet low in legumes	72·9 (35·1–99·1)	71·9 (35·2–98·4)	72·6 (36·5–98·6)	-1·31 (-13·9–6·98)	0·953 (-4·74–11·3)	69·3 (30·2–97·5)	67·9 (26·8–96·4)	68·6 (26·7–96·9)	-2 (-17·1–6·85)	1·03 (-7·55–14·9)
Short gestation	21·9 (17·6–27·6)	49·7 (40–61·8)	43·5 (33·4–57·5)	127 (68·7–193)	-12·4 (-33·7–14·3)	24·5 (21–28·2)	44·4 (37·4–52·2)	41·5 (33·7–50·7)	81·4 (53–116)	-6·56 (-24·2–15)
Low birth weight	11·2 (9·05–13·9)	23·1 (18·4–29)	21·3 (16·3–27·7)	106 (56·3–175)	-8·04 (-31–20·3)	12·9 (11·3–14·7)	21·2 (17·9–24·9)	20·2 (16·6–24·5)	64·9 (36–96·7)	-4·73 (-24·8–20)
High temperature	23·5 (11·4–36·5)	0 (0–0)	21·9 (10·1–35·4)	-100 (-100–-100)	--	23·5 (11·4–36·5)	0 (0–0)	21·9 (10·1–35·4)	-100 (-100–-100)	--
Low temperature	30·9 (26·2–35·9)	33·7 (29·7–38·1)	30·4 (25·8–35·3)	9·11 (5·85–13·4)	-9·87 (-13·4–-6·96)	30·9 (26·2–35·9)	33·7 (29·7–38·1)	30·4 (25·8–35·3)	9·11 (5·85–13·4)	-9·87 (-13·4–-6·96)
Kidney dysfunction	13·6 (9·1–20)	14·1 (9·56–20·5)	13·9 (9·4–20·2)	3·34 (-1·36–9·04)	-1·36 (-5·46–3·18)	14·6 (9·76–21·2)	14·8 (10–21·2)	14·8 (9·91–21·3)	1·82 (-2·29–6·43)	0·109 (-4·2–4·65)
Bullying victimization	6·02 (2·44–11·8)	6 (2·36–12·1)	5·58 (2·24–11·5)	-0·41 (-9·99–9·44)	-6·9 (-12·2–-2·43)	4·66 (1·91–9·52)	4·94 (1·97–10)	4·68 (1·85–9·58)	6·14 (-5·74–17·7)	-5·31 (-11·9–-0·522)
High LDL cholesterol	57·8 (53·3–62·3)	46·6 (42·8–50·6)	46·5 (42·3–50·9)	-19·2 (-26·2–-12·2)	-0·256 (-5·75–5·42)	--	--	--	--	--

Supplementary Results Table 1. Central Bedfordshire age-standardised Summary Exposure Values (SEVs) for most-detailed risk factors and percentage change, by sex, 1990, 2010, and 2019. Data in parentheses are 95% uncertainty intervals.

	Male					Female				
	1990	2010	2019	Percentage change 1990-2010	Percentage change 2010-2019	1990	2010	2019	Percentage change 1990-2010	Percentage change 2010-2019
Unsafe sanitation	0·526 (0·173–1·15)	0·318 (0·102–0·749)	0·239 (0·0815–0·534)	-39·5 (-82·4–107)	-25 (-68·5–69·6)	0·526 (0·173–1·15)	0·318 (0·102–0·749)	0·239 (0·0815–0·534)	-39·5 (-82·4–107)	-25 (-68·5–69·6)
Ambient particulate matter pollution	17·1 (6·08–31·4)	11·2 (7·5–15·5)	8·28 (4·98–12·1)	-34·4 (-65·4–64·4)	-26 (-35—21)	17·1 (6·09–31·4)	11·2 (7·51–15·5)	8·28 (4·98–12·1)	-34·4 (-65·4–64·5)	-26 (-35—21)
Household air pollution from solid fuels	0·0182 (0·00341–0·0537)	0·00652 (0·00108–0·0207)	0·00408 (0·000617–0·0139)	-64·1 (-90·7–34·6)	-37·5 (-72·2–42)	0·0239 (0·00503–0·0725)	0·00859 (0·00152–0·0272)	0·00539 (0·000841–0·0176)	-64 (-90·6–35·8)	-37·2 (-72·1–42·2)
Ambient ozone pollution	7·59 (1·28–19)	14·9 (2·86–34·8)	14·9 (3·07–35·8)	96·3 (-35·1–634)	-0·211 (-67·8–225)	7·59 (1·28–19)	14·9 (2·86–34·8)	14·9 (3·07–35·8)	96·3 (-35·1–634)	-0·211 (-67·8–225)
Residential radon	24 (4·15–70·7)	24 (4·15–70·7)	24 (4·15–70·7)	0 (0–0)	0 (0–0)	24 (4·15–70·7)	24 (4·15–70·7)	24 (4·15–70·7)	0 (0–0)	0 (0–0)
Lead exposure	23·7 (1·14–49·5)	15·3 (0·742–37·6)	10·7 (0·242–30·3)	-35·6 (-62·2—21·9)	-30·2 (-69—18·6)	10·2 (5·17e-12–30·9)	6·1 (4·57e-12–23·1)	4·26 (2·69e-12–18·9)	-40·3 (-100–6·18)	-30·1 (-100–17·1)
Child underweight	0·914 (0·527–1·38)	0·779 (0·413–1·19)	0·766 (0·405–1·18)	-14·8 (-32·6–7·75)	-1·61 (-16·9–18·3)	1·08 (0·621–1·6)	0·941 (0·538–1·42)	0·924 (0·527–1·39)	-12·7 (-30·6–8·29)	-1·76 (-14·8–14·5)
Iron deficiency	--	--	--	--	--	10·2 (8·17–12·6)	9·18 (7·35–11·2)	9·03 (7·14–11)	-9·96 (-24·6–6·57)	-1·63 (-10·9–10·2)
Vitamin A deficiency	4·29 (0·809–13·5)	2·02 (0·39–6·17)	1·65 (0·345–4·86)	-53 (-90·7–153)	-17·9 (-70–152)	3·17 (0·956–7·58)	2·19 (0·69–5·5)	1·8 (0·601–4·64)	-31·1 (-78–134)	-17·8 (-59·6–80·3)
Zinc deficiency	4·14 (0–12·4)	2·88 (0–9·01)	2·87 (0–8·68)	-30·4 (-100–NA)	-0·171 (-26·9–NA)	4·02 (0–11·6)	2·74 (0–9·03)	2·75 (0–8·68)	-31·8 (-100–NA)	0·302 (-28·4–NA)
Smoking	28·7 (25·1–33)	21·4 (18·5–24·9)	19 (16·2–22)	-25·6 (-32·4—18·2)	-11·4 (-16·9—5·66)	21·8 (18·3–26·1)	17·6 (14·7–21)	14 (11·5–17)	-19·3 (-31·7—5·63)	-20·2 (-27·8—12·3)
Secondhand smoke	45 (38·9–50·8)	34 (29·2–38·8)	33·7 (29–38·7)	-24·3 (-36·1—11·1)	-0·974 (-10·9–10)	46·8 (42·1–51·4)	34·6 (30·7–38·4)	34·3 (30·2–37·9)	-26·1 (-35—16·3)	-0·892 (-9–7·69)
Alcohol use	17·6 (9·45–25·5)	20·2 (11·1–29·5)	17·7 (9·32–26·2)	15 (-27·9–87·7)	-12·4 (-38·1–18·3)	--	--	--	--	--
Drug use	0·31 (0·151–0·639)	0·574 (0·325–1·02)	0·537 (0·299–0·984)	85·5 (44·1–143)	-6·56 (-15·6–2·55)	0·201 (0·109–0·387)	0·324 (0·194–0·553)	0·316 (0·18–0·559)	61·2 (20·9–114)	-2·32 (-13·3–7·95)
High fasting plasma glucose	8·34 (6·94–10·1)	18·2 (15·6–21·5)	23·2 (19·4–28·1)	118 (81·2–162)	27·2 (9·28–51)	7·64 (6·26–9·37)	13·6 (11·7–15·9)	17·7 (15·1–20·9)	78·5 (46·6–115)	29·8 (11·6–50·9)
High systolic blood pressure	44·2 (34·6–54·5)	27·4 (21·4–34·2)	28·9 (21–38·9)	-37·9 (-54·5—15·5)	5·31 (-21·1–37·7)	--	--	--	--	--
High body-mass index	21·7 (15·1–29·7)	28·2 (20·3–38)	30 (21·5–39·7)	30·3 (14–52·7)	6·3 (-0·618–15·1)	21·3 (13·1–35·7)	28·2 (18·7–43·7)	29·5 (20–45·7)	32·2 (-1·52–85·6)	4·93 (-12·4–25·5)
Low bone mineral density	8·85 (4·69–14·7)	7·61 (3·71–13·3)	9·2 (4·69–15·5)	-14·1 (-36–13·8)	21 (-7·26–61·9)	14·8 (9·28–21·8)	14 (8·86–20·7)	13·6 (8·58–20)	-5·6 (-26·5–22·7)	-2·56 (-24·5–26·5)
Diet low in fruits	66·7 (53·9–78·4)	60 (47·4–72·9)	59 (45·6–72·3)	-10 (-23·5–2·75)	-1·78 (-11·8–9·58)	67·6 (56–79·3)	60·9 (48·5–73·2)	60 (47·4–72·8)	-9·88 (-22·8–3·77)	-1·47 (-12·3–10·7)
Diet low in vegetables	52·5 (32·7–73·5)	48·8 (29·6–69·4)	47·7 (28·2–68·5)	-7·13 (-23·8–12·5)	-2·36 (-14·8–11·1)	49·8 (29·9–69·6)	45·5 (27·5–67·5)	44·1 (26·7–65·9)	-8·61 (-28·1–12·7)	-3·14 (-18·1–13)
Diet low in whole grains	90 (84·3–94·9)	89·7 (83·8–94·9)	89·8 (84–94·9)	-0·345 (-3·87–2·97)	0·122 (-1·95–2·13)	88·3 (82·2–93·9)	88·1 (81·9–93·7)	88·2 (81·4–93·9)	-0·209 (-3·72–3·58)	0·0725 (-2·29–2·63)
Diet low in nuts and seeds	82·7 (43–92·9)	75·3 (36·7–88·4)	77·4 (40–89·7)	-8·97 (-22·3–1·57)	2·76 (-5·4–13·6)	82·7 (43·9–93·2)	75·3 (38–88·3)	76·5 (39·8–89·5)	-8·95 (-23·6–2·96)	1·48 (-7·1–11·6)
Diet low in milk	59·1 (34–80·5)	61·1 (36–81·8)	61·1 (35·9–81·7)	3·43 (-7·32–20·8)	-0·0183 (-7·06–7·89)	61·5 (36·1–82·3)	64·5 (39·4–85·1)	64·4 (38·4–84·8)	4·96 (-3·55–21·9)	-0·14 (-7·04–6·33)
Diet high in red meat	65·7 (49·9–80·4)	50·9 (35·6–66·1)	53·2 (36·7–68·9)	-22·5 (-41·2—1·58)	4·36 (-12·7–27·5)	64·9 (47·9–79·8)	51·6 (36·5–67·4)	54·3 (38·4–69·8)	-20·4 (-40·9–2·24)	5·14 (-12·9–27·5)
Diet high in processed meat	76·6 (53·2–96·5)	81·2 (59·2–98·7)	81·4 (59·2–98·2)	5·99 (-4·25–22)	0·19 (-5·79–7)	79·8 (58·2–97·9)	84·7 (65·3–99·2)	85 (65·8–99·1)	6·14 (-2·8–22·3)	0·328 (-5·2–6·72)
Diet high in sugar-sweetened beverages	46·9 (28·3–72·6)	49·7 (30·8–73·9)	51·5 (31·6–75·3)	6 (-31·5–62)	3·65 (-21·8–42·1)	--	--	--	--	--
Diet low in fiber	42·8 (26·4–59)	36·2 (19·9–54·2)	35·9 (20·4–53·1)	-15·3 (-41·3–16·6)	-0·804 (-20·7–24·1)	42·9 (24·2–60·5)	36·4 (19·6–52·7)	36·2 (19·6–53·4)	-15·1 (-40·5–17·3)	-0·613 (-21·8–23·1)
Diet low in seafood omega-3 fatty acids	93·5 (83·5–100)	91·2 (78·5–100)	91 (78·3–100)	-2·52 (-13·4–5·28)	-0·173 (-9·31–9·95)	94·5 (84·5–100)	92·6 (81·8–100)	92·3 (81·1–100)	-2·03 (-11·4–5·39)	-0·266 (-8·68–9·18)
Diet low in polyunsaturated fatty acids	54·6 (17·3–94·6)	50·4 (14·6–92·3)	49·5 (14·2–91·8)	-7·61 (-24–0·997)	-1·84 (-11·6–6·92)	56·9 (18·4–96)	52·9 (15·9–94·2)	51·9 (15·8–94)	-7·02 (-24–1·34)	-1·98 (-11–6·18)
Diet high in trans fatty acids	57·9 (46·5–100)	51·1 (39·9–98·9)	51 (39·8–97·9)	-11·7 (-26·6–2·04)	-0·136 (-15–18·6)	56 (44·5–100)	47·9 (36–99·6)	47·9 (36·2–99·6)	-14·5 (-30–0)	0·0164 (-13·1–16·6)
Diet high in sodium	20·3 (4·33–42·8)	23·3 (4·95–45·7)	26·3 (5·82–50·6)	14·7 (-47·9–170)	12·8 (-29–105)	12·2 (3·19–32·1)	12·5 (3·31–30·9)	13·7 (3·38–34·3)	2·48 (-46·1–118)	9·32 (-35·7–81·8)
Low physical activity	4·4 (1·88–9·36)	5 (2·24–10·1)	5·12 (2·33–10·2)	13·6 (-1·26–36·6)	2·49 (-9·61–18·6)	6·94 (3·53–12·5)	7·92 (4·18–14·2)	8·04 (4·32–14·6)	14·2 (4·04–29·7)	1·49 (-6·79–11·6)
Occupational asthmagens	21·3 (17–26·6)	19·7 (15·7–24·9)	21·4 (16·5–29)	-7·36 (-26·1–15·7)	8·35 (-10·4–32·3)	11·9 (9·17–15·8)	10·5 (8·31–13·1)	11·5 (8·96–14·8)	-11·9 (-31·6–12·1)	9·53 (-5·82–29·5)
Occupational particulate matter, gases, and fumes	8·46 (5·84–12·2)	8·07 (5·72–11·6)	7·87 (5·61–11·4)	-4·59 (-13·6–6·11)	-2·43 (-7·17–2·55)	3·81 (2·5–6·01)	3·61 (2·46–5·54)	3·45 (2·37–5·41)	-5·2 (-19·6–13·9)	-4·31 (-10·8–3·02)
Occupational noise	8·29 (7·41–9·3)	8·1 (7·42–8·92)	7·97 (7·32–8·74)	-2·32 (-8·33–4·37)	-1·63 (-4·51–1·36)	4·93 (4·02–5·88)	5·4 (4·79–6·11)	5·53 (4·99–6·18)	9·61 (-0·559–23·6)	2·49 (-1·37–6·9)
Occupational injuries	--	--	--	--	--	--	--	--	--	--
Occupational ergonomic factors	11·8 (9·6–14·2)	11·2 (9·21–13·6)	11·6 (9·45–14·1)	-4·79 (-18·8–12·1)	3·33 (-8·69–18)	6·56 (4·98–8·5)	8·28 (6·24–10·8)	9·2 (6·77–12·6)	26·2 (-2·33–63·8)	11 (-10·8–38·3)
Childhood sexual abuse	10·6 (6·74–14·7)	11 (7·04–15·8)	10·7 (6·96–15·2)	4·38 (-2·5–12·8)	-2·79 (-11–6·54)	13·3 (9·72–18·3)	14·1 (10·3–19·6)	13·7 (10·1–18·8)	6·28 (-2·16–15·1)	-2·58 (-10·4–8·27)
Intimate partner violence	--	--	--	--	--	22·8 (12·5–31·3)	20 (11·3–28·2)	22·8 (12·5–31·3)	-12·2 (-19·2–0·696)	13·9 (2·98–28·7)
Non-exclusive breastfeeding	25·7 (19·1–34·1)	23·2 (17·3–30·8)	23·3 (17·4–30·9)	-9·89 (-25·4–9·21)	0·452 (-14·1–16·1)	25·7 (19·1–34·1)	23·2 (17·3–30·8)	23·3 (17·4–30·9)	-9·88 (-25·4–9·23)	0·46 (-14–16·1)
Discontinued breastfeeding	27·8 (26·7–28·7)	27·6 (26·4–28·6)	27·7 (26·5–28·7)	-0·539 (-5·73–4·81)	0·117 (-3·9–3·9)	27·9 (26·8–28·9)	27·5 (26·3–28·5)	27·5 (26·3–28·5)	-1·46 (-6·61–3·85)	0·098 (-3·94–3·89)
Diet low in calcium	20·2 (9·53–37·9)	15·1 (6·34–28·7)	14·9 (6·33–28·3)	-25·3 (-45·1—10·8)	-1·57 (-10·9–9·75)	14·7 (5·95–28·9)	9·7 (3·51–18·2)	9·45 (3·33–17·9)	-34·3 (-57·5—14·2)	-2·58 (-19·1–14·1)
Occupational exposure to asbestos	20·4 (18·4–22·5)	17·7 (16·4–19·1)	15·5 (12·8–18·4)	-13·3 (-23·7—1·68)	-12·4 (-28·1–5·32)	5·13 (2·79–7·13)	4·95 (2·33–6·08)	4·82 (2·09–7·03)	-3·5 (-37–39·9)	-2·62 (-29·6–31·7)
Occupational exposure to arsenic	0·463 (0–1·11)	0·495 (0–1·18)	0·509 (0–1·21)	6·83 (-15·4–NA)	3 (-11·1–NA)	0·402 (0–0·966)	0·42 (0–0·984)	0·442 (0–1·04)	4·41 (-16·8–NA)	5·32 (-7·93–NA)
Occupational exposure to benzene	0·893 (0·0478–2·49)	0·871 (0·0526–2·47)	0·915 (0·0551–2·6)	-2·54 (-12·6–13·6)	5·07 (-3·3–18·4)	1·19 (0·0654–3·44)	1·46 (0·0846–4·26)	1·59 (0·0932–4·77)	22·9 (6·89–51·3)	8·9 (-1·98–26·7)
Occupational exposure to beryllium	0·0243 (0·0227–0·026)	0·0256 (0·0237–0·0274)	0·0266 (0·0247–0·0285)	5·19 (-2·95–14·6)	4·02 (-1·96–10·5)	0·0255 (0·0229–0·0283)	0·0287 (0·0263–0·0312)	0·0309 (0·0282–0·0336)	12·4 (-0·246–26·3)	7·72 (-0·235–16·5)
Occupational exposure to cadmium	0·0545 (0·0472–0·0635)	0·0581 (0·0496–0·0674)	0·0601 (0·0508–0·0702)	6·66 (-13·9–29·3)	3·45 (-9·46–19·7)	0·0492 (0·0418–0·0584)	0·0519 (0·0455–0·06)	0·0549 (0·0478–0·0646)	5·41 (-13·9–30·4)	5·74 (-6–20·5)
Occupational exposure to chromium	0·13 (0·114–0·149)	0·137 (0·119–0·156)	0·142 (0·122–0·164)	5·53 (-12·6–25·5)	4·13 (-8·22–18·6)	0·126 (0·109–0·146)	0·136 (0·121–0·154)	0·145 (0·129–0·166)	7·71 (-9·64–28·5)	6·44 (-4·2–18·9)
Occupational exposure to diesel engine exhaust	0·723 (0·644–0·797)	0·749 (0·677–0·825)	0·794 (0·715–0·883)	3·59 (-8·74–18·4)	6 (-3·57–16·7)	0·659 (0·556–0·761)	0·68 (0·594–0·781)	0·732 (0·622–0·849)	3·2 (-14·1–25·6)	7·52 (-5·9–23)
Occupational exposure to formaldehyde	0·27 (0·23–0·319)	0·276 (0·231–0·322)	0·283 (0·236–0·334)	2·11 (-18·1–25·5)	2·77 (-11·7–20·4)	0·241 (0·203–0·284)	0·261 (0·23–0·299)	0·276 (0·242–0·32)	8·4 (-10·5–31·6)	5·95 (-5·49–19·4)
Occupational exposure to nickel	0·447 (0–1·98)	0·485 (0–2·1)	0·505 (0–2·16)	8·5 (-11·1–NA)	4·08 (-8·92–NA)	0·315 (0–1·44)	0·323 (0–1·36)	0·337 (0–1·48)	2·7 (-20·3–NA)	4·44 (-9·67–NA)
Occupational exposure to polycyclic aromatic hydrocarbons	0·273 (0·243–0·308)	0·286 (0·253–0·324)	0·299 (0·261–0·34)	4·83 (-10·8–21·9)	4·29 (-6·86–17)	--	--	--	--	--
Occupational exposure to silica	5·54 (0·75–17·3)	6·08 (0·833–20·4)	6·39 (0·938–20·6)	9·69 (-9·48–37·9)	5·21 (-8·51–22·1)	2·81 (0·391–9·1)	2·8 (0·383–8·7)	2·91 (0·392–9·31)	-0·369 (-21·7–33·3)	4·04 (-10·6–24·9)
Occupational exposure to sulfuric acid	0·837 (0·183–2·81)	0·837 (0·177–2·54)	0·846 (0·178–2·68)	0·0139 (-18·4–22·8)	0·972 (-11·9–15·4)	--	--	--	--	--
Unsafe sex	--	--	--	--	--	--	--	--	--	--
Occupational exposure to trichloroethylene	0·0746 (0·0678–0·0832)	0·078 (0·0702–0·0861)	0·0813 (0·0724–0·0906)	4·56 (-8·49–19)	4·31 (-4·81–15·1)	0·0772 (0·0675–0·0878)	0·0821 (0·0734–0·0916)	0·0878 (0·0779–0·0991)	6·32 (-9·39–25·1)	6·87 (-2·84–18·4)
No access to handwashing facility	3·33 (3·01–3·65)	3·2 (2·89–3·51)	3·15 (2·86–3·45)	-4·15 (-14·5–6·73)	-1·34 (-8·2–5·61)	3·33 (3·01–3·65)	3·2 (2·89–3·51)	3·15 (2·86–3·45)	-4·15 (-14·5–6·73)	-1·34 (-8·2–5·61)
Child wasting	1·26 (0·882–1·71)	1·22 (0·839–1·66)	1·21 (0·837–1·65)	-3·54 (-16·7–11·5)	-0·434 (-10–11·3)	--	--	--	--	--
Child stunting	1·64 (0·804–2·48)	1·22 (0·518–1·94)	1·21 (0·547–1·93)	-26 (-46·6–1·63)	-0·449 (-22·3–26·7)	1·94 (1–2·97)	1·59 (0·752–2·44)	1·56 (0·755–2·49)	-18 (-40·8–10·1)	-1·9 (-21·5–21)
Chewing tobacco	0·176 (0·109–0·266)	0·167 (0·107–0·252)	0·171 (0·109–0·267)	-5·04 (-43·6–55·6)	2·39 (-27·4–43·6)	0·136 (0·0708–0·24)	0·134 (0·0746–0·243)	0·135 (0·0731–0·237)	-1·43 (-50·2–109)	0·658 (-36·8–62·7)
Diet low in legumes	74·1 (38–99·1)	73·5 (37·3–98·9)	74·2 (39·6–98·8)	-0·825 (-11·1–7·94)	1·02 (-5·52–10·8)	70·5 (32·2–98·1)	69·6 (31–97·6)	70·3 (29·6–97·4)	-1·25 (-14·6–9·59)	1·01 (-7–12·5)
Short gestation	20 (15·9–25·3)	38·9 (30·4–49·1)	34·1 (26·1–43·7)	94·8 (43·5–157)	-12·4 (-34·2–14·5)	23 (19·6–27)	36·1 (29·9–42·8)	33·2 (27–40·4)	56·7 (27·1–91)	-7·87 (-25·1–14)
Low birth weight	11·1 (8·93–13·8)	20·4 (15·9–25·4)	19·6 (15·2–24·5)	84·4 (32·7–146)	-3·99 (-27·7–27·8)	12 (10·2–14·1)	19·9 (16·5–23·6)	18·8 (15·4–22·6)	65·3 (31·7–107)	-5·39 (-24·2–19·5)
High temperature	23 (7·1–40·3)	0 (0–0)	21·2 (6·11–37·9)	-100 (-100—100)	--	23 (7·1–40·3)	0 (0–0)	21·2 (6·11–37·9)	-100 (-100—100)	--
Low temperature	30·6 (25–36·6)	33·3 (28·5–38·8)	29·9 (24·5–36·1)	9·08 (5·24–13·8)	-10·3 (-14·4—6·31)	30·6 (25–36·6)	33·3 (28·5–38·8)	29·9 (24·5–36·1)	9·08 (5·24–13·8)	-10·3 (-14·4—6·31)
Kidney dysfunction	13·7 (9·11–20·1)	14·2 (9·76–20·5)	14·1 (9·58–20·5)	3·8 (-0·774–9·87)	-0·917 (-5·47–4)	14·8 (9·88–21·3)	15·2 (10·3–21·7)	15·1 (10·4–21·8)	2·63 (-1·48–7·85)	-0·143 (-4·36–4·34)
Bullying victimization	6·1 (2·41–12·2)	6·16 (2·41–12·5)	5·68 (2·24–11·8)	0·847 (-8·51–10·5)	-7·77 (-13·2—3·21)	4·71 (1·91–9·63)	5·05 (2–10·2)	4·77 (1·88–9·85)	7·25 (-4·54–18·6)	-5·63 (-12·4—0·813)
High LDL cholesterol	56·8 (51·9–61·5)	45·6 (41·8–49·4)	45·5 (41·3–49·6)	-19·8 (-27—12·9)	-0·233 (-6·02–5·77)	--	--	--	--	--

Supplementary Results Table 1. Essex age-standardised Summary Exposure Values (SEVs) for most-detailed risk factors and percentage change, by sex, 1990, 2010, and 2019. Data in parentheses are 95% uncertainty intervals.

	Male					Female				
	1990	2010	2019	Percentage change 1990-2010	Percentage change 2010-2019	1990	2010	2019	Percentage change 1990-2010	Percentage change 2010-2019
Unsafe sanitation	0·602 (0·221–1·26)	0·325 (0·124–0·692)	0·256 (0·0921–0·562)	-46 (-81·3–67·2)	-21·3 (-64·2–81·1)	0·602 (0·221–1·26)	0·325 (0·124–0·692)	0·256 (0·0921–0·562)	-46 (-81·3–67·2)	-21·3 (-64·2–81·1)
Ambient particulate matter pollution	18·5 (6·86–33·9)	12·2 (8·39–16·4)	9·5 (5·97–13·3)	-34·1 (-63·9–54·4)	-22·1 (-29·2—18·1)	18·5 (6·86–33·8)	12·2 (8·37–16·3)	9·5 (5·96–13·3)	-34·1 (-63·9–53·9)	-22·1 (-29·2—18·1)
Household air pollution from solid fuels	0·0208 (0·00451–0·0614)	0·00692 (0·00128–0·0218)	0·00441 (0·000741–0·0149)	-66·7 (-90·2–1·17)	-36·3 (-70·3–36·3)	0·0273 (0·00652–0·0774)	0·00911 (0·00174–0·0274)	0·00582 (0·00105–0·0196)	-66·6 (-90·1–2·52)	-36·1 (-70·2–36·9)
Ambient ozone pollution	15·2 (4·92–30·2)	12·6 (3–27·7)	11·8 (3·03–25·9)	-16·9 (-62·3–42·6)	-6·68 (-52·6–88·2)	15·2 (4·92–30·2)	12·6 (3–27·7)	11·8 (3·03–25·9)	-16·9 (-62·3–42·6)	-6·68 (-52·6–88·2)
Residential radon	13·3 (2·87–40)	13·3 (2·87–40)	13·3 (2·87–40)	0 (0–0)	0 (0–0)	13·3 (2·87–40)	13·3 (2·87–40)	13·3 (2·87–40)	0 (0–0)	0 (0–0)
Lead exposure	24·8 (1·32–49·7)	16·1 (0·835–38·2)	11·2 (0·289–31·2)	-35·1 (-61·8—19·8)	-30·1 (-68·3—18·3)	10·6 (5·52e-12–32·2)	6·37 (3·54e-12–23·9)	4·45 (2·8e-12–19·6)	-39·6 (-100–19·1)	-30·2 (-100–58·5)
Child underweight	0·912 (0·503–1·37)	0·777 (0·431–1·19)	0·766 (0·411–1·17)	-14·8 (-32–7·36)	-1·45 (-16·1–13·5)	1·07 (0·594–1·59)	0·938 (0·537–1·4)	0·923 (0·522–1·39)	-12·3 (-28·9–7·87)	-1·6 (-14·7–13·3)
Iron deficiency	--	--	--	--	--	9·97 (8·02–12)	9·12 (7·32–10·9)	9·09 (7·31–11·1)	-8·47 (-22·2–6·36)	-0·354 (-9·96–9·74)
Vitamin A deficiency	4·64 (0·927–13·5)	1·96 (0·421–5·87)	1·6 (0·326–4·56)	-57·7 (-90–89·5)	-18·2 (-66–127)	3·44 (1·06–8·01)	2·19 (0·711–4·93)	1·88 (0·639–4·43)	-36·4 (-76·7–99)	-13·9 (-54·6–91·5)
Zinc deficiency	4·14 (0–12·4)	2·76 (0–8·76)	2·76 (0–8·58)	-33·4 (-100–NA)	0·261 (-26·2–NA)	3·98 (0–12)	2·62 (0–8·38)	2·61 (0–8·41)	-34·2 (-100–NA)	-0·324 (-25·9–NA)
Smoking	28 (24·5–31·8)	20·8 (18·1–23·8)	18·4 (15·9–21·2)	-25·5 (-31·9—18·7)	-11·5 (-16·3—6·63)	21·4 (18·1–25·3)	17·2 (14·6–20·3)	13·6 (11·3–16·3)	-19·4 (-29·9—7·13)	-20·8 (-27·8—13·3)
Secondhand smoke	45·6 (39·9–51·6)	32·6 (28·3–37·4)	32·3 (27·9–37)	-28·5 (-39·3—16)	-0·909 (-10·1–9·62)	47·9 (43–52·5)	33·4 (29·6–36·7)	33·1 (29·7–36·4)	-30·2 (-38·4—21·1)	-1·01 (-8·57–7·43)
Alcohol use	17·6 (9·51–26·1)	21·3 (12·2–30·6)	20·5 (11·1–30·6)	21·3 (-17·4–79·9)	-3·77 (-28·2–24·5)	--	--	--	--	--
Drug use	0·313 (0·156–0·628)	0·579 (0·329–1·03)	0·561 (0·315–1·01)	85·3 (44·3–150)	-3·16 (-13·5–6·45)	0·201 (0·109–0·38)	0·322 (0·191–0·541)	0·324 (0·189–0·567)	60·3 (22·2–110)	0·606 (-9·41–10·5)
High fasting plasma glucose	8·48 (7·02–10·4)	17·1 (14·6–20·1)	21·7 (18·2–26·6)	102 (68·2–146)	27 (10·1–50·3)	7·27 (6·06–8·78)	12·3 (10·7–14·3)	15·7 (13·4–18·6)	69·6 (42·4–102)	27·7 (10·9–48·8)
High systolic blood pressure	43 (35·7–51·9)	26·7 (21·4–32·4)	28 (20·9–36·1)	-37·7 (-51·9—17·2)	4·72 (-19·2–32·4)	--	--	--	--	--
High body-mass index	19·5 (13·3–27)	26·3 (18·8–35·5)	28 (19·9–37·4)	34·9 (18·7–58·7)	6·45 (-0·9–15·3)	19·7 (12·3–33)	27 (18·3–42·6)	28·1 (18·8–43·6)	36·8 (4·5–87·2)	4·21 (-13·1–23·9)
Low bone mineral density	8·79 (4·67–15·2)	7·59 (3·78–13)	9·19 (4·96–15·4)	-13·6 (-35·5–13·6)	21 (-9·62–61·9)	14·7 (9·26–21·3)	13·9 (8·8–21·1)	13·7 (8·63–20·5)	-5·28 (-27·3–23·4)	-1·36 (-23·4–27·9)
Diet low in fruits	66·8 (55·3–78·7)	59·7 (47–71·9)	58·5 (45·6–71·1)	-10·6 (-23·7–0·926)	-1·93 (-11·9–8·03)	67·8 (56·4–79·1)	60·3 (47·8–73)	59·5 (46·8–72·3)	-11·1 (-23·3–0·0236)	-1·38 (-10·4–9·65)
Diet low in vegetables	52·5 (31·8–72·9)	47·9 (28·1–69·6)	46·9 (26·3–68·3)	-8·75 (-26–6·71)	-2·16 (-15·2–10·8)	49·7 (29·9–71·3)	44·7 (26·4–65·8)	43·6 (24·3–65·7)	-9·94 (-28·8–7·17)	-2·65 (-16·1–10·8)
Diet low in whole grains	90·1 (84·7–95·1)	89·7 (83·7–95)	89·8 (83·9–94·9)	-0·437 (-3·44–2·4)	0·121 (-1·76–1·91)	88·3 (82·1–93·9)	88·1 (81·6–93·7)	88·2 (81·8–93·7)	-0·287 (-3·41–3·05)	0·104 (-1·92–2·11)
Diet low in nuts and seeds	82·7 (42·4–93·3)	75·2 (39–88)	77·4 (40·4–89·4)	-9·05 (-22·4–1·35)	2·83 (-4·5–13·5)	82·9 (43·8–93·2)	75·1 (37·3–88·3)	76·2 (39·6–88·6)	-9·41 (-23·9–1·41)	1·43 (-6·5–11·2)
Diet low in milk	59·2 (33·6–80·3)	60·5 (35·5–81·2)	60·5 (34·9–81·5)	2·21 (-8·76–16·5)	0·0254 (-6·57–6·96)	61·7 (36·1–82·4)	64·3 (38–85·3)	64·2 (38·4–85·7)	4·2 (-4·06–17·8)	-0·198 (-5·8–5·59)
Diet high in red meat	66 (50·3–79·3)	51·1 (36·6–65·7)	53·2 (36·9–68)	-22·6 (-41·6—2·51)	4·26 (-14·1–26·8)	64·9 (48·1–79·3)	51·9 (38·1–66·2)	54·5 (39·8–70·3)	-20 (-38·2—0·058)	5·07 (-11·1–25·8)
Diet high in processed meat	77 (53·4–97)	81·5 (57·7–98·8)	81·7 (59·8–98·9)	5·91 (-2·49–21·5)	0·193 (-5·26–6·65)	80·1 (58·6–98·2)	84·8 (64·5–99·6)	85·1 (66·3–99·4)	5·89 (-2·28–20·8)	0·27 (-4·69–5·85)
Diet high in sugar-sweetened beverages	41·3 (26·7–59·8)	45·8 (29·7–65·4)	47·5 (30·9–65)	10·8 (-25·6–65)	3·71 (-22–38·7)	41·7 (25·7–60·6)	46·8 (29·4–65·3)	48·6 (31·6–67)	12·1 (-27·1–68·5)	3·91 (-21·1–39·9)
Diet low in fiber	42·7 (26·1–59·3)	35·4 (19·1–52·8)	35·3 (19·9–52·6)	-17 (-42·2–12·9)	-0·23 (-19·8–23·3)	42·9 (25·7–61·2)	35·5 (19·7–52·4)	35·3 (19·2–52)	-17·2 (-43–12·6)	-0·381 (-19·8–23·8)
Diet low in seafood omega-3 fatty acids	94·7 (84·8–100)	92·6 (80·4–100)	92 (79·5–100)	-2·21 (-12·6–5·29)	-0·648 (-10·8–10·6)	95·6 (87·5–100)	93·7 (83·9–100)	93·1 (81·7–100)	-1·97 (-10·5–3·67)	-0·686 (-8·78–6·4)
Diet low in polyunsaturated fatty acids	54·6 (16·5–94·9)	49·6 (14–92·3)	48·5 (13·3–91·7)	-9·17 (-26–0)	-2·14 (-12·3–5·81)	56·9 (17·8–96·2)	52·2 (15·2–94·2)	51·1 (15·1–93·5)	-8·28 (-23·6–0)	-2·04 (-11·1–5·6)
Diet high in trans fatty acids	57·5 (46·6–100)	50·7 (39·8–98·9)	50·7 (39·4–98·5)	-11·9 (-25·4–0·415)	0·00659 (-14·2–16·4)	55·7 (44·5–100)	47·6 (36·2–100)	47·5 (35·8–100)	-14·6 (-28·8–0)	-0·326 (-12·7–15)
Diet high in sodium	19·7 (4·26–42·7)	22·5 (4·74–44·6)	25·7 (6·06–50·3)	14·4 (-42·7–184)	14·1 (-26·6–103)	11·8 (3·2–30·4)	12·2 (3·16–30·5)	13·5 (3·36–33·6)	3·58 (-43·6–102)	9·91 (-31·1–80·3)
Low physical activity	4·36 (1·82–8·97)	4·98 (2·18–9·99)	5·08 (2·34–10·2)	14·1 (-1·4–37·1)	2·13 (-10·4–17)	6·83 (3·57–12·6)	7·88 (4·25–14·3)	8·05 (4·38–14·3)	15·4 (4·51–32·5)	2·22 (-6·32–12·8)
Occupational asthmagens	21·6 (17·4–26·6)	19·9 (15·8–24·8)	21·7 (16·9–28·6)	-7·55 (-24–16·1)	8·73 (-7·44–30·3)	11·6 (9·11–14·9)	10·3 (8·41–12·7)	11·3 (8·94–14·2)	-10·9 (-28·6–8·4)	9·67 (-4·78–26·4)
Occupational particulate matter, gases, and fumes	8·59 (6–12·4)	8·18 (5·82–11·9)	7·98 (5·68–11·5)	-4·72 (-12·8–3·82)	-2·52 (-6·77–1·52)	3·87 (2·54–6·04)	3·67 (2·48–5·49)	3·5 (2·39–5·09)	-5·29 (-17·9–11·1)	-4·58 (-10·5–2·18)
Occupational noise	8·39 (7·57–9·4)	8·18 (7·59–8·99)	8·04 (7·45–8·81)	-2·48 (-7·49–3·05)	-1·74 (-4·19–0·785)	4·91 (4·15–5·75)	5·4 (4·87–6·05)	5·53 (5·02–6·13)	9·96 (0·542–22·1)	2·51 (-1·17–6·23)
Occupational injuries	--	--	--	--	--	--	--	--	--	--
Occupational ergonomic factors	11·6 (9·64–14·1)	10·9 (9·19–13·1)	11·3 (9·38–13·7)	-5·67 (-17·8–7·63)	3·26 (-6·92–15·1)	--	--	--	--	--
Childhood sexual abuse	10·6 (6·74–14·7)	11 (7·04–15·8)	10·7 (6·96–15·2)	4·38 (-2·5–12·8)	-2·79 (-11–6·54)	13·3 (9·72–18·3)	14·1 (10·3–19·6)	13·7 (10·1–18·8)	6·28 (-2·16–15·1)	-2·58 (-10·4–8·27)
Intimate partner violence	--	--	--	--	--	22·8 (12·5–31·3)	20 (11·3–28·2)	22·8 (12·5–31·3)	-12·2 (-19·2–0·696)	13·9 (2·98–28·7)
Non-exclusive breastfeeding	26 (19·6–34)	23·3 (17·7–30·2)	23·4 (17·7–30·7)	-10·1 (-25·8–7·56)	0·491 (-11·5–15)	26 (19·6–34)	23·3 (17·7–30·2)	23·4 (17·7–30·7)	-10·1 (-25·8–7·58)	0·496 (-11·5–15)
Discontinued breastfeeding	27·9 (26·9–28·8)	27·5 (26·5–28·5)	27·7 (26·6–28·8)	-1·39 (-6·15–3·59)	0·708 (-3·07–4·55)	27·9 (26·9–28·8)	27·6 (26·5–28·5)	27·7 (26·6–28·8)	-1·31 (-6·07–3·68)	0·613 (-3·16–4·45)
Diet low in calcium	20·1 (9·31–38·3)	14·5 (6·14–27)	14·3 (6·12–26·4)	-27·6 (-47—12·9)	-1·39 (-10·7–9·35)	14·7 (5·72–28·6)	9·05 (3·38–17·3)	8·87 (3·09–16·4)	-38·3 (-61·3—17·8)	-2 (-17–15·2)
Occupational exposure to asbestos	22·7 (20·9–24·5)	21·2 (20·3–22·2)	19·1 (16·4–21·5)	-6·6 (-14·9–2·25)	-10·1 (-23·2–1·75)	4·77 (2·72–6·37)	4·73 (2·29–5·7)	5·13 (2·29–7·32)	-0·839 (-32·4–35·1)	8·63 (-20·5–44·3)
Occupational exposure to arsenic	0·473 (0–1·1)	0·505 (0–1·22)	0·522 (0–1·23)	6·73 (-13·6–NA)	3·38 (-9·39–NA)	0·408 (0–0·971)	0·421 (0–0·975)	0·445 (0–1·06)	3·29 (-17–NA)	5·66 (-7·76–NA)
Occupational exposure to benzene	0·899 (0·0512–2·49)	0·88 (0·0533–2·45)	0·919 (0·0573–2·64)	-2·11 (-11·6–13·1)	4·41 (-2·49–16·5)	1·17 (0·0614–3·34)	1·45 (0·0854–4·33)	1·58 (0·0937–4·56)	24·2 (8·79–48·6)	8·89 (-0·143–22·5)
Occupational exposure to beryllium	0·0245 (0·0231–0·0259)	0·0258 (0·0243–0·0271)	0·0268 (0·0251–0·0284)	5·29 (-1·78–12·6)	3·95 (-1·55–9·65)	0·0253 (0·023–0·0276)	0·0284 (0·0264–0·0302)	0·0306 (0·0284–0·033)	12·3 (0·86–25·2)	7·91 (1·34–15·5)
Occupational exposure to cadmium	0·0556 (0·0486–0·0636)	0·0592 (0·0515–0·0669)	0·0614 (0·0529–0·0702)	6·52 (-11·8–27)	3·68 (-8·21–16·9)	0·0497 (0·0425–0·0587)	0·052 (0·0458–0·0589)	0·055 (0·0481–0·0638)	4·54 (-13·9–26)	5·91 (-5·89–20·3)
Occupational exposure to chromium	0·132 (0·117–0·149)	0·139 (0·123–0·155)	0·145 (0·128–0·164)	5·49 (-10·6–23·7)	4·29 (-6·69–16·4)	0·127 (0·111–0·146)	0·136 (0·122–0·151)	0·145 (0·129–0·163)	7·03 (-9·23–25)	6·63 (-3·54–18·5)
Occupational exposure to diesel engine exhaust	0·725 (0·657–0·796)	0·754 (0·692–0·818)	0·796 (0·724–0·879)	4 (-7·5–16·6)	5·59 (-3·08–15·2)	0·651 (0·569–0·735)	0·667 (0·589–0·746)	0·718 (0·625–0·822)	2·49 (-12·4–21·2)	7·63 (-3·73–21·2)
Occupational exposure to formaldehyde	0·276 (0·24–0·32)	0·282 (0·243–0·321)	0·29 (0·248–0·333)	1·96 (-16·6–23·4)	2·96 (-9·49–17·2)	0·243 (0·207–0·285)	0·262 (0·231–0·296)	0·278 (0·244–0·316)	7·74 (-10·3–28·2)	6·2 (-4·97–19)
Occupational exposure to nickel	0·458 (0–2·04)	0·492 (0–2·19)	0·513 (0–2·29)	7·56 (-9·87–NA)	4·14 (-7·34–NA)	0·323 (0–1·46)	0·326 (0–1·47)	0·34 (0–1·48)	0·934 (-20·5–NA)	4·07 (-9·2–NA)
Occupational exposure to polycyclic aromatic hydrocarbons	0·278 (0·25–0·309)	0·291 (0·26–0·321)	0·304 (0·27–0·337)	4·81 (-9·9–21)	4·4 (-5·94–15·5)	--	--	--	--	--
Occupational exposure to silica	5·59 (0·773–17·6)	6·14 (0·836–19·9)	6·45 (0·899–20·3)	9·73 (-8·31–35)	5·14 (-7·03–19·3)	2·88 (0·421–9·53)	2·81 (0·391–8·55)	2·93 (0·399–9·46)	-2·72 (-23·5–25·6)	4·37 (-9·79–24·6)
Occupational exposure to sulfuric acid	0·848 (0·176–2·76)	0·853 (0·178–2·59)	0·862 (0·183–2·81)	0·585 (-17–18·6)	1·06 (-11·2–14·2)	--	--	--	--	--
Unsafe sex	--	--	--	--	--	--	--	--	--	--
Occupational exposure to trichloroethylene	0·0757 (0·0697–0·0831)	0·0792 (0·0725–0·0862)	0·0827 (0·0748–0·0908)	4·73 (-6·66–17·9)	4·33 (-4·05–13·6)	0·077 (0·0687–0·086)	0·0814 (0·0738–0·0895)	0·0871 (0·0785–0·097)	5·75 (-7·46–22·5)	7·04 (-1·99–18·3)
No access to handwashing facility	3·36 (3·08–3·69)	3·2 (2·92–3·49)	3·16 (2·89–3·46)	-4·72 (-14·2–5·06)	-1·2 (-7·39–6·15)	3·36 (3·08–3·69)	3·2 (2·92–3·49)	3·16 (2·89–3·46)	-4·72 (-14·2–5·06)	-1·2 (-7·39–6·15)
Child wasting	1·26 (0·864–1·67)	1·22 (0·85–1·64)	1·21 (0·837–1·63)	-3·18 (-15·7–10·2)	-0·533 (-9·25–9·25)	--	--	--	--	--
Child stunting	1·63 (0·789–2·53)	1·22 (0·538–1·92)	1·2 (0·539–1·9)	-25·3 (-44·5–0·903)	-1·35 (-22·3–23·9)	1·92 (0·969–2·92)	1·58 (0·797–2·42)	1·55 (0·784–2·41)	-17·8 (-37·5–8·38)	-1·71 (-18·7–18·4)
Chewing tobacco	0·173 (0·113–0·264)	0·163 (0·109–0·233)	0·167 (0·113–0·239)	-5·93 (-41·9–46·1)	2·34 (-26·2–38·1)	0·129 (0·0763–0·219)	0·127 (0·0762–0·206)	0·129 (0·0736–0·215)	-1·41 (-47·3–93·5)	1·53 (-33·6–59·1)
Diet low in legumes	74 (37·4–99·2)	73·2 (37–98·9)	73·9 (38·6–99·1)	-1·07 (-10·6–7·32)	0·944 (-5·32–11·7)	70·4 (30·7–98)	69·4 (29–97·4)	70·2 (30·2–97·6)	-1·53 (-15·6–6·19)	1·15 (-5·37–13)
Short gestation	19·8 (15·8–24·2)	47·2 (37·2–57·9)	41·8 (32·1–54·3)	139 (81·7–214)	-11·4 (-31·7–15·1)	21·7 (18·6–25·4)	41 (34·3–48·3)	36·2 (29·8–44·1)	88·4 (56·1–122)	-11·6 (-27·8–6·64)
Low birth weight	10·6 (8·7–12·9)	22·7 (17·9–27·9)	20 (15·3–25·6)	114 (63·3–187)	-12 (-33·5–16·8)	11·6 (9·93–13·4)	20·5 (17·3–24·3)	18·5 (15·3–22)	77·4 (46–115)	-9·7 (-27·3–10·3)
High temperature	21·1 (6·06–37·9)	0 (0–0)	21·2 (6·11–37·9)	-100 (-100—100)	--	21·1 (6·06–37·9)	0 (0–0)	21·2 (6·11–37·9)	-100 (-100—100)	--
Low temperature	31·2 (26–37)	34·1 (29·4–39·1)	30·5 (25·3–36·3)	9·2 (5·46–14)	-10·4 (-14·4—6·98)	31·2 (26–37)	34·1 (29·4–39·1)	30·5 (25·3–36·3)	9·2 (5·46–14)	-10·4 (-14·4—6·98)
Kidney dysfunction	13·7 (9·14–20·1)	14·2 (9·74–20·5)	14 (9·6–20·5)	3·66 (-0·842–9·73)	-1·45 (-6·41–3·74)	14·6 (9·73–21·2)	15 (10·2–21·4)	15 (10–21·8)	2·6 (-1·43–7·7)	0·335 (-4·08–4·45)
Bullying victimization	6·13 (2·43–12·3)	6·09 (2·37–12·2)	5·64 (2·24–11·6)	-0·665 (-10·2–8·78)	-7·34 (-13—2·72)	4·7 (1·92–9·62)	5·03 (2–10·2)	4·75 (1·86–9·76)	7·05 (-4·93–18·7)	-5·52 (-12·3—0·595)
High LDL cholesterol	55·9 (51·9–60)	45·2 (41·4–48·9)	45·1 (41·3–49·1)	-19·1 (-25·5—12·3)	-0·0628 (-5–5·12)	--	--	--	--	--

Supplementary Results Table 1. Hertfordshire age-standardised Summary Exposure Values (SEVs) for most-detailed risk factors and percentage change, by sex, 1990, 2010, and 2019. Data in parentheses are 95% uncertainty intervals.

	Male					Female				
	1990	2010	2019	Percentage change 1990-2010	Percentage change 2010-2019	1990	2010	2019	Percentage change 1990-2010	Percentage change 2010-2019
Unsafe sanitation	0·384 (0·136–0·872)	0·207 (0·0736–0·445)	0·171 (0·06–0·388)	-46 (-83·2–87·8)	-17·7 (-64·2–85·8)	0·384 (0·136–0·872)	0·207 (0·0736–0·445)	0·171 (0·06–0·388)	-46 (-83·2–87·8)	-17·7 (-64·2–85·8)
Ambient particulate matter pollution	17·8 (6·59–32·3)	11·9 (8·07–15·8)	8·99 (5·59–12·6)	-33·4 (-64·7–59·6)	-24·3 (-31·7–19·9)	17·8 (6·58–32·3)	11·9 (8·07–15·7)	8·99 (5·58–12·6)	-33·4 (-64·7–60)	-24·3 (-31·7–19·9)
Household air pollution from solid fuels	0·0106 (0·00205–0·0321)	0·00351 (0·000552–0·0115)	0·00238 (0·000331–0·00869)	-66·9 (-91–8·09)	-32·4 (-70·8–46·4)	0·014 (0·0029–0·0441)	0·00464 (0·000767–0·0146)	0·00314 (0·000475–0·0103)	-66·8 (-91–8·26)	-32·2 (-70·6–46·5)
Ambient ozone pollution	3·98 (0·683–10·1)	11·7 (2·64–26·9)	11·9 (2·39–28)	195 (23·6–746)	1·14 (-58·3–145)	3·98 (0·683–10·1)	11·7 (2·64–26·9)	11·9 (2·39–28)	195 (23·6–746)	1·14 (-58·3–145)
Residential radon	17·7 (3·17–50·7)	17·7 (3·17–50·7)	17·7 (3·17–50·7)	0 (0–0)	0 (0–0)	17·7 (3·17–50·7)	17·7 (3·17–50·7)	17·7 (3·17–50·7)	0 (0–0)	0 (0–0)
Lead exposure	19·3 (0·082–45·3)	12·2 (0·0754–33·6)	8·42 (0·0156–26·8)	-36·8 (-65·7–41·2)	-30·9 (-80·1–18·9)	8·68 (2·99e-12–29·2)	5·06 (2·45e-12–21·2)	3·47 (2·16e-12–17·2)	-41·8 (-100–57·6)	-31·4 (-100–107)
Child underweight	0·882 (0·492–1·33)	0·761 (0·402–1·16)	0·754 (0·392–1·17)	-13·7 (-31–8·98)	-0·974 (-15·8–17)	1·04 (0·601–1·54)	0·913 (0·523–1·36)	0·898 (0·508–1·35)	-11·9 (-28–8·73)	-1·6 (-14·5–14·8)
Iron deficiency	--	--	--	--	--	9·81 (7·89–12)	9·02 (7·29–10·9)	8·89 (7·2–10·8)	-8·1 (-22·8–8·46)	-1·4 (-11–9·04)
Vitamin A deficiency	3·27 (0·611–9·59)	1·41 (0·28–4·18)	1·21 (0·234–3·59)	-56·9 (-91·1–120)	-14·4 (-65·4–162)	2·68 (0·848–6·6)	1·64 (0·521–3·73)	1·46 (0·463–3·49)	-38·7 (-79·8–116)	-11·3 (-54·2–91·4)
Zinc deficiency	3·12 (0–9·9)	2·15 (0–6·93)	2·2 (0–7·08)	-31·1 (-100–NA)	2·36 (-22–NA)	3·04 (0–9·61)	2·06 (0–6·8)	2·1 (0–6·96)	-32·2 (-100–NA)	2·08 (-19·9–NA)
Smoking	27·8 (24·2–31·6)	21·2 (18·5–24·2)	18·9 (16·3–21·8)	-23·6 (-30–16·8)	-11·1 (-15·9–5·9)	21·2 (17·9–25·1)	17·5 (14·7–20·7)	13·9 (11·5–16·5)	-17·6 (-27·8–4·95)	-20·2 (-27·6–12·4)
Secondhand smoke	46·6 (41–53)	32·6 (28·1–37·3)	32·2 (27·8–37)	-30 (-41·3–17·6)	-1·16 (-11·2–9·96)	48·7 (43·6–53·5)	33·3 (29·6–36·7)	32·9 (29·5–36·5)	-31·7 (-40·8–22·4)	-1·01 (-8·68–6·9)
Alcohol use	19·3 (11–27·8)	21·5 (12·4–31·1)	21·6 (12·5–31·3)	11·4 (-23·8–65·9)	0·409 (-24·8–27)	--	--	--	--	--
Drug use	0·32 (0·159–0·655)	0·592 (0·338–1·04)	0·536 (0·29–0·967)	85·3 (46·3–144)	-9·47 (-19·6–0·621)	0·204 (0·113–0·391)	0·322 (0·189–0·547)	0·308 (0·174–0·553)	57·5 (21–106)	-4·33 (-15·7–5·44)
High fasting plasma glucose	8·16 (6·89–9·95)	16·8 (14·5–19·5)	22·6 (19·1–27)	106 (73·2–147)	34·7 (16–59·7)	6·99 (5·81–8·55)	12·3 (10·7–14·4)	16·8 (14·2–19·8)	76·3 (46·9–110)	36·2 (17·5–59·4)
High systolic blood pressure	44·9 (36·6–53·6)	28·6 (22·8–35)	30 (22·5–38·4)	-36·3 (-51–18·3)	4·71 (-18·7–33·3)	--	--	--	--	--
High body-mass index	23·4 (16·1–32)	31·1 (22·2–40·9)	32·2 (23·5–42·3)	32·8 (19·3–53·5)	3·5 (-3·55–11·4)	23·7 (15–38·1)	31·7 (22·1–47·5)	32·6 (22–50)	33·8 (4·19–78)	2·84 (-12·2–20·4)
Low bone mineral density	6·87 (3·31–12·3)	6·27 (2·99–11·3)	5·45 (2·42–10·1)	-8·67 (-32·4–20·2)	-13 (-38–15·5)	11·1 (6·64–17·2)	11·3 (6·93–17·5)	9·67 (5·6–15·2)	2·37 (-19·4–30·4)	-14·5 (-35·6–9·14)
Diet low in fruits	63·2 (50·9–75·3)	56·4 (42·5–69·5)	55·4 (42·3–68·9)	-10·8 (-24·7–3·34)	-1·76 (-12·7–9·83)	64·3 (52·1–76·2)	57·2 (43·8–70·1)	56·5 (42·8–70·1)	-11 (-25·2–1·52)	-1·32 (-12·1–10·1)
Diet low in vegetables	47·3 (27·9–69·5)	42·7 (24–63·9)	41·9 (23·6–63·7)	-9·78 (-29·3–10·4)	-1·86 (-14·7–12·3)	44·3 (25·4–65·8)	39·2 (21·5–61·4)	38·2 (21–59·6)	-11·5 (-33·3–8·47)	-2·64 (-19·4–15·4)
Diet low in whole grains	89·5 (84–94·5)	89·1 (82·8–94·6)	89·3 (83–94·6)	-0·421 (-3·45–3·04)	0·152 (-1·83–2·22)	--	--	--	--	--
Diet low in nuts and seeds	80·9 (41·7–92·2)	73 (34·8–85·8)	75·5 (38–88·1)	-9·83 (-22·7–1·9)	3·5 (-4·55–15)	80·9 (41·1–91·9)	72·5 (34·1–86·3)	73·8 (35·5–87·6)	-10·4 (-24·4–1·34)	1·86 (-7·51–13)
Diet low in milk	55·1 (30·7–74·7)	56·8 (33·1–76·8)	57 (33·3–77·7)	3·13 (-8·24–20·8)	0·377 (-8·16–9·76)	57·9 (33·8–78)	61 (35·2–81·6)	61 (35·2–81·4)	5·33 (-4·32–22·9)	0·0119 (-7·2–7·43)
Diet high in red meat	69·2 (53·4–83·3)	54 (39·2–68·2)	56·2 (40·8–71·9)	-21·9 (-40·4–1·92)	3·98 (-11–24)	68·4 (52·8–82·2)	54·7 (39·6–69)	57·2 (41–72)	-19·9 (-39·2–0·566)	4·41 (-12·5–24·5)
Diet high in processed meat	77·9 (54·6–97·2)	82·3 (61·3–99)	82·4 (62·4–98·9)	5·65 (-2·62–20·5)	0·0913 (-5·66–6·52)	80·9 (58–98·4)	85·8 (66·5–99·5)	85·8 (67·4–99·6)	5·98 (-2·22–22)	0·182 (-5·11–6·11)
Diet high in sugar-sweetened beverages	47·6 (28·7–74·5)	52·1 (31·9–73·7)	53·9 (33·4–75·7)	9·43 (-27·2–66·9)	3·44 (-20·4–35·7)	--	--	--	--	--
Diet low in fiber	37·7 (21·4–55·2)	30·9 (15·9–47·5)	31 (17·1–47·6)	-17·9 (-44–16·3)	0·179 (-21·8–28·7)	38 (20·5–56·3)	30·9 (16·5–48·9)	31 (16·1–48·5)	-18·6 (-46·3–16·5)	0·377 (-21–26·8)
Diet low in seafood omega-3 fatty acids	91·3 (78·3–100)	88·4 (73·2–100)	87·8 (71·6–100)	-3·17 (-15·9–8·21)	-0·709 (-14·5–13·9)	92·5 (81–100)	90·3 (77·2–100)	90 (76·1–100)	-2·33 (-13·2–5·34)	-0·377 (-11·5–11·1)
Diet low in polyunsaturated fatty acids	49·6 (14·5–91·9)	44·8 (12·4–88·1)	43·9 (11·8–87·3)	-9·71 (-26·9–0·747)	-1·96 (-12·5–8·19)	52·2 (14·9–93·7)	47·6 (13–90·8)	46·8 (13–90·4)	-8·72 (-26·4–0·321)	-1·74 (-11·6–8·35)
Diet high in trans fatty acids	57·6 (46·3–100)	50·9 (39·8–98)	50·7 (39·1–98·2)	-11·7 (-24·8–0·356)	-0·337 (-14·6–16)	55·9 (44·6–100)	47·6 (36·1–99·9)	47·6 (35·8–99·7)	-14·8 (-29·9–0)	-0·0672 (-12·5–16·8)
Diet high in sodium	19·6 (4·13–42·5)	22·6 (4·66–44·4)	25·9 (5·05–48·9)	15·1 (-41·5–169)	14·7 (-22·8–113)	12 (3·07–30·9)	12·4 (3·05–31)	13·5 (3·3–33·4)	2·74 (-44·7–95·8)	8·96 (-29·8–74·5)
Low physical activity	4·63 (1·97–9·31)	5·54 (2·52–10·9)	5·63 (2·58–11·4)	19·7 (2·4–44·2)	1·53 (-10·4–16·7)	7·18 (3·8–13·3)	8·38 (4·59–14·7)	8·47 (4·65–14·9)	16·7 (5·69–33·6)	1·15 (-6·87–10·7)
Occupational asthmagens	21·1 (16·9–26·8)	19·7 (15·7–24·8)	21·6 (16·7–28·7)	-6·31 (-24·5–17·4)	9·39 (-7·93–34)	11·7 (9·13–15)	10·4 (8·36–13)	11·4 (8·92–14·4)	-10·9 (-30·4–11)	9·78 (-5·09–28·3)
Occupational particulate matter, gases, and fumes	8·46 (5·85–12·3)	8·06 (5·7–11·7)	7·86 (5·53–11·5)	-4·73 (-12·9–3·97)	-2·46 (-6·84–2·03)	3·7 (2·44–5·63)	3·49 (2·4–5·11)	3·34 (2·31–4·91)	-5·77 (-18·4–9·89)	-4·33 (-10·5–2·63)
Occupational noise	8·24 (7·4–9·17)	8·06 (7·44–8·85)	7·94 (7·32–8·73)	-2·18 (-7·6–3·52)	-1·56 (-4·28–1·09)	4·9 (4·16–5·74)	5·4 (4·84–6·06)	5·55 (5·05–6·16)	10·2 (0·533–22·4)	2·76 (-0·747–7)
Occupational injuries	--	--	--	--	--	--	--	--	--	--
Occupational ergonomic factors	11 (9·21–13·4)	10·3 (8·67–12·5)	10·7 (8·93–13·1)	-6·38 (-18·7–6·36)	3·8 (-6·14–15·6)	--	--	--	--	--
Childhood sexual abuse	10·6 (6·74–14·7)	11 (7·04–15·8)	10·7 (6·96–15·2)	4·38 (-2·5–12·8)	-2·79 (-11–6·54)	13·3 (9·72–18·3)	14·1 (10·3–19·6)	13·7 (10·1–18·8)	6·28 (-2·16–15·1)	-2·58 (-10·4–8·27)
Intimate partner violence	--	--	--	--	--	22·8 (12·5–31·3)	20 (11·3–28·2)	22·8 (12·5–31·3)	-12·2 (-19·2–0·696)	13·9 (2·98–28·7)
Non-exclusive breastfeeding	25·7 (19·1–33·7)	23·2 (17·5–30·1)	23·3 (17·3–30·7)	-9·94 (-25·2–8·25)	0·35 (-13·5–16·5)	25·7 (19·1–33·7)	23·2 (17·5–30·1)	23·3 (17·3–30·6)	-9·93 (-25·2–8·26)	0·352 (-13·5–16·5)
Discontinued breastfeeding	28·2 (27·1–29·1)	27·7 (26·7–28·6)	27·5 (26·4–28·5)	-1·85 (-6·4–3·27)	-0·605 (-4·3–3·42)	28·2 (27·1–29·1)	27·7 (26·7–28·6)	27·5 (26·4–28·5)	-1·72 (-6·28–3·41)	-0·663 (-4·36–3·36)
Diet low in calcium	16·6 (7·57–31·2)	11·2 (4·61–20·7)	11·1 (4·7–20·7)	-32·8 (-50–18·3)	-0·0641 (-10·8–13)	11·1 (4·16–21·4)	5·66 (2·14–10·8)	5·69 (2·14–10·9)	-48·8 (-68·6–28·5)	0·43 (-19·8–25·6)
Occupational exposure to asbestos	20·6 (18·8–22·4)	18·4 (17·2–19·5)	16·3 (13·8–18·9)	-10·8 (-19·9–0·344)	-11·4 (-24·7–4·01)	4·5 (2·74–5·93)	4·48 (2·29–5·35)	4·51 (2·11–6·46)	-0·473 (-34·3–33·4)	0·786 (-26·1–38·9)
Occupational exposure to arsenic	0·464 (0–1·1)	0·493 (0–1·16)	0·51 (0–1·22)	6·2 (-12·5–NA)	3·52 (-9·4–NA)	0·401 (0–0·967)	0·413 (0–0·972)	0·437 (0–1·06)	3·01 (-15·8–NA)	5·66 (-7·74–NA)
Occupational exposure to benzene	0·888 (0·0513–2·56)	0·867 (0·0517–2·47)	0·915 (0·0524–2·59)	-2·41 (-11·7–11·8)	5·6 (-2·39–16·4)	1·22 (0·0766–3·6)	1·5 (0·093–4·25)	1·63 (0·104–4·85)	22·6 (7·03–49·5)	8·68 (-1·36–23·2)
Occupational exposure to beryllium	0·0242 (0·0228–0·0258)	0·0254 (0·024–0·0269)	0·0265 (0·0249–0·0283)	5·03 (-2·57–13·8)	4·21 (-1·07–10·5)	0·0258 (0·0233–0·0282)	0·0289 (0·0269–0·031)	0·0311 (0·0288–0·0338)	12·1 (1·19–25·2)	7·56 (0·152–15·3)
Occupational exposure to cadmium	0·0546 (0·0476–0·063)	0·0579 (0·0511–0·066)	0·0601 (0·0516–0·0693)	6·03 (-11·1–26·8)	3·88 (-7·95–18)	0·0492 (0·0417–0·0575)	0·0514 (0·0453–0·059)	0·0545 (0·0481–0·0627)	4·48 (-13–27·3)	5·86 (-5·8–18·8)
Occupational exposure to chromium	0·13 (0·115–0·148)	0·137 (0·122–0·154)	0·143 (0·125–0·162)	5·04 (-10·8–23·8)	4·5 (-6·3–16·9)	0·127 (0·11–0·145)	0·136 (0·122–0·152)	0·145 (0·13–0·163)	6·85 (-8·34–25·8)	6·51 (-3·4–16·9)
Occupational exposure to diesel engine exhaust	0·729 (0·66–0·807)	0·754 (0·69–0·826)	0·796 (0·72–0·876)	3·5 (-9·23–16·4)	5·61 (-3·13–14·6)	0·656 (0·569–0·755)	0·674 (0·596–0·763)	0·721 (0·624–0·84)	2·69 (-14·1–22)	6·97 (-5·75–21·4)
Occupational exposure to formaldehyde	0·271 (0·233–0·315)	0·275 (0·239–0·315)	0·284 (0·241–0·329)	1·52 (-15·9–23·4)	3·18 (-9·48–18·1)	0·243 (0·207–0·282)	0·261 (0·232–0·295)	0·277 (0·245–0·315)	7·44 (-9·8–29·5)	6·19 (-4·6–18·2)
Occupational exposure to nickel	0·448 (0–1·97)	0·481 (0–2·12)	0·501 (0–2·23)	7·38 (-9·36–NA)	4·29 (-7·29–NA)	0·312 (0–1·38)	0·315 (0–1·36)	0·33 (0–1·44)	1·03 (-19–NA)	4·78 (-9·2–NA)
Occupational exposure to polycyclic aromatic hydrocarbons	0·273 (0·244–0·306)	0·285 (0·257–0·317)	0·298 (0·264–0·333)	4·31 (-9·86–20·1)	4·66 (-5·08–15·9)	--	--	--	--	--
Occupational exposure to silica	5·55 (0·807–17·8)	6·03 (0·849–19·1)	6·34 (0·851–20·3)	8·77 (-8·97–35)	5·15 (-7·3–21·2)	2·74 (0·368–8·26)	2·68 (0·352–8·54)	2·8 (0·364–8·81)	-2·15 (-22·6–25·8)	4·46 (-9·84–24·7)
Occupational exposure to sulfuric acid	0·835 (0·18–2·66)	0·831 (0·171–2·62)	0·84 (0·177–2·67)	-0·55 (-18·8–19·5)	1·09 (-11·3–14·8)	--	--	--	--	--
Unsafe sex	--	--	--	--	--	--	--	--	--	--
Occupational exposure to trichloroethylene	0·075 (0·0682–0·0825)	0·0782 (0·0717–0·0859)	0·0817 (0·074–0·0903)	4·35 (-8·01–18·1)	4·47 (-3·78–14·5)	0·0775 (0·0683–0·0876)	0·0818 (0·0744–0·0906)	0·0873 (0·079–0·098)	5·64 (-8·13–22)	6·71 (-2·9–17·6)
No access to handwashing facility	3·29 (3·01–3·59)	3·15 (2·87–3·44)	3·12 (2·86–3·4)	-4·28 (-13·9–6·01)	-1·07 (-7·12–6·08)	3·29 (3·01–3·59)	3·15 (2·87–3·44)	3·12 (2·86–3·4)	-4·28 (-13·9–6·01)	-1·07 (-7·12–6·08)
Child wasting	1·24 (0·863–1·66)	1·2 (0·826–1·63)	1·2 (0·827–1·63)	-3·46 (-15·6–9·7)	-0·362 (-8·99–9·89)	--	--	--	--	--
Child stunting	1·59 (0·767–2·43)	1·19 (0·519–1·91)	1·19 (0·531–1·89)	-24·8 (-44·2–1·27)	-0·329 (-22·7–25)	1·86 (0·959–2·84)	1·55 (0·744–2·38)	1·53 (0·76–2·38)	-16·8 (-36·9–10·3)	-1·24 (-19·1–19·4)
Chewing tobacco	0·173 (0·115–0·259)	0·164 (0·11–0·241)	0·169 (0·112–0·244)	-4·89 (-44·1–56·1)	3·05 (-23·7–37·4)	0·131 (0·0745–0·221)	0·128 (0·0728–0·209)	0·131 (0·0736–0·222)	-2·29 (-49–89·2)	1·7 (-33·8–56·8)
Diet low in legumes	72·3 (34·5–99)	71·6 (33·6–98·6)	72·4 (35·2–98·6)	-1·06 (-12·5–7·92)	1·19 (-5·41–12)	68·5 (28·5–97·3)	67·5 (27·1–96·2)	68·4 (27·1–95·5)	-1·42 (-15–9·02)	1·36 (-7·54–16·2)
Short gestation	18·1 (14·6–22·5)	43·3 (33·7–54·7)	38·6 (30·2–49·1)	140 (81·5–217)	-11 (-32·3–15·1)	21·8 (17·7–26·6)	43·1 (35·4–53)	39·1 (31·9–47·4)	97·2 (47·4–168)	-9·25 (-28·4–12·9)
Low birth weight	9·98 (8·17–12·2)	20·8 (16·6–25·7)	18·7 (14·7–23·7)	109 (58·6–178)	-10·5 (-32·7–16·7)	12·2 (10·1–14·8)	21·2 (17·2–25·8)	19·7 (16·2–23·4)	72·9 (30·1–136)	-6·71 (-27·2–18·4)
High temperature	22·1 (6·52–39·2)	0 (0–0)	21·8 (6·35–38·4)	-100 (-100–100)	--	22·1 (6·52–39·2)	0 (0–0)	21·8 (6·35–38·4)	-100 (-100–100)	--
Low temperature	30·4 (24·9–36·5)	33·2 (28·3–38·7)	29·7 (24·3–35·7)	9·02 (5·18–13·8)	-10·5 (-14·8–6·73)	30·4 (24·9–36·5)	33·2 (28·3–38·7)	29·7 (24·3–35·7)	9·02 (5·18–13·8)	-10·5 (-14·8–6·73)
Kidney dysfunction	13·6 (9–19·9)	14 (9·63–20·2)	13·8 (9·4–20)	2·86 (-1·74–8·8)	-1·24 (-5·64–3·27)	14·6 (9·72–21·2)	14·9 (10·1–21·5)	14·9 (10–21·6)	1·8 (-2·42–6·96)	0·377 (-4·14–5·4)
Bullying victimization	6·15 (2·45–12·2)	6·12 (2·4–12·4)	5·67 (2·24–11·7)	-0·487 (-10–9·04)	-7·27 (-12·7–2·8)	4·7 (1·92–9·57)	5·03 (2–10·2)	4·75 (1·87–9·77)	7·03 (-5·03–19)	-5·55 (-12·3–0·711)
High LDL cholesterol	57·5 (53·2–61·7)	46·6 (42·7–50·6)	46·3 (42·2–50·4)	-18·9 (-25·8–12·2)	-0·664 (-6·26–5·07)	--	--	--	--	--

Supplementary Results Table 1. Luton age-standardised Summary Exposure Values (SEVs) for most-detailed risk factors and percentage change, by sex, 1990, 2010, and 2019. Data in parentheses are 95% uncertainty intervals.

	Male					Female				
	1990	2010	2019	Percentage change 1990-2010	Percentage change 2010-2019	1990	2010	2019	Percentage change 1990-2010	Percentage change 2010-2019
Unsafe sanitation	0·635 (0·216–1·41)	0·345 (0·115–0·792)	0·258 (0·0872–0·608)	-45·7 (-83·7–83·7)	-25·1 (-69–70·4)	0·635 (0·216–1·41)	0·345 (0·115–0·792)	0·258 (0·0872–0·608)	-45·7 (-83·7–83·7)	-25·1 (-69–70·4)
Ambient particulate matter pollution	17·6 (6·19–32·3)	11·6 (7·86–15·7)	8·78 (5·38–12·5)	-34·1 (-64·9–61·1)	-24·3 (-32–-19·7)	17·6 (6·2–32·3)	11·6 (7·84–15·7)	8·78 (5·37–12·5)	-34·1 (-64·9–60·2)	-24·3 (-31·9–-19·8)
Household air pollution from solid fuels	0·0117 (0·00247–0·0358)	0·00387 (0·000703–0·0121)	0·00246 (0·000374–0·00909)	-67·1 (-90·4–8·53)	-36·5 (-71·6–44·8)	0·0154 (0·00336–0·0456)	0·00511 (0·00098–0·0157)	0·00325 (0·00053–0·0115)	-66·9 (-90·4–9·78)	-36·4 (-71·6–45)
Ambient ozone pollution	2·34 (0–13)	14·6 (0·0691–50·7)	15·3 (0·154–49·4)	524 (-64·3–NA)	4·84 (-98–8090)	2·34 (0–13)	14·6 (0·0691–50·7)	15·3 (0·154–49·4)	524 (-64·3–NA)	4·84 (-98–8090)
Residential radon	18·9 (3·19–56·3)	18·9 (3·19–56·3)	18·9 (3·19–56·3)	0 (0–0)	0 (0–0)	18·9 (3·19–56·3)	18·9 (3·19–56·3)	18·9 (3·19–56·3)	0 (0–0)	0 (0–0)
Lead exposure	27·2 (2·69–53·4)	17·4 (1·44–40·5)	12 (0·491–32·3)	-36·1 (-61·6–-22·5)	-30·9 (-67·1–-19·1)	11·3 (0·00196–33·5)	6·76 (5·46e-12–24·6)	4·68 (3·41e-12–19·8)	-40·4 (-100–-23·2)	-30·8 (-100–-16·5)
Child underweight	0·95 (0·53–1·43)	0·798 (0·426–1·19)	0·779 (0·41–1·19)	-16 (-32·5–7·13)	-2·31 (-18·2–14·6)	1·11 (0·65–1·66)	0·959 (0·536–1·43)	0·936 (0·533–1·41)	-13·9 (-31–6·78)	-2·39 (-16·1–14·4)
Iron deficiency	--	--	--	--	--	10·8 (8·64–13·3)	9·89 (7·96–12·2)	9·58 (7·67–11·7)	-8·57 (-24·5–9·78)	-3·1 (-12·8–7·43)
Vitamin A deficiency	4·64 (0·885–13·7)	2·04 (0·403–6·26)	1·65 (0·3–4·98)	-56·1 (-90·5–129)	-18·9 (-69·8–150)	3·59 (1·02–9·02)	2·29 (0·696–5·38)	1·92 (0·589–4·39)	-36·3 (-78·7–114)	-16 (-58·8–76·2)
Zinc deficiency	3·63 (0–11·2)	2·44 (0–7·97)	2·54 (0–8·02)	-33 (-100–NA)	4·39 (-22·9–NA)	3·49 (0–10·7)	2·31 (0–7·6)	2·41 (0–7·84)	-33·7 (-100–NA)	4·32 (-19·2–NA)
Smoking	28·3 (24·7–32·2)	22·3 (19·3–25·8)	20 (17·3–23·3)	-21·2 (-28·3–-13·8)	-10·1 (-15·7–-4·18)	21·6 (17·9–25·6)	18·2 (15·2–21·8)	14·7 (12–17·9)	-15·6 (-28·4–-0·0136)	-19·1 (-27·5–-10·1)
Secondhand smoke	47 (41–53·2)	34·3 (29·5–39·5)	33·7 (28·6–38·5)	-27·1 (-38·2–-12·8)	-1·72 (-10·9–9·08)	48·9 (43·6–53·9)	35 (31–39·1)	34·4 (30·6–38·2)	-28·4 (-37·2–-18)	-1·45 (-9·45–6·58)
Alcohol use	16·9 (9·07–25·1)	17·8 (9·43–26·6)	17·1 (8·76–26)	5·75 (-34·6–69)	-4·18 (-32·3–30)	--	--	--	--	--
Drug use	0·316 (0·154–0·65)	0·618 (0·356–1·08)	0·554 (0·305–1·02)	95·7 (49·5–164)	-10·4 (-20·7–-1·88)	--	--	--	--	--
High fasting plasma glucose	10·1 (8·43–12·4)	20·1 (17·2–23·8)	27 (22·6–33·1)	98·3 (66–142)	34·3 (14·7–62·9)	8·47 (7·06–10·3)	14·8 (12·8–17·5)	19·6 (16·8–22·9)	75·2 (45·3–110)	32·3 (13·8–54·9)
High systolic blood pressure	43·6 (33·8–54·2)	27·3 (20·8–34·9)	28·8 (20·7–38·4)	-37·4 (-54·5–-12·6)	5·67 (-22·4–40·1)	--	--	--	--	--
High body-mass index	16·1 (10·6–22·8)	22·2 (15·2–30·3)	23·7 (16·7–31·7)	38·3 (20·3–65·2)	6·81 (-0·331–15·9)	17·2 (10·9–29·3)	24 (16–38·8)	25·2 (16·6–41·1)	39·4 (-2·02–95·8)	5·33 (-12·5–26·4)
Low bone mineral density	8·95 (4·83–14·9)	7·8 (4·18–13·7)	9·48 (5·09–15·8)	-12·9 (-33·8–12·3)	21·6 (-6·98–65)	14·8 (9·44–21·9)	14·2 (9·01–21)	14 (8·89–20·3)	-4·15 (-25·2–24·7)	-1·82 (-24·5–27·6)
Diet low in fruits	65·1 (52·6–77·4)	58·3 (45–71·5)	57·5 (43·7–70·4)	-10·5 (-25·1–2·9)	-1·34 (-12·5–10)	66·4 (54·8–78·3)	59 (46–71·9)	58·6 (44·7–72·1)	-11·3 (-24·7–1·88)	-0·638 (-11·5–10·7)
Diet low in vegetables	50·7 (30·6–72·5)	46·1 (27·9–66·9)	45·5 (26·4–67·4)	-9·06 (-27·6–9·19)	-1·35 (-15·1–13·3)	48·1 (28·4–70·3)	42·9 (24·1–63·9)	42·1 (23·3–61·8)	-10·8 (-31·2–10·3)	-1·81 (-17·8–15·3)
Diet low in whole grains	89·9 (84·1–94·9)	89·4 (83·5–94·8)	89·6 (83–94·9)	-0·461 (-4·01–2·76)	0·171 (-2·05–2·28)	88·1 (81·9–93·7)	87·9 (81·3–93·8)	88 (81–93·8)	-0·267 (-3·8–3·42)	0·138 (-1·98–2·52)
Diet low in nuts and seeds	82·1 (41·6–92·8)	74·7 (39·5–87·5)	76·5 (41·5–88·8)	-8·99 (-22·6–2·66)	2·44 (-5·76–13·3)	82·2 (42·8–93·3)	74·5 (37·4–88·3)	75·5 (37·9–88·9)	-9·36 (-24·5–2·05)	1·25 (-8·03–11·6)
Diet low in milk	57·7 (33·4–78·7)	59·1 (33·9–79·5)	59·4 (34·4–80·3)	2·38 (-9·83–18·5)	0·641 (-6·15–8·73)	60·2 (35·1–81)	62·9 (37·7–83·3)	63·1 (37·7–83·8)	4·41 (-4·59–20·5)	0·35 (-6·08–7·15)
Diet high in red meat	67 (51·1–81·5)	52·4 (37·9–67·3)	54·1 (37·7–69)	-21·8 (-40·8–-0·178)	3·3 (-14·5–24·5)	66·1 (50·2–80·2)	53·4 (38·4–68·6)	55·6 (39·2–71)	-19·2 (-38·7–3·31)	4·22 (-13·4–24·6)
Diet high in processed meat	77·1 (51·9–96·4)	81·8 (59·5–98·3)	81·8 (60·8–98·3)	6·05 (-2·96–22·6)	0·0252 (-5·85–6·13)	80·2 (58–98)	85·1 (65·9–99·3)	85·2 (66·7–99·4)	6·15 (-2·29–21·8)	0·139 (-5·48–6·13)
Diet high in sugar-sweetened beverages	47·9 (29·1–73·8)	52·3 (33·4–74·8)	53·1 (33·3–75·6)	9·21 (-27·5–67·1)	1·59 (-24·5–33·9)	--	--	--	--	--
Diet low in fiber	41·1 (24·9–58)	34·1 (18·9–51·4)	34·3 (19·3–51·8)	-17 (-45·4–17)	0·358 (-20·4–26·5)	41·4 (23·9–60·3)	34·1 (18·7–51)	34·4 (18·7–51·5)	-17·7 (-44·2–13·7)	0·788 (-19·3–27)
Diet low in seafood omega-3 fatty acids	92·8 (82·2–100)	90·8 (77·8–100)	89·7 (75·5–100)	-2·15 (-13·1–6·1)	-1·22 (-12·6–10·2)	94 (84·1–100)	92·1 (79·9–100)	91·7 (79·9–100)	-2·03 (-12–6·87)	-0·398 (-9·57–8·72)
Diet low in polyunsaturated fatty acids	52·8 (15·8–94·1)	47·9 (13·5–89·4)	47·3 (13–89·6)	-9·34 (-27–1·86)	-1·08 (-10·9–9·16)	55·2 (16·6–95)	50·5 (14·8–92·3)	50 (14·4–92·5)	-8·56 (-25·1–0·805)	-0·977 (-9·62–7·59)
Diet high in trans fatty acids	57·8 (46·1–100)	51 (40·1–96·6)	51 (39·3–98·6)	-11·7 (-24·8–2·36)	-0·0635 (-15·4–17·2)	56·1 (44·6–100)	47·9 (35·7–100)	47·8 (36–99·6)	-14·5 (-30–0)	-0·224 (-13·2–17·3)
Diet high in sodium	20 (4·31–43·2)	23·1 (5·26–46·3)	26·4 (6·01–49·9)	15·2 (-44·5–175)	14·3 (-28·2–104)	12·1 (3–31·2)	12·6 (3·32–30·6)	13·7 (3·36–33·5)	3·89 (-43·9–127)	8·81 (-32·6–70)
Low physical activity	4·37 (1·84–8·9)	5·06 (2·23–10·2)	5·17 (2·35–10·5)	15·8 (0·157–41·6)	2·3 (-9·87–18·4)	6·86 (3·58–12·8)	7·96 (4·28–14·3)	8·1 (4·34–14·6)	16·1 (5·41–33·8)	1·73 (-6·48–11·7)
Occupational asthmagens	22·5 (17·6–28·7)	21·1 (16·4–27·1)	22·8 (17·1–30·7)	-6·58 (-27·7–20·8)	8·49 (-11·3–34·8)	10·6 (8·19–14·1)	9·62 (7·59–12)	10·7 (8·26–13·7)	-9·48 (-32·6–17·2)	11·1 (-5·14–30·7)
Occupational particulate matter, gases, and fumes	8·72 (5·96–12·7)	8·27 (5·79–12·1)	8·06 (5·66–11·8)	-5·14 (-14·1–5·01)	-2·54 (-7·25–2·23)	3·84 (2·51–5·94)	3·55 (2·41–5·37)	3·37 (2·28–5·08)	-7·45 (-22·3–10)	-5·07 (-11·7–3·03)
Occupational noise	8·34 (7·43–9·33)	8·15 (7·43–8·93)	8·01 (7·36–8·82)	-2·29 (-8·13–4·41)	-1·63 (-4·37–1·21)	4·89 (3·98–5·79)	5·41 (4·81–6·14)	5·56 (5·01–6·21)	10·8 (0·261–24·6)	2·82 (-1·34–7·09)
Occupational injuries	--	--	--	--	--	--	--	--	--	--
Occupational ergonomic factors	10·3 (8·48–12·6)	9·81 (8·06–12)	10·2 (8·31–12·4)	-4·71 (-16·4–8·29)	3·59 (-5·82–14·7)	5·46 (4·2–7·2)	6·71 (5·14–8·52)	7·37 (5·5–9·95)	22·7 (-5·03–56·1)	9·94 (-9·34–35·6)
Childhood sexual abuse	10·6 (6·74–14·7)	11 (7·04–15·8)	10·7 (6·96–15·2)	4·38 (-2·5–12·8)	-2·79 (-11–6·54)	13·3 (9·72–18·3)	14·1 (10·3–19·6)	13·7 (10·1–18·8)	6·28 (-2·16–15·1)	-2·58 (-10·4–8·27)
Intimate partner violence	--	--	--	--	--	22·8 (12·5–31·3)	20 (11·3–28·2)	22·8 (12·5–31·3)	-12·2 (-19·2–0·696)	13·9 (2·98–28·7)
Non-exclusive breastfeeding	26·2 (19·2–34·3)	23·5 (17·8–31·1)	23·5 (17·7–30·9)	-10·1 (-25·2–8·14)	-0·0592 (-14–14·8)	26·2 (19·2–34·3)	23·5 (17·7–31·1)	23·5 (17·7–30·9)	-10·1 (-25·2–8·15)	-0·0538 (-14–14·8)
Discontinued breastfeeding	28·8 (27·7–29·8)	28·2 (27–29·2)	27·8 (26·6–28·8)	-2·26 (-7·14–2·9)	-1·3 (-5·22–2·56)	28·7 (27·6–29·7)	28·4 (27·2–29·4)	28 (26·8–29)	-1·32 (-6·25–3·88)	-1·47 (-5·36–2·39)
Diet low in calcium	19 (8·74–35·7)	13·5 (5·74–25·8)	13·6 (5·9–26)	-29·2 (-47·1–-14·7)	0·833 (-9·82–12·8)	13·6 (5·52–26)	7·84 (2·94–14·7)	8·01 (2·9–15·2)	-42·2 (-64·2–-20·6)	2·14 (-17·3–23·3)
Occupational exposure to asbestos	22·1 (20·2–24·2)	20·3 (18·9–21·8)	18·8 (15·7–21·7)	-8·13 (-17·4–3·51)	-7·56 (-22·2–7·7)	5·21 (2·83–6·89)	5·33 (2·6–6·47)	5·41 (2·41–7·97)	2·3 (-31–37·9)	1·57 (-25·8–36·7)
Occupational exposure to arsenic	0·51 (0–1·23)	0·538 (0–1·29)	0·553 (0–1·36)	5·6 (-14–NA)	2·66 (-10·8–NA)	0·41 (0–0·971)	0·422 (0–1)	0·443 (0–1·07)	2·88 (-19·4–NA)	5·04 (-8·57–NA)
Occupational exposure to benzene	0·921 (0·0504–2·57)	0·898 (0·0535–2·52)	0·939 (0·0563–2·68)	-2·51 (-13·15·4)	4·6 (-4·36–16·5)	1·2 (0·069–3·58)	1·49 (0·0853–4·33)	1·63 (0·0935–4·76)	23·6 (6·68–52)	9·68 (-0·836–25·6)
Occupational exposure to beryllium	0·0247 (0·0231–0·0264)	0·026 (0·0242–0·0278)	0·027 (0·0252–0·0291)	5·24 (-3·28–15·2)	4·01 (-2·35–10)	0·0247 (0·0218–0·0277)	0·0279 (0·0256–0·0303)	0·0302 (0·0273–0·033)	12·9 (-0·195–29·4)	8·07 (-0·527–16·5)
Occupational exposure to cadmium	0·0594 (0·0509–0·0688)	0·0626 (0·0543–0·0721)	0·0646 (0·0545–0·0753)	5·47 (-13–27·6)	3·22 (-9·4–18·1)	0·0498 (0·0411–0·0594)	0·0519 (0·0449–0·0605)	0·0547 (0·0476–0·0644)	4·17 (-16·2–30·4)	5·45 (-6·85–20·1)
Occupational exposure to chromium	0·141 (0·123–0·16)	0·147 (0·129–0·167)	0·153 (0·132–0·175)	4·48 (-11·7–24·1)	3·8 (-7·79–17·6)	0·127 (0·108–0·149)	0·136 (0·12–0·154)	0·144 (0·128–0·165)	6·53 (-10·9–28·5)	6·36 (-4·52–18·7)
Occupational exposure to diesel engine exhaust	0·721 (0·648–0·801)	0·749 (0·681–0·825)	0·792 (0·711–0·882)	3·89 (-9·47–19·2)	5·69 (-4–16)	0·604 (0·511–0·702)	0·616 (0·541–0·704)	0·661 (0·566–0·773)	2·04 (-15·7–22·6)	7·2 (-6·22–22·5)
Occupational exposure to formaldehyde	0·299 (0·253–0·346)	0·301 (0·258–0·35)	0·308 (0·259–0·364)	0·785 (-17·5–23·6)	2·21 (-11·3–17·9)	0·249 (0·208–0·296)	0·266 (0·233–0·304)	0·282 (0·248–0·326)	7·16 (-11·6–31·8)	5·93 (-6·33–19·4)
Occupational exposure to nickel	0·476 (0–2·04)	0·51 (0–2·19)	0·529 (0–2·3)	7·13 (-10·5–NA)	3·71 (-9·74–NA)	0·321 (0–1·41)	0·324 (0–1·4)	0·336 (0–1·45)	0·945 (-23·1–NA)	3·5 (-10·4–NA)
Occupational exposure to polycyclic aromatic hydrocarbons	0·294 (0·26–0·33)	0·305 (0·27–0·342)	0·317 (0·278–0·359)	3·98 (-10·5–21·3)	3·93 (-6·64–16·2)	0·273 (0·234–0·315)	0·301 (0·27–0·335)	0·322 (0·287–0·362)	10·5 (-6·28–30·8)	6·85 (-3·59–18·1)
Occupational exposure to silica	5·58 (0·774–17·9)	6·08 (0·851–19·5)	6·37 (0·928–19·8)	8·94 (-9·47–36·3)	4·8 (-8·34–20·7)	2·72 (0·408–8·52)	2·66 (0·363–8·4)	2·74 (0·357–8·45)	-2·01 (-25·5–33·5)	3·01 (-12–25·5)
Occupational exposure to sulfuric acid	0·885 (0·191–2·82)	0·888 (0·184–2·78)	0·894 (0·182–2·79)	0·357 (-17·7–21·2)	0·674 (-12·3–15·5)	--	--	--	--	--
Unsafe sex	--	--	--	--	--	--	--	--	--	--
Occupational exposure to trichloroethylene	0·0795 (0·0721–0·088)	0·0826 (0·0749–0·0916)	0·086 (0·0775–0·096)	3·99 (-8·6–19·3)	4·08 (-4·9–14·1)	0·0753 (0·0642–0·087)	0·0793 (0·0711–0·089)	0·0847 (0·0752–0·0959)	5·29 (-9·65–25·1)	6·72 (-3·44–17·8)
No access to handwashing facility	3·37 (3·04–3·69)	3·2 (2·9–3·54)	3·16 (2·88–3·46)	-4·85 (-15·2–6·34)	-1·41 (-8·15–6·3)	3·37 (3·04–3·69)	3·2 (2·9–3·54)	3·16 (2·88–3·46)	-4·85 (-15·2–6·34)	-1·41 (-8·15–6·3)
Child wasting	1·27 (0·878–1·71)	1·23 (0·851–1·66)	1·22 (0·834–1·66)	-3·57 (-16·5–10·8)	-0·818 (-10–9·31)	--	--	--	--	--
Child stunting	1·7 (0·82–2·61)	1·25 (0·569–1·97)	1·23 (0·563–1·98)	-26·7 (-46·4–0·359)	-1·43 (-22·3–23·9)	2·03 (1·01–3·12)	1·64 (0·789–2·54)	1·59 (0·794–2·48)	-18·9 (-39·8–8·44)	-2·95 (-21·7–18·2)
Chewing tobacco	0·177 (0·109–0·275)	0·169 (0·107–0·252)	0·173 (0·111–0·263)	-4·46 (-42·6–62·5)	2·17 (-28·8–41·8)	0·135 (0·0735–0·228)	0·133 (0·0726–0·232)	0·134 (0·0736–0·228)	-1·27 (-49·5–111)	0·539 (-35·8–62·6)
Diet low in legumes	73·4 (36·4–99·2)	72·6 (36·2–98·8)	73·5 (38·2–98·9)	-1·2 (-12·4–8·68)	1·36 (-4·72–12·3)	69·8 (30–97·4)	68·7 (28·4–97·2)	69·7 (29·9–97·2)	-1·58 (-16·2–8·98)	1·45 (-6·18–16·1)
Short gestation	28·1 (22·9–35·1)	57·4 (45–71·3)	46·9 (34·2–61·8)	104 (51·2–171)	-18·3 (-40·6–11·8)	30·7 (25·6–36·2)	54·1 (45–64·2)	44·9 (33·8–56·6)	75·9 (42·2–118)	-17 (-36·5–6·59)
Low birth weight	11·8 (9·62–14·7)	24·5 (19·3–30·8)	20·4 (14·8–26·7)	107 (50·5–175)	-16·9 (-39·6–13·3)	13·2 (11·1–15·5)	23·9 (20·1–28·3)	20·1 (15·4–25·2)	81·4 (45·4–123)	-16 (-36·3–7·45)
High temperature	22·3 (6·57–39·2)	0 (0–0)	0 (0–0)	-100 (-100–-100)	--	22·3 (6·57–39·2)	0 (0–0)	0 (0–0)	-100 (-100–-100)	--
Low temperature	30·5 (25–36·7)	33·3 (28·5–38·8)	30 (24·7–36)	9·09 (5·3–13·8)	-9·91 (-13·7–-6·39)	30·5 (25–36·7)	33·3 (28·5–38·8)	30 (24·7–36)	9·09 (5·3–13·8)	-9·91 (-13·7–-6·39)
Kidney dysfunction	14 (9·41–20·4)	14·6 (10·1–21·2)	14·5 (10–20·9)	4·71 (-0·449–11)	-0·68 (-5·65–4·46)	14·8 (10–21·5)	15·3 (10·4–21·6)	15·4 (10·4–22·1)	2·97 (-1·5–8·13)	0·93 (-3·33–5·68)
Bullying victimization	6·09 (2·43–12)	6·02 (2·37–12·2)	5·63 (2·22–11·7)	-1·06 (-10·4–8·3)	-6·53 (-11·8–-1·9)	4·62 (1·9–9·44)	4·93 (1·96–9·99)	4·72 (1·84–9·66)	6·8 (-5·13–18·6)	-4·42 (-11–0·585)
High LDL cholesterol	56·2 (51·5–60·8)	45·2 (41·4–49·2)	45·4 (41·5–49·6)	-19·5 (-26·9–-12·1)	0·319 (-5·57–6·54)	--	--	--	--	--

Supplementary Results Table 1. Albania age-standardised Summary Exposure Values (SEVs) for most-detailed risk factors and percentage change, by sex, 1990, 2010, and 2019. Data in parentheses are 95% uncertainty intervals.

	Male					Female				
	1990	2010	2019	Percentage change 1990-2010	Percentage change 2010-2019	1990	2010	2019	Percentage change 1990-2010	Percentage change 2010-2019
Unsafe sanitation	32·1 (23–42·6)	16·2 (12–20·7)	10·8 (6·53–16·4)	-49·6 (-66·3–-23·7)	-33·5 (-56–-4)	32·1 (23–42·6)	16·2 (12–20·7)	10·8 (6·53–16·4)	-49·6 (-66·3–-23·7)	-33·5 (-56–-4)
Ambient particulate matter pollution	14·8 (6·74–25·3)	18·2 (14·3–22·5)	16·7 (13·2–20·7)	23·6 (-26·1–144)	-8·59 (-13·7–-1·66)	12·8 (5·82–22·2)	16·9 (12·8–21·4)	15·8 (12·2–19·9)	32·5 (-22·7–159)	-6·49 (-12–1·17)
Household air pollution from solid fuels	20·8 (10·5–35·3)	6·86 (3·03–13·1)	4·42 (1·66–9·8)	-66·9 (-78·8–-53·3)	-35·6 (-54·9–-15·9)	23·2 (13·4–37·7)	8·4 (4·12–14·9)	5·52 (2·25–11·5)	-63·9 (-77·3–-50·1)	-34·3 (-53·9–-14·6)
Ambient ozone pollution	77 (34·3–100)	57·5 (24–96)	26·6 (9·77–49·7)	-25·3 (-39·2–-3·96)	-53·7 (-66·2–-40·3)	77 (34·3–100)	57·5 (24–96)	26·6 (9·77–49·7)	-25·3 (-39·2–-3·96)	-53·7 (-66·2–-40·3)
Residential radon	53 (21·9–96·9)	53 (21·9–96·9)	53 (21·9–96·9)	0 (0–0)	0 (0–0)	--	--	--	--	--
Lead exposure	82·8 (62·4–94·3)	71·9 (53·7–86)	62·7 (43·5–79·5)	-13·2 (-23–-6·06)	-12·8 (-20–-6·93)	52·2 (21·6–76·1)	48·3 (23·5–70·1)	41·4 (18·6–63·7)	-7·46 (-17·7–23·4)	-14·3 (-22·7–-8·01)
Child underweight	5·16 (3·82–6·39)	2·86 (2·01–3·69)	2·61 (1·8–3·45)	-44·5 (-54·2–-34·6)	-8·75 (-19·1–1·77)	6·02 (4·72–7·26)	3·47 (2·45–4·43)	3·13 (2·19–4·07)	-42·4 (-52·4–-33·5)	-9·56 (-18·4–0·268)
Iron deficiency	--	--	--	--	--	18·5 (16·3–20·9)	15·1 (13·3–17)	15·3 (13·4–17·3)	-18 (-25·9–-9·84)	1·05 (-4·44–7·36)
Vitamin A deficiency	50·2 (27·9–69·6)	21·3 (10·3–37)	16·3 (7·87–30·5)	-57·5 (-77·6–-20·1)	-23·7 (-52·1–21·2)	39·4 (23–54)	20·4 (11·7–31·6)	16·5 (9·29–26·9)	-48·2 (-67·2–-14·6)	-19·3 (-44·5–18·5)
Zinc deficiency	8·57 (0·808–21·4)	3·68 (0–11·2)	2·82 (0–9·23)	-57 (-100–-45·2)	-23·4 (-100–NA)	8·26 (0·773–20·6)	3·59 (0–10·9)	2·74 (0–9·01)	-56·6 (-100–-45·1)	-23·6 (-100–NA)
Smoking	28·1 (24·7–31·6)	27·4 (24·8–30·2)	29·3 (26·4–32·3)	-2·66 (-10·2–5·22)	7·09 (2·69–11·7)	2·8 (2·15–3·61)	3·19 (2·58–3·84)	4·37 (3·58–5·17)	14·2 (-9·98–45·5)	36·9 (19·9–57·5)
Secondhand smoke	39·4 (37–42)	36·5 (35·3–37·8)	36·6 (34·4–38·9)	-7·42 (-14·1–-0·475)	0·311 (-4·45–4·95)	58·3 (55·9–60·5)	53·3 (51·9–54·7)	53·7 (51·4–55·8)	-8·55 (-12·7–-3·66)	0·704 (-2·15–3·58)
Alcohol use	5·25 (3·01–8·66)	12·5 (8·87–16·8)	12·2 (8·16–17·1)	138 (82·2–225)	-2·13 (-18·6–16·4)	1·59 (0·853–2·87)	4·21 (2·82–6·16)	3·63 (2·22–5·53)	165 (101–283)	-13·8 (-31·2–6·2)
Drug use	0·093 (0·042–0·2)	0·106 (0·0534–0·217)	0·114 (0·0593–0·225)	14·4 (3·16–32·1)	6·71 (-2·78–17·1)	0·0581 (0·0272–0·124)	0·0656 (0·0336–0·13)	0·0686 (0·0366–0·13)	12·8 (2·42–29)	4·59 (-4·22–15·5)
High fasting plasma glucose	5·51 (4·65–6·48)	6·71 (5·76–7·77)	7·78 (6·68–8·87)	21·9 (11·1–34·1)	15·9 (6·44–26·7)	4·27 (3·67–4·93)	5·48 (4·82–6·28)	6·38 (5·54–7·36)	28·3 (17·1–39·9)	16·4 (7·66–26·5)
High systolic blood pressure	41·5 (34·6–49·4)	40·1 (35·1–45·4)	42·3 (34·4–51·3)	-3·39 (-22·9–21·2)	5·42 (-10·1–25)	--	--	--	--	--
High body-mass index	16·4 (11·1–23·2)	22·9 (16·4–31·2)	26·4 (19·1–35·6)	39·4 (23·3–63·7)	15·3 (8·15–24·4)	17·6 (12·4–24·5)	23 (17·3–30·8)	26·1 (19·6–34·6)	31·2 (16·4–51·1)	13·2 (5·09–22·5)
Low bone mineral density	7·53 (3·61–13·4)	7·06 (3·41–12·2)	6·88 (3·37–12)	-6·26 (-31·1–31·1)	-2·55 (-32·6–33·4)	17·9 (11·9–25·3)	17·1 (11·4–24·4)	16·7 (10·6–24)	-4·78 (-25·5–21·3)	-2·33 (-23·8–25·2)
Diet low in fruits	53·4 (41·5–67)	21·7 (13·7–30·6)	19 (10·9–28)	-59·3 (-70·5–-46·3)	-12·6 (-33·5–8·08)	54·9 (43·3–68·2)	24 (15·4–34·1)	21·5 (13·1–31·5)	-56·3 (-67·9–-43)	-10·7 (-29·8–8·32)
Diet low in vegetables	37·5 (20·5–59·1)	3·19 (1·63–9·21)	3·1 (1·53–9·36)	-91·5 (-95·6–-73·7)	-2·83 (-24·5–24·7)	--	--	--	--	--
Diet low in whole grains	99·9 (100–100)	99·9 (100–100)	99·9 (100–100)	0 (0–0)	0 (0–0)	99·9 (100–100)	99·9 (100–100)	99·9 (100–100)	0 (0–0)	0 (0–0)
Diet low in nuts and seeds	85·5 (44·3–93·9)	58·8 (27·3–76·4)	58·1 (26·7–76·2)	-31·2 (-52·7–-14·5)	-1·07 (-8·75–8·36)	82·9 (42·7–92·6)	53·8 (23·5–73·1)	52·9 (23·7–72·4)	-35 (-57·5–-17·7)	-1·67 (-11·5–8·24)
Diet low in milk	32·8 (12·3–51·3)	3·49 (0·825–12·1)	3·34 (0·81–11·4)	-89·4 (-97·1–-71·2)	-4·4 (-33·1–58·4)	31·7 (11–51·1)	3·12 (0·806–11·2)	3·01 (0·81–10·2)	-90·2 (-97·2–-73·6)	-3·6 (-35·4–51·5)
Diet high in red meat	32·2 (21·3–42·5)	59·4 (48·9–69·4)	63·6 (50·7–74·3)	84·5 (50·6–148)	7·01 (-2·9–20)	32·1 (21·3–42·7)	59·3 (48·3–69·4)	63·1 (51–74·3)	84·6 (50·6–149)	6·53 (-3·57–18·8)
Diet high in processed meat	81·7 (60·2–99·4)	83·3 (60·4–100)	81·3 (55–100)	1·89 (-8·73–15·5)	-2·39 (-9·71–0·574)	--	--	--	--	--
Diet high in sugar-sweetened beverages	29·4 (13·8–46·5)	37·1 (21·3–55)	48·1 (27·5–66·3)	26·3 (-22·3–110)	29·6 (3·15–77·3)	33·8 (16·1–53·6)	43·8 (25·9–61·3)	54·8 (34·2–73·2)	29·6 (-16·3–120)	25·1 (2·72–68·6)
Diet low in fiber	34·9 (21–49·1)	25·3 (14·4–37·6)	19·7 (10·5–30·6)	-27·6 (-35·7–-21·2)	-22·2 (-35·1–-9·53)	34·8 (20·5–49·7)	24·9 (13·6–37·3)	19·2 (9·75–29·8)	-28·5 (-36·9–-21·6)	-22·8 (-37·8–-8·93)
Diet low in seafood omega-3 fatty acids	99·4 (97·3–100)	92·6 (81·9–100)	90·8 (78·9–100)	-6·76 (-17·3–0)	-2 (-9·85–4·96)	99·7 (99–100)	93·9 (84·9–100)	92·4 (82–100)	-5·78 (-14·6–0)	-1·6 (-8·86–4·14)
Diet low in polyunsaturated fatty acids	78·8 (42–100)	80·7 (45·9–100)	78·3 (41·7–100)	2·43 (0–8·68)	-2·93 (-9·22–0)	80 (45·1–100)	81·8 (47·3–100)	79·6 (44–100)	2·26 (0–8·38)	-2·78 (-9·27–0)
Diet high in trans fatty acids	46·4 (35·8–100)	40·2 (29·7–100)	38·8 (28·2–99)	-13·5 (-25–0)	-3·46 (-12·6–5·28)	48·3 (36·9–100)	42·2 (30·4–100)	40·9 (29·1–100)	-12·6 (-23·8–0)	-3·02 (-11·2–4·64)
Diet high in sodium	96·8 (87·6–100)	89·9 (72–98·7)	88·9 (68·8–98·1)	-7·16 (-22·8–2·26)	-1·09 (-10·1–7·56)	67·9 (43·9–85·1)	45·3 (14·6–69·4)	42·8 (12·4–68·9)	-33·3 (-70·5–-11·8)	-5·57 (-25·9–8·98)
Low physical activity	1·64 (0·721–3·81)	1·79 (0·778–3·94)	1·9 (0·847–4·11)	9·25 (-0·721–25·5)	6·46 (-3·37–21·2)	2·27 (1·21–4·12)	2·47 (1·35–4·48)	2·63 (1·44–4·69)	8·79 (-0·216–20·4)	6·39 (-1·82–16·9)
Occupational asthmagens	20·6 (15·5–26·8)	21·4 (17·2–26·5)	19·9 (16·1–24·6)	3·63 (-9·45–20·6)	-6·84 (-15·2–2·1)	12·7 (8·87–17·8)	15·4 (11·7–20·4)	15 (11·1–20·2)	21·3 (2·99–46·1)	-2·74 (-14·8–9·28)
Occupational particulate matter, gases, and fumes	8·83 (6·35–12·6)	8·32 (6·04–11·9)	7·83 (5·7–11·1)	-5·79 (-10·3–-0·768)	-5·91 (-8·3–-3·7)	4·6 (3·18–6·68)	4·86 (3·52–6·96)	4·98 (3·6–7·08)	5·64 (-3·97–17·2)	2·45 (-1·42–6·68)
Occupational noise	8·63 (7·86–9·48)	8·27 (7·76–8·92)	7·89 (7·46–8·47)	-4·23 (-7·89–-0·0218)	-4·5 (-6·16–-2·76)	4·6 (3·78–5·24)	4·83 (4·43–5·31)	5·05 (4·73–5·45)	7·76 (-0·717–18·6)	4·61 (1·35–8·08)
Occupational injuries	--	--	--	--	--	--	--	--	--	--
Occupational ergonomic factors	27·9 (24·2–31·9)	25·2 (23·5–27)	22·3 (19·6–25·3)	-9·62 (-21·1–4·93)	-11·5 (-20·8–-2·06)	--	--	--	--	--
Childhood sexual abuse	8·36 (5·45–11·9)	8·36 (5·45–11·9)	8·36 (5·45–11·9)	0 (0–0)	0 (0–0)	7·02 (5·11–9·78)	7·02 (5·11–9·78)	7·02 (5·11–9·78)	0 (0–0)	0 (0–0)
Intimate partner violence	--	--	--	--	--	10·7 (5·87–15·4)	10·7 (5·87–15·4)	10·7 (5·87–15·4)	0 (0–0)	0 (0–0)
Non-exclusive breastfeeding	26·7 (16·2–40·8)	22·4 (13·9–33·8)	23·6 (14·4–35·4)	-16·1 (-22·6–-8·91)	5·08 (-1·94–13·1)	26·7 (16·2–40·8)	22·4 (13·9–33·8)	23·6 (14·4–35·3)	-16·1 (-22·6–-8·92)	5·02 (-2–13·1)
Discontinued breastfeeding	13·4 (11·8–15)	13·3 (12·1–14·5)	13·3 (11·7–15)	-1·01 (-13·7–14·7)	0·206 (-12·6–13·5)	13·6 (12–15·2)	13·3 (12·1–14·6)	13·4 (11·8–15)	-2·21 (-14·7–13·3)	0·302 (-12·5–13·6)
Diet low in calcium	22·9 (11·4–44)	10·2 (5·11–18·2)	8·69 (4·42–15·4)	-55·7 (-65·2–-45·2)	-14·3 (-20·9–-6·72)	--	--	--	--	--
Occupational exposure to asbestos	0·472 (0·191–1·82)	0·535 (0·159–2)	0·607 (0·209–2·09)	13·2 (-41·5–83·5)	13·6 (-25·2–79)	0·857 (0·457–1·43)	0·844 (0·342–1·46)	0·853 (0·337–1·57)	-1·58 (-43·9–81·1)	1·09 (-41·2–67·3)
Occupational exposure to arsenic	0·255 (0–0·604)	0·28 (0–0·653)	0·279 (0–0·654)	9·69 (0·515–NA)	-0·396 (-6·61–NA)	0·219 (0–0·532)	0·307 (0–0·736)	0·321 (0–0·758)	39·7 (21·2–NA)	4·74 (-3·84–NA)
Occupational exposure to benzene	0·475 (0·029–1·32)	0·592 (0·035–1·7)	0·608 (0·0377–1·76)	24·7 (16–39·9)	2·67 (-2·77–11·3)	0·385 (0·0231–1·1)	0·585 (0·0354–1·68)	0·685 (0·0407–1·98)	52 (34·6–79·4)	17 (8·38–30)
Occupational exposure to beryllium	0·0185 (0·0173–0·0196)	0·0198 (0·0195–0·0201)	0·0194 (0·0187–0·0201)	7·17 (0·701–14·3)	-1·88 (-5·84–2·03)	0·0153 (0·0136–0·0172)	0·0202 (0·0198–0·0207)	0·0211 (0·02–0·0221)	32·3 (18·2–50)	4·16 (-1·39–9·81)
Occupational exposure to cadmium	0·0306 (0·0283–0·0331)	0·034 (0·0331–0·035)	0·0339 (0·032–0·0359)	11·3 (2·39–21)	-0·312 (-5·92–5·66)	0·0262 (0·023–0·0297)	0·0366 (0·0353–0·0379)	0·0385 (0·0356–0·0415)	39·9 (22·3–60·3)	5·16 (-2·82–13)
Occupational exposure to chromium	0·0634 (0·0585–0·0687)	0·078 (0·0758–0·0802)	0·079 (0·0746–0·0835)	23 (13–34·1)	1·29 (-4·24–7·32)	0·0533 (0·0468–0·0606)	0·0788 (0·076–0·0817)	0·0857 (0·0793–0·0925)	48 (29–70)	8·7 (0·654–17·1)
Occupational exposure to diesel engine exhaust	0·411 (0·377–0·45)	0·616 (0·591–0·644)	0·615 (0·576–0·654)	49·7 (35·4–64·4)	-0·23 (-6·02–5·87)	0·228 (0·201–0·259)	0·342 (0·329–0·354)	0·373 (0·345–0·403)	49·8 (32·5–70·2)	9·19 (1·25–17·7)
Occupational exposure to formaldehyde	0·125 (0·099–0·14)	0·148 (0·121–0·156)	0·149 (0·119–0·162)	18·3 (7·34–31·1)	0·914 (-5·73–8·35)	--	--	--	--	--
Occupational exposure to nickel	0·302 (0–1·29)	0·34 (0–1·46)	0·33 (0–1·44)	12·6 (2·34–NA)	-2·79 (-8·43–NA)	0·209 (0–0·914)	0·275 (0–1·21)	0·281 (0–1·21)	31·8 (15·5–NA)	2·12 (-6·15–NA)
Occupational exposure to polycyclic aromatic hydrocarbons	0·139 (0·129–0·15)	0·174 (0·169–0·178)	0·176 (0·167–0·185)	25·1 (15·8–35·7)	1·05 (-3·98–6·53)	0·112 (0·099–0·127)	0·166 (0·16–0·171)	0·182 (0·169–0·195)	47·6 (29–69·1)	9·63 (1·81–17·9)
Occupational exposure to silica	3·94 (0·57–12·2)	5·26 (0·737–17·3)	5·07 (0·728–16·4)	33·6 (18·3–53·1)	-3·52 (-11·2–6·71)	2·1 (0·28–6·84)	2·61 (0·374–8·32)	2·59 (0·372–8·38)	24·2 (10·1–45)	-0·59 (-8·27–7·57)
Occupational exposure to sulfuric acid	0·561 (0·115–1·76)	0·611 (0·125–1·95)	0·6 (0·13–1·91)	8·75 (-0·107–18·6)	-1·67 (-7·92–5·3)	0·425 (0·088–1·36)	0·578 (0·123–1·78)	0·589 (0·122–1·81)	35·9 (19·9–54·7)	1·91 (-4·97–9·06)
Unsafe sex	--	--	--	--	--	--	--	--	--	--
Occupational exposure to trichloroethylene	0·038 (0·0354–0·0409)	0·0481 (0·0469–0·0496)	0·0488 (0·0465–0·0515)	26·6 (17·5–37·2)	1·32 (-3·67–7·02)	0·0315 (0·0278–0·0357)	0·0467 (0·0453–0·0483)	0·0506 (0·0475–0·0542)	48·2 (31·2–69·2)	8·49 (1·25–16·5)
No access to handwashing facility	9·58 (9·1–10·1)	6·64 (6·29–7)	5·51 (5·23–5·83)	-30·8 (-34·8–-26·4)	-16·9 (-20·3–-13·6)	9·58 (9·1–10·1)	6·64 (6·29–7)	5·51 (5·23–5·83)	-30·8 (-34·8–-26·4)	-16·9 (-20·3–-13·6)
Child wasting	1·99 (1·52–2·43)	1·81 (1·38–2·2)	1·76 (1·34–2·18)	-9·08 (-21·9–4·15)	-2·64 (-12·3–7·91)	1·67 (1·28–2·07)	1·48 (1·08–1·86)	1·43 (1·06–1·82)	-11 (-25·9–3·25)	-3·63 (-16·4–11·7)
Child stunting	29·1 (19·8–32·9)	17·2 (11·8–20·1)	15·7 (10·8–18·8)	-41·1 (-50–-32·4)	-8·7 (-19–1·7)	--	--	--	--	--
Chewing tobacco	0·941 (0·694–1·25)	1·02 (0·77–1·37)	1·03 (0·775–1·37)	8·56 (-21·2–51·8)	0·793 (-18·9–24·7)	0·236 (0·169–0·333)	0·249 (0·172–0·351)	0·267 (0·193–0·369)	5·54 (-30–52·8)	7·22 (-17·2–37·1)
Diet low in legumes	83·4 (61·6–100)	77·1 (46·5–99·9)	76·2 (44·2–99·7)	-7·49 (-25·2–0·103)	-1·16 (-6·64–1·38)	80·6 (54·7–100)	74·1 (38·2–99·6)	73·2 (36·2–99·3)	-8·1 (-30·1–0·305)	-1·17 (-7·09–1·59)
Short gestation	9·87 (7·88–12·6)	7·72 (6·1–9·97)	8·18 (6·13–10·3)	-21·9 (-42·3–6·06)	6·08 (-19·7–40·4)	10·2 (8·08–13·1)	6·09 (4·73–7·81)	6·77 (4·81–8·86)	-40·3 (-55·7–-16·1)	11·2 (-22·8–48·7)
Low birth weight	5·1 (4·14–6·3)	3·68 (2·93–4·54)	4·01 (3·08–5·05)	-28 (-46·4–-2·96)	9·06 (-18·3–44·3)	4·35 (3·5–5·45)	2·35 (1·86–2·98)	2·52 (1·87–3·28)	-46·1 (-59·7–-26·2)	7·41 (-24·6–46·3)
High temperature	19·5 (11·2–29·5)	20·7 (13·7–29·9)	20·4 (12·9–29·9)	6·26 (-7·96–30·6)	-1·51 (-10·1–5·36)	19·5 (11·2–29·5)	20·7 (13·7–29·9)	20·4 (12·9–29·9)	6·26 (-7·96–30·6)	-1·51 (-10·1–5·36)
Low temperature	32 (28·8–36·4)	32·2 (28·8–36·8)	32·3 (28·8–37·3)	0·637 (-1·54–3·06)	0·561 (-1·53–2·35)	32 (28·8–36·4)	32·2 (28·8–36·8)	32·3 (28·8–37·3)	0·637 (-1·54–3·06)	0·561 (-1·53–2·35)
Kidney dysfunction	16·3 (10·7–23·4)	18·5 (12·6–25·8)	19·2 (13·2–26·4)	13·3 (7·86–21·7)	3·52 (-0·121–8·25)	17·3 (11·4–24·8)	19·6 (13·5–27·3)	20·3 (14–28)	13·3 (8·53–21·2)	3·42 (-0·182–7·87)

Bullying victimization	4·79 (1·96–9·8)	5·07 (2·06–10·2)	5·04 (2·05–10·3)	5·71 (1·37–11·6)	-0·449 (-2·69–1·9)	3·58 (1·48–7·32)	3·92 (1·6–8·07)	3·91 (1·58–8·04)	9·56 (3·42–17·7)	-0·24 (-3·13–2·34)
High LDL cholesterol	44·6 (41–48·4)	40·1 (36·8–44)	40·5 (37–44·1)	-9·98 (-15·7–-3·88)	0·971 (-3·51–5·24)	43·2 (39·9–46·7)	39·9 (36·5–43·4)	40·4 (37·1–43·8)	-7·66 (-12·3–-2·92)	1·38 (-2·11–5·14)

Supplementary Results Table 1. Global age-standardised Summary Exposure Values (SEVs) for most-detailed risk factors and percentage change, by sex, 1990, 2010, and 2019. Data in parentheses are 95% uncertainty intervals.

	Male					Female				
	1990	2010	2019	Percentage change 1990-2010	Percentage change 2010-2019	1990	2010	2019	Percentage change 1990-2010	Percentage change 2010-2019
Unsafe sanitation	56·8 (54·6–58·9)	38·2 (36–40·8)	28·7 (26·6–31·1)	-32·7 (-36·2–-28·9)	-24·7 (-28·7–-20·3)	55·9 (53·7–58)	38·3 (36–40·8)	28·9 (26·8–31·2)	-31·5 (-34·9–-27·8)	-24·5 (-28·5–-20·3)
Ambient particulate matter pollution	16·4 (11·1–22·6)	24 (19·1–28·8)	27·2 (22·5–31·6)	46·6 (17–85·4)	13·2 (6·77–20·3)	14·9 (10·1–20·4)	21·9 (17·2–26·5)	25·2 (20·6–29·6)	46·9 (17·8–84·8)	15·1 (8·39–21·7)
Household air pollution from solid fuels	26·8 (15·5–38·4)	15·7 (8·87–24)	11·1 (6·07–17·6)	-41·5 (-49–-32·9)	-29·3 (-35·3–-23·7)	27·9 (16·8–39·2)	17·4 (10·4–26)	13 (7·3–20·3)	-37·6 (-44·7–-29·7)	-25·4 (-31·9–-20)
Ambient ozone pollution	50·9 (23·1–73·2)	60·3 (29·8–79·7)	61·7 (32·5–81)	18·5 (9·05–31·4)	2·41 (0·744–10·6)	50·5 (22·9–72·8)	59 (29·2–79)	60·7 (32–79·9)	16·8 (8·53–29·7)	2·93 (0·762–11)
Residential radon	18·8 (12·4–26·7)	18·5 (12·3–26·2)	18·4 (12·2–26·2)	-1·63 (-5·14–1·72)	-0·339 (-2·15–1·44)	18·9 (12·5–26·5)	18·6 (12·4–26·4)	18·6 (12·4–26·6)	-1·38 (-5·2–2·58)	-0·229 (-2·11–1·81)
Lead exposure	77·4 (64·4–87)	67·7 (52·6–80)	58 (42·3–72·6)	-12·6 (-18·4–-8·08)	-14·3 (-19·6–-9·27)	59·9 (42·1–75·2)	52·2 (34·8–68·9)	44·8 (28·2–62·2)	-12·8 (-17·7–-8·23)	-14·3 (-19–-9·36)
Child underweight	13·3 (11·8–14·8)	10·5 (8·95–12)	8·02 (6·42–9·53)	-21·1 (-24·3–-18·4)	-23·8 (-28·6–-20·1)	13·3 (11·7–14·7)	10·5 (8·93–12)	8·23 (6·54–9·77)	-21 (-24·6–-18·2)	-21·4 (-25·9–-18·1)
Iron deficiency	--	--	--	--	--	22·7 (21·6–24)	20·1 (18·8–21·6)	19·6 (18·1–21·1)	-11·2 (-14·2–-8·36)	-2·7 (-4·18–-1·25)
Vitamin A deficiency	39·1 (34·6–43·4)	24 (19·9–27·8)	16·3 (13·6–19·4)	-38·7 (-47·7–-27·5)	-32·1 (-38·8–-23·9)	27·4 (24–30·5)	19·7 (17·2–22·4)	13·6 (11·9–15·4)	-27·9 (-37·1–-16·1)	-31·1 (-37–-24·7)
Zinc deficiency	14 (6·06–24·3)	12·1 (5·17–21·5)	8·9 (3·02–17·8)	-13·8 (-21·3–-7·61)	-26·3 (-44·8–-16·6)	13·5 (5·68–23·8)	11·7 (4·6–21)	8·67 (2·77–17·5)	-13·3 (-21–-7·38)	-25·6 (-43·9–-16·8)
Smoking	23·8 (21·3–26·5)	20·4 (18·3–22·8)	18·6 (16·6–20·8)	-14·4 (-15·5–-13)	-9·04 (-9·83–-8·27)	6·26 (5·56–7·04)	4·7 (4·14–5·33)	4 (3·51–4·55)	-24·9 (-26·9–-23)	-15 (-16·4–-13·7)
Secondhand smoke	35·1 (34·4–35·7)	30·4 (29·8–31·1)	30·4 (29·6–31·1)	-13·2 (-15·3–-11·1)	-0·173 (-2·09–1·69)	51·5 (51–52·1)	45·3 (44·7–45·9)	44·8 (44·1–45·5)	-12·1 (-13·3–-10·8)	-1·1 (-2·34–0·194)
Alcohol use	9·55 (6·97–12·6)	10·2 (7·44–13·5)	10·8 (7·88–14·4)	6·4 (0·827–13·5)	6·7 (2·14–11·5)	3·44 (2·36–4·96)	3·18 (2·2–4·48)	3·12 (2·13–4·37)	-7·45 (-11·3–-2·07)	-2 (-5·64–1·66)
Drug use	0·197 (0·125–0·33)	0·202 (0·14–0·311)	0·208 (0·148–0·308)	2·75 (-6·34–12·6)	2·77 (-1·37–6·95)	0·156 (0·109–0·238)	0·162 (0·119–0·228)	0·174 (0·131–0·236)	4·21 (-4·94–12·3)	7·38 (2·27–11·7)
High fasting plasma glucose	8·4 (7·4–9·47)	11·3 (10·2–12·6)	12·9 (11·5–14·3)	35 (30–40·8)	13·7 (10·2–17·4)	7·7 (6·77–8·68)	9·98 (8·98–11)	11·1 (9·94–12·4)	29·7 (25·6–34·5)	11·4 (8·31–14·7)
High systolic blood pressure	26·3 (24·4–28·3)	27·2 (25–29·6)	28·6 (26·2–30·8)	3·42 (-2·83–9·7)	4·9 (0·0405–9·89)	27·6 (25·7–29·8)	25·6 (23·3–28·1)	26·8 (24·4–29·3)	-7·27 (-15–1·63)	4·53 (-3·01–12·7)
High body-mass index	9·7 (6·59–13·8)	15·1 (11·3–20)	18·3 (14·2–23·7)	55·7 (41·9–74·1)	21·5 (17·5–26·4)	12·7 (9·23–17·3)	18·1 (14·3–23·1)	20·8 (16·8–26·2)	43·2 (32·7–57)	15 (12·1–18·3)
Low bone mineral density	12·3 (7·75–18·7)	11·5 (7·18–17·9)	11·3 (6·96–17·6)	-6·08 (-9·07–-3·89)	-1·67 (-4·18–0·403)	21·2 (15·6–27·9)	20·8 (15·3–27·4)	20·7 (15–27·3)	-1·89 (-3·87–0·179)	-0·413 (-2·98–1·87)
Diet low in fruits	66·8 (59·5–75)	58·9 (51·1–67·7)	56·9 (49·3–65·6)	-11·8 (-15·3–-8·64)	-3·43 (-5·19–-1·87)	66·6 (59·2–75·1)	59·2 (51·3–68·1)	56·8 (49·2–65·6)	-11·1 (-14·6–-8·12)	-4·09 (-5·82–-2·62)
Diet low in vegetables	52 (38·8–66·6)	40·4 (30·1–52·6)	40·2 (29·6–52·3)	-22·3 (-23·8–-19·9)	-0·68 (-1·92–0·411)	50·7 (37·7–65·2)	40·2 (29·8–52·5)	40·3 (29·5–52·5)	-20·7 (-22·6–-18·4)	0·354 (-1·02–1·63)
Diet low in whole grains	80·7 (73·4–87·9)	80·6 (73·3–88)	80 (72·5–87·5)	-0·0986 (-0·447–0·241)	-0·776 (-1·27–-0·391)	79·1 (71·6–87)	78·6 (70·8–86·7)	77·7 (69·6–86)	-0·749 (-1·4–-0·236)	-1·11 (-1·73–-0·625)
Diet low in nuts and seeds	58·2 (30·9–73)	49·9 (26·1–67·4)	47·2 (24·6–65·6)	-14·2 (-20–-6·66)	-5·44 (-8·64–-2·15)	57·3 (28·1–73·5)	50·3 (24·4–68·8)	47·7 (23·1–67·5)	-12·2 (-17·6–-5·17)	-5·18 (-8·29–-2·1)
Diet low in milk	80·9 (69·5–89·7)	81·3 (70·9–89·8)	82·8 (72·3–91·3)	0·46 (-0·509–1·77)	1·9 (1·2–2·74)	79·4 (67·5–88·6)	80·4 (69·7–89)	82·2 (71·4–90·9)	1·28 (0·0325–3·08)	2·33 (1·56–3·27)
Diet high in red meat	39·8 (33·1–46·7)	43 (36·8–48·9)	43·9 (38·2–49·5)	7·93 (3·47–13·1)	2·19 (-0·852–6·08)	41·1 (34·3–47·5)	43·3 (36·8–49·3)	43·9 (37·9–49·6)	5·35 (1·4–10·6)	1·53 (-1·51–4·98)
Diet high in processed meat	29 (19–40·6)	28·9 (18·5–41·8)	28·2 (17·7–41·8)	-0·172 (-4·39–4·27)	-2·39 (-6·04–0·973)	32·8 (22·5–44)	32·1 (21·7–44·6)	31·4 (20·6–45)	-2 (-6·35–2·56)	-2·4 (-6·13–0·888)
Diet high in sugar-sweetened beverages	30·4 (23·1–43·5)	29·5 (22·3–42·2)	30·3 (22·6–43·4)	-3·08 (-12·7–4·89)	2·58 (-3·35–6·8)	29·6 (22·6–42)	29·2 (21·7–41·6)	30·5 (22·5–43·1)	-1·14 (-10·9–6·74)	4·3 (-1·69–8·52)
Diet low in fiber	39·5 (27·7–51·3)	33·4 (22·4–44·5)	29·4 (19·6–39·4)	-15·4 (-20·4–-11)	-12 (-15·6–-8·63)	40·2 (28–52·1)	34·7 (23·6–46·1)	30·5 (20·7–41)	-13·6 (-18·4–-9·71)	-12·1 (-15·5–-8·85)
Diet low in seafood omega-3 fatty acids	96·1 (92·8–99·9)	92·7 (88·4–98·4)	93 (87·9–99·4)	-3·51 (-4·95–-1·22)	0·309 (-0·949–1·35)	96·6 (93·6–99·9)	93·5 (89·7–98·5)	94 (89·5–99·5)	-3·19 (-4·39–-1·22)	0·525 (-0·484–1·37)
Diet low in polyunsaturated fatty acids	72·4 (51–91·1)	65·1 (38·5–88·6)	63·7 (35·8–88·6)	-10·2 (-24·3–-2·23)	-2·14 (-5·91–-0·029)	73 (51·7–91·6)	66·6 (39·9–89·6)	65·3 (37·8–89·7)	-8·85 (-22·1–-1·91)	-1·83 (-5·07–-0·0477)
Diet high in trans fatty acids	51·9 (43·7–95·1)	46·5 (38·1–95)	46 (37·5–95·6)	-10·4 (-15·5–-0·00141)	-1·14 (-4·57–1·82)	52 (43·6–98·1)	47·3 (38–97·8)	46·8 (37·5–97·7)	-9·17 (-14·8–0)	-0·977 (-3·94–1·79)
Diet high in sodium	52·7 (36·6–67·6)	51·7 (33·6–67·6)	50·6 (32·1–67)	-1·91 (-9·12–1·25)	-2·18 (-4·96–-0·494)	44·3 (28–60·9)	40·5 (23·9–58·1)	39·5 (22·7–57·4)	-8·41 (-16·2–-3·93)	-2·52 (-6·75–-0·244)
Low physical activity	2·92 (1·47–5·67)	3·04 (1·6–5·75)	3·24 (1·67–6·02)	4·16 (-3·35–15·6)	6·37 (1·06–12·5)	3·71 (2·06–6·5)	3·74 (2·19–6·29)	3·78 (2·16–6·47)	0·836 (-6·69–10·6)	1·01 (-3·53–5·46)
Occupational asthmagens	24·8 (20·7–29·9)	23·2 (19·4–27·8)	22·7 (19·1–27)	-6·73 (-10·9–-2·28)	-1·94 (-5·2–1·28)	12·5 (10–15·6)	11·8 (9·65–14·5)	11·5 (9·44–13·9)	-5·19 (-12·5–2·21)	-2·96 (-7·63–1·69)
Occupational particulate matter, gases, and fumes	13·5 (10·3–17·9)	13·2 (10·2–17·5)	12·9 (10–16·9)	-2 (-3·38–-0·788)	-2·58 (-3·52–-1·71)	7·58 (5·85–10·4)	7·72 (5·98–10·5)	7·54 (5·89–10·2)	1·89 (0·162–3·58)	-2·33 (-3·89–-0·942)
Occupational noise	13·7 (13·1–14·3)	13·7 (13·2–14·4)	13·6 (13–14·3)	0·355 (-0·126–0·85)	-1 (-1·23–-0·776)	7·77 (7·46–8·23)	8·23 (7·92–8·71)	8·33 (8–8·82)	6·02 (5–6·94)	1·11 (0·751–1·46)
Occupational injuries	--	--	--	--	--	--	--	--	--	--
Occupational ergonomic factors	26·6 (24·7–28·6)	23·5 (21·8–25·5)	21·5 (19·8–23·6)	-11·8 (-16·3–-6·94)	-8·13 (-11·2–-4·85)	16·3 (15–17·6)	14·6 (13·3–15·9)	13·3 (12–14·7)	-11 (-18·3–-3·23)	-8·35 (-12·9–-3·46)
Childhood sexual abuse	7·59 (5–10·8)	7·55 (4·96–10·5)	7·98 (5·21–11·3)	-0·511 (-2·79–1·95)	5·64 (2·89–8·41)	9·83 (7·25–13·3)	9·79 (7·49–13·2)	10·8 (8·04–14·7)	-0·347 (-3·61–3·11)	10·2 (6·53–14·5)
Intimate partner violence	--	--	--	--	--	22·5 (13–30·1)	22·2 (13·1–29·1)	23 (13·3–30·4)	-1·41 (-5·46–7·17)	3·66 (0·32–7·73)
Non-exclusive breastfeeding	21·3 (14·7–30)	19·4 (13·4–27·1)	18·4 (12·9–25·6)	-9·19 (-11·4–-6·96)	-5·2 (-7·23–-2·71)	21·4 (14·7–30)	19·4 (13·4–27·2)	18·4 (12·9–25·7)	-9·03 (-11·1–-6·86)	-5·25 (-7·21–-2·83)
Discontinued breastfeeding	12·3 (12·1–12·7)	10·7 (10·5–11)	10·3 (9·97–10·6)	-13 (-15·7–-10·2)	-4·55 (-7·57–-1·54)	12·3 (12–12·6)	10·7 (10·5–11)	10·3 (9·98–10·5)	-13·1 (-15·8–-10·4)	-4·24 (-7·27–-1·23)
Diet low in calcium	57·8 (48·2–70·6)	53·2 (43–67·4)	50·2 (39·6–65·3)	-8·01 (-11·5–-4·08)	-5·59 (-7·93–-2·97)	51·6 (42·2–64·7)	47·8 (37·5–62·4)	45·3 (34·7–60·7)	-7·46 (-11·4–-3·39)	-5·15 (-7·65–-2·58)
Occupational exposure to asbestos	3·89 (3·66–4·34)	3·91 (3·71–4·13)	3·53 (3·25–3·83)	0·299 (-5·77–5·05)	-9·54 (-15·5–-3·64)	1·47 (1·05–1·85)	1·2 (0·859–1·36)	1·12 (0·776–1·29)	-18·4 (-34–-3·09)	-6·26 (-15·7–3·66)
Occupational exposure to arsenic	0·48 (0·132–0·909)	0·48 (0·147–0·884)	0·487 (0·155–0·888)	0·0422 (-3·75–11·9)	1·51 (-0·967–5·67)	0·409 (0·105–0·791)	0·423 (0·126–0·784)	0·429 (0·131–0·794)	3·35 (-2·35–21·6)	1·54 (-1·86–7·49)
Occupational exposure to benzene	0·813 (0·396–1·67)	0·904 (0·474–1·77)	0·958 (0·514–1·86)	11·2 (7·02–19·8)	6·05 (4·5–8·95)	0·713 (0·303–1·53)	0·871 (0·409–1·79)	0·97 (0·48–1·95)	22·1 (16·7–34·6)	11·4 (8·8–16·8)
Occupational exposure to beryllium	0·0965 (0·0957–0·0973)	0·103 (0·102–0·104)	0·105 (0·104–0·106)	6·7 (5·66–7·76)	2·34 (1·39–3·3)	0·0761 (0·075–0·0771)	0·0887 (0·088–0·0894)	0·0936 (0·0923–0·095)	16·5 (14·7–18·4)	5·55 (3·98–7·25)
Occupational exposure to cadmium	0·192 (0·189–0·195)	0·209 (0·204–0·215)	0·218 (0·212–0·225)	8·9 (5·68–12·2)	4·09 (1·64–6·78)	0·155 (0·152–0·158)	0·18 (0·173–0·188)	0·19 (0·182–0·199)	16 (10·6–21·6)	5·51 (1·84–9·61)
Occupational exposure to chromium	0·411 (0·405–0·418)	0·467 (0·456–0·479)	0·497 (0·483–0·512)	13·7 (10·4–17)	6·42 (4·02–9·08)	0·323 (0·316–0·329)	0·392 (0·376–0·41)	0·424 (0·406–0·444)	21·4 (15·7–27·3)	8·07 (4·29–12·2)
Occupational exposure to diesel engine exhaust	2·23 (2·2–2·27)	2·78 (2·74–2·83)	3·05 (2·99–3·11)	24·6 (22–27·5)	9·57 (7·82–11·6)	1·27 (1·25–1·29)	1·59 (1·56–1·64)	1·76 (1·71–1·82)	25·8 (22·3–29·7)	10·7 (7·83–13·8)
Occupational exposure to formaldehyde	0·891 (0·852–0·911)	0·986 (0·942–1·02)	1·04 (0·99–1·07)	10·7 (6·88–14·6)	5·18 (2·45–8·22)	0·671 (0·647–0·688)	0·781 (0·744–0·823)	0·826 (0·784–0·871)	16·4 (10·7–22·7)	5·67 (1·76–9·9)
Occupational exposure to nickel	0·522 (0·124–1·62)	0·52 (0·139–1·54)	0·526 (0·144–1·52)	-0·374 (-5·16–11·8)	1·05 (-1·21–5·26)	0·393 (0·0892–1·25)	0·392 (0·0998–1·19)	0·392 (0·101–1·16)	-0·223 (-6·32–16·9)	0·05 (-3·09–7·17)
Occupational exposure to polycyclic aromatic hydrocarbons	0·844 (0·832–0·857)	0·964 (0·942–0·988)	1·03 (1–1·06)	14·3 (11·2–17·4)	6·63 (4·42–9·01)	0·677 (0·658–0·696)	0·818 (0·785–0·853)	0·885 (0·845–0·927)	20·8 (14·8–26·7)	8·2 (4·23–12·5)
Occupational exposure to silica	5·4 (2·23–13·5)	5·69 (2·53–13·8)	5·88 (2·68–14·2)	5·33 (1·4–13·6)	3·21 (1·03–6·92)	3·41 (1·36–8·6)	3·25 (1·4–7·89)	3·19 (1·42–7·64)	-4·74 (-9·13–3·98)	-1·83 (-4·66–2·06)
Occupational exposure to sulfuric acid	1·12 (0·68–2·29)	1·14 (0·724–2·26)	1·14 (0·743–2·24)	1·73 (-2·57–7·43)	0·783 (-1·32–3·45)	0·895 (0·523–1·89)	0·933 (0·583–1·87)	0·941 (0·602–1·86)	4·29 (-1·39–11·8)	0·877 (-2·34–5·16)
Unsafe sex	--	--	--	--	--	--	--	--	--	--
Occupational exposure to trichloroethylene	0·238 (0·235–0·242)	0·275 (0·271–0·281)	0·294 (0·29–0·301)	15·8 (13·5–18·4)	6·96 (5·12–8·87)	0·182 (0·179–0·186)	0·224 (0·218–0·232)	0·244 (0·237–0·254)	23·1 (19–27·6)	8·94 (6·05–12·2)
No access to handwashing facility	37 (36·8–37·3)	34 (33·7–34·2)	31·9 (31·6–32·2)	-8·28 (-9·08–-7·47)	-6·07 (-6·68–-5·51)	36·2 (36–36·5)	33·3 (33·1–33·6)	31·4 (31·2–31·7)	-7·88 (-8·7–-7·13)	-5·78 (-6·29–-5·29)
Child wasting	5·15 (4·37–5·86)	5·09 (4·28–5·82)	4·76 (3·96–5·47)	-1·27 (-2·89–0·261)	-6·48 (-7·92–-5·3)	5·44 (4·64–6·15)	5·44 (4·61–6·18)	5·08 (4·27–5·83)	-0·0146 (-1·72–1·48)	-6·68 (-8·17–-5·48)
Child stunting	23·8 (16·5–26·1)	19·5 (13·7–21·9)	16·1 (11·3–18·6)	-17·8 (-21–-15·4)	-17·5 (-21·9–-14·5)	24·3 (16·8–26·7)	19·8 (13·8–22·1)	16·4 (11·5–18·9)	-18·8 (-21·7–-16·4)	-17·2 (-21·3–-14·3)
Chewing tobacco	6·22 (5·55–6·88)	6·52 (6·15–6·92)	6·7 (6·21–7·22)	4·9 (-5·94–17·7)	2·68 (-4·81–11·2)	2·96 (2·55–3·38)	3·37 (3·09–3·68)	3·52 (3·16–3·88)	14·1 (-1·03–33·2)	4·34 (-6·35–15·3)
Diet low in legumes	70·8 (37·9–93)	62·6 (30·1–85·2)	61·3 (28·8–84·8)	-11·6 (-23–-7·67)	-2·07 (-6·18–-0·447)	68·1 (35·7–90·6)	59·8 (27·6–83·2)	58·1 (26·8–81·3)	-12·1 (-24·2–-8·02)	-2·93 (-6·29–-0·229)
Short gestation	14 (12·4–15·7)	13·2 (11·9–14·5)	13·3 (12·1–14·8)	-5·78 (-12·6–-0·134)	1·3 (-3·03–4·93)	13·8 (12·5–15·2)	12·9 (11·8–14)	13 (11·8–14·2)	-6·3 (-10·9–-2·07)	0·702 (-2·51–3·86)
Low birth weight	11·3 (10·5–12·4)	10·5 (9·95–11·1)	10·2 (9·56–10·9)	-6·55 (-13–-1·09)	-3·39 (-6·77–-0·132)	10·8 (10·1–11·5)	9·66 (9·12–10·2)	9·17 (8·66–9·75)	-10·4 (-14·7–-6·22)	-5·04 (-7·97–-2·41)
High temperature	26·1 (22·1–30·3)	29·3 (24·9–33·8)	29·6 (25·2–34·2)	12·3 (8·8–16)	1·08 (0·0176–2·09)	26·1 (22·1–30·3)	29·3 (24·9–33·8)	29·6 (25·2–34·2)	12·3 (8·8–16)	1·08 (0·0176–2·09)
Low temperature	33·2 (29·1–37·7)	33·5 (29–38·3)	33 (28·5–37·8)	0·867 (-0·969–2·53)	-1·49 (-2·48–-0·592)	33·2 (29·1–37·7)	33·5 (29–38·3)	33 (28·5–37·8)	0·867 (-0·969–2·53)	-1·49 (-2·48–-0·592)
Kidney dysfunction	19·7 (13·5–26·9)	21·4 (15–28·8)	21·7 (15·3–29·1)	8·99 (6·33–12·7)	1·47 (0·838–2·34)	21·4 (14·9–28·9)	23·2 (16·7–30·9)	23·7 (17–31·5)	8·6 (5·84–12·6)	2·09 (1·36–2·94)
Bullying victimization	6·44 (2·73–12·8)	7·72 (3·43–15·1)	8·15 (3·62–16)	19·8 (14·8–31·7)	5·68 (3·72–7·18)	4·51 (1·92–9·01)	5·85 (2·61–11·5)	6·34 (2·79–12·5)	29·8 (23·7–42·8)	8·49 (5·55–10·3)
High LDL cholesterol	34·1 (31·4–37·2)	31·3 (28·3–34·5)	31·1 (28·1–34·3)	-8·39 (-9·88–-7·13)	-0·669 (-1·14–-0·235)	37·1 (34·4–40·1)	33·9 (30·9–37·1)	33·7 (30·7–36·9)	-8·71 (-10–-7·49)	-0·591 (-1·02–-0·168)

Supplementary Results Table 1. Norfolk age-standardised Summary Exposure Values (SEVs) for most-detailed risk factors and percentage change, by sex, 1990, 2010, and 2019. Data in parentheses are 95% uncertainty intervals.

	Male					Female				
	1990	2010	2019	Percentage change 1990-2010	Percentage change 2010-2019	1990	2010	2019	Percentage change 1990-2010	Percentage change 2010-2019
Unsafe sanitation	0·631 (0·23–1·38)	0·367 (0·136–0·817)	0·277 (0·101–0·617)	-41·8 (-81·7–87·3)	-24·5 (-67·1–70·5)	0·631 (0·23–1·38)	0·367 (0·136–0·817)	0·277 (0·101–0·617)	-41·8 (-81·7–87·3)	-24·5 (-67·1–70·5)
Ambient particulate matter pollution	17·4 (5·85–32·2)	10·9 (6·9–15·7)	8·82 (5·11–13·3)	-37·3 (-66·9–50·4)	-19·1 (-26·2–15·1)	17·4 (5·85–32·2)	10·9 (6·9–15·7)	8·82 (5·13–13·3)	-37·2 (-66·9–50·9)	-19 (-26·2—15·1)
Household air pollution from solid fuels	0·0267 (0·0054–0·0766)	0·00884 (0·00171–0·0259)	0·00555 (0·000867–0·0189)	-66·9 (-89·8–8·16)	-37·2 (-73·2–33·8)	0·0351 (0·00749–0·0999)	0·0117 (0·00231–0·0329)	0·00734 (0·00122–0·0245)	-66·7 (-89·7–9·23)	-37·1 (-73·1–33·9)
Ambient ozone pollution	21·4 (6·87–41·8)	15·7 (5·07–31·6)	15 (4·3–31·5)	-26·5 (-59·1–19·5)	-4·74 (-47·3–66·1)	21·4 (6·87–41·8)	15·7 (5·07–31·6)	15 (4·3–31·5)	-26·5 (-59·1–19·5)	-4·74 (-47·3–66·1)
Residential radon	16·4 (3·39–48)	16·4 (3·39–48)	16·4 (3·39–48)	0 (0–0)	0 (0–0)	16·4 (3·39–48)	16·4 (3·39–48)	16·4 (3·39–48)	0 (0–0)	0 (0–0)
Lead exposure	25·3 (1·51–51·1)	16·5 (0·856–39·2)	11·5 (0·239–32·1)	-34·9 (-60·7—20·7)	-30 (-69·3—18·3)	10·7 (5·78e-12–31·9)	6·5 (4·37e-12–23·7)	4·56 (2·84e-12–19·1)	-39·4 (-100—2·32)	-29·8 (-100–9·3)
Child underweight	0·913 (0·508–1·38)	0·78 (0·43–1·2)	0·771 (0·405–1·19)	-14·5 (-32·1–6·49)	-1·14 (-16·9–16·9)	1·08 (0·619–1·61)	0·943 (0·55–1·44)	0·933 (0·517–1·43)	-12·5 (-29·4–8·92)	-1·13 (-15·3–14·9)
Iron deficiency	--	--	--	--	--	9·96 (8·01–12·2)	9·1 (7·27–10·9)	9·15 (7·29–11·1)	-8·59 (-23·2–7·84)	0·536 (-8·93–12·2)
Vitamin A deficiency	4·91 (1·06–14·9)	2·17 (0·452–6·47)	1·76 (0·345–4·97)	-55·8 (-90·5–112)	-18·7 (-66·2–138)	3·53 (1·04–8·08)	2·35 (0·733–5·11)	2·01 (0·635–4·6)	-33·2 (-79·1–139)	-14·7 (-57·8–80·6)
Zinc deficiency	4·21 (0–12·8)	2·83 (0–9·01)	2·83 (0–8·86)	-32·8 (-100–NA)	-0·0118 (-26·4–NA)	4·07 (0–12·1)	2·68 (0–8·88)	2·67 (0–8·51)	-34·2 (-100–NA)	-0·271 (-23·7–NA)
Smoking	27·4 (24–30·9)	20·3 (17·7–23·5)	18·1 (15·6–21)	-25·8 (-32·6—19·1)	-10·8 (-15·6—5·23)	21 (17·6–24·6)	16·9 (14·1–20)	13·4 (11·3–16·2)	-19·7 (-30·7—6·51)	-20·3 (-28—12·5)
Secondhand smoke	43·5 (37·9–49·6)	33 (28·4–37·8)	32·3 (27·8–37·1)	-24·2 (-36·2—10·4)	-2·05 (-11·7–8·59)	45·6 (41·1–50·2)	33·8 (30·2–37·4)	33 (29·3–36·9)	-25·9 (-35·1—16·1)	-2·36 (-9·78–6·08)
Alcohol use	17·9 (9·85–26)	21·1 (11·7–31·2)	19·1 (10·4–29·3)	17·5 (-26·3–86·6)	-9·31 (-34·6–21·3)	--	--	--	--	--
Drug use	0·33 (0·171–0·661)	0·697 (0·429–1·17)	0·654 (0·387–1·17)	111 (60·8–180)	-6·17 (-16·3–4·25)	--	--	--	--	--
High fasting plasma glucose	9·66 (8·08–11·8)	18·1 (15·4–21·2)	26·5 (22·2–32)	87·2 (54·7–125)	46·3 (25·2–72·1)	8·01 (6·68–9·8)	13·4 (11·5–15·5)	19 (16·2–22·3)	66·7 (39·6–101)	42·5 (23·2–66·4)
High systolic blood pressure	44·7 (36·4–54·2)	28·2 (22·7–35·1)	29·8 (22·1–38·2)	-36·9 (-51·3—17·9)	5·5 (-18·6–34·3)	--	--	--	--	--
High body-mass index	20·2 (13·9–27·7)	26·8 (18·9–36·3)	28·5 (20·4–38·4)	32·7 (17–54·5)	6·38 (-0·593–15·2)	20·4 (12·3–35·3)	27 (18·2–42·8)	28·4 (18·7–44·4)	32·6 (-0·92–84·3)	5·17 (-11·8–26·5)
Low bone mineral density	8·81 (4·68–14·6)	7·6 (3·9–13·3)	9·16 (4·77–15·4)	-13·8 (-36·4–14·1)	20·6 (-10·8–67·5)	14·6 (9·24–21·7)	13·9 (8·91–20·6)	13·5 (8·27–19·8)	-4·78 (-25·3–22·4)	-2·83 (-28–24·4)
Diet low in fruits	66·9 (55·2–78·6)	60·1 (47–72·9)	58·8 (45·4–71·1)	-10·2 (-23·3–1·76)	-2·08 (-11·2–9·07)	67·7 (55·5–79)	60·6 (47·9–73·5)	59·8 (47–73·6)	-10·5 (-23·4–1·67)	-1·34 (-10·9–9·97)
Diet low in vegetables	52·7 (32·7–73·6)	48·4 (28·7–69·3)	47·3 (27·1–68·7)	-8·12 (-24·9–9·17)	-2·23 (-15·2–11)	49·8 (30·4–70·7)	45·2 (25·8–66·2)	44 (25–65·5)	-9·3 (-28–8·15)	-2·71 (-16·1–12)
Diet low in whole grains	90·1 (83·9–95)	89·7 (83·7–94·9)	89·8 (83·7–95)	-0·373 (-3·57–2·76)	0·103 (-1·93–2·14)	88·3 (81·8–93·9)	88·1 (81·5–93·7)	88·2 (81·5–93·8)	-0·17 (-3·53–3·34)	0·132 (-2·1–2·19)
Diet low in nuts and seeds	83·3 (43·3–93·4)	75·9 (37·6–88·4)	77·9 (40·6–89·7)	-8·85 (-21·2–1·94)	2·64 (-4·95–13·3)	83·2 (42·6–93·5)	75·6 (38·8–88·2)	76·7 (37·6–89·5)	-9·12 (-22·3–2·18)	1·45 (-6·65–11·2)
Diet low in milk	59·3 (34·1–80·4)	60·9 (35·4–81·9)	60·9 (35·3–81·9)	2·82 (-7·79–20)	-0·118 (-6·8–7·03)	61·5 (35·2–82·5)	64·4 (38·3–84·9)	64·3 (38·2–84·7)	4·74 (-4·51–20·3)	-0·171 (-6·44–6·47)
Diet high in red meat	65·8 (50·4–81)	51·1 (36·8–66·1)	53·2 (37·8–68·7)	-22·3 (-41·2—0·403)	4·1 (-13·4–25·3)	65·2 (49·3–78·4)	52 (37·4–66·6)	54·6 (39·3–71)	-20·2 (-39·9–1·64)	4·88 (-12·7–26·2)
Diet high in processed meat	75·5 (51·4–95·4)	80·4 (58–98·5)	80·6 (58·6–98·1)	6·52 (-3·13–23)	0·249 (-5·4–7·08)	78·5 (56·6–97·5)	83·5 (63·1–99·1)	83·9 (64·6–99·1)	6·48 (-1·82–25·1)	0·41 (-5·27–6·2)
Diet high in sugar-sweetened beverages	46·3 (27·2–71·4)	49·4 (29·7–73·6)	51·4 (30·7–74·8)	6·69 (-32·3–69)	4·13 (-21·9–41·5)	--	--	--	--	--
Diet low in fiber	43·1 (27·1–60·7)	36·1 (19·8–54·1)	35·8 (19·7–53·5)	-16·3 (-41·3–12·2)	-0·808 (-21·6–23)	42·9 (25·2–60·6)	35·8 (19·8–52)	35·7 (19·8–52·3)	-16·6 (-42·1–13·6)	-0·391 (-20·1–23)
Diet low in seafood omega-3 fatty acids	95 (85·5–100)	92·8 (80·7–100)	92·5 (80·6–100)	-2·3 (-13·3–4·63)	-0·341 (-11·1–11)	96 (88·3–100)	94·1 (84·4–100)	93·8 (83·2–100)	-2·07 (-10–3·85)	-0·24 (-9·27–8·99)
Diet low in polyunsaturated fatty acids	54·7 (17·3–94·6)	50·1 (14–91)	49·1 (13·9–91)	-8·43 (-24·8–0·924)	-1·87 (-11·5–6·77)	56·9 (17·8–96·7)	52·5 (15·6–94·3)	51·5 (14·8–93·6)	-7·72 (-23·6–0·131)	-1·96 (-11·8–5·98)
Diet high in trans fatty acids	57·7 (46·7–100)	50·8 (39·8–98·6)	50·9 (39·5–98·4)	-11·9 (-25·9–1·02)	0·0447 (-14·2–16·3)	55·9 (44·4–100)	47·7 (36·1–99·6)	47·7 (36·5–100)	-14·6 (-30·3–0)	-0·0202 (-11·9–15·9)
Diet high in sodium	13·9 (3·71–33·9)	14·2 (3·66–33·6)	15 (3·86–35·2)	2·35 (-47·3–106)	5·44 (-39·6–81)	8·96 (2·75–20·6)	8·47 (2·92–18·8)	8·53 (2·99–18·8)	-5·39 (-40·7–80·6)	0·626 (-40·4–58·6)
Low physical activity	4·29 (1·82–8·84)	4·94 (2·19–10)	5·06 (2·26–10)	15 (-0·0798–37·5)	2·44 (-10·2–17·5)	6·84 (3·6–12·7)	7·76 (4·14–13·9)	7·99 (4·32–14·1)	13·6 (3·58–28·5)	2·88 (-5·29–12·9)
Occupational asthmagens	21·5 (17·4–27·1)	19·9 (15·8–25·3)	21·5 (16·6–28·2)	-7·78 (-26·3–14)	8·22 (-7·5–30·2)	11·8 (9·37–15·2)	10·5 (8·4–12·9)	11·5 (9·05–14·5)	-11·6 (-30·8–9·37)	9·61 (-4·79–26·4)
Occupational particulate matter, gases, and fumes	8·55 (5·98–12·5)	8·14 (5·75–11·9)	7·94 (5·55–11·5)	-4·75 (-13·2–4·76)	-2·5 (-7·02–2·48)	3·86 (2·61–5·94)	3·67 (2·51–5·47)	3·51 (2·39–5·21)	-4·79 (-17·2–13·2)	-4·34 (-10·6–2·77)
Occupational noise	8·4 (7·53–9·37)	8·18 (7·53–8·92)	8·04 (7·43–8·78)	-2·58 (-7·8–3·65)	-1·75 (-4·36–1·21)	4·9 (4·03–5·81)	5·38 (4·8–6·05)	5·51 (4·98–6·11)	9·75 (0·629–23·1)	2·54 (-1·16–6·69)
Occupational injuries	--	--	--	--	--	--	--	--	--	--
Occupational ergonomic factors	12·1 (10–14·6)	11·5 (9·49–13·9)	11·8 (9·57–14·3)	-5·29 (-20·1–11)	2·73 (-8·49–15·5)	--	--	--	--	--
Childhood sexual abuse	10·6 (6·74–14·7)	11 (7·04–15·8)	10·7 (6·96–15·2)	4·38 (-2·5–12·8)	-2·79 (-11–6·54)	13·3 (9·72–18·3)	14·1 (10·3–19·6)	13·7 (10·1–18·8)	6·28 (-2·16–15·1)	-2·58 (-10·4–8·27)
Intimate partner violence	--	--	--	--	--	22·8 (12·5–31·3)	20 (11·3–28·2)	22·8 (12·5–31·3)	-12·2 (-19·2–0·696)	13·9 (2·98–28·7)
Non-exclusive breastfeeding	26 (18·8–33·9)	23·4 (17·7–30·3)	23·4 (17·3–30·9)	-10·1 (-24·5–10·2)	0·00791 (-13·3–14·8)	26 (18·8–33·9)	23·4 (17·7–30·3)	23·4 (17·3–30·9)	-10·1 (-24·5–10·2)	0·0119 (-13·3–14·8)
Discontinued breastfeeding	27·9 (26·9–28·8)	27·8 (26·7–28·7)	27·5 (26·3–28·5)	-0·595 (-5·44–4·31)	-1·1 (-5·1–2·72)	27·9 (26·9–28·8)	27·8 (26·8–28·8)	27·5 (26·3–28·5)	-0·3 (-5·16–4·62)	-1·13 (-5·13–2·68)
Diet low in calcium	20·3 (9·52–38·5)	14·9 (6·22–28·5)	14·7 (6·17–27·9)	-26·5 (-46·4—11·4)	-1·41 (-11·1–9·66)	14·8 (5·95–28·7)	9·39 (3·51–17·6)	9·17 (3·27–17·8)	-36·4 (-59·3—16)	-2·33 (-18–15)
Occupational exposure to asbestos	20·8 (18·9–22·8)	19·5 (18·3–20·5)	18 (15·2–20·8)	-6·08 (-15·3–4·39)	-8·03 (-21·9–7·66)	3·78 (2·61–5·06)	3·85 (2·28–4·57)	3·85 (2·04–5·41)	1·95 (-31·6–38·5)	-0·0546 (-25·9–32·5)
Occupational exposure to arsenic	0·467 (0–1·09)	0·5 (0–1·2)	0·517 (0–1·21)	7·16 (-14·7–NA)	3·36 (-10·4–NA)	0·402 (0–0·96)	0·42 (0–0·98)	0·443 (0–1·06)	4·58 (-15·9–NA)	5·47 (-8·05–NA)
Occupational exposure to benzene	0·898 (0·0525–2·56)	0·876 (0·0496–2·42)	0·916 (0·0542–2·56)	-2·49 (-11·8–12·5)	4·52 (-3·11–15·7)	1·15 (0·0707–3·26)	1·43 (0·0826–4·07)	1·56 (0·0945–4·41)	24·8 (9·18–52·1)	9·14 (-1·29–24)
Occupational exposure to beryllium	0·0244 (0·023–0·026)	0·0257 (0·0242–0·0272)	0·0267 (0·025–0·0285)	5·23 (-2·2–12·9)	4·06 (-1·21–10·2)	0·0251 (0·0225–0·0277)	0·0283 (0·0261–0·0304)	0·0305 (0·028–0·033)	12·6 (0·852–27·1)	7·9 (0·525–16·1)
Occupational exposure to cadmium	0·0548 (0·0472–0·0632)	0·0586 (0·0511–0·0674)	0·0608 (0·0517–0·0704)	6·86 (-13–27·6)	3·81 (-8·9–18·1)	0·0491 (0·0416–0·0581)	0·0518 (0·0453–0·0592)	0·0549 (0·0482–0·0638)	5·52 (-12·9–29·7)	5·96 (-5·85–19·5)
Occupational exposure to chromium	0·13 (0·114–0·148)	0·138 (0·122–0·156)	0·144 (0·124–0·164)	5·8 (-11·9–24·6)	4·46 (-6·62–16·7)	0·125 (0·109–0·145)	0·135 (0·121–0·151)	0·144 (0·129–0·164)	7·9 (-8·13–27·8)	6·69 (-3·44–18·5)
Occupational exposure to diesel engine exhaust	0·72 (0·656–0·792)	0·749 (0·683–0·817)	0·795 (0·714–0·881)	3·98 (-8·3–17·2)	6·07 (-2·67–15·4)	0·65 (0·553–0·748)	0·67 (0·593–0·763)	0·723 (0·629–0·832)	2·95 (-12·8–23·7)	7·99 (-4·58–22·2)
Occupational exposure to formaldehyde	0·272 (0·231–0·316)	0·279 (0·24–0·324)	0·287 (0·242–0·335)	2·37 (-17·4–24·6)	3·09 (-10·2–17·9)	0·239 (0·204–0·283)	0·26 (0·229–0·294)	0·276 (0·242–0·316)	8·72 (-9·32–31·6)	6·19 (-4·68–18·8)
Occupational exposure to nickel	0·452 (0–2·09)	0·491 (0–2·16)	0·511 (0–2·34)	8·58 (-9·37–NA)	4·1 (-7·39–NA)	0·317 (0–1·42)	0·326 (0–1·46)	0·34 (0–1·44)	2·67 (-18·5–NA)	4·46 (-9·76–NA)
Occupational exposure to polycyclic aromatic hydrocarbons	0·274 (0·245–0·307)	0·288 (0·258–0·323)	0·301 (0·264–0·339)	5·09 (-10·3–21·1)	4·56 (-5·23–15·5)	0·265 (0·231–0·304)	0·296 (0·267–0·327)	0·317 (0·283–0·354)	11·7 (-3·72–30·6)	6·88 (-2·33–17·5)
Occupational exposure to silica	5·56 (0·789–17·4)	6·1 (0·863–19·6)	6·44 (0·988–21·4)	9·68 (-8·37–35·3)	5·47 (-6–21·5)	2·87 (0·379–8·8)	2·84 (0·384–9·21)	2·96 (0·406–9·46)	-1·17 (-21·8–30·1)	4·32 (-9·92–24·6)
Occupational exposure to sulfuric acid	0·839 (0·183–2·68)	0·847 (0·171–2·66)	0·856 (0·169–2·89)	0·953 (-16·4–22·4)	1·14 (-11–14·7)	--	--	--	--	--
Unsafe sex	--	--	--	--	--	--	--	--	--	--
Occupational exposure to trichloroethylene	0·0747 (0·068–0·0825)	0·0784 (0·0715–0·0862)	0·0819 (0·0734–0·091)	4·88 (-8·15–18·7)	4·57 (-3·43–14·4)	0·0763 (0·0665–0·0862)	0·0812 (0·0734–0·0905)	0·0871 (0·0783–0·0981)	6·43 (-7·35–25·7)	7·21 (-2·38–18·6)
No access to handwashing facility	3·37 (3·08–3·67)	3·21 (2·92–3·51)	3·17 (2·91–3·45)	-4·54 (-14·4–6·3)	-1·31 (-7·63–5·9)	3·37 (3·08–3·67)	3·21 (2·92–3·51)	3·17 (2·91–3·45)	-4·54 (-14·4–6·3)	-1·31 (-7·63–5·9)
Child wasting	1·26 (0·879–1·7)	1·22 (0·845–1·63)	1·22 (0·838–1·62)	-3·2 (-15·6–10·3)	-0·455 (-9·93–9·93)	--	--	--	--	--
Child stunting	1·63 (0·804–2·5)	1·22 (0·542–1·96)	1·22 (0·548–1·92)	-25·6 (-45·6–1·43)	0·0221 (-22·1–25·6)	1·93 (0·973–2·94)	1·59 (0·785–2·43)	1·58 (0·785–2·45)	-17·6 (-38·2–9·37)	-0·869 (-20·2–21·2)
Chewing tobacco	0·175 (0·109–0·267)	0·164 (0·108–0·242)	0·168 (0·109–0·245)	-5·78 (-42·3–53·6)	2·46 (-25·5–38·9)	0·133 (0·0739–0·224)	0·131 (0·0757–0·209)	0·133 (0·0759–0·222)	-1·31 (-48·2–98·1)	1·13 (-33·7–55·1)
Diet low in legumes	74·1 (36·8–99·4)	73·3 (36·7–98·9)	74·1 (37·7–98·9)	-1·02 (-13·6–7·4)	1·03 (-5·14–10·8)	70·4 (32·7–97·7)	69·5 (29·3–96·9)	70·3 (31·1–97·6)	-1·37 (-13·6–8·19)	1·19 (-6·02–12·7)
Short gestation	21·9 (17·4–27·3)	50·8 (40·2–63·5)	44·8 (35–57·8)	132 (73·7–203)	-11·8 (-33·6–15·7)	24·2 (19·5–30·1)	44·5 (36·1–54·5)	39·4 (31·8–47·8)	83·5 (32·7–160)	-11·4 (-30·5–14·1)
Low birth weight	11·1 (8·93–13·7)	24·3 (19·5–30·1)	21·8 (17·2–27·7)	119 (65·8–191)	-10 (-32·7–21·5)	12·5 (10·3–15·2)	20·8 (16·7–25·7)	19·2 (15·8–23·1)	66·9 (21·1–133)	-7·65 (-29–21·5)
High temperature	21·1 (6·05–37·9)	0 (0–0)	0 (0–0)	-100 (-100—100)	--	21·1 (6·05–37·9)	0 (0–0)	0 (0–0)	-100 (-100—100)	--
Low temperature	30·2 (24·7–36·4)	33·3 (28·4–38·9)	29·5 (24–35·6)	10·2 (6·2–15·2)	-11·3 (-15·6—7·44)	30·2 (24·7–36·4)	33·3 (28·4–38·9)	29·5 (24–35·6)	10·2 (6·2–15·2)	-11·3 (-15·6—7·44)
Kidney dysfunction	13·8 (9·16–20)	14·3 (9·84–20·7)	14 (9·56–20·3)	4·15 (-0·785–10·3)	-2·08 (-6·92–2·5)	14·7 (9·83–21·2)	15 (10·3–21·4)	15 (10–21·6)	2·07 (-1·81–6·81)	-0·248 (-4·72–4·19)
Bullying victimization	6·1 (2·44–12)	6·07 (2·38–12·3)	5·61 (2·25–11·6)	-0·507 (-9·6–8·8)	-7·48 (-12·8—3·01)	4·68 (1·93–9·55)	4·99 (1·99–10·1)	4·71 (1·86–9·68)	6·78 (-5·02–18·4)	-5·71 (-12·2—0·768)
High LDL cholesterol	55·5 (51·2–60)	44·7 (40·9–48·4)	44·8 (40·8–48·9)	-19·6 (-25·9—12·6)	0·337 (-4·95–5·8)	--	--	--	--	--

Supplementary Results Table 1. Peterborough age-standardised Summary Exposure Values (SEVs) for most-detailed risk factors and percentage change, by sex, 1990, 2010, and 2019. Data in parentheses are 95% uncertainty intervals.

	Male					Female				
	1990	2010	2019	Percentage change 1990-2010	Percentage change 2010-2019	1990	2010	2019	Percentage change 1990-2010	Percentage change 2010-2019
Unsafe sanitation	0·637 (0·208–1·44)	0·447 (0·151–1·05)	0·312 (0·113–0·74)	-29·8 (-78·5–148)	-30·1 (-69·4–72·3)	0·637 (0·208–1·44)	0·447 (0·151–1·05)	0·312 (0·113–0·74)	-29·8 (-78·5–148)	-30·1 (-69·4–72·3)
Ambient particulate matter pollution	17·5 (6·01–33·1)	12·1 (7·52–17·8)	9·5 (5·25–14·7)	-30·8 (-64–71·1)	-21·6 (-29·8–-17·4)	17·5 (6·01–33·1)	12·1 (7·53–17·8)	9·5 (5·24–14·7)	-30·8 (-64–71·8)	-21·6 (-29·8–-17·4)
Household air pollution from solid fuels	0·0207 (0·00413–0·0625)	0·00796 (0·00149–0·0256)	0·00487 (0·000785–0·0157)	-61·5 (-89·3–36·3)	-38·8 (-73·9–37·1)	0·0272 (0·00558–0·0806)	0·0105 (0·0019–0·0319)	0·00645 (0·00108–0·0219)	-61·3 (-89·2–37·5)	-38·7 (-73·9–37·5)
Ambient ozone pollution	14·1 (1·5–34·8)	16·8 (1·3–53·1)	17·3 (1·17–58·3)	19·1 (-86·6–652)	2·83 (-89·6–919)	14·1 (1·5–34·8)	16·8 (1·3–53·1)	17·3 (1·17–58·3)	19·1 (-86·6–652)	2·83 (-89·6–919)
Residential radon	25·1 (5·34–70·6)	25·1 (5·34–70·6)	25·1 (5·34–70·6)	0 (0–0)	0 (0–0)	25·1 (5·34–70·6)	25·1 (5·34–70·6)	25·1 (5·34–70·6)	0 (0–0)	0 (0–0)
Lead exposure	27 (2·88–52·4)	17·7 (1·44–40·3)	12·4 (0·535–32·6)	-34·6 (-58·9–-21)	-29·8 (-66·3–-17·9)	11·2 (0·00217–33·1)	6·89 (4·73e-12–24·8)	4·84 (3·7e-12–20·4)	-38·7 (-100–-5·81)	-29·7 (-100–3·87)
Child underweight	0·932 (0·526–1·39)	0·797 (0·431–1·21)	0·777 (0·425–1·2)	-14·5 (-32·7–7·75)	-2·44 (-18·2–14·8)	1·09 (0·627–1·61)	0·957 (0·55–1·45)	0·93 (0·537–1·42)	-11·9 (-29·3–10·3)	-2·83 (-17·7–13·8)
Iron deficiency	--	--	--	--	--	10·6 (8·42–12·9)	9·79 (7·87–12·1)	9·52 (7·58–11·8)	-7·28 (-22·5–9·65)	-2·82 (-12·9–8·54)
Vitamin A deficiency	4·71 (0·812–13·5)	2·45 (0·426–7·42)	1·88 (0·339–5·53)	-48 (-88·9–187)	-23·1 (-71·6–121)	3·51 (0·99–8·84)	2·57 (0·844–6·03)	2·07 (0·65–4·89)	-27 (-74·7–178)	-19·3 (-59·5–74·3)
Zinc deficiency	3·38 (0–10·6)	2·16 (0–6·95)	2·2 (0–7·19)	-36·1 (-100–NA)	2·18 (-22·9–NA)	3·26 (0–9·76)	2·05 (0–6·7)	2·09 (0–6·86)	-37·3 (-100–NA)	2·28 (-21·9–NA)
Smoking	27·9 (24·4–31·8)	21·7 (18·9–24·9)	19·5 (17–22·6)	-22·4 (-29·5–-14·8)	-9·93 (-15·2–-4·41)	21·4 (17·7–25·1)	17·8 (14·8–21·4)	14·5 (11·9–17·5)	-16·4 (-28·1–-2·37)	-19 (-26·6–-10·2)
[truncated: 891,069 more chars]
