# Supplementary material for: Global burden of 87 risk factors in 204 countries and territories, 1990–2019: a systematic analysis for the Global Burden of Disease Study 2019
Source: Lancet. 2020 Oct 17;396(10258):1223–49. doi: 10.1016/S0140-6736(20)30752-2 (PMC7566194; doi:10.1016/S0140-6736(20)30752-2)
Supplement: Supplementary appendix 2 [file mmc2b.pdf]

THE LANCET

Supplementary appendix 2b (Part 2 of 2)

This appendix formed part of the original submission and has been peer reviewed. We post it as supplied by the authors.

Supplement to: GBD 2019 Risk Factors Collaborators. Global burden of 87 risk factors in 204 countries and territories, 1990–2019: a systematic analysis for the Global Burden of Disease Study 2019. *Lancet* 2020; **396:** 1223–49.

Appendix 2B: Supplementary results appendix for “Global burden of 87 risk factors in 204 countries and territories, 1990–2019: a systematic analysis for the Global Burden of Disease Study 2019”

This appendix provides supplemental figures and more detailed results for "Global burden of 87 risk factors in 204 countries and territories, 1990–2019: a systematic analysis for the Global Burden of Disease Study 2019."

List of supplementary results appendix figures and tables

Appendix 2A

Figure S1 a-j: Leading 20 Level 4 risk factors for DALYs by SDI level and sex, 1990, 2010,and 2019. . . . 1
Figure S2: Leading 10 Level 3 risk factors for deaths in 2019 by location. 11
Figure S3: Leading 10 Level 3 risk factors for DALYs in 2019 by location. 32
Table S1: Age-standardised SEVs for Level 4 risk factors and percentage change, by sex, 1990, 2010, and 2019, by geography 53

Appendix 2B

Table S2: All-age PAFs attributable to the joint distribution of all risk factors, both sexes combined for each GBD Level 1, Level 2, and Level 3 cause and all causes in 2019, by geography. 1193
Table S3: All-age deaths, death PAFs, DALYs, and DALY PAFs, and percentage change for each risk factor and outcome for both sexes combined in 2010 and 2019, by geography. 3873

Supplementary Results Table 2. All-age PAFs attributable to the joint distribution of all risk factors for all ages, both sexes combined for each GBD level 1, level 2, and level 3 cause and all causes in 2019 for Alabama. Results are shown for deaths, YLLs, YLDs, and DALYs Data in parenthesis are 95% uncertainty intervals. PAFs=population attributable fractions. GBD=Global Burden of Disease. YLLs=years of life lost. YLDs=years lived with disability. DALYs=disability-adjusted life-years

	Deaths 2019	DALYs 2019	YLDs 2019	YLLs 2019
All causes	62·5 (60·2–64·8)	50·5 (47·5–53·4)	35·4 (32·5–37·9)	61·4 (59·5–63·2)
Communicable, maternal, neonatal, and nutritional diseases	44·1 (39·7–48·2)	58 (55·2–60·9)	52·7 (48·1–57·5)	60·7 (57·1–63·8)
HIV/AIDS and sexually transmitted infections	94·8 (93·9–95·5)	94·7 (93·7–95·5)	96·3 (95·4–97·3)	93·8 (92·7–94·5)
HIV/AIDS	94·7 (93·8–95·4)	94·2 (93·2–95)	95·1 (94·2–95·9)	93·7 (92·7–94·5)
Sexually transmitted infections excluding HIV	98·7 (98·2–99·1)	99·4 (99–99·7)	100 (100–100)	95·4 (93·5–96·9)
Respiratory infections and tuberculosis	35·5 (29·7–41)	34·3 (28·7–40·1)	3·06 (2·33–3·91)	41·6 (35·4–47·2)
Tuberculosis	55 (46·1–62·8)	58·6 (50·1–65·8)	53·9 (44–61·8)	59·2 (50·8–66·5)
Lower respiratory infections	35·3 (29·3–40·9)	41·2 (35–47)	34·2 (28·6–39·4)	41·3 (35–47·1)
Upper respiratory infections	0·934 (0·57–1·51)	0·0307 (0·0155–0·0547)	0·00536 (0·000206–0·0144)	1·93 (1·16–3·1)
Otitis media	2·11 (0·818–4·35)	4·29 (2·79–5·82)	4·26 (2·76–5·78)	5·29 (2·39–9·63)
Enteric infections	13·2 (8·06–20·1)	14·5 (9·45–21·3)	13·9 (8·97–20·9)	15·3 (10·2–21·9)
Diarrheal diseases	13·3 (8·11–20·2)	14·6 (9·52–21·5)	13·9 (8·98–20·9)	15·5 (10·4–22·2)
Other infectious diseases	0·936 (0·668–1·25)	1·64 (1·25–2·08)	0·716 (0·448–1·13)	1·83 (1·35–2·36)
Meningitis	1·95 (1·37–2·61)	4·09 (2·93–5·44)	0·00066 (1·8e-05–0·00189)	4·24 (3·03–5·65)
Encephalitis	0·781 (0·535–1·11)	2 (1·41–2·84)	0·000276 (6·92e-06–0·00076)	2·06 (1·45–2·93)
Measles	9·77 (-3·84–44·5)	9·93 (-3·9–45·2)	0·236 (0–1·09)	9·94 (-3·91–45·2)
Acute hepatitis	24·3 (15·7–38·2)	10·9 (7·12–16·1)	3·93 (2·48–6·09)	24·9 (16·1–38·2)
Maternal and neonatal disorders	86·7 (84·8–88·3)	85·1 (83·4–86·5)	77·5 (73·6–80·9)	88·5 (87·1–89·8)
Maternal disorders	12·4 (4·42–20·7)	12·2 (4·37–20·5)	12 (4·26–20·4)	12·2 (4·38–20·5)
Neonatal disorders	91·7 (90·6–92·7)	87·8 (86·2–89·2)	79·2 (75·2–82·7)	91·7 (90·6–92·7)
Nutritional deficiencies	94·9 (84·6–96·3)	91·2 (88–93·3)	90·1 (86·9–92·8)	94·4 (83·8–95·8)
Protein-energy malnutrition	100 (100–100)	100 (100–100)	100 (100–100)	100 (100–100)
Vitamin A deficiency	--	100 (100–100)	100 (100–100)	--
Dietary iron deficiency	--	100 (100–100)	100 (100–100)	--
Non-communicable diseases	66·4 (63·7–69)	53·3 (49·7–56·8)	35·1 (32–37·9)	67·4 (65·4–69·5)
Neoplasms	51·8 (48·1–55·8)	51·3 (47·7–55·1)	31·6 (27·7–36)	52·4 (48·9–56·1)
Lip and oral cavity cancer	67·5 (59·3–74·3)	70·2 (62·6–76·8)	69·4 (61·7–76·3)	70·3 (62·6–76·9)
Nasopharynx cancer	62·5 (52·4–71·9)	62·8 (52·1–72)	61·6 (51·6–71)	62·8 (52·2–72)
Other pharynx cancer	76·4 (69·9–82)	77·8 (71·7–83·1)	78·1 (71·6–83·3)	77·8 (71·6–83·1)
Esophageal cancer	81·1 (72·3–88·3)	82·3 (73·9–89·4)	81·4 (72·5–88·6)	82·3 (73·9–89·4)

Stomach cancer	26·4 (18·2–44·2)	27 (18·8–45·1)	27·2 (18·8–45)	27 (18·8–45·1)
Colon and rectum cancer	62·7 (53·8–71·5)	63·5 (55·1–71·8)	63·4 (55–72)	63·6 (55·1–71·8)
Liver cancer	79·8 (74·9–84·6)	79·5 (74·8–84)	79·8 (74·9–84·6)	79·5 (74·8–84)
Gallbladder and biliary tract cancer	27 (16–40)	28·2 (16·8–41·3)	26·1 (15–39)	28·2 (16·8–41·4)
Pancreatic cancer	45·5 (36–56·5)	45·6 (36·4–56·3)	45·6 (36·2–56·7)	45·6 (36·4–56·3)
Larynx cancer	82·3 (73·1–89)	83·6 (75·9–89·3)	83·1 (74·8–89·2)	83·6 (76–89·3)
Tracheal, bronchus, and lung cancer	84·8 (82–88)	84·4 (81·6–87·5)	84·7 (81·6–88·1)	84·4 (81·6–87·5)
Breast cancer	34·4 (24·7–46·3)	33·4 (24·3–44·2)	34 (24·6–45·2)	33·3 (24·3–44·1)
Cervical cancer	100 (100–100)	100 (100–100)	100 (100–100)	100 (100–100)
Uterine cancer	54·7 (39·1–68·4)	56·6 (41·2–69·8)	57·1 (41·8–70·2)	56·5 (41·1–69·8)
Ovarian cancer	20 (8·4–34·9)	18·4 (7·41–32·6)	17·9 (7·24–32)	18·4 (7·42–32·7)
Prostate cancer	6·51 (2·91–10·5)	7·31 (3·25–11·6)	7·76 (3·47–12·2)	7·21 (3·22–11·4)
Kidney cancer	43·8 (32·8–54·5)	44·6 (34·4–54·4)	44·7 (34·4–54·4)	44·6 (34·4–54·4)
Bladder cancer	43·1 (27·6–58·2)	46·7 (32–60·6)	47·2 (32·8–60·8)	46·7 (32–60·7)
Thyroid cancer	18·7 (9·74–29·3)	20 (10·5–31)	20·3 (11–31)	19·9 (10·4–30·9)
Mesothelioma	95·9 (94·3–97·1)	92·6 (90–94·6)	95·6 (93·8–97)	92·5 (89·9–94·6)
Non-Hodgkin lymphoma	8·9 (3·99–15·1)	9·44 (4·31–15·7)	9·19 (4·21–15·4)	9·45 (4·34–15·7)
Multiple myeloma	10·2 (4·71–16·9)	10·8 (5·13–17·7)	10·6 (5·05–17·8)	10·8 (5·1–17·7)
Leukemia	38·1 (23·7–52·2)	36·8 (24·3–48·9)	38·6 (24·4–52·3)	36·7 (24·3–48·8)
Cardiovascular diseases	83·4 (80·2–86·2)	83·9 (81·4–86·2)	73·5 (69·4–77·7)	85·4 (83·2–87·5)
Rheumatic heart disease	22·8 (13·4–42·2)	22·7 (14·9–38·6)	22·6 (14·1–38·4)	22·7 (15–38·2)
Ischemic heart disease	93·6 (90·3–96·4)	95·1 (92·9–96·9)	93·6 (90·7–96·2)	95·1 (93–97)
Stroke	81 (74·7–87·5)	84·6 (80·3–88·8)	83·5 (78·8–88·4)	84·9 (80·8–89·2)
Hypertensive heart disease	100 (100–100)	100 (100–100)	100 (100–100)	100 (100–100)
Non-rheumatic valvular heart disease	26·2 (16·6–38·7)	21·8 (15·1–30·4)	4·22 (2·78–6·06)	26·1 (18·2–36·3)
Cardiomyopathy and myocarditis	41·4 (34·1–49·2)	43·7 (37·5–49·3)	42·3 (35·9–48·1)	43·8 (37·4–49·6)
Atrial fibrillation and flutter	52·7 (43–62·7)	58·3 (49·6–67·1)	59·5 (50·9–68·1)	56·8 (48–65·6)
Aortic aneurysm	58·3 (52·2–64·4)	66 (61·2–70·3)	--	66 (61·2–70·3)
Endocarditis	32·9 (23·1–45)	34·1 (24·8–44·5)	32·1 (22–44)	34·1 (24·8–44·5)
Other cardiovascular and circulatory diseases	25·8 (20·6–32·4)	27·7 (22·7–33·5)	32·8 (25·7–40·1)	26·2 (21·3–32·4)
Chronic respiratory diseases	65·1 (60·3–69·8)	62 (58·3–65·7)	55·1 (50·7–59·4)	66·3 (62·3–70·5)
Chronic obstructive pulmonary disease	71·3 (66·9–75·4)	71·6 (68·1–75·1)	66·8 (63·1–70·4)	73·7 (70–77·3)
Pneumoconiosis	100 (100–100)	100 (100–100)	100 (100–100)	100 (100–100)

Asthma	52·2 (40·9–62·9)	42·6 (34·8–50·9)	41·6 (33·9–49·7)	51·3 (41·9–60·9)
Digestive diseases	46·5 (41·1–52·1)	48·9 (43·4–54·7)	19·7 (13·6–26·4)	55·7 (49·9–61·3)
Cirrhosis and other chronic liver diseases	79 (70·1–86·9)	81·5 (73·1–88·5)	76·7 (68·1–85·5)	81·5 (73·2–88·5)
Upper digestive system diseases	23·4 (19·5–27·2)	7·53 (4·71–11)	1·9 (1·17–2·89)	27·1 (23·1–31·2)
Gallbladder and biliary diseases	51·5 (35·8–66·3)	56·4 (41·9–69·1)	57·7 (43·6–70)	54·1 (39–67·9)
Pancreatitis	32·7 (18·3–49·8)	37·5 (21·9–55·2)	34·7 (19·3–52)	37·8 (22–55·3)
Neurological disorders	24·6 (9·09–40·8)	15·2 (7·44–27·2)	11·2 (5·61–21·5)	20·7 (7·09–37·2)
Alzheimer's disease and other dementias	42·4 (27·8–58·4)	46 (31·2–61·2)	46·8 (32·1–61·8)	45·4 (30·8–60·9)
Parkinson's disease	-10·3 (-16–-5·08)	-12·2 (-18·4–-6·16)	-12·9 (-19·2–-6·8)	-12·1 (-18·4–-6·06)
Idiopathic epilepsy	19·8 (13–26·7)	18·4 (12·1–24·6)	17·2 (11·1–23·3)	21 (13·8–27·8)
Multiple sclerosis	16·3 (12–21)	17·2 (12·7–22)	16·6 (12·4–21·4)	17·8 (13·1–22·8)
Mental disorders	--	11·7 (6·03–18·2)	11·7 (6·04–18·2)	--
Depressive disorders	--	22·2 (11·6–32·9)	22·2 (11·6–32·9)	--
Anxiety disorders	--	11 (3·04–22·4)	11 (3·04–22·4)	--
Idiopathic developmental intellectual disability	--	50·6 (25·9–69·8)	50·6 (25·9–69·8)	--
Substance use disorders	100 (100–100)	100 (100–100)	100 (100–100)	100 (100–100)
Alcohol use disorders	100 (100–100)	100 (100–100)	100 (100–100)	100 (100–100)
Drug use disorders	100 (100–100)	100 (100–100)	100 (100–100)	100 (100–100)
Diabetes and kidney diseases	99·9 (99·8–99·9)	99·9 (99·9–100)	100 (100–100)	99·9 (99·9–99·9)
Diabetes mellitus	100 (100–100)	100 (100–100)	100 (100–100)	100 (100–100)
Chronic kidney disease	100 (100–100)	100 (100–100)	100 (100–100)	100 (100–100)
Sense organ diseases	--	10·4 (9·26–11·7)	10·4 (9·26–11·7)	--
Blindness and vision loss	--	10·1 (6·59–14·8)	10·1 (6·59–14·8)	--
Age-related and other hearing loss	--	11·1 (10–12·3)	11·1 (10–12·3)	--
Musculoskeletal disorders	3·75 (1·07–6·83)	21·7 (18·3–25·5)	22·1 (18·5–26)	3·53 (1·05–6·39)
Rheumatoid arthritis	13·1 (3·89–23·2)	16·1 (5·15–28·5)	16·3 (5·27–28·9)	15·4 (4·83–27·4)
Osteoarthritis	--	15·4 (8·76–22·6)	15·4 (8·76–22·6)	--
Low back pain	--	42·9 (36·9–49·1)	42·9 (36·9–49·1)	--
Gout	--	61 (45·2–76·7)	61 (45·2–76·7)	--
Other non-communicable diseases	0·217 (0·152–0·301)	0·28 (0·192–0·389)	--	0·638 (0·454–0·856)
Sudden infant death syndrome	6·16 (4·27–8·51)	6·16 (4·27–8·5)	--	6·16 (4·27–8·5)
Injuries	24 (20·5–27·5)	22·9 (19·5–26·1)	28·6 (25·4–31·5)	20·5 (16·5–24·4)
Transport injuries	24·7 (20·4–29·5)	23·6 (19·3–28·6)	27·6 (24–31)	22·6 (17·7–28·1)

Road injuries	24·4 (20–29·1)	22·8 (18·4–27·9)	25·4 (22–28·9)	22·3 (17·4–27·8)
Other transport injuries	32 (27·4–37)	33 (28·7–37·4)	38·4 (34·2–42)	28·5 (23·5–34·3)
Unintentional injuries	26·9 (23·4–29·9)	23·4 (20·2–26·9)	29·3 (25·6–32·8)	16·1 (13·2–19·4)
Falls	52·1 (46·3–56·2)	42·3 (37·3–45·9)	41·8 (36·8–45·5)	43·4 (38·5–47·5)
Drowning	-7·55 (-13·9–-0·656)	-8·2 (-14·2–-1·65)	8·82 (5·61–13·1)	-8·51 (-14·6–-1·86)
Fire, heat, and hot substances	9·41 (6·31–13·5)	11·1 (7·69–15·4)	12·1 (8·26–16·9)	10·4 (7·15–14·4)
Poisonings	13·3 (9·21–18·3)	13·1 (9·11–18)	11 (7·51–15·4)	14 (9·8–19·1)
Exposure to mechanical forces	14·6 (9·13–19·8)	18·2 (14·9–21·7)	21·4 (18·7–24·3)	11·8 (5·93–17·5)
Animal contact	20·3 (16·4–24·8)	22·2 (18·4–26·6)	23·5 (19·4–28)	17·1 (13·5–21·4)
Foreign body	2·22 (1·47–3·18)	3·01 (2·04–4·28)	3·71 (2·55–5·29)	2·82 (1·88–4·07)
Environmental heat and cold exposure	6·19 (2·83–11·1)	6·69 (3·04–11·9)	6·15 (2·75–11)	7·07 (3·23–12·5)
Exposure to forces of nature	6 (2·68–10·6)	6·23 (2·83–10·9)	7·09 (3·26–12·4)	5·86 (2·66–10·3)
Other unintentional injuries	15·8 (11–21·4)	12·2 (8·46–16·9)	11·3 (7·71–16)	17·5 (12·4–23·6)
Self-harm and interpersonal violence	20·5 (13·3–28)	21·5 (14·6–28·7)	25·7 (19·7–31·6)	21·2 (14·2–28·4)
Self-harm	21·7 (10·7–32·2)	24·1 (12·7–34·9)	22·2 (12·8–31·7)	24·1 (12·7–34·9)
Interpersonal violence	18·8 (11·3–26·4)	18·7 (11·4–26·1)	26·7 (20·4–33·4)	17·5 (10·1–25·1)

Supplementary Results Table 2. All-age PAFs attributable to the joint distribution of all risk factors for all ages, both sexes combined for each GBD level 1, level 2, and level 3 cause and all causes in 2019 for Alaska. Results are shown for deaths, YLLs, YLDs, and DALYs Data in parenthesis are 95% uncertainty intervals. PAFs=population attributable fractions. GBD=Global Burden of Disease. YLLs=years of life lost. YLDs=years lived with disability. DALYs=disability-adjusted life-years				
	**Deaths 2019**	**DALYs 2019**	**YLDs 2019**	**YLLs 2019**
All causes	59·4 (57·1–61·6)	45·3 (42·5–48·2)	33·3 (30·5–35·8)	56·4 (54·1–58·6)
Communicable, maternal, neonatal, and nutritional diseases	49·7 (41·4–56·6)	53·4 (49·2–57·6)	44·2 (40–48·4)	60·4 (54·5–65·8)
HIV/AIDS and sexually transmitted infections	86 (84–87·8)	87·5 (85·3–89·8)	92 (89·1–95·2)	84 (81·7–86)
HIV/AIDS	85·2 (83–87·1)	84·3 (81·9–86·3)	85·7 (83·2–87·7)	83·6 (81·2–85·7)
Sexually transmitted infections excluding HIV	98 (97·1–98·7)	99·3 (98·8–99·7)	100 (100–100)	93·9 (91·3–96)
Respiratory infections and tuberculosis	45·9 (33·4–57·1)	34·5 (25·3–42·8)	2·61 (1·94–3·5)	50·2 (38·4–60·5)
Tuberculosis	56·3 (46·9–64·7)	57·8 (48·7–66)	55·5 (45·7–64·1)	58·2 (49·2–66·3)
Lower respiratory infections	45·6 (32·7–57·3)	49·8 (37·7–60·4)	33·8 (27·8–39·3)	50·1 (37·7–61)
Upper respiratory infections	0·703 (0·4–1·14)	0·0265 (0·0127–0·0469)	0·00701 (6·71e-06–0·0198)	1·25 (0·716–2·01)
Otitis media	2·88 (1·09–5·87)	4·22 (2·8–5·83)	4·19 (2·79–5·82)	5·8 (2·61–10·1)
Enteric infections	11·8 (7·44–17·8)	13·3 (9·04–19·4)	12·3 (7·94–18·4)	15·8 (11·4–21·8)
Diarrheal diseases	11·9 (7·55–18)	13·4 (9·1–19·5)	12·3 (7·95–18·4)	16·2 (11·6–22·4)
Other infectious diseases	0·938 (0·646–1·25)	1·41 (1·02–1·85)	0·687 (0·412–1·08)	1·57 (1·11–2·08)
Meningitis	1·51 (1·03–2·14)	2·77 (1·95–3·88)	0·000815 (5·63e-07–0·00258)	2·89 (2·02–4·05)
Encephalitis	0·715 (0·481–1·02)	1·54 (1·04–2·14)	0·000259 (1·72e-07–0·000792)	1·59 (1·07–2·21)
Measles	9·41 (-4·07–43·3)	9·56 (-4·13–44)	0·319 (0–0·964)	9·57 (-4·14–44·1)
Acute hepatitis	18·1 (11·2–30·5)	9·08 (5·64–14·5)	3·4 (2·07–5·42)	18·4 (11·1–30·6)
Maternal and neonatal disorders	81·8 (78·8–84·6)	75·4 (71·7–78·9)	63·7 (57·9–68·9)	83·3 (80·6–85·9)
Maternal disorders	11·3 (3·94–18·7)	11·2 (3·9–18·6)	11·2 (3·85–18·6)	11·2 (3·91–18·5)
Neonatal disorders	86·2 (83·8–88·4)	77·9 (74·3–81·2)	65·7 (59·7–71)	86·2 (83·8–88·4)
Nutritional deficiencies	93·7 (84·8–95·3)	87·8 (84·6–90·7)	86·4 (82·5–90)	92·9 (83·7–94·5)
Protein-energy malnutrition	100 (100–100)	100 (100–100)	100 (100–100)	100 (100–100)
Vitamin A deficiency	--	100 (100–100)	100 (100–100)	--
Dietary iron deficiency	--	100 (100–100)	100 (100–100)	--
Non-communicable diseases	64·7 (62·2–67·2)	49·3 (45·7–52·6)	33·2 (30–36)	65·8 (63·7–67·8)
Neoplasms	51·7 (48–55·6)	49·5 (46–53·3)	32 (27·8–36·1)	50·6 (47·1–54·4)
Lip and oral cavity cancer	68·4 (59·4–75·9)	69·8 (60·7–77)	69·3 (60·3–76·6)	69·8 (60·7–77·1)
Nasopharynx cancer	64·5 (52·7–73·8)	63·8 (51·9–73)	62·8 (50·9–72·1)	63·8 (51·9–73)
Other pharynx cancer	77·7 (70·3–83·1)	78·3 (71–83·7)	78·6 (71·4–83·7)	78·3 (71–83·7)
Esophageal cancer	79·7 (70·3–87·9)	80·4 (70·9–88·5)	79·8 (70·2–87·9)	80·4 (70·9–88·5)

Stomach cancer	26 (17·7–43·5)	25·7 (17·5–43·7)	26·3 (18–44·1)	25·7 (17·5–43·7)
Colon and rectum cancer	62·7 (54·2–70·9)	63·3 (55·2–70·8)	63·2 (55·3–71·1)	63·3 (55·2–70·8)
Liver cancer	79·9 (75·5–84·5)	78·9 (74·4–83·2)	79·4 (74·7–83·8)	78·9 (74·4–83·3)
Gallbladder and biliary tract cancer	26·9 (15·7–40)	27·8 (16·4–41·1)	25·8 (14·5–39·2)	27·9 (16·4–41·2)
Pancreatic cancer	43·8 (34·8–54·3)	43·1 (34·4–53·3)	43·7 (34·9–53·9)	43·1 (34·4–53·3)
Larynx cancer	83·2 (74·5–89·5)	83·3 (75·3–88·9)	83 (74·8–88·9)	83·4 (75·4–88·9)
Tracheal, bronchus, and lung cancer	84·5 (81·3–87·9)	83·3 (80–86·7)	83·9 (80·6–87·5)	83·2 (80–86·7)
Breast cancer	36 (27·1–46·5)	34·4 (25·7–43·6)	35·1 (26·1–44·9)	34·3 (25·7–43·5)
Cervical cancer	100 (100–100)	100 (100–100)	100 (100–100)	100 (100–100)
Uterine cancer	56 (40·6–69·2)	57·2 (41·8–70)	57·5 (41·8–70·2)	57·1 (41·8–69·9)
Ovarian cancer	18·5 (8·21–31·4)	16·4 (6·74–28·6)	15·6 (6·35–27·3)	16·4 (6·75–28·6)
Prostate cancer	6·3 (2·7–10·3)	6·88 (2·93–11·1)	7·15 (3·17–11·4)	6·79 (2·9–11)
Kidney cancer	44·1 (33·5–54·4)	43·8 (33·5–53·8)	43·9 (33·7–53·7)	43·8 (33·5–53·8)
Bladder cancer	42·8 (27·9–57·3)	45·4 (31·3–58·5)	45·7 (31·9–58·5)	45·4 (31·2–58·5)
Thyroid cancer	19·8 (10·2–30·8)	20·4 (10·8–31·7)	20·4 (11·1–31·1)	20·4 (10·6–31·6)
Mesothelioma	96·7 (95·5–97·6)	93·8 (91·8–95·4)	96·3 (94·7–97·5)	93·7 (91·8–95·4)
Non-Hodgkin lymphoma	9·64 (4·44–16)	9·87 (4·65–16·2)	9·75 (4·52–16·1)	9·87 (4·67–16·2)
Multiple myeloma	10·8 (5·05–17·7)	11·2 (5·31–18·5)	11·1 (5·22–18·4)	11·2 (5·3–18·5)
Leukemia	37·4 (23·8–50·7)	33·4 (22·5–44·2)	37·6 (24·4–50·7)	33·2 (22·5–44)
Cardiovascular diseases	80·7 (77·5–83·7)	81·3 (78·6–83·8)	73·1 (68·4–77·2)	82·5 (80–84·8)
Rheumatic heart disease	22·2 (13·5–38·1)	21·8 (14·2–34·2)	21·8 (13·7–35·8)	21·8 (14·1–34)
Ischemic heart disease	93·6 (90·6–96·3)	95 (92·7–96·9)	93·3 (90·5–96·1)	95 (92·7–96·9)
Stroke	81·9 (76–88·2)	85 (80·8–89·1)	83·7 (79·3–88·4)	85·4 (81·2–89·5)
Hypertensive heart disease	100 (100–100)	100 (100–100)	100 (100–100)	100 (100–100)
Non-rheumatic valvular heart disease	26·3 (17·1–37·9)	25·3 (17·6–34·5)	9·24 (5·96–13·3)	26·6 (18·6–36·1)
Cardiomyopathy and myocarditis	45·1 (38·8–51·3)	45·8 (40·2–50·9)	44·2 (38·5–50·1)	46 (40·3–51·2)
Atrial fibrillation and flutter	55·1 (45·3–64·5)	61 (52·9–69·2)	62·4 (54·1–70·5)	59·2 (50·6–67·6)
Aortic aneurysm	59·6 (54·2–65·4)	66·1 (61·5–70·5)	--	66·1 (61·5–70·5)
Endocarditis	32·3 (22·6–43·6)	33·1 (23·8–43·3)	31·6 (21·7–43)	33·2 (23·8–43·4)
Other cardiovascular and circulatory diseases	21·2 (16·3–28·5)	22·7 (17·6–29·1)	30·2 (22·8–38)	20·9 (16·2–27·5)
Chronic respiratory diseases	65·8 (60·6–71·1)	59·4 (55·4–63·4)	53 (48·2–57·6)	64·9 (60·1–69·7)
Chronic obstructive pulmonary disease	74·7 (69·5–79·4)	72·4 (68·4–76·1)	66·2 (62·6–69·9)	75·8 (71–80·2)
Pneumoconiosis	100 (100–100)	100 (100–100)	100 (100–100)	100 (100–100)

Asthma	52·4 (41·6–62·9)	42·3 (34·7–50·7)	41·4 (33·7–49·5)	49·4 (39·9–58·8)
Digestive diseases	52·5 (46·7–58)	51·2 (45·4–56·6)	16·5 (11·3–22·3)	59·9 (53·8–65·7)
Cirrhosis and other chronic liver diseases	81·2 (72·5–88·6)	82·7 (74·7–89·5)	79·2 (70·6–86·7)	82·8 (74·7–89·5)
Upper digestive system diseases	23·6 (19·8–27·4)	6·25 (3·74–9·52)	1·44 (0·839–2·33)	25·5 (21·6–29·4)
Gallbladder and biliary diseases	54·4 (38·8–68·8)	57·3 (42·9–70)	58·1 (43·9–70·3)	56·1 (41·1–70·2)
Pancreatitis	37 (18·8–54·9)	40·3 (21·6–58·4)	38·1 (19·1–55·3)	40·6 (21·9–58·8)
Neurological disorders	23·9 (9·16–39·8)	12·6 (5·97–23·3)	8·57 (4·11–17·9)	19·3 (7·42–35·4)
Alzheimer's disease and other dementias	44·3 (29·9–59)	47·6 (33·1–61·9)	48·2 (33·8–62·4)	47·1 (32·6–61·7)
Parkinson's disease	-10·8 (-17·3–-5·4)	-12·4 (-19·2–-6·37)	-13 (-19·7–-6·79)	-12·3 (-19–-6·24)
Idiopathic epilepsy	23·5 (15–32·4)	20·9 (13·4–28·7)	19·7 (12·3–27·3)	23 (14·8–31·4)
Multiple sclerosis	16·6 (12·1–21·6)	17·4 (12·7–22·5)	17·1 (12·6–21·9)	17·6 (12·8–22·7)
Mental disorders	--	9·65 (4·7–15·3)	9·65 (4·7–15·3)	--
Depressive disorders	--	20·2 (9·16–30·4)	20·2 (9·16–30·4)	--
Anxiety disorders	--	6·66 (1·45–15·3)	6·66 (1·45–15·3)	--
Idiopathic developmental intellectual disability	--	47·9 (23·4–68·3)	47·9 (23·4–68·3)	--
Substance use disorders	100 (100–100)	100 (100–100)	100 (100–100)	100 (100–100)
Alcohol use disorders	100 (100–100)	100 (100–100)	100 (100–100)	100 (100–100)
Drug use disorders	100 (100–100)	100 (100–100)	100 (100–100)	100 (100–100)
Diabetes and kidney diseases	99·9 (99·9–99·9)	100 (100–100)	100 (100–100)	99·9 (99·9–99·9)
Diabetes mellitus	100 (100–100)	100 (100–100)	100 (100–100)	100 (100–100)
Chronic kidney disease	100 (100–100)	100 (100–100)	100 (100–100)	100 (100–100)
Sense organ diseases	--	11·1 (10·1–12·3)	11·1 (10·1–12·3)	--
Blindness and vision loss	--	8·92 (5·88–12·8)	8·92 (5·88–12·8)	--
Age-related and other hearing loss	--	12·2 (11·1–13·5)	12·2 (11·1–13·5)	--
Musculoskeletal disorders	4·74 (1·4–8·44)	22·7 (19·2–26·5)	23·1 (19·6–26·9)	4·06 (1·23–7·22)
Rheumatoid arthritis	14·4 (4·69–25·9)	16·5 (5·33–29·1)	16·7 (5·37–29·6)	16 (5·19–28·5)
Osteoarthritis	--	13·3 (7·66–19·4)	13·3 (7·66–19·4)	--
Low back pain	--	44·8 (39·1–50·5)	44·8 (39·1–50·5)	--
Gout	--	60 (42·9–76·2)	60 (42·9–76·2)	--
Other non-communicable diseases	0·309 (0·214–0·44)	0·285 (0·19–0·416)	--	0·714 (0·5–0·991)
Sudden infant death syndrome	4·77 (3·34–6·77)	4·76 (3·33–6·76)	--	4·76 (3·33–6·76)
Injuries	17·4 (11·2–24·1)	18·1 (12·8–23·8)	29·2 (25·8–32·8)	13·2 (6·16–20·5)
Transport injuries	18·4 (11·1–25·9)	19·3 (13–26·2)	30·5 (26·6–34·6)	15·2 (7·13–23·2)

Road injuries	10 (0·46–19·8)	12·9 (4·99–21·1)	27 (23·1–31·4)	7·29 (-2·65–17·5)
Other transport injuries	32·6 (27·6–38)	31·2 (26·4–36·4)	38 (33·9–41·7)	29 (23·5–35)
Unintentional injuries	19·5 (14·8–24·1)	18·8 (14·3–23·5)	28·8 (25–33)	8·3 (2·92–13·8)
Falls	50·6 (45·2–55)	40·1 (35·4–44·6)	39·7 (35–44·2)	41 (36·3–45·9)
Drowning	-19 (-36·5–0·0727)	-20·3 (-37·4–-1·49)	11·9 (7·74–17·5)	-20·8 (-38·1–-1·81)
Fire, heat, and hot substances	12·1 (8·15–17·6)	13·8 (9·66–19·2)	16·1 (11·1–22·4)	12·1 (8·36–16·9)
Poisonings	16 (11·2–22·2)	15·6 (11·1–21·4)	13·9 (9·56–19·2)	15·9 (11·4–21·8)
Exposure to mechanical forces	-1·92 (-15·4–12·3)	11·8 (4·61–19)	21·4 (18·4–25·1)	-4·88 (-19·2–9·92)
Animal contact	25·3 (20·9–30·3)	23·1 (18·7–28)	24·7 (20·2–29·9)	20·3 (16·1–25)
Foreign body	3·44 (2·35–4·85)	4 (2·78–5·69)	4·96 (3·43–7·04)	3·82 (2·63–5·49)
Environmental heat and cold exposure	8·01 (3·56–14·4)	8·29 (3·73–14·8)	8·09 (3·64–14·4)	8·34 (3·75–14·8)
Exposure to forces of nature	7·05 (3·15–12·6)	8·01 (3·59–14·2)	8·54 (3·88–15·2)	6·53 (2·91–11·6)
Other unintentional injuries	18·4 (13·1–24·8)	16 (11·2–22·1)	15·5 (10·7–21·5)	19·4 (13·9–26)
Self-harm and interpersonal violence	14·7 (0·646–28·9)	16·3 (3·12–29·2)	29·2 (22·8–35·8)	15·5 (1·57–29·1)
Self-harm	14·8 (-2–32·2)	16·5 (-0·0324–33·6)	25·1 (15–36·1)	16·4 (-0·412–33·5)
Interpersonal violence	15·2 (1·53–29·4)	16·1 (4·12–29)	31 (23·5–38·6)	12·8 (-1·03–26·6)

Supplementary Results Table 2. All-age PAFs attributable to the joint distribution of all risk factors for all ages, both sexes combined for each GBD level 1, level 2, and level 3 cause and all causes in 2019 for Arizona. Results are shown for deaths, YLLs, YLDs, and DALYs Data in parenthesis are 95% uncertainty intervals. PAFs=population attributable fractions. GBD=Global Burden of Disease. YLLs=years of life lost. YLDs=years lived with disability. DALYs=disability-adjusted life-years				
	**Deaths 2019**	**DALYs 2019**	**YLDs 2019**	**YLLs 2019**
All causes	60·4 (57·7–62·8)	46·3 (43–49·1)	33 (30·3–35·4)	59·2 (57–61·2)
Communicable, maternal, neonatal, and nutritional diseases	43 (39·3–46·7)	52·9 (49·8–55·6)	48·3 (43·8–53·2)	56·2 (52·8–59·2)
HIV/AIDS and sexually transmitted infections	89·3 (87·4–90·6)	89·5 (87·6–91·2)	92·6 (90·7–94·9)	87·4 (85·2–89)
HIV/AIDS	88·9 (87–90·4)	87·9 (85·8–89·5)	89·3 (87·3–91)	87·3 (85–88·8)
Sexually transmitted infections excluding HIV	99 (98·5–99·3)	99·7 (99·4–99·8)	100 (100–100)	96·1 (94·4–97·3)
Respiratory infections and tuberculosis	40 (34·2–45·5)	32·7 (26·8–38·3)	2·38 (1·77–3·1)	44·7 (39·1–50·1)
Tuberculosis	55·5 (45·3–65·3)	57·6 (47·8–66·6)	53·6 (43·2–63·1)	58·3 (48·5–67·1)
Lower respiratory infections	39·8 (33·9–45·4)	44·3 (38·5–49·9)	31·1 (25·1–36·6)	44·6 (38·8–50·1)
Upper respiratory infections	0·559 (0·334–0·868)	0·0176 (0·00795–0·0313)	0·005 (0–0·0145)	1·23 (0·732–1·91)
Otitis media	1·43 (0·606–2·88)	3·41 (2·22–4·75)	3·4 (2·2–4·74)	3·86 (1·87–6·82)
Enteric infections	11·7 (7·41–18·2)	13 (8·73–19·4)	12·7 (8·44–19·1)	13·4 (9·01–19·7)
Diarrheal diseases	11·8 (7·45–18·3)	13·1 (8·79–19·5)	12·7 (8·45–19·1)	13·6 (9·14–19·9)
Other infectious diseases	0·655 (0·486–0·96)	1·12 (0·876–1·49)	0·801 (0·491–1·26)	1·18 (0·907–1·61)
Meningitis	1·28 (0·912–1·71)	2·74 (1·99–3·67)	0·000476 (0–0·00138)	2·89 (2·09–3·88)
Encephalitis	0·565 (0·397–0·779)	1·46 (1·04–2·01)	0·000119 (0–0·00036)	1·52 (1·08–2·08)
Measles	9·49 (-3·94–43·6)	6·57 (-3·38–42·3)	0·348 (0–1·15)	9·66 (-4·01–44·4)
Acute hepatitis	22·6 (15·3–34·3)	10·5 (6·87–15·1)	4·1 (2·55–6·22)	23·7 (16·1–35·6)
Maternal and neonatal disorders	82·3 (80–84·6)	80·6 (78·4–82·7)	75·5 (71·2–79·3)	84·1 (82·1–86·1)
Maternal disorders	11·4 (4·02–18·9)	11·2 (3·99–18·7)	11 (3·91–18·4)	11·3 (4·01–18·6)
Neonatal disorders	87·4 (85·5–89·2)	83·3 (81·1–85·4)	77·4 (73·2–81·2)	87·4 (85·5–89·2)
Nutritional deficiencies	94·3 (89·3–96·4)	88·8 (85·8–91·3)	87·7 (84·1–90·9)	93·6 (89–95·8)
Protein-energy malnutrition	100 (100–100)	100 (100–100)	100 (100–100)	100 (100–100)
Vitamin A deficiency	--	100 (100–100)	100 (100–100)	--
Dietary iron deficiency	--	100 (100–100)	100 (100–100)	--
Non-communicable diseases	63·7 (60·5–66·4)	48·2 (44·4–51·5)	32·4 (29·5–35·1)	64·9 (62·6–67)
Neoplasms	47 (43·4–51)	45·7 (42·3–49·4)	28·3 (24·4–32·3)	47 (43·7–50·8)
Lip and oral cavity cancer	66·2 (56·2–74·8)	67·9 (58·5–76)	67·5 (57·6–75·5)	68 (58·5–76)
Nasopharynx cancer	63·4 (51·8–73·3)	62·6 (51·3–72·4)	61·7 (50·4–71·6)	62·6 (51·4–72·5)
Other pharynx cancer	76·2 (69·2–82·2)	76·9 (69·9–82·8)	77·3 (70·1–83·2)	76·9 (70–82·8)

Esophageal cancer	76·5 (67·3–85)	77·3 (68–85·6)	76·6 (67·1–85·1)	77·3 (68–85·6)
Stomach cancer	24·4 (16·4–42·4)	24·6 (16·5–42·6)	25 (16·9–43)	24·6 (16·5–42·6)
Colon and rectum cancer	61·4 (53·2–70·1)	62·1 (54·1–70·2)	62·1 (54–70·5)	62·1 (54–70·2)
Liver cancer	80·2 (75·7–84·4)	79·7 (75·3–83·8)	79·9 (75·4–84·4)	79·7 (75·3–83·8)
Gallbladder and biliary tract cancer	24·8 (14·1–37·6)	26 (15·1–38·9)	23·8 (13·2–36·6)	26·1 (15·1–39)
Pancreatic cancer	42·3 (32·5–53·3)	41·9 (32·7–52·7)	42·2 (32·7–53·1)	41·9 (32·7–52·7)
Larynx cancer	80·8 (70·8–88·5)	81·6 (73·2–88·3)	81·3 (72·9–88·3)	81·6 (73·1–88·2)
Tracheal, bronchus, and lung cancer	82·4 (78·9–85·9)	81·5 (78·3–85·1)	82 (78·6–85·8)	81·5 (78·3–85·1)
Breast cancer	34·4 (25·7–44·7)	33·3 (25·2–42·7)	33·9 (25·5–43·9)	33·2 (25·2–42·6)
Cervical cancer	100 (100–100)	100 (100–100)	100 (100–100)	100 (100–100)
Uterine cancer	51·5 (36·6–66·1)	53·3 (38·4–67·2)	53·8 (38·8–67·7)	53·2 (38·4–67·2)
Ovarian cancer	18·4 (8·34–31·7)	16·6 (7·16–28·8)	16 (6·68–28)	16·6 (7·2–28·8)
Prostate cancer	5·62 (2·43–9·15)	6·28 (2·7–10·2)	6·61 (2·79–10·6)	6·18 (2·67–9·93)
Kidney cancer	40·5 (29·3–51·4)	40·9 (30·6–51·2)	41·1 (30·8–51·1)	40·9 (30·6–51·1)
Bladder cancer	39·4 (24·3–54·4)	42·7 (27·9–56·9)	43·3 (29–56·7)	42·7 (27·8–56·9)
Thyroid cancer	17·3 (8·32–27·8)	18·4 (9·09–29·2)	18·7 (9·59–29·1)	18·4 (8·96–29·2)
Mesothelioma	96·5 (95·2–97·5)	93·3 (91·2–95·2)	96·2 (94·6–97·4)	93·3 (91·1–95·1)
Non-Hodgkin lymphoma	8·09 (3·54–13·9)	8·56 (3·84–14·6)	8·32 (3·62–14·2)	8·57 (3·85–14·6)
Multiple myeloma	9·25 (4·2–15·8)	9·8 (4·44–16·5)	9·66 (4·44–16·5)	9·81 (4·46–16·5)
Leukemia	35·5 (21·6–49·6)	33·4 (21·5–45·3)	35·8 (22·1–49·7)	33·3 (21·5–45·1)
Cardiovascular diseases	81·8 (78·1–84·9)	81·5 (78·6–84·1)	70·2 (65·6–75)	83·5 (80·8–85·9)
Rheumatic heart disease	21·5 (12·4–40·2)	21·4 (13·5–34·8)	21·3 (13–35·6)	21·4 (13·5–34·7)
Ischemic heart disease	92 (88–95·2)	93·5 (90·7–95·9)	92 (88·5–95·1)	93·5 (90·8–95·9)
Stroke	78 (70·6–85·7)	81·5 (76·6–86·7)	80·7 (75·3–86·4)	81·9 (76·8–87)
Hypertensive heart disease	100 (100–100)	100 (100–100)	100 (100–100)	100 (100–100)
Non-rheumatic valvular heart disease	25 (15·2–38)	22·3 (15·1–31·4)	8·47 (5·4–12·2)	25·3 (17·1–35·9)
Cardiomyopathy and myocarditis	39·1 (32·2–46·8)	41·4 (35·6–47·2)	39·6 (33·3–45·7)	41·6 (35·6–47·5)
Atrial fibrillation and flutter	51·5 (41·7–61·5)	56·8 (47·9–65·6)	58 (49·4–66·4)	55·2 (46·1–64·3)
Aortic aneurysm	54·7 (48·9–60·8)	62·4 (57·6–67·2)	--	62·4 (57·6–67·2)
Endocarditis	31 (21·2–43·4)	32·4 (23·3–42·7)	30·5 (20·6–43)	32·5 (23·4–42·8)
Other cardiovascular and circulatory diseases	22 (16·8–28·4)	24·4 (19·2–29·5)	30·1 (23·8–37·5)	22·6 (17·6–28·2)
Chronic respiratory diseases	64·7 (59·5–69·2)	58·6 (54·6–62·4)	51·8 (46·8–56·5)	65 (60·9–68·9)
Chronic obstructive pulmonary disease	72·2 (67·2–76·6)	70 (66·1–73·6)	63·7 (59·6–67·7)	73·9 (69·8–77·7)

Pneumoconiosis	100 (100–100)	100 (100–100)	100 (100–100)	100 (100–100)
Asthma	48·5 (37·6–59·9)	40·3 (32·4–49·1)	39·6 (31·9–48·5)	47·7 (38·3–57·3)
Digestive diseases	52·5 (46·4–57·7)	53·1 (47–58·5)	18·1 (12·4–24·3)	61·9 (55·6–67·3)
Cirrhosis and other chronic liver diseases	81·8 (73·7–89·3)	83·3 (75·6–90·3)	79·4 (70·6–87·3)	83·3 (75·7–90·3)
Upper digestive system diseases	21·6 (17·8–25·3)	6·22 (3·69–9·38)	1·47 (0·874–2·3)	24·1 (20·2–27·9)
Gallbladder and biliary diseases	47·6 (32·5–62·6)	53 (39·3–66·4)	54·3 (40·6–67·5)	50·1 (35·4–64·4)
Pancreatitis	37·3 (19·4–55·6)	40·4 (22·2–58·6)	37·7 (20–55·5)	40·9 (22·5–59)
Neurological disorders	24·9 (10–40)	15·3 (7·43–27)	10·8 (5·48–21)	21·2 (7·9–37)
Alzheimer's disease and other dementias	40·8 (27·3–55·6)	43·9 (30·2–58·5)	44·5 (30·7–58·8)	43·5 (29·8–58·2)
Parkinson's disease	-9·56 (-15·1–-4·86)	-11·1 (-17–-5·75)	-11·7 (-17·6–-6·18)	-10·9 (-16·9–-5·67)
Idiopathic epilepsy	23·1 (14·1–31·9)	20·6 (13–28·3)	19·6 (12·1–27·3)	23·1 (14·6–31·8)
Multiple sclerosis	14·5 (10·4–18·9)	14·9 (10·7–19·4)	14·3 (10·3–18·5)	15·5 (11·1–20·1)
Mental disorders	--	8·92 (4·65–14·3)	8·92 (4·65–14·3)	--
Depressive disorders	--	17·9 (9·01–27·3)	17·9 (9·01–27·3)	--
Anxiety disorders	--	6·49 (1·48–14·7)	6·49 (1·48–14·7)	--
Idiopathic developmental intellectual disability	--	48·1 (24·3–67·6)	48·1 (24·3–67·6)	--
Substance use disorders	100 (100–100)	100 (100–100)	100 (100–100)	100 (100–100)
Alcohol use disorders	100 (100–100)	100 (100–100)	100 (100–100)	100 (100–100)
Drug use disorders	100 (100–100)	100 (100–100)	100 (100–100)	100 (100–100)
Diabetes and kidney diseases	99·9 (99·9–99·9)	100 (100–100)	100 (100–100)	99·9 (99·9–99·9)
Diabetes mellitus	100 (100–100)	100 (100–100)	100 (100–100)	100 (100–100)
Chronic kidney disease	100 (100–100)	100 (100–100)	100 (100–100)	100 (100–100)
Sense organ diseases	--	9·82 (8·74–11·1)	9·82 (8·74–11·1)	--
Blindness and vision loss	--	9·57 (6·07–14·1)	9·57 (6·07–14·1)	--
Age-related and other hearing loss	--	10·6 (9·55–11·8)	10·6 (9·55–11·8)	--
Musculoskeletal disorders	4·5 (1·38–8·3)	19·2 (16·1–22·7)	19·5 (16·2–23)	4·25 (1·35–7·68)
Rheumatoid arthritis	12·7 (3·99–23·2)	14·6 (4·55–26·2)	14·6 (4·56–26·3)	14·4 (4·51–26·1)
Osteoarthritis	--	14 (7·86–20·9)	14 (7·86–20·9)	--
Low back pain	--	38·8 (32·9–44·9)	38·8 (32·9–44·9)	--
Gout	--	58·3 (42·1–74·7)	58·3 (42·1–74·7)	--
Other non-communicable diseases	0·0972 (0·0666–0·136)	0·11 (0·0717–0·16)	--	0·303 (0·212–0·421)
Sudden infant death syndrome	6·46 (4·3–9·39)	6·46 (4·3–9·38)	--	6·46 (4·3–9·38)
Injuries	30·4 (25·5–34·7)	27·5 (23·3–31·6)	33·1 (29·5–36·6)	24·5 (19·2–29·5)

Transport injuries	26·9<br>(22·3–32·7)	26·9<br>(22·2–32·7)	30·1<br>(26·4–34·3)	25·6<br>(20·2–32·2)
Road injuries	26·3<br>(21·7–32·2)	25·9<br>(21·1–31·9)	28·1<br>(24·3–32·5)	25·1<br>(19·6–31·7)
Other transport injuries	35·6<br>(30·8–41·4)	36·3<br>(31·9–41·1)	40·1<br>(36–43·9)	32·5<br>(27–39)
Unintentional injuries	39·7<br>(34·4–43·8)	30·7<br>(27–34·8)	34·1<br>(30·1–38·3)	25·4<br>(21·3–29·5)
Falls	55·8<br>(49·1–60·2)	46·3<br>(40·9–50·6)	45·3<br>(40·1–49·6)	48·3<br>(42·8–52·6)
Drowning	-5·39<br>(-11·7–1·81)	-6·11<br>(-12·2–0·544)	11<br>(7·08–16·3)	-6·61<br>(-12·7—0·0272)
Fire, heat, and hot substances	12<br>(7·79–17·2)	15·4<br>(10·8–21·3)	16·4<br>(11·6–22·9)	13·5<br>(9·43–18·5)
Poisonings	17·6<br>(12·6–24)	17·2<br>(12·6–23·4)	14·2<br>(10·2–19·7)	18·7<br>(13·6–25·1)
Exposure to mechanical forces	16<br>(10·8–21·9)	22<br>(18·6–26·4)	24<br>(20·8–27·9)	13·1<br>(7·48–20·3)
Animal contact	24·2<br>(19·8–29·6)	24·9<br>(20·5–30·1)	25·9<br>(21·3–31)	20·7<br>(16·3–26)
Foreign body	3·18<br>(2·14–4·77)	4·34<br>(2·98–6·44)	3·89<br>(2·64–5·74)	4·54<br>(3·05–6·8)
Environmental heat and cold exposure	8·16<br>(3·46–14·5)	8·33<br>(3·53–14·6)	7·79<br>(3·31–13·9)	8·61<br>(3·65–15·2)
Exposure to forces of nature	7·09<br>(3–12·5)	7·09<br>(3·03–12·5)	8·16<br>(3·46–14·5)	6·52<br>(2·81–11·4)
Other unintentional injuries	20·6<br>(14·7–28)	15·8<br>(11·1–21·9)	15<br>(10·4–21)	23·3<br>(16·9–31·8)
Self-harm and interpersonal violence	22·7<br>(12·8–32·3)	24·1<br>(15·4–32·7)	32·2<br>(25·4–38·8)	23·4<br>(14·2–32·3)
Self-harm	22·6<br>(10·4–35)	24·6<br>(12·5–36·9)	23·9<br>(13·2–34·8)	24·6<br>(12·5–36·9)
Interpersonal violence	23·9<br>(16·3–31·7)	23·9<br>(16·4–31·3)	34·8<br>(27·5–42)	21·5<br>(13·8–29·1)

Supplementary Results Table 2. All-age PAFs attributable to the joint distribution of all risk factors for all ages, both sexes combined for each GBD level 1, level 2, and level 3 cause and all causes in 2019 for Arkansas. Results are shown for deaths, YLLs, YLDs, and DALYs Data in parenthesis are 95% uncertainty intervals. PAFs=population attributable fractions. GBD=Global Burden of Disease. YLLs=years of life lost. YLDs=years lived with disability. DALYs=disability-adjusted life-years

	Deaths 2019	DALYs 2019	YLDs 2019	YLLs 2019
All causes	62·9 (60·5–65·2)	50·1 (47·3–52·9)	34·6 (32·1–37·1)	61·1 (59·2–62·8)
Communicable, maternal, neonatal, and nutritional diseases	43·5 (39·5–46·9)	53·3 (50·6–56·1)	47·3 (43–51·6)	56·7 (53·4–60)
HIV/AIDS and sexually transmitted infections	84·7 (67·4–91·9)	84·1 (67·6–91·7)	88·5 (75·3–94·7)	81·3 (62·4–90)
HIV/AIDS	84·2 (66·3–91·7)	81·8 (63·4–90·3)	83·4 (65·9–91·6)	81 (61·7–89·8)
Sexually transmitted infections excluding HIV	99·1 (98·7–99·4)	99·6 (99·4–99·8)	100 (100–100)	96·8 (95·4–97·8)
Respiratory infections and tuberculosis	38·5 (33·3–43·4)	36·5 (31·1–41·8)	3 (2·27–3·89)	44·4 (39·1–49·4)
Tuberculosis	54·3 (45·5–62·4)	58 (49·6–65·4)	53·6 (44·7–61·7)	58·6 (50·2–66)
Lower respiratory infections	38·3 (33·1–43·3)	44·2 (38·7–49·2)	34·9 (29·3–40·1)	44·3 (38·8–49·3)
Upper respiratory infections	0·663 (0·407–1·06)	0·0237 (0·0113–0·0425)	0·00566 (9·7e-05–0·0169)	1·36 (0·841–2·18)
Otitis media	2·01 (0·813–4·45)	4·58 (3·05–6·19)	4·56 (3·04–6·19)	5·04 (2·38–9·13)
Enteric infections	14·2 (8·47–21·5)	15·6 (9·82–22·8)	15 (9·4–22·3)	16·5 (10·8–23·7)
Diarrheal diseases	14·4 (8·54–21·7)	15·7 (9·89–22·9)	15·1 (9·4–22·4)	16·8 (10·9–24·2)
Other infectious diseases	0·907 (0·642–1·3)	1·43 (1·08–1·87)	0·674 (0·405–1·08)	1·59 (1·19–2·09)
Meningitis	1·38 (0·987–1·82)	2·91 (2·14–3·83)	0·000647 (1·1e-05–0·00198)	3·02 (2·22–3·98)
Encephalitis	0·634 (0·443–0·886)	1·61 (1·15–2·23)	0·000191 (2·95e-06–0·000576)	1·67 (1·18–2·31)
Measles	9·8 (-3·8–44·2)	9·96 (-3·87–44·9)	0·264 (0–1·18)	9·97 (-3·87–44·9)
Acute hepatitis	21·8 (13·8–35·3)	11·5 (7·15–17·8)	3·76 (2·26–5·94)	22·7 (14·2–35·9)
Maternal and neonatal disorders	80·4 (77·2–83·1)	76·9 (74·1–79·5)	65·4 (60–70·4)	83·5 (81–85·6)
Maternal disorders	12·2 (4·33–20·5)	12 (4·26–20·2)	11·7 (4·2–19·8)	12·1 (4·26–20·3)
Neonatal disorders	89·3 (87·7–90·9)	81 (78·1–83·6)	66·9 (61·2–72·3)	89·3 (87·7–90·9)
Nutritional deficiencies	94·4 (84·3–95·9)	91 (87·7–93·1)	90 (86·8–92·7)	93·8 (83·5–95·3)
Protein-energy malnutrition	100 (100–100)	100 (100–100)	100 (100–100)	100 (100–100)
Vitamin A deficiency	--	100 (100–100)	100 (100–100)	--
Dietary iron deficiency	--	100 (100–100)	100 (100–100)	--
Non-communicable diseases	66·9 (64·1–69·3)	53·3 (49·9–56·5)	34·6 (31·7–37·3)	67·5 (65·3–69·4)
Neoplasms	52 (48·4–55·9)	51·5 (48·2–55)	32·6 (28·8–36·7)	52·5 (49·2–56)
Lip and oral cavity cancer	67·5 (59·2–74·8)	70·2 (62·5–76·9)	69·4 (61·6–76·4)	70·2 (62·7–76·8)
Nasopharynx cancer	62·6 (52·2–72·3)	62·7 (52·4–71·9)	61·6 (51–70·9)	62·7 (52·5–72)
Other pharynx cancer	76·8 (70·3–82·2)	78·1 (71·9–83·3)	78·3 (72·2–83·7)	78·1 (71·9–83·3)
Esophageal cancer	80·9 (72·1–88·3)	82·1 (73·7–89·3)	81·2 (72·5–88·5)	82·2 (73·7–89·4)

Stomach cancer	26·6 (18·4–43·8)	27·3 (19·2–44·7)	27·5 (19·2–45)	27·3 (19·2–44·7)
Colon and rectum cancer	61·8 (53–70·3)	62·8 (54·8–70·7)	62·7 (54·3–70·8)	62·8 (54·8–70·7)
Liver cancer	79·7 (74·9–84·2)	79·3 (74·7–83·7)	79·7 (75–84·1)	79·3 (74·7–83·7)
Gallbladder and biliary tract cancer	26·8 (15·6–39·5)	28·1 (16·7–40·5)	25·9 (14·9–38·6)	28·2 (16·7–40·6)
Pancreatic cancer	45·2 (35·7–56)	45·5 (36·6–55·9)	45·5 (36·1–56)	45·5 (36·6–55·9)
Larynx cancer	83·2 (73·3–90·1)	84·5 (76·5–90·3)	84 (75·5–90)	84·5 (76·6–90·4)
Tracheal, bronchus, and lung cancer	84·8 (81·9–87·9)	84·5 (81·8–87·6)	84·7 (81·8–88)	84·5 (81·7–87·6)
Breast cancer	33·4 (24·2–44·4)	32·5 (24·3–42·4)	33·1 (24·6–43·5)	32·4 (24·3–42·2)
Cervical cancer	100 (100–100)	100 (100–100)	100 (100–100)	100 (100–100)
Uterine cancer	53·9 (38·3–67·7)	55·9 (40·5–69)	56·5 (41·1–69·6)	55·8 (40·4–69)
Ovarian cancer	18·1 (7·79–31·7)	16·6 (6·55–29·7)	16·1 (6·44–28·7)	16·6 (6·56–29·7)
Prostate cancer	6·66 (2·88–11)	7·42 (3·23–12)	7·84 (3·49–12·5)	7·32 (3·2–11·8)
Kidney cancer	43·9 (32·6–54·5)	44·7 (34·1–54·6)	44·8 (34·4–54·6)	44·7 (34·2–54·6)
Bladder cancer	43·7 (28–58·8)	47·7 (32·5–61·4)	48·4 (33·3–61·7)	47·6 (32·5–61·4)
Thyroid cancer	18·4 (9·43–28·9)	19·8 (10·2–30·7)	20·1 (10·5–30·8)	19·7 (10·2–30·7)
Mesothelioma	95·3 (93·5–96·7)	91·8 (88·8–94·1)	95·1 (93–96·6)	91·7 (88·8–94)
Non-Hodgkin lymphoma	8·72 (3·86–14·7)	9·27 (4·16–15·4)	9·01 (4·1–15)	9·28 (4·17–15·4)
Multiple myeloma	9·96 (4·59–16·6)	10·6 (4·95–17·7)	10·5 (4·72–17·3)	10·6 (4·95–17·7)
Leukemia	38·2 (23·4–52·3)	36·6 (24·1–48·6)	38·7 (24·6–52·4)	36·5 (24·1–48·4)
Cardiovascular diseases	84 (80·9–86·9)	84·7 (82·3–87·1)	73·7 (69·4–78·1)	86·2 (84–88·3)
Rheumatic heart disease	21·9 (12·8–40·6)	22·1 (14·1–36·9)	22 (13·3–38·4)	22·1 (14·3–36·5)
Ischemic heart disease	93·3 (90–96·1)	94·9 (92·7–96·9)	93·3 (90·3–96·1)	94·9 (92·7–97)
Stroke	79·8 (73·4–87)	83·8 (79·4–88·3)	82·6 (77·7–88·2)	84·1 (79·8–88·5)
Hypertensive heart disease	100 (100–100)	100 (100–100)	100 (100–100)	100 (100–100)
Non-rheumatic valvular heart disease	25 (15·7–37·5)	22·1 (15–31·1)	5·54 (3·51–7·96)	25·6 (17·4–35·8)
Cardiomyopathy and myocarditis	38·2 (31·5–46·5)	40·1 (34·4–45·7)	39·4 (33·1–45·8)	40·2 (34·3–45·9)
Atrial fibrillation and flutter	51·1 (41·2–61·5)	57·4 (49·3–66·4)	58·7 (50·4–67·3)	55·7 (47·1–65)
Aortic aneurysm	57·7 (52·2–63·3)	65·5 (61·1–69·9)	--	65·5 (61·1–69·9)
Endocarditis	31·6 (22·1–43·9)	33·2 (24·2–44)	30·8 (21·5–43·7)	33·2 (24·1–44)
Other cardiovascular and circulatory diseases	24·6 (19·4–31·9)	26·5 (21·2–32·8)	31·9 (25·1–39)	25·1 (20–31·4)
Chronic respiratory diseases	67·5 (63·2–71·5)	64·1 (60·6–67·5)	57 (52·6–61·3)	68·6 (65–72)
Chronic obstructive pulmonary disease	73·8 (69·5–77·4)	73·5 (69·9–76·9)	68 (64·2–71·7)	75·9 (72·3–79·1)
Pneumoconiosis	100 (100–100)	100 (100–100)	100 (100–100)	100 (100–100)

Asthma	52·3 (41·4–62·9)	43·4 (35·5–51·9)	42·2 (34·6–50·6)	51·6 (42·6–61·2)
Digestive diseases	44·2 (38·5–49·9)	47·1 (41·5–52·5)	19 (13–25·6)	53·8 (47·7–59·6)
Cirrhosis and other chronic liver diseases	78·6 (69·5–87·2)	81·1 (72·6–88·7)	76 (66·8–84·2)	81·1 (72·6–88·8)
Upper digestive system diseases	24 (19·9–27·7)	7·5 (4·63–11)	1·76 (1·06–2·69)	27·7 (23·4–31·7)
Gallbladder and biliary diseases	50·7 (35·2–65·5)	56·2 (41·9–68·9)	57·7 (44–70)	53·7 (38·9–67·6)
Pancreatitis	31 (15·5–47·4)	35·8 (19·3–52·4)	33·2 (17·7–49·3)	36 (19·4–52·7)
Neurological disorders	25·6 (10·4–40·9)	15·7 (7·72–27·8)	11·3 (5·57–22·5)	21·5 (8·09–37·4)
Alzheimer's disease and other dementias	41·2 (27·6–56·4)	45 (31·3–60)	45·8 (31·7–60·7)	44·4 (30·7–59·2)
Parkinson's disease	-11 (-17·5–-5·61)	-12·9 (-19·8–-6·62)	-13·3 (-20·1–-6·97)	-12·9 (-19·9–-6·59)
Idiopathic epilepsy	19·9 (12·9–27·4)	18·1 (11·6–25·2)	16·7 (10·6–23·3)	20·9 (13·8–28·5)
Multiple sclerosis	18·1 (13·4–23·4)	19·4 (14·5–24·8)	18·9 (14·2–23·9)	19·8 (14·7–25·3)
Mental disorders	--	9·96 (4·94–15·6)	9·97 (4·94–15·6)	--
Depressive disorders	--	19·4 (8·77–29·6)	19·4 (8·77–29·6)	--
Anxiety disorders	--	6·29 (1·44–14·2)	6·29 (1·44–14·2)	--
Idiopathic developmental intellectual disability	--	52·1 (25·1–73·1)	52·1 (25·1–73·1)	--
Substance use disorders	100 (100–100)	100 (100–100)	100 (100–100)	100 (100–100)
Alcohol use disorders	100 (100–100)	100 (100–100)	100 (100–100)	100 (100–100)
Drug use disorders	100 (100–100)	100 (100–100)	100 (100–100)	100 (100–100)
Diabetes and kidney diseases	99·9 (99·8–99·9)	99·9 (99·9–99·9)	100 (100–100)	99·9 (99·8–99·9)
Diabetes mellitus	100 (100–100)	100 (100–100)	100 (100–100)	100 (100–100)
Chronic kidney disease	100 (100–100)	100 (100–100)	100 (100–100)	100 (100–100)
Sense organ diseases	--	10·6 (9·45–11·9)	10·6 (9·45–11·9)	--
Blindness and vision loss	--	10 (6·51–14·6)	10 (6·51–14·6)	--
Age-related and other hearing loss	--	11·2 (10·1–12·5)	11·2 (10·1–12·5)	--
Musculoskeletal disorders	4·21 (1·3–7·51)	23 (19·3–27)	23·4 (19·6–27·5)	3·98 (1·25–7·02)
Rheumatoid arthritis	14·6 (4·49–26)	17·9 (5·81–31·1)	18·2 (5·91–31·4)	17·1 (5·39–30·1)
Osteoarthritis	--	15·6 (8·7–22·8)	15·6 (8·7–22·8)	--
Low back pain	--	43·8 (37·6–50)	43·8 (37·6–50)	--
Gout	--	60·8 (45·1–76·6)	60·8 (45·1–76·6)	--
Other non-communicable diseases	0·192 (0·138–0·257)	0·251 (0·177–0·349)	--	0·547 (0·4–0·716)
Sudden infant death syndrome	3·79 (2·72–5·11)	3·78 (2·72–5·11)	--	3·78 (2·72–5·11)
Injuries	24·2 (20·2–28·2)	23·1 (19·3–26·7)	29·1 (25·9–32·1)	20·4 (15·8–24·7)
Transport injuries	24·8 (20·3–29·5)	23·9 (19·4–28·7)	28·1 (24·6–31·6)	22·7 (17·7–27·9)

Road injuries	24·2 (19·7–28·9)	23 (18·4–27·9)	26 (22·5–29·6)	22·2 (17·2–27·4)
Other transport injuries	32·1 (27·6–36·9)	32·6 (28·4–37·2)	38·6 (34·4–42)	28·5 (23·5–34·1)
Unintentional injuries	27·9 (24·4–31·1)	23·9 (20·8–27·3)	29·6 (26·1–33·2)	16·1 (13·1–19·4)
Falls	53·2 (47–57·4)	43 (38·5–46·8)	42·5 (37·7–46·3)	44·5 (39·7–48·4)
Drowning	-9·94 (-16·3–-3·15)	-10·5 (-16·4–-4·03)	9·09 (5·99–13·5)	-10·9 (-16·9–-4·33)
Fire, heat, and hot substances	9·32 (6·12–13·5)	11·4 (7·75–16·1)	12·7 (8·74–17·8)	10·3 (6·96–14·6)
Poisonings	13·8 (9·42–19)	13·8 (9·58–18·9)	11·5 (7·9–16)	14·5 (10·1–19·8)
Exposure to mechanical forces	12·8 (8·18–17·8)	18·1 (14·9–22)	21·7 (18·9–25·2)	9·89 (4·78–15·6)
Animal contact	20·9 (17·1–25·5)	21·4 (17·6–26)	22·4 (18·5–27·2)	17·8 (13·9–22·1)
Foreign body	2·46 (1·66–3·66)	3·2 (2·17–4·69)	2·82 (1·9–4·14)	3·3 (2·19–4·93)
Environmental heat and cold exposure	6·13 (2·66–10·7)	6·67 (2·93–11·7)	6·23 (2·76–10·9)	6·98 (3·05–12·2)
Exposure to forces of nature	5·94 (2·63–10·4)	6·62 (2·93–11·5)	7·07 (3·14–12·3)	5·77 (2·56–10)
Other unintentional injuries	16·8 (11·7–22·8)	12·8 (8·83–17·7)	12 (8·19–16·9)	18·6 (13·2–25)
Self-harm and interpersonal violence	20·2 (11·4–28·4)	21·5 (13·1–28·9)	27·4 (21·6–33)	21 (12·2–28·8)
Self-harm	20·5 (9·33–32·2)	22·7 (10·8–34·4)	20·9 (11·8–30·9)	22·7 (10·8–34·5)
Interpersonal violence	19·9 (12·5–26·9)	20 (13–26·6)	29·2 (23–35·6)	18·2 (10·9–25·2)

Supplementary Results Table 2. All-age PAFs attributable to the joint distribution of all risk factors for all ages, both sexes combined for each GBD level 1, level 2, and level 3 cause and all causes in 2019 for California. Results are shown for deaths, YLLs, YLDs, and DALYs Data in parenthesis are 95% uncertainty intervals. PAFs=population attributable fractions. GBD=Global Burden of Disease. YLLs=years of life lost. YLDs=years lived with disability. DALYs=disability-adjusted life-years

	Deaths 2019	DALYs 2019	YLDs 2019	YLLs 2019
All causes	59 (55·9–61·8)	43·3 (40·3–46·4)	30·4 (28–32·7)	57·5 (55·2–59·6)
Communicable, maternal, neonatal, and nutritional diseases	42·6 (38·6–46·3)	52·7 (49·5–55·6)	48 (43·3–53·1)	56·3 (52·5–59·7)
HIV/AIDS and sexually transmitted infections	92 (90·1–93·3)	91·9 (90·1–93·5)	94·3 (92·6–96·1)	90·5 (88·3–92·1)
HIV/AIDS	91·8 (90–93·2)	91 (89–92·6)	92·2 (90·4–93·7)	90·4 (88·2–92)
Sexually transmitted infections excluding HIV	99·2 (98·8–99·4)	99·8 (99·6–99·9)	100 (100–100)	96·8 (95·5–97·9)
Respiratory infections and tuberculosis	38·8 (33·6–43·8)	31·2 (25·5–36·8)	2·07 (1·52–2·74)	42·8 (37·2–47·8)
Tuberculosis	54·4 (44·4–63·5)	56·3 (46·5–64·8)	52·5 (41·7–61·2)	57·2 (47·5–65·5)
Lower respiratory infections	38·7 (33·3–43·7)	42·4 (36·9–47·5)	29·1 (23·5–34·2)	42·6 (37–47·7)
Upper respiratory infections	0·553 (0·344–0·877)	0·0198 (0·00962–0·0348)	0·00583 (0·000209–0·0155)	1·26 (0·783–1·97)
Otitis media	1·09 (0·469–2·25)	2·75 (1·77–3·85)	2·74 (1·76–3·84)	3·03 (1·46–5·49)
Enteric infections	9·76 (6·16–15·1)	10·8 (7·22–16)	10·9 (7·27–16·1)	10·7 (7·05–15·9)
Diarrheal diseases	9·82 (6·19–15·1)	10·9 (7·25–16·1)	10·9 (7·28–16·1)	10·8 (7·14–16)
Other infectious diseases	0·8 (0·467–1·1)	1·22 (0·796–1·6)	0·729 (0·448–1·16)	1·36 (0·817–1·79)
Meningitis	1·19 (0·872–1·65)	2·61 (1·92–3·58)	0·000576 (1·33e-05–0·00162)	2·79 (2·06–3·84)
Encephalitis	0·558 (0·388–0·778)	1·44 (1·04–2·04)	0·000124 (2·84e-06–0·000353)	1·52 (1·1–2·16)
Measles	9·08 (-3·93–42·5)	4·84 (-2·75–38·2)	0·331 (0·00849–0·821)	9·25 (-4·01–43·3)
Acute hepatitis	21 (13·9–31·9)	9·72 (6·26–14·2)	3·4 (2·1–5·45)	22·5 (14·7–33·3)
Maternal and neonatal disorders	81·1 (78·5–83·6)	80·6 (78·3–82·6)	76·6 (72·7–80·5)	83·4 (81·3–85·6)
Maternal disorders	10·9 (3·94–17·9)	10·8 (3·91–17·8)	10·8 (3·93–18)	10·8 (3·91–17·8)
Neonatal disorders	87·6 (85·7–89·6)	83·7 (81·5–85·7)	78·5 (74·3–82·3)	87·6 (85·7–89·6)
Nutritional deficiencies	93 (90·2–96·4)	86·6 (83·4–89·6)	85·9 (82·2–89·3)	91·5 (88·6–95·5)
Protein-energy malnutrition	100 (100–100)	100 (100–100)	100 (100–100)	100 (100–100)
Vitamin A deficiency	--	100 (100–100)	100 (100–100)	--
Dietary iron deficiency	--	100 (100–100)	100 (100–100)	--
Non-communicable diseases	61·8 (58·2–64·8)	44·5 (41·1–48·1)	29·5 (26·8–32·1)	62·2 (59·7–64·5)
Neoplasms	43·6 (40–47·9)	42·1 (38·5–46·1)	27·8 (24–31·7)	43·3 (39·8–47·3)
Lip and oral cavity cancer	63·3 (54·2–71·1)	65·4 (56·8–73)	64·7 (55·9–72·5)	65·5 (56·8–73·1)
Nasopharynx cancer	62 (50·4–71·5)	61·8 (50·1–71)	60·6 (48·5–70·3)	61·9 (50·1–71)
Other pharynx cancer	73·7 (65·4–80·2)	74·7 (66·9–81)	75 (67–81·3)	74·7 (66·9–81·1)
Esophageal cancer	73·6 (62·6–83·4)	74·5 (63·6–84·3)	73·5 (62·4–83·5)	74·5 (63·7–84·3)

Stomach cancer	21·3 (13·4–40·1)	21·1 (13·1–40·2)	21·8 (13·7–40·7)	21·1 (13·1–40·2)
Colon and rectum cancer	59·6 (51·2–68·4)	60·4 (52·1–68·6)	60·3 (52·3–68·8)	60·4 (52·2–68·5)
Liver cancer	79·9 (75·7–83·8)	79·4 (75·3–83·5)	79·9 (75·7–84)	79·4 (75·3–83·5)
Gallbladder and biliary tract cancer	26·1 (15·3–38·6)	27·1 (16·1–39·5)	25·2 (14·4–37·9)	27·1 (16·2–39·6)
Pancreatic cancer	37·8 (28·3–49·1)	37·2 (28·2–47·9)	37·7 (28·6–48·7)	37·2 (28·2–47·9)
Larynx cancer	77·5 (66·9–85·5)	77·7 (68·9–84·7)	77·1 (67·9–84·3)	77·7 (68·9–84·7)
Tracheal, bronchus, and lung cancer	77·5 (73·6–82·1)	76·2 (72·3–80·7)	76·9 (72·7–81·6)	76·2 (72·3–80·7)
Breast cancer	33·3 (24·6–43·2)	31·8 (23·7–40·7)	32·6 (24·4–41·9)	31·7 (23·6–40·5)
Cervical cancer	100 (100–100)	100 (100–100)	100 (100–100)	100 (100–100)
Uterine cancer	53·5 (37·7–67·2)	55·1 (39·6–68·4)	55·6 (40·6–68·6)	55 (39·4–68·3)
Ovarian cancer	18 (8·06–30·5)	15·9 (6·76–27·8)	15·4 (6·39–26·7)	15·9 (6·77–27·8)
Prostate cancer	5·06 (2·17–8·54)	5·58 (2·42–9·16)	5·83 (2·55–9·48)	5·5 (2·36–9·1)
Kidney cancer	39 (28·1–49·6)	39·4 (29·1–49·4)	39·6 (29·3–49·6)	39·4 (29·1–49·4)
Bladder cancer	36·1 (22·2–50·9)	38·7 (25–52·2)	38·9 (25·7–52)	38·6 (24·9–52·3)
Thyroid cancer	17·5 (9·06–27·5)	18·7 (9·77–29·1)	18·9 (10·3–28·5)	18·6 (9·61–29·2)
Mesothelioma	95·7 (94·2–96·9)	91·5 (88·9–93·7)	95·1 (93·3–96·6)	91·5 (88·8–93·6)
Non-Hodgkin lymphoma	8·37 (3·58–14·4)	8·87 (3·98–15)	8·62 (3·89–14·6)	8·89 (3·98–15)
Multiple myeloma	9·61 (4·42–16·1)	10·2 (4·74–16·9)	10 (4·57–16·8)	10·2 (4·75–17)
Leukemia	32·4 (20·6–45·6)	29·9 (19·6–40·5)	32·4 (20·6–45·3)	29·8 (19·5–40·3)
Cardiovascular diseases	80·9 (77·2–84·2)	80·9 (78–83·6)	70·5 (65·9–75)	82·7 (79·9–85·2)
Rheumatic heart disease	21·7 (12–43·1)	21·3 (13·4–35·2)	21·2 (12·9–36·6)	21·3 (13·5–35·3)
Ischemic heart disease	91·7 (87·6–95·5)	93·2 (90·3–95·8)	91·7 (88–94·9)	93·3 (90·3–95·8)
Stroke	77·8 (70·8–85·9)	81·4 (76·4–86·7)	80·3 (74·7–86·1)	81·9 (76·7–87)
Hypertensive heart disease	100 (100–100)	100 (100–100)	100 (100–100)	100 (100–100)
Non-rheumatic valvular heart disease	25·6 (15·3–39·3)	22·5 (15–31·8)	8·72 (5·57–12·4)	25·5 (17–36·4)
Cardiomyopathy and myocarditis	41·9 (35·5–49·4)	44·9 (39·6–50·5)	42 (35·9–48·6)	45·2 (39·6–50·7)
Atrial fibrillation and flutter	52 (42·3–61·9)	57·1 (48·6–65·4)	58·3 (50·1–66·5)	55·3 (46·8–64·1)
Aortic aneurysm	52·1 (46·1–58·4)	60·2 (55·6–64·9)	--	60·2 (55·6–64·9)
Endocarditis	31·3 (21·7–43·6)	32·5 (23·7–42·9)	30·6 (20·5–43)	32·6 (23·8–42·9)
Other cardiovascular and circulatory diseases	20·8 (16·4–27·4)	23·3 (18·8–28·6)	29·6 (23·4–36·7)	21·5 (17·2–26·9)
Chronic respiratory diseases	59·6 (54·7–64)	53·4 (49·6–57·1)	48·1 (43·4–52·6)	59·7 (55·8–63·6)
Chronic obstructive pulmonary disease	68·4 (63·6–72·7)	64·9 (60·8–68·4)	58·1 (54·1–61·8)	70·2 (66–73·9)
Pneumoconiosis	100 (100–100)	100 (100–100)	100 (100–100)	100 (100–100)
Asthma	47·5 (36·1–59·2)	39·7 (31·9–48·3)	39·1 (31·4–47·6)	47·7 (38–57·8)

Digestive diseases	52·9 (47·1–58·2)	52·1 (46–57·6)	15·8 (10·7–21·7)	63 (57·2–68·2)
Cirrhosis and other chronic liver diseases	83·1 (75·7–89·2)	84·5 (77·8–90·2)	80·2 (71·4–87·4)	84·6 (77·8–90·3)
Upper digestive system diseases	18·6 (15·4–22·2)	5·16 (3·18–7·77)	1·18 (0·702–1·8)	20·9 (17·4–24·4)
Gallbladder and biliary diseases	49·2 (33·6–63·8)	53·9 (39·1–67·2)	55·1 (40·7–68·3)	51·2 (36·1–65·3)
Pancreatitis	39·9 (24·2–57·8)	43·4 (27–60·8)	39·9 (24·5–57·5)	44·1 (27·5–61·6)
Neurological disorders	25·6 (11·3–39·8)	14·2 (6·68–25·3)	9·36 (4·46–19·2)	21·9 (8·55–36·4)
Alzheimer's disease and other dementias	38·5 (24·5–53·5)	41 (27·3–56·2)	41·6 (27·8–56·5)	40·6 (27–55·5)
Parkinson's disease	-7·81 (-12·9–-3·78)	-8·91 (-14·1–-4·44)	-9·25 (-14·4–-4·74)	-8·83 (-13·9–-4·42)
Idiopathic epilepsy	24·9 (16·7–33·6)	22 (14·5–29·5)	20·7 (13·6–28)	25·2 (17–33·4)
Multiple sclerosis	11·8 (8·4–15·5)	12·1 (8·7–15·7)	11·6 (8·5–15)	12·5 (8·91–16·5)
Mental disorders	--	7·21 (3·79–11·8)	7·22 (3·79–11·8)	--
Depressive disorders	--	15·5 (8·27–23·8)	15·5 (8·27–23·8)	--
Anxiety disorders	--	6·39 (1·39–14·6)	6·39 (1·39–14·6)	--
Idiopathic developmental intellectual disability	--	46 (22·9–62·8)	46 (22·9–62·8)	--
Substance use disorders	100 (100–100)	100 (100–100)	100 (100–100)	100 (100–100)
Alcohol use disorders	100 (100–100)	100 (100–100)	100 (100–100)	100 (100–100)
Drug use disorders	100 (100–100)	100 (100–100)	100 (100–100)	100 (100–100)
Diabetes and kidney diseases	99·9 (99·9–99·9)	100 (99·9–100)	100 (100–100)	99·9 (99·9–99·9)
Diabetes mellitus	100 (100–100)	100 (100–100)	100 (100–100)	100 (100–100)
Chronic kidney disease	100 (100–100)	100 (100–100)	100 (100–100)	100 (100–100)
Sense organ diseases	--	9·99 (8·92–11·2)	9·99 (8·92–11·2)	--
Blindness and vision loss	--	7·89 (4·92–11·8)	7·89 (4·92–11·8)	--
Age-related and other hearing loss	--	11·1 (10·1–12·3)	11·1 (10·1–12·3)	--
Musculoskeletal disorders	2·61 (0·771–4·82)	18·7 (15·6–22)	18·9 (15·9–22·3)	2·32 (0·69–4·26)
Rheumatoid arthritis	9·83 (2·93–18·3)	11·2 (3·31–20·5)	11·2 (3·33–20·7)	11·1 (3·32–20·4)
Osteoarthritis	--	15·2 (8·72–22·2)	15·2 (8·72–22·2)	--
Low back pain	--	37 (32·1–42·2)	37 (32·1–42·2)	--
Gout	--	59·6 (43·4–76·1)	59·6 (43·4–76·1)	--
Other non-communicable diseases	0·114 (0·0806–0·157)	0·124 (0·0853–0·179)	--	0·363 (0·262–0·488)
Sudden infant death syndrome	6·44 (4·55–8·74)	6·43 (4·54–8·74)	--	6·43 (4·54–8·74)
Injuries	27·9 (23·1–32·3)	27·5 (23·3–31·5)	32·8 (29–36·5)	23·7 (18·6–28·7)
Transport injuries	29·8 (24·2–35·8)	30·2 (24·9–36·1)	33 (28·8–37·4)	28·8 (22·6–35·5)
Road injuries	29·1 (23·4–35·1)	29·2 (23·7–35·2)	31·4 (27–36)	28·3 (22·1–35)
Other transport injuries	39·3 (33·8–45·3)	39 (34·5–43·8)	41·3 (37·3–45)	36·4 (30·4–42·9)

Unintentional injuries	36·9 (32–41)	31·2 (27·1–35·5)	33·6 (29·5–37·7)	24·8 (20·5–29·5)
Falls	55·1 (48·4–59·2)	45·4 (40·1–49·5)	44·7 (39·4–48·8)	47·8 (42·3–52·4)
Drowning	-2·6 (-9·77–5·31)	-2·76 (-9·3–4·74)	13 (8·95–18)	-3·49 (-10·3–4·29)
Fire, heat, and hot substances	13·9 (9·72–19)	18·8 (13·8–24·6)	19·6 (14·4–25·8)	16·6 (12·1–22)
Poisonings	22 (16·2–28·5)	20·4 (15–26·6)	17·3 (12·8–22·7)	23·5 (17·5–30·3)
Exposure to mechanical forces	18·6 (12·9–26)	25·5 (21·1–31·2)	26·8 (22·6–32·2)	16·8 (10·2–25·1)
Animal contact	27·7 (22·9–33)	28·5 (23·8–33·8)	28·9 (24·1–34·2)	24·7 (19·7–30)
Foreign body	5·12 (3·39–7·51)	6·81 (4·67–9·82)	6·36 (4·37–9·3)	7·22 (4·7–10·5)
Environmental heat and cold exposure	8·49 (3·94–14·6)	8·43 (3·93–14·6)	8·11 (3·76–14·2)	9·07 (4·25–15·4)
Exposure to forces of nature	7·44 (3·45–12·9)	7·5 (3·44–13)	8·72 (4–15·2)	6·87 (3·17–11·9)
Other unintentional injuries	25·4 (18·5–33·2)	19·3 (14–25·2)	18·7 (13·6–24·5)	28·4 (21–37·3)
Self-harm and interpersonal violence	20 (11·2–28·4)	20·6 (12·7–28·3)	25·8 (19·3–32·8)	20 (11·7–28)
Self-harm	21·8 (9·71–33·6)	23·4 (11·2–35·2)	23·2 (12·5–33·4)	23·4 (11·2–35·3)
Interpersonal violence	17·2 (9·34–25·7)	17·9 (10·3–25·9)	26·6 (19·4–34·2)	15·8 (8·08–24·1)

Supplementary Results Table 2. All-age PAFs attributable to the joint distribution of all risk factors for all ages, both sexes combined for each GBD level 1, level 2, and level 3 cause and all causes in 2019 for Colorado. Results are shown for deaths, YLLs, YLDs, and DALYs Data in parenthesis are 95% uncertainty intervals. PAFs=population attributable fractions. GBD=Global Burden of Disease. YLLs=years of life lost. YLDs=years lived with disability. DALYs=disability-adjusted life-years

	Deaths 2019	DALYs 2019	YLDs 2019	YLLs 2019
All causes	57·3 (54·4–59·9)	43·5 (40·5–46·4)	30·9 (28·4–33·3)	56·4 (54·1–58·6)
Communicable, maternal, neonatal, and nutritional diseases	45·9 (40·3–51·4)	54·2 (50·9–57·3)	48·3 (43·8–53·1)	58·9 (54·2–62·7)
HIV/AIDS and sexually transmitted infections	91·2 (89·3–92·7)	91·3 (89·1–93·1)	94·4 (92·4–96·5)	89 (86·6–90·8)
HIV/AIDS	90·9 (88·8–92·4)	89·5 (87·2–91·4)	91 (88·7–92·9)	88·8 (86·4–90·6)
Sexually transmitted infections excluding HIV	98·9 (98·4–99·2)	99·6 (99·3–99·8)	100 (100–100)	95·7 (94·1–97·1)
Respiratory infections and tuberculosis	41·6 (33·6–50)	32·9 (26–40)	2·05 (1·53–2·69)	46 (38·2–54·2)
Tuberculosis	54·1 (43·6–63·5)	56·9 (46·9–65·8)	54·2 (43·4–63·8)	57·4 (47·6–66·2)
Lower respiratory infections	41·6 (33·5–50·1)	45·9 (38–54·1)	30·6 (25–36·3)	46·1 (38·2–54·4)
Upper respiratory infections	0·572 (0·347–0·93)	0·0238 (0·012–0·0407)	0·00389 (0–0·0112)	1·28 (0·788–2·06)
Otitis media	1·27 (0·51–2·74)	3·18 (1·98–4·39)	3·17 (1·98–4·39)	3·44 (1·53–6·54)
Enteric infections	9·44 (6·04–14·8)	10·7 (7·33–15·9)	10·4 (7·04–15·7)	11·2 (7·66–16·5)
Diarrheal diseases	9·5 (6·07–14·9)	10·8 (7·37–16)	10·4 (7·05–15·7)	11·3 (7·76–16·7)
Other infectious diseases	0·629 (0·385–0·824)	1·08 (0·743–1·38)	0·606 (0·376–0·981)	1·2 (0·777–1·56)
Meningitis	1·22 (0·859–1·68)	2·7 (1·93–3·66)	0·000409 (0–0·00137)	2·83 (2·03–3·85)
Encephalitis	0·541 (0·391–0·733)	1·42 (1·03–1·92)	0·00014 (0–0·000425)	1·47 (1·07–1·99)
Measles	9·12 (-4·06–42·8)	9·28 (-4·14–43·7)	0·157 (0–0·692)	9·29 (-4·14–43·7)
Acute hepatitis	20·6 (12·1–30·1)	7·89 (5·12–11·5)	2·97 (1·85–4·93)	21·4 (12·9–31·1)
Maternal and neonatal disorders	85·4 (83·4–87·4)	82 (79·7–84)	75·3 (71–79·1)	87·1 (85·4–88·8)
Maternal disorders	11 (3·88–18·2)	10·9 (3·84–18)	10·8 (3·87–18·2)	10·9 (3·84–18)
Neonatal disorders	90·3 (88·7–91·7)	84·6 (82·3–86·6)	77·4 (73–81·2)	90·3 (88·8–91·7)
Nutritional deficiencies	93·1 (82·5–94·9)	88·1 (84·6–90·8)	86·8 (82·8–90·3)	92·5 (81·3–94·2)
Protein-energy malnutrition	100 (100–100)	100 (100–100)	100 (100–100)	100 (100–100)
Vitamin A deficiency	--	100 (100–100)	100 (100–100)	--
Dietary iron deficiency	--	100 (100–100)	100 (100–100)	--
Non-communicable diseases	60·6 (57·3–63·5)	45·1 (41·6–48·6)	30 (27·2–32·6)	62·2 (59·8–64·4)
Neoplasms	44·5 (41·2–48·4)	43·1 (39·9–46·8)	27·1 (23·4–30·8)	44·4 (41·2–48·1)
Lip and oral cavity cancer	66·3 (56·2–74·5)	68·4 (58·9–76·3)	68 (57·9–76·2)	68·5 (58·9–76·4)
Nasopharynx cancer	64·2 (52·6–74)	63·7 (52·3–73·1)	63 (51·3–72·8)	63·8 (52·3–73·2)

Other pharynx cancer	76·2 (68·5–82·2)	77·2 (69·8–83·2)	77·5 (70·2–83·4)	77·1 (69·8–83·2)
Esophageal cancer	77·1 (67–85·5)	78·1 (67·9–86·5)	77·3 (67·7–85·3)	78·1 (67·9–86·5)
Stomach cancer	23·6 (15·5–41·8)	23·8 (15·8–42·5)	24·2 (15·9–42·5)	23·8 (15·8–42·5)
Colon and rectum cancer	60·7 (52·2–69)	61·5 (53·4–69·2)	61·4 (53·5–69·4)	61·5 (53·3–69·2)
Liver cancer	78·3 (73·3–83)	78·3 (73·7–82·7)	78·3 (73·5–82·9)	78·3 (73·8–82·7)
Gallbladder and biliary tract cancer	25·2 (14·6–37·6)	26·1 (15·2–38·8)	24·1 (13·9–36·7)	26·1 (15·2–38·9)
Pancreatic cancer	39·7 (30·6–49·8)	39·1 (30·6–48·9)	39·6 (30·9–49·6)	39·1 (30·6–48·9)
Larynx cancer	80 (70·1–87·2)	80·8 (72·3–87)	80·5 (71·7–87)	80·8 (72·3–87)
Tracheal, bronchus, and lung cancer	80·1 (76·6–83·9)	79·2 (75·7–82·9)	79·7 (76–83·6)	79·2 (75·7–82·9)
Breast cancer	34·3 (25·9–44·1)	33·5 (25·6–42·4)	34 (26·1–43·2)	33·4 (25·6–42·2)
Cervical cancer	100 (100–100)	100 (100–100)	100 (100–100)	100 (100–100)
Uterine cancer	51·9 (36·6–66·3)	53·3 (38·7–67·1)	53·7 (39·2–67·5)	53·3 (38·6–67·1)
Ovarian cancer	16·5 (7·41–28·2)	14·7 (6·01–25·7)	14·2 (5·86–24·9)	14·7 (6·01–25·7)
Prostate cancer	5·3 (2·33–8·71)	5·89 (2·58–9·4)	6·19 (2·72–9·73)	5·79 (2·53–9·29)
Kidney cancer	40 (29·1–50·6)	40·3 (30·2–50·1)	40·5 (30·4–50·1)	40·3 (30·2–50·1)
Bladder cancer	37·8 (23·6–52·1)	40·9 (27·1–53·9)	41·5 (28·1–54·4)	40·8 (27–53·9)
Thyroid cancer	17·4 (8·53–27·9)	18·5 (9·34–29·3)	18·7 (9·59–29·3)	18·4 (9·06–29·4)
Mesothelioma	95·6 (94–96·9)	91·7 (89–93·9)	95·2 (93·2–96·7)	91·6 (88·9–93·8)
Non-Hodgkin lymphoma	8·23 (3·63–14·1)	8·64 (3·86–14·6)	8·44 (3·65–14·4)	8·66 (3·86–14·6)
Multiple myeloma	9·38 (4·24–16)	9·86 (4·55–16·6)	9·74 (4·39–16·5)	9·86 (4·55–16·6)
Leukemia	34·7 (21·5–48·8)	32·8 (21·3–44·8)	35·1 (21·8–48·8)	32·7 (21·3–44·5)
Cardiovascular diseases	79 (75·2–82·5)	79·2 (76·1–82·2)	69·8 (65·3–74·4)	80·9 (77·9–83·6)
Rheumatic heart disease	22·1 (12·3–42)	21·6 (13·6–36·9)	21·5 (12·8–37·2)	21·6 (13·7–36·8)
Ischemic heart disease	92 (88·1–95·4)	93·5 (90·8–96)	91·9 (88·2–95·2)	93·5 (90·9–96)
Stroke	78·5 (71·5–86)	81·8 (76·8–86·8)	80·7 (75·7–86)	82·2 (77·1–87·2)
Hypertensive heart disease	100 (100–100)	100 (100–100)	100 (100–100)	100 (100–100)
Non-rheumatic valvular heart disease	25·6 (15·9–39·1)	23·4 (15·7–33)	10·3 (6·72–14·7)	25·4 (17·1–35·8)
Cardiomyopathy and myocarditis	39·3 (32·4–47·4)	41·4 (35·3–47·1)	38·7 (32·6–45)	41·8 (35·4–47·6)
Atrial fibrillation and flutter	52·6 (42·7–63·2)	57·8 (49·4–66·8)	59·1 (50·6–67·8)	56 (47·2–65·6)
Aortic aneurysm	53·5 (47·4–59·9)	61 (56·2–65·8)	--	61 (56·2–65·8)
Endocarditis	31·3 (20·8–44·3)	32·3 (23–43·1)	30·9 (20·7–43·2)	32·4 (23·1–43·1)

Other cardiovascular and circulatory diseases	19·5 (14–29·1)	22·9 (17·8–30)	29·9 (23·2–37·1)	20·8 (15·9–28·8)
Chronic respiratory diseases	65·6 (61·1–70·1)	58·9 (55–62·6)	50·9 (46–55·4)	65·7 (61·8–69·8)
Chronic obstructive pulmonary disease	72·9 (68·2–77·3)	70·1 (66·2–73·8)	61·9 (57·8–65·8)	74·6 (70·4–78·5)
Pneumoconiosis	100 (100–100)	100 (100–100)	100 (100–100)	100 (100–100)
Asthma	47·9 (37·1–59·2)	40·4 (32·6–48·8)	39·8 (32·2–48·1)	47 (37·8–56·7)
Digestive diseases	49·7 (43·7–55·1)	51·6 (45·7–57·3)	16 (10·8–21·6)	60·5 (54·4–65·8)
Cirrhosis and other chronic liver diseases	82·4 (73·4–89·4)	84·4 (76·4–90·7)	80·4 (71–88·1)	84·5 (76·4–90·7)
Upper digestive system diseases	19·1 (15·8–22·4)	5·93 (3·6–9·12)	1·28 (0·745–2·07)	21·6 (18·2–25·2)
Gallbladder and biliary diseases	47·8 (32·7–63·1)	52·5 (38·5–65·8)	54·1 (40·5–67)	49·7 (35·5–64·2)
Pancreatitis	39·5 (20–56·7)	43·5 (24–60)	41·3 (21·7–58·1)	43·9 (24·3–60·4)
Neurological disorders	23·8 (9·97–38)	14·4 (7·16–25·2)	10·1 (4·97–19·7)	20·4 (8·36–34·7)
Alzheimer's disease and other dementias	38·8 (25·4–53·5)	41·7 (28·1–56·3)	42·4 (29·2–56·6)	41·2 (27·7–55·6)
Parkinson's disease	-8·38 (-13·8–-3·95)	-9·8 (-15·6–-4·85)	-10·6 (-16·3–-5·39)	-9·64 (-15·4–-4·74)
Idiopathic epilepsy	25·9 (16·6–34·8)	23·4 (14·9–31·3)	22 (14–29·7)	26·1 (16·9–34·7)
Multiple sclerosis	14·1 (10·3–18·4)	15 (11·1–19·1)	14·8 (10·8–19)	15·2 (11·1–19·7)
Mental disorders	--	9·26 (4·69–14·6)	9·27 (4·69–14·6)	--
Depressive disorders	--	19·1 (9·15–29·2)	19·1 (9·15–29·2)	--
Anxiety disorders	--	6·28 (1·39–14·5)	6·28 (1·39–14·5)	--
Idiopathic developmental intellectual disability	--	45·6 (22·9–63·2)	45·6 (22·9–63·2)	--
Substance use disorders	100 (100–100)	100 (100–100)	100 (100–100)	100 (100–100)
Alcohol use disorders	100 (100–100)	100 (100–100)	100 (100–100)	100 (100–100)
Drug use disorders	100 (100–100)	100 (100–100)	100 (100–100)	100 (100–100)
Diabetes and kidney diseases	99·9 (99·8–99·9)	99·9 (99·9–100)	100 (100–100)	99·9 (99·9–99·9)
Diabetes mellitus	100 (100–100)	100 (100–100)	100 (100–100)	100 (100–100)
Chronic kidney disease	100 (100–100)	100 (100–100)	100 (100–100)	100 (100–100)
Sense organ diseases	--	10·7 (9·58–11·9)	10·7 (9·58–11·9)	--
Blindness and vision loss	--	8·44 (5·41–12·3)	8·44 (5·41–12·3)	--
Age-related and other hearing loss	--	11·9 (10·8–13·2)	11·9 (10·8–13·2)	--
Musculoskeletal disorders	3·45 (1·04–6·31)	19·4 (16·1–22·7)	19·6 (16·3–23·1)	3·33 (1·02–6·13)
Rheumatoid arthritis	11·5 (3·51–20·6)	13·7 (4·34–24·6)	13·8 (4·36–24·8)	13·1 (4·15–23·5)
Osteoarthritis	--	14·9 (8·3–22·2)	14·9 (8·3–22·2)	--

Low back pain	--	39·9<br>(34·3–45·4)	39·9<br>(34·3–45·4)	--
Gout	--	57·6<br>(40·9–75·3)	57·6<br>(40·9–75·3)	--
Other non-communicable diseases	0·141<br>(0·0962–0·194)	0·149<br>(0·101–0·215)	--	0·438<br>(0·307–0·595)
Sudden infant death syndrome	8·21<br>(5·85–11·2)	8·2<br>(5·84–11·2)	--	8·2<br>(5·84–11·2)
Injuries	28·9<br>(23–34·7)	26·4<br>(21·2–31·7)	33·6<br>(29·9–37·5)	22·2<br>(15·2–29·1)
Transport injuries	22·4<br>(15·5–29·3)	24·3<br>(18·1–30·8)	31·6<br>(27·7–36)	21·1<br>(13·5–28·7)
Road injuries	21·2<br>(13·9–28·3)	22·7<br>(16·2–29·4)	29·6<br>(25·5–34·2)	20<br>(12·2–27·7)
Other transport injuries	36·4<br>(31·2–42·1)	36·8<br>(32·2–41·7)	40·2<br>(35·9–43·8)	33·3<br>(27·4–39·9)
Unintentional injuries	40·8<br>(35·4–45)	31·9<br>(27·7–36·4)	34·5<br>(30·1–39)	27·5<br>(23–32)
Falls	55·8<br>(49–60·5)	45·8<br>(40·4–50·6)	44·7<br>(39·5–49·5)	48·1<br>(42·5–53·1)
Drowning	-9·92<br>(-21·7–2·06)	-9·75<br>(-20·8–1·54)	12·4<br>(7·87–18)	-10·7<br>(-22·4–0·809)
Fire, heat, and hot substances	13<br>(8·51–18·5)	16·9<br>(11·6–22·9)	18<br>(12·4–24·5)	14·7<br>(10–20·1)
Poisonings	19·8<br>(13·9–26·6)	19·5<br>(13·7–25·9)	16<br>(11–21·6)	21·1<br>(14·9–28)
Exposure to mechanical forces	8·51<br>(-0·442–18·1)	20·6<br>(16·3–26)	24·2<br>(20·7–28·7)	5·73<br>(-3·82–16·4)
Animal contact	27·9<br>(23·2–33·6)	26·8<br>(21·8–32·5)	27·3<br>(22·2–32·9)	24·3<br>(19·5–30·3)
Foreign body	3·46<br>(2·27–5·23)	5·19<br>(3·49–7·59)	4·68<br>(3·21–6·96)	5·38<br>(3·51–8·03)
Environmental heat and cold exposure	8·67<br>(3·89–15·1)	8·91<br>(3·98–15·3)	8·59<br>(3·77–14·7)	9·35<br>(4·23–16·1)
Exposure to forces of nature	7·79<br>(3·44–13·4)	8·32<br>(3·68–14·3)	9·12<br>(4·04–15·7)	7·21<br>(3·22–12·4)
Other unintentional injuries	21·7<br>(15·1–29·3)	17·4<br>(12–23·7)	16·7<br>(11·4–23)	24·4<br>(17·3–32·9)
Self-harm and interpersonal violence	19·3<br>(6·66–31·8)	20·8<br>(9·28–32·4)	29·4<br>(22·5–36·6)	20·2<br>(7·69–32·1)
Self-harm	19·8<br>(4·85–33·8)	21·6<br>(7·48–35·3)	25·4<br>(14·1–37·1)	21·5<br>(7·32–35·3)
Interpersonal violence	18<br>(7·43–28·3)	19·4<br>(10·1–28·5)	31·4<br>(23·8–39·2)	15·9<br>(5·72–26)

Supplementary Results Table 2. All-age PAFs attributable to the joint distribution of all risk factors for all ages, both sexes combined for each GBD level 1, level 2, and level 3 cause and all causes in 2019 for Connecticut. Results are shown for deaths, YLLs, YLDs, and DALYs Data in parenthesis are 95% uncertainty intervals. PAFs=population attributable fractions. GBD=Global Burden of Disease. YLLs=years of life lost. YLDs=years lived with disability. DALYs=disability-adjusted life-years

	Deaths 2019	DALYs 2019	YLDs 2019	YLLs 2019
All causes	58·8 (55·4–61·7)	45·7 (42·3–48·9)	32·7 (30·2–35·2)	59·5 (57–61·8)
Communicable, maternal, neonatal, and nutritional diseases	44·2 (38·4–49·4)	54·1 (50·4–57·7)	47·3 (42·6–52·2)	58·9 (53·8–63·5)
HIV/AIDS and sexually transmitted infections	88·6 (86·6–90·4)	88·6 (86·5–90·6)	92·1 (90–94·8)	86·6 (84·3–88·7)
HIV/AIDS	88·3 (86·2–90·2)	87·2 (85–89·3)	89·1 (86·9–91)	86·4 (84·1–88·6)
Sexually transmitted infections excluding HIV	99·2 (98·9–99·5)	99·7 (99·5–99·9)	100 (100–100)	96·7 (95·2–97·9)
Respiratory infections and tuberculosis	43·3 (35·1–50·6)	35·5 (28·4–42·5)	2·19 (1·61–2·86)	47·3 (39·5–54·2)
Tuberculosis	56·9 (45·6–66)	59·4 (49·1–67·8)	56·6 (45·7–65·4)	60·1 (49·9–68·4)
Lower respiratory infections	43·3 (34·9–50·6)	47·1 (39·1–54)	32·2 (26·4–37·9)	47·3 (39·4–54·2)
Upper respiratory infections	0·466 (0·255–0·795)	0·0145 (0·00615–0·0272)	0·00433 (0–0·0127)	1·24 (0·686–2·05)
Otitis media	0·831 (0·332–1·83)	3·11 (1·99–4·3)	3·11 (1·99–4·31)	2·69 (1·19–5·07)
Enteric infections	8·04 (5·01–12·5)	9·21 (6·22–13·6)	9·02 (6·06–13·6)	9·45 (6·42–13·5)
Diarrheal diseases	8·09 (5·04–12·5)	9·25 (6·25–13·7)	9·03 (6·06–13·6)	9·54 (6·49–13·7)
Other infectious diseases	0·583 (0·357–0·806)	1·05 (0·691–1·42)	0·79 (0·48–1·23)	1·13 (0·702–1·56)
Meningitis	0·999 (0·637–1·53)	2·39 (1·55–3·58)	0·00038 (0–0·00125)	2·62 (1·69–3·94)
Encephalitis	0·427 (0·281–0·639)	1·29 (0·855–1·91)	0·000113 (0–0·000336)	1·35 (0·895–1·99)
Measles	8·95 (-4·09–42·4)	5·06 (-2·62–37·2)	0·254 (0–0·708)	9·13 (-4·18–43·2)
Acute hepatitis	24·2 (16·1–37·7)	9·87 (6·46–14·8)	4·09 (2·48–6·34)	26·3 (17·8–40·4)
Maternal and neonatal disorders	86·8 (84·3–88·7)	81·3 (78·5–83·9)	71·8 (66·7–76·2)	88·4 (86·5–90)
Maternal disorders	11·1 (3·85–18·6)	11 (3·77–18·6)	11 (3·73–18·7)	10·9 (3·76–18·5)
Neonatal disorders	91·3 (89·9–92·8)	83·6 (80·8–86·1)	73·3 (68·1–77·8)	91·3 (89·9–92·8)
Nutritional deficiencies	93 (89·4–96·5)	87·7 (84·5–90·6)	86·9 (83·2–90·4)	92·3 (89–95·7)
Protein-energy malnutrition	100 (100–100)	100 (100–100)	100 (100–100)	100 (100–100)
Vitamin A deficiency	--	100 (100–100)	100 (100–100)	--
Dietary iron deficiency	--	100 (100–100)	100 (100–100)	--
Non-communicable diseases	61 (57–64·2)	46·6 (42·8–50·2)	32 (29·1–34·7)	62·8 (60–65·5)
Neoplasms	47·1 (43·3–51·5)	46·6 (42·9–50·6)	30·7 (26·8–34·9)	47·7 (44·2–51·7)
Lip and oral cavity cancer	67·7 (58·6–76·1)	70 (61·5–77·5)	69·4 (60·8–77·1)	70·1 (61·6–77·6)
Nasopharynx cancer	66·9 (56–75·9)	66·6 (55·8–75·5)	67·1 (56·1–76)	66·6 (55·8–75·4)
Other pharynx cancer	77·2 (70·3–83·3)	78·3 (71·8–84·1)	78·6 (71·8–84·4)	78·2 (71·7–84·1)

Esophageal cancer	77·6 (68–86·3)	78·6 (69·4–87·1)	77·7 (68·5–86·2)	78·6 (69·4–87·1)
Stomach cancer	23·1 (15–40·9)	23·8 (15·7–41·8)	23·8 (15·7–42)	23·8 (15·7–41·8)
Colon and rectum cancer	61·9 (53·1–70·6)	62·9 (54·3–71)	62·9 (54·5–71·1)	62·9 (54·3–70·9)
Liver cancer	80·8 (76·4–85)	80·8 (76·5–85·1)	80·8 (76·4–85·1)	80·8 (76·5–85·1)
Gallbladder and biliary tract cancer	26·7 (15·3–39·6)	27·5 (15·9–40·3)	25·2 (14·1–38·1)	27·6 (15·9–40·3)
Pancreatic cancer	41 (31·3–52·3)	40·8 (31·5–51·9)	41 (31·7–52·2)	40·8 (31·5–51·9)
Larynx cancer	80·3 (69·6–88·2)	81·4 (72·3–88·5)	81·4 (72·5–88·2)	81·4 (72·3–88·5)
Tracheal, bronchus, and lung cancer	80·4 (76·6–84·5)	79·8 (76·1–83·6)	80·2 (76·4–84·3)	79·7 (76·1–83·6)
Breast cancer	36·9 (27·9–47·4)	36 (27·7–45·3)	36·4 (27·9–46·1)	36 (27·7–45·2)
Cervical cancer	100 (100–100)	100 (100–100)	100 (100–100)	100 (100–100)
Uterine cancer	53·9 (38·3–67·8)	55·3 (40·4–68·7)	55·8 (41–69)	55·2 (40·2–68·7)
Ovarian cancer	19·1 (8·7–32·3)	17 (7·19–29·4)	16·4 (6·94–28·7)	17 (7·19–29·5)
Prostate cancer	5·24 (2·22–8·98)	5·79 (2·46–9·49)	6·12 (2·62–9·92)	5·7 (2·45–9·43)
Kidney cancer	41·4 (30·3–52·4)	42 (31·4–52·1)	42·3 (31·6–52·1)	41·9 (31·4–52·2)
Bladder cancer	36·9 (22–52·2)	40·4 (25·9–54·6)	41·1 (26·7–54·7)	40·3 (25·8–54·5)
Thyroid cancer	18·5 (9·46–28·7)	19·7 (10·2–30·3)	20 (10·5–31·1)	19·6 (10–30·3)
Mesothelioma	96·7 (95·4–97·7)	93·6 (91·4–95·4)	96·2 (94·5–97·4)	93·6 (91·3–95·3)
Non-Hodgkin lymphoma	8·87 (3·99–14·9)	9·35 (4·38–15·5)	9·13 (4·25–15·3)	9·37 (4·39–15·6)
Multiple myeloma	10·2 (4·72–17·1)	10·7 (5·02–17·8)	10·6 (4·94–17·7)	10·7 (5·02–17·8)
Leukemia	35·3 (21·6–49·3)	34·3 (22·4–46·7)	35·7 (22·7–49·5)	34·2 (22·4–46·5)
Cardiovascular diseases	79·8 (75·7–83·5)	80 (77–82·9)	70·1 (65·4–74·6)	82 (79–84·7)
Rheumatic heart disease	21·8 (11·9–41)	21·7 (13·6–37·7)	21·6 (13–38·2)	21·7 (13·7–37·6)
Ischemic heart disease	91·9 (87·7–95·5)	93·4 (90·4–96·1)	92 (88·3–95·3)	93·5 (90·4–96·1)
Stroke	78·4 (70·9–86·6)	81·8 (76·5–87·3)	80·7 (75·1–86·7)	82·3 (76·8–87·7)
Hypertensive heart disease	100 (100–100)	100 (100–100)	100 (100–100)	100 (100–100)
Non-rheumatic valvular heart disease	25·2 (15·1–38·4)	22·4 (14·7–32·9)	9·23 (5·88–13·3)	25·3 (16·5–36·9)
Cardiomyopathy and myocarditis	37·6 (30–46·9)	41·6 (36–47·8)	39·7 (33·6–46·4)	41·9 (36–48·5)
Atrial fibrillation and flutter	53·9 (43·4–64·4)	59·2 (50·4–68·1)	60·5 (51·8–69·5)	57 (47·8–66·7)
Aortic aneurysm	51·2 (44·6–57·8)	59·4 (54·3–64·4)	--	59·4 (54·3–64·4)
Endocarditis	30·7 (20·5–43·9)	32·3 (23·2–43·4)	30·3 (20·3–43·6)	32·4 (23·2–43·4)
Other cardiovascular and circulatory diseases	21·1 (16·3–27·8)	24·3 (19·6–29·7)	30·8 (24–38)	22·2 (17·8–27·3)
Chronic respiratory diseases	61·3 (56·1–66·6)	56·2 (52·3–60)	51·6 (47–56)	61·8 (57·4–66·3)

Chronic obstructive pulmonary disease	70·3 (65·1–75·2)	67·4 (63·4–71·2)	61·2 (57·2–65·4)	72·5 (68·2–76·9)
Pneumoconiosis	100 (100–100)	100 (100–100)	100 (100–100)	100 (100–100)
Asthma	50·5 (38·8–62·2)	42·8 (34·8–51·6)	42·2 (34·3–50·9)	51·3 (41·3–61·7)
Digestive diseases	44·9 (39·9–49·6)	46·3 (40·8–51·2)	15·2 (10·4–20·5)	56·5 (51·5–61·3)
Cirrhosis and other chronic liver diseases	83·7 (75·8–90·2)	85·5 (78·9–91·3)	82·1 (74·4–89·2)	85·5 (78·9–91·4)
Upper digestive system diseases	19·3 (15·8–22·9)	5·19 (3·12–7·9)	1·36 (0·802–2·14)	22·4 (18·7–26·1)
Gallbladder and biliary diseases	50·9 (35·1–65·8)	55 (40·6–68)	56·4 (42·6–69·1)	52·6 (37·1–66·8)
Pancreatitis	43·5 (25·7–62)	47·1 (29·8–64·7)	45·1 (28–62·6)	47·5 (29·9–65·1)
Neurological disorders	28·7 (13·5–43·6)	18·1 (9·42–30·7)	12·7 (6·47–23·5)	25 (10·7–40·4)
Alzheimer's disease and other dementias	40·2 (26·2–55·9)	42·9 (28·6–58·4)	43·7 (29·5–59)	42·4 (28·2–58)
Parkinson's disease	-8·39 (-13·9–-3·97)	-9·7 (-15·6–-4·76)	-10·2 (-15·9–-5·1)	-9·59 (-15·5–-4·72)
Idiopathic epilepsy	27·4 (18·8–36·2)	24·8 (16·9–32·8)	24 (16·4–32)	27·6 (19·2–36·1)
Multiple sclerosis	13·3 (9·5–17·4)	14 (10·1–18·2)	13·7 (9·98–17·7)	14·3 (10·3–18·6)
Mental disorders	--	7·36 (3·68–11·8)	7·36 (3·68–11·8)	--
Depressive disorders	--	17·5 (8·29–26·9)	17·5 (8·29–26·9)	--
Anxiety disorders	--	5·91 (1·31–13·4)	5·91 (1·31–13·4)	--
Idiopathic developmental intellectual disability	--	44·6 (22·4–61·6)	44·6 (22·4–61·6)	--
Substance use disorders	100 (100–100)	100 (100–100)	100 (100–100)	100 (100–100)
Alcohol use disorders	100 (100–100)	100 (100–100)	100 (100–100)	100 (100–100)
Drug use disorders	100 (100–100)	100 (100–100)	100 (100–100)	100 (100–100)
Diabetes and kidney diseases	99·8 (99·8–99·9)	99·9 (99·9–100)	100 (100–100)	99·9 (99·8–99·9)
Diabetes mellitus	100 (100–100)	100 (100–100)	100 (100–100)	100 (100–100)
Chronic kidney disease	100 (100–100)	100 (100–100)	100 (100–100)	100 (100–100)
Sense organ diseases	--	10·3 (9·1–11·5)	10·3 (9·1–11·5)	--
Blindness and vision loss	--	9·58 (6·11–14·2)	9·58 (6·11–14·2)	--
Age-related and other hearing loss	--	11·3 (10·2–12·5)	11·3 (10·2–12·5)	--
Musculoskeletal disorders	2·66 (0·784–4·86)	19·5 (16·4–23·1)	19·8 (16·6–23·4)	2·6 (0·781–4·8)
Rheumatoid arthritis	10·6 (3·17–19·1)	13·2 (4·05–23·9)	13·4 (4·09–24·2)	12·3 (3·72–22·2)
Osteoarthritis	--	14·5 (8·23–21·3)	14·5 (8·23–21·3)	--
Low back pain	--	40·6 (35·1–46·4)	40·6 (35·1–46·4)	--
Gout	--	60·9 (45–76·4)	60·9 (45–76·4)	--
Other non-communicable diseases	0·065 (0·038–0·102)	0·0826 (0·0475–0·134)	--	0·256 (0·153–0·395)

Sudden infant death syndrome	5·11 (3·09–7·86)	5·1 (3·08–7·85)	--	5·1 (3·08–7·85)
Injuries	33·4 (28·8–37·8)	31 (26·8–35·2)	35·3 (31·2–39·5)	27·2 (22·1–32·5)
Transport injuries	28·8 (21·7–36·7)	30·3 (24–37·7)	34 (29·3–38·7)	28·2 (20·4–37·3)
Road injuries	28·1 (21–36·1)	29 (22·4–36·7)	31·8 (26·9–36·9)	27·6 (19·7–36·8)
Other transport injuries	40·5 (34·6–47·1)	41·5 (36·6–46·2)	43·1 (38·7–47·2)	38 (31·5–45·7)
Unintentional injuries	39·9 (34·9–44)	33·8 (29·5–38·4)	36 (31·7–40·8)	29·1 (25–33·8)
Falls	57·7 (50·8–62·7)	48·9 (43·4–53·7)	47·9 (42·5–52·6)	51·2 (45·5–56·2)
Drowning	-3·55 (-14·7–8·03)	-2·45 (-12·7–8·53)	13·9 (9·25–20·2)	-3·52 (-14·6–8·07)
Fire, heat, and hot substances	14·2 (9·42–20·3)	19 (13·4–25·7)	19·8 (14–26·6)	17·1 (12–23·5)
Poisonings	21·3 (15–29)	21·2 (15·1–28·4)	18·2 (13·1–24·3)	23·4 (16·7–31·2)
Exposure to mechanical forces	15 (6·46–24·5)	25·6 (21·3–31·4)	27·5 (23·4–32·7)	13 (3·03–23·9)
Animal contact	27·7 (22·8–33·8)	30·2 (24·9–35·9)	30·7 (25·3–36·5)	25·3 (20·1–32·1)
Foreign body	3·55 (2·28–5·44)	6·97 (4·7–10·3)	7·44 (5·1–10·7)	6·72 (4·3–10·1)
Environmental heat and cold exposure	9·03 (4·18–15·9)	9·42 (4·33–16·5)	9·21 (4·18–16·3)	9·78 (4·57–17·1)
Exposure to forces of nature	8·48 (3·91–14·8)	8·41 (3·93–14·6)	9·8 (4·55–17)	7·74 (3·57–13·5)
Other unintentional injuries	24·4 (17·1–32·8)	19·4 (13·6–26·1)	18·8 (13·3–25·6)	28·7 (20·3–37·7)
Self-harm and interpersonal violence	24·6 (14·9–34·9)	25·7 (17–34·3)	30·1 (23·4–37)	25·1 (15·9–34·4)
Self-harm	25·7 (13·4–38·9)	27·5 (15·1–40·5)	27·3 (16·4–38·9)	27·5 (15–40·4)
Interpersonal violence	21·4 (12·7–30·1)	22·8 (14·7–31·3)	31·3 (23·9–38·9)	19·5 (11–28·1)

Supplementary Results Table 2. All-age PAFs attributable to the joint distribution of all risk factors for all ages, both sexes combined for each GBD level 1, level 2, and level 3 cause and all causes in 2019 for Delaware. Results are shown for deaths, YLLs, YLDs, and DALYs Data in parenthesis are 95% uncertainty intervals. PAFs=population attributable fractions. GBD=Global Burden of Disease. YLLs=years of life lost. YLDs=years lived with disability. DALYs=disability-adjusted life-years

	Deaths 2019	DALYs 2019	YLDs 2019	YLLs 2019
All causes	62·4 (59·8–64·9)	49·2 (46·2–52·1)	34·6 (31·9–37·1)	62·3 (60·3–64·2)
Communicable, maternal, neonatal, and nutritional diseases	47·6 (43·4–51·9)	58·4 (54·7–61·9)	48·3 (43·9–52·6)	64·5 (60·4–68·6)
HIV/AIDS and sexually transmitted infections	76·9 (75·7–78·4)	77·2 (75·6–79·2)	81·2 (78·8–85)	74·9 (73·5–76·5)
HIV/AIDS	76·2 (75·1–77·7)	75·3 (73·9–77)	76·8 (75·2–78·5)	74·5 (73·2–76·2)
Sexually transmitted infections excluding HIV	99·1 (98·7–99·4)	99·6 (99·3–99·8)	100 (100–100)	96·5 (94·9–97·7)
Respiratory infections and tuberculosis	41·8 (35·7–48)	35·4 (29·3–41)	2·51 (1·9–3·25)	46·4 (40·5–52·4)
Tuberculosis	57·3 (47·5–66·2)	59·8 (50·6–68·2)	55·9 (45·8–65·1)	60·5 (51·4–68·6)
Lower respiratory infections	41·7 (35·6–47·9)	46·2 (40·1–52·2)	33·8 (28·1–39·6)	46·4 (40·3–52·4)
Upper respiratory infections	0·691 (0·414–1·07)	0·0278 (0·014–0·0472)	0·00493 (3·1e-05–0·0141)	1·6 (0·976–2·45)
Otitis media	1·62 (0·584–3·51)	3·78 (2·44–5·17)	3·75 (2·41–5·13)	4·43 (1·91–8·06)
Enteric infections	10·3 (6·39–15·8)	11·7 (7·76–17·1)	11 (7·2–16·4)	12·7 (8·75–18·1)
Diarrheal diseases	10·4 (6·43–15·9)	11·8 (7·8–17·2)	11 (7·21–16·5)	12·8 (8·83–18·3)
Other infectious diseases	0·802 (0·546–1·1)	1·34 (0·964–1·74)	0·797 (0·482–1·24)	1·47 (1·02–1·91)
Meningitis	1·51 (1·05–2·12)	3·4 (2·38–4·66)	0·000537 (2·21e-06–0·00174)	3·58 (2·52–4·94)
Encephalitis	0·616 (0·415–0·884)	1·71 (1·15–2·46)	0·000184 (9·62e-07–0·000559)	1·79 (1·21–2·56)
Measles	9·31 (-4·26–43·6)	9·48 (-4·33–44·4)	0·171 (0–0·798)	9·48 (-4·33–44·4)
Acute hepatitis	24·9 (15·5–38·8)	11·4 (7·12–16·9)	4·14 (2·57–6·53)	26·4 (16·4–40)
Maternal and neonatal disorders	90·3 (88·4–91·8)	84·4 (81·4–87·1)	70·3 (65·1–74·8)	91·3 (89·7–92·7)
Maternal disorders	11·8 (4·2–19·9)	11·6 (4·15–19·5)	11·5 (4·02–19·2)	11·7 (4·17–19·7)
Neonatal disorders	93·3 (92–94·4)	86·1 (83·1–88·6)	71·7 (66·4–76·4)	93·3 (92–94·4)
Nutritional deficiencies	94·7 (86·3–96·2)	89·7 (86·6–92)	88·2 (84·6–91·3)	94·3 (86·2–95·7)
Protein-energy malnutrition	100 (100–100)	100 (100–100)	100 (100–100)	100 (100–100)
Vitamin A deficiency	--	100 (100–100)	100 (100–100)	--
Dietary iron deficiency	--	100 (100–100)	100 (100–100)	--
Non-communicable diseases	65 (62–67·7)	50·6 (47·1–53·9)	34·1 (31·1–36·7)	66·1 (64–68·3)
Neoplasms	51·3 (47·5–55·5)	50·4 (46·9–54·4)	31·9 (27·8–36·1)	51·7 (48·1–55·6)
Lip and oral cavity cancer	67·8 (58·6–75·5)	69·9 (61·3–77)	69·2 (60·8–76·4)	69·9 (61·3–77)
Nasopharynx cancer	65 (52·9–74·5)	64·7 (52·8–73·9)	63·8 (51·8–73·1)	64·8 (52·8–73·9)
Other pharynx cancer	77·4 (70·3–83·1)	78·4 (71·6–84·1)	78·7 (72–84·4)	78·4 (71·5–84)
Esophageal cancer	80·1 (71–87·9)	81·1 (72·1–88·7)	80·3 (71·3–88·3)	81·1 (72·1–88·7)

Stomach cancer	25·5 (17·2–43·1)	26 (17·9–44·1)	26·2 (17·9–43·9)	26 (17·9–44·1)
Colon and rectum cancer	63·4 (54·6–72·1)	64·1 (55·9–72·3)	64 (55·4–72·2)	64·1 (55·9–72·3)
Liver cancer	81·5 (77–85·6)	81 (76·5–85·2)	81·2 (76·5–85·5)	81 (76·5–85·2)
Gallbladder and biliary tract cancer	27 (15·7–39·6)	28·1 (16·4–40·8)	25·9 (14·9–39·1)	28·2 (16·5–41)
Pancreatic cancer	44·4 (34·7–55·9)	44·2 (34·9–55·4)	44·5 (34·8–56·1)	44·2 (34·9–55·3)
Larynx cancer	82·6 (73·2–89·7)	83·5 (75·5–89·6)	83·2 (74·7–89·5)	83·5 (75·5–89·6)
Tracheal, bronchus, and lung cancer	84·4 (81·2–87·8)	83·5 (80·6–87)	84 (80·8–87·6)	83·5 (80·6–86·9)
Breast cancer	37 (27·3–48·2)	36 (27–46·2)	36·6 (27·3–47·1)	35·9 (27–46·1)
Cervical cancer	100 (100–100)	100 (100–100)	100 (100–100)	100 (100–100)
Uterine cancer	54·4 (38·9–68·3)	56·1 (40·7–69·3)	56·5 (41·1–69·5)	56 (40·5–69·3)
Ovarian cancer	20·3 (8·95–34·7)	18·3 (7·62–32)	17·8 (7·46–31·2)	18·3 (7·63–32·1)
Prostate cancer	5·92 (2·58–9·77)	6·57 (2·9–10·6)	6·9 (3·03–11)	6·47 (2·85–10·5)
Kidney cancer	43·1 (31·8–53·8)	43·7 (33·2–53·7)	44 (33·6–53·7)	43·7 (33·1–53·7)
Bladder cancer	41·8 (26–57)	45·1 (30·4–59·3)	45·7 (31·1–59·7)	45 (30·3–59·3)
Thyroid cancer	18·7 (9·63–29)	19·9 (10·4–30·5)	20·2 (10·7–30·5)	19·8 (10·3–30·6)
Mesothelioma	97·4 (96·5–98·1)	95 (93·5–96·3)	97 (95·7–98)	95 (93·4–96·3)
Non-Hodgkin lymphoma	9·08 (4·06–15·2)	9·56 (4·38–15·7)	9·34 (4·26–15·6)	9·58 (4·39–15·7)
Multiple myeloma	10·3 (4·8–17·1)	10·8 (5·07–18)	10·7 (5·01–17·9)	10·8 (5·13–18)
Leukemia	37·4 (23·1–52·1)	36 (23·6–48·1)	38·1 (23·8–52·1)	35·9 (23·6–48)
Cardiovascular diseases	81·6 (78·1–84·8)	81·9 (79–84·4)	72 (67·3–76·3)	83·5 (81–86)
Rheumatic heart disease	21·7 (12·3–41·8)	21·8 (13·5–36)	21·7 (13·2–36·9)	21·8 (13·6–36)
Ischemic heart disease	92·7 (88·9–95·9)	94·2 (91·7–96·5)	93 (89·8–95·9)	94·2 (91·7–96·5)
Stroke	79·5 (73–87·3)	83·2 (78·5–88·3)	82·5 (77·3–88·2)	83·5 (78·9–88·6)
Hypertensive heart disease	100 (100–100)	100 (100–100)	100 (100–100)	100 (100–100)
Non-rheumatic valvular heart disease	24·7 (15·4–37·1)	22·8 (15·6–32)	8·72 (5·73–12·3)	25·4 (17·4–35·3)
Cardiomyopathy and myocarditis	39·3 (32·1–47·1)	42·6 (36·6–48·8)	41 (35·1–47·4)	42·7 (36·8–49)
Atrial fibrillation and flutter	53 (43·3–63·4)	58·9 (49·8–67·7)	60·2 (51·3–68·8)	56·9 (47·9–66·3)
Aortic aneurysm	55·2 (49·4–61·6)	63 (58·1–67·8)	--	63 (58·1–67·8)
Endocarditis	31·2 (21·4–43·1)	33 (23·8–43·3)	30·8 (21·1–42·9)	33·1 (23·9–43·4)
Other cardiovascular and circulatory diseases	21·9 (16·8–29·4)	24·5 (19·4–30·9)	30·9 (23·8–38·5)	22·7 (17·8–29·5)
Chronic respiratory diseases	65 (60·1–69·5)	59·9 (55·9–63·6)	54·1 (49·4–58·5)	65·3 (60·8–69·5)
Chronic obstructive pulmonary disease	72·9 (68–77)	71·1 (66·9–74·5)	65·5 (61·5–69·3)	74·6 (70·3–78·2)
Pneumoconiosis	100 (100–100)	100 (100–100)	100 (100–100)	100 (100–100)

Asthma	52 (40·6–63·1)	43 (35·2–51·5)	42·4 (34·5–50·8)	51·6 (41·9–61·5)
Digestive diseases	46·5 (41·1–51·1)	47·3 (41·9–52·4)	16·8 (11·5–22·5)	56·3 (50·8–61·4)
Cirrhosis and other chronic liver diseases	81·8 (73·8–88·9)	83·7 (76·2–90·1)	79·8 (71·2–86·8)	83·8 (76·3–90·1)
Upper digestive system diseases	21·7 (18·1–25·3)	5·74 (3·53–8·59)	1·63 (0·999–2·45)	24·5 (20·7–28·1)
Gallbladder and biliary diseases	51·2 (35·9–66)	55·7 (41·2–68·5)	57 (42·6–69·5)	53·3 (38·2–67·3)
Pancreatitis	37·9 (20–56·2)	41·6 (22·8–59·5)	38·8 (20·3–56·9)	42·1 (23·3–60·2)
Neurological disorders	27·3 (11·3–43·7)	16·9 (8·46–29·4)	12·2 (6·15–22·7)	23·3 (8·93–39·3)
Alzheimer's disease and other dementias	43·5 (29·1–59)	46·4 (32–61·7)	47 (32·7–62·1)	45·9 (31·5–61·3)
Parkinson's disease	-9·89 (-15·9–-4·77)	-11·4 (-17·7–-5·76)	-12 (-18·3–-6·19)	-11·3 (-17·7–-5·68)
Idiopathic epilepsy	24·3 (15·3–32·7)	22·2 (14·1–30)	21·1 (13·4–28·6)	24·9 (15·7–33)
Multiple sclerosis	15·8 (11·5–20·3)	16·5 (12·1–21·2)	15·9 (11·8–20·5)	17 (12·5–22)
Mental disorders	--	8·97 (4·5–14)	8·97 (4·5–14)	--
Depressive disorders	--	19·2 (8·89–29·2)	19·2 (8·89–29·2)	--
Anxiety disorders	--	6·01 (1·34–13·8)	6·01 (1·34–13·8)	--
Idiopathic developmental intellectual disability	--	46·5 (23·1–65·1)	46·5 (23·1–65·1)	--
Substance use disorders	100 (100–100)	100 (100–100)	100 (100–100)	100 (100–100)
Alcohol use disorders	100 (100–100)	100 (100–100)	100 (100–100)	100 (100–100)
Drug use disorders	100 (100–100)	100 (100–100)	100 (100–100)	100 (100–100)
Diabetes and kidney diseases	99·8 (99·8–99·8)	99·9 (99·9–99·9)	100 (100–100)	99·8 (99·8–99·9)
Diabetes mellitus	100 (100–100)	100 (100–100)	100 (100–100)	100 (100–100)
Chronic kidney disease	100 (100–100)	100 (100–100)	100 (100–100)	100 (100–100)
Sense organ diseases	--	10·1 (8·98–11·3)	10·1 (8·98–11·3)	--
Blindness and vision loss	--	10·3 (6·61–15·2)	10·3 (6·61–15·2)	--
Age-related and other hearing loss	--	10·8 (9·78–12)	10·8 (9·78–12)	--
Musculoskeletal disorders	4·03 (1·2–7·36)	20·3 (16·9–23·9)	20·6 (17·1–24·2)	3·81 (1·16–6·84)
Rheumatoid arthritis	12·9 (3·92–22·9)	15·6 (5·03–27·9)	15·8 (5·13–28·1)	14·7 (4·52–26·1)
Osteoarthritis	--	16·2 (9·13–23·7)	16·2 (9·13–23·7)	--
Low back pain	--	41·9 (36·1–48)	41·9 (36·1–48)	--
Gout	--	61·3 (45·8–76·6)	61·3 (45·8–76·6)	--
Other non-communicable diseases	0·141 (0·0971–0·2)	0·19 (0·123–0·275)	--	0·464 (0·328–0·644)
Sudden infant death syndrome	5·43 (3·85–7·51)	5·42 (3·85–7·51)	--	5·42 (3·85–7·51)
Injuries	29·1 (24·9–33·5)	28·2 (24·6–32·2)	33 (29·4–36·7)	25·2 (20·4–30·3)
Transport injuries	27·7 (22·4–33·4)	27·7 (22·7–33·2)	31 (27·4–34·8)	26·3 (20·4–32·7)

Road injuries	27·2 (21·9–33·1)	26·7 (21·6–32·2)	28·7 (25–32·7)	25·9 (20–32·4)
Other transport injuries	36·9 (32·1–42·4)	38·3 (34·1–42·4)	40·9 (36·6–44·4)	34·2 (28·6–40·4)
Unintentional injuries	33·4 (29·3–37)	29·5 (25·7–33·8)	33·3 (29·1–37·6)	22·5 (18·9–26·5)
Falls	54·5 (48–58·9)	45·5 (40·2–50)	44·9 (39·6–49·5)	46·9 (41·8–51·7)
Drowning	-5·48 (-14·5–4·24)	-5·42 (-14·1–3·64)	11·4 (7·19–16·7)	-6·17 (-15·2–3·02)
Fire, heat, and hot substances	12·4 (8·23–17·7)	15·3 (10·8–20·9)	16·2 (11·3–22·2)	14·1 (9·86–19·6)
Poisonings	18·2 (13–24·6)	17·6 (12·6–23·8)	14·8 (10·5–20·3)	19·5 (14·2–26)
Exposure to mechanical forces	14·7 (8·69–21·6)	22·6 (19·1–26·8)	24·6 (21·4–28·4)	12·1 (5·49–19·9)
Animal contact	25·6 (21–31·1)	27 (22·3–32·4)	27·5 (22·8–32·9)	23·2 (18·8–29·2)
Foreign body	3·26 (2·21–4·66)	4·65 (3·21–6·54)	4·67 (3·34–6·52)	4·65 (3·17–6·72)
Environmental heat and cold exposure	7·74 (3·26–13·7)	8·15 (3·48–14·4)	7·87 (3·38–13·9)	8·47 (3·61–14·8)
Exposure to forces of nature	7·45 (3·17–13·1)	7·58 (3·27–13·3)	8·65 (3·75–15·1)	6·95 (2·98–12·1)
Other unintentional injuries	20·5 (14·6–27·6)	15·9 (11·2–21·9)	15·3 (10·6–21·2)	23·3 (16·8–31·1)
Self-harm and interpersonal violence	25·2 (15·5–34·3)	26·9 (18·1–35·1)	35·5 (29·1–42)	26 (16·7–34·7)
Self-harm	24·9 (12·4–36·6)	27·2 (14·1–38·9)	25·5 (14·2–35·7)	27·2 (14·1–39·1)
Interpersonal violence	27·2 (19·6–35·8)	27·4 (19·9–35·1)	38·1 (31·1–45·2)	24·5 (16·8–32·8)

Supplementary Results Table 2. All-age PAFs attributable to the joint distribution of all risk factors for all ages, both sexes combined for each GBD level 1, level 2, and level 3 cause and all causes in 2019 for Washington, DC. Results are shown for deaths, YLLs, YLDs, and DALYs Data in parenthesis are 95% uncertainty intervals. PAFs=population attributable fractions. GBD=Global Burden of Disease. YLLs=years of life lost. YLDs=years lived with disability. DALYs=disability-adjusted life-years

	Deaths 2019	DALYs 2019	YLDs 2019	YLLs 2019
All causes	60·9 (57·8–63·6)	47·9 (44·8–50·9)	33 (30·4–35·6)	59·3 (57·2–61·4)
Communicable, maternal, neonatal, and nutritional diseases	56 (51·8–60·1)	66·1 (62·7–69·4)	58·9 (53·3–64·4)	69·6 (65·8–73·2)
HIV/AIDS and sexually transmitted infections	88·2 (85·6–90·5)	87·8 (85·2–90·1)	90·6 (88·3–92·5)	86·5 (83·6–88·9)
HIV/AIDS	88·1 (85·5–90·4)	87·5 (84·9–89·8)	89·8 (87·6–91·8)	86·5 (83·5–88·9)
Sexually transmitted infections excluding HIV	97·9 (96·7–98·7)	98·7 (97·8–99·4)	100 (100–100)	93·3 (89·9–95·9)
Respiratory infections and tuberculosis	41·6 (36–47·3)	35·5 (29·4–41·6)	2·25 (1·61–3·11)	46·9 (41·4–52·7)
Tuberculosis	56·4 (46·2–64·8)	58·5 (48·3–66·4)	54·3 (42·5–63·2)	59 (49–66·9)
Lower respiratory infections	41·2 (35·5–47·1)	46·5 (40·7–52·3)	30·7 (24·8–36·3)	46·6 (40·9–52·5)
Upper respiratory infections	0·82 (0·478–1·33)	0·0251 (0·0116–0·0453)	0·00612 (0·000241–0·0162)	1·55 (0·923–2·46)
Otitis media	1·91 (0·701–4·03)	2·87 (1·86–4·03)	2·79 (1·77–3·95)	4·25 (1·83–7·67)
Enteric infections	10·1 (6·51–15)	12 (8·29–16·8)	10·3 (6·63–15·3)	14·8 (10·3–20·4)
Diarrheal diseases	10·2 (6·56–15·2)	12·2 (8·36–17)	10·3 (6·64–15·3)	15·2 (10·6–20·9)
Other infectious diseases	0·949 (0·66–1·43)	1·57 (1·15–2·18)	0·644 (0·387–1·04)	1·72 (1·24–2·42)
Meningitis	1·84 (1·22–2·77)	3·43 (2·38–4·96)	0·000953 (2·16e-05–0·0028)	3·52 (2·45–5·13)
Encephalitis	0·963 (0·545–1·48)	2·13 (1·25–3·19)	0·000636 (1·69e-05–0·00179)	2·17 (1·28–3·26)
Measles	9·49 (-4·12–44·3)	9·65 (-4·18–45·2)	0·164 (0–0·74)	9·65 (-4·18–45·2)
Acute hepatitis	20·2 (12·5–35·6)	9·8 (6·03–15·9)	3·29 (2·01–5·31)	20·2 (11·8–34·4)
Maternal and neonatal disorders	79·7 (73·8–84·1)	80·7 (76·7–83·9)	72·6 (67·6–77·1)	83·6 (79·2–86·9)
Maternal disorders	12·5 (4·32–20·7)	12·3 (4·29–20·4)	12·4 (4·37–20·5)	12·3 (4·3–20·4)
Neonatal disorders	91·8 (90·3–93·2)	86·7 (84·2–88·9)	74·1 (69·2–78·7)	91·8 (90·3–93·2)
Nutritional deficiencies	90·6 (84–94·3)	89·3 (86·2–92·1)	89·3 (85·8–92·3)	89·6 (81·8–93)
Protein-energy malnutrition	100 (100–100)	100 (100–100)	100 (100–100)	100 (100–100)
Vitamin A deficiency	--	100 (100–100)	100 (100–100)	--
Dietary iron deficiency	--	100 (100–100)	100 (100–100)	--
Non-communicable diseases	64·1 (60·6–67·2)	49·4 (45·6–53·2)	31·4 (28·2–34·2)	64·9 (62·3–67·4)
Neoplasms	46·2 (42·5–50·3)	45·5 (42·1–49·4)	31·2 (27·4–35·3)	46·3 (43–50·2)
Lip and oral cavity cancer	67·2 (58·5–74·7)	69·3 (60·8–76·4)	68·7 (60·2–76·2)	69·3 (60·8–76·4)
Nasopharynx cancer	64·3 (53·2–73·2)	64 (52·6–72·9)	63·1 (51·4–72·3)	64·1 (52·6–72·9)
Other pharynx cancer	77·2 (70·5–83)	77·9 (71·1–83·7)	77·9 (71·1–83·9)	77·9 (71·1–83·7)
Esophageal cancer	76·3 (66·8–84·8)	77·6 (68·5–86)	76·9 (67–85·4)	77·6 (68·5–86)

Stomach cancer	22·9 (15·2–41·5)	22·4 (14·5–41·4)	23·2 (15·2–41·5)	22·4 (14·5–41·4)
Colon and rectum cancer	60·6 (52·1–69)	61 (53·3–68·6)	61·1 (53·2–69·3)	61 (53·3–68·7)
Liver cancer	77·2 (72·3–82·1)	76·1 (71·2–80·9)	76·9 (71·8–81·8)	76·1 (71·2–80·9)
Gallbladder and biliary tract cancer	28·2 (16·9–40·7)	29·2 (17·8–41·8)	27·1 (16–40)	29·2 (17·9–41·8)
Pancreatic cancer	40·6 (32–50·8)	39·7 (31·3–49·2)	40·4 (31·9–50·4)	39·7 (31·3–49·2)
Larynx cancer	79 (70–86·3)	80·3 (72·2–86·4)	79·8 (71·8–86)	80·3 (72·2–86·4)
Tracheal, bronchus, and lung cancer	78·4 (74·9–82·4)	76·9 (73·4–80·9)	77·9 (74·3–82·1)	76·8 (73·4–80·9)
Breast cancer	34·6 (26·1–44·4)	32·2 (24·4–40·9)	33·4 (25·4–42·7)	32·1 (24·3–40·7)
Cervical cancer	100 (100–100)	100 (100–100)	100 (100–100)	100 (100–100)
Uterine cancer	55·1 (39·5–68·3)	56·5 (41·3–69·4)	56·9 (41·7–69·5)	56·4 (41·2–69·4)
Ovarian cancer	16·5 (6·58–29·4)	14·7 (5·65–26·4)	14 (5·29–25·5)	14·7 (5·67–26·4)
Prostate cancer	5·32 (2·35–8·65)	6·1 (2·71–9·56)	6·51 (2·87–10)	6 (2·66–9·48)
Kidney cancer	40·6 (30·5–50·5)	40·4 (30·5–49·9)	40·3 (30·6–49·7)	40·4 (30·5–50)
Bladder cancer	37·4 (25·1–50·7)	41·5 (29·4–53·7)	42 (30·5–53·6)	41·5 (29·3–53·8)
Thyroid cancer	17·8 (9·23–27·9)	18·8 (9·71–29·3)	18·9 (10·1–28·7)	18·7 (9·63–29·3)
Mesothelioma	91·4 (88·4–93·8)	84·1 (79·6–88·1)	90·7 (87·2–93·4)	83·9 (79·4–87·9)
Non-Hodgkin lymphoma	8·54 (3·71–14·4)	8·88 (3·96–14·7)	8·71 (3·87–14·5)	8·89 (3·99–14·8)
Multiple myeloma	9·86 (4·52–16·5)	10·3 (4·8–17·2)	10·2 (4·74–16·9)	10·3 (4·81–17·2)
Leukemia	34·4 (22·7–46·6)	31·2 (21·7–41·2)	35·2 (23·4–47·3)	31·1 (21·6–41)
Cardiovascular diseases	83·8 (80·6–86·6)	83·8 (81·2–86·2)	73·6 (69·2–78·2)	85·1 (82·6–87·4)
Rheumatic heart disease	21·8 (12·9–39·5)	21·7 (13·6–34·6)	21·5 (13·2–36·5)	21·7 (13·6–34·2)
Ischemic heart disease	92·8 (89–95·9)	94·4 (91·9–96·6)	92·6 (89–95·7)	94·4 (92–96·6)
Stroke	79·8 (73·4–87·1)	83·8 (79·5–88·6)	82·5 (77·5–87·9)	84·5 (80·1–88·9)
Hypertensive heart disease	100 (100–100)	100 (100–100)	100 (100–100)	100 (100–100)
Non-rheumatic valvular heart disease	24·6 (15·4–37·1)	21·5 (14·8–30·1)	8·05 (5·16–11·9)	24·7 (17–34·3)
Cardiomyopathy and myocarditis	42·3 (36–49·7)	44·3 (38·2–50·7)	42·4 (36·2–48·6)	44·5 (38·3–51)
Atrial fibrillation and flutter	52·5 (42·3–62·4)	57·9 (48·9–66·6)	58·8 (49·6–67·4)	56·9 (47·6–65·9)
Aortic aneurysm	54·6 (48·8–60·7)	62 (57·1–66·5)	--	62 (57·1–66·5)
Endocarditis	32·2 (22·4–44·2)	33·6 (24–43·9)	30·9 (20·7–43·7)	33·7 (24–44)
Other cardiovascular and circulatory diseases	22·3 (17·1–30·3)	23·5 (18·2–30·3)	29·2 (21·8–37·3)	22·6 (17·5–29·9)
Chronic respiratory diseases	57·7 (52·9–62·9)	52·4 (48·2–56·6)	47·9 (42·4–53·1)	57·4 (53–62·6)
Chronic obstructive pulmonary disease	70 (65·2–74·4)	67·7 (63·9–71·4)	60·7 (57·2–64·3)	72·2 (68·1–76·2)
Pneumoconiosis	100 (100–100)	100 (100–100)	100 (100–100)	100 (100–100)

Asthma	50·6 (39·6–61·7)	42·4 (34–51·5)	41·3 (33·2–50·2)	50·4 (40·5–60·8)
Digestive diseases	49·8 (44–55·8)	50·6 (44·6–56·6)	12·7 (8·36–17·6)	58·9 (53–64·7)
Cirrhosis and other chronic liver diseases	82·6 (74·4–89·3)	84·4 (77–90·5)	81 (73·4–87·7)	84·4 (77–90·5)
Upper digestive system diseases	20·3 (17–23·8)	6·48 (3·89–9·54)	1·27 (0·732–2)	22·5 (19·2–25·8)
Gallbladder and biliary diseases	50·3 (34·8–64·8)	53·8 (39·5–66·8)	55 (41·1–67·4)	52·4 (37·4–66·3)
Pancreatitis	42·3 (24·8–61)	45·7 (27·7–64·1)	42·8 (25·1–61)	46 (27·9–64·4)
Neurological disorders	28·9 (14·2–43·3)	16·2 (7·99–28·5)	10·8 (5·21–21·8)	24·7 (11·2–38·8)
Alzheimer's disease and other dementias	39 (24·9–54·2)	41·9 (27·4–56·7)	42·8 (28·4–57·4)	41·3 (27–56·3)
Parkinson's disease	-8·77 (-13·9–-4·38)	-10·3 (-15·8–-5·35)	-10·8 (-16·4–-5·52)	-10·2 (-15·7–-5·29)
Idiopathic epilepsy	25·9 (17·7–35·1)	23·9 (16·2–32·6)	22·7 (15–31)	26·2 (17·9–35·1)
Multiple sclerosis	15·3 (11·4–19·6)	15·2 (11·5–19·3)	14·2 (10·6–18)	16·1 (12–20·5)
Mental disorders	--	7·56 (3·4–12·6)	7·57 (3·4–12·6)	--
Depressive disorders	--	17·2 (7·43–26·7)	17·2 (7·43–26·7)	--
Anxiety disorders	--	6·06 (1·01–14·8)	6·06 (1·01–14·8)	--
Idiopathic developmental intellectual disability	--	44·7 (23·3–61·1)	44·7 (23·3–61·1)	--
Substance use disorders	100 (100–100)	100 (100–100)	100 (100–100)	100 (100–100)
Alcohol use disorders	100 (100–100)	100 (100–100)	100 (100–100)	100 (100–100)
Drug use disorders	100 (100–100)	100 (100–100)	100 (100–100)	100 (100–100)
Diabetes and kidney diseases	99·9 (99·9–99·9)	99·9 (99·9–100)	100 (100–100)	99·9 (99·9–99·9)
Diabetes mellitus	100 (100–100)	100 (100–100)	100 (100–100)	100 (100–100)
Chronic kidney disease	100 (100–100)	100 (100–100)	100 (100–100)	100 (100–100)
Sense organ diseases	--	8·95 (8·02–10·1)	8·95 (8·02–10·1)	--
Blindness and vision loss	--	8·68 (5·56–12·6)	8·68 (5·56–12·6)	--
Age-related and other hearing loss	--	9·78 (8·91–10·9)	9·78 (8·91–10·9)	--
Musculoskeletal disorders	2·3 (0·691–4·12)	19·5 (16·3–22·8)	20 (16·7–23·4)	1·92 (0·573–3·43)
Rheumatoid arthritis	12·4 (3·96–22·3)	14 (4·37–24·7)	13·9 (4·28–24·7)	14·1 (4·56–24·8)
Osteoarthritis	--	15 (8·41–21·9)	15 (8·41–21·9)	--
Low back pain	--	39·6 (34·6–44·7)	39·6 (34·6–44·7)	--
Gout	--	58·4 (42·6–74·3)	58·4 (42·6–74·3)	--
Other non-communicable diseases	0·155 (0·0964–0·238)	0·182 (0·108–0·279)	--	0·414 (0·27–0·6)
Sudden infant death syndrome	4·63 (3·06–6·81)	4·63 (3·05–6·8)	--	4·63 (3·05–6·8)
Injuries	26·3 (21·5–30·7)	24·8 (20·1–29·2)	33·3 (29·6–37·6)	21·5 (16–26·6)
Transport injuries	29·9 (23·2–37·6)	29·9 (23·9–36·7)	32·2 (28·1–37·2)	28·8 (21·7–36·9)

Road injuries	29·4<br>(22·6–37·2)	28·9<br>(22·6–36)	30<br>(25·5–35·3)	28·5<br>(21·3–36·7)
Other transport injuries	37·3<br>(31–44·6)	38·2<br>(33·4–43·3)	40·1<br>(35·9–44·2)	34·8<br>(27·8–42·6)
Unintentional injuries	34·4<br>(30–38·2)	29·7<br>(25·6–34·2)	34·2<br>(30–39)	23·3<br>(19·1–27·8)
Falls	54<br>(47·8–58·6)	44·9<br>(39·7–49·9)	44·7<br>(39·5–49·4)	45·4<br>(39·9–51·2)
Drowning	-2·91<br>(-13·6–6·98)	-3·69<br>(-14·1–6·01)	13·1<br>(8·44–18·8)	-4·22<br>(-14·8–5·75)
Fire, heat, and hot substances	15·2<br>(10·8–20·8)	18·3<br>(13·3–24·3)	19·9<br>(14·2–26·4)	16·9<br>(12·4–22·5)
Poisonings	22·6<br>(16·7–29·9)	21·6<br>(15·9–28·4)	18·9<br>(13·7–25)	23·4<br>(17·5–30·8)
Exposure to mechanical forces	18·5<br>(10·1–27·6)	25<br>(20·1–30·9)	26·5<br>(22·1–32·3)	16·6<br>(7·5–26·7)
Animal contact	27·8<br>(22·6–33·9)	28·3<br>(23·2–34·3)	29<br>(23·6–34·9)	24·3<br>(18·9–30·5)
Foreign body	5·58<br>(3·51–8·27)	6·82<br>(4·35–10)	6·78<br>(4·64–9·8)	6·84<br>(4·19–10·4)
Environmental heat and cold exposure	8·45<br>(3·61–14·6)	8·82<br>(3·78–15)	8·33<br>(3·58–14·4)	9·06<br>(3·89–15·3)
Exposure to forces of nature	7·59<br>(3·25–13)	7·66<br>(3·27–13·1)	9·04<br>(3·85–15·5)	7·09<br>(3·02–12·2)
Other unintentional injuries	24·7<br>(17·8–32·7)	19·9<br>(14·3–26·5)	19·2<br>(13·6–25·7)	27·7<br>(20·4–36·6)
Self-harm and interpersonal violence	20·4<br>(12·7–27·7)	20<br>(12·4–27·2)	28·7<br>(21·5–36)	19·5<br>(11·9–26·8)
Self-harm	25·3<br>(11·6–38·3)	26·6<br>(13·1–39·9)	25·9<br>(14·7–37·2)	26·6<br>(13–40)
Interpersonal violence	19·2<br>(10·2–27·7)	18·7<br>(10·1–27)	29·7<br>(22·3–37·7)	18<br>(9·3–26·3)

Supplementary Results Table 2. All-age PAFs attributable to the joint distribution of all risk factors for all ages, both sexes combined for each GBD level 1, level 2, and level 3 cause and all causes in 2019 for Florida. Results are shown for deaths, YLLs, YLDs, and DALYs Data in parenthesis are 95% uncertainty intervals. PAFs=population attributable fractions. GBD=Global Burden of Disease. YLLs=years of life lost. YLDs=years lived with disability. DALYs=disability-adjusted life-years

	Deaths 2019	DALYs 2019	YLDs 2019	YLLs 2019
All causes	60·3 (56·9–63)	47·7 (44·7–50·7)	33·8 (31·3–36·2)	60·3 (58–62·4)
Communicable, maternal, neonatal, and nutritional diseases	42·5 (37·7–47·1)	57·7 (54·5–60·7)	52·5 (47·2–57·7)	61·3 (57·5–64·8)
HIV/AIDS and sexually transmitted infections	95·2 (94·2–96)	94·9 (93·8–95·7)	96·2 (95·2–97)	94·2 (93–95)
HIV/AIDS	95·2 (94·2–95·9)	94·6 (93·5–95·4)	95·5 (94·5–96·3)	94·2 (93–95)
Sexually transmitted infections excluding HIV	98·7 (98·2–99·1)	99·5 (99·2–99·8)	100 (100–100)	95 (92·9–96·5)
Respiratory infections and tuberculosis	29·9 (22·4–36·6)	26·5 (20·6–32·4)	2·52 (1·91–3·31)	36·1 (28·9–42·4)
Tuberculosis	57·6 (48–66·3)	60·1 (50·7–68·4)	55·4 (45·6–64·4)	60·9 (51·7–69·1)
Lower respiratory infections	29·5 (21·9–36·2)	35·6 (28·2–42)	32·3 (26·5–37·8)	35·6 (28·3–42·1)
Upper respiratory infections	0·555 (0·332–0·882)	0·0162 (0·00767–0·0295)	0·00417 (0–0·0132)	1·29 (0·791–2·02)
Otitis media	1·39 (0·58–2·97)	3·34 (2·19–4·63)	3·32 (2·15–4·62)	3·94 (1·85–7·2)
Enteric infections	10·6 (6·54–15·9)	11·7 (7·69–17·1)	11·6 (7·5–17)	11·8 (7·82–17·2)
Diarrheal diseases	10·6 (6·57–16)	11·7 (7·72–17·2)	11·6 (7·51–17)	11·9 (7·89–17·4)
Other infectious diseases	0·725 (0·416–0·959)	1·21 (0·817–1·54)	0·699 (0·421–1·13)	1·34 (0·858–1·73)
Meningitis	1·31 (0·933–1·76)	2·87 (2·08–3·83)	0·000381 (0–0·00118)	3·07 (2·21–4·06)
Encephalitis	0·558 (0·387–0·813)	1·53 (1·08–2·14)	0·000101 (0–0·000306)	1·6 (1·13–2·24)
Measles	9·32 (-4·05–44)	5·23 (-2·99–40)	0·36 (0–0·979)	9·5 (-4·12–44·8)
Acute hepatitis	21·6 (13·8–33·5)	10 (6·48–14·8)	3·75 (2·24–5·96)	23 (14·7–34·8)
Maternal and neonatal disorders	83·1 (80·7–85·3)	81 (78·7–83)	73·9 (69·4–78·2)	85·4 (83·5–87·2)
Maternal disorders	11·5 (4·15–19·3)	11·3 (4·09–19·2)	11·3 (4·01–19·1)	11·3 (4·1–19·2)
Neonatal disorders	89·8 (88·1–91·2)	84·2 (81·9–86·2)	75·5 (70·8–79·7)	89·8 (88·1–91·2)
Nutritional deficiencies	93·1 (90·3–96·8)	89 (86–91·7)	88·3 (84·7–91·3)	92·4 (89·2–96)
Protein-energy malnutrition	100 (100–100)	100 (100–100)	100 (100–100)	100 (100–100)
Vitamin A deficiency	--	100 (100–100)	100 (100–100)	--
Dietary iron deficiency	--	100 (100–100)	100 (100–100)	--
Non-communicable diseases	63 (59·1–66)	49·3 (45·8–52·8)	33·1 (30·2–35·7)	64·9 (62·2–67·2)
Neoplasms	48·5 (44·8–52·6)	48·2 (44·6–52·1)	29·2 (25·4–33·3)	49·6 (46·2–53·4)
Lip and oral cavity cancer	67·9 (59·6–75·4)	70·3 (62·5–77·3)	69·7 (61·7–76·8)	70·4 (62·7–77·3)
Nasopharynx cancer	65·3 (54–74·8)	64·9 (54·1–74·1)	63·8 (52·8–73·4)	65 (54·1–74·2)
Other pharynx cancer	77·6 (70·3–83·1)	78·7 (71·9–84·1)	79 (72–84·1)	78·7 (71·9–84·1)

Esophageal cancer	77·9 (68·4–86·2)	79·3 (70·1–87·3)	78·2 (69–86·3)	79·3 (70·1–87·3)
Stomach cancer	24·4 (16·2–42·5)	25·3 (17·2–43·4)	25·5 (17·4–43·6)	25·3 (17·2–43·4)
Colon and rectum cancer	62·1 (53·2–70·8)	63 (54·8–71·1)	62·9 (54·6–71·4)	63 (54·9–71·1)
Liver cancer	79·7 (75·2–83·9)	79·6 (75·3–83·7)	79·8 (75–84·3)	79·6 (75·3–83·7)
Gallbladder and biliary tract cancer	25·2 (14·3–37·9)	26·4 (15·3–39·5)	24·2 (13·4–37·3)	26·4 (15·4–39·6)
Pancreatic cancer	43 (32·9–54·4)	43·2 (33·6–54·3)	43·2 (33·9–54·6)	43·2 (33·6–54·3)
Larynx cancer	81 (70·4–88·8)	82·5 (73·9–88·8)	82·3 (73·7–88·9)	82·5 (73·9–88·7)
Tracheal, bronchus, and lung cancer	81·9 (78·5–85·6)	81·7 (78·5–85·2)	81·9 (78·5–85·6)	81·7 (78·5–85·2)
Breast cancer	35·5 (26·5–46·2)	34·6 (26–44·2)	35·2 (26·4–45·3)	34·5 (25·9–44)
Cervical cancer	100 (100–100)	100 (100–100)	100 (100–100)	100 (100–100)
Uterine cancer	52·3 (36·9–66·8)	54·3 (39–67·9)	54·9 (39·2–68·3)	54·2 (38·6–67·8)
Ovarian cancer	18·8 (8·4–32·2)	17 (7·09–29·8)	16·5 (6·77–28·9)	17·1 (7·1–29·8)
Prostate cancer	5·73 (2·51–9·44)	6·53 (2·85–10·6)	6·97 (3·05–11·3)	6·4 (2·81–10·5)
Kidney cancer	41·3 (30·2–52·2)	42·3 (31·8–52·3)	42·6 (32·2–52·5)	42·3 (31·8–52·3)
Bladder cancer	38·8 (23·6–54·1)	42·8 (28·3–57·2)	43·7 (29·4–57·4)	42·7 (28·1–57·1)
Thyroid cancer	17·5 (8·98–27·7)	18·8 (9·74–29·3)	19·1 (10·2–29·5)	18·7 (9·68–29·3)
Mesothelioma	96·2 (94·8–97·4)	92·8 (90·4–94·8)	95·8 (94·1–97·1)	92·8 (90·3–94·8)
Non-Hodgkin lymphoma	8·36 (3·67–14·2)	8·91 (3·92–15·1)	8·66 (3·87–14·6)	8·93 (3·94–15·1)
Multiple myeloma	9·56 (4·3–16·1)	10·2 (4·67–17)	10 (4·61–16·9)	10·2 (4·67–17)
Leukemia	36·2 (22·3–50·6)	35·5 (23·1–48·1)	36·9 (22·4–51·1)	35·4 (23–47·9)
Cardiovascular diseases	80·8 (76·9–84·5)	81 (77·9–83·9)	70 (65·3–74·6)	83 (80·2–85·7)
Rheumatic heart disease	21·9 (12–41·7)	21·7 (13·6–37·1)	21·7 (13·3–37·6)	21·7 (13·8–36·8)
Ischemic heart disease	91·7 (87·5–95·5)	93·4 (90·5–96)	92·5 (89·3–95·6)	93·4 (90·5–96)
Stroke	76·9 (69–85·6)	81·3 (76–86·9)	81·1 (75·7–87)	81·4 (75·9–87)
Hypertensive heart disease	100 (100–100)	100 (100–100)	100 (100–100)	100 (100–100)
Non-rheumatic valvular heart disease	25·3 (15·3–38·4)	19·4 (12·9–27·9)	5·59 (3·53–8·13)	25·4 (16·9–35·9)
Cardiomyopathy and myocarditis	38·2 (30·6–47·3)	42·4 (36·5–48·6)	40·8 (34·1–47·2)	42·6 (36·6–48·7)
Atrial fibrillation and flutter	52 (41·8–61·9)	57·3 (48·8–65·7)	58·5 (49·9–66·8)	55·6 (46·6–64·5)
Aortic aneurysm	54·4 (48·4–60·6)	63·1 (58·3–67·8)	--	63·1 (58·3–67·8)
Endocarditis	31·1 (20·5–43·4)	32·6 (23·4–43·2)	30·7 (20·6–43·3)	32·6 (23·5–43·3)
Other cardiovascular and circulatory diseases	22·4 (17·6–28·5)	25 (20–30·3)	31 (24·1–37·9)	23·3 (18·8–28·5)
Chronic respiratory diseases	57·4 (52·4–62·5)	56·7 (52·8–60·5)	53·9 (49·3–58·2)	59·4 (55·1–63·7)

Chronic obstructive pulmonary disease	64·2 (58·7–69·2)	65·7 (61·3–69·8)	63·3 (59·1–67·4)	67·4 (62·8–71·7)
Pneumoconiosis	100 (100–100)	100 (100–100)	100 (100–100)	100 (100–100)
Asthma	49·7 (38·2–61·4)	42·1 (34–50·7)	41·5 (33·4–50·1)	49·7 (40–59·6)
Digestive diseases	50·3 (44·8–55·8)	50·4 (44·8–55·9)	17·2 (11·8–23·3)	60·4 (54·8–66)
Cirrhosis and other chronic liver diseases	82·1 (74·1–89·4)	83·7 (76·4–90·5)	79·9 (71·1–87·6)	83·7 (76·4–90·5)
Upper digestive system diseases	21·3 (17·8–24·9)	5·79 (3·75–8·52)	1·72 (1·09–2·58)	24·8 (20·9–28·3)
Gallbladder and biliary diseases	48·6 (33·4–64·1)	54·4 (39·9–67·7)	55·7 (41·5–68·7)	51·2 (36·8–65·8)
Pancreatitis	39·1 (21·7–56·8)	42·3 (25·1–59·8)	39·4 (22·4–56·8)	42·8 (25·4–60·4)
Neurological disorders	27·4 (12·7–42·2)	17·9 (9·09–30·2)	12·9 (6·52–23·8)	23·8 (9·84–39·1)
Alzheimer's disease and other dementias	39·7 (25·5–55·2)	43 (29·1–58·1)	44 (29·8–58·8)	42·4 (28·2–57·7)
Parkinson's disease	-9·17 (-14·9–-4·47)	-10·9 (-16·9–-5·47)	-11·6 (-17·7–-5·99)	-10·7 (-16·8–-5·45)
Idiopathic epilepsy	24 (15·4–32·2)	21·8 (14·1–29·5)	20·9 (13·3–28·5)	24·1 (15·6–32·1)
Multiple sclerosis	14·9 (10·8–19·4)	15·7 (11·6–20·1)	15·2 (11·2–19·3)	16·3 (11·8–21)
Mental disorders	--	8·96 (4·55–13·8)	8·96 (4·55–13·8)	--
Depressive disorders	--	18·4 (8·49–27·9)	18·4 (8·49–27·9)	--
Anxiety disorders	--	5·75 (1·25–13·2)	5·75 (1·25–13·2)	--
Idiopathic developmental intellectual disability	--	48·4 (24·7–66)	48·4 (24·7–66)	--
Substance use disorders	100 (100–100)	100 (100–100)	100 (100–100)	100 (100–100)
Alcohol use disorders	100 (100–100)	100 (100–100)	100 (100–100)	100 (100–100)
Drug use disorders	100 (100–100)	100 (100–100)	100 (100–100)	100 (100–100)
Diabetes and kidney diseases	99·9 (99·8–99·9)	99·9 (99·9–100)	100 (100–100)	99·9 (99·9–99·9)
Diabetes mellitus	100 (100–100)	100 (100–100)	100 (100–100)	100 (100–100)
Chronic kidney disease	100 (100–100)	100 (100–100)	100 (100–100)	100 (100–100)
Sense organ diseases	--	9·26 (8·2–10·5)	9·26 (8·2–10·5)	--
Blindness and vision loss	--	10·2 (6·44–15)	10·2 (6·44–15)	--
Age-related and other hearing loss	--	9·82 (8·85–10·9)	9·82 (8·85–10·9)	--
Musculoskeletal disorders	3·09 (0·926–5·51)	20·6 (17–24·4)	20·9 (17·3–24·8)	2·88 (0·874–5·13)
Rheumatoid arthritis	11·9 (3·62–21·6)	14·9 (4·67–26·5)	15·1 (4·75–26·9)	14 (4·32–25·2)
Osteoarthritis	--	14·7 (8·23–21·9)	14·7 (8·23–21·9)	--
Low back pain	--	40·2 (34·1–46·5)	40·2 (34·1–46·5)	--
Gout	--	59·9 (44·2–75·5)	59·9 (44·2–75·5)	--
Other non-communicable diseases	0·0835 (0·0575–0·117)	0·101 (0·0686–0·143)	--	0·284 (0·2–0·392)

Sudden infant death syndrome	5·26 (3·63–7·44)	5·26 (3·63–7·43)	--	5·26 (3·63–7·43)
Injuries	30·5 (26·1–34·7)	27·9 (24·1–31·7)	33·5 (29·8–37)	24·5 (19·8–29·1)
Transport injuries	27·8 (23–33)	27·5 (22·5–32·7)	30·3 (26·3–34·1)	26·4 (20·8–32·4)
Road injuries	27·4 (22·5–32·7)	26·5 (21·4–31·8)	27·9 (23·9–31·9)	26 (20·5–32)
Other transport injuries	35·1 (30–39·9)	36·9 (32·3–41·2)	41 (36·5–44·6)	31·8 (25·9–37·3)
Unintentional injuries	39·5 (34·2–43·8)	31·4 (27·4–35·2)	34·9 (30·6–38·8)	25 (21·1–29·3)
Falls	56·3 (49·5–61)	47·2 (41·9–51·6)	46·5 (41·1–50·8)	48·9 (43·1–53·4)
Drowning	2·07 (-5·55–11·3)	1·03 (-6·06–9·72)	11 (6·87–16·4)	0·767 (-6·46–9·69)
Fire, heat, and hot substances	11·3 (7·28–16·6)	14·2 (9·75–19·9)	15·1 (10·4–21·1)	12·7 (8·71–17·8)
Poisonings	16·6 (11·7–22·6)	16·1 (11·4–22)	13·7 (9·4–19·1)	17·6 (12·6–23·8)
Exposure to mechanical forces	21·2 (15·3–27)	22·8 (19·8–26·5)	23·7 (20·8–26·9)	18·1 (11·9–24·6)
Animal contact	23·9 (19·4–29·2)	25·7 (21·1–31)	26·4 (21·9–31·9)	20·7 (16·3–25·8)
Foreign body	2·45 (1·61–3·55)	3·19 (2·14–4·46)	3·21 (2·18–4·53)	3·19 (2·08–4·64)
Environmental heat and cold exposure	8·07 (3·68–13·8)	8·13 (3·75–14·1)	7·92 (3·68–13·9)	8·61 (3·97–14·7)
Exposure to forces of nature	7·36 (3·38–12·8)	6·89 (3·14–12)	8·39 (3·82–14·6)	6·76 (3·09–11·7)
Other unintentional injuries	20 (14·1–27·1)	15 (10·4–20·9)	14·3 (9·63–20·1)	22·2 (15·9–29·5)
Self-harm and interpersonal violence	22·4 (13·9–31·3)	23·3 (15·6–31·2)	28·3 (22–34·4)	22·9 (14·8–30·9)
Self-harm	23·2 (12–34·7)	25·2 (14·2–37)	23·7 (13·7–34·5)	25·3 (14·2–37·1)
Interpersonal violence	21 (13·3–29·1)	20·8 (13·5–28·6)	29·9 (22·6–36·8)	19 (11·5–27·1)

Supplementary Results Table 2. All-age PAFs attributable to the joint distribution of all risk factors for all ages, both sexes combined for each GBD level 1, level 2, and level 3 cause and all causes in 2019 for Azerbaijan. Results are shown for deaths, YLLs, YLDs, and DALYs Data in parenthesis are 95% uncertainty intervals. PAFs=population attributable fractions. GBD=Global Burden of Disease. YLLs=years of life lost. YLDs=years lived with disability. DALYs=disability-adjusted life-years				
	Deaths 2019	DALYs 2019	YLDs 2019	YLLs 2019
All causes	73·1 (70·2–75·4)	55·3 (52·1–58·6)	29·9 (27·5–32·3)	67·3 (65–69·4)
Communicable, maternal, neonatal, and nutritional diseases	68·8 (65·2–72·4)	70·5 (67·2–73·5)	62·4 (57·9–66·4)	72·6 (68·7–76·2)
HIV/AIDS and sexually transmitted infections	60·8 (44·8–72·7)	64·1 (47·1–77·7)	96·1 (94·3–97·7)	50·1 (32·4–65·5)
HIV/AIDS	76·5 (75·2–78·1)	74·6 (73·2–76·1)	78·2 (76·9–79·7)	74·1 (72·7–75·7)
Sexually transmitted infections excluding HIV	32·1 (13·4–59·3)	56 (31·5–81·1)	100 (100–100)	16·1 (5·55–35·6)
Respiratory infections and tuberculosis	68·3 (63·3–73·6)	70 (64·8–75·3)	18 (12·9–24·6)	73·5 (68·4–78·4)
Tuberculosis	57·4 (49·2–64·2)	52·7 (45·2–59·8)	48·4 (40·2–56)	53·3 (45·2–60·4)
Lower respiratory infections	71·5 (65·6–77·4)	77·6 (72–82·7)	44·8 (37·8–52·8)	77·7 (72·2–82·9)
Upper respiratory infections	8·29 (1·92–22·1)	2·03 (0·0634–7·35)	0·00818 (0·00154–0·0177)	8·55 (2·14–22·5)
Otitis media	5·87 (2·52–12·3)	5·7 (3·69–7·94)	5·7 (3·69–7·94)	11·5 (5·89–20·4)
Enteric infections	87·7 (77·7–94·3)	80·9 (68·9–90·1)	75·9 (62·1–87)	90·5 (82·3–95·7)
Diarrheal diseases	90·4 (81·2–96)	81·4 (69·5–90·7)	76 (62·2–87·1)	92·6 (85·7–96·9)
Other infectious diseases	2·88 (1·3–5·23)	3·1 (1·38–5·63)	0·14 (0·0727–0·255)	3·61 (1·61–6·69)
Meningitis	5·36 (3·14–8·28)	6·35 (3·83–9·69)	0·00137 (0·000235–0·00296)	7·64 (4·71–11·6)
Encephalitis	3·88 (1·14–8·13)	4·65 (1·49–9·79)	0·00128 (0·000245–0·00276)	4·82 (1·57–10·2)
Measles	32·3 (10·5–68·7)	32 (10·5–68·2)	1·25 (0·0358–2·93)	32·9 (10·7–69·7)
Acute hepatitis	0·879 (0·471–1·48)	0·789 (0·439–1·3)	0·898 (0·47–1·62)	0·733 (0·362–1·23)
Maternal and neonatal disorders	81·3 (76·6–85·3)	76·2 (71·8–80·2)	41·4 (34·7–47·7)	81·7 (77–85·8)
Maternal disorders	16·1 (5·8–26·1)	16·2 (5·8–26·3)	16·5 (5·91–26·9)	16·1 (5·79–26·1)
Neonatal disorders	82·4 (77·7–86·6)	77 (72·5–81)	42 (35–48·5)	82·4 (77·7–86·6)
Nutritional deficiencies	61·7 (56·8–66·8)	96·8 (95·7–97·6)	98 (97·4–98·6)	66·6 (61·5–72·2)
Protein-energy malnutrition	100 (100–100)	100 (100–100)	100 (100–100)	100 (100–100)
Vitamin A deficiency	--	100 (100–100)	100 (100–100)	--
Dietary iron deficiency	--	100 (100–100)	100 (100–100)	--
Non-communicable diseases	76·1 (73·1–78·7)	56·3 (52·3–60·2)	27 (24·1–29·8)	71·2 (68·5–73·7)
Neoplasms	44·2 (39·7–49·7)	41·5 (37·5–46·5)	36·1 (32–40·6)	41·6 (37·6–46·6)
Lip and oral cavity cancer	57·7 (49·2–65·4)	58 (49·1–65·7)	54·8 (45·1–63·1)	58·1 (49·2–65·8)
Nasopharynx cancer	52·7 (43·7–61·3)	49·3 (40·3–58)	50·6 (41·5–59)	49·3 (40·2–58)
Other pharynx cancer	69·8 (62·2–76·6)	69·1 (61–76·1)	65·4 (56·8–73·2)	69·1 (61·1–76·1)
Esophageal cancer	69 (56·3–79·5)	70 (57·8–80·4)	69·5 (57·3–80)	70·1 (57·8–80·4)

Stomach cancer	25·3 (16·7–43·4)	24·8 (16·3–42·8)	24·9 (16·2–43·1)	24·8 (16·3–42·8)
Colon and rectum cancer	58·1 (50·1–65·3)	57·4 (49·4–64·4)	57·5 (49·7–64·9)	57·4 (49·4–64·4)
Liver cancer	56·2 (46·6–65·7)	55·2 (46–64·6)	56 (46·5–65·4)	55·2 (46–64·6)
Gallbladder and biliary tract cancer	25·5 (14·7–38·7)	26·1 (15–39·6)	25·7 (14·9–39·1)	26·1 (15–39·6)
Pancreatic cancer	32·3 (24·2–41·6)	32 (24·4–41)	32·3 (24·2–41·5)	32 (24·4–41)
Larynx cancer	77·2 (71·3–82·4)	76·9 (70·7–82·1)	74·1 (67·1–79·6)	77 (70·8–82·2)
Tracheal, bronchus, and lung cancer	80 (75·5–84·7)	78·9 (74·3–83·5)	79·3 (74·2–84·3)	78·9 (74·3–83·5)
Breast cancer	22 (13·9–31)	19·4 (11·7–27·8)	19·5 (12–28)	19·4 (11·7–27·8)
Cervical cancer	100 (100–100)	100 (100–100)	100 (100–100)	100 (100–100)
Uterine cancer	52·8 (37·3–67·5)	53·7 (38–68·2)	54 (38·2–68·5)	53·6 (37·9–68·2)
Ovarian cancer	12·5 (4·39–22·9)	11·3 (3·68–20·6)	10·7 (3·52–19·8)	11·3 (3·69–20·6)
Prostate cancer	9·85 (4·62–15)	10·2 (4·88–15·6)	10·5 (5–15·9)	10·2 (4·88–15·5)
Kidney cancer	40·4 (30·3–50)	38·7 (28·8–48·1)	37·7 (28·1–47·3)	38·7 (28·8–48·1)
Bladder cancer	51·6 (41·2–61)	52·2 (41·9–61·3)	50·8 (40·5–60)	52·3 (42–61·4)
Thyroid cancer	16·6 (8·78–25·8)	16·6 (8·71–25·9)	16·3 (8·79–24·7)	16·6 (8·72–25·9)
Mesothelioma	71·7 (58·8–81·4)	62·5 (47·1–74·3)	69·6 (55·7–79·8)	62·4 (47–74·2)
Non-Hodgkin lymphoma	7·67 (3·31–13·3)	7·05 (3·08–12·3)	6·47 (2·77–11·4)	7·06 (3·08–12·3)
Multiple myeloma	9·69 (4·3–16·5)	9·91 (4·47–16·9)	9·96 (4·37–16·9)	9·91 (4·48–16·9)
Leukemia	21·8 (15·3–29·3)	15·3 (10·2–21·1)	15·1 (9·59–21·5)	15·3 (10·2–21·1)
Cardiovascular diseases	89·8 (87·2–92·4)	89·4 (86·4–91·6)	78·4 (74·2–82·5)	89·9 (86·9–92·1)
Rheumatic heart disease	27·5 (18·4–40·4)	26·2 (16·8–39·1)	24·9 (14·2–38·5)	27 (17·7–40·4)
Ischemic heart disease	94·9 (92·2–97·1)	95·8 (94–97·4)	95·6 (93·6–97·3)	95·8 (94–97·4)
Stroke	85·9 (81·3–90·1)	88·6 (85–91·8)	88·1 (84·7–91)	88·7 (85·1–91·8)
Hypertensive heart disease	100 (100–100)	100 (100–100)	100 (100–100)	100 (100–100)
Non-rheumatic valvular heart disease	25 (18·6–32)	18·4 (12·6–24·8)	4·03 (2·59–5·87)	25·8 (18·8–32·9)
Cardiomyopathy and myocarditis	38·5 (30·9–46·3)	38·9 (30·9–47)	37·1 (29·6–44)	38·9 (30·9–47)
Atrial fibrillation and flutter	53·7 (44–63·3)	63·4 (55·7–71·2)	66·7 (58·9–73·9)	57·6 (48·9–66·5)
Aortic aneurysm	63·4 (57·4–68·9)	67·8 (61·9–72·5)	--	67·8 (61·9–72·5)
Endocarditis	39·8 (29·9–49·8)	38·9 (27·7–49·9)	36·9 (28–46·8)	38·9 (27·7–49·9)
Other cardiovascular and circulatory diseases	39·8 (33·9–46·1)	38·3 (32·4–44·6)	33·2 (25·3–41)	38·7 (32·6–45·1)
Chronic respiratory diseases	65·2 (59·5–70·1)	57·5 (52·8–62·1)	44·8 (39·6–49·5)	63·9 (58·7–68·3)
Chronic obstructive pulmonary disease	74·6 (68·7–79·8)	69·5 (64·6–74·5)	56·6 (50·8–62·1)	74·7 (69·3–79·4)
Pneumoconiosis	100 (100–100)	100 (100–100)	100 (100–100)	100 (100–100)

Asthma	49·1 (37·6–61·2)	43·6 (34·7–53·4)	34 (26·7–42·2)	52·3 (40·9–63·8)
Digestive diseases	52·3 (42·3–62·9)	47·1 (38·3–56·5)	14·7 (9·86–20·2)	53·8 (43·7–64·4)
Cirrhosis and other chronic liver diseases	62·1 (49·8–75·5)	65·2 (52·5–78·5)	60·5 (48·9–72·9)	65·3 (52·5–78·6)
Upper digestive system diseases	32·3 (26·4–38)	13·1 (8–19)	0·968 (0·547–1·5)	33·1 (27·7–38·1)
Gallbladder and biliary diseases	51·4 (36·9–65·8)	52·3 (37·8–66)	52·4 (37·7–66·1)	52·3 (37·9–66·2)
Pancreatitis	39·2 (25·7–53·5)	42 (28·2–57)	33·4 (21·6–47·7)	42·8 (28·7–58·2)
Neurological disorders	20·6 (7·93–34·1)	8·68 (4·06–16·8)	4·94 (2·18–9·76)	17·8 (8·71–29·5)
Alzheimer's disease and other dementias	37·1 (24·4–51·2)	39·1 (26·4–53·1)	39·2 (26·1–53·4)	39 (26·2–52·9)
Parkinson's disease	-11·3 (-17–-5·86)	-13·1 (-19·3–-7)	-14·6 (-21·9–-7·93)	-12·8 (-19·1–-6·7)
Idiopathic epilepsy	15·9 (10·7–21·8)	13·3 (8·92–18·4)	12·8 (8·41–17·9)	13·9 (9·21–19·3)
Multiple sclerosis	17·4 (12·1–24·2)	15·5 (11·1–20·3)	14 (10·1–18·1)	17·6 (12·1–25·1)
Mental disorders	--	3·82 (1·64–6·46)	3·82 (1·64–6·46)	--
Depressive disorders	--	7·11 (1·69–14)	7·11 (1·69–14)	--
Anxiety disorders	--	2·34 (0·567–5·54)	2·34 (0·567–5·54)	--
Idiopathic developmental intellectual disability	--	51·3 (24·2–72·9)	51·3 (24·2–72·9)	--
Substance use disorders	100 (100–100)	100 (100–100)	100 (100–100)	100 (100–100)
Alcohol use disorders	100 (100–100)	100 (100–100)	100 (100–100)	100 (100–100)
Drug use disorders	100 (100–100)	100 (100–100)	100 (100–100)	100 (100–100)
Diabetes and kidney diseases	99·9 (99·8–100)	99·9 (99·8–100)	100 (100–100)	99·8 (99·7–99·9)
Diabetes mellitus	100 (100–100)	100 (100–100)	100 (100–100)	100 (100–100)
Chronic kidney disease	100 (100–100)	100 (100–100)	100 (100–100)	100 (100–100)
Sense organ diseases	--	13·9 (12·4–15·5)	13·9 (12·4–15·5)	--
Blindness and vision loss	--	7·44 (4·81–10·4)	7·44 (4·81–10·4)	--
Age-related and other hearing loss	--	18·4 (17·1–19·9)	18·4 (17·1–19·9)	--
Musculoskeletal disorders	6·16 (2·06–11·1)	33·3 (28·8–37·8)	33·4 (28·9–37·9)	5·43 (1·79–9·68)
Rheumatoid arthritis	17 (5·64–29·7)	10·6 (3·72–17·2)	10·1 (3·56–16·4)	17·7 (5·86–30·1)
Osteoarthritis	--	17 (9·18–25·5)	17 (9·18–25·5)	--
Low back pain	--	49·1 (44·6–53·6)	49·1 (44·6–53·6)	--
Gout	--	50·2 (34·1–67·6)	50·2 (34·1–67·6)	--
Other non-communicable diseases	0·0813 (0·0319–0·177)	0·0412 (0·015–0·0934)	--	0·114 (0·0436–0·254)
Sudden infant death syndrome	11·6 (5·63–20·3)	11·6 (5·63–20·3)	--	11·6 (5·63–20·3)
Injuries	15·2 (12–18·6)	15·9 (12·5–19·3)	20·4 (17·4–23·8)	13·1 (9·78–16·5)
Transport injuries	23·8 (18·9–29·2)	23·3 (18·6–28·5)	25·8 (22–30)	22·3 (17·2–28·1)

Road injuries	23·6 (18·7–29)	23 (18·4–28·3)	25·6 (21·7–29·8)	22·1 (17–27·9)
Other transport injuries	31·2 (26·2–36·4)	28·3 (23·7–33)	29·1 (25·4–33·2)	27·6 (22·5–33·1)
Unintentional injuries	10·7 (6·52–15·2)	13·6 (9·63–17·8)	20·2 (16·9–23·9)	7·79 (3·75–12·2)
Falls	29·2 (24·5–34·4)	25·7 (21·8–30·2)	27·6 (23·7–31·7)	21·7 (17·4–26·4)
Drowning	-7·22 (-13·3–-0·537)	-8·68 (-14·7–-2·44)	10·2 (7·45–13·4)	-9·15 (-15·2–-2·85)
Fire, heat, and hot substances	12·2 (8·57–16·5)	12·5 (8·89–16·7)	14·6 (11–18·8)	11·4 (7·84–15·6)
Poisonings	16 (11·7–21·2)	15·2 (11·2–19·8)	12·9 (9·73–16·7)	16 (11·7–21·1)
Exposure to mechanical forces	7·59 (2·2–13)	16·6 (12·7–20·7)	19·6 (16·3–23·4)	4·68 (-0·991–10·2)
Animal contact	18·4 (14·2–22·9)	16·8 (12·9–21·1)	18·4 (14·7–22·8)	13·8 (9·78–18·2)
Foreign body	8·65 (5·84–11·7)	7·71 (5·13–10·5)	7·25 (5·18–9·69)	7·89 (5·07–11·1)
Environmental heat and cold exposure	7·17 (3·11–12·6)	6·91 (3·02–12·2)	5·89 (2·55–10·3)	7·2 (3·17–12·6)
Exposure to forces of nature	0 (0–0)	6·74 (2·94–11·8)	6·74 (2·94–11·8)	0 (0–0)
Other unintentional injuries	17·7 (12·9–23·4)	15·3 (11·3–19·9)	14·5 (10·7–18·8)	16·4 (11·9–22)
Self-harm and interpersonal violence	15·1 (9·19–21·3)	14·1 (8·81–19·6)	13·2 (9·34–17·8)	14·4 (8·56–20·5)
Self-harm	14·8 (5·15–25·1)	14·9 (5·31–25·3)	14·8 (7·74–22·9)	15 (5·15–25·4)
Interpersonal violence	15·3 (9·1–22)	15·7 (9·95–22·4)	22·8 (17·2–29·5)	13·9 (7·71–20·6)

Supplementary Results Table 2. All-age PAFs attributable to the joint distribution of all risk factors for all ages, both sexes combined for each GBD level 1, level 2, and level 3 cause and all causes in 2019 for Georgia. Results are shown for deaths, YLLs, YLDs, and DALYs Data in parenthesis are 95% uncertainty intervals. PAFs=population attributable fractions. GBD=Global Burden of Disease. YLLs=years of life lost. YLDs=years lived with disability. DALYs=disability-adjusted life-years

	Deaths 2019	DALYs 2019	YLDs 2019	YLLs 2019
All causes	61·2 (58·9–63·6)	47·3 (44·3–50·4)	32·9 (30·4–35·4)	59·8 (58–61·8)
Communicable, maternal, neonatal, and nutritional diseases	44·9 (40·7–49)	56·1 (52·1–59·6)	51 (46·1–55·9)	59·1 (54·8–63·2)
HIV/AIDS and sexually transmitted infections	71·6 (47·2–86·2)	69 (45·6–84·4)	75·3 (55·4–88·3)	65·6 (39·9–82·4)
HIV/AIDS	71·1 (46·3–86)	66·8 (41·9–83·2)	69·8 (46·2–84·9)	65·4 (39·3–82·3)
Sexually transmitted infections excluding HIV	98·1 (97·4–98·7)	99·4 (98·9–99·7)	100 (100–100)	93·5 (91–95·6)
Respiratory infections and tuberculosis	35·3 (29·8–41·3)	31·4 (26–37·3)	2·5 (1·89–3·31)	40·4 (34·6–46·3)
Tuberculosis	55·8 (46·7–64·2)	58 (49·1–65·8)	53·1 (43·4–61·4)	58·8 (50–66·6)
Lower respiratory infections	35·1 (29·5–41·1)	40·1 (34·1–46·1)	31·8 (26·5–37·2)	40·2 (34·2–46·2)
Upper respiratory infections	0·845 (0·517–1·28)	0·023 (0·0112–0·0403)	0·00551 (2·3e-05–0·0151)	1·68 (1·02–2·53)
Otitis media	1·92 (0·835–4·05)	3·75 (2·4–5·21)	3·73 (2·38–5·17)	4·66 (2·33–8·44)
Enteric infections	11·8 (7·22–17·7)	13 (8·53–18·7)	12·7 (8·25–18·4)	13·5 (9·14–19·4)
Diarrheal diseases	11·8 (7·29–17·8)	13 (8·59–18·8)	12·7 (8·25–18·5)	13·7 (9·27–19·6)
Other infectious diseases	0·938 (0·646–1·24)	1·52 (1·14–1·93)	0·598 (0·374–0·956)	1·74 (1·25–2·27)
Meningitis	1·8 (1·29–2·5)	3·69 (2·69–5·05)	0·000646 (2·59e-06–0·00195)	3·87 (2·82–5·31)
Encephalitis	0·784 (0·559–1·07)	1·9 (1·36–2·57)	0·000233 (5·13e-07–0·000715)	1·96 (1·4–2·66)
Measles	9·43 (-3·84–43·6)	5·2 (-2·34–38·8)	0·401 (0–1)	9·6 (-3·92–44·4)
Acute hepatitis	22·1 (14·6–36·1)	9·54 (6·32–14·5)	3·11 (1·91–4·95)	23 (15–36·6)
Maternal and neonatal disorders	81·7 (78·4–84·2)	82·9 (80·9–84·6)	78 (74·1–81·4)	85·1 (82·8–87·1)
Maternal disorders	12 (4·18–20·1)	11·8 (4·13–19·9)	11·6 (4·01–19·8)	11·8 (4·15–20)
Neonatal disorders	91·8 (90·5–92·9)	87·9 (86·1–89·4)	79·9 (76·1–83·5)	91·8 (90·5–92·9)
Nutritional deficiencies	95·7 (86·2–96·9)	90·3 (87·4–92·6)	89 (85·5–92)	94·9 (86·1–96·1)
Protein-energy malnutrition	100 (100–100)	100 (100–100)	100 (100–100)	100 (100–100)
Vitamin A deficiency	--	100 (100–100)	100 (100–100)	--
Dietary iron deficiency	--	100 (100–100)	100 (100–100)	--
Non-communicable diseases	64·8 (62·3–67·5)	49·3 (45·7–52·9)	32·3 (29·5–35)	65·4 (63·3–67·7)
Neoplasms	49·6 (45·8–53·9)	48·4 (44·8–52·4)	30·6 (26·7–34·8)	49·5 (46–53·6)
Lip and oral cavity cancer	66·6 (58–74)	68·7 (60·2–75·7)	68·5 (59·4–75·7)	68·7 (60·1–75·7)
Nasopharynx cancer	62·2 (50·9–71·3)	61·9 (50·9–70·8)	60·9 (49·8–70·2)	62 (51–70·9)
Other pharynx cancer	75·7 (68·3–81·7)	76·7 (69·1–82·5)	77·3 (70·1–83·2)	76·6 (69–82·5)
Esophageal cancer	78·5 (69·1–86·4)	79·3 (70·5–87·1)	78·6 (69·3–86·5)	79·3 (70·5–87·1)

Stomach cancer	25·2 (17·1–42·9)	25·2 (17·1–43·5)	25·8 (17·5–43·6)	25·2 (17·1–43·5)
Colon and rectum cancer	62 (53·6–70·9)	62·6 (54·8–71·1)	62·6 (54·7–71·2)	62·6 (54·8–71·1)
Liver cancer	79·5 (75–84·2)	78·9 (74·5–83·6)	79·5 (74·8–84·3)	78·9 (74·5–83·6)
Gallbladder and biliary tract cancer	26·7 (15·6–39·5)	27·8 (16·5–40·7)	25·6 (14·8–38·4)	27·8 (16·6–40·7)
Pancreatic cancer	43·7 (34·1–55)	43·1 (34·1–54)	43·6 (34·2–54·8)	43·1 (34·1–53·9)
Larynx cancer	81·5 (72·7–88·4)	82·4 (74·7–88·4)	81·6 (73·6–88·2)	82·4 (74·7–88·4)
Tracheal, bronchus, and lung cancer	82·6 (79·2–86·4)	81·8 (78·5–85·4)	82·2 (78·8–86·2)	81·8 (78·5–85·4)
Breast cancer	34·4 (24·9–45·9)	33 (24·3–43·7)	33·8 (24·6–44·7)	32·9 (24·3–43·5)
Cervical cancer	100 (100–100)	100 (100–100)	100 (100–100)	100 (100–100)
Uterine cancer	54·4 (39·3–68·1)	56 (41·1–69)	56·5 (41·4–69·5)	56 (41·1–69)
Ovarian cancer	19·4 (8·29–33·7)	17·6 (7·23–31·2)	16·9 (6·76–30)	17·6 (7·25–31·2)
Prostate cancer	6·18 (2·73–10)	6·73 (2·99–10·7)	7·03 (3·16–11·1)	6·65 (2·93–10·5)
Kidney cancer	42·2 (31–52·8)	42·4 (32·3–52·3)	42·7 (32·3–52·4)	42·4 (32·3–52·3)
Bladder cancer	42·2 (26·7–57·3)	45·3 (30·8–58·9)	45·8 (31·8–59·1)	45·3 (30·7–58·9)
Thyroid cancer	18·3 (9·35–28·9)	19·4 (9·99–30·1)	19·8 (10·2–30·8)	19·3 (9·96–30·1)
Mesothelioma	94·3 (92·2–95·9)	89·7 (86·6–92·4)	93·8 (91·6–95·7)	89·6 (86·5–92·3)
Non-Hodgkin lymphoma	8·78 (3·9–15·1)	9·22 (4·16–15·5)	9·01 (4·05–15·3)	9·24 (4·17–15·6)
Multiple myeloma	10 (4·66–16·8)	10·6 (4·95–17·5)	10·4 (4·82–17·4)	10·6 (4·96–17·5)
Leukemia	36·5 (22·6–50·6)	34·2 (22·7–45·7)	36·8 (23·4–50·2)	34 (22·6–45·5)
Cardiovascular diseases	82·9 (79·8–85·7)	83·2 (80·7–85·6)	72·4 (68·2–76·7)	84·8 (82·5–86·9)
Rheumatic heart disease	22 (12·9–40·3)	22·1 (14–36·5)	22 (13·7–37)	22·1 (14·1–36)
Ischemic heart disease	93·3 (90·2–96·1)	94·7 (92·6–96·7)	93·4 (90·4–96)	94·8 (92·6–96·7)
Stroke	80·3 (74·2–86·9)	84 (79·8–88·4)	83·2 (78·5–87·9)	84·3 (79·9–88·6)
Hypertensive heart disease	100 (100–100)	100 (100–100)	100 (100–100)	100 (100–100)
Non-rheumatic valvular heart disease	25·5 (16·3–37·4)	21·1 (14·2–29·7)	5·3 (3·43–7·39)	25·7 (17·2–35·9)
Cardiomyopathy and myocarditis	41·9 (35·3–49·3)	43·9 (38·2–49·8)	42·3 (36·2–48·6)	44 (38·2–50·1)
Atrial fibrillation and flutter	52·4 (43·1–62·3)	58·6 (50·4–66·9)	59·8 (51·3–67·9)	56·7 (48·1–65·5)
Aortic aneurysm	57·1 (51·5–62·9)	64·5 (59·9–68·9)	--	64·5 (59·9–68·9)
Endocarditis	32·2 (22·9–43·8)	33·5 (24·6–43·7)	31·5 (21·7–42·6)	33·5 (24·6–43·8)
Other cardiovascular and circulatory diseases	24·2 (19·3–31·2)	26·2 (21·1–32·1)	31·2 (23·9–38·8)	24·7 (20·1–30·9)
Chronic respiratory diseases	62·9 (58·3–67·9)	58·3 (54·4–62·1)	52·1 (47·3–56·5)	63·3 (59·2–67·7)
Chronic obstructive pulmonary disease	70·1 (65·6–74·2)	69·4 (65·8–72·9)	64·8 (61·2–68·4)	71·9 (68–75·4)
Pneumoconiosis	100 (100–100)	100 (100–100)	100 (100–100)	100 (100–100)

Asthma	50·6 (39·7–61·9)	39·9 (32·3–48·3)	39 (31·6–47·3)	49 (39·7–58·9)
Digestive diseases	46·5 (41·1–51·7)	46·3 (40·7–51·7)	17·1 (11·6–23)	54·9 (49·2–60·2)
Cirrhosis and other chronic liver diseases	79·9 (71·5–87·6)	82·1 (74·1–88·8)	78 (69·1–85·8)	82·2 (74·1–88·9)
Upper digestive system diseases	22·2 (18·7–25·8)	5·96 (3·67–8·71)	1·49 (0·884–2·32)	24·7 (21–28·3)
Gallbladder and biliary diseases	51·3 (35·8–65·6)	55·6 (41·3–68·5)	56·7 (42·7–69·2)	53·3 (38·6–67)
Pancreatitis	34·7 (17·3–52·5)	38·8 (20·3–56·6)	36·1 (18·4–53·8)	39·1 (20·5–56·8)
Neurological disorders	25·2 (9·87–41·1)	14 (6·73–25·5)	9·64 (4·6–19·8)	21·2 (7·86–37·6)
Alzheimer's disease and other dementias	42·6 (28·8–58·7)	45·8 (31·6–61·6)	46·5 (32·3–61·8)	45·3 (30·9–60·9)
Parkinson's disease	-9·93 (-15·6–-4·91)	-11·3 (-17·5–-5·81)	-11·9 (-17·9–-6·17)	-11·2 (-17·4–-5·7)
Idiopathic epilepsy	21·6 (14–29·1)	19·4 (12·4–26·2)	18·3 (11·8–24·9)	22 (14·2–29·6)
Multiple sclerosis	15·3 (11–20·1)	15·8 (11·6–20·3)	15·1 (11·2–19·4)	16·4 (11·9–21·3)
Mental disorders	--	9·27 (4·58–14·6)	9·27 (4·58–14·6)	--
Depressive disorders	--	19·6 (9·13–29·7)	19·6 (9·13–29·7)	--
Anxiety disorders	--	6·51 (1·48–15)	6·51 (1·48–15)	--
Idiopathic developmental intellectual disability	--	48·9 (24·6–68·4)	48·9 (24·6–68·4)	--
Substance use disorders	100 (100–100)	100 (100–100)	100 (100–100)	100 (100–100)
Alcohol use disorders	100 (100–100)	100 (100–100)	100 (100–100)	100 (100–100)
Drug use disorders	100 (100–100)	100 (100–100)	100 (100–100)	100 (100–100)
Diabetes and kidney diseases	99·9 (99·8–99·9)	99·9 (99·9–100)	100 (100–100)	99·9 (99·9–99·9)
Diabetes mellitus	100 (100–100)	100 (100–100)	100 (100–100)	100 (100–100)
Chronic kidney disease	100 (100–100)	100 (100–100)	100 (100–100)	100 (100–100)
Sense organ diseases	--	10·7 (9·54–12)	10·7 (9·54–12)	--
Blindness and vision loss	--	9·86 (6·18–14·4)	9·86 (6·18–14·4)	--
Age-related and other hearing loss	--	11·5 (10·4–12·8)	11·5 (10·4–12·8)	--
Musculoskeletal disorders	3·37 (1·01–6·1)	20·9 (17·5–24·5)	21·2 (17·7–24·9)	3 (0·904–5·39)
Rheumatoid arthritis	12·7 (3·82–22·3)	14·8 (4·6–26·2)	14·9 (4·64–26·4)	14·4 (4·4–25·5)
Osteoarthritis	--	16·4 (9·3–24·1)	16·4 (9·3–24·1)	--
Low back pain	--	40·8 (35·1–46·7)	40·8 (35·1–46·7)	--
Gout	--	59·8 (43·7–75·9)	59·8 (43·7–75·9)	--
Other non-communicable diseases	0·238 (0·172–0·321)	0·272 (0·187–0·379)	--	0·669 (0·483–0·888)
Sudden infant death syndrome	5·7 (4·2–7·65)	5·69 (4·2–7·64)	--	5·69 (4·2–7·64)
Injuries	26·5 (22·3–30·6)	24·9 (21·2–28·9)	29·9 (26·4–33·3)	22·5 (18–26·9)
Transport injuries	27 (22·1–32·5)	26·2 (21·2–31·7)	29·2 (25·3–33·3)	25·2 (20–31·3)

Road injuries	26·6 (21·7–32·1)	25·4 (20·4–31·1)	27·2 (23·4–31·5)	24·9 (19·7–31)
Other transport injuries	34 (28·7–39·7)	34·6 (29·9–39·3)	38·3 (34–42·1)	30·7 (25·1–37)
Unintentional injuries	30·7 (26·4–34·2)	25·7 (22·2–29·4)	29·9 (26·1–33·7)	19·4 (16·2–23·2)
Falls	52·4 (46–56·7)	42·7 (37·7–46·7)	41·9 (37·1–46)	44·6 (39·2–49)
Drowning	-4 (-10·5–3·14)	-5·05 (-11–1·62)	10·5 (6·9–15·2)	-5·43 (-11·5–1·29)
Fire, heat, and hot substances	11·3 (7·67–16·1)	13·7 (9·67–19)	15 (10·6–20·5)	12·5 (8·8–17·4)
Poisonings	16·9 (12–23)	15·9 (11·4–21·6)	13·4 (9·46–18·4)	17·6 (12·8–23·8)
Exposure to mechanical forces	17·5 (12·2–23·3)	20·7 (17·3–25)	22·7 (19·6–26·6)	14·9 (9·2–21·4)
Animal contact	22·5 (18·4–27·3)	23·9 (19·7–28·8)	24·9 (20·6–29·7)	19·2 (15·2–24)
Foreign body	2·81 (1·87–4·19)	4·1 (2·81–5·98)	5·31 (3·74–7·49)	3·63 (2·44–5·32)
Environmental heat and cold exposure	6·95 (3·03–12·3)	7·32 (3·2–12·9)	6·99 (3·05–12·3)	7·64 (3·36–13·5)
Exposure to forces of nature	6·52 (2·84–11·4)	6·37 (2·79–11·1)	7·53 (3·32–13·2)	6·24 (2·72–10·9)
Other unintentional injuries	19 (13·4–25·9)	14·8 (10·5–20·3)	14·2 (9·92–19·6)	20·9 (15·1–28·3)
Self-harm and interpersonal violence	21·9 (13·8–30·5)	22·9 (15·3–30·6)	31·5 (25–38·2)	22·1 (14·3–30)
Self-harm	21·5 (9·93–32·9)	23·3 (11·5–34·9)	22·7 (12·5–33·2)	23·3 (11·5–34·9)
Interpersonal violence	23·2 (15·5–31·3)	22·9 (15·2–30·5)	33·8 (26·4–41·3)	21 (13·4–28·6)

Supplementary Results Table 2. All-age PAFs attributable to the joint distribution of all risk factors for all ages, both sexes combined for each GBD level 1, level 2, and level 3 cause and all causes in 2019 for Hawaii. Results are shown for deaths, YLLs, YLDs, and DALYs Data in parenthesis are 95% uncertainty intervals. PAFs=population attributable fractions. GBD=Global Burden of Disease. YLLs=years of life lost. YLDs=years lived with disability. DALYs=disability-adjusted life-years

	Deaths 2019	DALYs 2019	YLDs 2019	YLLs 2019
All causes	55·2 (51·4–58·5)	43·6 (40·5–46·6)	31·5 (29·1–33·8)	55·8 (53·2–58·4)
Communicable, maternal, neonatal, and nutritional diseases	34·8 (29·5–39·8)	50·9 (47–54·9)	46·1 (42–50·8)	53·8 (48·5–58·7)
HIV/AIDS and sexually transmitted infections	85·3 (66·6–93·2)	84·7 (67·9–92·9)	89·5 (76·6–95·8)	81·3 (61–91)
HIV/AIDS	84·9 (65·9–93)	82·3 (62·8–91·7)	84·5 (66·5–93·2)	81·2 (60·5–91)
Sexually transmitted infections excluding HIV	98·2 (97·1–98·8)	99·5 (99·1–99·8)	100 (100–100)	92·9 (89·4–95·4)
Respiratory infections and tuberculosis	28·2 (22·3–34·2)	27·5 (22·3–33·1)	2·59 (1·9–3·41)	34·3 (28·1–40·3)
Tuberculosis	51·2 (41·8–60)	55·5 (46·4–64·1)	53·2 (43·8–62·3)	56 (46·8–64·7)
Lower respiratory infections	28 (22–34)	33·9 (27·7–40)	30·5 (25–35·8)	33·9 (27·7–40·1)
Upper respiratory infections	0·55 (0·342–0·853)	0·0173 (0·00812–0·0312)	0·00408 (0–0·0131)	1·29 (0·808–2)
Otitis media	1·61 (0·594–3·67)	3·32 (2·17–4·57)	3·28 (2·14–4·52)	4·36 (1·95–8·25)
Enteric infections	10·4 (6·42–15·9)	11·6 (7·67–17·3)	11·2 (7·26–16·8)	13·3 (9·01–18·8)
Diarrheal diseases	10·6 (6·57–16·3)	11·7 (7·74–17·5)	11·3 (7·27–16·8)	13·9 (9·4–19·7)
Other infectious diseases	0·561 (0·36–0·837)	0·971 (0·687–1·27)	0·549 (0·32–0·92)	1·07 (0·725–1·43)
Meningitis	1·29 (0·902–1·77)	2·81 (2·02–3·77)	0·000362 (0–0·00126)	3·02 (2·17–4·07)
Encephalitis	0·554 (0·377–0·814)	1·51 (1·05–2·17)	0·000109 (0–0·000363)	1·59 (1·11–2·31)
Measles	9·24 (-3·8–42·8)	6·62 (-3·58–40·9)	0·301 (0–0·905)	9·41 (-3·88–43·7)
Acute hepatitis	14·6 (8·84–27·1)	7·52 (4·53–12·5)	2·85 (1·63–4·78)	15·9 (9·59–28·9)
Maternal and neonatal disorders	84·4 (81·6–86·8)	81·8 (79·1–84·2)	73·8 (69·5–77·7)	86·3 (84–88·4)
Maternal disorders	11·2 (4·02–18·5)	11 (3·95–18·3)	11 (3·88–18·4)	11 (3·95–18·2)
Neonatal disorders	89·8 (88·1–91·5)	84·7 (82–86·9)	75·7 (71·1–79·7)	89·9 (88·1–91·5)
Nutritional deficiencies	90·2 (84·9–94·6)	87·3 (84·3–90)	86·7 (83–90·1)	90 (84·9–94)
Protein-energy malnutrition	100 (100–100)	100 (100–100)	100 (100–100)	100 (100–100)
Vitamin A deficiency	--	100 (100–100)	100 (100–100)	--
Dietary iron deficiency	--	100 (100–100)	100 (100–100)	--
Non-communicable diseases	58·1 (53·7–61·9)	44·7 (41·1–48·2)	30·6 (27·9–33·1)	60 (56·9–62·8)
Neoplasms	45·5 (41·2–50·1)	45·2 (41·3–49·5)	29 (24·6–33·4)	46·4 (42·6–50·7)
Lip and oral cavity cancer	61·8 (52·1–70)	65·7 (56·7–73·2)	65·4 (56·5–73·3)	65·8 (56·7–73·2)
Nasopharynx cancer	62·8 (51·8–72·2)	63·4 (52·7–72·7)	62·3 (51·5–71·5)	63·4 (52·8–72·8)
Other pharynx cancer	74·2 (67·2–80·2)	75·9 (69·5–81·6)	76·4 (69·8–82·2)	75·8 (69·4–81·5)

Esophageal cancer	75·7 (66–84·6)	77·4 (67·5–86·1)	76·2 (66·1–85)	77·5 (67·5–86·1)
Stomach cancer	22·1 (14–40·9)	22·9 (14·8–41·8)	22·9 (14·7–41·4)	22·9 (14·8–41·8)
Colon and rectum cancer	59·8 (50·9–68·5)	61·2 (53–69·3)	61 (52·7–69·4)	61·2 (53–69·4)
Liver cancer	75·6 (69·1–81·1)	76·5 (70·4–81·5)	75·9 (69·6–81·3)	76·5 (70·4–81·6)
Gallbladder and biliary tract cancer	22·6 (12·7–35)	23·9 (13·6–36·4)	20·7 (10·7–33·4)	24 (13·7–36·5)
Pancreatic cancer	38·6 (29·4–49·6)	38·9 (30–49·5)	38·9 (29·8–49·7)	38·9 (30–49·5)
Larynx cancer	78·1 (67·8–86·5)	79·5 (70·6–86·7)	79·3 (70·1–86·7)	79·5 (70·6–86·7)
Tracheal, bronchus, and lung cancer	78·3 (74·4–82·7)	78 (74·3–82·1)	78·4 (74·5–83)	78 (74·3–82·1)
Breast cancer	32·2 (23·6–42·1)	31·9 (23·8–40·8)	32·2 (24–41·5)	31·8 (23·7–40·7)
Cervical cancer	100 (100–100)	100 (100–100)	100 (100–100)	100 (100–100)
Uterine cancer	50 (35–64·5)	52·3 (37·3–66)	52·8 (38–66·4)	52·2 (37·1–65·9)
Ovarian cancer	15·4 (6·21–27·6)	14·1 (5·36–25·7)	13·9 (5·19–25·3)	14·1 (5·37–25·7)
Prostate cancer	5·25 (2·29–8·7)	5·99 (2·6–9·79)	6·32 (2·75–10·2)	5·88 (2·53–9·62)
Kidney cancer	38·8 (28·2–49·3)	39·9 (29·8–49·6)	40·2 (30·1–49·8)	39·9 (29·8–49·6)
Bladder cancer	35·2 (21–49·8)	38·9 (25·5–52·7)	39·4 (26·3–52·9)	38·9 (25·3–52·7)
Thyroid cancer	16·1 (8·17–25·6)	17·8 (9·08–28)	18·6 (9·51–29·3)	17·5 (9·02–27·5)
Mesothelioma	95·2 (93·4–96·7)	91·4 (88·7–93·9)	94·9 (92·6–96·6)	91·3 (88·6–93·8)
Non-Hodgkin lymphoma	7·91 (3·43–13·7)	8·56 (3·81–14·4)	8·23 (3·67–13·9)	8·59 (3·83–14·5)
Multiple myeloma	8·96 (4·04–15·4)	9·63 (4·49–16·4)	9·33 (4·24–16)	9·64 (4·5–16·4)
Leukemia	33·2 (20·4–46·5)	32·1 (21–43)	33·3 (21–46·4)	32·1 (21–42·9)
Cardiovascular diseases	77·1 (72·8–81·3)	78·2 (74·6–81·5)	68·5 (63·7–73·4)	80 (76·6–83·2)
Rheumatic heart disease	21·2 (11·7–41)	21·1 (13·4–35·7)	20·9 (12·5–36·5)	21·2 (13·5–35·4)
Ischemic heart disease	91·5 (87·3–95·1)	93·2 (90·3–95·8)	91·7 (88–95)	93·3 (90·4–95·9)
Stroke	75·7 (67·8–84·4)	80·4 (74·8–86)	79·6 (73·9–85·9)	80·7 (75–86·3)
Hypertensive heart disease	100 (100–100)	100 (100–100)	100 (100–100)	100 (100–100)
Non-rheumatic valvular heart disease	24·8 (15–39)	21·9 (14·7–31·3)	7·38 (4·69–10·9)	25 (16·8–36)
Cardiomyopathy and myocarditis	41 (33·8–49·1)	45·4 (39·8–51·5)	41·7 (35·3–48·4)	45·8 (40–52·4)
Atrial fibrillation and flutter	48·8 (38·1–59·3)	54·9 (46·1–64·1)	56·3 (47·8–65·3)	52·8 (43·4–62·3)
Aortic aneurysm	51 (44·5–57·8)	59·8 (54·6–64·7)	--	59·8 (54·6–64·7)
Endocarditis	30·4 (20·7–42·9)	32 (23–42·6)	29·9 (19·5–42·8)	32·1 (23·1–42·6)
Other cardiovascular and circulatory diseases	21·3 (16·7–27·2)	24 (19·2–29)	30·9 (24·3–38·1)	22 (17·6–26·9)
Chronic respiratory diseases	50·9 (45·9–56·3)	50·5 (46·5–54·5)	49·1 (44·1–53·8)	52·5 (48·1–57·4)

Chronic obstructive pulmonary disease	62·4 (57·1–67·4)	62·6 (58·6–66·5)	59·4 (55·4–63·3)	65·7 (61·3–69·9)
Pneumoconiosis	100 (100–100)	100 (100–100)	100 (100–100)	100 (100–100)
Asthma	46·8 (35·7–58·6)	41·8 (33·5–51)	40·9 (32·9–49·8)	49·7 (39·5–60·4)
Digestive diseases	43·8 (37·5–49·9)	45·1 (39–50·7)	15·9 (10·7–21·8)	55·5 (49·1–60·9)
Cirrhosis and other chronic liver diseases	78·6 (68·7–87·3)	82·4 (74–89·4)	77·7 (68–86·5)	82·4 (74–89·5)
Upper digestive system diseases	17·7 (14·5–21·2)	5·26 (3·32–7·8)	1·49 (0·89–2·28)	21·4 (18–25·1)
Gallbladder and biliary diseases	45·2 (29·6–61·1)	51·8 (37·8–65·3)	53·9 (40·4–66·9)	48·1 (33·1–62·7)
Pancreatitis	35·2 (18·7–53·8)	41·2 (23·4–59·2)	39·2 (21·9–56·5)	41·6 (23·6–59·7)
Neurological disorders	26·2 (12·6–40·3)	17·3 (8·71–29·3)	12·1 (6·02–22·7)	23·5 (10·1–37·4)
Alzheimer's disease and other dementias	35·2 (21·9–50·6)	38·4 (25·3–53·7)	39·3 (25·8–54·4)	37·9 (24·6–52·8)
Parkinson's disease	-7·83 (-12·7–-3·92)	-9·55 (-15·1–-4·87)	-10·3 (-16–-5·3)	-9·4 (-14·8–-4·79)
Idiopathic epilepsy	23·4 (15·2–32·1)	21·2 (13·9–29·2)	19·9 (12·9–27·6)	24·6 (16·4–33·4)
Multiple sclerosis	12·3 (8·79–16·3)	13·2 (9·49–17·2)	12·8 (9·36–16·5)	13·5 (9·58–17·7)
Mental disorders	--	10·1 (5·01–16·3)	10·1 (5·01–16·3)	--
Depressive disorders	--	19·6 (10·2–30·1)	19·6 (10·2–30·1)	--
Anxiety disorders	--	10·5 (2·63–21·6)	10·5 (2·63–21·6)	--
Idiopathic developmental intellectual disability	--	48·1 (24·2–67·5)	48·1 (24·2–67·5)	--
Substance use disorders	100 (100–100)	100 (100–100)	100 (100–100)	100 (100–100)
Alcohol use disorders	100 (100–100)	100 (100–100)	100 (100–100)	100 (100–100)
Drug use disorders	100 (100–100)	100 (100–100)	100 (100–100)	100 (100–100)
Diabetes and kidney diseases	99·9 (99·9–99·9)	100 (99·9–100)	100 (100–100)	99·9 (99·9–99·9)
Diabetes mellitus	100 (100–100)	100 (100–100)	100 (100–100)	100 (100–100)
Chronic kidney disease	100 (100–100)	100 (100–100)	100 (100–100)	100 (100–100)
Sense organ diseases	--	9·18 (8·21–10·4)	9·18 (8·21–10·4)	--
Blindness and vision loss	--	8·99 (5·55–13·5)	8·99 (5·55–13·5)	--
Age-related and other hearing loss	--	9·99 (9·02–11·1)	9·99 (9·02–11·1)	--
Musculoskeletal disorders	2·69 (0·753–5·08)	20·9 (17·4–24·6)	21·2 (17·7–25)	2·73 (0·81–5·1)
Rheumatoid arthritis	9·7 (2·75–18)	12·5 (3·93–22·5)	12·7 (3·99–23)	11·7 (3·48–21·4)
Osteoarthritis	--	12·9 (7·21–19·4)	12·9 (7·21–19·4)	--
Low back pain	--	40·1 (34·6–45·8)	40·1 (34·6–45·8)	--
Gout	--	59·2 (42·9–75·3)	59·2 (42·9–75·3)	--
Other non-communicable diseases	0·0843 (0·0564–0·126)	0·0949 (0·061–0·138)	--	0·275 (0·187–0·389)

Sudden infant death syndrome	4·2 (2·73–6·17)	4·2 (2·73–6·17)	--	4·2 (2·73–6·17)
Injuries	31·2 (26·4–35·8)	29·5 (25·3–34·1)	34·9 (31·1–39·1)	25·5 (19·9–31)
Transport injuries	31·1 (25·7–38·1)	31·1 (25·8–37·8)	33·5 (29·2–38·4)	29·9 (23·7–37·9)
Road injuries	30·5 (25·1–37·5)	29·9 (24·5–36·9)	31·2 (26·7–36·5)	29·4 (23·1–37·4)
Other transport injuries	39 (33–45·9)	40 (35·4–45·2)	42·6 (38·4–46·4)	36·3 (29·7–44·2)
Unintentional injuries	37·3 (32·2–41·8)	31·7 (27·5–36·5)	35·8 (31·5–40·4)	24 (19·3–29·1)
Falls	56·1 (49·4–60·8)	47·4 (41·8–52·1)	47 (41·4–51·6)	48·4 (43·1–53·5)
Drowning	2·47 (-5·76–13·9)	1·89 (-5·98–12·9)	13·3 (9·19–18·8)	1·6 (-6·42–12·8)
Fire, heat, and hot substances	11·8 (8·03–16·7)	17·5 (12·6–23·6)	18·3 (13·1–24·9)	14·8 (10·4–20·1)
Poisonings	20·2 (14·5–27)	18·9 (13·8–25·4)	16·6 (11·9–22·4)	21·8 (15·8–29·1)
Exposure to mechanical forces	22·3 (16–30·2)	26·1 (22–31·9)	27·2 (23·3–32·5)	19·6 (12·4–28·6)
Animal contact	27·4 (22·6–33·2)	28·4 (23·5–34)	29 (24·2–34·5)	24·8 (19·6–31·1)
Foreign body	3·83 (2·45–5·75)	5·88 (3·89–8·66)	6·11 (4·26–8·75)	5·79 (3·78–8·74)
Environmental heat and cold exposure	7·06 (3·19–12·5)	7·6 (3·45–13·5)	7·47 (3·38–13·3)	8·1 (3·71–14·2)
Exposure to forces of nature	7·09 (3·21–12·6)	7·38 (3·34–13·1)	8·42 (3·79–14·8)	6·73 (3·05–11·9)
Other unintentional injuries	23·2 (16·3–31·5)	17·9 (12·8–24·3)	17·1 (12·2–23·4)	26·6 (19·2–36)
Self-harm and interpersonal violence	22·9 (12·6–33·5)	24·3 (14·7–33·8)	28·5 (22·1–34·6)	23·9 (13·8–34·1)
Self-harm	22·9 (10·9–34·9)	24·6 (12·5–36·6)	22·7 (12·5–33·2)	24·6 (12·5–36·7)
Interpersonal violence	24·5 (16·9–32·6)	24·6 (17·2–32·3)	30·5 (23·2–37·5)	22·1 (14·8–30·1)

Supplementary Results Table 2. All-age PAFs attributable to the joint distribution of all risk factors for all ages, both sexes combined for each GBD level 1, level 2, and level 3 cause and all causes in 2019 for Idaho. Results are shown for deaths, YLLs, YLDs, and DALYs Data in parenthesis are 95% uncertainty intervals. PAFs=population attributable fractions. GBD=Global Burden of Disease. YLLs=years of life lost. YLDs=years lived with disability. DALYs=disability-adjusted life-years

	Deaths 2019	DALYs 2019	YLDs 2019	YLLs 2019
All causes	57·6 (55–60·3)	43·4 (40·6–46·4)	31·5 (28·9–33·9)	55·2 (53·1–57·5)
Communicable, maternal, neonatal, and nutritional diseases	44·5 (38·2–50·3)	53·4 (49·7–56·9)	46·7 (42·2–51·4)	58·2 (53·2–62·6)
HIV/AIDS and sexually transmitted infections	85·7 (83·8–87·5)	88·3 (86–90·9)	93·2 (90·3–96·3)	83·4 (81·3–85·5)
HIV/AIDS	84·7 (82·7–86·6)	83·6 (81·4–85·7)	85·1 (82·8–87·1)	82·9 (80·7–85)
Sexually transmitted infections excluding HIV	98·8 (98·3–99·2)	99·7 (99·4–99·9)	100 (100–100)	95·6 (93·5–97·1)
Respiratory infections and tuberculosis	40·8 (32·1–49·1)	32·3 (25·5–39·4)	2·01 (1·52–2·6)	45·1 (36·3–52·8)
Tuberculosis	51·9 (42–61)	54·2 (44·9–62·8)	51·3 (40·8–60·2)	54·9 (45·5–63·2)
Lower respiratory infections	40·8 (32–49·1)	45 (36·2–52·7)	30·2 (25·1–35·4)	45·3 (36·4–53)
Upper respiratory infections	0·484 (0·286–0·745)	0·0236 (0·0107–0·0425)	0·00593 (0–0·0191)	1·08 (0·646–1·68)
Otitis media	1·61 (0·637–3·47)	3·7 (2·38–5·17)	3·69 (2·37–5·17)	4·2 (1·99–7·51)
Enteric infections	12·4 (7·87–19·2)	14 (9·48–20·7)	13·6 (8·94–20·3)	14·7 (10·3–21·4)
Diarrheal diseases	12·5 (7·93–19·4)	14·1 (9·53–20·8)	13·6 (8·95–20·4)	15 (10·5–21·8)
Other infectious diseases	0·591 (0·391–0·833)	0·983 (0·704–1·26)	0·7 (0·427–1·1)	1·06 (0·721–1·39)
Meningitis	1·13 (0·785–1·55)	2·45 (1·74–3·3)	0·000525 (0–0·00175)	2·6 (1·84–3·5)
Encephalitis	0·525 (0·349–0·787)	1·39 (0·948–2·02)	0·000153 (0–0·000476)	1·45 (0·994–2·11)
Measles	9·53 (-3·82–43·4)	5·79 (-2·75–38·8)	0·399 (0–1·1)	9·69 (-3·89–44·2)
Acute hepatitis	19·4 (12·2–33·4)	8·2 (5·24–13)	3·59 (2·18–5·55)	20·2 (12·7–34·4)
Maternal and neonatal disorders	80·4 (76·9–83·5)	78·8 (76–81·6)	71·4 (66·8–75·8)	83 (80·1–85·7)
Maternal disorders	11·3 (4·09–18·8)	11·1 (4·03–18·4)	10·9 (3·95–18·2)	11·2 (4·04–18·6)
Neonatal disorders	88·1 (85·8–90·2)	82·5 (79·8–85·2)	73·3 (68·4–77·7)	88·1 (85·8–90·2)
Nutritional deficiencies	92·3 (82·8–94·3)	88·9 (85·7–91·5)	88·2 (84·6–91·4)	92 (83·5–94·2)
Protein-energy malnutrition	100 (100–100)	100 (100–100)	100 (100–100)	100 (100–100)
Vitamin A deficiency	--	100 (100–100)	100 (100–100)	--
Dietary iron deficiency	--	100 (100–100)	100 (100–100)	--
Non-communicable diseases	61 (58–63·9)	45·4 (42·1–48·9)	30·9 (28–33·6)	61·1 (58·8–63·6)
Neoplasms	45·4 (41·7–49·5)	44·1 (40·5–48)	27·9 (24–31·9)	45·3 (41·8–49·1)
Lip and oral cavity cancer	63·7 (54·1–71·9)	66 (56·8–73·9)	65·5 (56·5–73·7)	66 (56·8–74)
Nasopharynx cancer	60·9 (49·6–71·1)	60·4 (49·2–70·4)	59·5 (47·8–69·7)	60·5 (49·2–70·5)
Other pharynx cancer	73·5 (65·5–79·8)	74·7 (66·8–80·8)	75·1 (67·1–81·2)	74·6 (66·8–80·8)

Esophageal cancer	77·9 (68·2–86·4)	78·9 (69·4–87·4)	78·1 (68·5–86·6)	78·9 (69·4–87·4)
Stomach cancer	23·5 (15·4–41·7)	23·9 (15·9–42·3)	24·2 (16·1–42·2)	23·9 (15·9–42·3)
Colon and rectum cancer	61 (52·3–69·9)	61·7 (53·5–69·9)	61·7 (53·4–70·1)	61·7 (53·6–69·9)
Liver cancer	78·6 (73·7–83·4)	78·1 (73·5–82·8)	78·3 (73·3–83)	78·1 (73·5–82·8)
Gallbladder and biliary tract cancer	26·3 (15·5–38·7)	27·4 (16·2–40·2)	25·1 (14–38)	27·4 (16·2–40·2)
Pancreatic cancer	41·2 (31·5–52·5)	40·7 (31·6–51·5)	41·1 (31·8–52·1)	40·7 (31·6–51·5)
Larynx cancer	79·2 (69–87)	79·8 (70·8–86·7)	79·6 (70·1–86·6)	79·8 (70·8–86·7)
Tracheal, bronchus, and lung cancer	81·8 (78·5–85·6)	80·8 (77·6–84·5)	81·4 (77·8–85·3)	80·8 (77·5–84·5)
Breast cancer	34·4 (24·9–45·5)	33·4 (24·5–43·5)	34 (25–44·5)	33·3 (24·5–43·3)
Cervical cancer	100 (100–100)	100 (100–100)	100 (100–100)	100 (100–100)
Uterine cancer	53 (37·8–67·1)	54·8 (39·6–68·3)	55·3 (39·9–68·7)	54·7 (39·5–68·2)
Ovarian cancer	20 (9·17–33·2)	17·7 (7·71–30)	17·1 (7·59–29)	17·7 (7·71–30)
Prostate cancer	5·51 (2·35–9·32)	6·06 (2·61–10·1)	6·35 (2·71–10·6)	5·97 (2·57–9·95)
Kidney cancer	40·7 (29·7–51·4)	41·2 (31–51·2)	41·4 (31·2–51·4)	41·2 (31–51·2)
Bladder cancer	38·3 (23·3–53·6)	41·1 (26·7–55)	41·6 (27·6–55·3)	41 (26·7–55)
Thyroid cancer	17·8 (9·08–27·8)	18·9 (9·88–29·1)	19·2 (10·1–29·4)	18·8 (9·72–29·1)
Mesothelioma	96·9 (95·8–97·8)	94 (92·1–95·5)	96·4 (95–97·6)	93·9 (92–95·5)
Non-Hodgkin lymphoma	8·56 (3·74–14·6)	8·97 (3·98–15·2)	8·79 (3·89–14·8)	8·98 (3·97–15·2)
Multiple myeloma	9·79 (4·48–16·4)	10·3 (4·81–17·2)	10·2 (4·65–17·2)	10·3 (4·82–17·2)
Leukemia	34·7 (21·2–48·6)	32·5 (21·1–44·3)	35·1 (21·9–48·8)	32·4 (21–44·1)
Cardiovascular diseases	79·5 (75·7–82·8)	79·7 (76·7–82·5)	70·1 (65·2–74·7)	81·3 (78·5–84)
Rheumatic heart disease	21·6 (12·2–41·6)	21·2 (13·1–35·5)	21·1 (12·6–36·2)	21·2 (13·3–35·3)
Ischemic heart disease	92·4 (88·7–95·6)	93·7 (91·1–96)	92·4 (89–95·3)	93·8 (91·1–96·1)
Stroke	79·2 (72·2–87)	82·1 (76·9–87·8)	81 (75·5–87·1)	82·5 (77·1–88·2)
Hypertensive heart disease	100 (100–100)	100 (100–100)	100 (100–100)	100 (100–100)
Non-rheumatic valvular heart disease	25·8 (15·9–38·9)	24 (15·8–34)	8·54 (5·43–12·3)	25·7 (17·1–36·4)
Cardiomyopathy and myocarditis	38·4 (31·4–46·5)	40·5 (34·4–46·5)	38·9 (32·8–45·2)	40·7 (34·5–46·9)
Atrial fibrillation and flutter	51·5 (41·5–61·9)	56·6 (47·9–65·5)	57·9 (49·3–66·7)	54·7 (45·7–63·9)
Aortic aneurysm	52·6 (46·7–58·7)	59·9 (55·3–64·8)	--	59·9 (55·3–64·8)
Endocarditis	30·7 (20·5–43·3)	31·6 (22·5–42·2)	30·4 (19·8–43)	31·7 (22·6–42·1)
Other cardiovascular and circulatory diseases	20·5 (15·3–28·7)	23 (18·2–29·4)	29·9 (23·5–36·5)	20·9 (16·3–27·8)
Chronic respiratory diseases	64·3 (59–69·6)	59·6 (55·6–63·4)	52·9 (48·3–57·2)	64·6 (60·1–69·3)

Chronic obstructive pulmonary disease	72·4 (67·7–76·6)	70 (65·9–73·5)	62·8 (58·7–66·7)	73·9 (69·7–77·6)
Pneumoconiosis	100 (100–100)	100 (100–100)	100 (100–100)	100 (100–100)
Asthma	48·6 (37·1–60·3)	41·9 (33·8–51)	41·2 (33·2–49·9)	47·3 (37·6–57)
Digestive diseases	43·9 (38·3–49·3)	46·1 (40·4–51·7)	16·9 (11·3–23)	54·3 (48·3–60)
Cirrhosis and other chronic liver diseases	80·2 (70·7–88·1)	82·4 (73·8–89·6)	77·9 (68·2–86)	82·4 (73·9–89·7)
Upper digestive system diseases	20 (16·6–23·5)	6·6 (3·95–9·74)	1·44 (0·849–2·25)	22·5 (18·9–26·1)
Gallbladder and biliary diseases	49·3 (33·7–64·2)	53·8 (39·3–66·9)	55·1 (41·2–67·9)	51·4 (36·5–65·8)
Pancreatitis	33·9 (16·1–51·4)	37·9 (19·9–55·7)	35·8 (18–53·2)	38·2 (20·1–56·2)
Neurological disorders	23·5 (8·97–39·5)	14·4 (7·03–25·8)	10·2 (5·01–20·3)	19·8 (7·14–35·7)
Alzheimer's disease and other dementias	41·6 (27·3–56·4)	44·5 (30·3–59·2)	45·2 (31·2–59·9)	44 (29·7–58·7)
Parkinson's disease	-9·11 (-14·3–-4·58)	-10·4 (-16·1–-5·42)	-11·2 (-17–-5·9)	-10·3 (-15·9–-5·31)
Idiopathic epilepsy	21·5 (13–28·9)	19·5 (12·1–26·5)	18·4 (11·4–24·7)	21·7 (13·4–29·1)
Multiple sclerosis	13·6 (9·84–17·9)	14·4 (10·6–19)	14·2 (10·5–18·6)	14·6 (10·6–19·2)
Mental disorders	--	9·33 (4·81–14·7)	9·33 (4·81–14·7)	--
Depressive disorders	--	18·7 (9·13–28·5)	18·7 (9·13–28·5)	--
Anxiety disorders	--	6·72 (1·6–14·9)	6·72 (1·6–14·9)	--
Idiopathic developmental intellectual disability	--	47·9 (23·2–67·2)	47·9 (23·2–67·2)	--
Substance use disorders	100 (100–100)	100 (100–100)	100 (100–100)	100 (100–100)
Alcohol use disorders	100 (100–100)	100 (100–100)	100 (100–100)	100 (100–100)
Drug use disorders	100 (100–100)	100 (100–100)	100 (100–100)	100 (100–100)
Diabetes and kidney diseases	99·9 (99·8–99·9)	99·9 (99·9–100)	100 (100–100)	99·9 (99·9–99·9)
Diabetes mellitus	100 (100–100)	100 (100–100)	100 (100–100)	100 (100–100)
Chronic kidney disease	100 (100–100)	100 (100–100)	100 (100–100)	100 (100–100)
Sense organ diseases	--	10·5 (9·41–11·8)	10·5 (9·41–11·8)	--
Blindness and vision loss	--	9·18 (5·82–13·4)	9·18 (5·82–13·4)	--
Age-related and other hearing loss	--	11·4 (10·4–12·7)	11·4 (10·4–12·7)	--
Musculoskeletal disorders	3·54 (1·03–6·52)	18·7 (15·7–22)	19 (15·9–22·4)	3·33 (0·988–6·05)
Rheumatoid arthritis	12 (3·48–22·1)	13·9 (4·31–25)	14·1 (4·39–25·3)	13·4 (3·99–24·4)
Osteoarthritis	--	14·2 (8·1–21·1)	14·2 (8·1–21·1)	--
Low back pain	--	39·8 (34·2–45·5)	39·8 (34·2–45·5)	--
Gout	--	59·8 (43·8–75·5)	59·8 (43·8–75·5)	--
Other non-communicable diseases	0·123 (0·085–0·176)	0·147 (0·096–0·214)	--	0·37 (0·256–0·521)

Sudden infant death syndrome	4·03 (2·87–5·63)	4·02 (2·86–5·62)	--	4·02 (2·86–5·62)
Injuries	24·6 (19·2–29·7)	23 (18·1–27·9)	31·7 (28·1–35·4)	18·3 (11·8–24·4)
Transport injuries	20 (13·7–26·8)	20·9 (14·9–27·5)	30·4 (26·6–34·6)	17·6 (10·5–25·2)
Road injuries	17·8 (11·3–24·9)	18·6 (12·3–25·5)	27·9 (24·1–32·3)	15·6 (8·34–23·5)
Other transport injuries	34·5 (29·7–40·3)	34 (29·5–39·5)	39·4 (35·2–43)	30·8 (25·4–37·3)
Unintentional injuries	34·7 (30–38·7)	27·9 (24·1–32)	32·4 (28·5–36·4)	20·7 (16·6–25)
Falls	55·2 (48·6–59·6)	44·9 (39·7–48·9)	43·8 (38·7–47·9)	47·5 (42·1–51·8)
Drowning	-12·2 (-24–0·185)	-12·5 (-24·3–-0·785)	10·8 (7·07–15·5)	-13·2 (-25·3–-1·12)
Fire, heat, and hot substances	11 (7·26–15·7)	14·2 (9·93–19·2)	15·5 (10·8–20·8)	12·3 (8·57–16·9)
Poisonings	16·9 (11·7–22·8)	16·5 (11·7–21·9)	13·6 (9·55–18·3)	17·9 (12·6–24)
Exposure to mechanical forces	7·13 (-1·49–16·3)	17·9 (12·7–23·5)	23·7 (20·3–28·1)	3·77 (-5·67–13·5)
Animal contact	24·7 (20·3–29·8)	24·2 (19·7–29)	25·2 (20·9–30)	21·4 (17·1–26·6)
Foreign body	2·97 (1·94–4·42)	4·49 (2·97–6·5)	4·62 (3·2–6·47)	4·44 (2·88–6·58)
Environmental heat and cold exposure	6·94 (2·85–12·1)	7·39 (3·05–12·9)	7·09 (2·97–12·6)	7·63 (3·13–13·3)
Exposure to forces of nature	6·63 (2·78–11·6)	7·04 (2·95–12·2)	7·83 (3·28–13·5)	6·17 (2·62–10·6)
Other unintentional injuries	19·4 (13·5–26·1)	15·5 (10·8–20·9)	14·7 (10·2–20)	21·9 (15·4–29·4)
Self-harm and interpersonal violence	16·3 (3·87–28·4)	18·1 (6·42–29·6)	27·4 (21–33·6)	17·4 (5·09–29·3)
Self-harm	16·3 (2·66–29·8)	18·1 (4·27–31·4)	23·1 (12·6–33·6)	18 (4·12–31·4)
Interpersonal violence	17·7 (7·74–27·8)	19·5 (11–28)	29·4 (22·5–36·5)	15·1 (5·12–25·1)

Supplementary Results Table 2. All-age PAFs attributable to the joint distribution of all risk factors for all ages, both sexes combined for each GBD level 1, level 2, and level 3 cause and all causes in 2019 for Illinois. Results are shown for deaths, YLLs, YLDs, and DALYs Data in parenthesis are 95% uncertainty intervals. PAFs=population attributable fractions. GBD=Global Burden of Disease. YLLs=years of life lost. YLDs=years lived with disability. DALYs=disability-adjusted life-years

	Deaths 2019	DALYs 2019	YLDs 2019	YLLs 2019
All causes	61·3 (58·5–63·9)	47·2 (44·3–50·3)	32·8 (30·2–35·1)	60·8 (58·8–62·7)
Communicable, maternal, neonatal, and nutritional diseases	45·8 (41·3–49·9)	56·1 (53–59·1)	49·1 (44·4–54)	60·4 (56·7–63·9)
HIV/AIDS and sexually transmitted infections	69·7 (63–75·1)	69·7 (63–75·5)	77·6 (70·9–85·1)	64·6 (57·4–70·8)
HIV/AIDS	68·8 (62–74·5)	65·4 (58·5–71·7)	68·3 (61·1–74·4)	64 (56·8–70·4)
Sexually transmitted infections excluding HIV	99·1 (98·7–99·4)	99·7 (99·4–99·9)	100 (100–100)	96·4 (95–97·5)
Respiratory infections and tuberculosis	43·5 (37–49·1)	37·3 (31·3–43·3)	2·47 (1·86–3·23)	48·4 (42·1–54)
Tuberculosis	55·6 (45·6–64·6)	58·7 (49·3–67·1)	54·9 (44·9–63·9)	59·4 (50–67·5)
Lower respiratory infections	43·4 (37–49)	48·1 (41·7–53·7)	33·5 (27·7–38·7)	48·3 (41·9–53·9)
Upper respiratory infections	0·682 (0·406–1·08)	0·0167 (0·00774–0·0301)	0·00544 (0·000199–0·0146)	1·51 (0·903–2·35)
Otitis media	1·48 (0·58–3·21)	3·59 (2·3–5·01)	3·58 (2·28–5·02)	4·05 (1·84–7·3)
Enteric infections	9·53 (5·94–15)	10·7 (7·11–16·1)	10·6 (7·01–15·9)	10·9 (7·35–16·1)
Diarrheal diseases	9·57 (5·96–15)	10·8 (7·14–16·1)	10·6 (7·01–15·9)	11 (7·41–16·2)
Other infectious diseases	0·703 (0·422–0·922)	1·24 (0·834–1·58)	0·644 (0·397–1·06)	1·39 (0·875–1·82)
Meningitis	1·5 (1·09–2·03)	3·33 (2·42–4·45)	0·000597 (1·84e-05–0·00168)	3·49 (2·52–4·67)
Encephalitis	0·64 (0·459–0·877)	1·7 (1·22–2·3)	0·000155 (4·85e-06–0·000433)	1·77 (1·29–2·4)
Measles	9·2 (-4·13–43·2)	4·64 (-2·32–37·7)	0·327 (0·00707–0·821)	9·37 (-4·2–44)
Acute hepatitis	22·2 (14–34·6)	8·49 (5·5–12·8)	3·2 (1·98–5·04)	23·4 (15–36·3)
Maternal and neonatal disorders	87 (85·1–88·9)	83·7 (81·6–85·5)	75 (70·7–78·9)	88·8 (87·2–90·4)
Maternal disorders	11·4 (4·15–19)	11·3 (4·08–18·8)	11·2 (3·92–18·7)	11·3 (4·13–18·9)
Neonatal disorders	92 (90·7–93·2)	86·4 (84·2–88·2)	76·9 (72·4–80·9)	92 (90·7–93·2)
Nutritional deficiencies	93·4 (86·7–95·3)	88·6 (85·4–91·2)	87·6 (84–90·8)	92·7 (86·5–94·8)
Protein-energy malnutrition	100 (100–100)	100 (100–100)	100 (100–100)	100 (100–100)
Vitamin A deficiency	--	100 (100–100)	100 (100–100)	--
Dietary iron deficiency	--	100 (100–100)	100 (100–100)	--
Non-communicable diseases	64·1 (60·9–66·9)	48·7 (45·3–52·2)	32 (29·2–34·7)	65·3 (63–67·6)
Neoplasms	49·3 (45·5–53·4)	48·6 (45–52·5)	31·6 (27·6–35·8)	49·8 (46·4–53·6)
Lip and oral cavity cancer	66·7 (58·1–74·3)	69·5 (61·5–76·4)	68·9 (60·7–76)	69·5 (61·4–76·5)
Nasopharynx cancer	64·7 (53·8–73·8)	64·6 (53·9–73·3)	63·6 (52·9–72·6)	64·7 (54–73·5)
Other pharynx cancer	77·2 (70·3–82·9)	78·5 (71·6–83·8)	78·8 (71·8–83·9)	78·4 (71·6–83·7)
Esophageal cancer	79·5 (70·4–87·7)	80·8 (71·8–88·7)	79·8 (70·8–87·9)	80·8 (71·8–88·7)

Stomach cancer	24·5 (16·5–42·4)	25 (17–43·4)	25·3 (17·1–43·6)	25 (17–43·4)
Colon and rectum cancer	61·4 (52·9–70·1)	62·6 (54·7–70·4)	62·5 (54·4–70·7)	62·6 (54·7–70·4)
Liver cancer	79·1 (74·5–83·6)	79 (74·5–83·4)	79·2 (74·6–83·4)	79 (74·5–83·4)
Gallbladder and biliary tract cancer	27·5 (16·3–40·6)	28·5 (17·2–41·5)	26·4 (15·4–39·2)	28·5 (17·2–41·7)
Pancreatic cancer	42·8 (33·4–54·1)	42·5 (33·6–53·4)	42·9 (33·8–53·8)	42·5 (33·6–53·4)
Larynx cancer	81·1 (71·5–88·4)	82·4 (74·3–88·6)	82·1 (73·8–88·5)	82·4 (74·3–88·6)
Tracheal, bronchus, and lung cancer	83·2 (79·9–86·6)	82·5 (79·4–85·9)	83 (79·6–86·5)	82·5 (79·4–85·9)
Breast cancer	35·2 (26·3–45·9)	34·5 (26–44)	35 (26·3–45·1)	34·4 (26–43·9)
Cervical cancer	100 (100–100)	100 (100–100)	100 (100–100)	100 (100–100)
Uterine cancer	54·4 (38·8–68·3)	56·1 (40·4–69·3)	56·6 (40·7–69·7)	56 (40·3–69·3)
Ovarian cancer	19·9 (8·9–33·2)	17·7 (7·56–30·2)	17·1 (7·43–29·4)	17·7 (7·58–30·2)
Prostate cancer	5·77 (2·55–9·47)	6·45 (2·82–10·4)	6·79 (3–10·9)	6·34 (2·77–10·2)
Kidney cancer	42·3 (31·5–52·9)	43 (32·7–53)	43·2 (32·9–52·9)	42·9 (32·7–53)
Bladder cancer	38·8 (24–53·7)	42·7 (28·6–57)	43·5 (29·7–57·2)	42·7 (28·5–57)
Thyroid cancer	18·5 (9·59–28·4)	19·6 (10·2–29·8)	19·9 (10·6–30·3)	19·5 (10·1–29·9)
Mesothelioma	96·5 (95·4–97·5)	93·4 (91·4–95·1)	96·1 (94·7–97·3)	93·3 (91·3–95)
Non-Hodgkin lymphoma	8·83 (3·96–14·9)	9·35 (4·32–15·6)	9·13 (4·11–15·2)	9·36 (4·35–15·6)
Multiple myeloma	10·2 (4·66–17)	10·7 (5·07–17·7)	10·6 (4·97–17·6)	10·7 (5·06–17·6)
Leukemia	36·2 (22·7–50·7)	34·8 (23–47·1)	36·8 (23·3–50·3)	34·7 (23–46·9)
Cardiovascular diseases	82·4 (78·8–85·4)	82·7 (80·1–85·1)	72·4 (67·9–76·6)	84·4 (81·9–86·6)
Rheumatic heart disease	22·5 (12·3–42·8)	22 (13·4–37·4)	22 (13·2–37·6)	21·9 (13·5–37·3)
Ischemic heart disease	93·1 (89·3–96·1)	94·5 (91·9–96·7)	93 (89·7–96)	94·5 (92–96·7)
Stroke	80·3 (73·1–88·1)	83·6 (78·7–88·5)	82·4 (77·3–88·1)	84·1 (79·3–89)
Hypertensive heart disease	100 (100–100)	100 (100–100)	100 (100–100)	100 (100–100)
Non-rheumatic valvular heart disease	26·5 (16·5–40)	23 (15·4–33·2)	7·7 (5·03–11·1)	26·2 (17·5–37·7)
Cardiomyopathy and myocarditis	39·7 (32·2–48·1)	42·5 (36·6–48·4)	40·6 (34–47·6)	42·7 (36·9–48·6)
Atrial fibrillation and flutter	53·2 (43·4–63·6)	58·6 (50–67·4)	59·9 (51·3–68·2)	56·8 (47·8–66)
Aortic aneurysm	54·7 (48·7–61·2)	62·8 (57·9–67·5)	--	62·8 (57·9–67·5)
Endocarditis	32·2 (22–45)	32·9 (23·8–43·6)	31·6 (21·4–45·2)	33 (23·9–43·5)
Other cardiovascular and circulatory diseases	22·6 (17·9–29·1)	24·8 (19·9–30·3)	30·3 (23·3–37·9)	23·1 (18·6–28·7)
Chronic respiratory diseases	64·3 (59·7–68·7)	59·4 (55·7–63)	53·8 (49·3–58·2)	64·9 (61·2–68·7)
Chronic obstructive pulmonary disease	72·7 (68·2–76·8)	70·8 (67–74·2)	64·7 (60·8–68·5)	75 (71·1–78·4)
Pneumoconiosis	100 (100–100)	100 (100–100)	100 (100–100)	100 (100–100)
Asthma	51·2 (40·4–61·8)	42·6 (34·7–51·1)	41·7 (33·8–50·1)	50·9 (41·4–60·7)

Digestive diseases	43·9 (38·8–48·8)	45·2 (40·1–50·3)	16·5 (11·4–22·3)	54·7 (49·3–59·6)
Cirrhosis and other chronic liver diseases	80·8 (72·2–88·2)	83·5 (75·8–89·9)	79·3 (71·1–87·1)	83·5 (75·9–89·9)
Upper digestive system diseases	20·7 (17·3–24·1)	5·61 (3·4–8·38)	1·46 (0·885–2·26)	23·9 (20·4–27·6)
Gallbladder and biliary diseases	50·6 (35·2–65·2)	55·4 (40·8–68·5)	56·7 (42·3–69·7)	52·7 (37·8–66·7)
Pancreatitis	37·6 (21–54·7)	42·5 (25·6–58·8)	39·6 (23–56·4)	43 (26·1–59·4)
Neurological disorders	26·7 (11·8–41·7)	16 (7·92–27·8)	11·1 (5·49–21·6)	22·9 (9·37–38·2)
Alzheimer's disease and other dementias	40·6 (26·9–56·3)	43·9 (29·7–59)	44·6 (30·5–59·4)	43·4 (29·2–58·7)
Parkinson's disease	-9·26 (-14·7–-4·5)	-10·9 (-16·9–-5·5)	-11·4 (-17·5–-5·86)	-10·8 (-16·7–-5·39)
Idiopathic epilepsy	24·4 (16·3–32·8)	21·9 (14·7–29·7)	20·8 (13·8–28·2)	25·1 (16·9–33·5)
Multiple sclerosis	14·6 (10·6–19·1)	15·3 (11·3–19·8)	14·9 (11–19·2)	15·7 (11·5–20·4)
Mental disorders	--	8·34 (4·17–13·2)	8·34 (4·17–13·2)	--
Depressive disorders	--	18·8 (8·8–28·6)	18·8 (8·8–28·6)	--
Anxiety disorders	--	6·25 (1·4–14·1)	6·25 (1·4–14·1)	--
Idiopathic developmental intellectual disability	--	46·1 (23·6–63)	46·1 (23·6–63)	--
Substance use disorders	100 (100–100)	100 (100–100)	100 (100–100)	100 (100–100)
Alcohol use disorders	100 (100–100)	100 (100–100)	100 (100–100)	100 (100–100)
Drug use disorders	100 (100–100)	100 (100–100)	100 (100–100)	100 (100–100)
Diabetes and kidney diseases	99·9 (99·8–99·9)	99·9 (99·9–100)	100 (100–100)	99·9 (99·9–99·9)
Diabetes mellitus	100 (100–100)	100 (100–100)	100 (100–100)	100 (100–100)
Chronic kidney disease	100 (100–100)	100 (100–100)	100 (100–100)	100 (100–100)
Sense organ diseases	--	10·5 (9·37–11·8)	10·5 (9·37–11·8)	--
Blindness and vision loss	--	9·54 (6·1–14)	9·54 (6·1–14)	--
Age-related and other hearing loss	--	11·4 (10·3–12·6)	11·4 (10·3–12·6)	--
Musculoskeletal disorders	3·12 (0·965–5·78)	20·1 (16·9–23·5)	20·4 (17·1–23·9)	2·96 (0·933–5·4)
Rheumatoid arthritis	11·9 (3·61–21·3)	14·5 (4·61–26)	14·7 (4·65–26·4)	13·8 (4·26–24·5)
Osteoarthritis	--	16·4 (9·44–24)	16·4 (9·44–24)	--
Low back pain	--	40·8 (35·1–46·6)	40·8 (35·1–46·6)	--
Gout	--	60·9 (45·2–76·3)	60·9 (45·2–76·3)	--
Other non-communicable diseases	0·105 (0·0734–0·142)	0·128 (0·0861–0·183)	--	0·345 (0·244–0·468)
Sudden infant death syndrome	7·7 (5·16–10·9)	7·69 (5·16–10·8)	--	7·69 (5·16–10·8)
Injuries	28·6 (24·2–32·8)	27·5 (23·6–31·9)	33·6 (29·7–37·6)	23·6 (18·9–28·7)
Transport injuries	28·4 (22·3–35·1)	29·2 (23·2–36)	32·8 (28·7–37·4)	27·4 (20·5–35·2)
Road injuries	27·6 (21·5–34·4)	27·9 (21·8–34·9)	30·6 (26·3–35·4)	26·8 (19·6–34·5)
Other transport injuries	38·7 (33–45·5)	39·2 (34·6–44·2)	41·6 (37·1–45·4)	36 (29·6–43·7)

Unintentional injuries	35·1 (30·7–38·8)	30·5 (26·4–35)	34 (29·7–38·5)	23·7 (19·7–28·3)
Falls	54·9 (48·4–59·5)	46·3 (41·2–50·9)	45·7 (40·6–50·3)	47·9 (42·6–52·7)
Drowning	-5·74 (-14·4–3·66)	-5·68 (-13·9–3·56)	12·6 (8·6–18)	-6·56 (-15–2·89)
Fire, heat, and hot substances	13·1 (8·81–18·5)	17·3 (12·3–23·3)	18·6 (13·4–25·3)	15·3 (10·6–20·8)
Poisonings	21·7 (15·6–29·5)	20·9 (15·1–28·1)	17·1 (12·3–23·1)	23·4 (16·9–31·6)
Exposure to mechanical forces	14·5 (6·94–22·4)	24·2 (19·6–29·7)	26·6 (22·5–31·6)	12·4 (3·74–21·6)
Animal contact	27·6 (22·9–33·2)	28·5 (23·7–34·1)	29 (24·1–34·5)	24·4 (19·4–30·4)
Foreign body	3·5 (2·31–5·22)	5·63 (3·84–8·27)	7·26 (5·12–10·2)	4·88 (3·25–7·43)
Environmental heat and cold exposure	8·08 (3·64–13·8)	8·45 (3·79–14·5)	8·01 (3·58–13·8)	8·92 (4·07–15·2)
Exposure to forces of nature	7·49 (3·36–12·9)	7·23 (3·23–12·4)	8·71 (3·92–15)	7·06 (3·16–12·1)
Other unintentional injuries	23·6 (16·8–32·1)	18·4 (13·2–24·7)	17·6 (12·6–23·8)	27·1 (19·6–36·8)
Self-harm and interpersonal violence	21·8 (13·3–29·7)	22·5 (15·1–29·8)	31·8 (25·4–38·5)	21·5 (13·7–29)
Self-harm	23·2 (10·8–35·3)	25 (12·5–37)	24·7 (13·7–35·8)	25 (12·5–37)
Interpersonal violence	20·1 (12·3–28·6)	20·3 (12·8–28·2)	33·3 (26·4–40·8)	18 (10·3–26·4)

Supplementary Results Table 2. All-age PAFs attributable to the joint distribution of all risk factors for all ages, both sexes combined for each GBD level 1, level 2, and level 3 cause and all causes in 2019 for Indiana. Results are shown for deaths, YLLs, YLDs, and DALYs Data in parenthesis are 95% uncertainty intervals. PAFs=population attributable fractions. GBD=Global Burden of Disease. YLLs=years of life lost. YLDs=years lived with disability. DALYs=disability-adjusted life-years

	Deaths 2019	DALYs 2019	YLDs 2019	YLLs 2019
All causes	62·4 (59·6–64·9)	49·4 (46·3–52·4)	34·5 (31·9–36·9)	61·6 (59·6–63·7)
Communicable, maternal, neonatal, and nutritional diseases	47·3 (42·7–51·5)	57·7 (54·7–60·6)	50 (45·4–54·6)	62·3 (58·8–65·6)
HIV/AIDS and sexually transmitted infections	89·9 (87·6–91·8)	90·3 (87·8–92·3)	93·5 (91·1–95·8)	88 (85·3–90·2)
HIV/AIDS	89·4 (86·9–91·4)	88·3 (85·6–90·6)	89·4 (86·7–91·7)	87·8 (85–90)
Sexually transmitted infections excluding HIV	98·7 (98·2–99·2)	99·5 (99·1–99·8)	100 (100–100)	95·3 (93·3–96·8)
Respiratory infections and tuberculosis	44·3 (37·7–50·3)	38·2 (32·1–44·5)	2·54 (1·94–3·28)	49·8 (43·3–55·4)
Tuberculosis	55·1 (46–63·9)	58·6 (49·8–66·4)	54·5 (44·4–63·2)	59·3 (50·6–67)
Lower respiratory infections	44·3 (37·5–50·3)	49·6 (43·1–55·3)	35·2 (29·9–40·6)	49·8 (43·2–55·5)
Upper respiratory infections	0·736 (0·436–1·17)	0·0226 (0·0113–0·0397)	0·00584 (0·000157–0·0149)	1·56 (0·941–2·48)
Otitis media	1·88 (0·752–4·14)	4·4 (2·85–5·97)	4·39 (2·82–5·98)	4·94 (2·33–9)
Enteric infections	11·7 (7·37–18·4)	13 (8·59–19·7)	12·7 (8·3–19·3)	13·2 (8·86–19·9)
Diarrheal diseases	11·8 (7·39–18·5)	13 (8·63–19·8)	12·7 (8·3–19·4)	13·3 (8·93–20·1)
Other infectious diseases	0·777 (0·521–1·04)	1·32 (0·964–1·69)	0·625 (0·373–0·962)	1·49 (1·03–1·96)
Meningitis	1·58 (1·16–2·2)	3·36 (2·52–4·6)	0·000615 (1·26e-05–0·0018)	3·54 (2·62–4·89)
Encephalitis	0·668 (0·477–0·931)	1·72 (1·25–2·37)	0·000172 (3·68e-06–0·000474)	1·79 (1·3–2·48)
Measles	9·45 (-4·01–43·1)	6·3 (-3·34–41·3)	0·343 (0–1·03)	9·62 (-4·09–43·9)
Acute hepatitis	21·3 (13·5–35·3)	8·99 (5·7–13·9)	3·29 (1·98–5·09)	22·1 (14·2–35·4)
Maternal and neonatal disorders	85·2 (82·8–87·2)	83·7 (81·8–85·4)	76·5 (72·5–80·4)	87·2 (85·4–88·8)
Maternal disorders	11·6 (4·11–19·4)	11·4 (4·02–19)	11·2 (3·89–18·9)	11·5 (4·07–19·2)
Neonatal disorders	91·1 (89·6–92·4)	86·9 (85–88·6)	78·6 (74·5–82·4)	91·1 (89·6–92·4)
Nutritional deficiencies	93·7 (84·6–95·4)	89·7 (86·8–92·1)	88·7 (85·1–91·6)	93·1 (84·5–94·7)
Protein-energy malnutrition	100 (100–100)	100 (100–100)	100 (100–100)	100 (100–100)
Vitamin A deficiency	--	100 (100–100)	100 (100–100)	--
Dietary iron deficiency	--	100 (100–100)	100 (100–100)	--
Non-communicable diseases	65·4 (62·3–68·2)	51·5 (48–55)	34·1 (31·3–36·8)	66·7 (64·5–68·9)
Neoplasms	52 (48·2–56·1)	51·5 (47·9–55·4)	32·8 (28·6–37·2)	52·6 (49·2–56·5)
Lip and oral cavity cancer	67·2 (59·2–74·5)	70 (62·5–76·8)	69·2 (61·4–76·5)	70·1 (62·6–76·9)
Nasopharynx cancer	64·2 (53·2–73·8)	64·2 (53·2–73·6)	63 (51·7–72·5)	64·2 (53·3–73·6)
Other pharynx cancer	77·7 (71·1–83·2)	79·1 (72·6–84·3)	79·4 (73·2–84·5)	79·1 (72·6–84·3)
Esophageal cancer	80·7 (72–88·5)	81·9 (73·6–89·4)	81 (72·7–88·8)	81·9 (73·6–89·4)

Stomach cancer	26·3 (18·1–43·9)	27·3 (19·1–45·1)	27·3 (19–44·9)	27·3 (19·1–45·1)
Colon and rectum cancer	62·8 (53·9–72)	63·7 (55·5–72·2)	63·6 (55–72·5)	63·7 (55·5–72·2)
Liver cancer	79·2 (74·3–84·2)	78·7 (73·9–83·4)	79 (74·1–83·8)	78·7 (73·9–83·5)
Gallbladder and biliary tract cancer	27·6 (16·3–40·4)	28·7 (17–41·4)	26·6 (15·2–39·4)	28·8 (17·1–41·5)
Pancreatic cancer	45·9 (36–57·7)	46 (36·7–57)	46·1 (36·7–57·5)	46 (36·7–57)
Larynx cancer	82·9 (73·5–89·5)	84·3 (76–90)	83·8 (75·3–89·8)	84·3 (76·1–90)
Tracheal, bronchus, and lung cancer	85·5 (82·6–88·6)	85·1 (82·2–88·1)	85·4 (82·3–88·6)	85·1 (82·2–88·1)
Breast cancer	35·8 (26–47·6)	34·9 (25·9–45·5)	35·5 (26–46·4)	34·9 (25·9–45·4)
Cervical cancer	100 (100–100)	100 (100–100)	100 (100–100)	100 (100–100)
Uterine cancer	55·1 (40·2–68·8)	57 (41·8–70)	57·5 (42·2–70·1)	56·9 (41·7–69·9)
Ovarian cancer	21·1 (9·53–35·7)	19 (8·12–32·8)	18·4 (7·81–32·2)	19·1 (8·15–32·8)
Prostate cancer	6·26 (2·78–10·2)	7·06 (3·11–11·3)	7·49 (3·27–11·8)	6·93 (3·03–11·2)
Kidney cancer	43·9 (32·8–54·4)	44·7 (34·2–54·5)	44·9 (34·5–54·6)	44·7 (34·2–54·6)
Bladder cancer	43 (27·4–57·8)	47 (31·7–60·8)	47·7 (33·3–61·5)	46·9 (31·7–60·7)
Thyroid cancer	18·8 (9·6–29·6)	20 (10·5–31)	20·3 (10·9–30·8)	20 (10·3–31·3)
Mesothelioma	96·7 (95·5–97·7)	93·8 (91·7–95·5)	96·3 (94·8–97·5)	93·7 (91·6–95·4)
Non-Hodgkin lymphoma	9·01 (3·98–15·1)	9·52 (4·31–15·6)	9·29 (4·11–15·6)	9·53 (4·34–15·6)
Multiple myeloma	10·3 (4·81–17)	10·9 (5·15–18·1)	10·7 (5–17·9)	10·9 (5·16–18·1)
Leukemia	37·9 (23·6–52·3)	36·6 (24·3–48·8)	38·5 (24·6–52·6)	36·5 (24·2–48·6)
Cardiovascular diseases	82·2 (78·9–85·3)	82·7 (80·1–85·2)	72·5 (68·4–76·9)	84·3 (81·8–86·6)
Rheumatic heart disease	22·3 (12·8–41·8)	22·2 (14·1–36·7)	22·2 (13·7–37·7)	22·2 (14·2–36·6)
Ischemic heart disease	93·3 (89·8–96·1)	94·8 (92·4–96·8)	93·3 (90·3–96)	94·9 (92·5–96·8)
Stroke	80·7 (73·8–88·2)	84·1 (79·6–88·9)	82·9 (78·1–88·4)	84·6 (79·9–89·2)
Hypertensive heart disease	100 (100–100)	100 (100–100)	100 (100–100)	100 (100–100)
Non-rheumatic valvular heart disease	25·5 (16–37·9)	22·9 (15·7–32)	6·84 (4·43–9·69)	25·8 (17·8–35·8)
Cardiomyopathy and myocarditis	39 (32·1–46·5)	41·6 (35·7–47·7)	40·6 (34·2–47·1)	41·7 (35·7–47·9)
Atrial fibrillation and flutter	52·4 (42·1–62·6)	58·5 (50·3–67·4)	59·9 (51·4–68·6)	56·6 (47·6–65·7)
Aortic aneurysm	56·8 (51·1–62·7)	65·1 (60·4–69·8)	--	65·1 (60·4–69·8)
Endocarditis	31·7 (21·9–44·2)	33 (24·1–43·9)	31·3 (21·3–43·3)	33·1 (24·1–44·1)
Other cardiovascular and circulatory diseases	23·1 (18–30·6)	25·7 (20·4–31·8)	31·6 (24·4–39·2)	23·8 (18·9–30·2)
Chronic respiratory diseases	68·1 (63·4–72·6)	63·6 (59·8–67·2)	56 (51·2–60·4)	69·1 (65·2–73·1)
Chronic obstructive pulmonary disease	75·2 (70·9–79)	74·2 (70·7–77·4)	67·7 (63·9–71·3)	77·4 (73·8–80·5)
Pneumoconiosis	100 (100–100)	100 (100–100)	100 (100–100)	100 (100–100)

Asthma	51·9 (41·3–62·8)	43·5 (35·7–51·8)	42·8 (35·2–51·3)	50·8 (41·8–60·2)
Digestive diseases	40·6 (32·7–47·4)	43 (35·3–49·9)	18·3 (12·6–24·5)	49·7 (40·6–57·1)
Cirrhosis and other chronic liver diseases	71 (56·2–82·9)	73·7 (59·2–84·4)	67·5 (53·6–79·4)	73·7 (59·2–84·5)
Upper digestive system diseases	22·9 (19·2–26·6)	6·97 (4·32–10·1)	1·85 (1·12–2·86)	26·8 (22·8–30·8)
Gallbladder and biliary diseases	51·4 (36–65·7)	56·5 (42·4–69)	57·8 (44·1–69·9)	53·7 (39·1–67·2)
Pancreatitis	33·4 (17–50·8)	38·2 (21·9–56·2)	35·6 (19·3–53·3)	38·7 (22·2–56·6)
Neurological disorders	27·5 (12–43·8)	17·3 (8·8–29·9)	12·5 (6·2–23·5)	23·3 (9·37–39·4)
Alzheimer's disease and other dementias	42·4 (28–58·2)	46·1 (31·6–61·4)	47 (32·6–62·3)	45·4 (31·1–60·7)
Parkinson's disease	-10·1 (-16–-4·99)	-12 (-18·5–-6·11)	-12·6 (-19–-6·6)	-11·9 (-18·4–-6·05)
Idiopathic epilepsy	21·6 (14–29·5)	19·6 (12·7–27)	18·5 (11·8–25·4)	22·4 (14·6–30·4)
Multiple sclerosis	16·7 (12·1–21·8)	18·1 (13·4–23)	17·7 (13·2–22·5)	18·3 (13·4–23·7)
Mental disorders	--	8·67 (4·02–13·3)	8·67 (4·02–13·3)	--
Depressive disorders	--	18·3 (7·99–28·4)	18·3 (7·99–28·4)	--
Anxiety disorders	--	4·2 (0·949–10)	4·2 (0·949–10)	--
Idiopathic developmental intellectual disability	--	47·9 (23·3–66·9)	47·9 (23·3–66·9)	--
Substance use disorders	100 (100–100)	100 (100–100)	100 (100–100)	100 (100–100)
Alcohol use disorders	100 (100–100)	100 (100–100)	100 (100–100)	100 (100–100)
Drug use disorders	100 (100–100)	100 (100–100)	100 (100–100)	100 (100–100)
Diabetes and kidney diseases	99·8 (99·8–99·9)	99·9 (99·9–99·9)	100 (100–100)	99·9 (99·8–99·9)
Diabetes mellitus	100 (100–100)	100 (100–100)	100 (100–100)	100 (100–100)
Chronic kidney disease	100 (100–100)	100 (100–100)	100 (100–100)	100 (100–100)
Sense organ diseases	--	11 (9·83–12·3)	11 (9·83–12·3)	--
Blindness and vision loss	--	10·2 (6·59–14·8)	10·2 (6·59–14·8)	--
Age-related and other hearing loss	--	11·9 (10·7–13·2)	11·9 (10·7–13·2)	--
Musculoskeletal disorders	3·92 (1·2–7·01)	21·6 (18·1–25·3)	21·9 (18·3–25·7)	3·9 (1·21–6·87)
Rheumatoid arthritis	13 (4–23·1)	16·7 (5·45–29·5)	17·1 (5·56–30·1)	15·5 (4·89–27·3)
Osteoarthritis	--	15·3 (8·82–22·2)	15·3 (8·82–22·2)	--
Low back pain	--	43·3 (37·1–49·7)	43·3 (37·1–49·7)	--
Gout	--	61·5 (46·1–76·9)	61·5 (46·1–76·9)	--
Other non-communicable diseases	0·146 (0·1–0·205)	0·193 (0·135–0·268)	--	0·452 (0·323–0·615)
Sudden infant death syndrome	6·01 (4·33–8·14)	6 (4·32–8·13)	--	6 (4·32–8·13)
Injuries	26·2 (21·8–30·5)	24·9 (20·7–28·9)	31·2 (27·7–34·6)	21·5 (16·4–26·5)
Transport injuries	25 (19·1–31·4)	25·2 (19·6–31·4)	30·4 (26·5–34·3)	23·2 (16·8–30·5)

Road injuries	24·4 (18·4–31)	24·1 (18·5–30·7)	28·3 (24·5–32·4)	22·7 (16·3–30·2)
Other transport injuries	35·4 (30·6–40·7)	36·3 (31·8–40·6)	40 (35·7–43·4)	31·9 (26·5–37·7)
Unintentional injuries	31·7 (27·5–35·3)	26·8 (23·2–30·5)	31·8 (27·8–35·7)	19·4 (15·9–23)
Falls	54·5 (47·8–58·9)	44·9 (39·7–48·7)	44·1 (38·8–48)	46·8 (41·4–51)
Drowning	-9·27 (-17·4–-0·85)	-9·66 (-17·4–-1·41)	10·8 (7·35–15·7)	-10·3 (-18·3–-1·84)
Fire, heat, and hot substances	11·6 (7·93–16·3)	14·5 (10·4–19·6)	15·9 (11·4–21·4)	13·1 (9·29–17·9)
Poisonings	17·8 (12·8–24)	17·6 (12·8–23·5)	14·4 (10·4–19·2)	19 (13·8–25·2)
Exposure to mechanical forces	11·2 (4·17–19)	20·3 (16·1–25·6)	24·4 (21·1–28·5)	8·47 (1·04–16·9)
Animal contact	23·6 (19·4–28·8)	25·5 (21·1–30·5)	26·4 (21·9–31·3)	20·7 (16·6–25·9)
Foreign body	3·17 (2·11–4·51)	4·47 (3–6·35)	5·81 (4·04–8·21)	4·05 (2·63–5·77)
Environmental heat and cold exposure	6·81 (3·02–12·2)	7·33 (3·27–12·9)	6·98 (3·11–12·4)	7·7 (3·35–13·5)
Exposure to forces of nature	6·64 (2·98–11·8)	6·9 (3·09–12·2)	7·87 (3·48–14)	6·36 (2·85–11·2)
Other unintentional injuries	20·3 (14·5–27·5)	15·8 (11·3–21·4)	14·9 (10·6–20·2)	22·9 (16·7–30·5)
Self-harm and interpersonal violence	21·1 (12·1–29·6)	22·1 (13·9–30·2)	27·5 (21·5–33·8)	21·7 (13·1–30·2)
Self-harm	22·1 (10·3–33·9)	24·2 (12·1–36·2)	23·4 (13·3–33·5)	24·2 (12·1–36·2)
Interpersonal violence	19 (11·3–26·8)	19·1 (11·9–26·8)	28·8 (22·2–35·8)	17·1 (9·68–24·8)

Supplementary Results Table 2. All-age PAFs attributable to the joint distribution of all risk factors for all ages, both sexes combined for each GBD level 1, level 2, and level 3 cause and all causes in 2019 for Iowa. Results are shown for deaths, YLLs, YLDs, and DALYs Data in parenthesis are 95% uncertainty intervals. PAFs=population attributable fractions. GBD=Global Burden of Disease. YLLs=years of life lost. YLDs=years lived with disability. DALYs=disability-adjusted life-years				
	Deaths 2019	DALYs 2019	YLDs 2019	YLLs 2019
All causes	59·6 (56·5–62·3)	45·4 (42·4–48·5)	31·5 (29·3–33·7)	58·1 (55·9–60·3)
Communicable, maternal, neonatal, and nutritional diseases	43·1 (38–47·7)	51·5 (47·9–54·8)	45·8 (41·4–50·3)	55·5 (50·6–59·7)
HIV/AIDS and sexually transmitted infections	91·2 (89·2–92·7)	92·3 (90·1–94·1)	95·6 (93·6–97·7)	89·2 (86·8–91)
HIV/AIDS	90·5 (88·3–92·1)	89·5 (87–91·4)	90·8 (88·4–92·6)	88·9 (86·3–90·8)
Sexually transmitted infections excluding HIV	99·4 (99·1–99·6)	99·8 (99·7–99·9)	100 (100–100)	97·5 (96·4–98·4)
Respiratory infections and tuberculosis	41·7 (35–47·9)	35·8 (29·7–41·7)	2·36 (1·82–3·04)	47 (40·4–53)
Tuberculosis	51·9 (42–61)	56·1 (46·7–64·4)	54 (43·3–63·2)	56·6 (47·2–64·6)
Lower respiratory infections	41·7 (34·9–47·9)	46·9 (40·1–52·9)	32·3 (26·6–37·7)	47·1 (40·3–53·2)
Upper respiratory infections	0·494 (0·271–0·83)	0·0192 (0·00843–0·035)	0·00618 (0–0·0181)	1·17 (0·648–1·93)
Otitis media	1·42 (0·561–2·9)	3·86 (2·5–5·24)	3·85 (2·51–5·24)	4·14 (1·91–7·31)
Enteric infections	10·2 (6·27–15·7)	11·6 (7·64–17·1)	11·2 (7·25–16·6)	12·3 (8·22–17·6)
Diarrheal diseases	10·3 (6·31–15·8)	11·7 (7·67–17·1)	11·2 (7·25–16·6)	12·5 (8·33–17·8)
Other infectious diseases	0·444 (0·303–0·665)	0·874 (0·606–1·2)	0·498 (0·298–0·858)	0·964 (0·646–1·35)
Meningitis	1·16 (0·714–1·8)	2·64 (1·65–4)	0·000553 (0–0·00161)	2·81 (1·77–4·26)
Encephalitis	0·464 (0·286–0·701)	1·31 (0·85–1·93)	0·000137 (0–0·000413)	1·36 (0·885–2)
Measles	9·39 (-4·16–43·8)	5·73 (-3·26–42·3)	0·326 (0–0·92)	9·55 (-4·23–44·6)
Acute hepatitis	15 (9·19–28·3)	5·97 (3·91–9·92)	2·59 (1·59–4·33)	16 (9·99–29·7)
Maternal and neonatal disorders	81·2 (77·8–84·1)	76·9 (73·7–79·8)	68·4 (63·5–73·1)	83·5 (80·7–85·9)
Maternal disorders	11·2 (4·06–18·7)	11 (3·96–18·4)	10·9 (3·83–18·3)	11·1 (4–18·5)
Neonatal disorders	87·8 (85·9–89·6)	80·1 (76·7–82·8)	70·4 (65·2–75·2)	87·8 (85·9–89·6)
Nutritional deficiencies	92·3 (85·5–94·6)	88·5 (85·6–91·2)	87·8 (84·2–91·1)	91·6 (84·7–94·2)
Protein-energy malnutrition	100 (100–100)	100 (100–100)	100 (100–100)	100 (100–100)
Vitamin A deficiency	--	100 (100–100)	100 (100–100)	--
Dietary iron deficiency	--	100 (100–100)	100 (100–100)	--
Non-communicable diseases	62·3 (58·8–65·2)	47·1 (43·6–50·8)	30·7 (28·2–33·3)	62·7 (60·3–65·2)
Neoplasms	48·2 (44·6–52·2)	48·1 (44·8–51·9)	32·3 (28·3–36·5)	49·1 (45·8–52·9)
Lip and oral cavity cancer	64·7 (55·5–72·5)	68·6 (60–75·7)	68·4 (59·6–76)	68·6 (60–75·7)
Nasopharynx cancer	64·3 (54·1–74·3)	64·5 (54·5–74)	63·3 (52·8–73)	64·6 (54·6–74)
Other pharynx cancer	76·3 (68·8–82·4)	78 (70·9–83·8)	78·5 (71·3–84·1)	78 (70·8–83·8)

Esophageal cancer	79<br>(69·6–87·4)	80·3<br>(70·8–88·6)	79·2<br>(69·8–87·4)	80·4<br>(70·8–88·6)
Stomach cancer	23·8<br>(15·8–41·4)	25<br>(16·9–43·1)	25<br>(16·9–42·7)	25<br>(16·9–43·1)
Colon and rectum cancer	62·5<br>(53·7–70·9)	63·6<br>(55·4–71·4)	63·4<br>(54·8–71·5)	63·6<br>(55·4–71·4)
Liver cancer	76·1<br>(69·2–81·5)	77·2<br>(71·1–82·2)	76·8<br>(70·2–82·3)	77·2<br>(71·1–82·2)
Gallbladder and biliary tract cancer	27·9<br>(16·3–40·4)	28·9<br>(17·1–41·8)	26·3<br>(14·9–39·2)	29<br>(17·3–41·9)
Pancreatic cancer	41·9<br>(32·3–52·9)	42·2<br>(33·2–52·7)	42·2<br>(32·9–52·9)	42·2<br>(33·2–52·7)
Larynx cancer	80·9<br>(70·5–88·7)	82·6<br>(73·8–89)	82·4<br>(73·5–89·1)	82·6<br>(73·7–89)
Tracheal, bronchus, and lung cancer	82·4<br>(78·9–86·1)	82·1<br>(78·9–85·8)	82·5<br>(79·2–86·2)	82·1<br>(78·9–85·8)
Breast cancer	35<br>(25·9–45·6)	34·5<br>(25·9–44)	34·9<br>(26–44·9)	34·5<br>(26–43·9)
Cervical cancer	100<br>(100–100)	100<br>(100–100)	100<br>(100–100)	100<br>(100–100)
Uterine cancer	54·6<br>(39–68·3)	56·3<br>(40·5–69·5)	56·8<br>(41–69·8)	56·2<br>(40·4–69·4)
Ovarian cancer	19·3<br>(8·75–32·2)	17·3<br>(7·44–29·7)	16·9<br>(7·18–28·8)	17·3<br>(7·43–29·7)
Prostate cancer	5·77<br>(2·45–9·53)	6·45<br>(2·77–10·4)	6·83<br>(2·99–10·9)	6·35<br>(2·71–10·3)
Kidney cancer	42·8<br>(31·6–53·6)	43·6<br>(33·1–53·6)	43·8<br>(33·3–53·5)	43·6<br>(33·1–53·6)
Bladder cancer	38·1<br>(23·7–52·7)	42·3<br>(27·8–55·7)	43·1<br>(29·3–56·4)	42·2<br>(27·8–55·7)
Thyroid cancer	19·2<br>(9·75–29·4)	20·4<br>(10·7–31·5)	20·9<br>(11–31·8)	20·3<br>(10·6–31·2)
Mesothelioma	96·4<br>(95·1–97·4)	93·3<br>(91·2–95·1)	96<br>(94·6–97·2)	93·2<br>(91·1–95)
Non-Hodgkin lymphoma	9·25<br>(4·16–15·6)	9·74<br>(4·53–16·1)	9·53<br>(4·43–15·8)	9·76<br>(4·52–16·2)
Multiple myeloma	10·5<br>(5·01–17·5)	11·1<br>(5·28–18·2)	11<br>(5·1–18·2)	11·1<br>(5·28–18·1)
Leukemia	35·9<br>(22·4–49·8)	34·8<br>(22·7–46·8)	36·9<br>(23·1–51)	34·7<br>(22·6–46·5)
Cardiovascular diseases	81·7<br>(78–84·9)	82·3<br>(79·3–84·9)	72<br>(67·3–76·5)	83·8<br>(81·1–86·3)
Rheumatic heart disease	22·2<br>(12·5–41·6)	22<br>(13·9–37·4)	21·9<br>(13·2–37·4)	22<br>(13·9–37·3)
Ischemic heart disease	92·5<br>(88·6–95·7)	94<br>(91·3–96·3)	92·6<br>(89·3–95·5)	94·1<br>(91·3–96·4)
Stroke	78·8<br>(71·5–86·7)	82·2<br>(76·9–87·8)	81·2<br>(75·8–87·1)	82·6<br>(77·2–88·1)
Hypertensive heart disease	100<br>(100–100)	100<br>(100–100)	100<br>(100–100)	100<br>(100–100)
Non-rheumatic valvular heart disease	25·4<br>(15·6–39·7)	24·1<br>(16·3–34·7)	10·4<br>(6·64–15)	25·5<br>(17·1–36·9)
Cardiomyopathy and myocarditis	38<br>(30·6–46·1)	41·2<br>(35·6–46·9)	39·2<br>(33·1–45·5)	41·5<br>(35·6–47)
Atrial fibrillation and flutter	52·3<br>(42–62·5)	58<br>(49–66·7)	59·5<br>(50·6–68)	55·9<br>(46·8–65·1)
Aortic aneurysm	52·6<br>(46·1–59·1)	61·3<br>(56·2–66·2)	--	61·3<br>(56·2–66·2)
Endocarditis	31<br>(20·9–44·2)	32·5<br>(23·5–43)	30·5<br>(20·5–43·2)	32·6<br>(23·6–43)
Other cardiovascular and circulatory diseases	20·9<br>(15·9–28·9)	23·8<br>(19·3–29·7)	31·3<br>(24·7–39)	21·8<br>(17·4–28·2)
Chronic respiratory diseases	64·3<br>(59·5–69·3)	61·1<br>(57·4–64·6)	55·2<br>(51–59·3)	65·6<br>(61·5–69·8)

Chronic obstructive pulmonary disease	72·2 (67·9–76·4)	70·8 (67–74·3)	64 (60·1–67·8)	74·7 (70·9–78·4)
Pneumoconiosis	100 (100–100)	100 (100–100)	100 (100–100)	100 (100–100)
Asthma	50·5 (39·1–62·1)	44 (36–52·6)	43·2 (35·2–51·8)	50·8 (41·3–60·4)
Digestive diseases	37·3 (32–42·2)	41·9 (36·7–46·6)	16·7 (11·3–22·6)	49·4 (43·6–54·4)
Cirrhosis and other chronic liver diseases	78·4 (68·4–87)	82·2 (73·3–89)	77 (67·3–85·2)	82·2 (73·3–89·1)
Upper digestive system diseases	18·4 (15·2–21·7)	6·69 (4·15–9·74)	1·42 (0·85–2·26)	22·1 (18·6–25·6)
Gallbladder and biliary diseases	51·3 (35·5–66·1)	55·8 (41·3–68·5)	57·4 (42·8–69·6)	53·3 (38·5–67·2)
Pancreatitis	34·4 (17·3–51·4)	40·1 (22·5–56·9)	38 (20·2–54·2)	40·4 (22·8–57·3)
Neurological disorders	25 (10·1–40·1)	15·6 (7·62–27·3)	11 (5·39–21·3)	21 (8·24–35·9)
Alzheimer's disease and other dementias	39·5 (25·4–54·7)	42·8 (28·9–57·5)	43·6 (29·5–58·3)	42·3 (28·3–57·2)
Parkinson's disease	-9·05 (-14·7–-4·41)	-10·6 (-16·5–-5·3)	-10·9 (-16·5–-5·68)	-10·5 (-16·5–-5·23)
Idiopathic epilepsy	23·4 (15·3–32)	21·6 (14·2–29·6)	20 (12·9–27·3)	24·3 (16·2–32·8)
Multiple sclerosis	14·7 (10·7–19·4)	16·1 (11·8–20·9)	16 (11·6–20·5)	16·2 (11·9–21·3)
Mental disorders	--	7·61 (3·96–12·3)	7·61 (3·96–12·3)	--
Depressive disorders	--	15·3 (7·74–23·8)	15·3 (7·74–23·8)	--
Anxiety disorders	--	6·24 (1·45–14·1)	6·24 (1·45–14·1)	--
Idiopathic developmental intellectual disability	--	46·3 (21·9–66)	46·3 (21·9–66)	--
Substance use disorders	100 (100–100)	100 (100–100)	100 (100–100)	100 (100–100)
Alcohol use disorders	100 (100–100)	100 (100–100)	100 (100–100)	100 (100–100)
Drug use disorders	100 (100–100)	100 (100–100)	100 (100–100)	100 (100–100)
Diabetes and kidney diseases	99·8 (99·8–99·9)	99·9 (99·9–99·9)	100 (100–100)	99·8 (99·8–99·9)
Diabetes mellitus	100 (100–100)	100 (100–100)	100 (100–100)	100 (100–100)
Chronic kidney disease	100 (100–100)	100 (100–100)	100 (100–100)	100 (100–100)
Sense organ diseases	--	10·8 (9·69–12·1)	10·8 (9·69–12·1)	--
Blindness and vision loss	--	9·75 (6·26–14·1)	9·75 (6·26–14·1)	--
Age-related and other hearing loss	--	11·7 (10·6–12·9)	11·7 (10·6–12·9)	--
Musculoskeletal disorders	3·46 (0·978–6·5)	21 (17·9–24·5)	21·3 (18·1–24·9)	3·62 (1·09–6·69)
Rheumatoid arthritis	11·4 (3·34–21)	14·7 (4·62–26·2)	15 (4·74–27·1)	13·6 (4·13–24·7)
Osteoarthritis	--	16 (9·23–23·5)	16 (9·23–23·5)	--
Low back pain	--	42·9 (37–48·6)	42·9 (37–48·6)	--
Gout	--	61·9 (45·8–77·2)	61·9 (45·8–77·2)	--
Other non-communicable diseases	0·13 (0·0791–0·211)	0·192 (0·112–0·304)	--	0·446 (0·276–0·708)

Sudden infant death syndrome	4·9 (3·01–7·38)	4·89 (3–7·37)	--	4·89 (3–7·37)
Injuries	30·8 (25·7–35·3)	27·6 (23·1–31·9)	34·5 (30·7–38·1)	23·1 (17·6–28·3)
Transport injuries	23·5 (17·4–29·8)	24·6 (18·6–30·7)	31·4 (27·2–35·6)	21·9 (14·8–29)
Road injuries	22·6 (16·3–29)	23·1 (16·9–29·3)	29·1 (25–33·4)	21·1 (13·8–28·3)
Other transport injuries	36·3 (31·2–41·9)	37·2 (32·5–41·9)	41·1 (36·8–44·7)	33·1 (27·3–39·6)
Unintentional injuries	40 (34·6–44)	32·1 (28–36·1)	35·6 (31·3–39·6)	26·3 (22·2–30·4)
Falls	56·2 (49·1–60·7)	47·3 (41·8–51·4)	46·6 (41–50·7)	48·9 (43·3–53·2)
Drowning	-9·6 (-19–-0·435)	-9·67 (-18·4–-0·884)	11·3 (7·33–16·4)	-10·5 (-19·7–-1·46)
Fire, heat, and hot substances	11·5 (7·56–16·5)	15·6 (10·7–21)	17·2 (11·9–23·2)	13·2 (9·09–17·8)
Poisonings	18·9 (13·3–25·3)	18·7 (13·2–24·8)	15·1 (10·4–20·6)	20·4 (14·7–26·8)
Exposure to mechanical forces	9·27 (2·11–17·3)	19·6 (15·3–24·6)	24·6 (21–28·8)	5·84 (-2·01–15)
Animal contact	25·9 (21·3–31·2)	26·4 (21·7–31·4)	27·1 (22·3–32·2)	23·1 (18·4–28·3)
Foreign body	2·95 (1·93–4·38)	4·59 (3·09–6·56)	4·47 (3·03–6·48)	4·65 (3·09–6·79)
Environmental heat and cold exposure	7·37 (3·14–12·9)	8·01 (3·52–13·8)	7·65 (3·31–13·3)	8·38 (3·66–14·4)
Exposure to forces of nature	7·35 (3·28–12·6)	7·16 (3·21–12·3)	8·33 (3·77–14·2)	6·99 (3·14–12)
Other unintentional injuries	20 (14·1–26·7)	16·4 (11·4–22·2)	15·4 (10·5–21·1)	23·2 (16·6–30·6)
Self-harm and interpersonal violence	20·5 (8·74–31·2)	22·2 (11·2–32·2)	29·2 (22·8–35·6)	21·7 (9·92–32)
Self-harm	20·6 (7·41–32·9)	22·4 (9·04–34·5)	22·9 (11·6–33·6)	22·3 (8·94–34·5)
Interpersonal violence	21·4 (12·9–30·3)	22·6 (14·5–30·9)	32·2 (25·1–39·4)	18·9 (10·3–28)

Supplementary Results Table 2. All-age PAFs attributable to the joint distribution of all risk factors for all ages, both sexes combined for each GBD level 1, level 2, and level 3 cause and all causes in 2019 for Kansas. Results are shown for deaths, YLLs, YLDs, and DALYs Data in parenthesis are 95% uncertainty intervals. PAFs=population attributable fractions. GBD=Global Burden of Disease. YLLs=years of life lost. YLDs=years lived with disability. DALYs=disability-adjusted life-years

	Deaths 2019	DALYs 2019	YLDs 2019	YLLs 2019
All causes	59·6 (56·8–62·1)	46 (43–48·8)	32·6 (30·2–35)	58·1 (56–60·1)
Communicable, maternal, neonatal, and nutritional diseases	44·6 (39·8–49·1)	55·2 (51·8–58·6)	49·5 (44·8–54)	59 (54·5–63·1)
HIV/AIDS and sexually transmitted infections	94·8 (92·5–96·3)	95·1 (93–96·5)	97·2 (95·3–98·4)	93·5 (90·9–95·2)
HIV/AIDS	94·6 (92·2–96·1)	94 (91·4–95·7)	95·1 (92·7–96·7)	93·4 (90·8–95·2)
Sexually transmitted infections excluding HIV	99 (98·6–99·4)	99·7 (99·5–99·9)	100 (100–100)	96·2 (94·6–97·5)
Respiratory infections and tuberculosis	41 (34·6–46·8)	35·5 (29·6–41·3)	2·4 (1·86–3·05)	46 (39·7–51·7)
Tuberculosis	53·1 (44·3–62·3)	56·7 (48·1–65·2)	53·4 (43·8–62·6)	57·4 (49·1–65·9)
Lower respiratory infections	41 (34·5–46·8)	45·8 (39·6–51·6)	31·7 (26·6–37)	46 (39·8–51·8)
Upper respiratory infections	0·648 (0·313–1·2)	0·0236 (0·0103–0·0446)	0·00538 (0–0·0171)	1·47 (0·72–2·71)
Otitis media	1·52 (0·645–3·25)	4·01 (2·63–5·49)	4 (2·61–5·53)	4·23 (2·05–7·85)
Enteric infections	10·7 (6·74–16·2)	12·1 (8·15–17·6)	11·7 (7·78–17·3)	12·9 (8·72–18·5)
Diarrheal diseases	10·8 (6·78–16·3)	12·2 (8·2–17·7)	11·7 (7·79–17·3)	13 (8·87–18·7)
Other infectious diseases	0·576 (0·39–0·832)	1·07 (0·76–1·45)	0·528 (0·315–0·868)	1·21 (0·822–1·7)
Meningitis	1·46 (0·926–2·25)	3·2 (2·08–4·79)	0·000543 (0–0·00179)	3·37 (2·18–5·02)
Encephalitis	0·614 (0·397–0·914)	1·63 (1·09–2·39)	0·000225 (0–0·000734)	1·69 (1·12–2·48)
Measles	9·32 (-3·94–43·5)	9·47 (-4·01–44·1)	0·182 (0–0·821)	9·49 (-4·01–44·3)
Acute hepatitis	17·3 (10·5–31·5)	6·89 (4·28–11·3)	2·93 (1·79–4·83)	18 (11–32·6)
Maternal and neonatal disorders	84·5 (81·5–87·2)	81·3 (78·5–83·8)	72·6 (68–76·7)	86·6 (84·2–88·8)
Maternal disorders	12·5 (4·54–20·9)	12·4 (4·48–21)	12·4 (4·45–20·9)	12·4 (4·49–20·8)
Neonatal disorders	90·6 (89–92·2)	84·5 (81·7–86·7)	74·7 (69·8–78·9)	90·6 (89–92·2)
Nutritional deficiencies	93·8 (84·4–95·5)	90·8 (87·9–93·1)	90·1 (86·9–92·9)	93·4 (84·4–94·9)
Protein-energy malnutrition	100 (100–100)	100 (100–100)	100 (100–100)	100 (100–100)
Vitamin A deficiency	--	100 (100–100)	100 (100–100)	--
Dietary iron deficiency	--	100 (100–100)	100 (100–100)	--
Non-communicable diseases	62·8 (59·6–65·6)	47·8 (44·2–51·1)	31·9 (29·2–34·6)	63·2 (60·9–65·5)
Neoplasms	48·1 (44·5–52·2)	47·5 (44–51·2)	30·7 (26·7–34·8)	48·6 (45·2–52·3)
Lip and oral cavity cancer	64·6 (55·5–72·9)	67·7 (59·3–75·5)	67·1 (58·6–74·7)	67·8 (59·3–75·6)
Nasopharynx cancer	62·1 (50·5–71·4)	62·2 (51·2–71·4)	61·1 (49·9–70·3)	62·3 (51·2–71·5)
Other pharynx cancer	75·3 (67·6–81·2)	76·7 (69·3–82·2)	77 (69·7–82·5)	76·6 (69·3–82·2)

Esophageal cancer	79·3 (69·8–87·7)	80·4 (71·3–88·7)	79·4 (70·1–87·9)	80·4 (71·3–88·7)
Stomach cancer	23·9 (15·8–41·9)	24·6 (16·6–42·8)	24·8 (16·6–42·9)	24·6 (16·6–42·8)
Colon and rectum cancer	62·2 (53·4–70·7)	63·3 (55–71·2)	63·2 (54·5–71·6)	63·3 (55–71·2)
Liver cancer	78 (72·5–82·9)	78·1 (72·8–83)	78·1 (72·5–83)	78·1 (72·8–82·9)
Gallbladder and biliary tract cancer	26·9 (16–39·7)	28 (16·7–41·1)	25·9 (14·4–38·2)	28·1 (16·7–41·2)
Pancreatic cancer	42·3 (32·6–53·4)	42·2 (33–52·9)	42·4 (33–53·6)	42·2 (33–52·8)
Larynx cancer	80·8 (70·1–88·2)	82·1 (73·3–88·5)	81·8 (72·8–88·2)	82·2 (73·3–88·5)
Tracheal, bronchus, and lung cancer	81·9 (78·5–85·5)	81·4 (78–84·9)	81·8 (78·2–85·6)	81·4 (78–84·9)
Breast cancer	35 (25·6–46·3)	34·4 (25·9–44·7)	34·9 (26–45·5)	34·3 (25·9–44·6)
Cervical cancer	100 (100–100)	100 (100–100)	100 (100–100)	100 (100–100)
Uterine cancer	54·5 (39·4–68·2)	56·5 (42·1–69·5)	57·1 (42·7–69·9)	56·4 (41·9–69·4)
Ovarian cancer	19·2 (8·32–32·5)	17·3 (7·02–30)	16·7 (6·78–29·2)	17·3 (7·02–30)
Prostate cancer	5·78 (2·52–9·5)	6·39 (2·76–10·4)	6·69 (2·9–10·9)	6·3 (2·71–10·2)
Kidney cancer	42·1 (31·4–52·7)	42·7 (32·5–52·6)	43 (32·8–52·8)	42·7 (32·5–52·7)
Bladder cancer	40·1 (25–55·4)	43·7 (28·5–58)	44·2 (29·7–57·9)	43·6 (28·4–58)
Thyroid cancer	19 (9·6–30)	20·4 (10·5–32)	20·7 (11–32·1)	20·3 (10·3–31·7)
Mesothelioma	95·8 (94·4–97)	92·3 (89·9–94·4)	95·4 (93·7–96·9)	92·2 (89·8–94·3)
Non-Hodgkin lymphoma	8·97 (3·97–15)	9·51 (4·4–15·7)	9·26 (4·22–15·4)	9·52 (4·42–15·7)
Multiple myeloma	10·3 (4·78–17)	10·9 (5·17–18·1)	10·7 (4·9–17·7)	10·9 (5·17–18·1)
Leukemia	35·5 (22·1–49·7)	33·9 (22·3–46·1)	36·2 (22·5–50·1)	33·8 (22·2–45·9)
Cardiovascular diseases	81 (77·3–84·4)	81·4 (78·6–84·2)	71·5 (66·8–75·9)	83·1 (80·3–85·5)
Rheumatic heart disease	21·9 (12·3–41·6)	21·8 (13·7–36·5)	21·7 (13–36·9)	21·8 (14–36·3)
Ischemic heart disease	92·8 (89–95·8)	94·3 (91·6–96·5)	92·8 (89·4–95·6)	94·3 (91·7–96·6)
Stroke	79·2 (71·7–86·9)	82·7 (77·6–87·8)	81·7 (76·4–87·4)	83·1 (77·8–88·1)
Hypertensive heart disease	100 (100–100)	100 (100–100)	100 (100–100)	100 (100–100)
Non-rheumatic valvular heart disease	25·6 (15·7–38·2)	23·8 (16·1–33·9)	8·6 (5·57–12·4)	25·9 (17·4–36·6)
Cardiomyopathy and myocarditis	38·5 (31·4–46·7)	41·3 (35·7–47·2)	39·5 (33·3–45·6)	41·6 (36–47·5)
Atrial fibrillation and flutter	51·6 (41·3–61·9)	57·5 (48·4–66·4)	58·9 (50–67·6)	55·5 (46·1–64·8)
Aortic aneurysm	53·9 (47·7–59·9)	62·1 (57·2–66·7)	--	62·1 (57·2–66·7)
Endocarditis	31 (21·3–42·8)	32·4 (23·6–42·8)	30·6 (20·8–43·1)	32·5 (23·6–43)
Other cardiovascular and circulatory diseases	22 (16·9–29·7)	24·8 (19·8–31)	30·4 (23·6–38·4)	22·9 (18·2–29·5)
Chronic respiratory diseases	65 (59·9–70)	60 (56–63·8)	52·6 (48·1–57)	65·8 (61·4–70·2)

Chronic obstructive pulmonary disease	72·5 (67·6–76·9)	70·7 (66·8–74·3)	63·5 (59·6–67·4)	74·6 (70·5–78·3)
Pneumoconiosis	100 (100–100)	100 (100–100)	100 (100–100)	100 (100–100)
Asthma	50·3 (39·3–61·3)	40·7 (33·3–48·7)	39·8 (32·7–47·7)	49·4 (40·1–58·9)
Digestive diseases	43·3 (37·2–48·8)	46 (40·1–51·4)	17·2 (11·8–23·1)	54·5 (47·8–60·2)
Cirrhosis and other chronic liver diseases	79·6 (70·2–88·1)	82·5 (74–89·6)	77·6 (68·7–86)	82·5 (74–89·7)
Upper digestive system diseases	20·2 (16·7–23·8)	6·28 (3·86–9·59)	1·48 (0·895–2·25)	23·5 (19·6–27·1)
Gallbladder and biliary diseases	50·2 (34·8–65·4)	55·5 (41·4–68·3)	56·9 (43·3–69·2)	52·6 (37·9–66·8)
Pancreatitis	34·5 (17·9–51·6)	39·7 (22·1–56·5)	37·1 (19·7–53·8)	40·1 (22·4–57)
Neurological disorders	25·3 (10·3–40·7)	15·7 (7·78–27·9)	11·3 (5·67–21·9)	21·3 (8·12–37·1)
Alzheimer's disease and other dementias	40·7 (26·4–56·3)	43·9 (29·8–58·9)	44·7 (30·5–59·3)	43·4 (29·3–58·4)
Parkinson's disease	-9·23 (-14·7–-4·63)	-10·8 (-16·7–-5·59)	-11·2 (-17·2–-5·89)	-10·7 (-16·7–-5·53)
Idiopathic epilepsy	22·5 (14·2–30·6)	20·4 (12·8–28·1)	19 (12·2–25·8)	23·4 (15–31·6)
Multiple sclerosis	14·9 (10·8–19·3)	16 (11·8–20·6)	15·7 (11·7–20·1)	16·2 (11·9–21)
Mental disorders	--	8·93 (4·57–14)	8·93 (4·58–14)	--
Depressive disorders	--	18·5 (8·99–27·9)	18·5 (8·99–27·9)	--
Anxiety disorders	--	6·52 (1·51–14·7)	6·52 (1·51–14·7)	--
Idiopathic developmental intellectual disability	--	46 (22·8–64·4)	46 (22·8–64·4)	--
Substance use disorders	100 (100–100)	100 (100–100)	100 (100–100)	100 (100–100)
Alcohol use disorders	100 (100–100)	100 (100–100)	100 (100–100)	100 (100–100)
Drug use disorders	100 (100–100)	100 (100–100)	100 (100–100)	100 (100–100)
Diabetes and kidney diseases	99·9 (99·8–99·9)	99·9 (99·9–100)	100 (100–100)	99·9 (99·9–99·9)
Diabetes mellitus	100 (100–100)	100 (100–100)	100 (100–100)	100 (100–100)
Chronic kidney disease	100 (100–100)	100 (100–100)	100 (100–100)	100 (100–100)
Sense organ diseases	--	10·8 (9·68–12·2)	10·8 (9·68–12·2)	--
Blindness and vision loss	--	9·55 (6·14–14·1)	9·55 (6·14–14·1)	--
Age-related and other hearing loss	--	11·7 (10·6–12·9)	11·7 (10·6–12·9)	--
Musculoskeletal disorders	3·38 (1·05–6·16)	21 (17·8–24·5)	21·3 (18·1–24·8)	3·33 (1·06–5·92)
Rheumatoid arthritis	12 (3·7–21)	14·7 (4·63–26·1)	14·9 (4·66–26·5)	14 (4·5–24·5)
Osteoarthritis	--	16·5 (9·5–24·2)	16·5 (9·5–24·2)	--
Low back pain	--	41·6 (35·9–47·4)	41·6 (35·9–47·4)	--
Gout	--	61·5 (45·6–76·8)	61·5 (45·6–76·8)	--
Other non-communicable diseases	0·175 (0·105–0·271)	0·218 (0·125–0·349)	--	0·531 (0·332–0·807)

Sudden infant death syndrome	5·76 (3·57–8·66)	5·76 (3·57–8·65)	--	5·76 (3·57–8·65)
Injuries	28·8 (24–33·2)	26·1 (21·7–30·4)	32·1 (28·3–35·5)	22·9 (17·6–28·2)
Transport injuries	25·6 (19·8–31·6)	25·5 (19·8–31·5)	30·4 (26·3–34·6)	23·8 (17·3–30·6)
Road injuries	25 (19·1–31)	24·4 (18·5–30·6)	28·3 (24·1–32·5)	23·3 (16·7–30·1)
Other transport injuries	35·1 (29·5–40·7)	35·8 (30·9–40·6)	40 (35·6–43·8)	31·6 (25·5–38·3)
Unintentional injuries	36·3 (31·5–40·2)	28·9 (25–33·1)	32·9 (28·6–37)	22·8 (19–26·9)
Falls	55·5 (48·5–60)	45·9 (40·8–50·2)	45·1 (39·9–49·5)	47·7 (42·4–52·3)
Drowning	-10·2 (-18·2–-1·37)	-10·5 (-18–-2·19)	10·9 (6·85–15·6)	-11·3 (-19–-2·72)
Fire, heat, and hot substances	11·5 (7·47–16·3)	14·5 (10·1–19·9)	15·7 (10·9–21·5)	13 (8·95–17·8)
Poisonings	17·4 (12·2–23·5)	16·9 (12–22·9)	14·2 (9·89–19·4)	18·5 (13·2–24·9)
Exposure to mechanical forces	11·1 (3·87–18·2)	19·4 (15·4–24·3)	23·8 (20·4–27·8)	7·58 (-0·0843–15·7)
Animal contact	25 (20·7–30·2)	25·2 (20·6–30·5)	25·9 (21·2–31·3)	22 (17·4–27·3)
Foreign body	3·02 (1·98–4·63)	4·7 (3·18–6·99)	5·48 (3·78–7·88)	4·39 (2·88–6·59)
Environmental heat and cold exposure	7·17 (2·99–12·5)	7·74 (3·29–13·2)	7·27 (3·1–12·6)	8·15 (3·45–14·1)
Exposure to forces of nature	6·97 (3·02–12·1)	6·9 (3·01–12)	8·16 (3·56–14·2)	6·68 (2·92–11·5)
Other unintentional injuries	19·6 (13·7–26·1)	15·9 (11–21·7)	14·9 (10·2–20·6)	22·1 (15·8–29·5)
Self-harm and interpersonal violence	21·4 (11·5–31·9)	22·7 (13·3–32·4)	28 (21·5–34·6)	22·3 (12·5–32·6)
Self-harm	21·9 (9·1–34·5)	23·8 (10·6–36·7)	23·2 (12·7–34·3)	23·8 (10·6–36·8)
Interpersonal violence	20·7 (13·1–29)	21 (13·7–29·1)	29·6 (22·6–37)	18·8 (11·2–27·2)

Supplementary Results Table 2. All-age PAFs attributable to the joint distribution of all risk factors for all ages, both sexes combined for each GBD level 1, level 2, and level 3 cause and all causes in 2019 for Kentucky. Results are shown for deaths, YLLs, YLDs, and DALYs Data in parenthesis are 95% uncertainty intervals. PAFs=population attributable fractions. GBD=Global Burden of Disease. YLLs=years of life lost. YLDs=years lived with disability. DALYs=disability-adjusted life-years

	Deaths 2019	DALYs 2019	YLDs 2019	YLLs 2019
All causes	64·6 (62·2–66·8)	52·6 (49·4–55·5)	36·8 (33·7–39·5)	64·5 (62·6–66·3)
Communicable, maternal, neonatal, and nutritional diseases	45·3 (41·3–49·2)	54·2 (51·4–57)	47·8 (43·1–52·4)	57·8 (54·4–61)
HIV/AIDS and sexually transmitted infections	89·7 (87·4–91·7)	90·2 (87·9–92·3)	93·4 (91·1–96)	88 (85·3–90·2)
HIV/AIDS	89·3 (86·9–91·3)	88·3 (85·6–90·6)	89·3 (86·8–91·6)	87·8 (85·1–90·1)
Sexually transmitted infections excluding HIV	99·1 (98·8–99·4)	99·7 (99·5–99·9)	100 (100–100)	96·9 (95·5–97·9)
Respiratory infections and tuberculosis	43·5 (38·5–48·6)	40·1 (34·7–45·6)	3·2 (2·5–4·15)	49 (44–54·3)
Tuberculosis	55 (46·1–63)	58·9 (50·8–66·4)	55 (45·8–63·2)	59·5 (51·4–66·7)
Lower respiratory infections	43·4 (38·4–48·5)	48·8 (43·6–54·1)	37·1 (31·4–42·4)	49 (43·8–54·3)
Upper respiratory infections	0·626 (0·371–0·991)	0·0222 (0·0105–0·038)	0·00552 (0·000114–0·0149)	1·34 (0·813–2·11)
Otitis media	1·74 (0·724–3·63)	5·06 (3·38–6·92)	5·07 (3·39–6·96)	4·7 (2·27–8·39)
Enteric infections	13·5 (8·3–20·4)	14·7 (9·38–21·5)	14·4 (9·14–21·1)	15·1 (9·85–22)
Diarrheal diseases	13·6 (8·34–20·5)	14·7 (9·42–21·5)	14·4 (9·15–21·1)	15·2 (9·94–22·2)
Other infectious diseases	0·853 (0·592–1·22)	1·39 (1·04–1·79)	1 (0·606–1·55)	1·48 (1·09–1·96)
Meningitis	1·31 (0·927–1·83)	2·85 (2·05–3·89)	0·000591 (1·09e-05–0·00177)	2·97 (2·13–4·09)
Encephalitis	0·6 (0·421–0·827)	1·57 (1·13–2·12)	0·00014 (2·95e-06–0·000396)	1·63 (1·17–2·2)
Measles	9·71 (-3·92–44·1)	5·91 (-2·62–41)	0·431 (0–1·19)	9·88 (-3·99–44·9)
Acute hepatitis	28 (17·7–44)	12·9 (8·11–19·1)	5·26 (3·17–8·26)	28·8 (18·5–44·4)
Maternal and neonatal disorders	83·3 (80·7–85·5)	81·8 (79·7–83·6)	75·1 (70·9–78·8)	85·6 (83·6–87·4)
Maternal disorders	12 (4·37–20)	11·7 (4·22–19·6)	11·4 (4·1–19·4)	11·8 (4·29–19·7)
Neonatal disorders	89·8 (88·2–91·3)	85·2 (83·1–87)	77·3 (73·1–80·9)	89·8 (88·2–91·3)
Nutritional deficiencies	93 (82·8–94·9)	90·1 (86·9–92·5)	89·3 (85·9–92·3)	92·6 (82·4–94·3)
Protein-energy malnutrition	100 (100–100)	100 (100–100)	100 (100–100)	100 (100–100)
Vitamin A deficiency	--	100 (100–100)	100 (100–100)	--
Dietary iron deficiency	--	100 (100–100)	100 (100–100)	--
Non-communicable diseases	68·1 (65·4–70·5)	55·3 (51·8–58·7)	36·9 (33·5–39·9)	70 (67·9–71·9)
Neoplasms	55 (51·5–59)	54·5 (51·1–58·2)	34·2 (30·3–38·5)	55·7 (52·5–59·3)
Lip and oral cavity cancer	69 (61·5–75·6)	71·7 (64·4–77·7)	71·5 (63·7–77·8)	71·7 (64·3–77·7)
Nasopharynx cancer	63·4 (53·7–72·2)	63·7 (53·8–72·2)	62·5 (52·3–71·5)	63·7 (53·8–72·3)
Other pharynx cancer	77·9 (71·7–82·8)	79·2 (73·5–83·9)	79·7 (73·8–84·3)	79·2 (73·4–83·9)
Esophageal cancer	81·9 (73·2–89)	82·9 (74·4–89·9)	82·2 (73·8–89·2)	82·9 (74·5–89·9)

Stomach cancer	28·1 (19·8–45·3)	29 (20·7–46·3)	29 (20·7–46·5)	29 (20·7–46·2)
Colon and rectum cancer	63·1 (54·1–72)	64·1 (55·8–72·4)	64 (55·6–72·4)	64·1 (55·8–72·3)
Liver cancer	81·5 (76·7–86)	81·1 (76·5–85·6)	81·2 (76·6–85·7)	81·1 (76·5–85·6)
Gallbladder and biliary tract cancer	27·2 (15·9–39·8)	28·4 (16·9–41·1)	26·5 (15·1–39·1)	28·4 (17–41·2)
Pancreatic cancer	47·8 (37·9–58·8)	48 (38·8–58·6)	48·1 (38·6–58·9)	48 (38·8–58·6)
Larynx cancer	83·7 (73·7–90·3)	84·9 (76·6–90·5)	84·6 (76·4–90·5)	84·9 (76·6–90·5)
Tracheal, bronchus, and lung cancer	86·5 (83·6–89·4)	86·2 (83·5–89·1)	86·4 (83·5–89·4)	86·2 (83·5–89·1)
Breast cancer	35·3 (25·3–47·2)	34·6 (25·4–45·4)	35 (25·7–46·5)	34·5 (25·4–45·4)
Cervical cancer	100 (100–100)	100 (100–100)	100 (100–100)	100 (100–100)
Uterine cancer	54·5 (39·1–68·2)	56·4 (41·1–69·5)	56·9 (41·6–69·9)	56·3 (41–69·4)
Ovarian cancer	20·6 (9·18–35·8)	18·8 (7·95–33·2)	18·4 (7·77–32·3)	18·9 (7·96–33·3)
Prostate cancer	7·02 (2·99–11·8)	7·83 (3·37–12·8)	8·3 (3·69–13·4)	7·68 (3·27–12·6)
Kidney cancer	44·8 (33·6–55·7)	45·7 (35·2–55·8)	46 (35·5–55·8)	45·7 (35·1–55·8)
Bladder cancer	45·2 (28·8–60·4)	48·8 (33·1–62·7)	49·5 (34·7–63·2)	48·7 (33–62·6)
Thyroid cancer	18·4 (9·6–28·5)	19·8 (10·5–30·4)	20·5 (11–31·3)	19·6 (10·4–30·2)
Mesothelioma	95·8 (94·4–97·1)	92·6 (90·2–94·6)	95·5 (93·9–96·8)	92·5 (90·1–94·5)
Non-Hodgkin lymphoma	8·85 (3·92–14·9)	9·38 (4·27–15·6)	9·13 (4·13–15·3)	9·39 (4·29–15·6)
Multiple myeloma	10·1 (4·75–16·8)	10·7 (5·12–17·8)	10·6 (4·86–17·7)	10·7 (5·12–17·8)
Leukemia	39·7 (24·4–54·3)	38·6 (25·7–51·2)	40·7 (25·4–55·4)	38·5 (25·7–51)
Cardiovascular diseases	84·1 (81–86·8)	84·7 (82·3–86·8)	73 (68·8–77·3)	86·4 (84·2–88·4)
Rheumatic heart disease	22·5 (13·1–41·2)	22·6 (14·3–36·8)	22·5 (13·4–37·8)	22·6 (14·4–36)
Ischemic heart disease	93·8 (90·8–96·4)	95·3 (93·4–97)	93·7 (91–96·2)	95·3 (93·4–97·1)
Stroke	81·1 (74·9–87·6)	84·7 (80·4–88·9)	83·8 (79·3–88·8)	85 (80·7–89·1)
Hypertensive heart disease	100 (100–100)	100 (100–100)	100 (100–100)	100 (100–100)
Non-rheumatic valvular heart disease	25·5 (16–38·1)	21·3 (14·3–29·8)	4·86 (3·1–6·97)	25·9 (17·7–36·1)
Cardiomyopathy and myocarditis	40·5 (33·6–48·6)	42·9 (37·2–48·5)	40·9 (34·5–47·4)	43·1 (37·2–48·7)
Atrial fibrillation and flutter	52·1 (42·5–62)	58·5 (50–67·2)	59·8 (51·4–68·3)	56·7 (48·1–65·5)
Aortic aneurysm	59·7 (54·1–65·1)	67·5 (62·7–71·6)	--	67·5 (62·7–71·6)
Endocarditis	32·4 (22·3–44·3)	33·9 (24·5–44·3)	31·8 (21·9–44·1)	34 (24·6–44·5)
Other cardiovascular and circulatory diseases	24·8 (19·7–32)	27 (21·8–32·7)	32·1 (25·1–39·3)	25·5 (20·5–31·4)
Chronic respiratory diseases	70·7 (66·2–74·7)	66·4 (62·6–69·7)	57·6 (52·9–61·8)	71·8 (67·8–75·3)
Chronic obstructive pulmonary disease	76·4 (72–80·1)	75·7 (72·1–78·9)	69·4 (65·6–72·9)	78·3 (74·7–81·6)
Pneumoconiosis	100 (100–100)	100 (100–100)	100 (100–100)	100 (100–100)

Asthma	52·6 (41·9–63·5)	44 (36·2–52·6)	43·2 (35·4–51·9)	51·6 (42·5–60·9)
Digestive diseases	45·2 (40·1–50·3)	47·5 (42·2–52·6)	19·4 (13·6–25·9)	54·6 (49·1–59·9)
Cirrhosis and other chronic liver diseases	78·7 (70·7–86·4)	81·2 (73·4–88·3)	76·4 (67·2–84·6)	81·3 (73·4–88·3)
Upper digestive system diseases	25·1 (21·1–29·3)	7·97 (4·97–11·5)	1·95 (1·22–2·92)	28·9 (24·5–33·2)
Gallbladder and biliary diseases	51·5 (36·6–65·7)	56·4 (42·7–69·2)	57·7 (44·3–69·8)	54·1 (39·4–67·8)
Pancreatitis	30·3 (16·5–44·9)	35·6 (20·5–50·5)	33·6 (18·9–48·8)	35·9 (20·6–50·8)
Neurological disorders	27·2 (11·3–43·5)	16·9 (8·4–29·3)	12·1 (6·24–23·3)	23·1 (8·7–40)
Alzheimer's disease and other dementias	43·9 (29·5–59·4)	47·6 (33·2–62·4)	48·5 (34–63·1)	47 (32·4–61·9)
Parkinson's disease	-12·1 (-18·8–-6·08)	-14·2 (-21·7–-7·33)	-15 (-22·6–-7·92)	-14 (-21·6–-7·19)
Idiopathic epilepsy	19·2 (12·2–26·6)	18 (11·6–24·7)	16·8 (10·7–23·2)	20·5 (13·4–27·8)
Multiple sclerosis	18·6 (13·6–24·2)	20·1 (14·8–25·5)	19·8 (14·7–25·1)	20·3 (14·9–26·2)
Mental disorders	--	9·53 (4·8–14·9)	9·53 (4·8–14·9)	--
Depressive disorders	--	19·3 (9·05–29·3)	19·3 (9·05–29·3)	--
Anxiety disorders	--	6·13 (1·43–14·1)	6·13 (1·43–14·1)	--
Idiopathic developmental intellectual disability	--	50·8 (25·2–70·4)	50·8 (25·2–70·4)	--
Substance use disorders	100 (100–100)	100 (100–100)	100 (100–100)	100 (100–100)
Alcohol use disorders	100 (100–100)	100 (100–100)	100 (100–100)	100 (100–100)
Drug use disorders	100 (100–100)	100 (100–100)	100 (100–100)	100 (100–100)
Diabetes and kidney diseases	99·8 (99·8–99·9)	99·9 (99·9–99·9)	100 (100–100)	99·8 (99·8–99·9)
Diabetes mellitus	100 (100–100)	100 (100–100)	100 (100–100)	100 (100–100)
Chronic kidney disease	100 (100–100)	100 (100–100)	100 (100–100)	100 (100–100)
Sense organ diseases	--	10·8 (9·68–12·1)	10·8 (9·68–12·1)	--
Blindness and vision loss	--	10·5 (6·81–15·3)	10·5 (6·81–15·3)	--
Age-related and other hearing loss	--	11·5 (10·5–12·8)	11·5 (10·5–12·8)	--
Musculoskeletal disorders	4·92 (1·55–8·61)	23 (19·2–27·2)	23·3 (19·5–27·6)	4·81 (1·55–8·51)
Rheumatoid arthritis	15·1 (4·7–26·4)	18·9 (6·22–32·5)	19·3 (6·38–33·2)	17·5 (5·6–30·5)
Osteoarthritis	--	16·3 (9·25–23·7)	16·3 (9·25–23·7)	--
Low back pain	--	44·8 (38·1–51·4)	44·8 (38·1–51·4)	--
Gout	--	60·9 (44·9–76·4)	60·9 (44·9–76·4)	--
Other non-communicable diseases	0·178 (0·125–0·246)	0·226 (0·155–0·321)	--	0·533 (0·379–0·714)
Sudden infant death syndrome	4·86 (3·53–6·58)	4·85 (3·52–6·58)	--	4·85 (3·52–6·58)
Injuries	26·4 (22·1–30·4)	24·9 (21–28·9)	30 (26·6–33·3)	22·3 (17·8–27·1)
Transport injuries	24·6 (19·6–30·1)	24·1 (19·2–29·7)	28·3 (24·9–32)	22·9 (16·9–29)

Road injuries	24 (18·8–29·5)	23·1 (18–28·8)	26·1 (22·6–29·9)	22·3 (16·4–28·5)
Other transport injuries	33·6 (28·9–38·9)	34·1 (29·8–38·8)	38·6 (34·3–41·9)	30·7 (25·5–36·7)
Unintentional injuries	30·4 (26·3–33·7)	25·5 (22·2–29·3)	30·6 (26·6–34·3)	18·5 (15·4–22·1)
Falls	53·2 (46·4–57·3)	43·3 (38·3–47·1)	42·6 (37·6–46·4)	45 (39·9–49)
Drowning	-10·3 (-18–-2·64)	-10·5 (-17·7–-3·17)	9·1 (6–13·3)	-11 (-18·3–-3·56)
Fire, heat, and hot substances	9·55 (6·39–13·6)	11·8 (8·31–16·3)	13 (9·08–18·1)	10·8 (7·61–15)
Poisonings	14·9 (10·6–20·5)	14·9 (10·7–20·1)	12 (8·43–16·4)	16·1 (11·6–22)
Exposure to mechanical forces	11 (5·3–17·2)	17·8 (14–22·2)	22·3 (19·5–25·7)	8·63 (2·54–15·7)
Animal contact	21·6 (17·8–26)	23·2 (19·1–27·7)	24·3 (20·1–28·6)	19·4 (15·4–23·9)
Foreign body	2·52 (1·72–3·66)	3·65 (2·53–5·2)	4·38 (3·01–6·22)	3·43 (2·33–4·94)
Environmental heat and cold exposure	6·03 (2·75–10·7)	6·55 (2·97–11·3)	6·05 (2·74–10·7)	6·94 (3·19–12)
Exposure to forces of nature	5·87 (2·65–10·2)	6·47 (2·92–11·2)	6·93 (3·14–12)	5·77 (2·6–9·95)
Other unintentional injuries	17·1 (12·2–23·2)	13·3 (9·34–18·5)	12·2 (8·52–17·2)	19·4 (14–26·3)
Self-harm and interpersonal violence	23 (13·8–31·6)	24·7 (15·8–33)	28·1 (22·2–34·1)	24·5 (15·2–33·1)
Self-harm	23·7 (11·9–35)	26·6 (14·4–38·1)	24·7 (15·1–34·7)	26·6 (14·3–38·1)
Interpersonal violence	21·3 (13·4–29·5)	21·4 (13·9–29·1)	29·2 (22·5–36·1)	19·5 (11·8–27·6)

Supplementary Results Table 2. All-age PAFs attributable to the joint distribution of all risk factors for all ages, both sexes combined for each GBD level 1, level 2, and level 3 cause and all causes in 2019 for Louisiana. Results are shown for deaths, YLLs, YLDs, and DALYs Data in parenthesis are 95% uncertainty intervals. PAFs=population attributable fractions. GBD=Global Burden of Disease. YLLs=years of life lost. YLDs=years lived with disability. DALYs=disability-adjusted life-years

	Deaths 2019	DALYs 2019	YLDs 2019	YLLs 2019
All causes	63·1 (60·6–65·4)	50·3 (47·3–53·2)	35·2 (32·5–37·8)	61·7 (59·8–63·5)
Communicable, maternal, neonatal, and nutritional diseases	45·8 (40·9–50·2)	59 (55·7–61·9)	54·2 (49·4–59·1)	61·7 (57·8–65)
HIV/AIDS and sexually transmitted infections	89 (86·6–91)	88·5 (85·9–90·8)	90·8 (88·4–93)	87·3 (84·5–89·6)
HIV/AIDS	88·8 (86·4–90·9)	87·8 (85·1–90·1)	89·1 (86·4–91·4)	87·2 (84·4–89·6)
Sexually transmitted infections excluding HIV	98 (97·1–98·7)	98·9 (98·2–99·5)	100 (100–100)	93 (90·1–95·4)
Respiratory infections and tuberculosis	34·1 (27·3–40·9)	32·2 (26·3–38·3)	2·87 (2·18–3·72)	40·4 (33·5–46·9)
Tuberculosis	57·5 (48·9–66)	60·3 (52·3–68·3)	55·2 (45·8–63·7)	61 (53·1–69)
Lower respiratory infections	33·8 (26·9–40·5)	40 (32·9–46·6)	33·3 (27·9–38·6)	40 (33–46·7)
Upper respiratory infections	0·736 (0·44–1·18)	0·0222 (0·011–0·0386)	0·00475 (0–0·0129)	1·41 (0·846–2·27)
Otitis media	2·08 (0·862–4·27)	4·55 (3·03–6·18)	4·53 (2·98–6·15)	5·04 (2·44–8·83)
Enteric infections	13·2 (8·16–20·3)	14·4 (9·52–21·5)	13·8 (8·9–21)	15·8 (10·6–22·5)
Diarrheal diseases	13·3 (8·22–20·4)	14·5 (9·57–21·6)	13·8 (8·91–21)	16 (10·8–22·9)
Other infectious diseases	1·12 (0·752–1·51)	1·71 (1·26–2·13)	0·73 (0·434–1·13)	1·9 (1·37–2·43)
Meningitis	1·6 (1·15–2·13)	3·24 (2·37–4·32)	0·000618 (0–0·00174)	3·33 (2·44–4·47)
Encephalitis	0·711 (0·501–0·987)	1·71 (1·21–2·32)	0·000172 (0–0·000493)	1·76 (1·25–2·39)
Measles	9·62 (-3·84–43·6)	9·77 (-3·89–44·4)	0·222 (0–1·01)	9·79 (-3·91–44·4)
Acute hepatitis	23·7 (15·6–36·1)	12·7 (8·19–18·7)	3·99 (2·44–6·06)	24·5 (15·9–36·8)
Maternal and neonatal disorders	84·1 (81·6–86·6)	84·2 (82·4–86)	79 (75–82·5)	86·8 (84·8–88·7)
Maternal disorders	12·3 (4·36–20·5)	12·1 (4·29–20·2)	11·9 (4·21–19·9)	12·1 (4·32–20·3)
Neonatal disorders	91·9 (90·6–93·1)	88 (86·3–89·5)	80·6 (76·8–84)	91·9 (90·6–93·1)
Nutritional deficiencies	96·1 (87–97·3)	91·3 (88·2–93·4)	89·7 (86·2–92·6)	95·5 (86·1–96·6)
Protein-energy malnutrition	100 (100–100)	100 (100–100)	100 (100–100)	100 (100–100)
Vitamin A deficiency	--	100 (100–100)	100 (100–100)	--
Dietary iron deficiency	--	100 (100–100)	100 (100–100)	--
Non-communicable diseases	67 (64·1–69·6)	53 (49·2–56·5)	34·7 (31·7–37·5)	68 (65·9–70·1)
Neoplasms	52·5 (48·6–56·8)	51·5 (47·9–55·5)	32·4 (28·4–36·7)	52·7 (49·2–56·7)
Lip and oral cavity cancer	67·9 (59·6–74·7)	70·1 (62–76·8)	69·1 (61–76·3)	70·2 (62·2–76·9)
Nasopharynx cancer	63·6 (52·7–72·5)	63·7 (53–72·6)	64·3 (53·8–73·2)	63·6 (52·9–72·6)

Other pharynx cancer	77·3 (71·2–82·6)	78·5 (72·3–83·7)	78·8 (72·6–84·1)	78·5 (72·2–83·7)
Esophageal cancer	81 (72·2–88·4)	82 (73·4–89·2)	81·2 (72·6–88·7)	82 (73·4–89·2)
Stomach cancer	26·2 (18–44)	26·6 (18·5–44·9)	26·7 (18·4–44·5)	26·6 (18·5–44·8)
Colon and rectum cancer	63·8 (54·9–72·5)	64·5 (56·3–72·5)	64·3 (56·1–72·8)	64·5 (56·2–72·5)
Liver cancer	80·6 (76·1–85·1)	80 (75·3–84·5)	80·6 (76–85)	80 (75·3–84·5)
Gallbladder and biliary tract cancer	28·1 (16·7–40·8)	29·2 (17·7–42·1)	27·3 (15·9–40·1)	29·3 (17·7–42·1)
Pancreatic cancer	45·6 (35·7–57·2)	45·3 (36·1–56·4)	45·7 (36–56·9)	45·3 (36·1–56·4)
Larynx cancer	83·8 (75·1–90·1)	84·8 (77·4–90)	85 (77·7–90·4)	84·8 (77·4–89·9)
Tracheal, bronchus, and lung cancer	85·1 (81·9–88·4)	84·3 (81·2–87·6)	84·7 (81·6–88·1)	84·3 (81·2–87·6)
Breast cancer	36 (25·8–47·7)	34·8 (25·4–45·3)	35·5 (25·7–46·9)	34·7 (25·4–45·1)
Cervical cancer	100 (100–100)	100 (100–100)	100 (100–100)	100 (100–100)
Uterine cancer	56·3 (41·2–69·6)	58 (43·2–70·9)	58·5 (43·8–71·2)	58 (43·1–70·9)
Ovarian cancer	22·6 (10·3–37·9)	20·3 (8·88–34·4)	19·7 (8·82–33·6)	20·3 (8·88–34·4)
Prostate cancer	6·35 (2·79–10·3)	7·18 (3·13–11·4)	7·61 (3·35–11·9)	7·04 (3·06–11·3)
Kidney cancer	44·1 (33·1–54·5)	44·6 (34·5–54)	44·9 (34·7–54·2)	44·5 (34·6–54)
Bladder cancer	44·5 (29·1–59·2)	48·3 (34–61·8)	48·9 (35–62·4)	48·3 (33·9–61·8)
Thyroid cancer	19 (10·2–28·8)	20·2 (10·9–30·4)	20·7 (11·4–31·1)	20·1 (10·8–30·6)
Mesothelioma	97·1 (96–97·9)	94·5 (92·6–96)	96·7 (95·3–97·7)	94·4 (92·6–95·9)
Non-Hodgkin lymphoma	9·22 (4·11–15·3)	9·7 (4·46–16·1)	9·47 (4·39–15·8)	9·71 (4·46–16·1)
Multiple myeloma	10·5 (4·95–17·4)	11·1 (5·32–18·2)	11 (5·09–18·2)	11·1 (5·31–18·2)
Leukemia	37·6 (24·1–51·8)	35·5 (23·8–46·9)	37·9 (24·7–51·2)	35·4 (23·7–46·8)
Cardiovascular diseases	84·1 (81·2–86·8)	84·4 (82·1–86·6)	72·9 (68·5–77·1)	86·1 (83·9–88·1)
Rheumatic heart disease	22·8 (13·9–40·7)	22·8 (14·9–36·7)	22·6 (14–38·3)	22·8 (15·1–36·2)
Ischemic heart disease	93·8 (90·8–96·4)	95·2 (93·2–97)	93·9 (91·1–96·3)	95·3 (93·3–97)
Stroke	81 (74·4–87·8)	84·7 (80·4–88·8)	83·6 (78·9–88·9)	85 (80·7–89·1)
Hypertensive heart disease	100 (100–100)	100 (100–100)	100 (100–100)	100 (100–100)
Non-rheumatic valvular heart disease	26·8 (17·2–40·1)	20·7 (14–29·1)	4·37 (2·74–6·19)	27·1 (18·7–37·8)
Cardiomyopathy and myocarditis	39·8 (32·4–48·3)	41·6 (35·6–47·5)	41·1 (34·4–47·5)	41·6 (35·6–47·7)
Atrial fibrillation and flutter	53·1 (43·1–63·7)	59·3 (50·6–68)	60·5 (51·8–68·9)	57·5 (48·6–66·6)
Aortic aneurysm	58 (52·4–63·8)	65·9 (61·4–70·3)	--	65·9 (61·4–70·3)
Endocarditis	33 (23·1–45·5)	34·4 (25·1–44·9)	32·2 (22·2–44·9)	34·5 (25·2–44·9)

Other cardiovascular and circulatory diseases	24·8 (19·5–32)	26·8 (21·6–32·7)	33·2 (26·4–40·7)	25·2 (20·1–31·4)
Chronic respiratory diseases	62·8 (57·8–67·7)	59·8 (56–63·6)	55·1 (50·8–59·3)	63·4 (59·1–67·9)
Chronic obstructive pulmonary disease	70·1 (65·3–74·4)	70·2 (66·4–73·8)	66·2 (62·4–69·8)	72·3 (68·4–76·1)
Pneumoconiosis	100 (100–100)	100 (100–100)	100 (100–100)	100 (100–100)
Asthma	52·4 (41·7–62·7)	42·7 (34·9–51·2)	41·6 (34–50·1)	50·4 (41·4–59·7)
Digestive diseases	48·2 (42·5–53·7)	49·1 (43·5–54·8)	19 (13–25·2)	57·2 (51–62·7)
Cirrhosis and other chronic liver diseases	80 (71·6–87·7)	82·2 (74·4–89·2)	77 (68·1–85·3)	82·2 (74·4–89·3)
Upper digestive system diseases	23·5 (19·6–27·2)	6·56 (4·16–9·74)	1·79 (1·1–2·75)	26·6 (22·6–30·5)
Gallbladder and biliary diseases	53 (37·1–67)	57·5 (43·6–69·8)	58·4 (44·7–70·6)	55·5 (40·5–68·7)
Pancreatitis	33·3 (16·6–50)	38·1 (20·5–54·8)	34·5 (17·7–50·5)	38·5 (20·9–55·3)
Neurological disorders	27·9 (11·8–43·9)	16·7 (8·4–29·1)	12 (6·06–22·9)	23·4 (8·91–39·7)
Alzheimer's disease and other dementias	43·4 (29·2–59·4)	47·1 (32·5–62·4)	48 (33·8–63·3)	46·4 (32·2–61·7)
Parkinson's disease	-10·4 (-16·1–-5·34)	-12·2 (-18·5–-6·39)	-12·7 (-18·8–-6·66)	-12·1 (-18·4–-6·31)
Idiopathic epilepsy	21·2 (13·8–28·9)	19·1 (12·2–26·3)	17·8 (11·3–24·5)	21·7 (14·4–29·3)
Multiple sclerosis	17·8 (12·9–23)	18·5 (13·8–23·6)	17·8 (13·4–22·5)	19·2 (14–24·5)
Mental disorders	--	8·51 (4·11–13·6)	8·51 (4·12–13·6)	--
Depressive disorders	--	17·7 (7·96–27·3)	17·7 (7·96–27·3)	--
Anxiety disorders	--	6·33 (1·38–14·4)	6·33 (1·38–14·4)	--
Idiopathic developmental intellectual disability	--	50·4 (26·1–70·4)	50·4 (26·1–70·4)	--
Substance use disorders	100 (100–100)	100 (100–100)	100 (100–100)	100 (100–100)
Alcohol use disorders	100 (100–100)	100 (100–100)	100 (100–100)	100 (100–100)
Drug use disorders	100 (100–100)	100 (100–100)	100 (100–100)	100 (100–100)
Diabetes and kidney diseases	99·9 (99·9–99·9)	99·9 (99·9–100)	100 (100–100)	99·9 (99·9–99·9)
Diabetes mellitus	100 (100–100)	100 (100–100)	100 (100–100)	100 (100–100)
Chronic kidney disease	100 (100–100)	100 (100–100)	100 (100–100)	100 (100–100)
Sense organ diseases	--	10·7 (9·52–12)	10·7 (9·52–12)	--
Blindness and vision loss	--	10·2 (6·49–14·9)	10·2 (6·49–14·9)	--
Age-related and other hearing loss	--	11·6 (10·5–12·9)	11·6 (10·5–12·9)	--
Musculoskeletal disorders	3·43 (1·05–6·08)	21·8 (18·4–25·7)	22·2 (18·7–26·2)	3·08 (0·945–5·49)
Rheumatoid arthritis	14·5 (4·49–25·9)	17·1 (5·59–29·7)	17·2 (5·61–29·9)	16·6 (5·31–29·3)
Osteoarthritis	--	16·2 (9·38–23·6)	16·2 (9·38–23·6)	--

Low back pain	--	43·1 (37·1–49·1)	43·1 (37·1–49·1)	--
Gout	--	62·1 (46·5–77·3)	62·1 (46·5–77·3)	--
Other non-communicable diseases	0·161 (0·114–0·219)	0·204 (0·137–0·283)	--	0·473 (0·337–0·625)
Sudden infant death syndrome	4·81 (3·4–6·63)	4·81 (3·39–6·62)	--	4·81 (3·39–6·62)
Injuries	24·2 (20·2–28·1)	22·8 (19·2–26·5)	28·6 (25·2–31·9)	20·5 (16·3–24·7)
Transport injuries	25·8 (21·2–31·2)	24·8 (20·1–30·2)	28·3 (24·4–32·2)	23·9 (18·6–29·8)
Road injuries	25·3 (20·5–30·6)	23·9 (19·2–29·4)	26·1 (22·2–30·1)	23·4 (18·2–29·4)
Other transport injuries	32·6 (27·5–38·3)	32·7 (28·1–37·7)	38·1 (33·6–41·8)	29·2 (23·6–35·7)
Unintentional injuries	26·4 (22·4–29·7)	22·4 (18·9–25·9)	28·8 (24·8–32·6)	14·9 (11·6–18·3)
Falls	51·6 (44·6–56·4)	41·9 (36·6–46·2)	41·4 (36·2–45·7)	43·1 (37·5–47·6)
Drowning	-4·65 (-11·9–3·36)	-5·88 (-12·8–1·29)	10·2 (6·58–14·9)	-6·13 (-13·1–1·13)
Fire, heat, and hot substances	10·2 (6·74–14·7)	12 (8·14–16·6)	13·7 (9·46–19·1)	10·7 (7·29–15)
Poisonings	14·8 (10·3–20)	14·4 (10·1–19·4)	12·3 (8·57–17·1)	15·3 (10·8–20·6)
Exposure to mechanical forces	16·7 (9·15–23·8)	19·2 (15·1–23·3)	21·5 (18·5–24·9)	14 (6·22–21·7)
Animal contact	21·1 (17–25·8)	23 (18·7–27·8)	24·2 (19·9–28·9)	17·8 (14–22·2)
Foreign body	2·52 (1·68–3·66)	3·1 (2·15–4·54)	4·52 (3·05–6·34)	2·76 (1·84–4·1)
Environmental heat and cold exposure	6·69 (3·07–11·7)	7·15 (3·25–12·6)	6·78 (3·03–12)	7·48 (3·43–13·1)
Exposure to forces of nature	6·44 (2·91–11·4)	7·81 (3·51–13·7)	7·96 (3·58–14)	6·21 (2·83–10·9)
Other unintentional injuries	18·1 (12·8–24·4)	13·9 (9·73–19·2)	13 (9·03–18·2)	19·5 (14–26·1)
Self-harm and interpersonal violence	21 (13·9–28·7)	21·6 (14·8–28·6)	28·1 (21·8–34·5)	21·1 (14·1–28·3)
Self-harm	22·6 (11·4–34·4)	24·8 (13·1–36·7)	23·5 (13·9–34·1)	24·8 (13·1–36·8)
Interpersonal violence	19·4 (12–27·1)	19 (11·9–26·4)	29·2 (22·4–36·2)	17·8 (10·7–25·2)

Supplementary Results Table 2. All-age PAFs attributable to the joint distribution of all risk factors for all ages, both sexes combined for each GBD level 1, level 2, and level 3 cause and all causes in 2019 for Maine. Results are shown for deaths, YLLs, YLDs, and DALYs Data in parenthesis are 95% uncertainty intervals. PAFs=population attributable fractions. GBD=Global Burden of Disease. YLLs=years of life lost. YLDs=years lived with disability. DALYs=disability-adjusted life-years

	Deaths 2019	DALYs 2019	YLDs 2019	YLLs 2019
All causes	59·7 (56·8–62·4)	47·9 (44·9–51)	33·8 (31·4–36·2)	60 (57·7–62·2)
Communicable, maternal, neonatal, and nutritional diseases	40·1 (32·5–47·2)	50·9 (46·9–55·1)	46·4 (41·8–51·1)	53·7 (47·8–59·4)
HIV/AIDS and sexually transmitted infections	88·4 (85·6–90·7)	88·8 (85·3–91·4)	92·6 (89·7–95·6)	85·6 (81·8–88·4)
HIV/AIDS	87·7 (84·6–90·2)	85·9 (82·2–88·7)	87·2 (83·4–90·1)	85·3 (81·4–88·1)
Sexually transmitted infections excluding HIV	99·1 (98·6–99·4)	99·6 (99·3–99·8)	100 (100–100)	96·1 (94·3–97·4)
Respiratory infections and tuberculosis	38·9 (28–49·1)	34·3 (26–42·8)	2·34 (1·76–3·05)	43·7 (33·6–53·4)
Tuberculosis	53·7 (43·1–63·4)	57·6 (47·9–66·3)	56·1 (45·8–65·2)	57·9 (48·3–66·6)
Lower respiratory infections	38·8 (27·9–49·3)	43·6 (33·5–53·5)	34·1 (28·5–39·6)	43·7 (33·5–53·7)
Upper respiratory infections	0·512 (0·298–0·861)	0·0327 (0·0161–0·0584)	0·00373 (0–0·012)	1·34 (0·799–2·2)
Otitis media	0·993 (0·388–2·11)	3·46 (2·26–4·73)	3·46 (2·24–4·75)	3·25 (1·41–6·16)
Enteric infections	10·2 (6·36–15·9)	11·4 (7·52–17)	11·1 (7·17–16·8)	11·7 (7·85–17·1)
Diarrheal diseases	10·3 (6·4–15·9)	11·5 (7·55–17·1)	11·1 (7·18–16·8)	11·8 (7·92–17·3)
Other infectious diseases	0·58 (0·374–0·799)	1·08 (0·764–1·38)	0·826 (0·513–1·33)	1·14 (0·758–1·49)
Meningitis	0·998 (0·7–1·41)	2·55 (1·81–3·53)	0·000364 (0–0·00123)	2·69 (1·92–3·75)
Encephalitis	0·401 (0·264–0·582)	1·24 (0·835–1·77)	8·57e-05 (0–0·000279)	1·3 (0·878–1·86)
Measles	9·16 (-3·86–43)	6·14 (-3·35–40·3)	0·299 (0–0·908)	9·34 (-3·94–43·8)
Acute hepatitis	21·9 (13·6–34·7)	10 (6·3–15)	4·37 (2·66–6·9)	23·6 (15·1–36·8)
Maternal and neonatal disorders	84·8 (82–87·1)	81·1 (78·3–83·6)	72·4 (67·5–76·6)	86·5 (84·2–88·5)
Maternal disorders	11·3 (4·11–19)	11·1 (3·98–18·8)	11 (3·9–18·7)	11·1 (4·05–18·9)
Neonatal disorders	89·7 (87·9–91·3)	83·8 (81·1–86·2)	74·6 (69·7–78·8)	89·7 (87·9–91·3)
Nutritional deficiencies	92·1 (84·4–94·6)	88·5 (85·5–91·2)	87·4 (83·7–90·9)	92·1 (84·7–94·4)
Protein-energy malnutrition	100 (100–100)	100 (100–100)	100 (100–100)	100 (100–100)
Vitamin A deficiency	--	100 (100–100)	100 (100–100)	--
Dietary iron deficiency	--	100 (100–100)	100 (100–100)	--
Non-communicable diseases	62·4 (59·3–65·3)	49·7 (46·3–53·1)	33·3 (30·6–35·9)	64·1 (61·7–66·6)
Neoplasms	51·3 (47·5–55·4)	51 (47·5–54·9)	32·5 (28·5–36·7)	52·3 (48·7–56·1)
Lip and oral cavity cancer	66·5 (56·5–74·4)	69·3 (59·9–76·6)	68·8 (59·4–76·3)	69·4 (60–76·6)
Nasopharynx cancer	66 (54·2–75·9)	66·2 (54·7–75·5)	65·2 (53·4–74·6)	66·3 (54·8–75·6)
Other pharynx cancer	77·7 (70·2–83·4)	79·1 (71·9–84·6)	79·4 (72·1–84·9)	79 (71·9–84·6)

Esophageal cancer	78·7 (69·1–86·8)	80 (70·7–87·9)	79 (69·7–87·1)	80 (70·7–88)
Stomach cancer	25·2 (16·9–42·8)	26·4 (18·2–44·2)	26·3 (18·1–44)	26·4 (18·2–44·2)
Colon and rectum cancer	61·9 (52·9–71)	63 (54·6–71·5)	62·9 (54·4–71·5)	63 (54·6–71·4)
Liver cancer	81·1 (76·4–85·6)	81·1 (76·6–85·3)	81·1 (76·5–85·5)	81·1 (76·6–85·3)
Gallbladder and biliary tract cancer	27·7 (16·2–40·1)	28·7 (17–41·6)	26·6 (15·1–39·5)	28·8 (17·1–41·6)
Pancreatic cancer	44·4 (34·3–55·5)	44·7 (35·4–55·4)	44·6 (35–55·6)	44·7 (35·4–55·4)
Larynx cancer	82·5 (72·2–89·9)	83·7 (75–90·2)	83·5 (74·6–90·1)	83·7 (75–90·2)
Tracheal, bronchus, and lung cancer	84·2 (80·8–87·8)	83·8 (80·6–87·2)	84 (80·6–87·5)	83·7 (80·5–87·2)
Breast cancer	36·6 (27·1–47·6)	36·1 (27·5–45·9)	36·5 (27·5–46·6)	36·1 (27·4–45·8)
Cervical cancer	100 (100–100)	100 (100–100)	100 (100–100)	100 (100–100)
Uterine cancer	54·2 (38·2–68)	55·9 (40·1–68·9)	56·4 (40·5–69·3)	55·8 (40·1–68·9)
Ovarian cancer	23·1 (10·8–37)	20·3 (9·17–33)	19·8 (8·98–32·1)	20·3 (9·17–33·1)
Prostate cancer	5·92 (2·52–10)	6·62 (2·79–10·8)	7·01 (3·01–11·5)	6·5 (2·75–10·7)
Kidney cancer	43·4 (31·7–54·5)	44·5 (33·9–55)	44·8 (34·3–55·1)	44·5 (33·9–55)
Bladder cancer	40·2 (24·6–55·6)	43·7 (28·3–58·3)	44·6 (29·3–58·7)	43·6 (28·3–58·2)
Thyroid cancer	18·7 (9·5–28·9)	19·9 (10·4–30·4)	20·2 (10·8–30·6)	19·8 (10·2–30·5)
Mesothelioma	98 (97·3–98·6)	96·2 (95–97·3)	97·6 (96·6–98·4)	96·2 (94·9–97·3)
Non-Hodgkin lymphoma	8·92 (3·88–15·2)	9·43 (4·21–15·9)	9·2 (4·14–15·6)	9·45 (4·24–15·9)
Multiple myeloma	10·3 (4·8–17)	10·8 (5·03–18)	10·7 (5·05–17·7)	10·8 (5·06–18)
Leukemia	37·6 (23·1–51·9)	37·3 (24–50·4)	38·5 (23·7–53·1)	37·2 (24·1–50·3)
Cardiovascular diseases	79·3 (75·3–82·8)	79·9 (76·7–82·8)	70·3 (65·5–75·1)	81·6 (78·7–84·3)
Rheumatic heart disease	21·9 (12–41·4)	21·8 (13·1–36)	21·5 (13–36·6)	21·8 (13·3–36)
Ischemic heart disease	92·1 (87·9–95·5)	93·5 (90·7–96)	92·1 (88·6–95·1)	93·5 (90·7–96)
Stroke	78·5 (71·3–86·6)	82 (76·7–87·5)	81·2 (75·8–87·4)	82·3 (76·9–87·8)
Hypertensive heart disease	100 (100–100)	100 (100–100)	100 (100–100)	100 (100–100)
Non-rheumatic valvular heart disease	25·5 (15·8–39·1)	23·7 (16·1–33·6)	8·07 (5·19–11·4)	25·8 (17·4–36·6)
Cardiomyopathy and myocarditis	40 (33·4–48·3)	43·8 (37·9–50·1)	41·4 (35·1–47·8)	44·1 (38·2–50·4)
Atrial fibrillation and flutter	52·9 (42·6–63·6)	58·5 (49·4–67·2)	59·8 (50·7–68·4)	56·4 (47–65·8)
Aortic aneurysm	53·3 (46·8–59·8)	61·3 (55·8–66·6)	--	61·3 (55·8–66·6)
Endocarditis	30·8 (21·1–43·5)	32·3 (23·3–43·5)	30·4 (20·4–43)	32·4 (23·4–43·5)
Other cardiovascular and circulatory diseases	21·1 (16·1–29)	24 (19–30·4)	31·1 (24·6–38·7)	22·1 (17·5–28·8)
Chronic respiratory diseases	63·8 (58–70·1)	61·2 (57·1–65·3)	56 (51·6–60·3)	65 (60–70·6)

Chronic obstructive pulmonary disease	71·3 (66·2–76·3)	70·5 (66·5–74·5)	65 (60·8–69·1)	73·4 (69·1–78·1)
Pneumoconiosis	100 (100–100)	100 (100–100)	100 (100–100)	100 (100–100)
Asthma	51·6 (39·9–63)	45·4 (37–54·1)	44·7 (36·5–53·2)	52·1 (42·1–61·7)
Digestive diseases	43·2 (37·6–48·2)	45·8 (40·4–51·2)	15·9 (11·1–21·5)	53·6 (47·5–59)
Cirrhosis and other chronic liver diseases	80·8 (71·4–88·5)	83 (75–89·9)	80 (70·8–87·9)	83 (75–90)
Upper digestive system diseases	20·8 (17–24·5)	6·99 (4·2–10·3)	1·6 (0·908–2·55)	24·3 (20·2–28·2)
Gallbladder and biliary diseases	51·7 (36–66·3)	56·1 (41·5–69)	57·8 (43·6–70·1)	53·8 (38·8–67·7)
Pancreatitis	36·1 (17·4–54·5)	40·2 (21·3–58·2)	38·8 (20·2–56·7)	40·4 (21·4–58·2)
Neurological disorders	25·7 (10·4–41)	17·6 (8·94–29·9)	13·2 (6·89–23·8)	22 (8·19–37·9)
Alzheimer's disease and other dementias	41·7 (27·6–56·8)	44·8 (30·5–59·9)	45·6 (31·3–60·6)	44·2 (29·9–59·3)
Parkinson's disease	-9·66 (-15·6–-4·67)	-11·4 (-17·8–-5·69)	-12·2 (-18·7–-6·21)	-11·2 (-17·6–-5·58)
Idiopathic epilepsy	24·1 (15–32·7)	22·4 (14·2–30·3)	21·4 (13·4–29·1)	25·1 (16–33·7)
Multiple sclerosis	15 (10·9–19·7)	16·9 (12·5–21·7)	17·2 (12·8–21·8)	16·7 (12·2–21·6)
Mental disorders	--	8·6 (4·33–13·3)	8·6 (4·33–13·3)	--
Depressive disorders	--	18·2 (8·39–27·8)	18·2 (8·39–27·8)	--
Anxiety disorders	--	5·37 (1·23–12·2)	5·37 (1·23–12·2)	--
Idiopathic developmental intellectual disability	--	48·5 (23·8–68·1)	48·5 (23·8–68·1)	--
Substance use disorders	100 (100–100)	100 (100–100)	100 (100–100)	100 (100–100)
Alcohol use disorders	100 (100–100)	100 (100–100)	100 (100–100)	100 (100–100)
Drug use disorders	100 (100–100)	100 (100–100)	100 (100–100)	100 (100–100)
Diabetes and kidney diseases	99·8 (99·8–99·9)	99·9 (99·9–99·9)	100 (100–100)	99·8 (99·8–99·9)
Diabetes mellitus	100 (100–100)	100 (100–100)	100 (100–100)	100 (100–100)
Chronic kidney disease	100 (100–100)	100 (100–100)	100 (100–100)	100 (100–100)
Sense organ diseases	--	10·6 (9·44–11·9)	10·6 (9·44–11·9)	--
Blindness and vision loss	--	10·6 (6·77–15·3)	10·6 (6·77–15·3)	--
Age-related and other hearing loss	--	11·3 (10·2–12·6)	11·3 (10·2–12·6)	--
Musculoskeletal disorders	3·93 (1·12–7·19)	20·4 (17–24·1)	20·7 (17·3–24·5)	4·02 (1·19–7·39)
Rheumatoid arthritis	11·9 (3·58–21·5)	15·4 (4·9–27·2)	15·9 (5·05–28·2)	14 (4·23–25·1)
Osteoarthritis	--	15·4 (8·86–22·5)	15·4 (8·86–22·5)	--
Low back pain	--	43·7 (37·5–49·9)	43·7 (37·5–49·9)	--
Gout	--	61·7 (46·3–77·1)	61·7 (46·3–77·1)	--
Other non-communicable diseases	0·0966 (0·0643–0·143)	0·138 (0·0902–0·199)	--	0·349 (0·24–0·499)

Sudden infant death syndrome	6·2 (4·39–8·63)	6·19 (4·38–8·62)	--	6·19 (4·38–8·62)
Injuries	27·8 (21·9–33·5)	26·9 (21·8–31·7)	34·7 (30·8–38·4)	21·2 (14·2–27·8)
Transport injuries	20·4 (12·9–27·5)	22·9 (16·2–29·2)	31·8 (27·6–35·7)	18·8 (10·4–26·8)
Road injuries	18·5 (10·7–25·9)	20·6 (13·6–27·2)	29·3 (25·2–33·4)	17 (8·18–25·1)
Other transport injuries	38·1 (32·7–43·8)	38·6 (33·8–43·4)	41·7 (37·6–45·3)	35·6 (29·2–42)
Unintentional injuries	37·7 (32·7–42)	32·1 (28–36·4)	35·7 (31·2–40)	25·6 (21·2–30)
Falls	56·5 (49·8–61·3)	47·7 (42·3–52·1)	46·7 (41·5–51)	49·9 (44·4–54·6)
Drowning	-10·3 (-25·1–3·23)	-9·54 (-23·8–3·3)	12 (7·74–17·6)	-10·4 (-25·1–2·84)
Fire, heat, and hot substances	12·2 (8·01–17·3)	15·7 (11·1–21·4)	16·6 (11·7–22·7)	14·5 (10·1–19·6)
Poisonings	18·8 (13·5–25·2)	18·3 (13·3–24·3)	15·2 (10·7–20·8)	20·7 (15·1–27·6)
Exposure to mechanical forces	8·97 (-1·72–19·4)	21·7 (16·7–26·9)	25·6 (21·9–29·9)	5·85 (-5·97–17·2)
Animal contact	25·3 (20·4–31·1)	28 (23·2–33·6)	28·6 (23·8–34·1)	24 (19·1–30)
Foreign body	3·05 (1·97–4·55)	4·98 (3·32–7·36)	4·78 (3·26–6·84)	5·06 (3·28–7·61)
Environmental heat and cold exposure	7·77 (3·17–13·7)	8·32 (3·44–14·6)	8·03 (3·31–14·3)	8·61 (3·56–15)
Exposure to forces of nature	7·57 (3·13–13·2)	7·93 (3·23–13·9)	8·93 (3·7–15·5)	7·06 (2·93–12·2)
Other unintentional injuries	19·4 (13·7–26·1)	16·2 (11·4–22)	15·6 (10·9–21·4)	23·2 (16·8–31)
Self-harm and interpersonal violence	17·6 (3·44–31·1)	20·5 (6·97–33·1)	30·1 (23·2–36·5)	19·7 (5·51–32·8)
Self-harm	17·4 (1·82–32·1)	20·1 (4·55–35·3)	25 (13·6–37)	20 (4·22–35·2)
Interpersonal violence	21·9 (10·8–32·8)	24 (14·9–33·3)	32·3 (24·9–39·7)	19 (7·34–30·2)

Supplementary Results Table 2. All-age PAFs attributable to the joint distribution of all risk factors for all ages, both sexes combined for each GBD level 1, level 2, and level 3 cause and all causes in 2019 for Georgia. Results are shown for deaths, YLLs, YLDs, and DALYs Data in parenthesis are 95% uncertainty intervals. PAFs=population attributable fractions. GBD=Global Burden of Disease. YLLs=years of life lost. YLDs=years lived with disability. DALYs=disability-adjusted life-years

	Deaths 2019	DALYs 2019	YLDs 2019	YLLs 2019
All causes	73 (69·7–75·5)	56·7 (53·2–59·7)	31·5 (29·1–33·8)	68·1 (65·9–70·2)
Communicable, maternal, neonatal, and nutritional diseases	56·6 (52·1–60·9)	60·7 (57·9–63·4)	60·9 (56·6–65)	60·6 (56·6–63·8)
HIV/AIDS and sexually transmitted infections	77·5 (77–78·1)	81·9 (79·7–84·8)	90·5 (86·7–93·7)	74·8 (74·2–75·5)
HIV/AIDS	76·1 (75·6–76·7)	75 (74·3–75·7)	77·9 (76·9–78·6)	73·9 (73·3–74·6)
Sexually transmitted infections excluding HIV	99·5 (98·9–99·8)	99·9 (99·7–100)	100 (100–100)	98·4 (96·6–99·4)
Respiratory infections and tuberculosis	58·2 (52·4–64·2)	56·6 (51·2–61·5)	18·5 (13·6–24·2)	62·9 (58·1–67·9)
Tuberculosis	63·5 (57·3–69·1)	61·5 (55·3–67)	52·5 (45·4–59)	62·8 (56·5–68·3)
Lower respiratory infections	57 (49·9–64·2)	62·6 (56·1–68·9)	45·6 (39·3–52·2)	63 (56·5–69·3)
Upper respiratory infections	0·474 (0·267–0·879)	0·0232 (0·0112–0·0444)	0·0078 (0·00182–0·0158)	1·39 (0·853–2·23)
Otitis media	0·875 (0·178–2·61)	5·45 (3·56–7·43)	5·46 (3·56–7·43)	4·5 (0·99–12·4)
Enteric infections	73 (61·4–82·7)	71·6 (56·6–84·2)	70·1 (54·3–83·5)	82·5 (73·9–89·2)
Diarrheal diseases	78·9 (67·1–88·6)	72 (56·9–84·7)	70·2 (54·3–83·5)	86 (77·7–92·8)
Other infectious diseases	1·36 (0·933–2·02)	1·6 (1·18–2·12)	0·257 (0·148–0·429)	2·21 (1·65–2·92)
Meningitis	1·54 (0·943–2·38)	2·21 (1·39–3·4)	0·000866 (0·000169–0·00171)	2·87 (1·81–4·37)
Encephalitis	1·21 (0·799–1·77)	2·14 (1·23–3·2)	0·00063 (0·000125–0·00123)	2·4 (1·36–3·56)
Measles	6·58 (1·05–22·3)	1·29 (0·209–2·98)	1·11 (0·0377–2·58)	8·54 (1·45–27·7)
Acute hepatitis	4·1 (2·08–7·23)	3·13 (1·7–5·34)	2·06 (1·18–3·39)	3·6 (1·81–6·56)
Maternal and neonatal disorders	63·8 (59·9–67·6)	58·9 (55·2–62·5)	41·3 (36·5–46)	64·9 (61·1–68·7)
Maternal disorders	16·8 (6·13–28)	16·7 (6·11–28)	16·6 (5·98–27·9)	16·8 (6·12–28)
Neonatal disorders	67·1 (63·1–71)	60·5 (56·6–64·4)	41·8 (36·9–46·5)	67·1 (63·1–70·9)
Nutritional deficiencies	42·5 (18·4–49·2)	96·8 (95·9–97·5)	97·9 (97·2–98·4)	42·4 (30·2–46·8)
Protein-energy malnutrition	100 (100–100)	100 (100–100)	100 (100–100)	100 (100–100)
Vitamin A deficiency	--	100 (100–100)	100 (100–100)	--
Dietary iron deficiency	--	100 (100–100)	100 (100–100)	--
Non-communicable diseases	76 (72·5–78·8)	60·9 (56·8–64·4)	29·9 (27·1–32·7)	74 (71·6–76·5)
Neoplasms	44·6 (40·8–49·2)	44·5 (40·8–48·6)	36·1 (31·6–41·1)	44·7 (41·1–48·8)
Lip and oral cavity cancer	65·3 (60·3–69·9)	69·4 (64·5–74·1)	65·5 (59·5–70·9)	69·5 (64·6–74·2)
Nasopharynx cancer	56·4 (49·2–63·1)	58·8 (51·4–65·4)	57·6 (50·3–64·3)	58·8 (51·4–65·4)
Other pharynx cancer	75·8 (71·3–79·7)	78·1 (73·5–81·9)	74·3 (69·7–78·2)	78·2 (73·6–81·9)
Esophageal cancer	73 (64–81·7)	75·1 (66·6–83·2)	74 (65·4–82·3)	75·2 (66·6–83·2)

Stomach cancer	23·7 (15·5–42·1)	24·3 (16–42·7)	23·8 (15·2–42·2)	24·3 (16–42·7)
Colon and rectum cancer	57·8 (49·6–65·7)	58·2 (50·3–65·8)	58·1 (50·2–65·9)	58·3 (50·3–65·8)
Liver cancer	65·6 (58·1–72·8)	66·5 (59–73·7)	66·2 (58·7–73·6)	66·5 (59–73·7)
Gallbladder and biliary tract cancer	23·3 (13·5–34·5)	23·3 (13·5–34·7)	23·3 (13·6–34·6)	23·3 (13·5–34·7)
Pancreatic cancer	33·6 (25·5–43·7)	34·3 (26·5–44·1)	33·8 (25·6–43·5)	34·3 (26·5–44·1)
Larynx cancer	84·7 (79·8–88·6)	85·4 (80·6–89)	83·8 (78·7–87·9)	85·4 (80·7–89·1)
Tracheal, bronchus, and lung cancer	84·7 (81·5–88·1)	84·4 (81·4–87·8)	84·4 (80·9–88)	84·4 (81·4–87·8)
Breast cancer	23·1 (14·4–34)	21·2 (13·1–31·1)	21·4 (13·3–31·4)	21·2 (13·1–31·1)
Cervical cancer	100 (100–100)	100 (100–100)	100 (100–100)	100 (100–100)
Uterine cancer	47·4 (33·8–61·3)	47·4 (33·8–61·5)	47·4 (33·7–61·5)	47·4 (33·8–61·5)
Ovarian cancer	13·6 (4·88–25·4)	12·5 (4·32–23·5)	12·2 (4–22·8)	12·5 (4·32–23·5)
Prostate cancer	8 (3·88–12·1)	8·84 (4·31–13·3)	9·2 (4·5–14)	8·82 (4·29–13·3)
Kidney cancer	38·6 (29·7–47·7)	38·6 (29·3–47·9)	37·8 (28·5–46·9)	38·6 (29·3–47·9)
Bladder cancer	49·2 (37·9–59·8)	51·7 (40·2–61·7)	51·2 (39·8–61·2)	51·7 (40·2–61·8)
Thyroid cancer	14·5 (7·64–23·2)	14·8 (7·77–23·7)	14·3 (8–22·2)	14·8 (7·79–23·8)
Mesothelioma	93 (90·1–95·1)	90·8 (87·1–93·5)	92·8 (89·8–95)	90·7 (87–93·5)
Non-Hodgkin lymphoma	7·2 (3·01–12·6)	7·09 (3·04–12·5)	6·91 (2·92–12·2)	7·09 (3·04–12·5)
Multiple myeloma	8·46 (3·67–14·8)	8·61 (3·78–15·1)	8·62 (3·74–15·2)	8·6 (3·78–15·1)
Leukemia	29·2 (20·8–38·3)	24·9 (17·2–33)	26·7 (18·5–35·1)	24·9 (17·1–33)
Cardiovascular diseases	89·2 (86·4–91·6)	89·8 (87·7–91·7)	79·4 (75·7–82·8)	90·4 (88·3–92·2)
Rheumatic heart disease	29·6 (19–51·2)	29·8 (21·1–43·6)	27·4 (17·6–39·5)	30·3 (21·2–44·6)
Ischemic heart disease	94·3 (91·2–97)	95·7 (93·6–97·5)	95 (92·5–97·1)	95·7 (93·6–97·5)
Stroke	85·3 (80·2–90·4)	88·7 (85–92·1)	88·7 (85·3–91·7)	88·7 (84·9–92·2)
Hypertensive heart disease	100 (100–100)	100 (100–100)	100 (100–100)	100 (100–100)
Non-rheumatic valvular heart disease	20·9 (13·6–31·7)	16·3 (10·9–24·4)	4·2 (2·79–5·84)	20·6 (13·9–30·7)
Cardiomyopathy and myocarditis	38·1 (28·7–48·4)	39·6 (29·2–51·5)	34·1 (27·4–40·6)	39·7 (29·2–51·6)
Atrial fibrillation and flutter	54·5 (44·8–63·9)	61·5 (53·3–69·3)	64·6 (56·9–72·2)	58·9 (49·6–67·3)
Aortic aneurysm	66·8 (61·4–72·3)	73·9 (69·8–77·8)	--	73·9 (69·8–77·8)
Endocarditis	43·5 (33–54·3)	44·8 (34·6–54·8)	41 (31·8–51·3)	44·9 (34·6–54·9)
Other cardiovascular and circulatory diseases	45·1 (39·6–50·4)	46·5 (41·1–51·7)	32·5 (24·3–40·3)	47·6 (42–53)
Chronic respiratory diseases	66·7 (62·5–70·3)	61·5 (57·9–65·2)	52·3 (47·6–56·8)	65·9 (62·2–69·4)
Chronic obstructive pulmonary disease	76·1 (71·1–80·3)	73·2 (69·4–77·1)	62·4 (57·8–67·4)	77·8 (73·3–81·7)
Pneumoconiosis	100 (100–100)	100 (100–100)	100 (100–100)	100 (100–100)
Asthma	44·1 (33·8–55·3)	39·6 (31·5–48·7)	34·8 (27·4–43·4)	45 (35·6–55·4)

Digestive diseases	55·8 (48·5–62·9)	56·2 (48·9–62·7)	15·6 (10·8–20·9)	63·3 (55·1–70·4)
Cirrhosis and other chronic liver diseases	71·7 (62·4–79·2)	75·9 (66·3–83·4)	73·3 (65·5–80·2)	75·9 (66·3–83·4)
Upper digestive system diseases	25 (21·3–28·5)	15·3 (10·4–20·6)	1·64 (0·962–2·57)	29·5 (25·6–33·1)
Gallbladder and biliary diseases	46·9 (32·4–60·2)	49·7 (36·4–63·1)	50·2 (36·6–63·6)	47·7 (34·3–61)
Pancreatitis	25·4 (17·8–34·3)	33·2 (24·6–43·2)	26·4 (19·3–35)	33·8 (25–44·1)
Neurological disorders	24·6 (11·9–38·9)	14·7 (7·12–25·5)	9·27 (4·73–17)	22·3 (10·1–36·1)
Alzheimer's disease and other dementias	33·4 (20·1–48·3)	35·2 (21·9–49·9)	35·1 (21·5–50)	35·3 (22–49·8)
Parkinson's disease	-7·65 (-11·4–-4·05)	-9·39 (-14–-5·08)	-10·6 (-15·9–-5·72)	-9·19 (-13·7–-4·89)
Idiopathic epilepsy	17·8 (13–23·1)	13·3 (9·43–17·7)	11·4 (7·89–15·1)	17·1 (12·2–22·2)
Multiple sclerosis	11·8 (8·04–18·6)	14·4 (10·8–18·5)	14·2 (10·7–17·7)	14·9 (10·4–22·1)
Mental disorders	--	2·55 (1·46–3·85)	2·55 (1·46–3·85)	--
Depressive disorders	--	3·11 (1·41–5·29)	3·11 (1·41–5·29)	--
Anxiety disorders	--	1·86 (0·455–4·5)	1·86 (0·455–4·5)	--
Idiopathic developmental intellectual disability	--	49·8 (23·1–69·6)	49·8 (23·1–69·6)	--
Substance use disorders	100 (100–100)	100 (100–100)	100 (100–100)	100 (100–100)
Alcohol use disorders	100 (100–100)	100 (100–100)	100 (100–100)	100 (100–100)
Drug use disorders	100 (100–100)	100 (100–100)	100 (100–100)	100 (100–100)
Diabetes and kidney diseases	100 (100–100)	100 (100–100)	100 (100–100)	100 (100–100)
Diabetes mellitus	100 (100–100)	100 (100–100)	100 (100–100)	100 (100–100)
Chronic kidney disease	100 (100–100)	100 (100–100)	100 (100–100)	100 (100–100)
Sense organ diseases	--	13·8 (11·6–16·1)	13·8 (11·6–16·1)	--
Blindness and vision loss	--	13·7 (8·88–19·1)	13·7 (8·88–19·1)	--
Age-related and other hearing loss	--	14·5 (13·4–15·9)	14·5 (13·4–15·9)	--
Musculoskeletal disorders	2·56 (0·708–5·61)	30·1 (25·1–34·8)	30·3 (25·4–35)	2·75 (0·802–5·71)
Rheumatoid arthritis	9·18 (2·4–20·7)	12·3 (4·42–20·2)	12·5 (4·51–20·7)	11·6 (3·27–24·3)
Osteoarthritis	--	14·2 (7·68–21·6)	14·2 (7·68–21·6)	--
Low back pain	--	46·4 (41·7–51·2)	46·4 (41·7–51·2)	--
Gout	--	49·3 (35·6–65·1)	49·3 (35·6–65·1)	--
Other non-communicable diseases	0·0722 (0·0402–0·146)	0·0272 (0·015–0·0458)	--	0·132 (0·0744–0·26)
Sudden infant death syndrome	8·29 (4·89–12·7)	8·28 (4·88–12·7)	--	8·28 (4·88–12·7)
Injuries	15·7 (12·4–19·3)	16·1 (13·1–19·3)	21·7 (19–24·5)	12·8 (9·3–16·7)
Transport injuries	15·8 (10·3–22·1)	15·8 (10·5–21·6)	23·5 (20·1–26·8)	13·9 (7·73–20·5)
Road injuries	14·7 (9·01–20·9)	15 (9·61–21)	23·1 (19·7–26·4)	12·9 (6·5–19·6)
Other transport injuries	29·9 (25·6–34·6)	26·7 (22·2–31·6)	31·4 (27·2–35·3)	25·9 (21·2–31)

Unintentional injuries	16·9 (13·2–20·7)	16·9 (13·6–20·7)	22·1 (19–25·4)	11 (7·13–15·1)
Falls	36·1 (31·3–40·2)	29·1 (25–33·3)	31·3 (26·9–35·4)	25·4 (21·4–29·8)
Drowning	-14·2 (-25·1–-3·7)	-13·5 (-23·8–-3·31)	5·95 (4·07–8·5)	-14·3 (-25·2–-3·82)
Fire, heat, and hot substances	9·53 (6·29–13·5)	10·2 (6·83–14·1)	9·44 (6·45–13·3)	10·8 (7·22–15)
Poisonings	9·61 (6·51–13·8)	9·24 (6·36–13·1)	8·21 (5·62–11·6)	9·98 (6·77–14·2)
Exposure to mechanical forces	1·13 (-6·21–8·62)	10·7 (6·82–14·2)	16·6 (14·4–18·3)	-2·38 (-9·72–5·54)
Animal contact	29·2 (25–32·9)	19·4 (16–22·8)	16·3 (12·8–20·3)	22·1 (18·6–25·7)
Foreign body	4·3 (3·33–5·38)	4·04 (3·16–5·07)	2·9 (2·2–3·79)	4·38 (3·4–5·5)
Environmental heat and cold exposure	8·63 (3·94–14·3)	7·82 (3·59–13·1)	5·93 (2·77–10)	8·95 (4·07–15)
Exposure to forces of nature	0 (0–0)	6·53 (3·09–11·1)	6·53 (3·09–11·1)	0 (0–0)
Other unintentional injuries	13·5 (9·12–19·2)	12·3 (8·37–17·2)	10·5 (7·07–14·8)	14 (9·48–19·6)
Self-harm and interpersonal violence	13·2 (4·68–21·6)	13·9 (6·38–21·2)	11·7 (8·45–15·3)	14·3 (5·6–22·6)
Self-harm	13·1 (2·21–23·6)	15·1 (3·87–25·9)	15·4 (8·34–22·9)	15·1 (3·74–26·1)
Interpersonal violence	13·6 (5·1–22·6)	14·6 (6·86–23·2)	23 (17·4–28·9)	12·7 (4·06–21·9)

Supplementary Results Table 2. All-age PAFs attributable to the joint distribution of all risk factors for all ages, both sexes combined for each GBD level 1, level 2, and level 3 cause and all causes in 2019 for Maryland. Results are shown for deaths, YLLs, YLDs, and DALYs Data in parenthesis are 95% uncertainty intervals. PAFs=population attributable fractions. GBD=Global Burden of Disease. YLLs=years of life lost. YLDs=years lived with disability. DALYs=disability-adjusted life-years				
	**Deaths 2019**	**DALYs 2019**	**YLDs 2019**	**YLLs 2019**
All causes	60·3 (57·4–62·9)	46·5 (43·4–49·5)	32 (29·5–34·5)	59·2 (57·2–61·2)
Communicable, maternal, neonatal, and nutritional diseases	46·1 (42–49·9)	58·5 (55·6–61·5)	51·5 (46·8–56)	62·6 (59·2–65·9)
HIV/AIDS and sexually transmitted infections	87·8 (84·1–90·7)	86·8 (83·2–90)	90·2 (87·1–93·1)	85·1 (81–88·5)
HIV/AIDS	87·6 (83·9–90·6)	85·9 (81·9–89·3)	87·8 (84·3–90·9)	85 (80·8–88·5)
Sexually transmitted infections excluding HIV	98·6 (98–99)	99·5 (99·1–99·8)	100 (100–100)	94·8 (92·6–96·4)
Respiratory infections and tuberculosis	41·4 (35·5–46·9)	35·4 (29·6–41·2)	2·36 (1·76–3·08)	45·7 (39·7–51·2)
Tuberculosis	57·2 (47·6–66·4)	59·2 (49·7–67·5)	55·2 (44·8–64·2)	59·9 (50·6–68·1)
Lower respiratory infections	41·3 (35·3–46·8)	45·4 (39·3–51)	32 (25·9–37·4)	45·6 (39·6–51·2)
Upper respiratory infections	0·704 (0·434–1·1)	0·023 (0·0114–0·0402)	0·0053 (5·23e-05–0·0147)	1·54 (0·932–2·37)
Otitis media	1·32 (0·562–2·85)	3·42 (2·21–4·72)	3·42 (2·21–4·73)	3·61 (1·75–6·59)
Enteric infections	8·83 (5·48–13·5)	10·1 (6·75–14·8)	9·83 (6·44–14·6)	10·4 (7–15)
Diarrheal diseases	8·87 (5·51–13·6)	10·1 (6·78–14·9)	9·84 (6·45–14·6)	10·5 (7·07–15·2)
Other infectious diseases	0·838 (0·574–1·13)	1·39 (1·02–1·75)	0·684 (0·408–1·08)	1·55 (1·11–1·98)
Meningitis	1·58 (1·15–2·15)	3·42 (2·54–4·54)	0·000625 (5·7e-06–0·00196)	3·57 (2·64–4·71)
Encephalitis	0·69 (0·488–0·937)	1·78 (1·26–2·4)	0·000187 (1·53e-06–0·000526)	1·85 (1·31–2·49)
Measles	9·15 (-4·08–43·1)	4·65 (-2·77–38·4)	0·296 (0–0·766)	9·32 (-4·17–43·8)
Acute hepatitis	22·4 (13·9–34·4)	10·4 (6·49–15·6)	3·56 (2·2–5·49)	23·8 (14·6–36·4)
Maternal and neonatal disorders	86·8 (84·5–88·7)	85·1 (83·3–86·8)	77·3 (73·3–80·9)	89 (87·4–90·4)
Maternal disorders	11·7 (4·03–19·8)	11·6 (4·01–19·6)	11·7 (4·1–19·7)	11·6 (4–19·6)
Neonatal disorders	93 (91·9–94)	88·3 (86·6–89·9)	79·2 (75–82·8)	93 (91·9–94)
Nutritional deficiencies	92 (88·1–95·6)	89·2 (86·4–91·8)	88·9 (85·4–91·8)	91·4 (87·3–94·8)
Protein-energy malnutrition	100 (100–100)	100 (100–100)	100 (100–100)	100 (100–100)
Vitamin A deficiency	--	100 (100–100)	100 (100–100)	--
Dietary iron deficiency	--	100 (100–100)	100 (100–100)	--
Non-communicable diseases	63·3 (60·2–66·3)	48 (44·4–51·4)	31·1 (28·4–33·6)	64 (61·7–66·4)
Neoplasms	48·1 (44·2–52·3)	47 (43·4–51·1)	31 (26·9–35·2)	48·1 (44·5–52·1)
Lip and oral cavity cancer	67·5 (58·7–75)	69·3 (60·7–76·4)	68·8 (60–76·2)	69·4 (60·8–76·4)
Nasopharynx cancer	64·7 (54·5–73·7)	64·3 (54·4–73·2)	63·5 (53·4–72·6)	64·4 (54·4–73·3)
Other pharynx cancer	76·8 (69·5–82·4)	77·6 (70·6–83·3)	77·9 (71·1–83·4)	77·6 (70·6–83·3)
Esophageal cancer	76·8 (66·5–85·5)	77·5 (67·5–86·5)	76·8 (66·4–85·7)	77·5 (67·5–86·5)

Stomach cancer	23·4 (15·3–41·5)	23·5 (15·4–42·2)	23·9 (15·8–42·3)	23·5 (15·4–42·2)
Colon and rectum cancer	61·9 (53·3–70·8)	62·5 (54·3–70·6)	62·5 (54·3–70·8)	62·5 (54·3–70·5)
Liver cancer	80·3 (75·6–84·6)	80 (75·5–84·1)	80·2 (75·6–84·4)	80 (75·5–84·1)
Gallbladder and biliary tract cancer	27·7 (16·3–40·4)	28·7 (17·1–41·4)	26·5 (15·3–39·6)	28·7 (17·1–41·5)
Pancreatic cancer	41·8 (32·1–53·3)	41·2 (32·1–52·3)	41·7 (32·1–52·6)	41·2 (32·1–52·3)
Larynx cancer	80·3 (70·9–87·8)	81·1 (72·6–87·7)	80·9 (72·4–87·4)	81·2 (72·5–87·7)
Tracheal, bronchus, and lung cancer	81·1 (77·5–85·1)	80·1 (76·6–84·1)	80·7 (77·1–84·8)	80·1 (76·6–84·1)
Breast cancer	36·1 (26·9–46·8)	34·7 (26·3–44·3)	35·4 (26·7–45·5)	34·6 (26·3–44·1)
Cervical cancer	100 (100–100)	100 (100–100)	100 (100–100)	100 (100–100)
Uterine cancer	55·4 (39·8–68·9)	56·9 (41·3–69·7)	57·3 (41·8–70)	56·8 (41·2–69·7)
Ovarian cancer	19·6 (8·75–33·4)	17·6 (7·36–30·6)	17·1 (7·09–29·7)	17·7 (7·35–30·6)
Prostate cancer	5·42 (2·35–8·98)	5·88 (2·54–9·62)	6·09 (2·61–9·84)	5·81 (2·49–9·52)
Kidney cancer	41·6 (30·6–52·4)	41·9 (31·6–52)	42·1 (31·7–52·1)	41·9 (31·6–52)
Bladder cancer	38·5 (23·7–54·2)	41·6 (27–55·7)	42·1 (27·9–55·6)	41·6 (27–55·7)
Thyroid cancer	18·9 (9·78–29·1)	20 (10·5–30·7)	20·2 (11–30·7)	19·9 (10·3–30·7)
Mesothelioma	96·1 (94·7–97·3)	92·6 (90·1–94·6)	95·7 (94–97)	92·5 (90–94·5)
Non-Hodgkin lymphoma	9·06 (4·02–15·2)	9·54 (4·42–15·8)	9·3 (4·17–15·5)	9·55 (4·44–15·9)
Multiple myeloma	10·4 (4·79–17·6)	10·9 (5·23–18·2)	10·8 (5·03–18)	10·9 (5·23–18·1)
Leukemia	35·3 (22·3–49)	33·4 (22·2–45)	35·6 (22·8–48·9)	33·3 (22·1–44·8)
Cardiovascular diseases	81·8 (78·5–84·8)	82·2 (79·5–84·8)	71·9 (67·3–76·1)	83·8 (81·3–86·2)
Rheumatic heart disease	21·4 (12·3–40·1)	21·4 (13·5–36)	21·4 (12·6–37·6)	21·4 (13·7–35·8)
Ischemic heart disease	92·5 (88·6–95·8)	93·9 (91·4–96·3)	92·5 (89–95·5)	94 (91·5–96·3)
Stroke	79·2 (72·4–87)	82·8 (78–87·9)	81·7 (76·4–87·2)	83·3 (78·6–88·3)
Hypertensive heart disease	100 (100–100)	100 (100–100)	100 (100–100)	100 (100–100)
Non-rheumatic valvular heart disease	24·5 (15·3–36·9)	21·6 (14·6–30·5)	7·49 (4·9–10·8)	24·8 (16·9–35·2)
Cardiomyopathy and myocarditis	39·6 (32·5–47·5)	42·5 (36·8–48·6)	40·9 (34·3–47·4)	42·7 (36·7–48·8)
Atrial fibrillation and flutter	53·4 (43·4–63·4)	59 (50–67·3)	60·2 (51·4–68·5)	57·2 (47·8–65·9)
Aortic aneurysm	53·6 (47·6–59·6)	61·2 (56·3–65·9)	--	61·2 (56·3–65·9)
Endocarditis	31 (21·8–42·5)	32·5 (23·5–42·6)	30·4 (20·7–42·3)	32·6 (23·6–42·7)
Other cardiovascular and circulatory diseases	21·3 (16·5–28·9)	23·9 (18·8–29·9)	30·3 (23·1–37·9)	22·2 (17·4–28·6)
Chronic respiratory diseases	60·8 (55·7–65·6)	55·7 (51·8–59·6)	51·3 (46·8–55·8)	60·8 (56·6–65)
Chronic obstructive pulmonary disease	70·1 (65·2–74·2)	67·5 (63·7–71·1)	61·9 (58·1–65·9)	72 (67·9–75·7)
Pneumoconiosis	100 (100–100)	100 (100–100)	100 (100–100)	100 (100–100)

Asthma	51·4 (40·1–62·6)	42 (34–50·6)	41·2 (33·4–49·7)	50·7 (40·9–60·8)
Digestive diseases	45·6 (40·6–50·4)	45·6 (40·3–50·8)	15·8 (10·8–21·5)	55·1 (50–60)
Cirrhosis and other chronic liver diseases	82·9 (75·3–89·5)	84·6 (77·6–90·6)	80·9 (72·7–88)	84·7 (77·6–90·6)
Upper digestive system diseases	20·4 (17–23·9)	5·4 (3·25–8·04)	1·33 (0·786–2·03)	22·7 (19·3–26·4)
Gallbladder and biliary diseases	51·6 (36–66)	55·9 (41·3–68·7)	57·3 (43–70)	53·6 (38·6–67·2)
Pancreatitis	42·2 (25·1–59·4)	45·6 (28·7–62·7)	43·5 (26·9–60·7)	45·9 (29·1–63)
Neurological disorders	27·3 (11·9–42·7)	16·4 (8–28·4)	11·7 (5·74–22·9)	23·1 (9·21–38·8)
Alzheimer's disease and other dementias	41·8 (27·5–57·5)	44·7 (30·2–60·2)	45·4 (30·9–60·5)	44·3 (29·8–59·4)
Parkinson's disease	-8·97 (-14·5–-4·41)	-10·1 (-15·9–-5·08)	-10·5 (-16–-5·38)	-10·1 (-15·9–-5·04)
Idiopathic epilepsy	25·7 (17·4–33·3)	23·1 (15·7–30·5)	22·1 (15–29·3)	25·8 (17·6–33·4)
Multiple sclerosis	13·8 (9·91–18·2)	14·4 (10·4–18·7)	14 (10·1–18·2)	14·7 (10·7–19·1)
Mental disorders	--	11·1 (5·45–17·2)	11·1 (5·45–17·2)	--
Depressive disorders	--	23·5 (11·9–35)	23·5 (11·9–35)	--
Anxiety disorders	--	11·2 (2·94–22·2)	11·2 (2·94–22·2)	--
Idiopathic developmental intellectual disability	--	44·6 (22·4–62·4)	44·6 (22·4–62·4)	--
Substance use disorders	100 (100–100)	100 (100–100)	100 (100–100)	100 (100–100)
Alcohol use disorders	100 (100–100)	100 (100–100)	100 (100–100)	100 (100–100)
Drug use disorders	100 (100–100)	100 (100–100)	100 (100–100)	100 (100–100)
Diabetes and kidney diseases	99·8 (99·8–99·8)	99·9 (99·9–99·9)	100 (100–100)	99·8 (99·8–99·9)
Diabetes mellitus	100 (100–100)	100 (100–100)	100 (100–100)	100 (100–100)
Chronic kidney disease	100 (100–100)	100 (100–100)	100 (100–100)	100 (100–100)
Sense organ diseases	--	10 (8·9–11·2)	10 (8·9–11·2)	--
Blindness and vision loss	--	9·42 (5·94–13·8)	9·42 (5·94–13·8)	--
Age-related and other hearing loss	--	10·9 (9·89–12·1)	10·9 (9·89–12·1)	--
Musculoskeletal disorders	2·95 (0·866–5·4)	20·4 (17–23·9)	20·7 (17·2–24·4)	2·68 (0·814–4·97)
Rheumatoid arthritis	11·7 (3·49–21·3)	13·6 (4·22–24·3)	13·7 (4·25–24·6)	13·2 (4·05–23·5)
Osteoarthritis	--	16 (9·04–23·5)	16 (9·04–23·5)	--
Low back pain	--	41 (35·3–46·6)	41 (35·3–46·6)	--
Gout	--	60·9 (44·8–76·7)	60·9 (44·8–76·7)	--
Other non-communicable diseases	0·179 (0·126–0·244)	0·227 (0·153–0·323)	--	0·571 (0·398–0·764)
Sudden infant death syndrome	6·23 (4·52–8·29)	6·22 (4·52–8·28)	--	6·22 (4·52–8·28)
Injuries	28·6 (23·9–33·2)	26·8 (22·7–31·1)	32·4 (28·8–36·4)	23·8 (18·5–28·8)
Transport injuries	28·8 (22·6–34·9)	28·9 (23·2–34·8)	31·8 (27·7–36·2)	27·5 (21–34·3)

Road injuries	28·3 (22·1–34·4)	28 (22·1–34·1)	30 (25·9–34·8)	27·1 (20·6–34)
Other transport injuries	37·4 (31·8–43·7)	38·3 (33·8–43)	40·9 (36·5–44·6)	34·7 (28·4–41·8)
Unintentional injuries	36·7 (32–40·6)	30·3 (26·4–34·5)	33·2 (29–37·7)	24·8 (20·8–29·2)
Falls	54·7 (48·1–59·4)	45·5 (40·5–50·1)	44·7 (39·7–49·3)	47·3 (42–52·1)
Drowning	-4·47 (-12·7–4·66)	-4·94 (-12·6–3·72)	12·3 (8·13–17·8)	-5·63 (-13·5–3·18)
Fire, heat, and hot substances	13·4 (9·02–18·9)	16·8 (12–22·7)	18·4 (13–24·9)	14·6 (10·4–19·9)
Poisonings	19·6 (14·3–26·2)	19·1 (13·9–25·2)	16·6 (11·9–22·5)	20·6 (15·3–27·1)
Exposure to mechanical forces	15·5 (9·24–23·2)	23·2 (19·3–28·6)	25·1 (21·4–29·9)	12·8 (5·72–21·3)
Animal contact	25·3 (20·4–31·1)	27·7 (22·9–33·7)	28·4 (23·5–34·1)	22·5 (17·6–28·4)
Foreign body	4·14 (2·69–6·11)	5·68 (3·85–8·22)	5·17 (3·56–7·35)	5·93 (3·89–8·7)
Environmental heat and cold exposure	8·5 (3·82–14·8)	8·78 (3·93–15·1)	8·47 (3·8–14·5)	9·15 (4·13–15·8)
Exposure to forces of nature	7·78 (3·52–13·4)	7·84 (3·57–13·5)	9 (4·04–15·3)	7·21 (3·29–12·4)
Other unintentional injuries	22·9 (16·5–30·8)	18 (12·8–24·3)	17·5 (12·4–23·7)	25·5 (18·5–33·8)
Self-harm and interpersonal violence	22 (13·7–30·6)	22·2 (14·2–30·1)	27·4 (20·3–34·5)	21·7 (13·5–29·9)
Self-harm	23·8 (11·5–36·2)	25 (12·9–37·4)	24 (13·6–35·1)	25·1 (12·8–37·5)
Interpersonal violence	19·6 (11·6–28·2)	19·7 (12·1–27·9)	28·3 (20·9–36·4)	18·1 (10·2–26·8)

Supplementary Results Table 2. All-age PAFs attributable to the joint distribution of all risk factors for all ages, both sexes combined for each GBD level 1, level 2, and level 3 cause and all causes in 2019 for Massachusetts. Results are shown for deaths, YLLs, YLDs, and DALYs Data in parenthesis are 95% uncertainty intervals. PAFs=population attributable fractions. GBD=Global Burden of Disease. YLLs=years of life lost. YLDs=years lived with disability. DALYs=disability-adjusted life-years

	Deaths 2019	DALYs 2019	YLDs 2019	YLLs 2019
All causes	59·4 (56·4–62·4)	46·7 (43·4–50)	32·8 (30·2–35·2)	61 (58·7–63·2)
Communicable, maternal, neonatal, and nutritional diseases	43·4 (37·7–48·6)	52·1 (48·9–55·4)	46·4 (41·8–50·9)	56·1 (51·2–60·7)
HIV/AIDS and sexually transmitted infections	87·7 (85·7–89·5)	88·1 (86–90·2)	92·3 (90–95·1)	85·5 (83·2–87·5)
HIV/AIDS	87·4 (85·4–89·3)	86·2 (84–88·2)	88·3 (86·4–90·1)	85·3 (83–87·4)
Sexually transmitted infections excluding HIV	99·5 (99·2–99·7)	99·8 (99·7–99·9)	100 (100–100)	97·7 (96·6–98·6)
Respiratory infections and tuberculosis	43·9 (36·3–50·6)	37·4 (30·6–44·2)	2·17 (1·6–2·86)	47·8 (40·6–54·6)
Tuberculosis	56·7 (46–65·8)	59·4 (49–67·9)	56·6 (45·9–65·2)	60·1 (49·8–68·5)
Lower respiratory infections	43·8 (36·3–50·6)	47·6 (40·3–54·4)	32·7 (26·6–38·5)	47·8 (40·4–54·6)
Upper respiratory infections	0·402 (0·234–0·63)	0·0123 (0·00541–0·0229)	0·00436 (0–0·0131)	1·09 (0·641–1·65)
Otitis media	0·721 (0·284–1·59)	3·04 (1·97–4·2)	3·05 (1·98–4·24)	2·38 (0·994–4·72)
Enteric infections	7·81 (4·91–12)	8·85 (5·98–12·9)	8·79 (5·96–12·9)	8·9 (6·04–12·8)
Diarrheal diseases	7·85 (4·94–12)	8·89 (6–13)	8·8 (5·96–12·9)	8·98 (6·08–13)
Other infectious diseases	0·748 (0·424–1·02)	1·2 (0·766–1·58)	0·85 (0·504–1·31)	1·3 (0·782–1·73)
Meningitis	0·925 (0·623–1·29)	2·19 (1·52–3·02)	0·000378 (0–0·00122)	2·41 (1·65–3·27)
Encephalitis	0·398 (0·269–0·561)	1·19 (0·82–1·63)	0·000104 (0–0·000314)	1·24 (0·856–1·68)
Measles	8·89 (-4·17–43·3)	5 (-3·07–38·8)	0·235 (0–0·665)	9·06 (-4·25–44·2)
Acute hepatitis	28·4 (18·2–39·9)	12·1 (7·92–17·1)	4·38 (2·75–6·76)	31 (19·9–42)
Maternal and neonatal disorders	87·9 (85·8–89·6)	78·5 (75·1–81·7)	66·7 (61–71·9)	89·2 (87·5–90·7)
Maternal disorders	11·3 (4·11–18·6)	11·2 (4·03–18·7)	11·3 (3·99–19·2)	11·1 (4·04–18·4)
Neonatal disorders	91·6 (90·2–92·9)	80·5 (77–83·7)	68·3 (62·5–73·7)	91·7 (90·2–92·9)
Nutritional deficiencies	93·1 (89·4–96·1)	88·3 (85·4–91·1)	87·5 (84·1–90·8)	92·4 (88·5–95·6)
Protein-energy malnutrition	100 (100–100)	100 (100–100)	100 (100–100)	100 (100–100)
Vitamin A deficiency	--	100 (100–100)	100 (100–100)	--
Dietary iron deficiency	--	100 (100–100)	100 (100–100)	--
Non-communicable diseases	61·6 (58·3–64·7)	47·7 (44·1–51·3)	32 (29–34·8)	64·2 (61·8–66·6)
Neoplasms	48·3 (44·7–52·2)	47·5 (44·1–51·4)	30·1 (26·1–34·1)	48·9 (45·6–52·6)
Lip and oral cavity cancer	68·6 (59·1–76·2)	70·8 (61·8–78)	70·4 (61·3–77·8)	70·8 (61·7–78)
Nasopharynx cancer	67·2 (56·5–76·4)	67 (56·7–75·7)	66·3 (55·1–74·9)	67·1 (56·8–75·7)
Other pharynx cancer	77·9 (70·8–83·8)	79 (72·4–84·5)	79·2 (72·7–84·7)	78·9 (72·3–84·5)

Esophageal cancer	78·3 (68·3–86·9)	79·2 (69·4–87·8)	78·4 (69–86·6)	79·2 (69·4–87·8)
Stomach cancer	23·7 (15·7–42)	24·3 (16·1–42·6)	24·5 (16·3–42·9)	24·3 (16·1–42·7)
Colon and rectum cancer	60·7 (52–69·2)	61·7 (53·8–69·4)	61·6 (53·3–69·7)	61·7 (53·8–69·5)
Liver cancer	81·9 (77·5–85·8)	81·9 (77·8–85·9)	81·9 (77·3–86)	81·9 (77·8–85·9)
Gallbladder and biliary tract cancer	26·4 (15·1–39·3)	27·2 (15·6–40·3)	25·3 (14·2–38·4)	27·3 (15·6–40·3)
Pancreatic cancer	41·6 (31·7–52·7)	41·3 (32·2–51·8)	41·7 (32·2–52·3)	41·3 (32·2–51·8)
Larynx cancer	81·4 (71·3–89·1)	82·2 (73·2–88·8)	81·9 (72·8–88·8)	82·2 (73·2–88·8)
Tracheal, bronchus, and lung cancer	81·8 (78·5–85·4)	81 (77·9–84·7)	81·5 (78·1–85·3)	81 (77·9–84·7)
Breast cancer	36 (27·3–45·9)	35·2 (27·3–44·1)	35·7 (27·6–45)	35·1 (27·2–44)
Cervical cancer	100 (100–100)	100 (100–100)	100 (100–100)	100 (100–100)
Uterine cancer	53·2 (37·7–67·4)	54·7 (40–68·3)	55·1 (40·4–68·7)	54·6 (39·8–68·3)
Ovarian cancer	19·1 (8·89–31·3)	16·8 (7·24–28·4)	16·3 (7–27·6)	16·8 (7·23–28·4)
Prostate cancer	5·32 (2·29–8·76)	5·93 (2·52–9·66)	6·25 (2·65–10·1)	5·82 (2·47–9·49)
Kidney cancer	41·4 (29·8–52·6)	42·1 (31·4–52·2)	42·4 (31·6–52·5)	42·1 (31·3–52·3)
Bladder cancer	37·8 (22·5–52·6)	41 (26·4–55·1)	41·7 (27·8–55·7)	40·9 (26·4–55·1)
Thyroid cancer	18·3 (9·35–28·8)	19·5 (9·98–30·7)	19·8 (10·5–30·7)	19·4 (9·75–30·7)
Mesothelioma	97·1 (96·1–98)	94·4 (92·4–95·9)	96·6 (95·1–97·8)	94·3 (92·3–95·9)
Non-Hodgkin lymphoma	8·58 (3·64–14·5)	9·11 (4–15·3)	8·86 (3·9–14·8)	9·13 (4·02–15·3)
Multiple myeloma	9·9 (4·59–16·7)	10·4 (4·85–17·5)	10·3 (4·65–17·3)	10·4 (4·86–17·5)
Leukemia	35·7 (21·9–49·9)	34·9 (22·5–47·2)	36·2 (22·4–50·2)	34·8 (22·6–47·1)
Cardiovascular diseases	79·4 (75·5–83·1)	80·1 (76·9–83·2)	70·5 (65·9–75·2)	82 (79–84·7)
Rheumatic heart disease	21·4 (11·6–42·7)	21·5 (13·5–36·6)	21·4 (13–37·1)	21·5 (13·6–36·9)
Ischemic heart disease	91·9 (87·5–95·3)	93·5 (90·4–96·1)	92·2 (88·7–95·4)	93·6 (90·5–96·1)
Stroke	77·8 (70·3–85·9)	81·5 (76·1–87·2)	80·7 (75·3–86·9)	81·8 (76·4–87·3)
Hypertensive heart disease	100 (100–100)	100 (100–100)	100 (100–100)	100 (100–100)
Non-rheumatic valvular heart disease	25·2 (14·8–39·5)	23 (15–33·8)	9·49 (6·02–13·7)	25·5 (16·7–37·4)
Cardiomyopathy and myocarditis	36·8 (29·3–45·7)	41·2 (35·1–47·7)	38·7 (32·4–45·4)	41·6 (35·5–48·2)
Atrial fibrillation and flutter	53·1 (42·9–63·3)	58·7 (50·4–67·7)	60·1 (51·9–68·6)	56·6 (47·8–66·2)
Aortic aneurysm	50·7 (44·3–57·8)	59·4 (54·2–64·6)	--	59·4 (54·2–64·6)
Endocarditis	30·4 (20·4–43·7)	32·1 (23–43·4)	30 (19·6–43·2)	32·2 (23–43·5)
Other cardiovascular and circulatory diseases	20 (15·3–26·8)	23·5 (18·8–28·6)	30·2 (23·5–37)	21·3 (17–26·7)
Chronic respiratory diseases	61·6 (56·5–67)	57·1 (53·3–61·2)	52·3 (47·6–56·9)	62·2 (57·9–67)

Chronic obstructive pulmonary disease	70·6 (65·8–75·5)	68·3 (64·3–72·2)	62·5 (58·2–66·7)	72·7 (68·4–76·8)
Pneumoconiosis	100 (100–100)	100 (100–100)	100 (100–100)	100 (100–100)
Asthma	49·9 (38·4–62·1)	42·6 (34·6–51·4)	41·9 (34·1–50·5)	51·1 (41–61·6)
Digestive diseases	45 (39·7–50·4)	46·7 (41·5–52)	14·9 (10·3–20·2)	56·5 (51·2–61·6)
Cirrhosis and other chronic liver diseases	83·8 (75·9–90·2)	85·7 (78·7–91·5)	82·4 (74·4–89)	85·7 (78·7–91·5)
Upper digestive system diseases	19·8 (16·2–23·4)	5·22 (3·07–7·8)	1·33 (0·775–2·08)	22·6 (18·9–26·4)
Gallbladder and biliary diseases	49·6 (33·5–64·8)	54·2 (39·5–67·5)	56 (41·5–68·8)	51·3 (35·6–65·8)
Pancreatitis	42·9 (23·8–60·5)	47 (28·3–63·9)	45·3 (26·8–62·5)	47·4 (28·4–64·1)
Neurological disorders	27·9 (13–43·1)	17·5 (8·92–29·9)	12·4 (6·28–23·3)	24 (10·1–39·1)
Alzheimer's disease and other dementias	40·2 (26·3–55·2)	42·8 (28·6–57·6)	43·5 (29·5–58·2)	42·3 (28·3–57·2)
Parkinson's disease	-8·67 (-14–-4·14)	-10·1 (-15·7–-5·04)	-10·8 (-16·6–-5·59)	-9·97 (-15·6–-4·91)
Idiopathic epilepsy	27·5 (18·1–36·2)	25·1 (17·1–33·2)	24·2 (16·1–32·3)	28·1 (19·1–36·5)
Multiple sclerosis	13·9 (9·96–18·2)	14·7 (10·7–19)	14·4 (10·5–18·7)	14·9 (10·8–19·5)
Mental disorders	--	8·16 (4–12·9)	8·16 (4–12·9)	--
Depressive disorders	--	18·3 (8·62–27·9)	18·3 (8·62–27·9)	--
Anxiety disorders	--	5·94 (1·25–13·8)	5·94 (1·25–13·8)	--
Idiopathic developmental intellectual disability	--	43·8 (22·1–60)	43·8 (22·1–60)	--
Substance use disorders	100 (100–100)	100 (100–100)	100 (100–100)	100 (100–100)
Alcohol use disorders	100 (100–100)	100 (100–100)	100 (100–100)	100 (100–100)
Drug use disorders	100 (100–100)	100 (100–100)	100 (100–100)	100 (100–100)
Diabetes and kidney diseases	99·8 (99·8–99·9)	99·9 (99·9–99·9)	100 (100–100)	99·8 (99·8–99·9)
Diabetes mellitus	100 (100–100)	100 (100–100)	100 (100–100)	100 (100–100)
Chronic kidney disease	100 (100–100)	100 (100–100)	100 (100–100)	100 (100–100)
Sense organ diseases	--	10·2 (9·17–11·5)	10·2 (9·17–11·5)	--
Blindness and vision loss	--	9·41 (5·89–13·7)	9·41 (5·89–13·7)	--
Age-related and other hearing loss	--	11·2 (10·1–12·4)	11·2 (10·1–12·4)	--
Musculoskeletal disorders	3·28 (1–6·06)	19 (15·8–22·4)	19·2 (16–22·6)	3·16 (0·986–5·78)
Rheumatoid arthritis	11·4 (3·36–20·8)	13·9 (4·33–25·1)	14·1 (4·39–25·3)	13 (3·94–23·5)
Osteoarthritis	--	14 (7·76–20·8)	14 (7·76–20·8)	--
Low back pain	--	40·5 (34·7–46·3)	40·5 (34·7–46·3)	--
Gout	--	60·2 (43·8–76·6)	60·2 (43·8–76·6)	--
Other non-communicable diseases	0·0693 (0·0459–0·101)	0·0857 (0·055–0·127)	--	0·268 (0·178–0·375)

Sudden infant death syndrome	6·73 (4·66–9·27)	6·72 (4·65–9·26)	--	6·72 (4·65–9·26)
Injuries	33·2 (28·2–38)	32 (27·2–36·6)	36·1 (31·7–40·6)	28·1 (22·1–34·1)
Transport injuries	29·1 (22·2–36·7)	31·5 (25·4–38·7)	34·6 (30–39·5)	29·3 (21·2–38·2)
Road injuries	28·3 (21·3–36)	30 (23·7–37·5)	32·4 (27·9–37·6)	28·6 (20·4–37·7)
Other transport injuries	42·1 (36–49·1)	42·4 (37·2–47·3)	43·1 (38·2–47·3)	40·7 (33·5–48·7)
Unintentional injuries	39·6 (34–43·9)	34·8 (30–39·8)	36·9 (32·1–41·7)	29·9 (25–35·3)
Falls	56·3 (48·7–61·4)	48·5 (42·7–53·3)	47·8 (41·8–52·6)	50·5 (44·4–55·7)
Drowning	-1·8 (-13·2–9·14)	-0·503 (-11·4–10·6)	14·8 (9·8–20·8)	-1·5 (-13–9·92)
Fire, heat, and hot substances	15 (9·89–20·9)	20·4 (14·7–27·1)	21·3 (15·1–28·4)	18·6 (13–25·2)
Poisonings	24·3 (17·5–32·6)	23·4 (16·9–30·9)	19·7 (14·4–26·3)	27·1 (19·7–36)
Exposure to mechanical forces	16·4 (6·87–26·3)	27·2 (22·2–33·4)	28·7 (24·1–34·4)	15·3 (4·49–27·5)
Animal contact	28 (22·8–34·4)	31·3 (25·9–37·7)	31·7 (26·3–38·1)	26·2 (20·8–32·9)
Foreign body	4·23 (2·65–6·33)	8·18 (5·47–11·6)	8·43 (5·88–11·8)	8·02 (5·14–11·7)
Environmental heat and cold exposure	8·83 (3·9–15·1)	9·4 (4·16–16)	9·18 (4·03–15·6)	9·74 (4·36–16·5)
Exposure to forces of nature	8·5 (3·75–14·4)	8·63 (3·8–14·5)	9·91 (4·41–16·7)	7·84 (3·47–13·3)
Other unintentional injuries	26·8 (19·4–36·6)	20·7 (14·7–27·9)	20·1 (14·2–26·8)	32 (23·4–42·5)
Self-harm and interpersonal violence	25·3 (14·4–36·1)	26·7 (16·8–36·4)	30·5 (23·3–37·2)	26·2 (15·4–36·6)
Self-harm	26·6 (13·3–39·4)	28·7 (15·4–41·7)	28·3 (16·3–40·3)	28·7 (15·3–41·7)
Interpersonal violence	21·1 (12·1–30·3)	22·9 (14·3–31·6)	31·8 (24·4–39·3)	19·2 (9·94–28·3)

Supplementary Results Table 2. All-age PAFs attributable to the joint distribution of all risk factors for all ages, both sexes combined for each GBD level 1, level 2, and level 3 cause and all causes in 2019 for Michigan. Results are shown for deaths, YLLs, YLDs, and DALYs Data in parenthesis are 95% uncertainty intervals. PAFs=population attributable fractions. GBD=Global Burden of Disease. YLLs=years of life lost. YLDs=years lived with disability. DALYs=disability-adjusted life-years

	Deaths 2019	DALYs 2019	YLDs 2019	YLLs 2019
All causes	62·8 (60–65·4)	49·6 (46·7–52·6)	34·4 (31·9–36·8)	62·5 (60·4–64·4)
Communicable, maternal, neonatal, and nutritional diseases	47·1 (41·3–52·2)	56 (52·8–59·1)	47·7 (43·1–52·3)	61·4 (56·9–65·2)
HIV/AIDS and sexually transmitted infections	87·6 (86–89)	88·4 (86·7–90·2)	92 (89·9–94·8)	85·8 (84–87·3)
HIV/AIDS	87·1 (85·4–88·5)	86·2 (84·5–87·7)	87·5 (85·9–89)	85·5 (83·7–87)
Sexually transmitted infections excluding HIV	99·2 (98·9–99·5)	99·7 (99·5–99·9)	100 (100–100)	97 (95·6–98)
Respiratory infections and tuberculosis	43·2 (35·4–50·3)	37·5 (30·8–44·3)	2·44 (1·83–3·23)	48·3 (40·6–54·9)
Tuberculosis	56·7 (47·4–65·4)	59·9 (51·3–67·5)	56·1 (46·6–64·6)	60·6 (52·2–68·1)
Lower respiratory infections	43·2 (35·3–50·2)	48·1 (40·4–54·7)	34 (27·9–39·6)	48·3 (40·5–55)
Upper respiratory infections	0·639 (0·384–1·02)	0·0178 (0·00897–0·0324)	0·00502 (0–0·0144)	1·48 (0·895–2·37)
Otitis media	1·54 (0·636–3·18)	3·88 (2·54–5·3)	3·87 (2·53–5·28)	4·32 (2·06–7·83)
Enteric infections	10·2 (6·21–15·8)	11·4 (7·52–16·9)	11·2 (7·22–16·7)	11·6 (7·75–17)
Diarrheal diseases	10·3 (6·23–15·9)	11·4 (7·54–17)	11·2 (7·22–16·7)	11·7 (7·81–17·2)
Other infectious diseases	0·753 (0·467–1·01)	1·26 (0·848–1·64)	0·697 (0·418–1·12)	1·41 (0·895–1·85)
Meningitis	1·4 (1–1·91)	3·19 (2·32–4·36)	0·000539 (0–0·00163)	3·34 (2·42–4·56)
Encephalitis	0·58 (0·414–0·786)	1·59 (1·15–2·15)	0·00011 (0–0·00032)	1·67 (1·2–2·25)
Measles	9·41 (-3·98–44)	4·41 (-2·71–34·9)	0·364 (0·0108–0·927)	9·58 (-4·05–44·7)
Acute hepatitis	23·4 (15·8–37·3)	9·99 (6·54–15)	3·7 (2·23–5·89)	24·4 (16·2–38·4)
Maternal and neonatal disorders	85·3 (82·9–87·5)	80·8 (78–83·2)	69·6 (64·3–74·5)	87·5 (85·7–89·3)
Maternal disorders	11·7 (4·15–19·6)	11·5 (4·09–19·3)	11·4 (4·07–19·4)	11·6 (4·1–19·3)
Neonatal disorders	91·7 (90·3–93)	83·9 (81·1–86·3)	71·2 (65·8–76·3)	91·7 (90·3–93)
Nutritional deficiencies	95·8 (88·7–97)	90·2 (87·5–92·4)	88·6 (85·4–91·6)	95·3 (89·1–96·4)
Protein-energy malnutrition	100 (100–100)	100 (100–100)	100 (100–100)	100 (100–100)
Vitamin A deficiency	--	100 (100–100)	100 (100–100)	--
Dietary iron deficiency	--	100 (100–100)	100 (100–100)	--
Non-communicable diseases	65·6 (62·5–68·5)	51·6 (48·2–55)	34 (31·2–36·7)	67·2 (64·9–69·4)
Neoplasms	50·7 (47–54·8)	50·4 (46·8–54·1)	32·5 (28·6–36·8)	51·5 (48–55·3)
Lip and oral cavity cancer	68 (58·7–75·4)	70·8 (62–77·7)	69·8 (60·6–77)	70·9 (62·1–77·8)
Nasopharynx cancer	65·8 (54·9–75·1)	65·8 (55·3–74·5)	64·6 (54·2–73·7)	65·9 (55·4–74·5)
Other pharynx cancer	78·3 (71·5–83·8)	79·7 (73·5–85)	80·2 (73·6–85·4)	79·7 (73·5–84·9)

Esophageal cancer	79·9 (71–87·8)	81·2 (72·6–88·7)	80 (71–87·9)	81·3 (72·6–88·8)
Stomach cancer	25·3 (17·2–43)	26·3 (18·2–44·2)	26·3 (18·1–43·9)	26·3 (18·2–44·2)
Colon and rectum cancer	62·9 (54–71·9)	63·9 (55·6–72·1)	63·7 (55·2–72·4)	63·9 (55·6–72·1)
Liver cancer	82·2 (78–86·2)	82 (78–85·7)	82·1 (78–85·8)	82 (78–85·7)
Gallbladder and biliary tract cancer	27·6 (16·3–40·3)	28·6 (17·2–41·1)	26·9 (16–39·9)	28·6 (17·1–41·1)
Pancreatic cancer	45 (35·2–56·5)	45·2 (35·8–56·3)	45·2 (35·5–56·6)	45·2 (35·8–56·3)
Larynx cancer	82·9 (73·5–89·9)	84·2 (76·2–90·3)	83·9 (75·6–90·1)	84·2 (76·2–90·3)
Tracheal, bronchus, and lung cancer	84 (80·7–87·5)	83·8 (80·7–87·1)	84·1 (80·7–87·6)	83·7 (80·7–87·1)
Breast cancer	37·2 (27·4–48·4)	36·4 (27·5–46·5)	37 (27·8–47·6)	36·3 (27·5–46·3)
Cervical cancer	100 (100–100)	100 (100–100)	100 (100–100)	100 (100–100)
Uterine cancer	55·9 (41–69·3)	57·6 (43·1–70·3)	58 (43·6–70·6)	57·5 (42·9–70·2)
Ovarian cancer	21·1 (9·31–35·7)	19 (7·93–32·5)	18·6 (7·78–31·9)	19 (7·93–32·5)
Prostate cancer	6 (2·65–9·87)	6·77 (3–10·8)	7·18 (3·21–11·4)	6·65 (2·95–10·7)
Kidney cancer	43·6 (32·5–54·1)	44·5 (34·1–54·4)	44·7 (34·2–54·8)	44·5 (34·1–54·4)
Bladder cancer	41·9 (25·9–57·2)	45·9 (31–59·8)	46·8 (32–61)	45·9 (30·9–59·7)
Thyroid cancer	19·1 (9·93–29·7)	20·1 (10·6–30·8)	19·8 (11–29·3)	20·2 (10·5–31·1)
Mesothelioma	96·9 (95·7–97·8)	94·1 (92·1–95·7)	96·4 (95–97·6)	94 (92–95·7)
Non-Hodgkin lymphoma	9·15 (4·1–15·3)	9·64 (4·42–15·9)	9·44 (4·22–15·7)	9·65 (4·43–15·8)
Multiple myeloma	10·5 (4·89–17·5)	11 (5·24–18·1)	10·8 (4·99–17·8)	11 (5·24–18·2)
Leukemia	37·4 (23·2–51·7)	36·6 (24·2–49·3)	38·2 (23·9–52·4)	36·5 (24·2–49·1)
Cardiovascular diseases	82·5 (78·9–85·6)	83·2 (80·5–85·7)	72·8 (68·4–77·1)	84·9 (82·4–87·1)
Rheumatic heart disease	21 (11·8–40·3)	21·6 (13·5–36·4)	21·6 (12·9–37·4)	21·6 (13·5–35·9)
Ischemic heart disease	92·4 (88·4–95·7)	94·1 (91·4–96·3)	92·6 (89·2–95·6)	94·1 (91·5–96·4)
Stroke	79·1 (72·1–87·4)	83 (78·3–88·3)	82 (76·8–87·8)	83·5 (78·8–88·6)
Hypertensive heart disease	100 (100–100)	100 (100–100)	100 (100–100)	100 (100–100)
Non-rheumatic valvular heart disease	24·1 (15–37·1)	22·4 (15·2–32·2)	8 (5·22–11·6)	25·3 (17·2–36)
Cardiomyopathy and myocarditis	39·2 (32·3–46·9)	43·5 (37·4–49)	41·3 (34·8–47·8)	43·7 (37·5–49·4)
Atrial fibrillation and flutter	52·1 (42·2–62·2)	58·8 (50·4–67·2)	60·3 (51·6–68·5)	56·7 (47·9–65·5)
Aortic aneurysm	54·6 (48·6–60·6)	63·3 (58·5–68)	--	63·3 (58·5–68)
Endocarditis	30·3 (20·5–42·9)	32·5 (23·2–42·8)	30·1 (20·2–42·8)	32·7 (23·4–42·9)
Other cardiovascular and circulatory diseases	22·2 (17·3–29·3)	25·6 (20·6–31·2)	31·7 (24·9–38·3)	23·7 (19–29·5)
Chronic respiratory diseases	65·7 (60·9–70·6)	61·2 (57·4–64·7)	54·8 (50·1–59·1)	66·9 (62·6–71·2)

Chronic obstructive pulmonary disease	73·7 (69·2–77·8)	72·1 (68·4–75·5)	65·6 (61·8–69·4)	76 (72·2–79·6)
Pneumoconiosis	100 (100–100)	100 (100–100)	100 (100–100)	100 (100–100)
Asthma	52 (41·2–63·3)	43·6 (35·6–51·9)	42·9 (34·9–51·2)	51·3 (42·2–61·2)
Digestive diseases	45·7 (40·3–50·9)	47 (41·7–52·2)	16·9 (11·6–22·6)	55·8 (50·3–61·2)
Cirrhosis and other chronic liver diseases	81·5 (73·3–89)	83·5 (75·9–90·3)	80 (71·8–87)	83·5 (75·9–90·3)
Upper digestive system diseases	21·5 (17·8–25·3)	6·81 (4·28–9·84)	1·79 (1·1–2·75)	25·1 (21·1–29·2)
Gallbladder and biliary diseases	51·8 (36·2–66·6)	56·8 (42·5–69·6)	58·2 (44·1–70·6)	54·1 (39–68·2)
Pancreatitis	36·9 (19·4–55·3)	41·3 (23·9–59·1)	39·1 (22·1–56·9)	41·6 (24–59·7)
Neurological disorders	27·5 (12·2–42·8)	18·3 (9·75–30·5)	13·8 (7·35–25)	23·7 (10·4–39·2)
Alzheimer's disease and other dementias	42·5 (28–58·3)	45·9 (31·3–61·5)	46·7 (31·9–62·3)	45·3 (31·1–60·9)
Parkinson's disease	-9·56 (-15·4–-4·62)	-11·4 (-17·6–-5·73)	-12·1 (-18·7–-6·23)	-11·2 (-17·6–-5·55)
Idiopathic epilepsy	24·3 (15·9–33·4)	22·4 (14·9–30·7)	21·2 (13·7–29·5)	24·5 (16·4–33·3)
Multiple sclerosis	16·3 (12–21·1)	17·8 (13·4–22·8)	17·7 (13·3–22·4)	17·8 (13·3–23)
Mental disorders	--	9·12 (4·53–14·1)	9·12 (4·54–14·1)	--
Depressive disorders	--	19·7 (9·05–29·8)	19·7 (9·05–29·8)	--
Anxiety disorders	--	6·09 (1·37–13·7)	6·09 (1·37–13·7)	--
Idiopathic developmental intellectual disability	--	46·8 (24·1–65·1)	46·8 (24·1–65·1)	--
Substance use disorders	100 (100–100)	100 (100–100)	100 (100–100)	100 (100–100)
Alcohol use disorders	100 (100–100)	100 (100–100)	100 (100–100)	100 (100–100)
Drug use disorders	100 (100–100)	100 (100–100)	100 (100–100)	100 (100–100)
Diabetes and kidney diseases	99·9 (99·8–99·9)	99·9 (99·9–100)	100 (100–100)	99·9 (99·8–99·9)
Diabetes mellitus	100 (100–100)	100 (100–100)	100 (100–100)	100 (100–100)
Chronic kidney disease	100 (100–100)	100 (100–100)	100 (100–100)	100 (100–100)
Sense organ diseases	--	10·4 (9·25–11·8)	10·4 (9·25–11·8)	--
Blindness and vision loss	--	10·4 (6·63–15·3)	10·4 (6·63–15·3)	--
Age-related and other hearing loss	--	11·1 (10·1–12·4)	11·1 (10·1–12·4)	--
Musculoskeletal disorders	3·66 (1·07–6·54)	20·3 (17–23·8)	20·6 (17·2–24·2)	3·59 (1·11–6·36)
Rheumatoid arthritis	12·7 (3·87–22·5)	16·1 (5·18–28·2)	16·5 (5·31–28·9)	14·9 (4·71–26)
Osteoarthritis	--	16·6 (9·54–24·1)	16·6 (9·54–24·1)	--
Low back pain	--	41·6 (35·5–47·8)	41·6 (35·5–47·8)	--
Gout	--	62 (46·2–77·2)	62 (46·2–77·2)	--
Other non-communicable diseases	0·128 (0·0898–0·175)	0·166 (0·113–0·233)	--	0·43 (0·306–0·567)

Sudden infant death syndrome	7·55 (5·3–10·2)	7·54 (5·29–10·2)	--	7·54 (5·29–10·2)
Injuries	26·5 (21·2–31·2)	24·9 (20·1–29·3)	32·9 (29·2–36·5)	20·3 (14·3–25·7)
Transport injuries	21·6 (15·3–28·5)	23·8 (17·9–30·2)	31·7 (27·5–35·6)	20·3 (13·1–28)
Road injuries	20·5 (14–27·5)	22 (15·8–28·8)	29·1 (25·1–33·3)	19·3 (12–27·1)
Other transport injuries	37·2 (31·8–42·4)	38·3 (33·4–42·9)	41 (36·6–44·6)	34·5 (28·5–40·9)
Unintentional injuries	36 (31–39·9)	29·7 (25·7–33·8)	33·7 (29·6–37·8)	23·3 (19·4–27·3)
Falls	55·2 (48·2–59·8)	46·5 (41·3–50·7)	45·7 (40·6–50)	48·1 (42·4–52·6)
Drowning	-9·86 (-21·2–0·642)	-9·92 (-20·7–0·212)	11·5 (7·31–16·7)	-10·7 (-21·7—0·356)
Fire, heat, and hot substances	12 (8·1–17·3)	15·6 (11–21·2)	17 (11·8–23·3)	13·9 (9·77–19·1)
Poisonings	19·3 (13·8–25·7)	19·3 (14–25·4)	15·7 (11·2–21·1)	20·8 (15·1–27·8)
Exposure to mechanical forces	9·39 (1·03–18)	21·6 (17·1–26·7)	25·1 (21·3–29·5)	6·97 (-2·26–16·5)
Animal contact	24·8 (20–30·1)	27 (22·2–32·2)	27·9 (23·1–33·2)	22·1 (17·4–27·6)
Foreign body	2·94 (1·91–4·39)	4·08 (2·77–6)	4·62 (3·14–6·82)	3·89 (2·53–5·82)
Environmental heat and cold exposure	7·85 (3·48–13·7)	8·29 (3·64–14·7)	7·94 (3·44–13·9)	8·62 (3·78–15·1)
Exposure to forces of nature	7·42 (3·24–13)	7·5 (3·3–13·1)	8·65 (3·78–14·9)	6·92 (3·02–12·1)
Other unintentional injuries	20·8 (14·8–28)	16·5 (11·5–22·6)	15·7 (10·8–22)	24·2 (17·6–32·6)
Self-harm and interpersonal violence	18·5 (8·25–28·5)	19·6 (10·3–28·9)	28·6 (21·9–35)	18·8 (8·96–28·4)
Self-harm	19·9 (6·28–33·4)	21·8 (7·91–35·1)	24·6 (13·7–35·8)	21·8 (7·8–35·1)
Interpersonal violence	15·9 (5·84–25·6)	16·6 (7·6–25·6)	29·9 (22·5–37·4)	13·8 (3·98–23·6)

Supplementary Results Table 2. All-age PAFs attributable to the joint distribution of all risk factors for all ages, both sexes combined for each GBD level 1, level 2, and level 3 cause and all causes in 2019 for Minnesota. Results are shown for deaths, YLLs, YLDs, and DALYs Data in parenthesis are 95% uncertainty intervals. PAFs=population attributable fractions. GBD=Global Burden of Disease. YLLs=years of life lost. YLDs=years lived with disability. DALYs=disability-adjusted life-years

	Deaths 2019	DALYs 2019	YLDs 2019	YLLs 2019
All causes	56·8 (53·8–59·6)	43·1 (40·1–46·1)	30·9 (28·5–33·1)	55·7 (53·5–58)
Communicable, maternal, neonatal, and nutritional diseases	41·9 (34·3–48·8)	52·8 (49·1–56·3)	45·9 (41·3–50·7)	58·4 (53·3–62·9)
HIV/AIDS and sexually transmitted infections	94 (91·7–95·4)	94·2 (91·9–95·7)	96·7 (94·9–98·2)	92 (89·2–93·7)
HIV/AIDS	93·6 (91·1–95·1)	92·6 (89·6–94·3)	93·9 (91·3–95·7)	91·9 (88·9–93·7)
Sexually transmitted infections excluding HIV	98·9 (98·5–99·3)	99·6 (99·2–99·8)	100 (100–100)	95·6 (93·8–97)
Respiratory infections and tuberculosis	38·9 (27·7–49·3)	30·6 (22·8–38·5)	2·07 (1·56–2·68)	44·1 (33·3–53·7)
Tuberculosis	54·2 (44–63·3)	57·3 (47·6–65·7)	55·4 (45·1–64·4)	57·8 (48·3–66·1)
Lower respiratory infections	38·9 (27·5–49·3)	43·9 (33·1–53·6)	31·6 (25·6–37·1)	44·2 (33·1–53·9)
Upper respiratory infections	0·653 (0·403–1·04)	0·0223 (0·0104–0·0402)	0·00494 (0–0·0152)	1·54 (0·959–2·4)
Otitis media	1·27 (0·56–2·71)	3·62 (2·33–5·01)	3·61 (2·32–5·01)	3·81 (1·85–6·85)
Enteric infections	8·97 (5·64–13·8)	10·5 (7·21–15·2)	10·2 (6·86–15·1)	11 (7·66–15·8)
Diarrheal diseases	9·04 (5·68–14)	10·6 (7·26–15·3)	10·2 (6·87–15·1)	11·2 (7·79–16)
Other infectious diseases	0·526 (0·35–0·715)	1 (0·716–1·28)	0·555 (0·323–0·935)	1·11 (0·76–1·46)
Meningitis	1·48 (1·06–2·07)	3·33 (2·38–4·53)	0·000503 (0–0·00172)	3·54 (2·5–4·88)
Encephalitis	0·58 (0·423–0·782)	1·58 (1·19–2·09)	0·000113 (0–0·000362)	1·67 (1·26–2·24)
Measles	9·16 (-4·14–44·1)	9·3 (-4·19–44·6)	0·151 (0–0·693)	9·32 (-4·2–44·8)
Acute hepatitis	17·9 (11·3–29·6)	6·75 (4·16–10·4)	2·61 (1·59–4·5)	18·9 (11·9–31·2)
Maternal and neonatal disorders	85·2 (83·1–87·2)	79·1 (76·5–81·6)	68·8 (63·7–73·7)	86·8 (85–88·5)
Maternal disorders	10·8 (3·87–18·1)	10·7 (3·79–18·1)	10·6 (3·72–18·1)	10·7 (3·82–18·1)
Neonatal disorders	89·8 (88–91·4)	81·5 (78·8–84·1)	70·6 (65·4–75·5)	89·8 (88–91·4)
Nutritional deficiencies	88·4 (82·5–93·5)	86·3 (83–89·2)	85·8 (82·1–89·4)	88·4 (82·3–93·3)
Protein-energy malnutrition	100 (100–100)	100 (100–100)	100 (100–100)	100 (100–100)
Vitamin A deficiency	--	100 (100–100)	100 (100–100)	--
Dietary iron deficiency	--	100 (100–100)	100 (100–100)	--
Non-communicable diseases	59·3 (56·2–62·4)	44·4 (40·9–47·9)	29·8 (27·2–32·3)	60·1 (57·7–62·6)
Neoplasms	46·9 (43·3–50·9)	46·4 (42·8–50·1)	30·4 (26·3–34·5)	47·7 (44·4–51·3)
Lip and oral cavity cancer	67·2 (58·4–75·3)	70 (61·7–77·4)	69·6 (61–77·1)	70·1 (61·7–77·4)
Nasopharynx cancer	65·2 (52·8–74·9)	65·1 (53·5–74·4)	64·3 (52·6–73·9)	65·2 (53·6–74·4)
Other pharynx cancer	77·3 (69·8–83·3)	78·6 (71·4–84·3)	79 (71·8–84·7)	78·6 (71·4–84·3)

Esophageal cancer	79·2 (69·5–87·3)	80·3 (71·1–88)	79·3 (70–87·1)	80·4 (71·1–88)
Stomach cancer	23·7 (15·6–41·8)	24·5 (16·4–42·9)	24·7 (16·5–43)	24·5 (16·4–42·9)
Colon and rectum cancer	61·7 (53–70·2)	62·8 (54·7–70·6)	62·7 (54·7–70·7)	62·8 (54·7–70·7)
Liver cancer	73·9 (67·8–80)	74·7 (68·8–80·1)	74·6 (68·6–80·2)	74·7 (68·8–80·1)
Gallbladder and biliary tract cancer	27·1 (15·8–39·6)	28·1 (16·6–41·2)	26 (14·8–38·9)	28·1 (16·7–41·3)
Pancreatic cancer	41·3 (31·7–52·1)	41·3 (32·3–51·9)	41·5 (32·4–52·2)	41·3 (32·3–51·9)
Larynx cancer	81·4 (71·6–88·8)	82·5 (73·7–88·8)	82·3 (73·8–88·8)	82·5 (73·6–88·9)
Tracheal, bronchus, and lung cancer	82·2 (78·7–85·6)	81·6 (78·2–84·9)	82 (78·5–85·6)	81·6 (78·2–84·9)
Breast cancer	35·8 (26·9–45·8)	35·4 (27–44·6)	35·8 (27·3–45·5)	35·3 (26·9–44·4)
Cervical cancer	100 (100–100)	100 (100–100)	100 (100–100)	100 (100–100)
Uterine cancer	53·5 (38·1–67·4)	55·2 (39·6–68·7)	55·7 (40·4–69·1)	55·1 (39·5–68·6)
Ovarian cancer	20·1 (9·45–32·6)	17·7 (7·73–29·4)	17·1 (7·48–28·4)	17·7 (7·73–29·4)
Prostate cancer	5·53 (2·4–9·27)	6·18 (2·71–10)	6·52 (2·85–10·4)	6·06 (2·67–9·94)
Kidney cancer	42 (30·7–53)	42·7 (32·3–52·8)	43 (32·7–53·1)	42·7 (32·2–52·8)
Bladder cancer	37·2 (23·1–52·2)	41·1 (27·3–54·7)	42 (28·1–55·1)	41·1 (27·2–54·7)
Thyroid cancer	18·6 (9·53–29·1)	19·8 (10·3–30·5)	20 (10·7–30·8)	19·7 (10·1–30·4)
Mesothelioma	97·3 (96·3–98·1)	94·8 (93–96·2)	96·9 (95·5–97·9)	94·7 (92·9–96·1)
Non-Hodgkin lymphoma	8·94 (3·94–15)	9·43 (4·27–15·7)	9·22 (4·16–15·4)	9·44 (4·28–15·7)
Multiple myeloma	10·2 (4·64–17)	10·8 (5·04–17·9)	10·6 (4·9–17·8)	10·8 (5·06–17·9)
Leukemia	35·8 (22·2–49·8)	34·7 (22·9–47)	36·6 (23–50·7)	34·6 (22·9–46·8)
Cardiovascular diseases	76·5 (72·5–80·6)	77·9 (74·8–81·2)	70·5 (65·9–75·2)	79·4 (76·4–82·4)
Rheumatic heart disease	20·7 (11·4–41·7)	21 (12·8–36)	20·9 (12·5–35·9)	21 (12·9–36·2)
Ischemic heart disease	91·7 (87·3–95·3)	93·4 (90·5–96)	92 (88·3–95·1)	93·5 (90·6–96)
Stroke	77·1 (69·8–85·8)	81 (75·6–86·9)	80·4 (75–86·5)	81·3 (76–87·2)
Hypertensive heart disease	100 (100–100)	100 (100–100)	100 (100–100)	100 (100–100)
Non-rheumatic valvular heart disease	24·1 (14·2–38·4)	23·6 (15·6–34·2)	11 (6·89–15·8)	24·8 (16·3–36)
Cardiomyopathy and myocarditis	37·3 (30·2–45·9)	41·2 (35·1–47·3)	39·2 (32·8–46)	41·5 (35·5–47·9)
Atrial fibrillation and flutter	51·8 (41·6–62·1)	58 (49–66·7)	59·6 (50·8–68·3)	55·6 (46·1–65·1)
Aortic aneurysm	50·6 (44·6–57·4)	59·5 (54·4–64·6)	--	59·5 (54·4–64·6)
Endocarditis	29·2 (19·3–42·1)	31 (21·8–41·9)	29·3 (19·4–42·4)	31·1 (21·8–41·9)
Other cardiovascular and circulatory diseases	17·7 (12·7–26·7)	21·5 (16·6–28)	29·3 (22·5–36·3)	19 (14·4–26·1)
Chronic respiratory diseases	61·1 (55·6–67)	57·7 (53·6–61·6)	52·8 (48·2–57·2)	62 (57·5–67·1)

Chronic obstructive pulmonary disease	69·9 (64·8–75·1)	68·9 (65–72·8)	63·2 (59–67·3)	72·3 (67·8–77)
Pneumoconiosis	100 (100–100)	100 (100–100)	100 (100–100)	100 (100–100)
Asthma	49·8 (38·2–61·2)	42·5 (34·8–51·3)	41·7 (34·1–50·2)	50·2 (40·7–59·9)
Digestive diseases	43·3 (37·4–48·4)	44·7 (38·9–50)	14·8 (9·83–20·2)	54·9 (49·2–59·7)
Cirrhosis and other chronic liver diseases	81·2 (72–88·8)	83·8 (75·8–90·3)	80·1 (70·7–87·9)	83·8 (75·9–90·3)
Upper digestive system diseases	19·9 (16·3–23·5)	5·15 (2·91–8·08)	1·15 (0·664–1·86)	23·3 (19·5–26·8)
Gallbladder and biliary diseases	50·3 (34·9–65·4)	54·8 (40–68·1)	56·3 (42·2–69·1)	52·2 (37–66·6)
Pancreatitis	40 (22·4–57·7)	44·5 (26·6–61·4)	42·5 (24·3–59·8)	44·8 (27–61·6)
Neurological disorders	24·2 (9·83–39·6)	15·1 (7·46–26·7)	11·2 (5·68–21·6)	20·2 (7·49–35·3)
Alzheimer's disease and other dementias	40·7 (26·5–55·4)	43·7 (29·7–58·1)	44·3 (30·2–58·8)	43·2 (29·1–57·8)
Parkinson's disease	-8·93 (-14·4–-4·32)	-10·3 (-16·4–-5·24)	-10·9 (-16·8–-5·67)	-10·2 (-16·1–-5·1)
Idiopathic epilepsy	25·7 (16·5–34·3)	22·8 (14·8–30·5)	21·8 (14·2–29·3)	25·9 (17·3–34·4)
Multiple sclerosis	14·2 (10·2–18·8)	15·5 (11·3–20·1)	15·5 (11·4–20)	15·5 (11·3–20·2)
Mental disorders	--	6·49 (2·97–10·2)	6·49 (2·97–10·2)	--
Depressive disorders	--	14·9 (6·11–23·9)	14·9 (6·11–23·9)	--
Anxiety disorders	--	3·16 (0·724–7·62)	3·16 (0·724–7·62)	--
Idiopathic developmental intellectual disability	--	45·1 (22·3–64·1)	45·1 (22·3–64·1)	--
Substance use disorders	100 (100–100)	100 (100–100)	100 (100–100)	100 (100–100)
Alcohol use disorders	100 (100–100)	100 (100–100)	100 (100–100)	100 (100–100)
Drug use disorders	100 (100–100)	100 (100–100)	100 (100–100)	100 (100–100)
Diabetes and kidney diseases	99·9 (99·8–99·9)	99·9 (99·9–100)	100 (100–100)	99·9 (99·8–99·9)
Diabetes mellitus	100 (100–100)	100 (100–100)	100 (100–100)	100 (100–100)
Chronic kidney disease	100 (100–100)	100 (100–100)	100 (100–100)	100 (100–100)
Sense organ diseases	--	11 (9·95–12·4)	11 (9·95–12·4)	--
Blindness and vision loss	--	9·32 (6·02–13·6)	9·32 (6·02–13·6)	--
Age-related and other hearing loss	--	12 (10·9–13·4)	12 (10·9–13·4)	--
Musculoskeletal disorders	3·85 (1·12–6·9)	19·9 (16·7–23·1)	20·2 (17–23·5)	3·92 (1·2–7·02)
Rheumatoid arthritis	11·4 (3·27–20·9)	14·2 (4·31–25·1)	14·4 (4·44–25·6)	13·3 (3·95–23·9)
Osteoarthritis	--	15·4 (8·72–22·6)	15·4 (8·72–22·6)	--
Low back pain	--	41·7 (36·2–47·6)	41·7 (36·2–47·6)	--
Gout	--	60·7 (44·5–76·8)	60·7 (44·5–76·8)	--
Other non-communicable diseases	0·155 (0·108–0·212)	0·185 (0·127–0·265)	--	0·486 (0·345–0·648)

Sudden infant death syndrome	7·82 (5·49–10·5)	7·81 (5·48–10·5)	--	7·81 (5·48–10·5)
Injuries	32·5 (27·2–37·5)	28·2 (23·5–32·9)	35·5 (31·6–39·4)	22·7 (16·1–29·1)
Transport injuries	22·6 (15·1–30·8)	25·4 (18·7–33)	32·9 (28·9–37·5)	21·5 (13–31)
Road injuries	20·8 (13–29·4)	23·2 (16–31·4)	30·6 (26·4–35·6)	19·9 (10·7–29·5)
Other transport injuries	38·9 (33·4–45·9)	39·2 (34·4–44·7)	41·9 (37·8–45·5)	36·3 (30·2–44·3)
Unintentional injuries	43·8 (38·4–47·9)	34·1 (29·9–38·3)	36·5 (31·9–40·9)	29·8 (25·7–34·2)
Falls	58 (51·2–62·5)	48·8 (43·5–52·9)	47·6 (42·3–51·8)	51·2 (45·6–55·6)
Drowning	-8·21 (-22·7–6·21)	-7·89 (-21·8–5·78)	13·5 (9·03–19·2)	-9 (-23·2–5·24)
Fire, heat, and hot substances	13·3 (9·09–18·8)	17·6 (12·6–24·1)	18·8 (13·4–25·4)	15·3 (11·1–20·9)
Poisonings	21·5 (15·8–29·3)	21·1 (15·6–28·4)	17·3 (12·4–23·1)	23·3 (17·3–31·4)
Exposure to mechanical forces	9·72 (-0·828–20·8)	22·6 (17·5–28·8)	26·3 (22·4–31·4)	7·24 (-4·09–19·9)
Animal contact	26·8 (22·2–32·1)	28·3 (23·3–33·6)	29·1 (24·3–34·6)	23·6 (19–29·5)
Foreign body	3·4 (2·22–5·24)	5·81 (3·9–8·36)	6·91 (4·75–9·84)	5·26 (3·4–7·93)
Environmental heat and cold exposure	8·46 (3·96–14·4)	8·91 (4·19–15)	8·65 (4·07–14·6)	9·21 (4·35–15·5)
Exposure to forces of nature	7·98 (3·75–13·5)	8·12 (3·82–13·6)	9·38 (4·39–15·7)	7·35 (3·48–12·4)
Other unintentional injuries	22·3 (16·1–30·5)	18·5 (13·3–25·2)	17·8 (12·5–24·5)	26·1 (19·1–35·3)
Self-harm and interpersonal violence	17·2 (3·12–30·6)	18·8 (6·16–30·9)	29·4 (23·1–36·3)	17·7 (4·09–30·7)
Self-harm	17·4 (1·58–33·2)	18·9 (3·22–34·6)	24·9 (13·2–36·4)	18·7 (3·03–34·6)
Interpersonal violence	16·3 (5·3–28·3)	19·1 (9·95–29·1)	31·1 (24–38·8)	13·8 (2·72–26)

Supplementary Results Table 2. All-age PAFs attributable to the joint distribution of all risk factors for all ages, both sexes combined for each GBD level 1, level 2, and level 3 cause and all causes in 2019 for Mississippi. Results are shown for deaths, YLLs, YLDs, and DALYs Data in parenthesis are 95% uncertainty intervals. PAFs=population attributable fractions. GBD=Global Burden of Disease. YLLs=years of life lost. YLDs=years lived with disability. DALYs=disability-adjusted life-years

	Deaths 2019	DALYs 2019	YLDs 2019	YLLs 2019
All causes	63·4 (61·2–65·5)	51·6 (48·9–54·2)	36·3 (33·4–38·8)	61·5 (59·7–63·3)
Communicable, maternal, neonatal, and nutritional diseases	43·5 (39·2–47·9)	58·2 (55·3–61·3)	54·7 (50·2–59)	60·1 (56·1–63·8)
HIV/AIDS and sexually transmitted infections	89 (86·6–91·1)	88·7 (86–90·9)	91·1 (88·6–93·7)	87·3 (84·5–89·7)
HIV/AIDS	88·7 (86·2–90·9)	87·7 (84·8–90·1)	88·9 (86·3–91·2)	87·2 (84·3–89·6)
Sexually transmitted infections excluding HIV	98·5 (97·7–99)	99·1 (98·4–99·6)	100 (100–100)	94·7 (92·1–96·6)
Respiratory infections and tuberculosis	35·3 (29·5–41)	34·4 (29–40)	2·91 (2·16–3·72)	41·4 (35·5–47·1)
Tuberculosis	54·6 (46·6–62·5)	58·1 (50·3–65·3)	53 (43·5–61·1)	58·8 (51–65·9)
Lower respiratory infections	35·1 (29·3–40·9)	41·1 (35·1–46·9)	33·8 (28·4–38·6)	41·2 (35·2–47)
Upper respiratory infections	0·868 (0·504–1·42)	0·0286 (0·014–0·0514)	0·00482 (6·41e-05–0·0133)	1·7 (0·998–2·74)
Otitis media	2·33 (0·913–5·05)	4·38 (2·87–5·96)	4·34 (2·82–5·93)	5·49 (2·58–10)
Enteric infections	14·8 (9·23–22·4)	16·2 (10·6–23·7)	15·5 (9·86–23·2)	17·4 (11·6–24·9)
Diarrheal diseases	15 (9·34–22·7)	16·3 (10·7–23·9)	15·5 (9·87–23·2)	17·8 (11·9–25·5)
Other infectious diseases	0·921 (0·655–1·35)	1·56 (1·16–2·07)	0·618 (0·373–1·01)	1·74 (1·28–2·37)
Meningitis	1·79 (1·2–2·5)	3·65 (2·54–5)	0·000614 (5·92e-06–0·00178)	3·77 (2·62–5·17)
Encephalitis	0·77 (0·526–1·09)	1·9 (1·33–2·66)	0·000387 (4·12e-06–0·00112)	1·94 (1·36–2·71)
Measles	9·81 (-3·78–44·8)	9·98 (-3·83–45·5)	0·244 (0–1·14)	9·99 (-3·85–45·6)
Acute hepatitis	18·9 (11·3–35·1)	9·59 (6–16·1)	3·46 (2·13–5·59)	19·2 (11·7–34·7)
Maternal and neonatal disorders	85·1 (82·7–87·3)	84·6 (82·7–86·4)	78·6 (74·7–82)	87·3 (85·4–89)
Maternal disorders	12·7 (4·58–20·6)	12·4 (4·58–20·2)	12·2 (4·48–20·2)	12·5 (4·59–20·4)
Neonatal disorders	91·3 (90·1–92·6)	87·9 (86·2–89·4)	80·5 (76·7–83·9)	91·3 (90·1–92·6)
Nutritional deficiencies	93 (84·4–94·8)	91·5 (88·6–93·6)	91·2 (88·5–93·7)	92·7 (84·6–94·3)
Protein-energy malnutrition	100 (100–100)	100 (100–100)	100 (100–100)	100 (100–100)
Vitamin A deficiency	--	100 (100–100)	100 (100–100)	--
Dietary iron deficiency	--	100 (100–100)	100 (100–100)	--
Non-communicable diseases	67·8 (65·2–70·2)	55·1 (51·8–58·3)	36 (32·8–38·8)	68·4 (66·3–70·4)
Neoplasms	52·5 (48·8–56·3)	51·7 (48·3–55·4)	33·2 (29·4–37·7)	52·7 (49·3–56·3)
Lip and oral cavity cancer	68·6 (61·4–75·2)	71·2 (64·1–77·3)	70·3 (63–76·9)	71·3 (64·2–77·3)
Nasopharynx cancer	62·8 (52·1–71·7)	63 (52·1–71·5)	61·9 (51–70·6)	63 (52·2–71·5)
Other pharynx cancer	76·6 (70·6–81·7)	78 (71·9–82·9)	78·2 (72·1–83·4)	77·9 (71·9–82·9)
Esophageal cancer	81·3 (72·8–88·6)	82·4 (74·1–89·7)	81·5 (72·7–88·9)	82·4 (74·1–89·7)

Stomach cancer	26·5 (18·5–44·1)	27·1 (18·8–45)	27·3 (19–45·3)	27·1 (18·8–45)
Colon and rectum cancer	63·4 (54·6–72·2)	64·2 (56·1–72·3)	64·1 (55·8–72·4)	64·2 (56·1–72·3)
Liver cancer	79·5 (74·6–84·1)	78·7 (74–83·3)	79·3 (74·5–84·1)	78·7 (74–83·3)
Gallbladder and biliary tract cancer	27·5 (16·4–40·2)	28·7 (17·1–41·8)	26·6 (15·3–39·4)	28·8 (17·1–41·8)
Pancreatic cancer	45·5 (35·9–56·7)	45·5 (36·4–56·1)	45·6 (36·3–56·6)	45·5 (36·4–56·1)
Larynx cancer	82·8 (73·8–89·4)	83·9 (76·3–89·5)	83·5 (75·7–89·4)	84 (76·3–89·5)
Tracheal, bronchus, and lung cancer	84·8 (81·7–88)	84·3 (81·3–87·5)	84·6 (81·5–87·9)	84·3 (81·3–87·5)
Breast cancer	34·2 (24·7–45·7)	33 (24–43·4)	33·7 (24·4–44·7)	33 (24–43·3)
Cervical cancer	100 (100–100)	100 (100–100)	100 (100–100)	100 (100–100)
Uterine cancer	55·5 (40·1–68·9)	57·4 (42·3–70·3)	57·9 (43–70·6)	57·3 (42·3–70·2)
Ovarian cancer	19·9 (8·68–34·5)	18·2 (7·67–31·9)	17·7 (7·55–30·9)	18·2 (7·7–31·9)
Prostate cancer	6·75 (3·01–10·8)	7·46 (3·31–11·7)	7·83 (3·53–12·2)	7·38 (3·27–11·6)
Kidney cancer	44·2 (33·5–54·3)	44·8 (34·7–54·3)	44·8 (34·8–54·1)	44·8 (34·7–54·2)
Bladder cancer	44 (28·4–58·4)	47·7 (32·6–61·2)	48·1 (33·8–61·2)	47·6 (32·5–61·2)
Thyroid cancer	18·6 (9·8–28·6)	19·8 (10·7–30·2)	20·2 (11·2–30·3)	19·7 (10·5–30·2)
Mesothelioma	95·7 (94·1–97)	92·4 (89·7–94·6)	95·4 (93·7–96·9)	92·3 (89·6–94·5)
Non-Hodgkin lymphoma	9·03 (4·01–15·1)	9·56 (4·36–15·8)	9·31 (4·28–15·5)	9·57 (4·36–15·8)
Multiple myeloma	10·3 (4·78–17·2)	10·9 (5·1–18·1)	10·8 (5–17·8)	10·9 (5·1–18·1)
Leukemia	38·2 (24·2–51·9)	36·2 (24–48·1)	38·4 (25·2–51·7)	36·1 (24–47·9)
Cardiovascular diseases	84·7 (81·8–87·4)	85·3 (83–87·4)	75 (70·9–79)	86·6 (84·5–88·6)
Rheumatic heart disease	22·5 (13·6–41·2)	22·7 (14·8–36·8)	22·5 (14·1–37·1)	22·7 (14·9–36·4)
Ischemic heart disease	93·8 (90·7–96·3)	95·3 (93·2–97·1)	93·7 (90·8–96·3)	95·3 (93·3–97·1)
Stroke	81·1 (74·9–87·8)	84·8 (80·7–89·2)	83·5 (78·6–88·6)	85·3 (81·1–89·4)
Hypertensive heart disease	100 (100–100)	100 (100–100)	100 (100–100)	100 (100–100)
Non-rheumatic valvular heart disease	25·7 (16·6–38·4)	22·9 (15·8–31·7)	5·06 (3·37–7·13)	26·3 (18·4–36·4)
Cardiomyopathy and myocarditis	41·3 (34·8–49·1)	43·3 (37·1–49)	41·8 (35·5–48·5)	43·4 (37·2–49·4)
Atrial fibrillation and flutter	51·8 (42·3–62·7)	58·2 (49·9–67·1)	59·4 (50·9–67·9)	56·6 (48·1–65·9)
Aortic aneurysm	58·3 (52·7–64)	66 (61·6–70·3)	--	66 (61·6–70·3)
Endocarditis	32·6 (23·5–44·5)	34·2 (25·2–44·2)	31·8 (22·2–44·1)	34·2 (25·3–44·3)
Other cardiovascular and circulatory diseases	26 (20·9–33)	27·5 (22·3–33·3)	32·6 (25·6–40·1)	26·4 (21·4–32·6)
Chronic respiratory diseases	65·4 (60·9–69·6)	62·5 (59–65·9)	56·1 (51·8–60·3)	66·3 (62·6–70)
Chronic obstructive pulmonary disease	71·5 (67·1–75·7)	71·9 (68·2–75·3)	67·2 (63·5–70·8)	73·9 (70·1–77·4)
Pneumoconiosis	100 (100–100)	100 (100–100)	100 (100–100)	100 (100–100)

Asthma	52·9 (42·5–63·5)	43·2 (35·3–51·6)	41·7 (33·8–49·9)	51·7 (42·4–61)
Digestive diseases	46·7 (41·4–52·3)	48·6 (43·2–54·3)	18·9 (12·8–25·5)	55·5 (49·3–61·3)
Cirrhosis and other chronic liver diseases	78·6 (69·8–86·5)	81·2 (72·8–88·3)	75·9 (66·7–84·1)	81·2 (72·8–88·4)
Upper digestive system diseases	23·4 (19·8–26·9)	6·9 (4·37–10·1)	1·52 (0·942–2·33)	26·7 (22·7–30·5)
Gallbladder and biliary diseases	52·3 (37·4–66·5)	57·1 (43–69·6)	58·3 (44·5–70·4)	54·9 (40·3–68·4)
Pancreatitis	31·4 (17·2–47)	36·5 (21·2–52·9)	32·8 (18·3–48·7)	36·8 (21·7–53·4)
Neurological disorders	25·1 (9·82–41)	14·7 (7·08–26·4)	10·5 (5·07–21·1)	20·8 (7·52–36·6)
Alzheimer's disease and other dementias	41·9 (27·9–57·5)	45·7 (31·4–60·8)	46·6 (32·2–61·6)	45 (30·6–60·4)
Parkinson's disease	-10·7 (-17–-5·29)	-12·5 (-19·2–-6·3)	-12·7 (-19·4–-6·59)	-12·5 (-19·3–-6·26)
Idiopathic epilepsy	20 (12·6–27·7)	18·3 (11·5–25·2)	16·5 (10·3–22·5)	21 (13·4–28·4)
Multiple sclerosis	17 (12·5–22)	17·8 (13·2–22·6)	17·1 (12·8–21·7)	18·4 (13·4–23·7)
Mental disorders	--	10·6 (5·39–16·3)	10·6 (5·39–16·3)	--
Depressive disorders	--	20·9 (9·92–31·6)	20·9 (9·92–31·6)	--
Anxiety disorders	--	7·41 (1·85–16·3)	7·41 (1·85–16·3)	--
Idiopathic developmental intellectual disability	--	52·8 (26·1–73)	52·8 (26·1–73)	--
Substance use disorders	100 (100–100)	100 (100–100)	100 (100–100)	100 (100–100)
Alcohol use disorders	100 (100–100)	100 (100–100)	100 (100–100)	100 (100–100)
Drug use disorders	100 (100–100)	100 (100–100)	100 (100–100)	100 (100–100)
Diabetes and kidney diseases	99·9 (99·8–99·9)	99·9 (99·9–99·9)	100 (100–100)	99·9 (99·8–99·9)
Diabetes mellitus	100 (100–100)	100 (100–100)	100 (100–100)	100 (100–100)
Chronic kidney disease	100 (100–100)	100 (100–100)	100 (100–100)	100 (100–100)
Sense organ diseases	--	10·4 (9·3–11·7)	10·4 (9·3–11·7)	--
Blindness and vision loss	--	10 (6·47–14·7)	10 (6·47–14·7)	--
Age-related and other hearing loss	--	11·1 (10–12·3)	11·1 (10–12·3)	--
Musculoskeletal disorders	2·92 (0·917–5·26)	22·9 (19·3–26·7)	23·4 (19·7–27·2)	2·65 (0·837–4·82)
Rheumatoid arthritis	13·7 (4·4–24·3)	16·4 (5·26–28·8)	16·5 (5·3–29)	15·9 (5·16–28·3)
Osteoarthritis	--	17·6 (10·1–25·7)	17·6 (10·1–25·7)	--
Low back pain	--	42·9 (36·8–49)	42·9 (36·8–49)	--
Gout	--	61·4 (45·9–76·6)	61·4 (45·9–76·6)	--
Other non-communicable diseases	0·216 (0·149–0·294)	0·285 (0·19–0·404)	--	0·612 (0·433–0·818)
Sudden infant death syndrome	7·17 (4·95–9·8)	7·16 (4·94–9·79)	--	7·16 (4·94–9·79)
Injuries	23·4 (20·2–26·6)	21·7 (18·5–24·9)	27·9 (24·7–30·6)	19·4 (15·7–22·9)
Transport injuries	23·6 (19·5–28)	22·2 (18–26·8)	26·7 (23·1–29·9)	21·3 (16·7–26·1)

Road injuries	23·2<br>(19·1–27·5)	21·5<br>(17·2–26·1)	24·5<br>(20·9–27·8)	20·9<br>(16·4–25·8)
Other transport injuries	30·5<br>(25·8–35)	31<br>(26·8–34·9)	37·6<br>(33·5–40·8)	26·8<br>(22–31·8)
Unintentional injuries	25·8<br>(22·2–28·7)	21·6<br>(18·3–24·7)	28·4<br>(24·9–31·6)	14·4<br>(11·6–17·4)
Falls	52·1<br>(45·7–56·1)	42·1<br>(37·1–45·7)	41·6<br>(36·7–45·3)	43·2<br>(38·2–46·9)
Drowning	-8·68<br>(-15·2–-1·2)	-9·51<br>(-15·6–-2·61)	8·45<br>(5·3–12·6)	-9·77<br>(-16–-2·8)
Fire, heat, and hot substances	8·62<br>(5·51–12·7)	10<br>(6·63–14·2)	11·2<br>(7·46–15·8)	9·34<br>(6·16–13·4)
Poisonings	12·7<br>(8·59–17·9)	12·2<br>(8·38–17·1)	10·1<br>(6·6–14·2)	13·2<br>(9·02–18·3)
Exposure to mechanical forces	13<br>(7·47–18·6)	16·4<br>(13–19·6)	20·2<br>(17·5–22·9)	9·92<br>(4·15–15·8)
Animal contact	18·1<br>(14·5–22·2)	20·6<br>(16·7–24·9)	22·3<br>(18·2–26·6)	15·3<br>(11·7–19·4)
Foreign body	1·93<br>(1·26–2·81)	2·47<br>(1·61–3·62)	3·28<br>(2·24–4·74)	2·3<br>(1·48–3·42)
Environmental heat and cold exposure	5·91<br>(2·5–10·4)	6·48<br>(2·78–11·3)	5·87<br>(2·5–10·4)	6·83<br>(2·88–11·8)
Exposure to forces of nature	5·81<br>(2·54–10·1)	6·84<br>(3·01–11·8)	7·09<br>(3·12–12·2)	5·75<br>(2·52–9·94)
Other unintentional injuries	14·8<br>(10·2–20·4)	11·5<br>(7·65–16)	10·5<br>(6·91–14·8)	16·1<br>(11·3–22)
Self-harm and interpersonal violence	20·4<br>(12·9–27·5)	21·2<br>(14·2–27·9)	26·7<br>(21–32·8)	20·8<br>(13·6–27·7)
Self-harm	21·1<br>(10·3–31·6)	23·5<br>(12·1–34·4)	21·5<br>(12·8–30·7)	23·5<br>(12·1–34·5)
Interpersonal violence	19·9<br>(12·8–27·4)	19·5<br>(12·4–26·5)	27·9<br>(21·4–34·8)	18·4<br>(11·3–25·4)

Supplementary Results Table 2. All-age PAFs attributable to the joint distribution of all risk factors for all ages, both sexes combined for each GBD level 1, level 2, and level 3 cause and all causes in 2019 for Missouri. Results are shown for deaths, YLLs, YLDs, and DALYs Data in parenthesis are 95% uncertainty intervals. PAFs=population attributable fractions. GBD=Global Burden of Disease. YLLs=years of life lost. YLDs=years lived with disability. DALYs=disability-adjusted life-years

	Deaths 2019	DALYs 2019	YLDs 2019	YLLs 2019
All causes	62·5 (59·9–64·9)	49·3 (46·2–52·5)	34·1 (31·5–36·6)	61·7 (59·8–63·7)
Communicable, maternal, neonatal, and nutritional diseases	45·5 (41·6–49·6)	56 (53·2–59)	50·2 (45·3–55)	59·5 (56·1–62·7)
HIV/AIDS and sexually transmitted infections	92·4 (90·9–93·5)	92·8 (91·2–94·1)	95·3 (93·6–97·1)	90·9 (89·2–92·2)
HIV/AIDS	92·1 (90·5–93·3)	91·3 (89·7–92·7)	92·5 (90·9–93·8)	90·8 (89–92·1)
Sexually transmitted infections excluding HIV	98·9 (98·4–99·2)	99·6 (99·3–99·8)	100 (100–100)	95·6 (93·9–97)
Respiratory infections and tuberculosis	43·3 (37·5–48·8)	38·6 (32·7–44·6)	2·56 (1·93–3·29)	48·6 (42·7–54)
Tuberculosis	54·9 (45·8–63·4)	58·5 (49·6–66·6)	54·7 (45–63·1)	59·2 (50·4–67·1)
Lower respiratory infections	43·3 (37·4–48·8)	48·5 (42·5–54·1)	35·5 (29·8–40·9)	48·6 (42·6–54·2)
Upper respiratory infections	0·547 (0·34–0·843)	0·0179 (0·00829–0·0338)	0·00556 (0·000103–0·0158)	1·21 (0·739–1·85)
Otitis media	1·58 (0·631–3·35)	4·44 (2·89–6·13)	4·44 (2·87–6·14)	4·36 (1·99–7·87)
Enteric infections	11·4 (6·97–17)	12·6 (8·25–18·1)	12·4 (8·02–17·9)	12·9 (8·54–18·4)
Diarrheal diseases	11·4 (6·99–17·1)	12·7 (8·27–18·2)	12·4 (8·03–17·9)	13 (8·59–18·5)
Other infectious diseases	0·662 (0·446–0·883)	1·12 (0·822–1·4)	0·633 (0·38–1·02)	1·24 (0·867–1·58)
Meningitis	1·2 (0·853–1·65)	2·65 (1·91–3·55)	0·000561 (8·88e-06–0·00169)	2·78 (1·99–3·72)
Encephalitis	0·549 (0·389–0·759)	1·45 (1·06–1·96)	0·000128 (1·86e-06–0·000405)	1·51 (1·1–2·04)
Measles	9·47 (-3·92–44·5)	6·21 (-3·37–40·8)	0·345 (0–1·02)	9·63 (-3·98–45·2)
Acute hepatitis	22·9 (14·7–35·7)	9·29 (6–14·1)	3·43 (2·11–5·49)	23·8 (15·6–36·5)
Maternal and neonatal disorders	85·1 (82·5–87·2)	83·7 (81·6–85·5)	77·1 (72·8–81·1)	87·5 (85·5–89·1)
Maternal disorders	11·6 (4·14–19·1)	11·4 (4·06–18·8)	11·2 (3·99–18·7)	11·4 (4·09–18·9)
Neonatal disorders	91·9 (90·6–93·1)	87·1 (85·1–88·8)	79 (74·8–83)	91·9 (90·6–93·1)
Nutritional deficiencies	94·8 (87·9–96·2)	90 (86·9–92·3)	88·6 (85–91·6)	94·4 (86·8–95·7)
Protein-energy malnutrition	100 (100–100)	100 (100–100)	100 (100–100)	100 (100–100)
Vitamin A deficiency	--	100 (100–100)	100 (100–100)	--
Dietary iron deficiency	--	100 (100–100)	100 (100–100)	--
Non-communicable diseases	65·9 (62·9–68·6)	51·6 (47·9–55·4)	33·6 (30·8–36·3)	67·4 (65·2–69·5)
Neoplasms	51·9 (48·4–55·8)	51·5 (48–55·1)	32·9 (28·8–37·2)	52·6 (49·3–56·2)
Lip and oral cavity cancer	68 (59·7–75·2)	70·8 (62·8–77·6)	70·1 (61·8–76·7)	70·9 (63–77·7)
Nasopharynx cancer	64·8 (53·5–74·1)	64·8 (53·9–73·9)	63·6 (52·4–72·9)	64·9 (53·9–73·9)
Other pharynx cancer	78·2 (71·8–83·3)	79·5 (73·4–84·6)	79·8 (73·6–84·8)	79·4 (73·3–84·6)
Esophageal cancer	80·6 (71·5–88)	81·5 (72·8–89)	80·7 (71·7–88·2)	81·5 (72·8–89)

Stomach cancer	26·6 (18·5–44)	27·5 (19·4–45·2)	27·6 (19·5–45·2)	27·5 (19·4–45·2)
Colon and rectum cancer	63·1 (54·5–71·9)	64·2 (56–72·3)	64 (55·8–72·2)	64·2 (56–72·2)
Liver cancer	80·2 (75·5–84·7)	79·9 (75·4–84·3)	80·1 (75·3–84·6)	79·9 (75·4–84·3)
Gallbladder and biliary tract cancer	27·2 (16·1–40·4)	28·4 (17·1–41·4)	26·2 (14·9–39)	28·5 (17·1–41·4)
Pancreatic cancer	45·6 (36·2–56·7)	45·6 (36·5–56·2)	45·7 (36·4–56·4)	45·6 (36·5–56·2)
Larynx cancer	83·1 (73·1–89·9)	84·3 (76·1–90)	84 (75·5–89·9)	84·4 (76·1–90·1)
Tracheal, bronchus, and lung cancer	85 (82·1–88·3)	84·7 (81·9–87·8)	85 (81·8–88·1)	84·7 (81·9–87·8)
Breast cancer	35·7 (26·6–46·9)	35·2 (26·5–45·3)	35·6 (26·6–46·2)	35·2 (26·5–45·2)
Cervical cancer	100 (100–100)	100 (100–100)	100 (100–100)	100 (100–100)
Uterine cancer	54·5 (38·8–68·6)	56·4 (41·1–69·7)	57 (41·4–70·1)	56·3 (40·9–69·6)
Ovarian cancer	19·7 (8·71–34)	17·9 (7·43–31·1)	17·3 (7·14–30·6)	17·9 (7·45–31·1)
Prostate cancer	6·47 (2·79–10·6)	7·25 (3·12–11·7)	7·65 (3·33–12·3)	7·13 (3·08–11·5)
Kidney cancer	44 (32·8–54·9)	44·8 (34·5–54·8)	45·1 (34·7–54·9)	44·8 (34·6–54·8)
Bladder cancer	42·6 (26·9–57·3)	46·3 (31·4–60)	47·1 (32–60·4)	46·3 (31·3–59·9)
Thyroid cancer	18·7 (9·5–28·9)	19·9 (10·4–30·9)	20·2 (10·9–30·9)	19·8 (10·3–30·9)
Mesothelioma	96·1 (94·8–97·2)	92·9 (90·6–94·8)	95·8 (94·2–97·1)	92·8 (90·5–94·7)
Non-Hodgkin lymphoma	8·91 (3·97–15·1)	9·43 (4·3–15·6)	9·21 (4·16–15·4)	9·44 (4·32–15·6)
Multiple myeloma	10·2 (4·68–17)	10·8 (4·97–17·9)	10·6 (4·85–17·7)	10·8 (4·98–18)
Leukemia	38·4 (23·8–53)	37·2 (24·3–49·8)	39·1 (24·3–53·7)	37·1 (24·4–49·7)
Cardiovascular diseases	82·7 (79·4–85·7)	83·3 (80·6–85·7)	72 (67·6–76·3)	85 (82·6–87·2)
Rheumatic heart disease	22·1 (12·5–41·5)	22·1 (13·9–37·4)	22·1 (13·4–37·5)	22·1 (14–37·1)
Ischemic heart disease	93·3 (89·8–96·3)	94·8 (92·6–96·9)	93·2 (90–96)	94·8 (92·6–96·9)
Stroke	80·3 (73·8–87·9)	83·9 (79·4–88·7)	83 (78–88·3)	84·3 (79·7–89)
Hypertensive heart disease	100 (100–100)	100 (100–100)	100 (100–100)	100 (100–100)
Non-rheumatic valvular heart disease	25·2 (15·5–37·7)	21·8 (14·6–30·5)	6·04 (3·86–8·79)	25·6 (17·2–36)
Cardiomyopathy and myocarditis	37 (29·9–44·6)	39·4 (33·5–45·3)	39·7 (33·7–46·5)	39·4 (33·5–45·3)
Atrial fibrillation and flutter	51·8 (41·9–62·2)	58·2 (49·5–67)	59·5 (50·8–68·4)	56·2 (47·1–65·5)
Aortic aneurysm	56·8 (51·1–62·4)	64·9 (60·4–69·6)	--	64·9 (60·4–69·6)
Endocarditis	31·4 (21–44·1)	32·9 (24–43·7)	31 (20·8–43·8)	33 (24–43·7)
Other cardiovascular and circulatory diseases	23·3 (18·5–30·3)	25·9 (20·9–31·8)	31·6 (24·9–38·7)	24·1 (19·5–30·1)
Chronic respiratory diseases	68·3 (63·7–72·5)	63·6 (59·9–67·1)	56·4 (51·8–60·7)	69·2 (65·2–72·8)
Chronic obstructive pulmonary disease	75·5 (71·1–79·4)	74·2 (70·5–77·5)	68·1 (64·1–71·7)	77·5 (73·7–80·8)
Pneumoconiosis	100 (100–100)	100 (100–100)	100 (100–100)	100 (100–100)
Asthma	52·3 (41·4–63·2)	43·1 (35·3–51·4)	42·3 (34·4–50·4)	51·7 (42·5–61·4)

Digestive diseases	43<br>(37·8–48·2)	45·4<br>(40·2–50·7)	19<br>(13·3–25·6)	53·5<br>(47·7–58·9)
Cirrhosis and other chronic liver diseases	79·8<br>(71·3–87·3)	82·4<br>(74·8–89·3)	77·8<br>(69·1–85·7)	82·5<br>(74·8–89·3)
Upper digestive system diseases	23·7<br>(19·9–27·8)	7·25<br>(4·51–10·7)	1·91<br>(1·15–2·88)	27·5<br>(23·4–31·7)
Gallbladder and biliary diseases	50·7<br>(35·2–65·6)	56·4<br>(42·5–69·4)	57·9<br>(44·1–70·4)	53·4<br>(38·5–67·3)
Pancreatitis	33·3<br>(17·3–49·1)	38·3<br>(21·1–55·1)	35·8<br>(19·2–52)	38·8<br>(21·4–55·6)
Neurological disorders	27·4<br>(12·1–43)	17·5<br>(9·01–30·1)	12·6<br>(6·46–23·4)	23·6<br>(9·56–39·2)
Alzheimer's disease and other dementias	42·2<br>(27·9–57·6)	45·9<br>(31·6–60·8)	46·8<br>(32·8–61·6)	45·2<br>(30·9–60·1)
Parkinson's disease	-10·8<br>(-16·8–-5·42)	-12·7<br>(-19·4–-6·55)	-13·3<br>(-19·9–-7·04)	-12·6<br>(-19·2–-6·46)
Idiopathic epilepsy	22·3<br>(14·2–30·4)	20·6<br>(13·3–28·1)	19·2<br>(12·3–26·1)	23·3<br>(15·1–31·3)
Multiple sclerosis	17·1<br>(12·4–22·2)	18·4<br>(13·6–23·6)	18<br>(13·4–22·9)	18·7<br>(13·6–24)
Mental disorders	--	7·95<br>(4·16–12·7)	7·95<br>(4·17–12·7)	--
Depressive disorders	--	17·3<br>(8·53–26·3)	17·3<br>(8·53–26·3)	--
Anxiety disorders	--	6·19<br>(1·42–14·2)	6·19<br>(1·42–14·2)	--
Idiopathic developmental intellectual disability	--	48·2<br>(24·7–67·1)	48·2<br>(24·7–67·1)	--
Substance use disorders	100<br>(100–100)	100<br>(100–100)	100<br>(100–100)	100<br>(100–100)
Alcohol use disorders	100<br>(100–100)	100<br>(100–100)	100<br>(100–100)	100<br>(100–100)
Drug use disorders	100<br>(100–100)	100<br>(100–100)	100<br>(100–100)	100<br>(100–100)
Diabetes and kidney diseases	99·9<br>(99·8–99·9)	99·9<br>(99·9–100)	100<br>(100–100)	99·9<br>(99·9–99·9)
Diabetes mellitus	100<br>(100–100)	100<br>(100–100)	100<br>(100–100)	100<br>(100–100)
Chronic kidney disease	100<br>(100–100)	100<br>(100–100)	100<br>(100–100)	100<br>(100–100)
Sense organ diseases	--	10·5<br>(9·37–11·8)	10·5<br>(9·37–11·8)	--
Blindness and vision loss	--	10·2<br>(6·62–14·9)	10·2<br>(6·62–14·9)	--
Age-related and other hearing loss	--	11·2<br>(10·2–12·5)	11·2<br>(10·2–12·5)	--
Musculoskeletal disorders	3·99<br>(1·23–7·24)	21·3<br>(17·9–24·9)	21·6<br>(18·1–25·4)	3·87<br>(1·22–6·99)
Rheumatoid arthritis	13·9<br>(4·42–24·7)	17·2<br>(5·56–30·5)	17·5<br>(5·66–30·7)	16·2<br>(5·16–28·6)
Osteoarthritis	--	15·8<br>(8·89–23)	15·8<br>(8·89–23)	--
Low back pain	--	43·6<br>(37·4–49·9)	43·6<br>(37·4–49·9)	--
Gout	--	61·3<br>(45·8–76·5)	61·3<br>(45·8–76·5)	--
Other non-communicable diseases	0·111<br>(0·0798–0·158)	0·14<br>(0·0949–0·198)	--	0·356<br>(0·259–0·488)
Sudden infant death syndrome	5·81<br>(4·08–8·11)	5·81<br>(4·08–8·1)	--	5·81<br>(4·08–8·1)
Injuries	28·2<br>(23·9–32·5)	26<br>(22–29·9)	32·4<br>(28·7–36·1)	22·7<br>(17·8–27·7)
Transport injuries	25·4<br>(20–31·2)	25·2<br>(20–31)	29·8<br>(26·2–34)	23·6<br>(17·7–30·1)
Road injuries	24·8<br>(19·4–30·8)	24·2<br>(18·9–30·1)	27·6<br>(24–31·9)	23·2<br>(17·2–29·6)
Other transport injuries	34·6<br>(29·9–40·2)	35·8<br>(31·5–40·8)	40<br>(35·8–43·6)	31·4<br>(26–37·7)

Unintentional injuries	35·4 (30·8–39·3)	28·6 (24·9–32·8)	33·2 (29·2–37·2)	21·7 (18·2–25·6)
Falls	55·2 (48·7–59·6)	45·5 (40·1–49·8)	44·6 (39·4–48·9)	47·6 (42–52·3)
Drowning	-9·51 (-17·6–-1·57)	-9·69 (-17·2–-1·99)	10·4 (6·75–15·3)	-10·3 (-18–-2·41)
Fire, heat, and hot substances	11·1 (7·35–15·7)	13·7 (9·59–19·1)	14·9 (10·2–20·7)	12·5 (8·73–17·4)
Poisonings	16·7 (11·8–22·9)	16·5 (11·8–22·4)	13·5 (9·47–18·7)	17·9 (12·9–24·2)
Exposure to mechanical forces	11·5 (5·5–18·3)	19·5 (15·8–24·3)	23·5 (20·4–27·2)	8·86 (2·26–16·5)
Animal contact	24·3 (20·1–29·6)	24·9 (20·7–30)	25·8 (21·4–31·1)	21·4 (17·1–26·7)
Foreign body	2·73 (1·8–3·99)	3·86 (2·62–5·47)	5·03 (3·48–7·06)	3·47 (2·28–5·01)
Environmental heat and cold exposure	6·92 (3·12–12·2)	7·51 (3·45–13·2)	7·04 (3·16–12·5)	7·85 (3·53–13·6)
Exposure to forces of nature	6·77 (3·08–12)	6·74 (3·06–11·9)	7·82 (3·53–13·8)	6·5 (2·96–11·4)
Other unintentional injuries	18·7 (13·3–25·3)	14·8 (10·2–20·5)	13·9 (9·58–19·4)	21·4 (15·4–28·7)
Self-harm and interpersonal violence	22·1 (13·3–31·1)	23·3 (15·1–31·7)	31·3 (24·9–37·7)	22·6 (14·1–31·3)
Self-harm	22·6 (10·7–35·1)	24·7 (12·4–37·6)	23·4 (13·5–34·4)	24·7 (12·4–37·6)
Interpersonal violence	21·5 (13·7–30)	21·7 (14·2–29·4)	33·1 (25·9–40·6)	19·5 (11·9–27·6)

Supplementary Results Table 2. All-age PAFs attributable to the joint distribution of all risk factors for all ages, both sexes combined for each GBD level 1, level 2, and level 3 cause and all causes in 2019 for Montana. Results are shown for deaths, YLLs, YLDs, and DALYs Data in parenthesis are 95% uncertainty intervals. PAFs=population attributable fractions. GBD=Global Burden of Disease. YLLs=years of life lost. YLDs=years lived with disability. DALYs=disability-adjusted life-years

	Deaths 2019	DALYs 2019	YLDs 2019	YLLs 2019
All causes	58·2 (55·3–60·8)	45·5 (42·6–48·4)	33·1 (30·6–35·5)	56·2 (54–58·5)
Communicable, maternal, neonatal, and nutritional diseases	43·4 (36·1–50·1)	50·8 (46·8–54·5)	46·1 (41·5–50·7)	53·9 (48–59·2)
HIV/AIDS and sexually transmitted infections	79·1 (67·2–84·5)	81·5 (70·5–87·3)	88·6 (80·2–94)	74·9 (61·5–81·4)
HIV/AIDS	77·1 (63·9–83)	74·7 (60·8–81·3)	76·7 (62·3–83)	73·8 (59·7–80·6)
Sexually transmitted infections excluding HIV	99·2 (98·7–99·5)	99·7 (99·4–99·9)	100 (100–100)	96·6 (95–97·8)
Respiratory infections and tuberculosis	41 (30·8–50·4)	35·2 (27–42·9)	2·14 (1·59–2·82)	45·6 (35·9–54·5)
Tuberculosis	54·5 (44·4–63·6)	57·6 (48–66·1)	55·1 (45–64·5)	58·1 (48·5–66·4)
Lower respiratory infections	41 (30·7–50·5)	45·6 (35·9–54·5)	32·4 (26·5–38·3)	45·8 (36–54·8)
Upper respiratory infections	0·47 (0·277–0·766)	0·0351 (0·0169–0·0614)	0·00476 (0–0·0138)	1·08 (0·642–1·7)
Otitis media	1·28 (0·548–2·77)	3·44 (2·21–4·73)	3·43 (2·19–4·72)	3·72 (1·74–6·98)
Enteric infections	10·7 (6·65–16·2)	12·1 (8·19–17·5)	11·7 (7·62–17·2)	12·8 (8·86–18)
Diarrheal diseases	10·8 (6·73–16·4)	12·2 (8·26–17·7)	11·7 (7·63–17·2)	13·1 (9·06–18·5)
Other infectious diseases	0·596 (0·412–0·848)	1·04 (0·776–1·34)	0·682 (0·42–1·09)	1·12 (0·798–1·48)
Meningitis	1·06 (0·739–1·57)	2·36 (1·65–3·42)	0·00043 (0–0·00142)	2·5 (1·76–3·6)
Encephalitis	0·451 (0·307–0·646)	1·24 (0·854–1·71)	0·000147 (0–0·000474)	1·28 (0·885–1·79)
Measles	9·45 (-3·84–43·5)	9·62 (-3·9–44·2)	0·177 (0–0·831)	9·62 (-3·9–44·2)
Acute hepatitis	18·6 (12·4–31·4)	9 (5·88–13·9)	3·68 (2·25–5·95)	19·4 (12·8–32·3)
Maternal and neonatal disorders	78·1 (74–82)	76·8 (73·5–80)	70·5 (65·5–74·9)	81·3 (78–84·5)
Maternal disorders	11·5 (4·13–19)	11·3 (4·12–18·7)	11·2 (4·08–18·5)	11·3 (4·12–18·8)
Neonatal disorders	87·9 (85·9–89·8)	81·2 (78·1–84)	72·3 (67·1–77·1)	87·9 (85·9–89·8)
Nutritional deficiencies	94·2 (85·6–95·8)	89·7 (86·7–92·1)	88·3 (85–91·4)	93·9 (85·5–95·4)
Protein-energy malnutrition	100 (100–100)	100 (100–100)	100 (100–100)	100 (100–100)
Vitamin A deficiency	--	100 (100–100)	100 (100–100)	--
Dietary iron deficiency	--	100 (100–100)	100 (100–100)	--
Non-communicable diseases	61·7 (58·6–64·7)	48·3 (44·8–51·7)	32·7 (30–35·4)	62·7 (60·4–65·1)
Neoplasms	48 (44·5–52)	47 (43·8–50·6)	30 (26·3–34·1)	48·2 (45–51·9)
Lip and oral cavity cancer	66·4 (56·8–74·2)	69 (59·6–76·2)	68·5 (59–75·9)	69 (59·6–76·3)
Nasopharynx cancer	64·4 (52·8–74·4)	64·1 (52·6–73·6)	63·2 (51·8–72·7)	64·2 (52·7–73·6)
Other pharynx cancer	76·6 (68·9–83·1)	77·7 (69·8–83·8)	78 (70·4–84·1)	77·7 (69·8–83·8)

Esophageal cancer	79·4 (70·3–87·4)	80·5 (71·7–88·3)	79·7 (70·5–87·4)	80·5 (71·7–88·3)
Stomach cancer	25 (16·9–42·4)	25·4 (17·2–43·1)	25·7 (17·5–43·3)	25·4 (17·2–43·1)
Colon and rectum cancer	61·9 (53·3–70·6)	62·7 (54·4–70·8)	62·7 (54·6–71·1)	62·7 (54·5–70·9)
Liver cancer	79·6 (75·1–84·1)	79·1 (74·5–83·6)	79·4 (74·7–83·8)	79·1 (74·5–83·6)
Gallbladder and biliary tract cancer	27·1 (15·7–40·2)	28·2 (16·9–41·4)	26·1 (15·4–39)	28·3 (16·9–41·5)
Pancreatic cancer	42·6 (33·2–53·2)	42·4 (33·4–52·6)	42·7 (33·7–53·3)	42·4 (33·4–52·6)
Larynx cancer	81·7 (72·3–89)	82·7 (74·4–88·9)	82·5 (74·1–88·9)	82·7 (74·4–89)
Tracheal, bronchus, and lung cancer	83·3 (79·9–86·9)	82·5 (79·3–86)	83 (79·5–86·5)	82·5 (79·3–86)
Breast cancer	35·3 (26·3–45·1)	34·5 (26·2–43·5)	35·1 (26·4–44·4)	34·5 (26·2–43·4)
Cervical cancer	100 (100–100)	100 (100–100)	100 (100–100)	100 (100–100)
Uterine cancer	53 (37·5–67·3)	54·7 (39·5–68·3)	55·2 (40·2–68·6)	54·6 (39·4–68·2)
Ovarian cancer	20·2 (9·53–32·7)	17·7 (8·22–29·3)	17·1 (7·99–28·6)	17·7 (8·22–29·3)
Prostate cancer	5·86 (2·5–9·72)	6·37 (2·73–10·3)	6·63 (2·84–10·6)	6·29 (2·7–10·3)
Kidney cancer	42·2 (30·8–53·2)	42·6 (31·9–52·7)	42·8 (32·4–52·8)	42·6 (31·9–52·6)
Bladder cancer	39·8 (24·7–55·3)	43·2 (29–57·1)	43·9 (30–57·3)	43·1 (28·9–57·2)
Thyroid cancer	18·3 (9·35–28·5)	19·4 (10·1–29·9)	19·6 (10·3–30·1)	19·3 (9·93–29·9)
Mesothelioma	97·4 (96·4–98·2)	95·1 (93·4–96·5)	97 (95·8–98)	95 (93·4–96·5)
Non-Hodgkin lymphoma	8·78 (3·82–14·8)	9·22 (4·16–15·3)	9·05 (4·06–15·2)	9·23 (4·15–15·3)
Multiple myeloma	9·96 (4·63–16·9)	10·5 (4·92–17·6)	10·4 (4·74–17·8)	10·5 (4·9–17·6)
Leukemia	36·6 (22·4–51·1)	34·7 (22·8–47)	37·3 (23·1–51·5)	34·5 (22·6–46·7)
Cardiovascular diseases	79·1 (75·1–82·7)	80 (76·7–82·8)	70·8 (65·6–75·5)	81·5 (78·5–84·3)
Rheumatic heart disease	21·5 (12·2–40·3)	21·4 (13·2–36·7)	21·3 (12·8–37·3)	21·4 (13·3–36·8)
Ischemic heart disease	92·2 (88·4–95·6)	93·7 (90·9–96·2)	92·2 (88·6–95·5)	93·8 (91–96·3)
Stroke	78·5 (71·3–86·9)	81·8 (76·4–87·5)	80·9 (75·3–87)	82·2 (76·7–88)
Hypertensive heart disease	100 (100–100)	100 (100–100)	100 (100–100)	100 (100–100)
Non-rheumatic valvular heart disease	25·1 (15·5–38·6)	23·8 (15·8–34)	8·47 (5·5–12·2)	25·6 (17·2–36·6)
Cardiomyopathy and myocarditis	37·9 (31–45·8)	40·7 (34·9–47)	39·1 (32·6–45·7)	40·9 (34·9–47·2)
Atrial fibrillation and flutter	51·8 (41·3–62·2)	57·5 (48·6–66·2)	59 (50·2–67·5)	55·5 (46–64·8)
Aortic aneurysm	54·2 (48·1–60·4)	61·6 (56·7–66·5)	--	61·6 (56·7–66·5)
Endocarditis	30·3 (20·4–43·5)	31·8 (22·6–42·4)	30 (20·2–42·7)	31·9 (22·8–42·4)
Other cardiovascular and circulatory diseases	21·3 (15·9–29·2)	24 (18·7–30)	30·4 (23·7–37·2)	22·1 (17–28·8)
Chronic respiratory diseases	66·2 (61·1–71·2)	62·1 (58·3–66)	55·5 (51·2–59·8)	66·6 (62·2–71)

Chronic obstructive pulmonary disease	73·1 (68·4–77·8)	71·5 (67·6–75·3)	64·8 (60·7–69)	74·9 (70·8–79·1)
Pneumoconiosis	100 (100–100)	100 (100–100)	100 (100–100)	100 (100–100)
Asthma	50 (38·8–60·8)	43·6 (35·5–52·6)	42·8 (34·7–51·6)	48·9 (39·7–58·4)
Digestive diseases	45·3 (39·5–50·7)	48·9 (43–54·6)	17·1 (11·7–22·9)	56·4 (50·3–62)
Cirrhosis and other chronic liver diseases	80·8 (71·8–88·9)	83 (74·6–90·3)	79·3 (70·1–87·5)	83·1 (74·6–90·4)
Upper digestive system diseases	21 (17·5–24·7)	6·82 (4·24–10)	1·46 (0·834–2·36)	23·8 (20·1–27·6)
Gallbladder and biliary diseases	49·9 (34·6–64·9)	54·2 (39·7–67·7)	55·6 (41·4–68·3)	52·1 (37·1–66·4)
Pancreatitis	34·7 (17·2–52·6)	39·3 (21·2–57·1)	37·5 (19·5–56·3)	39·5 (21·5–57·3)
Neurological disorders	25·3 (10·5–40·2)	16·7 (8·68–28·7)	12·4 (6·38–22·6)	21·5 (8·66–36·6)
Alzheimer's disease and other dementias	41·2 (27·6–55·7)	44·3 (30·6–58·6)	45·1 (31·5–59·3)	43·8 (30–58·2)
Parkinson's disease	-9·89 (-15·8–-4·81)	-11·2 (-17·5–-5·67)	-11·8 (-18·1–-6·11)	-11·1 (-17·5–-5·6)
Idiopathic epilepsy	23·7 (14·9–32·4)	21·7 (13·8–29·8)	20·5 (13–28·2)	24·2 (15·4–33)
Multiple sclerosis	15·5 (11·3–20)	16·8 (12·4–21·5)	16·8 (12·4–21·3)	16·9 (12·5–21·7)
Mental disorders	--	9·19 (4·73–14·2)	9·2 (4·73–14·2)	--
Depressive disorders	--	19·2 (9·09–29·1)	19·2 (9·09–29·1)	--
Anxiety disorders	--	5·87 (1·33–13·7)	5·87 (1·33–13·7)	--
Idiopathic developmental intellectual disability	--	48·1 (23–68·9)	48·1 (23–68·9)	--
Substance use disorders	100 (100–100)	100 (100–100)	100 (100–100)	100 (100–100)
Alcohol use disorders	100 (100–100)	100 (100–100)	100 (100–100)	100 (100–100)
Drug use disorders	100 (100–100)	100 (100–100)	100 (100–100)	100 (100–100)
Diabetes and kidney diseases	99·9 (99·8–99·9)	99·9 (99·9–100)	100 (100–100)	99·9 (99·8–99·9)
Diabetes mellitus	100 (100–100)	100 (100–100)	100 (100–100)	100 (100–100)
Chronic kidney disease	100 (100–100)	100 (100–100)	100 (100–100)	100 (100–100)
Sense organ diseases	--	10·5 (9·4–11·7)	10·5 (9·4–11·7)	--
Blindness and vision loss	--	9·84 (6·4–14·3)	9·84 (6·4–14·3)	--
Age-related and other hearing loss	--	11·2 (10·2–12·5)	11·2 (10·2–12·5)	--
Musculoskeletal disorders	4·37 (1·26–7·98)	20·4 (17–23·8)	20·8 (17·4–24·3)	4·27 (1·27–7·84)
Rheumatoid arthritis	12·8 (3·83–22·9)	15·5 (4·76–27·6)	15·7 (4·88–28·1)	14·7 (4·4–26·1)
Osteoarthritis	--	13·4 (7·71–19·8)	13·4 (7·71–19·8)	--
Low back pain	--	42·3 (36·2–48·3)	42·3 (36·2–48·3)	--
Gout	--	60·4 (44·7–76)	60·4 (44·7–76)	--
Other non-communicable diseases	0·157 (0·107–0·23)	0·204 (0·135–0·295)	--	0·495 (0·349–0·696)

Sudden infant death syndrome	5·24 (3·64–7·3)	5·23 (3·64–7·3)	--	5·23 (3·64–7·3)
Injuries	25·2 (19·4–30·6)	22·6 (17·5–27·6)	32 (28·4–35·7)	17·8 (11–24·4)
Transport injuries	16·6 (9·4–23·8)	17·6 (11·5–24·4)	29·5 (25·7–33·4)	13·9 (6·26–21·8)
Road injuries	14·5 (6·94–22·2)	15·3 (8·73–22·3)	26·7 (23·1–30·9)	12 (3·83–20·2)
Other transport injuries	33·1 (28·5–38·5)	33·2 (28·9–38·3)	39·9 (35·8–43·2)	29·1 (24–35·3)
Unintentional injuries	37 (32–41·2)	28·4 (24·5–32·5)	33 (28·9–37·3)	21·8 (17·6–26·2)
Falls	55·7 (48·7–60·4)	45·5 (40·3–49·8)	44·6 (39·5–49·1)	47·1 (41·8–51·7)
Drowning	-15·6 (-29·7–-2·43)	-16·2 (-29·5–-3·8)	10·4 (6·21–16·1)	-16·9 (-30·8–-4·22)
Fire, heat, and hot substances	10·6 (6·46–16·2)	12·7 (8·26–18·8)	13·8 (8·89–20·3)	11·3 (7·27–16·7)
Poisonings	15·2 (9·94–22)	15 (9·97–21·4)	12·5 (8·05–18·2)	15·8 (10·6–22·5)
Exposure to mechanical forces	3·13 (-6·62–12·7)	15 (10–19·8)	21·9 (19·3–25·2)	-1·22 (-11·4–8·95)
Animal contact	23·3 (18·9–28·8)	23·4 (18·9–28·8)	25 (20·3–30·4)	20·3 (15·9–25·6)
Foreign body	2·28 (1·48–3·48)	3·35 (2·25–5·08)	3·25 (2·2–4·77)	3·39 (2·2–5·21)
Environmental heat and cold exposure	7·99 (3·32–14·2)	8·45 (3·59–14·8)	7·97 (3·29–14·1)	8·71 (3·71–15·3)
Exposure to forces of nature	7·44 (3·08–13·2)	8·19 (3·43–14·5)	8·54 (3·6–15·1)	6·97 (2·88–12·2)
Other unintentional injuries	16 (10·6–22·9)	13·8 (8·99–20·2)	13·2 (8·35–19·5)	17·9 (12·4–25·1)
Self-harm and interpersonal violence	17·2 (3·65–30·3)	19·3 (6·4–31·4)	28·8 (21·9–35·3)	18·7 (5·3–31·4)
Self-harm	17·2 (2·44–31·8)	19·4 (4·63–34·2)	24·2 (13·2–35·7)	19·3 (4·45–34·2)
Interpersonal violence	18·8 (7·87–29·9)	20·1 (10·9–29·5)	30·8 (23·6–38·1)	16·2 (5·27–27·3)

Supplementary Results Table 2. All-age PAFs attributable to the joint distribution of all risk factors for all ages, both sexes combined for each GBD level 1, level 2, and level 3 cause and all causes in 2019 for Nebraska. Results are shown for deaths, YLLs, YLDs, and DALYs Data in parenthesis are 95% uncertainty intervals. PAFs=population attributable fractions. GBD=Global Burden of Disease. YLLs=years of life lost. YLDs=years lived with disability. DALYs=disability-adjusted life-years

	Deaths 2019	DALYs 2019	YLDs 2019	YLLs 2019
All causes	58·2 (55·2–61)	44·4 (41·5–47·2)	32·1 (29·8–34·6)	56·1 (53·9–58·3)
Communicable, maternal, neonatal, and nutritional diseases	45·5 (40·4–50·3)	54·6 (51·2–58·2)	47 (42·3–51·7)	59·8 (55·6–64)
HIV/AIDS and sexually transmitted infections	82·8 (79·3–85·6)	83·7 (79·8–87·1)	89·5 (85·7–93·7)	79 (74·7–82·4)
HIV/AIDS	81·7 (78–84·7)	79·3 (75–82·8)	81 (76·3–84·6)	78·5 (74–82)
Sexually transmitted infections excluding HIV	99 (98·6–99·4)	99·6 (99·4–99·8)	100 (100–100)	96·1 (94·3–97·4)
Respiratory infections and tuberculosis	42·3 (35·4–48·6)	35·4 (28·9–41·8)	2·29 (1·72–2·97)	47·5 (40·8–53·7)
Tuberculosis	52·4 (42·3–61·6)	56·3 (47·1–65·1)	53·8 (43·3–62·8)	56·8 (47·8–65·5)
Lower respiratory infections	42·2 (35·3–48·6)	47·3 (40·6–53·5)	31·8 (26·1–36·8)	47·6 (40·8–53·9)
Upper respiratory infections	0·64 (0·354–1·08)	0·0225 (0·0104–0·041)	0·00596 (1·81e-05–0·0172)	1·41 (0·789–2·35)
Otitis media	1·66 (0·662–3·67)	4·08 (2·68–5·61)	4·08 (2·68–5·61)	4·52 (2·13–8·23)
Enteric infections	10·4 (6·55–16)	12 (8·28–17·5)	11·5 (7·85–17·1)	13·1 (9·11–18·5)
Diarrheal diseases	10·5 (6·59–16·1)	12 (8·31–17·6)	11·5 (7·86–17·1)	13·3 (9·25–18·8)
Other infectious diseases	0·524 (0·354–0·756)	1·01 (0·706–1·37)	0·497 (0·286–0·832)	1·13 (0·753–1·59)
Meningitis	1·47 (0·938–2·22)	3·21 (2·08–4·84)	0·000571 (1·65e-06–0·00185)	3·4 (2·19–5·16)
Encephalitis	0·597 (0·386–0·893)	1·61 (1·07–2·38)	0·000194 (4·67e-07–0·000597)	1·67 (1·11–2·46)
Measles	9·33 (-4·12–43·8)	9·47 (-4·19–44·6)	0·174 (0–0·819)	9·49 (-4·19–44·6)
Acute hepatitis	14·3 (8·76–26·1)	5·97 (3·74–9·82)	2·51 (1·51–4·11)	15·1 (9·14–27·4)
Maternal and neonatal disorders	83·7 (81·2–86)	80·9 (78·2–83·3)	72·9 (68·3–77)	85·7 (83·7–87·7)
Maternal disorders	11·3 (4·12–18·6)	11·1 (4·11–18·5)	10·9 (4·07–18·4)	11·1 (4·1–18·5)
Neonatal disorders	89·5 (87·8–91·2)	83·9 (81·4–86)	74·8 (70·2–79·1)	89·5 (87·8–91·3)
Nutritional deficiencies	92·1 (84–94·4)	88·3 (85·3–91)	87·4 (83·7–90·9)	91·6 (83·4–93·9)
Protein-energy malnutrition	100 (100–100)	100 (100–100)	100 (100–100)	100 (100–100)
Vitamin A deficiency	--	100 (100–100)	100 (100–100)	--
Dietary iron deficiency	--	100 (100–100)	100 (100–100)	--
Non-communicable diseases	60·9 (57·4–64)	45·9 (42·6–49·3)	31·4 (28·8–34)	60·7 (58·2–63·1)
Neoplasms	47·5 (43·6–51·8)	47 (43·4–51)	30·7 (26·8–34·9)	48·1 (44·6–52)
Lip and oral cavity cancer	64·2 (54–72·6)	67·7 (57·8–75·5)	67·1 (56·9–75·1)	67·8 (58–75·5)
Nasopharynx cancer	63·5 (52·3–72·8)	63·6 (52·8–72·6)	62·4 (51·4–71·8)	63·6 (52·9–72·6)
Other pharynx cancer	75·7 (68·5–81·8)	77·3 (70–83·2)	77·7 (70·4–83·4)	77·3 (70–83·1)
Esophageal cancer	78 (68·6–86·6)	79·7 (70·3–87·7)	78·5 (69·1–86·7)	79·7 (70·3–87·7)

Stomach cancer	23·6 (15·7–42·2)	24·7 (16·7–43·1)	24·8 (16·6–43)	24·7 (16·7–43·1)
Colon and rectum cancer	62·4 (53·8–71·3)	63·6 (55·5–71·9)	63·5 (55·3–71·8)	63·6 (55·5–71·9)
Liver cancer	75·6 (69·3–81·5)	75·8 (69·5–81·4)	75·7 (68·9–81·6)	75·8 (69·5–81·4)
Gallbladder and biliary tract cancer	27·6 (15·9–40·5)	28·7 (17–41·8)	26·6 (15–39·5)	28·8 (17·1–41·9)
Pancreatic cancer	42 (32·4–53·2)	42·2 (33·1–53)	42·3 (32·8–53·6)	42·2 (33·1–53)
Larynx cancer	80 (69·5–88)	81·5 (72·5–88·2)	81·2 (72–88·1)	81·5 (72·6–88·3)
Tracheal, bronchus, and lung cancer	82 (78·4–85·7)	81·6 (78·4–85·2)	81·9 (78·4–85·7)	81·6 (78·4–85·2)
Breast cancer	35·6 (26–46·4)	35 (26·4–44·9)	35·5 (26·5–45·8)	34·9 (26·4–44·8)
Cervical cancer	100 (100–100)	100 (100–100)	100 (100–100)	100 (100–100)
Uterine cancer	54·4 (39·2–67·6)	56·3 (41·4–69)	56·8 (41·7–69·6)	56·2 (41·1–68·9)
Ovarian cancer	20·3 (9·18–33·5)	18 (8·04–30·2)	17·4 (7·69–29·3)	18 (8·04–30·3)
Prostate cancer	5·54 (2·41–9·04)	6·28 (2·71–10)	6·68 (2·92–10·6)	6·16 (2·67–9·86)
Kidney cancer	42·4 (31–53·2)	43·2 (32·6–53·1)	43·4 (33–53·4)	43·2 (32·5–53·1)
Bladder cancer	37·5 (22·5–52·4)	41·6 (27·1–55·5)	42·3 (28–56·4)	41·5 (26·9–55·4)
Thyroid cancer	18·9 (9·65–29·5)	20·2 (10·5–31·4)	20·5 (10·9–31·5)	20·1 (10·4–31·2)
Mesothelioma	96·5 (95·3–97·5)	93·4 (91·2–95·1)	96·2 (94·8–97·4)	93·3 (91·1–95·1)
Non-Hodgkin lymphoma	9·03 (4·06–15·2)	9·54 (4·43–15·9)	9·31 (4·21–15·5)	9·55 (4·41–15·9)
Multiple myeloma	10·4 (4·81–17·5)	11 (5·16–18·3)	10·8 (5–18·3)	11 (5·17–18·3)
Leukemia	35·6 (22·3–49·7)	34·2 (22·7–46·3)	36·4 (22·8–50)	34·1 (22·6–46·1)
Cardiovascular diseases	79·1 (75–82·6)	79·6 (76·5–82·4)	70·9 (66·3–75·5)	81·1 (78·3–83·8)
Rheumatic heart disease	22 (12·2–42·9)	21·9 (13·6–37·9)	21·7 (13·2–37·9)	21·9 (13·7–38)
Ischemic heart disease	92·3 (88–95·8)	93·8 (90·9–96·3)	92·5 (89·1–95·5)	93·9 (91–96·3)
Stroke	79 (71·8–87)	82·4 (77·2–88)	81·4 (75·9–87·5)	82·8 (77·6–88·2)
Hypertensive heart disease	100 (100–100)	100 (100–100)	100 (100–100)	100 (100–100)
Non-rheumatic valvular heart disease	25·6 (15·1–39·8)	24·4 (16–34·4)	9·94 (6·43–14·8)	25·9 (17–36·6)
Cardiomyopathy and myocarditis	36·6 (29·2–45·3)	39·4 (33·9–44·9)	38·2 (31·9–44·6)	39·5 (33·9–45·2)
Atrial fibrillation and flutter	51·6 (41·1–62·2)	57·5 (48·6–66·4)	59·1 (50·5–67·6)	55·3 (45·5–64·5)
Aortic aneurysm	52·5 (46·4–59)	60·9 (55·9–65·6)	--	60·9 (55·9–65·6)
Endocarditis	30·8 (20·2–43·3)	32·2 (23·1–42·8)	30·6 (20·3–43·5)	32·3 (23·1–43)
Other cardiovascular and circulatory diseases	21·2 (16–29·5)	24·4 (19·5–30·3)	30·7 (23·9–37·7)	22·1 (17·5–28·4)
Chronic respiratory diseases	64·8 (59·8–69·7)	60·7 (57–64·4)	53·9 (49·6–58·1)	66 (61·9–70·3)
Chronic obstructive pulmonary disease	72·2 (67·6–76·5)	70·5 (66·7–74·2)	63·2 (59·2–67·1)	74·7 (70·6–78·5)
Pneumoconiosis	100 (100–100)	100 (100–100)	100 (100–100)	100 (100–100)

Asthma	50·1 (38·8–61·6)	43·1 (35·1–51·7)	42 (34–50·6)	50·5 (41·2–60·6)
Digestive diseases	38·9 (33·5–44·6)	42·8 (37·2–48·5)	18 (12·3–23·9)	50·6 (44·3–56·6)
Cirrhosis and other chronic liver diseases	78·3 (67·6–87·4)	81·4 (71·9–89·1)	76·9 (66–86·2)	81·4 (71·9–89·2)
Upper digestive system diseases	18·5 (15·1–21·8)	6·03 (3·66–8·82)	1·39 (0·837–2·18)	22·6 (18·8–26·1)
Gallbladder and biliary diseases	50·9 (35–65·8)	55·6 (41·1–68·5)	56·8 (42·7–69·4)	53·1 (37·8–67·3)
Pancreatitis	34·9 (17·4–52·8)	40·1 (21·3–57·8)	38·1 (20·1–55·9)	40·4 (21·5–58·2)
Neurological disorders	24·9 (10·1–40·2)	15·6 (7·7–27·4)	11·2 (5·5–22·1)	21·2 (8·26–36·4)
Alzheimer's disease and other dementias	39·4 (25·2–54·6)	42·8 (28·7–57·7)	43·7 (29·4–58·4)	42·3 (28·2–57·3)
Parkinson's disease	-8·25 (-13·4–-4·03)	-9·96 (-15·5–-5·07)	-10·5 (-16–-5·44)	-9·87 (-15·4–-5·02)
Idiopathic epilepsy	23·2 (14·6–31·5)	21 (13·1–29)	19·7 (12·3–27·4)	23·9 (15·6–32)
Multiple sclerosis	14·1 (10·2–18·3)	15·6 (11·4–20·1)	15·5 (11·5–19·9)	15·7 (11·3–20·5)
Mental disorders	--	8·55 (4·3–13·5)	8·56 (4·3–13·5)	--
Depressive disorders	--	18·5 (8·7–28·1)	18·5 (8·7–28·1)	--
Anxiety disorders	--	6·45 (1·48–14·6)	6·45 (1·48–14·6)	--
Idiopathic developmental intellectual disability	--	45·4 (22·2–64·2)	45·4 (22·2–64·2)	--
Substance use disorders	100 (100–100)	100 (100–100)	100 (100–100)	100 (100–100)
Alcohol use disorders	100 (100–100)	100 (100–100)	100 (100–100)	100 (100–100)
Drug use disorders	100 (100–100)	100 (100–100)	100 (100–100)	100 (100–100)
Diabetes and kidney diseases	99·9 (99·9–99·9)	100 (99·9–100)	100 (100–100)	99·9 (99·9–99·9)
Diabetes mellitus	100 (100–100)	100 (100–100)	100 (100–100)	100 (100–100)
Chronic kidney disease	100 (100–100)	100 (100–100)	100 (100–100)	100 (100–100)
Sense organ diseases	--	10·9 (9·76–12·2)	10·9 (9·76–12·2)	--
Blindness and vision loss	--	9·49 (6·01–13·9)	9·49 (6·01–13·9)	--
Age-related and other hearing loss	--	11·8 (10·7–13·1)	11·8 (10·7–13·1)	--
Musculoskeletal disorders	2·93 (0·838–5·28)	22·7 (19·2–26·6)	23·1 (19·4–27)	3·05 (0·904–5·52)
Rheumatoid arthritis	10·8 (3·13–19·5)	14·3 (4·48–25·6)	14·6 (4·6–26·1)	13·1 (3·97–23·6)
Osteoarthritis	--	16·3 (9·44–23·8)	16·3 (9·44–23·8)	--
Low back pain	--	42·6 (36·7–48·2)	42·6 (36·7–48·2)	--
Gout	--	61·5 (45·2–77·4)	61·5 (45·2–77·4)	--
Other non-communicable diseases	0·177 (0·104–0·286)	0·229 (0·13–0·375)	--	0·537 (0·32–0·838)
Sudden infant death syndrome	5·11 (3·08–7·54)	5·1 (3·08–7·53)	--	5·1 (3·08–7·53)
Injuries	28·5 (23·7–33·1)	26·2 (21·7–30·6)	33·4 (29·3–37·4)	21·5 (16·1–26·8)
Transport injuries	23·5 (16·8–30·8)	24·3 (18·1–31·5)	31·4 (27·3–36·3)	21·5 (14–29·5)

Road injuries	22·5 (15·8–30)	22·8 (16·2–30·2)	29·3 (25·1–34·4)	20·6 (13–28·8)
Other transport injuries	36·3 (30·8–42·7)	36·8 (32–42·4)	40·7 (36·1–44·8)	32·8 (26·9–40)
Unintentional injuries	37 (31·7–41·2)	30·3 (26·1–34·6)	34·3 (29·9–38·7)	23·6 (19·4–28·1)
Falls	55·9 (48·9–60·8)	46·4 (40·8–50·9)	45·6 (40·2–50·1)	48·2 (42·4–53·1)
Drowning	-10·9 (-20·6–-0·785)	-11·3 (-20·5–-1·95)	11·8 (7·78–17·1)	-12·3 (-21·7–-2·57)
Fire, heat, and hot substances	11·9 (7·87–16·9)	15·4 (10·8–21·3)	16·8 (11·7–23·4)	13·2 (9·15–18·4)
Poisonings	18·3 (13·1–24·8)	18·1 (13–24·2)	15·1 (10·6–20·7)	19·4 (14–25·9)
Exposure to mechanical forces	8·97 (0·468–18·2)	19·7 (14·9–25·3)	24·5 (20·9–29·1)	5·5 (-3·67–15·6)
Animal contact	26·3 (21·5–32·1)	26·1 (21·4–31·2)	26·8 (22–32·3)	23·2 (18·5–29·1)
Foreign body	3·16 (2·11–4·72)	4·84 (3·28–7·09)	5·03 (3·43–7·45)	4·78 (3·21–7·08)
Environmental heat and cold exposure	7·58 (3·32–13·3)	8·13 (3·72–14·2)	7·83 (3·48–13·9)	8·49 (3·88–14·9)
Exposure to forces of nature	7·4 (3·33–13·1)	7·16 (3·25–12·5)	8·53 (3·89–15)	7 (3·17–12·3)
Other unintentional injuries	20·7 (14·6–28·4)	17 (11·9–23·3)	16·1 (11·3–22·5)	23·3 (16·6–31·8)
Self-harm and interpersonal violence	19·3 (8·27–30·1)	20·7 (10·2–30·7)	28·2 (21·1–35·2)	19·9 (8·95–30·5)
Self-harm	19·6 (6·15–32·8)	21·1 (7·66–34·5)	23·1 (11·6–34·9)	21·1 (7·57–34·5)
Interpersonal violence	18·9 (10·4–27·6)	20·1 (12–28·3)	29·7 (22·1–37·3)	16·8 (8·3–25·3)

Supplementary Results Table 2. All-age PAFs attributable to the joint distribution of all risk factors for all ages, both sexes combined for each GBD level 1, level 2, and level 3 cause and all causes in 2019 for Nevada. Results are shown for deaths, YLLs, YLDs, and DALYs Data in parenthesis are 95% uncertainty intervals. PAFs=population attributable fractions. GBD=Global Burden of Disease. YLLs=years of life lost. YLDs=years lived with disability. DALYs=disability-adjusted life-years

	Deaths 2019	DALYs 2019	YLDs 2019	YLLs 2019
All causes	63·6 (61·4–65·8)	48·7 (45·5–51·7)	34·1 (31·3–36·7)	62 (60·2–64)
Communicable, maternal, neonatal, and nutritional diseases	50·1 (45·4–54·4)	56·2 (52·6–59·6)	49·9 (45·6–54·8)	60·1 (55·9–64·5)
HIV/AIDS and sexually transmitted infections	84·6 (69–90·8)	84 (69·4–90·5)	88·1 (75·7–93·6)	81·5 (64·8–88·8)
HIV/AIDS	84·2 (68·1–90·5)	82 (65·2–89·2)	83·6 (68·3–90·5)	81·3 (64·2–88·7)
Sexually transmitted infections excluding HIV	98·7 (98–99·1)	99·5 (99·1–99·8)	100 (100–100)	95·2 (92·9–96·8)
Respiratory infections and tuberculosis	48·6 (42·3–54·3)	40·6 (34·1–46·9)	2·4 (1·77–3·12)	51·7 (45·5–57·2)
Tuberculosis	57·9 (48·3–66·6)	59·1 (49·7–67·2)	54·3 (44·1–63·5)	60·1 (51–68)
Lower respiratory infections	48·6 (42·2–54·3)	51·5 (45·3–57)	34·2 (28·1–39·8)	51·7 (45·4–57·2)
Upper respiratory infections	0·519 (0·305–0·832)	0·0161 (0·00709–0·0306)	0·00503 (2·51e-05–0·0148)	1·09 (0·635–1·77)
Otitis media	1·58 (0·676–3·28)	3·59 (2·31–4·91)	3·58 (2·3–4·93)	4·05 (1·97–7·23)
Enteric infections	12·1 (7·54–18·5)	13·6 (9·18–20)	13·2 (8·71–19·4)	14·3 (9·87–20·7)
Diarrheal diseases	12·2 (7·59–18·6)	13·7 (9·24–20·1)	13·2 (8·72–19·5)	14·4 (10–20·9)
Other infectious diseases	0·782 (0·489–1·05)	1·21 (0·834–1·56)	0·736 (0·447–1·12)	1·34 (0·873–1·75)
Meningitis	1·12 (0·781–1·56)	2·34 (1·65–3·21)	0·000451 (1·11e-06–0·00135)	2·5 (1·76–3·44)
Encephalitis	0·549 (0·375–0·79)	1·4 (0·981–1·96)	0·000149 (4·52e-07–0·000467)	1·45 (1·01–2·02)
Measles	9·62 (-3·99–44·1)	6·93 (-2·95–42·2)	0·359 (0–1·03)	9·78 (-4·06–44·8)
Acute hepatitis	21·9 (14·5–35·4)	9·86 (6·28–14·8)	3·85 (2·39–5·86)	22·8 (15·1–36)
Maternal and neonatal disorders	84 (81·2–86·6)	81·6 (78·9–84·1)	75·7 (71·6–79·6)	85·5 (83·2–87·8)
Maternal disorders	11·7 (4·18–19·4)	11·4 (4·05–19·3)	11·3 (3·94–19·2)	11·5 (4·1–19·3)
Neonatal disorders	88·3 (86·3–90·3)	84 (81·5–86·3)	77·6 (73·5–81·4)	88·3 (86·4–90·3)
Nutritional deficiencies	95·4 (88·4–96·6)	89·5 (86·7–92)	88·3 (85–91·4)	94·6 (87·6–95·8)
Protein-energy malnutrition	100 (100–100)	100 (100–100)	100 (100–100)	100 (100–100)
Vitamin A deficiency	--	100 (100–100)	100 (100–100)	--
Dietary iron deficiency	--	100 (100–100)	100 (100–100)	--
Non-communicable diseases	67·2 (64·7–69·6)	50·8 (47·2–54·4)	33·6 (30·5–36·5)	67·9 (65·9–69·9)
Neoplasms	50·8 (47·3–54·7)	48·8 (45·4–52·5)	30·8 (27–35)	50 (46·7–53·7)
Lip and oral cavity cancer	69 (60·6–76·6)	70 (61·8–77·4)	69·7 (61·4–77·2)	70 (61·8–77·4)
Nasopharynx cancer	65 (53·9–74·4)	63·9 (52·9–73·2)	63·1 (52–72·8)	64 (52·9–73·2)
Other pharynx cancer	78·1 (71·6–83·9)	78·4 (71·6–84·1)	78·7 (72–84·3)	78·3 (71·5–84)
Esophageal cancer	79·4 (70·6–87·3)	79·7 (70·8–87·8)	79·3 (70·4–86·9)	79·7 (70·8–87·8)

Stomach cancer	27·5 (19·3–45·1)	27·1 (18·9–44·8)	27·7 (19·3–45·4)	27 (18·9–44·8)
Colon and rectum cancer	62·6 (53·6–71·2)	62·9 (54·6–71·1)	62·9 (54·2–71·2)	62·9 (54·5–71·1)
Liver cancer	80·3 (76·1–84·7)	79·4 (75–83·8)	79·8 (75·3–84·2)	79·4 (75–83·8)
Gallbladder and biliary tract cancer	24·2 (13·6–36·6)	25·1 (14·3–37·8)	23·2 (12·4–35·8)	25·2 (14·3–37·9)
Pancreatic cancer	45·4 (36·4–56)	44·3 (35·7–54·7)	45 (36·2–55·3)	44·3 (35·7–54·6)
Larynx cancer	83·3 (74·4–89·9)	83·5 (75·7–89·4)	83·1 (75·1–89·1)	83·5 (75·8–89·5)
Tracheal, bronchus, and lung cancer	85·1 (82·3–88·1)	83·8 (81·1–86·9)	84·5 (81·5–87·7)	83·8 (81·1–86·9)
Breast cancer	35·4 (26·5–45·7)	34 (25·7–43·5)	34·7 (26·2–44·5)	33·9 (25·6–43·4)
Cervical cancer	100 (100–100)	100 (100–100)	100 (100–100)	100 (100–100)
Uterine cancer	51·5 (35·7–65·6)	52·9 (37·1–66·8)	53·3 (37·8–67·1)	52·8 (36·9–66·7)
Ovarian cancer	19·1 (8·64–32)	16·9 (7·2–29·1)	16·3 (7·01–28·2)	16·9 (7·21–29·2)
Prostate cancer	6·68 (2·9–10·9)	7·23 (3·19–11·5)	7·5 (3·34–11·9)	7·15 (3·15–11·5)
Kidney cancer	42·5 (31·5–52·8)	42·4 (31·8–52·5)	42·5 (32–52·2)	42·4 (31·8–52·4)
Bladder cancer	44·3 (28·6–59·3)	46·6 (31·7–60)	47 (32·2–60·1)	46·6 (31·6–60)
Thyroid cancer	17·2 (8·59–27·4)	18·2 (9·25–28·8)	18·4 (9·6–28·9)	18·1 (9·12–28·8)
Mesothelioma	95·9 (94·5–97)	92·3 (90·1–94·2)	95·5 (93·9–96·8)	92·3 (90–94·2)
Non-Hodgkin lymphoma	8·2 (3·67–14)	8·61 (3·81–14·5)	8·39 (3·71–14·3)	8·62 (3·82–14·5)
Multiple myeloma	9·25 (4·18–15·9)	9·73 (4·51–16·7)	9·6 (4·24–16·4)	9·73 (4·51–16·7)
Leukemia	38·7 (23·4–53·3)	35·8 (23·5–48·1)	38·6 (24–53)	35·7 (23·5–48)
Cardiovascular diseases	84·1 (81·2–86·7)	84 (81·6–86·3)	72·2 (68·1–76·3)	85·7 (83·5–87·7)
Rheumatic heart disease	21·5 (12·8–38·5)	21·4 (13·7–34·3)	21·3 (13·1–35·9)	21·4 (13·9–33·6)
Ischemic heart disease	93·1 (89·9–96)	94·5 (92·1–96·6)	93·4 (90·4–96)	94·5 (92·1–96·6)
Stroke	81·2 (74·9–88)	84·3 (80–88·7)	82·9 (78·1–87·9)	84·8 (80·6–89)
Hypertensive heart disease	100 (100–100)	100 (100–100)	100 (100–100)	100 (100–100)
Non-rheumatic valvular heart disease	25·5 (16·5–38)	21·6 (14·8–30·5)	6·24 (4·01–8·84)	25·8 (17·7–36·1)
Cardiomyopathy and myocarditis	44·8 (38·1–51·5)	47·3 (41·6–53·1)	42·6 (36·4–49)	47·8 (42–53·7)
Atrial fibrillation and flutter	52·5 (43·3–62·4)	58·3 (50·2–67)	59·5 (51·3–68·1)	56·5 (48·2–65·5)
Aortic aneurysm	59 (53·5–64·5)	65·7 (61·3–69·7)	--	65·7 (61·3–69·7)
Endocarditis	31·6 (22·4–43·5)	32·9 (23·8–43·3)	31 (21·2–42·9)	32·9 (23·7–43·4)
Other cardiovascular and circulatory diseases	23·5 (19·1–29·8)	25·1 (20·3–30·5)	29·9 (22·8–37·1)	23·8 (19·5–29·3)
Chronic respiratory diseases	72·2 (68·3–75·5)	64 (60·5–67·4)	55·2 (50·3–59·9)	71·3 (68·2–74·5)
Chronic obstructive pulmonary disease	78·2 (74·1–81·7)	75·3 (72–78·4)	68·3 (64·6–71·7)	79 (75·7–82)
Pneumoconiosis	100 (100–100)	100 (100–100)	100 (100–100)	100 (100–100)
Asthma	50 (39·4–60·7)	41·2 (33·1–50·1)	40·6 (32·8–49·6)	47 (37·9–56·9)

Digestive diseases	52·3 (46·5–57·7)	52·1 (46·1–57·8)	18·1 (12·4–24·6)	60·7 (54·3–66·5)
Cirrhosis and other chronic liver diseases	81·4 (72·7–89)	83 (75·3–90)	79·1 (69·4–87)	83 (75·4–90)
Upper digestive system diseases	26·1 (22·1–30)	7·04 (4·32–10·4)	1·7 (1·02–2·56)	27·6 (23·4–31·5)
Gallbladder and biliary diseases	49 (34·1–63·8)	53·7 (39·7–67·1)	54·8 (40·8–67·8)	51·1 (36·5–65)
Pancreatitis	36·1 (18·5–54·3)	39·2 (20·8–57·1)	36·6 (19·2–55)	39·6 (21–57·5)
Neurological disorders	25·7 (9·45–42·6)	14·1 (6·6–26·3)	9·48 (4·51–19·5)	21·9 (7·64–39·4)
Alzheimer's disease and other dementias	46 (32·5–60·4)	48·7 (35·4–62·6)	49·4 (36–63·3)	48·3 (35–62·2)
Parkinson's disease	-12·7 (-19·6–-6·3)	-14·1 (-21·6–-7·28)	-14·7 (-22·2–-7·65)	-14 (-21·4–-7·22)
Idiopathic epilepsy	22·2 (13·5–31)	20·2 (12·5–28·2)	19·5 (11·9–27·1)	22·3 (13·9–30·8)
Multiple sclerosis	16·9 (12·2–22·1)	16·6 (12–21·4)	15·7 (11·4–20·4)	17·5 (12·6–22·7)
Mental disorders	--	9·86 (5·05–15·4)	9·87 (5·05–15·4)	--
Depressive disorders	--	20 (9·73–29·9)	20 (9·73–29·9)	--
Anxiety disorders	--	6·38 (1·42–14·6)	6·38 (1·42–14·6)	--
Idiopathic developmental intellectual disability	--	46·9 (24–64)	46·9 (24–64)	--
Substance use disorders	100 (100–100)	100 (100–100)	100 (100–100)	100 (100–100)
Alcohol use disorders	100 (100–100)	100 (100–100)	100 (100–100)	100 (100–100)
Drug use disorders	100 (100–100)	100 (100–100)	100 (100–100)	100 (100–100)
Diabetes and kidney diseases	99·8 (99·7–99·8)	99·9 (99·9–99·9)	100 (100–100)	99·8 (99·8–99·8)
Diabetes mellitus	100 (100–100)	100 (100–100)	100 (100–100)	100 (100–100)
Chronic kidney disease	100 (100–100)	100 (100–100)	100 (100–100)	100 (100–100)
Sense organ diseases	--	10·7 (9·54–11·9)	10·7 (9·54–11·9)	--
Blindness and vision loss	--	9·45 (6·26–13·7)	9·45 (6·26–13·7)	--
Age-related and other hearing loss	--	11·7 (10·6–13)	11·7 (10·6–13)	--
Musculoskeletal disorders	4·36 (1·35–7·88)	21 (17·5–24·7)	21·2 (17·6–25)	3·75 (1·18–6·73)
Rheumatoid arthritis	15·6 (4·89–27·8)	16·6 (5·23–29·4)	16·6 (5·25–29·3)	16·8 (5·33–29·9)
Osteoarthritis	--	14·2 (7·86–21·3)	14·2 (7·86–21·3)	--
Low back pain	--	41·8 (35·7–48)	41·8 (35·7–48)	--
Gout	--	57·4 (40·7–74·3)	57·4 (40·7–74·3)	--
Other non-communicable diseases	0·106 (0·0681–0·158)	0·105 (0·0652–0·161)	--	0·307 (0·2–0·457)
Sudden infant death syndrome	5·66 (3·75–8·35)	5·65 (3·75–8·34)	--	5·65 (3·75–8·34)
Injuries	25·5 (20·2–30·7)	25·8 (21·1–30·3)	32·5 (29·2–36·4)	22·4 (16·6–27·9)
Transport injuries	26·2 (20·1–32·5)	27·2 (21·5–33·5)	32·3 (28·1–36·7)	25 (18·2–32·2)
Road injuries	25·2 (19–31·7)	25·9 (19·9–32·4)	30·3 (26–35)	24·2 (17·2–31·4)
Other transport injuries	37·8 (32·3–43·9)	37·7 (33·3–42·7)	39·9 (35·9–43·5)	35·1 (28·7–42·1)

Unintentional injuries	32·2 (28·1–35·9)	28·3 (24·2–32·7)	32·6 (28·6–37·1)	20·9 (16·9–25·4)
Falls	53·5 (47·5–57·6)	44·1 (39·3–48·5)	43·2 (38·4–47·7)	46·3 (41·3–51)
Drowning	-7·16 (-15·1–1·51)	-7·82 (-15·4–0·242)	12·3 (8·27–17·7)	-8·4 (-16·1—0·111)
Fire, heat, and hot substances	13·1 (9·04–18)	17·1 (12·1–22·9)	18·8 (13·5–25·3)	14·5 (10·3–19·8)
Poisonings	20·1 (14·5–26·7)	19·6 (14·2–26·1)	16·6 (12–22·2)	21·4 (15·6–28·4)
Exposure to mechanical forces	13·5 (6·56–20·8)	23·1 (18·4–28·7)	25·9 (21·8–31·2)	11·4 (3·69–19·6)
Animal contact	28·3 (24–33·3)	27·2 (22·4–32·8)	27·7 (22·8–33·5)	24 (19·7–29·2)
Foreign body	4·44 (2·94–6·69)	5·61 (3·75–8·22)	5·23 (3·49–7·56)	5·78 (3·72–8·66)
Environmental heat and cold exposure	7·72 (3·29–13·4)	8·02 (3·46–14)	7·71 (3·35–13·4)	8·26 (3·55–14·4)
Exposure to forces of nature	6·98 (3–12·1)	7·29 (3·19–12·7)	8·41 (3·62–14·5)	6·48 (2·8–11·3)
Other unintentional injuries	22·8 (16·3–30·5)	18·1 (13–24·4)	17·4 (12·5–23·6)	25·8 (18·6–34·7)
Self-harm and interpersonal violence	20·9 (11·1–30·6)	22·5 (13·7–31·3)	32·3 (26–39·2)	21·7 (12·4–30·7)
Self-harm	20·8 (8·5–33)	22·8 (10·5–34·6)	24·2 (13·6–34·7)	22·8 (10·4–34·6)
Interpersonal violence	22·5 (14·3–31·2)	22·9 (15·2–31·1)	34·6 (27·3–42·4)	20 (11·9–28·8)

Supplementary Results Table 2. All-age PAFs attributable to the joint distribution of all risk factors for all ages, both sexes combined for each GBD level 1, level 2, and level 3 cause and all causes in 2019 for New Hampshire. Results are shown for deaths, YLLs, YLDs, and DALYs Data in parenthesis are 95% uncertainty intervals. PAFs=population attributable fractions. GBD=Global Burden of Disease. YLLs=years of life lost. YLDs=years lived with disability. DALYs=disability-adjusted life-years

	Deaths 2019	DALYs 2019	YLDs 2019	YLLs 2019
All causes	60·3 (57·5–63)	48·3 (45·1–51·4)	34·6 (31·9–37·2)	61·1 (58·9–63·3)
Communicable, maternal, neonatal, and nutritional diseases	41·1 (35·4–46·5)	49·6 (45·7–53·3)	45·4 (40·9–49·9)	52·7 (47·4–57·9)
HIV/AIDS and sexually transmitted infections	90 (87·8–91·9)	91 (88·7–93·2)	94·3 (91·8–96·8)	88·2 (85·6–90·5)
HIV/AIDS	89·4 (87–91·4)	88·3 (85·6–90·6)	89·1 (86·4–91·4)	87·9 (85·2–90·2)
Sexually transmitted infections excluding HIV	99·4 (99·1–99·6)	99·8 (99·7–99·9)	100 (100–100)	97·5 (96·3–98·4)
Respiratory infections and tuberculosis	41·7 (33·7–49·5)	34·3 (27·3–41·3)	2·11 (1·56–2·82)	46 (38–53·3)
Tuberculosis	57·8 (47·7–66·9)	60·4 (50·7–69)	57·9 (48·1–67·2)	61 (51·3–69·5)
Lower respiratory infections	41·7 (33·6–49·6)	45·8 (37·8–53·1)	34·1 (28·1–39·8)	46 (37·9–53·4)
Upper respiratory infections	0·336 (0·198–0·553)	0·0118 (0·00529–0·0221)	0·00395 (0–0·0118)	0·909 (0·545–1·47)
Otitis media	0·925 (0·316–2·19)	3·18 (2·03–4·43)	3·19 (2·03–4·46)	2·78 (1·07–5·79)
Enteric infections	8·27 (5·13–13)	9·28 (6·06–14)	9·16 (6·02–14)	9·39 (6·11–14·1)
Diarrheal diseases	8·29 (5·14–13)	9·31 (6·08–14)	9·16 (6·03–14)	9·44 (6·16–14·2)
Other infectious diseases	0·627 (0·379–0·871)	1·01 (0·673–1·32)	0·864 (0·537–1·33)	1·05 (0·656–1·41)
Meningitis	0·75 (0·509–1·07)	1·8 (1·25–2·52)	0·000316 (0–0·00108)	1·98 (1·37–2·76)
Encephalitis	0·327 (0·219–0·476)	0·97 (0·657–1·35)	6·46e-05 (0–0·000206)	1·03 (0·692–1·44)
Measles	8·94 (-4·14–42·3)	9·11 (-4·22–43·1)	0·231 (0–0·7)	9·12 (-4·23–43·1)
Acute hepatitis	25·1 (16·7–39)	10·8 (7·29–15·8)	4·48 (2·85–6·75)	27·1 (18·3–41·4)
Maternal and neonatal disorders	81·2 (77·4–84·5)	77·5 (74–80·8)	70 (64·6–74·6)	84 (81·1–86·7)
Maternal disorders	11·1 (3·94–18·3)	10·9 (3·88–18·2)	10·9 (3·85–18·5)	10·9 (3·9–18)
Neonatal disorders	89·3 (87·4–91·1)	81 (77·6–84·1)	71·9 (66·2–76·9)	89·3 (87·4–91·1)
Nutritional deficiencies	91·9 (84·1–94·3)	87·6 (84·7–90·4)	86·5 (83–89·9)	91·9 (84·4–94·4)
Protein-energy malnutrition	100 (100–100)	100 (100–100)	100 (100–100)	100 (100–100)
Vitamin A deficiency	--	100 (100–100)	100 (100–100)	--
Dietary iron deficiency	--	100 (100–100)	100 (100–100)	--
Non-communicable diseases	62·9 (59·8–65·8)	50 (46·4–53·4)	34·2 (31·2–37)	65·2 (62·8–67·5)
Neoplasms	50·4 (46·8–54·5)	49·7 (46·3–53·6)	31·8 (27·8–35·9)	51 (47·6–54·8)
Lip and oral cavity cancer	69·4 (60·1–76·6)	71·4 (62·8–78·3)	71 (62–78)	71·4 (62·8–78·2)
Nasopharynx cancer	68 (57·3–77·2)	67·8 (57·4–76·7)	67 (56·5–76·1)	67·8 (57·4–76·7)
Other pharynx cancer	78·8 (72–84·5)	79·8 (72·9–85·1)	80 (73·2–85·3)	79·7 (72·8–85·1)

Esophageal cancer	79·6 (70·3–87·7)	80·5 (71·3–88·7)	79·7 (70·5–88)	80·6 (71·3–88·7)
Stomach cancer	24·9 (16·6–42·6)	25·6 (17·4–43·4)	25·7 (17·4–43·2)	25·6 (17·4–43·4)
Colon and rectum cancer	62·3 (53·7–71·1)	63·3 (55·1–71·4)	63·2 (55–71·4)	63·3 (55·1–71·4)
Liver cancer	82·6 (78·2–86·8)	82·5 (78·2–86·5)	82·5 (77·9–86·5)	82·5 (78·2–86·5)
Gallbladder and biliary tract cancer	27 (15·3–40)	27·9 (16–41·1)	25·8 (14·9–39)	27·9 (16–41·2)
Pancreatic cancer	43·4 (33·6–54·8)	43·2 (33·9–54·3)	43·5 (34–54·6)	43·2 (33·9–54·3)
Larynx cancer	82 (72·1–89·6)	83 (74·4–89·6)	82·8 (74·2–89·6)	83 (74·3–89·6)
Tracheal, bronchus, and lung cancer	83 (79·5–86·6)	82·3 (79·1–85·8)	82·7 (79·3–86·4)	82·3 (79–85·8)
Breast cancer	37·4 (28·5–47·7)	36·6 (28·6–46·2)	37·1 (28·8–47·1)	36·5 (28·6–46)
Cervical cancer	100 (100–100)	100 (100–100)	100 (100–100)	100 (100–100)
Uterine cancer	54·8 (39·8–68·3)	56·2 (41·3–69·3)	56·6 (41·6–69·6)	56·1 (41·2–69·3)
Ovarian cancer	19·1 (8·52–32·1)	17 (7·14–29·3)	16·5 (7·01–28·7)	17·1 (7·16–29·3)
Prostate cancer	5·77 (2·43–9·77)	6·38 (2·66–10·6)	6·69 (2·83–11)	6·27 (2·63–10·3)
Kidney cancer	43·3 (31·8–54·5)	44·1 (33·5–54·2)	44·4 (33·6–54·5)	44 (33·4–54·2)
Bladder cancer	39·4 (24–54·7)	42·7 (28–57)	43·4 (29·2–56·9)	42·6 (27·8–57)
Thyroid cancer	19·3 (9·93–30·1)	20·5 (10·6–31·5)	20·7 (10·9–31·6)	20·4 (10·5–31·6)
Mesothelioma	97·2 (96·1–98)	94·7 (92·9–96·2)	96·7 (95·2–97·9)	94·7 (92·9–96·2)
Non-Hodgkin lymphoma	9·28 (4·22–15·5)	9·8 (4·54–16·1)	9·56 (4·41–15·8)	9·82 (4·55–16·1)
Multiple myeloma	10·5 (4·91–17·4)	11·1 (5·25–18·3)	11 (5·23–18·2)	11·1 (5·25–18·3)
Leukemia	37·2 (22·8–51·9)	36·4 (24·1–49·3)	38 (23·5–52·6)	36·4 (24·1–49·1)
Cardiovascular diseases	80·2 (76·3–83·5)	80·8 (77·7–83·5)	71·5 (66·8–76·3)	82·4 (79·6–85)
Rheumatic heart disease	21·8 (12·3–42·3)	21·6 (13·6–36·1)	21·6 (13–36·4)	21·7 (13·7–35·9)
Ischemic heart disease	92 (87·9–95·4)	93·5 (90·6–96)	92·2 (88·6–95·3)	93·5 (90·7–96·1)
Stroke	78·8 (72–86·7)	82·2 (77·3–87·4)	81·4 (75·9–87·4)	82·6 (77·6–87·9)
Hypertensive heart disease	100 (100–100)	100 (100–100)	100 (100–100)	100 (100–100)
Non-rheumatic valvular heart disease	25·6 (16–39·2)	24 (16·5–33·6)	9·2 (5·88–12·9)	25·9 (17·7–36·4)
Cardiomyopathy and myocarditis	40 (32·9–47·8)	43·4 (37·7–49)	40·7 (34·6–47)	43·7 (37·8–49·4)
Atrial fibrillation and flutter	54·5 (43·7–64·2)	60 (51·3–68·5)	61·5 (52·7–69·5)	57·8 (48·4–67)
Aortic aneurysm	53·7 (47·4–60·5)	61·6 (56·5–66·7)	--	61·6 (56·5–66·7)
Endocarditis	30·6 (21·1–43)	32·1 (23·1–42·5)	30·3 (20·6–42·5)	32·2 (23·2–42·5)
Other cardiovascular and circulatory diseases	19·7 (14·8–27·7)	22·6 (18–28·8)	30 (23·4–37·2)	20·7 (16·2–27·4)
Chronic respiratory diseases	63·5 (58·2–68·9)	60·3 (56·3–64·2)	55·7 (51·3–59·9)	64·3 (59·6–69·2)

Chronic obstructive pulmonary disease	71·6 (67–75·9)	70·1 (66·2–73·8)	64·6 (60·6–68·6)	73·5 (69·4–77·5)
Pneumoconiosis	100 (100–100)	100 (100–100)	100 (100–100)	100 (100–100)
Asthma	52·6 (41·5–64)	46·3 (38·3–55)	45·7 (37·6–54·3)	53·2 (43·5–62·8)
Digestive diseases	45·5 (40·8–50·1)	46·8 (41·3–51·8)	16·6 (11·6–22·1)	56 (51–60·6)
Cirrhosis and other chronic liver diseases	83·8 (76·8–90·4)	85·4 (78·5–91·3)	82·3 (74·9–88·9)	85·4 (78·5–91·3)
Upper digestive system diseases	20·5 (16·9–24·2)	6·37 (3·76–9·63)	1·5 (0·885–2·35)	23·4 (19·7–27·2)
Gallbladder and biliary diseases	51·4 (36–66)	56·3 (42·1–69·1)	58 (43·8–70·5)	53·5 (38·5–67·6)
Pancreatitis	43·6 (25·3–63·4)	46·7 (28·3–65·5)	44·7 (26·8–63·5)	47·1 (28·6–65·9)
Neurological disorders	27·3 (11·7–42·9)	17·7 (9·02–30·5)	12·7 (6·57–23·7)	23·5 (9·04–39·1)
Alzheimer's disease and other dementias	42·4 (28·5–57·1)	45·4 (31·1–59·9)	46·1 (31·9–60·8)	44·9 (30·8–59·5)
Parkinson's disease	-9·68 (-15·7–-4·63)	-11·2 (-17·7–5·63)	-12·1 (-18·6–6·32)	-11·1 (-17·5–-5·52)
Idiopathic epilepsy	27·3 (17·8–36·3)	25 (16·4–33)	24 (15·8–32)	27·8 (18·6–36·5)
Multiple sclerosis	14·6 (10·7–19)	16 (11·8–20·5)	15·9 (11·8–20·3)	16 (11·7–20·6)
Mental disorders	--	9·07 (4·46–13·9)	9·07 (4·46–13·9)	--
Depressive disorders	--	19 (8·78–29·1)	19 (8·78–29·1)	--
Anxiety disorders	--	5·61 (1·25–13)	5·61 (1·25–13)	--
Idiopathic developmental intellectual disability	--	45·8 (22·2–64·8)	45·8 (22·2–64·8)	--
Substance use disorders	100 (100–100)	100 (100–100)	100 (100–100)	100 (100–100)
Alcohol use disorders	100 (100–100)	100 (100–100)	100 (100–100)	100 (100–100)
Drug use disorders	100 (100–100)	100 (100–100)	100 (100–100)	100 (100–100)
Diabetes and kidney diseases	99·9 (99·8–99·9)	99·9 (99·9–100)	100 (100–100)	99·9 (99·9–99·9)
Diabetes mellitus	100 (100–100)	100 (100–100)	100 (100–100)	100 (100–100)
Chronic kidney disease	100 (100–100)	100 (100–100)	100 (100–100)	100 (100–100)
Sense organ diseases	--	11·3 (10·1–12·6)	11·3 (10·1–12·6)	--
Blindness and vision loss	--	10·1 (6·66–14·6)	10·1 (6·66–14·6)	--
Age-related and other hearing loss	--	12·3 (11·1–13·7)	12·3 (11·1–13·7)	--
Musculoskeletal disorders	3·77 (1·13–6·93)	21·2 (17·7–24·7)	21·4 (18–25·1)	3·69 (1·11–6·68)
Rheumatoid arthritis	12·1 (3·62–21·9)	15 (4·71–27)	15·3 (4·82–27·5)	13·9 (4·21–25·1)
Osteoarthritis	--	15·5 (8·91–22·5)	15·5 (8·91–22·5)	--
Low back pain	--	43·5 (37·5–49·7)	43·5 (37·5–49·7)	--
Gout	--	61·8 (45·5–77·9)	61·8 (45·5–77·9)	--
Other non-communicable diseases	0·0686 (0·0455–0·103)	0·09 (0·0583–0·132)	--	0·255 (0·169–0·369)

Sudden infant death syndrome	6·7 (4·55–9·39)	6·7 (4·55–9·38)	--	6·7 (4·55–9·38)
Injuries	31·8 (26·6–37)	29·8 (25·1–34·3)	35·2 (31·4–39·4)	25·7 (18·9–32·1)
Transport injuries	23·5 (16·7–30·8)	25·9 (19·8–32·6)	32·6 (28·5–37·1)	22·4 (14·7–30·6)
Road injuries	22·2 (15·2–29·6)	24 (17·8–31)	30·3 (26·2–35·2)	21·2 (13·3–29·5)
Other transport injuries	38·9 (33·4–45·6)	39·7 (35–44·7)	42·2 (37·9–46·2)	36·5 (30–44·2)
Unintentional injuries	41·2 (35·8–45·7)	33·9 (29·5–38·5)	36·1 (31·6–40·9)	29·4 (24·7–34)
Falls	57 (50·2–61·7)	47·9 (42·3–52·6)	46·8 (41·4–51·6)	50·3 (44·6–55·4)
Drowning	-5·63 (-17·1–6·19)	-4·63 (-15·8–6·6)	13·5 (8·73–19·3)	-5·54 (-17·1–6·11)
Fire, heat, and hot substances	14 (9·2–19·9)	17·7 (12·3–24·2)	18·4 (12·7–25·3)	16·5 (11·5–22·7)
Poisonings	21 (14·9–28·3)	20·3 (14·4–27·2)	16·8 (11·7–22·9)	22·8 (16·5–30·3)
Exposure to mechanical forces	10·8 (2·11–20)	22·6 (17·8–28)	25·8 (22·1–30·7)	8·49 (-1·08–19)
Animal contact	26 (20·8–31·9)	29·1 (24·2–35·1)	29·6 (24·5–35·6)	24·7 (19·4–31·2)
Foreign body	3·74 (2·4–5·59)	6·12 (4·01–9·01)	5·41 (3·6–7·83)	6·49 (4·25–9·65)
Environmental heat and cold exposure	9·07 (4·01–15·6)	9·5 (4·25–16·2)	9·18 (4·06–15·8)	9·86 (4·49–16·6)
Exposure to forces of nature	8·41 (3·75–14·5)	8·48 (3·79–14·4)	9·67 (4·3–16·5)	7·71 (3·47–13·1)
Other unintentional injuries	21·5 (15·1–29)	18 (12·6–24·8)	17·5 (11·9–24·3)	25·1 (18–33·3)
Self-harm and interpersonal violence	23·4 (10·1–35·7)	25·8 (13·6–37·3)	31·4 (24·7–38·3)	25·3 (12·2–37·7)
Self-harm	23·6 (9·19–36·8)	26·1 (11·8–39·4)	28·2 (16–39·7)	26·1 (11·8–39·4)
Interpersonal violence	23·6 (13·2–34·4)	25·8 (16·9–35·1)	33·3 (25·8–41·6)	21·2 (10·8–31·7)

Supplementary Results Table 2. All-age PAFs attributable to the joint distribution of all risk factors for all ages, both sexes combined for each GBD level 1, level 2, and level 3 cause and all causes in 2019 for Kazakhstan. Results are shown for deaths, YLLs, YLDs, and DALYs Data in parenthesis are 95% uncertainty intervals. PAFs=population attributable fractions. GBD=Global Burden of Disease. YLLs=years of life lost. YLDs=years lived with disability. DALYs=disability-adjusted life-years

	Deaths 2019	DALYs 2019	YLDs 2019	YLLs 2019
All causes	67·4 (65·1–69·6)	50·4 (47·4–53)	31·9 (29·4–34·3)	59·4 (57·2–61·6)
Communicable, maternal, neonatal, and nutritional diseases	59·9 (56·2–63·5)	64 (61·4–66·4)	62·2 (58·5–65·7)	64·7 (61·5–67·7)
HIV/AIDS and sexually transmitted infections	79·3 (78·4–80·3)	80·9 (79·5–82·7)	91·4 (88·5–94·3)	77·9 (77–78·9)
HIV/AIDS	77·8 (76·9–78·7)	76·9 (76–77·8)	77·9 (76·9–78·8)	76·8 (75·9–77·7)
Sexually transmitted infections excluding HIV	97·6 (96·3–98·5)	98·6 (97·7–99·3)	100 (100–100)	95 (92·4–96·9)
Respiratory infections and tuberculosis	58·5 (52·9–63·4)	58·2 (53·2–63·2)	14·9 (10·5–20·4)	63·5 (58·6–68·3)
Tuberculosis	56·8 (49·4–63·7)	53·8 (46·5–60·7)	48·5 (40·8–55·9)	54·6 (47·2–61·5)
Lower respiratory infections	59 (52·5–65)	65·6 (59·7–71·1)	40·8 (34·5–47·3)	65·8 (59·9–71·3)
Upper respiratory infections	0·569 (0·338–0·993)	0·066 (0·0331–0·126)	0·00907 (0·00125–0·0195)	0·844 (0·511–1·49)
Otitis media	5·07 (1·9–11·7)	7·25 (4·86–9·85)	7·25 (4·85–9·85)	10·4 (4·58–21)
Enteric infections	82·1 (73·7–89)	75·2 (62·3–86·4)	72·8 (58–85·1)	87·4 (81·2–92·6)
Diarrheal diseases	87·2 (79·6–93·4)	75·8 (62·7–86·9)	72·9 (58–85·2)	90·8 (85–95·4)
Other infectious diseases	1·65 (1·26–2·18)	1·8 (1·37–2·35)	0·337 (0·205–0·532)	2·15 (1·64–2·83)
Meningitis	2·14 (1·48–3·07)	2·91 (2·06–4·08)	0·00146 (0·000164–0·00327)	3·34 (2·38–4·61)
Encephalitis	1·52 (1·06–2·13)	2·04 (1·45–2·84)	0·0013 (0·000151–0·00282)	2·12 (1·5–2·95)
Measles	12·9 (3·98–36·7)	4·7 (0·728–9·94)	4·03 (0·122–8·93)	15·7 (5·09–43·3)
Acute hepatitis	6·45 (3·88–11·7)	4 (2·47–6·5)	2·4 (1·46–3·77)	5·31 (2·98–9·53)
Maternal and neonatal disorders	76·7 (73·3–79·8)	70·5 (66·6–74·1)	44·3 (39·7–48·7)	77·4 (74·2–80·5)
Maternal disorders	17 (6·26–27·9)	16·8 (6·15–27·7)	16·8 (6·06–27·7)	16·9 (6·2–27·8)
Neonatal disorders	78·8 (75·6–81·9)	72 (68·1–75·5)	45·5 (40·7–50)	78·8 (75·6–81·9)
Nutritional deficiencies	69·7 (58–78·1)	96·3 (95·1–97·1)	97·6 (97–98·2)	71·1 (65·4–81·6)
Protein-energy malnutrition	100 (100–100)	100 (100–100)	100 (100–100)	100 (100–100)
Vitamin A deficiency	--	100 (100–100)	100 (100–100)	--
Dietary iron deficiency	--	100 (100–100)	100 (100–100)	--
Non-communicable diseases	74·3 (71·6–77)	55·8 (51·5–59·5)	29·4 (26·2–32·4)	69·8 (67·1–72·8)
Neoplasms	46·7 (42·8–51·5)	44·5 (40·9–48·8)	39·5 (35·3–44)	44·6 (41·1–48·9)
Lip and oral cavity cancer	59 (52·7–64·8)	61·4 (54·9–67·4)	57·7 (50·6–64·2)	61·5 (55–67·5)
Nasopharynx cancer	53·1 (44·4–60·5)	52·9 (44·3–60·3)	52·7 (43·8–60·5)	52·9 (44·3–60·3)
Other pharynx cancer	71·7 (65·8–76·5)	72·7 (66·7–77·8)	67·9 (61·6–73·4)	72·7 (66·8–77·8)

Esophageal cancer	67·3 (54·1–79·4)	69·6 (57·2–81)	68·5 (55·1–80·2)	69·6 (57·2–81)
Stomach cancer	23·1 (15–41·9)	23 (14·8–41·9)	22·9 (14·5–41·7)	23 (14·8–41·9)
Colon and rectum cancer	58·6 (50·3–66·4)	58·7 (50·8–65·9)	58·7 (50·6–65·9)	58·7 (50·8–66)
Liver cancer	73·1 (65·7–79·5)	71·1 (63·8–77·9)	72·6 (65·2–79·2)	71·1 (63·7–77·9)
Gallbladder and biliary tract cancer	28·7 (17·3–41·3)	28·6 (17·3–41·4)	28·7 (17·3–41·4)	28·6 (17·3–41·4)
Pancreatic cancer	33·2 (24·6–43·3)	33·1 (24·9–42·7)	33·2 (24·7–43·1)	33·1 (24·9–42·7)
Larynx cancer	78·2 (71·8–82·9)	78·7 (72·4–83·3)	76·2 (69·6–81·3)	78·7 (72·5–83·3)
Tracheal, bronchus, and lung cancer	80·9 (77·2–85·6)	80·4 (76·6–85)	80·5 (76·5–85·4)	80·4 (76·6–85)
Breast cancer	29·4 (20·4–40)	27·1 (18·7–36·4)	27·1 (18·6–36·4)	27·1 (18·7–36·4)
Cervical cancer	100 (100–100)	100 (100–100)	100 (100–100)	100 (100–100)
Uterine cancer	56 (41·2–70·2)	56·2 (41·7–70·4)	56·4 (41·8–70·7)	56·2 (41·6–70·4)
Ovarian cancer	16·7 (6·44–29·1)	14·8 (5·66–25·9)	14 (5·42–24·6)	14·8 (5·67–26)
Prostate cancer	7·7 (3·76–11·7)	8·52 (4·18–12·9)	8·88 (4·37–13·4)	8·49 (4·17–12·9)
Kidney cancer	39·9 (29·9–49·6)	38·6 (28·9–48)	37·8 (28–47·3)	38·7 (28·9–48)
Bladder cancer	43·3 (32·2–52·9)	45·8 (34·6–54·6)	45·2 (34·2–54·1)	45·8 (34·6–54·7)
Thyroid cancer	17·9 (10·1–26·9)	17·9 (10·1–26·9)	17·3 (10–25·7)	18 (10·1–27)
Mesothelioma	88·6 (81·8–94·5)	83·5 (74·3–91·7)	87·6 (80·7–93·8)	83·4 (74·2–91·7)
Non-Hodgkin lymphoma	8·2 (3·71–14·1)	7·36 (3·38–12·7)	7·22 (3·23–12·3)	7·37 (3·39–12·7)
Multiple myeloma	10·6 (4·69–17·6)	10·7 (4·71–17·6)	10·8 (4·74–17·9)	10·7 (4·71–17·6)
Leukemia	25·9 (18·2–34)	19·6 (13–26·2)	24·2 (16·6–32)	19·6 (12·9–26·1)
Cardiovascular diseases	89·3 (86·6–92·1)	88·1 (85·3–91·6)	85·3 (82·7–87·8)	88·2 (85·4–91·9)
Rheumatic heart disease	36·7 (26·1–52·4)	36·6 (25·9–51·8)	35·5 (25·6–50·8)	36·7 (25·7–51·7)
Ischemic heart disease	96 (93·6–97·9)	96·8 (95·3–98·1)	96·5 (94·7–98)	96·8 (95·3–98·2)
Stroke	89·9 (86–93·6)	92·1 (89·3–94·5)	90·7 (88–92·9)	92·2 (89·4–94·7)
Hypertensive heart disease	100 (100–100)	100 (100–100)	100 (100–100)	100 (100–100)
Non-rheumatic valvular heart disease	30 (23·3–38·4)	21·8 (15·9–29·2)	8·5 (5·66–11·9)	29·5 (22·5–38·4)
Cardiomyopathy and myocarditis	50·4 (41·9–57·8)	51·2 (41·4–59·7)	46·4 (39–52·9)	51·2 (41·4–59·7)
Atrial fibrillation and flutter	62 (52·5–71·6)	69·1 (62–76·2)	71·9 (65·1–78·4)	65·6 (57·4–73·7)
Aortic aneurysm	71·5 (66·4–75·9)	74·7 (70·2–78·4)	--	74·7 (70·2–78·4)
Endocarditis	50·4 (40·7–59·6)	49·3 (37·6–59·8)	43·3 (34·6–52)	49·3 (37·6–59·8)
Other cardiovascular and circulatory diseases	52·7 (46·4–58·1)	50·9 (43·9–56·6)	37·7 (29·6–45·6)	52·2 (44·8–58·2)
Chronic respiratory diseases	68 (64·1–71·8)	65·1 (61·5–68·4)	48·1 (42·9–52·7)	68·9 (65·3–72·3)
Chronic obstructive pulmonary disease	74 (70–77·8)	73·1 (69·6–76·7)	58·7 (53·7–63·1)	75·8 (72·2–79·4)

Pneumoconiosis	100 (100–100)	100 (100–100)	100 (100–100)	100 (100–100)
Asthma	48·5 (36·7–60·3)	45·8 (36–56·1)	32·5 (25·1–40·9)	51·6 (40·1–62·7)
Digestive diseases	58·2 (51·7–64·2)	54·9 (48·4–61·1)	17·9 (12·8–23·4)	60·5 (53·8–67)
Cirrhosis and other chronic liver diseases	69·7 (61·9–77·6)	72 (63·6–80·2)	69·2 (61·6–76·6)	72·1 (63·5–80·3)
Upper digestive system diseases	27 (23·3–30·8)	12·9 (8·44–17·7)	1·2 (0·709–1·85)	28·6 (25–32·6)
Gallbladder and biliary diseases	54·5 (40·2–68·2)	54·6 (40·5–67·6)	54·6 (40·4–67·7)	54·6 (40·4–68)
Pancreatitis	32·7 (22·1–43·7)	37·5 (26·5–49·4)	33·9 (23·3–45·2)	37·7 (26·6–49·5)
Neurological disorders	22·5 (9·96–37)	10·4 (5·15–18·7)	6·15 (2·93–12·1)	20 (11·2–32)
Alzheimer's disease and other dementias	36·4 (21–53·4)	38·4 (23·2–54·8)	38·5 (23·2–54·9)	38·3 (23·2–54·8)
Parkinson's disease	-6·14 (-9·63–-3)	-7·99 (-12·6–-3·91)	-9·24 (-14·7–-4·5)	-7·78 (-12·2–-3·82)
Idiopathic epilepsy	20·2 (14·3–25·8)	16·3 (11·5–21·3)	14·2 (10·1–19)	18·8 (13·3–24·2)
Multiple sclerosis	11·4 (7·39–17·1)	14·4 (10·8–18·4)	14·8 (11·2–18·3)	13·8 (8·93–19·2)
Mental disorders	--	3·8 (1·69–6·41)	3·8 (1·69–6·42)	--
Depressive disorders	--	6·47 (1·98–12·2)	6·47 (1·98–12·2)	--
Anxiety disorders	--	2·59 (0·658–6·1)	2·59 (0·658–6·1)	--
Idiopathic developmental intellectual disability	--	47·1 (20·6–68·2)	47·1 (20·6–68·2)	--
Substance use disorders	100 (100–100)	100 (100–100)	100 (100–100)	100 (100–100)
Alcohol use disorders	100 (100–100)	100 (100–100)	100 (100–100)	100 (100–100)
Drug use disorders	100 (100–100)	100 (100–100)	100 (100–100)	100 (100–100)
Diabetes and kidney diseases	99·6 (99·4–99·7)	99·7 (99·7–99·8)	100 (100–100)	99·5 (99·3–99·6)
Diabetes mellitus	100 (100–100)	100 (100–100)	100 (100–100)	100 (100–100)
Chronic kidney disease	100 (100–100)	100 (100–100)	100 (100–100)	100 (100–100)
Sense organ diseases	--	14·8 (13·2–16·5)	14·8 (13·2–16·5)	--
Blindness and vision loss	--	8·77 (5·66–12·4)	8·77 (5·66–12·4)	--
Age-related and other hearing loss	--	19·2 (17·9–20·7)	19·2 (17·9–20·7)	--
Musculoskeletal disorders	6·04 (1·9–11·8)	30·6 (26·6–34·6)	30·8 (26·7–34·8)	6·13 (2·02–11·8)
Rheumatoid arthritis	17·4 (5·35–29·7)	9·27 (2·68–16·1)	8·6 (2·48–14·7)	19·3 (6·23–32·6)
Osteoarthritis	--	18·9 (10·7–27·8)	18·9 (10·7–27·8)	--
Low back pain	--	44·6 (40·5–48·8)	44·6 (40·5–48·8)	--
Gout	--	53·7 (38–70·5)	53·7 (38–70·5)	--
Other non-communicable diseases	0·207 (0·123–0·344)	0·125 (0·0743–0·202)	--	0·309 (0·176–0·519)
Sudden infant death syndrome	7·59 (5·08–10·8)	7·58 (5·07–10·8)	--	7·58 (5·07–10·8)
Injuries	12·3 (7·83–16·6)	13·2 (9·34–17)	21·5 (18·8–24·1)	11 (6·45–15·4)

Transport injuries	15·1 (9·45–20·7)	15·4 (10·3–20·6)	23·8 (20·3–27·2)	13·2 (7·35–18·9)
Road injuries	14·7 (8·94–20·3)	15·1 (9·86–20·3)	23·6 (20·2–27)	12·8 (6·76–18·5)
Other transport injuries	29·8 (25·8–34·1)	27·2 (23·3–31·4)	28·3 (24·8–31·9)	26·8 (22·6–31·3)
Unintentional injuries	9 (5·32–12·9)	11·4 (7·94–15·1)	20·8 (17·7–24)	6·09 (2·49–9·86)
Falls	29 (24·9–33)	25·9 (22–30)	28·2 (24·1–32·1)	22·3 (18·6–26·4)
Drowning	-10·6 (-20·4—1·6)	-12·2 (-21·8—3·67)	9·39 (6·57–13·1)	-12·5 (-22·2—3·96)
Fire, heat, and hot substances	12·7 (9·25–16·8)	12·6 (9·31–16·5)	12·8 (9·35–16·9)	12·5 (9·27–16·2)
Poisonings	14·6 (10·8–19·4)	14·4 (10·8–18·7)	11·7 (8·58–15·6)	14·7 (11–19·1)
Exposure to mechanical forces	2·34 (-4·75–9·25)	11 (6·72–14·9)	17·6 (15·2–19·9)	-0·139 (-7·21–6·87)
Animal contact	17·5 (14–21·3)	15·3 (12·1–18·8)	17 (13·6–20·9)	14 (10·8–17·6)
Foreign body	6·23 (4·91–7·71)	5·39 (4·15–6·71)	4·97 (3·75–6·46)	5·43 (4·17–6·81)
Environmental heat and cold exposure	8·1 (3·73–13·8)	8·2 (3·81–13·9)	6·79 (3·12–11·6)	8·35 (3·87–14·2)
Exposure to forces of nature	0 (0–0)	7·05 (3·28–12·1)	7·05 (3·28–12·1)	0 (0–0)
Other unintentional injuries	17 (12·8–22)	14·2 (10·8–18·5)	12·4 (9·04–16·4)	16·6 (12·6–21·3)
Self-harm and interpersonal violence	13·7 (4·23–22·6)	14·2 (4·92–22·8)	20·8 (15·7–26)	13·9 (4·29–22·7)
Self-harm	13·6 (1·15–25·1)	14·3 (1·93–25·8)	17·6 (8·79–26·7)	14·2 (1·61–25·8)
Interpersonal violence	14 (5·46–22·8)	14 (5·95–22·5)	23·6 (17·6–29·6)	13·1 (4·57–22·1)

Supplementary Results Table 2. All-age PAFs attributable to the joint distribution of all risk factors for all ages, both sexes combined for each GBD level 1, level 2, and level 3 cause and all causes in 2019 for New Jersey. Results are shown for deaths, YLLs, YLDs, and DALYs Data in parenthesis are 95% uncertainty intervals. PAFs=population attributable fractions. GBD=Global Burden of Disease. YLLs=years of life lost. YLDs=years lived with disability. DALYs=disability-adjusted life-years

	Deaths 2019	DALYs 2019	YLDs 2019	YLLs 2019
All causes	60·7 (57·5–63·4)	46·2 (42·9–49·6)	31·9 (29·4–34·3)	60·8 (58·5–63·1)
Communicable, maternal, neonatal, and nutritional diseases	46·1 (41·6–50·5)	55·6 (52·5–58·7)	49·7 (44·9–54·8)	60 (55·9–63·6)
HIV/AIDS and sexually transmitted infections	90·6 (88·8–92·3)	90·2 (88·3–92)	92·9 (91·1–94·8)	88·9 (86·8–90·7)
HIV/AIDS	90·5 (88·6–92·1)	89·5 (87·4–91·4)	91·2 (89·4–92·9)	88·8 (86·7–90·7)
Sexually transmitted infections excluding HIV	99·2 (98·9–99·5)	99·7 (99·5–99·9)	100 (100–100)	96·9 (95·6–97·9)
Respiratory infections and tuberculosis	41·9 (35·5–48·3)	34·2 (28·1–40·3)	2·33 (1·74–3·04)	46·2 (39·9–52·3)
Tuberculosis	56·7 (46·6–65·8)	59·1 (49·3–67·4)	55·5 (45·5–64·2)	59·8 (50·1–67·9)
Lower respiratory infections	41·8 (35·2–48·2)	45·8 (39·4–52)	31·5 (26–36·8)	46·1 (39·6–52·2)
Upper respiratory infections	0·492 (0·305–0·767)	0·0137 (0·00583–0·0255)	0·00523 (0–0·0152)	1·22 (0·757–1·87)
Otitis media	1·07 (0·477–2·36)	3·36 (2·14–4·67)	3·36 (2·13–4·69)	3·16 (1·55–5·96)
Enteric infections	8·53 (5·17–13·1)	9·66 (6·34–14·1)	9·53 (6·24–14·1)	9·89 (6·55–14·3)
Diarrheal diseases	8·58 (5·2–13·2)	9·7 (6·36–14·2)	9·53 (6·25–14·1)	9·99 (6·62–14·5)
Other infectious diseases	0·725 (0·421–0·967)	1·18 (0·774–1·54)	0·718 (0·441–1·14)	1·31 (0·788–1·75)
Meningitis	1·1 (0·751–1·5)	2·59 (1·83–3·5)	0·000544 (0–0·00163)	2·71 (1·9–3·66)
Encephalitis	0·491 (0·344–0·676)	1·38 (0·983–1·89)	0·000108 (0–0·000319)	1·44 (1·02–1·98)
Measles	9·07 (-4·14–42·8)	4·32 (-2·47–36·5)	0·287 (0·00949–0·772)	9·24 (-4·21–43·6)
Acute hepatitis	23·9 (14·7–34·8)	10·2 (6·5–15)	3·63 (2·26–5·78)	25·9 (16·3–37·5)
Maternal and neonatal disorders	80·7 (77·2–83·8)	80·3 (78–82·6)	75·5 (71·3–79·2)	84·1 (81·5–86·4)
Maternal disorders	11·4 (4·12–18·9)	11·2 (4·1–18·7)	11·3 (4·12–18·8)	11·2 (4·06–18·6)
Neonatal disorders	90·5 (89–91·9)	84·4 (82·2–86·5)	77 (72·6–80·8)	90·5 (89–92)
Nutritional deficiencies	94·6 (90·5–96·5)	88·5 (85·4–91·1)	87·4 (83·7–90·7)	93·7 (89·5–95·9)
Protein-energy malnutrition	100 (100–100)	100 (100–100)	100 (100–100)	100 (100–100)
Vitamin A deficiency	--	100 (100–100)	100 (100–100)	--
Dietary iron deficiency	--	100 (100–100)	100 (100–100)	--
Non-communicable diseases	62·9 (59·4–66)	47·1 (43·3–50·8)	30·9 (28·2–33·5)	64·1 (61·6–66·5)
Neoplasms	47·3 (43·4–51·9)	46·3 (42·5–50·4)	29·9 (25·8–34·2)	47·6 (43·9–51·8)
Lip and oral cavity cancer	67·1 (57·6–74·8)	69·3 (60·2–76·4)	68·9 (59·6–76·1)	69·3 (60·1–76·5)
Nasopharynx cancer	65·2 (53·8–74·6)	64·9 (53·7–73·8)	65 (53·9–74·1)	64·9 (53·6–73·8)
Other pharynx cancer	76·7 (69·3–82·8)	77·6 (70·6–83·4)	77·7 (70·8–83·6)	77·6 (70·5–83·4)

Esophageal cancer	77 (66·6–85·8)	78 (68–86·6)	77·2 (67·1–85·8)	78 (68–86·6)
Stomach cancer	23 (15·1–41·6)	23·4 (15·3–42·2)	23·5 (15·4–42)	23·4 (15·3–42·2)
Colon and rectum cancer	60·9 (51·8–69·5)	61·6 (53·1–69·7)	61·5 (53–70)	61·6 (53·1–69·7)
Liver cancer	79·9 (75·3–84·2)	79·7 (75·2–83·9)	79·6 (75·3–84·2)	79·7 (75·2–84)
Gallbladder and biliary tract cancer	26·7 (15·6–39)	27·6 (16·4–40·3)	26·2 (15·5–39)	27·6 (16·4–40·3)
Pancreatic cancer	41·6 (31·7–52·5)	41·1 (31·9–52)	41·6 (31·9–52·7)	41·1 (31·9–52)
Larynx cancer	80·2 (70·4–87·9)	81 (72·6–87·8)	80·8 (72·5–87·7)	81 (72·7–87·9)
Tracheal, bronchus, and lung cancer	81·4 (77·6–85·2)	80·4 (76·8–84·2)	80·9 (77·1–84·8)	80·4 (76·7–84·2)
Breast cancer	35·8 (26·6–46·4)	34·8 (26·4–44·6)	35·3 (26·7–45·3)	34·7 (26·3–44·4)
Cervical cancer	100 (100–100)	100 (100–100)	100 (100–100)	100 (100–100)
Uterine cancer	53·9 (38·6–68)	55·3 (40·3–69)	55·7 (40·7–69·1)	55·2 (40·2–69)
Ovarian cancer	19·8 (9·07–33·2)	17·5 (7·44–29·9)	16·9 (7·17–29)	17·5 (7·45–29·9)
Prostate cancer	5 (2·21–8·09)	5·57 (2·46–8·94)	5·86 (2·58–9·25)	5·47 (2·41–8·81)
Kidney cancer	40·9 (29·7–51·7)	41·3 (30·9–51·5)	41·6 (31·1–51·8)	41·3 (30·8–51·5)
Bladder cancer	37·2 (22·4–52·1)	40·6 (25·9–54·5)	41·3 (27·1–54·6)	40·5 (25·9–54·5)
Thyroid cancer	18·3 (9·29–28·6)	19·3 (10·1–29·9)	19·4 (10·4–29·5)	19·3 (9·88–30·1)
Mesothelioma	96·9 (95·8–97·8)	93·8 (91·7–95·4)	96·3 (94·8–97·6)	93·7 (91·7–95·3)
Non-Hodgkin lymphoma	8·72 (3·89–14·9)	9·18 (4·18–15·4)	8·99 (4·06–15)	9·2 (4·18–15·5)
Multiple myeloma	10·1 (4·63–16·9)	10·6 (4·91–17·6)	10·7 (4·9–17·6)	10·6 (4·91–17·6)
Leukemia	34·9 (21·7–48·6)	33·6 (22·1–45·7)	35·4 (22·3–49)	33·5 (22–45·6)
Cardiovascular diseases	81 (76·9–84·3)	80·9 (77·9–83·6)	70 (65·5–74·7)	82·9 (80·1–85·3)
Rheumatic heart disease	21·6 (11·8–41·8)	21·7 (13·6–36·8)	21·6 (13–37)	21·8 (13·8–37·1)
Ischemic heart disease	92 (87·6–95·6)	93·5 (90·5–96)	92·3 (88·8–95·4)	93·6 (90·5–96·1)
Stroke	78·9 (71·5–86·4)	82·5 (77·6–87·7)	81·1 (75·8–87)	83·1 (78·2–88·2)
Hypertensive heart disease	100 (100–100)	100 (100–100)	100 (100–100)	100 (100–100)
Non-rheumatic valvular heart disease	25·4 (15·2–40)	21·6 (14·2–31·1)	7·03 (4·45–10·1)	25·8 (17–37·5)
Cardiomyopathy and myocarditis	38·2 (30·9–46·3)	42·5 (37–48·6)	41·6 (35·2–48)	42·7 (36·9–48·9)
Atrial fibrillation and flutter	53·1 (42·4–63·2)	58·6 (50–67·6)	59·9 (51·4–68·6)	56·7 (47·2–65·9)
Aortic aneurysm	51·6 (45·1–58·4)	59·9 (55–65)	--	59·9 (55–65)
Endocarditis	31 (20·7–44)	32·7 (23·5–43·8)	30·7 (20·4–43·4)	32·8 (23·6–43·9)
Other cardiovascular and circulatory diseases	21·9 (17·1–27·9)	25 (20·3–30·3)	31 (24–38·2)	22·9 (18·6–28·1)
Chronic respiratory diseases	61 (56·3–65·4)	55·4 (51·6–59·3)	50·7 (46·1–55·3)	61·4 (57·5–65·4)
Chronic obstructive pulmonary disease	69·7 (64·4–74·1)	67·3 (63·2–71)	61·8 (57·9–65·8)	71·9 (67·7–75·8)

Pneumoconiosis	100 (100–100)	100 (100–100)	100 (100–100)	100 (100–100)
Asthma	50·3 (38·9–61·9)	40·5 (32·6–48·9)	39·7 (32–48)	50·7 (40·9–60·6)
Digestive diseases	45·9 (40·7–50·7)	46 (40·4–51·3)	15·3 (10·4–20·7)	56·7 (51·5–61·5)
Cirrhosis and other chronic liver diseases	83·1 (75–90·1)	84·9 (77·8–91)	81·5 (73·6–88·3)	84·9 (77·8–91·1)
Upper digestive system diseases	18·8 (15·5–22·1)	4·7 (2·89–6·85)	1·41 (0·892–2·07)	21·6 (18·2–25·1)
Gallbladder and biliary diseases	50·8 (34·4–65·7)	54·8 (39·9–68·3)	55·9 (41·3–69)	52·6 (37–67)
Pancreatitis	42·5 (24·3–60)	46·1 (28·1–62·9)	43·6 (25·4–60·1)	46·5 (28·3–63·3)
Neurological disorders	27·4 (12·6–42·4)	16·6 (8·28–28·5)	11·6 (5·87–22·3)	23·8 (10·1–39)
Alzheimer's disease and other dementias	40 (25·9–55·7)	42·8 (28·5–58·4)	43·5 (29·2–58·9)	42·4 (28·3–58)
Parkinson's disease	-7·89 (-13–-3·8)	-9·19 (-14·4–-4·56)	-9·73 (-15–-4·87)	-9·07 (-14·3–-4·5)
Idiopathic epilepsy	26 (17·2–34·4)	23·3 (15·4–31·1)	22·5 (14·6–30·1)	26·3 (17·6–34·3)
Multiple sclerosis	13·6 (9·82–17·6)	14·3 (10·4–18·5)	13·9 (10·2–17·9)	14·7 (10·6–19·1)
Mental disorders	--	7·14 (3·62–11·4)	7·14 (3·62–11·4)	--
Depressive disorders	--	16·7 (8·23–25·5)	16·7 (8·23–25·5)	--
Anxiety disorders	--	5·98 (1·32–13·9)	5·98 (1·32–13·9)	--
Idiopathic developmental intellectual disability	--	44·2 (22·8–61·4)	44·2 (22·8–61·4)	--
Substance use disorders	100 (100–100)	100 (100–100)	100 (100–100)	100 (100–100)
Alcohol use disorders	100 (100–100)	100 (100–100)	100 (100–100)	100 (100–100)
Drug use disorders	100 (100–100)	100 (100–100)	100 (100–100)	100 (100–100)
Diabetes and kidney diseases	99·8 (99·8–99·9)	99·9 (99·9–100)	100 (100–100)	99·9 (99·8–99·9)
Diabetes mellitus	100 (100–100)	100 (100–100)	100 (100–100)	100 (100–100)
Chronic kidney disease	100 (100–100)	100 (100–100)	100 (100–100)	100 (100–100)
Sense organ diseases	--	9·95 (8·86–11·2)	9·95 (8·86–11·2)	--
Blindness and vision loss	--	9·47 (5·91–13·9)	9·47 (5·91–13·9)	--
Age-related and other hearing loss	--	11 (9·91–12·1)	11 (9·91–12·1)	--
Musculoskeletal disorders	2·64 (0·75–4·81)	19·3 (16·2–22·9)	19·6 (16·5–23·2)	2·45 (0·722–4·46)
Rheumatoid arthritis	10·5 (3·01–19·2)	13·2 (4·13–23·7)	13·4 (4·21–23·9)	12·3 (3·58–22·1)
Osteoarthritis	--	16·4 (9·2–24·1)	16·4 (9·2–24·1)	--
Low back pain	--	39·6 (33·8–45·1)	39·6 (33·8–45·1)	--
Gout	--	60·5 (44·8–76·3)	60·5 (44·8–76·3)	--
Other non-communicable diseases	0·0677 (0·0463–0·0945)	0·0849 (0·0556–0·124)	--	0·248 (0·169–0·344)
Sudden infant death syndrome	5·49 (3·79–7·59)	5·48 (3·79–7·58)	--	5·48 (3·79–7·58)
Injuries	30·9 (26·5–35·2)	30·4 (26·3–34·5)	35 (31–39·4)	26·5 (21·6–31·3)

Transport injuries	31·3 (25–38·2)	32·4 (26·3–39·2)	34·9 (30·2–40)	31 (24–39)
Road injuries	30·7 (24·2–37·5)	31·3 (25·1–38·4)	32·9 (28·2–38·3)	30·5 (23·4–38·5)
Other transport injuries	41·7 (35·5–48·5)	41·8 (36·8–46·8)	43·1 (38·7–47·1)	39·5 (32·3–47)
Unintentional injuries	36·3 (31·7–40·3)	33 (28·3–37·5)	35·8 (31·1–40·4)	26·7 (22·4–31·4)
Falls	56·4 (49·5–61·4)	47·8 (42·4–52·7)	47 (41·5–51·9)	49·9 (44·1–55·1)
Drowning	-1·3 (-11·2–8·56)	-0·976 (-10·4–8·32)	13·9 (9·48–19·8)	-1·94 (-11·7–7·78)
Fire, heat, and hot substances	14·1 (9·61–19·8)	19·5 (14·3–25·9)	21·2 (15·5–28)	16·8 (12–22·6)
Poisonings	23 (16·7–30·3)	22·3 (16·5–29·3)	19 (13·9–25)	25 (18·5–32·8)
Exposure to mechanical forces	19 (11·1–27·9)	27·6 (22·5–34·1)	28·9 (24·3–35)	17·4 (8·31–27·5)
Animal contact	28·7 (23·6–34·5)	30·8 (25·7–36·6)	31·3 (26·1–37·1)	25·7 (20·8–31·6)
Foreign body	4·06 (2·64–5·93)	7·1 (4·83–10·1)	6·98 (4·72–10·1)	7·17 (4·7–10·3)
Environmental heat and cold exposure	8·44 (3·92–14·7)	8·9 (4·09–15·3)	8·69 (4·02–15·2)	9·18 (4·23–15·8)
Exposure to forces of nature	8·02 (3·69–13·8)	7·98 (3·7–13·7)	9·35 (4·3–16·1)	7·39 (3·44–12·6)
Other unintentional injuries	26·5 (19·1–35·5)	21 (15·3–27·8)	20·3 (14·8–27·1)	30·9 (22·8–41·1)
Self-harm and interpersonal violence	23·6 (14·2–32·3)	24·1 (16–32·3)	28·1 (21·8–35)	23·6 (15–32·1)
Self-harm	25·3 (12·8–37·5)	27·1 (14·8–39·4)	25·6 (14·6–36·3)	27·2 (14·8–39·5)
Interpersonal violence	20·6 (12·8–29·3)	21·2 (13·8–29·6)	30·1 (23–37·8)	18·9 (11–27·4)

Supplementary Results Table 2. All-age PAFs attributable to the joint distribution of all risk factors for all ages, both sexes combined for each GBD level 1, level 2, and level 3 cause and all causes in 2019 for New Mexico. Results are shown for deaths, YLLs, YLDs, and DALYs Data in parenthesis are 95% uncertainty intervals. PAFs=population attributable fractions. GBD=Global Burden of Disease. YLLs=years of life lost. YLDs=years lived with disability. DALYs=disability-adjusted life-years				
	**Deaths 2019**	**DALYs 2019**	**YLDs 2019**	**YLLs 2019**
All causes	60·3 (57·5–62·8)	47·7 (44·7–50·4)	34·1 (31·4–36·7)	59·3 (57·1–61·4)
Communicable, maternal, neonatal, and nutritional diseases	43·8 (39·6–47·5)	52·1 (48·9–55·3)	46·5 (42·2–50·9)	55·8 (51·6–59·7)
HIV/AIDS and sexually transmitted infections	82·8 (66·9–89·2)	83·1 (68·6–89·5)	88·1 (76·4–93·7)	79·6 (62·8–87·2)
HIV/AIDS	82 (65·4–88·7)	80·1 (63·5–87·6)	82 (66·5–89)	79·2 (61·9–86·9)
Sexually transmitted infections excluding HIV	99·1 (98·7–99·5)	99·7 (99·4–99·8)	100 (100–100)	96·9 (95·4–98)
Respiratory infections and tuberculosis	40·7 (34·8–45·9)	34·8 (29·4–40·4)	2·26 (1·68–2·94)	45·3 (39·6–50·5)
Tuberculosis	53·4 (44·1–62·1)	56·4 (47·4–64·2)	53·3 (43·9–61·7)	56·8 (47·8–64·6)
Lower respiratory infections	40·5 (34·7–45·9)	45 (39·1–50·4)	30·1 (24·8–35·6)	45·2 (39·3–50·5)
Upper respiratory infections	0·612 (0·333–1·02)	0·0297 (0·0132–0·0525)	0·00443 (0–0·0138)	1·32 (0·724–2·2)
Otitis media	1·3 (0·561–2·68)	3·31 (2·12–4·58)	3·3 (2·1–4·58)	3·53 (1·69–6·63)
Enteric infections	13 (7·79–19·6)	14·3 (9·16–20·7)	13·9 (8·75–20·4)	14·9 (9·73–21·3)
Diarrheal diseases	13·1 (7·85–19·7)	14·4 (9·23–20·9)	13·9 (8·76–20·5)	15·1 (9·91–21·7)
Other infectious diseases	0·844 (0·528–1·15)	1·41 (1–1·86)	0·829 (0·514–1·33)	1·53 (1·03–2·09)
Meningitis	1·29 (0·822–1·9)	2·78 (1·77–4·11)	0·000487 (0–0·00151)	2·88 (1·83–4·24)
Encephalitis	0·546 (0·353–0·815)	1·38 (0·893–2·07)	0·000116 (0–0·000382)	1·45 (0·93–2·14)
Measles	9·69 (-3·85–43·8)	6·14 (-3·47–39·6)	0·376 (0–1·12)	9·85 (-3·92–44·5)
Acute hepatitis	26·7 (15·4–35·8)	11·7 (7·54–16·1)	4·39 (2·83–6·83)	27·5 (15·9–36·7)
Maternal and neonatal disorders	81·7 (78·5–84·6)	76·6 (73·3–79·9)	65·1 (59·5–70·3)	84·6 (82–86·7)
Maternal disorders	11·8 (4·42–19·8)	11·7 (4·27–19·6)	11·5 (4·27–19·4)	11·7 (4·34–19·7)
Neonatal disorders	90·1 (88·6–91·4)	80·2 (76·9–83·2)	66·6 (60·7–71·9)	90·1 (88·6–91·4)
Nutritional deficiencies	92·5 (81·9–94·6)	90 (86·8–92·4)	89·3 (85·8–92·4)	92·3 (82·1–94)
Protein-energy malnutrition	100 (100–100)	100 (100–100)	100 (100–100)	100 (100–100)
Vitamin A deficiency	--	100 (100–100)	100 (100–100)	--
Dietary iron deficiency	--	100 (100–100)	100 (100–100)	--
Non-communicable diseases	64 (60·9–66·8)	50·3 (46·9–53·6)	33·8 (30·8–36·6)	65·8 (63·4–68·2)
Neoplasms	46·7 (42·6–51·1)	45·4 (41·7–49·6)	28·9 (24·9–33·2)	46·5 (42·8–50·7)
Lip and oral cavity cancer	65·4 (56·2–73·6)	67·5 (58·6–75·1)	66·8 (57·9–74·6)	67·5 (58·6–75·1)
Nasopharynx cancer	61·5 (50·2–70·9)	61·1 (50·2–70·5)	60·2 (48·7–69·5)	61·2 (50·2–70·5)
Other pharynx cancer	75·4 (68·3–81·6)	76·4 (69·4–82·4)	76·7 (69·6–82·6)	76·4 (69·3–82·3)

Esophageal cancer	77·4 (68·2–85·8)	78·3 (69–86·4)	77·7 (68·3–85·9)	78·3 (69–86·4)
Stomach cancer	25·1 (17–42·9)	25 (16·9–43·2)	25·3 (17·2–43·2)	25 (16·9–43·2)
Colon and rectum cancer	61·9 (52·7–70·8)	62·4 (54·1–70·7)	62·4 (53·7–71)	62·4 (54·1–70·7)
Liver cancer	79·4 (74·8–83·8)	78·8 (74·2–83·3)	79·1 (74·5–83·6)	78·8 (74·2–83·3)
Gallbladder and biliary tract cancer	25·3 (14·6–37·7)	26·4 (15·5–39·2)	23·3 (12·9–36)	26·5 (15·6–39·2)
Pancreatic cancer	43·4 (33·5–54·7)	42·7 (33·6–53·5)	43·2 (33·8–54·3)	42·7 (33·6–53·5)
Larynx cancer	80·7 (71·3–87·7)	81·1 (72·7–87·3)	80·5 (71·9–86·9)	81·1 (72·8–87·3)
Tracheal, bronchus, and lung cancer	82·4 (78·9–86·1)	81·4 (78–85)	81·9 (78·4–85·6)	81·4 (78–85)
Breast cancer	34·2 (24·9–45·8)	33 (24·3–43·5)	33·8 (24·7–44·6)	32·9 (24·2–43·2)
Cervical cancer	100 (100–100)	100 (100–100)	100 (100–100)	100 (100–100)
Uterine cancer	52 (36·8–66·5)	53·8 (38·2–67·7)	54·4 (38·6–68)	53·7 (38·2–67·6)
Ovarian cancer	19·3 (8·51–33·4)	17·4 (7·22–30·7)	16·8 (6·94–29·8)	17·4 (7·23–30·7)
Prostate cancer	5·87 (2·62–9·41)	6·46 (2·86–10·2)	6·82 (3·03–10·7)	6·37 (2·84–10·1)
Kidney cancer	40·9 (30–51·2)	41·2 (31·1–51)	41·3 (31·4–50·9)	41·2 (31·1–51)
Bladder cancer	42·5 (27·5–57·3)	45·1 (31–59·1)	45·4 (31·3–58·6)	45·1 (30·9–59·1)
Thyroid cancer	16·8 (8·5–26·5)	18·1 (9·37–28·1)	18·5 (9·92–28·4)	18 (9·19–28·2)
Mesothelioma	95·9 (94·3–97·1)	92·4 (89·8–94·5)	95·5 (93·7–96·9)	92·3 (89·8–94·4)
Non-Hodgkin lymphoma	8·12 (3·55–14)	8·6 (3·82–14·6)	8·35 (3·71–14·2)	8·61 (3·83–14·6)
Multiple myeloma	9·27 (4·2–15·6)	9·83 (4·59–16·6)	9·57 (4·26–16·1)	9·85 (4·59–16·6)
Leukemia	36·1 (22·4–50·4)	33·5 (22·2–45)	36·7 (23·2–50·6)	33·3 (22·1–44·8)
Cardiovascular diseases	81·5 (77·9–84·7)	81·4 (78·5–84·2)	70·9 (66·1–75·4)	83·1 (80·4–85·6)
Rheumatic heart disease	21·3 (12·3–40·1)	21·1 (13·3–34·5)	20·9 (12·7–36·3)	21·2 (13·4–34·4)
Ischemic heart disease	92·1 (88·1–95·4)	93·5 (90·7–96)	91·9 (88·3–95)	93·6 (90·7–96)
Stroke	78·3 (70·9–86·6)	81·6 (76·4–87·1)	80·6 (75·2–86·9)	82·1 (76·7–87·4)
Hypertensive heart disease	100 (100–100)	100 (100–100)	100 (100–100)	100 (100–100)
Non-rheumatic valvular heart disease	24·3 (15·4–36·8)	22·1 (15–31·2)	7·12 (4·56–10·3)	24·6 (16·8–34·7)
Cardiomyopathy and myocarditis	39·5 (32·9–47)	42·1 (36–47·6)	40·3 (34·3–46·5)	42·3 (36·2–48)
Atrial fibrillation and flutter	50·2 (40·3–60·4)	55·7 (46·9–64·4)	56·8 (48·4–65·3)	54·1 (45·4–63·2)
Aortic aneurysm	54·2 (48·3–60·2)	61·8 (57·3–66·4)	--	61·8 (57·3–66·4)
Endocarditis	30·7 (21–43)	32·2 (23·1–42·7)	29·9 (19·8–42·5)	32·3 (23·1–42·6)
Other cardiovascular and circulatory diseases	22·3 (17·4–29·5)	24·7 (19·8–30·9)	30·3 (23·5–37·5)	23 (18·2–29·2)
Chronic respiratory diseases	65·3 (60·6–70·1)	59·4 (55·5–63·1)	52·5 (47·8–56·9)	65·2 (61·1–69·4)

Chronic obstructive pulmonary disease	73·6 (69·2–77·5)	71 (67·4–74·5)	63·8 (60·2–67·6)	75·1 (71·5–78·8)
Pneumoconiosis	100 (100–100)	100 (100–100)	100 (100–100)	100 (100–100)
Asthma	48·5 (37·8–59·7)	40·8 (32·8–49·4)	39·9 (32·2–48·5)	47·7 (38·4–57·5)
Digestive diseases	55·1 (48·9–60·9)	57·9 (51·6–63·8)	18·3 (12·3–24·9)	64·6 (58·3–70·4)
Cirrhosis and other chronic liver diseases	80·5 (72·4–88·4)	82·6 (75·2–89·6)	77·9 (69·1–85·9)	82·6 (75·2–89·6)
Upper digestive system diseases	21·8 (18·3–25·3)	7·14 (4·34–10·6)	1·42 (0·857–2·23)	24·6 (21–28·1)
Gallbladder and biliary diseases	47·9 (32·7–63·2)	52·8 (38·4–66·3)	54·6 (40·6–67·6)	50·3 (35·5–64·8)
Pancreatitis	33·9 (17·2–51·2)	38·2 (21·1–55·8)	34·8 (18·2–52·6)	38·7 (21·4–56·6)
Neurological disorders	25·7 (10·9–41·3)	16·1 (7·86–28·2)	11·3 (5·72–21·5)	21·9 (8·43–37·6)
Alzheimer's disease and other dementias	40·6 (26·8–55·8)	43·6 (29·5–58·4)	44·2 (29·9–59·1)	43·2 (29·3–57·7)
Parkinson's disease	-9·26 (-14·8–-4·42)	-10·7 (-16·6–-5·24)	-11·5 (-17·4–-5·78)	-10·6 (-16·4–-5·15)
Idiopathic epilepsy	22 (14·3–30·7)	19·4 (12·7–27·3)	18·2 (11·9–25·5)	22·7 (15–31·2)
Multiple sclerosis	14·8 (10·8–19·1)	15·5 (11·5–19·7)	15 (11·2–18·9)	15·9 (11·7–20·4)
Mental disorders	--	9·81 (4·95–15·3)	9·81 (4·95–15·3)	--
Depressive disorders	--	19 (8·97–28·6)	19 (8·97–28·6)	--
Anxiety disorders	--	6·32 (1·46–13·8)	6·32 (1·46–13·8)	--
Idiopathic developmental intellectual disability	--	50·2 (25·7–69·7)	50·2 (25·7–69·7)	--
Substance use disorders	100 (100–100)	100 (100–100)	100 (100–100)	100 (100–100)
Alcohol use disorders	100 (100–100)	100 (100–100)	100 (100–100)	100 (100–100)
Drug use disorders	100 (100–100)	100 (100–100)	100 (100–100)	100 (100–100)
Diabetes and kidney diseases	99·9 (99·8–99·9)	99·9 (99·9–100)	100 (100–100)	99·9 (99·9–99·9)
Diabetes mellitus	100 (100–100)	100 (100–100)	100 (100–100)	100 (100–100)
Chronic kidney disease	100 (100–100)	100 (100–100)	100 (100–100)	100 (100–100)
Sense organ diseases	--	10 (8·95–11·3)	10 (8·95–11·3)	--
Blindness and vision loss	--	10·2 (6·41–14·8)	10·2 (6·41–14·8)	--
Age-related and other hearing loss	--	10·7 (9·67–11·9)	10·7 (9·67–11·9)	--
Musculoskeletal disorders	4·07 (1·2–7·43)	20·1 (16·8–23·6)	20·5 (17·2–24)	3·79 (1·17–6·9)
Rheumatoid arthritis	12 (3·51–21·5)	14·1 (4·39–24·9)	14·3 (4·45–25·4)	13·7 (4·19–24·2)
Osteoarthritis	--	12·7 (7·12–19·1)	12·7 (7·12–19·1)	--
Low back pain	--	40·2 (34·5–45·8)	40·2 (34·5–45·8)	--
Gout	--	58 (41·4–74·6)	58 (41·4–74·6)	--
Other non-communicable diseases	0·119 (0·0689–0·19)	0·139 (0·0786–0·228)	--	0·364 (0·216–0·568)

Sudden infant death syndrome	9·47 (5·58–14·7)	9·46 (5·57–14·7)	--	9·46 (5·57–14·7)
Injuries	28·4 (23·7–32·8)	25·2 (21–29·3)	32·1 (28·6–35·6)	22 (17·1–27·1)
Transport injuries	22·6 (17·4–28·1)	22·4 (17·2–27·9)	28·8 (25–32·5)	20·5 (14·7–26·5)
Road injuries	22 (16·7–27·5)	21·3 (16–27)	26·5 (22·7–30·5)	20 (14·1–26·1)
Other transport injuries	32·8 (27·8–37·8)	34 (29·5–38·4)	39·4 (35·2–42·9)	29·1 (23·8–34·8)
Unintentional injuries	38 (33·3–41·8)	29·4 (25·5–33·3)	33·4 (29·2–37·2)	23·9 (20·2–27·8)
Falls	54·9 (48·4–59·1)	45 (39·9–49)	44·2 (39·1–48·3)	46·5 (41·2–50·6)
Drowning	-11 (-18·2–-3·28)	-11·5 (-18·2–-4·16)	9·59 (6·06–14·4)	-12 (-19–-4·46)
Fire, heat, and hot substances	11·2 (7·44–16·4)	13·1 (9·18–18·4)	13·7 (9·54–19·1)	12·4 (8·55–17·6)
Poisonings	16·2 (11·4–22·6)	15·5 (11·1–21·4)	12·3 (8·54–17·1)	16·9 (12·1–23·3)
Exposure to mechanical forces	9·79 (4·32–15·5)	18·3 (14·7–22·4)	22·4 (19·6–25·9)	6·39 (0·525–13)
Animal contact	25·5 (21·3–30·3)	24 (19·9–29·1)	24·7 (20·4–29·6)	21·8 (17·7–26·7)
Foreign body	3 (1·98–4·45)	4·12 (2·78–5·96)	3·61 (2·4–5·15)	4·32 (2·87–6·42)
Environmental heat and cold exposure	7·61 (3·24–13·4)	7·94 (3·4–13·9)	7·17 (3·04–12·6)	8·29 (3·62–14·5)
Exposure to forces of nature	6·63 (2·83–11·6)	6·85 (2·94–11·9)	7·8 (3·33–13·5)	6·26 (2·75–11)
Other unintentional injuries	18 (12·7–24·9)	14·1 (9·89–19·7)	13 (8·94–18·3)	20 (14·3–27·3)
Self-harm and interpersonal violence	21·1 (11·9–30·3)	22·4 (13·7–31)	27·4 (21·4–33·8)	22 (13·1–30·9)
Self-harm	22·1 (10·6–33·5)	24·4 (12·9–36·1)	23·6 (13·9–33)	24·4 (12·8–36·2)
Interpersonal violence	19·3 (11·4–27·6)	19·4 (12·1–27·5)	28·7 (22–35·9)	17·7 (10·1–25·8)

Supplementary Results Table 2. All-age PAFs attributable to the joint distribution of all risk factors for all ages, both sexes combined for each GBD level 1, level 2, and level 3 cause and all causes in 2019 for New York. Results are shown for deaths, YLLs, YLDs, and DALYs Data in parenthesis are 95% uncertainty intervals. PAFs=population attributable fractions. GBD=Global Burden of Disease. YLLs=years of life lost. YLDs=years lived with disability. DALYs=disability-adjusted life-years

	Deaths 2019	DALYs 2019	YLDs 2019	YLLs 2019
All causes	61·8 (58·5–64·8)	45·8 (42·6–49·2)	31·7 (29·3–34)	61·5 (59·1–63·8)
Communicable, maternal, neonatal, and nutritional diseases	46·9 (41·5–52·2)	56·5 (53·2–59·7)	50·9 (46–56·1)	60·2 (56·1–64·3)
HIV/AIDS and sexually transmitted infections	93·6 (92·1–94·6)	93·1 (91·5–94·3)	95·2 (93·9–96·5)	91·9 (90·1–93·2)
HIV/AIDS	93·5 (92–94·6)	92·5 (90·9–93·8)	94 (92·7–95·2)	91·8 (90–93·1)
Sexually transmitted infections excluding HIV	99·3 (99–99·5)	99·8 (99·6–99·9)	100 (100–100)	97 (95·8–98)
Respiratory infections and tuberculosis	43·1 (35·9–50·2)	37·4 (30·8–43·7)	2·6 (1·9–3·41)	47·5 (40·1–54·3)
Tuberculosis	57·6 (48–66·5)	59·9 (50–68·1)	55·7 (45·2–64·6)	60·8 (51·2–68·8)
Lower respiratory infections	43 (35·6–50·2)	47·2 (39·7–54)	32·5 (26·6–38)	47·4 (39·8–54·4)
Upper respiratory infections	0·542 (0·333–0·849)	0·0174 (0·00787–0·0311)	0·00516 (0–0·0157)	1·29 (0·796–1·97)
Otitis media	1·26 (0·485–2·67)	3·41 (2·15–4·69)	3·41 (2·14–4·71)	3·66 (1·67–6·93)
Enteric infections	8·86 (5·52–13·7)	9·94 (6·62–14·8)	9·88 (6·52–14·8)	10·1 (6·65–14·9)
Diarrheal diseases	8·93 (5·55–13·8)	9·99 (6·65–14·9)	9·89 (6·53–14·8)	10·2 (6·73–15·1)
Other infectious diseases	0·741 (0·428–0·999)	1·22 (0·793–1·59)	0·687 (0·427–1·07)	1·37 (0·818–1·84)
Meningitis	1·24 (0·877–1·74)	2·79 (2·04–3·83)	0·000498 (0–0·00163)	2·96 (2·15–4·05)
Encephalitis	0·538 (0·386–0·753)	1·44 (1·05–2)	7·91e-05 (0–0·000247)	1·54 (1·1–2·14)
Measles	9·11 (-4·19–42·9)	2·58 (-1·04–22·2)	0·303 (0·00802–0·784)	9·28 (-4·27–43·7)
Acute hepatitis	22·5 (14·2–34·4)	9·65 (6·05–14·1)	3·5 (2·13–5·34)	24·4 (15·5–36·4)
Maternal and neonatal disorders	80·3 (77·1–83·1)	80·1 (77·7–82·4)	75·7 (71·3–79·6)	83·4 (80·9–85·6)
Maternal disorders	11·4 (4·03–19)	11·3 (4·01–18·8)	11·3 (4·02–18·9)	11·3 (4–18·8)
Neonatal disorders	89·2 (87·7–90·8)	84 (81·6–86·1)	77·2 (72·8–81·2)	89·2 (87·7–90·8)
Nutritional deficiencies	93 (91·2–97·4)	87·8 (84·5–90·9)	87·5 (83·9–90·8)	91·1 (89·6–96·1)
Protein-energy malnutrition	100 (100–100)	100 (100–100)	100 (100–100)	100 (100–100)
Vitamin A deficiency	--	100 (100–100)	100 (100–100)	--
Dietary iron deficiency	--	100 (100–100)	100 (100–100)	--
Non-communicable diseases	64·2 (60·5–67·6)	46·6 (42·9–50·3)	30·6 (27·9–33·1)	65·1 (62·3–67·7)
Neoplasms	47·4 (43·3–51·9)	46·7 (42·8–50·9)	30·4 (26·2–34·8)	47·9 (44–52)
Lip and oral cavity cancer	67·5 (58·5–75·3)	69·8 (61·6–77)	69·4 (60·7–76·8)	69·8 (61·6–77)
Nasopharynx cancer	65·9 (55–74·8)	65·6 (54·5–74·3)	64·7 (54·1–73·6)	65·6 (54·6–74·3)
Other pharynx cancer	77·6 (70·4–83·3)	78·6 (71·9–84)	78·9 (72·4–84·3)	78·6 (71·8–84)

Esophageal cancer	77·3 (67·4–85·9)	78·5 (68·4–86·9)	77·6 (67·6–86·2)	78·5 (68·4–86·9)
Stomach cancer	23·8 (15·8–42)	24·5 (16·3–43)	24·7 (16·7–42·7)	24·4 (16·3–43)
Colon and rectum cancer	61·9 (53–70·7)	62·7 (54·3–70·8)	62·6 (54–71·2)	62·7 (54·3–70·8)
Liver cancer	76·9 (71·4–82·1)	76·7 (71·3–81·9)	76·9 (71·2–82·3)	76·7 (71·3–81·9)
Gallbladder and biliary tract cancer	26·9 (15·6–39·8)	27·8 (16·6–41·1)	25·6 (14·6–38·6)	27·9 (16·6–41·2)
Pancreatic cancer	42·5 (32·5–54·3)	42·3 (32·7–53·4)	42·6 (32·9–54·1)	42·3 (32·7–53·4)
Larynx cancer	79·8 (69·2–87·8)	80·9 (71·4–87·7)	80·6 (70·9–87·6)	81 (71·5–87·8)
Tracheal, bronchus, and lung cancer	81·2 (77·5–85·3)	80·6 (77·2–84·5)	81 (77·3–85·3)	80·6 (77·2–84·5)
Breast cancer	36·4 (27·1–47)	35·1 (26·5–44·6)	35·8 (26·8–46)	35 (26·4–44·3)
Cervical cancer	100 (100–100)	100 (100–100)	100 (100–100)	100 (100–100)
Uterine cancer	54·4 (39·3–68·3)	55·8 (40·7–69·1)	56·2 (40·9–69·5)	55·7 (40·7–69)
Ovarian cancer	18·8 (8·09–32·4)	17 (7·12–29·9)	16·5 (6·82–29)	17 (7·14–29·9)
Prostate cancer	5·17 (2·3–8·39)	5·87 (2·66–9·35)	6·25 (2·82–9·78)	5·75 (2·6–9·19)
Kidney cancer	41·3 (30·4–52·1)	42·1 (31·7–52·1)	42·4 (31·9–52·2)	42 (31·6–52·1)
Bladder cancer	37·9 (22·4–52·5)	41·5 (27·1–55·5)	42·2 (28·4–55·7)	41·4 (27–55·5)
Thyroid cancer	18 (9·39–28)	19·2 (10·2–29·5)	19·5 (10·5–29·6)	19·1 (10·1–29·6)
Mesothelioma	95·6 (94·1–96·9)	91·6 (89·1–93·8)	95·1 (93·3–96·6)	91·5 (89–93·7)
Non-Hodgkin lymphoma	8·72 (3·88–14·8)	9·24 (4·17–15·5)	8·98 (3·97–15·2)	9·26 (4·2–15·5)
Multiple myeloma	10 (4·5–16·7)	10·6 (4·95–17·5)	10·4 (4·88–17·5)	10·6 (4·94–17·5)
Leukemia	35·6 (21·8–49·9)	34·4 (22·8–46·5)	36·1 (22·6–50·2)	34·3 (22·9–46·4)
Cardiovascular diseases	83·5 (79·7–86·6)	83 (80–85·4)	70·6 (66·2–75·1)	85·2 (82·6–87·5)
Rheumatic heart disease	21·4 (11·8–41·4)	21·3 (13·5–36·1)	21·4 (13·4–36·3)	21·3 (13·6–36·1)
Ischemic heart disease	91·9 (87·7–95·4)	93·4 (90·5–96)	92·3 (88·8–95·4)	93·5 (90·6–96)
Stroke	78·9 (72·1–86·4)	82·4 (77·7–87·5)	81·2 (76–87·3)	83·1 (78·4–88)
Hypertensive heart disease	100 (100–100)	100 (100–100)	100 (100–100)	100 (100–100)
Non-rheumatic valvular heart disease	24·8 (14·9–38·7)	19·6 (12·5–28·8)	6·92 (4·35–9·93)	25·1 (16·5–36·6)
Cardiomyopathy and myocarditis	37·8 (30·8–45·8)	41 (35·3–46·5)	40·3 (34·1–46·7)	41·1 (35–46·9)
Atrial fibrillation and flutter	52·7 (42·6–62·6)	58·3 (49·5–66·7)	59·6 (51·1–68)	56·3 (47·1–65·2)
Aortic aneurysm	52·8 (46·5–58·9)	61·6 (56·3–66·1)	--	61·6 (56·3–66·1)
Endocarditis	30·7 (20·8–43·1)	32·1 (22·9–42·9)	30·2 (20·6–42·8)	32·3 (23–43·1)
Other cardiovascular and circulatory diseases	21·6 (16·9–27·4)	24·5 (19·9–29·4)	30·7 (24·3–37·4)	22·4 (18–27)
Chronic respiratory diseases	61·6 (57·4–65·7)	55·7 (52–59·5)	51·4 (46·7–56)	62·1 (58·7–65·6)
Chronic obstructive pulmonary disease	70·7 (66–75)	67·6 (63·9–71·2)	62·2 (58·3–66)	73·1 (69·2–76·8)

Pneumoconiosis	100 (100–100)	100 (100–100)	100 (100–100)	100 (100–100)
Asthma	51·2 (40–62·6)	42·4 (34·3–51·1)	41·7 (33·7–50·4)	51·7 (42–61·9)
Digestive diseases	44·3 (38·9–49·4)	43·5 (37·6–48·8)	16 (10·9–21·7)	54·8 (49·1–59·6)
Cirrhosis and other chronic liver diseases	82·8 (74·7–89·9)	84·5 (77·3–91)	80·7 (72·9–87·9)	84·6 (77·3–91·1)
Upper digestive system diseases	20·3 (16·8–24)	5·58 (3·5–8·2)	1·52 (0·963–2·27)	23·5 (19·9–27·2)
Gallbladder and biliary diseases	50·5 (34·6–65·2)	55·2 (40·8–68·3)	56·4 (42·4–69·1)	52·4 (37·4–66·6)
Pancreatitis	41·9 (24·8–58·3)	45·3 (28–61·8)	42·8 (25·9–59·5)	45·8 (28·5–62·2)
Neurological disorders	28·9 (13·6–44·3)	17·5 (8·8–29·8)	12 (6·1–22·6)	25·4 (11–40·7)
Alzheimer's disease and other dementias	40·4 (26–56·2)	43·3 (28·6–58·7)	44 (29·3–59)	42·8 (28·4–58·2)
Parkinson's disease	-8 (-13·1–-3·86)	-9·44 (-14·8–-4·78)	-10·1 (-15·6–-5·13)	-9·29 (-14·7–-4·64)
Idiopathic epilepsy	25·7 (17–34·7)	23·2 (15·4–31·3)	22·4 (14·8–30·3)	25·9 (17·4–34·8)
Multiple sclerosis	13·7 (10–17·8)	14·5 (10·8–18·9)	14·2 (10·6–18·1)	14·8 (10·9–19·2)
Mental disorders	--	6·57 (2·98–10·2)	6·57 (2·98–10·2)	--
Depressive disorders	--	15·4 (6·71–24·5)	15·4 (6·71–24·5)	--
Anxiety disorders	--	2·07 (0·425–5·42)	2·07 (0·425–5·42)	--
Idiopathic developmental intellectual disability	--	44·9 (23·1–61·2)	44·9 (23·1–61·2)	--
Substance use disorders	100 (100–100)	100 (100–100)	100 (100–100)	100 (100–100)
Alcohol use disorders	100 (100–100)	100 (100–100)	100 (100–100)	100 (100–100)
Drug use disorders	100 (100–100)	100 (100–100)	100 (100–100)	100 (100–100)
Diabetes and kidney diseases	99·9 (99·8–99·9)	100 (99·9–100)	100 (100–100)	99·9 (99·9–99·9)
Diabetes mellitus	100 (100–100)	100 (100–100)	100 (100–100)	100 (100–100)
Chronic kidney disease	100 (100–100)	100 (100–100)	100 (100–100)	100 (100–100)
Sense organ diseases	--	9·53 (8·52–10·8)	9·53 (8·52–10·8)	--
Blindness and vision loss	--	9·7 (6·07–14·4)	9·7 (6·07–14·4)	--
Age-related and other hearing loss	--	10·5 (9·49–11·6)	10·5 (9·49–11·6)	--
Musculoskeletal disorders	2·35 (0·705–4·23)	19·3 (16·1–22·7)	19·5 (16·3–22·9)	2·15 (0·654–3·82)
Rheumatoid arthritis	10·9 (3·24–19·8)	13·7 (4·33–24·7)	13·9 (4·37–25)	12·7 (3·88–22·6)
Osteoarthritis	--	15·6 (8·8–23)	15·6 (8·8–23)	--
Low back pain	--	40·4 (34·9–46·1)	40·4 (34·9–46·1)	--
Gout	--	60·1 (44·3–75·8)	60·1 (44·3–75·8)	--
Other non-communicable diseases	0·0643 (0·0429–0·0937)	0·0673 (0·0443–0·101)	--	0·221 (0·147–0·312)
Sudden infant death syndrome	7·3 (4·93–10·3)	7·29 (4·93–10·3)	--	7·29 (4·93–10·3)
Injuries	30·1 (25·2–34·4)	29·6 (25·1–34·1)	35·7 (31·4–40·4)	24·3 (18·7–29·4)

Transport injuries	28·1 (21·2–36)	30·7 (24·1–38·1)	35·4 (30·6–40·7)	28·1 (20·1–37·4)
Road injuries	27 (19·9–34·9)	29·1 (22·3–36·9)	33·3 (28·2–39·2)	27·1 (19–36·4)
Other transport injuries	42·2 (35·7–50)	42 (36·8–47·5)	43·2 (38·5–47·2)	40·2 (33–49)
Unintentional injuries	38·9 (33·7–43·2)	34·1 (29·5–38·9)	36·6 (31·7–41·6)	28·2 (23·3–33·3)
Falls	56·3 (49·4–61·2)	48 (42·5–52·9)	47·4 (41·8–52·2)	49·8 (44·1–55·2)
Drowning	-2·41 (-14·3–8·93)	-1·93 (-12·9–8·56)	14 (9·36–19·8)	-2·98 (-14·6–7·96)
Fire, heat, and hot substances	14·9 (10·2–20·5)	20·4 (14·7–27·2)	21·8 (15·7–29·2)	17·7 (12·7–24·2)
Poisonings	24·1 (17·6–32·2)	23·3 (17·2–30·8)	19·8 (14·6–26·4)	26·4 (19·4–35·2)
Exposure to mechanical forces	16 (7·14–26)	27·5 (22·2–34·6)	29·5 (24·7–36)	14·8 (4·46–27·1)
Animal contact	28 (23–34)	31·1 (25·8–37·4)	31·6 (26·2–37·8)	25·5 (20·2–32·3)
Foreign body	4·89 (3·12–7·16)	7·33 (4·93–10·7)	7·12 (4·82–10·3)	7·47 (4·76–11·1)
Environmental heat and cold exposure	8·51 (3·67–14·8)	8·72 (3·8–15)	8·47 (3·63–14·8)	9·14 (3·97–15·8)
Exposure to forces of nature	7·87 (3·41–13·6)	7·83 (3·39–13·6)	9·06 (3·94–15·6)	7·26 (3·16–12·6)
Other unintentional injuries	27·1 (19·3–36·4)	21 (15–28·2)	20·4 (14·5–27·4)	31·7 (22·9–42·8)
Self-harm and interpersonal violence	20·2 (10·5–29·7)	21·1 (12·8–29·2)	28·2 (21·9–34·6)	20·1 (11·2–29)
Self-harm	21·6 (8·17–34·1)	23·4 (10·1–35·9)	24·8 (13·8–35·7)	23·3 (10–35·9)
Interpersonal violence	17·8 (8·67–26·7)	18·9 (10·5–27·2)	31·2 (24·1–38·6)	15·6 (6·52–24·7)

Supplementary Results Table 2. All-age PAFs attributable to the joint distribution of all risk factors for all ages, both sexes combined for each GBD level 1, level 2, and level 3 cause and all causes in 2019 for North Carolina. Results are shown for deaths, YLLs, YLDs, and DALYs Data in parenthesis are 95% uncertainty intervals. PAFs=population attributable fractions. GBD=Global Burden of Disease. YLLs=years of life lost. YLDs=years lived with disability. DALYs=disability-adjusted life-years	Deaths 2019	DALYs 2019	YLDs 2019	YLLs 2019
All causes	61 (58·6–63·4)	48·7 (45·6–51·6)	34·3 (31·7–36·7)	60·7 (58·9–62·6)
Communicable, maternal, neonatal, and nutritional diseases	44·4 (40·7–47·9)	57·2 (54·2–60·1)	51·4 (46·8–55·9)	60·5 (56·8–63·8)
HIV/AIDS and sexually transmitted infections	85·8 (69·8–91·9)	84·6 (69·4–91·2)	88·4 (76·4–93·9)	82·3 (65–89·8)
HIV/AIDS	85·5 (69·3–91·8)	83 (66·3–90·3)	84·8 (70·2–91·7)	82·1 (64·6–89·7)
Sexually transmitted infections excluding HIV	98·8 (98·3–99·2)	99·6 (99·3–99·8)	100 (100–100)	95·7 (93·9–97·1)
Respiratory infections and tuberculosis	37·7 (32·9–42·6)	34·4 (29·2–40)	2·47 (1·89–3·27)	43·3 (38–48·1)
Tuberculosis	54·3 (45–63)	57·7 (48·7–66·3)	53·7 (43·8–62·5)	58·3 (49·3–66·9)
Lower respiratory infections	37·6 (32·7–42·5)	43 (37·6–48)	33·1 (27·8–38·1)	43·2 (37·8–48·1)
Upper respiratory infections	0·748 (0·45–1·22)	0·0211 (0·0101–0·0381)	0·00505 (1·81e-05–0·0151)	1·58 (0·937–2·51)
Otitis media	1·7 (0·645–3·65)	3·74 (2·43–5·11)	3·72 (2·41–5·08)	4·45 (2·01–8·3)
Enteric infections	11·7 (7·36–18·4)	12·9 (8·67–19·7)	12·8 (8·55–19·4)	13·1 (8·85–19·6)
Diarrheal diseases	11·7 (7·39–18·5)	13 (8·71–19·8)	12·8 (8·55–19·4)	13·2 (8·92–19·8)
Other infectious diseases	0·762 (0·506–1·03)	1·32 (0·956–1·65)	0·663 (0·415–1·05)	1·48 (1·03–1·88)
Meningitis	1·53 (1·12–2·06)	3·34 (2·47–4·41)	0·000567 (1·27e-06–0·00182)	3·5 (2·58–4·64)
Encephalitis	0·674 (0·475–0·967)	1·77 (1·27–2·52)	0·000219 (6·54e-07–0·000672)	1·83 (1·32–2·6)
Measles	9·42 (-3·98–43·5)	9·58 (-4·05–44·2)	0·216 (0–0·932)	9·59 (-4·05–44·2)
Acute hepatitis	21·6 (14·2–34·2)	9·33 (5·95–14)	3·48 (2·19–5·43)	22·8 (14·6–34·9)
Maternal and neonatal disorders	87·3 (85·4–88·9)	84·7 (82·9–86·4)	76·6 (72·2–80·3)	88·9 (87·4–90·2)
Maternal disorders	11·7 (4·3–19·4)	11·5 (4·23–19·3)	11·4 (4·15–19·2)	11·6 (4·24–19·2)
Neonatal disorders	91·8 (90·6–93)	87·2 (85·3–88·9)	78·4 (73·9–82·2)	91·8 (90·6–93)
Nutritional deficiencies	94·6 (84·5–96)	89·8 (86·5–92·1)	88·4 (84·9–91·5)	94 (84·3–95·4)
Protein-energy malnutrition	100 (100–100)	100 (100–100)	100 (100–100)	100 (100–100)
Vitamin A deficiency	--	100 (100–100)	100 (100–100)	--
Dietary iron deficiency	--	100 (100–100)	100 (100–100)	--
Non-communicable diseases	64·3 (61·4–66·9)	50·6 (46·9–54)	33·7 (30·9–36·5)	65·6 (63·6–67·8)
Neoplasms	50·6 (46·9–54·6)	50 (46·4–53·9)	31·3 (27·5–35·6)	51·2 (47·8–55)
Lip and oral cavity cancer	67·5 (59·6–74·6)	70 (62·6–76·9)	69·4 (61·7–76·2)	70·1 (62·7–76·9)
Nasopharynx cancer	63 (52·2–72·5)	63·1 (52·7–71·8)	62 (51·6–70·9)	63·2 (52·7–71·8)
Other pharynx cancer	76·7 (70–82·2)	78·1 (71·6–83·3)	78·3 (71·6–83·7)	78 (71·6–83·3)
Esophageal cancer	80·2 (71·2–87·9)	81·3 (72·5–88·9)	80·3 (71·6–88·1)	81·3 (72·5–88·9)

Stomach cancer	25·8 (17·7–43·3)	26·5 (18·2–44·3)	26·7 (18·4–44·4)	26·5 (18·2–44·3)
Colon and rectum cancer	62·8 (53·9–71·7)	63·8 (55·5–72)	63·7 (55·2–72·3)	63·8 (55·5–71·9)
Liver cancer	79·5 (74·6–84·3)	79·5 (74·7–84·1)	79·6 (74·7–84·2)	79·5 (74·7–84·1)
Gallbladder and biliary tract cancer	26·9 (15·9–40)	28·2 (16·8–41·4)	25·9 (14·8–39·2)	28·2 (16·9–41·4)
Pancreatic cancer	44·8 (35·1–56·2)	44·8 (35·7–55·5)	45 (35·4–56·1)	44·8 (35·7–55·5)
Larynx cancer	81·6 (71·6–89·1)	82·9 (73·9–89·1)	82·5 (73·4–89)	82·9 (74·1–89·1)
Tracheal, bronchus, and lung cancer	83·8 (80·6–87·3)	83·4 (80·2–86·8)	83·7 (80·4–87·3)	83·4 (80·2–86·8)
Breast cancer	35·1 (25·6–46·3)	34·2 (25·3–44·2)	34·7 (25·6–45·2)	34·1 (25·3–44)
Cervical cancer	100 (100–100)	100 (100–100)	100 (100–100)	100 (100–100)
Uterine cancer	54·5 (39·1–68·2)	56·4 (41–69·7)	56·9 (41·7–70·1)	56·3 (40·9–69·6)
Ovarian cancer	19·8 (8·71–34·3)	18 (7·43–31·8)	17·5 (7·12–30·9)	18·1 (7·44–31·9)
Prostate cancer	6·25 (2·77–10·3)	6·97 (3·06–11·2)	7·35 (3·25–11·7)	6·86 (3–11·1)
Kidney cancer	43·1 (31·9–53·7)	43·9 (33·8–53·8)	44·2 (34·2–53·7)	43·9 (33·8–53·8)
Bladder cancer	41·8 (26·2–57)	45·3 (30–59·4)	45·9 (31·4–59·6)	45·2 (30–59·4)
Thyroid cancer	18·4 (9·62–28·8)	19·7 (10·3–30·5)	20·1 (10·6–30·4)	19·6 (10·3–30·6)
Mesothelioma	95·5 (93·9–96·8)	91·7 (88·9–93·9)	95·1 (93·3–96·7)	91·6 (88·8–93·8)
Non-Hodgkin lymphoma	8·75 (3·93–14·9)	9·3 (4·33–15·6)	9·05 (4·08–15·2)	9·32 (4·34–15·6)
Multiple myeloma	10 (4·68–16·7)	10·6 (5·05–17·6)	10·5 (4·92–17·3)	10·7 (5·04–17·6)
Leukemia	37·5 (23·5–51·9)	36·3 (23·9–48·9)	38·2 (23·7–52·5)	36·2 (24–48·7)
Cardiovascular diseases	81·4 (77·8–84·5)	82·3 (79·6–84·6)	73 (68·8–77·2)	83·7 (81·2–86)
Rheumatic heart disease	21·5 (12·1–39·7)	21·6 (13·7–35·9)	21·5 (13·2–36·4)	21·6 (13·8–35·8)
Ischemic heart disease	93·1 (89·4–96·1)	94·6 (92·2–96·6)	93·1 (90–95·8)	94·6 (92·3–96·6)
Stroke	79·5 (72·7–87)	83·3 (78·8–88·1)	82·6 (77·9–87·9)	83·5 (78·9–88·3)
Hypertensive heart disease	100 (100–100)	100 (100–100)	100 (100–100)	100 (100–100)
Non-rheumatic valvular heart disease	24·8 (15·3–37·8)	22·7 (15·3–32)	7·13 (4·63–10·3)	25·1 (17–35·8)
Cardiomyopathy and myocarditis	40·4 (33·1–47·9)	43·5 (38–49·1)	40·9 (34·9–47·2)	43·7 (38–49·6)
Atrial fibrillation and flutter	51·2 (41·6–61·4)	57·5 (49·2–65·8)	58·9 (50·4–67·1)	55·4 (46·9–64·3)
Aortic aneurysm	55·5 (49·8–61·1)	63·6 (59·2–68·1)	--	63·6 (59·2–68·1)
Endocarditis	30·9 (21·2–43·2)	32·3 (23·6–43·1)	30·3 (20·4–42·6)	32·4 (23·7–43·2)
Other cardiovascular and circulatory diseases	20·7 (15·5–29·5)	23·5 (18·5–30·1)	30·5 (23·6–38·4)	21·7 (17–28·8)
Chronic respiratory diseases	64·2 (59·2–69·2)	60·6 (56·6–64·5)	54·9 (50·3–59·3)	65 (60·5–69·6)
Chronic obstructive pulmonary disease	72·5 (68·2–76·4)	71·7 (68·1–75·2)	66·5 (62·5–70·3)	74·5 (71–78)
Pneumoconiosis	100 (100–100)	100 (100–100)	100 (100–100)	100 (100–100)

Asthma	51·9 (40·8–63)	42·6 (34·8–51·2)	41·6 (33·8–50·1)	51·7 (42·4–61·1)
Digestive diseases	45·4 (40–50·8)	47·3 (41·5–53)	17·2 (11·8–23·3)	55·1 (49·2–60·7)
Cirrhosis and other chronic liver diseases	79·5 (70·6–87·6)	82·1 (74·2–89·6)	77·7 (68·7–85·7)	82·1 (74·2–89·7)
Upper digestive system diseases	22·1 (18·5–25·7)	6·73 (4·14–10·2)	1·55 (0·927–2·49)	25·5 (21·7–29·2)
Gallbladder and biliary diseases	50·9 (35·4–65·5)	56 (42·1–69)	57·7 (43·8–70·3)	53·3 (38·3–67·1)
Pancreatitis	33·8 (18·3–50·7)	38·8 (21·8–56·2)	36·6 (19·8–53·6)	39·1 (22·1–56·5)
Neurological disorders	25 (9·53–40·8)	14·9 (7·31–26·3)	10·6 (5·11–21·3)	21·1 (7·55–37·1)
Alzheimer's disease and other dementias	41·8 (27·8–57·6)	45·2 (30·7–60·2)	45·9 (31·4–60·7)	44·7 (30·5–59·9)
Parkinson's disease	-10 (-15·8–-4·9)	-11·8 (-18·1–-5·98)	-12·4 (-18·6–-6·48)	-11·6 (-18–-5·82)
Idiopathic epilepsy	21·2 (13·8–28·8)	19·5 (12·3–26·4)	18·4 (11·7–25·1)	22·1 (14·5–29·8)
Multiple sclerosis	15·3 (11–20·1)	16·2 (11·8–20·8)	15·7 (11·7–19·9)	16·6 (12–21·6)
Mental disorders	--	8·24 (4–13·1)	8·24 (4–13·1)	--
Depressive disorders	--	18·7 (8·09–28·7)	18·7 (8·09–28·7)	--
Anxiety disorders	--	6·27 (1·42–14·3)	6·27 (1·42–14·3)	--
Idiopathic developmental intellectual disability	--	48·7 (23–69·2)	48·7 (23–69·2)	--
Substance use disorders	100 (100–100)	100 (100–100)	100 (100–100)	100 (100–100)
Alcohol use disorders	100 (100–100)	100 (100–100)	100 (100–100)	100 (100–100)
Drug use disorders	100 (100–100)	100 (100–100)	100 (100–100)	100 (100–100)
Diabetes and kidney diseases	99·8 (99·8–99·9)	99·9 (99·9–99·9)	100 (100–100)	99·8 (99·8–99·9)
Diabetes mellitus	100 (100–100)	100 (100–100)	100 (100–100)	100 (100–100)
Chronic kidney disease	100 (100–100)	100 (100–100)	100 (100–100)	100 (100–100)
Sense organ diseases	--	10·6 (9·51–11·9)	10·6 (9·51–11·9)	--
Blindness and vision loss	--	9·88 (6·3–14·5)	9·88 (6·3–14·5)	--
Age-related and other hearing loss	--	11·4 (10·3–12·7)	11·4 (10·3–12·7)	--
Musculoskeletal disorders	3·87 (1·18–7·12)	21·3 (17·9–25)	21·7 (18·2–25·4)	3·69 (1·17–6·66)
Rheumatoid arthritis	12·5 (3·82–22·2)	15·3 (4·74–27)	15·6 (4·88–27·6)	14·5 (4·49–25·9)
Osteoarthritis	--	16·6 (9·41–24·3)	16·6 (9·41–24·3)	--
Low back pain	--	42·3 (36·2–48·3)	42·3 (36·2–48·3)	--
Gout	--	61 (45·2–76·7)	61 (45·2–76·7)	--
Other non-communicable diseases	0·107 (0·0732–0·15)	0·144 (0·0998–0·203)	--	0·348 (0·244–0·471)
Sudden infant death syndrome	7·46 (5·08–10·4)	7·45 (5·07–10·4)	--	7·45 (5·07–10·4)
Injuries	28·4 (24·4–32·6)	26·4 (22·7–30·2)	31·8 (28·4–35)	23·5 (19–27·9)
Transport injuries	26·8 (21·9–32)	26·6 (21·7–31·8)	30·3 (26·5–34·3)	25·3 (19·8–31·1)

Road injuries	26·4 (21·5–31·6)	25·5 (20·6–30·8)	27·7 (23·9–31·9)	24·9 (19·4–30·7)
Other transport injuries	35·2 (30·3–40·5)	36·5 (32·3–40·8)	39·2 (35·2–42·9)	32·2 (26·6–38·2)
Unintentional injuries	34·7 (30·3–38·2)	28·5 (24·8–32·3)	32·4 (28·4–36·1)	22·4 (18·8–26·1)
Falls	54 (47·4–58·1)	44·8 (39·6–48·6)	44 (38·9–47·8)	46·7 (41·3–50·8)
Drowning	-7·1 (-14–0·509)	-7·4 (-14–-0·274)	10·3 (6·79–14·8)	-7·97 (-14·7–-0·623)
Fire, heat, and hot substances	10·7 (7·18–15·5)	13·9 (9·81–19)	15·2 (10·7–20·8)	12·4 (8·57–17·2)
Poisonings	16·7 (11·9–22·5)	16·4 (11·9–22)	13·6 (9·59–18·6)	17·9 (12·8–24·1)
Exposure to mechanical forces	14·8 (9·81–20·3)	20·7 (17·1–25·2)	23·4 (20·2–27·2)	12·5 (6·91–19·1)
Animal contact	21·5 (17·3–26·5)	24 (19·7–28·9)	25·2 (20·8–30·2)	19·1 (15–24·3)
Foreign body	2·56 (1·69–3·78)	3·53 (2·39–5·02)	3·71 (2·47–5·32)	3·47 (2·27–5·08)
Environmental heat and cold exposure	6·68 (2·81–11·4)	7·19 (3·05–12·3)	6·82 (2·9–11·8)	7·57 (3·2–12·8)
Exposure to forces of nature	6·51 (2·79–11·2)	6·9 (2·98–11·9)	7·65 (3·28–13·2)	6·29 (2·72–10·8)
Other unintentional injuries	18·5 (13–25·6)	14·6 (10·3–20·1)	13·9 (9·71–19·2)	21·2 (15–28·9)
Self-harm and interpersonal violence	22 (13–30·4)	23·4 (14·9–31·3)	30·4 (24·2–36·7)	22·7 (14–31)
Self-harm	21·7 (10–33·3)	23·9 (11·7–35·8)	22·6 (12·4–33)	23·9 (11·8–35·9)
Interpersonal violence	23·1 (15–30·8)	23 (15·5–30·7)	32·5 (25·3–39·9)	21·1 (13·3–28·9)

Supplementary Results Table 2. All-age PAFs attributable to the joint distribution of all risk factors for all ages, both sexes combined for each GBD level 1, level 2, and level 3 cause and all causes in 2019 for North Dakota. Results are shown for deaths, YLLs, YLDs, and DALYs Data in parenthesis are 95% uncertainty intervals. PAFs=population attributable fractions. GBD=Global Burden of Disease. YLLs=years of life lost. YLDs=years lived with disability. DALYs=disability-adjusted life-years

	Deaths 2019	DALYs 2019	YLDs 2019	YLLs 2019
All causes	57·8 (54·5–60·6)	43·8 (40·9–46·8)	30·8 (28·5–33·2)	55·4 (53·1–57·6)
Communicable, maternal, neonatal, and nutritional diseases	41·2 (32·6–49·4)	53·5 (48·9–57·8)	47·7 (42·9–52·7)	57·2 (50·7–63·2)
HIV/AIDS and sexually transmitted infections	89·1 (86·7–91·2)	91·2 (88·8–93·5)	95·4 (93·1–97·7)	86·8 (83·9–89·2)
HIV/AIDS	88·1 (85·4–90·4)	86·9 (83·8–89·4)	88·2 (85·1–90·8)	86·3 (83·3–88·9)
Sexually transmitted infections excluding HIV	99·1 (98·6–99·4)	99·7 (99·5–99·9)	100 (100–100)	96·3 (94·6–97·6)
Respiratory infections and tuberculosis	37 (24·9–48·7)	32·6 (24–41·1)	2·44 (1·86–3·18)	42·7 (31·7–53·1)
Tuberculosis	52·2 (42·1–61·7)	56·7 (47·2–65·6)	55 (45–64·6)	57 (47·7–65·7)
Lower respiratory infections	36·9 (24·7–48·8)	42·7 (31·6–53)	31·8 (26·3–37·2)	42·9 (31·6–53·4)
Upper respiratory infections	0·493 (0·301–0·768)	0·0347 (0·0175–0·0606)	0·00538 (0–0·0173)	1·12 (0·686–1·73)
Otitis media	1·5 (0·541–3·45)	4·12 (2·67–5·61)	4·12 (2·68–5·62)	4·15 (1·83–7·69)
Enteric infections	9·75 (6·07–15·3)	11·4 (7·68–16·9)	10·8 (7·07–16·5)	12·8 (8·85–18·1)
Diarrheal diseases	9·89 (6·14–15·5)	11·5 (7·73–17)	10·8 (7·07–16·5)	13·1 (9·09–18·6)
Other infectious diseases	0·51 (0·333–0·75)	0·95 (0·69–1·26)	0·47 (0·272–0·819)	1·06 (0·742–1·45)
Meningitis	1·21 (0·829–1·72)	2·6 (1·82–3·58)	0·000506 (0–0·0016)	2·75 (1·94–3·81)
Encephalitis	0·551 (0·375–0·765)	1·47 (1·01–2·03)	0·000197 (0–0·000622)	1·52 (1·05–2·1)
Measles	9·27 (-4·19–43·3)	9·43 (-4·27–44)	0·16 (0–0·762)	9·43 (-4·27–44)
Acute hepatitis	13·4 (6·31–26·7)	6·32 (3·47–11·1)	2·42 (1·43–4·12)	14·7 (7·11–28·2)
Maternal and neonatal disorders	81·8 (78·2–84·9)	80·8 (78·1–83·4)	74·3 (69·7–78·1)	84·3 (81·3–86·9)
Maternal disorders	11·2 (4·05–18·4)	11·1 (3·97–18·3)	11 (3·93–18·3)	11·1 (4–18·3)
Neonatal disorders	89·5 (87·6–91·3)	84·7 (82·2–86·8)	76·3 (71·7–80·2)	89·5 (87·6–91·4)
Nutritional deficiencies	90·7 (84–93·9)	88·4 (85·3–91·2)	88 (84·2–91·2)	90·3 (83·5–93·5)
Protein-energy malnutrition	100 (100–100)	100 (100–100)	100 (100–100)	100 (100–100)
Vitamin A deficiency	--	100 (100–100)	100 (100–100)	--
Dietary iron deficiency	--	100 (100–100)	100 (100–100)	--
Non-communicable diseases	61·1 (57·4–64·3)	45·9 (42·5–49·5)	30 (27·5–32·7)	61·3 (58·7–63·6)
Neoplasms	47·3 (43·6–51·5)	46·8 (43·2–50·7)	30·7 (26·9–34·7)	47·9 (44·5–51·7)
Lip and oral cavity cancer	65·3 (56·3–73·3)	68·8 (60·2–76·3)	68·3 (59·6–76·2)	68·8 (60·2–76·4)
Nasopharynx cancer	63·5 (51·9–73·2)	63·8 (52·6–73·3)	62·6 (51·6–72·1)	63·9 (52·6–73·3)
Other pharynx cancer	76·6 (68·8–82·4)	78·1 (71·1–83·6)	78·5 (71·4–84)	78·1 (71–83·6)

Esophageal cancer	79·5 (70·3–87·5)	80·8 (71·3–88·7)	79·7 (70·3–87·4)	80·8 (71·3–88·7)
Stomach cancer	23·9 (15·7–41·9)	24·7 (16·6–43)	25 (16·9–43·1)	24·7 (16·6–43)
Colon and rectum cancer	62·8 (53·9–71·3)	63·8 (55·3–71·9)	63·7 (55·1–71·9)	63·8 (55·3–71·9)
Liver cancer	75·4 (68·6–81·5)	75·9 (69·5–81·4)	75·8 (69·1–81·6)	75·9 (69·5–81·4)
Gallbladder and biliary tract cancer	28·4 (16·8–41·8)	29·5 (17·7–42·4)	27·3 (15·6–40·4)	29·5 (17·8–42·5)
Pancreatic cancer	42·4 (32·8–53·6)	42·5 (33·5–53·5)	42·6 (33·2–53·6)	42·5 (33·5–53·5)
Larynx cancer	81·8 (71·8–88·8)	83·2 (74·7–89·2)	82·9 (74·3–89·2)	83·2 (74·7–89·2)
Tracheal, bronchus, and lung cancer	82·9 (79·5–86·6)	82·3 (79–85·9)	82·7 (79·3–86·4)	82·3 (79–85·9)
Breast cancer	35·3 (26·3–45·7)	34·5 (26·3–43·8)	35·1 (26·5–44·9)	34·4 (26·3–43·6)
Cervical cancer	100 (100–100)	100 (100–100)	100 (100–100)	100 (100–100)
Uterine cancer	54·7 (39·2–69·2)	56·5 (41·2–70·2)	57·1 (41·9–70·5)	56·4 (41·1–70·1)
Ovarian cancer	19·4 (8·75–32·1)	17·2 (7·44–29·1)	16·5 (7·2–28·3)	17·2 (7·45–29·1)
Prostate cancer	5·67 (2·45–9·23)	6·46 (2·82–10·4)	6·88 (3·02–11)	6·33 (2·75–10·2)
Kidney cancer	43·4 (32·4–54·4)	44·1 (33·6–54·3)	44·4 (34–54·4)	44·1 (33·6–54·2)
Bladder cancer	39·6 (24·6–54·7)	43·7 (29·1–57·5)	44·3 (29·7–57·8)	43·6 (29·1–57·5)
Thyroid cancer	19·3 (9·98–29·5)	20·6 (10·8–31·4)	20·9 (11·4–31·3)	20·6 (10·7–31·5)
Mesothelioma	97·1 (96·1–97·9)	94·5 (92·7–95·9)	96·8 (95·4–97·8)	94·4 (92·6–95·8)
Non-Hodgkin lymphoma	9·49 (4·27–15·9)	10 (4·63–16·6)	9·79 (4·46–16·3)	10 (4·64–16·7)
Multiple myeloma	10·8 (5·1–17·9)	11·4 (5·49–18·8)	11·3 (5·24–18·6)	11·4 (5·49–18·8)
Leukemia	36·3 (23–50)	34·6 (23–46·2)	37·4 (23·9–51)	34·5 (22·9–46)
Cardiovascular diseases	80·4 (76·6–83·8)	81·1 (78·2–83·8)	70·5 (65·9–75·3)	82·8 (79·9–85·3)
Rheumatic heart disease	21·2 (12–40·6)	21·7 (13·6–36·8)	21·7 (13·1–38)	21·7 (13·6–36·1)
Ischemic heart disease	92·1 (87·9–95·5)	93·8 (91–96·1)	92·4 (88·8–95·3)	93·9 (91·1–96·1)
Stroke	77·8 (70·1–86·2)	81·8 (76·5–87·5)	80·7 (75–86·8)	82·2 (77–87·8)
Hypertensive heart disease	100 (100–100)	100 (100–100)	100 (100–100)	100 (100–100)
Non-rheumatic valvular heart disease	23·8 (14·3–36·7)	22·3 (14·9–31·8)	8·82 (5·57–13·1)	24·7 (16·4–34·8)
Cardiomyopathy and myocarditis	37·2 (30·2–45·1)	40 (34·3–45·5)	38·2 (31·9–44·6)	40·2 (34·3–45·9)
Atrial fibrillation and flutter	51·4 (41–61·9)	57·7 (48·8–66·6)	59·4 (50·6–68·2)	55·6 (46·2–64·8)
Aortic aneurysm	52·4 (46·4–58·5)	61·5 (56·6–66·1)	--	61·5 (56·6–66·1)
Endocarditis	30 (20·2–42·1)	32·1 (23·1–42·4)	29·7 (19·7–41·8)	32·2 (23·2–42·6)
Other cardiovascular and circulatory diseases	22·7 (17·9–28·9)	25·1 (20·3–30·2)	30·5 (23·9–37·7)	23·5 (18·8–28·8)
Chronic respiratory diseases	62·6 (57·5–67·6)	58·9 (55–62·7)	53·8 (49·4–58·1)	63·7 (59·3–68·1)

Chronic obstructive pulmonary disease	70·5 (65·2–75·9)	69 (64·7–73·1)	63 (58·9–67·2)	73·1 (68·4–77·7)
Pneumoconiosis	100 (100–100)	100 (100–100)	100 (100–100)	100 (100–100)
Asthma	51·7 (40·3–63·3)	43·8 (35·7–52·7)	42·6 (34·7–51)	52·6 (42·8–62·4)
Digestive diseases	39·9 (34·3–45·5)	44·2 (38·5–49·7)	16·4 (11·3–22)	52·2 (46·2–58·1)
Cirrhosis and other chronic liver diseases	78·6 (68–87·7)	82·2 (72·6–89·6)	77·2 (67·1–85·5)	82·3 (72·7–89·7)
Upper digestive system diseases	18·8 (15·4–22·3)	6·3 (3·88–9·2)	1·47 (0·852–2·29)	22·3 (18·8–25·8)
Gallbladder and biliary diseases	51·6 (35·3–66·5)	55·9 (41·1–69·2)	57·5 (43–70·3)	53·8 (38·4–68)
Pancreatitis	35·8 (18·8–53·8)	41·7 (23·6–59·6)	39·2 (21·8–56·7)	42 (24–59·9)
Neurological disorders	27·2 (12·1–42·7)	17·8 (9·48–29·8)	13 (6·68–23·9)	23·1 (9·89–37·6)
Alzheimer's disease and other dementias	39·5 (25·2–55·5)	42·7 (28·5–58·3)	43·8 (29·2–59·2)	42·1 (27·7–57·5)
Parkinson's disease	-8·31 (-13·3–-4·05)	-10·1 (-15·6–-5·13)	-10·6 (-16·2–-5·48)	-10 (-15·5–-5·05)
Idiopathic epilepsy	24·7 (16·4–33·7)	22·9 (15·2–31·2)	21·1 (13·7–28·8)	26 (17·5–34·8)
Multiple sclerosis	14·8 (10·7–19·2)	15·9 (11·5–20·5)	15·4 (11·1–19·8)	16·2 (11·7–21)
Mental disorders	--	7·68 (3·93–12·4)	7·69 (3·93–12·4)	--
Depressive disorders	--	16·3 (8·11–24·7)	16·3 (8·11–24·7)	--
Anxiety disorders	--	6·25 (1·35–14·6)	6·25 (1·35–14·6)	--
Idiopathic developmental intellectual disability	--	46·5 (22·4–66·5)	46·5 (22·4–66·5)	--
Substance use disorders	100 (100–100)	100 (100–100)	100 (100–100)	100 (100–100)
Alcohol use disorders	100 (100–100)	100 (100–100)	100 (100–100)	100 (100–100)
Drug use disorders	100 (100–100)	100 (100–100)	100 (100–100)	100 (100–100)
Diabetes and kidney diseases	99·9 (99·9–99·9)	100 (100–100)	100 (100–100)	99·9 (99·9–99·9)
Diabetes mellitus	100 (100–100)	100 (100–100)	100 (100–100)	100 (100–100)
Chronic kidney disease	100 (100–100)	100 (100–100)	100 (100–100)	100 (100–100)
Sense organ diseases	--	10·9 (9·77–12·2)	10·9 (9·77–12·2)	--
Blindness and vision loss	--	9·92 (6·31–14·5)	9·92 (6·31–14·5)	--
Age-related and other hearing loss	--	11·7 (10·6–12·9)	11·7 (10·6–12·9)	--
Musculoskeletal disorders	3·74 (1·1–6·8)	18·9 (15·8–22·2)	19·2 (16–22·5)	3·67 (1·09–6·62)
Rheumatoid arthritis	11·1 (3·31–20)	14·4 (4·49–25·3)	14·7 (4·57–26)	13·4 (4·14–23·8)
Osteoarthritis	--	10·5 (6·04–15·4)	10·5 (6·04–15·4)	--
Low back pain	--	44 (38·3–49·6)	44 (38·3–49·6)	--
Gout	--	62·7 (46·4–78·2)	62·7 (46·4–78·2)	--
Other non-communicable diseases	0·143 (0·0968–0·206)	0·185 (0·119–0·269)	--	0·411 (0·297–0·584)

Sudden infant death syndrome	3·48 (2·49–4·74)	3·48 (2·48–4·73)	--	3·48 (2·48–4·73)
Injuries	25·2 (18·7–30·8)	22·8 (17–28)	32·2 (28·2–35·7)	17·7 (9·79–24·5)
Transport injuries	17 (8·8–24·7)	18·4 (10·9–25·6)	30·3 (26·1–34·5)	14·5 (5·63–22·6)
Road injuries	15·2 (6·29–23·2)	16·3 (8·25–23·7)	28 (23·8–32·4)	12·9 (3·48–21·4)
Other transport injuries	33·8 (28·3–39·8)	34·4 (29·4–39·6)	40·3 (35·8–44·3)	30·5 (24·4–37·1)
Unintentional injuries	35·9 (30·5–40·3)	28·1 (23·9–32·1)	33 (28·6–37·1)	21 (16·2–25·4)
Falls	56·1 (48·8–60·8)	45·8 (40·1–50·2)	45·2 (39·5–49·5)	47·1 (41·2–51·7)
Drowning	-12·2 (-28·4–2·83)	-12·3 (-28·1–2·24)	11·1 (7·03–16·6)	-13·1 (-29·3–1·93)
Fire, heat, and hot substances	12 (7·71–17·4)	14·6 (9·82–20·3)	15·4 (10·3–21·5)	13·6 (9·32–19)
Poisonings	18 (12·4–25)	17·6 (12·3–24)	13·8 (9·38–19·3)	18·9 (13·2–25·6)
Exposure to mechanical forces	4·31 (-7·04–15·8)	15·7 (10·3–20·8)	22·5 (19·5–26·1)	0·352 (-11·4–11·8)
Animal contact	23·5 (18·4–29)	24·7 (20·1–30)	25·6 (20·9–30·8)	21·1 (16·2–26·8)
Foreign body	2·69 (1·81–3·95)	3·88 (2·63–5·65)	3·9 (2·69–5·4)	3·89 (2·61–5·81)
Environmental heat and cold exposure	8·06 (3·43–14·2)	8·7 (3·87–15·1)	8·04 (3·59–14·1)	9·1 (4·11–15·8)
Exposure to forces of nature	7·65 (3·44–13·2)	8·04 (3·64–13·8)	8·93 (4·01–15·3)	7·3 (3·33–12·5)
Other unintentional injuries	18 (12·4–24·7)	15·2 (10·3–21·2)	14·4 (9·62–20·3)	20·3 (14·2–27·4)
Self-harm and interpersonal violence	17 (1·47–31·3)	19·1 (4·76–32·5)	29·1 (22·2–35·6)	18·3 (3·02–32·3)
Self-harm	16·9 (-0·619–32·4)	18·8 (1·56–34·2)	23·8 (12·3–35·7)	18·7 (1·25–34·2)
Interpersonal violence	18·6 (6·19–30·3)	21 (10·9–30·9)	31·3 (24–38·4)	16·5 (4·15–28·6)

Supplementary Results Table 2. All-age PAFs attributable to the joint distribution of all risk factors for all ages, both sexes combined for each GBD level 1, level 2, and level 3 cause and all causes in 2019 for Ohio. Results are shown for deaths, YLLs, YLDs, and DALYs Data in parenthesis are 95% uncertainty intervals. PAFs=population attributable fractions. GBD=Global Burden of Disease. YLLs=years of life lost. YLDs=years lived with disability. DALYs=disability-adjusted life-years				
	Deaths 2019	DALYs 2019	YLDs 2019	YLLs 2019
All causes	63 (60·2–65·5)	51·2 (48·1–54·2)	36 (33·2–38·7)	63·5 (61·5–65·4)
Communicable, maternal, neonatal, and nutritional diseases	47·1 (42·1–51·6)	57·1 (54·1–60·1)	48·9 (44·5–53·3)	61·9 (58·2–65·4)
HIV/AIDS and sexually transmitted infections	90·3 (88·3–92·1)	90·6 (88·4–92·5)	93·6 (91·4–95·9)	88·5 (86·2–90·5)
HIV/AIDS	89·9 (87·8–91·7)	88·9 (86·5–90·9)	90·1 (87·7–92)	88·3 (85·9–90·3)
Sexually transmitted infections excluding HIV	98·9 (98·4–99·3)	99·5 (99·2–99·8)	100 (100–100)	95·7 (93·9–97·1)
Respiratory infections and tuberculosis	44 (36·7–50·6)	38·9 (32·3–45·4)	2·6 (1·98–3·35)	49·1 (41·9–55·7)
Tuberculosis	55·1 (46–64·3)	58·9 (50·1–66·8)	55·3 (45·7–64·2)	59·5 (50·9–67·3)
Lower respiratory infections	43·9 (36·6–50·7)	49 (41·7–55·6)	35·3 (29·8–40·8)	49·2 (41·9–55·8)
Upper respiratory infections	0·659 (0·397–1·05)	0·0328 (0·0168–0·0578)	0·00529 (0·000124–0·0151)	1·51 (0·91–2·39)
Otitis media	1·65 (0·698–3·59)	4·27 (2·8–5·87)	4·26 (2·78–5·87)	4·6 (2·16–8·38)
Enteric infections	11·1 (6·89–16·9)	12·2 (8·01–17·9)	12·1 (7·99–17·8)	12·3 (8·12–18)
Diarrheal diseases	11·1 (6·91–17)	12·2 (8·05–18)	12·1 (8–17·8)	12·4 (8·19–18·1)
Other infectious diseases	0·821 (0·514–1·1)	1·36 (0·955–1·76)	0·734 (0·429–1·16)	1·53 (1·03–1·99)
Meningitis	1·49 (1·08–2·04)	3·23 (2·42–4·35)	0·000459 (1·05e-05–0·00138)	3·51 (2·6–4·72)
Encephalitis	0·63 (0·46–0·862)	1·71 (1·27–2·34)	0·000133 (2·74e-06–0·000394)	1·79 (1·32–2·46)
Measles	9·42 (-3·87–43·9)	6·7 (-3·7–41·4)	0·338 (0–1·02)	9·58 (-3·93–44·6)
Acute hepatitis	28·8 (18·3–43·1)	11·5 (7·07–17·1)	4·28 (2·45–6·67)	29·6 (18·8–43·5)
Maternal and neonatal disorders	86·8 (84·8–88·5)	83·1 (81–85)	72·5 (67·9–76·5)	88·5 (87–90)
Maternal disorders	11·7 (4·19–20)	11·5 (4·12–19·5)	11·4 (4·09–19·3)	11·6 (4·13–19·7)
Neonatal disorders	91·7 (90·5–92·9)	85·8 (83·6–87·7)	74·2 (69·4–78·4)	91·7 (90·5–92·9)
Nutritional deficiencies	94·5 (86·2–95·9)	90·5 (87·5–92·8)	89·4 (85·8–92·4)	94 (85·2–95·4)
Protein-energy malnutrition	100 (100–100)	100 (100–100)	100 (100–100)	100 (100–100)
Vitamin A deficiency	--	100 (100–100)	100 (100–100)	--
Dietary iron deficiency	--	100 (100–100)	100 (100–100)	--
Non-communicable diseases	65·7 (62·5–68·5)	53 (49·5–56·4)	35·8 (32·7–38·5)	67·7 (65·5–69·8)
Neoplasms	51·7 (47·9–55·8)	51·5 (47·9–55·4)	33·2 (29–37·8)	52·6 (49·1–56·5)
Lip and oral cavity cancer	67·2 (58·6–74·7)	70·1 (61·7–77·2)	69·5 (60·6–76·5)	70·2 (61·6–77·2)
Nasopharynx cancer	64·6 (53·2–73·6)	64·8 (53·9–73·4)	63·5 (52·5–72·2)	64·8 (54–73·4)
Other pharynx cancer	77·6 (70·7–82·8)	79 (72·4–84)	79·2 (72·5–84·3)	79 (72·3–84)
Esophageal cancer	78·9 (69·7–86·8)	80·4 (71·6–88)	79·3 (70·2–87·2)	80·4 (71·7–88)

Stomach cancer	25·5 (17·4–43·3)	26·7 (18·8–44·4)	26·7 (18·4–44·1)	26·7 (18·8–44·4)
Colon and rectum cancer	63·2 (54·3–72)	64·1 (55·9–72·2)	64 (55·4–72·4)	64·1 (55·8–72·2)
Liver cancer	81·2 (76·5–85·5)	80·9 (76·2–85·2)	81·1 (76·5–85·4)	80·9 (76·2–85·2)
Gallbladder and biliary tract cancer	27·3 (16·2–40·2)	28·4 (17–41·1)	26·3 (15·3–39)	28·4 (17–41·1)
Pancreatic cancer	45·5 (35·5–56·9)	45·8 (36·3–56·8)	45·7 (36–56·9)	45·8 (36·3–56·8)
Larynx cancer	82·9 (73·3–90)	84·2 (76·1–90·3)	83·9 (75·6–90·2)	84·2 (76·1–90·3)
Tracheal, bronchus, and lung cancer	85·2 (82–88·5)	84·9 (81·9–88·1)	85·2 (82·1–88·5)	84·9 (81·9–88·1)
Breast cancer	36·9 (26·9–49·3)	36·3 (27·1–47·1)	36·7 (27·2–47·8)	36·2 (27·1–47)
Cervical cancer	100 (100–100)	100 (100–100)	100 (100–100)	100 (100–100)
Uterine cancer	55 (39·5–68·6)	56·7 (41·6–69·6)	57·2 (42·2–69·9)	56·7 (41·5–69·5)
Ovarian cancer	21·5 (9·93–36·6)	19·4 (8·36–33·4)	18·8 (7·94–32·5)	19·4 (8·35–33·5)
Prostate cancer	5·88 (2·65–9·58)	6·71 (2·92–10·7)	7·16 (3·13–11·2)	6·58 (2·89–10·5)
Kidney cancer	43·4 (32·1–54·1)	44·3 (33·8–54·3)	44·6 (34·2–54·3)	44·3 (33·8–54·2)
Bladder cancer	42·1 (26·1–57·8)	46·1 (30·6–60·4)	46·9 (31·7–60·6)	46 (30·5–60·4)
Thyroid cancer	18·7 (9·37–29·4)	20 (10·3–31·1)	20·3 (10·8–31·1)	19·9 (10·1–31·1)
Mesothelioma	97 (96–97·9)	94·3 (92·4–95·9)	96·5 (95–97·7)	94·3 (92·4–95·9)
Non-Hodgkin lymphoma	8·92 (3·9–15)	9·45 (4·32–15·7)	9·24 (4·13–15·6)	9·47 (4·33–15·7)
Multiple myeloma	10·2 (4·81–17)	10·8 (5·16–17·7)	10·7 (4·96–17·7)	10·8 (5·16–17·7)
Leukemia	37·5 (23·1–52)	36·8 (24·1–49·8)	38·4 (23·8–52·7)	36·7 (24·1–49·7)
Cardiovascular diseases	82·4 (78·6–85·5)	82·8 (79·9–85·2)	72·2 (67·8–76·5)	84·4 (81·8–86·8)
Rheumatic heart disease	22·2 (12·7–42·4)	22·1 (14–37·1)	22·1 (13·4–37·7)	22·1 (14·2–36·7)
Ischemic heart disease	92·8 (89·1–96)	94·3 (91·8–96·6)	92·9 (89·6–95·8)	94·3 (91·9–96·6)
Stroke	80·2 (73·5–88·2)	83·5 (78·9–88·5)	82·4 (77·5–87·8)	83·9 (79·1–88·8)
Hypertensive heart disease	100 (100–100)	100 (100–100)	100 (100–100)	100 (100–100)
Non-rheumatic valvular heart disease	25·8 (16·1–39·6)	22·6 (15·4–32·4)	6·88 (4·42–9·9)	26·1 (17·8–37·3)
Cardiomyopathy and myocarditis	39·6 (32·6–48·1)	42·8 (37·4–48·3)	40·9 (34·7–47·4)	43 (37·3–48·8)
Atrial fibrillation and flutter	52·5 (42·8–62·8)	58·4 (50–67·3)	59·8 (51·4–68·4)	56·5 (48·1–65·7)
Aortic aneurysm	55 (49–61·3)	63·8 (59·1–68·3)	--	63·8 (59·1–68·3)
Endocarditis	31·7 (22–44·9)	33·1 (24·2–43·7)	31·4 (21·3–44·1)	33·2 (24·3–43·7)
Other cardiovascular and circulatory diseases	22·5 (17·2–30·3)	25·4 (20·4–31·5)	31·6 (24·9–38·8)	23·5 (18·7–29·5)
Chronic respiratory diseases	67·2 (62·5–71·8)	63 (59·3–66·7)	56 (51·4–60·3)	68·5 (64·5–72·6)
Chronic obstructive pulmonary disease	74·6 (70·2–78·7)	73·4 (69·7–76·8)	66·8 (62·7–70·7)	76·9 (73·1–80·5)
Pneumoconiosis	100 (100–100)	100 (100–100)	100 (100–100)	100 (100–100)

Asthma	51·9 (41·1–62·7)	44·2 (36·3–52·5)	43·5 (35·6–51·9)	52·1 (42·9–61·3)
Digestive diseases	42·3 (36·1–48·1)	44·6 (38·2–50·3)	17·7 (12·3–23·8)	52·1 (45·1–58·3)
Cirrhosis and other chronic liver diseases	76·5 (66·1–85·8)	79·1 (69·2–87·6)	73·6 (62·4–83·8)	79·1 (69·2–87·7)
Upper digestive system diseases	21·7 (18·2–25·5)	7·39 (4·53–10·7)	1·88 (1·14–2·84)	25·8 (21·9–29·6)
Gallbladder and biliary diseases	51 (35·5–65·8)	56·1 (41·9–69·1)	57·6 (44–69·9)	53·4 (38·5–67·5)
Pancreatitis	33·8 (16·9–50·6)	38·6 (20·9–55·1)	36·3 (19·4–52·8)	39 (21–55·5)
Neurological disorders	27·5 (12–43·3)	17·8 (9·22–30·8)	13 (6·55–23·7)	23·6 (9·46–39·2)
Alzheimer's disease and other dementias	41·8 (27·2–57·5)	45·2 (30·6–60·6)	46·1 (31·2–61·4)	44·6 (30–59·8)
Parkinson's disease	-9·16 (-14·6–-4·41)	-11·1 (-17–-5·61)	-12 (-18·2–-6·23)	-10·9 (-16·8–-5·46)
Idiopathic epilepsy	22·5 (14·8–30·6)	20·7 (13·9–28·2)	19·6 (13–27)	23·4 (15·9–31·5)
Multiple sclerosis	16·1 (12–21)	17·8 (13·2–22·6)	17·8 (13·3–22·4)	17·8 (13·1–22·9)
Mental disorders	--	10·7 (5·64–16·6)	10·7 (5·64–16·6)	--
Depressive disorders	--	21·2 (10·8–31·7)	21·2 (10·8–31·7)	--
Anxiety disorders	--	9·56 (2·65–19·5)	9·56 (2·65–19·5)	--
Idiopathic developmental intellectual disability	--	47·8 (23·9–66·2)	47·8 (23·9–66·2)	--
Substance use disorders	100 (100–100)	100 (100–100)	100 (100–100)	100 (100–100)
Alcohol use disorders	100 (100–100)	100 (100–100)	100 (100–100)	100 (100–100)
Drug use disorders	100 (100–100)	100 (100–100)	100 (100–100)	100 (100–100)
Diabetes and kidney diseases	99·9 (99·8–99·9)	99·9 (99·9–100)	100 (100–100)	99·9 (99·9–99·9)
Diabetes mellitus	100 (100–100)	100 (100–100)	100 (100–100)	100 (100–100)
Chronic kidney disease	100 (100–100)	100 (100–100)	100 (100–100)	100 (100–100)
Sense organ diseases	--	10·6 (9·49–11·9)	10·6 (9·49–11·9)	--
Blindness and vision loss	--	10·4 (6·6–15·2)	10·4 (6·6–15·2)	--
Age-related and other hearing loss	--	11·4 (10·3–12·7)	11·4 (10·3–12·7)	--
Musculoskeletal disorders	3·69 (1·05–6·6)	21 (17·6–24·6)	21·3 (17·8–25·1)	3·74 (1·17–6·58)
Rheumatoid arthritis	12·2 (3·57–21·6)	16·2 (5·29–28·3)	16·7 (5·52–29)	14·7 (4·66–25·6)
Osteoarthritis	--	16 (9·11–23·2)	16 (9·11–23·2)	--
Low back pain	--	42·7 (36·6–49)	42·7 (36·6–49)	--
Gout	--	61·7 (46·3–76·7)	61·7 (46·3–76·7)	--
Other non-communicable diseases	0·123 (0·0848–0·172)	0·172 (0·12–0·247)	--	0·407 (0·288–0·559)
Sudden infant death syndrome	5·57 (3·9–7·76)	5·57 (3·9–7·75)	--	5·57 (3·9–7·75)
Injuries	29·4 (25–33·5)	27·7 (23·7–31·8)	33·4 (29·8–36·9)	24·1 (19·2–29)
Transport injuries	26·3 (20·4–32·6)	27·1 (21·4–33·2)	31·4 (27·3–35·5)	25·2 (18·5–32·5)

Road injuries	25·6 (19·7–32)	25·8 (20–32·2)	29·1 (25·1–33·3)	24·6 (17·9–31·9)
Other transport injuries	37·8 (32·4–43·8)	38·6 (34–43·2)	41 (36·6–44·6)	35·3 (29·5–42·2)
Unintentional injuries	36·5 (31·7–40·1)	30·6 (26·9–34·5)	34·3 (30·2–38·2)	24·4 (20·6–28·2)
Falls	54·8 (47·9–59·1)	46·5 (41·2–50·6)	45·8 (40·5–49·9)	48 (42·6–52·1)
Drowning	-8·68 (-17·4–0·726)	-8·85 (-17–-0·0402)	11·2 (7·36–16·2)	-9·63 (-18–-0·604)
Fire, heat, and hot substances	11·7 (7·68–16·5)	15·2 (10·7–20·2)	16·6 (11·7–22·2)	13·5 (9·3–18·4)
Poisonings	19·1 (13·5–25·6)	19 (13·6–25)	15·5 (10·9–20·7)	20·9 (15·1–27·8)
Exposure to mechanical forces	12·4 (4·61–20·8)	22·3 (18·1–27·7)	25·6 (22–30·5)	10·4 (1·71–19·8)
Animal contact	24·1 (19·9–29)	26·8 (22·1–32·1)	27·8 (23–33·2)	21·4 (17·1–26·8)
Foreign body	3·02 (1·96–4·45)	4·72 (3·19–6·81)	6·13 (4·22–8·73)	4·17 (2·7–6·12)
Environmental heat and cold exposure	7·08 (3·17–12·2)	7·6 (3·45–13)	7·31 (3·3–12·5)	7·96 (3·64–13·6)
Exposure to forces of nature	6·99 (3·14–11·9)	7·22 (3·31–12·3)	8·29 (3·77–14)	6·68 (3·02–11·5)
Other unintentional injuries	21·5 (15·3–29·2)	16·4 (11·6–22·1)	15·6 (10·9–21)	25·2 (18·1–33·7)
Self-harm and interpersonal violence	22·3 (13·4–31·5)	23·6 (15·1–32)	28·3 (21·8–34·7)	23·1 (14·5–31·9)
Self-harm	23·8 (11·8–35·7)	26·4 (14·2–38)	25·4 (14·4–35·7)	26·4 (14·2–38)
Interpersonal violence	19·3 (11·4–27·9)	19·8 (12·1–27·9)	29·4 (22·4–36·6)	17·5 (9·62–25·9)

Supplementary Results Table 2. All-age PAFs attributable to the joint distribution of all risk factors for all ages, both sexes combined for each GBD level 1, level 2, and level 3 cause and all causes in 2019 for Oklahoma. Results are shown for deaths, YLLs, YLDs, and DALYs Data in parenthesis are 95% uncertainty intervals. PAFs=population attributable fractions. GBD=Global Burden of Disease. YLLs=years of life lost. YLDs=years lived with disability. DALYs=disability-adjusted life-years

	Deaths 2019	DALYs 2019	YLDs 2019	YLLs 2019
All causes	64·2 (61·7–66·5)	51 (47·8–54)	35·2 (32·4–37·8)	63 (61·1–64·8)
Communicable, maternal, neonatal, and nutritional diseases	45·6 (42–49)	56 (53·1–58·6)	49·1 (44·4–53·4)	60 (56·9–63)
HIV/AIDS and sexually transmitted infections	89·5 (87·1–91·4)	89·7 (87·3–91·9)	92·9 (90·6–95·5)	87·7 (84·9–90)
HIV/AIDS	89·2 (86·7–91·2)	88·2 (85·5–90·5)	89·5 (86·9–91·8)	87·5 (84·7–89·9)
Sexually transmitted infections excluding HIV	98·8 (98·2–99·2)	99·6 (99·3–99·8)	100 (100–100)	95·6 (93·7–97)
Respiratory infections and tuberculosis	40·8 (35·9–45·6)	36·8 (31·4–42·2)	2·85 (2·19–3·7)	46·6 (41·4–51·4)
Tuberculosis	54·3 (45·8–62·7)	57·9 (49·7–65·8)	53·6 (44·1–62·1)	58·6 (50·6–66·3)
Lower respiratory infections	40·7 (35·6–45·6)	46·3 (41·2–51·3)	34·8 (29·3–39·6)	46·5 (41·3–51·5)
Upper respiratory infections	0·639 (0·391–1·02)	0·0223 (0·0107–0·0402)	0·0057 (0·000179–0·0163)	1·32 (0·81–2·07)
Otitis media	2·11 (0·884–4·24)	4·64 (3·03–6·32)	4·62 (3·01–6·31)	5·25 (2·54–8·95)
Enteric infections	13 (7·91–19·8)	14·3 (9·34–21)	13·8 (8·84–20·5)	15·1 (10–21·9)
Diarrheal diseases	13·1 (7·97–19·9)	14·3 (9·39–21·1)	13·8 (8·85–20·5)	15·3 (10·2–22·2)
Other infectious diseases	0·943 (0·659–1·31)	1·46 (1·11–1·85)	0·753 (0·457–1·22)	1·62 (1·19–2·09)
Meningitis	1·42 (1·04–1·89)	2·99 (2·24–3·91)	0·000692 (1·4e-05–0·00208)	3·09 (2·31–4·09)
Encephalitis	0·628 (0·453–0·862)	1·58 (1·15–2·18)	0·000202 (4·81e-06–0·000588)	1·63 (1·19–2·25)
Measles	9·59 (-4·02–43·5)	5·09 (-2·15–39·9)	0·441 (0–1·17)	9·75 (-4·09–44·3)
Acute hepatitis	21·9 (14·2–35·9)	11·1 (7–17·1)	4·03 (2·46–6·32)	22·2 (14·1–35·9)
Maternal and neonatal disorders	83·5 (81–85·7)	82·4 (80·3–84·2)	75·5 (71·1–79·2)	85·8 (83·9–87·5)
Maternal disorders	12 (4·35–19·7)	11·9 (4·29–19·6)	11·6 (4·2–19·3)	11·9 (4·31–19·5)
Neonatal disorders	90 (88·7–91·4)	85·9 (84–87·6)	77·4 (73–81·3)	90 (88·7–91·4)
Nutritional deficiencies	95·1 (88·1–96·4)	90·8 (88–93·1)	89·8 (86·9–92·7)	94·4 (86·8–95·6)
Protein-energy malnutrition	100 (100–100)	100 (100–100)	100 (100–100)	100 (100–100)
Vitamin A deficiency	--	100 (100–100)	100 (100–100)	--
Dietary iron deficiency	--	100 (100–100)	100 (100–100)	--
Non-communicable diseases	67·9 (64·8–70·4)	53·7 (50–57·3)	35 (32–37·8)	69 (66·8–71)
Neoplasms	51·8 (48·2–55·8)	51·1 (47·6–54·9)	32·8 (28·7–37·1)	52·1 (48·7–55·8)
Lip and oral cavity cancer	67 (58·8–74·5)	69·7 (62·1–76·7)	69 (61·4–76)	69·8 (62·1–76·7)
Nasopharynx cancer	62·8 (52·3–72·2)	62·9 (52·3–72·1)	61·8 (51·2–71·2)	62·9 (52·3–72·1)
Other pharynx cancer	76·7 (70·7–81·9)	78 (71·9–83·2)	78·3 (72·4–83·6)	78 (71·9–83·2)
Esophageal cancer	80·4 (71·5–88·5)	81·5 (72·7–89·3)	80·6 (71·8–88·4)	81·5 (72·7–89·3)

Stomach cancer	27·1 (18·9–44·4)	27·8 (19·6–45·4)	28 (19·8–45·2)	27·8 (19·6–45·4)
Colon and rectum cancer	63·2 (54·1–71·9)	64·2 (55·9–72·1)	64 (55·3–72·3)	64·2 (55·9–72·1)
Liver cancer	80·4 (75·7–84·9)	79·6 (75–84)	80·2 (75·5–84·6)	79·6 (75–84)
Gallbladder and biliary tract cancer	27·4 (16·3–39·8)	28·7 (17·3–41·1)	26·4 (15·6–39·2)	28·7 (17·3–41·2)
Pancreatic cancer	46·5 (37·1–57·5)	46·6 (37·5–57·4)	46·7 (37·4–57·4)	46·6 (37·5–57·4)
Larynx cancer	81·5 (71·1–88·7)	82·8 (74·4–89)	82·4 (73·3–88·6)	82·9 (74·3–89·1)
Tracheal, bronchus, and lung cancer	85·3 (82·4–88·6)	84·9 (82·1–88)	85·2 (82·4–88·5)	84·9 (82·1–88)
Breast cancer	34·8 (25·3–46·3)	34·1 (25·3–44·4)	34·6 (25·5–45·5)	34 (25·3–44·2)
Cervical cancer	100 (100–100)	100 (100–100)	100 (100–100)	100 (100–100)
Uterine cancer	54·5 (39·2–68)	56·4 (41–69·4)	56·9 (41·5–69·9)	56·3 (40·9–69·3)
Ovarian cancer	19·9 (8·86–34·1)	18·1 (7·68–31·3)	17·6 (7·43–30·6)	18·2 (7·68–31·3)
Prostate cancer	6·77 (2·88–11·1)	7·53 (3·27–12)	7·93 (3·5–12·6)	7·43 (3·21–11·9)
Kidney cancer	44·4 (33·5–54·8)	45 (34·6–54·5)	45·2 (35–54·7)	45 (34·6–54·6)
Bladder cancer	43 (27·5–57·4)	46·6 (31·4–60·3)	47·2 (32·1–60·5)	46·6 (31·4–60·3)
Thyroid cancer	18·7 (9·64–29·2)	20 (10·5–30·9)	20·3 (11–30·8)	19·9 (10·4–31)
Mesothelioma	95·6 (93·9–96·9)	92·1 (89·5–94·2)	95·3 (93·4–96·6)	92·1 (89·4–94·2)
Non-Hodgkin lymphoma	8·91 (3·89–14·9)	9·44 (4·37–15·5)	9·22 (4·22–15·2)	9·45 (4·39–15·5)
Multiple myeloma	10·2 (4·75–17·1)	10·8 (5·18–17·9)	10·7 (4·97–18)	10·8 (5·18–17·9)
Leukemia	38·7 (24·1–52·5)	36·8 (24·6–49·1)	39·4 (25·1–53·4)	36·7 (24·6–48·8)
Cardiovascular diseases	85 (82–87·7)	85·5 (83·1–87·7)	73·6 (69·4–77·7)	87·1 (84·8–89·1)
Rheumatic heart disease	22·2 (12·9–40·4)	22·5 (14·2–37·1)	22·2 (13·6–38)	22·5 (14·5–36·6)
Ischemic heart disease	93·5 (90·2–96·3)	95·1 (92·9–96·9)	93·5 (90·5–96)	95·1 (92·9–96·9)
Stroke	80·4 (74–87·4)	84·1 (79·8–88·8)	83·1 (78·3–88·7)	84·5 (80·2–89)
Hypertensive heart disease	100 (100–100)	100 (100–100)	100 (100–100)	100 (100–100)
Non-rheumatic valvular heart disease	24·8 (15·7–37·1)	21·7 (14·9–30·4)	6·18 (3·97–8·64)	25·7 (17·7–35·7)
Cardiomyopathy and myocarditis	39·9 (33·1–47·3)	41·7 (35·7–47·4)	40·6 (34·4–46·7)	41·8 (35·7–47·7)
Atrial fibrillation and flutter	51·1 (41·1–61·4)	57·7 (49·2–66·4)	59·1 (50·8–67·5)	55·9 (47–65)
Aortic aneurysm	58·3 (53·1–63·4)	66·2 (61·9–70·3)	--	66·2 (61·9–70·3)
Endocarditis	32 (22·6–43·9)	33·8 (24·7–44·2)	31·2 (21·2–43·1)	33·9 (24·8–44·3)
Other cardiovascular and circulatory diseases	24·6 (19·7–31·2)	26·8 (21·8–32·5)	32·8 (26·1–40·4)	25 (20·3–30·6)
Chronic respiratory diseases	69·2 (65·2–73·1)	64·3 (60·7–67·8)	55·8 (51–60)	70·1 (66·7–73·5)
Chronic obstructive pulmonary disease	75·3 (71·3–79)	74·4 (71·1–77·6)	68·1 (64·3–71·8)	77·2 (73·9–80·4)
Pneumoconiosis	100 (100–100)	100 (100–100)	100 (100–100)	100 (100–100)

Asthma	51·7 (41·4–62·7)	42·3 (34·7–50·4)	41·5 (33·9–49·6)	49·8 (41–58·9)
Digestive diseases	47·2 (41·9–52·5)	50·1 (44·5–55·5)	19·9 (13·9–26·4)	56·9 (51–62·2)
Cirrhosis and other chronic liver diseases	79·2 (71·2–87)	81·5 (73·8–88·8)	76·5 (67·5–84·6)	81·5 (73·9–88·8)
Upper digestive system diseases	24·6 (20·4–28·3)	8·02 (4·95–11·7)	1·9 (1·17–2·91)	28 (23·6–32·2)
Gallbladder and biliary diseases	51·7 (36·7–65·6)	56·6 (42·5–68·9)	57·6 (43·8–69·6)	54·4 (39·9–67·5)
Pancreatitis	31·2 (17·1–47·2)	36·4 (21·2–52·8)	33·7 (19·1–49·6)	36·7 (21·5–53·2)
Neurological disorders	27·5 (11·6–43·5)	16·9 (8·37–29·8)	11·8 (5·88–22·7)	23·5 (8·9–40·2)
Alzheimer's disease and other dementias	42·5 (28–58·1)	46·3 (31·9–61·2)	47·3 (32·7–61·8)	45·6 (31·2–60·6)
Parkinson's disease	-11·4 (-17·7–-5·94)	-13·4 (-20·2–-7·2)	-14·1 (-21·2–-7·57)	-13·2 (-20–-7·11)
Idiopathic epilepsy	20 (12·9–27·2)	18·2 (11·8–24·7)	17 (11–23·4)	21 (13·8–28·2)
Multiple sclerosis	16·6 (12–21·8)	17·5 (12·7–22·7)	16·8 (12·3–21·6)	17·9 (13–23·3)
Mental disorders	--	9·43 (4·88–14·7)	9·44 (4·88–14·7)	--
Depressive disorders	--	18·5 (9·14–27·9)	18·5 (9·14–27·9)	--
Anxiety disorders	--	6·44 (1·49–14·6)	6·44 (1·49–14·6)	--
Idiopathic developmental intellectual disability	--	50 (24·8–70)	50 (24·8–70)	--
Substance use disorders	100 (100–100)	100 (100–100)	100 (100–100)	100 (100–100)
Alcohol use disorders	100 (100–100)	100 (100–100)	100 (100–100)	100 (100–100)
Drug use disorders	100 (100–100)	100 (100–100)	100 (100–100)	100 (100–100)
Diabetes and kidney diseases	99·8 (99·8–99·9)	99·9 (99·9–99·9)	100 (100–100)	99·9 (99·8–99·9)
Diabetes mellitus	100 (100–100)	100 (100–100)	100 (100–100)	100 (100–100)
Chronic kidney disease	100 (100–100)	100 (100–100)	100 (100–100)	100 (100–100)
Sense organ diseases	--	10·6 (9·55–11·9)	10·6 (9·55–11·9)	--
Blindness and vision loss	--	10·2 (6·54–14·8)	10·2 (6·54–14·8)	--
Age-related and other hearing loss	--	11·5 (10·4–12·7)	11·5 (10·4–12·7)	--
Musculoskeletal disorders	3·99 (1·22–7·11)	21·8 (18·4–25·6)	22·2 (18·7–25·9)	3·72 (1·19–6·62)
Rheumatoid arthritis	13·9 (4·23–24·8)	17 (5·53–29·9)	17·2 (5·63–30·3)	16·1 (5·13–28·3)
Osteoarthritis	--	15·8 (8·87–23·1)	15·8 (8·87–23·1)	--
Low back pain	--	44·1 (38–50·2)	44·1 (38–50·2)	--
Gout	--	61·1 (45·2–76·4)	61·1 (45·2–76·4)	--
Other non-communicable diseases	0·173 (0·124–0·236)	0·214 (0·148–0·293)	--	0·497 (0·363–0·66)
Sudden infant death syndrome	4·62 (3·23–6·17)	4·62 (3·22–6·16)	--	4·62 (3·22–6·16)
Injuries	27·5 (23·5–31·4)	25 (21·2–28·7)	30·5 (27·2–33·5)	22·5 (18·1–27·1)
Transport injuries	25·5 (20·7–30·5)	24·7 (19·9–29·9)	28·5 (24·9–32)	23·6 (18·4–29·4)

Road injuries	25·1 (20·2–30·1)	23·9 (19–29·2)	26·4 (22·8–30·1)	23·3 (18·1–29)
Other transport injuries	33·3 (28·6–38·5)	33·9 (29·6–38·2)	38·6 (34·4–42)	29·8 (24·7–35·7)
Unintentional injuries	33·7 (29·1–37·2)	26·3 (22·8–29·9)	31·3 (27·4–34·9)	20 (16·7–23·4)
Falls	53·7 (47–57·7)	44·2 (39·2–47·8)	43·3 (38·6–47·1)	45·8 (40·6–49·7)
Drowning	-11·1 (-17·5–-4·54)	-11·9 (-17·9–-5·8)	9·54 (6·19–13·7)	-12·3 (-18·2–-6·12)
Fire, heat, and hot substances	9·96 (6·71–13·9)	12·2 (8·59–16·4)	13·6 (9·47–18·5)	11·1 (7·77–15·1)
Poisonings	15·1 (10·8–20·2)	15·1 (10·8–20)	12·4 (8·7–16·9)	16·1 (11·6–21·2)
Exposure to mechanical forces	13 (8·19–18·4)	18·8 (15·4–22·9)	22·4 (19·5–25·9)	10·1 (4·82–16·3)
Animal contact	21·8 (18–26·1)	22·5 (18·7–27)	24 (19·8–28·4)	18·8 (15–23·3)
Foreign body	2·51 (1·67–3·66)	3·67 (2·46–5·31)	4·59 (3·22–6·63)	3·33 (2·16–4·93)
Environmental heat and cold exposure	6·24 (2·76–10·7)	6·81 (2·97–11·6)	6·24 (2·72–10·8)	7·18 (3·15–12·2)
Exposure to forces of nature	6·11 (2·7–10·5)	6·3 (2·82–10·8)	7·27 (3·25–12·4)	6·03 (2·67–10·3)
Other unintentional injuries	17·9 (12·8–23·9)	14 (9·74–18·8)	12·8 (8·9–17·5)	20·1 (14·4–26·6)
Self-harm and interpersonal violence	22·1 (13·6–30·9)	23·5 (15·2–31·8)	27·4 (21·6–33·1)	23·2 (14·7–31·9)
Self-harm	22·8 (11·6–34·1)	25·2 (13·5–37·2)	23·5 (14·4–33·4)	25·3 (13·5–37·3)
Interpersonal violence	21·4 (13·6–29·6)	21·1 (13·7–28·8)	29·3 (22·6–35·9)	19·5 (12–27·5)

Supplementary Results Table 2. All-age PAFs attributable to the joint distribution of all risk factors for all ages, both sexes combined for each GBD level 1, level 2, and level 3 cause and all causes in 2019 for Oregon. Results are shown for deaths, YLLs, YLDs, and DALYs Data in parenthesis are 95% uncertainty intervals. PAFs=population attributable fractions. GBD=Global Burden of Disease. YLLs=years of life lost. YLDs=years lived with disability. DALYs=disability-adjusted life-years

	Deaths 2019	DALYs 2019	YLDs 2019	YLLs 2019
All causes	58·5 (55·8–61·2)	45·8 (42·7–48·7)	33 (30·2–35·4)	58·1 (55·9–60·3)
Communicable, maternal, neonatal, and nutritional diseases	45·1 (40–49·5)	52·3 (48·9–55·6)	46·4 (41·8–51)	57·1 (52·6–61·4)
HIV/AIDS and sexually transmitted infections	85·1 (71·7–90·1)	85·4 (73·1–90·5)	90·1 (80·5–94·5)	81·8 (66·9–87·7)
HIV/AIDS	84·4 (70·3–89·6)	82·4 (67·9–88·3)	84·3 (71·4–90)	81·4 (66–87·4)
Sexually transmitted infections excluding HIV	99·4 (99·2–99·6)	99·8 (99·6–99·9)	100 (100–100)	97·6 (96·5–98·4)
Respiratory infections and tuberculosis	43·9 (36·5–50·5)	33·7 (27·2–40·5)	2·06 (1·54–2·71)	48·1 (41–54·4)
Tuberculosis	55·8 (45·6–64·6)	58·4 (49·1–66·7)	55·5 (45·3–64·6)	59 (49·6–67·3)
Lower respiratory infections	43·8 (36·3–50·5)	47·8 (40·7–54·2)	32·6 (27–38·1)	48 (40·9–54·5)
Upper respiratory infections	0·448 (0·246–0·807)	0·0143 (0·00596–0·0274)	0·00457 (0–0·0144)	1·15 (0·634–2·03)
Otitis media	0·973 (0·399–2·14)	3·21 (2·07–4·47)	3·21 (2·06–4·5)	2·98 (1·35–5·68)
Enteric infections	9·95 (6–15·2)	11·1 (7·2–16·3)	10·9 (7–16·2)	11·3 (7·45–16·5)
Diarrheal diseases	10 (6·04–15·3)	11·1 (7·23–16·3)	10·9 (7·01–16·2)	11·4 (7·52–16·5)
Other infectious diseases	0·678 (0·413–0·922)	1·14 (0·776–1·51)	0·887 (0·542–1·36)	1·2 (0·767–1·64)
Meningitis	0·956 (0·615–1·46)	2·34 (1·53–3·46)	0·000429 (0–0·0014)	2·48 (1·63–3·69)
Encephalitis	0·407 (0·262–0·621)	1·19 (0·785–1·8)	0·000109 (0–0·000344)	1·25 (0·817–1·89)
Measles	9·21 (-4·02–42·3)	3·93 (-2·34–32·6)	0·348 (0·00875–0·875)	9·38 (-4·09–43·2)
Acute hepatitis	25·5 (15·4–37·4)	11·5 (7·3–16·5)	4·54 (2·81–7·06)	27·5 (16·9–39·2)
Maternal and neonatal disorders	82·9 (80·1–85·4)	79·3 (76·5–81·9)	72·3 (67·5–76·7)	84·8 (82·5–87)
Maternal disorders	11·1 (4·05–18·4)	10·9 (3·96–18)	10·8 (3·92–18·1)	10·9 (4–18·1)
Neonatal disorders	88·3 (86·4–90·2)	82 (79·3–84·5)	74·1 (69·1–78·7)	88·3 (86·4–90·2)
Nutritional deficiencies	91 (82·7–93·5)	87·4 (84·4–90·2)	86·6 (82·9–90·1)	90·5 (82·5–92·9)
Protein-energy malnutrition	100 (100–100)	100 (100–100)	100 (100–100)	100 (100–100)
Vitamin A deficiency	--	100 (100–100)	100 (100–100)	--
Dietary iron deficiency	--	100 (100–100)	100 (100–100)	--
Non-communicable diseases	61 (58–64)	47·5 (44–50·8)	32·5 (29·3–35·3)	62·5 (60·1–64·9)
Neoplasms	48·1 (44·5–52)	47·1 (43·6–50·7)	29·5 (25·8–33·3)	48·4 (45–52·1)
Lip and oral cavity cancer	67·1 (58·5–74·9)	69·2 (60·7–76·7)	68·8 (59·9–76·4)	69·3 (60·8–76·7)
Nasopharynx cancer	65·1 (53·5–74)	64·6 (53·4–73·3)	63·9 (52·2–72·9)	64·7 (53·4–73·4)
Other pharynx cancer	76·8 (69·4–82·8)	77·8 (70·8–83·4)	78·1 (70·6–83·7)	77·8 (70·7–83·4)

Esophageal cancer	78·5 (68·7–87)	79·5 (69·7–87·6)	78·7 (68·9–86·8)	79·5 (69·7–87·6)
Stomach cancer	24·3 (16·2–42·2)	24·9 (16·8–43·1)	25·1 (16·9–43)	24·9 (16·8–43·1)
Colon and rectum cancer	61·5 (52·7–70·1)	62·4 (54–70·3)	62·3 (53·9–70·6)	62·4 (54–70·3)
Liver cancer	81·2 (77–85·4)	81 (76·8–85·2)	81·1 (76·8–85·4)	81 (76·8–85·2)
Gallbladder and biliary tract cancer	26·5 (15·4–39·1)	27·5 (16·3–40·3)	25·3 (14·5–38·5)	27·6 (16·3–40·3)
Pancreatic cancer	42·1 (32·4–53)	41·9 (32·8–52·5)	42·2 (32·5–53)	41·9 (32·8–52·5)
Larynx cancer	81 (71·1–88·7)	81·9 (73·4–88·6)	81·7 (72·8–88·5)	81·9 (73·4–88·6)
Tracheal, bronchus, and lung cancer	82·5 (79–85·9)	81·7 (78·5–85·1)	82·1 (78·7–85·7)	81·7 (78·5–85·1)
Breast cancer	36·1 (27·1–46·5)	35·4 (27–44·8)	35·8 (27·5–45·4)	35·3 (27–44·7)
Cervical cancer	100 (100–100)	100 (100–100)	100 (100–100)	100 (100–100)
Uterine cancer	54 (38·5–67·5)	55·6 (40·6–68·6)	56 (41·1–69)	55·5 (40·5–68·5)
Ovarian cancer	20·3 (9·71–33·2)	18 (8·25–30·1)	17·5 (7·92–29·5)	18 (8·26–30·1)
Prostate cancer	5·55 (2·4–9·11)	6·18 (2·66–9·98)	6·5 (2·83–10·5)	6·07 (2·61–9·73)
Kidney cancer	41·8 (30·4–52·7)	42·5 (32·1–52·7)	42·8 (32·3–52·9)	42·5 (32·1–52·7)
Bladder cancer	38·1 (23·2–53·4)	41·7 (27·1–55·9)	42·5 (28·5–56·4)	41·6 (26·9–55·9)
Thyroid cancer	18·3 (9·37–28·8)	19·4 (10·1–30·1)	19·7 (10·7–30·1)	19·3 (9·87–30·3)
Mesothelioma	97·2 (96·1–98)	94·6 (92·9–96·1)	96·8 (95·5–97·8)	94·5 (92·8–96)
Non-Hodgkin lymphoma	8·7 (3·81–14·8)	9·2 (4·13–15·4)	8·98 (4·03–15)	9·21 (4·14–15·5)
Multiple myeloma	9·95 (4·61–16·7)	10·5 (4·96–17·6)	10·3 (4·71–17·5)	10·5 (4·95–17·6)
Leukemia	36·2 (22·3–50·8)	35·2 (23–48)	36·9 (22·8–51·5)	35·1 (23·1–47·8)
Cardiovascular diseases	77·4 (73·4–81·2)	78·8 (75·6–81·8)	72 (67·2–76·4)	80 (76·9–82·9)
Rheumatic heart disease	22·1 (12·1–43·2)	22 (13·5–37·6)	21·8 (13·2–38·3)	22 (13·6–37·5)
Ischemic heart disease	92·1 (88·1–95·5)	93·5 (90·6–96·1)	92·2 (88·7–95·4)	93·6 (90·7–96·1)
Stroke	79 (72–86·5)	82·3 (77·1–87·6)	81·3 (75·7–87·3)	82·6 (77·5–87·8)
Hypertensive heart disease	100 (100–100)	100 (100–100)	100 (100–100)	100 (100–100)
Non-rheumatic valvular heart disease	26·6 (16·4–41·5)	26·1 (17·4–37·5)	12·6 (8·2–18·1)	26·9 (18–38·7)
Cardiomyopathy and myocarditis	43 (35·6–51)	46·9 (40·7–53)	43 (36·5–48·9)	47·5 (40·7–53·5)
Atrial fibrillation and flutter	53·3 (43·3–63·3)	58·4 (49·8–66·8)	59·9 (51·7–68·1)	56·5 (47·3–65·4)
Aortic aneurysm	53·9 (47·9–60)	61·4 (56·5–66·3)	--	61·4 (56·5–66·3)
Endocarditis	31·3 (21·1–44·6)	32·7 (23·6–43·6)	31 (20·8–43·6)	32·8 (23·7–43·7)
Other cardiovascular and circulatory diseases	19·5 (14·3–28·5)	23·1 (18–29·9)	31·3 (24·7–38)	20·7 (16·1–28·1)
Chronic respiratory diseases	64 (59–68·9)	60·3 (56·4–64·2)	54·6 (49·8–59·2)	64·8 (60·6–69·3)

Chronic obstructive pulmonary disease	72·5 (68·2–76·7)	70·7 (66·8–74·3)	64 (59·8–68)	74·3 (70·6–77·8)
Pneumoconiosis	100 (100–100)	100 (100–100)	100 (100–100)	100 (100–100)
Asthma	50·7 (39·5–62·2)	44·6 (36·3–53·8)	43·8 (35·4–52·7)	51·5 (41·6–61·9)
Digestive diseases	47·8 (42·1–52·7)	50·6 (44·7–56)	15·2 (10·3–20·5)	59 (53–64·4)
Cirrhosis and other chronic liver diseases	82·8 (74·2–90)	84·3 (76·3–90·7)	80·5 (71·4–87·9)	84·3 (76·3–90·7)
Upper digestive system diseases	20·5 (17–24·1)	6·5 (3·94–9·75)	1·52 (0·904–2·4)	23·4 (19·7–27)
Gallbladder and biliary diseases	50·3 (34·7–64·9)	54·8 (40·7–67·9)	56·5 (42·6–69)	52·4 (37·9–66·5)
Pancreatitis	39·4 (21·1–57·1)	42·6 (24·1–59·9)	40·5 (22·3–58)	43 (24·3–60·2)
Neurological disorders	25 (10·5–40·4)	16 (8·01–27·9)	11·7 (5·91–22·6)	21·3 (8·08–37)
Alzheimer's disease and other dementias	41·6 (27·2–56·7)	44·6 (30·3–59·5)	45·3 (31·2–59·9)	44·1 (29·7–58·9)
Parkinson's disease	-9·38 (-15–-4·65)	-10·8 (-16·6–-5·49)	-11·6 (-17·8–-6·04)	-10·7 (-16·4–-5·38)
Idiopathic epilepsy	25·3 (16·1–33·7)	22·6 (14·7–30·3)	21·3 (13·8–28·8)	25·6 (16·7–33·8)
Multiple sclerosis	14·5 (10·5–19·4)	15·7 (11·6–20·3)	15·6 (11·5–20)	15·8 (11·5–20·8)
Mental disorders	--	9·65 (4·88–15)	9·66 (4·88–15)	--
Depressive disorders	--	19·2 (8·89–29·1)	19·2 (8·89–29·1)	--
Anxiety disorders	--	5·91 (1·34–13·7)	5·91 (1·34–13·7)	--
Idiopathic developmental intellectual disability	--	47·4 (23·2–66)	47·4 (23·2–66)	--
Substance use disorders	100 (100–100)	100 (100–100)	100 (100–100)	100 (100–100)
Alcohol use disorders	100 (100–100)	100 (100–100)	100 (100–100)	100 (100–100)
Drug use disorders	100 (100–100)	100 (100–100)	100 (100–100)	100 (100–100)
Diabetes and kidney diseases	99·9 (99·9–99·9)	100 (99·9–100)	100 (100–100)	99·9 (99·9–99·9)
Diabetes mellitus	100 (100–100)	100 (100–100)	100 (100–100)	100 (100–100)
Chronic kidney disease	100 (100–100)	100 (100–100)	100 (100–100)	100 (100–100)
Sense organ diseases	--	10·2 (9·13–11·4)	10·2 (9·13–11·4)	--
Blindness and vision loss	--	9·67 (6·29–14)	9·67 (6·29–14)	--
Age-related and other hearing loss	--	10·9 (9·89–12·1)	10·9 (9·89–12·1)	--
Musculoskeletal disorders	4·01 (1·22–7·31)	19·4 (16·2–23)	19·6 (16·4–23·3)	4·03 (1·27–7·38)
Rheumatoid arthritis	12·4 (3·78–22·2)	14·7 (4·61–26·7)	15 (4·77–27)	14·1 (4·38–25·4)
Osteoarthritis	--	14·2 (8·12–20·9)	14·2 (8·12–20·9)	--
Low back pain	--	40·5 (34·6–46·5)	40·5 (34·6–46·5)	--
Gout	--	60·3 (44·2–76·4)	60·3 (44·2–76·4)	--
Other non-communicable diseases	0·126 (0·0741–0·206)	0·174 (0·0955–0·291)	--	0·449 (0·266–0·716)

Sudden infant death syndrome	6·3 (3·87–9·46)	6·3 (3·86–9·45)	--	6·3 (3·86–9·45)
Injuries	30·6 (25·4–35·3)	27·9 (23·1–32·1)	33·8 (30·1–37·6)	23·8 (18–29·5)
Transport injuries	24·1 (18–30·5)	25·8 (20·1–31·9)	31·4 (27·7–35·5)	23·2 (16·3–30·2)
Road injuries	22·6 (16·5–29·1)	23·9 (18–30·2)	28·8 (25–33·1)	21·9 (14·9–29·1)
Other transport injuries	37·6 (32·7–42·9)	38·2 (33·9–42·9)	41·2 (37–44·9)	35·2 (29·4–41·5)
Unintentional injuries	41 (35·4–45·1)	32·2 (28–36·2)	34·8 (30·5–38·9)	27·3 (22·8–31·6)
Falls	57·1 (50·4–61·6)	47·6 (42·1–51·9)	46·5 (41·2–50·7)	50 (44·2–54·4)
Drowning	-8·49 (-18·9–1·67)	-7·73 (-17·6–2·28)	12·4 (8·25–18)	-8·48 (-18·7–1·81)
Fire, heat, and hot substances	12·4 (8·31–17·6)	15·9 (11·2–21·5)	16·7 (11·7–22·9)	14·6 (10·2–19·9)
Poisonings	18·5 (13·3–25·1)	18·3 (13·3–24·5)	15·5 (11·1–21·1)	20·5 (15–27·3)
Exposure to mechanical forces	10·2 (2·62–17·4)	21·2 (17·3–25·8)	24·9 (21·6–28·9)	7·89 (-0·0376–16·1)
Animal contact	27·8 (23·3–33·2)	27·3 (22·8–32·4)	27·8 (23·2–32·9)	24·4 (20–29·7)
Foreign body	2·93 (1·93–4·29)	5·27 (3·61–7·52)	5·73 (3·87–8·12)	4·98 (3·32–7·21)
Environmental heat and cold exposure	8·27 (3·67–13·9)	8·57 (3·74–14·4)	8·3 (3·66–14)	8·9 (3·93–14·7)
Exposure to forces of nature	7·64 (3·39–12·8)	7·9 (3·51–13·3)	8·99 (3·95–15·1)	6·99 (3·09–11·8)
Other unintentional injuries	19·3 (13·7–26·2)	16·3 (11·5–22·3)	15·8 (11·1–21·8)	23·2 (16·9–31·2)
Self-harm and interpersonal violence	20·8 (9·58–32·4)	22·6 (12·4–33·2)	29·3 (22·8–36·3)	22 (11–33·3)
Self-harm	21·1 (8·54–34·1)	23·1 (10·2–35·9)	25·5 (14·3–37)	23·1 (9·99–35·9)
Interpersonal violence	21·1 (12·1–30·4)	22·2 (14–30·9)	30·9 (23·7–38·7)	18·6 (9·77–27·8)

Supplementary Results Table 2. All-age PAFs attributable to the joint distribution of all risk factors for all ages, both sexes combined for each GBD level 1, level 2, and level 3 cause and all causes in 2019 for Pennsylvania. Results are shown for deaths, YLLs, YLDs, and DALYs Data in parenthesis are 95% uncertainty intervals. PAFs=population attributable fractions. GBD=Global Burden of Disease. YLLs=years of life lost. YLDs=years lived with disability. DALYs=disability-adjusted life-years

	Deaths 2019	DALYs 2019	YLDs 2019	YLLs 2019
All causes	61·3 (58·2–63·9)	49·1 (45·9–52·1)	34·3 (31·4–36·8)	62 (60–64)
Communicable, maternal, neonatal, and nutritional diseases	45·5 (40·7–50)	56·3 (53·3–59·3)	49·4 (45·1–53·9)	60·6 (56·5–64·3)
HIV/AIDS and sexually transmitted infections	91·1 (89·7–92·3)	91·4 (89·9–92·9)	94·1 (92·5–96·1)	89·5 (87·8–90·8)
HIV/AIDS	90·9 (89·4–92·1)	90 (88·3–91·4)	91·3 (89·7–92·7)	89·3 (87·6–90·7)
Sexually transmitted infections excluding HIV	99·1 (98·7–99·4)	99·7 (99·5–99·9)	100 (100–100)	96·2 (94·6–97·4)
Respiratory infections and tuberculosis	43·6 (37–50·1)	38·2 (32·1–44·1)	2·62 (1·99–3·39)	48·6 (41·8–54·7)
Tuberculosis	55·6 (44·9–64·8)	59·2 (49–67·7)	55·6 (44·9–64·6)	59·9 (49·7–68·3)
Lower respiratory infections	43·6 (36·9–50)	48·4 (41·6–54·4)	35·2 (29·5–40·7)	48·6 (41·7–54·7)
Upper respiratory infections	0·522 (0·325–0·809)	0·017 (0·00825–0·0305)	0·00511 (0·00018–0·0146)	1·29 (0·811–1·99)
Otitis media	1·26 (0·503–2·71)	3·98 (2·62–5·41)	3·99 (2·63–5·45)	3·78 (1·72–7·08)
Enteric infections	9·6 (6·06–14·9)	10·6 (7·09–15·9)	10·6 (7–15·8)	10·8 (7·21–15·9)
Diarrheal diseases	9·65 (6·09–15)	10·7 (7·12–16)	10·6 (7–15·8)	10·9 (7·28–16)
Other infectious diseases	0·78 (0·48–1·04)	1·29 (0·877–1·65)	0·794 (0·485–1·25)	1·42 (0·911–1·83)
Meningitis	1·19 (0·85–1·6)	2·82 (2·07–3·73)	0·000525 (1·23e-05–0·00158)	2·96 (2·16–3·94)
Encephalitis	0·505 (0·362–0·703)	1·44 (1·03–2)	9·99e-05 (2·62e-06–0·000301)	1·52 (1·09–2·12)
Measles	9·26 (-4·19–44)	4·4 (-3·27–36)	0·335 (0·012–0·87)	9·42 (-4·27–44·8)
Acute hepatitis	25 (16·1–38)	11·6 (7·38–16·7)	4·29 (2·69–6·55)	26·7 (16·9–39·4)
Maternal and neonatal disorders	86·3 (84·2–88·2)	83·3 (81·1–85·1)	74·7 (70·2–78·7)	88·1 (86·5–89·7)
Maternal disorders	11·9 (4·29–19·8)	11·8 (4·2–19·5)	11·7 (4·16–19·5)	11·8 (4·22–19·6)
Neonatal disorders	91·5 (90·1–92·7)	86 (84–87·9)	76·4 (72–80·6)	91·5 (90·1–92·7)
Nutritional deficiencies	93·4 (88·9–96·2)	89·8 (87–92·3)	89·1 (85·7–92)	93·1 (88·2–95·6)
Protein-energy malnutrition	100 (100–100)	100 (100–100)	100 (100–100)	100 (100–100)
Vitamin A deficiency	--	100 (100–100)	100 (100–100)	--
Dietary iron deficiency	--	100 (100–100)	100 (100–100)	--
Non-communicable diseases	63·9 (60·4–66·8)	50·7 (47–54·2)	33·8 (30·7–36·5)	66·2 (63·8–68·5)
Neoplasms	49·7 (46–53·8)	49·6 (46·2–53·4)	31·8 (28–36·2)	50·8 (47·5–54·6)
Lip and oral cavity cancer	67·4 (58·6–74·8)	70·5 (62–77·2)	69·8 (61·5–76·8)	70·5 (62–77·2)
Nasopharynx cancer	65·3 (54·1–74·3)	65·5 (54·7–74·1)	64·4 (53·3–73·1)	65·6 (54·9–74·2)
Other pharynx cancer	77·8 (71·3–83·4)	79·2 (72·9–84·5)	79·5 (73·1–84·7)	79·2 (72·8–84·5)
Esophageal cancer	79·1 (70–87·2)	80·5 (71·4–88·4)	79·4 (70·2–87·2)	80·6 (71·4–88·4)

Stomach cancer	24·6 (16·6–42·3)	25·7 (17·7–43·8)	25·8 (17·7–43·6)	25·7 (17·7–43·8)
Colon and rectum cancer	61·7 (53·1–70·4)	63 (54·8–71)	62·9 (54·7–71·1)	63 (54·8–71)
Liver cancer	81·1 (76·7–85·4)	81 (76·7–85·2)	81·1 (76·4–85·6)	81 (76·7–85·3)
Gallbladder and biliary tract cancer	27·7 (16·4–40·2)	28·6 (17·1–41·6)	26·5 (15·6–39·7)	28·7 (17·1–41·6)
Pancreatic cancer	43·4 (33·9–54·4)	43·8 (34·6–54·5)	43·8 (34·6–54·7)	43·8 (34·6–54·5)
Larynx cancer	82 (72·6–89·3)	83·3 (75·1–89·5)	83 (74·6–89·4)	83·3 (75·1–89·5)
Tracheal, bronchus, and lung cancer	84 (81·1–87·4)	83·7 (80·9–87·1)	84 (80·9–87·4)	83·7 (80·9–87·1)
Breast cancer	35·8 (26·6–46·9)	35·5 (27–45·4)	35·9 (27–46·3)	35·4 (27–45·3)
Cervical cancer	100 (100–100)	100 (100–100)	100 (100–100)	100 (100–100)
Uterine cancer	55·4 (39·9–68·8)	57 (41·9–69·8)	57·4 (42·7–70·1)	56·9 (41·7–69·7)
Ovarian cancer	20·7 (9·58–33·9)	18·4 (8·3–30·9)	17·9 (8·13–30·3)	18·5 (8·33–30·9)
Prostate cancer	5·71 (2·53–9·27)	6·46 (2·83–10·4)	6·85 (3·06–11)	6·34 (2·79–10·3)
Kidney cancer	42·9 (31·7–53·6)	43·9 (33·4–54)	44·2 (33·7–54·2)	43·9 (33·4–53·9)
Bladder cancer	39·2 (24·7–54)	43·2 (28·6–57)	44·1 (30–57·7)	43·2 (28·5–57)
Thyroid cancer	18·8 (9·9–29·2)	20·1 (10·6–30·8)	20·4 (10·9–30·8)	20 (10·6–30·8)
Mesothelioma	97·3 (96·4–98·1)	94·7 (93–96·1)	96·8 (95·6–97·9)	94·6 (92·9–96)
Non-Hodgkin lymphoma	9·05 (4·09–15·2)	9·6 (4·44–16)	9·37 (4·22–15·5)	9·62 (4·47–16)
Multiple myeloma	10·4 (4·92–17·3)	11 (5·25–18)	10·9 (5·15–17·9)	11 (5·29–18)
Leukemia	36·9 (23·1–51·2)	36·5 (24·1–49·2)	37·7 (23·6–52)	36·5 (24·1–49)
Cardiovascular diseases	80 (76–83·6)	80·8 (77·6–83·6)	70·1 (65·5–74·7)	82·6 (79·6–85·2)
Rheumatic heart disease	21·2 (11·4–42·3)	21·5 (13·2–37·9)	21·4 (12·8–37·3)	21·5 (13·3–38·1)
Ischemic heart disease	92·1 (88–95·5)	93·8 (90·9–96·2)	92·4 (88·8–95·6)	93·8 (90·9–96·2)
Stroke	78·5 (71·3–86·5)	82·4 (77·6–87·7)	81·4 (75·9–87·3)	82·8 (78–88·1)
Hypertensive heart disease	100 (100–100)	100 (100–100)	100 (100–100)	100 (100–100)
Non-rheumatic valvular heart disease	24·7 (14·7–39)	21·8 (14·3–31·8)	6·66 (4·2–9·54)	25·3 (16·6–36·3)
Cardiomyopathy and myocarditis	36·6 (28·8–45)	40·2 (34·4–46·7)	39·3 (32·8–46·1)	40·3 (34·4–46·9)
Atrial fibrillation and flutter	52 (42·1–62·8)	58·2 (49·4–67·3)	59·6 (51–68·2)	56 (47·2–65·6)
Aortic aneurysm	52·3 (46–58·5)	61·4 (56·4–66·1)	--	61·4 (56·4–66·1)
Endocarditis	30·2 (20–42·7)	32·1 (23·1–42·8)	29·8 (19·5–42·2)	32·2 (23·2–43·1)
Other cardiovascular and circulatory diseases	21·5 (16·5–28·9)	24·8 (20·1–30·7)	31·1 (24·7–37·6)	23 (18·4–29·4)
Chronic respiratory diseases	64·8 (60·2–69·5)	60·6 (56·8–64·4)	55 (50·5–59·3)	66 (62·2–70)
Chronic obstructive pulmonary disease	72·9 (68·4–77)	71·4 (67·8–74·8)	65·5 (61·4–69·2)	75·4 (71·8–79)
Pneumoconiosis	100 (100–100)	100 (100–100)	100 (100–100)	100 (100–100)

Asthma	51·6 (40·2–62·7)	43·7 (35·7–52)	43 (35·2–51·2)	52·2 (42·4–61·8)
Digestive diseases	44·4 (39·1–49·3)	46·2 (40·8–51·2)	17·2 (12·1–23)	54·8 (49·1–60·1)
Cirrhosis and other chronic liver diseases	81 (72·6–88·7)	83·3 (75·4–90·1)	79·7 (71·1–87·4)	83·3 (75·4–90·1)
Upper digestive system diseases	20·6 (17·2–24·1)	6·27 (3·91–9·2)	1·67 (1–2·59)	24·6 (20·9–28·3)
Gallbladder and biliary diseases	51·6 (35·6–66·1)	56·1 (41·5–69)	57·4 (43·4–70)	53·7 (38·2–67·7)
Pancreatitis	36·8 (19·1–54·3)	41·7 (23·1–59·4)	39·4 (21·4–56·7)	42·1 (23·5–59·7)
Neurological disorders	27 (11·8–42)	17·6 (9·11–29·8)	12·8 (6·66–23·5)	23·4 (9·44–38·5)
Alzheimer's disease and other dementias	40·2 (26·7–55·8)	43·5 (29·5–58·7)	44·2 (30·4–59·1)	43 (29·2–58·2)
Parkinson's disease	-8·85 (-14·1–-4·26)	-10·6 (-16·3–-5·27)	-11·2 (-17–-5·78)	-10·5 (-16·3–-5·17)
Idiopathic epilepsy	23·9 (15–32·4)	22·2 (14–29·9)	21·2 (13·3–28·5)	24·8 (16–33·3)
Multiple sclerosis	15 (10·9–19·4)	16·3 (12–20·9)	16·2 (11·9–20·6)	16·4 (12–21·3)
Mental disorders	--	8·5 (4·21–13·5)	8·5 (4·21–13·5)	--
Depressive disorders	--	18·8 (8·67–28·7)	18·8 (8·67–28·7)	--
Anxiety disorders	--	5·92 (1·34–13·6)	5·92 (1·34–13·6)	--
Idiopathic developmental intellectual disability	--	47 (24·3–66·1)	47 (24·3–66·1)	--
Substance use disorders	100 (100–100)	100 (100–100)	100 (100–100)	100 (100–100)
Alcohol use disorders	100 (100–100)	100 (100–100)	100 (100–100)	100 (100–100)
Drug use disorders	100 (100–100)	100 (100–100)	100 (100–100)	100 (100–100)
Diabetes and kidney diseases	99·9 (99·8–99·9)	99·9 (99·9–100)	100 (100–100)	99·9 (99·9–99·9)
Diabetes mellitus	100 (100–100)	100 (100–100)	100 (100–100)	100 (100–100)
Chronic kidney disease	100 (100–100)	100 (100–100)	100 (100–100)	100 (100–100)
Sense organ diseases	--	10·2 (9·07–11·4)	10·2 (9·07–11·4)	--
Blindness and vision loss	--	10 (6·47–14·5)	10 (6·47–14·5)	--
Age-related and other hearing loss	--	11 (9·92–12·2)	11 (9·92–12·2)	--
Musculoskeletal disorders	3·04 (0·88–5·52)	20·9 (17·5–24·4)	21·2 (17·8–24·8)	3·11 (0·946–5·57)
Rheumatoid arthritis	11·5 (3·32–20·5)	15·2 (4·85–26·8)	15·5 (5·01–27·4)	13·7 (4·2–24·1)
Osteoarthritis	--	16·4 (9·21–23·7)	16·4 (9·21–23·7)	--
Low back pain	--	42·5 (36·5–48·5)	42·5 (36·5–48·5)	--
Gout	--	62 (46·1–77·5)	62 (46·1–77·5)	--
Other non-communicable diseases	0·099 (0·0674–0·136)	0·134 (0·0879–0·188)	--	0·359 (0·249–0·487)
Sudden infant death syndrome	5·92 (4·08–8·07)	5·91 (4·07–8·06)	--	5·91 (4·07–8·06)
Injuries	29·7 (25·4–34·2)	27·9 (24–32·1)	33·3 (29·3–37)	24·3 (19·5–29·4)
Transport injuries	25·5 (19·7–32)	26·8 (21·4–33)	31·4 (27·3–35·8)	24·6 (17·8–32·1)

Road injuries	24·8 (18·9–31·4)	25·5 (19·8–31·8)	29·2 (25·1–33·5)	24 (17·1–31·4)
Other transport injuries	37·7 (32·5–43·5)	38·8 (34·2–43·6)	41·1 (36·3–45)	35·3 (29–42)
Unintentional injuries	36·8 (31·6–40·7)	31·1 (26·8–35·3)	34·1 (29·6–38·5)	25·7 (21·8–29·7)
Falls	54·8 (47·4–59·8)	46·7 (40·7–51·4)	46 (40·1–50·7)	48·3 (42·3–53)
Drowning	-7·64 (-16·9–1·69)	-7·28 (-16–1·38)	11·5 (7·42–16·9)	-8·28 (-17·3–0·357)
Fire, heat, and hot substances	12 (7·86–17·3)	15·5 (10·9–21·4)	16·8 (11·9–22·9)	13·9 (9·54–19·2)
Poisonings	19·3 (13·8–26)	19·1 (13·8–25·4)	15·7 (11·2–21·2)	21·2 (15·4–28·1)
Exposure to mechanical forces	11·9 (4·19–19·9)	22·2 (17·9–27·5)	25·4 (21·7–29·8)	9·83 (1·44–19·1)
Animal contact	23·9 (19–29·6)	27·3 (22·4–32·8)	28·1 (23·1–33·5)	22·4 (17·5–28·4)
Foreign body	2·89 (1·87–4·21)	5·16 (3·46–7·42)	5·88 (4·02–8·27)	4·84 (3·17–7·1)
Environmental heat and cold exposure	7·67 (3·38–13·3)	8·23 (3·69–14)	7·91 (3·53–13·5)	8·62 (3·87–14·8)
Exposure to forces of nature	7·53 (3·36–12·7)	7·7 (3·43–13)	8·86 (3·96–14·9)	7·08 (3·2–11·9)
Other unintentional injuries	21·6 (15·4–28·9)	16·6 (11·8–22·6)	15·8 (11·2–21·8)	25·3 (18·4–33·6)
Self-harm and interpersonal violence	22·7 (13·2–32·1)	23·8 (15·1–32·4)	28·4 (22–35)	23·4 (14·1–32·5)
Self-harm	24·4 (12·3–36·2)	26·8 (14·7–38·9)	26 (15·9–36·8)	26·8 (14·7–39)
Interpersonal violence	18·4 (10·3–27·6)	19·1 (11·4–27·7)	29·4 (22·3–36·8)	16·6 (8·66–25·6)

Supplementary Results Table 2. All-age PAFs attributable to the joint distribution of all risk factors for all ages, both sexes combined for each GBD level 1, level 2, and level 3 cause and all causes in 2019 for Rhode Island. Results are shown for deaths, YLLs, YLDs, and DALYs Data in parenthesis are 95% uncertainty intervals. PAFs=population attributable fractions. GBD=Global Burden of Disease. YLLs=years of life lost. YLDs=years lived with disability. DALYs=disability-adjusted life-years

	Deaths 2019	DALYs 2019	YLDs 2019	YLLs 2019
All causes	61·1 (57·6–63·9)	48·5 (45·4–51·5)	34·5 (31·8–37·1)	62·1 (59·7–64·1)
Communicable, maternal, neonatal, and nutritional diseases	42·6 (35·8–48·3)	53·5 (49·3–57·5)	46·6 (42–51·2)	58·4 (52·5–63·4)
HIV/AIDS and sexually transmitted infections	85·7 (81·2–88·9)	84·9 (79·9–88·7)	89 (84·5–92·8)	82·2 (76·5–86·2)
HIV/AIDS	85·2 (80·6–88·6)	82·8 (76·9–86·9)	84·5 (78·6–88·7)	82 (76·1–86·1)
Sexually transmitted infections excluding HIV	99·4 (99·1–99·6)	99·8 (99·6–99·9)	100 (100–100)	97·3 (95·9–98·3)
Respiratory infections and tuberculosis	43·5 (33·8–52·1)	35·7 (28·2–43·3)	2·33 (1·76–3·03)	47·8 (38·8–55·8)
Tuberculosis	56·6 (46·2–65·9)	59·5 (49·6–68·1)	56·3 (45·6–65·2)	60·2 (50·5–68·6)
Lower respiratory infections	43·5 (33·6–52·2)	47·6 (38·6–55·6)	33·1 (27·1–38·8)	47·8 (38·7–56)
Upper respiratory infections	0·401 (0·239–0·654)	0·0211 (0·0105–0·037)	0·0043 (0–0·0133)	1·13 (0·692–1·8)
Otitis media	0·839 (0·342–1·88)	3·26 (2·09–4·5)	3·26 (2·09–4·53)	2·79 (1·25–5·45)
Enteric infections	9·06 (5·56–14·4)	10·3 (6·78–15·5)	9·9 (6·43–15·4)	10·7 (7·12–15·9)
Diarrheal diseases	9·1 (5·58–14·4)	10·3 (6·8–15·6)	9·9 (6·43–15·4)	10·8 (7·16–16)
Other infectious diseases	0·731 (0·474–1·04)	1·23 (0·866–1·59)	0·873 (0·526–1·35)	1·32 (0·893–1·73)
Meningitis	0·968 (0·671–1·32)	2·36 (1·66–3·13)	0·000364 (0–0·00109)	2·55 (1·78–3·39)
Encephalitis	0·426 (0·29–0·587)	1·3 (0·908–1·81)	0·000166 (0–0·000514)	1·36 (0·942–1·89)
Measles	9·04 (-4·12–42·8)	9·22 (-4·22–43·6)	0·163 (0–0·751)	9·22 (-4·22–43·6)
Acute hepatitis	21·7 (13·2–34·4)	11·3 (6·98–17)	4·41 (2·62–6·9)	23·5 (14·5–36·6)
Maternal and neonatal disorders	86·9 (84·3–89·2)	81 (77·8–83·8)	70·8 (65·8–75·4)	88·5 (86·2–90·3)
Maternal disorders	11·2 (3·99–18·6)	11 (3·96–18·4)	11 (3·88–18·4)	11 (3·95–18·3)
Neonatal disorders	91·3 (89·7–92·7)	83·1 (80–85·9)	72·1 (66·9–76·6)	91·3 (89·7–92·7)
Nutritional deficiencies	91·6 (88·2–95·9)	87·4 (84–90·4)	86·7 (82·8–90·2)	91 (87·8–95·3)
Protein-energy malnutrition	100 (100–100)	100 (100–100)	100 (100–100)	100 (100–100)
Vitamin A deficiency	--	100 (100–100)	100 (100–100)	--
Dietary iron deficiency	--	100 (100–100)	100 (100–100)	--
Non-communicable diseases	63·3 (59·4–66·4)	49·6 (45·9–52·9)	33·8 (30·8–36·5)	65·2 (62·6–67·5)
Neoplasms	49·7 (46·1–53·7)	49·1 (45·6–52·9)	31·4 (27·2–35·5)	50·4 (47–54·1)
Lip and oral cavity cancer	67·6 (57·5–75·6)	69·9 (60·1–77·1)	69·5 (60–76·9)	69·9 (60·1–77·2)
Nasopharynx cancer	66·4 (54·7–75·9)	66·1 (54·7–75)	65·3 (54–74·5)	66·2 (54·8–75·1)
Other pharynx cancer	77·8 (69·6–83·6)	78·9 (71·4–84·4)	79·2 (72–84·6)	78·9 (71·3–84·4)

Esophageal cancer	79·3 (70·4–87·4)	80·4 (71·4–88·3)	79·5 (70·6–87·7)	80·4 (71·4–88·3)
Stomach cancer	24·3 (16·1–42)	25 (16·9–43·2)	25·2 (17·1–43·4)	25 (16·9–43·2)
Colon and rectum cancer	62·2 (53·4–70·8)	63·2 (55·1–71·3)	63·1 (54·7–71·5)	63·2 (55·1–71·3)
Liver cancer	81·4 (76·8–85·7)	81·2 (77–85·3)	81·2 (77–85·4)	81·2 (77–85·3)
Gallbladder and biliary tract cancer	26·5 (15·2–39·1)	27·5 (15·9–40·1)	25·4 (14·7–38·3)	27·5 (16–40·2)
Pancreatic cancer	42·9 (33·3–53·7)	42·8 (33·8–53·2)	43·1 (33·6–53·6)	42·8 (33·8–53·2)
Larynx cancer	81·8 (71·7–89·1)	82·9 (74·4–89·2)	82·6 (73·9–89)	82·9 (74·3–89·3)
Tracheal, bronchus, and lung cancer	82·5 (79·1–85·9)	81·9 (78·6–85·3)	82·3 (78·7–86·1)	81·9 (78·6–85·3)
Breast cancer	36·7 (27·7–46·4)	36 (27·6–44·9)	36·4 (27·9–45·8)	35·9 (27·5–44·7)
Cervical cancer	100 (100–100)	100 (100–100)	100 (100–100)	100 (100–100)
Uterine cancer	53·3 (37·7–67·7)	55 (39·8–68·7)	55·5 (40·5–68·9)	54·9 (39·7–68·7)
Ovarian cancer	18·6 (8·4–31·5)	16·7 (6·94–28·7)	16·2 (6·93–28·1)	16·7 (6·95–28·8)
Prostate cancer	5·71 (2·4–9·62)	6·41 (2·72–10·7)	6·77 (2·93–11·3)	6·29 (2·67–10·5)
Kidney cancer	42·2 (30·6–53·2)	42·9 (32·3–53·2)	43·2 (32·4–53·4)	42·9 (32·3–53·2)
Bladder cancer	38·9 (24–54·6)	42·7 (28·1–56·9)	43·5 (29·1–57·3)	42·6 (28–56·9)
Thyroid cancer	18 (9·23–28·3)	19·3 (10–30)	19·6 (10·4–30·1)	19·2 (9·92–29·9)
Mesothelioma	97 (96–97·9)	94·5 (92·6–96)	96·7 (95·2–97·8)	94·4 (92·6–95·9)
Non-Hodgkin lymphoma	8·69 (3·76–14·8)	9·21 (4·11–15·5)	8·97 (3·91–15·1)	9·23 (4·12–15·5)
Multiple myeloma	10 (4·57–16·9)	10·6 (4·99–17·4)	10·4 (4·94–17·5)	10·6 (4·99–17·4)
Leukemia	36·6 (22·5–51)	35·7 (23·6–48·5)	37·3 (22·9–51·7)	35·6 (23·6–48·3)
Cardiovascular diseases	82·1 (78·1–85·6)	82·1 (79·1–84·9)	70·8 (66·2–75·2)	84 (81–86·5)
Rheumatic heart disease	21·6 (11·9–40·8)	21·7 (13·1–36·3)	21·5 (12·6–37·5)	21·8 (13·4–36·4)
Ischemic heart disease	91·9 (87·6–95·5)	93·5 (90·3–96·1)	92·3 (88·7–95·4)	93·5 (90·4–96·1)
Stroke	78·4 (70·7–86)	82·1 (76·7–87·5)	81·1 (75·6–87)	82·6 (76·8–87·8)
Hypertensive heart disease	100 (100–100)	100 (100–100)	100 (100–100)	100 (100–100)
Non-rheumatic valvular heart disease	24·3 (14·8–37·3)	22 (14·9–31·6)	8·97 (5·74–12·9)	24·9 (16·8–35·5)
Cardiomyopathy and myocarditis	38 (31·2–45·9)	41·3 (35·4–47·1)	39·3 (33·5–45·2)	41·5 (35·5–47·5)
Atrial fibrillation and flutter	52·5 (41·8–62·7)	58·5 (49·5–67·3)	60 (51·1–68·7)	56·3 (46·7–65·5)
Aortic aneurysm	52·9 (46·5–60)	61·5 (56·4–66·8)	--	61·5 (56·4–66·8)
Endocarditis	30·7 (21–43·8)	32·5 (23·7–43·3)	30 (20·3–42·6)	32·7 (23·8–43·4)
Other cardiovascular and circulatory diseases	19·7 (14·8–27·4)	22·5 (17·9–28·7)	29·7 (23·1–37·2)	20·4 (16–27)
Chronic respiratory diseases	63·9 (58·7–68·8)	59·1 (55·4–62·9)	54·1 (49·3–58·5)	64·6 (60·1–68·9)

Chronic obstructive pulmonary disease	72·3 (66·9–77·5)	70 (66–74)	64·1 (60·1–67·8)	74·3 (69·7–78·9)
Pneumoconiosis	100 (100–100)	100 (100–100)	100 (100–100)	100 (100–100)
Asthma	50·4 (38·7–61·9)	43·9 (35·8–52·6)	43·4 (35·4–52·1)	51 (41·2–61·1)
Digestive diseases	44 (38·7–49·3)	46 (40·6–51·3)	14·7 (10·1–19·9)	55·1 (49·6–60·5)
Cirrhosis and other chronic liver diseases	82·3 (73·6–89·6)	84·3 (76·5–90·9)	81·2 (72·5–88·3)	84·4 (76·5–90·9)
Upper digestive system diseases	20·2 (16·6–23·7)	6·04 (3·77–8·94)	1·51 (0·882–2·33)	23·4 (19·5–27·1)
Gallbladder and biliary diseases	50·1 (34·3–65·2)	54·6 (39·9–67·9)	56·3 (41·8–69)	52·2 (36·9–66·4)
Pancreatitis	40·2 (20·3–58·5)	44·1 (24·1–61·2)	42·4 (22·2–59·6)	44·4 (24·3–61·5)
Neurological disorders	28·8 (13·7–43·8)	18·7 (9·85–31·4)	13·3 (7·03–24)	25·1 (11–40·2)
Alzheimer's disease and other dementias	40·4 (26·3–55·5)	43·4 (29·2–57·8)	44·3 (30–58·7)	42·8 (28·7–57·5)
Parkinson's disease	-9·28 (-15·2–-4·48)	-11 (-17·4–-5·55)	-11·7 (-18·1–-6·08)	-10·8 (-17·3–-5·46)
Idiopathic epilepsy	25·5 (16·4–34·5)	23·7 (15·5–32)	23 (14·8–30·8)	26 (17·2–34·6)
Multiple sclerosis	14·8 (10·6–19·4)	15·6 (11·4–20·2)	15·3 (11·1–19·7)	16 (11·6–20·8)
Mental disorders	--	8·64 (4·49–13·5)	8·65 (4·49–13·5)	--
Depressive disorders	--	17·5 (8·76–26·7)	17·5 (8·76–26·7)	--
Anxiety disorders	--	5·9 (1·31–13·7)	5·9 (1·31–13·7)	--
Idiopathic developmental intellectual disability	--	46·5 (23·7–64·3)	46·5 (23·7–64·3)	--
Substance use disorders	100 (100–100)	100 (100–100)	100 (100–100)	100 (100–100)
Alcohol use disorders	100 (100–100)	100 (100–100)	100 (100–100)	100 (100–100)
Drug use disorders	100 (100–100)	100 (100–100)	100 (100–100)	100 (100–100)
Diabetes and kidney diseases	99·8 (99·8–99·9)	99·9 (99·9–99·9)	100 (100–100)	99·8 (99·8–99·9)
Diabetes mellitus	100 (100–100)	100 (100–100)	100 (100–100)	100 (100–100)
Chronic kidney disease	100 (100–100)	100 (100–100)	100 (100–100)	100 (100–100)
Sense organ diseases	--	10·2 (9·11–11·5)	10·2 (9·11–11·5)	--
Blindness and vision loss	--	9·78 (6·25–14·3)	9·78 (6·25–14·3)	--
Age-related and other hearing loss	--	11·1 (10·1–12·4)	11·1 (10·1–12·4)	--
Musculoskeletal disorders	2·92 (0·861–5·34)	20·2 (16·8–23·9)	20·5 (17–24·3)	2·87 (0·862–5·2)
Rheumatoid arthritis	11·9 (3·6–21·5)	14·8 (4·72–26·2)	15 (4·79–26·6)	13·7 (4·15–24·7)
Osteoarthritis	--	14·5 (8·07–21·4)	14·5 (8·07–21·4)	--
Low back pain	--	41·1 (35·1–47·1)	41·1 (35·1–47·1)	--
Gout	--	60·1 (44–76·3)	60·1 (44–76·3)	--
Other non-communicable diseases	0·0495 (0·0331–0·0736)	0·0716 (0·0456–0·107)	--	0·19 (0·13–0·271)

Sudden infant death syndrome	4·48 (2·92–6·4)	4·48 (2·92–6·4)	--	4·48 (2·92–6·4)
Injuries	36·3 (31·2–41)	33·5 (28·9–38·2)	37·6 (33·3–42·1)	29·8 (23·9–35·4)
Transport injuries	30·1 (22·5–38)	31·7 (25·1–39·2)	34·7 (30–39·6)	29·8 (21·2–38·9)
Road injuries	29·2 (21·6–37·5)	30·3 (23·1–38·1)	32·4 (27·6–37·6)	29·1 (20·3–38·5)
Other transport injuries	41·5 (35·2–48·8)	41·9 (37·1–46·9)	43·2 (38·7–47·1)	39·2 (32·1–47·6)
Unintentional injuries	43·3 (37·9–47·7)	36·2 (31·6–41·1)	38·2 (33·3–43)	32·1 (27·4–37·4)
Falls	58·1 (50·9–63)	49·5 (43·9–54·4)	48·5 (43–53·3)	51·9 (46·2–56·8)
Drowning	-2·05 (-14·1–10·7)	-1·1 (-12·6–11·2)	14·2 (9·58–20)	-1·98 (-14–10·8)
Fire, heat, and hot substances	14·7 (9·8–21)	19·7 (14·1–26·5)	20·6 (14·8–27·5)	17·9 (12·5–24·6)
Poisonings	23 (16·3–31·3)	22·7 (16·5–30·6)	19·3 (14·1–25·6)	25·7 (18·5–34·7)
Exposure to mechanical forces	17·3 (6·3–29)	27·4 (22·2–34·1)	28·9 (24·1–35)	14·8 (3·01–27·5)
Animal contact	27·5 (22·4–33·5)	30·9 (25·6–37·1)	31·5 (26·1–37·4)	25·5 (20·2–32·2)
Foreign body	4·11 (2·69–6·13)	7·11 (4·76–10·5)	6·83 (4·65–9·98)	7·25 (4·76–10·9)
Environmental heat and cold exposure	8·38 (3·7–14·6)	8·92 (3·96–15·5)	8·77 (3·88–15·3)	9·18 (4·07–15·8)
Exposure to forces of nature	8·12 (3·59–13·9)	7·98 (3·54–13·6)	9·39 (4·14–16)	7·48 (3·31–12·7)
Other unintentional injuries	25·4 (17·8–34·9)	20 (14·4–27)	19·4 (14–26·2)	30·5 (21·4–41·4)
Self-harm and interpersonal violence	26·8 (15·5–37·1)	29·2 (19–38·5)	38·6 (31·6–45·5)	28 (17·3–38)
Self-harm	25·9 (12·5–38·5)	28·4 (15–41)	27·5 (16–38·9)	28·4 (15–41·1)
Interpersonal violence	31·2 (21·7–40·1)	32 (23·2–40·5)	41·8 (34·7–49·5)	28 (18·6–37)

Supplementary Results Table 2. All-age PAFs attributable to the joint distribution of all risk factors for all ages, both sexes combined for each GBD level 1, level 2, and level 3 cause and all causes in 2019 for Kyrgyzstan. Results are shown for deaths, YLLs, YLDs, and DALYs Data in parenthesis are 95% uncertainty intervals. PAFs=population attributable fractions. GBD=Global Burden of Disease. YLLs=years of life lost. YLDs=years lived with disability. DALYs=disability-adjusted life-years

	Deaths 2019	DALYs 2019	YLDs 2019	YLLs 2019
All causes	67·7 (65–70·1)	49·1 (45·9–51·8)	28·1 (25·9–30·3)	60·2 (58·2–62·1)
Communicable, maternal, neonatal, and nutritional diseases	68·4 (65·3–71·4)	69·4 (67·1–71·8)	64 (60·2–67·4)	71·2 (68·4–74)
HIV/AIDS and sexually transmitted infections	73·5 (72·4–74·8)	71·6 (69·9–73·6)	89·6 (84·7–93·5)	68·6 (67·1–70·1)
HIV/AIDS	73·4 (72·3–74·8)	69 (67·7–70·6)	75·6 (73–77·4)	68·6 (67·1–70·1)
Sexually transmitted infections excluding HIV	82·3 (75·6–87·7)	97·6 (95·8–98·8)	100 (100–100)	68·3 (58·9–76·6)
Respiratory infections and tuberculosis	63·4 (57·8–68·8)	60·8 (54·9–66·3)	15·9 (11·5–21)	66·3 (59·9–71·7)
Tuberculosis	52·2 (45·8–58·3)	47·6 (41·1–53·6)	40·8 (33·7–47·2)	48·5 (42–54·7)
Lower respiratory infections	71·2 (63·3–79·1)	75·3 (67·5–82·6)	44·2 (37·3–51·6)	75·5 (67·7–82·8)
Upper respiratory infections	1·18 (0·692–1·97)	0·497 (0·277–0·808)	0·0137 (0·00339–0·027)	1·25 (0·736–2·1)
Otitis media	12 (7·3–19·1)	6·77 (4·52–9·24)	6·77 (4·52–9·24)	15·6 (10·1–24·6)
Enteric infections	92·8 (88·2–96)	89·6 (82·7–94·8)	86 (76·8–93)	94·1 (90·1–96·8)
Diarrheal diseases	94·1 (89·7–97·2)	90 (83–95·2)	86 (76·9–93·1)	94·8 (90·9–97·5)
Other infectious diseases	1·7 (1·32–2·27)	1·56 (1·21–2·03)	0·176 (0·103–0·295)	2·12 (1·63–2·82)
Meningitis	3·6 (2·74–4·6)	3·63 (2·74–4·68)	0·00131 (3e-04–0·00254)	5·04 (3·86–6·4)
Encephalitis	0·635 (0·338–1·43)	0·78 (0·423–1·7)	0·000611 (0·000153–0·00123)	0·846 (0·459–1·81)
Measles	7·52 (1·37–22·9)	3·17 (0·789–7·01)	2·2 (0·0838–5·3)	10·3 (2·16–28·6)
Acute hepatitis	2·27 (1·28–3·66)	1·68 (1·02–2·66)	1·58 (0·938–2·55)	1·72 (0·971–2·81)
Maternal and neonatal disorders	77·1 (73·8–80·2)	73·7 (70·6–76·9)	41·5 (35·8–47·1)	78 (74·7–81)
Maternal disorders	18·2 (6·61–29·9)	18·1 (6·55–29·8)	18 (6·45–29·7)	18·1 (6·57–29·8)
Neonatal disorders	79·7 (76·5–82·8)	75·4 (72·2–78·5)	42·4 (36·6–48·1)	79·7 (76·5–82·8)
Nutritional deficiencies	82·5 (71·5–85·4)	98·3 (97·7–98·7)	98·5 (98–98·9)	80·9 (70·6–84)
Protein-energy malnutrition	100 (100–100)	100 (100–100)	100 (100–100)	100 (100–100)
Vitamin A deficiency	--	100 (100–100)	100 (100–100)	--
Dietary iron deficiency	--	100 (100–100)	100 (100–100)	--
Non-communicable diseases	73·9 (70·6–76·7)	51·3 (46·7–55·3)	23·7 (21–26·4)	68·7 (66–71·2)
Neoplasms	40·5 (37·1–45·3)	38·1 (35–42·6)	35·8 (32·4–39·7)	38·1 (35–42·6)
Lip and oral cavity cancer	58·5 (53–63·3)	60·6 (54·8–65·6)	57·1 (51·3–62·7)	60·7 (54·9–65·7)
Nasopharynx cancer	50·2 (43·1–56·6)	49·1 (41·8–55·7)	49·2 (41·7–55·7)	49·1 (41·8–55·7)
Other pharynx cancer	66·4 (61·2–71)	65·5 (60–70·6)	62 (56·2–67)	65·5 (60–70·6)

Esophageal cancer	69·1 (59·2–78·6)	70·9 (61·6–79·8)	69·8 (60·2–79·1)	70·9 (61·6–79·8)
Stomach cancer	25·6 (17·3–44)	25 (16·5–43·7)	25·2 (16·8–43·4)	25 (16·5–43·7)
Colon and rectum cancer	52 (44·5–58·9)	52·2 (45–58·8)	52·1 (44·8–58·6)	52·2 (45–58·8)
Liver cancer	66·3 (58·8–73·5)	62·7 (55·1–69·8)	65·5 (57·8–72·5)	62·7 (55·1–69·7)
Gallbladder and biliary tract cancer	20·7 (11·4–32)	21·1 (11·8–32·6)	20·8 (11·6–32)	21·1 (11·8–32·6)
Pancreatic cancer	28·7 (22·9–36)	28·8 (22·9–35·9)	28·9 (23·2–36)	28·8 (22·9–35·9)
Larynx cancer	80·4 (75·4–84·6)	79·9 (75–84·1)	77·2 (71·8–81·6)	80 (75·1–84·2)
Tracheal, bronchus, and lung cancer	80·5 (77–84·9)	79·5 (75·9–84·2)	80 (75·8–84·7)	79·5 (75·9–84·2)
Breast cancer	19·7 (13·3–26·8)	18 (12–24·4)	18 (12·1–24·6)	18 (12–24·4)
Cervical cancer	100 (100–100)	100 (100–100)	100 (100–100)	100 (100–100)
Uterine cancer	45 (30·9–60·3)	45·5 (30·9–61)	45·8 (30·8–61·2)	45·5 (30·9–60·9)
Ovarian cancer	8·72 (2·86–16·3)	7·98 (2·51–14·9)	7·6 (2·33–14·7)	7·99 (2·52–14·9)
Prostate cancer	8·34 (4·04–12·8)	9·3 (4·5–14·2)	9·73 (4·69–14·9)	9·28 (4·49–14·2)
Kidney cancer	34·2 (26–43·1)	31·6 (23·6–40·3)	30·6 (22·5–39·5)	31·6 (23·7–40·3)
Bladder cancer	45·8 (36·2–54·1)	48·2 (38·9–56·3)	47·4 (37·9–55·2)	48·3 (38·9–56·4)
Thyroid cancer	12·7 (6·76–20·2)	12·7 (6·69–20·2)	12·5 (6·66–19·7)	12·7 (6·68–20·2)
Mesothelioma	69·1 (61·6–75·4)	58·2 (49·7–65·6)	67·4 (59·6–73·9)	58 (49·6–65·5)
Non-Hodgkin lymphoma	5·84 (2·38–10·4)	5·26 (2·19–9·51)	4·88 (1·97–8·79)	5·27 (2·2–9·53)
Multiple myeloma	7·66 (3·25–13·8)	7·85 (3·36–14)	7·89 (3·36–14·1)	7·85 (3·36–14)
Leukemia	19·9 (13·6–26·6)	14 (9·16–18·9)	17·2 (11·5–23)	13·9 (9·13–18·8)
Cardiovascular diseases	88·6 (85·9–91)	88·4 (86·2–90·4)	70·3 (65·3–74·8)	89·4 (87·3–91·3)
Rheumatic heart disease	19·8 (12·4–31·4)	17·9 (10·5–28·1)	13·9 (6·96–22·7)	19·3 (11·6–29·8)
Ischemic heart disease	91·9 (88·5–94·7)	93·6 (91·3–95·6)	92·8 (90·1–95·1)	93·6 (91·4–95·6)
Stroke	84·7 (80·9–88·9)	87·4 (84·2–90·6)	83 (79·3–86·6)	87·8 (84·6–91)
Hypertensive heart disease	100 (100–100)	100 (100–100)	100 (100–100)	100 (100–100)
Non-rheumatic valvular heart disease	19·9 (13·4–27·3)	14·6 (9·35–20·8)	1·19 (0·716–1·85)	19·7 (13·3–27·2)
Cardiomyopathy and myocarditis	90·8 (86·6–92·9)	89·7 (86·4–92·2)	76·7 (69·5–82·4)	89·9 (86·5–92·3)
Atrial fibrillation and flutter	44 (34·3–53·8)	53·3 (44·9–61·6)	56 (47·5–64·3)	47·8 (39·3–56·8)
Aortic aneurysm	56·1 (50·8–61·7)	61·6 (57·2–66)	--	61·6 (57·2–66)
Endocarditis	31 (21·8–41·6)	29·2 (19·9–40·3)	25·8 (17·9–34·9)	29·2 (19·9–40·3)
Other cardiovascular and circulatory diseases	30·4 (24·3–36·4)	27·6 (20·9–33·6)	19 (12·8–25·9)	28·9 (22·2–35·2)
Chronic respiratory diseases	76·7 (71·9–80·9)	64·9 (60·3–69·4)	43·2 (37·5–48·7)	76·1 (71·3–80·1)
Chronic obstructive pulmonary disease	79·1 (74·3–83·3)	75·7 (71·4–79·6)	61·4 (56·3–66·3)	79·9 (75·2–83·9)

Pneumoconiosis	100 (100–100)	100 (100–100)	100 (100–100)	100 (100–100)
Asthma	44·4 (34·2–55·5)	30·4 (23·3–38·8)	26·3 (20–33·9)	46·2 (36·2–57)
Digestive diseases	64 (56·8–71·4)	58·9 (51·4–66·2)	14·4 (10·1–19·1)	65·5 (57·6–73·4)
Cirrhosis and other chronic liver diseases	70·6 (62·3–79)	71·8 (63·1–80·5)	63·7 (55·7–72·1)	71·9 (63·2–80·7)
Upper digestive system diseases	29·9 (26·1–33·8)	11·3 (6·99–16·2)	0·827 (0·481–1·26)	31 (27·3–34·7)
Gallbladder and biliary diseases	43·4 (30·7–56·7)	43·1 (30·4–56·5)	42·8 (29·9–56·4)	44 (31·5–56·9)
Pancreatitis	36·7 (26·4–49·2)	39·2 (28·3–52·5)	30·4 (21·3–41·1)	39·6 (28·4–53)
Neurological disorders	21 (12·2–31·8)	8·98 (4·55–15·7)	4·29 (1·93–8·75)	18 (12·1–26·1)
Alzheimer's disease and other dementias	27·4 (16·8–39·8)	30·2 (19·1–42·5)	31·3 (20·6–43·5)	29·7 (18·8–41·6)
Parkinson's disease	-9·54 (-14·6–-5·22)	-11·9 (-18·1–-6·47)	-13·4 (-20·8–-7·17)	-11·5 (-17·7–-6·27)
Idiopathic epilepsy	17·3 (12·1–22·8)	13·7 (9·36–18·2)	10·4 (7·08–14·1)	15·9 (11·1–21)
Multiple sclerosis	17·2 (11·5–22·7)	16 (11·8–20·3)	14·7 (10·8–18·7)	17·9 (12·3–23·4)
Mental disorders	--	6·06 (3·16–9·55)	6·07 (3·16–9·55)	--
Depressive disorders	--	8·37 (2·1–15·9)	8·37 (2·1–15·9)	--
Anxiety disorders	--	2·94 (0·762–6·8)	2·94 (0·762–6·8)	--
Idiopathic developmental intellectual disability	--	52·6 (25·3–72·7)	52·6 (25·3–72·7)	--
Substance use disorders	100 (100–100)	100 (100–100)	100 (100–100)	100 (100–100)
Alcohol use disorders	100 (100–100)	100 (100–100)	100 (100–100)	100 (100–100)
Drug use disorders	100 (100–100)	100 (100–100)	100 (100–100)	100 (100–100)
Diabetes and kidney diseases	99·2 (99·1–99·4)	99·4 (99·2–99·5)	100 (100–100)	98·9 (98·6–99·1)
Diabetes mellitus	100 (100–100)	100 (100–100)	100 (100–100)	100 (100–100)
Chronic kidney disease	100 (100–100)	100 (100–100)	100 (100–100)	100 (100–100)
Sense organ diseases	--	15 (13·4–16·7)	15 (13·4–16·7)	--
Blindness and vision loss	--	9·4 (5·82–13·3)	9·4 (5·82–13·3)	--
Age-related and other hearing loss	--	18·6 (17·4–20·1)	18·6 (17·4–20·1)	--
Musculoskeletal disorders	5·66 (1·79–10·5)	29·9 (26·1–34)	30·7 (26·7–34·8)	4·07 (1·3–7·32)
Rheumatoid arthritis	8·81 (2·69–16·3)	9·26 (3·05–15·9)	9·33 (3·12–15·9)	9·13 (2·9–16·4)
Osteoarthritis	--	14·2 (7·39–22)	14·2 (7·39–22)	--
Low back pain	--	45·1 (40·9–49·4)	45·1 (40·9–49·4)	--
Gout	--	42·9 (29–59·3)	42·9 (29–59·3)	--
Other non-communicable diseases	0·189 (0·108–0·278)	0·104 (0·0638–0·156)	--	0·245 (0·131–0·36)
Sudden infant death syndrome	9·35 (6·24–13·3)	9·34 (6·23–13·3)	--	9·34 (6·23–13·3)
Injuries	4·6 (-0·955–9·42)	6·14 (1·58–10·1)	17·4 (15·5–19·4)	2·35 (-3·31–7·21)

Transport injuries	7·24 (-0·912–15·2)	8·05 (0·857–15·3)	21·8 (18·7–24·8)	5·29 (-3·11–13·5)
Road injuries	6·49 (-1·93–14·7)	7·42 (-0·0989–15)	21·7 (18·5–24·6)	4·58 (-4·21–13·1)
Other transport injuries	25·1 (21·4–28·8)	22·2 (18·7–25·9)	25·1 (21·8–28·1)	21·6 (18–25·4)
Unintentional injuries	1·54 (-3·94–7·35)	5·32 (0·885–9·68)	16·1 (13·8–18·5)	-2·39 (-8·31–3·61)
Falls	25·4 (22–29·3)	21·2 (18·2–24·5)	23 (19·9–26·2)	18·1 (15·2–21·6)
Drowning	-24·8 (-40·7–-7·83)	-26·3 (-41·8–-9·23)	7·1 (5·29–9·65)	-26·8 (-42·7–-9·44)
Fire, heat, and hot substances	9·82 (7·23–13·1)	9·42 (7·12–12·3)	10·1 (7·64–13·3)	8·72 (6·47–11·4)
Poisonings	12·5 (9·16–16·7)	12 (8·99–15·8)	9·23 (7·09–12·1)	12·6 (9·44–16·7)
Exposure to mechanical forces	-7·42 (-19·8–6·32)	9·45 (5·31–13·3)	15 (13·1–16·8)	-9·58 (-22·1–4·34)
Animal contact	13·1 (10·2–16·7)	12·5 (9·98–15·6)	14 (11·2–17·2)	10·3 (7·89–13·4)
Foreign body	3·5 (2·76–4·39)	2·88 (2·26–3·65)	4·42 (3·38–5·58)	2·58 (1·98–3·33)
Environmental heat and cold exposure	7·1 (3·23–12·1)	6·93 (3·14–11·7)	5·1 (2·31–8·75)	7·3 (3·32–12·4)
Exposure to forces of nature	0 (0–0)	5·42 (2·49–9·22)	5·42 (2·49–9·22)	0 (0–0)
Other unintentional injuries	14 (10·5–18·3)	11·7 (8·85–15·3)	10·4 (7·81–13·6)	13·2 (10·1–17·2)
Self-harm and interpersonal violence	4·75 (-6·38–15·1)	5·07 (-5·32–14·7)	17 (13·2–21·4)	4·1 (-7·01–14·4)
Self-harm	4·01 (-10·7–18·5)	3·86 (-10·5–17·9)	13·7 (7·27–20·5)	3·58 (-11·1–18)
Interpersonal violence	6·25 (-5·95–19·4)	7·4 (-3·73–19·4)	21·4 (16·2–27)	5·16 (-7·18–18·4)

Supplementary Results Table 2. All-age PAFs attributable to the joint distribution of all risk factors for all ages, both sexes combined for each GBD level 1, level 2, and level 3 cause and all causes in 2019 for South Carolina. Results are shown for deaths, YLLs, YLDs, and DALYs Data in parenthesis are 95% uncertainty intervals. PAFs=population attributable fractions. GBD=Global Burden of Disease. YLLs=years of life lost. YLDs=years lived with disability. DALYs=disability-adjusted life-years	Deaths 2019	DALYs 2019	YLDs 2019	YLLs 2019
All causes	61·2 (58·6–63·6)	49·4 (46·4–52·4)	35 (32·4–37·5)	60·7 (58·7–62·7)
Communicable, maternal, neonatal, and nutritional diseases	44·3 (40·5–47·9)	57·6 (54·7–60·3)	53·5 (48·9–58)	60·1 (56·7–63·2)
HIV/AIDS and sexually transmitted infections	92·7 (89·7–94·8)	92·1 (88·9–94·3)	94·2 (91·4–96·1)	91 (87·4–93·4)
HIV/AIDS	92·6 (89·4–94·7)	91·5 (88–93·8)	92·7 (89·5–94·9)	90·9 (87·2–93·3)
Sexually transmitted infections excluding HIV	98·8 (98·3–99·2)	99·4 (99–99·7)	100 (100–100)	95·6 (93·7–97·1)
Respiratory infections and tuberculosis	35·2 (29·6–40·3)	32·3 (27–38)	2·69 (2·05–3·47)	41 (35·3–46·3)
Tuberculosis	56·1 (47·1–64·4)	59·4 (50·8–67·1)	54·5 (45–63·3)	60 (51·6–67·8)
Lower respiratory infections	34·9 (29·3–40·1)	40·6 (34·7–46)	33·2 (27·5–38·6)	40·7 (34·8–46·1)
Upper respiratory infections	0·733 (0·444–1·21)	0·022 (0·0106–0·0398)	0·00463 (7·01e-05–0·0127)	1·58 (0·936–2·56)
Otitis media	1·59 (0·663–3·37)	3·97 (2·59–5·41)	3·97 (2·58–5·47)	4·19 (1·98–7·7)
Enteric infections	12·1 (7·48–19)	13·3 (8·75–20)	13 (8·36–19·9)	13·6 (9·08–20·3)
Diarrheal diseases	12·2 (7·52–19·1)	13·4 (8·8–20·1)	13 (8·37–19·9)	13·8 (9·19–20·5)
Other infectious diseases	0·878 (0·612–1·2)	1·46 (1·1–1·87)	0·747 (0·464–1·16)	1·61 (1·18–2·06)
Meningitis	1·47 (1·07–2·03)	3·24 (2·41–4·4)	0·000511 (5·49e-06–0·00164)	3·36 (2·49–4·57)
Encephalitis	0·64 (0·465–0·901)	1·69 (1·24–2·36)	0·000182 (1·63e-06–0·000537)	1·74 (1·28–2·42)
Measles	9·58 (-3·97–43·8)	9·73 (-4·04–44·5)	0·221 (0–0·928)	9·75 (-4·04–44·5)
Acute hepatitis	25·1 (15·7–38·3)	12 (7·5–17·4)	4·01 (2·51–6·12)	26·7 (16·8–39·4)
Maternal and neonatal disorders	83·9 (81·2–86·3)	83·7 (81·9–85·5)	78·6 (74·6–82)	86·5 (84·5–88·4)
Maternal disorders	12 (4·37–19·9)	11·8 (4·3–19·7)	11·7 (4·16–19·7)	11·9 (4·33–19·8)
Neonatal disorders	91·5 (90·2–92·6)	87·5 (85·8–89)	80·4 (76·5–83·8)	91·5 (90·2–92·6)
Nutritional deficiencies	93·9 (81–95·6)	90·4 (86·7–92·5)	89·1 (85·8–92)	93·6 (81·4–95·2)
Protein-energy malnutrition	100 (100–100)	100 (100–100)	100 (100–100)	100 (100–100)
Vitamin A deficiency	--	100 (100–100)	100 (100–100)	--
Dietary iron deficiency	--	100 (100–100)	100 (100–100)	--
Non-communicable diseases	64·6 (61·8–67·3)	51·7 (48·3–55·3)	34·7 (31·7–37·4)	66·2 (64–68·5)
Neoplasms	50·6 (46·9–54·9)	50·2 (46·6–54·1)	31·5 (27·6–35·8)	51·3 (47·7–55·2)
Lip and oral cavity cancer	67·7 (59·1–75)	70·1 (62–77·2)	69·3 (60·8–76·5)	70·1 (62–77·3)
Nasopharynx cancer	63·7 (52·8–73·1)	63·7 (53·3–73·1)	62·6 (51·9–71·8)	63·7 (53·3–73·1)
Other pharynx cancer	77·2 (70·8–82·5)	78·4 (72·2–83·5)	78·7 (72·3–83·7)	78·4 (72·2–83·5)
Esophageal cancer	79·6 (70·6–87·3)	80·9 (72·2–88·1)	79·9 (70·6–87·7)	80·9 (72·2–88·2)

Stomach cancer	25·9 (17·9–43·5)	26·5 (18·4–44·4)	26·6 (18·4–44·2)	26·5 (18·4–44·4)
Colon and rectum cancer	62·7 (53·6–71·3)	63·7 (55·2–71·9)	63·5 (54·6–71·9)	63·7 (55·2–71·9)
Liver cancer	80·3 (75·6–84·7)	79·9 (75·3–84·4)	80·1 (75·5–84·6)	79·9 (75·2–84·4)
Gallbladder and biliary tract cancer	27·1 (16·2–39·8)	28·3 (17·2–40·9)	26·1 (15·4–38·7)	28·4 (17·3–41)
Pancreatic cancer	44·6 (34·6–56·1)	44·6 (35·4–55·5)	44·8 (35·2–56)	44·6 (35·4–55·5)
Larynx cancer	82·2 (73–89·2)	83·5 (75·5–89·3)	83·1 (75·1–89·3)	83·5 (75·5–89·4)
Tracheal, bronchus, and lung cancer	83·9 (80·6–87·4)	83·4 (80·4–86·8)	83·7 (80·2–87·4)	83·4 (80·4–86·8)
Breast cancer	35·1 (25·5–46·6)	34·3 (25·2–45)	34·8 (25·3–45·6)	34·2 (25·2–44·9)
Cervical cancer	100 (100–100)	100 (100–100)	100 (100–100)	100 (100–100)
Uterine cancer	54·8 (39·5–68·4)	56·5 (41·6–69·4)	57 (42–69·7)	56·4 (41·4–69·4)
Ovarian cancer	20·3 (8·99–35)	18·6 (7·84–32·5)	18 (7·48–31·7)	18·6 (7·84–32·5)
Prostate cancer	6·31 (2·72–10·2)	7·05 (3·02–11·2)	7·46 (3·31–11·8)	6·94 (2·99–11)
Kidney cancer	43·1 (32–54·2)	43·9 (33·8–54)	44 (33·8–53·9)	43·9 (33·8–53·9)
Bladder cancer	41·8 (26·8–57·4)	45·5 (30·8–59·4)	46 (31·8–59·9)	45·4 (30·7–59·4)
Thyroid cancer	18·7 (9·56–29·1)	19·9 (10·3–30·8)	20·3 (10·9–31·7)	19·9 (10·2–30·9)
Mesothelioma	96·3 (94·9–97·5)	93·3 (91·1–95·2)	96 (94·5–97·3)	93·3 (91–95·2)
Non-Hodgkin lymphoma	8·81 (3·92–14·8)	9·36 (4·22–15·5)	9·11 (4·15–15·3)	9·37 (4·23–15·6)
Multiple myeloma	10·1 (4·56–16·6)	10·7 (4·94–17·6)	10·5 (4·84–17·6)	10·7 (4·92–17·6)
Leukemia	37·6 (23·5–51·8)	36·4 (24·1–48·8)	38·3 (24·3–52·6)	36·3 (24–48·7)
Cardiovascular diseases	81·9 (78·4–84·9)	82·7 (80–85·2)	72·7 (68·4–76·9)	84·2 (81·7–86·5)
Rheumatic heart disease	22·8 (13·4–42·2)	22·8 (14·5–37·9)	22·7 (13·8–38·6)	22·8 (14·7–37·6)
Ischemic heart disease	93·4 (90·2–96·1)	94·9 (92·7–96·7)	93·3 (90·4–95·9)	94·9 (92·7–96·7)
Stroke	80·1 (73·1–87·5)	84 (79·4–88·6)	83·1 (78·3–88·5)	84·3 (79·6–88·8)
Hypertensive heart disease	100 (100–100)	100 (100–100)	100 (100–100)	100 (100–100)
Non-rheumatic valvular heart disease	26·1 (16·4–38·6)	23·3 (15·9–31·7)	6·58 (4·29–9·6)	26·5 (18·1–36·7)
Cardiomyopathy and myocarditis	40·5 (33·6–48·2)	42·8 (37–48·7)	41·8 (35·6–47·8)	42·9 (37–48·9)
Atrial fibrillation and flutter	52·9 (42·9–62·5)	58·8 (50·5–66·9)	60 (51·5–68)	57·1 (48·4–65·8)
Aortic aneurysm	57·4 (51·9–63·1)	65 (60·3–69·3)	--	65 (60·3–69·3)
Endocarditis	32·4 (22·7–44)	33·9 (24·9–43·8)	32 (22·3–43·9)	34 (25–43·8)
Other cardiovascular and circulatory diseases	23·9 (18·2–32·6)	26·6 (21·3–33·3)	32·8 (25·9–40·2)	24·8 (19·5–31·8)
Chronic respiratory diseases	62·9 (57·7–68·4)	60·1 (56·4–64·2)	54·7 (50·3–59)	64 (59·4–68·9)
Chronic obstructive pulmonary disease	70·7 (66·3–74·8)	70·7 (67·1–74·2)	66 (62·3–69·6)	73 (69·4–76·5)
Pneumoconiosis	100 (100–100)	100 (100–100)	100 (100–100)	100 (100–100)

Asthma	52·2 (41·4–63·3)	43 (35·1–51·5)	41·8 (34·2–50·4)	52·2 (42·7–61·9)
Digestive diseases	47·2 (41·5–52·5)	49·1 (43·3–54·7)	18 (12·4–24·3)	56·4 (50·6–62·1)
Cirrhosis and other chronic liver diseases	80·3 (71·7–87·9)	82·3 (74·5–89·1)	77·8 (68·2–86·2)	82·4 (74·5–89·2)
Upper digestive system diseases	22·2 (18·7–26)	7·19 (4·59–10·5)	1·68 (1·03–2·6)	25·6 (21·8–29·4)
Gallbladder and biliary diseases	51·2 (35·9–65·7)	56·2 (42·4–69)	57·7 (44–70)	53·8 (39·4–67·5)
Pancreatitis	34·9 (19·9–52·5)	39·2 (23·4–56·5)	35·7 (20·5–51·9)	39·6 (23·6–56·9)
Neurological disorders	25·2 (9·73–40·5)	15·5 (7·52–27·3)	11·1 (5·42–21·4)	21·5 (8·18–37·5)
Alzheimer's disease and other dementias	41·7 (27·4–57·8)	45·2 (30·4–60·5)	46·1 (31·6–61·2)	44·7 (29·9–59·8)
Parkinson's disease	-10·1 (-15·9–-4·82)	-11·8 (-18·1–-6·03)	-12·5 (-18·7–-6·5)	-11·7 (-18–-5·87)
Idiopathic epilepsy	22·2 (14·1–30·5)	20·3 (12·9–28)	18·9 (11·9–26)	23 (14·9–31·3)
Multiple sclerosis	15·6 (11·4–20·1)	16·8 (12·3–21·4)	16·3 (12–20·8)	17·2 (12·6–21·9)
Mental disorders	--	9·75 (4·84–15·1)	9·75 (4·84–15·1)	--
Depressive disorders	--	19·9 (8·9–30·1)	19·9 (8·9–30·1)	--
Anxiety disorders	--	6·1 (1·4–14)	6·1 (1·4–14)	--
Idiopathic developmental intellectual disability	--	49·4 (24·7–68·8)	49·4 (24·7–68·8)	--
Substance use disorders	100 (100–100)	100 (100–100)	100 (100–100)	100 (100–100)
Alcohol use disorders	100 (100–100)	100 (100–100)	100 (100–100)	100 (100–100)
Drug use disorders	100 (100–100)	100 (100–100)	100 (100–100)	100 (100–100)
Diabetes and kidney diseases	99·8 (99·8–99·8)	99·9 (99·9–99·9)	100 (100–100)	99·8 (99·8–99·9)
Diabetes mellitus	100 (100–100)	100 (100–100)	100 (100–100)	100 (100–100)
Chronic kidney disease	100 (100–100)	100 (100–100)	100 (100–100)	100 (100–100)
Sense organ diseases	--	10·4 (9·26–11·6)	10·4 (9·26–11·6)	--
Blindness and vision loss	--	10 (6·38–14·8)	10 (6·38–14·8)	--
Age-related and other hearing loss	--	11·1 (10·1–12·3)	11·1 (10·1–12·3)	--
Musculoskeletal disorders	3·34 (1·02–6)	21·5 (18·1–25·3)	21·9 (18·4–25·7)	3·18 (1·01–5·61)
Rheumatoid arthritis	12·3 (3·77–22·4)	15·4 (4·89–27·3)	15·7 (5·02–27·7)	14·5 (4·59–25·7)
Osteoarthritis	--	16·4 (9·4–24)	16·4 (9·4–24)	--
Low back pain	--	42 (36–48·1)	42 (36–48·1)	--
Gout	--	61·1 (45·4–76·8)	61·1 (45·4–76·8)	--
Other non-communicable diseases	0·145 (0·103–0·198)	0·197 (0·135–0·273)	--	0·473 (0·339–0·621)
Sudden infant death syndrome	6·65 (4·6–8·98)	6·64 (4·59–8·97)	--	6·64 (4·59–8·97)
Injuries	26·3 (22·4–29·9)	24·6 (21·2–28·1)	29·6 (26·4–32·8)	22·3 (18·2–26·4)
Transport injuries	25·8 (21·4–30·7)	25 (20·6–30)	28·2 (24·5–31·7)	24·1 (19·1–29·7)

Road injuries	25·5 (21–30·3)	24·2 (19·7–29·4)	26 (22·4–29·7)	23·8 (18·8–29·5)
Other transport injuries	32·8 (28–37·7)	34 (29·7–38·2)	38·9 (34·7–42·3)	29·6 (24–35·3)
Unintentional injuries	29·7 (25·7–33)	24·9 (21·4–28·3)	30 (26·3–33·7)	18·1 (14·9–21·4)
Falls	53·2 (46·6–57·5)	43·5 (38·6–47·4)	42·9 (38·1–46·7)	45·1 (39·9–49·2)
Drowning	-6·1 (-12·6–1·31)	-6·53 (-12·5–0·188)	9·57 (6·07–14·6)	-6·89 (-12·9–-0·0133)
Fire, heat, and hot substances	10·2 (6·56–14·9)	12 (8·04–17)	12·9 (8·63–18·3)	11·4 (7·54–15·9)
Poisonings	14·9 (10·2–20·7)	14·3 (9·78–19·6)	11·7 (7·99–16·4)	15·8 (10·9–21·8)
Exposure to mechanical forces	14·8 (10·1–19·5)	19 (16·1–22·2)	21·4 (18·9–24·2)	12 (7·18–17·4)
Animal contact	20·5 (16·3–25·4)	23 (18·8–27·7)	24·2 (19·9–28·9)	18 (14–23)
Foreign body	2·19 (1·45–3·18)	3·11 (2·13–4·42)	3·86 (2·66–5·42)	2·89 (1·94–4·25)
Environmental heat and cold exposure	7·08 (3·12–12·4)	7·48 (3·33–13·3)	6·9 (3·03–12·5)	7·89 (3·51–13·9)
Exposure to forces of nature	6·58 (2·93–11·7)	6·39 (2·85–11·4)	7·57 (3·32–13·5)	6·29 (2·81–11·2)
Other unintentional injuries	16·2 (11·2–22·2)	12·7 (8·59–18)	12·1 (8·11–17·2)	18·2 (12·7–24·9)
Self-harm and interpersonal violence	22·8 (14·3–31)	23·9 (15·9–31·3)	30·4 (24·3–36·9)	23·3 (15·2–31·1)
Self-harm	22·7 (10·8–33·9)	24·8 (12·8–36·4)	23·4 (13–33·6)	24·9 (12·8–36·5)
Interpersonal violence	23·4 (15·3–31·8)	23 (15·3–30·8)	32·1 (24·9–39·3)	21·4 (13·7–29·3)

Supplementary Results Table 2. All-age PAFs attributable to the joint distribution of all risk factors for all ages, both sexes combined for each GBD level 1, level 2, and level 3 cause and all causes in 2019 for South Dakota. Results are shown for deaths, YLLs, YLDs, and DALYs Data in parenthesis are 95% uncertainty intervals. PAFs=population attributable fractions. GBD=Global Burden of Disease. YLLs=years of life lost. YLDs=years lived with disability. DALYs=disability-adjusted life-years

	Deaths 2019	DALYs 2019	YLDs 2019	YLLs 2019
All causes	58·6 (55·8–61·1)	44·2 (41·3–47·1)	31·2 (28·9–33·5)	55·3 (53·2–57·5)
Communicable, maternal, neonatal, and nutritional diseases	42·8 (37·5–47·9)	53·2 (49·4–57·1)	47·9 (42·9–53·2)	56·4 (51·6–61·2)
HIV/AIDS and sexually transmitted infections	89·3 (86·9–91·3)	90·5 (88·1–92·8)	94·6 (92·1–97·1)	86·8 (84–89·2)
HIV/AIDS	88·1 (85·4–90·3)	87 (84–89·4)	88·6 (85·6–91·1)	86·2 (83·2–88·7)
Sexually transmitted infections excluding HIV	99·5 (99·2–99·7)	99·8 (99·6–99·9)	100 (100–100)	97·9 (96·9–98·6)
Respiratory infections and tuberculosis	40·9 (33·8–47·9)	36·1 (29·8–42·3)	2·29 (1·73–2·98)	46·5 (39·5–53·1)
Tuberculosis	51·6 (42–61·3)	55·8 (46·1–64·7)	54·1 (43·7–63)	56·1 (46·5–64·9)
Lower respiratory infections	40·9 (33·7–47·9)	46·3 (39·2–53·1)	31 (25·7–36·5)	46·5 (39·4–53·3)
Upper respiratory infections	0·514 (0·301–0·825)	0·0208 (0·00932–0·0388)	0·0057 (0–0·0179)	1·11 (0·658–1·76)
Otitis media	1·97 (0·834–4·16)	4·07 (2·64–5·55)	4·03 (2·57–5·56)	4·95 (2·47–8·68)
Enteric infections	11·3 (6·92–17·2)	13·1 (8·92–19)	12·3 (7·97–18·3)	14·4 (10–20·3)
Diarrheal diseases	11·3 (6·97–17·3)	13·2 (8·98–19·1)	12·3 (7·98–18·3)	14·6 (10·2–20·6)
Other infectious diseases	0·492 (0·349–0·725)	0·965 (0·701–1·3)	0·45 (0·26–0·762)	1·07 (0·761–1·48)
Meningitis	1·22 (0·858–1·69)	2·52 (1·81–3·43)	0·000576 (0–0·00202)	2·63 (1·9–3·58)
Encephalitis	0·577 (0·393–0·805)	1·47 (1·03–2)	0·000277 (0–0·000987)	1·51 (1·06–2·06)
Measles	9·51 (-4·03–44·4)	9·66 (-4·1–45·1)	0·196 (0–0·918)	9·66 (-4·1–45·1)
Acute hepatitis	11·9 (6·46–25·6)	5·36 (3·09–9·87)	2·44 (1·44–4·21)	12·4 (6·75–26·2)
Maternal and neonatal disorders	81·8 (78·1–84·9)	80·6 (77·7–83·2)	74·2 (69·5–78·2)	84·2 (81·2–86·8)
Maternal disorders	11·4 (4·08–19·1)	11·2 (4·06–18·8)	11·1 (3·99–18·9)	11·3 (4·07–18·8)
Neonatal disorders	88·8 (86·7–90·6)	84·2 (81·5–86·5)	76·4 (71·5–80·4)	88·8 (86·7–90·6)
Nutritional deficiencies	89·7 (82·1–93·2)	89 (85·9–91·6)	89 (85·3–92·1)	89·1 (82–92·7)
Protein-energy malnutrition	100 (100–100)	100 (100–100)	100 (100–100)	100 (100–100)
Vitamin A deficiency	--	100 (100–100)	100 (100–100)	--
Dietary iron deficiency	--	100 (100–100)	100 (100–100)	--
Non-communicable diseases	62·1 (59–64·9)	46·7 (43·1–50)	30·5 (28–32·9)	61·7 (59·4–64·1)
Neoplasms	47 (43·3–51)	46·2 (42·7–50)	29·8 (26–33·8)	47·2 (43·9–51)
Lip and oral cavity cancer	63·1 (54·2–71·3)	67 (58–74·5)	66·4 (57·4–74)	67 (58·1–74·5)
Nasopharynx cancer	63·2 (52·2–73·1)	63·1 (52·3–72·5)	61·9 (51–71·7)	63·2 (52·3–72·5)
Other pharynx cancer	75·7 (68·7–81·6)	77·2 (70·5–82·9)	77·5 (70·6–83·1)	77·2 (70·5–82·8)

Esophageal cancer	78·4 (68·4–87·3)	79·8 (70·2–88·1)	78·7 (68·6–87·2)	79·8 (70·2–88·1)
Stomach cancer	23·9 (15·8–42)	24·7 (16·6–43)	24·9 (16·9–42·9)	24·7 (16·6–43)
Colon and rectum cancer	61·3 (52·5–69·9)	62·4 (54·4–70·4)	62·3 (54·1–70·4)	62·4 (54·4–70·4)
Liver cancer	74·9 (67·6–81·3)	75·3 (68·5–81·1)	75·2 (68·1–81·3)	75·3 (68·5–81·1)
Gallbladder and biliary tract cancer	27·3 (16·1–40·5)	28·4 (16·8–41·5)	26·3 (14·7–39·3)	28·5 (17–41·5)
Pancreatic cancer	41·8 (32·1–52·9)	41·9 (32·7–52·5)	42 (32·7–52·7)	41·9 (32·7–52·5)
Larynx cancer	81·7 (71·5–89·1)	82·9 (74·3–89·3)	82·7 (73·8–89·2)	83 (74·4–89·3)
Tracheal, bronchus, and lung cancer	82·2 (78·6–85·7)	81·6 (78·4–85·1)	82 (78·4–85·6)	81·6 (78·4–85·1)
Breast cancer	33·8 (25·2–43·9)	33·3 (25·1–42·4)	33·8 (25·5–43·3)	33·2 (25·1–42·4)
Cervical cancer	100 (100–100)	100 (100–100)	100 (100–100)	100 (100–100)
Uterine cancer	53·8 (37·9–68)	55·8 (39·8–69·3)	56·4 (40·4–69·6)	55·7 (39·6–69·2)
Ovarian cancer	17 (7·14–29·9)	15·4 (6·05–27·5)	14·8 (5·89–26·6)	15·4 (6·07–27·6)
Prostate cancer	6·02 (2·56–10·2)	6·59 (2·8–11·1)	6·9 (2·94–11·4)	6·51 (2·78–10·9)
Kidney cancer	42·5 (31·3–53·2)	42·9 (32·7–52·8)	43·2 (32·9–53)	42·9 (32·7–52·8)
Bladder cancer	39·9 (25·2–55)	43·7 (28·8–57·5)	44·3 (29·9–57·8)	43·6 (28·7–57·6)
Thyroid cancer	18·8 (9·72–29·2)	20 (10·5–30·9)	20·2 (10·9–30·7)	19·9 (10·4–30·9)
Mesothelioma	96·4 (95·1–97·5)	93·4 (91·1–95·2)	96·2 (94·5–97·4)	93·4 (91–95·1)
Non-Hodgkin lymphoma	9·17 (4·06–15·4)	9·65 (4·47–16)	9·46 (4·23–15·7)	9·67 (4·46–16)
Multiple myeloma	10·4 (4·91–17·3)	11 (5·33–18·2)	10·9 (5·15–18·2)	11 (5·33–18·2)
Leukemia	35·5 (22·2–49)	33 (21·7–44·3)	36·2 (23–49·5)	32·8 (21·5–44·1)
Cardiovascular diseases	80·9 (76·9–84·4)	81·3 (78·2–84·1)	70·6 (65·6–75·3)	82·9 (80–85·6)
Rheumatic heart disease	21·6 (12·1–39·6)	21·5 (13·6–36·2)	21·5 (13·1–37·5)	21·5 (13·6–35·4)
Ischemic heart disease	91·9 (87·7–95·4)	93·5 (90·6–96)	91·9 (88·1–95·3)	93·5 (90·7–96·1)
Stroke	77·3 (69·7–85·8)	80·8 (75·5–86·5)	79·8 (73·8–86·1)	81·2 (75·7–86·9)
Hypertensive heart disease	100 (100–100)	100 (100–100)	100 (100–100)	100 (100–100)
Non-rheumatic valvular heart disease	24·2 (14·5–37·4)	22·8 (15·3–32·7)	9·77 (6·22–14·1)	24·6 (16·5–35·4)
Cardiomyopathy and myocarditis	36·2 (29·6–44·2)	37·9 (32·2–43·6)	36·9 (30·5–43·4)	38 (32·2–43·7)
Atrial fibrillation and flutter	51·1 (39·7–61·4)	57 (47·5–65·9)	58·6 (49·2–67·2)	55 (45·2–64·2)
Aortic aneurysm	53·1 (47–59·3)	61·6 (56·5–66·2)	--	61·6 (56·5–66·2)
Endocarditis	30·4 (20·4–43·1)	31·9 (23–42·6)	30·1 (19·9–42·8)	32 (23·2–42·8)
Other cardiovascular and circulatory diseases	21·8 (17–28·8)	24·1 (19·3–29·5)	30·4 (23·6–37·6)	22·2 (17·8–28)
Chronic respiratory diseases	64·6 (60·1–68·8)	60·1 (56·4–63·6)	54 (49·8–58·1)	65·2 (61·3–68·8)

Chronic obstructive pulmonary disease	72·4 (68·1–76·3)	70·3 (66·5–73·6)	63·1 (59·2–66·8)	74·7 (70·9–78·3)
Pneumoconiosis	100 (100–100)	100 (100–100)	100 (100–100)	100 (100–100)
Asthma	49·9 (38·5–61·3)	42·8 (34·7–51·4)	41·7 (33·9–50·3)	49·3 (39·7–58·5)
Digestive diseases	41·3 (35–46·7)	46·1 (40·1–51·5)	16·6 (11·4–22·3)	53·6 (46·5–59·4)
Cirrhosis and other chronic liver diseases	77 (66·1–86·2)	80·7 (70·1–88·5)	75·7 (64·7–84·9)	80·8 (70·1–88·6)
Upper digestive system diseases	18·5 (15·2–21·9)	6·27 (3·94–9·36)	1·52 (0·892–2·39)	21·9 (18·3–25·3)
Gallbladder and biliary diseases	50·2 (34·2–65·1)	54·9 (40·4–68·1)	56·6 (42·5–69·3)	52·4 (36·9–66·6)
Pancreatitis	32·5 (15·6–49·8)	38·2 (20·3–55·6)	35·4 (18·1–52·8)	38·6 (20·5–56·1)
Neurological disorders	24·4 (9·96–39·4)	15·2 (7·74–26·6)	11·1 (5·67–21·1)	20·3 (8·15–35·6)
Alzheimer's disease and other dementias	39·8 (26·4–55·2)	42·9 (29·1–58·2)	43·6 (29·9–58·6)	42·5 (28·7–57·7)
Parkinson's disease	-9·23 (-14·7–-4·54)	-10·7 (-16·5–-5·43)	-10·6 (-16·3–-5·46)	-10·7 (-16·6–-5·42)
Idiopathic epilepsy	22·6 (14·1–31·2)	20·9 (13·2–29)	19·5 (12·1–27·1)	23·2 (14·9–31·3)
Multiple sclerosis	15·4 (11·2–19·8)	16·6 (12·1–21)	16·2 (12–20·4)	16·8 (12·2–21·6)
Mental disorders	--	8·19 (4·14–12·9)	8·19 (4·15–12·9)	--
Depressive disorders	--	17·6 (8·5–26·8)	17·6 (8·5–26·8)	--
Anxiety disorders	--	6·34 (1·5–14·3)	6·34 (1·5–14·3)	--
Idiopathic developmental intellectual disability	--	47·2 (22·3–66·1)	47·2 (22·3–66·1)	--
Substance use disorders	100 (100–100)	100 (100–100)	100 (100–100)	100 (100–100)
Alcohol use disorders	100 (100–100)	100 (100–100)	100 (100–100)	100 (100–100)
Drug use disorders	100 (100–100)	100 (100–100)	100 (100–100)	100 (100–100)
Diabetes and kidney diseases	99·9 (99·8–99·9)	99·9 (99·9–100)	100 (100–100)	99·9 (99·8–99·9)
Diabetes mellitus	100 (100–100)	100 (100–100)	100 (100–100)	100 (100–100)
Chronic kidney disease	100 (100–100)	100 (100–100)	100 (100–100)	100 (100–100)
Sense organ diseases	--	10·7 (9·64–12)	10·7 (9·64–12)	--
Blindness and vision loss	--	9·74 (6·26–14·3)	9·74 (6·26–14·3)	--
Age-related and other hearing loss	--	11·6 (10·5–12·8)	11·6 (10·5–12·8)	--
Musculoskeletal disorders	4·01 (1·15–7·32)	20·2 (17–23·5)	20·5 (17·2–23·9)	3·92 (1·16–7·05)
Rheumatoid arthritis	11·9 (3·51–21·5)	14·9 (4·59–26·6)	15·2 (4·77–27·2)	14·1 (4·25–25·3)
Osteoarthritis	--	14·2 (8·21–20·7)	14·2 (8·21–20·7)	--
Low back pain	--	43 (37·3–48·8)	43 (37·3–48·8)	--
Gout	--	61·5 (45·3–77·3)	61·5 (45·3–77·3)	--
Other non-communicable diseases	0·165 (0·113–0·236)	0·208 (0·137–0·302)	--	0·472 (0·331–0·649)

Sudden infant death syndrome	4·04 (2·85–5·67)	4·03 (2·84–5·67)	--	4·03 (2·84–5·67)
Injuries	26·4 (21·2–31·2)	22·7 (18·2–27·4)	32·3 (28·9–35·9)	17·9 (12·4–23·5)
Transport injuries	18·5 (12·7–24·7)	18·6 (13·1–24·3)	29·6 (25·8–33·3)	15·4 (9·06–22)
Road injuries	16·3 (10·1–23)	16·4 (10·5–22·6)	27·3 (23·5–31·2)	13·5 (6·72–20·4)
Other transport injuries	33 (28·3–38·2)	32·1 (27·4–37·1)	39·8 (35·5–43·4)	29 (23·6–34·8)
Unintentional injuries	37·4 (32–41·6)	28·1 (24·1–32·3)	33·4 (29·2–37·5)	20·7 (16·6–24·7)
Falls	55·8 (48·5–60·4)	45·5 (40·1–49·8)	44·9 (39·6–49·4)	46·6 (41·1–51·4)
Drowning	-14 (-24·7–-3·64)	-15 (-25·1–-4·91)	10·4 (6·49–15·6)	-15·6 (-25·8–-5·33)
Fire, heat, and hot substances	10·8 (7·05–15·8)	12·9 (8·83–18·1)	14·4 (9·74–20·3)	11·4 (7·82–16)
Poisonings	15·7 (10·9–22)	15·1 (10·7–21·2)	12·7 (8·69–18)	16 (11·3–22·3)
Exposure to mechanical forces	3·57 (-4·45–11·8)	14·3 (9·51–18·7)	22·1 (19·1–25·4)	-0·901 (-9·45–8·18)
Animal contact	25·3 (21·2–30·3)	23·2 (19·1–28)	24·7 (20·4–29·8)	20·2 (16·4–25)
Foreign body	2·73 (1·79–3·96)	3·51 (2·33–4·94)	3·68 (2·53–5·15)	3·45 (2·25–4·97)
Environmental heat and cold exposure	7·7 (3·34–13·5)	8·22 (3·58–14·3)	7·53 (3·24–13·6)	8·56 (3·72–14·9)
Exposure to forces of nature	7·18 (3·11–12·7)	7·65 (3·3–13·5)	8·48 (3·66–15)	6·88 (2·99–12·1)
Other unintentional injuries	17·2 (12–23·8)	14·3 (9·8–20·1)	13·5 (9·11–19·3)	18·7 (13·4–25·5)
Self-harm and interpersonal violence	17·4 (5·96–28·7)	19 (8·06–29·5)	27·9 (21·5–34·5)	18·3 (7·05–29·3)
Self-harm	17·6 (4·54–30·8)	19·2 (5·93–32·3)	22·3 (11·4–33·6)	19·2 (5·83–32·3)
Interpersonal violence	17·8 (8·39–27·4)	19·2 (10·5–27·8)	30·1 (23·1–37·4)	15·6 (6·51–25·1)

Supplementary Results Table 2. All-age PAFs attributable to the joint distribution of all risk factors for all ages, both sexes combined for each GBD level 1, level 2, and level 3 cause and all causes in 2019 for Tennessee. Results are shown for deaths, YLLs, YLDs, and DALYs Data in parenthesis are 95% uncertainty intervals. PAFs=population attributable fractions. GBD=Global Burden of Disease. YLLs=years of life lost. YLDs=years lived with disability. DALYs=disability-adjusted life-years				
	**Deaths 2019**	**DALYs 2019**	**YLDs 2019**	**YLLs 2019**
All causes	63·4 (60·8–65·7)	51·2 (48–54·1)	35·6 (32·7–38·2)	63 (61·1–64·7)
Communicable, maternal, neonatal, and nutritional diseases	44·9 (41·1–48·4)	56·2 (53·6–58·8)	51·2 (46·8–55·8)	59 (55·5–62·1)
HIV/AIDS and sexually transmitted infections	96·9 (95·3–98)	96·8 (95·3–97·8)	98 (96·7–98·8)	96·1 (94·3–97·3)
HIV/AIDS	96·9 (95·2–97·9)	96·5 (94·8–97·6)	97·3 (95·7–98·4)	96·1 (94·3–97·3)
Sexually transmitted infections excluding HIV	98·9 (98·4–99·3)	99·5 (99·2–99·8)	100 (100–100)	95·9 (94·2–97·2)
Respiratory infections and tuberculosis	40·4 (35·5–44·8)	37·8 (32·6–43)	2·97 (2·27–3·83)	45·8 (40·7–50·4)
Tuberculosis	54·5 (45·8–62·6)	58·1 (50·1–65·7)	54 (44·9–62·1)	58·7 (50·7–66)
Lower respiratory infections	40·3 (35·3–44·7)	45·6 (40·3–50·3)	34·9 (29·1–40·1)	45·7 (40·4–50·4)
Upper respiratory infections	0·64 (0·393–1·01)	0·0215 (0·0107–0·0372)	0·00479 (9·82e-05–0·0132)	1·37 (0·834–2·13)
Otitis media	1·84 (0·765–4)	4·44 (2·93–6·06)	4·43 (2·92–6·08)	4·79 (2·26–8·67)
Enteric infections	12·5 (7·36–19·1)	13·6 (8·66–20·2)	13·3 (8·34–19·9)	14 (9·02–20·4)
Diarrheal diseases	12·5 (7·4–19·2)	13·7 (8·71–20·3)	13·3 (8·35–19·9)	14·2 (9·13–20·6)
Other infectious diseases	0·903 (0·595–1·26)	1·4 (1·04–1·78)	0·847 (0·504–1·34)	1·52 (1·1–1·96)
Meningitis	1·39 (0·995–1·87)	2·93 (2·12–3·87)	0·000438 (6·15e-06–0·00123)	3·12 (2·24–4·14)
Encephalitis	0·6 (0·436–0·839)	1·56 (1·15–2·18)	0·00013 (2·36e-06–0·000383)	1·62 (1·19–2·25)
Measles	9·49 (-3·98–43·7)	5·13 (-3·02–38·6)	0·429 (0–1·16)	9·66 (-4·05–44·6)
Acute hepatitis	29·5 (17·1–44·2)	13·2 (8·16–19·1)	4·66 (2·76–7·2)	30·1 (17·6–44·3)
Maternal and neonatal disorders	84·9 (82·6–86·8)	82·6 (80·5–84·5)	75·7 (71·3–79·5)	86·8 (85·1–88·4)
Maternal disorders	11·9 (4·31–19·8)	11·7 (4·28–19·6)	11·5 (4·21–19·4)	11·8 (4·28–19·6)
Neonatal disorders	90·3 (88·8–91·6)	85·4 (83·3–87·2)	77·3 (72·9–81·3)	90·3 (88·9–91·6)
Nutritional deficiencies	93·3 (83·9–95)	90·2 (87·2–92·6)	89·4 (86–92·4)	92·8 (83·4–94·5)
Protein-energy malnutrition	100 (100–100)	100 (100–100)	100 (100–100)	100 (100–100)
Vitamin A deficiency	--	100 (100–100)	100 (100–100)	--
Dietary iron deficiency	--	100 (100–100)	100 (100–100)	--
Non-communicable diseases	67 (64·2–69·5)	53·8 (50·1–57·2)	35·4 (32·2–38·3)	68·6 (66·4–70·6)
Neoplasms	52·6 (49–56·7)	52·1 (48·7–55·9)	32·6 (28·7–36·8)	53·2 (49·9–56·9)
Lip and oral cavity cancer	67·7 (60·2–74·2)	70·3 (63·2–76·5)	69·5 (62·1–76)	70·3 (63·1–76·5)
Nasopharynx cancer	62·2 (52·1–71·4)	62·4 (52·2–71·2)	61·2 (50·8–70·2)	62·4 (52·2–71·3)
Other pharynx cancer	77 (70·8–81·9)	78·2 (72·2–83)	78·4 (72·5–83·1)	78·2 (72·2–83)
Esophageal cancer	80·5 (71·7–88·2)	81·7 (72·8–89)	80·7 (72·2–88·3)	81·7 (72·8–89)

Stomach cancer	26·5 (18·3–43·8)	27·3 (19–45)	27·4 (19·2–44·8)	27·2 (19–45)
Colon and rectum cancer	63·5 (54·5–72·3)	64·2 (56·1–72·2)	64·2 (55·6–72·5)	64·2 (56·1–72·3)
Liver cancer	81·2 (77–85·6)	80·8 (76·4–85·3)	81 (76·5–85·5)	80·8 (76·4–85·3)
Gallbladder and biliary tract cancer	27·2 (16–40·2)	28·5 (17·2–41·5)	26·2 (15·3–38·7)	28·6 (17·3–41·6)
Pancreatic cancer	45·8 (36–57)	45·9 (36·9–56·9)	46 (36·1–57·1)	45·9 (36·9–56·9)
Larynx cancer	83·2 (73·9–90)	84·5 (76·3–90·2)	84·1 (76·3–90)	84·5 (76·5–90·2)
Tracheal, bronchus, and lung cancer	85·1 (82·1–88·3)	84·7 (81·8–87·8)	84·9 (81·9–88·4)	84·7 (81·8–87·8)
Breast cancer	35·1 (25·7–46·5)	34·3 (25·6–44·8)	34·8 (25·7–45·6)	34·3 (25·5–44·7)
Cervical cancer	100 (100–100)	100 (100–100)	100 (100–100)	100 (100–100)
Uterine cancer	54·5 (39–68·6)	56·4 (41·5–69·8)	56·9 (41·9–70·1)	56·3 (41·4–69·7)
Ovarian cancer	20 (8·72–34·3)	18·3 (7·51–31·8)	17·8 (7·22–31·2)	18·3 (7·52–31·8)
Prostate cancer	6·41 (2·79–10·5)	7·18 (3·15–11·5)	7·59 (3·36–12)	7·07 (3·1–11·4)
Kidney cancer	43·6 (32·7–54·3)	44·5 (34·3–54·5)	44·7 (34·6–54·3)	44·5 (34·3–54·5)
Bladder cancer	44·1 (27·9–59)	47·9 (32·5–61·4)	48·5 (33·7–61·6)	47·8 (32·4–61·5)
Thyroid cancer	18·5 (9·65–28·8)	19·8 (10·5–30·3)	20·1 (11·1–30·9)	19·7 (10·3–30·3)
Mesothelioma	96 (94·7–97·2)	92·8 (90·6–94·8)	95·7 (94·1–97·1)	92·8 (90·5–94·8)
Non-Hodgkin lymphoma	8·81 (3·89–14·9)	9·36 (4·26–15·6)	9·11 (4·07–15·3)	9·38 (4·3–15·6)
Multiple myeloma	10·1 (4·7–16·9)	10·7 (5·11–17·8)	10·6 (4·92–17·5)	10·7 (5·12–17·8)
Leukemia	38·1 (23·8–52·6)	37·1 (24·7–49·6)	38·9 (24·2–53·2)	37 (24·7–49·5)
Cardiovascular diseases	84·4 (81·3–87·3)	84·9 (82·5–87·1)	73·8 (69·3–78)	86·4 (84·1–88·5)
Rheumatic heart disease	22·6 (13·1–41·1)	22·5 (13·9–36·9)	22·4 (13·5–38)	22·5 (14·2–36·2)
Ischemic heart disease	93·6 (90·3–96·2)	95 (92·9–96·9)	93·5 (90·6–96)	95 (92·9–96·9)
Stroke	80·7 (74·5–87·5)	84·2 (79·7–88·7)	83·4 (78·6–88·6)	84·5 (79·9–88·9)
Hypertensive heart disease	100 (100–100)	100 (100–100)	100 (100–100)	100 (100–100)
Non-rheumatic valvular heart disease	25·5 (16·6–37·6)	22·2 (15·2–30·9)	6·03 (4·02–8·45)	25·8 (17·9–35·9)
Cardiomyopathy and myocarditis	43·4 (36·9–51·1)	45·9 (40·1–51·8)	42·8 (36·5–49·2)	46·2 (40·1–52·2)
Atrial fibrillation and flutter	52·1 (41·7–61·9)	58·4 (49·8–67)	59·7 (50·9–68·2)	56·5 (47·5–65·4)
Aortic aneurysm	57·9 (52·3–63·7)	65·7 (61·2–70·1)	--	65·7 (61·2–70·1)
Endocarditis	32·3 (22·8–44)	33·7 (24·6–44·5)	31·7 (22·1–43·3)	33·7 (24·7–44·4)
Other cardiovascular and circulatory diseases	24 (18·9–31·2)	26 (20·9–32·2)	31·3 (24·5–38·6)	24·6 (19·8–30·8)
Chronic respiratory diseases	67·1 (62·8–71·4)	63·5 (59·9–66·9)	56·8 (52·4–61·1)	68·1 (64·4–71·9)
Chronic obstructive pulmonary disease	74 (70–77·7)	73·3 (69·9–76·6)	67·6 (63·9–71·3)	76 (72·5–79·2)
Pneumoconiosis	100 (100–100)	100 (100–100)	100 (100–100)	100 (100–100)

Asthma	52·1 (41·4–62·9)	43·9 (35·8–52·3)	43·1 (35·1–51·5)	51·3 (42–61)
Digestive diseases	46·5 (41·3–51·6)	48·5 (43·4–53·6)	18·9 (13·1–25·4)	55·7 (50·5–60·6)
Cirrhosis and other chronic liver diseases	80 (73–86·6)	81·9 (75·1–88·1)	77·4 (69·5–84·7)	81·9 (75·1–88·1)
Upper digestive system diseases	23·8 (20–27·7)	7·65 (4·76–11·3)	1·74 (1·06–2·72)	27·1 (23–31·3)
Gallbladder and biliary diseases	50·9 (35·9–65·6)	56·1 (42·2–69·1)	57·6 (43·8–69·9)	53·6 (39·2–67·6)
Pancreatitis	32·8 (18·5–50)	37·4 (22·2–55·2)	35·3 (20·4–52·4)	37·7 (22·3–55·6)
Neurological disorders	26 (10·4–41·7)	16·1 (7·82–28·1)	11·6 (5·84–23·1)	22·1 (8–39·1)
Alzheimer's disease and other dementias	42·6 (28·5–57·8)	46·2 (32–61·2)	47 (32·6–61·9)	45·5 (31·3–60·4)
Parkinson's disease	-10·5 (-16·3–-5·35)	-12·3 (-18·6–-6·52)	-13 (-19·3–-6·83)	-12·2 (-18·5–-6·45)
Idiopathic epilepsy	19·8 (12·9–26·3)	18·4 (12–24·4)	17·4 (11·4–23·3)	20·8 (13·6–27·5)
Multiple sclerosis	17·6 (13–23·2)	18·6 (13·8–24·1)	18·1 (13·3–23)	19·1 (14·1–24·8)
Mental disorders	--	9·15 (4·44–14·3)	9·15 (4·44–14·3)	--
Depressive disorders	--	18·9 (8·4–28·7)	18·9 (8·4–28·7)	--
Anxiety disorders	--	6·16 (1·4–13·9)	6·16 (1·4–13·9)	--
Idiopathic developmental intellectual disability	--	50·5 (25–72·1)	50·5 (25–72·1)	--
Substance use disorders	100 (100–100)	100 (100–100)	100 (100–100)	100 (100–100)
Alcohol use disorders	100 (100–100)	100 (100–100)	100 (100–100)	100 (100–100)
Drug use disorders	100 (100–100)	100 (100–100)	100 (100–100)	100 (100–100)
Diabetes and kidney diseases	99·8 (99·8–99·9)	99·9 (99·9–99·9)	100 (100–100)	99·9 (99·8–99·9)
Diabetes mellitus	100 (100–100)	100 (100–100)	100 (100–100)	100 (100–100)
Chronic kidney disease	100 (100–100)	100 (100–100)	100 (100–100)	100 (100–100)
Sense organ diseases	--	10·6 (9·45–11·9)	10·6 (9·45–11·9)	--
Blindness and vision loss	--	10·2 (6·48–14·9)	10·2 (6·48–14·9)	--
Age-related and other hearing loss	--	11·3 (10·3–12·6)	11·3 (10·3–12·6)	--
Musculoskeletal disorders	4·61 (1·41–8·14)	22·2 (18·6–25·9)	22·5 (18·8–26·4)	4·37 (1·37–7·68)
Rheumatoid arthritis	14 (4·29–24·8)	17·3 (5·61–30·1)	17·6 (5·68–30·7)	16·3 (5·19–28·7)
Osteoarthritis	--	16·7 (9·51–24·4)	16·7 (9·51–24·4)	--
Low back pain	--	43·2 (36·9–49·5)	43·2 (36·9–49·5)	--
Gout	--	60·7 (44·8–76·1)	60·7 (44·8–76·1)	--
Other non-communicable diseases	0·135 (0·0936–0·187)	0·182 (0·124–0·254)	--	0·429 (0·301–0·582)
Sudden infant death syndrome	6·26 (4·29–8·74)	6·26 (4·28–8·73)	--	6·26 (4·28–8·73)
Injuries	26·3 (22·4–30)	24·6 (21·1–28·1)	29·8 (26·2–33)	22·2 (18–26·3)
Transport injuries	25·1 (19·9–30·1)	24·6 (19·6–29·7)	28 (24·1–31·8)	23·5 (17·9–29·2)

Road injuries	24·7 (19·4–29·7)	23·8 (18·7–29)	25·9 (22·1–29·6)	23·2 (17·5–28·8)
Other transport injuries	33·2 (28·3–38·3)	34·2 (29·6–38·5)	38·1 (33·3–41·8)	30·2 (25·1–35·9)
Unintentional injuries	30·5 (25·9–34)	25·3 (21·7–28·9)	30·2 (26·1–33·8)	19 (15·6–22·5)
Falls	51·6 (44·5–56·2)	43·1 (37·5–47·2)	42·4 (37–46·6)	44·4 (38·5–48·7)
Drowning	-8·79 (-15·3–-1·18)	-8·92 (-15·2–-1·82)	9·29 (6·27–13·6)	-9·37 (-15·7–-2·15)
Fire, heat, and hot substances	9·53 (6·52–13·8)	11·9 (8·52–16·5)	13·1 (9·27–18)	10·9 (7·74–15·3)
Poisonings	14·7 (10·5–20·1)	14·7 (10·7–19·7)	12·1 (8·74–16·6)	15·9 (11·4–21·6)
Exposure to mechanical forces	12·3 (7·39–17·5)	18·6 (14·9–22·6)	22·3 (19–26)	10·3 (4·97–16·2)
Animal contact	20 (16·3–24·6)	22·6 (18·6–27·2)	24·1 (19·9–28·8)	17·9 (14·2–22·7)
Foreign body	2·25 (1·51–3·3)	3·22 (2·24–4·75)	4·54 (3·21–6·65)	2·87 (1·93–4·2)
Environmental heat and cold exposure	5·95 (2·73–10·6)	6·46 (2·97–11·4)	6·05 (2·82–10·7)	6·81 (3·12–12)
Exposure to forces of nature	5·88 (2·73–10·3)	6·47 (3–11·2)	7·09 (3·3–12·3)	5·76 (2·67–10)
Other unintentional injuries	17 (12·1–23)	13·1 (9·32–18)	12·3 (8·77–17·1)	19·5 (14–26·2)
Self-harm and interpersonal violence	22·3 (14·4–30·2)	23·8 (16·5–31·2)	31 (25–37·4)	23·2 (15·6–31)
Self-harm	22·3 (11·4–32·9)	24·9 (13·5–36·2)	23·3 (14·4–32·4)	24·9 (13·5–36·3)
Interpersonal violence	22·4 (15·2–30·3)	22·4 (15·6–29·9)	32·8 (26·3–40·3)	20·4 (13·3–28)

Supplementary Results Table 2. All-age PAFs attributable to the joint distribution of all risk factors for all ages, both sexes combined for each GBD level 1, level 2, and level 3 cause and all causes in 2019 for Texas. Results are shown for deaths, YLLs, YLDs, and DALYs Data in parenthesis are 95% uncertainty intervals. PAFs=population attributable fractions. GBD=Global Burden of Disease. YLLs=years of life lost. YLDs=years lived with disability. DALYs=disability-adjusted life-years

	Deaths 2019	DALYs 2019	YLDs 2019	YLLs 2019
All causes	60·3 (57·4–62·7)	45·5 (42·3–48·4)	31·9 (29·4–34·3)	58·5 (56·4–60·4)
Communicable, maternal, neonatal, and nutritional diseases	45·4 (41·6–49·3)	55·7 (52·5–58·7)	49·5 (44·6–54·3)	60·2 (57–63·5)
HIV/AIDS and sexually transmitted infections	93·1 (91·4–94·3)	92·9 (91·3–94·2)	94·9 (93·3–96·3)	91·9 (90–93·2)
HIV/AIDS	93 (91·3–94·2)	92·3 (90·5–93·6)	93·3 (91·6–94·5)	91·9 (90–93·2)
Sexually transmitted infections excluding HIV	98·3 (97·6–98·8)	99·5 (99·1–99·8)	100 (100–100)	94·1 (91·8–95·9)
Respiratory infections and tuberculosis	35·4 (29·4–41·1)	29·6 (23·8–35·1)	2·31 (1·73–3·03)	40·5 (34·6–46·1)
Tuberculosis	54·7 (45·6–63·3)	56·8 (47·8–65·1)	52 (41·5–61·2)	57·7 (48·8–65·8)
Lower respiratory infections	35·1 (29·1–40·9)	40 (34–45·7)	29·6 (24·4–34·5)	40·2 (34·3–45·9)
Upper respiratory infections	0·759 (0·472–1·18)	0·0192 (0·00889–0·0343)	0·0059 (9·25e-05–0·0164)	1·5 (0·917–2·27)
Otitis media	1·76 (0·751–3·46)	3·65 (2·39–5·02)	3·63 (2·37–4·99)	4·29 (2·11–7·58)
Enteric infections	12·3 (7·62–19·4)	13·4 (8·72–20·5)	13·4 (8·56–20·5)	13·6 (8·96–20·7)
Diarrheal diseases	12·4 (7·67–19·5)	13·5 (8·77–20·6)	13·4 (8·57–20·5)	13·8 (9·08–20·9)
Other infectious diseases	0·888 (0·599–1·16)	1·38 (1·01–1·7)	0·604 (0·367–1)	1·56 (1·1–2)
Meningitis	1·63 (1·19–2·21)	3·37 (2·51–4·47)	0·00066 (7·83e-06–0·00198)	3·52 (2·61–4·67)
Encephalitis	0·733 (0·532–1)	1·74 (1·28–2·33)	0·000162 (1·69e-06–0·000446)	1·81 (1·33–2·43)
Measles	9·38 (-3·89–43·9)	4·71 (-2·35–37)	0·423 (0·0125–1·08)	9·55 (-3·95–44·7)
Acute hepatitis	19·7 (12·3–31)	8·83 (5·76–13·1)	2·89 (1·78–4·69)	20·1 (12·6–31·3)
Maternal and neonatal disorders	81·1 (78·4–83·6)	81·1 (78·9–83·1)	76·4 (72·2–80)	84·1 (82–86)
Maternal disorders	10·9 (3·94–18·3)	10·8 (3·88–18)	10·6 (3·81–17·7)	10·8 (3·88–18·1)
Neonatal disorders	89·6 (87·9–90·9)	85 (83–86·8)	78·1 (74·1–81·9)	89·6 (88–91)
Nutritional deficiencies	94·7 (84·2–96·2)	88·5 (85·5–91·2)	86·8 (83·2–90·2)	94·1 (84·3–95·4)
Protein-energy malnutrition	100 (100–100)	100 (100–100)	100 (100–100)	100 (100–100)
Vitamin A deficiency	--	100 (100–100)	100 (100–100)	--
Dietary iron deficiency	--	100 (100–100)	100 (100–100)	--
Non-communicable diseases	63·7 (60·5–66·5)	47·3 (43·7–50·7)	31·1 (28·4–33·8)	64·3 (62–66·5)
Neoplasms	47·9 (44·2–52·1)	46·6 (43–50·6)	30·1 (26·3–34·3)	47·7 (44·2–51·6)
Lip and oral cavity cancer	64·6 (55·8–72·5)	66·8 (58·5–74·3)	66·1 (57·4–73·7)	66·8 (58·5–74·4)
Nasopharynx cancer	61·1 (49·5–71·2)	60·7 (49·2–70·7)	59·7 (48·3–69·7)	60·8 (49·2–70·7)
Other pharynx cancer	74·8 (68–80·8)	75·8 (69·1–81·7)	76·1 (69·5–82·2)	75·7 (69–81·7)
Esophageal cancer	76·8 (66·9–85·3)	77·9 (68·3–86·3)	77 (66·9–85·5)	77·9 (68·3–86·3)

Stomach cancer	23·8 (15·9–42·1)	23·6 (15·4–42·2)	24·1 (15·8–42·4)	23·6 (15·4–42·3)
Colon and rectum cancer	62·1 (53·5–70·9)	62·8 (54·6–70·8)	62·7 (54·4–70·9)	62·8 (54·6–70·8)
Liver cancer	81·3 (76·9–85·6)	80·9 (76·4–85·1)	81·2 (76·7–85·5)	80·9 (76·4–85·1)
Gallbladder and biliary tract cancer	27 (15·8–39·6)	28·2 (16·6–40·9)	25·9 (15·3–38·5)	28·2 (16·7–41)
Pancreatic cancer	42 (32–53·5)	41·3 (32·1–52·4)	41·8 (32·2–53·3)	41·3 (32·1–52·4)
Larynx cancer	79·9 (69·7–87·3)	80·5 (72–87)	80·2 (71·5–86·7)	80·5 (72·1–87·1)
Tracheal, bronchus, and lung cancer	81·4 (77·9–85·4)	80·4 (77–84·3)	81 (77·3–85·1)	80·4 (77–84·3)
Breast cancer	33·9 (24·6–45·1)	32·3 (23·6–42·6)	33·1 (24·2–43·8)	32·2 (23·6–42·4)
Cervical cancer	100 (100–100)	100 (100–100)	100 (100–100)	100 (100–100)
Uterine cancer	54·8 (39·2–68·4)	56·5 (41·1–69·7)	57 (41·8–70)	56·5 (41–69·6)
Ovarian cancer	19 (8·2–32·9)	17·2 (6·98–30·4)	16·5 (6·73–29·3)	17·2 (7·01–30·4)
Prostate cancer	5·74 (2·56–9·24)	6·35 (2·82–10)	6·64 (2·97–10·4)	6·26 (2·79–9·99)
Kidney cancer	41·4 (30·6–51·7)	41·7 (31·5–51·4)	41·8 (31·6–51·4)	41·7 (31·5–51·5)
Bladder cancer	40 (25–55·1)	43 (28·8–56·9)	43·4 (29·4–56·5)	43 (28·8–56·9)
Thyroid cancer	18·4 (9·47–28·9)	19·5 (10·3–30·6)	19·8 (10·7–30·3)	19·5 (10·2–30·5)
Mesothelioma	95·2 (93·5–96·5)	91 (88·5–93·2)	94·8 (92·9–96·3)	91 (88·4–93·1)
Non-Hodgkin lymphoma	8·72 (3·84–14·7)	9·23 (4·12–15·4)	8·99 (3·97–15·1)	9·25 (4·14–15·4)
Multiple myeloma	10 (4·71–16·8)	10·6 (5·03–17·7)	10·4 (4·86–17·6)	10·6 (5·05–17·7)
Leukemia	35 (22–49)	32·2 (21·4–43·3)	35 (22·4–48·3)	32 (21·4–43·1)
Cardiovascular diseases	82·5 (79·2–85·5)	82·6 (79·9–85·1)	72·3 (67·9–76·4)	84·4 (81·9–86·6)
Rheumatic heart disease	21·6 (12·5–40·6)	21·6 (13·8–35·2)	21·5 (13·3–35·1)	21·6 (13·9–35·2)
Ischemic heart disease	92·9 (89·1–95·8)	94·4 (91·9–96·5)	92·9 (89·8–95·8)	94·4 (92–96·6)
Stroke	79·2 (72·3–86·5)	82·9 (78·1–87·6)	82·1 (77·2–87·1)	83·3 (78·3–87·9)
Hypertensive heart disease	100 (100–100)	100 (100–100)	100 (100–100)	100 (100–100)
Non-rheumatic valvular heart disease	25 (15·9–38·2)	21·7 (14·7–31·1)	6·34 (4·19–9·14)	25·5 (17·7–35·6)
Cardiomyopathy and myocarditis	37·5 (30·6–45·4)	38·7 (32·8–44·4)	38·5 (32·5–45·4)	38·7 (32·8–44·4)
Atrial fibrillation and flutter	51·3 (41·5–61·5)	57·4 (48·8–66·4)	58·7 (50·1–67·4)	55·5 (46·6–64·9)
Aortic aneurysm	55·8 (50·4–61·5)	63·3 (59·1–67·6)	--	63·3 (59·1–67·6)
Endocarditis	31·8 (22·4–44·1)	33·1 (24·1–43·3)	30·9 (21·4–43·4)	33·2 (24·1–43·3)
Other cardiovascular and circulatory diseases	23·5 (18·4–30·1)	25·6 (20·3–31·1)	30·2 (23·6–37·7)	23·9 (19·1–29·7)
Chronic respiratory diseases	61·2 (56·5–65·8)	55·6 (51·7–59·4)	49·9 (45·3–54·6)	61·4 (57·6–65·4)
Chronic obstructive pulmonary disease	68·8 (64·1–73·3)	67·5 (63·9–71·1)	62·8 (59–66·5)	70·5 (66·8–74·2)
Pneumoconiosis	100 (100–100)	100 (100–100)	100 (100–100)	100 (100–100)

Asthma	49·5 (38·9–60·3)	38·4 (31–46·5)	37·6 (30·3–45·9)	47·8 (39·1–57·3)
Digestive diseases	51 (44·5–56·8)	51·1 (45–56·8)	18 (12·2–24·4)	60·1 (53·7–65·5)
Cirrhosis and other chronic liver diseases	79·6 (70·8–87·5)	82·1 (74·4–89)	77·5 (68·6–85·9)	82·1 (74·4–89)
Upper digestive system diseases	21·2 (17·8–24·8)	5·05 (3·05–7·64)	1·29 (0·787–2·04)	23·6 (20–27·3)
Gallbladder and biliary diseases	50·1 (35–65·5)	55·2 (40·8–68·1)	56·4 (42·3–68·9)	52·5 (37·6–66·9)
Pancreatitis	33·3 (17·1–50·2)	37·9 (21–55·1)	35·2 (18·9–53)	38·4 (21·4–55·5)
Neurological disorders	26·6 (11·6–41·8)	14·9 (7·09–26·3)	10 (4·7–20·1)	22·8 (9·14–38·2)
Alzheimer's disease and other dementias	41·2 (27·2–56·7)	44·2 (30·1–59·5)	44·9 (30·6–60)	43·7 (29·4–59·1)
Parkinson's disease	-8·85 (-14·1–-4·28)	-10·2 (-15·9–-5·13)	-10·7 (-16·4–-5·49)	-10·1 (-15·8–-5·05)
Idiopathic epilepsy	21·5 (13·4–30)	19·2 (11·9–26·8)	18·1 (11·2–25)	21·9 (13·9–30)
Multiple sclerosis	13·7 (9·94–18)	13·9 (10·1–17·9)	13·3 (9·68–17)	14·5 (10·5–18·9)
Mental disorders	--	9·14 (4·59–14·5)	9·14 (4·59–14·5)	--
Depressive disorders	--	19·3 (9·18–29·3)	19·3 (9·18–29·3)	--
Anxiety disorders	--	6·8 (1·56–15·4)	6·8 (1·56–15·4)	--
Idiopathic developmental intellectual disability	--	48·4 (24·3–66·8)	48·4 (24·3–66·8)	--
Substance use disorders	100 (100–100)	100 (100–100)	100 (100–100)	100 (100–100)
Alcohol use disorders	100 (100–100)	100 (100–100)	100 (100–100)	100 (100–100)
Drug use disorders	100 (100–100)	100 (100–100)	100 (100–100)	100 (100–100)
Diabetes and kidney diseases	99·8 (99·8–99·8)	99·9 (99·9–99·9)	100 (100–100)	99·8 (99·8–99·9)
Diabetes mellitus	100 (100–100)	100 (100–100)	100 (100–100)	100 (100–100)
Chronic kidney disease	100 (100–100)	100 (100–100)	100 (100–100)	100 (100–100)
Sense organ diseases	--	11 (9·82–12·3)	11 (9·82–12·3)	--
Blindness and vision loss	--	8·75 (5·49–12·9)	8·75 (5·49–12·9)	--
Age-related and other hearing loss	--	12·2 (11·1–13·4)	12·2 (11·1–13·4)	--
Musculoskeletal disorders	3·04 (0·892–5·49)	20·1 (16·9–23·5)	20·5 (17·2–23·9)	2·67 (0·81–4·78)
Rheumatoid arthritis	11·3 (3·4–20·4)	13·1 (4·11–23·7)	13·1 (4·15–23·9)	12·7 (3·99–22·6)
Osteoarthritis	--	16·2 (9·16–23·7)	16·2 (9·16–23·7)	--
Low back pain	--	40 (34·6–45·4)	40 (34·6–45·4)	--
Gout	--	60 (43·6–76·2)	60 (43·6–76·2)	--
Other non-communicable diseases	0·147 (0·105–0·205)	0·16 (0·109–0·226)	--	0·417 (0·305–0·566)
Sudden infant death syndrome	5·33 (3·77–7·38)	5·33 (3·77–7·37)	--	5·33 (3·77–7·37)
Injuries	26·5 (22–30·5)	25 (20·9–28·9)	30·4 (26·9–34·1)	22·1 (17·5–26·9)
Transport injuries	28·2 (22·9–34)	27·3 (22·1–33·2)	30·2 (26·2–34·4)	26·5 (20·6–32·7)

Road injuries	27·8 (22·6–33·7)	26·7 (21·3–32·6)	28·4 (24·4–32·9)	26·2 (20·4–32·4)
Other transport injuries	34·9 (29·4–40·5)	35·2 (30·7–39·9)	38·7 (34·4–42·2)	31·3 (25·2–37·6)
Unintentional injuries	32·9 (28·3–36·3)	27·2 (23·5–31·2)	31·1 (27·2–35·2)	20·5 (16·7–24·6)
Falls	53·6 (47·2–58)	43·4 (38·2–47·5)	42·4 (37·3–46·4)	46 (40·6–50·2)
Drowning	-3·65 (-10·5–3·93)	-4·92 (-11·2–2·19)	11·2 (7·47–15·6)	-5·34 (-11·7–1·83)
Fire, heat, and hot substances	11·9 (7·94–16·7)	15·1 (10·6–20·4)	16·3 (11·5–21·9)	13·2 (9·27–17·9)
Poisonings	18·3 (13·1–24·4)	17 (12·3–22·3)	14·4 (10·2–19·3)	18·9 (13·7–25·1)
Exposure to mechanical forces	18·8 (12·7–25·1)	22 (18·1–26·7)	23·8 (20·3–28)	16·3 (9·77–23·3)
Animal contact	23·5 (18·9–28·6)	24·4 (20–29·4)	25·5 (21–30·3)	20·4 (15·9–26·1)
Foreign body	3·28 (2·24–4·81)	4·86 (3·36–6·88)	6·29 (4·43–8·7)	4·17 (2·8–6·01)
Environmental heat and cold exposure	7·28 (3·21–12·7)	7·49 (3·29–12·9)	7·14 (3·16–12·3)	7·93 (3·52–13·5)
Exposure to forces of nature	6·6 (2·94–11·3)	6·43 (2·86–10·9)	7·89 (3·43–13·5)	6·27 (2·79–10·6)
Other unintentional injuries	22·1 (15·8–29·4)	16·6 (11·8–22·3)	15·7 (11–21·2)	23·8 (17·2–31·4)
Self-harm and interpersonal violence	19·3 (11·2–28)	19·9 (12·5–27·9)	24·7 (18·5–31·3)	19·5 (11·8–27·8)
Self-harm	20·6 (9·57–32·7)	22 (11–34·2)	22·1 (12·6–32·7)	22 (10·9–34·3)
Interpersonal violence	17·5 (10·2–25·4)	17·5 (10·6–24·9)	25·6 (18·7–33)	16 (9·01–23·6)

Supplementary Results Table 2. All-age PAFs attributable to the joint distribution of all risk factors for all ages, both sexes combined for each GBD level 1, level 2, and level 3 cause and all causes in 2019 for Utah. Results are shown for deaths, YLLs, YLDs, and DALYs Data in parenthesis are 95% uncertainty intervals. PAFs=population attributable fractions. GBD=Global Burden of Disease. YLLs=years of life lost. YLDs=years lived with disability. DALYs=disability-adjusted life-years

	Deaths 2019	DALYs 2019	YLDs 2019	YLLs 2019
All causes	55·4 (52·8–58·2)	41·6 (38·8–44·4)	30·8 (28·3–33·3)	53·6 (51·4–55·8)
Communicable, maternal, neonatal, and nutritional diseases	44·4 (39·8–48·7)	53·7 (50·2–57·2)	46·3 (41·7–51·2)	59·2 (55·1–63·2)
HIV/AIDS and sexually transmitted infections	81 (78–83·5)	83·7 (80·3–86·9)	90·1 (86·3–94·3)	78·2 (74·7–81)
HIV/AIDS	80·1 (76·9–82·7)	78·5 (75·1–81·4)	80·1 (76·8–83·1)	77·7 (74·1–80·5)
Sexually transmitted infections excluding HIV	98·2 (97·3–98·9)	99·6 (99·2–99·8)	100 (100–100)	93·8 (90·9–95·9)
Respiratory infections and tuberculosis	37·3 (31·4–43·1)	29·4 (24–35·3)	1·67 (1·25–2·15)	41·7 (35·9–47·2)
Tuberculosis	46·7 (37·5–55·6)	49·5 (40·5–57·6)	45·9 (36·4–54·3)	50·4 (41·4–58·5)
Lower respiratory infections	37·3 (31·4–43·1)	41·5 (35·7–47)	24·8 (20·2–29·1)	41·8 (35·9–47·4)
Upper respiratory infections	0·868 (0·506–1·41)	0·0289 (0·0137–0·0512)	0·00668 (0·000115–0·0185)	1·69 (1·01–2·76)
Otitis media	1·47 (0·64–3·12)	3·13 (2·03–4·34)	3·12 (2·04–4·34)	3·64 (1·75–6·51)
Enteric infections	11·3 (7·2–17·2)	13·1 (9·04–18·7)	12·5 (8·4–18·3)	14·4 (10·3–19·9)
Diarrheal diseases	11·4 (7·25–17·3)	13·1 (9·08–18·8)	12·5 (8·41–18·3)	14·6 (10·5–20·2)
Other infectious diseases	0·902 (0·632–1·22)	1·47 (1·08–1·9)	0·691 (0·409–1·09)	1·66 (1·19–2·22)
Meningitis	1·95 (1·36–2·81)	3·78 (2·7–5·3)	0·000722 (7·85e-06–0·0022)	3·98 (2·83–5·57)
Encephalitis	0·859 (0·577–1·27)	1·99 (1·37–2·89)	0·000311 (3·98e-06–0·00094)	2·05 (1·42–2·97)
Measles	9·44 (-4·01–43·5)	9·58 (-4·05–44·2)	0·19 (0–0·833)	9·59 (-4·07–44·3)
Acute hepatitis	20·7 (13·4–32·3)	8·68 (5·6–13·1)	3·43 (2·04–5·35)	21·7 (14·1–33)
Maternal and neonatal disorders	83·3 (80·8–85·7)	79·2 (76·4–81·7)	69·3 (64·4–73·6)	85·2 (83–87·3)
Maternal disorders	11·3 (4·1–19)	11·1 (4·03–18·6)	10·9 (3·87–18·6)	11·1 (4·04–18·7)
Neonatal disorders	88·8 (86·9–90·5)	82·2 (79·5–84·7)	71·3 (66·4–75·8)	88·8 (86·9–90·5)
Nutritional deficiencies	92 (75·6–94·3)	88·6 (84·6–91·3)	87·8 (84·2–91·1)	91·5 (78–93·5)
Protein-energy malnutrition	100 (100–100)	100 (100–100)	100 (100–100)	100 (100–100)
Vitamin A deficiency	--	100 (100–100)	100 (100–100)	--
Dietary iron deficiency	--	100 (100–100)	100 (100–100)	--
Non-communicable diseases	59 (55·9–62·2)	43·1 (39·7–46·4)	30·1 (27·1–32·9)	59·4 (56·9–61·9)
Neoplasms	38 (33·9–42·5)	36·7 (32·9–40·8)	23·2 (19·5–27·2)	37·8 (34–42)
Lip and oral cavity cancer	53·9 (45·7–61·8)	57·5 (49·8–64·9)	56·7 (48·6–64·8)	57·6 (50–64·8)
Nasopharynx cancer	53·4 (43·2–62·5)	53·8 (43·9–62·3)	52·7 (42–61·6)	53·8 (44–62·4)
Other pharynx cancer	65·5 (57·3–72·1)	67·6 (59·5–73·9)	68 (59·7–74·6)	67·5 (59·5–73·9)
Esophageal cancer	70·9 (58·4–82·1)	72·6 (60·4–83·5)	71·6 (59·5–82·5)	72·6 (60·4–83·5)

Stomach cancer	18·2 (10·6–37·6)	18·6 (10·8–38·3)	18·7 (11–38·2)	18·6 (10·8–38·3)
Colon and rectum cancer	57·7 (48·5–67·2)	58·6 (50·2–67·2)	58·6 (49·8–67·3)	58·6 (50·2–67·2)
Liver cancer	77·2 (72·6–81·7)	76 (71·5–80·6)	76·4 (71·7–81·2)	76 (71·5–80·6)
Gallbladder and biliary tract cancer	26·5 (15·5–39·3)	27·5 (16·3–40·5)	25·2 (14·5–38·1)	27·6 (16·3–40·6)
Pancreatic cancer	35 (24·9–47·1)	34·8 (25·3–46·2)	35 (25·3–47·1)	34·8 (25·3–46·2)
Larynx cancer	70·6 (59·3–80·1)	72 (62–80·6)	71·3 (60·6–80·2)	72 (62·1–80·5)
Tracheal, bronchus, and lung cancer	74·8 (70–79·8)	73·7 (69·3–78·5)	74·1 (68·9–79·4)	73·7 (69·3–78·5)
Breast cancer	30·4 (20·8–42)	28·8 (20–39·1)	29·7 (20·5–40·7)	28·7 (20–38·9)
Cervical cancer	100 (100–100)	100 (100–100)	100 (100–100)	100 (100–100)
Uterine cancer	52·6 (37·3–67)	54·3 (39·4–68·1)	54·8 (39·8–68·3)	54·2 (39·1–68)
Ovarian cancer	19·4 (8·66–33)	17·2 (7·15–29·9)	16·5 (6·92–29·1)	17·2 (7·16–30)
Prostate cancer	3·84 (1·65–6·5)	4·34 (1·85–7·19)	4·64 (1·95–7·51)	4·25 (1·82–7·05)
Kidney cancer	36·4 (25·8–46·9)	36·9 (26·8–46·9)	37 (27–46·9)	36·9 (26·8–46·8)
Bladder cancer	31·1 (17·4–45·9)	33·8 (20·6–47·7)	34·2 (21–47·6)	33·8 (20·5–47·7)
Thyroid cancer	17·9 (9·05–28·3)	19·1 (9·77–30·1)	19·9 (9·85–32·2)	18·8 (9·72–29·7)
Mesothelioma	95·8 (94·2–97)	91·6 (88·9–93·7)	95·3 (93·4–96·7)	91·5 (88·8–93·6)
Non-Hodgkin lymphoma	8·43 (3·61–14·3)	8·79 (3·83–14·6)	8·61 (3·76–14·5)	8·8 (3·84–14·6)
Multiple myeloma	9·65 (4·37–16·4)	10·2 (4·7–17·2)	10 (4·6–17)	10·2 (4·69–17·3)
Leukemia	27·8 (17·4–39·5)	25·6 (17–34·8)	28 (17·8–39·2)	25·5 (16·9–34·7)
Cardiovascular diseases	78·1 (74–81·8)	77·4 (73·9–80·6)	68·8 (63·7–73·3)	79 (75·7–81·9)
Rheumatic heart disease	21·2 (11·8–39·4)	20·9 (13·1–35·2)	20·7 (12·7–34·8)	20·9 (13·2–35)
Ischemic heart disease	91·8 (87·4–95·4)	93·1 (90·1–96)	92 (88·5–95·3)	93·2 (90·2–96)
Stroke	77·9 (70·7–86·2)	80·7 (75·4–86·2)	79·6 (74·4–85·4)	81·2 (75·6–86·5)
Hypertensive heart disease	100 (100–100)	100 (100–100)	100 (100–100)	100 (100–100)
Non-rheumatic valvular heart disease	25·2 (15·8–38·6)	24·3 (16·5–34·1)	11·1 (7·19–15·8)	25·5 (17·4–35·9)
Cardiomyopathy and myocarditis	36·4 (29·6–44)	37·5 (31·9–43·1)	35·8 (29·8–42)	37·8 (32·1–43·5)
Atrial fibrillation and flutter	48·8 (38·7–59·5)	54·2 (45·3–63·5)	55·7 (46·8–64·7)	52·2 (42·9–62·1)
Aortic aneurysm	47·7 (41·8–54·1)	55 (49·8–59·9)	--	55 (49·8–59·9)
Endocarditis	30·6 (20·7–42·8)	31·5 (22·1–41·9)	30·1 (20·3–41·9)	31·6 (22·1–42·1)
Other cardiovascular and circulatory diseases	19·5 (14–28·6)	22·2 (17·1–29·4)	28·3 (21·1–37·3)	19·9 (14·9–27·5)
Chronic respiratory diseases	56·8 (51·3–62·5)	50·6 (46·5–54·7)	43·4 (38·8–48)	57·3 (52·5–62·4)
Chronic obstructive pulmonary disease	66 (61·2–70·3)	62·5 (58·4–66·1)	52·7 (48·9–56·6)	68·1 (63·7–72·1)
Pneumoconiosis	100 (100–100)	100 (100–100)	100 (100–100)	100 (100–100)

Asthma	45·2 (34·2–56·7)	37·6 (29·8–46·2)	36·8 (29·2–45·2)	44·4 (34·9–54·6)
Digestive diseases	41·9 (36·7–47·2)	42·7 (37·3–47·7)	16·4 (10·9–22·5)	51·6 (45·9–57·2)
Cirrhosis and other chronic liver diseases	79·2 (70·6–87·2)	81·7 (73·6–89)	76·9 (67·7–85·5)	81·7 (73·6–89·1)
Upper digestive system diseases	13·7 (11·2–16·4)	3·98 (2·41–6·03)	0·997 (0·569–1·6)	16·2 (13·3–19·1)
Gallbladder and biliary diseases	46·8 (31·2–61·9)	51·7 (37–65)	52·9 (38·7–65·8)	49·1 (33·8–63·6)
Pancreatitis	33·1 (19·7–49·8)	38·4 (24·5–55·2)	35·9 (22·5–51·6)	38·8 (24·7–55·6)
Neurological disorders	18·3 (5·98–33)	9·43 (4·17–18·1)	6·41 (2·86–13·8)	14·7 (5·02–29·2)
Alzheimer's disease and other dementias	36·9 (23–53·3)	39·4 (25·3–55·8)	40 (25·6–55·9)	39·1 (24·9–55·2)
Parkinson's disease	-5·6 (-8·89–-2·75)	-6·5 (-10·2–-3·29)	-6·95 (-10·4–-3·63)	-6·43 (-10·1–-3·23)
Idiopathic epilepsy	19 (12·7–25·6)	17 (11·3–23·1)	15·8 (10·4–21·4)	19·5 (13·4–26·1)
Multiple sclerosis	9·15 (6·43–12·3)	9·72 (7·01–12·8)	9·46 (6·75–12·4)	10 (7·16–13·4)
Mental disorders	--	10·9 (5·37–17·2)	10·9 (5·37–17·2)	--
Depressive disorders	--	21 (9·89–31·7)	21 (9·89–31·7)	--
Anxiety disorders	--	7·64 (1·84–17·1)	7·64 (1·84–17·1)	--
Idiopathic developmental intellectual disability	--	43·7 (20·9–62·1)	43·7 (20·9–62·1)	--
Substance use disorders	100 (100–100)	100 (100–100)	100 (100–100)	100 (100–100)
Alcohol use disorders	100 (100–100)	100 (100–100)	100 (100–100)	100 (100–100)
Drug use disorders	100 (100–100)	100 (100–100)	100 (100–100)	100 (100–100)
Diabetes and kidney diseases	100 (99·9–100)	100 (100–100)	100 (100–100)	100 (99·9–100)
Diabetes mellitus	100 (100–100)	100 (100–100)	100 (100–100)	100 (100–100)
Chronic kidney disease	100 (100–100)	100 (100–100)	100 (100–100)	100 (100–100)
Sense organ diseases	--	10·8 (9·74–12·1)	10·8 (9·74–12·1)	--
Blindness and vision loss	--	7·41 (4·41–11·3)	7·41 (4·41–11·3)	--
Age-related and other hearing loss	--	12·2 (11·2–13·5)	12·2 (11·2–13·5)	--
Musculoskeletal disorders	2·23 (0·644–4·29)	18·4 (15·5–21·5)	18·8 (15·8–21·9)	2·15 (0·617–4·08)
Rheumatoid arthritis	7·37 (1·99–13·8)	8·93 (2·64–16·6)	9·03 (2·72–17)	8·66 (2·37–16·3)
Osteoarthritis	--	14·7 (8·31–21·7)	14·7 (8·31–21·7)	--
Low back pain	--	35·5 (30·9–40·3)	35·5 (30·9–40·3)	--
Gout	--	58·4 (41·7–75·4)	58·4 (41·7–75·4)	--
Other non-communicable diseases	0·204 (0·135–0·306)	0·229 (0·145–0·341)	--	0·541 (0·376–0·781)
Sudden infant death syndrome	6·67 (4·6–9·33)	6·66 (4·59–9·32)	--	6·66 (4·59–9·32)
Injuries	26·2 (21·5–31·2)	24·8 (20·5–29·5)	31·7 (27·9–35·7)	21·4 (16–27·6)
Transport injuries	24·7 (18·8–31·8)	25·3 (19·7–32·2)	31·4 (27·3–36·1)	23 (16·4–30·8)

Road injuries	23·3 (17·2–30·4)	23·7 (17·9–30·8)	29·6 (25·4–34·6)	21·7 (15–29·8)
Other transport injuries	36·8 (31·3–43·7)	35·7 (31·1–41·5)	38·4 (34·3–42·2)	33·6 (27·6–41·5)
Unintentional injuries	35·9 (31·5–39·5)	28·6 (24·8–33·1)	32 (28–36·2)	23·1 (19–27·7)
Falls	54·2 (47·6–58·3)	43·7 (38·8–47·9)	42·4 (37·6–46·5)	46·6 (41·4–51·2)
Drowning	-7·89 (-16·8–1·66)	-8·44 (-17–0·615)	11·2 (8·02–15·3)	-9·1 (-17·8–0·214)
Fire, heat, and hot substances	12 (8·58–16·3)	16·9 (12·5–22·5)	18·3 (13·6–24·3)	13·9 (10–18·7)
Poisonings	20·1 (14·9–26·7)	20·1 (15·1–26·5)	16·1 (12·1–21·5)	21·4 (16–28·4)
Exposure to mechanical forces	14·2 (5·82–24·1)	23·6 (18–30·8)	26·5 (21·8–32·5)	12·2 (2·84–23·4)
Animal contact	24·9 (20·7–29·9)	25·3 (20·8–30·7)	26·1 (21·5–31·6)	21·6 (17·2–27·3)
Foreign body	4·46 (2·93–6·65)	6·92 (4·62–10·1)	8·3 (5·67–11·8)	6·28 (4·08–9·42)
Environmental heat and cold exposure	5·58 (2·4–9·61)	6·13 (2·66–10·5)	5·8 (2·54–10)	6·41 (2·77–11·1)
Exposure to forces of nature	5·45 (2·37–9·36)	5·91 (2·58–10·1)	6·49 (2·84–11·2)	5·28 (2·29–9·04)
Other unintentional injuries	24·6 (17·8–33·4)	18·5 (13·8–24·7)	17·3 (12·7–23·1)	27·8 (20·3–38)
Self-harm and interpersonal violence	18·8 (9·33–28·5)	20·5 (11·4–29·7)	28·5 (22·7–34·6)	20 (10·6–29·7)
Self-harm	18·7 (8·62–29·6)	20·5 (10·2–31·3)	22·8 (14·2–32)	20·4 (10·2–31·3)
Interpersonal violence	21 (13·3–29·2)	21·9 (14·7–29·5)	31 (24·5–38·2)	18·3 (10·8–26·4)

Supplementary Results Table 2. All-age PAFs attributable to the joint distribution of all risk factors for all ages, both sexes combined for each GBD level 1, level 2, and level 3 cause and all causes in 2019 for Vermont. Results are shown for deaths, YLLs, YLDs, and DALYs Data in parenthesis are 95% uncertainty intervals. PAFs=population attributable fractions. GBD=Global Burden of Disease. YLLs=years of life lost. YLDs=years lived with disability. DALYs=disability-adjusted life-years

	Deaths 2019	DALYs 2019	YLDs 2019	YLLs 2019
All causes	58·4 (55·3–61)	46·5 (43·6–49·3)	33·2 (30·8–35·6)	58·2 (56–60·3)
Communicable, maternal, neonatal, and nutritional diseases	38·5 (32·3–44·2)	48·2 (44·5–51·9)	45·6 (41–50·3)	50·3 (44·8–55·4)
HIV/AIDS and sexually transmitted infections	90·3 (88–92·1)	91·2 (88·7–93·3)	94·4 (92–96·8)	88·3 (85·5–90·5)
HIV/AIDS	89·5 (87–91·4)	88·4 (85·6–90·7)	89·5 (86·8–91·7)	87·9 (85–90·2)
Sexually transmitted infections excluding HIV	99·5 (99·2–99·7)	99·8 (99·6–99·9)	100 (100–100)	97·7 (96·6–98·5)
Respiratory infections and tuberculosis	39·1 (29·1–48·3)	31·8 (24·1–39·6)	2·01 (1·49–2·65)	44 (34·7–52·7)
Tuberculosis	56 (46·2–65·6)	59·1 (49·7–68)	56·9 (46·8–66·1)	59·5 (50·2–68·4)
Lower respiratory infections	39 (28·9–48·3)	43·9 (34·4–52·6)	32·9 (26·5–39)	44·1 (34·5–52·8)
Upper respiratory infections	0·425 (0·238–0·747)	0·0198 (0·00919–0·0362)	0·00408 (0–0·0126)	1·12 (0·621–1·95)
Otitis media	0·795 (0·3–1·77)	3·02 (1·95–4·23)	3·03 (1·94–4·25)	2·66 (1·11–5·11)
Enteric infections	8·69 (5·48–13·6)	9·74 (6·52–14·4)	9·62 (6·32–14·5)	9·85 (6·6–14·6)
Diarrheal diseases	8·75 (5·51–13·6)	9·79 (6·56–14·5)	9·63 (6·33–14·5)	9·95 (6·66–14·7)
Other infectious diseases	0·562 (0·361–0·807)	1·02 (0·707–1·34)	0·758 (0·465–1·17)	1·08 (0·715–1·46)
Meningitis	0·899 (0·614–1·3)	2·2 (1·53–3·09)	0·000282 (0–0·000918)	2·46 (1·7–3·48)
Encephalitis	0·357 (0·245–0·51)	1·11 (0·773–1·56)	7·08e-05 (0–0·000234)	1·17 (0·812–1·65)
Measles	9·17 (-4·05–43·5)	9·35 (-4·13–44·4)	0·14 (0–0·66)	9·35 (-4·13–44·4)
Acute hepatitis	22·3 (13·3–36·4)	10 (6·12–15·2)	4·15 (2·51–6·4)	24·1 (14·6–38·9)
Maternal and neonatal disorders	80·6 (77·5–83·5)	77·4 (74·3–80·3)	71·1 (66·2–75·6)	83·1 (80·6–85·6)
Maternal disorders	11 (3·91–17·9)	10·9 (3·94–17·8)	10·9 (3·93–17·9)	10·9 (3·91–17·8)
Neonatal disorders	87·9 (85·9–89·7)	80·9 (77·8–83·7)	73·4 (68·4–77·8)	87·9 (85·9–89·8)
Nutritional deficiencies	79 (67·8–85·9)	85·1 (81·7–88·2)	86·7 (83·1–90·3)	79·7 (69·5–86·7)
Protein-energy malnutrition	100 (100–100)	100 (100–100)	100 (100–100)	100 (100–100)
Vitamin A deficiency	--	100 (100–100)	100 (100–100)	--
Dietary iron deficiency	--	100 (100–100)	100 (100–100)	--
Non-communicable diseases	61 (57·7–64)	48·3 (45–51·5)	32·6 (29·9–35·3)	62·6 (60·1–64·9)
Neoplasms	49 (45·4–53)	48·8 (45·4–52·6)	31·3 (27·3–35·2)	50·1 (46·7–53·8)
Lip and oral cavity cancer	68·4 (58·2–76·7)	70·7 (61·3–78·2)	70·3 (60·8–78·1)	70·8 (61·3–78·3)
Nasopharynx cancer	66·2 (54·1–75·8)	66·2 (54·2–75·3)	65·4 (53·1–74·6)	66·3 (54·2–75·4)
Other pharynx cancer	78·1 (70·1–83·8)	79·2 (71·7–84·7)	79·5 (72–84·9)	79·1 (71·6–84·7)

Esophageal cancer	79·6 (69·9–87·7)	80·7 (71·2–88·6)	79·8 (70·5–87·7)	80·7 (71·2–88·6)
Stomach cancer	24·2 (16–42·3)	25·3 (17–43·4)	25·3 (17·1–43·6)	25·3 (17–43·4)
Colon and rectum cancer	61·9 (53·5–70·2)	62·9 (54·9–70·6)	62·8 (55–70·8)	62·9 (54·9–70·6)
Liver cancer	81·6 (77·2–85·9)	81·5 (77·2–85·9)	81·6 (77–86)	81·5 (77·2–85·9)
Gallbladder and biliary tract cancer	27 (15·9–39·7)	27·8 (16·5–40·5)	25·8 (14·9–38·4)	27·9 (16·5–40·6)
Pancreatic cancer	41·8 (32·3–52·4)	41·8 (32·7–52·3)	41·9 (32·4–52·6)	41·8 (32·7–52·3)
Larynx cancer	81·1 (70·6–88·9)	82·5 (73·4–89·4)	82·3 (73·5–89·2)	82·5 (73·4–89·5)
Tracheal, bronchus, and lung cancer	81·7 (78·3–85·5)	81·2 (78–85·1)	81·5 (78–85·5)	81·2 (78–85)
Breast cancer	36·5 (27·6–46·8)	36 (27·7–45·3)	36·4 (27·8–46·1)	35·9 (27·7–45·2)
Cervical cancer	100 (100–100)	100 (100–100)	100 (100–100)	100 (100–100)
Uterine cancer	54·6 (39·9–68·2)	56·1 (40·7–69·2)	56·5 (41·2–69·6)	56 (40·7–69·2)
Ovarian cancer	18·2 (8·19–30·6)	16·4 (7–28·1)	16 (6·7–27·6)	16·4 (6·99–28·1)
Prostate cancer	5·54 (2·31–9·26)	6·2 (2·64–10·1)	6·55 (2·81–10·5)	6·09 (2·57–9·97)
Kidney cancer	42·9 (31·5–53·9)	43·7 (33–54)	44 (33·4–54·2)	43·7 (32·9–54)
Bladder cancer	37·2 (22·5–52·3)	41 (26·6–55)	41·8 (27·9–55·3)	40·9 (26·5–54·9)
Thyroid cancer	19 (10·2–29·5)	20·1 (10·7–30·9)	20·3 (10·9–31·4)	20 (10·7–30·9)
Mesothelioma	96·9 (95·9–97·8)	94·4 (92·7–95·9)	96·5 (95·2–97·6)	94·4 (92·6–95·8)
Non-Hodgkin lymphoma	9·31 (4·2–15·4)	9·78 (4·53–16)	9·58 (4·4–15·9)	9·8 (4·54–16·1)
Multiple myeloma	10·5 (4·91–17·6)	11 (5·16–18·3)	10·9 (5·19–18·3)	11 (5·17–18·3)
Leukemia	36·6 (22·7–50·6)	36·2 (23·7–48·8)	37·5 (23·3–52)	36·1 (23·7–48·6)
Cardiovascular diseases	79·4 (75·7–83)	80·5 (77·6–83·3)	71·4 (66·8–76·2)	82 (79·1–84·6)
Rheumatic heart disease	21·6 (11·9–41·8)	21·6 (13·2–36·7)	21·4 (12·6–36·7)	21·6 (13·3–36·6)
Ischemic heart disease	91·7 (87·7–95·1)	93·3 (90·4–95·7)	92·1 (88·7–95·1)	93·3 (90·5–95·7)
Stroke	77·3 (69·9–86)	81·2 (75·6–87)	80·5 (74·9–86·6)	81·5 (75·9–87·4)
Hypertensive heart disease	100 (100–100)	100 (100–100)	100 (100–100)	100 (100–100)
Non-rheumatic valvular heart disease	24·9 (15–38·5)	24·4 (16·4–35·4)	11·1 (7·14–16·1)	25·5 (17·3–36·7)
Cardiomyopathy and myocarditis	39·4 (32·4–48·2)	43·1 (37–48·8)	40·4 (34·1–47)	43·3 (37·3–49·3)
Atrial fibrillation and flutter	53·7 (43·4–63·8)	59·3 (50·6–67·4)	60·7 (52·2–68·6)	57·2 (48·2–66·2)
Aortic aneurysm	53·2 (46·8–59·5)	61·2 (55·8–66·2)	--	61·2 (55·8–66·2)
Endocarditis	30·4 (20·4–43·3)	32·1 (23–43·1)	30·1 (20·3–42·6)	32·1 (23·1–43)
Other cardiovascular and circulatory diseases	19·6 (14·8–27·8)	22·4 (17·6–29)	30·1 (23·6–37·2)	20·6 (15·9–27·7)
Chronic respiratory diseases	61·7 (56·5–67·3)	59·2 (55·3–63·2)	54·6 (50·4–58·8)	62·9 (58·3–68·1)

Chronic obstructive pulmonary disease	69·9 (65–74·7)	68·7 (64·6–72·7)	62·9 (58·8–66·9)	72·2 (68–76·6)
Pneumoconiosis	100 (100–100)	100 (100–100)	100 (100–100)	100 (100–100)
Asthma	51·8 (40·2–63·6)	45·4 (37·3–54·3)	44·6 (36·6–53·6)	53·1 (43·2–62·9)
Digestive diseases	41·3 (36·4–46·1)	44·2 (38·8–49·1)	16·1 (10·9–21·6)	52 (46·7–56·8)
Cirrhosis and other chronic liver diseases	82·9 (74·6–90)	84·7 (76·9–91)	81·5 (73·4–88·5)	84·7 (76·9–91·1)
Upper digestive system diseases	19·4 (16–23)	7·02 (4·42–10·4)	1·42 (0·805–2·27)	22·8 (19·1–26·5)
Gallbladder and biliary diseases	51·4 (35·6–66·3)	55·5 (40·8–68·6)	57 (42·6–69·7)	53·3 (38–67·6)
Pancreatitis	41·9 (24·5–59·6)	45·2 (27·5–62·7)	43·7 (26·3–61·5)	45·5 (27·8–62·9)
Neurological disorders	24·2 (9·64–39·3)	16 (8·08–27·9)	11·9 (6·17–22·4)	20·3 (7·61–35·3)
Alzheimer's disease and other dementias	39·7 (26·2–54·6)	43 (29·2–57·7)	43·9 (30·1–58·3)	42·4 (28·7–56·7)
Parkinson's disease	-8·99 (-14·7–-4·22)	-10·6 (-16·7–-5·3)	-11·3 (-17·6–-5·83)	-10·4 (-16·5–-5·19)
Idiopathic epilepsy	26·6 (16·7–35·9)	24·6 (15·6–33·3)	23·4 (14·8–31·7)	27·3 (17·3–36·5)
Multiple sclerosis	14·2 (10·4–18·5)	15·4 (11·2–19·8)	15·2 (11·1–19·6)	15·5 (11·3–20·2)
Mental disorders	--	8·03 (4·01–12·7)	8·03 (4·01–12·7)	--
Depressive disorders	--	16·2 (7·84–24·7)	16·2 (7·84–24·7)	--
Anxiety disorders	--	5·58 (1·23–12·9)	5·58 (1·23–12·9)	--
Idiopathic developmental intellectual disability	--	45·3 (21·1–65·1)	45·3 (21·1–65·1)	--
Substance use disorders	100 (100–100)	100 (100–100)	100 (100–100)	100 (100–100)
Alcohol use disorders	100 (100–100)	100 (100–100)	100 (100–100)	100 (100–100)
Drug use disorders	100 (100–100)	100 (100–100)	100 (100–100)	100 (100–100)
Diabetes and kidney diseases	99·9 (99·8–99·9)	99·9 (99·9–100)	100 (100–100)	99·9 (99·9–99·9)
Diabetes mellitus	100 (100–100)	100 (100–100)	100 (100–100)	100 (100–100)
Chronic kidney disease	100 (100–100)	100 (100–100)	100 (100–100)	100 (100–100)
Sense organ diseases	--	10·9 (9·82–12·2)	10·9 (9·82–12·2)	--
Blindness and vision loss	--	9·91 (6·57–14·3)	9·91 (6·57–14·3)	--
Age-related and other hearing loss	--	11·8 (10·7–13·2)	11·8 (10·7–13·2)	--
Musculoskeletal disorders	3·48 (1·04–6·39)	21·5 (17·8–25·2)	21·8 (18·1–25·7)	3·52 (1·08–6·37)
Rheumatoid arthritis	11·4 (3·33–20·8)	14·4 (4·48–25·8)	14·8 (4·58–26·6)	13·3 (3·96–24)
Osteoarthritis	--	13·8 (8·02–20·5)	13·8 (8·02–20·5)	--
Low back pain	--	43·2 (37·2–49·2)	43·2 (37·2–49·2)	--
Gout	--	61·2 (45–76·9)	61·2 (45–76·9)	--
Other non-communicable diseases	0·0724 (0·0467–0·114)	0·104 (0·065–0·161)	--	0·27 (0·177–0·414)

Sudden infant death syndrome	7·48 (5·01–10·6)	7·47 (5–10·6)	--	7·47 (5–10·6)
Injuries	31·8 (26·2–37·2)	28·9 (24·1–33·6)	35·3 (31·3–39·4)	24·1 (17·4–30·5)
Transport injuries	21·9 (15–28·7)	24·4 (18·2–31)	32·1 (28–36)	20·6 (13–28·3)
Road injuries	20·4 (13·3–27·5)	22·4 (15·9–29·2)	29·5 (25·5–33·7)	19·3 (11·3–27·1)
Other transport injuries	38·1 (32·9–43·4)	39·2 (34·5–43·7)	42·1 (37·8–45·8)	35·5 (29·7–41·8)
Unintentional injuries	41·8 (36·3–46·2)	33·9 (29·7–38·4)	36·4 (32–41)	29·8 (25·5–34·1)
Falls	57·2 (49·5–62)	48·2 (42·5–52·8)	47·2 (41·6–51·8)	50·2 (44·4–54·9)
Drowning	-6·6 (-19·9–5·54)	-5·54 (-18–5·71)	12·8 (8·29–18·7)	-6·44 (-19·7–5·16)
Fire, heat, and hot substances	12·8 (8·36–18·4)	16·2 (11·4–22·4)	17·2 (12·1–23·8)	15 (10·6–20·5)
Poisonings	18·1 (12·9–24·8)	18·3 (13·4–24·4)	15·9 (11·4–21·9)	19·9 (14·5–26·7)
Exposure to mechanical forces	9·17 (0·0997–18·4)	20·8 (16·4–25·6)	25·2 (21·8–29·1)	6·81 (-3·02–16·5)
Animal contact	25·8 (20·9–31·9)	28·1 (23·4–33·9)	28·9 (24·2–34·5)	24·4 (19·4–30·4)
Foreign body	3·19 (2·17–4·64)	5·21 (3·67–7·5)	4·76 (3·37–6·73)	5·43 (3·76–7·8)
Environmental heat and cold exposure	8·77 (3·74–15)	9·23 (4–16·1)	8·91 (3·86–15·6)	9·53 (4·12–16·5)
Exposure to forces of nature	8·27 (3·57–14·2)	8·4 (3·7–14·2)	9·46 (4·03–16·1)	7·62 (3·39–13)
Other unintentional injuries	18 (12·6–24·7)	16·7 (11·6–23·1)	16·2 (11·3–22·5)	22 (16–29·3)
Self-harm and interpersonal violence	20·1 (5·82–32·3)	22·5 (9·31–33·9)	30·7 (24·2–37·7)	21·8 (7·61–34)
Self-harm	20 (4·33–33·6)	22·5 (6·58–36·1)	26·2 (14·5–37·2)	22·4 (6·42–36)
Interpersonal violence	21·7 (11–32)	24 (15–32·9)	32·6 (25·4–40·5)	19·2 (8·32–29·9)

Supplementary Results Table 2. All-age PAFs attributable to the joint distribution of all risk factors for all ages, both sexes combined for each GBD level 1, level 2, and level 3 cause and all causes in 2019 for Virginia. Results are shown for deaths, YLLs, YLDs, and DALYs Data in parenthesis are 95% uncertainty intervals. PAFs=population attributable fractions. GBD=Global Burden of Disease. YLLs=years of life lost. YLDs=years lived with disability. DALYs=disability-adjusted life-years				
	Deaths 2019	DALYs 2019	YLDs 2019	YLLs 2019
All causes	59·8 (57–62·4)	46·1 (43–49·2)	32·3 (29·7–34·6)	59·3 (57·3–61·4)
Communicable, maternal, neonatal, and nutritional diseases	44·8 (40·7–48·8)	55·1 (52·1–58·2)	47·8 (43·3–52·7)	60 (56·2–63·6)
HIV/AIDS and sexually transmitted infections	89·9 (87·9–91·7)	90 (87·8–91·9)	93·1 (90·9–95·3)	88 (85·6–90)
HIV/AIDS	89·6 (87·5–91·5)	88·6 (86·2–90·6)	89·9 (87·6–92)	87·9 (85·5–90)
Sexually transmitted infections excluding HIV	98·6 (98–99)	99·5 (99·2–99·8)	100 (100–100)	94·8 (92·7–96·4)
Respiratory infections and tuberculosis	40·8 (35·2–46·3)	34·9 (29·3–40·5)	2·49 (1·89–3·26)	45·5 (39·9–51)
Tuberculosis	55·7 (46·6–64·4)	58·5 (49·6–66·7)	55·3 (45·7–64·2)	59·1 (50·3–67·3)
Lower respiratory infections	40·7 (35·1–46·3)	45·3 (39·7–50·8)	32·8 (27·5–38·3)	45·5 (39·8–51)
Upper respiratory infections	0·713 (0·445–1·1)	0·0195 (0·0093–0·0339)	0·00485 (0–0·0152)	1·57 (0·997–2·45)
Otitis media	1·56 (0·612–3·3)	3·81 (2·47–5·24)	3·8 (2·45–5·24)	4·25 (1·92–7·74)
Enteric infections	9·34 (5·9–14·3)	10·5 (7·06–15·4)	10·3 (6·81–15·2)	10·8 (7·39–15·5)
Diarrheal diseases	9·39 (5·93–14·3)	10·5 (7·09–15·4)	10·3 (6·82–15·3)	10·9 (7·46–15·7)
Other infectious diseases	0·744 (0·467–0·951)	1·3 (0·906–1·67)	0·62 (0·37–1·01)	1·47 (0·977–1·93)
Meningitis	1·58 (1·12–2·14)	3·46 (2·54–4·56)	0·000553 (0–0·00186)	3·61 (2·63–4·77)
Encephalitis	0·665 (0·483–0·911)	1·75 (1·28–2·39)	0·000155 (0–0·000496)	1·82 (1·33–2·48)
Measles	9·21 (-4·26–43·9)	6·04 (-3·88–40)	0·265 (0–0·822)	9·38 (-4·34–44·7)
Acute hepatitis	21·3 (13·8–32·7)	8·47 (5·47–12·3)	3·12 (1·9–5·02)	22·4 (14·4–33·8)
Maternal and neonatal disorders	86·5 (84·4–88·3)	83·4 (81·3–85·4)	75·3 (71–79·2)	88·3 (86·6–89·8)
Maternal disorders	11·4 (4·12–19·3)	11·3 (4·03–18·9)	11·2 (3·97–18·9)	11·3 (4·07–19)
Neonatal disorders	91·6 (90·3–92·7)	86·1 (84–88)	77·2 (72·9–81·2)	91·6 (90·3–92·7)
Nutritional deficiencies	95 (87·5–96·3)	89 (86·1–91·6)	87·5 (83·9–90·8)	94·4 (87·2–95·6)
Protein-energy malnutrition	100 (100–100)	100 (100–100)	100 (100–100)	100 (100–100)
Vitamin A deficiency	--	100 (100–100)	100 (100–100)	--
Dietary iron deficiency	--	100 (100–100)	100 (100–100)	--
Non-communicable diseases	62·8 (59·7–65·6)	47·5 (44–51·1)	31·6 (28·8–34·3)	63·8 (61·5–66·1)
Neoplasms	49·1 (45·3–53·3)	48 (44·6–52)	30·7 (26·8–35)	49·3 (45·8–53·3)
Lip and oral cavity cancer	68 (59·5–75·3)	70·1 (62–77·1)	69·5 (60·8–76·5)	70·1 (61·9–77·1)
Nasopharynx cancer	64·3 (53·7–73·6)	64·1 (54–73)	63·2 (52·8–72·2)	64·1 (54·2–73)
Other pharynx cancer	77·3 (70·4–82·9)	78·2 (71·7–83·7)	78·5 (72·1–84)	78·2 (71·6–83·6)

Esophageal cancer	79·1 (70·2–87)	80 (71·1–87·8)	79·1 (69·9–87·1)	80 (71·1–87·8)
Stomach cancer	24·6 (16·5–42·4)	24·9 (16·7–43·1)	25·2 (17–43·1)	24·9 (16·7–43·1)
Colon and rectum cancer	61·7 (53–70·3)	62·5 (54·4–70·5)	62·4 (53·9–70·9)	62·5 (54·4–70·5)
Liver cancer	79·1 (74·1–83·7)	78·8 (74–83·3)	78·9 (73·9–83·5)	78·8 (73·9–83·3)
Gallbladder and biliary tract cancer	27·1 (16–39·8)	28·2 (16·8–41)	26 (15·2–38·7)	28·3 (16·8–41·1)
Pancreatic cancer	43 (33·2–53·9)	42·7 (33·4–53·4)	43 (33·4–53·9)	42·7 (33·4–53·4)
Larynx cancer	82·1 (72·6–89·3)	82·8 (74·6–89)	82·5 (74–88·9)	82·8 (74·6–89)
Tracheal, bronchus, and lung cancer	83 (79·8–86·4)	82·1 (79–85·5)	82·6 (79·3–86·3)	82·1 (79–85·5)
Breast cancer	35·5 (26·6–46·7)	34·6 (25·9–44·5)	35·1 (26·5–45·4)	34·5 (25·9–44·4)
Cervical cancer	100 (100–100)	100 (100–100)	100 (100–100)	100 (100–100)
Uterine cancer	54·6 (39·8–68·2)	56·3 (41·5–69·4)	56·7 (41·8–69·6)	56·2 (41·4–69·3)
Ovarian cancer	20·1 (8·99–33·9)	18·1 (7·81–31·3)	17·5 (7·54–30·3)	18·1 (7·8–31·3)
Prostate cancer	5·72 (2·47–9·34)	6·3 (2·73–10·1)	6·6 (2·87–10·5)	6·2 (2·67–10)
Kidney cancer	42·2 (31·2–53)	42·7 (32·2–52·7)	43 (32·6–52·8)	42·7 (32·2–52·7)
Bladder cancer	40·7 (25·1–55·8)	43·7 (29·2–57·5)	44·2 (30–57·3)	43·6 (29·2–57·5)
Thyroid cancer	18·5 (9·65–28·5)	19·7 (10·4–30·1)	19·9 (10·7–30·3)	19·6 (10·3–30·2)
Mesothelioma	96·7 (95·4–97·6)	93·6 (91·5–95·3)	96·3 (94·7–97·4)	93·6 (91·5–95·3)
Non-Hodgkin lymphoma	8·87 (3·93–14·9)	9·38 (4·26–15·6)	9·15 (4·15–15·4)	9·4 (4·29–15·6)
Multiple myeloma	10·1 (4·72–17)	10·7 (5·1–17·7)	10·6 (5–17·6)	10·7 (5·09–17·7)
Leukemia	36·3 (22·3–50·3)	34·7 (22·9–46·9)	36·7 (23·5–50·7)	34·6 (22·9–46·7)
Cardiovascular diseases	80·7 (77·1–83·9)	81·3 (78·6–84)	71·4 (67·1–75·6)	83 (80·4–85·4)
Rheumatic heart disease	21·7 (12·3–42)	21·8 (13·9–37·5)	21·6 (13–37·6)	21·8 (14·1–37)
Ischemic heart disease	92·8 (89–95·9)	94·3 (91·7–96·4)	92·9 (89·6–95·7)	94·3 (91·7–96·4)
Stroke	79·4 (72·5–86·8)	83·1 (78·5–87·8)	82·3 (77·2–87·6)	83·4 (78·8–88·1)
Hypertensive heart disease	100 (100–100)	100 (100–100)	100 (100–100)	100 (100–100)
Non-rheumatic valvular heart disease	25·2 (15·6–38·4)	22·5 (15·3–31·7)	7·58 (4·78–11)	25·5 (17·3–35·8)
Cardiomyopathy and myocarditis	39·6 (32·3–47·9)	43·2 (37·6–49)	41 (35–47·2)	43·5 (37·5–49·3)
Atrial fibrillation and flutter	52·9 (43–62·9)	58·7 (50·2–67·3)	60·1 (51·6–68·4)	56·7 (47·8–65·5)
Aortic aneurysm	53·8 (48·1–59·8)	61·6 (57–66·3)	--	61·6 (57–66·3)
Endocarditis	31·4 (21·7–43·2)	32·8 (23·8–43·1)	30·8 (20·8–42·3)	32·9 (23·9–43·2)
Other cardiovascular and circulatory diseases	21·4 (16·5–28·9)	24·6 (20–30·7)	31·7 (25·3–39)	22·4 (18–28·9)
Chronic respiratory diseases	63·1 (58·1–68)	58·1 (54·3–62)	52·8 (48·3–57·3)	63·3 (59·1–67·7)

Chronic obstructive pulmonary disease	71·4 (66·8–75·6)	69·5 (65·7–73·2)	64·1 (60·2–67·9)	73·2 (69·1–76·8)
Pneumoconiosis	100 (100–100)	100 (100–100)	100 (100–100)	100 (100–100)
Asthma	51·1 (40·2–62·5)	42·4 (34·5–51·1)	41·7 (33·8–50·4)	50·9 (41·3–60·9)
Digestive diseases	46·6 (41·3–51·8)	47·1 (41·3–52·1)	16·7 (11·4–22·6)	56·5 (50·9–61·6)
Cirrhosis and other chronic liver diseases	81·7 (73·2–88·8)	83·8 (76–90)	80 (71·2–87·3)	83·8 (76·1–90)
Upper digestive system diseases	21·1 (17·7–24·6)	5·43 (3·27–8·17)	1·43 (0·854–2·27)	24·1 (20·4–27·7)
Gallbladder and biliary diseases	50·9 (35·2–65·5)	55·8 (41·3–68·8)	57·3 (43·1–70·2)	53·1 (37·8–67)
Pancreatitis	39·6 (22·3–56·5)	43·6 (26·2–60·9)	41·2 (24·2–59)	43·9 (26·4–61·3)
Neurological disorders	26·8 (11·5–42·1)	16 (7·9–27·8)	11 (5·37–21·7)	23·1 (9·38–39)
Alzheimer's disease and other dementias	41·4 (27–56·8)	44·5 (30·3–59·7)	45·3 (30·8–60·1)	44 (29·8–59·4)
Parkinson's disease	-9·15 (-14·7–-4·43)	-10·6 (-16·2–-5·29)	-11·1 (-16·8–-5·7)	-10·5 (-16·2–-5·18)
Idiopathic epilepsy	24·3 (16·1–32·7)	22·2 (14·9–29·7)	21·1 (14–28·4)	24·8 (16·8–33·1)
Multiple sclerosis	14·5 (10·6–19·1)	15·3 (11·1–20)	15 (11–19·4)	15·7 (11·3–20·5)
Mental disorders	--	7·78 (4·06–12·1)	7·78 (4·06–12·1)	--
Depressive disorders	--	16·7 (8·2–25·4)	16·7 (8·2–25·4)	--
Anxiety disorders	--	4·8 (1·07–11·2)	4·8 (1·07–11·2)	--
Idiopathic developmental intellectual disability	--	45·8 (22·6–63·8)	45·8 (22·6–63·8)	--
Substance use disorders	100 (100–100)	100 (100–100)	100 (100–100)	100 (100–100)
Alcohol use disorders	100 (100–100)	100 (100–100)	100 (100–100)	100 (100–100)
Drug use disorders	100 (100–100)	100 (100–100)	100 (100–100)	100 (100–100)
Diabetes and kidney diseases	99·8 (99·8–99·9)	99·9 (99·9–100)	100 (100–100)	99·9 (99·8–99·9)
Diabetes mellitus	100 (100–100)	100 (100–100)	100 (100–100)	100 (100–100)
Chronic kidney disease	100 (100–100)	100 (100–100)	100 (100–100)	100 (100–100)
Sense organ diseases	--	10·3 (9·21–11·5)	10·3 (9·21–11·5)	--
Blindness and vision loss	--	9·56 (6·17–14)	9·56 (6·17–14)	--
Age-related and other hearing loss	--	11·2 (10·2–12·4)	11·2 (10·2–12·4)	--
Musculoskeletal disorders	3·54 (1·03–6·49)	21 (17·6–24·5)	21·3 (17·9–24·9)	3·35 (1·02–6·01)
Rheumatoid arthritis	12 (3·59–21·6)	14·6 (4·64–26·1)	14·9 (4·73–26·5)	13·9 (4·27–24·9)
Osteoarthritis	--	15·8 (9·04–23·2)	15·8 (9·04–23·2)	--
Low back pain	--	41·8 (35·9–47·6)	41·8 (35·9–47·6)	--
Gout	--	60·8 (44·6–76·8)	60·8 (44·6–76·8)	--
Other non-communicable diseases	0·158 (0·113–0·226)	0·198 (0·138–0·28)	--	0·505 (0·368–0·681)

Sudden infant death syndrome	5·54 (4–7·65)	5·53 (3·99–7·64)	--	5·53 (3·99–7·64)
Injuries	29·4 (24·8–33·9)	27·6 (23·6–31·6)	32·4 (29–36·1)	24·6 (19·4–29·8)
Transport injuries	28·8 (23·4–35·2)	29·1 (23·7–35·4)	31·8 (27·8–36·4)	27·9 (21·7–35)
Road injuries	28·3 (22·8–34·7)	28·1 (22·5–34·6)	29·8 (25·7–34·6)	27·4 (21·3–34·5)
Other transport injuries	38·2 (32·6–44·6)	38·7 (34·1–43·6)	40·7 (36·4–44·5)	35·8 (29·5–42·9)
Unintentional injuries	35·4 (31·2–39)	29·6 (25·8–33·8)	33·1 (29·1–37·2)	23·8 (19·9–28)
Falls	55 (48·7–59·2)	46 (41–50·1)	45 (40·1–49·3)	48·1 (42·9–52·4)
Drowning	-5·19 (-13·5–3·63)	-5·37 (-13·2–2·89)	12·4 (8·23–17·5)	-6·06 (-14–2·3)
Fire, heat, and hot substances	12·6 (8·62–17·8)	16·3 (11·7–21·9)	17·8 (12·9–23·9)	14·4 (10·2–19·6)
Poisonings	20·2 (14·6–26·9)	19·5 (14·2–25·9)	16·3 (11·7–21·9)	21·6 (15·8–28·9)
Exposure to mechanical forces	15·4 (9·03–22·6)	22·9 (18·6–28·1)	25·4 (21·8–30·1)	13·5 (6·11–21·8)
Animal contact	23·7 (18·9–29·5)	27·1 (22·6–32·6)	28 (23·5–33·4)	21·6 (16·8–27·5)
Foreign body	3·27 (2·15–4·7)	5·49 (3·74–7·91)	6·7 (4·71–9·47)	4·96 (3·32–7·13)
Environmental heat and cold exposure	7·93 (3·61–13·4)	8·33 (3·87–14·3)	7·96 (3·68–13·4)	8·67 (4–14·5)
Exposure to forces of nature	7·41 (3·45–12·5)	7·61 (3·52–12·9)	8·45 (3·91–14·3)	6·99 (3·23–11·8)
Other unintentional injuries	22·5 (16·2–30·1)	17·6 (12·7–23·6)	16·9 (12–22·8)	25·6 (18·5–34·3)
Self-harm and interpersonal violence	22·5 (12·9–32·2)	23·5 (14·7–32)	27·9 (21·5–34·5)	23 (13·8–32·1)
Self-harm	23·3 (10·7–36)	25·2 (12·3–38·1)	24·5 (13·7–35·5)	25·2 (12·2–38·1)
Interpersonal violence	21·2 (13·2–29·8)	21·4 (13·8–29·5)	29·7 (22·7–37·5)	19·3 (11·6–27·7)

Supplementary Results Table 2. All-age PAFs attributable to the joint distribution of all risk factors for all ages, both sexes combined for each GBD level 1, level 2, and level 3 cause and all causes in 2019 for Washington. Results are shown for deaths, YLLs, YLDs, and DALYs Data in parenthesis are 95% uncertainty intervals. PAFs=population attributable fractions. GBD=Global Burden of Disease. YLLs=years of life lost. YLDs=years lived with disability. DALYs=disability-adjusted life-years

	Deaths 2019	DALYs 2019	YLDs 2019	YLLs 2019
All causes	58·2 (55·4–60·9)	44·3 (41·1–47·1)	31·6 (29·1–34)	57·9 (55·7–60·1)
Communicable, maternal, neonatal, and nutritional diseases	43·8 (38·6–48·5)	51·8 (48·4–54·9)	46 (41·2–50·8)	56·8 (52·7–60·7)
HIV/AIDS and sexually transmitted infections	89·2 (86·8–91)	89·4 (86·8–91·7)	93·2 (90·7–96)	86·6 (83·5–88·8)
HIV/AIDS	88·8 (86·3–90·7)	87·2 (84–89·4)	88·9 (85·9–91·1)	86·3 (83·3–88·6)
Sexually transmitted infections excluding HIV	99·1 (98·7–99·4)	99·7 (99·5–99·9)	100 (100–100)	96·4 (95–97·6)
Respiratory infections and tuberculosis	42·1 (34·2–49·1)	31·8 (25·3–38·5)	2·06 (1·53–2·73)	46·5 (38·8–53·3)
Tuberculosis	55·4 (44·9–64·8)	57·8 (47·8–66·7)	54·9 (44·4–64·3)	58·5 (48·4–67·3)
Lower respiratory infections	42 (34–49)	46·2 (38·5–53·1)	31·1 (25·3–36·6)	46·5 (38·8–53·5)
Upper respiratory infections	0·503 (0·31–0·77)	0·0154 (0·00698–0·0289)	0·00499 (0–0·0152)	1·22 (0·758–1·83)
Otitis media	1·17 (0·479–2·51)	3·23 (2·07–4·54)	3·23 (2·06–4·53)	3·37 (1·57–6·41)
Enteric infections	9·39 (5·84–14·3)	10·6 (7·07–15·4)	10·4 (6·84–15·2)	10·8 (7·22–15·6)
Diarrheal diseases	9·43 (5·85–14·3)	10·6 (7·09–15·5)	10·4 (6·84–15·3)	10·9 (7·26–15·7)
Other infectious diseases	0·699 (0·433–0·946)	1·13 (0·763–1·45)	0·763 (0·462–1·25)	1·23 (0·78–1·6)
Meningitis	1·11 (0·808–1·54)	2·54 (1·87–3·46)	0·00047 (0–0·00154)	2·74 (2–3·74)
Encephalitis	0·479 (0·338–0·655)	1·32 (0·967–1·76)	0·000105 (0–0·000344)	1·39 (1·02–1·85)
Measles	9·1 (-4·08–43·1)	3·65 (-2·23–28·8)	0·315 (0·00845–0·817)	9·26 (-4·16–43·9)
Acute hepatitis	21·2 (13·9–32·2)	9·71 (6·18–14·8)	3·8 (2·33–5·9)	22·5 (14·6–34·2)
Maternal and neonatal disorders	83·3 (80·9–85·6)	79·5 (77–81·8)	72·9 (68·4–77)	85·1 (83·1–87·2)
Maternal disorders	11 (4·04–18·4)	10·9 (3·97–18·3)	10·8 (3·92–18·5)	10·9 (4·01–18·2)
Neonatal disorders	88·5 (86·6–90·4)	82·3 (79·5–84·6)	75 (70·4–79·2)	88·5 (86·6–90·4)
Nutritional deficiencies	92·4 (83·8–94·6)	87·1 (84·2–90)	86 (82·6–89·4)	91·9 (84–94·1)
Protein-energy malnutrition	100 (100–100)	100 (100–100)	100 (100–100)	100 (100–100)
Vitamin A deficiency	--	100 (100–100)	100 (100–100)	--
Dietary iron deficiency	--	100 (100–100)	100 (100–100)	--
Non-communicable diseases	60·7 (57·6–63·7)	45·5 (42–48·9)	30·8 (28·1–33·4)	62·1 (59·8–64·5)
Neoplasms	47·3 (43·6–51·4)	46·3 (42·8–50)	29·7 (25·9–33·8)	47·6 (44·1–51·3)
Lip and oral cavity cancer	65·8 (55·8–74·2)	68 (58·6–75·8)	67·6 (58·2–75·4)	68·1 (58·7–75·9)
Nasopharynx cancer	64·4 (53–74·1)	64 (52·7–73·2)	63·3 (51·8–72·5)	64 (52·7–73·2)
Other pharynx cancer	76·2 (68·4–82·6)	77·2 (69·6–83·3)	77·4 (69·9–83·5)	77·2 (69·6–83·3)

Esophageal cancer	77·2 (67·1–86·3)	78·4 (68·5–87·3)	77·5 (67·4–86·5)	78·5 (68·5–87·3)
Stomach cancer	23·3 (15·2–41·4)	23·9 (15·7–42·3)	24·1 (15·8–42·4)	23·9 (15·7–42·3)
Colon and rectum cancer	61·3 (52·3–70·3)	62·3 (53·8–70·4)	62·1 (53·6–70·4)	62·3 (53·8–70·4)
Liver cancer	80·1 (75·6–84·5)	79·8 (75·3–84·2)	79·5 (74·7–84)	79·8 (75·3–84·2)
Gallbladder and biliary tract cancer	26·9 (15·6–39·4)	27·9 (16·7–40·5)	25·6 (14·4–38·7)	28 (16·7–40·6)
Pancreatic cancer	40·9 (31·2–51·9)	40·9 (31·6–51·5)	41 (31·5–51·7)	40·9 (31·6–51·5)
Larynx cancer	79·6 (69·5–87·8)	80·6 (72·1–87·9)	80·4 (71·5–87·5)	80·7 (72·2–88)
Tracheal, bronchus, and lung cancer	81·2 (77·7–85·1)	80·5 (76·9–84·3)	81 (77·3–85)	80·5 (76·9–84·3)
Breast cancer	36·2 (27·2–46·9)	35·3 (27–45·1)	35·8 (27·3–45·8)	35·3 (27–44·9)
Cervical cancer	100 (100–100)	100 (100–100)	100 (100–100)	100 (100–100)
Uterine cancer	54·4 (38·8–67·7)	56·1 (41–69·2)	56·6 (41·6–69·6)	56 (40·8–69·1)
Ovarian cancer	20·4 (9·57–33·4)	18 (8·03–30·3)	17·3 (7·68–29·1)	18 (8·03–30·4)
Prostate cancer	5·36 (2·23–9·11)	6·02 (2·48–10·2)	6·38 (2·67–10·7)	5·9 (2·44–9·92)
Kidney cancer	41·4 (30·3–52·5)	42·1 (31·4–52·2)	42·3 (31·7–52·4)	42·1 (31·4–52·2)
Bladder cancer	36·1 (22–51·1)	39·8 (25·9–54)	40·6 (26·8–54·5)	39·7 (25·8–54)
Thyroid cancer	18·4 (9·52–28·6)	19·6 (10·4–30·6)	20·2 (10·7–31·5)	19·5 (10·2–30·4)
Mesothelioma	97·3 (96·3–98·1)	94·7 (92·8–96·1)	96·8 (95·4–97·8)	94·6 (92·8–96·1)
Non-Hodgkin lymphoma	8·85 (3·93–14·9)	9·34 (4·2–15·4)	9·11 (4·05–15·2)	9·36 (4·21–15·4)
Multiple myeloma	10·1 (4·76–16·9)	10·7 (5·06–17·9)	10·6 (4·91–17·7)	10·7 (5·1–17·9)
Leukemia	35·1 (22–49·1)	33·8 (22·2–46)	35·6 (22·1–49·2)	33·7 (22·2–45·7)
Cardiovascular diseases	78·8 (74·8–82·3)	79·5 (76·4–82·4)	71·6 (67·1–76·3)	81 (78·1–83·7)
Rheumatic heart disease	21·5 (11·9–41·1)	21·5 (13·3–36·1)	21·3 (13–36)	21·5 (13·3–35·9)
Ischemic heart disease	91·9 (87·9–95·3)	93·4 (90·6–95·9)	92·1 (88·7–95·2)	93·5 (90·6–96)
Stroke	78·3 (71·1–86·2)	81·8 (76·8–87)	80·9 (75·7–86·5)	82·3 (77–87·3)
Hypertensive heart disease	100 (100–100)	100 (100–100)	100 (100–100)	100 (100–100)
Non-rheumatic valvular heart disease	25·5 (15·3–38·6)	24·7 (16·5–34·6)	11·8 (7·51–16·9)	25·9 (17·5–36·3)
Cardiomyopathy and myocarditis	42·6 (35·9–50·4)	46·2 (40·3–52·3)	42·9 (37·2–49)	46·6 (40·3–52·9)
Atrial fibrillation and flutter	53 (43–63·3)	58·6 (49·9–67)	59·9 (51·4–68·3)	56·6 (47·7–65·6)
Aortic aneurysm	53·3 (47·1–59·6)	61·1 (56·2–65·9)	--	61·1 (56·2–65·9)
Endocarditis	30·6 (20·4–43)	32·2 (23·3–42·5)	30·2 (20·4–42·2)	32·3 (23·4–42·6)
Other cardiovascular and circulatory diseases	19·9 (14·9–28·4)	23·3 (18·5–29·6)	30·4 (23·5–37·9)	21·2 (16·8–27·8)
Chronic respiratory diseases	61·3 (56·1–66·4)	57·3 (53·5–61·1)	52 (47·3–56·5)	62·1 (57·7–66·7)

Chronic obstructive pulmonary disease	69·7 (65–74)	67·9 (64–71·5)	61·5 (57·3–65·5)	71·8 (67·7–75·6)
Pneumoconiosis	100 (100–100)	100 (100–100)	100 (100–100)	100 (100–100)
Asthma	50 (38·5–61·8)	43·3 (35·1–52·2)	42·6 (34·4–51·5)	50·1 (40·6–60·2)
Digestive diseases	47·3 (41·9–52·5)	49·1 (43·3–54·4)	16·3 (11·1–22·1)	58·3 (52·6–63·4)
Cirrhosis and other chronic liver diseases	82·9 (74·5–89·7)	84·4 (76·4–90·6)	81·4 (72·9–88·4)	84·4 (76·4–90·7)
Upper digestive system diseases	19·6 (16–23·3)	6·09 (3·77–9·15)	1·36 (0·795–2·16)	22·7 (18·9–26·5)
Gallbladder and biliary diseases	49·8 (34·3–64·7)	55·2 (40·6–68·4)	56·8 (42·8–69·4)	52·1 (36·9–66·5)
Pancreatitis	40·6 (21·6–59·4)	43·8 (24·3–61·8)	41·8 (22·8–60·4)	44·1 (24·7–62·1)
Neurological disorders	24 (9·55–39·3)	14·5 (7·09–25·9)	10·3 (5·08–20·5)	20·3 (7·75–35·5)
Alzheimer's disease and other dementias	40·1 (26·4–55·1)	43·3 (29·7–58)	44 (30·3–58·4)	42·8 (29·2–57·2)
Parkinson's disease	-8·84 (-14·7–-4·21)	-10·3 (-16·3–-5·12)	-11·1 (-17·2–-5·59)	-10·2 (-16·2–-5·01)
Idiopathic epilepsy	25·5 (16·9–34·8)	22·9 (15·4–31·1)	21·7 (14·5–29·7)	25·5 (17·1–34·4)
Multiple sclerosis	13·6 (9·69–18·1)	14·9 (10·8–19·5)	14·9 (10·9–19·3)	14·9 (10·7–19·7)
Mental disorders	--	9·96 (5·1–15·2)	9·96 (5·1–15·2)	--
Depressive disorders	--	21·1 (10·2–31·2)	21·1 (10·2–31·2)	--
Anxiety disorders	--	6·08 (1·36–14·1)	6·08 (1·36–14·1)	--
Idiopathic developmental intellectual disability	--	45·7 (23–63·8)	45·7 (23–63·8)	--
Substance use disorders	100 (100–100)	100 (100–100)	100 (100–100)	100 (100–100)
Alcohol use disorders	100 (100–100)	100 (100–100)	100 (100–100)	100 (100–100)
Drug use disorders	100 (100–100)	100 (100–100)	100 (100–100)	100 (100–100)
Diabetes and kidney diseases	99·9 (99·8–99·9)	99·9 (99·9–100)	100 (100–100)	99·9 (99·9–99·9)
Diabetes mellitus	100 (100–100)	100 (100–100)	100 (100–100)	100 (100–100)
Chronic kidney disease	100 (100–100)	100 (100–100)	100 (100–100)	100 (100–100)
Sense organ diseases	--	10·4 (9·26–11·6)	10·4 (9·26–22·6)	--
Blindness and vision loss	--	9·19 (5·99–13·4)	9·19 (5·99–13·4)	--
Age-related and other hearing loss	--	11·3 (10·3–12·6)	11·3 (10·3–12·6)	--
Musculoskeletal disorders	3·32 (0·974–6·13)	17·9 (14·8–21·1)	18·1 (15–21·4)	3·38 (1·06–6·29)
Rheumatoid arthritis	11·4 (3·36–20·4)	14 (4·42–25·2)	14·2 (4·51–25·6)	13·3 (4·05–23·8)
Osteoarthritis	--	13·8 (7·77–20·2)	13·8 (7·77–20·2)	--
Low back pain	--	40·2 (34·5–46)	40·2 (34·5–46)	--
Gout	--	60·2 (43·6–76·7)	60·2 (43·6–76·7)	--
Other non-communicable diseases	0·141 (0·0992–0·199)	0·183 (0·125–0·256)	--	0·483 (0·341–0·65)

Sudden infant death syndrome	5·83 (4·2–7·75)	5·82 (4·2–7·75)	--	5·82 (4·2–7·75)
Injuries	30·7 (25–35·4)	29 (24–33·4)	35·3 (31·2–39·5)	24·4 (17·8–30·2)
Transport injuries	26·3 (19·9–33)	28·3 (22·2–34·8)	33·4 (29–38·1)	25·6 (18·3–33·4)
Road injuries	24·8 (18·3–31·6)	26·4 (20·1–33·3)	31·2 (26·7–36·1)	24·3 (16·9–32·2)
Other transport injuries	39·5 (33·9–45·3)	39·4 (34·5–44·5)	41·7 (37–45·7)	37·2 (30·8–44)
Unintentional injuries	40·3 (34·9–44·6)	33·4 (29–38·1)	36·2 (31·7–40·9)	27·8 (23·2–32·6)
Falls	56·4 (49·4–61·1)	47·4 (41·8–51·8)	46·5 (40·8–51)	49·8 (43·9–54·5)
Drowning	-5·45 (-15·6–5·31)	-4·82 (-14·6–5·54)	13·9 (9·36–19·4)	-5·6 (-15·7–5·02)
Fire, heat, and hot substances	13·5 (9·12–19·3)	18·6 (13·3–25)	20·2 (14·5–27·2)	15·8 (11·2–21·5)
Poisonings	22·2 (16·1–29·7)	21·6 (15·8–28·6)	18 (13·1–24·2)	24·3 (17·7–32·1)
Exposure to mechanical forces	13 (4·71–21·9)	24·4 (19·4–30·3)	27·2 (22·9–32·4)	11·3 (2·23–22·3)
Animal contact	26·6 (21·6–32·1)	28·6 (23·5–34·4)	29·4 (24·1–35·3)	24·3 (19·2–30·2)
Foreign body	3·69 (2·46–5·42)	6·03 (4·1–8·63)	5·72 (3·81–8·32)	6·21 (4·2–9·17)
Environmental heat and cold exposure	8·49 (3·81–14·7)	8·75 (3·93–15)	8·53 (3·87–14·9)	9·04 (4·05–15·5)
Exposure to forces of nature	7·77 (3·56–13·3)	8·1 (3·67–13·9)	9·04 (4·14–15·5)	7·12 (3·27–12·2)
Other unintentional injuries	24·1 (17·3–32·1)	19·4 (13·9–26·3)	18·8 (13·4–25·5)	28·2 (20·6–37·8)
Self-harm and interpersonal violence	20·8 (8·79–31·7)	22·4 (11·3–32)	29·4 (22·9–36·4)	21·7 (9·96–32·1)
Self-harm	21·3 (7·05–33·8)	23·2 (9·11–36)	25·9 (14·3–37)	23·1 (8·99–36·1)
Interpersonal violence	19·7 (10·6–29)	20·8 (12·6–29·4)	31 (23·7–38·6)	17·3 (8·37–26·2)

Supplementary Results Table 2. All-age PAFs attributable to the joint distribution of all risk factors for all ages, both sexes combined for each GBD level 1, level 2, and level 3 cause and all causes in 2019 for West Virginia. Results are shown for deaths, YLLs, YLDs, and DALYs Data in parenthesis are 95% uncertainty intervals. PAFs=population attributable fractions. GBD=Global Burden of Disease. YLLs=years of life lost. YLDs=years lived with disability. DALYs=disability-adjusted life-years				
	Deaths 2019	DALYs 2019	YLDs 2019	YLLs 2019
All causes	65 (62·4–67·3)	54·3 (51·3–57·2)	38·4 (35·4–41·1)	65·4 (63·5–67·3)
Communicable, maternal, neonatal, and nutritional diseases	44·6 (39·9–48·9)	53·6 (50·4–56·9)	48 (43·1–52·3)	56·7 (52·5–60·7)
HIV/AIDS and sexually transmitted infections	86·9 (84·9–88·6)	87·9 (85·7–90)	91·9 (89·3–95)	84·9 (82·6–86·9)
HIV/AIDS	86 (83·8–87·8)	85 (82·7–87)	86·1 (83·9–88·2)	84·4 (82·1–86·5)
Sexually transmitted infections excluding HIV	99 (98·5–99·4)	99·6 (99·3–99·8)	100 (100–100)	96·1 (94·2–97·6)
Respiratory infections and tuberculosis	43·9 (37·4–49·7)	40·8 (35–46·7)	3·04 (2·34–3·94)	49·2 (42·9–55)
Tuberculosis	54·2 (45·8–62·2)	58·7 (51–65·7)	54·6 (46·1–62·7)	59·3 (51·6–66·2)
Lower respiratory infections	43·8 (37·3–49·8)	49 (42·6–54·8)	37 (31·5–42·5)	49·1 (42·7–55)
Upper respiratory infections	0·552 (0·306–0·929)	0·0181 (0·00822–0·0337)	0·0042 (0·000111–0·0126)	1·32 (0·733–2·19)
Otitis media	1·61 (0·619–3·77)	4·61 (3·06–6·23)	4·6 (3·03–6·22)	4·56 (2·03–9·28)
Enteric infections	13·8 (8·76–21·3)	14·8 (9·76–22·2)	14·4 (9·34–21·9)	15·3 (10·2–22·7)
Diarrheal diseases	13·9 (8·81–21·4)	14·9 (9·79–22·3)	14·4 (9·35–21·9)	15·5 (10·2–22·9)
Other infectious diseases	0·764 (0·509–1·09)	1·27 (0·944–1·71)	0·919 (0·542–1·42)	1·35 (0·972–1·84)
Meningitis	1·17 (0·697–1·89)	2·64 (1·62–4·18)	0·000327 (6·28e-06–0·00103)	2·86 (1·74–4·52)
Encephalitis	0·487 (0·309–0·747)	1·38 (0·895–2·06)	0·000108 (2·01e-06–0·000345)	1·44 (0·924–2·17)
Measles	9·69 (-3·73–42·7)	9·85 (-3·78–43·3)	0·24 (0–1·06)	9·86 (-3·8–43·4)
Acute hepatitis	26·5 (16·7–42·5)	12·9 (8·2–19·6)	5·38 (3·27–8·24)	27·3 (17–42·5)
Maternal and neonatal disorders	85·1 (82·4–87·3)	81·9 (79·3–84·1)	73·9 (69·5–77·8)	86·8 (84·7–88·7)
Maternal disorders	12·3 (4·4–20·5)	12 (4·33–20·3)	11·7 (4·22–19·8)	12·1 (4·33–20·3)
Neonatal disorders	89·8 (88·3–91·2)	84·5 (81·9–86·5)	75·7 (71·2–79·9)	89·8 (88·3–91·2)
Nutritional deficiencies	92·3 (83·4–94·4)	90·8 (88–93)	90·3 (87·2–93)	92·3 (83·4–94·1)
Protein-energy malnutrition	100 (100–100)	100 (100–100)	100 (100–100)	100 (100–100)
Vitamin A deficiency	--	100 (100–100)	100 (100–100)	--
Dietary iron deficiency	--	100 (100–100)	100 (100–100)	--
Non-communicable diseases	68·2 (65·3–70·7)	57 (53·4–60·3)	38·6 (35·4–41·6)	70·4 (68·3–72·4)
Neoplasms	54·3 (50·4–58·4)	54·1 (50·3–57·9)	34·4 (30·3–38·9)	55·1 (51·6–58·9)
Lip and oral cavity cancer	67·5 (60·6–74·5)	70·7 (63·9–76·9)	69·8 (62·6–76·3)	70·7 (63·9–77)
Nasopharynx cancer	62·7 (53–71·4)	63·3 (53·7–71·9)	62·1 (52·2–70·9)	63·4 (53·9–71·9)
Other pharynx cancer	76·4 (70·5–81·7)	78·1 (72·6–83)	78·4 (72·5–83·2)	78·1 (72·6–83)
Esophageal cancer	81·4 (73–88·6)	82·6 (74·4–89·8)	81·6 (73·4–88·7)	82·6 (74·4–89·8)

Stomach cancer	27·2 (19·1–44·5)	28·4 (20–45·9)	28·3 (20–45·5)	28·4 (20–45·9)
Colon and rectum cancer	63·5 (54·1–72·8)	64·5 (55·9–73·2)	64·4 (55·6–73·3)	64·6 (55·9–73·2)
Liver cancer	81·8 (77–86·4)	81·5 (76·8–86·1)	81·7 (76·8–86·2)	81·5 (76·8–86·1)
Gallbladder and biliary tract cancer	27·7 (16·7–40·7)	29 (17·8–41·7)	26·7 (15·5–39·8)	29 (17·8–41·8)
Pancreatic cancer	47·7 (37·7–59·2)	48·1 (38·7–59)	48 (38·3–59·5)	48·1 (38·7–59)
Larynx cancer	83·3 (73·5–90·2)	84·6 (76–90·4)	84·3 (75·5–90·4)	84·6 (76·1–90·4)
Tracheal, bronchus, and lung cancer	86·9 (83·6–90·1)	86·6 (83·6–89·7)	86·8 (83·7–90·2)	86·6 (83·6–89·7)
Breast cancer	35·5 (25·2–48)	34·8 (25·5–46·1)	35·2 (25·5–47·1)	34·8 (25·4–46)
Cervical cancer	100 (100–100)	100 (100–100)	100 (100–100)	100 (100–100)
Uterine cancer	54·7 (39–68·6)	56·5 (41·1–69·7)	57·1 (41·7–70·3)	56·5 (41–69·6)
Ovarian cancer	22 (9·78–37·6)	20·2 (8·56–35·1)	19·7 (8·51–34·3)	20·2 (8·56–35·1)
Prostate cancer	6·62 (2·84–10·9)	7·43 (3·23–11·9)	7·89 (3·46–12·6)	7·32 (3·17–11·9)
Kidney cancer	44·7 (33·5–55·5)	45·9 (35·6–55·8)	46·1 (35·7–55·9)	45·9 (35·6–55·8)
Bladder cancer	44·8 (28·1–60·7)	48·5 (32·8–63)	49·1 (33·8–63·2)	48·4 (32·8–63)
Thyroid cancer	18·5 (9·62–28·8)	19·9 (10·5–30·4)	20·3 (11–30·9)	19·8 (10·4–30·7)
Mesothelioma	97·7 (96·8–98·4)	95·9 (94·3–97)	97·5 (96·3–98·3)	95·8 (94·2–97)
Non-Hodgkin lymphoma	9 (4·02–15·3)	9·59 (4·43–15·9)	9·33 (4·22–15·6)	9·6 (4·44–16)
Multiple myeloma	10·2 (4·8–17·2)	10·9 (5·05–18)	10·7 (4·92–17·9)	10·9 (5·05–18)
Leukemia	39·2 (24·2–53·7)	38·8 (25·6–52)	40·3 (25·3–54·9)	38·8 (25·6–51·8)
Cardiovascular diseases	83·3 (80·1–86·3)	83·6 (80·9–86)	72·2 (67·8–76·8)	85·2 (82·8–87·4)
Rheumatic heart disease	21·9 (13–39·1)	22·1 (14·2–36·3)	22 (13·6–37·3)	22·1 (14·4–36·2)
Ischemic heart disease	93·1 (89·7–96)	94·5 (92·2–96·6)	93·2 (90·3–95·9)	94·6 (92·3–96·6)
Stroke	80·3 (73·7–87·6)	83·8 (79·1–88·7)	82·7 (77·8–88·3)	84·1 (79·5–88·9)
Hypertensive heart disease	100 (100–100)	100 (100–100)	100 (100–100)	100 (100–100)
Non-rheumatic valvular heart disease	25·3 (16–37·9)	22·3 (15·3–31·6)	5·67 (3·62–8·08)	25·9 (17·8–36)
Cardiomyopathy and myocarditis	40·4 (33·7–48)	43·6 (37·8–49·3)	41·4 (35·2–48)	43·8 (37·8–49·6)
Atrial fibrillation and flutter	51·1 (41·2–61·4)	57·2 (48·4–66)	58·4 (49·5–66·8)	55·4 (46·4–64·7)
Aortic aneurysm	57·4 (51·6–63)	65·3 (60·3–69·5)	--	65·3 (60·3–69·5)
Endocarditis	31·5 (21·6–43·9)	33·1 (23·9–43·6)	30·9 (20·5–43·3)	33·2 (24·1–43·6)
Other cardiovascular and circulatory diseases	24·3 (19–31·6)	27 (21·5–32·9)	32·8 (25·8–39·7)	25·3 (20–31·7)
Chronic respiratory diseases	70·7 (66·5–74·8)	66·8 (63·1–70·1)	58·2 (53·8–62·5)	71·7 (68·1–75·3)
Chronic obstructive pulmonary disease	75·6 (71·4–79·4)	74·8 (71·3–78)	68 (64–71·7)	77·6 (74–80·8)
Pneumoconiosis	100 (100–100)	100 (100–100)	100 (100–100)	100 (100–100)

Asthma	52·9 (41·9–63·8)	45·7 (37·4–54·3)	44·9 (36·6–53·5)	52·7 (43·3–62·1)
Digestive diseases	45·2 (40·1–50·8)	48·1 (42·9–53·6)	19·9 (14·1–26·4)	54·9 (49·5–60·5)
Cirrhosis and other chronic liver diseases	77·7 (70·1–85·7)	80·6 (73·2–87·7)	75·5 (66·9–84)	80·6 (73·2–87·8)
Upper digestive system diseases	23·7 (19·7–27·7)	8·01 (5·08–11·5)	2·02 (1·24–3·04)	27·5 (23·2–31·7)
Gallbladder and biliary diseases	51·5 (36·2–65·6)	56·8 (43·2–69·4)	58·3 (44·9–70·4)	54·1 (39·8–67·5)
Pancreatitis	28·6 (16–43)	34·2 (20·3–49·8)	32·1 (18·7–47·3)	34·5 (20·4–50)
Neurological disorders	28·1 (12–44·3)	18·6 (9·39–31·8)	13·7 (7·08–25·1)	24·1 (9·09–40·9)
Alzheimer's disease and other dementias	44·2 (29·6–59·8)	47·6 (32·8–63)	48·4 (33·4–63·7)	47 (32·6–62·3)
Parkinson's disease	-11 (-17·4–-5·59)	-13 (-20–-6·8)	-13·8 (-20·6–-7·35)	-12·9 (-19·8–-6·7)
Idiopathic epilepsy	18·2 (11·8–25)	17·3 (11·2–24·1)	16·1 (10·4–22·5)	20·1 (13·4–27·5)
Multiple sclerosis	17·6 (12·7–23·1)	19·4 (14·2–25·2)	19·4 (14·3–24·6)	19·5 (14·2–25·4)
Mental disorders	--	9·25 (4·71–14·3)	9·25 (4·71–14·3)	--
Depressive disorders	--	18·3 (8·58–27·9)	18·3 (8·58–27·9)	--
Anxiety disorders	--	5·61 (1·29–12·9)	5·61 (1·29–12·9)	--
Idiopathic developmental intellectual disability	--	52·1 (25·9–72·4)	52·1 (25·9–72·4)	--
Substance use disorders	100 (100–100)	100 (100–100)	100 (100–100)	100 (100–100)
Alcohol use disorders	100 (100–100)	100 (100–100)	100 (100–100)	100 (100–100)
Drug use disorders	100 (100–100)	100 (100–100)	100 (100–100)	100 (100–100)
Diabetes and kidney diseases	99·8 (99·8–99·9)	99·9 (99·9–99·9)	100 (100–100)	99·9 (99·8–99·9)
Diabetes mellitus	100 (100–100)	100 (100–100)	100 (100–100)	100 (100–100)
Chronic kidney disease	100 (100–100)	100 (100–100)	100 (100–100)	100 (100–100)
Sense organ diseases	--	10·5 (9·33–11·8)	10·5 (9·33–11·8)	--
Blindness and vision loss	--	11·3 (7·3–16·5)	11·3 (7·3–16·5)	--
Age-related and other hearing loss	--	11 (9·97–12·3)	11 (9·97–12·3)	--
Musculoskeletal disorders	5·09 (1·55–9·01)	23·3 (19·4–27·3)	23·7 (19·7–27·8)	5·06 (1·58–9·01)
Rheumatoid arthritis	14·2 (4·54–24·9)	18·1 (5·9–31·4)	18·7 (6·11–32·3)	16·6 (5·36–28·8)
Osteoarthritis	--	15·8 (9·11–23·1)	15·8 (9·11–23·1)	--
Low back pain	--	44·7 (38–51·4)	44·7 (38–51·4)	--
Gout	--	62·1 (46·8–77·2)	62·1 (46·8–77·2)	--
Other non-communicable diseases	0·156 (0·093–0·249)	0·224 (0·133–0·356)	--	0·521 (0·32–0·811)
Sudden infant death syndrome	6·14 (3·77–9·39)	6·13 (3·77–9·38)	--	6·13 (3·77–9·38)
Injuries	27·1 (23–30·6)	25·3 (21·5–28·7)	30·8 (27·2–33·8)	22·5 (18–26·8)
Transport injuries	22·8 (17·5–28·3)	22·8 (18·1–28·3)	28·4 (24·8–31·8)	21 (15·1–27·2)

Road injuries	21·7 (16·1–27·3)	21·3 (16·1–26·8)	25·7 (22·3–29·2)	20 (13·9–26·3)
Other transport injuries	33·8 (29·3–38·8)	34·3 (30·1–39)	39·3 (35·1–42·5)	31 (25·8–36·9)
Unintentional injuries	32·7 (28·2–36)	27 (23·3–30·5)	31·7 (27·8–35·2)	21 (17·4–24·4)
Falls	53·2 (47–57·4)	44·6 (39·5–48)	43·9 (39–47·5)	45·8 (40·6–49·6)
Drowning	-11·7 (-19·4–-3·76)	-11·2 (-18·9–-3·67)	8·33 (5·33–12·3)	-11·7 (-19·4–-3·96)
Fire, heat, and hot substances	8·56 (5·48–12·6)	11·1 (7·56–15·6)	11·9 (8·05–16·8)	10·4 (7·09–14·8)
Poisonings	14 (9·64–19·6)	14·4 (10·1–19·9)	11·3 (7·68–15·8)	15·6 (11–21·3)
Exposure to mechanical forces	8·76 (2·21–15·9)	17·1 (13·1–21·4)	22·4 (19·5–25·6)	6·47 (-0·703–14·3)
Animal contact	19 (15–23·8)	22·3 (18·3–26·8)	24·1 (20–28·6)	17·7 (13·5–22·7)
Foreign body	2·23 (1·45–3·29)	3·45 (2·31–4·94)	3·28 (2·23–4·65)	3·5 (2·29–5·18)
Environmental heat and cold exposure	5·6 (2·51–10·2)	6·15 (2·78–11)	5·56 (2·52–9·96)	6·69 (3·05–11·9)
Exposure to forces of nature	5·64 (2·56–9·91)	6·08 (2·77–10·6)	6·55 (2·99–11·5)	5·64 (2·55–9·78)
Other unintentional injuries	16·6 (11·5–22·9)	12·5 (8·45–17·6)	11·2 (7·57–16)	19·4 (13·7–26·2)
Self-harm and interpersonal violence	22·6 (13·9–31)	25 (16·6–32·9)	27 (21·7–32·3)	24·8 (16·1–33·4)
Self-harm	23·3 (13·2–34·3)	27 (16·1–38·3)	23·9 (15·1–33·4)	27·1 (16·1–38·4)
Interpersonal violence	20·9 (13·7–28·5)	21 (13·9–28·2)	28·2 (22·3–34·7)	19·1 (12–26·8)

Supplementary Results Table 2. All-age PAFs attributable to the joint distribution of all risk factors for all ages, both sexes combined for each GBD level 1, level 2, and level 3 cause and all causes in 2019 for Wisconsin. Results are shown for deaths, YLLs, YLDs, and DALYs Data in parenthesis are 95% uncertainty intervals. PAFs=population attributable fractions. GBD=Global Burden of Disease. YLLs=years of life lost. YLDs=years lived with disability. DALYs=disability-adjusted life-years

	Deaths 2019	DALYs 2019	YLDs 2019	YLLs 2019
All causes	59·2 (56·3–61·9)	46·4 (43·2–49·2)	33·1 (30·5–35·5)	58·7 (56·6–60·9)
Communicable, maternal, neonatal, and nutritional diseases	43·7 (37·8–49·3)	54·9 (51·6–58·2)	48·4 (43·9–53·1)	59·3 (54·8–63·3)
HIV/AIDS and sexually transmitted infections	90·4 (88·4–92·1)	91·3 (89·1–93·3)	94·7 (92·5–97·1)	88·4 (86–90·4)
HIV/AIDS	89·8 (87·7–91·6)	88·7 (86·2–90·8)	90 (87·6–92)	88 (85·6–90·1)
Sexually transmitted infections excluding HIV	99·3 (99–99·5)	99·8 (99·6–99·9)	100 (100–100)	97·2 (96–98·1)
Respiratory infections and tuberculosis	40·8 (32·2–48·8)	34·4 (27·7–41·4)	2·16 (1·63–2·76)	46 (37·5–53·2)
Tuberculosis	54·5 (43·3–64)	57·8 (46·9–66·7)	55·2 (44·4–64·5)	58·3 (47·3–66·9)
Lower respiratory infections	40·7 (32·1–48·8)	45·8 (37·2–53)	33·4 (27·6–39·1)	46 (37·4–53·3)
Upper respiratory infections	0·6 (0·372–0·917)	0·0168 (0·00774–0·0312)	0·00527 (3·52e-06–0·0156)	1·41 (0·886–2·13)
Otitis media	1·39 (0·546–2·89)	3·55 (2·27–4·85)	3·54 (2·27–4·85)	4·03 (1·78–7·47)
Enteric infections	9·71 (6·1–14·9)	11·1 (7·4–16·2)	10·8 (7–15·9)	11·5 (7·91–16·6)
Diarrheal diseases	9·77 (6·13–15)	11·2 (7·44–16·3)	10·8 (7·01–15·9)	11·7 (8·04–16·8)
Other infectious diseases	0·536 (0·374–0·785)	1·03 (0·754–1·3)	0·669 (0·405–1·04)	1·12 (0·791–1·44)
Meningitis	1·34 (0·982–1·79)	3·04 (2·25–3·98)	0·000531 (2·08e-07–0·00165)	3·22 (2·39–4·21)
Encephalitis	0·55 (0·4–0·735)	1·53 (1·13–2·02)	0·000138 (4·88e-08–0·00041)	1·6 (1·17–2·12)
Measles	9·27 (-4·19–43·9)	9·41 (-4·24–44·6)	0·175 (0–0·773)	9·43 (-4·27–44·7)
Acute hepatitis	17·8 (10·9–32·4)	7·67 (4·73–12·9)	3·44 (2·08–5·25)	19 (11·7–34·8)
Maternal and neonatal disorders	85·8 (83·6–87·6)	81·8 (79·5–84)	72·5 (67·6–76·8)	87·6 (85·8–89·1)
Maternal disorders	11 (3·98–18·6)	10·9 (3·94–18·4)	10·8 (3·92–18·4)	10·9 (3·95–18·3)
Neonatal disorders	91·1 (89·7–92·5)	84·6 (82·2–86·9)	74·3 (69·2–78·7)	91·1 (89·7–92·5)
Nutritional deficiencies	91·7 (82·7–94·4)	88·1 (85–90·9)	87·3 (83·6–90·5)	91·3 (83·1–94·2)
Protein-energy malnutrition	100 (100–100)	100 (100–100)	100 (100–100)	100 (100–100)
Vitamin A deficiency	--	100 (100–100)	100 (100–100)	--
Dietary iron deficiency	--	100 (100–100)	100 (100–100)	--
Non-communicable diseases	61·9 (58·4–64·9)	47·9 (44·4–51·3)	32·3 (29·6–34·9)	63·2 (60·8–65·5)
Neoplasms	48·1 (44·5–52)	47·6 (44·2–51·3)	31 (27·2–35·1)	48·8 (45·5–52·5)
Lip and oral cavity cancer	67·4 (57·2–75·1)	70 (60·5–77·3)	69·5 (59·5–76·7)	70·1 (60·6–77·3)
Nasopharynx cancer	65·6 (53·5–75)	65·4 (54–74·5)	64·5 (52·5–74·1)	65·5 (54–74·5)
Other pharynx cancer	77·7 (70·5–83·5)	79 (71·7–84·5)	79·3 (72·1–84·8)	79 (71·6–84·4)
Esophageal cancer	79·1 (69·2–87·5)	80·3 (70·7–88·5)	79·3 (69·8–87·3)	80·3 (70·7–88·5)

Stomach cancer	24·3 (16·3–42·3)	25·3 (17·2–43·4)	25·4 (17·3–43·7)	25·3 (17·2–43·4)
Colon and rectum cancer	61·5 (52·8–70·2)	62·7 (54·6–70·8)	62·6 (54·1–70·7)	62·7 (54·6–70·8)
Liver cancer	80·1 (75·5–84·5)	79·9 (75·2–84·4)	80 (75–84·4)	79·9 (75·2–84·3)
Gallbladder and biliary tract cancer	27·5 (16·4–40·7)	28·5 (17·3–41·7)	26·5 (15·4–39·2)	28·6 (17·3–41·7)
Pancreatic cancer	42·4 (32·9–53·4)	42·6 (33·8–53)	42·6 (33·7–53·3)	42·6 (33·8–53)
Larynx cancer	81·2 (71·5–88·7)	82·5 (73·6–88·8)	82·3 (73·5–88·8)	82·5 (73·6–88·8)
Tracheal, bronchus, and lung cancer	83·3 (79·9–86·9)	82·9 (79·7–86·3)	83·2 (79·9–86·7)	82·9 (79·7–86·2)
Breast cancer	35·8 (26·7–45·8)	35·2 (26·7–44·2)	35·7 (26·9–45·1)	35·1 (26·6–43·9)
Cervical cancer	100 (100–100)	100 (100–100)	100 (100–100)	100 (100–100)
Uterine cancer	54·3 (38·9–68·1)	55·9 (40·7–69·3)	56·4 (41·3–69·6)	55·8 (40·6–69·3)
Ovarian cancer	20·4 (9·5–33·3)	18 (8·11–29·8)	17·4 (7·84–29·1)	18 (8·12–29·8)
Prostate cancer	5·64 (2·48–9·37)	6·36 (2·77–10·5)	6·76 (2·94–10·9)	6·23 (2·72–10·3)
Kidney cancer	42·8 (31·7–53·7)	43·6 (33–53·6)	43·8 (33·3–53·8)	43·5 (33–53·6)
Bladder cancer	37 (22·7–51·7)	41·1 (26·9–54·8)	42 (28·2–55·6)	41 (26·8–54·7)
Thyroid cancer	18·7 (9·91–28·8)	19·8 (10·7–30·5)	20 (11·1–30·3)	19·7 (10·5–30·6)
Mesothelioma	97·2 (96·1–98)	94·6 (92·7–96)	96·7 (95·4–97·8)	94·5 (92·6–96)
Non-Hodgkin lymphoma	9·03 (3·94–15·2)	9·5 (4·38–15·7)	9·31 (4·3–15·5)	9·52 (4·35–15·8)
Multiple myeloma	10·4 (4·73–17·5)	10·9 (5·11–18·1)	10·8 (4·95–18)	10·9 (5·12–18·2)
Leukemia	36·6 (22·8–50·5)	35·5 (23·3–47·8)	37·1 (23·3–51)	35·4 (23·2–47·6)
Cardiovascular diseases	79·3 (75·4–82·8)	80·5 (77·4–83·3)	70·8 (66–75·2)	82 (79·1–84·6)
Rheumatic heart disease	21·6 (11·8–41·9)	21·8 (13·1–37·5)	21·8 (12·8–37·7)	21·8 (13·2–37·5)
Ischemic heart disease	92·1 (88–95·5)	93·8 (91–96·2)	92·4 (88·9–95·5)	93·8 (91·1–96·2)
Stroke	78·1 (70·6–85·9)	82·1 (76·8–87·4)	81·2 (75·6–87)	82·4 (77·1–87·7)
Hypertensive heart disease	100 (100–100)	100 (100–100)	100 (100–100)	100 (100–100)
Non-rheumatic valvular heart disease	24·8 (15–38·2)	23·9 (16–34·4)	9·48 (6·25–13·6)	25·6 (17·1–36·8)
Cardiomyopathy and myocarditis	38·7 (31·2–47·8)	43·3 (37·4–49·8)	41·2 (34·8–47·7)	43·5 (37·6–50·2)
Atrial fibrillation and flutter	52·7 (43–62·9)	58·9 (50·3–67·3)	60·4 (51·8–68·6)	56·7 (47·8–65·6)
Aortic aneurysm	52·8 (46·4–59·4)	61·2 (56–66·1)	--	61·2 (56–66·1)
Endocarditis	30·2 (20·4–43·7)	32·1 (23–42·6)	30·2 (20–43·1)	32·2 (23·1–42·6)
Other cardiovascular and circulatory diseases	20 (15·1–28·4)	23·4 (19–29·8)	31·1 (24·7–38·6)	21·3 (17–27·9)
Chronic respiratory diseases	62·9 (58–68·4)	59·8 (56·1–63·6)	55 (50·6–59·2)	63·9 (59·7–68·8)
Chronic obstructive pulmonary disease	71·9 (67·4–76·4)	70·8 (67–74·3)	65·1 (61–69)	74·4 (70·4–78·2)
Pneumoconiosis	100 (100–100)	100 (100–100)	100 (100–100)	100 (100–100)

Asthma	50·9 (39·5–62·6)	44·8 (36·6–53·5)	44·2 (36–52·8)	51·2 (41·6–60·8)
Digestive diseases	42·5 (37·3–47·4)	44·5 (39·2–49·6)	15·4 (10·5–20·9)	53·6 (48·2–58·5)
Cirrhosis and other chronic liver diseases	81·3 (72·4–88·5)	83·6 (75·7–89·9)	79·8 (70·4–87·5)	83·6 (75·7–89·9)
Upper digestive system diseases	20 (16·5–23·6)	6·3 (3·81–9·31)	1·44 (0·843–2·31)	23·7 (19·9–27·6)
Gallbladder and biliary diseases	51·4 (35·7–66·1)	55·6 (41·2–68·9)	57 (42·9–69·7)	53·3 (38·2–67·5)
Pancreatitis	37·5 (19–55·9)	42 (23·5–60·3)	40·5 (21·9–58·7)	42·2 (23·7–60·5)
Neurological disorders	25·6 (10·8–40·4)	16·1 (8·11–28)	11·5 (5·86–21·8)	21·8 (8·62–37·1)
Alzheimer's disease and other dementias	40·2 (26·4–55·4)	43·3 (29·3–58·1)	44·1 (30·1–58·8)	42·8 (28·9–57·8)
Parkinson's disease	-8·91 (-14·2–-4·36)	-10·6 (-16·3–-5·3)	-11·4 (-17·5–-5·74)	-10·4 (-16·1–-5·19)
Idiopathic epilepsy	25 (15·8–33·8)	22·3 (14·5–30·1)	21·2 (13·6–28·9)	25·3 (16·4–33·9)
Multiple sclerosis	14·3 (10·3–18·5)	15·7 (11·5–20·1)	15·7 (11·5–20·1)	15·7 (11·4–20·2)
Mental disorders	--	8·55 (4·22–13·4)	8·56 (4·23–13·4)	--
Depressive disorders	--	17·6 (8·27–26·9)	17·6 (8·27–26·9)	--
Anxiety disorders	--	6·06 (1·4–13·6)	6·06 (1·4–13·6)	--
Idiopathic developmental intellectual disability	--	46·2 (22·4–64·5)	46·2 (22·4–64·5)	--
Substance use disorders	100 (100–100)	100 (100–100)	100 (100–100)	100 (100–100)
Alcohol use disorders	100 (100–100)	100 (100–100)	100 (100–100)	100 (100–100)
Drug use disorders	100 (100–100)	100 (100–100)	100 (100–100)	100 (100–100)
Diabetes and kidney diseases	99·9 (99·8–99·9)	99·9 (99·9–100)	100 (100–100)	99·9 (99·8–99·9)
Diabetes mellitus	100 (100–100)	100 (100–100)	100 (100–100)	100 (100–100)
Chronic kidney disease	100 (100–100)	100 (100–100)	100 (100–100)	100 (100–100)
Sense organ diseases	--	11·2 (10–12·5)	11·2 (10–12·5)	--
Blindness and vision loss	--	9·41 (6·06–13·6)	9·41 (6·06–13·6)	--
Age-related and other hearing loss	--	12·1 (11–13·5)	12·1 (11–13·5)	--
Musculoskeletal disorders	3·58 (1·04–6·58)	21·3 (18–24·9)	21·6 (18·2–25·3)	3·67 (1·13–6·57)
Rheumatoid arthritis	11·3 (3·27–20·1)	14·6 (4·56–25·9)	15 (4·69–26·7)	13·4 (4·09–23·7)
Osteoarthritis	--	15·7 (9–22·9)	15·7 (9–22·9)	--
Low back pain	--	42·6 (36·9–48·4)	42·6 (36·9–48·4)	--
Gout	--	62·1 (46·8–77·6)	62·1 (46·8–77·6)	--
Other non-communicable diseases	0·128 (0·0905–0·173)	0·172 (0·118–0·246)	--	0·426 (0·305–0·564)
Sudden infant death syndrome	6·97 (4·87–9·55)	6·96 (4·86–9·54)	--	6·96 (4·86–9·54)
Injuries	32·8 (27·3–37·5)	28·4 (23·5–32·7)	35·2 (31·4–39·3)	23·6 (17·2–29·2)
Transport injuries	22·4 (15·5–29·4)	24·5 (18·1–31·1)	32·1 (27·8–36·4)	21·2 (13·6–29)

Road injuries	21·2 (13·8–28·3)	22·8 (16·1–29·7)	30·1 (25·8–34·5)	20 (12–27·9)
Other transport injuries	38 (32·4–43·9)	38·4 (33·5–43·2)	41·3 (36·9–45)	35·4 (29·1–42·1)
Unintentional injuries	44·4 (38·5–49)	34·4 (30–38·8)	36·4 (31·9–40·9)	30·8 (26·2–35·5)
Falls	57·1 (49·9–61·8)	48·3 (42·9–52·8)	47·2 (41·8–51·7)	50·4 (44·5–55·1)
Drowning	-8·16 (-19·2–2·8)	-7·94 (-18·4–2·54)	12·8 (8·32–18·6)	-8·94 (-19·9–1·74)
Fire, heat, and hot substances	12·8 (8·34–18·3)	16·9 (11·7–23·3)	18·5 (13–25·2)	14·6 (10·1–20·1)
Poisonings	19·9 (14·2–26·7)	19·8 (14·3–26·7)	16·7 (11·8–22·9)	21·5 (15·6–28·6)
Exposure to mechanical forces	9·53 (1·25–18·5)	21·7 (17·1–27·5)	25·5 (21·6–30·4)	6·93 (-2·2–17)
Animal contact	25·4 (20·3–31·3)	27·3 (22·2–33)	28·4 (23·2–34·1)	23·4 (18·3–29·3)
Foreign body	3·37 (2·21–4·95)	5·08 (3·39–7·25)	5·25 (3·61–7·49)	5·01 (3·28–7·23)
Environmental heat and cold exposure	8·41 (3·68–14·6)	8·85 (3·94–15·4)	8·65 (3·82–15)	9·08 (4·07–15·9)
Exposure to forces of nature	7·93 (3·51–13·8)	7·58 (3·37–13·2)	9·13 (4·01–15·9)	7·34 (3·25–12·8)
Other unintentional injuries	21·5 (15·2–28·9)	17·9 (12·6–24·6)	17·3 (12–24)	25 (18–33·4)
Self-harm and interpersonal violence	19·2 (6·35–30·4)	20·7 (8·99–30·9)	29·7 (23–36·4)	19·9 (7·72–30·7)
Self-harm	19·8 (4·58–33·4)	21·6 (6·69–35·1)	25·2 (13·6–36·7)	21·5 (6·44–35·1)
Interpersonal violence	17·3 (6·68–28·1)	19·1 (9·38–28·8)	31·6 (23·9–39·4)	15·2 (4·85–25·9)

Supplementary Results Table 2. All-age PAFs attributable to the joint distribution of all risk factors for all ages, both sexes combined for each GBD level 1, level 2, and level 3 cause and all causes in 2019 for Mongolia. Results are shown for deaths, YLLs, YLDs, and DALYs Data in parenthesis are 95% uncertainty intervals. PAFs=population attributable fractions. GBD=Global Burden of Disease. YLLs=years of life lost. YLDs=years lived with disability. DALYs=disability-adjusted life-years

	Deaths 2019	DALYs 2019	YLDs 2019	YLLs 2019
All causes	63·6 (61·2–66·1)	49·5 (46·6–52·4)	28·1 (25·8–30·4)	57·5 (55·4–59·9)
Communicable, maternal, neonatal, and nutritional diseases	60·4 (56–64·6)	62·3 (58·1–66·1)	61·4 (57·2–65)	62·5 (57·5–67·1)
HIV/AIDS and sexually transmitted infections	8·82 (3·42–21·6)	12·4 (4·58–29)	97·7 (94·4–99·4)	4·72 (1·74–12·4)
HIV/AIDS	70·7 (68·1–74·7)	66·2 (63·2–70·2)	77·8 (74·8–81·9)	64·1 (61·5–67·6)
Sexually transmitted infections excluding HIV	3·33 (0·978–9·13)	9·57 (3·2–23·9)	100 (100–100)	1·69 (0·489–4·71)
Respiratory infections and tuberculosis	64·7 (59·1–69·9)	63·9 (58·1–69·2)	21·4 (15·6–28)	67·9 (62·1–73·2)
Tuberculosis	52·5 (44·7–59·4)	49·4 (41·7–56·6)	44·5 (36·4–52·1)	50 (42·3–57·2)
Lower respiratory infections	73·5 (65·5–80·6)	76·9 (69·3–83·6)	49·3 (41·6–57)	77 (69·4–83·7)
Upper respiratory infections	4·04 (1·51–10·8)	0·456 (0·0478–1·57)	0·0205 (0·00822–0·0343)	4·2 (1·6–11)
Otitis media	3·45 (0·902–8·8)	7·33 (4·77–9·95)	7·33 (4·77–9·96)	6·19 (2·05–12·9)
Enteric infections	94·3 (90·3–96·9)	92·6 (86·4–96·3)	90·6 (82–95·7)	95·2 (91·7–97·5)
Diarrheal diseases	97 (93·8–98·7)	93·4 (87·2–97·1)	90·7 (82·1–95·8)	97·2 (94·1–98·8)
Other infectious diseases	2·95 (0·932–5·8)	3·13 (1·01–6·22)	0·245 (0·127–0·427)	3·64 (1·11–7·37)
Meningitis	1·6 (0·934–2·69)	2 (1·16–3·32)	0·00205 (0·000657–0·00382)	2·41 (1·42–4·01)
Encephalitis	6·02 (1·23–11·9)	7·23 (1·59–14·3)	0·00326 (0·00107–0·00575)	7·5 (1·63–14·8)
Measles	26·2 (5·6–57·7)	26 (5·61–57·4)	0·679 (0–1·65)	26·5 (5·72–58·5)
Acute hepatitis	1·95 (0·913–3·48)	1·43 (0·693–2·48)	1·24 (0·646–2·13)	1·48 (0·675–2·69)
Maternal and neonatal disorders	72 (67·2–76·4)	68·5 (64·1–72·8)	38·5 (32·3–43·9)	73·3 (68·4–77·6)
Maternal disorders	19·2 (6·83–32·7)	19·2 (6·88–32·4)	19·3 (6·84–32·1)	19·2 (6·86–32·5)
Neonatal disorders	75·9 (70·6–80·3)	70·9 (66·1–75·2)	39·3 (32·9–45·2)	75·9 (70·6–80·3)
Nutritional deficiencies	2·44 (1·71–3·74)	92·1 (89·1–94·5)	97·2 (96·4–98)	3·51 (2·31–5·83)
Protein-energy malnutrition	100 (100–100)	100 (100–100)	100 (100–100)	100 (100–100)
Vitamin A deficiency	--	100 (100–100)	100 (100–100)	--
Dietary iron deficiency	--	100 (100–100)	100 (100–100)	--
Non-communicable diseases	71·2 (68·6–74)	55·6 (51·4–59·5)	24·7 (21·7–27·6)	68·6 (66·1–71·4)
Neoplasms	48·3 (43·2–54·4)	46·5 (41·2–52·5)	44 (39·8–49·1)	46·5 (41·2–52·6)
Lip and oral cavity cancer	59·4 (52·6–65·7)	60·3 (53·4–66·8)	56·8 (49·2–64)	60·4 (53·5–66·8)
Nasopharynx cancer	59·8 (50·8–67·6)	58·9 (49·9–66·8)	59·8 (50·8–67·6)	58·9 (49·9–66·8)
Other pharynx cancer	63·3 (56·3–69·8)	66·7 (59·3–72·8)	62·7 (55·7–69·1)	66·7 (59·3–72·8)
Esophageal cancer	72 (61·4–81)	73·8 (63·6–82)	73 (62·4–81·6)	73·8 (63·6–82)

Stomach cancer	20·8 (12·1–40·7)	20 (10·9–40·3)	20·4 (11·4–40·3)	20 (10·9–40·3)
Colon and rectum cancer	61·1 (54–66·9)	60·9 (53·8–66·8)	60·8 (53·4–66·6)	60·9 (53·8–66·8)
Liver cancer	55·3 (46·2–63·9)	55·7 (46–64·9)	55·3 (45·9–64·4)	55·7 (46–64·9)
Gallbladder and biliary tract cancer	20·6 (10·7–32·5)	20·8 (11–32·9)	20·7 (10·8–32·6)	20·8 (11–32·9)
Pancreatic cancer	25·3 (19·6–32·2)	24·4 (18·4–31·6)	25 (19·1–32·2)	24·4 (18·4–31·6)
Larynx cancer	74·6 (67·9–79·8)	75·3 (68·7–80·2)	71·9 (65–77·5)	75·4 (68·7–80·4)
Tracheal, bronchus, and lung cancer	79·3 (75·2–84·1)	78·2 (73·9–83·3)	78·7 (74·3–83·9)	78·1 (73·9–83·3)
Breast cancer	20·5 (14·8–26·4)	19 (13–24·9)	19·2 (13·3–25)	19 (13–24·9)
Cervical cancer	100 (100–100)	100 (100–100)	100 (100–100)	100 (100–100)
Uterine cancer	40·3 (26–55·3)	40·4 (26·3–55·5)	40·5 (26·3–55·7)	40·4 (26·3–55·4)
Ovarian cancer	6·93 (2·24–12·8)	6·5 (2·1–12)	6·31 (1·97–11·8)	6·5 (2·1–12)
Prostate cancer	8·24 (3·74–12·9)	8·67 (3·89–13·5)	8·88 (4·01–13·8)	8·66 (3·89–13·5)
Kidney cancer	30·4 (21·3–39·9)	28·8 (19·9–38·7)	28·1 (19·2–38·1)	28·8 (19·9–38·7)
Bladder cancer	36·1 (27–43·7)	36·1 (26·2–43·7)	34·4 (24·7–41·7)	36·2 (26·3–43·8)
Thyroid cancer	13 (6·08–21·5)	13·3 (6·16–22·3)	12·9 (6·44–21·2)	13·3 (6·16–22·4)
Mesothelioma	78·4 (66·7–85·9)	72·4 (57·2–82)	77·9 (65·9–85·5)	72·4 (57–82)
Non-Hodgkin lymphoma	6·02 (2·63–10·7)	5·82 (2·56–10·3)	5·43 (2·39–9·78)	5·82 (2·56–10·3)
Multiple myeloma	7·21 (3·14–12·8)	7·28 (3·18–12·9)	7·31 (3·19–13)	7·28 (3·18–12·9)
Leukemia	19·3 (10·7–28)	14·4 (7·69–21·3)	17 (9·34–24·9)	14·4 (7·67–21·3)
Cardiovascular diseases	91·7 (89·8–93·2)	91·6 (90·1–92·9)	79·8 (75·4–83·4)	92·1 (90·6–93·4)
Rheumatic heart disease	33·3 (23·7–48·3)	32 (21·4–45·6)	26·2 (14·6–39·1)	33·8 (23–48·7)
Ischemic heart disease	96·2 (94·4–97·6)	97·1 (96·1–98)	96·5 (95–97·7)	97·1 (96·1–98·1)
Stroke	92·4 (89·7–94·6)	94 (92·1–95·6)	91·4 (89·3–93·2)	94·2 (92·2–95·7)
Hypertensive heart disease	100 (100–100)	100 (100–100)	100 (100–100)	100 (100–100)
Non-rheumatic valvular heart disease	13·5 (8·78–23·1)	12·1 (8–20·1)	1·16 (0·752–1·66)	13·6 (8·97–22·7)
Cardiomyopathy and myocarditis	45·2 (36·8–55)	46·2 (38·2–55)	39·3 (32·8–45·3)	46·3 (38·3–55·1)
Atrial fibrillation and flutter	54·7 (45·7–63·6)	65·1 (58–72)	67·9 (60·9–74·5)	60·1 (52·3–67·7)
Aortic aneurysm	64·6 (59·1–69·4)	67·3 (62·3–72·1)	--	67·3 (62·3–72·1)
Endocarditis	47·4 (37·3–57·9)	46·2 (35·1–57·1)	39·3 (30·6–48·1)	46·2 (35·1–57·1)
Other cardiovascular and circulatory diseases	44·9 (37·9–51·2)	41·2 (32·6–47·8)	32·7 (24·5–41)	42·7 (32·6–49·5)
Chronic respiratory diseases	63·8 (59·3–68)	53·3 (48·6–57·8)	37·6 (32·1–42·7)	60·5 (56·1–64·6)
Chronic obstructive pulmonary disease	78·4 (73·6–82·7)	72·8 (68·1–77·2)	57·7 (52·2–63·4)	77 (71·9–81·4)
Pneumoconiosis	100 (100–100)	100 (100–100)	100 (100–100)	100 (100–100)
Asthma	43·6 (33·1–55)	37·1 (28·9–46)	28·4 (22–35·8)	46·4 (36·1–57·3)

Digestive diseases	51·8 (44·9–58·3)	49·7 (42·4–56·4)	15·2 (10·6–20·2)	53·4 (45·8–60·7)
Cirrhosis and other chronic liver diseases	62·4 (53·8–70·9)	65·6 (56·2–74·1)	62·2 (54·3–70·3)	65·6 (56·2–74·2)
Upper digestive system diseases	22·6 (18·8–26·4)	15·5 (11·4–19·7)	1·41 (0·837–2·14)	24·3 (20·5–28·2)
Gallbladder and biliary diseases	41·5 (28·7–54·8)	42·4 (29·5–55·9)	42·6 (29·3–56·5)	42·1 (29·4–55·6)
Pancreatitis	35·3 (24·9–46·9)	41 (29·8–53·9)	35 (25–45·8)	41·2 (29·9–54)
Neurological disorders	18·9 (11·3–28·3)	7·97 (4·14–13·8)	4·34 (1·9–8·62)	17·6 (12–24·7)
Alzheimer's disease and other dementias	28·9 (19–41·2)	31·2 (21–43·1)	31·3 (21–43·1)	31·1 (20·9–43)
Parkinson's disease	-9·94 (-15·6–-4·61)	-11·1 (-17·4–-5·15)	-10·9 (-17·1–-5·04)	-11·1 (-17·4–-5·15)
Idiopathic epilepsy	21 (14·6–27·8)	16·6 (11·6–22·2)	13·3 (9·18–18·1)	20 (13·9–26·5)
Multiple sclerosis	16·2 (11·8–20·1)	14·9 (11·3–18·4)	14·1 (10·4–17·5)	16·5 (12·1–20·7)
Mental disorders	--	5·76 (2·92–9·38)	5·77 (2·92–9·38)	--
Depressive disorders	--	8·44 (3·17–14·7)	8·44 (3·17–14·7)	--
Anxiety disorders	--	6·49 (1·83–13·8)	6·49 (1·83–13·8)	--
Idiopathic developmental intellectual disability	--	53·7 (26·2–73·8)	53·7 (26·2–73·8)	--
Substance use disorders	100 (100–100)	100 (100–100)	100 (100–100)	100 (100–100)
Alcohol use disorders	100 (100–100)	100 (100–100)	100 (100–100)	100 (100–100)
Drug use disorders	100 (100–100)	100 (100–100)	100 (100–100)	100 (100–100)
Diabetes and kidney diseases	100 (100–100)	100 (100–100)	100 (100–100)	100 (100–100)
Diabetes mellitus	100 (100–100)	100 (100–100)	100 (100–100)	100 (100–100)
Chronic kidney disease	100 (100–100)	100 (100–100)	100 (100–100)	100 (100–100)
Sense organ diseases	--	13·9 (12·5–15·4)	13·9 (12·5–15·4)	--
Blindness and vision loss	--	6·45 (4·04–8·9)	6·45 (4·04–8·9)	--
Age-related and other hearing loss	--	19·2 (17·9–20·9)	19·2 (17·9–20·9)	--
Musculoskeletal disorders	3·12 (0·819–6·16)	30·3 (26·3–34·5)	30·9 (26·9–35·2)	2·55 (0·641–5·22)
Rheumatoid arthritis	8·87 (2·45–16·1)	9·75 (2·67–16·7)	9·75 (2·54–17)	9·75 (2·68–17·7)
Osteoarthritis	--	13·3 (6·77–21·2)	13·3 (6·77–21·2)	--
Low back pain	--	44·8 (40·7–49·1)	44·8 (40·7–49·1)	--
Gout	--	43·3 (28·6–60·5)	43·3 (28·6–60·5)	--
Other non-communicable diseases	0·0498 (0·0191–0·0904)	0·0273 (0·0111–0·0506)	--	0·0653 (0·0239–0·12)
Sudden infant death syndrome	8·81 (4·25–15·3)	8·8 (4·25–15·3)	--	8·8 (4·25–15·3)
Injuries	8·15 (2·76–13·1)	8·8 (4·17–13·1)	17·9 (15·3–20·7)	6·51 (0·969–11·5)
Transport injuries	7·27 (-1·2–15·6)	8·03 (0·437–15·6)	21·5 (18–25·2)	5·52 (-3·14–14·3)
Road injuries	6·54 (-2·19–15·1)	7·39 (-0·532–15·4)	21·3 (17·9–25·1)	4·8 (-4·32–13·7)
Other transport injuries	23·9 (19·4–28·3)	21·7 (17·3–26)	24·1 (20·5–27·8)	21·2 (16·6–25·7)

Unintentional injuries	6·84 (2·05–12·2)	8·17 (3·77–12·8)	16·7 (13·7–20·1)	4·33 (-0·44–9·65)
Falls	22·9 (18·6–27·6)	20·9 (16·8–25·4)	23·4 (19·3–27·8)	18·4 (14–23·1)
Drowning	-18·9 (-35·9–-3·21)	-20·3 (-36·7–-4·56)	8·29 (5·49–12·1)	-20·6 (-37·3–-4·76)
Fire, heat, and hot substances	10·4 (6·31–17·1)	9·54 (5·86–15·5)	10·8 (7·19–15·2)	9·03 (5·01–16·2)
Poisonings	12·5 (8·21–17·7)	12·3 (8·13–17·2)	9·42 (6·27–13·3)	12·6 (8·31–17·7)
Exposure to mechanical forces	-10·7 (-22·9–3·1)	2·16 (-5·55–9·47)	13 (11·2–15·1)	-12·8 (-25·3–0·832)
Animal contact	7·6 (4·96–15·6)	8·73 (5·89–13·3)	14 (10·4–18)	4·58 (2·7–12·1)
Foreign body	3·29 (2·31–4·63)	2·56 (1·79–3·65)	3·2 (2·26–4·48)	2·49 (1·71–3·58)
Environmental heat and cold exposure	9·82 (4·6–17)	9·74 (4·56–16·8)	8·29 (3·85–14·1)	9·83 (4·59–17)
Exposure to forces of nature	5·64 (2·68–9·64)	5·28 (2·51–9·05)	7·29 (3·49–12·4)	4·99 (2·37–8·54)
Other unintentional injuries	14 (9·2–19·5)	11·9 (8·11–16·7)	10·8 (7·25–15·3)	12·9 (8·3–18·1)
Self-harm and interpersonal violence	10·5 (-1·67–21·4)	10·5 (-1·18–21·1)	20·8 (15·8–26·2)	9·98 (-2·23–20·9)
Self-harm	10·8 (-5·18–25·8)	10·6 (-5·11–25·3)	17·7 (8·94–26·4)	10·4 (-5·47–25·2)
Interpersonal violence	10 (-2·59–23)	10·6 (-1·02–22·5)	23·7 (17·4–30·1)	9·19 (-3·47–22·3)

Supplementary Results Table 2. All-age PAFs attributable to the joint distribution of all risk factors for all ages, both sexes combined for each GBD level 1, level 2, and level 3 cause and all causes in 2019 for Wyoming. Results are shown for deaths, YLLs, YLDs, and DALYs Data in parenthesis are 95% uncertainty intervals. PAFs=population attributable fractions. GBD=Global Burden of Disease. YLLs=years of life lost. YLDs=years lived with disability. DALYs=disability-adjusted life-years	Deaths 2019	DALYs 2019	YLDs 2019	YLLs 2019
All causes	58·4 (55·8–60·9)	45·3 (42·5–48·1)	32·8 (30·2–35·4)	56·7 (54·6–58·8)
Communicable, maternal, neonatal, and nutritional diseases	44 (36·3–51)	50·7 (46·5–54·7)	42·1 (37·9–46·3)	56·4 (50·2–62)
HIV/AIDS and sexually transmitted infections	81·1 (69·5–86·4)	83·1 (72·5–88·8)	90·1 (82·2–95·1)	76·7 (63·6–83·4)
HIV/AIDS	78·1 (64·6–84·2)	75·7 (61·9–82·6)	77·4 (63·5–84·3)	75 (60·8–82·1)
Sexually transmitted infections excluding HIV	99·2 (98·8–99·5)	99·5 (99·2–99·8)	100 (100–100)	96·8 (95·4–97·9)
Respiratory infections and tuberculosis	40·9 (30·3–50·4)	35·2 (27·7–43·1)	2·21 (1·67–2·9)	45·7 (35·8–54·7)
Tuberculosis	52·9 (43·2–61·9)	55·9 (46·8–64·3)	54·1 (43·6–63)	56·4 (47·3–64·8)
Lower respiratory infections	40·9 (30·2–50·5)	45·7 (35·7–54·5)	31·9 (26·1–37·2)	45·8 (35·8–54·9)
Upper respiratory infections	0·547 (0·334–0·887)	0·0195 (0·0093–0·0356)	0·00453 (0–0·0147)	1·22 (0·76–1·99)
Otitis media	1·72 (0·699–3·74)	4·16 (2·72–5·64)	4·15 (2·71–5·65)	4·44 (2·04–8·37)
Enteric infections	10·8 (6·67–16·7)	12 (7·87–17·9)	11·7 (7·57–17·6)	13 (8·92–19)
Diarrheal diseases	10·9 (6·73–16·9)	12·1 (7·9–18)	11·7 (7·58–17·6)	13·2 (9·07–19·3)
Other infectious diseases	0·555 (0·381–0·825)	1 (0·744–1·33)	0·528 (0·32–0·889)	1·11 (0·79–1·51)
Meningitis	1·23 (0·857–1·73)	2·66 (1·89–3·66)	0·000441 (0–0·0015)	2·81 (1·99–3·86)
Encephalitis	0·553 (0·373–0·785)	1·45 (1–2·05)	0·000271 (0–0·000912)	1·49 (1·03–2·09)
Measles	9·44 (-4·09–43·7)	9·6 (-4·16–44·4)	0·185 (0–0·769)	9·6 (-4·16–44·4)
Acute hepatitis	14·7 (8·62–28·3)	6·63 (4·1–11·4)	2·77 (1·68–4·6)	15·3 (8·91–29·3)
Maternal and neonatal disorders	80·4 (76·9–83·6)	74·6 (70·6–78·2)	60·9 (55·3–66·4)	82·8 (80–85·5)
Maternal disorders	11·4 (4–19·1)	11·2 (3·94–19)	11·1 (3·98–18·6)	11·3 (3·96–19)
Neonatal disorders	87·4 (85·2–89·4)	77·8 (73·9–81·2)	62·4 (56·4–67·9)	87·4 (85·2–89·5)
Nutritional deficiencies	91·1 (80·6–93·4)	89·1 (86·1–91·6)	88·5 (85–91·6)	90·9 (80·5–92·9)
Protein-energy malnutrition	100 (100–100)	100 (100–100)	100 (100–100)	100 (100–100)
Vitamin A deficiency	--	100 (100–100)	100 (100–100)	--
Dietary iron deficiency	--	100 (100–100)	100 (100–100)	--
Non-communicable diseases	62·4 (59·4–65)	48·1 (44·9–51·4)	32·6 (29·8–35·4)	63·6 (61·4–65·8)
Neoplasms	47·5 (44–51·3)	46·3 (42·9–49·9)	28·8 (25·2–32·7)	47·5 (44·1–51·1)
Lip and oral cavity cancer	67·1 (58·4–75)	69·3 (61·1–76·8)	68·7 (60·2–76·5)	69·3 (61·1–76·8)
Nasopharynx cancer	63·7 (51·7–73·1)	63·1 (51·2–72·5)	62·3 (50·3–71·8)	63·2 (51·3–72·5)
Other pharynx cancer	75·9 (68·2–81·3)	76·9 (69·6–82·2)	77·2 (70·2–82·6)	76·9 (69·5–82·2)

Esophageal cancer	79·4 (70–87·4)	80·4 (71·2–88·3)	79·6 (70·1–87·5)	80·4 (71·2–88·3)
Stomach cancer	25 (16·9–43·1)	25·3 (17·3–43·6)	25·7 (17·5–43·5)	25·3 (17·2–43·6)
Colon and rectum cancer	60·6 (52·1–69·3)	61·4 (53·5–69·5)	61·4 (52·9–69·5)	61·5 (53·5–69·4)
Liver cancer	77·5 (71·2–82·9)	77·7 (71·9–82·6)	77·6 (71·5–82·9)	77·7 (71·9–82·6)
Gallbladder and biliary tract cancer	26·3 (15–39·3)	27·5 (15·9–40·4)	25·3 (14·3–37·6)	27·5 (15·9–40·5)
Pancreatic cancer	42·4 (33–52·7)	42·1 (33·4–51·9)	42·4 (33·5–52·5)	42·1 (33·4–51·9)
Larynx cancer	82·2 (72·5–89·3)	82·8 (74·5–89)	82·6 (73·9–88·9)	82·8 (74·6–89)
Tracheal, bronchus, and lung cancer	83·5 (80·4–86·9)	82·5 (79·5–85·9)	83·1 (80·1–86·4)	82·5 (79·5–85·9)
Breast cancer	34·3 (25·7–44·1)	33·4 (25·4–42)	34 (25·6–43·2)	33·3 (25·4–41·9)
Cervical cancer	100 (100–100)	100 (100–100)	100 (100–100)	100 (100–100)
Uterine cancer	53·8 (38·4–67·7)	55·5 (39·3–69·1)	55·9 (40–69·4)	55·4 (39·2–69)
Ovarian cancer	19·1 (8·8–31·5)	16·8 (7·51–28·2)	16·2 (7·06–27·4)	16·9 (7·52–28·3)
Prostate cancer	5·96 (2·51–10)	6·52 (2·78–10·7)	6·8 (2·93–11·2)	6·44 (2·76–10·6)
Kidney cancer	42·5 (31·3–53·2)	43 (32·6–53)	43·2 (32·9–53·1)	43 (32·7–53)
Bladder cancer	39·8 (24·6–54·9)	42·9 (28–56·5)	43·5 (29·6–56·3)	42·9 (28–56·4)
Thyroid cancer	18·2 (9·53–27·9)	19·3 (10·4–29·3)	19·6 (10·6–29·6)	19·2 (10·2–29·4)
Mesothelioma	97·4 (96·5–98·1)	94·9 (93·4–96·2)	97 (95·7–97·9)	94·8 (93·4–96·2)
Non-Hodgkin lymphoma	8·88 (3·92–15)	9·36 (4·26–15·7)	9·15 (4·14–15·3)	9·38 (4·28–15·7)
Multiple myeloma	10·1 (4·7–17·2)	10·7 (5·05–17·9)	10·6 (4·79–18)	10·7 (5·07–17·9)
Leukemia	36·7 (22·8–50·7)	34·5 (22·9–46·3)	37·2 (23·3–50·8)	34·4 (22·9–46·1)
Cardiovascular diseases	79·5 (75·9–82·9)	80 (77–82·9)	70·4 (65·8–74·9)	81·6 (78·7–84·3)
Rheumatic heart disease	21·7 (12·8–39·9)	21·6 (13·6–35·9)	21·5 (13·2–36·2)	21·6 (13·8–35·8)
Ischemic heart disease	92·2 (88·3–95·3)	93·7 (91–96·2)	92·2 (88·6–95·3)	93·7 (91·1–96·2)
Stroke	78·4 (71·6–86·3)	82 (77·3–87·3)	80·8 (75·4–86·5)	82·5 (77·7–87·7)
Hypertensive heart disease	100 (100–100)	100 (100–100)	100 (100–100)	100 (100–100)
Non-rheumatic valvular heart disease	25 (15·8–37·8)	23·8 (16·3–33·1)	8·5 (5·41–12·2)	25·6 (17·6–35·5)
Cardiomyopathy and myocarditis	38·3 (31·5–45·7)	40·6 (34·8–45·9)	38·6 (32·6–44·7)	40·8 (35–46·3)
Atrial fibrillation and flutter	52 (42–62·3)	57·8 (49·3–66·5)	59·3 (50·7–67·7)	56 (47·2–64·8)
Aortic aneurysm	55·6 (49·7–61·5)	63·2 (58·2–67·7)	--	63·2 (58·2–67·7)
Endocarditis	30·9 (21·1–43·2)	32·4 (23·4–42·9)	30·5 (20·6–42·5)	32·5 (23·5–43)
Other cardiovascular and circulatory diseases	20·9 (15·5–30)	23·8 (18·7–31·5)	30·5 (23·4–38·7)	21·7 (16·7–30·1)
Chronic respiratory diseases	68·4 (64–72·9)	62·9 (59·3–66·4)	55·3 (51·2–59·5)	68·4 (64·5–72·3)

Chronic obstructive pulmonary disease	74·8 (70·3–79·4)	72·3 (68·5–76)	64·6 (60·6–68·3)	76·3 (72·3–80·5)
Pneumoconiosis	100 (100–100)	100 (100–100)	100 (100–100)	100 (100–100)
Asthma	51·1 (40·5–62·3)	43·8 (35·6–52·6)	42·9 (34·9–51·6)	49·9 (40·7–59·7)
Digestive diseases	44·6 (38·7–50·2)	48·2 (42·4–53·6)	18·8 (12·9–25·5)	55·4 (49·2–61·2)
Cirrhosis and other chronic liver diseases	79·8 (70·4–88·4)	82·4 (74·5–89·6)	78 (68·6–86·3)	82·5 (74·5–89·7)
Upper digestive system diseases	21·6 (17·7–25·4)	7·34 (4·66–10·7)	1·69 (1–2·63)	24·3 (20·5–28·4)
Gallbladder and biliary diseases	50·3 (35–65)	55·4 (41·1–68·5)	56·9 (42·7–69·5)	52·8 (37·5–66·7)
Pancreatitis	35·8 (18·3–53·3)	40·1 (22·5–57·5)	37·5 (20·1–55·2)	40·4 (22·6–57·8)
Neurological disorders	24·6 (9·84–39·6)	14·9 (7·42–26)	10·6 (5·41–20·3)	20·5 (8·24–35·8)
Alzheimer's disease and other dementias	40·8 (27·2–55·3)	44·2 (30·2–58·6)	45·1 (31·3–59·3)	43·6 (29·8–57·8)
Parkinson's disease	-10·1 (-15·8–-5·11)	-11·7 (-17·8–-6·04)	-12·3 (-18·5–-6·47)	-11·5 (-17·8–-5·97)
Idiopathic epilepsy	23·7 (14·7–31·8)	21·5 (13·7–29)	20·1 (12·6–27·1)	24·3 (15·3–32·5)
Multiple sclerosis	15·5 (11·2–20·4)	16·6 (12·1–21·4)	16·4 (12·1–21·1)	16·8 (12·1–21·9)
Mental disorders	--	9·37 (4·69–14·7)	9·37 (4·69–14·7)	--
Depressive disorders	--	19 (8·92–28·7)	19 (8·92–28·7)	--
Anxiety disorders	--	6·24 (1·44–14·3)	6·24 (1·44–14·3)	--
Idiopathic developmental intellectual disability	--	46·8 (21·8–67·3)	46·8 (21·8–67·3)	--
Substance use disorders	100 (100–100)	100 (100–100)	100 (100–100)	100 (100–100)
Alcohol use disorders	100 (100–100)	100 (100–100)	100 (100–100)	100 (100–100)
Drug use disorders	100 (100–100)	100 (100–100)	100 (100–100)	100 (100–100)
Diabetes and kidney diseases	99·9 (99·9–99·9)	100 (100–100)	100 (100–100)	99·9 (99·9–99·9)
Diabetes mellitus	100 (100–100)	100 (100–100)	100 (100–100)	100 (100–100)
Chronic kidney disease	100 (100–100)	100 (100–100)	100 (100–100)	100 (100–100)
Sense organ diseases	--	11·8 (10·6–13·1)	11·8 (10·6–13·1)	--
Blindness and vision loss	--	9·28 (6·1–13·4)	9·28 (6·1–13·4)	--
Age-related and other hearing loss	--	12·9 (11·7–14·2)	12·9 (11·7–14·2)	--
Musculoskeletal disorders	4·8 (1·44–8·58)	22·9 (19·3–26·8)	23·3 (19·6–27·3)	4·53 (1·38–8·14)
Rheumatoid arthritis	13·2 (4·01–23·5)	15·9 (5–28·3)	16·2 (5·17–28·6)	15·1 (4·74–27·1)
Osteoarthritis	--	12·9 (7·33–19·1)	12·9 (7·33–19·1)	--
Low back pain	--	43·8 (38–49·8)	43·8 (38–49·8)	--
Gout	--	60 (43·3–76·8)	60 (43·3–76·8)	--
Other non-communicable diseases	0·164 (0·112–0·239)	0·195 (0·129–0·279)	--	0·505 (0·354–0·714)

Sudden infant death syndrome	6·91<br>(5·01–9·44)	6·9<br>(5–9·43)	--	6·9<br>(5–9·43)
Injuries	23·1<br>(17·3–28·5)	22·1<br>(17·1–26·7)	31·2<br>(27·8–34·5)	17·6<br>(10·8–24)
Transport injuries	17<br>(9·68–24·2)	18<br>(11·2–24·9)	29·6<br>(25·8–33·3)	14·5<br>(6·41–22·7)
Road injuries	15·1<br>(7·36–22·8)	15·9<br>(8·6–23·3)	27·2<br>(23·4–31·1)	12·7<br>(4·31–21·3)
Other transport injuries	33·2<br>(28·3–38·3)	33·2<br>(28·5–37·8)	39·3<br>(35·4–42·6)	29·6<br>(24·3–35·5)
Unintentional injuries	32<br>(27·4–35·6)	26·1<br>(22·2–29·9)	31·5<br>(27·7–35·5)	18·5<br>(14·2–22·7)
Falls	54·3<br>(47·9–58·6)	43·7<br>(38·7–47·8)	43·1<br>(38·1–47·3)	45·1<br>(39·9–49·4)
Drowning	-14·6<br>(-29–-0·963)	-15·1<br>(-29·3–-2·04)	10·5<br>(6·5–15·7)	-15·8<br>(-30·3–-2·45)
Fire, heat, and hot substances	11·2<br>(7·24–16·5)	13·6<br>(9·12–19·1)	14·5<br>(9·67–20·3)	12·1<br>(8·23–17·1)
Poisonings	16·7<br>(11·6–23)	16·3<br>(11·4–22·3)	13<br>(8·74–18·2)	17·4<br>(12·3–23·9)
Exposure to mechanical forces	3·28<br>(-6·61–13·4)	14·6<br>(9·16–19·7)	21·9<br>(19·1–25·2)	-0·391<br>(-11·1–10·6)
Animal contact	25·1<br>(20·6–30·2)	23·6<br>(19·1–28·7)	24·6<br>(19·9–29·5)	21·4<br>(17·1–26·4)
Foreign body	2·92<br>(1·97–4·27)	4·09<br>(2·81–5·88)	3·84<br>(2·61–5·49)	4·16<br>(2·85–6·07)
Environmental heat and cold exposure	7·77<br>(3·4–13·6)	8·23<br>(3·57–14·2)	7·69<br>(3·38–13·4)	8·53<br>(3·68–14·8)
Exposure to forces of nature	7·16<br>(3·15–12·4)	7·96<br>(3·41–13·7)	8·45<br>(3·63–14·5)	6·78<br>(2·99–11·7)
Other unintentional injuries	17·5<br>(12·2–24)	14·7<br>(9·98–20·8)	13·9<br>(9·23–19·8)	19·4<br>(13·6–26·1)
Self-harm and interpersonal violence	18<br>(4·14–30·6)	20·7<br>(7·75–32·5)	33·6<br>(27·1–40)	19·8<br>(5·92–32·2)
Self-harm	17·1<br>(1·47–31·2)	19·6<br>(3·9–34)	24·1<br>(13·3–35·1)	19·5<br>(3·7–34)
Interpersonal violence	25·8<br>(15·1–36·6)	26·6<br>(17·5–36·1)	36·9<br>(29·6–44·2)	22·6<br>(11·9–33·6)

Supplementary Results Table 2. All-age PAFs attributable to the joint distribution of all risk factors for all ages, both sexes combined for each GBD level 1, level 2, and level 3 cause and all causes in 2019 for Southern Latin America. Results are shown for deaths, YLLs, YLDs, and DALYs Data in parenthesis are 95% uncertainty intervals. PAFs=population attributable fractions. GBD=Global Burden of Disease. YLLs=years of life lost. YLDs=years lived with disability. DALYs=disability-adjusted life-years

	Deaths 2019	DALYs 2019	YLDs 2019	YLLs 2019
All causes	56·3 (54–58·8)	41·8 (39·2–44·4)	25·3 (23·4–27·3)	53·6 (51·9–55·5)
Communicable, maternal, neonatal, and nutritional diseases	50·9 (45·5–56)	58·6 (55·4–61·7)	51·2 (47·2–55·1)	61 (57·1–64·5)
HIV/AIDS and sexually transmitted infections	90·9 (89·6–92·3)	90 (88·7–91·5)	93 (91–95·5)	89·1 (87·7–90·6)
HIV/AIDS	91·2 (90–92·6)	90·2 (88·9–91·5)	90·9 (89·2–92·7)	90 (88·7–91·5)
Sexually transmitted infections excluding HIV	76·9 (70–83)	87·3 (79·9–93·5)	100 (100–100)	56·8 (47·8–65·7)
Respiratory infections and tuberculosis	46·6 (39·8–52·6)	47·2 (41·3–52·6)	5·53 (4·07–7·42)	52 (45·5–57·5)
Tuberculosis	56·9 (48·5–64)	57 (48·7–63·9)	51·7 (42–59·8)	57·6 (49·3–64·4)
Lower respiratory infections	46·4 (39·3–52·5)	51·6 (44·9–57·4)	38·1 (32–43·8)	51·7 (45–57·5)
Upper respiratory infections	0·226 (0·122–0·383)	0·0109 (0·00462–0·0201)	0·00843 (0·00248–0·0171)	0·501 (0·279–0·827)
Otitis media	1·11 (0·404–2·81)	5·97 (3·94–8·11)	5·98 (3·94–8·14)	3·64 (1·4–8·89)
Enteric infections	47·5 (32·4–63·5)	51 (36·6–66·6)	46·7 (31·1–63·8)	59·1 (46·6–71·9)
Diarrheal diseases	47·7 (32·6–63·9)	51·2 (36·7–66·9)	46·8 (31·1–63·9)	59·7 (47–72·5)
Other infectious diseases	0·951 (0·738–1·22)	1·27 (1–1·62)	0·264 (0·168–0·423)	1·53 (1·21–1·93)
Meningitis	1·13 (0·828–1·51)	2·21 (1·64–2·91)	0·000929 (0·000261–0·00184)	2·39 (1·8–3·13)
Encephalitis	0·396 (0·286–0·539)	0·72 (0·528–0·976)	0·000176 (4·56e-05–0·000396)	0·794 (0·584–1·07)
Measles	5·34 (0·725–18·6)	6·78 (1·04–22·5)	3·51 (0·13–7·58)	6·95 (0·952–23·5)
Acute hepatitis	12·4 (7·58–18)	5·33 (3·46–7·63)	1·54 (0·993–2·42)	11·6 (7·05–17)
Maternal and neonatal disorders	77·1 (74·4–79·5)	74·3 (71·4–76·8)	57·1 (53–61·1)	79·4 (76·9–81·5)
Maternal disorders	11·7 (4·31–19·2)	11·7 (4·29–19·2)	11·6 (4·23–18·9)	11·7 (4·3–19·2)
Neonatal disorders	84·3 (82·2–86·3)	78·6 (76–81)	59·9 (55·5–64)	84·3 (82·2–86·3)
Nutritional deficiencies	93·6 (90·2–96·2)	94·7 (93·3–95·9)	95 (93·3–96·3)	93·9 (90·4–96·2)
Protein-energy malnutrition	100 (100–100)	100 (100–100)	100 (100–100)	100 (100–100)
Vitamin A deficiency	--	100 (100–100)	100 (100–100)	--
Dietary iron deficiency	--	100 (100–100)	100 (100–100)	--
Non-communicable diseases	59·6 (56·7–62·6)	42·1 (39–45·3)	22·8 (20·7–25·1)	57·9 (55·7–60·3)
Neoplasms	42·3 (38·7–46·9)	42·4 (39·1–46·4)	35·6 (32·1–39·6)	42·6 (39·3–46·6)
Lip and oral cavity cancer	67·3 (61·2–72·9)	70·4 (64·5–76)	69·8 (63·7–75·3)	70·4 (64·5–76)
Nasopharynx cancer	63·7 (55·2–71·3)	64·1 (55·8–71·5)	61·7 (53·1–69·3)	64·2 (55·8–71·5)
Other pharynx cancer	78·4 (72·4–83·2)	80·1 (74·3–84·7)	78·9 (73–83·6)	80·1 (74·4–84·7)
Esophageal cancer	69·1 (59·2–78·5)	72 (62·6–80·6)	70·4 (60·5–79·3)	72 (62·7–80·6)

Stomach cancer	22·7 (13·7–42·4)	23·5 (14·4–43·3)	23·3 (14·1–42·8)	23·5 (14·4–43·3)
Colon and rectum cancer	62·9 (55·4–70)	63·4 (56·3–70)	63·6 (56·5–70·5)	63·4 (56·3–70)
Liver cancer	69·6 (63–76·3)	70 (63·3–76·5)	70 (63·4–76·6)	70 (63·3–76·5)
Gallbladder and biliary tract cancer	24 (13·4–36·4)	24·7 (13·9–37·3)	24 (13·4–36·2)	24·7 (13·9–37·3)
Pancreatic cancer	36·2 (28·5–46)	36·7 (29·4–46)	36·6 (29·3–46·2)	36·7 (29·4–46)
Larynx cancer	75·6 (67·5–82·1)	77·9 (70·5–84)	76·4 (68·8–82·5)	78 (70·6–84)
Tracheal, bronchus, and lung cancer	78·4 (75·1–81·8)	78·2 (75·1–81·4)	78·4 (75·2–81·8)	78·2 (75·1–81·4)
Breast cancer	34 (26·3–42·8)	32·6 (25·8–40·3)	33 (25·9–41)	32·6 (25·7–40·3)
Cervical cancer	100 (100–100)	100 (100–100)	100 (100–100)	100 (100–100)
Uterine cancer	44·8 (30–60·8)	45·8 (30·8–61·8)	46·6 (31·7–62·2)	45·8 (30·8–61·8)
Ovarian cancer	15 (6·38–26·3)	12·8 (5·29–22·4)	12·3 (5·03–21·6)	12·8 (5·29–22·5)
Prostate cancer	5·11 (2·06–8·19)	6·06 (2·62–9·49)	6·59 (2·92–10·3)	6·02 (2·6–9·41)
Kidney cancer	36·1 (26·5–46)	36 (26–45·8)	35·5 (25·7–45·3)	36 (26·1–45·8)
Bladder cancer	34 (23·1–44·8)	38·5 (27·6–49)	39·2 (28·2–49·8)	38·4 (27·6–48·9)
Thyroid cancer	14·1 (7·04–22·8)	14·5 (7·19–23·4)	14·3 (7·3–22·6)	14·5 (7·17–23·5)
Mesothelioma	91·9 (88·8–94·3)	86·4 (81·6–90·2)	91·3 (88·1–93·8)	86·3 (81·5–90·1)
Non-Hodgkin lymphoma	6·8 (2·79–12·1)	6·65 (2·8–11·7)	6·72 (2·82–12)	6·65 (2·8–11·7)
Multiple myeloma	8·19 (3·52–14·5)	8·42 (3·7–14·8)	8·46 (3·7–14·9)	8·42 (3·71–14·8)
Leukemia	30·3 (20·3–40·4)	24·5 (15·7–33)	28·8 (19–38·7)	24·3 (15·6–32·9)
Cardiovascular diseases	80·4 (77·5–83·1)	81 (78·5–83·3)	71 (66·6–74·8)	81·9 (79·5–84·1)
Rheumatic heart disease	23·5 (13·7–44·1)	22·6 (15·2–34·2)	19·8 (11·6–30·8)	24·2 (16·1–38·8)
Ischemic heart disease	93·5 (90·3–96·2)	94·7 (92·6–96·6)	94 (91·4–96·4)	94·8 (92·6–96·6)
Stroke	83·8 (78·7–89·1)	86·6 (83·1–90·1)	85·1 (81·2–89)	86·8 (83·4–90·3)
Hypertensive heart disease	100 (100–100)	100 (100–100)	100 (100–100)	100 (100–100)
Non-rheumatic valvular heart disease	30·9 (21–44·3)	31·7 (23·4–41·9)	22·7 (15·9–30·9)	32·1 (23·7–42·5)
Cardiomyopathy and myocarditis	32·7 (24·9–41·6)	34·3 (28–41·4)	34·7 (27·7–42·6)	34·3 (28–41·4)
Atrial fibrillation and flutter	52·9 (43·8–62·8)	58·8 (51·2–66·8)	62·5 (55·2–69·9)	56·3 (48·4–65)
Aortic aneurysm	58·9 (53·3–64·2)	65·9 (61·6–70)	--	65·9 (61·6–70)
Endocarditis	35·6 (25·9–47·5)	36·6 (27·8–47·3)	35·5 (25·5–46·8)	36·7 (27·8–47·3)
Other cardiovascular and circulatory diseases	34 (28–40·5)	35·1 (29·9–40·4)	35·1 (29·3–41·3)	35·1 (29·8–40·4)
Chronic respiratory diseases	58·7 (55–62·6)	53·4 (49·7–57·3)	43·9 (38·3–49·9)	59·5 (56·2–63·2)
Chronic obstructive pulmonary disease	69·7 (66–73·5)	70·8 (67·5–73·9)	62·7 (59·1–66·3)	73·1 (69·7–76·3)
Pneumoconiosis	100 (100–100)	100 (100–100)	100 (100–100)	100 (100–100)
Asthma	42·8 (31·8–54·9)	36·9 (28·7–45·9)	35·9 (27·8–45·1)	45·4 (35·3–55·9)

Digestive diseases	47·2 (42·4–52)	46·5 (41–51·3)	7·95 (5·06–11)	55·3 (50·4–59·9)
Cirrhosis and other chronic liver diseases	79·6 (71·5–86·8)	81·9 (74·3–88·6)	76·8 (70·3–83·1)	82 (74·3–88·6)
Upper digestive system diseases	19·7 (16·6–22·6)	5·92 (3·49–8·83)	0·403 (0·225–0·655)	23·8 (20·6–26·8)
Gallbladder and biliary diseases	43·1 (28·9–58·1)	45·7 (31·8–60·2)	48·8 (34·9–63)	44·7 (30·8–59·2)
Pancreatitis	42 (31·4–53·7)	44·2 (33·4–56·3)	41·1 (30·6–52·8)	44·3 (33·5–56·5)
Neurological disorders	21·5 (9·32–35·3)	13·3 (6·71–23·3)	8·96 (4·64–16·7)	19·7 (9·1–32·6)
Alzheimer's disease and other dementias	32·8 (20·2–48·1)	36·1 (23–51)	36·6 (23·7–51·1)	35·7 (22·7–50·5)
Parkinson's disease	-6·59 (-10·4–-3·09)	-8·47 (-13·2–-4·04)	-9·81 (-15·3–-4·76)	-8·24 (-12·8–-3·91)
Idiopathic epilepsy	25·9 (19·2–32·5)	21·8 (16·2–27·6)	20·3 (14·9–26)	24·7 (18·2–30·9)
Multiple sclerosis	15·1 (11·3–18·9)	16·3 (12·5–20·1)	15·9 (12·1–19·6)	16·6 (12·5–20·5)
Mental disorders	--	6·92 (3·75–11)	6·92 (3·75–11)	--
Depressive disorders	--	14·7 (7·21–22·7)	14·7 (7·21–22·7)	--
Anxiety disorders	--	7·24 (2·06–15·6)	7·24 (2·06–15·6)	--
Idiopathic developmental intellectual disability	--	57·6 (29·6–77·6)	57·6 (29·6–77·6)	--
Substance use disorders	100 (100–100)	100 (100–100)	100 (100–100)	100 (100–100)
Alcohol use disorders	100 (100–100)	100 (100–100)	100 (100–100)	100 (100–100)
Drug use disorders	100 (100–100)	100 (100–100)	100 (100–100)	100 (100–100)
Diabetes and kidney diseases	100 (100–100)	100 (100–100)	100 (100–100)	100 (100–100)
Diabetes mellitus	100 (100–100)	100 (100–100)	100 (100–100)	100 (100–100)
Chronic kidney disease	100 (100–100)	100 (100–100)	100 (100–100)	100 (100–100)
Sense organ diseases	--	11·7 (10·5–13·1)	11·7 (10·5–13·1)	--
Blindness and vision loss	--	8·41 (5·6–11·8)	8·41 (5·6–11·8)	--
Age-related and other hearing loss	--	14·4 (13·1–15·8)	14·4 (13·1–15·8)	--
Musculoskeletal disorders	3·57 (0·949–6·5)	17·7 (14·6–21·3)	18 (14·7–21·6)	3·55 (0·971–6·3)
Rheumatoid arthritis	9·5 (2·56–17·6)	13·7 (3·62–24·4)	14·2 (3·65–25·2)	12·4 (3·42–22)
Osteoarthritis	--	15·8 (8·26–24·5)	15·8 (8·26–24·5)	--
Low back pain	--	41·6 (36·9–46·2)	41·6 (36·9–46·2)	--
Gout	--	49·5 (34·4–66·2)	49·5 (34·4–66·2)	--
Other non-communicable diseases	0·129 (0·0918–0·183)	0·12 (0·0776–0·176)	--	0·316 (0·232–0·445)
Sudden infant death syndrome	7·24 (5·25–9·48)	7·23 (5·24–9·47)	--	7·23 (5·24–9·47)
Injuries	25 (20·9–29·4)	25·6 (21·1–29·9)	31·7 (27·6–36·3)	22·5 (17·7–27·4)
Transport injuries	31·9 (25·5–38·6)	31·6 (25–38·3)	36·2 (31·1–41·6)	30·6 (23·6–37·6)
Road injuries	31·1 (24·6–38)	31 (24·3–37·7)	35·9 (30·8–41·3)	29·9 (22·8–37·1)
Other transport injuries	43 (37·3–48·7)	40·3 (34·2–46·2)	41·6 (36·9–46·3)	40·1 (33·8–46·4)

Unintentional injuries	25·5 (21·3–29·8)	26·5 (21·3–31·8)	31·7 (27–36·9)	19·4 (14·4–24·7)
Falls	54·5 (47·9–59·1)	41·8 (36·6–46·8)	40·5 (35·4–45·3)	45·4 (39·8–50·6)
Drowning	0·419 (-8·74–10·4)	-1·49 (-10–7·83)	17·1 (12·8–22·2)	-1·92 (-10·6–7·47)
Fire, heat, and hot substances	17·5 (13·1–22·7)	22·5 (17–28·2)	24 (18·5–30·2)	20·5 (15·6–25·9)
Poisonings	21·1 (16–27)	20·6 (15·8–26·2)	20·3 (15·8–25·7)	20·8 (15·9–26·5)
Exposure to mechanical forces	18·9 (11·4–26·8)	27·9 (22·4–34·1)	30·5 (25·4–36·6)	18·1 (10·2–26·3)
Animal contact	26·1 (21·3–31·4)	26·4 (21·2–32·1)	27·9 (22·8–33·6)	22·2 (17·5–27·6)
Foreign body	12 (8·65–16·2)	12·8 (9·02–17·5)	13·6 (10–18)	12·6 (8·74–17·6)
Environmental heat and cold exposure	10·2 (4·95–17·3)	9·47 (4·47–16·1)	8·83 (4·15–15)	10·3 (4·93–17·3)
Exposure to forces of nature	8·06 (3·83–13·6)	8·64 (4·11–14·5)	9·43 (4·5–15·8)	7·37 (3·5–12·5)
Other unintentional injuries	29·8 (23·1–37·1)	26 (20·3–32·6)	23·8 (18·3–30·1)	29·5 (22·8–36·6)
Self-harm and interpersonal violence	18·7 (9·47–27·8)	19·1 (10·7–27·7)	25·8 (19·7–32)	18·3 (9·48–27·2)
Self-harm	19 (5·7–32·1)	19·1 (5·89–32·1)	21·8 (11·2–32·2)	19·1 (5·67–32)
Interpersonal violence	18·3 (9·44–27)	19·5 (11·2–28)	29·2 (21·7–36·7)	17·1 (8·33–25·8)

Supplementary Results Table 2. All-age PAFs attributable to the joint distribution of all risk factors for all ages, both sexes combined for each GBD level 1, level 2, and level 3 cause and all causes in 2019 for Argentina. Results are shown for deaths, YLLs, YLDs, and DALYs Data in parenthesis are 95% uncertainty intervals. PAFs=population attributable fractions. GBD=Global Burden of Disease. YLLs=years of life lost. YLDs=years lived with disability. DALYs=disability-adjusted life-years

	Deaths 2019	DALYs 2019	YLDs 2019	YLLs 2019
All causes	56·8 (54·5–59·1)	42·6 (40–45·2)	25·1 (23·2–27)	54·2 (52·4–56)
Communicable, maternal, neonatal, and nutritional diseases	50·7 (45·1–55·8)	58·8 (55·5–62)	52·6 (48·4–56·8)	60·5 (56·3–64·1)
HIV/AIDS and sexually transmitted infections	89·9 (88·4–92)	88·9 (87·3–90·7)	92 (89·7–94·9)	87·9 (86·2–89·9)
HIV/AIDS	90·5 (88·9–92·5)	89·3 (87·6–91·2)	89·8 (87·9–92·1)	89·2 (87·5–91·2)
Sexually transmitted infections excluding HIV	71·7 (63·9–78·9)	83·2 (74·4–91·1)	100 (100–100)	51·4 (42·3–60·7)
Respiratory infections and tuberculosis	47 (40·1–52·9)	48·2 (42·2–53·5)	5·61 (4·11–7·55)	52·1 (45·5–57·6)
Tuberculosis	58·5 (50·2–65·7)	56·6 (48·1–63·5)	50·8 (40·7–59·8)	57·2 (48·8–64·1)
Lower respiratory infections	46·8 (39·7–52·7)	51·8 (45–57·5)	38·2 (32–43·9)	51·9 (45·1–57·6)
Upper respiratory infections	0·293 (0·146–0·523)	0·00997 (0·00229–0·0228)	0·00772 (0·000366–0·02)	0·552 (0·279–0·971)
Otitis media	1·95 (0·588–5·2)	5·99 (3·95–8·26)	5·99 (3·92–8·27)	5·94 (2·01–14·7)
Enteric infections	54·9 (38·3–71·1)	56·6 (40·8–72·3)	51 (33–68·7)	67·2 (54–79)
Diarrheal diseases	55·3 (38·5–71·6)	56·9 (40·9–72·7)	51·1 (33·1–68·8)	67·8 (54·5–79·7)
Other infectious diseases	1·06 (0·792–1·36)	1·33 (1·03–1·72)	0·226 (0·142–0·362)	1·62 (1·26–2·09)
Meningitis	1·09 (0·784–1·51)	2·13 (1·55–2·91)	0·00082 (3·1e-05–0·00208)	2·28 (1·67–3·09)
Encephalitis	0·445 (0·315–0·624)	0·809 (0·579–1·14)	0·000167 (6·03e-06–0·000448)	0·883 (0·634–1·24)
Measles	5·71 (0·84–20·2)	7·3 (1·34–24·4)	3·69 (0·126–8·31)	7·53 (1·22–25·1)
Acute hepatitis	11·4 (6·66–16·9)	5·42 (3·34–8)	1·43 (0·887–2·25)	10·6 (6·2–15·6)
Maternal and neonatal disorders	76·3 (73·5–79)	73·3 (70·3–76)	51·8 (47·6–56)	78·6 (76–81)
Maternal disorders	11·9 (4·34–19·6)	11·9 (4·33–19·5)	11·9 (4·35–19·6)	11·9 (4·33–19·5)
Neonatal disorders	83·8 (81·3–86)	77·9 (75·1–80·6)	54·5 (49·9–58·8)	83·8 (81·3–86)
Nutritional deficiencies	94·4 (92·4–97·3)	96·4 (95·3–97·3)	97 (95·8–97·8)	94·4 (92·1–97)
Protein-energy malnutrition	100 (100–100)	100 (100–100)	100 (100–100)	100 (100–100)
Vitamin A deficiency	--	100 (100–100)	100 (100–100)	--
Dietary iron deficiency	--	100 (100–100)	100 (100–100)	--
Non-communicable diseases	60·4 (57·5–63·2)	42·7 (39·6–45·9)	22·2 (20·1–24·4)	58·4 (56·2–60·7)
Neoplasms	44·1 (40·6–48·5)	43·8 (40·5–47·6)	37·6 (34–41·6)	44 (40·7–47·8)
Lip and oral cavity cancer	68·6 (62·4–74·4)	71·1 (64·9–76·6)	70·8 (64·2–76·1)	71·1 (64·9–76·6)
Nasopharynx cancer	64·4 (55·3–72·2)	64·4 (55·7–71·8)	62·6 (53·7–70·2)	64·5 (55·7–71·9)
Other pharynx cancer	79·9 (74–84·5)	80·9 (75–85·4)	80·2 (74·2–84·8)	80·9 (75–85·4)
Esophageal cancer	70·5 (61·3–79·1)	72·8 (64·3–80·9)	71·5 (62·2–79·9)	72·8 (64·3–80·9)

Stomach cancer	24·4 (15·4–43·6)	24·4 (15·3–43·5)	24·5 (15·5–43·6)	24·4 (15·3–43·5)
Colon and rectum cancer	62·7 (55·3–69·9)	63·1 (55·9–69·9)	63·2 (55·9–70·1)	63·1 (55·9–69·9)
Liver cancer	69·8 (63·4–76)	69·8 (63–75·9)	70 (63·5–76·2)	69·8 (63·1–75·9)
Gallbladder and biliary tract cancer	21·8 (12–33·7)	22·1 (12·1–33·8)	21·7 (12–33·6)	22·1 (12·1–33·8)
Pancreatic cancer	36·8 (29·4–46·6)	36·8 (29·6–46)	37 (29·8–46·7)	36·8 (29·6–46)
Larynx cancer	77·9 (70·1–84·1)	79·6 (72·2–85·3)	78·8 (71·4–84·5)	79·6 (72·2–85·4)
Tracheal, bronchus, and lung cancer	79·7 (76·5–82·9)	78·8 (75·7–81·9)	79·4 (76·2–82·6)	78·8 (75·7–81·9)
Breast cancer	34 (26·4–42·9)	32·5 (25·6–40·2)	32·9 (25·8–41)	32·5 (25·6–40·2)
Cervical cancer	100 (100–100)	100 (100–100)	100 (100–100)	100 (100–100)
Uterine cancer	43·6 (28·7–59·6)	44·5 (29·6–60·4)	44·7 (29·9–60·8)	44·5 (29·6–60·4)
Ovarian cancer	15·1 (6·57–26·2)	12·8 (5·32–22·2)	12·2 (5·09–21·4)	12·8 (5·32–22·2)
Prostate cancer	5·61 (2·34–8·87)	6·53 (2·84–10·2)	7·1 (3·08–11·1)	6·49 (2·81–10·1)
Kidney cancer	36·2 (26·8–45·8)	35·6 (25·9–45·3)	35 (25·3–44·5)	35·6 (26–45·3)
Bladder cancer	36·5 (25·6–47·6)	40·8 (29·7–51·1)	41·8 (30·5–52·2)	40·8 (29·6–51·1)
Thyroid cancer	13·5 (6·58–22·1)	13·6 (6·59–22·5)	13·2 (6·57–21·2)	13·7 (6·55–22·6)
Mesothelioma	92·5 (89·4–94·8)	87·1 (82·5–90·8)	91·8 (88·8–94·2)	87·1 (82·4–90·7)
Non-Hodgkin lymphoma	6·46 (2·64–11·6)	6·25 (2·57–11·1)	6·18 (2·53–11·1)	6·25 (2·57–11·1)
Multiple myeloma	7·66 (3·29–13·8)	7·8 (3·38–14)	7·8 (3·33–14)	7·8 (3·37–14)
Leukemia	30·7 (20·8–40·8)	24·1 (15·8–32·3)	28·9 (19·6–38·6)	24 (15·7–32·2)
Cardiovascular diseases	78·8 (75·6–82)	79·4 (76·7–81·9)	67 (62·1–71·4)	80·5 (77·9–82·9)
Rheumatic heart disease	22·8 (12·9–44·1)	22 (14·4–33·7)	19·5 (11–30·6)	23·6 (15·2–39·1)
Ischemic heart disease	92·9 (89·6–95·8)	94·3 (92·1–96·3)	93·3 (90·4–95·8)	94·3 (92·1–96·3)
Stroke	83 (78–88·2)	85·9 (82·2–89·5)	84 (79·8–88·2)	86·2 (82·5–89·6)
Hypertensive heart disease	100 (100–100)	100 (100–100)	100 (100–100)	100 (100–100)
Non-rheumatic valvular heart disease	30·5 (20·3–44·1)	31·6 (22·7–42·4)	20·1 (13·6–28)	32·1 (23–43)
Cardiomyopathy and myocarditis	30·8 (23·1–40·1)	32·2 (25·6–39·6)	31·2 (23·7–39·8)	32·3 (25·6–39·6)
Atrial fibrillation and flutter	49·9 (40·3–60·2)	56·4 (48·3–64·9)	60·6 (53–68·1)	53·6 (44·9–62·8)
Aortic aneurysm	58·9 (53·6–64·4)	65·9 (61·1–70·2)	--	65·9 (61·1–70·2)
Endocarditis	34·3 (24·2–46·6)	35·5 (26·4–46·2)	33·6 (23·1–45·2)	35·5 (26·4–46·3)
Other cardiovascular and circulatory diseases	33·5 (27·4–40·1)	34·9 (29·1–40·5)	34·8 (28·6–41·4)	34·9 (29·2–40·4)
Chronic respiratory diseases	61·7 (58·3–64·9)	54·7 (51–58·5)	43·4 (37·7–49·5)	62·1 (58·9–64·9)
Chronic obstructive pulmonary disease	71·2 (67·5–74·8)	71·8 (68·5–75)	63·7 (59·9–67·3)	74·1 (70·6–77·1)
Pneumoconiosis	100 (100–100)	100 (100–100)	100 (100–100)	100 (100–100)
Asthma	43·8 (33·1–55·4)	35·5 (27·5–44·4)	34·5 (26·7–43·5)	45·1 (35·5–55·4)

Digestive diseases	45·1 (40·2–50·3)	44·2 (38·8–49)	7·49 (4·64–10·5)	52·8 (47·6–57·8)
Cirrhosis and other chronic liver diseases	79·4 (71·2–86·9)	81·7 (74–88·5)	75·7 (68·4–82·5)	81·8 (74–88·6)
Upper digestive system diseases	21·2 (17·9–24·3)	6·61 (3·85–9·97)	0·411 (0·225–0·675)	24·9 (21·4–28·1)
Gallbladder and biliary diseases	42·3 (27·7–57·5)	44·5 (30·6–58·9)	47 (33·1–61·6)	43·7 (29·7–58)
Pancreatitis	42·1 (30·9–54·9)	44·1 (32·9–56·7)	41 (29·9–53)	44·2 (33–56·9)
Neurological disorders	21·7 (9·22–35·6)	13·1 (6·48–23·4)	8·76 (4·45–16·6)	19·7 (8·79–32·9)
Alzheimer's disease and other dementias	33·2 (20·6–48·5)	36·6 (24–51·3)	37·2 (24·8–51·6)	36·2 (23·5–50·9)
Parkinson's disease	-7·56 (-11·7–-3·59)	-9·55 (-14·6–-4·63)	-10·9 (-16·8–-5·39)	-9·32 (-14·3–-4·49)
Idiopathic epilepsy	25·4 (18·7–31·6)	21·1 (15·2–26·9)	19·7 (14·1–25·5)	23·7 (17·2–29·7)
Multiple sclerosis	15·4 (11·5–19·4)	16·3 (12·4–20·1)	15·9 (12–19·5)	16·6 (12·5–20·7)
Mental disorders	--	5·93 (3·36–9·38)	5·93 (3·36–9·38)	--
Depressive disorders	--	12·3 (6·68–18·8)	12·3 (6·68–18·8)	--
Anxiety disorders	--	7·01 (2·09–14·8)	7·01 (2·09–14·8)	--
Idiopathic developmental intellectual disability	--	58·4 (29·7–78·1)	58·4 (29·7–78·1)	--
Substance use disorders	100 (100–100)	100 (100–100)	100 (100–100)	100 (100–100)
Alcohol use disorders	100 (100–100)	100 (100–100)	100 (100–100)	100 (100–100)
Drug use disorders	100 (100–100)	100 (100–100)	100 (100–100)	100 (100–100)
Diabetes and kidney diseases	100 (100–100)	100 (100–100)	100 (100–100)	100 (100–100)
Diabetes mellitus	100 (100–100)	100 (100–100)	100 (100–100)	100 (100–100)
Chronic kidney disease	100 (100–100)	100 (100–100)	100 (100–100)	100 (100–100)
Sense organ diseases	--	11·5 (10·3–12·8)	11·5 (10·3–12·8)	--
Blindness and vision loss	--	7·26 (4·87–10·4)	7·26 (4·87–10·4)	--
Age-related and other hearing loss	--	14·6 (13·3–16·1)	14·6 (13·3–16·1)	--
Musculoskeletal disorders	3·95 (1·09–7·16)	17·2 (14–20·7)	17·5 (14·2–21)	3·67 (1·04–6·58)
Rheumatoid arthritis	11·9 (3·34–22)	14·6 (4·17–25·7)	14·8 (4·29–26·1)	14·1 (4·07–25·1)
Osteoarthritis	--	14·9 (7·66–23·5)	14·9 (7·66–23·5)	--
Low back pain	--	40·4 (35·7–45·2)	40·4 (35·7–45·2)	--
Gout	--	47·6 (32·7–64·5)	47·6 (32·7–64·5)	--
Other non-communicable diseases	0·15 (0·104–0·217)	0·138 (0·0889–0·204)	--	0·35 (0·247–0·499)
Sudden infant death syndrome	7·1 (4·9–9·75)	7·09 (4·9–9·74)	--	7·09 (4·9–9·74)
Injuries	25·2 (20·6–29·7)	26·3 (21·5–31·2)	32·9 (28·2–38)	23·3 (18·1–28·4)
Transport injuries	34·2 (27·1–41·5)	33·5 (26·4–41·1)	38·3 (32·7–44·5)	32·5 (25·1–40·4)
Road injuries	33·5 (26·3–40·8)	32·9 (25·8–40·6)	38 (32·4–44·4)	31·9 (24·3–39·7)
Other transport injuries	44·7 (38·5–51·5)	41·7 (35·1–48·8)	42·6 (37·7–47·8)	41·5 (34·7–49)

Unintentional injuries	23·4 (19–27·8)	26·9 (21·3–32·6)	32·9 (27·5–38·9)	19·3 (14·2–24·9)
Falls	54·8 (48·4–59·7)	42·3 (36·8–47·8)	41·1 (35·6–46·4)	46·1 (40·3–52)
Drowning	3·18 (-5·66–13·6)	-0·278 (-8·55–8·88)	17·9 (13·4–23·3)	-0·7 (-9·11–8·6)
Fire, heat, and hot substances	18·8 (13·9–24·2)	24·1 (18·4–30·2)	25·7 (19·7–32·1)	21·9 (16·6–27·9)
Poisonings	21·4 (16·1–27·4)	20·9 (15·9–26·5)	21·4 (16·4–27·2)	20·6 (15·5–26·3)
Exposure to mechanical forces	21·1 (12·5–29·9)	30·4 (24–37·7)	33·2 (27·1–40·7)	20 (11·1–29·4)
Animal contact	26·9 (21·6–32·7)	27·3 (21·8–33·1)	29·2 (23·5–35·5)	22 (16·9–27·6)
Foreign body	13·5 (9·53–18·7)	13·7 (9·53–19)	15·2 (11·1–20·5)	13·5 (9·22–18·9)
Environmental heat and cold exposure	10·1 (4·85–17·2)	9·27 (4·41–15·6)	8·91 (4·24–15·1)	9·87 (4·69–16·5)
Exposure to forces of nature	0 (0–0)	9·77 (4·6–16·4)	9·77 (4·6–16·4)	0 (0–0)
Other unintentional injuries	30·8 (23·5–38·5)	27·6 (21·3–34·7)	25·7 (19·8–32·3)	30·1 (23·1–37·8)
Self-harm and interpersonal violence	19·2 (10·1–28·5)	19·4 (11·1–27·8)	25·3 (19·1–31·8)	18·7 (9·78–27·6)
Self-harm	19·4 (5·85–32·4)	19·4 (6·07–32·2)	21·9 (11–32·4)	19·3 (5·87–32·3)
Interpersonal violence	18·9 (9·95–27·8)	19·9 (11·5–28·5)	29·6 (22–37·5)	17·7 (8·85–26·7)

Supplementary Results Table 2. All-age PAFs attributable to the joint distribution of all risk factors for all ages, both sexes combined for each GBD level 1, level 2, and level 3 cause and all causes in 2019 for Chile. Results are shown for deaths, YLLs, YLDs, and DALYs Data in parenthesis are 95% uncertainty intervals. PAFs=population attributable fractions. GBD=Global Burden of Disease. YLLs=years of life lost. YLDs=years lived with disability. DALYs=disability-adjusted life-years

	Deaths 2019	DALYs 2019	YLDs 2019	YLLs 2019
All causes	55·7 (52·9–58·4)	39·7 (37–42·4)	25·9 (23·8–28·1)	52·4 (50·2–54·7)
Communicable, maternal, neonatal, and nutritional diseases	53·9 (48·7–59·3)	58·1 (54·4–61·3)	46·5 (41·9–51·4)	64·6 (61·1–68)
HIV/AIDS and sexually transmitted infections	97·4 (97·1–97·7)	97 (96·6–97·4)	98·4 (97·7–99)	96·6 (96·2–97)
HIV/AIDS	97·4 (97·1–97·7)	96·9 (96·5–97·2)	97·7 (97–98·1)	96·7 (96·3–97)
Sexually transmitted infections excluding HIV	97·1 (95·6–98·2)	98·9 (97·9–99·5)	100 (100–100)	92 (88·3–95)
Respiratory infections and tuberculosis	47·4 (39·7–55·4)	42·6 (36·2–48·9)	5·31 (3·76–7·34)	53·2 (46·3–60·2)
Tuberculosis	54·4 (45·1–62·7)	57·8 (48·9–65·3)	53·4 (43·4–61·6)	58·3 (49·4–65·7)
Lower respiratory infections	46·7 (38·5–55·2)	52·3 (44·5–60·1)	38·4 (31·9–45·1)	52·5 (44·7–60·3)
Upper respiratory infections	0·188 (0·07–0·384)	0·0141 (0·00494–0·0256)	0·0107 (0·00227–0·0213)	0·491 (0·184–0·963)
Otitis media	0·307 (0·106–0·729)	5·96 (3·92–8·17)	6 (3·95–8·22)	1·06 (0·358–2·5)
Enteric infections	37·3 (24·5–54)	38·7 (26–55·1)	38 (25·3–54·8)	40·4 (27·6–56·4)
Diarrheal diseases	37·5 (24·6–54·3)	38·8 (26–55·3)	38 (25·3–54·8)	40·7 (27·9–56·9)
Other infectious diseases	0·735 (0·543–1·04)	1·14 (0·832–1·56)	0·389 (0·234–0·637)	1·33 (0·959–1·87)
Meningitis	1·32 (0·856–1·88)	2·52 (1·69–3·47)	0·00125 (0·000189–0·00259)	2·83 (1·9–3·92)
Encephalitis	0·22 (0·141–0·363)	0·368 (0·238–0·601)	0·000207 (3·03e-05–0·000446)	0·435 (0·283–0·715)
Measles	4·49 (0·124–16·3)	5·7 (0·157–20·2)	2·38 (0·064–5·57)	5·75 (0·167–20·5)
Acute hepatitis	18·7 (12·7–28·4)	5·08 (3·38–7·34)	1·78 (1·07–2·81)	17·5 (11·3–25·9)
Maternal and neonatal disorders	81·3 (78·6–83·7)	78 (75·2–80·4)	68·5 (64·2–72·5)	83·2 (80·9–85·3)
Maternal disorders	10·4 (3·62–17·1)	10·3 (3·59–17)	10·3 (3·62–16·9)	10·3 (3·59–17)
Neonatal disorders	87·2 (85·2–89)	81·5 (78·9–83·6)	71·3 (66·7–75·1)	87·2 (85·2–89)
Nutritional deficiencies	95·4 (90·4–96·7)	88·4 (85·9–90·6)	84·9 (81·7–88·3)	94·8 (90·2–96·3)
Protein-energy malnutrition	100 (100–100)	100 (100–100)	100 (100–100)	100 (100–100)
Vitamin A deficiency	--	100 (100–100)	100 (100–100)	--
Dietary iron deficiency	--	100 (100–100)	100 (100–100)	--
Non-communicable diseases	58·5 (55·2–61·7)	40·5 (37·4–43·7)	24·4 (22–26·9)	56·8 (54·2–59·5)
Neoplasms	37·4 (33–42·9)	37·8 (33·8–42·9)	30·8 (27·2–35)	38 (34–43·1)
Lip and oral cavity cancer	63 (56–69·7)	67·5 (60·9–73·8)	66·8 (60·1–72·9)	67·6 (60·9–73·8)
Nasopharynx cancer	61·7 (52·3–69·7)	62·7 (53·6–70·3)	59·6 (49·8–67·8)	62·9 (53·7–70·4)
Other pharynx cancer	74 (67·1–79·9)	77·3 (70·8–82·8)	75·7 (68·9–81·4)	77·4 (70·8–82·8)
Esophageal cancer	65·4 (53·5–76·8)	68·9 (57·8–79·3)	67 (55·4–77·9)	68·9 (57·9–79·4)

Stomach cancer	20·7 (11·6–41)	22·2 (12·8–42·4)	21·7 (12·3–41·7)	22·2 (12·8–42·4)
Colon and rectum cancer	65·1 (57·2–72·7)	65·6 (58–72·7)	65·6 (57·9–72·8)	65·6 (58–72·7)
Liver cancer	69·5 (62·1–77·2)	70·5 (63·1–77·6)	70 (62·7–77·3)	70·5 (63·1–77·6)
Gallbladder and biliary tract cancer	26·4 (15–39·3)	27·4 (15·6–40·3)	26·4 (15·1–39·4)	27·4 (15·6–40·3)
Pancreatic cancer	35·7 (26·6–46·8)	37·1 (28·6–47·7)	36·6 (27·7–47·3)	37·1 (28·6–47·7)
Larynx cancer	62·1 (51·9–71·2)	65·7 (56–73·9)	64·5 (54·4–72·9)	65·7 (56·1–74)
Tracheal, bronchus, and lung cancer	73·9 (69·2–79·1)	75·5 (71·4–80)	74·9 (70·3–79·8)	75·5 (71·4–80)
Breast cancer	35·6 (27·1–45·6)	33·9 (25·9–42·5)	34·1 (26·1–43·1)	33·9 (25·9–42·5)
Cervical cancer	100 (100–100)	100 (100–100)	100 (100–100)	100 (100–100)
Uterine cancer	48·7 (32·9–64·6)	50·2 (34–65·6)	50·9 (34·6–66·3)	50·1 (33·9–65·6)
Ovarian cancer	15·7 (6·28–28·4)	13·7 (5·08–25)	13·1 (4·95–23·8)	13·8 (5·08–25·1)
Prostate cancer	3·83 (1·33–6·41)	4·78 (1·78–7·88)	5·48 (2·1–8·95)	4·71 (1·75–7·71)
Kidney cancer	36·1 (25·4–46·7)	36·9 (26·2–47·5)	36·7 (26–47·1)	36·9 (26·2–47·5)
Bladder cancer	27·6 (16·5–39·7)	31·6 (20·4–43·2)	32·9 (21·4–44·4)	31·5 (20·4–43·2)
Thyroid cancer	15·5 (7·92–24·3)	16·2 (8·3–25·3)	16·1 (8·42–25)	16·2 (8·28–25·3)
Mesothelioma	90·1 (86·3–93·1)	83·9 (78·3–88·4)	89·5 (85·5–92·7)	83·8 (78·2–88·3)
Non-Hodgkin lymphoma	7·8 (3·23–13·8)	7·82 (3·32–13·7)	7·74 (3·29–13·5)	7·82 (3·32–13·7)
Multiple myeloma	9·11 (3·93–15·9)	9·48 (4·23–16·5)	9·42 (4·14–16·3)	9·48 (4·23–16·5)
Leukemia	29 (18·1–39·7)	24·4 (14·5–34·1)	28·1 (17–38·8)	24·3 (14·4–33·9)
Cardiovascular diseases	86·4 (83·6–88·9)	86 (83·9–87·9)	79·8 (76·6–82·9)	86·7 (84·6–88·6)
Rheumatic heart disease	29 (19·2–45·7)	26·8 (18·7–40·8)	26·9 (18·6–40·4)	26·8 (18·6–40·5)
Ischemic heart disease	95·6 (93–97·7)	96·4 (94·6–97·9)	95·5 (93·1–97·5)	96·4 (94·6–97·9)
Stroke	87·7 (82·5–93·1)	89·6 (86·2–93)	88·2 (84·9–91·6)	89·9 (86·3–93·3)
Hypertensive heart disease	100 (100–100)	100 (100–100)	100 (100–100)	100 (100–100)
Non-rheumatic valvular heart disease	32·8 (24–42·8)	31·5 (24·2–39·9)	27·6 (19·8–37)	31·9 (24·4–40·4)
Cardiomyopathy and myocarditis	42·7 (35·2–51·1)	42·3 (35·3–49·1)	41·2 (33·7–48·8)	42·4 (35·4–49·2)
Atrial fibrillation and flutter	60·1 (50·5–69·6)	64·3 (56·1–71·9)	66·8 (59·8–73·9)	62·6 (54·1–70·9)
Aortic aneurysm	59·3 (52·5–65·7)	65·7 (60·4–70·3)	--	65·7 (60·4–70·3)
Endocarditis	42·9 (32·7–53·9)	41·3 (31·6–51·8)	41·7 (31·2–53·1)	41·3 (31·6–51·8)
Other cardiovascular and circulatory diseases	40 (33·5–46·5)	36·6 (31·2–42·2)	36 (29·2–42·9)	36·9 (31·5–42·3)
Chronic respiratory diseases	49·5 (43·1–58·2)	47·6 (42·5–53·3)	44·8 (39·2–50·7)	49·5 (43·5–58)
Chronic obstructive pulmonary disease	66 (61·4–70·8)	67·3 (63·2–71·3)	60 (55·9–64)	70·1 (65·9–74·4)
Pneumoconiosis	100 (100–100)	100 (100–100)	100 (100–100)	100 (100–100)
Asthma	41·8 (29·3–55·1)	41·1 (32–50·8)	40·4 (31·4–50)	46·5 (34·8–58·4)

Digestive diseases	54·6 (49·1–59·9)	53·4 (47·6–58·7)	9·18 (6·01–12·6)	62·9 (57·1–68)
Cirrhosis and other chronic liver diseases	80·1 (71·6–87·7)	82·3 (74·3–89·2)	78·7 (71·8–85·5)	82·4 (74·3–89·3)
Upper digestive system diseases	15·5 (12·7–18·4)	4·18 (2·41–6·43)	0·383 (0·209–0·638)	20·4 (17·2–23·5)
Gallbladder and biliary diseases	45·8 (30·4–61·3)	49·4 (34·4–64·1)	53·6 (38·9–67·4)	47·9 (32·9–63·3)
Pancreatitis	41·9 (31·2–54·6)	44·8 (33·9–58·3)	41·8 (31·1–54·4)	44·9 (34–58·4)
Neurological disorders	21·8 (9·84–35·7)	13·5 (6·83–23·2)	9·15 (4·55–16·6)	20·2 (10·2–32·9)
Alzheimer's disease and other dementias	33·3 (19·4–49·6)	36·1 (22·3–52·1)	36·5 (22·5–52·6)	35·8 (21·6–51·6)
Parkinson's disease	-4·29 (-7·48–-1·74)	-5·83 (-9·71–-2·41)	-7·38 (-12·2–-3·2)	-5·55 (-9·3–-2·31)
Idiopathic epilepsy	27·1 (20·1–34·1)	23·3 (17–29·5)	21·2 (15·4–27·3)	26·7 (19·9–33·7)
Multiple sclerosis	13·2 (9·81–16·5)	15·7 (11·7–19·5)	15·6 (11·7–19·5)	15·8 (11·9–19·6)
Mental disorders	--	9·24 (4·22–14·7)	9·24 (4·22–14·7)	--
Depressive disorders	--	19 (7·68–29·8)	19 (7·68–29·8)	--
Anxiety disorders	--	8·25 (2·11–17·5)	8·25 (2·11–17·5)	--
Idiopathic developmental intellectual disability	--	52·9 (26·3–73·6)	52·9 (26·3–73·6)	--
Substance use disorders	100 (100–100)	100 (100–100)	100 (100–100)	100 (100–100)
Alcohol use disorders	100 (100–100)	100 (100–100)	100 (100–100)	100 (100–100)
Drug use disorders	100 (100–100)	100 (100–100)	100 (100–100)	100 (100–100)
Diabetes and kidney diseases	100 (100–100)	100 (100–100)	100 (100–100)	100 (100–100)
Diabetes mellitus	100 (100–100)	100 (100–100)	100 (100–100)	100 (100–100)
Chronic kidney disease	100 (100–100)	100 (100–100)	100 (100–100)	100 (100–100)
Sense organ diseases	--	12·5 (11·1–14·1)	12·5 (11·1–14·1)	--
Blindness and vision loss	--	12·3 (8·02–17)	12·3 (8·02–17)	--
Age-related and other hearing loss	--	14·1 (12·9–15·4)	14·1 (12·9–15·4)	--
Musculoskeletal disorders	2·98 (0·596–5·7)	18·7 (15·3–22·4)	19 (15·6–22·8)	3·25 (0·652–5·99)
Rheumatoid arthritis	6·9 (1·4–13·1)	12·6 (2·9–22·4)	13·4 (3·03–23·5)	10·1 (2·08–18·3)
Osteoarthritis	--	17·7 (9·4–26·9)	17·7 (9·4–26·9)	--
Low back pain	--	44·3 (39·9–48·8)	44·3 (39·9–48·8)	--
Gout	--	53·9 (37·8–71·1)	53·9 (37·8–71·1)	--
Other non-communicable diseases	0·0913 (0·0543–0·145)	0·0816 (0·0455–0·135)	--	0·242 (0·151–0·38)
Sudden infant death syndrome	8·45 (5·25–12·6)	8·44 (5·24–12·6)	--	8·44 (5·24–12·6)
Injuries	24·7 (19·8–29·1)	23·7 (19–27·9)	29·5 (25·5–33·7)	20·2 (14·8–25·4)
Transport injuries	25·4 (18·9–32)	26 (19·4–32·7)	31·8 (27·4–36·7)	24·7 (17·5–31·9)
Road injuries	24·5 (17·9–31·1)	25·2 (18·5–32)	31·4 (27–36·3)	23·8 (16·5–31·1)
Other transport injuries	39 (33·7–44·7)	37·1 (31·4–43)	39·2 (34·8–43·5)	36·7 (30·8–42·9)

Unintentional injuries	31·2 (26·1–36·2)	26·1 (21·3–31·2)	29·4 (24·9–34)	20·3 (15–26·4)
Falls	53·7 (47·2–58·5)	40·8 (35·7–45·8)	39·4 (34·4–44·4)	44·1 (38·7–49·5)
Drowning	-3·58 (-15·2–7·82)	-3·8 (-14·8–7·59)	16 (11·7–21·3)	-4·3 (-15·5–7·2)
Fire, heat, and hot substances	16 (11·5–21·7)	19·5 (14·5–25·3)	20·5 (15·3–26·5)	18·5 (13·6–24·2)
Poisonings	21 (15·8–27·1)	19·7 (14·7–25·3)	18·1 (13·4–23·7)	22·6 (17·1–29·1)
Exposure to mechanical forces	12·2 (3·42–21·5)	22·6 (17·6–27·9)	25·1 (20·6–29·7)	10·7 (1·29–20·6)
Animal contact	24 (19·2–29·8)	24·3 (19·3–29·7)	25 (20–30·4)	22·1 (17·3–27·7)
Foreign body	10·5 (7·09–14·4)	10·7 (7·23–14·8)	10·2 (7·24–13·8)	11 (7·21–15·5)
Environmental heat and cold exposure	10·5 (5·05–17·8)	10 (4·77–16·9)	8·86 (4·22–15·1)	10·9 (5·21–18·4)
Exposure to forces of nature	8·06 (3·83–13·6)	8·49 (4·07–14·3)	9·37 (4·45–15·8)	7·37 (3·5–12·5)
Other unintentional injuries	25·4 (19·2–32·8)	21·2 (16–27·5)	19·6 (14·6–26)	26·6 (20·2–33·8)
Self-harm and interpersonal violence	17 (6·73–27)	17·7 (8·22–26·7)	27 (20·5–33·6)	16·7 (6·46–26·3)
Self-harm	17·8 (3·74–31·4)	18 (4·08–31·4)	21·8 (11·4–32·2)	17·9 (3·69–31·4)
Interpersonal violence	15·2 (5·64–24·9)	17·5 (8·62–26·3)	28·5 (21·2–36·2)	14·2 (4·65–23·8)

Supplementary Results Table 2. All-age PAFs attributable to the joint distribution of all risk factors for all ages, both sexes combined for each GBD level 1, level 2, and level 3 cause and all causes in 2019 for Uruguay. Results are shown for deaths, YLLs, YLDs, and DALYs Data in parenthesis are 95% uncertainty intervals. PAFs=population attributable fractions. GBD=Global Burden of Disease. YLLs=years of life lost. YLDs=years lived with disability. DALYs=disability-adjusted life-years	Deaths 2019	DALYs 2019	YLDs 2019	YLLs 2019
All causes	53·3 (50·6–56)	41·1 (38·5–43·8)	24·4 (22·6–26·3)	51·9 (49·9–53·9)
Communicable, maternal, neonatal, and nutritional diseases	44·9 (40·5–49·1)	57·3 (54·2–60·4)	52·5 (47·9–56·8)	59·4 (55·8–62·8)
HIV/AIDS and sexually transmitted infections	82 (80·9–83·2)	80·6 (79·3–82·1)	85·5 (82·6–89)	79·6 (78·4–80·9)
HIV/AIDS	81·7 (80·6–82·9)	79·6 (78·3–80·8)	79·9 (78·6–81·5)	79·5 (78·3–80·8)
Sexually transmitted infections excluding HIV	96·7 (95–98)	97·9 (95·9–99)	100 (100–100)	89·3 (84·5–93)
Respiratory infections and tuberculosis	37 (31·3–42·5)	39 (33·8–44·5)	5·63 (4–7·72)	44·8 (39·5–50·2)
Tuberculosis	55·3 (46·4–62·4)	57·4 (48·9–64·5)	52·7 (43·3–60·3)	58·2 (49·8–65·1)
Lower respiratory infections	36·4 (30·6–42)	44 (38·2–49·5)	35·1 (29·4–40·4)	44 (38·3–49·6)
Upper respiratory infections	0·0546 (0·0295–0·0953)	0·00691 (0·00112–0·0164)	0·006 (0·00021–0·0156)	0·147 (0·0815–0·257)
Otitis media	0·552 (0·123–1·57)	5·65 (3·68–7·74)	5·66 (3·68–7·76)	1·61 (0·41–4·19)
Enteric infections	40·8 (23·7–61·3)	44·3 (28·5–63·7)	40·3 (23–61·1)	48·2 (33·1–66·1)
Diarrheal diseases	40·8 (23·7–61·4)	44·4 (28·6–63·9)	40·4 (23·1–61·2)	48·3 (33·2–66·3)
Other infectious diseases	0·575 (0·436–0·762)	0·877 (0·661–1·14)	0·274 (0·165–0·43)	1·09 (0·82–1·43)
Meningitis	0·699 (0·451–1·04)	1·92 (1·29–2·81)	0·000442 (1·12e-05–0·00125)	2·34 (1·59–3·35)
Encephalitis	0·298 (0·194–0·437)	0·633 (0·426–0·913)	0·000141 (3·31e-06–0·000388)	0·672 (0·452–0·96)
Measles	4·11 (0·603–14·2)	5·03 (0·904–16·4)	3·21 (0·104–7·28)	5·27 (0·747–17·8)
Acute hepatitis	18·6 (13–27·7)	4·98 (3·27–7·25)	1·64 (1·01–2·55)	18·3 (12·7–28)
Maternal and neonatal disorders	80·1 (77·6–82·6)	77·3 (74·5–79·9)	67·1 (62·7–71·2)	81·8 (79·5–84)
Maternal disorders	11·4 (4·13–18·9)	11·3 (4·16–18·7)	11·3 (4·11–18·8)	11·3 (4·16–18·7)
Neonatal disorders	85·1 (83·1–87·1)	80·6 (77·9–83)	70·3 (65·6–74·3)	85·1 (83·1–87·1)
Nutritional deficiencies	78 (66·9–84·5)	91·1 (88·1–93·4)	94·2 (92·2–95·9)	83·7 (75·4–88·1)
Protein-energy malnutrition	100 (100–100)	100 (100–100)	100 (100–100)	100 (100–100)
Vitamin A deficiency	--	100 (100–100)	100 (100–100)	--
Dietary iron deficiency	--	100 (100–100)	100 (100–100)	--
Non-communicable diseases	56·4 (53·2–59·5)	42·2 (39–45·4)	22·1 (20·1–24·2)	56·2 (53·9–58·6)
Neoplasms	42·3 (38·9–46·4)	43·8 (40·7–47·2)	35·4 (32·1–39)	44·1 (41–47·6)
Lip and oral cavity cancer	66 (59·5–71·9)	69·8 (63·5–75·4)	69·2 (63–75)	69·9 (63·5–75·5)
Nasopharynx cancer	63·1 (54·4–71)	64·1 (55·7–71·7)	61·3 (52·4–69·3)	64·2 (55·7–71·8)
Other pharynx cancer	79·3 (73·2–83·9)	81·1 (75·5–85·8)	80·2 (74·4–84·8)	81·2 (75·5–85·8)
Esophageal cancer	69 (59·1–78·5)	72·8 (63·5–81·3)	70·7 (60·7–79·8)	72·8 (63·5–81·3)
Stomach cancer	23·3 (14·4–42·8)	24·8 (15·8–44)	24·3 (15·4–43·5)	24·9 (15·8–44)

Colon and rectum cancer	59·4 (51·8–66·7)	60·7 (53–67·5)	60·6 (53–67·5)	60·7 (53–67·5)
Liver cancer	68·6 (62·1–75)	70·1 (63·4–76·4)	69·3 (62·8–75·8)	70·1 (63·4–76·4)
Gallbladder and biliary tract cancer	21·8 (12–33·5)	22·5 (12·6–34·7)	21·8 (12–33·9)	22·5 (12·6–34·7)
Pancreatic cancer	32·8 (25·8–41·1)	34·6 (28–42·8)	33·7 (26·7–42·1)	34·7 (28–42·8)
Larynx cancer	76·1 (68·1–82·5)	79·5 (72·1–84·9)	78·3 (70·4–84·1)	79·5 (72·2–84·9)
Tracheal, bronchus, and lung cancer	78·4 (75·3–81·7)	78·9 (76·1–82)	78·7 (75·6–82)	78·9 (76·1–82)
Breast cancer	30·9 (24·1–38·1)	30·2 (23·8–36·8)	30·4 (24·1–37·3)	30·2 (23·8–36·8)
Cervical cancer	100 (100–100)	100 (100–100)	100 (100–100)	100 (100–100)
Uterine cancer	43·3 (28·7–59·2)	44·9 (30·2–60·6)	45·6 (30·9–61·2)	44·8 (30·2–60·6)
Ovarian cancer	11·7 (4·79–20·5)	10·1 (3·97–17·9)	9·88 (3·73–17·7)	10·2 (3·98–17·9)
Prostate cancer	5·73 (2·55–9·13)	6·79 (3·05–10·6)	7·47 (3·45–11·5)	6·74 (3·03–10·5)
Kidney cancer	35·6 (26·2–45·4)	36·3 (26·8–45·8)	35·8 (26·3–45·4)	36·3 (26·8–45·8)
Bladder cancer	31·2 (21·1–41·6)	37·4 (26·5–47·6)	39·2 (28·3–49·4)	37·3 (26·4–47·5)
Thyroid cancer	13·3 (6·56–21·7)	14·1 (6·82–22·9)	13·9 (6·99–22·3)	14·1 (6·79–22·9)
Mesothelioma	91·4 (88–94·2)	85·8 (80·5–90·2)	90·7 (87·2–93·6)	85·7 (80·4–90·1)
Non-Hodgkin lymphoma	6·46 (2·64–11·6)	6·5 (2·67–11·7)	6·39 (2·59–11·4)	6·5 (2·67–11·7)
Multiple myeloma	7·55 (3·08–13·6)	7·94 (3·34–14)	7·89 (3·21–13·9)	7·94 (3·34–14)
Leukemia	31·1 (21·1–41·8)	28·5 (19·2–38·2)	31·4 (21·5–41·7)	28·4 (19·1–38·1)
Cardiovascular diseases	79·3 (75·3–83·2)	81·2 (78·3–84·1)	74·5 (70·3–78·5)	81·9 (79–84·6)
Rheumatic heart disease	24·5 (14·8–42·9)	25·4 (17·3–39·2)	25 (16·4–39·2)	25·5 (17·5–39·1)
Ischemic heart disease	93 (89·5–96)	94·5 (92–96·6)	93·1 (90·1–95·9)	94·5 (92·1–96·6)
Stroke	78·7 (71·3–86·5)	82·8 (77·7–87·8)	82·5 (77·4–87·5)	82·8 (77·7–87·9)
Hypertensive heart disease	100 (100–100)	100 (100–100)	100 (100–100)	100 (100–100)
Non-rheumatic valvular heart disease	31 (20–45)	32·4 (22·8–43·8)	22·1 (14·8–30·9)	32·7 (23·1–44·5)
Cardiomyopathy and myocarditis	41·1 (33·9–49·6)	45·3 (39–51·8)	41·6 (34·3–49·5)	45·7 (39·1–52)
Atrial fibrillation and flutter	49·5 (39·6–59·9)	55·7 (47·3–64·3)	59·8 (51·6–67·4)	52·7 (43·4–62·1)
Aortic aneurysm	58·6 (52·9–64·7)	66·8 (62·2–71·2)	--	66·8 (62·2–71·2)
Endocarditis	36·9 (26–50·2)	39·2 (29·3–50)	35·8 (24·8–49)	39·3 (29·3–50·2)
Other cardiovascular and circulatory diseases	33·8 (26·8–41·4)	36 (30·1–42)	36·7 (30·5–43·5)	35·8 (29·6–42·2)
Chronic respiratory diseases	59·4 (55·8–63·1)	57·8 (54·1–61·4)	46·8 (41·5–52)	63·1 (59·8–66·3)
Chronic obstructive pulmonary disease	65·4 (61·6–69·4)	68·8 (65·5–72·1)	62 (58·1–65·8)	70·5 (67·1–74·1)
Pneumoconiosis	100 (100–100)	100 (100–100)	100 (100–100)	100 (100–100)
Asthma	40·8 (29·9–53·5)	38·2 (29·8–47·2)	36·1 (28·4–44·9)	44·7 (34·6–55·7)
Digestive diseases	30·6 (26·8–34·3)	32 (27·6–35·9)	6·93 (4·22–9·97)	38·8 (34·6–42·6)
Cirrhosis and other chronic liver diseases	78·1 (69·3–86·7)	80·6 (72·8–87·8)	73·5 (65·7–80·9)	80·7 (72·8–88)

Upper digestive system diseases	17·7 (14·9–20·6)	5·8 (3·38–8·66)	0·416 (0·235–0·67)	22·9 (19·7–26·2)
Gallbladder and biliary diseases	40·7 (27–55·4)	44·3 (30·9–58·4)	48·3 (34·7–62·4)	43·1 (29·6–57·3)
Pancreatitis	39·8 (28·8–53·6)	41·4 (30·4–55·1)	38·6 (27·9–51·5)	41·6 (30·5–55·3)
Neurological disorders	19 (8·23–31·5)	13·6 (7·26–22·9)	10 (5·37–17·7)	17·5 (7·69–29·2)
Alzheimer's disease and other dementias	28·2 (16·5–42·5)	31·5 (19·6–45·2)	32·1 (20·2–45·8)	31·1 (19·3–45)
Parkinson's disease	-6·89 (-11–-3·31)	-9·02 (-14·2–-4·49)	-10·2 (-16–-5·11)	-8·84 (-13·8–-4·43)
Idiopathic epilepsy	23·4 (16·5–30)	21·1 (15–27·1)	19·9 (14·1–26)	22·8 (16·2–29)
Multiple sclerosis	15·7 (11·9–19·5)	17·5 (13·5–21·6)	17·1 (13·2–21·2)	17·8 (13·5–22·1)
Mental disorders	--	5·21 (2·88–8·13)	5·21 (2·88–8·13)	--
Depressive disorders	--	10·7 (5·02–17·1)	10·7 (5·02–17·1)	--
Anxiety disorders	--	4·49 (1·24–10·1)	4·49 (1·24–10·1)	--
Idiopathic developmental intellectual disability	--	68·2 (36·2–87·2)	68·2 (36·2–87·2)	--
Substance use disorders	100 (100–100)	100 (100–100)	100 (100–100)	100 (100–100)
Alcohol use disorders	100 (100–100)	100 (100–100)	100 (100–100)	100 (100–100)
Drug use disorders	100 (100–100)	100 (100–100)	100 (100–100)	100 (100–100)
Diabetes and kidney diseases	100 (100–100)	100 (100–100)	100 (100–100)	100 (100–100)
Diabetes mellitus	100 (100–100)	100 (100–100)	100 (100–100)	100 (100–100)
Chronic kidney disease	100 (100–100)	100 (100–100)	100 (100–100)	100 (100–100)
Sense organ diseases	--	10·1 (9·09–11·2)	10·1 (9·09–11·2)	--
Blindness and vision loss	--	4·14 (2·67–6·02)	4·14 (2·67–6·02)	--
Age-related and other hearing loss	--	13 (11·8–14·5)	13 (11·8–14·5)	--
Musculoskeletal disorders	3·4 (0·916–6·18)	18·4 (15·1–22)	18·5 (15·2–22·4)	4·1 (1·21–7·47)
Rheumatoid arthritis	6·8 (1·89–12·5)	13·3 (4·07–23·5)	14·3 (4·43–25·1)	10·4 (2·97–18·7)
Osteoarthritis	--	15·2 (7·84–23·9)	15·2 (7·84–23·9)	--
Low back pain	--	42·2 (37·5–47·4)	42·2 (37·5–47·4)	--
Gout	--	48·5 (33·8–65·1)	48·5 (33·8–65·1)	--
Other non-communicable diseases	0·0584 (0·0348–0·0891)	0·0706 (0·0407–0·113)	--	0·187 (0·122–0·268)
Sudden infant death syndrome	6·39 (4·12–9·23)	6·38 (4·11–9·22)	--	6·38 (4·11–9·22)
Injuries	25 (20·7–29·3)	24·9 (20·9–29·4)	29·3 (25·5–33·5)	22·8 (18·2–27·6)
Transport injuries	32·8 (27·9–38·2)	31·5 (26–37·5)	31·6 (27·5–36·5)	31·5 (25·6–37·8)
Road injuries	32·2 (27·4–37·6)	31·1 (25·6–37·1)	31·2 (27–36·1)	31·1 (25·2–37·4)
Other transport injuries	39·6 (34·9–45)	36·8 (31·6–42·9)	40·1 (35·8–44·6)	36·3 (30·7–42·7)
Unintentional injuries	24·5 (20·7–28·4)	24·1 (19·5–29)	29·3 (24·9–33·9)	17·5 (12·9–22·6)
Falls	55·9 (48·4–60·7)	42·3 (37–47)	40·8 (35·8–45·4)	45·7 (39·9–50·9)
Drowning	-1·56 (-9·93–7·52)	-1·61 (-9·98–7·21)	14·5 (10·6–19·7)	-1·91 (-10·3–6·91)

Fire, heat, and hot substances	12·6 (8·63–17·6)	17·2 (12·3–23·1)	18·9 (14·1–25·1)	15·3 (10·8–20·7)
Poisonings	16·3 (11·7–22)	18·1 (13·5–23·7)	16·4 (12·2–21·7)	19·6 (14·5–25·8)
Exposure to mechanical forces	18·5 (11·5–26·5)	24·5 (19·6–30·6)	26·2 (21·7–31·8)	19·7 (12–28·3)
Animal contact	27·3 (22·3–32·9)	24·5 (19·5–29·9)	24·4 (19·7–29·7)	24·5 (18·8–30·5)
Foreign body	4·7 (3·22–6·54)	7·39 (4·83–10·7)	9·47 (6·69–13·1)	7·12 (4·6–10·4)
Environmental heat and cold exposure	8·54 (3·94–14·5)	8·41 (3·89–14·3)	7·84 (3·64–13·4)	9·1 (4·23–15·4)
Exposure to forces of nature	0 (0–0)	8·16 (3·8–13·8)	8·16 (3·8–13·8)	0 (0–0)
Other unintentional injuries	23·4 (17·2–30·7)	20·5 (15·3–27)	18·4 (13·6–24·4)	24·6 (18·1–32·2)
Self-harm and interpersonal violence	20·1 (11–29)	21·2 (12·6–29·3)	26 (19·8–32·2)	20·7 (11·8–29·3)
Self-harm	20 (8·2–31·3)	21 (9·11–32·3)	20·2 (10·6–29·9)	21 (9·04–32·4)
Interpersonal violence	20·6 (12·5–28·7)	21·6 (13·6–29·2)	27·7 (20·7–34·9)	20 (11·8–27·6)

Supplementary Results Table 2. All-age PAFs attributable to the joint distribution of all risk factors for all ages, both sexes combined for each GBD level 1, level 2, and level 3 cause and all causes in 2019 for Western Europe. Results are shown for deaths, YLLs, YLDs, and DALYs Data in parenthesis are 95% uncertainty intervals. PAFs=population attributable fractions. GBD=Global Burden of Disease. YLLs=years of life lost. YLDs=years lived with disability. DALYs=disability-adjusted life-years

	Deaths 2019	DALYs 2019	YLDs 2019	YLLs 2019
All causes	57·4 (54·2–60·4)	42·2 (39·1–45·3)	26·9 (24·8–29·1)	56·4 (54·1–58·7)
Communicable, maternal, neonatal, and nutritional diseases	44·2 (39·1–49·1)	49·7 (46·8–52·7)	45·6 (41·8–49·6)	52·7 (48·6–56·6)
HIV/AIDS and sexually transmitted infections	89·9 (88·1–91·1)	89·3 (86·7–91·2)	94·8 (93·5–96·2)	86·1 (82·8–88·2)
HIV/AIDS	90·5 (89·4–91·4)	89·5 (88·3–90·5)	92·3 (91·1–93·2)	88·4 (87·2–89·4)
Sexually transmitted infections excluding HIV	81·9 (66–93·7)	87·6 (72·6–96·6)	100 (100–100)	50·8 (29·4–75·9)
Respiratory infections and tuberculosis	44·7 (38·4–50·6)	39·6 (33·8–45·3)	3·56 (2·65–4·76)	48·5 (42·4–54·2)
Tuberculosis	54·5 (45·3–61·8)	58·7 (50·1–65·4)	57·6 (48·7–64·5)	58·9 (50·4–65·8)
Lower respiratory infections	44·5 (38·1–50·5)	48·2 (41·9–54)	36·6 (30·9–41·8)	48·3 (42–54·1)
Upper respiratory infections	0·145 (0·0945–0·203)	0·0111 (0·00727–0·0162)	0·00564 (0·00348–0·00871)	0·515 (0·334–0·707)
Otitis media	1·66 (0·991–2·72)	3·71 (2·39–5·13)	3·68 (2·37–5·13)	5·59 (3·58–8·28)
Enteric infections	6·83 (4·5–10·2)	9·79 (7·4–13)	10·1 (7·56–13·5)	9·2 (6·84–12·4)
Diarrheal diseases	6·88 (4·54–10·3)	9·84 (7·44–13·1)	10·1 (7·56–13·5)	9·31 (6·91–12·6)
Other infectious diseases	0·554 (0·432–0·746)	1·05 (0·861–1·26)	0·401 (0·254–0·678)	1·32 (1·08–1·59)
Meningitis	1·13 (0·916–1·39)	2·46 (2·01–2·98)	0·000508 (0·000282–0·000811)	3·19 (2·64–3·81)
Encephalitis	0·225 (0·177–0·273)	0·619 (0·469–0·751)	0·000148 (8·23e-05–0·000243)	0·764 (0·596–0·919)
Measles	2·61 (-0·778–12·2)	2·89 (-0·666–12·5)	0·953 (0·0411–2·13)	3·38 (-1·02–15·3)
Acute hepatitis	8·73 (6·28–15·2)	4·99 (3·64–7·49)	2·42 (1·55–4·03)	9·88 (7·17–16·6)
Maternal and neonatal disorders	80·4 (79·1–81·7)	75·9 (73·8–78)	70·7 (66·8–74·1)	81·2 (79·9–82·5)
Maternal disorders	8·86 (3·21–14·4)	8·9 (3·23–14·5)	8·99 (3·27–14·7)	8·77 (3·18–14·3)
Neonatal disorders	82·7 (81·3–83·9)	77·6 (75·4–79·6)	72·5 (68·5–76·1)	82·7 (81·3–83·9)
Nutritional deficiencies	87·7 (74·1–89·7)	74·1 (70·9–76·5)	72·7 (69·4–75·1)	84·7 (71·3–86·8)
Protein-energy malnutrition	100 (100–100)	100 (100–100)	100 (100–100)	100 (100–100)
Vitamin A deficiency	--	100 (100–100)	100 (100–100)	--
Dietary iron deficiency	--	100 (100–100)	100 (100–100)	--
Non-communicable diseases	59·3 (55·6–62·6)	42·8 (39·3–46·2)	25·2 (22·9–27·7)	59 (56·4–61·7)
Neoplasms	45·8 (42·4–49·7)	46·6 (43·5–50·1)	34 (30·5–37·9)	47·4 (44·3–51)
Lip and oral cavity cancer	70·3 (64·7–75·1)	73·8 (68·5–78·4)	72·7 (67·2–77·5)	73·9 (68·5–78·5)
Nasopharynx cancer	68·1 (60·1–75·3)	68·8 (60·9–75·8)	67·3 (59·2–74·3)	69 (61·1–76)
Other pharynx cancer	82·6 (77·8–86·4)	83·7 (79–87·4)	82·4 (77·4–86·3)	83·8 (79·1–87·5)
Esophageal cancer	75·2 (67·1–82·8)	77·1 (69·3–84·3)	76 (67·9–83·4)	77·1 (69·3–84·3)

Stomach cancer	23 (15·9–40·1)	24·5 (16·8–41·8)	24·3 (16·8–41·5)	24·5 (16·8–41·8)
Colon and rectum cancer	63·3 (55·2–71·1)	64·1 (56·7–71·4)	64·2 (56·6–71·5)	64·1 (56·7–71·4)
Liver cancer	72·2 (66·5–77·8)	74·5 (69·1–79·8)	73·8 (68·2–79·1)	74·5 (69·1–79·9)
Gallbladder and biliary tract cancer	20·2 (11–31·9)	20·3 (11·1–32)	19·9 (10·7–31·6)	20·3 (11·1–32)
Pancreatic cancer	37·7 (29·6–47·6)	38·3 (30·7–47·6)	38·1 (30·1–47·5)	38·3 (30·7–47·6)
Larynx cancer	83 (76·4–88·2)	83·8 (77·5–88·4)	83·2 (76·8–87·9)	83·9 (77·5–88·5)
Tracheal, bronchus, and lung cancer	84·4 (81·8–87·2)	83·6 (81·1–86·3)	84 (81·4–86·8)	83·6 (81·1–86·3)
Breast cancer	35·1 (27·3–44·5)	34·7 (27·7–42·7)	34·8 (27·7–42·8)	34·7 (27·7–42·7)
Cervical cancer	100 (100–100)	100 (100–100)	100 (100–100)	100 (100–100)
Uterine cancer	42·6 (28·9–56·4)	43·5 (29·7–57·5)	43·6 (29·9–57·6)	43·5 (29·7–57·6)
Ovarian cancer	20·3 (10·3–32·2)	17·2 (8·26–27·9)	16·4 (7·91–26·9)	17·2 (8·27–27·9)
Prostate cancer	6 (2·75–9·48)	6·7 (3·06–10·6)	7·24 (3·31–11·3)	6·61 (3·02–10·4)
Kidney cancer	36·3 (26·9–46·1)	37·2 (28–46·6)	37·2 (28·2–46·7)	37·2 (28–46·6)
Bladder cancer	38·4 (25·4–51·1)	42·1 (29·8–54)	43·4 (31·4–54·5)	42 (29·7–53·9)
Thyroid cancer	13·4 (6·39–22·3)	14 (6·62–23·3)	13·7 (6·71–22·4)	14·1 (6·62–23·4)
Mesothelioma	98·2 (97·7–98·7)	96·7 (95·7–97·4)	97·8 (97·2–98·4)	96·6 (95·7–97·4)
Non-Hodgkin lymphoma	6·54 (2·7–11·7)	6·7 (2·81–11·9)	6·63 (2·74–11·8)	6·7 (2·81–11·9)
Multiple myeloma	7·46 (3·21–13·3)	7·72 (3·38–13·7)	7·76 (3·38–13·8)	7·72 (3·38–13·7)
Leukemia	33·7 (21·4–46·8)	33 (22·2–44·3)	32·7 (22·1–43·9)	33·1 (22·2–44·3)
Cardiovascular diseases	79·9 (76·7–82·9)	81 (78·3–83·5)	74 (70·5–77·6)	81·9 (79·4–84·3)
Rheumatic heart disease	25·7 (14·1–50·7)	26·5 (16·6–45·8)	26·5 (16·9–43·6)	26·5 (16·4–46·1)
Ischemic heart disease	93·3 (89·5–96·5)	94·5 (91·7–96·9)	94 (91·2–96·5)	94·5 (91·7–96·9)
Stroke	80·3 (73·1–88·6)	83·4 (78–89·5)	83·4 (78·2–88·9)	83·4 (77·9–89·6)
Hypertensive heart disease	100 (100–100)	100 (100–100)	100 (100–100)	100 (100–100)
Non-rheumatic valvular heart disease	28·6 (19·1–42·7)	28·4 (20·1–39·2)	13·9 (9·65–18·9)	29·7 (21·2–41·1)
Cardiomyopathy and myocarditis	38·3 (30·4–48·9)	45·5 (39·8–52·3)	42·4 (35·3–49·9)	45·8 (39·8–52·7)
Atrial fibrillation and flutter	53·4 (45·1–62·6)	60·2 (53–67·8)	64·5 (57·9–71·3)	56·4 (48·7–64·9)
Aortic aneurysm	54·8 (48·8–60·9)	62·8 (58·2–67·7)	--	62·8 (58·2–67·7)
Endocarditis	34·9 (24·6–48·8)	37·6 (28·5–48·6)	35·8 (25·7–48·2)	37·7 (28·7–48·6)
Other cardiovascular and circulatory diseases	33·3 (27·5–39·9)	36·9 (32·4–41·7)	40·3 (35·7–45·1)	35·7 (31·1–41)
Chronic respiratory diseases	63·4 (59·2–67·8)	59·1 (55·6–62·8)	52·3 (48·1–56·6)	64·8 (60·8–68·8)
Chronic obstructive pulmonary disease	70·4 (66·6–73·9)	69·6 (66·5–72·5)	62·7 (59·5–65·9)	73·5 (70·2–76·5)
Pneumoconiosis	100 (100–100)	100 (100–100)	100 (100–100)	100 (100–100)
Asthma	39·8 (28·9–51·9)	41·3 (33–50·6)	41·1 (32·9–50·3)	43·7 (33·5–54·9)

Digestive diseases	35·7 (31·5–39·7)	39·5 (34·7–43·8)	17·4 (12–23·7)	47·4 (42·3–51·9)
Cirrhosis and other chronic liver diseases	76·2 (65·7–84·1)	79·4 (69·8–86·7)	75·4 (67·9–82)	79·4 (69·8–86·9)
Upper digestive system diseases	19 (16·2–21·8)	7·73 (5·01–10·9)	0·846 (0·495–1·34)	23·2 (20·2–26·2)
Gallbladder and biliary diseases	41·7 (28–56)	45·7 (32·4–59·4)	47·3 (34–61)	42·7 (29·3–56·8)
Pancreatitis	42·8 (33·5–53·6)	47·7 (38·1–58·6)	45·2 (35·8–56·1)	47·9 (38·3–58·8)
Neurological disorders	23·6 (10·8–37·6)	15·6 (7·99–26·6)	10·7 (5·55–20·2)	21·7 (10·3–35)
Alzheimer's disease and other dementias	35 (22·4–49·7)	37·5 (24·8–52·1)	37·9 (25·2–52·2)	37·2 (24·4–51·8)
Parkinson's disease	-8·49 (-13·3–-4·19)	-9·93 (-15·5–-5)	-10·4 (-16·1–-5·35)	-9·81 (-15·4–-4·87)
Idiopathic epilepsy	28·4 (21·2–35·6)	26·6 (20·2–33·1)	25·2 (18·8–31·5)	28·9 (21·9–35·9)
Multiple sclerosis	15·1 (11·4–18·8)	16·7 (12·7–20·4)	16·2 (12·6–19·9)	17·1 (13–21·1)
Mental disorders	--	6·08 (3·23–9·52)	6·09 (3·23–9·53)	--
Depressive disorders	--	11·6 (5·18–18·3)	11·6 (5·18–18·3)	--
Anxiety disorders	--	5·44 (1·7–11·6)	5·44 (1·7–11·6)	--
Idiopathic developmental intellectual disability	--	50·9 (25·5–69·1)	50·9 (25·5–69·1)	--
Substance use disorders	100 (100–100)	100 (100–100)	100 (100–100)	100 (100–100)
Alcohol use disorders	100 (100–100)	100 (100–100)	100 (100–100)	100 (100–100)
Drug use disorders	100 (100–100)	100 (100–100)	100 (100–100)	100 (100–100)
Diabetes and kidney diseases	100 (100–100)	100 (100–100)	100 (100–100)	100 (99·9–100)
Diabetes mellitus	100 (100–100)	100 (100–100)	100 (100–100)	100 (100–100)
Chronic kidney disease	100 (100–100)	100 (100–100)	100 (100–100)	100 (100–100)
Sense organ diseases	--	7·33 (6·23–8·68)	7·33 (6·23–8·68)	--
Blindness and vision loss	--	9·35 (6·12–13·5)	9·35 (6·12–13·5)	--
Age-related and other hearing loss	--	7·64 (6·89–8·49)	7·64 (6·89–8·49)	--
Musculoskeletal disorders	2·56 (0·687–4·57)	22·4 (18·6–26·4)	22·8 (18·8–26·9)	3·25 (0·898–5·68)
Rheumatoid arthritis	9·69 (2·67–17·4)	14·2 (4·18–24·7)	14·7 (4·35–25·7)	11·9 (3·37–21)
Osteoarthritis	--	15·6 (8·36–24·2)	15·6 (8·36–24·2)	--
Low back pain	--	39·8 (34·6–45·1)	39·8 (34·6–45·1)	--
Gout	--	49·5 (35·2–65·9)	49·5 (35·2–65·9)	--
Other non-communicable diseases	0·0516 (0·041–0·0666)	0·0406 (0·0279–0·056)	--	0·209 (0·165–0·258)
Sudden infant death syndrome	6·46 (5·42–7·78)	6·46 (5·42–7·77)	--	6·46 (5·42–7·77)
Injuries	34·3 (29·3–38·2)	32 (28·1–35·8)	36·4 (32·1–40·3)	27·5 (22·5–32·3)
Transport injuries	28·7 (24·1–33·1)	29·3 (24·6–33·7)	32·2 (28·6–35·7)	28·1 (22·7–33·1)
Road injuries	27·5 (22·7–32·1)	28·3 (23·5–32·7)	31·2 (27·7–34·7)	27 (21·5–32·2)
Other transport injuries	39·9 (35·7–43·6)	38·6 (34·2–42·5)	41·1 (36·8–44·7)	37·5 (32·6–41·7)

Unintentional injuries	40·8 (34·8–45)	35·5 (30·8–39·7)	37·1 (32·1–41·5)	31·7 (27·1–35·7)
Falls	56·9 (48·7–62·2)	46·3 (40·3–50·9)	44·8 (39·2–49·4)	50·5 (44·1–55·4)
Drowning	-6·79 (-15·1–2·04)	-4·03 (-11·8–4·4)	13·9 (9·72–19·4)	-4·85 (-12·9–3·81)
Fire, heat, and hot substances	12·6 (8·39–18·4)	18·4 (13·9–24·3)	19 (14·4–24·9)	17·1 (12·8–22·6)
Poisonings	17·1 (12·6–23·1)	18·6 (14·4–23·8)	17·4 (13·2–22·5)	20·4 (16–26·1)
Exposure to mechanical forces	18·1 (12·4–23·5)	23·8 (20·8–26·6)	25·3 (22·5–27·8)	13·4 (7·95–18·9)
Animal contact	27·7 (22·8–33·1)	26·8 (22·2–31·8)	27 (22·2–32)	26·3 (21·8–31·6)
Foreign body	2·85 (2·31–3·58)	7·01 (5·78–8·52)	7·7 (6·42–9·26)	6·74 (5·48–8·29)
Environmental heat and cold exposure	10·3 (4·86–17·4)	10·6 (4·97–17·8)	9·36 (4·4–15·8)	11·3 (5·26–19)
Exposure to forces of nature	9·69 (4·6–16·4)	9·32 (4·37–15·7)	9·5 (4·46–16)	8·96 (4·24–15·1)
Other unintentional injuries	21·7 (16·3–28·4)	18·7 (14·2–24·3)	18 (13·5–23·7)	25·8 (20·4–32·5)
Self-harm and interpersonal violence	22·9 (11·5–34·5)	24·2 (13·6–34·5)	32·3 (26·3–38)	23·2 (11·7–34·6)
Self-harm	22·6 (10·3–35·1)	23 (10·7–35·5)	24·3 (13·6–34·4)	23 (10·5–35·6)
Interpersonal violence	28·7 (21·3–36·3)	31·9 (24·6–39·3)	37·6 (30·2–45·2)	26·2 (18·8–34)

Supplementary Results Table 2. All-age PAFs attributable to the joint distribution of all risk factors for all ages, both sexes combined for each GBD level 1, level 2, and level 3 cause and all causes in 2019 for Andorra. Results are shown for deaths, YLLs, YLDs, and DALYs Data in parenthesis are 95% uncertainty intervals. PAFs=population attributable fractions. GBD=Global Burden of Disease. YLLs=years of life lost. YLDs=years lived with disability. DALYs=disability-adjusted life-years

	Deaths 2019	DALYs 2019	YLDs 2019	YLLs 2019
All causes	55·5 (51·7–58·9)	39·3 (35·9–42·7)	25·1 (22·9–27·4)	54 (51·1–57)
Communicable, maternal, neonatal, and nutritional diseases	42·9 (30·2–61·2)	50·1 (42–65)	46·3 (41·1–52·9)	52·5 (38·8–73)
HIV/AIDS and sexually transmitted infections	91·1 (83·1–96·1)	90·2 (82·3–95·3)	95·5 (89·9–99·2)	88·4 (74·9–95·1)
HIV/AIDS	92·4 (85·1–96·8)	92 (84·6–96·5)	92·7 (86·1–96·5)	91·9 (84–96·5)
Sexually transmitted infections excluding HIV	73 (52·5–89·5)	74·8 (51·6–91·8)	100 (100–100)	43 (22·8–68·5)
Respiratory infections and tuberculosis	40·3 (27·6–53·8)	34·1 (24·1–44·2)	2·78 (2·05–3·7)	45·2 (32·9–57·5)
Tuberculosis	54·3 (44·6–62·5)	59·1 (49·8–66·4)	58·3 (49·2–66)	59·3 (50·1–66·7)
Lower respiratory infections	40·2 (27·5–53·8)	44·9 (32·6–57·4)	37·1 (30·7–42·7)	45 (32·6–57·6)
Upper respiratory infections	0·0178 (0·00719–0·0366)	0·00369 (0·000473–0·00973)	0·0031 (0–0·0089)	0·0586 (0·0218–0·11)
Otitis media	0·0961 (0·044–0·199)	3·08 (1·95–4·42)	3·09 (1·95–4·43)	0·476 (0·225–0·929)
Enteric infections	5·66 (3·92–8·33)	7·79 (5·92–10·5)	7·41 (5·53–10)	9·22 (6·25–13·6)
Diarrheal diseases	5·78 (4·01–8·51)	7·89 (5·98–10·6)	7·42 (5·53–10)	9·74 (6·51–14·5)
Other infectious diseases	3·73 (2·45–5·54)	3·5 (2·34–5·13)	0·603 (0·362–1·02)	4·24 (2·84–6·14)
Meningitis	0·593 (0·378–0·928)	1·03 (0·637–1·63)	0·000237 (0–0·000738)	1·63 (1·05–2·54)
Encephalitis	0·132 (0·0758–0·238)	0·354 (0·203–0·617)	0·000102 (0–0·000304)	0·399 (0·231–0·71)
Measles	0 (0–0)	0·202 (0–0·769)	0·202 (0–0·769)	0 (0–0)
Acute hepatitis	9·99 (6·58–14·5)	10·1 (6·75–14·4)	3·17 (1·98–5·12)	11 (7·41–15·8)
Maternal and neonatal disorders	82·5 (78·8–85·9)	75·1 (71·9–78·1)	72·6 (68·7–76·3)	83·5 (79·9–87)
Maternal disorders	8·92 (3·23–14·8)	8·86 (3·18–14·9)	8·87 (3·19–15)	8·82 (3·18–14·7)
Neonatal disorders	85·2 (81·5–88·5)	76·7 (73·3–79·6)	74·1 (70·2–77·8)	85·2 (81·5–88·5)
Nutritional deficiencies	68·3 (58·9–75·3)	73·1 (69·4–76·3)	73·3 (69·4–76·6)	67·6 (60·6–72·6)
Protein-energy malnutrition	100 (100–100)	100 (100–100)	100 (100–100)	100 (100–100)
Vitamin A deficiency	--	100 (100–100)	100 (100–100)	--
Dietary iron deficiency	--	100 (100–100)	100 (100–100)	--
Non-communicable diseases	57·5 (53·3–61·2)	40 (36–44)	23·4 (21–25·9)	57·3 (54·2–60·4)
Neoplasms	47·4 (44·1–51·1)	47·6 (44·5–51·1)	35 (31·5–38·6)	48·3 (45·4–51·8)
Lip and oral cavity cancer	73·9 (68–79)	76·3 (70·6–81·3)	75·8 (70–80·9)	76·3 (70·7–81·3)
Nasopharynx cancer	71·1 (62·5–78·2)	71·3 (63–78·5)	70·5 (62·2–77·6)	71·4 (63·1–78·5)
Other pharynx cancer	83·5 (78·6–87·5)	84 (78·8–88)	83·5 (78·3–87·6)	84·1 (78·9–88)
Esophageal cancer	77·3 (69·1–85·3)	78·5 (70·4–86·3)	77·7 (69·6–85·4)	78·5 (70·4–86·3)

Stomach cancer	21·5 (14·6–38)	22·8 (15·2–39·9)	22·8 (15·3–39·8)	22·8 (15·2–39·9)
Colon and rectum cancer	63·7 (55·8–71·1)	64·4 (57·3–71·4)	64·5 (57·2–71·6)	64·4 (57·3–71·4)
Liver cancer	76·2 (69·4–81·8)	77·9 (71·7–83)	76·6 (70·4–82·1)	78 (71·7–83)
Gallbladder and biliary tract cancer	18·8 (9·75–29·9)	19·3 (10–31·1)	18·7 (9·46–30·2)	19·3 (10–31·1)
Pancreatic cancer	35·1 (27·3–44·3)	35·6 (27·9–44·3)	35·4 (27·7–44·3)	35·6 (27·9–44·3)
Larynx cancer	85·3 (78·7–90·3)	85·2 (78·7–90·1)	85·4 (78·9–90·2)	85·2 (78·7–90·1)
Tracheal, bronchus, and lung cancer	84·7 (81·1–88·2)	83·1 (79·2–86·9)	83·7 (79·7–87·5)	83·1 (79·2–86·9)
Breast cancer	33·1 (26·3–40·6)	32·5 (26·1–39·1)	32·5 (26·1–39·1)	32·5 (26·2–39·1)
Cervical cancer	100 (100–100)	100 (100–100)	100 (100–100)	100 (100–100)
Uterine cancer	43·7 (29·5–58·7)	44·9 (30·6–59·6)	45·3 (30·8–60·4)	44·8 (30·6–59·5)
Ovarian cancer	16 (7·17–26·2)	13·1 (5·64–21·8)	12·3 (5·21–20·9)	13·1 (5·66–21·8)
Prostate cancer	6·05 (2·71–9·69)	6·89 (3·18–10·9)	7·47 (3·52–11·6)	6·78 (3·13–10·7)
Kidney cancer	36·9 (27·4–47)	37·7 (27·6–47·7)	37·5 (27·6–47·3)	37·7 (27·6–47·8)
Bladder cancer	40·8 (28·2–53·1)	44·4 (32·1–55·8)	45·5 (33·3–56·6)	44·3 (32–55·7)
Thyroid cancer	14·9 (6·64–25·5)	15·7 (6·98–27)	15·7 (7·3–26·3)	15·8 (6·96–27·1)
Mesothelioma	98·2 (97–99)	96·8 (94·9–98)	97·9 (96·5–98·8)	96·8 (94·9–98)
Non-Hodgkin lymphoma	7·19 (3·04–13)	7·41 (3·08–13·4)	7·27 (3–13·2)	7·42 (3·1–13·4)
Multiple myeloma	7·95 (3·35–14·3)	8·26 (3·55–14·7)	8·25 (3·43–14·6)	8·26 (3·55–14·7)
Leukemia	35·8 (22·9–48·7)	34·4 (22·5–45·7)	34 (22·3–45·6)	34·4 (22·6–45·7)
Cardiovascular diseases	80·6 (77–84·2)	82·4 (79·4–85·2)	78 (74·1–81·7)	83·1 (80·1–85·8)
Rheumatic heart disease	27·6 (16·3–49·6)	29 (19·3–45·5)	29·2 (19·5–45·7)	28·9 (19·3–45·5)
Ischemic heart disease	93·5 (89·7–96·6)	94·9 (92·4–97·1)	94·5 (92·1–96·8)	94·9 (92·4–97·1)
Stroke	81·5 (73·9–88·7)	85 (79·9–89·9)	84·8 (80·2–89·4)	85·1 (79·7–90·3)
Hypertensive heart disease	100 (100–100)	100 (100–100)	100 (100–100)	100 (100–100)
Non-rheumatic valvular heart disease	29·9 (20·2–43·9)	31·7 (23·2–42·3)	32·4 (22·6–43·3)	31·6 (23–42·6)
Cardiomyopathy and myocarditis	43·7 (34·5–54·5)	52·4 (45·6–60)	52·1 (45·1–59·6)	52·5 (45·5–60·3)
Atrial fibrillation and flutter	55·1 (45·5–65)	63·5 (55·9–71)	68·2 (61·3–74·6)	59 (50·2–67·4)
Aortic aneurysm	56·2 (49·1–64)	65 (59·1–70·7)	--	65 (59·1–70·7)
Endocarditis	38·1 (27·3–51·7)	42·1 (32·7–52·4)	38·7 (28·8–50·7)	42·2 (32·8–52·6)
Other cardiovascular and circulatory diseases	35·6 (28·7–43·5)	39·6 (33·7–46)	44·2 (37·2–51·2)	38 (32·2–44·7)
Chronic respiratory diseases	62·9 (55·9–69·7)	58·4 (53·1–63·1)	52·7 (48–57·3)	63·3 (56·1–70·4)
Chronic obstructive pulmonary disease	71·5 (66–77·2)	70 (65·3–74·3)	62·2 (57·9–66)	74·7 (69·6–79·5)
Pneumoconiosis	100 (100–100)	100 (100–100)	100 (100–100)	100 (100–100)

Asthma	42·5 (31·5–54·9)	44·7 (35·9–54·6)	44·5 (35·7–54·2)	47·1 (37·4–58)
Digestive diseases	42·5 (35·5–49·4)	44·3 (37–50·8)	12·8 (8·63–17·9)	55·3 (47·6–62·4)
Cirrhosis and other chronic liver diseases	78·8 (68·5–87·2)	81·4 (71·9–89)	75·9 (67·8–83·7)	81·5 (71·9–89·2)
Upper digestive system diseases	19·8 (16·2–23·6)	4·44 (2·51–6·88)	0·823 (0·468–1·31)	24·4 (20·4–28·3)
Gallbladder and biliary diseases	40·7 (27·1–55·4)	47 (33·5–61·5)	50·3 (36·4–64·5)	42·3 (29–56·8)
Pancreatitis	45·9 (34·7–59)	50·3 (39·1–63·5)	45·7 (34·3–58·8)	50·7 (39·5–63·9)
Neurological disorders	24·2 (11·8–37·4)	14·4 (6·89–25·5)	8·37 (3·82–17·1)	22·2 (10·6–35·2)
Alzheimer's disease and other dementias	33·5 (21·3–47·8)	36·5 (24·2–50·7)	36·8 (24·4–50·8)	36·3 (23·8–50·4)
Parkinson's disease	-8·63 (-13·8–-4·06)	-10·5 (-16·4–-4·93)	-11·3 (-17·5–-5·33)	-10·3 (-16·2–-4·86)
Idiopathic epilepsy	29·8 (22·3–37·4)	27·5 (20·5–34·4)	25·5 (18·9–32·4)	30·6 (23·2–38·1)
Multiple sclerosis	16·8 (12·2–21·5)	18·4 (13·9–23·3)	18 (13·3–22·7)	18·7 (13·8–23·8)
Mental disorders	--	5 (2·58–7·97)	5 (2·58–7·97)	--
Depressive disorders	--	10·4 (4·7–16·7)	10·4 (4·7–16·7)	--
Anxiety disorders	--	4·59 (1·21–10·1)	4·59 (1·21–10·1)	--
Idiopathic developmental intellectual disability	--	51·5 (25·2–71·4)	51·5 (25·2–71·4)	--
Substance use disorders	100 (100–100)	100 (100–100)	100 (100–100)	100 (100–100)
Alcohol use disorders	100 (100–100)	100 (100–100)	100 (100–100)	100 (100–100)
Drug use disorders	100 (100–100)	100 (100–100)	100 (100–100)	100 (100–100)
Diabetes and kidney diseases	100 (99·9–100)	100 (100–100)	100 (100–100)	100 (99·9–100)
Diabetes mellitus	100 (100–100)	100 (100–100)	100 (100–100)	100 (100–100)
Chronic kidney disease	100 (100–100)	100 (100–100)	100 (100–100)	100 (100–100)
Sense organ diseases	--	7·6 (6·66–8·71)	7·6 (6·66–8·71)	--
Blindness and vision loss	--	7·55 (5·11–10·9)	7·55 (5·11–10·9)	--
Age-related and other hearing loss	--	8·99 (8·14–10)	8·99 (8·14–10)	--
Musculoskeletal disorders	3·55 (1·01–6·75)	23·1 (18·9–27·7)	23·2 (19·1–27·8)	4·19 (1·24–7·83)
Rheumatoid arthritis	11·6 (3·14–21·7)	15·8 (4·8–27·7)	15·9 (4·88–27·8)	14·1 (4–25·8)
Osteoarthritis	--	13·1 (6·68–20·5)	13·1 (6·68–20·5)	--
Low back pain	--	42·9 (37·3–48·8)	42·9 (37·3–48·8)	--
Gout	--	48·9 (32·9–66·2)	48·9 (32·9–66·2)	--
Other non-communicable diseases	0·0471 (0·0208–0·0931)	0·0257 (0·0107–0·0521)	--	0·209 (0·0969–0·389)
Sudden infant death syndrome	7·72 (4·7–13)	7·71 (4·69–13)	--	7·71 (4·69–13)
Injuries	36·5 (29·7–42)	29 (23·9–33·7)	33·4 (28·9–37·8)	24·4 (17·2–30·4)
Transport injuries	18·6 (10·4–26·3)	19·9 (12·7–27)	28·2 (23·9–32·7)	16·8 (8·14–25)

Road injuries	17·2 (8·56–25·3)	18·9 (11·4–26·3)	27·6 (23·4–32·2)	15·5 (6·35–24)
Other transport injuries	35·4 (30·1–40·4)	33·5 (28·1–38·6)	37·4 (32·5–41·6)	32·4 (26·7–37·7)
Unintentional injuries	51 (42·8–56·5)	35·6 (30·1–40·3)	34·3 (29–39·1)	38·9 (32·5–44·1)
Falls	56·3 (47·5–62·1)	43·3 (36·7–48·4)	41·7 (35·5–46·7)	47 (39·8–52·3)
Drowning	-8·89 (-28·8–8·89)	-7·17 (-25·7–9·65)	12·8 (8·05–19)	-8·46 (-28·7–9·2)
Fire, heat, and hot substances	12·9 (7·96–19·7)	16·1 (10·8–22·9)	16·1 (10·7–23·1)	16·2 (10·9–22·6)
Poisonings	14·6 (9·62–20·2)	15·5 (10·5–21·7)	14·7 (9·88–21·2)	16·7 (11·4–22·6)
Exposure to mechanical forces	6·81 (-5·92–19·6)	18·5 (14·8–22·7)	20·1 (16·9–23·7)	2·01 (-11·6–15·6)
Animal contact	27·1 (21·7–33)	23·8 (18·9–30)	23·8 (18·7–29·9)	23·9 (18·7–29·5)
Foreign body	3·65 (2·52–5·17)	5·57 (3·84–8·08)	5·13 (3·41–7·7)	6·08 (4·22–8·7)
Environmental heat and cold exposure	9·28 (4·28–16·1)	9·99 (4·83–17)	9·91 (4·79–16·6)	10·2 (4·82–17·2)
Exposure to forces of nature	0 (0–0)	0 (0–0)	0 (0–0)	0 (0–0)
Other unintentional injuries	17·4 (11·6–24·1)	15·8 (10·6–22·5)	15·7 (10·4–22·4)	19·6 (13·5–27·1)
Self-harm and interpersonal violence	16·5 (-0·0302–31·6)	19·3 (5·4–32·5)	33·2 (26·9–39·9)	17·3 (0·881–32·2)
Self-harm	16·1 (-1·18–32·1)	17·3 (0·197–33·1)	23·2 (12·3–34)	17 (-0·261–33)
Interpersonal violence	23·4 (11·4–34·9)	31 (22·5–40)	37·2 (29·4–45·6)	21·7 (9·41–33·6)

Supplementary Results Table 2. All-age PAFs attributable to the joint distribution of all risk factors for all ages, both sexes combined for each GBD level 1, level 2, and level 3 cause and all causes in 2019 for Austria. Results are shown for deaths, YLLs, YLDs, and DALYs Data in parenthesis are 95% uncertainty intervals. PAFs=population attributable fractions. GBD=Global Burden of Disease. YLLs=years of life lost. YLDs=years lived with disability. DALYs=disability-adjusted life-years

	Deaths 2019	DALYs 2019	YLDs 2019	YLLs 2019
All causes	61·8 (58–64·8)	43·2 (40–46·6)	26·5 (24·3–28·8)	59·2 (56·7–61·3)
Communicable, maternal, neonatal, and nutritional diseases	44·8 (38·7–50·6)	48·8 (45·4–52·4)	42·7 (38·8–47·1)	57 (52·4–61)
HIV/AIDS and sexually transmitted infections	86·3 (85·2–87·2)	87·3 (85·8–89·1)	90·7 (88·9–93·1)	83·7 (82·5–84·7)
HIV/AIDS	85·6 (84·5–86·6)	85·1 (83·8–86·3)	87·2 (85·8–88·5)	83·3 (82·1–84·4)
Sexually transmitted infections excluding HIV	99·8 (99·8–99·9)	99·9 (99·9–100)	100 (100–100)	99·3 (99·1–99·6)
Respiratory infections and tuberculosis	47·9 (40–55·7)	35 (28·3–42·2)	3·84 (2·79–5·16)	53 (45·7–60)
Tuberculosis	60·7 (51·7–67·9)	64·9 (56·5–71·4)	61·1 (52·5–68·6)	65·7 (57·6–72·2)
Lower respiratory infections	47·5 (39·3–55·5)	52·2 (44·4–59·5)	42·5 (36·6–48·2)	52·5 (44·6–59·9)
Upper respiratory infections	0·236 (0·0785–0·452)	0·0117 (0·00394–0·0225)	0·00585 (0·000251–0·0149)	0·572 (0·189–1·08)
Otitis media	0·0158 (0·00675–0·0324)	3·59 (2·25–5·24)	3·6 (2·25–5·26)	0·0889 (0·0375–0·188)
Enteric infections	10·5 (7·39–14·3)	13·7 (10·5–17·5)	13·3 (10–17)	15·2 (11·5–19·5)
Diarrheal diseases	10·6 (7·45–14·5)	13·7 (10·6–17·6)	13·3 (10–17·1)	15·4 (11·7–19·7)
Other infectious diseases	0·599 (0·43–0·966)	1·18 (0·898–1·61)	0·445 (0·271–0·77)	1·55 (1·13–2·23)
Meningitis	1·34 (1–1·76)	3·19 (2·42–4·1)	0·000724 (2·01e-05–0·00202)	3·94 (3·01–5·06)
Encephalitis	0·188 (0·119–0·281)	0·415 (0·259–0·641)	0·000138 (3·57e-06–0·000386)	0·557 (0·352–0·857)
Measles	2·52 (-0·736–13·5)	2·29 (-0·439–11·8)	0·609 (0·0198–1·53)	3·34 (-0·962–18·5)
Acute hepatitis	8·21 (4·55–17·1)	4·16 (2·65–6·75)	2·77 (1·67–4·76)	8·5 (4·79–17)
Maternal and neonatal disorders	82·3 (79·3–85)	73·2 (70–76)	65·7 (61·2–69·5)	82·9 (80–85·7)
Maternal disorders	8·9 (3·19–14·8)	8·89 (3·2–14·8)	8·93 (3·21–14·9)	8·81 (3·16–14·7)
Neonatal disorders	84·1 (81·2–86·8)	74·7 (71·4–77·7)	67·4 (62·7–71·4)	84·1 (81·2–86·8)
Nutritional deficiencies	20·1 (15·9–31·4)	72·7 (69·2–75·8)	73·6 (70–76·7)	25·5 (22·3–31·6)
Protein-energy malnutrition	100 (100–100)	100 (100–100)	100 (100–100)	100 (100–100)
Vitamin A deficiency	--	100 (100–100)	100 (100–100)	--
Dietary iron deficiency	--	100 (100–100)	100 (100–100)	--
Non-communicable diseases	63·7 (59·6–67)	44·1 (40·5–47·7)	24·7 (22·3–27·2)	62·4 (59·7–64·8)
Neoplasms	44 (40·6–48)	45·4 (42·4–48·9)	31·3 (27·6–35·1)	46·3 (43·3–49·8)
Lip and oral cavity cancer	73 (66·9–78·2)	76·2 (70·5–80·8)	74·4 (68·5–79·3)	76·2 (70·6–80·9)
Nasopharynx cancer	69·7 (61·4–76·6)	70·8 (63·1–77·3)	67·9 (59·6–74·9)	71 (63·3–77·5)
Other pharynx cancer	83·6 (78·8–87·3)	84·6 (80–88·3)	82·9 (77·9–86·9)	84·7 (80–88·4)
Esophageal cancer	75·9 (68·1–83·1)	77·7 (70·5–84·8)	75·8 (68–83·2)	77·7 (70·5–84·8)
Stomach cancer	24·6 (16·1–43·8)	26 (17·2–44·8)	26 (17·1–44·9)	26 (17·3–44·8)

Colon and rectum cancer	61·4 (53·5–68·9)	62·5 (55·1–69·5)	62·5 (55–69·5)	62·5 (55·1–69·5)
Liver cancer	83·7 (78·5–88·2)	84·9 (80·1–88·8)	83·7 (78·5–88·2)	84·9 (80·1–88·8)
Gallbladder and biliary tract cancer	20·1 (10·6–31·5)	20·3 (10·8–32)	19·6 (10·4–31·1)	20·3 (10·9–32)
Pancreatic cancer	37 (29·7–46·1)	38·1 (31·2–46·6)	37·4 (30·3–46·3)	38·1 (31·2–46·6)
Larynx cancer	84·6 (77·9–89·4)	85·7 (79·6–90)	85 (78·9–89·4)	85·7 (79·7–90·1)
Tracheal, bronchus, and lung cancer	83·7 (80·9–86·6)	83·5 (80·8–86·3)	83·2 (80·3–86·2)	83·5 (80·8–86·3)
Breast cancer	34·4 (27·1–42·7)	34·3 (27·8–41·4)	34·3 (27·8–41·5)	34·3 (27·8–41·4)
Cervical cancer	100 (100–100)	100 (100–100)	100 (100–100)	100 (100–100)
Uterine cancer	41·8 (27·5–56·5)	42·8 (28·5–57·7)	43 (29–58)	42·7 (28·4–57·6)
Ovarian cancer	15·4 (7·35–26·1)	13·4 (6·11–22·8)	12·9 (5·78–22·2)	13·4 (6·13–22·8)
Prostate cancer	6·38 (2·89–10·1)	7·51 (3·49–11·8)	8·57 (4·04–13·3)	7·3 (3·37–11·5)
Kidney cancer	35·7 (26·4–45·4)	37·1 (27·9–46·8)	37·2 (28·2–47)	37·1 (27·9–46·8)
Bladder cancer	36·8 (24·9–49·1)	41·6 (30·3–52·9)	43·4 (31·8–54·7)	41·4 (30–52·6)
Thyroid cancer	13·3 (6·23–22·5)	13·9 (6·52–23·5)	13·6 (6·69–22·5)	13·9 (6·44–23·7)
Mesothelioma	96·5 (95·2–97·5)	93·8 (91·7–95·5)	96 (94·5–97·2)	93·7 (91·7–95·5)
Non-Hodgkin lymphoma	6·42 (2·56–11·6)	6·58 (2·7–11·9)	6·51 (2·66–11·8)	6·58 (2·7–11·9)
Multiple myeloma	7·41 (3·11–13·3)	7·63 (3·26–13·7)	7·61 (3·18–13·7)	7·63 (3·26–13·7)
Leukemia	34·1 (22–46·5)	34·1 (23·2–45·6)	34 (23–45·4)	34·1 (23·1–45·6)
Cardiovascular diseases	82 (78·3–85·4)	82·9 (79·9–85·8)	75·4 (71·1–79·4)	84 (81·2–86·8)
Rheumatic heart disease	26·5 (13·8–52·9)	27·7 (17–48·9)	28·7 (18·5–47·8)	27·6 (16·8–49·2)
Ischemic heart disease	93·3 (89·3–96·7)	94·5 (91·5–97)	94·2 (91·2–96·8)	94·5 (91·6–97)
Stroke	81·5 (73·9–89·3)	84·6 (79·1–90·4)	84·6 (79·3–90)	84·6 (79·1–90·7)
Hypertensive heart disease	100 (100–100)	100 (100–100)	100 (100–100)	100 (100–100)
Non-rheumatic valvular heart disease	28·6 (17·9–42·7)	29·3 (20·1–40·7)	29·5 (20·1–40·2)	29·2 (19·9–41)
Cardiomyopathy and myocarditis	36·4 (25·4–49·8)	41·7 (32·7–52·6)	39·7 (31–50·5)	41·9 (32·8–53)
Atrial fibrillation and flutter	54·4 (43·9–64·2)	62·4 (54·6–70)	66·8 (59·5–73·8)	57·5 (48·6–66·3)
Aortic aneurysm	60 (53·6–66·9)	68·9 (64·2–73·4)	--	68·9 (64·2–73·4)
Endocarditis	38·4 (27·1–52·8)	41·4 (31·3–52·5)	38·8 (27·8–52·4)	41·5 (31·3–52·5)
Other cardiovascular and circulatory diseases	35·9 (28·2–44)	39·5 (33–45·7)	42·4 (36–49·2)	38·5 (31·4–45)
Chronic respiratory diseases	68·8 (64·9–72·5)	63 (59·7–66·5)	54·1 (49·5–58·5)	70·9 (67·4–74·1)
Chronic obstructive pulmonary disease	73·6 (69·5–77·4)	72·8 (69·5–76·1)	65·4 (61·9–68·8)	76·9 (73·3–80·2)
Pneumoconiosis	100 (100–100)	100 (100–100)	100 (100–100)	100 (100–100)
Asthma	41·9 (30·4–54·1)	43·9 (35·2–53·4)	43·8 (35·1–53·3)	45·9 (35·4–57·8)
Digestive diseases	50·1 (44–55·3)	47·2 (41·1–52·6)	15·4 (10·6–21·1)	61·4 (54·6–66·9)
Cirrhosis and other chronic liver diseases	79·8 (69·2–87·9)	82·1 (72·3–89·5)	78·9 (71–86)	82·1 (72·3–89·6)

Upper digestive system diseases	20·4 (17·3–23·8)	6·33 (3·91–9·34)	1·12 (0·625–1·81)	25·9 (22·2–29·7)
Gallbladder and biliary diseases	41·3 (26·8–55·9)	46·9 (33·2–60·5)	48·2 (34·8–62)	42·6 (28·4–57·1)
Pancreatitis	46·9 (36–59·7)	51·5 (40·6–63·9)	48·5 (37·4–60·8)	52 (41–64·4)
Neurological disorders	23·1 (10·2–36·7)	15·2 (7·58–26·1)	10·2 (5·23–18·9)	21·6 (9·94–35)
Alzheimer's disease and other dementias	34·2 (21·9–48·3)	37·6 (25·3–51·2)	38·3 (26–51·8)	37·2 (25·2–50·8)
Parkinson's disease	-9·59 (-14·4–-5·15)	-11·9 (-17·7–-6·46)	-12·9 (-19·3–-7·06)	-11·7 (-17·4–-6·29)
Idiopathic epilepsy	29·4 (22·1–36·9)	27·4 (20·4–34·2)	25·9 (19·4–32·3)	30·1 (22·9–37·3)
Multiple sclerosis	17·7 (12·9–22·9)	20·5 (15·3–25·5)	20·3 (15·4–25·2)	20·6 (15·1–25·9)
Mental disorders	--	5·19 (2·81–8·1)	5·2 (2·82–8·11)	--
Depressive disorders	--	9·48 (5·03–14·6)	9·48 (5·03–14·6)	--
Anxiety disorders	--	6·94 (2·15–14)	6·94 (2·15–14)	--
Idiopathic developmental intellectual disability	--	50·5 (24–70·6)	50·5 (24–70·6)	--
Substance use disorders	100 (100–100)	100 (100–100)	100 (100–100)	100 (100–100)
Alcohol use disorders	100 (100–100)	100 (100–100)	100 (100–100)	100 (100–100)
Drug use disorders	100 (100–100)	100 (100–100)	100 (100–100)	100 (100–100)
Diabetes and kidney diseases	100 (100–100)	100 (100–100)	100 (100–100)	100 (100–100)
Diabetes mellitus	100 (100–100)	100 (100–100)	100 (100–100)	100 (100–100)
Chronic kidney disease	100 (100–100)	100 (100–100)	100 (100–100)	100 (100–100)
Sense organ diseases	--	7·15 (6·22–8·32)	7·15 (6·22–8·32)	--
Blindness and vision loss	--	8·57 (5·82–12·1)	8·57 (5·82–12·1)	--
Age-related and other hearing loss	--	7·83 (7·01–8·74)	7·83 (7·01–8·74)	--
Musculoskeletal disorders	2·95 (0·92–5·28)	22 (17·6–26·4)	22·2 (17·8–26·8)	3·79 (1·2–6·67)
Rheumatoid arthritis	11·6 (3·58–20·9)	18·5 (6·01–31·5)	19·2 (6·29–32·8)	15 (4·79–26·3)
Osteoarthritis	--	12·9 (6·72–20·3)	12·9 (6·72–20·3)	--
Low back pain	--	43·2 (36·7–49·7)	43·2 (36·7–49·7)	--
Gout	--	49·4 (35·1–65·5)	49·4 (35·1–65·5)	--
Other non-communicable diseases	0·0339 (0·0207–0·0536)	0·022 (0·0129–0·0351)	--	0·112 (0·0683–0·182)
Sudden infant death syndrome	5·07 (3·42–7·21)	5·07 (3·42–7·2)	--	5·07 (3·42–7·2)
Injuries	33·7 (28·1–38·8)	32·8 (28·4–37·1)	38·2 (33·8–42·5)	27·6 (21·3–33·4)
Transport injuries	28·1 (22–34·4)	30·3 (24·5–36·5)	33·7 (29·5–38·2)	28·8 (21·9–36·2)
Road injuries	26 (19·6–32·5)	28·8 (22·9–35·2)	32·7 (28·5–37·2)	27·1 (19·7–34·5)
Other transport injuries	42·9 (37·9–47·8)	42 (36·6–47·3)	42·8 (38·2–47)	41·7 (36–47·6)
Unintentional injuries	43·3 (37·1–47·5)	37·2 (32·3–41·6)	38·7 (33·8–43·4)	33·5 (28·9–37·9)
Falls	56 (48–60·9)	46·7 (40·9–51·5)	45·7 (40–50·5)	49·4 (43–54·1)
Drowning	-6·12 (-19·2–5·93)	-2·28 (-14·3–9·09)	15·2 (10·7–21·2)	-3·26 (-15·9–8·45)

Fire, heat, and hot substances	14·2<br>(9·73–20·2)	21<br>(15·7–27·3)	21·4<br>(16·2–27·8)	19·5<br>(14·2–25·5)
Poisonings	23·8<br>(17·9–30·5)	25·4<br>(19·9–32·3)	21·2<br>(16–27·4)	29·1<br>(22·7–36·5)
Exposure to mechanical forces	13·1<br>(4·51–22)	25·3<br>(20·8–30·3)	27·6<br>(23·7–32)	12·4<br>(2·86–22)
Animal contact	28·5<br>(23·6–34·7)	29·1<br>(24·1–35)	30<br>(24·7–35·7)	27·9<br>(22·7–34·3)
Foreign body	7·29<br>(5·41–9·67)	10·9<br>(8·16–14·4)	9·69<br>(7·27–12·7)	11·5<br>(8·56–15·3)
Environmental heat and cold exposure	10<br>(4·76–17)	10·6<br>(5·01–17·8)	9·9<br>(4·67–16·8)	11·1<br>(5·22–18·6)
Exposure to forces of nature	9·53<br>(4·51–16·1)	9·21<br>(4·35–15·5)	10·6<br>(5·04–17·8)	8·97<br>(4·24–15·1)
Other unintentional injuries	28<br>(21·5–35·5)	21·2<br>(15·7–27·3)	20·5<br>(15·2–26·6)	30·5<br>(23·7–38·4)
Self-harm and interpersonal violence	21·3<br>(7·9–34·1)	23·7<br>(11·1–35)	36·9<br>(30·1–43·2)	22·4<br>(8·88–34·8)
Self-harm	20·6<br>(6·66–34·2)	21·9<br>(7·72–35·3)	24·9<br>(13·5–35)	21·8<br>(7·48–35·3)
Interpersonal violence	34·5<br>(25·7–43·6)	37·2<br>(29·3–45·5)	42·8<br>(35·2–50·6)	30·9<br>(21·8–40·4)

Supplementary Results Table 2. All-age PAFs attributable to the joint distribution of all risk factors for all ages, both sexes combined for each GBD level 1, level 2, and level 3 cause and all causes in 2019 for Belgium. Results are shown for deaths, YLLs, YLDs, and DALYs Data in parenthesis are 95% uncertainty intervals. PAFs=population attributable fractions. GBD=Global Burden of Disease. YLLs=years of life lost. YLDs=years lived with disability. DALYs=disability-adjusted life-years

	Deaths 2019	DALYs 2019	YLDs 2019	YLLs 2019
All causes	55·7 (52·6–58·4)	41·6 (38·6–44·7)	26·7 (24·1–29·2)	55 (52·9–57·2)
Communicable, maternal, neonatal, and nutritional diseases	45·5 (39·6–51·1)	48·4 (44·8–52)	43·5 (39·5–47·7)	51·1 (46·1–55·8)
HIV/AIDS and sexually transmitted infections	93·9 (93·3–94·4)	93·5 (92·4–94·6)	97·2 (96·5–97·9)	90·1 (89·3–90·8)
HIV/AIDS	93·4 (92·8–93·9)	92·6 (91·3–93·7)	96·3 (95·6–96·9)	89·7 (88·9–90·5)
Sexually transmitted infections excluding HIV	99·9 (99·9–100)	100 (99·9–100)	100 (100–100)	99·7 (99·6–99·8)
Respiratory infections and tuberculosis	48·9 (41·4–55·9)	45·4 (38·9–52)	4·5 (3·33–5·96)	52·4 (45·2–59·2)
Tuberculosis	59·4 (49·8–67·3)	62·2 (53·4–69·2)	60·2 (50·9–67·5)	62·6 (54–69·7)
Lower respiratory infections	48·8 (41·2–55·9)	52·2 (44·7–59·1)	39·6 (33·8–45·3)	52·3 (44·9–59·3)
Upper respiratory infections	0·0821 (0·041–0·134)	0·00993 (0·00342–0·0197)	0·00615 (0·000314–0·0156)	0·335 (0·18–0·536)
Otitis media	0·268 (0·0774–0·796)	3·43 (2·16–4·88)	3·45 (2·16–4·91)	1·42 (0·417–3·84)
Enteric infections	6·78 (4·25–10·2)	8·92 (6·37–12·1)	9·12 (6·51–12·3)	8·67 (5·96–12·2)
Diarrheal diseases	6·8 (4·26–10·3)	8·94 (6·39–12·2)	9·13 (6·52–12·3)	8·71 (5·98–12·2)
Other infectious diseases	0·682 (0·467–0·881)	1·15 (0·915–1·41)	0·531 (0·327–0·87)	1·3 (1·02–1·66)
Meningitis	1·12 (0·813–1·52)	2·42 (1·75–3·22)	0·000574 (2·52e-05–0·00149)	2·96 (2·17–3·93)
Encephalitis	0·283 (0·202–0·384)	0·815 (0·591–1·1)	0·000252 (1·08e-05–0·000653)	0·895 (0·65–1·2)
Measles	3·47 (-0·914–19·7)	2·42 (-0·364–12·9)	0·531 (0·0247–1·28)	4·29 (-1·14–23·7)
Acute hepatitis	26·6 (11·8–36·1)	9·72 (5·25–13·7)	2·87 (1·78–4·65)	27·2 (12·5–36)
Maternal and neonatal disorders	77·7 (74·9–80·4)	73·6 (70·9–75·9)	68·7 (64·5–72·3)	78·5 (75·5–81·2)
Maternal disorders	8·67 (3·09–14·3)	8·58 (3·06–14·1)	8·58 (3·06–14·2)	8·57 (3·07–14)
Neonatal disorders	79·9 (76·8–82·6)	75·4 (72·7–77·7)	70·9 (66·8–74·7)	79·9 (76·8–82·6)
Nutritional deficiencies	91·1 (68·2–94·2)	76·6 (72·1–79·2)	73·3 (70·5–75·8)	89·8 (67·4–92·7)
Protein-energy malnutrition	100 (100–100)	100 (100–100)	100 (100–100)	100 (100–100)
Vitamin A deficiency	--	100 (100–100)	100 (100–100)	--
Dietary iron deficiency	--	100 (100–100)	100 (100–100)	--
Non-communicable diseases	58·3 (54·8–61·5)	42·6 (39·1–46·1)	24·8 (22–27·7)	59·1 (56·6–61·6)
Neoplasms	48 (44·5–51·7)	49·1 (46–52·5)	35·5 (31·5–39·4)	49·9 (46·8–53·2)
Lip and oral cavity cancer	74·6 (69–79·4)	76·7 (71·2–81·3)	76·2 (70·7–81)	76·8 (71·3–81·3)
Nasopharynx cancer	71·1 (62·9–78·2)	71·3 (63·1–78·1)	69·9 (61·6–77·1)	71·4 (63·3–78·2)
Other pharynx cancer	83·1 (78·1–87·1)	83·9 (79–87·8)	83·3 (78·3–87·2)	83·9 (79–87·8)
Esophageal cancer	75·4 (67·6–82·8)	77 (69·5–83·9)	75·7 (67·9–83·1)	77 (69·5–83·9)
Stomach cancer	24·5 (16·2–43·6)	25·8 (17·6–44·5)	25·9 (17·5–44·3)	25·8 (17·6–44·5)

Colon and rectum cancer	62·7 (54·8–70·2)	63·9 (56·5–71)	64 (56·7–71·1)	63·8 (56·5–70·9)
Liver cancer	77·5 (71·7–82·8)	78·9 (73·6–84)	77·9 (72·6–83)	78·9 (73·7–84)
Gallbladder and biliary tract cancer	19·3 (10·2–30·2)	19·3 (10·6–30·6)	19 (10·1–30·1)	19·3 (10·6–30·6)
Pancreatic cancer	37 (29·5–46)	37·7 (30·8–46·4)	37·4 (30·2–46·2)	37·7 (30·8–46·4)
Larynx cancer	84·9 (78·1–89·7)	85·5 (79·4–89·9)	85·5 (79·6–89·9)	85·5 (79·4–89·9)
Tracheal, bronchus, and lung cancer	86·1 (83·5–88·6)	85·1 (82·5–87·5)	85·5 (82·7–88)	85·1 (82·5–87·5)
Breast cancer	36 (28·7–44·4)	35·9 (29·4–43·3)	36 (29·4–43·5)	35·9 (29·4–43·3)
Cervical cancer	100 (100–100)	100 (100–100)	100 (100–100)	100 (100–100)
Uterine cancer	40·8 (26·6–55·2)	41·7 (27·8–55·9)	42·1 (28·4–56·4)	41·6 (27·8–55·9)
Ovarian cancer	18·5 (9·06–29·4)	15·9 (7·58–26)	15·1 (7·33–25)	15·9 (7·59–26)
Prostate cancer	6·59 (2·87–10·6)	7·35 (3·21–11·8)	7·91 (3·49–12·6)	7·26 (3·17–11·6)
Kidney cancer	35·4 (26·2–45·1)	36·3 (27·2–45·6)	36·4 (27·4–45·8)	36·3 (27·2–45·6)
Bladder cancer	37·4 (24·5–49·8)	41·3 (28·6–52·8)	42·8 (30·4–54)	41·2 (28·5–52·6)
Thyroid cancer	12·8 (5·93–21·8)	13·5 (6·04–23·5)	13·6 (6·22–23·5)	13·5 (6·01–23·5)
Mesothelioma	98·4 (97·7–98·9)	97 (95·9–97·9)	98·1 (97·2–98·7)	97 (95·9–97·9)
Non-Hodgkin lymphoma	6·01 (2·43–10·9)	6·17 (2·53–11·1)	6·09 (2·5–11)	6·18 (2·53–11·2)
Multiple myeloma	6·89 (2·96–12·3)	7·14 (3·05–12·7)	7·12 (3·04–12·9)	7·14 (3·05–12·7)
Leukemia	33·3 (20·3–46·6)	33·2 (21·5–44·9)	33 (21·7–44·6)	33·2 (21·5–44·9)
Cardiovascular diseases	77·6 (73·6–81·3)	80·3 (77·2–83·2)	76·4 (72·8–79·7)	80·9 (77·8–83·8)
Rheumatic heart disease	26·8 (14–53·7)	28·2 (17–50·2)	29·4 (18·1–47·6)	28·1 (16·6–50·7)
Ischemic heart disease	93·2 (89·4–96·6)	94·5 (91·7–96·9)	94·1 (91·3–96·6)	94·5 (91·7–96·9)
Stroke	82·1 (75·4–88·7)	85·1 (80·1–90)	84·7 (79·8–89·4)	85·1 (80·1–90·2)
Hypertensive heart disease	100 (100–100)	100 (100–100)	100 (100–100)	100 (100–100)
Non-rheumatic valvular heart disease	31·9 (20–49·3)	33·1 (22–48)	30·7 (20·9–41·5)	33·1 (22·1–48)
Cardiomyopathy and myocarditis	37·5 (27·7–51·1)	44·8 (36·7–54·4)	43·9 (35·9–53·1)	44·9 (36·7–54·7)
Atrial fibrillation and flutter	55·1 (46–64·3)	62 (54·7–69·3)	67·1 (60·4–73·5)	58·3 (50·3–66·4)
Aortic aneurysm	56·8 (49·6–63·9)	65·2 (59·5–70·4)	--	65·2 (59·5–70·4)
Endocarditis	37·9 (25·3–54·4)	40·6 (29·4–53·7)	38·6 (26·7–53·8)	40·8 (29·5–53·7)
Other cardiovascular and circulatory diseases	36·7 (29·9–44·3)	41·3 (35·8–47·1)	44·7 (38·7–50·3)	40 (34·4–46)
Chronic respiratory diseases	67·3 (62·7–71·6)	63·2 (59·8–66·7)	55 (51–58·8)	69 (65·1–72·7)
Chronic obstructive pulmonary disease	72·1 (67·8–76·1)	71·1 (67·7–74·3)	63·9 (60·5–67·3)	75 (71·5–78·5)
Pneumoconiosis	100 (100–100)	100 (100–100)	100 (100–100)	100 (100–100)
Asthma	41·2 (30·1–52·8)	40·4 (31·9–49·8)	39·8 (31·5–49·1)	44·9 (34·6–55·8)
Digestive diseases	37·1 (32·9–41·8)	41·5 (36·8–46·2)	12·3 (8·54–16·7)	49·6 (44·7–54·5)
Cirrhosis and other chronic liver diseases	82·1 (73·3–89·4)	84·4 (76·2–90·7)	79·8 (73·1–86·1)	84·5 (76·2–90·8)

Upper digestive system diseases	19·9 (16·5–23·1)	7·49 (4·8–10·7)	0·848 (0·482–1·35)	24·7 (20·8–28·4)
Gallbladder and biliary diseases	41 (26·9–55·1)	44·3 (31·1–57·7)	47·5 (34·3–60·8)	41·7 (28–55·3)
Pancreatitis	48·2 (37·4–60·8)	52 (41·2–63·5)	49 (38–60·5)	52·4 (41·6–64)
Neurological disorders	23·4 (11·1–36·8)	14·7 (7·34–25·6)	9·86 (4·65–20·4)	21·5 (10·4–34·7)
Alzheimer's disease and other dementias	35·4 (22·9–49·2)	38·2 (25·8–51·8)	38·7 (26·5–52·1)	37·9 (25·7–51·4)
Parkinson's disease	-10·5 (-15·9–-5·52)	-12·2 (-18·3–-6·53)	-12·7 (-18·6–-7·02)	-12·1 (-18·2–-6·46)
Idiopathic epilepsy	29·4 (21·7–37·1)	27·9 (20·8–34·8)	26·5 (20–33·2)	29·8 (22·4–37)
Multiple sclerosis	16 (11·5–21·3)	17·9 (13·1–22·9)	17·7 (13–22·3)	18·1 (13·1–23·6)
Mental disorders	--	6·73 (3·61–10·4)	6·73 (3·61–10·4)	--
Depressive disorders	--	12·3 (5·77–19·4)	12·3 (5·77–19·4)	--
Anxiety disorders	--	7·64 (2·48–15·5)	7·64 (2·48–15·5)	--
Idiopathic developmental intellectual disability	--	64·7 (34·5–84·9)	64·7 (34·5–84·9)	--
Substance use disorders	100 (100–100)	100 (100–100)	100 (100–100)	100 (100–100)
Alcohol use disorders	100 (100–100)	100 (100–100)	100 (100–100)	100 (100–100)
Drug use disorders	100 (100–100)	100 (100–100)	100 (100–100)	100 (100–100)
Diabetes and kidney diseases	100 (100–100)	100 (100–100)	100 (100–100)	100 (100–100)
Diabetes mellitus	100 (100–100)	100 (100–100)	100 (100–100)	100 (100–100)
Chronic kidney disease	100 (100–100)	100 (100–100)	100 (100–100)	100 (100–100)
Sense organ diseases	--	6·58 (5·68–7·7)	6·58 (5·68–7·7)	--
Blindness and vision loss	--	8·48 (5·68–12)	8·48 (5·68–12)	--
Age-related and other hearing loss	--	6·91 (6·21–7·74)	6·91 (6·21–7·74)	--
Musculoskeletal disorders	2·36 (0·672–4·35)	22·1 (17·6–26·7)	22·4 (17·9–27)	3·11 (0·91–5·62)
Rheumatoid arthritis	11·8 (3·41–21·4)	16·9 (5·49–29·6)	17·4 (5·65–30·4)	14·5 (4·39–25·8)
Osteoarthritis	--	13·1 (6·79–20·7)	13·1 (6·79–20·7)	--
Low back pain	--	38·3 (32–44·6)	38·3 (32–44·6)	--
Gout	--	47·5 (33·6–63·9)	47·5 (33·6–63·9)	--
Other non-communicable diseases	0·0585 (0·0389–0·102)	0·0468 (0·0287–0·0739)	--	0·257 (0·18–0·394)
Sudden infant death syndrome	5·38 (3·76–7·53)	5·38 (3·76–7·52)	--	5·38 (3·76–7·52)
Injuries	32·7 (27·3–36·8)	30·7 (26·3–34·7)	36·1 (31·3–40·4)	26·1 (20·2–31·7)
Transport injuries	24·5 (18·8–30·4)	24·4 (18·7–30·2)	29 (25–33·1)	22·8 (16·3–29·2)
Road injuries	23·6 (17·7–29·7)	23·6 (17·8–29·4)	28 (24–32·2)	22 (15·4–28·6)
Other transport injuries	36·2 (31·2–40·9)	34·2 (29·2–39·1)	38·6 (33·8–42·4)	32·2 (26·6–37·4)
Unintentional injuries	39·1 (33·3–43·3)	34·7 (29·8–39·1)	36·9 (31·6–41·6)	30·1 (25·4–34·2)
Falls	56·8 (48·8–62·1)	45·3 (39–50·3)	43·9 (37·8–48·8)	48·9 (42·3–54·1)
Drowning	-10·2 (-20·9–0·992)	-9·27 (-19–0·85)	12·7 (8·02–18·8)	-10·2 (-20·1–-0·0387)

Fire, heat, and hot substances	11·8 (7·09–17·9)	15·3 (10·3–21·8)	16·3 (11·1–22·9)	13·9 (9·23–19·6)
Poisonings	14·4 (9·21–21·1)	15·3 (10·5–21·5)	14·6 (9·83–20·7)	16·1 (10·9–22·3)
Exposure to mechanical forces	12·5 (3·85–21·1)	19·1 (15·8–22·4)	21 (18·2–23·9)	4·75 (-3·59–13·8)
Animal contact	26·3 (20·9–32·4)	25·1 (20·1–31)	25·7 (20·7–31·4)	23·9 (18·6–30)
Foreign body	1·23 (0·865–1·74)	3·1 (2·21–4·25)	4·2 (2·95–5·85)	2·94 (2·08–4·07)
Environmental heat and cold exposure	8·91 (4·18–15·2)	10 (4·75–17)	10·3 (4·84–17·5)	9·74 (4·61–16·4)
Exposure to forces of nature	0 (0–0)	11 (5·29–18·5)	11 (5·29–18·5)	0 (0–0)
Other unintentional injuries	15·5 (10·2–22·2)	15·6 (10·5–22)	15·3 (10·3–21·7)	18·6 (13·1–25·2)
Self-harm and interpersonal violence	24·3 (12–35·4)	25·2 (13·7–35·7)	33·7 (26·9–40·3)	24·5 (12·4–35·5)
Self-harm	23·7 (10·3–35·9)	24·1 (10·8–36·2)	25·5 (14·2–36·1)	24 (10·7–36·2)
Interpersonal violence	32·6 (24·5–41·1)	33·5 (26·1–41·5)	39 (31·3–47·1)	29·8 (21·9–38)

Supplementary Results Table 2. All-age PAFs attributable to the joint distribution of all risk factors for all ages, both sexes combined for each GBD level 1, level 2, and level 3 cause and all causes in 2019 for Cyprus. Results are shown for deaths, YLLs, YLDs, and DALYs Data in parenthesis are 95% uncertainty intervals. PAFs=population attributable fractions. GBD=Global Burden of Disease. YLLs=years of life lost. YLDs=years lived with disability. DALYs=disability-adjusted life-years

	Deaths 2019	DALYs 2019	YLDs 2019	YLLs 2019
All causes	60·2 (57–63·8)	40·8 (37·4–44·3)	24·8 (22·6–27·1)	57·5 (55–60·4)
Communicable, maternal, neonatal, and nutritional diseases	42·3 (36·9–47·8)	49·1 (45·6–52·5)	41·7 (37·8–45·8)	57·2 (53·1–61·3)
HIV/AIDS and sexually transmitted infections	88·3 (81–92·7)	83·9 (72·3–91·4)	98·4 (97·1–99·4)	78·4 (65·2–87·4)
HIV/AIDS	94 (93·2–94·8)	91·4 (90·3–92·4)	94·2 (93–95·1)	91·1 (89·9–92)
Sexually transmitted infections excluding HIV	52 (27·7–78·2)	69·7 (42·1–90·9)	100 (100–100)	26·4 (10·9–51·1)
Respiratory infections and tuberculosis	40·2 (33·7–46·5)	30·1 (24–36·7)	2·83 (2·09–3·75)	45·8 (39·4–52)
Tuberculosis	55·2 (46·5–63·2)	59·4 (51·4–66·4)	59·2 (50·8–66·8)	59·5 (51·4–66·6)
Lower respiratory infections	39·8 (33–46·3)	45·3 (38·7–51·8)	42·9 (36·8–48·8)	45·4 (38·7–51·8)
Upper respiratory infections	0·117 (0·0301–0·297)	0·00672 (0·00101–0·0149)	0·00617 (0·000454–0·0143)	0·356 (0·107–0·875)
Otitis media	0·786 (0·382–1·52)	3·99 (2·6–5·64)	4 (2·6–5·64)	2·18 (1·02–4·14)
Enteric infections	8·53 (5·28–13·4)	11·5 (8·21–16·3)	11·7 (8·24–16·5)	11·2 (7·49–16·5)
Diarrheal diseases	8·65 (5·36–13·6)	11·6 (8·28–16·4)	11·7 (8·24–16·5)	11·7 (7·71–17·2)
Other infectious diseases	0·789 (0·573–1·06)	1·49 (1·05–2·03)	0·172 (0·101–0·302)	2·23 (1·63–2·96)
Meningitis	2·66 (1·74–3·79)	5·69 (3·8–7·95)	0·000714 (3·79e-05–0·00178)	7·33 (5·01–9·96)
Encephalitis	0·181 (0·0917–0·401)	0·457 (0·251–0·835)	0·000145 (7·56e-06–0·000365)	0·572 (0·319–1·04)
Measles	12·5 (-3·01–51·7)	12 (-2·89–49)	0·683 (0·023–1·65)	12·8 (-3·07–52·7)
Acute hepatitis	2·01 (1·21–3·58)	1·37 (0·862–2·1)	0·76 (0·431–1·34)	2·77 (1·67–4·56)
Maternal and neonatal disorders	81·3 (78·7–84·1)	72·3 (68·9–75·3)	61·8 (57·3–66)	81·7 (79–84·4)
Maternal disorders	9·04 (3·21–14·7)	9·04 (3·22–14·7)	9·09 (3·3–14·9)	8·95 (3·18–14·5)
Neonatal disorders	82·3 (79·5–85·1)	73·1 (69·5–76)	62·7 (58·1–67)	82·3 (79·5–85·1)
Nutritional deficiencies	87·5 (81·8–90·1)	72·9 (69·5–75·7)	72·1 (68·5–75·1)	85 (80·1–87·3)
Protein-energy malnutrition	100 (100–100)	100 (100–100)	100 (100–100)	100 (100–100)
Vitamin A deficiency	--	100 (100–100)	100 (100–100)	--
Dietary iron deficiency	--	100 (100–100)	100 (100–100)	--
Non-communicable diseases	62·8 (59·2–66·7)	42 (38·1–45·9)	23·6 (21·2–26·1)	61·8 (59·1–65·3)
Neoplasms	44·1 (40·4–48·5)	43·1 (39·8–47)	31·9 (28·1–36·2)	43·9 (40·5–47·8)
Lip and oral cavity cancer	66 (59·5–71·4)	70 (64·1–75·4)	69·4 (62·9–75·5)	70 (64·1–75·5)
Nasopharynx cancer	69·1 (61·3–76)	68·4 (60·6–75·4)	66·5 (57·7–74·2)	68·6 (60·8–75·5)
Other pharynx cancer	79·2 (74·2–83·7)	80·4 (75·3–84·8)	78·4 (72·4–83·2)	80·5 (75·4–84·9)
Esophageal cancer	73·7 (66·1–81)	75·9 (68·5–82·6)	73·4 (65·5–80·8)	76 (68·6–82·6)

Stomach cancer	25·8<br>(18–43)	26·9<br>(19–44·1)	27<br>(18·8–44·3)	26·9<br>(18·9–44·1)
Colon and rectum cancer	63·7<br>(55–72·1)	63·7<br>(55·8–71·6)	63·8<br>(55·6–72·1)	63·7<br>(55·8–71·6)
Liver cancer	62·1<br>(53·6–69·7)	64·1<br>(55·8–71·7)	62·8<br>(54·5–70·3)	64·1<br>(55·8–71·8)
Gallbladder and biliary tract cancer	15·9<br>(8·16–25·8)	16·4<br>(8·44–26·7)	15·7<br>(7·96–25·5)	16·4<br>(8·45–26·7)
Pancreatic cancer	40·3<br>(31·4–50·9)	40·7<br>(32·6–50·7)	40·5<br>(31·7–50·9)	40·7<br>(32·6–50·7)
Larynx cancer	86·2<br>(80·1–90·6)	86·7<br>(81·4–90·6)	84·9<br>(79·2–89)	86·8<br>(81·6–90·8)
Tracheal, bronchus, and lung cancer	86·9<br>(84·3–89·5)	86·1<br>(83·8–88·6)	86·4<br>(83·9–89·1)	86·1<br>(83·8–88·6)
Breast cancer	30·8<br>(22–41·4)	29·7<br>(21·8–38·9)	29·7<br>(21·8–38·9)	29·7<br>(21·9–38·8)
Cervical cancer	100<br>(100–100)	100<br>(100–100)	100<br>(100–100)	100<br>(100–100)
Uterine cancer	35·8<br>(23·3–49·2)	37·3<br>(24·8–51·4)	37·9<br>(25·1–52·3)	37·2<br>(24·7–51·2)
Ovarian cancer	18<br>(7·68–31·4)	14·9<br>(6·01–26·7)	13·9<br>(5·66–24·9)	14·9<br>(6·02–26·8)
Prostate cancer	8·41<br>(3·96–12·9)	9·19<br>(4·34–14)	9·65<br>(4·52–14·6)	9·1<br>(4·27–13·9)
Kidney cancer	35·9<br>(27·5–44·6)	35·9<br>(27·7–44·6)	35·2<br>(27·1–43·7)	35·9<br>(27·7–44·6)
Bladder cancer	48·4<br>(34·1–61·4)	51·5<br>(38·2–63·5)	53·1<br>(39·7–64·6)	51·3<br>(38·1–63·3)
Thyroid cancer	10·7<br>(5·03–18·1)	11·3<br>(5·15–19·3)	11·3<br>(5·29–19·6)	11·3<br>(5·15–19·2)
Mesothelioma	96·5<br>(95·2–97·4)	93·7<br>(91·6–95·2)	96<br>(94·2–97·2)	93·6<br>(91·5–95·2)
Non-Hodgkin lymphoma	5·06<br>(1·99–9·32)	5·19<br>(2·09–9·54)	5·14<br>(2·04–9·44)	5·19<br>(2·08–9·54)
Multiple myeloma	5·9<br>(2·52–10·8)	6·15<br>(2·54–11·2)	6·14<br>(2·56–11·3)	6·15<br>(2·54–11·2)
Leukemia	35·1<br>(21·8–47·8)	33·1<br>(22·3–44·6)	33·2<br>(22·2–45·1)	33·1<br>(22·4–44·6)
Cardiovascular diseases	82·1<br>(78·5–85·4)	83·8<br>(81–86·2)	78·4<br>(74·7–81·8)	84·4<br>(81·6–86·8)
Rheumatic heart disease	26·6<br>(14·9–47·4)	27·8<br>(17·3–45·8)	28·3<br>(17·5–44·7)	27·7<br>(17·2–45·7)
Ischemic heart disease	94·9<br>(91·8–97·4)	96<br>(94–97·7)	95·5<br>(93·2–97·4)	96<br>(94–97·7)
Stroke	82·4<br>(74·7–90·6)	85·2<br>(79·7–90·8)	86·1<br>(82–90·3)	85·1<br>(79·3–91)
Hypertensive heart disease	100<br>(100–100)	100<br>(100–100)	100<br>(100–100)	100<br>(100–100)
Non-rheumatic valvular heart disease	31·6<br>(21·3–45·2)	33·1<br>(23·7–44·8)	35·9<br>(24·9–47·8)	33<br>(23·7–44·7)
Cardiomyopathy and myocarditis	37·7<br>(29–47·8)	41·2<br>(34–48·4)	40·4<br>(33·1–47·7)	41·2<br>(34–48·5)
Atrial fibrillation and flutter	51<br>(41·5–60·2)	59·6<br>(52·1–67·1)	65·9<br>(59·2–72·4)	54·4<br>(46–62·7)
Aortic aneurysm	67·1<br>(61·8–72·4)	73·2<br>(68·8–77·3)	--	73·2<br>(68·8–77·3)
Endocarditis	37<br>(26·4–50·7)	39·8<br>(29·9–50·7)	38·8<br>(28·7–50·1)	39·8<br>(29·9–50·7)
Other cardiovascular and circulatory diseases	28·8<br>(21·5–40)	31·9<br>(25·5–41·1)	40·7<br>(33·1–48·6)	29·9<br>(23·3–40·1)
Chronic respiratory diseases	59·4<br>(53·2–67·7)	54·7<br>(49·9–60·1)	49·6<br>(44·4–54·9)	59·6<br>(53·6–67·5)
Chronic obstructive pulmonary disease	71·3<br>(66·1–76·4)	71·9<br>(68·3–75·4)	67·2<br>(64–70·6)	74·4<br>(70–78·7)
Pneumoconiosis	100<br>(100–100)	100<br>(100–100)	100<br>(100–100)	100<br>(100–100)
Asthma	33·9<br>(24·1–45·8)	38·7<br>(30·8–47·8)	38·8<br>(31·1–47·6)	38·1<br>(28·1–49·8)

Digestive diseases	33·9 (28·7–39·6)	33·9 (28·6–39·3)	10·2 (6·58–14·6)	42·6 (36·7–48·4)
Cirrhosis and other chronic liver diseases	68·3 (56·3–79·3)	72·3 (61·5–82·1)	67·2 (58·7–76·2)	72·4 (61·5–82·3)
Upper digestive system diseases	21·4 (18–25·2)	6·39 (3·74–9·56)	0·603 (0·323–1·01)	26·2 (22·3–30·1)
Gallbladder and biliary diseases	34·7 (21·7–48·4)	38·9 (26·8–52·1)	42·5 (30–56)	36·2 (24–49·5)
Pancreatitis	34·3 (24·2–47·2)	39·9 (29·4–52·7)	36·4 (26·4–47·5)	40·2 (29·6–53)
Neurological disorders	21 (7·37–36·5)	11·5 (4·92–22·4)	7·05 (3·16–15·5)	19·7 (6·72–35·3)
Alzheimer's disease and other dementias	36·4 (23·5–51·5)	40·2 (27·3–54·4)	41·2 (28·3–55·6)	39·6 (27–53·9)
Parkinson's disease	-13·1 (-20·9–-6·47)	-15·3 (-23·6–-7·77)	-15·9 (-24·3–-8·28)	-15·1 (-23·5–-7·64)
Idiopathic epilepsy	22·6 (15·9–29·3)	22·3 (15·8–28·8)	21 (14·9–27·5)	25 (18·1–32·2)
Multiple sclerosis	18·9 (14·2–24)	20·3 (15·4–25·3)	19·2 (14·6–23·7)	21·4 (16·1–26·9)
Mental disorders	--	4·77 (2·51–7·87)	4·77 (2·51–7·87)	--
Depressive disorders	--	9·41 (4·7–14·9)	9·41 (4·7–14·9)	--
Anxiety disorders	--	5·09 (1·27–11·4)	5·09 (1·27–11·4)	--
Idiopathic developmental intellectual disability	--	54·8 (27·7–74·3)	54·8 (27·7–74·3)	--
Substance use disorders	100 (100–100)	100 (100–100)	100 (100–100)	100 (100–100)
Alcohol use disorders	100 (100–100)	100 (100–100)	100 (100–100)	100 (100–100)
Drug use disorders	100 (100–100)	100 (100–100)	100 (100–100)	100 (100–100)
Diabetes and kidney diseases	100 (100–100)	100 (100–100)	100 (100–100)	100 (100–100)
Diabetes mellitus	100 (100–100)	100 (100–100)	100 (100–100)	100 (100–100)
Chronic kidney disease	100 (100–100)	100 (100–100)	100 (100–100)	100 (100–100)
Sense organ diseases	--	7·44 (6·47–8·63)	7·44 (6·47–8·63)	--
Blindness and vision loss	--	8·21 (5·31–12)	8·21 (5·31–12)	--
Age-related and other hearing loss	--	8·54 (7·73–9·44)	8·54 (7·73–9·44)	--
Musculoskeletal disorders	3·65 (1·07–6·53)	22·7 (18·7–26·9)	23·1 (18·9–27·2)	4·07 (1·23–7·18)
Rheumatoid arthritis	10·8 (3·06–19·5)	15·3 (4·72–26·3)	15·9 (5·01–26·9)	13·1 (3·9–23·1)
Osteoarthritis	--	11·1 (5·38–17·9)	11·1 (5·38–17·9)	--
Low back pain	--	42 (36·3–47·9)	42 (36·3–47·9)	--
Gout	--	43·8 (30·5–60)	43·8 (30·5–60)	--
Other non-communicable diseases	0·106 (0·0502–0·192)	0·0826 (0·036–0·162)	--	0·435 (0·219–0·77)
Sudden infant death syndrome	8·59 (5·31–14·5)	8·58 (5·3–14·5)	--	8·58 (5·3–14·5)
Injuries	30·9 (27·1–34·3)	27·9 (24·7–31)	30·3 (26·9–34·2)	26·1 (22·5–30)
Transport injuries	29·7 (25·1–34·6)	27·8 (22·5–33)	29 (25·1–33)	27·6 (22·1–33·1)
Road injuries	27·3 (22·7–32)	26·1 (20·8–31·4)	28·1 (24·3–32·2)	25·7 (20–31·2)
Other transport injuries	33·9 (29–38·8)	30·9 (25·7–36·4)	37 (33–40·7)	30·7 (25·4–36·2)

Unintentional injuries	33·9<br>(28·8–37·9)	28·2<br>(24·3–32·5)	30·3<br>(26·4–34·6)	23·7<br>(19·4–28·2)
Falls	53·7<br>(47·1–58·3)	39·3<br>(34·2–44)	37·5<br>(32·8–42·3)	44·7<br>(39·2–49·4)
Drowning	-2·14<br>(-9·82–7·24)	-0·17<br>(-7·69–8·8)	11·6<br>(7·78–16·9)	-0·464<br>(-8·11–8·61)
Fire, heat, and hot substances	10·9<br>(6·9–16·4)	15·9<br>(11·6–21·8)	16·6<br>(12·4–22·6)	14·2<br>(9·87–19·8)
Poisonings	17·6<br>(12·2–24·7)	17·4<br>(12·7–23·8)	15·5<br>(11·6–21)	19·6<br>(14–27·2)
Exposure to mechanical forces	18·9<br>(12·7–25·3)	18·6<br>(16·1–21)	19·7<br>(17·5–21·7)	13·8<br>(7·63–20·4)
Animal contact	24·3<br>(20–29)	22·5<br>(18·3–27·4)	23·7<br>(19·3–28·7)	19·5<br>(15·5–24·4)
Foreign body	3·77<br>(2·97–4·69)	6·4<br>(5·11–7·83)	6·24<br>(5–7·68)	6·47<br>(5·13–7·89)
Environmental heat and cold exposure	6·88<br>(3–13·3)	8·3<br>(3·98–14·4)	8·44<br>(4·04–14·6)	8·16<br>(3·7–15·2)
Exposure to forces of nature	0<br>(0–0)	9·81<br>(4·68–16·8)	9·81<br>(4·68–16·8)	0<br>(0–0)
Other unintentional injuries	17·5<br>(12·2–24·4)	16·4<br>(12·2–22·4)	16<br>(11·8–21·8)	20<br>(14·2–27·2)
Self-harm and interpersonal violence	25·3<br>(15·9–34·2)	26·9<br>(18·4–34·9)	33·4<br>(26·6–40·6)	25·7<br>(16·2–34·6)
Self-harm	23·9<br>(12·2–34·9)	24·6<br>(12·6–35·9)	20·8<br>(11–30·1)	24·8<br>(12·7–36·1)
Interpersonal violence	30<br>(21·8–38·7)	31·8<br>(24–40·4)	36·9<br>(29–45·3)	28·9<br>(20·4–37·6)

Supplementary Results Table 2. All-age PAFs attributable to the joint distribution of all risk factors for all ages, both sexes combined for each GBD level 1, level 2, and level 3 cause and all causes in 2019 for Tajikistan. Results are shown for deaths, YLLs, YLDs, and DALYs Data in parenthesis are 95% uncertainty intervals. PAFs=population attributable fractions. GBD=Global Burden of Disease. YLLs=years of life lost. YLDs=years lived with disability. DALYs=disability-adjusted life-years				
	**Deaths 2019**	**DALYs 2019**	**YLDs 2019**	**YLLs 2019**
All causes	65·7 (63·6–67·8)	48·2 (45·2–51·6)	26·1 (24–28·3)	57·4 (54·8–61)
Communicable, maternal, neonatal, and nutritional diseases	70·1 (66·1–73·4)	72 (68·4–75·1)	64·1 (59·9–67·7)	73·4 (69·2–76·7)
HIV/AIDS and sexually transmitted infections	72·9 (64·5–78·2)	72·1 (61·5–79·5)	94·1 (92–96·1)	66·9 (54·7–75·3)
HIV/AIDS	77·4 (76·5–78·4)	76·8 (75·9–77·8)	77·3 (76·3–78·4)	76·8 (75·9–77·8)
Sexually transmitted infections excluding HIV	58·4 (33·4–82)	64·7 (40·9–85·3)	100 (100–100)	41·8 (19–68·7)
Respiratory infections and tuberculosis	68·7 (62·6–74)	72 (66–77)	13·2 (9·72–17·4)	74·5 (68·7–79·4)
Tuberculosis	32·7 (27·3–38·2)	28·2 (22·9–33·8)	29·2 (23·2–35·5)	28·1 (22·7–33·8)
Lower respiratory infections	77·5 (70·8–83·3)	82·7 (76·4–87·5)	44·9 (35·6–54·6)	82·9 (76·5–87·7)
Upper respiratory infections	3·29 (1·04–9·09)	1·23 (0·337–3·5)	0·0214 (0·00861–0·0367)	3·43 (1·07–9·47)
Otitis media	7·59 (3·58–15·9)	4·07 (2·6–5·81)	4·07 (2·59–5·81)	11·5 (6·3–22·9)
Enteric infections	95·5 (91·7–97·9)	94·5 (90·1–97·5)	87·7 (78–94·3)	96·3 (93·3–98·3)
Diarrheal diseases	96·1 (92·4–98·4)	95 (90·6–97·8)	87·8 (78·1–94·4)	96·9 (93·9–98·8)
Other infectious diseases	2·74 (1·38–4·57)	2·48 (1·24–4·22)	0·127 (0·071–0·218)	2·93 (1·35–5·12)
Meningitis	5·55 (3·49–8·27)	5·94 (3·72–8·89)	0·00275 (0·00108–0·00472)	7·01 (4·42–10·2)
Encephalitis	0·72 (0·245–2·31)	0·727 (0·258–2·26)	0·00099 (0·000408–0·00167)	1·07 (0·425–2·81)
Measles	49·8 (28·3–78·7)	50 (28·4–79·1)	4·56 (0·146–10·1)	50·4 (28·8–79·7)
Acute hepatitis	4·28 (2·39–7·45)	3·36 (1·79–5·91)	1·15 (0·668–1·9)	3·65 (1·86–6·49)
Maternal and neonatal disorders	74·5 (70·1–78·6)	72·3 (68·2–76·3)	44·5 (36·9–51·4)	74·9 (70·5–79)
Maternal disorders	17·9 (6·46–29·1)	17·9 (6·4–29)	17·8 (6·31–28·7)	17·9 (6·42–29)
Neonatal disorders	75·7 (71·3–79·8)	73·2 (69–77·4)	45·9 (38–54)	75·7 (71·3–79·8)
Nutritional deficiencies	6·8 (4·7–9·28)	92·3 (89·2–94·4)	95·8 (94·6–96·9)	7·08 (4·37–10·4)
Protein-energy malnutrition	100 (100–100)	100 (100–100)	100 (100–100)	100 (100–100)
Vitamin A deficiency	--	100 (100–100)	100 (100–100)	--
Dietary iron deficiency	--	100 (100–100)	100 (100–100)	--
Non-communicable diseases	69·8 (67·1–72·8)	44·8 (40·5–49·7)	21·1 (18·6–23·7)	57·6 (53·6–63·7)
Neoplasms	30·5 (26·6–36·6)	26·6 (23·1–32·1)	25·8 (22–30·6)	26·6 (23·1–32·1)
Lip and oral cavity cancer	39·8 (34·3–45·1)	39·6 (34·1–44·8)	37 (31·5–42·5)	39·6 (34·2–44·9)
Nasopharynx cancer	33·7 (26·2–41·3)	32·9 (25·4–40·5)	33·6 (26–41·2)	32·9 (25·4–40·5)
Other pharynx cancer	40 (34·3–46·1)	39 (33·2–45·4)	37·3 (31·6–43·5)	39 (33·2–45·4)
Esophageal cancer	57·5 (47·5–68)	57·6 (47·1–67·9)	57·5 (47–68·4)	57·6 (47·1–67·9)
Stomach cancer	17·9 (10·5–37·8)	17·1 (9·63–36·9)	17·5 (9·86–37·6)	17·1 (9·62–36·9)

Colon and rectum cancer	56·1 (48·6–62·2)	54·3 (47·3–60·1)	54·7 (47·6–60·6)	54·3 (47·2–60·1)
Liver cancer	55·9 (47·2–63·6)	51·1 (43·2–58·5)	54·5 (46·2–61·9)	51 (43·2–58·4)
Gallbladder and biliary tract cancer	12·4 (6·13–20·6)	12·7 (6·39–21·1)	12·5 (6·18–20·9)	12·7 (6·39–21·1)
Pancreatic cancer	23 (16–32·1)	22·1 (15·6–30·7)	22·9 (16·1–31·9)	22·1 (15·6–30·7)
Larynx cancer	56·3 (48·4–63·6)	53·7 (46·7–60·7)	51·8 (44·2–59)	53·8 (46·8–60·8)
Tracheal, bronchus, and lung cancer	73·5 (68·6–79·2)	70·7 (65·6–76·6)	72·1 (66·6–78·5)	70·7 (65·6–76·6)
Breast cancer	14·7 (8·39–22·9)	12·8 (7·11–20·2)	13·1 (7·13–20·8)	12·8 (7·11–20·2)
Cervical cancer	100 (100–100)	100 (100–100)	100 (100–100)	100 (100–100)
Uterine cancer	31·6 (18·7–45·2)	31·2 (18·5–44·8)	31·3 (18·3–45·3)	31·2 (18·5–44·7)
Ovarian cancer	10·4 (3·58–20·6)	9·3 (3·22–18·3)	8·88 (3·13–17·6)	9·31 (3·22–18·3)
Prostate cancer	5·27 (2·17–9·05)	5·66 (2·31–9·67)	5·84 (2·4–9·86)	5·65 (2·3–9·66)
Kidney cancer	22·3 (15·6–30)	19·6 (13·6–26·4)	18·8 (12·6–25·9)	19·6 (13·6–26·5)
Bladder cancer	31·5 (21–42·4)	31·3 (22·2–40·5)	30·5 (21·7–39·4)	31·3 (22·2–40·6)
Thyroid cancer	7·55 (3·17–13·9)	7·28 (3·03–13·5)	7·2 (3·34–12·8)	7·28 (3·01–13·6)
Mesothelioma	74·8 (64·6–83·7)	70·9 (57·7–82·7)	74·1 (63·5–83·8)	70·9 (57·6–82·7)
Non-Hodgkin lymphoma	3·05 (1·11–6·03)	2·59 (0·92–5·23)	2·2 (0·743–4·55)	2·6 (0·923–5·24)
Multiple myeloma	4·28 (1·65–8·27)	4·36 (1·71–8·43)	4·41 (1·74–8·44)	4·35 (1·7–8·42)
Leukemia	12·3 (7·78–17·8)	8·16 (5·09–12)	9·44 (5·63–14)	8·15 (5·07–12)
Cardiovascular diseases	91·4 (89·3–93·4)	90·8 (88·9–92·5)	71 (65·4–75·9)	91·6 (89·8–93·1)
Rheumatic heart disease	28·1 (18·5–39·8)	25·4 (15·6–37·1)	20·6 (10·8–32·5)	27·1 (17·2–39·7)
Ischemic heart disease	94·8 (92·3–96·9)	95·7 (94–97·3)	95·5 (93·6–97·3)	95·7 (94–97·3)
Stroke	88·6 (84·2–92·1)	89·9 (86·3–92·6)	84·4 (80·9–87·4)	90·3 (86·6–93·1)
Hypertensive heart disease	100 (100–100)	100 (100–100)	100 (100–100)	100 (100–100)
Non-rheumatic valvular heart disease	25 (18·9–31·1)	19·2 (13·6–24·9)	0·761 (0·47–1·16)	24 (17·8–30·1)
Cardiomyopathy and myocarditis	39·2 (31·2–47)	37·2 (28·5–45·7)	26·1 (19·2–32·9)	37·4 (28·7–45·9)
Atrial fibrillation and flutter	44·9 (35·5–54·8)	54·9 (47·2–62)	58·8 (51·3–65·9)	49·4 (40·9–57·7)
Aortic aneurysm	53·7 (47–59·9)	57·6 (51·5–63·6)	--	57·6 (51·5–63·6)
Endocarditis	40·8 (31·1–51·2)	37·8 (26·7–48·6)	33 (24–42·5)	37·8 (26·7–48·6)
Other cardiovascular and circulatory diseases	40·1 (33·2–46)	37·7 (27·7–44·4)	39·5 (31·4–47·2)	37·6 (27–44·3)
Chronic respiratory diseases	55·5 (50·6–60·3)	46·5 (41·5–51·7)	30·1 (24·6–35·4)	52·8 (47·6–57·4)
Chronic obstructive pulmonary disease	75·7 (70·1–81·4)	69·6 (64·2–75·2)	52 (45·6–59·3)	73·8 (68·3–79·1)
Pneumoconiosis	100 (100–100)	100 (100–100)	100 (100–100)	100 (100–100)
Asthma	28·2 (19·3–38·6)	22·7 (16·5–30·7)	16·5 (11·8–22·3)	32·1 (23·1–42)
Digestive diseases	48·8 (42·4–55·4)	41·3 (35–47·5)	9·19 (6·04–13·2)	47 (40·3–54·1)

Cirrhosis and other chronic liver diseases	57·1 (49·6–64·7)	56·2 (48·3–64·1)	49·9 (42·7–56·6)	56·3 (48·3–64·3)
Upper digestive system diseases	17·4 (14–20·8)	7·51 (4·77–10·7)	0·645 (0·37–1·03)	16·3 (13·3–19·6)
Gallbladder and biliary diseases	30·3 (18·7–43·3)	28·7 (17·5–41·6)	28·3 (17–41·3)	30·2 (18·9–43·2)
Pancreatitis	13·3 (6·48–22·3)	16·3 (8·67–25·7)	17 (9·86–24·8)	16·2 (8·55–26)
Neurological disorders	12·2 (6·67–21)	5·31 (2·86–9·02)	2·59 (1·12–5·35)	10·4 (6·8–16)
Alzheimer's disease and other dementias	25·2 (15·3–37·4)	27·7 (17·6–39·7)	27·7 (17·7–39·8)	27·7 (17·5–39·5)
Parkinson's disease	-6·02 (-8·82–-3·37)	-7·08 (-10·3–-4·07)	-7·86 (-11·5–-4·54)	-6·96 (-10·1–-3·97)
Idiopathic epilepsy	10·1 (6·54–13·8)	8·39 (5·44–11·6)	7·04 (4·47–9·73)	9·12 (5·93–12·4)
Multiple sclerosis	9·72 (6·37–13·2)	8·91 (6·11–11·9)	8·23 (5·65–11)	9·81 (6·44–13·4)
Mental disorders	--	5·48 (2·94–8·37)	5·48 (2·94–8·37)	--
Depressive disorders	--	6·95 (1·6–13·8)	6·95 (1·6–13·8)	--
Anxiety disorders	--	1·69 (0·449–4·01)	1·69 (0·449–4·01)	--
Idiopathic developmental intellectual disability	--	60·2 (30·5–79·3)	60·2 (30·5–79·3)	--
Substance use disorders	100 (100–100)	100 (100–100)	100 (100–100)	100 (100–100)
Alcohol use disorders	100 (100–100)	100 (100–100)	100 (100–100)	100 (100–100)
Drug use disorders	100 (100–100)	100 (100–100)	100 (100–100)	100 (100–100)
Diabetes and kidney diseases	99·9 (99·9–100)	99·9 (99·9–100)	100 (100–100)	99·9 (99·8–99·9)
Diabetes mellitus	100 (100–100)	100 (100–100)	100 (100–100)	100 (100–100)
Chronic kidney disease	100 (100–100)	100 (100–100)	100 (100–100)	100 (100–100)
Sense organ diseases	--	11·7 (10·2–13·3)	11·7 (10·2–13·3)	--
Blindness and vision loss	--	8·32 (5·09–12)	8·32 (5·09–12)	--
Age-related and other hearing loss	--	14·2 (13–15·6)	14·2 (13–15·6)	--
Musculoskeletal disorders	3·57 (1·05–6·74)	25·6 (21·8–29·7)	26·1 (22·2–30·1)	2·98 (0·868–5·74)
Rheumatoid arthritis	6·65 (1·88–12·8)	6·2 (1·84–11·6)	6·09 (1·83–11·6)	6·51 (1·8–12·6)
Osteoarthritis	--	8·93 (4·12–15·1)	8·93 (4·12–15·1)	--
Low back pain	--	37·6 (33·4–41·9)	37·6 (33·4–41·9)	--
Gout	--	27·8 (16·8–42·6)	27·8 (16·8–42·6)	--
Other non-communicable diseases	0·0308 (0·0139–0·0574)	0·0236 (0·0106–0·0456)	--	0·0355 (0·0158–0·0659)
Sudden infant death syndrome	7·6 (3·27–13·8)	7·59 (3·27–13·8)	--	7·59 (3·27–13·8)
Injuries	1·72 (-3·47–6·9)	3·84 (-0·656–8·1)	15 (12·4–18)	-1·62 (-7·05–3·85)
Transport injuries	10·4 (2·67–17·8)	10·7 (3·83–17·5)	24 (20·5–27·8)	7·53 (-0·24–15·2)
Road injuries	8·87 (0·759–16·8)	9·48 (2·17–16·6)	23·9 (20·3–27·8)	5·99 (-2·28–14·3)
Other transport injuries	26·6 (22·1–31·4)	23·8 (19·8–28·4)	25·2 (21·9–28·7)	23·5 (19·2–28·4)
Unintentional injuries	-1·2 (-7·65–6·1)	2·93 (-3·09–8·7)	17·3 (14·4–20·6)	-5·6 (-12·5–2·07)

Falls	22·3 (18·6–26·4)	20 (16·3–23·7)	23·4 (20·1–27·2)	16·1 (12·6–20·1)
Drowning	-24·2 (-37–-9·32)	-26·7 (-39·5–-11·6)	9·98 (7·5–13)	-27 (-39·7–-11·7)
Fire, heat, and hot substances	10·2 (6·42–14·3)	9·97 (6·6–13·7)	13·1 (9·93–16·7)	8·38 (5·05–12·2)
Poisonings	16·7 (12·4–21·5)	14·1 (10·5–18)	10·8 (8·05–14)	16·1 (11·8–20·9)
Exposure to mechanical forces	-2·12 (-14·4–10·3)	14·6 (10·1–19)	18 (14·8–21·7)	-4·75 (-17·6–8·33)
Animal contact	10·3 (7·93–13)	13·5 (10·6–17)	15·6 (12·3–19·1)	6·3 (4·55–8·52)
Foreign body	9·86 (5·42–13·1)	8·22 (4·7–10·9)	6·99 (4·97–9·48)	8·73 (4·05–11·7)
Environmental heat and cold exposure	5·06 (2·23–8·81)	4·11 (1·85–7·18)	3·73 (1·63–6·63)	5·04 (2·24–8·73)
Exposure to forces of nature	0 (0–0)	4·36 (1·95–7·67)	4·36 (1·95–7·67)	0 (0–0)
Other unintentional injuries	16·6 (12–21·6)	13·6 (10·2–17·5)	12·6 (9·59–16·2)	14·8 (10·6–19·5)
Self-harm and interpersonal violence	-1·44 (-11·6–8·08)	-0·0861 (-6·76–6·7)	3·48 (2·12–5·19)	-1·87 (-11·9–7·69)
Self-harm	-3·28 (-16·7–10·4)	-3·11 (-16–10·1)	7·62 (3·13–12·4)	-3·48 (-16·6–10·1)
Interpersonal violence	2·19 (-9·61–15·1)	4·67 (-5·02–15·7)	18·6 (13·7–24·7)	1·24 (-10·5–14·1)

Supplementary Results Table 2. All-age PAFs attributable to the joint distribution of all risk factors for all ages, both sexes combined for each GBD level 1, level 2, and level 3 cause and all causes in 2019 for Denmark. Results are shown for deaths, YLLs, YLDs, and DALYs Data in parenthesis are 95% uncertainty intervals. PAFs=population attributable fractions. GBD=Global Burden of Disease. YLLs=years of life lost. YLDs=years lived with disability. DALYs=disability-adjusted life-years

	Deaths 2019	DALYs 2019	YLDs 2019	YLLs 2019
All causes	59·1 (56·5–61·5)	43·5 (40·4–46·7)	26·2 (24·1–28·5)	58·5 (56·5–60·4)
Communicable, maternal, neonatal, and nutritional diseases	46·2 (39·5–52·6)	49·7 (45·9–53·7)	41·1 (37–45·4)	54·9 (49·8–60·1)
HIV/AIDS and sexually transmitted infections	93·1 (92·3–93·8)	92·8 (91·6–94)	95·6 (94·3–97·1)	90·4 (89·3–91·3)
HIV/AIDS	92·2 (91·3–93)	91·2 (90·2–92·2)	93·4 (92·4–94·4)	90 (88·8–90·9)
Sexually transmitted infections excluding HIV	100 (99·9–100)	100 (99·9–100)	100 (100–100)	99·8 (99·7–99·9)
Respiratory infections and tuberculosis	51·3 (42·9–59·1)	44·9 (37·8–52·1)	3·68 (2·71–5·02)	54 (46·1–61·4)
Tuberculosis	62·4 (53·3–69·8)	63·2 (54·5–70·4)	58·8 (49·6–66·3)	64·4 (56–71·4)
Lower respiratory infections	51·2 (42·7–59·2)	53·8 (45·7–61·2)	39·6 (33·5–45·6)	53·9 (45·9–61·5)
Upper respiratory infections	0·0334 (0·0154–0·0647)	0·00703 (0·0011–0·0174)	0·00582 (0·00014–0·0159)	0·135 (0·0632–0·261)
Otitis media	0·00978 (0·00343–0·0216)	3·33 (2·07–4·73)	3·37 (2·09–4·79)	0·0443 (0·016–0·0992)
Enteric infections	5·73 (3·83–8·8)	8·09 (6·04–11·1)	8·08 (6·02–11·1)	8·12 (5·81–11·1)
Diarrheal diseases	5·75 (3·84–8·83)	8·12 (6·06–11·2)	8·09 (6·02–11·1)	8·17 (5·86–11·1)
Other infectious diseases	0·486 (0·359–0·683)	0·956 (0·741–1·25)	0·454 (0·265–0·758)	1·16 (0·885–1·54)
Meningitis	0·667 (0·466–0·933)	1·66 (1·17–2·28)	0·000469 (7·31e-06–0·00137)	2·2 (1·57–3)
Encephalitis	0·218 (0·103–0·351)	0·5 (0·213–0·796)	0·000187 (3·12e-06–0·00052)	0·679 (0·32–1·06)
Measles	1·29 (-0·293–8·7)	1·91 (-0·383–11·5)	0·445 (0·0138–1·1)	2·03 (-0·484–12·5)
Acute hepatitis	12·7 (8·12–19·4)	6·38 (4·23–9·56)	3·47 (2·11–5·54)	12·1 (7·71–18·8)
Maternal and neonatal disorders	86·9 (84·5–89·4)	79·3 (76·3–82)	68·4 (64–72·5)	87·2 (84·8–89·7)
Maternal disorders	7·67 (2·72–12·7)	7·52 (2·62–12·6)	7·51 (2·6–12·6)	7·53 (2·67–12·6)
Neonatal disorders	87·9 (85·5–90·3)	80·6 (77·4–83·2)	70·3 (65·8–74·2)	87·9 (85·5–90·3)
Nutritional deficiencies	59·7 (13·9–69·1)	73·9 (68·3–77)	75·9 (73·3–79)	59·1 (16·8–67·4)
Protein-energy malnutrition	100 (100–100)	100 (100–100)	100 (100–100)	100 (100–100)
Vitamin A deficiency	--	100 (100–100)	100 (100–100)	--
Dietary iron deficiency	--	100 (100–100)	100 (100–100)	--
Non-communicable diseases	61·1 (58·1–63·7)	44·2 (40·7–47·7)	24·8 (22·5–27·2)	60·9 (58·8–63·1)
Neoplasms	48·6 (45·8–51·9)	48 (45·1–51·2)	35·3 (32–39·1)	48·6 (45·9–51·9)
Lip and oral cavity cancer	74 (68–79·1)	74·9 (69·3–80)	74·9 (69–80·1)	74·9 (69·3–80)
Nasopharynx cancer	69·7 (60·7–76·9)	68·3 (59·4–75·5)	67·6 (58·5–75)	68·3 (59·5–75·6)
Other pharynx cancer	82·8 (77·5–87)	82·7 (77·5–87)	82 (76·6–86·4)	82·7 (77·5–87)
Esophageal cancer	76·3 (69·1–83·3)	76·5 (69·3–83·6)	76·1 (68·7–83)	76·5 (69·3–83·6)
Stomach cancer	27·9 (19·9–45)	27·3 (19–44·8)	27·9 (19·6–45·4)	27·3 (18·9–44·8)

Colon and rectum cancer	62·8 (55·2–70·2)	63 (55·7–69·9)	63·2 (56·1–70·2)	63 (55·7–69·9)
Liver cancer	80·2 (74·3–85·5)	79·8 (74–84·9)	79·8 (74·1–84·9)	79·8 (74–84·9)
Gallbladder and biliary tract cancer	18·6 (10·2–29)	18·9 (10·4–29·6)	17·3 (9·15–27·9)	19 (10·4–29·7)
Pancreatic cancer	40·8 (33·9–48·8)	39·6 (33·1–47·2)	40·4 (33·7–48·2)	39·6 (33·1–47·1)
Larynx cancer	86·2 (80·3–90·7)	85·7 (79·7–90·1)	85·2 (79·3–89·7)	85·7 (79·7–90·1)
Tracheal, bronchus, and lung cancer	85·3 (82·9–87·7)	83·7 (81·2–86·2)	84·4 (81·9–87)	83·7 (81·2–86·2)
Breast cancer	37·2 (30·2–45·2)	36·6 (30·2–43·8)	36·6 (30·1–43·8)	36·6 (30·2–43·8)
Cervical cancer	100 (100–100)	100 (100–100)	100 (100–100)	100 (100–100)
Uterine cancer	39·4 (25·9–53·6)	40·7 (27·7–54·8)	41·3 (28·3–55·3)	40·6 (27·5–54·7)
Ovarian cancer	16·5 (8·53–26·5)	14·3 (7·04–23·3)	14 (6·97–22·8)	14·3 (7·06–23·3)
Prostate cancer	7·82 (3·48–12·4)	8·18 (3·74–13)	8·44 (3·84–13·3)	8·15 (3·73–12·9)
Kidney cancer	37·8 (28·5–47·4)	37·5 (28·6–47)	37·2 (28·3–46·6)	37·5 (28·6–47)
Bladder cancer	44·8 (32·2–56·5)	46·7 (34·9–57·9)	47·4 (35·5–58·6)	46·7 (34·8–57·9)
Thyroid cancer	11·8 (5·84–19·7)	12·4 (6·12–20·6)	12·6 (6·29–20·8)	12·3 (6·07–20·5)
Mesothelioma	98·2 (97·5–98·7)	96·8 (95·7–97·7)	97·9 (97–98·6)	96·8 (95·7–97·7)
Non-Hodgkin lymphoma	5·83 (2·4–10·6)	6 (2·44–10·9)	5·92 (2·41–10·8)	6·01 (2·44–10·9)
Multiple myeloma	6·61 (2·79–11·9)	6·88 (2·95–12·4)	6·87 (2·95–12·4)	6·88 (2·95–12·4)
Leukemia	38·9 (23·7–53·3)	36 (23·8–48·7)	36·8 (23·9–49·6)	35·9 (23·7–48·6)
Cardiovascular diseases	81 (77·5–84·4)	82·6 (79·8–85·3)	77·9 (74–81·6)	83·2 (80·5–85·9)
Rheumatic heart disease	28·6 (15·9–53·4)	29·9 (18·5–49·2)	30·2 (19·1–47)	29·9 (18·4–49·7)
Ischemic heart disease	93·5 (89·9–96·6)	94·7 (92·1–97)	94·5 (92–96·8)	94·7 (92·1–97)
Stroke	82·7 (75·8–89·3)	85·6 (80·6–90·3)	84·7 (80–89·1)	85·8 (80·8–90·5)
Hypertensive heart disease	100 (100–100)	100 (100–100)	100 (100–100)	100 (100–100)
Non-rheumatic valvular heart disease	31·6 (20·9–45·4)	32·8 (23·1–44·3)	36·7 (26–48·7)	32·7 (23–44·2)
Cardiomyopathy and myocarditis	44·4 (35·3–51·6)	49·8 (42·3–55·1)	44·8 (37·8–51·5)	50 (42·2–55·5)
Atrial fibrillation and flutter	57·2 (48·3–66)	63·9 (56·7–71·1)	68·2 (61·6–74·6)	60·7 (53–68·6)
Aortic aneurysm	64 (57·7–70·3)	69·4 (64·1–74·6)	--	69·4 (64·1–74·6)
Endocarditis	40·5 (29·4–52·8)	43·5 (33·3–54·3)	40·5 (29·5–52·5)	43·5 (33·5–54·3)
Other cardiovascular and circulatory diseases	37·9 (30·3–45·6)	41·1 (34·4–47·2)	43·2 (35·9–51·1)	40·4 (32·8–46·8)
Chronic respiratory diseases	73·6 (70·4–76·7)	68 (65–70·9)	59·1 (55·2–62·8)	73·3 (70·4–76·1)
Chronic obstructive pulmonary disease	77·6 (74·3–80·8)	75·4 (72·4–78·3)	69 (65·8–72)	78·3 (75·2–81·1)
Pneumoconiosis	100 (100–100)	100 (100–100)	100 (100–100)	100 (100–100)
Asthma	44·4 (33·3–56)	40·6 (32·4–49·5)	39·9 (31·6–48·7)	46·7 (36·3–57·3)
Digestive diseases	38·4 (33·8–42·5)	41·3 (36–45·9)	14 (9·51–19·2)	49·5 (43·7–54·5)
Cirrhosis and other chronic liver diseases	80 (69·7–88·9)	81·2 (71·1–89·6)	76·1 (67·8–83·7)	81·3 (71·1–89·8)

Upper digestive system diseases	26·1 (22·3–29·9)	13·8 (9·51–18·5)	1·04 (0·607–1·63)	28·8 (24·8–32·8)
Gallbladder and biliary diseases	40·7 (27·1–54·5)	45·3 (32·6–58·4)	47·9 (35·1–60·9)	41·8 (28·8–55·5)
Pancreatitis	45·7 (34–59·1)	48·2 (36·5–61·4)	45 (33–58·2)	48·4 (36·7–61·7)
Neurological disorders	25·5 (11·8–38·9)	16·5 (8·6–28·2)	11·4 (5·84–20·6)	22·8 (10·7–36·8)
Alzheimer's disease and other dementias	40·3 (28·6–52·2)	43·1 (30·9–54·9)	43·8 (32·2–55·4)	42·7 (30·5–54·5)
Parkinson's disease	-14·8 (-22·6–-7·87)	-16·1 (-24·4–-8·72)	-16·3 (-24·5–-8·83)	-16·1 (-24·4–-8·65)
Idiopathic epilepsy	30·2 (22·1–37·7)	27·3 (19·7–34·4)	25·6 (18·6–32·2)	29·7 (21·9–37·4)
Multiple sclerosis	18·9 (13·9–23·7)	19·7 (14·6–24·7)	19·1 (14·3–24·1)	20·1 (14·9–25·3)
Mental disorders	--	5·16 (2·22–8·52)	5·16 (2·22–8·53)	--
Depressive disorders	--	10·7 (3·74–18·7)	10·7 (3·74–18·7)	--
Anxiety disorders	--	4·92 (1·5–10·6)	4·92 (1·5–10·6)	--
Idiopathic developmental intellectual disability	--	43·2 (19·2–73)	43·2 (19·2–73)	--
Substance use disorders	100 (100–100)	100 (100–100)	100 (100–100)	100 (100–100)
Alcohol use disorders	100 (100–100)	100 (100–100)	100 (100–100)	100 (100–100)
Drug use disorders	100 (100–100)	100 (100–100)	100 (100–100)	100 (100–100)
Diabetes and kidney diseases	100 (100–100)	100 (100–100)	100 (100–100)	100 (100–100)
Diabetes mellitus	100 (100–100)	100 (100–100)	100 (100–100)	100 (100–100)
Chronic kidney disease	100 (100–100)	100 (100–100)	100 (100–100)	100 (100–100)
Sense organ diseases	--	7·28 (6·35–8·38)	7·28 (6·35–8·38)	--
Blindness and vision loss	--	8·09 (5·52–11·1)	8·09 (5·52–11·1)	--
Age-related and other hearing loss	--	8·34 (7·52–9·25)	8·34 (7·52–9·25)	--
Musculoskeletal disorders	4·41 (1·25–8·09)	22·8 (18·6–27·3)	23·2 (19–27·8)	5·13 (1·53–8·93)
Rheumatoid arthritis	15·2 (4·5–26·8)	17·9 (5·8–30·9)	18·2 (5·94–31·7)	17·1 (5·37–29·5)
Osteoarthritis	--	12·2 (6·23–19·2)	12·2 (6·23–19·2)	--
Low back pain	--	41·6 (35·3–47·7)	41·6 (35·3–47·7)	--
Gout	--	47·1 (33·3–63·1)	47·1 (33·3–63·1)	--
Other non-communicable diseases	0·0226 (0·0132–0·0361)	0·0173 (0·00925–0·0299)	--	0·0914 (0·0518–0·145)
Sudden infant death syndrome	5·44 (3·21–8·64)	5·43 (3·21–8·63)	--	5·43 (3·21–8·63)
Injuries	35·4 (29·5–40·2)	31·5 (27–36)	35·8 (31·2–40·1)	26·8 (20·3–32·5)
Transport injuries	26·1 (19·2–32·7)	27·7 (21·5–34)	31·9 (27·5–36·1)	25·7 (18·2–33·3)
Road injuries	24·8 (17·7–31·7)	26·6 (20·1–33·1)	30·9 (26·5–35·2)	24·6 (16·8–32·4)
Other transport injuries	41·1 (35·9–46)	39·7 (34·4–44·7)	41·1 (36·2–45·4)	38·8 (33·1–44·5)
Unintentional injuries	45·8 (38·5–50·9)	36 (31–40·5)	36·2 (31·2–40·8)	35·4 (30–40·2)
Falls	57·9 (49·3–64)	45·9 (39·4–51·1)	43·6 (37·5–48·7)	53·2 (45·8–58·6)
Drowning	-5·15 (-18–7·77)	-3·57 (-15·8–8·53)	15 (9·99–21·5)	-4·56 (-17·1–7·9)

Fire, heat, and hot substances	15·1 (9·93–21·9)	20·1 (14·6–27·2)	20·3 (14·8–27·4)	19·8 (14·4–26·8)
Poisonings	22·3 (16·6–29·8)	21·4 (16–28·2)	18·8 (13·7–25)	25·2 (19·2–32·8)
Exposure to mechanical forces	15·7 (6·03–25·7)	24·4 (19·9–29·2)	25·9 (21·9–30·2)	11·5 (0·887–22·7)
Animal contact	28·4 (23·1–34·4)	27·6 (22·4–33·6)	28·2 (22·9–34·2)	26·3 (21·3–32·2)
Foreign body	6·83 (4·93–9·3)	9·96 (7·19–13·5)	8·53 (6·11–11·7)	11 (7·81–15·3)
Environmental heat and cold exposure	10·2 (4·66–17·4)	10·2 (4·77–17·4)	9·98 (4·62–17)	10·6 (5·02–17·8)
Exposure to forces of nature	0 (0–0)	10·8 (5·07–18·2)	10·8 (5·07–18·2)	0 (0–0)
Other unintentional injuries	19·8 (14–27·2)	19·7 (14·2–26·5)	19·5 (14·1–26·4)	23·3 (17·4–30·7)
Self-harm and interpersonal violence	20·6 (7·58–32·5)	22·4 (10·6–32·9)	36·2 (29·1–43·1)	20·7 (7·87–32·2)
Self-harm	19·9 (5·9–32·7)	20·2 (6·18–32·9)	23·4 (11·5–34)	20 (6·03–32·9)
Interpersonal violence	31·9 (23·3–40·7)	35·2 (27·4–43·3)	40·9 (33–49)	28·9 (20·6–37·6)

Supplementary Results Table 2. All-age PAFs attributable to the joint distribution of all risk factors for all ages, both sexes combined for each GBD level 1, level 2, and level 3 cause and all causes in 2019 for Finland. Results are shown for deaths, YLLs, YLDs, and DALYs Data in parenthesis are 95% uncertainty intervals. PAFs=population attributable fractions. GBD=Global Burden of Disease. YLLs=years of life lost. YLDs=years lived with disability. DALYs=disability-adjusted life-years

	Deaths 2019	DALYs 2019	YLDs 2019	YLLs 2019
All causes	60·2 (56·2–63·6)	43·5 (40·2–46·7)	27·1 (24·9–29·3)	58·1 (55·3–60·7)
Communicable, maternal, neonatal, and nutritional diseases	33·6 (23·7–44·3)	42·3 (38·2–46·3)	41·8 (37·3–46·4)	43·1 (35·9–50·5)
HIV/AIDS and sexually transmitted infections	91·4 (90·4–92·3)	92·1 (89·6–94·8)	97·5 (95·9–98·8)	84·2 (82·9–85·4)
HIV/AIDS	87·7 (86·5–88·8)	84·9 (83·3–86·5)	91·3 (89·6–92·8)	81·9 (80·4–83·2)
Sexually transmitted infections excluding HIV	100 (100–100)	100 (100–100)	100 (100–100)	99·8 (99·7–99·9)
Respiratory infections and tuberculosis	35·8 (23·9–49)	27·2 (19·3–36·2)	2·97 (2·11–4·06)	40·8 (29·6–52·9)
Tuberculosis	46·6 (37–55·5)	52 (43·2–60·3)	54·2 (45·2–62·1)	51·7 (42·7–59·9)
Lower respiratory infections	35·1 (22·5–49·4)	39·7 (27·7–52·7)	28·8 (23·4–33·8)	40 (27·8–53·2)
Upper respiratory infections	0·0153 (0·0086–0·0243)	0·00379 (0·000229–0·011)	0·00353 (0–0·0108)	0·0474 (0·0266–0·0766)
Otitis media	0·00986 (0·00364–0·0232)	2·96 (1·81–4·17)	2·98 (1·83–4·19)	0·0572 (0·0208–0·133)
Enteric infections	6·35 (4·26–9·72)	9·33 (7·1–12·7)	9·33 (7·06–12·8)	9·35 (6·98–12·8)
Diarrheal diseases	6·42 (4·32–9·81)	9·36 (7·13–12·8)	9·34 (7·06–12·8)	9·5 (7·06–13)
Other infectious diseases	0·297 (0·193–0·469)	0·557 (0·413–0·743)	0·547 (0·33–0·887)	0·562 (0·406–0·775)
Meningitis	0·319 (0·2–0·453)	0·772 (0·489–1·1)	0·000262 (0–0·000878)	1·04 (0·66–1·46)
Encephalitis	0·0942 (0·0615–0·182)	0·262 (0·172–0·453)	8·21e-05 (0–0·00026)	0·322 (0·215–0·59)
Measles	1·79 (-0·429–9·16)	2·35 (-0·485–11·5)	0·539 (0·0189–1·24)	2·57 (-0·618–12·7)
Acute hepatitis	11 (6·15–20)	5·91 (3·74–8·88)	3·99 (2·46–6·33)	10·8 (5·96–19·3)
Maternal and neonatal disorders	75·6 (72·6–78·4)	71·9 (69·1–74·5)	69·1 (65·2–72·8)	76·6 (73·6–79·5)
Maternal disorders	10·3 (3·66–17·2)	10·5 (3·69–17·6)	10·7 (3·73–18)	10·2 (3·64–17)
Neonatal disorders	78·5 (75·4–81·5)	73·9 (71·1–76·7)	71·2 (67–74·9)	78·6 (75·4–81·5)
Nutritional deficiencies	44·8 (28·7–51·1)	73·2 (69·7–76·6)	74·6 (71–77·9)	36 (25·1–40·7)
Protein-energy malnutrition	100 (100–100)	100 (100–100)	100 (100–100)	100 (100–100)
Vitamin A deficiency	--	100 (100–100)	100 (100–100)	--
Dietary iron deficiency	--	100 (100–100)	100 (100–100)	--
Non-communicable diseases	62·5 (58·2–66·1)	45·3 (41·7–48·9)	25·6 (23·3–28·1)	62·1 (59·2–65)
Neoplasms	39·7 (35·9–44·3)	39·8 (36·1–43·9)	27·1 (23·2–31·2)	40·7 (37·1–44·9)
Lip and oral cavity cancer	57·5 (50·3–64)	63·9 (57·2–70)	63·4 (55·9–69·9)	63·9 (57·2–70)
Nasopharynx cancer	61 (51·9–69·2)	62·7 (54–70·6)	60·3 (51·4–68·7)	62·9 (54·4–70·8)
Other pharynx cancer	75·5 (68·9–80·9)	78 (72·1–83·1)	75·4 (69·1–80·8)	78·2 (72·2–83·2)
Esophageal cancer	68·7 (58·5–78·1)	72 (62·5–80·8)	69·7 (59·9–78·7)	72 (62·5–80·9)

Stomach cancer	19·6 (12·1–38)	20·8 (13·2–39·3)	20·4 (12·9–38·7)	20·9 (13·2–39·3)
Colon and rectum cancer	58·4 (49·3–67·2)	59·3 (51·1–67·7)	59·3 (50·8–67·7)	59·3 (51·1–67·7)
Liver cancer	78 (72·2–83·5)	79 (73·5–84)	77·8 (72·2–83·2)	79 (73·5–84)
Gallbladder and biliary tract cancer	20·2 (11–32·2)	20·6 (11·3–32·5)	20 (10·9–31·7)	20·6 (11·3–32·5)
Pancreatic cancer	34·4 (25·5–45·1)	35·3 (26·8–45·6)	34·7 (26–45·3)	35·3 (26·9–45·6)
Larynx cancer	76 (67·8–82·8)	77·7 (70·1–83·7)	76·3 (68·7–82·7)	77·8 (70·2–83·9)
Tracheal, bronchus, and lung cancer	80·2 (76–84·3)	79·9 (76·1–83·9)	79·9 (75·7–84·1)	79·9 (76·1–83·9)
Breast cancer	33·6 (24·6–44·3)	33·6 (25·7–42·8)	33·8 (25·5–43·2)	33·5 (25·7–42·8)
Cervical cancer	100 (100–100)	100 (100–100)	100 (100–100)	100 (100–100)
Uterine cancer	41·2 (27·4–56·6)	42·7 (28·8–57·7)	43·4 (29·4–58·4)	42·6 (28·7–57·6)
Ovarian cancer	20·6 (9·95–34·2)	18·1 (8·44–30·7)	17·5 (8·12–29·4)	18·1 (8·44–30·8)
Prostate cancer	5·1 (2·23–8·14)	5·83 (2·64–9·17)	6·23 (2·81–9·7)	5·74 (2·61–9·02)
Kidney cancer	32·8 (23·8–42·7)	34·2 (25–43·8)	34·3 (25·1–44·1)	34·2 (24·9–43·8)
Bladder cancer	31·5 (19·8–44·1)	35·3 (23·5–46·9)	36·9 (25–48·7)	35·2 (23·4–46·8)
Thyroid cancer	12·8 (6·21–21·3)	13·5 (6·52–22·4)	13·6 (6·97–22)	13·5 (6·46–22·5)
Mesothelioma	97·9 (97·2–98·6)	96·4 (95·1–97·4)	97·6 (96·5–98·5)	96·3 (95–97·4)
Non-Hodgkin lymphoma	6·23 (2·53–11·1)	6·45 (2·62–11·5)	6·34 (2·56–11·4)	6·46 (2·62–11·5)
Multiple myeloma	7·2 (3·02–13)	7·51 (3·22–13·4)	7·44 (3·18–13·4)	7·51 (3·22–13·4)
Leukemia	30 (19·4–41·6)	29·2 (19·5–39·8)	30 (20–41·4)	29·1 (19·4–39·6)
Cardiovascular diseases	84·2 (80·5–87·5)	84·9 (81·9–87·6)	77·7 (73·6–81·9)	85·7 (82·8–88·3)
Rheumatic heart disease	27·8 (15·6–51·3)	29·8 (18·7–48·8)	30·3 (18·8–48·2)	29·7 (18·6–49)
Ischemic heart disease	93·2 (88·9–96·8)	94·2 (91–97)	93·8 (90·7–96·7)	94·2 (91–97)
Stroke	80·6 (72·2–89·8)	83·7 (77·3–90·2)	83·3 (77·6–89·3)	83·8 (77·2–90·4)
Hypertensive heart disease	100 (100–100)	100 (100–100)	100 (100–100)	100 (100–100)
Non-rheumatic valvular heart disease	28·6 (19·1–42)	30·7 (22·3–41·8)	33·2 (23·4–44·3)	30·5 (22–41·7)
Cardiomyopathy and myocarditis	55·7 (41·5–63·6)	63·1 (54·5–68·2)	55·1 (48·3–61·8)	63·6 (54·6–68·7)
Atrial fibrillation and flutter	51·1 (41·3–61·3)	59·9 (51·8–67·9)	64·5 (57·2–71·6)	55 (45·5–64)
Aortic aneurysm	55·6 (48·4–62·8)	63·3 (57·4–68·7)	--	63·3 (57·4–68·7)
Endocarditis	40·4 (29·6–52·4)	44·2 (34·2–54·6)	39·9 (29·9–51·4)	44·3 (34·3–54·8)
Other cardiovascular and circulatory diseases	39·2 (31·7–46·5)	43·1 (36·4–49·4)	44·1 (38·1–50·3)	42·9 (35·4–49·2)
Chronic respiratory diseases	55 (48·9–62·2)	52·3 (47·6–57·2)	47 (42–52)	57·5 (51·8–64·2)
Chronic obstructive pulmonary disease	65·8 (60·8–71·5)	64·6 (60·9–68·6)	57·1 (53·8–60·6)	69·1 (64·3–74·5)
Pneumoconiosis	100 (100–100)	100 (100–100)	100 (100–100)	100 (100–100)
Asthma	35·2 (23·7–48)	40·5 (31·4–50·4)	40·6 (31·7–50·5)	39·1 (28·1–51·3)

Digestive diseases	42 (37–46·6)	45·6 (39·7–50·9)	12·1 (8·22–16·4)	55·3 (49–61·1)
Cirrhosis and other chronic liver diseases	77·5 (67·7–86·3)	80·2 (70·6–88·3)	76·1 (68·3–83·7)	80·2 (70·6–88·5)
Upper digestive system diseases	17·8 (14·9–20·8)	7·94 (5·09–11·4)	0·687 (0·39–1·12)	21·9 (18·5–25·4)
Gallbladder and biliary diseases	39·3 (25·2–54·2)	44 (30·7–58·1)	47·5 (33·8–61·2)	40·2 (26·6–54·9)
Pancreatitis	41·6 (30·7–53·7)	48·2 (37·2–60·8)	43·1 (31·9–55·4)	48·6 (37·4–61·2)
Neurological disorders	21·3 (9·09–35·8)	14·4 (7·11–25·3)	10·3 (5·14–19·6)	19 (8·24–32·7)
Alzheimer's disease and other dementias	32·9 (19·4–48·2)	35·7 (22·4–50·7)	36·3 (23–51·2)	35·3 (22·2–50·4)
Parkinson's disease	-6·71 (-10·7–-3·31)	-8·11 (-12·5–-4·06)	-8·73 (-13·5–-4·35)	-7·95 (-12·4–-3·98)
Idiopathic epilepsy	26·7 (19·7–33·4)	25·4 (18·7–32·3)	23 (16·6–29·4)	28·9 (21·7–36)
Multiple sclerosis	13·1 (9·77–17)	14·7 (11·2–18·4)	14·2 (10·8–17·8)	15·1 (11·4–19·4)
Mental disorders	--	6·14 (2·91–9·94)	6·14 (2·91–9·94)	--
Depressive disorders	--	11·6 (4·46–19·6)	11·6 (4·46–19·6)	--
Anxiety disorders	--	5·58 (1·83–11·5)	5·58 (1·83–11·5)	--
Idiopathic developmental intellectual disability	--	38 (17·4–56·6)	38 (17·4–56·6)	--
Substance use disorders	100 (100–100)	100 (100–100)	100 (100–100)	100 (100–100)
Alcohol use disorders	100 (100–100)	100 (100–100)	100 (100–100)	100 (100–100)
Drug use disorders	100 (100–100)	100 (100–100)	100 (100–100)	100 (100–100)
Diabetes and kidney diseases	100 (99·9–100)	100 (100–100)	100 (100–100)	100 (99·9–100)
Diabetes mellitus	100 (100–100)	100 (100–100)	100 (100–100)	100 (100–100)
Chronic kidney disease	100 (100–100)	100 (100–100)	100 (100–100)	100 (100–100)
Sense organ diseases	--	6·96 (5·92–8·23)	6·96 (5·92–8·23)	--
Blindness and vision loss	--	8·27 (5·1–12·4)	8·27 (5·1–12·4)	--
Age-related and other hearing loss	--	7·61 (6·74–8·56)	7·61 (6·74–8·56)	--
Musculoskeletal disorders	4·07 (1·12–7·56)	20·7 (16·8–24·6)	20·9 (17·1–24·9)	5·19 (1·46–9·52)
Rheumatoid arthritis	8·15 (2·21–14·6)	12·2 (3·47–21·9)	12·7 (3·64–23)	10·3 (2·85–18·6)
Osteoarthritis	--	16·2 (8·49–25·4)	16·2 (8·49–25·4)	--
Low back pain	--	38·5 (33·3–43·8)	38·5 (33·3–43·8)	--
Gout	--	46·9 (32·3–63·9)	46·9 (32·3–63·9)	--
Other non-communicable diseases	0·0558 (0·0348–0·0778)	0·0249 (0·0143–0·0389)	--	0·188 (0·113–0·258)
Sudden infant death syndrome	4·35 (3·03–6·09)	4·34 (3·03–6·09)	--	4·34 (3·03–6·09)
Injuries	31·4 (25·2–37)	28·8 (24–33·4)	34·4 (29·8–38·4)	22·8 (14·6–29·9)
Transport injuries	20·1 (13·3–26·6)	22·2 (16·2–27·9)	27·8 (23·9–31·3)	19·3 (11·5–26·4)
Road injuries	15·5 (7·26–23·1)	19·4 (12·8–25·4)	26·7 (22·8–30·2)	14·9 (5·76–23·1)
Other transport injuries	36·2 (31·6–40)	36 (31–40·3)	38 (33·5–41·4)	35·5 (30·2–40)

Unintentional injuries	41·4 (35·1–46)	33·5 (28·5–38)	35 (30·1–39·5)	30 (24·7–35)
Falls	53·6 (46·3–58·4)	42·7 (36·6–47·4)	41·4 (35·5–45·9)	46·4 (40·2–51·3)
Drowning	-13·8 (-32·7–3·45)	-10·7 (-28·1–6·03)	11·3 (7·2–17·1)	-11·4 (-29·4–5·71)
Fire, heat, and hot substances	13·8 (8·97–20·1)	15·8 (10·9–22·1)	14·9 (10·4–21·1)	16·9 (11·7–23·8)
Poisonings	16·5 (11·3–23·4)	16·4 (11·7–22·4)	14·2 (9·98–19·8)	19 (13·4–25·9)
Exposure to mechanical forces	4·2 (-7·96–16·6)	19 (15·6–22·3)	21·2 (18·6–23·6)	0·751 (-12·4–13·9)
Animal contact	25·1 (20·1–31·2)	24·7 (19·9–30)	25 (20·4–30·1)	24 (18·8–30·4)
Foreign body	3·08 (2·24–4·11)	4·98 (3·69–6·61)	4·86 (3·56–6·46)	5·05 (3·66–6·69)
Environmental heat and cold exposure	9·23 (4·14–15·7)	9·84 (4·56–16·7)	8·83 (4·06–14·9)	10·1 (4·68–17·1)
Exposure to forces of nature	0 (0–0)	9·71 (4·5–16·3)	9·71 (4·5–16·3)	0 (0–0)
Other unintentional injuries	15·5 (10·5–22·1)	14·3 (9·85–20·1)	14·1 (9·7–19·9)	18·9 (13·3–25·9)
Self-harm and interpersonal violence	16·3 (-0·0834–30·7)	19·2 (4·21–32·3)	32·5 (26·6–38·2)	17·8 (1·79–32·1)
Self-harm	15·4 (-2·39–31·2)	17·2 (-0·0192–32·9)	21·1 (10·4–31·7)	17·1 (-0·545–32·9)
Interpersonal violence	25·8 (14·4–36·7)	31 (22·1–40·3)	39·7 (32·9–46·8)	25 (13·7–36·2)

Supplementary Results Table 2. All-age PAFs attributable to the joint distribution of all risk factors for all ages, both sexes combined for each GBD level 1, level 2, and level 3 cause and all causes in 2019 for France. Results are shown for deaths, YLLs, YLDs, and DALYs Data in parenthesis are 95% uncertainty intervals. PAFs=population attributable fractions. GBD=Global Burden of Disease. YLLs=years of life lost. YLDs=years lived with disability. DALYs=disability-adjusted life-years

	Deaths 2019	DALYs 2019	YLDs 2019	YLLs 2019
All causes	52·1 (48·6–55)	38·5 (35·5–41·3)	24·1 (22·1–26·1)	51·9 (49·5–54)
Communicable, maternal, neonatal, and nutritional diseases	49·5 (44·3–54·5)	51 (47·9–54)	45·8 (41·4–50·6)	54·4 (50·5–58·2)
HIV/AIDS and sexually transmitted infections	96·3 (95·9–96·7)	95·9 (95·3–96·5)	98·2 (97·7–98·8)	94·5 (94–95)
HIV/AIDS	96·1 (95·6–96·5)	95·3 (94·7–95·8)	97·4 (97–97·8)	94·4 (93·8–94·9)
Sexually transmitted infections excluding HIV	99·9 (99·9–99·9)	99·9 (99·9–100)	100 (100–100)	99·6 (99·4–99·7)
Respiratory infections and tuberculosis	43·8 (37–50·4)	37·8 (31·9–43·8)	3·86 (2·82–5·26)	47·4 (40·7–53·9)
Tuberculosis	51·8 (40·7–60·5)	56·2 (46·2–64·2)	58·2 (48·5–66·4)	55·9 (45·9–64)
Lower respiratory infections	43·5 (36·2–50·4)	47 (39·9–53·6)	34·7 (28·7–40·2)	47·1 (40–53·8)
Upper respiratory infections	0·441 (0·215–0·732)	0·0253 (0·0113–0·0451)	0·00611 (2·52e-05–0·0163)	1·34 (0·65–2·2)
Otitis media	1·02 (0·281–2·56)	4·45 (2·89–6·2)	4·45 (2·88–6·22)	3·95 (1·34–8·01)
Enteric infections	9·52 (5·65–15·3)	13 (9·16–18·8)	12·8 (8·75–18·8)	13·5 (9·33–19·5)
Diarrheal diseases	9·68 (5·77–15·6)	13·1 (9·22–18·9)	12·8 (8·76–18·8)	13·8 (9·63–19·9)
Other infectious diseases	0·436 (0·29–0·573)	1·15 (0·838–1·48)	0·386 (0·238–0·66)	1·43 (0·984–1·87)
Meningitis	1·61 (1·17–2·18)	3·55 (2·57–4·75)	0·000639 (1·08e-06–0·00177)	4·79 (3·54–6·22)
Encephalitis	0·325 (0·204–0·458)	0·959 (0·591–1·34)	0·000241 (5·45e-07–0·00067)	1·08 (0·661–1·5)
Measles	2·11 (-0·572–12·2)	1·63 (-0·312–8·56)	0·16 (0·00471–0·402)	3·19 (-0·912–17·7)
Acute hepatitis	8·77 (4·72–15·6)	3·53 (2·29–5·24)	1·92 (1·19–3·26)	10·2 (5·23–17·3)
Maternal and neonatal disorders	69·6 (65·8–73·3)	69·6 (66·7–72·3)	68·3 (63·8–72·2)	70·4 (66·7–74·1)
Maternal disorders	8·13 (2·95–13·2)	7·98 (2·89–13·1)	7·92 (2·87–13·1)	8·03 (2·91–13·1)
Neonatal disorders	72 (68·2–75·7)	71·5 (68·5–74·3)	70·6 (66–74·7)	72 (68·2–75·7)
Nutritional deficiencies	96·8 (89·8–97·7)	79·1 (76–81·9)	72·1 (69·4–74·2)	95·9 (89·8–96·9)
Protein-energy malnutrition	100 (100–100)	100 (100–100)	100 (100–100)	100 (100–100)
Vitamin A deficiency	--	100 (100–100)	100 (100–100)	--
Dietary iron deficiency	--	100 (100–100)	100 (100–100)	--
Non-communicable diseases	53·9 (49·8–57·1)	38·4 (35–41·6)	21·3 (19·1–23·5)	54·9 (52–57·4)
Neoplasms	45 (42·1–48)	47·2 (44·6–49·9)	34·1 (30·7–37·3)	47·9 (45·4–50·6)
Lip and oral cavity cancer	73·6 (67·3–79)	76·4 (70·6–81·4)	75·9 (69·8–81)	76·4 (70·7–81·4)
Nasopharynx cancer	71 (62·2–78·1)	71·6 (62·9–78·5)	70·1 (61·5–77·2)	71·7 (63·1–78·6)
Other pharynx cancer	83·3 (77·8–87·5)	84·2 (78·9–88·3)	83·4 (77·8–87·7)	84·2 (79–88·4)
Esophageal cancer	76·2 (68·6–83·7)	78·1 (70·7–85·1)	76·6 (68·7–83·8)	78·1 (70·7–85·1)

Stomach cancer	21·2 (13·9–39·5)	22·5 (14·9–40·4)	22·4 (14·8–40·3)	22·6 (14·9–40·4)
Colon and rectum cancer	63·3 (55·7–69·8)	64·5 (57·4–71)	64·7 (57·5–71·3)	64·5 (57·3–71)
Liver cancer	71·2 (63·8–78)	74·3 (67·6–80·5)	72·5 (65·4–78·8)	74·3 (67·6–80·5)
Gallbladder and biliary tract cancer	19 (10·6–30·2)	19·2 (10·6–30·2)	18·7 (10·3–29·6)	19·2 (10·6–30·2)
Pancreatic cancer	31·4 (25–39·1)	32·7 (26·6–39·8)	32 (25·9–39·3)	32·7 (26·7–39·8)
Larynx cancer	82·9 (75·9–87·9)	83·4 (76·9–88·3)	83·5 (77·1–88·5)	83·4 (76·8–88·3)
Tracheal, bronchus, and lung cancer	82 (78·8–84·8)	80·9 (77·8–83·8)	81·4 (78·1–84·3)	80·9 (77·8–83·8)
Breast cancer	33·5 (27·2–40·1)	33·6 (27·9–39·5)	33·7 (27·9–39·7)	33·6 (27·9–39·5)
Cervical cancer	100 (100–100)	100 (100–100)	100 (100–100)	100 (100–100)
Uterine cancer	41·4 (27·6–55·6)	42·7 (29·3–56·2)	43·2 (29·7–56·8)	42·6 (29·2–56·1)
Ovarian cancer	16·3 (7·82–25·3)	13·6 (6·44–21·4)	13 (6·21–20·5)	13·6 (6·45–21·5)
Prostate cancer	5·61 (2·5–8·95)	6·41 (2·92–10·2)	7·04 (3·28–11)	6·31 (2·86–9·99)
Kidney cancer	33·7 (24·8–43)	34·5 (25·8–44·2)	34·4 (25·5–44)	34·5 (25·8–44·2)
Bladder cancer	33·3 (23–43·7)	38·2 (27·6–47·9)	39·9 (29·3–49·5)	38 (27·5–47·8)
Thyroid cancer	12·6 (6·01–21·1)	13·3 (6·2–22·5)	13·3 (6·37–22·4)	13·3 (6·16–22·5)
Mesothelioma	98·4 (97·8–98·9)	97·2 (96·2–98)	98·2 (97·4–98·8)	97·1 (96·2–97·9)
Non-Hodgkin lymphoma	5·96 (2·41–10·7)	6·12 (2·54–10·8)	6·06 (2·5–10·8)	6·12 (2·54–10·8)
Multiple myeloma	6·84 (2·96–12·2)	7·09 (3·1–12·5)	7·08 (3·08–12·5)	7·09 (3·1–12·6)
Leukemia	31·1 (20·3–43·5)	30·8 (20·7–41·5)	30·6 (20·6–41·1)	30·8 (20·7–41·6)
Cardiovascular diseases	75·1 (71·2–79)	76·6 (73·5–79·7)	71·1 (67·1–74·9)	77·7 (74·6–80·7)
Rheumatic heart disease	25·5 (13·5–51·2)	26·2 (16·3–47·9)	26·5 (16·9–44·6)	26·2 (16–48·3)
Ischemic heart disease	92·4 (88·2–95·8)	93·8 (90·8–96·2)	93·2 (90·4–95·7)	93·8 (90·9–96·3)
Stroke	79·6 (72·2–86·6)	82·6 (77·2–87·7)	81·9 (76·8–86·7)	82·7 (77·1–88)
Hypertensive heart disease	100 (100–100)	100 (100–100)	100 (100–100)	100 (100–100)
Non-rheumatic valvular heart disease	32·5 (20·2–49·2)	33·8 (22·9–47·9)	28·8 (19·8–39·4)	33·9 (23–47·9)
Cardiomyopathy and myocarditis	35·9 (25·9–48·9)	42 (34·5–51·2)	41·4 (33·5–50·2)	42·1 (34·6–51·4)
Atrial fibrillation and flutter	53·6 (44·4–63·5)	60·7 (53·2–68·1)	65·1 (58·3–71·8)	56·3 (48·1–65·3)
Aortic aneurysm	53·2 (46·3–60·6)	62·4 (56·9–67·9)	--	62·4 (56·9–67·9)
Endocarditis	35·2 (23·5–49·7)	37·7 (27·7–49·3)	35·8 (25·2–49·2)	37·8 (27·9–49·2)
Other cardiovascular and circulatory diseases	33·5 (26·3–41·9)	37·5 (31·9–43·6)	41·2 (35·1–47·2)	35·9 (30·2–42·5)
Chronic respiratory diseases	58·3 (54–62·5)	51·6 (47·4–55·7)	44·7 (39·3–50·3)	58·8 (55·1–62·7)
Chronic obstructive pulmonary disease	67 (62·5–71·4)	66 (62·5–69·3)	57·2 (53·4–60·9)	70·6 (66·6–74·2)
Pneumoconiosis	100 (100–100)	100 (100–100)	100 (100–100)	100 (100–100)
Asthma	37·4 (26·7–49·4)	39·4 (31·1–48·6)	39·1 (30·9–48·1)	42·2 (32·4–53)

Digestive diseases	35 (30·3–39·3)	41·2 (36–45·7)	12·8 (8·96–17·3)	48·7 (43·1–53·6)
Cirrhosis and other chronic liver diseases	80·6 (69·6–88·9)	82·8 (72·9–90)	77·8 (69·6–85)	82·9 (72·8–90·1)
Upper digestive system diseases	16·9 (14–20)	5·9 (3·67–8·59)	0·755 (0·435–1·21)	22·3 (18·8–25·8)
Gallbladder and biliary diseases	39·5 (25·1–53·8)	43·3 (30·3–56·8)	46·4 (33·7–59·3)	40·3 (26·9–54·2)
Pancreatitis	49·8 (37·7–64)	53·9 (42·4–67·1)	49·7 (38·5–62·8)	54·2 (42·8–67·6)
Neurological disorders	20·9 (10·6–32·9)	14·5 (7·83–24)	10 (5·16–18·1)	19·8 (10·5–31·1)
Alzheimer's disease and other dementias	29·1 (18·3–41·8)	31·4 (20·5–43·9)	31·9 (20·9–44·4)	31·2 (20·4–43·5)
Parkinson's disease	-7·16 (-11·6–-3·38)	-8·52 (-13·6–-4·06)	-9·01 (-14·4–-4·41)	-8·39 (-13·5–-3·99)
Idiopathic epilepsy	30·4 (22·2–38)	28·5 (21·2–35·5)	26·6 (19·6–33·2)	31·1 (23·2–38·3)
Multiple sclerosis	15·2 (11·4–18·9)	17·6 (13·5–21·3)	17·3 (13·2–21·2)	17·9 (13·7–21·9)
Mental disorders	--	6·19 (3·05–10)	6·2 (3·06–10)	--
Depressive disorders	--	10·2 (4·07–17·1)	10·2 (4·07–17·1)	--
Anxiety disorders	--	7·48 (2·51–14·8)	7·48 (2·51–14·8)	--
Idiopathic developmental intellectual disability	--	50·7 (25·4–69)	50·7 (25·4–69)	--
Substance use disorders	100 (100–100)	100 (100–100)	100 (100–100)	100 (100–100)
Alcohol use disorders	100 (100–100)	100 (100–100)	100 (100–100)	100 (100–100)
Drug use disorders	100 (100–100)	100 (100–100)	100 (100–100)	100 (100–100)
Diabetes and kidney diseases	100 (100–100)	100 (100–100)	100 (100–100)	100 (100–100)
Diabetes mellitus	100 (100–100)	100 (100–100)	100 (100–100)	100 (100–100)
Chronic kidney disease	100 (100–100)	100 (100–100)	100 (100–100)	100 (100–100)
Sense organ diseases	--	5·99 (5·29–6·83)	5·99 (5·29–6·83)	--
Blindness and vision loss	--	6·46 (4·41–9·14)	6·46 (4·41–9·14)	--
Age-related and other hearing loss	--	6·78 (6·06–7·61)	6·78 (6·06–7·61)	--
Musculoskeletal disorders	1·11 (0·26–2·16)	22·6 (18·9–26·5)	23·1 (19·3–27·1)	1·61 (0·39–2·98)
Rheumatoid arthritis	8·04 (1·95–14·9)	13·7 (3·61–23·8)	14·3 (3·82–25)	10·6 (2·76–18·9)
Osteoarthritis	--	14·6 (7·69–22·7)	14·6 (7·69–22·7)	--
Low back pain	--	38·2 (33·5–43·2)	38·2 (33·5–43·2)	--
Gout	--	47·7 (33·9–63·5)	47·7 (33·9–63·5)	--
Other non-communicable diseases	0·0724 (0·0441–0·109)	0·0561 (0·0327–0·0896)	--	0·281 (0·173–0·417)
Sudden infant death syndrome	5·33 (3·35–7·93)	5·32 (3·35–7·92)	--	5·32 (3·35–7·92)
Injuries	34 (28·6–38·6)	34·3 (29·1–39·3)	40·6 (35·1–45·6)	29 (23·4–34·5)
Transport injuries	34 (27·6–40·3)	34·5 (28–41)	36 (31–41·2)	33·9 (26·6–41·3)
Road injuries	32·9 (26·4–39·3)	33·6 (27–40·3)	35·1 (30·1–40·4)	33 (25·7–40·4)
Other transport injuries	44 (37·9–49·8)	42·6 (36·4–48·9)	44·3 (38·9–49·1)	42 (35·2–48·8)

Unintentional injuries	37·5 (31·2–42·4)	37·3 (31·8–42·8)	41·3 (35·5–46·9)	30·4 (25·4–35·5)
Falls	57·6 (48·5–63·7)	49·3 (42·4–54·9)	48·1 (41·5–53·8)	52·2 (44·7–57·9)
Drowning	-3·68 (-13·1–6·72)	-0·501 (-9·83–9·31)	15·7 (11–22)	-1·09 (-10·5–9·03)
Fire, heat, and hot substances	15·6 (10·7–21·9)	23·4 (17·7–30·6)	23·9 (18–31·3)	22·4 (16·8–29·2)
Poisonings	17·1 (12·1–23·5)	22·6 (17·3–29·2)	21·8 (16·7–28·4)	23·5 (17·9–30·3)
Exposure to mechanical forces	20·9 (13·3–28·6)	28·4 (23·1–34·7)	29·9 (25·2–36)	19·7 (10·7–28·8)
Animal contact	28·8 (22·9–36·1)	30·7 (25–37·5)	31·4 (25·7–37·8)	29·4 (23·3–36·7)
Foreign body	2·99 (2·09–4·17)	8·63 (6·21–11·9)	11 (7·91–15·1)	8·2 (5·86–11·2)
Environmental heat and cold exposure	11·3 (5·3–18·9)	11·8 (5·52–19·7)	10·2 (4·92–17·2)	11·9 (5·59–20)
Exposure to forces of nature	0 (0–0)	11·2 (5·32–18·8)	11·2 (5·32–18·8)	0 (0–0)
Other unintentional injuries	24 (17·7–31·8)	23·6 (17·8–31)	22·2 (16·5–29·4)	30·2 (23–38·7)
Self-harm and interpersonal violence	24·9 (12·3–36·9)	25·7 (14–36·9)	34 (27·2–40·6)	24·9 (12·7–36·9)
Self-harm	24·8 (11·8–37·4)	24·9 (12·1–37·6)	25·8 (14–36·9)	24·9 (12–37·6)
Interpersonal violence	29·1 (21·1–37·3)	32·4 (24·9–40·3)	39·2 (31·5–47·5)	25·8 (18·4–33·8)

Supplementary Results Table 2. All-age PAFs attributable to the joint distribution of all risk factors for all ages, both sexes combined for each GBD level 1, level 2, and level 3 cause and all causes in 2019 for Germany. Results are shown for deaths, YLLs, YLDs, and DALYs Data in parenthesis are 95% uncertainty intervals. PAFs=population attributable fractions. GBD=Global Burden of Disease. YLLs=years of life lost. YLDs=years lived with disability. DALYs=disability-adjusted life-years				
	**Deaths 2019**	**DALYs 2019**	**YLDs 2019**	**YLLs 2019**
All causes	62 (58·7–64·9)	46·1 (42·8–49·5)	28·9 (26·6–31·3)	60·5 (58·2–63)
Communicable, maternal, neonatal, and nutritional diseases	43·9 (38·5–49·3)	49·5 (46·1–52·6)	45·7 (41·6–50·1)	52·6 (48–56·9)
HIV/AIDS and sexually transmitted infections	91·8 (90·7–92·5)	91·5 (90·1–93)	95·8 (94·3–97·4)	88·7 (87·5–89·6)
HIV/AIDS	91·2 (90·1–92)	89·6 (88·4–90·6)	92·8 (91·7–93·9)	88·3 (87–89·3)
Sexually transmitted infections excluding HIV	99·9 (99·9–99·9)	100 (99·9–100)	100 (100–100)	99·7 (99·6–99·8)
Respiratory infections and tuberculosis	49·1 (41·9–56)	42·5 (35·9–49·1)	3·84 (2·77–5·2)	52·7 (45·6–59·4)
Tuberculosis	63·8 (53·8–71·3)	66·1 (57·1–72·8)	62·7 (52·8–69·8)	67 (58–73·7)
Lower respiratory infections	48·9 (41·5–55·8)	52·4 (45–59)	40 (33·8–45·7)	52·5 (45·2–59·2)
Upper respiratory infections	0·0535 (0·0252–0·105)	0·0077 (0·00192–0·0178)	0·00552 (0·000222–0·0152)	0·222 (0·108–0·421)
Otitis media	4·34 (1·97–8·25)	3·25 (2·08–4·69)	3·13 (1·96–4·54)	10·5 (5·98–16·2)
Enteric infections	5·25 (3·35–8·23)	7·07 (5·11–9·9)	7·7 (5·7–10·6)	6·05 (4·14–8·91)
Diarrheal diseases	5·28 (3·37–8·27)	7·09 (5·13–9·93)	7·71 (5·71–10·6)	6·1 (4·18–8·98)
Other infectious diseases	0·311 (0·217–0·535)	0·629 (0·482–0·833)	0·352 (0·201–0·617)	0·755 (0·571–1·03)
Meningitis	0·649 (0·449–0·915)	1·54 (1·09–2·1)	0·000515 (1·5e-05–0·00141)	2 (1·4–2·74)
Encephalitis	0·126 (0·0896–0·198)	0·316 (0·226–0·462)	0·000125 (3·26e-06–0·000356)	0·44 (0·317–0·658)
Measles	2·32 (-1·07–13·3)	2·76 (-1·23–16·1)	0·263 (0·00679–0·641)	2·93 (-1·35–16·9)
Acute hepatitis	6·85 (3·89–14·7)	4·23 (2·72–7·39)	2·45 (1·45–4·04)	8·09 (4·8–16·7)
Maternal and neonatal disorders	83·3 (81·4–85·2)	78·9 (76·8–81)	74·8 (71·4–78)	84·1 (82·3–86)
Maternal disorders	9·05 (3·25–15·2)	9·04 (3·22–15·3)	9·05 (3·21–15·4)	9·01 (3·23–15·2)
Neonatal disorders	85·7 (83·8–87·6)	80·7 (78·6–82·7)	76·8 (73·5–80·1)	85·7 (83·8–87·6)
Nutritional deficiencies	78·8 (49·1–83·6)	73·5 (70·6–75·7)	73·5 (70·8–75·7)	72·9 (44·6–77·7)
Protein-energy malnutrition	100 (100–100)	100 (100–100)	100 (100–100)	100 (100–100)
Vitamin A deficiency	--	100 (100–100)	100 (100–100)	--
Dietary iron deficiency	--	100 (100–100)	100 (100–100)	--
Non-communicable diseases	63·8 (60·3–67)	47 (43·4–50·8)	27·5 (24·9–30·1)	63·1 (60·4–65·8)
Neoplasms	46·5 (42·7–50·8)	47·6 (44·2–51·6)	35·4 (31·6–39·8)	48·3 (45–52·2)
Lip and oral cavity cancer	76·6 (71–81·3)	78·9 (73·7–83·2)	78 (72·5–82·4)	78·9 (73·8–83·2)
Nasopharynx cancer	73 (65·1–79·8)	73·4 (65·6–79·9)	72·4 (64·7–79·1)	73·5 (65·7–80)
Other pharynx cancer	84·9 (80·4–88·6)	85·8 (81·3–89·3)	84·9 (80·1–88·7)	85·8 (81·3–89·3)
Esophageal cancer	78·7 (71–85·7)	80·2 (72·5–87·2)	79·3 (71·4–86·3)	80·3 (72·5–87·2)
Stomach cancer	23·2 (15·8–40·8)	24·8 (16·9–42·5)	24·5 (16·6–42·3)	24·8 (16·9–42·5)

Colon and rectum cancer	64·3 (55·6–72·8)	65·2 (56·8–72·9)	65·2 (56·9–73·1)	65·2 (56·8–72·9)
Liver cancer	74·5 (68–80·8)	77 (70·9–82·8)	75·9 (69·7–82)	77 (70·9–82·9)
Gallbladder and biliary tract cancer	21·5 (11·9–33·6)	21·7 (11·8–33·8)	21 (11·5–33·2)	21·7 (11·8–33·9)
Pancreatic cancer	39·9 (30·7–51)	40·4 (31·8–50·7)	40·2 (31·2–51)	40·4 (31·8–50·7)
Larynx cancer	85·3 (78·5–90·2)	85·8 (79·7–90·3)	85·6 (79·5–90·2)	85·8 (79·7–90·3)
Tracheal, bronchus, and lung cancer	84·5 (81·6–87·7)	83·8 (80·8–87)	83·9 (80·9–87·1)	83·8 (80·8–87)
Breast cancer	39·5 (30·4–50·4)	39·1 (30·9–48·5)	39·2 (31–48·7)	39·1 (30·9–48·5)
Cervical cancer	100 (100–100)	100 (100–100)	100 (100–100)	100 (100–100)
Uterine cancer	44·2 (29·8–58·7)	45·3 (31·3–59·8)	45·7 (31·6–60·5)	45·2 (31·2–59·8)
Ovarian cancer	21·4 (10–35·6)	18·4 (8·57–31·3)	17·6 (8·21–30·4)	18·5 (8·59–31·4)
Prostate cancer	5·98 (2·68–9·66)	6·69 (3·11–10·5)	7·24 (3·39–11·3)	6·59 (3·04–10·4)
Kidney cancer	37·1 (27·3–47·2)	38·4 (28·9–47·9)	38·7 (29·3–48·3)	38·4 (28·9–47·9)
Bladder cancer	40·2 (26–53·9)	43·5 (29·7–56·6)	45 (31·6–57·9)	43·4 (29·6–56·5)
Thyroid cancer	14·5 (6·92–23·9)	15·1 (7·3–24·8)	14·6 (7·46–23·3)	15·1 (7·24–24·9)
Mesothelioma	97·7 (96·9–98·4)	95·7 (94·3–96·9)	97·3 (96·1–98·2)	95·6 (94·2–96·8)
Non-Hodgkin lymphoma	6·93 (2·87–12·5)	7·13 (3–12·7)	7·06 (2·94–12·6)	7·14 (3·01–12·7)
Multiple myeloma	7·96 (3·39–14)	8·22 (3·57–14·5)	8·2 (3·52–14·6)	8·22 (3·58–14·5)
Leukemia	34·7 (21·8–48·3)	34·6 (23·1–46·8)	34·2 (22·5–46·3)	34·6 (23·2–46·8)
Cardiovascular diseases	83·5 (80·3–86·3)	84·1 (81·6–86·6)	78·7 (75·1–82·3)	84·8 (82·2–87·1)
Rheumatic heart disease	28·6 (15–56)	29 (17·4–51·7)	29·3 (18·3–48·5)	29 (17·3–51·9)
Ischemic heart disease	94·5 (90·6–97·6)	95·3 (92·4–97·6)	95 (92·2–97·4)	95·3 (92·4–97·6)
Stroke	84·2 (76·7–91·9)	86·5 (80·8–92·4)	86 (80·6–91·9)	86·6 (80·9–92·6)
Hypertensive heart disease	100 (100–100)	100 (100–100)	100 (100–100)	100 (100–100)
Non-rheumatic valvular heart disease	30·6 (20·4–45)	31·4 (22·1–43·1)	34·9 (24·5–46·8)	31·3 (22–43)
Cardiomyopathy and myocarditis	53·3 (44·8–63·9)	61·5 (55·4–68·3)	60·4 (53·7–66·9)	61·6 (55·2–68·4)
Atrial fibrillation and flutter	59·6 (50·9–68·9)	64·8 (57·7–72·3)	68·9 (62·5–75·4)	61·9 (54–70·4)
Aortic aneurysm	58·5 (51·8–65·3)	66·2 (60·9–71·2)	--	66·2 (60·9–71·2)
Endocarditis	38·7 (27·5–53)	40·6 (30·9–52·2)	39·2 (28·5–52·1)	40·7 (30·9–52·2)
Other cardiovascular and circulatory diseases	36·7 (30·1–44·9)	40·1 (34·5–46·3)	43·3 (36·9–49·6)	39·1 (33·2–45·5)
Chronic respiratory diseases	65·7 (61·8–69·5)	62·5 (59·4–65·6)	56·1 (52·7–59·8)	67·7 (64·2–71·3)
Chronic obstructive pulmonary disease	71·6 (67·7–75·5)	70 (66·8–73·2)	63 (59·5–66·4)	74·7 (71·3–78·1)
Pneumoconiosis	100 (100–100)	100 (100–100)	100 (100–100)	100 (100–100)
Asthma	43·5 (32·5–55·7)	44·6 (36·2–54·1)	44·1 (35·8–53·4)	47·9 (37·7–59)
Digestive diseases	41·8 (36·6–46·9)	46 (40·7–50·7)	17·3 (12·2–23·4)	53·5 (47·6–58·7)

Cirrhosis and other chronic liver diseases	82·9 (72·2–89·9)	84·5 (74·8–90·8)	80·7 (72·6–86·9)	84·6 (74·8–90·9)
Upper digestive system diseases	18 (15·3–20·8)	9·27 (6·28–12·8)	0·953 (0·567–1·47)	22·4 (19·3–25·6)
Gallbladder and biliary diseases	43·2 (28·8–57·7)	48·3 (34·7–62·1)	51·1 (37·4–64·9)	44·2 (30·4–58·5)
Pancreatitis	56·3 (44·8–69·5)	59·1 (48·1–72·1)	56 (44·9–68·8)	59·4 (48·3–72·2)
Neurological disorders	24·7 (11·3–39·3)	17 (9·02–28·5)	12·7 (6·4–23·4)	22·6 (10·8–37·2)
Alzheimer's disease and other dementias	37·8 (24·2–53·6)	40·3 (26·5–56)	40·8 (26·6–56·5)	40 (26·2–55·5)
Parkinson's disease	-8·2 (-12·9–-3·86)	-9·62 (-15·1–-4·64)	-10·4 (-16·3–-5·06)	-9·43 (-14·8–-4·54)
Idiopathic epilepsy	32·8 (24·8–40·9)	30·8 (23·4–38·1)	29·6 (22·6–36·8)	32·9 (25·1–40·8)
Multiple sclerosis	15·3 (11·5–19·2)	17·3 (13·2–21·5)	17 (13–21·2)	17·6 (13·3–21·8)
Mental disorders	--	6·64 (3·51–10·3)	6·65 (3·51–10·3)	--
Depressive disorders	--	13·7 (6·5–20·9)	13·7 (6·5–20·9)	--
Anxiety disorders	--	5·77 (1·67–12·3)	5·77 (1·67–12·3)	--
Idiopathic developmental intellectual disability	--	54·6 (27–77·1)	54·6 (27–77·1)	--
Substance use disorders	100 (100–100)	100 (100–100)	100 (100–100)	100 (100–100)
Alcohol use disorders	100 (100–100)	100 (100–100)	100 (100–100)	100 (100–100)
Drug use disorders	100 (100–100)	100 (100–100)	100 (100–100)	100 (100–100)
Diabetes and kidney diseases	100 (100–100)	100 (100–100)	100 (100–100)	100 (100–100)
Diabetes mellitus	100 (100–100)	100 (100–100)	100 (100–100)	100 (100–100)
Chronic kidney disease	100 (100–100)	100 (100–100)	100 (100–100)	100 (100–100)
Sense organ diseases	--	7·39 (6·3–8·79)	7·39 (6·3–8·79)	--
Blindness and vision loss	--	9·62 (5·97–14·2)	9·62 (5·97–14·2)	--
Age-related and other hearing loss	--	7·79 (6·96–8·73)	7·79 (6·96–8·73)	--
Musculoskeletal disorders	2·53 (0·714–4·63)	24·1 (19·9–28·3)	24·3 (20–28·6)	3·19 (0·923–5·7)
Rheumatoid arthritis	9·9 (2·84–18)	15·2 (4·59–26·5)	15·7 (4·77–27·5)	12·5 (3·68–22·5)
Osteoarthritis	--	18 (9·74–27·5)	18 (9·74–27·5)	--
Low back pain	--	40·6 (35–46·3)	40·6 (35–46·3)	--
Gout	--	52·6 (37·8–68·9)	52·6 (37·8–68·9)	--
Other non-communicable diseases	0·0509 (0·0363–0·0699)	0·036 (0·0232–0·0536)	--	0·198 (0·139–0·272)
Sudden infant death syndrome	6·26 (4·41–8·51)	6·25 (4·41–8·5)	--	6·25 (4·41–8·5)
Injuries	36·2 (30·5–41)	33·6 (29·1–37·9)	37·4 (32·4–42)	29·6 (23·7–35·2)
Transport injuries	27·2 (21·3–33·2)	28·7 (22·9–34·5)	32·2 (27·7–36·4)	27·1 (20·3–33·7)
Road injuries	26·1 (20·1–32·3)	27·7 (21·7–33·6)	31·2 (26·6–35·4)	26·1 (19·3–32·9)
Other transport injuries	40 (34·8–44·8)	38·9 (33·6–43·7)	41·6 (36·6–45·8)	37·5 (31·4–42·7)
Unintentional injuries	43·6 (36·7–48·3)	37·1 (31·6–41·9)	38·1 (32·6–43·2)	34·7 (29·2–39·2)

Falls	56·7 (48·1–62·4)	47·3 (40·4–52·5)	45·9 (39·3–51·2)	51·2 (43·7–56·7)
Drowning	-5·91 (-17·6–5·23)	-3·68 (-14·5–6·39)	15·5 (10·3–22·2)	-4·85 (-16·1–5·65)
Fire, heat, and hot substances	14·6 (9·2–21·6)	19·8 (14–26·7)	20·3 (14·5–27·5)	18·5 (13·2–25·1)
Poisonings	20·6 (14·7–27·8)	20·8 (15·1–27·4)	19 (13·6–25·5)	25 (18·7–32·4)
Exposure to mechanical forces	13·4 (5·99–21·7)	23·3 (19·6–27·5)	24·8 (21·2–28·9)	10·2 (1·95–19·7)
Animal contact	28·9 (23·2–35·8)	28·6 (23·1–34·5)	29 (23·5–34·8)	27·8 (22·2–34·4)
Foreign body	3·49 (2·51–4·79)	7·17 (5·17–9·7)	7·55 (5·44–10·2)	7 (4·99–9·59)
Environmental heat and cold exposure	11·7 (5·4–19·6)	11·7 (5·39–19·7)	11·4 (5·24–19·1)	12·4 (5·68–20·6)
Exposure to forces of nature	10·8 (4·98–18·2)	10·9 (5·06–18·4)	12 (5·48–20)	9·9 (4·56–16·5)
Other unintentional injuries	19·9 (14·1–27·2)	19·5 (13·8–26·4)	19·2 (13·6–26·1)	23·1 (17·1–30·3)
Self-harm and interpersonal violence	26·3 (13·9–38·5)	27·1 (15·6–38·6)	34·4 (28–40·6)	26·3 (14·3–38·6)
Self-harm	26 (13·3–39·2)	26·4 (13·7–39·5)	28·5 (17–39·8)	26·3 (13·6–39·5)
Interpersonal violence	30·8 (22·6–39)	32·9 (25·3–41)	37·2 (29·5–44·9)	27·9 (20–36·2)

Supplementary Results Table 2. All-age PAFs attributable to the joint distribution of all risk factors for all ages, both sexes combined for each GBD level 1, level 2, and level 3 cause and all causes in 2019 for Greece. Results are shown for deaths, YLLs, YLDs, and DALYs Data in parenthesis are 95% uncertainty intervals. PAFs=population attributable fractions. GBD=Global Burden of Disease. YLLs=years of life lost. YLDs=years lived with disability. DALYs=disability-adjusted life-years

	Deaths 2019	DALYs 2019	YLDs 2019	YLLs 2019
All causes	62·8 (59·1–65·8)	46·7 (43·3–50·1)	26·9 (24·7–29·3)	61·9 (59·4–64·3)
Communicable, maternal, neonatal, and nutritional diseases	45·4 (40·2–50·2)	50·3 (46·9–54·1)	44·6 (39·8–49·4)	53·4 (48·8–57·6)
HIV/AIDS and sexually transmitted infections	76·8 (75·9–77·8)	81·5 (78·2–85·9)	90·9 (87–94·8)	73·4 (72·3–74·4)
HIV/AIDS	75·1 (74–76·1)	73·5 (72·4–74·6)	77 (75·7–78·4)	72·3 (71·3–73·4)
Sexually transmitted infections excluding HIV	99·9 (99·8–99·9)	100 (99·9–100)	100 (100–100)	99·6 (99·4–99·7)
Respiratory infections and tuberculosis	45·6 (40·2–50·7)	43·6 (38·4–48·6)	4·49 (3·34–5·91)	49·7 (44·1–54·8)
Tuberculosis	52·2 (44·6–59·1)	58·1 (51·1–64·4)	58·8 (50·7–65·8)	58 (50·9–64·2)
Lower respiratory infections	45·5 (40–50·7)	49·4 (43·7–54·6)	43·8 (37·5–49·6)	49·5 (43·7–54·7)
Upper respiratory infections	0·0102 (0·00546–0·0213)	0·00609 (0·00115–0·0139)	0·00551 (0·00062–0·0132)	0·0555 (0·031–0·103)
Otitis media	0·159 (0·0521–0·352)	4·09 (2·62–5·74)	4·09 (2·62–5·74)	0·954 (0·347–2)
Enteric infections	8·5 (6–11·7)	11·3 (8·65–14·6)	10·8 (8·16–14·2)	15·7 (11·3–20·8)
Diarrheal diseases	8·67 (6·14–11·9)	11·3 (8·67–14·7)	10·9 (8·17–14·2)	16·1 (11·6–21·4)
Other infectious diseases	0·994 (0·639–1·86)	1·36 (0·967–1·95)	0·188 (0·11–0·327)	1·96 (1·42–2·87)
Meningitis	1·57 (1·1–2·17)	3·11 (2·25–4·25)	0·000589 (5·21e-05–0·0014)	4·09 (3–5·55)
Encephalitis	0·0941 (0·0549–0·199)	0·235 (0·142–0·475)	0·000107 (8·1e-06–0·000265)	0·307 (0·184–0·664)
Measles	2·08 (-0·479–10·3)	2·57 (-0·361–12)	0·817 (0·0328–2)	2·96 (-0·683–14·5)
Acute hepatitis	2·14 (1·12–4·88)	2·12 (1·24–4·42)	0·854 (0·517–1·46)	2·73 (1·49–6·06)
Maternal and neonatal disorders	88·7 (87·1–90·2)	82·8 (80·5–84·7)	77·1 (73·8–80·2)	89·7 (88·2–91·1)
Maternal disorders	9·33 (3·39–15·2)	9·2 (3·28–14·9)	9·17 (3·24–15·1)	9·24 (3·35–15·1)
Neonatal disorders	91·3 (89·7–92·7)	84·3 (82–86·2)	78·5 (75·1–81·5)	91·3 (89·8–92·7)
Nutritional deficiencies	36·5 (30·7–60·6)	77·4 (72·9–81·6)	78·1 (73·5–82·2)	38·9 (34·6–56)
Protein-energy malnutrition	100 (100–100)	100 (100–100)	100 (100–100)	100 (100–100)
Vitamin A deficiency	--	100 (100–100)	100 (100–100)	--
Dietary iron deficiency	--	100 (100–100)	100 (100–100)	--
Non-communicable diseases	65 (60·9–68·4)	48·1 (44·4–51·9)	26 (23·5–28·6)	65·3 (62·4–68)
Neoplasms	47·5 (44·2–51·1)	48·9 (45·9–52·3)	36·2 (32·6–39·9)	49·5 (46·6–52·9)
Lip and oral cavity cancer	65·6 (60·1–70·3)	71·3 (66·3–75·7)	70·6 (65·1–75·4)	71·4 (66·3–75·7)
Nasopharynx cancer	68·7 (60·9–75·5)	69·9 (62–76·5)	66·2 (57·7–73·4)	70·2 (62·3–76·9)
Other pharynx cancer	79·7 (75·3–83·3)	82·8 (78·7–86·2)	81·2 (77–84·8)	82·9 (78·8–86·2)
Esophageal cancer	74·2 (66·8–82·2)	76·9 (70·3–83·7)	74·4 (67·1–82·4)	76·9 (70·4–83·7)
Stomach cancer	28·9 (21·2–45·4)	30·7 (22·7–46·9)	30·4 (22·5–46·5)	30·7 (22·7–47)

Colon and rectum cancer	62 (53·7–69·7)	63·2 (55·8–70·5)	63·2 (55·8–70·4)	63·2 (55·8–70·5)
Liver cancer	60·7 (51·5–69·1)	62·5 (53·4–71·1)	61·3 (52·3–69·7)	62·5 (53·5–71·1)
Gallbladder and biliary tract cancer	19·3 (10·2–30·8)	19·6 (10·3–31·5)	19·1 (10–30·7)	19·6 (10·3–31·5)
Pancreatic cancer	42·2 (34·9–50·8)	43·8 (36·9–51·7)	42·8 (35·6–51·2)	43·8 (36·9–51·7)
Larynx cancer	87·3 (79·8–92·3)	88·5 (82·7–92·4)	88·3 (82·5–92·4)	88·5 (82·7–92·4)
Tracheal, bronchus, and lung cancer	87·9 (85·7–90·2)	88 (85·9–90·1)	87·9 (85·7–90·3)	88 (85·9–90·1)
Breast cancer	30·3 (22·6–39·3)	30·9 (24–38·7)	30·9 (23·9–38·8)	30·9 (24–38·7)
Cervical cancer	100 (100–100)	100 (100–100)	100 (100–100)	100 (100–100)
Uterine cancer	42·6 (27·4–58·1)	43·5 (28·8–58·6)	43·8 (29·1–59)	43·5 (28·7–58·5)
Ovarian cancer	13·5 (5·03–24·5)	11·9 (4·31–21·9)	11·4 (4·07–21·1)	11·9 (4·33–22)
Prostate cancer	8·3 (3·98–12·8)	9·09 (4·37–13·9)	9·77 (4·71–14·9)	9 (4·31–13·8)
Kidney cancer	40·3 (30·1–51·1)	41·6 (32·2–51·4)	41·4 (32·2–51·2)	41·6 (32·2–51·4)
Bladder cancer	48·4 (33·3–61·5)	52·3 (38·5–64·3)	53·7 (40·2–65·6)	52·2 (38·4–64·2)
Thyroid cancer	13·1 (6·17–21·9)	13·9 (6·45–23·5)	14·1 (6·68–23·5)	13·9 (6·4–23·5)
Mesothelioma	94 (91·3–96·4)	90·4 (86–94·3)	93·2 (90·1–95·8)	90·3 (85·9–94·2)
Non-Hodgkin lymphoma	6·52 (2·61–11·8)	6·65 (2·68–11·9)	6·6 (2·64–11·9)	6·66 (2·69–12)
Multiple myeloma	7·43 (3·06–13·5)	7·72 (3·19–14)	7·71 (3·2–13·9)	7·72 (3·19–14)
Leukemia	38·4 (23·8–52·3)	38·6 (25·7–51)	38 (25·2–50·3)	38·6 (25·8–51·1)
Cardiovascular diseases	81·5 (77·4–85·3)	83·7 (80·5–86·8)	76·6 (72·5–80·8)	84·3 (81·1–87·3)
Rheumatic heart disease	23·1 (12·5–44·7)	24·1 (14·5–41·9)	24·6 (15·4–41·5)	24 (14·4–42·3)
Ischemic heart disease	92·3 (88·3–95·8)	94·1 (91·3–96·6)	93 (89·8–95·9)	94·1 (91·3–96·6)
Stroke	77·7 (69·9–85·9)	81 (74·8–87·5)	81·8 (75·9–87·8)	80·9 (74·6–87·5)
Hypertensive heart disease	100 (100–100)	100 (100–100)	100 (100–100)	100 (100–100)
Non-rheumatic valvular heart disease	25·9 (16·6–38·8)	26·8 (18·3–38·1)	28·2 (19–39·6)	26·7 (18·2–38·1)
Cardiomyopathy and myocarditis	29·8 (20·8–41·2)	32·9 (25·7–41·4)	30·9 (22·9–39·7)	33 (25·7–41·6)
Atrial fibrillation and flutter	49·8 (40·6–59·9)	57·1 (48·8–65·4)	61·9 (54·3–69·5)	53·3 (44·7–62·7)
Aortic aneurysm	61·2 (55·9–66·3)	69·9 (65·6–73·7)	--	69·9 (65·6–73·7)
Endocarditis	31·7 (20·9–45·9)	34 (24·4–45·6)	32·8 (22·4–46·5)	34 (24·4–45·6)
Other cardiovascular and circulatory diseases	29·4 (22·5–37·5)	32 (26·2–38·4)	36·1 (30–42·1)	31·1 (25·1–37·7)
Chronic respiratory diseases	70·7 (65·7–76·5)	65·7 (61·8–69·7)	60·2 (56–64·4)	71·3 (66·3–76·9)
Chronic obstructive pulmonary disease	75·3 (71·1–79·5)	74·1 (70·6–77·2)	69·4 (65·6–72·9)	77·6 (73·9–81·2)
Pneumoconiosis	100 (100–100)	100 (100–100)	100 (100–100)	100 (100–100)
Asthma	42·6 (31·6–54·9)	45·1 (36·1–54·7)	45·1 (36·1–54·6)	46·6 (36·1–57·9)
Digestive diseases	33·3 (29·3–37)	34·8 (30–39·2)	12·3 (7·91–17·7)	43·5 (39·1–47·6)
Cirrhosis and other chronic liver diseases	66·4 (57–74·5)	72·5 (63·9–79·8)	65·7 (58·4–72·7)	72·7 (64–80·2)

Upper digestive system diseases	24·2 (20·6–27·7)	12·1 (8·2–16·6)	0·982 (0·558–1·56)	29·3 (25·3–33·2)
Gallbladder and biliary diseases	42·1 (27·4–56·9)	46·4 (32·7–60·7)	49·7 (36·4–63·4)	43·4 (28·9–57·8)
Pancreatitis	28 (19·7–37·7)	33·6 (24·7–43·7)	30·9 (22·4–41)	33·8 (24·7–43·9)
Neurological disorders	24·5 (10·2–39·5)	16·5 (7·75–28·8)	11·3 (5·67–21·3)	22·6 (8·56–37·5)
Alzheimer's disease and other dementias	37·8 (25·6–51·5)	40·1 (27·9–53·8)	40·4 (28–53·9)	40 (27·8–53·5)
Parkinson's disease	-13·3 (-20·7—6·57)	-15·4 (-23·4—7·77)	-16·2 (-24·5—8·27)	-15·2 (-23·2—7·66)
Idiopathic epilepsy	21·6 (15·4–27·7)	20·4 (14·6–26·4)	19·1 (13·5–25)	23·3 (16·7–29·7)
Multiple sclerosis	23·2 (17·2–28·4)	24·8 (18·9–29·9)	22·9 (17·9–28·3)	25·7 (19·5–31·2)
Mental disorders	--	6·11 (3·3–9·46)	6·11 (3·31–9·46)	--
Depressive disorders	--	10·4 (4·82–16·6)	10·4 (4·82–16·6)	--
Anxiety disorders	--	4·49 (1·3–9·66)	4·49 (1·3–9·66)	--
Idiopathic developmental intellectual disability	--	57·7 (29·9–77·9)	57·7 (29·9–77·9)	--
Substance use disorders	100 (100–100)	100 (100–100)	100 (100–100)	100 (100–100)
Alcohol use disorders	100 (100–100)	100 (100–100)	100 (100–100)	100 (100–100)
Drug use disorders	100 (100–100)	100 (100–100)	100 (100–100)	100 (100–100)
Diabetes and kidney diseases	100 (100–100)	100 (100–100)	100 (100–100)	100 (100–100)
Diabetes mellitus	100 (100–100)	100 (100–100)	100 (100–100)	100 (100–100)
Chronic kidney disease	100 (100–100)	100 (100–100)	100 (100–100)	100 (100–100)
Sense organ diseases	--	6·78 (5·86–7·91)	6·78 (5·86–7·91)	--
Blindness and vision loss	--	9·94 (6·84–13·9)	9·94 (6·84–13·9)	--
Age-related and other hearing loss	--	6·71 (5·95–7·55)	6·71 (5·95–7·55)	--
Musculoskeletal disorders	3·84 (1·1–6·96)	25·7 (21·1–30·5)	25·8 (21·2–30·7)	4·06 (1·22–7·07)
Rheumatoid arthritis	11·9 (3·64–20·8)	20·5 (6·88–33·8)	21·4 (7·16–35)	14·7 (4·51–25)
Osteoarthritis	--	18·6 (9·58–28·7)	18·6 (9·58–28·7)	--
Low back pain	--	46·5 (40·2–52·9)	46·5 (40·2–52·9)	--
Gout	--	50·6 (36·2–66·7)	50·6 (36·2–66·7)	--
Other non-communicable diseases	0·0305 (0·0206–0·0443)	0·0165 (0·00992–0·0265)	--	0·111 (0·0772–0·164)
Sudden infant death syndrome	6·93 (4·31–10·2)	6·92 (4·3–10·2)	--	6·92 (4·3–10·2)
Injuries	23·1 (19·7–26·1)	25·1 (21·7–28·1)	30·2 (26·9–33·6)	20·2 (16·6–23·8)
Transport injuries	25·5 (20·8–30·3)	24·7 (19·8–29·7)	28·1 (24·5–31·6)	23·7 (18·1–29·1)
Road injuries	25·2 (20·5–29·9)	24·4 (19·4–29·4)	27·4 (23·8–30·9)	23·5 (17·9–29)
Other transport injuries	31·8 (27·3–36·4)	30·3 (25·7–34·9)	38 (34–41·5)	27·2 (22·2–32·3)
Unintentional injuries	21·4 (17·4–25·3)	25·3 (21·5–29·4)	30·5 (26·7–34·3)	14·1 (10·3–18·4)
Falls	52·8 (46·8–56·9)	39·7 (34·7–43·9)	39 (34·2–43·1)	42·4 (37·4–47·1)
Drowning	-12·6 (-20·2—4·59)	-9·52 (-17·1—1·41)	8·82 (5·19–13·6)	-10 (-17·7—1·73)

Fire, heat, and hot substances	9·05 (5·44–13·5)	13·3 (8·98–18·7)	13·5 (9·13–18·8)	13 (8·61–18·2)
Poisonings	13·5 (8·79–19)	14 (9·53–19·4)	12·4 (8·43–17·3)	16·4 (11·3–22·4)
Exposure to mechanical forces	11·9 (7·19–16·4)	19·1 (16·4–21·9)	21·3 (18·9–23·8)	8·37 (3·46–13·2)
Animal contact	25·6 (21–31)	23·1 (18·7–28)	23·5 (19–28·1)	22·4 (17·7–28·1)
Foreign body	3·72 (2·59–5·06)	5 (3·5–6·82)	4·45 (3·13–6·05)	5·36 (3·67–7·36)
Environmental heat and cold exposure	7·18 (3·31–12·2)	7·99 (3·67–13·5)	7·73 (3·57–13)	8·5 (3·95–14·4)
Exposure to forces of nature	0 (0–0)	9·02 (4·17–15·1)	9·02 (4·17–15·1)	0 (0–0)
Other unintentional injuries	15·9 (10·8–21·7)	13·3 (9·08–18·5)	12·8 (8·68–17·9)	18 (12·6–24·2)
Self-harm and interpersonal violence	22·7 (12·9–32)	24·7 (15·8–32·7)	32·7 (26·5–39·7)	22·9 (13·1–32·2)
Self-harm	21·3 (9·29–32·8)	21·7 (9·25–33)	17·3 (8·4–26·2)	21·9 (9·33–33·3)
Interpersonal violence	28·4 (21·1–36)	31 (23·8–38·8)	36·7 (29·6–44·5)	26·5 (18·9–34·5)

Supplementary Results Table 2. All-age PAFs attributable to the joint distribution of all risk factors for all ages, both sexes combined for each GBD level 1, level 2, and level 3 cause and all causes in 2019 for Iceland. Results are shown for deaths, YLLs, YLDs, and DALYs Data in parenthesis are 95% uncertainty intervals. PAFs=population attributable fractions. GBD=Global Burden of Disease. YLLs=years of life lost. YLDs=years lived with disability. DALYs=disability-adjusted life-years

	Deaths 2019	DALYs 2019	YLDs 2019	YLLs 2019
All causes	56 (52·4–58·9)	37·9 (34·8–41)	25 (22·8–27·3)	53·5 (51–55·9)
Communicable, maternal, neonatal, and nutritional diseases	41 (29·2–52·7)	47·4 (41·9–52·8)	43 (38·4–47·6)	51·2 (41·9–59·7)
HIV/AIDS and sexually transmitted infections	77·2 (75·8–79·6)	80·6 (77·5–84·8)	88·8 (84·6–93·1)	74·8 (73·3–77·4)
HIV/AIDS	75·7 (74·3–78·2)	74·9 (73·4–77·6)	77·4 (75·9–79·9)	74 (72·5–76·8)
Sexually transmitted infections excluding HIV	99·8 (99·7–99·9)	99·9 (99·9–100)	100 (100–100)	99·3 (99–99·6)
Respiratory infections and tuberculosis	41·6 (28·5–55·2)	35·5 (25·6–45·4)	2·83 (2·11–3·7)	45·9 (33·2–58·6)
Tuberculosis	48·2 (38·8–56·5)	52·1 (43·5–59·6)	51·5 (42·7–59·6)	52·3 (43·5–59·9)
Lower respiratory infections	41·5 (28·3–55·3)	45·6 (32·8–58·3)	32·5 (26·8–37·5)	45·8 (32·9–58·7)
Upper respiratory infections	0·132 (0·0629–0·228)	0·00802 (0·00204–0·0185)	0·00499 (0–0·0152)	0·513 (0·256–0·807)
Otitis media	0·416 (0·209–0·749)	3·71 (2·39–5·32)	3·71 (2·39–5·33)	1·44 (0·741–2·52)
Enteric infections	6·22 (4·32–8·93)	9·81 (7·69–12·6)	9·41 (7·29–12·4)	11·5 (8·3–15·7)
Diarrheal diseases	6·28 (4·36–9)	9·84 (7·71–12·7)	9·42 (7·29–12·4)	11·7 (8·38–15·9)
Other infectious diseases	0·536 (0·366–0·77)	1·08 (0·738–1·56)	0·265 (0·156–0·438)	1·56 (1·07–2·2)
Meningitis	1·34 (0·853–2·05)	2·3 (1·47–3·48)	0·000427 (0–0·00134)	3·46 (2·35–4·95)
Encephalitis	0·306 (0·181–0·481)	0·512 (0·297–0·808)	0·000128 (0–0·000402)	0·887 (0·552–1·34)
Measles	2·47 (-0·604–11·6)	1·56 (-0·0946–6·76)	0·484 (0·0155–1·17)	3·4 (-0·837–16)
Acute hepatitis	5·54 (2·96–9·74)	3·75 (2·3–5·79)	2·78 (1·65–4·48)	6·01 (3·21–10·7)
Maternal and neonatal disorders	85·9 (83·9–87·9)	79·5 (76·4–82·1)	73·6 (70·1–77)	86·5 (84·5–88·5)
Maternal disorders	8·25 (2·89–13·7)	8·23 (2·87–13·8)	8·26 (2·89–13·9)	8·18 (2·87–13·6)
Neonatal disorders	87·6 (85·7–89·5)	81·1 (78·1–83·6)	75·6 (71·9–79)	87·6 (85·7–89·5)
Nutritional deficiencies	86·7 (71·7–90·1)	76·8 (74·7–79·4)	76·5 (74·5–79·3)	81·1 (68·4–85·1)
Protein-energy malnutrition	100 (100–100)	100 (100–100)	100 (100–100)	100 (100–100)
Vitamin A deficiency	--	100 (100–100)	100 (100–100)	--
Dietary iron deficiency	--	100 (100–100)	100 (100–100)	--
Non-communicable diseases	58·6 (54·5–62)	38·4 (35–41·7)	23·4 (21–25·8)	57·2 (54·5–59·8)
Neoplasms	42·7 (39·4–46·4)	42·2 (39·2–45·4)	29·7 (26·5–33·4)	43 (40–46·3)
Lip and oral cavity cancer	63·9 (57–69·7)	67 (60·5–72·3)	66·7 (60·1–72·3)	67 (60·5–72·3)
Nasopharynx cancer	63·7 (54·5–71·8)	63·4 (54·3–71·2)	62·6 (53·3–70·8)	63·5 (54·4–71·3)
Other pharynx cancer	77·1 (71·3–82·1)	77·9 (72·2–82·8)	76·9 (71·4–82)	77·9 (72·2–82·9)

Esophageal cancer	75·8 (66·8–84·2)	77·1 (68·4–85·3)	75·9 (67·2–84)	77·1 (68·4–85·3)
Stomach cancer	23·4 (15·2–41·9)	24 (15·8–42·4)	24·1 (16–42·6)	24 (15·8–42·4)
Colon and rectum cancer	61·9 (53·8–69·9)	62·6 (54·9–70)	62·6 (55·1–70)	62·6 (54·9–70)
Liver cancer	76·3 (69·9–82·1)	76·8 (70·6–82)	76 (70–81·4)	76·8 (70·7–82)
Gallbladder and biliary tract cancer	19·2 (9·98–31·2)	19·7 (10·5–32·3)	19 (9·98–30·9)	19·7 (10·5–32·3)
Pancreatic cancer	35·8 (27·8–45·1)	35·4 (28·1–44·4)	35·8 (28·3–45)	35·4 (28·1–44·4)
Larynx cancer	80·9 (71·9–87·8)	81·7 (74·3–87·4)	81·5 (74·1–87·3)	81·7 (74·3–87·4)
Tracheal, bronchus, and lung cancer	79·4 (76·1–82·5)	78·1 (74·9–81)	79·2 (75·9–82·3)	78·1 (74·9–81)
Breast cancer	32·1 (25–39·9)	31·2 (24·9–38·1)	31·5 (25·2–38·8)	31·1 (24·8–38)
Cervical cancer	100 (100–100)	100 (100–100)	100 (100–100)	100 (100–100)
Uterine cancer	44·5 (30·5–59·7)	46·2 (32–61·5)	46·9 (32·6–62·3)	46·1 (31·9–61·3)
Ovarian cancer	14·7 (6·6–25)	12·4 (5·32–21·4)	11·7 (4·81–20·5)	12·5 (5·33–21·4)
Prostate cancer	5·77 (2·3–9·82)	6·33 (2·53–10·7)	6·65 (2·68–11·2)	6·27 (2·51–10·6)
Kidney cancer	36·5 (26·3–47·4)	37 (27·2–47·2)	36·9 (27·2–47)	37 (27·2–47·3)
Bladder cancer	35·2 (21·8–49·2)	39·1 (26·1–51·9)	40·4 (27·3–53)	39 (25·9–51·8)
Thyroid cancer	15·4 (6·96–26·2)	16·3 (7·21–27·8)	16·4 (7·49–27·2)	16·3 (7·19–27·8)
Mesothelioma	97·3 (96·5–98)	95·1 (93·8–96·2)	96·9 (95·7–97·8)	95·1 (93·7–96·2)
Non-Hodgkin lymphoma	7·27 (3·12–13·1)	7·43 (3·2–13·1)	7·35 (3·12–13·2)	7·44 (3·2–13·1)
Multiple myeloma	8·03 (3·39–14·5)	8·43 (3·57–15·1)	8·43 (3·62–15·2)	8·43 (3·57–15·1)
Leukemia	32·4 (19·5–45·5)	30 (19·3–40·8)	30·6 (19·8–41·8)	29·9 (19·3–40·7)
Cardiovascular diseases	82·8 (78·9–86)	83·8 (80·7–86·4)	76·2 (71·9–80·1)	85 (82–87·5)
Rheumatic heart disease	25·2 (14·4–48·5)	25·9 (16·8–43·9)	26 (16·9–41·1)	25·9 (16·7–44·2)
Ischemic heart disease	93·2 (89–96·5)	94·5 (91·5–96·9)	93·8 (90·7–96·5)	94·5 (91·6–96·9)
Stroke	80·6 (72·8–88·6)	83·4 (78–89)	82·8 (77·5–87·9)	83·6 (77·8–89·5)
Hypertensive heart disease	100 (100–100)	100 (100–100)	100 (100–100)	100 (100–100)
Non-rheumatic valvular heart disease	30·4 (19·9–45·3)	32·3 (23–44·2)	33·8 (23·4–45·5)	32·3 (23–44·2)
Cardiomyopathy and myocarditis	32·3 (25·1–41·3)	35·5 (29·9–41·9)	34·4 (27·9–41·9)	35·6 (30–42)
Atrial fibrillation and flutter	51·6 (41·6–61)	59·5 (51·6–67·4)	64·2 (57–71·5)	55·2 (46·6–64)
Aortic aneurysm	55·6 (48·9–62·4)	63·7 (58·6–68·9)	--	63·7 (58·6–68·9)
Endocarditis	35·3 (25–49·1)	37·3 (27·8–47·9)	35·3 (25·2–47·4)	37·4 (27·9–47·9)
Other cardiovascular and circulatory diseases	33·2 (25·5–41)	34·5 (27·3–40·4)	35·1 (28·5–42)	34·2 (25·2–40·3)
Chronic respiratory diseases	63·7 (58·2–69·1)	55·1 (51–59·5)	49 (44·4–53·9)	63·8 (58·9–69)

Chronic obstructive pulmonary disease	69·9 (63·9–75·5)	65·7 (61·5–69·6)	58·4 (54·5–62·5)	72 (66·9–76·9)
Pneumoconiosis	100 (100–100)	100 (100–100)	100 (100–100)	100 (100–100)
Asthma	43·3 (32·1–55·6)	41·9 (33·9–51·7)	41·7 (33·7–51·3)	46·4 (36·3–57·6)
Digestive diseases	27·1 (22·8–31·6)	27·6 (23·1–32·2)	9·08 (6·24–12·7)	37·6 (31·9–42·9)
Cirrhosis and other chronic liver diseases	73·6 (61·9–83·9)	75·3 (63·2–85·4)	63·2 (53·6–73·5)	75·7 (63·4–85·9)
Upper digestive system diseases	19·3 (15·7–23)	5·57 (3·4–8·33)	0·689 (0·385–1·09)	22·6 (18·6–26·5)
Gallbladder and biliary diseases	41·4 (27·9–56·3)	47·5 (34·1–61·8)	51·8 (38·1–65·6)	43 (29·3–58)
Pancreatitis	35·2 (24·6–47·1)	39·8 (28·5–51·8)	36·6 (26–48·6)	40·1 (28·8–52·4)
Neurological disorders	22 (9·13–35·8)	12·9 (6·13–23·2)	8·71 (4·19–17·5)	19 (7·57–32·7)
Alzheimer's disease and other dementias	35·8 (23·2–50)	38·6 (26–52·2)	39·2 (26·6–52·8)	38·2 (25·7–52)
Parkinson's disease	-9·38 (-14·4–-4·96)	-10·8 (-16·3–-5·95)	-11·2 (-16·5–-6·31)	-10·7 (-16·3–-5·8)
Idiopathic epilepsy	24·4 (17·5–30·9)	22·4 (16·1–28·6)	20·8 (15–26·6)	25·1 (18–31·8)
Multiple sclerosis	13·9 (9·56–18·6)	14·4 (10–19·1)	13·8 (9·58–18·3)	15 (10·4–20·1)
Mental disorders	--	3·31 (1·83–5·23)	3·31 (1·83–5·23)	--
Depressive disorders	--	6·56 (3·53–10·1)	6·56 (3·53–10·1)	--
Anxiety disorders	--	3·96 (1·18–8·61)	3·96 (1·18–8·61)	--
Idiopathic developmental intellectual disability	--	45 (21·9–64·4)	45 (21·9–64·4)	--
Substance use disorders	100 (100–100)	100 (100–100)	100 (100–100)	100 (100–100)
Alcohol use disorders	100 (100–100)	100 (100–100)	100 (100–100)	100 (100–100)
Drug use disorders	100 (100–100)	100 (100–100)	100 (100–100)	100 (100–100)
Diabetes and kidney diseases	99·9 (99·9–99·9)	100 (99·9–100)	100 (100–100)	99·9 (99·8–99·9)
Diabetes mellitus	100 (100–100)	100 (100–100)	100 (100–100)	100 (100–100)
Chronic kidney disease	100 (100–100)	100 (100–100)	100 (100–100)	100 (100–100)
Sense organ diseases	--	8·58 (7·52–9·79)	8·58 (7·52–9·79)	--
Blindness and vision loss	--	8·52 (5·74–11·9)	8·52 (5·74–11·9)	--
Age-related and other hearing loss	--	10·1 (9·2–11·2)	10·1 (9·2–11·2)	--
Musculoskeletal disorders	2·62 (0·744–5·04)	22·9 (18·7–27)	23·1 (18·9–27·2)	3·41 (0·991–6·37)
Rheumatoid arthritis	10·5 (3·05–19·7)	14 (4·32–25·8)	14·2 (4·42–26·3)	12·5 (3·76–23·5)
Osteoarthritis	--	12·2 (6·31–19·1)	12·2 (6·31–19·1)	--
Low back pain	--	42·8 (37·4–48·3)	42·8 (37·4–48·3)	--
Gout	--	49·8 (33·8–67·4)	49·8 (33·8–67·4)	--
Other non-communicable diseases	0·087 (0·0475–0·149)	0·0423 (0·02–0·0795)	--	0·286 (0·161–0·453)

Sudden infant death syndrome	7·47 (4·65–10·9)	7·46 (4·64–10·9)	--	7·46 (4·64–10·9)
Injuries	28 (21·1–34·5)	27·8 (22·5–32·8)	34·5 (30·6–38·4)	20·5 (11·7–28·9)
Transport injuries	25·8 (19·5–32·7)	26·9 (20·9–33·1)	32·6 (28·4–36·4)	24·4 (17·2–31·9)
Road injuries	19 (10·4–27·3)	22·5 (15·5–29·4)	30·9 (26·8–34·7)	18 (8·71–26·8)
Other transport injuries	40 (34·8–45·1)	38·6 (32·9–44·2)	41 (36·4–45)	38 (32–44)
Unintentional injuries	40·3 (34·2–44·9)	33·5 (29·1–38)	34·8 (30·4–39·2)	29·3 (24·3–34·3)
Falls	55·8 (47·7–61)	42·9 (37·6–47·5)	41·6 (36·4–46·2)	47·7 (41·6–52·5)
Drowning	-10·2 (-29·7–8·05)	-8·25 (-27–9·33)	15·7 (11·7–21·1)	-9·23 (-28·8–8·94)
Fire, heat, and hot substances	14·2 (9·92–19·9)	20·3 (15·7–26·3)	20·7 (16·1–26·6)	19·5 (14·7–25·3)
Poisonings	20·8 (15·8–27·1)	22·3 (17·4–28·2)	19 (14·9–24·2)	25·1 (19·5–31·7)
Exposure to mechanical forces	9·47 (-3·35–23·8)	23·9 (19·8–28·3)	26·2 (22·6–29·9)	6·54 (-7·34–21·7)
Animal contact	28·8 (24·2–33·9)	27·4 (23–32·2)	27·6 (23·2–32·3)	27·1 (22·6–32·5)
Foreign body	4·45 (3·42–5·72)	9·42 (7·26–11·9)	9·97 (7·73–12·5)	8·98 (6·82–11·5)
Environmental heat and cold exposure	8·51 (3·85–14·6)	9·12 (4·24–15·6)	8·52 (3·93–14·6)	9·61 (4·49–16·4)
Exposure to forces of nature	0 (0–0)	8·82 (4·11–15)	8·82 (4·11–15)	0 (0–0)
Other unintentional injuries	21·3 (16–27·7)	20·1 (15·7–26)	19·6 (15·1–25·4)	25·8 (20·1–32·7)
Self-harm and interpersonal violence	12 (-5·39–28·5)	15 (-1·11–29·8)	32·2 (25·6–38·8)	12·9 (-4·37–29·4)
Self-harm	11·6 (-7·03–29·4)	12·9 (-5·38–30·2)	21·1 (10·6–31·6)	12·6 (-5·97–30·5)
Interpersonal violence	18·8 (6·39–31)	27·4 (18·4–36·8)	36·2 (28·4–44·5)	17 (4·77–29·6)

Supplementary Results Table 2. All-age PAFs attributable to the joint distribution of all risk factors for all ages, both sexes combined for each GBD level 1, level 2, and level 3 cause and all causes in 2019 for Ireland. Results are shown for deaths, YLLs, YLDs, and DALYs Data in parenthesis are 95% uncertainty intervals. PAFs=population attributable fractions. GBD=Global Burden of Disease. YLLs=years of life lost. YLDs=years lived with disability. DALYs=disability-adjusted life-years

	Deaths 2019	DALYs 2019	YLDs 2019	YLLs 2019
All causes	57·7 (54·8–60·5)	39·7 (36·7–42·7)	25·7 (23·5–27·9)	55·4 (53·2–57·5)
Communicable, maternal, neonatal, and nutritional diseases	47·1 (38·7–54·6)	50·3 (46·1–54·5)	43·7 (38·8–48·6)	55 (48·6–60·5)
HIV/AIDS and sexually transmitted infections	90 (89–90·9)	91 (89·2–93·2)	95·7 (93·7–97·7)	86·7 (85·5–87·7)
HIV/AIDS	87·7 (86·5–88·8)	87 (85·7–88·2)	90·4 (89·1–91·4)	85·6 (84·2–86·6)
Sexually transmitted infections excluding HIV	99·9 (99·9–100)	100 (99·9–100)	100 (100–100)	99·8 (99·6–99·8)
Respiratory infections and tuberculosis	47·6 (38·4–56)	40·2 (32·1–47·6)	3·17 (2·31–4·23)	50·3 (41·3–58·3)
Tuberculosis	55·3 (45·9–63·1)	58·1 (49–65·3)	57·7 (48·4–65)	58·2 (49·1–65·5)
Lower respiratory infections	47·5 (38·1–56)	49·9 (40·6–58·1)	33·5 (27·7–38·7)	50·1 (40·8–58·3)
Upper respiratory infections	0·0695 (0·0419–0·112)	0·0055 (0·000365–0·0157)	0·00519 (0–0·0154)	0·196 (0·121–0·314)
Otitis media	1·1 (0·42–2·43)	4·44 (2·84–6·22)	4·44 (2·84–6·22)	5·15 (2·51–9·18)
Enteric infections	9·47 (6·77–13·6)	13·9 (10·9–17·9)	12·9 (9·96–17)	17·7 (13·4–23)
Diarrheal diseases	9·55 (6·82–13·7)	14 (11–18)	12·9 (9·96–17)	17·9 (13·6–23·3)
Other infectious diseases	1·07 (0·772–1·4)	1·54 (1·15–2)	0·393 (0·234–0·67)	2·09 (1·58–2·7)
Meningitis	2·37 (1·68–3·22)	3·59 (2·55–4·87)	0·000618 (0–0·00193)	4·72 (3·42–6·26)
Encephalitis	0·472 (0·303–0·695)	0·943 (0·618–1·42)	0·000183 (0–0·00057)	1·26 (0·843–1·84)
Measles	2·49 (-0·761–13·9)	2·22 (-0·502–11·5)	0·45 (0·0139–1·07)	3·18 (-0·965–17·7)
Acute hepatitis	18·9 (13·1–31·2)	6·31 (4·18–9·4)	2·21 (1·33–3·75)	18·9 (13·2–30·2)
Maternal and neonatal disorders	82·1 (79·5–84·6)	77·2 (74·6–79·4)	71 (67·1–74·8)	82·9 (80·3–85·4)
Maternal disorders	8·8 (3·21–14·6)	8·8 (3·25–14·8)	8·84 (3·27–14·8)	8·73 (3·21–14·6)
Neonatal disorders	84·2 (81·5–86·6)	78·9 (76·2–81·1)	73 (68·9–76·8)	84·2 (81·5–86·6)
Nutritional deficiencies	59 (51–73·4)	73·4 (69·9–76·6)	73·9 (70·3–77·2)	57·8 (49·1–68·6)
Protein-energy malnutrition	100 (100–100)	100 (100–100)	100 (100–100)	100 (100–100)
Vitamin A deficiency	--	100 (100–100)	100 (100–100)	--
Dietary iron deficiency	--	100 (100–100)	100 (100–100)	--
Non-communicable diseases	59·5 (56·3–62·5)	39·9 (36·5–43·2)	24·2 (21·9–26·6)	57·8 (55·5–60·2)
Neoplasms	45·2 (41·9–48·9)	44·1 (41·1–47·6)	32·2 (28·8–35·9)	44·9 (41·8–48·4)
Lip and oral cavity cancer	69·3 (62·9–74·9)	71·2 (64·9–76·5)	70·8 (64·7–76·2)	71·2 (65–76·5)
Nasopharynx cancer	66·9 (58·3–74·5)	66·7 (58·1–74)	66·2 (57·6–73·8)	66·7 (58·2–74)
Other pharynx cancer	79·4 (73·9–83·9)	80 (74·6–84·6)	79·4 (73·6–84·1)	80·1 (74·6–84·6)

Esophageal cancer	73·2 (64–81·9)	74·1 (65·2–82·7)	73·1 (64–81·8)	74·1 (65·2–82·7)
Stomach cancer	23·2 (16·5–39·9)	23·1 (16–40·1)	23·4 (16·2–40·2)	23·1 (16–40·1)
Colon and rectum cancer	62·8 (55·2–70·5)	63·2 (55·8–70·6)	63·3 (55·9–70·8)	63·2 (55·8–70·6)
Liver cancer	74·8 (68·6–80·6)	75·7 (69·7–81·3)	74·6 (68·7–80·4)	75·7 (69·8–81·3)
Gallbladder and biliary tract cancer	21·2 (11·7–33)	21·7 (12·1–33·5)	21 (11·5–33)	21·7 (12·1–33·5)
Pancreatic cancer	38·4 (30·8–47·6)	37·5 (30·4–46·3)	38·1 (30·7–47·1)	37·5 (30·4–46·3)
Larynx cancer	81·2 (73·8–86·9)	80·6 (73·5–86·3)	80·6 (73·3–86·1)	80·6 (73·5–86·4)
Tracheal, bronchus, and lung cancer	81·6 (78·4–85·2)	80·2 (76·9–83·6)	80·7 (77·5–84·2)	80·2 (76·9–83·6)
Breast cancer	35·4 (28·2–43·4)	34·4 (27·8–41·6)	34·5 (27·7–41·8)	34·4 (27·8–41·5)
Cervical cancer	100 (100–100)	100 (100–100)	100 (100–100)	100 (100–100)
Uterine cancer	43·2 (29·2–57·7)	44·3 (30·3–58·7)	44·7 (30·8–59·2)	44·2 (30·3–58·6)
Ovarian cancer	13·4 (5·26–24)	11·9 (4·47–21·2)	11·3 (4·32–20)	11·9 (4·47–21·3)
Prostate cancer	5·76 (2·68–9·21)	6·11 (2·83–9·56)	6·34 (2·96–9·85)	6·06 (2·8–9·53)
Kidney cancer	36·9 (27·3–46·7)	36·7 (27·2–46·7)	36·5 (27–46·2)	36·7 (27·2–46·6)
Bladder cancer	39·3 (26·7–51·9)	40·8 (29·1–52·6)	41·4 (29·6–53)	40·8 (29–52·5)
Thyroid cancer	14·2 (6·77–23·7)	14·7 (7·21–24·3)	14·5 (7·35–23·4)	14·7 (7·24–24·4)
Mesothelioma	96·4 (94·8–97·7)	93·7 (91–96)	96 (94–97·4)	93·7 (90·9–96)
Non-Hodgkin lymphoma	6·93 (2·83–12·6)	7·08 (2·95–12·6)	6·97 (2·89–12·6)	7·09 (2·96–12·6)
Multiple myeloma	7·83 (3·38–13·9)	8·14 (3·48–14·5)	8·12 (3·54–14·4)	8·14 (3·49–14·5)
Leukemia	35 (22·7–48·6)	32·1 (21·4–43·4)	32·5 (21·6–44)	32·1 (21·3–43·4)
Cardiovascular diseases	82·5 (79–85·7)	83·4 (80·6–85·9)	75·7 (71·9–79·4)	84·4 (81·6–86·8)
Rheumatic heart disease	27·6 (16·6–48·7)	27·8 (18–42·7)	27·6 (18–42·7)	27·8 (18·1–42·9)
Ischemic heart disease	93·7 (90–96·7)	94·8 (92·2–96·9)	94·3 (91·7–96·6)	94·8 (92·2–97)
Stroke	82·6 (75·5–89·9)	85 (80·1–90·1)	84·3 (80·2–88·5)	85·2 (79·8–90·6)
Hypertensive heart disease	100 (100–100)	100 (100–100)	100 (100–100)	100 (100–100)
Non-rheumatic valvular heart disease	31·9 (21·7–45·7)	33·3 (24·4–44·3)	33·3 (23·6–44·8)	33·3 (24·3–44·2)
Cardiomyopathy and myocarditis	40·3 (32–50·1)	47 (40·8–53·9)	44·3 (37·3–51·5)	47·2 (40·9–54·1)
Atrial fibrillation and flutter	56·2 (47·2–66·1)	62·8 (55·2–70·4)	66·5 (59·1–73)	59·2 (50·9–68)
Aortic aneurysm	57·6 (50·7–64·4)	63·4 (57·5–68·8)	--	63·4 (57·5–68·8)
Endocarditis	38·3 (27·5–51·6)	40·5 (31·1–51·2)	37·8 (27·8–50·4)	40·6 (31·3–51·3)
Other cardiovascular and circulatory diseases	35·9 (29·9–42·7)	38·3 (32·8–44·1)	41·9 (34·4–49·5)	36·9 (31–43)
Chronic respiratory diseases	62·9 (56·9–69·5)	56·6 (52·1–61·5)	50·6 (46·1–55·3)	61·8 (56·1–68·2)

Chronic obstructive pulmonary disease	72·2 (67·8–76·6)	69·9 (66·4–73·3)	63·2 (59·9–66·6)	73·6 (69·6–77·6)
Pneumoconiosis	100 (100–100)	100 (100–100)	100 (100–100)	100 (100–100)
Asthma	43·8 (32·8–55·8)	40·2 (32·1–49·2)	39·7 (31·8–48·7)	45·3 (35·2–56)
Digestive diseases	32·8 (28·6–36·9)	35·1 (30·1–39·6)	12·8 (8·45–18)	44·5 (39·1–49·4)
Cirrhosis and other chronic liver diseases	77·7 (67·3–87)	80·1 (70·3–88·6)	73·7 (64·8–81·7)	80·3 (70·3–88·8)
Upper digestive system diseases	20·9 (17·7–24·1)	6·8 (4·23–9·95)	0·736 (0·422–1·15)	23·2 (20–26·5)
Gallbladder and biliary diseases	42·8 (29–57·6)	47·5 (34–61·7)	50 (36·3–63·7)	43·8 (30·6–58·3)
Pancreatitis	43·5 (32·6–57·8)	47·9 (37–61·6)	45·9 (34·6–59·3)	48·1 (37·3–61·9)
Neurological disorders	23·5 (10·5–38·2)	13·4 (6·51–23·6)	8·76 (4·09–17·5)	20·9 (10–34·7)
Alzheimer's disease and other dementias	38·2 (25·6–52·4)	40·4 (27·6–54·2)	40·7 (28·1–54·4)	40·2 (27·2–54·1)
Parkinson's disease	-9·28 (-14·5–-4·66)	-10·3 (-15·8–-5·26)	-10·6 (-16·3–-5·41)	-10·2 (-15·7–-5·18)
Idiopathic epilepsy	28·6 (21·2–35·6)	26·7 (19·7–33·5)	24·7 (18·1–31·2)	29·9 (22·4–37·4)
Multiple sclerosis	15·2 (11·4–19·1)	15·7 (11·9–19·6)	15·2 (11·5–19)	16·2 (12·2–20·2)
Mental disorders	--	5·57 (2·93–8·68)	5·57 (2·94–8·68)	--
Depressive disorders	--	10·4 (5·1–16·5)	10·4 (5·1–16·5)	--
Anxiety disorders	--	5·58 (1·71–11·5)	5·58 (1·71–11·5)	--
Idiopathic developmental intellectual disability	--	48·7 (22·4–69)	48·7 (22·4–69)	--
Substance use disorders	100 (100–100)	100 (100–100)	100 (100–100)	100 (100–100)
Alcohol use disorders	100 (100–100)	100 (100–100)	100 (100–100)	100 (100–100)
Drug use disorders	100 (100–100)	100 (100–100)	100 (100–100)	100 (100–100)
Diabetes and kidney diseases	100 (100–100)	100 (100–100)	100 (100–100)	100 (100–100)
Diabetes mellitus	100 (100–100)	100 (100–100)	100 (100–100)	100 (100–100)
Chronic kidney disease	100 (100–100)	100 (100–100)	100 (100–100)	100 (100–100)
Sense organ diseases	--	7·08 (6·23–8·14)	7·08 (6·23–8·14)	--
Blindness and vision loss	--	7·63 (5·14–10·7)	7·63 (5·14–10·7)	--
Age-related and other hearing loss	--	8·17 (7·4–9·02)	8·17 (7·4–9·02)	--
Musculoskeletal disorders	4·55 (1·25–8·25)	23·4 (19·5–27·3)	23·8 (19·7–27·8)	5·05 (1·4–9·08)
Rheumatoid arthritis	11·8 (3·37–21·4)	14·4 (4·12–25·9)	14·7 (4·16–26·4)	13·4 (3·88–23·8)
Osteoarthritis	--	15·8 (8·49–24·3)	15·8 (8·49–24·3)	--
Low back pain	--	41·3 (36–46·6)	41·3 (36–46·6)	--
Gout	--	51·2 (35·9–68·1)	51·2 (35·9–68·1)	--
Other non-communicable diseases	0·145 (0·0979–0·208)	0·0885 (0·0544–0·14)	--	0·413 (0·285–0·577)

Sudden infant death syndrome	6·52 (4·5–9·09)	6·51 (4·5–9·08)	--	6·51 (4·5–9·08)
Injuries	32 (26·4–37·5)	31·8 (26·9–36·5)	35·8 (31–40·9)	27·3 (20·7–33·8)
Transport injuries	30·2 (23·3–37·3)	32 (25·3–38·8)	35·2 (30·3–40·4)	30·2 (22·3–38·2)
Road injuries	29·1 (22–36·2)	30·9 (24–37·9)	34·3 (29·4–39·6)	29·2 (21–37·2)
Other transport injuries	44·4 (38·4–50·8)	42·7 (37–48·6)	42·8 (37·9–47·8)	42·6 (36–49·7)
Unintentional injuries	39·4 (33·4–44·3)	34·9 (29·5–40·5)	36·2 (31·1–41·8)	30·3 (24·4–36·3)
Falls	54·6 (47·2–59·8)	43·8 (37·9–49·2)	42·6 (36·9–48·1)	48·6 (42·5–54·1)
Drowning	2·64 (-10·8–15·7)	5·2 (-8·09–17·6)	18·6 (13·7–24·8)	4·77 (-9·02–17·5)
Fire, heat, and hot substances	15·4 (11–20·9)	22·8 (17·2–29·3)	23·9 (18·2–30·9)	20·4 (15·3–26·2)
Poisonings	26·5 (20·2–33·8)	25·6 (19·7–32·6)	21·6 (16·3–27·8)	29·9 (23–37·8)
Exposure to mechanical forces	17·1 (5·66–29·1)	28·2 (22·1–34·6)	30·1 (24·8–36·2)	14·3 (2·03–26·6)
Animal contact	31·3 (26·2–37)	30·6 (25·2–36·9)	30·2 (24·7–36·5)	31·2 (25·8–37·6)
Foreign body	12·8 (9·24–17·4)	15·1 (10·8–20·3)	12·2 (8·63–16·6)	17 (12·1–23·2)
Environmental heat and cold exposure	8·29 (3·82–14·1)	9·3 (4·33–15·8)	9·38 (4·35–15·9)	9·08 (4·23–15·4)
Exposure to forces of nature	0 (0–0)	10·2 (4·76–17·2)	10·2 (4·76–17·2)	0 (0–0)
Other unintentional injuries	26·2 (19·9–33·3)	22·4 (16·8–29·1)	22·2 (16·6–29)	30·1 (23·1–38·1)
Self-harm and interpersonal violence	23·7 (12·2–35·3)	24·8 (14·1–35·7)	28·6 (22·4–34·7)	24·4 (13–36)
Self-harm	23·8 (11·7–36·6)	24·7 (12·5–37·6)	25 (14·2–36·2)	24·7 (12·4–37·7)
Interpersonal violence	21·5 (13·1–30)	25·8 (18·3–33·7)	30·5 (23·1–38·4)	20·4 (12–29)

Supplementary Results Table 2. All-age PAFs attributable to the joint distribution of all risk factors for all ages, both sexes combined for each GBD level 1, level 2, and level 3 cause and all causes in 2019 for Israel. Results are shown for deaths, YLLs, YLDs, and DALYs Data in parenthesis are 95% uncertainty intervals. PAFs=population attributable fractions. GBD=Global Burden of Disease. YLLs=years of life lost. YLDs=years lived with disability. DALYs=disability-adjusted life-years

	Deaths 2019	DALYs 2019	YLDs 2019	YLLs 2019
All causes	52·1 (49–54·9)	34·1 (31·3–37)	22·1 (20·1–24·3)	48·4 (46·1–50·5)
Communicable, maternal, neonatal, and nutritional diseases	33·1 (28·3–38)	48·4 (45·3–51·8)	46·1 (41·7–51)	50·5 (46·3–54·9)
HIV/AIDS and sexually transmitted infections	80·5 (79·4–81·5)	80·6 (78–83·2)	90 (87·9–92·7)	72·9 (71·5–74·1)
HIV/AIDS	79·9 (78·8–80·9)	77·6 (75·3–79·8)	86·1 (84·6–87·5)	72·4 (71–73·7)
Sexually transmitted infections excluding HIV	99·6 (99·4–99·7)	99·9 (99·8–100)	100 (100–100)	98·6 (98·1–99)
Respiratory infections and tuberculosis	32·1 (25·4–38·6)	26·3 (20·7–32·3)	2·45 (1·88–3·15)	37·2 (30·8–43·7)
Tuberculosis	35·1 (28–42·8)	39·2 (31·7–45·9)	39·4 (31·4–47·3)	39·1 (32·1–45·7)
Lower respiratory infections	32 (25·2–38·7)	37·2 (30·7–43·8)	32·6 (27·2–38)	37·2 (30·7–43·8)
Upper respiratory infections	0·654 (0·289–1·3)	0·0208 (0·00643–0·0408)	0·0155 (0·0018–0·0344)	2·21 (1·02–4·26)
Otitis media	0·203 (0·0833–0·469)	5·61 (3·63–7·75)	5·62 (3·63–7·77)	1·02 (0·421–2·24)
Enteric infections	8·32 (5·74–12·2)	13·1 (10·2–16·9)	12·9 (10–16·9)	13·2 (9·96–17·7)
Diarrheal diseases	8·34 (5·76–12·3)	13·1 (10·3–17)	12·9 (10–16·9)	13·3 (10–17·7)
Other infectious diseases	1·06 (0·734–1·62)	1·75 (1·27–2·35)	0·304 (0·181–0·504)	2·35 (1·68–3·25)
Meningitis	2·61 (1·86–3·53)	4·84 (3·47–6·43)	0·00139 (0·000119–0·00343)	5·98 (4·29–7·93)
Encephalitis	0·777 (0·547–1·08)	1·8 (1·2–2·54)	0·000506 (4·39e-05–0·00124)	2·23 (1·51–3·12)
Measles	4·98 (-0·387–21·6)	4·23 (0·534–9·82)	3·8 (0·176–8·64)	6·09 (-0·471–26)
Acute hepatitis	11·3 (7·09–20·5)	4·35 (2·71–7·16)	1·84 (1·09–3·18)	10·4 (6·45–19·4)
Maternal and neonatal disorders	80·3 (77·7–82·6)	78 (75·6–80)	73·7 (70·1–76·9)	81·3 (78·7–83·5)
Maternal disorders	9·55 (3·42–16·1)	9·51 (3·43–15·9)	9·55 (3·44–15·9)	9·46 (3·4–15·9)
Neonatal disorders	83·1 (80·5–85·5)	80·4 (78·2–82·3)	76·8 (73·2–80)	83·1 (80·5–85·5)
Nutritional deficiencies	82·4 (60·9–86·7)	79 (75·6–83·1)	79·2 (75·4–83·3)	76·7 (60·1–80·4)
Protein-energy malnutrition	100 (100–100)	100 (100–100)	100 (100–100)	100 (100–100)
Vitamin A deficiency	--	100 (100–100)	100 (100–100)	--
Dietary iron deficiency	--	100 (100–100)	100 (100–100)	--
Non-communicable diseases	54·9 (51·4–58·2)	34·4 (31·1–37·7)	20·4 (18·2–22·8)	51·7 (49·1–54·3)
Neoplasms	38 (34·1–42·4)	37 (33·6–40·8)	27·9 (24·2–32·3)	37·4 (34·1–41·3)
Lip and oral cavity cancer	45·1 (38·8–51·4)	49·6 (43·1–55·8)	49·1 (42·6–55·7)	49·6 (43·1–55·9)
Nasopharynx cancer	45·9 (36·9–54·6)	46·2 (37·2–55·2)	43·3 (34–51·9)	46·4 (37·4–55·3)
Other pharynx cancer	63·9 (57·7–69·4)	65·7 (59·1–71·5)	63·8 (57·3–69·7)	65·8 (59·2–71·5)
Esophageal cancer	57·9 (46·5–69·8)	60·9 (50·4–72·2)	58·7 (47·5–70·7)	61 (50·4–72·2)

Stomach cancer	21·5 (14·2–39·6)	22 (14·4–40·3)	22·1 (14·7–40·3)	22 (14·3–40·3)
Colon and rectum cancer	55·5 (46–64·2)	55·6 (46·7–63·8)	55·8 (46·8–64·1)	55·6 (46·7–63·8)
Liver cancer	69·6 (62·5–76·4)	70·3 (63·3–76·7)	69·7 (62·7–76·1)	70·3 (63·3–76·7)
Gallbladder and biliary tract cancer	21·1 (11·3–32·8)	21·4 (11·5–33·3)	20·9 (11·2–32·6)	21·4 (11·5–33·3)
Pancreatic cancer	34·7 (26·8–44·5)	34·9 (27·3–44·2)	34·9 (27–44·2)	34·9 (27·3–44·2)
Larynx cancer	73 (63·3–80·9)	74·8 (66·4–81·8)	74·6 (66·1–81·3)	74·9 (66·4–81·8)
Tracheal, bronchus, and lung cancer	79·1 (75·9–82·4)	78·7 (75·6–81·9)	78·9 (75·8–82·4)	78·7 (75·6–81·9)
Breast cancer	26·1 (17·9–36·2)	24·2 (17·1–32·3)	24·5 (17·2–32·7)	24·2 (17·1–32·2)
Cervical cancer	100 (100–100)	100 (100–100)	100 (100–100)	100 (100–100)
Uterine cancer	44·3 (29·3–59·7)	45·3 (30·2–60·8)	45·6 (30·4–61·1)	45·2 (30·1–60·7)
Ovarian cancer	15·2 (6·51–26·9)	13·2 (5·12–23·7)	12·6 (4·84–22·9)	13·3 (5·13–23·7)
Prostate cancer	5·59 (2·49–8·74)	6·35 (2·8–9·94)	6·86 (3·04–10·7)	6·28 (2·77–9·8)
Kidney cancer	34·5 (25·1–44·3)	34·7 (25·6–44·4)	34·3 (25·1–43·9)	34·7 (25·6–44·4)
Bladder cancer	36·2 (23·7–48·8)	40·1 (28·3–51·7)	41·5 (29·8–52·7)	40 (28·1–51·6)
Thyroid cancer	13·7 (6·62–22·4)	14·2 (6·84–23·3)	13·9 (6·92–22·6)	14·2 (6·84–23·3)
Mesothelioma	92·6 (89·9–94·8)	87·4 (83·1–90·9)	91·8 (88·4–94·5)	87·3 (83–90·8)
Non-Hodgkin lymphoma	6·64 (2·77–11·9)	6·63 (2·77–11·9)	6·66 (2·82–11·8)	6·62 (2·77–11·9)
Multiple myeloma	7·74 (3·29–13·8)	8·01 (3·38–14·3)	7·99 (3·36–14·3)	8·01 (3·37–14·3)
Leukemia	30·8 (19·6–42·5)	28·4 (19·1–38·1)	29·6 (19·8–40·2)	28·3 (19–38)
Cardiovascular diseases	78·8 (75–82·3)	79·8 (76·9–82·6)	75 (71–78·9)	80·6 (77·7–83·4)
Rheumatic heart disease	28·6 (17·4–50·5)	29·2 (20–44·2)	29 (18·7–43·6)	29·2 (20·1–44·3)
Ischemic heart disease	93 (89·2–96·3)	94·3 (91·5–96·8)	94·2 (91·5–96·6)	94·3 (91·5–96·8)
Stroke	80 (72·7–87·1)	83·3 (78·1–88·1)	82·9 (78·3–87·4)	83·4 (77·9–88·3)
Hypertensive heart disease	100 (100–100)	100 (100–100)	100 (100–100)	100 (100–100)
Non-rheumatic valvular heart disease	30·1 (19·6–44·3)	30·4 (21·1–41·4)	22·2 (12·8–31·7)	30·5 (21·1–41·6)
Cardiomyopathy and myocarditis	35·4 (27·1–45)	34·7 (28·4–41·2)	32·4 (25–39·2)	34·9 (28·5–41·4)
Atrial fibrillation and flutter	50·8 (40·6–60·9)	58·3 (49·6–66·9)	62·1 (54–70)	54·3 (45·1–63·8)
Aortic aneurysm	56·5 (49·7–63·4)	64·4 (58·9–69·5)	--	64·4 (58·9–69·5)
Endocarditis	39·3 (28·6–51·9)	41·3 (32–51·4)	38·7 (28·8–50·5)	41·3 (32–51·4)
Other cardiovascular and circulatory diseases	30 (23·5–39·7)	32·2 (27·3–38)	39 (32·2–46·1)	30 (25–36·6)
Chronic respiratory diseases	60·3 (56·4–64·1)	51·8 (47·6–56·1)	44·2 (39·3–49·4)	60·7 (57·1–64)
Chronic obstructive pulmonary disease	67·8 (63·2–72·3)	66·5 (63–69·9)	59·5 (56–63)	70·9 (66·8–74·9)
Pneumoconiosis	100 (100–100)	100 (100–100)	100 (100–100)	100 (100–100)
Asthma	40 (28·8–52·2)	35·3 (27·8–43·9)	34·3 (27·1–42·8)	42·5 (32·5–53·2)

Digestive diseases	25·5 (21·5–29·5)	25·9 (21·7–29·7)	11·3 (7·07–16·6)	32·5 (28·5–36·6)
Cirrhosis and other chronic liver diseases	50·9 (43·2–58·7)	57·6 (50·6–64·7)	48·6 (41·2–55·5)	57·8 (50·7–65)
Upper digestive system diseases	16·9 (14·2–19·7)	2·98 (1·7–4·63)	0·309 (0·17–0·502)	20·3 (17·4–23·3)
Gallbladder and biliary diseases	41·5 (27·2–56·7)	44·8 (31–59·4)	47·5 (33·4–61·5)	42·3 (28·2–57)
Pancreatitis	8·63 (2·14–16·1)	12·2 (3·91–21·4)	12·2 (3·79–21·1)	12·3 (3·9–21·4)
Neurological disorders	22·8 (9·58–36·6)	11·1 (4·56–21·4)	6·38 (2·91–14·2)	19·6 (7·64–33·5)
Alzheimer's disease and other dementias	34·4 (22–49·3)	37·1 (24·3–51·9)	37·5 (24·5–52)	36·9 (24·2–51·3)
Parkinson's disease	-7·9 (-12·2–-4·14)	-9·28 (-13·9–-4·92)	-9·66 (-14·2–-5·21)	-9·18 (-13·8–-4·87)
Idiopathic epilepsy	10·7 (5·99–15·6)	9·78 (5·64–14·4)	8·8 (5–12·9)	11·4 (6·69–16·6)
Multiple sclerosis	14·2 (10·4–18)	14·4 (10·7–17·9)	13·4 (10·1–16·8)	15·6 (11·3–19·5)
Mental disorders	--	7·68 (3·95–11·9)	7·68 (3·95–11·9)	--
Depressive disorders	--	14·6 (6·59–23·2)	14·6 (6·59–23·2)	--
Anxiety disorders	--	8·29 (2·81–16·9)	8·29 (2·81–16·9)	--
Idiopathic developmental intellectual disability	--	42·1 (19·9–61·9)	42·1 (19·9–61·9)	--
Substance use disorders	100 (100–100)	100 (100–100)	100 (100–100)	100 (100–100)
Alcohol use disorders	100 (100–100)	100 (100–100)	100 (100–100)	100 (100–100)
Drug use disorders	100 (100–100)	100 (100–100)	100 (100–100)	100 (100–100)
Diabetes and kidney diseases	100 (100–100)	100 (100–100)	100 (100–100)	100 (100–100)
Diabetes mellitus	100 (100–100)	100 (100–100)	100 (100–100)	100 (100–100)
Chronic kidney disease	100 (100–100)	100 (100–100)	100 (100–100)	100 (100–100)
Sense organ diseases	--	6·53 (5·67–7·57)	6·53 (5·67–7·57)	--
Blindness and vision loss	--	6·84 (4·41–10)	6·84 (4·41–10)	--
Age-related and other hearing loss	--	7·58 (6·9–8·37)	7·58 (6·9–8·37)	--
Musculoskeletal disorders	1·6 (0·419–3·05)	20·3 (16·6–24·1)	20·6 (16·8–24·3)	1·65 (0·458–3·07)
Rheumatoid arthritis	7·94 (2·2–14·6)	11·7 (3·44–21·1)	12 (3·49–21·6)	10·1 (2·87–18·2)
Osteoarthritis	--	13·9 (7·21–21·8)	13·9 (7·21–21·8)	--
Low back pain	--	36 (31·2–41·1)	36 (31·2–41·1)	--
Gout	--	50 (35·6–66)	50 (35·6–66)	--
Other non-communicable diseases	0·0707 (0·046–0·115)	0·0649 (0·0395–0·099)	--	0·234 (0·16–0·343)
Sudden infant death syndrome	5·8 (3·85–8·38)	5·79 (3·84–8·37)	--	5·79 (3·84–8·37)
Injuries	22·4 (18·8–25·6)	21·8 (18·6–25·3)	26·2 (22·9–29·9)	17·4 (13·8–21·1)
Transport injuries	27·1 (22·8–31·4)	25·5 (20·9–30·5)	28·5 (24·3–33·3)	24·5 (19·7–29·7)
Road injuries	26·8 (22·6–31·1)	25·1 (20·5–30·1)	27·8 (23·6–32·6)	24·2 (19·4–29·4)
Other transport injuries	35·6 (30·6–40·8)	33 (28·1–38·3)	36·1 (31·7–40·5)	30·6 (24·8–36·8)

Unintentional injuries	28·2<br>(23·1–31·8)	25·8<br>(22–29·8)	28·2<br>(24·4–32·4)	19·1<br>(15·2–22·7)
Falls	53·3<br>(45·5–58)	36·2<br>(31·7–40·5)	34·7<br>(30·4–39)	43·1<br>(37·5–47·5)
Drowning	0·32<br>(-7·39–11·1)	0·239<br>(-7·27–10·4)	11·3<br>(8·3–14·6)	-0·0977<br>(-7·86–10·4)
Fire, heat, and hot substances	9·59<br>(7·01–12·7)	15·6<br>(11·6–19·9)	16·3<br>(12·2–21)	14<br>(10·4–18·3)
Poisonings	13·5<br>(10·1–17·7)	14·4<br>(10·7–18·5)	14<br>(10·4–18)	15·2<br>(11·3–20)
Exposure to mechanical forces	29·1<br>(21·8–36·2)	24·7<br>(20·4–29·8)	25·1<br>(21–30·4)	20·9<br>(13·9–28·4)
Animal contact	26·3<br>(21·8–30·1)	20·5<br>(16·9–24·5)	21·7<br>(17·7–26·1)	17·2<br>(13·9–20·9)
Foreign body	3·06<br>(2·16–4·3)	7<br>(4·9–9·77)	9·15<br>(6·57–12·5)	6·2<br>(4·3–8·75)
Environmental heat and cold exposure	2·74<br>(1·03–5·22)	3·79<br>(1·47–7·24)	4·09<br>(1·58–7·79)	3·25<br>(1·27–6·17)
Exposure to forces of nature	0<br>(0–0)	4·68<br>(1·89–8·62)	4·68<br>(1·89–8·62)	0<br>(0–0)
Other unintentional injuries	16<br>(11·8–20·8)	15·4<br>(11·6–19·9)	14·7<br>(11–19)	20·2<br>(15–26·3)
Self-harm and interpersonal violence	9·97<br>(3·32–17·4)	11·2<br>(5·87–17·3)	12·4<br>(8·26–17·2)	10·8<br>(4·18–18·5)
Self-harm	7·63<br>(-1·05–16·6)	8·77<br>(-0·635–18·4)	9·37<br>(2·73–16·8)	8·75<br>(-0·754–18·4)
Interpersonal violence	17·3<br>(10·6–24)	19·7<br>(13·2–26·6)	27<br>(19·9–34·3)	16·3<br>(9·61–23·2)

Supplementary Results Table 2. All-age PAFs attributable to the joint distribution of all risk factors for all ages, both sexes combined for each GBD level 1, level 2, and level 3 cause and all causes in 2019 for Italy. Results are shown for deaths, YLLs, YLDs, and DALYs Data in parenthesis are 95% uncertainty intervals. PAFs=population attributable fractions. GBD=Global Burden of Disease. YLLs=years of life lost. YLDs=years lived with disability. DALYs=disability-adjusted life-years

	Deaths 2019	DALYs 2019	YLDs 2019	YLLs 2019
All causes	59 (54·8–62·4)	41·6 (38·4–45)	25·9 (23·7–28·3)	57 (54–59·8)
Communicable, maternal, neonatal, and nutritional diseases	43·8 (39·4–47·8)	49·5 (45·7–52·7)	45·9 (40·5–51·4)	53·4 (50–56·6)
HIV/AIDS and sexually transmitted infections	91·2 (88·8–93)	91·2 (88·8–93·1)	94·8 (92·9–96·5)	89·4 (86·7–91·4)
HIV/AIDS	90·8 (88·3–92·7)	89·7 (87·1–91·7)	91·5 (89·4–93·3)	89·2 (86·4–91·3)
Sexually transmitted infections excluding HIV	99·7 (99·6–99·8)	99·9 (99·8–99·9)	100 (100–100)	98·6 (98·3–98·9)
Respiratory infections and tuberculosis	44·2 (38·8–49·2)	34·2 (28·7–39·8)	3·27 (2·48–4·26)	48 (42·6–52·9)
Tuberculosis	52·5 (43·2–60·8)	56 (47·3–63·6)	54 (45·4–61·6)	56·4 (47·7–63·8)
Lower respiratory infections	44 (38·3–49·2)	47·5 (41·9–52·6)	38·3 (32·5–43·8)	47·7 (42·1–52·8)
Upper respiratory infections	0·153 (0·108–0·198)	0·00821 (0·00558–0·0113)	0·00494 (0·00304–0·00746)	0·635 (0·461–0·794)
Otitis media	0·602 (0·399–0·856)	2·98 (1·9–4·17)	2·99 (1·9–4·18)	2·4 (1·63–3·32)
Enteric infections	9 (5·69–14)	12·9 (9·39–17·7)	13·3 (9·73–18·3)	12 (8·58–16·8)
Diarrheal diseases	9·12 (5·76–14·2)	13 (9·49–17·8)	13·4 (9·74–18·3)	12·2 (8·74–17·1)
Other infectious diseases	0·839 (0·589–1·51)	1·2 (0·912–1·79)	0·816 (0·495–1·38)	1·34 (1·01–2·12)
Meningitis	0·529 (0·425–0·652)	1·54 (1·25–1·85)	0·000278 (0·00015–0·000464)	1·81 (1·48–2·17)
Encephalitis	0·134 (0·11–0·164)	0·421 (0·35–0·5)	9·34e-05 (5·19e-05–0·000146)	0·477 (0·397–0·562)
Measles	2·59 (-0·589–11)	2·52 (-0·248–10·2)	0·837 (0·0297–2)	3·44 (-0·783–14·6)
Acute hepatitis	8·35 (5·35–17·2)	6·91 (4·62–11·9)	3·89 (2·41–6·32)	9·72 (6·06–19·4)
Maternal and neonatal disorders	78·9 (77·3–80·2)	74·8 (71·8–77·3)	71·6 (66·6–75·6)	79·7 (78·1–81)
Maternal disorders	8·84 (3·19–14·6)	8·83 (3·17–14·5)	8·88 (3·16–14·6)	8·74 (3·15–14·4)
Neonatal disorders	81 (79·6–82·3)	76·1 (72·9–78·8)	72·9 (67·9–77·1)	81 (79·6–82·3)
Nutritional deficiencies	73·1 (34·8–77·9)	60·4 (51·8–67·4)	59·4 (50·4–66·7)	72·2 (36·6–76·8)
Protein-energy malnutrition	100 (100–100)	100 (100–100)	100 (100–100)	100 (100–100)
Vitamin A deficiency	--	100 (100–100)	100 (100–100)	--
Dietary iron deficiency	--	100 (100–100)	100 (100–100)	--
Non-communicable diseases	60·4 (55·7–63·9)	41·8 (38·2–45·5)	24·1 (21·7–26·7)	58·8 (55·5–62)
Neoplasms	44·5 (40·9–48·8)	44·6 (41·2–48·6)	32·2 (28·4–36·3)	45·4 (42·1–49·5)
Lip and oral cavity cancer	65·4 (58·9–71·5)	69·2 (63·3–74·7)	67·8 (61·4–73·7)	69·3 (63·3–74·8)
Nasopharynx cancer	64·9 (56·2–72·5)	65·4 (56·9–72·8)	64·6 (55·6–72·4)	65·5 (57·1–72·9)
Other pharynx cancer	78·9 (73·8–83·3)	80·4 (75·1–84·6)	79·8 (74·6–84·2)	80·4 (75·1–84·6)
Esophageal cancer	69 (60·7–77·5)	71·4 (63·4–79·5)	69 (60·2–77·8)	71·4 (63·4–79·5)
Stomach cancer	22·8 (15·1–41)	24·1 (15·8–42·5)	23·9 (15·7–42·3)	24·1 (15·8–42·5)
Colon and rectum cancer	63·9 (55·7–71·9)	64·4 (56·7–71·9)	64·6 (56·9–72·4)	64·4 (56·6–71·9)

Liver cancer	75 (70·1–80)	77·2 (72·9–81·7)	76·3 (71–81·5)	77·2 (72·9–81·7)
Gallbladder and biliary tract cancer	18·5 (9·83–29·5)	18·4 (9·71–29·8)	18·6 (10·1–29·8)	18·4 (9·71–29·8)
Pancreatic cancer	36·4 (28–46·8)	36·9 (29·1–46·7)	36·7 (28·5–46·7)	36·9 (29·1–46·7)
Larynx cancer	81·7 (74·3–87·3)	82·7 (75·7–87·7)	81·7 (74·7–86·9)	82·8 (75·7–87·8)
Tracheal, bronchus, and lung cancer	85·3 (82·2–88·2)	84·6 (81·6–87·4)	84·6 (81·6–87·8)	84·6 (81·6–87·4)
Breast cancer	32·3 (24–41·9)	31·8 (24·5–40·3)	31·8 (24·5–40·5)	31·8 (24·5–40·3)
Cervical cancer	100 (100–100)	100 (100–100)	100 (100–100)	100 (100–100)
Uterine cancer	39·7 (26·4–53·4)	39·7 (26·5–53·6)	39·7 (26·5–53·9)	39·7 (26·5–53·6)
Ovarian cancer	22·3 (11·4–34·7)	18·3 (9·17–29·2)	16·7 (8·27–26·8)	18·4 (9·19–29·3)
Prostate cancer	5·77 (2·63–9·15)	6·47 (2·96–10·1)	7·12 (3·37–10·9)	6·34 (2·88–9·9)
Kidney cancer	34 (24·8–43·4)	34·5 (25·6–43·6)	34·5 (25·8–43·9)	34·5 (25·6–43·6)
Bladder cancer	37·9 (24·9–50·9)	41·7 (29·2–54)	42·8 (30·7–54·8)	41·6 (29–54)
Thyroid cancer	12·2 (5·71–20·6)	12·4 (5·73–21·4)	12·1 (5·72–20·7)	12·5 (5·73–21·5)
Mesothelioma	98·4 (97·8–98·8)	96·9 (95·9–97·8)	97·9 (97·2–98·6)	96·9 (95·9–97·8)
Non-Hodgkin lymphoma	5·94 (2·41–10·8)	5·96 (2·42–10·8)	6·01 (2·42–11)	5·96 (2·42–10·8)
Multiple myeloma	6·82 (2·88–12·4)	6·95 (2·93–12·6)	6·93 (2·94–12·8)	6·95 (2·93–12·6)
Leukemia	31·9 (20·1–44·4)	31·1 (20·7–41·9)	30·6 (20·4–41)	31·1 (20·7–41·9)
Cardiovascular diseases	80·1 (76·6–83·6)	80·1 (77–83)	69·3 (64·9–73·5)	81·6 (78·7–84·5)
Rheumatic heart disease	22·8 (12·4–44·5)	23·9 (14·5–40·3)	24·5 (14·8–40·6)	23·8 (14·4–40·6)
Ischemic heart disease	92·5 (88·2–96·3)	93·7 (90·4–96·6)	93·5 (90·3–96·3)	93·7 (90·4–96·6)
Stroke	78·6 (70·6–87·8)	81·5 (75·4–88·5)	82·1 (76·5–88·5)	81·5 (75·1–88·4)
Hypertensive heart disease	100 (100–100)	100 (100–100)	100 (100–100)	100 (100–100)
Non-rheumatic valvular heart disease	23·1 (14·4–35·5)	18·6 (12·2–26·5)	6·54 (4·42–9·11)	24·2 (16–34·6)
Cardiomyopathy and myocarditis	24·8 (16·7–36·8)	29·8 (22·6–38·8)	32·8 (24·8–41·9)	29·2 (22–38·4)
Atrial fibrillation and flutter	48·1 (39·1–58)	55 (47·3–63·1)	60 (52·4–67·2)	51·1 (42·7–60·2)
Aortic aneurysm	52·6 (46·7–58·9)	60·8 (56–65·7)	--	60·8 (56–65·7)
Endocarditis	32 (21·5–45·6)	34·8 (25·3–46·4)	33·5 (23–46·3)	34·8 (25·4–46·3)
Other cardiovascular and circulatory diseases	28 (22·2–34·7)	31·2 (26·2–36·2)	35·4 (30–41·4)	29·7 (24·6–35·1)
Chronic respiratory diseases	65·3 (60·5–69·8)	59·5 (55·7–63·3)	51·5 (47·1–55·8)	65·9 (61·3–70·4)
Chronic obstructive pulmonary disease	70·4 (66·2–74·3)	68·9 (65·3–72·3)	61·6 (58–65·3)	73·2 (69·3–76·6)
Pneumoconiosis	100 (100–100)	100 (100–100)	100 (100–100)	100 (100–100)
Asthma	36·1 (25·3–48·5)	37·6 (29·1–47·1)	37·4 (29·1–46·9)	39·2 (29–50·7)
Digestive diseases	36·7 (31·5–41·9)	36·1 (30·8–40·7)	19 (12·8–26·5)	46·1 (40·6–51·3)
Cirrhosis and other chronic liver diseases	70·2 (59·8–79·7)	74·4 (65·3–82·4)	72·3 (64·3–80·3)	74·5 (65·3–82·7)
Upper digestive system diseases	16 (13·4–18·5)	4·13 (2·49–6·15)	0·808 (0·46–1·31)	19·6 (16·7–22·3)

Gallbladder and biliary diseases	39·6<br>(26·3–53·5)	41<br>(28·4–54·7)	41·3<br>(28·6–54·9)	39·9<br>(26·7–54)
Pancreatitis	39·4<br>(28·4–51·8)	43·5<br>(32·4–56)	43·5<br>(32·8–55·9)	43·5<br>(32·3–56)
Neurological disorders	25<br>(12·1–39·1)	16·8<br>(8·22–28·8)	11·1<br>(5·3–21·9)	23·5<br>(11–37·4)
Alzheimer's disease and other dementias	34·6<br>(22·1–49·1)	36·7<br>(24·1–51·2)	36·8<br>(24–51·2)	36·7<br>(23·9–50·9)
Parkinson's disease	-8·13<br>(-12·6–-4·07)	-9·39<br>(-14·4–-4·72)	-9·34<br>(-14·3–-4·74)	-9·41<br>(-14·5–-4·72)
Idiopathic epilepsy	22·8<br>(16·3–29·4)	22·1<br>(16·2–28·4)	21·1<br>(15·5–27·3)	24·1<br>(17·6–30·8)
Multiple sclerosis	13·8<br>(10·2–17·4)	14·7<br>(11·2–18·4)	14·2<br>(10·8–17·7)	15·6<br>(11·7–19·7)
Mental disorders	--	6·22<br>(2·81–9·75)	6·22<br>(2·81–9·75)	--
Depressive disorders	--	13·7<br>(4·86–22·8)	13·7<br>(4·86–22·8)	--
Anxiety disorders	--	3·65<br>(0·98–8·2)	3·65<br>(0·98–8·2)	--
Idiopathic developmental intellectual disability	--	54<br>(27·4–72·9)	54<br>(27·4–72·9)	--
Substance use disorders	100<br>(100–100)	100<br>(100–100)	100<br>(100–100)	100<br>(100–100)
Alcohol use disorders	100<br>(100–100)	100<br>(100–100)	100<br>(100–100)	100<br>(100–100)
Drug use disorders	100<br>(100–100)	100<br>(100–100)	100<br>(100–100)	100<br>(100–100)
Diabetes and kidney diseases	100<br>(100–100)	100<br>(100–100)	100<br>(100–100)	100<br>(100–100)
Diabetes mellitus	100<br>(100–100)	100<br>(100–100)	100<br>(100–100)	100<br>(100–100)
Chronic kidney disease	100<br>(100–100)	100<br>(100–100)	100<br>(100–100)	100<br>(100–100)
Sense organ diseases	--	7·45<br>(6·08–9·24)	7·45<br>(6·08–9·24)	--
Blindness and vision loss	--	11·1<br>(7·13–16·1)	11·1<br>(7·13–16·1)	--
Age-related and other hearing loss	--	6·68<br>(5·97–7·49)	6·68<br>(5·97–7·49)	--
Musculoskeletal disorders	2·66<br>(0·736–4·79)	20·1<br>(16·3–24)	20·4<br>(16·5–24·3)	3·15<br>(0·878–5·53)
Rheumatoid arthritis	7·91<br>(2·2–14·4)	12·3<br>(3·49–21·6)	13<br>(3·69–22·8)	10·1<br>(2·79–17·8)
Osteoarthritis	--	14·2<br>(7·28–22·6)	14·2<br>(7·28–22·6)	--
Low back pain	--	36·6<br>(31·1–41·8)	36·6<br>(31·1–41·8)	--
Gout	--	46·1<br>(32·3–62·1)	46·1<br>(32·3–62·1)	--
Other non-communicable diseases	0·0189<br>(0·0147–0·0238)	0·0114<br>(0·0077–0·0161)	--	0·0735<br>(0·0577–0·0906)
Sudden infant death syndrome	7·71<br>(6·23–9·35)	7·7<br>(6·22–9·34)	--	7·7<br>(6·22–9·34)
Injuries	38·8<br>(33·7–42·5)	35·5<br>(31·4–39·2)	39·5<br>(34·6–43·6)	31<br>(26·6–35·2)
Transport injuries	31·2<br>(26·5–35·8)	32·5<br>(27·7–37·7)	35·3<br>(31–39·9)	31·2<br>(25·9–36·9)
Road injuries	30·5<br>(25·7–35)	31·8<br>(26·9–37·1)	34·6<br>(30·2–39·2)	30·5<br>(25·2–36·2)
Other transport injuries	41·8<br>(37–46·7)	41·3<br>(36·4–46·1)	43·6<br>(38·8–47·5)	40<br>(34·7–45·4)
Unintentional injuries	47·3<br>(40·7–51·6)	40<br>(34·7–44·3)	40·5<br>(35·3–45)	38·6<br>(33·2–42·8)
Falls	58·1<br>(50–63·3)	48·9<br>(42·7–53·4)	47·3<br>(41·4–51·9)	53<br>(46·4–57·7)
Drowning	-3·01<br>(-11·2–5·65)	-0·0176<br>(-7·88–8·48)	14·9<br>(11–19·5)	-0·953<br>(-9·21–7·72)
Fire, heat, and hot substances	9·96<br>(6·3–14·4)	19·5<br>(14·7–25·2)	21·8<br>(16·7–27·7)	15·8<br>(11·6–20·7)

Poisonings	17·8 (13·1–23·2)	20·7 (16·1–26·2)	19·4 (15–24·4)	23·1 (17·7–29·1)
Exposure to mechanical forces	25 (18·7–30·6)	27·4 (22·9–32·7)	29 (24·6–34·1)	19·9 (13·8–26·7)
Animal contact	29·1 (24·3–34·2)	26·9 (22·3–32·1)	25·2 (20·7–30·6)	29·1 (24·1–34·7)
Foreign body	4·31 (3·18–5·81)	9·78 (7·29–12·9)	10 (7·51–13·3)	9·44 (7·05–12·5)
Environmental heat and cold exposure	6·06 (2·73–10·5)	7·76 (3·65–13·3)	8·02 (3·77–13·7)	7·14 (3·33–12·3)
Exposure to forces of nature	0 (0–0)	8·77 (4·05–14·9)	8·77 (4·05–14·9)	0 (0–0)
Other unintentional injuries	24·4 (18·4–31·1)	21·3 (16·3–27·1)	20·7 (15·9–26·5)	26·9 (20·6–34)
Self-harm and interpersonal violence	20·8 (10·3–31·1)	22·5 (12·6–31·8)	31·7 (25·2–38·3)	21·2 (10·7–31·4)
Self-harm	20 (8·38–31·4)	20·6 (8·83–32·2)	21·5 (11·8–31·1)	20·6 (8·73–32·3)
Interpersonal violence	27·7 (19·9–35·2)	30·1 (22·7–37·9)	34·8 (27·5–42·8)	25·9 (18·1–33·6)

Supplementary Results Table 2. All-age PAFs attributable to the joint distribution of all risk factors for all ages, both sexes combined for each GBD level 1, level 2, and level 3 cause and all causes in 2019 for Luxembourg. Results are shown for deaths, YLLs, YLDs, and DALYs Data in parenthesis are 95% uncertainty intervals. PAFs=population attributable fractions. GBD=Global Burden of Disease. YLLs=years of life lost. YLDs=years lived with disability. DALYs=disability-adjusted life-years

	Deaths 2019	DALYs 2019	YLDs 2019	YLLs 2019
All causes	57·7 (54·4–60·7)	40·2 (37·2–43·4)	27 (24·7–29·2)	55·4 (53–57·8)
Communicable, maternal, neonatal, and nutritional diseases	43·2 (36·9–49·3)	45 (41·9–48)	41·4 (37·8–45·4)	48·5 (43·4–53·3)
HIV/AIDS and sexually transmitted infections	83·7 (81·6–85·5)	84·7 (82–87·3)	91 (87·7–94·4)	80·6 (78·2–82·7)
HIV/AIDS	81·8 (79·6–83·8)	80·9 (78·5–83)	84·1 (81·8–86·3)	79·7 (77·2–81·8)
Sexually transmitted infections excluding HIV	99·8 (99·7–99·9)	99·9 (99·7–99·9)	100 (100–100)	99·1 (98·6–99·5)
Respiratory infections and tuberculosis	47·6 (39·1–55·7)	38·1 (30·8–45·3)	3·24 (2·33–4·4)	51·6 (43·5–59)
Tuberculosis	59·1 (49·5–67·4)	61·7 (52·7–69·2)	59·7 (50·4–68·2)	62·5 (53·7–69·9)
Lower respiratory infections	47·5 (39–55·7)	51·3 (43–58·7)	38·1 (32–43·7)	51·5 (43·1–59)
Upper respiratory infections	0·157 (0·0765–0·27)	0·00878 (0·00242–0·0187)	0·00533 (0–0·0149)	0·581 (0·29–0·957)
Otitis media	3·54 (1·52–7·08)	3·87 (2·46–5·52)	3·87 (2·46–5·52)	8·61 (4·62–14·2)
Enteric infections	6·24 (4·31–9·19)	9·5 (7·3–12·7)	8·62 (6·47–11·7)	11·5 (8·04–15·5)
Diarrheal diseases	6·36 (4·38–9·38)	9·58 (7·36–12·8)	8·62 (6·47–11·7)	11·8 (8·36–16)
Other infectious diseases	0·217 (0·146–0·304)	0·588 (0·401–0·808)	0·638 (0·386–1·03)	0·576 (0·373–0·835)
Meningitis	1·19 (0·762–1·81)	2·39 (1·55–3·53)	0·000577 (0–0·00175)	3·17 (2·14–4·56)
Encephalitis	0·266 (0·162–0·404)	0·67 (0·417–1·01)	0·000182 (0–0·000519)	0·78 (0·503–1·16)
Measles	2·03 (-0·626–10·8)	2·23 (-0·527–11·1)	0·422 (0·0155–0·973)	2·69 (-0·806–14·3)
Acute hepatitis	8·9 (5·78–16)	4·59 (3–6·96)	3·35 (2·06–5·33)	9·26 (5·9–16·4)
Maternal and neonatal disorders	73·1 (69·7–76·4)	64·4 (60·1–68·1)	58·4 (53·7–62·9)	74·5 (71·2–77·6)
Maternal disorders	8·81 (3·33–14·3)	8·81 (3·33–14·3)	8·91 (3·38–14·6)	8·73 (3·3–14·1)
Neonatal disorders	77·2 (73·9–80·3)	65·9 (61·5–69·6)	59·3 (54·4–63·8)	77·2 (73·9–80·3)
Nutritional deficiencies	92·5 (82·2–94·6)	75 (72·3–77·5)	73·4 (70·5–76)	89·7 (80·4–92·3)
Protein-energy malnutrition	100 (100–100)	100 (100–100)	100 (100–100)	100 (100–100)
Vitamin A deficiency	--	100 (100–100)	100 (100–100)	--
Dietary iron deficiency	--	100 (100–100)	100 (100–100)	--
Non-communicable diseases	60·2 (56·6–63·6)	41·1 (37·5–44·8)	25·6 (23·1–28·2)	59·4 (56·7–62·1)
Neoplasms	47·1 (43·1–51·4)	46·4 (42·6–50·3)	33·8 (29·8–38)	47·1 (43·3–50·9)
Lip and oral cavity cancer	73·1 (67–78·4)	75·2 (69·4–80·3)	74·9 (68·9–80·2)	75·2 (69·4–80·4)
Nasopharynx cancer	71·1 (62·5–78)	71 (62·7–77·7)	70·2 (61·7–77·2)	71·1 (62·8–77·8)
Other pharynx cancer	82 (76·5–86·3)	82·6 (76·9–86·7)	81·8 (76–86·1)	82·6 (76·9–86·7)

Esophageal cancer	75·5 (67·2–83·4)	77 (68·7–84·4)	75·6 (67·1–83·4)	77 (68·7–84·4)
Stomach cancer	23·7 (15·8–41·7)	24·3 (16·3–42·2)	24·6 (16·5–42·6)	24·3 (16·3–42·2)
Colon and rectum cancer	63·9 (55·5–72·5)	64·3 (56·5–72·1)	64·5 (56·5–72·5)	64·3 (56·5–72·1)
Liver cancer	79·5 (73·9–84·8)	79·7 (74·4–84·4)	79·2 (73·9–84)	79·7 (74·4–84·4)
Gallbladder and biliary tract cancer	19·5 (10·6–30·6)	19·6 (10·8–31)	19 (10·4–30·4)	19·6 (10·8–31)
Pancreatic cancer	39·5 (30·8–50·7)	38·7 (30·5–49·2)	39·3 (30·7–50)	38·7 (30·5–49·2)
Larynx cancer	82·4 (75·1–88·1)	82·9 (75·8–88·3)	83 (76·2–88·4)	82·9 (75·7–88·3)
Tracheal, bronchus, and lung cancer	84 (80·1–87·8)	82·1 (78–86·2)	83 (78·9–86·9)	82·1 (78–86·2)
Breast cancer	36·9 (28·4–46·9)	35·5 (27·8–44)	35·6 (28–44)	35·5 (27·8–44)
Cervical cancer	100 (100–100)	100 (100–100)	100 (100–100)	100 (100–100)
Uterine cancer	41·6 (27·8–56·4)	42·6 (29·3–57·1)	42·9 (29·6–56·9)	42·5 (29·3–57·1)
Ovarian cancer	17·1 (7·1–30·6)	14·5 (5·72–26·3)	13·5 (5·36–24·9)	14·5 (5·73–26·3)
Prostate cancer	6·26 (2·84–9·91)	6·86 (3·14–10·8)	7·25 (3·35–11·4)	6·79 (3·1–10·8)
Kidney cancer	35·6 (26·6–45·3)	35·5 (26·9–45·2)	35·2 (26·6–44·9)	35·6 (26·9–45·2)
Bladder cancer	39·8 (26–53·9)	43·6 (31·1–56·5)	45 (32·4–58)	43·5 (30·9–56·3)
Thyroid cancer	13·4 (6·15–22·9)	14 (6·19–24·1)	13·8 (6·19–23·4)	14 (6·2–24·2)
Mesothelioma	97 (96–97·8)	94·5 (92·8–96)	96·6 (95·2–97·7)	94·5 (92·8–95·9)
Non-Hodgkin lymphoma	6·18 (2·62–10·9)	6·22 (2·6–11)	6·24 (2·63–11·1)	6·22 (2·59–11)
Multiple myeloma	7·18 (3·04–12·7)	7·4 (3·17–13·1)	7·39 (3·21–13·1)	7·4 (3·17–13·1)
Leukemia	33·7 (21·4–46·4)	30·9 (20·6–42)	31·2 (20·9–42·3)	30·9 (20·5–42)
Cardiovascular diseases	79·9 (76·1–83·6)	81 (78–83·9)	75·1 (71·2–78·6)	82·1 (79·1–84·9)
Rheumatic heart disease	28·1 (16·2–51·4)	29·7 (19·4–47·4)	29·6 (19·2–46·7)	29·7 (19·5–47·4)
Ischemic heart disease	94·6 (91·1–97·6)	95·6 (93–97·8)	95·3 (92·6–97·6)	95·6 (93·1–97·8)
Stroke	83·6 (76·4–91·5)	86·4 (81·2–91·8)	85·9 (81·4–90·8)	86·5 (81·1–92)
Hypertensive heart disease	100 (100–100)	100 (100–100)	100 (100–100)	100 (100–100)
Non-rheumatic valvular heart disease	30·7 (20–46·2)	32·6 (23–45·5)	31·9 (22·4–43·6)	32·6 (23–45·6)
Cardiomyopathy and myocarditis	37·8 (28·8–50·3)	44 (37·3–52·4)	43·3 (35·9–52·2)	44·1 (37·2–52·5)
Atrial fibrillation and flutter	55·2 (45·6–65·6)	63·7 (56·4–71·5)	68·5 (62–74·9)	58·5 (50–67·9)
Aortic aneurysm	59·6 (52·4–66·3)	67·4 (61·9–72·4)	--	67·4 (61·9–72·4)
Endocarditis	37·7 (26·6–53·4)	41 (31·1–52·8)	37·6 (26·3–52·9)	41·2 (31·3–52·8)
Other cardiovascular and circulatory diseases	36·5 (29–45)	40·6 (34·9–47·3)	43·2 (37·2–49·8)	39·3 (33–45·9)
Chronic respiratory diseases	65·5 (61·4–69·6)	57·6 (53·3–61·5)	50·4 (45·3–55·3)	66·5 (62·7–70·2)

Chronic obstructive pulmonary disease	71·2 (67–75·5)	68·8 (65·2–72·5)	62 (58·2–66)	73·9 (70·1–77·5)
Pneumoconiosis	100 (100–100)	100 (100–100)	100 (100–100)	100 (100–100)
Asthma	40·9 (30·1–52·8)	40·3 (32–50·1)	40 (31·7–49·5)	43·3 (33·4–54·2)
Digestive diseases	40 (34·9–44·5)	42·1 (36·6–46·9)	12·4 (8·59–16·7)	52·9 (47·2–58·1)
Cirrhosis and other chronic liver diseases	80·4 (70·7–87·9)	82·4 (73·2–89·3)	76·7 (68·9–83·5)	82·5 (73·2–89·5)
Upper digestive system diseases	19·3 (16–23)	5·27 (3·23–8·02)	0·779 (0·453–1·21)	23·3 (19·7–27·1)
Gallbladder and biliary diseases	41·7 (27·8–55·6)	45·2 (32·4–58·6)	47 (33·9–60·3)	42·6 (29·4–56·7)
Pancreatitis	48·4 (37·2–61·2)	52·8 (41·6–65·1)	50·1 (38·9–62·3)	53·1 (41·9–65·6)
Neurological disorders	24·5 (11·1–39·5)	14·3 (7·09–24·5)	9·02 (4·35–17·5)	22·6 (11·6–36·2)
Alzheimer's disease and other dementias	37·4 (23·1–53·2)	40·5 (26·7–55·4)	41·4 (27·6–56·2)	40·1 (26·2–55·1)
Parkinson's disease	-9·5 (-15·2—4·85)	-11 (-17·3—5·73)	-11·5 (-17·9—6·04)	-10·9 (-17·2—5·62)
Idiopathic epilepsy	29·8 (22·1–38)	28·3 (21·2–35·5)	26·8 (20–33·7)	30·4 (22·7–38·1)
Multiple sclerosis	15 (10·9–19·3)	16·1 (11·7–20·5)	15·8 (11·6–20·1)	16·3 (11·9–20·9)
Mental disorders	--	5·43 (2·74–8·74)	5·43 (2·74–8·75)	--
Depressive disorders	--	10·9 (5·35–17·1)	10·9 (5·35–17·1)	--
Anxiety disorders	--	6·91 (2·06–14·4)	6·91 (2·06–14·4)	--
Idiopathic developmental intellectual disability	--	46·5 (21·5–66·9)	46·5 (21·5–66·9)	--
Substance use disorders	100 (100–100)	100 (100–100)	100 (100–100)	100 (100–100)
Alcohol use disorders	100 (100–100)	100 (100–100)	100 (100–100)	100 (100–100)
Drug use disorders	100 (100–100)	100 (100–100)	100 (100–100)	100 (100–100)
Diabetes and kidney diseases	100 (100–100)	100 (100–100)	100 (100–100)	100 (100–100)
Diabetes mellitus	100 (100–100)	100 (100–100)	100 (100–100)	100 (100–100)
Chronic kidney disease	100 (100–100)	100 (100–100)	100 (100–100)	100 (100–100)
Sense organ diseases	--	6·99 (5·99–8·31)	6·99 (5·99–8·31)	--
Blindness and vision loss	--	8·64 (5·39–12·7)	8·64 (5·39–12·7)	--
Age-related and other hearing loss	--	7·63 (6·95–8·43)	7·63 (6·95–8·43)	--
Musculoskeletal disorders	2·88 (0·792–5·51)	21·6 (17·5–25·9)	21·7 (17·6–26·1)	3·67 (1·03–6·76)
Rheumatoid arthritis	10·9 (3·1–20·2)	14·7 (4·18–27)	15 (4·35–27·4)	13 (3·64–24·2)
Osteoarthritis	--	14·6 (7·65–22·7)	14·6 (7·65–22·7)	--
Low back pain	--	37·9 (32·2–43·8)	37·9 (32·2–43·8)	--
Gout	--	47·3 (32·7–64·1)	47·3 (32·7–64·1)	--
Other non-communicable diseases	0·189 (0·114–0·285)	0·081 (0·0421–0·141)	--	0·613 (0·375–0·902)

Sudden infant death syndrome	8·65 (6–11·7)	8·64 (5·99–11·7)	--	8·64 (5·99–11·7)
Injuries	32·5 (27·4–36·7)	30·1 (25·9–34)	34·2 (29·9–38·6)	26 (20·5–31·1)
Transport injuries	24·2 (17·6–30·8)	24·9 (18·9–31)	30·8 (26·8–34·9)	22·6 (15·5–29·6)
Road injuries	23·4 (16·5–30·1)	24·2 (18·2–30·3)	30 (26–34·2)	21·9 (14·6–29·2)
Other transport injuries	37·1 (32·4–41·7)	35·6 (31–40·2)	39·2 (35·2–43·3)	33·6 (28·4–38·6)
Unintentional injuries	38·9 (33·2–43·2)	32·8 (28·1–37·5)	34·5 (29·8–39·4)	28·8 (24·1–33·3)
Falls	56 (48·3–61·3)	43 (37·1–48·1)	41·7 (36·1–47)	46·8 (40·7–52·2)
Drowning	-6·88 (-19·9–5·87)	-5·13 (-17·4–6·68)	14·5 (9·81–21·1)	-6·19 (-19–6·1)
Fire, heat, and hot substances	13 (8·12–19·1)	18·5 (13·2–25·3)	19 (13·6–26)	16·8 (11·7–23·1)
Poisonings	17·4 (12–24)	18·3 (13·2–24·6)	17·2 (12·2–23·1)	20·3 (14·7–26·9)
Exposure to mechanical forces	11·9 (2·42–21·5)	20·2 (16·6–23·8)	22·6 (19·8–25·6)	6·02 (-3·65–16·1)
Animal contact	26·9 (21·2–33·7)	26·3 (20·8–32·5)	26·2 (21–31·9)	26·3 (20·7–32·9)
Foreign body	2·7 (1·97–3·53)	6·18 (4·59–7·99)	7·09 (5·37–9·15)	5·92 (4·34–7·77)
Environmental heat and cold exposure	11 (5·02–18·6)	10·6 (4·95–18)	10·4 (4·86–17·7)	11·5 (5·26–19·2)
Exposure to forces of nature	0 (0–0)	11·6 (5·36–19·3)	11·6 (5·36–19·3)	0 (0–0)
Other unintentional injuries	17·9 (12·2–24·6)	18·4 (13·3–25)	17·7 (12·6–24·2)	21·1 (15·2–28)
Self-harm and interpersonal violence	24·9 (12·8–36·4)	26·6 (15·3–37·1)	35·6 (28·6–42·4)	25·5 (13·5–36·9)
Self-harm	24·1 (10·8–36·8)	24·9 (11·5–37·4)	25·9 (14·4–36·6)	24·8 (11·4–37·5)
Interpersonal violence	33·3 (24·8–42·3)	35 (27·1–43·4)	39·4 (31·3–47·7)	31·6 (23·2–40·6)

Supplementary Results Table 2. All-age PAFs attributable to the joint distribution of all risk factors for all ages, both sexes combined for each GBD level 1, level 2, and level 3 cause and all causes in 2019 for Turkmenistan. Results are shown for deaths, YLLs, YLDs, and DALYs Data in parenthesis are 95% uncertainty intervals. PAFs=population attributable fractions. GBD=Global Burden of Disease. YLLs=years of life lost. YLDs=years lived with disability. DALYs=disability-adjusted life-years

	Deaths 2019	DALYs 2019	YLDs 2019	YLLs 2019
All causes	72·2 (70·1–74·1)	54·4 (51–57·5)	28·5 (26·2–30·8)	64·9 (62·6–67)
Communicable, maternal, neonatal, and nutritional diseases	66·2 (62·6–69·4)	68·2 (65·1–71·1)	61·5 (57·2–65·4)	69·5 (65·7–72·8)
HIV/AIDS and sexually transmitted infections	78·3 (75·5–83·3)	79·8 (76·9–84·5)	90·2 (86–94·1)	77·6 (74·9–82·6)
HIV/AIDS	77·7 (74·9–82·9)	77·1 (74·4–82·2)	77·8 (75·5–82)	77·1 (74·3–82·3)
Sexually transmitted infections excluding HIV	99·8 (99·6–99·9)	99·9 (99·9–100)	100 (100–100)	99·5 (99·2–99·7)
Respiratory infections and tuberculosis	67·5 (62·2–72·2)	69·4 (63·5–74·5)	16·5 (11·8–22·3)	72·2 (66·2–77·1)
Tuberculosis	48·9 (41·3–55·7)	45·6 (38·2–52·3)	42·2 (34·3–49·1)	45·9 (38·6–52·6)
Lower respiratory infections	74·3 (67·6–79·8)	78·3 (71·2–83·5)	40·9 (33·4–48·7)	78·4 (71·4–83·6)
Upper respiratory infections	2·49 (1·55–3·85)	0·587 (0·288–1·01)	0·0116 (0·00158–0·0265)	2·68 (1·64–4·2)
Otitis media	3·62 (1·73–6·9)	7·07 (4·64–9·7)	7·07 (4·64–9·7)	8·1 (4·32–13·8)
Enteric infections	91·3 (85·4–95·3)	87·3 (78·9–93·5)	81·6 (69·6–90·8)	93·1 (88·4–96·3)
Diarrheal diseases	93·1 (87·8–96·9)	88 (79·6–94·2)	81·7 (69·6–90·9)	94·6 (90·1–97·6)
Other infectious diseases	3·52 (2·78–4·53)	3·56 (2·83–4·5)	0·187 (0·102–0·319)	4·1 (3·27–5·15)
Meningitis	4·81 (3·46–6·42)	5·62 (4·12–7·43)	0·00262 (0·000291–0·00585)	6·25 (4·59–8·27)
Encephalitis	2·96 (1·51–4·18)	3·55 (1·84–4·9)	0·00198 (0·00022–0·00455)	3·66 (1·91–5·1)
Measles	0 (0–0)	0·535 (0–2·18)	0·535 (0–2·18)	0 (0–0)
Acute hepatitis	3·87 (2·06–6·66)	2·97 (1·58–5·14)	1·25 (0·718–2·11)	3·18 (1·68–5·56)
Maternal and neonatal disorders	71·2 (65·9–78)	68·5 (63·6–74·8)	34·8 (29·2–40·1)	72·1 (66·8–79)
Maternal disorders	16·7 (6·04–27·6)	16·6 (6·05–27·6)	16·5 (6·08–27·6)	16·6 (6·04–27·6)
Neonatal disorders	73·9 (68·4–80·8)	70·2 (65·2–76·6)	35·5 (29·7–40·9)	73·9 (68·4–80·8)
Nutritional deficiencies	52·6 (43·6–62·5)	96·9 (96–97·7)	98·3 (97·7–98·7)	54·1 (46·9–63·1)
Protein-energy malnutrition	100 (100–100)	100 (100–100)	100 (100–100)	100 (100–100)
Vitamin A deficiency	--	100 (100–100)	100 (100–100)	--
Dietary iron deficiency	--	100 (100–100)	100 (100–100)	--
Non-communicable diseases	76·3 (73·8–78·5)	55·5 (51–59·6)	25·3 (22·5–28·1)	69·4 (66·6–72·4)
Neoplasms	39·6 (36–43·8)	35·8 (32·5–39·6)	32·6 (29–36·4)	35·9 (32·6–39·7)
Lip and oral cavity cancer	53 (47·3–58·7)	53·6 (47·6–59·3)	50 (44–56·1)	53·7 (47·7–59·4)
Nasopharynx cancer	40·4 (33·4–46·9)	35 (28·4–41·1)	37·7 (30·8–44·4)	35 (28·4–41·1)
Other pharynx cancer	67·9 (62·5–72·7)	68 (62·4–73)	64·7 (59–69·8)	68 (62·5–73)
Esophageal cancer	61·8 (47·4–75·7)	63 (48·6–76·3)	62·5 (47·3–75·9)	63 (48·7–76·3)

Stomach cancer	21·4 (13·6–40·2)	21·2 (13·3–40)	21·1 (13·3–40)	21·2 (13·3–40)
Colon and rectum cancer	57·7 (49·8–64·3)	57·6 (50·1–63·8)	57·6 (49·9–63·8)	57·6 (50·1–63·8)
Liver cancer	62·3 (54·1–69·9)	58·4 (50·4–66·1)	61·2 (53·1–69)	58·4 (50·4–66·1)
Gallbladder and biliary tract cancer	26·3 (15–39·4)	26·5 (15·2–39·4)	26·4 (15·1–39·6)	26·5 (15·2–39·4)
Pancreatic cancer	28 (20·6–37·1)	27·8 (20·7–36·6)	28 (20·6–37)	27·8 (20·7–36·6)
Larynx cancer	62·7 (55·4–68·7)	63·1 (55·9–69·1)	59·9 (52·7–66)	63·2 (56–69·2)
Tracheal, bronchus, and lung cancer	76 (71·8–80·9)	74·4 (70·3–79·4)	75 (70·2–80·2)	74·4 (70·2–79·4)
Breast cancer	21·4 (14·5–29·3)	18·8 (12·1–26)	19 (12·1–26·1)	18·8 (12–25·9)
Cervical cancer	100 (100–100)	100 (100–100)	100 (100–100)	100 (100–100)
Uterine cancer	52·2 (36·8–66·8)	52·3 (37·1–67·1)	52·5 (37·3–67·4)	52·3 (37·1–67·1)
Ovarian cancer	11·8 (4·31–21·1)	10·5 (3·77–19)	9·93 (3·53–17·5)	10·5 (3·77–19)
Prostate cancer	5·56 (2·56–8·71)	6·02 (2·74–9·32)	6·29 (2·9–9·76)	6 (2·73–9·3)
Kidney cancer	37·4 (27·6–47·1)	35 (25·9–44·3)	34·2 (25·2–43·7)	35 (25·9–44·4)
Bladder cancer	32·8 (23·1–42·2)	34·7 (25·5–43·3)	34·2 (25·3–42·5)	34·7 (25·5–43·3)
Thyroid cancer	17·7 (9·36–27·8)	17·5 (9·17–27·5)	16·6 (9·07–25·4)	17·5 (9·15–27·7)
Mesothelioma	82·7 (66·3–96·4)	78·5 (57·6–96·8)	81·6 (64·4–96·6)	78·4 (57·5–96·8)
Non-Hodgkin lymphoma	8·09 (3·56–13·9)	7·32 (3·29–12·6)	6·72 (2·99–11·5)	7·33 (3·3–12·6)
Multiple myeloma	9·8 (4·47–16·6)	9·51 (4·34–16)	9·8 (4·4–16·5)	9·51 (4·34–16)
Leukemia	17·9 (12·3–23·8)	12·4 (8·52–16·8)	14·5 (9·86–19·8)	12·4 (8·5–16·8)
Cardiovascular diseases	90·4 (88·2–92·3)	88·9 (87–90·6)	80·5 (76·3–84·1)	89·3 (87·3–91)
Rheumatic heart disease	30·3 (20·5–43·9)	27·7 (17–40·8)	24·8 (14·1–37·3)	28·7 (17·6–42·4)
Ischemic heart disease	95·2 (93–97·1)	96·3 (94·8–97·6)	95·6 (93·7–97·3)	96·3 (94·9–97·6)
Stroke	89·5 (85·9–92·3)	91·5 (88·9–93·5)	89·9 (87·2–92·1)	91·6 (89·1–93·7)
Hypertensive heart disease	100 (100–100)	100 (100–100)	100 (100–100)	100 (100–100)
Non-rheumatic valvular heart disease	28·2 (21·3–35·8)	19·5 (13·7–26·2)	4·23 (2·73–6·15)	27 (19·9–34·3)
Cardiomyopathy and myocarditis	44·8 (36·5–54)	42·3 (33–51·4)	41 (34·1–47·4)	42·3 (33–51·5)
Atrial fibrillation and flutter	56·5 (46·7–65·9)	64·7 (56·9–72·2)	67·2 (59·4–74·3)	61·4 (52·8–69·4)
Aortic aneurysm	65 (59·7–69·9)	70·1 (65·7–74·1)	--	70·1 (65·7–74·1)
Endocarditis	44 (33·7–54)	41·8 (30·2–52·7)	37·1 (28·6–46·4)	41·8 (30·2–52·7)
Other cardiovascular and circulatory diseases	38·3 (32·2–44·8)	32·3 (25·6–39·9)	28·5 (20·4–36·7)	32·6 (25·6–40·3)
Chronic respiratory diseases	63·2 (56·1–68)	50·1 (45·2–55·3)	36·9 (31·3–42)	60·2 (54·4–64·7)
Chronic obstructive pulmonary disease	70 (61·8–75·3)	64·1 (58·7–69·4)	51·1 (45·6–56·9)	69·4 (62·9–74·3)
Pneumoconiosis	100 (100–100)	100 (100–100)	100 (100–100)	100 (100–100)
Asthma	46·4 (35–58·3)	35·1 (27·2–44·5)	30·8 (23·7–39·4)	46·3 (35·9–56·9)

Digestive diseases	60·5 (53·1–67·1)	56·3 (48·9–63)	16·9 (12–21·9)	60·5 (52·6–67·6)
Cirrhosis and other chronic liver diseases	66·9 (58·1–74·6)	67·1 (58·2–74·9)	62·3 (55·4–69·3)	67·1 (58·2–75)
Upper digestive system diseases	26·6 (22·6–30·6)	13·5 (8·95–18·5)	1·2 (0·707–1·89)	26·7 (22·7–30·6)
Gallbladder and biliary diseases	50·5 (35·9–64·6)	50·6 (36·7–64·4)	50·7 (36·4–64·5)	50·4 (36·4–64·1)
Pancreatitis	32·6 (22·7–44·5)	35·8 (25·4–48·7)	28·4 (20·2–38·6)	36·2 (25·6–49·2)
Neurological disorders	21 (10·6–33·7)	8·39 (4·18–15)	4·77 (2·22–9·44)	17 (9·78–26·7)
Alzheimer's disease and other dementias	31·2 (18·6–46·1)	33·7 (21–48·5)	33·5 (20·8–48·4)	33·8 (21·1–48·4)
Parkinson's disease	-6·64 (-9·63–-3·77)	-8·32 (-11·9–-4·87)	-8·89 (-12·9–-5)	-8·2 (-11·9–-4·68)
Idiopathic epilepsy	15·6 (10·5–20·7)	12·5 (8·5–16·9)	10·8 (7·42–14·9)	14·2 (9·59–19·2)
Multiple sclerosis	8·99 (5·21–14·1)	10·1 (6·81–14)	9·87 (6·75–13·1)	10·4 (6·28–15·8)
Mental disorders	--	3·65 (1·8–5·83)	3·65 (1·8–5·83)	--
Depressive disorders	--	6·21 (2·01–11·5)	6·21 (2·01–11·5)	--
Anxiety disorders	--	2·96 (0·746–6·94)	2·96 (0·746–6·94)	--
Idiopathic developmental intellectual disability	--	48·6 (23·4–69)	48·6 (23·4–69)	--
Substance use disorders	100 (100–100)	100 (100–100)	100 (100–100)	100 (100–100)
Alcohol use disorders	100 (100–100)	100 (100–100)	100 (100–100)	100 (100–100)
Drug use disorders	100 (100–100)	100 (100–100)	100 (100–100)	100 (100–100)
Diabetes and kidney diseases	99·8 (99·8–99·9)	99·8 (99·8–99·9)	100 (100–100)	99·8 (99·7–99·8)
Diabetes mellitus	100 (100–100)	100 (100–100)	100 (100–100)	100 (100–100)
Chronic kidney disease	100 (100–100)	100 (100–100)	100 (100–100)	100 (100–100)
Sense organ diseases	--	12·1 (10·6–13·8)	12·1 (10·6–13·8)	--
Blindness and vision loss	--	8·69 (5·77–12·5)	8·69 (5·77–12·5)	--
Age-related and other hearing loss	--	14·9 (13·8–16·3)	14·9 (13·8–16·3)	--
Musculoskeletal disorders	3·35 (0·944–7·05)	29·4 (25·3–33·6)	29·5 (25·4–33·8)	3·27 (0·973–6·45)
Rheumatoid arthritis	7·38 (1·81–16·6)	8·53 (2·62–15)	8·55 (2·63–14·8)	8·71 (2·32–18)
Osteoarthritis	--	19·5 (11–28·6)	19·5 (11–28·6)	--
Low back pain	--	42·2 (37·7–46·7)	42·2 (37·7–46·7)	--
Gout	--	53 (36·7–70)	53 (36·7–70)	--
Other non-communicable diseases	0·0262 (0·0145–0·0547)	0·0176 (0·0096–0·034)	--	0·034 (0·0176–0·0755)
Sudden infant death syndrome	7·57 (4·39–12)	7·56 (4·39–12)	--	7·56 (4·39–12)
Injuries	14·6 (11·3–18·1)	14·9 (12–18)	19·9 (17·1–23·1)	12·7 (9·39–16·1)
Transport injuries	24·2 (19·3–29·3)	23·1 (18·4–28)	25·7 (21·7–29·9)	22·1 (17·1–27·3)
Road injuries	24·1 (19·1–29·2)	23 (18·2–27·9)	25·5 (21·4–29·7)	22·1 (17·1–27·3)
Other transport injuries	26·9 (22·9–31·6)	25·4 (21·6–29·6)	28 (24·5–32)	22·5 (18·4–27·2)

Unintentional injuries	10·3 (7·25–13·9)	12·5 (9·24–16·2)	19·3 (16·2–22·8)	7·29 (4·37–10·7)
Falls	29·3 (25–33·6)	25 (21·2–29)	26·4 (22·8–30·4)	21·7 (17·8–26)
Drowning	-6·52 (-11·6–-1·21)	-9·04 (-13·6–-4·17)	9·16 (6·81–12·2)	-9·42 (-14·1–-4·47)
Fire, heat, and hot substances	12·8 (9·58–16·5)	12·5 (9·32–16·1)	13·7 (10·4–17·7)	11·7 (8·61–15·1)
Poisonings	15·5 (11·4–20)	13·7 (10·2–17·6)	12 (9–15·6)	14·3 (10·5–18·5)
Exposure to mechanical forces	9·75 (5·19–14·6)	15·7 (11·9–20·1)	19·4 (16·2–23·5)	6·81 (2·34–11·5)
Animal contact	15·4 (12·2–19·2)	14·7 (11·5–18·3)	17·8 (14–21·8)	11·4 (8·81–14·8)
Foreign body	9·84 (7·37–13)	7·97 (5·98–10·6)	6·68 (4·64–9·24)	8·21 (6·11–11)
Environmental heat and cold exposure	7·18 (3·33–12·4)	7·02 (3·25–12·2)	6 (2·77–10·5)	7·19 (3·33–12·5)
Exposure to forces of nature	0 (0–0)	5·9 (2·67–10·3)	5·9 (2·67–10·3)	0 (0–0)
Other unintentional injuries	16·7 (12·6–21·4)	14·4 (10·9–18·3)	14 (10·4–18)	14·9 (11·3–19·2)
Self-harm and interpersonal violence	15·2 (8·74–21·7)	14·9 (8·85–21·1)	15·9 (11·8–19·9)	14·8 (8·48–21·4)
Self-harm	14·6 (5·38–23·6)	14·5 (5·55–23·3)	13·1 (6·48–19·9)	14·5 (5·48–23·4)
Interpersonal violence	16·6 (10·1–23·1)	16·4 (10·1–22·9)	21·3 (15·7–27·3)	15·6 (8·99–22·2)

Supplementary Results Table 2. All-age PAFs attributable to the joint distribution of all risk factors for all ages, both sexes combined for each GBD level 1, level 2, and level 3 cause and all causes in 2019 for Malta. Results are shown for deaths, YLLs, YLDs, and DALYs Data in parenthesis are 95% uncertainty intervals. PAFs=population attributable fractions. GBD=Global Burden of Disease. YLLs=years of life lost. YLDs=years lived with disability. DALYs=disability-adjusted life-years

	Deaths 2019	DALYs 2019	YLDs 2019	YLLs 2019
All causes	60·2 (56·6–63)	43 (39·7–46·3)	26·9 (24·7–29·3)	58·7 (56·2–61)
Communicable, maternal, neonatal, and nutritional diseases	32·8 (24·4–40·9)	48·6 (43·7–53·5)	48·1 (43·5–52·5)	48·9 (41·7–55·6)
HIV/AIDS and sexually transmitted infections	86·5 (84·2–88·3)	87·3 (84·7–90·1)	91·8 (88·8–95)	83·5 (80·6–85·6)
HIV/AIDS	85·6 (83·1–87·5)	84·3 (81·5–86·4)	86·8 (84·5–88·8)	83 (80–85·1)
Sexually transmitted infections excluding HIV	99·9 (99·8–99·9)	100 (99·9–100)	100 (100–100)	99·6 (99·3–99·7)
Respiratory infections and tuberculosis	30·4 (21–39·3)	30·4 (22·8–37·8)	3·92 (2·85–5·26)	35·7 (26·6–44·2)
Tuberculosis	53·4 (44·4–61·6)	56·5 (48·4–64)	54·4 (44·5–62·2)	57·7 (49·4–65·2)
Lower respiratory infections	30·3 (20·9–39·3)	35·6 (26·5–44·2)	35·9 (30·1–41·1)	35·6 (26·4–44·2)
Upper respiratory infections	0·269 (0·14–0·437)	0·0252 (0·0117–0·0483)	0·00578 (0·000369–0·0154)	0·868 (0·475–1·38)
Otitis media	4·96 (2·24–9·36)	3·16 (1·98–4·58)	3·15 (1·97–4·58)	11·1 (6·62–17)
Enteric infections	8·85 (5·97–13)	12·1 (9·04–16·4)	11·3 (8·23–15·6)	16·1 (11·3–22)
Diarrheal diseases	9·16 (6·24–13·4)	12·2 (9·1–16·4)	11·4 (8·24–15·6)	16·9 (11·8–23)
Other infectious diseases	0·816 (0·567–1·12)	1·64 (1·13–2·23)	0·353 (0·214–0·604)	2·22 (1·57–3·05)
Meningitis	2·11 (1·36–3·01)	3·99 (2·62–5·71)	0·000552 (2·72e-05–0·00142)	5·32 (3·64–7·24)
Encephalitis	0·401 (0·239–0·621)	1·02 (0·6–1·56)	0·000226 (1·09e-05–0·000587)	1·19 (0·708–1·8)
Measles	3·24 (-0·555–15·3)	2·61 (-0·142–11·1)	0·817 (0·0258–2·01)	4·16 (-0·727–19·4)
Acute hepatitis	6·27 (3·74–10·6)	3·58 (2·35–5·3)	2·24 (1·36–3·74)	7·03 (4·11–11·7)
Maternal and neonatal disorders	87·2 (85–89·3)	80·6 (77·2–83·7)	70 (66·1–73·6)	88·4 (86·4–90·3)
Maternal disorders	9·18 (3·36–15·1)	9·15 (3·34–15·1)	9·22 (3·39–15·3)	9·09 (3·33–15)
Neonatal disorders	90·6 (88·8–92·2)	82·7 (79·3–85·5)	71·8 (67·7–75·5)	90·6 (88·8–92·2)
Nutritional deficiencies	62 (41·9–68·9)	75·1 (72·1–78·4)	75·6 (72·5–79)	58·8 (44·1–64·9)
Protein-energy malnutrition	100 (100–100)	100 (100–100)	100 (100–100)	100 (100–100)
Vitamin A deficiency	--	100 (100–100)	100 (100–100)	--
Dietary iron deficiency	--	100 (100–100)	100 (100–100)	--
Non-communicable diseases	63 (58·8–66·3)	43·6 (39·9–47·2)	25·2 (22·8–27·8)	61·5 (58·7–64·1)
Neoplasms	44·3 (40·5–49·2)	44 (40·6–48·3)	32·5 (28·5–37·3)	44·7 (41·2–49)
Lip and oral cavity cancer	62·4 (55·7–68·1)	66·4 (60–72)	65·6 (58·8–71·3)	66·4 (60·1–72)
Nasopharynx cancer	63·9 (54·7–71·6)	64·6 (55·7–72·2)	61·8 (52·6–69·7)	64·8 (55·9–72·4)
Other pharynx cancer	77·1 (71·5–81·9)	78·6 (73·2–83·1)	77·2 (71·5–81·9)	78·7 (73·2–83·1)
Esophageal cancer	69·4 (60·7–78·1)	72·2 (64–80·3)	70 (61·2–78·6)	72·2 (64–80·3)
Stomach cancer	23 (15–41·5)	24·8 (16·5–43·3)	24·5 (16·2–43·1)	24·8 (16·5–43·3)

Colon and rectum cancer	64 (55·6–72·1)	64·6 (56·5–72·3)	64·4 (56·2–72·1)	64·6 (56·5–72·3)
Liver cancer	74·3 (67·4–80·7)	75·6 (69–81·5)	74·7 (68·3–80·8)	75·6 (69–81·5)
Gallbladder and biliary tract cancer	17·2 (8·85–28)	17·6 (9·23–28·6)	17 (8·58–27·7)	17·6 (9·24–28·7)
Pancreatic cancer	36·1 (27·2–47·2)	36·9 (28·6–47·1)	36·5 (27·6–47·1)	36·9 (28·6–47·1)
Larynx cancer	81·1 (72·8–87·2)	83·1 (76–88·3)	83·1 (76·1–88·6)	83·1 (76–88·3)
Tracheal, bronchus, and lung cancer	85·9 (82·9–88·7)	85·3 (82·5–88)	85·4 (82·4–88·3)	85·3 (82·5–88)
Breast cancer	32·6 (23·7–43·6)	31·7 (23·5–41·3)	31·7 (23·5–41·7)	31·6 (23·5–41·3)
Cervical cancer	100 (100–100)	100 (100–100)	100 (100–100)	100 (100–100)
Uterine cancer	39·8 (25·7–55)	41 (26·8–56·3)	41·4 (27·1–56·5)	40·9 (26·8–56·2)
Ovarian cancer	18·3 (7·76–32·5)	16·2 (6·7–29)	15·4 (6·37–27·7)	16·2 (6·72–29)
Prostate cancer	5·65 (2·42–9·03)	6·54 (2·82–10·4)	6·99 (3·09–11·1)	6·45 (2·78–10·3)
Kidney cancer	32·8 (23·7–42·6)	33·9 (24·8–43·3)	33·9 (24·9–43·3)	33·9 (24·8–43·3)
Bladder cancer	35·3 (22·2–48·9)	39·8 (26·8–52·7)	41·2 (28–54·2)	39·6 (26·6–52·5)
Thyroid cancer	11·8 (5·68–19·9)	12·3 (5·84–20·8)	12·3 (6·01–20·4)	12·3 (5·8–20·8)
Mesothelioma	97·8 (97·1–98·4)	96·1 (94·9–97)	97·5 (96·6–98·3)	96 (94·8–97)
Non-Hodgkin lymphoma	5·7 (2·28–10·4)	5·78 (2·31–10·6)	5·74 (2·29–10·6)	5·78 (2·31–10·6)
Multiple myeloma	6·61 (2·75–12·1)	6·86 (2·87–12·5)	6·84 (2·9–12·5)	6·86 (2·87–12·5)
Leukemia	29·7 (17·8–42·4)	28·7 (18·5–39·6)	28·9 (18·3–40·2)	28·7 (18·5–39·6)
Cardiovascular diseases	85·4 (82·1–88·4)	86 (83·3–88·3)	78·3 (74·4–81·9)	86·9 (84·3–89·2)
Rheumatic heart disease	27·6 (16·7–48·9)	29·5 (19·3–47·3)	29·7 (19·2–47)	29·4 (19·2–47·2)
Ischemic heart disease	94·6 (91–97·4)	95·4 (92·9–97·5)	95 (92·3–97·3)	95·4 (92·9–97·5)
Stroke	82·1 (74·2–90·6)	84·8 (79–90·8)	85·8 (81·2–90·8)	84·7 (78·6–90·8)
Hypertensive heart disease	100 (100–100)	100 (100–100)	100 (100–100)	100 (100–100)
Non-rheumatic valvular heart disease	29·8 (20·2–42·2)	32 (23·1–41·8)	33·6 (23·2–45·1)	32 (23–41·7)
Cardiomyopathy and myocarditis	29·7 (22·4–38·2)	32·4 (26·3–38·6)	34 (26·7–41·5)	32·2 (26–38·4)
Atrial fibrillation and flutter	51 (41·6–60·7)	59·8 (52–67·7)	63·8 (56·5–70·8)	54·7 (46–63·4)
Aortic aneurysm	60 (53·6–66·4)	67·3 (62–72·5)	--	67·3 (62–72·5)
Endocarditis	39·3 (27·9–53)	42·8 (32·4–54·1)	39·6 (28·2–52·9)	42·9 (32·6–54·2)
Other cardiovascular and circulatory diseases	36·7 (28·8–44)	40·9 (33·9–46·8)	45 (38–51·5)	39·3 (30·9–45·5)
Chronic respiratory diseases	55·5 (49·5–62·1)	51·8 (47·1–56·8)	47·9 (42·6–53·4)	56·3 (50·6–62·8)
Chronic obstructive pulmonary disease	68·9 (63·1–74)	67·6 (63·2–71·4)	60·7 (56·4–64·6)	72·4 (67·2–76·9)
Pneumoconiosis	100 (100–100)	100 (100–100)	100 (100–100)	100 (100–100)
Asthma	36·8 (26·1–49)	40·2 (31·3–50·6)	40·2 (31·4–50·5)	40·2 (30·2–51·4)
Digestive diseases	30·8 (26·5–35·5)	31·5 (26·5–36·1)	7·28 (4·8–10·4)	41 (36–46)
Cirrhosis and other chronic liver diseases	73·2 (62·9–83·4)	75·9 (66·4–84·6)	70·1 (62–78·2)	76 (66·5–84·8)

Upper digestive system diseases	16·5 (13·6–20)	5·93 (3·72–8·59)	0·5 (0·295–0·76)	20·5 (17·1–24·3)
Gallbladder and biliary diseases	37·7 (24·6–52)	40·9 (28·2–55·2)	43·7 (30·9–57·7)	39·1 (26·3–53·4)
Pancreatitis	34 (23·7–46·2)	38 (27·2–50)	35·1 (24·4–46·5)	38·3 (27·4–50·2)
Neurological disorders	21 (8·07–35·3)	13·3 (6·07–24·4)	9·32 (4·44–18·8)	18·5 (6·65–33)
Alzheimer's disease and other dementias	33·5 (20·2–49·7)	36·8 (23·8–52)	37·4 (24·4–52·3)	36·4 (23·5–51·8)
Parkinson's disease	-8·15 (-12·4–-4·21)	-10 (-15–-5·45)	-10·7 (-15·9–-5·84)	-9·82 (-14·8–-5·27)
Idiopathic epilepsy	22·1 (15·9–28·7)	21 (15·2–27)	19·9 (14·2–26·1)	23·1 (16·7–29·4)
Multiple sclerosis	14·5 (10·5–18·9)	16·6 (12·2–21·1)	16·1 (12–20·4)	16·8 (12·2–21·6)
Mental disorders	--	3·88 (2·15–6·07)	3·89 (2·15–6·07)	--
Depressive disorders	--	8·31 (3·99–13·3)	8·31 (3·99–13·3)	--
Anxiety disorders	--	3·18 (0·824–7·27)	3·18 (0·824–7·27)	--
Idiopathic developmental intellectual disability	--	59·5 (29·6–79·8)	59·5 (29·6–79·8)	--
Substance use disorders	100 (100–100)	100 (100–100)	100 (100–100)	100 (100–100)
Alcohol use disorders	100 (100–100)	100 (100–100)	100 (100–100)	100 (100–100)
Drug use disorders	100 (100–100)	100 (100–100)	100 (100–100)	100 (100–100)
Diabetes and kidney diseases	100 (100–100)	100 (100–100)	100 (100–100)	100 (100–100)
Diabetes mellitus	100 (100–100)	100 (100–100)	100 (100–100)	100 (100–100)
Chronic kidney disease	100 (100–100)	100 (100–100)	100 (100–100)	100 (100–100)
Sense organ diseases	--	6·86 (5·85–8·22)	6·86 (5·85–8·22)	--
Blindness and vision loss	--	8·6 (5·27–13)	8·6 (5·27–13)	--
Age-related and other hearing loss	--	7·34 (6·59–8·2)	7·34 (6·59–8·2)	--
Musculoskeletal disorders	2·21 (0·65–4·14)	21·6 (17·3–25·9)	21·8 (17·4–26·2)	2·95 (0·882–5·45)
Rheumatoid arthritis	9·03 (2·7–16·4)	14·5 (4·72–25·5)	15·2 (4·95–26·7)	11·3 (3·47–20·2)
Osteoarthritis	--	13·8 (6·96–22·2)	13·8 (6·96–22·2)	--
Low back pain	--	36·2 (30–42·7)	36·2 (30–42·7)	--
Gout	--	46·3 (32·8–62·4)	46·3 (32·8–62·4)	--
Other non-communicable diseases	0·0189 (0·0113–0·0299)	0·0117 (0·00605–0·0192)	--	0·0569 (0·0364–0·0861)
Sudden infant death syndrome	6·02 (3·53–9·18)	6·01 (3·52–9·17)	--	6·01 (3·52–9·17)
Injuries	34·3 (30–37·6)	32·6 (29–36·1)	35·9 (32–39·6)	27·9 (23·9–31·7)
Transport injuries	34·1 (29·3–39)	32·9 (27·8–37·9)	32·5 (28·6–36·4)	33·1 (27·5–38·8)
Road injuries	31·3 (26·3–36·3)	31·3 (26·3–36·3)	31·5 (27·7–35·6)	31·1 (25·4–37·1)
Other transport injuries	39·3 (34·7–43·9)	37·4 (32·4–42·5)	41·7 (37·4–45·4)	36·9 (31·8–42·1)
Unintentional injuries	39·5 (34·1–43·5)	34·8 (30·5–38·8)	36·4 (32·1–40·4)	30·1 (25·5–34·3)
Falls	54·6 (47·4–59·3)	44 (38·8–48·2)	43·2 (38·1–47·5)	47·2 (41·5–51·5)
Drowning	-1·47 (-12·3–9)	0·821 (-9·26–11·1)	13·9 (10·3–18·7)	0·335 (-10·1–10·9)

Fire, heat, and hot substances	13 (9·18–18·1)	19·7 (15·3–25·1)	20·1 (15·7–25·5)	18·4 (14·2–23·7)
Poisonings	19·2 (14·7–25·1)	20·7 (16·2–26·1)	19·5 (15·1–24·5)	23·3 (18·3–29·4)
Exposure to mechanical forces	22·8 (13·2–31·6)	25·1 (21·2–29·1)	26·4 (23·1–30)	20·3 (10·1–29·2)
Animal contact	28·9 (24·5–34·1)	28·7 (24·3–33·7)	28·8 (24·4–33·5)	28·5 (24–33·9)
Foreign body	3·68 (2·87–4·81)	8·15 (6·28–10·6)	9·95 (7·89–12·6)	7·21 (5·45–9·34)
Environmental heat and cold exposure	6·67 (3·05–11·7)	7·73 (3·59–13·4)	7·6 (3·54–13·4)	7·92 (3·73–13·5)
Exposure to forces of nature	0 (0–0)	8·75 (4·04–15)	8·75 (4·04–15)	0 (0–0)
Other unintentional injuries	20·7 (15·8–26·9)	19·8 (15·4–25·1)	18·8 (14·7–24·1)	24·6 (19·2–31)
Self-harm and interpersonal violence	20 (10·7–28·6)	22·8 (15·1–30·3)	32·1 (25·7–38·6)	20·7 (11·5–29·2)
Self-harm	19·2 (7·78–30·1)	20·4 (8·43–31·4)	18·9 (9·56–28·4)	20·4 (8·39–31·5)
Interpersonal violence	28·1 (20·5–35·6)	30·5 (23–37·9)	34·7 (27·1–42·1)	26·4 (18·7–33·9)

Supplementary Results Table 2. All-age PAFs attributable to the joint distribution of all risk factors for all ages, both sexes combined for each GBD level 1, level 2, and level 3 cause and all causes in 2019 for Monaco. Results are shown for deaths, YLLs, YLDs, and DALYs Data in parenthesis are 95% uncertainty intervals. PAFs=population attributable fractions. GBD=Global Burden of Disease. YLLs=years of life lost. YLDs=years lived with disability. DALYs=disability-adjusted life-years

	Deaths 2019	DALYs 2019	YLDs 2019	YLLs 2019
All causes	54·9 (51·2–58·4)	42·7 (39–46·1)	26·2 (23·9–28·6)	54 (50·5–57·1)
Communicable, maternal, neonatal, and nutritional diseases	39·5 (32·5–46·5)	45·3 (40·1–51·9)	42·7 (37·8–47·9)	46·6 (39·1–54·7)
HIV/AIDS and sexually transmitted infections	90·3 (84·8–93·4)	87·7 (79·8–91·7)	94·8 (91·2–97·9)	85·9 (76·2–91·2)
HIV/AIDS	92·1 (87·5–95·2)	90·5 (84·5–93·5)	91·2 (87·1–94)	90·4 (83·7–94·2)
Sexually transmitted infections excluding HIV	46·1 (24·4–71·7)	67·3 (40·7–89)	100 (100–100)	19·4 (7·9–40·5)
Respiratory infections and tuberculosis	38·1 (31–44·9)	37·8 (30·8–44·9)	4·89 (3·27–7·3)	42·1 (34·7–49·1)
Tuberculosis	46·5 (26·4–63·1)	51·1 (31·2–67)	49·2 (29·7–65·5)	51·4 (31·4–67·1)
Lower respiratory infections	37·8 (30·9–44·6)	41·6 (34·3–48·3)	34·7 (26·6–42·2)	41·6 (34·3–48·4)
Upper respiratory infections	0·031 (0·00558–0·0764)	0·00535 (0·000332–0·0135)	0·00514 (0·000136–0·0134)	0·11 (0·025–0·255)
Otitis media	0·021 (0·00468–0·0795)	4·11 (2·6–5·88)	4·12 (2·61–5·89)	0·134 (0·0405–0·441)
Enteric infections	5·48 (3·75–8·13)	8·06 (6·17–10·8)	7·77 (5·89–10·5)	10·1 (6·69–14·8)
Diarrheal diseases	5·66 (3·87–8·37)	8·14 (6·22–10·9)	7·78 (5·89–10·5)	10·8 (7·18–16·6)
Other infectious diseases	0·644 (0·43–0·935)	0·975 (0·692–1·31)	0·607 (0·338–1·08)	1·16 (0·804–1·61)
Meningitis	0·365 (0·216–0·569)	1·01 (0·616–1·57)	0·000775 (1·53e-05–0·0022)	1·16 (0·718–1·78)
Encephalitis	0·0983 (0·0467–0·188)	0·282 (0·136–0·537)	0·000219 (4·53e-06–0·000599)	0·341 (0·163–0·66)
Measles	0 (0–0)	0·161 (0–0·661)	0·161 (0–0·661)	0 (0–0)
Acute hepatitis	8·33 (4·99–12·7)	6·01 (3·74–9·09)	3·16 (1·79–5·47)	10·6 (6·25–16·8)
Maternal and neonatal disorders	68·1 (63·3–72·4)	71·4 (68·3–74·4)	73 (69·1–76·9)	68·5 (63·7–72·8)
Maternal disorders	8·64 (3·03–14·1)	8·67 (3·05–14·2)	8·7 (3·07–14·4)	8·54 (2·99–14)
Neonatal disorders	69·1 (64·3–73·6)	72·6 (69·6–75·5)	74·6 (70·6–78·4)	69·1 (64·3–73·6)
Nutritional deficiencies	89·4 (87–91·7)	74·9 (71·6–77·6)	74·4 (71–77·2)	84·9 (82·2–87·5)
Protein-energy malnutrition	100 (100–100)	100 (100–100)	100 (100–100)	100 (100–100)
Vitamin A deficiency	--	100 (100–100)	100 (100–100)	--
Dietary iron deficiency	--	100 (100–100)	100 (100–100)	--
Non-communicable diseases	57·5 (53·5–61·1)	44·1 (40–47·9)	25·1 (22·7–27·7)	57·1 (53·7–60·3)
Neoplasms	46·2 (41·7–50·5)	47 (42·6–50·8)	32·2 (27·8–36·6)	47·8 (43·5–51·7)
Lip and oral cavity cancer	56·6 (36·1–72·6)	60·9 (39·7–76·2)	60·3 (39·5–75·8)	60·9 (39·7–76·2)
Nasopharynx cancer	49·2 (26·2–67·2)	51·3 (27·2–69·5)	49·4 (26–67·8)	51·6 (27·3–69·8)
Other pharynx cancer	71·1 (55·1–82·7)	73·3 (56·5–84·5)	72 (55·2–83·6)	73·4 (56·5–84·6)
Esophageal cancer	63·7 (48–77·2)	67·1 (52·3–79·5)	64·9 (49·6–78·1)	67·2 (52·3–79·5)
Stomach cancer	23·1 (15·4–41·2)	24·6 (16·7–42·4)	24·5 (16·6–42·4)	24·6 (16·7–42·4)

Colon and rectum cancer	60 (50·4–69)	61·2 (51·9–69·9)	61·2 (52–70·1)	61·1 (51·9–69·9)
Liver cancer	72 (64·1–79·1)	74·8 (67·3–81·4)	73·1 (65·2–79·9)	74·8 (67·3–81·4)
Gallbladder and biliary tract cancer	21·7 (11·3–34·2)	22 (11·6–34·8)	21·4 (11–34)	22·1 (11·6–34·8)
Pancreatic cancer	36·5 (28·1–45·9)	37·8 (30–46·5)	37 (28·9–46·3)	37·8 (30–46·5)
Larynx cancer	81·3 (71·4–88·8)	82 (72·5–89·1)	82·4 (73–89·4)	82 (72·5–89·1)
Tracheal, bronchus, and lung cancer	82 (77·8–85·7)	81·6 (77·4–85·3)	81·8 (77·7–85·4)	81·6 (77·4–85·3)
Breast cancer	32·2 (22·1–41·9)	31·7 (21·9–40·8)	31·7 (21·8–40·9)	31·7 (21·9–40·8)
Cervical cancer	100 (100–100)	100 (100–100)	100 (100–100)	100 (100–100)
Uterine cancer	46·9 (30·9–62·7)	48 (32·2–63·9)	48·5 (32·1–64·3)	47·9 (32·2–63·9)
Ovarian cancer	14·5 (5·67–25·7)	12·9 (4·9–23·1)	12·4 (4·67–22·3)	12·9 (4·91–23·1)
Prostate cancer	5·94 (2·62–9·49)	6·7 (3·03–10·7)	7·27 (3·39–11·5)	6·6 (2·97–10·5)
Kidney cancer	38·5 (28·1–48·6)	39·6 (29·1–50·2)	39·7 (29·1–50·4)	39·6 (29·1–50·2)
Bladder cancer	37 (24·7–49·3)	40·9 (28·9–53·1)	42·6 (30·4–54·3)	40·7 (28·7–52·9)
Thyroid cancer	17·2 (7·18–30)	18·2 (7·48–31·6)	18·4 (7·75–31·7)	18·2 (7·43–31·5)
Mesothelioma	98·1 (97·4–98·7)	96·8 (95·5–97·8)	97·8 (96·8–98·6)	96·7 (95·4–97·7)
Non-Hodgkin lymphoma	7·8 (3·17–13·9)	8·03 (3·37–14·2)	7·92 (3·29–14)	8·04 (3·38–14·2)
Multiple myeloma	8·81 (3·69–16)	9·15 (3·87–16·4)	9·14 (3·87–16·3)	9·15 (3·85–16·4)
Leukemia	34·4 (22·2–46·9)	34·2 (23·1–45·7)	34 (23–45·6)	34·2 (23·1–45·7)
Cardiovascular diseases	80·4 (76·3–84·2)	81·7 (78·2–84·9)	75·9 (71·1–80·5)	82·5 (79·1–85·6)
Rheumatic heart disease	27·6 (15·9–50·7)	29·2 (18·5–46·7)	29·3 (18·5–47·1)	29·1 (18·6–46·8)
Ischemic heart disease	93·1 (89–96·5)	94·3 (91·2–97)	93·9 (91–96·6)	94·4 (91·2–97)
Stroke	79·3 (70·4–88·1)	82·6 (75·9–88·7)	83·1 (77·1–89·1)	82·5 (75·4–88·9)
Hypertensive heart disease	100 (100–100)	100 (100–100)	100 (100–100)	100 (100–100)
Non-rheumatic valvular heart disease	29 (19·5–42)	31·5 (23·1–42·4)	34·8 (25·3–47·2)	30·9 (22·4–41·6)
Cardiomyopathy and myocarditis	36 (26·5–49·1)	40·6 (32·8–50)	40·1 (32–49·4)	40·6 (32·6–50·1)
Atrial fibrillation and flutter	53·4 (41·6–65·1)	60·7 (50·8–69·9)	65·1 (55·6–73·6)	56·5 (45·9–67·3)
Aortic aneurysm	53·7 (46·5–61·5)	61·9 (55·7–68·1)	--	61·9 (55·7–68·1)
Endocarditis	37·9 (27·4–52)	41·5 (31·9–52·8)	38·7 (28·3–51·9)	41·6 (32–52·8)
Other cardiovascular and circulatory diseases	37·5 (29·3–45·9)	41·5 (34·1–48·3)	44·2 (37·3–51·6)	40·8 (33·3–47·8)
Chronic respiratory diseases	61 (56·3–65·4)	58·3 (54·4–62)	54·6 (49·9–59·2)	62 (58–66·1)
Chronic obstructive pulmonary disease	69·2 (63·8–73·9)	67·8 (63·7–72)	61·9 (57·2–66·1)	72·6 (67·9–76·7)
Pneumoconiosis	100 (100–100)	100 (100–100)	100 (100–100)	100 (100–100)
Asthma	45 (32·5–58·7)	46·5 (36·9–56·9)	46·3 (36·9–56·4)	48·8 (36·7–61·3)
Digestive diseases	32·7 (24·9–42)	37·2 (29·3–46·2)	14·2 (9·38–19·7)	44 (35·1–54·5)
Cirrhosis and other chronic liver diseases	65·3 (50·1–83·6)	70·3 (56·4–85·9)	67·8 (53·6–83·5)	70·4 (56·3–85·9)

Upper digestive system diseases	16·6 (13·2–20·4)	5·5 (3·33–8·22)	0·731 (0·412–1·2)	21·3 (17·5–25·3)
Gallbladder and biliary diseases	46·1 (30·6–61·9)	50·1 (35–64·8)	52·9 (38·3–67·2)	47·1 (31·4–62·7)
Pancreatitis	19·6 (-1·75–47·8)	23·2 (-1·81–52·5)	24·6 (-1·92–53·9)	23·1 (-1·79–52·4)
Neurological disorders	23·2 (9·3–37·4)	15·7 (7·69–27·1)	11·3 (5·69–20·5)	20·8 (7·34–35·3)
Alzheimer's disease and other dementias	35·3 (22·3–50)	38 (24·5–52·3)	38·7 (25·1–52·8)	37·6 (24·3–52·2)
Parkinson's disease	-8·56 (-13·6–-4·07)	-10·2 (-15·7–-4·97)	-10·8 (-16·3–-5·28)	-10·1 (-15·7–-4·85)
Idiopathic epilepsy	20·3 (0·692–36·8)	18·1 (0·559–33·4)	17·2 (0·503–31·6)	20·6 (0·736–37)
Multiple sclerosis	13·2 (9·62–17·3)	15·9 (11·8–20·5)	16·1 (12–20·5)	15·6 (11·6–20·4)
Mental disorders	--	4·85 (2·38–7·89)	4·85 (2·38–7·89)	--
Depressive disorders	--	9·98 (4·37–16·1)	9·98 (4·37–16·1)	--
Anxiety disorders	--	4·16 (1·14–9·07)	4·16 (1·14–9·07)	--
Idiopathic developmental intellectual disability	--	45·3 (19·6–71·9)	45·3 (19·6–71·9)	--
Substance use disorders	100 (100–100)	100 (100–100)	100 (100–100)	100 (100–100)
Alcohol use disorders	100 (100–100)	100 (100–100)	100 (100–100)	100 (100–100)
Drug use disorders	100 (100–100)	100 (100–100)	100 (100–100)	100 (100–100)
Diabetes and kidney diseases	100 (100–100)	100 (100–100)	100 (100–100)	100 (100–100)
Diabetes mellitus	100 (100–100)	100 (100–100)	100 (100–100)	100 (100–100)
Chronic kidney disease	100 (100–100)	100 (100–100)	100 (100–100)	100 (100–100)
Sense organ diseases	--	6·68 (5·7–7·87)	6·68 (5·7–7·87)	--
Blindness and vision loss	--	8·94 (5·99–12·7)	8·94 (5·99–12·7)	--
Age-related and other hearing loss	--	6·93 (6·16–7·8)	6·93 (6·16–7·8)	--
Musculoskeletal disorders	1·97 (0·551–3·64)	22·5 (18·1–27)	22·6 (18·2–27·1)	2·63 (0·728–4·74)
Rheumatoid arthritis	10·9 (3·09–20·1)	16 (4·52–28·1)	16·3 (4·57–28·3)	13·5 (3·89–24·3)
Osteoarthritis	--	17·5 (9·16–26·6)	17·5 (9·16–26·6)	--
Low back pain	--	39·4 (33–46)	39·4 (33–46)	--
Gout	--	53·8 (38–70·4)	53·8 (38–70·4)	--
Other non-communicable diseases	0·0886 (0·0348–0·164)	0·0285 (0·0101–0·0587)	--	0·312 (0·128–0·563)
Sudden infant death syndrome	4·06 (2·01–7·36)	4·05 (2·01–7·35)	--	4·05 (2·01–7·35)
Injuries	18·8 (11·5–25·9)	23·3 (15·9–29·9)	32·2 (26·9–37·1)	15·3 (5·61–24·1)
Transport injuries	24·4 (16·8–32·1)	23·9 (16·2–31·6)	26·9 (21·6–32·5)	22 (12·4–31·1)
Road injuries	23·1 (15·2–31·1)	22·9 (15·3–30·9)	26 (20·6–31·6)	21 (11·3–30·4)
Other transport injuries	33·2 (25·9–40·4)	31·8 (24–39·6)	37·8 (32–43)	29·3 (20·5–37·9)
Unintentional injuries	20·1 (15·7–24·2)	25·7 (20·6–30·8)	33·1 (27·7–38·2)	13·2 (9·5–17·4)
Falls	55·3 (46·4–61·3)	42 (35·5–47·7)	40·8 (34·5–46·6)	47 (39·5–53)
Drowning	-14·1 (-25·2–-1·54)	-12·1 (-23·2–-0·0814)	8·16 (3·01–14·5)	-12·8 (-23·9–-0·558)

Fire, heat, and hot substances	8·3 (3·14–14·8)	12·3 (6·33–19·4)	12·6 (6·5–19·8)	11·5 (5·85–18·2)
Poisonings	14·5 (7·29–22·7)	15·5 (8·93–23·5)	11·9 (6·28–18·7)	16·8 (9·75–25·3)
Exposure to mechanical forces	17·8 (12·1–23·1)	21·1 (17·8–24·3)	22·3 (19·3–25·1)	9·39 (4·05–14·6)
Animal contact	20·4 (14·4–27·4)	23·1 (16·7–29·9)	25·6 (19·7–32·1)	19·9 (13·7–27·1)
Foreign body	1·59 (1·02–2·28)	3·95 (2·75–5·48)	4·64 (3·21–6·44)	3·72 (2·51–5·22)
Environmental heat and cold exposure	5·99 (0·23–13)	6·48 (0·25–13·6)	6·38 (0·24–13·3)	6·81 (0·284–14·5)
Exposure to forces of nature	0 (0–0)	0 (0–0)	0 (0–0)	0 (0–0)
Other unintentional injuries	14 (7·1–22·1)	12·2 (6·22–19·4)	11·8 (5·95–18·9)	16·3 (9·33–24·6)
Self-harm and interpersonal violence	13·3 (-6·57–32·2)	15·7 (-3·01–34)	30 (18·5–40·9)	14·4 (-5·77–33·8)
Self-harm	12·9 (-7·42–32·3)	14 (-6·56–33·8)	14·8 (0·62–30·6)	14 (-6·74–33·9)
Interpersonal violence	25·2 (12·9–36·9)	30·3 (18·6–41·2)	34 (22·4–44·9)	23·9 (11·4–35·5)

Supplementary Results Table 2. All-age PAFs attributable to the joint distribution of all risk factors for all ages, both sexes combined for each GBD level 1, level 2, and level 3 cause and all causes in 2019 for Netherlands. Results are shown for deaths, YLLs, YLDs, and DALYs Data in parenthesis are 95% uncertainty intervals. PAFs=population attributable fractions. GBD=Global Burden of Disease. YLLs=years of life lost. YLDs=years lived with disability. DALYs=disability-adjusted life-years

	Deaths 2019	DALYs 2019	YLDs 2019	YLLs 2019
All causes	55 (52·1–57·7)	40·6 (37·5–43·6)	25·8 (23·7–28·1)	54 (51·9–56·3)
Communicable, maternal, neonatal, and nutritional diseases	45 (37·8–51·8)	49·9 (46·2–53·5)	44·9 (40·6–49·4)	53·3 (47·8–58·5)
HIV/AIDS and sexually transmitted infections	95·6 (95·1–95·9)	95·8 (95·1–96·7)	98·2 (97·7–98·8)	92·9 (92·3–93·4)
HIV/AIDS	95·2 (94·7–95·6)	94·8 (94–95·5)	97·4 (96·9–97·8)	92·6 (91·9–93·1)
Sexually transmitted infections excluding HIV	99·9 (99·9–100)	100 (99·9–100)	100 (100–100)	99·7 (99·6–99·8)
Respiratory infections and tuberculosis	47·7 (39–55·8)	41 (33·6–48·2)	2·93 (2·16–3·88)	50·5 (41·9–58·4)
Tuberculosis	54·4 (45·3–62·2)	56·7 (47·9–64)	55·1 (46·3–62·6)	57 (48·2–64·3)
Lower respiratory infections	47·6 (38·8–55·9)	50·4 (41·8–58·5)	36·9 (30·5–43·1)	50·5 (41·9–58·6)
Upper respiratory infections	0·435 (0·206–0·728)	0·0259 (0·0113–0·0466)	0·00589 (0·000247–0·0159)	1·17 (0·61–1·93)
Otitis media	0·365 (0·122–0·994)	3·33 (2·14–4·8)	3·36 (2·15–4·84)	1·46 (0·515–3·71)
Enteric infections	5·63 (3·76–8·77)	9·06 (6·93–12·2)	9·39 (7·2–12·7)	8·35 (6·01–11·5)
Diarrheal diseases	5·67 (3·78–8·85)	9·1 (6·97–12·2)	9·39 (7·2–12·7)	8·44 (6·08–11·6)
Other infectious diseases	0·561 (0·418–0·745)	1·49 (1·12–1·92)	0·35 (0·218–0·566)	1·82 (1·36–2·37)
Meningitis	1·52 (1·08–2·11)	3·72 (2·74–4·99)	0·000749 (2·26e-05–0·00209)	4·47 (3·28–5·99)
Encephalitis	0·331 (0·22–0·467)	0·973 (0·629–1·35)	0·000227 (5·34e-06–0·000641)	1·11 (0·704–1·54)
Measles	4·29 (-1·18–23·4)	4·93 (-1·28–25·7)	0·497 (0·0193–1·18)	5·44 (-1·51–28·9)
Acute hepatitis	11·8 (5·83–16·3)	4·32 (2·62–6·21)	2 (1·27–3·19)	11·3 (5·84–15·6)
Maternal and neonatal disorders	80·5 (77·9–83)	74·3 (71·6–76·8)	66·6 (62·4–70·5)	81·1 (78·5–83·5)
Maternal disorders	8·66 (3·17–14·4)	8·66 (3·12–14·4)	8·7 (3·15–14·5)	8·59 (3·12–14·3)
Neonatal disorders	82·1 (79·6–84·6)	75·6 (72·9–78·1)	68 (63·7–72·1)	82·1 (79·6–84·6)
Nutritional deficiencies	59·8 (45·4–69·4)	71·8 (69–74·4)	73·2 (70·4–75·9)	58·5 (46·3–68·4)
Protein-energy malnutrition	100 (100–100)	100 (100–100)	100 (100–100)	100 (100–100)
Vitamin A deficiency	--	100 (100–100)	100 (100–100)	--
Dietary iron deficiency	--	100 (100–100)	100 (100–100)	--
Non-communicable diseases	56·4 (53·3–59·3)	40·8 (37·4–44·2)	24·1 (21·7–26·6)	56 (53·5–58·6)
Neoplasms	47·9 (44·8–51·2)	47·4 (44·6–50·5)	33·1 (29·8–37·1)	48·3 (45·5–51·4)
Lip and oral cavity cancer	71·4 (65·4–76·8)	73·2 (67·3–78·3)	73·1 (67·1–78·3)	73·2 (67·3–78·3)
Nasopharynx cancer	68·2 (59·4–75·6)	67·7 (59–74·9)	67·1 (58·1–74·5)	67·8 (59·1–74·9)
Other pharynx cancer	81·8 (76·4–86)	82·2 (76·7–86·3)	81·6 (75·9–85·9)	82·3 (76·8–86·4)
Esophageal cancer	75·2 (67·4–82·7)	76 (68–83·5)	75·2 (67·1–82·7)	76 (68–83·5)
Stomach cancer	25·7 (17·9–42·7)	25·9 (17·8–43·1)	26·2 (18·2–43·3)	25·9 (17·8–43·1)

Colon and rectum cancer	59·6 (51·5–66·9)	60·3 (52·5–67·2)	60·5 (52·8–67·6)	60·3 (52·5–67·2)
Liver cancer	76·8 (70·5–82·5)	77·3 (71·5–82·5)	76·5 (70·4–82)	77·3 (71·5–82·5)
Gallbladder and biliary tract cancer	18·9 (9·85–30·5)	19·2 (10·3–30·8)	18·7 (9·78–30·3)	19·2 (10·3–30·8)
Pancreatic cancer	39·2 (32·2–47·3)	38·7 (31·9–46·6)	39 (32·1–47)	38·7 (31·9–46·6)
Larynx cancer	84·4 (77·6–89·4)	84 (77·5–88·8)	84·1 (77·7–88·8)	84 (77·5–88·8)
Tracheal, bronchus, and lung cancer	85·4 (83·1–87·7)	83·5 (81·1–86)	84·2 (81·7–86·6)	83·5 (81·1–86)
Breast cancer	34·8 (28·1–42·3)	34·4 (28·1–41·2)	34·5 (28·4–41·5)	34·4 (28·1–41·1)
Cervical cancer	100 (100–100)	100 (100–100)	100 (100–100)	100 (100–100)
Uterine cancer	39·5 (25·1–54·4)	40·8 (26·6–56·2)	41·3 (27·1–56·7)	40·7 (26·5–56)
Ovarian cancer	18·5 (9·48–28·3)	15·7 (8·02–24·7)	15 (7·54–23·7)	15·7 (8·04–24·8)
Prostate cancer	6·53 (2·89–10·2)	6·99 (3·1–10·8)	7·31 (3·27–11·4)	6·93 (3·06–10·7)
Kidney cancer	37 (27·4–47·1)	37·1 (27·8–47)	37 (27·6–46·9)	37·1 (27·8–47·1)
Bladder cancer	40·9 (28·6–52·4)	43·3 (32–54·3)	44·2 (32·7–55·1)	43·2 (31·8–54·2)
Thyroid cancer	12·2 (5·65–20·8)	12·7 (5·94–21·6)	12·7 (6·12–21·2)	12·7 (5·91–21·6)
Mesothelioma	98·9 (98·5–99·3)	98 (97·2–98·6)	98·7 (98·1–99·2)	98 (97·2–98·6)
Non-Hodgkin lymphoma	5·9 (2·31–10·8)	6·07 (2·44–11·1)	5·98 (2·36–11)	6·08 (2·44–11·1)
Multiple myeloma	6·74 (2·8–12·3)	7·01 (2·88–12·8)	6·98 (2·85–12·9)	7·01 (2·89–12·8)
Leukemia	37·6 (23·6–51·8)	35·6 (23·7–47·8)	34·1 (22·8–45·8)	35·7 (23·8–48)
Cardiovascular diseases	76·3 (72·2–80)	79·1 (75·8–82·1)	76·5 (72·3–80·3)	79·5 (76·3–82·4)
Rheumatic heart disease	26 (14·1–52·1)	27·2 (16·8–46·6)	28 (17·5–45·6)	27·1 (16·6–46·8)
Ischemic heart disease	93·1 (89·3–96·3)	94·4 (91·7–96·8)	94·2 (91·5–96·6)	94·4 (91·6–96·8)
Stroke	81 (73·3–88·4)	84·1 (78·4–89·5)	83·8 (78·7–88·8)	84·2 (78·3–89·7)
Hypertensive heart disease	100 (100–100)	100 (100–100)	100 (100–100)	100 (100–100)
Non-rheumatic valvular heart disease	25 (15·6–37·9)	25·9 (17·4–36·6)	16·9 (8·84–24)	26 (17·5–36·8)
Cardiomyopathy and myocarditis	38·1 (29·2–49·2)	45·7 (38·4–54·1)	47·1 (39·7–54·6)	45·6 (37·9–54)
Atrial fibrillation and flutter	54·3 (45·1–63·8)	61·9 (54·3–69·6)	66·2 (59·1–72·9)	57·9 (49·7–66·5)
Aortic aneurysm	57·5 (51·1–64·4)	64 (58·5–69·6)	--	64 (58·5–69·6)
Endocarditis	35·3 (23·2–50)	37·9 (27·5–50·4)	36·2 (25–49·5)	38 (27·5–50·6)
Other cardiovascular and circulatory diseases	37·8 (30·3–45·4)	39·9 (32·9–46)	40 (33·2–46·4)	39·9 (32·1–46·4)
Chronic respiratory diseases	70·3 (65·5–75)	63·8 (60·1–67·5)	56·6 (52–60·9)	70·7 (66·1–75·2)
Chronic obstructive pulmonary disease	75 (70·9–78·7)	72·9 (69·7–76)	67·3 (64·1–70·5)	76·5 (72·8–79·8)
Pneumoconiosis	100 (100–100)	100 (100–100)	100 (100–100)	100 (100–100)
Asthma	40·1 (28·9–52·7)	42·5 (33·8–52·1)	42·5 (33·8–52·1)	43 (32·7–54·2)
Digestive diseases	27·7 (23·7–31·4)	30·5 (26·4–34·3)	13·8 (9·28–19·1)	35·8 (31·2–39·9)

Cirrhosis and other chronic liver diseases	75·6 (64·2–85·9)	79 (68·7–87·5)	72·2 (63·5–79·9)	79·2 (68·7–87·8)
Upper digestive system diseases	20·5 (17·1–23·6)	7·19 (4·58–10·1)	0·94 (0·546–1·47)	23·9 (20·2–27·2)
Gallbladder and biliary diseases	39·2 (25·6–53·7)	43·3 (29·6–57·3)	46·4 (32·9–60·4)	40·2 (26·8–54·3)
Pancreatitis	43·6 (32·1–56·6)	46·8 (35·5–59·5)	44·8 (33·3–57·6)	47 (35·6–59·6)
Neurological disorders	22 (9·95–35)	14·7 (7·53–24·8)	10·4 (5·26–19·9)	19·7 (8·71–32·4)
Alzheimer's disease and other dementias	34·9 (23·4–47·5)	37·5 (25·9–50·2)	37·8 (26·4–50·5)	37·2 (25·7–49·8)
Parkinson's disease	-9·7 (-15·8–-4·28)	-10·9 (-17·5–-4·94)	-11·4 (-18–-5·31)	-10·8 (-17·3–-4·83)
Idiopathic epilepsy	27·2 (19·7–34·4)	24·9 (18·4–31·5)	24 (17·7–30·5)	26·4 (19·4–33·2)
Multiple sclerosis	16·2 (12·3–20·2)	17 (12·9–21)	16·4 (12·6–20·3)	17·4 (13·3–21·6)
Mental disorders	--	5·98 (3·22–9·32)	5·98 (3·22–9·33)	--
Depressive disorders	--	13·1 (6·64–19·8)	13·1 (6·64–19·8)	--
Anxiety disorders	--	5·37 (1·53–11·6)	5·37 (1·53–11·6)	--
Idiopathic developmental intellectual disability	--	47·9 (23·1–69·7)	47·9 (23·1–69·7)	--
Substance use disorders	100 (100–100)	100 (100–100)	100 (100–100)	100 (100–100)
Alcohol use disorders	100 (100–100)	100 (100–100)	100 (100–100)	100 (100–100)
Drug use disorders	100 (100–100)	100 (100–100)	100 (100–100)	100 (100–100)
Diabetes and kidney diseases	100 (100–100)	100 (100–100)	100 (100–100)	100 (100–100)
Diabetes mellitus	100 (100–100)	100 (100–100)	100 (100–100)	100 (100–100)
Chronic kidney disease	100 (100–100)	100 (100–100)	100 (100–100)	100 (100–100)
Sense organ diseases	--	6·5 (5·72–7·4)	6·5 (5·72–7·4)	--
Blindness and vision loss	--	6·41 (4·27–9·17)	6·41 (4·27–9·17)	--
Age-related and other hearing loss	--	7·72 (6·95–8·62)	7·72 (6·95–8·62)	--
Musculoskeletal disorders	3·43 (0·91–6·22)	21·9 (18·1–26)	22·4 (18·3–26·6)	3·78 (1·03–6·69)
Rheumatoid arthritis	11·3 (3–20·4)	14·7 (4·28–26·3)	15 (4·41–27·2)	13·2 (3·7–23·4)
Osteoarthritis	--	15·7 (7·95–24·7)	15·7 (7·95–24·7)	--
Low back pain	--	40·5 (35·2–45·7)	40·5 (35·2–45·7)	--
Gout	--	45·7 (31·5–62·6)	45·7 (31·5–62·6)	--
Other non-communicable diseases	0·0243 (0·0157–0·038)	0·0216 (0·0132–0·0338)	--	0·107 (0·0704–0·154)
Sudden infant death syndrome	7·21 (4·65–10·9)	7·2 (4·65–10·9)	--	7·2 (4·65–10·9)
Injuries	40 (34·2–44·7)	33 (28·5–37·1)	36·8 (32·1–41)	28·9 (22·9–34·6)
Transport injuries	25·5 (19·6–31·7)	26·9 (21·1–32·4)	30·1 (25·9–34·1)	25·1 (18·3–31·9)
Road injuries	24·8 (18·8–31·2)	26·2 (20·4–31·9)	29·2 (25·1–33·2)	24·4 (17·6–31·4)
Other transport injuries	38·5 (33·6–43·2)	37·7 (32·8–42·4)	40·1 (35·4–44·2)	35·9 (30·1–41·3)
Unintentional injuries	51·3 (43·9–56·3)	38·9 (33·3–43·4)	37·8 (32·6–42·5)	41·6 (35·6–46·1)

Falls	58·3 (50–63·7)	48·9 (42·5–53·7)	46·9 (40·4–51·8)	53·2 (46–58)
Drowning	-9·48 (-21·1–2·07)	-8·23 (-18·8–1·88)	13·7 (8·94–19·7)	-9·73 (-20·9–0·861)
Fire, heat, and hot substances	10·8 (6·33–16·7)	17·1 (12–23·3)	17·9 (12·6–24·1)	14·5 (9·98–19·9)
Poisonings	17·7 (12·6–24)	17·6 (12·8–23·7)	16·8 (11·9–22·5)	20·4 (15–26·8)
Exposure to mechanical forces	17·8 (8·96–27·4)	23·1 (19·3–27·1)	24·4 (21–28·3)	10 (0·542–20·4)
Animal contact	26·5 (21·3–32·8)	25·8 (20·8–31·1)	26·1 (21–31·4)	25 (20·1–31·2)
Foreign body	2·68 (1·86–3·7)	6·38 (4·43–8·78)	7·76 (5·54–10·6)	5·2 (3·58–7·25)
Environmental heat and cold exposure	8·88 (4·05–15·3)	9·56 (4·37–16·3)	9·67 (4·4–16·5)	9·09 (4·13–15·7)
Exposure to forces of nature	0 (0–0)	10·2 (4·61–17·4)	10·2 (4·61–17·4)	0 (0–0)
Other unintentional injuries	16·9 (11·7–23·3)	17·8 (12·6–24·1)	17·7 (12·5–24)	21·6 (16–28·6)
Self-harm and interpersonal violence	20·9 (9·14–31·9)	21·7 (10·7–31·7)	31·7 (24·8–38)	20·6 (9·07–31·3)
Self-harm	20·4 (7·86–32·4)	20·2 (7·83–32·1)	22·4 (11·4–33·1)	20·1 (7·7–32)
Interpersonal violence	28·7 (20·5–36·9)	31·4 (23·6–39·3)	37·2 (29·1–45·3)	26·3 (17·9–34·6)

Supplementary Results Table 2. All-age PAFs attributable to the joint distribution of all risk factors for all ages, both sexes combined for each GBD level 1, level 2, and level 3 cause and all causes in 2019 for Norway. Results are shown for deaths, YLLs, YLDs, and DALYs Data in parenthesis are 95% uncertainty intervals. PAFs=population attributable fractions. GBD=Global Burden of Disease. YLLs=years of life lost. YLDs=years lived with disability. DALYs=disability-adjusted life-years

	Deaths 2019	DALYs 2019	YLDs 2019	YLLs 2019
All causes	51·1 (47·5–54·5)	36·7 (33·8–39·7)	24·9 (22·8–27·1)	49·9 (47·4–52·5)
Communicable, maternal, neonatal, and nutritional diseases	32 (23·4–42·9)	40·6 (36·4–45·6)	42·4 (38·4–46·5)	39·2 (32–47·8)
HIV/AIDS and sexually transmitted infections	94·2 (92·7–95·1)	93·4 (91·6–94·9)	96·8 (95–98·1)	90·1 (88–91·4)
HIV/AIDS	91·1 (89·1–92·4)	89·6 (87–91·3)	92·2 (89·2–93·9)	88·4 (86·1–90)
Sexually transmitted infections excluding HIV	99·9 (99·8–99·9)	99·9 (99·8–99·9)	100 (100–100)	99·4 (99·1–99·6)
Respiratory infections and tuberculosis	33·9 (22·5–47·9)	29·3 (20·6–40)	2·55 (1·95–3·3)	37·1 (25·8–50·4)
Tuberculosis	43·6 (33·5–52·8)	47·4 (37·9–55·7)	47·7 (38·8–55·6)	47·4 (37·7–55·7)
Lower respiratory infections	33·8 (22·1–47·9)	36·7 (25·2–50)	26·6 (21·2–31·3)	36·9 (25·2–50·4)
Upper respiratory infections	0·0892 (0·0543–0·121)	0·00732 (0·00457–0·0107)	0·00462 (0·00238–0·00737)	0·345 (0·221–0·458)
Otitis media	0·537 (0·36–0·73)	4·06 (2·6–5·6)	4·07 (2·6–5·6)	2·03 (1·4–2·71)
Enteric infections	4·83 (3·14–7·3)	6·5 (4·78–8·93)	7·25 (5·38–9·76)	5·6 (3·9–8·1)
Diarrheal diseases	4·85 (3·15–7·32)	6·51 (4·79–8·96)	7·26 (5·39–9·77)	5·62 (3·92–8·15)
Other infectious diseases	0·252 (0·173–0·452)	0·638 (0·51–0·825)	0·383 (0·228–0·657)	0·743 (0·581–1·04)
Meningitis	0·707 (0·516–0·873)	1·35 (0·99–1·68)	0·000262 (0·000127–0·000482)	1·97 (1·5–2·37)
Encephalitis	0·114 (0·0806–0·242)	0·364 (0·272–0·558)	0·000122 (6·06e-05–0·000212)	0·466 (0·352–0·722)
Measles	1·19 (-0·331–5·47)	1·84 (-0·47–8·27)	0·398 (0·0157–0·978)	2·04 (-0·564–9·36)
Acute hepatitis	6·85 (4·75–10·4)	4·12 (2·94–5·84)	2·66 (1·66–4·47)	7·55 (5·27–11)
Maternal and neonatal disorders	75·5 (73·6–77·3)	69·8 (66·3–72·6)	65·7 (60·4–70·5)	76 (74·1–77·8)
Maternal disorders	8·91 (3·29–14·5)	9·02 (3·29–14·9)	9·06 (3·3–15·1)	8·86 (3·26–14·4)
Neonatal disorders	76·8 (74·8–78·6)	71·3 (67·8–74·4)	67·7 (62·2–72·6)	76·8 (74·8–78·6)
Nutritional deficiencies	91·9 (63·2–94·9)	75·4 (71·9–78·2)	73·5 (70·9–76·2)	88·9 (60·8–91·9)
Protein-energy malnutrition	100 (100–100)	100 (100–100)	100 (100–100)	100 (100–100)
Vitamin A deficiency	--	100 (100–100)	100 (100–100)	--
Dietary iron deficiency	--	100 (100–100)	100 (100–100)	--
Non-communicable diseases	53·6 (49·3–57·3)	37·3 (34·1–40·7)	23·1 (20·8–25·5)	53·5 (50·7–56·4)
Neoplasms	38·1 (34·5–42·3)	38·2 (34·9–42)	25·9 (22·8–29·5)	39·1 (35·8–42·9)
Lip and oral cavity cancer	57·5 (50·6–64·3)	62·6 (55·8–68·8)	62·2 (55–68·6)	62·7 (55·9–68·9)
Nasopharynx cancer	58·3 (48·9–67)	59·1 (49·8–67·5)	58 (48·5–66·7)	59·3 (50–67·6)
Other pharynx cancer	71·8 (65–77·8)	74·3 (67·7–80)	74·7 (68·1–80·4)	74·3 (67·6–80)
Esophageal cancer	63·4 (53·4–73·4)	66·6 (57–76·2)	64·1 (54–73·9)	66·6 (57·1–76·2)
Stomach cancer	16·8 (10·3–35·2)	18·4 (11·5–36·9)	18·1 (11·3–36·6)	18·5 (11·5–36·9)

Colon and rectum cancer	59·1 (50·2–67·4)	59·8 (51·5–67·6)	59·8 (51·5–68)	59·8 (51·5–67·6)
Liver cancer	76·6 (72·3–81)	76·3 (72·3–80·4)	76·3 (72·3–80·5)	76·3 (72·4–80·4)
Gallbladder and biliary tract cancer	17 (8·91–27·6)	17·3 (8·99–28·1)	15·7 (8·05–26)	17·3 (9·02–28·1)
Pancreatic cancer	30·4 (22·3–40·8)	31·6 (24–41·5)	30·9 (23·1–41)	31·6 (24–41·5)
Larynx cancer	65·9 (57–73·3)	68·4 (59·8–75·4)	69·3 (60·3–76·5)	68·3 (59·8–75·3)
Tracheal, bronchus, and lung cancer	75·6 (71·5–80)	75·3 (71·6–79·6)	75·8 (71·5–80·1)	75·3 (71·6–79·6)
Breast cancer	31·3 (23·8–40·4)	30·9 (24–38·8)	31·1 (24·2–39·2)	30·9 (24–38·7)
Cervical cancer	100 (100–100)	100 (100–100)	100 (100–100)	100 (100–100)
Uterine cancer	36·2 (22·8–49·7)	37·5 (24·3–51·1)	38 (24·9–51·6)	37·4 (24·2–50·9)
Ovarian cancer	20·1 (10·7–33·1)	16·9 (8·79–28·4)	16·2 (8·47–27·1)	17 (8·8–28·4)
Prostate cancer	3·67 (1·69–5·79)	4·62 (2·14–7·17)	5·45 (2·5–8·37)	4·48 (2·07–6·96)
Kidney cancer	28·2 (20·2–37)	29·7 (21·1–38·8)	30 (21·3–39·2)	29·7 (21·1–38·8)
Bladder cancer	25·2 (14·9–36·8)	28·1 (18·1–39·3)	29·2 (19·1–40·4)	28 (18–39·3)
Thyroid cancer	10·8 (5·09–18·3)	11·4 (5·26–19·5)	11·7 (5·41–20)	11·4 (5·24–19·4)
Mesothelioma	98 (97·3–98·5)	96·3 (95·2–97·2)	97·5 (96·6–98·2)	96·2 (95·2–97·2)
Non-Hodgkin lymphoma	5·29 (2·12–9·75)	5·47 (2·17–10)	5·38 (2·17–9·9)	5·48 (2·17–10·1)
Multiple myeloma	6·04 (2·52–11)	6·3 (2·63–11·4)	6·29 (2·64–11·6)	6·3 (2·63–11·4)
Leukemia	26 (17·1–36·2)	26·1 (17·1–35·4)	26·9 (17·8–36·7)	26 (17·1–35·3)
Cardiovascular diseases	78·5 (74·4–82)	80·2 (77–83·1)	75 (70·9–78·9)	81·2 (78–84)
Rheumatic heart disease	27·2 (14·9–51·9)	28·8 (17·7–48·7)	29·7 (18·9–48·3)	28·7 (17·4–49·1)
Ischemic heart disease	93·4 (89·3–96·8)	94·5 (91·7–97)	94·2 (91·5–96·7)	94·6 (91·6–97·1)
Stroke	80·1 (71·4–89·2)	83 (76·9–89·1)	82·8 (77–88·6)	83·1 (76·6–89·5)
Hypertensive heart disease	100 (100–100)	100 (100–100)	100 (100–100)	100 (100–100)
Non-rheumatic valvular heart disease	30·8 (19·8–45·1)	28·9 (20·2–39·7)	2·13 (1·38–3·12)	32·7 (22·9–44·7)
Cardiomyopathy and myocarditis	36·4 (28·4–45·2)	42·1 (35·9–47·9)	43·1 (36·5–49·2)	41·9 (35·5–48)
Atrial fibrillation and flutter	49·5 (40·3–59·2)	57·3 (49·8–65)	63 (55·8–69·6)	53 (44·6–61·8)
Aortic aneurysm	51·3 (44–58·9)	59·3 (53–65·2)	--	59·3 (53–65·2)
Endocarditis	37 (26·1–50·5)	40·8 (31·3–51·3)	38·1 (27·8–50·4)	41 (31·5–51·3)
Other cardiovascular and circulatory diseases	36·4 (28·1–43·9)	40·2 (32·4–45·7)	43 (37·1–49·4)	38·7 (28·1–44·9)
Chronic respiratory diseases	56·2 (51·3–62·2)	52 (48·1–56·1)	43·7 (39·4–47·9)	59·1 (54·5–64·7)
Chronic obstructive pulmonary disease	61·1 (55·8–67·6)	60·9 (56·6–65·2)	52·8 (48·8–56·6)	65·1 (60·1–71·1)
Pneumoconiosis	100 (100–100)	100 (100–100)	100 (100–100)	100 (100–100)
Asthma	33 (23·1–44·3)	35·8 (28·3–44·6)	35·6 (28·2–44)	37·8 (28–48·6)
Digestive diseases	24·9 (21·4–28·3)	25·7 (21·9–29·7)	15·6 (10·7–21·8)	34·4 (30·3–38·3)

Cirrhosis and other chronic liver diseases	68·9 (59·9–77)	72·9 (64·3–80·7)	64·3 (57·2–71·6)	73·2 (64·5–81·1)
Upper digestive system diseases	12·4 (10·3–14·5)	7·63 (5·47–10)	1·66 (1·04–2·48)	16·9 (14·4–19·4)
Gallbladder and biliary diseases	33·6 (20·6–47·6)	39·2 (26·7–52·5)	40·5 (28–53·7)	34·5 (21·7–48·5)
Pancreatitis	30·3 (19·7–42·1)	34·8 (23·7–46·4)	35·6 (24·3–47·1)	34·7 (23·7–46·4)
Neurological disorders	20 (9·36–32·8)	12·1 (5·88–21·8)	7·86 (3·7–15·9)	18·2 (8·67–30)
Alzheimer's disease and other dementias	28·7 (16·8–43·5)	31·5 (19·4–45·9)	32·1 (19·8–46·4)	31·2 (19·2–45·4)
Parkinson's disease	-5·11 (-7·88–-2·6)	-6·96 (-10·6–-3·59)	-8·2 (-12·3–-4·3)	-6·62 (-10–-3·39)
Idiopathic epilepsy	23 (16·3–29·5)	21·4 (15·2–27·4)	20 (14·1–26)	23·7 (16·7–30)
Multiple sclerosis	10·7 (7·7–13·7)	13 (9·66–16·3)	13·1 (9·78–16·2)	13 (9·46–16·5)
Mental disorders	--	4·99 (2·59–8·18)	4·99 (2·59–8·18)	--
Depressive disorders	--	10·9 (5·39–17)	10·9 (5·39–17)	--
Anxiety disorders	--	4·74 (1·3–10·5)	4·74 (1·3–10·5)	--
Idiopathic developmental intellectual disability	--	47·8 (23·2–68·3)	47·8 (23·2–68·3)	--
Substance use disorders	100 (100–100)	100 (100–100)	100 (100–100)	100 (100–100)
Alcohol use disorders	100 (100–100)	100 (100–100)	100 (100–100)	100 (100–100)
Drug use disorders	100 (100–100)	100 (100–100)	100 (100–100)	100 (100–100)
Diabetes and kidney diseases	100 (100–100)	100 (100–100)	100 (100–100)	100 (100–100)
Diabetes mellitus	100 (100–100)	100 (100–100)	100 (100–100)	100 (100–100)
Chronic kidney disease	100 (100–100)	100 (100–100)	100 (100–100)	100 (100–100)
Sense organ diseases	--	7·74 (6·77–8·87)	7·74 (6·77–8·87)	--
Blindness and vision loss	--	7·27 (4·5–10·8)	7·27 (4·5–10·8)	--
Age-related and other hearing loss	--	9·18 (8·32–10·2)	9·18 (8·32–10·2)	--
Musculoskeletal disorders	1·82 (0·501–3·38)	21·6 (17·7–25·5)	22 (18–26)	2·53 (0·722–4·63)
Rheumatoid arthritis	5·75 (1·6–10·9)	10·7 (3·16–19)	11·4 (3·39–20·4)	7·94 (2·29–14·6)
Osteoarthritis	--	11·1 (5·52–17·9)	11·1 (5·52–17·9)	--
Low back pain	--	38·4 (33·7–43·4)	38·4 (33·7–43·4)	--
Gout	--	42·4 (28·7–58·8)	42·4 (28·7–58·8)	--
Other non-communicable diseases	0·0688 (0·0459–0·101)	0·0459 (0·0292–0·0663)	--	0·291 (0·182–0·368)
Sudden infant death syndrome	7·6 (6·58–8·77)	7·59 (6·57–8·76)	--	7·59 (6·57–8·76)
Injuries	35·8 (29·4–40·8)	28·5 (23·9–32·6)	34·5 (30·1–38·4)	21·6 (14·1–28·4)
Transport injuries	20·3 (13·4–26·5)	22·3 (16·5–27·8)	29·1 (25·3–32·7)	18·8 (11·1–25·5)
Road injuries	16·4 (8·4–23·7)	19·5 (13·1–25·5)	27·6 (24–31·3)	15 (6·35–23·1)
Other transport injuries	35·8 (31·2–40·2)	34·5 (29·8–39·1)	37·7 (33·4–41·4)	33·4 (28·3–38·4)
Unintentional injuries	47·5 (40–52·3)	34·7 (29·9–38·8)	35·1 (30·4–39·2)	33·8 (28·4–38·1)

Falls	57·4 (49·1–62·7)	45·7 (39·3–50·2)	43·6 (37·4–48·1)	51·6 (44·4–56·5)
Drowning	-11·2 (-28·1–4·66)	-8·91 (-24·6–6·18)	12·3 (8·23–17·7)	-9·72 (-26·3–5·97)
Fire, heat, and hot substances	11·4 (7·34–16·5)	16 (11·6–21·6)	16·3 (11·9–21·9)	15·2 (11·1–20·5)
Poisonings	9·15 (6·68–12·6)	12·2 (9·06–16·3)	15·1 (11·2–19·9)	6·89 (5·21–9·38)
Exposure to mechanical forces	8·27 (-2·7–19·6)	20·4 (17·1–23·6)	22·7 (19·9–25·6)	2·62 (-8·66–14·5)
Animal contact	24·5 (20–29·2)	25·4 (21·3–30·2)	26·6 (22·2–31·4)	22·3 (18·3–26·8)
Foreign body	2·95 (2·25–3·87)	6·12 (4·74–7·9)	6·18 (4·71–8·03)	6·07 (4·73–7·81)
Environmental heat and cold exposure	7·95 (3·55–13·8)	8·25 (3·68–14·3)	7·9 (3·43–13·9)	8·45 (3·81–14·6)
Exposure to forces of nature	0 (0–0)	8·69 (4–15)	8·69 (4–15)	0 (0–0)
Other unintentional injuries	17·6 (13–23·7)	15·6 (11·3–20·9)	15·4 (11–20·7)	20·3 (15·3–26·4)
Self-harm and interpersonal violence	11·3 (-4·53–25·7)	13·3 (-1·32–26·5)	31·1 (24·3–37·7)	11·6 (-4·12–26)
Self-harm	10·5 (-5·97–25·8)	11·1 (-5·32–26·2)	18·7 (7·99–29·1)	10·9 (-5·7–26·1)
Interpersonal violence	23·9 (12·5–35)	29·5 (21·3–38·6)	35·1 (27·4–43·2)	22·5 (10·4–34·1)

Supplementary Results Table 2. All-age PAFs attributable to the joint distribution of all risk factors for all ages, both sexes combined for each GBD level 1, level 2, and level 3 cause and all causes in 2019 for Portugal. Results are shown for deaths, YLLs, YLDs, and DALYs Data in parenthesis are 95% uncertainty intervals. PAFs=population attributable fractions. GBD=Global Burden of Disease. YLLs=years of life lost. YLDs=years lived with disability. DALYs=disability-adjusted life-years

	Deaths 2019	DALYs 2019	YLDs 2019	YLLs 2019
All causes	54·6 (51·1–57·9)	41·7 (38·7–44·8)	26·9 (24·7–29·3)	54·6 (52·1–57·4)
Communicable, maternal, neonatal, and nutritional diseases	39·6 (35·1–43·9)	48·9 (45·9–51·8)	48 (43·8–52·6)	49·3 (45·5–53)
HIV/AIDS and sexually transmitted infections	87·9 (87–88·7)	87·1 (86·1–88)	89·5 (88–91·3)	86·7 (85·7–87·6)
HIV/AIDS	87·7 (86·8–88·6)	86·7 (85·8–87·6)	87·5 (86·5–88·5)	86·6 (85·6–87·5)
Sexually transmitted infections excluding HIV	99·5 (99·3–99·7)	99·6 (99·3–99·9)	100 (100–100)	98·3 (97·5–98·8)
Respiratory infections and tuberculosis	36·9 (31·8–41·7)	37·6 (33–42·2)	5·63 (3·86–7·91)	41·3 (36·3–46·2)
Tuberculosis	56·7 (47–64·6)	61·2 (52·5–68·3)	57·7 (49–65·1)	61·7 (52·9–68·7)
Lower respiratory infections	36·4 (31·3–41·4)	40·3 (35·2–45·4)	32·4 (26·8–37·5)	40·4 (35·2–45·5)
Upper respiratory infections	0·0446 (0·0251–0·0742)	0·00456 (0·00105–0·0104)	0·00327 (0–0·00901)	0·248 (0·14–0·411)
Otitis media	3·01 (0·856–8·89)	2·81 (1·76–4·07)	2·67 (1·64–3·85)	9·98 (3·33–24·5)
Enteric infections	21·3 (13·6–31·3)	24·4 (17·2–34·2)	23·9 (16·5–33·5)	25·3 (18·1–35)
Diarrheal diseases	21·4 (13·7–31·6)	24·5 (17·2–34·4)	24 (16·5–33·6)	25·6 (18·3–35·5)
Other infectious diseases	0·493 (0·274–0·681)	0·786 (0·54–1·04)	0·3 (0·18–0·534)	1·03 (0·66–1·38)
Meningitis	0·642 (0·413–0·987)	1·21 (0·789–1·81)	0·000171 (0–0·000502)	1·86 (1·22–2·8)
Encephalitis	0·17 (0·11–0·256)	0·454 (0·295–0·663)	6·84e-05 (0–0·000217)	0·534 (0·352–0·782)
Measles	1·37 (-0·288–6·05)	1·9 (-0·337–8·07)	1·08 (0·0398–2·55)	1·96 (-0·399–8·87)
Acute hepatitis	12·6 (6·48–18·4)	5·21 (2·97–7·66)	1·99 (1·19–3·51)	14 (6·97–19·7)
Maternal and neonatal disorders	76·1 (73·6–78·7)	73·9 (71·4–76·2)	71·5 (67·7–74·9)	77·3 (74·8–79·9)
Maternal disorders	9·27 (3·35–15·4)	9·16 (3·27–15·2)	9·17 (3·23–15·3)	9·15 (3·31–15·2)
Neonatal disorders	79·6 (77·1–82)	75·7 (73–78·1)	73·1 (69–76·6)	79·6 (77·1–82)
Nutritional deficiencies	76 (69·2–86·5)	72·7 (69–76·1)	72·4 (68·5–76)	75·1 (69–85·6)
Protein-energy malnutrition	100 (100–100)	100 (100–100)	100 (100–100)	100 (100–100)
Vitamin A deficiency	--	100 (100–100)	100 (100–100)	--
Dietary iron deficiency	--	100 (100–100)	100 (100–100)	--
Non-communicable diseases	57·2 (53–61)	42·1 (38·7–45·5)	25·6 (23·2–28·2)	57·4 (54·4–60·7)
Neoplasms	39·6 (35·6–44·6)	42·1 (38·5–46·6)	32·7 (28·8–37·2)	42·6 (39–47·2)
Lip and oral cavity cancer	68·6 (62·6–74·1)	74·2 (68·7–79)	70·1 (64·1–75·4)	74·4 (68·9–79·2)
Nasopharynx cancer	67·5 (60–74·3)	68·9 (61·3–75·6)	64·7 (57·2–72·1)	69·2 (61·6–75·9)
Other pharynx cancer	82·7 (78·1–86·6)	84·3 (79·4–88·1)	82·8 (77·8–86·9)	84·3 (79·5–88·1)
Esophageal cancer	70 (61·6–78·4)	73·7 (65·8–81·5)	70·7 (62·3–79·2)	73·8 (65·8–81·6)

Stomach cancer	18·1 (11·4–36·1)	20·5 (12·9–39·1)	19·7 (12·2–38)	20·5 (12·9–39·1)
Colon and rectum cancer	62·5 (53·3–71)	63·5 (54·8–71·4)	63·9 (55·5–71·8)	63·4 (54·8–71·4)
Liver cancer	71 (63·5–77·7)	73·8 (66·8–80)	72·2 (65–78·6)	73·8 (66·8–80)
Gallbladder and biliary tract cancer	17·6 (9·08–28·9)	17·8 (9·21–29·1)	17·2 (8·67–28·5)	17·8 (9·21–29·1)
Pancreatic cancer	31·2 (21·9–43·3)	32·7 (24–44)	32 (23–43·9)	32·7 (24·1–44)
Larynx cancer	78·2 (70·8–84·1)	80·7 (73·4–86)	78·7 (71·3–84·5)	80·8 (73·4–86·1)
Tracheal, bronchus, and lung cancer	75·7 (71·4–80·5)	76·6 (72·8–80·9)	76·2 (71·7–80·9)	76·6 (72·8–80·9)
Breast cancer	31·8 (23–42·8)	31 (23·3–40·3)	31·1 (23·2–40·6)	30·9 (23·2–40·3)
Cervical cancer	100 (100–100)	100 (100–100)	100 (100–100)	100 (100–100)
Uterine cancer	39·5 (25·3–54·2)	40·4 (26·2–55·3)	40·8 (26·6–55·3)	40·4 (26·1–55·3)
Ovarian cancer	16·3 (6·24–30·2)	14·2 (5·37–26·6)	13·2 (4·94–24·9)	14·2 (5·37–26·7)
Prostate cancer	4·61 (2·07–7·38)	5·51 (2·53–8·69)	6·42 (2·95–10·1)	5·37 (2·48–8·46)
Kidney cancer	30·3 (21·6–39·9)	31·7 (23·1–41·2)	32 (23–41·6)	31·7 (23·1–41·2)
Bladder cancer	31·3 (19·4–45·1)	36·1 (24·5–48·7)	38·5 (27·1–50·6)	35·9 (24·4–48·6)
Thyroid cancer	12 (5·55–20·4)	12·6 (5·72–21·5)	12 (5·98–19·6)	12·7 (5·62–21·9)
Mesothelioma	93·2 (90·4–95·2)	88·8 (84·7–92)	92·5 (89·5–95)	88·7 (84·6–92)
Non-Hodgkin lymphoma	5·87 (2·32–10·9)	5·98 (2·39–10·9)	5·93 (2·34–10·9)	5·98 (2·4–10·9)
Multiple myeloma	6·74 (2·74–12·3)	6·98 (2·89–12·7)	6·98 (2·83–12·6)	6·98 (2·89–12·7)
Leukemia	26·1 (17·1–36·7)	26·7 (17·8–36·4)	27·2 (18·1–36·8)	26·7 (17·8–36·3)
Cardiovascular diseases	78·8 (74·1–83·6)	80·3 (76·7–83·8)	74·4 (70–78·8)	81·1 (77·5–84·8)
Rheumatic heart disease	23·4 (13·6–44·2)	25·3 (16·8–41·4)	25·5 (16·4–41·7)	25·3 (16·8–41·7)
Ischemic heart disease	92·7 (88·4–96·3)	94 (90·8–96·7)	93·2 (89·9–96·1)	94·1 (90·8–96·7)
Stroke	78·9 (70·1–88·3)	82 (75·3–89·1)	81·7 (75·5–88·4)	82 (75·3–89)
Hypertensive heart disease	100 (100–100)	100 (100–100)	100 (100–100)	100 (100–100)
Non-rheumatic valvular heart disease	26·3 (17·1–39·4)	28·1 (19·9–39)	27·4 (18·9–38·4)	28·1 (19·9–39·1)
Cardiomyopathy and myocarditis	35·2 (27·3–45·3)	43·1 (36·4–50·5)	38·3 (31·1–46·5)	43·4 (36·6–50·9)
Atrial fibrillation and flutter	47·9 (38·9–57·8)	56·6 (48·6–64·2)	60·9 (53·1–68·3)	51·3 (42·5–60·4)
Aortic aneurysm	53·5 (47·4–59·3)	62·8 (57·7–67·5)	--	62·8 (57·7–67·5)
Endocarditis	33 (23·1–46·2)	36·9 (27·8–48·3)	33·4 (23·7–46·3)	37 (27·8–48·3)
Other cardiovascular and circulatory diseases	30·4 (24·3–37·2)	34·7 (29·3–40·4)	38·5 (32·1–44·8)	33·6 (28·2–39·6)
Chronic respiratory diseases	52·2 (47·4–57·7)	49·1 (44·8–53·3)	43 (37·9–48·2)	54·9 (50·6–60·2)
Chronic obstructive pulmonary disease	57 (52·2–61·7)	57·6 (53·9–61·4)	49·6 (45·8–53·3)	61·7 (57·5–65·9)
Pneumoconiosis	100 (100–100)	100 (100–100)	100 (100–100)	100 (100–100)
Asthma	33·3 (22·4–46)	39·1 (30·4–49·3)	39·1 (30·6–49·3)	37·8 (27·4–49·9)

Digestive diseases	36·9 (32·3–41·3)	42·7 (37·4–47·7)	9·36 (6·18–13)	50 (44·5–55·1)
Cirrhosis and other chronic liver diseases	77·9 (67·6–86·7)	81·1 (71·6–88·8)	77 (68·7–84·4)	81·2 (71·6–88·8)
Upper digestive system diseases	12·9 (10·7–15·1)	5·38 (3·33–7·84)	0·439 (0·254–0·702)	18·3 (15·6–21·2)
Gallbladder and biliary diseases	37·2 (23·2–52·2)	39·8 (26·2–54·7)	43·8 (29·9–58·4)	38·1 (24·6–53)
Pancreatitis	40·3 (29·6–53)	46·3 (35·2–58·6)	44·4 (33·4–56·3)	46·4 (35·3–58·7)
Neurological disorders	21·9 (9·94–36·6)	14·6 (6·88–26·2)	9·65 (4·4–19·8)	20·7 (9·62–34·2)
Alzheimer's disease and other dementias	30·5 (16·9–47·6)	32·6 (19·1–49·3)	33 (19·7–49·5)	32·4 (18·9–49·1)
Parkinson's disease	-5·11 (-8·11–-2·34)	-6·34 (-10–-3·04)	-6·82 (-10·9–-3·28)	-6·22 (-9·82–-2·96)
Idiopathic epilepsy	25·4 (18·5–32·9)	25·4 (18·8–32·1)	23·3 (17·4–29·6)	27·8 (20·7–35)
Multiple sclerosis	11·9 (8·67–15·9)	14 (10·6–17·7)	13·2 (9·98–16·5)	14·6 (11–19)
Mental disorders	--	5·4 (2·96–8·4)	5·4 (2·96–8·4)	--
Depressive disorders	--	8·98 (4·64–13·9)	8·98 (4·64–13·9)	--
Anxiety disorders	--	4·9 (1·33–10·7)	4·9 (1·33–10·7)	--
Idiopathic developmental intellectual disability	--	63·6 (33·5–83·1)	63·6 (33·5–83·1)	--
Substance use disorders	100 (100–100)	100 (100–100)	100 (100–100)	100 (100–100)
Alcohol use disorders	100 (100–100)	100 (100–100)	100 (100–100)	100 (100–100)
Drug use disorders	100 (100–100)	100 (100–100)	100 (100–100)	100 (100–100)
Diabetes and kidney diseases	99·8 (99·8–99·9)	99·9 (99·9–100)	100 (100–100)	99·9 (99·8–99·9)
Diabetes mellitus	100 (100–100)	100 (100–100)	100 (100–100)	100 (100–100)
Chronic kidney disease	100 (100–100)	100 (100–100)	100 (100–100)	100 (100–100)
Sense organ diseases	--	7·4 (6·27–8·84)	7·4 (6·27–8·84)	--
Blindness and vision loss	--	8·21 (4·79–13)	8·21 (4·79–13)	--
Age-related and other hearing loss	--	8·25 (7·32–9·22)	8·25 (7·32–9·22)	--
Musculoskeletal disorders	1·85 (0·477–3·52)	21·7 (18·1–25·6)	21·9 (18·2–25·9)	2·44 (0·648–4·49)
Rheumatoid arthritis	4·83 (1·25–9·37)	9·93 (2·61–17·8)	10·9 (2·87–19·5)	7·06 (1·84–13·4)
Osteoarthritis	--	13·6 (6·81–21·9)	13·6 (6·81–21·9)	--
Low back pain	--	38 (33·6–42·7)	38 (33·6–42·7)	--
Gout	--	46·2 (32–62·6)	46·2 (32–62·6)	--
Other non-communicable diseases	0·00837 (0·00521–0·0144)	0·0102 (0·00618–0·0158)	--	0·0452 (0·0294–0·0704)
Sudden infant death syndrome	9·52 (6·02–14)	9·51 (6·02–14)	--	9·51 (6·02–14)
Injuries	29·5 (25·3–33·6)	30·9 (26·7–35)	34·7 (30·5–38·9)	28·1 (23·3–32·9)
Transport injuries	33·2 (28·2–38·1)	33·7 (28·1–39·2)	33·5 (29–37·9)	33·7 (27·8–39·7)
Road injuries	31·8 (26·8–36·7)	32·5 (26·9–38)	31·3 (27–35·9)	32·8 (26·8–39)
Other transport injuries	42·4 (37·4–47·3)	41·5 (36–46·7)	42·7 (37·6–46·8)	41 (35·1–46·8)

Unintentional injuries	31·2 (26·8–35·1)	32·2 (27·7–37)	35·5 (30·7–40·3)	26·3 (21·9–30·9)
Falls	54·4 (47·3–59·3)	46 (40·3–51)	45·4 (39·5–50·3)	47·5 (41·5–52·8)
Drowning	-4·33 (-12·6–4·75)	-0·639 (-8·94–8·5)	12·8 (9·04–17·8)	-1·18 (-9·62–8·16)
Fire, heat, and hot substances	10 (6·24–15·1)	16·9 (12·2–22·8)	18·4 (13·4–24·5)	14·9 (10·5–20·3)
Poisonings	18·7 (13·5–25·2)	19·9 (14·7–26·4)	17·6 (12·9–23·6)	23·1 (17·1–30·2)
Exposure to mechanical forces	21·4 (15·8–27·4)	26·1 (21·7–31·6)	27·1 (23–32·4)	18·1 (11·6–25·7)
Animal contact	28·9 (23·8–33·8)	28·7 (23·6–34)	27·9 (22·9–33·5)	29·6 (24·5–35·4)
Foreign body	4·03 (2·74–5·74)	8·45 (5·83–11·9)	8·42 (5·86–11·8)	8·46 (5·79–12)
Environmental heat and cold exposure	7·21 (3·44–12·3)	8·63 (4·02–14·6)	8·46 (3·93–14·5)	8·82 (4·14–15)
Exposure to forces of nature	0 (0–0)	9·94 (4·61–16·7)	9·94 (4·61–16·7)	0 (0–0)
Other unintentional injuries	20·4 (14·9–27·4)	18·7 (13·7–25)	17·4 (12·6–23·6)	26·4 (19·8–34·5)
Self-harm and interpersonal violence	24·3 (13·9–34·3)	25·6 (16·2–34·4)	28·3 (22·9–34·1)	25·2 (14·9–35·1)
Self-harm	23·3 (11·4–34·5)	24·2 (12·1–35·8)	21·2 (11·8–29·9)	24·3 (12·2–36·1)
Interpersonal violence	33·3 (25·5–41·2)	35·1 (27·8–42·7)	42·5 (34·9–50·6)	30·8 (23·3–38·8)

Supplementary Results Table 2. All-age PAFs attributable to the joint distribution of all risk factors for all ages, both sexes combined for each GBD level 1, level 2, and level 3 cause and all causes in 2019 for San Marino. Results are shown for deaths, YLLs, YLDs, and DALYs Data in parenthesis are 95% uncertainty intervals. PAFs=population attributable fractions. GBD=Global Burden of Disease. YLLs=years of life lost. YLDs=years lived with disability. DALYs=disability-adjusted life-years

	Deaths 2019	DALYs 2019	YLDs 2019	YLLs 2019
All causes	52·1 (48–55·9)	38 (34·1–42)	25·3 (23·1–27·7)	50·3 (46·4–53·6)
Communicable, maternal, neonatal, and nutritional diseases	37·9 (31·2–45)	45·2 (40·6–51·6)	43·2 (38·4–48)	46·6 (39·7–56·7)
HIV/AIDS and sexually transmitted infections	89·4 (82·9–93)	86·6 (77·4–91·2)	94·6 (90·2–98)	84·6 (73·3–90·4)
HIV/AIDS	91·4 (85·5–94·8)	89·6 (82–93·2)	90·5 (86–93·8)	89·6 (81·5–93·8)
Sexually transmitted infections excluding HIV	47·9 (25–74·1)	66·1 (39·6–88·6)	100 (100–100)	19·8 (7·81–40·9)
Respiratory infections and tuberculosis	39·8 (32·2–46·1)	32·1 (25–39·1)	3·02 (2·09–4·11)	43·1 (35·5–49·2)
Tuberculosis	47·1 (23·9–59·5)	51·4 (27·7–63·1)	51·8 (26·6–64)	51·3 (28–63·1)
Lower respiratory infections	40·2 (32·6–46·5)	43·9 (36·1–50·4)	35·6 (27·2–42·5)	44·1 (36·2–50·6)
Upper respiratory infections	0·0803 (0·0164–0·271)	0·0305 (0·00637–0·101)	0·0049 (6·71e-05–0·013)	0·305 (0·0737–0·958)
Otitis media	2·59 (0·273–7·31)	3·78 (2·45–5·38)	3·7 (2·39–5·27)	7·01 (1·38–13·6)
Enteric infections	6·51 (4·37–9·4)	9·44 (7·16–12·3)	8·8 (6·65–11·8)	14 (8·93–20·9)
Diarrheal diseases	6·72 (4·46–9·68)	9·53 (7·25–12·4)	8·81 (6·66–11·8)	15·2 (9·61–23·2)
Other infectious diseases	1·08 (0·66–1·54)	1·44 (0·95–1·95)	0·416 (0·247–0·696)	1·6 (1·04–2·17)
Meningitis	0·808 (0·467–1·3)	1·77 (1·08–2·71)	0·000498 (5·32e-06–0·00147)	2·33 (1·39–3·67)
Encephalitis	0·221 (0·123–0·363)	0·661 (0·379–1·05)	0·00024 (2·51e-06–0·000689)	0·718 (0·404–1·13)
Measles	0 (0–0)	0·238 (0–0·941)	0·238 (0–0·941)	0 (0–0)
Acute hepatitis	6·11 (3·46–8·7)	7·16 (4·1–10·2)	2·54 (1·48–4·18)	7·91 (4·47–11·2)
Maternal and neonatal disorders	75 (71·3–78·9)	73·4 (70·6–76·1)	71·4 (67·6–75·2)	75·4 (71·7–79·3)
Maternal disorders	8·76 (3·08–14·4)	8·78 (3·09–14·5)	8·83 (3·08–14·6)	8·67 (3·07–14·3)
Neonatal disorders	76·1 (72·4–79·9)	74·7 (71·8–77·3)	73·1 (69–76·9)	76·1 (72·4–79·9)
Nutritional deficiencies	51·6 (44·4–58·3)	72·3 (69·2–75·5)	74·1 (70·7–77·2)	48·9 (42·6–55·1)
Protein-energy malnutrition	100 (100–100)	100 (100–100)	100 (100–100)	100 (100–100)
Vitamin A deficiency	--	100 (100–100)	100 (100–100)	--
Dietary iron deficiency	--	100 (100–100)	100 (100–100)	--
Non-communicable diseases	53·9 (49·6–57·9)	38·6 (34·2–43·1)	23·9 (21·6–26·5)	52·9 (49·3–56·4)
Neoplasms	38·5 (34·3–43·2)	38·8 (35–43·2)	28·8 (24·7–32·9)	39·4 (35·6–43·8)
Lip and oral cavity cancer	62·3 (34·3–73·3)	66·5 (37·9–76·8)	66·5 (37·6–76·8)	66·5 (37·9–76·8)
Nasopharynx cancer	61·5 (29·4–73·4)	62·8 (30·2–74·4)	61·4 (28·7–73·1)	62·9 (30·4–74·5)
Other pharynx cancer	77·5 (55·4–84·9)	78·8 (56·2–85·9)	78·1 (55·3–85·4)	78·8 (56·3–86)
Esophageal cancer	73·4 (59·3–83·1)	74·9 (60·2–84·3)	73·8 (59·5–83·3)	74·9 (60·2–84·4)

Stomach cancer	21 (13·3–39·3)	22·3 (14·4–40·8)	22·4 (14·5–40·9)	22·3 (14·4–40·7)
Colon and rectum cancer	61·2 (51·5–69·9)	61·9 (52·4–70·3)	62·2 (52·6–70·4)	61·9 (52·3–70·2)
Liver cancer	72·1 (64·8–78·7)	73·7 (66·7–79·9)	72·6 (65·7–78·9)	73·8 (66·7–79·9)
Gallbladder and biliary tract cancer	19·1 (9·72–31·4)	19·6 (10–32·3)	18·9 (9·6–31·2)	19·6 (10–32·3)
Pancreatic cancer	33·8 (25·2–44)	34·4 (26·1–44·3)	34·1 (25·7–44·2)	34·4 (26·1–44·3)
Larynx cancer	83·1 (71·8–89·9)	83·5 (73·2–89·9)	83·7 (73·7–90)	83·5 (73·2–89·8)
Tracheal, bronchus, and lung cancer	81·4 (76·9–85·5)	80·3 (75·9–84·5)	81 (76·7–85·1)	80·3 (75·9–84·5)
Breast cancer	32·9 (22·4–42·8)	32 (20·9–40·8)	31·9 (21–40·8)	32 (20·9–40·8)
Cervical cancer	100 (100–100)	100 (100–100)	100 (100–100)	100 (100–100)
Uterine cancer	44·3 (29·5–59·1)	45·5 (30·5–60·7)	46 (31–61·2)	45·4 (30·3–60·6)
Ovarian cancer	18·7 (8·45–30·2)	15 (6·52–24·8)	13·8 (6·06–23·6)	15·1 (6·53–24·8)
Prostate cancer	5·53 (2·33–9·16)	6·14 (2·64–10·1)	6·62 (2·85–11·1)	6·06 (2·6–10)
Kidney cancer	35·4 (25·5–45·9)	36·1 (26·3–46·6)	36·1 (26·3–46·5)	36·1 (26·3–46·6)
Bladder cancer	35 (22–47·8)	38·6 (26–50·7)	40·2 (27·7–52)	38·4 (25·8–50·6)
Thyroid cancer	14·9 (6·91–25)	15·8 (7·25–26·5)	15·9 (7·67–26·3)	15·8 (7·23–26·5)
Mesothelioma	97·7 (96·4–98·7)	95·7 (93·5–97·4)	97·2 (95·5–98·5)	95·6 (93·4–97·4)
Non-Hodgkin lymphoma	7·04 (2·9–12·6)	7·21 (3·03–12·7)	7·17 (2·98–12·7)	7·21 (3·05–12·7)
Multiple myeloma	8·16 (3·38–14·5)	8·49 (3·56–15)	8·47 (3·56–15·1)	8·49 (3·56–15)
Leukemia	31·7 (19·4–44·7)	30·2 (20–41·1)	28·7 (18·9–38·5)	30·3 (20–41·4)
Cardiovascular diseases	76 (72–79·9)	77 (73·6–80·3)	71·4 (66·5–75·5)	77·9 (74·7–81·2)
Rheumatic heart disease	26·9 (14·8–51·9)	28·3 (17·6–45·9)	28·6 (18–46·2)	28·3 (17·5–46)
Ischemic heart disease	93·4 (89·4–96·8)	94·6 (91·6–97·1)	94·3 (91·4–96·9)	94·6 (91·6–97·1)
Stroke	80·3 (72·3–89)	83·2 (77–89·9)	83·7 (78·1–89)	83·1 (76·7–90)
Hypertensive heart disease	100 (100–100)	100 (100–100)	100 (100–100)	100 (100–100)
Non-rheumatic valvular heart disease	28·1 (18·3–43·1)	28·9 (20·1–40·6)	30·3 (20·9–41·9)	28·9 (19·9–40·8)
Cardiomyopathy and myocarditis	38·2 (28·8–49·6)	44·5 (37·3–53·1)	45 (37·7–53·2)	44·4 (37–53·2)
Atrial fibrillation and flutter	53·7 (43·1–64·9)	60·7 (51·5–69·8)	65·5 (56·5–73·3)	56·3 (46·3–66·6)
Aortic aneurysm	57·7 (50·7–65·2)	65·2 (59·1–70·8)	--	65·2 (59·1–70·8)
Endocarditis	35·6 (24·2–51·7)	38·3 (27·9–50·9)	36·8 (26·1–51)	38·4 (28·1–51)
Other cardiovascular and circulatory diseases	37 (29·8–45·5)	41 (35·1–47·3)	43·3 (36·7–49·7)	40·2 (33·9–46·9)
Chronic respiratory diseases	60 (55·2–64·8)	54·7 (50·6–59·1)	50·3 (45·5–55·1)	61·3 (57–65·4)
Chronic obstructive pulmonary disease	66·4 (61·1–71·7)	63·7 (59·3–68·2)	57·8 (53·3–62·4)	69·9 (65·1–74·4)
Pneumoconiosis	100 (100–100)	100 (100–100)	100 (100–100)	100 (100–100)
Asthma	40·8 (29·1–53·6)	42·2 (33·6–52·1)	42 (33·3–51·6)	45·2 (34·2–57·2)

Digestive diseases	45·6 (30·4–55·5)	46·6 (33·9–56·2)	13·9 (9·39–18·7)	56·7 (41·4–66)
Cirrhosis and other chronic liver diseases	71·1 (47·9–85·2)	75·3 (55·8–86·9)	74 (53·9–84·5)	75·3 (55·7–87)
Upper digestive system diseases	15·6 (12·4–19·2)	4·46 (2·54–7·03)	0·673 (0·37–1·11)	19 (15·5–23)
Gallbladder and biliary diseases	42·5 (27·7–57·6)	48·6 (34·4–62·8)	50·8 (36·6–65)	43·5 (29·2–58·5)
Pancreatitis	29·6 (-0·00487–50·7)	33·3 (0–54·1)	38·4 (0–59·4)	32·8 (0–53·7)
Neurological disorders	27·2 (14·2–41·3)	16·1 (7·36–28·4)	9·81 (4·6–19·9)	26·4 (13·4–40·7)
Alzheimer's disease and other dementias	34·4 (21·7–49·2)	36·9 (24·2–51·4)	37·4 (24·7–51·8)	36·5 (23·7–51)
Parkinson's disease	-7·86 (-12·7–-3·87)	-9·25 (-14·7–-4·74)	-9·83 (-15·6–-5·08)	-9·04 (-14·4–-4·57)
Idiopathic epilepsy	26·1 (1·96–37·3)	23·1 (1·59–33·7)	22·1 (1·48–32·1)	26·4 (1·96–37·8)
Multiple sclerosis	14·5 (10·5–18·8)	15·1 (10·9–19·6)	14·6 (10·5–19)	16·6 (12–21·4)
Mental disorders	--	5·74 (2·94–9·19)	5·75 (2·94–9·19)	--
Depressive disorders	--	11 (4·95–17·7)	11 (4·95–17·7)	--
Anxiety disorders	--	5·28 (1·43–11·6)	5·28 (1·43–11·6)	--
Idiopathic developmental intellectual disability	--	49·2 (23·3–70·3)	49·2 (23·3–70·3)	--
Substance use disorders	100 (100–100)	100 (100–100)	100 (100–100)	100 (100–100)
Alcohol use disorders	100 (100–100)	100 (100–100)	100 (100–100)	100 (100–100)
Drug use disorders	100 (100–100)	100 (100–100)	100 (100–100)	100 (100–100)
Diabetes and kidney diseases	99·4 (99·1–99·6)	99·8 (99·7–99·9)	100 (100–100)	99·3 (99·1–99·5)
Diabetes mellitus	100 (100–100)	100 (100–100)	100 (100–100)	100 (100–100)
Chronic kidney disease	100 (100–100)	100 (100–100)	100 (100–100)	100 (100–100)
Sense organ diseases	--	6·9 (5·95–8·06)	6·9 (5·95–8·06)	--
Blindness and vision loss	--	8·12 (5·31–11·8)	8·12 (5·31–11·8)	--
Age-related and other hearing loss	--	7·61 (6·79–8·48)	7·61 (6·79–8·48)	--
Musculoskeletal disorders	0·686 (0·19–1·4)	22·4 (18·1–26·7)	22·5 (18·3–26·9)	0·853 (0·251–1·67)
Rheumatoid arthritis	7·97 (2·31–15·8)	14·2 (4·33–26·1)	14·4 (4·44–26·6)	9·77 (2·91–18·9)
Osteoarthritis	--	16·3 (8·55–25·1)	16·3 (8·55–25·1)	--
Low back pain	--	39·5 (33·7–45·7)	39·5 (33·7–45·7)	--
Gout	--	50·4 (34·8–67·8)	50·4 (34·8–67·8)	--
Other non-communicable diseases	0·0779 (0·0346–0·14)	0·0373 (0·0161–0·0696)	--	0·293 (0·14–0·517)
Sudden infant death syndrome	5·49 (3·28–9·22)	5·48 (3·27–9·21)	--	5·48 (3·27–9·21)
Injuries	29·4 (18·5–36·1)	28·6 (18·2–33·9)	32·7 (26·8–37)	24·4 (10·7–32)
Transport injuries	27·9 (19·6–34·4)	26·8 (17·4–33·1)	29·7 (22·9–34·5)	25·5 (15·5–32·7)
Road injuries	27·4 (19·2–33·9)	26·3 (16·9–32·7)	28·9 (22·1–33·8)	25·2 (15·1–32·6)
Other transport injuries	36·4 (28·7–42)	34·3 (26–39·9)	38·9 (32·7–43·4)	31·6 (22·6–37·8)

Unintentional injuries	35·2 (29·3–40·6)	30·9 (25·1–35·6)	33 (27·2–37·7)	25 (19·8–29·6)
Falls	56·6 (48·3–62·3)	42 (35·5–47·2)	40·4 (33·7–45·7)	48·3 (40·8–54·1)
Drowning	-8·63 (-21·1–2·69)	-7·59 (-18·9–2·9)	12·4 (5·15–18·4)	-8·75 (-20·6–2·06)
Fire, heat, and hot substances	11·3 (4·05–17·3)	16·3 (8·11–22·8)	16·6 (8·25–23·2)	15 (7·33–21·2)
Poisonings	13·4 (6·21–19·7)	15·6 (8·43–21·6)	14·7 (7·54–20·4)	16 (8·75–22·1)
Exposure to mechanical forces	16·2 (10·4–21·8)	20·6 (16·4–25·2)	22·7 (19·3–26·5)	9·84 (4·5–15·1)
Animal contact	24·4 (17·5–30)	25·5 (18–31·1)	26·7 (19·3–32·4)	22·5 (15·7–28·2)
Foreign body	1·77 (1·32–2·39)	5·03 (3·51–7·08)	5·97 (4·02–8·52)	4·27 (3·22–5·56)
Environmental heat and cold exposure	7·6 (0·609–13·8)	8·71 (0·702–15·4)	8·73 (0·67–15·3)	8·66 (0·736–15·3)
Exposure to forces of nature	0 (0–0)	0 (0–0)	0 (0–0)	0 (0–0)
Other unintentional injuries	14·4 (6·71–20·8)	15·6 (7·82–21·9)	15·3 (7·34–21·6)	17·7 (9·53–24·2)
Self-harm and interpersonal violence	21·9 (-2·94–38·3)	24·1 (0·259–39·2)	36·1 (23·1–45·2)	23 (-2·16–39·2)
Self-harm	21·2 (-4·37–37·9)	22·2 (-3·61–39·2)	22·3 (1·35–36·6)	22·2 (-3·68–39·3)
Interpersonal violence	41·1 (29·9–52·3)	38·8 (27·2–48·4)	38·8 (26·6–48·5)	38·7 (27·1–49·9)

Supplementary Results Table 2. All-age PAFs attributable to the joint distribution of all risk factors for all ages, both sexes combined for each GBD level 1, level 2, and level 3 cause and all causes in 2019 for Spain. Results are shown for deaths, YLLs, YLDs, and DALYs Data in parenthesis are 95% uncertainty intervals. PAFs=population attributable fractions. GBD=Global Burden of Disease. YLLs=years of life lost. YLDs=years lived with disability. DALYs=disability-adjusted life-years

	Deaths 2019	DALYs 2019	YLDs 2019	YLLs 2019
All causes	54·7 (51·2–58)	40·5 (37·5–43·7)	26·1 (24–28·3)	54·8 (52·2–57·4)
Communicable, maternal, neonatal, and nutritional diseases	39·5 (35·2–43·5)	47·1 (44·3–50)	43·3 (39·3–47·4)	50·3 (46·7–54)
HIV/AIDS and sexually transmitted infections	89·1 (88–90·1)	88·6 (87·4–89·8)	91·9 (90·4–93·6)	87·4 (86·2–88·5)
HIV/AIDS	88·8 (87·6–89·8)	87·8 (86·6–88·8)	89·6 (88·5–90·6)	87·2 (86–88·3)
Sexually transmitted infections excluding HIV	99·8 (99·7–99·9)	99·9 (99·8–99·9)	100 (100–100)	99·2 (98·9–99·5)
Respiratory infections and tuberculosis	39·5 (34·3–44·4)	35·5 (30·2–40·7)	3·29 (2·44–4·34)	44·9 (39·6–49·9)
Tuberculosis	51·2 (42·4–59)	57·4 (49·4–64·2)	56·5 (47·9–64·1)	57·6 (49·7–64·4)
Lower respiratory infections	39·3 (33·9–44·3)	44·4 (38·8–49·5)	36·6 (31·1–42)	44·5 (38·9–49·6)
Upper respiratory infections	0·0229 (0·0111–0·0432)	0·00484 (0·000936–0·0111)	0·00382 (7·16e-05–0·0103)	0·134 (0·067–0·246)
Otitis media	0·0595 (0·0211–0·175)	3·68 (2·33–5·19)	3·7 (2·34–5·22)	0·291 (0·103–0·833)
Enteric infections	7·23 (4·34–11·9)	9·88 (6·84–14·6)	9·81 (6·67–14·6)	10·1 (6·94–14·8)
Diarrheal diseases	7·29 (4·37–12)	9·93 (6·86–14·7)	9·82 (6·67–14·6)	10·2 (7·02–15)
Other infectious diseases	0·424 (0·279–0·578)	0·744 (0·529–0·967)	0·124 (0·0693–0·219)	1·07 (0·748–1·41)
Meningitis	0·723 (0·49–1·07)	1·51 (1·02–2·18)	0·000273 (3·51e-06–0·000778)	2·14 (1·45–3·1)
Encephalitis	0·264 (0·157–0·387)	0·684 (0·41–1·01)	9·47e-05 (1·19e-06–0·000264)	0·855 (0·512–1·23)
Measles	1·74 (-0·504–9·91)	1·91 (-0·444–9·9)	0·395 (0·0128–0·958)	2·57 (-0·763–14·2)
Acute hepatitis	8·77 (5·09–14·5)	3·07 (1·73–5·05)	0·812 (0·449–1·41)	10·3 (5·95–17·1)
Maternal and neonatal disorders	75·5 (71·9–79)	73·3 (70·5–75·9)	70·9 (66·9–74·4)	76·3 (72·8–79·7)
Maternal disorders	9·04 (3·36–14·9)	9·03 (3·35–14·9)	9·05 (3·35–14·9)	8·99 (3·34–14·9)
Neonatal disorders	77·5 (74–81)	74·9 (72·1–77·5)	72·8 (68·5–76·4)	77·6 (74–81)
Nutritional deficiencies	64·6 (55·8–77·8)	76·1 (72·9–79·5)	77·2 (73·7–80·7)	64·1 (56–77)
Protein-energy malnutrition	100 (100–100)	100 (100–100)	100 (100–100)	100 (100–100)
Vitamin A deficiency	--	100 (100–100)	100 (100–100)	--
Dietary iron deficiency	--	100 (100–100)	100 (100–100)	--
Non-communicable diseases	56·5 (52·6–60)	41·1 (37·7–44·6)	24·8 (22·4–27·3)	57·2 (54·2–60·2)
Neoplasms	46 (42·4–50·3)	48·3 (45·1–52)	38 (34·2–42·3)	48·9 (45·8–52·6)
Lip and oral cavity cancer	66·6 (60·5–72·2)	72·8 (67·1–77·7)	72·1 (66·1–77·2)	72·9 (67·2–77·7)
Nasopharynx cancer	67·2 (59·2–74·7)	69·4 (61·4–76·5)	66·6 (58·2–74·4)	69·7 (61·7–76·8)
Other pharynx cancer	83·6 (79·3–87·2)	85·2 (80·9–88·6)	83·6 (79–87·1)	85·2 (80·9–88·7)
Esophageal cancer	74·1 (65·9–82)	76·8 (69–84·3)	74·6 (66·5–82·6)	76·9 (69–84·3)
Stomach cancer	22·3 (16·7–35·8)	25·7 (19·1–40·2)	25 (18·6–39·1)	25·8 (19·1–40·3)

Colon and rectum cancer	63·8 (55·5–71·9)	65·2 (57·6–72·7)	65·2 (57·6–72·7)	65·2 (57·5–72·7)
Liver cancer	57·4 (48·3–66·1)	61·9 (52·8–70·3)	60·4 (51·2–69·1)	61·9 (52·8–70·3)
Gallbladder and biliary tract cancer	21·8 (12–33·8)	21·8 (12·1–34)	20·6 (11·1–32·8)	21·9 (12·1–34)
Pancreatic cancer	38·1 (29·4–48·5)	40·5 (32·7–50)	38·9 (30·8–49)	40·5 (32·7–50)
Larynx cancer	83·1 (76·1–88·6)	84·6 (78·5–89·4)	84·5 (78·2–89·3)	84·6 (78·5–89·4)
Tracheal, bronchus, and lung cancer	85·2 (82·4–88·1)	85·5 (82·9–88)	85·7 (83·1–88·3)	85·5 (82·9–88)
Breast cancer	32·1 (23·7–42·1)	32·2 (24·9–40·6)	32·2 (24·7–41)	32·3 (24·9–40·6)
Cervical cancer	100 (100–100)	100 (100–100)	100 (100–100)	100 (100–100)
Uterine cancer	45·7 (31·3–61)	46·8 (32·5–61·9)	47·2 (32·8–62·2)	46·7 (32·5–61·9)
Ovarian cancer	17·2 (7·93–29·8)	14·6 (6·39–25·7)	14·1 (6·18–24·5)	14·6 (6·38–25·8)
Prostate cancer	6·68 (3·02–10·5)	7·65 (3·56–11·7)	8·38 (3·87–12·8)	7·54 (3·5–11·6)
Kidney cancer	38·6 (28·8–48·5)	40·3 (31–49·7)	40·6 (31·4–50)	40·3 (31–49·7)
Bladder cancer	39·5 (26–53·5)	44 (30·8–56·6)	45·8 (33·1–57·8)	43·8 (30·7–56·5)
Thyroid cancer	14·5 (7·05–23·7)	15·1 (7·29–24·8)	15·1 (7·38–24·6)	15·1 (7·3–24·8)
Mesothelioma	95·8 (94·2–97·1)	92·5 (89·8–94·7)	95·2 (93·1–96·7)	92·4 (89·7–94·6)
Non-Hodgkin lymphoma	6·98 (2·92–12·4)	7·1 (3·03–12·4)	7·05 (3·01–12·4)	7·1 (3·03–12·5)
Multiple myeloma	8·04 (3·4–14·4)	8·3 (3·62–14·7)	8·25 (3·52–14·7)	8·3 (3·64–14·7)
Leukemia	33·6 (21·8–45·7)	33·8 (22·9–44·9)	33·4 (22·7–43·9)	33·9 (22·9–44·9)
Cardiovascular diseases	76 (72·2–79·8)	78·1 (74·9–81·3)	74·2 (70·3–78·3)	78·6 (75·5–81·7)
Rheumatic heart disease	24·7 (13·3–48·9)	26·4 (16·1–46·1)	27·1 (17–44·9)	26·4 (16–46)
Ischemic heart disease	92·7 (88·3–96·6)	94·2 (91·1–96·9)	93·6 (90·5–96·4)	94·2 (91·2–96·9)
Stroke	78·9 (70·9–87·7)	82·5 (76·6–89)	83 (77·3–89·3)	82·4 (76·4–89·1)
Hypertensive heart disease	100 (100–100)	100 (100–100)	100 (100–100)	100 (100–100)
Non-rheumatic valvular heart disease	26·7 (17·5–39)	28·7 (20·5–39·4)	26·6 (17·9–36·8)	28·7 (20·5–39·5)
Cardiomyopathy and myocarditis	32·9 (24·4–44·6)	39·1 (32·1–48·1)	38·2 (30·5–47·1)	39·2 (32·2–48·1)
Atrial fibrillation and flutter	50 (40·1–60·2)	57·8 (49·5–66)	63·2 (55·8–70·5)	53·2 (44–62·5)
Aortic aneurysm	58 (52·2–64·6)	67·3 (62·7–72·1)	--	67·3 (62·7–72·1)
Endocarditis	33·5 (23–47·9)	37 (27·7–48·3)	35 (25–47·8)	37·1 (27·8–48·3)
Other cardiovascular and circulatory diseases	29·7 (23·6–37)	34·6 (29·5–39·9)	40·4 (34·5–46·4)	32·8 (27·8–38·7)
Chronic respiratory diseases	59·5 (54·2–66·1)	58·1 (54·3–62·2)	52·7 (48·4–57)	61·5 (56·9–67·1)
Chronic obstructive pulmonary disease	67·3 (62·6–72·4)	68·4 (64·7–71·9)	61·3 (57·4–65·3)	71·6 (67·4–75·8)
Pneumoconiosis	100 (100–100)	100 (100–100)	100 (100–100)	100 (100–100)
Asthma	36·8 (24·3–51·1)	43 (34·1–53·1)	43·4 (34·7–53·1)	40·5 (29–53·5)
Digestive diseases	32·9 (27–38·7)	37·4 (31·4–43·4)	11·6 (7·99–15·6)	44·2 (37·2–51)
Cirrhosis and other chronic liver diseases	68·7 (54·1–82·5)	74·3 (60·9–86)	71·6 (60·9–82·5)	74·3 (60·9–86·1)

Upper digestive system diseases	17·2 (14·5–20·1)	5·78 (3·65–8·61)	0·877 (0·49–1·41)	23·8 (20·3–27·1)
Gallbladder and biliary diseases	43·5 (28·7–58·7)	46·5 (32·1–61·1)	51·7 (37·9–65·3)	44·2 (29·7–59·2)
Pancreatitis	35·1 (25·2–47·6)	41·2 (30·6–53·7)	38·4 (27·8–50·9)	41·3 (30·8–54)
Neurological disorders	23·1 (9·68–38·2)	14·9 (6·94–26·8)	9·74 (4·66–19·1)	21·1 (8·39–35·6)
Alzheimer's disease and other dementias	34·1 (19·8–50·3)	36·7 (22·8–52·5)	37·1 (23·2–52·7)	36·4 (22·4–52·2)
Parkinson's disease	-8·29 (-13·2–-4·04)	-10·1 (-15·8–-5·06)	-10·9 (-17·1–-5·58)	-9·93 (-15·7–-4·94)
Idiopathic epilepsy	21·4 (15·2–28·4)	22·1 (16·3–28·7)	21·1 (15·4–27·6)	23·8 (17·4–31·2)
Multiple sclerosis	15·5 (11·4–19·6)	17·3 (13·2–21·6)	16·8 (12·8–21)	18·1 (13·6–22·9)
Mental disorders	--	4·6 (2·71–6·88)	4·6 (2·71–6·89)	--
Depressive disorders	--	6·42 (3·08–10·4)	6·42 (3·08–10·4)	--
Anxiety disorders	--	3·47 (1·01–7·67)	3·47 (1·01–7·67)	--
Idiopathic developmental intellectual disability	--	51·8 (25·5–70·2)	51·8 (25·5–70·2)	--
Substance use disorders	100 (100–100)	100 (100–100)	100 (100–100)	100 (100–100)
Alcohol use disorders	100 (100–100)	100 (100–100)	100 (100–100)	100 (100–100)
Drug use disorders	100 (100–100)	100 (100–100)	100 (100–100)	100 (100–100)
Diabetes and kidney diseases	100 (100–100)	100 (100–100)	100 (100–100)	100 (100–100)
Diabetes mellitus	100 (100–100)	100 (100–100)	100 (100–100)	100 (100–100)
Chronic kidney disease	100 (100–100)	100 (100–100)	100 (100–100)	100 (100–100)
Sense organ diseases	--	7·49 (6·05–9·47)	7·49 (6·05–9·47)	--
Blindness and vision loss	--	10·1 (6·59–14·5)	10·1 (6·59–14·5)	--
Age-related and other hearing loss	--	6·74 (6·04–7·55)	6·74 (6·04–7·55)	--
Musculoskeletal disorders	2·07 (0·558–3·81)	21·7 (17·8–26·1)	22·1 (18·1–26·5)	3·31 (0·946–5·88)
Rheumatoid arthritis	8·22 (2·31–14·9)	15 (4·73–25·4)	16 (5·04–27·3)	11·3 (3·38–19·7)
Osteoarthritis	--	15·2 (7·48–24·1)	15·2 (7·48–24·1)	--
Low back pain	--	41·8 (36·3–47·1)	41·8 (36·3–47·1)	--
Gout	--	50·5 (35·2–67·2)	50·5 (35·2–67·2)	--
Other non-communicable diseases	0·0253 (0·0172–0·0368)	0·0266 (0·0165–0·0409)	--	0·13 (0·0904–0·184)
Sudden infant death syndrome	7·03 (3·96–11·8)	7·03 (3·95–11·7)	--	7·03 (3·95–11·7)
Injuries	27·8 (23·7–31·2)	29·7 (25·9–33·4)	33·2 (29·3–37)	25·4 (20·9–29·9)
Transport injuries	30·6 (25·7–34·8)	29·9 (25–34·5)	29·9 (26–33·6)	29·9 (24·3–35)
Road injuries	28·7 (23·7–33·2)	28·5 (23·5–33·1)	28·9 (25–32·5)	28·4 (22·5–33·6)
Other transport injuries	38·9 (34·1–43·2)	37·2 (32–41·8)	39·8 (35·2–43·5)	36·6 (31·3–41·5)
Unintentional injuries	29·6 (25·3–33)	31·1 (27–35·2)	33·6 (29·3–37·7)	24·1 (20·3–27·7)
Falls	54·4 (47·4–59·3)	42·1 (37·2–46·4)	41·2 (36·3–45·6)	45·6 (40·3–50·3)
Drowning	-7·03 (-14·6–1·25)	-3·79 (-11·5–4·35)	11·7 (7·84–16·5)	-4·47 (-12·3–3·92)

Fire, heat, and hot substances	10·4 (6·77–14·9)	16·1 (11·5–21·3)	16·4 (11·8–21·7)	15·2 (10·9–20·4)
Poisonings	14·9 (10·5–20·2)	16·6 (12·1–21·9)	15 (10·8–20)	18·6 (13·6–24·3)
Exposure to mechanical forces	18·1 (12·4–23·6)	23·2 (19·7–27)	24·3 (21–27·9)	14·1 (8·03–20·4)
Animal contact	27·4 (22·4–32·7)	25·9 (21–30·9)	25·4 (20·7–30·4)	26·8 (21·5–32·2)
Foreign body	1·56 (1·1–2·17)	5·03 (3·56–6·82)	6·69 (4·69–9·03)	4·62 (3·28–6·36)
Environmental heat and cold exposure	7·5 (3·46–12·5)	8·33 (3·87–13·9)	8·08 (3·76–13·5)	9·03 (4·21–15·1)
Exposure to forces of nature	0 (0–0)	9·24 (4·28–15·3)	9·24 (4·28–15·3)	0 (0–0)
Other unintentional injuries	22·9 (17–29·6)	16·3 (11·7–21·5)	15·5 (11·2–20·7)	24·8 (18·5–31·8)
Self-harm and interpersonal violence	21·6 (11–31·7)	24·4 (14·5–33·6)	33·4 (27·5–38·8)	23·1 (12·4–33·2)
Self-harm	20·9 (9·3–31·6)	22·4 (10·3–33·3)	20·5 (10·8–29·3)	22·5 (10·3–33·5)
Interpersonal violence	30·5 (23·4–37·4)	34·6 (27·8–41·6)	39 (32·3–45·9)	29·9 (22·7–36·9)

Supplementary Results Table 2. All-age PAFs attributable to the joint distribution of all risk factors for all ages, both sexes combined for each GBD level 1, level 2, and level 3 cause and all causes in 2019 for Sweden. Results are shown for deaths, YLLs, YLDs, and DALYs Data in parenthesis are 95% uncertainty intervals. PAFs=population attributable fractions. GBD=Global Burden of Disease. YLLs=years of life lost. YLDs=years lived with disability. DALYs=disability-adjusted life-years

	Deaths 2019	DALYs 2019	YLDs 2019	YLLs 2019
All causes	57·1 (53·9–60)	41 (38·1–44)	27 (24·8–29·3)	54·7 (52·3–57·1)
Communicable, maternal, neonatal, and nutritional diseases	35·3 (27·2–42·6)	42 (37·9–45·8)	40·3 (36·1–44·4)	43·5 (36·9–49·2)
HIV/AIDS and sexually transmitted infections	88·4 (86·4–90·1)	89·8 (87–92·6)	95·2 (92·5–97·6)	83·7 (81·3–85·8)
HIV/AIDS	86·4 (84·2–88·4)	84·3 (81·6–86·6)	87·8 (84·9–90·2)	82·6 (79·9–84·9)
Sexually transmitted infections excluding HIV	99·9 (99·9–100)	100 (99·9–100)	100 (100–100)	99·7 (99·6–99·8)
Respiratory infections and tuberculosis	41·3 (30·3–51·6)	33·4 (25·2–41·9)	2·99 (2·2–4·04)	44·4 (33·7–54·3)
Tuberculosis	54·8 (44·7–63·6)	56·2 (46·7–64)	53·4 (43·6–61·5)	56·8 (47–64·9)
Lower respiratory infections	41·1 (29·6–51·5)	43·9 (32·8–53·9)	30·7 (25·1–36)	44·1 (33–54·3)
Upper respiratory infections	0·0086 (0·00506–0·0143)	0·00596 (0·00104–0·0152)	0·00547 (5e-04–0·0148)	0·0417 (0·0257–0·0686)
Otitis media	0·286 (0·094–0·595)	2·63 (1·66–3·77)	2·64 (1·66–3·78)	1·88 (0·641–3·78)
Enteric infections	5·7 (3·86–8·71)	7·66 (5·69–10·6)	8·22 (6·07–11·1)	6·94 (4·97–9·93)
Diarrheal diseases	5·72 (3·86–8·72)	7·68 (5·7–10·6)	8·22 (6·08–11·1)	6·97 (4·99–9·98)
Other infectious diseases	0·213 (0·139–0·385)	0·597 (0·461–0·781)	0·28 (0·157–0·514)	0·717 (0·538–0·999)
Meningitis	0·768 (0·552–1·05)	1·98 (1·44–2·72)	0·000307 (2·18e-05–0·000867)	2·65 (1·97–3·51)
Encephalitis	0·156 (0·114–0·228)	0·371 (0·262–0·5)	0·000112 (6·03e-06–0·000314)	0·606 (0·457–0·798)
Measles	2·46 (-0·582–12·1)	3·07 (-0·686–14·8)	0·507 (0·0163–1·26)	3·37 (-0·813–16·6)
Acute hepatitis	6·97 (3·89–14·2)	3·71 (2·37–5·97)	2·21 (1·24–3·94)	7·33 (4·26–14·9)
Maternal and neonatal disorders	72·7 (69·3–75·9)	72 (68·9–74·6)	70·5 (65·6–75)	73·3 (69·9–76·6)
Maternal disorders	8·62 (3·14–14·2)	8·72 (3·17–14·4)	8·89 (3·21–14·7)	8·44 (3·03–13·8)
Neonatal disorders	74·4 (71–77·7)	73·5 (70·4–76·3)	72·4 (67·4–77)	74·4 (71–77·8)
Nutritional deficiencies	84·4 (70·5–87·7)	75·3 (72·2–77·9)	74·3 (71·2–77·1)	83·9 (70–86·7)
Protein-energy malnutrition	100 (100–100)	100 (100–100)	100 (100–100)	100 (100–100)
Vitamin A deficiency	--	100 (100–100)	100 (100–100)	--
Dietary iron deficiency	--	100 (100–100)	100 (100–100)	--
Non-communicable diseases	59·5 (55·7–62·7)	42 (38·7–45·6)	25·4 (23–28)	58·2 (55·5–60·9)
Neoplasms	40 (36·7–43·7)	39·6 (36·4–43·1)	28·4 (24·9–32·2)	40·3 (37·2–43·8)
Lip and oral cavity cancer	67·1 (59·6–73·4)	68·9 (61·7–74·9)	69·2 (62–75·2)	68·9 (61·7–74·9)
Nasopharynx cancer	65·9 (56·3–74)	64·3 (55–71·9)	63·4 (54–71·2)	64·3 (55·1–72)
Other pharynx cancer	77·8 (71·6–82·9)	78·2 (72·1–83·2)	77·4 (71·3–82·6)	78·2 (72·2–83·3)
Esophageal cancer	73·7 (65·5–81·8)	74·7 (66·7–82·7)	74·1 (65·9–82·2)	74·7 (66·7–82·7)
Stomach cancer	22·6 (15·1–40·5)	22·7 (14·9–40·7)	23 (15·3–41·1)	22·7 (14·9–40·7)
Colon and rectum cancer	61 (52·6–68·7)	61·3 (53·4–68·6)	61·5 (53·8–69)	61·3 (53·4–68·6)

Liver cancer	74·9 (69·1–80·7)	76·9 (71·5–82)	75·4 (69·6–81·1)	76·9 (71·5–82)
Gallbladder and biliary tract cancer	20·4 (11·1–31·5)	20·9 (11·4–32·1)	20·4 (11·1–31·8)	20·9 (11·4–32·1)
Pancreatic cancer	37·2 (29·5–46·6)	36·6 (29·1–45·5)	37 (29·1–46·3)	36·6 (29·1–45·5)
Larynx cancer	80·5 (71·6–87·1)	80·8 (73–86·8)	80·8 (73·1–86·9)	80·8 (73–86·8)
Tracheal, bronchus, and lung cancer	80·5 (77·1–84·2)	79 (75·6–82·8)	79·6 (76·1–83·4)	79 (75·6–82·7)
Breast cancer	36·3 (29–44·8)	35·6 (28·7–42·9)	35·9 (29·1–43·6)	35·5 (28·7–42·8)
Cervical cancer	100 (100–100)	100 (100–100)	100 (100–100)	100 (100–100)
Uterine cancer	41·5 (27·6–56·8)	43 (29–58·1)	43·6 (29·4–58·7)	42·9 (29–58)
Ovarian cancer	16·5 (7·75–26·8)	14·2 (6·43–23·6)	13·2 (5·93–22·3)	14·2 (6·43–23·6)
Prostate cancer	5·72 (2·27–9·99)	6·13 (2·49–10·6)	6·36 (2·64–10·7)	6·09 (2·47–10·5)
Kidney cancer	34·8 (25·1–44·9)	34·9 (25·5–44·7)	34·6 (25·3–44·6)	34·9 (25·5–44·7)
Bladder cancer	35·7 (22·9–49)	38·7 (25·9–51)	39·7 (27·3–51·9)	38·5 (25·9–51)
Thyroid cancer	12·7 (6·06–21·2)	13·4 (6·35–22·4)	13·4 (6·63–22)	13·4 (6·3–22·5)
Mesothelioma	97·6 (96·8–98·2)	95·4 (94–96·5)	97·1 (96–98·1)	95·3 (93·9–96·5)
Non-Hodgkin lymphoma	6·15 (2·44–11·1)	6·4 (2·59–11·5)	6·28 (2·55–11·3)	6·42 (2·6–11·6)
Multiple myeloma	7·08 (2·99–12·7)	7·4 (3·15–13·3)	7·46 (3·23–13·7)	7·4 (3·15–13·3)
Leukemia	32·7 (20–46·6)	30·6 (19·8–42·2)	31·1 (19·9–43·2)	30·6 (19·7–42)
Cardiovascular diseases	81 (77·2–84·4)	81·8 (78·7–84·5)	76·1 (72·1–79·7)	82·8 (79·8–85·5)
Rheumatic heart disease	26·5 (14·3–51·9)	27·7 (16·5–49·3)	28·5 (17·5–46·4)	27·6 (16·4–49·9)
Ischemic heart disease	92·7 (88·3–96·3)	93·7 (90·5–96·5)	93·4 (90·2–96·2)	93·7 (90·5–96·6)
Stroke	80·3 (72·2–88·9)	83 (77·1–89·2)	82·6 (77·6–87·7)	83·1 (76·8–89·7)
Hypertensive heart disease	100 (100–100)	100 (100–100)	100 (100–100)	100 (100–100)
Non-rheumatic valvular heart disease	30·2 (19·2–44·7)	31·4 (21·5–44·1)	28·6 (20–38·4)	31·6 (21·7–44·6)
Cardiomyopathy and myocarditis	40·9 (32–49·3)	46·1 (39·7–52·1)	45 (38·1–51·6)	46·3 (39·4–52·6)
Atrial fibrillation and flutter	53·9 (44·6–63·8)	60·9 (53·1–68·9)	65·7 (59·2–72·6)	56·4 (47·5–65·7)
Aortic aneurysm	57 (50·2–63·7)	63·5 (57·7–69·1)	--	63·5 (57·7–69·1)
Endocarditis	36 (24·5–50·3)	38·7 (28·6–50·7)	36·8 (25·8–50·4)	38·8 (28·8–50·7)
Other cardiovascular and circulatory diseases	33·5 (26·6–41·5)	38·8 (32·7–44·8)	43·8 (37·4–50·3)	35·3 (28·9–42·1)
Chronic respiratory diseases	59·6 (54·5–65·1)	54 (50·1–58·1)	48·9 (44·4–53·7)	60·5 (55·6–65·5)
Chronic obstructive pulmonary disease	68·2 (63–72·9)	65·2 (61·5–68·9)	58·9 (55·4–62·3)	70·4 (65·7–74·8)
Pneumoconiosis	100 (100–100)	100 (100–100)	100 (100–100)	100 (100–100)
Asthma	39·7 (28·3–52·5)	39·7 (31·1–49·8)	39·5 (31–49·6)	42·4 (31·5–54·3)
Digestive diseases	35 (30·6–39·2)	34·4 (29·8–38·9)	13·8 (9·51–19)	44·3 (39·2–49·1)
Cirrhosis and other chronic liver diseases	77·9 (67–87·6)	79·9 (70·2–88·7)	72·9 (64·3–81·2)	80·1 (70·3–88·9)
Upper digestive system diseases	21·4 (17·7–25·1)	9·83 (6·45–13·6)	1·16 (0·654–1·83)	23·9 (20–27·7)

Gallbladder and biliary diseases	39·9 (26–54)	45·9 (32·2–60·1)	49·2 (35·2–63·5)	41 (27·7–55·1)
Pancreatitis	44·8 (32·8–58)	46·9 (35·6–59)	44·7 (33·3–56·7)	47·2 (35·8–59·4)
Neurological disorders	23·7 (10·7–37·3)	15·2 (7·36–26·7)	10·2 (5·1–19·7)	21·2 (9·26–35·1)
Alzheimer's disease and other dementias	36·7 (24·8–50·2)	39·5 (27·6–52·8)	40·3 (28·2–53·5)	39·1 (27·3–52·3)
Parkinson's disease	-10·3 (-16·4—5·31)	-11·5 (-18—6·03)	-11·7 (-17·9—6·15)	-11·5 (-18—6·03)
Idiopathic epilepsy	27·8 (19·9–35·4)	24·5 (17·5–31·6)	23·3 (16·4–30·2)	26·5 (18·8–33·6)
Multiple sclerosis	14·6 (10·4–19·5)	15·1 (10·7–19·8)	14·8 (10·5–19·2)	15·8 (11·2–20·7)
Mental disorders	--	6·5 (3·4–10)	6·5 (3·4–10)	--
Depressive disorders	--	13·1 (6·09–20·6)	13·1 (6·09–20·6)	--
Anxiety disorders	--	4·69 (1·31–10·3)	4·69 (1·31–10·3)	--
Idiopathic developmental intellectual disability	--	41·1 (18·4–58·9)	41·1 (18·4–58·9)	--
Substance use disorders	100 (100–100)	100 (100–100)	100 (100–100)	100 (100–100)
Alcohol use disorders	100 (100–100)	100 (100–100)	100 (100–100)	100 (100–100)
Drug use disorders	100 (100–100)	100 (100–100)	100 (100–100)	100 (100–100)
Diabetes and kidney diseases	100 (100–100)	100 (100–100)	100 (100–100)	100 (100–100)
Diabetes mellitus	100 (100–100)	100 (100–100)	100 (100–100)	100 (100–100)
Chronic kidney disease	100 (100–100)	100 (100–100)	100 (100–100)	100 (100–100)
Sense organ diseases	--	7·69 (6·57–9)	7·69 (6·57–9)	--
Blindness and vision loss	--	9·12 (6·03–13)	9·12 (6·03–13)	--
Age-related and other hearing loss	--	9·08 (8·25–9·97)	9·08 (8·25–9·97)	--
Musculoskeletal disorders	4·39 (1·32–8·3)	20·4 (16·7–24·2)	20·7 (16·9–24·6)	4·85 (1·48–9·16)
Rheumatoid arthritis	11·7 (3·47–21·6)	14·7 (4·56–26·9)	15 (4·69–27·4)	13·5 (4·04–25)
Osteoarthritis	--	15·7 (8·08–24·4)	15·7 (8·08–24·4)	--
Low back pain	--	37·9 (32·3–43·7)	37·9 (32·3–43·7)	--
Gout	--	47·7 (34·2–63·5)	47·7 (34·2–63·5)	--
Other non-communicable diseases	0·0916 (0·0606–0·132)	0·0624 (0·0377–0·0961)	--	0·361 (0·228–0·5)
Sudden infant death syndrome	6·86 (5·05–9·2)	6·85 (5·04–9·19)	--	6·85 (5·04–9·19)
Injuries	34·2 (28–39·4)	30·6 (25·6–34·9)	37·7 (33·3–41·5)	22·6 (14·9–29·9)
Transport injuries	22·9 (16·2–29·7)	25·9 (19·8–31·9)	32·3 (28·2–36·4)	21·9 (14·1–29·5)
Road injuries	20·5 (13·3–27·7)	24·1 (17·6–30·3)	31 (26·9–35·1)	19·8 (11·3–28)
Other transport injuries	39·6 (34·8–44·1)	39·1 (34·1–43·8)	41·2 (36·8–44·8)	37·7 (32–43·1)
Unintentional injuries	46·5 (40·3–50·6)	37·7 (32·9–41·8)	38·5 (33·6–42·8)	35·5 (30·3–39·8)
Falls	59·3 (51·8–63·9)	48·5 (42·6–52·9)	46·8 (41·2–51·1)	53·7 (47·3–58·3)
Drowning	-9·36 (-25·1–5·08)	-6·07 (-21–7·48)	15 (10·3–21·3)	-7·26 (-22·9–6·85)
Fire, heat, and hot substances	14·2 (9·47–20·6)	19·1 (14·1–25·5)	19·6 (14·6–26·2)	17·6 (12·9–23·7)

Poisonings	22·3 (16·9–29·3)	21·7 (16·7–28·3)	18·4 (13·9–24·5)	25 (19·2–32·1)
Exposure to mechanical forces	13·2 (2·99–23·1)	23·8 (20·4–27·7)	25·8 (22·5–29·3)	6·74 (-4·36–18)
Animal contact	26·9 (21·8–32·9)	29 (24–34·4)	30 (25–35·6)	25·1 (20·5–30·8)
Foreign body	3·04 (2·29–3·94)	7·16 (5·45–9·15)	7·52 (5·66–9·74)	6·73 (5·11–8·65)
Environmental heat and cold exposure	9·23 (4·51–16)	9·07 (4·4–15·7)	9·48 (4·52–16·3)	8·71 (4·21–15·1)
Exposure to forces of nature	0 (0–0)	10 (4·76–17·3)	10 (4·76–17·3)	0 (0–0)
Other unintentional injuries	19·1 (13·8–25·7)	18·6 (13·7–25)	18·2 (13·3–24·5)	22·3 (16·8–29·1)
Self-harm and interpersonal violence	14·6 (-0·486–28·3)	15·6 (2·33–27·8)	31·9 (25·1–38·4)	13·8 (-0·88–27·2)
Self-harm	13·9 (-2·02–28·6)	13·5 (-2·19–28·2)	22·4 (11·4–33·4)	13·3 (-2·47–28·2)
Interpersonal violence	22·1 (11·4–32·8)	26·1 (18–35·1)	34·1 (26·5–41·7)	18·9 (7·99–29·7)

Supplementary Results Table 2. All-age PAFs attributable to the joint distribution of all risk factors for all ages, both sexes combined for each GBD level 1, level 2, and level 3 cause and all causes in 2019 for Stockholm. Results are shown for deaths, YLLs, YLDs, and DALYs Data in parenthesis are 95% uncertainty intervals. PAFs=population attributable fractions. GBD=Global Burden of Disease. YLLs=years of life lost. YLDs=years lived with disability. DALYs=disability-adjusted life-years

	Deaths 2019	DALYs 2019	YLDs 2019	YLLs 2019
All causes	55·3 (51·9–58·3)	38·2 (35·1–41·4)	25 (22·9–27·2)	52·7 (50·1–55·2)
Communicable, maternal, neonatal, and nutritional diseases	37·4 (29·8–44·4)	43·3 (39·6–46·8)	42·2 (37·5–46·8)	44·6 (39·1–49·8)
HIV/AIDS and sexually transmitted infections	87·9 (85·8–89·7)	87·7 (85–90·4)	93·2 (90·2–96·1)	83·5 (81–85·7)
HIV/AIDS	86·3 (84–88·3)	84·2 (81·4–86·6)	87·6 (84·7–90·1)	82·6 (80–84·9)
Sexually transmitted infections excluding HIV	100 (100–100)	100 (100–100)	100 (100–100)	99·8 (99·8–99·9)
Respiratory infections and tuberculosis	42·3 (30·9–51·7)	31 (23·1–38·7)	3·43 (2·45–4·65)	45·2 (34·2–54·1)
Tuberculosis	55 (43–65·5)	56·1 (44·5–66·2)	52·4 (39·3–64)	57 (45·5–67)
Lower respiratory infections	41·9 (30·1–51·4)	44·3 (32·8–53·4)	29·3 (23·2–34·9)	44·8 (33·4–54)
Upper respiratory infections	0·0127 (0·00744–0·0203)	0·00577 (0·000397–0·0173)	0·00534 (0–0·0169)	0·057 (0·034–0·0899)
Otitis media	0·741 (0·223–1·93)	2·95 (1·9–4·2)	2·94 (1·89–4·2)	4·55 (1·49–10·7)
Enteric infections	5·95 (3·77–9·45)	8·44 (6·12–11·7)	9 (6·58–12·4)	7·45 (5·16–10·9)
Diarrheal diseases	5·96 (3·78–9·48)	8·45 (6·13–11·8)	9·01 (6·58–12·4)	7·48 (5·18–11)
Other infectious diseases	0·279 (0·164–0·523)	0·752 (0·523–1·09)	0·318 (0·171–0·582)	0·888 (0·585–1·35)
Meningitis	0·715 (0·483–1·02)	2·04 (1·42–2·88)	0·00038 (0–0·00123)	2·39 (1·67–3·32)
Encephalitis	0·156 (0·104–0·294)	0·42 (0·292–0·628)	0·000138 (0–0·00045)	0·58 (0·41–0·872)
Measles	2·06 (-0·463–9·88)	2·69 (-0·555–12·4)	0·558 (0·0151–1·53)	2·95 (-0·682–14·1)
Acute hepatitis	7·08 (4·15–13·9)	3·94 (2·53–6·05)	2·28 (1·25–4·07)	7·77 (4·54–14·3)
Maternal and neonatal disorders	68·4 (64·5–72·5)	69·8 (66·5–73·2)	69·7 (64·4–75)	69·9 (66·1–74)
Maternal disorders	8·79 (3·13–14·6)	9 (3·22–15)	9·5 (3·4–16·1)	8·61 (3·07–14·2)
Neonatal disorders	73 (68·9–77·2)	72·3 (68·9–75·7)	71·7 (66·2–76·9)	73 (68·9–77·2)
Nutritional deficiencies	84·7 (62·2–88·6)	75·8 (72–79·9)	74·7 (70·4–79·2)	84·2 (62·2–87·7)
Protein-energy malnutrition	100 (100–100)	100 (100–100)	100 (100–100)	100 (100–100)
Vitamin A deficiency	--	100 (100–100)	100 (100–100)	--
Dietary iron deficiency	--	100 (100–100)	100 (100–100)	--
Non-communicable diseases	57·4 (53·8–60·7)	38·7 (35·1–42·2)	22·9 (20·6–25·4)	55·8 (53·2–58·4)
Neoplasms	40·6 (37·3–44·4)	39·8 (36·7–43·3)	28·1 (24·7–31·9)	40·5 (37·4–44·1)
Lip and oral cavity cancer	67·2 (56·1–76·5)	68·8 (57·7–77·4)	68·9 (57·6–77·8)	68·8 (57·7–77·3)
Nasopharynx cancer	64·6 (52·7–74·6)	63·1 (50·8–73·1)	62·3 (49·7–72·6)	63·2 (50·8–73·1)
Other pharynx cancer	76·9 (68·9–83·8)	77·3 (69·4–83·9)	76·5 (68·5–83·3)	77·4 (69·5–84)
Esophageal cancer	72·8 (64–81·2)	73·6 (65–81·9)	73·2 (64·2–81·3)	73·6 (64·9–81·9)

Stomach cancer	22 (14·5–40)	21·7 (13·9–39·7)	22·1 (14·4–39·9)	21·7 (13·9–39·7)
Colon and rectum cancer	60·6 (51·8–68·5)	60·7 (52–68·2)	60·9 (52·1–68·6)	60·6 (52·1–68·1)
Liver cancer	73·6 (66·4–80·1)	75·5 (68·8–81·2)	74 (66·8–80·2)	75·5 (68·8–81·2)
Gallbladder and biliary tract cancer	18·7 (10·1–29·4)	19·3 (10·6–30·1)	18·8 (9·9–29·9)	19·3 (10·6–30·1)
Pancreatic cancer	35·4 (27·8–44·4)	34·6 (27·4–43·4)	35·2 (27·7–44·1)	34·6 (27·3–43·4)
Larynx cancer	80·1 (71·3–86·7)	80 (72–86·1)	79·9 (72·1–86)	80 (72–86·1)
Tracheal, bronchus, and lung cancer	79·7 (75·8–84·2)	78 (73·9–82·7)	78·7 (74·4–83·5)	78 (73·9–82·7)
Breast cancer	34·4 (26·9–42·7)	33·6 (26·5–40·9)	33·9 (26·7–41·4)	33·5 (26·5–40·8)
Cervical cancer	100 (100–100)	100 (100–100)	100 (100–100)	100 (100–100)
Uterine cancer	39 (24·7–54)	40·7 (26·5–55·3)	41·3 (27·2–56)	40·5 (26·4–55·2)
Ovarian cancer	15·8 (7·39–25·7)	13·3 (6·18–22·1)	12·3 (5·63–20·8)	13·3 (6·19–22·2)
Prostate cancer	5·86 (2·34–10·3)	6·22 (2·55–10·6)	6·41 (2·67–11)	6·19 (2·52–10·6)
Kidney cancer	33·1 (23·8–43·1)	33 (24–42·8)	32·6 (23·7–42·4)	33 (24–42·8)
Bladder cancer	37·2 (24·5–49·6)	39·8 (27·7–51·4)	40·7 (28·6–52·1)	39·7 (27·6–51·4)
Thyroid cancer	11·8 (5·21–20·3)	12·4 (5·49–21·3)	12·4 (5·81–21·2)	12·4 (5·36–21·4)
Mesothelioma	97 (95·9–97·8)	94·2 (92·3–95·7)	96·4 (94·9–97·6)	94·1 (92·2–95·7)
Non-Hodgkin lymphoma	5·55 (2·22–10·2)	5·77 (2·34–10·5)	5·66 (2·29–10·3)	5·78 (2·36–10·6)
Multiple myeloma	6·38 (2·68–11·6)	6·68 (2·83–12·2)	6·74 (2·87–12·3)	6·68 (2·82–12·2)
Leukemia	32 (20–45·1)	29·1 (18·9–39·7)	29·7 (19·3–41)	29 (18·9–39·6)
Cardiovascular diseases	79·3 (74·9–83·2)	79·8 (76·2–83·1)	72·2 (67·5–76·9)	81·2 (77·7–84·4)
Rheumatic heart disease	23·2 (12·1–46)	23·8 (13·7–42)	24 (14·5–40·5)	23·8 (13·7–42·3)
Ischemic heart disease	92·1 (87·7–95·7)	93·2 (89·8–96)	92·9 (89·4–95·8)	93·2 (89·9–96·1)
Stroke	77·8 (68·5–86·5)	80·5 (74–86·9)	80·6 (75·2–86)	80·5 (73·4–87·4)
Hypertensive heart disease	100 (100–100)	100 (100–100)	100 (100–100)	100 (100–100)
Non-rheumatic valvular heart disease	27·5 (16·4–42·8)	28·4 (18·8–40·8)	26 (16·7–37·1)	28·6 (18·9–41·4)
Cardiomyopathy and myocarditis	37·9 (29·6–46·2)	42·9 (36·6–49·4)	42 (35·9–48·6)	43 (36·5–49·7)
Atrial fibrillation and flutter	50·5 (39·6–61·9)	57·4 (48·1–66·1)	62·1 (53·7–70·2)	53 (42·9–63·4)
Aortic aneurysm	55·1 (48·2–61·9)	61·6 (55·9–67·2)	--	61·6 (55·9–67·2)
Endocarditis	32·4 (21·2–46·9)	34·4 (24·5–46)	32·6 (21·9–46)	34·6 (24·6–46·2)
Other cardiovascular and circulatory diseases	30·4 (23·1–39·1)	33·7 (26·8–41·4)	36·4 (28–46·3)	31·7 (25–39)
Chronic respiratory diseases	60·4 (56–64·8)	53·2 (49·3–57·2)	47·3 (42·6–52·3)	60·6 (56·7–64·6)
Chronic obstructive pulmonary disease	67·3 (62·8–71·9)	64·3 (60·4–68)	57·9 (54·1–61·7)	69·2 (65–73·2)
Pneumoconiosis	100 (100–100)	100 (100–100)	100 (100–100)	100 (100–100)

Asthma	39·4 (28·5–51·5)	38·2 (29·8–48·2)	38 (29·8–47·9)	41·7 (31·5–52·7)
Digestive diseases	36·7 (29·4–42·8)	34·4 (28·2–39·8)	12·4 (8·38–17·2)	46·5 (37·9–53·1)
Cirrhosis and other chronic liver diseases	75·8 (60·1–87·7)	78 (63·6–88·6)	72 (59–82)	78·1 (63·8–88·9)
Upper digestive system diseases	22 (17·9–26·2)	8·59 (5·38–12·3)	1·05 (0·58–1·71)	24·2 (20–28·4)
Gallbladder and biliary diseases	37·3 (23·5–51·7)	44·2 (30·8–58)	46·7 (33–60·8)	38·4 (25–52·2)
Pancreatitis	44·4 (21·6–62·5)	46·1 (23·2–63·8)	43·2 (21·4–60·8)	46·5 (23·5–64·4)
Neurological disorders	22·3 (9·71–35·9)	13·2 (6·19–23·6)	8·79 (4·24–18·1)	19·2 (8·17–33)
Alzheimer's disease and other dementias	35·7 (23·8–49)	38·5 (26·4–51·1)	39·2 (27·2–51·8)	38·1 (26·2–50·7)
Parkinson's disease	-10·6 (-16·7–-5·38)	-11·7 (-18·2–-6·02)	-11·8 (-18·1–-6·1)	-11·6 (-18·2–-5·98)
Idiopathic epilepsy	27·6 (16·6–37·7)	24·3 (14·3–33·9)	23 (13·2–32)	26·3 (15·5–36)
Multiple sclerosis	15·5 (11–20·3)	15·7 (11·2–20·3)	15·3 (10·9–19·7)	16·5 (11·8–21·6)
Mental disorders	--	5·12 (2·51–8·07)	5·12 (2·51–8·07)	--
Depressive disorders	--	12·2 (5·44–19·1)	12·2 (5·44–19·1)	--
Anxiety disorders	--	4·09 (1·07–9·29)	4·09 (1·07–9·29)	--
Idiopathic developmental intellectual disability	--	23·4 (8·04–42·9)	23·4 (8·04–42·9)	--
Substance use disorders	100 (100–100)	100 (100–100)	100 (100–100)	100 (100–100)
Alcohol use disorders	100 (100–100)	100 (100–100)	100 (100–100)	100 (100–100)
Drug use disorders	100 (100–100)	100 (100–100)	100 (100–100)	100 (100–100)
Diabetes and kidney diseases	100 (100–100)	100 (100–100)	100 (100–100)	100 (100–100)
Diabetes mellitus	100 (100–100)	100 (100–100)	100 (100–100)	100 (100–100)
Chronic kidney disease	100 (100–100)	100 (100–100)	100 (100–100)	100 (100–100)
Sense organ diseases	--	8·16 (7·12–9·37)	8·16 (7·12–9·37)	--
Blindness and vision loss	--	8·04 (5·36–11·4)	8·04 (5·36–11·4)	--
Age-related and other hearing loss	--	10·4 (9·52–11·4)	10·4 (9·52–11·4)	--
Musculoskeletal disorders	4·31 (1·26–8·09)	17·2 (14–20·7)	17·5 (14·2–21)	4·53 (1·38–8·33)
Rheumatoid arthritis	12·2 (3·58–22·2)	14·9 (4·64–27·1)	15·1 (4·75–27·4)	14 (4·3–25·5)
Osteoarthritis	--	15·7 (7·94–24·9)	15·7 (7·94–24·9)	--
Low back pain	--	37·6 (32–43·3)	37·6 (32–43·3)	--
Gout	--	44·4 (30·9–60·5)	44·4 (30·9–60·5)	--
Other non-communicable diseases	0·0617 (0·0384–0·107)	0·0401 (0·0237–0·0646)	--	0·204 (0·126–0·339)
Sudden infant death syndrome	6·24 (4·13–8·88)	6·24 (4·12–8·87)	--	6·24 (4·12–8·87)
Injuries	34·4 (27·4–41·3)	31·5 (25·7–37·6)	37·7 (32·9–43·2)	24·2 (15·1–33)
Transport injuries	28·4 (20·3–36·5)	30·7 (23–38·4)	35 (29·4–41·1)	27·7 (18·6–37)

Road injuries	26 (17·4–34·5)	29·2 (21·2–37·3)	33·9 (28·3–40·3)	25·8 (15·9–35·5)
Other transport injuries	41·9 (35·9–48·3)	40·8 (34·7–47·2)	42·5 (37·2–47·6)	39·6 (32·5–47·2)
Unintentional injuries	45·8 (39·7–50·6)	37·7 (32·5–43·1)	38·3 (33–44)	35·7 (30·2–40·7)
Falls	58·6 (51·2–63·9)	47·6 (41·6–53·2)	46 (40–51·6)	53 (46·5–58·5)
Drowning	-3·35 (-19·1–11·1)	0·127 (-15·2–13·2)	17·3 (11·9–24·4)	-0·934 (-16·8–12·9)
Fire, heat, and hot substances	15·2 (10–22·2)	22·1 (16·2–29·6)	23·1 (17–31)	19·1 (13·7–26)
Poisonings	25·3 (18·5–33·9)	24·9 (18·6–32·8)	21·4 (15·9–28·5)	28·2 (20·6–37·1)
Exposure to mechanical forces	20·2 (10–30·5)	27·7 (22·4–34·5)	28·7 (23·5–35·2)	12·8 (1·85–25·2)
Animal contact	26·2 (21–32·9)	30·6 (24·9–37·1)	32 (26·1–38·5)	23·8 (18·7–30·3)
Foreign body	4·17 (2·81–6·04)	9·69 (6·73–13·6)	10·3 (7·07–14·6)	8·88 (6·13–12·6)
Environmental heat and cold exposure	9·04 (3·87–15·9)	8·85 (3·83–15·7)	9·32 (4·05–16·5)	8·44 (3·61–15)
Exposure to forces of nature	0 (0–0)	9·96 (4·33–17·8)	9·96 (4·33–17·8)	0 (0–0)
Other unintentional injuries	21·3 (15·1–29·1)	21·8 (15·9–29·1)	21·5 (15·6–28·9)	24·7 (17·9–32·8)
Self-harm and interpersonal violence	16·4 (0·278–32·1)	17·4 (2·95–31·6)	31·7 (23·9–39·9)	15·6 (-0·355–31)
Self-harm	15·7 (-2·13–33)	15·3 (-2·39–32·5)	21·9 (8·71–34·7)	15·2 (-2·62–32·6)
Interpersonal violence	22·1 (11·2–32·6)	25·8 (16·5–35·3)	33·6 (25·1–42·6)	19·2 (8·16–29·7)

Supplementary Results Table 2. All-age PAFs attributable to the joint distribution of all risk factors for all ages, both sexes combined for each GBD level 1, level 2, and level 3 cause and all causes in 2019 for Sweden except Stockholm. Results are shown for deaths, YLLs, YLDs, and DALYs Data in parenthesis are 95% uncertainty intervals. PAFs=population attributable fractions. GBD=Global Burden of Disease. YLLs=years of life lost. YLDs=years lived with disability. DALYs=disability-adjusted life-years

	Deaths 2019	DALYs 2019	YLDs 2019	YLLs 2019
All causes	57·5 (54·1–60·6)	41·7 (38·8–44·7)	27·6 (25·3–29·9)	55·1 (52·7–57·6)
Communicable, maternal, neonatal, and nutritional diseases	34·8 (26·4–42·9)	41·7 (37·4–45·8)	39·8 (35·5–44)	43·2 (36·2–49·7)
HIV/AIDS and sexually transmitted infections	88·9 (87–90·6)	91·3 (88·5–94·1)	96·2 (94–98·3)	84 (81·5–86·1)
HIV/AIDS	86·5 (84·3–88·5)	84·4 (81·7–86·9)	87·9 (85–90·3)	82·6 (79·9–84·9)
Sexually transmitted infections excluding HIV	99·9 (99·9–100)	100 (99·9–100)	100 (100–100)	99·6 (99·5–99·8)
Respiratory infections and tuberculosis	41·2 (29·4–52·4)	34 (24·8–42·7)	2·86 (2·07–3·89)	44·2 (32·9–55)
Tuberculosis	54·8 (44·2–63·6)	56·2 (45·6–64·2)	53·7 (42·9–62·5)	56·8 (46·2–65)
Lower respiratory infections	40·9 (28·6–52·3)	43·8 (32·5–54·7)	31·3 (25·3–36·8)	44 (32·6–55)
Upper respiratory infections	0·00799 (0·00439–0·0145)	0·00602 (0·000506–0·0179)	0·00552 (0–0·0175)	0·0392 (0·0224–0·0707)
Otitis media	0·175 (0·043–0·439)	2·55 (1·58–3·68)	2·55 (1·58–3·69)	1·16 (0·293–2·87)
Enteric infections	5·67 (3·77–8·74)	7·5 (5·52–10·4)	8·03 (5·91–11)	6·86 (4·86–9·89)
Diarrheal diseases	5·68 (3·78–8·76)	7·51 (5·53–10·4)	8·03 (5·91–11)	6·89 (4·87–9·93)
Other infectious diseases	0·195 (0·123–0·361)	0·547 (0·409–0·73)	0·27 (0·148–0·498)	0·659 (0·478–0·916)
Meningitis	0·802 (0·529–1·17)	1·95 (1·3–2·84)	0·000288 (0–0·000943)	2·82 (1·93–4·01)
Encephalitis	0·157 (0·112–0·223)	0·359 (0·248–0·493)	0·000108 (0–0·000334)	0·616 (0·443–0·831)
Measles	2·6 (-0·619–12·9)	3·19 (-0·73–15·7)	0·489 (0·0149–1·23)	3·51 (-0·853–17·4)
Acute hepatitis	6·94 (3·57–14·7)	3·64 (2·2–6)	2·19 (1·21–3·96)	7·19 (3·85–15·3)
Maternal and neonatal disorders	73·7 (69·7–77·5)	72·5 (69·4–75·4)	70·8 (65·8–75·2)	74·1 (70·1–77·9)
Maternal disorders	8·44 (3·06–13·8)	8·56 (3·12–14·1)	8·68 (3·15–14·4)	8·26 (2·98–13·5)
Neonatal disorders	74·7 (70·8–78·6)	73·8 (70·6–76·7)	72·6 (67·6–77·2)	74·7 (70·8–78·6)
Nutritional deficiencies	84·2 (73·7–89)	75·1 (72–77·9)	74·2 (71·1–77·2)	83·7 (73–87·9)
Protein-energy malnutrition	100 (100–100)	100 (100–100)	100 (100–100)	100 (100–100)
Vitamin A deficiency	--	100 (100–100)	100 (100–100)	--
Dietary iron deficiency	--	100 (100–100)	100 (100–100)	--
Non-communicable diseases	59·9 (56·2–63·2)	42·8 (39·6–46·3)	26 (23·6–28·6)	58·7 (56–61·4)
Neoplasms	39·8 (36·5–43·6)	39·5 (36·3–43)	28·4 (24·9–32·4)	40·2 (37–43·8)
Lip and oral cavity cancer	67·1 (59·1–73·7)	69·1 (61·7–75·2)	69·4 (61·9–75·3)	69 (61·7–75·2)
Nasopharynx cancer	66·2 (56–74·3)	64·6 (54·7–72·4)	63·7 (53·5–71·9)	64·7 (54·8–72·5)
Other pharynx cancer	78·3 (71·4–83·6)	78·7 (72·2–83·9)	77·9 (71·1–83·1)	78·7 (72·2–83·9)
Esophageal cancer	73·9 (65·4–82·1)	75 (66·7–83·2)	74·3 (66–82·4)	75 (66·8–83·2)

Stomach cancer	22·7 (15·1–40·8)	22·9 (15·1–41·1)	23·2 (15·4–41·2)	22·9 (15·1–41·1)
Colon and rectum cancer	61 (52·4–68·9)	61·4 (53·5–68·8)	61·6 (53·6–69·2)	61·4 (53·5–68·8)
Liver cancer	75·3 (69·1–80·9)	77·3 (71·7–82·3)	75·8 (69·7–81·3)	77·3 (71·7–82·3)
Gallbladder and biliary tract cancer	20·7 (11·1–32)	21·2 (11·6–32·5)	20·7 (11·2–32·2)	21·2 (11·6–32·5)
Pancreatic cancer	37·7 (29·7–47·2)	37 (29·5–46)	37·5 (29·5–46·7)	37 (29·5–46)
Larynx cancer	80·6 (71·1–87·4)	81 (73–87·1)	81 (72·9–87·2)	81 (73–87·1)
Tracheal, bronchus, and lung cancer	80·7 (77·2–84·5)	79·3 (75·8–83·2)	79·9 (76·2–83·7)	79·3 (75·7–83·2)
Breast cancer	36·8 (29·1–45·4)	36 (28·9–43·7)	36·4 (29·2–44·2)	36 (28·9–43·6)
Cervical cancer	100 (100–100)	100 (100–100)	100 (100–100)	100 (100–100)
Uterine cancer	42 (27·9–57·5)	43·5 (29·1–59)	44·1 (29·7–59·4)	43·4 (29–58·9)
Ovarian cancer	16·6 (7·74–27·1)	14·3 (6·44–24·1)	13·4 (5·9–22·8)	14·4 (6·45–24·1)
Prostate cancer	5·7 (2·25–9·99)	6·11 (2·48–10·4)	6·35 (2·63–10·8)	6·07 (2·47–10·4)
Kidney cancer	35·1 (25·2–45·2)	35·3 (25·7–45·2)	35·1 (25·6–45)	35·3 (25·7–45·2)
Bladder cancer	35·4 (22·4–48·8)	38·4 (25·6–50·9)	39·4 (27–51·7)	38·3 (25·5–50·8)
Thyroid cancer	12·9 (6·25–21·5)	13·6 (6·55–22·6)	13·7 (6·81–22·1)	13·6 (6·5–22·7)
Mesothelioma	97·7 (96·9–98·3)	95·6 (94·3–96·7)	97·3 (96·1–98·2)	95·6 (94·2–96·7)
Non-Hodgkin lymphoma	6·28 (2·47–11·3)	6·54 (2·67–11·8)	6·42 (2·57–11·6)	6·56 (2·68–11·8)
Multiple myeloma	7·22 (3·06–12·9)	7·55 (3·24–13·5)	7·61 (3·3–14)	7·55 (3·24–13·5)
Leukemia	32·9 (19·9–46·9)	30·9 (19·9–42·6)	31·4 (20·1–43·5)	30·9 (19·9–42·5)
Cardiovascular diseases	81·3 (77·5–84·8)	82·2 (79–84·9)	76·8 (72·8–80·5)	83·2 (80·2–85·9)
Rheumatic heart disease	27·1 (14·5–53·3)	28·4 (17·2–50·8)	29·5 (18·1–48)	28·3 (17·1–51·2)
Ischemic heart disease	92·8 (88·4–96·4)	93·8 (90·5–96·7)	93·5 (90·3–96·4)	93·8 (90·5–96·7)
Stroke	80·8 (72·8–89·6)	83·4 (77·5–89·7)	83 (77·8–88·2)	83·6 (77·5–90·2)
Hypertensive heart disease	100 (100–100)	100 (100–100)	100 (100–100)	100 (100–100)
Non-rheumatic valvular heart disease	30·7 (19·5–45·7)	32 (22–44·8)	29·2 (20·5–39·3)	32·2 (22·1–45·3)
Cardiomyopathy and myocarditis	41·4 (32·2–49·7)	46·7 (40·2–52·9)	45·4 (38·5–52·3)	46·9 (39·8–53·4)
Atrial fibrillation and flutter	54·5 (45–64·9)	61·5 (53·5–69·6)	66·4 (59·4–73·4)	57 (48·3–66·6)
Aortic aneurysm	57·3 (50·4–64·3)	63·9 (58–69·4)	--	63·9 (58–69·4)
Endocarditis	36·6 (24·8–51·7)	39·5 (29·2–51·9)	37·5 (26·3–51·3)	39·7 (29·5–52)
Other cardiovascular and circulatory diseases	34·2 (27·1–42·7)	40·2 (33·8–46·5)	45·8 (38·9–52·4)	36·2 (29·7–43·3)
Chronic respiratory diseases	59·5 (54–65·4)	54·2 (50·1–58·3)	49·2 (44·6–54)	60·4 (55·4–65·8)
Chronic obstructive pulmonary disease	68·4 (63·1–73·3)	65·4 (61·6–69·1)	59·1 (55·5–62·6)	70·7 (65·9–75·1)
Pneumoconiosis	100 (100–100)	100 (100–100)	100 (100–100)	100 (100–100)
Asthma	39·7 (28·1–52·6)	40·1 (31·3–50·4)	39·9 (31·2–50)	42·5 (31·6–54·5)

Digestive diseases	34·6 (30–38·9)	34·4 (29·7–38·9)	14·2 (9·77–19·5)	43·8 (38·3–48·6)
Cirrhosis and other chronic liver diseases	78·4 (67–88·1)	80·5 (69·8–89·2)	73·1 (63·6–81·9)	80·6 (69·9–89·4)
Upper digestive system diseases	21·2 (17·6–24·9)	10·2 (6·65–14)	1·2 (0·667–1·88)	23·8 (20–27·7)
Gallbladder and biliary diseases	40·3 (26·5–54·5)	46·2 (32·5–60·4)	49·8 (35·6–64·3)	41·3 (28·1–55·7)
Pancreatitis	44·9 (31·7–58·7)	47·1 (34·2–60·2)	45·1 (32·6–58·4)	47·3 (34·4–60·5)
Neurological disorders	24·1 (10·9–37·6)	15·7 (7·67–27·2)	10·6 (5·34–20·2)	21·7 (9·47–35·6)
Alzheimer's disease and other dementias	36·9 (24·8–50·5)	39·8 (27·6–53)	40·5 (28·3–53·8)	39·4 (27·3–52·7)
Parkinson's disease	-10·3 (-16·3–-5·31)	-11·5 (-18–-6·01)	-11·7 (-17·9–-6·14)	-11·4 (-17·9–-5·97)
Idiopathic epilepsy	27·9 (19·6–35·9)	24·7 (17·3–32·2)	23·4 (16·2–30·8)	26·6 (18·4–34·3)
Multiple sclerosis	14·4 (10·2–19·3)	15 (10·6–19·7)	14·6 (10·4–19·1)	15·6 (11–20·6)
Mental disorders	--	6·88 (3·67–10·5)	6·88 (3·67–10·6)	--
Depressive disorders	--	13·4 (6·23–20·9)	13·4 (6·23–20·9)	--
Anxiety disorders	--	4·87 (1·38–10·5)	4·87 (1·38–10·5)	--
Idiopathic developmental intellectual disability	--	42·1 (19·1–59·9)	42·1 (19·1–59·9)	--
Substance use disorders	100 (100–100)	100 (100–100)	100 (100–100)	100 (100–100)
Alcohol use disorders	100 (100–100)	100 (100–100)	100 (100–100)	100 (100–100)
Drug use disorders	100 (100–100)	100 (100–100)	100 (100–100)	100 (100–100)
Diabetes and kidney diseases	100 (100–100)	100 (100–100)	100 (100–100)	100 (100–100)
Diabetes mellitus	100 (100–100)	100 (100–100)	100 (100–100)	100 (100–100)
Chronic kidney disease	100 (100–100)	100 (100–100)	100 (100–100)	100 (100–100)
Sense organ diseases	--	7·57 (6·42–8·92)	7·57 (6·42–8·92)	--
Blindness and vision loss	--	9·39 (6·16–13·4)	9·39 (6·16–13·4)	--
Age-related and other hearing loss	--	8·76 (7·94–9·65)	8·76 (7·94–9·65)	--
Musculoskeletal disorders	4·41 (1·33–8·3)	21·2 (17·3–25·1)	21·5 (17·6–25·6)	4·92 (1·5–9·22)
Rheumatoid arthritis	11·6 (3·46–21·3)	14·7 (4·57–26·9)	15 (4·71–27·4)	13·5 (4·07–25·1)
Osteoarthritis	--	15·7 (8·14–24·3)	15·7 (8·14–24·3)	--
Low back pain	--	38 (32·3–43·8)	38 (32·3–43·8)	--
Gout	--	48·5 (34·9–64·2)	48·5 (34·9–64·2)	--
Other non-communicable diseases	0·101 (0·0636–0·145)	0·0691 (0·04–0·107)	--	0·421 (0·249–0·608)
Sudden infant death syndrome	7·01 (4·91–9·78)	7 (4·91–9·77)	--	7 (4·91–9·77)
Injuries	34·2 (27·9–39·6)	30·3 (25·2–34·7)	37·6 (33·2–41·5)	22·2 (14·1–29·8)
Transport injuries	21·7 (14·8–28·7)	24·8 (18·6–31·2)	31·6 (27·4–35·7)	20·6 (12·5–29)
Road injuries	19·4 (11·9–26·9)	22·9 (16·2–29·6)	30·3 (26·1–34·4)	18·4 (9·84–27·1)
Other transport injuries	39 (34·3–43·6)	38·7 (33·6–43·6)	40·9 (36·6–44·7)	37·2 (31·4–42·7)

Unintentional injuries	46·6 (40·4–50·8)	37·7 (32·5–42·1)	38·5 (33·4–42·8)	35·5 (30·2–39·9)
Falls	59·4 (51·9–63·9)	48·7 (42·6–53·2)	47 (41–51·5)	53·8 (47·3–58·4)
Drowning	-10·7 (-28·3–5·2)	-7·57 (-23·9–7·01)	14·3 (9·65–20·8)	-8·77 (-25·5–6·63)
Fire, heat, and hot substances	14 (9·29–20·5)	18·3 (13·4–24·7)	18·7 (13·7–25·3)	17·2 (12·5–23·4)
Poisonings	21·4 (16·1–28·3)	20·7 (15·7–27·1)	17·5 (13–23·4)	24 (18·3–30·9)
Exposure to mechanical forces	12·3 (1·76–22·8)	22·8 (19·4–26·7)	25 (21·8–28·2)	5·93 (-5·67–17·6)
Animal contact	27 (21·8–33·2)	28·6 (23·7–34·1)	29·6 (24·5–35·2)	25·4 (20·5–31·2)
Foreign body	2·78 (2·03–3·71)	6·5 (4·9–8·46)	6·75 (5·07–8·79)	6·22 (4·62–8·19)
Environmental heat and cold exposure	9·27 (4·47–16·2)	9·13 (4·47–15·6)	9·52 (4·62–16·2)	8·79 (4·32–15·2)
Exposure to forces of nature	0 (0–0)	10·1 (4·94–17·3)	10·1 (4·94–17·3)	0 (0–0)
Other unintentional injuries	18·5 (13·3–25·4)	17·8 (12·9–24·2)	17·3 (12·5–23·7)	21·6 (16·2–28·3)
Self-harm and interpersonal violence	14·1 (-1·27–28·8)	15·2 (1·3–28·3)	31·9 (25·2–38·4)	13·4 (-1·59–27·7)
Self-harm	13·5 (-2·77–29·4)	13·1 (-3·08–28·8)	22·6 (11·1–33·7)	12·9 (-3·32–28·6)
Interpersonal violence	22·1 (10·5–33·3)	26·2 (17·5–35·2)	34·2 (26·7–41·8)	18·7 (6·87–29·9)

Supplementary Results Table 2. All-age PAFs attributable to the joint distribution of all risk factors for all ages, both sexes combined for each GBD level 1, level 2, and level 3 cause and all causes in 2019 for Uzbekistan. Results are shown for deaths, YLLs, YLDs, and DALYs Data in parenthesis are 95% uncertainty intervals. PAFs=population attributable fractions. GBD=Global Burden of Disease. YLLs=years of life lost. YLDs=years lived with disability. DALYs=disability-adjusted life-years

	Deaths 2019	DALYs 2019	YLDs 2019	YLLs 2019
All causes	71·6 (70·1–73·3)	54 (51·2–56·7)	29·8 (27·7–32)	64·3 (62·7–65·8)
Communicable, maternal, neonatal, and nutritional diseases	64·8 (61·4–68·3)	68·6 (65·7–71·2)	66·9 (62·8–70·5)	69 (65·7–72·2)
HIV/AIDS and sexually transmitted infections	78·3 (74·6–83·8)	79·5 (76–84·6)	92·5 (89·4–95·4)	76·6 (73–82)
HIV/AIDS	77·8 (74·2–83·5)	76·3 (72·7–81·7)	76·9 (73·6–81·8)	76·2 (72·6–81·7)
Sexually transmitted infections excluding HIV	99·8 (99·8–99·9)	100 (99·9–100)	100 (100–100)	99·7 (99·5–99·8)
Respiratory infections and tuberculosis	66·1 (61·5–70·7)	69 (64·1–73·3)	13·7 (9·93–18·2)	71·4 (66·6–75·9)
Tuberculosis	42·5 (36·1–48·6)	38·9 (32·8–44·8)	35·4 (28·4–41·8)	39·3 (33·2–45·3)
Lower respiratory infections	72·2 (66·8–77·3)	76·9 (71·5–81·4)	36·7 (28·7–46·2)	77 (71·6–81·6)
Upper respiratory infections	8·64 (5·54–12·2)	4·18 (2·4–6·35)	0·015 (0·00415–0·0279)	9 (5·74–12·7)
Otitis media	4·32 (2·14–8·06)	4·44 (2·85–6·17)	4·44 (2·85–6·17)	7·51 (4·26–13·6)
Enteric infections	88·7 (83·3–93·5)	83·5 (73·6–91·1)	81 (69·3–89·8)	91·9 (87·9–95·1)
Diarrheal diseases	93 (88·2–96·5)	84·1 (74·3–91·8)	81·1 (69·3–89·9)	94·7 (91–97·4)
Other infectious diseases	1·62 (1·27–2·07)	1·67 (1·31–2·15)	0·183 (0·101–0·302)	2·07 (1·62–2·69)
Meningitis	2·63 (1·91–3·56)	2·92 (2·13–3·88)	0·00172 (0·000386–0·00349)	3·75 (2·76–4·93)
Encephalitis	1·59 (1·16–2·2)	2·04 (1·49–2·8)	0·00173 (0·000389–0·00312)	2·12 (1·55–2·91)
Measles	15·2 (3·88–40)	19·3 (6·22–44·8)	1·57 (0·0432–3·63)	21·1 (6·86–48·2)
Acute hepatitis	1·55 (0·867–2·4)	1·27 (0·722–1·91)	1·59 (0·926–2·59)	1·18 (0·629–1·86)
Maternal and neonatal disorders	72·9 (68·8–76·7)	68·3 (64·4–72·3)	36·8 (31·4–42·2)	73·7 (69·6–77·3)
Maternal disorders	18·3 (6·62–30·2)	18·3 (6·6–30·2)	18·3 (6·62–30)	18·3 (6·61–30·2)
Neonatal disorders	75·3 (71·2–79·1)	69·9 (65·8–73·8)	37·5 (32–43·1)	75·3 (71·2–79·1)
Nutritional deficiencies	21·3 (18·1–25·3)	96·7 (95·8–97·5)	97·4 (96·7–98·1)	25·8 (21·6–30·6)
Protein-energy malnutrition	100 (100–100)	100 (100–100)	100 (100–100)	100 (100–100)
Vitamin A deficiency	--	100 (100–100)	100 (100–100)	--
Dietary iron deficiency	--	100 (100–100)	100 (100–100)	--
Non-communicable diseases	77·9 (76–79·7)	56·5 (52·2–60·3)	24·1 (21·5–26·7)	72·2 (70·4–74·2)
Neoplasms	35·6 (32·3–40·2)	32·1 (29·1–36)	30·9 (27·4–35)	32·1 (29·1–36)
Lip and oral cavity cancer	44·9 (39·3–50·1)	44·6 (39–50)	41 (35·3–46·9)	44·7 (39·1–50)
Nasopharynx cancer	38·5 (31–45·5)	37·1 (29·7–44)	37·6 (30·1–44·7)	37·1 (29·7–43·9)
Other pharynx cancer	52·4 (46·6–57·7)	51·3 (45·3–57)	47·2 (41·4–52·5)	51·4 (45·3–57)
Esophageal cancer	56·3 (41·9–70·2)	57·4 (43·2–71·3)	56·8 (42·1–71·2)	57·4 (43·2–71·3)

Stomach cancer	17·3 (9·51–37·1)	16·6 (8·83–36·8)	17 (8·88–36·8)	16·6 (8·83–36·8)
Colon and rectum cancer	53·2 (45·1–60·8)	52·5 (44·7–59·8)	52·7 (44·6–59·9)	52·5 (44·7–59·8)
Liver cancer	61·9 (53·5–70·2)	58·5 (50·1–66·8)	61 (52·4–69·3)	58·4 (50·1–66·7)
Gallbladder and biliary tract cancer	22·3 (12·7–34·1)	22·5 (12·8–34·4)	22·4 (12·7–34·2)	22·5 (12·8–34·4)
Pancreatic cancer	24·7 (17·1–34·5)	24·1 (16·7–33·5)	24·7 (17·1–34·4)	24·1 (16·7–33·5)
Larynx cancer	51·8 (45·7–57·3)	50·4 (44·3–55·9)	47·4 (41·1–53·1)	50·5 (44·4–56)
Tracheal, bronchus, and lung cancer	71·4 (66·2–77·5)	69·4 (64·1–75·7)	70·4 (64·7–76·6)	69·4 (64·1–75·7)
Breast cancer	18 (10·9–26·2)	15·5 (8·7–22·9)	15·6 (8·66–22·8)	15·5 (8·67–22·9)
Cervical cancer	100 (100–100)	100 (100–100)	100 (100–100)	100 (100–100)
Uterine cancer	48·5 (33·2–63·1)	48·7 (33·7–63·2)	48·8 (33·7–63·4)	48·7 (33·7–63·2)
Ovarian cancer	11·3 (3·9–20·9)	10·1 (3·48–18·9)	9·58 (3·25–18·1)	10·1 (3·48–19)
Prostate cancer	5·45 (2·5–8·64)	5·87 (2·72–9·14)	6·06 (2·83–9·4)	5·86 (2·71–9·14)
Kidney cancer	31 (22·1–40·6)	28·2 (19·7–37)	27·6 (19·3–36·2)	28·2 (19·7–37)
Bladder cancer	36·6 (26·2–45·8)	37·1 (26·7–46·1)	35·6 (25·6–44·6)	37·2 (26·8–46·2)
Thyroid cancer	14·9 (7·58–24)	14·8 (7·54–23·7)	14·4 (7·55–22·5)	14·8 (7·49–23·9)
Mesothelioma	63·9 (54·8–72·1)	53 (42·8–63)	61·4 (51·8–70)	52·9 (42·7–62·9)
Non-Hodgkin lymphoma	6·44 (2·78–11·4)	5·69 (2·43–10·1)	5·12 (2·22–9·13)	5·7 (2·44–10·1)
Multiple myeloma	8·52 (3·77–14·8)	8·62 (3·8–14·9)	8·68 (3·82–15)	8·62 (3·8–14·9)
Leukemia	16·4 (10·7–22·3)	12 (7·53–16·5)	13 (8·25–18)	11·9 (7·52–16·5)
Cardiovascular diseases	92·1 (90·2–93·9)	91·5 (89·8–93)	77·1 (72·5–81·1)	92·1 (90·4–93·6)
Rheumatic heart disease	30·3 (19·7–44·3)	28·3 (18·4–40·6)	23 (12·6–35)	29·3 (19–42·8)
Ischemic heart disease	95·4 (93·3–97·2)	96·2 (94·6–97·6)	96·4 (94·7–97·8)	96·2 (94·6–97·6)
Stroke	89·5 (86–92·4)	91·3 (88·4–93·6)	88·7 (85·8–91·2)	91·5 (88·6–93·9)
Hypertensive heart disease	100 (100–100)	100 (100–100)	100 (100–100)	100 (100–100)
Non-rheumatic valvular heart disease	17·7 (12·3–25)	15·3 (10·5–21·4)	3·18 (2·02–4·62)	17·3 (12·1–23·9)
Cardiomyopathy and myocarditis	31·4 (24·2–38·4)	29·3 (21·9–36·7)	17·2 (12–22·7)	29·4 (22–36·8)
Atrial fibrillation and flutter	52·3 (43·8–61·8)	61·2 (53·4–68·8)	64·3 (56·4–71·9)	56·9 (48·8–65·5)
Aortic aneurysm	59 (53·4–64·2)	62·6 (57·8–67·1)	--	62·6 (57·8–67·1)
Endocarditis	40·5 (31–50·3)	37·3 (27·2–48·5)	30 (22·1–39·3)	37·3 (27·2–48·5)
Other cardiovascular and circulatory diseases	41·7 (35·3–47·4)	36·8 (29·6–43)	17 (10·3–25·5)	38·5 (30·5–44·8)
Chronic respiratory diseases	55·4 (50·6–60)	46·4 (41·6–51·3)	35·7 (30·5–41·3)	53 (48·4–57·4)
Chronic obstructive pulmonary disease	71·5 (65·1–76·7)	64·4 (58·9–69·9)	49 (42·6–55·6)	70·5 (64·9–75·6)
Pneumoconiosis	100 (100–100)	100 (100–100)	100 (100–100)	100 (100–100)
Asthma	46·6 (36·4–57·9)	39·8 (31·6–48·9)	32·3 (25·5–40·3)	48·6 (38·7–59·3)

Digestive diseases	56·5 (49·9–63)	51·6 (45·2–57·6)	14·8 (10·1–20)	56·1 (49·4–62·6)
Cirrhosis and other chronic liver diseases	62·4 (55–69·8)	62·1 (54·4–69·4)	57·5 (50·2–64·8)	62·1 (54·4–69·4)
Upper digestive system diseases	21 (17·9–24)	10·1 (6·79–13·6)	0·762 (0·454–1·19)	20·4 (17·4–23·4)
Gallbladder and biliary diseases	46·1 (31·8–60·2)	46·9 (33·1–60·5)	47·1 (33·1–60·6)	46·4 (32·4–60·3)
Pancreatitis	26·3 (17·4–36·7)	28·9 (19·3–40)	21·8 (13·9–30·8)	29·3 (19·6–40·4)
Neurological disorders	15·2 (8·69–24·1)	6 (3·11–10·7)	2·97 (1·25–6·26)	13·3 (8·78–19·8)
Alzheimer's disease and other dementias	30·5 (18·2–44·9)	32·8 (20–46·9)	32·9 (20·3–47·6)	32·7 (20–46·7)
Parkinson's disease	-5·26 (-8·22–-2·54)	-6·21 (-9·59–-3·13)	-6·77 (-10·9–-3·3)	-6·09 (-9·42–-3·02)
Idiopathic epilepsy	13·5 (9·17–18·4)	11 (7·44–15)	8·85 (5·91–12·2)	12·5 (8·47–17)
Multiple sclerosis	11·1 (7·85–15·4)	10·4 (7·69–13·6)	9·61 (7·2–12·3)	11·4 (8·07–15·9)
Mental disorders	--	4·41 (2·29–6·95)	4·41 (2·29–6·95)	--
Depressive disorders	--	6·34 (2·04–11·7)	6·34 (2·04–11·7)	--
Anxiety disorders	--	2·98 (0·8–6·94)	2·98 (0·8–6·94)	--
Idiopathic developmental intellectual disability	--	55·1 (27·4–74·7)	55·1 (27·4–74·7)	--
Substance use disorders	100 (100–100)	100 (100–100)	100 (100–100)	100 (100–100)
Alcohol use disorders	100 (100–100)	100 (100–100)	100 (100–100)	100 (100–100)
Drug use disorders	100 (100–100)	100 (100–100)	100 (100–100)	100 (100–100)
Diabetes and kidney diseases	99·4 (99·2–99·5)	99·4 (99·2–99·5)	100 (100–100)	99·1 (98·9–99·3)
Diabetes mellitus	100 (100–100)	100 (100–100)	100 (100–100)	100 (100–100)
Chronic kidney disease	100 (100–100)	100 (100–100)	100 (100–100)	100 (100–100)
Sense organ diseases	--	15·8 (14·3–17·5)	15·8 (14·3–17·5)	--
Blindness and vision loss	--	6·23 (3·88–9·2)	6·23 (3·88–9·2)	--
Age-related and other hearing loss	--	21·9 (20·4–23·5)	21·9 (20·4–23·5)	--
Musculoskeletal disorders	3·04 (0·801–5·69)	30·9 (27–34·9)	31·7 (27·6–35·7)	2·6 (0·672–4·9)
Rheumatoid arthritis	6·1 (1·64–11·2)	6 (1·7–10·6)	5·93 (1·69–10·5)	6·32 (1·67–11·5)
Osteoarthritis	--	15·8 (8·38–23·9)	15·8 (8·38–23·9)	--
Low back pain	--	47·4 (43·3–51·8)	47·4 (43·3–51·8)	--
Gout	--	47·9 (32·9–64·7)	47·9 (32·9–64·7)	--
Other non-communicable diseases	0·0489 (0·0294–0·0769)	0·0242 (0·0137–0·0396)	--	0·0671 (0·0389–0·113)
Sudden infant death syndrome	13 (7·27–19·8)	13 (7·26–19·8)	--	13 (7·26–19·8)
Injuries	12·3 (9·46–15·7)	12·1 (9·5–15·1)	18·7 (16·2–21·7)	9·98 (7·27–13·1)
Transport injuries	20·2 (15·9–25)	19·1 (15–23·6)	23·3 (19·8–26·8)	18·2 (13·9–23·1)
Road injuries	20 (15·7–24·9)	18·9 (14·8–23·5)	23·2 (19·7–26·8)	18 (13·7–23)
Other transport injuries	26·4 (22·2–30·6)	24 (20·1–28·1)	25·2 (21·8–28·5)	23·6 (19·5–28)

Unintentional injuries	6·67 (3·49–10·1)	8·78 (5·43–12·4)	17·7 (15–21)	3·31 (0·325–6·32)
Falls	24·6 (21·1–28·5)	21·9 (18·6–25·7)	23·5 (20·2–27·4)	19·3 (16–23)
Drowning	-12·8 (-18–-7·68)	-14·9 (-19·8–-10·1)	8·09 (6·17–10·8)	-15·2 (-20·1–-10·3)
Fire, heat, and hot substances	11·4 (8·65–14·7)	11·2 (8·43–14·4)	12·6 (9·72–16·2)	10·3 (7·85–13·3)
Poisonings	15 (11·5–19·4)	13·8 (10·7–17·8)	11·1 (8·7–14·4)	14·4 (11–18·5)
Exposure to mechanical forces	6·07 (0·822–11·5)	14·7 (11·4–18·4)	17·2 (14·3–20·3)	3·86 (-1·34–9·36)
Animal contact	13·2 (10·6–16·5)	13·8 (11–17·3)	15·9 (12·8–19·8)	10·4 (8·09–13·1)
Foreign body	5·11 (3·7–6·73)	4·18 (3–5·5)	6·15 (4·46–8·36)	3·84 (2·71–5·15)
Environmental heat and cold exposure	6·34 (2·99–11·1)	5·74 (2·76–10·2)	4·65 (2·22–8·5)	6·34 (2·99–11·3)
Exposure to forces of nature	0 (0–0)	5·24 (2·48–9·36)	5·24 (2·48–9·36)	0 (0–0)
Other unintentional injuries	17·5 (13·4–22·4)	15·5 (11·9–19·8)	13·9 (10·7–17·9)	16·5 (12·6–21)
Self-harm and interpersonal violence	10·4 (4·1–17·1)	10·1 (4·27–16·5)	15·3 (11·3–19·6)	9·71 (3·65–16·4)
Self-harm	9·42 (1·51–17·5)	8·8 (1·3–16·5)	9·28 (3·96–14·8)	8·78 (1·2–16·6)
Interpersonal violence	14·1 (8·31–20·3)	14·7 (9·15–20·9)	20 (14·8–25·9)	13·6 (7·77–20)

Supplementary Results Table 2. All-age PAFs attributable to the joint distribution of all risk factors for all ages, both sexes combined for each GBD level 1, level 2, and level 3 cause and all causes in 2019 for Switzerland. Results are shown for deaths, YLLs, YLDs, and DALYs Data in parenthesis are 95% uncertainty intervals. PAFs=population attributable fractions. GBD=Global Burden of Disease. YLLs=years of life lost. YLDs=years lived with disability. DALYs=disability-adjusted life-years

	Deaths 2019	DALYs 2019	YLDs 2019	YLLs 2019
All causes	57·3 (53·4–60·4)	40·1 (37·1–43)	27·3 (25·1–29·5)	54·9 (52·4–57·4)
Communicable, maternal, neonatal, and nutritional diseases	43 (36·4–49·4)	51·3 (47·8–54·6)	47·1 (42·6–51·8)	55·3 (50·7–59·6)
HIV/AIDS and sexually transmitted infections	92·8 (92–93·5)	93·4 (92·3–94·5)	95·5 (94·6–96·6)	90·4 (89·4–91·3)
HIV/AIDS	92·1 (91·1–92·8)	92·2 (91·2–93·1)	94·2 (93·3–95)	90 (89–90·9)
Sexually transmitted infections excluding HIV	100 (99·9–100)	100 (100–100)	100 (100–100)	99·8 (99·7–99·8)
Respiratory infections and tuberculosis	45·7 (36·7–54·3)	35·2 (27·9–42·5)	2·93 (2·06–4·06)	48·8 (40·4–56·8)
Tuberculosis	60 (49·9–67·7)	61·3 (52·1–68·7)	58·1 (48·5–66·1)	62·3 (53·1–69·6)
Lower respiratory infections	45·5 (36·5–54·2)	48·5 (39·8–56·5)	36·2 (29·8–41·9)	48·6 (39·9–56·9)
Upper respiratory infections	0·0133 (0·00784–0·0224)	0·00563 (0·000555–0·0147)	0·00518 (7·56e-05–0·0144)	0·0561 (0·0338–0·0941)
Otitis media	0·00579 (0·00251–0·0117)	3·45 (2·13–4·85)	3·48 (2·14–4·91)	0·0297 (0·0124–0·0608)
Enteric infections	5·42 (3·93–7·76)	9·55 (7·66–12)	8·11 (6·26–10·6)	12·5 (9·79–15·9)
Diarrheal diseases	5·51 (3·99–7·94)	9·63 (7·72–12·1)	8·12 (6·27–10·6)	12·8 (10–16·2)
Other infectious diseases	1·32 (0·845–1·64)	1·7 (1·13–2·07)	0·555 (0·339–0·909)	2·13 (1·38–2·58)
Meningitis	0·604 (0·43–0·825)	1·86 (1·35–2·47)	0·000566 (6·31e-06–0·00176)	2·51 (1·83–3·29)
Encephalitis	0·277 (0·182–0·409)	0·928 (0·546–1·37)	0·000149 (1·83e-06–0·000463)	1·17 (0·732–1·72)
Measles	2·06 (-0·686–10·8)	1·27 (-0·118–6·3)	0·342 (0·0139–0·837)	2·92 (-0·946–14·9)
Acute hepatitis	50·1 (26·9–59·4)	18·2 (10·2–24·8)	3·34 (2·08–5·38)	47·2 (25·6–55·9)
Maternal and neonatal disorders	82·7 (80·3–85·1)	78·1 (75·8–80·4)	72·5 (68·7–76)	83·5 (81·1–85·8)
Maternal disorders	8·88 (3·08–14·6)	8·86 (3·05–14·7)	8·89 (3·08–14·7)	8·81 (3·06–14·5)
Neonatal disorders	84·8 (82·4–87·2)	79·7 (77·2–81·9)	74·3 (70·6–77·9)	84·8 (82·4–87·2)
Nutritional deficiencies	79·4 (29–86·6)	74·5 (69·9–77·4)	74·6 (72·1–77·3)	73·7 (29·7–80·3)
Protein-energy malnutrition	100 (100–100)	100 (100–100)	100 (100–100)	100 (100–100)
Vitamin A deficiency	--	100 (100–100)	100 (100–100)	--
Dietary iron deficiency	--	100 (100–100)	100 (100–100)	--
Non-communicable diseases	59 (54·6–62·4)	39·6 (36·2–43·1)	24·2 (22–26·7)	57·3 (54·4–60·1)
Neoplasms	44·5 (41·3–48·2)	44·7 (41·8–48)	30·8 (27·5–34·3)	45·7 (42·8–49)
Lip and oral cavity cancer	73·3 (67–78·7)	74·8 (68·8–79·7)	74·6 (68·3–79·6)	74·8 (68·9–79·7)
Nasopharynx cancer	69·8 (60·8–77·1)	69·5 (60·6–76·8)	68·9 (59·9–76·2)	69·6 (60·8–76·8)
Other pharynx cancer	82·2 (77·1–86·5)	82·6 (77·6–86·9)	82·1 (76·8–86·3)	82·7 (77·6–87)
Esophageal cancer	76·7 (69·2–84·1)	77·2 (69·6–84·5)	76·5 (68·9–83·8)	77·2 (69·6–84·5)
Stomach cancer	23·5 (16·2–41·4)	24·1 (16·4–42·3)	24·3 (16·5–42·3)	24·1 (16·4–42·3)
Colon and rectum cancer	63·9 (56·2–71·3)	64·1 (56·8–71·1)	63·9 (56·3–70·9)	64·1 (56·8–71·1)

Liver cancer	80·3 (74·5–85·3)	80·2 (74·7–85)	80·5 (75–85·2)	80·2 (74·7–85)
Gallbladder and biliary tract cancer	18·3 (9·97–28·7)	18·4 (9·97–29)	18·1 (9·71–28·5)	18·4 (9·97–29)
Pancreatic cancer	37 (29·6–46·3)	36·8 (30–45·4)	37 (29·8–46)	36·8 (30–45·3)
Larynx cancer	85·8 (79·8–90·3)	85·1 (79·1–89·3)	85·1 (79·2–89·2)	85·1 (79·1–89·4)
Tracheal, bronchus, and lung cancer	84·3 (81·3–87)	82·7 (79·6–85·6)	83·3 (80·1–86·2)	82·7 (79·6–85·6)
Breast cancer	37·4 (30·3–45·9)	36·8 (30–44·2)	36·9 (30–44·4)	36·8 (30·1–44·2)
Cervical cancer	100 (100–100)	100 (100–100)	100 (100–100)	100 (100–100)
Uterine cancer	39·1 (25·4–53·3)	39·7 (26·7–53·5)	40 (26·9–53·7)	39·7 (26·5–53·5)
Ovarian cancer	17·3 (8·41–28·7)	14·7 (6·84–24·6)	14·1 (6·54–24)	14·7 (6·85–24·7)
Prostate cancer	5·91 (2·71–9·13)	6·39 (2·97–9·91)	6·7 (3·12–10·4)	6·33 (2·92–9·78)
Kidney cancer	34·2 (25–43·4)	34·4 (25·8–43·4)	34·2 (25·9–43·3)	34·4 (25·8–43·4)
Bladder cancer	43·2 (29·7–55·9)	45·1 (32·5–56·6)	45·4 (32·9–56·2)	45·1 (32·5–56·6)
Thyroid cancer	11·9 (5·71–20)	12·2 (5·72–20·9)	12 (5·86–20·2)	12·3 (5·73–21)
Mesothelioma	98·2 (97·5–98·8)	96·7 (95·6–97·7)	97·9 (96·9–98·6)	96·7 (95·5–97·7)
Non-Hodgkin lymphoma	5·7 (2·26–10·3)	5·79 (2·32–10·5)	5·77 (2·28–10·4)	5·79 (2·32–10·5)
Multiple myeloma	6·58 (2·86–11·9)	6·72 (2·91–12·1)	6·65 (2·86–12)	6·72 (2·9–12·1)
Leukemia	33·5 (20·8–46·9)	31·6 (21·1–43)	31·4 (20·8–42·6)	31·6 (21·1–43)
Cardiovascular diseases	79·9 (76·1–83·7)	80·2 (77–83·3)	73·5 (69·4–77·6)	81·1 (78–84·2)
Rheumatic heart disease	21·6 (10·8–45·3)	21·9 (13–39·8)	21·7 (13·3–36·6)	21·9 (12·6–40·6)
Ischemic heart disease	92·2 (87·8–95·9)	93·3 (90–96·2)	93 (89·9–95·7)	93·3 (90–96·2)
Stroke	78·9 (71–88·1)	81·4 (75·2–88·1)	81·2 (75·8–86·9)	81·5 (75·3–88·5)
Hypertensive heart disease	100 (100–100)	100 (100–100)	100 (100–100)	100 (100–100)
Non-rheumatic valvular heart disease	23·9 (14·2–38)	24·5 (15·9–36·4)	25·1 (16·3–36·4)	24·5 (15·8–36·7)
Cardiomyopathy and myocarditis	36·9 (27·9–48·2)	43·5 (36·3–51)	46·5 (40·1–53·9)	43·2 (36–50·9)
Atrial fibrillation and flutter	51·4 (42·4–61·2)	58·1 (50·5–65·8)	61·5 (54·4–68·6)	54 (45·6–62·8)
Aortic aneurysm	50·9 (44·7–57·9)	58·7 (53·2–64·3)	--	58·7 (53·2–64·3)
Endocarditis	29·7 (18·3–45·9)	31·7 (21·2–45·1)	30·6 (20·3–44·8)	31·8 (21·2–45·1)
Other cardiovascular and circulatory diseases	27 (20·2–34·3)	28·3 (22·4–34·1)	29·6 (23·7–35·7)	27·9 (21·7–34·3)
Chronic respiratory diseases	65·7 (61·6–69·9)	57·5 (53·6–61·2)	50·6 (46·1–55·4)	65·8 (62·1–69·7)
Chronic obstructive pulmonary disease	74·7 (70·2–78·5)	70·8 (67·1–74·1)	62·9 (59·3–66·4)	76·5 (72·6–79·9)
Pneumoconiosis	100 (100–100)	100 (100–100)	100 (100–100)	100 (100–100)
Asthma	37·1 (26·5–48·8)	40·5 (32·5–49·7)	40·4 (32·5–49·5)	40·7 (30·5–51·6)
Digestive diseases	34·9 (30–40·3)	38·5 (33·7–43·3)	13·3 (9·53–18)	47·3 (41·9–52·7)
Cirrhosis and other chronic liver diseases	80·6 (70·1–89·2)	82·2 (72·6–90)	77·4 (69·4–84·5)	82·3 (72·6–90·1)
Upper digestive system diseases	17 (14·2–19·8)	7·34 (4·79–10·2)	1·08 (0·628–1·66)	20·1 (16·9–23·1)
Gallbladder and biliary diseases	38·8 (25·3–52·8)	42·1 (29·1–55·4)	44 (30·8–57·3)	39·3 (26·2–52·6)

Pancreatitis	48·4 (36·9–61·8)	50·7 (39–63·9)	49·9 (38·1–62·7)	50·8 (39·2–64)
Neurological disorders	23·7 (11·5–37·4)	15·7 (8·14–26·4)	10·7 (5·59–19·8)	21·5 (10·4–34·7)
Alzheimer's disease and other dementias	34·2 (22·1–48·3)	36·5 (24·4–50·2)	37·1 (25·1–50·5)	36·2 (24·1–49·9)
Parkinson's disease	-8·67 (-13·4–-4·37)	-9·82 (-14·9–-5·02)	-10·2 (-15·6–-5·25)	-9·71 (-14·9–-4·93)
Idiopathic epilepsy	30·2 (22·5–38·1)	27·5 (20·5–34·6)	26·5 (19·7–33·2)	29·3 (21·9–36·5)
Multiple sclerosis	14·4 (10·7–18·2)	16·6 (12·4–20·7)	16·8 (12·6–21)	16·3 (12·1–20·4)
Mental disorders	--	5·18 (2·85–8·16)	5·19 (2·86–8·17)	--
Depressive disorders	--	9·37 (5·22–14)	9·37 (5·22–14)	--
Anxiety disorders	--	6·1 (1·82–12·6)	6·1 (1·82–12·6)	--
Idiopathic developmental intellectual disability	--	52·8 (26–73)	52·8 (26–73)	--
Substance use disorders	100 (100–100)	100 (100–100)	100 (100–100)	100 (100–100)
Alcohol use disorders	100 (100–100)	100 (100–100)	100 (100–100)	100 (100–100)
Drug use disorders	100 (100–100)	100 (100–100)	100 (100–100)	100 (100–100)
Diabetes and kidney diseases	100 (100–100)	100 (100–100)	100 (100–100)	100 (100–100)
Diabetes mellitus	100 (100–100)	100 (100–100)	100 (100–100)	100 (100–100)
Chronic kidney disease	100 (100–100)	100 (100–100)	100 (100–100)	100 (100–100)
Sense organ diseases	--	7·51 (6·52–8·72)	7·51 (6·52–8·72)	--
Blindness and vision loss	--	8·03 (5·24–11·6)	8·03 (5·24–11·6)	--
Age-related and other hearing loss	--	8·72 (7·89–9·67)	8·72 (7·89–9·67)	--
Musculoskeletal disorders	2·13 (0·561–4·05)	23·6 (19·6–27·5)	23·9 (19·9–27·9)	2·62 (0·705–4·8)
Rheumatoid arthritis	9·65 (2·39–17·9)	14·2 (4·24–24·9)	14·7 (4·42–25·7)	11·6 (3·03–21·3)
Osteoarthritis	--	13·3 (6·77–21·1)	13·3 (6·77–21·1)	--
Low back pain	--	40·7 (35·6–45·9)	40·7 (35·6–45·9)	--
Gout	--	45·8 (32·1–62)	45·8 (32·1–62)	--
Other non-communicable diseases	0·0336 (0·0221–0·0471)	0·0208 (0·0128–0·0315)	--	0·115 (0·0757–0·159)
Sudden infant death syndrome	5·49 (3·74–7·86)	5·49 (3·73–7·85)	--	5·49 (3·73–7·85)
Injuries	41·3 (35·2–46·5)	39·7 (34·4–45·8)	45·4 (39·6–51·7)	32·3 (25·3–38·8)
Transport injuries	35·7 (28·3–43·7)	39·5 (32–47·9)	40·4 (34·9–47)	38·9 (29·8–48·9)
Road injuries	32·6 (24·7–40·7)	37·6 (29·9–46·1)	39·5 (33·9–46·1)	36·3 (26·6–46·4)
Other transport injuries	50·8 (44–58·8)	50·6 (43·3–58·8)	48·9 (43–55)	51·2 (43–60·6)
Unintentional injuries	52·5 (45·3–57·6)	46 (39·9–52·3)	46·3 (40·1–52·8)	45·2 (39·2–51·1)
Falls	59·6 (51·3–65·2)	52·6 (46·1–58·4)	51·5 (45·2–57·7)	55·7 (48·7–61·1)
Drowning	2·4 (-11·2–15·1)	6·66 (-6·93–19·1)	22 (16·6–29·1)	5·84 (-8·25–18·8)
Fire, heat, and hot substances	18·2 (13–25·3)	30·1 (23·1–38·9)	31·1 (24–40)	26·4 (20·2–33·9)
Poisonings	27·8 (21·4–35·8)	30·1 (23·2–38·4)	28·9 (22–36·9)	32·4 (24·9–41·4)
Exposure to mechanical forces	26·3 (15·1–38·9)	37 (29·5–46·4)	38 (31–46·9)	27·3 (14·4–42·1)

Animal contact	34·8 (28·5–42)	37·5 (30·9–45·3)	38·2 (31·6–45·7)	36 (29–44·7)
Foreign body	7·19 (5·14–9·95)	15·2 (10·9–20·8)	17 (12·1–23·5)	14·2 (10·2–19·3)
Environmental heat and cold exposure	10·9 (5·2–18·4)	10·5 (5–17·9)	10·5 (5–18)	10·4 (5·01–17·5)
Exposure to forces of nature	9·83 (4·74–16·9)	9·34 (4·51–16)	10·8 (5·15–18·4)	8·7 (4·22–14·9)
Other unintentional injuries	24·9 (18·6–32·8)	29·6 (22·7–38·2)	29·4 (22·5–38)	35·1 (26·9–44·7)
Self-harm and interpersonal violence	19·1 (4·64–32·5)	20·5 (7·51–32·2)	35·3 (28·3–41·7)	18·8 (4·68–31·9)
Self-harm	18·4 (3·33–32·6)	18·4 (3·46–32·3)	25·1 (13·7–36·2)	18·1 (2·98–32·2)
Interpersonal violence	34 (24·2–43·6)	36·4 (28·3–44·8)	40·6 (32·3–48·4)	30·9 (21·2–40·8)

Supplementary Results Table 2. All-age PAFs attributable to the joint distribution of all risk factors for all ages, both sexes combined for each GBD level 1, level 2, and level 3 cause and all causes in 2019 for UK. Results are shown for deaths, YLLs, YLDs, and DALYs Data in parenthesis are 95% uncertainty intervals. PAFs=population attributable fractions. GBD=Global Burden of Disease. YLLs=years of life lost. YLDs=years lived with disability. DALYs=disability-adjusted life-years

	Deaths 2019	DALYs 2019	YLDs 2019	YLLs 2019
All causes	55·9 (53·1–58·8)	42·9 (40–45·8)	29·2 (26·9–31·5)	55·8 (53·6–58·1)
Communicable, maternal, neonatal, and nutritional diseases	45·1 (37·9–52·3)	52·4 (48·3–56·3)	48·9 (45·1–52·6)	54·2 (48·2–60)
HIV/AIDS and sexually transmitted infections	80·1 (68·1–88·6)	80·1 (66·9–88·8)	94·9 (92·8–96·6)	65·9 (49·1–80·1)
HIV/AIDS	90·9 (88·7–92·3)	89·6 (87–91·5)	92·1 (89·6–93·9)	87·3 (84·6–88·8)
Sexually transmitted infections excluding HIV	56·4 (34·1–80·1)	62·5 (37·3–85·8)	100 (100–100)	23·5 (10·3–46·5)
Respiratory infections and tuberculosis	45·7 (37·7–53·8)	43 (36–50·1)	3·39 (2·57–4·42)	49·2 (41·3–56·8)
Tuberculosis	56 (47·1–63·8)	58·7 (50·2–65·7)	55·6 (46·5–63)	59·3 (51·1–66·4)
Lower respiratory infections	45·6 (37·6–53·8)	49 (41–56·7)	34 (27·8–39·7)	49·1 (41·1–56·9)
Upper respiratory infections	0·0471 (0·0364–0·0598)	0·00788 (0·00586–0·0104)	0·0062 (0·00441–0·00854)	0·114 (0·0873–0·145)
Otitis media	2·4 (1·62–3·27)	4·29 (2·79–5·89)	4·25 (2·76–5·87)	6·01 (4·18–7·89)
Enteric infections	5·32 (3·3–8·74)	9·02 (6·77–12·5)	9·37 (6·91–12·8)	8·21 (5·9–11·3)
Diarrheal diseases	5·38 (3·34–8·8)	9·08 (6·81–12·6)	9·38 (6·92–12·8)	8·35 (6·05–11·5)
Other infectious diseases	0·668 (0·49–0·854)	1·26 (1–1·53)	0·412 (0·257–0·674)	1·6 (1·26–1·96)
Meningitis	1·63 (1·29–2·02)	2·79 (2·19–3·46)	0·000576 (0·000372–0·000855)	3·55 (2·83–4·37)
Encephalitis	0·326 (0·251–0·411)	0·858 (0·566–1·11)	2e-04 (0·000137–0·000286)	1·08 (0·803–1·37)
Measles	2·91 (-0·774–12·8)	3·45 (-0·852–14·8)	0·562 (0·0192–1·29)	3·63 (-1·04–16)
Acute hepatitis	11·3 (6·78–22)	5·09 (3·29–8·18)	2·96 (1·86–4·71)	10·6 (6·43–20·1)
Maternal and neonatal disorders	88·8 (87·3–90·1)	79·8 (77–82·6)	67·3 (62·4–71·9)	89·6 (88·2–90·8)
Maternal disorders	9·28 (3·34–15·1)	9·45 (3·45–15·5)	9·65 (3·51–15·9)	9·16 (3·31–14·9)
Neonatal disorders	91 (89·7–92·2)	81·5 (78·6–84·3)	69·2 (63·9–73·9)	91 (89·7–92·2)
Nutritional deficiencies	74·6 (71·9–87·5)	79·6 (77–82·5)	79·7 (77·1–82·7)	75 (68·3–82·1)
Protein-energy malnutrition	100 (100–100)	100 (100–100)	100 (100–100)	100 (100–100)
Vitamin A deficiency	--	100 (100–100)	100 (100–100)	--
Dietary iron deficiency	--	100 (100–100)	100 (100–100)	--
Non-communicable diseases	57·7 (54·6–60·9)	43·5 (40·3–46·6)	28·3 (25·9–31)	58 (55·6–60·6)
Neoplasms	49·1 (45·6–53·1)	48·5 (45·2–52·1)	34·7 (30·8–39)	49·3 (46–53)
Lip and oral cavity cancer	67·1 (60·6–73·1)	70·1 (64·1–75·7)	69·3 (63–75·2)	70·2 (64·1–75·8)
Nasopharynx cancer	65·3 (55·3–73·7)	65·6 (56–73·6)	63·7 (53·6–72)	65·8 (56·2–73·7)
Other pharynx cancer	79 (73·1–83·7)	80·4 (74·7–85)	79·8 (73·7–84·6)	80·4 (74·8–85)
Esophageal cancer	75·6 (66·7–83·7)	77·1 (68·5–84·8)	76·2 (67·5–84·3)	77·1 (68·5–84·8)
Stomach cancer	25·1 (17·9–41·5)	25·5 (18·2–41·8)	25·7 (18·4–41·9)	25·5 (18·2–41·8)

Colon and rectum cancer	64·2 (55·7–72·7)	64·7 (56·8–72·8)	64·9 (56·8–72·9)	64·7 (56·7–72·7)
Liver cancer	76·4 (71·1–81·7)	77·1 (72–82)	77·4 (72·1–82·4)	77·1 (72–82)
Gallbladder and biliary tract cancer	22·8 (12·4–35·2)	23·4 (12·8–35·8)	21·4 (11·3–33·9)	23·4 (12·8–35·9)
Pancreatic cancer	42·9 (33·8–53·8)	42·7 (33·9–53·2)	42·9 (33·9–53·6)	42·7 (33·9–53·2)
Larynx cancer	82·1 (74·8–88·4)	82·2 (75·3–87·7)	80·5 (73·6–86·2)	82·4 (75·5–87·8)
Tracheal, bronchus, and lung cancer	86·4 (83·6–89·3)	85·8 (83–88·6)	86·3 (83·6–89·2)	85·8 (83–88·6)
Breast cancer	36·9 (27·9–47·8)	36 (28–45·5)	36·3 (28·2–46)	35·9 (28–45·4)
Cervical cancer	100 (100–100)	100 (100–100)	100 (100–100)	100 (100–100)
Uterine cancer	44·7 (30·1–60)	46·1 (30·9–61·5)	46·6 (31·4–62·1)	46 (30·9–61·4)
Ovarian cancer	26·4 (13·6–40)	22·3 (11·4–34·9)	21·3 (11–33·4)	22·4 (11·5–34·9)
Prostate cancer	5·91 (2·58–9·44)	6·47 (2·88–10·3)	6·87 (3·1–10·8)	6·41 (2·85–10·2)
Kidney cancer	40 (29·2–50·5)	40·4 (30·2–50·4)	40·4 (30·2–50·4)	40·4 (30·2–50·4)
Bladder cancer	40·4 (26·1–54·5)	42·9 (29·3–56)	43·7 (30–56·2)	42·8 (29·3–55·9)
Thyroid cancer	15·3 (7·51–24·9)	15·9 (7·87–25·7)	15·8 (8–25·2)	16 (7·84–25·9)
Mesothelioma	99 (98·7–99·3)	98 (97·3–98·5)	98·9 (98·5–99·2)	97·9 (97·3–98·5)
Non-Hodgkin lymphoma	7·59 (3·2–13·5)	7·83 (3·34–13·7)	7·76 (3·32–13·7)	7·83 (3·34–13·7)
Multiple myeloma	8·57 (3·75–15·1)	8·93 (3·9–15·7)	8·88 (3·88–15·8)	8·94 (3·9–15·7)
Leukemia	37·5 (23·2–52·1)	35·5 (23·6–47·6)	35 (23·2–47)	35·6 (23·6–47·6)
Cardiovascular diseases	79·2 (75·9–82·4)	80·5 (77·9–83)	71·7 (67·6–75·8)	81·6 (79–84·1)
Rheumatic heart disease	23·4 (13·6–44·7)	24·3 (15·5–40·6)	24 (15·7–39·1)	24·3 (15·6–40·8)
Ischemic heart disease	93·6 (90–96·7)	94·8 (92·3–97)	94 (91·3–96·5)	94·8 (92·3–97)
Stroke	79·8 (72·2–88·6)	82·9 (77·3–89·2)	82·5 (77·4–88·4)	82·9 (77·3–89·5)
Hypertensive heart disease	100 (100–100)	100 (100–100)	100 (100–100)	100 (100–100)
Non-rheumatic valvular heart disease	26·5 (17·8–39)	26·9 (19·5–36·6)	18·6 (13–25·2)	27·9 (20·3–38·2)
Cardiomyopathy and myocarditis	35·1 (28·6–43)	41·3 (36·6–46·3)	36·7 (30·6–42·7)	41·6 (36·7–46·7)
Atrial fibrillation and flutter	51·1 (42·4–60·8)	58·6 (51·2–66·6)	62·5 (55·4–69·7)	54·2 (46·2–63·4)
Aortic aneurysm	51·1 (44·9–57·6)	58 (52·9–63·1)	--	58 (52·9–63·1)
Endocarditis	32·1 (22·9–44·8)	34·6 (26·7–44·4)	31·7 (22·6–43·6)	34·7 (26·9–44·5)
Other cardiovascular and circulatory diseases	32·3 (27·3–37·4)	34·7 (30·6–39·1)	34·8 (30–39·3)	34·7 (30·5–39·3)
Chronic respiratory diseases	63·7 (58–69·8)	59·5 (55·2–64·2)	52·9 (48–57·8)	64·5 (59·4–70·1)
Chronic obstructive pulmonary disease	72·5 (68·5–76·5)	71·8 (68·5–75)	65·5 (62·2–68·7)	74·7 (71·1–78)
Pneumoconiosis	100 (100–100)	100 (100–100)	100 (100–100)	100 (100–100)
Asthma	43·1 (31·6–56)	42·6 (33·8–52·3)	42·3 (33·8–51·9)	45·2 (35·2–56·1)
Digestive diseases	29·3 (25·8–32·8)	35·8 (31·1–40·2)	24·1 (16·8–32·7)	41·2 (36·7–45·9)
Cirrhosis and other chronic liver diseases	71·6 (62·6–80·7)	74·7 (65·9–83·7)	72·6 (65·2–80·4)	74·8 (65·9–83·8)

Upper digestive system diseases	21·3 (18–24·3)	9·68 (6·51–13·2)	0·764 (0·448–1·19)	24·2 (20·8–27·4)
Gallbladder and biliary diseases	44·8 (30·1–60)	49·7 (35·3–63·8)	50·8 (36·4–64·9)	46·2 (31·7–61·3)
Pancreatitis	34·1 (23·7–45·4)	40·1 (29·5–51·9)	40·6 (30–52·4)	40·1 (29·4–51·9)
Neurological disorders	25·4 (11·1–40·5)	15·7 (7·83–27·4)	10·1 (5·05–19·1)	22·2 (10·4–36·9)
Alzheimer's disease and other dementias	40·8 (26·8–56·5)	42·9 (29–58)	43·2 (29·3–58·2)	42·7 (28·6–57·9)
Parkinson's disease	-9·21 (-15·3–-4·2)	-10·5 (-17·1–-4·88)	-11 (-17·8–-5·08)	-10·4 (-17–-4·79)
Idiopathic epilepsy	26·2 (19·1–33·8)	24·7 (18·3–31·8)	22·9 (16·9–29·4)	27·4 (20·1–34·9)
Multiple sclerosis	14·8 (11·2–18·6)	15·8 (12·1–19·5)	15·6 (12–19·2)	16 (12·1–20)
Mental disorders	--	6·6 (3·41–10·1)	6·61 (3·41–10·1)	--
Depressive disorders	--	13·5 (6·27–21·1)	13·5 (6·27–21·1)	--
Anxiety disorders	--	5 (1·36–11·2)	5 (1·36–11·2)	--
Idiopathic developmental intellectual disability	--	41·3 (19·1–59·1)	41·3 (19·1–59·1)	--
Substance use disorders	100 (100–100)	100 (100–100)	100 (100–100)	100 (100–100)
Alcohol use disorders	100 (100–100)	100 (100–100)	100 (100–100)	100 (100–100)
Drug use disorders	100 (100–100)	100 (100–100)	100 (100–100)	100 (100–100)
Diabetes and kidney diseases	99·7 (99·6–99·7)	99·9 (99·9–99·9)	100 (100–100)	99·7 (99·6–99·7)
Diabetes mellitus	100 (100–100)	100 (100–100)	100 (100–100)	100 (100–100)
Chronic kidney disease	100 (100–100)	100 (100–100)	100 (100–100)	100 (100–100)
Sense organ diseases	--	8·56 (7·4–9·88)	8·56 (7·4–9·88)	--
Blindness and vision loss	--	9·4 (6·09–13·5)	9·4 (6·09–13·5)	--
Age-related and other hearing loss	--	9·59 (8·7–10·6)	9·59 (8·7–10·6)	--
Musculoskeletal disorders	3·5 (0·863–6·32)	23 (19·2–26·9)	23·5 (19·5–27·6)	4·22 (1·03–7·4)
Rheumatoid arthritis	11·3 (2·8–19·9)	13·9 (3·52–24·3)	14·1 (3·51–24·7)	13 (3·29–22·7)
Osteoarthritis	--	17·5 (9·37–26·6)	17·5 (9·37–26·6)	--
Low back pain	--	41·9 (36·7–47·3)	41·9 (36·7–47·3)	--
Gout	--	52·8 (37·3–69·6)	52·8 (37·3–69·6)	--
Other non-communicable diseases	0·0841 (0·0634–0·108)	0·0779 (0·0524–0·108)	--	0·333 (0·238–0·419)
Sudden infant death syndrome	8·21 (5·46–12)	8·2 (5·45–12)	--	8·2 (5·45–12)
Injuries	32·8 (27·8–37)	27·2 (23·2–30·7)	30 (25·9–33·8)	23·8 (18·3–29)
Transport injuries	20·1 (14·5–26·2)	20·5 (15·4–25·8)	25·9 (22·1–29·5)	17·9 (11·6–24·4)
Road injuries	19·3 (13·4–25·5)	19·5 (14·2–25·1)	24·8 (21–28·4)	17·1 (10·6–23·8)
Other transport injuries	32 (27·4–36·1)	31·3 (26·9–35·3)	35·2 (31·1–38·9)	28·3 (23·3–33·1)
Unintentional injuries	41 (34·8–45·1)	29·6 (25–33·6)	30·2 (25·6–34·4)	27·8 (23·2–31·6)
Falls	54·9 (47·1–59·9)	40·9 (35–45·5)	39·2 (33·6–43·9)	46·2 (39·7–50·9)
Drowning	-14·8 (-25·7–-2·98)	-13·7 (-23·9–-2·81)	10·4 (6·14–16·1)	-14·9 (-25·3–-3·58)

Fire, heat, and hot substances	10·4 (6·06–16)	12·5 (7·86–18·3)	12·8 (8–18·6)	11·8 (7·4–17·3)
Poisonings	13·7 (8·82–19·8)	12·9 (8·45–18·2)	11·7 (7·37–17)	14·4 (9·64–20·4)
Exposure to mechanical forces	9·28 (1·39–18·2)	16·2 (13·7–18·2)	17·7 (15·4–19·6)	-0·832 (-9·13–8·34)
Animal contact	23·4 (18·9–28·6)	21 (16·6–26·1)	21·7 (17–26·9)	19·2 (15·1–23·8)
Foreign body	2·45 (1·82–3·16)	3·4 (2·55–4·42)	2·85 (2·13–3·78)	3·77 (2·83–4·85)
Environmental heat and cold exposure	8·08 (3·81–13·9)	9·22 (4·37–15·7)	9·21 (4·36–15·7)	9·26 (4·36–15·6)
Exposure to forces of nature	0 (0–0)	10 (4·78–16·8)	10 (4·78–16·8)	0 (0–0)
Other unintentional injuries	13·5 (8·57–19·6)	12·2 (7·73–17·9)	12·1 (7·61–17·8)	15 (10·1–21·2)
Self-harm and interpersonal violence	22·1 (10·2–33·5)	24·4 (13·6–34·6)	32·8 (26·4–39·6)	23·2 (11·3–34·6)
Self-harm	21·9 (9·34–33·7)	23·1 (10·7–35·1)	22·7 (11·6–33·4)	23·2 (10·6–35·2)
Interpersonal violence	27·4 (19·5–35·5)	33 (25·5–41)	38·6 (30·5–47·2)	24·6 (17–32·6)

Supplementary Results Table 2. All-age PAFs attributable to the joint distribution of all risk factors for all ages, both sexes combined for each GBD level 1, level 2, and level 3 cause and all causes in 2019 for East Midlands. Results are shown for deaths, YLLs, YLDs, and DALYs Data in parenthesis are 95% uncertainty intervals. PAFs=population attributable fractions. GBD=Global Burden of Disease. YLLs=years of life lost. YLDs=years lived with disability. DALYs=disability-adjusted life-years

	Deaths 2019	DALYs 2019	YLDs 2019	YLLs 2019
All causes	55·3 (52·1–58·4)	42·6 (39·3–45·7)	29·1 (26·8–31·4)	55·2 (52·6–57·8)
Communicable, maternal, neonatal, and nutritional diseases	44·4 (35·3–53·1)	51·7 (46·7–56·6)	46·7 (42·3–50·6)	54·2 (47–60·9)
HIV/AIDS and sexually transmitted infections	75·6 (59·6–87·7)	76·2 (59·5–88·8)	97·5 (96·3–98·5)	57·7 (38–76·6)
HIV/AIDS	92·1 (90·3–93·3)	91·3 (89–93)	94·8 (92·8–96·1)	88·5 (86·2–89·9)
Sexually transmitted infections excluding HIV	52 (29·8–77·3)	60·9 (35·9–84·6)	100 (100–100)	20·3 (8·41–42·2)
Respiratory infections and tuberculosis	44·8 (34·7–54·5)	42·3 (33·7–50·7)	3·44 (2·57–4·44)	48·3 (38·5–57·7)
Tuberculosis	54·1 (42·9–64·3)	57·3 (47–66·4)	54·6 (43·9–64·1)	57·8 (47·6–67·1)
Lower respiratory infections	44·8 (34·6–54·4)	48·2 (38·3–57·6)	33·1 (26·7–39·3)	48·3 (38·3–57·8)
Upper respiratory infections	0·0444 (0·031–0·0605)	0·00804 (0·00479–0·0123)	0·00601 (0·0029–0·01)	0·108 (0·076–0·149)
Otitis media	2·28 (1·41–3·57)	4·17 (2·65–5·8)	4·15 (2·64–5·77)	5·84 (3·82–8·45)
Enteric infections	5·51 (3·3–9·5)	9 (6·56–12·9)	9·51 (6·89–13·5)	7·91 (5·53–11·9)
Diarrheal diseases	5·57 (3·34–9·63)	9·05 (6·6–13)	9·52 (6·89–13·5)	8·03 (5·65–12)
Other infectious diseases	0·68 (0·463–0·871)	1·32 (1·03–1·62)	0·456 (0·272–0·765)	1·66 (1·25–2·05)
Meningitis	1·6 (1·24–2)	2·89 (2·25–3·66)	0·000575 (0·000258–0·00105)	3·65 (2·87–4·53)
Encephalitis	0·325 (0·232–0·428)	0·882 (0·554–1·18)	0·000197 (8·96e-05–0·000346)	1·12 (0·757–1·48)
Measles	2·88 (-0·749–13·2)	3·48 (-0·857–15·4)	0·652 (0·0221–1·69)	3·61 (-0·954–16·2)
Acute hepatitis	12·2 (6·96–23·5)	5·22 (3·31–8·47)	3·07 (1·88–5)	11·4 (6·64–21·9)
Maternal and neonatal disorders	90·7 (89·5–91·8)	80·3 (77·4–83·2)	65·2 (59·7–70·3)	91·3 (90·2–92·4)
Maternal disorders	9·61 (3·43–16)	9·69 (3·49–16·2)	9·81 (3·54–16·5)	9·49 (3·4–15·8)
Neonatal disorders	92·4 (91·3–93·4)	81·7 (78·6–84·7)	66·9 (61–72·1)	92·4 (91·3–93·4)
Nutritional deficiencies	80·1 (69·5–86·8)	77·8 (74·3–81·6)	77·7 (74·2–81·7)	80·7 (66·7–83·4)
Protein-energy malnutrition	100 (100–100)	100 (100–100)	100 (100–100)	100 (100–100)
Vitamin A deficiency	--	100 (100–100)	100 (100–100)	--
Dietary iron deficiency	--	100 (100–100)	100 (100–100)	--
Non-communicable diseases	57·1 (53·6–60·4)	43·2 (39·8–46·5)	28·4 (25·9–31)	57·3 (54·4–60)
Neoplasms	47·8 (44–52·1)	47·5 (44–51·5)	34 (30–38·5)	48·3 (44·8–52·3)
Lip and oral cavity cancer	64·1 (53·9–72·6)	67·7 (58·1–75·7)	66·2 (56·3–74·5)	67·8 (58·3–75·8)
Nasopharynx cancer	63·2 (51·2–72·8)	63·8 (52–72·8)	60·2 (48·1–70)	64·1 (52·4–73·1)
Other pharynx cancer	77·2 (69·9–82·9)	79 (71·9–84·3)	78·7 (71·5–84·2)	79 (71·9–84·3)
Esophageal cancer	74·1 (63·6–82·8)	75·8 (65·6–84·1)	74·8 (64·8–83·3)	75·9 (65·6–84·1)
Stomach cancer	24·2 (16·8–41·2)	25 (17·7–42)	25 (17·7–41·5)	25 (17·7–42)

Colon and rectum cancer	63·5 (54·2–72·5)	64·1 (55·5–72·5)	64·2 (55·5–72·6)	64·1 (55·5–72·5)
Liver cancer	75·1 (69·1–80·9)	76 (70·4–81·3)	76·4 (70·7–81·7)	76 (70·4–81·3)
Gallbladder and biliary tract cancer	22·5 (12·1–34·7)	23·1 (12·4–35·6)	21 (10·9–33·7)	23·2 (12·4–35·6)
Pancreatic cancer	42·2 (32·7–53·3)	42·4 (33·4–52·9)	42·4 (32·8–53·4)	42·4 (33·4–52·9)
Larynx cancer	80·5 (72–87·5)	80·9 (73·2–87·1)	79·3 (71·4–85·7)	81·1 (73·4–87·3)
Tracheal, bronchus, and lung cancer	86 (82·9–89·1)	85·7 (82·7–88·7)	86·2 (83·1–89·2)	85·7 (82·7–88·7)
Breast cancer	36·6 (27·1–47·9)	36 (27–45·9)	36·3 (27·2–46·3)	36 (27–45·8)
Cervical cancer	100 (100–100)	100 (100–100)	100 (100–100)	100 (100–100)
Uterine cancer	44·3 (29·3–59·9)	45·8 (30·3–61·9)	46·4 (30·9–62·5)	45·7 (30·2–61·8)
Ovarian cancer	25·5 (13·5–39·4)	21·8 (11·1–34·6)	21 (10·9–33·5)	21·8 (11·1–34·7)
Prostate cancer	5·63 (2·42–9·06)	6·17 (2·76–9·78)	6·52 (2·98–10·4)	6·12 (2·73–9·71)
Kidney cancer	39 (28·4–49·9)	39·7 (29·2–49·9)	39·6 (29·5–49·6)	39·7 (29·2–49·9)
Bladder cancer	39 (24·9–53·4)	41·6 (27·9–55)	42·7 (28·9–55·8)	41·5 (27·8–55)
Thyroid cancer	15 (7·24–24·5)	15·8 (7·57–25·8)	16·5 (7·59–27·3)	15·7 (7·61–25·6)
Mesothelioma	98·9 (98·5–99·2)	97·8 (97·1–98·4)	98·8 (98·3–99·1)	97·8 (97·1–98·4)
Non-Hodgkin lymphoma	7·42 (3·08–13·2)	7·67 (3·24–13·5)	7·59 (3·22–13·5)	7·68 (3·24–13·5)
Multiple myeloma	8·4 (3·55–14·9)	8·77 (3·73–15·4)	8·67 (3·69–15·3)	8·78 (3·74–15·4)
Leukemia	36·5 (22·5–51)	35·3 (23·4–47·5)	34·8 (22·9–47·2)	35·3 (23·5–47·6)
Cardiovascular diseases	79·9 (76·2–83·3)	80·8 (78–83·5)	71 (66·3–75·3)	82 (79·2–84·6)
Rheumatic heart disease	23·8 (13·2–45·8)	24·1 (15·1–39·3)	23·8 (15–39·5)	24·1 (15·1–39·7)
Ischemic heart disease	93·9 (90·1–96·9)	94·9 (92·2–97·1)	94·1 (91·4–96·6)	94·9 (92·2–97·1)
Stroke	80·3 (72·5–88·7)	83·1 (77·4–89·1)	82·4 (77·1–87·9)	83·2 (77·1–89·3)
Hypertensive heart disease	100 (100–100)	100 (100–100)	100 (100–100)	100 (100–100)
Non-rheumatic valvular heart disease	27·9 (18·1–40·7)	27·6 (19·4–37·6)	17·5 (11·8–23·8)	28·9 (20·4–39·4)
Cardiomyopathy and myocarditis	38·9 (30·9–47·5)	44·1 (38–49·2)	38·1 (31·9–45·2)	44·5 (38·2–49·7)
Atrial fibrillation and flutter	51·5 (41·7–62·2)	58·4 (50·3–67)	62·2 (54·4–70·1)	54·2 (45·3–64)
Aortic aneurysm	51·6 (44·6–58·9)	57·9 (52·1–63·5)	--	57·9 (52·1–63·5)
Endocarditis	33·1 (23·1–46)	35·1 (26·7–45·6)	32·8 (23·1–45)	35·2 (26·7–45·5)
Other cardiovascular and circulatory diseases	32·8 (27·2–38·8)	34·7 (29·8–39·6)	34·7 (29·1–40·2)	34·7 (29·7–39·7)
Chronic respiratory diseases	62·6 (56·4–69·6)	59 (54·3–64·1)	52·9 (47·8–57·9)	63·4 (57·6–70)
Chronic obstructive pulmonary disease	71·3 (66·5–76·1)	71 (67·3–74·5)	65·1 (61·7–68·5)	73·6 (69·5–77·9)
Pneumoconiosis	100 (100–100)	100 (100–100)	100 (100–100)	100 (100–100)
Asthma	41·8 (30·2–55)	42·7 (33·8–52·6)	42·5 (33·9–52·3)	44·4 (34·2–55·6)
Digestive diseases	27·3 (23–31·8)	34·3 (28·9–39·4)	25·1 (17·4–33·9)	38·7 (33·2–44·7)

Cirrhosis and other chronic liver diseases	70·2 (59·4–82·9)	73·8 (63·4–85·9)	71·7 (61·6–82·8)	73·9 (63·5–85·9)
Upper digestive system diseases	20·3 (17·1–23·3)	9·74 (6·57–13·4)	0·741 (0·43–1·18)	23·5 (20·3–26·7)
Gallbladder and biliary diseases	43·9 (28·4–58·8)	49·3 (34·9–64)	50·6 (36·2–64·7)	45·2 (30·2–60·5)
Pancreatitis	31·4 (14·9–49·4)	37·7 (19·9–55·6)	38·4 (20·5–57·4)	37·6 (19·8–55·5)
Neurological disorders	24·6 (10·5–40·3)	15·4 (7·74–27·1)	9·83 (4·96–18·8)	21·7 (9·8–36·6)
Alzheimer's disease and other dementias	39·9 (25·6–55·9)	42 (27·9–57·5)	42·3 (28·4–57·8)	41·8 (27·6–57·3)
Parkinson's disease	-8·53 (-14·1–-3·82)	-9·75 (-15·8–-4·46)	-10·3 (-16·7–-4·75)	-9·61 (-15·6–-4·39)
Idiopathic epilepsy	24·8 (15·2–34)	23·7 (14·8–32·3)	21·9 (13·6–30·2)	26·4 (16·8–35·6)
Multiple sclerosis	13·8 (10·4–17·6)	15·1 (11·5–18·8)	15 (11·5–18·6)	15·2 (11·4–19·1)
Mental disorders	--	6·45 (3·27–9·88)	6·46 (3·27–9·88)	--
Depressive disorders	--	13·3 (6·07–20·9)	13·3 (6·07–20·9)	--
Anxiety disorders	--	4·83 (1·29–10·9)	4·83 (1·29–10·9)	--
Idiopathic developmental intellectual disability	--	38·5 (17·3–56)	38·5 (17·3–56)	--
Substance use disorders	100 (100–100)	100 (100–100)	100 (100–100)	100 (100–100)
Alcohol use disorders	100 (100–100)	100 (100–100)	100 (100–100)	100 (100–100)
Drug use disorders	100 (100–100)	100 (100–100)	100 (100–100)	100 (100–100)
Diabetes and kidney diseases	99·6 (99·6–99·7)	99·9 (99·9–99·9)	100 (100–100)	99·6 (99·6–99·7)
Diabetes mellitus	100 (100–100)	100 (100–100)	100 (100–100)	100 (100–100)
Chronic kidney disease	100 (100–100)	100 (100–100)	100 (100–100)	100 (100–100)
Sense organ diseases	--	8·99 (7·81–10·4)	8·99 (7·81–10·4)	--
Blindness and vision loss	--	9·38 (6–13·7)	9·38 (6–13·7)	--
Age-related and other hearing loss	--	10·2 (9·25–11·4)	10·2 (9·25–11·4)	--
Musculoskeletal disorders	3·76 (0·901–6·8)	23·3 (19·4–27·4)	23·8 (19·7–27·9)	4·56 (1·11–8·04)
Rheumatoid arthritis	10·1 (2·44–18·1)	13 (3·24–22·6)	13·4 (3·32–23·4)	11·8 (2·95–20·8)
Osteoarthritis	--	17·4 (9·19–26·6)	17·4 (9·19–26·6)	--
Low back pain	--	41·9 (36·6–47·3)	41·9 (36·6–47·3)	--
Gout	--	52·2 (36·5–68·9)	52·2 (36·5–68·9)	--
Other non-communicable diseases	0·062 (0·0457–0·0824)	0·0607 (0·0409–0·085)	--	0·255 (0·181–0·325)
Sudden infant death syndrome	7·82 (5·11–11·5)	7·81 (5·11–11·5)	--	7·81 (5·11–11·5)
Injuries	32·6 (26·9–37·5)	27 (22·5–31·2)	29·9 (25·6–34)	23·4 (17·1–29·3)
Transport injuries	20 (13–27·5)	20·2 (14·1–26·6)	25·9 (21·9–29·9)	17·7 (9·96–25·7)
Road injuries	19·3 (12–27·1)	19·4 (13–26)	24·7 (20·8–28·8)	17·1 (9·08–25·4)
Other transport injuries	32·2 (27·3–36·8)	31·5 (26·8–36)	35·4 (30·8–39·4)	28·3 (22·9–33·5)
Unintentional injuries	40·8 (34·2–45·1)	29·7 (25·1–33·8)	30·2 (25·6–34·6)	28·2 (23·6–32·2)

Falls	55<br>(46·9–60·2)	41·4<br>(35·6–46·2)	39·7<br>(34–44·5)	46·7<br>(40·1–51·8)
Drowning	-15<br>(-27·7–-1·47)	-13·5<br>(-25·6–-0·75)	10·1<br>(5·63–16·2)	-14·8<br>(-27·2–-1·63)
Fire, heat, and hot substances	9·95<br>(5·53–16·2)	12·2<br>(7·33–18·8)	12·4<br>(7·41–19·2)	11·6<br>(6·92–17·9)
Poisonings	13·1<br>(7·91–19·7)	12·4<br>(7·6–18·7)	11·3<br>(6·71–17·4)	13·9<br>(8·74–20·3)
Exposure to mechanical forces	9·47<br>(-1·28–20·7)	16·4<br>(13·7–18·9)	18<br>(15·4–20·2)	-0·23<br>(-11·5–12)
Animal contact	23·3<br>(18·4–28·9)	20·9<br>(16·2–26·2)	21·3<br>(16·3–26·6)	19·8<br>(15·2–25·1)
Foreign body	2·33<br>(1·58–3·38)	3·33<br>(2·3–4·68)	2·8<br>(1·92–3·97)	3·67<br>(2·5–5·26)
Environmental heat and cold exposure	7·41<br>(3·13–13·5)	8·86<br>(3·88–15·9)	8·92<br>(3·9–15·9)	8·64<br>(3·79–15·5)
Exposure to forces of nature	0<br>(0–0)	9·52<br>(4·26–16·9)	9·52<br>(4·26–16·9)	0<br>(0–0)
Other unintentional injuries	13·1<br>(7·86–19·8)	11·8<br>(7·16–18·2)	11·7<br>(7·08–18·1)	14·8<br>(9·24–21·4)
Self-harm and interpersonal violence	20·8<br>(7·21–33·4)	23·4<br>(11·2–34·6)	32·3<br>(24·7–39·8)	22<br>(8·53–34·2)
Self-harm	20·4<br>(6·36–33·6)	21·8<br>(7·72–34·8)	21·6<br>(8·89–33·3)	21·8<br>(7·64–35)
Interpersonal violence	29<br>(20–38·1)	34·1<br>(25·7–43·1)	38·2<br>(29·6–47·1)	26·2<br>(17·4–34·9)

Supplementary Results Table 2. All-age PAFs attributable to the joint distribution of all risk factors for all ages, both sexes combined for each GBD level 1, level 2, and level 3 cause and all causes in 2019 for Derby. Results are shown for deaths, YLLs, YLDs, and DALYs Data in parenthesis are 95% uncertainty intervals. PAFs=population attributable fractions. GBD=Global Burden of Disease. YLLs=years of life lost. YLDs=years lived with disability. DALYs=disability-adjusted life-years

	Deaths 2019	DALYs 2019	YLDs 2019	YLLs 2019
All causes	55·7 (52·4–59)	42·3 (39–45·5)	28·8 (26·4–31·1)	55·5 (52·8–58·2)
Communicable, maternal, neonatal, and nutritional diseases	45·9 (35·9–55·4)	54·4 (49·3–59·4)	47 (42·1–51·6)	58 (50·6–65)
HIV/AIDS and sexually transmitted infections	76·8 (61·9–88·1)	75·4 (58·4–88·1)	97·4 (96·1–98·4)	58·4 (39–76·7)
HIV/AIDS	91·5 (89·7–92·7)	90·4 (88·1–92·2)	94·8 (92·7–96·1)	87·2 (84·9–88·7)
Sexually transmitted infections excluding HIV	58·4 (35·7–81·9)	59·7 (35·2–83·8)	100 (100–100)	25·3 (10·6–49·7)
Respiratory infections and tuberculosis	45·3 (34·1–56)	42·8 (33·4–52·3)	3·52 (2·52–4·77)	49·2 (38·8–59·4)
Tuberculosis	53·8 (40·6–66)	57·2 (44·7–67·7)	54·4 (41·4–65·8)	57·6 (45·1–68·3)
Lower respiratory infections	45·3 (34–55·9)	49·1 (38·5–59·5)	33 (26·1–40)	49·2 (38·5–59·6)
Upper respiratory infections	0·039 (0·0236–0·0635)	0·00783 (0·00169–0·0177)	0·00621 (0·000162–0·0163)	0·0892 (0·0534–0·151)
Otitis media	4·04 (1·94–7·59)	4·83 (3·03–6·91)	4·79 (3·01–6·9)	9·29 (5·15–15·5)
Enteric infections	5·35 (3·28–9·19)	9·64 (7·17–13·6)	9·9 (7·16–14·1)	8·99 (6·24–12·9)
Diarrheal diseases	5·42 (3·33–9·27)	9·71 (7·22–13·8)	9·91 (7·16–14·1)	9·18 (6·5–13·2)
Other infectious diseases	0·937 (0·643–1·23)	1·68 (1·25–2·12)	0·431 (0·241–0·725)	2·13 (1·53–2·72)
Meningitis	2·26 (1·66–2·98)	3·66 (2·7–4·79)	0·000677 (1·07e-05–0·00194)	4·46 (3·29–5·78)
Encephalitis	0·472 (0·292–0·718)	1·17 (0·672–1·78)	0·000244 (3·37e-06–0·000658)	1·45 (0·871–2·17)
Measles	3·7 (-0·969–17·6)	3·75 (-0·88–16·9)	0·582 (0·0158–1·62)	4·58 (-1·25–21·7)
Acute hepatitis	10·4 (4·15–21·6)	4·78 (2·48–8·61)	2·81 (1·64–4·63)	8·79 (3·39–18·5)
Maternal and neonatal disorders	91·7 (90·1–93·3)	81·3 (78–84·4)	62·7 (56·7–67·9)	92·1 (90·5–93·8)
Maternal disorders	9·86 (3·46–16·4)	9·97 (3·52–16·9)	10·1 (3·52–17·4)	9·72 (3·4–16·2)
Neonatal disorders	92·8 (91·3–94·4)	82·5 (79·2–85·6)	64·5 (58–70)	92·9 (91·3–94·5)
Nutritional deficiencies	73·1 (67·3–85·9)	78·2 (73·8–83·1)	78·3 (73·9–83·3)	74·1 (64·5–79·9)
Protein-energy malnutrition	100 (100–100)	100 (100–100)	100 (100–100)	100 (100–100)
Vitamin A deficiency	--	100 (100–100)	100 (100–100)	--
Dietary iron deficiency	--	100 (100–100)	100 (100–100)	--
Non-communicable diseases	57·5 (54·1–60·9)	42·7 (39·2–46·1)	28 (25·4–30·5)	57·5 (54·7–60·4)
Neoplasms	48·3 (44·4–52·6)	47·6 (44–51·8)	34·3 (30–38·8)	48·3 (44·7–52·3)
Lip and oral cavity cancer	64·7 (52·5–74·9)	68·4 (56–77·5)	67 (54·6–76·9)	68·5 (56·1–77·6)
Nasopharynx cancer	64·6 (51–75·4)	64·8 (51·7–74·8)	61·2 (47·6–73)	65·1 (52–75)
Other pharynx cancer	77·3 (68·7–84)	79·2 (70·7–85·5)	79 (70·9–85·5)	79·2 (70·7–85·5)
Esophageal cancer	73·8 (63·4–82·9)	75·7 (65·5–84)	74·6 (64·6–83·2)	75·7 (65·5–84·1)
Stomach cancer	24·1 (16·6–41·2)	24·8 (17·5–41·2)	24·9 (17·5–41·5)	24·8 (17·5–41·2)

Colon and rectum cancer	63·2 (54·5–72·2)	63·7 (55·3–72·1)	63·8 (55·4–72·3)	63·7 (55·3–72·2)
Liver cancer	73·3 (66·6–79·9)	74·3 (68–80)	74·5 (68·2–80·5)	74·3 (68·1–79·9)
Gallbladder and biliary tract cancer	22·2 (11·6–34·5)	22·8 (12·1–35·1)	20·7 (10·7–33)	22·9 (12·1–35·1)
Pancreatic cancer	42 (32·3–52·8)	42·1 (33·4–52·5)	42·2 (32·7–53)	42·1 (33·4–52·5)
Larynx cancer	80·6 (72·6–87·5)	81 (73·5–87·1)	79 (71·2–85·6)	81·2 (73·8–87·3)
Tracheal, bronchus, and lung cancer	85·8 (82·2–89·1)	85·3 (81·9–88·5)	85·9 (82·4–89·3)	85·3 (81·9–88·5)
Breast cancer	36·4 (26·8–47·6)	35·6 (26·7–45·5)	35·9 (26·9–45·9)	35·6 (26·7–45·4)
Cervical cancer	100 (100–100)	100 (100–100)	100 (100–100)	100 (100–100)
Uterine cancer	43·8 (27·5–59·6)	45·4 (29·4–61·4)	46·1 (30·5–62·2)	45·3 (29·2–61·2)
Ovarian cancer	24 (12·7–37·4)	20·1 (10·3–32·4)	19·3 (9·61–31·2)	20·2 (10·3–32·4)
Prostate cancer	5·7 (2·5–9·32)	6·27 (2·86–9·99)	6·64 (3·08–10·5)	6·22 (2·82–9·96)
Kidney cancer	38·5 (28·3–49·1)	38·9 (28·9–49·1)	38·6 (28·8–48·7)	38·9 (28·9–49·1)
Bladder cancer	38·8 (25·4–53·6)	41·4 (28·8–54·6)	42·6 (30–55·4)	41·4 (28·7–54·6)
Thyroid cancer	14·6 (6·74–24·2)	15·4 (7·1–25·4)	16 (7·17–26·6)	15·3 (7·1–25·3)
Mesothelioma	99·1 (98·8–99·4)	98·1 (97·5–98·6)	98·9 (98·5–99·3)	98·1 (97·4–98·6)
Non-Hodgkin lymphoma	7·11 (2·9–12·8)	7·34 (3·02–13)	7·25 (2·94–13)	7·35 (3·03–13)
Multiple myeloma	8·15 (3·33–14·7)	8·53 (3·65–15·2)	8·41 (3·49–14·9)	8·54 (3·65–15·2)
Leukemia	36·1 (22·5–50·1)	33·7 (22·2–45·4)	33·1 (21·6–44·5)	33·8 (22·3–45·4)
Cardiovascular diseases	80·1 (75·8–83·9)	81·1 (77·8–84)	71·6 (66·4–76·3)	82·2 (78·9–84·9)
Rheumatic heart disease	24·1 (13–45·4)	24·4 (15–41·6)	24 (14·4–40·9)	24·4 (15–41·5)
Ischemic heart disease	94 (89·9–97·1)	95·1 (92·2–97·3)	94·2 (91·1–96·7)	95·1 (92·3–97·4)
Stroke	80·7 (72·5–89·6)	83·4 (77·4–89·3)	82·4 (77·1–87·8)	83·6 (77·4–89·6)
Hypertensive heart disease	100 (100–100)	100 (100–100)	100 (100–100)	100 (100–100)
Non-rheumatic valvular heart disease	28·6 (18·4–43)	29 (20·2–39·8)	18·8 (12·4–26·9)	29·9 (20·8–41·2)
Cardiomyopathy and myocarditis	40·1 (31·2–49·5)	45·6 (39·5–51·4)	39 (32·4–46·2)	46 (39·6–51·9)
Atrial fibrillation and flutter	51·5 (40·2–62·8)	57·9 (48·2–67·1)	61·7 (52·6–70·1)	54·2 (43·9–64·4)
Aortic aneurysm	51·7 (44·3–60·3)	58·6 (52·2–65·2)	--	58·6 (52·2–65·2)
Endocarditis	33·6 (22·7–47·3)	35·6 (26·7–45·9)	32·9 (21·9–45·9)	35·7 (26·8–45·9)
Other cardiovascular and circulatory diseases	33·4 (26·2–41·1)	35 (28·3–41·6)	34·2 (26·9–42·2)	35·1 (28·5–41·6)
Chronic respiratory diseases	63·1 (56·2–70·8)	58·6 (53·7–64·2)	51·5 (46·3–57)	63·9 (57·7–70·7)
Chronic obstructive pulmonary disease	71·7 (66–77·3)	71·3 (67–75·5)	65 (61–69)	74 (69·2–78·6)
Pneumoconiosis	100 (100–100)	100 (100–100)	100 (100–100)	100 (100–100)
Asthma	41·6 (29·9–54·4)	41·4 (32·9–50·8)	41·2 (32·5–50·4)	43·7 (33·6–54·9)
Digestive diseases	29·4 (23·8–35·3)	37·9 (31·3–44·3)	30·5 (21·2–40·6)	42·1 (34·8–49·3)
Cirrhosis and other chronic liver diseases	71·7 (59–85·8)	75·1 (62·7–88·2)	72·5 (59·6–85·3)	75·1 (62·8–88·4)

Upper digestive system diseases	20·9 (17·4–24·4)	9·87 (6·47–13·8)	0·652 (0·372–1·07)	24·4 (20·8–28)
Gallbladder and biliary diseases	43·7 (27·5–58·6)	51·3 (36·5–65·5)	52·5 (37·6–66·7)	45 (29·6–59·8)
Pancreatitis	34·5 (12·2–58·1)	41 (17·7–62·9)	41·1 (17·8–63·5)	41 (17·5–62·9)
Neurological disorders	24·4 (10·5–40·4)	14·5 (7·06–25·9)	9·07 (4·27–18·2)	21·5 (10·1–35·8)
Alzheimer's disease and other dementias	39·1 (25·1–55·2)	41·2 (27·2–56·9)	41·6 (27·9–57)	41 (27–56·5)
Parkinson's disease	-8·54 (-14·1–-3·78)	-9·84 (-16–-4·41)	-10·5 (-17·1–-4·78)	-9·68 (-15·8–-4·36)
Idiopathic epilepsy	25·7 (13·7–37·3)	24·2 (13·2–34·7)	22·2 (11·9–32·3)	26·6 (14·8–37·5)
Multiple sclerosis	14·3 (10·4–18·3)	15·6 (11·5–19·6)	15·4 (11·5–19·2)	15·7 (11·5–20)
Mental disorders	--	6·62 (3·34–10·2)	6·62 (3·34–10·2)	--
Depressive disorders	--	13·7 (6·25–21·4)	13·7 (6·25–21·4)	--
Anxiety disorders	--	5·29 (1·4–11·9)	5·29 (1·4–11·9)	--
Idiopathic developmental intellectual disability	--	37·2 (15·6–56·5)	37·2 (15·6–56·5)	--
Substance use disorders	100 (100–100)	100 (100–100)	100 (100–100)	100 (100–100)
Alcohol use disorders	100 (100–100)	100 (100–100)	100 (100–100)	100 (100–100)
Drug use disorders	100 (100–100)	100 (100–100)	100 (100–100)	100 (100–100)
Diabetes and kidney diseases	99·9 (99·8–99·9)	100 (100–100)	100 (100–100)	99·9 (99·8–99·9)
Diabetes mellitus	100 (100–100)	100 (100–100)	100 (100–100)	100 (100–100)
Chronic kidney disease	100 (100–100)	100 (100–100)	100 (100–100)	100 (100–100)
Sense organ diseases	--	9 (7·85–10·4)	9 (7·85–10·4)	--
Blindness and vision loss	--	8·83 (5·63–13)	8·83 (5·63–13)	--
Age-related and other hearing loss	--	10·5 (9·42–11·7)	10·5 (9·42–11·7)	--
Musculoskeletal disorders	3·85 (0·891–7·17)	22·6 (18·6–26·6)	23 (19–27·1)	4·68 (1·16–8·5)
Rheumatoid arthritis	10·4 (2·45–18·9)	13·3 (3·44–23·6)	13·7 (3·46–24·1)	12·3 (3·1–22)
Osteoarthritis	--	17·1 (8·85–26·2)	17·1 (8·85–26·2)	--
Low back pain	--	41·3 (35·8–46·8)	41·3 (35·8–46·8)	--
Gout	--	50·5 (35–67·7)	50·5 (35–67·7)	--
Other non-communicable diseases	0·118 (0·0801–0·171)	0·111 (0·0697–0·17)	--	0·409 (0·276–0·574)
Sudden infant death syndrome	8·6 (4·91–14)	8·59 (4·91–14)	--	8·59 (4·91–14)
Injuries	32·5 (26·3–38·4)	26·3 (21·2–31·4)	28·9 (24·3–33·9)	23·3 (15·8–30·6)
Transport injuries	19·8 (11·5–27·9)	20 (13·1–26·8)	25·9 (21·3–30·4)	17·5 (8·78–26·2)
Road injuries	19·1 (10·6–27·5)	19·2 (12·1–26·3)	24·8 (20·2–29·3)	17 (7·98–26)
Other transport injuries	31·5 (25·9–36·6)	31 (25·6–35·9)	34·4 (29·8–38·7)	27·9 (21·5–33·6)
Unintentional injuries	40·3 (33·7–45·2)	28·3 (23·5–33)	28·9 (24–34)	26·6 (21·8–31·2)
Falls	54·5 (46·3–60·1)	39·9 (34–45·5)	38·2 (32·2–43·7)	45·2 (38·7–51)
Drowning	-15 (-29·1–-0·541)	-14·5 (-27·8–-0·828)	10·6 (5·91–17·7)	-15·6 (-29·6–-1·23)

Fire, heat, and hot substances	10·3 (5·51–17·2)	12·5 (7·38–19·8)	12·9 (7·71–20·5)	11·5 (6·75–17·9)
Poisonings	13·8 (8·29–21·2)	12·8 (7·75–19·7)	11·8 (7·03–18·4)	14·3 (8·89–21·4)
Exposure to mechanical forces	7·88 (-4·43–20·1)	15·7 (12·7–18·5)	17·2 (14·5–19·7)	-2·16 (-14·7–11·6)
Animal contact	23·9 (19·2–29·6)	19·8 (15·2–25·8)	20·7 (15·9–27)	16·4 (12·5–21·5)
Foreign body	2·57 (1·62–3·99)	3·43 (2·22–5·14)	3·02 (1·95–4·55)	3·69 (2·36–5·7)
Environmental heat and cold exposure	8·07 (3·16–15·1)	9·25 (4·01–17·1)	9·19 (3·97–17·2)	9·38 (3·96–17·4)
Exposure to forces of nature	0 (0–0)	9·93 (4·39–18·7)	9·93 (4·39–18·7)	0 (0–0)
Other unintentional injuries	13·9 (8·13–21·5)	12·3 (7·22–19·6)	12·2 (7·14–19·4)	15 (9·25–22·3)
Self-harm and interpersonal violence	22·3 (7·05–36)	24·7 (10·9–36·9)	33·3 (24·7–41·7)	23·4 (8·13–36·9)
Self-harm	22 (5·86–36·3)	23·3 (7·19–37·6)	22·8 (9·04–36·2)	23·3 (7·06–37·6)
Interpersonal violence	30 (20·9–39·8)	34·2 (25·2–43·2)	38·7 (29·2–48·5)	25·7 (17·2–35)

Supplementary Results Table 2. All-age PAFs attributable to the joint distribution of all risk factors for all ages, both sexes combined for each GBD level 1, level 2, and level 3 cause and all causes in 2019 for Derbyshire. Results are shown for deaths, YLLs, YLDs, and DALYs Data in parenthesis are 95% uncertainty intervals. PAFs=population attributable fractions. GBD=Global Burden of Disease. YLLs=years of life lost. YLDs=years lived with disability. DALYs=disability-adjusted life-years

	Deaths 2019	DALYs 2019	YLDs 2019	YLLs 2019
All causes	55·3 (52·1–58·5)	42·8 (39·4–46)	29·4 (27·1–31·9)	55·3 (52·3–58·1)
Communicable, maternal, neonatal, and nutritional diseases	43·2 (35·1–51·2)	49·2 (44·5–54·2)	46 (41·5–50·5)	50·8 (43·7–57·6)
HIV/AIDS and sexually transmitted infections	74·7 (57·5–87·9)	76·4 (58·7–89·6)	98·2 (97·2–98·9)	54·6 (34·1–74·8)
HIV/AIDS	92·7 (91·1–93·8)	92 (89·9–93·6)	95·3 (93·5–96·5)	89·1 (87·2–90·5)
Sexually transmitted infections excluding HIV	55·2 (32·3–79·3)	64·7 (40–87·2)	100 (100–100)	22 (9–45)
Respiratory infections and tuberculosis	44·1 (35·2–52·9)	42 (34·1–50)	3·37 (2·47–4·39)	47·3 (38·6–55·8)
Tuberculosis	53·6 (40·6–65)	56·9 (44·8–67·3)	54·2 (41·9–64·9)	57·4 (45·5–67·8)
Lower respiratory infections	44·1 (35·1–52·9)	47·2 (38·5–55·7)	32·8 (25·8–39·4)	47·3 (38·5–55·8)
Upper respiratory infections	0·0358 (0·0192–0·0594)	0·00639 (0·00117–0·015)	0·00522 (9·69e-05–0·0138)	0·0938 (0·0503–0·154)
Otitis media	1·52 (0·716–2·79)	3·78 (2·39–5·42)	3·77 (2·38–5·39)	4·43 (2·35–7·42)
Enteric infections	5·6 (3·2–10·1)	8·5 (5·95–13·2)	9·17 (6·43–13·9)	7·2 (4·71–11·7)
Diarrheal diseases	5·66 (3·24–10·2)	8·56 (6–13·3)	9·18 (6·43–13·9)	7·32 (4·84–11·8)
Other infectious diseases	0·474 (0·333–0·643)	1·04 (0·775–1·32)	0·463 (0·268–0·774)	1·29 (0·918–1·69)
Meningitis	1·12 (0·794–1·49)	2·15 (1·56–2·84)	0·000459 (6·26e-06–0·00129)	2·84 (2·08–3·76)
Encephalitis	0·203 (0·134–0·289)	0·582 (0·35–0·849)	0·00014 (2·12e-06–0·000417)	0·767 (0·494–1·1)
Measles	2·45 (-0·641–12·7)	2·99 (-0·714–14·5)	0·7 (0·0202–1·85)	3·13 (-0·817–16·2)
Acute hepatitis	8·57 (3·12–22·9)	4·54 (2·49–8·27)	3·3 (1·92–5·42)	8·39 (3·13–21·8)
Maternal and neonatal disorders	89·6 (88·1–91·2)	78·4 (74·8–81·7)	65·5 (59·7–70·8)	90·4 (88·9–91·8)
Maternal disorders	9·55 (3·39–16)	9·62 (3·35–16·2)	9·72 (3·41–16·5)	9·43 (3·33–15·9)
Neonatal disorders	91·7 (90·2–93·1)	80 (76·3–83·3)	67·2 (61·1–72·7)	91·7 (90·2–93·1)
Nutritional deficiencies	82·5 (74·4–89·2)	77·8 (73·8–82·3)	77·6 (73·3–82·3)	82·9 (70·8–85·8)
Protein-energy malnutrition	100 (100–100)	100 (100–100)	100 (100–100)	100 (100–100)
Vitamin A deficiency	--	100 (100–100)	100 (100–100)	--
Dietary iron deficiency	--	100 (100–100)	100 (100–100)	--
Non-communicable diseases	57·1 (53·6–60·5)	43·5 (39·9–47)	28·6 (26·1–31·3)	57·4 (54·4–60·4)
Neoplasms	47·9 (44–52·4)	47·7 (43·9–51·8)	33·9 (29·8–38·5)	48·5 (44·8–52·5)
Lip and oral cavity cancer	63·1 (50·9–73·5)	66·7 (54·2–76·1)	65·2 (52·8–75)	66·8 (54·4–76·2)
Nasopharynx cancer	61·7 (48·3–73·4)	62·7 (49·1–73·8)	58·9 (45·6–70·9)	63 (49·5–74·1)
Other pharynx cancer	76·3 (67·9–83·4)	78·2 (69·9–84·8)	78 (69·4–84·6)	78·2 (69·9–84·7)
Esophageal cancer	73·8 (62·4–82·5)	75·6 (64·9–83·8)	74·6 (63·3–83·1)	75·6 (64·9–83·8)
Stomach cancer	24 (16·5–41·4)	24·8 (17·3–41·7)	24·9 (17·2–42)	24·8 (17·3–41·6)

Colon and rectum cancer	63·1 (53·9–71·9)	63·7 (55–72·1)	63·8 (55–72·2)	63·7 (55–72·1)
Liver cancer	75·1 (68·6–81·3)	76·2 (70·3–82·1)	76·5 (70·5–82·3)	76·2 (70·3–82·1)
Gallbladder and biliary tract cancer	22 (11·6–34·7)	22·6 (12·2–35)	20·6 (10·5–33·2)	22·7 (12·2–35)
Pancreatic cancer	41·6 (31·8–52·8)	41·7 (32·6–52·5)	41·7 (32·2–52·9)	41·7 (32·6–52·5)
Larynx cancer	80·2 (71·3–87·4)	80·7 (72·8–87·1)	79·2 (71·3–86·1)	80·9 (73–87·3)
Tracheal, bronchus, and lung cancer	86 (82·4–89·4)	85·7 (82·3–89)	86·2 (82·7–89·5)	85·7 (82·3–89)
Breast cancer	36·1 (26·2–47·6)	35·7 (26·6–45·8)	36 (26·9–46·1)	35·7 (26·6–45·8)
Cervical cancer	100 (100–100)	100 (100–100)	100 (100–100)	100 (100–100)
Uterine cancer	43·6 (28·4–59·4)	45·1 (29·9–60·9)	45·7 (30·4–61·7)	45 (29·6–60·8)
Ovarian cancer	24·9 (13–38·5)	21·5 (11·1–34·1)	20·8 (10·8–33)	21·5 (11·1–34·2)
Prostate cancer	5·63 (2·45–9·28)	6·15 (2·73–10)	6·48 (2·89–10·5)	6·1 (2·69–9·94)
Kidney cancer	38·6 (27·9–49·6)	39·5 (28·8–49·9)	39·4 (28·9–49·8)	39·5 (28·8–49·9)
Bladder cancer	38·4 (24–52·8)	40·9 (26·9–54·6)	42 (28·5–55·1)	40·8 (26·8–54·6)
Thyroid cancer	14·4 (7–23·7)	15·2 (7·48–24·9)	15·9 (7·51–26·5)	15·2 (7·44–24·8)
Mesothelioma	98·9 (98·5–99·3)	97·9 (97·2–98·5)	98·8 (98·2–99·2)	97·9 (97·2–98·5)
Non-Hodgkin lymphoma	7·25 (2·95–12·9)	7·53 (3·17–13·4)	7·41 (3·01–13·2)	7·54 (3·18–13·4)
Multiple myeloma	8·21 (3·42–14·7)	8·57 (3·64–15·3)	8·47 (3·47–15·2)	8·58 (3·65–15·3)
Leukemia	36·5 (22–51·4)	35·8 (23·5–48·5)	35·3 (23·1–48)	35·8 (23·5–48·5)
Cardiovascular diseases	80 (76–83·7)	81 (77·7–84)	71·4 (66·3–76·1)	82·1 (78·8–85)
Rheumatic heart disease	23·5 (12·8–43·6)	23·8 (14·6–40·5)	23·5 (14·7–39·4)	23·8 (14·6–40·9)
Ischemic heart disease	93·7 (90–96·9)	94·7 (91·8–97)	94 (90·9–96·5)	94·7 (91·8–97·1)
Stroke	80·1 (71·8–88·7)	82·8 (76·8–89·3)	82·2 (76·6–88·3)	82·9 (76·8–89·5)
Hypertensive heart disease	100 (100–100)	100 (100–100)	100 (100–100)	100 (100–100)
Non-rheumatic valvular heart disease	28 (17·7–42·1)	28·1 (19·1–39·6)	20·8 (13·6–29)	29 (19·6–40·9)
Cardiomyopathy and myocarditis	39·8 (30·7–49·4)	45·7 (37·9–51·8)	38·8 (32·4–46·4)	46·2 (38–52·3)
Atrial fibrillation and flutter	50·9 (39·5–62·2)	57·8 (48·4–67)	61·4 (52·2–70)	53·6 (43·5–63·8)
Aortic aneurysm	50·9 (43·6–58·3)	57 (50·9–63·4)	--	57 (50·9–63·4)
Endocarditis	32·7 (21·7–47·7)	34·7 (24·9–46·5)	32·4 (21·8–46·6)	34·7 (25–46·3)
Other cardiovascular and circulatory diseases	32·8 (25·7–40)	34·9 (28·2–40·8)	34·6 (27·7–42)	34·9 (28·3–40·8)
Chronic respiratory diseases	62·9 (56·6–70·1)	59·7 (55–64·9)	53·7 (48·7–58·7)	63·7 (57·7–70·5)
Chronic obstructive pulmonary disease	71·2 (66·5–75·5)	70·8 (66·9–74·6)	65·1 (61·3–69·1)	73·4 (69·4–77·3)
Pneumoconiosis	100 (100–100)	100 (100–100)	100 (100–100)	100 (100–100)
Asthma	41·2 (28·6–54·8)	42·9 (33·7–53·2)	42·8 (33·7–53)	44·2 (33·4–55·9)
Digestive diseases	26·4 (21·6–32·1)	33·9 (27·8–40·3)	28·1 (19·5–37·3)	37·1 (30·9–44·3)

Cirrhosis and other chronic liver diseases	69·3 (57·3–84·8)	72·9 (61·3–87·4)	71·3 (59·2–84·7)	73 (61·2–87·5)
Upper digestive system diseases	20·5 (17·2–23·9)	9·88 (6·52–13·7)	0·773 (0·443–1·25)	23·8 (20·3–27·3)
Gallbladder and biliary diseases	43·3 (27·9–58·7)	49·9 (35–64·5)	51·2 (36·5–65·5)	44·6 (30–59·8)
Pancreatitis	30·1 (9·92–53·5)	36·2 (14·6–58·4)	37·1 (15·1–59·4)	36 (14·5–58·3)
Neurological disorders	24·1 (10·2–39·6)	15·6 (7·7–27·6)	10·2 (5·15–18·9)	21·4 (9·1–36·3)
Alzheimer's disease and other dementias	39·2 (24·6–54·7)	41·3 (27·1–56·5)	41·6 (28–56·7)	41·1 (26·8–56·4)
Parkinson's disease	-8·47 (-14–-3·81)	-9·62 (-15·6–-4·4)	-10·2 (-16·3–-4·72)	-9·49 (-15·5–-4·36)
Idiopathic epilepsy	23·8 (12·7–35·2)	23·4 (12·6–34·3)	21·5 (11·5–31·7)	25·9 (14·5–37·3)
Multiple sclerosis	13·7 (10–17·3)	14·9 (11·1–18·7)	14·9 (11·1–18·5)	15 (11–18·8)
Mental disorders	--	6·33 (3·18–9·91)	6·34 (3·18–9·92)	--
Depressive disorders	--	12·9 (5·91–20·3)	12·9 (5·91–20·3)	--
Anxiety disorders	--	4·48 (1·19–9·85)	4·48 (1·19–9·85)	--
Idiopathic developmental intellectual disability	--	40·3 (18–59·6)	40·3 (18–59·6)	--
Substance use disorders	100 (100–100)	100 (100–100)	100 (100–100)	100 (100–100)
Alcohol use disorders	100 (100–100)	100 (100–100)	100 (100–100)	100 (100–100)
Drug use disorders	100 (100–100)	100 (100–100)	100 (100–100)	100 (100–100)
Diabetes and kidney diseases	99·7 (99·6–99·7)	99·9 (99·9–99·9)	100 (100–100)	99·7 (99·6–99·7)
Diabetes mellitus	100 (100–100)	100 (100–100)	100 (100–100)	100 (100–100)
Chronic kidney disease	100 (100–100)	100 (100–100)	100 (100–100)	100 (100–100)
Sense organ diseases	--	8·86 (7·62–10·3)	8·86 (7·62–10·3)	--
Blindness and vision loss	--	9·43 (6·05–13·8)	9·43 (6·05–13·8)	--
Age-related and other hearing loss	--	10 (8·89–11·2)	10 (8·89–11·2)	--
Musculoskeletal disorders	3·63 (0·847–6·73)	24·8 (20·6–29·2)	25·2 (20·9–29·7)	4·4 (1·08–7·93)
Rheumatoid arthritis	10 (2·4–18·3)	12·8 (3·34–22·6)	13·3 (3·37–23·2)	11·7 (2·85–21)
Osteoarthritis	--	17·1 (9·01–26·3)	17·1 (9·01–26·3)	--
Low back pain	--	41·9 (36·3–47·6)	41·9 (36·3–47·6)	--
Gout	--	51·7 (36·1–68·8)	51·7 (36·1–68·8)	--
Other non-communicable diseases	0·0442 (0·0287–0·0641)	0·042 (0·0245–0·064)	--	0·2 (0·123–0·283)
Sudden infant death syndrome	7·37 (4·08–12·2)	7·37 (4·08–12·2)	--	7·37 (4·08–12·2)
Injuries	35 (28·7–40·1)	28·7 (23·7–33·2)	31·4 (26·7–35·9)	24·9 (18·4–31·2)
Transport injuries	19·8 (13·1–26·4)	20·7 (14·5–26·8)	26·1 (21·6–30·4)	17·9 (10·5–25·6)
Road injuries	19 (12·1–25·8)	19·7 (13·4–25·9)	24·8 (20·4–29·1)	17·2 (9·55–25·1)
Other transport injuries	33·1 (27·8–38)	32·6 (27·4–37·5)	36·2 (31·2–40·3)	29·2 (23·1–34·8)
Unintentional injuries	42·6 (35·9–47·2)	31·7 (26·6–36·1)	31·9 (26·8–36·6)	31·1 (26–35·4)

Falls	55·1<br>(46·7–60·4)	42·8<br>(36·4–47·7)	41·1<br>(34·9–45·9)	47·7<br>(40·8–52·7)
Drowning	-15·1<br>(-26·6–-3·09)	-13<br>(-23·6–-1·47)	9·87<br>(5·31–16)	-14·3<br>(-25·4–-2·27)
Fire, heat, and hot substances	9·6<br>(4·96–15·8)	12<br>(7·05–18·4)	12·2<br>(7·17–18·4)	11·6<br>(6·67–17·9)
Poisonings	12·8<br>(7·6–19·6)	12·5<br>(7·44–18·8)	11·2<br>(6·53–17·1)	14<br>(8·71–20·9)
Exposure to mechanical forces	10<br>(1·07–19·3)	17·1<br>(14–19·8)	18·8<br>(15·9–21·2)	0·25<br>(-8·81–10·3)
Animal contact	24·6<br>(19·3–30·5)	21·8<br>(16·8–27·6)	21·8<br>(16·7–27·6)	21·7<br>(16·6–27·4)
Foreign body	2·26<br>(1·42–3·37)	3·44<br>(2·2–5·08)	2·82<br>(1·82–4·15)	3·84<br>(2·45–5·66)
Environmental heat and cold exposure	7·11<br>(2·63–13·3)	8·6<br>(3·48–15·4)	8·69<br>(3·5–15·6)	8·33<br>(3·35–14·8)
Exposure to forces of nature	0<br>(0–0)	9·35<br>(3·94–16·9)	9·35<br>(3·94–16·9)	0<br>(0–0)
Other unintentional injuries	12·8<br>(7·48–19·6)	11·7<br>(6·84–17·8)	11·5<br>(6·79–17·7)	14·9<br>(9·3–21·8)
Self-harm and interpersonal violence	19·4<br>(4·24–34·7)	22·4<br>(9·06–35·8)	31·6<br>(23·4–39·9)	20·8<br>(5·67–35·8)
Self-harm	19·1<br>(3·57–35)	20·6<br>(5·04–36·2)	21·2<br>(7·82–35·6)	20·6<br>(4·88–36·4)
Interpersonal violence	27·2<br>(17·7–36·8)	33·7<br>(25·2–42·8)	37·6<br>(28·7–46·8)	24·4<br>(15·1–33·4)

Supplementary Results Table 2. All-age PAFs attributable to the joint distribution of all risk factors for all ages, both sexes combined for each GBD level 1, level 2, and level 3 cause and all causes in 2019 for Leicester. Results are shown for deaths, YLLs, YLDs, and DALYs Data in parenthesis are 95% uncertainty intervals. PAFs=population attributable fractions. GBD=Global Burden of Disease. YLLs=years of life lost. YLDs=years lived with disability. DALYs=disability-adjusted life-years

	Deaths 2019	DALYs 2019	YLDs 2019	YLLs 2019
All causes	56·8 (53·5–59·8)	42 (38·6–45·2)	28·4 (25·9–30·8)	56·1 (53·3–58·7)
Communicable, maternal, neonatal, and nutritional diseases	48·4 (39·5–57·2)	58·1 (53·5–62·2)	49·5 (44·5–54·1)	62·5 (56·5–68·2)
HIV/AIDS and sexually transmitted infections	78·7 (65·9–87·6)	78 (63·8–87·6)	95·8 (93·9–97·2)	65·6 (48·4–79·5)
HIV/AIDS	90·7 (88·5–92)	89·6 (86·9–91·5)	93·9 (91·4–95·6)	86·5 (83·9–88·2)
Sexually transmitted infections excluding HIV	41 (20·3–68)	52·8 (27·4–79·6)	100 (100–100)	16 (6·08–35)
Respiratory infections and tuberculosis	47 (36·7–57·3)	43·7 (34·8–52·5)	3·81 (2·68–5·25)	51·6 (41·6–61·1)
Tuberculosis	54·6 (41·8–66·2)	57·3 (45·1–67·9)	53·5 (40·8–64·9)	58 (46–68·5)
Lower respiratory infections	46·9 (36·5–57·5)	51·3 (41·2–61)	34·7 (28–41·5)	51·5 (41·2–61·2)
Upper respiratory infections	0·0894 (0·049–0·152)	0·00954 (0·00183–0·0229)	0·00788 (0·000206–0·0214)	0·168 (0·0896–0·286)
Otitis media	4·1 (1·9–7·46)	4·83 (3·08–6·73)	4·77 (3·04–6·69)	8·46 (4·46–13·6)
Enteric infections	6 (3·66–10·1)	10·9 (8·11–15)	10·9 (7·96–15·1)	11 (7·59–15·6)
Diarrheal diseases	6·05 (3·69–10·2)	11 (8·14–15·2)	10·9 (7·97–15·1)	11·1 (7·77–15·7)
Other infectious diseases	1·3 (0·916–1·88)	1·87 (1·42–2·39)	0·399 (0·231–0·685)	2·37 (1·78–3·11)
Meningitis	2·52 (1·88–3·29)	3·73 (2·81–4·84)	0·000793 (1·34e-05–0·00226)	4·51 (3·38–5·89)
Encephalitis	0·586 (0·376–0·851)	1·29 (0·675–1·95)	0·000328 (5·08e-06–0·000909)	1·59 (0·97–2·33)
Measles	3·45 (-0·88–16·1)	4·1 (-1·02–18·9)	0·655 (0·0173–1·79)	4·21 (-1·11–19·6)
Acute hepatitis	12·2 (4·89–25·5)	5·73 (2·71–10·9)	2·5 (1·47–4·21)	10·2 (3·96–22·2)
Maternal and neonatal disorders	91·8 (90·3–93)	83·7 (81·1–86·3)	66·9 (61·4–72·2)	92·3 (90·8–93·5)
Maternal disorders	9·79 (3·53–16·5)	9·91 (3·61–16·8)	10·1 (3·69–17·1)	9·68 (3·51–16·3)
Neonatal disorders	93·2 (91·8–94·3)	85 (82·3–87·6)	68·7 (62·9–74·1)	93·2 (91·8–94·3)
Nutritional deficiencies	75·2 (69·9–86·6)	77·7 (73·1–82·7)	77·8 (73–82·8)	74·8 (66–79·3)
Protein-energy malnutrition	100 (100–100)	100 (100–100)	100 (100–100)	100 (100–100)
Vitamin A deficiency	--	100 (100–100)	100 (100–100)	--
Dietary iron deficiency	--	100 (100–100)	100 (100–100)	--
Non-communicable diseases	58·9 (55·3–62·1)	42·3 (38·6–45·8)	27·5 (25–30·2)	58·4 (55·6–61)
Neoplasms	48·5 (44·8–52·8)	47·3 (43·9–51·1)	34·2 (30·1–38·5)	48 (44·5–51·8)
Lip and oral cavity cancer	66 (53–76·6)	69 (56–78·7)	67·6 (54·5–77·8)	69·1 (56–78·8)
Nasopharynx cancer	64·3 (50·1–75·4)	63·7 (49·8–74·4)	60·2 (45·6–72)	64 (50·1–74·6)
Other pharynx cancer	78·9 (70·6–85·3)	80·2 (71·8–86·2)	80 (71·2–86·2)	80·3 (71·9–86·2)
Esophageal cancer	74·4 (63·5–83·2)	76·1 (65·4–84·3)	75·1 (64·3–83·6)	76·2 (65·4–84·4)
Stomach cancer	25·5 (17·9–42)	26 (18·4–42·4)	26·1 (18·4–42·5)	26 (18·4–42·4)

Colon and rectum cancer	63·5 (54·6–72·2)	63·9 (55·3–71·8)	64 (55·4–72)	63·9 (55·4–71·8)
Liver cancer	74·8 (68·5–81)	74·7 (69–80·1)	75·2 (69·6–80·8)	74·7 (69–80·1)
Gallbladder and biliary tract cancer	21·3 (11·1–33·3)	21·7 (11·4–33·8)	19·7 (9·98–32·1)	21·8 (11·5–33·9)
Pancreatic cancer	42·6 (33·5–53·7)	42·4 (33·7–52·6)	42·7 (33·6–53·4)	42·4 (33·7–52·6)
Larynx cancer	82·4 (73·7–89·1)	82·3 (74·2–88·5)	80·3 (71·8–86·9)	82·5 (74·5–88·7)
Tracheal, bronchus, and lung cancer	86·3 (83·2–89·5)	85·5 (82·5–88·8)	86·3 (83–89·5)	85·5 (82·5–88·8)
Breast cancer	35·8 (26·1–46·6)	34·4 (25·6–43·9)	34·8 (25·9–44·4)	34·3 (25·6–43·9)
Cervical cancer	100 (100–100)	100 (100–100)	100 (100–100)	100 (100–100)
Uterine cancer	43 (27·5–58·9)	44·5 (29–60·5)	45·1 (29·6–61·1)	44·4 (28·9–60·4)
Ovarian cancer	21·3 (10·5–34·4)	17·8 (8·51–29·7)	16·7 (8·04–28·2)	17·8 (8·51–29·8)
Prostate cancer	6·01 (2·62–9·68)	6·66 (2·99–10·6)	7·06 (3·26–11·1)	6·61 (2·96–10·5)
Kidney cancer	38·8 (28·3–49·4)	38·8 (28·8–49)	38·3 (28·7–48·5)	38·8 (28·9–49)
Bladder cancer	41 (26·7–55·3)	43·8 (30·3–56·4)	44·8 (31·8–56·9)	43·7 (30·3–56·5)
Thyroid cancer	14·6 (6·7–24·5)	15·2 (6·85–25·8)	15·6 (6·71–26·8)	15·2 (6·91–25·7)
Mesothelioma	98 (97·3–98·5)	95·9 (94·7–96·9)	97·7 (96·7–98·4)	95·9 (94·7–96·9)
Non-Hodgkin lymphoma	6·98 (2·84–12·6)	7·11 (2·94–12·7)	7·07 (2·93–12·9)	7·11 (2·95–12·8)
Multiple myeloma	7·93 (3·25–14·3)	8·27 (3·45–14·9)	8·18 (3·36–14·8)	8·28 (3·43–14·9)
Leukemia	36·4 (23·2–49·8)	32·8 (22·1–43·6)	31·9 (21·2–42·9)	32·9 (22·2–43·7)
Cardiovascular diseases	81·4 (77·4–84·9)	82·3 (79·3–84·9)	71·3 (66·4–75·6)	83·5 (80·6–86)
Rheumatic heart disease	24·2 (13·8–44·4)	24·8 (15·9–40·8)	24 (15·1–38·1)	24·8 (16·1–40·8)
Ischemic heart disease	94·3 (90·9–97·2)	95·5 (93·2–97·5)	94·4 (91·7–96·9)	95·6 (93·2–97·5)
Stroke	81·4 (73·3–89·6)	84·5 (79·2–89·7)	82·9 (77·8–87·8)	84·7 (79·2–90·2)
Hypertensive heart disease	100 (100–100)	100 (100–100)	100 (100–100)	100 (100–100)
Non-rheumatic valvular heart disease	28·3 (18–41·4)	29 (20·4–39·4)	21·1 (14·4–29·6)	29·7 (21–40·2)
Cardiomyopathy and myocarditis	37·3 (29·6–45·6)	40·4 (34·7–45·8)	36·2 (29·8–42·9)	40·7 (34·9–46·1)
Atrial fibrillation and flutter	51·2 (40–62·2)	58·8 (49·6–67·6)	62·7 (53·8–70·6)	54·5 (44·5–64·3)
Aortic aneurysm	53·6 (46·5–60·8)	60·7 (55–66·2)	--	60·7 (55–66·2)
Endocarditis	34·1 (23·9–46·6)	36·3 (27·2–46·3)	33·1 (22·8–45·9)	36·4 (27·3–46·3)
Other cardiovascular and circulatory diseases	32·4 (25·7–39·5)	33·8 (27·4–40·3)	34·5 (26·6–42·9)	33·6 (27·4–40)
Chronic respiratory diseases	63·4 (56·5–71·1)	57·3 (52·2–63)	49·7 (44–55·4)	63·7 (57·2–70·8)
Chronic obstructive pulmonary disease	73·3 (68·1–78·2)	72·8 (68·7–76·5)	66·2 (62·3–69·9)	75·7 (70·9–79·9)
Pneumoconiosis	100 (100–100)	100 (100–100)	100 (100–100)	100 (100–100)
Asthma	42 (30·6–54·8)	40·3 (31·7–49·5)	40 (31·6–49·1)	43·2 (33·6–53·7)
Digestive diseases	32·9 (27·2–39·5)	38·6 (32–45·1)	25·9 (17·7–35·1)	45·2 (37·6–53·4)

Cirrhosis and other chronic liver diseases	72·3 (60·4–86·7)	75 (63·2–88·3)	71·8 (59·6–84·2)	75·1 (63·2–88·4)
Upper digestive system diseases	21·2 (17·7–24·9)	9·6 (6·23–13·5)	0·694 (0·403–1·11)	24·1 (20·7–27·7)
Gallbladder and biliary diseases	42·8 (27·1–58)	48·9 (34·4–63·5)	49·8 (35·3–64·3)	44·2 (29·2–59)
Pancreatitis	33 (11–56·3)	39·2 (15·3–62·2)	39·6 (15–62·1)	39·1 (15·3–61·9)
Neurological disorders	24·2 (10·5–39·3)	12·7 (6·11–23·3)	7·53 (3·34–15·9)	20·7 (9·98–34·4)
Alzheimer's disease and other dementias	39·6 (25–55·3)	41·8 (27·4–57·2)	42·1 (27·9–57·5)	41·7 (27·4–57)
Parkinson's disease	-9·33 (-15·4–-4·24)	-10·8 (-17·3–-5·01)	-11·4 (-18·3–-5·22)	-10·7 (-17·1–-4·97)
Idiopathic epilepsy	25·6 (14·2–37·3)	23·8 (13·1–34·4)	21·7 (11·8–32)	25·9 (14·5–37·1)
Multiple sclerosis	15·3 (11·3–19·7)	16·1 (12·1–20·2)	15·7 (11·9–19·7)	16·4 (12·2–20·7)
Mental disorders	--	6·95 (3·48–10·9)	6·95 (3·48–10·9)	--
Depressive disorders	--	14·3 (6·59–22·6)	14·3 (6·59–22·6)	--
Anxiety disorders	--	5·87 (1·48–13·1)	5·87 (1·48–13·1)	--
Idiopathic developmental intellectual disability	--	36·7 (16·1–54·8)	36·7 (16·1–54·8)	--
Substance use disorders	100 (100–100)	100 (100–100)	100 (100–100)	100 (100–100)
Alcohol use disorders	100 (100–100)	100 (100–100)	100 (100–100)	100 (100–100)
Drug use disorders	100 (100–100)	100 (100–100)	100 (100–100)	100 (100–100)
Diabetes and kidney diseases	99·6 (99·5–99·7)	99·9 (99·9–99·9)	100 (100–100)	99·6 (99·5–99·7)
Diabetes mellitus	100 (100–100)	100 (100–100)	100 (100–100)	100 (100–100)
Chronic kidney disease	100 (100–100)	100 (100–100)	100 (100–100)	100 (100–100)
Sense organ diseases	--	9·54 (8·4–10·9)	9·54 (8·4–10·9)	--
Blindness and vision loss	--	8·04 (5·19–11·7)	8·04 (5·19–11·7)	--
Age-related and other hearing loss	--	11·6 (10·5–12·8)	11·6 (10·5–12·8)	--
Musculoskeletal disorders	4·56 (1·09–8·38)	22·9 (19·1–26·9)	23·2 (19·3–27·3)	5·36 (1·32–9·65)
Rheumatoid arthritis	11·3 (2·67–20·4)	13·9 (3·41–24·5)	14·1 (3·48–24·8)	13·3 (3·22–23·5)
Osteoarthritis	--	16·6 (8·62–25·5)	16·6 (8·62–25·5)	--
Low back pain	--	41·9 (36·9–47·2)	41·9 (36·9–47·2)	--
Gout	--	48·8 (32·6–66·5)	48·8 (32·6–66·5)	--
Other non-communicable diseases	0·125 (0·0844–0·18)	0·111 (0·0714–0·164)	--	0·371 (0·255–0·508)
Sudden infant death syndrome	7·18 (4·11–11·5)	7·17 (4·11–11·4)	--	7·17 (4·11–11·4)
Injuries	29·1 (22·3–35·4)	24 (18·6–29·4)	26·7 (22·5–31·5)	21·1 (13·1–28·5)
Transport injuries	18·6 (10·5–27·4)	18·6 (11·6–26·4)	24·8 (20·2–29·7)	16·3 (7·57–25·5)
Road injuries	18 (9·6–27)	17·9 (10·6–25·9)	23·9 (19·3–28·8)	15·8 (6·74–25·3)
Other transport injuries	29·9 (24·6–35·4)	28·8 (23·9–34·2)	32·1 (27·2–36·6)	26·1 (20·2–32·1)
Unintentional injuries	37·2 (31·2–41·7)	25·6 (21·1–30·3)	26·5 (22–31·5)	23·2 (18·8–27·4)

Falls	52 (44·3–57·5)	36·5 (31–42)	35 (29·7–40·7)	40·9 (34·9–46·5)
Drowning	-14·5 (-27·5–-0·646)	-14·3 (-26·5–-1·39)	10·5 (5·81–17·1)	-15·2 (-27·8–-1·94)
Fire, heat, and hot substances	10·6 (5·99–17·1)	12·5 (7·64–19·3)	12·9 (7·85–19·9)	11·6 (7·1–17·7)
Poisonings	13·6 (8·45–20·7)	12·7 (7·98–18·9)	11·7 (7·12–18·1)	13·9 (8·84–20·7)
Exposure to mechanical forces	4·06 (-7·92–16·9)	14·5 (11·7–17·6)	15·8 (13·4–18·6)	-3·04 (-15·5–10·5)
Animal contact	20·9 (16·5–26·3)	18·7 (14–24·6)	19·5 (14·6–25·5)	15 (11·2–19·9)
Foreign body	3·08 (1·98–4·74)	3·76 (2·46–5·71)	3·22 (2·11–4·88)	4·05 (2·61–6·21)
Environmental heat and cold exposure	8·78 (3·71–16·1)	9·09 (3·88–16·8)	8·98 (3·84–16·7)	9·53 (4·1–17·3)
Exposure to forces of nature	0 (0–0)	9·61 (4·12–17·7)	9·61 (4·12–17·7)	0 (0–0)
Other unintentional injuries	13·8 (8·46–20·9)	12·3 (7·6–18·9)	12·2 (7·57–18·8)	14·7 (9·34–21·6)
Self-harm and interpersonal violence	21 (5·74–35·4)	23·2 (9·19–36·1)	33·7 (24·3–42·7)	21·8 (6·55–35·8)
Self-harm	20·8 (4·64–35·6)	21·7 (5·8–36·6)	21·8 (8·07–35·4)	21·7 (5·74–36·7)
Interpersonal violence	26·7 (17·1–36·4)	33·5 (24·2–42·8)	39·3 (29·2–49·2)	24 (14·6–33·2)

Supplementary Results Table 2. All-age PAFs attributable to the joint distribution of all risk factors for all ages, both sexes combined for each GBD level 1, level 2, and level 3 cause and all causes in 2019 for Leicestershire. Results are shown for deaths, YLLs, YLDs, and DALYs Data in parenthesis are 95% uncertainty intervals. PAFs=population attributable fractions. GBD=Global Burden of Disease. YLLs=years of life lost. YLDs=years lived with disability. DALYs=disability-adjusted life-years

	Deaths 2019	DALYs 2019	YLDs 2019	YLLs 2019
All causes	54·4 (50·9–57·7)	41·4 (38·2–44·4)	28·5 (26·1–30·7)	54·3 (51·3–57·1)
Communicable, maternal, neonatal, and nutritional diseases	44 (34–53·7)	50·1 (44·6–55·7)	44·7 (40·1–49)	52·9 (44·7–60·6)
HIV/AIDS and sexually transmitted infections	75·6 (59–88·4)	76·6 (59·1–89·6)	98 (97–98·8)	56·6 (36·6–76·6)
HIV/AIDS	93 (91·3–94·1)	92·2 (90·1–93·7)	95·2 (93·3–96·4)	89·8 (87·6–91·2)
Sexually transmitted infections excluding HIV	52·8 (29·1–77·5)	63·2 (38·1–86·2)	100 (100–100)	20 (7·81–42·1)
Respiratory infections and tuberculosis	45 (34–55·5)	42·3 (33–51·6)	3·41 (2·52–4·44)	48·3 (37·6–58·2)
Tuberculosis	53·2 (39·7–64·4)	56·6 (44·3–67·1)	55 (42·4–65·5)	57 (44·6–67·3)
Lower respiratory infections	44·9 (33·9–55·5)	48·1 (37·4–58·2)	33·3 (26·4–40·3)	48·2 (37·5–58·3)
Upper respiratory infections	0·0657 (0·0357–0·114)	0·00672 (0·00105–0·0172)	0·00568 (3·59e-05–0·0153)	0·163 (0·0906–0·291)
Otitis media	1·83 (0·91–3·35)	4 (2·53–5·63)	3·99 (2·52–5·59)	5·3 (2·91–8·82)
Enteric infections	5 (2·95–8·95)	8·16 (5·88–12)	8·77 (6·35–12·8)	6·89 (4·6–10·7)
Diarrheal diseases	5·04 (3·01–8·99)	8·19 (5·9–12·1)	8·78 (6·36–12·8)	6·97 (4·65–10·9)
Other infectious diseases	0·487 (0·335–0·653)	1·07 (0·799–1·33)	0·487 (0·271–0·831)	1·33 (0·943–1·71)
Meningitis	1·49 (1·09–1·95)	2·69 (1·98–3·49)	0·000532 (3·33e-06–0·00159)	3·6 (2·71–4·67)
Encephalitis	0·272 (0·181–0·396)	0·799 (0·474–1·19)	0·000192 (1·36e-06–0·000581)	1·02 (0·652–1·48)
Measles	2·88 (-0·846–14·1)	3·44 (-0·995–16·7)	0·568 (0·0155–1·59)	3·56 (-1·04–17)
Acute hepatitis	8·71 (3·48–23)	4·4 (2·52–8·04)	3·15 (1·88–5·25)	8·58 (3·54–21·9)
Maternal and neonatal disorders	90·8 (89·4–92·1)	77·2 (73·3–80·9)	60·7 (54·9–66·3)	91·3 (89·9–92·6)
Maternal disorders	9·28 (3·35–15·7)	9·36 (3·38–16)	9·44 (3·38–16·3)	9·15 (3·35–15·5)
Neonatal disorders	92·2 (90·9–93·5)	78·5 (74·5–82·3)	62·2 (56–68)	92·2 (90·9–93·5)
Nutritional deficiencies	76·8 (72·4–91·2)	77 (72·9–81·6)	77 (72·9–81·8)	75·8 (72·4–85)
Protein-energy malnutrition	100 (100–100)	100 (100–100)	100 (100–100)	100 (100–100)
Vitamin A deficiency	--	100 (100–100)	100 (100–100)	--
Dietary iron deficiency	--	100 (100–100)	100 (100–100)	--
Non-communicable diseases	56 (52·3–59·5)	41·8 (38·3–45·1)	27·6 (25·2–30·2)	56·1 (53–59·1)
Neoplasms	46·6 (42·6–50·9)	46·3 (42·6–50·4)	33·3 (29·1–37·9)	47·2 (43·3–51·3)
Lip and oral cavity cancer	64·7 (52·1–74·9)	68·4 (56·5–77·5)	67 (54·7–76·8)	68·5 (56·7–77·7)
Nasopharynx cancer	63·4 (49·4–74·2)	64·4 (50·5–74·3)	61·2 (46·8–72)	64·7 (50·9–74·5)
Other pharynx cancer	77·2 (69·2–83·8)	79·2 (71·4–85·1)	79 (71·1–85·1)	79·2 (71·4–85·1)
Esophageal cancer	74·2 (63–83·2)	76·1 (65·4–84·6)	75 (63·7–83·8)	76·1 (65·4–84·6)
Stomach cancer	24 (16·3–40·9)	24·9 (17·4–41·6)	24·9 (17·2–41·7)	24·9 (17·4–41·6)

Colon and rectum cancer	63·2 (54·2–72·1)	63·9 (55·1–72·1)	64 (55·1–72·5)	63·9 (55·1–72)
Liver cancer	74·4 (67·2–80·7)	75·7 (69–81·4)	76·2 (69·1–82)	75·7 (69–81·5)
Gallbladder and biliary tract cancer	23·5 (12·7–36·4)	24·1 (13·2–36·8)	22 (11·6–34·9)	24·2 (13·3–36·9)
Pancreatic cancer	41·5 (32–52·4)	41·7 (33–52)	41·6 (32·1–52·4)	41·7 (33–52)
Larynx cancer	80·5 (71·4–87·5)	81 (72·9–87·6)	79·5 (71·3–86·2)	81·2 (73·1–87·7)
Tracheal, bronchus, and lung cancer	85·4 (81·8–88·7)	85·1 (81·5–88·4)	85·6 (82·1–88·9)	85·1 (81·5–88·4)
Breast cancer	36·5 (26·8–47·6)	36 (27·3–45·9)	36·3 (27·4–46·4)	36 (27·2–45·8)
Cervical cancer	100 (100–100)	100 (100–100)	100 (100–100)	100 (100–100)
Uterine cancer	45·7 (29·9–61·3)	47·2 (31·4–62·5)	47·8 (31·9–63·4)	47·1 (31·2–62·3)
Ovarian cancer	24·6 (12·9–37·7)	21·1 (10·9–33·5)	20·4 (10·4–32·7)	21·2 (10·9–33·5)
Prostate cancer	5·54 (2·35–8·97)	6·07 (2·66–9·66)	6·43 (2·91–10·1)	6·02 (2·63–9·54)
Kidney cancer	39·6 (29·3–50·2)	40·5 (29·9–50·7)	40·5 (30–50·4)	40·5 (29·9–50·7)
Bladder cancer	37·6 (24–51·7)	40·4 (26·7–54)	41·5 (27·8–54·7)	40·3 (26·6–53·9)
Thyroid cancer	15·7 (7·62–25·3)	16·5 (8·08–26·8)	17·3 (8·19–28·5)	16·5 (8·05–26·7)
Mesothelioma	98·9 (98·5–99·2)	97·9 (97·1–98·4)	98·8 (98·2–99·2)	97·9 (97·1–98·4)
Non-Hodgkin lymphoma	7·76 (3·26–13·8)	8·05 (3·43–14·1)	7·93 (3·39–13·9)	8·06 (3·43–14·1)
Multiple myeloma	8·8 (3·81–15·4)	9·19 (4·01–16)	9·07 (3·91–16)	9·2 (4–16)
Leukemia	36·6 (23·1–50·6)	35·8 (23·9–48·1)	35·1 (23–47·2)	35·9 (23·9–48·2)
Cardiovascular diseases	79·6 (75·3–83·5)	80·4 (76·9–83·5)	71·5 (66·5–76·2)	81·7 (78·3–84·6)
Rheumatic heart disease	24·1 (12·7–47·6)	24·4 (14·4–41·2)	24·1 (14·8–38·8)	24·4 (14·4–41·7)
Ischemic heart disease	93·8 (89·9–97)	94·8 (91·8–97·1)	94·1 (91·2–96·6)	94·8 (91·9–97·1)
Stroke	80·2 (71·8–89·1)	82·9 (76·5–89·1)	82·3 (76·7–88·1)	83 (76·4–89·5)
Hypertensive heart disease	100 (100–100)	100 (100–100)	100 (100–100)	100 (100–100)
Non-rheumatic valvular heart disease	27·8 (17·2–41·5)	27·4 (18·5–38)	18·2 (11·9–25·4)	28·7 (19·3–39·8)
Cardiomyopathy and myocarditis	37·3 (28·8–47)	42·6 (35·7–48·6)	36·9 (29·5–44·8)	43 (36–49·1)
Atrial fibrillation and flutter	52·4 (41–63·5)	59·4 (50·1–68·1)	63·2 (54·5–71·4)	55 (44·6–64·9)
Aortic aneurysm	50·9 (43·2–58·8)	57·1 (50·5–64)	--	57·1 (50·5–64)
Endocarditis	33 (21·8–46·9)	35 (25·6–45·9)	32·9 (21·8–45·4)	35·1 (25·7–46)
Other cardiovascular and circulatory diseases	32·5 (25·6–40·4)	34·6 (28·5–41·3)	35·1 (28–42·3)	34·4 (28·3–41·3)
Chronic respiratory diseases	60·7 (53·6–68·3)	57·9 (53–63·1)	53·5 (48·4–58·7)	61·5 (55–68·6)
Chronic obstructive pulmonary disease	70·6 (64·9–76·1)	70 (65·8–74·2)	64·6 (60·6–68·5)	73 (67·9–78)
Pneumoconiosis	100 (100–100)	100 (100–100)	100 (100–100)	100 (100–100)
Asthma	42·5 (30·2–55·8)	43·7 (34·5–53·8)	43·5 (34·5–53·5)	45·4 (34·7–57·1)
Digestive diseases	26·2 (21·1–31·3)	31·9 (26·4–37·4)	19·9 (13·5–27·5)	37·6 (31·1–44)

Cirrhosis and other chronic liver diseases	70·9 (57·8–85·5)	74·7 (62–87·7)	72·5 (59·8–84·9)	74·7 (62–87·8)
Upper digestive system diseases	18·9 (15·7–22·2)	8·6 (5·53–12·2)	0·726 (0·411–1·18)	22·1 (18·8–25·6)
Gallbladder and biliary diseases	45·5 (29·5–60·6)	48·8 (34·4–63·5)	49·6 (35·4–63·8)	46·7 (31·2–61·5)
Pancreatitis	33 (11·6–54·5)	39·5 (16·8–61·1)	40·5 (17·1–61·6)	39·3 (16·6–60·9)
Neurological disorders	25·1 (11–40·5)	16·1 (7·95–28·2)	10·5 (5·24–20·1)	22·2 (10–36·8)
Alzheimer's disease and other dementias	39·2 (25–54·9)	41·4 (27·4–56·5)	41·7 (27·6–56·8)	41·2 (27·2–56·4)
Parkinson's disease	-8·33 (-13·5–-3·74)	-9·56 (-15·2–-4·32)	-10·2 (-16·2–-4·67)	-9·4 (-15–-4·23)
Idiopathic epilepsy	25·8 (14·2–36·8)	24·7 (13·6–35·1)	22·8 (12·5–32·4)	27·7 (15·8–38·5)
Multiple sclerosis	13·4 (9·77–17·3)	14·8 (11–18·5)	14·8 (11·1–18·5)	14·8 (10·8–18·8)
Mental disorders	--	6·33 (3·23–9·68)	6·33 (3·23–9·69)	--
Depressive disorders	--	13·1 (5·96–20·4)	13·1 (5·96–20·4)	--
Anxiety disorders	--	4·67 (1·26–10·3)	4·67 (1·26–10·3)	--
Idiopathic developmental intellectual disability	--	37·3 (15·7–57·1)	37·3 (15·7–57·1)	--
Substance use disorders	100 (100–100)	100 (100–100)	100 (100–100)	100 (100–100)
Alcohol use disorders	100 (100–100)	100 (100–100)	100 (100–100)	100 (100–100)
Drug use disorders	100 (100–100)	100 (100–100)	100 (100–100)	100 (100–100)
Diabetes and kidney diseases	99·5 (99·4–99·6)	99·9 (99·8–99·9)	100 (100–100)	99·5 (99·4–99·6)
Diabetes mellitus	100 (100–100)	100 (100–100)	100 (100–100)	100 (100–100)
Chronic kidney disease	100 (100–100)	100 (100–100)	100 (100–100)	100 (100–100)
Sense organ diseases	--	8·76 (7·6–10·2)	8·76 (7·6–10·2)	--
Blindness and vision loss	--	9·39 (6·14–13·5)	9·39 (6·14–13·5)	--
Age-related and other hearing loss	--	9·88 (8·85–11·1)	9·88 (8·85–11·1)	--
Musculoskeletal disorders	3·65 (0·852–6·81)	23·2 (19·1–27·3)	23·5 (19·4–27·7)	4·53 (1·12–8·13)
Rheumatoid arthritis	9·66 (2·23–17·5)	12·8 (3·07–22·8)	13·3 (3·28–23·6)	11·4 (2·79–20·6)
Osteoarthritis	--	18·2 (9·59–27·6)	18·2 (9·59–27·6)	--
Low back pain	--	41·9 (36·3–47·5)	41·9 (36·3–47·5)	--
Gout	--	53·8 (37·9–70·8)	53·8 (37·9–70·8)	--
Other non-communicable diseases	0·0488 (0·032–0·0716)	0·049 (0·0297–0·0781)	--	0·224 (0·141–0·318)
Sudden infant death syndrome	9·22 (5·46–14·3)	9·21 (5·45–14·3)	--	9·21 (5·45–14·3)
Injuries	34·5 (28·5–39·7)	28·6 (23·7–33·1)	30·9 (26·2–35·2)	25·2 (18·1–31·6)
Transport injuries	21·3 (13·3–29·3)	21·8 (15·2–28·5)	26·7 (22·6–31)	19·3 (10·6–28)
Road injuries	20·6 (12·3–28·9)	20·9 (14·1–27·9)	25·5 (21·4–29·9)	18·6 (9·64–27·7)
Other transport injuries	33·5 (28·4–38·6)	32·9 (27·9–37·8)	36·6 (31·9–40·7)	29·5 (23·4–35·3)
Unintentional injuries	42·9 (36–47·7)	31·2 (26·4–35·7)	31·2 (26·3–35·9)	31·2 (26·1–35·7)

Falls	56 (47·4–61·5)	42·8 (36·5–47·7)	41 (35·1–46)	48·7 (41·6–53·9)
Drowning	-14·6 (-28·9–0·0676)	-12·5 (-25·5–1·18)	10·6 (5·76–17·3)	-14·4 (-28·1–0·0372)
Fire, heat, and hot substances	9·94 (5·11–16·3)	12·7 (7·46–19·4)	12·9 (7·59–19·7)	11·9 (6·91–18·2)
Poisonings	13·1 (7·73–19·7)	12·7 (7·68–19)	11·8 (7–18·1)	14·2 (8·81–20·8)
Exposure to mechanical forces	9·67 (-2·15–21·8)	17·7 (14·8–20·6)	18·9 (16·2–21·5)	0·671 (-12·3–14·3)
Animal contact	24·9 (19·9–31)	21·9 (16·8–27·8)	22·2 (16·9–27·9)	21 (16·3–26·8)
Foreign body	2·37 (1·5–3·61)	3·5 (2·24–5·26)	3·01 (1·92–4·48)	3·99 (2·56–5·97)
Environmental heat and cold exposure	7·98 (3·22–14·7)	9·29 (4·14–16·8)	9·33 (4·19–16·9)	9·1 (3·91–16·5)
Exposure to forces of nature	0 (0–0)	10·1 (4·49–18·1)	10·1 (4·49–18·1)	0 (0–0)
Other unintentional injuries	13·5 (7·97–20·4)	12·4 (7·24–18·8)	12·3 (7·18–18·7)	15·5 (9·7–22·3)
Self-harm and interpersonal violence	21·8 (7·02–35·6)	24·6 (11·4–36·8)	32·7 (24·9–41·1)	23·1 (8·32–36·9)
Self-harm	21·4 (5·99–35·8)	22·9 (7·84–37·3)	22·6 (8·79–35·1)	22·9 (7·82–37·3)
Interpersonal violence	31·3 (22–41·2)	35·8 (27·1–45·1)	38·2 (29·7–47·4)	28·3 (19·1–38)

Supplementary Results Table 2. All-age PAFs attributable to the joint distribution of all risk factors for all ages, both sexes combined for each GBD level 1, level 2, and level 3 cause and all causes in 2019 for Lincolnshire. Results are shown for deaths, YLLs, YLDs, and DALYs Data in parenthesis are 95% uncertainty intervals. PAFs=population attributable fractions. GBD=Global Burden of Disease. YLLs=years of life lost. YLDs=years lived with disability. DALYs=disability-adjusted life-years

	Deaths 2019	DALYs 2019	YLDs 2019	YLLs 2019
All causes	55·8 (52·5–59)	43·7 (40·2–46·9)	29·1 (26·7–31·5)	55·7 (52·9–58·3)
Communicable, maternal, neonatal, and nutritional diseases	43·6 (33·7–52·5)	49·3 (43·7–54·8)	45·6 (40·8–50·2)	51 (43·2–58·6)
HIV/AIDS and sexually transmitted infections	74·2 (57–87·8)	75·1 (56·7–89·2)	98·1 (97·1–98·9)	53·2 (33·1–74·1)
HIV/AIDS	92·8 (91–93·9)	92·2 (90–93·8)	95·2 (93·4–96·4)	89·6 (87·3–91)
Sexually transmitted infections excluding HIV	57·1 (34·4–80·4)	63 (38·1–86·4)	100 (100–100)	23·1 (9·82–45·9)
Respiratory infections and tuberculosis	44·4 (33·8–54·2)	42·2 (33·1–50·8)	3·37 (2·45–4·43)	47·5 (37·5–56·9)
Tuberculosis	52·7 (38·9–64·9)	56 (42·9–66·5)	54 (40·5–65)	56·4 (43·6–66·8)
Lower respiratory infections	44·4 (33·7–54·2)	47·4 (37·4–56·9)	32·4 (25·8–39·1)	47·5 (37·4–57)
Upper respiratory infections	0·0284 (0·0154–0·0501)	0·00777 (0·00173–0·0183)	0·0058 (5·69e-05–0·0162)	0·0789 (0·043–0·137)
Otitis media	1·48 (0·66–3·17)	3·81 (2·41–5·35)	3·81 (2·4–5·33)	4·2 (2·09–7·84)
Enteric infections	5·61 (3·26–10·3)	8·72 (6·12–13·2)	9·3 (6·54–13·8)	7·37 (4·8–12)
Diarrheal diseases	5·69 (3·29–10·4)	8·78 (6·17–13·3)	9·3 (6·55–13·8)	7·54 (4·98–12·3)
Other infectious diseases	0·517 (0·321–0·705)	1·02 (0·743–1·31)	0·474 (0·269–0·815)	1·24 (0·846–1·64)
Meningitis	1·08 (0·769–1·45)	2·16 (1·56–2·89)	0·00054 (3·99e-06–0·00165)	2·71 (1·98–3·62)
Encephalitis	0·211 (0·142–0·314)	0·625 (0·392–0·923)	0·000156 (1·21e-06–0·000473)	0·8 (0·523–1·17)
Measles	2·43 (-0·606–11·7)	2·93 (-0·709–13·9)	0·709 (0·0206–1·9)	3·06 (-0·742–14·9)
Acute hepatitis	14·7 (7·52–27·3)	5·83 (3·43–9·24)	3·29 (1·95–5·65)	14·2 (7·43–26·3)
Maternal and neonatal disorders	89·8 (88·2–91·3)	79·2 (75·7–82·4)	65·5 (59·6–70·7)	90·5 (88·9–92)
Maternal disorders	9·57 (3·41–16·2)	9·63 (3·45–16·1)	9·72 (3·48–16·5)	9·46 (3·4–15·9)
Neonatal disorders	91·8 (90·2–93·2)	80·9 (77·3–84)	67·4 (61·3–72·9)	91·8 (90·2–93·2)
Nutritional deficiencies	74·5 (68·2–87·3)	77·7 (73·5–82·2)	77·7 (73·4–82·5)	76 (68·3–83·7)
Protein-energy malnutrition	100 (100–100)	100 (100–100)	100 (100–100)	100 (100–100)
Vitamin A deficiency	--	100 (100–100)	100 (100–100)	--
Dietary iron deficiency	--	100 (100–100)	100 (100–100)	--
Non-communicable diseases	57·5 (53·9–60·8)	44·5 (40·8–47·9)	28·4 (25·9–31)	57·8 (54·9–60·8)
Neoplasms	47·5 (43·5–51·9)	47·5 (43·7–51·5)	33·1 (29–37·6)	48·3 (44·5–52·3)
Lip and oral cavity cancer	63·2 (50·7–73·6)	66·7 (54·6–76·5)	65·2 (53·1–75·4)	66·8 (54·7–76·6)
Nasopharynx cancer	62·8 (48·8–73·8)	63·5 (49·7–74)	59·7 (45·5–71·3)	63·8 (50·1–74·2)
Other pharynx cancer	76·4 (67–83·2)	78·2 (69·2–84·7)	78 (69·2–84·4)	78·2 (69·2–84·7)
Esophageal cancer	74·3 (63·2–83·3)	76 (65·2–84·7)	75 (63·7–83·9)	76 (65·2–84·7)
Stomach cancer	23·9 (16·3–41·1)	24·7 (17·1–41·7)	24·7 (17·1–41·6)	24·7 (17·1–41·7)

Colon and rectum cancer	63·6 (54·1–72·5)	64·2 (54·7–72·6)	64·3 (54·8–72·8)	64·2 (54·7–72·6)
Liver cancer	75·6 (69·4–81·7)	76·6 (70·9–82·1)	77 (71–82·5)	76·5 (70·8–82·1)
Gallbladder and biliary tract cancer	22·9 (12·2–35·6)	23·6 (12·8–36·2)	21·4 (10·8–34·1)	23·6 (12·9–36·3)
Pancreatic cancer	41·6 (31·9–52·7)	41·9 (32·5–52·5)	41·8 (32·2–52·7)	41·9 (32·5–52·5)
Larynx cancer	80·3 (70·9–87·5)	80·8 (72·6–87·4)	79·4 (71·3–86·2)	81 (72·8–87·5)
Tracheal, bronchus, and lung cancer	86·3 (82·7–89·8)	86 (82·5–89·4)	86·5 (82·7–90)	86 (82·5–89·4)
Breast cancer	36·3 (26·3–48·2)	35·9 (26·7–46·5)	36·2 (26·6–46·7)	35·9 (26·7–46·5)
Cervical cancer	100 (100–100)	100 (100–100)	100 (100–100)	100 (100–100)
Uterine cancer	45·1 (29·3–61·3)	46·5 (30·8–62·8)	47·1 (31·4–63·3)	46·4 (30·7–62·7)
Ovarian cancer	25·7 (13·4–39·2)	22·2 (11·4–34·9)	21·5 (11·1–33·7)	22·2 (11·4–34·9)
Prostate cancer	5·54 (2·38–8·92)	6·04 (2·64–9·66)	6·36 (2·89–10·1)	5·99 (2·61–9·63)
Kidney cancer	39·3 (28·3–50·7)	40·1 (29·3–50·7)	40 (29·7–50·5)	40·1 (29·3–50·7)
Bladder cancer	38·4 (23·4–52·8)	40·9 (27·3–54·5)	42·1 (28·5–55·2)	40·9 (27·1–54·3)
Thyroid cancer	15·4 (7·39–25·2)	16·2 (7·71–26·5)	17 (7·99–28·2)	16·2 (7·77–26·3)
Mesothelioma	99 (98·6–99·3)	98·1 (97·4–98·6)	98·9 (98·4–99·2)	98·1 (97·4–98·6)
Non-Hodgkin lymphoma	7·74 (3·27–13·7)	8·02 (3·39–14·1)	7·92 (3·34–13·9)	8·03 (3·41–14·1)
Multiple myeloma	8·68 (3·72–15·2)	9·06 (3·87–15·7)	8·96 (3·78–15·8)	9·06 (3·91–15·8)
Leukemia	36·5 (22·5–51·3)	35·7 (23·2–48·8)	35·3 (23·1–48)	35·8 (23·2–48·8)
Cardiovascular diseases	80·4 (76·4–84)	81·2 (78–84)	69·9 (64·6–74·4)	82·5 (79·5–85·3)
Rheumatic heart disease	23·6 (12·8–45·9)	23·8 (14·5–41·3)	23·6 (14·5–39·8)	23·8 (14·5–41·3)
Ischemic heart disease	93·8 (90–96·9)	94·7 (91·8–97·1)	94 (90·9–96·7)	94·7 (91·8–97·1)
Stroke	80·3 (71·7–89·3)	82·9 (76·3–89·4)	82·1 (76–88·1)	83 (76·2–89·4)
Hypertensive heart disease	100 (100–100)	100 (100–100)	100 (100–100)	100 (100–100)
Non-rheumatic valvular heart disease	28 (17·9–40·7)	26·4 (18·2–36·3)	12·5 (8·25–17·3)	29 (19·9–39·4)
Cardiomyopathy and myocarditis	39·4 (30·8–48·5)	45 (38–50·8)	38·5 (31·6–45·5)	45·3 (38·2–51·2)
Atrial fibrillation and flutter	51·8 (40·7–62·8)	58·3 (48·9–67·7)	61·8 (53–70·4)	54·6 (44·4–64·6)
Aortic aneurysm	51·8 (44·7–59·5)	57·6 (51·4–63·8)	--	57·6 (51·4–63·8)
Endocarditis	32·8 (22·4–45·9)	34·7 (25·5–45·1)	32·6 (22·4–45·7)	34·7 (25·7–45·1)
Other cardiovascular and circulatory diseases	33 (26·7–39·9)	34·9 (29·1–40·9)	34·5 (27·6–41·5)	35 (29·2–41·2)
Chronic respiratory diseases	61·6 (54·7–68·9)	59·2 (53·9–64·5)	53·9 (48·7–58·8)	62·6 (56·1–69·6)
Chronic obstructive pulmonary disease	70·9 (65·5–75·9)	70·5 (66·2–74·6)	64·4 (60·3–68·4)	73·2 (68·4–77·8)
Pneumoconiosis	100 (100–100)	100 (100–100)	100 (100–100)	100 (100–100)
Asthma	42 (29·5–55·9)	43·8 (34·6–54·2)	43·7 (34·6–54·1)	44·8 (33·9–56·8)
Digestive diseases	25·3 (20·8–30·4)	31·9 (26·5–37·6)	23·5 (16–32·2)	35·4 (29·4–41·6)
Cirrhosis and other chronic liver diseases	69·4 (57–84·4)	73·1 (61·2–86·8)	71·1 (58·3–83·8)	73·2 (61·1–86·9)

Upper digestive system diseases	20·2 (16·7–23·6)	9·94 (6·75–13·6)	0·75 (0·433–1·19)	23·3 (19·8–26·7)
Gallbladder and biliary diseases	44·3 (28·3–60)	48·9 (34·1–64)	50·4 (35·8–65)	45·6 (30·2–61)
Pancreatitis	31·1 (9·52–53·5)	36·8 (13·1–58·7)	36·9 (13·5–58·5)	36·8 (13·1–58·7)
Neurological disorders	24·8 (10·5–40·6)	16·5 (8·34–29·4)	10·8 (5·56–19·9)	22 (9·82–37·3)
Alzheimer's disease and other dementias	39·9 (26–56)	42 (28–57·4)	42·1 (28–57·8)	41·9 (27·9–57·3)
Parkinson's disease	-8·48 (-14·2–-3·82)	-9·56 (-15·7–-4·36)	-9·94 (-16·5–-4·64)	-9·48 (-15·6–-4·3)
Idiopathic epilepsy	24·1 (13·3–35·1)	23·5 (12·8–34)	21·6 (11·8–31·5)	26 (15–36·8)
Multiple sclerosis	13·4 (9·95–17)	14·6 (11–18·2)	14·6 (11–18·2)	14·6 (11–18·4)
Mental disorders	--	6·33 (3·17–9·79)	6·33 (3·17–9·79)	--
Depressive disorders	--	12·9 (5·86–20·4)	12·9 (5·86–20·4)	--
Anxiety disorders	--	4·41 (1·16–9·99)	4·41 (1·16–9·99)	--
Idiopathic developmental intellectual disability	--	39·5 (17·1–58·7)	39·5 (17·1–58·7)	--
Substance use disorders	100 (100–100)	100 (100–100)	100 (100–100)	100 (100–100)
Alcohol use disorders	100 (100–100)	100 (100–100)	100 (100–100)	100 (100–100)
Drug use disorders	100 (100–100)	100 (100–100)	100 (100–100)	100 (100–100)
Diabetes and kidney diseases	99·7 (99·6–99·7)	99·9 (99·9–99·9)	100 (100–100)	99·6 (99·6–99·7)
Diabetes mellitus	100 (100–100)	100 (100–100)	100 (100–100)	100 (100–100)
Chronic kidney disease	100 (100–100)	100 (100–100)	100 (100–100)	100 (100–100)
Sense organ diseases	--	8·77 (7·56–10·2)	8·77 (7·56–10·2)	--
Blindness and vision loss	--	9·74 (6·13–14·3)	9·74 (6·13–14·3)	--
Age-related and other hearing loss	--	9·77 (8·72–11)	9·77 (8·72–11)	--
Musculoskeletal disorders	3·99 (1·01–7·52)	23 (19–27)	23·5 (19·4–27·7)	4·88 (1·22–8·83)
Rheumatoid arthritis	9·91 (2·52–18·2)	12·6 (3·35–22·4)	13 (3·49–23·2)	11·6 (2·95–20·7)
Osteoarthritis	--	17·8 (9·39–27·1)	17·8 (9·39–27·1)	--
Low back pain	--	41·7 (36–47·1)	41·7 (36–47·1)	--
Gout	--	54·2 (38·5–70·7)	54·2 (38·5–70·7)	--
Other non-communicable diseases	0·044 (0·0286–0·0655)	0·0452 (0·0278–0·0691)	--	0·197 (0·126–0·285)
Sudden infant death syndrome	6·56 (3·69–10·7)	6·55 (3·69–10·7)	--	6·55 (3·69–10·7)
Injuries	31·3 (25·1–36·8)	27 (22–31·7)	30·6 (26·1–34·9)	22·8 (15·6–29·5)
Transport injuries	20·2 (12·8–27·9)	20·2 (13·4–27)	25·8 (21·5–29·8)	17·9 (9·63–26·3)
Road injuries	19·6 (12–27·6)	19·4 (12·3–26·3)	24·6 (20·4–28·6)	17·3 (8·83–26)
Other transport injuries	32·3 (27·1–37·1)	31·9 (26·7–36·5)	36·3 (31·5–40·3)	28·5 (22·4–34·2)
Unintentional injuries	39·5 (33·2–43·9)	30·1 (25·4–34·4)	31 (26·2–35·5)	27·6 (22·9–31·8)
Falls	55·3 (46·9–60·7)	42·6 (36·4–47·6)	41 (35–46)	47·8 (40·9–52·9)
Drowning	-15·4 (-29·6–-1·24)	-13·6 (-27·1–-0·166)	9·65 (4·96–15·9)	-14·8 (-28·7–-0·951)

Fire, heat, and hot substances	9·4<br>(4·67–15·6)	11·6<br>(6·52–18·2)	11·8<br>(6·6–18·6)	11·2<br>(6·24–17·5)
Poisonings	12·2<br>(7·01–19)	11·9<br>(6·95–18·2)	10·9<br>(6·12–16·9)	13·2<br>(7·89–20)
Exposure to mechanical forces	10·4<br>(-0·612–22·2)	16·9<br>(13·9–19·6)	18·7<br>(16–21)	0·599<br>(-11–13·2)
Animal contact	22·4<br>(17·3–28·7)	21·2<br>(16·1–27)	21·7<br>(16·5–27·3)	19·7<br>(14·7–25·7)
Foreign body	2·02<br>(1·24–3·08)	3·01<br>(1·92–4·62)	2·46<br>(1·6–3·77)	3·4<br>(2·17–5·17)
Environmental heat and cold exposure	7·06<br>(2·66–12·8)	8·59<br>(3·48–15·6)	8·72<br>(3·54–15·8)	8·23<br>(3·3–14·8)
Exposure to forces of nature	0<br>(0–0)	9·55<br>(3·95–17·1)	9·55<br>(3·95–17·1)	0<br>(0–0)
Other unintentional injuries	11·8<br>(6·57–18·6)	11·3<br>(6·39–17·8)	11·2<br>(6·32–17·7)	13·7<br>(7·9–20·6)
Self-harm and interpersonal violence	20·3<br>(5·61–34·7)	23<br>(9·76–35·9)	31·9<br>(23·2–39·8)	21·7<br>(6·67–35·8)
Self-harm	19·9<br>(4·44–35)	21·4<br>(5·78–36·1)	20·8<br>(7·47–34·3)	21·5<br>(5·59–36·2)
Interpersonal violence	28·2<br>(18·7–38)	33·7<br>(24·3–42·6)	38·3<br>(28·7–47·3)	25·6<br>(16·4–35·1)

Supplementary Results Table 2. All-age PAFs attributable to the joint distribution of all risk factors for all ages, both sexes combined for each GBD level 1, level 2, and level 3 cause and all causes in 2019 for Northamptonshire. Results are shown for deaths, YLLs, YLDs, and DALYs Data in parenthesis are 95% uncertainty intervals. PAFs=population attributable fractions. GBD=Global Burden of Disease. YLLs=years of life lost. YLDs=years lived with disability. DALYs=disability-adjusted life-years

	Deaths 2019	DALYs 2019	YLDs 2019	YLLs 2019
All causes	55·3 (51·8–58·6)	43 (39·8–46)	30·5 (28·2–32·9)	55·2 (52·1–58·1)
Communicable, maternal, neonatal, and nutritional diseases	44·3 (34·3–54)	53·1 (47·7–58·2)	48·4 (43·4–52·9)	55·4 (47·4–63)
HIV/AIDS and sexually transmitted infections	76 (60·6–87·9)	76·2 (59·1–88·8)	97·4 (96·2–98·4)	58·3 (38·8–76·8)
HIV/AIDS	92·3 (90·4–93·5)	91·6 (89·3–93·2)	94·9 (92·9–96·2)	88·9 (86·5–90·3)
Sexually transmitted infections excluding HIV	53 (30·8–77·9)	59·8 (34·3–83·9)	100 (100–100)	21·7 (8·92–44·3)
Respiratory infections and tuberculosis	44·6 (33·5–55·7)	42·2 (32·6–51·3)	3·58 (2·62–4·7)	48·4 (38–59)
Tuberculosis	56·1 (42·7–67·9)	59·2 (47·1–69·6)	56·7 (44·4–67·5)	59·7 (47·6–69·8)
Lower respiratory infections	44·6 (33·4–55·8)	48·3 (37·7–58·8)	33·3 (26·7–40·1)	48·4 (37·7–59)
Upper respiratory infections	0·0489 (0·0266–0·0854)	0·00822 (0·00211–0·0193)	0·00591 (4·51e-05–0·017)	0·114 (0·0618–0·2)
Otitis media	2·36 (1·15–4·56)	4·55 (2·86–6·46)	4·52 (2·83–6·44)	5·73 (3·1–9·67)
Enteric infections	5·51 (3·31–9·55)	9·22 (6·61–13·3)	9·83 (7·1–14)	8·09 (5·53–12·3)
Diarrheal diseases	5·56 (3·34–9·6)	9·27 (6·7–13·3)	9·84 (7·11–14·1)	8·2 (5·63–12·5)
Other infectious diseases	0·755 (0·511–0·991)	1·43 (1·09–1·8)	0·44 (0·249–0·734)	1·8 (1·35–2·31)
Meningitis	1·79 (1·29–2·37)	3·12 (2·32–4·1)	0·000598 (3·99e-06–0·00185)	3·92 (2·89–5·13)
Encephalitis	0·363 (0·235–0·529)	0·957 (0·559–1·44)	0·00021 (1·47e-06–0·000623)	1·21 (0·765–1·79)
Measles	3·18 (-0·831–15·1)	3·87 (-0·937–17·7)	0·645 (0·0232–1·78)	3·98 (-1·03–18·1)
Acute hepatitis	15·5 (8·77–26·4)	5·67 (3·47–8·88)	2·96 (1·69–4·85)	15 (8·59–25·1)
Maternal and neonatal disorders	91 (89·6–92·4)	83·7 (81–86·4)	71·1 (66·1–75·6)	91·7 (90·3–93)
Maternal disorders	9·53 (3·38–15·8)	9·6 (3·37–16·1)	9·73 (3·41–16·5)	9·41 (3·34–15·6)
Neonatal disorders	92·8 (91·5–94)	85·2 (82·5–87·9)	73·1 (67·8–77·9)	92·8 (91·5–94)
Nutritional deficiencies	75·1 (69·6–86·9)	77·6 (73·5–82·2)	77·6 (73·4–82·4)	76·3 (66·6–82·3)
Protein-energy malnutrition	100 (100–100)	100 (100–100)	100 (100–100)	100 (100–100)
Vitamin A deficiency	--	100 (100–100)	100 (100–100)	--
Dietary iron deficiency	--	100 (100–100)	100 (100–100)	--
Non-communicable diseases	57·2 (53·5–60·6)	43·6 (40·2–46·9)	30 (27·4–32·6)	57·3 (54·3–60·4)
Neoplasms	48·9 (44·4–53·7)	48·4 (44·2–52·8)	35·1 (30·4–40·1)	49·1 (45·1–53·6)
Lip and oral cavity cancer	64·2 (51·9–74·7)	67·7 (56·3–77·2)	66·3 (54·3–76·2)	67·8 (56·4–77·4)
Nasopharynx cancer	63·8 (49·5–74·6)	64·3 (50·6–74·7)	61 (47–72·3)	64·6 (51–74·9)
Other pharynx cancer	77·6 (68·8–84)	79·3 (71·2–85·2)	79·2 (70·6–85)	79·3 (71·2–85·1)
Esophageal cancer	74·3 (63·5–83·2)	76·1 (65·6–84·5)	75·1 (64·6–83·8)	76·1 (65·6–84·5)
Stomach cancer	24·5 (17–41·4)	25·2 (17·6–42)	25·3 (17·7–41·9)	25·2 (17·6–42)

Colon and rectum cancer	65 (55·3–74·7)	65·3 (56·1–74·5)	65·5 (56·4–74·7)	65·3 (56·1–74·4)
Liver cancer	75·6 (69·1–81·7)	76·4 (70·5–81·9)	76·8 (70·6–82·3)	76·4 (70·5–82)
Gallbladder and biliary tract cancer	23·3 (12·3–36·2)	23·9 (12·9–36·6)	21·8 (11·3–34·2)	24 (12·9–36·7)
Pancreatic cancer	44·8 (34·2–57·4)	44·7 (34·8–56·8)	44·9 (34·3–57·1)	44·7 (34·8–56·8)
Larynx cancer	80·7 (72·1–87·7)	81 (73·1–87·4)	79·5 (71·3–86·3)	81·2 (73·5–87·5)
Tracheal, bronchus, and lung cancer	86·7 (83·2–90·2)	86·2 (82·9–89·7)	86·8 (83·2–90·3)	86·2 (82·9–89·7)
Breast cancer	38·9 (27·6–51·8)	37·7 (27·4–49·2)	38·1 (27·5–49·8)	37·7 (27·3–49·1)
Cervical cancer	100 (100–100)	100 (100–100)	100 (100–100)	100 (100–100)
Uterine cancer	45 (28·9–60·9)	46·4 (30·5–62·3)	47·1 (31·1–63)	46·3 (30·3–62·2)
Ovarian cancer	28·9 (14·7–44·9)	24·7 (12·1–39·3)	23·6 (11·6–37·9)	24·8 (12·1–39·4)
Prostate cancer	5·69 (2·41–9·17)	6·26 (2·73–9·95)	6·63 (2·99–10·4)	6·2 (2·71–9·9)
Kidney cancer	39·6 (29–50·4)	40·3 (29·7–50·4)	40·1 (29·7–50·2)	40·3 (29·6–50·4)
Bladder cancer	41·7 (26·7–57·1)	44 (29·6–58·2)	45 (31·3–59·1)	44 (29·6–58·2)
Thyroid cancer	15·3 (7·25–24·8)	16·1 (7·74–26·1)	16·7 (7·71–27·3)	16 (7·65–26)
Mesothelioma	98·9 (98·5–99·2)	97·8 (97–98·4)	98·7 (98·2–99·2)	97·8 (97–98·4)
Non-Hodgkin lymphoma	7·61 (3·15–13·4)	7·86 (3·34–13·8)	7·77 (3·26–13·7)	7·87 (3·35–13·8)
Multiple myeloma	8·61 (3·55–15·1)	8·98 (3·81–15·7)	8·87 (3·83–15·6)	8·99 (3·8–15·7)
Leukemia	36·9 (23·1–51)	35·5 (23·7–47·6)	35 (23–46·6)	35·6 (23·7–47·7)
Cardiovascular diseases	79·1 (75·2–82·6)	80·1 (76·8–82·9)	71 (66·1–75·6)	81·3 (78·2–83·9)
Rheumatic heart disease	24 (13·5–44·4)	24·4 (15·3–39·3)	23·9 (14·9–38·3)	24·4 (15·2–39·7)
Ischemic heart disease	94 (90·4–97)	95·1 (92·5–97·2)	94·3 (91·6–96·9)	95·1 (92·5–97·3)
Stroke	80·5 (71·9–88·6)	83·5 (77·5–89·2)	82·9 (77·5–88·2)	83·6 (77·4–89·5)
Hypertensive heart disease	100 (100–100)	100 (100–100)	100 (100–100)	100 (100–100)
Non-rheumatic valvular heart disease	27·9 (17·7–40·8)	27·7 (19·3–38·4)	19·6 (13·3–27·2)	28·8 (20·2–39·7)
Cardiomyopathy and myocarditis	39·6 (31·7–47·9)	45 (39–50·8)	38·7 (32·4–45·6)	45·3 (39·2–51·2)
Atrial fibrillation and flutter	52 (41·1–63·5)	59·4 (50·5–68·6)	63·1 (54·7–71·6)	54·9 (45–65·2)
Aortic aneurysm	52·2 (44·4–59·7)	58·6 (52·5–64·7)	--	58·6 (52·5–64·7)
Endocarditis	33·3 (23·1–46·3)	35·4 (26·1–46·4)	33 (22·8–45·4)	35·4 (26·2–46·3)
Other cardiovascular and circulatory diseases	32·4 (25·6–40·3)	34·3 (27·8–40·7)	34·7 (27·2–42·4)	34·2 (27·9–40·6)
Chronic respiratory diseases	62·9 (56·3–70·2)	59·1 (54·2–64·3)	52·6 (47·2–57·6)	63·8 (57·4–70·4)
Chronic obstructive pulmonary disease	71·5 (66·2–76·7)	71·3 (67·1–75·4)	65·3 (61·5–69·2)	73·9 (69·1–78·5)
Pneumoconiosis	100 (100–100)	100 (100–100)	100 (100–100)	100 (100–100)
Asthma	42·8 (30·6–55·8)	43·2 (34·2–53)	42·9 (33·9–52·7)	45·6 (35–56·9)
Digestive diseases	28·3 (22·9–34)	35·2 (29·1–41·2)	27·1 (18·9–36·4)	39·8 (32·9–47·2)

Cirrhosis and other chronic liver diseases	70·6 (58–85·4)	74 (61·9–87·5)	71·4 (58·5–84·1)	74·1 (61·9–87·7)
Upper digestive system diseases	20·6 (17·2–24)	9·38 (6·19–13·2)	0·744 (0·427–1·19)	24 (20·7–27·5)
Gallbladder and biliary diseases	44·4 (28·6–59·7)	50 (35·4–64·6)	50·9 (36·4–65·3)	45·9 (30·4–60·9)
Pancreatitis	31·7 (11·4–52·5)	37·9 (14·7–58·3)	38·5 (15–59·9)	37·8 (14·7–58)
Neurological disorders	26·3 (11·6–42·6)	15·6 (7·59–28)	9·68 (4·72–18·4)	22·9 (10·5–39·1)
Alzheimer's disease and other dementias	42·9 (27·5–60)	44·9 (29·9–61·3)	45·3 (30–61·9)	44·7 (29·8–61·2)
Parkinson's disease	-8·69 (-14·4–-3·88)	-9·99 (-16·3–-4·51)	-10·7 (-17·6–-4·97)	-9·82 (-15·9–-4·44)
Idiopathic epilepsy	25·2 (13·4–36·1)	23·8 (12·8–34·2)	21·7 (11·6–31·5)	26·6 (14·9–37·2)
Multiple sclerosis	14·4 (10·7–18·3)	15·5 (11·7–19·5)	15·4 (11·7–19·3)	15·6 (11·6–19·8)
Mental disorders	--	6·46 (3·31–9·96)	6·46 (3·31–9·96)	--
Depressive disorders	--	13·4 (6·15–21·1)	13·4 (6·15–21·1)	--
Anxiety disorders	--	4·87 (1·33–10·7)	4·87 (1·33–10·7)	--
Idiopathic developmental intellectual disability	--	38·1 (16·7–56)	38·1 (16·7–56)	--
Substance use disorders	100 (100–100)	100 (100–100)	100 (100–100)	100 (100–100)
Alcohol use disorders	100 (100–100)	100 (100–100)	100 (100–100)	100 (100–100)
Drug use disorders	100 (100–100)	100 (100–100)	100 (100–100)	100 (100–100)
Diabetes and kidney diseases	99·6 (99·5–99·7)	99·9 (99·9–100)	100 (100–100)	99·6 (99·5–99·7)
Diabetes mellitus	100 (100–100)	100 (100–100)	100 (100–100)	100 (100–100)
Chronic kidney disease	100 (100–100)	100 (100–100)	100 (100–100)	100 (100–100)
Sense organ diseases	--	9·38 (8·11–10·9)	9·38 (8·11–10·9)	--
Blindness and vision loss	--	10 (6·32–14·8)	10 (6·32–14·8)	--
Age-related and other hearing loss	--	10·6 (9·6–11·9)	10·6 (9·6–11·9)	--
Musculoskeletal disorders	3·65 (0·864–6·81)	23·4 (19·3–27·4)	23·8 (19·6–28)	4·36 (1·02–7·8)
Rheumatoid arthritis	10·3 (2·48–18·4)	13·2 (3·32–22·8)	13·6 (3·38–23·9)	12·1 (3·01–21·2)
Osteoarthritis	--	17·8 (9·23–27·2)	17·8 (9·23–27·2)	--
Low back pain	--	42·8 (37·5–48·3)	42·8 (37·5–48·3)	--
Gout	--	52·4 (36·4–69·4)	52·4 (36·4–69·4)	--
Other non-communicable diseases	0·0712 (0·046–0·106)	0·0699 (0·0443–0·108)	--	0·286 (0·187–0·416)
Sudden infant death syndrome	7·98 (4·49–13)	7·97 (4·49–13)	--	7·97 (4·49–13)
Injuries	31·1 (24·7–36·5)	25·8 (20·7–30·5)	28·7 (24·4–32·9)	22·5 (14·8–29·3)
Transport injuries	20 (12·2–28·2)	20 (13–27·2)	25·5 (21·3–29·7)	17·7 (9·05–26·2)
Road injuries	19·3 (11·3–27·8)	19·2 (11·9–26·5)	24·5 (20·3–28·8)	17·1 (8·29–26)
Other transport injuries	31·8 (26·5–36·8)	31·1 (26–36)	34·8 (30·1–38·8)	28·1 (22–33·9)
Unintentional injuries	39 (32·4–43·5)	28 (23·6–32·4)	28·8 (24·3–33·4)	26 (21·1–30·3)

Falls	54·6 (46·3–60)	40 (34·1–45)	38·2 (32·5–43·4)	45·7 (39·1–50·9)
Drowning	-14·7 (-28·3–-0·0933)	-13·5 (-26·7–0·464)	10·1 (5·51–16·8)	-14·8 (-28·2–-0·272)
Fire, heat, and hot substances	10·3 (5·52–16·9)	12·2 (7–18·7)	12·3 (7·07–19)	11·8 (6·82–18·3)
Poisonings	13·4 (7·9–20·5)	12·4 (7·33–19)	11·1 (6·32–17·2)	14·1 (8·54–21·2)
Exposure to mechanical forces	8·74 (-2·43–21)	16·1 (13·3–18·8)	17·5 (15·1–20)	-0·249 (-12·6–13)
Animal contact	21·2 (16·5–26·9)	20·2 (15·2–26)	20·9 (15·7–26·8)	16·4 (12·2–21·7)
Foreign body	2·36 (1·5–3·63)	3·26 (2·1–4·92)	2·7 (1·72–4·06)	3·57 (2·28–5·38)
Environmental heat and cold exposure	7·21 (2·83–13·8)	8·83 (3·57–16·3)	8·96 (3·62–16·5)	8·51 (3·46–15·7)
Exposure to forces of nature	0 (0–0)	9·75 (4·01–17·8)	9·75 (4·01–17·8)	0 (0–0)
Other unintentional injuries	14 (8·24–21·4)	11·8 (6·7–18·4)	11·6 (6·59–18·3)	15·2 (9·3–22·5)
Self-harm and interpersonal violence	21·2 (5·32–35·5)	23·7 (9·14–36·9)	32·4 (23·6–40·9)	22·4 (6·42–36·5)
Self-harm	20·9 (4·47–35·8)	22·2 (5·92–37·2)	22 (8·01–35·1)	22·2 (5·75–37·2)
Interpersonal violence	28·7 (19·3–38·4)	33·9 (25·3–43·2)	38·1 (29·1–47·5)	25·9 (16·5–35·3)

Supplementary Results Table 2. All-age PAFs attributable to the joint distribution of all risk factors for all ages, both sexes combined for each GBD level 1, level 2, and level 3 cause and all causes in 2019 for Central Europe. Results are shown for deaths, YLLs, YLDs, and DALYs Data in parenthesis are 95% uncertainty intervals. PAFs=population attributable fractions. GBD=Global Burden of Disease. YLLs=years of life lost. YLDs=years lived with disability. DALYs=disability-adjusted life-years

	Deaths 2019	DALYs 2019	YLDs 2019	YLLs 2019
All causes	69 (66·1–71·7)	54·4 (51·1–57·6)	33·7 (31·1–36·3)	66·6 (64·5–68·6)
Communicable, maternal, neonatal, and nutritional diseases	55·2 (49·9–60)	57·5 (54·4–60·6)	50·5 (46·4–55)	62·8 (58·9–66·3)
HIV/AIDS and sexually transmitted infections	79·2 (76·6–81)	82·6 (78·5–86·7)	95·4 (93·4–97·3)	74·5 (70·1–77·3)
HIV/AIDS	79·9 (79·1–80·7)	78·1 (77·2–79)	81·9 (80·7–83·1)	77·4 (76·6–78·3)
Sexually transmitted infections excluding HIV	74 (54·3–89·8)	90·9 (80·1–97·4)	100 (100–100)	49 (27·1–73·8)
Respiratory infections and tuberculosis	55·7 (49·5–61·1)	53·6 (48·1–59·1)	8·12 (5·84–11·1)	61·3 (55·8–66·2)
Tuberculosis	63·8 (56·4–69·7)	65·9 (58·5–71·7)	60·3 (52·6–66·3)	66·6 (59·2–72·4)
Lower respiratory infections	55·2 (48·6–61)	60·7 (54·6–66·1)	43·7 (38–49·7)	60·9 (54·7–66·3)
Upper respiratory infections	0·278 (0·117–0·717)	0·0188 (0·00881–0·0428)	0·00531 (0·00347–0·00746)	0·774 (0·312–1·99)
Otitis media	3·17 (1·24–6·51)	4·45 (2·94–6·08)	4·42 (2·89–6·05)	10·3 (5·18–18·5)
Enteric infections	33·5 (20·4–49·2)	39·8 (25·2–55·9)	39·5 (24·4–56·1)	42·1 (30·6–55·6)
Diarrheal diseases	34·3 (20·8–50·2)	40 (25·3–56·2)	39·5 (24·5–56·1)	43·5 (31·5–57·3)
Other infectious diseases	1·01 (0·8–1·28)	1·52 (1·21–1·96)	0·286 (0·185–0·456)	2·06 (1·63–2·64)
Meningitis	1·45 (1·19–1·77)	2·84 (2·34–3·47)	0·000631 (0·000406–0·000942)	3·52 (2·95–4·21)
Encephalitis	0·581 (0·443–0·718)	1·21 (0·935–1·49)	0·000288 (0·000173–0·000434)	1·31 (1·01–1·61)
Measles	25 (9·84–57)	24·2 (9·66–55·3)	3·56 (0·132–7·53)	25·6 (10·1–58·6)
Acute hepatitis	6·72 (4·85–9·79)	3·19 (2·31–4·39)	1·59 (1·04–2·52)	6·97 (5·01–9·69)
Maternal and neonatal disorders	83·4 (82–84·6)	71·5 (68·3–74·4)	58·4 (54·1–62·2)	84·4 (83–85·5)
Maternal disorders	12·6 (4·54–20·6)	12·5 (4·46–20·5)	12·6 (4·49–20·6)	12·5 (4·51–20·5)
Neonatal disorders	86·1 (84·8–87·2)	73·2 (70–76·1)	59·9 (55·6–63·8)	86·1 (84·8–87·2)
Nutritional deficiencies	78·2 (56·4–81·2)	93·3 (91·5–94·8)	94 (92·4–95·4)	79 (59·4–81·9)
Protein-energy malnutrition	100 (100–100)	100 (100–100)	100 (100–100)	100 (100–100)
Vitamin A deficiency	--	100 (100–100)	100 (100–100)	--
Dietary iron deficiency	--	100 (100–100)	100 (100–100)	--
Non-communicable diseases	71·4 (68·2–74·3)	57·6 (54·2–61·1)	33·6 (30·6–36·6)	70·5 (68·3–72·8)
Neoplasms	50·5 (47–54·5)	51·3 (48·2–55)	39·3 (35·4–43·4)	51·7 (48·7–55·3)
Lip and oral cavity cancer	73·5 (68·5–77·6)	76·7 (71·9–80·8)	74·3 (69·3–78·7)	76·7 (72–80·8)
Nasopharynx cancer	66·9 (59·1–73·4)	68·3 (60·7–75)	67·8 (60·1–74·3)	68·3 (60·7–75)
Other pharynx cancer	83·9 (79·9–87·2)	84·8 (80·6–88·2)	82 (77·4–85·7)	84·8 (80·7–88·2)
Esophageal cancer	79 (70·8–86·1)	80·8 (73–87·8)	79·8 (71·5–86·9)	80·9 (73–87·8)

Stomach cancer	28·5 (18·9–47·6)	29·2 (19·4–48·1)	28·8 (19·1–47·8)	29·2 (19·5–48·1)
Colon and rectum cancer	62·1 (54·2–69·7)	63·1 (55·7–70·3)	63·2 (55·8–70·4)	63·1 (55·7–70·3)
Liver cancer	75·1 (69·2–80·7)	75·4 (69·2–81·1)	75·2 (69·1–80·9)	75·4 (69·2–81·1)
Gallbladder and biliary tract cancer	27·4 (16·4–39·9)	27·6 (16·6–40·4)	27·3 (16·3–39·9)	27·6 (16·6–40·4)
Pancreatic cancer	40·8 (32·8–50·5)	41·3 (33·5–50·6)	41·1 (33·1–50·7)	41·3 (33·5–50·6)
Larynx cancer	84·5 (78·2–89·1)	85·6 (79·7–89·8)	84·6 (78·7–89)	85·6 (79·8–89·8)
Tracheal, bronchus, and lung cancer	85·1 (82·8–87·7)	85·1 (82·8–87·6)	85 (82·7–87·6)	85·1 (82·8–87·6)
Breast cancer	32·6 (24·2–42·3)	31·8 (24·3–40·2)	31·9 (24·4–40·3)	31·7 (24·3–40·2)
Cervical cancer	100 (100–100)	100 (100–100)	100 (100–100)	100 (100–100)
Uterine cancer	52·2 (38·1–66·6)	52·8 (38·8–67·2)	53 (38·7–67·5)	52·8 (38·8–67·2)
Ovarian cancer	15·2 (5·95–26·8)	13·6 (5·21–24·2)	13·2 (5·06–23·3)	13·6 (5·22–24·2)
Prostate cancer	7·3 (3·48–11·2)	8·03 (3·97–12·2)	8·38 (4·16–12·8)	8 (3·95–12·2)
Kidney cancer	43·3 (33·4–52·9)	43·8 (33·9–53·4)	43·5 (33·6–53)	43·8 (33·9–53·4)
Bladder cancer	43·2 (31·1–54·7)	47·2 (35·8–57·9)	48·2 (36·6–58·8)	47·1 (35·8–57·8)
Thyroid cancer	17·5 (9·63–26·7)	17·9 (9·77–27·4)	17 (9·56–25·5)	18 (9·79–27·6)
Mesothelioma	92 (89·7–93·9)	87·9 (84·7–90·6)	91·3 (88·8–93·3)	87·9 (84·7–90·5)
Non-Hodgkin lymphoma	8·78 (4–14·9)	8·7 (3·98–14·8)	8·81 (4·02–14·7)	8·69 (3·97–14·8)
Multiple myeloma	10·3 (4·69–17·2)	10·5 (4·94–17·5)	10·5 (4·83–17·4)	10·5 (4·95–17·5)
Leukemia	38·1 (26·4–50·1)	36·2 (25·6–46·7)	38·8 (27·4–50·4)	36 (25·5–46·5)
Cardiovascular diseases	86·6 (83·7–89·1)	87·7 (85·4–89·6)	81 (77·7–84·1)	88·3 (86–90·2)
Rheumatic heart disease	28·9 (18·4–47·7)	30·4 (20·7–45·4)	30·4 (20·2–44·8)	30·4 (20·6–45·4)
Ischemic heart disease	94 (90·7–96·8)	95·3 (93–97·3)	95·2 (93–97·1)	95·3 (93–97·3)
Stroke	84·8 (79·1–90·8)	87·9 (84·1–92)	87·7 (84–91·3)	88 (84–92·1)
Hypertensive heart disease	100 (100–100)	100 (100–100)	100 (100–100)	100 (100–100)
Non-rheumatic valvular heart disease	28·9 (20·2–38·9)	28·3 (21–35·9)	22·6 (16·4–29·4)	29·6 (21·9–37·9)
Cardiomyopathy and myocarditis	43·5 (35·9–52·8)	51·6 (45·8–57·5)	46·7 (39·4–54·4)	51·8 (45·9–57·7)
Atrial fibrillation and flutter	56·7 (48·6–65·7)	65·4 (58·4–72·7)	68·8 (62·1–75·4)	61 (53·7–69·1)
Aortic aneurysm	65·3 (60–70·2)	72·3 (68·2–76)	--	72·3 (68·2–76)
Endocarditis	46·9 (36·8–57·7)	48·4 (38·3–58·3)	44·5 (34·2–55·7)	48·4 (38·4–58·4)
Other cardiovascular and circulatory diseases	42·7 (37·6–47·8)	45·3 (40·7–49·6)	42·4 (37·3–47·4)	45·8 (41·2–50·2)
Chronic respiratory diseases	73·1 (70·3–75·7)	67·5 (64·3–70·7)	57·4 (52·7–61·8)	74·7 (72·2–77)
Chronic obstructive pulmonary disease	77·7 (74·7–80·6)	77·2 (74·5–79·7)	70·2 (67·2–73·2)	80·3 (77·6–82·8)
Pneumoconiosis	100 (100–100)	100 (100–100)	100 (100–100)	100 (100–100)
Asthma	49·2 (38·3–61·3)	45·3 (36·7–54·6)	44 (35·7–53·2)	52·5 (41·9–64)

Digestive diseases	51·2 (46·7–55·4)	51·2 (45·9–55·9)	21·2 (15–27·9)	59·5 (54·3–64·2)
Cirrhosis and other chronic liver diseases	78·5 (70·7–85·6)	80 (72·2–86·9)	75·2 (69·1–81·1)	80·1 (72·2–87)
Upper digestive system diseases	24·1 (21·2–27)	12·9 (8·91–17·3)	1·54 (0·942–2·31)	29 (25·8–32·3)
Gallbladder and biliary diseases	51·5 (37·4–65·6)	54·8 (41·4–67·7)	55·5 (42·5–68·3)	52·8 (39·1–66·5)
Pancreatitis	42·4 (33·8–52·4)	48·5 (39·3–59)	43·3 (34·6–52·8)	48·8 (39·6–59·3)
Neurological disorders	26·7 (12·9–41·2)	17·6 (9·58–29·3)	11·8 (6·43–20·3)	25·4 (12·7–38·8)
Alzheimer's disease and other dementias	38·3 (24·4–53·5)	41·1 (27·2–56)	41·1 (27·1–56)	41 (27·2–55·9)
Parkinson's disease	-9·3 (-14·2–-4·68)	-11·3 (-17·1–-5·71)	-12·3 (-18·6–-6·22)	-11·1 (-16·8–-5·58)
Idiopathic epilepsy	29·2 (22·3–36·3)	25·1 (19–31·4)	22·7 (17·1–28·5)	29·5 (22·7–36·7)
Multiple sclerosis	18·9 (14·4–22·9)	20·4 (15·7–24·6)	19·3 (15·1–23·3)	20·8 (16–25·1)
Mental disorders	--	4·63 (2·34–7·24)	4·63 (2·34–7·24)	--
Depressive disorders	--	9·07 (3·37–15·5)	9·07 (3·37–15·5)	--
Anxiety disorders	--	3·78 (1·07–8·27)	3·78 (1·07–8·27)	--
Idiopathic developmental intellectual disability	--	55·9 (27·8–75·4)	55·9 (27·8–75·4)	--
Substance use disorders	100 (100–100)	100 (100–100)	100 (100–100)	100 (100–100)
Alcohol use disorders	100 (100–100)	100 (100–100)	100 (100–100)	100 (100–100)
Drug use disorders	100 (100–100)	100 (100–100)	100 (100–100)	100 (100–100)
Diabetes and kidney diseases	99·9 (99·9–99·9)	99·9 (99·9–100)	100 (100–100)	99·9 (99·9–99·9)
Diabetes mellitus	100 (100–100)	100 (100–100)	100 (100–100)	100 (100–100)
Chronic kidney disease	100 (100–100)	100 (100–100)	100 (100–100)	100 (100–100)
Sense organ diseases	--	8·7 (7·67–9·75)	8·7 (7·67–9·75)	--
Blindness and vision loss	--	6·58 (4·2–9·23)	6·58 (4·2–9·23)	--
Age-related and other hearing loss	--	9·86 (9·05–10·8)	9·86 (9·05–10·8)	--
Musculoskeletal disorders	4·86 (1·28–8·43)	36·1 (30·9–41·3)	36·6 (31·3–41·9)	5·12 (1·39–8·79)
Rheumatoid arthritis	12·1 (3·24–21)	17 (4·98–28·9)	17·6 (5·17–29·9)	14·7 (4·04–25·3)
Osteoarthritis	--	22·3 (12·9–32·2)	22·3 (12·9–32·2)	--
Low back pain	--	46·9 (41·4–52·4)	46·9 (41·4–52·4)	--
Gout	--	56·4 (41·4–72)	56·4 (41·4–72)	--
Other non-communicable diseases	0·0841 (0·0638–0·102)	0·0395 (0·0258–0·0568)	--	0·209 (0·16–0·248)
Sudden infant death syndrome	7·21 (6·24–8·32)	7·21 (6·23–8·31)	--	7·21 (6·23–8·31)
Injuries	26·8 (22·3–30·6)	25·6 (22·2–28·8)	28·3 (25·3–31·2)	22·7 (18·3–27)
Transport injuries	24·1 (19·1–28·8)	25·4 (21–29·4)	27·5 (24–30·8)	23·6 (18·3–28·7)
Road injuries	22·3 (17·3–27·3)	24·6 (20·2–28·7)	27·2 (23·7–30·5)	22·1 (16·7–27·5)
Other transport injuries	36·7 (32·4–40·4)	34·6 (29·9–38·5)	35·8 (31·9–39·3)	34·3 (29·5–38·2)

Unintentional injuries	30·9 (25·9–35·4)	26·6 (22·8–30·4)	28·8 (25·2–32·4)	21·7 (17·4–26·4)
Falls	48·9 (42·2–53·9)	39·1 (33·7–43·6)	38·6 (33·5–42·9)	40·4 (34·7–45·6)
Drowning	-6·57 (-15·8–3·12)	-5·29 (-13·9–3·7)	10·6 (7·41–14·9)	-5·86 (-14·8–3·35)
Fire, heat, and hot substances	13·4 (8·88–19·1)	16·2 (11·8–21·7)	16·1 (11·9–21·7)	16·3 (11·5–22·4)
Poisonings	15·1 (10·4–20·9)	15·9 (11·7–21·1)	14·3 (10·7–18·9)	17·3 (12·5–22·9)
Exposure to mechanical forces	8·13 (2·43–14·2)	19·6 (17·1–21·8)	21·1 (18·8–23)	5·04 (-0·597–11·2)
Animal contact	27·3 (22·3–33·2)	22·2 (18·1–27·2)	21·1 (17–25·9)	25·8 (20·8–31·6)
Foreign body	6·94 (6·02–8·09)	7·8 (6·76–9·06)	6·04 (5·02–7·22)	8·39 (7·24–9·79)
Environmental heat and cold exposure	11·1 (5·2–18·5)	11·3 (5·28–18·9)	8·12 (3·91–13·8)	11·8 (5·51–19·7)
Exposure to forces of nature	5·2 (2·45–9·17)	5·3 (2·52–9·18)	9·26 (4·42–15·4)	4·84 (2·28–8·51)
Other unintentional injuries	20 (14·6–26·8)	16·4 (12·1–21·7)	15 (11·1–20·1)	21·5 (16–28·1)
Self-harm and interpersonal violence	22·5 (11·8–32·8)	23·1 (13·4–32·5)	23·3 (18·4–28·5)	23·1 (12·2–33·4)
Self-harm	22·3 (10·5–33·9)	23·3 (11·2–35)	22·6 (13·1–31·7)	23·3 (11·2–35·1)
Interpersonal violence	23·8 (15·9–31·9)	25·5 (18·4–33)	30·1 (23·6–37·2)	22 (14–30·3)

Supplementary Results Table 2. All-age PAFs attributable to the joint distribution of all risk factors for all ages, both sexes combined for each GBD level 1, level 2, and level 3 cause and all causes in 2019 for Nottingham. Results are shown for deaths, YLLs, YLDs, and DALYs Data in parenthesis are 95% uncertainty intervals. PAFs=population attributable fractions. GBD=Global Burden of Disease. YLLs=years of life lost. YLDs=years lived with disability. DALYs=disability-adjusted life-years

	Deaths 2019	DALYs 2019	YLDs 2019	YLLs 2019
All causes	56·5 (53·2–59·5)	42 (38·7–45·4)	28·3 (25·8–30·6)	55·6 (52·8–58·3)
Communicable, maternal, neonatal, and nutritional diseases	47·7 (38·3–56·4)	56·2 (51·7–60·4)	48·3 (43·2–53·3)	60·5 (54·1–66·5)
HIV/AIDS and sexually transmitted infections	72·2 (55·7–85·5)	74·2 (56·7–87·1)	96·9 (95·4–98)	56·3 (36·9–75·6)
HIV/AIDS	91·4 (89·3–92·7)	90·5 (87·9–92·3)	94·2 (91·8–95·8)	87·7 (85·1–89·3)
Sexually transmitted infections excluding HIV	32·3 (14·8–59·1)	53·9 (28·7–80·5)	100 (100–100)	11·3 (4·22–26·3)
Respiratory infections and tuberculosis	46·7 (35·9–56·6)	43 (34·3–51·6)	3·36 (2·37–4·62)	51·1 (41–60·5)
Tuberculosis	56·6 (43·8–67·5)	59·4 (47·6–69)	55·4 (42–66·3)	60·1 (48·5–69·6)
Lower respiratory infections	46·7 (35·6–56·7)	51·2 (40·7–60·6)	34·5 (27·7–40·9)	51·3 (40·8–60·8)
Upper respiratory infections	0·0663 (0·0362–0·113)	0·0127 (0·00446–0·0257)	0·00717 (0·000261–0·0196)	0·132 (0·0708–0·234)
Otitis media	3·29 (1·52–6·63)	4·63 (2·95–6·54)	4·56 (2·89–6·44)	6·48 (3·25–11·4)
Enteric infections	5·41 (3·36–9·01)	10·1 (7·66–13·8)	10·1 (7·46–14)	10·2 (7·31–14·2)
Diarrheal diseases	5·46 (3·38–9·11)	10·2 (7·69–13·9)	10·1 (7·46–14)	10·4 (7·43–14·5)
Other infectious diseases	1·1 (0·783–1·42)	1·9 (1·44–2·42)	0·421 (0·234–0·709)	2·37 (1·77–3·14)
Meningitis	2·73 (2–3·55)	4·2 (3·11–5·49)	0·000802 (2·14e-05–0·00227)	4·95 (3·68–6·47)
Encephalitis	0·647 (0·408–0·984)	1·49 (0·779–2·29)	0·00034 (8·78e-06–0·000917)	1·76 (1·04–2·66)
Measles	3·03 (-0·867–15·1)	3·05 (-0·719–14·7)	0·528 (0·0221–1·5)	3·79 (-1·08–19)
Acute hepatitis	15·3 (9·97–23·9)	4·98 (3·16–7·55)	2·57 (1·47–4·37)	14·1 (9–22·2)
Maternal and neonatal disorders	90·8 (89·2–92·2)	82 (78·9–84·9)	65·4 (59·4–71·3)	91·3 (89·8–92·8)
Maternal disorders	9·86 (3·6–16·6)	9·97 (3·62–16·9)	10·1 (3·66–17·2)	9·77 (3·58–16·5)
Neonatal disorders	92·4 (90·9–93·8)	83·4 (80·3–86·4)	67·2 (60·8–73·4)	92·4 (90·9–93·8)
Nutritional deficiencies	89·7 (64·6–92·9)	79 (74·6–83·7)	78·3 (73·7–83·4)	89·3 (60·7–92·6)
Protein-energy malnutrition	100 (100–100)	100 (100–100)	100 (100–100)	100 (100–100)
Vitamin A deficiency	--	100 (100–100)	100 (100–100)	--
Dietary iron deficiency	--	100 (100–100)	100 (100–100)	--
Non-communicable diseases	58·6 (55·3–61·7)	42·7 (39–46·2)	27·5 (24·8–30·1)	58·4 (55·6–61·2)
Neoplasms	50·1 (46·4–54·4)	49·1 (45·7–52·9)	35·6 (31·6–40)	49·8 (46·4–53·6)
Lip and oral cavity cancer	68·5 (56·6–78)	71·4 (59·7–79·9)	70 (57·9–79)	71·5 (59·7–80)
Nasopharynx cancer	65·8 (53·3–76·9)	65·7 (53·5–76·2)	61·9 (48·7–73·6)	66 (53·8–76·4)
Other pharynx cancer	79·9 (71·9–86·2)	81·2 (73·5–87·1)	80·9 (72·9–87)	81·2 (73·6–87·1)
Esophageal cancer	75·1 (64·8–83·4)	76·9 (66·7–84·9)	75·9 (65·6–84·3)	76·9 (66·8–84·9)
Stomach cancer	25·3 (17·7–42·2)	25·8 (18·4–42·5)	25·9 (18·4–42·7)	25·8 (18·4–42·5)

Colon and rectum cancer	64 (54·8–72·8)	64·5 (55·9–72·6)	64·6 (55·6–72·9)	64·5 (55·9–72·6)
Liver cancer	75·5 (69·2–81·4)	75·6 (69·8–80·9)	76 (70·3–81·6)	75·6 (69·8–80·9)
Gallbladder and biliary tract cancer	22·8 (12·2–35·2)	23·3 (12·4–35·6)	21·3 (11–33·9)	23·3 (12·5–35·7)
Pancreatic cancer	43·1 (33·5–53·9)	42·8 (34·2–53·1)	43·1 (33·9–53·8)	42·7 (34·2–53·1)
Larynx cancer	81·7 (73·8–88·5)	81·8 (74·3–88)	79·3 (71·4–85·9)	82·1 (74·6–88·1)
Tracheal, bronchus, and lung cancer	86·4 (82·9–89·7)	85·7 (82·3–88·9)	86·4 (83–89·7)	85·7 (82·3–88·9)
Breast cancer	36·8 (27–48·1)	35·4 (26·9–45·6)	35·9 (27·1–46·2)	35·4 (26·8–45·5)
Cervical cancer	100 (100–100)	100 (100–100)	100 (100–100)	100 (100–100)
Uterine cancer	44·7 (28·9–60·9)	46·2 (30·4–62·1)	46·9 (31·3–62·7)	46·1 (30·3–62·1)
Ovarian cancer	24·4 (12·8–37·8)	20 (10·1–31·9)	18·7 (9·46–30·6)	20 (10·1–32·1)
Prostate cancer	6·09 (2·68–9·88)	6·7 (3–10·6)	7·08 (3·22–11·1)	6·65 (2·97–10·6)
Kidney cancer	39·8 (29·2–50·5)	39·9 (29·7–50)	39·4 (29·4–49·4)	39·9 (29·7–50)
Bladder cancer	41·3 (27·2–55·5)	44 (30·5–56·8)	45 (32·2–57·6)	44 (30·4–56·7)
Thyroid cancer	15·2 (7·2–25·1)	15·9 (7·52–26·3)	16·4 (7·43–27·5)	15·9 (7·5–26·3)
Mesothelioma	98·4 (97·9–98·9)	96·6 (95·6–97·5)	98·2 (97·4–98·7)	96·6 (95·6–97·4)
Non-Hodgkin lymphoma	7·44 (3·15–13·3)	7·62 (3·26–13·6)	7·57 (3·16–13·5)	7·63 (3·26–13·6)
Multiple myeloma	8·45 (3·6–14·9)	8·81 (3·64–15·5)	8·72 (3·68–15·6)	8·81 (3·63–15·6)
Leukemia	36·7 (23·5–50·7)	33·2 (22·8–44·3)	32·4 (21·5–43·6)	33·2 (22·8–44·4)
Cardiovascular diseases	80·3 (76·5–83·8)	81·3 (78·4–84·1)	70·9 (66·1–75·5)	82·5 (79·7–85·2)
Rheumatic heart disease	24·1 (13·6–44·8)	24·7 (15·8–40·5)	24·1 (15·4–39·2)	24·7 (15·8–40·3)
Ischemic heart disease	94·3 (90·7–97·2)	95·5 (93·2–97·6)	94·6 (91·9–97)	95·6 (93·2–97·6)
Stroke	81·3 (73·3–89·5)	84·4 (78·9–90·1)	83 (78·1–88·1)	84·7 (79–90·5)
Hypertensive heart disease	100 (100–100)	100 (100–100)	100 (100–100)	100 (100–100)
Non-rheumatic valvular heart disease	27·7 (18–40·9)	28·3 (20·1–38·5)	21·8 (14·9–30·7)	28·9 (20·5–39·5)
Cardiomyopathy and myocarditis	40 (31·9–49·1)	44·3 (38–50·1)	39·9 (33·8–46·7)	44·6 (38·1–50·7)
Atrial fibrillation and flutter	51·9 (40·7–63·9)	59·4 (50·3–69·1)	63·7 (55·4–72·3)	55·1 (45–65·9)
Aortic aneurysm	53·3 (45·9–61)	60·6 (54·5–66·5)	--	60·6 (54·5–66·5)
Endocarditis	34·1 (24·1–47·1)	36·4 (27·6–46·4)	33·3 (23·2–45·7)	36·5 (27·6–46·5)
Other cardiovascular and circulatory diseases	34·8 (28·3–41·8)	36·4 (30·1–42·7)	34·9 (27·1–43)	36·7 (30·6–43)
Chronic respiratory diseases	64·9 (58·5–72·1)	59·1 (54–64·3)	50·3 (44·8–55·8)	65·5 (59·7–72·3)
Chronic obstructive pulmonary disease	73 (68–78·2)	72·8 (68·9–76·7)	65·8 (62–69·4)	75·4 (70·9–79·8)
Pneumoconiosis	100 (100–100)	100 (100–100)	100 (100–100)	100 (100–100)
Asthma	43 (31·7–55·4)	41·5 (33–50·5)	41·1 (32·8–50·2)	44·2 (34·8–54·4)
Digestive diseases	32·9 (26·6–39·3)	39·6 (32·9–46·3)	24 (16·6–32·3)	45·7 (37·5–53·5)

Cirrhosis and other chronic liver diseases	73·8 (60·8–87·9)	76·6 (64·3–89·4)	73·5 (60·4–85·7)	76·7 (64·4–89·5)
Upper digestive system diseases	22·2 (18·8–25·9)	11·5 (7·5–15·9)	0·735 (0·417–1·18)	25·6 (22–29·1)
Gallbladder and biliary diseases	44·8 (28·8–60·5)	51 (36·2–65·3)	52·6 (37·6–66·9)	45·9 (30·7–60·6)
Pancreatitis	35·1 (13·4–57·7)	41·9 (19·3–62·8)	42·7 (19·2–64)	41·8 (19·3–62·8)
Neurological disorders	25·1 (11–40·2)	13·3 (6·39–23·8)	7·91 (3·64–16·4)	21·7 (10·6–36·4)
Alzheimer's disease and other dementias	40·3 (25·9–56·1)	42·5 (28·3–57·9)	42·9 (28·5–58·4)	42·4 (28–57·7)
Parkinson's disease	-9·26 (-15–-4·14)	-10·8 (-17·4–-5·05)	-11·4 (-18·2–-5·28)	-10·6 (-17–-4·95)
Idiopathic epilepsy	27·1 (15·5–38·6)	25·4 (14·6–35·5)	23·4 (13·3–33·4)	27·9 (16·4–38·8)
Multiple sclerosis	15·6 (11·6–19·8)	16·5 (12·6–20·5)	15·9 (12·2–19·9)	16·8 (12·7–21·1)
Mental disorders	--	6·88 (3·41–10·8)	6·89 (3·42–10·9)	--
Depressive disorders	--	14·3 (6·6–22·1)	14·3 (6·6–22·1)	--
Anxiety disorders	--	5·93 (1·48–13·5)	5·93 (1·48–13·5)	--
Idiopathic developmental intellectual disability	--	33·5 (14–53·1)	33·5 (14–53·1)	--
Substance use disorders	100 (100–100)	100 (100–100)	100 (100–100)	100 (100–100)
Alcohol use disorders	100 (100–100)	100 (100–100)	100 (100–100)	100 (100–100)
Drug use disorders	100 (100–100)	100 (100–100)	100 (100–100)	100 (100–100)
Diabetes and kidney diseases	99·7 (99·7–99·8)	99·9 (99·9–100)	100 (100–100)	99·7 (99·7–99·8)
Diabetes mellitus	100 (100–100)	100 (100–100)	100 (100–100)	100 (100–100)
Chronic kidney disease	100 (100–100)	100 (100–100)	100 (100–100)	100 (100–100)
Sense organ diseases	--	9·5 (8·34–10·8)	9·5 (8·34–10·8)	--
Blindness and vision loss	--	8·26 (5·27–12)	8·26 (5·27–12)	--
Age-related and other hearing loss	--	11·4 (10·3–12·6)	11·4 (10·3–12·6)	--
Musculoskeletal disorders	3·28 (0·772–5·99)	22·8 (19–26·6)	23·2 (19·3–27·1)	3·86 (0·938–6·79)
Rheumatoid arthritis	10·8 (2·52–19·3)	13·7 (3·48–24·1)	13·9 (3·5–24·3)	12·8 (3·26–22·8)
Osteoarthritis	--	17·5 (9·07–26·8)	17·5 (9·07–26·8)	--
Low back pain	--	41·8 (36·8–47)	41·8 (36·8–47)	--
Gout	--	50·6 (34–68·4)	50·6 (34–68·4)	--
Other non-communicable diseases	0·0714 (0·047–0·113)	0·062 (0·0395–0·098)	--	0·221 (0·149–0·344)
Sudden infant death syndrome	9·29 (5·26–15)	9·28 (5·26–15)	--	9·28 (5·26–15)
Injuries	29·3 (22·5–35·3)	23·7 (18·3–28·8)	26·5 (22·2–31·3)	21·2 (13·9–28·6)
Transport injuries	17·8 (9·26–26·5)	17·6 (9·97–25·7)	24·8 (19·9–30)	15·4 (6·24–24·7)
Road injuries	17·2 (8·55–26·1)	17 (9·08–25·2)	24 (19–29·3)	14·9 (5·56–24·4)
Other transport injuries	28·1 (22·2–34·4)	27·6 (22–33·5)	31·9 (27·2–36·9)	24·7 (18·2–31·4)
Unintentional injuries	36·9 (30·6–41·5)	24·9 (20·4–29·6)	26·2 (21·4–31·3)	21·9 (17·2–26·3)

Falls	53·1 (44·9–58·7)	36·8 (30·7–42·5)	35 (29·3–40·8)	41·9 (35·3–47·6)
Drowning	-14·1 (-27·8–0·313)	-14 (-27·2–0·00169)	10·5 (5·54–17·5)	-14·7 (-28·1—0·405)
Fire, heat, and hot substances	11·4 (6·18–18·7)	12·5 (7·1–19·6)	12·6 (7·08–20)	12·3 (6·98–19·1)
Poisonings	13·4 (7·66–20·7)	12·5 (7·37–19·3)	11·3 (6·33–18·1)	13·7 (7·95–20·9)
Exposure to mechanical forces	5·76 (-6·19–18·4)	12·6 (10–15·3)	14·5 (12·3–16·6)	-4·59 (-17·7–9·12)
Animal contact	18·6 (13·8–24·7)	18·2 (13·2–24·7)	19·1 (13·9–25·4)	14·2 (9·75–19·8)
Foreign body	2·54 (1·58–3·82)	3·07 (1·94–4·5)	2·46 (1·58–3·68)	3·33 (2·08–4·97)
Environmental heat and cold exposure	8·72 (3·57–16·2)	9·58 (4·03–17·6)	9·51 (4·01–17·4)	9·83 (4·17–17·9)
Exposure to forces of nature	0 (0–0)	10·1 (4·32–18·2)	10·1 (4·32–18·2)	0 (0–0)
Other unintentional injuries	13·3 (7·64–20·6)	11·9 (6·71–18·8)	11·8 (6·59–18·6)	14·3 (8·59–21·5)
Self-harm and interpersonal violence	22·9 (8·52–36·9)	24·8 (11·7–37·5)	34·9 (25·8–43·9)	23·7 (9·23–37·4)
Self-harm	22·5 (7·33–37·4)	23·4 (8·05–38·3)	23·3 (9·85–37·2)	23·4 (7·89–38·4)
Interpersonal violence	29·1 (19–38·6)	33·5 (23·8–42·6)	40·3 (30·2–50·4)	26·7 (16·8–35·9)

Supplementary Results Table 2. All-age PAFs attributable to the joint distribution of all risk factors for all ages, both sexes combined for each GBD level 1, level 2, and level 3 cause and all causes in 2019 for Nottinghamshire. Results are shown for deaths, YLLs, YLDs, and DALYs Data in parenthesis are 95% uncertainty intervals. PAFs=population attributable fractions. GBD=Global Burden of Disease. YLLs=years of life lost. YLDs=years lived with disability. DALYs=disability-adjusted life-years

	Deaths 2019	DALYs 2019	YLDs 2019	YLLs 2019
All causes	55 (51·8–58)	42·5 (39·3–45·6)	28·9 (26·5–31·2)	54·7 (52–57·4)
Communicable, maternal, neonatal, and nutritional diseases	44 (33·7–53·7)	50·6 (45·5–55·9)	46 (41·4–50·3)	52·9 (44·7–60·4)
HIV/AIDS and sexually transmitted infections	75·2 (58·7–87·6)	76·5 (59·3–89·2)	97·9 (96·7–98·7)	56·6 (36·5–76·2)
HIV/AIDS	93 (91·2–94·1)	92·3 (90–93·8)	95·2 (93·3–96·4)	89·8 (87·6–91·2)
Sexually transmitted infections excluding HIV	51·3 (28·9–76·2)	62·2 (37·3–85·7)	100 (100–100)	19·7 (7·75–40·6)
Respiratory infections and tuberculosis	44·6 (33·2–55·4)	41·9 (32·2–51)	3·29 (2·42–4·27)	47·8 (37–58)
Tuberculosis	52·7 (39·8–64·9)	56·1 (44·3–67)	54 (41·3–65·5)	56·6 (44·8–67·4)
Lower respiratory infections	44·5 (33·1–55·4)	47·7 (36·8–57·9)	32·9 (26·2–39·9)	47·9 (36·8–58·1)
Upper respiratory infections	0·0404 (0·0219–0·0707)	0·00818 (0·00208–0·0176)	0·00592 (0·000242–0·0152)	0·103 (0·0565–0·179)
Otitis media	1·99 (0·959–3·62)	4·01 (2·52–5·64)	3·99 (2·52–5·63)	5·46 (2·99–8·98)
Enteric infections	5·74 (3·26–10·4)	8·96 (6·29–13·5)	9·53 (6·72–14·3)	7·76 (5·04–12·6)
Diarrheal diseases	5·79 (3·28–10·5)	9·01 (6·31–13·5)	9·54 (6·72–14·3)	7·88 (5·2–12·7)
Other infectious diseases	0·673 (0·427–0·884)	1·34 (0·963–1·73)	0·477 (0·273–0·808)	1·69 (1·17–2·21)
Meningitis	1·5 (1·07–2)	2·84 (2·06–3·8)	0·000556 (1·89e-05–0·00148)	3·59 (2·59–4·79)
Encephalitis	0·269 (0·182–0·391)	0·748 (0·447–1·1)	0·000166 (4·99e-06–0·000472)	0·989 (0·645–1·44)
Measles	2·93 (-0·763–14·2)	3·58 (-0·906–17)	0·721 (0·0188–1·94)	3·71 (-0·972–18)
Acute hepatitis	13·3 (6·98–24·6)	5·56 (3·39–8·93)	3·28 (1·9–5·42)	12·9 (6·9–23·2)
Maternal and neonatal disorders	90·5 (89–91·8)	77·8 (74·1–81·5)	62·7 (56·6–68·4)	91·1 (89·6–92·4)
Maternal disorders	9·61 (3·53–16·4)	9·68 (3·63–16·7)	9·77 (3·63–16·8)	9·51 (3·5–16·2)
Neonatal disorders	92·1 (90·7–93·4)	79·2 (75·1–82·8)	64·2 (58–69·9)	92·1 (90·7–93·4)
Nutritional deficiencies	82·7 (66·1–86·8)	77·9 (74–82·1)	77·6 (73·4–82)	83·6 (63–86·8)
Protein-energy malnutrition	100 (100–100)	100 (100–100)	100 (100–100)	100 (100–100)
Vitamin A deficiency	--	100 (100–100)	100 (100–100)	--
Dietary iron deficiency	--	100 (100–100)	100 (100–100)	--
Non-communicable diseases	56·6 (53·1–59·8)	43·2 (39·7–46·5)	28·1 (25·5–30·7)	56·7 (53·7–59·6)
Neoplasms	47·5 (43·5–52)	47·4 (43·6–51·5)	34·1 (29·7–38·7)	48·1 (44·4–52·3)
Lip and oral cavity cancer	63·1 (51–73·5)	66·7 (55·3–76·7)	65·2 (53·3–75·4)	66·8 (55·6–76·8)
Nasopharynx cancer	62·6 (49·4–74·2)	63·3 (50·2–74·4)	59·6 (45·6–71·6)	63·6 (50·6–74·7)
Other pharynx cancer	76·4 (68·3–83·5)	78·3 (70·4–84·8)	78·1 (69·9–84·7)	78·3 (70·4–84·9)
Esophageal cancer	73·4 (62·9–82·4)	75·2 (65·1–83·7)	74·1 (63·6–82·9)	75·2 (65·1–83·8)
Stomach cancer	24·2 (16·5–41·6)	24·9 (17·5–41·9)	25 (17·3–42·2)	24·9 (17·5–41·9)

Colon and rectum cancer	63 (53·6–72)	63·5 (54·5–72·1)	63·7 (54·7–72·3)	63·5 (54·5–72·1)
Liver cancer	74·9 (68·4–81·1)	75·9 (70–81·6)	76·3 (70·4–82·1)	75·9 (70–81·6)
Gallbladder and biliary tract cancer	21·7 (11·5–34·1)	22·3 (12–34·8)	20·2 (10·5–32·7)	22·4 (12·1–34·9)
Pancreatic cancer	41·8 (32·4–53·1)	42 (33·2–52·6)	41·9 (32·4–53)	42 (33·2–52·6)
Larynx cancer	79·7 (70·4–87·1)	80·2 (71·8–86·8)	78·7 (70·3–85·4)	80·4 (72·1–86·9)
Tracheal, bronchus, and lung cancer	85·7 (82·1–89·1)	85·4 (82–88·6)	85·9 (82·5–89)	85·4 (82–88·6)
Breast cancer	36 (25·8–47·7)	35·5 (26·6–45·7)	35·8 (26·6–46·1)	35·5 (26·5–45·7)
Cervical cancer	100 (100–100)	100 (100–100)	100 (100–100)	100 (100–100)
Uterine cancer	42·8 (27·3–59·1)	44·3 (28·7–60·4)	44·9 (29·1–61·1)	44·2 (28·7–60·2)
Ovarian cancer	25·3 (13·3–39·2)	21·7 (11·1–34·6)	21 (10·6–33·5)	21·7 (11·1–34·6)
Prostate cancer	5·58 (2·37–9·11)	6·12 (2·71–9·77)	6·48 (2·98–10·3)	6·07 (2·68–9·7)
Kidney cancer	38·2 (27·5–48·8)	38·9 (28·7–49·2)	38·9 (28·4–48·9)	38·9 (28·7–49·2)
Bladder cancer	38·4 (24·5–52·8)	40·9 (27·1–53·9)	42 (28·6–54·8)	40·8 (27–54)
Thyroid cancer	14·1 (6·61–23·4)	14·9 (6·95–24·7)	15·6 (7·06–26)	14·8 (6·96–24·5)
Mesothelioma	99 (98·6–99·3)	98 (97·3–98·5)	98·8 (98·3–99·2)	98 (97·2–98·5)
Non-Hodgkin lymphoma	6·98 (2·86–12·7)	7·24 (3–13)	7·13 (2·94–12·9)	7·25 (3·01–13)
Multiple myeloma	7·95 (3·32–14·2)	8·31 (3·48–14·9)	8·2 (3·4–14·7)	8·31 (3·46–14·9)
Leukemia	36·1 (22·1–50·9)	35·3 (23–47·9)	34·9 (22·7–47·5)	35·3 (23·1–47·9)
Cardiovascular diseases	79·7 (75·6–83·4)	80·5 (77·2–83·4)	70·7 (65·6–75·4)	81·7 (78·5–84·5)
Rheumatic heart disease	23·6 (12·9–45·1)	23·8 (14·6–41·4)	23·6 (14·6–39·7)	23·9 (14·7–41·5)
Ischemic heart disease	93·8 (89·8–96·9)	94·8 (91·9–97·2)	94 (91·1–96·7)	94·8 (92–97·2)
Stroke	80·1 (71·2–88·7)	82·8 (76·3–89)	82·2 (76·6–87·9)	82·8 (76·2–89·2)
Hypertensive heart disease	100 (100–100)	100 (100–100)	100 (100–100)	100 (100–100)
Non-rheumatic valvular heart disease	27·5 (17·2–40·3)	27·3 (18·6–37·5)	18 (11·9–25·2)	28·4 (19·4–39·2)
Cardiomyopathy and myocarditis	38·2 (29·8–47·9)	43·5 (36·6–49·7)	37·9 (31–45·7)	43·9 (36·6–50·2)
Atrial fibrillation and flutter	50·3 (39·5–61·7)	57·4 (48·1–66·7)	61·4 (52·6–70)	53 (43·2–63·3)
Aortic aneurysm	51·2 (43·5–59·3)	57·3 (50·8–63·7)	--	57·3 (50·8–63·7)
Endocarditis	33 (22·3–46·4)	34·9 (25·8–45·8)	32·7 (22·2–45·6)	35 (25·8–45·8)
Other cardiovascular and circulatory diseases	32·7 (26·4–40)	34·7 (28·8–40·6)	34·7 (27·9–41·6)	34·7 (29–40·7)
Chronic respiratory diseases	63·4 (56·3–70·6)	59·8 (54·7–65·1)	53·3 (48–58·4)	64·2 (57·8–71·1)
Chronic obstructive pulmonary disease	71·1 (65·5–76·4)	70·9 (66·7–75·1)	65·4 (61·5–69·1)	73·3 (68·3–77·9)
Pneumoconiosis	100 (100–100)	100 (100–100)	100 (100–100)	100 (100–100)
Asthma	40·6 (29·1–53·8)	42·3 (33·3–52·4)	42·1 (33·3–52·1)	43·5 (33·2–54·7)
Digestive diseases	26·4 (21·6–31·9)	33·1 (27·4–39·1)	23·2 (16–31·8)	37·3 (31·3–44·4)
Cirrhosis and other chronic liver diseases	68·4 (56·8–84)	72·4 (61·3–86)	71·2 (59·3–85·1)	72·4 (61·4–86·1)

Upper digestive system diseases	19·8 (16·3–23·1)	9·96 (6·49–13·7)	0·767 (0·428–1·26)	22·9 (19·4–26·2)
Gallbladder and biliary diseases	42·6 (27–58·6)	47·4 (33–62)	48·8 (34·7–63·4)	43·8 (29–59)
Pancreatitis	29·1 (10–50·7)	35·3 (14·6–57·3)	36·6 (15·3–59·8)	35·1 (14·5–57·2)
Neurological disorders	23·4 (9·9–38·9)	15·2 (7·55–26·8)	9·89 (5·03–18·9)	20·7 (8·98–35·6)
Alzheimer's disease and other dementias	39·3 (25–55)	41·3 (27–56·5)	41·7 (27·9–57)	41·1 (26·9–56·4)
Parkinson's disease	-8·38 (-13·9–-3·67)	-9·56 (-15·6–-4·24)	-10·2 (-16·8–-4·57)	-9·4 (-15·4–-4·19)
Idiopathic epilepsy	23·7 (12·9–35)	23 (12·9–33·4)	21·1 (11·7–30·7)	25·5 (14·7–36·6)
Multiple sclerosis	13·6 (10·1–17·3)	14·9 (11·2–18·8)	14·9 (11·3–18·7)	14·9 (11·1–19)
Mental disorders	--	6·36 (3·24–9·72)	6·36 (3·24–9·73)	--
Depressive disorders	--	13 (5·98–20·4)	13 (5·98–20·4)	--
Anxiety disorders	--	4·57 (1·22–10·3)	4·57 (1·22–10·3)	--
Idiopathic developmental intellectual disability	--	39·8 (17·2–59·5)	39·8 (17·2–59·5)	--
Substance use disorders	100 (100–100)	100 (100–100)	100 (100–100)	100 (100–100)
Alcohol use disorders	100 (100–100)	100 (100–100)	100 (100–100)	100 (100–100)
Drug use disorders	100 (100–100)	100 (100–100)	100 (100–100)	100 (100–100)
Diabetes and kidney diseases	99·6 (99·5–99·7)	99·9 (99·9–99·9)	100 (100–100)	99·6 (99·5–99·7)
Diabetes mellitus	100 (100–100)	100 (100–100)	100 (100–100)	100 (100–100)
Chronic kidney disease	100 (100–100)	100 (100–100)	100 (100–100)	100 (100–100)
Sense organ diseases	--	8·92 (7·72–10·3)	8·92 (7·72–10·3)	--
Blindness and vision loss	--	9·32 (5·94–13·6)	9·32 (5·94–13·6)	--
Age-related and other hearing loss	--	10·2 (9·05–11·4)	10·2 (9·05–11·4)	--
Musculoskeletal disorders	3·72 (0·875–6·87)	22·7 (18·8–26·7)	23·2 (19·2–27·2)	4·52 (1·1–8·11)
Rheumatoid arthritis	9·92 (2·4–18·5)	12·9 (3·21–23)	13·4 (3·31–23·9)	11·6 (2·9–21)
Osteoarthritis	--	16·7 (8·6–25·8)	16·7 (8·6–25·8)	--
Low back pain	--	41·5 (35·8–47)	41·5 (35·8–47)	--
Gout	--	50·9 (35–67·3)	50·9 (35–67·3)	--
Other non-communicable diseases	0·0582 (0·0388–0·087)	0·0591 (0·0366–0·0932)	--	0·252 (0·163–0·367)
Sudden infant death syndrome	8·61 (4·82–14)	8·6 (4·82–13·9)	--	8·6 (4·82–13·9)
Injuries	33·9 (27·9–39·2)	27·6 (22·9–32·4)	30·6 (26–34·8)	23·9 (17–30·6)
Transport injuries	20·3 (12·4–28·4)	20·5 (13·9–27·6)	26 (21·8–30·2)	18 (9·47–27)
Road injuries	19·7 (11·5–28)	19·7 (12·8–26·9)	24·8 (20·6–29·1)	17·4 (8·55–26·9)
Other transport injuries	32·7 (27·6–37·5)	32·1 (27·3–36·9)	35·9 (31·3–39·9)	28·6 (22·8–34·2)
Unintentional injuries	42·3 (35·8–46·8)	30·7 (25·8–35)	31 (26·2–35·5)	30 (25–34·3)
Falls	55·5 (47·2–60·9)	42·3 (36·1–47·2)	40·4 (34·5–45·3)	48 (41–53·2)
Drowning	-15·6 (-29·2–-1·57)	-13·7 (-26·7–0·578)	9·78 (5·26–16·1)	-15·1 (-28·5–-1·26)

Fire, heat, and hot substances	9·47 (4·93–15·7)	12 (6·98–18·4)	12·2 (7·09–18·7)	11·3 (6·61–17·4)
Poisonings	13·2 (7·71–19·8)	12·4 (7·47–18·6)	11·1 (6·49–17·3)	14·2 (8·57–20·9)
Exposure to mechanical forces	10·9 (-0·77–22·8)	16·8 (13·7–20)	18·6 (15·9–21·3)	0·784 (-11·7–14·2)
Animal contact	22·4 (17·3–28·4)	21·1 (16–26·9)	21·5 (16·3–27·3)	19·9 (14·9–25·8)
Foreign body	2·23 (1·42–3·45)	3·37 (2·16–5·17)	2·89 (1·86–4·48)	3·68 (2·37–5·62)
Environmental heat and cold exposure	7·13 (2·88–13·2)	8·56 (3·61–15·7)	8·64 (3·65–15·9)	8·26 (3·45–15·1)
Exposure to forces of nature	0 (0–0)	9·41 (4·08–17)	9·41 (4·08–17)	0 (0–0)
Other unintentional injuries	12·6 (7·25–19·2)	11·6 (6·71–17·9)	11·5 (6·64–17·9)	14·4 (8·68–21)
Self-harm and interpersonal violence	19·7 (4·92–33·4)	22·4 (9·23–34·7)	31·2 (23–39·2)	20·9 (5·97–34·6)
Self-harm	19·1 (3·63–33·5)	20·5 (4·79–34·6)	20·7 (7·49–33·6)	20·5 (4·61–34·7)
Interpersonal violence	30·9 (21·6–40·4)	34·3 (25·7–43·1)	37·3 (28·5–46·2)	28 (18·8–37·3)

Supplementary Results Table 2. All-age PAFs attributable to the joint distribution of all risk factors for all ages, both sexes combined for each GBD level 1, level 2, and level 3 cause and all causes in 2019 for Rutland. Results are shown for deaths, YLLs, YLDs, and DALYs Data in parenthesis are 95% uncertainty intervals. PAFs=population attributable fractions. GBD=Global Burden of Disease. YLLs=years of life lost. YLDs=years lived with disability. DALYs=disability-adjusted life-years

	Deaths 2019	DALYs 2019	YLDs 2019	YLLs 2019
All causes	53·4 (49·5–57)	41·3 (37·9–44·6)	29·1 (26·9–31·5)	53·2 (50·1–56·4)
Communicable, maternal, neonatal, and nutritional diseases	42·1 (31·7–51·8)	46 (40·3–52·1)	45·6 (40·7–49·9)	46·3 (36·9–55)
HIV/AIDS and sexually transmitted infections	74·5 (57·2–88·7)	73·5 (54–88·7)	98·4 (97·4–99·2)	50·6 (30·4–73·3)
HIV/AIDS	92·7 (90·9–93·8)	92 (89·7–93·7)	95·4 (93·5–96·6)	89·1 (86·7–90·6)
Sexually transmitted infections excluding HIV	62·3 (39–84·8)	63·1 (36·9–86·4)	100 (100–100)	25·3 (10·8–50·6)
Respiratory infections and tuberculosis	43·6 (32·3–54·1)	40·5 (31·2–50·2)	3·38 (2·47–4·39)	46·3 (35·4–56·6)
Tuberculosis	46·3 (32·3–59·9)	50·8 (37·9–62·8)	53·2 (40·2–64·6)	50·3 (37·2–62·6)
Lower respiratory infections	43·6 (32·2–54·1)	46·1 (35·2–56·6)	31·7 (24·6–38·7)	46·3 (35·3–56·7)
Upper respiratory infections	0·022 (0·0112–0·0412)	0·005 (0·000304–0·013)	0·00478 (0·000101–0·0128)	0·092 (0·0478–0·172)
Otitis media	0·576 (0·327–0·912)	3·63 (2·3–5·12)	3·63 (2·3–5·12)	2·14 (1·34–3·15)
Enteric infections	4·83 (2·69–8·79)	7·45 (5·14–11·4)	8·12 (5·72–12·1)	5·68 (3·51–9·67)
Diarrheal diseases	4·92 (2·73–8·89)	7·51 (5·18–11·5)	8·13 (5·72–12·1)	5·83 (3·62–9·9)
Other infectious diseases	0·346 (0·206–0·513)	0·699 (0·486–0·988)	0·377 (0·213–0·658)	0·888 (0·565–1·31)
Meningitis	0·663 (0·404–1·04)	1·31 (0·807–2·07)	0·000329 (5·95e-06–0·00094)	1·96 (1·2–3·05)
Encephalitis	0·129 (0·0709–0·254)	0·327 (0·177–0·517)	0·000109 (1·35e-06–0·000324)	0·553 (0·311–0·946)
Measles	1·63 (-0·411–9·6)	1·82 (-0·275–9·62)	0·577 (0·0135–1·59)	2·4 (-0·625–13·7)
Acute hepatitis	12·6 (5·43–25·8)	5·3 (2·99–8·8)	3·26 (1·94–5·56)	13 (5·7–25·7)
Maternal and neonatal disorders	86·8 (82·9–90)	73 (68·4–77·3)	66·7 (61–71·7)	88·7 (85·5–91·6)
Maternal disorders	9·33 (3·22–15·8)	9·36 (3·29–16)	9·45 (3·26–16·1)	9·24 (3·19–15·6)
Neonatal disorders	92·3 (89·7–94·9)	74·9 (70·2–79·2)	68 (61·9–73·1)	92·3 (89·7–94·9)
Nutritional deficiencies	71·3 (65·8–87·8)	76·9 (73–81·4)	77·1 (73–81·5)	72·1 (67·3–84·4)
Protein-energy malnutrition	100 (100–100)	100 (100–100)	100 (100–100)	100 (100–100)
Vitamin A deficiency	--	100 (100–100)	100 (100–100)	--
Dietary iron deficiency	--	100 (100–100)	100 (100–100)	--
Non-communicable diseases	55 (50·6–58·8)	41·9 (38·2–45·4)	28·2 (25·7–30·8)	55·3 (52–58·7)
Neoplasms	44 (39·8–48·6)	44·4 (40·3–48·6)	33 (28·8–37·7)	45·1 (41·2–49·4)
Lip and oral cavity cancer	62·9 (50·4–74·1)	67 (54·8–76·9)	65·7 (53·2–75·8)	67·1 (54·9–77·1)
Nasopharynx cancer	62 (48·3–73·8)	63·5 (50·4–74·3)	60·1 (46·3–72)	63·9 (51–74·6)
Other pharynx cancer	73·8 (64·8–81·7)	76·5 (67·5–83·5)	76·5 (67·6–83·6)	76·5 (67·5–83·5)
Esophageal cancer	74·6 (62·8–83·8)	76·4 (65·1–85)	75·3 (63·7–84·4)	76·5 (65·1–85)
Stomach cancer	23·3 (15·8–40·5)	24·3 (16·8–41)	24·3 (16·7–41·4)	24·3 (16·8–41)

Colon and rectum cancer	63·5 (54·6–72·5)	64·2 (55·7–72·8)	64·3 (55·4–72·7)	64·2 (55·7–72·8)
Liver cancer	77·8 (71·7–83·7)	79 (73·4–84·1)	79·3 (73·6–84·6)	78·9 (73·3–84·1)
Gallbladder and biliary tract cancer	23·3 (12·5–36·2)	23·9 (12·9–37)	21·8 (11·1–34·7)	24 (12·9–37·2)
Pancreatic cancer	40·9 (31·5–52·2)	41·3 (32·5–51·7)	41·1 (31·6–52)	41·3 (32·5–51·7)
Larynx cancer	78·4 (68·1–86·6)	79·2 (70–86·5)	77·9 (68·8–85·5)	79·4 (70·2–86·7)
Tracheal, bronchus, and lung cancer	85 (80·8–88·7)	84·7 (80·9–88·3)	85·2 (81–88·8)	84·7 (80·9–88·3)
Breast cancer	36·5 (26·4–47·8)	36·3 (27–46·1)	36·5 (27·3–46·3)	36·2 (27–46·1)
Cervical cancer	100 (100–100)	100 (100–100)	100 (100–100)	100 (100–100)
Uterine cancer	47 (30·4–62·7)	48·4 (32·3–63·9)	49·1 (33–64·7)	48·3 (31·9–63·8)
Ovarian cancer	24·8 (12·3–38·2)	21·5 (10·4–33·8)	21 (10·2–33·3)	21·5 (10·4–33·8)
Prostate cancer	5·32 (2·2–8·73)	5·84 (2·54–9·51)	6·2 (2·77–10·1)	5·78 (2·5–9·35)
Kidney cancer	40·1 (29·3–51·1)	41·2 (30·3–51·9)	41·3 (30·7–51·7)	41·2 (30·3–51·9)
Bladder cancer	36 (22·4–50·4)	38·6 (25·3–52·2)	39·9 (26·4–53·2)	38·5 (25·2–52·1)
Thyroid cancer	16·8 (7·98–27·3)	17·8 (8·54–28·8)	18·8 (8·69–30·8)	17·7 (8·52–28·6)
Mesothelioma	98·9 (98·4–99·2)	97·7 (96·9–98·4)	98·7 (98–99·1)	97·7 (96·8–98·4)
Non-Hodgkin lymphoma	8·38 (3·57–14·6)	8·73 (3·72–15·1)	8·57 (3·66–15·1)	8·74 (3·74–15·1)
Multiple myeloma	9·28 (3·91–16·4)	9·68 (4·15–17·1)	9·56 (3·96–16·9)	9·69 (4·17–17·1)
Leukemia	36·5 (22·6–50·8)	36·5 (23·7–49·1)	36 (23·4–48·2)	36·5 (23·7–49·2)
Cardiovascular diseases	79·1 (74·4–83·3)	80·4 (76·6–83·9)	73·2 (67·6–78·3)	81·4 (77·7–84·7)
Rheumatic heart disease	23·4 (11·6–44·4)	23·5 (13·5–41·1)	23·8 (14·4–39·3)	23·5 (13·4–41·1)
Ischemic heart disease	93·6 (89·5–96·9)	94·5 (91·3–97)	93·9 (90·8–96·7)	94·5 (91·3–97)
Stroke	79·5 (70·5–89·2)	81·9 (74·9–89·4)	81·4 (75–87·9)	82 (74·9–89·6)
Hypertensive heart disease	100 (100–100)	100 (100–100)	100 (100–100)	100 (100–100)
Non-rheumatic valvular heart disease	28·8 (17·6–43·8)	29·6 (19·7–41·8)	24·6 (16·3–34·2)	30 (20–42·5)
Cardiomyopathy and myocarditis	34·3 (25·3–46)	39·7 (32·4–47·5)	33·7 (25·7–42·2)	40·1 (32·5–47·9)
Atrial fibrillation and flutter	52·5 (40·8–64·2)	59 (49·1–68·7)	63 (53·7–72·1)	54·8 (43·7–65·6)
Aortic aneurysm	50 (42–58·4)	56·1 (49–62·9)	--	56·1 (49–62·9)
Endocarditis	31·6 (19·1–46·8)	33·1 (22·5–45·4)	31·9 (20·3–46·4)	33·2 (22·6–45·3)
Other cardiovascular and circulatory diseases	31·6 (23·6–40·6)	33·6 (26·7–40·6)	33·9 (26·8–41·3)	33·5 (26·4–40·8)
Chronic respiratory diseases	58·5 (51·3–66·3)	56·7 (51·8–61·8)	53·9 (48·9–58·9)	59·3 (52·9–66·5)
Chronic obstructive pulmonary disease	69·4 (63·3–75·3)	68·3 (63·8–72·9)	63 (58·7–67·4)	71·8 (66·6–77)
Pneumoconiosis	100 (100–100)	100 (100–100)	100 (100–100)	100 (100–100)
Asthma	43·4 (30·2–56·9)	44·8 (35·4–54·9)	44·6 (35·3–54·6)	47·1 (35–59·2)
Digestive diseases	22·3 (17·5–27·5)	28·4 (22·9–33·8)	15·3 (10·1–21·7)	33·4 (27–39·8)

Cirrhosis and other chronic liver diseases	69·8 (56–85·1)	74·4 (61·5–87·7)	71·6 (58·5–84·2)	74·4 (61·4–87·7)
Upper digestive system diseases	18·9 (15·6–22·3)	7·51 (4·63–10·8)	0·648 (0·364–1·05)	21·9 (18·5–25·3)
Gallbladder and biliary diseases	46·1 (30·2–61·3)	51·4 (36·1–66)	53·8 (38·8–68·2)	47·3 (31·7–62·3)
Pancreatitis	30·4 (8·97–52·9)	37 (14·1–57·6)	38·9 (14·9–60·1)	36·7 (13·9–57·6)
Neurological disorders	25·1 (10·5–40·7)	17·4 (8·8–29·7)	11·9 (6·24–21·5)	22·5 (9·21–37·4)
Alzheimer's disease and other dementias	39·6 (25·4–55·1)	41·5 (27·2–56·4)	41·8 (27·5–56·9)	41·3 (27·1–56·5)
Parkinson's disease	-7·71 (-13·1–-3·41)	-8·88 (-15–-4)	-9·56 (-15·8–-4·36)	-8·72 (-14·8–-3·92)
Idiopathic epilepsy	24·4 (12·9–35·4)	24·2 (13·3–34·1)	22·8 (12·3–32·3)	27·3 (15·5–38·4)
Multiple sclerosis	12·6 (9·12–16·4)	14 (10·4–17·8)	14·1 (10·5–17·8)	14 (10·3–18)
Mental disorders	--	6·12 (3·13–9·35)	6·13 (3·14–9·38)	--
Depressive disorders	--	12·5 (5·8–19·6)	12·5 (5·8–19·6)	--
Anxiety disorders	--	4·62 (1·27–10·2)	4·62 (1·27–10·2)	--
Idiopathic developmental intellectual disability	--	37·7 (16·9–56·8)	37·7 (16·9–56·8)	--
Substance use disorders	100 (100–100)	100 (100–100)	100 (100–100)	100 (100–100)
Alcohol use disorders	100 (100–100)	100 (100–100)	100 (100–100)	100 (100–100)
Drug use disorders	100 (100–100)	100 (100–100)	100 (100–100)	100 (100–100)
Diabetes and kidney diseases	99·9 (99·9–100)	100 (100–100)	100 (100–100)	99·9 (99·9–100)
Diabetes mellitus	100 (100–100)	100 (100–100)	100 (100–100)	100 (100–100)
Chronic kidney disease	100 (100–100)	100 (100–100)	100 (100–100)	100 (100–100)
Sense organ diseases	--	8·33 (7·15–9·7)	8·33 (7·15–9·7)	--
Blindness and vision loss	--	9·89 (6·45–14·3)	9·89 (6·45–14·3)	--
Age-related and other hearing loss	--	9·03 (8–10·2)	9·03 (8–10·2)	--
Musculoskeletal disorders	3·96 (0·94–7·36)	23·4 (19·2–27·7)	23·8 (19·5–28·1)	4·98 (1·2–8·98)
Rheumatoid arthritis	9·2 (2·23–17·1)	12·3 (3·29–21·8)	12·7 (3·46–22·6)	10·9 (2·78–19·6)
Osteoarthritis	--	18·8 (10·3–28·5)	18·8 (10·3–28·5)	--
Low back pain	--	41·7 (36·2–47·1)	41·7 (36·2–47·1)	--
Gout	--	56·7 (41·5–72·9)	56·7 (41·5–72·9)	--
Other non-communicable diseases	0·0396 (0·0236–0·0626)	0·0482 (0·027–0·0773)	--	0·22 (0·126–0·338)
Sudden infant death syndrome	17·7 (8·82–28·6)	17·7 (8·81–28·6)	--	17·7 (8·81–28·6)
Injuries	35·8 (29·4–40·8)	30 (24·8–34·5)	32·4 (27·5–36·9)	26·6 (19·4–32·9)
Transport injuries	22·3 (15–29·6)	22·3 (15·6–28·9)	26·7 (22·5–30·9)	20·3 (11·9–28·4)
Road injuries	20·9 (13·1–28·7)	21 (14–27·8)	25·4 (21·2–29·5)	18·9 (10·2–27·4)
Other transport injuries	34 (28·5–38·7)	32·3 (26·8–37·3)	37·2 (32·3–41·2)	30·2 (24·1–36)
Unintentional injuries	44·2 (37·1–48·9)	33·3 (28·2–37·8)	33·1 (27·9–37·8)	34·1 (28·6–38·6)

Falls	57·1 (48·2–62·6)	44·8 (38·2–49·8)	43 (36·5–48·1)	50·7 (43·5–55·9)
Drowning	-16·9 (-31·6–-2·51)	-13·2 (-26·6–0·208)	10 (5·19–16·8)	-15·4 (-30–-1·57)
Fire, heat, and hot substances	8·98 (4·2–15·6)	12·2 (6·79–19·5)	12·3 (6·97–19·7)	11·2 (6·19–18·4)
Poisonings	13·4 (7·8–21)	12·4 (7·13–19·4)	11·5 (6·56–18·3)	15·2 (9·27–23)
Exposure to mechanical forces	11·9 (0·509–23·5)	18·3 (15·4–21·1)	19·1 (16·2–21·7)	2·83 (-8·85–15·4)
Animal contact	22·3 (16·7–29·4)	21·8 (16·5–28·2)	22·3 (16·9–28·6)	21 (15·4–27·8)
Foreign body	1·5 (0·907–2·44)	2·79 (1·73–4·47)	2·48 (1·53–3·97)	3·15 (1·97–5·1)
Environmental heat and cold exposure	7·53 (3·02–14·1)	9·13 (3·91–16·4)	9·26 (3·94–16·7)	8·78 (3·71–15·9)
Exposure to forces of nature	0 (0–0)	10 (4·25–17·9)	10 (4·25–17·9)	0 (0–0)
Other unintentional injuries	14·1 (8·22–22·2)	11·9 (6·68–19)	11·8 (6·58–18·8)	16·2 (9·81–24·2)
Self-harm and interpersonal violence	21·7 (7·04–36·4)	24·5 (11·4–37·5)	30·3 (22·3–38·1)	23·3 (8·41–38)
Self-harm	21·3 (6·26–36·3)	23 (7·74–37·9)	22·3 (8·95–35·8)	23 (7·61–38)
Interpersonal violence	33·7 (23·8–43·7)	36·3 (27·5–45)	37·5 (28·6–46·3)	32 (21·6–41·9)

Supplementary Results Table 2. All-age PAFs attributable to the joint distribution of all risk factors for all ages, both sexes combined for each GBD level 1, level 2, and level 3 cause and all causes in 2019 for East of England. Results are shown for deaths, YLLs, YLDs, and DALYs Data in parenthesis are 95% uncertainty intervals. PAFs=population attributable fractions. GBD=Global Burden of Disease. YLLs=years of life lost. YLDs=years lived with disability. DALYs=disability-adjusted life-years

	Deaths 2019	DALYs 2019	YLDs 2019	YLLs 2019
All causes	54·3 (51·2–57·5)	41·7 (38·7–44·8)	29·3 (26·9–31·6)	54 (51·5–56·8)
Communicable, maternal, neonatal, and nutritional diseases	44 (35·3–53)	50·4 (45·7–55·3)	47·1 (43–51·4)	52·2 (45·1–59·2)
HIV/AIDS and sexually transmitted infections	76·9 (62·3–88·1)	76·9 (60·3–89)	97·5 (96·3–98·4)	59·2 (40·2–77·5)
HIV/AIDS	92·3 (90·4–93·4)	91·4 (89·2–93)	94·9 (92·8–96·2)	88·6 (86·3–90)
Sexually transmitted infections excluding HIV	54·5 (31·7–78·3)	61·2 (36·1–84·9)	100 (100–100)	21·3 (8·67–42·7)
Respiratory infections and tuberculosis	44·8 (35·4–54·8)	42 (34–51)	3·27 (2·44–4·26)	48·1 (39·1–57·6)
Tuberculosis	54·8 (43·2–65·3)	57·9 (46·9–67·2)	55·5 (44·9–65·1)	58·4 (47·4–67·6)
Lower respiratory infections	44·8 (35·4–54·7)	47·9 (39–57·6)	33·5 (26·8–40)	48 (39·1–57·7)
Upper respiratory infections	0·044 (0·0305–0·0598)	0·00787 (0·00454–0·0122)	0·00638 (0·00325–0·0106)	0·11 (0·0776–0·149)
Otitis media	2·18 (1·34–3·32)	4·29 (2·74–5·99)	4·27 (2·7–6)	5·7 (3·75–8·1)
Enteric infections	5·12 (3·11–8·72)	8·76 (6·37–12·2)	9·32 (6·82–12·8)	7·4 (5·14–10·8)
Diarrheal diseases	5·18 (3·14–8·8)	8·8 (6·41–12·2)	9·33 (6·83–12·8)	7·52 (5·23–11·1)
Other infectious diseases	0·528 (0·374–0·68)	1·08 (0·831–1·32)	0·462 (0·28–0·762)	1·32 (0·996–1·64)
Meningitis	1·36 (1·03–1·74)	2·47 (1·88–3·12)	0·000587 (0·000261–0·00104)	3·16 (2·45–3·98)
Encephalitis	0·267 (0·192–0·352)	0·753 (0·495–1·01)	0·000203 (9·32e-05–0·000351)	0·964 (0·671–1·26)
Measles	2·79 (-0·712–12·8)	3·31 (-0·817–14·7)	0·569 (0·014–1·41)	3·5 (-0·891–16·2)
Acute hepatitis	11·1 (6·23–22·8)	4·91 (3·04–7·93)	3·03 (1·83–4·87)	10·7 (6–21·3)
Maternal and neonatal disorders	88·7 (87·2–90)	78·4 (75·3–81·5)	66·9 (61·8–71·7)	89·6 (88·1–90·8)
Maternal disorders	9·29 (3·33–15·6)	9·35 (3·42–15·7)	9·48 (3·46–15·9)	9·14 (3·32–15·3)
Neonatal disorders	91·1 (89·8–92·3)	80·2 (76·9–83·3)	68·7 (63·3–73·7)	91·1 (89·8–92·3)
Nutritional deficiencies	73·3 (70·1–88·5)	76·9 (73·6–80·6)	77 (73·6–80·8)	73·9 (69·7–82·9)
Protein-energy malnutrition	100 (100–100)	100 (100–100)	100 (100–100)	100 (100–100)
Vitamin A deficiency	--	100 (100–100)	100 (100–100)	--
Dietary iron deficiency	--	100 (100–100)	100 (100–100)	--
Non-communicable diseases	56 (52·4–59·5)	42·2 (39·1–45·5)	28·5 (26–31·1)	56·1 (53·3–59)
Neoplasms	47·4 (43·5–51·6)	46·9 (43·2–50·8)	33 (28·8–37·6)	47·7 (44–51·8)
Lip and oral cavity cancer	64·9 (54·4–73·4)	68·4 (58·7–76·1)	68 (58·1–76·2)	68·5 (58·8–76·1)
Nasopharynx cancer	64·3 (51·8–74·1)	64·8 (52·7–74·1)	62 (49·2–72·1)	65·1 (53–74·4)
Other pharynx cancer	77·8 (70·4–83·9)	79·5 (72·5–85·1)	79·4 (72·2–85·2)	79·6 (72·4–85·1)
Esophageal cancer	74·9 (64·2–83·7)	76·8 (66·9–85)	75·8 (65–84·2)	76·8 (66·9–85)
Stomach cancer	24·7 (17·3–41·5)	25·4 (17·9–41·8)	25·9 (18·4–42·4)	25·4 (17·9–41·8)

Colon and rectum cancer	64·2 (55–73·1)	64·7 (56–73·2)	65 (56·4–73·3)	64·6 (56–73·2)
Liver cancer	76·1 (70·4–81·7)	77 (71·7–82·1)	77·8 (72·3–82·8)	77 (71·7–82·1)
Gallbladder and biliary tract cancer	23 (12·7–35·3)	23·5 (13·1–36·1)	21·6 (11·6–34·2)	23·6 (13·2–36·1)
Pancreatic cancer	43·1 (33·5–54·4)	43 (33·9–53·9)	43·1 (33·7–54·2)	43 (33·9–53·9)
Larynx cancer	81·1 (72·7–87·7)	81·4 (74·3–87·6)	79·3 (71·6–85·8)	81·6 (74·6–87·8)
Tracheal, bronchus, and lung cancer	86·3 (83·1–89·4)	85·7 (82·7–88·7)	86·3 (83·1–89·3)	85·7 (82·7–88·7)
Breast cancer	37·8 (28·1–49·4)	36·9 (28·1–47·1)	37·1 (28·2–47·6)	36·8 (28–47)
Cervical cancer	100 (100–100)	100 (100–100)	100 (100–100)	100 (100–100)
Uterine cancer	45·3 (29·9–61·1)	46·5 (30·9–62)	47 (31·5–62·5)	46·4 (30·8–61·9)
Ovarian cancer	28·8 (14·1–43·3)	24·4 (11·9–38)	22·9 (11·1–35·6)	24·5 (11·9–38·1)
Prostate cancer	5·89 (2·5–9·49)	6·49 (2·84–10·4)	6·98 (3·15–11·1)	6·41 (2·8–10·3)
Kidney cancer	40·1 (29·2–50·7)	40·7 (30·1–51)	40·7 (30·2–50·9)	40·7 (30·1–51)
Bladder cancer	39·2 (25·1–53·9)	41·9 (28·2–55·3)	43·1 (29·6–56·3)	41·9 (28·1–55·4)
Thyroid cancer	15·5 (7·53–25·3)	16·2 (7·82–26·3)	16·7 (7·98–27·4)	16·2 (7·8–26·2)
Mesothelioma	99·2 (98·9–99·4)	98·2 (97·6–98·7)	99·1 (98·8–99·4)	98·2 (97·6–98·7)
Non-Hodgkin lymphoma	7·72 (3·19–13·7)	7·96 (3·35–14)	7·9 (3·3–14)	7·96 (3·36–14)
Multiple myeloma	8·71 (3·74–15·6)	9·06 (3·93–16)	9·03 (3·89–16·2)	9·06 (3·93–16)
Leukemia	37·6 (22·9–52·3)	36·2 (23·9–48·6)	35·4 (23·5–47·6)	36·2 (24·1–48·6)
Cardiovascular diseases	78·2 (74·5–81·8)	79·2 (76·2–82·2)	70·1 (65·5–74·7)	80·4 (77·5–83·3)
Rheumatic heart disease	22 (12·3–43·3)	22·9 (14·2–38·4)	22·9 (14·4–37·2)	22·9 (14·2–38·4)
Ischemic heart disease	93·3 (89·5–96·7)	94·4 (91·5–96·9)	93·8 (90·9–96·4)	94·5 (91·5–97)
Stroke	78·8 (70·6–88·3)	81·9 (75·9–88·8)	81·7 (76·2–87·7)	81·9 (75·8–89)
Hypertensive heart disease	100 (100–100)	100 (100–100)	100 (100–100)	100 (100–100)
Non-rheumatic valvular heart disease	25 (16·1–38)	24·9 (17·4–34·9)	15·8 (10·7–22·1)	26·3 (18·4–36·9)
Cardiomyopathy and myocarditis	34·8 (27·6–43·9)	41·2 (35·6–46·6)	35·4 (29·4–42·1)	41·6 (35·9–47)
Atrial fibrillation and flutter	50·1 (40·3–61·4)	57·5 (49·2–66·5)	61·5 (53·7–69·4)	52·9 (43·6–63·3)
Aortic aneurysm	49·1 (42·8–56·2)	56·1 (50·7–61·7)	--	56·1 (50·7–61·7)
Endocarditis	30·3 (21–43·5)	33 (24·7–43·7)	30·1 (20·7–43)	33·1 (24·8–43·7)
Other cardiovascular and circulatory diseases	30·8 (25–37·2)	33·6 (28·4–38·7)	33·7 (27·9–39·4)	33·5 (28·3–38·7)
Chronic respiratory diseases	62 (56·2–68·3)	58·3 (53·8–62·9)	52·9 (47·8–57·8)	62·8 (57·5–68·7)
Chronic obstructive pulmonary disease	71·4 (66·9–75·8)	70·8 (67·3–74·3)	65·1 (61·8–68·4)	73·8 (69·7–77·5)
Pneumoconiosis	100 (100–100)	100 (100–100)	100 (100–100)	100 (100–100)
Asthma	42·5 (30·4–55·8)	42·9 (33·8–52·8)	42·7 (33·8–52·5)	44·9 (34·1–56·5)
Digestive diseases	26·1 (22·1–30·3)	32·8 (27·5–37·8)	24·5 (17–33·2)	37·6 (32·4–43)

Cirrhosis and other chronic liver diseases	70·9 (59·9–82·5)	74·4 (63·9–85·3)	71·9 (61·7–82·6)	74·5 (63·9–85·4)
Upper digestive system diseases	20 (16·6–23·1)	8·9 (5·79–12·3)	0·715 (0·404–1·14)	23 (19·6–26·2)
Gallbladder and biliary diseases	44·9 (29·3–60·7)	49·5 (35·7–63·9)	50·3 (36·4–64·6)	46·2 (31–61·6)
Pancreatitis	33·4 (15·9–51)	39·6 (21·6–57·1)	40·3 (22–57·3)	39·5 (21·5–56·9)
Neurological disorders	25·5 (10·8–41·2)	16·3 (8·09–28·5)	10·5 (5·28–20·3)	22·5 (9·95–38·2)
Alzheimer's disease and other dementias	41·1 (26·6–57·2)	43·1 (28·8–59)	43·5 (29·4–59)	43 (28·7–58·6)
Parkinson's disease	-8·86 (-15–-3·89)	-10·1 (-16·8–-4·54)	-10·7 (-17·6–-4·82)	-10 (-16·6–-4·48)
Idiopathic epilepsy	25·8 (16–34·8)	24·6 (15·6–33)	22·7 (14·1–31·1)	27·3 (17·4–36·4)
Multiple sclerosis	13·8 (10·3–17·6)	15 (11·3–18·9)	15 (11·4–18·7)	15·1 (11·3–19)
Mental disorders	--	6·39 (3·21–9·73)	6·4 (3·21–9·73)	--
Depressive disorders	--	13·3 (6·09–20·8)	13·3 (6·09–20·8)	--
Anxiety disorders	--	4·8 (1·26–10·7)	4·8 (1·26–10·7)	--
Idiopathic developmental intellectual disability	--	35·6 (15·8–53·6)	35·6 (15·8–53·6)	--
Substance use disorders	100 (100–100)	100 (100–100)	100 (100–100)	100 (100–100)
Alcohol use disorders	100 (100–100)	100 (100–100)	100 (100–100)	100 (100–100)
Drug use disorders	100 (100–100)	100 (100–100)	100 (100–100)	100 (100–100)
Diabetes and kidney diseases	99·7 (99·7–99·8)	99·9 (99·9–100)	100 (100–100)	99·7 (99·7–99·7)
Diabetes mellitus	100 (100–100)	100 (100–100)	100 (100–100)	100 (100–100)
Chronic kidney disease	100 (100–100)	100 (100–100)	100 (100–100)	100 (100–100)
Sense organ diseases	--	8·46 (7·28–9·84)	8·46 (7·28–9·84)	--
Blindness and vision loss	--	9·81 (6·29–14·3)	9·81 (6·29–14·3)	--
Age-related and other hearing loss	--	9·32 (8·42–10·3)	9·32 (8·42–10·3)	--
Musculoskeletal disorders	3·3 (0·789–6·09)	23·1 (19·2–27)	23·6 (19·5–27·6)	4·08 (1·01–7·28)
Rheumatoid arthritis	10·3 (2·44–18·5)	13 (3·21–22·8)	13·3 (3·28–23·4)	12 (3·05–21·4)
Osteoarthritis	--	18·1 (9·63–27·4)	18·1 (9·63–27·4)	--
Low back pain	--	42·1 (36·8–47·4)	42·1 (36·8–47·4)	--
Gout	--	53·6 (37·9–70·4)	53·6 (37·9–70·4)	--
Other non-communicable diseases	0·0612 (0·0424–0·0814)	0·0617 (0·0399–0·0877)	--	0·269 (0·176–0·353)
Sudden infant death syndrome	7·39 (4·88–10·8)	7·39 (4·87–10·7)	--	7·39 (4·87–10·7)
Injuries	33·2 (27·5–37·9)	27·8 (23·1–31·9)	30·5 (26·3–34·5)	24·3 (17·3–30·4)
Transport injuries	21·6 (14·8–28·4)	21·8 (15·8–27·8)	26·6 (22·4–30·6)	19·5 (12·1–26·8)
Road injuries	20·8 (13·7–27·8)	20·9 (14·8–27)	25·4 (21·2–29·4)	18·8 (11·2–26·4)
Other transport injuries	33 (27·8–37·7)	32·2 (27·2–36·8)	36 (31·4–40·3)	29·2 (23·7–34·6)
Unintentional injuries	41·1 (34·8–45·5)	30·1 (25·4–34·4)	30·7 (25·9–35·2)	28·3 (23·4–32·3)

Falls	55·7<br>(47·4–61·3)	41·9<br>(35·9–46·8)	40·2<br>(34·4–45·1)	48<br>(41·2–53)
Drowning	-14·3<br>(-27·2–-1·39)	-12·7<br>(-24·8–-0·662)	10·6<br>(6·01–16·8)	-14·1<br>(-26·5–-1·53)
Fire, heat, and hot substances	10·1<br>(5·62–16·3)	12·8<br>(7·99–19·2)	13·1<br>(8·12–19·6)	12<br>(7·44–18·1)
Poisonings	13·9<br>(8·7–20·4)	13·1<br>(8·39–19·3)	11·9<br>(7·4–17·9)	14·9<br>(9·64–21·5)
Exposure to mechanical forces	11·8<br>(2·11–21·9)	17·3<br>(14·5–19·7)	18·5<br>(15·9–20·9)	1·49<br>(-8·38–12)
Animal contact	23·1<br>(18·3–28·7)	21·1<br>(16·5–26·4)	21·9<br>(17–27·2)	19·3<br>(14·9–24·7)
Foreign body	2·45<br>(1·7–3·49)	3·66<br>(2·55–5·1)	3·09<br>(2·16–4·41)	4·03<br>(2·81–5·69)
Environmental heat and cold exposure	7·95<br>(3·43–14·2)	9·28<br>(4·13–16·2)	9·32<br>(4·13–16·3)	9·17<br>(4·1–15·9)
Exposure to forces of nature	0<br>(0–0)	10·1<br>(4·62–17·5)	10·1<br>(4·62–17·5)	0<br>(0–0)
Other unintentional injuries	13·7<br>(8·46–20·4)	12·5<br>(7·74–18·6)	12·3<br>(7·63–18·4)	15·6<br>(10·1–22·4)
Self-harm and interpersonal violence	22·2<br>(8·44–35·2)	24·8<br>(11·7–36·4)	32·9<br>(25·3–40·9)	23·5<br>(9·46–36·1)
Self-harm	21·9<br>(7·59–35·5)	23·3<br>(8·88–36·5)	22·4<br>(9·35–34·1)	23·4<br>(8·77–36·7)
Interpersonal violence	29·1<br>(20·2–38)	34·5<br>(26·3–43·4)	38·5<br>(29·8–47·3)	26·1<br>(17·4–34·9)

Supplementary Results Table 2. All-age PAFs attributable to the joint distribution of all risk factors for all ages, both sexes combined for each GBD level 1, level 2, and level 3 cause and all causes in 2019 for Bedford. Results are shown for deaths, YLLs, YLDs, and DALYs Data in parenthesis are 95% uncertainty intervals. PAFs=population attributable fractions. GBD=Global Burden of Disease. YLLs=years of life lost. YLDs=years lived with disability. DALYs=disability-adjusted life-years

	Deaths 2019	DALYs 2019	YLDs 2019	YLLs 2019
All causes	55 (51·6–58·6)	42·9 (39·8–46·1)	30·3 (27·9–32·8)	54·6 (51·8–57·8)
Communicable, maternal, neonatal, and nutritional diseases	44·5 (35·1–54)	51·8 (46·8–56·9)	46·6 (41·9–51·4)	54·4 (46·8–61·7)
HIV/AIDS and sexually transmitted infections	74·8 (59·6–86·3)	75·2 (57·9–87·4)	97·2 (95·8–98·3)	57·6 (38·7–75·1)
HIV/AIDS	91 (89·2–92·1)	89·9 (87·7–91·8)	94·8 (92·8–96·1)	86·2 (84–87·7)
Sexually transmitted infections excluding HIV	45·1 (23·4–71·5)	56·6 (30·9–82·9)	100 (100–100)	16·4 (6·29–35·1)
Respiratory infections and tuberculosis	45·3 (34·9–55·8)	42·4 (33·2–51·4)	3·58 (2·61–4·84)	48·9 (38·9–58·7)
Tuberculosis	56 (43–67·7)	58·8 (47–69·3)	55·9 (43·5–66·6)	59·3 (47·4–69·7)
Lower respiratory infections	45·2 (34·7–55·9)	48·6 (38·5–58·6)	33·8 (26·7–41·2)	48·8 (38·6–58·8)
Upper respiratory infections	0·0634 (0·0331–0·117)	0·00838 (0·00161–0·0199)	0·00659 (7·95e-05–0·0179)	0·136 (0·0697–0·245)
Otitis media	2·52 (1·13–4·93)	4·47 (2·86–6·41)	4·44 (2·83–6·42)	6·5 (3·41–11)
Enteric infections	5·25 (3·1–9·02)	9 (6·48–12·9)	9·49 (6·88–13·4)	8·03 (5·45–11·9)
Diarrheal diseases	5·29 (3·16–9·07)	9·05 (6·54–13)	9·5 (6·89–13·4)	8·14 (5·58–12·1)
Other infectious diseases	0·555 (0·394–0·854)	1·09 (0·838–1·37)	0·463 (0·262–0·774)	1·32 (1–1·73)
Meningitis	1·54 (1·11–2·04)	2·55 (1·85–3·38)	0·000531 (3·67e-06–0·00165)	3·17 (2·33–4·21)
Encephalitis	0·32 (0·219–0·462)	0·848 (0·536–1·25)	0·000211 (1·98e-06–0·000595)	1·07 (0·726–1·55)
Measles	2·83 (-0·755–14)	3·01 (-0·618–14·4)	0·572 (0·014–1·61)	3·63 (-0·967–18)
Acute hepatitis	7·69 (3·49–23·7)	4·36 (2·5–8·84)	2·97 (1·75–4·87)	7·4 (3·45–20·9)
Maternal and neonatal disorders	87·6 (85·2–89·7)	78·9 (75·5–82)	64·9 (59·4–69·8)	88·8 (86·5–90·8)
Maternal disorders	9·46 (3·46–15·9)	9·48 (3·43–16)	9·65 (3·49–16·6)	9·32 (3·4–15·7)
Neonatal disorders	90·9 (88·8–92·9)	81 (77·4–84·1)	66·8 (60·9–72)	90·9 (88·8–92·9)
Nutritional deficiencies	67·7 (61·2–89·5)	77·4 (73·1–82·3)	77·5 (73·2–82·5)	68·8 (64·7–83)
Protein-energy malnutrition	100 (100–100)	100 (100–100)	100 (100–100)	100 (100–100)
Vitamin A deficiency	--	100 (100–100)	100 (100–100)	--
Dietary iron deficiency	--	100 (100–100)	100 (100–100)	--
Non-communicable diseases	56·8 (53·1–60·5)	43·7 (40·3–47)	29·7 (27–32·5)	56·9 (53·9–59·9)
Neoplasms	46·9 (42·5–51·7)	46·3 (42–50·6)	33·5 (29·1–38·4)	47 (42·8–51·4)
Lip and oral cavity cancer	67·1 (54·5–77)	70·3 (58·7–79·1)	69·7 (58·1–79·2)	70·3 (58·8–79·1)
Nasopharynx cancer	67·6 (52·7–77·7)	67·6 (53·2–77·3)	65 (50·1–75·4)	67·8 (53·4–77·5)
Other pharynx cancer	79·1 (70·2–85·7)	80·5 (71·7–86·6)	80·3 (71·7–86·4)	80·5 (71·8–86·6)
Esophageal cancer	75 (64·6–84)	76·9 (66·6–85·2)	75·9 (65·9–84·5)	76·9 (66·6–85·2)
Stomach cancer	25·2 (17·7–41·9)	25·8 (18·2–42·2)	26·2 (18·7–43)	25·8 (18·2–42·2)

Colon and rectum cancer	65·3 (55·8–74·5)	65·7 (56·6–74·3)	66 (56·7–74·6)	65·7 (56·5–74·3)
Liver cancer	77·1 (71·2–83)	77·7 (72·2–83)	78·4 (72·9–83·6)	77·7 (72·2–83)
Gallbladder and biliary tract cancer	22·8 (12·5–35·5)	23·2 (12·7–35·7)	21·3 (11·1–34·1)	23·3 (12·8–35·8)
Pancreatic cancer	44·3 (34·2–56·5)	44 (34·6–55·7)	44·3 (34·3–56·1)	44 (34·6–55·7)
Larynx cancer	81·7 (73·1–88·6)	81·9 (74·1–88·4)	79·8 (71·8–86·8)	82·1 (74·4–88·5)
Tracheal, bronchus, and lung cancer	86·5 (82·7–89·9)	85·7 (82·2–89)	86·4 (82·7–89·9)	85·7 (82·2–89)
Breast cancer	38·7 (28·5–51)	37·4 (28·2–48·1)	37·6 (28·3–48·5)	37·3 (28·2–48)
Cervical cancer	100 (100–100)	100 (100–100)	100 (100–100)	100 (100–100)
Uterine cancer	45·2 (29·7–61)	46·5 (30·9–62·3)	46·9 (31·4–62·8)	46·4 (30·8–62·3)
Ovarian cancer	27·6 (14·5–42·9)	23·4 (11·7–37·5)	21·8 (11–34·9)	23·4 (11·7–37·6)
Prostate cancer	6·07 (2·65–9·77)	6·68 (2·94–10·6)	7·16 (3·16–11·4)	6·6 (2·9–10·5)
Kidney cancer	40·4 (29·6–51·2)	40·7 (30·3–50·7)	40·6 (30·1–50·7)	40·7 (30·3–50·7)
Bladder cancer	41·1 (26·7–56·3)	43·6 (30·1–57·7)	44·7 (31–58·1)	43·6 (29·9–57·7)
Thyroid cancer	15·6 (7·61–25·5)	16·3 (7·92–26·6)	16·8 (7·97–27·4)	16·3 (7·93–26·6)
Mesothelioma	99 (98·6–99·3)	97·9 (97·2–98·5)	98·9 (98·4–99·3)	97·9 (97·2–98·5)
Non-Hodgkin lymphoma	7·59 (3·15–13·5)	7·81 (3·25–13·8)	7·75 (3·2–13·7)	7·82 (3·25–13·8)
Multiple myeloma	8·7 (3·65–15·5)	9·04 (3·94–16·1)	9 (3·8–16·1)	9·04 (3·95–16·1)
Leukemia	37·9 (23·8–52·7)	35·8 (23·9–48·1)	34·8 (23·3–46·9)	35·9 (23·9–48·2)
Cardiovascular diseases	78·9 (74·7–83)	80·2 (76·7–83·5)	70·7 (65·4–75·9)	81·4 (78·1–84·6)
Rheumatic heart disease	22·1 (12·2–41·8)	23·1 (13·9–38·6)	22·9 (13·8–37·6)	23·1 (13·9–38·6)
Ischemic heart disease	93·6 (89·7–96·8)	94·8 (92–97)	93·9 (91–96·6)	94·8 (92–97·1)
Stroke	79·3 (71–88·8)	82·5 (76·3–89·4)	82 (76·2–88·2)	82·6 (76·1–89·5)
Hypertensive heart disease	100 (100–100)	100 (100–100)	100 (100–100)	100 (100–100)
Non-rheumatic valvular heart disease	25·1 (16·2–38·1)	26·2 (18–36·6)	19·3 (12·7–27·1)	26·8 (18·7–37·5)
Cardiomyopathy and myocarditis	35·5 (28·2–44·4)	41·8 (35·7–47·9)	36·7 (30–44·2)	42·1 (35·7–48·3)
Atrial fibrillation and flutter	50·1 (39·3–62·6)	57·7 (48·3–67·5)	61·8 (52·3–70·5)	53·1 (42·9–64·2)
Aortic aneurysm	51 (44·3–58·1)	58·2 (52·3–63·8)	--	58·2 (52·3–63·8)
Endocarditis	30·6 (20·7–44)	33·3 (24·3–44·2)	30·4 (20·2–43·7)	33·4 (24·4–44·3)
Other cardiovascular and circulatory diseases	30·5 (23·8–38·2)	33·2 (27·2–39·8)	34·4 (27·2–41·8)	33 (27–39·6)
Chronic respiratory diseases	63·1 (56·5–70·1)	59 (54–64·1)	52·6 (47·4–57·8)	63·8 (57·6–70·4)
Chronic obstructive pulmonary disease	72·3 (67–77·5)	71·6 (67·5–75·5)	65·4 (61·4–69·3)	74·5 (69·8–79·2)
Pneumoconiosis	100 (100–100)	100 (100–100)	100 (100–100)	100 (100–100)
Asthma	43·3 (31·2–56·8)	42·4 (33·6–52·2)	42·1 (33·5–51·8)	45·1 (34·6–56·5)
Digestive diseases	28·5 (23·3–34·4)	36·2 (30·1–42·7)	28·1 (19·5–37·6)	40·3 (33·4–47·7)

Cirrhosis and other chronic liver diseases	72·1 (59·3–86)	75·2 (62·9–87·9)	72·1 (59·5–84·1)	75·3 (63–88)
Upper digestive system diseases	19·1 (15·9–22·5)	8·56 (5·57–11·8)	0·632 (0·368–0·999)	21·5 (18·2–25·3)
Gallbladder and biliary diseases	45 (29·3–61)	51·4 (36·6–66)	52·5 (37·5–67)	46·3 (31·5–61·7)
Pancreatitis	33·5 (10·8–54·6)	39·5 (15·5–59·8)	39·6 (15·6–60·7)	39·5 (15·4–59·8)
Neurological disorders	25 (10·3–41·7)	15·6 (7·77–28·1)	10·1 (4·92–19·7)	21·7 (9·4–37·8)
Alzheimer's disease and other dementias	42·3 (27·3–58·9)	44·3 (29·7–60·3)	44·6 (30·1–60·6)	44·2 (29·5–60·2)
Parkinson's disease	-9·13 (-15·4–-3·87)	-10·5 (-17·4–-4·51)	-11 (-18·2–-4·88)	-10·4 (-17·2–-4·49)
Idiopathic epilepsy	26·2 (14·3–37·2)	24·9 (13·8–34·9)	22·9 (12·3–32·8)	27·2 (15·4–37·8)
Multiple sclerosis	14·4 (10·7–18·3)	15·5 (11·6–19·4)	15·2 (11·4–19·2)	15·7 (11·6–19·8)
Mental disorders	--	6·54 (3·28–10)	6·54 (3·29–10)	--
Depressive disorders	--	13·5 (6·12–21·3)	13·5 (6·12–21·3)	--
Anxiety disorders	--	5·01 (1·34–11·1)	5·01 (1·34–11·1)	--
Idiopathic developmental intellectual disability	--	34·6 (14·6–53·4)	34·6 (14·6–53·4)	--
Substance use disorders	100 (100–100)	100 (100–100)	100 (100–100)	100 (100–100)
Alcohol use disorders	100 (100–100)	100 (100–100)	100 (100–100)	100 (100–100)
Drug use disorders	100 (100–100)	100 (100–100)	100 (100–100)	100 (100–100)
Diabetes and kidney diseases	99·6 (99·5–99·7)	99·9 (99·9–99·9)	100 (100–100)	99·6 (99·5–99·6)
Diabetes mellitus	100 (100–100)	100 (100–100)	100 (100–100)	100 (100–100)
Chronic kidney disease	100 (100–100)	100 (100–100)	100 (100–100)	100 (100–100)
Sense organ diseases	--	8·69 (7·46–10·1)	8·69 (7·46–10·1)	--
Blindness and vision loss	--	9·95 (6·28–14·6)	9·95 (6·28–14·6)	--
Age-related and other hearing loss	--	9·63 (8·63–10·7)	9·63 (8·63–10·7)	--
Musculoskeletal disorders	3·69 (0·876–6·85)	23·1 (19·1–27·1)	23·6 (19·4–27·7)	4·49 (1·07–8·01)
Rheumatoid arthritis	10·7 (2·63–19·6)	13·2 (3·33–23·6)	13·5 (3·34–24·1)	12·5 (3·1–22·4)
Osteoarthritis	--	17·8 (9·47–27)	17·8 (9·47–27)	--
Low back pain	--	42·3 (36·9–47·8)	42·3 (36·9–47·8)	--
Gout	--	53·4 (37·8–70·2)	53·4 (37·8–70·2)	--
Other non-communicable diseases	0·08 (0·0532–0·114)	0·0836 (0·0512–0·123)	--	0·305 (0·198–0·43)
Sudden infant death syndrome	8·08 (4·6–13·2)	8·07 (4·59–13·2)	--	8·07 (4·59–13·2)
Injuries	31·7 (25·1–37·3)	26·4 (20·9–31·6)	29·8 (25·5–34·6)	22·6 (14·7–29·4)
Transport injuries	20·2 (12–28·2)	20·2 (13–27·3)	25·7 (21·3–30)	17·7 (8·51–26·5)
Road injuries	19·5 (11·1–27·8)	19·3 (11·9–26·7)	24·6 (20·2–28·9)	17·1 (7·75–26·1)
Other transport injuries	32·6 (27·3–37·4)	31·9 (26·8–36·7)	35·1 (30·2–39·6)	28·8 (22·7–34·4)
Unintentional injuries	38·9 (32·6–43·7)	28·4 (23·8–33·3)	30 (25·3–35·1)	24·3 (19·5–29)

Falls	55·1<br>(46·9–60·7)	41·1<br>(34·8–46·4)	39·4<br>(33·4–44·8)	46·4<br>(39·6–51·9)
Drowning	-14·7<br>(-29·4–-0·315)	-14·4<br>(-28·1–-0·388)	10·2<br>(5·3–16·7)	-15·1<br>(-29·6–-0·777)
Fire, heat, and hot substances	9·55<br>(4·88–15·6)	11·9<br>(6·82–18·7)	12·4<br>(7·11–19·5)	10·6<br>(6–16·4)
Poisonings	14·6<br>(8·84–22)	13·1<br>(8·07–19·9)	11·4<br>(6·57–17·8)	15·3<br>(9·46–22·7)
Exposure to mechanical forces	11·3<br>(0·123–23·6)	15·8<br>(12·9–18·3)	17·4<br>(14·9–19·6)	0·216<br>(-11·9–13·1)
Animal contact	21·4<br>(16·3–27·9)	20·3<br>(15·4–26·1)	21<br>(15·8–26·9)	18·4<br>(13·6–24·4)
Foreign body	2·37<br>(1·47–3·55)	3·23<br>(2·07–4·78)	2·56<br>(1·65–3·82)	3·52<br>(2·25–5·21)
Environmental heat and cold exposure	8·07<br>(3·19–14·6)	9·31<br>(3·98–16·7)	9·27<br>(3·96–16·7)	9·4<br>(4·02–16·6)
Exposure to forces of nature	0<br>(0–0)	10·1<br>(4·44–18·1)	10·1<br>(4·44–18·1)	0<br>(0–0)
Other unintentional injuries	12·8<br>(7·47–19·6)	11·8<br>(6·79–18·4)	11·7<br>(6·7–18·3)	14·3<br>(8·76–21·5)
Self-harm and interpersonal violence	22·3<br>(7·2–36)	24·6<br>(10·7–37·3)	33·2<br>(24·7–42·2)	23·4<br>(8·51–36·9)
Self-harm	22·1<br>(6·63–36·3)	23·4<br>(7·93–37·5)	22·6<br>(8·55–35·7)	23·4<br>(7·9–37·6)
Interpersonal violence	28·1<br>(18·7–37·9)	34·1<br>(25·5–43·3)	39·1<br>(30·1–48·7)	25<br>(16·1–34·7)

Supplementary Results Table 2. All-age PAFs attributable to the joint distribution of all risk factors for all ages, both sexes combined for each GBD level 1, level 2, and level 3 cause and all causes in 2019 for Cambridgeshire. Results are shown for deaths, YLLs, YLDs, and DALYs Data in parenthesis are 95% uncertainty intervals. PAFs=population attributable fractions. GBD=Global Burden of Disease. YLLs=years of life lost. YLDs=years lived with disability. DALYs=disability-adjusted life-years				
	Deaths 2019	DALYs 2019	YLDs 2019	YLLs 2019
All causes	54·4 (51·1–57·7)	41·3 (38·3–44·4)	30 (27·5–32·3)	54 (51·3–56·8)
Communicable, maternal, neonatal, and nutritional diseases	44·3 (35·4–52·9)	51·4 (46·8–56·1)	48 (42·9–52·9)	53·4 (46·1–60·3)
HIV/AIDS and sexually transmitted infections	75·5 (59·2–88·1)	75·8 (58–88·8)	97·7 (96·6–98·7)	56·5 (36·9–76·1)
HIV/AIDS	92·5 (90·5–93·7)	91·7 (89·4–93·3)	94·9 (93–96·3)	89·2 (86·8–90·6)
Sexually transmitted infections excluding HIV	55·7 (32·6–79·5)	61·7 (36·7–85·5)	100 (100–100)	22 (9·15–43·9)
Respiratory infections and tuberculosis	45·5 (35·7–55·3)	41·4 (33·3–49·7)	3·09 (2·29–4·16)	48·8 (39·6–58)
Tuberculosis	57·7 (45·1–68·8)	60·5 (49·2–70·6)	58·2 (46·4–68·4)	61·1 (49·6–71·2)
Lower respiratory infections	45·4 (35·5–55·3)	48·6 (39·3–57·9)	34·6 (27·5–42·1)	48·8 (39·4–58·1)
Upper respiratory infections	0·0461 (0·0252–0·0816)	0·0069 (0·00123–0·0168)	0·00584 (0·000213–0·016)	0·117 (0·0643–0·202)
Otitis media	2·21 (1·04–4·5)	4·2 (2·64–6·02)	4·16 (2·6–5·96)	5·51 (3–9·57)
Enteric infections	4·54 (2·7–7·7)	7·94 (5·89–11)	8·57 (6·28–11·9)	6·64 (4·54–9·61)
Diarrheal diseases	4·57 (2·71–7·72)	7·97 (5·9–11·1)	8·58 (6·28–11·9)	6·71 (4·61–9·74)
Other infectious diseases	0·527 (0·354–0·739)	1·08 (0·788–1·39)	0·46 (0·269–0·777)	1·36 (0·945–1·83)
Meningitis	1·49 (1·04–2·06)	2·65 (1·87–3·62)	0·000546 (1·48e-05–0·00163)	3·49 (2·51–4·72)
Encephalitis	0·265 (0·167–0·399)	0·699 (0·42–1·07)	0·000176 (5e-06–0·000495)	0·965 (0·601–1·46)
Measles	2·46 (-0·745–12·1)	2·32 (-0·518–10·9)	0·448 (0·0118–1·21)	3·28 (-0·993–15·8)
Acute hepatitis	8·57 (3·23–21·8)	4·33 (2·27–8·13)	2·97 (1·74–4·8)	8·21 (3·14–20·5)
Maternal and neonatal disorders	90·1 (88·3–91·6)	81·7 (78·7–84·3)	72·2 (67·5–76·5)	90·7 (89–92·2)
Maternal disorders	9·09 (3·31–15·1)	9·2 (3·35–15·5)	9·32 (3·37–15·7)	8·93 (3·26–14·8)
Neonatal disorders	91·7 (90·1–93·2)	83·3 (80·3–85·9)	74·2 (69·5–78·7)	91·7 (90·1–93·2)
Nutritional deficiencies	81 (76·1–90·8)	76·7 (72·4–81·5)	76·5 (72·2–81·5)	80·6 (72·8–85·1)
Protein-energy malnutrition	100 (100–100)	100 (100–100)	100 (100–100)	100 (100–100)
Vitamin A deficiency	--	100 (100–100)	100 (100–100)	--
Dietary iron deficiency	--	100 (100–100)	100 (100–100)	--
Non-communicable diseases	56 (52·3–59·6)	41·7 (38·5–45·2)	29·2 (26·6–31·9)	56·1 (53·1–59)
Neoplasms	47·5 (43·3–52·1)	46·8 (42·9–51)	32 (27·8–36·8)	47·8 (44–52·1)
Lip and oral cavity cancer	66·8 (54·8–76·9)	70·2 (59–79·2)	69·9 (57·6–79)	70·3 (59–79·3)
Nasopharynx cancer	65·5 (52–76·6)	66·1 (53·4–76·3)	63·7 (50·6–74·7)	66·3 (53·7–76·5)
Other pharynx cancer	79 (70·6–85·6)	80·7 (72·6–86·8)	80·6 (72·4–86·6)	80·7 (72·6–86·8)
Esophageal cancer	76·4 (65·3–85·4)	78·1 (67·6–86·3)	77·2 (66·6–85·9)	78·1 (67·6–86·3)
Stomach cancer	25·1 (17·6–42·2)	25·8 (18·4–42·5)	26·4 (18·6–43·2)	25·8 (18·4–42·5)

Colon and rectum cancer	65·7 (56·1–75·1)	66·2 (57·2–75·2)	66·5 (57·5–75·6)	66·2 (57·2–75·1)
Liver cancer	77·4 (71·1–83·2)	78·3 (72·6–83·4)	79·1 (73·2–84·3)	78·3 (72·6–83·4)
Gallbladder and biliary tract cancer	24·2 (13·2–37·3)	24·7 (13·7–37·6)	22·7 (11·9–36·1)	24·8 (13·8–37·7)
Pancreatic cancer	44·9 (34·9–56·7)	44·7 (35·2–55·9)	44·9 (34·8–56·4)	44·7 (35·2–55·9)
Larynx cancer	81·4 (72·6–88·3)	81·6 (73·4–87·8)	79·4 (70·9–86·1)	81·9 (73·8–88)
Tracheal, bronchus, and lung cancer	86·7 (83·1–90)	86·1 (82·5–89·4)	86·7 (83–90·1)	86 (82·5–89·3)
Breast cancer	40 (29·1–52·1)	39 (29·1–49·6)	39·2 (29·4–49·7)	39 (29·1–49·5)
Cervical cancer	100 (100–100)	100 (100–100)	100 (100–100)	100 (100–100)
Uterine cancer	47·8 (31·5–64·8)	49 (33·1–65·2)	49·4 (33·7–65·5)	48·9 (33·1–65·1)
Ovarian cancer	29·2 (14·7–44·3)	24·9 (12·3–38·9)	23·3 (11·6–36·6)	25 (12·3–38·9)
Prostate cancer	6·02 (2·51–9·87)	6·65 (2·85–10·7)	7·15 (3·11–11·5)	6·56 (2·82–10·6)
Kidney cancer	41·9 (30·8–53)	42·5 (31·5–52·9)	42·4 (31·7–52·7)	42·5 (31·5–52·9)
Bladder cancer	40·8 (25·9–56)	43·4 (29·1–57·3)	44·5 (30·4–58)	43·4 (29–57·3)
Thyroid cancer	16·9 (8·25–26·9)	17·7 (8·66–28·2)	18·1 (8·66–29·5)	17·6 (8·68–28·1)
Mesothelioma	99 (98·6–99·3)	97·9 (97·2–98·5)	98·9 (98·4–99·3)	97·9 (97·2–98·5)
Non-Hodgkin lymphoma	8·45 (3·61–15)	8·71 (3·76–15·3)	8·61 (3·65–15·1)	8·72 (3·79–15·3)
Multiple myeloma	9·49 (4·11–16·8)	9·85 (4·35–17·3)	9·8 (4·35–17·2)	9·85 (4·35–17·3)
Leukemia	38·7 (24·3–53)	37·4 (25·2–49·7)	36·4 (24·3–48·4)	37·4 (25·2–49·8)
Cardiovascular diseases	78·2 (73·9–82·3)	79·3 (76·1–82·3)	71·1 (66·1–75·9)	80·5 (77·3–83·5)
Rheumatic heart disease	22·3 (12·3–43·6)	23·3 (13·9–39·4)	23·4 (14·2–38·1)	23·3 (13·9–39·7)
Ischemic heart disease	93·6 (89·6–96·8)	94·7 (91·9–97·1)	94·1 (91·1–96·8)	94·7 (91·9–97·2)
Stroke	79·3 (70·8–88·8)	82·3 (76–89·2)	82·5 (76·7–88·6)	82·3 (75·8–89·6)
Hypertensive heart disease	100 (100–100)	100 (100–100)	100 (100–100)	100 (100–100)
Non-rheumatic valvular heart disease	25·6 (16–39·9)	25·6 (17·6–36·3)	16·2 (10·6–22·9)	27 (18·4–38·3)
Cardiomyopathy and myocarditis	36·6 (28·5–46·7)	43·7 (36·9–50·2)	37·4 (30·7–44·3)	44·1 (37–50·6)
Atrial fibrillation and flutter	52·2 (41·1–63·1)	59·7 (50·7–68·5)	63·7 (55–71·7)	54·9 (44·6–64·7)
Aortic aneurysm	49·5 (42·7–57·1)	56·6 (50·8–62·6)	--	56·6 (50·8–62·6)
Endocarditis	30·7 (20·3–44·7)	33·4 (24–44·3)	30·6 (20·2–44·5)	33·5 (24·1–44·4)
Other cardiovascular and circulatory diseases	30·8 (24·1–38·1)	33·7 (27·7–39·9)	34·2 (26·8–41·9)	33·6 (27·4–39·9)
Chronic respiratory diseases	62·1 (56–68·6)	58·6 (54·1–63·4)	53·7 (48·6–58·9)	63 (57·4–68·9)
Chronic obstructive pulmonary disease	72·1 (67·3–77)	71·2 (67·4–75·2)	65·4 (61·7–69·2)	74·4 (70·1–78·5)
Pneumoconiosis	100 (100–100)	100 (100–100)	100 (100–100)	100 (100–100)
Asthma	44·7 (31·9–58·2)	45 (36–54·9)	44·8 (35·8–54·3)	47·3 (35·9–58·9)
Digestive diseases	26·1 (21·2–31)	32·4 (26·7–37·7)	24·3 (16·8–32·7)	37·4 (31·2–43·5)

Cirrhosis and other chronic liver diseases	73 (59·5–86·4)	76·4 (63·7–88·1)	74·4 (61·9–85·3)	76·5 (63·6–88·3)
Upper digestive system diseases	20·3 (16·7–23·9)	8·05 (4·99–11·6)	0·728 (0·417–1·18)	23·5 (19·5–27)
Gallbladder and biliary diseases	47·7 (31·2–63·7)	51·6 (36·5–66)	52·3 (37·4–66·6)	49 (33–64·5)
Pancreatitis	37·7 (16·5–61·1)	43·8 (21·7–64·7)	44·6 (22·5–65·2)	43·7 (21·8–64·9)
Neurological disorders	27·6 (12·2–44·1)	17 (8·38–29·9)	10·6 (5·09–20·4)	24·3 (11–40·4)
Alzheimer's disease and other dementias	43·2 (28·2–59·7)	45·3 (30·7–61·5)	45·7 (31·1–61·7)	45·2 (30·5–61·2)
Parkinson's disease	-9·08 (-15·2–-3·98)	-10·4 (-17·3–-4·57)	-11·1 (-18·3–-4·93)	-10·3 (-17–-4·49)
Idiopathic epilepsy	28 (16·7–38·7)	26·6 (16·1–36·3)	24·7 (14·6–34·1)	29·6 (18·3–39·7)
Multiple sclerosis	14·1 (10·5–18·1)	15·4 (11·6–19·2)	15·3 (11·7–19·1)	15·4 (11·6–19·3)
Mental disorders	--	6·37 (3·17–9·84)	6·37 (3·17–9·84)	--
Depressive disorders	--	13·3 (6·11–20·8)	13·3 (6·11–20·8)	--
Anxiety disorders	--	4·84 (1·25–10·9)	4·84 (1·25–10·9)	--
Idiopathic developmental intellectual disability	--	33·8 (14·4–52·3)	33·8 (14·4–52·3)	--
Substance use disorders	100 (100–100)	100 (100–100)	100 (100–100)	100 (100–100)
Alcohol use disorders	100 (100–100)	100 (100–100)	100 (100–100)	100 (100–100)
Drug use disorders	100 (100–100)	100 (100–100)	100 (100–100)	100 (100–100)
Diabetes and kidney diseases	100 (100–100)	100 (100–100)	100 (100–100)	100 (100–100)
Diabetes mellitus	100 (100–100)	100 (100–100)	100 (100–100)	100 (100–100)
Chronic kidney disease	100 (100–100)	100 (100–100)	100 (100–100)	100 (100–100)
Sense organ diseases	--	8·71 (7·52–10·1)	8·71 (7·52–10·1)	--
Blindness and vision loss	--	10·3 (6·65–15)	10·3 (6·65–15)	--
Age-related and other hearing loss	--	9·55 (8·57–10·6)	9·55 (8·57–10·6)	--
Musculoskeletal disorders	3·77 (0·914–6·99)	24 (20–28·1)	24·4 (20·3–28·7)	4·69 (1·15–8·5)
Rheumatoid arthritis	10·5 (2·57–19·6)	13·3 (3·28–23·5)	13·6 (3·36–24)	12·3 (3·16–22·5)
Osteoarthritis	--	19·2 (10·4–28·8)	19·2 (10·4–28·8)	--
Low back pain	--	43·4 (37·8–48·7)	43·4 (37·8–48·7)	--
Gout	--	55·7 (39·6–73)	55·7 (39·6–73)	--
Other non-communicable diseases	0·0678 (0·0422–0·101)	0·0592 (0·0349–0·0934)	--	0·287 (0·172–0·417)
Sudden infant death syndrome	8·36 (4·66–13·9)	8·35 (4·65–13·9)	--	8·35 (4·65–13·9)
Injuries	35·2 (29·3–40·3)	28·7 (24·1–33·2)	30·9 (26·6–35·3)	25·6 (18·1–31·9)
Transport injuries	22·2 (15–29)	22·2 (15·5–28·4)	26·7 (22·2–31·3)	20 (11·7–27·5)
Road injuries	21·6 (14·1–28·5)	21·3 (14·4–27·7)	25·6 (21·1–30·3)	19·3 (10·7–27·1)
Other transport injuries	33·3 (27·7–38·7)	32·7 (27·6–38)	36·1 (31·6–40·4)	29·8 (23·5–35·8)
Unintentional injuries	42·9 (36·5–47·5)	30·8 (26·2–35·4)	31·2 (26·5–36)	29·5 (24·8–34·1)

Falls	57·3 (49·4–62·6)	42·6 (36·9–47·6)	40·7 (35·1–45·9)	49·1 (42·8–54·3)
Drowning	-14·6 (-26·9–-2·21)	-12·8 (-24·5–-1·46)	10·9 (5·64–17·7)	-14·1 (-26·4–-2·29)
Fire, heat, and hot substances	10·6 (5·48–17·8)	13·2 (7·63–20·4)	13·3 (7·71–20·5)	12·6 (7·15–19·6)
Poisonings	14·1 (8·14–21·8)	13·2 (7·7–19·9)	12·3 (6·92–19)	15 (9·04–22·5)
Exposure to mechanical forces	13·6 (4·44–23·1)	16·8 (14·2–19·4)	17·9 (15·5–20·3)	1·97 (-7·5–12·1)
Animal contact	21·3 (16·1–28·5)	20·9 (15·8–27)	21·9 (16·6–27·8)	19·2 (13·9–25·9)
Foreign body	2·07 (1·36–3·2)	3·23 (2·1–4·82)	2·77 (1·76–4·19)	3·53 (2·31–5·38)
Environmental heat and cold exposure	8·63 (3·58–15·9)	9·96 (4·36–17·8)	9·97 (4·39–17·7)	9·92 (4·33–17·8)
Exposure to forces of nature	0 (0–0)	10·6 (4·75–18·8)	10·6 (4·75–18·8)	0 (0–0)
Other unintentional injuries	13·3 (7·56–20·7)	12·7 (7·36–19·8)	12·6 (7·3–19·7)	15·2 (9·16–22·6)
Self-harm and interpersonal violence	24 (9·44–38)	26·7 (13·4–39·5)	34 (25·7–42·2)	25·3 (10·6–39·1)
Self-harm	23·8 (8·7–38·2)	25·2 (10–39·4)	24·3 (10·4–37·3)	25·2 (10–39·6)
Interpersonal violence	32 (23–41·2)	36·8 (28–46)	39·1 (30·2–48·3)	29·1 (20·2–38·6)

Supplementary Results Table 2. All-age PAFs attributable to the joint distribution of all risk factors for all ages, both sexes combined for each GBD level 1, level 2, and level 3 cause and all causes in 2019 for Central Bedfordshire. Results are shown for deaths, YLLs, YLDs, and DALYs Data in parenthesis are 95% uncertainty intervals. PAFs=population attributable fractions. GBD=Global Burden of Disease. YLLs=years of life lost. YLDs=years lived with disability. DALYs=disability-adjusted life-years

	Deaths 2019	DALYs 2019	YLDs 2019	YLLs 2019
All causes	53·8 (50·5–57·2)	40·5 (37·6–43·7)	29·3 (27–31·7)	53·2 (50·3–56)
Communicable, maternal, neonatal, and nutritional diseases	43·7 (33·6–53·9)	48·3 (42·7–53·7)	43·9 (39·2–48·8)	50·7 (42·6–58·7)
HIV/AIDS and sexually transmitted infections	71·4 (53·9–85·9)	73·6 (54–88)	98 (97–98·8)	51·6 (32–72·7)
HIV/AIDS	91·9 (90·1–93·1)	91·1 (88·9–92·9)	95·2 (93·2–96·4)	87·8 (85·7–89·3)
Sexually transmitted infections excluding HIV	47·6 (25·8–73·6)	59·8 (34·3–84·2)	100 (100–100)	17·9 (7·03–37·8)
Respiratory infections and tuberculosis	45·1 (34–56·3)	41·3 (31·7–50·7)	3·08 (2·26–4·05)	48·4 (37·5–59·1)
Tuberculosis	54·4 (41·5–66·5)	57·1 (44·5–67·7)	55·7 (42·9–66·9)	57·4 (45–68)
Lower respiratory infections	45·1 (33·9–56·3)	48·3 (37·3–59·2)	34 (26·9–41·1)	48·4 (37·4–59·3)
Upper respiratory infections	0·0397 (0·0211–0·0721)	0·00774 (0·00186–0·0173)	0·00576 (0·000125–0·0152)	0·0895 (0·0472–0·16)
Otitis media	0·656 (0·37–1·07)	4·22 (2·66–6·16)	4·22 (2·66–6·16)	2·34 (1·4–3·57)
Enteric infections	5·19 (3·08–9·16)	8·83 (6·36–13)	9·28 (6·65–13·6)	7·79 (5·25–11·9)
Diarrheal diseases	5·24 (3·13–9·2)	8·87 (6·43–13·1)	9·29 (6·66–13·6)	7·91 (5·39–12)
Other infectious diseases	0·46 (0·31–0·76)	0·837 (0·629–1·11)	0·39 (0·217–0·672)	1·03 (0·763–1·38)
Meningitis	1·15 (0·773–1·56)	1·87 (1·29–2·51)	0·000426 (6·56e-06–0·00121)	2·46 (1·68–3·29)
Encephalitis	0·222 (0·149–0·319)	0·478 (0·282–0·71)	0·000139 (2·07e-06–0·000441)	0·767 (0·514–1·09)
Measles	3·57 (-1·08–16·7)	3·77 (-0·931–17·4)	0·58 (0·0184–1·58)	4·6 (-1·42–21·8)
Acute hepatitis	8·6 (2·7–22·8)	4·52 (2·25–8·85)	2·95 (1·69–4·98)	8·03 (2·61–20·9)
Maternal and neonatal disorders	86·2 (83·9–88·2)	76·2 (72·3–79·6)	63·8 (58·2–68·9)	87·4 (85·4–89·3)
Maternal disorders	9·2 (3·28–15·2)	9·26 (3·28–15·5)	9·42 (3·3–15·9)	9·06 (3·22–15)
Neonatal disorders	89·7 (87·9–91·3)	78·5 (74·6–82·1)	66·1 (59·9–71·3)	89·7 (87·9–91·3)
Nutritional deficiencies	61 (54·7–85·8)	76·5 (72·2–81·2)	76·8 (72·5–81·5)	62·4 (57·5–80·2)
Protein-energy malnutrition	100 (100–100)	100 (100–100)	100 (100–100)	100 (100–100)
Vitamin A deficiency	--	100 (100–100)	100 (100–100)	--
Dietary iron deficiency	--	100 (100–100)	100 (100–100)	--
Non-communicable diseases	55·6 (52–59·1)	41·1 (37·9–44·5)	28·7 (26·1–31·3)	55·3 (52·3–58·4)
Neoplasms	47·1 (43·1–51·5)	46·2 (42·3–50·4)	33 (28·5–37·9)	47·1 (43·2–51·3)
Lip and oral cavity cancer	64·6 (52·3–75·6)	67·8 (56·1–77·6)	67·4 (55·3–77·7)	67·8 (56·1–77·7)
Nasopharynx cancer	64·9 (51·1–76·3)	64·7 (51·5–75·3)	62·4 (48·3–74·1)	64·9 (51·8–75·6)
Other pharynx cancer	78·1 (69·5–85)	79·6 (71·3–86·1)	79·4 (70·9–86·1)	79·6 (71·4–86·1)
Esophageal cancer	75·5 (65·2–84·4)	77·2 (67·4–85·5)	76·3 (66·1–85)	77·2 (67·4–85·6)
Stomach cancer	25·1 (17·4–42)	25·7 (18–42·2)	26·2 (18·3–43)	25·7 (18–42·2)

Colon and rectum cancer	64·8 (55·2–73·9)	65·1 (56·2–73·9)	65·4 (56·4–74·1)	65·1 (56·1–73·9)
Liver cancer	77·5 (71·2–83·2)	77·7 (72–83)	78·6 (73–83·6)	77·7 (72–83)
Gallbladder and biliary tract cancer	23·5 (13–35·9)	24·2 (13·4–36·6)	22·2 (11·9–34·9)	24·3 (13·4–36·7)
Pancreatic cancer	44 (34·3–55·4)	43·9 (34·9–54·6)	44 (34·1–55·1)	43·9 (34·9–54·6)
Larynx cancer	80·7 (72–87·8)	80·7 (73–87)	78·6 (70·7–85·5)	81 (73·3–87·3)
Tracheal, bronchus, and lung cancer	86·4 (82·7–90)	85·5 (81·9–89·1)	86·2 (82·5–89·6)	85·5 (81·9–89·1)
Breast cancer	38·3 (28·2–49·7)	37·1 (27·7–47·2)	37·3 (28–47·5)	37 (27·6–47·2)
Cervical cancer	100 (100–100)	100 (100–100)	100 (100–100)	100 (100–100)
Uterine cancer	45·8 (29·9–61·6)	46·9 (31·3–62·9)	47·4 (31·5–63·1)	46·9 (31·2–62·8)
Ovarian cancer	28·4 (13·8–43·4)	23·9 (11·3–37·7)	22·2 (10·7–35·2)	24 (11·3–37·8)
Prostate cancer	5·98 (2·49–9·89)	6·6 (2·87–10·6)	7·1 (3·16–11·1)	6·51 (2·81–10·4)
Kidney cancer	40·6 (30·1–51·3)	41 (30·6–51·1)	41 (30·4–51·1)	41 (30·7–51·1)
Bladder cancer	40 (25·4–54·4)	42·6 (28·5–55·7)	43·6 (30–56·3)	42·5 (28·4–55·6)
Thyroid cancer	15 (7·52–24·1)	15·6 (7·92–24·8)	16 (8·17–25·6)	15·6 (7·87–24·8)
Mesothelioma	98·9 (98·5–99·2)	97·7 (97–98·4)	98·8 (98·4–99·2)	97·7 (96·9–98·4)
Non-Hodgkin lymphoma	7·93 (3·29–13·8)	8·11 (3·43–14·2)	8·06 (3·35–14·1)	8·11 (3·43–14·2)
Multiple myeloma	8·83 (3·81–15·7)	9·19 (4·04–16·3)	9·14 (3·89–16·2)	9·19 (4–16·3)
Leukemia	38 (24·1–52·8)	35·7 (23·8–47·2)	34·9 (22·9–46·7)	35·7 (23·9–47·5)
Cardiovascular diseases	78·4 (74·4–82·3)	79·4 (75·9–82·6)	71 (65·8–75·6)	80·7 (77·4–83·8)
Rheumatic heart disease	22 (11·9–41·4)	22·8 (13·9–37·4)	22·9 (13·9–36·6)	22·8 (13·9–37·5)
Ischemic heart disease	93·4 (89·5–96·6)	94·6 (91·7–97)	94 (91·1–96·6)	94·6 (91·7–97)
Stroke	79·2 (70·5–88·4)	82·3 (76–88·7)	82·4 (76·9–88·1)	82·3 (75·7–88·8)
Hypertensive heart disease	100 (100–100)	100 (100–100)	100 (100–100)	100 (100–100)
Non-rheumatic valvular heart disease	25 (15·8–38·2)	25·7 (17·8–35·9)	20·4 (13·5–28·2)	26·3 (18·2–36·9)
Cardiomyopathy and myocarditis	34·7 (27–43·9)	39·7 (33·5–45·7)	34 (27·2–41)	40·1 (33·6–46·1)
Atrial fibrillation and flutter	50·5 (39·6–62·1)	58·7 (49·6–67·8)	62·4 (53·7–70·8)	53·4 (43·7–63·9)
Aortic aneurysm	50·4 (43·6–57·6)	57·4 (51·3–63·4)	--	57·4 (51·3–63·4)
Endocarditis	30·5 (20·5–43·5)	33·1 (24·2–44·1)	30·3 (19·8–42·9)	33·2 (24·3–44·1)
Other cardiovascular and circulatory diseases	31 (24·3–38)	33·4 (27·4–39·6)	33·4 (26·5–40·4)	33·3 (27·3–39·7)
Chronic respiratory diseases	62·9 (56·7–69·4)	58·6 (54·1–63·4)	53·3 (48–58·6)	63·5 (58–69·1)
Chronic obstructive pulmonary disease	71·9 (66·5–77·1)	71·1 (67·1–75·3)	65·9 (62·2–69·7)	74·1 (69·4–78·7)
Pneumoconiosis	100 (100–100)	100 (100–100)	100 (100–100)	100 (100–100)
Asthma	43·4 (30·7–56·2)	43·5 (34·5–53·2)	43·3 (34·5–53·1)	44·9 (34·1–56·2)
Digestive diseases	26·8 (21·8–32·2)	30·9 (25·3–36·7)	17·6 (12·1–24·3)	37·6 (31·2–44·6)
Cirrhosis and other chronic liver diseases	71·3 (58·9–85·9)	74·2 (62·1–87·8)	71·9 (59·6–84·4)	74·3 (62·2–87·9)

Upper digestive system diseases	20·1 (16·7–23·5)	7·97 (4·98–11·2)	0·644 (0·363–1·05)	22·8 (19·3–26·3)
Gallbladder and biliary diseases	45·6 (29·3–61·2)	49·5 (34·8–64)	50·5 (36·4–64·7)	47·1 (31·4–62·3)
Pancreatitis	34·6 (12·6–56·6)	39·9 (16·7–60·4)	39·8 (16·1–60·2)	39·9 (16·7–60·5)
Neurological disorders	26·5 (11·7–42·8)	16·2 (7·96–28·7)	10·1 (5–19·5)	23·5 (10·6–39·3)
Alzheimer's disease and other dementias	42·1 (27·1–58·1)	44·2 (29·4–59·7)	44·5 (29·5–59·9)	44 (29·3–59·6)
Parkinson's disease	-9 (-15·3–-3·97)	-10·4 (-17·1–-4·67)	-11·1 (-17·9–-4·99)	-10·1 (-16·9–-4·53)
Idiopathic epilepsy	25 (13·2–36·3)	23·9 (13·1–34)	22·3 (12·2–32·2)	25·8 (14·5–36·5)
Multiple sclerosis	14·3 (10·5–18·4)	15·5 (11·6–19·5)	15·6 (11·7–19·6)	15·5 (11·5–19·6)
Mental disorders	--	6·38 (3·24–9·73)	6·38 (3·24–9·74)	--
Depressive disorders	--	13·2 (6·06–20·5)	13·2 (6·06–20·5)	--
Anxiety disorders	--	4·64 (1·23–10·2)	4·64 (1·23–10·2)	--
Idiopathic developmental intellectual disability	--	37 (16·5–55·6)	37 (16·5–55·6)	--
Substance use disorders	100 (100–100)	100 (100–100)	100 (100–100)	100 (100–100)
Alcohol use disorders	100 (100–100)	100 (100–100)	100 (100–100)	100 (100–100)
Drug use disorders	100 (100–100)	100 (100–100)	100 (100–100)	100 (100–100)
Diabetes and kidney diseases	99·8 (99·7–99·8)	100 (99·9–100)	100 (100–100)	99·8 (99·7–99·8)
Diabetes mellitus	100 (100–100)	100 (100–100)	100 (100–100)	100 (100–100)
Chronic kidney disease	100 (100–100)	100 (100–100)	100 (100–100)	100 (100–100)
Sense organ diseases	--	8·72 (7·57–10·2)	8·72 (7·57–10·2)	--
Blindness and vision loss	--	9·83 (6·3–14·3)	9·83 (6·3–14·3)	--
Age-related and other hearing loss	--	9·74 (8·73–10·9)	9·74 (8·73–10·9)	--
Musculoskeletal disorders	4·05 (1·01–7·57)	23·7 (19·6–27·7)	24·1 (19·9–28·2)	4·91 (1·24–8·81)
Rheumatoid arthritis	10·5 (2·57–18·9)	13·3 (3·33–23·6)	13·7 (3·45–24·1)	12·2 (3·02–21·8)
Osteoarthritis	--	18·1 (9·84–27·6)	18·1 (9·84–27·6)	--
Low back pain	--	43·3 (37·9–49)	43·3 (37·9–49)	--
Gout	--	53·9 (38–70·8)	53·9 (38–70·8)	--
Other non-communicable diseases	0·0581 (0·0351–0·088)	0·0561 (0·0325–0·0886)	--	0·246 (0·143–0·362)
Sudden infant death syndrome	6·63 (3·8–10·9)	6·63 (3·79–10·9)	--	6·63 (3·79–10·9)
Injuries	33 (26·9–38·3)	27·7 (22·7–32·3)	30·5 (26·1–34·9)	23·7 (16·6–30·5)
Transport injuries	21 (13·1–29·1)	21·3 (14·3–28·3)	26·7 (22·1–31·5)	18·6 (9·63–27·4)
Road injuries	20·1 (11·7–28·6)	20·3 (13·1–27·6)	25·6 (21·2–30·4)	17·7 (8·46–27·1)
Other transport injuries	32·5 (26·9–38)	31·6 (26·2–37·1)	36·1 (31·6–40·6)	28·4 (22·1–34·4)
Unintentional injuries	40·3 (34–44·7)	29·9 (25·4–34·6)	30·8 (26·1–35·7)	27·4 (22·6–31·6)
Falls	55·5 (47–60·8)	41·6 (35·5–46·9)	40·1 (34·3–45·4)	47·1 (40·2–52·4)
Drowning	-14·5 (-28–-0·265)	-13·7 (-26·3–-0·535)	10·8 (5·88–17·5)	-15·5 (-28·7–-1·71)

Fire, heat, and hot substances	9·88 (5·04–16·3)	13 (7·74–19·9)	13·4 (8·03–20·6)	11·3 (6·58–17·7)
Poisonings	13·1 (7·71–20·1)	12·7 (7·72–19·1)	12·1 (7·35–18·5)	13·6 (8·32–20·4)
Exposure to mechanical forces	10·8 (-0·655–22·4)	17·6 (14·5–20·6)	19 (16·2–21·8)	0·529 (-11·7–13·3)
Animal contact	25·3 (20·3–31)	21·6 (16·6–27·3)	22·2 (17–28·2)	19·5 (15·3–25·1)
Foreign body	2·47 (1·57–3·74)	3·62 (2·39–5·45)	3·41 (2·23–5·14)	3·75 (2·41–5·69)
Environmental heat and cold exposure	8·16 (3·35–14·7)	9·29 (3·92–16·4)	9·3 (3·89–16·6)	9·27 (4·02–16·5)
Exposure to forces of nature	0 (0–0)	10·1 (4·35–18)	10·1 (4·35–18)	0 (0–0)
Other unintentional injuries	13·8 (8·21–21·3)	12·8 (7·63–19·5)	12·7 (7·58–19·4)	14·9 (9·22–22·2)
Self-harm and interpersonal violence	22 (7·19–35·4)	24·8 (11·5–36·7)	32·7 (24·2–41)	23·2 (8·51–36·3)
Self-harm	21·7 (6·24–35·6)	23·1 (8·02–36·6)	22·7 (8·65–36·1)	23·1 (7·98–36·8)
Interpersonal violence	29·8 (20·3–39·6)	34·7 (26·3–43·9)	37·9 (28·7–47·1)	25·9 (16·7–35·1)

Supplementary Results Table 2. All-age PAFs attributable to the joint distribution of all risk factors for all ages, both sexes combined for each GBD level 1, level 2, and level 3 cause and all causes in 2019 for Essex. Results are shown for deaths, YLLs, YLDs, and DALYs Data in parenthesis are 95% uncertainty intervals. PAFs=population attributable fractions. GBD=Global Burden of Disease. YLLs=years of life lost. YLDs=years lived with disability. DALYs=disability-adjusted life-years

	Deaths 2019	DALYs 2019	YLDs 2019	YLLs 2019
All causes	54·3 (51·1–57·6)	41·7 (38·7–44·7)	28·9 (26·5–31·2)	54·1 (51·4–56·9)
Communicable, maternal, neonatal, and nutritional diseases	43·9 (35·2–52·9)	49·7 (44·7–54·7)	45·6 (41·2–50·2)	51·7 (44·3–58·6)
HIV/AIDS and sexually transmitted infections	78·9 (64–89·5)	78·2 (62·7–89·8)	97·6 (96·5–98·5)	61·1 (41–79·2)
HIV/AIDS	92·9 (91·2–94)	92·1 (90·1–93·6)	95·1 (93·2–96·3)	89·7 (87·6–91·1)
Sexually transmitted infections excluding HIV	60·1 (36·2–82·3)	63·8 (39·3–86·4)	100 (100–100)	25·4 (10·6–48·6)
Respiratory infections and tuberculosis	44·7 (35·1–54·6)	42·1 (33·8–50·5)	3·29 (2·42–4·35)	47·8 (38·5–57·1)
Tuberculosis	54 (40·6–65·3)	57·1 (45·3–67·6)	55 (43–66)	57·5 (45·6–68)
Lower respiratory infections	44·6 (35–54·6)	47·6 (38·3–57)	33·4 (26·3–40·3)	47·7 (38·4–57·1)
Upper respiratory infections	0·0457 (0·0253–0·0795)	0·00801 (0·00151–0·019)	0·00656 (0·000297–0·0177)	0·12 (0·067–0·206)
Otitis media	1·8 (0·862–3·38)	4·12 (2·62–5·82)	4·1 (2·6–5·82)	4·95 (2·64–8·21)
Enteric infections	5·17 (3–8·8)	8·76 (6·37–12·6)	9·34 (6·78–13·3)	7·18 (4·75–10·8)
Diarrheal diseases	5·24 (3·04–8·88)	8·81 (6·4–12·6)	9·35 (6·79–13·3)	7·32 (4·92–11·1)
Other infectious diseases	0·512 (0·349–0·689)	1·07 (0·797–1·34)	0·472 (0·272–0·777)	1·29 (0·949–1·65)
Meningitis	1·31 (0·936–1·71)	2·46 (1·79–3·18)	0·000631 (2·19e-05–0·00174)	3·16 (2·32–4·07)
Encephalitis	0·256 (0·169–0·375)	0·782 (0·473–1·17)	0·000215 (6·5e-06–0·000612)	0·961 (0·62–1·42)
Measles	2·79 (-0·72–14)	3·4 (-0·864–16·5)	0·587 (0·0125–1·54)	3·53 (-0·912–17·4)
Acute hepatitis	13·8 (6·9–24·6)	5·41 (3·08–8·73)	3·11 (1·74–5·04)	13 (6·5–23·3)
Maternal and neonatal disorders	88·7 (87–90·3)	75·8 (71·7–79·6)	62·1 (56·2–67·8)	89·6 (88–91·2)
Maternal disorders	9·21 (3·28–15·4)	9·26 (3·28–15·6)	9·41 (3·36–15·9)	9·05 (3·21–15·2)
Neonatal disorders	91·4 (89·8–92·9)	77·6 (73·2–81·6)	63·7 (57·4–69·6)	91·4 (89·8–92·9)
Nutritional deficiencies	71·8 (66·1–88)	76·7 (72·9–81·2)	76·8 (72·9–81·5)	73·1 (67·3–82·9)
Protein-energy malnutrition	100 (100–100)	100 (100–100)	100 (100–100)	100 (100–100)
Vitamin A deficiency	--	100 (100–100)	100 (100–100)	--
Dietary iron deficiency	--	100 (100–100)	100 (100–100)	--
Non-communicable diseases	55·9 (52·4–59·4)	42·2 (39–45·5)	28·2 (25·6–30·8)	55·9 (53–58·9)
Neoplasms	47·7 (43·7–52·1)	47·2 (43·4–51·3)	33·2 (28·9–37·7)	48 (44·3–52·1)
Lip and oral cavity cancer	64·2 (52·6–73·7)	67·8 (56·2–76·6)	67·3 (55·7–76·4)	67·8 (56·3–76·6)
Nasopharynx cancer	64 (49·5–74·3)	64·6 (50·5–74·7)	61·7 (46·8–72·7)	64·9 (50·7–74·9)
Other pharynx cancer	77·3 (68·3–84·1)	79·1 (70·8–85·2)	78·9 (70·4–85·2)	79·1 (70·9–85·2)
Esophageal cancer	74·7 (64·2–83·5)	76·5 (66·4–84·6)	75·5 (65·3–84·1)	76·5 (66·4–84·6)

Stomach cancer	24·4 (17–41·1)	25·1 (17·6–41·3)	25·6 (18–42·3)	25·1 (17·6–41·3)
Colon and rectum cancer	63·6 (54·3–72·8)	64·1 (55·5–72·8)	64·4 (55·9–73)	64 (55·5–72·7)
Liver cancer	75·4 (69–81·4)	76·4 (70·6–81·9)	77·1 (71·5–82·4)	76·4 (70·6–81·9)
Gallbladder and biliary tract cancer	22·7 (12·5–35·8)	23·3 (12·9–36·2)	21·3 (11·3–34·5)	23·4 (12·9–36·5)
Pancreatic cancer	42·7 (33·2–53·9)	42·6 (33·7–53·2)	42·7 (33·4–53·7)	42·6 (33·7–53·2)
Larynx cancer	80·8 (72·1–87·8)	81·2 (73·5–87·5)	79·1 (71·1–85·8)	81·4 (73·8–87·7)
Tracheal, bronchus, and lung cancer	86 (82·6–89·3)	85·5 (82·3–88·7)	86 (82·7–89·3)	85·5 (82·3–88·7)
Breast cancer	37·2 (27·3–48·7)	36·3 (27·4–46·6)	36·6 (27·5–46·9)	36·3 (27·4–46·6)
Cervical cancer	100 (100–100)	100 (100–100)	100 (100–100)	100 (100–100)
Uterine cancer	44·8 (29·3–60·6)	46 (30·7–61·8)	46·5 (30·6–62)	45·9 (30·6–61·7)
Ovarian cancer	29·5 (14·2–44)	25 (11·9–38·3)	23·4 (11·2–36)	25 (11·9–38·3)
Prostate cancer	5·86 (2·48–9·47)	6·46 (2·8–10·3)	6·94 (3·08–11)	6·37 (2·77–10·2)
Kidney cancer	39·7 (28·9–50·7)	40·4 (30–50·5)	40·3 (29·8–50·5)	40·4 (29·9–50·5)
Bladder cancer	38·6 (24·3–53·2)	41·2 (27·5–54·9)	42·4 (29–55·7)	41·2 (27·3–54·9)
Thyroid cancer	15·1 (7·3–24·5)	15·8 (7·62–25·6)	16·3 (7·84–26·9)	15·8 (7·58–25·5)
Mesothelioma	99·3 (99–99·5)	98·5 (98–98·9)	99·2 (98·9–99·5)	98·5 (98–98·9)
Non-Hodgkin lymphoma	7·52 (3·08–13·4)	7·77 (3·28–13·7)	7·7 (3·21–13·6)	7·77 (3·29–13·8)
Multiple myeloma	8·52 (3·54–15·2)	8·87 (3·8–15·7)	8·84 (3·72–16)	8·87 (3·8–15·7)
Leukemia	37·5 (22·6–52·7)	36·2 (23·9–48·7)	35·3 (23·1–47·8)	36·3 (23·9–48·7)
Cardiovascular diseases	78·4 (74·3–82·4)	79·2 (75·9–82·4)	69·9 (64·8–74·6)	80·5 (77·3–83·6)
Rheumatic heart disease	21·8 (12–43·2)	22·7 (14–39·5)	22·7 (14–38·2)	22·7 (13·9–40)
Ischemic heart disease	93·3 (89·2–96·8)	94·3 (91·3–97)	93·8 (90·6–96·5)	94·4 (91·4–97)
Stroke	79 (70·3–88·6)	81·9 (75·4–88·8)	81·6 (75·5–87·7)	82 (75·2–89)
Hypertensive heart disease	100 (100–100)	100 (100–100)	100 (100–100)	100 (100–100)
Non-rheumatic valvular heart disease	24·9 (15·1–39·2)	24·1 (16·3–34·4)	14·2 (9·38–20·4)	25·9 (17·3–37·1)
Cardiomyopathy and myocarditis	33·2 (25·9–42·1)	39·1 (33·6–45·2)	34·3 (27·8–41·4)	39·4 (33·7–45·4)
Atrial fibrillation and flutter	49·7 (39–61·5)	57 (48·3–66·5)	61 (52·4–69·5)	52·4 (42·6–62·8)
Aortic aneurysm	48·7 (41·9–56·6)	55·5 (49·2–61·8)	--	55·5 (49·2–61·8)
Endocarditis	30·2 (20·2–44)	32·8 (24·1–43·7)	30·1 (20·6–43·4)	32·9 (24·2–43·8)
Other cardiovascular and circulatory diseases	30·8 (24·5–37·9)	33·5 (27·6–39·2)	33·8 (27·2–40·9)	33·4 (27·6–39·2)
Chronic respiratory diseases	62·5 (56·4–68·8)	58·9 (54·4–63·5)	53·2 (48·2–58·3)	63·3 (57·7–69·1)
Chronic obstructive pulmonary disease	71·2 (66·4–75·9)	70·8 (67–74·5)	65·2 (61·3–68·8)	73·5 (69–77·6)
Pneumoconiosis	100 (100–100)	100 (100–100)	100 (100–100)	100 (100–100)
Asthma	41·8 (29·3–55·1)	42·6 (33·5–52·5)	42·4 (33·5–52·1)	44·4 (33·5–55·9)

Digestive diseases	25·7 (21·1–30·9)	33·2 (27·5–38·8)	26·8 (18·6–36·2)	37·1 (31–43·7)
Cirrhosis and other chronic liver diseases	69·9 (57·2–84·1)	73·4 (61·4–86·2)	71·3 (58·3–83·9)	73·4 (61·4–86·3)
Upper digestive system diseases	19·8 (16·2–23)	9·04 (5·94–12·6)	0·72 (0·41–1·16)	22·9 (19·3–26·2)
Gallbladder and biliary diseases	44·6 (28·7–60·8)	49·4 (35·2–63·8)	50 (35·8–64·4)	45·9 (30·8–61·6)
Pancreatitis	31·7 (11·5–53·7)	38·1 (16·2–59·1)	39·8 (17·3–60·5)	37·8 (16–59)
Neurological disorders	24·9 (10·7–41·1)	16·3 (7·99–28·6)	10·7 (5·25–20·3)	22·1 (9·78–37·9)
Alzheimer's disease and other dementias	40·4 (25·9–56·3)	42·5 (28·1–58·1)	42·9 (28·6–58·3)	42·3 (28–57·7)
Parkinson's disease	-8·81 (-15·2–-3·89)	-10 (-16·9–-4·55)	-10·6 (-17·4–-4·78)	-9·9 (-16·7–-4·49)
Idiopathic epilepsy	25·2 (14·4–35·9)	24·5 (14–34·1)	22·5 (12·4–31·5)	27 (15·6–37·6)
Multiple sclerosis	13·6 (9·98–17·5)	14·8 (11–18·8)	14·7 (11·1–18·6)	14·8 (10·9–19)
Mental disorders	--	6·36 (3·2–9·83)	6·36 (3·2–9·84)	--
Depressive disorders	--	13·1 (6·04–20·7)	13·1 (6·04–20·7)	--
Anxiety disorders	--	4·66 (1·25–10·5)	4·66 (1·25–10·5)	--
Idiopathic developmental intellectual disability	--	36·4 (15·6–55·9)	36·4 (15·6–55·9)	--
Substance use disorders	100 (100–100)	100 (100–100)	100 (100–100)	100 (100–100)
Alcohol use disorders	100 (100–100)	100 (100–100)	100 (100–100)	100 (100–100)
Drug use disorders	100 (100–100)	100 (100–100)	100 (100–100)	100 (100–100)
Diabetes and kidney diseases	99·7 (99·6–99·7)	99·9 (99·9–99·9)	100 (100–100)	99·7 (99·6–99·7)
Diabetes mellitus	100 (100–100)	100 (100–100)	100 (100–100)	100 (100–100)
Chronic kidney disease	100 (100–100)	100 (100–100)	100 (100–100)	100 (100–100)
Sense organ diseases	--	8·36 (7·19–9·79)	8·36 (7·19–9·79)	--
Blindness and vision loss	--	9·72 (6·21–14·2)	9·72 (6·21–14·2)	--
Age-related and other hearing loss	--	9·21 (8·23–10·3)	9·21 (8·23–10·3)	--
Musculoskeletal disorders	2·99 (0·696–5·68)	22·7 (18·8–26·7)	23·2 (19·2–27·4)	3·68 (0·912–6·74)
Rheumatoid arthritis	10·1 (2·38–18·4)	12·8 (3·21–22·8)	13·2 (3·29–23·5)	11·8 (2·99–21·2)
Osteoarthritis	--	17·6 (9·37–26·9)	17·6 (9·37–26·9)	--
Low back pain	--	41·6 (36·1–46·9)	41·6 (36·1–46·9)	--
Gout	--	53·2 (37·4–70)	53·2 (37·4–70)	--
Other non-communicable diseases	0·0538 (0·0347–0·0789)	0·0551 (0·0333–0·0825)	--	0·246 (0·153–0·354)
Sudden infant death syndrome	7·82 (4·32–13)	7·81 (4·32–13)	--	7·81 (4·32–13)
Injuries	32·9 (26·7–38·1)	28 (22·9–32·6)	30·6 (26·3–34·9)	24·5 (16·8–31·1)
Transport injuries	22·4 (15·7–29·2)	22·8 (16·5–29)	27 (22·7–31·2)	20·5 (13–28)
Road injuries	21·6 (14·6–28·6)	21·8 (15·4–28·1)	25·7 (21·4–30)	19·7 (11·8–27·4)
Other transport injuries	33·6 (28·1–38·8)	32·9 (27·6–37·9)	36·8 (32–40·9)	29·8 (23·4–35·6)

Unintentional injuries	40·9 (34·6–45·4)	30·4 (25·8–35·1)	30·8 (26·2–35·7)	28·9 (24·2–33·2)
Falls	56·1 (47·7–61·9)	42·3 (36·3–47·5)	40·6 (34·6–45·7)	48·8 (42–54·1)
Drowning	-14·5 (-26·4–-2·88)	-12·3 (-23·5–-1·21)	10·5 (5·84–16·9)	-14 (-25·6–-2·44)
Fire, heat, and hot substances	10 (5·43–16·5)	12·9 (7·89–20)	13·1 (8·03–20·2)	12·1 (7·42–18·7)
Poisonings	13·9 (8·66–21·1)	13·2 (8·34–19·9)	11·9 (7·34–18·3)	15 (9·78–22·3)
Exposure to mechanical forces	13·4 (4·09–23)	18·3 (15·1–21·2)	19·4 (16·5–22·1)	3 (-6·59–13)
Animal contact	26·2 (21·6–31·9)	22 (17·2–27·8)	22·4 (17·3–28·5)	20·8 (16·7–26·5)
Foreign body	2·65 (1·69–4·1)	3·93 (2·57–5·83)	3·28 (2·11–4·98)	4·44 (2·87–6·69)
Environmental heat and cold exposure	7·79 (3·25–14·1)	9·11 (3·91–16·1)	9·16 (3·89–16·2)	8·93 (3·93–15·7)
Exposure to forces of nature	0 (0–0)	10 (4·34–17·6)	10 (4·34–17·6)	0 (0–0)
Other unintentional injuries	13·6 (8·35–20·9)	12·5 (7·77–19·3)	12·4 (7·62–19·1)	15·9 (10·3–23·3)
Self-harm and interpersonal violence	21·8 (7·17–35·6)	24·4 (10·7–36·9)	32·6 (24·4–40·9)	23 (8·14–36·9)
Self-harm	21·4 (6·07–35·9)	22·8 (7·37–37·4)	21·7 (7·87–34·9)	22·8 (7·37–37·5)
Interpersonal violence	30·2 (20·9–39·8)	34·9 (26·4–44·3)	38·4 (29·3–47·4)	27·7 (18·6–37·5)

Supplementary Results Table 2. All-age PAFs attributable to the joint distribution of all risk factors for all ages, both sexes combined for each GBD level 1, level 2, and level 3 cause and all causes in 2019 for Hertfordshire. Results are shown for deaths, YLLs, YLDs, and DALYs Data in parenthesis are 95% uncertainty intervals. PAFs=population attributable fractions. GBD=Global Burden of Disease. YLLs=years of life lost. YLDs=years lived with disability. DALYs=disability-adjusted life-years

	Deaths 2019	DALYs 2019	YLDs 2019	YLLs 2019
All causes	53·7 (50·2–57·2)	40·5 (37·3–43·6)	28·8 (26·5–31·2)	53·6 (50·6–56·6)
Communicable, maternal, neonatal, and nutritional diseases	44·2 (33·9–54·7)	50·6 (44·7–56·5)	47·7 (43·1–52·5)	52·1 (43·7–60·4)
HIV/AIDS and sexually transmitted infections	74 (56·9–86·9)	75·6 (58–88·6)	97·7 (96·6–98·6)	55·6 (35·2–75)
HIV/AIDS	92·2 (90·3–93·3)	91·3 (89·1–93)	95 (93·1–96·3)	88·4 (86·1–89·9)
Sexually transmitted infections excluding HIV	48·1 (25·7–74)	60·8 (36–84·2)	100 (100–100)	17·2 (6·73–37·3)
Respiratory infections and tuberculosis	45·1 (33·8–56·4)	42·3 (32·9–52·4)	3·19 (2·34–4·23)	48·4 (37·4–59·2)
Tuberculosis	56·5 (43·2–68)	59·6 (47·9–69·7)	57 (44·7–67·7)	60·2 (48·7–70·3)
Lower respiratory infections	45·1 (33·7–56·4)	48·3 (37·2–59·1)	34·2 (26·8–41·6)	48·4 (37·2–59·3)
Upper respiratory infections	0·0514 (0·028–0·0853)	0·00824 (0·00149–0·0199)	0·0068 (0·000118–0·0187)	0·13 (0·0704–0·209)
Otitis media	2·04 (0·971–4·07)	4·63 (2·92–6·49)	4·61 (2·87–6·46)	5·39 (2·92–9·18)
Enteric infections	4·63 (2·75–7·84)	8·52 (6·24–11·9)	9·17 (6·68–12·6)	7 (4·85–10·1)
Diarrheal diseases	4·67 (2·78–7·88)	8·56 (6·28–11·9)	9·18 (6·69–12·6)	7·1 (4·92–10·3)
Other infectious diseases	0·632 (0·405–0·863)	1·2 (0·882–1·56)	0·458 (0·264–0·761)	1·53 (1·07–1·98)
Meningitis	1·57 (1·15–2·12)	2·76 (2·01–3·71)	0·000668 (7·14e-06–0·002)	3·65 (2·72–4·89)
Encephalitis	0·297 (0·194–0·441)	0·833 (0·524–1·24)	0·000217 (2·28e-06–0·000654)	1·07 (0·702–1·6)
Measles	2·47 (-0·777–12·8)	2·73 (-0·749–13·3)	0·449 (0·0105–1·25)	3·23 (-1·03–16·6)
Acute hepatitis	13·3 (5·99–23·8)	5·1 (2·81–8·35)	2·88 (1·7–4·71)	12·4 (5·59–22·1)
Maternal and neonatal disorders	89 (87·3–90·6)	80 (76·7–83)	70·3 (65·4–74·8)	89·8 (88·2–91·3)
Maternal disorders	9·23 (3·35–15·6)	9·24 (3·36–15·6)	9·31 (3·39–15·7)	9·08 (3·28–15·4)
Neonatal disorders	91·1 (89·6–92·6)	81·8 (78·5–84·7)	72·4 (67·3–77·2)	91·1 (89·7–92·6)
Nutritional deficiencies	65 (57·9–90·5)	76·1 (72·1–80·4)	76·2 (72·1–80·6)	65·2 (60·8–82·2)
Protein-energy malnutrition	100 (100–100)	100 (100–100)	100 (100–100)	100 (100–100)
Vitamin A deficiency	--	100 (100–100)	100 (100–100)	--
Dietary iron deficiency	--	100 (100–100)	100 (100–100)	--
Non-communicable diseases	55·4 (51·7–59·1)	40·7 (37·4–44·1)	28 (25·5–30·7)	55·5 (52·5–58·6)
Neoplasms	47·4 (43·2–51·8)	46·8 (42·8–51)	33·5 (28·9–38·4)	47·6 (43·7–51·9)
Lip and oral cavity cancer	66·5 (54·5–76·5)	70·2 (58·7–78·8)	69·8 (57·9–79)	70·2 (58·7–78·8)
Nasopharynx cancer	66·3 (52·7–77·3)	66·8 (54·2–76·9)	64·5 (51·1–75·5)	67·1 (54·4–77)
Other pharynx cancer	79·2 (71·1–85·9)	80·9 (73·1–87·1)	80·8 (73·1–86·9)	80·9 (73·1–87·2)
Esophageal cancer	75·7 (65·2–85)	77·6 (67·4–86·1)	76·6 (66·1–85·6)	77·7 (67·4–86·1)
Stomach cancer	24·7 (17·2–40·9)	25·5 (18–41·5)	26 (18·2–42·2)	25·5 (18–41·5)

Colon and rectum cancer	64·6 (55·2–73·7)	65·2 (56·3–73·9)	65·5 (56·8–74·1)	65·2 (56·3–73·8)
Liver cancer	76·4 (70–82·2)	77·5 (71·7–82·7)	78·2 (72·2–83·4)	77·5 (71·7–82·7)
Gallbladder and biliary tract cancer	24·4 (13·5–37·2)	24·8 (13·9–37·6)	22·9 (12·1–35·9)	24·9 (13·9–37·7)
Pancreatic cancer	43·6 (33·7–54·9)	43·5 (34·3–54·2)	43·6 (33·9–54·8)	43·5 (34·3–54·2)
Larynx cancer	81·6 (73·3–88·2)	81·8 (74–87·8)	79·8 (71·8–86·2)	82·1 (74·1–88·1)
Tracheal, bronchus, and lung cancer	85·8 (82·3–89·4)	85·2 (81·9–88·5)	85·8 (82·4–89·1)	85·2 (81·9–88·5)
Breast cancer	38·8 (28·6–50·5)	37·8 (28·6–48·2)	38 (28·8–48·5)	37·8 (28·5–48·2)
Cervical cancer	100 (100–100)	100 (100–100)	100 (100–100)	100 (100–100)
Uterine cancer	47 (30·3–63·1)	48·2 (31·9–64)	48·7 (32·7–64·5)	48·2 (31·8–63·9)
Ovarian cancer	28·8 (14·3–43·3)	24·3 (11·7–37·7)	22·7 (11·2–35·5)	24·3 (11·7–37·8)
Prostate cancer	5·89 (2·51–9·49)	6·54 (2·85–10·4)	7·06 (3·19–11·3)	6·44 (2·82–10·3)
Kidney cancer	41·2 (30·1–51·9)	41·8 (31·1–51·9)	41·8 (30·9–51·8)	41·8 (31·1–51·9)
Bladder cancer	39·2 (24·9–53·8)	42 (28·5–55·4)	43·2 (30–56·4)	41·9 (28·4–55·3)
Thyroid cancer	16·1 (8·16–25·9)	16·9 (8·59–27)	17·4 (8·64–28·1)	16·9 (8·58–26·9)
Mesothelioma	99 (98·6–99·3)	97·8 (97·1–98·4)	98·9 (98·4–99·2)	97·8 (97·1–98·4)
Non-Hodgkin lymphoma	8·11 (3·51–14·2)	8·38 (3·61–14·6)	8·29 (3·55–14·5)	8·39 (3·61–14·6)
Multiple myeloma	9·23 (4·05–16·3)	9·6 (4·32–16·8)	9·56 (4·2–16·7)	9·61 (4·31–16·8)
Leukemia	38 (23·6–52·5)	36·6 (24·6–48·9)	35·9 (24–47·6)	36·7 (24·7–49)
Cardiovascular diseases	77·5 (73·2–81·6)	78·6 (75·3–82)	70·3 (65·2–75·1)	79·8 (76·5–83)
Rheumatic heart disease	22·2 (11·8–43·2)	23·2 (14·4–38·8)	23·2 (14·6–35·9)	23·2 (14·3–39)
Ischemic heart disease	93·4 (89·3–96·9)	94·6 (91·6–97·1)	94·1 (91·2–96·7)	94·6 (91·7–97·1)
Stroke	79 (70·3–88·3)	82·1 (75·7–88·8)	82·2 (76·7–88·1)	82·1 (75·3–89·1)
Hypertensive heart disease	100 (100–100)	100 (100–100)	100 (100–100)	100 (100–100)
Non-rheumatic valvular heart disease	25·1 (15·5–38·8)	24·7 (16·8–35·1)	14·5 (9·58–20·4)	26·4 (18–37·9)
Cardiomyopathy and myocarditis	33·3 (25·5–44)	39·8 (34–46·3)	34 (27·6–41·1)	40·2 (34·3–46·7)
Atrial fibrillation and flutter	51·4 (40·2–63·7)	59·2 (49·8–68·4)	63·3 (54·4–71·7)	54·2 (43·8–64·9)
Aortic aneurysm	48·6 (41·6–56·3)	55·9 (50–62·1)	--	55·9 (50–62·1)
Endocarditis	30·5 (20·6–44·3)	33·4 (24·8–45)	30·5 (20·2–44)	33·5 (24·9–45)
Other cardiovascular and circulatory diseases	30·3 (23·5–37·5)	33·2 (27·1–39·1)	33·7 (26·7–41·2)	33·1 (27·1–39·4)
Chronic respiratory diseases	61·4 (54·5–68·7)	57·8 (52·9–62·9)	52·7 (47·3–57·6)	62·4 (56·3–69)
Chronic obstructive pulmonary disease	71 (65·4–76·5)	70·5 (66·3–74·6)	65 (61·2–68·6)	73·5 (68·5–78·1)
Pneumoconiosis	100 (100–100)	100 (100–100)	100 (100–100)	100 (100–100)
Asthma	43·5 (31·4–57·2)	43·6 (34·7–53·4)	43·4 (34·5–53)	46 (35·2–57·6)
Digestive diseases	27 (22·1–31·9)	33·5 (27·6–38·9)	25 (17·2–33·9)	38·9 (32·6–45)

Cirrhosis and other chronic liver diseases	73·8 (60·8–86·9)	76·9 (65–88·8)	73·5 (61–84·9)	77 (65–89)
Upper digestive system diseases	20·1 (16·6–23·6)	8·54 (5·55–12·1)	0·754 (0·429–1·2)	23·3 (19·6–26·6)
Gallbladder and biliary diseases	47 (30·4–63)	52 (37·2–66·5)	52·7 (38·1–67·1)	48·3 (32·4–63·8)
Pancreatitis	37·4 (14·3–58·5)	43·7 (20·7–64·2)	44·3 (21–65)	43·6 (20·7–64)
Neurological disorders	26·7 (12·1–42·8)	16·2 (7·98–29·1)	10·1 (4·99–19·8)	23·6 (10·8–39·5)
Alzheimer's disease and other dementias	41·7 (27·3–58·2)	43·8 (29·2–59·5)	44·2 (29·8–59·8)	43·6 (29–59·5)
Parkinson's disease	-8·76 (-14·8–-3·62)	-10·1 (-16·6–-4·39)	-10·8 (-17·5–-4·85)	-9·94 (-16·3–-4·24)
Idiopathic epilepsy	27 (15·4–38·5)	25·6 (14·7–35·5)	23·7 (13·4–33·4)	28·7 (17·2–39·7)
Multiple sclerosis	14 (10·5–18)	15·2 (11·5–19·2)	15·2 (11·4–19·1)	15·2 (11·4–19·4)
Mental disorders	--	6·45 (3·22–9·88)	6·45 (3·23–9·88)	--
Depressive disorders	--	13·5 (6·22–21·2)	13·5 (6·22–21·2)	--
Anxiety disorders	--	4·98 (1·33–11·3)	4·98 (1·33–11·3)	--
Idiopathic developmental intellectual disability	--	31·9 (13–50·6)	31·9 (13–50·6)	--
Substance use disorders	100 (100–100)	100 (100–100)	100 (100–100)	100 (100–100)
Alcohol use disorders	100 (100–100)	100 (100–100)	100 (100–100)	100 (100–100)
Drug use disorders	100 (100–100)	100 (100–100)	100 (100–100)	100 (100–100)
Diabetes and kidney diseases	99·8 (99·7–99·8)	100 (99·9–100)	100 (100–100)	99·8 (99·7–99·8)
Diabetes mellitus	100 (100–100)	100 (100–100)	100 (100–100)	100 (100–100)
Chronic kidney disease	100 (100–100)	100 (100–100)	100 (100–100)	100 (100–100)
Sense organ diseases	--	8·63 (7·47–10)	8·63 (7·47–10)	--
Blindness and vision loss	--	9·67 (6·22–14)	9·67 (6·22–14)	--
Age-related and other hearing loss	--	9·65 (8·68–10·7)	9·65 (8·68–10·7)	--
Musculoskeletal disorders	3·76 (0·881–6·93)	23·6 (19·7–27·8)	24 (19·9–28·2)	4·74 (1·23–8·6)
Rheumatoid arthritis	10·1 (2·52–18·4)	13·2 (3·42–23·2)	13·5 (3·47–23·9)	12·1 (3·22–21·3)
Osteoarthritis	--	19·8 (10·7–29·5)	19·8 (10·7–29·5)	--
Low back pain	--	42·8 (37·5–48·3)	42·8 (37·5–48·3)	--
Gout	--	54·7 (39·1–71·6)	54·7 (39·1–71·6)	--
Other non-communicable diseases	0·0674 (0·0434–0·0995)	0·068 (0·0409–0·106)	--	0·313 (0·189–0·442)
Sudden infant death syndrome	9·07 (5·21–14·2)	9·07 (5·21–14·2)	--	9·07 (5·21–14·2)
Injuries	34·4 (27·6–40)	28·1 (22·9–33·1)	29·9 (25·3–34·7)	25·4 (17·7–32·1)
Transport injuries	21·6 (14–29·3)	22·2 (15·8–28·7)	26·5 (21·8–31·1)	19·9 (11·5–28·2)
Road injuries	20·8 (12·8–28·8)	21·3 (14·6–28)	25·4 (20·8–30)	19·1 (10·5–27·8)
Other transport injuries	33 (27·4–38·4)	32·1 (26·7–37·3)	35·1 (30·1–39·8)	29·6 (23·2–35·5)
Unintentional injuries	41·3 (33·4–47)	29·8 (24·8–34·8)	30 (24·9–35·3)	29·2 (23·6–34·1)

Falls	54·4 (44·3–61)	40·8 (34–46·2)	39 (32·5–44·7)	47·4 (39·3–53·3)
Drowning	-13·1 (-27·3–2·41)	-11·3 (-24·5–2·83)	11·4 (6·42–18·3)	-13·1 (-26·8–1·92)
Fire, heat, and hot substances	10·6 (5·71–17·5)	13·6 (8·41–20·6)	13·9 (8·59–21)	12·5 (7·56–19·2)
Poisonings	14·5 (8·92–21·8)	13·7 (8·5–20·3)	12·6 (7·82–19)	15·5 (9·9–22·6)
Exposure to mechanical forces	12·1 (0·476–24·2)	16·8 (13·7–19·8)	17·8 (14·7–20·7)	1·69 (-10·1–14·6)
Animal contact	21·3 (16·4–27·5)	20·6 (15·7–26·5)	21·8 (16·5–27·7)	17·9 (13·1–23·8)
Foreign body	2·42 (1·59–3·68)	3·74 (2·51–5·61)	3·27 (2·19–4·91)	4·08 (2·72–6·07)
Environmental heat and cold exposure	8·48 (3·86–15·6)	9·9 (4·32–17·4)	9·96 (4·33–17·5)	9·61 (4·25–17)
Exposure to forces of nature	0 (0–0)	10·8 (4·67–18·7)	10·8 (4·67–18·7)	0 (0–0)
Other unintentional injuries	14·8 (9–22·4)	13·2 (8·16–20·1)	13·1 (8·08–20)	16·6 (10·6–23·9)
Self-harm and interpersonal violence	23·8 (8·09–38)	26·5 (12·5–39·2)	34·1 (25·9–42·7)	25 (9·53–39)
Self-harm	23·5 (7·15–38·5)	24·9 (8·65–39·5)	24·5 (9·11–37·9)	24·9 (8·64–39·7)
Interpersonal violence	29·9 (20·5–39·7)	35·6 (27–44·6)	39·1 (30·1–48·1)	26·9 (17·6–36·4)

Supplementary Results Table 2. All-age PAFs attributable to the joint distribution of all risk factors for all ages, both sexes combined for each GBD level 1, level 2, and level 3 cause and all causes in 2019 for Luton. Results are shown for deaths, YLLs, YLDs, and DALYs Data in parenthesis are 95% uncertainty intervals. PAFs=population attributable fractions. GBD=Global Burden of Disease. YLLs=years of life lost. YLDs=years lived with disability. DALYs=disability-adjusted life-years

	Deaths 2019	DALYs 2019	YLDs 2019	YLLs 2019
All causes	55·3 (52·1–58·6)	41·7 (38·7–45)	29 (26·6–31·4)	54·5 (51·9–57·1)
Communicable, maternal, neonatal, and nutritional diseases	47·6 (38·5–56·8)	57·7 (53·4–62·1)	51·3 (46·2–56·3)	61·1 (54·7–67·2)
HIV/AIDS and sexually transmitted infections	76·7 (63·4–86)	75·6 (60·6–85·8)	95·7 (93·7–97·2)	62·1 (44·9–76·3)
HIV/AIDS	89·1 (86·8–90·5)	87·8 (85·2–90)	93·6 (90·9–95·5)	83·8 (81·2–85·6)
Sexually transmitted infections excluding HIV	47·5 (25·4–73·3)	52·3 (28·1–78·9)	100 (100–100)	19·3 (7·69–40·5)
Respiratory infections and tuberculosis	46·1 (35·4–57)	42·8 (34–52·2)	3·53 (2·51–4·85)	50·6 (40·6–60·6)
Tuberculosis	56·3 (43·7–68·2)	58·5 (46·8–69·4)	54·6 (41·9–66·2)	59·2 (47·4–69·9)
Lower respiratory infections	46 (35–57·2)	50·5 (40·2–60·7)	34 (27·1–41·1)	50·6 (40·2–60·9)
Upper respiratory infections	0·0707 (0·0371–0·127)	0·00974 (0·00219–0·0219)	0·0075 (9·02e-05–0·0193)	0·123 (0·0633–0·221)
Otitis media	4·37 (2·17–8·1)	5·44 (3·48–7·53)	5·3 (3·42–7·4)	7·96 (4·53–12·8)
Enteric infections	5·96 (3·74–9·9)	11·3 (8·4–15·4)	11·2 (8·2–15·4)	11·7 (8·21–15·8)
Diarrheal diseases	6·03 (3·78–9·98)	11·4 (8·45–15·5)	11·2 (8·21–15·4)	12 (8·41–16·1)
Other infectious diseases	0·986 (0·663–1·31)	1·57 (1·2–2·03)	0·393 (0·229–0·666)	1·99 (1·47–2·54)
Meningitis	2·36 (1·75–3·16)	3·39 (2·5–4·52)	0·000709 (9·5e-06–0·00199)	4·09 (3·02–5·47)
Encephalitis	0·594 (0·365–0·915)	1·25 (0·739–1·93)	0·000302 (2·89e-06–0·000833)	1·52 (0·943–2·33)
Measles	3·54 (-0·923–18·5)	4·01 (-0·968–20·2)	0·602 (0·0161–1·64)	4·19 (-1·05–21·6)
Acute hepatitis	15·1 (9·08–26·2)	4·9 (3–7·77)	2·48 (1·46–4·12)	14·1 (8·52–24)
Maternal and neonatal disorders	88·6 (85·6–91·1)	83·1 (80·2–86)	72 (66·7–76·6)	89·4 (86·4–91·8)
Maternal disorders	9·66 (3·5–16·3)	9·73 (3·52–16·6)	9·95 (3·57–16·9)	9·51 (3·46–16·1)
Neonatal disorders	90·8 (87·8–93·1)	84·7 (81·7–87·6)	73·9 (68·4–78·6)	90·8 (87·8–93·1)
Nutritional deficiencies	85·3 (71·2–88·9)	78 (73·5–84)	77·8 (73·1–83·9)	84·1 (65·8–87·2)
Protein-energy malnutrition	100 (100–100)	100 (100–100)	100 (100–100)	100 (100–100)
Vitamin A deficiency	--	100 (100–100)	100 (100–100)	--
Dietary iron deficiency	--	100 (100–100)	100 (100–100)	--
Non-communicable diseases	57·1 (53·8–60·5)	42 (38·7–45·5)	28·1 (25·5–30·6)	56·6 (53·8–59·4)
Neoplasms	48·9 (44·9–53·2)	47·7 (43·9–51·6)	34·5 (30·2–39·1)	48·3 (44·5–52·3)
Lip and oral cavity cancer	67·4 (55·9–77·7)	70·2 (58·7–79·7)	69·7 (57·7–79·3)	70·2 (58·7–79·7)
Nasopharynx cancer	67·2 (53·3–77·8)	66·4 (53·1–76·8)	63·5 (49·5–75)	66·5 (53·4–76·8)
Other pharynx cancer	79·5 (71–86·4)	80·7 (72·5–87·1)	80·5 (72–87)	80·7 (72·5–87·1)
Esophageal cancer	74·8 (64·9–83·4)	76·6 (67–84·8)	75·6 (65·5–84·1)	76·6 (67·1–84·8)
Stomach cancer	26 (18·2–42·7)	26·3 (18·5–42·8)	26·8 (18·9–43·4)	26·3 (18·5–42·8)

Colon and rectum cancer	64·8 (55·2–74·1)	64·9 (56·2–73·7)	65·2 (56·3–74·1)	64·9 (56·2–73·7)
Liver cancer	75·6 (69·3–81·3)	75·2 (69·8–80·5)	76·2 (70·4–81·5)	75·2 (69·8–80·5)
Gallbladder and biliary tract cancer	20·5 (10·7–32·8)	20·9 (10·9–33·3)	19·1 (9·57–31·2)	20·9 (11·1–33·4)
Pancreatic cancer	44·6 (34·9–56·4)	43·9 (34·7–55·2)	44·4 (34·6–55·9)	43·9 (34·7–55·2)
Larynx cancer	81·7 (73·3–87·8)	81·6 (74·4–87·6)	78·9 (70·7–85·6)	81·9 (74·7–87·8)
Tracheal, bronchus, and lung cancer	87·2 (83·8–90·4)	86 (82·7–89·3)	86·9 (83·6–90·1)	86 (82·7–89·3)
Breast cancer	37·9 (27·5–49·9)	36 (26·3–46·4)	36·3 (26·9–46·9)	36 (26·3–46·3)
Cervical cancer	100 (100–100)	100 (100–100)	100 (100–100)	100 (100–100)
Uterine cancer	42·8 (27·7–58·4)	44 (28·9–59·5)	44·4 (29·4–60)	43·9 (28·8–59·5)
Ovarian cancer	29·2 (14·8–44·5)	23·8 (11·9–37·3)	21·6 (10·6–34·2)	23·9 (11·9–37·5)
Prostate cancer	6·36 (2·82–10·3)	7·03 (3·14–11·2)	7·56 (3·42–11·9)	6·95 (3·12–11)
Kidney cancer	39·2 (28·9–49·5)	38·8 (29–48·6)	38·6 (29·1–48·4)	38·8 (29–48·7)
Bladder cancer	42·6 (28·2–57·5)	45·1 (31·8–58·2)	46 (32·4–58·5)	45·1 (31·7–58·1)
Thyroid cancer	14·1 (6·67–23·6)	14·7 (6·88–24·5)	15 (6·88–25·2)	14·7 (6·89–24·4)
Mesothelioma	98·7 (98·3–99·1)	97·1 (96·2–97·9)	98·6 (98–99·1)	97·1 (96·2–97·9)
Non-Hodgkin lymphoma	6·91 (2·78–12·4)	7·01 (2·94–12·5)	7 (2·82–12·5)	7·01 (2·95–12·5)
Multiple myeloma	7·86 (3·31–14·1)	8·18 (3·47–14·6)	8·14 (3·39–14·6)	8·18 (3·48–14·6)
Leukemia	37·1 (23·5–50·6)	32·8 (22–44)	31·9 (21·3–43·1)	32·9 (22·1–43·9)
Cardiovascular diseases	79·5 (75·5–83·2)	80·8 (77·5–83·7)	70·1 (64·6–75)	82 (79–84·7)
Rheumatic heart disease	22·2 (12·1–42·6)	23·3 (14·6–38·3)	22·8 (14–37·1)	23·3 (14·7–38·4)
Ischemic heart disease	93·8 (90·1–96·8)	95·2 (92·6–97·2)	94·1 (91–96·6)	95·3 (92·7–97·3)
Stroke	79·8 (72·1–88·6)	83·2 (77·6–89·3)	81·9 (76·7–87·5)	83·4 (77·5–89·6)
Hypertensive heart disease	100 (100–100)	100 (100–100)	100 (100–100)	100 (100–100)
Non-rheumatic valvular heart disease	25·1 (16–38·3)	26·1 (18·2–36·2)	18·3 (11·8–26·1)	26·8 (18·6–36·9)
Cardiomyopathy and myocarditis	36·3 (29–44·6)	40·6 (35–46·1)	34·3 (28–40·9)	40·9 (35·2–46·7)
Atrial fibrillation and flutter	49·2 (37·7–60·4)	56·9 (47·1–66·2)	60·9 (51·7–69·5)	52·5 (41·7–62·5)
Aortic aneurysm	52·4 (45·7–59·6)	60·1 (54·5–65·8)	--	60·1 (54·5–65·8)
Endocarditis	31 (21·1–44·2)	33·8 (24·7–44·1)	30·2 (19·6–43·4)	33·9 (24·9–44·2)
Other cardiovascular and circulatory diseases	31·7 (25·1–39·2)	33·7 (26·8–40·3)	32·7 (24·7–40·9)	33·9 (27·1–40·5)
Chronic respiratory diseases	63·6 (56·6–71·1)	56·8 (51·4–62·4)	48·7 (43·1–54·4)	63·6 (57·1–70·6)
Chronic obstructive pulmonary disease	73·2 (68–78·4)	72·5 (68·4–76·4)	65·8 (62–69·6)	75·4 (70·7–79·9)
Pneumoconiosis	100 (100–100)	100 (100–100)	100 (100–100)	100 (100–100)
Asthma	42·2 (31·2–54·5)	39·8 (31·4–48·8)	39·4 (31·2–48·4)	43 (33·6–53·1)
Digestive diseases	30·5 (25–37)	35·6 (29·6–42·2)	17·8 (12–24·8)	42·8 (35·4–50·8)

Cirrhosis and other chronic liver diseases	71·3 (59·1–86)	74·2 (62·3–87·6)	70·2 (57–83·5)	74·3 (62·2–87·8)
Upper digestive system diseases	22·2 (18·6–25·5)	9·52 (6·05–13·2)	0·649 (0·369–1·05)	25·2 (21·5–28·6)
Gallbladder and biliary diseases	42·2 (27·2–57·6)	47·2 (33·4–61·5)	48·6 (34·7–63·1)	43·6 (29–58·4)
Pancreatitis	34·6 (12·9–58·2)	40·7 (17·8–62·7)	40·8 (17·9–62·5)	40·7 (17·7–62·7)
Neurological disorders	24·2 (10–40·3)	13·6 (6·6–24·3)	8·27 (3·81–16·8)	20·8 (9·81–36·3)
Alzheimer's disease and other dementias	42·2 (27·6–58·4)	44·3 (29·9–59·7)	44·6 (30–60·2)	44·1 (29·8–59·6)
Parkinson's disease	-9·56 (-16–-4·16)	-11 (-18·3–-4·97)	-11·7 (-19·2–-5·28)	-10·9 (-18·1–-4·83)
Idiopathic epilepsy	25·6 (14·3–36·8)	23·8 (13·3–34·3)	21·7 (11·6–32·2)	25·7 (14·7–36·5)
Multiple sclerosis	15·2 (11·1–19·6)	16·1 (12–20·4)	15·9 (11·9–20)	16·3 (12–20·8)
Mental disorders	--	6·87 (3·46–10·9)	6·87 (3·46–10·9)	--
Depressive disorders	--	14·3 (6·53–22)	14·3 (6·53–22)	--
Anxiety disorders	--	5·88 (1·58–13·2)	5·88 (1·58–13·2)	--
Idiopathic developmental intellectual disability	--	34·9 (14·8–53·6)	34·9 (14·8–53·6)	--
Substance use disorders	100 (100–100)	100 (100–100)	100 (100–100)	100 (100–100)
Alcohol use disorders	100 (100–100)	100 (100–100)	100 (100–100)	100 (100–100)
Drug use disorders	100 (100–100)	100 (100–100)	100 (100–100)	100 (100–100)
Diabetes and kidney diseases	99·9 (99·8–99·9)	100 (100–100)	100 (100–100)	99·9 (99·8–99·9)
Diabetes mellitus	100 (100–100)	100 (100–100)	100 (100–100)	100 (100–100)
Chronic kidney disease	100 (100–100)	100 (100–100)	100 (100–100)	100 (100–100)
Sense organ diseases	--	9·02 (7·86–10·4)	9·02 (7·86–10·4)	--
Blindness and vision loss	--	8·81 (5·56–13)	8·81 (5·56–13)	--
Age-related and other hearing loss	--	10·6 (9·61–11·7)	10·6 (9·61–11·7)	--
Musculoskeletal disorders	4·07 (0·983–7·41)	22·6 (18·9–26·6)	23 (19·2–27)	4·72 (1·2–8·48)
Rheumatoid arthritis	11·3 (2·82–20·1)	13·8 (3·5–24·4)	14 (3·46–24·7)	13·1 (3·4–23·4)
Osteoarthritis	--	16·5 (8·55–25·7)	16·5 (8·55–25·7)	--
Low back pain	--	41·7 (36·3–46·9)	41·7 (36·3–46·9)	--
Gout	--	49·2 (33·5–66·3)	49·2 (33·5–66·3)	--
Other non-communicable diseases	0·0966 (0·0626–0·151)	0·0987 (0·0611–0·154)	--	0·289 (0·192–0·425)
Sudden infant death syndrome	6·2 (3·5–10·2)	6·2 (3·49–10·2)	--	6·2 (3·49–10·2)
Injuries	30·7 (24·3–36·3)	24·4 (19–29·4)	27 (23–31·3)	21·7 (14·2–28·7)
Transport injuries	19 (10·8–27·3)	18·8 (11·5–26·3)	25·1 (20·3–30·3)	16·4 (7·74–25·4)
Road injuries	18·1 (9·63–26·9)	17·9 (10·3–25·7)	24·2 (19·4–29·5)	15·7 (6·57–25)
Other transport injuries	29·6 (24–35·5)	28·2 (23–34·1)	32·3 (27·4–37·2)	25·9 (20·2–32·3)
Unintentional injuries	38·8 (32·9–43·4)	25·9 (21·9–30·5)	26·8 (22·4–31·5)	23·9 (19·7–28·2)

Falls	53·4 (45·6–58·8)	37·5 (32–42·6)	35·7 (30·2–41)	42·5 (36·5–47·8)
Drowning	-14·7 (-28·9–-0·285)	-14·9 (-28·3–-1·3)	10·2 (5·71–16·6)	-15·9 (-29·6–-1·86)
Fire, heat, and hot substances	10·2 (5·76–16·3)	12·2 (7·34–18·5)	12·8 (7·84–19·7)	10·7 (6·53–16·2)
Poisonings	14·5 (8·95–21·3)	12·8 (7·99–18·8)	11·5 (6·99–17·1)	14·6 (9·21–21·3)
Exposure to mechanical forces	7·52 (-4–19·3)	14·1 (11·2–16·9)	15·9 (13·4–18·5)	-2·67 (-15–10·6)
Animal contact	18·9 (14·5–24·4)	18·5 (13·8–24)	19·4 (14·5–25·1)	14·2 (10·4–19·3)
Foreign body	2·69 (1·73–4·05)	3·33 (2·17–4·93)	3·01 (1·94–4·53)	3·49 (2·28–5·15)
Environmental heat and cold exposure	8·9 (3·89–16)	9·38 (4·16–16·4)	9·06 (3·95–16)	10 (4·47–17·7)
Exposure to forces of nature	0 (0–0)	9·85 (4·37–17·1)	9·85 (4·37–17·1)	0 (0–0)
Other unintentional injuries	13·4 (8·18–20·2)	12 (7·4–18·4)	11·9 (7·27–18·2)	14·2 (8·91–20·7)
Self-harm and interpersonal violence	22 (7·17–36·3)	24 (10·4–37·1)	33·9 (25·2–43·2)	22·7 (7·89–36·3)
Self-harm	21·8 (5·94–37)	22·8 (6·85–37·9)	22·2 (8·42–36)	22·9 (6·78–38)
Interpersonal violence	24·7 (15–34·2)	30·5 (21·4–39·8)	39 (29·4–48·8)	21·5 (12·3–30·3)

Supplementary Results Table 2. All-age PAFs attributable to the joint distribution of all risk factors for all ages, both sexes combined for each GBD level 1, level 2, and level 3 cause and all causes in 2019 for Albania. Results are shown for deaths, YLLs, YLDs, and DALYs Data in parenthesis are 95% uncertainty intervals. PAFs=population attributable fractions. GBD=Global Burden of Disease. YLLs=years of life lost. YLDs=years lived with disability. DALYs=disability-adjusted life-years

	Deaths 2019	DALYs 2019	YLDs 2019	YLLs 2019
All causes	68·3 (64·7–71·3)	48·8 (45·2–52·5)	30·3 (27·9–32·7)	61·8 (59·2–64·2)
Communicable, maternal, neonatal, and nutritional diseases	56·7 (52·7–60·9)	62 (58·6–65·2)	60·4 (55·2–64·7)	63 (58·7–67·3)
HIV/AIDS and sexually transmitted infections	43·6 (23·6–67)	60·1 (36·4–82·7)	99·8 (99·6–99·9)	25·8 (11·8–46·9)
HIV/AIDS	85·5 (84·1–86·6)	77·1 (75·1–78·9)	91·6 (89·9–93·4)	75·9 (73·9–77·6)
Sexually transmitted infections excluding HIV	23·5 (9·29–48·4)	57·7 (31·7–83·3)	100 (100–100)	9·71 (3·36–23·1)
Respiratory infections and tuberculosis	59·5 (54·4–64·8)	55·6 (49·3–61·4)	5·06 (3·89–6·51)	66 (60·7–71)
Tuberculosis	49·8 (42·9–56·6)	50·3 (43·4–56·6)	49·6 (41–57·5)	50·5 (43·8–56·7)
Lower respiratory infections	61·1 (55·7–66·4)	68·2 (62·8–73·4)	44·9 (38·8–51·2)	68·4 (63·1–73·7)
Upper respiratory infections	0·548 (0·0526–2·12)	0·26 (0·0182–0·979)	0·00864 (0·00207–0·0168)	1·27 (0·086–4·71)
Otitis media	2·89 (0·908–7·34)	4·39 (2·84–6·05)	4·39 (2·82–6·05)	12·9 (5·41–26·9)
Enteric infections	62·2 (46·5–75·1)	65·9 (46·8–81·2)	65·8 (46·3–81·5)	67 (53·9–77·8)
Diarrheal diseases	71·1 (54·3–84)	66·5 (47·3–81·8)	65·9 (46·3–81·6)	79·4 (65·8–89·2)
Other infectious diseases	1·56 (0·953–2·47)	2·58 (1·54–4·12)	0·194 (0·112–0·337)	3·07 (1·76–5·03)
Meningitis	1·72 (1·03–2·59)	2·81 (1·76–4·01)	0·00112 (0·000227–0·00227)	3·27 (2·06–4·67)
Encephalitis	1·39 (0·455–2·63)	2·34 (0·831–4·33)	0·000588 (0·000124–0·00118)	2·49 (0·864–4·55)
Measles	36·8 (20·8–63·1)	36·8 (20·9–63)	5·17 (0·209–11·5)	37·5 (21·2–64·2)
Acute hepatitis	12·4 (5·42–18·4)	3·26 (1·68–5·06)	1·04 (0·604–1·83)	11·3 (5·16–17·2)
Maternal and neonatal disorders	73·6 (68·5–78·1)	68·1 (63·4–72·4)	51·9 (47·7–55·9)	74 (68·9–78·6)
Maternal disorders	13·7 (4·95–22·5)	13·4 (4·8–22·1)	13·3 (4·73–22·1)	13·6 (4·9–22·4)
Neonatal disorders	74·7 (69·5–79·2)	69·2 (64·7–73·5)	53·9 (49·4–58)	74·7 (69·5–79·2)
Nutritional deficiencies	71·8 (66·6–77·5)	92·7 (90·4–94·6)	92·9 (90·5–94·9)	85·3 (81·4–89·4)
Protein-energy malnutrition	100 (100–100)	100 (100–100)	100 (100–100)	100 (100–100)
Vitamin A deficiency	--	100 (100–100)	100 (100–100)	--
Dietary iron deficiency	--	100 (100–100)	100 (100–100)	--
Non-communicable diseases	70·6 (66·7–73·9)	51·2 (46·6–55·8)	28·2 (25·2–31)	66·2 (63·1–68·9)
Neoplasms	46 (42·6–49·8)	43 (39·9–46·6)	30·7 (26·6–34·8)	43·4 (40·2–47)
Lip and oral cavity cancer	59·8 (54–65)	60·5 (54·9–65·9)	58·8 (52·4–64·9)	60·6 (54·9–65·9)
Nasopharynx cancer	56·5 (48·1–63·7)	56·3 (48·4–63·4)	56·2 (48·2–63·5)	56·3 (48·4–63·4)
Other pharynx cancer	72·8 (67·8–77·5)	73·5 (68·4–78·2)	66·3 (60·7–71·5)	73·6 (68·6–78·2)
Esophageal cancer	73·9 (65·3–82)	73·9 (65·4–81·9)	74·1 (65·6–82·2)	73·9 (65·4–81·9)

Stomach cancer	31 (21·5–49·6)	29·8 (20·2–48·5)	30·2 (20·4–48·9)	29·8 (20·1–48·5)
Colon and rectum cancer	56·5 (48·7–63·9)	55·9 (48·6–63·1)	56·1 (48·7–63·2)	55·9 (48·6–63)
Liver cancer	71·2 (63·6–78·3)	70·3 (62·8–77·3)	71·1 (63·7–78)	70·3 (62·8–77·3)
Gallbladder and biliary tract cancer	25 (14·1–38·1)	24·8 (14·1–37·2)	25 (14·1–37·8)	24·8 (14·1–37·2)
Pancreatic cancer	36·9 (30·4–44·6)	35·7 (29·5–43·2)	36·3 (29·7–43·9)	35·6 (29·5–43·2)
Larynx cancer	85·8 (80·6–89·8)	85·4 (80·6–88·9)	83·9 (78·9–87·7)	85·4 (80·7–89)
Tracheal, bronchus, and lung cancer	85·4 (82·6–88·4)	83·8 (80·8–87)	84·4 (81·2–87·6)	83·8 (80·8–87)
Breast cancer	22·9 (16·4–29·9)	20·8 (14·8–26·9)	20·9 (14·8–27·2)	20·8 (14·8–26·9)
Cervical cancer	100 (100–100)	100 (100–100)	100 (100–100)	100 (100–100)
Uterine cancer	46·9 (32·3–62)	46·3 (32–61·8)	46 (31·7–61·6)	46·3 (32·1–61·8)
Ovarian cancer	10·5 (3·98–18·5)	9·03 (3·23–16·1)	8·44 (2·93–15·2)	9·04 (3·24–16·2)
Prostate cancer	9·41 (4·63–14·1)	9·6 (4·65–14·4)	9·75 (4·79–14·6)	9·59 (4·64–14·4)
Kidney cancer	41·3 (31·6–51·4)	39·1 (30·2–48·1)	37·6 (28·7–46·6)	39·1 (30·2–48·2)
Bladder cancer	46·9 (35·3–56·8)	47·6 (37–56·5)	46·7 (36·7–55·3)	47·6 (37·1–56·6)
Thyroid cancer	15·6 (7·48–25·8)	15·4 (7·23–25·6)	14·4 (7·14–23·3)	15·5 (7·23–25·9)
Mesothelioma	81·3 (70·4–90·6)	72·5 (57·8–85·8)	78·8 (66·6–89·7)	72·4 (57·6–85·7)
Non-Hodgkin lymphoma	7·22 (3·08–12·8)	6·66 (2·81–11·8)	6·92 (2·96–12·3)	6·64 (2·81–11·8)
Multiple myeloma	8·8 (3·81–15·3)	8·83 (3·88–15·4)	8·88 (3·85–15·4)	8·83 (3·88–15·4)
Leukemia	30·4 (20·5–40·3)	22·3 (15·4–30)	27·2 (18·3–36·3)	22·1 (15·4–29·9)
Cardiovascular diseases	85·5 (81·8–88·7)	85·3 (82·5–87·8)	76·3 (72·2–79·9)	86·2 (83·2–88·7)
Rheumatic heart disease	30·9 (20·3–48·4)	30·3 (20·9–41·5)	30·2 (19·7–42)	30·4 (21·5–42·8)
Ischemic heart disease	94·1 (91·1–96·6)	94·7 (92·6–96·6)	95·1 (92·9–96·9)	94·7 (92·6–96·6)
Stroke	83·2 (76·5–88·9)	84·9 (80·1–89)	87 (83·4–90·2)	84·7 (79·5–89·1)
Hypertensive heart disease	100 (100–100)	100 (100–100)	100 (100–100)	100 (100–100)
Non-rheumatic valvular heart disease	26·1 (18·7–34·4)	23·5 (17–29·8)	15·3 (10·7–20·7)	27·2 (19·9–34·6)
Cardiomyopathy and myocarditis	39·2 (31·2–48·9)	40·8 (34·2–48·3)	44 (36·6–51·4)	40·6 (33·9–48·3)
Atrial fibrillation and flutter	57·2 (47·9–66·6)	66 (58·7–73·2)	68·9 (61·8–75·5)	61·1 (53–69·5)
Aortic aneurysm	65·1 (59–70·9)	69·5 (64·7–74)	--	69·5 (64·7–74)
Endocarditis	46·3 (34·8–59·3)	44·4 (33·7–55·9)	47·9 (36·5–59·9)	44·3 (33·6–55·9)
Other cardiovascular and circulatory diseases	43·2 (37·1–49·6)	42·9 (37·4–48·2)	44·7 (38·5–50·6)	42·4 (37–48)
Chronic respiratory diseases	69·6 (65·5–73·3)	61·9 (57·6–65·9)	52·5 (47·2–57·5)	67·5 (63·3–71·5)
Chronic obstructive pulmonary disease	80·6 (76·9–84)	77·1 (73·5–80·4)	68·8 (64·7–73)	80·6 (77·1–83·8)
Pneumoconiosis	100 (100–100)	100 (100–100)	100 (100–100)	100 (100–100)
Asthma	49·4 (37·5–62·3)	43·6 (34·4–53·9)	38·1 (30·1–47·5)	50·8 (39·2–62·8)

Digestive diseases	46·8 (41–52·3)	38·6 (32·6–44·6)	20 (13·5–27·8)	50·1 (44–55·9)
Cirrhosis and other chronic liver diseases	66·4 (58–74·3)	68·3 (60·3–75·6)	61·8 (54·1–69·9)	68·4 (60·3–75·8)
Upper digestive system diseases	24·8 (20·8–28·6)	6·41 (3·74–9·79)	1·27 (0·759–1·97)	26·4 (22·1–30·3)
Gallbladder and biliary diseases	47·6 (33·2–62·1)	47·8 (33·7–62·2)	47·9 (33·7–62·4)	47·2 (33·5–61·2)
Pancreatitis	23·2 (14·6–33·3)	28·5 (18·7–39·5)	27 (18·1–37·8)	28·6 (18·7–39·7)
Neurological disorders	23·5 (8·42–38·6)	14·1 (6·98–24·5)	9·42 (5·06–16·2)	20·1 (6·99–34·8)
Alzheimer's disease and other dementias	40 (28·1–53·1)	41·5 (29·6–54·5)	41·1 (29·1–54·1)	41·7 (30–54·7)
Parkinson's disease	-15·5 (-23·2–-8·25)	-16·3 (-24·1–-8·69)	-15·9 (-23·7–-8·35)	-16·4 (-24·3–-8·71)
Idiopathic epilepsy	13·3 (8·75–18·7)	13 (8·87–17·4)	13·6 (9·02–18·2)	12·3 (8·12–17·3)
Multiple sclerosis	18·9 (13·5–26·7)	19·7 (14·6–25·9)	19·2 (14·7–23·8)	19·8 (14·4–28·2)
Mental disorders	--	4·61 (2·56–7·15)	4·61 (2·56–7·15)	--
Depressive disorders	--	7·92 (3·34–13·4)	7·92 (3·34–13·4)	--
Anxiety disorders	--	4·07 (1·03–9·38)	4·07 (1·03–9·38)	--
Idiopathic developmental intellectual disability	--	63·5 (33·5–82·9)	63·5 (33·5–82·9)	--
Substance use disorders	100 (100–100)	100 (100–100)	100 (100–100)	100 (100–100)
Alcohol use disorders	100 (100–100)	100 (100–100)	100 (100–100)	100 (100–100)
Drug use disorders	100 (100–100)	100 (100–100)	100 (100–100)	100 (100–100)
Diabetes and kidney diseases	99·7 (99·1–99·8)	99·8 (99·6–99·9)	100 (100–100)	99·6 (98·9–99·8)
Diabetes mellitus	100 (100–100)	100 (100–100)	100 (100–100)	100 (100–100)
Chronic kidney disease	100 (100–100)	100 (100–100)	100 (100–100)	100 (100–100)
Sense organ diseases	--	9·12 (7·9–10·3)	9·12 (7·9–10·3)	--
Blindness and vision loss	--	6·73 (4·14–9·69)	6·73 (4·14–9·69)	--
Age-related and other hearing loss	--	10·5 (9·53–11·6)	10·5 (9·53–11·6)	--
Musculoskeletal disorders	4·23 (1·26–8·22)	38·9 (33·6–43·9)	39·4 (34–44·4)	4·15 (1·34–7·32)
Rheumatoid arthritis	16·6 (5–30·4)	15·5 (5·37–25·6)	15·4 (5·28–25·4)	16·6 (5·26–29·8)
Osteoarthritis	--	19·4 (10·5–29·1)	19·4 (10·5–29·1)	--
Low back pain	--	50·7 (45·8–55·7)	50·7 (45·8–55·7)	--
Gout	--	50·2 (35·2–66·8)	50·2 (35·2–66·8)	--
Other non-communicable diseases	0·0822 (0·0392–0·142)	0·0378 (0·0178–0·0686)	--	0·126 (0·0625–0·216)
Sudden infant death syndrome	4·31 (1·95–8·2)	4·31 (1·94–8·19)	--	4·31 (1·94–8·19)
Injuries	18·3 (15–21·5)	22·2 (17·9–27·2)	28·3 (23·9–33·7)	14·5 (11·2–17·7)
Transport injuries	24·4 (18·9–29·9)	25·4 (19·7–31·4)	31 (25·6–36·9)	21·3 (15·4–27·2)
Road injuries	23·3 (17·7–29·1)	25·1 (19·3–31·2)	30·8 (25·4–36·7)	20·6 (14·5–26·7)
Other transport injuries	33·6 (28·4–38·6)	29·3 (24–34·7)	36·2 (31·3–41·2)	27·5 (22·1–32·8)

Unintentional injuries	17·2 (13·2–21·7)	23 (18·2–28·5)	28·6 (23·9–34·3)	10 (6·73–13·9)
Falls	43·9 (37·2–50·3)	36 (30·8–41·1)	36·7 (31·7–41·7)	32·7 (26·7–40·4)
Drowning	-10·9 (-17·5–-3·45)	-12·8 (-19·1–-6·09)	12·1 (8·56–16·2)	-13·7 (-20·2–-6·92)
Fire, heat, and hot substances	10·6 (7·51–14·4)	16·8 (11·9–22·6)	18·8 (13·6–25·1)	11·7 (7·94–16·1)
Poisonings	14·6 (10·7–19·5)	14·6 (10·6–19·4)	15·4 (11·1–20·6)	13·5 (9·9–18·1)
Exposure to mechanical forces	12·2 (4·75–19·5)	23·7 (17·5–31·1)	26·6 (21·3–33·5)	7·19 (-0·295–14·3)
Animal contact	26·1 (21·8–30·9)	21·9 (17·3–27·3)	22 (17·4–27·5)	20·1 (16–24·9)
Foreign body	6·96 (4·86–9·74)	7·59 (5·3–10·6)	10·4 (6·92–15·2)	6·21 (4·29–8·7)
Environmental heat and cold exposure	6·71 (3·03–12·1)	5·99 (2·82–10·6)	4·95 (2·31–8·76)	6·93 (3·21–12·4)
Exposure to forces of nature	5·2 (2·45–9·17)	4·85 (2·28–8·54)	6·42 (3·01–11·2)	4·84 (2·28–8·51)
Other unintentional injuries	21·2 (15·5–28·1)	19 (14–25·1)	18·2 (13·1–24·4)	20·7 (15·1–27·5)
Self-harm and interpersonal violence	12·3 (5·71–19·5)	13·1 (7·23–19·3)	16 (11·9–20·6)	12·4 (5·72–19·5)
Self-harm	11 (2·17–20·6)	11·5 (2·72–21)	12 (5·5–19·1)	11·5 (2·61–21·1)
Interpersonal violence	15·7 (9·25–22·4)	17·9 (12·1–24·6)	24·6 (18·8–31·1)	14·5 (8·04–21·4)

**Supplementary Results Table 2. All-age PAFs attributable to the joint distribution of all risk factors for all ages, both sexes combined for each GBD level 1, level 2, and level 3 cause and all causes in 2019 for Global. Results are shown for deaths, YLLs, YLDs, and DALYs Data in parenthesis are 95% uncertainty intervals. PAFs=population attributable fractions. GBD=Global Burden of Disease. YLLs=years of life lost. YLDs=years lived with disability. DALYs=disability-adjusted life-years**				
	**Deaths 2019**	**DALYs 2019**	**YLDs 2019**	**YLLs 2019**
All causes	61·9 (59·8–63·8)	47·8 (45·3–50)	28·6 (26·7–30·6)	57·6 (56·2–59·1)
Communicable, maternal, neonatal, and nutritional diseases	60·3 (56·9–63·5)	62·2 (59–65·1)	56·5 (54·4–58·5)	63·3 (59·4–66·6)
HIV/AIDS and sexually transmitted infections	77·3 (70·3–81·9)	70·5 (61·5–76·9)	90·3 (88·8–92·1)	68·6 (59–75·4)
HIV/AIDS	84·3 (82·9–85·6)	80·1 (78·5–81·5)	87·9 (86·6–89·1)	79·4 (77·8–80·8)
Sexually transmitted infections excluding HIV	10·6 (4·39–21·7)	17·6 (7·3–36·2)	100 (100–100)	4·88 (1·92–10·6)
Respiratory infections and tuberculosis	55·1 (51·6–58·6)	60·2 (56·6–63·6)	13·1 (9·94–16·9)	64·6 (61·4–67·7)
Tuberculosis	36·3 (31–41·3)	32·2 (27·3–36·8)	29·2 (24·3–33·8)	32·5 (27·7–37·2)
Lower respiratory infections	64·2 (59·6–68·9)	79·1 (75·4–82·6)	51·5 (45·3–57·6)	79·2 (75·6–82·8)
Upper respiratory infections	0·893 (0·517–1·22)	0·142 (0·0767–0·235)	0·0245 (0·0183–0·0326)	1·62 (0·99–2·31)
Otitis media	23·7 (3·97–49·4)	5·66 (3·6–7·99)	5·04 (3·32–6·71)	35·5 (7·7–63·4)
Enteric infections	80·7 (73·3–86·5)	78·3 (71–84·2)	78·3 (69–85·3)	78·3 (70·4–84·5)
Diarrheal diseases	92·2 (87–95·5)	93·8 (89·8–96·5)	79·1 (69·7–86·1)	96·1 (92·7–98)
Other infectious diseases	8·68 (4·6–15·5)	10·5 (5·73–19)	0·156 (0·0845–0·267)	11·3 (6·13–20·5)
Meningitis	4·9 (4·32–5·7)	6·25 (5·57–7·13)	0·00951 (0·00699–0·0127)	6·52 (5·8–7·4)
Encephalitis	1·31 (0·993–1·59)	2·16 (1·67–2·56)	0·00135 (0·00103–0·00177)	2·4 (1·82–2·86)
Measles	59·1 (44·2–76·9)	61·1 (46·1–78·9)	6·61 (0·274–12·8)	61·4 (46·4–79·2)
Acute hepatitis	2·44 (1·23–3·81)	1·8 (0·914–2·88)	0·858 (0·571–1·33)	1·9 (0·926–3·09)
Maternal and neonatal disorders	81·2 (79·4–83)	79·8 (78·1–81·6)	49·9 (43·7–55·9)	83·2 (81·7–84·6)
Maternal disorders	21·5 (7·91–34·5)	21·5 (7·92–34·4)	20·5 (7·5–32·8)	21·6 (7·95–34·6)
Neonatal disorders	87·5 (86·3–88·5)	83·9 (82·2–85·4)	51·9 (45·1–58·2)	87·5 (86·3–88·5)
Nutritional deficiencies	84·3 (82·2–86·3)	90·3 (88·8–91·7)	90·6 (88·5–92·2)	89·6 (87·9–91·4)
Protein-energy malnutrition	100 (100–100)	100 (100–100)	100 (100–100)	100 (100–100)
Vitamin A deficiency	--	100 (100–100)	100 (100–100)	--
Dietary iron deficiency	--	100 (100–100)	100 (100–100)	--
Non-communicable diseases	66·3 (63·4–68·8)	46·1 (42·6–49·4)	24·5 (22–26·8)	62·1 (60–64·1)
Neoplasms	44·2 (41–48·2)	41·8 (38·9–45·4)	33·6 (30·4–37·3)	42·1 (39·2–45·7)
Lip and oral cavity cancer	62·2 (57·5–66·5)	61·4 (56·5–65·8)	61·9 (57·5–66·2)	61·4 (56·5–65·8)
Nasopharynx cancer	48·8 (41·8–55·1)	47·7 (41–53·9)	53·7 (46·3–60·5)	47·4 (40·7–53·5)
Other pharynx cancer	61·9 (56·2–66·8)	60·7 (55–65·8)	67·9 (62·8–72·4)	60·6 (54·8–65·7)

Esophageal cancer	68·2 (60·8–75·2)	69·4 (62·2–76·2)	67·8 (60·5–75·1)	69·4 (62·2–76·2)
Stomach cancer	24·2 (15·7–43·1)	23·6 (14·9–42·7)	25·4 (16·7–44·4)	23·5 (14·8–42·7)
Colon and rectum cancer	58·2 (51·1–65·2)	57·6 (50·9–64·3)	59·1 (52·2–65·9)	57·5 (50·8–64·2)
Liver cancer	52·6 (45·6–59·4)	50·1 (43–57)	52·6 (45·7–59·5)	50 (42·9–56·9)
Gallbladder and biliary tract cancer	15·2 (8·05–24·7)	15·7 (8·45–25·2)	15·6 (8·2–25·2)	15·7 (8·45–25·2)
Pancreatic cancer	32·7 (25·6–41·6)	31·9 (25·2–40·7)	32·5 (25·5–41·2)	31·9 (25·2–40·7)
Larynx cancer	70·9 (65–75·6)	69·9 (63·7–74·7)	73·8 (68·2–78·2)	69·8 (63·5–74·6)
Tracheal, bronchus, and lung cancer	80·3 (77·5–83·2)	79·1 (76·2–81·9)	80 (77·4–82·9)	79·1 (76·2–81·9)
Breast cancer	25·2 (18·2–33·7)	22·4 (16·3–29·7)	26·7 (20–34·6)	22·1 (16–29·4)
Cervical cancer	100 (100–100)	100 (100–100)	100 (100–100)	100 (100–100)
Uterine cancer	39·8 (27·6–52·7)	40·2 (28–53·1)	43·5 (30·5–57)	39·8 (27·7–52·7)
Ovarian cancer	13·7 (5·8–23·9)	11·4 (4·64–20·3)	11·1 (4·54–20)	11·4 (4·64–20·4)
Prostate cancer	6·01 (2·71–9·29)	6·61 (3·02–10·1)	7·22 (3·35–10·9)	6·53 (3–10)
Kidney cancer	33·8 (25·8–42·3)	32·3 (24·5–40·5)	33·9 (25·6–42·5)	32·2 (24·5–40·4)
Bladder cancer	40·6 (29·6–51·2)	42·6 (32·3–52)	44·6 (34·3–54·3)	42·4 (32·1–51·9)
Thyroid cancer	10·2 (4·97–17)	10·4 (5·05–17)	11·8 (6·23–18·7)	10·2 (4·94–16·9)
Mesothelioma	91·7 (89·7–93·4)	85·2 (82·1–88·1)	91·4 (89·3–93·3)	85·1 (82–88)
Non-Hodgkin lymphoma	5·42 (2·3–9·6)	5·08 (2·18–8·96)	5·74 (2·41–10·1)	5·06 (2·17–8·92)
Multiple myeloma	7·07 (3·11–12·4)	7·2 (3·2–12·5)	7·52 (3·35–13·1)	7·19 (3·18–12·5)
Leukemia	25 (17·1–33·2)	18 (12·2–24·1)	24·3 (16·8–32·1)	17·8 (12·1–23·9)
Cardiovascular diseases	86 (83·7–88·2)	85·8 (84–87·5)	75·5 (72·1–78·8)	86·8 (85·1–88·4)
Rheumatic heart disease	25·1 (17·1–39·2)	23 (16–32·5)	20·1 (12·4–29·5)	23·6 (16·6–34·2)
Ischemic heart disease	93·5 (90·8–95·9)	94·5 (92·7–96·2)	94 (91·7–96)	94·6 (92·8–96·2)
Stroke	85 (81·2–88·9)	87 (84·2–89·8)	84·7 (81·4–88·1)	87·4 (84·5–90)
Hypertensive heart disease	100 (100–100)	100 (100–100)	100 (100–100)	100 (100–100)
Non-rheumatic valvular heart disease	26·6 (18·6–37·5)	24·9 (19–32·2)	12·1 (8·72–16)	26·5 (20·3–34·4)
Cardiomyopathy and myocarditis	45·2 (39·7–51·4)	48·3 (43·9–52·7)	39·7 (33·9–45·5)	48·7 (44·2–53·2)
Atrial fibrillation and flutter	49·6 (41·8–58·1)	56·7 (50·1–63·6)	59·4 (52·9–66·1)	53·4 (46·5–61)
Aortic aneurysm	56·2 (50·9–61·4)	62·9 (58·6–66·8)	--	62·9 (58·6–66·8)
Endocarditis	36·7 (27·7–47·2)	35·1 (27·1–44)	35·4 (26·3–46·4)	35·1 (27–43·9)
Other cardiovascular and circulatory diseases	35·1 (30·8–39·6)	32·7 (29·1–36·7)	33·7 (28·4–39·4)	32·4 (28·8–36·3)
Chronic respiratory diseases	69·6 (66·5–72·6)	62 (58·7–65·2)	51 (46·7–55)	67 (63·9–70·2)

Chronic obstructive pulmonary disease	79·2 (76–82·1)	76·1 (73·1–79)	66·1 (62·6–69·5)	79·7 (76·5–82·6)
Pneumoconiosis	100 (100–100)	100 (100–100)	100 (100–100)	100 (100–100)
Asthma	31·6 (24·2–40·2)	30·7 (24·8–37·7)	28·8 (23–35·7)	32·5 (25·7–40·3)
Digestive diseases	36·7 (32·2–41·5)	34·6 (29·9–39·4)	11·9 (7·93–16·9)	39·9 (34·8–45·3)
Cirrhosis and other chronic liver diseases	55·5 (48·1–63·2)	56·5 (48·9–64·3)	54 (47·5–61·3)	56·5 (48·9–64·4)
Upper digestive system diseases	16·3 (14–18·4)	7·73 (5·38–10·2)	0·875 (0·536–1·32)	16·3 (14–18·5)
Gallbladder and biliary diseases	34·8 (23·8–47·2)	36·2 (25·2–48·4)	37 (25·9–49·7)	34·8 (24·3–46·5)
Pancreatitis	30·2 (23·2–38·8)	33 (25·7–42)	31·2 (23·8–39·9)	33·1 (25·7–42·1)
Neurological disorders	20·4 (9·43–33·1)	9·74 (4·45–18·3)	5·52 (2·63–11·6)	17·4 (7·68–29·4)
Alzheimer's disease and other dementias	31·1 (20·1–44·2)	33·2 (22·2–46·2)	33·8 (22·7–46·8)	32·9 (21·9–45·8)
Parkinson's disease	-8·75 (-13·3–-4·45)	-10·3 (-15·5–-5·31)	-11·6 (-17·3–-6·14)	-9·95 (-15–-5·08)
Idiopathic epilepsy	13·1 (9·46–16·6)	10·7 (7·69–13·7)	10·3 (7·25–13·5)	11·2 (8·06–14·2)
Multiple sclerosis	13·6 (10·2–17·1)	13·9 (10·6–17·4)	13·5 (10·3–16·7)	14·1 (10·6–17·8)
Mental disorders	--	8·99 (5·32–13·1)	8·99 (5·32–13·1)	--
Depressive disorders	--	13·9 (6·08–21·8)	13·9 (6·08–21·8)	--
Anxiety disorders	--	7·07 (2·15–14·4)	7·07 (2·15–14·4)	--
Idiopathic developmental intellectual disability	--	62·5 (32·5–80·6)	62·5 (32·5–80·6)	--
Substance use disorders	100 (100–100)	100 (100–100)	100 (100–100)	100 (100–100)
Alcohol use disorders	100 (100–100)	100 (100–100)	100 (100–100)	100 (100–100)
Drug use disorders	100 (100–100)	100 (100–100)	100 (100–100)	100 (100–100)
Diabetes and kidney diseases	99·6 (99·6–99·8)	99·7 (99·7–99·8)	100 (100–100)	99·5 (99·5–99·7)
Diabetes mellitus	100 (100–100)	100 (100–100)	100 (100–100)	100 (100–100)
Chronic kidney disease	100 (100–100)	100 (100–100)	100 (100–100)	100 (100–100)
Sense organ diseases	--	15·5 (13·5–17·5)	15·5 (13·5–17·5)	--
Blindness and vision loss	--	14·5 (9·37–19·5)	14·5 (9·37–19·5)	--
Age-related and other hearing loss	--	17·4 (16·3–18·7)	17·4 (16·3–18·7)	--
Musculoskeletal disorders	3·22 (0·969–5·65)	19·3 (16·4–22·5)	19·7 (16·6–22·9)	2·78 (0·845–4·85)
Rheumatoid arthritis	8·53 (2·52–15)	9·62 (2·89–16·8)	9·71 (2·91–16·9)	9·39 (2·83–16·5)
Osteoarthritis	--	14·1 (7·63–21·4)	14·1 (7·63–21·4)	--
Low back pain	--	39·8 (36·1–43·6)	39·8 (36·1–43·6)	--
Gout	--	41 (28·6–56·2)	41 (28·6–56·2)	--
Other non-communicable diseases	0·181 (0·103–0·338)	0·12 (0·0619–0·236)	--	0·277 (0·156–0·508)

Sudden infant death syndrome	7·97 (6·14–10·6)	7·96 (6·13–10·6)	--	7·96 (6·13–10·6)
Injuries	22·5 (20·1–24·8)	20·9 (18·7–23·1)	28·4 (26·1–30·6)	18·3 (16·1–20·6)
Transport injuries	27 (24–30·2)	26·4 (23·7–29·6)	30·3 (27·6–33·2)	25·3 (22·4–28·7)
Road injuries	26·4 (23·5–29·7)	26·1 (23·3–29·3)	29·9 (27·3–32·8)	24·9 (22–28·4)
Other transport injuries	34·7 (31·6–37·8)	31·9 (29–35·2)	36·6 (33·6–39·7)	30·8 (27·8–34·1)
Unintentional injuries	25·4 (22·5–28·1)	21·3 (18·7–24)	30·1 (27·3–32·8)	16·3 (14·1–18·8)
Falls	47·3 (42·4–50·7)	37·4 (33·6–40·5)	39 (35·1–42·1)	35·4 (32–38·6)
Drowning	2·24 (-2·03–7·01)	2·73 (-1·03–7·45)	14 (11·7–17)	2·59 (-1·25–7·33)
Fire, heat, and hot substances	11 (8·73–14)	13·9 (11·2–17·3)	19·7 (17·2–23)	11 (8·66–13·8)
Poisonings	15·2 (12·8–18·5)	15·1 (12·7–18·1)	17·5 (14·9–21·1)	14·8 (12·5–17·8)
Exposure to mechanical forces	18·2 (14·8–21·7)	19·8 (17·1–22·6)	24·7 (22·8–26·3)	15·2 (11·7–19)
Animal contact	12·7 (10·4–15·9)	13·6 (11–16·7)	25·1 (22·6–28)	11·7 (9·29–14·7)
Foreign body	5·99 (5·41–6·69)	7·27 (6·47–8·2)	9·29 (7·47–11·3)	6·65 (5·9–7·47)
Environmental heat and cold exposure	6·69 (3·09–12·2)	6·01 (2·75–10·5)	4·7 (2·19–8·19)	6·8 (3·1–12·2)
Exposure to forces of nature	2·49 (1·15–4·39)	2·94 (1·38–5·06)	3·41 (1·57–5·92)	2·1 (0·966–3·69)
Other unintentional injuries	19·7 (17–23·4)	18·7 (16·3–22·1)	17·3 (14·6–20·9)	19·6 (17–23)
Self-harm and interpersonal violence	13·8 (8·9–18·9)	13·9 (9·73–18·3)	17 (12·4–22·4)	13·5 (8·96–18·4)
Self-harm	13·5 (6·03–21·1)	13·4 (6·29–20·6)	11·8 (6·46–17·1)	13·5 (6·26–20·7)
Interpersonal violence	16·9 (11·7–22·6)	18 (13–23·6)	28·1 (21·9–35·4)	15·9 (10·8–21·6)

Supplementary Results Table 2. All-age PAFs attributable to the joint distribution of all risk factors for all ages, both sexes combined for each GBD level 1, level 2, and level 3 cause and all causes in 2019 for Norfolk. Results are shown for deaths, YLLs, YLDs, and DALYs Data in parenthesis are 95% uncertainty intervals. PAFs=population attributable fractions. GBD=Global Burden of Disease. YLLs=years of life lost. YLDs=years lived with disability. DALYs=disability-adjusted life-years

	Deaths 2019	DALYs 2019	YLDs 2019	YLLs 2019
All causes	54·7 (51·3–58·3)	43·1 (40·1–46·4)	30·5 (28·2–33)	54·6 (51·7–57·5)
Communicable, maternal, neonatal, and nutritional diseases	43·2 (32·6–53·1)	49·2 (43·4–55)	46·5 (42·3–50·9)	50·6 (42·2–58·7)
HIV/AIDS and sexually transmitted infections	80·9 (67·8–90·4)	79·7 (64·2–90·2)	97·4 (96·1–98·4)	64·2 (45·4–81·1)
HIV/AIDS	93·2 (91·3–94·3)	92·5 (90·3–94)	95·1 (93–96·4)	90·4 (87·9–91·8)
Sexually transmitted infections excluding HIV	62·5 (39·9–84·3)	63·7 (38·7–86·8)	100 (100–100)	26·3 (11·3–50·6)
Respiratory infections and tuberculosis	44·2 (32·8–55)	41·7 (31·7–51·2)	3·43 (2·51–4·54)	47·1 (36·1–57·7)
Tuberculosis	54·4 (40·9–66·8)	57·6 (45·9–68·7)	55·4 (43·6–66·6)	58 (46·1–69·2)
Lower respiratory infections	44·1 (32·7–55·1)	47 (36–57·6)	32·4 (25·3–39·6)	47·1 (36–57·8)
Upper respiratory infections	0·0316 (0·0167–0·0532)	0·00707 (0·00163–0·0178)	0·00549 (0·000132–0·0158)	0·0888 (0·0458–0·147)
Otitis media	1·48 (0·651–2·9)	3·82 (2·38–5·5)	3·81 (2·37–5·5)	4·36 (2·13–7·65)
Enteric infections	5·33 (3·04–9·23)	8·34 (5·83–12·3)	8·97 (6·32–13·1)	6·96 (4·49–10·8)
Diarrheal diseases	5·39 (3·08–9·41)	8·39 (5·85–12·4)	8·98 (6·32–13·1)	7·08 (4·58–11·1)
Other infectious diseases	0·385 (0·268–0·545)	0·945 (0·696–1·21)	0·506 (0·284–0·865)	1·11 (0·789–1·46)
Meningitis	1·1 (0·771–1·5)	2·21 (1·58–2·97)	0·000497 (8·2e-06–0·00152)	2·82 (2·01–3·8)
Encephalitis	0·206 (0·129–0·306)	0·658 (0·386–0·986)	0·000197 (3·74e-06–0·000575)	0·814 (0·507–1·2)
Measles	2·28 (-0·532–13·1)	2·73 (-0·614–15)	0·635 (0·0165–1·74)	2·86 (-0·672–15·8)
Acute hepatitis	8 (2·74–23·1)	4·39 (2·35–8·34)	3·33 (1·92–5·49)	7·87 (2·83–22·5)
Maternal and neonatal disorders	89 (87·1–90·6)	76·2 (72–79·8)	63·8 (57·8–69·4)	90 (88·4–91·5)
Maternal disorders	9·29 (3·27–15·6)	9·33 (3·28–15·5)	9·49 (3·4–15·8)	9·13 (3·23–15·3)
Neonatal disorders	91·8 (90·2–93·2)	77·8 (73·6–81·4)	65·2 (58·8–70·7)	91·9 (90·2–93·3)
Nutritional deficiencies	77·6 (72·5–89·5)	77·3 (73·4–81·8)	77·3 (73·2–81·9)	78·1 (71–85·2)
Protein-energy malnutrition	100 (100–100)	100 (100–100)	100 (100–100)	100 (100–100)
Vitamin A deficiency	--	100 (100–100)	100 (100–100)	--
Dietary iron deficiency	--	100 (100–100)	100 (100–100)	--
Non-communicable diseases	56·4 (52·8–60·2)	43·8 (40·6–47·4)	29·9 (27·3–32·6)	56·6 (53·6–59·7)
Neoplasms	47 (42·4–51·6)	46·9 (42·6–51·2)	33·1 (28·4–38·1)	47·7 (43·4–52)
Lip and oral cavity cancer	63·8 (51·5–74·7)	67·5 (55·9–77·3)	67 (54·9–77·2)	67·6 (55·9–77·3)
Nasopharynx cancer	62·6 (48·1–74·2)	63·4 (49·9–74·6)	60·3 (46·2–72·4)	63·7 (50·4–74·8)
Other pharynx cancer	76·3 (67·3–83·5)	78·2 (69·7–85)	78·1 (69·1–84·9)	78·2 (69·7–85)
Esophageal cancer	74·4 (63·5–83·5)	76·3 (65·9–85)	75·3 (64·7–84·2)	76·3 (66–85)
Stomach cancer	24·2 (16·7–41·2)	24·9 (17·4–41·5)	25·4 (17·9–42)	24·9 (17·4–41·5)

Colon and rectum cancer	64·7 (54·5–74·4)	65·2 (55·7–74·3)	65·5 (55·7–74·5)	65·1 (55·7–74·3)
Liver cancer	75·9 (69·9–82)	77·1 (71·4–82·6)	77·7 (71·6–83·3)	77 (71·4–82·6)
Gallbladder and biliary tract cancer	22·8 (12·3–35·1)	23·3 (12·7–35·8)	21·3 (11–33·9)	23·4 (12·8–35·9)
Pancreatic cancer	43·5 (33·1–55·6)	43·4 (33·7–55·1)	43·5 (33·1–55·6)	43·4 (33·7–55·1)
Larynx cancer	80·7 (71·8–88·1)	81·1 (73·3–87·7)	79·1 (70·8–86·1)	81·3 (73·5–88)
Tracheal, bronchus, and lung cancer	86·3 (82·6–89·7)	85·9 (82·5–89·3)	86·4 (82·8–89·8)	85·9 (82·5–89·2)
Breast cancer	38·4 (27·7–51·4)	37·6 (28–49·1)	37·9 (28·2–49·3)	37·6 (28–49·1)
Cervical cancer	100 (100–100)	100 (100–100)	100 (100–100)	100 (100–100)
Uterine cancer	44·8 (28·7–60·6)	46 (30·6–61·9)	46·5 (31·1–62·2)	46 (30·5–61·9)
Ovarian cancer	28·2 (14·1–43·7)	24·4 (11·9–38·8)	23·1 (11·3–36·9)	24·4 (11·9–38·9)
Prostate cancer	5·76 (2·42–9·45)	6·3 (2·76–10·1)	6·75 (3·03–10·8)	6·23 (2·73–9·96)
Kidney cancer	39·5 (28·7–50·3)	40·2 (29·3–50·6)	40·2 (29·2–50·7)	40·2 (29·4–50·6)
Bladder cancer	39·9 (24·7–55·4)	42·3 (28·3–56·7)	43·4 (29·2–57·4)	42·3 (28·2–56·7)
Thyroid cancer	15·5 (7·47–25·9)	16·4 (7·6–26·8)	16·9 (7·77–28·1)	16·3 (7·66–26·8)
Mesothelioma	99·2 (98·9–99·5)	98·4 (97·9–98·9)	99·2 (98·8–99·4)	98·4 (97·8–98·8)
Non-Hodgkin lymphoma	7·65 (3·18–13·5)	7·92 (3·35–14)	7·84 (3·29–14)	7·93 (3·36–14)
Multiple myeloma	8·55 (3·52–15·1)	8·9 (3·69–15·8)	8·87 (3·75–15·9)	8·9 (3·69–15·8)
Leukemia	37·2 (22·8–52·5)	36·3 (23·6–49)	35·7 (23–48·7)	36·3 (23·6–49)
Cardiovascular diseases	78·5 (74·4–82·5)	79·4 (76–82·6)	70·1 (64·6–75·2)	80·6 (77·4–83·7)
Rheumatic heart disease	21·8 (11·8–44)	22·7 (13·8–39)	22·7 (13·7–38·3)	22·8 (13·8–39·1)
Ischemic heart disease	93·1 (88·8–96·7)	94·2 (90·7–96·9)	93·5 (90·3–96·4)	94·2 (90·8–96·9)
Stroke	78·5 (70–88·4)	81·3 (74·4–88·5)	81·2 (75·2–88)	81·4 (74·3–88·7)
Hypertensive heart disease	100 (100–100)	100 (100–100)	100 (100–100)	100 (100–100)
Non-rheumatic valvular heart disease	24·6 (15–38)	24·7 (16·6–35·9)	17·2 (11·2–24·7)	25·8 (17·3–37·6)
Cardiomyopathy and myocarditis	35·6 (27·5–45·2)	42·2 (35·6–48·4)	35·9 (28·6–43·6)	42·7 (36–48·8)
Atrial fibrillation and flutter	49·5 (38·3–61)	56·3 (46·8–66·2)	60·2 (51·3–69·2)	52·2 (41·9–62·9)
Aortic aneurysm	48·5 (41·2–56·4)	55 (48·8–61·5)	--	55 (48·8–61·5)
Endocarditis	30 (19·7–44·4)	32·5 (23·4–43·9)	29·7 (19·4–44·3)	32·6 (23·4–44)
Other cardiovascular and circulatory diseases	31 (24·8–38·4)	33·9 (27·9–40·4)	33·7 (26·3–40·9)	34 (27·9–40·3)
Chronic respiratory diseases	60·7 (54–68·1)	58 (53·3–63·1)	53·3 (48·1–58·4)	61·8 (55·8–68·6)
Chronic obstructive pulmonary disease	71 (65·5–76·4)	70·2 (65·8–74·3)	64·4 (60·1–68·5)	73·3 (68·4–78)
Pneumoconiosis	100 (100–100)	100 (100–100)	100 (100–100)	100 (100–100)
Asthma	41·8 (29·6–55·6)	43·2 (33·8–53·6)	43 (33·8–53·2)	44·8 (33·8–56·5)
Digestive diseases	24·7 (20·1–29·9)	30·9 (25·5–36·5)	21 (14·4–28·8)	35·7 (29·4–42·2)
Cirrhosis and other chronic liver diseases	69·8 (56·9–84·6)	73·8 (61·6–87)	71·5 (59·3–84·3)	73·9 (61·7–87·1)

Upper digestive system diseases	19·4 (15·9–22·9)	9·17 (5·85–12·7)	0·726 (0·41–1·17)	22·2 (18·6–25·7)
Gallbladder and biliary diseases	43·9 (28–60·3)	46·9 (32·4–61·7)	47·5 (33·5–62·1)	45·1 (29·7–60·7)
Pancreatitis	32 (10·3–54·8)	38 (15·4–59)	37·5 (15·3–58·9)	38·1 (15·7–59·6)
Neurological disorders	26 (11·1–42·4)	17·6 (8·85–30·7)	12 (6·01–22·2)	22·9 (9·73–39·2)
Alzheimer's disease and other dementias	41·8 (26·8–58·4)	43·7 (28·7–59·9)	44·1 (29·2–60·1)	43·5 (28·7–59·6)
Parkinson's disease	-8·78 (-14·7–-3·79)	-9·95 (-16·6–-4·35)	-10·4 (-17·4–-4·6)	-9·85 (-16·4–-4·33)
Idiopathic epilepsy	25·2 (13·5–36·1)	24·4 (13·4–34·6)	22·4 (12·1–32)	27·3 (15·7–37·9)
Multiple sclerosis	13·3 (9·69–17)	14·5 (10·8–18·3)	14·3 (10·8–18·1)	14·6 (10·7–18·6)
Mental disorders	--	6·25 (3·18–9·6)	6·25 (3·18–9·61)	--
Depressive disorders	--	12·8 (5·81–20·1)	12·8 (5·81–20·1)	--
Anxiety disorders	--	4·46 (1·17–9·98)	4·46 (1·17–9·98)	--
Idiopathic developmental intellectual disability	--	37·7 (17–56·6)	37·7 (17–56·6)	--
Substance use disorders	100 (100–100)	100 (100–100)	100 (100–100)	100 (100–100)
Alcohol use disorders	100 (100–100)	100 (100–100)	100 (100–100)	100 (100–100)
Drug use disorders	100 (100–100)	100 (100–100)	100 (100–100)	100 (100–100)
Diabetes and kidney diseases	99·6 (99·5–99·7)	99·9 (99·9–99·9)	100 (100–100)	99·6 (99·5–99·7)
Diabetes mellitus	100 (100–100)	100 (100–100)	100 (100–100)	100 (100–100)
Chronic kidney disease	100 (100–100)	100 (100–100)	100 (100–100)	100 (100–100)
Sense organ diseases	--	8·24 (6·98–9·76)	8·24 (6·98–9·76)	--
Blindness and vision loss	--	10·7 (6·62–15·8)	10·7 (6·62–15·8)	--
Age-related and other hearing loss	--	8·64 (7·69–9·66)	8·64 (7·69–9·66)	--
Musculoskeletal disorders	3·42 (0·79–6·45)	22·7 (18·7–26·6)	23·2 (19·1–27·2)	4·26 (1·04–7·74)
Rheumatoid arthritis	9·95 (2·34–18·2)	12·4 (3·12–22·1)	12·7 (3·21–22·8)	11·7 (2·84–20·9)
Osteoarthritis	--	17·6 (9·34–26·7)	17·6 (9·34–26·7)	--
Low back pain	--	41 (35·9–46·6)	41 (35·9–46·6)	--
Gout	--	54 (38·7–70·4)	54 (38·7–70·4)	--
Other non-communicable diseases	0·0504 (0·0323–0·0757)	0·0527 (0·0317–0·0797)	--	0·234 (0·148–0·342)
Sudden infant death syndrome	6·29 (3·67–10·1)	6·28 (3·66–10·1)	--	6·28 (3·66–10·1)
Injuries	33·6 (27–39·3)	28·4 (23·1–33)	31·5 (26·8–35·8)	24·6 (16·7–31·2)
Transport injuries	21·1 (13·2–29·2)	21·3 (14·4–28·2)	26·3 (21·9–30·7)	18·9 (10·5–27·8)
Road injuries	20·4 (12·2–28·8)	20·4 (13·4–27·6)	25·1 (20·8–29·4)	18·3 (9·57–27·5)
Other transport injuries	33·1 (27·5–38·1)	32·6 (27·2–37·7)	36·8 (32–40·8)	29·1 (22·7–35)
Unintentional injuries	41·8 (35·3–46·6)	31·3 (26·3–35·8)	31·9 (26·9–36·8)	29·4 (24·3–34)
Falls	56·1 (47·6–61·7)	43·6 (37·1–48·8)	41·9 (35·6–47)	48·8 (41·6–54·2)
Drowning	-14·2 (-28·6–1·03)	-12·5 (-26·1–1·98)	9·83 (5·12–16·3)	-13·5 (-27·7–1·4)

Fire, heat, and hot substances	9·69 (4·9–16)	12 (6·94–18·7)	12·2 (6·97–18·9)	11·6 (6·59–18·2)
Poisonings	13·6 (7·88–20·9)	12·8 (7·61–19·6)	11·3 (6·49–17·5)	14·8 (9·05–22·1)
Exposure to mechanical forces	11·4 (0·265–23·5)	17·5 (14·6–19·9)	18·9 (16·2–21·1)	1·11 (-11·3–14·2)
Animal contact	23 (18–29·3)	21·1 (16·2–26·7)	22 (16·9–27·6)	19·5 (14·7–25·4)
Foreign body	2·04 (1·32–2·98)	3·17 (2·08–4·62)	2·54 (1·65–3·64)	3·61 (2·38–5·26)
Environmental heat and cold exposure	7·45 (2·9–13·7)	8·91 (3·74–16)	8·98 (3·78–16·1)	8·68 (3·66–15·4)
Exposure to forces of nature	0 (0–0)	9·88 (4·3–17·5)	9·88 (4·3–17·5)	0 (0–0)
Other unintentional injuries	12·7 (7·28–19·9)	11·6 (6·55–18·1)	11·5 (6·51–18)	14·8 (9·06–22·2)
Self-harm and interpersonal violence	21·8 (6·87–35·8)	24·5 (10·8–37·7)	32·4 (24·2–40·8)	23·3 (8·17–37·2)
Self-harm	21·5 (5·81–36)	23·1 (7·55–37·3)	21·6 (8·09–34·9)	23·2 (7·47–37·5)
Interpersonal violence	30·6 (21·4–40·3)	35·1 (26·5–44·5)	38·3 (29·6–47·5)	27·7 (18·4–37·5)

Supplementary Results Table 2. All-age PAFs attributable to the joint distribution of all risk factors for all ages, both sexes combined for each GBD level 1, level 2, and level 3 cause and all causes in 2019 for Peterborough. Results are shown for deaths, YLLs, YLDs, and DALYs Data in parenthesis are 95% uncertainty intervals. PAFs=population attributable fractions. GBD=Global Burden of Disease. YLLs=years of life lost. YLDs=years lived with disability. DALYs=disability-adjusted life-years				
	Deaths 2019	DALYs 2019	YLDs 2019	YLLs 2019
All causes	55·6 (52·2–59)	42·6 (39·4–45·7)	30·1 (27·7–32·7)	54·7 (51·8–57·7)
Communicable, maternal, neonatal, and nutritional diseases	45·2 (35·1–54·5)	54·1 (48·8–59)	49 (44·2–53·8)	56·7 (49·7–63·6)
HIV/AIDS and sexually transmitted infections	71·9 (54·9–85·1)	72·8 (54·3–86·2)	96·9 (95·4–98·1)	54·9 (35·4–73·8)
HIV/AIDS	90·6 (88·3–92)	89·7 (87–91·7)	94·3 (91·8–95·8)	86·4 (83·7–88)
Sexually transmitted infections excluding HIV	40·3 (20·7–67·6)	52·8 (28·3–80·6)	100 (100–100)	14·5 (5·61–32·3)
Respiratory infections and tuberculosis	45·2 (33·7–55·9)	42·2 (32·7–51·2)	3·29 (2·42–4·5)	49·1 (38·5–59·1)
Tuberculosis	55·4 (42·2–67·8)	57·8 (45·9–69·2)	54·3 (41·7–66·2)	58·6 (46·9–70)
Lower respiratory infections	45·1 (33·6–56)	49·1 (38·3–59·2)	33·6 (26·5–41)	49·2 (38·4–59·4)
Upper respiratory infections	0·0539 (0·029–0·0931)	0·0109 (0·00285–0·0238)	0·00801 (0·00037–0·0211)	0·105 (0·056–0·183)
Otitis media	3·68 (2·05–6·49)	5·15 (3·24–7·35)	5·05 (3·15–7·26)	7·85 (4·81–12·3)
Enteric infections	6·01 (3·58–11)	10·7 (7·94–15·3)	10·9 (8–15·7)	10·1 (7·06–15·1)
Diarrheal diseases	6·08 (3·65–11·1)	10·8 (8·01–15·4)	11 (8·01–15·7)	10·3 (7·24–15·4)
Other infectious diseases	0·764 (0·502–1·01)	1·27 (0·941–1·62)	0·443 (0·261–0·733)	1·55 (1·12–2·01)
Meningitis	1·88 (1·33–2·56)	2·82 (2·02–3·77)	0·000742 (2·46e-05–0·00203)	3·44 (2·45–4·57)
Encephalitis	0·433 (0·259–0·698)	1·02 (0·581–1·61)	0·000327 (9·44e-06–0·000962)	1·2 (0·707–1·86)
Measles	3·54 (-1·08–18)	3·69 (-0·964–18)	0·675 (0·0159–1·84)	4·31 (-1·26–21·5)
Acute hepatitis	16·2 (10·4–25·3)	5·49 (3·57–8·27)	2·74 (1·6–4·5)	15·4 (9·85–23·1)
Maternal and neonatal disorders	88·6 (86·6–90·4)	81·6 (78·7–84·4)	69·3 (63·9–74·2)	89·5 (87·5–91·1)
Maternal disorders	9·59 (3·55–15·9)	9·67 (3·46–16·1)	9·83 (3·51–16·5)	9·46 (3·51–15·6)
Neonatal disorders	90·9 (89·1–92·6)	83·4 (80·5–86·1)	71·4 (65·8–76·4)	90·9 (89·1–92·6)
Nutritional deficiencies	76·6 (70·9–87·5)	78 (73·6–83·1)	78·1 (73·5–83·2)	76·4 (66·8–80·8)
Protein-energy malnutrition	100 (100–100)	100 (100–100)	100 (100–100)	100 (100–100)
Vitamin A deficiency	--	100 (100–100)	100 (100–100)	--
Dietary iron deficiency	--	100 (100–100)	100 (100–100)	--
Non-communicable diseases	57·7 (54·2–61·2)	43·3 (39·9–46·8)	29·5 (26·8–32·2)	57·5 (54·4–60·5)
Neoplasms	48·6 (44·2–53·4)	47·5 (43·5–51·7)	34·2 (29·6–39)	48·2 (44·3–52·5)
Lip and oral cavity cancer	64·7 (52·8–76·2)	67·9 (56–78·3)	67·5 (55·7–78·4)	68 (55·9–78·3)
Nasopharynx cancer	62·5 (47·3–75·3)	62·3 (47·4–74·4)	59·1 (43·7–72·7)	62·6 (47·7–74·7)
Other pharynx cancer	78·6 (69·6–85·8)	79·9 (71–86·7)	79·7 (70·8–86·4)	79·9 (71–86·7)
Esophageal cancer	74·2 (63·1–83)	76·1 (65·3–84·3)	75·1 (64·4–83·8)	76·1 (65·3–84·4)

Stomach cancer	25·6 (18–42·4)	26 (18·4–42·2)	26·5 (18·7–42·9)	26 (18·3–42·2)
Colon and rectum cancer	64·7 (54·9–74·1)	64·9 (55·6–73·8)	65·2 (55·8–74·2)	64·9 (55·6–73·8)
Liver cancer	75·3 (68·7–81·4)	75·4 (69·2–81)	76·2 (70·2–81·7)	75·4 (69·2–81)
Gallbladder and biliary tract cancer	22 (11·9–34·6)	22·3 (12·1–34·8)	20·6 (10·7–33)	22·4 (12·2–34·9)
Pancreatic cancer	44·9 (34·8–57·4)	44·3 (34·9–56·4)	44·7 (34·7–57·1)	44·3 (34·9–56·4)
Larynx cancer	80·9 (72·4–87·6)	80·9 (73·1–87)	78·3 (69·9–85·2)	81·2 (73·4–87·2)
Tracheal, bronchus, and lung cancer	87·3 (83·8–90·6)	86·4 (83·1–89·7)	87·1 (83·7–90·5)	86·3 (83·1–89·7)
Breast cancer	38·3 (27·7–51·2)	36·5 (26·9–47·6)	36·9 (27·1–47·9)	36·5 (26·9–47·5)
Cervical cancer	100 (100–100)	100 (100–100)	100 (100–100)	100 (100–100)
Uterine cancer	43·7 (28·3–60·3)	44·9 (29·5–61·1)	45·4 (29·9–61·4)	44·9 (29·4–61·1)
Ovarian cancer	29·8 (14·9–45·5)	24·7 (12·2–39·1)	22·7 (11·2–36·2)	24·8 (12·2–39·2)
Prostate cancer	6·22 (2·68–10)	6·85 (3·05–10·9)	7·35 (3·32–11·4)	6·78 (2·99–10·8)
Kidney cancer	39·7 (29·3–50·6)	39·6 (29·4–49·9)	39·5 (29·3–49·6)	39·6 (29·4–49·9)
Bladder cancer	43 (27·5–58)	45·5 (31·3–59·4)	46·4 (32·3–59·6)	45·5 (31·3–59·4)
Thyroid cancer	14·4 (6·93–23·6)	15·1 (7·27–24·5)	15·4 (7·28–25·1)	15 (7·23–24·5)
Mesothelioma	98·9 (98·5–99·2)	97·5 (96·7–98·3)	98·8 (98·3–99·2)	97·5 (96·7–98·2)
Non-Hodgkin lymphoma	7·29 (2·9–13·2)	7·44 (3·09–13·2)	7·41 (2·97–13·2)	7·44 (3·1–13·2)
Multiple myeloma	8·25 (3·47–14·8)	8·58 (3·7–15·5)	8·55 (3·64–15·3)	8·58 (3·69–15·5)
Leukemia	37·4 (23·3–51·3)	33·6 (22·6–44·6)	33·1 (21·9–44·2)	33·7 (22·7–44·7)
Cardiovascular diseases	79·8 (76–83·5)	81·1 (78–84)	70·8 (65·3–75·8)	82·3 (79·4–85)
Rheumatic heart disease	22·2 (11·9–43·4)	23·3 (14·7–37·7)	22·9 (14·3–36·1)	23·3 (14·8–38·1)
Ischemic heart disease	93·6 (89·8–96·8)	95 (92·4–97·2)	94 (91·1–96·6)	95 (92·4–97·2)
Stroke	79·3 (71·2–88·3)	82·8 (77·1–88·8)	82·2 (76·7–87·8)	82·9 (76·9–89·2)
Hypertensive heart disease	100 (100–100)	100 (100–100)	100 (100–100)	100 (100–100)
Non-rheumatic valvular heart disease	25·5 (16–38·5)	26·5 (18·7–37)	19·4 (12·7–28·3)	27·1 (19·2–38)
Cardiomyopathy and myocarditis	38·2 (30·6–47·3)	43·8 (38·2–50·4)	37·3 (30·6–44·3)	44·2 (38·4–50·9)
Atrial fibrillation and flutter	49·1 (37·2–60·7)	56·9 (46·8–66·2)	60·9 (51·4–69·7)	52·4 (41·6–62·8)
Aortic aneurysm	51·1 (44–58·4)	58·6 (52·7–64·6)	--	58·6 (52·7–64·6)
Endocarditis	30·6 (20·5–44·5)	33·2 (24·4–44·4)	30 (20·1–43·3)	33·3 (24·4–44·4)
Other cardiovascular and circulatory diseases	31·6 (24·9–39)	34·1 (27·5–40·2)	33·5 (25·7–41·5)	34·3 (27·8–40·5)
Chronic respiratory diseases	64·4 (57·9–71·2)	58·7 (53·9–64·1)	51·1 (45·9–56·6)	64·7 (58·9–70·9)
Chronic obstructive pulmonary disease	73 (67·5–78·1)	72·4 (68·1–76·6)	65·9 (61·9–69·7)	75·2 (70·3–79·8)
Pneumoconiosis	100 (100–100)	100 (100–100)	100 (100–100)	100 (100–100)
Asthma	42·4 (31–55)	41 (32·4–50·5)	40·7 (32·2–50·1)	43·7 (33·8–54·2)

Digestive diseases	29·7 (24·2–35·8)	36·3 (29·9–43·1)	25·7 (17·6–34·7)	41·8 (34·9–49·6)
Cirrhosis and other chronic liver diseases	70·2 (58·4–85·4)	73·4 (62–87·4)	69·7 (56·8–82·8)	73·5 (62–87·5)
Upper digestive system diseases	22 (18·2–25·6)	9·75 (6·38–13·6)	0·64 (0·355–1·04)	25·2 (21·4–28·6)
Gallbladder and biliary diseases	43·8 (28·2–59·9)	50 (35·6–64·3)	51·2 (36·6–65·7)	45 (29·9–60·8)
Pancreatitis	30·4 (8·57–54·5)	36·7 (12·7–59·8)	37 (12·8–60·3)	36·6 (12·8–59·8)
Neurological disorders	25·4 (10·5–42·1)	14·5 (6·99–26)	8·95 (4·13–17·6)	21·7 (10·1–37·4)
Alzheimer's disease and other dementias	42·6 (27·8–59)	44·7 (29·7–60·4)	45 (30·3–61)	44·5 (29·7–60·3)
Parkinson's disease	-9·32 (-15·7–-4·05)	-10·8 (-17·8–-4·77)	-11·5 (-18·6–-5·12)	-10·7 (-17·5–-4·7)
Idiopathic epilepsy	25·1 (13·6–36·7)	23·4 (12·4–34·1)	21·2 (11–31·9)	25·7 (14·4–36·5)
Multiple sclerosis	15·1 (11·2–19·3)	16·2 (12·2–20·4)	15·9 (11·9–19·9)	16·3 (12·2–20·8)
Mental disorders	--	6·63 (3·3–10·3)	6·63 (3·3–10·3)	--
Depressive disorders	--	13·9 (6·33–21·7)	13·9 (6·33–21·7)	--
Anxiety disorders	--	5·34 (1·35–12·1)	5·34 (1·35–12·1)	--
Idiopathic developmental intellectual disability	--	33 (13–53·1)	33 (13–53·1)	--
Substance use disorders	100 (100–100)	100 (100–100)	100 (100–100)	100 (100–100)
Alcohol use disorders	100 (100–100)	100 (100–100)	100 (100–100)	100 (100–100)
Drug use disorders	100 (100–100)	100 (100–100)	100 (100–100)	100 (100–100)
Diabetes and kidney diseases	99·7 (99·7–99·8)	99·9 (99·9–100)	100 (100–100)	99·7 (99·7–99·8)
Diabetes mellitus	100 (100–100)	100 (100–100)	100 (100–100)	100 (100–100)
Chronic kidney disease	100 (100–100)	100 (100–100)	100 (100–100)	100 (100–100)
Sense organ diseases	--	8·97 (7·81–10·4)	8·97 (7·81–10·4)	--
Blindness and vision loss	--	9·48 (5·97–14)	9·48 (5·97–14)	--
Age-related and other hearing loss	--	10·2 (9·28–11·4)	10·2 (9·28–11·4)	--
Musculoskeletal disorders	4·13 (0·973–7·62)	23·3 (19·3–27·2)	23·6 (19·6–27·8)	4·94 (1·22–8·85)
Rheumatoid arthritis	11·1 (2·68–20·1)	13·7 (3·47–24·2)	13·9 (3·44–24·7)	13 (3·34–23·3)
Osteoarthritis	--	17·1 (8·93–26·2)	17·1 (8·93–26·2)	--
Low back pain	--	42·7 (37·3–48·2)	42·7 (37·3–48·2)	--
Gout	--	50·9 (35·2–68·3)	50·9 (35·2–68·3)	--
Other non-communicable diseases	0·101 (0·0647–0·148)	0·1 (0·0605–0·152)	--	0·338 (0·217–0·474)
Sudden infant death syndrome	6·54 (3·74–10·8)	6·53 (3·73–10·8)	--	6·53 (3·73–10·8)
Injuries	29·1 (21·7–35·4)	24·2 (18·2–29·6)	27·7 (23·6–32·4)	21 (12·4–28·6)
Transport injuries	19 (10·5–27·6)	18·8 (11·2–26·6)	25·2 (20·7–30·2)	16·4 (7·02–25·4)
Road injuries	18·3 (9·43–27·2)	18 (10·2–25·9)	24·2 (19·7–29·3)	15·7 (5·97–25·2)
Other transport injuries	29·5 (23·7–35·5)	28·6 (23·2–34·4)	33·3 (28·6–38)	25·9 (19·4–32·6)

Unintentional injuries	37·4 (31·2–42·2)	26·1 (21·8–30·9)	27·7 (23·2–32·6)	22·2 (17·7–26·6)
Falls	54·1 (46·1–59·8)	38·5 (32·8–44)	36·8 (31·4–42·4)	43·8 (37·4–49·2)
Drowning	-13·9 (-27·8–1·48)	-14·1 (-27·6–0·663)	10·2 (5·36–16·8)	-14·8 (-28·5–0·227)
Fire, heat, and hot substances	10·2 (5·36–17·2)	12·2 (7·01–19·2)	12·5 (7·2–19·8)	11·3 (6·55–17·7)
Poisonings	14 (8·26–21·2)	12·8 (7·67–19·4)	11·2 (6·34–17·7)	14·2 (8·7–21·1)
Exposure to mechanical forces	5·8 (-6·04–18)	14·4 (11·3–17·4)	16·6 (14·1–19·2)	-3·01 (-15·6–10·1)
Animal contact	16·6 (11·6–23·3)	18·4 (13·4–24·3)	19·9 (14·9–25·8)	14·1 (9·46–19·7)
Foreign body	2·96 (1·91–4·58)	3·61 (2·4–5·51)	2·99 (1·96–4·51)	3·89 (2·56–5·9)
Environmental heat and cold exposure	7·77 (2·79–14·9)	8·9 (3·52–16·3)	8·81 (3·49–16·3)	9·11 (3·63–16·8)
Exposure to forces of nature	0 (0–0)	9·62 (3·85–17·5)	9·62 (3·85–17·5)	0 (0–0)
Other unintentional injuries	14·3 (8·46–21·9)	11·9 (6·81–18·6)	11·7 (6·69–18·3)	15 (9·22–22·3)
Self-harm and interpersonal violence	21·3 (6·53–35·7)	23·2 (9·35–36·6)	32·5 (23·8–41·5)	22·2 (7·35–36·3)
Self-harm	21·1 (5·45–36·3)	22·3 (6·54–37·3)	21·5 (7·5–35·6)	22·4 (6·39–37·3)
Interpersonal violence	24 (14·2–33)	30·1 (21–39·1)	38·2 (28·7–47·8)	21 (11·6–29·7)

Supplementary Results Table 2. All-age PAFs attributable to the joint distribution of all risk factors for all ages, both sexes combined for each GBD level 1, level 2, and level 3 cause and all causes in 2019 for Southend-on-Sea. Results are shown for deaths, YLLs, YLDs, and DALYs Data in parenthesis are 95% uncertainty intervals. PAFs=population attributable fractions. GBD=Global Burden of Disease. YLLs=years of life lost. YLDs=years lived with disability. DALYs=disability-adjusted life-years	Deaths 2019	DALYs 2019	YLDs 2019	YLLs 2019
All causes	54 (50·6–57·6)	42·4 (39·2–45·5)	29·2 (26·8–31·6)	54·2 (51·3–57·2)
Communicable, maternal, neonatal, and nutritional diseases	43·8 (32·9–55·2)	50 (43·6–56·1)	47·7 (43·1–52·3)	51 (42·3–60)
HIV/AIDS and sexually transmitted infections	79·1 (66·1–88·5)	79·2 (64·2–89)	96·8 (95·4–97·9)	64·7 (47·2–79·7)
HIV/AIDS	92 (90–93·1)	90·9 (88·7–92·7)	94·7 (92·6–96·1)	88·1 (85·6–89·6)
Sexually transmitted infections excluding HIV	43·1 (21·8–70·2)	59·1 (33·6–84·3)	100 (100–100)	15 (5·6–33·1)
Respiratory infections and tuberculosis	44·5 (32·9–56·6)	42·7 (33–52·9)	3·43 (2·51–4·59)	47·9 (36·9–59·5)
Tuberculosis	54·5 (41·6–66·6)	57·8 (45·7–68·9)	54·6 (41·6–65·7)	58·4 (46·3–69·5)
Lower respiratory infections	44·4 (32·7–56·6)	47·8 (36·6–59·5)	33·5 (26·4–41)	47·9 (36·7–59·6)
Upper respiratory infections	0·0351 (0·0193–0·0582)	0·00766 (0·00106–0·0189)	0·00678 (2·17e-05–0·0181)	0·0842 (0·0466–0·137)
Otitis media	2·05 (0·984–3·71)	4·21 (2·62–6·05)	4·19 (2·6–6·07)	5·29 (2·82–8·55)
Enteric infections	5·63 (3·2–9·83)	9·37 (6·66–13·2)	9·89 (7·05–13·9)	8·09 (5·44–12·4)
Diarrheal diseases	5·71 (3·26–9·99)	9·43 (6·73–13·3)	9·9 (7·06–13·9)	8·26 (5·62–12·6)
Other infectious diseases	0·543 (0·385–0·721)	1·06 (0·78–1·36)	0·451 (0·255–0·743)	1·28 (0·922–1·66)
Meningitis	1·15 (0·779–1·58)	2·08 (1·44–2·85)	0·000552 (1·31e-06–0·00168)	2·54 (1·77–3·46)
Encephalitis	0·209 (0·133–0·316)	0·578 (0·35–0·857)	0·000199 (4·62e-07–0·000564)	0·73 (0·465–1·07)
Measles	3·83 (-0·97–18·3)	4·41 (-1·09–20·8)	0·705 (0·0145–1·95)	4·79 (-1·25–23·2)
Acute hepatitis	11·2 (6·03–23·4)	5·27 (3·22–8·87)	3·06 (1·74–4·99)	11 (6·19–21·9)
Maternal and neonatal disorders	87·5 (85·3–89·5)	77·5 (73·9–81)	66·8 (61·2–71·9)	88·3 (86·2–90·1)
Maternal disorders	9·48 (3·41–16)	9·55 (3·43–16·2)	9·66 (3·42–16·6)	9·34 (3·37–15·7)
Neonatal disorders	89·7 (87·6–91·3)	79·1 (75·3–82·7)	68·6 (62·8–73·8)	89·7 (87·6–91·3)
Nutritional deficiencies	73·9 (68·5–89·7)	77·4 (73·1–82)	77·4 (73·1–82·1)	74·8 (71–83·5)
Protein-energy malnutrition	100 (100–100)	100 (100–100)	100 (100–100)	100 (100–100)
Vitamin A deficiency	--	100 (100–100)	100 (100–100)	--
Dietary iron deficiency	--	100 (100–100)	100 (100–100)	--
Non-communicable diseases	55·8 (52·1–59·5)	43 (39·6–46·4)	28·4 (25·8–31)	56·4 (53·4–59·6)
Neoplasms	47·6 (43·5–52)	47 (43·1–51)	33·2 (28·9–37·6)	47·8 (44–51·7)
Lip and oral cavity cancer	63·9 (51–75·3)	67·6 (55·1–77·7)	67 (54·4–77·6)	67·6 (55·2–77·7)
Nasopharynx cancer	65·2 (50·2–76·9)	65·5 (50·5–76·6)	62·4 (47·7–74·5)	65·7 (50·8–76·8)
Other pharynx cancer	78·2 (69·4–85·7)	79·7 (71·1–86·7)	79·5 (70·5–86·4)	79·7 (71·1–86·7)
Esophageal cancer	73·4 (62·8–82·5)	75·4 (65·4–83·9)	74·4 (63·9–83·4)	75·4 (65·4–84)
Stomach cancer	24·7 (17·1–41·6)	25·5 (17·9–41·9)	25·9 (18·1–42·4)	25·4 (17·8–41·9)

Colon and rectum cancer	63·1 (52·7–72·1)	63·5 (54–71·9)	63·8 (54·4–72·2)	63·5 (54·1–71·9)
Liver cancer	74·8 (67·9–80·7)	75·8 (69·8–81·3)	76·4 (70·3–81·9)	75·7 (69·7–81·3)
Gallbladder and biliary tract cancer	20·5 (10·6–32·5)	20·9 (11–32·8)	19·1 (9·79–31)	21 (11–33)
Pancreatic cancer	42·2 (32·4–53·1)	41·9 (32·7–52·3)	42·2 (32·5–52·7)	41·9 (32·7–52·3)
Larynx cancer	81·5 (73·1–88·5)	81·8 (74·5–88·1)	79·6 (71·9–86·4)	82 (74·7–88·2)
Tracheal, bronchus, and lung cancer	86·5 (83·1–89·6)	85·8 (82·5–89)	86·4 (82·8–89·7)	85·8 (82·4–89)
Breast cancer	36 (25·8–47·1)	35·1 (25·9–44·8)	35·3 (26–45·1)	35 (25·9–44·8)
Cervical cancer	100 (100–100)	100 (100–100)	100 (100–100)	100 (100–100)
Uterine cancer	40·9 (25·3–56·6)	42·2 (26·9–58)	42·7 (27·5–58·4)	42·1 (26·8–58)
Ovarian cancer	29·3 (14·1–43·9)	24·5 (11·7–37·5)	22·6 (10·8–34·5)	24·5 (11·7–37·6)
Prostate cancer	6 (2·59–9·85)	6·61 (2·88–10·6)	7·08 (3·19–11·3)	6·53 (2·84–10·5)
Kidney cancer	37·7 (27·3–48·5)	38·1 (28·3–48·3)	38 (28·3–48·1)	38·1 (28·3–48·3)
Bladder cancer	39·1 (25–53·6)	42 (28·8–55·3)	43·1 (29·8–55·9)	41·9 (28·7–55·3)
Thyroid cancer	13·2 (5·94–22·7)	13·9 (6·28–23·7)	14·3 (6·25–24·5)	13·9 (6·27–23·8)
Mesothelioma	99·2 (98·8–99·4)	98·2 (97·6–98·7)	99·1 (98·7–99·4)	98·2 (97·6–98·7)
Non-Hodgkin lymphoma	6·47 (2·56–11·9)	6·68 (2·73–12·1)	6·61 (2·69–12·1)	6·69 (2·75–12·1)
Multiple myeloma	7·35 (2·99–13·5)	7·66 (3·23–14)	7·62 (3·16–14)	7·66 (3·19–14)
Leukemia	36·7 (22·7–51·1)	35·1 (23·2–47·7)	34 (22·2–46·4)	35·2 (23·2–47·9)
Cardiovascular diseases	77·4 (73–81·5)	78·6 (75–81·9)	69·3 (63·7–74·4)	79·8 (76·3–82·9)
Rheumatic heart disease	22 (12·1–41·8)	23·1 (14·5–38·3)	22·8 (14·3–37)	23·1 (14·5–38·3)
Ischemic heart disease	93·3 (89·3–96·7)	94·5 (91·7–97·1)	93·7 (90·6–96·4)	94·6 (91·7–97·2)
Stroke	79 (69·9–88·7)	82·1 (75·6–88·9)	81·6 (75·5–87·5)	82·2 (75·4–89·1)
Hypertensive heart disease	100 (100–100)	100 (100–100)	100 (100–100)	100 (100–100)
Non-rheumatic valvular heart disease	25·6 (16–38·7)	26·3 (18·1–37·2)	17·3 (11–24·5)	27·2 (18·6–38·6)
Cardiomyopathy and myocarditis	35·1 (27·5–44·1)	42·1 (35·5–49·2)	36·2 (29·1–44·6)	42·4 (35·7–49·7)
Atrial fibrillation and flutter	47·7 (36–59·4)	55·4 (45·7–64·6)	59·6 (50·3–68)	50·7 (39·7–61·3)
Aortic aneurysm	50 (42·9–57·8)	57·2 (51·3–63·1)	--	57·2 (51·3–63·1)
Endocarditis	30·3 (19·9–43·8)	33·1 (23·8–44·3)	30·1 (19·4–43·7)	33·2 (23·9–44·4)
Other cardiovascular and circulatory diseases	31·2 (24·2–38·7)	34·1 (27·8–40·7)	34·3 (27·1–41·4)	34·1 (27·7–40·8)
Chronic respiratory diseases	63·5 (57·1–70·1)	59 (54·3–64)	51·9 (46·7–56·9)	64·3 (58·4–70·5)
Chronic obstructive pulmonary disease	71·8 (65·4–77·6)	71·5 (66·9–76)	65·5 (61·6–69·5)	74·1 (68·4–79·2)
Pneumoconiosis	100 (100–100)	100 (100–100)	100 (100–100)	100 (100–100)
Asthma	39·5 (27·5–52·7)	40·1 (31·4–49·9)	39·9 (31·3–49·4)	42·2 (32–53·5)
Digestive diseases	27·5 (22·2–33·4)	34·8 (28·9–41)	23·8 (15·8–33·3)	39·8 (33–47·3)
Cirrhosis and other chronic liver diseases	70·3 (58·1–84·9)	73·8 (62·2–87·5)	70·4 (57·7–83·7)	73·8 (62·2–87·6)

Upper digestive system diseases	19·9 (16·5–23·4)	9·81 (6·48–13·7)	0·681 (0·38–1·09)	22·8 (19·2–26·5)
Gallbladder and biliary diseases	40·3 (25·2–55·8)	46·3 (31·9–61·2)	47·5 (33·2–62·4)	41·7 (27·1–56·8)
Pancreatitis	30 (7·61–53·1)	36·2 (12–57·9)	36·7 (12·6–59·2)	36·1 (11·9–57·7)
Neurological disorders	23·7 (10·3–39·5)	15·3 (7·6–26·5)	9·83 (4·94–18·7)	21·1 (9·77–36·3)
Alzheimer's disease and other dementias	38·6 (24·6–53·8)	40·7 (26·8–55·8)	41 (27·2–56)	40·6 (26·6–55·5)
Parkinson's disease	-8·87 (-14·9–-3·95)	-10·2 (-16·7–-4·68)	-10·8 (-17·7–-4·97)	-10·1 (-16·6–-4·6)
Idiopathic epilepsy	25·9 (14·3–37·1)	24·6 (13·6–34·6)	22·1 (11·8–32·1)	27·1 (15·5–37·6)
Multiple sclerosis	14·3 (10·5–18·4)	15·5 (11·6–19·7)	15·4 (11·6–19·4)	15·5 (11·5–19·9)
Mental disorders	--	6·44 (3·26–9·99)	6·45 (3·26–10)	--
Depressive disorders	--	13·3 (6·11–20·8)	13·3 (6·11–20·8)	--
Anxiety disorders	--	4·74 (1·23–10·8)	4·74 (1·23–10·8)	--
Idiopathic developmental intellectual disability	--	37·6 (15·9–58·9)	37·6 (15·9–58·9)	--
Substance use disorders	100 (100–100)	100 (100–100)	100 (100–100)	100 (100–100)
Alcohol use disorders	100 (100–100)	100 (100–100)	100 (100–100)	100 (100–100)
Drug use disorders	100 (100–100)	100 (100–100)	100 (100–100)	100 (100–100)
Diabetes and kidney diseases	99·7 (99·7–99·8)	99·9 (99·9–100)	100 (100–100)	99·8 (99·7–99·8)
Diabetes mellitus	100 (100–100)	100 (100–100)	100 (100–100)	100 (100–100)
Chronic kidney disease	100 (100–100)	100 (100–100)	100 (100–100)	100 (100–100)
Sense organ diseases	--	8·51 (7·3–9·97)	8·51 (7·3–9·97)	--
Blindness and vision loss	--	9·21 (5·83–13·6)	9·21 (5·83–13·6)	--
Age-related and other hearing loss	--	9·61 (8·59–10·7)	9·61 (8·59–10·7)	--
Musculoskeletal disorders	1·73 (0·423–3·29)	21·7 (17·6–25·6)	22·4 (18·3–26·5)	2·14 (0·55–3·96)
Rheumatoid arthritis	11·1 (2·77–20·4)	13·5 (3·57–24)	13·6 (3·55–24·2)	12·8 (3·41–22·9)
Osteoarthritis	--	15·6 (7·85–24·6)	15·6 (7·85–24·6)	--
Low back pain	--	40·7 (35·3–45·9)	40·7 (35·3–45·9)	--
Gout	--	48·4 (33·5–65·7)	48·4 (33·5–65·7)	--
Other non-communicable diseases	0·0523 (0·0323–0·0789)	0·0639 (0·0377–0·101)	--	0·237 (0·143–0·343)
Sudden infant death syndrome	7·6 (4·25–12·4)	7·6 (4·24–12·3)	--	7·6 (4·24–12·3)
Injuries	30 (23·1–36·1)	26·6 (20·8–31·8)	29·6 (25·3–33·8)	23·1 (13·9–31·2)
Transport injuries	22·8 (14·5–30·8)	22·9 (15·6–29·6)	26·9 (22·3–31·5)	20·8 (11·7–29·5)
Road injuries	21·8 (13·2–30·3)	21·8 (14·2–28·9)	25·6 (21·1–30·3)	19·9 (10·4–29)
Other transport injuries	33·5 (28–39·1)	32·8 (27·3–38·2)	36 (31·2–40·5)	30·6 (24·3–37)
Unintentional injuries	38·1 (32–42·6)	28·7 (24·3–33·4)	29·7 (25·3–34·3)	25·7 (21·2–30)
Falls	54·9 (46·7–60·4)	40·7 (34·8–45·8)	39·1 (33·6–44·2)	46·5 (39·7–51·8)
Drowning	-14·3 (-28·6–-0·399)	-13 (-26·6–0·117)	10·5 (5·95–17·2)	-14·3 (-28·6–-0·58)

Fire, heat, and hot substances	10·3 (5·59–16·4)	13·2 (8·05–20·2)	13·5 (8·33–20·6)	12·2 (7·41–18·4)
Poisonings	14 (8·58–20·9)	13·6 (8·53–20·1)	12·2 (7·48–18·7)	15·1 (9·57–21·8)
Exposure to mechanical forces	12·2 (-0·127–24·9)	18·2 (15·2–21·4)	19·3 (16·5–22·3)	2·98 (-9·93–16·7)
Animal contact	21·5 (16·8–27·4)	21·3 (16·2–26·9)	22 (16·7–27·8)	17 (12·7–22·2)
Foreign body	3·21 (2·04–4·96)	4·55 (2·91–6·89)	3·79 (2·41–5·74)	5·11 (3·25–7·69)
Environmental heat and cold exposure	7·49 (2·9–13·6)	8·85 (3·63–15·7)	8·88 (3·63–15·9)	8·7 (3·57–15·6)
Exposure to forces of nature	0 (0–0)	9·72 (4·06–17·1)	9·72 (4·06–17·1)	0 (0–0)
Other unintentional injuries	14·7 (8·9–22·1)	12·9 (7·84–19·7)	12·8 (7·74–19·5)	16·8 (10·8–24·2)
Self-harm and interpersonal violence	21·1 (4·73–35·4)	23·4 (7·96–36·8)	31·7 (23·2–40·3)	22·4 (5·87–36·5)
Self-harm	20·9 (4·09–35·6)	22·3 (5·35–36·9)	21 (6·95–34·7)	22·3 (5·21–37)
Interpersonal violence	26·6 (16·7–36·3)	32·2 (23–41·5)	37·9 (28·7–47·4)	23·9 (14·4–33·5)

Supplementary Results Table 2. All-age PAFs attributable to the joint distribution of all risk factors for all ages, both sexes combined for each GBD level 1, level 2, and level 3 cause and all causes in 2019 for Suffolk. Results are shown for deaths, YLLs, YLDs, and DALYs Data in parenthesis are 95% uncertainty intervals. PAFs=population attributable fractions. GBD=Global Burden of Disease. YLLs=years of life lost. YLDs=years lived with disability. DALYs=disability-adjusted life-years

	Deaths 2019	DALYs 2019	YLDs 2019	YLLs 2019
All causes	53·8 (50·5–57·1)	41·4 (38·4–44·6)	28·3 (25·9–30·6)	53·7 (50·9–56·6)
Communicable, maternal, neonatal, and nutritional diseases	43·4 (34·5–52·5)	49 (43·7–54·3)	47·1 (42·2–51·6)	49·9 (42·5–57·5)
HIV/AIDS and sexually transmitted infections	76·6 (60·5–88·9)	76·8 (59–89·4)	97·8 (96·6–98·7)	57·7 (37·3–77·4)
HIV/AIDS	93·1 (91·2–94·2)	92·3 (90·1–93·9)	95·1 (93·1–96·4)	90·1 (87·7–91·5)
Sexually transmitted infections excluding HIV	56·6 (34·3–81·1)	62·6 (37·9–86·4)	100 (100–100)	22·1 (9·16–46)
Respiratory infections and tuberculosis	44·5 (35–54·4)	41·9 (34–50·7)	3·25 (2·42–4·3)	47·5 (38·2–56·9)
Tuberculosis	51·1 (37·6–63·7)	54·5 (42–66)	52·9 (40–64·4)	54·9 (42·5–66·5)
Lower respiratory infections	44·5 (35–54·4)	47·4 (38–56·8)	32·9 (26–40·3)	47·5 (38·1–57)
Upper respiratory infections	0·0336 (0·0183–0·0552)	0·00682 (0·00103–0·0171)	0·00584 (0·00011–0·016)	0·0969 (0·0553–0·158)
Otitis media	1·75 (0·805–3·42)	4·01 (2·51–5·65)	4 (2·51–5·65)	5·3 (2·69–9·35)
Enteric infections	5·39 (3·11–9·61)	8·52 (6·05–12·6)	9·11 (6·42–13·2)	7·09 (4·7–11·3)
Diarrheal diseases	5·45 (3·15–9·66)	8·57 (6·08–12·7)	9·12 (6·43–13·2)	7·22 (4·79–11·4)
Other infectious diseases	0·425 (0·285–0·593)	0·923 (0·685–1·17)	0·48 (0·266–0·809)	1·1 (0·778–1·44)
Meningitis	1·05 (0·716–1·44)	2·07 (1·45–2·84)	0·000512 (7·61e-06–0·00153)	2·68 (1·86–3·63)
Encephalitis	0·201 (0·122–0·312)	0·59 (0·351–0·909)	0·000173 (2·49e-06–0·000524)	0·782 (0·475–1·19)
Measles	2·28 (-0·621–11·4)	2·55 (-0·555–12·2)	0·622 (0·0138–1·63)	2·94 (-0·795–14·7)
Acute hepatitis	9·45 (3·66–22·6)	4·85 (2·53–8·57)	3·23 (1·84–5·33)	9·18 (3·64–21·1)
Maternal and neonatal disorders	88·6 (86·6–90·3)	77·6 (74–81·1)	68·1 (62·8–73·2)	89·3 (87·4–90·9)
Maternal disorders	9·16 (3·32–15·5)	9·28 (3·31–15·9)	9·4 (3·32–16·1)	9·01 (3·22–15·1)
Neonatal disorders	90·5 (88·8–92·1)	79·2 (75·5–82·8)	69·8 (64·1–75)	90·5 (88·8–92·1)
Nutritional deficiencies	70·9 (64·7–90·1)	76·9 (72·9–81·3)	77 (73–81·5)	71·5 (67·5–84·6)
Protein-energy malnutrition	100 (100–100)	100 (100–100)	100 (100–100)	100 (100–100)
Vitamin A deficiency	--	100 (100–100)	100 (100–100)	--
Dietary iron deficiency	--	100 (100–100)	100 (100–100)	--
Non-communicable diseases	55·3 (51·8–58·9)	41·9 (38·7–45·4)	27·3 (24·8–29·9)	55·6 (52·7–58·7)
Neoplasms	46·5 (42·7–50·7)	46·3 (42·7–50·2)	32·3 (28·2–36·6)	47·2 (43·6–51·1)
Lip and oral cavity cancer	63·8 (51–74·8)	67·3 (55·2–77·4)	66·9 (54·6–77)	67·4 (55·3–77·4)
Nasopharynx cancer	62·8 (47·6–74·7)	63·5 (49–75)	60·6 (45·1–73·2)	63·8 (49·3–75·1)
Other pharynx cancer	76·6 (67·8–84·4)	78·4 (69·8–85·5)	78·3 (70–85·5)	78·4 (69·9–85·5)
Esophageal cancer	74·6 (64·2–83·6)	76·4 (66·3–85·1)	75·5 (65–84·2)	76·4 (66·4–85·1)
Stomach cancer	24·5 (17–42)	25·3 (17·6–42·3)	25·8 (18–42·7)	25·3 (17·6–42·3)

Colon and rectum cancer	62·8 (53·7–71·1)	63·4 (54·8–71·3)	63·7 (55·1–71·6)	63·4 (54·8–71·2)
Liver cancer	76 (69·8–82·1)	77·1 (71·2–82·5)	77·8 (72–83·1)	77 (71·2–82·5)
Gallbladder and biliary tract cancer	23·1 (12·5–35·6)	23·6 (12·9–36)	21·6 (11·4–34·1)	23·7 (12·9–36·1)
Pancreatic cancer	40·9 (31·9–51·6)	41 (32·4–51·1)	41 (32–51·4)	41 (32·4–51·1)
Larynx cancer	80·9 (72·1–88·2)	81·3 (73·6–88·2)	79·4 (71·6–86·5)	81·5 (73·8–88·4)
Tracheal, bronchus, and lung cancer	86·3 (83–89·3)	85·8 (82·6–88·9)	86·3 (82·8–89·3)	85·8 (82·6–88·9)
Breast cancer	35·7 (26·5–46·2)	35·3 (26·5–44·9)	35·5 (26·8–45·3)	35·3 (26·5–44·9)
Cervical cancer	100 (100–100)	100 (100–100)	100 (100–100)	100 (100–100)
Uterine cancer	45 (29·5–61·1)	46·3 (30·5–62·2)	46·8 (30·6–62·5)	46·2 (30·5–62·1)
Ovarian cancer	28 (13·1–41·6)	23·8 (11·1–36·1)	22·3 (10·4–34)	23·9 (11·1–36·2)
Prostate cancer	5·76 (2·51–9·41)	6·33 (2·8–10·2)	6·8 (3·04–10·8)	6·25 (2·78–10)
Kidney cancer	39·9 (29–51)	40·6 (29·7–51·3)	40·6 (29·8–51·2)	40·6 (29·8–51·3)
Bladder cancer	36·8 (23·1–50·6)	39·6 (26·2–53)	40·8 (27·5–53·6)	39·5 (26·1–52·9)
Thyroid cancer	15·6 (7·46–25·4)	16·4 (7·94–26·5)	16·9 (7·82–27·6)	16·3 (7·92–26·4)
Mesothelioma	99·2 (98·9–99·5)	98·4 (97·8–98·9)	99·2 (98·8–99·5)	98·4 (97·8–98·8)
Non-Hodgkin lymphoma	7·75 (3·17–13·7)	8·01 (3·42–14·1)	7·93 (3·38–14)	8·02 (3·42–14·2)
Multiple myeloma	8·67 (3·74–15·6)	9·02 (3·95–15·9)	8·97 (3·91–15·9)	9·02 (3·95–16)
Leukemia	37·4 (22·9–52·4)	36·5 (23·8–49·2)	35·7 (23·2–48·2)	36·5 (23·8–49·3)
Cardiovascular diseases	77·6 (73·5–81·8)	78·7 (75·2–82)	69·3 (64·3–74·9)	79·9 (76·5–83·1)
Rheumatic heart disease	21·8 (11·5–43·8)	22·6 (13·4–39·3)	22·7 (13·7–37·4)	22·6 (13·5–39·4)
Ischemic heart disease	93 (89·1–96·4)	94 (90·9–96·7)	93·4 (90·2–96·3)	94·1 (91–96·7)
Stroke	78·2 (69·6–87·8)	81 (74·6–88·4)	80·7 (74·8–87·3)	81·1 (74·6–88·5)
Hypertensive heart disease	100 (100–100)	100 (100–100)	100 (100–100)	100 (100–100)
Non-rheumatic valvular heart disease	25·1 (15·6–38·1)	25 (16·9–36·3)	16·7 (10·9–23·8)	26·2 (17·7–38·1)
Cardiomyopathy and myocarditis	35·6 (27·6–45)	42·5 (36–49·1)	36 (29·1–43·5)	42·9 (36·2–49·4)
Atrial fibrillation and flutter	49·7 (38·8–61·5)	56·5 (47·3–65·9)	60·4 (51·7–69·1)	52·3 (42–62·9)
Aortic aneurysm	49 (42·1–56·3)	55·6 (49·4–61·2)	--	55·6 (49·4–61·2)
Endocarditis	30·1 (19·7–43·4)	32·6 (23·4–44·1)	29·9 (19·2–43)	32·7 (23·5–44·1)
Other cardiovascular and circulatory diseases	30·6 (23·9–38·1)	33·4 (27·1–39·8)	33·4 (26·4–40·5)	33·4 (27·2–39·9)
Chronic respiratory diseases	60·9 (54·8–67·7)	57·9 (53–62·8)	53·2 (47·8–58·6)	61·8 (56·3–68)
Chronic obstructive pulmonary disease	71·3 (66·3–76·2)	70·4 (66·2–74·4)	64·6 (60·4–68·8)	73·6 (69·2–78)
Pneumoconiosis	100 (100–100)	100 (100–100)	100 (100–100)	100 (100–100)
Asthma	42·2 (29·5–55·9)	43·1 (33·7–53·3)	42·9 (33·6–53·1)	44·9 (33·5–56·7)
Digestive diseases	24·5 (20–29·5)	31·5 (26–37)	24·7 (17–33·6)	35·2 (29·2–41·4)
Cirrhosis and other chronic liver diseases	69·3 (56·3–84·4)	73·2 (60·8–86·8)	70·8 (58·2–83·9)	73·3 (60·8–86·9)

Upper digestive system diseases	19·6 (16·3–23)	9·17 (6·06–12·7)	0·718 (0·4–1·16)	22·4 (18·8–25·9)
Gallbladder and biliary diseases	44·2 (28·7–60)	48·8 (34·2–63·4)	49·8 (35·3–64·3)	45·5 (30·3–60·7)
Pancreatitis	31·7 (9·27–53·7)	37·4 (13·8–58·6)	37·4 (13·1–58·9)	37·4 (13·8–58·5)
Neurological disorders	23·4 (9·91–38·9)	16 (8·12–27·9)	10·8 (5·46–19·9)	20·8 (8·62–36)
Alzheimer's disease and other dementias	38·7 (25·2–53·1)	40·9 (27·5–55·1)	41·2 (27·4–55·5)	40·7 (27·4–55)
Parkinson's disease	-8·67 (-14·7–-3·83)	-9·88 (-16·4–-4·43)	-10·4 (-17·1–-4·75)	-9·76 (-16·3–-4·36)
Idiopathic epilepsy	24·9 (13·1–36·3)	23·8 (12·5–34·5)	21·8 (11·4–32·1)	26·9 (14·7–38·3)
Multiple sclerosis	13·3 (9·77–17·1)	14·5 (10·8–18·2)	14·5 (11–18·3)	14·5 (10·8–18·4)
Mental disorders	--	6·26 (3·17–9·59)	6·26 (3·17–9·59)	--
Depressive disorders	--	12·9 (5·92–20·2)	12·9 (5·92–20·2)	--
Anxiety disorders	--	4·6 (1·21–10·3)	4·6 (1·21–10·3)	--
Idiopathic developmental intellectual disability	--	37·7 (16·3–56·5)	37·7 (16·3–56·5)	--
Substance use disorders	100 (100–100)	100 (100–100)	100 (100–100)	100 (100–100)
Alcohol use disorders	100 (100–100)	100 (100–100)	100 (100–100)	100 (100–100)
Drug use disorders	100 (100–100)	100 (100–100)	100 (100–100)	100 (100–100)
Diabetes and kidney diseases	99·8 (99·7–99·8)	99·9 (99·9–100)	100 (100–100)	99·8 (99·7–99·8)
Diabetes mellitus	100 (100–100)	100 (100–100)	100 (100–100)	100 (100–100)
Chronic kidney disease	100 (100–100)	100 (100–100)	100 (100–100)	100 (100–100)
Sense organ diseases	--	8·07 (6·93–9·34)	8·07 (6·93–9·34)	--
Blindness and vision loss	--	9·28 (6·1–13·3)	9·28 (6·1–13·3)	--
Age-related and other hearing loss	--	8·89 (7·91–9·94)	8·89 (7·91–9·94)	--
Musculoskeletal disorders	2·9 (0·728–5·3)	23 (19–27·1)	23·5 (19·3–27·7)	3·56 (0·908–6·36)
Rheumatoid arthritis	10·1 (2·39–18·1)	12·7 (3·26–22·4)	13 (3·35–23)	11·8 (3·04–21·2)
Osteoarthritis	--	17·8 (9·41–27·1)	17·8 (9·41–27·1)	--
Low back pain	--	41·8 (36·5–47·5)	41·8 (36·5–47·5)	--
Gout	--	53·9 (38·4–70·5)	53·9 (38·4–70·5)	--
Other non-communicable diseases	0·0553 (0·0338–0·0816)	0·0558 (0·0322–0·0881)	--	0·261 (0·158–0·381)
Sudden infant death syndrome	7·02 (3·86–11·5)	7·02 (3·86–11·5)	--	7·02 (3·86–11·5)
Injuries	33·6 (27–38·8)	28·7 (23·6–33·2)	31·6 (27–35·9)	24·7 (16·7–31·3)
Transport injuries	21·9 (14·8–28·9)	22·2 (16–28·7)	26·7 (22·6–31·1)	20 (12–27·9)
Road injuries	21·1 (13·7–28·4)	21·3 (14·8–27·8)	25·5 (21·3–29·8)	19·3 (11·1–27·3)
Other transport injuries	33·5 (28·6–38·5)	32·8 (27·7–38)	36·8 (32·3–41·3)	29·5 (23·8–35·5)
Unintentional injuries	41·7 (35·1–46·6)	31·4 (26·5–36)	32 (27–36·8)	29·6 (24·7–34)
Falls	56·4 (47·8–62)	43·4 (37·1–48·6)	41·7 (35·5–46·9)	49·4 (42·5–54·9)
Drowning	-15·3 (-27·5–-3·17)	-13 (-24–-1·6)	10·2 (5·34–16·6)	-14·5 (-26·4–-2·82)

Fire, heat, and hot substances	9·96 (5·09–16·4)	12·6 (7·35–19·3)	12·8 (7·49–19·6)	12·1 (6·99–18·4)
Poisonings	13·4 (7·88–20)	12·8 (7·77–19·1)	11·7 (6·87–17·9)	14·7 (9·2–21·4)
Exposure to mechanical forces	12 (2·48–21·4)	18 (15–20·7)	19·4 (16·7–21·9)	2·5 (-7·32–12·9)
Animal contact	24·7 (19·8–30·9)	22 (17–27·8)	22·3 (17–28)	21·1 (16·5–27)
Foreign body	2·42 (1·51–3·59)	3·81 (2·44–5·54)	3·11 (2·01–4·6)	4·25 (2·73–6·25)
Environmental heat and cold exposure	7·59 (2·76–13·8)	8·86 (3·54–15·7)	8·89 (3·58–15·9)	8·77 (3·58–15·5)
Exposure to forces of nature	0 (0–0)	9·76 (4·14–17·3)	9·76 (4·14–17·3)	0 (0–0)
Other unintentional injuries	13·6 (7·94–20·4)	12·2 (7·11–18·9)	12·1 (7·02–18·7)	15·8 (9·97–22·8)
Self-harm and interpersonal violence	21·4 (6·21–35·1)	24·1 (9·99–36·8)	31·8 (23·5–40·1)	22·8 (7·07–36·5)
Self-harm	21·1 (5·1–35·1)	22·6 (6·66–36·8)	21·4 (7·55–34·3)	22·7 (6·71–36·9)
Interpersonal violence	28·3 (19–37·6)	34·1 (25·4–43)	37·7 (28·5–46·9)	25·5 (16·4–34·7)

Supplementary Results Table 2. All-age PAFs attributable to the joint distribution of all risk factors for all ages, both sexes combined for each GBD level 1, level 2, and level 3 cause and all causes in 2019 for Thurrock. Results are shown for deaths, YLLs, YLDs, and DALYs Data in parenthesis are 95% uncertainty intervals. PAFs=population attributable fractions. GBD=Global Burden of Disease. YLLs=years of life lost. YLDs=years lived with disability. DALYs=disability-adjusted life-years

	Deaths 2019	DALYs 2019	YLDs 2019	YLLs 2019
All causes	55·4 (52·2–58·5)	40·7 (37·5–43·8)	28·4 (26–30·7)	54·6 (51·8–57·2)
Communicable, maternal, neonatal, and nutritional diseases	45·3 (34·9–55·7)	52·8 (47·7–57·7)	50·3 (45·4–55·4)	54·5 (46·4–62)
HIV/AIDS and sexually transmitted infections	74·1 (58·8–85·5)	75·8 (58·9–87·7)	97 (95·6–98·1)	58·6 (39·7–75·3)
HIV/AIDS	90·8 (88·9–92·1)	89·8 (87·4–91·7)	94·7 (92·6–96·1)	86·2 (83·8–87·8)
Sexually transmitted infections excluding HIV	31·9 (14·5–57·6)	56·3 (30·6–82·7)	100 (100–100)	11·2 (4·07–25·1)
Respiratory infections and tuberculosis	45·6 (33·9–57·1)	41·3 (32·4–51)	3·06 (2·26–4·06)	49·6 (38·4–60·2)
Tuberculosis	55·5 (43·4–66·9)	58·1 (46·9–68·8)	54 (42–65·2)	58·8 (47·7–69·4)
Lower respiratory infections	45·5 (33·7–57·1)	49·4 (38·1–60·3)	34·3 (27·1–41·3)	49·6 (38·2–60·4)
Upper respiratory infections	0·0646 (0·0328–0·119)	0·00998 (0·00213–0·0236)	0·00769 (2·53e-05–0·0219)	0·129 (0·066–0·235)
Otitis media	1·88 (1·02–3·55)	4·83 (3·02–6·86)	4·83 (3·02–6·86)	4·42 (2·48–8·08)
Enteric infections	6·25 (3·65–11)	11 (7·98–15·9)	11·1 (7·84–16·1)	10·6 (7·22–15·5)
Diarrheal diseases	6·33 (3·74–11·1)	11 (8·03–16·1)	11·1 (7·85–16·1)	10·9 (7·49–15·8)
Other infectious diseases	0·801 (0·517–1·07)	1·34 (0·954–1·78)	0·393 (0·226–0·664)	1·7 (1·18–2·31)
Meningitis	1·69 (1·13–2·42)	2·66 (1·82–3·71)	0·000661 (1·69e-06–0·00193)	3·21 (2·21–4·48)
Encephalitis	0·374 (0·244–0·555)	0·779 (0·506–1·14)	0·00021 (5·79e-07–0·000594)	1·09 (0·724–1·57)
Measles	3·85 (-0·911–18·9)	3·71 (-0·622–16·9)	0·738 (0·0205–2·08)	5·03 (-1·22–23·3)
Acute hepatitis	13·6 (7·54–25·1)	4·97 (2·95–8·06)	2·69 (1·59–4·47)	13·1 (7·42–23·5)
Maternal and neonatal disorders	86·6 (83·9–88·9)	80·2 (77·2–83·1)	72·6 (67·7–76·9)	87·9 (85·6–89·9)
Maternal disorders	9·44 (3·49–15·7)	9·49 (3·53–15·9)	9·63 (3·61–16·3)	9·3 (3·5–15·5)
Neonatal disorders	90·1 (88·1–91·9)	82·5 (79·5–85·3)	74·9 (69·9–79·3)	90·1 (88·1–91·9)
Nutritional deficiencies	64·5 (58–87·2)	77·3 (72·8–82·4)	77·5 (72·9–82·7)	65·4 (61·3–79·2)
Protein-energy malnutrition	100 (100–100)	100 (100–100)	100 (100–100)	100 (100–100)
Vitamin A deficiency	--	100 (100–100)	100 (100–100)	--
Dietary iron deficiency	--	100 (100–100)	100 (100–100)	--
Non-communicable diseases	57·2 (53·8–60·6)	41·1 (37·8–44·4)	27·4 (24·9–30·1)	56·9 (54–59·6)
Neoplasms	49·1 (45·3–53·6)	47·8 (44·3–51·6)	33·3 (29·1–37·8)	48·6 (45·2–52·5)
Lip and oral cavity cancer	64 (52–75·2)	67·2 (55·2–77·5)	66·8 (54·9–77·3)	67·2 (55·3–77·5)
Nasopharynx cancer	61·2 (47·3–73·2)	61·2 (47·4–72·9)	58·4 (43·9–71·2)	61·5 (47·7–73)
Other pharynx cancer	79·6 (70·7–86·4)	80·7 (72·3–87·2)	80·5 (72–87)	80·7 (72·3–87·2)
Esophageal cancer	74·5 (63·9–83·6)	76·2 (66–84·8)	75·3 (64·9–84·3)	76·3 (66–84·8)
Stomach cancer	25·5 (17·9–42·2)	25·9 (18·3–42·1)	26·4 (18·6–42·7)	25·9 (18·3–42·1)

Colon and rectum cancer	63·5 (53·9–72·5)	63·7 (54·8–72)	64 (55·2–72·4)	63·7 (54·9–72)
Liver cancer	76·1 (69·7–81·7)	76 (70·4–81·2)	77 (71·4–82·2)	76 (70·5–81·2)
Gallbladder and biliary tract cancer	20·1 (10·4–32·1)	20·6 (10·9–32·5)	18·8 (9·22–31·1)	20·6 (10·9–32·6)
Pancreatic cancer	43·2 (33·5–54·3)	42·8 (34·1–53)	43·1 (33·5–53·7)	42·8 (34·1–53)
Larynx cancer	82·5 (74·2–89)	82·4 (74·9–88·4)	80·4 (72·7–86·8)	82·6 (75·1–88·5)
Tracheal, bronchus, and lung cancer	87·2 (83·8–90·2)	86·1 (83–89)	87 (83·7–90)	86·1 (83–89)
Breast cancer	36 (26·6–46·7)	34·4 (26·2–43·8)	34·7 (26·3–44·3)	34·3 (26·2–43·8)
Cervical cancer	100 (100–100)	100 (100–100)	100 (100–100)	100 (100–100)
Uterine cancer	42·4 (27·2–58·4)	43·6 (28·6–59·5)	44 (28·9–59·6)	43·5 (28·5–59·5)
Ovarian cancer	30·2 (13·6–45)	24·5 (11·3–37·7)	22·2 (10·1–34·2)	24·6 (11·4–37·8)
Prostate cancer	6·27 (2·78–10·2)	6·93 (3·15–11·1)	7·42 (3·42–11·7)	6·85 (3·08–10·9)
Kidney cancer	39 (28·9–49·7)	38·9 (29–48·8)	38·7 (28·9–48·6)	38·9 (29–48·8)
Bladder cancer	40·8 (27–54·8)	43·6 (30·4–56·5)	44·5 (31·3–56·7)	43·5 (30·4–56·5)
Thyroid cancer	14·1 (6·57–23·4)	14·7 (6·82–24·5)	15 (6·82–25)	14·6 (6·81–24·4)
Mesothelioma	99·2 (98·9–99·4)	98·2 (97·6–98·8)	99·1 (98·7–99·4)	98·2 (97·6–98·8)
Non-Hodgkin lymphoma	6·91 (2·79–12·7)	7·06 (2·92–12·7)	7·03 (2·86–12·7)	7·06 (2·92–12·8)
Multiple myeloma	7·88 (3·18–14·4)	8·18 (3·43–14·8)	8·15 (3·31–14·7)	8·19 (3·42–14·8)
Leukemia	37·4 (23·7–51·1)	34·2 (22·9–45·7)	32·9 (21·8–44·4)	34·3 (23–45·9)
Cardiovascular diseases	79·1 (75·2–82·8)	80·4 (77·2–83·4)	70·4 (65·4–75·3)	81·6 (78·8–84·5)
Rheumatic heart disease	22 (12·2–40·2)	23 (14·4–37·7)	22·8 (13·9–36·8)	23 (14·4–37·6)
Ischemic heart disease	93·6 (89·8–96·7)	95 (92·5–97·2)	94 (91·1–96·6)	95 (92·5–97·2)
Stroke	79·5 (71–88·4)	82·9 (77–88·9)	82·2 (77·4–87·4)	83 (76·9–89·3)
Hypertensive heart disease	100 (100–100)	100 (100–100)	100 (100–100)	100 (100–100)
Non-rheumatic valvular heart disease	24·9 (15·7–36·6)	25·3 (17·6–34·8)	15·9 (10·3–22·6)	26·3 (18·3–36·4)
Cardiomyopathy and myocarditis	39·5 (31·9–47·9)	45·6 (40–51·4)	38·1 (31·7–45·1)	46·1 (40·3–51·9)
Atrial fibrillation and flutter	48·4 (37–60·1)	57 (47·1–66·1)	60·9 (51·7–69·4)	51·9 (41·7–62·6)
Aortic aneurysm	52 (45·3–59·1)	59·6 (54–65·2)	--	59·6 (54–65·2)
Endocarditis	31·1 (21·5–43·9)	34·1 (24·9–44·7)	30·3 (20·6–43)	34·2 (25–44·7)
Other cardiovascular and circulatory diseases	32·2 (25·1–39·6)	34·1 (27·9–40·8)	32·8 (25·5–40·7)	34·4 (27·8–41·2)
Chronic respiratory diseases	64·8 (58·7–70·7)	58·4 (53·8–63·1)	50·9 (45·5–56·7)	65·2 (59·7–70·6)
Chronic obstructive pulmonary disease	72·9 (67·1–78·1)	72·4 (68·1–76·4)	66·8 (63·1–70·3)	75·1 (69·9–80)
Pneumoconiosis	100 (100–100)	100 (100–100)	100 (100–100)	100 (100–100)
Asthma	42·1 (30·5–54·6)	40·6 (32·1–49·9)	40·3 (31·9–49·4)	43·9 (34·1–54·6)
Digestive diseases	28·3 (23·5–34)	34 (28·2–40·2)	24·6 (16·8–33·8)	40·1 (33·8–47·7)

Cirrhosis and other chronic liver diseases	70 (58·7–84·5)	72·8 (62·1–86·6)	68·7 (56·7–81·9)	72·9 (62·1–86·7)
Upper digestive system diseases	22·7 (18·9–26·6)	8·98 (5·68–12·5)	0·668 (0·384–1·09)	26 (22·1–29·7)
Gallbladder and biliary diseases	42·7 (27·4–58·4)	47·2 (33·2–61·5)	47·6 (33·6–62·1)	44·3 (29·4–59·3)
Pancreatitis	31·5 (10·5–55)	37 (14·3–60·1)	35·9 (13·3–59·2)	37·2 (14·4–60·2)
Neurological disorders	24·4 (10·7–40·3)	13·6 (6·6–24·5)	8·19 (3·86–16·5)	21·4 (10–36·5)
Alzheimer's disease and other dementias	40·1 (26–55·9)	42·5 (28·5–57·8)	42·9 (28·8–58·2)	42·3 (28–57·6)
Parkinson's disease	-9·38 (-15·6–-4·15)	-10·9 (-18–-4·91)	-11·7 (-18·7–-5·21)	-10·7 (-17·8–-4·82)
Idiopathic epilepsy	24·1 (12·1–35·9)	22·5 (10·9–33·3)	20·4 (9·84–30·7)	24·9 (12·6–36·2)
Multiple sclerosis	15·9 (12–20)	16·8 (12·7–20·8)	16·4 (12·3–20·3)	17·1 (12·9–21·4)
Mental disorders	--	6·66 (3·4–10·3)	6·66 (3·4–10·3)	--
Depressive disorders	--	13·8 (6·38–21·5)	13·8 (6·38–21·5)	--
Anxiety disorders	--	5·26 (1·4–11·8)	5·26 (1·4–11·8)	--
Idiopathic developmental intellectual disability	--	35·9 (15·5–54·4)	35·9 (15·5–54·4)	--
Substance use disorders	100 (100–100)	100 (100–100)	100 (100–100)	100 (100–100)
Alcohol use disorders	100 (100–100)	100 (100–100)	100 (100–100)	100 (100–100)
Drug use disorders	100 (100–100)	100 (100–100)	100 (100–100)	100 (100–100)
Diabetes and kidney diseases	99·5 (99·3–99·6)	99·9 (99·8–99·9)	100 (100–100)	99·5 (99·4–99·6)
Diabetes mellitus	100 (100–100)	100 (100–100)	100 (100–100)	100 (100–100)
Chronic kidney disease	100 (100–100)	100 (100–100)	100 (100–100)	100 (100–100)
Sense organ diseases	--	8·99 (7·86–10·3)	8·99 (7·86–10·3)	--
Blindness and vision loss	--	8·65 (5·53–12·6)	8·65 (5·53–12·6)	--
Age-related and other hearing loss	--	10·6 (9·55–11·8)	10·6 (9·55–11·8)	--
Musculoskeletal disorders	3·84 (0·971–6·96)	23 (19·2–27)	23·4 (19·5–27·4)	4·53 (1·17–8·06)
Rheumatoid arthritis	11·3 (2·88–20·7)	13·9 (3·57–24·4)	14·1 (3·55–24·9)	13·1 (3·47–23·4)
Osteoarthritis	--	16·3 (8·4–25·4)	16·3 (8·4–25·4)	--
Low back pain	--	42·6 (37·3–47·8)	42·6 (37·3–47·8)	--
Gout	--	49 (32·9–66·3)	49 (32·9–66·3)	--
Other non-communicable diseases	0·0701 (0·0421–0·106)	0·0615 (0·0351–0·0981)	--	0·248 (0·146–0·361)
Sudden infant death syndrome	7·08 (3·71–11·6)	7·08 (3·71–11·6)	--	7·08 (3·71–11·6)
Injuries	29·5 (22·7–35·2)	25·2 (20·2–30·1)	27·8 (23·6–32·1)	21·7 (13·5–28·9)
Transport injuries	21·6 (12·8–29·8)	21·6 (14·3–28·8)	26·3 (21·8–31·1)	19·5 (10·3–28·3)
Road injuries	20·9 (11·7–29·3)	20·8 (13·1–28·3)	25·3 (20·8–30·2)	18·9 (9·29–28·1)
Other transport injuries	31·6 (26·3–37·5)	30·7 (25·5–36·4)	34·4 (30–39)	28 (22·3–34·5)
Unintentional injuries	37·2 (31·4–41·7)	26·7 (22·6–31·3)	27·7 (23·4–32·4)	23·6 (19·2–28)

Falls	54 (46·1–59·4)	38·1 (32·6–43)	36·6 (31·4–41·6)	44·1 (37·8–49·2)
Drowning	-13·3 (-27·2–0·881)	-12·8 (-25·8–0·209)	10·7 (6·13–16·8)	-14·1 (-27·6–-0·508)
Fire, heat, and hot substances	10·9 (6·28–17)	13·2 (8·34–19·7)	13·5 (8·42–20·1)	12·4 (7·79–18·5)
Poisonings	13·9 (8·82–20·5)	13·1 (8·39–19·1)	12 (7·42–17·9)	14·4 (9·23–20·7)
Exposure to mechanical forces	6·58 (-6·12–19·8)	16·6 (13·4–20·3)	18·1 (15·2–21·3)	-0·218 (-13·6–14·1)
Animal contact	22 (17·1–28·4)	20·4 (15·6–26)	20·8 (15·9–26·6)	19·4 (14·5–25·8)
Foreign body	4·2 (2·64–6·52)	4·82 (3·09–7·42)	3·92 (2·52–5·98)	5·5 (3·49–8·66)
Environmental heat and cold exposure	6·97 (2·57–13·3)	8·61 (3·52–15·7)	8·72 (3·55–15·9)	8·1 (3·32–15)
Exposure to forces of nature	0 (0–0)	9·48 (3·91–17·1)	9·48 (3·91–17·1)	0 (0–0)
Other unintentional injuries	13·4 (8·22–19·8)	12·7 (7·85–18·9)	12·6 (7·77–18·9)	14·9 (9·48–21·4)
Self-harm and interpersonal violence	20·8 (4·16–35·2)	23·1 (8·48–36·1)	32·3 (23·3–40·6)	21·6 (5·09–35·8)
Self-harm	20·4 (3–35·3)	21·4 (4·14–36·5)	20·6 (6·04–34·4)	21·5 (4·03–36·6)
Interpersonal violence	26·3 (16·2–35·7)	32 (23·2–41)	37·5 (28·2–47)	23·6 (14–32·6)

Supplementary Results Table 2. All-age PAFs attributable to the joint distribution of all risk factors for all ages, both sexes combined for each GBD level 1, level 2, and level 3 cause and all causes in 2019 for Greater London. Results are shown for deaths, YLLs, YLDs, and DALYs Data in parenthesis are 95% uncertainty intervals. PAFs=population attributable fractions. GBD=Global Burden of Disease. YLLs=years of life lost. YLDs=years lived with disability. DALYs=disability-adjusted life-years

	Deaths 2019	DALYs 2019	YLDs 2019	YLLs 2019
All causes	54·7 (51·8–57·7)	39·2 (36·3–42·1)	27·7 (25·4–30·1)	54·2 (52–56·5)
Communicable, maternal, neonatal, and nutritional diseases	48 (39·2–57)	55·5 (51·4–59·6)	49·7 (45·7–53·8)	59·3 (53·1–65·3)
HIV/AIDS and sexually transmitted infections	81·8 (71·8–88·6)	80·3 (68·5–88·1)	95·7 (93·7–97·1)	70·6 (56–81·6)
HIV/AIDS	90·9 (88·7–92·2)	89·3 (86·7–91·1)	93·8 (91·2–95·4)	86·8 (84·2–88·3)
Sexually transmitted infections excluding HIV	39·4 (19·5–66·1)	55·8 (30·3–81·6)	100 (100–100)	14·5 (5·47–32·1)
Respiratory infections and tuberculosis	47·6 (37·3–57·7)	42·4 (34·1–51·1)	3·42 (2·54–4·57)	51·5 (42·1–60·9)
Tuberculosis	59·2 (49·6–67·7)	60·6 (51·4–68)	55·8 (45·7–63·9)	61·6 (52·6–68·9)
Lower respiratory infections	47·5 (37–57·8)	51·3 (41·5–60·7)	35·5 (29·4–41·5)	51·4 (41·6–60·9)
Upper respiratory infections	0·0745 (0·0553–0·0961)	0·01 (0·00724–0·0136)	0·0084 (0·00578–0·012)	0·15 (0·112–0·196)
Otitis media	3·66 (2·43–5·11)	5·07 (3·23–6·94)	5·03 (3·19–6·91)	7·87 (5·53–10·6)
Enteric infections	4·86 (3·16–7·63)	10 (7·79–13)	10·1 (7·64–13·4)	9·81 (7·31–12·8)
Diarrheal diseases	4·91 (3·19–7·68)	10·1 (7·83–13·1)	10·1 (7·65–13·4)	9·99 (7·58–13)
Other infectious diseases	1 (0·709–1·28)	1·55 (1·21–1·9)	0·351 (0·216–0·559)	2·08 (1·62–2·58)
Meningitis	2·39 (1·87–2·97)	3·4 (2·64–4·28)	0·000763 (0·000474–0·00123)	4·44 (3·53–5·46)
Encephalitis	0·549 (0·398–0·695)	1·15 (0·698–1·52)	0·000267 (0·000172–0·000396)	1·55 (1·08–1·98)
Measles	3·33 (-1·01–15·1)	3·74 (-1·08–16·4)	0·382 (0·0112–0·951)	4·06 (-1·23–18·4)
Acute hepatitis	12·1 (7·8–20·9)	4·91 (3·23–7·47)	2·54 (1·58–4·01)	10·6 (6·79–18·7)
Maternal and neonatal disorders	88·5 (86·6–90)	79·2 (76·2–82·2)	66 (60·8–70·9)	89·2 (87·5–90·6)
Maternal disorders	9·47 (3·37–15·6)	9·56 (3·45–15·9)	9·7 (3·48–16·3)	9·34 (3·29–15·4)
Neonatal disorders	90·5 (88·8–91·8)	80·9 (77·8–83·8)	68 (62·4–73·1)	90·5 (88·8–91·8)
Nutritional deficiencies	71·8 (69·2–87)	79 (75·5–82·9)	79·2 (75·6–83·1)	71·6 (66·4–78·4)
Protein-energy malnutrition	100 (100–100)	100 (100–100)	100 (100–100)	100 (100–100)
Vitamin A deficiency	--	100 (100–100)	100 (100–100)	--
Dietary iron deficiency	--	100 (100–100)	100 (100–100)	--
Non-communicable diseases	56·4 (53·1–59·6)	39·2 (36–42·4)	26·8 (24·3–29·4)	56·2 (53·7–58·8)
Neoplasms	47·9 (44·4–52·1)	46·4 (43·2–50·1)	32·9 (29·1–37·2)	47·2 (44–51)
Lip and oral cavity cancer	68·5 (60·2–75·3)	71 (63·4–77·4)	70·3 (62·3–77·1)	71·1 (63·5–77·4)
Nasopharynx cancer	66·8 (56·4–75·4)	66·2 (56·3–74·5)	64·1 (53·5–73·1)	66·4 (56·6–74·5)
Other pharynx cancer	79·9 (73·3–85·1)	80·8 (74·5–85·9)	79·7 (73·2–85·2)	80·9 (74·6–86)
Esophageal cancer	75·6 (66·8–83·8)	76·8 (68·2–84·8)	75·9 (67·2–84·1)	76·8 (68·3–84·8)

Stomach cancer	24·2 (16·9–40·9)	24·1 (16·9–40·5)	24·3 (17–40·8)	24·1 (16·9–40·5)
Colon and rectum cancer	64 (55·2–72·8)	64·2 (56·2–72·1)	64·2 (55·9–72·5)	64·2 (56·1–72·1)
Liver cancer	77·1 (71·6–82·2)	76·9 (72–81·6)	77·3 (72·2–82)	76·9 (72–81·6)
Gallbladder and biliary tract cancer	22·7 (12·6–35·1)	23 (12·9–35·3)	21·4 (11·6–33·8)	23·1 (12·9–35·4)
Pancreatic cancer	42·4 (32·9–53·6)	41·4 (32·6–51·9)	42·1 (32·8–53·1)	41·4 (32·6–51·9)
Larynx cancer	83 (75·6–88·7)	82·2 (75·1–87·7)	79·6 (72·6–85·5)	82·5 (75·4–87·9)
Tracheal, bronchus, and lung cancer	85·6 (82·6–88·7)	84·2 (81·2–87·3)	85 (82–88)	84·1 (81·2–87·3)
Breast cancer	36·8 (27·6–47·7)	35 (27·1–44·3)	35·3 (27·1–44·7)	34·9 (27–44·2)
Cervical cancer	100 (100–100)	100 (100–100)	100 (100–100)	100 (100–100)
Uterine cancer	44·7 (30·3–59·8)	45·8 (31·3–60·8)	46·1 (31·7–61·2)	45·7 (31·2–60·7)
Ovarian cancer	25·8 (13–39·6)	21·2 (10·5–33·9)	20 (9·71–32)	21·2 (10·5–33·9)
Prostate cancer	5·63 (2·43–9·06)	6·11 (2·73–9·7)	6·45 (2·96–10·1)	6·05 (2·68–9·62)
Kidney cancer	38·7 (28·6–49·2)	38·3 (28·5–48)	38 (28·4–47·6)	38·4 (28·5–48)
Bladder cancer	42·5 (28·1–56·9)	44·4 (30·9–57·2)	44·6 (31·4–57)	44·3 (30·8–57·4)
Thyroid cancer	14·9 (7·19–24·2)	15·5 (7·53–25·2)	15·6 (7·65–25·3)	15·5 (7·5–25·3)
Mesothelioma	98·5 (98–98·9)	96·8 (95·9–97·5)	98·3 (97·7–98·7)	96·8 (95·8–97·5)
Non-Hodgkin lymphoma	7·42 (3·14–13·1)	7·6 (3·28–13·3)	7·56 (3·23–13·4)	7·6 (3·29–13·3)
Multiple myeloma	8·42 (3·7–14·9)	8·77 (3·85–15·4)	8·72 (3·81–15·5)	8·78 (3·86–15·4)
Leukemia	35·8 (22·7–49·6)	32·1 (21·7–43·1)	31·1 (20·9–41·8)	32·2 (21·7–43·2)
Cardiovascular diseases	77·1 (73·5–80·7)	78·5 (75·6–81·4)	69·7 (65·2–73·9)	79·8 (76·8–82·7)
Rheumatic heart disease	22 (12·6–41·7)	22·9 (14·7–37·7)	22·9 (14·9–36·4)	22·9 (14·6–38)
Ischemic heart disease	93·6 (89·9–96·6)	94·9 (92·4–97·1)	94·1 (91·3–96·6)	95 (92·5–97·1)
Stroke	79·7 (71·9–88·2)	83·3 (78·1–88·9)	82·8 (78·4–88)	83·3 (77·8–89·3)
Hypertensive heart disease	100 (100–100)	100 (100–100)	100 (100–100)	100 (100–100)
Non-rheumatic valvular heart disease	25·2 (16·6–37·4)	25·7 (18·5–35·1)	16·6 (11·4–22·8)	27 (19·5–36·9)
Cardiomyopathy and myocarditis	32·6 (25·3–41·2)	39 (33·3–45·5)	35·8 (30·2–41·6)	39·2 (33·4–46·1)
Atrial fibrillation and flutter	50·3 (41·2–60·4)	58·7 (50·6–66·9)	62·7 (55–70·3)	53·8 (45·7–63)
Aortic aneurysm	50 (43·8–56·7)	57·6 (52·2–62·7)	--	57·6 (52·2–62·7)
Endocarditis	30·4 (21·7–42·2)	33·1 (25–42·8)	30·2 (21·5–41·5)	33·2 (25·1–42·9)
Other cardiovascular and circulatory diseases	30·5 (25·1–35·9)	32·7 (27·7–37·6)	32·7 (26·8–38·6)	32·7 (27·6–37·6)
Chronic respiratory diseases	63·5 (57·8–69·1)	55·3 (50·8–59·8)	47·9 (42·5–53·6)	63·5 (58·5–68·4)
Chronic obstructive pulmonary disease	72 (66·9–77·2)	70·4 (66·5–74·1)	63·8 (60·6–67·1)	73·9 (69·2–78·4)
Pneumoconiosis	100 (100–100)	100 (100–100)	100 (100–100)	100 (100–100)
Asthma	42·5 (31·3–54·8)	39·4 (31·3–49·1)	39·1 (31·1–48·7)	42·8 (33·3–53·2)

Digestive diseases	33·8<br>(28·9–38·5)	38·2<br>(32·1–43·6)	25·9<br>(17·9–35)	47<br>(41–52·9)
Cirrhosis and other chronic liver diseases	76·5<br>(65·9–86·7)	78·9<br>(68·7–88·5)	74·9<br>(65·7–83·7)	79<br>(68·7–88·5)
Upper digestive system diseases	21·3<br>(18–24·5)	7·66<br>(4·93–11)	0·653<br>(0·379–1·04)	23·8<br>(20·4–27)
Gallbladder and biliary diseases	44·9<br>(30·6–59·8)	49·2<br>(34·9–63·3)	49·6<br>(35·3–63·8)	45·9<br>(32–60·8)
Pancreatitis	41·6<br>(26·7–56·6)	47·6<br>(32·3–62·2)	47·6<br>(32–62·5)	47·6<br>(32·3–62·3)
Neurological disorders	26·9<br>(12·6–41·9)	13·7<br>(6·26–24·7)	7·95<br>(3·77–16·2)	23·2<br>(11·6–37·8)
Alzheimer's disease and other dementias	41·4<br>(27·3–56·8)	43·2<br>(29·3–58·3)	43·4<br>(29·6–58·6)	43<br>(29·1–58·1)
Parkinson's disease	-8·8<br>(-14·6–-3·97)	-9·9<br>(-16–-4·61)	-10·5<br>(-16·8–-4·94)	-9·73<br>(-15·8–-4·54)
Idiopathic epilepsy	28·4<br>(19·7–37·4)	25·5<br>(17·6–33·4)	23·7<br>(16·2–31·6)	28·4<br>(20–37·1)
Multiple sclerosis	14·9<br>(11·3–18·8)	15·1<br>(11·5–18·8)	14·5<br>(11·1–18·2)	15·6<br>(11·8–19·4)
Mental disorders	--	6·8<br>(3·32–10·6)	6·81<br>(3·32–10·6)	--
Depressive disorders	--	14·4<br>(6·5–22·4)	14·4<br>(6·5–22·4)	--
Anxiety disorders	--	5·41<br>(1·33–12·4)	5·41<br>(1·33–12·4)	--
Idiopathic developmental intellectual disability	--	37·2<br>(16·9–54·3)	37·2<br>(16·9–54·3)	--
Substance use disorders	100<br>(100–100)	100<br>(100–100)	100<br>(100–100)	100<br>(100–100)
Alcohol use disorders	100<br>(100–100)	100<br>(100–100)	100<br>(100–100)	100<br>(100–100)
Drug use disorders	100<br>(100–100)	100<br>(100–100)	100<br>(100–100)	100<br>(100–100)
Diabetes and kidney diseases	99·7<br>(99·7–99·7)	99·9<br>(99·9–100)	100<br>(100–100)	99·7<br>(99·7–99·7)
Diabetes mellitus	100<br>(100–100)	100<br>(100–100)	100<br>(100–100)	100<br>(100–100)
Chronic kidney disease	100<br>(100–100)	100<br>(100–100)	100<br>(100–100)	100<br>(100–100)
Sense organ diseases	--	8·53<br>(7·55–9·71)	8·53<br>(7·55–9·71)	--
Blindness and vision loss	--	8·27<br>(5·29–12)	8·27<br>(5·29–12)	--
Age-related and other hearing loss	--	9·93<br>(9·09–10·9)	9·93<br>(9·09–10·9)	--
Musculoskeletal disorders	2·68<br>(0·647–4·87)	21·3<br>(17·7–25)	21·6<br>(17·9–25·5)	3·11<br>(0·757–5·47)
Rheumatoid arthritis	11·6<br>(2·83–20·4)	13·1<br>(3·13–23)	13·1<br>(3·1–23)	13<br>(3·27–23)
Osteoarthritis	--	17·4<br>(9·35–26·4)	17·4<br>(9·35–26·4)	--
Low back pain	--	39·5<br>(34·4–44·5)	39·5<br>(34·4–44·5)	--
Gout	--	50·8<br>(35·1–68)	50·8<br>(35·1–68)	--
Other non-communicable diseases	0·123<br>(0·0896–0·163)	0·0911<br>(0·0609–0·128)	--	0·412<br>(0·273–0·526)
Sudden infant death syndrome	8·44<br>(5·57–12·4)	8·43<br>(5·57–12·4)	--	8·43<br>(5·57–12·4)
Injuries	30·4<br>(25·2–35·4)	25·1<br>(21·2–29·2)	26·8<br>(22·8–30·9)	22·8<br>(17·2–28·9)
Transport injuries	21·2<br>(13·5–28·6)	21·2<br>(14·9–27·5)	25·8<br>(21·6–30·3)	18·8<br>(10·7–26·7)
Road injuries	20·3<br>(12·2–28)	20·3<br>(13·8–27)	24·9<br>(20·6–29·4)	18·1<br>(9·61–26·3)
Other transport injuries	31·2<br>(26·2–36)	29·8<br>(25·1–34·5)	33·1<br>(28·7–37·3)	27·5<br>(22·1–32·7)

Unintentional injuries	37 (31–41·4)	25·5 (21·2–30·1)	26·4 (22–31)	22·4 (18·1–26·4)
Falls	53·7 (45·6–59)	36·4 (30·6–41·6)	34·9 (29·1–40·2)	43 (36·6–48·3)
Drowning	-14·4 (-27·1–-0·811)	-14·3 (-26·3–-1·38)	11·2 (6·67–17·3)	-15·5 (-28·1–-1·96)
Fire, heat, and hot substances	11·2 (6·65–17·5)	13·3 (8·54–19·8)	13·9 (8·92–20·6)	12 (7·48–17·8)
Poisonings	14·2 (9·09–20·6)	13·2 (8·54–19·1)	12·4 (7·92–18·2)	14·4 (9·34–20·6)
Exposure to mechanical forces	8·3 (-3·26–20·1)	15·2 (12·6–17·7)	16·3 (14·1–18·6)	-1·25 (-13·6–11·9)
Animal contact	20·7 (16·2–26·2)	19·4 (14·8–25·1)	20·4 (15·4–26·1)	16·2 (12·2–21·2)
Foreign body	3·06 (2·07–4·27)	3·83 (2·68–5·28)	3·45 (2·43–4·82)	4·13 (2·83–5·78)
Environmental heat and cold exposure	9·05 (4·4–15·6)	9·75 (4·74–16·9)	9·67 (4·63–16·8)	10·1 (4·91–17·2)
Exposure to forces of nature	0 (0–0)	10·6 (5·12–18·1)	10·6 (5·12–18·1)	0 (0–0)
Other unintentional injuries	14·8 (9·46–21·4)	13·2 (8·33–19·4)	13·1 (8·3–19·4)	15·6 (10·4–22·3)
Self-harm and interpersonal violence	24·7 (13·2–36·4)	26·6 (16·1–36·8)	35·1 (27·8–42·7)	25·1 (13·8–36·6)
Self-harm	24·7 (12·2–37·4)	25·5 (13–38·1)	25·1 (14–36·8)	25·5 (13·1–38·1)
Interpersonal violence	26 (18–34·5)	32·2 (24·5–40·4)	39·7 (31·2–49·2)	23 (15·2–31·3)

Supplementary Results Table 2. All-age PAFs attributable to the joint distribution of all risk factors for all ages, both sexes combined for each GBD level 1, level 2, and level 3 cause and all causes in 2019 for Barking and Dagenham. Results are shown for deaths, YLLs, YLDs, and DALYs Data in parenthesis are 95% uncertainty intervals. PAFs=population attributable fractions. GBD=Global Burden of Disease. YLLs=years of life lost. YLDs=years lived with disability. DALYs=disability-adjusted life-years

	Deaths 2019	DALYs 2019	YLDs 2019	YLLs 2019
All causes	54·9 (51·9–57·8)	39·5 (36·3–42·5)	26·3 (24–28·6)	53·8 (51·2–56·2)
Communicable, maternal, neonatal, and nutritional diseases	47·8 (37·3–57·7)	56·5 (52–60·9)	50·7 (45·8–55·7)	59·9 (52·9–66·1)
HIV/AIDS and sexually transmitted infections	76·3 (63·1–85·5)	74·3 (59·1–84·8)	96·1 (94·3–97·5)	61·5 (44·6–75·3)
HIV/AIDS	89 (87·1–90·3)	86·8 (84·4–89)	93·6 (91·1–95·3)	83·2 (80·9–84·8)
Sexually transmitted infections excluding HIV	41·7 (21–68·2)	52·2 (27·6–78·8)	100 (100–100)	16·3 (6·25–35·3)
Respiratory infections and tuberculosis	46·7 (34·8–57·9)	42·5 (33·2–51·9)	3·3 (2·46–4·38)	50·9 (39·7–61·3)
Tuberculosis	56 (43·9–66·6)	56·8 (45·6–66·5)	50·8 (38·4–61·2)	58·1 (47·3–67·6)
Lower respiratory infections	46·6 (34·7–57·9)	50·7 (39·5–61·2)	33·8 (27·6–40·2)	50·9 (39·5–61·4)
Upper respiratory infections	0·109 (0·0569–0·202)	0·0126 (0·00217–0·0301)	0·0106 (0·000202–0·0277)	0·192 (0·0993–0·353)
Otitis media	6·78 (2·61–15·6)	6·05 (3·9–8·44)	6·05 (3·9–8·44)	12·2 (5·48–25·7)
Enteric infections	6·29 (3·92–10·8)	12·2 (9·25–16·8)	12·2 (9·04–16·8)	12·3 (8·59–17·3)
Diarrheal diseases	6·35 (3·96–10·9)	12·3 (9·32–16·9)	12·2 (9·05–16·9)	12·5 (8·87–17·5)
Other infectious diseases	1·07 (0·697–1·41)	1·54 (1·12–2·03)	0·322 (0·181–0·54)	2·06 (1·5–2·72)
Meningitis	2·33 (1·6–3·17)	3·13 (2·24–4·27)	0·00097 (1·7e-05–0·00275)	3·84 (2·75–5·19)
Encephalitis	0·645 (0·424–0·967)	1·17 (0·756–1·76)	0·000321 (4·62e-06–0·000901)	1·58 (1·07–2·35)
Measles	3·08 (-0·884–16·4)	3·53 (-0·968–17·8)	0·657 (0·0138–1·85)	3·65 (-1·05–19·3)
Acute hepatitis	13·6 (7·67–24·8)	4·32 (2·58–6·87)	2·38 (1·38–3·9)	12·5 (7·15–23)
Maternal and neonatal disorders	88·4 (86·2–90·2)	81·5 (78·6–84·2)	68·5 (63·4–73·4)	89·1 (87–90·8)
Maternal disorders	9·93 (3·45–16·8)	9·99 (3·52–16·8)	10·1 (3·59–17·2)	9·8 (3·44–16·5)
Neonatal disorders	90·4 (88·5–92·1)	83·5 (80·5–86·1)	71·1 (65·7–76·1)	90·5 (88·5–92·1)
Nutritional deficiencies	75·4 (69·2–86·4)	81·8 (77·2–86·9)	82 (77·3–87·1)	74·9 (64·6–79·1)
Protein-energy malnutrition	100 (100–100)	100 (100–100)	100 (100–100)	100 (100–100)
Vitamin A deficiency	--	100 (100–100)	100 (100–100)	--
Dietary iron deficiency	--	100 (100–100)	100 (100–100)	--
Non-communicable diseases	56·7 (53·4–59·6)	39·4 (35·9–42·8)	24·9 (22·5–27·6)	55·7 (53–58·2)
Neoplasms	49·3 (45·6–53·5)	47·2 (43·8–50·9)	33·3 (29·2–37·6)	47·8 (44·5–51·5)
Lip and oral cavity cancer	65·4 (53·6–75·3)	67·8 (56·2–77)	66·9 (55–76·3)	67·8 (56·2–77·1)
Nasopharynx cancer	64·1 (51–75)	62·7 (49·6–73·3)	59·6 (45·9–71·5)	62·9 (49·8–73·5)
Other pharynx cancer	78·4 (70·1–85·4)	79·2 (70·8–85·9)	78·1 (69·2–84·9)	79·3 (70·9–85·9)
Esophageal cancer	73·7 (64·8–82·1)	74·9 (66·2–83·1)	74·1 (64·9–82·3)	74·9 (66·2–83·1)
Stomach cancer	23·9 (16·3–40·5)	23·6 (16·3–40·3)	23·8 (16·6–40·2)	23·6 (16·3–40·3)

Colon and rectum cancer	62·4 (53·3–71·2)	62·2 (53·9–70·4)	62·3 (53·8–70·5)	62·2 (53·9–70·4)
Liver cancer	73·5 (67–79·4)	73·1 (67·1–78·4)	73·5 (67·8–78·9)	73·1 (67·1–78·4)
Gallbladder and biliary tract cancer	18·5 (9·45–30·1)	18·8 (9·73–30·3)	17·4 (8·64–28·5)	18·9 (9·77–30·4)
Pancreatic cancer	40·8 (31·4–51·9)	39·6 (30·7–50·1)	40·4 (30·9–51·3)	39·6 (30·7–50·1)
Larynx cancer	82·8 (74·9–89)	82 (74·9–87·7)	79·4 (71·7–85·7)	82·2 (75·1–87·9)
Tracheal, bronchus, and lung cancer	86·8 (83·2–90)	85·3 (81·8–88·5)	86·2 (82·7–89·4)	85·3 (81·8–88·5)
Breast cancer	34 (24·6–44·8)	32·1 (23·8–41·4)	32·4 (24–42)	32 (23·7–41·4)
Cervical cancer	100 (100–100)	100 (100–100)	100 (100–100)	100 (100–100)
Uterine cancer	38·8 (24·4–54)	40 (25·7–55·3)	40·3 (26–55·8)	40 (25·7–55·2)
Ovarian cancer	30·5 (13–44·8)	23·9 (10·7–36·3)	22·1 (10·1–34·4)	24 (10·7–36·4)
Prostate cancer	5·59 (2·47–9·1)	6·03 (2·76–9·7)	6·35 (2·95–10)	5·98 (2·75–9·66)
Kidney cancer	35·3 (25·7–45·6)	34·6 (25·6–44·1)	34 (25·1–43·8)	34·6 (25·6–44·2)
Bladder cancer	42·1 (27·9–56·5)	43·7 (30·8–56·5)	44 (31·3–56·2)	43·7 (30·8–56·5)
Thyroid cancer	11·6 (5·57–19·2)	12 (5·67–19·8)	12 (5·69–19·7)	12 (5·68–19·8)
Mesothelioma	99·1 (98·7–99·4)	98 (97·3–98·6)	98·9 (98·4–99·2)	98 (97·3–98·5)
Non-Hodgkin lymphoma	5·96 (2·37–11)	6·01 (2·38–11)	6·02 (2·38–11·1)	6 (2·38–11)
Multiple myeloma	6·82 (2·8–12·6)	7·12 (2·91–13)	7·07 (2·8–13)	7·12 (2·91–13)
Leukemia	33·4 (20·4–46·6)	28·2 (18·6–38·3)	27 (17·7–36·5)	28·3 (18·6–38·3)
Cardiovascular diseases	77·9 (73·9–81·3)	79·1 (75·9–81·9)	66·8 (61·6–71·3)	80·4 (77·4–83·1)
Rheumatic heart disease	21·2 (11·7–41·5)	21·7 (13·7–35·5)	21·6 (13·6–33·7)	21·7 (13·6–35·6)
Ischemic heart disease	93·5 (89·9–96·6)	95 (92·5–97·2)	93·9 (91–96·5)	95 (92·6–97·2)
Stroke	79·2 (70·9–88·2)	82·5 (76·9–88·2)	80·8 (76·2–85·8)	82·8 (76·9–88·7)
Hypertensive heart disease	100 (100–100)	100 (100–100)	100 (100–100)	100 (100–100)
Non-rheumatic valvular heart disease	24·4 (15·5–36·1)	23·7 (16·4–32·9)	10·6 (6·6–15·1)	26 (17·9–36)
Cardiomyopathy and myocarditis	34·8 (27·6–43·2)	39 (33·8–45)	35·2 (29·3–41·6)	39·3 (33·8–45·2)
Atrial fibrillation and flutter	45·9 (35·4–57·3)	54·4 (45·2–63·2)	58·9 (50·2–67·4)	49·8 (40·4–59·9)
Aortic aneurysm	50·5 (44·1–57·3)	58·2 (52·4–63·9)	--	58·2 (52·4–63·9)
Endocarditis	29·2 (19·6–41·4)	30·8 (22·2–41)	28·5 (18·9–40·3)	30·9 (22·3–41·1)
Other cardiovascular and circulatory diseases	29·6 (23·2–36·1)	30·9 (25·1–36·8)	30·4 (22·7–38·1)	31 (25·2–36·8)
Chronic respiratory diseases	65·9 (59·9–71·8)	55·1 (50·2–60·1)	42·7 (37·3–48·3)	65·6 (60·2–71·1)
Chronic obstructive pulmonary disease	72·1 (66·4–77·8)	71 (66·7–75·4)	63·3 (59·5–67·2)	73·8 (68·7–79)
Pneumoconiosis	100 (100–100)	100 (100–100)	100 (100–100)	100 (100–100)
Asthma	37·4 (27·1–48·8)	32 (24·7–40·2)	31·7 (24·5–40·1)	34·8 (27–43·7)
Digestive diseases	30·8 (25·2–37)	35·4 (29·1–42·1)	20·1 (13·2–28·4)	42·7 (35·4–50·6)

Cirrhosis and other chronic liver diseases	72·3 (60·1–86·3)	74·5 (62·6–88)	69·2 (57·1–81·7)	74·6 (62·6–88·1)
Upper digestive system diseases	20·7 (17·3–23·9)	9·54 (6·32–13·4)	0·683 (0·391–1·1)	22·8 (19·4–26·1)
Gallbladder and biliary diseases	38·7 (24·7–53·6)	42·6 (28·8–57·2)	43·3 (29·3–57·9)	39·7 (26·3–54·1)
Pancreatitis	35·5 (14·9–55·3)	40·9 (19–59·9)	40·2 (17·7–59·7)	40·9 (19·1–60)
Neurological disorders	24·2 (11–39)	11·8 (5·37–21·6)	6·59 (3·04–14·3)	20·3 (10·3–33·3)
Alzheimer's disease and other dementias	38·8 (25·1–54·1)	40·6 (26·8–55·6)	40·8 (26·9–56)	40·5 (26·6–55·5)
Parkinson's disease	-8·64 (-14·4–-3·93)	-9·74 (-15·7–-4·54)	-10·3 (-16·3–-4·83)	-9·6 (-15·5–-4·47)
Idiopathic epilepsy	24·3 (13·8–34·9)	20·9 (12·2–30·4)	18·7 (10·4–27·5)	23·5 (13·6–33·7)
Multiple sclerosis	15·1 (11·1–19·1)	15·1 (11·3–19)	14·5 (10·9–18·3)	15·6 (11·5–19·8)
Mental disorders	--	7·12 (3·68–11)	7·13 (3·69–11)	--
Depressive disorders	--	14·7 (6·8–22·6)	14·7 (6·8–22·6)	--
Anxiety disorders	--	6·1 (1·66–13·5)	6·1 (1·66–13·5)	--
Idiopathic developmental intellectual disability	--	38·5 (16·4–57·1)	38·5 (16·4–57·1)	--
Substance use disorders	100 (100–100)	100 (100–100)	100 (100–100)	100 (100–100)
Alcohol use disorders	100 (100–100)	100 (100–100)	100 (100–100)	100 (100–100)
Drug use disorders	100 (100–100)	100 (100–100)	100 (100–100)	100 (100–100)
Diabetes and kidney diseases	99·7 (99·5–99·8)	99·9 (99·9–99·9)	100 (100–100)	99·7 (99·6–99·7)
Diabetes mellitus	100 (100–100)	100 (100–100)	100 (100–100)	100 (100–100)
Chronic kidney disease	100 (100–100)	100 (100–100)	100 (100–100)	100 (100–100)
Sense organ diseases	--	8·38 (7·28–9·59)	8·38 (7·28–9·59)	--
Blindness and vision loss	--	7·17 (4·49–10·6)	7·17 (4·49–10·6)	--
Age-related and other hearing loss	--	10·1 (9·2–11·2)	10·1 (9·2–11·2)	--
Musculoskeletal disorders	3·5 (0·841–6·34)	20·3 (16·9–23·9)	20·6 (17·1–24·3)	3·97 (1·02–7·08)
Rheumatoid arthritis	11·8 (2·87–20·8)	12·9 (3·29–22·5)	12·8 (3·28–22·4)	13·2 (3·35–23·1)
Osteoarthritis	--	14·5 (7·17–23·1)	14·5 (7·17–23·1)	--
Low back pain	--	37·6 (32·7–42·6)	37·6 (32·7–42·6)	--
Gout	--	44·3 (29·3–61·7)	44·3 (29·3–61·7)	--
Other non-communicable diseases	0·117 (0·0747–0·175)	0·109 (0·0661–0·163)	--	0·373 (0·232–0·531)
Sudden infant death syndrome	7·85 (4·57–12·7)	7·84 (4·56–12·7)	--	7·84 (4·56–12·7)
Injuries	29·6 (23·7–35)	23·4 (18·8–28)	26·3 (22·4–30·6)	20·1 (13·9–26·8)
Transport injuries	18·8 (10·6–26·7)	18·3 (10·8–25·5)	26 (21·1–31·3)	15·6 (6·86–24·1)
Road injuries	17·7 (8·97–26·1)	17·3 (9·52–24·7)	25·1 (20·3–30·5)	14·6 (5·36–23·3)
Other transport injuries	29·2 (23·8–35·1)	27·3 (22–33·1)	32·3 (27·5–36·8)	25·1 (19·4–31·3)
Unintentional injuries	37·7 (31·5–42·3)	24·9 (20·9–29·2)	25·9 (21·7–30·5)	22 (17·7–26·1)

Falls	53·4<br>(45·1–58·9)	35·7<br>(30–40·7)	33·9<br>(28·7–39)	41·8<br>(35·7–46·9)
Drowning	-17·2<br>(-30·2–-3·76)	-17·8<br>(-30·3–-5·07)	10·2<br>(6–16·2)	-18·9<br>(-31·8–-5·75)
Fire, heat, and hot substances	9·93<br>(5·76–15·7)	12·7<br>(8·2–18·6)	13·7<br>(8·9–20)	10·2<br>(6·34–15·3)
Poisonings	14·2<br>(9·16–20·7)	12·6<br>(8·26–18·1)	11·8<br>(7·69–17·1)	13·9<br>(9·16–20·2)
Exposure to mechanical forces	9·21<br>(-3·45–22)	15·8<br>(12·4–19·4)	17·5<br>(14·5–20·9)	-2·01<br>(-15·8–12)
Animal contact	19·7<br>(15·3–25·3)	18·3<br>(13·8–23·5)	19·3<br>(14·6–24·8)	15·1<br>(11·2–20)
Foreign body	3·73<br>(2·31–5·52)	4·44<br>(2·8–6·46)	4·38<br>(2·81–6·53)	4·48<br>(2·8–6·61)
Environmental heat and cold exposure	7·71<br>(3·4–14·1)	8·32<br>(3·78–14·9)	8·27<br>(3·73–14·7)	8·48<br>(3·8–15·1)
Exposure to forces of nature	0<br>(0–0)	9·34<br>(4·26–16·4)	9·34<br>(4·26–16·4)	0<br>(0–0)
Other unintentional injuries	14·4<br>(9·2–21·1)	12·7<br>(8·26–18·6)	12·6<br>(8·14–18·5)	14·5<br>(9·46–21)
Self-harm and interpersonal violence	21·5<br>(8·72–35)	23·1<br>(11·6–35·3)	33·3<br>(25·4–41·7)	21·5<br>(8·95–34·8)
Self-harm	21·2<br>(7·2–35·7)	21·7<br>(7·95–36·2)	22·2<br>(10·3–35·2)	21·7<br>(7·91–36·2)
Interpersonal violence	24·7<br>(16·5–33·2)	29·8<br>(21·7–38·2)	37·8<br>(28·7–47·5)	21·1<br>(13·5–29·2)

Supplementary Results Table 2. All-age PAFs attributable to the joint distribution of all risk factors for all ages, both sexes combined for each GBD level 1, level 2, and level 3 cause and all causes in 2019 for Barnet. Results are shown for deaths, YLLs, YLDs, and DALYs Data in parenthesis are 95% uncertainty intervals. PAFs=population attributable fractions. GBD=Global Burden of Disease. YLLs=years of life lost. YLDs=years lived with disability. DALYs=disability-adjusted life-years

	Deaths 2019	DALYs 2019	YLDs 2019	YLLs 2019
All causes	53·4 (49·5–57·1)	38·1 (34·9–41·4)	27·6 (25·2–29·9)	52·6 (49·5–55·7)
Communicable, maternal, neonatal, and nutritional diseases	47 (35·6–57·7)	51·8 (46·6–56·8)	46·9 (42·3–51·7)	55·2 (46·8–63·1)
HIV/AIDS and sexually transmitted infections	78·6 (66·4–87·4)	77·8 (63·4–87·5)	96·6 (94·9–97·8)	65 (48·5–78·9)
HIV/AIDS	90·9 (88·9–92·1)	89·2 (86·8–90·9)	94 (91·5–95·6)	86·5 (84·1–88)
Sexually transmitted infections excluding HIV	37·6 (17·7–63·6)	57·4 (31·7–82·8)	100 (100–100)	12·4 (4·5–28)
Respiratory infections and tuberculosis	47·5 (35·1–59·1)	42·3 (32·2–52)	3·43 (2·52–4·58)	50·8 (39·1–61·6)
Tuberculosis	57·5 (46–67·4)	59·1 (48·5–67·9)	55·4 (43·7–64·9)	60 (49·5–68·9)
Lower respiratory infections	47·4 (34·8–59·1)	50·6 (38·7–61·4)	35·2 (28·1–42·2)	50·7 (38·7–61·6)
Upper respiratory infections	0·0901 (0·0456–0·16)	0·00913 (0·00157–0·0218)	0·0077 (0·000173–0·02)	0·189 (0·099–0·339)
Otitis media	2·74 (0·959–6·45)	5·04 (3·19–7·12)	5·03 (3·19–7·12)	6·02 (2·56–11·5)
Enteric infections	4·56 (2·91–7·53)	9·39 (7·2–12·6)	9·57 (7·22–12·9)	8·73 (6·15–12·1)
Diarrheal diseases	4·61 (2·94–7·66)	9·44 (7·26–12·6)	9·58 (7·22–12·9)	8·91 (6·32–12·3)
Other infectious diseases	0·763 (0·484–1·03)	1·21 (0·872–1·6)	0·345 (0·191–0·583)	1·65 (1·16–2·19)
Meningitis	1·76 (1·22–2·43)	2·6 (1·83–3·61)	0·00062 (8·15e-06–0·00174)	3·56 (2·52–4·84)
Encephalitis	0·426 (0·261–0·631)	0·965 (0·561–1·47)	0·000215 (3·06e-06–0·000571)	1·36 (0·834–2·03)
Measles	3·38 (-1·11–17·2)	3·62 (-1·14–18)	0·367 (0·0128–1·06)	4·04 (-1·37–20)
Acute hepatitis	13 (6·99–22·7)	4·97 (2·89–8·1)	2·58 (1·46–4·28)	11·4 (6·03–20·4)
Maternal and neonatal disorders	86·5 (84·2–88·5)	75·1 (71–78·8)	62·8 (57·2–68·2)	87·4 (85·3–89·3)
Maternal disorders	9·2 (3·31–15·3)	9·27 (3·36–15·5)	9·38 (3·4–15·9)	9·06 (3·28–15·1)
Neonatal disorders	89 (87·1–90·7)	76·9 (72·9–80·7)	64·7 (58·7–70·6)	89 (87·1–90·7)
Nutritional deficiencies	66·5 (59·9–89·2)	78·6 (74·6–83·4)	78·8 (74·7–83·6)	66 (62–80·4)
Protein-energy malnutrition	100 (100–100)	100 (100–100)	100 (100–100)	100 (100–100)
Vitamin A deficiency	--	100 (100–100)	100 (100–100)	--
Dietary iron deficiency	--	100 (100–100)	100 (100–100)	--
Non-communicable diseases	54·8 (50·7–58·8)	38·1 (34·7–41·6)	26·7 (24·2–29·3)	54·3 (51·1–57·6)
Neoplasms	46·2 (42·1–50·6)	44·7 (40·9–48·8)	32 (27·8–36·6)	45·5 (41·8–49·6)
Lip and oral cavity cancer	67·4 (56·1–75·9)	70·3 (60·1–78·1)	69·7 (59–77·7)	70·3 (60·1–78·1)
Nasopharynx cancer	66·7 (54·4–76·1)	66·3 (54·5–75·4)	64·7 (52·6–74·7)	66·5 (54·9–75·5)
Other pharynx cancer	79·8 (72·5–85·6)	80·9 (74–86·3)	79·8 (72·9–85·9)	80·9 (74·1–86·3)
Esophageal cancer	75·8 (65·5–84·3)	77·1 (67·6–85·2)	76·2 (66·3–84·7)	77·2 (67·6–85·2)

Stomach cancer	23·9 (16·5–40·7)	24 (16·5–40·7)	24·1 (16·6–40·8)	24 (16·5–40·7)
Colon and rectum cancer	64·3 (55·3–73·3)	64·6 (56·2–73)	64·6 (56·1–72·8)	64·6 (56·3–73)
Liver cancer	77·5 (71·4–83·1)	77·8 (72·4–82·8)	78 (72·5–82·9)	77·8 (72·4–82·7)
Gallbladder and biliary tract cancer	22·7 (12·4–35·1)	23·1 (12·9–35·6)	21·5 (11·5–33·5)	23·1 (12·9–35·7)
Pancreatic cancer	42·4 (32·4–54)	41·6 (32·2–52·5)	42·2 (32·4–53·5)	41·6 (32·2–52·4)
Larynx cancer	82·6 (74·7–88·8)	82 (74·3–87·8)	79·6 (71·7–85·8)	82·3 (74·6–88)
Tracheal, bronchus, and lung cancer	84·8 (81·1–88·5)	83·6 (80–87·4)	84·3 (80·4–88·1)	83·6 (80–87·4)
Breast cancer	37·2 (27·4–48·6)	35·7 (27·2–45·7)	36 (27·3–46·1)	35·6 (27·1–45·6)
Cervical cancer	100 (100–100)	100 (100–100)	100 (100–100)	100 (100–100)
Uterine cancer	45·8 (30·9–61)	46·8 (31·8–62·1)	47·1 (32–62·5)	46·8 (31·8–62·1)
Ovarian cancer	23·4 (11·8–38·3)	19·8 (9·61–32·9)	18·9 (9·05–31·2)	19·8 (9·66–33·1)
Prostate cancer	5·5 (2·36–8·97)	5·94 (2·63–9·39)	6·27 (2·82–9·88)	5·88 (2·61–9·32)
Kidney cancer	39·1 (28·7–49·9)	39 (29–49)	38·7 (28·7–48·4)	39 (29–49)
Bladder cancer	41·9 (27–56)	43·8 (30–57·5)	44·2 (30·6–57·5)	43·8 (29·9–57·5)
Thyroid cancer	15·7 (7·48–25·7)	16·4 (8·04–26·6)	16·5 (8·15–27·1)	16·4 (8·06–26·7)
Mesothelioma	98·3 (97·6–98·7)	96·5 (95·4–97·4)	98 (97·2–98·6)	96·5 (95·4–97·4)
Non-Hodgkin lymphoma	7·7 (3·22–13·5)	7·9 (3·35–13·8)	7·83 (3·36–13·8)	7·91 (3·38–13·8)
Multiple myeloma	8·69 (3·75–15·3)	9·05 (4·03–15·9)	9 (3·92–15·9)	9·06 (4·04–15·9)
Leukemia	36 (22·5–50·1)	33·1 (21·9–44·6)	32·2 (21·2–43·9)	33·2 (21·9–44·8)
Cardiovascular diseases	73·9 (69–80·4)	76·1 (71·9–81·4)	69·6 (64·9–74·3)	76·9 (72·9–82·6)
Rheumatic heart disease	21·7 (11·7–42·5)	22·7 (13·7–39·6)	22·7 (14·3–35·3)	22·7 (13·7–40)
Ischemic heart disease	93·3 (89·2–96·8)	94·6 (91·6–97·1)	94 (91·2–96·6)	94·6 (91·7–97·1)
Stroke	79·3 (71·1–88·5)	82·5 (76·2–89·2)	82·3 (77·6–88)	82·5 (75·8–89·4)
Hypertensive heart disease	100 (100–100)	100 (100–100)	100 (100–100)	100 (100–100)
Non-rheumatic valvular heart disease	25 (15·5–38·5)	23·9 (16–34·2)	11·4 (7·13–16·4)	26·7 (17·9–38)
Cardiomyopathy and myocarditis	27·5 (17·9–40)	32·2 (23·9–42·7)	30·9 (24–38·5)	32·3 (23·9–43·4)
Atrial fibrillation and flutter	50·4 (40–61·6)	58·3 (49·5–67·3)	62·5 (53·6–71·1)	53·5 (43·8–63·7)
Aortic aneurysm	48·1 (40·8–55·8)	55·6 (49·4–61·9)	--	55·6 (49·4–61·9)
Endocarditis	29·7 (19·6–43·4)	32·4 (23·5–43·6)	29·9 (20·3–43)	32·5 (23·5–43·6)
Other cardiovascular and circulatory diseases	30 (23·4–37·3)	32·9 (26·8–39·1)	33·7 (26·4–41·5)	32·7 (26·6–39)
Chronic respiratory diseases	60·6 (53·6–67·7)	53·7 (48·9–58·5)	48·3 (42·9–54·1)	60·6 (54·4–66·9)
Chronic obstructive pulmonary disease	71·4 (65·2–77·5)	69·2 (64·8–73·7)	63·5 (59·5–67·3)	73·2 (67·7–78·7)
Pneumoconiosis	100 (100–100)	100 (100–100)	100 (100–100)	100 (100–100)
Asthma	43 (31–55·5)	39·4 (31–49·1)	39·1 (30·7–48·5)	43·8 (33·6–54·6)

Digestive diseases	29·4 (24·3–34·4)	34·9 (28·7–40·5)	27 (18·7–36·2)	42·3 (35·8–48·5)
Cirrhosis and other chronic liver diseases	76·4 (62·7–88·1)	79 (66·5–89·8)	74·7 (62·8–84·8)	79·2 (66·6–90)
Upper digestive system diseases	20·6 (17·1–24·1)	6·66 (4·18–9·81)	0·676 (0·38–1·1)	23·2 (19·7–26·5)
Gallbladder and biliary diseases	45·5 (30·3–60·7)	51·1 (36·5–65·2)	51·6 (36·9–65·8)	46·5 (31·7–61·5)
Pancreatitis	41·4 (21·1–59·5)	47·4 (26·7–64·4)	47 (26·8–64·5)	47·5 (26·8–64·3)
Neurological disorders	27·6 (13·1–42·9)	15·1 (7·2–27)	9·04 (4·26–18·3)	23·9 (11·4–38·6)
Alzheimer's disease and other dementias	41·8 (27·7–58·1)	43·5 (29·1–59·1)	43·6 (29·2–59·2)	43·4 (29·1–59)
Parkinson's disease	-8·62 (-14·2–-3·88)	-9·63 (-15·4–-4·45)	-10·1 (-15·9–-4·7)	-9·5 (-15·2–-4·37)
Idiopathic epilepsy	28·3 (17·6–38·2)	25 (15·4–33·9)	23·1 (14–31·9)	28·4 (18·4–37·5)
Multiple sclerosis	14·5 (10·8–18·3)	14·9 (11·1–18·7)	14·3 (10·9–18·1)	15·3 (11·3–19·4)
Mental disorders	--	6·79 (3·36–10·5)	6·8 (3·36–10·5)	--
Depressive disorders	--	14·2 (6·41–22·3)	14·2 (6·41–22·3)	--
Anxiety disorders	--	5·41 (1·39–12·2)	5·41 (1·39–12·2)	--
Idiopathic developmental intellectual disability	--	36·1 (15·7–54·4)	36·1 (15·7–54·4)	--
Substance use disorders	100 (100–100)	100 (100–100)	100 (100–100)	100 (100–100)
Alcohol use disorders	100 (100–100)	100 (100–100)	100 (100–100)	100 (100–100)
Drug use disorders	100 (100–100)	100 (100–100)	100 (100–100)	100 (100–100)
Diabetes and kidney diseases	99·9 (99·9–99·9)	100 (100–100)	100 (100–100)	99·9 (99·9–99·9)
Diabetes mellitus	100 (100–100)	100 (100–100)	100 (100–100)	100 (100–100)
Chronic kidney disease	100 (100–100)	100 (100–100)	100 (100–100)	100 (100–100)
Sense organ diseases	--	8·2 (7·08–9·44)	8·2 (7·08–9·44)	--
Blindness and vision loss	--	8·93 (5·74–13)	8·93 (5·74–13)	--
Age-related and other hearing loss	--	9·18 (8·27–10·2)	9·18 (8·27–10·2)	--
Musculoskeletal disorders	3·27 (0·806–6·05)	21·4 (17·6–25·1)	21·6 (17·8–25·3)	3·93 (1·02–7·22)
Rheumatoid arthritis	11·2 (2·88–20·3)	12·8 (3·31–23·2)	12·8 (3·31–23·3)	12·7 (3·29–22·6)
Osteoarthritis	--	17·8 (9·63–27)	17·8 (9·63–27)	--
Low back pain	--	39·2 (34–44·3)	39·2 (34–44·3)	--
Gout	--	52·6 (36·5–69·7)	52·6 (36·5–69·7)	--
Other non-communicable diseases	0·097 (0·0603–0·143)	0·0688 (0·0388–0·109)	--	0·362 (0·204–0·53)
Sudden infant death syndrome	7·37 (4·25–11·8)	7·36 (4·24–11·8)	--	7·36 (4·24–11·8)
Injuries	31·5 (25·8–36·7)	26·5 (22·1–30·7)	28·2 (23·9–32·6)	23·6 (17–30·2)
Transport injuries	22·5 (14·1–30·2)	22·5 (15·7–28·8)	26·8 (22·6–31·4)	19·9 (10·9–28·1)
Road injuries	21·6 (12·8–29·7)	21·5 (14·4–28·2)	25·7 (21·5–30·3)	19·2 (9·6–27·8)
Other transport injuries	33 (28·1–38)	31·7 (27–36·5)	34·9 (30·4–39·1)	28·9 (23·4–34·4)

Unintentional injuries	38·3 (32·5–42·7)	27·2 (22·8–31·8)	27·9 (23·2–32·8)	24·3 (19·8–28·6)
Falls	55·8 (47·3–61·3)	38·4 (32·5–43·6)	36·8 (31–42·1)	46·5 (40·1–52·1)
Drowning	-14·5 (-28·2–-0·408)	-13·7 (-26·4–-1·33)	11·4 (6·64–17·7)	-15·7 (-29–-2·67)
Fire, heat, and hot substances	10·7 (5·95–16·8)	13·6 (8·51–20·1)	14·1 (8·98–20·8)	11·8 (7·18–17·6)
Poisonings	14·1 (8·74–20·7)	13·3 (8·42–19·4)	12·6 (7·87–18·5)	14·5 (9·42–20·8)
Exposure to mechanical forces	10·4 (-2·37–23·1)	16·9 (14–19·9)	17·8 (15·2–20·7)	0·516 (-12·7–15)
Animal contact	21 (16–26·7)	20·3 (15·3–26·1)	21·3 (16·2–27·3)	17·1 (12·6–22·5)
Foreign body	2·9 (1·79–4·46)	4·01 (2·5–5·95)	3·66 (2·32–5·28)	4·34 (2·68–6·62)
Environmental heat and cold exposure	8·82 (3·91–15·3)	9·64 (4·43–16·7)	9·62 (4·43–16·7)	9·74 (4·34–16·6)
Exposure to forces of nature	0 (0–0)	10·6 (4·88–18·1)	10·6 (4·88–18·1)	0 (0–0)
Other unintentional injuries	14·9 (9·19–21·9)	13·4 (8·34–19·8)	13·3 (8·26–19·7)	16·2 (10·6–23·3)
Self-harm and interpersonal violence	24·5 (11·8–37·9)	26·7 (15·5–38·4)	35·2 (27·8–43·2)	25 (12·3–38·1)
Self-harm	24·5 (10·6–39·3)	25·5 (11·6–40)	24·8 (12·6–38·1)	25·5 (11·6–40·1)
Interpersonal violence	25·4 (17·1–33·9)	33 (25·4–41·8)	39·8 (31·4–49)	22 (14·1–30·1)

Supplementary Results Table 2. All-age PAFs attributable to the joint distribution of all risk factors for all ages, both sexes combined for each GBD level 1, level 2, and level 3 cause and all causes in 2019 for Bexley. Results are shown for deaths, YLLs, YLDs, and DALYs Data in parenthesis are 95% uncertainty intervals. PAFs=population attributable fractions. GBD=Global Burden of Disease. YLLs=years of life lost. YLDs=years lived with disability. DALYs=disability-adjusted life-years

	Deaths 2019	DALYs 2019	YLDs 2019	YLLs 2019
All causes	53·9 (50·3–57·1)	39·8 (36·7–43)	27·5 (25·3–29·8)	53·6 (50·8–56·3)
Communicable, maternal, neonatal, and nutritional diseases	44·8 (33·9–55)	50·4 (45–55·4)	46·3 (41·9–50·8)	53 (44·3–60·8)
HIV/AIDS and sexually transmitted infections	75 (58·9–86·6)	75 (57·7–87·5)	97·5 (96·2–98·5)	58·3 (38·6–76·1)
HIV/AIDS	91·5 (89·8–92·7)	89·7 (87·5–91·4)	94·5 (92·3–95·9)	87 (84·9–88·4)
Sexually transmitted infections excluding HIV	37·4 (17·4–65·1)	58·5 (32·9–83·8)	100 (100–100)	12 (4·28–28·2)
Respiratory infections and tuberculosis	45·3 (33·6–56·6)	40·7 (31·1–50·3)	3·19 (2·36–4·2)	48·2 (37·1–59)
Tuberculosis	56 (43·7–66·3)	58 (46·7–67·4)	54·2 (42·3–64·2)	58·8 (47·7–68·1)
Lower respiratory infections	45·3 (33·4–56·6)	48·1 (36·8–59)	32·9 (25·9–40)	48·2 (36·8–59·2)
Upper respiratory infections	0·052 (0·0278–0·0898)	0·00981 (0·00209–0·0228)	0·00774 (0·000194–0·0207)	0·116 (0·0611–0·2)
Otitis media	2·51 (1·06–5·28)	4·75 (3·01–6·65)	4·69 (2·96–6·61)	5·91 (2·89–10·3)
Enteric infections	4·93 (2·99–8·2)	9·33 (6·92–12·7)	9·65 (7·06–13·1)	8·24 (5·72–11·7)
Diarrheal diseases	4·99 (3·02–8·34)	9·37 (6·99–12·8)	9·66 (7·07–13·1)	8·4 (5·91–11·9)
Other infectious diseases	0·605 (0·397–0·803)	1·14 (0·832–1·46)	0·356 (0·206–0·602)	1·53 (1·08–2·03)
Meningitis	1·56 (1·09–2·11)	2·47 (1·79–3·31)	0·000616 (7·69e-06–0·00181)	3·37 (2·44–4·51)
Encephalitis	0·308 (0·202–0·434)	0·773 (0·443–1·14)	0·000216 (3·31e-06–0·00062)	1·07 (0·709–1·52)
Measles	3·34 (-0·915–16·7)	3·02 (-0·605–14·3)	0·485 (0·0136–1·33)	4·35 (-1·22–21·2)
Acute hepatitis	8·25 (3·18–22·4)	3·89 (2·08–7·18)	2·77 (1·64–4·51)	7·61 (2·93–20·4)
Maternal and neonatal disorders	86·3 (84–88·2)	74·5 (70·6–78·1)	60·6 (54·6–66·3)	87·3 (85·3–89·1)
Maternal disorders	9·36 (3·37–15·6)	9·4 (3·32–16)	9·52 (3·31–16·4)	9·23 (3·32–15·4)
Neonatal disorders	89 (87·2–90·9)	76·4 (72·3–80)	62·4 (56·1–68·3)	89·1 (87·2–90·9)
Nutritional deficiencies	59·9 (52·3–90·2)	79·2 (75·1–84)	79·4 (75·3–84·2)	61·2 (56·3–81·4)
Protein-energy malnutrition	100 (100–100)	100 (100–100)	100 (100–100)	100 (100–100)
Vitamin A deficiency	--	100 (100–100)	100 (100–100)	--
Dietary iron deficiency	--	100 (100–100)	100 (100–100)	--
Non-communicable diseases	55·3 (51·6–58·8)	40·1 (36·5–43·4)	26·5 (24·2–29·1)	55·2 (52·2–58·1)
Neoplasms	47·7 (43·9–52·1)	46·8 (43·2–50·8)	32·5 (28·4–37)	47·6 (44–51·7)
Lip and oral cavity cancer	63·8 (51·8–73·3)	67·1 (55·3–75·7)	66·4 (54·3–75·4)	67·2 (55·4–75·7)
Nasopharynx cancer	63·2 (49·2–73·7)	63·2 (50·3–73·5)	60·9 (47·6–71·9)	63·5 (50·5–73·6)
Other pharynx cancer	77·2 (68·7–84)	78·6 (70·4–85)	77·4 (68·7–84·2)	78·7 (70·5–85·1)
Esophageal cancer	73·6 (64·3–82·4)	75 (66–83·6)	74·1 (64·7–82·9)	75·1 (66–83·7)
Stomach cancer	23·3 (15·9–40·1)	23·5 (16·1–40)	23·6 (16·2–40·5)	23·5 (16·1–40)

Colon and rectum cancer	62·9 (53·6–71·8)	63·1 (54·4–71·5)	63·1 (54·3–71·7)	63·1 (54·4–71·5)
Liver cancer	74·9 (68·7–81·1)	75·3 (69·8–80·8)	75·6 (69·7–81·2)	75·3 (69·8–80·8)
Gallbladder and biliary tract cancer	21 (11–33·2)	21·5 (11·5–34·1)	19·9 (10·5–32)	21·5 (11·5–34·2)
Pancreatic cancer	41·2 (31·8–52·8)	40·4 (31·6–51·4)	41 (31·6–52·6)	40·4 (31·5–51·4)
Larynx cancer	81·8 (73·1–88·2)	81·4 (73·3–87·3)	79·2 (70·8–85·5)	81·6 (73·5–87·5)
Tracheal, bronchus, and lung cancer	85·6 (81·8–89·2)	84·5 (80·9–87·9)	85·1 (81·6–88·5)	84·5 (80·9–87·9)
Breast cancer	35·9 (26·2–47·3)	34·8 (26–44·9)	35 (26·2–45·2)	34·8 (26–44·8)
Cervical cancer	100 (100–100)	100 (100–100)	100 (100–100)	100 (100–100)
Uterine cancer	41·7 (27·1–56·8)	42·9 (28·8–58)	43·3 (29·1–58·5)	42·9 (28·8–57·9)
Ovarian cancer	29·1 (13·9–43·8)	24·1 (11·4–37·6)	23·2 (11–36·1)	24·2 (11·4–37·7)
Prostate cancer	5·3 (2·29–8·77)	5·7 (2·5–9·21)	6·02 (2·66–9·5)	5·64 (2·46–9·16)
Kidney cancer	36·5 (26·7–47)	36·5 (27–46·5)	36·3 (26·9–46·3)	36·5 (27·1–46·5)
Bladder cancer	40·9 (25·9–55·6)	42·9 (28·8–56·1)	43·4 (29·1–56·7)	42·9 (28·8–56·1)
Thyroid cancer	13·1 (6·34–21·7)	13·8 (6·6–23·1)	14 (6·78–23)	13·8 (6·57–23)
Mesothelioma	99·2 (98·8–99·4)	98·2 (97·6–98·7)	99 (98·6–99·3)	98·2 (97·6–98·7)
Non-Hodgkin lymphoma	6·57 (2·65–11·9)	6·8 (2·82–12·2)	6·72 (2·79–12·1)	6·8 (2·81–12·2)
Multiple myeloma	7·48 (3·17–13·6)	7·82 (3·28–14·1)	7·75 (3·24–14)	7·82 (3·29–14·2)
Leukemia	34·6 (20·7–49)	32·1 (20·7–44·3)	31·1 (19·8–42·8)	32·2 (20·7–44·3)
Cardiovascular diseases	76·5 (72–80·6)	77·7 (74·2–80·9)	68·7 (63·5–73·6)	78·9 (75·3–82)
Rheumatic heart disease	21·3 (11·5–43·7)	22·1 (13·1–39·1)	22·3 (13·6–37·5)	22·1 (13·1–39·4)
Ischemic heart disease	92·9 (88·7–96·5)	94·2 (91–96·8)	93·5 (90·3–96·3)	94·2 (91·1–96·9)
Stroke	78·2 (69·2–87·7)	81·3 (74·7–88·1)	80·9 (75·1–86·9)	81·4 (74·7–88·3)
Hypertensive heart disease	100 (100–100)	100 (100–100)	100 (100–100)	100 (100–100)
Non-rheumatic valvular heart disease	24·4 (14·7–37)	24·4 (16·5–34·1)	16·3 (10·7–23·1)	25·8 (17·5–36·1)
Cardiomyopathy and myocarditis	31·6 (24–41·3)	37·6 (31·5–44·3)	32·6 (25·9–39·6)	37·9 (31·9–44·9)
Atrial fibrillation and flutter	47·5 (37·2–58·4)	55·3 (46·1–64·3)	59·7 (51–68·3)	50·6 (41·3–60·3)
Aortic aneurysm	47·3 (40·6–54·6)	54·4 (48·5–60·6)	--	54·4 (48·5–60·6)
Endocarditis	29·2 (19·1–42·1)	31·5 (22·8–42·1)	28·8 (19·1–41·5)	31·6 (22·8–42·1)
Other cardiovascular and circulatory diseases	29·3 (22·3–36·9)	31·6 (25·5–38·2)	31·3 (23·8–39·4)	31·7 (25·3–38·4)
Chronic respiratory diseases	62·1 (55·4–68·8)	55·7 (51–60·6)	48·4 (43–53·8)	62·4 (56·3–68·3)
Chronic obstructive pulmonary disease	70·3 (63·5–76·5)	68·9 (64·2–73·7)	62·8 (58·6–66·7)	72·1 (66·4–77·6)
Pneumoconiosis	100 (100–100)	100 (100–100)	100 (100–100)	100 (100–100)
Asthma	39·3 (27·4–52·6)	36·7 (28·5–46·2)	36·4 (28·3–45·8)	40 (30·2–51·2)
Digestive diseases	26·8 (22·2–31·9)	32·2 (26·4–37·8)	20·2 (13·4–28·1)	38·9 (32·5–45)

Cirrhosis and other chronic liver diseases	72·8 (59·4–86·2)	76·1 (63·6–88·3)	72·3 (59·9–83·8)	76·2 (63·6–88·4)
Upper digestive system diseases	19·5 (15·9–23·2)	8·2 (5·33–11·6)	0·693 (0·39–1·11)	21·8 (18·4–25·1)
Gallbladder and biliary diseases	42·1 (27·1–57)	46·2 (32–60·7)	47·1 (32·8–61·9)	43·1 (28·7–57·9)
Pancreatitis	36·3 (16–55·8)	42·3 (21–61·1)	41·9 (20–61·6)	42·3 (21·2–61·2)
Neurological disorders	26·1 (11·6–41·2)	15·6 (7·43–27·6)	9·66 (4·64–18·4)	22·8 (10·2–37·3)
Alzheimer's disease and other dementias	39·6 (25·7–55·3)	41·2 (27·3–56·8)	41·4 (27·5–56·9)	41 (27·1–56·6)
Parkinson's disease	-8·14 (-13·6–-3·62)	-9·07 (-15–-4·15)	-9·65 (-15·6–-4·57)	-8·92 (-14·8–-4·07)
Idiopathic epilepsy	25·2 (14·2–36)	22·8 (13·2–32·3)	21·1 (11·5–30·2)	26 (15·2–36·1)
Multiple sclerosis	14·1 (10·6–17·8)	14·5 (11–18·3)	14 (10·6–17·8)	14·8 (11·1–18·8)
Mental disorders	--	6·8 (3·49–10·4)	6·8 (3·49–10·4)	--
Depressive disorders	--	13·7 (6·26–21·5)	13·7 (6·26–21·5)	--
Anxiety disorders	--	5·25 (1·38–11·6)	5·25 (1·38–11·6)	--
Idiopathic developmental intellectual disability	--	40·7 (18·6–58·7)	40·7 (18·6–58·7)	--
Substance use disorders	100 (100–100)	100 (100–100)	100 (100–100)	100 (100–100)
Alcohol use disorders	100 (100–100)	100 (100–100)	100 (100–100)	100 (100–100)
Drug use disorders	100 (100–100)	100 (100–100)	100 (100–100)	100 (100–100)
Diabetes and kidney diseases	100 (100–100)	100 (100–100)	100 (100–100)	100 (100–100)
Diabetes mellitus	100 (100–100)	100 (100–100)	100 (100–100)	100 (100–100)
Chronic kidney disease	100 (100–100)	100 (100–100)	100 (100–100)	100 (100–100)
Sense organ diseases	--	7·82 (6·67–9·13)	7·82 (6·67–9·13)	--
Blindness and vision loss	--	9·07 (5·69–13·5)	9·07 (5·69–13·5)	--
Age-related and other hearing loss	--	8·53 (7·62–9·53)	8·53 (7·62–9·53)	--
Musculoskeletal disorders	2·45 (0·586–4·69)	20·1 (16·4–23·8)	20·6 (16·7–24·3)	2·94 (0·7–5·53)
Rheumatoid arthritis	10·6 (2·49–19·1)	12·4 (3·24–22·3)	12·5 (3·23–22·4)	11·9 (2·99–21·5)
Osteoarthritis	--	15·8 (8·28–24·7)	15·8 (8·28–24·7)	--
Low back pain	--	37 (31·7–42)	37 (31·7–42)	--
Gout	--	49·4 (35–65·9)	49·4 (35–65·9)	--
Other non-communicable diseases	0·0609 (0·0369–0·0934)	0·0586 (0·0339–0·0908)	--	0·276 (0·161–0·405)
Sudden infant death syndrome	8·21 (4·74–13·4)	8·2 (4·74–13·4)	--	8·2 (4·74–13·4)
Injuries	33·6 (27·5–39)	27·4 (22·8–31·9)	29·4 (25·1–33·6)	24·2 (17·4–31·1)
Transport injuries	22·2 (14–29·7)	22·2 (15·3–28·7)	26·9 (22·4–31·4)	19·6 (10·5–27·9)
Road injuries	21·3 (12·7–29·1)	21·3 (14·1–27·9)	25·7 (21·3–30·3)	18·9 (9·44–27·4)
Other transport injuries	33·5 (28·1–38·9)	32·6 (27·5–37·7)	35·9 (31·2–40·1)	29·5 (23·5–35·5)
Unintentional injuries	41·4 (34·8–46)	29 (24·5–33·3)	29·3 (24·8–33·8)	28 (23·1–32·1)

Falls	56<br>(47·4–61·4)	40·4<br>(34·2–45·5)	38·8<br>(32·8–43·8)	47·3<br>(40·4–52·7)
Drowning	-15·2<br>(-28·5–-2·05)	-14·4<br>(-26·6–-2·13)	10·4<br>(5·99–16·7)	-16·3<br>(-29·2–-3·18)
Fire, heat, and hot substances	9·82<br>(5·33–15·9)	12·8<br>(7·96–19·4)	13·3<br>(8·28–20·2)	11·2<br>(6·85–17)
Poisonings	12·6<br>(7·79–19·2)	12·3<br>(7·78–18·4)	11·8<br>(7·3–17·7)	13·3<br>(8·49–19·6)
Exposure to mechanical forces	13·7<br>(1·6–26·4)	17·8<br>(14·8–20·8)	18·8<br>(16·2–21·6)	2·2<br>(-10·8–16)
Animal contact	19·1<br>(14·6–25·2)	19·8<br>(15·2–25·4)	21·4<br>(16·5–27·1)	15·8<br>(11·7–21·3)
Foreign body	2·94<br>(1·88–4·44)	3·98<br>(2·6–5·9)	3·51<br>(2·34–5·15)	4·53<br>(2·93–6·72)
Environmental heat and cold exposure	7·61<br>(3·22–13·8)	8·91<br>(3·95–15·7)	8·99<br>(3·98–16)	8·27<br>(3·66–14·5)
Exposure to forces of nature	0<br>(0–0)	9·97<br>(4·49–17·5)	9·97<br>(4·49–17·5)	0<br>(0–0)
Other unintentional injuries	14<br>(8·76–20·8)	12·5<br>(7·77–18·9)	12·4<br>(7·67–18·8)	15·6<br>(10·1–22·4)
Self-harm and interpersonal violence	22·9<br>(9·65–36·1)	25·4<br>(13·4–36·7)	34·1<br>(26·4–41·9)	23·6<br>(10·3–36·5)
Self-harm	22·8<br>(8·34–37)	23·8<br>(9·62–37·9)	23<br>(10·2–35·9)	23·8<br>(9·56–37·9)
Interpersonal violence	26·4<br>(17·7–35·4)	33·4<br>(25·2–42)	39<br>(30·2–48)	23·1<br>(14·8–31·7)

Supplementary Results Table 2. All-age PAFs attributable to the joint distribution of all risk factors for all ages, both sexes combined for each GBD level 1, level 2, and level 3 cause and all causes in 2019 for Brent. Results are shown for deaths, YLLs, YLDs, and DALYs Data in parenthesis are 95% uncertainty intervals. PAFs=population attributable fractions. GBD=Global Burden of Disease. YLLs=years of life lost. YLDs=years lived with disability. DALYs=disability-adjusted life-years

	Deaths 2019	DALYs 2019	YLDs 2019	YLLs 2019
All causes	55·6 (52·4–58·8)	39·4 (36·3–42·5)	27·6 (25·3–30)	54·7 (52·1–57·3)
Communicable, maternal, neonatal, and nutritional diseases	49·8 (40–59)	56·5 (52·3–60·6)	49·6 (45·1–54·4)	61·1 (54·9–67·4)
HIV/AIDS and sexually transmitted infections	79·3 (68·1–87·1)	77·9 (64·7–86·8)	96·1 (94·3–97·4)	66·5 (51·1–78·6)
HIV/AIDS	89·7 (87·6–91·1)	87·9 (85·3–89·9)	94 (91·6–95·5)	84·5 (82–86·2)
Sexually transmitted infections excluding HIV	38 (18·6–64·8)	54·9 (29·9–81·1)	100 (100–100)	14 (5·36–31·2)
Respiratory infections and tuberculosis	49 (37·4–59·8)	43·1 (34·1–52·2)	3·78 (2·74–5·31)	53·1 (42·5–62·8)
Tuberculosis	58·9 (47·5–68·9)	60·1 (49·5–69·1)	54·5 (42·4–64·1)	61·2 (50·4–70·1)
Lower respiratory infections	48·8 (36·9–59·7)	52·7 (42–62·6)	36·3 (29·9–43·1)	52·9 (42·1–62·9)
Upper respiratory infections	0·131 (0·0718–0·228)	0·0101 (0·00181–0·024)	0·00846 (0·00024–0·0224)	0·247 (0·13–0·425)
Otitis media	4·55 (2·28–8·2)	5·07 (3·26–7·16)	5·02 (3·21–7·1)	9·03 (5·14–14·3)
Enteric infections	5·28 (3·36–8·46)	10·6 (8·06–13·7)	10·3 (7·69–13·7)	11·4 (8·16–15·1)
Diarrheal diseases	5·37 (3·45–8·56)	10·6 (8·12–13·8)	10·4 (7·7–13·8)	11·8 (8·55–15·4)
Other infectious diseases	1·14 (0·778–1·48)	1·68 (1·26–2·16)	0·328 (0·19–0·531)	2·3 (1·7–2·99)
Meningitis	2·87 (2·08–3·86)	3·77 (2·75–5·11)	0·000729 (2·05e-05–0·00198)	5·03 (3·68–6·68)
Encephalitis	0·715 (0·422–1·09)	1·36 (0·666–2·08)	0·000245 (5·28e-06–0·000672)	1·89 (1·1–2·85)
Measles	2·84 (-0·769–15·1)	2·92 (-0·653–15·1)	0·44 (0·00901–1·26)	3·54 (-0·943–18·7)
Acute hepatitis	12 (7·7–19·9)	4·81 (3·15–7·18)	2·49 (1·45–4·02)	10·4 (6·56–17·2)
Maternal and neonatal disorders	88 (85·8–89·8)	79·6 (76·5–82·6)	66·6 (60·9–71·6)	88·7 (86·7–90·5)
Maternal disorders	9·55 (3·46–16·1)	9·7 (3·6–16·5)	9·87 (3·64–17·1)	9·45 (3·37–16)
Neonatal disorders	90·1 (88·1–91·8)	81·3 (78·1–84·2)	68·5 (62·5–73·9)	90·1 (88·2–91·8)
Nutritional deficiencies	75·1 (70·5–88·4)	79·1 (75–84)	79·2 (75–84·1)	74·1 (68–79·8)
Protein-energy malnutrition	100 (100–100)	100 (100–100)	100 (100–100)	100 (100–100)
Vitamin A deficiency	--	100 (100–100)	100 (100–100)	--
Dietary iron deficiency	--	100 (100–100)	100 (100–100)	--
Non-communicable diseases	57·3 (53·9–60·8)	39·4 (36–42·8)	26·7 (24·2–29·3)	56·9 (54·1–59·7)
Neoplasms	47·3 (43·6–51·5)	45·8 (42·4–49·7)	32·5 (28·6–36·9)	46·6 (43·2–50·6)
Lip and oral cavity cancer	68·8 (58·5–77·3)	71 (60·7–79·1)	70·2 (59·2–78·7)	71·1 (60·9–79·2)
Nasopharynx cancer	67·3 (55·2–76·8)	66·4 (54·8–75·6)	64·5 (52–74·5)	66·5 (55–75·7)
Other pharynx cancer	80·1 (72·7–86·1)	80·9 (73·6–86·7)	79·8 (72·2–86·1)	81 (73·6–86·8)
Esophageal cancer	75·5 (66·5–83·9)	76·7 (67·5–84·7)	75·8 (66·3–83·6)	76·7 (67·6–84·7)

Stomach cancer	24·7 (17·2–41·7)	24·6 (17·3–41·4)	24·7 (17·3–41·4)	24·5 (17·3–41·4)
Colon and rectum cancer	64·3 (55·1–73·3)	64·3 (55·6–72·5)	64·3 (55·6–72·9)	64·3 (55·7–72·5)
Liver cancer	77·9 (72·2–83·3)	77·2 (71·8–82·3)	77·5 (72–82·5)	77·2 (71·8–82·3)
Gallbladder and biliary tract cancer	21·4 (11·6–33·8)	21·7 (11·9–34·1)	20·2 (10·9–32·5)	21·8 (11·9–34·2)
Pancreatic cancer	42·7 (33·1–54·8)	41·5 (32·5–52·9)	42·3 (32·6–54·1)	41·5 (32·5–52·9)
Larynx cancer	82·9 (74·8–88·7)	82·1 (74·9–87·6)	79·7 (72·1–85·8)	82·3 (75·1–87·8)
Tracheal, bronchus, and lung cancer	85·9 (82·6–89·2)	84·6 (81·2–87·9)	85·3 (81·8–88·8)	84·6 (81·2–87·9)
Breast cancer	36·4 (27–47·8)	34·6 (26·2–44·5)	34·9 (26·4–45·1)	34·6 (26·2–44·5)
Cervical cancer	100 (100–100)	100 (100–100)	100 (100–100)	100 (100–100)
Uterine cancer	43·5 (28·5–58·7)	44·4 (29·7–59·6)	44·7 (29·9–60·3)	44·4 (29·6–59·7)
Ovarian cancer	22·2 (10·9–36·8)	18·6 (8·77–31·7)	17·5 (8·26–29·9)	18·7 (8·79–31·8)
Prostate cancer	5·79 (2·48–9·24)	6·3 (2·76–9·91)	6·66 (2·91–10·5)	6·23 (2·73–9·82)
Kidney cancer	38·2 (28·3–48·4)	37·6 (28–47·1)	37·1 (27·5–46·8)	37·6 (28–47·2)
Bladder cancer	43·7 (28·4–58·6)	45·4 (31·4–58·6)	45·6 (32·2–58·5)	45·4 (31·4–58·6)
Thyroid cancer	14·3 (6·86–23·6)	14·8 (7·07–24·4)	14·9 (6·85–24·5)	14·8 (7·07–24·5)
Mesothelioma	97·9 (97·1–98·5)	95·8 (94·5–96·9)	97·6 (96·6–98·3)	95·7 (94·4–96·8)
Non-Hodgkin lymphoma	7·06 (2·99–12·8)	7·19 (3·08–12·8)	7·15 (3·04–12·9)	7·19 (3·08–12·8)
Multiple myeloma	7·96 (3·41–14·3)	8·28 (3·59–14·9)	8·23 (3·55–14·8)	8·29 (3·59–14·9)
Leukemia	35·7 (22·5–49·1)	31·4 (21·1–41·9)	30·4 (20·4–40·9)	31·5 (21·1–41·9)
Cardiovascular diseases	76·8 (72·6–81·4)	78·3 (75–82·3)	69·2 (64·5–73·7)	79·5 (76·2–83·7)
Rheumatic heart disease	22 (12·2–42·4)	23 (14·5–38·7)	22·7 (14·2–37·8)	23 (14·4–38·9)
Ischemic heart disease	93·8 (90·1–96·7)	95·1 (92·7–97·3)	94·2 (91·3–96·7)	95·2 (92·7–97·3)
Stroke	80·4 (72·4–88·4)	83·9 (78·5–89·5)	82·8 (77·8–87·5)	84·1 (78·4–90)
Hypertensive heart disease	100 (100–100)	100 (100–100)	100 (100–100)	100 (100–100)
Non-rheumatic valvular heart disease	25·6 (16·4–38·6)	26·3 (18·2–37·1)	16·5 (11–23·6)	27·5 (19–38·9)
Cardiomyopathy and myocarditis	33·8 (25·7–44·4)	40·8 (33·3–50·4)	36·4 (29·9–43·9)	41·2 (33·5–51·9)
Atrial fibrillation and flutter	49·4 (39·4–60·5)	57·8 (48·9–66·8)	61·7 (53·1–69·9)	53·1 (43·9–63·2)
Aortic aneurysm	50·8 (43·8–57·8)	58·6 (52·5–64·3)	--	58·6 (52·5–64·3)
Endocarditis	30·5 (21·1–43·6)	33·1 (24·2–44·2)	30 (20·7–43·1)	33·2 (24·3–44)
Other cardiovascular and circulatory diseases	30·1 (23·6–37)	32·8 (26·5–38·8)	33·7 (26·1–41)	32·5 (26·4–38·6)
Chronic respiratory diseases	61·9 (55·1–69·2)	53·1 (48·2–58·2)	46·4 (40·7–52·3)	61·6 (55·2–68·3)
Chronic obstructive pulmonary disease	73·2 (67·9–78·7)	71·2 (67·1–75·3)	64·7 (60·8–68·2)	75·1 (70·1–80)
Pneumoconiosis	100 (100–100)	100 (100–100)	100 (100–100)	100 (100–100)

Asthma	42·1 (31–54·5)	38·1 (30·1–47·4)	37·8 (29·8–47·1)	42·3 (32·9–52·9)
Digestive diseases	36·5 (30·5–42·6)	38·6 (32–45)	25·4 (17·3–34·9)	49·1 (41·5–56·3)
Cirrhosis and other chronic liver diseases	75·8 (63·4–87·6)	78 (66·3–89·1)	74·2 (62·2–85)	78·1 (66·4–89·2)
Upper digestive system diseases	22·8 (19·1–26·3)	7·68 (4·92–11·2)	0·676 (0·387–1·07)	25·4 (21·7–28·7)
Gallbladder and biliary diseases	42·8 (27·9–57·7)	46·5 (32·6–61)	46·8 (32·8–61·2)	43·8 (29·5–58·4)
Pancreatitis	40·7 (21·1–60·9)	46·2 (26–65·5)	45·7 (24·7–64·6)	46·3 (26·3–65·7)
Neurological disorders	27 (12·3–42·5)	13·3 (6·07–24·2)	7·64 (3·49–15·8)	22·9 (10·9–37·2)
Alzheimer's disease and other dementias	41·5 (27·6–57·8)	43·5 (29·3–59·2)	43·7 (29·8–59·2)	43·3 (29–59)
Parkinson's disease	-9·21 (-15·2–-4·1)	-10·3 (-16·7–-4·73)	-10·8 (-17·6–-5·04)	-10·2 (-16·6–-4·66)
Idiopathic epilepsy	28·7 (18·4–39·6)	24·9 (15·6–34·9)	22·5 (13·5–32·1)	28·1 (18·2–38·4)
Multiple sclerosis	15·1 (11·2–19·2)	15 (11·3–19·2)	14·3 (10·9–18·2)	15·6 (11·7–20)
Mental disorders	--	6·88 (3·44–10·8)	6·89 (3·44–10·8)	--
Depressive disorders	--	14·3 (6·58–22·2)	14·3 (6·58–22·2)	--
Anxiety disorders	--	5·69 (1·41–12·9)	5·69 (1·41–12·9)	--
Idiopathic developmental intellectual disability	--	37·8 (16–56·6)	37·8 (16–56·6)	--
Substance use disorders	100 (100–100)	100 (100–100)	100 (100–100)	100 (100–100)
Alcohol use disorders	100 (100–100)	100 (100–100)	100 (100–100)	100 (100–100)
Drug use disorders	100 (100–100)	100 (100–100)	100 (100–100)	100 (100–100)
Diabetes and kidney diseases	99·7 (99·5–99·8)	99·9 (99·9–100)	100 (100–100)	99·7 (99·5–99·7)
Diabetes mellitus	100 (100–100)	100 (100–100)	100 (100–100)	100 (100–100)
Chronic kidney disease	100 (100–100)	100 (100–100)	100 (100–100)	100 (100–100)
Sense organ diseases	--	8·61 (7·54–9·87)	8·61 (7·54–9·87)	--
Blindness and vision loss	--	8·24 (5·2–12·1)	8·24 (5·2–12·1)	--
Age-related and other hearing loss	--	10·1 (9·07–11·1)	10·1 (9·07–11·1)	--
Musculoskeletal disorders	3·17 (0·81–5·79)	21·1 (17·6–24·9)	21·4 (17·8–25·3)	3·67 (0·962–6·49)
Rheumatoid arthritis	12·2 (3·09–21·7)	13·3 (3·36–23·4)	13·2 (3·29–23·4)	13·6 (3·48–24·1)
Osteoarthritis	--	16·5 (8·58–25·4)	16·5 (8·58–25·4)	--
Low back pain	--	39·2 (34·1–44·2)	39·2 (34·1–44·2)	--
Gout	--	49 (33·1–66·4)	49 (33·1–66·4)	--
Other non-communicable diseases	0·174 (0·117–0·249)	0·107 (0·0671–0·161)	--	0·485 (0·316–0·685)
Sudden infant death syndrome	8·77 (4·9–14·2)	8·76 (4·9–14·2)	--	8·76 (4·9–14·2)
Injuries	27·7 (21·9–33·1)	23·5 (19–28)	25·8 (21·7–30·2)	20·7 (14·3–27·3)
Transport injuries	20·5 (12–28·5)	20·4 (13·3–27·5)	25·4 (20·8–30·3)	18·2 (9·15–26·5)

Road injuries	19·6 (10·7–28)	19·6 (12·1–26·9)	24·5 (19·8–29·6)	17·5 (8·04–26·3)
Other transport injuries	30·5 (25·4–36·3)	29 (24–34·3)	32·6 (28·2–37·1)	26·6 (21–32·8)
Unintentional injuries	33·4 (27·9–38)	23·9 (19·6–28·4)	25·3 (21–29·9)	19·3 (15·1–23·7)
Falls	51·9 (44·4–57·1)	34·9 (29·2–40·2)	33·6 (28·2–38·8)	40·6 (34·5–46·2)
Drowning	-15·1 (-28·7–-1·48)	-15·1 (-28·3–-1·79)	10·7 (6·22–16·7)	-16·1 (-29·6–-2·66)
Fire, heat, and hot substances	10·6 (6·04–16·8)	12·6 (7·7–18·5)	13·4 (8·31–19·7)	11·1 (6·7–16·5)
Poisonings	14·1 (8·79–20·7)	12·9 (8·24–18·8)	12 (7·51–17·6)	14 (8·96–20·4)
Exposure to mechanical forces	7·22 (-5·6–20·5)	14·7 (11·9–17·5)	16 (13·7–18·6)	-1·66 (-14·9–12·6)
Animal contact	19·9 (15–25·8)	18·4 (13·6–24)	19·7 (14·6–25·5)	16·1 (11·7–21·5)
Foreign body	3·15 (2·03–4·59)	3·78 (2·48–5·46)	3·43 (2·28–4·98)	4·04 (2·63–5·91)
Environmental heat and cold exposure	9·6 (4·26–16·9)	9·31 (4·23–16·6)	9·19 (4·16–16·4)	9·94 (4·52–17·5)
Exposure to forces of nature	0 (0–0)	10 (4·48–17·5)	10 (4·48–17·5)	0 (0–0)
Other unintentional injuries	15 (9·36–22)	12·8 (7·89–18·8)	12·7 (7·72–18·6)	15·6 (9·89–22·2)
Self-harm and interpersonal violence	23·1 (11·2–35·5)	24·6 (13·9–35·7)	33·8 (25·9–41·7)	23·1 (11·6–35·1)
Self-harm	23·4 (10·2–37·6)	24 (11–38)	23·7 (11·9–36·7)	24 (10·9–37·9)
Interpersonal violence	22 (13·2–31·4)	27·6 (19·8–36·3)	38 (29·3–47)	19·1 (10·6–28)

Supplementary Results Table 2. All-age PAFs attributable to the joint distribution of all risk factors for all ages, both sexes combined for each GBD level 1, level 2, and level 3 cause and all causes in 2019 for Bosnia and Herzegovina. Results are shown for deaths, YLLs, YLDs, and DALYs Data in parenthesis are 95% uncertainty intervals. PAFs=population attributable fractions. GBD=Global Burden of Disease. YLLs=years of life lost. YLDs=years lived with disability. DALYs=disability-adjusted life-years

	Deaths 2019	DALYs 2019	YLDs 2019	YLLs 2019
All causes	72·2 (69·3–74·9)	55·9 (52–59·4)	34·3 (31·5–36·8)	69·6 (67·4–71·8)
Communicable, maternal, neonatal, and nutritional diseases	59·9 (55·7–64·1)	58·2 (54·8–61·6)	52·6 (47·8–57·5)	65·8 (61·7–69·4)
HIV/AIDS and sexually transmitted infections	59·3 (37·6–80·4)	75·4 (53·3–91·2)	99·9 (99·8–99·9)	38·5 (19·8–62·7)
HIV/AIDS	92·5 (91·4–93·3)	88·6 (87·1–89·9)	97·4 (96·4–98·2)	86·6 (85·2–88)
Sexually transmitted infections excluding HIV	37·8 (18–66·5)	73·3 (48·9–91·4)	100 (100–100)	17·1 (6·84–37·6)
Respiratory infections and tuberculosis	62·2 (57–67·4)	51·3 (44–58)	9·18 (6·49–12·7)	65·7 (60·8–70·3)
Tuberculosis	59·4 (52·4–65·8)	63·8 (57·5–69·4)	60·3 (53·4–67·1)	64·2 (57·8–69·9)
Lower respiratory infections	63·1 (56·7–69·2)	66 (59·9–71·7)	52·3 (45·7–59)	66·3 (60·2–72)
Upper respiratory infections	0·0805 (0·03–0·192)	0·00857 (0·00335–0·015)	0·00787 (0·00287–0·0143)	0·266 (0·105–0·644)
Otitis media	0·198 (0·014–1·09)	4·31 (2·83–6·03)	4·31 (2·83–6·04)	1·2 (0·141–4·83)
Enteric infections	46·6 (31·9–62·6)	43·5 (26·6–62·2)	42·6 (25·1–61·8)	60·6 (47–72·4)
Diarrheal diseases	52·6 (35·9–69)	43·8 (26·7–62·6)	42·6 (25·1–61·9)	68·5 (54–80·6)
Other infectious diseases	2·07 (1·18–3·77)	3·04 (1·62–5·97)	0·259 (0·153–0·429)	4·12 (2·03–8·4)
Meningitis	4·92 (3·14–7·59)	7·31 (4·68–11·2)	0·00092 (0·000294–0·00167)	11 (7·37–16·3)
Encephalitis	0·215 (0·108–0·395)	0·487 (0·257–0·897)	0·000256 (8·38e-05–0·000477)	0·568 (0·3–1·04)
Measles	22·1 (8–53·7)	21·9 (7·96–53·3)	3·48 (0·137–8·24)	22·7 (8·24–55·3)
Acute hepatitis	4·33 (2·58–6·53)	3·29 (1·97–4·94)	1·65 (0·968–2·74)	4·95 (2·71–7·47)
Maternal and neonatal disorders	87·6 (84·9–89·9)	70·8 (67·2–74·1)	55·6 (51·5–59·5)	87·9 (85·2–90·3)
Maternal disorders	13·2 (4·68–22·1)	13·1 (4·6–21·6)	13 (4·56–21·6)	13·1 (4·67–21·9)
Neonatal disorders	88·5 (85·8–90·9)	71·7 (67·9–75·2)	56·6 (52·3–60·5)	88·5 (85·8–90·9)
Nutritional deficiencies	45·7 (39·2–50·2)	91·2 (88·9–93·3)	91·9 (89·7–93·8)	42·7 (37·4–47)
Protein-energy malnutrition	100 (100–100)	100 (100–100)	100 (100–100)	100 (100–100)
Vitamin A deficiency	--	100 (100–100)	100 (100–100)	--
Dietary iron deficiency	--	100 (100–100)	100 (100–100)	--
Non-communicable diseases	74·1 (71–77)	59·4 (55·3–63·1)	35 (31·6–38·2)	72·8 (70·4–75·2)
Neoplasms	52·6 (48·7–57)	52·5 (48·9–56·5)	40·7 (36·2–45·6)	52·8 (49·2–56·9)
Lip and oral cavity cancer	69·8 (64·7–74·5)	73·5 (68·5–77·9)	71·1 (66·1–75·9)	73·6 (68·5–78)
Nasopharynx cancer	61·6 (53·8–68·8)	63·2 (55·4–69·9)	62·2 (54·5–69·1)	63·2 (55·4–69·9)
Other pharynx cancer	82·3 (78·1–85·8)	83·6 (79·6–87)	80·4 (76·2–84·1)	83·7 (79·6–87)
Esophageal cancer	77·6 (69·5–85)	79·3 (71·8–86·2)	78·4 (70·6–85·6)	79·3 (71·8–86·2)

Stomach cancer	32·6 (23–50·9)	32·6 (22·9–50·8)	32·5 (22·6–50·4)	32·6 (22·9–50·8)
Colon and rectum cancer	59 (50·1–67·9)	59·3 (51·1–67·8)	59·3 (51–67·7)	59·3 (51·1–67·8)
Liver cancer	70·1 (62·3–77·4)	71·2 (63·2–78·4)	70·5 (62·9–77·8)	71·2 (63·2–78·4)
Gallbladder and biliary tract cancer	24·1 (14·1–35·8)	24·5 (14·1–36·7)	24·2 (14–36·1)	24·5 (14·1–36·7)
Pancreatic cancer	45·9 (37·2–55·9)	45·9 (37·8–55·6)	46 (37·5–55·9)	45·9 (37·8–55·6)
Larynx cancer	87·2 (81·1–91·4)	87·8 (82·7–91·7)	86·7 (81·2–90·7)	87·9 (82·7–91·7)
Tracheal, bronchus, and lung cancer	89·4 (87·2–91·6)	89 (86·8–91·2)	89·1 (87–91·4)	89 (86·8–91·2)
Breast cancer	30·1 (20·7–41·5)	28·6 (20·1–38·9)	28·5 (19·9–38·8)	28·6 (20·1–38·9)
Cervical cancer	100 (100–100)	100 (100–100)	100 (100–100)	100 (100–100)
Uterine cancer	48·8 (35–63·8)	49·2 (35·1–64·2)	49·4 (35–64·4)	49·2 (35·1–64·2)
Ovarian cancer	15·2 (5·15–28·9)	13·7 (4·59–26·2)	13·1 (4·35–25·1)	13·7 (4·59–26·2)
Prostate cancer	9·24 (4·61–14)	9·87 (4·89–14·9)	10·2 (4·96–15·5)	9·85 (4·88–14·9)
Kidney cancer	43·8 (34·5–52·9)	44 (34·6–53·3)	43·5 (34·2–52·8)	44 (34·6–53·3)
Bladder cancer	54·2 (39·5–66·4)	56·2 (43·1–67·3)	56·3 (43·5–67·1)	56·2 (43·1–67·3)
Thyroid cancer	15·2 (8·16–23·8)	15·4 (8·22–24·2)	14·8 (8·15–22·5)	15·5 (8·22–24·3)
Mesothelioma	84·1 (77·3–89·6)	77·5 (68·3–85)	83·4 (75·8–89·3)	77·4 (68·2–84·9)
Non-Hodgkin lymphoma	7·65 (3·3–13·2)	7·36 (3·18–12·8)	7·38 (3·23–12·8)	7·36 (3·18–12·8)
Multiple myeloma	9·11 (4·08–15·8)	9·31 (4·14–16·1)	9·32 (4·12–16·1)	9·31 (4·15–16·1)
Leukemia	40·7 (28–53·2)	37 (26–47·9)	40·9 (27·9–53·5)	36·8 (25·9–47·8)
Cardiovascular diseases	86·7 (83·3–90)	88 (85·3–90·5)	83·7 (80·3–86·8)	88·5 (85·8–90·9)
Rheumatic heart disease	31·3 (20·7–51)	32·8 (22–48·9)	33·7 (22·3–50)	32·7 (22–48·8)
Ischemic heart disease	95·1 (92–97·6)	96·2 (94·1–97·9)	96·2 (94·3–97·9)	96·2 (94–97·9)
Stroke	86·6 (80·8–93·3)	89·3 (85·2–93·8)	90·4 (87–94)	89·1 (84·8–93·8)
Hypertensive heart disease	100 (100–100)	100 (100–100)	100 (100–100)	100 (100–100)
Non-rheumatic valvular heart disease	20·7 (14·4–28·4)	18·8 (13·7–24·5)	15·3 (10·5–20·7)	19·4 (14–25·6)
Cardiomyopathy and myocarditis	42·3 (32·8–53·5)	47·9 (39·2–56·4)	47 (39·2–54·8)	48 (39·2–56·5)
Atrial fibrillation and flutter	56·9 (47·6–66)	66 (58·3–73)	69·7 (62·4–76·2)	61·6 (53·1–69·6)
Aortic aneurysm	68·2 (62·9–73·2)	75·2 (71·1–78·9)	--	75·2 (71·1–78·9)
Endocarditis	48·9 (37·5–60·9)	50·5 (39·2–61·2)	48·4 (37·5–59·5)	50·5 (39·3–61·3)
Other cardiovascular and circulatory diseases	43·4 (36·4–50)	46·9 (40·6–52·7)	46·7 (40–52·9)	46·9 (40·4–52·6)
Chronic respiratory diseases	80·4 (77·6–83·1)	72 (68–75·8)	59·5 (54–64·8)	80·9 (78·4–83·3)
Chronic obstructive pulmonary disease	84·5 (81·7–87·4)	83 (80·3–85·8)	76·5 (73·2–80·1)	85·6 (83·1–88·1)
Pneumoconiosis	100 (100–100)	100 (100–100)	100 (100–100)	100 (100–100)
Asthma	48·7 (37·2–60·7)	45·3 (36–55·1)	44·4 (35·4–54·1)	51·4 (39·8–63·3)

Digestive diseases	43·8 (38·8–50·1)	42·6 (36·9–48·7)	22·1 (15·3–29·7)	51·1 (45·6–57·7)
Cirrhosis and other chronic liver diseases	73·5 (65·5–80·5)	76 (67·7–82·7)	67·8 (60·1–74·7)	76·1 (67·8–82·9)
Upper digestive system diseases	29·9 (25·8–33·7)	12·5 (7·97–17·9)	1·53 (0·901–2·32)	33·8 (29·5–37·9)
Gallbladder and biliary diseases	49 (35·9–62·5)	52·6 (39·6–65·7)	53·3 (40·1–66·3)	50·5 (37·6–63·8)
Pancreatitis	27·5 (19·5–37·4)	32·7 (24·2–42·7)	30·7 (23·1–40·1)	32·9 (24·3–43)
Neurological disorders	27·3 (10·3–44)	16·8 (8·36–29·9)	11 (5·93–19·4)	25·1 (9·3–42·1)
Alzheimer's disease and other dementias	45·1 (31·2–60·2)	48 (34·2–62·3)	48·1 (34·4–62·1)	47·9 (34·2–62·5)
Parkinson's disease	-14·7 (-21·9–-7·74)	-17 (-25·5–-9·03)	-18·2 (-27·6–-9·8)	-16·8 (-25·1–-8·98)
Idiopathic epilepsy	19·9 (14·6–25·5)	18 (13·1–23·4)	16·3 (11·8–20·9)	20·4 (15·1–26)
Multiple sclerosis	23·8 (18–30)	25·3 (19·3–31·5)	23·9 (18·4–29)	25·9 (19·8–32·5)
Mental disorders	--	5·13 (2·58–8·01)	5·13 (2·58–8·01)	--
Depressive disorders	--	9·28 (3·19–16·4)	9·28 (3·19–16·4)	--
Anxiety disorders	--	3·53 (0·865–8·14)	3·53 (0·865–8·14)	--
Idiopathic developmental intellectual disability	--	64·2 (34·1–83·4)	64·2 (34·1–83·4)	--
Substance use disorders	100 (100–100)	100 (100–100)	100 (100–100)	100 (100–100)
Alcohol use disorders	100 (100–100)	100 (100–100)	100 (100–100)	100 (100–100)
Drug use disorders	100 (100–100)	100 (100–100)	100 (100–100)	100 (100–100)
Diabetes and kidney diseases	100 (100–100)	100 (100–100)	100 (100–100)	100 (100–100)
Diabetes mellitus	100 (100–100)	100 (100–100)	100 (100–100)	100 (100–100)
Chronic kidney disease	100 (100–100)	100 (100–100)	100 (100–100)	100 (100–100)
Sense organ diseases	--	7·32 (6·23–8·45)	7·32 (6·23–8·45)	--
Blindness and vision loss	--	8·2 (5–11·6)	8·2 (5–11·6)	--
Age-related and other hearing loss	--	7·29 (6·56–8·17)	7·29 (6·56–8·17)	--
Musculoskeletal disorders	6·02 (1·83–10·6)	36 (29·9–42·2)	36·4 (30·2–42·7)	6·17 (1·98–10·6)
Rheumatoid arthritis	14 (4·31–24·5)	22 (7·68–36·1)	23·1 (7·86–37·5)	17·1 (5·61–29·6)
Osteoarthritis	--	20·4 (11·3–30·6)	20·4 (11·3–30·6)	--
Low back pain	--	47·2 (39·9–54·2)	47·2 (39·9–54·2)	--
Gout	--	52·2 (37·7–68·4)	52·2 (37·7–68·4)	--
Other non-communicable diseases	0·284 (0·0931–0·572)	0·0809 (0·0245–0·169)	--	0·603 (0·195–1·21)
Sudden infant death syndrome	28·6 (6·49–47)	28·6 (6·49–47)	--	28·6 (6·49–47)
Injuries	22·2 (17·9–26·5)	22·3 (18·7–25·6)	23·5 (20–27)	20·2 (15·7–24·5)
Transport injuries	25·5 (19·6–30·8)	27 (22·2–31·7)	27·7 (23·9–31·9)	25·9 (19·3–32·1)
Road injuries	23·9 (18–29·4)	26·3 (21·4–31)	27·4 (23·6–31·6)	24·6 (17·7–31)
Other transport injuries	38·5 (33·4–43·1)	36·3 (31·1–41·3)	36·4 (31·9–40·3)	36·2 (30·7–41·8)

Unintentional injuries	25·7 (19·8–30·6)	25·7 (21·6–30)	27·5 (23·8–31·6)	20·5 (15·5–25·4)
Falls	45·4 (39·6–50·5)	36·9 (32·3–41·1)	36·2 (31·8–40·4)	39·1 (33·8–44·3)
Drowning	-8·09 (-17–1·6)	-5·29 (-14·1–4·18)	9·03 (6·68–12·1)	-5·98 (-14·9–3·86)
Fire, heat, and hot substances	13 (8·99–17·5)	16·8 (12·4–21·9)	16·8 (12·5–21·8)	16·8 (12·2–22·1)
Poisonings	14·9 (11–20·3)	15·8 (12–20·6)	14·3 (10·9–18·6)	18·2 (13·6–24·3)
Exposure to mechanical forces	9·84 (2·71–17·5)	22·2 (18·5–26·8)	23·4 (19·9–27·8)	7·64 (-0·0897–15·7)
Animal contact	27·2 (23·1–31·5)	21·3 (17·3–25·9)	21·2 (17·1–25·9)	22·4 (18·7–26·3)
Foreign body	9·77 (7·1–13·3)	10·7 (7·94–14·3)	8·33 (5·97–11·4)	12·1 (8·82–16·6)
Environmental heat and cold exposure	8·33 (3·89–14·3)	7·79 (3·71–12·8)	5·67 (2·63–9·7)	8·84 (4·13–15·1)
Exposure to forces of nature	0 (0–0)	6·95 (3·26–11·7)	6·95 (3·26–11·7)	0 (0–0)
Other unintentional injuries	20·3 (14·9–26·6)	17·2 (13·1–22·1)	15·5 (11·5–20·3)	23 (17·2–29·9)
Self-harm and interpersonal violence	16·4 (7·98–24·6)	12·2 (7·03–17·8)	5·47 (3·35–7·84)	17·1 (8·69–25·4)
Self-harm	15·5 (4·94–26·1)	16·5 (5·89–27·2)	15·8 (8·15–23·4)	16·6 (5·8–27·4)
Interpersonal violence	20·7 (13·5–28·8)	22·2 (15·5–29·5)	26·7 (20·8–33·1)	19·4 (11·9–27·7)

Supplementary Results Table 2. All-age PAFs attributable to the joint distribution of all risk factors for all ages, both sexes combined for each GBD level 1, level 2, and level 3 cause and all causes in 2019 for Bromley. Results are shown for deaths, YLLs, YLDs, and DALYs Data in parenthesis are 95% uncertainty intervals. PAFs=population attributable fractions. GBD=Global Burden of Disease. YLLs=years of life lost. YLDs=years lived with disability. DALYs=disability-adjusted life-years

	Deaths 2019	DALYs 2019	YLDs 2019	YLLs 2019
All causes	53·5 (49·8–56·9)	39·3 (36·2–42·3)	27·7 (25·5–30)	53·3 (50·4–56·1)
Communicable, maternal, neonatal, and nutritional diseases	44·6 (34·4–54·9)	50·4 (45·1–55·9)	47·7 (43·2–52·2)	52·1 (43·7–60)
HIV/AIDS and sexually transmitted infections	79·6 (66·1–89·5)	78·3 (62·3–89·4)	97·4 (95·9–98·4)	63·9 (45·2–80·4)
HIV/AIDS	93 (91·2–94·1)	91·7 (89·4–93·1)	94·6 (92·2–95·9)	90 (87·7–91·4)
Sexually transmitted infections excluding HIV	50·9 (28–76·6)	61·5 (35·5–85·4)	100 (100–100)	18·2 (7·01–39·1)
Respiratory infections and tuberculosis	45·6 (34·6–56·8)	41·6 (32·3–51·5)	3·29 (2·43–4·38)	48·5 (37·8–59·1)
Tuberculosis	55·6 (43·2–66·5)	57·9 (46·3–68)	55·1 (43–65)	58·6 (47–68·7)
Lower respiratory infections	45·5 (34·4–56·8)	48·3 (37·5–59)	33·7 (26·8–40·7)	48·4 (37·6–59·1)
Upper respiratory infections	0·0554 (0·0325–0·0934)	0·00771 (0·00106–0·0183)	0·00694 (0·000322–0·0176)	0·146 (0·0853–0·239)
Otitis media	2·27 (1·03–4·38)	4·56 (2·86–6·46)	4·55 (2·84–6·46)	6·47 (3·37–10·6)
Enteric infections	4·46 (2·66–7·59)	8·49 (6·28–11·8)	9·1 (6·73–12·6)	6·8 (4·69–9·95)
Diarrheal diseases	4·49 (2·69–7·68)	8·53 (6·33–11·8)	9·11 (6·73–12·6)	6·88 (4·8–10)
Other infectious diseases	0·735 (0·462–1·03)	1·26 (0·906–1·65)	0·375 (0·219–0·616)	1·68 (1·16–2·24)
Meningitis	1·5 (1·03–2·04)	2·6 (1·86–3·51)	0·00055 (1·79e-05–0·00149)	3·54 (2·56–4·74)
Encephalitis	0·28 (0·175–0·428)	0·732 (0·453–1·13)	0·000168 (5·12e-06–0·000452)	1·04 (0·657–1·57)
Measles	2·19 (-0·638–11·9)	2·51 (-0·656–13·2)	0·414 (0·0108–1·21)	2·73 (-0·802–15·4)
Acute hepatitis	12·7 (5·97–23·9)	5·86 (3·26–10·1)	2·95 (1·75–4·92)	11·7 (5·51–21·9)
Maternal and neonatal disorders	87·9 (85·8–89·7)	77·5 (73·9–80·6)	66·8 (61·6–71·6)	88·9 (86·8–90·5)
Maternal disorders	9·22 (3·27–15·3)	9·3 (3·33–15·9)	9·42 (3·25–16·1)	9·06 (3·21–15)
Neonatal disorders	90·4 (88·4–92)	79·4 (75·7–82·6)	68·8 (63·3–73·8)	90·4 (88·4–92·1)
Nutritional deficiencies	65·4 (57·9–91·3)	79 (74·9–83·5)	79·1 (75–83·6)	64·6 (59·8–82·5)
Protein-energy malnutrition	100 (100–100)	100 (100–100)	100 (100–100)	100 (100–100)
Vitamin A deficiency	--	100 (100–100)	100 (100–100)	--
Dietary iron deficiency	--	100 (100–100)	100 (100–100)	--
Non-communicable diseases	54·9 (50·9–58·6)	39·4 (36·1–42·7)	26·7 (24·3–29·1)	54·9 (51·8–58)
Neoplasms	46·2 (42·3–50·6)	45·4 (41·8–49·5)	32 (27·9–36·7)	46·3 (42·7–50·4)
Lip and oral cavity cancer	66·2 (54·4–75)	69·4 (58·1–77·4)	68·8 (56·8–77·2)	69·5 (58·1–77·4)
Nasopharynx cancer	66·9 (54·7–76·4)	66·8 (55–75·8)	64·9 (52·7–74·8)	67 (55·2–75·9)
Other pharynx cancer	78 (70·3–84·4)	79·4 (71·9–85·3)	78·3 (70·4–84·7)	79·5 (72–85·4)
Esophageal cancer	74·6 (65·3–83·4)	76 (66·9–84·4)	75 (66–83·5)	76 (66·9–84·4)
Stomach cancer	23·3 (15·8–40·2)	23·5 (16·1–40·1)	23·6 (16·3–40·4)	23·5 (16·1–40·1)

Colon and rectum cancer	63·3 (53·8–72·2)	63·6 (55·1–72·2)	63·7 (55·1–72·4)	63·6 (55·1–72·2)
Liver cancer	75·8 (69·3–81·9)	76·9 (71–82·3)	77·1 (71·2–82·4)	76·9 (71–82·3)
Gallbladder and biliary tract cancer	21·8 (11·6–34·2)	22·2 (12·1–34·6)	20·7 (11–33)	22·3 (12·1–34·7)
Pancreatic cancer	41·3 (31·6–52·7)	40·6 (31·5–51·7)	41·2 (31·4–52·7)	40·6 (31·5–51·6)
Larynx cancer	82·5 (74·3–89·1)	82·1 (74·7–87·9)	80·2 (72·4–86·3)	82·3 (75–88·1)
Tracheal, bronchus, and lung cancer	85·6 (81·4–89·2)	84·5 (80·5–88·1)	85·2 (81·3–88·6)	84·5 (80·5–88)
Breast cancer	36·5 (26·5–48·2)	35·5 (26·7–45·8)	35·7 (26·7–46·3)	35·5 (26·7–45·7)
Cervical cancer	100 (100–100)	100 (100–100)	100 (100–100)	100 (100–100)
Uterine cancer	43·6 (28·6–59·2)	44·8 (30–60·1)	45·1 (30·2–60·8)	44·7 (30·1–60)
Ovarian cancer	29·8 (13·7–44·6)	24·9 (11·8–38·2)	24 (11·4–37)	25 (11·8–38·3)
Prostate cancer	5·29 (2·32–8·7)	5·68 (2·53–9·21)	5·98 (2·73–9·61)	5·62 (2·49–9·17)
Kidney cancer	37·5 (27·2–48·5)	37·7 (27·8–48·3)	37·5 (27·7–47·7)	37·7 (27·8–48·4)
Bladder cancer	40·5 (25·6–55·2)	42·6 (28·8–56·6)	43·2 (29·5–56·6)	42·6 (28·7–56·6)
Thyroid cancer	14·1 (6·94–23·2)	14·8 (7·08–24·4)	15·1 (7·34–24·7)	14·8 (7·07–24·4)
Mesothelioma	99·1 (98·7–99·3)	98 (97·3–98·6)	98·9 (98·4–99·2)	98 (97·3–98·6)
Non-Hodgkin lymphoma	7·01 (2·93–12·6)	7·28 (3·07–12·9)	7·18 (3·02–12·8)	7·29 (3·07–12·9)
Multiple myeloma	8 (3·43–14·4)	8·36 (3·68–14·8)	8·3 (3·62–14·9)	8·37 (3·69–14·8)
Leukemia	35·1 (21·3–49·7)	33·3 (21·8–45·9)	32·3 (21·3–44·3)	33·4 (21·8–46·1)
Cardiovascular diseases	77 (72·7–81·3)	78·4 (74·8–81·6)	70·1 (64·8–75)	79·6 (76·3–82·7)
Rheumatic heart disease	21·5 (11·2–42·4)	22·5 (13·6–39·4)	22·7 (13·9–37·1)	22·5 (13·5–39·6)
Ischemic heart disease	93 (88·7–96·6)	94·2 (91–96·8)	93·6 (90·6–96·4)	94·2 (91–96·9)
Stroke	78·2 (69·5–88·3)	81·4 (74·9–88·5)	81·5 (75·7–87·7)	81·3 (74·5–88·8)
Hypertensive heart disease	100 (100–100)	100 (100–100)	100 (100–100)	100 (100–100)
Non-rheumatic valvular heart disease	24·5 (15·1–39)	24·6 (16·8–35·7)	16·1 (10·4–23·2)	26·1 (17·8–37·6)
Cardiomyopathy and myocarditis	31·5 (23·9–42·7)	38·2 (32·6–45)	32·8 (26·6–40·5)	38·6 (32·8–45·4)
Atrial fibrillation and flutter	48·7 (38·1–59·8)	56·6 (47·6–65·5)	60·9 (52·2–69·4)	51·8 (41·6–61·9)
Aortic aneurysm	47 (39·7–54·8)	54·3 (48–60·7)	--	54·3 (48–60·7)
Endocarditis	29·4 (19·1–44·1)	31·9 (22·6–43)	29·4 (19·4–43)	32 (22·7–43·1)
Other cardiovascular and circulatory diseases	27·3 (20·8–35·1)	30·2 (24·2–37·1)	32·2 (24·6–39·8)	29·8 (23·8–36·6)
Chronic respiratory diseases	60·9 (54·8–67·3)	55·5 (50·9–60·1)	49·9 (44·5–55·2)	61·2 (55·8–66·9)
Chronic obstructive pulmonary disease	69·6 (63·9–75·2)	68·2 (63·9–72·3)	62·9 (58·8–66·9)	71·6 (66·4–76·4)
Pneumoconiosis	100 (100–100)	100 (100–100)	100 (100–100)	100 (100–100)
Asthma	40·6 (29–54·1)	39 (30·6–49)	38·8 (30·4–48·7)	42·1 (31·5–53·5)
Digestive diseases	27·5 (22·6–32·6)	33·7 (27·7–39·3)	24·4 (16·5–33·2)	40·5 (34·1–46·7)
Cirrhosis and other chronic liver diseases	73·8 (60·4–86·8)	77·3 (65·1–88·6)	73·7 (61·3–84·6)	77·4 (65·3–88·8)

Upper digestive system diseases	19·4 (16–23)	7·03 (4·47–10·3)	0·654 (0·372–1·06)	21·9 (18·5–25·1)
Gallbladder and biliary diseases	44 (28·8–59·1)	47·3 (33–61·9)	47·7 (33·4–62·2)	44·9 (30·3–59·9)
Pancreatitis	36·8 (16·6–56·6)	43·6 (23·2–62)	44·7 (23·7–62·8)	43·3 (23·3–61·9)
Neurological disorders	26·4 (12–41·5)	16·3 (8·12–28·2)	10·4 (5·19–19·4)	23·1 (10·6–37·8)
Alzheimer's disease and other dementias	40 (25·9–55·7)	41·6 (27·4–57·2)	41·8 (27·6–57·3)	41·5 (27·5–57·1)
Parkinson's disease	-8·13 (-13·6–-3·58)	-9·07 (-15–-4·15)	-9·58 (-15·3–-4·39)	-8·93 (-14·8–-4·01)
Idiopathic epilepsy	26·2 (16–36·8)	24·5 (15·3–34·1)	22·7 (13·7–31·5)	27·4 (17·2–37·7)
Multiple sclerosis	13·9 (10·1–17·7)	14·4 (10·7–18·4)	14·1 (10·5–18)	14·7 (10·8–18·7)
Mental disorders	--	6·6 (3·31–10·2)	6·6 (3·31–10·2)	--
Depressive disorders	--	13·5 (6·09–21·2)	13·5 (6·09–21·2)	--
Anxiety disorders	--	4·77 (1·28–10·5)	4·77 (1·28–10·5)	--
Idiopathic developmental intellectual disability	--	38·5 (18·1–57·1)	38·5 (18·1–57·1)	--
Substance use disorders	100 (100–100)	100 (100–100)	100 (100–100)	100 (100–100)
Alcohol use disorders	100 (100–100)	100 (100–100)	100 (100–100)	100 (100–100)
Drug use disorders	100 (100–100)	100 (100–100)	100 (100–100)	100 (100–100)
Diabetes and kidney diseases	99·8 (99·7–99·8)	100 (99·9–100)	100 (100–100)	99·8 (99·7–99·8)
Diabetes mellitus	100 (100–100)	100 (100–100)	100 (100–100)	100 (100–100)
Chronic kidney disease	100 (100–100)	100 (100–100)	100 (100–100)	100 (100–100)
Sense organ diseases	--	7·83 (6·68–9·11)	7·83 (6·68–9·11)	--
Blindness and vision loss	--	9·41 (5·93–13·7)	9·41 (5·93–13·7)	--
Age-related and other hearing loss	--	8·44 (7·49–9·48)	8·44 (7·49–9·48)	--
Musculoskeletal disorders	2·45 (0·585–4·62)	20·6 (16·8–24·4)	21 (17·1–24·9)	3·05 (0·731–5·54)
Rheumatoid arthritis	10·6 (2·59–19·4)	12·5 (3·19–22·3)	12·6 (3·23–22·5)	12 (3·02–21·7)
Osteoarthritis	--	16·7 (8·86–25·9)	16·7 (8·86–25·9)	--
Low back pain	--	37·8 (32·3–43·1)	37·8 (32·3–43·1)	--
Gout	--	51·3 (36·3–68·1)	51·3 (36·3–68·1)	--
Other non-communicable diseases	0·0462 (0·0281–0·0719)	0·0386 (0·0225–0·0601)	--	0·22 (0·123–0·33)
Sudden infant death syndrome	9·7 (5·39–15·6)	9·69 (5·39–15·6)	--	9·69 (5·39–15·6)
Injuries	33 (27·2–38·3)	28·4 (24–32·8)	30·4 (25·9–34·7)	24·6 (17·8–31)
Transport injuries	22·7 (15·5–30·2)	23·2 (17·2–29·4)	27·5 (23·2–31·6)	20·6 (12·5–28·7)
Road injuries	21·6 (13·9–29·5)	22 (15·8–28·6)	26·2 (21·9–30·3)	19·5 (11–28·1)
Other transport injuries	34·5 (29·5–39·4)	33·7 (28·8–38·3)	36·8 (32·2–40·9)	31·1 (25–36·7)
Unintentional injuries	41·3 (34·6–46·2)	30·1 (25·2–34·7)	30·4 (25·6–35·1)	28·4 (23·5–33)
Falls	56·7 (48–62·3)	41·2 (34·7–46·4)	39·9 (33·5–45·1)	48·6 (41·7–54)
Drowning	-13·6 (-27·2–0·388)	-12 (-24–0·85)	10·9 (6·52–16·9)	-14·3 (-27·5–0·39)

Fire, heat, and hot substances	9·71 (5·35–15·9)	13·3 (8·34–19·5)	13·8 (8·76–20)	11·4 (6·81–17·2)
Poisonings	13·6 (8·34–20)	13 (8·36–19)	12·4 (7·87–18·2)	14·4 (9·26–20·8)
Exposure to mechanical forces	16 (4·75–27·6)	18·5 (15·4–21·4)	19·5 (16·6–22·2)	3·76 (-7·84–16·4)
Animal contact	22·1 (17·3–27·7)	21·7 (16·7–27·4)	22·5 (17·3–28·4)	17·3 (13·1–22·4)
Foreign body	2·89 (1·81–4·29)	4·16 (2·67–6·05)	3·63 (2·34–5·28)	4·77 (3–6·99)
Environmental heat and cold exposure	9·15 (4·3–15·9)	9·37 (4·41–16)	9·36 (4·39–16)	9·5 (4·56–16·2)
Exposure to forces of nature	0 (0–0)	10·4 (4·99–17·5)	10·4 (4·99–17·5)	0 (0–0)
Other unintentional injuries	14·6 (9–21·5)	13·1 (8·29–19·2)	13 (8·23–19·1)	16·4 (10·6–23·4)
Self-harm and interpersonal violence	23·2 (10·3–36·4)	25·8 (14·4–37·2)	33·9 (26·5–42·1)	24 (11–37·1)
Self-harm	23·1 (9·54–37·6)	24·2 (10·6–38·6)	24 (11·4–36·9)	24·2 (10·4–38·6)
Interpersonal violence	26·3 (17·5–35)	34·1 (26–43)	39·1 (30·8–48·2)	23 (14·8–31·7)

Supplementary Results Table 2. All-age PAFs attributable to the joint distribution of all risk factors for all ages, both sexes combined for each GBD level 1, level 2, and level 3 cause and all causes in 2019 for Camden. Results are shown for deaths, YLLs, YLDs, and DALYs Data in parenthesis are 95% uncertainty intervals. PAFs=population attributable fractions. GBD=Global Burden of Disease. YLLs=years of life lost. YLDs=years lived with disability. DALYs=disability-adjusted life-years				
	**Deaths 2019**	**DALYs 2019**	**YLDs 2019**	**YLLs 2019**
All causes	55·7 (52·7–58·6)	40·9 (37·8–43·9)	29·5 (27–31·9)	55·6 (53–58)
Communicable, maternal, neonatal, and nutritional diseases	49 (39·1–58·7)	54·2 (49·8–58·6)	51·1 (46·3–55·5)	56·4 (49·2–63·3)
HIV/AIDS and sexually transmitted infections	86·1 (78·9–90·9)	85 (76–90·4)	95·4 (93·3–96·7)	77·7 (66·3–85·8)
HIV/AIDS	92·1 (89·8–93·5)	91·1 (88·5–92·9)	94 (91·5–95·6)	89·3 (86·5–91)
Sexually transmitted infections excluding HIV	47·5 (25·9–73·5)	59·8 (33·9–84·4)	100 (100–100)	18·2 (7·3–38·4)
Respiratory infections and tuberculosis	50 (38·7–60·7)	44·5 (35·7–53·4)	3·7 (2·62–5·15)	53·1 (42·5–63·2)
Tuberculosis	63·1 (52·1–72·3)	64·7 (54·9–72·9)	60·7 (49·7–69·7)	65·3 (55·5–73·3)
Lower respiratory infections	49·7 (38·2–60·8)	52·8 (41·8–63·2)	38 (31·2–44·8)	52·9 (41·9–63·3)
Upper respiratory infections	0·041 (0·0207–0·0749)	0·00982 (0·00226–0·0214)	0·00734 (0·000214–0·0188)	0·0892 (0·0438–0·165)
Otitis media	2·37 (0·688–6·36)	4·73 (2·99–6·73)	4·72 (2·98–6·63)	5·15 (1·79–11·1)
Enteric infections	3·97 (2·52–6·27)	8·09 (6·1–10·7)	8·48 (6·33–11·3)	7·04 (4·94–9·66)
Diarrheal diseases	4·02 (2·54–6·35)	8·14 (6·14–10·8)	8·49 (6·34–11·4)	7·18 (5·06–9·81)
Other infectious diseases	0·836 (0·521–1·38)	1·26 (0·912–1·72)	0·425 (0·25–0·704)	1·57 (1·11–2·27)
Meningitis	1·51 (1·06–2·1)	2·48 (1·79–3·39)	0·000807 (1·88e-05–0·00221)	3·14 (2·21–4·32)
Encephalitis	0·342 (0·209–0·525)	0·874 (0·515–1·37)	3e-04 (6·32e-06–0·00081)	1·06 (0·655–1·64)
Measles	3·37 (-1·22–18·4)	3·92 (-1·42–22·9)	0·219 (0·00571–0·63)	4·26 (-1·54–23·9)
Acute hepatitis	11 (4·64–21·8)	5·69 (2·77–10·2)	2·64 (1·54–4·36)	9·81 (3·9–19·9)
Maternal and neonatal disorders	86·5 (83·6–88·9)	75·9 (72–79·6)	66·9 (61·4–72·2)	87·5 (84·8–89·8)
Maternal disorders	9·23 (3·33–15·5)	9·33 (3·34–15·8)	9·43 (3·37–16)	9·13 (3·28–15·5)
Neonatal disorders	89·1 (86·5–91·4)	77·7 (73·6–81·6)	68·7 (63·1–74·2)	89·1 (86·5–91·4)
Nutritional deficiencies	81·4 (76·5–89·2)	77·8 (73·7–82·1)	77·8 (73·6–82·1)	79 (71·2–82·3)
Protein-energy malnutrition	100 (100–100)	100 (100–100)	100 (100–100)	100 (100–100)
Vitamin A deficiency	--	100 (100–100)	100 (100–100)	--
Dietary iron deficiency	--	100 (100–100)	100 (100–100)	--
Non-communicable diseases	57·3 (54·1–60·6)	41·1 (37·8–44·5)	28·6 (26–31·4)	58 (55·2–60·8)
Neoplasms	49·9 (45·9–54·1)	48·5 (44·7–52·4)	34·8 (30·6–39·3)	49·4 (45·7–53·3)
Lip and oral cavity cancer	72·3 (63·2–79·2)	74·5 (66·3–80·7)	73·8 (65·2–80·5)	74·5 (66·5–80·7)
Nasopharynx cancer	70·4 (59·6–78·5)	70·1 (59·7–77·9)	68·4 (57·2–76·9)	70·3 (59·9–78)
Other pharynx cancer	81·2 (74·6–86·4)	82·1 (75·8–87·1)	81 (74–86·4)	82·2 (75·9–87·1)
Esophageal cancer	76·7 (67·1–85·7)	77·9 (68·7–86·3)	77 (67·4–85·9)	78 (68·7–86·3)
Stomach cancer	24·4 (16·8–41·1)	24·2 (16·8–40·6)	24·4 (17·1–40·9)	24·2 (16·8–40·6)

Colon and rectum cancer	65·8 (56·9–74·4)	66·1 (57·5–74·1)	66·2 (57·7–74·5)	66·1 (57·5–74·1)
Liver cancer	79·9 (74–84·8)	80·1 (74·6–84·7)	80·1 (74·3–84·8)	80·1 (74·6–84·7)
Gallbladder and biliary tract cancer	27·7 (15·8–41·7)	28 (16–41·7)	26·2 (14·9–39·9)	28·1 (16·1–41·7)
Pancreatic cancer	44·8 (34·6–56·5)	43·8 (34·5–54·7)	44·5 (34·7–55·8)	43·8 (34·5–54·6)
Larynx cancer	83·7 (76–89·5)	83 (76–88·5)	80·6 (73–86·5)	83·3 (76·2–88·7)
Tracheal, bronchus, and lung cancer	85·5 (81·8–89)	84·1 (80·5–87·6)	84·9 (81·3–88·6)	84·1 (80·5–87·6)
Breast cancer	40 (30·2–50·9)	37·7 (29·1–47·3)	37·9 (29·1–47·8)	37·6 (29·1–47·2)
Cervical cancer	100 (100–100)	100 (100–100)	100 (100–100)	100 (100–100)
Uterine cancer	50·6 (34·6–65·7)	51·5 (35·6–66·7)	51·8 (36·1–67)	51·5 (35·5–66·7)
Ovarian cancer	27·3 (13·9–41·7)	22·8 (11·3–36)	21·5 (10·8–33·9)	22·8 (11·3–36·1)
Prostate cancer	5·74 (2·42–9·18)	6·2 (2·76–9·79)	6·55 (2·93–10·2)	6·14 (2·72–9·71)
Kidney cancer	42·8 (31·9–53·6)	42·5 (31·8–52·8)	42·2 (31·4–52·6)	42·6 (31·8–52·8)
Bladder cancer	43·3 (29–58·3)	44·9 (31·3–58·6)	45·2 (31·8–58·3)	44·9 (31·2–58·7)
Thyroid cancer	18·3 (9·29–28·9)	19 (9·51–30)	19·2 (9·57–30·2)	19 (9·57–30)
Mesothelioma	98·4 (97·8–98·8)	96·6 (95·6–97·4)	98·2 (97·5–98·8)	96·6 (95·5–97·4)
Non-Hodgkin lymphoma	9·01 (3·89–15·4)	9·23 (4·1–15·6)	9·17 (3·95–15·7)	9·23 (4·12–15·6)
Multiple myeloma	10·3 (4·66–17·9)	10·7 (4·82–18·4)	10·6 (4·73–18·3)	10·7 (4·81–18·5)
Leukemia	38·2 (24·7–52)	35·2 (23·6–46·7)	33·8 (23–45·1)	35·3 (23·7–46·7)
Cardiovascular diseases	77·5 (73·6–81·6)	78·8 (75·4–82)	72 (67·1–76·4)	79·8 (76·5–82·9)
Rheumatic heart disease	22·9 (12·5–45·2)	24 (14·7–40·7)	24·1 (14·9–38·7)	24 (14·6–41)
Ischemic heart disease	93·8 (90·3–97)	95 (92·5–97·3)	94·3 (91·6–96·8)	95 (92·5–97·4)
Stroke	80·8 (73–88·9)	84·2 (78·6–89·9)	84·2 (79·5–89·1)	84·1 (78·3–90·1)
Hypertensive heart disease	100 (100–100)	100 (100–100)	100 (100–100)	100 (100–100)
Non-rheumatic valvular heart disease	26·1 (16·7–38·5)	27·1 (18·9–37·3)	19·6 (13·1–27·7)	27·9 (19·4–38·4)
Cardiomyopathy and myocarditis	36·9 (29·4–46·6)	44·1 (38·3–50·7)	39·1 (32·7–46)	44·5 (38·6–51·1)
Atrial fibrillation and flutter	55·2 (44·8–65·8)	62·7 (54·2–71·1)	66·6 (58·4–74·3)	58·4 (49·2–68)
Aortic aneurysm	50·6 (43·2–58·4)	57·4 (51·2–63·5)	--	57·4 (51·2–63·5)
Endocarditis	31·5 (22–44·4)	34·4 (25·8–45)	31·4 (21·6–43·9)	34·5 (25·9–45·1)
Other cardiovascular and circulatory diseases	31·8 (24·6–39·2)	34·1 (27·4–40·5)	33·8 (25·8–42·7)	34·1 (27·6–40·6)
Chronic respiratory diseases	64·5 (58–70·7)	57·9 (53·2–62·8)	51·3 (45·5–57)	64·5 (58·9–70·1)
Chronic obstructive pulmonary disease	72·5 (66·3–78·1)	71 (66·3–75·4)	64·4 (60·6–68·2)	74·2 (68·5–79·4)
Pneumoconiosis	100 (100–100)	100 (100–100)	100 (100–100)	100 (100–100)
Asthma	47·2 (34·4–60·3)	44·7 (35·6–54·9)	44·5 (35·5–54·6)	47·9 (37–59·1)
Digestive diseases	36 (30·3–41)	41·9 (35·6–47·3)	30·7 (21·7–40·7)	49·6 (42·4–55·6)

Cirrhosis and other chronic liver diseases	80 (67·4–89·5)	82·3 (70·6–90·9)	79·6 (68·2–88·1)	82·3 (70·6–91·1)
Upper digestive system diseases	20·7 (17·1–24·1)	7·79 (4·87–11·2)	0·646 (0·376–1·05)	22·7 (19·2–26·1)
Gallbladder and biliary diseases	50·6 (34·7–65·9)	53·7 (38·7–68·1)	54 (38·9–68·2)	51·4 (35·9–66·7)
Pancreatitis	48·1 (30–65·2)	54 (36·9–70·2)	55·8 (38·1–71·8)	53·8 (36·6–70·1)
Neurological disorders	29 (13·2–44·9)	14·9 (6·93–27·2)	8·59 (3·85–17·9)	25·5 (12·2–40·3)
Alzheimer's disease and other dementias	45 (29·6–61·1)	46·6 (31·6–62·3)	46·8 (31·7–62·3)	46·5 (31·6–62·2)
Parkinson's disease	-9·02 (-14·9–-4·08)	-10·1 (-16·6–-4·61)	-10·7 (-17·5–-4·97)	-9·9 (-16·3–-4·56)
Idiopathic epilepsy	31·5 (21·6–41·1)	29·7 (20·5–39)	28·3 (19·5–37·3)	32·2 (22·5–41·4)
Multiple sclerosis	14·8 (10·9–19·1)	14·8 (11·1–19)	14·3 (10·7–18·3)	15·4 (11·5–19·7)
Mental disorders	--	6·53 (3·06–10·4)	6·53 (3·06–10·4)	--
Depressive disorders	--	14·4 (6·51–22·8)	14·4 (6·51–22·8)	--
Anxiety disorders	--	5·18 (1·18–12)	5·18 (1·18–12)	--
Idiopathic developmental intellectual disability	--	25·7 (8·38–48·4)	25·7 (8·38–48·4)	--
Substance use disorders	100 (100–100)	100 (100–100)	100 (100–100)	100 (100–100)
Alcohol use disorders	100 (100–100)	100 (100–100)	100 (100–100)	100 (100–100)
Drug use disorders	100 (100–100)	100 (100–100)	100 (100–100)	100 (100–100)
Diabetes and kidney diseases	99·5 (99·4–99·6)	99·9 (99·9–99·9)	100 (100–100)	99·5 (99·4–99·6)
Diabetes mellitus	100 (100–100)	100 (100–100)	100 (100–100)	100 (100–100)
Chronic kidney disease	100 (100–100)	100 (100–100)	100 (100–100)	100 (100–100)
Sense organ diseases	--	8·64 (7·61–9·83)	8·64 (7·61–9·83)	--
Blindness and vision loss	--	9·12 (5·93–13·1)	9·12 (5·93–13·1)	--
Age-related and other hearing loss	--	9·79 (8·85–10·8)	9·79 (8·85–10·8)	--
Musculoskeletal disorders	2·37 (0·568–4·52)	22·1 (18·3–26)	22·5 (18·7–26·5)	2·75 (0·677–5)
Rheumatoid arthritis	11·7 (2·74–20·9)	12·9 (3·11–22·7)	12·9 (3·11–22·8)	12·9 (3·11–22·9)
Osteoarthritis	--	20·5 (11·3–30·2)	20·5 (11·3–30·2)	--
Low back pain	--	40·6 (35·2–45·8)	40·6 (35·2–45·8)	--
Gout	--	57 (40·3–73·4)	57 (40·3–73·4)	--
Other non-communicable diseases	0·0844 (0·051–0·131)	0·0638 (0·0369–0·0995)	--	0·336 (0·19–0·508)
Sudden infant death syndrome	7·93 (4·58–12·6)	7·92 (4·57–12·6)	--	7·92 (4·57–12·6)
Injuries	34·4 (28·9–39·6)	27·1 (22·7–32·1)	27·9 (23·5–32·7)	26·2 (19·9–32·2)
Transport injuries	22·1 (14·1–30·1)	22·1 (15·6–28·4)	25·9 (21·2–30·3)	20 (11·2–28·2)
Road injuries	21·2 (12·6–29·4)	21·2 (14·3–27·8)	24·9 (20·3–29·4)	19·2 (10–27·9)
Other transport injuries	31·8 (26·7–36·7)	29·9 (25–34·6)	33·2 (28·5–37·4)	27·5 (22–32·8)
Unintentional injuries	40·3 (33·9–45·4)	27·3 (22·5–32·4)	27·5 (22·6–32·8)	26·7 (21·8–31·6)

Falls	54·7 (46·6–60·3)	38·3 (32·1–44·1)	36·2 (30·1–42·1)	44·9 (38·3–50·6)
Drowning	-14·6 (-28·4–0·301)	-13·6 (-26·7–0·00403)	11·7 (6·51–18·7)	-15 (-28·8—0·779)
Fire, heat, and hot substances	11·5 (6·29–18·5)	13·5 (7·85–20·6)	14·2 (8·28–21·5)	12·6 (7·35–19·2)
Poisonings	14·7 (8·76–21·8)	14 (8·44–20·9)	13 (7·76–19·7)	15·3 (9·39–22·3)
Exposure to mechanical forces	12·6 (0·299–25·3)	14·5 (11·9–16·9)	15·5 (13·1–17·6)	0·0656 (-12·9–14·6)
Animal contact	21·8 (16·5–28·5)	19·9 (14·6–26·4)	20·6 (15·1–27·2)	17·7 (12·9–23·7)
Foreign body	2·02 (1·25–2·96)	2·98 (1·92–4·3)	2·7 (1·73–3·93)	3·2 (2·01–4·7)
Environmental heat and cold exposure	9·57 (4·4–16·6)	10·9 (5·13–18·5)	10·9 (5·13–18·5)	10·9 (5·18–18·5)
Exposure to forces of nature	0 (0–0)	11·7 (5·61–19·7)	11·7 (5·61–19·7)	0 (0–0)
Other unintentional injuries	14·3 (8·3–21·6)	13·6 (7·99–20·3)	13·5 (7·9–20·3)	15·8 (9·52–22·9)
Self-harm and interpersonal violence	27·5 (14·9–40)	29·6 (18·3–40·5)	37·1 (29·3–45·1)	28·3 (15·9–40·6)
Self-harm	27·4 (13·8–40·6)	28·5 (15·1–41·7)	28·2 (16·1–40·4)	28·5 (14·9–41·7)
Interpersonal violence	31·1 (22·5–40·5)	36·4 (28–45·5)	42·5 (33·5–52·1)	28·2 (20–37·2)

Supplementary Results Table 2. All-age PAFs attributable to the joint distribution of all risk factors for all ages, both sexes combined for each GBD level 1, level 2, and level 3 cause and all causes in 2019 for Croydon. Results are shown for deaths, YLLs, YLDs, and DALYs Data in parenthesis are 95% uncertainty intervals. PAFs=population attributable fractions. GBD=Global Burden of Disease. YLLs=years of life lost. YLDs=years lived with disability. DALYs=disability-adjusted life-years

	Deaths 2019	DALYs 2019	YLDs 2019	YLLs 2019
All causes	54·3 (51–57·4)	39·7 (36·8–42·8)	27·3 (25·1–29·6)	53·8 (51·2–56·5)
Communicable, maternal, neonatal, and nutritional diseases	47 (37·2–56·6)	55·2 (50·6–59·8)	49·3 (44·6–54·1)	58·6 (51·7–65·3)
HIV/AIDS and sexually transmitted infections	81·2 (70·4–88·7)	79·6 (66·7–88·1)	96·1 (94·3–97·4)	68·9 (53·5–81·3)
HIV/AIDS	91·2 (89·3–92·4)	89·6 (87·1–91·4)	94 (91·6–95·5)	87·1 (84·7–88·6)
Sexually transmitted infections excluding HIV	42·4 (21·9–69·1)	56·8 (31–82·4)	100 (100–100)	15·7 (6·08–34·1)
Respiratory infections and tuberculosis	46·1 (35–57)	42 (32·8–50·9)	3·47 (2·58–4·52)	49·7 (39·2–59·8)
Tuberculosis	56·3 (45·4–66·4)	57·6 (47·1–67·2)	53·4 (41·8–63·7)	58·8 (48·3–68·3)
Lower respiratory infections	46·1 (34·9–57)	49·5 (38·9–59·7)	33·9 (27·5–40·4)	49·6 (39–59·9)
Upper respiratory infections	0·0778 (0·0435–0·13)	0·0101 (0·00169–0·0252)	0·00851 (0·000114–0·0239)	0·16 (0·0896–0·266)
Otitis media	3·48 (1·63–6·57)	5·23 (3·31–7·24)	5·16 (3·27–7·24)	7·7 (4·26–12·6)
Enteric infections	5·04 (3·19–8·27)	10·2 (7·77–13·7)	10·4 (7·8–14·1)	9·41 (6·58–13·1)
Diarrheal diseases	5·11 (3·24–8·37)	10·3 (7·82–13·8)	10·5 (7·81–14·1)	9·65 (6·83–13·3)
Other infectious diseases	1 (0·653–1·33)	1·61 (1·23–2·05)	0·332 (0·192–0·536)	2·21 (1·65–2·88)
Meningitis	2·44 (1·81–3·25)	3·62 (2·73–4·77)	0·000732 (8·97e-06–0·00213)	4·77 (3·63–6·27)
Encephalitis	0·525 (0·335–0·787)	1·09 (0·585–1·67)	0·000234 (2·22e-06–0·00068)	1·58 (0·967–2·37)
Measles	3·42 (-1·07–17·8)	3·89 (-1·06–19·4)	0·463 (0·0132–1·22)	4·14 (-1·3–21·3)
Acute hepatitis	15·8 (9·17–25·9)	5·31 (3·15–8·19)	2·67 (1·53–4·26)	13·9 (8·14–22·9)
Maternal and neonatal disorders	89·5 (87·7–91·2)	80·3 (77·2–83·3)	64·9 (59·3–70·4)	90·3 (88·6–91·9)
Maternal disorders	9·5 (3·38–15·8)	9·57 (3·41–16·2)	9·72 (3·46–16·5)	9·37 (3·39–15·7)
Neonatal disorders	91·7 (90–93·1)	82 (78·8–85)	66·9 (60·9–72·4)	91·7 (90–93·1)
Nutritional deficiencies	70·6 (64·7–89·3)	79·9 (75·2–84·6)	80·1 (75·3–84·9)	70 (66·6–80·3)
Protein-energy malnutrition	100 (100–100)	100 (100–100)	100 (100–100)	100 (100–100)
Vitamin A deficiency	--	100 (100–100)	100 (100–100)	--
Dietary iron deficiency	--	100 (100–100)	100 (100–100)	--
Non-communicable diseases	55·9 (52·4–59·1)	39·7 (36·4–42·9)	26·3 (23·8–28·9)	55·5 (52·8–58·4)
Neoplasms	46·8 (42·9–51·3)	45·5 (41·9–49·5)	32·6 (28·6–37·1)	46·2 (42·6–50·3)
Lip and oral cavity cancer	65·9 (54·4–75·3)	68·6 (57·7–77·1)	67·9 (56·6–76·6)	68·7 (57·7–77·1)
Nasopharynx cancer	66 (53·6–75·9)	65·5 (53·1–74·9)	63·1 (50·3–73·4)	65·7 (53·2–75·1)
Other pharynx cancer	78·4 (71·1–84·7)	79·4 (72·3–85·5)	78·2 (70·5–84·6)	79·5 (72·3–85·5)
Esophageal cancer	74·7 (65·5–83)	76 (66·7–84)	75·1 (65·5–83·2)	76 (66·7–84·1)

Stomach cancer	24 (16·5–40·8)	23·9 (16·5–40·5)	24·1 (16·7–40·9)	23·9 (16·5–40·5)
Colon and rectum cancer	63·5 (54·7–72·5)	63·6 (55·4–71·9)	63·6 (55·3–72·2)	63·6 (55·4–71·9)
Liver cancer	76·2 (69·8–81·9)	76 (70·4–81·2)	76·3 (70·8–81·9)	76 (70·4–81·2)
Gallbladder and biliary tract cancer	21·2 (11·3–33·1)	21·6 (11·7–33·8)	20·1 (10·7–31·7)	21·7 (11·7–33·9)
Pancreatic cancer	41·9 (32·2–53·4)	40·9 (31·8–51·4)	41·6 (32·1–52·8)	40·9 (31·8–51·4)
Larynx cancer	82·1 (73·4–88·3)	81·4 (73·4–87·1)	78·9 (71–85·2)	81·6 (73·6–87·3)
Tracheal, bronchus, and lung cancer	85 (81·4–88·6)	83·8 (80·1–87·4)	84·5 (80·8–88·1)	83·7 (80·1–87·3)
Breast cancer	36·2 (26·6–47·1)	34·7 (26·3–44·4)	34·9 (26·3–44·4)	34·7 (26·2–44·4)
Cervical cancer	100 (100–100)	100 (100–100)	100 (100–100)	100 (100–100)
Uterine cancer	42·7 (27·7–57·9)	43·7 (28·8–59·1)	44 (29·3–59·6)	43·7 (28·8–59)
Ovarian cancer	23·6 (12·4–37·4)	19·8 (9·91–32·1)	18·8 (9·6–31)	19·8 (9·91–32·2)
Prostate cancer	5·54 (2·36–9·07)	5·98 (2·66–9·57)	6·3 (2·83–9·94)	5·93 (2·64–9·56)
Kidney cancer	37·3 (27·5–47·8)	37·1 (27·6–46·8)	36·8 (27·2–46·3)	37·1 (27·6–46·8)
Bladder cancer	42·1 (27·6–57·3)	43·9 (30·4–57·4)	44·3 (31·3–57·1)	43·9 (30·4–57·5)
Thyroid cancer	13·9 (6·54–23·3)	14·5 (6·78–24·2)	14·5 (6·66–24·5)	14·4 (6·77–24·2)
Mesothelioma	98·5 (98–98·9)	97·1 (96·1–97·8)	98·3 (97·6–98·9)	97 (96–97·8)
Non-Hodgkin lymphoma	6·79 (2·75–12·1)	6·94 (2·89–12·3)	6·9 (2·88–12·5)	6·95 (2·9–12·3)
Multiple myeloma	7·7 (3·24–13·6)	8·03 (3·39–14·2)	7·97 (3·32–14·2)	8·04 (3·36–14·2)
Leukemia	35·2 (22·1–49·8)	31·9 (21–43·8)	30·3 (20–41·6)	32 (21·1–44)
Cardiovascular diseases	77·6 (73·8–81·1)	79 (75·7–81·8)	69·5 (64·3–73·9)	80·2 (77–82·8)
Rheumatic heart disease	21·6 (11·9–42·3)	22·4 (13·4–38·2)	22·4 (13·6–37·7)	22·4 (13·4–38·2)
Ischemic heart disease	93·4 (89·6–96·6)	94·7 (91·9–96·9)	93·9 (90·9–96·5)	94·7 (92–96·9)
Stroke	79·3 (71·6–88)	82·7 (77–88·4)	81·8 (76·5–87·2)	82·8 (77–88·8)
Hypertensive heart disease	100 (100–100)	100 (100–100)	100 (100–100)	100 (100–100)
Non-rheumatic valvular heart disease	24·7 (15·5–37·6)	25·5 (17·5–35·5)	18·4 (11·9–26·3)	26·3 (17·9–36·8)
Cardiomyopathy and myocarditis	36·7 (29·3–44·9)	44 (37·8–49·9)	38·6 (32·2–45)	44·3 (38·1–50·4)
Atrial fibrillation and flutter	48·8 (38·7–60·1)	56·8 (47·7–65·8)	60·8 (52·1–69)	52·4 (43·1–62·3)
Aortic aneurysm	50 (43·3–57·2)	57·3 (51·1–63·3)	--	57·3 (51·1–63·3)
Endocarditis	30·1 (20·2–42·7)	32·4 (23·3–42·9)	29·6 (19·8–42)	32·4 (23·4–42·9)
Other cardiovascular and circulatory diseases	29·8 (23·4–36·7)	31·7 (25·5–38·2)	31·8 (24·2–40·3)	31·7 (25·5–38)
Chronic respiratory diseases	62·7 (56·6–68·7)	55·1 (50·5–59·8)	46·8 (41·4–52·5)	63 (57·8–68·6)
Chronic obstructive pulmonary disease	71·1 (65·6–76·3)	69·9 (65·8–73·8)	63·4 (59·7–67·4)	72·9 (68·2–77·5)
Pneumoconiosis	100 (100–100)	100 (100–100)	100 (100–100)	100 (100–100)
Asthma	40·8 (29·2–53·7)	37·4 (29·3–46·9)	37 (29·1–46·4)	41·3 (31·5–52)

Digestive diseases	31·4 (26–36·8)	36·6 (30·5–42·8)	26·5 (18·2–35·8)	43·5 (36·4–50·1)
Cirrhosis and other chronic liver diseases	74·3 (61·2–86·8)	76·9 (64·4–88·6)	72·8 (60·8–84·1)	77 (64·3–88·8)
Upper digestive system diseases	21·5 (17·8–25)	8·42 (5·45–11·9)	0·723 (0·413–1·14)	23·9 (20·3–27·2)
Gallbladder and biliary diseases	43·2 (28·3–58·2)	46·8 (33–61·3)	47·1 (33·4–61·8)	44·3 (30·1–59)
Pancreatitis	38·1 (18–57·2)	44 (23–62·6)	44·4 (23–63·1)	43·9 (22·9–62·6)
Neurological disorders	24·9 (11·3–40)	13·7 (6·42–24·7)	8·24 (3·82–16·6)	21·4 (10·3–35·3)
Alzheimer's disease and other dementias	40·5 (26·3–56·1)	42·2 (28·1–57·4)	42·3 (28·1–57·5)	42·1 (27·9–57·2)
Parkinson's disease	-8·71 (-14·4–-4·05)	-9·7 (-15·7–-4·55)	-10·2 (-16·2–-4·72)	-9·58 (-15·6–-4·45)
Idiopathic epilepsy	27·2 (17–37·7)	24·3 (14·8–33·9)	21·9 (13·1–31)	27·2 (17–37·1)
Multiple sclerosis	14·6 (10·7–18·7)	14·8 (11·1–18·7)	14·3 (10·7–18·1)	15·2 (11·3–19·4)
Mental disorders	--	6·78 (3·41–10·5)	6·78 (3·42–10·5)	--
Depressive disorders	--	14·2 (6·38–22·2)	14·2 (6·38–22·2)	--
Anxiety disorders	--	5·43 (1·42–12·2)	5·43 (1·42–12·2)	--
Idiopathic developmental intellectual disability	--	37·6 (16·1–56·1)	37·6 (16·1–56·1)	--
Substance use disorders	100 (100–100)	100 (100–100)	100 (100–100)	100 (100–100)
Alcohol use disorders	100 (100–100)	100 (100–100)	100 (100–100)	100 (100–100)
Drug use disorders	100 (100–100)	100 (100–100)	100 (100–100)	100 (100–100)
Diabetes and kidney diseases	99·7 (99·6–99·7)	99·9 (99·9–100)	100 (100–100)	99·7 (99·6–99·7)
Diabetes mellitus	100 (100–100)	100 (100–100)	100 (100–100)	100 (100–100)
Chronic kidney disease	100 (100–100)	100 (100–100)	100 (100–100)	100 (100–100)
Sense organ diseases	--	8·3 (7·25–9·52)	8·3 (7·25–9·52)	--
Blindness and vision loss	--	8·41 (5·36–12·3)	8·41 (5·36–12·3)	--
Age-related and other hearing loss	--	9·53 (8·58–10·6)	9·53 (8·58–10·6)	--
Musculoskeletal disorders	2·08 (0·494–3·9)	20·6 (16·9–24·3)	21·1 (17·3–24·9)	2·42 (0·597–4·4)
Rheumatoid arthritis	11·5 (2·63–21)	12·9 (2·98–23)	12·9 (3·02–22·9)	12·8 (2·95–22·9)
Osteoarthritis	--	16·2 (8·37–25·1)	16·2 (8·37–25·1)	--
Low back pain	--	38·3 (33·2–43·6)	38·3 (33·2–43·6)	--
Gout	--	48·9 (33·6–65·8)	48·9 (33·6–65·8)	--
Other non-communicable diseases	0·0935 (0·0624–0·138)	0·0795 (0·049–0·12)	--	0·333 (0·219–0·453)
Sudden infant death syndrome	8·87 (5·11–14·6)	8·86 (5·1–14·6)	--	8·86 (5·1–14·6)
Injuries	29·3 (23·5–34·8)	24·9 (20·4–29·3)	27·3 (23·1–31·5)	21·5 (14·9–28·1)
Transport injuries	20 (12·7–28·3)	20·2 (13·9–27·1)	25·8 (21·6–30·3)	17·5 (9·59–26·2)
Road injuries	19·1 (11·4–27·9)	19·2 (12·7–26·5)	24·8 (20·5–29·4)	16·7 (8·4–26)
Other transport injuries	31·7 (26·5–36·9)	30·5 (25·5–35·4)	33·7 (29–37·9)	27·9 (22·5–33·7)

Unintentional injuries	36·2 (30·2–40·7)	26·1 (21·6–30·4)	27·1 (22·6–31·6)	22·7 (18·5–26·7)
Falls	54·2 (45·9–59·5)	37·5 (31·5–42·4)	35·9 (30·1–40·9)	44·2 (37·6–49·4)
Drowning	-15 (-28–-1·19)	-14·5 (-26·6–-1·59)	10·4 (6·03–16·5)	-16·1 (-28·8–-2·45)
Fire, heat, and hot substances	10·7 (6–16·9)	12·7 (7·83–18·8)	13·1 (8·08–19·5)	11·6 (7·13–17·3)
Poisonings	13·2 (8·05–19·6)	12·3 (7·61–18)	11·4 (7·09–16·9)	13·4 (8·47–19·6)
Exposure to mechanical forces	7·1 (-4·15–18·8)	15·8 (13–18·6)	17 (14·5–19·5)	-0·868 (-12·9–12)
Animal contact	23·8 (19·2–29·1)	19·8 (15·2–25·1)	20·4 (15·5–26·1)	17·5 (13·5–22·3)
Foreign body	3·17 (1·98–4·71)	3·89 (2·5–5·78)	3·3 (2·18–4·89)	4·3 (2·68–6·41)
Environmental heat and cold exposure	8·2 (3·76–14·7)	9·05 (4·32–16)	9·08 (4·34–16)	8·81 (4·16–15·4)
Exposure to forces of nature	0 (0–0)	10·1 (4·84–17·5)	10·1 (4·84–17·5)	0 (0–0)
Other unintentional injuries	13·8 (8·46–20·4)	12·3 (7·43–18·2)	12·2 (7·34–18·1)	14·8 (9·43–21·2)
Self-harm and interpersonal violence	22·4 (9·11–35·1)	24·4 (12·2–35·9)	34 (26·2–41·5)	22·8 (9·3–35·4)
Self-harm	22·2 (7·74–35·9)	23 (8·43–36·7)	23·3 (10·6–36·1)	23 (8·41–36·7)
Interpersonal violence	25·2 (16·5–33·8)	31·6 (23·5–40·3)	39·1 (30·3–48·3)	22·1 (13·8–30·6)

Supplementary Results Table 2. All-age PAFs attributable to the joint distribution of all risk factors for all ages, both sexes combined for each GBD level 1, level 2, and level 3 cause and all causes in 2019 for Ealing. Results are shown for deaths, YLLs, YLDs, and DALYs Data in parenthesis are 95% uncertainty intervals. PAFs=population attributable fractions. GBD=Global Burden of Disease. YLLs=years of life lost. YLDs=years lived with disability. DALYs=disability-adjusted life-years				
	**Deaths 2019**	**DALYs 2019**	**YLDs 2019**	**YLLs 2019**
All causes	55 (51·7–58·1)	39·1 (36·1–42·1)	27·7 (25·4–30·1)	54·7 (52·2–57·2)
Communicable, maternal, neonatal, and nutritional diseases	48·1 (37·8–57·9)	53·8 (49·2–58·4)	48·2 (43·8–52·4)	57·5 (50·1–64·4)
HIV/AIDS and sexually transmitted infections	80·2 (68·7–88)	79·4 (66·3–88)	96·1 (94·2–97·4)	68·4 (52·4–80·9)
HIV/AIDS	91·3 (89·2–92·6)	89·8 (87·2–91·6)	94 (91·5–95·6)	87·4 (84·9–88·9)
Sexually transmitted infections excluding HIV	22·1 (9·38–45·1)	54·4 (28·8–81)	100 (100–100)	7·18 (2·58–17·4)
Respiratory infections and tuberculosis	48·5 (37·2–59·7)	43·6 (34·3–53·1)	3·76 (2·73–5·13)	52·3 (41·6–62·9)
Tuberculosis	58·5 (47·2–68·4)	59·8 (49·3–68·3)	55·3 (43·7–64·8)	60·8 (50·4–69·5)
Lower respiratory infections	48·4 (36·8–59·7)	52 (41·3–62·7)	36·2 (29·5–42·9)	52·1 (41·3–62·8)
Upper respiratory infections	0·0807 (0·0478–0·129)	0·00868 (0·00108–0·0221)	0·00815 (0·000573–0·0218)	0·171 (0·0983–0·277)
Otitis media	3·39 (1·73–5·93)	5·06 (3·27–7·03)	5·02 (3·23–7)	7·4 (4·21–11·5)
Enteric infections	4·72 (3·06–7·84)	9·66 (7·39–12·8)	9·8 (7·31–13)	9·23 (6·63–12·4)
Diarrheal diseases	4·76 (3·08–7·87)	9·7 (7·41–12·9)	9·81 (7·31–13)	9·37 (6·75–12·6)
Other infectious diseases	0·779 (0·532–1·15)	1·16 (0·876–1·52)	0·35 (0·196–0·594)	1·53 (1·13–2·09)
Meningitis	1·99 (1·44–2·66)	2·56 (1·85–3·48)	0·000646 (3·28e-05–0·00171)	3·72 (2·72–4·96)
Encephalitis	0·441 (0·279–0·637)	0·979 (0·529–1·46)	0·000273 (1·34e-05–0·000721)	1·26 (0·789–1·8)
Measles	3·19 (-0·985–15·2)	2·61 (-0·615–11·8)	0·377 (0·0144–1·08)	4·02 (-1·27–18·9)
Acute hepatitis	12·7 (6·2–22·9)	5 (2·75–8·41)	2·55 (1·48–4·39)	10·8 (5·03–20·4)
Maternal and neonatal disorders	88·4 (86·3–90·3)	77·4 (73·8–80·6)	64·5 (58·9–69·4)	89 (86·9–90·8)
Maternal disorders	9·25 (3·34–15·4)	9·41 (3·26–15·9)	9·53 (3·2–16·2)	9·08 (3·25–15·1)
Neonatal disorders	89·9 (87·9–91·8)	79 (75·5–82·3)	66·5 (60·5–71·7)	90 (87·9–91·8)
Nutritional deficiencies	67·3 (61·1–88·3)	78·7 (74·4–83·4)	78·8 (74·5–83·6)	68·1 (64·1–79·8)
Protein-energy malnutrition	100 (100–100)	100 (100–100)	100 (100–100)	100 (100–100)
Vitamin A deficiency	--	100 (100–100)	100 (100–100)	--
Dietary iron deficiency	--	100 (100–100)	100 (100–100)	--
Non-communicable diseases	56·7 (53·2–60·2)	39·1 (35·8–42·5)	26·9 (24·3–29·5)	56·8 (54·2–59·6)
Neoplasms	47·3 (43·5–51·6)	45·8 (42·1–49·8)	31·9 (27·8–36·6)	46·7 (43–50·6)
Lip and oral cavity cancer	69·6 (59·1–78)	72 (62·1–79·6)	71·1 (60·8–79·2)	72 (62·1–79·6)
Nasopharynx cancer	66·9 (55·5–75·7)	66·1 (54·6–74·9)	64·3 (52·5–73·5)	66·3 (54·8–75)
Other pharynx cancer	79·8 (72·1–85·6)	80·8 (73·4–86·4)	79·6 (72–85·6)	80·8 (73·5–86·4)
Esophageal cancer	76·3 (67·7–84·6)	77·4 (69·1–85·5)	76·5 (67·9–84·9)	77·4 (69·1–85·5)
Stomach cancer	24·5 (17·2–41)	24·4 (17·1–40·8)	24·6 (17·2–41)	24·4 (17·1–40·8)

Colon and rectum cancer	64·4 (55·1–73·3)	64·5 (56–72·8)	64·5 (55·7–73)	64·5 (56–72·8)
Liver cancer	76·3 (70·4–82)	76·2 (70·7–81·4)	76·5 (70·9–81·7)	76·2 (70·7–81·4)
Gallbladder and biliary tract cancer	22·9 (12·5–35·4)	23·2 (12·7–35·7)	21·7 (11·6–34·3)	23·2 (12·7–35·9)
Pancreatic cancer	42·6 (32·7–54·5)	41·7 (32·4–52·8)	42·4 (32·6–54·1)	41·7 (32·4–52·7)
Larynx cancer	82·8 (75·1–88·8)	82 (74·8–87·8)	79·7 (72·3–85·9)	82·3 (75–88)
Tracheal, bronchus, and lung cancer	85·4 (81·8–89)	84·2 (80·6–87·8)	84·9 (81·1–88·5)	84·2 (80·6–87·8)
Breast cancer	37·3 (27·3–48·3)	35·8 (27·1–45·9)	36 (27·2–46·2)	35·8 (27·1–45·7)
Cervical cancer	100 (100–100)	100 (100–100)	100 (100–100)	100 (100–100)
Uterine cancer	44·7 (29·5–59·7)	45·6 (30·5–60·8)	45·9 (30·8–61·3)	45·6 (30·5–60·7)
Ovarian cancer	23·2 (11·5–37·9)	19·7 (9·67–32·9)	18·8 (9·01–31·4)	19·7 (9·68–33)
Prostate cancer	5·74 (2·53–9·27)	6·23 (2·83–9·99)	6·59 (3·01–10·5)	6·17 (2·8–9·89)
Kidney cancer	38·8 (28·9–49·4)	38·4 (28·5–48·3)	38 (28·1–47·7)	38·4 (28·5–48·3)
Bladder cancer	43·1 (28·4–57·4)	44·9 (31·6–57·6)	45·1 (32·1–57·5)	44·8 (31·6–57·6)
Thyroid cancer	15·1 (7·14–25·2)	15·7 (7·41–26·2)	15·7 (7·38–26·2)	15·6 (7·37–26·1)
Mesothelioma	97·9 (97·2–98·5)	95·9 (94·6–96·9)	97·7 (96·7–98·4)	95·8 (94·6–96·9)
Non-Hodgkin lymphoma	7·32 (3·05–13)	7·48 (3·18–13·1)	7·43 (3·16–13·3)	7·48 (3·18–13·1)
Multiple myeloma	8·29 (3·57–14·8)	8·62 (3·77–15·2)	8·54 (3·66–15·1)	8·62 (3·79–15·3)
Leukemia	36·1 (22·6–49·9)	32·3 (21·8–43·5)	31·4 (21–42·5)	32·4 (21·8–43·6)
Cardiovascular diseases	77·8 (73·8–81·8)	79·1 (75·8–82·2)	69·3 (64·2–73·9)	80·5 (77·4–83·7)
Rheumatic heart disease	22·2 (12·1–42·4)	23·1 (14·2–39·2)	22·9 (14·4–38)	23·1 (14·2–39·4)
Ischemic heart disease	93·9 (90·4–96·8)	95·2 (92·7–97·3)	94·2 (91·4–96·7)	95·2 (92·7–97·3)
Stroke	80·3 (72·3–88·8)	83·7 (78·4–89·2)	83 (78·3–87·8)	83·9 (78·1–89·6)
Hypertensive heart disease	100 (100–100)	100 (100–100)	100 (100–100)	100 (100–100)
Non-rheumatic valvular heart disease	25·6 (16·6–38·3)	26·1 (18·3–36)	17·3 (11·3–24·4)	27·4 (19·1–37·9)
Cardiomyopathy and myocarditis	32·2 (24·3–42·3)	37·6 (30·7–45·3)	33·7 (27·3–40·4)	37·9 (30·9–46)
Atrial fibrillation and flutter	50·6 (39·9–62·2)	58·9 (49·6–67·7)	62·4 (53·4–70·7)	54·2 (44·5–64·4)
Aortic aneurysm	51·2 (44·8–58·2)	58·8 (53–64·5)	--	58·8 (53–64·5)
Endocarditis	30·6 (21–42·9)	33·1 (24·4–43·5)	30·4 (20·5–42·5)	33·2 (24·5–43·5)
Other cardiovascular and circulatory diseases	30·7 (24·3–37·7)	33·1 (26·7–39·2)	33·4 (25·3–41·2)	32·9 (26·8–39)
Chronic respiratory diseases	62·4 (55·7–69·1)	54·1 (49–59·2)	47·4 (41·6–53·3)	62·3 (56·4–68·4)
Chronic obstructive pulmonary disease	72·7 (67–78·4)	70·8 (66·5–74·9)	64·5 (60·6–68·3)	74·6 (69·4–79·6)
Pneumoconiosis	100 (100–100)	100 (100–100)	100 (100–100)	100 (100–100)
Asthma	42·6 (31–55·4)	39·1 (30·9–48·6)	38·8 (30·6–48·3)	43 (33·2–53·6)
Digestive diseases	36·7 (30·4–42·8)	39·5 (33–45·9)	28·2 (19·5–37·8)	49·7 (41·8–56·5)

Cirrhosis and other chronic liver diseases	77·2 (64·3–88·6)	79·3 (67·4–90·1)	75·1 (63·2–85·1)	79·4 (67·4–90·2)
Upper digestive system diseases	21·7 (18·2–25·4)	6·63 (4·11–9·73)	0·609 (0·351–0·95)	24 (20·7–27·5)
Gallbladder and biliary diseases	45 (29·9–60·6)	48·6 (34·4–63·1)	48·9 (34·6–63·6)	46 (31·3–61)
Pancreatitis	43·4 (22·3–62·5)	48·8 (27·5–67·2)	48·4 (26·9–67)	48·9 (27·7–67·2)
Neurological disorders	27·4 (13·2–42·8)	13·8 (6·39–25·1)	7·96 (3·61–16·4)	23·4 (11·5–37·7)
Alzheimer's disease and other dementias	41·6 (27·2–57·3)	43·5 (29·3–59)	43·7 (29·4–59·1)	43·4 (29·1–58·9)
Parkinson's disease	-9·09 (-15·5–-3·98)	-10·2 (-16·9–-4·67)	-10·8 (-17·6–-4·91)	-10 (-16·8–-4·59)
Idiopathic epilepsy	28·2 (18·2–38·6)	25·2 (16·1–34·2)	23·1 (14·5–32·1)	28 (18·3–37·5)
Multiple sclerosis	15 (11·3–19)	15·1 (11·5–19·1)	14·5 (11–18·4)	15·7 (11·7–19·9)
Mental disorders	--	6·82 (3·41–10·6)	6·82 (3·41–10·6)	--
Depressive disorders	--	14·3 (6·45–22·3)	14·3 (6·45–22·3)	--
Anxiety disorders	--	5·47 (1·41–12·4)	5·47 (1·41–12·4)	--
Idiopathic developmental intellectual disability	--	37·4 (16·2–56)	37·4 (16·2–56)	--
Substance use disorders	100 (100–100)	100 (100–100)	100 (100–100)	100 (100–100)
Alcohol use disorders	100 (100–100)	100 (100–100)	100 (100–100)	100 (100–100)
Drug use disorders	100 (100–100)	100 (100–100)	100 (100–100)	100 (100–100)
Diabetes and kidney diseases	99·5 (99·4–99·6)	99·9 (99·9–99·9)	100 (100–100)	99·5 (99·4–99·6)
Diabetes mellitus	100 (100–100)	100 (100–100)	100 (100–100)	100 (100–100)
Chronic kidney disease	100 (100–100)	100 (100–100)	100 (100–100)	100 (100–100)
Sense organ diseases	--	8·55 (7·47–9·81)	8·55 (7·47–9·81)	--
Blindness and vision loss	--	8·42 (5·38–12·4)	8·42 (5·38–12·4)	--
Age-related and other hearing loss	--	9·91 (8·96–10·9)	9·91 (8·96–10·9)	--
Musculoskeletal disorders	2·84 (0·668–5·3)	21·2 (17·6–25)	21·6 (17·9–25·4)	3·32 (0·838–6·06)
Rheumatoid arthritis	12 (2·98–21·6)	13·3 (3·09–23·5)	13·2 (3·08–23·6)	13·4 (3·52–23·7)
Osteoarthritis	--	17·1 (9·12–26·2)	17·1 (9·12–26·2)	--
Low back pain	--	39·5 (34·5–44·6)	39·5 (34·5–44·6)	--
Gout	--	50·5 (34·6–67·7)	50·5 (34·6–67·7)	--
Other non-communicable diseases	0·111 (0·0699–0·161)	0·0779 (0·0465–0·118)	--	0·392 (0·234–0·549)
Sudden infant death syndrome	8·57 (4·94–13·6)	8·56 (4·93–13·6)	--	8·56 (4·93–13·6)
Injuries	28·6 (22·8–34·2)	24·6 (20·3–29·4)	26·5 (22·5–31·1)	22 (15·2–28·7)
Transport injuries	21·3 (13–29)	21·4 (14·4–28)	25·9 (21·4–30·7)	19·1 (10·1–27·1)
Road injuries	20·5 (11·9–28·6)	20·5 (13·2–27·3)	25 (20·4–29·8)	18·4 (9·06–26·8)
Other transport injuries	31·4 (25·7–36·8)	30·2 (25–35·3)	33·4 (29–37·8)	27·7 (21·7–33·2)
Unintentional injuries	33·9 (28·2–38·6)	24·7 (20·5–29·5)	26 (21·7–31)	19·9 (15·7–24·7)

Falls	52·9<br>(45–58·6)	35·8<br>(30·2–41·5)	34·5<br>(28·9–40·1)	42·3<br>(35·9–48)
Drowning	-15<br>(-28·1–-0·658)	-14·9<br>(-27·3–-1·14)	11·3<br>(6·7–18·1)	-16<br>(-28·9–-1·99)
Fire, heat, and hot substances	11·3<br>(6·33–17·7)	13·4<br>(8·37–20·3)	14·1<br>(8·7–21·3)	11·8<br>(7·19–17·9)
Poisonings	15·1<br>(9·48–22·5)	13·7<br>(8·53–20·4)	12·6<br>(7·95–19·2)	15·1<br>(9·72–22)
Exposure to mechanical forces	5·93<br>(-6·76–19·1)	15·4<br>(12·5–18·2)	16·5<br>(14–19·3)	-1·64<br>(-15·2–12·8)
Animal contact	19·3<br>(14·9–25·1)	19·6<br>(14·7–26)	20·6<br>(15·5–27)	14·4<br>(10·7–19·5)
Foreign body	3<br>(1·94–4·41)	3·86<br>(2·54–5·67)	3·59<br>(2·37–5·26)	4·08<br>(2·63–6·02)
Environmental heat and cold exposure	8·78<br>(4–15·6)	9·6<br>(4·45–17)	9·57<br>(4·36–17·2)	9·72<br>(4·54–17·3)
Exposure to forces of nature	0<br>(0–0)	10·4<br>(4·84–18·5)	10·4<br>(4·84–18·5)	0<br>(0–0)
Other unintentional injuries	14·5<br>(8·74–21·9)	13·3<br>(8·15–20)	13·2<br>(8·11–19·9)	15·4<br>(9·77–22·7)
Self-harm and interpersonal violence	24·6<br>(11·7–38)	26·5<br>(14·9–38·2)	35·1<br>(27·2–42·9)	25<br>(12·2–38·2)
Self-harm	24·6<br>(10·6–38·9)	25·4<br>(11·4–39·6)	25<br>(12·2–37·9)	25·4<br>(11·4–39·7)
Interpersonal violence	26·3<br>(18·2–35·3)	32·9<br>(25–41·5)	39·5<br>(30·7–48·6)	22·7<br>(15·2–30·8)

Supplementary Results Table 2. All-age PAFs attributable to the joint distribution of all risk factors for all ages, both sexes combined for each GBD level 1, level 2, and level 3 cause and all causes in 2019 for Enfield. Results are shown for deaths, YLLs, YLDs, and DALYs Data in parenthesis are 95% uncertainty intervals. PAFs=population attributable fractions. GBD=Global Burden of Disease. YLLs=years of life lost. YLDs=years lived with disability. DALYs=disability-adjusted life-years

	Deaths 2019	DALYs 2019	YLDs 2019	YLLs 2019
All causes	53·7 (50·1–57·4)	38·4 (35·3–41·6)	26·7 (24·4–28·9)	53·2 (50·4–56)
Communicable, maternal, neonatal, and nutritional diseases	47 (36·3–56·8)	54·9 (50–59·4)	49·3 (44·4–53·8)	58·6 (51–65·4)
HIV/AIDS and sexually transmitted infections	76·1 (62·9–86)	75·4 (60–86·4)	96·6 (94·9–97·8)	61·5 (44·4–76·4)
HIV/AIDS	89·9 (87·9–91·2)	88 (85·6–89·9)	94·1 (91·7–95·6)	84·6 (82·2–86·2)
Sexually transmitted infections excluding HIV	31·9 (14·7–59·2)	54·2 (28·1–80·6)	100 (100–100)	10·4 (3·75–24·5)
Respiratory infections and tuberculosis	46·5 (34·8–57·8)	41·5 (31·9–51)	3·24 (2·37–4·35)	50·2 (38·8–60·6)
Tuberculosis	58·1 (46·6–68)	58·9 (48·5–67·9)	52·9 (40·9–63)	60·7 (50·3–69·7)
Lower respiratory infections	46·5 (34·6–57·8)	50 (38·5–60·6)	33·6 (27·1–40)	50·2 (38·6–60·7)
Upper respiratory infections	0·0493 (0·0282–0·0811)	0·0102 (0·00162–0·0241)	0·00887 (0·000276–0·0229)	0·11 (0·0615–0·188)
Otitis media	4·01 (2·07–6·97)	5·22 (3·3–7·33)	5·19 (3·27–7·31)	8·49 (5·05–12·9)
Enteric infections	5·17 (3·23–8·33)	10·2 (7·77–13·4)	10·3 (7·73–13·6)	10·1 (6·93–14·2)
Diarrheal diseases	5·23 (3·28–8·46)	10·3 (7·82–13·5)	10·3 (7·74–13·6)	10·3 (7·14–14·4)
Other infectious diseases	1·1 (0·635–1·47)	1·74 (1·23–2·27)	0·323 (0·184–0·559)	2·46 (1·67–3·28)
Meningitis	2·61 (1·9–3·53)	3·68 (2·7–4·97)	0·000793 (1·46e-05–0·00226)	4·97 (3·66–6·63)
Encephalitis	0·564 (0·354–0·842)	1·22 (0·674–1·87)	0·00026 (5·16e-06–0·000707)	1·68 (1·03–2·52)
Measles	3·17 (-0·823–15·3)	3·2 (-0·679–15·1)	0·475 (0·015–1·27)	3·87 (-1·01–18·3)
Acute hepatitis	13·8 (8·48–23)	4·76 (2·94–7·28)	2·52 (1·51–4·18)	12·5 (7·61–20)
Maternal and neonatal disorders	88·8 (86·6–90·9)	79·7 (76·4–83)	66·8 (61·3–72·1)	89·5 (87·4–91·5)
Maternal disorders	9·31 (3·32–15·8)	9·36 (3·3–16·1)	9·48 (3·33–16·3)	9·17 (3·27–15·8)
Neonatal disorders	90·9 (88·7–92·7)	81·2 (77·9–84·6)	68·6 (62·8–74·2)	90·9 (88·7–92·7)
Nutritional deficiencies	69·2 (62·8–89·7)	78·9 (74·6–84)	79 (74·7–84·2)	67·4 (64–79)
Protein-energy malnutrition	100 (100–100)	100 (100–100)	100 (100–100)	100 (100–100)
Vitamin A deficiency	--	100 (100–100)	100 (100–100)	--
Dietary iron deficiency	--	100 (100–100)	100 (100–100)	--
Non-communicable diseases	55·2 (51·5–59·4)	38·2 (34·9–41·8)	25·5 (23·1–28·1)	54·7 (51·7–57·9)
Neoplasms	46·6 (42·6–51)	45·3 (41·7–49·2)	31·6 (27·7–35·9)	46·1 (42·7–50·1)
Lip and oral cavity cancer	66·8 (55·8–76·2)	69·5 (59·4–78·1)	68·7 (58·2–77·7)	69·6 (59·4–78·1)
Nasopharynx cancer	66·1 (53·3–76·4)	65·5 (53·3–75)	63·6 (51·2–74·4)	65·7 (53·4–75·1)
Other pharynx cancer	78·1 (70–84·7)	79·2 (71–85·7)	78 (69·8–84·7)	79·3 (71·1–85·7)
Esophageal cancer	74·5 (65–83)	75·8 (66·6–84)	74·9 (65·2–83·3)	75·8 (66·6–84·1)

Stomach cancer	23·9 (16·3–40·6)	23·9 (16·5–40·4)	24 (16·6–40·9)	23·9 (16·5–40·5)
Colon and rectum cancer	63·2 (54·1–72·1)	63·3 (54·9–71·4)	63·4 (54·9–71·7)	63·3 (54·9–71·4)
Liver cancer	76·2 (70·3–82)	75·9 (70·6–81·1)	76·2 (70·6–81·5)	75·9 (70·5–81·1)
Gallbladder and biliary tract cancer	20·7 (11·1–32·7)	20·9 (11·2–32·9)	19·5 (9·96–31·9)	21 (11·3–32·9)
Pancreatic cancer	40·8 (31·2–52·1)	39·9 (31·1–50·6)	40·6 (31·3–51·7)	39·9 (31·1–50·6)
Larynx cancer	81·7 (73·3–88·5)	81·1 (73·1–87·2)	78·5 (70·3–85·2)	81·4 (73·5–87·5)
Tracheal, bronchus, and lung cancer	85·1 (81·6–88·7)	84 (80·4–87·7)	84·6 (80·9–88·2)	84 (80·4–87·7)
Breast cancer	35·3 (25·8–46·1)	34 (25·6–43·2)	34·2 (25·6–43·5)	34 (25·5–43·2)
Cervical cancer	100 (100–100)	100 (100–100)	100 (100–100)	100 (100–100)
Uterine cancer	42·3 (27·4–57·9)	43·3 (28·8–58·4)	43·6 (29–59)	43·3 (28·7–58·4)
Ovarian cancer	21·6 (10·8–35·3)	18 (8·55–30·4)	17·1 (8·31–29·2)	18·1 (8·54–30·5)
Prostate cancer	5·52 (2·39–8·89)	5·97 (2·66–9·45)	6·3 (2·84–9·81)	5·91 (2·64–9·33)
Kidney cancer	37 (26·8–47·2)	36·8 (26·9–46·6)	36·4 (26·8–46·3)	36·8 (26·9–46·6)
Bladder cancer	41·5 (26·6–56·1)	43·4 (29·6–57)	43·8 (30·2–57)	43·4 (29·6–57)
Thyroid cancer	13·7 (6·47–22·7)	14·2 (6·7–23·7)	14·3 (6·7–23·7)	14·2 (6·7–23·7)
Mesothelioma	98·5 (97·9–98·9)	96·9 (95·9–97·8)	98·2 (97·4–98·8)	96·9 (95·8–97·8)
Non-Hodgkin lymphoma	6·77 (2·77–12·2)	6·95 (2·9–12·4)	6·88 (2·82–12·4)	6·95 (2·91–12·4)
Multiple myeloma	7·59 (3·17–13·7)	7·93 (3·38–14·2)	7·86 (3·32–14·2)	7·93 (3·39–14·2)
Leukemia	34·9 (21·9–49)	31·6 (21–43)	30·4 (19·9–41·1)	31·7 (21·1–43)
Cardiovascular diseases	74·5 (69·6–80·4)	76·6 (72·8–81·6)	69·3 (64·1–74)	77·6 (73·7–82·8)
Rheumatic heart disease	21·5 (11·5–43·4)	22·5 (13·9–38·4)	22·2 (14–36·6)	22·5 (13·8–38·4)
Ischemic heart disease	93·3 (89·4–96·6)	94·6 (91·9–97)	93·9 (91–96·5)	94·6 (91·9–97)
Stroke	79·1 (70–88·2)	82·3 (76–88·6)	81·4 (76–86·9)	82·5 (75·9–89)
Hypertensive heart disease	100 (100–100)	100 (100–100)	100 (100–100)	100 (100–100)
Non-rheumatic valvular heart disease	24·8 (15·6–38·1)	25·9 (17·8–36·8)	21·8 (14·3–30·6)	26·5 (18·1–37·7)
Cardiomyopathy and myocarditis	27·6 (18·1–39·3)	32 (24·2–42·3)	30·5 (23·8–38·2)	32·2 (24·1–43·5)
Atrial fibrillation and flutter	48·1 (37·8–59·6)	56·3 (47–65·5)	60·5 (51·5–69·2)	51·5 (41·8–62·1)
Aortic aneurysm	48·9 (42·2–56)	56·4 (50·6–62)	--	56·4 (50·6–62)
Endocarditis	29·7 (19·4–42·7)	32·3 (23·1–43)	29·8 (20·5–42·4)	32·4 (23·1–42·9)
Other cardiovascular and circulatory diseases	29·6 (23·2–36·2)	32·5 (26·5–38·1)	33·6 (26·7–40·8)	32·3 (26·2–37·8)
Chronic respiratory diseases	61·7 (55·3–67·9)	53·2 (48·5–58·2)	46 (40·3–51·7)	61·6 (56·3–66·9)
Chronic obstructive pulmonary disease	71·4 (65·2–77·2)	69·4 (65·1–73·8)	63 (59·1–66·8)	73·3 (67·8–78·5)
Pneumoconiosis	100 (100–100)	100 (100–100)	100 (100–100)	100 (100–100)
Asthma	40·3 (28·7–53)	36·1 (28·2–45·5)	35·7 (28–44·9)	40·8 (31–51·4)

Digestive diseases	30 (24·4–35·1)	34·2 (28·2–39·9)	24 (16·4–33·4)	42·7 (35·7–49)
Cirrhosis and other chronic liver diseases	74·2 (61·4–87·2)	76·9 (64·8–88·6)	71·4 (59·1–82·8)	77·1 (64·9–88·8)
Upper digestive system diseases	20·5 (17–24·1)	6·84 (4·34–10)	0·623 (0·355–0·984)	22·8 (19·2–26·1)
Gallbladder and biliary diseases	42·2 (27·8–57·3)	45·1 (31·8–59·5)	45·4 (32–59·7)	43·2 (29·4–58·3)
Pancreatitis	37·3 (16·7–58)	43·5 (22·4–63·4)	43·8 (23·7–63·5)	43·4 (22·5–63·4)
Neurological disorders	25·9 (12–40·4)	13·7 (6·29–24·6)	8·05 (3·71–16·3)	22·2 (10·6–35·6)
Alzheimer's disease and other dementias	39·3 (25·3–55)	41·2 (27–56·6)	41·4 (27·3–56·5)	41·1 (27·1–56·5)
Parkinson's disease	-8·59 (-14·1–-3·86)	-9·63 (-15·7–-4·45)	-10·1 (-16·2–-4·65)	-9·5 (-15·5–-4·34)
Idiopathic epilepsy	25·5 (15·2–35·9)	22·8 (13·5–32·4)	21 (12·3–30·6)	25·7 (15·8–35·6)
Multiple sclerosis	14·5 (10·8–18·3)	14·7 (11·1–18·7)	14·1 (10·7–17·9)	15·2 (11·4–19·2)
Mental disorders	--	6·97 (3·53–10·9)	6·97 (3·53–10·9)	--
Depressive disorders	--	14·4 (6·55–22·6)	14·4 (6·55–22·6)	--
Anxiety disorders	--	5·74 (1·54–12·8)	5·74 (1·54–12·8)	--
Idiopathic developmental intellectual disability	--	37·3 (16·3–55·1)	37·3 (16·3–55·1)	--
Substance use disorders	100 (100–100)	100 (100–100)	100 (100–100)	100 (100–100)
Alcohol use disorders	100 (100–100)	100 (100–100)	100 (100–100)	100 (100–100)
Drug use disorders	100 (100–100)	100 (100–100)	100 (100–100)	100 (100–100)
Diabetes and kidney diseases	99·6 (99·5–99·7)	99·9 (99·9–99·9)	100 (100–100)	99·6 (99·5–99·7)
Diabetes mellitus	100 (100–100)	100 (100–100)	100 (100–100)	100 (100–100)
Chronic kidney disease	100 (100–100)	100 (100–100)	100 (100–100)	100 (100–100)
Sense organ diseases	--	8·14 (7·13–9·35)	8·14 (7·13–9·35)	--
Blindness and vision loss	--	8·16 (5·23–12)	8·16 (5·23–12)	--
Age-related and other hearing loss	--	9·38 (8·5–10·3)	9·38 (8·5–10·3)	--
Musculoskeletal disorders	3·29 (0·752–6·13)	20·5 (17–24·2)	20·8 (17·2–24·6)	3·88 (0·937–6·97)
Rheumatoid arthritis	11 (2·48–20·1)	12·6 (3·13–22·3)	12·7 (3·16–22·3)	12·4 (2·98–22·3)
Osteoarthritis	--	16 (8·31–24·9)	16 (8·31–24·9)	--
Low back pain	--	37·7 (32·7–42·8)	37·7 (32·7–42·8)	--
Gout	--	48·6 (33·2–65·5)	48·6 (33·2–65·5)	--
Other non-communicable diseases	0·144 (0·0928–0·211)	0·108 (0·0646–0·169)	--	0·492 (0·301–0·707)
Sudden infant death syndrome	8·1 (4·52–13)	8·09 (4·51–13)	--	8·09 (4·51–13)
Injuries	29·3 (23·5–35·1)	24·9 (20·7–29·6)	27·2 (23·3–31·7)	21·6 (14·8–28·6)
Transport injuries	21 (12·6–29·4)	21 (14·1–28·3)	26 (21·5–30·8)	18·5 (9·47–27·5)
Road injuries	20·3 (11·4–28·9)	20·1 (12·9–27·7)	25 (20·4–29·8)	17·9 (8·52–27·1)
Other transport injuries	31·3 (25·6–36·8)	30·5 (25·4–35·8)	33·8 (29·3–38·4)	27·7 (21·3–34)

Unintentional injuries	36·4<br>(30·5–41)	25·9<br>(21·7–30·6)	26·8<br>(22·5–31·6)	22·1<br>(17·9–26·4)
Falls	54·2<br>(46·1–59·6)	36·9<br>(31·3–42·4)	35·5<br>(30·1–41)	44<br>(37·7–49·6)
Drowning	-14·9<br>(-28·8–-0·219)	-14·4<br>(-27·5–-0·817)	10·7<br>(6·31–16·7)	-15·9<br>(-29·6–-1·76)
Fire, heat, and hot substances	11<br>(6·39–17·5)	13<br>(8·25–19·6)	13·3<br>(8·46–20·2)	12·1<br>(7·58–18·2)
Poisonings	12·9<br>(8·17–19·5)	12·2<br>(7·77–18·2)	11·7<br>(7·35–17·7)	13<br>(8·56–19·4)
Exposure to mechanical forces	6·11<br>(-6·79–19·6)	16<br>(13–18·9)	17<br>(14·5–19·7)	-1·16<br>(-15–13·2)
Animal contact	17·9<br>(13·2–24)	19·2<br>(14·4–25)	20·3<br>(15·4–26·2)	14·4<br>(10·3–19·8)
Foreign body	3·11<br>(1·96–4·71)	3·86<br>(2·5–5·67)	3·48<br>(2·3–5·11)	4·21<br>(2·65–6·31)
Environmental heat and cold exposure	8·08<br>(3·65–14·8)	8·98<br>(4·06–15·8)	8·94<br>(4·06–15·9)	9·11<br>(4·24–15·9)
Exposure to forces of nature	0<br>(0–0)	9·96<br>(4·66–17·4)	9·96<br>(4·66–17·4)	0<br>(0–0)
Other unintentional injuries	14·5<br>(9·13–21·6)	12·5<br>(7·85–18·9)	12·4<br>(7·78–18·8)	15·3<br>(9·94–22·3)
Self-harm and interpersonal violence	22·6<br>(9·43–36·6)	24·9<br>(13·1–36·7)	34·4<br>(26·5–42·5)	23<br>(9·98–36·6)
Self-harm	22·7<br>(8·12–38·1)	23·5<br>(8·84–38·8)	23·2<br>(10·4–36·9)	23·5<br>(8·76–38·8)
Interpersonal violence	23·6<br>(15·2–32·5)	31·4<br>(23·1–39·8)	39·4<br>(30·6–48·6)	20·6<br>(12·7–29·4)

Supplementary Results Table 2. All-age PAFs attributable to the joint distribution of all risk factors for all ages, both sexes combined for each GBD level 1, level 2, and level 3 cause and all causes in 2019 for Greenwich. Results are shown for deaths, YLLs, YLDs, and DALYs Data in parenthesis are 95% uncertainty intervals. PAFs=population attributable fractions. GBD=Global Burden of Disease. YLLs=years of life lost. YLDs=years lived with disability. DALYs=disability-adjusted life-years

	Deaths 2019	DALYs 2019	YLDs 2019	YLLs 2019
All causes	55·6 (52·4–58·6)	39·8 (36·8–43)	27·3 (25–29·6)	55·2 (52·7–57·8)
Communicable, maternal, neonatal, and nutritional diseases	49·2 (39·3–58·8)	58·6 (54·1–62·9)	49·1 (44·2–53·6)	64·4 (58–70)
HIV/AIDS and sexually transmitted infections	80·6 (69·8–88·3)	78·9 (66·1–87·6)	95·9 (94–97·2)	68·3 (53·2–80·6)
HIV/AIDS	90·7 (88·7–92)	89·1 (86·5–90·8)	93·8 (91·4–95·5)	86·3 (83·9–87·9)
Sexually transmitted infections excluding HIV	43·2 (22·3–69·4)	54·8 (30–81·5)	100 (100–100)	16·9 (6·55–36·5)
Respiratory infections and tuberculosis	46·8 (35·3–58·3)	40·8 (32–50·1)	3·25 (2·35–4·42)	50·8 (40–61·5)
Tuberculosis	56·4 (44·6–66·2)	57·6 (46·4–66·6)	53·5 (41·7–63·9)	58·6 (47·6–67·5)
Lower respiratory infections	46·7 (35·1–58·4)	50·7 (39·7–61·7)	34·4 (27·7–41)	50·9 (39·8–61·9)
Upper respiratory infections	0·117 (0·059–0·204)	0·0137 (0·00437–0·0296)	0·00899 (0·000417–0·0238)	0·222 (0·111–0·392)
Otitis media	4·25 (2·26–7·28)	5·12 (3·26–7·27)	5·04 (3·17–7·17)	9·05 (5·35–14·2)
Enteric infections	5·35 (3·42–8·76)	11 (8·45–14·8)	11·1 (8·38–15)	10·7 (7·57–14·6)
Diarrheal diseases	5·4 (3·49–8·81)	11·1 (8·49–14·9)	11·1 (8·39–15)	10·9 (7·81–14·8)
Other infectious diseases	1·53 (0·996–2·02)	2·31 (1·67–3·01)	0·339 (0·185–0·572)	3·19 (2·31–4·12)
Meningitis	3·4 (2·43–4·47)	4·88 (3·57–6·41)	0·000843 (2·66e-05–0·00234)	6·04 (4·42–7·84)
Encephalitis	0·711 (0·47–1·02)	1·21 (0·707–1·83)	0·000247 (6·97e-06–0·000668)	1·89 (1·19–2·74)
Measles	3·49 (-1·05–18·1)	4·05 (-1·17–21·2)	0·497 (0·0126–1·35)	4·22 (-1·26–21·7)
Acute hepatitis	13 (6·44–24·3)	4·49 (2·52–7·44)	2·49 (1·43–4·15)	11·4 (5·6–21·9)
Maternal and neonatal disorders	91·1 (89·3–92·7)	83·2 (80·1–86·2)	65 (59–70·4)	91·8 (90·1–93·2)
Maternal disorders	9·54 (3·43–16)	9·59 (3·5–16·5)	9·72 (3·56–16·6)	9·4 (3·38–15·9)
Neonatal disorders	92·8 (91·3–94·2)	84·9 (81·8–87·8)	67·3 (61·2–72·9)	92·9 (91·3–94·2)
Nutritional deficiencies	72·6 (67·6–86·9)	79·4 (74·8–84·5)	79·5 (74·9–84·6)	72·1 (66·9–79·1)
Protein-energy malnutrition	100 (100–100)	100 (100–100)	100 (100–100)	100 (100–100)
Vitamin A deficiency	--	100 (100–100)	100 (100–100)	--
Dietary iron deficiency	--	100 (100–100)	100 (100–100)	--
Non-communicable diseases	57·3 (53·8–60·4)	39·7 (36·4–43·1)	26·4 (23·9–29)	56·8 (54–59·4)
Neoplasms	49·8 (45·9–54)	48·1 (44·6–52)	33·6 (29·4–38·2)	48·9 (45·4–52·8)
Lip and oral cavity cancer	67·7 (56·1–77)	70 (59·2–78·6)	69·2 (58–78·1)	70·1 (59·3–78·6)
Nasopharynx cancer	66·7 (54·1–76·5)	65·7 (53·1–75·2)	63·5 (50·3–73·4)	65·9 (53·3–75·4)
Other pharynx cancer	79·1 (70·8–85·5)	80 (71·6–86·1)	78·8 (70·1–85·2)	80 (71·6–86·1)
Esophageal cancer	75·4 (66·9–83·6)	76·6 (67·8–84·6)	75·7 (66·9–83·6)	76·6 (67·8–84·7)

Stomach cancer	24·4 (16·9–41·5)	24·3 (16·9–40·8)	24·5 (17·1–41·2)	24·3 (16·9–40·8)
Colon and rectum cancer	63·8 (54·6–72·6)	63·8 (55·1–72·1)	63·8 (54·9–72·4)	63·8 (55·2–72·2)
Liver cancer	77 (71–82·5)	76·3 (70·8–81·3)	76·8 (71·4–81·9)	76·3 (70·8–81·3)
Gallbladder and biliary tract cancer	20·4 (10·9–32·4)	20·7 (11·1–32·6)	19·2 (10·1–31·1)	20·7 (11·1–32·7)
Pancreatic cancer	42·2 (32·7–54)	41 (32·1–51·8)	41·8 (32·3–53·1)	41 (32·1–51·8)
Larynx cancer	83·3 (75·8–88·9)	82·5 (75·1–87·8)	79·9 (72–85·6)	82·7 (75·4–88)
Tracheal, bronchus, and lung cancer	86·5 (82·8–89·8)	85 (81·5–88·3)	85·8 (82·2–89·2)	85 (81·5–88·3)
Breast cancer	35·5 (25·9–46·9)	33·5 (24·7–43·3)	33·8 (24·9–43·9)	33·5 (24·6–43·2)
Cervical cancer	100 (100–100)	100 (100–100)	100 (100–100)	100 (100–100)
Uterine cancer	41·8 (27–57·7)	42·8 (28·3–58·7)	43·1 (28·7–58·7)	42·8 (28·3–58·7)
Ovarian cancer	28·8 (13·7–43·4)	23·1 (11–36·3)	21·6 (10·4–34·2)	23·2 (11·1–36·4)
Prostate cancer	5·71 (2·48–9·28)	6·19 (2·74–9·95)	6·53 (2·92–10·3)	6·13 (2·71–9·89)
Kidney cancer	37·2 (27·2–47·6)	36·6 (26·8–46·4)	36·1 (26·5–45·7)	36·6 (26·8–46·4)
Bladder cancer	43·4 (28·7–58·2)	45·1 (31·9–58·2)	45·4 (32·6–58)	45·1 (31·8–58·2)
Thyroid cancer	13·4 (6·35–22·3)	14 (6·47–23·3)	14 (6·47–23)	14 (6·44–23·3)
Mesothelioma	98·7 (98·3–99·1)	97·3 (96·4–98)	98·5 (97·9–99)	97·2 (96·3–97·9)
Non-Hodgkin lymphoma	6·6 (2·72–11·8)	6·71 (2·78–12)	6·69 (2·82–12)	6·72 (2·78–11·9)
Multiple myeloma	7·49 (3·12–13·4)	7·81 (3·26–14)	7·77 (3·22–14·4)	7·81 (3·25–14)
Leukemia	35·1 (21·9–49)	30·9 (20·7–41·5)	29·9 (19·6–40·8)	31 (20·8–41·6)
Cardiovascular diseases	77·9 (74·1–81·4)	79·2 (75·9–82)	68·9 (63·6–73·5)	80·5 (77·5–83·2)
Rheumatic heart disease	21·5 (12·2–40·3)	22·4 (14·1–37·5)	22·3 (14·1–34·9)	22·4 (14·1–37·4)
Ischemic heart disease	93·6 (90·2–96·6)	95·1 (92·5–97·2)	94·1 (91·3–96·6)	95·1 (92·6–97·2)
Stroke	79·6 (71·1–88·3)	83·3 (77·8–89·1)	82·4 (77·7–87·1)	83·5 (77·7–89·5)
Hypertensive heart disease	100 (100–100)	100 (100–100)	100 (100–100)	100 (100–100)
Non-rheumatic valvular heart disease	24·9 (15·9–38·1)	25·7 (17·8–35·5)	17·8 (11·8–25·4)	26·7 (18·5–36·9)
Cardiomyopathy and myocarditis	35·2 (28·5–43·7)	40·4 (34·2–47·4)	35·2 (29·3–41·5)	40·7 (34·3–47·9)
Atrial fibrillation and flutter	48·2 (37·8–59·4)	57·2 (47·9–65·5)	61·3 (52·2–69·6)	52·3 (42·7–61·9)
Aortic aneurysm	50·8 (44·2–57·6)	58·5 (52·8–64)	--	58·5 (52·8–64)
Endocarditis	30·5 (21·1–42·9)	33 (23·9–43)	29·8 (20·7–41·6)	33·1 (24·1–43·2)
Other cardiovascular and circulatory diseases	30·5 (24·4–37·6)	32 (25·8–38·2)	30·6 (23–39·2)	32·3 (26·3–38·7)
Chronic respiratory diseases	65·2 (59·3–71·1)	55·9 (51·5–60·7)	46·2 (40·6–51·9)	65·1 (60–70·4)
Chronic obstructive pulmonary disease	72·5 (66·3–78·1)	71·1 (66·8–75·4)	64 (60·2–67·5)	74·2 (68·9–79·3)
Pneumoconiosis	100 (100–100)	100 (100–100)	100 (100–100)	100 (100–100)

Asthma	40·3 (29·4–52·2)	36·3 (28·3–45·4)	36·1 (28·2–45·1)	39·4 (30·3–49·4)
Digestive diseases	33·4 (27·5–39·5)	37·4 (30·8–43·5)	23 (15·3–31·9)	45·9 (38·3–53·4)
Cirrhosis and other chronic liver diseases	74·5 (61·6–87·3)	76·8 (64·5–88·9)	72·7 (61–84)	76·9 (64·6–89)
Upper digestive system diseases	19·7 (16·5–23·4)	8·13 (5·18–11·6)	0·672 (0·383–1·07)	21·4 (18·2–25·2)
Gallbladder and biliary diseases	42·5 (27·6–58)	45·9 (31·5–60·6)	46·3 (31·8–61)	43·5 (28·9–58·3)
Pancreatitis	38·6 (18·4–58·4)	44·3 (23·4–62·7)	44·1 (22–63·1)	44·3 (23·3–62·8)
Neurological disorders	25·9 (11·9–41·8)	12·4 (5·56–23·1)	7 (3·05–14·6)	21·9 (10·4–36·1)
Alzheimer's disease and other dementias	40·7 (26·3–56·6)	42·6 (28·5–58·1)	42·7 (28·3–58·4)	42·5 (28·4–57·9)
Parkinson's disease	-8·95 (-15–-4·04)	-10·1 (-16·3–-4·77)	-10·7 (-17·3–-5·06)	-9·93 (-16·2–-4·67)
Idiopathic epilepsy	26·7 (16·2–36·7)	23·5 (14·3–33)	21·4 (12·6–30·6)	26·2 (16·3–36)
Multiple sclerosis	15 (10·9–19·3)	15·1 (11·1–19·2)	14·5 (10·7–18·5)	15·5 (11·5–19·9)
Mental disorders	--	7·06 (3·56–10·9)	7·06 (3·56–10·9)	--
Depressive disorders	--	14·4 (6·54–22·5)	14·4 (6·54–22·5)	--
Anxiety disorders	--	5·57 (1·4–12·6)	5·57 (1·4–12·6)	--
Idiopathic developmental intellectual disability	--	40·1 (18·5–57·4)	40·1 (18·5–57·4)	--
Substance use disorders	100 (100–100)	100 (100–100)	100 (100–100)	100 (100–100)
Alcohol use disorders	100 (100–100)	100 (100–100)	100 (100–100)	100 (100–100)
Drug use disorders	100 (100–100)	100 (100–100)	100 (100–100)	100 (100–100)
Diabetes and kidney diseases	99·7 (99·7–99·8)	99·9 (99·9–100)	100 (100–100)	99·7 (99·7–99·8)
Diabetes mellitus	100 (100–100)	100 (100–100)	100 (100–100)	100 (100–100)
Chronic kidney disease	100 (100–100)	100 (100–100)	100 (100–100)	100 (100–100)
Sense organ diseases	--	8·69 (7·58–9·91)	8·69 (7·58–9·91)	--
Blindness and vision loss	--	7·77 (4·94–11·4)	7·77 (4·94–11·4)	--
Age-related and other hearing loss	--	10·4 (9·38–11·5)	10·4 (9·38–11·5)	--
Musculoskeletal disorders	2·42 (0·576–4·55)	20·8 (17·2–24·6)	21·2 (17·5–25·1)	2·76 (0·685–5·07)
Rheumatoid arthritis	11·8 (2·83–21·2)	13·2 (3·19–23·4)	13·2 (3·19–23·4)	13·1 (3·23–23·6)
Osteoarthritis	--	15·8 (8·08–24·7)	15·8 (8·08–24·7)	--
Low back pain	--	39 (34·1–44)	39 (34·1–44)	--
Gout	--	47·1 (31·8–64·6)	47·1 (31·8–64·6)	--
Other non-communicable diseases	0·181 (0·117–0·269)	0·14 (0·0854–0·217)	--	0·566 (0·354–0·808)
Sudden infant death syndrome	12 (7·04–18·7)	12 (7·04–18·7)	--	12 (7·04–18·7)
Injuries	27·7 (21·7–33·3)	23·7 (19·3–28·5)	25·8 (21·8–30·2)	21 (14·1–28·1)
Transport injuries	20·8 (12·7–28·6)	20·7 (13·9–27·5)	26 (21·6–30·8)	18·4 (9·86–26·5)

Road injuries	20 (11·5–28·1)	19·9 (12·8–26·9)	25·1 (20·6–30)	17·7 (8·65–26·1)
Other transport injuries	31·3 (26·2–36·5)	29·8 (24·9–34·9)	32·7 (28·4–37·1)	27·6 (21·9–33·3)
Unintentional injuries	34·2 (29–38·7)	24·2 (20–28·9)	25·2 (21–30)	20·1 (16–24·4)
Falls	52·5 (44·5–58·1)	34·7 (29·4–40·2)	33·3 (28·1–38·9)	41·6 (35·8–47)
Drowning	-14·8 (-27·7–-1·17)	-15 (-27·2–-2·19)	11 (6·66–17·4)	-16·3 (-29–-2·94)
Fire, heat, and hot substances	11 (6·43–17·5)	13·4 (8·59–19·8)	14·2 (9·28–21·1)	11·5 (7·21–17·1)
Poisonings	15·2 (9·78–22·2)	13·5 (8·88–19·6)	12·6 (8·2–18·7)	15·2 (10–21·6)
Exposure to mechanical forces	7·29 (-5·62–20·7)	15·7 (12·5–18·9)	17 (14·2–20·1)	-1·09 (-14·7–13·3)
Animal contact	18·3 (14·2–23·8)	19·1 (14·5–25)	20·1 (15·3–26·1)	13·6 (10·1–18·4)
Foreign body	3·72 (2·39–5·48)	4·5 (2·92–6·57)	4·18 (2·72–6·15)	4·77 (3·06–7·06)
Environmental heat and cold exposure	9·55 (4·44–17·1)	9·12 (4·3–16·3)	9·03 (4·26–16·3)	9·62 (4·58–17)
Exposure to forces of nature	0 (0–0)	9·96 (4·69–17·6)	9·96 (4·69–17·6)	0 (0–0)
Other unintentional injuries	14·8 (9·42–21·8)	13·4 (8·61–19·9)	13·3 (8·54–19·7)	15·4 (10·2–22)
Self-harm and interpersonal violence	22·7 (9·33–35·1)	24·5 (12·7–35·7)	34·1 (26·3–42·6)	23 (9·84–35·4)
Self-harm	22·5 (8·07–36·4)	23·2 (8·76–36·6)	23·3 (11·2–35·8)	23·2 (8·71–36·7)
Interpersonal violence	25·8 (17·6–34·6)	31·8 (24–40·8)	38·9 (30–48·6)	22·5 (14·6–31·1)

Supplementary Results Table 2. All-age PAFs attributable to the joint distribution of all risk factors for all ages, both sexes combined for each GBD level 1, level 2, and level 3 cause and all causes in 2019 for Hackney. Results are shown for deaths, YLLs, YLDs, and DALYs Data in parenthesis are 95% uncertainty intervals. PAFs=population attributable fractions. GBD=Global Burden of Disease. YLLs=years of life lost. YLDs=years lived with disability. DALYs=disability-adjusted life-years

	Deaths 2019	DALYs 2019	YLDs 2019	YLLs 2019
All causes	56·8 (53·8–59·8)	39·7 (36·6–42·8)	28 (25·5–30·4)	55·5 (52·6–58)
Communicable, maternal, neonatal, and nutritional diseases	52·5 (43·7–60·8)	60·2 (56·1–63·9)	51·2 (46·6–55·8)	65·9 (60·6–70·4)
HIV/AIDS and sexually transmitted infections	81·4 (71·8–87·8)	80·1 (69·2–87·3)	95·3 (93–96·8)	71 (57·3–80·9)
HIV/AIDS	89·9 (87·5–91·4)	88·4 (85·5–90·4)	93·4 (90·6–95·2)	85·6 (82·8–87·4)
Sexually transmitted infections excluding HIV	31·6 (14·4–57·7)	53·9 (29·2–80·4)	100 (100–100)	12·6 (4·74–28·3)
Respiratory infections and tuberculosis	50 (38·7–60·4)	43 (34·3–51·7)	3·35 (2·36–4·79)	54·6 (44·4–64)
Tuberculosis	61·8 (51–70·9)	62·4 (52·2–71·1)	56·9 (45–67·1)	63·5 (53·3–71·9)
Lower respiratory infections	49·8 (38·3–60·5)	54·5 (44–64·1)	37·5 (30·9–44·6)	54·6 (44·1–64·3)
Upper respiratory infections	0·092 (0·0499–0·156)	0·0136 (0·00359–0·0294)	0·0101 (0·000648–0·0266)	0·157 (0·0822–0·275)
Otitis media	6·13 (3·06–10·6)	6·24 (3·97–8·83)	6·09 (3·82–8·67)	10·8 (6·12–17·2)
Enteric infections	5·43 (3·68–8·29)	11·5 (8·98–14·8)	10·9 (8·27–14)	13·2 (9·55–17·8)
Diarrheal diseases	5·51 (3·73–8·39)	11·6 (9·08–14·9)	10·9 (8·28–14)	13·6 (9·83–18·2)
Other infectious diseases	1·47 (1·07–1·96)	2·13 (1·61–2·82)	0·41 (0·231–0·692)	2·68 (2–3·56)
Meningitis	3·31 (2·4–4·37)	4·8 (3·52–6·28)	0·0018 (8·1e-05–0·00483)	5·4 (3·95–7·04)
Encephalitis	0·928 (0·581–1·36)	1·83 (0·971–2·7)	0·000622 (2·77e-05–0·00164)	2·13 (1·29–3·09)
Measles	3·97 (-1·44–20·2)	4·39 (-1·62–21·8)	0·279 (0·00711–0·766)	4·96 (-1·77–24·6)
Acute hepatitis	16·5 (9·6–26)	5·44 (3·25–8·46)	2·37 (1·35–3·98)	14·3 (8·23–23)
Maternal and neonatal disorders	88·5 (86·6–90·4)	81·1 (77·9–84)	64·8 (58·9–70·1)	89·3 (87·4–91·1)
Maternal disorders	9·76 (3·48–16·5)	9·86 (3·48–16·7)	10 (3·57–17·2)	9·66 (3·45–16·4)
Neonatal disorders	90·7 (88·9–92·3)	82·8 (79·6–85·7)	66·9 (61–72·4)	90·7 (89–92·3)
Nutritional deficiencies	76·4 (64·7–82·1)	79·3 (74·8–83·7)	79·3 (74·7–83·9)	77·2 (62·2–80·3)
Protein-energy malnutrition	100 (100–100)	100 (100–100)	100 (100–100)	100 (100–100)
Vitamin A deficiency	--	100 (100–100)	100 (100–100)	--
Dietary iron deficiency	--	100 (100–100)	100 (100–100)	--
Non-communicable diseases	58·9 (55·5–62)	39·6 (36·2–43)	27·2 (24·5–30)	57·9 (54·4–60·6)
Neoplasms	49·2 (44·8–53·5)	46·7 (41·3–50·8)	33·5 (29·4–37·8)	47·4 (41·7–51·6)
Lip and oral cavity cancer	71·7 (61·5–79·5)	73·1 (63·3–80·4)	72·2 (62·2–79·7)	73·1 (63·3–80·4)
Nasopharynx cancer	66·9 (54·3–75·9)	65·9 (54–74·8)	63·4 (50·5–73·1)	66·1 (54·2–75)
Other pharynx cancer	81·7 (74·9–87)	82·1 (75·3–87·3)	81·1 (74·2–86·7)	82·1 (75·3–87·4)
Esophageal cancer	75·6 (65·1–84·4)	76·6 (66·8–85·1)	75·9 (65·8–84·6)	76·7 (66·9–85·1)

Stomach cancer	24·7 (17·2–40·9)	24 (16·7–40·8)	24·3 (16·9–40·9)	24 (16·7–40·8)
Colon and rectum cancer	65·9 (57·4–74·4)	65·7 (57·4–73·6)	65·8 (57·5–73·8)	65·7 (57·4–73·6)
Liver cancer	78 (72·3–83·3)	76·7 (71·3–81·8)	77·2 (71·9–82·2)	76·7 (71·3–81·7)
Gallbladder and biliary tract cancer	26·6 (15–40)	26·9 (15·4–40·1)	25·1 (13·9–38·4)	26·9 (15·4–40·2)
Pancreatic cancer	43·9 (34·1–55·6)	42·4 (33·1–53·4)	43·4 (33·4–54·9)	42·4 (33·1–53·4)
Larynx cancer	83·2 (75·5–88·8)	81·8 (74·7–87·3)	78·5 (71·2–84·7)	82·1 (75–87·7)
Tracheal, bronchus, and lung cancer	85·4 (82–88·8)	83·4 (79·8–87·1)	84·6 (81–88·1)	83·4 (79·7–87·1)
Breast cancer	37·6 (28·7–48·4)	34·6 (26·4–43·7)	35 (26·7–44·6)	34·6 (26·4–43·7)
Cervical cancer	100 (100–100)	100 (100–100)	100 (100–100)	100 (100–100)
Uterine cancer	50·2 (33·6–65·6)	51 (34·6–66·5)	51·1 (34·7–66·3)	50·9 (34·5–66·5)
Ovarian cancer	23·3 (11·9–36·9)	19·1 (9·15–31·4)	17·5 (8·24–29·2)	19·2 (9·16–31·4)
Prostate cancer	5·88 (2·54–9·43)	6·38 (2·86–10·1)	6·73 (3·09–10·7)	6·32 (2·83–9·99)
Kidney cancer	42·1 (31·3–52·7)	41 (30·5–51)	40·4 (29·9–50·4)	41·1 (30·5–51·1)
Bladder cancer	44·5 (30·4–58·8)	45·7 (32·8–58·3)	45·6 (33·1–57·7)	45·7 (32·7–58·3)
Thyroid cancer	18·1 (9·02–28·4)	18·4 (9·23–29)	18·3 (9·35–29·1)	18·5 (9·22–29·1)
Mesothelioma	97·3 (96·6–98)	94·4 (93–95·7)	97 (96–97·8)	94·3 (92·9–95·6)
Non-Hodgkin lymphoma	9·09 (4–15·6)	9·11 (4·07–15·6)	9·14 (4·02–15·8)	9·11 (4·07–15·6)
Multiple myeloma	10·2 (4·53–17·7)	10·6 (4·74–18·2)	10·5 (4·76–18·4)	10·6 (4·75–18·2)
Leukemia	36·9 (24·5–49·7)	31·8 (21·8–41·9)	30·6 (21·1–40·7)	31·8 (21·9–41·9)
Cardiovascular diseases	80·3 (77·1–83·6)	80·9 (78·1–83·5)	70·1 (65·4–74·4)	82·4 (79·7–84·8)
Rheumatic heart disease	23·3 (13·7–41·1)	24·1 (15·7–36·7)	24 (15·4–36·4)	24·1 (15·6–36·8)
Ischemic heart disease	94·3 (91·2–97)	95·7 (93·6–97·5)	94·6 (92·1–96·9)	95·7 (93·6–97·6)
Stroke	81·9 (75·1–89·4)	85·8 (81·4–90·3)	85·1 (81·2–89·2)	86 (81·2–90·7)
Hypertensive heart disease	100 (100–100)	100 (100–100)	100 (100–100)	100 (100–100)
Non-rheumatic valvular heart disease	27·3 (18·2–39·6)	26·8 (19·2–36·5)	11·2 (7·46–16)	29·3 (21–39·4)
Cardiomyopathy and myocarditis	41·1 (33·9–48·1)	45·5 (39·9–51·8)	40·9 (35·2–46·3)	45·8 (40–52·3)
Atrial fibrillation and flutter	54·6 (44·4–65·2)	63·1 (54·3–71·5)	66·7 (58·1–74·7)	58·6 (49·2–68·1)
Aortic aneurysm	54·5 (47·8–61·2)	61·8 (56·4–67)	--	61·8 (56·4–67)
Endocarditis	32·7 (23·3–44)	35 (25·7–45·3)	31·8 (22·6–43)	35·1 (25·8–45·4)
Other cardiovascular and circulatory diseases	32·2 (26·3–38·4)	33·1 (26·7–39·3)	32·7 (24·2–42·2)	33·2 (26·8–39·1)
Chronic respiratory diseases	66 (60·3–71·6)	56·1 (51·2–61·1)	48·5 (42·3–55)	65·3 (60·3–70·3)
Chronic obstructive pulmonary disease	74 (68·2–79·4)	71·9 (67·5–75·8)	64·1 (60·5–67·8)	75·5 (70·4–80·3)
Pneumoconiosis	100 (100–100)	100 (100–100)	100 (100–100)	100 (100–100)
Asthma	48 (36·4–60·7)	43·7 (35–53·4)	43·4 (34·7–53·1)	47·4 (37·3–57·9)

Digestive diseases	40 (33·4–46·2)	42 (35·3–48·7)	28 (19·5–37·5)	51·7 (43·8–59·2)
Cirrhosis and other chronic liver diseases	77·5 (65·2–88·3)	79·3 (67·3–89·7)	75·5 (62·9–85·8)	79·3 (67·2–89·8)
Upper digestive system diseases	22·5 (18·8–26·1)	7·19 (4·48–10·6)	0·597 (0·35–0·937)	24·4 (20·7–28)
Gallbladder and biliary diseases	50·9 (34·9–66·3)	54·8 (39·8–69)	55·2 (40·2–69·5)	51·8 (36–66·8)
Pancreatitis	44·8 (24·6–62·2)	50 (30–67·2)	49·4 (29·2–67·2)	50 (29·8–67·3)
Neurological disorders	28·3 (13·7–44)	11·7 (5·35–21·6)	6·52 (2·68–14·3)	23·6 (12·9–37·8)
Alzheimer's disease and other dementias	44·7 (29·6–60·8)	46·7 (31·9–62·3)	47·1 (32·1–62·5)	46·5 (31·7–62·2)
Parkinson's disease	-9·4 (-15·6–-4·31)	-10·6 (-17–-4·92)	-11·3 (-18·2–-5·27)	-10·4 (-16·9–-4·82)
Idiopathic epilepsy	29·6 (19·9–39·7)	26·7 (17·9–36·1)	25·1 (16·5–34·1)	29·2 (19·8–38·9)
Multiple sclerosis	16 (11·9–20·1)	15·7 (11·7–19·7)	14·8 (11–19)	16·4 (12·2–20·6)
Mental disorders	--	6·62 (3–10·7)	6·62 (3–10·7)	--
Depressive disorders	--	15 (6·73–23·3)	15 (6·73–23·3)	--
Anxiety disorders	--	5·7 (1·25–13·3)	5·7 (1·25–13·3)	--
Idiopathic developmental intellectual disability	--	23·3 (8·89–49·2)	23·3 (8·89–49·2)	--
Substance use disorders	100 (100–100)	100 (100–100)	100 (100–100)	100 (100–100)
Alcohol use disorders	100 (100–100)	100 (100–100)	100 (100–100)	100 (100–100)
Drug use disorders	100 (100–100)	100 (100–100)	100 (100–100)	100 (100–100)
Diabetes and kidney diseases	99·8 (99·7–99·8)	99·9 (99·9–100)	100 (100–100)	99·8 (99·7–99·8)
Diabetes mellitus	100 (100–100)	100 (100–100)	100 (100–100)	100 (100–100)
Chronic kidney disease	100 (100–100)	100 (100–100)	100 (100–100)	100 (100–100)
Sense organ diseases	--	9·36 (8·34–10·5)	9·36 (8·34–10·5)	--
Blindness and vision loss	--	7·08 (4·7–10·2)	7·08 (4·7–10·2)	--
Age-related and other hearing loss	--	11·6 (10·6–12·7)	11·6 (10·6–12·7)	--
Musculoskeletal disorders	1·84 (0·439–3·38)	22·5 (18·7–26·4)	22·8 (18·9–26·8)	1·95 (0·461–3·54)
Rheumatoid arthritis	12·8 (3·04–22·8)	13·2 (3·14–23·3)	13·1 (3·13–23)	14 (3·47–25·4)
Osteoarthritis	--	20·2 (11–29·9)	20·2 (11–29·9)	--
Low back pain	--	41·7 (36·6–46·7)	41·7 (36·6–46·7)	--
Gout	--	54·2 (36·8–72)	54·2 (36·8–72)	--
Other non-communicable diseases	0·242 (0·16–0·342)	0·146 (0·0898–0·223)	--	0·589 (0·365–0·839)
Sudden infant death syndrome	8·05 (4·57–12·9)	8·04 (4·56–12·9)	--	8·04 (4·56–12·9)
Injuries	27·1 (21·2–32·7)	22·4 (17·7–27·2)	23·2 (19·2–27·6)	21·5 (15–28)
Transport injuries	19·7 (11·7–27·7)	19·6 (12·4–26·5)	23·9 (19·2–29)	17·7 (9·29–25·9)
Road injuries	18·9 (10·5–27·3)	18·9 (11·4–26·1)	23·2 (18·5–28·4)	17 (8·17–25·6)
Other transport injuries	27·9 (22·6–33·2)	26·1 (21–31·1)	29·2 (24·8–34)	24·4 (18·9–29·8)

Unintentional injuries	30·7 (24·9–35·4)	20·9 (16·6–25·8)	22·2 (17·7–27·2)	17·4 (13·2–22·3)
Falls	49·4 (41·7–54·9)	31·1 (25·4–36·8)	29·6 (24–35·3)	37 (30·7–43·1)
Drowning	-13·6 (-27·1–0·754)	-14 (-27–-0·107)	11·1 (6·34–17·4)	-14·8 (-28·2–-0·496)
Fire, heat, and hot substances	12·2 (7·09–18·6)	13·2 (8·03–19·7)	13·8 (8·49–20·5)	12·5 (7·56–18·6)
Poisonings	15·3 (9·55–22·4)	13·8 (8·64–20·3)	12·3 (7·57–18·3)	15·2 (9·66–22·1)
Exposure to mechanical forces	3·68 (-9·08–17·6)	12 (9·45–14·7)	13·4 (11·4–15·9)	-3·8 (-17·5–10·8)
Animal contact	19·9 (15–25·8)	17·7 (12·8–23·4)	18·4 (13·3–24·5)	16·1 (11·6–21·5)
Foreign body	3·19 (2·03–4·83)	3·51 (2·29–5·29)	3·11 (2·02–4·64)	3·73 (2·39–5·66)
Environmental heat and cold exposure	10·2 (4·76–17·5)	10·2 (4·82–17·3)	9·88 (4·75–16·8)	11 (5·16–18·7)
Exposure to forces of nature	0 (0–0)	10·7 (5·09–18·1)	10·7 (5·09–18·1)	0 (0–0)
Other unintentional injuries	15 (9·35–22)	13·1 (7·95–19·3)	12·9 (7·84–19·1)	15·4 (9·73–22·3)
Self-harm and interpersonal violence	25·8 (13·2–38)	27·2 (15·8–38·4)	35·8 (27·6–44·2)	26 (13·6–38·1)
Self-harm	25·9 (11·5–39·9)	26·5 (12·2–40·5)	26 (13·7–38·9)	26·5 (12·2–40·5)
Interpersonal violence	25·8 (17·3–34·6)	30·3 (22·2–38·9)	39·6 (30·3–49·4)	23·2 (15–31·8)

Supplementary Results Table 2. All-age PAFs attributable to the joint distribution of all risk factors for all ages, both sexes combined for each GBD level 1, level 2, and level 3 cause and all causes in 2019 for Hammersmith and Fulham. Results are shown for deaths, YLLs, YLDs, and DALYs Data in parenthesis are 95% uncertainty intervals. PAFs=population attributable fractions. GBD=Global Burden of Disease. YLLs=years of life lost. YLDs=years lived with disability. DALYs=disability-adjusted life-years

	Deaths 2019	DALYs 2019	YLDs 2019	YLLs 2019
All causes	56·4 (53·3–59·2)	39·5 (36·3–42·7)	28·7 (26·2–31·3)	55·8 (53·3–58·2)
Communicable, maternal, neonatal, and nutritional diseases	50·2 (40·5–59·3)	56 (51·8–60)	50·2 (45·4–54·5)	60·5 (54–66·5)
HIV/AIDS and sexually transmitted infections	87 (80·5–91·2)	85·7 (77·7–90·7)	95·2 (93–96·6)	79·7 (69·2–86·8)
HIV/AIDS	92·3 (90–93·7)	91·2 (88·5–93)	94 (91·3–95·6)	89·6 (86·9–91·2)
Sexually transmitted infections excluding HIV	37·8 (18·2–64·3)	58·1 (32·2–83·7)	100 (100–100)	14·4 (5·52–31·1)
Respiratory infections and tuberculosis	50·3 (38·6–61·4)	42·8 (33·8–51·6)	3·32 (2·35–4·67)	54·3 (43·7–64·5)
Tuberculosis	64 (53·9–72·4)	65·1 (54·9–72·8)	60 (48·4–69·2)	66·1 (56·6–73·8)
Lower respiratory infections	50·1 (38–61·2)	53·8 (42·8–64·2)	38·5 (31·9–45·3)	54 (42·9–64·4)
Upper respiratory infections	0·125 (0·0678–0·22)	0·00816 (0·00126–0·0194)	0·00704 (0·000273–0·0185)	0·239 (0·129–0·428)
Otitis media	3·57 (1·65–7·35)	4·59 (2·9–6·62)	4·59 (2·9–6·62)	8·11 (4·24–15·5)
Enteric infections	4·18 (2·72–6·79)	8·99 (6·92–11·7)	9·31 (7–12·3)	8·24 (5·83–11·3)
Diarrheal diseases	4·21 (2·73–6·82)	9·04 (6·97–11·8)	9·32 (7·01–12·3)	8·35 (5·98–11·4)
Other infectious diseases	0·966 (0·692–1·31)	1·52 (1·14–2·01)	0·359 (0·206–0·596)	2 (1·48–2·65)
Meningitis	2·28 (1·64–3·1)	3·31 (2·4–4·43)	0·000718 (1·52e-05–0·00199)	4·2 (3·05–5·6)
Encephalitis	0·545 (0·363–0·803)	1·18 (0·672–1·75)	0·000306 (5·9e-06–0·000816)	1·51 (1–2·19)
Measles	2·55 (-0·986–13·5)	2·9 (-1·08–14·5)	0·245 (0·00665–0·685)	3·23 (-1·28–17·4)
Acute hepatitis	10·2 (4·77–19·1)	4·45 (2·48–7·25)	2·48 (1·41–4·21)	8·93 (4·12–17·1)
Maternal and neonatal disorders	89 (86·8–90·9)	77·2 (73·3–80·9)	63·7 (58–69)	89·5 (87·3–91·3)
Maternal disorders	9·34 (3·29–15·8)	9·5 (3·31–16·3)	9·58 (3·33–16·4)	9·23 (3·28–15·6)
Neonatal disorders	90·3 (88·3–92)	78·9 (74·8–82·5)	65·8 (59·6–71·5)	90·3 (88·3–92·1)
Nutritional deficiencies	80·2 (71·2–87)	78·2 (74–82·7)	78·2 (73·9–82·8)	79·1 (67·1–82·1)
Protein-energy malnutrition	100 (100–100)	100 (100–100)	100 (100–100)	100 (100–100)
Vitamin A deficiency	--	100 (100–100)	100 (100–100)	--
Dietary iron deficiency	--	100 (100–100)	100 (100–100)	--
Non-communicable diseases	58·2 (55·1–61·3)	39·7 (36·3–43·3)	28 (25·3–30·8)	58·5 (55·9–61)
Neoplasms	50·6 (46·7–54·9)	48·7 (45·1–52·6)	34·8 (30·7–39·2)	49·5 (46–53·5)
Lip and oral cavity cancer	72·4 (63–79·3)	74·4 (65·5–81)	73·7 (64·6–80·8)	74·5 (65·6–81)
Nasopharynx cancer	71·5 (60·9–79·4)	70·8 (61·2–78·4)	69·3 (59·2–77·4)	71 (61·3–78·6)
Other pharynx cancer	82·4 (76·5–87·3)	83·1 (77·1–87·8)	82·1 (75·6–87·2)	83·2 (77·2–87·9)
Esophageal cancer	78 (68·8–86)	79·1 (70·4–86·8)	78·2 (69·4–86·2)	79·1 (70·4–86·8)

Stomach cancer	24·8 (17·2–41·2)	24·4 (17·1–41)	24·6 (17·2–41·3)	24·4 (17·1–41)
Colon and rectum cancer	65·7 (56·7–74·3)	65·8 (57·3–73·8)	65·8 (57·4–73·9)	65·8 (57·4–73·7)
Liver cancer	79 (73·1–84·3)	78·8 (73·5–83·6)	79 (73·6–83·8)	78·8 (73·5–83·6)
Gallbladder and biliary tract cancer	25·3 (14–38·6)	25·5 (14·2–38·9)	23·9 (12·9–36·9)	25·6 (14·3–39)
Pancreatic cancer	44·5 (34·7–55·6)	43·2 (34·1–53·9)	44·1 (34·6–55·4)	43·2 (34·1–53·9)
Larynx cancer	83·7 (76·2–89·2)	82·7 (75·6–88·1)	79·6 (72·2–85·4)	83 (76–88·3)
Tracheal, bronchus, and lung cancer	85·3 (81·7–88·8)	83·6 (80–87·2)	84·6 (80·9–88·1)	83·6 (80–87·2)
Breast cancer	38·9 (29·6–49·5)	36·5 (28·6–45·9)	36·9 (28·8–46·3)	36·5 (28·5–45·8)
Cervical cancer	100 (100–100)	100 (100–100)	100 (100–100)	100 (100–100)
Uterine cancer	49·2 (32·8–64·3)	50·1 (34·2–65·3)	50·3 (34·5–65·6)	50·1 (34·2–65·2)
Ovarian cancer	24·4 (12·5–38·6)	20·2 (9·82–33·1)	18·8 (9·25–31·1)	20·2 (9·85–33·2)
Prostate cancer	5·91 (2·57–9·56)	6·41 (2·89–10·2)	6·77 (3·14–10·7)	6·35 (2·83–10·1)
Kidney cancer	42·1 (31·4–52·9)	41·4 (31–51·4)	40·9 (30·8–51·1)	41·4 (31·1–51·5)
Bladder cancer	44·2 (29·5–58·8)	45·8 (32·2–58·3)	46 (32·7–58·3)	45·8 (32·2–58·4)
Thyroid cancer	17 (8·65–26·7)	17·6 (8·96–27·5)	17·6 (8·96–27·7)	17·6 (8·92–27·5)
Mesothelioma	97·9 (97·2–98·5)	95·6 (94·3–96·7)	97·6 (96·6–98·4)	95·6 (94·3–96·6)
Non-Hodgkin lymphoma	8·68 (3·81–14·9)	8·82 (3·88–15·2)	8·8 (3·87–15·1)	8·82 (3·88–15·2)
Multiple myeloma	9·81 (4·49–16·9)	10·2 (4·67–17·6)	10·1 (4·5–17·6)	10·2 (4·69–17·5)
Leukemia	37·5 (24·9–51)	33·4 (22·9–44·1)	32·2 (22·1–42·7)	33·5 (22·9–44·1)
Cardiovascular diseases	76·9 (73–80·7)	78·1 (75·1–81·3)	71·9 (67·4–76·2)	79·1 (76·1–82·2)
Rheumatic heart disease	22·9 (12·6–42·4)	24 (15·1–37·9)	24 (15·2–37·1)	24 (15·1–38·5)
Ischemic heart disease	94·1 (90·8–97)	95·4 (93·2–97·5)	94·7 (92·1–97)	95·5 (93·2–97·5)
Stroke	81 (73·4–89·1)	84·8 (80–90·1)	85·1 (81–89·5)	84·7 (79·5–90·3)
Hypertensive heart disease	100 (100–100)	100 (100–100)	100 (100–100)	100 (100–100)
Non-rheumatic valvular heart disease	26 (16·4–38·4)	27·3 (18·8–37)	22·2 (15–31·1)	27·8 (19–37·6)
Cardiomyopathy and myocarditis	34·7 (27·4–44·1)	40·5 (34·4–46·7)	37·8 (31·8–44·5)	40·7 (34·5–47·1)
Atrial fibrillation and flutter	54·8 (45–65·2)	63·1 (55–71·3)	66·7 (59–74·2)	58·4 (49·4–67·7)
Aortic aneurysm	51·9 (44·7–58·8)	59·2 (53·2–65)	--	59·2 (53·2–65)
Endocarditis	31·6 (21·5–43·9)	34·4 (25·5–44)	31·4 (21·5–44·1)	34·5 (25·6–44·2)
Other cardiovascular and circulatory diseases	32·6 (26·1–39·6)	34·9 (28·5–41·3)	34·7 (26·9–43·1)	34·9 (28·6–41·1)
Chronic respiratory diseases	66·2 (60·4–71·8)	57·6 (53–62·5)	50·3 (44·3–56·5)	66·2 (61·3–71)
Chronic obstructive pulmonary disease	73·2 (67·3–78·8)	71·4 (67–75·4)	64·5 (60·9–68·2)	74·9 (69·7–79·9)
Pneumoconiosis	100 (100–100)	100 (100–100)	100 (100–100)	100 (100–100)

Asthma	46·8 (34·9–59·3)	44·1 (35·4–54·4)	43·9 (35·2–54·1)	47 (37·2–57·5)
Digestive diseases	41·6 (35·5–47·3)	44·2 (37·4–50·3)	29·8 (21–39·6)	55·3 (47·8–61·6)
Cirrhosis and other chronic liver diseases	80·7 (68·6–90)	82·5 (71·1–91·2)	79·6 (68·9–87·6)	82·6 (71·1–91·4)
Upper digestive system diseases	22·8 (18·9–26·6)	7·67 (4·68–11·2)	0·589 (0·333–0·952)	25·4 (21·6–29)
Gallbladder and biliary diseases	50·3 (34·4–65·5)	54·2 (39·4–68·1)	54·4 (39·8–68·5)	51·2 (35·9–65·9)
Pancreatitis	49·2 (30·2–66·4)	54·7 (36·4–70·3)	54·8 (36·1–70·3)	54·7 (36·5–70·4)
Neurological disorders	29·8 (14·6–45·4)	13·4 (6·07–24·9)	7·47 (3·19–16·2)	26·1 (14–41·2)
Alzheimer's disease and other dementias	44·2 (28·9–59·8)	46·1 (31–61·5)	46·5 (31·2–61·6)	46 (30·9–61·2)
Parkinson's disease	-9·39 (-15·3–-4·16)	-10·6 (-16·9–-4·77)	-11·2 (-17·9–-5·09)	-10·4 (-16·7–-4·74)
Idiopathic epilepsy	33·5 (23·1–43·3)	30·4 (20·8–39·4)	28·4 (19–37·1)	33·4 (23·3–43)
Multiple sclerosis	15·5 (11·4–19·8)	15·3 (11·4–19·4)	14·6 (11–18·5)	16 (11·7–20·3)
Mental disorders	--	6·85 (3·21–10·7)	6·85 (3·21–10·7)	--
Depressive disorders	--	14·8 (6·51–23·2)	14·8 (6·51–23·2)	--
Anxiety disorders	--	5·16 (1·09–12·1)	5·16 (1·09–12·1)	--
Idiopathic developmental intellectual disability	--	31·3 (12·1–52)	31·3 (12·1–52)	--
Substance use disorders	100 (100–100)	100 (100–100)	100 (100–100)	100 (100–100)
Alcohol use disorders	100 (100–100)	100 (100–100)	100 (100–100)	100 (100–100)
Drug use disorders	100 (100–100)	100 (100–100)	100 (100–100)	100 (100–100)
Diabetes and kidney diseases	99·5 (99·4–99·6)	99·9 (99·9–99·9)	100 (100–100)	99·5 (99·4–99·6)
Diabetes mellitus	100 (100–100)	100 (100–100)	100 (100–100)	100 (100–100)
Chronic kidney disease	100 (100–100)	100 (100–100)	100 (100–100)	100 (100–100)
Sense organ diseases	--	9·05 (8·02–10·2)	9·05 (8·02–10·2)	--
Blindness and vision loss	--	8·09 (5·36–11·6)	8·09 (5·36–11·6)	--
Age-related and other hearing loss	--	10·8 (9·84–11·9)	10·8 (9·84–11·9)	--
Musculoskeletal disorders	2·29 (0·584–4·28)	22·1 (18·6–25·9)	22·5 (18·8–26·3)	2·59 (0·675–4·79)
Rheumatoid arthritis	12·2 (3·08–21·7)	13·4 (3·08–23·8)	13·4 (3·06–23·8)	13·6 (3·61–24·1)
Osteoarthritis	--	19·6 (10·6–29·1)	19·6 (10·6–29·1)	--
Low back pain	--	41·3 (36·3–46·3)	41·3 (36·3–46·3)	--
Gout	--	54·3 (37·4–72·2)	54·3 (37·4–72·2)	--
Other non-communicable diseases	0·18 (0·115–0·258)	0·106 (0·0645–0·167)	--	0·56 (0·342–0·794)
Sudden infant death syndrome	10·6 (5·97–17·4)	10·6 (5·96–17·4)	--	10·6 (5·96–17·4)
Injuries	31·6 (25·4–36·7)	25·2 (20·8–29·5)	25·9 (21·7–30·7)	24·3 (17·5–30·6)
Transport injuries	20·9 (12·3–28·9)	21 (13·8–28)	25·3 (20·5–30·2)	19 (9·79–27·2)

Road injuries	20·3 (11·3–28·7)	20·3 (12·8–27·4)	24·5 (19·7–29·4)	18·4 (8·89–27)
Other transport injuries	30 (24·1–35·6)	29·1 (23·8–34·3)	31·8 (27·3–36·2)	26·8 (20·5–32·8)
Unintentional injuries	36·3 (29·9–41·4)	24·2 (19·6–29·3)	25·2 (20·4–30·6)	21·4 (16·4–26·4)
Falls	52·9 (44·7–58·5)	35 (28·9–40·8)	33·3 (27·2–39·1)	41·5 (34·6–47·4)
Drowning	-14·4 (-27·8–0·543)	-14·3 (-27–-0·519)	11·9 (6·66–19)	-15·4 (-28·3–-1·36)
Fire, heat, and hot substances	12·2 (6·8–19·3)	13·6 (8–20·9)	14·3 (8·32–22)	12·5 (7·34–19)
Poisonings	14·5 (8·59–22·1)	13·7 (8·21–20·6)	13 (7·74–19·7)	14·7 (8·94–21·8)
Exposure to mechanical forces	9·15 (-3·66–22·2)	13 (10·4–15·5)	14·3 (12·1–16·5)	-2·8 (-16·4–12)
Animal contact	23·7 (19·1–29)	19·1 (14·1–25·2)	20·1 (14·6–26·7)	16·1 (11·9–20·8)
Foreign body	2·8 (1·73–4·17)	3·31 (2·11–4·81)	2·75 (1·79–4·04)	3·69 (2·32–5·46)
Environmental heat and cold exposure	10·1 (4·6–17·7)	11·1 (5·35–18·9)	10·9 (5·25–18·7)	11·5 (5·44–19·5)
Exposure to forces of nature	0 (0–0)	11·8 (5·69–20)	11·8 (5·69–20)	0 (0–0)
Other unintentional injuries	14·8 (8·61–22·5)	13·6 (7·91–21)	13·6 (7·85–20·9)	15·7 (9·48–23·1)
Self-harm and interpersonal violence	28·5 (16·5–40·8)	30·2 (19·4–41)	37·7 (29·8–46)	29 (17·2–41·2)
Self-harm	28·5 (15·3–41·9)	29·3 (16·1–42·7)	28·7 (17–40·9)	29·4 (16–42·8)
Interpersonal violence	30 (21·4–39·1)	35·5 (27·1–44·4)	42·5 (33·1–52·4)	27·1 (18·8–35·8)

Supplementary Results Table 2. All-age PAFs attributable to the joint distribution of all risk factors for all ages, both sexes combined for each GBD level 1, level 2, and level 3 cause and all causes in 2019 for Haringey. Results are shown for deaths, YLLs, YLDs, and DALYs Data in parenthesis are 95% uncertainty intervals. PAFs=population attributable fractions. GBD=Global Burden of Disease. YLLs=years of life lost. YLDs=years lived with disability. DALYs=disability-adjusted life-years

	Deaths 2019	DALYs 2019	YLDs 2019	YLLs 2019
All causes	55·5 (52·3–58·4)	38·7 (35·6–41·8)	27·6 (25·2–30)	54·2 (51·7–56·7)
Communicable, maternal, neonatal, and nutritional diseases	50·3 (41·4–59·2)	56·8 (52·6–60·8)	51·5 (46·9–56)	60·6 (54·4–66·2)
HIV/AIDS and sexually transmitted infections	85·1 (78·6–89·3)	83·5 (75·3–88·7)	94·8 (92·6–96·3)	76·6 (66·4–83·6)
HIV/AIDS	90·5 (88·2–91·9)	89 (86·2–90·9)	93·4 (90·8–95·2)	86·4 (83·7–88)
Sexually transmitted infections excluding HIV	35·6 (16·8–62·9)	56·5 (31·5–82·1)	100 (100–100)	13·9 (5·21–31·2)
Respiratory infections and tuberculosis	49·6 (38·6–60·4)	42·6 (33·6–51·7)	3·41 (2·51–4·72)	53·5 (43·3–63·2)
Tuberculosis	60·2 (49·4–69·2)	60·9 (50·2–69·2)	55·2 (43·2–64·7)	62·2 (51·6–70·4)
Lower respiratory infections	49·5 (38·3–60·4)	53·3 (43·1–63·2)	37·5 (30·7–44·1)	53·5 (43·1–63·5)
Upper respiratory infections	0·0825 (0·0419–0·154)	0·0117 (0·00315–0·024)	0·00889 (0·000566–0·0214)	0·15 (0·0745–0·277)
Otitis media	3·56 (1·79–6·36)	4·69 (3–6·6)	4·65 (2·95–6·55)	7·15 (3·9–11·6)
Enteric infections	5·2 (3·37–8·13)	10·3 (7·99–13·4)	10 (7·51–13·3)	11 (7·82–14·7)
Diarrheal diseases	5·25 (3·43–8·2)	10·3 (8·03–13·4)	10 (7·52–13·3)	11·2 (8·02–15)
Other infectious diseases	1·11 (0·773–1·74)	1·58 (1·18–2·15)	0·343 (0·197–0·563)	2·08 (1·51–2·91)
Meningitis	2·5 (1·76–3·4)	3·31 (2·36–4·53)	0·000757 (3·52e-05–0·00196)	4·32 (3·06–5·81)
Encephalitis	0·631 (0·387–0·936)	1·17 (0·6–1·8)	0·000264 (1·19e-05–0·000707)	1·62 (0·972–2·42)
Measles	4·38 (-1·35–21·8)	5 (-1·53–25·3)	0·418 (0·0126–1·14)	5·37 (-1·68–26·6)
Acute hepatitis	6·91 (2·43–19·9)	4·2 (2·01–9·23)	2·53 (1·49–4·14)	6·14 (2·25–17·2)
Maternal and neonatal disorders	87·2 (85·1–89·2)	78·8 (75·6–82·1)	67·9 (62·4–72·6)	88·1 (86·1–90)
Maternal disorders	9·46 (3·28–15·8)	9·53 (3·34–16)	9·67 (3·42–16·4)	9·33 (3·24–15·6)
Neonatal disorders	89·8 (87·9–91·4)	80·6 (77·3–83·8)	69·8 (64·1–74·7)	89·8 (87·9–91·5)
Nutritional deficiencies	69·5 (62·7–83·5)	78·4 (74·1–83·1)	78·5 (74·2–83·3)	71·3 (61·5–77)
Protein-energy malnutrition	100 (100–100)	100 (100–100)	100 (100–100)	100 (100–100)
Vitamin A deficiency	--	100 (100–100)	100 (100–100)	--
Dietary iron deficiency	--	100 (100–100)	100 (100–100)	--
Non-communicable diseases	57·3 (54–60·3)	38·7 (35·2–42·1)	26·6 (24–29·3)	56·5 (53·8–59·2)
Neoplasms	47·2 (43·7–51·4)	45·3 (41·9–49·3)	32·2 (28·3–36·5)	46·1 (42·7–50·1)
Lip and oral cavity cancer	69·9 (59·2–78·2)	71·6 (61·5–79·5)	70·7 (60·3–79·1)	71·7 (61·5–79·6)
Nasopharynx cancer	68·2 (57·2–77·4)	67·3 (56·4–76·1)	65·4 (53·7–74·9)	67·4 (56·6–76·3)
Other pharynx cancer	78·8 (71·1–85·1)	79·6 (71·8–85·5)	78·2 (70·2–84·5)	79·6 (71·9–85·6)
Esophageal cancer	76·3 (66·8–84·5)	77·2 (68·1–85·6)	76·5 (67·2–84·5)	77·3 (68·1–85·6)

Stomach cancer	24·8 (17·3–41·6)	24·3 (17–40·8)	24·6 (17·3–41·1)	24·3 (17–40·7)
Colon and rectum cancer	64·1 (54·8–72·8)	63·8 (55·1–71·8)	63·9 (55·1–72·1)	63·8 (55·1–71·9)
Liver cancer	77·1 (70·9–82·3)	76·3 (70·7–81·2)	76·8 (71·4–81·9)	76·3 (70·7–81·2)
Gallbladder and biliary tract cancer	20·6 (10·9–33·1)	21·1 (11·2–33·4)	19·4 (9·57–31·4)	21·2 (11·3–33·6)
Pancreatic cancer	42·7 (33·3–53·8)	41·4 (32·5–52)	42·3 (32·8–53·3)	41·4 (32·5–52)
Larynx cancer	83·2 (75·2–88·9)	82·1 (74·3–87·8)	79·5 (71·6–85·5)	82·3 (74·6–88·1)
Tracheal, bronchus, and lung cancer	85·5 (82·1–89)	83·8 (80·3–87·4)	84·7 (81·1–88·3)	83·8 (80·2–87·3)
Breast cancer	36·3 (27·3–46·8)	34·4 (26·2–43·7)	34·7 (26·2–44)	34·4 (26·2–43·7)
Cervical cancer	100 (100–100)	100 (100–100)	100 (100–100)	100 (100–100)
Uterine cancer	43·9 (29–59·6)	44·8 (29·9–60·4)	45 (29·9–60·7)	44·7 (29·8–60·3)
Ovarian cancer	20·9 (10·4–34·7)	17·5 (8·32–30)	16·4 (7·66–28·6)	17·6 (8·34–30·1)
Prostate cancer	5·87 (2·59–9·42)	6·35 (2·89–10·1)	6·7 (3·11–10·7)	6·29 (2·83–10)
Kidney cancer	38·4 (28·5–48·6)	37·7 (28·2–47·4)	37·1 (27·6–47)	37·7 (28·2–47·4)
Bladder cancer	44·2 (29·6–58·3)	45·5 (32–58·4)	45·6 (32·4–57·9)	45·5 (32·1–58·4)
Thyroid cancer	14 (6·86–23)	14·5 (7·05–23·6)	14·5 (7·03–23·6)	14·5 (7·04–23·7)
Mesothelioma	97·5 (96·7–98·2)	94·9 (93·4–96·2)	97·2 (96–98·1)	94·9 (93·3–96·2)
Non-Hodgkin lymphoma	7 (2·87–12·5)	7·09 (2·94–12·6)	7·09 (2·95–12·7)	7·09 (2·93–12·6)
Multiple myeloma	8 (3·38–14·1)	8·31 (3·57–14·8)	8·26 (3·49–14·8)	8·31 (3·58–14·7)
Leukemia	35·9 (23–48·8)	31·5 (21·4–41·4)	30·5 (20·5–40·7)	31·5 (21·5–41·5)
Cardiovascular diseases	78·1 (74·2–81·8)	79 (75·5–82·2)	70·1 (65·1–74·5)	80·2 (76·9–83·4)
Rheumatic heart disease	22 (12·1–40·9)	22·7 (14·2–36·5)	22·7 (14·5–35·3)	22·8 (14·1–37)
Ischemic heart disease	94 (90·5–96·8)	95·3 (92·9–97·4)	94·4 (91·6–96·8)	95·4 (92·9–97·4)
Stroke	81·2 (74–88·8)	84·6 (79·7–89·5)	83·7 (79·4–88·3)	84·8 (79·7–90)
Hypertensive heart disease	100 (100–100)	100 (100–100)	100 (100–100)	100 (100–100)
Non-rheumatic valvular heart disease	25·5 (16·5–37·6)	26·3 (18·6–36·1)	18·2 (12·2–25·3)	27·2 (19·3–37·5)
Cardiomyopathy and myocarditis	32·7 (25·2–41·4)	37 (30·4–44·5)	34 (27·8–40·6)	37·3 (30·4–45)
Atrial fibrillation and flutter	50·2 (39·9–60·7)	58·7 (49·8–67·3)	62·4 (53·6–70·6)	54 (44·4–63·5)
Aortic aneurysm	52·2 (45·3–59·2)	59·4 (53·6–65)	--	59·4 (53·6–65)
Endocarditis	30·9 (21·5–43·2)	33·3 (24·3–43·2)	30·5 (21·1–42·4)	33·4 (24·4–43·2)
Other cardiovascular and circulatory diseases	30·6 (24·3–37·1)	32·5 (26·2–38·7)	33·1 (25·5–41·3)	32·3 (26·2–38·4)
Chronic respiratory diseases	64·7 (59·1–70·2)	54·4 (49·4–59·3)	46·9 (41·2–53)	64 (59·1–68·7)
Chronic obstructive pulmonary disease	74 (68·1–79·3)	71·7 (67·4–75·8)	65·1 (61·4–68·8)	75·4 (70·3–80·3)
Pneumoconiosis	100 (100–100)	100 (100–100)	100 (100–100)	100 (100–100)
Asthma	43 (31·8–55)	38·6 (30·5–48·1)	38·3 (30·4–47·7)	42·8 (32·9–53)

Digestive diseases	38·3 (32·1–44·4)	39·3 (33·1–45·7)	23·9 (16·2–33·1)	50·4 (42·7–58)
Cirrhosis and other chronic liver diseases	76·4 (64·3–88·5)	78·3 (66·1–89·6)	74·9 (63·1–85·3)	78·3 (66·1–89·7)
Upper digestive system diseases	23·4 (19·6–26·9)	8·15 (5·09–11·8)	0·708 (0·405–1·12)	25·7 (22–29·1)
Gallbladder and biliary diseases	44 (29·4–59)	45·8 (32·3–60·4)	45·9 (32·5–60·3)	45·2 (31·3–60)
Pancreatitis	41 (20·2–59·2)	46·3 (24·2–63·9)	46·2 (24·1–64·2)	46·3 (24·4–63·8)
Neurological disorders	25·4 (11·5–41)	11·8 (5·2–22·6)	6·61 (2·79–14·6)	22·1 (11·2–36·6)
Alzheimer's disease and other dementias	42·3 (28·2–57·8)	44·1 (29·9–59·1)	44·2 (30·2–59·2)	44 (29·9–59)
Parkinson's disease	-9·4 (-15·6–-4·44)	-10·5 (-17–-5·01)	-11·2 (-18·1–-5·35)	-10·3 (-16·7–-4·94)
Idiopathic epilepsy	28·6 (18·3–38·6)	25·3 (16·1–34·4)	23 (14·2–31·9)	28·3 (18·2–37·9)
Multiple sclerosis	15·6 (11·7–19·9)	15·6 (11·8–19·6)	15 (11·3–19)	16·1 (12–20·3)
Mental disorders	--	6·99 (3·49–10·8)	6·99 (3·5–10·8)	--
Depressive disorders	--	14·5 (6·62–22·7)	14·5 (6·62–22·7)	--
Anxiety disorders	--	5·59 (1·35–12·9)	5·59 (1·35–12·9)	--
Idiopathic developmental intellectual disability	--	37·6 (17·2–55·9)	37·6 (17·2–55·9)	--
Substance use disorders	100 (100–100)	100 (100–100)	100 (100–100)	100 (100–100)
Alcohol use disorders	100 (100–100)	100 (100–100)	100 (100–100)	100 (100–100)
Drug use disorders	100 (100–100)	100 (100–100)	100 (100–100)	100 (100–100)
Diabetes and kidney diseases	99·6 (99·5–99·7)	99·9 (99·9–99·9)	100 (100–100)	99·6 (99·6–99·7)
Diabetes mellitus	100 (100–100)	100 (100–100)	100 (100–100)	100 (100–100)
Chronic kidney disease	100 (100–100)	100 (100–100)	100 (100–100)	100 (100–100)
Sense organ diseases	--	8·94 (7·89–10·1)	8·94 (7·89–10·1)	--
Blindness and vision loss	--	7·61 (4·95–11·1)	7·61 (4·95–11·1)	--
Age-related and other hearing loss	--	10·8 (9·81–12)	10·8 (9·81–12)	--
Musculoskeletal disorders	3·28 (0·792–5·88)	21·4 (17·8–25·2)	21·7 (18·1–25·6)	3·55 (0·887–6·27)
Rheumatoid arthritis	12·5 (2·97–22·3)	13·5 (3·13–24)	13·5 (3·16–24)	13·7 (3·24–24·1)
Osteoarthritis	--	16·7 (8·84–25·6)	16·7 (8·84–25·6)	--
Low back pain	--	40·1 (34·8–45·3)	40·1 (34·8–45·3)	--
Gout	--	48·7 (32·6–66·4)	48·7 (32·6–66·4)	--
Other non-communicable diseases	0·199 (0·13–0·292)	0·12 (0·0737–0·185)	--	0·554 (0·346–0·802)
Sudden infant death syndrome	9·3 (5·34–14·6)	9·29 (5·33–14·6)	--	9·29 (5·33–14·6)
Injuries	27·6 (21·8–33·6)	23·6 (19–28·6)	25·3 (21·5–30)	21·5 (14·5–28·2)
Transport injuries	20·4 (12·1–28·5)	20·5 (13·4–27·5)	25·4 (20·9–30·4)	18·3 (9·25–26·9)
Road injuries	19·7 (10·8–28·1)	19·7 (12·3–27)	24·5 (20–29·6)	17·6 (8·41–26·5)
Other transport injuries	30·1 (24·6–35·8)	28·9 (23·8–34·4)	32·1 (27·7–36·7)	26·7 (21–32·8)

Unintentional injuries	33·3 (28·1–37·7)	23·6 (19·4–28·4)	24·7 (20·3–29·9)	19·7 (15·7–24·4)
Falls	51·6 (44·4–56·9)	34·1 (28·8–39·9)	32·7 (27·3–38·3)	40·7 (34·8–46·4)
Drowning	-14·3 (-27·5–-0·404)	-14·3 (-26·6–-1·27)	11 (6·44–17·4)	-15·4 (-28·4–-1·97)
Fire, heat, and hot substances	11·3 (6·56–17·8)	13·1 (8·13–19·7)	13·8 (8·59–20·8)	11·7 (7·28–17·9)
Poisonings	13·3 (8·03–19·9)	12·7 (7·82–18·9)	12·2 (7·59–18·2)	13·3 (8·27–19·8)
Exposure to mechanical forces	4·08 (-8·9–17·7)	14·7 (11·9–17·8)	15·8 (13·4–18·7)	-2·05 (-15·4–12·4)
Animal contact	20·5 (15·6–26·7)	19·2 (14·5–25)	19·9 (15–25·7)	16·6 (12·1–22)
Foreign body	3·65 (2·32–5·54)	4·08 (2·63–6·04)	3·55 (2·32–5·37)	4·46 (2·85–6·72)
Environmental heat and cold exposure	9·37 (4·31–16·3)	9·56 (4·42–16·5)	9·4 (4·32–16·4)	10·2 (4·78–17·4)
Exposure to forces of nature	0 (0–0)	10·3 (4·91–17·7)	10·3 (4·91–17·7)	0 (0–0)
Other unintentional injuries	15 (9·13–22·1)	13·1 (8·15–19·8)	13 (8·08–19·7)	15·6 (9·88–22·6)
Self-harm and interpersonal violence	23·9 (11·1–36·4)	25·5 (14·1–36·5)	34·6 (26·7–42·9)	24·2 (11·5–36·4)
Self-harm	23·9 (9·3–37·8)	24·6 (9·73–38·3)	24·4 (11·7–37·2)	24·6 (9·68–38·3)
Interpersonal violence	25·4 (17·1–34·6)	30 (21·7–38·7)	39·1 (30–48·5)	22·4 (14·5–31·2)

Supplementary Results Table 2. All-age PAFs attributable to the joint distribution of all risk factors for all ages, both sexes combined for each GBD level 1, level 2, and level 3 cause and all causes in 2019 for Harrow. Results are shown for deaths, YLLs, YLDs, and DALYs Data in parenthesis are 95% uncertainty intervals. PAFs=population attributable fractions. GBD=Global Burden of Disease. YLLs=years of life lost. YLDs=years lived with disability. DALYs=disability-adjusted life-years

	Deaths 2019	DALYs 2019	YLDs 2019	YLLs 2019
All causes	52·9 (49·2–56·5)	38 (35–41·1)	27·2 (24·9–29·6)	52·2 (49·2–55)
Communicable, maternal, neonatal, and nutritional diseases	47·2 (36·9–56·7)	55·1 (50·7–59·6)	47·5 (42·9–52)	59·8 (53–66·2)
HIV/AIDS and sexually transmitted infections	74·3 (58·8–85·9)	73·5 (55·7–86·3)	97·2 (95·6–98·3)	57·6 (38·6–75)
HIV/AIDS	90·4 (88·5–91·7)	88·6 (86·2–90·5)	94·2 (91·8–95·7)	85·5 (83·1–87)
Sexually transmitted infections excluding HIV	43 (22·1–69·5)	55·5 (29·8–81·3)	100 (100–100)	14·7 (5·38–32·4)
Respiratory infections and tuberculosis	46·8 (35·1–57·9)	41·4 (32·2–50·9)	3·58 (2·58–4·81)	50·1 (39·2–60·2)
Tuberculosis	55·9 (43·1–66·9)	57·3 (46·3–66·6)	54 (42·5–63·3)	58·2 (46·8–67·5)
Lower respiratory infections	46·7 (34·9–57·9)	50 (38·8–60·2)	34·3 (27·9–41·3)	50·1 (38·9–60·5)
Upper respiratory infections	0·0983 (0·0518–0·169)	0·0107 (0·00272–0·0234)	0·00788 (0·000309–0·0207)	0·2 (0·107–0·345)
Otitis media	4·22 (1·89–8·44)	4·91 (3·13–6·91)	4·83 (3·05–6·82)	9·41 (5·12–15)
Enteric infections	4·96 (3·12–7·94)	9·78 (7·51–12·8)	9·68 (7·15–13·1)	10·1 (7·25–13·7)
Diarrheal diseases	5·01 (3·14–8·11)	9·83 (7·54–12·9)	9·69 (7·16–13·2)	10·3 (7·38–13·9)
Other infectious diseases	1·22 (0·731–1·66)	1·98 (1·44–2·55)	0·317 (0·183–0·544)	2·79 (2·01–3·66)
Meningitis	3·16 (2·31–4·22)	4·16 (3·01–5·49)	0·00066 (2·06e-05–0·002)	5·77 (4·2–7·57)
Encephalitis	0·666 (0·373–1)	1·44 (0·629–2·23)	0·000236 (7·02e-06–0·000668)	2·04 (1·04–3·06)
Measles	4·27 (-1·4–21·6)	4·74 (-1·64–24)	0·431 (0·0128–1·24)	4·99 (-1·69–25·4)
Acute hepatitis	14·1 (7·43–26·1)	4·86 (2·79–7·91)	2·67 (1·54–4·37)	11·9 (6·07–21·8)
Maternal and neonatal disorders	89·2 (87–91·1)	80·3 (77·1–83·5)	64·6 (58·5–70·2)	89·8 (87·7–91·6)
Maternal disorders	9·09 (3·09–15·2)	9·19 (3·06–15·4)	9·37 (3·15–15·8)	8·92 (3·06–14·9)
Neonatal disorders	90·8 (88·8–92·6)	81·6 (78·3–84·8)	66·2 (60·1–72)	90·8 (88·8–92·6)
Nutritional deficiencies	56·3 (48·4–88·8)	78·4 (74·4–83·5)	78·7 (74·7–83·8)	57·9 (52·8–79·1)
Protein-energy malnutrition	100 (100–100)	100 (100–100)	100 (100–100)	100 (100–100)
Vitamin A deficiency	--	100 (100–100)	100 (100–100)	--
Dietary iron deficiency	--	100 (100–100)	100 (100–100)	--
Non-communicable diseases	54·2 (50·1–58)	37·6 (34·3–40·8)	26·2 (23·7–28·7)	53·2 (50·1–56·5)
Neoplasms	44·9 (40·9–49·5)	43·6 (39·7–47·7)	30·9 (26·7–35·7)	44·4 (40·6–48·7)
Lip and oral cavity cancer	65 (53·6–74·8)	68 (57·4–77·3)	67·5 (56·4–77·2)	68 (57·6–77·2)
Nasopharynx cancer	65·5 (53·1–75·9)	65 (52·8–74·9)	63·3 (50·4–73·8)	65·2 (53–75)
Other pharynx cancer	78·3 (69·8–84·9)	79·5 (71·2–85·7)	78·4 (70–85·1)	79·6 (71·3–85·8)
Esophageal cancer	74·4 (65–82·8)	75·7 (66·9–84·1)	74·8 (65·1–83·1)	75·7 (66·9–84·1)

Stomach cancer	23·7 (16·4–40·5)	23·8 (16·7–40·7)	24 (16·7–40·8)	23·8 (16·7–40·7)
Colon and rectum cancer	63·4 (53·9–72·6)	63·5 (54·8–72·2)	63·6 (54·6–72·4)	63·5 (54·8–72·2)
Liver cancer	75·7 (69·1–81·8)	76 (70–81·5)	76·4 (70·2–81·9)	76 (69·9–81·4)
Gallbladder and biliary tract cancer	21·5 (11·6–34·1)	21·9 (11·9–34·6)	20·3 (10·7–32·7)	22 (11·9–34·7)
Pancreatic cancer	42 (32·1–53·4)	41·3 (32·2–51·9)	41·8 (32·1–53)	41·3 (32·2–51·9)
Larynx cancer	82·1 (73·7–88·9)	81·6 (73·6–87·8)	79·5 (71·4–85·9)	81·8 (73·9–88·1)
Tracheal, bronchus, and lung cancer	85 (81·3–88·4)	83·8 (80·2–87·1)	84·5 (80·8–88·1)	83·7 (80·2–87·1)
Breast cancer	36·3 (26·6–47·6)	35 (26·3–45·1)	35·2 (26·5–45·5)	35 (26·3–45·1)
Cervical cancer	100 (100–100)	100 (100–100)	100 (100–100)	100 (100–100)
Uterine cancer	43·2 (28·7–59·3)	44·2 (29·8–60·1)	44·5 (29·8–60·4)	44·2 (29·8–60·1)
Ovarian cancer	23·8 (11·8–38·5)	20·1 (9·74–33·4)	19·2 (9·26–31·9)	20·2 (9·75–33·5)
Prostate cancer	5·43 (2·26–8·91)	5·88 (2·56–9·42)	6·22 (2·72–10)	5·81 (2·53–9·35)
Kidney cancer	37·3 (27·2–48)	37·1 (27·3–47)	36·8 (27–46·7)	37·1 (27·3–47·1)
Bladder cancer	41·3 (26·8–56·3)	43·3 (29·5–57·3)	43·8 (30·3–57·2)	43·3 (29·4–57·2)
Thyroid cancer	13·8 (6·57–22·8)	14·4 (6·82–23·8)	14·5 (6·87–23·9)	14·4 (6·8–23·8)
Mesothelioma	98·3 (97·6–98·8)	96·5 (95·3–97·4)	98 (97·2–98·6)	96·4 (95·3–97·3)
Non-Hodgkin lymphoma	6·84 (2·82–12·3)	7·01 (2·94–12·7)	6·96 (2·87–12·6)	7·01 (2·95–12·6)
Multiple myeloma	7·77 (3·26–14)	8·13 (3·41–14·5)	8·05 (3·39–14·5)	8·13 (3·4–14·5)
Leukemia	35 (21·2–49)	31·5 (20·6–43·1)	30·3 (19·8–41·5)	31·6 (20·7–43·2)
Cardiovascular diseases	74·6 (70–80)	76·3 (72·7–81)	69·6 (64·8–74·2)	77·2 (73·6–82·3)
Rheumatic heart disease	21·5 (11·2–43·1)	22·4 (13·1–39·5)	22·5 (13·5–36·9)	22·4 (13–39·6)
Ischemic heart disease	93·1 (88·8–96·5)	94·3 (91·3–96·9)	93·7 (90·5–96·4)	94·3 (91·3–96·9)
Stroke	78·8 (70·4–87·9)	81·9 (75·8–88·5)	81·6 (76·6–87·3)	81·9 (75·4–88·7)
Hypertensive heart disease	100 (100–100)	100 (100–100)	100 (100–100)	100 (100–100)
Non-rheumatic valvular heart disease	24·4 (15·4–37·3)	24·9 (16·9–35)	17·3 (11·5–24·4)	25·9 (17·6–36·6)
Cardiomyopathy and myocarditis	27·8 (18·8–39·7)	32·4 (24·8–42·1)	31·1 (24·2–39)	32·6 (24·7–42·6)
Atrial fibrillation and flutter	48·5 (37·7–59·8)	56·2 (47·4–65·5)	60·4 (51·4–69·3)	51·5 (42·1–61·9)
Aortic aneurysm	47·9 (41·1–55·4)	55·2 (49·1–61·3)	--	55·2 (49·1–61·3)
Endocarditis	29·4 (19·7–43·2)	32 (23–42·6)	29·9 (20·1–42·2)	32 (23·1–42·7)
Other cardiovascular and circulatory diseases	29·1 (22·4–36·6)	31·9 (25·9–38·2)	33·4 (26·1–41·1)	31·5 (25·6–38)
Chronic respiratory diseases	59·5 (52·2–67·1)	52·8 (47·8–57·9)	47·4 (41·9–53)	59·5 (52·9–66·4)
Chronic obstructive pulmonary disease	71·2 (64·9–77·3)	69·2 (64·7–73·6)	63·4 (59·3–67·5)	73·1 (67·4–78·5)
Pneumoconiosis	100 (100–100)	100 (100–100)	100 (100–100)	100 (100–100)
Asthma	40·4 (28·7–53·4)	37·5 (29·4–47·3)	37·3 (29·1–46·8)	40·9 (31–52·1)

Digestive diseases	28·8 (23·7–34·2)	30·6 (25·2–35·8)	14·6 (9·73–20·5)	41 (34·6–47·6)
Cirrhosis and other chronic liver diseases	73·8 (60·7–86·3)	76·7 (65–88·2)	72·3 (60·3–83·4)	76·9 (65–88·3)
Upper digestive system diseases	20·4 (16·6–24)	6·91 (4·36–10)	0·678 (0·383–1·08)	22·7 (19·2–26)
Gallbladder and biliary diseases	44·1 (29·3–59·7)	45·1 (31·8–59·7)	45·1 (31·8–59·6)	45·1 (30·9–60·4)
Pancreatitis	36 (16·4–55·4)	42·4 (22·1–59·7)	43·8 (23·6–61·6)	42·1 (21·9–59·8)
Neurological disorders	27·7 (13·4–43·2)	15·5 (7·29–27·7)	9·15 (4·48–18·2)	24·1 (11·5–38·9)
Alzheimer's disease and other dementias	40·3 (25·9–55·6)	42·1 (28–57)	42·4 (28·2–57·4)	41·9 (27·9–56·9)
Parkinson's disease	-8·46 (-14–-3·8)	-9·48 (-15·3–-4·28)	-10·1 (-15·9–-4·7)	-9·31 (-15·1–-4·22)
Idiopathic epilepsy	26·5 (16·7–37)	23·6 (14·7–32·5)	21·9 (13·1–30·7)	26·3 (17·1–35·9)
Multiple sclerosis	14·4 (10·7–18·2)	14·7 (11–18·5)	14·2 (10·7–18)	15·1 (11·3–19)
Mental disorders	--	6·71 (3·37–10·4)	6·71 (3·37–10·4)	--
Depressive disorders	--	13·9 (6·31–21·8)	13·9 (6·31–21·8)	--
Anxiety disorders	--	5·37 (1·38–12·4)	5·37 (1·38–12·4)	--
Idiopathic developmental intellectual disability	--	37·5 (16·8–56·2)	37·5 (16·8–56·2)	--
Substance use disorders	100 (100–100)	100 (100–100)	100 (100–100)	100 (100–100)
Alcohol use disorders	100 (100–100)	100 (100–100)	100 (100–100)	100 (100–100)
Drug use disorders	100 (100–100)	100 (100–100)	100 (100–100)	100 (100–100)
Diabetes and kidney diseases	100 (100–100)	100 (100–100)	100 (100–100)	100 (100–100)
Diabetes mellitus	100 (100–100)	100 (100–100)	100 (100–100)	100 (100–100)
Chronic kidney disease	100 (100–100)	100 (100–100)	100 (100–100)	100 (100–100)
Sense organ diseases	--	8·05 (6·98–9·31)	8·05 (6·98–9·31)	--
Blindness and vision loss	--	8·93 (5·65–13·2)	8·93 (5·65–13·2)	--
Age-related and other hearing loss	--	8·95 (8·07–9·96)	8·95 (8·07–9·96)	--
Musculoskeletal disorders	3·48 (0·877–6·48)	20·7 (16·9–24·6)	21 (17·1–24·9)	4·18 (1·07–7·52)
Rheumatoid arthritis	11·1 (2·7–20)	12·8 (3·24–22·5)	12·9 (3·25–22·6)	12·5 (3·2–22)
Osteoarthritis	--	16·4 (8·36–25·1)	16·4 (8·36–25·1)	--
Low back pain	--	38 (32·9–43·4)	38 (32·9–43·4)	--
Gout	--	49·8 (34·6–66·8)	49·8 (34·6–66·8)	--
Other non-communicable diseases	0·159 (0·107–0·234)	0·12 (0·0756–0·18)	--	0·516 (0·351–0·723)
Sudden infant death syndrome	9·37 (5·15–15·9)	9·36 (5·15–15·8)	--	9·36 (5·15–15·8)
Injuries	31·2 (25·6–36·7)	26·4 (22·1–30·9)	28·5 (24·3–32·8)	22·7 (16·3–29·7)
Transport injuries	22 (13·7–29·6)	22·2 (15·3–28·6)	26·7 (22·2–31·2)	19·6 (10·7–27·8)
Road injuries	21·1 (12·1–29)	21·3 (14·1–28)	25·6 (21·1–30·2)	18·9 (9·38–27·4)
Other transport injuries	32 (26·6–37·1)	30·3 (25·3–35·1)	34·6 (30–39)	27·1 (21·3–32·6)

Unintentional injuries	39<br>(32·1–43·9)	27·6<br>(23·2–32·2)	28·3<br>(23·8–33·1)	24·5<br>(19·6–28·6)
Falls	54·9<br>(45·8–60·8)	38·6<br>(32·5–43·9)	37·2<br>(31·3–42·5)	46<br>(39–51·5)
Drowning	-15·7<br>(-29·2–-2·04)	-15<br>(-27·6–-2·35)	11·1<br>(6·51–17·4)	-16·9<br>(-30·1–-3·6)
Fire, heat, and hot substances	9·88<br>(5·49–15·9)	13·2<br>(8·19–19·4)	14·1<br>(8·96–20·7)	10·5<br>(6·5–16·1)
Poisonings	14<br>(8·67–20·7)	13·1<br>(8·34–19·2)	12·5<br>(8–18·7)	14·2<br>(9·18–20·6)
Exposure to mechanical forces	9·71<br>(-2·99–23)	17·4<br>(14·2–20·6)	18·3<br>(15·4–21·5)	0·693<br>(-12·7–14·6)
Animal contact	20·8<br>(16–26·7)	20·5<br>(15·6–26·2)	21·4<br>(16·4–27·3)	16·3<br>(12·2–21·5)
Foreign body	3·41<br>(2·13–5·1)	4·38<br>(2·83–6·43)	4·08<br>(2·65–6·04)	4·73<br>(2·99–7·07)
Environmental heat and cold exposure	7·84<br>(3·39–14·3)	9·12<br>(4·12–16·4)	9·21<br>(4·18–16·5)	8·42<br>(3·79–15·1)
Exposure to forces of nature	0<br>(0–0)	10·1<br>(4·67–18·1)	10·1<br>(4·67–18·1)	0<br>(0–0)
Other unintentional injuries	13·9<br>(8·62–20·8)	13·3<br>(8·32–19·8)	13·3<br>(8·28–19·8)	15·3<br>(9·92–21·7)
Self-harm and interpersonal violence	22·7<br>(9·65–36·2)	25·1<br>(13·8–36·7)	33·7<br>(25·7–41·9)	23·2<br>(10·3–36·4)
Self-harm	22·6<br>(8·81–37·2)	23·7<br>(9·79–38·2)	23·4<br>(11·4–36·7)	23·7<br>(9·76–38·3)
Interpersonal violence	24·7<br>(16·6–33·4)	32<br>(24·2–40·7)	38·7<br>(29·8–48·1)	20·7<br>(12·8–28·9)

Supplementary Results Table 2. All-age PAFs attributable to the joint distribution of all risk factors for all ages, both sexes combined for each GBD level 1, level 2, and level 3 cause and all causes in 2019 for Bulgaria. Results are shown for deaths, YLLs, YLDs, and DALYs Data in parenthesis are 95% uncertainty intervals. PAFs=population attributable fractions. GBD=Global Burden of Disease. YLLs=years of life lost. YLDs=years lived with disability. DALYs=disability-adjusted life-years				
	**Deaths 2019**	**DALYs 2019**	**YLDs 2019**	**YLLs 2019**
All causes	75·1 (72·1–77·9)	61·6 (57·9–65)	33·9 (31·1–36·5)	73·1 (71–75)
Communicable, maternal, neonatal, and nutritional diseases	57·8 (52·8–62·5)	60·4 (57·1–63·6)	52·9 (47–58·2)	64·9 (61–68·3)
HIV/AIDS and sexually transmitted infections	77·4 (76·9–77·9)	81·7 (79·5–85)	96 (93·9–97·7)	76 (75·4–76·6)
HIV/AIDS	76·9 (76·4–77·4)	75·9 (75·3–76·5)	78·9 (77·9–80·2)	75·7 (75·1–76·2)
Sexually transmitted infections excluding HIV	98·5 (97·9–98·9)	99·8 (99·6–99·9)	100 (100–100)	95·7 (94–96·9)
Respiratory infections and tuberculosis	59·3 (53·5–64·7)	58·6 (53·2–64·2)	9·55 (6·82–13)	65·6 (60–70·4)
Tuberculosis	67 (59·4–72·9)	69·7 (62·4–75·1)	62·8 (54·8–69·4)	70·9 (63·9–76·2)
Lower respiratory infections	59 (52·7–64·7)	65·2 (59·3–70·3)	47·6 (41·6–53·4)	65·3 (59·4–70·4)
Upper respiratory infections	0·17 (0·0891–0·299)	0·0156 (0·00803–0·0278)	0·00458 (0·000755–0·00949)	0·542 (0·284–0·925)
Otitis media	0·21 (0·0581–0·625)	4·75 (3·1–6·63)	4·76 (3·1–6·65)	1·01 (0·293–3·01)
Enteric infections	49·5 (33·8–66·5)	42·1 (23·9–62·3)	39·5 (20·2–60·9)	65·5 (52·7–78)
Diarrheal diseases	51 (34·9–68·3)	42·2 (24–62·4)	39·5 (20·3–61)	66·9 (53·9–79·6)
Other infectious diseases	1·3 (0·994–1·75)	1·84 (1·42–2·33)	0·299 (0·177–0·489)	2·28 (1·76–2·83)
Meningitis	1·52 (1·08–2·05)	2·93 (2·07–3·93)	7e-04 (1e-04–0·00151)	3·3 (2·35–4·38)
Encephalitis	0·857 (0·458–1·19)	1·69 (0·949–2·33)	0·000388 (5·1e-05–0·000838)	1·78 (1·01–2·44)
Measles	8·07 (1·99–22·7)	4·36 (0·975–9·6)	3·42 (0·112–7·86)	10·2 (2·69–28·9)
Acute hepatitis	6·77 (3·72–14·7)	4·16 (2·33–8·41)	1·47 (0·896–2·41)	6·43 (3·4–13·7)
Maternal and neonatal disorders	83·7 (81·5–85·6)	69·4 (65·3–73·3)	48·9 (44·1–53·2)	85 (82·9–86·8)
Maternal disorders	13·3 (4·88–22·2)	13·2 (4·8–22)	13·2 (4·76–22)	13·2 (4·83–22)
Neonatal disorders	87·3 (85·2–89·2)	71·3 (67·1–75·4)	50·1 (45·2–54·5)	87·3 (85·2–89·2)
Nutritional deficiencies	34·5 (25·2–44)	93·7 (91·6–95·5)	95·2 (93·3–96·6)	34·2 (27·9–41·3)
Protein-energy malnutrition	100 (100–100)	100 (100–100)	100 (100–100)	100 (100–100)
Vitamin A deficiency	--	100 (100–100)	100 (100–100)	--
Dietary iron deficiency	--	100 (100–100)	100 (100–100)	--
Non-communicable diseases	77 (73·8–79·9)	65 (61·2–68·7)	34 (30·8–37·2)	76·4 (74·2–78·5)
Neoplasms	50·2 (46·7–54·4)	51·7 (48·5–55·3)	42·2 (38·3–46·6)	52 (48·8–55·6)
Lip and oral cavity cancer	76·3 (71·1–80·6)	79·9 (75·2–83·9)	78·3 (73·3–82·8)	79·9 (75·3–84)
Nasopharynx cancer	70·5 (62·6–76·9)	72·9 (65·4–79·2)	72·8 (64·8–79·3)	72·9 (65·4–79·3)
Other pharynx cancer	85·7 (81·9–88·8)	87·2 (83·5–90·2)	85 (81–88·3)	87·2 (83·5–90·2)
Esophageal cancer	79·8 (72·5–86·5)	82·2 (75·3–88·4)	81 (73·9–87·5)	82·2 (75·3–88·4)
Stomach cancer	29·7 (20·2–48·7)	31·4 (21·9–50)	30·7 (20·9–48·9)	31·4 (21·9–50)

Colon and rectum cancer	63·5 (56·3–70·6)	64·9 (57·9–71·6)	64·7 (57·7–71·4)	64·9 (57·9–71·6)
Liver cancer	75·5 (68·9–81·6)	76·1 (69·4–82)	75·9 (69·2–81·9)	76·1 (69·4–82)
Gallbladder and biliary tract cancer	24·5 (13·9–36·9)	25·2 (14·5–37·8)	25 (14·3–37·5)	25·2 (14·5–37·8)
Pancreatic cancer	40·5 (33·1–49·4)	42·2 (35–50·7)	41·4 (33·7–50·2)	42·2 (35·1–50·7)
Larynx cancer	85·8 (79·4–90·6)	87·8 (82·1–91·8)	87·2 (81·5–91·3)	87·8 (82·1–91·8)
Tracheal, bronchus, and lung cancer	86 (83·9–88·3)	86·6 (84·7–88·8)	86·5 (84·3–88·8)	86·6 (84·7–88·8)
Breast cancer	32·8 (25·2–41·5)	33 (26–40·8)	33 (25·9–40·9)	33 (26–40·8)
Cervical cancer	100 (100–100)	100 (100–100)	100 (100–100)	100 (100–100)
Uterine cancer	49·2 (34·8–63·9)	50·5 (36–65)	51·1 (36·7–65·7)	50·5 (36–64·9)
Ovarian cancer	12·5 (4·4–23·3)	11·3 (3·84–21·1)	11·1 (3·8–20·9)	11·3 (3·85–21·1)
Prostate cancer	7·71 (3·77–11·9)	8·67 (4·38–13·2)	8·87 (4·45–13·5)	8·65 (4·38–13·2)
Kidney cancer	43·7 (34–53·1)	44·4 (35–53·6)	43·5 (34·1–52·8)	44·5 (35–53·7)
Bladder cancer	41·1 (28·8–52·9)	46·4 (34·4–57)	47·5 (35·7–58·1)	46·3 (34·4–57)
Thyroid cancer	16·3 (8·6–25·5)	16·9 (8·92–26·2)	16·5 (8·89–25·2)	16·9 (8·91–26·3)
Mesothelioma	77 (69–83·4)	69·8 (60·7–77·5)	75·6 (67·1–82·5)	69·7 (60·6–77·4)
Non-Hodgkin lymphoma	8·09 (3·53–14)	7·9 (3·55–13·5)	7·91 (3·54–13·7)	7·9 (3·55–13·5)
Multiple myeloma	9·48 (4·31–16·3)	9·73 (4·44–16·5)	9·74 (4·46–16·6)	9·73 (4·44–16·5)
Leukemia	37·8 (26–49·3)	35·8 (25·8–45·9)	37·6 (26·6–48·6)	35·8 (25·7–45·8)
Cardiovascular diseases	88·2 (84·8–91·2)	89·1 (86·7–91·4)	81·9 (78·1–85·5)	89·6 (87·1–91·8)
Rheumatic heart disease	29·1 (18·2–43)	29·6 (19·5–44·8)	28·7 (18·2–43·2)	29·6 (19·6–45)
Ischemic heart disease	93·7 (90·1–96·7)	95·1 (92·6–97·2)	94·7 (92·1–97)	95·1 (92·6–97·2)
Stroke	84·5 (78·5–90·6)	87·6 (83·1–92)	87·6 (83·3–91·8)	87·6 (83·1–92)
Hypertensive heart disease	100 (100–100)	100 (100–100)	100 (100–100)	100 (100–100)
Non-rheumatic valvular heart disease	22·6 (15·3–30·2)	21·1 (15–27·4)	18·3 (12–25·5)	22·4 (15·8–28·9)
Cardiomyopathy and myocarditis	29·4 (22·2–38·7)	31·7 (25·2–38·7)	29 (22·2–36·3)	31·7 (25·2–38·8)
Atrial fibrillation and flutter	56·2 (46·8–65·1)	63·6 (56–71·1)	66·6 (59·2–73·8)	61 (52·2–69·2)
Aortic aneurysm	70·5 (65·7–75)	76·3 (72·5–79·6)	--	76·3 (72·5–79·6)
Endocarditis	45·2 (34·4–56·3)	45·1 (34·1–56·2)	43 (32–53·9)	45·1 (34·1–56·2)
Other cardiovascular and circulatory diseases	42·3 (35·8–48·5)	44·7 (38·6–50·8)	39·9 (32·9–46·5)	45·1 (38·9–51·1)
Chronic respiratory diseases	73·4 (69·6–76·6)	70·1 (66·7–73·3)	59·7 (55·5–64)	76·5 (73·2–79·2)
Chronic obstructive pulmonary disease	75·6 (71·8–78·9)	76·9 (73·9–79·8)	70·5 (67·1–73·8)	79·6 (76·4–82·4)
Pneumoconiosis	100 (100–100)	100 (100–100)	100 (100–100)	100 (100–100)
Asthma	46·5 (35·3–58·7)	45·5 (36·7–55)	44·8 (36·3–54·4)	51·5 (40·8–62·6)
Digestive diseases	55·7 (49·9–60·8)	56·3 (50·1–62)	22·8 (16·2–30·4)	63·7 (57·4–69)

Cirrhosis and other chronic liver diseases	82·2 (73·2–89·1)	83·7 (74·8–90·4)	80·1 (73·1–86·5)	83·7 (74·8–90·5)
Upper digestive system diseases	23·7 (20·5–27·7)	14·4 (10–19·4)	1·55 (0·929–2·36)	28·9 (25·4–32·9)
Gallbladder and biliary diseases	45·8 (31·3–60·5)	52·7 (39·3–66·2)	53·7 (40·5–67)	48·9 (34·6–63·1)
Pancreatitis	45·4 (35·7–57·5)	50·6 (40·4–62·8)	46·3 (36–57·9)	50·8 (40·6–63·2)
Neurological disorders	21·2 (8·53–35·1)	16·2 (8·9–27)	11·8 (6·35–20·3)	21·4 (9·59–34·8)
Alzheimer's disease and other dementias	33·9 (21·2–48·9)	37·3 (24·4–51·9)	37·3 (24·4–52)	37·2 (24·3–51·7)
Parkinson's disease	-9·33 (-14–-4·79)	-11·8 (-17·8–-6·11)	-13 (-19·9–-6·8)	-11·6 (-17·6–-5·97)
Idiopathic epilepsy	31·1 (23·9–38·7)	27·3 (20·4–34)	25·1 (18·8–31·4)	30·5 (23·5–38)
Multiple sclerosis	17·1 (12·8–22·4)	21·4 (16·6–26·2)	20·8 (16–25·4)	21·7 (16·6–26·9)
Mental disorders	--	5·97 (2·79–9·55)	5·97 (2·79–9·55)	--
Depressive disorders	--	11 (3·6–19·5)	11 (3·6–19·5)	--
Anxiety disorders	--	5·27 (1·48–11·2)	5·27 (1·48–11·2)	--
Idiopathic developmental intellectual disability	--	56·8 (28·3–76·9)	56·8 (28·3–76·9)	--
Substance use disorders	100 (100–100)	100 (100–100)	100 (100–100)	100 (100–100)
Alcohol use disorders	100 (100–100)	100 (100–100)	100 (100–100)	100 (100–100)
Drug use disorders	100 (100–100)	100 (100–100)	100 (100–100)	100 (100–100)
Diabetes and kidney diseases	100 (99·9–100)	100 (100–100)	100 (100–100)	99·9 (99·9–100)
Diabetes mellitus	100 (100–100)	100 (100–100)	100 (100–100)	100 (100–100)
Chronic kidney disease	100 (100–100)	100 (100–100)	100 (100–100)	100 (100–100)
Sense organ diseases	--	7·59 (6·62–8·6)	7·59 (6·62–8·6)	--
Blindness and vision loss	--	4·53 (2·71–6·58)	4·53 (2·71–6·58)	--
Age-related and other hearing loss	--	9·13 (8·32–10·1)	9·13 (8·32–10·1)	--
Musculoskeletal disorders	3·48 (1·02–6·22)	36·2 (30·2–42·1)	36·5 (30·5–42·4)	3·86 (1·16–6·73)
Rheumatoid arthritis	10·1 (2·91–17·9)	18·5 (5·98–30·8)	19·4 (6·35–32·5)	13·7 (4·14–23·7)
Osteoarthritis	--	21 (11·7–31·1)	21 (11·7–31·1)	--
Low back pain	--	46·4 (39·8–52·6)	46·4 (39·8–52·6)	--
Gout	--	55·5 (40·7–71)	55·5 (40·7–71)	--
Other non-communicable diseases	0·0412 (0·0227–0·058)	0·0177 (0·0108–0·0268)	--	0·0842 (0·0574–0·114)
Sudden infant death syndrome	6·88 (4·28–10·7)	6·87 (4·28–10·7)	--	6·87 (4·28–10·7)
Injuries	22·2 (17·8–26·4)	23·7 (20–27·2)	26·7 (23·5–29·9)	20·5 (15·9–24·8)
Transport injuries	24·9 (19·5–29·7)	25·3 (20·6–29·7)	27·2 (23·4–30·7)	23·8 (17·8–29)
Road injuries	23·3 (17·7–28·3)	24·7 (19·9–29·1)	26·9 (23·1–30·4)	22·6 (16·5–28)
Other transport injuries	37 (32·4–41·2)	33·9 (29·2–38·2)	35·8 (31·6–39·5)	33·4 (28·6–37·8)
Unintentional injuries	20·6 (16–25·8)	23·1 (18·7–27·6)	26·7 (23·1–30·4)	15·8 (11·3–20·9)

Falls	41·5 (35·8–46·9)	36 (30·8–41·3)	36·9 (31·8–41·9)	33·7 (28·3–39·6)
Drowning	-9·12 (-18·1–0·887)	-8·75 (-17·1–0·493)	10·1 (6·56–14·8)	-9·33 (-17·9–0·03)
Fire, heat, and hot substances	13 (8·28–19)	15·4 (10·4–21·3)	15·4 (10·3–21·3)	15·3 (10·3–21·4)
Poisonings	14·2 (9·24–20·3)	14·8 (10–20·3)	13·5 (9·22–18·8)	16·1 (11–22·1)
Exposure to mechanical forces	8·19 (1·5–14·4)	17·9 (14·7–20·9)	20·1 (17·4–22·6)	4·06 (-2·68–10·6)
Animal contact	26·7 (21·3–32·8)	22·3 (17·5–28)	20·9 (16·2–26·2)	25·3 (19·8–31·6)
Foreign body	6·53 (4·7–8·64)	6·52 (4·75–8·62)	4·95 (3·55–6·64)	7·09 (5·11–9·48)
Environmental heat and cold exposure	11·5 (5·41–19)	11·7 (5·47–19·4)	8·84 (4·1–14·8)	12·1 (5·72–20·1)
Exposure to forces of nature	0 (0–0)	10·3 (4·82–17·3)	10·3 (4·82–17·3)	0 (0–0)
Other unintentional injuries	18·2 (12·2–25·3)	16·2 (11·2–22·2)	14·6 (9·72–20·2)	19·5 (13·6–26·5)
Self-harm and interpersonal violence	22·5 (12·2–32·4)	23·4 (14·1–32·8)	23·9 (18–29·9)	23·4 (13·1–33·4)
Self-harm	22·8 (10·9–34·4)	24·3 (11·9–36·1)	22·8 (12·6–32·7)	24·3 (11·9–36·2)
Interpersonal violence	20·8 (13·1–29·3)	20·9 (13·4–28·9)	24·3 (17·8–31·8)	18·9 (11·1–27·7)

Supplementary Results Table 2. All-age PAFs attributable to the joint distribution of all risk factors for all ages, both sexes combined for each GBD level 1, level 2, and level 3 cause and all causes in 2019 for Havering. Results are shown for deaths, YLLs, YLDs, and DALYs Data in parenthesis are 95% uncertainty intervals. PAFs=population attributable fractions. GBD=Global Burden of Disease. YLLs=years of life lost. YLDs=years lived with disability. DALYs=disability-adjusted life-years

	Deaths 2019	DALYs 2019	YLDs 2019	YLLs 2019
All causes	53·7 (50·2–57·1)	40·5 (37·4–43·7)	27·4 (25·2–29·6)	53·8 (51–56·6)
Communicable, maternal, neonatal, and nutritional diseases	44·5 (33·4–55·4)	49·4 (43·1–55·4)	45·9 (41·6–50·4)	51·1 (41·9–59·7)
HIV/AIDS and sexually transmitted infections	70·6 (52·5–85·3)	72 (52–87·2)	98·1 (97–98·9)	50·9 (30·7–71·7)
HIV/AIDS	92·2 (90·6–93·3)	90·4 (88·4–92·1)	94·5 (92·4–95·9)	88·1 (86–89·5)
Sexually transmitted infections excluding HIV	41·1 (20·6–67·8)	58·8 (33·1–83·7)	100 (100–100)	13·2 (4·94–29·4)
Respiratory infections and tuberculosis	45·2 (33·1–56·9)	42·5 (32–52·3)	3·57 (2·64–4·7)	48·1 (36·7–59·3)
Tuberculosis	54 (41·2–64·9)	56·5 (44·5–66·1)	53·1 (40·8–62·8)	57·3 (45·5–66·9)
Lower respiratory infections	45·2 (33–57)	48 (36·5–59·2)	32·7 (25·9–39·6)	48·1 (36·5–59·4)
Upper respiratory infections	0·0349 (0·0183–0·06)	0·00872 (0·00168–0·0206)	0·00716 (0·000117–0·0191)	0·0894 (0·0486–0·15)
Otitis media	1·78 (0·568–4·5)	4·51 (2·9–6·35)	4·51 (2·89–6·38)	4·47 (1·69–9·55)
Enteric infections	5·11 (3·04–8·81)	9·26 (6·82–13)	9·76 (7·14–13·6)	7·73 (5·27–11·5)
Diarrheal diseases	5·16 (3·09–8·85)	9·31 (6·86–13)	9·77 (7·15–13·6)	7·88 (5·47–11·7)
Other infectious diseases	0·609 (0·356–0·817)	1·15 (0·791–1·51)	0·382 (0·223–0·648)	1·51 (0·975–2·03)
Meningitis	1·35 (0·932–1·81)	2·39 (1·67–3·19)	0·000577 (5·67e-06–0·00167)	3·11 (2·19–4·14)
Encephalitis	0·268 (0·166–0·41)	0·695 (0·422–1·06)	0·000176 (1·61e-06–0·000498)	0·951 (0·593–1·43)
Measles	3·42 (-0·934–17·8)	3·63 (-0·812–18·2)	0·531 (0·014–1·46)	4·37 (-1·21–22·6)
Acute hepatitis	13·6 (7·7–24·7)	5·1 (3·18–8·03)	2·95 (1·73–4·95)	13·2 (7·76–23·3)
Maternal and neonatal disorders	87·6 (85·2–89·7)	75·9 (71·8–79·4)	62 (56·3–67·6)	88·4 (86·1–90·4)
Maternal disorders	9·4 (3·36–15·7)	9·5 (3·46–16)	9·61 (3·49–16·3)	9·25 (3·3–15·5)
Neonatal disorders	89·8 (87·6–91·8)	77·9 (73·8–81·5)	64·3 (58·3–70)	89·8 (87·6–91·8)
Nutritional deficiencies	73·8 (68·5–89·6)	79·8 (75·9–84·1)	79·9 (75·9–84·2)	75·1 (70·2–84·3)
Protein-energy malnutrition	100 (100–100)	100 (100–100)	100 (100–100)	100 (100–100)
Vitamin A deficiency	--	100 (100–100)	100 (100–100)	--
Dietary iron deficiency	--	100 (100–100)	100 (100–100)	--
Non-communicable diseases	55·3 (51·5–58·7)	40·7 (37·4–44·2)	26·4 (24–28·9)	55·5 (52·5–58·5)
Neoplasms	47·4 (43·6–51·7)	46·6 (43·2–50·6)	32·9 (28·9–37·4)	47·4 (43·9–51·5)
Lip and oral cavity cancer	62·8 (51·1–73)	66·4 (55·4–75·4)	65·7 (53·9–75·3)	66·5 (55·4–75·5)
Nasopharynx cancer	63 (50·4–74)	63·3 (51·1–73·4)	60·3 (47·8–72·1)	63·6 (51·4–73·7)
Other pharynx cancer	75·3 (66·4–82·5)	76·9 (68·5–83·9)	75·5 (66·6–82·9)	77 (68·6–83·9)
Esophageal cancer	73·7 (63·8–82·3)	75·2 (65·6–83·5)	74·2 (64–82·9)	75·2 (65·6–83·5)
Stomach cancer	23·5 (16·1–40·4)	23·7 (16·3–40·5)	23·8 (16·4–40·8)	23·7 (16·3–40·5)

Colon and rectum cancer	62·6 (53·5–71·4)	63 (54·3–71·3)	63 (54·3–71·3)	63 (54·3–71·3)
Liver cancer	74·7 (68·3–80·9)	75·9 (69·9–81·3)	75·9 (70–81·8)	75·9 (69·9–81·3)
Gallbladder and biliary tract cancer	20·8 (11·1–33·2)	21·1 (11·3–33·5)	19·7 (10–31·6)	21·2 (11·4–33·7)
Pancreatic cancer	40·4 (31–51·6)	39·6 (30·8–50·3)	40·2 (31–51·4)	39·6 (30·8–50·3)
Larynx cancer	82·4 (74·1–88·7)	82·2 (74·4–88·2)	80·2 (72·2–86·5)	82·4 (74·6–88·4)
Tracheal, bronchus, and lung cancer	86·2 (82·1–89·6)	85·3 (81·6–88·8)	85·8 (82–89·3)	85·3 (81·6–88·8)
Breast cancer	35·3 (25·5–46·8)	34·4 (25·5–44·4)	34·6 (25·6–44·7)	34·3 (25·5–44·4)
Cervical cancer	100 (100–100)	100 (100–100)	100 (100–100)	100 (100–100)
Uterine cancer	41·6 (26·7–56·8)	42·8 (28·2–58)	43·2 (28·6–58·3)	42·8 (28·1–58)
Ovarian cancer	32 (13·9–47·2)	26·7 (11·6–40·3)	25·7 (11·2–39)	26·7 (11·6–40·4)
Prostate cancer	5·33 (2·25–8·71)	5·71 (2·5–9·14)	6·01 (2·67–9·5)	5·66 (2·47–9·08)
Kidney cancer	36·5 (26·4–47·3)	36·6 (27·1–46·6)	36·4 (26·7–46·4)	36·7 (27·1–46·6)
Bladder cancer	40·3 (25·5–55·4)	42·3 (28·5–56)	42·9 (29·6–55·9)	42·3 (28·5–56)
Thyroid cancer	13·2 (6·42–21·7)	13·9 (6·61–23·1)	14·2 (6·73–23·4)	13·9 (6·59–23·1)
Mesothelioma	99·4 (99·1–99·6)	98·7 (98·2–99·1)	99·2 (98·9–99·5)	98·7 (98·2–99)
Non-Hodgkin lymphoma	6·61 (2·69–11·9)	6·87 (2·79–12·3)	6·77 (2·77–12·3)	6·88 (2·8–12·3)
Multiple myeloma	7·5 (3·16–13·5)	7·85 (3·25–14·2)	7·79 (3·27–14·2)	7·86 (3·27–14·2)
Leukemia	34·7 (21–49·2)	32·7 (21·3–45·2)	31·8 (20·6–43·8)	32·8 (21·3–45·3)
Cardiovascular diseases	77·7 (73·2–81·5)	79 (75·4–82)	69 (63·9–73·8)	80·3 (77–83·2)
Rheumatic heart disease	21·2 (11·4–41)	22·1 (13·3–38·6)	22·2 (13·5–38·2)	22·1 (13·3–38·6)
Ischemic heart disease	93 (88·5–96·3)	94·2 (91·2–96·7)	93·4 (90·1–96·2)	94·2 (91·3–96·8)
Stroke	78·2 (68·8–88)	81·3 (74·8–88·3)	80·7 (74·5–87·4)	81·4 (74·6–88·5)
Hypertensive heart disease	100 (100–100)	100 (100–100)	100 (100–100)	100 (100–100)
Non-rheumatic valvular heart disease	24 (14·9–36·7)	24·6 (16·7–35·3)	18·1 (11·7–25·4)	25·6 (17·3–36·9)
Cardiomyopathy and myocarditis	33·9 (25·7–43·9)	41·1 (34·4–48·4)	34·6 (28·1–41·8)	41·6 (34·5–48·9)
Atrial fibrillation and flutter	47·2 (36·7–59·2)	55 (46–64·3)	59·2 (50·7–67·7)	50·4 (40·8–60·9)
Aortic aneurysm	47·9 (40·9–55·2)	55 (49–61)	--	55 (49–61)
Endocarditis	29·4 (19·6–42·3)	31·9 (23·3–42·8)	28·9 (19·1–41·6)	32 (23·4–42·9)
Other cardiovascular and circulatory diseases	30·2 (23·3–36·8)	32·8 (26·8–38·7)	32·2 (25·2–39·4)	33 (26·8–38·9)
Chronic respiratory diseases	63·4 (56·9–69·3)	57·3 (52·9–62·1)	49·6 (44·6–54·8)	63·8 (58·2–69·2)
Chronic obstructive pulmonary disease	70·2 (63·5–76·5)	68·9 (64·1–73·9)	62·8 (58·4–67·1)	72 (66·1–77·6)
Pneumoconiosis	100 (100–100)	100 (100–100)	100 (100–100)	100 (100–100)
Asthma	39·2 (27·8–52·7)	37·2 (28·8–47)	37 (28·6–46·8)	40·2 (30·5–51·8)
Digestive diseases	25·9 (20·9–31)	31·2 (25·5–36·3)	16·7 (11·2–23·3)	38 (31·5–44·5)
Cirrhosis and other chronic liver diseases	72·4 (59·4–85·9)	75·9 (63·8–87·9)	72·2 (59·9–83·6)	76 (63·8–88·1)

Upper digestive system diseases	19·4 (16–22·9)	8·27 (5·34–11·8)	0·691 (0·391–1·11)	21·6 (18·3–25·1)
Gallbladder and biliary diseases	41·5 (27·2–56·8)	45 (31–59·7)	46·1 (32·1–60·9)	42·4 (28·4–57·7)
Pancreatitis	34·9 (15·3–55·4)	41·3 (21·1–61·1)	40·8 (20–61·3)	41·3 (21–61)
Neurological disorders	25·6 (11·8–40·4)	16 (7·87–28·2)	10·3 (5·08–19·7)	22·5 (10·3–37)
Alzheimer's disease and other dementias	39 (25·2–54·6)	40·6 (26·9–55·5)	40·8 (26·9–55·7)	40·5 (26·7–55·5)
Parkinson's disease	-8·13 (-13·5–-3·61)	-9·05 (-14·8–-4·17)	-9·51 (-15·3–-4·49)	-8·94 (-14·7–-4·11)
Idiopathic epilepsy	26·4 (15·6–36·8)	24 (14–33·6)	21·6 (12·3–30·7)	27·4 (16·7–37·7)
Multiple sclerosis	13·8 (10·1–17·7)	14·2 (10·5–18)	13·7 (10·1–17·4)	14·5 (10·7–18·7)
Mental disorders	--	6·57 (3·33–10·2)	6·58 (3·34–10·2)	--
Depressive disorders	--	13·5 (6·12–21·3)	13·5 (6·12–21·3)	--
Anxiety disorders	--	4·89 (1·28–11)	4·89 (1·28–11)	--
Idiopathic developmental intellectual disability	--	40·5 (18–59)	40·5 (18–59)	--
Substance use disorders	100 (100–100)	100 (100–100)	100 (100–100)	100 (100–100)
Alcohol use disorders	100 (100–100)	100 (100–100)	100 (100–100)	100 (100–100)
Drug use disorders	100 (100–100)	100 (100–100)	100 (100–100)	100 (100–100)
Diabetes and kidney diseases	99·6 (99·5–99·7)	99·9 (99·9–99·9)	100 (100–100)	99·6 (99·6–99·7)
Diabetes mellitus	100 (100–100)	100 (100–100)	100 (100–100)	100 (100–100)
Chronic kidney disease	100 (100–100)	100 (100–100)	100 (100–100)	100 (100–100)
Sense organ diseases	--	7·66 (6·57–8·93)	7·66 (6·57–8·93)	--
Blindness and vision loss	--	9·15 (5·65–13·4)	9·15 (5·65–13·4)	--
Age-related and other hearing loss	--	8·25 (7·34–9·18)	8·25 (7·34–9·18)	--
Musculoskeletal disorders	3·34 (0·767–6·31)	20·4 (16·9–24·3)	20·8 (17·1–24·8)	4·03 (0·992–7·3)
Rheumatoid arthritis	10·4 (2·38–18·9)	12·3 (3·09–21·7)	12·4 (3·18–22·1)	11·7 (2·87–21·2)
Osteoarthritis	--	15·9 (8·3–24·9)	15·9 (8·3–24·9)	--
Low back pain	--	37·2 (31·9–42·4)	37·2 (31·9–42·4)	--
Gout	--	49·7 (34·9–66·3)	49·7 (34·9–66·3)	--
Other non-communicable diseases	0·0403 (0·0252–0·0632)	0·0455 (0·027–0·0698)	--	0·197 (0·117–0·292)
Sudden infant death syndrome	7·84 (4·38–12·6)	7·83 (4·38–12·6)	--	7·83 (4·38–12·6)
Injuries	34·1 (28·2–39·3)	28·5 (23·7–33·1)	30·7 (26·2–35·1)	24·8 (18·3–30·9)
Transport injuries	22·5 (14·3–29·9)	22·4 (15·1–29·1)	26·7 (22·3–31·3)	20·1 (11·3–28·1)
Road injuries	21·5 (13–29·2)	21·3 (13·8–28·2)	25·4 (21–30)	19·1 (9·86–27·5)
Other transport injuries	33·2 (27·8–38·2)	32·1 (26·8–37·2)	36·2 (31·6–40·6)	29·2 (23–34·9)
Unintentional injuries	41·3 (34·6–45·8)	30·5 (25·7–34·9)	31 (26·1–35·6)	28·8 (23·9–33·1)
Falls	55·8 (47·3–61·3)	41·8 (35·3–47)	40·3 (34–45·5)	47·5 (40·4–52·7)
Drowning	-15·5 (-28·8–-1·24)	-14·3 (-26·6–-1·53)	10 (5·85–15·9)	-16·3 (-29·3–-2·69)

Fire, heat, and hot substances	9·53 (5·13–15·8)	12·6 (7·77–18·6)	12·9 (8–19)	11·5 (6·73–17·4)
Poisonings	14·6 (9·01–21·5)	13 (8·14–19·2)	11·7 (7·33–17·5)	15·5 (9·9–22·2)
Exposure to mechanical forces	14 (1·21–26·2)	17·8 (14·9–20·8)	19 (16·3–21·7)	2·38 (-11–15·9)
Animal contact	20·3 (15·7–25·7)	20·8 (16–26·3)	21·8 (16·9–27·4)	15·2 (11·2–20·1)
Foreign body	2·81 (1·76–4·31)	4 (2·59–5·89)	3·34 (2·23–4·97)	4·53 (2·9–6·83)
Environmental heat and cold exposure	7·22 (3·2–13·1)	8·68 (3·87–15·6)	8·76 (3·87–15·7)	8·21 (3·71–14·6)
Exposure to forces of nature	0 (0–0)	9·74 (4·35–17·1)	9·74 (4·35–17·1)	0 (0–0)
Other unintentional injuries	13·9 (8·26–20·7)	12·3 (7·61–18·1)	12·1 (7·53–18)	15·6 (9·81–22·3)
Self-harm and interpersonal violence	22·8 (8·93–36·2)	25·2 (12·6–36·7)	33·2 (24·9–41·2)	23·6 (9·66–37)
Self-harm	22·7 (8·21–37)	23·7 (9·03–37·9)	22·7 (9·88–35·6)	23·7 (9·04–38)
Interpersonal violence	27·1 (18·1–36·1)	34·5 (26–43·1)	38·6 (29·8–47·7)	23·8 (15·5–32·8)

Supplementary Results Table 2. All-age PAFs attributable to the joint distribution of all risk factors for all ages, both sexes combined for each GBD level 1, level 2, and level 3 cause and all causes in 2019 for Hillingdon. Results are shown for deaths, YLLs, YLDs, and DALYs Data in parenthesis are 95% uncertainty intervals. PAFs=population attributable fractions. GBD=Global Burden of Disease. YLLs=years of life lost. YLDs=years lived with disability. DALYs=disability-adjusted life-years

	Deaths 2019	DALYs 2019	YLDs 2019	YLLs 2019
All causes	54·7 (51·5–57·9)	39·8 (36·8–42·9)	28·1 (25·8–30·3)	54 (51·3–56·7)
Communicable, maternal, neonatal, and nutritional diseases	47 (36·3–57·2)	53·9 (48·9–58·6)	48·8 (44·1–53·4)	57·1 (49·4–64·1)
HIV/AIDS and sexually transmitted infections	74·8 (60·5–85·1)	74·7 (58·9–85·7)	96·5 (94·8–97·8)	60·2 (42·7–75·1)
HIV/AIDS	89·3 (87·2–90·6)	87·5 (85–89·5)	93·8 (91·3–95·4)	83·9 (81·3–85·5)
Sexually transmitted infections excluding HIV	31·1 (13·8–57·6)	53·9 (28·3–81)	100 (100–100)	10·4 (3·75–24·9)
Respiratory infections and tuberculosis	47·5 (35·5–59·1)	42·4 (32·8–52·6)	3·55 (2·55–4·8)	50·8 (39·6–61·7)
Tuberculosis	58·7 (47·1–68·4)	59·6 (48·7–68)	55·3 (43·4–64·6)	60·8 (49·8–69·1)
Lower respiratory infections	47·4 (35·3–59·1)	50·6 (39·2–61·5)	34·8 (27·9–41·6)	50·7 (39·3–61·8)
Upper respiratory infections	0·07 (0·041–0·11)	0·00944 (0·00153–0·0218)	0·00818 (2e-04–0·0208)	0·147 (0·0857–0·236)
Otitis media	3·87 (2–6·7)	5·38 (3·49–7·39)	5·35 (3·46–7·37)	8·66 (5·03–13·5)
Enteric infections	4·59 (2·91–7·49)	9·48 (7·34–12·5)	9·82 (7·46–12·9)	8·74 (6·29–12·2)
Diarrheal diseases	4·62 (2·93–7·51)	9·52 (7·38–12·6)	9·83 (7·46–13)	8·84 (6·36–12·3)
Other infectious diseases	0·98 (0·653–1·33)	1·43 (1·08–1·85)	0·352 (0·206–0·592)	1·97 (1·45–2·53)
Meningitis	2·26 (1·6–2·98)	3·22 (2·3–4·31)	0·000776 (1·27e-05–0·00221)	4·3 (3·08–5·64)
Encephalitis	0·499 (0·336–0·725)	1·02 (0·668–1·51)	0·000272 (4·64e-06–0·000766)	1·46 (0·995–2·12)
Measles	3·45 (-1·19–16·3)	2·79 (-0·751–12·9)	0·332 (0·00878–0·898)	4·27 (-1·47–19·8)
Acute hepatitis	12·1 (5·15–23·4)	4·99 (2·63–8·62)	2·53 (1·5–4·26)	10·4 (4·28–19·9)
Maternal and neonatal disorders	87·8 (85·5–89·9)	79·2 (75·8–82·5)	67·4 (62·3–72·3)	88·7 (86·4–90·6)
Maternal disorders	9·3 (3·29–15·3)	9·37 (3·33–15·8)	9·51 (3·35–16)	9·16 (3·27–15·2)
Neonatal disorders	90·2 (88·1–92)	81 (77·6–84·2)	69·5 (63·8–74·6)	90·2 (88·1–92)
Nutritional deficiencies	73 (67·1–85·6)	79·6 (75·5–84·7)	79·8 (75·5–85)	73·4 (65·1–78·9)
Protein-energy malnutrition	100 (100–100)	100 (100–100)	100 (100–100)	100 (100–100)
Vitamin A deficiency	--	100 (100–100)	100 (100–100)	--
Dietary iron deficiency	--	100 (100–100)	100 (100–100)	--
Non-communicable diseases	56·3 (53·1–59·8)	39·9 (36·6–43·3)	27·3 (24·8–29·8)	55·9 (53·1–58·9)
Neoplasms	47·9 (44–52·2)	46·4 (42·8–50·4)	33·1 (29–37·6)	47·2 (43·7–51·2)
Lip and oral cavity cancer	69 (57·9–77·3)	71·3 (60·8–79)	70·6 (59·4–78·4)	71·4 (60·8–79)
Nasopharynx cancer	65·1 (53·2–74·8)	64·5 (52·4–73·8)	62·6 (50·6–73)	64·7 (52·7–73·9)
Other pharynx cancer	78·2 (69·7–84·7)	79·3 (71·3–85·4)	78 (69·2–84·4)	79·4 (71·4–85·5)
Esophageal cancer	75·6 (65·9–84)	76·8 (67·3–85·1)	75·9 (66·1–84·4)	76·8 (67·3–85·2)
Stomach cancer	23·8 (16·3–40·6)	23·7 (16·4–40·1)	23·9 (16·6–40·4)	23·7 (16·3–40·1)

Colon and rectum cancer	64·3 (55·2–73·2)	64·3 (55·6–72·7)	64·4 (55·4–73·1)	64·3 (55·7–72·7)
Liver cancer	77 (70·4–82·7)	76·9 (71·1–82)	77·2 (71·8–82·5)	76·8 (71·1–82)
Gallbladder and biliary tract cancer	23·3 (12·7–36·3)	23·7 (13·1–36·6)	22 (12–35)	23·8 (13·1–36·7)
Pancreatic cancer	42·5 (32·6–54·3)	41·6 (32·2–53)	42·2 (32·3–53·9)	41·5 (32·2–53)
Larynx cancer	83·1 (74·8–89)	82·4 (75·2–88·1)	80·1 (72·9–86)	82·6 (75·4–88·3)
Tracheal, bronchus, and lung cancer	85·7 (81·9–89·2)	84·3 (80·3–87·8)	85·1 (81·3–88·6)	84·3 (80·3–87·8)
Breast cancer	37·8 (28·1–49·5)	36 (27·1–46)	36·3 (27·6–46·5)	35·9 (27–46·1)
Cervical cancer	100 (100–100)	100 (100–100)	100 (100–100)	100 (100–100)
Uterine cancer	46·5 (31·5–62)	47·4 (32·2–63)	47·7 (32·4–63·1)	47·4 (32·2–63)
Ovarian cancer	29·4 (14·2–44·1)	24·2 (11·8–37·6)	22·9 (11·1–35·8)	24·3 (11·8–37·7)
Prostate cancer	5·51 (2·35–8·96)	5·94 (2·58–9·46)	6·25 (2·8–9·83)	5·88 (2·53–9·37)
Kidney cancer	39·3 (28·9–49·8)	39·1 (28·8–49)	38·7 (28·5–48·7)	39·1 (28·8–49)
Bladder cancer	42·8 (27·6–57·8)	44·5 (30·5–58)	44·8 (31·5–57·7)	44·5 (30·4–58)
Thyroid cancer	15·8 (7·7–25·6)	16·4 (8·08–26·3)	16·5 (8·17–26·6)	16·4 (8·02–26·4)
Mesothelioma	98·9 (98·4–99·2)	97·6 (96·8–98·2)	98·6 (98·1–99·1)	97·6 (96·8–98·2)
Non-Hodgkin lymphoma	7·8 (3·39–13·8)	7·95 (3·46–14)	7·91 (3·34–14·1)	7·96 (3·47–14)
Multiple myeloma	8·84 (3·75–15·6)	9·2 (4–16·1)	9·13 (3·94–16)	9·21 (4·02–16·1)
Leukemia	35·9 (23–49·5)	32·3 (21·6–42·9)	31·4 (20·9–42·6)	32·3 (21·6–43)
Cardiovascular diseases	76·3 (72·3–80·5)	77·6 (74–81·3)	69·8 (64·4–74·7)	78·8 (75·2–82·6)
Rheumatic heart disease	22·1 (11·9–42·6)	23 (14·1–37·2)	23·1 (14·5–36·9)	23 (14–37·5)
Ischemic heart disease	93·6 (89·9–96·6)	94·8 (92·2–97·1)	94 (91·2–96·7)	94·8 (92·2–97·1)
Stroke	79·6 (71·1–88·3)	82·9 (77–89·2)	82·2 (77–87·9)	83 (76·8–89·4)
Hypertensive heart disease	100 (100–100)	100 (100–100)	100 (100–100)	100 (100–100)
Non-rheumatic valvular heart disease	25·1 (16·2–37·9)	25·9 (18·2–35·9)	20·1 (13·6–27·9)	26·8 (19–37·4)
Cardiomyopathy and myocarditis	30·3 (22–39·9)	35·3 (28·3–44·1)	33·9 (27·5–40·9)	35·4 (28·3–44·6)
Atrial fibrillation and flutter	51·4 (40·7–62·3)	59·7 (50·6–68·2)	63·3 (55·1–71·4)	54·7 (45·1–64·6)
Aortic aneurysm	49·7 (42·9–57·2)	56·8 (51·1–62·8)	--	56·8 (51·1–62·8)
Endocarditis	30·4 (20·6–42·9)	32·8 (24·3–42·9)	30·4 (20·8–42·5)	32·9 (24·4–42·9)
Other cardiovascular and circulatory diseases	31·5 (24·8–38·3)	34 (27·7–39·9)	34·6 (27·2–41·6)	33·9 (27·6–39·9)
Chronic respiratory diseases	62·7 (56·3–69)	54·9 (50·1–59·9)	47·9 (42·3–53·5)	62·7 (56·8–68·5)
Chronic obstructive pulmonary disease	71·5 (65·4–77·4)	69·5 (64·7–74·2)	63 (58·9–67·1)	73·1 (67·3–78·7)
Pneumoconiosis	100 (100–100)	100 (100–100)	100 (100–100)	100 (100–100)
Asthma	43·5 (31·8–55·9)	39·4 (31–48·8)	39·1 (30·7–48·5)	43·2 (33·4–53·9)
Digestive diseases	34·4 (28·4–40·3)	38·1 (32–44·1)	24·8 (17–33·9)	47·2 (39·7–54)

Cirrhosis and other chronic liver diseases	76·6 (63·6–88·3)	79 (66·5–89·6)	75·6 (64·2–86·1)	79·1 (66·5–89·8)
Upper digestive system diseases	21 (17·4–24·5)	7·13 (4·55–10·4)	0·639 (0·366–1·03)	23·1 (19·6–26·4)
Gallbladder and biliary diseases	46·5 (31·3–61·8)	51·6 (36·8–66·4)	52·2 (37·4–67·2)	47·6 (32·8–62·6)
Pancreatitis	42·2 (21·7–60·1)	48 (27–65·3)	49·4 (27·8–66·7)	47·8 (27·1–65)
Neurological disorders	26·7 (12·2–42·4)	14·3 (6·61–26·2)	8·67 (4·06–17·6)	22·8 (10·8–38·3)
Alzheimer's disease and other dementias	42·9 (28·5–59)	44·4 (29·9–60·1)	44·6 (30·1–60·2)	44·3 (29·8–60·2)
Parkinson's disease	-8·74 (-14·5–-3·92)	-9·68 (-15·9–-4·52)	-10·2 (-16·5–-4·82)	-9·55 (-15·9–-4·47)
Idiopathic epilepsy	28 (17·6–37·5)	25·1 (15·7–34·1)	23·5 (14·4–32·2)	27·8 (17·9–36·9)
Multiple sclerosis	14·6 (10·7–18·6)	14·7 (10·9–18·9)	14·2 (10·5–17·9)	15·2 (11·2–19·5)
Mental disorders	--	6·64 (3·29–10·3)	6·64 (3·29–10·3)	--
Depressive disorders	--	14·1 (6·45–21·9)	14·1 (6·45–21·9)	--
Anxiety disorders	--	5·58 (1·45–12·3)	5·58 (1·45–12·3)	--
Idiopathic developmental intellectual disability	--	31·6 (12·5–52·5)	31·6 (12·5–52·5)	--
Substance use disorders	100 (100–100)	100 (100–100)	100 (100–100)	100 (100–100)
Alcohol use disorders	100 (100–100)	100 (100–100)	100 (100–100)	100 (100–100)
Drug use disorders	100 (100–100)	100 (100–100)	100 (100–100)	100 (100–100)
Diabetes and kidney diseases	100 (100–100)	100 (100–100)	100 (100–100)	100 (100–100)
Diabetes mellitus	100 (100–100)	100 (100–100)	100 (100–100)	100 (100–100)
Chronic kidney disease	100 (100–100)	100 (100–100)	100 (100–100)	100 (100–100)
Sense organ diseases	--	8·31 (7·28–9·51)	8·31 (7·28–9·51)	--
Blindness and vision loss	--	8·79 (5·64–12·8)	8·79 (5·64–12·8)	--
Age-related and other hearing loss	--	9·41 (8·5–10·4)	9·41 (8·5–10·4)	--
Musculoskeletal disorders	2·82 (0·605–5·24)	21 (17·4–24·5)	21·4 (17·7–25·1)	3·23 (0·749–5·87)
Rheumatoid arthritis	11·5 (2·68–20·8)	12·8 (3·17–22·5)	12·8 (3·25–22·7)	12·8 (3·04–22·8)
Osteoarthritis	--	18·1 (9·63–27·4)	18·1 (9·63–27·4)	--
Low back pain	--	38·6 (33·5–43·6)	38·6 (33·5–43·6)	--
Gout	--	52·6 (36·5–70)	52·6 (36·5–70)	--
Other non-communicable diseases	0·104 (0·0695–0·158)	0·0761 (0·0471–0·118)	--	0·345 (0·225–0·506)
Sudden infant death syndrome	9·43 (5·14–15·4)	9·42 (5·13–15·3)	--	9·42 (5·13–15·3)
Injuries	30 (24–35·5)	25·3 (20·8–30)	27·3 (23·1–31·9)	22·5 (15·7–29·5)
Transport injuries	20·7 (12·1–28·8)	20·9 (13·9–27·8)	26·4 (21·8–31·2)	18·2 (9·02–26·8)
Road injuries	20 (11–28·4)	20·1 (12·7–27·3)	25·4 (20·9–30·4)	17·6 (8·18–26·5)
Other transport injuries	31·5 (26·4–36·8)	30·6 (25·7–35·7)	33·9 (29·2–38·4)	27·7 (21·8–33·8)
Unintentional injuries	36·6 (30·9–41·1)	25·9 (21·5–30·7)	26·9 (22·3–31·9)	22 (17·5–26·6)

Falls	54·1 (46·4–59·6)	37·2 (31·3–42·8)	35·7 (29·9–41·5)	44·1 (37·7–49·8)
Drowning	-15·3 (-28·6–-0·783)	-15·3 (-27·6–-2·08)	11·1 (6·48–17·9)	-16·4 (-29·4–-3·13)
Fire, heat, and hot substances	10·5 (5·82–17·4)	13·1 (8·09–20·3)	13·8 (8·64–21·1)	10·9 (6·55–16·9)
Poisonings	13·4 (8·2–20·5)	12·7 (8·02–19·2)	12·3 (7·69–18·7)	13·5 (8·47–20·2)
Exposure to mechanical forces	6·66 (-6·19–19·8)	15·7 (12·8–18·7)	16·9 (14·5–19·7)	-1·62 (-14·9–12·5)
Animal contact	20·7 (15·8–27·2)	19·7 (14·8–25·9)	20·6 (15·6–27)	16·5 (12·1–22·3)
Foreign body	2·68 (1·72–4·13)	3·53 (2·33–5·15)	3·37 (2·21–5·01)	3·69 (2·4–5·6)
Environmental heat and cold exposure	8·33 (3·71–15·2)	9·62 (4·32–17·2)	9·69 (4·32–17·2)	9·37 (4·22–16·8)
Exposure to forces of nature	0 (0–0)	10·7 (4·8–18·6)	10·7 (4·8–18·6)	0 (0–0)
Other unintentional injuries	14·3 (8·67–22·1)	13·1 (8·06–19·9)	13 (8–19·8)	15·1 (9·56–22·3)
Self-harm and interpersonal violence	24·8 (11·4–37·7)	26·7 (14·5–38·5)	34·7 (26·9–42·5)	25·3 (12–38·2)
Self-harm	24·6 (10·5–38·1)	25·4 (11·5–38·8)	25·3 (12·6–38)	25·4 (11·4–38·8)
Interpersonal violence	29·8 (21·9–38·4)	35 (26·9–43·5)	39·3 (30·6–48·2)	26 (18–34·2)

Supplementary Results Table 2. All-age PAFs attributable to the joint distribution of all risk factors for all ages, both sexes combined for each GBD level 1, level 2, and level 3 cause and all causes in 2019 for Hounslow. Results are shown for deaths, YLLs, YLDs, and DALYs Data in parenthesis are 95% uncertainty intervals. PAFs=population attributable fractions. GBD=Global Burden of Disease. YLLs=years of life lost. YLDs=years lived with disability. DALYs=disability-adjusted life-years				
	Deaths 2019	DALYs 2019	YLDs 2019	YLLs 2019
All causes	55 (51·8–58)	39·5 (36·5–42·4)	28·2 (25·8–30·6)	54·7 (52·1–57·1)
Communicable, maternal, neonatal, and nutritional diseases	48·4 (38·4–58·3)	56·9 (52·6–61·2)	49·4 (45–54·2)	61·6 (55·1–67·6)
HIV/AIDS and sexually transmitted infections	79·4 (67·8–87·6)	78·1 (64·2–87·7)	96·1 (94·4–97·4)	66·9 (50·7–79·6)
HIV/AIDS	91·1 (88·9–92·4)	89·5 (86·9–91·3)	94·1 (91·6–95·7)	86·9 (84·5–88·5)
Sexually transmitted infections excluding HIV	27·1 (11·6–52·7)	51·8 (26·7–79·2)	100 (100–100)	8·79 (3·07–21)
Respiratory infections and tuberculosis	47·9 (36·1–59·4)	42·4 (32·9–51·4)	3·73 (2·7–5·08)	51·9 (41·1–62·4)
Tuberculosis	59·9 (48·5–69·5)	61·1 (50·9–69·4)	56·5 (45·2–65·7)	62·1 (51·8–70·6)
Lower respiratory infections	47·7 (35·6–59·4)	51·6 (40·5–62·3)	35·5 (28·8–42)	51·7 (40·5–62·5)
Upper respiratory infections	0·0964 (0·0509–0·168)	0·0109 (0·00258–0·026)	0·00841 (0·000519–0·0239)	0·178 (0·0915–0·317)
Otitis media	4·06 (2·15–6·91)	5·29 (3·4–7·39)	5·29 (3·4–7·39)	8·69 (5·25–13)
Enteric infections	4·76 (3·11–7·63)	10·1 (7·87–13·3)	10·1 (7·59–13·4)	10·1 (7·19–13·5)
Diarrheal diseases	4·79 (3·14–7·66)	10·1 (7·9–13·3)	10·1 (7·6–13·5)	10·3 (7·32–13·6)
Other infectious diseases	1·06 (0·748–1·4)	1·61 (1·22–2·06)	0·342 (0·192–0·575)	2·21 (1·64–2·88)
Meningitis	2·8 (2·06–3·69)	3·74 (2·73–4·93)	0·000815 (3·66e-05–0·00247)	4·94 (3·64–6·43)
Encephalitis	0·636 (0·399–0·947)	1·16 (0·679–1·76)	0·000266 (1·2e-05–0·000722)	1·73 (1·06–2·58)
Measles	3·54 (-1·24–17·8)	3 (-0·917–15)	0·34 (0·00913–0·963)	4·39 (-1·52–22·4)
Acute hepatitis	9·49 (4·95–19·2)	4·14 (2·5–6·84)	2·54 (1·46–4·16)	8·37 (4·46–16·7)
Maternal and neonatal disorders	90·3 (88·5–91·9)	80·9 (77·8–83·8)	65·5 (60–70·5)	90·7 (88·9–92·2)
Maternal disorders	9·48 (3·42–16)	9·62 (3·38–16·6)	9·71 (3·44–16·8)	9·33 (3·34–15·6)
Neonatal disorders	91·2 (89·4–92·7)	82·1 (79–85·1)	67·4 (61·7–72·6)	91·2 (89·4–92·7)
Nutritional deficiencies	75·9 (70·3–87)	79·8 (75·4–84·6)	79·8 (75·3–84·7)	76·3 (67·9–79·9)
Protein-energy malnutrition	100 (100–100)	100 (100–100)	100 (100–100)	100 (100–100)
Vitamin A deficiency	--	100 (100–100)	100 (100–100)	--
Dietary iron deficiency	--	100 (100–100)	100 (100–100)	--
Non-communicable diseases	56·7 (53·4–60)	39·4 (36–42·5)	27·3 (24·7–30)	56·5 (53·8–59·1)
Neoplasms	48·1 (44·1–52·3)	46·4 (42·5–50·3)	32·9 (28·5–37·2)	47·2 (43·3–51·1)
Lip and oral cavity cancer	68·5 (57·6–77·3)	71 (61·1–79·1)	70·3 (59·8–78·7)	71·1 (61·2–79)
Nasopharynx cancer	64·3 (51·7–74·6)	63·8 (51·5–73·6)	61·8 (48·8–72·3)	64 (51·9–73·7)
Other pharynx cancer	81·1 (74·1–86·7)	81·9 (75·1–87·2)	80·9 (73·8–86·6)	81·9 (75·2–87·2)
Esophageal cancer	76·3 (66·8–84·5)	77·5 (68·1–85·6)	76·6 (67·5–84·7)	77·5 (68·1–85·6)

Stomach cancer	24·6 (17·1–41·7)	24·5 (17–41·3)	24·7 (17·2–41·7)	24·5 (17–41·3)
Colon and rectum cancer	64·5 (55·3–73·3)	64·6 (55·6–72·9)	64·7 (55·7–73·2)	64·6 (55·6–72·9)
Liver cancer	77·1 (71·2–82·7)	77 (71·5–82·1)	77·4 (71·9–82·6)	77 (71·5–82·1)
Gallbladder and biliary tract cancer	23·8 (13–36·4)	24·1 (13·5–36·8)	22·5 (12·2–35·1)	24·2 (13·5–36·9)
Pancreatic cancer	43·1 (33·4–54·7)	42 (33·1–53)	42·8 (33·4–54·2)	42 (33–53)
Larynx cancer	83·2 (75·2–89·2)	82·4 (74·8–88·1)	79·8 (71·9–86·1)	82·6 (75–88·4)
Tracheal, bronchus, and lung cancer	85·2 (81·8–88·9)	83·7 (80·1–87·3)	84·6 (81·1–88·1)	83·7 (80·1–87·3)
Breast cancer	37·7 (27·7–49·3)	35·9 (27·2–45·8)	36·2 (27·3–46·4)	35·8 (27·1–45·8)
Cervical cancer	100 (100–100)	100 (100–100)	100 (100–100)	100 (100–100)
Uterine cancer	46 (31–61·4)	47 (31·8–62·3)	47·2 (32·2–62·7)	46·9 (31·8–62·3)
Ovarian cancer	23·8 (12–38·4)	19·9 (9·7–33)	18·8 (9·23–31·2)	19·9 (9·71–33)
Prostate cancer	5·74 (2·41–9·23)	6·22 (2·66–9·83)	6·56 (2·87–10·3)	6·16 (2·63–9·76)
Kidney cancer	39·6 (29·4–50·2)	39 (29·1–48·8)	38·6 (28·7–48·5)	39·1 (29·2–48·9)
Bladder cancer	43·6 (28·7–58·6)	45·3 (31·1–58·7)	45·5 (31·7–58·6)	45·3 (31·1–58·7)
Thyroid cancer	15·8 (7·49–25·7)	16·4 (7·79–27·1)	16·5 (7·96–27·4)	16·4 (7·73–27·1)
Mesothelioma	97·8 (97–98·4)	95·5 (94·1–96·6)	97·5 (96·6–98·3)	95·5 (94·1–96·6)
Non-Hodgkin lymphoma	7·67 (3·17–13·5)	7·82 (3·32–13·6)	7·79 (3·3–13·6)	7·82 (3·32–13·6)
Multiple myeloma	8·71 (3·8–15·4)	9·06 (3·96–16·1)	9·01 (3·85–16·2)	9·06 (3·97–16·1)
Leukemia	36·3 (23·1–50·4)	32·2 (21·9–42·6)	30·4 (20·5–40·7)	32·3 (22–42·8)
Cardiovascular diseases	77·9 (73·9–81·6)	79 (75·9–82·1)	70·1 (65·4–74·5)	80·3 (77·3–83·4)
Rheumatic heart disease	22·3 (11·9–42·5)	23·2 (14·5–38·8)	23·2 (14·7–37·8)	23·2 (14·4–38·9)
Ischemic heart disease	93·8 (90·1–96·7)	95·1 (92·5–97·2)	94·2 (91·4–96·8)	95·1 (92·6–97·2)
Stroke	80·1 (72–88·8)	83·6 (78·3–89·6)	83·1 (78·6–88)	83·7 (77·9–89·8)
Hypertensive heart disease	100 (100–100)	100 (100–100)	100 (100–100)	100 (100–100)
Non-rheumatic valvular heart disease	25·1 (16·3–38·1)	25·9 (18·3–35·8)	19·4 (13–27·7)	26·8 (19–37)
Cardiomyopathy and myocarditis	32·8 (25–41·7)	37·8 (31·4–44·3)	34·4 (28·5–40·7)	38 (31·4–44·9)
Atrial fibrillation and flutter	51·5 (40·7–62·2)	60 (50·8–68·6)	63·7 (54·9–71·6)	55·1 (44·9–64·7)
Aortic aneurysm	51·3 (44·6–58·4)	58·5 (52·7–64·4)	--	58·5 (52·7–64·4)
Endocarditis	30·6 (20·7–44·4)	33·1 (24·2–43·1)	30·7 (20·8–42·6)	33·2 (24·3–43·3)
Other cardiovascular and circulatory diseases	31·5 (24·9–37·9)	33·7 (27·6–40)	33·5 (26·3–41·6)	33·8 (27·6–39·9)
Chronic respiratory diseases	62·8 (56·2–69·4)	54·9 (50–59·9)	48·2 (42·5–54·1)	62·7 (56·7–68·8)
Chronic obstructive pulmonary disease	72·2 (66·2–77·9)	70·4 (65·9–74·6)	64·1 (60·4–68)	73·9 (68·5–79)
Pneumoconiosis	100 (100–100)	100 (100–100)	100 (100–100)	100 (100–100)
Asthma	43·4 (31·6–56)	40·2 (31·8–50·3)	39·9 (31·6–49·7)	43·6 (33·4–54·3)

Digestive diseases	37·3 (31–42·8)	40·2 (33·4–45·9)	24·9 (17·2–33·9)	50·5 (43–57·1)
Cirrhosis and other chronic liver diseases	78 (65·6–89)	80·1 (68·1–90·2)	76 (64·2–85·8)	80·2 (68·1–90·4)
Upper digestive system diseases	21·6 (17·8–25·1)	7·08 (4·4–10·3)	0·647 (0·366–1·03)	23·9 (20·2–27·4)
Gallbladder and biliary diseases	45·8 (30·8–61·1)	50 (35·4–64·3)	50·5 (36–64·8)	46·7 (31·9–61·7)
Pancreatitis	43·2 (23·3–62·5)	49·1 (29·3–67·1)	49·1 (29·6–67·4)	49·1 (29·1–67·2)
Neurological disorders	27·5 (12·9–43)	13·7 (6·3–25·1)	7·93 (3·61–16·6)	23·8 (11·3–39·2)
Alzheimer's disease and other dementias	42·7 (28–58·4)	44·5 (29·9–60)	44·7 (30·1–60·1)	44·4 (29·8–60)
Parkinson's disease	-8·98 (-14·7–-4·03)	-10·1 (-16·2–-4·58)	-10·7 (-17·1–-4·91)	-9·93 (-16·1–-4·55)
Idiopathic epilepsy	29·2 (18·5–39·4)	25·7 (16–34·8)	23·7 (14·3–32·7)	28·9 (18·7–38·4)
Multiple sclerosis	15·1 (11–19)	15·2 (11·4–19·2)	14·7 (11–18·9)	15·7 (11·6–19·6)
Mental disorders	--	6·67 (3·29–10·5)	6·67 (3·29–10·5)	--
Depressive disorders	--	14·2 (6·43–22)	14·2 (6·43–22)	--
Anxiety disorders	--	5·35 (1·34–12·5)	5·35 (1·34–12·5)	--
Idiopathic developmental intellectual disability	--	35·2 (15–55·4)	35·2 (15–55·4)	--
Substance use disorders	100 (100–100)	100 (100–100)	100 (100–100)	100 (100–100)
Alcohol use disorders	100 (100–100)	100 (100–100)	100 (100–100)	100 (100–100)
Drug use disorders	100 (100–100)	100 (100–100)	100 (100–100)	100 (100–100)
Diabetes and kidney diseases	99·9 (99·8–99·9)	100 (100–100)	100 (100–100)	99·8 (99·8–99·9)
Diabetes mellitus	100 (100–100)	100 (100–100)	100 (100–100)	100 (100–100)
Chronic kidney disease	100 (100–100)	100 (100–100)	100 (100–100)	100 (100–100)
Sense organ diseases	--	8·6 (7·53–9·87)	8·6 (7·53–9·87)	--
Blindness and vision loss	--	8·46 (5·45–12·3)	8·46 (5·45–12·3)	--
Age-related and other hearing loss	--	9·99 (9–11·1)	9·99 (9–11·1)	--
Musculoskeletal disorders	2·15 (0·518–3·99)	21·4 (17·7–25·2)	21·9 (18·1–25·7)	2·51 (0·632–4·6)
Rheumatoid arthritis	12·1 (3·01–21·7)	13·3 (3·3–23·6)	13·3 (3·29–23·5)	13·4 (3·4–24)
Osteoarthritis	--	17·9 (9·61–27·1)	17·9 (9·61–27·1)	--
Low back pain	--	39·9 (34·8–45)	39·9 (34·8–45)	--
Gout	--	51·8 (35·8–69·4)	51·8 (35·8–69·4)	--
Other non-communicable diseases	0·13 (0·0854–0·2)	0·102 (0·0642–0·154)	--	0·415 (0·271–0·589)
Sudden infant death syndrome	8·3 (4·74–13·6)	8·29 (4·74–13·6)	--	8·29 (4·74–13·6)
Injuries	28·8 (23·2–34·4)	24·4 (20·1–29·2)	26·3 (22·3–30·9)	21·7 (15–28·4)
Transport injuries	21·1 (13–29·2)	21·1 (14·4–28·1)	25·7 (21·3–30·4)	18·8 (10·2–27·4)
Road injuries	20·4 (12–28·8)	20·3 (13·3–27·5)	24·7 (20·3–29·6)	18·2 (9·24–27·2)
Other transport injuries	31·3 (25·9–36·5)	30·3 (25·4–35·3)	33 (28·4–37·6)	27·7 (21·9–33·5)

Unintentional injuries	33·7 (27·8–38·4)	24·3 (19·9–29·1)	25·8 (21·3–30·8)	18·9 (14·4–23·7)
Falls	53·1 (45–58·7)	35·8 (29·9–41·3)	34·4 (28·7–39·8)	42·3 (35·8–48·2)
Drowning	-14·3 (-27·4–-0·266)	-14·7 (-27·2–-1·35)	11·2 (6·36–17·7)	-15·5 (-28·3–-1·89)
Fire, heat, and hot substances	11·8 (6·63–18·4)	13·5 (8·18–20·1)	14 (8·41–20·9)	12·3 (7·37–18·3)
Poisonings	14·6 (8·91–21·5)	13·3 (8·09–19·6)	12·2 (7·32–18·4)	14·7 (9·17–21·2)
Exposure to mechanical forces	8·02 (-4·86–21·1)	14·9 (12·2–17·7)	16·1 (13·7–18·7)	-1·96 (-15·3–12·6)
Animal contact	22·9 (18–28·7)	19·4 (14·4–25)	20·3 (15–26·2)	16·7 (12·7–21·8)
Foreign body	3·04 (1·9–4·57)	3·68 (2·39–5·39)	3·36 (2·22–5)	3·98 (2·5–5·9)
Environmental heat and cold exposure	8·84 (3·74–15·7)	9·86 (4·36–17·1)	9·83 (4·3–17·2)	9·96 (4·55–17·2)
Exposure to forces of nature	0 (0–0)	10·7 (4·79–18·4)	10·7 (4·79–18·4)	0 (0–0)
Other unintentional injuries	14·5 (8·64–21·7)	13·1 (7·87–19·6)	13 (7·84–19·5)	15·2 (9·48–21·9)
Self-harm and interpersonal violence	25 (12·1–38·4)	26·9 (15·4–38·6)	35 (27–43·6)	25·5 (12·5–38·6)
Self-harm	25·2 (11·4–39·5)	26 (12·2–40·1)	25·7 (13·6–38·7)	26 (12·1–40·2)
Interpersonal violence	24·8 (15·6–33·6)	32·7 (24·2–41·4)	39·4 (30·6–49·3)	21·6 (13·1–29·8)

Supplementary Results Table 2. All-age PAFs attributable to the joint distribution of all risk factors for all ages, both sexes combined for each GBD level 1, level 2, and level 3 cause and all causes in 2019 for Islington. Results are shown for deaths, YLLs, YLDs, and DALYs Data in parenthesis are 95% uncertainty intervals. PAFs=population attributable fractions. GBD=Global Burden of Disease. YLLs=years of life lost. YLDs=years lived with disability. DALYs=disability-adjusted life-years

	Deaths 2019	DALYs 2019	YLDs 2019	YLLs 2019
All causes	57·3 (54·4–60)	40·6 (37·4–43·7)	29·1 (26·6–31·5)	56·4 (53·9–58·7)
Communicable, maternal, neonatal, and nutritional diseases	50·3 (40·8–59·6)	55·6 (51·4–59·8)	51·2 (46·4–55·6)	58·9 (52·3–65·3)
HIV/AIDS and sexually transmitted infections	84·9 (77–90·1)	83·6 (74·4–89·6)	95·4 (93·1–96·8)	76 (63·7–84·7)
HIV/AIDS	91·4 (88·9–92·9)	90·2 (87·3–92·1)	93·7 (90·9–95·4)	88·1 (85·2–89·8)
Sexually transmitted infections excluding HIV	47 (25·3–73·9)	60·1 (34·7–84·1)	100 (100–100)	20·5 (8·13–43·1)
Respiratory infections and tuberculosis	50·7 (39·5–61·7)	44·3 (35·2–53·3)	3·5 (2·5–4·97)	54·6 (44·3–64·7)
Tuberculosis	63 (51·7–72)	63·9 (53·4–71·9)	59·2 (47·4–68·1)	64·9 (54·7–72·9)
Lower respiratory infections	50·5 (39·1–61·8)	54·3 (43·6–64·8)	38·9 (32·1–45·6)	54·4 (43·7–64·9)
Upper respiratory infections	0·0541 (0·0287–0·0943)	0·0102 (0·00197–0·0244)	0·00869 (0·000481–0·0226)	0·103 (0·0522–0·185)
Otitis media	4·56 (1·7–11·1)	4·72 (3–6·71)	4·71 (3–6·71)	8·63 (3·67–18·5)
Enteric infections	4·42 (2·87–6·78)	9·39 (7·25–12·1)	9·48 (7·09–12·4)	9·16 (6·57–12·2)
Diarrheal diseases	4·46 (2·89–6·83)	9·44 (7·3–12·2)	9·49 (7·1–12·5)	9·32 (6·67–12·4)
Other infectious diseases	1·03 (0·66–1·68)	1·3 (0·927–1·81)	0·406 (0·229–0·666)	1·68 (1·18–2·42)
Meningitis	1·74 (1·24–2·37)	2·45 (1·78–3·29)	0·000834 (2·8e-05–0·00228)	3·21 (2·33–4·28)
Encephalitis	0·436 (0·298–0·616)	0·872 (0·559–1·26)	0·000315 (1·19e-05–0·00088)	1·17 (0·786–1·64)
Measles	3·02 (-1·02–16·6)	3·61 (-1·19–18·1)	0·247 (0·00704–0·676)	3·86 (-1·35–20·3)
Acute hepatitis	11·7 (5·37–22·5)	5·8 (2·98–10·3)	2·5 (1·42–4·07)	10·3 (4·58–20·3)
Maternal and neonatal disorders	86·4 (83·8–88·7)	76·7 (73·4–80)	67·3 (61·8–72·4)	87 (84·5–89·3)
Maternal disorders	9·44 (3·34–15·9)	9·57 (3·35–16·3)	9·66 (3·34–16·4)	9·34 (3·29–15·7)
Neonatal disorders	88·2 (85·7–90·5)	78·5 (75–81·7)	69·4 (63·9–74·7)	88·2 (85·7–90·5)
Nutritional deficiencies	55·6 (48·8–81·8)	77·4 (73·2–81·9)	77·8 (73·5–82·3)	57·5 (52·6–73·7)
Protein-energy malnutrition	100 (100–100)	100 (100–100)	100 (100–100)	100 (100–100)
Vitamin A deficiency	--	100 (100–100)	100 (100–100)	--
Dietary iron deficiency	--	100 (100–100)	100 (100–100)	--
Non-communicable diseases	59·4 (56·3–62·3)	41·1 (37·7–44·5)	28·4 (25·6–31·1)	59·5 (56·8–62)
Neoplasms	50·8 (46·9–55)	48·8 (45·2–52·7)	34·9 (30·7–39·4)	49·5 (46–53·4)
Lip and oral cavity cancer	72 (62·1–79·3)	73·8 (64·8–80·6)	73 (63·5–79·9)	73·9 (64·9–80·6)
Nasopharynx cancer	70·8 (59·6–79·4)	70·2 (59·5–78·2)	68·2 (56·7–77·3)	70·3 (59·6–78·3)
Other pharynx cancer	81·7 (74·4–87)	82·4 (75·2–87·6)	81·2 (73·5–86·7)	82·4 (75·3–87·6)
Esophageal cancer	77·4 (68·2–85·6)	78·4 (69·7–86·5)	77·6 (68·4–85·6)	78·4 (69·7–86·6)
Stomach cancer	24·9 (17·3–41·3)	24·4 (16·9–40·9)	24·6 (17·4–41·6)	24·4 (16·9–40·9)

Colon and rectum cancer	65·9 (57·2–74·5)	65·9 (57·6–74·1)	65·9 (57·5–74·3)	65·9 (57·6–74·1)
Liver cancer	78·5 (72·5–83·9)	78 (72·3–83·1)	78·3 (72·9–83·4)	78 (72·3–83·1)
Gallbladder and biliary tract cancer	25·5 (14·4–38·5)	25·8 (14·7–38·9)	24·1 (13·3–37·6)	25·9 (14·7–39)
Pancreatic cancer	44·3 (34·1–56·3)	43 (33·7–54·3)	43·8 (34·1–55·5)	42·9 (33·6–54·3)
Larynx cancer	84·4 (77·8–89·8)	83·4 (77–88·6)	80·3 (72·9–86·2)	83·7 (77·3–88·8)
Tracheal, bronchus, and lung cancer	85·8 (82·2–89·3)	84 (80·3–87·5)	85 (81·4–88·6)	84 (80·3–87·5)
Breast cancer	39 (29·7–49·9)	36·3 (27·8–45·8)	36·7 (28–46·4)	36·3 (27·8–45·9)
Cervical cancer	100 (100–100)	100 (100–100)	100 (100–100)	100 (100–100)
Uterine cancer	49·7 (33·5–65·1)	50·5 (34·6–65·9)	50·7 (34·8–65·9)	50·5 (34·5–65·9)
Ovarian cancer	24 (12·1–38·7)	19·9 (9·77–32·9)	18·5 (8·84–30·6)	20 (9·82–33·1)
Prostate cancer	5·92 (2·64–9·49)	6·41 (2·96–10)	6·76 (3·16–10·5)	6·35 (2·91–9·89)
Kidney cancer	42·1 (31·8–52·6)	41·5 (31·3–51·6)	41 (30·9–51·2)	41·5 (31·3–51·7)
Bladder cancer	45·1 (30·1–60·2)	46·4 (32·9–59·7)	46·5 (33·3–59)	46·4 (32·8–59·7)
Thyroid cancer	17·4 (8·87–27·3)	17·9 (9·16–28)	17·8 (9·03–27·9)	17·9 (9·13–28·2)
Mesothelioma	97·8 (97–98·4)	95·3 (93·9–96·4)	97·5 (96·5–98·3)	95·3 (93·8–96·4)
Non-Hodgkin lymphoma	8·79 (3·82–15·3)	8·91 (4·03–15·3)	8·9 (3·85–15·4)	8·91 (4·04–15·3)
Multiple myeloma	10 (4·37–17·4)	10·3 (4·62–17·9)	10·3 (4·54–18)	10·3 (4·63–17·8)
Leukemia	37·5 (24·7–50·8)	33·3 (23–43·5)	31·6 (21·3–41·7)	33·4 (23·1–43·7)
Cardiovascular diseases	79·4 (75·9–82·9)	80·4 (77·5–83·1)	71·4 (66·7–75·9)	81·7 (78·9–84·3)
Rheumatic heart disease	23 (12·9–42·2)	24 (15·4–38·5)	24 (15·5–37·6)	24 (15·4–38·5)
Ischemic heart disease	94·2 (90·6–97)	95·5 (93–97·6)	94·6 (91·9–97)	95·6 (93·1–97·6)
Stroke	81·4 (73·9–89·1)	85·2 (80·2–90·4)	85·3 (81–89·7)	85·2 (79·8–90·5)
Hypertensive heart disease	100 (100–100)	100 (100–100)	100 (100–100)	100 (100–100)
Non-rheumatic valvular heart disease	26·6 (17·3–38·6)	27·9 (19·5–37·2)	20·4 (13·9–27·7)	28·6 (20–38·3)
Cardiomyopathy and myocarditis	41·6 (33·9–50·1)	48·1 (42·3–54·2)	41·9 (35·6–48·1)	48·6 (42·6–54·6)
Atrial fibrillation and flutter	54·8 (45·2–65·6)	63 (54·8–71·5)	66·6 (58·9–74·6)	58·4 (49·3–68)
Aortic aneurysm	52·7 (45·9–59·8)	59·8 (54·1–65·4)	--	59·8 (54·1–65·4)
Endocarditis	32 (22·5–43·8)	34·7 (25·9–45·2)	31·2 (21·9–43·7)	34·8 (26–45·2)
Other cardiovascular and circulatory diseases	32·5 (25·7–39·2)	33·9 (27·4–40·6)	32·6 (23·9–42)	34·3 (27·9–40·8)
Chronic respiratory diseases	66·3 (60·7–72)	57·8 (53–62·6)	50·3 (44·3–56·4)	66 (60·9–71)
Chronic obstructive pulmonary disease	73·9 (68·4–79·1)	71·9 (67·9–75·9)	64·7 (61–68·1)	75·3 (70·4–80)
Pneumoconiosis	100 (100–100)	100 (100–100)	100 (100–100)	100 (100–100)
Asthma	47·5 (35·8–60)	44·2 (35·2–54·3)	44 (35·1–54)	47·4 (37·4–58·1)
Digestive diseases	39·2 (33·3–44·5)	42·8 (36·5–48·8)	29·2 (20·4–39·1)	51·9 (44·8–58·2)

Cirrhosis and other chronic liver diseases	79·6 (67·6–89·7)	81·4 (70–90·8)	78·3 (66·2–87·4)	81·5 (70–90·9)
Upper digestive system diseases	23·5 (19·7–27·3)	8·07 (5·05–12)	0·654 (0·373–1·05)	26 (22·2–29·5)
Gallbladder and biliary diseases	49·6 (33·7–65)	54·3 (39·3–68·2)	54·8 (39·9–68·8)	50·5 (35·4–65·3)
Pancreatitis	49·6 (30·2–66·9)	54·4 (36·1–70·8)	52·5 (33·3–69·4)	54·7 (36·1–70·9)
Neurological disorders	28·2 (13·3–43·5)	12·7 (5·78–23·7)	7·27 (3·1–16·2)	24·5 (13·3–39)
Alzheimer's disease and other dementias	45·4 (30·1–61·4)	47·1 (31·8–62·6)	47·2 (32·1–62·6)	46·9 (31·8–62·6)
Parkinson's disease	-9·39 (-15·6–-4·19)	-10·5 (-17·3–-4·8)	-11·2 (-18·3–-5·1)	-10·3 (-17–-4·73)
Idiopathic epilepsy	31·9 (21·7–41·6)	29·5 (20·1–38·4)	27·7 (18·6–36·5)	32 (22·2–41·6)
Multiple sclerosis	15·8 (11·7–20·2)	15·6 (11·6–19·8)	14·8 (11·1–18·9)	16·3 (12·1–20·6)
Mental disorders	--	6·77 (3·13–10·8)	6·77 (3·13–10·8)	--
Depressive disorders	--	14·9 (6·54–23·3)	14·9 (6·54–23·3)	--
Anxiety disorders	--	5·31 (1·08–12·7)	5·31 (1·08–12·7)	--
Idiopathic developmental intellectual disability	--	30·7 (11–52)	30·7 (11–52)	--
Substance use disorders	100 (100–100)	100 (100–100)	100 (100–100)	100 (100–100)
Alcohol use disorders	100 (100–100)	100 (100–100)	100 (100–100)	100 (100–100)
Drug use disorders	100 (100–100)	100 (100–100)	100 (100–100)	100 (100–100)
Diabetes and kidney diseases	99·5 (99·4–99·6)	99·9 (99·9–99·9)	100 (100–100)	99·5 (99·4–99·6)
Diabetes mellitus	100 (100–100)	100 (100–100)	100 (100–100)	100 (100–100)
Chronic kidney disease	100 (100–100)	100 (100–100)	100 (100–100)	100 (100–100)
Sense organ diseases	--	9·19 (8·17–10·3)	9·19 (8·17–10·3)	--
Blindness and vision loss	--	7·98 (5·27–11·5)	7·98 (5·27–11·5)	--
Age-related and other hearing loss	--	11 (10·1–12·1)	11 (10·1–12·1)	--
Musculoskeletal disorders	2·84 (0·663–5·2)	22·3 (18·6–26·1)	22·6 (18·9–26·6)	3·11 (0·753–5·6)
Rheumatoid arthritis	12·6 (3·08–22·7)	13·5 (3·37–23·9)	13·4 (3·33–23·7)	14 (3·51–24·7)
Osteoarthritis	--	19·9 (11–29·3)	19·9 (11–29·3)	--
Low back pain	--	41·3 (36·4–46·2)	41·3 (36·4–46·2)	--
Gout	--	54·5 (37·4–71·9)	54·5 (37·4–71·9)	--
Other non-communicable diseases	0·157 (0·0997–0·235)	0·0913 (0·053–0·144)	--	0·474 (0·282–0·7)
Sudden infant death syndrome	7·8 (4·27–12·5)	7·79 (4·27–12·5)	--	7·79 (4·27–12·5)
Injuries	30·6 (24·8–36·3)	24·8 (20–29·5)	25·2 (21–30)	24·3 (17·4–30·8)
Transport injuries	20·6 (12·2–28·6)	20·6 (13·6–27·3)	24·7 (20–30)	18·6 (9·64–27)
Road injuries	19·7 (10·9–28·3)	19·8 (12·4–26·8)	24 (19·2–29·3)	17·9 (8·37–26·6)
Other transport injuries	29·4 (23·6–35·4)	28 (22·7–33·6)	30·7 (26·2–35·5)	26·4 (20·2–32·5)
Unintentional injuries	35·4 (29·9–39·8)	23·7 (19·3–28·9)	24·4 (19·9–29·9)	21·8 (17·5–26·4)

Falls	51·7 (44·2–57·1)	34·1 (28·2–39·9)	32·3 (26·5–38·5)	40·2 (34·1–46·3)
Drowning	-13·7 (-27·5–1·24)	-13·1 (-25·9–1·26)	11·6 (6·59–18·4)	-14·3 (-27·7–0·563)
Fire, heat, and hot substances	12·3 (6·92–19·5)	13·6 (8·09–20·6)	14 (8·32–21·2)	12·9 (7·55–19·7)
Poisonings	13·7 (8·16–20·8)	13·3 (8·07–20)	12·7 (7·56–19·3)	13·9 (8·29–20·9)
Exposure to mechanical forces	4·33 (-8·57–17·8)	12·3 (9·79–14·8)	13·6 (11·6–16)	-3·62 (-17·2–10·9)
Animal contact	19·5 (13·9–26·6)	18·5 (13·1–25·1)	19·4 (14·1–25·9)	16·9 (11·7–23·6)
Foreign body	2·46 (1·58–3·57)	3·08 (2·01–4·5)	2·7 (1·75–4·02)	3·28 (2·14–4·79)
Environmental heat and cold exposure	9·88 (4·75–17·3)	10·7 (5·11–18·1)	10·6 (5·03–18)	11·1 (5·39–18·8)
Exposure to forces of nature	0 (0–0)	11·4 (5·41–19·2)	11·4 (5·41–19·2)	0 (0–0)
Other unintentional injuries	15·1 (9·06–22·7)	13·4 (8·03–20·6)	13·3 (7·92–20·5)	15·9 (9·73–23·5)
Self-harm and interpersonal violence	27·8 (15–40·6)	29·3 (17·6–40·6)	37·2 (28·6–45·3)	28·2 (15·6–40·7)
Self-harm	27·9 (13·7–41·9)	28·7 (14·6–42·7)	28·3 (15·3–41·4)	28·7 (14·6–42·7)
Interpersonal violence	28·6 (19·5–37·9)	33·4 (24·7–42·4)	41·5 (32–51·3)	26·2 (17·6–35·4)

Supplementary Results Table 2. All-age PAFs attributable to the joint distribution of all risk factors for all ages, both sexes combined for each GBD level 1, level 2, and level 3 cause and all causes in 2019 for Kensington and Chelsea. Results are shown for deaths, YLLs, YLDs, and DALYs Data in parenthesis are 95% uncertainty intervals. PAFs=population attributable fractions. GBD=Global Burden of Disease. YLLs=years of life lost. YLDs=years lived with disability. DALYs=disability-adjusted life-years

	Deaths 2019	DALYs 2019	YLDs 2019	YLLs 2019
All causes	55 (51·9–58·1)	40·2 (37·1–43·2)	29·6 (27–32·1)	55 (52·4–57·5)
Communicable, maternal, neonatal, and nutritional diseases	48·6 (39·1–57·8)	55·2 (50·8–59·3)	50·5 (46–55)	58·3 (51·5–64·7)
HIV/AIDS and sexually transmitted infections	87·9 (82·1–91·7)	86·8 (79·4–91·3)	95·1 (92·8–96·6)	81·3 (72·1–87·8)
HIV/AIDS	92·4 (89·7–93·9)	91·6 (88·3–93·4)	94 (91·1–95·6)	90·1 (86·8–91·8)
Sexually transmitted infections excluding HIV	40·5 (20·2–67·9)	59·3 (32·6–84·1)	100 (100–100)	14·3 (5·4–32·1)
Respiratory infections and tuberculosis	49·4 (38·5–60·2)	43·6 (34·6–52·7)	3·51 (2·48–4·89)	53·1 (42·8–63·1)
Tuberculosis	63·1 (52·1–71·7)	64·7 (54·5–72·2)	61·8 (51·1–70·2)	65·2 (55·2–72·9)
Lower respiratory infections	49·1 (38·1–60·2)	52·7 (42·2–63)	38·5 (31·7–45·3)	52·8 (42·2–63·2)
Upper respiratory infections	0·0241 (0·0135–0·0409)	0·00703 (0·000635–0·0183)	0·00655 (5·5e-05–0·0177)	0·0525 (0·0276–0·0922)
Otitis media	3·41 (1·69–5·98)	4·32 (2·75–6·09)	4·27 (2·69–6·04)	7·74 (4·46–11·8)
Enteric infections	4·01 (2·58–6·36)	8·24 (6·31–10·9)	8·43 (6·42–11·3)	7·85 (5·54–10·8)
Diarrheal diseases	4·04 (2·61–6·4)	8·29 (6·35–10·9)	8·43 (6·42–11·3)	7·96 (5·66–10·9)
Other infectious diseases	0·584 (0·425–0·773)	1·03 (0·781–1·32)	0·412 (0·232–0·701)	1·27 (0·946–1·68)
Meningitis	1·44 (0·994–2·05)	2·32 (1·6–3·25)	0·000614 (2·55e-06–0·00188)	2·92 (2·01–4·16)
Encephalitis	0·31 (0·194–0·474)	0·769 (0·454–1·19)	0·00022 (1e-06–0·000601)	0·983 (0·614–1·49)
Measles	3·84 (-1·46–20·2)	4·21 (-1·61–22·7)	0·222 (0·00596–0·604)	4·61 (-1·76–23·9)
Acute hepatitis	14 (8·82–21·7)	5·52 (3·48–8·17)	2·82 (1·64–4·56)	13 (7·78–19·7)
Maternal and neonatal disorders	87·8 (84·8–90·1)	78·4 (74·5–81·9)	67·2 (61·8–72·2)	88·5 (85·7–90·7)
Maternal disorders	9·15 (3·31–15·5)	9·23 (3·26–15·9)	9·31 (3·29–16·1)	9·04 (3·24–15·3)
Neonatal disorders	89·6 (87–91·7)	80 (76·3–83·5)	69·3 (64–74·4)	89·6 (87·1–91·7)
Nutritional deficiencies	74·6 (69·3–89·2)	76·9 (73–81·5)	77 (73–81·7)	73·3 (68·6–82)
Protein-energy malnutrition	100 (100–100)	100 (100–100)	100 (100–100)	100 (100–100)
Vitamin A deficiency	--	100 (100–100)	100 (100–100)	--
Dietary iron deficiency	--	100 (100–100)	100 (100–100)	--
Non-communicable diseases	56·6 (53·3–59·9)	40·3 (37–43·7)	28·8 (26·1–31·6)	57·2 (54·3–60)
Neoplasms	49·3 (45·3–53·5)	48·2 (44·5–52·1)	35 (30·7–39·4)	49 (45·3–53)
Lip and oral cavity cancer	72 (61·7–79·3)	74·4 (65·3–80·8)	73·9 (64·6–80·6)	74·5 (65·3–80·9)
Nasopharynx cancer	70·2 (58·9–78·7)	70·1 (59·2–78·1)	68·7 (57·3–77·4)	70·3 (59·4–78·2)
Other pharynx cancer	81·7 (75–86·8)	82·6 (76·5–87·5)	81·6 (74·6–86·8)	82·7 (76·6–87·5)
Esophageal cancer	77·4 (68·6–85·4)	78·6 (69·8–86·4)	77·7 (68·8–85·8)	78·6 (69·8–86·4)
Stomach cancer	24·5 (17·1–40·9)	24·5 (17·1–40·6)	24·7 (17·2–41)	24·5 (17·1–40·6)

Colon and rectum cancer	65·8 (56·9–74·4)	66·1 (57·4–74·4)	66·2 (57·6–74·6)	66·1 (57·5–74·4)
Liver cancer	82·4 (77·5–86·8)	82·4 (78·1–86·5)	82·5 (78–86·7)	82·4 (78–86·5)
Gallbladder and biliary tract cancer	26·5 (15·1–39·9)	26·9 (15·3–40·3)	25·2 (14–38·5)	27 (15·3–40·4)
Pancreatic cancer	44·1 (34·3–55·4)	43·3 (33·8–54·3)	43·9 (34·2–55·1)	43·3 (33·8–54·3)
Larynx cancer	83·8 (76–89·6)	83·1 (75·7–88·6)	81 (73·3–86·9)	83·3 (75·9–88·9)
Tracheal, bronchus, and lung cancer	85·3 (81·6–88·8)	83·8 (80·2–87·4)	84·7 (81–88·3)	83·8 (80·2–87·4)
Breast cancer	39·8 (30·3–51·1)	38 (29·5–47·6)	38·3 (29·7–48)	38 (29·4–47·5)
Cervical cancer	100 (100–100)	100 (100–100)	100 (100–100)	100 (100–100)
Uterine cancer	50·3 (34·3–65·5)	51·2 (35·4–66·1)	51·4 (35·8–66·4)	51·1 (35·3–66)
Ovarian cancer	26 (13·4–40·1)	21·8 (10·6–34·6)	20·7 (10·1–33)	21·9 (10·6–34·7)
Prostate cancer	5·69 (2·38–9·26)	6·18 (2·69–9·91)	6·54 (2·9–10·2)	6·11 (2·66–9·83)
Kidney cancer	42·4 (31·6–52·9)	42·2 (31·5–52·2)	41·8 (31·3–52·1)	42·2 (31·5–52·2)
Bladder cancer	43 (28–57·9)	45 (31·5–58·4)	45·4 (31·9–58·6)	44·9 (31·4–58·4)
Thyroid cancer	17·7 (9–27·7)	18·3 (9·35–28·7)	18·3 (9·25–28·7)	18·3 (9·34–28·6)
Mesothelioma	98·2 (97·5–98·7)	96·3 (95–97·3)	97·9 (97·1–98·6)	96·2 (95–97·2)
Non-Hodgkin lymphoma	8·96 (3·93–15·7)	9·17 (4·09–15·8)	9·12 (3·98–15·9)	9·18 (4·08–15·8)
Multiple myeloma	10·1 (4·45–17·5)	10·5 (4·67–17·9)	10·4 (4·67–18·1)	10·5 (4·68–17·9)
Leukemia	38 (24·3–51·3)	35·2 (24·2–46·6)	35 (23·9–46·9)	35·2 (24·2–46·6)
Cardiovascular diseases	76·4 (72·4–80·3)	77·4 (74·2–80·7)	69·1 (63·8–74)	78·8 (75·7–82)
Rheumatic heart disease	22·9 (12·5–42·8)	23·9 (14·9–39·1)	24 (15·3–37·5)	23·9 (14·9–39·4)
Ischemic heart disease	93·7 (89·9–96·8)	95 (92·4–97·3)	94·5 (91·9–96·9)	95 (92·4–97·3)
Stroke	80·6 (72·5–89·1)	84·2 (78·8–90)	84·7 (80·2–89·8)	84·1 (78·5–90·1)
Hypertensive heart disease	100 (100–100)	100 (100–100)	100 (100–100)	100 (100–100)
Non-rheumatic valvular heart disease	26·2 (16·7–39·3)	21·7 (14·6–30·5)	5·38 (3·43–7·7)	27·9 (19·3–38·4)
Cardiomyopathy and myocarditis	34·2 (26·2–44)	41·1 (35·1–47·5)	37·5 (30·9–44·2)	41·3 (35·1–47·9)
Atrial fibrillation and flutter	55·4 (44·9–66·2)	63·3 (54·3–71·9)	67 (59·1–74·9)	58·7 (48·8–68·1)
Aortic aneurysm	50 (42·5–57·4)	57·3 (50·8–63·4)	--	57·3 (50·8–63·4)
Endocarditis	31·4 (21·3–44·4)	34·2 (24·9–44·9)	31·7 (22–43·8)	34·3 (25–45)
Other cardiovascular and circulatory diseases	30·4 (23·4–37·4)	32·7 (26·1–39·6)	33 (24·7–42·2)	32·6 (26·1–39·5)
Chronic respiratory diseases	64·6 (58·8–70·1)	58·2 (53·9–62·6)	52·4 (46·6–58)	64·9 (59·9–69·7)
Chronic obstructive pulmonary disease	72·1 (66·2–78)	70·5 (66·1–74·9)	64·5 (60·7–68·1)	74 (68·7–79·3)
Pneumoconiosis	100 (100–100)	100 (100–100)	100 (100–100)	100 (100–100)
Asthma	46·6 (34·1–60·4)	45·7 (36·6–55·8)	45·6 (36·5–55·7)	47·7 (36·9–58·8)
Digestive diseases	38·6 (32·5–43·9)	43·6 (37·2–49·8)	33·1 (23·5–43·7)	52·6 (45·1–58·7)
Cirrhosis and other chronic liver diseases	80·3 (67·6–89·6)	82·5 (71·3–90·9)	79·9 (69·1–88·2)	82·5 (71·3–90·9)

Upper digestive system diseases	20·5 (17–24·3)	6·71 (4·19–9·87)	0·554 (0·315–0·904)	23·1 (19·6–26·6)
Gallbladder and biliary diseases	49·7 (33·8–65·4)	54·2 (39·6–68·3)	54·5 (39·9–68·8)	50·6 (35·3–66·2)
Pancreatitis	53·5 (36·5–70·7)	57·9 (41·6–73·2)	57 (40·4–72·6)	58 (41·9–73·4)
Neurological disorders	29·2 (13·4–45·1)	15·1 (6·83–27·1)	8·93 (4·09–18·1)	25·4 (12–41·3)
Alzheimer's disease and other dementias	44 (29·5–59·9)	45·9 (31·5–61·4)	46·2 (32·1–61·5)	45·8 (31·4–61·3)
Parkinson's disease	-8·92 (-14·7–-4·06)	-10·1 (-16·3–-4·64)	-10·7 (-17·3–-5·09)	-9·9 (-16·3–-4·59)
Idiopathic epilepsy	32·6 (22·5–42·8)	30·3 (20·9–39·7)	29 (19·4–38·4)	32·8 (22·9–42·5)
Multiple sclerosis	15·1 (11·2–18·9)	15·2 (11·3–18·9)	14·7 (10·8–18·3)	15·8 (11·7–19·8)
Mental disorders	--	6·45 (3·03–10·1)	6·45 (3·03–10·1)	--
Depressive disorders	--	14·1 (6·23–22·2)	14·1 (6·23–22·2)	--
Anxiety disorders	--	4·5 (0·981–10·9)	4·5 (0·981–10·9)	--
Idiopathic developmental intellectual disability	--	29·9 (12–50·5)	29·9 (12–50·5)	--
Substance use disorders	100 (100–100)	100 (100–100)	100 (100–100)	100 (100–100)
Alcohol use disorders	100 (100–100)	100 (100–100)	100 (100–100)	100 (100–100)
Drug use disorders	100 (100–100)	100 (100–100)	100 (100–100)	100 (100–100)
Diabetes and kidney diseases	100 (100–100)	100 (100–100)	100 (100–100)	100 (100–100)
Diabetes mellitus	100 (100–100)	100 (100–100)	100 (100–100)	100 (100–100)
Chronic kidney disease	100 (100–100)	100 (100–100)	100 (100–100)	100 (100–100)
Sense organ diseases	--	8·77 (7·68–10)	8·77 (7·68–10)	--
Blindness and vision loss	--	9·16 (6·06–13·2)	9·16 (6·06–13·2)	--
Age-related and other hearing loss	--	10 (9–11·1)	10 (9–11·1)	--
Musculoskeletal disorders	1·8 (0·411–3·38)	22·5 (18·6–26·3)	22·8 (18·8–26·8)	2·17 (0·512–3·89)
Rheumatoid arthritis	11·8 (2·72–21·3)	13·3 (3·36–23·3)	13·3 (3·4–23·4)	13·1 (3·1–23·4)
Osteoarthritis	--	20·2 (11·2–29·7)	20·2 (11·2–29·7)	--
Low back pain	--	41·5 (36·1–46·7)	41·5 (36·1–46·7)	--
Gout	--	56 (39·4–73)	56 (39·4–73)	--
Other non-communicable diseases	0·0815 (0·0484–0·125)	0·0585 (0·0334–0·0893)	--	0·317 (0·174–0·476)
Sudden infant death syndrome	6·63 (3·66–10·8)	6·62 (3·65–10·8)	--	6·62 (3·65–10·8)
Injuries	35 (28·9–40·4)	27·7 (23–32·5)	28·4 (23·8–33·1)	26·8 (20·2–33)
Transport injuries	22·9 (15·1–30·1)	22·9 (16·5–28·8)	26·1 (21·6–30·5)	20·9 (12·2–28·4)
Road injuries	21·9 (13·4–29·5)	21·9 (15·1–28·3)	25 (20·5–29·4)	20 (10·8–28)
Other transport injuries	32·7 (27·4–37·1)	31 (25·9–35·5)	34·1 (29·6–38·4)	28·8 (23·2–33·8)
Unintentional injuries	41 (34·3–46·1)	27·7 (22·9–32·8)	28 (23–33·1)	26·6 (21·5–31·3)
Falls	55·2 (46·7–61)	38·8 (32·5–44·5)	36·9 (30·8–42·8)	45·3 (38·4–51·3)
Drowning	-14 (-28–1·21)	-13·5 (-26·5–0·253)	12·2 (6·63–19·3)	-15 (-28·4–0·58)

Fire, heat, and hot substances	11·6<br>(6·09–18·7)	13·7<br>(7·86–20·9)	14·5<br>(8·4–21·9)	12·2<br>(7·06–18·9)
Poisonings	12·8<br>(7·34–19·6)	13·2<br>(7·76–20·1)	13·3<br>(7·71–20·2)	13·1<br>(7·88–19·8)
Exposure to mechanical forces	10·5<br>(-2·52–23·7)	14·9<br>(12·2–17·5)	15·9<br>(13·5–18·4)	-0·755<br>(-14·4–13·7)
Animal contact	23·1<br>(17·3–29·7)	20·9<br>(15·5–27·4)	21·5<br>(16·1–28·1)	19·1<br>(13·7–25·3)
Foreign body	1·88<br>(1·2–2·79)	2·87<br>(1·89–4·26)	2·75<br>(1·83–4·12)	2·95<br>(1·9–4·4)
Environmental heat and cold exposure	9·51<br>(4·33–16·2)	11·1<br>(5·1–19)	11·2<br>(5·1–19)	10·8<br>(5·01–18·3)
Exposure to forces of nature	0<br>(0–0)	12·1<br>(5·76–20·6)	12·1<br>(5·76–20·6)	0<br>(0–0)
Other unintentional injuries	15·3<br>(8·9–22·9)	14<br>(8·17–21·1)	13·9<br>(8·12–21)	16·4<br>(10–23·9)
Self-harm and interpersonal violence	28·2<br>(15·5–40·8)	30·1<br>(18·4–41·3)	36·9<br>(29·1–44·9)	29·1<br>(16·4–41·3)
Self-harm	28·2<br>(14·6–41·8)	29·4<br>(15·7–42·7)	28·9<br>(16·3–41·1)	29·4<br>(15·7–42·7)
Interpersonal violence	30·1<br>(21·7–39·7)	35·6<br>(27·2–44·7)	41·9<br>(33–51·3)	26·7<br>(18·7–35·8)

Supplementary Results Table 2. All-age PAFs attributable to the joint distribution of all risk factors for all ages, both sexes combined for each GBD level 1, level 2, and level 3 cause and all causes in 2019 for Kingston upon Thames. Results are shown for deaths, YLLs, YLDs, and DALYs Data in parenthesis are 95% uncertainty intervals. PAFs=population attributable fractions. GBD=Global Burden of Disease. YLLs=years of life lost. YLDs=years lived with disability. DALYs=disability-adjusted life-years

	Deaths 2019	DALYs 2019	YLDs 2019	YLLs 2019
All causes	53·8 (50·1–57·2)	38·7 (35·6–41·7)	27·6 (25·2–29·9)	53·6 (50·8–56·3)
Communicable, maternal, neonatal, and nutritional diseases	46·5 (35·1–57·4)	52 (46·5–57·3)	44·1 (39·9–48·9)	56·6 (48·5–64·3)
HIV/AIDS and sexually transmitted infections	76·8 (62·2–87·5)	75·2 (58·1–87·3)	96·9 (95·3–98·1)	60·8 (41·5–77·3)
HIV/AIDS	91·4 (89·4–92·5)	89·8 (87·3–91·6)	94·2 (91·9–95·7)	87·2 (84·7–88·5)
Sexually transmitted infections excluding HIV	46·8 (24·6–74)	55·4 (28·8–81·5)	100 (100–100)	16·7 (6·44–36·5)
Respiratory infections and tuberculosis	47 (34–59)	42·4 (32·4–51·8)	3·21 (2·36–4·28)	50·5 (38·5–61·5)
Tuberculosis	59·5 (47·7–69·3)	60·5 (49·7–69·4)	56·6 (44·3–66·2)	61·8 (51·3–70·5)
Lower respiratory infections	46·9 (33·9–59)	50·3 (38·1–61·3)	35 (28·1–41·7)	50·4 (38·2–61·5)
Upper respiratory infections	0·142 (0·075–0·227)	0·00775 (0·000702–0·0199)	0·00715 (0·000156–0·0191)	0·329 (0·18–0·532)
Otitis media	3·12 (1·67–5·37)	4·85 (3·14–6·76)	4·8 (3·05–6·7)	7·45 (4·42–11·2)
Enteric infections	4·39 (2·88–7·09)	9·26 (7·09–12)	9·29 (7·04–12·2)	9·19 (6·54–12·6)
Diarrheal diseases	4·43 (2·9–7·13)	9·3 (7·11–12·1)	9·3 (7·05–12·2)	9·31 (6·73–12·7)
Other infectious diseases	0·815 (0·502–1·1)	1·3 (0·916–1·73)	0·339 (0·19–0·563)	1·86 (1·24–2·49)
Meningitis	2·05 (1·43–2·78)	2·76 (1·93–3·79)	0·000601 (7·26e-06–0·00179)	3·87 (2·75–5·26)
Encephalitis	0·397 (0·268–0·576)	0·721 (0·413–1·1)	2e-04 (3·3e-06–0·000609)	1·27 (0·821–1·88)
Measles	4·59 (-1·41–23·6)	5·17 (-1·58–26·8)	0·317 (0·00719–0·889)	5·47 (-1·74–27·4)
Acute hepatitis	9·94 (5·72–20·3)	4·49 (2·79–7·18)	2·69 (1·56–4·36)	9·33 (5·35–18)
Maternal and neonatal disorders	88·2 (85·9–90·2)	76 (72–79·8)	57·3 (51·5–62·8)	88·7 (86·3–90·6)
Maternal disorders	9·21 (3·31–15·3)	9·34 (3·38–15·7)	9·42 (3·38–16·1)	9·06 (3·24–15·2)
Neonatal disorders	89·3 (87·1–91·2)	77·6 (73·6–81·3)	59·6 (53·5–65·4)	89·4 (87·1–91·2)
Nutritional deficiencies	83·1 (79·1–92·4)	79·1 (74·7–84·3)	79·1 (74·7–84·4)	77·6 (74·1–82·5)
Protein-energy malnutrition	100 (100–100)	100 (100–100)	100 (100–100)	100 (100–100)
Vitamin A deficiency	--	100 (100–100)	100 (100–100)	--
Dietary iron deficiency	--	100 (100–100)	100 (100–100)	--
Non-communicable diseases	55·4 (51·5–59)	38·7 (35·4–42)	26·8 (24·3–29·5)	55·3 (52·5–58·2)
Neoplasms	47 (43–51·3)	45·8 (42·1–49·8)	33·4 (29·1–38·1)	46·6 (43–50·5)
Lip and oral cavity cancer	67·7 (55·6–76·4)	70·8 (60–78·7)	70·4 (59·6–78·4)	70·8 (60–78·8)
Nasopharynx cancer	65·3 (53–75·3)	65·3 (53·1–74·5)	63·6 (51·6–73·9)	65·5 (53·4–74·6)
Other pharynx cancer	80·3 (73–86·1)	81·5 (74·6–86·8)	80·4 (73–86·2)	81·5 (74·7–86·9)
Esophageal cancer	75·9 (66·3–84·6)	77·3 (67·8–85·5)	76·4 (67·1–84·9)	77·3 (67·8–85·6)

Stomach cancer	23·9<br>(16·6–40·4)	24·1<br>(16·9–40·7)	24·2<br>(17–40·8)	24·1<br>(16·9–40·7)
Colon and rectum cancer	63·8<br>(54·8–72·6)	64·2<br>(55·7–72·4)	64·2<br>(55·6–72·8)	64·2<br>(55·7–72·3)
Liver cancer	77·3<br>(71·7–83·2)	77·8<br>(72·6–83)	78·1<br>(72·8–83·2)	77·8<br>(72·6–83)
Gallbladder and biliary tract cancer	24<br>(13·4–36·6)	24·2<br>(13·3–36·8)	22·6<br>(12·1–35·4)	24·2<br>(13·4–36·8)
Pancreatic cancer	42·4<br>(33–53·3)	41·6<br>(32·8–52)	42·2<br>(32·8–53·1)	41·6<br>(32·8–52)
Larynx cancer	83·1<br>(74·6–89·3)	82·4<br>(74·9–88·1)	79·9<br>(72·2–86)	82·7<br>(75·3–88·4)
Tracheal, bronchus, and lung cancer	85·6<br>(82–89)	84·3<br>(80·8–87·6)	85·1<br>(81·5–88·4)	84·3<br>(80·8–87·6)
Breast cancer	37·1<br>(27·6–48·4)	35·6<br>(27·2–45·3)	35·9<br>(27·4–45·7)	35·6<br>(27·2–45·2)
Cervical cancer	100<br>(100–100)	100<br>(100–100)	100<br>(100–100)	100<br>(100–100)
Uterine cancer	46·1<br>(30·5–61·3)	47·2<br>(32·1–62)	47·5<br>(32·4–62·9)	47·1<br>(32·1–61·9)
Ovarian cancer	29·2<br>(13·6–43·7)	24·1<br>(11·3–37)	22·9<br>(10·7–35·4)	24·2<br>(11·4–37)
Prostate cancer	5·52<br>(2·38–8·83)	5·97<br>(2·63–9·43)	6·31<br>(2·84–9·79)	5·91<br>(2·58–9·35)
Kidney cancer	39·5<br>(29·2–50·2)	39·3<br>(29·5–49·1)	38·9<br>(29·1–48·9)	39·3<br>(29·5–49·1)
Bladder cancer	40·8<br>(26·4–55·2)	43<br>(29·4–55·7)	43·5<br>(30·3–55·8)	42·9<br>(29·3–55·7)
Thyroid cancer	15·6<br>(7·57–25·6)	16·4<br>(7·93–26·7)	16·5<br>(8·02–26·9)	16·3<br>(7·93–26·8)
Mesothelioma	98·7<br>(98·2–99·1)	97·3<br>(96·3–98)	98·5<br>(97·8–99)	97·2<br>(96·3–98)
Non-Hodgkin lymphoma	7·71<br>(3·19–13·6)	7·93<br>(3·39–14)	7·88<br>(3·3–13·9)	7·94<br>(3·39–14)
Multiple myeloma	8·75<br>(3·82–15·5)	9·12<br>(4·13–16·1)	9·06<br>(3·99–16)	9·13<br>(4·13–16·1)
Leukemia	36·1<br>(22·3–50·5)	32·7<br>(21·4–43·9)	31·6<br>(20·9–42·8)	32·8<br>(21·5–44·1)
Cardiovascular diseases	76·6<br>(72·3–80·6)	77·9<br>(74·3–81·2)	71·1<br>(66–75·6)	79·1<br>(75·7–82·3)
Rheumatic heart disease	21·9<br>(11·4–43·5)	22·7<br>(13·9–40·8)	23<br>(14·5–38·1)	22·7<br>(13·7–41·2)
Ischemic heart disease	93·3<br>(89·2–96·7)	94·5<br>(91·8–97)	93·9<br>(90·8–96·6)	94·6<br>(91·8–97)
Stroke	79·1<br>(70·3–88·7)	82·4<br>(76·4–88·7)	82·4<br>(77·5–88·1)	82·4<br>(76–89)
Hypertensive heart disease	100<br>(100–100)	100<br>(100–100)	100<br>(100–100)	100<br>(100–100)
Non-rheumatic valvular heart disease	24·8<br>(15·5–37·9)	25·9<br>(17·4–36·5)	20·1<br>(13·5–28·6)	26·5<br>(17·8–37·3)
Cardiomyopathy and myocarditis	29·8<br>(21·4–40·4)	34·5<br>(28·2–41·9)	31·4<br>(25–38·7)	34·7<br>(28·3–42·5)
Atrial fibrillation and flutter	51·2<br>(40·2–62·3)	59·7<br>(50·4–68·3)	63·9<br>(55·4–71·9)	54·4<br>(44·3–64·2)
Aortic aneurysm	48·6<br>(41·8–55·6)	56·1<br>(50·4–61·9)	--	56·1<br>(50·4–61·9)
Endocarditis	29·9<br>(19·8–43)	32·4<br>(23·7–43·3)	29·7<br>(20–42·4)	32·5<br>(23·8–43·3)
Other cardiovascular and circulatory diseases	30·5<br>(23·2–37·8)	32·8<br>(26·5–38·9)	32·8<br>(25–40·6)	32·9<br>(26·2–39·1)
Chronic respiratory diseases	60·8<br>(54·1–67·6)	54·5<br>(49·7–59·4)	49·3<br>(43·8–54·8)	60·9<br>(55–67·1)
Chronic obstructive pulmonary disease	70·7<br>(64·1–77·2)	68·9<br>(64·2–73·4)	63·3<br>(59·2–67·3)	72·7<br>(67–78·3)
Pneumoconiosis	100<br>(100–100)	100<br>(100–100)	100<br>(100–100)	100<br>(100–100)
Asthma	42·8<br>(30·3–56·3)	40·6<br>(32–50·4)	40·3<br>(31·9–49·9)	43·8<br>(33·4–55·2)

Digestive diseases	32·8 (26·8–38·4)	36·8 (30·7–42·6)	21·6 (14·4–29·9)	46·7 (39·3–53·1)
Cirrhosis and other chronic liver diseases	76·8 (62·8–88·1)	79·8 (67·4–89·9)	76·3 (64·8–86)	79·9 (67·4–90)
Upper digestive system diseases	20·4 (16·7–24·1)	6·76 (4·22–10·1)	0·551 (0·313–0·892)	23·1 (19·4–26·4)
Gallbladder and biliary diseases	46·2 (30·6–61·5)	51·2 (36·1–65·7)	52·2 (36·9–66·5)	47 (31·9–62·2)
Pancreatitis	42·7 (21·4–62·7)	49·1 (28·4–68·2)	50·3 (29·7–68·9)	48·9 (28·2–68)
Neurological disorders	28·1 (13·5–43·2)	15·5 (7·32–27·3)	9·17 (4·26–18·6)	24·6 (11·8–39·7)
Alzheimer's disease and other dementias	40·7 (26·6–56·2)	42·5 (28·5–57·8)	42·8 (28·6–57·9)	42·4 (28·5–57·6)
Parkinson's disease	-8·51 (-14–-3·91)	-9·61 (-15·6–-4·46)	-10·3 (-16·4–-4·69)	-9·44 (-15·5–-4·41)
Idiopathic epilepsy	29 (18·6–39·4)	26·5 (17–35·9)	24·9 (15·8–34·1)	29 (18·9–38·6)
Multiple sclerosis	14·6 (10·8–18·6)	14·8 (11·1–18·6)	14·3 (10·7–17·9)	15·3 (11·4–19·4)
Mental disorders	--	6·71 (3·28–10·5)	6·71 (3·28–10·5)	--
Depressive disorders	--	14·1 (6·32–21·9)	14·1 (6·32–21·9)	--
Anxiety disorders	--	5·14 (1·28–11·5)	5·14 (1·28–11·5)	--
Idiopathic developmental intellectual disability	--	33·5 (14–53·5)	33·5 (14–53·5)	--
Substance use disorders	100 (100–100)	100 (100–100)	100 (100–100)	100 (100–100)
Alcohol use disorders	100 (100–100)	100 (100–100)	100 (100–100)	100 (100–100)
Drug use disorders	100 (100–100)	100 (100–100)	100 (100–100)	100 (100–100)
Diabetes and kidney diseases	99·8 (99·7–99·8)	100 (99·9–100)	100 (100–100)	99·8 (99·7–99·8)
Diabetes mellitus	100 (100–100)	100 (100–100)	100 (100–100)	100 (100–100)
Chronic kidney disease	100 (100–100)	100 (100–100)	100 (100–100)	100 (100–100)
Sense organ diseases	--	8·17 (7·12–9·38)	8·17 (7·12–9·38)	--
Blindness and vision loss	--	8·82 (5·75–12·7)	8·82 (5·75–12·7)	--
Age-related and other hearing loss	--	9·18 (8·26–10·2)	9·18 (8·26–10·2)	--
Musculoskeletal disorders	2·31 (0·544–4·39)	21·1 (17·3–25)	21·5 (17·6–25·5)	2·81 (0·656–5·08)
Rheumatoid arthritis	11 (2·59–20·2)	12·8 (3·1–22·5)	12·9 (3·14–22·7)	12·4 (3·07–22·1)
Osteoarthritis	--	18 (9·69–27·1)	18 (9·69–27·1)	--
Low back pain	--	39 (33·6–44·4)	39 (33·6–44·4)	--
Gout	--	52·9 (36·9–69·7)	52·9 (36·9–69·7)	--
Other non-communicable diseases	0·117 (0·0747–0·173)	0·101 (0·0602–0·153)	--	0·444 (0·265–0·642)
Sudden infant death syndrome	7·4 (4·21–12·1)	7·39 (4·21–12)	--	7·39 (4·21–12)
Injuries	30·8 (25·3–36·3)	26·3 (22·1–31)	28·4 (24·1–33·1)	22·8 (16·4–29·6)
Transport injuries	22·4 (14–30·2)	22·5 (15·5–28·9)	26·8 (22·4–31·7)	19·9 (11·1–28·2)
Road injuries	21·4 (12·5–29·5)	21·4 (14·2–28·3)	25·7 (21·3–30·7)	19·1 (9·8–27·6)
Other transport injuries	32·9 (27·6–38·2)	31·8 (26·5–36·8)	35 (30·4–39·3)	29·2 (23·3–35·1)

Unintentional injuries	36·8<br>(30·5–41·7)	26·7<br>(22–31·8)	28<br>(23·1–33·2)	20·5<br>(15·9–25·3)
Falls	56·2<br>(47·5–61·8)	38·4<br>(32·4–44)	37<br>(31–42·8)	46·5<br>(39·9–52·1)
Drowning	-14·8<br>(-28·5–-0·685)	-15·4<br>(-28·4–-2·33)	11·6<br>(6·45–18·2)	-16·6<br>(-30–-3·08)
Fire, heat, and hot substances	10·5<br>(5·48–16·7)	13·5<br>(8·01–20·3)	14·2<br>(8·43–21·2)	11<br>(6·36–16·6)
Poisonings	13·3<br>(7·67–19·8)	12·9<br>(7·72–19·2)	12·5<br>(7·55–18·9)	13·4<br>(8·04–19·8)
Exposure to mechanical forces	7·79<br>(-5·22–21·3)	16·5<br>(13·6–19·5)	17·5<br>(14·8–20·3)	-1·07<br>(-15·1–13·4)
Animal contact	19·8<br>(14·8–26)	19·3<br>(14·2–25·3)	21·2<br>(15·7–27·5)	16<br>(11·5–21·6)
Foreign body	1·97<br>(1·25–2·92)	3·14<br>(2·04–4·55)	3·32<br>(2·15–4·9)	2·98<br>(1·88–4·4)
Environmental heat and cold exposure	8·32<br>(3·64–14·3)	10<br>(4·52–17·2)	10·1<br>(4·54–17·5)	9·48<br>(4·3–16·2)
Exposure to forces of nature	0<br>(0–0)	11<br>(5·07–18·8)	11<br>(5·07–18·8)	0<br>(0–0)
Other unintentional injuries	14<br>(8·01–21·1)	13·4<br>(7·89–20)	13·3<br>(7·87–20)	15·1<br>(9·18–21·9)
Self-harm and interpersonal violence	25·4<br>(12·2–39)	27·8<br>(16·6–39·5)	36<br>(28·4–43·8)	26·1<br>(13·1–39·4)
Self-harm	25·4<br>(11·5–39·8)	26·3<br>(12·8–40·6)	26·2<br>(14·3–39)	26·3<br>(12·6–40·6)
Interpersonal violence	28·9<br>(20·2–38)	36·4<br>(28–45·4)	40·9<br>(32·2–50·3)	24·9<br>(16·8–33·3)

Supplementary Results Table 2. All-age PAFs attributable to the joint distribution of all risk factors for all ages, both sexes combined for each GBD level 1, level 2, and level 3 cause and all causes in 2019 for Lambeth. Results are shown for deaths, YLLs, YLDs, and DALYs Data in parenthesis are 95% uncertainty intervals. PAFs=population attributable fractions. GBD=Global Burden of Disease. YLLs=years of life lost. YLDs=years lived with disability. DALYs=disability-adjusted life-years

	Deaths 2019	DALYs 2019	YLDs 2019	YLLs 2019
All causes	56·1 (53·1–58·9)	39·5 (36·4–42·6)	28·2 (25·9–30·6)	55·4 (53–57·7)
Communicable, maternal, neonatal, and nutritional diseases	52·2 (43·2–60·7)	60·9 (57·2–64·6)	54·7 (50·3–59·2)	65·4 (60–70·5)
HIV/AIDS and sexually transmitted infections	86·4 (79·9–90·5)	84·9 (77·3–89·7)	94·9 (92·6–96·4)	78·6 (68·4–85·3)
HIV/AIDS	91·3 (89–92·7)	89·9 (87·1–91·7)	93·6 (91–95·3)	87·8 (85·1–89·4)
Sexually transmitted infections excluding HIV	41·3 (20·4–68·7)	58·8 (33·3–83·9)	100 (100–100)	17·2 (6·55–37·9)
Respiratory infections and tuberculosis	49·5 (38–60·1)	42·8 (33·9–51·4)	3·25 (2·29–4·61)	54·5 (43·9–64·2)
Tuberculosis	63·7 (53–72·1)	64·6 (54·3–72·2)	58·6 (47·3–67·6)	65·8 (55·8–73·2)
Lower respiratory infections	49·3 (37·5–60)	54 (43·2–64·1)	37·3 (30·4–43·7)	54·1 (43·3–64·3)
Upper respiratory infections	0·114 (0·0651–0·195)	0·00924 (0·00156–0·0218)	0·00791 (0·000359–0·0204)	0·212 (0·119–0·357)
Otitis media	4·26 (2·05–7·84)	4·74 (2·99–6·72)	4·67 (2·91–6·64)	7·95 (4·45–12·6)
Enteric infections	4·88 (3·27–7·65)	10·5 (8·23–13·5)	10·2 (7·72–13·3)	11·6 (8·36–15·8)
Diarrheal diseases	4·94 (3·3–7·75)	10·5 (8·31–13·6)	10·2 (7·73–13·4)	11·9 (8·6–16·1)
Other infectious diseases	1·14 (0·833–1·56)	1·7 (1·29–2·27)	0·356 (0·205–0·596)	2·21 (1·6–3·07)
Meningitis	2·95 (2·18–3·91)	3·91 (2·9–5·23)	0·000737 (2·75e-05–0·00204)	5·06 (3·71–6·71)
Encephalitis	0·797 (0·482–1·15)	1·63 (0·763–2·37)	0·000332 (1·13e-05–0·000896)	1·99 (1·15–2·83)
Measles	2·95 (-0·912–14)	2·74 (-0·755–13·1)	0·3 (0·00508–0·798)	3·87 (-1·21–18·2)
Acute hepatitis	17·5 (8·49–24·1)	5·6 (3·24–8·08)	2·42 (1·41–3·93)	15·9 (7·48–22·4)
Maternal and neonatal disorders	89·9 (87·5–91·9)	81·7 (78·7–84·6)	70·1 (64·9–75·1)	90·4 (88–92·3)
Maternal disorders	9·49 (3·42–16)	9·65 (3·4–16·3)	9·79 (3·46–16·6)	9·37 (3·34–15·7)
Neonatal disorders	91·3 (89–93·1)	82·9 (79·8–85·8)	71·8 (66·3–76·7)	91·3 (89–93·1)
Nutritional deficiencies	75·3 (63·5–82·1)	78·7 (74·2–83·4)	78·7 (74·1–83·6)	76·5 (61·9–80)
Protein-energy malnutrition	100 (100–100)	100 (100–100)	100 (100–100)	100 (100–100)
Vitamin A deficiency	--	100 (100–100)	100 (100–100)	--
Dietary iron deficiency	--	100 (100–100)	100 (100–100)	--
Non-communicable diseases	57·8 (54·9–60·8)	39·1 (35·8–42·6)	27 (24·5–29·6)	57·4 (54·9–59·9)
Neoplasms	49·9 (46·2–54)	48 (44·7–51·8)	33·6 (29·7–38)	48·8 (45·5–52·6)
Lip and oral cavity cancer	71·8 (61·5–79·6)	73·7 (64·1–81)	73 (63–80·7)	73·8 (64·3–81·1)
Nasopharynx cancer	69·1 (57·4–78)	68·2 (56·8–76·6)	65·9 (53·4–75·3)	68·4 (57–76·7)
Other pharynx cancer	81·9 (75·2–86·9)	82·6 (75·9–87·5)	81·5 (74·1–87)	82·7 (76–87·6)
Esophageal cancer	77·5 (68·6–85·7)	78·6 (70·2–86·2)	77·8 (68·7–85·7)	78·6 (70·2–86·3)

Stomach cancer	25·1 (17·7–41·4)	24·7 (17·5–40·9)	24·9 (17·8–41·4)	24·7 (17·5–40·9)
Colon and rectum cancer	65·2 (56·5–74·3)	65·3 (57·2–73·5)	65·3 (57·1–73·6)	65·3 (57·1–73·6)
Liver cancer	78·8 (73·4–83·8)	78·2 (72·9–83)	78·3 (72·8–83·1)	78·2 (72·9–83·1)
Gallbladder and biliary tract cancer	24 (13·5–36·8)	24·1 (13·5–37)	22·7 (12·4–35·1)	24·2 (13·6–37·1)
Pancreatic cancer	44·1 (34·1–55·5)	42·6 (33·3–53·2)	43·6 (33·9–54·5)	42·6 (33·2–53·2)
Larynx cancer	83·8 (76·8–89·1)	82·8 (75·9–88·2)	79·9 (72·6–85·5)	83·1 (76·2–88·4)
Tracheal, bronchus, and lung cancer	85·6 (81·9–88·9)	83·7 (80·2–87·2)	84·8 (81·1–88·2)	83·7 (80·1–87·2)
Breast cancer	37·7 (28·1–48·4)	35·3 (26·8–44·4)	35·7 (27·1–45·3)	35·3 (26·7–44·4)
Cervical cancer	100 (100–100)	100 (100–100)	100 (100–100)	100 (100–100)
Uterine cancer	47 (32·2–62·2)	47·8 (33·3–63·3)	48·1 (33·4–63·6)	47·8 (33·2–63·2)
Ovarian cancer	24·7 (12·7–38·5)	19·8 (9·74–32·2)	18·3 (8·96–30)	19·9 (9·74–32·4)
Prostate cancer	6·03 (2·65–9·62)	6·58 (2·96–10·2)	6·95 (3·16–10·9)	6·52 (2·9–10·1)
Kidney cancer	40·7 (30·4–50·9)	40 (30–49·7)	39·4 (29·5–49·1)	40 (30–49·7)
Bladder cancer	44·7 (29·8–59)	46·1 (33·1–59·1)	46·2 (33·8–58·4)	46·1 (33–59·2)
Thyroid cancer	15·7 (8·01–24·9)	16·3 (8·39–25·8)	16·3 (8·36–25·8)	16·3 (8·36–25·8)
Mesothelioma	97·5 (96·6–98·1)	94·8 (93·3–96)	97·2 (96–98)	94·8 (93·3–96)
Non-Hodgkin lymphoma	8·18 (3·54–14·3)	8·29 (3·66–14·3)	8·25 (3·56–14·4)	8·29 (3·66–14·3)
Multiple myeloma	9·09 (4·07–15·9)	9·41 (4·23–16·6)	9·35 (4·06–16·4)	9·42 (4·23–16·5)
Leukemia	37 (24·5–49·9)	32·4 (22·5–42·6)	31·3 (21·5–41·3)	32·5 (22·5–42·7)
Cardiovascular diseases	78 (74·4–81·5)	78·8 (75·8–81·7)	69·6 (64·7–73·9)	80·2 (77·3–83)
Rheumatic heart disease	22·7 (13·2–41·2)	23·6 (15·6–37·4)	23·4 (15·5–35·5)	23·6 (15·6–37·7)
Ischemic heart disease	94·3 (90·9–97)	95·7 (93·5–97·6)	94·7 (92·1–97)	95·7 (93·6–97·6)
Stroke	81·1 (73·6–89·1)	85 (80·3–90·1)	84·7 (80·3–89·1)	85·1 (80·1–90·4)
Hypertensive heart disease	100 (100–100)	100 (100–100)	100 (100–100)	100 (100–100)
Non-rheumatic valvular heart disease	25·6 (16·7–37·8)	26·7 (19·2–36·2)	20·8 (14·2–28·6)	27·4 (19·6–37·1)
Cardiomyopathy and myocarditis	39·1 (32·2–47·1)	44·6 (39–50·9)	40 (34·4–46)	45 (39·3–51·4)
Atrial fibrillation and flutter	53 (43–63·8)	61·9 (53·2–70·4)	65·6 (57·4–73·6)	57 (47·7–66·6)
Aortic aneurysm	53·1 (46–59·8)	60·9 (55·1–66·2)	--	60·9 (55·1–66·2)
Endocarditis	31·9 (22·5–43·9)	34·5 (25·7–44·5)	31·1 (21·9–42·8)	34·6 (25·7–44·6)
Other cardiovascular and circulatory diseases	31·4 (25·1–38·4)	32·9 (26·3–39·4)	32·6 (24–41·4)	33 (27–39·6)
Chronic respiratory diseases	65·3 (59·4–71)	55·8 (50·8–60·5)	48·1 (42·2–54·1)	65 (59·7–70·1)
Chronic obstructive pulmonary disease	73·3 (67·5–79·1)	71·4 (67·1–75·6)	64·2 (60·4–67·7)	75·1 (70–80·1)
Pneumoconiosis	100 (100–100)	100 (100–100)	100 (100–100)	100 (100–100)
Asthma	45·1 (33·8–57·3)	41·7 (33·3–51·6)	41·5 (33·2–51·4)	44·4 (34·9–54·7)

Digestive diseases	40·8 (34·3–46·4)	42·7 (36·2–48·5)	25·6 (17·8–34·7)	53·2 (45·3–59·6)
Cirrhosis and other chronic liver diseases	79·8 (67·2–89·9)	81·4 (69·3–90·8)	77·4 (66–87)	81·5 (69·3–91)
Upper digestive system diseases	23·1 (19·5–26·7)	9·06 (5·71–13)	0·71 (0·413–1·12)	25·5 (21·7–29)
Gallbladder and biliary diseases	46·7 (31–61·5)	50·6 (36·5–64·9)	51 (36·9–65·4)	47·6 (33–62·4)
Pancreatitis	48·6 (29·7–65·8)	53·6 (35–70·3)	52·9 (33·9–69·5)	53·7 (35–70·3)
Neurological disorders	28 (13·8–43)	12·1 (5·66–22·9)	6·73 (2·88–15)	23·5 (12·7–37·4)
Alzheimer's disease and other dementias	43·3 (29·1–59·3)	45·3 (31–60·7)	45·7 (31·5–61·1)	45·1 (30·7–60·5)
Parkinson's disease	-9·55 (-15·7–-4·27)	-10·9 (-17·5–-4·97)	-11·6 (-18·6–-5·2)	-10·7 (-17·2–-4·89)
Idiopathic epilepsy	31·1 (21·1–41·3)	27·9 (18·3–37·5)	26·1 (17–35·7)	30·4 (20·7–40)
Multiple sclerosis	16·2 (12–20·4)	16·1 (12·1–20·2)	15·3 (11·4–19·4)	16·7 (12·4–21)
Mental disorders	--	6·98 (3·35–11)	6·98 (3·35–11)	--
Depressive disorders	--	14·9 (6·64–23·2)	14·9 (6·64–23·2)	--
Anxiety disorders	--	5·42 (1·2–12·6)	5·42 (1·2–12·6)	--
Idiopathic developmental intellectual disability	--	36·8 (15·5–56)	36·8 (15·5–56)	--
Substance use disorders	100 (100–100)	100 (100–100)	100 (100–100)	100 (100–100)
Alcohol use disorders	100 (100–100)	100 (100–100)	100 (100–100)	100 (100–100)
Drug use disorders	100 (100–100)	100 (100–100)	100 (100–100)	100 (100–100)
Diabetes and kidney diseases	99·4 (99·2–99·5)	99·9 (99·8–99·9)	100 (100–100)	99·4 (99·2–99·5)
Diabetes mellitus	100 (100–100)	100 (100–100)	100 (100–100)	100 (100–100)
Chronic kidney disease	100 (100–100)	100 (100–100)	100 (100–100)	100 (100–100)
Sense organ diseases	--	9·26 (8·24–10·5)	9·26 (8·24–10·5)	--
Blindness and vision loss	--	7·61 (4·88–11)	7·61 (4·88–11)	--
Age-related and other hearing loss	--	11·3 (10·3–12·5)	11·3 (10·3–12·5)	--
Musculoskeletal disorders	2·73 (0·644–5·02)	22 (18·4–25·8)	22·3 (18·6–26·3)	3·02 (0·735–5·42)
Rheumatoid arthritis	12·6 (3·12–22·6)	13·7 (3·35–24·1)	13·6 (3·27–23·9)	14 (3·64–24·9)
Osteoarthritis	--	18·4 (9·85–27·6)	18·4 (9·85–27·6)	--
Low back pain	--	41·2 (36·1–46·2)	41·2 (36·1–46·2)	--
Gout	--	51·6 (35–69)	51·6 (35–69)	--
Other non-communicable diseases	0·193 (0·129–0·275)	0·119 (0·0751–0·179)	--	0·528 (0·332–0·758)
Sudden infant death syndrome	7·52 (4·2–12·5)	7·51 (4·19–12·5)	--	7·51 (4·19–12·5)
Injuries	29·6 (23·7–35·2)	24·2 (19·6–28·6)	25·2 (20·9–30)	22·9 (16·5–29·5)
Transport injuries	20·8 (13·3–28·6)	20·8 (14·2–27·1)	25·1 (20·4–30·3)	18·7 (10·5–26·6)
Road injuries	19·9 (11·9–28·2)	20 (13·1–26·6)	24·4 (19·6–29·6)	17·9 (9·53–26·4)
Other transport injuries	30·2 (24·7–35·4)	28·6 (23·4–33·6)	31·1 (26·5–35·7)	26·9 (21·2–32·3)

Unintentional injuries	34·5 (28·7–39·3)	23·4 (19·1–28·3)	24·4 (19·9–29·6)	20·5 (16·3–25·1)
Falls	51·4 (43·7–56·9)	33·9 (28–39·8)	32·3 (26·7–38·4)	39·7 (33·4–45·6)
Drowning	-13·9 (-27–0·62)	-14·1 (-26·7—0·449)	11·7 (6·72–18·4)	-15·2 (-28·1—1·05)
Fire, heat, and hot substances	11·7 (6·58–18·4)	13·5 (8·13–20·3)	14·3 (8·82–21·5)	12 (7·18–18·2)
Poisonings	14 (8·55–21·1)	13·3 (8·26–20)	12·9 (7·94–19·4)	13·9 (8·6–20·6)
Exposure to mechanical forces	5·41 (-7·36–18·6)	13·6 (10·8–16·5)	14·8 (12·5–17·5)	-2·58 (-15·6–11·9)
Animal contact	19·7 (14·8–26·1)	18·8 (13·8–25)	19·8 (14·6–26·1)	16·1 (11·5–21·8)
Foreign body	3·06 (1·92–4·59)	3·56 (2·31–5·32)	3·33 (2·16–4·94)	3·72 (2·32–5·58)
Environmental heat and cold exposure	9·28 (4·32–16·2)	10·4 (4·88–18)	10·3 (4·81–18)	10·6 (5·03–18)
Exposure to forces of nature	0 (0–0)	11·1 (5·18–19·2)	11·1 (5·18–19·2)	0 (0–0)
Other unintentional injuries	14·7 (8·93–22·1)	13·7 (8·47–20·4)	13·6 (8·41–20·3)	15 (9·41–22·1)
Self-harm and interpersonal violence	26·4 (13·8–39)	27·7 (16·4–39)	36·6 (28·6–44·9)	26·4 (13·9–39)
Self-harm	26·7 (12·7–40·9)	27·2 (13·3–41·3)	27·1 (14·2–40·2)	27·2 (13·2–41·3)
Interpersonal violence	25·6 (16·6–35)	30·8 (22·6–39·7)	40·7 (31·4–50·4)	22·7 (14·5–31·6)

Supplementary Results Table 2. All-age PAFs attributable to the joint distribution of all risk factors for all ages, both sexes combined for each GBD level 1, level 2, and level 3 cause and all causes in 2019 for Lewisham. Results are shown for deaths, YLLs, YLDs, and DALYs Data in parenthesis are 95% uncertainty intervals. PAFs=population attributable fractions. GBD=Global Burden of Disease. YLLs=years of life lost. YLDs=years lived with disability. DALYs=disability-adjusted life-years

	Deaths 2019	DALYs 2019	YLDs 2019	YLLs 2019
All causes	55·5 (52·5–58·6)	40 (36·9–43·1)	27·4 (25·2–29·8)	55·3 (52·7–57·9)
Communicable, maternal, neonatal, and nutritional diseases	49·9 (39·5–59·3)	58·7 (54·2–63·2)	52·8 (48·1–57·6)	62·3 (55·6–68·7)
HIV/AIDS and sexually transmitted infections	84·8 (77·5–89·7)	82·8 (73·4–88·8)	95·2 (93–96·6)	75·3 (63·8–83·6)
HIV/AIDS	91·1 (89·1–92·4)	89·4 (87–91·2)	93·7 (91·2–95·4)	87 (84·6–88·6)
Sexually transmitted infections excluding HIV	42·5 (21·3–69·6)	56 (30·6–81·7)	100 (100–100)	16·7 (6·2–36)
Respiratory infections and tuberculosis	48·2 (36·7–59·2)	43·9 (34·6–53)	3·28 (2·36–4·47)	52·3 (41·2–62·4)
Tuberculosis	59·2 (48–68·8)	60·5 (50·2–69·3)	54·9 (43·1–64·7)	61·7 (51·3–70·3)
Lower respiratory infections	48·1 (36·5–59·2)	52·1 (40·9–62·3)	36·2 (29·1–42·9)	52·2 (40·9–62·5)
Upper respiratory infections	0·0613 (0·0354–0·101)	0·0111 (0·00196–0·0264)	0·00977 (0·00052–0·0254)	0·129 (0·0738–0·221)
Otitis media	3·19 (1·53–6·14)	5 (3·22–7·06)	4·98 (3·2–7·05)	6·66 (3·65–11·1)
Enteric infections	5·1 (3·26–8·26)	10·7 (8·16–14·1)	10·9 (8·22–14·6)	9·86 (6·95–13·6)
Diarrheal diseases	5·16 (3·31–8·37)	10·8 (8·2–14·2)	11 (8·23–14·6)	10·1 (7·19–13·9)
Other infectious diseases	1·04 (0·731–1·37)	1·54 (1·14–1·97)	0·329 (0·187–0·54)	2·14 (1·59–2·75)
Meningitis	2·74 (1·95–3·67)	3·49 (2·55–4·68)	0·000783 (2·7e-05–0·00223)	5·02 (3·7–6·69)
Encephalitis	0·673 (0·415–1·02)	1·35 (0·677–2·08)	0·000296 (1·15e-05–0·000817)	1·82 (1·08–2·71)
Measles	2·85 (-0·768–14·5)	2·33 (-0·313–11·1)	0·471 (0·00975–1·25)	3·76 (-0·995–18·8)
Acute hepatitis	12·4 (6·64–22·8)	5·02 (2·96–8·27)	2·57 (1·53–4·2)	11·1 (5·8–20·8)
Maternal and neonatal disorders	90·1 (88·2–91·7)	82·3 (79·4–85·1)	70·1 (64·4–75·2)	91 (89·3–92·5)
Maternal disorders	9·63 (3·4–16)	9·72 (3·35–16·3)	9·9 (3·44–16·8)	9·47 (3·3–15·7)
Neonatal disorders	92·5 (90·9–93·8)	84·2 (81·2–86·9)	72·2 (66·6–77·4)	92·5 (90·9–93·8)
Nutritional deficiencies	85·9 (72–89)	80·1 (75·6–85·2)	80 (75·3–85·2)	84·1 (66·4–86·8)
Protein-energy malnutrition	100 (100–100)	100 (100–100)	100 (100–100)	100 (100–100)
Vitamin A deficiency	--	100 (100–100)	100 (100–100)	--
Dietary iron deficiency	--	100 (100–100)	100 (100–100)	--
Non-communicable diseases	57·2 (53·9–60·4)	39·7 (36·3–43·2)	26·2 (23·8–28·9)	57·1 (54·5–59·8)
Neoplasms	49·6 (45·9–53·9)	48 (44·5–52)	33·8 (29·9–38·2)	48·7 (45·3–52·8)
Lip and oral cavity cancer	69·1 (58·7–78·1)	71·3 (61·2–79·7)	70·4 (59·9–78·9)	71·4 (61·3–79·7)
Nasopharynx cancer	65·9 (53·8–75·9)	65·2 (53·4–74·8)	62·6 (49·7–73·2)	65·5 (53·7–74·9)
Other pharynx cancer	79·8 (72·3–85·8)	80·6 (73–86·2)	79·5 (71·8–85·6)	80·7 (73·1–86·3)
Esophageal cancer	76·1 (67·3–83·9)	77·2 (68·5–85)	76·4 (67·6–83·9)	77·2 (68·6–85)
Stomach cancer	24·7 (17·2–41·7)	24·5 (17·2–41)	24·7 (17·3–41·3)	24·5 (17·2–41)

Colon and rectum cancer	64·3 (55·2–73)	64·3 (55·8–72·2)	64·3 (55·8–72·6)	64·3 (55·8–72·2)
Liver cancer	76·6 (70·7–82)	76·1 (70·5–81·2)	76·4 (70·8–81·5)	76·1 (70·4–81·2)
Gallbladder and biliary tract cancer	20·4 (10·7–32·5)	20·7 (11·1–33)	19·2 (10–30·9)	20·7 (11·1–33·1)
Pancreatic cancer	42·6 (33–54)	41·3 (32·7–52·1)	42·2 (32·9–53·5)	41·3 (32·7–52·1)
Larynx cancer	83·4 (75·7–89)	82·6 (75·1–88·1)	80 (72·5–85·9)	82·9 (75·4–88·3)
Tracheal, bronchus, and lung cancer	86·2 (82·8–89·5)	84·8 (81·4–88·2)	85·6 (82·1–88·8)	84·8 (81·4–88·2)
Breast cancer	36·1 (26·5–47·5)	34·3 (25·8–44·1)	34·6 (26–44·3)	34·2 (25·8–44·1)
Cervical cancer	100 (100–100)	100 (100–100)	100 (100–100)	100 (100–100)
Uterine cancer	42·3 (27·7–57)	43·3 (28·7–58·2)	43·6 (29·2–58·5)	43·3 (28·6–58·1)
Ovarian cancer	22·9 (11·6–37·1)	18·8 (9·01–31·5)	17·7 (8·42–29·9)	18·9 (9·02–31·5)
Prostate cancer	5·83 (2·51–9·4)	6·31 (2·78–10·1)	6·66 (2·98–10·5)	6·26 (2·72–9·98)
Kidney cancer	37·8 (28–47·8)	37·2 (28·1–46·9)	36·7 (27·6–46·4)	37·2 (28·2–46·9)
Bladder cancer	44 (29·2–58·4)	45·6 (32·1–58·6)	45·8 (32·6–58·3)	45·6 (32–58·6)
Thyroid cancer	14·2 (6·5–24·1)	14·7 (6·7–25·4)	14·7 (6·65–25·4)	14·7 (6·68–25·4)
Mesothelioma	98·4 (97·8–98·8)	96·7 (95·6–97·6)	98·1 (97·3–98·7)	96·7 (95·6–97·5)
Non-Hodgkin lymphoma	6·73 (2·76–12·1)	6·86 (2·87–12·4)	6·83 (2·85–12·3)	6·87 (2·87–12·4)
Multiple myeloma	7·61 (3·26–13·7)	7·92 (3·36–14·2)	7·87 (3·27–14·1)	7·92 (3·34–14·2)
Leukemia	35·6 (22·3–48·9)	31·6 (21·1–42·8)	30·3 (20–41·2)	31·7 (21·2–42·9)
Cardiovascular diseases	77·9 (73·9–81·4)	79·1 (75·9–82)	68·7 (63·7–73)	80·5 (77·4–83·3)
Rheumatic heart disease	21·7 (12–42)	22·5 (13·9–38·2)	22·4 (13·9–36·7)	22·5 (13·9–38·1)
Ischemic heart disease	93·8 (90·4–96·7)	95·2 (92·7–97·2)	94·2 (91·3–96·7)	95·2 (92·7–97·3)
Stroke	80·2 (72–88·9)	83·8 (78·3–89·5)	83 (78·2–87·9)	84 (78·1–90)
Hypertensive heart disease	100 (100–100)	100 (100–100)	100 (100–100)	100 (100–100)
Non-rheumatic valvular heart disease	24·9 (15·7–36·5)	25·7 (18·1–34·9)	16·9 (11–23·8)	26·8 (18·8–36·3)
Cardiomyopathy and myocarditis	36·9 (29·8–45·1)	43·4 (37·2–50·9)	38·7 (32·4–45·2)	43·8 (37·3–51·7)
Atrial fibrillation and flutter	48·9 (38·8–59·3)	57·3 (48·4–65·9)	61·4 (52·8–69·6)	52·7 (43·3–62·1)
Aortic aneurysm	51·2 (45–58)	58·9 (53·1–64·6)	--	58·9 (53·1–64·6)
Endocarditis	30·3 (20·5–42·2)	32·7 (23·9–42·5)	29·9 (20·3–41·5)	32·8 (23·9–42·6)
Other cardiovascular and circulatory diseases	31 (25–37·5)	32·7 (26·6–38·9)	31·9 (24·3–40·5)	33 (27–39)
Chronic respiratory diseases	66·2 (60·5–71·7)	56·8 (52·1–61·4)	46·8 (41·3–52·7)	66·2 (61·1–71·1)
Chronic obstructive pulmonary disease	73·3 (67·6–78·8)	72 (67·8–76)	65 (61·1–68·6)	75·1 (69·9–80)
Pneumoconiosis	100 (100–100)	100 (100–100)	100 (100–100)	100 (100–100)
Asthma	41·3 (30·4–53·3)	36·9 (28·8–46·3)	36·6 (28·6–46)	40·7 (31·8–50·6)
Digestive diseases	35·4 (29·3–41)	39 (32·3–45)	26·2 (17·8–35·9)	48 (40·4–54·8)

Cirrhosis and other chronic liver diseases	76·4 (63·8–88·3)	78·4 (66·1–89·4)	74·2 (62·5–84·7)	78·5 (66·3–89·5)
Upper digestive system diseases	22·3 (18·8–25·9)	9·52 (6·3–13·5)	0·692 (0·415–1·08)	24·7 (21·2–28·1)
Gallbladder and biliary diseases	42 (27·4–56·5)	45·9 (32–60)	46·2 (32–60·4)	43 (28·9–57·3)
Pancreatitis	41·8 (20·8–61·9)	47·1 (24·9–66·3)	45·6 (23·8–65·7)	47·3 (25·4–66·4)
Neurological disorders	25·6 (11·5–40·5)	12·4 (5·7–22·8)	6·99 (3·19–14·9)	21·8 (10·5–36·2)
Alzheimer's disease and other dementias	41·1 (26·5–57·4)	42·9 (28·5–58·6)	43·1 (28·8–58·6)	42·8 (28·3–58·6)
Parkinson's disease	-9·18 (-15·1–-4·12)	-10·3 (-16·6–-4·7)	-10·9 (-17·4–-5·14)	-10·2 (-16·4–-4·56)
Idiopathic epilepsy	27·3 (16·8–37·2)	24·3 (14·6–33·5)	22 (12·8–31·2)	27·3 (17–37·1)
Multiple sclerosis	15·4 (11·4–19·7)	15·4 (11·6–19·6)	14·8 (11·1–18·7)	15·9 (11·8–20·4)
Mental disorders	--	7·15 (3·59–11·2)	7·15 (3·59–11·2)	--
Depressive disorders	--	14·6 (6·58–22·9)	14·6 (6·58–22·9)	--
Anxiety disorders	--	5·36 (1·33–12·2)	5·36 (1·33–12·2)	--
Idiopathic developmental intellectual disability	--	40·2 (18·3–57·3)	40·2 (18·3–57·3)	--
Substance use disorders	100 (100–100)	100 (100–100)	100 (100–100)	100 (100–100)
Alcohol use disorders	100 (100–100)	100 (100–100)	100 (100–100)	100 (100–100)
Drug use disorders	100 (100–100)	100 (100–100)	100 (100–100)	100 (100–100)
Diabetes and kidney diseases	99·6 (99·6–99·7)	99·9 (99·9–100)	100 (100–100)	99·6 (99·6–99·7)
Diabetes mellitus	100 (100–100)	100 (100–100)	100 (100–100)	100 (100–100)
Chronic kidney disease	100 (100–100)	100 (100–100)	100 (100–100)	100 (100–100)
Sense organ diseases	--	8·78 (7·68–10)	8·78 (7·68–10)	--
Blindness and vision loss	--	7·87 (4·99–11·6)	7·87 (4·99–11·6)	--
Age-related and other hearing loss	--	10·5 (9·42–11·6)	10·5 (9·42–11·6)	--
Musculoskeletal disorders	2·55 (0·622–4·79)	21·1 (17·4–25)	21·4 (17·7–25·5)	2·9 (0·708–5·19)
Rheumatoid arthritis	12·1 (2·8–22·2)	13·4 (3·2–23·7)	13·4 (3·2–23·7)	13·5 (3·38–24·1)
Osteoarthritis	--	16 (8·29–24·8)	16 (8·29–24·8)	--
Low back pain	--	39·6 (34·5–44·8)	39·6 (34·5–44·8)	--
Gout	--	47·2 (31·8–64·6)	47·2 (31·8–64·6)	--
Other non-communicable diseases	0·138 (0·0871–0·203)	0·105 (0·062–0·166)	--	0·469 (0·277–0·684)
Sudden infant death syndrome	9·77 (5·31–15·4)	9·76 (5·31–15·4)	--	9·76 (5·31–15·4)
Injuries	28·4 (21·5–34·8)	23·9 (19·1–29·1)	25·6 (21·6–29·9)	21·9 (14·4–29·6)
Transport injuries	20·7 (12·4–29)	20·6 (13·6–27·5)	25·4 (21–30·1)	18·5 (9·48–27·1)
Road injuries	19·9 (11·1–28·6)	19·8 (12·4–27·1)	24·4 (20–29·2)	17·8 (8·39–26·7)
Other transport injuries	30·4 (25·4–35·6)	28·9 (24–33·9)	32·3 (27·8–36·7)	26·6 (21·2–32·3)
Unintentional injuries	35·3 (29·4–39·7)	24·2 (20–28·8)	25 (20·7–29·7)	21·3 (16·8–25·9)

Falls	52·5 (44·8–57·8)	34·8 (29·1–40·2)	33·2 (27·7–38·7)	41·6 (35·4–47·1)
Drowning	-14·3 (-27·7–0·0484)	-14·3 (-26·9–-0·764)	10·9 (6·51–17·2)	-15·4 (-28·5–-1·47)
Fire, heat, and hot substances	11·6 (6·86–18·2)	13·2 (8·38–19·7)	13·7 (8·69–20·4)	12·3 (7·67–18·5)
Poisonings	14·6 (9·31–21·7)	13·1 (8·49–19·3)	12·2 (7·83–18·2)	14·7 (9·61–21·5)
Exposure to mechanical forces	5·24 (-7·66–18·7)	14·8 (12·1–17·8)	16 (13·7–18·8)	-1·79 (-15·1–12·6)
Animal contact	20·6 (15·8–26·9)	19·2 (14·6–25)	19·9 (15·1–25·8)	16·6 (12·3–22)
Foreign body	3·65 (2·4–5·56)	4·15 (2·81–6·16)	3·56 (2·4–5·22)	4·67 (3·07–7·03)
Environmental heat and cold exposure	8·22 (3·8–14·7)	9·22 (4·22–16·4)	9·26 (4·23–16·4)	8·96 (4·19–15·6)
Exposure to forces of nature	0 (0–0)	10·1 (4·7–17·7)	10·1 (4·7–17·7)	0 (0–0)
Other unintentional injuries	15 (9·54–22·3)	12·9 (8·15–19·6)	12·8 (8·05–19·5)	15·7 (10·3–22·8)
Self-harm and interpersonal violence	23·5 (9·84–36·8)	25·2 (12·8–37)	34·7 (26·9–42·9)	23·8 (10·3–36·8)
Self-harm	23·6 (8·99–38)	24·2 (9·79–38·4)	23·9 (11·5–37·2)	24·2 (9·73–38·5)
Interpersonal violence	24 (14·9–32·9)	30·5 (22·4–39)	39·6 (30·4–49·2)	21·4 (12·5–29·9)

Supplementary Results Table 2. All-age PAFs attributable to the joint distribution of all risk factors for all ages, both sexes combined for each GBD level 1, level 2, and level 3 cause and all causes in 2019 for Merton. Results are shown for deaths, YLLs, YLDs, and DALYs Data in parenthesis are 95% uncertainty intervals. PAFs=population attributable fractions. GBD=Global Burden of Disease. YLLs=years of life lost. YLDs=years lived with disability. DALYs=disability-adjusted life-years

	Deaths 2019	DALYs 2019	YLDs 2019	YLLs 2019
All causes	53·8 (50·4–57)	38·5 (35·4–41·6)	27·2 (25–29·6)	53·1 (50·3–55·7)
Communicable, maternal, neonatal, and nutritional diseases	46·5 (35·8–56·4)	53·2 (48·8–57·7)	47·9 (43·4–52·7)	56·8 (49·6–63·6)
HIV/AIDS and sexually transmitted infections	79·1 (66·5–87·7)	77·2 (62·4–87·1)	96·5 (94·7–97·7)	65·3 (48–79·1)
HIV/AIDS	91 (89–92·3)	89·3 (86·8–91·1)	94·2 (91·8–95·7)	86·6 (84·2–88·2)
Sexually transmitted infections excluding HIV	40·1 (19·4–67·6)	54·2 (28·4–80·9)	100 (100–100)	14 (5·16–31·9)
Respiratory infections and tuberculosis	46·8 (34·7–58)	41·3 (31·9–50·7)	3·41 (2·45–4·59)	50·7 (39·8–60·9)
Tuberculosis	56·5 (45·1–66·7)	58·4 (48·3–67·5)	55·4 (44·2–64·8)	59 (49·1–67·9)
Lower respiratory infections	46·6 (34·4–57·9)	50·4 (39·2–60·8)	34·9 (27·7–41·7)	50·5 (39·4–60·9)
Upper respiratory infections	0·0777 (0·0421–0·141)	0·00867 (0·00109–0·022)	0·00784 (0·00025–0·0212)	0·147 (0·0796–0·262)
Otitis media	3·22 (1·08–7·66)	5·02 (3·21–7·11)	4·99 (3·19–7·09)	6·44 (2·66–12·4)
Enteric infections	4·74 (3·04–7·79)	9·98 (7·62–13·1)	10·1 (7·59–13·4)	9·5 (6·54–13·1)
Diarrheal diseases	4·79 (3·08–7·84)	10 (7·67–13·1)	10·2 (7·6–13·4)	9·69 (6·72–13·3)
Other infectious diseases	0·709 (0·451–0·938)	1·14 (0·838–1·46)	0·346 (0·202–0·582)	1·49 (1·1–1·92)
Meningitis	1·72 (1·2–2·33)	2·44 (1·73–3·31)	0·000653 (1·5e-05–0·00192)	3·17 (2·28–4·29)
Encephalitis	0·401 (0·242–0·595)	0·846 (0·46–1·27)	0·000229 (4·83e-06–0·000636)	1·17 (0·696–1·72)
Measles	3·27 (-1·04–17·8)	3·74 (-1·1–20·5)	0·392 (0·0154–1·11)	3·98 (-1·24–21·8)
Acute hepatitis	16·2 (9·72–26·2)	5·04 (3·11–7·6)	2·62 (1·58–4·2)	15·1 (8·77–23·9)
Maternal and neonatal disorders	85·4 (82·7–87·8)	76·4 (72·6–79·8)	63·4 (58–68·7)	86·7 (84·2–89)
Maternal disorders	9·5 (3·37–16)	9·53 (3·4–16·1)	9·64 (3·4–16·4)	9·38 (3·36–15·7)
Neonatal disorders	88·9 (86·5–90·9)	78·7 (75–82·1)	65·8 (59·9–71·3)	88·9 (86·6–90·9)
Nutritional deficiencies	62·3 (55·1–89·1)	79·2 (74·8–84)	79·4 (74·9–84·2)	61·7 (57·2–78·7)
Protein-energy malnutrition	100 (100–100)	100 (100–100)	100 (100–100)	100 (100–100)
Vitamin A deficiency	--	100 (100–100)	100 (100–100)	--
Dietary iron deficiency	--	100 (100–100)	100 (100–100)	--
Non-communicable diseases	55·4 (51·9–58·8)	38·5 (35·2–41·9)	26·2 (23·8–28·9)	55·1 (52·1–57·9)
Neoplasms	46·6 (42·9–50·8)	45·2 (41·8–49·1)	32·2 (28·3–36·5)	46 (42·5–49·9)
Lip and oral cavity cancer	68·4 (57·8–77)	71·1 (61–78·9)	70·4 (60·1–78·4)	71·2 (61·1–78·9)
Nasopharynx cancer	63·3 (50·6–73·7)	63·2 (50·7–72·9)	61·3 (47·9–72·1)	63·4 (51·1–73)
Other pharynx cancer	79·7 (72·4–85·6)	80·7 (73·6–86·4)	79·6 (71·9–85·7)	80·8 (73·6–86·5)
Esophageal cancer	75·7 (66·5–83·7)	77 (68–84·8)	76·1 (66·6–84·1)	77 (68–84·8)
Stomach cancer	24·4 (16·8–41·2)	24·3 (17·1–40·7)	24·5 (17·3–41)	24·3 (17·1–40·6)

Colon and rectum cancer	63·7 (54·7–72·4)	63·9 (55·6–72·2)	63·9 (55·4–72·3)	63·9 (55·6–72·2)
Liver cancer	77·2 (71·3–82·6)	77·2 (71·8–82)	77·5 (72·3–82·5)	77·2 (71·8–82)
Gallbladder and biliary tract cancer	22 (11·7–34·9)	22·4 (12·1–34·9)	20·8 (10·9–33·1)	22·5 (12·2–35·1)
Pancreatic cancer	42 (32·7–53)	41 (31·9–51·4)	41·7 (32·3–52·5)	41 (31·9–51·4)
Larynx cancer	82·6 (74·6–88·6)	81·8 (74·5–87·4)	79·2 (71·7–85·4)	82·1 (74·8–87·7)
Tracheal, bronchus, and lung cancer	85·3 (81·6–88·7)	83·8 (80·3–87·4)	84·7 (81·1–88·1)	83·8 (80·3–87·4)
Breast cancer	36·2 (26·7–47)	34·4 (26·1–44·3)	34·8 (26·3–44·5)	34·4 (26·1–44·2)
Cervical cancer	100 (100–100)	100 (100–100)	100 (100–100)	100 (100–100)
Uterine cancer	44·2 (29·4–60·2)	45·2 (30·2–60·7)	45·6 (30·7–61)	45·2 (30·3–60·6)
Ovarian cancer	25·5 (12·7–39·3)	20·9 (10–33·5)	19·7 (9·75–31·9)	21 (10–33·6)
Prostate cancer	5·63 (2·43–9·11)	6·12 (2·68–9·82)	6·48 (2·93–10·1)	6·05 (2·65–9·79)
Kidney cancer	38·4 (28·4–48·8)	38 (28·1–48·1)	37·6 (27·9–47·2)	38 (28·2–48·1)
Bladder cancer	41·7 (27·3–56·4)	43·7 (30·2–57)	44·1 (30·8–56·8)	43·6 (30·2–57)
Thyroid cancer	14·9 (6·92–25·2)	15·6 (7·23–26·2)	15·7 (7·14–26·4)	15·6 (7·17–26·3)
Mesothelioma	98·3 (97·8–98·8)	96·5 (95·4–97·4)	98·1 (97·3–98·7)	96·4 (95·4–97·3)
Non-Hodgkin lymphoma	7·11 (2·98–12·6)	7·29 (3·05–12·9)	7·25 (3–13)	7·3 (3·05–12·9)
Multiple myeloma	8·13 (3·55–14·5)	8·48 (3·68–15·2)	8·42 (3·62–15·3)	8·49 (3·66–15·2)
Leukemia	35·9 (22·2–49·5)	32·4 (21·2–43·3)	31·2 (20·7–42·2)	32·4 (21·3–43·4)
Cardiovascular diseases	76·9 (72·8–80·5)	78·2 (74·9–81·3)	70·1 (65·1–75)	79·4 (76·3–82·4)
Rheumatic heart disease	21·8 (11·6–40·9)	22·7 (14–36·8)	22·7 (14·6–35·8)	22·7 (13·9–36·8)
Ischemic heart disease	93·4 (89·4–96·6)	94·8 (91·9–97·1)	93·9 (91–96·6)	94·8 (91·9–97·2)
Stroke	79 (70·3–88·5)	82·6 (76·7–88·8)	82·4 (77·5–87·6)	82·6 (76·4–89·1)
Hypertensive heart disease	100 (100–100)	100 (100–100)	100 (100–100)	100 (100–100)
Non-rheumatic valvular heart disease	25·1 (15·6–38·6)	26·2 (18–36·4)	19·9 (13·1–28)	26·8 (18·3–37·2)
Cardiomyopathy and myocarditis	36·1 (28·5–45·9)	43·8 (38·1–50)	38·6 (32·4–45·9)	44·2 (38·3–50·4)
Atrial fibrillation and flutter	49·9 (39·3–61·3)	58·5 (49·2–67·5)	62·6 (53·8–70·8)	53·3 (43·7–63·8)
Aortic aneurysm	49·4 (42·6–56·6)	56·9 (51·1–62·8)	--	56·9 (51·1–62·8)
Endocarditis	29·9 (19·9–42·7)	32·4 (23·7–42·9)	29·6 (19·8–42·2)	32·5 (23·6–42·9)
Other cardiovascular and circulatory diseases	30·1 (23·6–36·9)	32·4 (26·1–38·6)	32·5 (25·3–40·2)	32·4 (26·1–38·3)
Chronic respiratory diseases	62·8 (56·5–68·9)	55·2 (50·4–60)	48·3 (42·6–54·2)	62·9 (57·3–68·5)
Chronic obstructive pulmonary disease	71·4 (65·4–77·1)	69·9 (65·6–74)	63·8 (60–67·5)	73·3 (67·9–78·2)
Pneumoconiosis	100 (100–100)	100 (100–100)	100 (100–100)	100 (100–100)
Asthma	41·9 (29·9–54·9)	39·4 (31·1–49·1)	39·1 (30·9–48·8)	42·5 (32·3–53·4)
Digestive diseases	31·2 (25·9–36·5)	34·9 (28·8–40·5)	17·9 (11·9–25·3)	44·9 (38·2–51·3)

Cirrhosis and other chronic liver diseases	76·1 (63·8–88)	78·6 (67·1–89·1)	74 (62·8–84·6)	78·8 (67·3–89·3)
Upper digestive system diseases	21·2 (17·6–25)	7·32 (4·52–10·7)	0·605 (0·349–0·96)	24 (20·3–27·4)
Gallbladder and biliary diseases	44·1 (29·5–59·6)	47·1 (33·2–61·5)	47·7 (33·8–61·9)	45·1 (31·2–60·2)
Pancreatitis	41·6 (22·2–60·3)	47·6 (28–64·6)	47·3 (27·5–64·6)	47·7 (28–64·6)
Neurological disorders	27 (12·9–41·6)	14 (6·41–25·4)	8·05 (3·67–16·5)	23·2 (11·2–37·5)
Alzheimer's disease and other dementias	40·4 (25·9–55·9)	42·3 (27·9–57·3)	42·6 (28·1–57·6)	42·1 (27·8–57·2)
Parkinson's disease	-8·75 (-14·5–-3·84)	-9·87 (-15·9–-4·45)	-10·5 (-17·1–-4·83)	-9·7 (-15·6–-4·38)
Idiopathic epilepsy	27·6 (17·6–37·3)	24·9 (15·9–33·9)	23 (14·4–32·2)	27·7 (18·1–37·4)
Multiple sclerosis	15 (11·1–18·8)	15·2 (11·3–18·9)	14·6 (11–18·4)	15·7 (11·7–19·5)
Mental disorders	--	6·79 (3·35–10·6)	6·79 (3·35–10·6)	--
Depressive disorders	--	14·3 (6·41–22·5)	14·3 (6·41–22·5)	--
Anxiety disorders	--	5·08 (1·25–11·8)	5·08 (1·25–11·8)	--
Idiopathic developmental intellectual disability	--	37·4 (15·7–56·2)	37·4 (15·7–56·2)	--
Substance use disorders	100 (100–100)	100 (100–100)	100 (100–100)	100 (100–100)
Alcohol use disorders	100 (100–100)	100 (100–100)	100 (100–100)	100 (100–100)
Drug use disorders	100 (100–100)	100 (100–100)	100 (100–100)	100 (100–100)
Diabetes and kidney diseases	99·8 (99·8–99·9)	100 (100–100)	100 (100–100)	99·8 (99·8–99·9)
Diabetes mellitus	100 (100–100)	100 (100–100)	100 (100–100)	100 (100–100)
Chronic kidney disease	100 (100–100)	100 (100–100)	100 (100–100)	100 (100–100)
Sense organ diseases	--	8·44 (7·4–9·63)	8·44 (7·4–9·63)	--
Blindness and vision loss	--	8·33 (5·33–12·1)	8·33 (5·33–12·1)	--
Age-related and other hearing loss	--	9·78 (8·85–10·9)	9·78 (8·85–10·9)	--
Musculoskeletal disorders	2·64 (0·588–5·01)	21·3 (17·6–25·1)	21·7 (17·8–25·6)	3·17 (0·754–5·71)
Rheumatoid arthritis	11·6 (2·62–21·2)	13·2 (3·3–23·2)	13·2 (3·36–23·1)	13·1 (3·13–23·3)
Osteoarthritis	--	17 (8·98–25·8)	17 (8·98–25·8)	--
Low back pain	--	39·6 (34·5–44·8)	39·6 (34·5–44·8)	--
Gout	--	50 (33·9–67·3)	50 (33·9–67·3)	--
Other non-communicable diseases	0·0921 (0·0569–0·136)	0·0732 (0·0431–0·116)	--	0·317 (0·187–0·463)
Sudden infant death syndrome	6·4 (3·51–10·2)	6·39 (3·5–10·2)	--	6·39 (3·5–10·2)
Injuries	32·4 (26·7–37·7)	26 (21·3–30·8)	27·6 (23·3–32·1)	23·7 (16·7–30·7)
Transport injuries	21·8 (13·8–29·5)	21·6 (15·1–28·3)	25·9 (21·4–30·3)	19·4 (10·9–27·7)
Road injuries	20·7 (12·4–29)	20·7 (13·7–27·6)	24·8 (20·2–29·4)	18·5 (9·43–27·3)
Other transport injuries	31·6 (25·9–36·9)	30·2 (25–35·2)	33·5 (28·9–37·7)	28·1 (22·2–33·8)
Unintentional injuries	39·8 (33·1–44·7)	26·8 (22·1–31·7)	27·3 (22·8–32·4)	24·7 (20–29·3)

Falls	54·8 (46·6–60·6)	37·6 (31·5–43·1)	35·9 (30·2–41·4)	44·4 (37·6–50·2)
Drowning	-15·4 (-29—0·766)	-15·2 (-27·7—1·68)	11 (6·33–17·3)	-16·8 (-29·7—2·72)
Fire, heat, and hot substances	10·1 (5·71–16·4)	12·7 (7·85–19·1)	13·7 (8·57–20·4)	10·5 (6·4–16·2)
Poisonings	14·4 (8·94–21·3)	13·2 (8·4–19·5)	12·3 (7·73–18·5)	14·6 (9·35–21·1)
Exposure to mechanical forces	10·4 (-2·34–23·3)	15·6 (12·7–18·7)	16·8 (14·3–19·6)	-0·912 (-14·3–13·7)
Animal contact	21·4 (16·9–27·4)	19·1 (14·4–24·7)	20·5 (15·6–26·3)	15·6 (11·7–20·9)
Foreign body	2·85 (1·77–4·16)	3·71 (2·37–5·41)	3·39 (2·22–5·02)	3·99 (2·53–5·87)
Environmental heat and cold exposure	8·97 (3·95–15·8)	9·59 (4·26–16·8)	9·58 (4·25–16·9)	9·69 (4·31–16·8)
Exposure to forces of nature	0 (0–0)	10·4 (4·71–17·9)	10·4 (4·71–17·9)	0 (0–0)
Other unintentional injuries	13·5 (8·34–20·3)	12·9 (8–19·4)	12·9 (7·92–19·3)	14·8 (9·48–21·6)
Self-harm and interpersonal violence	24·4 (11–37·9)	26·3 (14·2–38·2)	34·6 (27–42·6)	24·9 (11·4–38·2)
Self-harm	24·3 (10–38·7)	25·1 (11–39·2)	24·8 (12–37·2)	25·1 (10·9–39·5)
Interpersonal violence	27·9 (19·7–37)	34·4 (26·2–43·1)	39·7 (31·3–49·3)	24·2 (16·3–32·9)

Supplementary Results Table 2. All-age PAFs attributable to the joint distribution of all risk factors for all ages, both sexes combined for each GBD level 1, level 2, and level 3 cause and all causes in 2019 for Newham. Results are shown for deaths, YLLs, YLDs, and DALYs Data in parenthesis are 95% uncertainty intervals. PAFs=population attributable fractions. GBD=Global Burden of Disease. YLLs=years of life lost. YLDs=years lived with disability. DALYs=disability-adjusted life-years

	Deaths 2019	DALYs 2019	YLDs 2019	YLLs 2019
All causes	56·2 (53·2–59·1)	38·2 (35·1–41·2)	27·1 (24·7–29·6)	54·2 (51·6–56·6)
Communicable, maternal, neonatal, and nutritional diseases	50·6 (41·7–58·9)	57·7 (53·5–61·3)	51·5 (46·7–55·9)	62·3 (56·7–67·1)
HIV/AIDS and sexually transmitted infections	78·2 (67·6–85·5)	76·7 (64·8–84·9)	94·8 (92·2–96·4)	66·5 (52·5–77·2)
HIV/AIDS	87·9 (85·4–89·5)	86 (83·2–88·3)	92·9 (90–94·9)	82·2 (79·4–84·1)
Sexually transmitted infections excluding HIV	27·1 (11·6–51·8)	47·7 (23·6–75·2)	100 (100–100)	10·4 (3·67–23·9)
Respiratory infections and tuberculosis	49 (37·6–59·6)	41·6 (33·2–50·4)	3·4 (2·36–4·8)	54 (43·5–63·5)
Tuberculosis	58 (46·8–68·3)	58·4 (47·2–67·8)	53·3 (41·1–64·4)	59·6 (48·5–68·8)
Lower respiratory infections	48·8 (37–59·6)	53·7 (42·8–63·5)	35·3 (29–41·9)	53·9 (42·8–63·8)
Upper respiratory infections	0·0868 (0·05–0·136)	0·0109 (0·00154–0·0272)	0·00975 (0·000509–0·026)	0·141 (0·0795–0·231)
Otitis media	5·83 (3·07–9·67)	5·2 (3·31–7·28)	5·14 (3·28–7·22)	9·81 (5·72–14·8)
Enteric infections	6·57 (4·34–10·4)	12·5 (9·67–16·4)	11·4 (8·57–15·4)	16 (11·5–21·3)
Diarrheal diseases	6·64 (4·42–10·4)	12·6 (9·74–16·5)	11·5 (8·58–15·5)	16·4 (11·9–21·7)
Other infectious diseases	1·33 (0·953–1·87)	1·7 (1·25–2·27)	0·329 (0·19–0·555)	2·19 (1·58–2·99)
Meningitis	2·74 (1·93–3·72)	3·43 (2·42–4·61)	0·000912 (3·66e-05–0·00257)	4·21 (3·01–5·73)
Encephalitis	0·854 (0·503–1·29)	1·45 (0·781–2·19)	0·000327 (1·56e-05–0·000916)	1·82 (1·06–2·7)
Measles	4·59 (-1·12–21·9)	5·15 (-1·18–24)	0·507 (0·012–1·37)	5·64 (-1·36–26·5)
Acute hepatitis	11·3 (5·66–21·3)	4·72 (2·5–8·14)	2·21 (1·3–3·57)	9·24 (4·42–17·4)
Maternal and neonatal disorders	86·8 (84·2–88·9)	79·8 (76·8–82·7)	68·8 (63·4–73·8)	87·5 (84·9–89·6)
Maternal disorders	9·63 (3·45–16·3)	9·75 (3·55–16·6)	9·91 (3·65–17)	9·5 (3·45–16)
Neonatal disorders	88·8 (86·4–90·8)	81·5 (78·5–84·4)	70·9 (65·2–76·2)	88·8 (86·4–90·8)
Nutritional deficiencies	58·4 (52·4–82)	78·5 (73·6–83·6)	78·7 (73·8–83·9)	60·2 (56·5–71·9)
Protein-energy malnutrition	100 (100–100)	100 (100–100)	100 (100–100)	100 (100–100)
Vitamin A deficiency	--	100 (100–100)	100 (100–100)	--
Dietary iron deficiency	--	100 (100–100)	100 (100–100)	--
Non-communicable diseases	58·4 (55·4–61·4)	38·1 (34·8–41·5)	26 (23·4–28·8)	56·9 (54·2–59·6)
Neoplasms	49 (45·4–53·2)	46·1 (42·9–49·9)	33·2 (29·1–37·5)	46·8 (43·5–50·4)
Lip and oral cavity cancer	67·6 (56–77·2)	69·2 (57·8–78)	68·3 (56·5–77·1)	69·2 (57·8–78·1)
Nasopharynx cancer	65·6 (52·3–75·4)	64·2 (50·9–73·8)	62 (48·3–72·3)	64·4 (51·3–74)
Other pharynx cancer	79·5 (71·3–85·5)	80 (71·7–85·8)	78·8 (70·2–85·1)	80 (71·8–85·8)
Esophageal cancer	75·4 (66·6–83·6)	76·3 (67·8–84·3)	75·6 (66·5–83·6)	76·3 (67·8–84·3)
Stomach cancer	24·8 (17·3–41·7)	24 (16·7–40·7)	24·3 (16·9–41·1)	24 (16·7–40·7)

Colon and rectum cancer	63·4 (54·7–71·9)	63 (55–71)	63·1 (55–71·2)	63 (54·9–71)
Liver cancer	75 (69–80·6)	73 (67·5–78·3)	73·8 (68·5–79·2)	73 (67·4–78·3)
Gallbladder and biliary tract cancer	21·2 (11·4–33·2)	21·4 (11·5–33·6)	19·9 (10·5–31·6)	21·4 (11·6–33·6)
Pancreatic cancer	42·4 (33·3–53·4)	40·8 (32·3–51·2)	41·8 (32·8–52·5)	40·8 (32·3–51·2)
Larynx cancer	83·1 (75·6–88·7)	81·6 (74·4–87)	78·3 (70·6–84·5)	81·9 (74·8–87·4)
Tracheal, bronchus, and lung cancer	86·9 (83·5–90·1)	84·6 (81·4–88·1)	86 (82·8–89·3)	84·6 (81·4–88·1)
Breast cancer	34·9 (25·8–45·7)	32·7 (24·3–42)	33 (24·7–42·5)	32·6 (24·3–41·9)
Cervical cancer	100 (100–100)	100 (100–100)	100 (100–100)	100 (100–100)
Uterine cancer	42·3 (27·1–57·7)	43·1 (28·5–58·6)	43·2 (28·7–58·7)	43·1 (28·3–58·5)
Ovarian cancer	29·2 (12·8–43·5)	23·1 (10·5–35·5)	21 (9·65–32·4)	23·2 (10·5–35·7)
Prostate cancer	5·93 (2·6–9·59)	6·43 (2·89–10·2)	6·79 (3·15–10·8)	6·38 (2·85–10·1)
Kidney cancer	37·3 (27·8–47·1)	35·9 (26·4–45·4)	35·2 (25·9–44·7)	35·9 (26·5–45·5)
Bladder cancer	44·8 (30·3–59·1)	46 (32·8–58·4)	45·9 (33·1–57·7)	46 (32·7–58·4)
Thyroid cancer	13·2 (6·36–21·8)	13·4 (6·48–22·2)	13·3 (6·34–21·9)	13·4 (6·49–22·3)
Mesothelioma	98 (97·3–98·6)	95·6 (94·1–96·7)	97·7 (96·7–98·4)	95·5 (94·1–96·6)
Non-Hodgkin lymphoma	6·63 (2·75–11·9)	6·61 (2·71–11·8)	6·65 (2·7–12)	6·6 (2·71–11·8)
Multiple myeloma	7·57 (3·2–13·6)	7·84 (3·3–14)	7·79 (3·23–14·1)	7·84 (3·32–14)
Leukemia	34·3 (21·8–46·8)	28·5 (19·2–37·8)	26·9 (17·8–36·2)	28·6 (19·3–38)
Cardiovascular diseases	79·8 (76·3–83)	80·4 (77·5–83)	68·6 (63·6–73)	82 (79·3–84·5)
Rheumatic heart disease	22·2 (13–39·3)	23 (15·1–36)	22·6 (14·9–34·6)	23·1 (15–36·3)
Ischemic heart disease	94·3 (91·2–97)	95·8 (93·7–97·5)	94·6 (91·9–96·8)	95·8 (93·8–97·5)
Stroke	81·2 (74–88·6)	84·8 (80·2–89·4)	83·2 (78·9–87·4)	85·1 (80·2–90·1)
Hypertensive heart disease	100 (100–100)	100 (100–100)	100 (100–100)	100 (100–100)
Non-rheumatic valvular heart disease	26·2 (17·3–38·4)	27·2 (19·6–37)	19·4 (13·5–26·7)	28·1 (20·1–38·2)
Cardiomyopathy and myocarditis	38·7 (32·7–45·4)	42·5 (36·6–49)	39·6 (33·7–45·4)	42·8 (36·6–49·7)
Atrial fibrillation and flutter	49·2 (38·6–60·4)	57·9 (48·9–66·9)	61·5 (52·6–70)	53·4 (44–63·5)
Aortic aneurysm	53·6 (47·1–60·3)	61·2 (55·8–66·5)	--	61·2 (55·8–66·5)
Endocarditis	31·8 (22·9–43)	34·2 (25·3–44·6)	30·9 (21·9–42·4)	34·3 (25·3–44·6)
Other cardiovascular and circulatory diseases	30·9 (25–37·8)	31·9 (25·9–38·6)	32·1 (24·5–40)	31·8 (26·2–38·5)
Chronic respiratory diseases	66·4 (60·5–72·2)	53·2 (48–58·3)	43·5 (37·8–49·8)	65·2 (60·1–70·3)
Chronic obstructive pulmonary disease	74 (68·3–79·1)	71·7 (67·5–75·8)	63·8 (60·3–67·4)	75·4 (70·3–79·9)
Pneumoconiosis	100 (100–100)	100 (100–100)	100 (100–100)	100 (100–100)
Asthma	41·6 (31·6–53·3)	36·4 (28·5–45·4)	36·1 (28·2–45·2)	40·2 (31·6–49·9)
Digestive diseases	38·1 (31·5–45·1)	39·2 (32–46·1)	24·8 (16·4–34·1)	49·6 (41·3–57·8)

Cirrhosis and other chronic liver diseases	74·4 (62·3–87·4)	76·4 (64·5–88·4)	72·2 (60·2–84·2)	76·5 (64·5–88·6)
Upper digestive system diseases	23·8 (20·2–27·4)	7·46 (4·62–11)	0·605 (0·356–0·951)	25·7 (22·1–29·1)
Gallbladder and biliary diseases	41·7 (27·4–56·9)	45·6 (32·1–60·5)	45·9 (32·2–61)	42·6 (29·2–57·2)
Pancreatitis	39·5 (17·9–60)	44·7 (22·3–63·8)	43·9 (22·6–62·7)	44·8 (22·5–64)
Neurological disorders	25·8 (12·4–40·6)	10·1 (4·48–19·8)	5·45 (2·3–12·6)	21·4 (11·3–35·2)
Alzheimer's disease and other dementias	41·1 (27·3–56·9)	43·2 (29·2–58·3)	43·5 (29·5–58·5)	43 (29·2–58·2)
Parkinson's disease	-9·56 (-15·4–-4·44)	-10·8 (-17·2–-5·01)	-11·4 (-18·2–-5·35)	-10·6 (-16·8–-4·91)
Idiopathic epilepsy	27·1 (16·3–37·6)	23·7 (13·5–33·2)	21·4 (12·2–30·2)	26·6 (16·3–36·6)
Multiple sclerosis	16·2 (12·1–20·4)	15·8 (12–19·9)	15·1 (11·5–19)	16·6 (12·5–20·8)
Mental disorders	--	7·05 (3·5–11·1)	7·05 (3·5–11·1)	--
Depressive disorders	--	14·6 (6·81–22·4)	14·6 (6·81–22·4)	--
Anxiety disorders	--	6·1 (1·41–14)	6·1 (1·41–14)	--
Idiopathic developmental intellectual disability	--	40·1 (17·1–59·2)	40·1 (17·1–59·2)	--
Substance use disorders	100 (100–100)	100 (100–100)	100 (100–100)	100 (100–100)
Alcohol use disorders	100 (100–100)	100 (100–100)	100 (100–100)	100 (100–100)
Drug use disorders	100 (100–100)	100 (100–100)	100 (100–100)	100 (100–100)
Diabetes and kidney diseases	99·9 (99·9–99·9)	100 (100–100)	100 (100–100)	99·9 (99·8–99·9)
Diabetes mellitus	100 (100–100)	100 (100–100)	100 (100–100)	100 (100–100)
Chronic kidney disease	100 (100–100)	100 (100–100)	100 (100–100)	100 (100–100)
Sense organ diseases	--	9·19 (8·15–10·3)	9·19 (8·15–10·3)	--
Blindness and vision loss	--	6·39 (4·12–9·38)	6·39 (4·12–9·38)	--
Age-related and other hearing loss	--	11·6 (10·6–12·8)	11·6 (10·6–12·8)	--
Musculoskeletal disorders	3·8 (0·864–6·89)	21·3 (17·7–25)	21·5 (17·8–25·3)	3·95 (0·919–7·09)
Rheumatoid arthritis	12·9 (3·05–23·2)	13·7 (3·32–24·2)	13·5 (3·27–24)	14·3 (3·55–25·3)
Osteoarthritis	--	15·8 (8·19–24·3)	15·8 (8·19–24·3)	--
Low back pain	--	39·8 (34·8–44·7)	39·8 (34·8–44·7)	--
Gout	--	45·5 (29·7–63·2)	45·5 (29·7–63·2)	--
Other non-communicable diseases	0·134 (0·0861–0·201)	0·0871 (0·0535–0·134)	--	0·328 (0·206–0·487)
Sudden infant death syndrome	6·49 (3·6–10·9)	6·48 (3·6–10·9)	--	6·48 (3·6–10·9)
Injuries	25·7 (19·5–32·1)	21·9 (17·3–27)	23·4 (19·5–28·2)	20·3 (13·4–27·7)
Transport injuries	19·9 (11·2–28·6)	19·9 (12·4–27·6)	25 (19·9–30·5)	18 (9·07–26·9)
Road injuries	19·1 (10·1–28·1)	19·2 (11·4–27·2)	24·4 (19·2–30)	17·3 (8·05–26·5)
Other transport injuries	28·8 (22·9–35·1)	27·4 (21·9–33·2)	30 (25·4–35)	26 (19·9–32·6)
Unintentional injuries	31 (26·1–35·6)	21·4 (17·2–26·2)	22·5 (18·3–27·5)	17·6 (13·6–22·1)

Falls	49·4 (42·1–54·8)	30·9 (25·8–36·9)	29·6 (24·5–35·6)	36·9 (31·7–42·9)
Drowning	-13·5 (-26·5–0·327)	-13·6 (-26–-0·631)	11·1 (6·83–17·6)	-14·6 (-27·3–-1·33)
Fire, heat, and hot substances	12·1 (7·49–18·4)	13·8 (8·93–20·2)	14·4 (9·33–21·1)	12·5 (7·94–18·4)
Poisonings	14·7 (9·46–21·5)	13·5 (8·85–19·9)	12·7 (8·19–18·9)	14·6 (9·49–21·1)
Exposure to mechanical forces	5·23 (-7·19–18·7)	13·3 (10·2–17·1)	14·9 (12·3–18·2)	-3·03 (-16·2–11·4)
Animal contact	19·3 (14·5–25)	18·2 (13·6–24·1)	18·9 (14–24·9)	15·5 (11·3–20·8)
Foreign body	4·67 (2·95–7)	4·79 (3·07–7·07)	4·3 (2·74–6·29)	5·15 (3·27–7·76)
Environmental heat and cold exposure	8·96 (3·99–16·2)	9·22 (4·15–16·3)	9·04 (4·02–16·1)	9·78 (4·43–17·3)
Exposure to forces of nature	0 (0–0)	9·78 (4·44–17·4)	9·78 (4·44–17·4)	0 (0–0)
Other unintentional injuries	16·2 (10·5–23·6)	13·6 (8·74–19·9)	13·5 (8·62–19·7)	16·5 (10·9–23·8)
Self-harm and interpersonal violence	22·9 (9·87–36·3)	24·3 (12·5–35·9)	33·4 (25·1–41·4)	23 (10·3–36)
Self-harm	23·1 (8·6–37·7)	23·7 (9·39–38·3)	23·8 (11·6–37)	23·7 (9·37–38·3)
Interpersonal violence	21·9 (13–30·6)	27·2 (19–36)	37·3 (28–47·4)	19·4 (10·9–27·7)

Supplementary Results Table 2. All-age PAFs attributable to the joint distribution of all risk factors for all ages, both sexes combined for each GBD level 1, level 2, and level 3 cause and all causes in 2019 for Croatia. Results are shown for deaths, YLLs, YLDs, and DALYs Data in parenthesis are 95% uncertainty intervals. PAFs=population attributable fractions. GBD=Global Burden of Disease. YLLs=years of life lost. YLDs=years lived with disability. DALYs=disability-adjusted life-years

	Deaths 2019	DALYs 2019	YLDs 2019	YLLs 2019
All causes	68·4 (65–71·2)	52·5 (48·7–56)	32·7 (30–35·3)	65·9 (63·4–68·1)
Communicable, maternal, neonatal, and nutritional diseases	53·8 (48·5–58·5)	49·4 (45·4–53·3)	41·5 (36·3–47·2)	60·9 (57·2–64·2)
HIV/AIDS and sexually transmitted infections	89·6 (88·5–90·6)	93·4 (91·4–95·5)	98·5 (97·6–99·2)	86 (84·8–87·1)
HIV/AIDS	88·9 (87·8–89·9)	86·5 (85·3–87·7)	90·9 (89·5–92·2)	85·4 (84·2–86·6)
Sexually transmitted infections excluding HIV	99·8 (99·7–99·8)	100 (99·9–100)	100 (100–100)	99·2 (98·9–99·5)
Respiratory infections and tuberculosis	55·6 (49–61·4)	44·4 (37·1–51·5)	6·22 (4·54–8·49)	59·6 (53·3–65·2)
Tuberculosis	59·9 (52–66·6)	63·6 (56–69·9)	58·1 (49·5–65·3)	64·5 (57–70·8)
Lower respiratory infections	55·3 (48·3–61·4)	58·7 (51·8–64·8)	44·6 (38·2–50·9)	59 (52·1–65·1)
Upper respiratory infections	0·0659 (0·041–0·1)	0·00481 (0·000788–0·0109)	0·00439 (0·000358–0·0106)	0·189 (0·118–0·289)
Otitis media	0·0878 (0·0214–0·24)	4·3 (2·79–6·02)	4·32 (2·8–6·05)	0·29 (0·0681–0·815)
Enteric infections	34·5 (18·6–54·9)	34·8 (19·1–55)	34 (17·8–54·5)	42·7 (28·6–60·5)
Diarrheal diseases	34·9 (18·9–55·3)	34·8 (19·1–55·1)	34 (17·8–54·6)	43·4 (29·2–61·3)
Other infectious diseases	0·479 (0·351–0·639)	0·892 (0·636–1·22)	0·0783 (0·0432–0·141)	1·44 (1·08–1·94)
Meningitis	0·975 (0·703–1·35)	2·28 (1·64–3·13)	0·000516 (2·97e-05–0·00126)	2·94 (2·15–4·01)
Encephalitis	0·143 (0·0745–0·404)	0·324 (0·19–0·709)	0·000171 (8·86e-06–0·000427)	0·444 (0·267–0·939)
Measles	3·55 (0·742–11·8)	4·69 (1·2–14·3)	2·93 (0·118–6·49)	5·04 (1·08–16·2)
Acute hepatitis	1·05 (0·571–1·75)	0·558 (0·337–0·896)	0·419 (0·232–0·752)	1·09 (0·627–1·85)
Maternal and neonatal disorders	75·2 (71·6–78·7)	57·3 (52·6–61·9)	45·1 (39·8–49·9)	75·8 (72·3–79·3)
Maternal disorders	11·4 (4·11–18·9)	11·4 (4·13–18·8)	11·4 (4·11–18·9)	11·3 (4·1–18·8)
Neonatal disorders	76·9 (73·3–80·4)	58·2 (53·4–62·7)	45·8 (40·4–50·7)	76·9 (73·3–80·4)
Nutritional deficiencies	22·6 (15·3–26·6)	92·7 (90·2–94·7)	93·6 (91·4–95·4)	28·2 (24·8–31·7)
Protein-energy malnutrition	100 (100–100)	100 (100–100)	100 (100–100)	100 (100–100)
Vitamin A deficiency	--	100 (100–100)	100 (100–100)	--
Dietary iron deficiency	--	100 (100–100)	100 (100–100)	--
Non-communicable diseases	70·5 (66·8–73·5)	55·5 (51·2–59·3)	32·7 (29·6–35·8)	69·3 (66·7–71·8)
Neoplasms	48·8 (45–52·9)	49·9 (46·4–53·6)	37·2 (33·1–41·4)	50·5 (47–54·1)
Lip and oral cavity cancer	74·2 (68·6–79·1)	76·7 (71·1–81·4)	71·5 (65·2–77·1)	77 (71·4–81·6)
Nasopharynx cancer	69·2 (60·4–76·3)	69·7 (61·5–76·7)	70·4 (62–77·1)	69·7 (61·4–76·7)
Other pharynx cancer	84·6 (79·9–88·1)	85·3 (80·8–88·7)	81·8 (76·5–85·9)	85·3 (80·8–88·7)
Esophageal cancer	80·5 (72·4–87·5)	81·7 (74·1–88·6)	79·9 (71·5–87·1)	81·7 (74·1–88·6)
Stomach cancer	29·2 (19·4–48·3)	30·1 (20·3–48·8)	29·3 (19·4–48·2)	30·1 (20·3–48·8)

Colon and rectum cancer	61·4 (53·2–69·5)	62 (54·2–69·6)	61·5 (53·5–69·2)	62 (54·2–69·6)
Liver cancer	68·6 (60–76·2)	69·9 (61·7–77·5)	68·8 (60·1–76·3)	69·9 (61·7–77·5)
Gallbladder and biliary tract cancer	25·8 (14·9–38·7)	26·1 (15–39·2)	26 (14·9–39·1)	26·1 (15–39·3)
Pancreatic cancer	42·3 (34·1–52·2)	42·9 (34·9–52·4)	42·5 (34·5–52·1)	42·9 (34·9–52·4)
Larynx cancer	88·7 (83·5–92·7)	88·7 (83·8–92·5)	87·9 (82·9–91·8)	88·8 (83·8–92·5)
Tracheal, bronchus, and lung cancer	88·2 (85·8–90·6)	87·9 (85·6–90·3)	87·7 (85·2–90·2)	87·9 (85·6–90·3)
Breast cancer	33·5 (24·7–43·7)	32·9 (25–42)	32·8 (24·8–41·9)	32·9 (25–42)
Cervical cancer	100 (100–100)	100 (100–100)	100 (100–100)	100 (100–100)
Uterine cancer	50·5 (35·5–65·8)	51·7 (37·1–66·5)	52·1 (37·3–66·7)	51·6 (37–66·5)
Ovarian cancer	17 (7·24–28·9)	14·9 (6·31–25·8)	14·3 (6·05–25·1)	14·9 (6·32–25·8)
Prostate cancer	7·3 (3·59–11·3)	7·8 (3·83–12)	8·05 (4·04–12·4)	7·77 (3·81–12)
Kidney cancer	43·2 (33–53)	44 (33·9–53·6)	43·6 (33·6–53·5)	44 (34–53·7)
Bladder cancer	49·6 (35·1–62·6)	51·3 (37·8–63·1)	52·1 (39·4–63·4)	51·3 (37·6–63·1)
Thyroid cancer	17·2 (9·12–26·6)	17·7 (9·43–27·4)	16·6 (9·07–25·1)	17·9 (9·38–27·7)
Mesothelioma	97·3 (96·3–98·1)	95·7 (94·2–97)	97·2 (96·1–98·1)	95·7 (94·1–96·9)
Non-Hodgkin lymphoma	8·56 (3·78–14·5)	8·63 (3·87–14·6)	8·67 (3·83–14·6)	8·63 (3·9–14·6)
Multiple myeloma	9·89 (4·53–16·7)	10·2 (4·69–17·1)	10·2 (4·64–17·5)	10·2 (4·69–17)
Leukemia	38·5 (25·5–51·9)	37·6 (25·6–49·3)	39 (26·5–51·2)	37·5 (25·5–49·1)
Cardiovascular diseases	88·2 (84·9–91·1)	89·1 (86·5–91·4)	83 (79·7–86·3)	89·7 (87·2–92)
Rheumatic heart disease	30·3 (18·5–52·4)	31·6 (20·3–48·8)	32·2 (20·9–48·7)	31·5 (20·3–48·6)
Ischemic heart disease	94·5 (90·8–97·4)	95·5 (92·9–97·7)	95·7 (93·5–97·7)	95·5 (92·8–97·7)
Stroke	85·2 (78·6–92·1)	87·9 (83·1–92·7)	88 (83·6–92·2)	87·8 (83–92·8)
Hypertensive heart disease	100 (100–100)	100 (100–100)	100 (100–100)	100 (100–100)
Non-rheumatic valvular heart disease	35·5 (23·7–49·2)	33·8 (23·8–44·2)	23·7 (17·1–31·3)	36·1 (24·8–47·3)
Cardiomyopathy and myocarditis	49·1 (41·5–57·8)	59·3 (52·9–65·8)	54·7 (47·6–61·7)	59·5 (52·9–66)
Atrial fibrillation and flutter	59·3 (50·2–69)	67·5 (60·3–74·8)	70·7 (63·9–77·2)	62·7 (54·6–71·2)
Aortic aneurysm	66·1 (59·8–71·7)	72·9 (68·1–77·1)	--	72·9 (68·1–77·1)
Endocarditis	48·5 (37·3–60·3)	50·3 (39·4–60·2)	47·8 (36·9–58·5)	50·4 (39·5–60·3)
Other cardiovascular and circulatory diseases	43·1 (35·9–49·9)	45·1 (38·9–50·8)	43·3 (36·3–50·3)	45·5 (39·1–51·4)
Chronic respiratory diseases	76·9 (73·2–80·1)	69·9 (66·3–73·3)	59·4 (54·6–63·8)	78 (74·7–80·8)
Chronic obstructive pulmonary disease	79 (75·1–82·3)	77·4 (74–80·5)	70·9 (67·4–74·3)	80·7 (77·4–83·7)
Pneumoconiosis	100 (100–100)	100 (100–100)	100 (100–100)	100 (100–100)
Asthma	47·2 (35·1–59·9)	45·5 (36·4–55·5)	44·9 (36·1–54·6)	50·2 (38·9–62·3)
Digestive diseases	45·8 (39·5–52·1)	46 (38·6–52·4)	21·2 (15·2–28·1)	54·4 (46·5–61·4)

Cirrhosis and other chronic liver diseases	73·8 (62·3–83·9)	75·4 (63·8–85·7)	70·9 (61·5–80·5)	75·5 (63·9–85·8)
Upper digestive system diseases	23·5 (20·4–26·8)	12·5 (8·45–17·2)	1·67 (1–2·55)	28·2 (24·7–31·8)
Gallbladder and biliary diseases	50·3 (35·6–65)	54·1 (40·4–67·7)	55 (41·7–68·4)	51·4 (37·1–65·8)
Pancreatitis	37·3 (26·1–49·3)	42 (30·3–54·3)	39·7 (28·4–52)	42·2 (30·5–54·7)
Neurological disorders	28·4 (13·9–43·3)	19·5 (10·7–32·3)	13·4 (7·24–22·8)	26·6 (13·3–41)
Alzheimer's disease and other dementias	41·6 (27·1–56·8)	43·7 (29·2–58·5)	43·7 (29·2–58·6)	43·7 (29·3–58·6)
Parkinson's disease	-9·72 (-15·2–-4·86)	-11·5 (-17·6–-5·82)	-12·5 (-19·2–-6·37)	-11·2 (-17·3–-5·62)
Idiopathic epilepsy	27·8 (20·2–35·3)	24·1 (17·4–31·1)	22·1 (15·8–28·4)	27·2 (19·7–34·7)
Multiple sclerosis	20·5 (15·6–25·2)	22·7 (17·5–27·6)	22 (17·2–26·7)	22·9 (17·6–27·8)
Mental disorders	--	4·91 (2·31–7·95)	4·91 (2·31–7·95)	--
Depressive disorders	--	9·37 (3·33–16·4)	9·37 (3·33–16·4)	--
Anxiety disorders	--	3·39 (0·945–7·64)	3·39 (0·945–7·64)	--
Idiopathic developmental intellectual disability	--	50·5 (23·6–71·1)	50·5 (23·6–71·1)	--
Substance use disorders	100 (100–100)	100 (100–100)	100 (100–100)	100 (100–100)
Alcohol use disorders	100 (100–100)	100 (100–100)	100 (100–100)	100 (100–100)
Drug use disorders	100 (100–100)	100 (100–100)	100 (100–100)	100 (100–100)
Diabetes and kidney diseases	100 (100–100)	100 (100–100)	100 (100–100)	100 (100–100)
Diabetes mellitus	100 (100–100)	100 (100–100)	100 (100–100)	100 (100–100)
Chronic kidney disease	100 (100–100)	100 (100–100)	100 (100–100)	100 (100–100)
Sense organ diseases	--	6·92 (5·98–7·89)	6·92 (5·98–7·89)	--
Blindness and vision loss	--	5·74 (3·48–8·35)	5·74 (3·48–8·35)	--
Age-related and other hearing loss	--	7·68 (6·96–8·51)	7·68 (6·96–8·51)	--
Musculoskeletal disorders	5·1 (1·43–9·16)	34·8 (29·5–40·2)	35·5 (29·9–40·9)	5·17 (1·5–9·08)
Rheumatoid arthritis	12·1 (3·3–21·6)	18 (5·39–30·2)	19·1 (5·8–31·8)	14·8 (4·16–25·9)
Osteoarthritis	--	22·1 (12·3–32·3)	22·1 (12·3–32·3)	--
Low back pain	--	45·6 (39·4–51·8)	45·6 (39·4–51·8)	--
Gout	--	57·8 (42·6–73·2)	57·8 (42·6–73·2)	--
Other non-communicable diseases	0·0831 (0·0562–0·12)	0·0541 (0·0313–0·089)	--	0·302 (0·21–0·42)
Sudden infant death syndrome	10·4 (6·74–14·9)	10·4 (6·73–14·9)	--	10·4 (6·73–14·9)
Injuries	35·3 (30–39·9)	28·7 (24·8–32·2)	30·5 (26·8–33·7)	26·4 (21·5–31·2)
Transport injuries	24·7 (19·4–29·6)	24·7 (20–29·1)	25·6 (21·8–29·1)	23·7 (17·5–29·2)
Road injuries	22·9 (17·2–28·2)	24·1 (19·3–28·5)	25·4 (21·6–28·9)	22·4 (15·9–28·3)
Other transport injuries	35·6 (30·9–39·6)	32·9 (28–37·2)	34·5 (30·3–38·2)	32·6 (27·6–37·1)
Unintentional injuries	44·8 (38·1–49·7)	32·4 (27·7–36·4)	32·4 (28–36·3)	32·5 (26·9–37·2)

Falls	55·2 (47·6–60·5)	44 (38·2–48·4)	41·8 (36·2–46·2)	49·1 (42·7–54·2)
Drowning	-11 (-20·1–-1·56)	-8·66 (-17·5–0·211)	8·82 (5·51–13·3)	-9·68 (-18·8–-0·559)
Fire, heat, and hot substances	9·77 (5·66–15·3)	13·3 (8·7–19·1)	13·7 (9·01–19·5)	12·1 (7·74–17·7)
Poisonings	11·3 (7·15–16·9)	12·6 (8·59–17·6)	12 (8·08–17)	13·4 (9·1–19·2)
Exposure to mechanical forces	6·51 (-0·576–13·7)	17·2 (14·2–20)	18·8 (16·3–21·2)	3·04 (-4–10·4)
Animal contact	24·6 (19·3–30·6)	19·5 (15–24·8)	18·1 (13·7–23·1)	23 (17·7–29·1)
Foreign body	3·83 (2·77–5·32)	4·81 (3·53–6·55)	4·01 (2·85–5·58)	5·28 (3·78–7·26)
Environmental heat and cold exposure	9·69 (4·64–16·3)	9·51 (4·5–16)	7·67 (3·66–13)	10·4 (4·88–17·4)
Exposure to forces of nature	0 (0–0)	9·02 (4·29–15)	9·02 (4·29–15)	0 (0–0)
Other unintentional injuries	14·7 (9·39–21·2)	13·2 (8·92–18·6)	12·9 (8·62–18·3)	17 (11·5–23·7)
Self-harm and interpersonal violence	21 (9·82–31·4)	21·9 (12·2–31·3)	23·7 (17·9–29·3)	21·7 (10·4–32)
Self-harm	20·1 (8·12–31·4)	20·8 (8·61–32)	20·1 (10·1–29·8)	20·8 (8·57–32·1)
Interpersonal violence	34·8 (26·7–43)	34·8 (27·1–42·8)	37·3 (29·4–45·2)	32·1 (24·1–40)

Supplementary Results Table 2. All-age PAFs attributable to the joint distribution of all risk factors for all ages, both sexes combined for each GBD level 1, level 2, and level 3 cause and all causes in 2019 for Redbridge. Results are shown for deaths, YLLs, YLDs, and DALYs Data in parenthesis are 95% uncertainty intervals. PAFs=population attributable fractions. GBD=Global Burden of Disease. YLLs=years of life lost. YLDs=years lived with disability. DALYs=disability-adjusted life-years

	Deaths 2019	DALYs 2019	YLDs 2019	YLLs 2019
All causes	53·6 (50·3–56·9)	37·5 (34·4–40·7)	26·7 (24·4–28·9)	52·6 (49·9–55·4)
Communicable, maternal, neonatal, and nutritional diseases	46·8 (36·2–56·9)	53·7 (48·9–58·4)	47·7 (43·1–52)	57·8 (50·4–64·8)
HIV/AIDS and sexually transmitted infections	76·9 (64·1–86·1)	75·5 (60·8–86·2)	96·4 (94·5–97·6)	62·4 (45·5–76·6)
HIV/AIDS	89·9 (88–91·2)	88 (85·5–89·9)	93·8 (91·4–95·5)	84·7 (82·3–86·3)
Sexually transmitted infections excluding HIV	38·5 (18·9–64·7)	53·8 (28·8–80·1)	100 (100–100)	13 (4·84–29)
Respiratory infections and tuberculosis	46·5 (34·7–58·1)	41·3 (32·3–50·7)	3·61 (2·68–4·92)	50·1 (39–60·7)
Tuberculosis	54·6 (42·9–65·2)	56·2 (44·9–65·9)	52·6 (40·5–62·5)	57·2 (46·3–66·8)
Lower respiratory infections	46·4 (34·5–58·1)	49·9 (38·6–60·5)	33·8 (27–40·3)	50 (38·7–60·8)
Upper respiratory infections	0·0633 (0·0386–0·0988)	0·00954 (0·00129–0·0222)	0·00875 (0·000507–0·0215)	0·136 (0·0808–0·221)
Otitis media	1·8 (1·08–2·72)	5·6 (3·64–7·74)	5·6 (3·64–7·74)	4·61 (2·95–6·55)
Enteric infections	5·25 (3·27–8·77)	10·5 (7·95–14·1)	10·6 (7·94–14·3)	10·5 (7·44–14·6)
Diarrheal diseases	5·29 (3·33–8·86)	10·6 (8–14·1)	10·6 (7·95–14·3)	10·7 (7·58–14·7)
Other infectious diseases	1·16 (0·636–1·57)	1·69 (1·24–2·19)	0·321 (0·184–0·531)	2·43 (1·69–3·21)
Meningitis	2·56 (1·84–3·41)	3·57 (2·63–4·78)	0·000714 (2·88e-05–0·00199)	4·68 (3·43–6·19)
Encephalitis	0·593 (0·378–0·89)	1·01 (0·637–1·56)	0·000216 (9·38e-06–0·000575)	1·67 (1·05–2·52)
Measles	3·29 (-0·997–16·9)	3·22 (-0·822–16)	0·496 (0·0139–1·32)	3·99 (-1·21–20·3)
Acute hepatitis	13·9 (7·26–24·2)	4·53 (2·62–7·4)	2·44 (1·47–4·05)	11·8 (6·14–21·2)
Maternal and neonatal disorders	87·8 (85·8–89·7)	77·3 (73·8–80·6)	63 (57·4–68·5)	88·6 (86·7–90·4)
Maternal disorders	9·33 (3·31–15·5)	9·41 (3·36–16)	9·53 (3·39–16·3)	9·19 (3·27–15·3)
Neonatal disorders	89·9 (88–91·6)	79 (75·6–82·4)	65·1 (59·1–70·7)	89·9 (88–91·6)
Nutritional deficiencies	63·3 (56·1–88·2)	79·2 (75–83·9)	79·4 (75·2–84·1)	63·7 (59·7–78·5)
Protein-energy malnutrition	100 (100–100)	100 (100–100)	100 (100–100)	100 (100–100)
Vitamin A deficiency	--	100 (100–100)	100 (100–100)	--
Dietary iron deficiency	--	100 (100–100)	100 (100–100)	--
Non-communicable diseases	55·3 (51·7–58·8)	37·3 (33·8–40·6)	25·5 (23·1–28·1)	54·3 (51·4–57·3)
Neoplasms	45 (41–49·3)	43·3 (39·6–47·3)	30·2 (26·1–34·8)	44·1 (40·5–48·2)
Lip and oral cavity cancer	65·3 (53·6–74·9)	68·3 (57–77·2)	67·7 (55·7–76·7)	68·4 (57·1–77·3)
Nasopharynx cancer	65·4 (51·4–75·7)	64·7 (51·6–74·6)	62·7 (48·7–73·5)	64·9 (51·8–74·8)
Other pharynx cancer	76 (67·2–83·1)	77·4 (68·5–84·1)	76 (66·7–83·3)	77·5 (68·5–84·1)
Esophageal cancer	74·3 (64·5–82·5)	75·7 (66–83·8)	74·8 (64·8–83)	75·7 (66–83·8)
Stomach cancer	23·7 (16·3–40)	23·8 (16·4–39·9)	23·9 (16·5–40·4)	23·8 (16·4–39·9)

Colon and rectum cancer	62·9 (53·6–72·1)	63·1 (54·3–71·5)	63·1 (54·3–71·9)	63·1 (54·4–71·5)
Liver cancer	75·5 (69·5–81·4)	75·5 (70–80·7)	75·9 (70·1–81)	75·5 (70–80·7)
Gallbladder and biliary tract cancer	20·6 (11–32·7)	21 (11·4–33·1)	19·4 (10·3–31·3)	21 (11·4–33·1)
Pancreatic cancer	41·2 (31·9–52·5)	40·3 (31·4–50·8)	40·9 (31·5–52)	40·3 (31·4–50·8)
Larynx cancer	82 (73·5–88·5)	81·3 (73·5–87·4)	78·8 (71·2–85·4)	81·6 (73·7–87·7)
Tracheal, bronchus, and lung cancer	85·3 (81·5–88·8)	84·1 (80·4–87·5)	84·8 (81·2–88·4)	84·1 (80·4–87·5)
Breast cancer	35·3 (26·2–46·5)	33·9 (25·6–43·2)	34·1 (25·9–43·4)	33·9 (25·6–43·2)
Cervical cancer	100 (100–100)	100 (100–100)	100 (100–100)	100 (100–100)
Uterine cancer	41·7 (27·2–56·9)	42·9 (28·5–58·1)	43·3 (28·8–58·4)	42·9 (28·4–58)
Ovarian cancer	23·1 (11·4–36·7)	19 (9·18–30·9)	17·9 (8·7–29·4)	19 (9·2–31)
Prostate cancer	5·47 (2·37–8·81)	5·94 (2·6–9·5)	6·29 (2·8–9·9)	5·88 (2·55–9·38)
Kidney cancer	36·7 (26·5–47·2)	36·3 (26·8–46·1)	35·9 (26·5–45·5)	36·3 (26·8–46·2)
Bladder cancer	41·1 (26·4–55·5)	43·2 (29·3–56·5)	43·6 (30·5–56)	43·1 (29·3–56·7)
Thyroid cancer	13·1 (6·47–21·6)	13·7 (6·65–22·6)	13·8 (6·63–22·9)	13·7 (6·65–22·7)
Mesothelioma	98·6 (98–99)	96·9 (95·9–97·8)	98·3 (97·6–98·8)	96·9 (95·8–97·7)
Non-Hodgkin lymphoma	6·55 (2·67–12)	6·7 (2·79–12·1)	6·67 (2·76–12·1)	6·7 (2·81–12·1)
Multiple myeloma	7·47 (3·24–13·4)	7·82 (3·38–13·8)	7·76 (3·31–13·8)	7·82 (3·38–13·8)
Leukemia	34·4 (21–48·3)	30·4 (20·1–41·4)	29·2 (19·2–39·7)	30·5 (20·2–41·5)
Cardiovascular diseases	78 (73·7–81·8)	78·8 (75·4–81·9)	67·8 (62·6–72·3)	80·5 (77–83·5)
Rheumatic heart disease	21·3 (11·6–41)	22·2 (14–37·5)	22·1 (13·9–35·4)	22·2 (13·9–37·7)
Ischemic heart disease	93·2 (89·1–96·6)	94·5 (91·6–97)	93·8 (90·6–96·5)	94·6 (91·6–97)
Stroke	78·8 (70·2–88·1)	82·1 (76·1–88·4)	81·4 (76·3–86·6)	82·2 (75·8–88·9)
Hypertensive heart disease	100 (100–100)	100 (100–100)	100 (100–100)	100 (100–100)
Non-rheumatic valvular heart disease	24·6 (15·2–37·8)	23·6 (16·2–33·6)	11·7 (7·46–17)	26·3 (17·9–37·4)
Cardiomyopathy and myocarditis	33·9 (26·2–44·1)	40·3 (35–46·7)	36 (29·6–42·8)	40·7 (35·2–47·1)
Atrial fibrillation and flutter	47·5 (37·1–58·8)	55·7 (46·4–64·7)	60 (51·2–68·1)	50·9 (41·2–61·1)
Aortic aneurysm	48·4 (41·5–55·3)	56·2 (50·3–62·3)	--	56·2 (50·3–62·3)
Endocarditis	29·3 (19·6–42·4)	31·6 (22·8–42·2)	29 (19·4–41·4)	31·7 (22·9–42·3)
Other cardiovascular and circulatory diseases	29·1 (22·3–36·3)	31·1 (24·9–37·1)	31 (23·5–39·2)	31·1 (25·1–37·1)
Chronic respiratory diseases	61·2 (54·4–68·1)	52 (47·1–57·3)	45·3 (39·8–51·1)	60·9 (55–67·2)
Chronic obstructive pulmonary disease	71·2 (64·6–77)	69·2 (64·7–73·4)	63·3 (59·4–67·1)	73·1 (67·1–78·3)
Pneumoconiosis	100 (100–100)	100 (100–100)	100 (100–100)	100 (100–100)
Asthma	39·3 (27·8–52·4)	35·6 (27·7–44·5)	35·3 (27·4–44·2)	39·7 (29·7–50·3)
Digestive diseases	28·9 (23·4–34·3)	33·7 (27·8–39·4)	22·9 (15·2–31·7)	41·9 (34·5–48·7)
Cirrhosis and other chronic liver diseases	73·6 (60·5–86·9)	76·6 (64·3–88·2)	72·3 (61–83·2)	76·7 (64·4–88·4)

Upper digestive system diseases	19·8 (16·2–23·2)	6·96 (4·34–9·95)	0·697 (0·39–1·09)	21·9 (18·3–25·3)
Gallbladder and biliary diseases	42·2 (27·4–57·2)	46·3 (31·5–60·8)	46·7 (31·9–61·5)	43·3 (29·1–57·8)
Pancreatitis	35·6 (15·8–56·7)	42·2 (20·3–62·2)	42·9 (21·2–63·3)	42 (20·1–62·2)
Neurological disorders	26·1 (12·4–41·2)	13·5 (6·12–24·5)	7·93 (3·69–16·2)	22·7 (11·1–36·9)
Alzheimer's disease and other dementias	39·4 (25·3–54·5)	41·2 (27·4–56·1)	41·4 (27·5–56·3)	41·1 (27·3–56)
Parkinson's disease	-8·48 (-14·1–-3·8)	-9·58 (-15·5–-4·42)	-10·2 (-16·4–-4·74)	-9·41 (-15·4–-4·31)
Idiopathic epilepsy	27·5 (16·4–37·7)	23·6 (13·8–33·2)	21·2 (12·2–30·5)	27·2 (16·8–36·8)
Multiple sclerosis	14·7 (11·1–18·6)	14·9 (11·2–18·8)	14·4 (10·8–18·2)	15·4 (11·6–19·4)
Mental disorders	--	6·96 (3·56–10·8)	6·97 (3·56–10·8)	--
Depressive disorders	--	14·3 (6·62–22·2)	14·3 (6·62–22·2)	--
Anxiety disorders	--	5·83 (1·55–13·1)	5·83 (1·55–13·1)	--
Idiopathic developmental intellectual disability	--	38·2 (16·8–55·9)	38·2 (16·8–55·9)	--
Substance use disorders	100 (100–100)	100 (100–100)	100 (100–100)	100 (100–100)
Alcohol use disorders	100 (100–100)	100 (100–100)	100 (100–100)	100 (100–100)
Drug use disorders	100 (100–100)	100 (100–100)	100 (100–100)	100 (100–100)
Diabetes and kidney diseases	99·6 (99·5–99·7)	99·9 (99·9–99·9)	100 (100–100)	99·6 (99·5–99·6)
Diabetes mellitus	100 (100–100)	100 (100–100)	100 (100–100)	100 (100–100)
Chronic kidney disease	100 (100–100)	100 (100–100)	100 (100–100)	100 (100–100)
Sense organ diseases	--	8·24 (7·23–9·51)	8·24 (7·23–9·51)	--
Blindness and vision loss	--	8·03 (5·03–11·8)	8·03 (5·03–11·8)	--
Age-related and other hearing loss	--	9·58 (8·63–10·6)	9·58 (8·63–10·6)	--
Musculoskeletal disorders	3·31 (0·767–6·19)	20·7 (17–24·3)	20·9 (17·2–24·7)	3·9 (0·957–7·01)
Rheumatoid arthritis	11 (2·63–19·9)	12·7 (3·15–22·8)	12·8 (3·18–22·9)	12·5 (2·99–22·1)
Osteoarthritis	--	15·8 (8·11–24·5)	15·8 (8·11–24·5)	--
Low back pain	--	38·1 (33–42·9)	38·1 (33–42·9)	--
Gout	--	48·4 (33·4–65·2)	48·4 (33·4–65·2)	--
Other non-communicable diseases	0·101 (0·0666–0·153)	0·0811 (0·0502–0·121)	--	0·362 (0·229–0·519)
Sudden infant death syndrome	8·95 (4·97–14·5)	8·94 (4·96–14·5)	--	8·94 (4·96–14·5)
Injuries	32·1 (26·4–37·6)	25·7 (21·3–30·4)	27·8 (23·7–32)	22·6 (16·3–29·2)
Transport injuries	21·2 (12·5–29·1)	21·2 (14·2–28)	26·4 (21·8–31·2)	18·7 (9·61–27)
Road injuries	20·4 (11·3–28·7)	20·3 (13–27·3)	25·5 (21–30·4)	17·9 (8·55–26·7)
Other transport injuries	31·3 (25·9–37)	30·1 (24·9–35·5)	33·6 (29·1–38)	27·5 (21·4–33·6)
Unintentional injuries	40·3 (33·7–45·1)	27 (22·6–31·3)	27·6 (23·2–31·9)	24·9 (20·3–29)
Falls	55·6 (46·9–61·6)	37·9 (32–43)	36·3 (30·6–41·3)	45·3 (38·3–50·9)
Drowning	-15·3 (-28·8–-1·33)	-15 (-27·4–-2·15)	11 (6·57–17·1)	-16·8 (-30·1–-3·03)

Fire, heat, and hot substances	10·1 (5·86–15·9)	13·2 (8·36–19·4)	13·9 (8·91–20·3)	11 (6·76–16·4)
Poisonings	13·5 (8·46–19·8)	12·7 (8·14–18·4)	12·1 (7·8–17·6)	13·6 (8·61–19·6)
Exposure to mechanical forces	10·6 (-2·34–23·1)	16·1 (13–19·6)	17·4 (14·9–20·6)	-0·577 (-14·1–13·2)
Animal contact	19·3 (14·9–24·6)	19·5 (14·7–24·8)	20·4 (15·3–26)	14·2 (10·4–19·1)
Foreign body	3·74 (2·34–5·64)	4·49 (2·87–6·77)	3·98 (2·64–6·01)	4·96 (3·13–7·52)
Environmental heat and cold exposure	8·02 (3·55–14·4)	9·04 (4·01–16·1)	9·01 (3·99–16)	9·15 (4·11–15·9)
Exposure to forces of nature	0 (0–0)	9·97 (4·39–17·6)	9·97 (4·39–17·6)	0 (0–0)
Other unintentional injuries	14·4 (9·05–21·2)	13 (8·27–19)	13 (8·2–18·9)	15·7 (10·1–22·3)
Self-harm and interpersonal violence	22·7 (9·81–35·5)	24·9 (13·2–36·3)	34·1 (25·8–42)	23·1 (10·4–35·9)
Self-harm	22·5 (8·67–36·3)	23·3 (9·67–37·2)	23·2 (11·4–36·1)	23·3 (9·62–37·2)
Interpersonal violence	26·3 (18·3–35)	32·2 (24·2–40·5)	38·8 (29·9–48·1)	22·6 (14·9–30·8)

Supplementary Results Table 2. All-age PAFs attributable to the joint distribution of all risk factors for all ages, both sexes combined for each GBD level 1, level 2, and level 3 cause and all causes in 2019 for Richmond upon Thames. Results are shown for deaths, YLLs, YLDs, and DALYs Data in parenthesis are 95% uncertainty intervals. PAFs=population attributable fractions. GBD=Global Burden of Disease. YLLs=years of life lost. YLDs=years lived with disability. DALYs=disability-adjusted life-years

	Deaths 2019	DALYs 2019	YLDs 2019	YLLs 2019
All causes	52·9 (49·5–56·4)	38·7 (35·7–41·9)	28·4 (26–30·8)	53 (50·5–55·8)
Communicable, maternal, neonatal, and nutritional diseases	45·8 (35·4–55·9)	50·8 (46–55·8)	47·5 (42·8–52·3)	53·1 (44·9–61·1)
HIV/AIDS and sexually transmitted infections	83·8 (73·7–90·7)	81 (67·8–89·5)	96·5 (94·9–97·8)	70·7 (54·8–83·1)
HIV/AIDS	92·4 (90·3–93·6)	91·1 (88·5–92·7)	94·4 (92·1–95·9)	89 (86·4–90·5)
Sexually transmitted infections excluding HIV	62·7 (40·4–83·8)	61 (35·5–84·9)	100 (100–100)	27 (11·7–51·3)
Respiratory infections and tuberculosis	47·2 (35·7–58·4)	42 (32·7–51·6)	3·1 (2·35–4·07)	50·3 (39·7–61·2)
Tuberculosis	60·2 (48·9–69·5)	62·1 (52–70·6)	58·6 (47·4–67·1)	62·9 (52·9–71·4)
Lower respiratory infections	47·2 (35·4–58·4)	50·3 (39·5–61·1)	35·9 (29·1–42·5)	50·4 (39·6–61·3)
Upper respiratory infections	0·0376 (0·02–0·0685)	0·00864 (0·00169–0·0198)	0·00711 (0·000226–0·0183)	0·0864 (0·0465–0·157)
Otitis media	2·27 (1·11–4·08)	4·9 (3·11–6·88)	4·86 (3·08–6·88)	5·77 (3·17–9·13)
Enteric infections	3·95 (2·51–6·49)	8·14 (6·15–10·7)	8·73 (6·55–11·5)	6·67 (4·6–9·26)
Diarrheal diseases	3·99 (2·54–6·53)	8·18 (6·19–10·8)	8·74 (6·56–11·5)	6·78 (4·72–9·4)
Other infectious diseases	0·612 (0·392–0·82)	1·08 (0·789–1·37)	0·37 (0·213–0·615)	1·41 (1·02–1·83)
Meningitis	1·45 (1·02–1·99)	2·27 (1·56–3·11)	0·000584 (1·15e-05–0·00165)	3·14 (2·24–4·26)
Encephalitis	0·265 (0·173–0·384)	0·715 (0·441–1·05)	0·000231 (5·37e-06–0·00061)	0·916 (0·604–1·3)
Measles	2·77 (-0·841–14·6)	1·78 (-0·364–8·93)	0·279 (0·00639–0·816)	3·77 (-1·12–19·5)
Acute hepatitis	14 (7·94–24·4)	5·34 (3·21–8·28)	2·85 (1·65–4·65)	13 (7·43–22)
Maternal and neonatal disorders	87·4 (84·9–89·5)	74·8 (70·7–78·7)	64·2 (58·4–69·1)	88 (85·6–90)
Maternal disorders	9·27 (3·27–15·4)	9·42 (3·39–16)	9·5 (3·43–16·2)	9·12 (3·22–15·2)
Neonatal disorders	89 (86·7–90·9)	76·5 (72·3–80·4)	66·2 (60·4–71·2)	89 (86·7–91)
Nutritional deficiencies	59·3 (51–90·7)	78·7 (74·5–83·5)	78·9 (74·6–83·7)	59·9 (54·9–81·3)
Protein-energy malnutrition	100 (100–100)	100 (100–100)	100 (100–100)	100 (100–100)
Vitamin A deficiency	--	100 (100–100)	100 (100–100)	--
Dietary iron deficiency	--	100 (100–100)	100 (100–100)	--
Non-communicable diseases	54·3 (50·5–57·9)	38·8 (35·6–42·1)	27·5 (24·9–30·2)	54·7 (52–57·7)
Neoplasms	46·6 (42·9–50·9)	45·7 (42·2–49·7)	32·9 (29–37·5)	46·6 (43–50·5)
Lip and oral cavity cancer	70·2 (60·4–77·9)	73·1 (64·1–79·9)	72·5 (63·1–79·7)	73·1 (64·2–80)
Nasopharynx cancer	66·9 (55·9–76·2)	67·1 (57–75·3)	65·6 (54·8–75)	67·3 (57·1–75·5)
Other pharynx cancer	80·9 (74·5–86·5)	82·1 (75·9–87·1)	81·1 (74·8–86·3)	82·1 (75·9–87·2)
Esophageal cancer	76·4 (67·2–84·5)	77·8 (69–85·3)	76·9 (67·5–84·5)	77·9 (69–85·3)
Stomach cancer	23·8 (16·3–40·7)	23·9 (16·7–40·6)	24·1 (16·6–41·1)	23·9 (16·7–40·6)

Colon and rectum cancer	64 (55·1–72·9)	64·4 (56·3–72·7)	64·5 (55·8–72·7)	64·4 (56·2–72·7)
Liver cancer	79 (73·2–84·4)	79·7 (74·7–84·5)	79·9 (75–84·6)	79·7 (74·6–84·5)
Gallbladder and biliary tract cancer	25 (13·8–38·1)	25·2 (14·2–38·3)	23·8 (13–37·1)	25·3 (14·2–38·4)
Pancreatic cancer	42·5 (33–53·9)	41·8 (32·9–52·7)	42·3 (32·6–53·5)	41·8 (32·9–52·7)
Larynx cancer	83·4 (75·3–89·5)	83 (75·7–88·7)	81·1 (73·5–87)	83·2 (75·8–88·9)
Tracheal, bronchus, and lung cancer	84·1 (80·3–87·6)	83·1 (79·5–86·6)	83·7 (79·9–87·2)	83·1 (79·5–86·6)
Breast cancer	37·7 (28·3–48·8)	36·3 (28–45·7)	36·6 (28·3–46·2)	36·3 (28–45·7)
Cervical cancer	100 (100–100)	100 (100–100)	100 (100–100)	100 (100–100)
Uterine cancer	47·1 (31·6–62·4)	48·2 (33·1–63·5)	48·5 (33·4–63·8)	48·1 (33–63·4)
Ovarian cancer	23·8 (11·9–37·6)	20 (9·81–32·6)	19·2 (9·36–31·4)	20·1 (9·82–32·5)
Prostate cancer	5·51 (2·37–9·02)	5·96 (2·65–9·57)	6·29 (2·84–9·92)	5·9 (2·61–9·51)
Kidney cancer	40·1 (29·6–50·9)	40·1 (29·8–50·2)	39·9 (29·7–49·9)	40·1 (29·8–50·2)
Bladder cancer	40·6 (26–55·4)	42·8 (29·6–56·2)	43·3 (30·3–56·3)	42·8 (29·5–56·2)
Thyroid cancer	16·1 (7·7–25·8)	16·8 (8·29–27·1)	17 (8·38–27·3)	16·8 (8·26–27·2)
Mesothelioma	98·6 (98–99)	97·1 (96·2–97·9)	98·4 (97·6–98·9)	97·1 (96·1–97·9)
Non-Hodgkin lymphoma	7·93 (3·34–14)	8·19 (3·51–14·1)	8·1 (3·44–14·3)	8·2 (3·53–14·2)
Multiple myeloma	9·07 (3·92–15·8)	9·44 (4·19–16·4)	9·38 (4·1–16·4)	9·44 (4·17–16·4)
Leukemia	36·7 (22·8–50·7)	34·4 (22·9–46·1)	33·1 (22·4–44·8)	34·4 (22·9–46·2)
Cardiovascular diseases	76·6 (72·3–80·8)	78 (74·6–81·2)	71·7 (66·8–76·3)	79·1 (75·8–82·2)
Rheumatic heart disease	22·1 (11·5–42·8)	23 (13·6–39·6)	23·3 (14·7–38·3)	22·9 (13·5–39·9)
Ischemic heart disease	93·4 (89·4–96·8)	94·6 (91·6–97·1)	94·1 (91·2–96·7)	94·6 (91·7–97·1)
Stroke	79·3 (70·8–88·5)	82·6 (76·7–88·8)	82·8 (78–87·9)	82·6 (76·4–89·1)
Hypertensive heart disease	100 (100–100)	100 (100–100)	100 (100–100)	100 (100–100)
Non-rheumatic valvular heart disease	25 (15·7–38·8)	26·1 (18·1–37·2)	21·9 (14·6–31)	26·7 (18·4–38)
Cardiomyopathy and myocarditis	33·9 (26·6–44·6)	41·4 (35·5–47·7)	36 (29·7–43·3)	41·7 (35·8–48·2)
Atrial fibrillation and flutter	52·1 (41·9–63·1)	60·7 (52–69·5)	64·9 (56·8–72·9)	55·3 (45·7–65·3)
Aortic aneurysm	48·3 (41·1–55·8)	55·9 (49·5–62·1)	--	55·9 (49·5–62·1)
Endocarditis	29·9 (20–43·9)	32·5 (23·9–43·7)	29·9 (20·1–42·7)	32·6 (23·9–43·7)
Other cardiovascular and circulatory diseases	30·1 (23–37·3)	32·6 (26·4–39)	32·7 (25·5–40·9)	32·6 (26·4–38·8)
Chronic respiratory diseases	61·7 (55·5–67·7)	55·9 (51·3–60·5)	50·9 (45·3–56·3)	62·1 (56·4–67·6)
Chronic obstructive pulmonary disease	70·7 (64–76·7)	69·1 (64·5–73·6)	63·8 (59·9–67·7)	72·8 (67–78·1)
Pneumoconiosis	100 (100–100)	100 (100–100)	100 (100–100)	100 (100–100)
Asthma	43·6 (31·4–56·8)	41·8 (33–51·7)	41·6 (33–51·4)	45 (34·4–56·3)
Digestive diseases	31·1 (25·9–36·4)	39 (32·6–45·1)	31·7 (22·2–42)	45·2 (38·7–51·4)

Cirrhosis and other chronic liver diseases	77·9 (65–88·7)	80·8 (69·2–90·4)	77·6 (66·5–86·8)	80·9 (69·2–90·5)
Upper digestive system diseases	19·8 (16·1–23·5)	7·18 (4·35–10·4)	0·594 (0·328–0·959)	22·5 (18·9–26)
Gallbladder and biliary diseases	47·9 (32·7–63·1)	52·9 (38·2–67·2)	53·3 (38·8–67·5)	48·7 (34·1–63·7)
Pancreatitis	43·1 (23·7–62·1)	49·8 (30·9–66·6)	51·9 (32·8–68·4)	49·4 (30·4–66·4)
Neurological disorders	28·2 (13·7–43·1)	16 (7·74–28·1)	9·65 (4·74–18·4)	24·9 (11·8–39·4)
Alzheimer's disease and other dementias	41·1 (27·1–56·5)	42·9 (29·2–57·9)	43·3 (29·4–58·1)	42·7 (29·1–57·8)
Parkinson's disease	-8·39 (-13·8–-3·75)	-9·5 (-15·6–-4·42)	-10·2 (-16·4–-4·67)	-9·31 (-15·3–-4·29)
Idiopathic epilepsy	28·6 (18·7–38·6)	26·5 (17·8–35·1)	25·1 (16·6–33·7)	29·2 (19·9–38·7)
Multiple sclerosis	14·5 (10·6–18·4)	14·9 (11·1–18·6)	14·5 (10·9–18·2)	15·3 (11·2–19·2)
Mental disorders	--	6·45 (3·15–9·99)	6·45 (3·15–10)	--
Depressive disorders	--	13·7 (6·22–21·5)	13·7 (6·22–21·5)	--
Anxiety disorders	--	4·67 (1·22–10·5)	4·67 (1·22–10·5)	--
Idiopathic developmental intellectual disability	--	33·9 (13·8–53·5)	33·9 (13·8–53·5)	--
Substance use disorders	100 (100–100)	100 (100–100)	100 (100–100)	100 (100–100)
Alcohol use disorders	100 (100–100)	100 (100–100)	100 (100–100)	100 (100–100)
Drug use disorders	100 (100–100)	100 (100–100)	100 (100–100)	100 (100–100)
Diabetes and kidney diseases	99·6 (99·5–99·7)	99·9 (99·9–100)	100 (100–100)	99·6 (99·5–99·7)
Diabetes mellitus	100 (100–100)	100 (100–100)	100 (100–100)	100 (100–100)
Chronic kidney disease	100 (100–100)	100 (100–100)	100 (100–100)	100 (100–100)
Sense organ diseases	--	8·23 (7·14–9·5)	8·23 (7·14–9·5)	--
Blindness and vision loss	--	9·17 (6·03–13·3)	9·17 (6·03–13·3)	--
Age-related and other hearing loss	--	9·17 (8·24–10·2)	9·17 (8·24–10·2)	--
Musculoskeletal disorders	1·72 (0·392–3·37)	21·5 (17·6–25·5)	22 (17·9–25·9)	2·14 (0·543–3·95)
Rheumatoid arthritis	10·9 (2·54–19·9)	12·8 (3·03–22·8)	12·9 (3·02–22·9)	12·2 (3·12–21·9)
Osteoarthritis	--	18·6 (10–27·9)	18·6 (10–27·9)	--
Low back pain	--	39·8 (34·4–45·2)	39·8 (34·4–45·2)	--
Gout	--	53·7 (37·6–70·6)	53·7 (37·6–70·6)	--
Other non-communicable diseases	0·0525 (0·0321–0·079)	0·0466 (0·0268–0·0716)	--	0·235 (0·137–0·347)
Sudden infant death syndrome	8·46 (4·62–13·9)	8·45 (4·62–13·9)	--	8·45 (4·62–13·9)
Injuries	34·1 (28·2–39·2)	28 (23·6–32·3)	29·5 (24·9–33·9)	25·2 (18·8–31·2)
Transport injuries	23·5 (15·7–30·7)	23·7 (17·6–29·4)	26·9 (22·6–31·1)	21·4 (13·2–28·9)
Road injuries	22·6 (14·3–30·1)	22·6 (16·4–28·7)	25·7 (21·4–29·9)	20·6 (11·8–28·3)
Other transport injuries	34·1 (28·9–38·5)	33·2 (28·3–37·5)	35·7 (31–39·9)	30·7 (25·1–35·5)
Unintentional injuries	40·3 (33·5–45·3)	28·6 (23·7–33·4)	29·4 (24·3–34·2)	25·4 (20·1–30·2)

Falls	56·5 (47·5–62·4)	40·1 (33·7–45·7)	38·6 (32·3–44)	47·8 (40·5–53·3)
Drowning	-14·6 (-28·3–0·139)	-14·5 (-27–-0·7)	11·7 (6·72–18·2)	-15·9 (-29·2–-1·48)
Fire, heat, and hot substances	10·5 (5·78–17)	13·5 (8·23–20·3)	14·2 (8·8–21·2)	11·3 (6·65–17)
Poisonings	13·7 (8·29–20·5)	13·2 (8·17–19·5)	12·6 (7·7–18·6)	14·2 (8·8–20·7)
Exposure to mechanical forces	12·1 (-0·445–25·1)	17 (14·1–19·8)	17·8 (15·1–20·6)	1·24 (-12–15·1)
Animal contact	19·3 (13·7–25·7)	20·7 (15·4–26·5)	21·9 (16·6–28)	15·5 (10·8–21)
Foreign body	2·36 (1·47–3·63)	3·45 (2·2–5·23)	3·11 (2–4·67)	3·76 (2·37–5·73)
Environmental heat and cold exposure	8·87 (4·07–15·1)	10·4 (4·97–17·6)	10·4 (5·02–17·7)	10 (4·91–16·9)
Exposure to forces of nature	0 (0–0)	11·5 (5·64–19·5)	11·5 (5·64–19·5)	0 (0–0)
Other unintentional injuries	13·6 (8·15–20·4)	13·4 (8·24–20)	13·3 (8·21–19·9)	15·2 (9·47–21·9)
Self-harm and interpersonal violence	26·2 (13·4–38·9)	28·6 (17·3–39·2)	35·4 (28–43·2)	27 (14·3–39·7)
Self-harm	26·1 (12·6–39·6)	27·1 (13·7–40·6)	26·9 (14·8–39·6)	27·2 (13·6–40·6)
Interpersonal violence	31·6 (22·9–40·2)	37·1 (28·9–46)	40·1 (31·5–49·2)	27·4 (19·2–36·2)

Supplementary Results Table 2. All-age PAFs attributable to the joint distribution of all risk factors for all ages, both sexes combined for each GBD level 1, level 2, and level 3 cause and all causes in 2019 for Southwark. Results are shown for deaths, YLLs, YLDs, and DALYs Data in parenthesis are 95% uncertainty intervals. PAFs=population attributable fractions. GBD=Global Burden of Disease. YLLs=years of life lost. YLDs=years lived with disability. DALYs=disability-adjusted life-years				
	Deaths<br>2019	DALYs<br>2019	YLDs<br>2019	YLLs<br>2019
All causes	56·7<br>(53·8–59·3)	39·7<br>(36·8–42·7)	28·2<br>(25·7–30·6)	56<br>(53·5–58·3)
Communicable, maternal, neonatal, and nutritional diseases	52·4<br>(43·6–60·5)	61·2<br>(57·3–65)	55·3<br>(50·6–59·7)	65·7<br>(60·1–70·6)
HIV/AIDS and sexually transmitted infections	84·9<br>(77·9–89·6)	83·5<br>(75·1–88·9)	94·9<br>(92·6–96·4)	76·3<br>(65·4–83·9)
HIV/AIDS	90·8<br>(88·4–92·1)	89·3<br>(86·5–91·1)	93·5<br>(90·8–95·3)	86·8<br>(84–88·4)
Sexually transmitted infections excluding HIV	36·3<br>(17·3–63·1)	57<br>(31·5–82·7)	100<br>(100–100)	14·4<br>(5·42–31·6)
Respiratory infections and tuberculosis	50·2<br>(39·1–60·7)	43·2<br>(34·9–51·9)	3·26<br>(2·26–4·62)	55·1<br>(44·7–64·4)
Tuberculosis	63·8<br>(53–72·1)	64·6<br>(54·6–72·3)	58·7<br>(46·7–67·7)	65·8<br>(56–73·3)
Lower respiratory infections	50<br>(38·7–60·5)	54·7<br>(44·1–64·2)	38·5<br>(31·6–44·9)	54·8<br>(44·2–64·4)
Upper respiratory infections	0·114<br>(0·0631–0·189)	0·0117<br>(0·00193–0·0256)	0·00992<br>(0·000411–0·0238)	0·212<br>(0·117–0·352)
Otitis media	2·54<br>(0·892–6·21)	5·07<br>(3·23–7·31)	5·06<br>(3·21–7·29)	4·97<br>(2·03–10·3)
Enteric infections	4·68<br>(3·11–7·23)	10·5<br>(8·25–13·6)	10·6<br>(7·9–13·9)	10·5<br>(7·63–14)
Diarrheal diseases	4·73<br>(3·15–7·27)	10·6<br>(8·29–13·7)	10·6<br>(7·91–14)	10·7<br>(7·88–14·2)
Other infectious diseases	1·32<br>(0·951–1·81)	1·99<br>(1·48–2·64)	0·381<br>(0·219–0·632)	2·62<br>(1·9–3·52)
Meningitis	3·51<br>(2·56–4·75)	4·94<br>(3·6–6·59)	0·00111<br>(3·38e-05–0·00302)	6·13<br>(4·5–8·16)
Encephalitis	0·915<br>(0·538–1·36)	1·8<br>(0·883–2·72)	0·000455<br>(1·53e-05–0·00117)	2·24<br>(1·25–3·33)
Measles	2·91<br>(-0·96–14·5)	3·4<br>(-1·06–16·7)	0·281<br>(0·00702–0·829)	3·57<br>(-1·17–17·9)
Acute hepatitis	11·5<br>(5·63–21·4)	4·45<br>(2·46–7·29)	2·42<br>(1·4–3·91)	10·1<br>(4·78–19·2)
Maternal and neonatal disorders	89·9<br>(88·1–91·5)	81·8<br>(78·6–84·6)	70·6<br>(65·1–75·3)	91<br>(89·3–92·5)
Maternal disorders	9·64<br>(3·45–16·2)	9·73<br>(3·45–16·5)	9·93<br>(3·46–17·1)	9·53<br>(3·39–16)
Neonatal disorders	92·8<br>(91·3–94·1)	83·6<br>(80·4–86·5)	72·3<br>(66·8–77·4)	92·8<br>(91·3–94·1)
Nutritional deficiencies	68·8<br>(63·2–85·5)	78·8<br>(74·1–83·7)	78·9<br>(74·2–83·9)	68·2<br>(62·8–75·9)
Protein-energy malnutrition	100<br>(100–100)	100<br>(100–100)	100<br>(100–100)	100<br>(100–100)
Vitamin A deficiency	--	100<br>(100–100)	100<br>(100–100)	--
Dietary iron deficiency	--	100<br>(100–100)	100<br>(100–100)	--
Non-communicable diseases	58·6<br>(55·4–61·5)	39·4<br>(36·3–42·8)	26·9<br>(24·3–29·6)	58·3<br>(55·8–60·8)
Neoplasms	51·5<br>(47·6–55·7)	49·2<br>(45·7–53)	35·4<br>(31·4–40)	50<br>(46·5–53·8)
Lip and oral cavity cancer	73·1<br>(64–80·4)	74·9<br>(66·1–81·9)	74·1<br>(64·4–81·2)	75<br>(66·3–81·9)
Nasopharynx cancer	69·4<br>(57·8–78·1)	68·7<br>(56·9–77)	66·5<br>(55·2–75·5)	68·9<br>(57·2–77·1)
Other pharynx cancer	82·9<br>(76·1–88·2)	83·4<br>(76·9–88·6)	82·5<br>(75·6–87·8)	83·4<br>(77–88·6)
Esophageal cancer	77·7<br>(69·3–85·6)	78·8<br>(70·5–86·6)	78<br>(69·4–85·9)	78·8<br>(70·5–86·6)

Stomach cancer	24·7 (17·4–41·3)	24·3 (17–40·6)	24·5 (17·4–41·1)	24·3 (17–40·6)
Colon and rectum cancer	65·2 (56·3–73·9)	65·3 (56·9–73·2)	65·4 (56·9–73·5)	65·3 (56·9–73·2)
Liver cancer	78·1 (72·4–83·3)	77·8 (72·8–82·7)	77·9 (72·5–82·9)	77·8 (72·8–82·7)
Gallbladder and biliary tract cancer	24 (13·3–36·7)	24·1 (13·5–37·1)	22·6 (12·2–35·6)	24·2 (13·5–37·3)
Pancreatic cancer	43·4 (33·8–54·8)	41·9 (33–52·9)	42·9 (33·3–54·2)	41·9 (33–52·9)
Larynx cancer	83·8 (76·3–89)	82·7 (75·8–88)	79·7 (72·4–85·7)	83 (76·1–88·2)
Tracheal, bronchus, and lung cancer	85·9 (82·5–89·3)	84·1 (80·6–87·6)	85·2 (81·8–88·5)	84·1 (80·5–87·6)
Breast cancer	38·1 (28·7–48·4)	35·6 (27·3–44·8)	36 (27·4–45·3)	35·6 (27·3–44·7)
Cervical cancer	100 (100–100)	100 (100–100)	100 (100–100)	100 (100–100)
Uterine cancer	48·1 (32·8–63·4)	49 (33·6–64·3)	49·2 (33·9–64·5)	49 (33·6–64·3)
Ovarian cancer	25 (12·4–38·9)	20·1 (9·72–32·5)	18·6 (9·05–30·3)	20·1 (9·76–32·6)
Prostate cancer	5·97 (2·54–9·57)	6·51 (2·83–10·3)	6·86 (3·08–10·8)	6·44 (2·79–10·2)
Kidney cancer	41·2 (30·8–51·9)	40·5 (30·5–50·5)	39·9 (29·8–49·9)	40·5 (30·5–50·6)
Bladder cancer	44·3 (30·2–58·4)	45·8 (33·1–58·3)	45·8 (33·8–57·6)	45·8 (33·1–58·4)
Thyroid cancer	16·5 (8·43–26·2)	17 (8·65–26·9)	17 (8·46–26·9)	17 (8·69–27)
Mesothelioma	97·8 (97·1–98·4)	95·5 (94·1–96·5)	97·5 (96·5–98·3)	95·4 (94–96·5)
Non-Hodgkin lymphoma	8·46 (3·67–14·6)	8·57 (3·76–14·7)	8·55 (3·7–14·7)	8·58 (3·76–14·7)
Multiple myeloma	9·5 (4·28–16·6)	9·83 (4·37–17·2)	9·8 (4·4–17·4)	9·83 (4·36–17·2)
Leukemia	37 (24·2–50·1)	32·6 (22·3–43)	31·4 (21·3–41·3)	32·7 (22·4–43)
Cardiovascular diseases	78·2 (74·5–81·4)	79·3 (76·3–82·1)	70·1 (65·2–74·4)	80·6 (77·9–83·3)
Rheumatic heart disease	22·8 (13·1–41·7)	23·7 (15·2–37·1)	23·6 (15·2–36·2)	23·8 (15·3–37·2)
Ischemic heart disease	94·4 (91·2–97·1)	95·9 (93·8–97·7)	95 (92·5–97·1)	95·9 (93·8–97·7)
Stroke	81·4 (74·1–88·9)	85·6 (81–90·2)	85·2 (81·4–89·2)	85·7 (80·7–90·5)
Hypertensive heart disease	100 (100–100)	100 (100–100)	100 (100–100)	100 (100–100)
Non-rheumatic valvular heart disease	26·2 (17–38·3)	26·9 (18·8–36·3)	16·6 (11–23·2)	28·3 (19·8–38·1)
Cardiomyopathy and myocarditis	40·1 (33·1–48)	46 (39·2–53·4)	41·1 (34·8–47·2)	46·3 (39·4–54·2)
Atrial fibrillation and flutter	53·6 (43·1–64)	62·8 (53·8–70·9)	66·4 (58·1–73·9)	57·8 (48·1–67·1)
Aortic aneurysm	52·6 (46·2–59·5)	60·7 (55·5–66·1)	--	60·7 (55·5–66·1)
Endocarditis	32 (22–43·5)	34·8 (25·6–44·6)	31·4 (21·7–42·7)	34·9 (25·7–44·7)
Other cardiovascular and circulatory diseases	31·9 (25·1–38·3)	33·4 (27·1–40)	32·9 (24·4–41·8)	33·6 (27·1–40·3)
Chronic respiratory diseases	66·6 (61–72·2)	57·3 (52·4–62·1)	48·8 (42·9–55)	66·3 (61·3–71·4)
Chronic obstructive pulmonary disease	73·8 (68·2–79·1)	72·3 (68·1–76·1)	64·9 (61–68·4)	75·6 (70·6–80·2)
Pneumoconiosis	100 (100–100)	100 (100–100)	100 (100–100)	100 (100–100)

Asthma	46·1 (34·8–58·3)	42·1 (33·7–51·8)	41·9 (33·6–51·5)	45·5 (36·1–55·9)
Digestive diseases	39·4 (33·2–45·1)	43·1 (36·3–49)	30·5 (21·6–40·4)	52·3 (44·7–58·9)
Cirrhosis and other chronic liver diseases	80·4 (68·5–90·2)	82 (70·4–91·3)	78·3 (66·6–87·4)	82·1 (70·4–91·5)
Upper digestive system diseases	22·4 (18·7–26·1)	8·61 (5·57–12·4)	0·67 (0·385–1·07)	24·6 (21–28)
Gallbladder and biliary diseases	48·3 (33·3–63·4)	52·5 (38·3–66·8)	52·9 (38·8–67·1)	49·1 (34·9–63·9)
Pancreatitis	49·6 (30·7–68·3)	54·7 (36·6–71·8)	53·4 (35·1–70·3)	54·8 (36·7–72·1)
Neurological disorders	28·5 (14·2–43·8)	12 (5·35–22·6)	6·63 (2·78–14·3)	24·3 (13·2–38·5)
Alzheimer's disease and other dementias	43·7 (28·9–59·6)	45·6 (31–61·2)	45·8 (30·9–61·2)	45·5 (30·9–61·1)
Parkinson's disease	-9·34 (-15·5–-4·23)	-10·7 (-17·4–-4·85)	-11·4 (-18·3–-5·19)	-10·5 (-17·2–-4·75)
Idiopathic epilepsy	32·5 (21·9–42·4)	28·8 (19·2–37·9)	26·5 (17·6–35·6)	32·2 (21·8–41·8)
Multiple sclerosis	16·3 (12·3–20·6)	15·9 (12–19·9)	15·1 (11·3–18·9)	16·7 (12·6–21·1)
Mental disorders	--	6·68 (3·15–10·5)	6·68 (3·15–10·5)	--
Depressive disorders	--	14·9 (6·61–23·4)	14·9 (6·61–23·4)	--
Anxiety disorders	--	5·35 (1·23–12·6)	5·35 (1·23–12·6)	--
Idiopathic developmental intellectual disability	--	33·2 (13·3–53·6)	33·2 (13·3–53·6)	--
Substance use disorders	100 (100–100)	100 (100–100)	100 (100–100)	100 (100–100)
Alcohol use disorders	100 (100–100)	100 (100–100)	100 (100–100)	100 (100–100)
Drug use disorders	100 (100–100)	100 (100–100)	100 (100–100)	100 (100–100)
Diabetes and kidney diseases	99·8 (99·7–99·8)	100 (99·9–100)	100 (100–100)	99·7 (99·7–99·8)
Diabetes mellitus	100 (100–100)	100 (100–100)	100 (100–100)	100 (100–100)
Chronic kidney disease	100 (100–100)	100 (100–100)	100 (100–100)	100 (100–100)
Sense organ diseases	--	9·32 (8·33–10·5)	9·32 (8·33–10·5)	--
Blindness and vision loss	--	7·45 (4·85–10·8)	7·45 (4·85–10·8)	--
Age-related and other hearing loss	--	11·4 (10·4–12·6)	11·4 (10·4–12·6)	--
Musculoskeletal disorders	2·36 (0·567–4·3)	22·1 (18·4–25·8)	22·4 (18·7–26·3)	2·64 (0·662–4·78)
Rheumatoid arthritis	12·6 (3·01–22·5)	13·5 (3·28–24·1)	13·4 (3·18–24)	14·1 (3·41–25·2)
Osteoarthritis	--	19·1 (10·5–28·5)	19·1 (10·5–28·5)	--
Low back pain	--	41·2 (36·4–45·9)	41·2 (36·4–45·9)	--
Gout	--	52·5 (35·6–70·5)	52·5 (35·6–70·5)	--
Other non-communicable diseases	0·248 (0·161–0·365)	0·153 (0·0941–0·239)	--	0·697 (0·432–0·997)
Sudden infant death syndrome	11·8 (7·02–19)	11·8 (7·01–19)	--	11·8 (7·01–19)
Injuries	29·7 (23·7–35·4)	24 (19·1–28·8)	24·5 (20·3–29·1)	23·5 (16·5–30·2)
Transport injuries	20·5 (12·2–28·9)	20·5 (13·3–27·5)	24·6 (19·9–29·7)	18·7 (9·75–27·3)

Road injuries	19·7 (11–28·4)	19·7 (12·3–27)	23·8 (19·1–28·9)	18 (8·69–27·1)
Other transport injuries	30 (24·4–35·6)	28·6 (23·2–34)	30·7 (26·1–35·4)	27·1 (21·2–33)
Unintentional injuries	34·5 (28·4–39·4)	22·8 (18·2–27·9)	23·6 (19–29)	20·4 (15·8–25·6)
Falls	50·8 (43–56·4)	32·9 (26·9–39)	31·3 (25·6–37·4)	38·5 (32·2–44·5)
Drowning	-13·2 (-27·2–1·4)	-13·3 (-26·2–0·705)	11·6 (6·54–18·6)	-14·2 (-27·6–0·358)
Fire, heat, and hot substances	12·3 (6·96–19·9)	13·5 (7·9–20·8)	13·9 (8·24–21·5)	12·7 (7·46–19·5)
Poisonings	15·2 (9·21–22·9)	13·8 (8·4–21·1)	12·6 (7·57–19·3)	15·2 (9·39–22·7)
Exposure to mechanical forces	4·46 (-8·67–17·8)	12·5 (9·91–14·9)	13·7 (11·6–15·8)	-3·71 (-17·2–10·8)
Animal contact	20·1 (14·9–26·9)	18·6 (13·5–25·1)	19·3 (14·1–25·9)	16·3 (11·7–22·4)
Foreign body	2·91 (1·86–4·35)	3·24 (2·13–4·78)	2·75 (1·81–4·06)	3·6 (2·31–5·31)
Environmental heat and cold exposure	10·1 (4·65–17·6)	10·8 (5·1–18·5)	10·5 (4·96–18·1)	11·4 (5·44–19·3)
Exposure to forces of nature	0 (0–0)	11·3 (5·32–19·3)	11·3 (5·32–19·3)	0 (0–0)
Other unintentional injuries	15·3 (9·35–23·1)	13·3 (7·85–20·4)	13·2 (7·78–20·3)	15·7 (9·8–23·3)
Self-harm and interpersonal violence	27·4 (15·2–39·7)	28·7 (17·4–39·8)	37 (28·7–45·4)	27·6 (15·6–39·7)
Self-harm	27·7 (14·2–41·4)	28·3 (14·7–42)	28 (15·4–40·2)	28·3 (14·8–42·1)
Interpersonal violence	26·6 (17·5–35·6)	31·9 (23·3–40·9)	41·3 (32·1–51·7)	24·1 (15·7–32·9)

Supplementary Results Table 2. All-age PAFs attributable to the joint distribution of all risk factors for all ages, both sexes combined for each GBD level 1, level 2, and level 3 cause and all causes in 2019 for Sutton. Results are shown for deaths, YLLs, YLDs, and DALYs Data in parenthesis are 95% uncertainty intervals. PAFs=population attributable fractions. GBD=Global Burden of Disease. YLLs=years of life lost. YLDs=years lived with disability. DALYs=disability-adjusted life-years

	Deaths 2019	DALYs 2019	YLDs 2019	YLLs 2019
All causes	53·6 (50·1–56·9)	39·5 (36·5–42·7)	27·4 (25·2–29·7)	53·3 (50·5–56)
Communicable, maternal, neonatal, and nutritional diseases	45·4 (34·8–55·2)	51 (45·7–56·1)	46·9 (42·7–51·3)	53·4 (45·1–61)
HIV/AIDS and sexually transmitted infections	75·7 (61·2–86·3)	74·8 (58–86·7)	97·2 (95·8–98·4)	59·3 (41–75·5)
HIV/AIDS	90·6 (88·8–91·8)	88·7 (86·4–90·6)	94·5 (92·1–95·9)	85·5 (83·2–87)
Sexually transmitted infections excluding HIV	43·3 (22·1–70·7)	56·9 (31·1–82·5)	100 (100–100)	15 (5·81–33·9)
Respiratory infections and tuberculosis	46·3 (34·7–57·1)	42·6 (33·1–51·9)	3·4 (2·52–4·52)	49·6 (38·5–59·6)
Tuberculosis	55 (43·3–65·2)	57·2 (46·3–66·7)	54·5 (43·5–64·6)	57·8 (47·1–67)
Lower respiratory infections	46·2 (34·6–57·1)	49·4 (38–59·5)	34·2 (27·3–41·3)	49·5 (38·1–59·7)
Upper respiratory infections	0·158 (0·0758–0·253)	0·0075 (0·000777–0·0187)	0·00694 (0·000277–0·018)	0·423 (0·214–0·687)
Otitis media	2·78 (1·4–5·04)	4·74 (2·97–6·64)	4·66 (2·9–6·58)	6·87 (3·98–10·7)
Enteric infections	4·82 (3·02–7·92)	9·23 (6·92–12·4)	9·42 (6·93–12·7)	8·75 (6·21–12·2)
Diarrheal diseases	4·85 (3·06–7·99)	9·27 (6·97–12·4)	9·43 (6·94–12·7)	8·86 (6·36–12·3)
Other infectious diseases	0·665 (0·471–0·895)	1·12 (0·851–1·44)	0·348 (0·193–0·581)	1·48 (1·1–1·92)
Meningitis	1·59 (1·14–2·14)	2·4 (1·71–3·24)	0·000561 (1·36e-05–0·00158)	3·17 (2·31–4·25)
Encephalitis	0·339 (0·213–0·522)	0·69 (0·433–1·07)	0·000167 (3·89e-06–0·000446)	1·08 (0·69–1·64)
Measles	3·83 (-1·17–19·2)	3·66 (-1–17·6)	0·449 (0·0112–1·28)	4·75 (-1·51–23·2)
Acute hepatitis	9·83 (4·93–20·6)	4·77 (2·76–8·37)	2·84 (1·65–4·67)	8·94 (4·54–18·1)
Maternal and neonatal disorders	87·9 (85·9–89·6)	76·6 (73–79·9)	64 (58·6–69·2)	88·2 (86·3–89·9)
Maternal disorders	9·38 (3·32–15·5)	9·44 (3·33–16·2)	9·47 (3·36–16·5)	9·25 (3·3–15·4)
Neonatal disorders	88·8 (86·9–90·5)	78·1 (74·3–81·4)	66·1 (60·2–71·4)	88·8 (86·9–90·5)
Nutritional deficiencies	75·6 (70·2–91)	79·2 (75·1–83·8)	79·3 (75·2–83·9)	74·2 (70·8–83·2)
Protein-energy malnutrition	100 (100–100)	100 (100–100)	100 (100–100)	100 (100–100)
Vitamin A deficiency	--	100 (100–100)	100 (100–100)	--
Dietary iron deficiency	--	100 (100–100)	100 (100–100)	--
Non-communicable diseases	55·1 (51·4–58·5)	39·7 (36·4–43·2)	26·4 (24·1–29)	55 (52·2–57·8)
Neoplasms	47·3 (43·5–51·5)	46·1 (42·7–50·1)	33 (29·1–37·6)	46·9 (43·5–50·8)
Lip and oral cavity cancer	65·3 (53·7–75·1)	68·5 (57·4–77·4)	67·8 (56·4–77)	68·5 (57·4–77·5)
Nasopharynx cancer	63·7 (50·5–74·4)	63·8 (51·1–74)	61·5 (48·5–72·6)	64 (51·3–74·1)
Other pharynx cancer	78·5 (70·5–84·9)	79·7 (72·2–85·7)	78·6 (70·8–85)	79·8 (72·3–85·7)
Esophageal cancer	74·8 (65·2–83·3)	76·1 (67–84·5)	75·2 (65·7–83·6)	76·1 (67–84·6)

Stomach cancer	23·8 (16·3–40·6)	23·8 (16·6–40·5)	24 (16·7–40·7)	23·8 (16·6–40·5)
Colon and rectum cancer	62·9 (53·7–71·8)	63·2 (54·4–71·4)	63·2 (54·5–71·6)	63·2 (54·4–71·3)
Liver cancer	75·6 (69·2–81·5)	76·2 (70·5–81·4)	76·5 (70·7–81·9)	76·2 (70·5–81·4)
Gallbladder and biliary tract cancer	21·5 (11·5–33·9)	21·9 (11·9–34·4)	20·3 (10·8–32·2)	21·9 (11·9–34·4)
Pancreatic cancer	41 (31·6–51·9)	40·2 (31·5–50·5)	40·8 (31·3–51·5)	40·2 (31·5–50·5)
Larynx cancer	82·9 (74·5–89·4)	82·4 (74·7–88·4)	80·5 (72·7–86·6)	82·6 (74·8–88·5)
Tracheal, bronchus, and lung cancer	85·8 (82·1–89·4)	84·6 (80·9–88·2)	85·3 (81·5–88·9)	84·6 (80·9–88·2)
Breast cancer	35·7 (26·3–46·7)	34·5 (25·9–44)	34·7 (26·1–44·5)	34·5 (25·9–44)
Cervical cancer	100 (100–100)	100 (100–100)	100 (100–100)	100 (100–100)
Uterine cancer	43 (27·5–58·5)	44·1 (29–59·5)	44·5 (29·4–59·6)	44·1 (28·9–59·5)
Ovarian cancer	29·1 (13·6–43·6)	24 (11·1–36·9)	22·9 (10·7–35·7)	24 (11·1–37)
Prostate cancer	5·43 (2·38–9·01)	5·86 (2·67–9·55)	6·19 (2·84–9·93)	5·8 (2·64–9·55)
Kidney cancer	37·3 (27·4–48·3)	37·3 (27·5–47·3)	37 (27·5–46·9)	37·3 (27·5–47·4)
Bladder cancer	40·9 (26·2–54·9)	42·9 (29·2–55·6)	43·3 (30·2–55·5)	42·8 (29·1–55·7)
Thyroid cancer	14·2 (6·74–23·8)	15 (6·94–24·9)	15·2 (6·96–25·5)	14·9 (6·99–24·9)
Mesothelioma	99 (98·6–99·3)	97·9 (97·1–98·5)	98·8 (98·3–99·2)	97·9 (97·1–98·5)
Non-Hodgkin lymphoma	6·93 (2·79–12·4)	7·17 (3–12·7)	7·08 (3–12·6)	7·17 (3·02–12·7)
Multiple myeloma	7·76 (3·29–13·9)	8·12 (3·41–14·5)	8·04 (3·36–14·3)	8·12 (3·41–14·6)
Leukemia	35·3 (21·6–49·6)	32·7 (21·5–44·4)	32 (20·9–43·8)	32·7 (21·6–44·3)
Cardiovascular diseases	76·5 (72·2–80·7)	78 (74·6–81·3)	69·4 (63·8–74·2)	79·3 (76–82·4)
Rheumatic heart disease	21·6 (11·6–43·1)	22·5 (13·5–39·1)	22·6 (14–38·2)	22·5 (13·5–39·7)
Ischemic heart disease	93·2 (89–96·5)	94·4 (91·7–96·8)	93·6 (90·6–96·3)	94·5 (91·7–96·9)
Stroke	78·6 (70·1–88·1)	81·9 (75·8–88·5)	81·7 (76·4–87·6)	81·9 (75·5–88·7)
Hypertensive heart disease	100 (100–100)	100 (100–100)	100 (100–100)	100 (100–100)
Non-rheumatic valvular heart disease	24·5 (14·7–37·6)	25·3 (17–35·9)	17·4 (11·1–25)	26·2 (17·6–37·2)
Cardiomyopathy and myocarditis	34·8 (27·3–44·4)	42·2 (36·6–48·3)	36·9 (30·1–44·5)	42·5 (36·9–48·8)
Atrial fibrillation and flutter	48·7 (38·2–59·7)	57·4 (48·4–66·5)	61·4 (52·6–69·7)	52·1 (42·4–62·5)
Aortic aneurysm	48·8 (41·9–56·2)	56 (49·8–62·3)	--	56 (49·8–62·3)
Endocarditis	29·7 (19·1–43·2)	32·2 (23·7–43·3)	29·4 (19·3–42·4)	32·3 (23·7–43·4)
Other cardiovascular and circulatory diseases	30 (23·6–37·1)	32·4 (26·3–38·5)	32·3 (25·2–39·5)	32·4 (26·1–38·8)
Chronic respiratory diseases	63·4 (57–69·3)	56·8 (52·3–61·4)	49·3 (43·9–54·5)	63·8 (58·1–69)
Chronic obstructive pulmonary disease	70·9 (64·8–76·8)	69·6 (64·9–74·2)	63·6 (59·6–67·5)	72·8 (67·2–78·2)
Pneumoconiosis	100 (100–100)	100 (100–100)	100 (100–100)	100 (100–100)
Asthma	40·7 (29·2–53·5)	38·4 (29·8–47·9)	38·1 (29·8–47·5)	41·5 (31·6–52·1)

Digestive diseases	28 (22·9–33·1)	32·8 (27–38·1)	16·6 (11–23·4)	40·9 (34·1–47·1)
Cirrhosis and other chronic liver diseases	74·8 (61·9–87·3)	77·7 (65·5–88·8)	73·5 (62–84·7)	77·8 (65·6–88·9)
Upper digestive system diseases	20·4 (16·8–24·2)	7·76 (5–11·2)	0·628 (0·358–0·995)	22·8 (19·3–26·3)
Gallbladder and biliary diseases	42·5 (27·7–57·7)	46·5 (31·7–61·2)	47·5 (32·5–62·1)	43·5 (28·8–58·7)
Pancreatitis	37·7 (16·8–56·6)	44·1 (22·4–62·2)	45 (23·7–63·6)	44 (22·4–62·2)
Neurological disorders	25·8 (11·9–40·7)	15 (7·34–26·7)	9·21 (4·38–18)	22·3 (10·3–36·9)
Alzheimer's disease and other dementias	39·4 (25·2–54·9)	41·2 (27·2–56·3)	41·5 (27·5–56·1)	41 (26·9–56·2)
Parkinson's disease	-8·46 (-14·2–-3·75)	-9·45 (-15·2–-4·37)	-10 (-16–-4·59)	-9·31 (-15–-4·31)
Idiopathic epilepsy	26·2 (15·9–36·1)	24·1 (14·6–33·5)	22·4 (13·4–31·5)	26·7 (16·6–36)
Multiple sclerosis	14·3 (10·4–18·3)	14·6 (10·9–18·3)	14·1 (10·6–17·7)	15 (11–19)
Mental disorders	--	6·65 (3·36–10·2)	6·65 (3·36–10·2)	--
Depressive disorders	--	13·8 (6·2–21·7)	13·8 (6·2–21·7)	--
Anxiety disorders	--	4·93 (1·27–10·9)	4·93 (1·27–10·9)	--
Idiopathic developmental intellectual disability	--	39·2 (17·1–58·1)	39·2 (17·1–58·1)	--
Substance use disorders	100 (100–100)	100 (100–100)	100 (100–100)	100 (100–100)
Alcohol use disorders	100 (100–100)	100 (100–100)	100 (100–100)	100 (100–100)
Drug use disorders	100 (100–100)	100 (100–100)	100 (100–100)	100 (100–100)
Diabetes and kidney diseases	100 (99·9–100)	100 (100–100)	100 (100–100)	100 (99·9–100)
Diabetes mellitus	100 (100–100)	100 (100–100)	100 (100–100)	100 (100–100)
Chronic kidney disease	100 (100–100)	100 (100–100)	100 (100–100)	100 (100–100)
Sense organ diseases	--	8·06 (7·03–9·36)	8·06 (7·03–9·36)	--
Blindness and vision loss	--	8·82 (5·67–12·8)	8·82 (5·67–12·8)	--
Age-related and other hearing loss	--	9 (8·03–10·1)	9 (8·03–10·1)	--
Musculoskeletal disorders	2·53 (0·596–4·62)	20·8 (17·1–24·6)	21·1 (17·3–25·1)	3·08 (0·788–5·44)
Rheumatoid arthritis	11·3 (2·71–20·3)	12·8 (3·08–22·7)	12·9 (3·1–22·7)	12·6 (3·12–22·2)
Osteoarthritis	--	16·4 (8·47–25·2)	16·4 (8·47–25·2)	--
Low back pain	--	38·3 (32·9–43·5)	38·3 (32·9–43·5)	--
Gout	--	49·6 (33·9–66·4)	49·6 (33·9–66·4)	--
Other non-communicable diseases	0·072 (0·048–0·11)	0·0651 (0·041–0·0969)	--	0·28 (0·182–0·408)
Sudden infant death syndrome	7·19 (3·93–11·9)	7·18 (3·93–11·9)	--	7·18 (3·93–11·9)
Injuries	32·6 (26·4–38·2)	26·8 (22·4–31·5)	29 (24·5–33·2)	23·5 (16·8–30·4)
Transport injuries	22·1 (14·1–29·6)	22 (15·4–28·2)	26·6 (22·4–30·9)	19·6 (10·8–27·7)
Road injuries	21·4 (13·1–29)	21·1 (14·1–27·4)	25·4 (21·2–29·8)	18·9 (9·72–27·4)
Other transport injuries	33·2 (28·2–38)	32·1 (27·4–36·7)	35·4 (31–39·5)	29 (23·5–34·5)

Unintentional injuries	41·1 (34·3–46)	28·5 (23·8–32·9)	28·9 (24·3–33·6)	26·7 (21·7–30·9)
Falls	56 (47–61·6)	39·9 (33·7–45·2)	38·2 (32·3–43·5)	47·1 (39·9–52·4)
Drowning	-16·6 (-29·9–-2·31)	-15·9 (-28·4–-2·53)	10·5 (5·72–16·7)	-17·6 (-30·8–-3·76)
Fire, heat, and hot substances	10·2 (5·46–16·2)	12·8 (7·62–19·1)	13·3 (8–19·9)	11·1 (6·5–16·8)
Poisonings	12·9 (7·64–19·4)	12·4 (7·48–18·3)	11·9 (7·17–17·8)	13·3 (8·26–19·7)
Exposure to mechanical forces	11·5 (-1·23–24·5)	17·2 (14·2–20·2)	18·3 (15·5–21·2)	0·795 (-12·8–15·1)
Animal contact	25·3 (20·6–31·1)	20·6 (15·9–26·1)	21·4 (16·3–27)	18·7 (14·6–23·8)
Foreign body	2·82 (1·76–4·3)	3·77 (2·42–5·61)	3·36 (2·18–4·95)	4·11 (2·57–6·14)
Environmental heat and cold exposure	7·93 (3·48–14·3)	9·18 (4·18–16·2)	9·25 (4·28–16·4)	8·59 (3·9–14·8)
Exposure to forces of nature	0 (0–0)	10·2 (4·8–18)	10·2 (4·8–18)	0 (0–0)
Other unintentional injuries	15·3 (9·3–22·7)	12·7 (7·62–18·9)	12·6 (7·53–18·8)	16·1 (10–23·4)
Self-harm and interpersonal violence	22·6 (9·4–36)	24·9 (12·8–36·6)	33·9 (26–41·7)	23·3 (10·1–36·3)
Self-harm	22·4 (8·65–36·5)	23·4 (9·57–37·3)	23·1 (11·1–36)	23·4 (9·5–37·4)
Interpersonal violence	26·5 (18·1–35·6)	34 (26·2–42·9)	39·2 (30·5–48·2)	23·1 (14·9–31·6)

Supplementary Results Table 2. All-age PAFs attributable to the joint distribution of all risk factors for all ages, both sexes combined for each GBD level 1, level 2, and level 3 cause and all causes in 2019 for Tower Hamlets. Results are shown for deaths, YLLs, YLDs, and DALYs Data in parenthesis are 95% uncertainty intervals. PAFs=population attributable fractions. GBD=Global Burden of Disease. YLLs=years of life lost. YLDs=years lived with disability. DALYs=disability-adjusted life-years

	Deaths 2019	DALYs 2019	YLDs 2019	YLLs 2019
All causes	57·6 (54·9–60·3)	38·8 (35·8–41·9)	27·6 (25–30·2)	55·9 (53·4–58·2)
Communicable, maternal, neonatal, and nutritional diseases	53·7 (45·5–62)	60·3 (56·3–64·1)	49·9 (45·2–54·5)	67·6 (62·7–72)
HIV/AIDS and sexually transmitted infections	78·5 (66·3–86·9)	77·6 (63·7–86·8)	95·4 (93·1–96·9)	67 (50·5–80)
HIV/AIDS	90 (87·4–91·6)	88·6 (85·5–90·7)	93·1 (90–95·1)	86·1 (83·1–87·9)
Sexually transmitted infections excluding HIV	31·8 (14·3–58·7)	51·7 (26·3–78·8)	100 (100–100)	13·2 (4·86–30·6)
Respiratory infections and tuberculosis	50·5 (39·4–61·5)	41·5 (33·1–50·6)	3·27 (2·27–4·77)	55·3 (45·4–64·9)
Tuberculosis	61·8 (50·4–71·1)	62·4 (51·5–70·9)	57·8 (45·6–67·7)	63·3 (53–71·5)
Lower respiratory infections	50·2 (38·8–61·3)	55 (44·6–64·8)	37·5 (30·6–44·3)	55·2 (44·7–65)
Upper respiratory infections	0·0811 (0·0491–0·129)	0·0107 (0·00197–0·0248)	0·00937 (0·000607–0·0235)	0·134 (0·0794–0·228)
Otitis media	5·57 (3–9·45)	6·03 (3·87–8·47)	5·81 (3·64–8·17)	9·52 (5·62–15)
Enteric infections	5·36 (3·71–7·91)	11·8 (9·26–14·8)	11·3 (8·64–14·4)	13·4 (9·78–17·8)
Diarrheal diseases	5·42 (3·77–7·99)	11·9 (9·32–14·9)	11·3 (8·66–14·5)	13·8 (10–18·2)
Other infectious diseases	1·47 (1·08–1·94)	1·93 (1·44–2·48)	0·367 (0·209–0·607)	2·55 (1·9–3·3)
Meningitis	3·21 (2·33–4·29)	4·25 (3·11–5·63)	0·00127 (5·41e-05–0·00346)	5·03 (3·68–6·68)
Encephalitis	0·813 (0·517–1·3)	1·32 (0·84–2·09)	0·00043 (2·01e-05–0·00118)	1·73 (1·13–2·73)
Measles	2·81 (-1–15·4)	3·16 (-1·02–15·6)	0·272 (0·00655–0·752)	3·49 (-1·24–19·2)
Acute hepatitis	11·5 (5·93–21·3)	4·17 (2·37–6·68)	2·22 (1·3–3·65)	9·96 (4·96–18·7)
Maternal and neonatal disorders	88·7 (86·5–90·7)	81 (77·7–83·8)	64·7 (58·7–70·1)	89·3 (87·1–91·3)
Maternal disorders	9·52 (3·39–16)	9·63 (3·4–16·3)	9·76 (3·41–16·7)	9·42 (3·37–15·8)
Neonatal disorders	90·3 (88·3–92·2)	82·6 (79·3–85·4)	67 (60·8–72·4)	90·4 (88·3–92·2)
Nutritional deficiencies	81·5 (72·8–86·3)	78·7 (74·1–83·2)	78·6 (74–83·3)	79·1 (66·2–81·9)
Protein-energy malnutrition	100 (100–100)	100 (100–100)	100 (100–100)	100 (100–100)
Vitamin A deficiency	--	100 (100–100)	100 (100–100)	--
Dietary iron deficiency	--	100 (100–100)	100 (100–100)	--
Non-communicable diseases	59·8 (56·9–62·7)	38·7 (35·3–42·1)	26·9 (24·1–29·7)	58·5 (55·8–61)
Neoplasms	51·7 (47·5–55·9)	48·4 (44·7–52·2)	34·8 (30·7–39·1)	49·1 (45·5–52·9)
Lip and oral cavity cancer	70·9 (61·1–79)	72·5 (62·8–80)	71·7 (61·8–79·5)	72·6 (62·9–80·1)
Nasopharynx cancer	68·7 (57·8–78·2)	67·5 (56·2–76·8)	65·6 (53·9–75·7)	67·7 (56·6–76·9)
Other pharynx cancer	81 (73·2–86·6)	81·5 (73·8–87)	80·3 (72·2–86·2)	81·6 (73·9–87)
Esophageal cancer	76·4 (67·2–84·9)	77·5 (68·8–85·7)	76·7 (67·3–85)	77·5 (68·9–85·7)

Stomach cancer	24·6 (17·1–41·1)	23·7 (16·6–40·3)	24 (16·6–40·6)	23·7 (16·6–40·3)
Colon and rectum cancer	65·1 (56·3–73·6)	64·9 (56·4–72·6)	64·9 (56·4–73)	64·8 (56·4–72·6)
Liver cancer	76·8 (71·1–82·3)	74·9 (69·7–80·1)	75·4 (70–80·9)	74·9 (69·7–80)
Gallbladder and biliary tract cancer	26·1 (14·8–39·2)	26·2 (14·7–39)	24·6 (13·7–37·7)	26·2 (14·7–39·1)
Pancreatic cancer	43·8 (34–55)	42 (33·1–52·5)	43·2 (33·6–54·1)	41·9 (33·1–52·5)
Larynx cancer	83·6 (76·4–89·2)	82·2 (75·6–87·8)	78·7 (71·2–85)	82·6 (75·9–88·1)
Tracheal, bronchus, and lung cancer	86·2 (82·8–89·6)	83·8 (80·3–87·4)	85·2 (81·9–88·7)	83·8 (80·3–87·4)
Breast cancer	37 (28–47·1)	33·5 (25·5–42·6)	34 (25·8–43)	33·5 (25·5–42·6)
Cervical cancer	100 (100–100)	100 (100–100)	100 (100–100)	100 (100–100)
Uterine cancer	49·1 (32·9–64·6)	49·9 (34·1–65·2)	50·1 (34·4–65·3)	49·9 (34·1–65·2)
Ovarian cancer	26·9 (13·5–40·3)	20·9 (10·1–32·7)	18·7 (9·03–29·8)	21 (10·1–32·9)
Prostate cancer	6·02 (2·62–9·68)	6·58 (2·91–10·3)	6·96 (3·21–10·9)	6·51 (2·89–10·2)
Kidney cancer	41·5 (31·1–52)	40·1 (29·9–49·8)	39·3 (29·2–49·2)	40·1 (29·9–49·9)
Bladder cancer	44·6 (30·2–58·5)	45·8 (33–58·3)	45·6 (33·2–57·7)	45·8 (32·9–58·4)
Thyroid cancer	17·6 (8·72–28)	17·9 (8·85–29)	17·8 (8·61–28·7)	17·9 (8·89–29)
Mesothelioma	97·7 (97–98·3)	94·8 (93·4–96)	97·3 (96·2–98·1)	94·8 (93·3–96)
Non-Hodgkin lymphoma	8·66 (3·79–15)	8·63 (3·84–14·8)	8·67 (3·79–15)	8·63 (3·85–14·9)
Multiple myeloma	9·87 (4·36–17·1)	10·2 (4·62–17·6)	10·1 (4·44–17·7)	10·2 (4·61–17·5)
Leukemia	35·9 (23·8–48·1)	29·9 (20·1–39)	28·6 (19·4–37·8)	29·9 (20·2–39)
Cardiovascular diseases	80 (76·4–83·1)	80·4 (77·5–83)	69·8 (64·8–74·2)	81·9 (79·2–84·3)
Rheumatic heart disease	23 (13·6–41·7)	23·8 (15·5–37·7)	23·8 (15·6–36·7)	23·8 (15·5–37·7)
Ischemic heart disease	94·4 (91·3–97·1)	95·9 (93·8–97·7)	94·8 (92·4–97)	95·9 (93·9–97·7)
Stroke	81·7 (74·3–89·2)	85·8 (81·4–90·3)	85 (80·8–88·9)	86 (81·3–90·8)
Hypertensive heart disease	100 (100–100)	100 (100–100)	100 (100–100)	100 (100–100)
Non-rheumatic valvular heart disease	26·4 (17·2–39)	27·2 (19·4–36·5)	16·6 (11·3–23·3)	28·4 (20·1–38·3)
Cardiomyopathy and myocarditis	39·6 (33·3–46·9)	43·3 (37·8–49)	39 (33·1–45·1)	43·7 (38·1–49·5)
Atrial fibrillation and flutter	53·8 (43·4–64·9)	62·8 (54–71·1)	66·7 (58·3–74·6)	57·8 (48·4–67·4)
Aortic aneurysm	53·3 (46·5–59·9)	61·3 (55·8–66·5)	--	61·3 (55·8–66·5)
Endocarditis	32·5 (23·3–44·1)	35·1 (25·7–45·3)	31·4 (22·6–42·4)	35·2 (25·8–45·4)
Other cardiovascular and circulatory diseases	32·5 (26·1–38·9)	33·3 (26·8–40)	32·5 (23·5–42·7)	33·6 (27·1–40·2)
Chronic respiratory diseases	66·8 (61·1–72·2)	55·1 (50·1–59·8)	46·8 (40·6–53·1)	65·7 (60·7–70·4)
Chronic obstructive pulmonary disease	73·9 (68·1–79·1)	71·7 (67·3–75·5)	63·5 (59·9–66·7)	75·4 (70·1–80·1)
Pneumoconiosis	100 (100–100)	100 (100–100)	100 (100–100)	100 (100–100)
Asthma	46·4 (35·4–58·2)	41·7 (33·4–51·3)	41·5 (33·2–51·1)	44·9 (35·9–54·6)

Digestive diseases	40·9 (34·3–47)	43·4 (36·1–50)	30·9 (21·7–41·3)	53·6 (45·6–61)
Cirrhosis and other chronic liver diseases	79·1 (66·7–90·1)	80·6 (68·7–90·9)	76·7 (64·4–85·9)	80·7 (68·8–91)
Upper digestive system diseases	23·5 (19·7–27·4)	6·9 (4·3–10)	0·589 (0·339–0·912)	25·9 (22–29·3)
Gallbladder and biliary diseases	49 (33·6–64·4)	52·9 (38·1–67·1)	53·2 (38·5–67·4)	49·8 (34·7–64·9)
Pancreatitis	48 (27–65·8)	52·9 (31·9–69·9)	52·2 (31·6–69·5)	53 (31·9–70)
Neurological disorders	28·4 (14·1–43·8)	10·6 (4·69–20·8)	5·93 (2·25–13·8)	23·7 (13–37·4)
Alzheimer's disease and other dementias	44·4 (29·4–60·1)	46·3 (31·4–61·6)	46·7 (31·7–61·8)	46·2 (31·3–61·3)
Parkinson's disease	-9·48 (-15·6–-4·3)	-10·8 (-17·6–-4·88)	-11·7 (-18·8–-5·38)	-10·6 (-17·1–-4·78)
Idiopathic epilepsy	31 (19·7–41·9)	27·7 (17·7–37·2)	25·9 (16·2–35·6)	30·6 (19·7–40·9)
Multiple sclerosis	15·8 (11·8–20·1)	15·3 (11·6–19·5)	14·6 (11–18·6)	16 (11·9–20·5)
Mental disorders	--	6·82 (3·15–11·1)	6·82 (3·15–11·1)	--
Depressive disorders	--	15·2 (6·88–23·4)	15·2 (6·88–23·4)	--
Anxiety disorders	--	6·08 (1·36–14·5)	6·08 (1·36–14·5)	--
Idiopathic developmental intellectual disability	--	23·7 (7·93–51·1)	23·7 (7·93–51·1)	--
Substance use disorders	100 (100–100)	100 (100–100)	100 (100–100)	100 (100–100)
Alcohol use disorders	100 (100–100)	100 (100–100)	100 (100–100)	100 (100–100)
Drug use disorders	100 (100–100)	100 (100–100)	100 (100–100)	100 (100–100)
Diabetes and kidney diseases	99·3 (99·2–99·5)	99·9 (99·8–99·9)	100 (100–100)	99·3 (99·2–99·4)
Diabetes mellitus	100 (100–100)	100 (100–100)	100 (100–100)	100 (100–100)
Chronic kidney disease	100 (100–100)	100 (100–100)	100 (100–100)	100 (100–100)
Sense organ diseases	--	9·45 (8·43–10·5)	9·45 (8·43–10·5)	--
Blindness and vision loss	--	6·38 (4·19–9·22)	6·38 (4·19–9·22)	--
Age-related and other hearing loss	--	12 (11–13·2)	12 (11–13·2)	--
Musculoskeletal disorders	2·52 (0·605–4·64)	22·2 (18·6–26)	22·5 (18·8–26·3)	2·62 (0·695–4·64)
Rheumatoid arthritis	12·9 (3·17–23·1)	13·3 (3·33–23·3)	13·2 (3·23–23)	14·3 (3·72–25·4)
Osteoarthritis	--	19·6 (10·6–29)	19·6 (10·6–29)	--
Low back pain	--	41·2 (36·2–46)	41·2 (36·2–46)	--
Gout	--	52·6 (34·6–71)	52·6 (34·6–71)	--
Other non-communicable diseases	0·187 (0·123–0·278)	0·104 (0·0658–0·162)	--	0·422 (0·275–0·613)
Sudden infant death syndrome	8·23 (4·53–13·4)	8·22 (4·53–13·3)	--	8·22 (4·53–13·3)
Injuries	27·7 (21–33·8)	22·2 (17·1–27·4)	22·6 (18·6–27·4)	21·7 (14·4–28·8)
Transport injuries	19·5 (10·9–28·1)	19·5 (11·8–26·9)	24 (18·9–29·4)	17·6 (8·44–26·4)
Road injuries	18·9 (9·67–27·8)	18·9 (11–26·6)	23·5 (18·4–29)	17 (7·71–26·2)
Other transport injuries	27·9 (22–33·8)	26·2 (20·9–31·8)	28·3 (23·5–33·4)	25 (19·1–31)

Unintentional injuries	31·5<br>(25·9–36·4)	20·2<br>(15·6–25·4)	21·4<br>(17–26·9)	16·5<br>(11·6–22)
Falls	49·6<br>(42·4–55·1)	30<br>(24–36·1)	28·3<br>(22·6–34·5)	36·2<br>(30–42·4)
Drowning	-13·2<br>(-26·6–0·649)	-13·6<br>(-26·6–-0·0224)	11·7<br>(6·72–18·5)	-14·2<br>(-27·2–-0·329)
Fire, heat, and hot substances	13·1<br>(7·72–20)	14<br>(8·49–21)	14·4<br>(8·71–21·7)	13·3<br>(8·1–19·8)
Poisonings	15·8<br>(9·82–23·4)	14·1<br>(8·75–21)	12·9<br>(7·82–19·3)	15·7<br>(9·79–23)
Exposure to mechanical forces	1·38<br>(-11·6–15·1)	11·4<br>(8·92–14·1)	12·6<br>(10·4–15·1)	-4·74<br>(-18·3–9·92)
Animal contact	19·4<br>(15–25)	16·6<br>(11·6–22·9)	18<br>(12·5–24·6)	13·5<br>(9·62–18·6)
Foreign body	3·55<br>(2·28–5·34)	3·7<br>(2·42–5·53)	3·18<br>(2·03–4·86)	4·02<br>(2·57–5·98)
Environmental heat and cold exposure	9·4<br>(4·17–16·3)	10·5<br>(4·79–18·2)	10·5<br>(4·8–18·3)	10·5<br>(4·83–17·9)
Exposure to forces of nature	0<br>(0–0)	11·1<br>(5·08–19·3)	11·1<br>(5·08–19·3)	0<br>(0–0)
Other unintentional injuries	16·1<br>(9·91–23·8)	13·7<br>(8·26–20·5)	13·5<br>(8·16–20·3)	16·2<br>(10·2–23·7)
Self-harm and interpersonal violence	26·8<br>(13·4–40·1)	28·1<br>(16·2–40)	36·7<br>(27·6–45·3)	26·9<br>(13·7–40)
Self-harm	27·1<br>(12·5–41·6)	27·6<br>(12·9–42)	27·6<br>(15·2–40·8)	27·6<br>(12·9–42)
Interpersonal violence	24·9<br>(16·3–33·9)	31·2<br>(22·6–40)	40·3<br>(30·6–50·3)	22·8<br>(14·4–31·4)

Supplementary Results Table 2. All-age PAFs attributable to the joint distribution of all risk factors for all ages, both sexes combined for each GBD level 1, level 2, and level 3 cause and all causes in 2019 for Waltham Forest. Results are shown for deaths, YLLs, YLDs, and DALYs Data in parenthesis are 95% uncertainty intervals. PAFs=population attributable fractions. GBD=Global Burden of Disease. YLLs=years of life lost. YLDs=years lived with disability. DALYs=disability-adjusted life-years

	Deaths 2019	DALYs 2019	YLDs 2019	YLLs 2019
All causes	54·8 (51·7–57·7)	38·5 (35·6–41·6)	26·9 (24·5–29·3)	53·9 (51·3–56·4)
Communicable, maternal, neonatal, and nutritional diseases	48·6 (38·6–58·1)	55·5 (50·8–59·8)	49 (44·6–53·7)	59·6 (52·8–66)
HIV/AIDS and sexually transmitted infections	81·3 (71–88·4)	78·8 (65·9–87·2)	95·6 (93·7–97)	69 (54·2–80·5)
HIV/AIDS	90·2 (88–91·6)	88·5 (85·7–90·4)	93·7 (90·9–95·4)	85·6 (83·1–87·4)
Sexually transmitted infections excluding HIV	51·2 (28·7–76·7)	55·1 (30·3–81·1)	100 (100–100)	22·4 (9·3–45·3)
Respiratory infections and tuberculosis	48 (36·3–59)	42·8 (33·7–52)	3·32 (2·38–4·51)	52·1 (41·2–62·1)
Tuberculosis	57·3 (45·5–67·4)	58·5 (47·7–67·7)	53·1 (40·6–63·1)	59·9 (48·7–69)
Lower respiratory infections	47·9 (36·1–59·1)	51·9 (40·8–62)	35·7 (29–42·3)	52 (40·9–62·2)
Upper respiratory infections	0·0661 (0·039–0·107)	0·0106 (0·00192–0·0249)	0·00937 (0·000647–0·0237)	0·132 (0·0759–0·22)
Otitis media	4·03 (1·94–7·52)	5 (3·14–7·08)	4·97 (3·1–7·02)	8·07 (4·44–13·1)
Enteric infections	5·57 (3·48–9·07)	11 (8·35–14·8)	11 (8·08–14·9)	11·2 (7·93–15·5)
Diarrheal diseases	5·62 (3·51–9·16)	11·1 (8·41–14·8)	11 (8·09–14·9)	11·4 (8·07–15·7)
Other infectious diseases	1·36 (0·919–1·99)	1·62 (1·22–2·15)	0·306 (0·179–0·526)	2·35 (1·71–3·16)
Meningitis	2·58 (1·85–3·49)	3·21 (2·3–4·35)	0·000673 (3·23e-05–0·00178)	4·64 (3·32–6·21)
Encephalitis	0·623 (0·406–0·919)	1·08 (0·579–1·68)	0·000253 (1·24e-05–0·000665)	1·64 (1·04–2·43)
Measles	2·99 (-0·764–14·6)	2·21 (-0·285–10·1)	0·532 (0·0117–1·48)	3·96 (-1·02–18·9)
Acute hepatitis	11·7 (4·6–24·4)	5·5 (2·55–10·5)	2·43 (1·43–3·96)	9·72 (3·64–20·9)
Maternal and neonatal disorders	86·2 (83·5–88·5)	78·1 (74·7–81·3)	65·2 (59·7–70·5)	87 (84·3–89·3)
Maternal disorders	9·62 (3·36–16·5)	9·69 (3·47–16·8)	9·8 (3·57–16·9)	9·51 (3·33–16·4)
Neonatal disorders	88·5 (85·9–90·6)	80·1 (76·8–83·3)	67·7 (61·8–73)	88·5 (85·9–90·6)
Nutritional deficiencies	67·3 (61·6–87·1)	79 (74·4–83·7)	79·2 (74·5–83·9)	67·9 (64·2–78·1)
Protein-energy malnutrition	100 (100–100)	100 (100–100)	100 (100–100)	100 (100–100)
Vitamin A deficiency	--	100 (100–100)	100 (100–100)	--
Dietary iron deficiency	--	100 (100–100)	100 (100–100)	--
Non-communicable diseases	56·6 (53·3–59·8)	38·4 (35·1–41·9)	25·8 (23·3–28·4)	56 (53·3–58·6)
Neoplasms	47·6 (44–51·7)	45·8 (42·5–49·5)	31·8 (28·1–36·1)	46·6 (43·3–50·4)
Lip and oral cavity cancer	66·2 (55·2–75·3)	68·6 (57·8–77·1)	67·8 (56·6–76·9)	68·7 (58–77·2)
Nasopharynx cancer	62·6 (49·5–73·5)	61·9 (49·5–72·2)	59·5 (45·6–71·3)	62·1 (49·6–72·3)
Other pharynx cancer	78·5 (70·1–85·1)	79·4 (71·3–85·9)	78·1 (69·6–85·1)	79·5 (71·4–86)
Esophageal cancer	74·9 (65·3–82·7)	76·1 (66·9–83·6)	75·3 (65·3–83)	76·1 (66·9–83·6)
Stomach cancer	24·5 (17·1–41)	24·2 (16·8–40·7)	24·4 (17·2–41)	24·2 (16·8–40·7)

Colon and rectum cancer	63·2 (54·6–71·5)	63·1 (55·1–71)	63·2 (54·9–71·1)	63·1 (55·2–71)
Liver cancer	76·2 (70·2–81·7)	75·2 (69·7–80·2)	75·7 (70·2–81)	75·2 (69·6–80·2)
Gallbladder and biliary tract cancer	20·3 (10·9–32·5)	20·5 (11·1–32·6)	19·1 (9·86–30·9)	20·6 (11·1–32·7)
Pancreatic cancer	41·7 (32·6–52·7)	40·5 (31·8–50·9)	41·3 (32·3–51·8)	40·5 (31·8–50·9)
Larynx cancer	82·5 (74·9–88·6)	81·5 (74·5–87·2)	78·6 (70·5–84·7)	81·8 (74·9–87·4)
Tracheal, bronchus, and lung cancer	86·3 (83–89·5)	84·7 (81·4–87·9)	85·7 (82·3–88·8)	84·7 (81·4–87·9)
Breast cancer	35·1 (25·9–45·9)	33·4 (25·3–42·7)	33·6 (25·6–43·2)	33·3 (25·3–42·7)
Cervical cancer	100 (100–100)	100 (100–100)	100 (100–100)	100 (100–100)
Uterine cancer	41 (26·8–56·4)	42 (27·7–57·1)	42·3 (28–57·8)	42 (27·7–57)
Ovarian cancer	25·2 (12·2–38·8)	20·4 (9·84–32·4)	19 (9·2–30·1)	20·4 (9·88–32·5)
Prostate cancer	5·78 (2·49–9·25)	6·24 (2·77–9·86)	6·59 (2·98–10·4)	6·19 (2·73–9·79)
Kidney cancer	36·9 (27·2–47·1)	36·3 (26·8–46)	35·8 (26·5–45·6)	36·3 (26·8–46)
Bladder cancer	42·9 (28·3–56·9)	44·5 (31·5–57·5)	44·7 (31·9–57)	44·5 (31·5–57·5)
Thyroid cancer	13·2 (6·14–22)	13·7 (6·37–23·1)	13·7 (6·44–22·8)	13·6 (6·38–23)
Mesothelioma	98·4 (97·8–98·8)	96·5 (95·4–97·4)	98·1 (97·3–98·7)	96·5 (95·4–97·4)
Non-Hodgkin lymphoma	6·42 (2·67–11·7)	6·55 (2·78–11·8)	6·51 (2·76–12)	6·55 (2·78–11·8)
Multiple myeloma	7·27 (2·95–13·2)	7·58 (3·17–13·7)	7·51 (3·09–13·8)	7·58 (3·18–13·7)
Leukemia	35·2 (21·9–49·2)	31·1 (20·7–41·7)	30·1 (19·5–41·1)	31·2 (20·8–41·9)
Cardiovascular diseases	78·5 (74·9–81·8)	79·5 (76·4–82·2)	68·5 (63·4–73)	81 (78·1–83·5)
Rheumatic heart disease	21·5 (11·7–41·2)	22·3 (14–37·4)	22·2 (14·1–35·6)	22·3 (14–37·6)
Ischemic heart disease	93·7 (90–96·7)	95·1 (92·6–97·3)	94·2 (91·3–96·7)	95·2 (92·7–97·3)
Stroke	80 (72·1–88·8)	83·5 (78–89)	82·6 (77·9–87·5)	83·7 (77·8–89·5)
Hypertensive heart disease	100 (100–100)	100 (100–100)	100 (100–100)	100 (100–100)
Non-rheumatic valvular heart disease	25·3 (16·1–37·9)	26·4 (18·4–36·9)	20·5 (13·9–29·2)	27·1 (18·9–37·9)
Cardiomyopathy and myocarditis	36·2 (29·2–44·2)	41·7 (36·1–47·7)	37·2 (31·3–43·7)	42 (36·2–48·2)
Atrial fibrillation and flutter	47·7 (37·7–58·5)	56·4 (47·4–64·8)	60·6 (51·5–69)	51·5 (42·1–61)
Aortic aneurysm	51·2 (44·6–58·5)	58·6 (53–64·4)	--	58·6 (53–64·4)
Endocarditis	30·1 (20·6–42·6)	32·4 (23·7–43·2)	29·7 (20·8–42·2)	32·4 (24–43·2)
Other cardiovascular and circulatory diseases	31·1 (24·9–37·8)	32·6 (26–39·2)	31·3 (23·4–39·7)	33·1 (26·6–39)
Chronic respiratory diseases	64·4 (58·4–70·6)	54·2 (49·3–59·2)	45·7 (39·9–51·4)	63·9 (58·4–69·6)
Chronic obstructive pulmonary disease	73·4 (67·7–78·6)	71·5 (67·2–75·6)	64·9 (61·3–68·5)	75 (69·7–79·8)
Pneumoconiosis	100 (100–100)	100 (100–100)	100 (100–100)	100 (100–100)
Asthma	40·5 (29·8–52·7)	36 (28·1–45·1)	35·6 (27·8–44·8)	40·7 (31·3–51·1)
Digestive diseases	32·9 (27·2–38·6)	36·2 (29·6–42·1)	24·2 (16·4–33·7)	45·5 (38·3–52·3)

Cirrhosis and other chronic liver diseases	74·4 (62–86·6)	76·7 (65–88·1)	72·2 (60–83·5)	76·8 (65·2–88·3)
Upper digestive system diseases	23 (19·2–26·5)	8·11 (5·06–11·7)	0·679 (0·379–1·11)	25·5 (22–29·1)
Gallbladder and biliary diseases	41·1 (27·2–55·7)	43·7 (30·2–58·3)	43·9 (30·3–58·5)	42·2 (28·8–56·5)
Pancreatitis	37·7 (17·3–57·2)	43·4 (21·6–62·7)	43·2 (21·4–62·6)	43·5 (21·5–62·8)
Neurological disorders	25·6 (12·5–40·3)	12·2 (5·6–22·6)	6·82 (3·11–14·1)	22 (11·1–35·9)
Alzheimer's disease and other dementias	40·2 (26·6–55·3)	41·9 (28·3–56·6)	42·2 (28·4–56·8)	41·8 (28·3–56·6)
Parkinson's disease	-9·1 (-15·1–-4·24)	-10·2 (-16·5–-4·85)	-10·9 (-17·4–-5·18)	-10 (-16·3–-4·74)
Idiopathic epilepsy	26·2 (16·2–36·5)	23·5 (14·2–32·6)	21·2 (12·4–29·9)	26·4 (16·4–36·2)
Multiple sclerosis	15·3 (11·4–19·5)	15·2 (11·5–19·3)	14·6 (11–18·6)	15·8 (11·9–20)
Mental disorders	--	7·01 (3·51–10·8)	7·02 (3·51–10·9)	--
Depressive disorders	--	14·5 (6·59–22·4)	14·5 (6·59–22·4)	--
Anxiety disorders	--	5·62 (1·38–12·6)	5·62 (1·38–12·6)	--
Idiopathic developmental intellectual disability	--	41·3 (18·8–60·3)	41·3 (18·8–60·3)	--
Substance use disorders	100 (100–100)	100 (100–100)	100 (100–100)	100 (100–100)
Alcohol use disorders	100 (100–100)	100 (100–100)	100 (100–100)	100 (100–100)
Drug use disorders	100 (100–100)	100 (100–100)	100 (100–100)	100 (100–100)
Diabetes and kidney diseases	99·9 (99·8–99·9)	100 (100–100)	100 (100–100)	99·8 (99·8–99·9)
Diabetes mellitus	100 (100–100)	100 (100–100)	100 (100–100)	100 (100–100)
Chronic kidney disease	100 (100–100)	100 (100–100)	100 (100–100)	100 (100–100)
Sense organ diseases	--	8·75 (7·69–9·95)	8·75 (7·69–9·95)	--
Blindness and vision loss	--	7·48 (4·8–10·9)	7·48 (4·8–10·9)	--
Age-related and other hearing loss	--	10·6 (9·57–11·8)	10·6 (9·57–11·8)	--
Musculoskeletal disorders	3·48 (0·857–6·35)	21·1 (17·4–24·9)	21·3 (17·6–25·2)	3·88 (0·985–6·85)
Rheumatoid arthritis	12 (2·89–21·7)	13·4 (3·39–23·6)	13·4 (3·44–23·5)	13·5 (3·33–23·9)
Osteoarthritis	--	15·4 (7·85–23·9)	15·4 (7·85–23·9)	--
Low back pain	--	39·2 (34·2–44·3)	39·2 (34·2–44·3)	--
Gout	--	46·3 (30·8–63·7)	46·3 (30·8–63·7)	--
Other non-communicable diseases	0·125 (0·0804–0·19)	0·0891 (0·0536–0·135)	--	0·379 (0·236–0·542)
Sudden infant death syndrome	7·21 (3·86–11·8)	7·21 (3·85–11·8)	--	7·21 (3·85–11·8)
Injuries	29·3 (23·4–34·9)	24·2 (19·6–29)	25·9 (21·8–30·7)	22 (15·1–28·8)
Transport injuries	20·7 (12·4–28·9)	20·7 (13·3–27·8)	25·4 (20·7–30·3)	18·6 (9·82–27·1)
Road injuries	20 (11·4–28·5)	19·9 (12·3–27·3)	24·5 (19·9–29·5)	18 (8·89–26·8)
Other transport injuries	30·5 (24·8–36·2)	29·6 (24·4–34·9)	32·2 (27·8–36·8)	27·4 (21·3–33·5)
Unintentional injuries	36·3 (30·7–40·9)	24·7 (20·4–29·6)	25·4 (21·1–30·4)	22·4 (18·2–27·1)

Falls	52·7 (45–58·2)	35·2 (29·7–40·8)	33·6 (28·3–39·3)	41·7 (35·6–47·3)
Drowning	-14 (-27·5–0·139)	-13·5 (-26·4—0·181)	10·7 (6·42–17·2)	-14·9 (-28·2—0·751)
Fire, heat, and hot substances	11·5 (6·88–18·4)	13·3 (8·46–20)	13·7 (8·8–20·7)	12·4 (7·82–18·7)
Poisonings	15·2 (9·73–22·5)	13·5 (8·86–20·1)	12·2 (7·92–18·5)	15·4 (10–22·7)
Exposure to mechanical forces	6·93 (-5·8–19·9)	15·1 (12·3–18·2)	16·2 (13·6–19·1)	-1·23 (-14·4–13)
Animal contact	20·6 (16–27)	19·1 (14·5–25·2)	19·7 (14·9–25·9)	16·7 (12·4–22·2)
Foreign body	4·01 (2·46–6·02)	4·43 (2·82–6·6)	3·75 (2·44–5·54)	4·97 (3·09–7·43)
Environmental heat and cold exposure	8·87 (4·15–16·4)	9·12 (4·26–16·6)	9 (4·18–16·4)	9·67 (4·42–17·5)
Exposure to forces of nature	0 (0–0)	9·82 (4·56–17·7)	9·82 (4·56–17·7)	0 (0–0)
Other unintentional injuries	14·5 (9·14–21·7)	12·9 (8·26–19·4)	12·8 (8·2–19·3)	15·5 (9·99–22·7)
Self-harm and interpersonal violence	23·1 (10·3–36·4)	25 (13·1–37·1)	34·1 (25·6–41·9)	23·5 (10·8–36·9)
Self-harm	22·9 (8·8–37·6)	23·7 (9·57–38·3)	23·1 (10·8–35·9)	23·7 (9·49–38·4)
Interpersonal violence	26 (17·6–35)	31·4 (23–39·9)	38·7 (29·1–48·3)	23·1 (14·8–31·5)

Supplementary Results Table 2. All-age PAFs attributable to the joint distribution of all risk factors for all ages, both sexes combined for each GBD level 1, level 2, and level 3 cause and all causes in 2019 for Wandsworth. Results are shown for deaths, YLLs, YLDs, and DALYs Data in parenthesis are 95% uncertainty intervals. PAFs=population attributable fractions. GBD=Global Burden of Disease. YLLs=years of life lost. YLDs=years lived with disability. DALYs=disability-adjusted life-years

	Deaths 2019	DALYs 2019	YLDs 2019	YLLs 2019
All causes	54·9 (51·8–57·8)	37·8 (34·7–40·6)	27·6 (25·2–30·1)	53·7 (51·2–56)
Communicable, maternal, neonatal, and nutritional diseases	49·2 (39·8–57·9)	56·1 (51·9–60·2)	49·9 (45·6–54·2)	60·7 (54·2–66·8)
HIV/AIDS and sexually transmitted infections	84·1 (75·4–90)	82·8 (71·9–89·6)	95·6 (93·5–96·9)	74·6 (61–84·4)
HIV/AIDS	91·9 (89·6–93·2)	90·6 (87·9–92·3)	93·8 (91·2–95·5)	88·7 (85·9–90·3)
Sexually transmitted infections excluding HIV	38·3 (18·7–65·6)	57·6 (31·5–82·8)	100 (100–100)	14·8 (5·77–32·9)
Respiratory infections and tuberculosis	48·7 (37–59)	42 (33·1–50·9)	3·27 (2·34–4·52)	52·9 (42·4–62·5)
Tuberculosis	61·1 (50·2–70·3)	62·2 (52–70·6)	58·4 (47·2–67·5)	63·1 (53·2–71·5)
Lower respiratory infections	48·5 (36·7–59)	52·6 (41·9–62·5)	37·3 (30·6–43·9)	52·7 (42–62·7)
Upper respiratory infections	0·095 (0·0485–0·169)	0·00971 (0·00159–0·0248)	0·00802 (3·45e-05–0·0228)	0·179 (0·0931–0·313)
Otitis media	3·72 (1·83–6·5)	4·79 (3·06–6·96)	4·72 (2·97–6·82)	7·84 (4·36–12·5)
Enteric infections	4·37 (2·87–6·81)	9·82 (7·6–12·6)	10·1 (7·6–13·1)	9·02 (6·48–12·2)
Diarrheal diseases	4·4 (2·89–6·83)	9·86 (7·65–12·6)	10·1 (7·61–13·1)	9·13 (6·58–12·3)
Other infectious diseases	1·09 (0·784–1·45)	1·67 (1·29–2·18)	0·352 (0·2–0·58)	2·24 (1·72–2·9)
Meningitis	2·55 (1·85–3·36)	3·68 (2·67–4·82)	0·000778 (3·81e-06–0·00247)	4·72 (3·46–6·18)
Encephalitis	0·621 (0·398–0·939)	1·21 (0·693–1·88)	0·000262 (1·14e-06–0·000797)	1·63 (1·02–2·45)
Measles	3·41 (-1·15–17·4)	3·86 (-1·26–20·2)	0·286 (0·00624–0·749)	4·16 (-1·37–20·9)
Acute hepatitis	13·5 (8·2–22)	4·62 (2·87–7·08)	2·48 (1·46–4·03)	12·3 (7·36–19·7)
Maternal and neonatal disorders	88·7 (86·5–90·8)	78·4 (74·6–82)	64·8 (59·4–69·9)	89·5 (87·4–91·4)
Maternal disorders	9·33 (3·37–15·3)	9·45 (3·44–15·9)	9·57 (3·43–16·4)	9·22 (3·28–15)
Neonatal disorders	90·8 (88·9–92·6)	80·3 (76·5–83·7)	67 (61·1–72·2)	90·8 (88·9–92·6)
Nutritional deficiencies	74·9 (69·9–87·7)	78·1 (73·8–83·1)	78·1 (73·9–83·3)	72·3 (66·5–78·1)
Protein-energy malnutrition	100 (100–100)	100 (100–100)	100 (100–100)	100 (100–100)
Vitamin A deficiency	--	100 (100–100)	100 (100–100)	--
Dietary iron deficiency	--	100 (100–100)	100 (100–100)	--
Non-communicable diseases	56·6 (53·3–59·7)	37·6 (34·3–40·7)	26·7 (24–29·4)	55·6 (53–58·1)
Neoplasms	48·2 (44·4–52·4)	45·8 (42·4–49·6)	33·5 (29·7–37·8)	46·6 (43·3–50·3)
Lip and oral cavity cancer	72 (62·5–79·4)	74 (65–80·7)	73·2 (64·1–80·5)	74 (65–80·8)
Nasopharynx cancer	69·7 (58·9–78·3)	69·2 (58·8–77·6)	67·5 (56·6–76·9)	69·4 (59–77·7)
Other pharynx cancer	81·1 (74·5–86·6)	81·9 (75·4–87·1)	80·6 (73·6–86·5)	81·9 (75·5–87·1)
Esophageal cancer	77·2 (68·7–85·3)	78·4 (70–86)	77·5 (68·5–85·4)	78·4 (70–86)
Stomach cancer	24·7 (17·5–41·5)	24·3 (17·2–40·7)	24·6 (17·3–41·3)	24·3 (17·2–40·7)

Colon and rectum cancer	64·5 (55·3–73)	64·5 (56·3–72·2)	64·6 (56·3–72·7)	64·5 (56·3–72·2)
Liver cancer	76·6 (70·3–82)	76·5 (70·9–81·3)	76·9 (71·1–82)	76·5 (70·9–81·3)
Gallbladder and biliary tract cancer	25·3 (14·3–38·4)	25·5 (14·5–38·2)	24 (13·5–36·9)	25·6 (14·6–38·2)
Pancreatic cancer	43·9 (34·3–54·7)	42·6 (33·8–53)	43·4 (33·9–54·3)	42·6 (33·8–53)
Larynx cancer	83·7 (76·7–89·3)	82·5 (75·5–87·7)	79·2 (72–85)	82·9 (75·9–88·1)
Tracheal, bronchus, and lung cancer	85 (81·5–88·4)	83·1 (79·6–86·8)	84·2 (80·6–87·8)	83·1 (79·6–86·7)
Breast cancer	37·4 (28·2–48)	35 (26·7–44·2)	35·3 (27–44·7)	35 (26·7–44·1)
Cervical cancer	100 (100–100)	100 (100–100)	100 (100–100)	100 (100–100)
Uterine cancer	47·3 (32·5–62·6)	48·1 (33·5–63·2)	48·3 (33·4–63·4)	48·1 (33·5–63·2)
Ovarian cancer	24·1 (12·3–37·6)	19·6 (9·59–31·8)	18·1 (8·94–29·4)	19·7 (9·62–31·9)
Prostate cancer	5·94 (2·58–9·64)	6·49 (2·94–10·1)	6·88 (3·16–10·8)	6·42 (2·85–10·1)
Kidney cancer	40·8 (30·4–51)	40·1 (29·9–50)	39·6 (29·3–49·4)	40·1 (29·9–50)
Bladder cancer	43·6 (28·9–57·7)	45·2 (32–57·6)	45·3 (32·7–57·4)	45·2 (31·8–57·6)
Thyroid cancer	15·7 (7·89–25·1)	16·2 (8·16–25·8)	16·2 (8·1–25·5)	16·2 (8·16–25·8)
Mesothelioma	97·6 (96·8–98·2)	94·8 (93·4–96·1)	97·3 (96·2–98·1)	94·8 (93·3–96)
Non-Hodgkin lymphoma	7·92 (3·32–13·9)	8·05 (3·47–14·1)	8·04 (3·45–14·2)	8·05 (3·46–14)
Multiple myeloma	9·1 (3·98–15·9)	9·43 (4·17–16·3)	9·39 (4·13–16·2)	9·43 (4·16–16·3)
Leukemia	37 (24·3–50·3)	32·9 (22·5–43·3)	31·4 (21·3–41·8)	33 (22·6–43·4)
Cardiovascular diseases	76·5 (72·3–80·3)	77·5 (74·1–81·1)	70·9 (66·1–75·1)	78·6 (75·3–82·1)
Rheumatic heart disease	22·4 (12·4–42·2)	23·3 (14·9–38)	23·4 (15·1–36·1)	23·3 (14·9–38·7)
Ischemic heart disease	93·9 (90·3–96·8)	95·2 (92·7–97·4)	94·5 (92–96·9)	95·3 (92·8–97·4)
Stroke	80·2 (72·2–88·7)	84·1 (78·9–89·5)	84·5 (80·2–89)	84 (78·2–89·9)
Hypertensive heart disease	100 (100–100)	100 (100–100)	100 (100–100)	100 (100–100)
Non-rheumatic valvular heart disease	25·5 (16·3–37·9)	26·3 (18·3–35·5)	19 (12·6–26·2)	27·2 (18·9–36·6)
Cardiomyopathy and myocarditis	33·9 (26·2–43)	40·6 (34·3–47·9)	37·6 (31·5–44)	40·9 (34·4–48·3)
Atrial fibrillation and flutter	52·5 (42–63·1)	61·4 (52·7–70)	65·5 (57·3–73·2)	56 (46·6–65·5)
Aortic aneurysm	51·1 (44·1–57·9)	58·7 (52·8–64·3)	--	58·7 (52·8–64·3)
Endocarditis	31 (21·1–42·9)	33·9 (24·5–44)	30·9 (21·6–42·6)	34 (24·6–44·2)
Other cardiovascular and circulatory diseases	30·8 (24·2–37·4)	32·9 (26·3–38·9)	33·5 (25·3–41·6)	32·7 (26·5–38·8)
Chronic respiratory diseases	64·3 (58·2–70·2)	55·3 (50·5–60·4)	48·7 (42·7–54·8)	64 (58·8–69·5)
Chronic obstructive pulmonary disease	72·5 (66·7–78·1)	70·5 (66·3–74·8)	64·1 (60·2–67·9)	74·2 (68·9–79·3)
Pneumoconiosis	100 (100–100)	100 (100–100)	100 (100–100)	100 (100–100)
Asthma	44·9 (33–57·5)	42·2 (33·6–52)	42 (33·5–51·8)	45·2 (35·3–56·1)
Digestive diseases	36·3 (30·3–41·6)	40·3 (33·7–46·3)	29·5 (20·3–39·5)	49·8 (42·3–56·1)

Cirrhosis and other chronic liver diseases	79 (66·5–89·1)	81 (69·5–90·5)	77·6 (66·8–86·5)	81·1 (69·5–90·7)
Upper digestive system diseases	23 (19·1–26·6)	7·1 (4·31–10·5)	0·671 (0·383–1·11)	25·8 (21·9–29·4)
Gallbladder and biliary diseases	48·5 (32·8–63·3)	51·3 (37·2–65·5)	51·5 (37·6–65·6)	49·4 (34·7–63·8)
Pancreatitis	47·7 (28·6–65·4)	53·4 (35–70·1)	53·6 (35·6–69·9)	53·4 (35–70)
Neurological disorders	27·1 (13·4–42·3)	12·5 (5·99–22·8)	7·17 (3·18–15·1)	23·1 (13–36·4)
Alzheimer's disease and other dementias	42·8 (28·2–58·4)	44·8 (30·7–60)	45·1 (31–60·3)	44·6 (30·5–59·9)
Parkinson's disease	-9·3 (-15·6–-4·19)	-10·6 (-17·4–-4·72)	-11·4 (-18·4–-5·22)	-10·3 (-17·2–-4·65)
Idiopathic epilepsy	30·9 (20·8–40·5)	28·7 (19·2–38)	26·7 (17·7–35·5)	31·2 (21·4–40·8)
Multiple sclerosis	15·8 (11·8–19·9)	15·8 (11·8–19·8)	15·3 (11·4–19·3)	16·3 (12·1–20·3)
Mental disorders	--	7 (3·31–11)	7·01 (3·31–11)	--
Depressive disorders	--	15 (6·58–23·5)	15 (6·58–23·5)	--
Anxiety disorders	--	5·22 (1·09–12·5)	5·22 (1·09–12·5)	--
Idiopathic developmental intellectual disability	--	34·8 (14·7–55·3)	34·8 (14·7–55·3)	--
Substance use disorders	100 (100–100)	100 (100–100)	100 (100–100)	100 (100–100)
Alcohol use disorders	100 (100–100)	100 (100–100)	100 (100–100)	100 (100–100)
Drug use disorders	100 (100–100)	100 (100–100)	100 (100–100)	100 (100–100)
Diabetes and kidney diseases	99·8 (99·7–99·8)	100 (99·9–100)	100 (100–100)	99·8 (99·7–99·8)
Diabetes mellitus	100 (100–100)	100 (100–100)	100 (100–100)	100 (100–100)
Chronic kidney disease	100 (100–100)	100 (100–100)	100 (100–100)	100 (100–100)
Sense organ diseases	--	9·05 (8·03–10·2)	9·05 (8·03–10·2)	--
Blindness and vision loss	--	7·78 (5·11–11·3)	7·78 (5·11–11·3)	--
Age-related and other hearing loss	--	10·9 (9·9–12·1)	10·9 (9·9–12·1)	--
Musculoskeletal disorders	2·98 (0·708–5·51)	21·9 (18·2–25·8)	22·2 (18·4–26·2)	3·35 (0·808–5·93)
Rheumatoid arthritis	12·3 (2·98–22)	13·5 (3·39–23·7)	13·5 (3·39–23·6)	13·7 (3·45–24·3)
Osteoarthritis	--	18·5 (10·1–27·9)	18·5 (10·1–27·9)	--
Low back pain	--	41·1 (35·9–46·1)	41·1 (35·9–46·1)	--
Gout	--	52 (35·2–69·5)	52 (35·2–69·5)	--
Other non-communicable diseases	0·136 (0·085–0·2)	0·0852 (0·05–0·13)	--	0·431 (0·251–0·625)
Sudden infant death syndrome	10·5 (5·81–17·1)	10·5 (5·8–17)	--	10·5 (5·8–17)
Injuries	31·6 (26·1–37·4)	25·4 (21–30·2)	26·1 (21·7–30·7)	24·5 (17·9–31·6)
Transport injuries	22·1 (14·1–29·8)	22·1 (15·1–28·6)	25·6 (20·7–30·6)	20·3 (11·7–28·5)
Road injuries	21·1 (12·7–29·4)	21·3 (13·9–28)	24·8 (19·7–29·9)	19·5 (10·4–28)
Other transport injuries	30·9 (25·1–36·6)	29·3 (23·8–34·6)	31·8 (27·3–36·5)	27·6 (21·6–33·5)
Unintentional injuries	38·1 (31·8–42·8)	24·9 (20·4–29·7)	25·4 (20·7–30·3)	22·9 (17·9–27·8)

Falls	54<br>(45·8–59·6)	35<br>(29·1–40·8)	33·3<br>(27·7–39·1)	42·9<br>(36·1–48·4)
Drowning	-12·8<br>(-26·4–1·79)	-12·6<br>(-25·7–1·03)	12·2<br>(7·07–18·7)	-14<br>(-27·4–0·285)
Fire, heat, and hot substances	11·9<br>(6·84–18·5)	14·1<br>(8·61–20·8)	14·7<br>(8·96–21·7)	12·7<br>(7·71–18·9)
Poisonings	14·7<br>(9·04–21·7)	13·9<br>(8·57–20·3)	13·4<br>(8·26–19·7)	15<br>(9·39–21·8)
Exposure to mechanical forces	8·5<br>(-4·24–21·8)	14·3<br>(11·6–17·3)	15·3<br>(12·8–18·1)	-1·06<br>(-14·5–13·2)
Animal contact	20·7<br>(15·1–27·2)	19·6<br>(14·1–25·9)	20·3<br>(14·8–26·8)	17·4<br>(12·4–23·6)
Foreign body	3·4<br>(2·15–5·17)	4·03<br>(2·62–6)	3·45<br>(2·2–5·22)	4·49<br>(2·85–6·64)
Environmental heat and cold exposure	10·1<br>(4·8–17·1)	10·7<br>(5·04–18·2)	10·6<br>(5·03–18)	11·1<br>(5·26–18·9)
Exposure to forces of nature	0<br>(0–0)	11·5<br>(5·37–19·3)	11·5<br>(5·37–19·3)	0<br>(0–0)
Other unintentional injuries	15·9<br>(9·7–23·3)	14·1<br>(8·78–20·8)	14<br>(8·64–20·6)	16·8<br>(10·6–24·4)
Self-harm and interpersonal violence	26·8<br>(14·1–39·8)	28·8<br>(17–40·3)	37·5<br>(29·3–46)	27·3<br>(14·6–40·2)
Self-harm	26·7<br>(12·8–40·7)	27·5<br>(13·8–41·3)	27·4<br>(15·5–40·5)	27·5<br>(13·7–41·4)
Interpersonal violence	29·8<br>(20·4–38·8)	36·1<br>(27·1–45·5)	42·4<br>(33–52·6)	27·3<br>(18·2–35·8)

Supplementary Results Table 2. All-age PAFs attributable to the joint distribution of all risk factors for all ages, both sexes combined for each GBD level 1, level 2, and level 3 cause and all causes in 2019 for Westminster. Results are shown for deaths, YLLs, YLDs, and DALYs Data in parenthesis are 95% uncertainty intervals. PAFs=population attributable fractions. GBD=Global Burden of Disease. YLLs=years of life lost. YLDs=years lived with disability. DALYs=disability-adjusted life-years				
	**Deaths 2019**	**DALYs 2019**	**YLDs 2019**	**YLLs 2019**
All causes	55·3 (51·7–58·8)	40·1 (36·8–43·2)	29·7 (27·1–32·3)	55·4 (51·3–58·3)
Communicable, maternal, neonatal, and nutritional diseases	49·4 (39·5–59)	56·2 (51·9–60·5)	52·2 (47·6–56·6)	59·2 (52·3–65·7)
HIV/AIDS and sexually transmitted infections	86·8 (80·3–90·8)	85·8 (78·4–90·4)	95·1 (92·8–96·5)	79·6 (69·4–86·3)
HIV/AIDS	91·8 (89·3–93·2)	90·7 (87·8–92·5)	93·7 (90·9–95·4)	88·9 (85·9–90·6)
Sexually transmitted infections excluding HIV	31·3 (14·1–57·7)	60·5 (34·3–85·2)	100 (100–100)	10·3 (3·81–24·4)
Respiratory infections and tuberculosis	50·1 (38·8–61·4)	43·7 (34·7–52·8)	3·63 (2·57–5·05)	53·8 (43·5–64·1)
Tuberculosis	63·3 (52·3–72·1)	65·1 (55·1–72·9)	61 (50–69·6)	65·8 (56–73·6)
Lower respiratory infections	49·8 (38·3–61·2)	53·2 (42·4–63·8)	38·8 (31·8–45·4)	53·4 (42·5–64)
Upper respiratory infections	0·0608 (0·0343–0·103)	0·00797 (0·00114–0·019)	0·00719 (0·000343–0·0184)	0·135 (0·0778–0·224)
Otitis media	2·57 (1·29–4·55)	5·06 (3·16–7·14)	5·02 (3·13–7·12)	6·28 (3·56–10·1)
Enteric infections	4·04 (2·58–6·32)	8·25 (6·33–10·8)	8·53 (6·42–11·4)	7·59 (5·45–10·4)
Diarrheal diseases	4·08 (2·62–6·37)	8·3 (6·38–10·9)	8·54 (6·42–11·4)	7·73 (5·57–10·6)
Other infectious diseases	0·839 (0·595–1·17)	1·36 (1·04–1·79)	0·412 (0·245–0·686)	1·75 (1·3–2·38)
Meningitis	1·88 (1·36–2·5)	3·12 (2·3–4·14)	0·000991 (4·09e-05–0·00276)	3·79 (2·8–4·99)
Encephalitis	0·407 (0·25–0·628)	1·01 (0·608–1·56)	0·000356 (1·44e-05–0·000949)	1·25 (0·78–1·87)
Measles	3·02 (-1·13–15·5)	3·34 (-1·21–16·6)	0·197 (0·00484–0·538)	3·72 (-1·36–19·3)
Acute hepatitis	11·3 (5·89–20·1)	5·17 (3·02–8·44)	2·61 (1·56–4·26)	10·4 (5·44–18·5)
Maternal and neonatal disorders	88·9 (86·8–90·7)	78·2 (74·4–81·4)	68·1 (62·6–73·1)	89·5 (87·5–91·2)
Maternal disorders	9·3 (3·34–15·4)	9·42 (3·37–15·6)	9·51 (3·4–15·7)	9·18 (3·29–15·2)
Neonatal disorders	90·4 (88·5–92)	79·6 (75·9–83)	69·9 (64·2–75·1)	90·4 (88·5–92)
Nutritional deficiencies	81·7 (76·7–90)	77·8 (73·6–82·7)	77·8 (73·5–82·8)	79·4 (70·6–82·3)
Protein-energy malnutrition	100 (100–100)	100 (100–100)	100 (100–100)	100 (100–100)
Vitamin A deficiency	--	100 (100–100)	100 (100–100)	--
Dietary iron deficiency	--	100 (100–100)	100 (100–100)	--
Non-communicable diseases	57 (52·9–60·6)	40·3 (36·7–43·7)	28·9 (26·1–31·7)	57·9 (52·6–61·1)
Neoplasms	49 (43·1–53·6)	47·6 (39·6–51·9)	33·8 (29·5–38·1)	48·4 (40·1–52·8)
Lip and oral cavity cancer	72 (61·7–79·5)	74·5 (65·3–81·1)	73·9 (63·9–80·8)	74·6 (65·3–81·1)
Nasopharynx cancer	71·4 (61·2–79·8)	71·1 (61·3–79·1)	69·5 (59·2–78·3)	71·3 (61·4–79·2)
Other pharynx cancer	82 (75·3–87·1)	82·9 (76·7–87·8)	81·9 (74·9–87·2)	83 (76·8–87·8)
Esophageal cancer	76·8 (67·3–85·4)	78·1 (68·8–86·6)	77·2 (67·3–85·7)	78·1 (68·9–86·6)
Stomach cancer	24·6 (17·2–41)	24·5 (17·1–41·1)	24·7 (17·3–41·3)	24·5 (17·1–41·1)

Colon and rectum cancer	67 (58·6–75·4)	67·4 (59·2–75·4)	67·4 (59–75·3)	67·4 (59·2–75·4)
Liver cancer	80·5 (74·6–85·4)	80·6 (75·3–85·3)	80·7 (75·4–85·4)	80·6 (75·3–85·3)
Gallbladder and biliary tract cancer	27·7 (15·9–41·1)	28 (16–41·6)	26·2 (14·3–40)	28·1 (16–41·6)
Pancreatic cancer	44·3 (33·8–55·9)	43·4 (33·7–54·6)	44·1 (33·8–55·4)	43·4 (33·7–54·6)
Larynx cancer	83·9 (76–89·4)	83·1 (75·8–88·4)	80·6 (73·1–86·4)	83·3 (76·1–88·6)
Tracheal, bronchus, and lung cancer	85·2 (81·7–88·8)	83·7 (80·1–87·3)	84·6 (80·9–88·1)	83·7 (80–87·3)
Breast cancer	40·2 (30·8–51)	38 (29·4–47·7)	38·4 (29·6–47·9)	38 (29·4–47·7)
Cervical cancer	100 (100–100)	100 (100–100)	100 (100–100)	100 (100–100)
Uterine cancer	52·3 (35·5–67·8)	53·2 (36·7–68·5)	53·4 (36·9–68·8)	53·1 (36·6–68·4)
Ovarian cancer	25·3 (12·8–39·8)	21·3 (10·2–34·5)	20·2 (9·99–32·6)	21·3 (10·2–34·6)
Prostate cancer	5·74 (2·5–9·31)	6·22 (2·83–9·82)	6·6 (3·06–10·3)	6·16 (2·79–9·76)
Kidney cancer	43·5 (32·6–54·4)	43·3 (32·2–53·7)	43 (32–53·3)	43·3 (32·2–53·7)
Bladder cancer	42·9 (28·8–57·3)	44·8 (31·6–58·1)	45·2 (32·2–58)	44·8 (31·5–58)
Thyroid cancer	19·1 (9·79–29·5)	19·8 (10·1–30·6)	19·9 (10·2–31)	19·8 (10·1–30·7)
Mesothelioma	98 (97·2–98·5)	95·8 (94·4–96·8)	97·7 (96·7–98·4)	95·7 (94·3–96·8)
Non-Hodgkin lymphoma	9·76 (4·32–16·7)	9·97 (4·5–17·1)	9·91 (4·43–17)	9·98 (4·49–17)
Multiple myeloma	11 (4·88–18·6)	11·3 (5·14–19)	11·3 (5·1–18·9)	11·3 (5·12–19)
Leukemia	38·6 (25–52·1)	35·5 (24·3–46·7)	34·9 (24–46·3)	35·5 (24·3–46·7)
Cardiovascular diseases	77 (73·2–80·7)	78·5 (75·2–81·5)	72·9 (67·8–77·2)	79·5 (76·3–82·5)
Rheumatic heart disease	23·3 (12·6–45·4)	24·3 (15·2–39·7)	24·5 (15·4–40·3)	24·3 (15·1–40·3)
Ischemic heart disease	93·8 (90·2–96·8)	95 (92·4–97·3)	94·4 (91·5–96·9)	95·1 (92·4–97·3)
Stroke	80·9 (73·4–89)	84·4 (79–89·9)	84·6 (80·1–89·4)	84·3 (78·7–90·3)
Hypertensive heart disease	100 (100–100)	100 (100–100)	100 (100–100)	100 (100–100)
Non-rheumatic valvular heart disease	26·9 (17·1–39·9)	28·2 (19·5–39·5)	22·1 (14·4–30·7)	28·7 (19·9–40·4)
Cardiomyopathy and myocarditis	36·6 (28·8–46·4)	44·1 (37·6–51·1)	39·8 (33–46·8)	44·4 (37·9–51·4)
Atrial fibrillation and flutter	56·4 (45·9–67·1)	64·2 (55·3–72·6)	67·8 (59·5–75·6)	59·5 (49·8–69·5)
Aortic aneurysm	51 (44–58·3)	58·2 (51·9–64·3)	--	58·2 (51·9–64·3)
Endocarditis	32 (21·6–45·5)	35 (25·8–46·1)	32 (22·1–44·3)	35·1 (25·8–46·2)
Other cardiovascular and circulatory diseases	32·3 (25·1–39·5)	35·1 (28·5–41·4)	35·3 (27·5–43·5)	35·1 (28·6–41·5)
Chronic respiratory diseases	63·5 (57·3–69·6)	57·2 (52·5–62)	52 (45·9–57·9)	63·6 (58·1–69·2)
Chronic obstructive pulmonary disease	72·5 (66·5–78)	70·7 (66·3–74·8)	64·6 (60·9–68·2)	74·4 (68·9–79·2)
Pneumoconiosis	100 (100–100)	100 (100–100)	100 (100–100)	100 (100–100)
Asthma	48·6 (35·6–61·5)	46·3 (37·1–56·7)	46·2 (37–56·4)	49·5 (38·1–60·7)
Digestive diseases	37·6 (31·3–43)	43·4 (36·6–49·5)	34·7 (24·4–45·2)	51·6 (44–57·7)

Cirrhosis and other chronic liver diseases	79·6 (66·4–89·2)	82·1 (70·2–90·6)	79·4 (68·2–87·6)	82·1 (70·1–90·8)
Upper digestive system diseases	21·8 (17·8–25·7)	7·19 (4·37–10·7)	0·644 (0·358–1·05)	24·6 (20·9–28·2)
Gallbladder and biliary diseases	52·6 (35·9–68)	55·5 (40·4–69·7)	55·7 (40·5–69·7)	53·4 (37·4–68·2)
Pancreatitis	49 (29·6–65·8)	54·9 (36·7–71·1)	55·5 (37·3–71·5)	54·8 (36·4–71)
Neurological disorders	31·1 (15·3–47·5)	15·4 (7·26–27·8)	8·89 (3·98–18·4)	27·4 (13·7–43)
Alzheimer's disease and other dementias	45·2 (30·2–61·1)	47 (31·8–62)	47·3 (32·1–62·3)	46·8 (31·7–61·9)
Parkinson's disease	-9·09 (-15·1–-4·11)	-10·2 (-16·5–-4·66)	-10·9 (-17·4–-4·96)	-10 (-16·2–-4·56)
Idiopathic epilepsy	32 (21·9–42·2)	30 (20·6–39·1)	28·8 (19·2–38·1)	32·5 (22·7–42·5)
Multiple sclerosis	15·2 (11·3–19·3)	15·3 (11·4–19·3)	14·7 (10·9–18·5)	15·9 (11·9–20·1)
Mental disorders	--	6·26 (2·97–9·95)	6·26 (2·97–9·95)	--
Depressive disorders	--	14·2 (6·38–22·1)	14·2 (6·38–22·1)	--
Anxiety disorders	--	4·99 (1·12–11·9)	4·99 (1·12–11·9)	--
Idiopathic developmental intellectual disability	--	21·5 (7·18–46·9)	21·5 (7·18–46·9)	--
Substance use disorders	100 (100–100)	100 (100–100)	100 (100–100)	100 (100–100)
Alcohol use disorders	100 (100–100)	100 (100–100)	100 (100–100)	100 (100–100)
Drug use disorders	100 (100–100)	100 (100–100)	100 (100–100)	100 (100–100)
Diabetes and kidney diseases	99·8 (99·7–99·9)	100 (100–100)	100 (100–100)	99·8 (99·7–99·8)
Diabetes mellitus	100 (100–100)	100 (100–100)	100 (100–100)	100 (100–100)
Chronic kidney disease	100 (100–100)	100 (100–100)	100 (100–100)	100 (100–100)
Sense organ diseases	--	8·85 (7·8–10)	8·85 (7·8–10)	--
Blindness and vision loss	--	8·97 (5·94–12·8)	8·97 (5·94–12·8)	--
Age-related and other hearing loss	--	10·2 (9·24–11·2)	10·2 (9·24–11·2)	--
Musculoskeletal disorders	2·04 (0·518–3·79)	22·9 (19–26·8)	23·2 (19·3–27·3)	2·44 (0·594–4·36)
Rheumatoid arthritis	12·1 (2·94–21·8)	13·3 (3·13–23·8)	13·3 (3·07–23·8)	13·5 (3·3–24)
Osteoarthritis	--	21·4 (12·1–31·2)	21·4 (12·1–31·2)	--
Low back pain	--	41·9 (36·7–46·9)	41·9 (36·7–46·9)	--
Gout	--	57·7 (40·8–74·6)	57·7 (40·8–74·6)	--
Other non-communicable diseases	0·0973 (0·0579–0·146)	0·0607 (0·0355–0·0958)	--	0·365 (0·207–0·545)
Sudden infant death syndrome	9·11 (5·2–14·9)	9·1 (5·19–14·9)	--	9·1 (5·19–14·9)
Injuries	33·7 (27·7–39·1)	26·9 (22·1–31·5)	27·4 (23·1–32·4)	26·2 (19·6–32·6)
Transport injuries	22·7 (14·3–30·5)	22·9 (15·8–29·1)	26·1 (21·5–30·8)	21 (11·9–29·1)
Road injuries	21·9 (12·9–29·9)	22·1 (14·7–28·5)	25·2 (20·5–29·8)	20·3 (10·5–28·6)
Other transport injuries	32·1 (26·7–37·2)	30·7 (25·6–35·5)	33·2 (28·4–37·7)	28·8 (22·9–34·1)
Unintentional injuries	39·4 (32·9–44·3)	26·7 (22–31·6)	27 (22·2–32·2)	25·6 (20·6–30·3)

Falls	54·5 (46·2–60·4)	37·6 (31·2–43·4)	35·6 (29·4–41·5)	44·4 (37·8–50·4)
Drowning	-13·1 (-26·8–1·73)	-12·4 (-25·8–1·51)	12·2 (6·9–19·2)	-13·7 (-27·3–0·595)
Fire, heat, and hot substances	11·9 (6·57–18·9)	14 (8·3–21·1)	14·6 (8·9–22·1)	12·7 (7·48–19·3)
Poisonings	14·5 (8·8–21·9)	14 (8·58–20·9)	13·5 (8·18–20·3)	15 (9·22–22)
Exposure to mechanical forces	8·02 (-4·28–21·2)	14·4 (11·9–17·2)	15·3 (13–18)	-1·48 (-15·2–13)
Animal contact	21·9 (16·4–28·2)	20·2 (14·8–26·4)	20·9 (15·2–27·5)	18·1 (13·1–24)
Foreign body	2·03 (1·25–3·11)	3·07 (1·95–4·61)	2·92 (1·87–4·43)	3·17 (1·96–4·8)
Environmental heat and cold exposure	10·4 (4·69–17·9)	11·2 (5·13–19·1)	11·1 (5·05–18·9)	11·7 (5·33–19·8)
Exposure to forces of nature	0 (0–0)	11·9 (5·37–20·2)	11·9 (5·37–20·2)	0 (0–0)
Other unintentional injuries	14·5 (8·68–21·8)	14 (8·49–21·1)	13·9 (8·43–21)	15·8 (9·8–23·2)
Self-harm and interpersonal violence	27·9 (15·2–40·3)	29·6 (18–40·7)	35·8 (28·3–43·4)	28·6 (15·9–40·8)
Self-harm	27·9 (14·2–41·5)	29 (15·2–42·4)	28·5 (15·8–41·3)	29·1 (15·2–42·5)
Interpersonal violence	28·9 (20·4–38·4)	33·8 (25·7–42·8)	41 (32–50·7)	26 (17·6–34·9)

Supplementary Results Table 2. All-age PAFs attributable to the joint distribution of all risk factors for all ages, both sexes combined for each GBD level 1, level 2, and level 3 cause and all causes in 2019 for North East England. Results are shown for deaths, YLLs, YLDs, and DALYs Data in parenthesis are 95% uncertainty intervals. PAFs=population attributable fractions. GBD=Global Burden of Disease. YLLs=years of life lost. YLDs=years lived with disability. DALYs=disability-adjusted life-years

	Deaths 2019	DALYs 2019	YLDs 2019	YLLs 2019
All causes	58·3 (55·4–61·2)	46 (43–49)	31·5 (29·2–33·9)	58·2 (55·7–60·8)
Communicable, maternal, neonatal, and nutritional diseases	45·2 (37–52·7)	50·5 (45·8–54·9)	48·2 (44·1–52·3)	51·6 (44·8–58)
HIV/AIDS and sexually transmitted infections	74·1 (57·1–87·5)	75 (56·8–88·7)	97·8 (96·5–98·7)	54·8 (34·5–75·6)
HIV/AIDS	92·8 (90·8–94)	92·1 (89·8–93·6)	94·9 (92·9–96·2)	89·8 (87·3–91·2)
Sexually transmitted infections excluding HIV	53·4 (31·3–78·3)	61 (35·9–84·9)	100 (100–100)	21·6 (9·27–43·8)
Respiratory infections and tuberculosis	46·5 (37·7–54·6)	44·3 (36·7–51·4)	3·4 (2·56–4·47)	49·8 (41·2–57·6)
Tuberculosis	57·5 (47·9–67·6)	60·4 (51·4–69·2)	57·2 (47·6–66·4)	61 (52–69·9)
Lower respiratory infections	46·5 (37·6–54·6)	49·7 (40·9–57·5)	34·4 (27·8–40·3)	49·8 (40·9–57·6)
Upper respiratory infections	0·0328 (0·0238–0·0444)	0·00644 (0·00393–0·0096)	0·00519 (0·00279–0·00836)	0·0807 (0·0583–0·11)
Otitis media	1·62 (1·02–2·44)	4·18 (2·68–5·84)	4·17 (2·69–5·85)	4·39 (2·84–6·35)
Enteric infections	5·5 (3·23–9·53)	8·61 (6·16–12·7)	9·25 (6·67–13·3)	7·39 (4·97–11·3)
Diarrheal diseases	5·57 (3·27–9·61)	8·67 (6·19–12·8)	9·26 (6·68–13·3)	7·52 (5·12–11·5)
Other infectious diseases	0·479 (0·358–0·644)	0·904 (0·717–1·1)	0·397 (0·237–0·662)	1·13 (0·884–1·39)
Meningitis	1·02 (0·763–1·3)	1·72 (1·29–2·18)	0·000399 (0·000198–0·000679)	2·36 (1·8–2·96)
Encephalitis	0·216 (0·166–0·27)	0·544 (0·393–0·7)	0·000131 (6·6e-05–0·00022)	0·738 (0·56–0·93)
Measles	2·32 (-0·592–11·5)	2·9 (-0·673–13·9)	0·671 (0·0189–1·71)	2·98 (-0·741–14·9)
Acute hepatitis	9·69 (4·9–22·7)	4·88 (2·93–8·55)	3·22 (2·01–5·22)	9·23 (4·74–21·1)
Maternal and neonatal disorders	86·6 (84·7–88·3)	77 (73·7–80)	66·8 (61·6–71·5)	87·6 (85·7–89·2)
Maternal disorders	9·55 (3·43–16·1)	9·51 (3·43–16·3)	9·58 (3·48–16·5)	9·4 (3·38–16)
Neonatal disorders	89·3 (87·6–90·9)	78·7 (75·5–81·9)	68·5 (63–73·4)	89·4 (87·6–90·9)
Nutritional deficiencies	71·7 (68·5–86·4)	80 (76·3–83·8)	80·2 (76·5–84·1)	73·4 (66·2–81·1)
Protein-energy malnutrition	100 (100–100)	100 (100–100)	100 (100–100)	100 (100–100)
Vitamin A deficiency	--	100 (100–100)	100 (100–100)	--
Dietary iron deficiency	--	100 (100–100)	100 (100–100)	--
Non-communicable diseases	60·3 (57·2–63·5)	47·1 (43·8–50·3)	31 (28·5–33·6)	60·8 (58·2–63·6)
Neoplasms	53·8 (50–58·3)	52·9 (49·2–57)	38·2 (33·7–43·1)	53·7 (50·1–57·9)
Lip and oral cavity cancer	66·8 (57·7–75·1)	69·4 (60·5–77·3)	68 (58·9–76·3)	69·5 (60·6–77·4)
Nasopharynx cancer	64·5 (53·1–74·9)	64·6 (53·3–74·8)	61 (49–72)	64·9 (53·5–74·9)
Other pharynx cancer	79 (72·4–84·7)	80·1 (73·2–85·5)	79·8 (73–85·5)	80·1 (73·2–85·5)
Esophageal cancer	75·9 (66·2–84·2)	77·3 (67·5–85·2)	76·3 (66·4–84·4)	77·3 (67·5–85·2)
Stomach cancer	26·8 (19·4–43·3)	26·9 (19·5–43·1)	27·1 (19·7–43·5)	26·9 (19·5–43·1)

Colon and rectum cancer	66·5 (57·1–76)	66·7 (57·5–75·7)	66·8 (57·6–75·8)	66·7 (57·6–75·7)
Liver cancer	76·6 (70·8–82·4)	77·1 (71·5–82·4)	77·4 (71·7–82·8)	77·1 (71·5–82·4)
Gallbladder and biliary tract cancer	22·8 (12·3–35·5)	23·7 (12·9–36·5)	21·4 (11·1–33·9)	23·7 (13–36·5)
Pancreatic cancer	47·9 (37·7–60)	47 (37·4–58·9)	47·5 (37·4–59·8)	47 (37·4–58·9)
Larynx cancer	82·8 (75·3–88·7)	82·6 (75·9–87·9)	81·5 (74·4–87)	82·8 (76–88)
Tracheal, bronchus, and lung cancer	88·4 (85·4–91·4)	87·4 (84·5–90·4)	88·2 (85·3–91·2)	87·4 (84·5–90·4)
Breast cancer	39·4 (28·6–53)	38 (28·3–50)	38·2 (28·4–50·5)	38 (28·3–49·9)
Cervical cancer	100 (100–100)	100 (100–100)	100 (100–100)	100 (100–100)
Uterine cancer	44·2 (28·5–60·3)	46·1 (30·3–62)	46·9 (31·2–62·9)	45·9 (30·2–61·9)
Ovarian cancer	32·7 (16·3–49·2)	28 (14–43·5)	26·1 (13–40·9)	28·1 (14–43·6)
Prostate cancer	6·41 (3·01–10)	7·06 (3·31–11)	7·54 (3·6–11·6)	7 (3·26–10·9)
Kidney cancer	41·2 (30·5–51·5)	41·5 (31·3–51·4)	41·5 (31·3–51·4)	41·5 (31·3–51·4)
Bladder cancer	45·4 (29·4–61)	47 (31·8–61·5)	47·4 (33·2–61·3)	47 (31·8–61·5)
Thyroid cancer	15·3 (7·33–24·9)	16·2 (7·99–26·3)	16·4 (8·13–26·2)	16·2 (7·96–26·3)
Mesothelioma	99·3 (99·1–99·5)	98·6 (98·1–99)	99·1 (98·8–99·4)	98·6 (98·1–99)
Non-Hodgkin lymphoma	7·65 (3·2–13·4)	7·97 (3·4–13·8)	7·86 (3·3–13·9)	7·98 (3·39–13·9)
Multiple myeloma	8·64 (3·74–15·4)	9·08 (4·02–16)	8·95 (3·68–16·1)	9·09 (4·04–16·1)
Leukemia	39·4 (24·8–54·1)	37·4 (25·2–49·6)	37·1 (24·8–49·5)	37·4 (25·2–49·6)
Cardiovascular diseases	80·5 (77·2–83·7)	82·1 (79·5–84·7)	72·6 (68·2–76·8)	83·1 (80·6–85·6)
Rheumatic heart disease	24·2 (14–47)	25·6 (16·5–42·3)	25·7 (16·6–43·1)	25·6 (16·5–42·7)
Ischemic heart disease	94 (90·6–96·9)	95·2 (92·9–97·3)	94·5 (91·8–96·9)	95·2 (92·9–97·3)
Stroke	80·4 (73·1–88·9)	83·9 (78·6–89·8)	83·5 (78·6–89)	83·9 (78·5–89·9)
Hypertensive heart disease	100 (100–100)	100 (100–100)	100 (100–100)	100 (100–100)
Non-rheumatic valvular heart disease	27·1 (18·3–39·6)	28 (20·1–38·2)	19·3 (13·5–26·5)	29·2 (20·9–39·6)
Cardiomyopathy and myocarditis	38·5 (30·4–47·4)	44·7 (38·5–50)	38·3 (31·9–44·9)	45·1 (38·6–50·4)
Atrial fibrillation and flutter	50·7 (41–61·1)	59·3 (51–67·7)	63·8 (55·7–71·4)	54·5 (45·4–64·1)
Aortic aneurysm	53·7 (47·4–60·8)	60·6 (55·3–66·3)	--	60·6 (55·3–66·3)
Endocarditis	33·2 (23·4–45·8)	36·3 (27·7–46·5)	32·9 (23·3–45·3)	36·4 (27·7–46·5)
Other cardiovascular and circulatory diseases	34 (28·4–40·2)	37·1 (31·7–42·1)	37·3 (31·1–43·2)	37 (31·6–42·1)
Chronic respiratory diseases	67·1 (61·8–72·8)	62·8 (58·5–67·2)	54·7 (49·6–59·6)	67·5 (62·7–72·7)
Chronic obstructive pulmonary disease	74·3 (70·4–78·2)	73·7 (70·4–76·9)	67·3 (64–70·5)	76 (72·7–79·4)
Pneumoconiosis	100 (100–100)	100 (100–100)	100 (100–100)	100 (100–100)
Asthma	44·2 (32·4–57·2)	44 (35·2–53·9)	43·7 (35–53·6)	46·3 (36·2–57·4)
Digestive diseases	29·7 (25·5–34·5)	36·2 (31·3–41·8)	22·9 (15·9–30·8)	41·3 (36·1–47·3)
Cirrhosis and other chronic liver diseases	69·7 (59·8–81·1)	73·5 (63·9–84·6)	70·9 (60·6–81·3)	73·6 (63·9–84·7)

Upper digestive system diseases	24·7 (21·1–28)	11·4 (7·77–15·3)	0·957 (0·574–1·48)	27·2 (23·6–30·5)
Gallbladder and biliary diseases	44·3 (29·3–59·8)	50 (35·5–64·5)	51·8 (37·2–66·2)	46 (31·2–61·2)
Pancreatitis	30·1 (13·2–47)	37 (19–53·7)	38 (19·7–55·2)	36·8 (18·9–53·6)
Neurological disorders	26·8 (11·1–43·8)	16·7 (8·25–30·1)	10·8 (5·5–20·1)	23 (9·93–39·9)
Alzheimer's disease and other dementias	45·8 (30·3–62)	47·7 (32·5–63·3)	48 (33·1–63·8)	47·5 (32·3–63·2)
Parkinson's disease	-10·7 (-17·5–-5)	-12·3 (-19·6–-5·85)	-12·9 (-20·5–-6·02)	-12·2 (-19·4–-5·8)
Idiopathic epilepsy	25·2 (15·9–34·5)	24·2 (15·2–33·2)	21·8 (13·3–30·2)	27·2 (17·5–37)
Multiple sclerosis	15·5 (11·6–19·5)	16·2 (12·3–20·4)	15·8 (12·1–19·9)	16·4 (12·4–20·7)
Mental disorders	--	6·49 (3·33–9·97)	6·5 (3·33–9·97)	--
Depressive disorders	--	13·2 (6·01–20·9)	13·2 (6·01–20·9)	--
Anxiety disorders	--	4·74 (1·26–10·7)	4·74 (1·26–10·7)	--
Idiopathic developmental intellectual disability	--	42·2 (19·9–60)	42·2 (19·9–60)	--
Substance use disorders	100 (100–100)	100 (100–100)	100 (100–100)	100 (100–100)
Alcohol use disorders	100 (100–100)	100 (100–100)	100 (100–100)	100 (100–100)
Drug use disorders	100 (100–100)	100 (100–100)	100 (100–100)	100 (100–100)
Diabetes and kidney diseases	99·6 (99·6–99·7)	99·9 (99·9–100)	100 (100–100)	99·6 (99·6–99·7)
Diabetes mellitus	100 (100–100)	100 (100–100)	100 (100–100)	100 (100–100)
Chronic kidney disease	100 (100–100)	100 (100–100)	100 (100–100)	100 (100–100)
Sense organ diseases	--	9·3 (8·05–10·8)	9·3 (8·05–10·8)	--
Blindness and vision loss	--	11·2 (7·09–16·2)	11·2 (7·09–16·2)	--
Age-related and other hearing loss	--	9·97 (9·01–11·1)	9·97 (9·01–11·1)	--
Musculoskeletal disorders	4·81 (1·27–8·5)	23·6 (19·5–27·6)	24 (19·8–28·2)	5·62 (1·5–9·81)
Rheumatoid arthritis	12·6 (3·31–22·3)	14·8 (3·82–25·8)	15·1 (3·95–26·3)	14·2 (3·84–24·8)
Osteoarthritis	--	18 (9·73–27·3)	18 (9·73–27·3)	--
Low back pain	--	42·3 (36·8–47·9)	42·3 (36·8–47·9)	--
Gout	--	53·5 (37·9–70·2)	53·5 (37·9–70·2)	--
Other non-communicable diseases	0·0751 (0·0552–0·0957)	0·0726 (0·05–0·101)	--	0·313 (0·219–0·394)
Sudden infant death syndrome	8·04 (5·26–11·8)	8·03 (5·26–11·8)	--	8·03 (5·26–11·8)
Injuries	32·5 (26·4–37·2)	26·8 (22–30·9)	30 (25·8–34)	23·4 (16–29·8)
Transport injuries	18·3 (11·6–25·3)	18·9 (12·9–25·4)	25·3 (20·9–29·3)	16·1 (8·55–23·7)
Road injuries	17·4 (10·4–24·6)	17·9 (11·6–24·6)	24 (19·8–28·1)	15·3 (7·44–23)
Other transport injuries	31·4 (26·1–36·3)	30·8 (25·8–35·7)	35 (30·4–39)	27·7 (21·7–33·3)
Unintentional injuries	40·5 (34·3–44·7)	29·6 (25–33·5)	30·3 (25·5–34·5)	28 (23·2–31·9)
Falls	54·2 (46·4–59·3)	41·1 (35·1–45·8)	39·4 (33·6–44·3)	45·5 (39–50·5)
Drowning	-15·7 (-27·5–-3·37)	-14·5 (-26–-2·69)	9·34 (5·15–14·7)	-15·5 (-27·4–-3·28)

Fire, heat, and hot substances	9·63<br>(5·17–15·1)	11·5<br>(6·81–17·3)	11·6<br>(6·82–17·4)	11·4<br>(6·73–16·9)
Poisonings	13·3<br>(8·19–19·6)	12·5<br>(7·69–18·3)	10·7<br>(6·39–16)	14·2<br>(8·99–20·7)
Exposure to mechanical forces	8·21<br>(-0·437–17·7)	15·4<br>(12·8–17·7)	17·4<br>(14·9–19·3)	-2·04<br>(-10·6–7·48)
Animal contact	22·1<br>(17·6–27·1)	20·2<br>(15·5–25·3)	20·9<br>(16–26·1)	17·9<br>(13·8–22·7)
Foreign body	2·24<br>(1·52–3·22)	3·1<br>(2·11–4·46)	2·37<br>(1·61–3·38)	3·54<br>(2·41–5·08)
Environmental heat and cold exposure	7·65<br>(3·27–13)	8·73<br>(3·98–14·7)	8·62<br>(3·85–14·6)	9·06<br>(4·18–15·1)
Exposure to forces of nature	0<br>(0–0)	9·64<br>(4·36–16)	9·64<br>(4·36–16)	0<br>(0–0)
Other unintentional injuries	12·6<br>(7·61–18·7)	11·1<br>(6·52–16·6)	11<br>(6·47–16·5)	14·3<br>(9·03–20·9)
Self-harm and interpersonal violence	20·9<br>(6·15–34·6)	23·3<br>(9·45–35·7)	32·1<br>(24·5–39·5)	22·3<br>(7·23–35·9)
Self-harm	20·6<br>(5·21–35)	22·2<br>(6·63–36·7)	21·5<br>(9·17–33·6)	22·2<br>(6·55–36·8)
Interpersonal violence	26·4<br>(17·4–35·6)	32·4<br>(23·9–41·5)	38·4<br>(30–47·5)	23·7<br>(14·8–32·9)

Supplementary Results Table 2. All-age PAFs attributable to the joint distribution of all risk factors for all ages, both sexes combined for each GBD level 1, level 2, and level 3 cause and all causes in 2019 for County Durham. Results are shown for deaths, YLLs, YLDs, and DALYs Data in parenthesis are 95% uncertainty intervals. PAFs=population attributable fractions. GBD=Global Burden of Disease. YLLs=years of life lost. YLDs=years lived with disability. DALYs=disability-adjusted life-years

	Deaths 2019	DALYs 2019	YLDs 2019	YLLs 2019
All causes	58·6 (55·7–61·6)	46·2 (43·1–49·3)	31·8 (29·4–34·1)	58·2 (55·7–61)
Communicable, maternal, neonatal, and nutritional diseases	44·8 (37·1–53)	50·5 (46–55·3)	47·9 (43·6–52·4)	51·9 (45·4–58·5)
HIV/AIDS and sexually transmitted infections	74 (56·8–87·7)	75·2 (56·7–89)	97·9 (96·7–98·8)	54·1 (33·6–74·8)
HIV/AIDS	93 (91·1–94·2)	92·3 (90·1–93·9)	94·9 (92·9–96·2)	90·1 (87·7–91·5)
Sexually transmitted infections excluding HIV	55·1 (32·7–79·7)	62·7 (37·9–86)	100 (100–100)	23 (9·88–47)
Respiratory infections and tuberculosis	45·9 (37·5–54·6)	43·6 (36·2–51·1)	3·31 (2·48–4·41)	49·1 (41–57·5)
Tuberculosis	56·8 (45·5–67·9)	59·6 (49–69·5)	56·9 (46–67·6)	60·1 (49·4–70·1)
Lower respiratory infections	45·8 (37·4–54·6)	48·9 (40·7–57·4)	34·1 (27·3–40·3)	49 (40·8–57·5)
Upper respiratory infections	0·0476 (0·0257–0·0839)	0·00642 (0·00153–0·015)	0·00468 (0–0·0135)	0·116 (0·0635–0·197)
Otitis media	1·57 (0·679–3·27)	3·97 (2·51–5·58)	3·97 (2·49–5·59)	4·1 (1·91–7·53)
Enteric infections	5·64 (3·22–10·1)	8·72 (6·22–13·1)	9·26 (6·47–13·6)	7·57 (5·02–12)
Diarrheal diseases	5·72 (3·28–10·2)	8·79 (6·3–13·2)	9·26 (6·48–13·7)	7·72 (5·13–12·2)
Other infectious diseases	0·462 (0·32–0·634)	0·95 (0·705–1·26)	0·414 (0·235–0·71)	1·19 (0·849–1·61)
Meningitis	1·11 (0·759–1·58)	1·89 (1·31–2·64)	0·000367 (0–0·00113)	2·61 (1·81–3·68)
Encephalitis	0·244 (0·16–0·358)	0·639 (0·393–0·951)	0·000121 (0–0·000363)	0·846 (0·548–1·25)
Measles	2·25 (-0·507–12)	2·76 (-0·539–14·4)	0·706 (0·0157–2·04)	2·94 (-0·648–15·9)
Acute hepatitis	9·35 (3·93–24·4)	4·69 (2·64–8·81)	3·33 (1·95–5·58)	9·12 (3·95–23·2)
Maternal and neonatal disorders	87·9 (85·6–89·9)	77·4 (73·8–80·7)	65·7 (59·9–70·7)	88·7 (86·4–90·6)
Maternal disorders	9·44 (3·42–15·6)	9·39 (3·35–16·2)	9·45 (3·35–16·3)	9·28 (3·32–15·5)
Neonatal disorders	90·2 (87·8–92)	78·9 (75·1–82·4)	67·3 (61·3–72·3)	90·2 (87·8–92·1)
Nutritional deficiencies	62·4 (56·4–84·7)	79·2 (74·9–84·2)	79·7 (75·3–84·7)	64·6 (60–80)
Protein-energy malnutrition	100 (100–100)	100 (100–100)	100 (100–100)	100 (100–100)
Vitamin A deficiency	--	100 (100–100)	100 (100–100)	--
Dietary iron deficiency	--	100 (100–100)	100 (100–100)	--
Non-communicable diseases	60·5 (57·4–63·7)	47·3 (44–50·6)	31·3 (28·7–33·9)	60·8 (58–63·6)
Neoplasms	53·1 (48·9–57·8)	52·2 (48·2–56·5)	37·2 (32·3–42·5)	52·9 (49·1–57·3)
Lip and oral cavity cancer	66·5 (55·8–76·3)	69 (58–78·4)	67·6 (56·1–77·6)	69·1 (58·1–78·5)
Nasopharynx cancer	63·4 (49·9–75)	63·5 (50·1–74·8)	59·7 (46·5–72·4)	63·8 (50·5–74·9)
Other pharynx cancer	77·9 (70·1–84·4)	79·1 (70·8–85·3)	78·9 (70·6–85·3)	79·1 (70·7–85·4)
Esophageal cancer	76·2 (65·9–84·5)	77·5 (67·4–85·8)	76·5 (65·7–84·8)	77·5 (67·4–85·8)

Stomach cancer	26·8 (19·1–43·1)	27 (19·3–43)	27·2 (19·5–43·1)	27 (19·3–43)
Colon and rectum cancer	66·7 (56·9–76·7)	66·9 (57·5–76·2)	66·9 (57·4–76·1)	66·9 (57·5–76·2)
Liver cancer	77 (70·5–83·1)	77·4 (71·6–83)	77·8 (71·9–83·5)	77·4 (71·6–83)
Gallbladder and biliary tract cancer	23·4 (12·5–36·2)	24·3 (13·3–37)	22 (11·5–34·4)	24·4 (13·4–37·1)
Pancreatic cancer	48·2 (37·7–60·5)	47·4 (37·4–59·3)	47·9 (37·5–59·9)	47·4 (37·4–59·3)
Larynx cancer	82·2 (74–88·3)	82 (74·7–87·7)	80·9 (73·6–86·8)	82·2 (74·8–87·9)
Tracheal, bronchus, and lung cancer	88 (84·5–91·4)	87·2 (83·7–90·6)	87·8 (84·4–91·2)	87·2 (83·7–90·6)
Breast cancer	39·5 (28·1–53·8)	38·2 (27·6–50·7)	38·4 (27·7–51·1)	38·2 (27·6–50·6)
Cervical cancer	100 (100–100)	100 (100–100)	100 (100–100)	100 (100–100)
Uterine cancer	44·8 (28·9–60·6)	46·6 (31·2–62·2)	47·4 (31·9–62·7)	46·5 (31–62·2)
Ovarian cancer	30·3 (15·8–46·9)	26·4 (13·2–42·1)	24·8 (12·1–39·8)	26·5 (13·2–42·2)
Prostate cancer	6·43 (2·88–10·1)	7·08 (3·27–11·1)	7·54 (3·49–11·8)	7·01 (3·23–10·9)
Kidney cancer	41·6 (31–52·1)	42 (31·3–52)	42 (31·9–52·1)	42 (31·3–52)
Bladder cancer	45·6 (29·6–61·2)	47·1 (32·1–61·7)	47·5 (33·2–61·7)	47·1 (32–61·7)
Thyroid cancer	15·5 (7·58–25·1)	16·4 (8·1–26·4)	16·5 (8·25–26·3)	16·4 (8·04–26·2)
Mesothelioma	99 (98·6–99·3)	97·9 (97·2–98·6)	98·7 (98·2–99·1)	97·9 (97·2–98·5)
Non-Hodgkin lymphoma	7·82 (3·29–13·7)	8·12 (3·47–14)	8·01 (3·34–14)	8·13 (3·47–14)
Multiple myeloma	8·83 (3·78–15·6)	9·27 (4·08–16·3)	9·15 (3·87–16·1)	9·27 (4·08–16·4)
Leukemia	39·7 (25·4–54·2)	37·7 (25·7–50·1)	37·4 (25–49·8)	37·7 (25·8–50)
Cardiovascular diseases	80·6 (77·1–84·1)	82·1 (79·3–84·9)	72·8 (68·3–77·3)	83·1 (80·5–85·9)
Rheumatic heart disease	24·2 (13·5–48·8)	25·5 (16·1–42·7)	25·7 (16·2–41·5)	25·5 (16–43·1)
Ischemic heart disease	93·9 (90·6–97)	95·2 (92·7–97·3)	94·5 (91·9–97)	95·2 (92·8–97·3)
Stroke	80·7 (73·2–88·9)	84·1 (78·6–89·9)	83·7 (78·5–89·2)	84·1 (78·6–90)
Hypertensive heart disease	100 (100–100)	100 (100–100)	100 (100–100)	100 (100–100)
Non-rheumatic valvular heart disease	26·9 (17·7–39·4)	27·8 (19·7–38)	19·6 (13·5–26·6)	28·9 (20·5–39·7)
Cardiomyopathy and myocarditis	37·7 (30·1–46)	43·1 (37·3–48·7)	37·7 (31–44·3)	43·5 (37·4–49·2)
Atrial fibrillation and flutter	50·9 (40·6–61·4)	59·4 (50·6–68·1)	63·8 (55·3–71·5)	54·7 (45·4–64·6)
Aortic aneurysm	53·7 (47·2–60·7)	60·3 (54·5–66·4)	--	60·3 (54·5–66·4)
Endocarditis	33·3 (23·6–46·2)	36·3 (27·5–47)	33·1 (23–45·5)	36·4 (27·6–47·1)
Other cardiovascular and circulatory diseases	34·3 (27·9–41·2)	37·2 (31·3–43)	37·1 (29·9–44·5)	37·2 (31·3–42·9)
Chronic respiratory diseases	68·2 (62·9–73·5)	63·9 (59·8–68·2)	55·3 (50·1–60·5)	68·7 (64·1–73·7)
Chronic obstructive pulmonary disease	74·4 (70·3–78·5)	73·9 (70·4–77·3)	67·4 (63·7–71)	76·1 (72·4–79·7)
Pneumoconiosis	100 (100–100)	100 (100–100)	100 (100–100)	100 (100–100)

Asthma	44·6 (32·7–57·7)	44·8 (35·8–54·9)	44·6 (35·6–54·7)	46·6 (36·2–57·8)
Digestive diseases	29·2 (24·2–35·2)	35·9 (30·2–42·1)	26·1 (18·2–35)	39·9 (33·5–47·5)
Cirrhosis and other chronic liver diseases	68 (56·8–82·2)	71·9 (60·7–85·1)	70 (57·6–82·9)	71·9 (60·7–85·2)
Upper digestive system diseases	24·3 (20·3–27·9)	11·2 (7·53–15·3)	0·98 (0·564–1·52)	26·7 (22·8–30·2)
Gallbladder and biliary diseases	44·9 (29·5–60)	50·8 (36·5–65·2)	52·3 (37·8–66·7)	46·7 (32·2–61·5)
Pancreatitis	28·1 (8·91–48·3)	34·7 (13·5–54·9)	36·7 (14·7–57·1)	34·5 (13·3–54·6)
Neurological disorders	27·2 (11·4–44·3)	17 (8·39–30·6)	11 (5·51–20)	23·2 (9·88–40)
Alzheimer's disease and other dementias	46·2 (30·6–62·5)	48·1 (32·8–63·7)	48·5 (33·2–64)	47·9 (32·7–63·7)
Parkinson's disease	-10·8 (-17·8–-5·09)	-12·3 (-19·9–-5·91)	-12·9 (-20·9–-6·13)	-12·2 (-19·7–-5·85)
Idiopathic epilepsy	24·2 (13·5–34·9)	23·6 (13·4–33·7)	21·3 (11·9–30·9)	26·2 (15·2–37·2)
Multiple sclerosis	15·4 (11·4–19·6)	16·1 (12·1–20·6)	15·8 (11·8–20·1)	16·3 (12·1–20·7)
Mental disorders	--	6·45 (3·31–9·87)	6·45 (3·31–9·87)	--
Depressive disorders	--	13·1 (5·91–20·8)	13·1 (5·91–20·8)	--
Anxiety disorders	--	4·58 (1·21–10·3)	4·58 (1·21–10·3)	--
Idiopathic developmental intellectual disability	--	42·8 (20–61·3)	42·8 (20–61·3)	--
Substance use disorders	100 (100–100)	100 (100–100)	100 (100–100)	100 (100–100)
Alcohol use disorders	100 (100–100)	100 (100–100)	100 (100–100)	100 (100–100)
Drug use disorders	100 (100–100)	100 (100–100)	100 (100–100)	100 (100–100)
Diabetes and kidney diseases	99·6 (99·5–99·6)	99·9 (99·9–99·9)	100 (100–100)	99·6 (99·5–99·6)
Diabetes mellitus	100 (100–100)	100 (100–100)	100 (100–100)	100 (100–100)
Chronic kidney disease	100 (100–100)	100 (100–100)	100 (100–100)	100 (100–100)
Sense organ diseases	--	9·31 (8·05–10·8)	9·31 (8·05–10·8)	--
Blindness and vision loss	--	11·4 (7·27–16·5)	11·4 (7·27–16·5)	--
Age-related and other hearing loss	--	9·93 (8·9–11·1)	9·93 (8·9–11·1)	--
Musculoskeletal disorders	5·05 (1·33–9)	24·2 (20·2–28·4)	24·7 (20·5–29)	5·86 (1·6–10·2)
Rheumatoid arthritis	12·7 (3·51–22·6)	14·9 (3·94–26·4)	15·1 (3·89–26·5)	14·2 (4·08–25)
Osteoarthritis	--	18·2 (9·85–27·4)	18·2 (9·85–27·4)	--
Low back pain	--	42·7 (37·3–48·4)	42·7 (37·3–48·4)	--
Gout	--	54·4 (38·8–71)	54·4 (38·8–71)	--
Other non-communicable diseases	0·0816 (0·0544–0·116)	0·0738 (0·0459–0·111)	--	0·329 (0·213–0·461)
Sudden infant death syndrome	7·69 (4·54–12·6)	7·68 (4·53–12·6)	--	7·68 (4·53–12·6)
Injuries	31·5 (25·2–37·2)	26·5 (21·2–31·6)	30·1 (25·8–34·3)	22·7 (15·1–30·4)
Transport injuries	17·6 (10·6–25·1)	18·5 (11·6–25·4)	25·4 (21·1–29·6)	15·5 (7·19–23·9)

Road injuries	16·8 (9·66–24·5)	17·5 (10·3–24·7)	24·2 (19·9–28·4)	14·8 (6·26–23·4)
Other transport injuries	31·7 (26·2–36·7)	31·3 (25·9–36·2)	35·3 (30·5–39·4)	27·9 (21·7–33·8)
Unintentional injuries	40·2 (33·8–44·4)	29·8 (25·1–34)	30·4 (25·6–34·9)	28·1 (23·2–32·2)
Falls	54·2 (46·2–59·3)	41·4 (35·3–46·5)	39·7 (33·8–45)	46 (39·6–51)
Drowning	-16·1 (-28·7–-3·07)	-14·8 (-27–-2·42)	9·19 (4·82–14·9)	-16 (-28·7–-3·16)
Fire, heat, and hot substances	9·35 (4·78–15·2)	11·4 (6·54–17·5)	11·5 (6·55–17·6)	11·1 (6·35–17·3)
Poisonings	12·6 (7·43–19·3)	11·9 (7·06–18·2)	10·7 (6–16·5)	13·5 (8·29–20·4)
Exposure to mechanical forces	8·73 (-0·0642–18)	15·7 (12·9–18·1)	17·9 (15·4–20)	-1·85 (-10·5–7·42)
Animal contact	20·3 (15·3–26·3)	20·4 (15·4–26·3)	21·1 (16–27)	17 (12·3–22·7)
Foreign body	2·1 (1·4–3·18)	2·99 (2–4·43)	2·45 (1·61–3·62)	3·37 (2·24–5·01)
Environmental heat and cold exposure	7·22 (2·7–12·8)	8·5 (3·54–14·8)	8·48 (3·51–14·7)	8·58 (3·59–14·8)
Exposure to forces of nature	0 (0–0)	9·44 (4·01–16·2)	9·44 (4·01–16·2)	0 (0–0)
Other unintentional injuries	12·2 (7·13–19·1)	11 (6·21–17·3)	10·9 (6·13–17·1)	14 (8·5–21·1)
Self-harm and interpersonal violence	19·8 (4·56–35·2)	22·3 (8·2–36·5)	31·6 (23·7–39·2)	21·2 (5·92–36·5)
Self-harm	19·4 (3·71–35·3)	21 (5·35–36·9)	21·1 (8·44–34·7)	21 (5·12–37)
Interpersonal violence	27·8 (18–37·5)	33·4 (24·5–42·3)	38·3 (29·7–47·2)	25 (15·3–34·5)

Supplementary Results Table 2. All-age PAFs attributable to the joint distribution of all risk factors for all ages, both sexes combined for each GBD level 1, level 2, and level 3 cause and all causes in 2019 for Czech Republic. Results are shown for deaths, YLLs, YLDs, and DALYs Data in parenthesis are 95% uncertainty intervals. PAFs=population attributable fractions. GBD=Global Burden of Disease. YLLs=years of life lost. YLDs=years lived with disability. DALYs=disability-adjusted life-years

	Deaths 2019	DALYs 2019	YLDs 2019	YLLs 2019
All causes	67·4 (64·1–70·5)	52·9 (49·6–56·2)	36·9 (34–39·6)	65·1 (62·5–67·6)
Communicable, maternal, neonatal, and nutritional diseases	52·3 (46·2–58·2)	51·6 (47·8–55·7)	43·8 (38·9–48·8)	58 (52·9–62·6)
HIV/AIDS and sexually transmitted infections	90·8 (90–91·6)	92·7 (90·8–94·9)	98·9 (98·1–99·4)	85·8 (84·7–86·8)
HIV/AIDS	88·6 (87·8–89·4)	85 (83·9–86·2)	91·9 (90·7–92·9)	83·8 (82·6–84·9)
Sexually transmitted infections excluding HIV	99·9 (99·9–100)	100 (100–100)	100 (100–100)	99·8 (99·7–99·9)
Respiratory infections and tuberculosis	53·8 (47–60·4)	50·3 (44–56·7)	6·08 (4·54–8·13)	58·1 (51·4–64·3)
Tuberculosis	66·8 (58·7–73·9)	69·8 (62·2–75·9)	64·9 (56·4–71·9)	71·2 (63·9–77·2)
Lower respiratory infections	53·8 (46·9–60·5)	58 (51·2–64·4)	43·1 (37·2–48·9)	58·2 (51·3–64·5)
Upper respiratory infections	0·116 (0·059–0·204)	0·0205 (0·00959–0·0379)	0·00474 (0·000416–0·0111)	0·385 (0·204–0·667)
Otitis media	1·93 (0·669–4·83)	4·3 (2·79–5·96)	4·25 (2·72–5·89)	6·37 (2·5–13·4)
Enteric infections	21·5 (9·43–37·3)	23·2 (11·2–39)	23 (10·5–38·8)	24·2 (12·6–39·4)
Diarrheal diseases	22·2 (9·79–38·4)	23·4 (11·3–39·2)	23 (10·5–38·8)	25 (13–41)
Other infectious diseases	0·913 (0·66–1·21)	1·36 (0·977–1·87)	0·324 (0·203–0·522)	1·94 (1·41–2·66)
Meningitis	1·36 (0·908–1·99)	2·79 (1·84–4·05)	0·000582 (3·63e-05–0·00144)	3·51 (2·38–5·05)
Encephalitis	0·362 (0·237–0·566)	0·768 (0·499–1·14)	0·000216 (1·27e-05–0·000539)	0·933 (0·615–1·4)
Measles	3·26 (0·301–12·5)	2·53 (0·471–5·65)	2·08 (0·0658–4·87)	4·7 (0·446–17·9)
Acute hepatitis	7·65 (4·36–10·6)	3·34 (2·09–4·72)	1·82 (1·13–2·87)	8·05 (4·59–11)
Maternal and neonatal disorders	77·4 (75–79·8)	60·2 (55·6–64·2)	49·9 (45·2–54·3)	78 (75·5–80·4)
Maternal disorders	11·8 (4·13–19·4)	11·8 (4·13–19·5)	11·8 (4·14–19·5)	11·7 (4·1–19·3)
Neonatal disorders	78·9 (76·4–81·3)	61·4 (56·7–65·3)	51 (46·1–55·7)	78·9 (76·4–81·3)
Nutritional deficiencies	88·6 (47·1–93·6)	93·3 (87·2–95·4)	94·3 (92·3–95·9)	87·7 (49·4–92·6)
Protein-energy malnutrition	100 (100–100)	100 (100–100)	100 (100–100)	100 (100–100)
Vitamin A deficiency	--	100 (100–100)	100 (100–100)	--
Dietary iron deficiency	--	100 (100–100)	100 (100–100)	--
Non-communicable diseases	69·8 (66–73·1)	55·3 (51·4–59)	36·4 (33–39·6)	68·5 (65·7–71·3)
Neoplasms	49·9 (45·7–54·8)	50·1 (46·2–54·5)	38 (33·5–42·8)	50·6 (46·7–55·1)
Lip and oral cavity cancer	76 (70·6–80·4)	78·2 (73·2–82·4)	75·6 (70–80·4)	78·3 (73·3–82·5)
Nasopharynx cancer	71·5 (63·5–78·3)	72·2 (64·6–78·7)	71·4 (63·2–78·1)	72·3 (64·6–78·8)
Other pharynx cancer	85·3 (81–88·7)	85·8 (81·5–89·1)	82·6 (77·6–86·5)	85·9 (81·6–89·2)
Esophageal cancer	82·4 (74·4–89·3)	83·6 (76·1–90·3)	82·3 (74·5–89·1)	83·6 (76·1–90·3)

Stomach cancer	27·7 (17·9–47·2)	28·1 (18·1–47·3)	27·9 (18–47·3)	28·1 (18·1–47·3)
Colon and rectum cancer	65·8 (57·2–74·5)	66·5 (58·1–74·6)	66·5 (58·1–74·5)	66·5 (58·1–74·6)
Liver cancer	80·8 (74·8–85·9)	80·4 (74·2–85·5)	80·8 (74·6–85·9)	80·4 (74·2–85·5)
Gallbladder and biliary tract cancer	27·7 (16·1–41)	28 (16·4–41·4)	27·7 (16–40·7)	28 (16·4–41·4)
Pancreatic cancer	44·6 (34·4–56·5)	44·4 (34·6–55·9)	44·5 (34·5–56·2)	44·4 (34·6–55·8)
Larynx cancer	85·4 (78·9–90)	86·2 (80·3–90·5)	85 (78·9–89·4)	86·3 (80·4–90·5)
Tracheal, bronchus, and lung cancer	85·4 (81·8–89·2)	85·1 (81·6–88·8)	85·1 (81·5–88·9)	85·1 (81·6–88·8)
Breast cancer	39·5 (29·4–51·2)	38·5 (29·2–48·6)	38·3 (29·3–48·8)	38·5 (29·2–48·7)
Cervical cancer	100 (100–100)	100 (100–100)	100 (100–100)	100 (100–100)
Uterine cancer	53 (37·5–67·6)	53·6 (37·9–68·2)	53·6 (38·1–68·4)	53·5 (37·8–68·1)
Ovarian cancer	19·5 (7·85–34·7)	17·5 (6·82–31·1)	17·2 (6·8–31·3)	17·5 (6·83–31·1)
Prostate cancer	7·26 (3·43–11·1)	7·95 (3·83–12·1)	8·36 (4·04–12·8)	7·9 (3·8–12·1)
Kidney cancer	43·8 (33·3–53·6)	44·3 (33·9–54·3)	44·2 (33·9–53·9)	44·3 (33·9–54·3)
Bladder cancer	45·9 (32–59·4)	49·2 (36·5–61·9)	50·1 (37·3–62·6)	49·1 (36·2–61·9)
Thyroid cancer	18·4 (9·95–28·1)	18·7 (10·1–28·5)	17·4 (9·84–26·3)	18·9 (10·1–28·9)
Mesothelioma	91 (88–93·7)	85·9 (81·3–89·8)	91·5 (88·2–94·1)	85·8 (81·2–89·7)
Non-Hodgkin lymphoma	9·27 (4·19–15·7)	9·29 (4·18–15·7)	9·34 (4·17–15·8)	9·29 (4·17–15·7)
Multiple myeloma	10·7 (4·92–18)	10·9 (5·02–18·3)	10·9 (5·12–18·1)	10·9 (5·02–18·4)
Leukemia	39·2 (26·7–51·9)	37·9 (26·7–49·5)	39·7 (27·6–52·2)	37·8 (26·6–49·4)
Cardiovascular diseases	86·2 (82·6–89·2)	86·2 (83·4–88·7)	78 (73·9–81·8)	87·3 (84·5–89·7)
Rheumatic heart disease	23·6 (13·5–42·5)	25·6 (16·4–40·5)	25·9 (16–41·3)	25·6 (16·2–40·5)
Ischemic heart disease	93·5 (89·5–96·7)	94·7 (91·9–97·1)	94·7 (92–97)	94·7 (91·9–97·1)
Stroke	82·6 (75·5–90·9)	86 (81·2–91·6)	86·4 (81·8–91)	85·9 (80·7–91·8)
Hypertensive heart disease	100 (100–100)	100 (100–100)	100 (100–100)	100 (100–100)
Non-rheumatic valvular heart disease	26·7 (16·8–38·1)	27·1 (18·5–36·8)	21 (14·2–28·7)	28·4 (19–38·4)
Cardiomyopathy and myocarditis	39·7 (29·3–47·4)	45·2 (37·1–51·7)	36·9 (29·7–44·4)	45·8 (37·5–52·4)
Atrial fibrillation and flutter	56·5 (47–66·5)	65·1 (56·8–72·9)	68·1 (60·3–75·3)	60·2 (51·2–69·2)
Aortic aneurysm	61 (55·6–66·7)	68·2 (63·5–72·9)	--	68·2 (63·5–72·9)
Endocarditis	44 (32·3–55·6)	46·2 (35–57·2)	42 (30·2–53·9)	46·3 (35·1–57·3)
Other cardiovascular and circulatory diseases	35·3 (28·6–41·7)	39·5 (33·1–45·4)	40·7 (34·1–47·1)	39·1 (32·6–45)
Chronic respiratory diseases	73·4 (69·5–77·4)	68·2 (64·6–71·9)	58·8 (54·4–62·6)	74·1 (70·3–78·2)
Chronic obstructive pulmonary disease	78·6 (75·2–81·6)	77 (74·2–79·7)	69·5 (66·5–72·5)	80·5 (77·6–83·3)
Pneumoconiosis	100 (100–100)	100 (100–100)	100 (100–100)	100 (100–100)
Asthma	51·1 (39·1–63)	45·4 (36·6–55)	43·9 (35·4–53)	53·2 (42·5–64·2)

Digestive diseases	51·7 (46·7–56·3)	51·4 (45·5–56·6)	24 (17·1–31·4)	60·9 (55·3–65·7)
Cirrhosis and other chronic liver diseases	84 (75·6–91·1)	85·1 (76·7–91·8)	80·5 (73·3–86·8)	85·2 (76·7–91·9)
Upper digestive system diseases	25·6 (22·1–28·9)	13·4 (9·07–18·1)	1·48 (0·87–2·32)	30·1 (26·5–33·8)
Gallbladder and biliary diseases	52·6 (37·4–67·2)	55·1 (41–68·5)	55·7 (41·8–68·8)	53·7 (39–68·1)
Pancreatitis	52·1 (42·1–64·5)	56·5 (46·2–68·3)	53·3 (43–64·8)	56·7 (46·5–68·8)
Neurological disorders	31·3 (15·3–47·4)	21 (11·6–34·4)	14·6 (8·23–24·7)	29·2 (14·6–45·1)
Alzheimer's disease and other dementias	44·2 (28·8–61·1)	46·9 (31·5–63·4)	46·9 (31·7–62·9)	46·8 (31·4–63·3)
Parkinson's disease	-10·3 (-16–-5·26)	-12·1 (-18·7–-6·23)	-12·7 (-19·4–-6·59)	-12 (-18·5–-6·15)
Idiopathic epilepsy	33·2 (25·3–41)	29·9 (22·7–37·1)	28·2 (21·2–35·3)	33·2 (25·4–41)
Multiple sclerosis	17·9 (13·3–22·8)	19·6 (14·7–24·6)	19·5 (14·9–24·4)	19·6 (14·7–24·7)
Mental disorders	--	5·55 (2·52–8·86)	5·55 (2·52–8·87)	--
Depressive disorders	--	12·3 (4·54–20·8)	12·3 (4·54–20·8)	--
Anxiety disorders	--	2·33 (0·624–5·41)	2·33 (0·624–5·41)	--
Idiopathic developmental intellectual disability	--	47·6 (21·1–68·2)	47·6 (21·1–68·2)	--
Substance use disorders	100 (100–100)	100 (100–100)	100 (100–100)	100 (100–100)
Alcohol use disorders	100 (100–100)	100 (100–100)	100 (100–100)	100 (100–100)
Drug use disorders	100 (100–100)	100 (100–100)	100 (100–100)	100 (100–100)
Diabetes and kidney diseases	99·9 (99·9–100)	100 (100–100)	100 (100–100)	99·9 (99·9–99·9)
Diabetes mellitus	100 (100–100)	100 (100–100)	100 (100–100)	100 (100–100)
Chronic kidney disease	100 (100–100)	100 (100–100)	100 (100–100)	100 (100–100)
Sense organ diseases	--	9·02 (7·89–10·3)	9·02 (7·89–10·3)	--
Blindness and vision loss	--	5·69 (3·38–8·75)	5·69 (3·38–8·75)	--
Age-related and other hearing loss	--	10·7 (9·81–11·8)	10·7 (9·81–11·8)	--
Musculoskeletal disorders	3·87 (1·05–6·9)	35·5 (30–41)	35·8 (30·3–41·3)	3·93 (1·13–6·96)
Rheumatoid arthritis	13 (3·5–23·2)	17 (4·92–29·6)	17·3 (5·04–30·2)	15·3 (4·28–26·9)
Osteoarthritis	--	23·3 (13·1–33·8)	23·3 (13·1–33·8)	--
Low back pain	--	44·9 (38·9–50·7)	44·9 (38·9–50·7)	--
Gout	--	57·8 (42·2–73·6)	57·8 (42·2–73·6)	--
Other non-communicable diseases	0·0772 (0·0462–0·117)	0·0429 (0·0226–0·0727)	--	0·276 (0·156–0·421)
Sudden infant death syndrome	7·54 (4·3–11·8)	7·53 (4·29–11·7)	--	7·53 (4·29–11·7)
Injuries	35·1 (29·5–39·9)	34·7 (30·1–39·4)	37·2 (32·7–41·7)	31·2 (25·1–36·7)
Transport injuries	33·1 (27–39·3)	35·5 (30–41)	35·9 (31·2–40·6)	35 (28·1–42·2)
Road injuries	30·7 (24·6–37·1)	34·5 (29·1–40·1)	35·6 (31–40·3)	33 (25·5–40·6)
Other transport injuries	45·6 (40·1–51·1)	44·8 (39·2–50·6)	44 (38·9–48·6)	45·1 (39–51·4)

Unintentional injuries	40·2 (34·1–45·1)	36·2 (30·8–41·1)	37·4 (32·3–42·2)	33 (27·6–38·1)
Falls	55 (47·4–60·4)	47·7 (41·6–52·9)	46·7 (40·9–51·9)	50·2 (43·7–55·8)
Drowning	3·57 (-7·68–15·2)	7·4 (-3·33–18·2)	17·5 (12·7–23·2)	6·71 (-4·65–18)
Fire, heat, and hot substances	17·8 (12·4–23·9)	25·7 (19·7–32·8)	26 (19·9–33)	24·6 (18·5–31·6)
Poisonings	24·7 (18·2–31·6)	27·1 (21·2–34·1)	23·7 (18·4–30·1)	30·9 (23·8–39)
Exposure to mechanical forces	19·9 (11·1–28·4)	29·4 (24·2–35·3)	30·1 (25·5–35·7)	18·8 (8·96–28·4)
Animal contact	32·9 (26·7–39·4)	31 (25·3–37·4)	30·3 (24·8–36·7)	33·7 (27·2–40·6)
Foreign body	12·6 (9·56–16·5)	15·9 (12·1–20·6)	13·2 (9·75–17·3)	16·9 (12·9–22·3)
Environmental heat and cold exposure	12·2 (5·76–20·5)	12·5 (5·87–20·9)	10·5 (4·98–17·6)	13 (6·12–21·7)
Exposure to forces of nature	0 (0–0)	11·6 (5·46–19·4)	11·6 (5·46–19·4)	0 (0–0)
Other unintentional injuries	26·3 (19·5–33·9)	25·7 (19·9–32·6)	24·5 (18·8–31·2)	31·9 (24·7–40·4)
Self-harm and interpersonal violence	26·4 (13·9–38·6)	28·7 (17·3–39·6)	40·2 (33·4–47)	26·9 (14·5–38·8)
Self-harm	25·5 (12·1–38·5)	26·3 (13–39·1)	27·6 (16·1–38·4)	26·2 (12·9–39·2)
Interpersonal violence	38·9 (30·1–48)	40·8 (32·9–49)	43·9 (35·9–51·5)	35·9 (27–44·8)

Supplementary Results Table 2. All-age PAFs attributable to the joint distribution of all risk factors for all ages, both sexes combined for each GBD level 1, level 2, and level 3 cause and all causes in 2019 for Darlington. Results are shown for deaths, YLLs, YLDs, and DALYs Data in parenthesis are 95% uncertainty intervals. PAFs=population attributable fractions. GBD=Global Burden of Disease. YLLs=years of life lost. YLDs=years lived with disability. DALYs=disability-adjusted life-years

	Deaths 2019	DALYs 2019	YLDs 2019	YLLs 2019
All causes	57·3 (54·1–60·6)	45·4 (42·2–48·6)	31·9 (29·6–34·4)	57·2 (54·4–59·9)
Communicable, maternal, neonatal, and nutritional diseases	44·9 (35·1–54·9)	50·2 (44·7–55·4)	48·5 (43·8–52·9)	51·1 (43·1–58·9)
HIV/AIDS and sexually transmitted infections	74·1 (57·2–88·1)	73·1 (53·7–88·3)	98 (96·9–98·9)	52·3 (32·1–74·4)
HIV/AIDS	92·5 (90·3–93·7)	91·9 (89·5–93·5)	94·9 (92·9–96·2)	89·3 (86·6–90·9)
Sexually transmitted infections excluding HIV	60·9 (38·9–83·3)	61 (36·1–84·6)	100 (100–100)	26·8 (12–51·8)
Respiratory infections and tuberculosis	46·1 (35·4–56·9)	43·6 (34·7–52·3)	3·3 (2·43–4·42)	49·3 (39–59·2)
Tuberculosis	58·8 (46·9–68·9)	61·7 (50·7–70·9)	57·9 (45·9–67·5)	62·3 (51·4–71·4)
Lower respiratory infections	46·1 (35·2–57·1)	49·1 (38·8–59·3)	34·4 (27·5–41)	49·2 (38·8–59·5)
Upper respiratory infections	0·0284 (0·0151–0·0491)	0·0068 (0·0013–0·0159)	0·00548 (0–0·0147)	0·0754 (0·0398–0·134)
Otitis media	1·12 (0·429–2·36)	4·41 (2·81–6·24)	4·42 (2·81–6·27)	3·47 (1·47–6·76)
Enteric infections	5·33 (3·09–9·6)	8·65 (6·12–13)	9·25 (6·57–13·6)	7·06 (4·6–11·2)
Diarrheal diseases	5·41 (3·17–9·75)	8·72 (6·18–13·1)	9·26 (6·58–13·7)	7·26 (4·81–11·6)
Other infectious diseases	0·512 (0·362–0·731)	1·03 (0·772–1·36)	0·377 (0·208–0·662)	1·29 (0·944–1·72)
Meningitis	1·11 (0·744–1·57)	1·99 (1·36–2·78)	0·000458 (0–0·00124)	2·56 (1·76–3·57)
Encephalitis	0·21 (0·139–0·302)	0·543 (0·338–0·79)	0·000147 (0–0·000404)	0·746 (0·496–1·04)
Measles	2·05 (-0·518–11)	2·32 (-0·424–11·5)	0·621 (0·0177–1·86)	2·89 (-0·739–15·1)
Acute hepatitis	9·11 (3·72–21)	4·87 (2·65–8·75)	3·15 (1·84–5·38)	8·56 (3·66–19·5)
Maternal and neonatal disorders	89·3 (87·4–91)	78·2 (74·8–81·4)	67·7 (62·3–72·6)	89·9 (88–91·5)
Maternal disorders	9·48 (3·38–16·1)	9·45 (3·4–16·2)	9·49 (3·37–16·5)	9·32 (3·38–15·8)
Neonatal disorders	90·9 (89·1–92·5)	79·7 (75·9–82·9)	69·5 (63·6–74·4)	90·9 (89·1–92·5)
Nutritional deficiencies	72·2 (67–88·6)	80·1 (75·9–84·8)	80·3 (76–85)	73·3 (68·2–83·5)
Protein-energy malnutrition	100 (100–100)	100 (100–100)	100 (100–100)	100 (100–100)
Vitamin A deficiency	--	100 (100–100)	100 (100–100)	--
Dietary iron deficiency	--	100 (100–100)	100 (100–100)	--
Non-communicable diseases	59·3 (55·9–62·6)	46·5 (43·2–50)	31·4 (28·9–34·1)	59·7 (56·9–62·6)
Neoplasms	52 (47·8–56·7)	51·3 (47·3–55·8)	36 (31·2–41·3)	52·2 (48·2–56·6)
Lip and oral cavity cancer	66·5 (54·5–76·3)	69·3 (57·4–78·3)	67·9 (55·8–77·5)	69·4 (57·4–78·4)
Nasopharynx cancer	62·1 (48·7–73·6)	62·5 (49·4–73·6)	59·1 (45·7–71·4)	62·8 (49·6–73·8)
Other pharynx cancer	78·8 (70·8–85·2)	79·9 (72·1–86)	79·7 (71·2–86·1)	79·9 (72·1–86·1)

Esophageal cancer	75·5 (65·7–84·3)	77 (66·9–85·5)	75·9 (65·8–84·7)	77 (67–85·5)
Stomach cancer	26·4 (18·7–43·2)	26·6 (19·3–42·7)	26·8 (19·2–43)	26·6 (19·3–42·7)
Colon and rectum cancer	66·4 (56·6–76·2)	66·6 (57·6–75·7)	66·7 (57·4–76·1)	66·6 (57·6–75·7)
Liver cancer	75·5 (68·6–82)	76·4 (70·3–82·2)	76·7 (70·2–82·6)	76·4 (70·2–82·2)
Gallbladder and biliary tract cancer	23·1 (12·2–35·8)	23·9 (13–36·9)	21·6 (11·5–34·2)	24 (13–37)
Pancreatic cancer	48·1 (37·4–60·6)	47·2 (37·3–59·2)	47·7 (37–60·2)	47·2 (37·3–59·2)
Larynx cancer	82·8 (75·2–89·1)	82·8 (75·9–88·4)	81·8 (74·6–87·5)	82·9 (75·9–88·5)
Tracheal, bronchus, and lung cancer	88·1 (84·7–91·3)	87·1 (83·7–90·4)	87·9 (84·5–91·2)	87·1 (83·7–90·4)
Breast cancer	39·6 (28·1–53·2)	38·1 (27·9–50·1)	38·3 (27·9–50·5)	38·1 (27·8–50·1)
Cervical cancer	100 (100–100)	100 (100–100)	100 (100–100)	100 (100–100)
Uterine cancer	45·2 (28·6–60·9)	47·1 (30·5–62·5)	48 (31·7–63·4)	47 (30·4–62·4)
Ovarian cancer	30·5 (15·6–47)	26·1 (13–42)	24·3 (12·2–38·9)	26·2 (13–42)
Prostate cancer	6·3 (2·85–10·1)	6·93 (3·24–10·9)	7·41 (3·48–11·4)	6·86 (3·22–10·7)
Kidney cancer	41·7 (30·9–52·3)	42 (31·6–52·2)	42 (31·6–51·9)	42 (31·6–52·2)
Bladder cancer	45·1 (28·9–60·4)	46·6 (31·3–60·6)	47·1 (32·1–60·9)	46·6 (31·3–60·6)
Thyroid cancer	15·7 (7·64–25·2)	16·6 (8·26–26·6)	16·8 (8·44–26·5)	16·6 (8·24–26·6)
Mesothelioma	99·3 (99·1–99·6)	98·6 (98·1–99·1)	99·2 (98·8–99·4)	98·6 (98·1–99·1)
Non-Hodgkin lymphoma	7·96 (3·32–14)	8·3 (3·55–14·4)	8·16 (3·44–14·3)	8·31 (3·55–14·4)
Multiple myeloma	8·97 (3·75–16)	9·42 (4·04–16·7)	9·28 (3·76–16·9)	9·43 (4·06–16·6)
Leukemia	39·4 (24·5–54·1)	37·5 (25·2–49·9)	36·8 (24·7–49·1)	37·6 (25·2–50)
Cardiovascular diseases	79·9 (76·1–83·6)	81·5 (78·5–84·4)	72·6 (67·5–77)	82·5 (79·5–85·3)
Rheumatic heart disease	24·2 (13·4–46·8)	25·7 (16·2–43·3)	25·7 (16–41·9)	25·7 (16·2–43·1)
Ischemic heart disease	93·9 (90·5–96·9)	95·2 (92·7–97·3)	94·5 (91·7–96·9)	95·2 (92·7–97·3)
Stroke	80·2 (71·9–88·6)	83·7 (77·8–89·7)	83·5 (78–89·4)	83·7 (77·4–90)
Hypertensive heart disease	100 (100–100)	100 (100–100)	100 (100–100)	100 (100–100)
Non-rheumatic valvular heart disease	27·4 (17·6–41)	27·5 (19·2–38·1)	13·4 (8·64–19·1)	29·4 (20·8–40·8)
Cardiomyopathy and myocarditis	35·8 (27·9–45·9)	41·8 (35·7–48·5)	36·8 (30–44·1)	42 (35·8–48·8)
Atrial fibrillation and flutter	51·1 (40·5–62·2)	59·4 (50·7–68·4)	64·1 (55·7–72·3)	54·6 (44·9–65·1)
Aortic aneurysm	53·5 (46·8–61·3)	60·5 (54·6–66·7)	--	60·5 (54·6–66·7)
Endocarditis	33·2 (22·6–47·3)	36·5 (27–47·6)	33·3 (23·3–46·7)	36·6 (27·1–47·7)
Other cardiovascular and circulatory diseases	34·5 (27·4–42·3)	37·8 (31·7–44·3)	37·8 (30·4–45)	37·8 (31·4–44·4)
Chronic respiratory diseases	65·8 (59·9–71·9)	61·6 (57·3–66·1)	54·8 (49·6–59·8)	66·3 (61·2–71·9)
Chronic obstructive pulmonary disease	73·9 (69·2–78·5)	73·1 (69·2–77)	67·1 (63·1–70·9)	75·6 (71·4–79·5)

Pneumoconiosis	100 (100–100)	100 (100–100)	100 (100–100)	100 (100–100)
Asthma	44·6 (32·5–57·7)	44·5 (35·3–54·5)	44·2 (35·3–54·1)	46·9 (36·3–58·4)
Digestive diseases	26·9 (22·3–31·9)	35·5 (29·7–41·7)	27·9 (19·1–37·4)	38·7 (32·6–45·4)
Cirrhosis and other chronic liver diseases	70·3 (58·6–83·9)	74·2 (62·4–87)	70·5 (57·3–82·8)	74·3 (62·3–87·1)
Upper digestive system diseases	24·9 (20·9–28·5)	11·3 (7·31–15·5)	0·764 (0·442–1·19)	27·7 (23·7–31·4)
Gallbladder and biliary diseases	44·7 (29–61·4)	52·9 (38·2–67·5)	55 (39·9–69·4)	46·4 (31·2–62)
Pancreatitis	29·9 (8·77–49·5)	36·9 (13·5–55·9)	37·3 (13·6–57·5)	36·9 (13·4–55·9)
Neurological disorders	26·9 (10·8–44·2)	17 (8·33–30·5)	11·1 (5·58–20·2)	23 (9·95–40)
Alzheimer's disease and other dementias	46·3 (30·5–62·4)	48·1 (32·6–63·9)	48·4 (32·9–64·3)	47·9 (32·2–63·8)
Parkinson's disease	-10·5 (-17·1–-5)	-12·1 (-19·4–-5·72)	-12·7 (-20·4–-6·03)	-11·9 (-19·3–-5·71)
Idiopathic epilepsy	25·2 (13–36·4)	24·5 (12·8–35·4)	21·9 (11·3–31·9)	27·1 (14·6–38·7)
Multiple sclerosis	15·2 (11·1–19·1)	16 (11·7–20·2)	15·7 (11·6–19·7)	16·1 (11·8–20·4)
Mental disorders	--	6·43 (3·22–9·95)	6·43 (3·22–9·95)	--
Depressive disorders	--	13·3 (6·11–20·9)	13·3 (6·11–20·9)	--
Anxiety disorders	--	4·84 (1·31–10·9)	4·84 (1·31–10·9)	--
Idiopathic developmental intellectual disability	--	39·8 (17·7–60·9)	39·8 (17·7–60·9)	--
Substance use disorders	100 (100–100)	100 (100–100)	100 (100–100)	100 (100–100)
Alcohol use disorders	100 (100–100)	100 (100–100)	100 (100–100)	100 (100–100)
Drug use disorders	100 (100–100)	100 (100–100)	100 (100–100)	100 (100–100)
Diabetes and kidney diseases	99·7 (99·6–99·8)	100 (99·9–100)	100 (100–100)	99·7 (99·7–99·8)
Diabetes mellitus	100 (100–100)	100 (100–100)	100 (100–100)	100 (100–100)
Chronic kidney disease	100 (100–100)	100 (100–100)	100 (100–100)	100 (100–100)
Sense organ diseases	--	9·19 (7·89–10·7)	9·19 (7·89–10·7)	--
Blindness and vision loss	--	11·4 (7·16–16·6)	11·4 (7·16–16·6)	--
Age-related and other hearing loss	--	9·75 (8·77–11)	9·75 (8·77–11)	--
Musculoskeletal disorders	3·86 (1–7·01)	23·5 (19·4–27·8)	24 (19·7–28·4)	4·58 (1·21–8·09)
Rheumatoid arthritis	12·4 (3·34–22·3)	14·7 (3·99–25·8)	14·9 (4·07–26·2)	14 (3·77–24·7)
Osteoarthritis	--	18·5 (10·1–27·9)	18·5 (10·1–27·9)	--
Low back pain	--	42·6 (36·9–48·5)	42·6 (36·9–48·5)	--
Gout	--	54·6 (38·5–71·4)	54·6 (38·5–71·4)	--
Other non-communicable diseases	0·109 (0·0709–0·153)	0·108 (0·0681–0·161)	--	0·45 (0·296–0·623)
Sudden infant death syndrome	12·7 (7·19–19·7)	12·7 (7·19–19·7)	--	12·7 (7·19–19·7)
Injuries	31·5 (24·8–37·3)	26·7 (20·9–31·6)	29·7 (25·2–34)	23·3 (14·3–31·1)

Transport injuries	19·1 (11·4–26·8)	19·6 (12·5–26·5)	25·5 (21·2–29·7)	16·9 (8·03–25·3)
Road injuries	17·8 (9·63–25·9)	18·3 (10·9–25·4)	24·2 (19·9–28·5)	15·6 (6·24–24·5)
Other transport injuries	31·5 (25·7–36·8)	30·5 (24·9–35·8)	35·1 (30·2–39·2)	28·2 (21·9–34·1)
Unintentional injuries	40·2 (33·8–44·8)	29·5 (24·6–33·7)	30 (24·9–34·6)	28 (22·9–32·3)
Falls	54·3 (46·1–59·6)	40·8 (34·6–46)	39·2 (33–44·1)	45·7 (38·7–50·9)
Drowning	-15·4 (-29·9—0·936)	-14 (-27·7—0·287)	9·5 (5–15)	-15·3 (-29·5—1)
Fire, heat, and hot substances	8·84 (4·38–14·3)	11·6 (6·71–17·7)	11·9 (6·85–18·3)	10·6 (5·96–16·1)
Poisonings	12·3 (7·19–18·3)	12 (7·02–18)	10·8 (6·17–16·6)	13·4 (8·05–19·7)
Exposure to mechanical forces	9·23 (-1·8–20·8)	16·4 (13·6–18·9)	17·7 (15·1–20·1)	-0·904 (-12·4–11·6)
Animal contact	24·2 (19·2–28·6)	19·3 (15–24·3)	20·9 (16·1–26·3)	16·5 (12·6–20·9)
Foreign body	2·44 (1·49–3·8)	3·32 (2·08–5·15)	2·59 (1·59–4·05)	3·92 (2·44–6·09)
Environmental heat and cold exposure	8·23 (3·29–14·3)	8·8 (3·66–15·1)	8·66 (3·57–14·9)	9·38 (4·04–16)
Exposure to forces of nature	0 (0–0)	9·66 (4·01–16·5)	9·66 (4·01–16·5)	0 (0–0)
Other unintentional injuries	12·8 (7·31–19·1)	11·4 (6·54–17·3)	11·2 (6·43–17·2)	14·6 (8·93–21·4)
Self-harm and interpersonal violence	21·1 (4·18–35·6)	23·5 (7·6–37·2)	31·6 (22·8–39·5)	22·5 (5·31–37·1)
Self-harm	20·7 (3·23–35·6)	22·3 (4·72–37·3)	21·8 (7·55–34·8)	22·3 (4·73–37·3)
Interpersonal violence	30·3 (20·5–40·1)	34·6 (25·3–44·3)	38·3 (29–48)	27·5 (17·6–37·5)

Supplementary Results Table 2. All-age PAFs attributable to the joint distribution of all risk factors for all ages, both sexes combined for each GBD level 1, level 2, and level 3 cause and all causes in 2019 for Gateshead. Results are shown for deaths, YLLs, YLDs, and DALYs Data in parenthesis are 95% uncertainty intervals. PAFs=population attributable fractions. GBD=Global Burden of Disease. YLLs=years of life lost. YLDs=years lived with disability. DALYs=disability-adjusted life-years

	Deaths 2019	DALYs 2019	YLDs 2019	YLLs 2019
All causes	58·2 (55·2–61·4)	46·2 (43·1–49·2)	31·5 (29·1–34)	58·4 (55·7–61·2)
Communicable, maternal, neonatal, and nutritional diseases	45·7 (36–55·3)	52·5 (47·2–57·7)	51·3 (46·8–56)	53·2 (45·7–60·6)
HIV/AIDS and sexually transmitted infections	72·3 (54·1–87·5)	73·1 (52·9–88·3)	98·1 (97–99)	51·1 (30·3–73·7)
HIV/AIDS	93·2 (91·3–94·4)	92·6 (90·2–94·1)	95·1 (93–96·4)	90·5 (88–91·9)
Sexually transmitted infections excluding HIV	54·7 (32·7–79·6)	60·6 (35·1–84·7)	100 (100–100)	22·9 (9·92–47)
Respiratory infections and tuberculosis	46·9 (36·1–57·3)	44·6 (35·7–53·3)	3·45 (2·57–4·69)	50·3 (40·1–59·8)
Tuberculosis	58 (46–69·3)	61·1 (49·8–70·9)	58 (46·2–68·6)	61·6 (50·4–71·3)
Lower respiratory infections	46·8 (36–57·2)	50 (39·8–59·6)	34·8 (27·6–41·5)	50·1 (39·9–59·8)
Upper respiratory infections	0·0554 (0·0281–0·101)	0·00722 (0·0015–0·0172)	0·00556 (0–0·0156)	0·133 (0·0666–0·242)
Otitis media	3·24 (1·32–8·25)	4·22 (2·68–5·96)	4·22 (2·68–5·96)	7·72 (3·54–17·9)
Enteric infections	5·33 (3·07–9·45)	8·56 (6·08–12·8)	9·18 (6·52–13·6)	7·3 (4·83–11·2)
Diarrheal diseases	5·42 (3·12–9·58)	8·64 (6·15–12·9)	9·19 (6·53–13·6)	7·49 (5–11·5)
Other infectious diseases	0·559 (0·369–1)	1·05 (0·783–1·44)	0·398 (0·23–0·677)	1·35 (0·964–1·91)
Meningitis	1·37 (0·923–2)	2·18 (1·46–3·13)	0·000445 (0–0·00139)	3·12 (2·13–4·54)
Encephalitis	0·263 (0·181–0·381)	0·685 (0·432–0·997)	0·00016 (0–0·000494)	0·899 (0·61–1·3)
Measles	3·57 (-1·1–18·4)	4·34 (-1·24–22·1)	0·63 (0·0157–1·75)	4·49 (-1·32–22·5)
Acute hepatitis	6·43 (1·72–25)	4·44 (1·99–11·5)	3·2 (1·9–5·4)	6·2 (1·69–24·1)
Maternal and neonatal disorders	90·2 (88·3–91·8)	80·3 (77·1–83·4)	71 (65·7–75·8)	90·8 (89–92·3)
Maternal disorders	9·67 (3·41–16·3)	9·62 (3·42–16·5)	9·65 (3·42–16·6)	9·52 (3·37–16·2)
Neonatal disorders	91·7 (89·9–93·3)	81·7 (78·3–84·9)	72·7 (67·2–77·6)	91·8 (89·9–93·3)
Nutritional deficiencies	60·2 (54–84·3)	80·1 (75·8–84·6)	80·5 (76·2–85·1)	64 (58·9–79·4)
Protein-energy malnutrition	100 (100–100)	100 (100–100)	100 (100–100)	100 (100–100)
Vitamin A deficiency	--	100 (100–100)	100 (100–100)	--
Dietary iron deficiency	--	100 (100–100)	100 (100–100)	--
Non-communicable diseases	60·2 (57–63·4)	47·2 (43·9–50·5)	30·9 (28·3–33·5)	61 (58·2–63·9)
Neoplasms	54·4 (50·3–58·9)	53·5 (49·7–57·7)	39·1 (34·3–44·3)	54·1 (50·4–58·3)
Lip and oral cavity cancer	68·2 (56·4–78·1)	70·7 (59·7–80·1)	69·4 (58·1–79·1)	70·8 (59·9–80·2)
Nasopharynx cancer	66·7 (53·7–77·8)	66·6 (54·2–77·3)	63·2 (50·5–75)	66·8 (54·4–77·4)
Other pharynx cancer	78·2 (70·4–84·8)	79·4 (71·5–85·8)	79·2 (71·3–85·7)	79·4 (71·4–85·8)

Esophageal cancer	75·8 (65·5–84·1)	77·2 (67·6–85·2)	76·3 (65·9–84·4)	77·3 (67·7–85·2)
Stomach cancer	26·7 (18·9–43·4)	26·7 (18·9–43)	26·9 (19·1–43·7)	26·7 (18·9–43)
Colon and rectum cancer	66·4 (56·9–75·8)	66·6 (57·5–75·2)	66·7 (57·5–75·4)	66·6 (57·5–75·2)
Liver cancer	75·6 (69·2–81·8)	75·7 (69·9–81·5)	76·2 (69·7–82·3)	75·7 (69·9–81·5)
Gallbladder and biliary tract cancer	22·3 (11·9–34·9)	23·1 (12·4–35·5)	20·8 (10·7–33·5)	23·2 (12·5–35·6)
Pancreatic cancer	47·4 (36·8–59·7)	46·5 (36·4–58·4)	47 (36·7–59·3)	46·5 (36·4–58·4)
Larynx cancer	83·1 (75·5–89·3)	82·9 (75·7–88·6)	81·8 (74·3–87·7)	83 (75·8–88·7)
Tracheal, bronchus, and lung cancer	88·4 (85–91·6)	87·3 (83·9–90·5)	88·1 (84·8–91·3)	87·3 (83·9–90·5)
Breast cancer	39·2 (28–52·8)	37·6 (27·5–49·4)	37·9 (27·8–50·1)	37·6 (27·5–49·4)
Cervical cancer	100 (100–100)	100 (100–100)	100 (100–100)	100 (100–100)
Uterine cancer	44·2 (27·9–59·9)	46 (30·3–62·1)	46·9 (31·4–63)	45·9 (30·2–61·9)
Ovarian cancer	32·7 (16·2–49·1)	27·8 (13·9–43)	25·7 (12·7–40·1)	27·8 (13·9–43·1)
Prostate cancer	6·35 (2·92–10·2)	6·99 (3·29–11)	7·47 (3·52–11·7)	6·92 (3·25–10·9)
Kidney cancer	40·8 (30·2–51·3)	41·1 (30·5–51·2)	41 (30·4–51)	41·1 (30·5–51·2)
Bladder cancer	45·2 (29·6–60·6)	46·8 (32·5–61·1)	47·2 (33·5–61·1)	46·7 (32·5–61)
Thyroid cancer	15·1 (7·18–24·6)	16 (7·78–25·8)	16·2 (7·91–26·3)	16 (7·76–26)
Mesothelioma	99·2 (98·9–99·5)	98·3 (97·7–98·8)	99 (98·6–99·3)	98·3 (97·7–98·8)
Non-Hodgkin lymphoma	7·56 (3·11–13·5)	7·87 (3·3–13·8)	7·76 (3·23–13·7)	7·88 (3·3–13·8)
Multiple myeloma	8·52 (3·62–15·3)	8·96 (3·93–16)	8·79 (3·51–15·8)	8·97 (3·95–15·9)
Leukemia	39·3 (24·7–53·9)	37·1 (24·8–49·4)	37·3 (24·5–49·8)	37·1 (24·8–49·4)
Cardiovascular diseases	80·4 (76·6–83·9)	82·1 (79·1–84·9)	72·9 (67·8–77·4)	83·1 (80·3–85·8)
Rheumatic heart disease	24·2 (13·7–46·5)	25·8 (16·3–42·9)	25·9 (16·5–43·1)	25·8 (16·3–43·1)
Ischemic heart disease	94 (90·7–96·8)	95·3 (92·9–97·4)	94·5 (91·7–96·9)	95·3 (93–97·4)
Stroke	80·4 (72·5–89·2)	83·9 (77·9–90)	83·6 (78·2–89·3)	84 (77·8–90·3)
Hypertensive heart disease	100 (100–100)	100 (100–100)	100 (100–100)	100 (100–100)
Non-rheumatic valvular heart disease	26·8 (17·5–40·2)	28·3 (20·2–39)	23·1 (15·8–32·4)	28·9 (20·4–39·6)
Cardiomyopathy and myocarditis	39·4 (30·7–49)	45·8 (38·9–52·1)	39·1 (32·5–46·4)	46·3 (39–52·6)
Atrial fibrillation and flutter	50·6 (39·4–62·2)	59·1 (49·7–68)	63·6 (54·9–71·6)	54·2 (44–64·7)
Aortic aneurysm	53·2 (46·2–61)	60·3 (54–66·5)	--	60·3 (54–66·5)
Endocarditis	33·2 (22·9–47·8)	36·4 (27–47·2)	32·8 (22·3–46·7)	36·5 (27–47·2)
Other cardiovascular and circulatory diseases	33·8 (26·9–41·1)	36·9 (30·5–43·3)	37·4 (29·7–45)	36·8 (30·4–43·3)
Chronic respiratory diseases	66·6 (60·6–72·9)	62·4 (57·6–67·4)	54·7 (49·3–59·7)	66·9 (61·2–73)

Chronic obstructive pulmonary disease	74·2 (69·3–78·6)	73·6 (69·6–77·3)	67·3 (63·6–71·1)	75·8 (71·6–79·6)
Pneumoconiosis	100 (100–100)	100 (100–100)	100 (100–100)	100 (100–100)
Asthma	44 (31·8–56·9)	44·3 (35·3–54·2)	44 (35·2–54·1)	46·3 (35·8–57·3)
Digestive diseases	29·5 (24·5–34·9)	35·9 (30·5–41·8)	19·3 (13·2–26·2)	41·2 (35–48·1)
Cirrhosis and other chronic liver diseases	71 (60·1–84·3)	74·6 (63·8–87·2)	71·9 (59·4–84·1)	74·7 (63·9–87·3)
Upper digestive system diseases	25·6 (21·5–29·5)	12·7 (8·68–17·1)	1·05 (0·619–1·63)	28·4 (24·4–32·1)
Gallbladder and biliary diseases	44·2 (28·6–60·8)	49·1 (34·8–64·1)	51·2 (37–65·6)	46 (31–62·1)
Pancreatitis	29·8 (9·76–51·5)	36·8 (13·9–58·2)	38·6 (15·2–59·9)	36·6 (13·7–58)
Neurological disorders	25·9 (9·89–43)	16·2 (8·01–29·6)	10·6 (5·23–20·3)	22·2 (8·87–39·7)
Alzheimer's disease and other dementias	45·6 (30·4–61·9)	47·4 (32·4–63·3)	47·8 (32·7–63·8)	47·2 (32·1–63·1)
Parkinson's disease	-10·7 (-17·6–-4·79)	-12·2 (-19·6–5·62)	-12·9 (-20·9–-5·95)	-12·1 (-19·4–5·58)
Idiopathic epilepsy	25·6 (14·1–37)	24·5 (13·3–35·3)	21·9 (11·8–31·8)	27·5 (15·7–38·8)
Multiple sclerosis	15·7 (11·8–20·2)	16·4 (12·4–20·7)	16 (12–20·5)	16·7 (12·6–21·2)
Mental disorders	--	6·46 (3·26–10)	6·47 (3·26–10)	--
Depressive disorders	--	13·3 (6·05–20·9)	13·3 (6·05–20·9)	--
Anxiety disorders	--	4·63 (1·16–10·5)	4·63 (1·16–10·5)	--
Idiopathic developmental intellectual disability	--	42·7 (19·1–62·1)	42·7 (19·1–62·1)	--
Substance use disorders	100 (100–100)	100 (100–100)	100 (100–100)	100 (100–100)
Alcohol use disorders	100 (100–100)	100 (100–100)	100 (100–100)	100 (100–100)
Drug use disorders	100 (100–100)	100 (100–100)	100 (100–100)	100 (100–100)
Diabetes and kidney diseases	99·7 (99·7–99·8)	100 (99·9–100)	100 (100–100)	99·7 (99·7–99·8)
Diabetes mellitus	100 (100–100)	100 (100–100)	100 (100–100)	100 (100–100)
Chronic kidney disease	100 (100–100)	100 (100–100)	100 (100–100)	100 (100–100)
Sense organ diseases	--	9·28 (8·03–10·8)	9·28 (8·03–10·8)	--
Blindness and vision loss	--	11·1 (7·09–16·2)	11·1 (7·09–16·2)	--
Age-related and other hearing loss	--	9·97 (8·89–11·2)	9·97 (8·89–11·2)	--
Musculoskeletal disorders	4·59 (1·18–8·42)	23·3 (19·2–27·4)	23·8 (19·6–28)	5·35 (1·46–9·49)
Rheumatoid arthritis	12·6 (3·39–22·4)	14·9 (4·23–25·7)	15·2 (4·35–26)	14·3 (3·97–24·7)
Osteoarthritis	--	17·8 (9·6–27)	17·8 (9·6–27)	--
Low back pain	--	42·4 (36·7–48·4)	42·4 (36·7–48·4)	--
Gout	--	53 (37·2–69·8)	53 (37·2–69·8)	--
Other non-communicable diseases	0·126 (0·0779–0·191)	0·12 (0·0689–0·197)	--	0·515 (0·304–0·788)

Sudden infant death syndrome	10·6<br>(5·94–17·9)	10·6<br>(5·93–17·8)	--	10·6<br>(5·93–17·8)
Injuries	33<br>(26·8–38·5)	26·8<br>(21·8–31·8)	29·8<br>(25·3–34·2)	23·5<br>(16·4–30·4)
Transport injuries	17·9<br>(9·93–26·3)	18·6<br>(11·6–25·9)	25<br>(20·6–29·8)	15·7<br>(7·04–24·6)
Road injuries	17<br>(8·73–25·8)	17·6<br>(10·3–25·2)	23·8<br>(19·4–28·5)	14·9<br>(5·96–24·2)
Other transport injuries	31·2<br>(25·6–36·3)	30·3<br>(25–35·6)	34·5<br>(29·5–38·8)	26·9<br>(21–33)
Unintentional injuries	40·2<br>(33·8–44·7)	29·3<br>(24·4–33·9)	30·1<br>(25·3–34·9)	27·7<br>(22·8–32·1)
Falls	53·8<br>(45·9–59)	40·7<br>(34·6–45·9)	39·1<br>(32·9–44·3)	44·8<br>(38·4–50)
Drowning	-14·9<br>(-29·5–-0·241)	-14<br>(-27·9–-0·114)	9·44<br>(5·14–15)	-14·9<br>(-29·3–-0·615)
Fire, heat, and hot substances	9·45<br>(4·75–15)	11·5<br>(6·52–17·5)	11·7<br>(6·65–17·7)	11·1<br>(6·33–17)
Poisonings	14·6<br>(8·63–21·9)	13·3<br>(7·98–20)	10·9<br>(6·25–16·5)	15·4<br>(9·44–22·7)
Exposure to mechanical forces	8·15<br>(-3·35–19·8)	15·1<br>(12·3–17·7)	17·1<br>(14·6–19·1)	-2·47<br>(-14·2–10·2)
Animal contact	21·4<br>(16·2–27·8)	20·1<br>(15·3–25·9)	20·6<br>(15·7–26·2)	18·9<br>(13·8–25·1)
Foreign body	2·28<br>(1·41–3·38)	3·04<br>(1·91–4·47)	2·31<br>(1·48–3·47)	3·51<br>(2·17–5·21)
Environmental heat and cold exposure	7·55<br>(3·02–13·3)	8·81<br>(3·82–15)	8·73<br>(3·79–14·8)	9·02<br>(3·93–15·3)
Exposure to forces of nature	0<br>(0–0)	9·73<br>(4·14–16·6)	9·73<br>(4·14–16·6)	0<br>(0–0)
Other unintentional injuries	12·6<br>(7·24–19·2)	11·2<br>(6·33–17·2)	11·1<br>(6·25–17)	14·2<br>(8·53–21·1)
Self-harm and interpersonal violence	21·4<br>(6·33–35·9)	23·8<br>(9·88–37·3)	32·3<br>(23·6–40·4)	22·7<br>(7·45–37·3)
Self-harm	21·2<br>(5·15–36·4)	22·8<br>(6·39–37·9)	22·1<br>(8·5–34·7)	22·8<br>(6·34–38·1)
Interpersonal violence	24·3<br>(14·1–34·8)	31·4<br>(22·6–41·1)	38·4<br>(29·4–48)	21·8<br>(11·9–32·1)

Supplementary Results Table 2. All-age PAFs attributable to the joint distribution of all risk factors for all ages, both sexes combined for each GBD level 1, level 2, and level 3 cause and all causes in 2019 for Hartlepool. Results are shown for deaths, YLLs, YLDs, and DALYs Data in parenthesis are 95% uncertainty intervals. PAFs=population attributable fractions. GBD=Global Burden of Disease. YLLs=years of life lost. YLDs=years lived with disability. DALYs=disability-adjusted life-years				
	**Deaths 2019**	**DALYs 2019**	**YLDs 2019**	**YLLs 2019**
All causes	59·5 (56·4–62·7)	47·8 (44·6–50·9)	31·9 (29·6–34·2)	59·6 (56·9–62·4)
Communicable, maternal, neonatal, and nutritional diseases	45·5 (35·7–54·8)	50·1 (44·3–55·6)	46·2 (41·6–50·7)	51·8 (43·8–59·5)
HIV/AIDS and sexually transmitted infections	73·9 (56·6–87·2)	75·1 (57·5–88·4)	97·5 (96·2–98·5)	56 (35·6–76·4)
HIV/AIDS	92·7 (90·9–93·8)	91·9 (89·8–93·5)	95 (93·2–96·2)	89·4 (87·1–90·8)
Sexually transmitted infections excluding HIV	45·4 (23·6–73·8)	58 (33–83·7)	100 (100–100)	17·1 (6·58–38·6)
Respiratory infections and tuberculosis	46·7 (35·9–56·8)	45 (36·1–53·7)	3·6 (2·63–4·8)	50 (39·8–59·7)
Tuberculosis	56 (44·7–67·8)	59 (48·2–69·6)	56·3 (44·5–67·5)	59·4 (48·5–69·9)
Lower respiratory infections	46·6 (35·7–56·8)	49·8 (39·5–59·4)	34·9 (28·3–41·4)	49·9 (39·5–59·5)
Upper respiratory infections	0·0546 (0·0303–0·0907)	0·00564 (0·00043–0·0159)	0·00528 (5·96e-05–0·0157)	0·136 (0·0767–0·228)
Otitis media	1·3 (0·576–2·5)	4·21 (2·66–5·92)	4·23 (2·65–6·01)	3·52 (1·74–6·47)
Enteric infections	6·09 (3·46–11·1)	9·06 (6·26–14·3)	9·86 (6·99–15)	7·75 (4·95–12·7)
Diarrheal diseases	6·15 (3·5–11·1)	9·12 (6·32–14·4)	9·87 (6·99–15)	7·87 (4·99–12·9)
Other infectious diseases	0·501 (0·327–0·681)	0·832 (0·626–1·09)	0·368 (0·208–0·619)	1·07 (0·754–1·44)
Meningitis	0·881 (0·597–1·28)	1·42 (0·981–2·03)	0·000375 (3·31e-06–0·00117)	1·97 (1·35–2·83)
Encephalitis	0·209 (0·13–0·329)	0·466 (0·29–0·744)	0·000124 (8·59e-07–0·00037)	0·692 (0·439–1·1)
Measles	2·01 (-0·589–11·7)	2·37 (-0·583–13·4)	0·805 (0·0185–2·28)	2·56 (-0·751–15·1)
Acute hepatitis	13 (6·66–25)	5·36 (3·2–8·7)	3·16 (1·82–5·14)	12·5 (6·57–23·8)
Maternal and neonatal disorders	85·9 (81·6–89·6)	73·9 (69·3–77·8)	59·5 (53·4–65)	86·8 (82·6–90·6)
Maternal disorders	9·73 (3·49–16·6)	9·65 (3·48–16·9)	9·67 (3·51–16·8)	9·61 (3·44–16·5)
Neonatal disorders	88·4 (84·2–92·3)	75·6 (70·9–79·6)	61·1 (54·7–66·9)	88·5 (84·2–92·3)
Nutritional deficiencies	88·5 (80·6–91·6)	81·3 (77·1–85·8)	81·1 (76·6–85·9)	87·6 (75·7–90)
Protein-energy malnutrition	100 (100–100)	100 (100–100)	100 (100–100)	100 (100–100)
Vitamin A deficiency	--	100 (100–100)	100 (100–100)	--
Dietary iron deficiency	--	100 (100–100)	100 (100–100)	--
Non-communicable diseases	61·6 (58·4–64·8)	49·2 (45·6–52·5)	31·6 (29–34·2)	62·2 (59·4–65·1)
Neoplasms	54·9 (50·8–59·6)	54·2 (50·4–58·6)	39·9 (35·2–45·5)	54·9 (51·1–59·3)
Lip and oral cavity cancer	64·7 (52·9–75)	67·5 (55·6–77·2)	66 (53·6–76·7)	67·6 (55·8–77·3)
Nasopharynx cancer	65·9 (52·1–76·8)	65·6 (51·8–76·1)	62·1 (48·3–73·6)	65·8 (52·1–76·3)
Other pharynx cancer	79·3 (71·8–86)	80·2 (72·4–86·6)	79·9 (71·9–86·4)	80·2 (72·4–86·6)
Esophageal cancer	75·3 (65·2–84·1)	76·7 (66·7–85·3)	75·7 (65·3–84·4)	76·7 (66·7–85·3)
Stomach cancer	26·7 (19·1–43·3)	26·8 (19·3–43·2)	27 (19·3–43·5)	26·8 (19·3–43·2)

Colon and rectum cancer	66·5 (57–76·3)	66·6 (57·6–75·6)	66·7 (57·6–76)	66·6 (57·5–75·6)
Liver cancer	75·6 (69·2–81·5)	76·1 (70·2–81·6)	76·3 (70·1–82·2)	76·1 (70·1–81·6)
Gallbladder and biliary tract cancer	22·5 (11·7–35·3)	23·3 (12·4–36·1)	21·2 (10·6–33·4)	23·4 (12·5–36·2)
Pancreatic cancer	47·6 (36·6–60·6)	46·6 (35·9–58·9)	47·2 (36·3–59·8)	46·6 (35·9–58·9)
Larynx cancer	82·8 (75–88·9)	82·7 (75·3–88·5)	81·6 (73·9–87·5)	82·8 (75·4–88·6)
Tracheal, bronchus, and lung cancer	88·6 (85·2–91·6)	87·5 (84·2–90·5)	88·3 (85–91·3)	87·5 (84·2–90·5)
Breast cancer	39·1 (27·5–53)	37·6 (26·8–50·2)	37·8 (27·1–50·7)	37·6 (26·8–50·2)
Cervical cancer	100 (100–100)	100 (100–100)	100 (100–100)	100 (100–100)
Uterine cancer	42·3 (26·6–57·9)	44·2 (28·6–60·3)	45 (29·2–61·3)	44·1 (28·5–60·2)
Ovarian cancer	34·5 (16·3–51)	29·2 (14·1–44·8)	27·1 (13·2–42·2)	29·3 (14·1–44·8)
Prostate cancer	6·44 (3·02–10·2)	7·09 (3·41–11)	7·54 (3·63–11·7)	7·03 (3·36–10·9)
Kidney cancer	40·1 (29·8–50·5)	40·3 (30·1–50·1)	40·2 (29·9–49·9)	40·3 (30·1–50·1)
Bladder cancer	46 (30·1–61·7)	47·5 (32·6–62·2)	47·9 (33·5–62·3)	47·5 (32·6–62·3)
Thyroid cancer	14·1 (6·64–23·3)	15 (7·23–24·5)	15·2 (7·46–24·7)	15 (7·22–24·5)
Mesothelioma	99·5 (99·3–99·7)	99 (98·6–99·3)	99·4 (99·1–99·6)	99 (98·6–99·3)
Non-Hodgkin lymphoma	7·24 (2·91–12·8)	7·55 (3·12–13·2)	7·43 (3·04–13·2)	7·55 (3·12–13·2)
Multiple myeloma	8·05 (3·45–14·6)	8·48 (3·66–15·2)	8·34 (3·19–15·2)	8·49 (3·69–15·1)
Leukemia	38·9 (24·5–54·1)	36·7 (24·3–49·5)	37·2 (24·4–50·5)	36·7 (24·3–49·5)
Cardiovascular diseases	81·4 (77·7–84·9)	83·2 (80·3–86)	74 (69–78·5)	84·1 (81·3–86·7)
Rheumatic heart disease	24 (13·1–48·5)	25·2 (15·9–42·8)	25·4 (15·8–41·5)	25·2 (15·8–42·7)
Ischemic heart disease	93·9 (90·6–96·9)	95·2 (92·7–97·4)	94·4 (91·7–96·9)	95·2 (92·8–97·4)
Stroke	80·4 (72·5–88·8)	83·9 (78·3–89·8)	83·3 (78·1–88·9)	84 (78·2–90)
Hypertensive heart disease	100 (100–100)	100 (100–100)	100 (100–100)	100 (100–100)
Non-rheumatic valvular heart disease	26·7 (17–40·4)	28·2 (19·1–39·4)	22·6 (15·2–32·1)	28·7 (19·3–40·3)
Cardiomyopathy and myocarditis	39·7 (31·9–49)	46·2 (39·5–52·7)	37·5 (30·4–45·5)	46·6 (39·7–53·1)
Atrial fibrillation and flutter	49·4 (38·9–61·6)	57·4 (48·4–66·8)	62·3 (53·7–70·9)	53·1 (43·3–64·1)
Aortic aneurysm	53·9 (46·8–61·5)	60·7 (54·6–67·1)	--	60·7 (54·6–67·1)
Endocarditis	33 (22·6–46·5)	36 (26·7–47·2)	32·5 (22·2–45·9)	36·1 (26·7–47·3)
Other cardiovascular and circulatory diseases	32·8 (26·2–40·2)	35·5 (29–42·1)	36·2 (28·4–43·7)	35·4 (29–42·1)
Chronic respiratory diseases	67·6 (61·7–73·3)	62·9 (58·1–67·7)	53·7 (48·2–59)	67·9 (62·6–73·2)
Chronic obstructive pulmonary disease	74·1 (69·3–78·6)	73·7 (69·9–77·4)	67·4 (63·5–71·5)	75·8 (71·7–79·6)
Pneumoconiosis	100 (100–100)	100 (100–100)	100 (100–100)	100 (100–100)
Asthma	43·2 (31·2–55·8)	42·7 (33·7–52·3)	42·4 (33·5–52)	45 (34·8–55·5)
Digestive diseases	31·3 (25·8–37·6)	37·8 (31·7–45)	19·1 (12·7–26·4)	43·1 (36–51·1)

Cirrhosis and other chronic liver diseases	69 (57·9–82·7)	72·8 (61·7–85·8)	69·6 (58·4–83·3)	72·8 (61·7–85·8)
Upper digestive system diseases	23·6 (19·6–27·2)	10·6 (6·98–14·6)	0·8 (0·47–1·28)	25·8 (21·9–29·3)
Gallbladder and biliary diseases	41·9 (27·3–57·5)	47·3 (33·3–61·9)	50 (35·9–64·4)	43·7 (29·5–59)
Pancreatitis	28·7 (8·72–49·9)	35·1 (12·7–56·1)	35·3 (12·7–56·4)	35·1 (12·8–56)
Neurological disorders	25·8 (9·99–43·9)	16·3 (8·12–29·9)	10·6 (5·24–20·2)	22·2 (9·31–39·2)
Alzheimer's disease and other dementias	45·7 (30·3–62·7)	47·5 (32·2–63·6)	47·8 (32·3–64·1)	47·3 (31·9–63·6)
Parkinson's disease	-10·9 (-17·5–-5·22)	-12·4 (-20–-6·04)	-12·9 (-20·3–-6·13)	-12·3 (-19·8–-6·05)
Idiopathic epilepsy	24·8 (12·5–36·4)	23·7 (12·1–34·6)	20·8 (9·83–31·4)	26·3 (13·7–38)
Multiple sclerosis	15·9 (11·7–20)	16·5 (12·2–20·7)	16 (11·9–20·1)	16·8 (12·3–21·1)
Mental disorders	--	6·58 (3·38–10·1)	6·58 (3·38–10·1)	--
Depressive disorders	--	13·3 (6·02–21)	13·3 (6·02–21)	--
Anxiety disorders	--	4·84 (1·32–10·8)	4·84 (1·32–10·8)	--
Idiopathic developmental intellectual disability	--	42·5 (18·6–62·2)	42·5 (18·6–62·2)	--
Substance use disorders	100 (100–100)	100 (100–100)	100 (100–100)	100 (100–100)
Alcohol use disorders	100 (100–100)	100 (100–100)	100 (100–100)	100 (100–100)
Drug use disorders	100 (100–100)	100 (100–100)	100 (100–100)	100 (100–100)
Diabetes and kidney diseases	99·6 (99·4–99·6)	99·9 (99·9–99·9)	100 (100–100)	99·6 (99·5–99·6)
Diabetes mellitus	100 (100–100)	100 (100–100)	100 (100–100)	100 (100–100)
Chronic kidney disease	100 (100–100)	100 (100–100)	100 (100–100)	100 (100–100)
Sense organ diseases	--	9·37 (8·07–10·9)	9·37 (8·07–10·9)	--
Blindness and vision loss	--	11·2 (7·01–16·5)	11·2 (7·01–16·5)	--
Age-related and other hearing loss	--	10·1 (8·99–11·3)	10·1 (8·99–11·3)	--
Musculoskeletal disorders	5·2 (1·3–9·14)	23 (18·9–27·1)	23·4 (19·2–27·6)	6·03 (1·55–10·6)
Rheumatoid arthritis	12·7 (3·15–22·5)	14·8 (3·96–25·8)	15·1 (4·05–26·2)	14·3 (3·79–25)
Osteoarthritis	--	17 (9·04–26·1)	17 (9·04–26·1)	--
Low back pain	--	41·8 (35·9–47·4)	41·8 (35·9–47·4)	--
Gout	--	52 (36·6–69)	52 (36·6–69)	--
Other non-communicable diseases	0·0612 (0·0401–0·0909)	0·067 (0·0421–0·105)	--	0·256 (0·167–0·372)
Sudden infant death syndrome	8·22 (4·75–13·6)	8·22 (4·74–13·6)	--	8·22 (4·74–13·6)
Injuries	29·4 (23·1–35·2)	25·1 (19·7–30·2)	28·9 (24·7–33·1)	21·3 (13·2–28·6)
Transport injuries	17·9 (9·89–26)	18·3 (11·2–25·8)	25·1 (20·6–29·4)	15·5 (6·38–24·4)
Road injuries	16·8 (8·36–25·3)	17·2 (9·87–24·9)	23·8 (19·5–28·2)	14·5 (4·99–23·8)
Other transport injuries	30·9 (25·5–36·2)	29·8 (24·3–35·1)	34·7 (30·1–39)	26·9 (20·8–32·9)
Unintentional injuries	37·1 (31·3–41·6)	27·7 (23·1–32)	29·1 (24·5–33·5)	24·3 (19·9–28·6)

Falls	53·2<br>(45·4–58·6)	40<br>(34–45)	38·4<br>(32·5–43·3)	44·4<br>(37·7–49·7)
Drowning	-16·4<br>(-30·5–-1·69)	-15·4<br>(-29·2–-1·26)	9·07<br>(4·74–14·5)	-16·3<br>(-30·3–-1·85)
Fire, heat, and hot substances	9·84<br>(5·16–15·9)	11·5<br>(6·6–17·6)	11·4<br>(6·54–17·6)	11·6<br>(6·83–17·8)
Poisonings	12·5<br>(7·48–19·1)	11·9<br>(7·02–18)	10·4<br>(5·91–16·1)	13·4<br>(8·25–19·9)
Exposure to mechanical forces	2·09<br>(-9·23–14·4)	15<br>(11·9–17·8)	17·5<br>(14·9–19·7)	-4·02<br>(-15·8–9·07)
Animal contact	19·5<br>(15·2–24·6)	19·8<br>(15·2–25)	20·7<br>(15·9–26)	15<br>(11–19·8)
Foreign body	2·55<br>(1·61–3·95)	3·47<br>(2·23–5·26)	2·52<br>(1·61–3·78)	3·83<br>(2·42–5·95)
Environmental heat and cold exposure	7·66<br>(2·96–13·3)	8·4<br>(3·36–14·6)	8·21<br>(3·23–14·4)	8·95<br>(3·65–15·4)
Exposure to forces of nature	0<br>(0–0)	9·18<br>(3·78–15·9)	9·18<br>(3·78–15·9)	0<br>(0–0)
Other unintentional injuries	13·4<br>(8–20·3)	10·9<br>(6·27–16·8)	10·8<br>(6·2–16·7)	14·6<br>(9·1–21·5)
Self-harm and interpersonal violence	20<br>(4·87–34·7)	22·4<br>(8·28–36)	31·8<br>(23·5–40·3)	21·2<br>(5·97–35·9)
Self-harm	19·7<br>(3·87–35·1)	21·2<br>(5·14–36·5)	20·4<br>(7·75–33·3)	21·2<br>(5·06–36·6)
Interpersonal violence	24·4<br>(14–35)	30·8<br>(21·5–40·4)	38·2<br>(28·8–47·7)	21·9<br>(11·6–32·3)

Supplementary Results Table 2. All-age PAFs attributable to the joint distribution of all risk factors for all ages, both sexes combined for each GBD level 1, level 2, and level 3 cause and all causes in 2019 for Middlesbrough. Results are shown for deaths, YLLs, YLDs, and DALYs Data in parenthesis are 95% uncertainty intervals. PAFs=population attributable fractions. GBD=Global Burden of Disease. YLLs=years of life lost. YLDs=years lived with disability. DALYs=disability-adjusted life-years

	Deaths 2019	DALYs 2019	YLDs 2019	YLLs 2019
All causes	59·4 (56·4–62·6)	47 (43·9–50)	31·2 (28·8–33·4)	58·8 (56·2–61·5)
Communicable, maternal, neonatal, and nutritional diseases	46·6 (37·2–55·7)	51·8 (46·7–57)	45·5 (41–50·2)	54·2 (47–61·3)
HIV/AIDS and sexually transmitted infections	73·1 (56·5–85·8)	74·6 (57·6–87·6)	96·8 (95·3–98)	57·2 (37·5–75·9)
HIV/AIDS	92 (90·1–93·2)	91 (88·7–92·7)	94·4 (92·2–95·9)	88·4 (85·9–89·9)
Sexually transmitted infections excluding HIV	31 (14–56·7)	53 (28·7–79·9)	100 (100–100)	10·4 (3·8–23·7)
Respiratory infections and tuberculosis	47·3 (36·8–57·4)	45·7 (36·9–54·4)	3·63 (2·66–4·82)	51 (40·7–60·6)
Tuberculosis	55·9 (44·6–67·3)	58·5 (48·1–68·7)	55·3 (43·5–65·9)	59·1 (48·8–69)
Lower respiratory infections	47·2 (36·6–57·4)	50·9 (40·5–60·5)	35·1 (28·4–41·4)	51 (40·6–60·6)
Upper respiratory infections	0·0475 (0·0264–0·0836)	0·00719 (0·00134–0·0176)	0·00579 (0–0·0164)	0·1 (0·0562–0·175)
Otitis media	3·17 (1·59–5·75)	4·92 (3·17–6·93)	4·91 (3·17–6·93)	7·14 (4·16–11·7)
Enteric infections	6·27 (3·63–11·1)	10·5 (7·51–15·4)	10·7 (7·59–15·9)	9·93 (6·73–14·8)
Diarrheal diseases	6·35 (3·7–11·3)	10·6 (7·59–15·5)	10·7 (7·6–15·9)	10·1 (6·94–15·1)
Other infectious diseases	0·575 (0·409–0·792)	0·995 (0·739–1·31)	0·324 (0·178–0·535)	1·29 (0·937–1·72)
Meningitis	1·37 (0·949–1·93)	2·04 (1·42–2·88)	0·000469 (0–0·00141)	2·75 (1·94–3·8)
Encephalitis	0·33 (0·203–0·505)	0·675 (0·391–1·04)	0·000146 (0–0·000469)	0·969 (0·608–1·47)
Measles	3·81 (-1·04–18·5)	4·01 (-0·791–19)	0·754 (0·018–2)	4·81 (-1·25–22·7)
Acute hepatitis	12 (5·05–25)	4·84 (2·57–8·17)	2·99 (1·72–4·87)	11·2 (4·91–23·4)
Maternal and neonatal disorders	83·6 (81–85·9)	75·9 (72·5–79·1)	57·5 (51·4–63)	84·8 (82·4–87)
Maternal disorders	9·74 (3·53–16·5)	9·67 (3·49–16·5)	9·78 (3·57–16·9)	9·57 (3·49–16·3)
Neonatal disorders	87 (84·6–89·2)	78·2 (74·8–81·6)	59·9 (53·2–65·6)	87 (84·6–89·2)
Nutritional deficiencies	66·2 (60·5–84·4)	81·1 (76·8–85·8)	81·4 (77–86·1)	68·8 (62·1–78·8)
Protein-energy malnutrition	100 (100–100)	100 (100–100)	100 (100–100)	100 (100–100)
Vitamin A deficiency	--	100 (100–100)	100 (100–100)	--
Dietary iron deficiency	--	100 (100–100)	100 (100–100)	--
Non-communicable diseases	61·6 (58·4–64·9)	48·4 (44·9–51·7)	30·9 (28·3–33·4)	61·8 (59–64·6)
Neoplasms	55·1 (50·9–59·7)	54 (50·2–58·3)	40 (35·3–45·2)	54·7 (50·9–59)
Lip and oral cavity cancer	67·4 (55·7–77)	69·7 (57·8–78·8)	68·3 (56·1–78)	69·8 (57·9–79)
Nasopharynx cancer	64·6 (51·1–76·1)	64·2 (49·7–75·6)	60·3 (46–72·9)	64·5 (50–75·8)
Other pharynx cancer	80 (71·8–86·2)	80·7 (72·6–86·8)	80·5 (72·1–87)	80·8 (72·6–86·8)

Esophageal cancer	75·4 (65·5–83·8)	76·8 (67·2–84·9)	75·8 (65·6–84·2)	76·8 (67·2–84·9)
Stomach cancer	27·1 (19·7–43·7)	27·1 (19·6–43·3)	27·3 (19·9–43·8)	27·1 (19·6–43·3)
Colon and rectum cancer	66·6 (57–76·3)	66·7 (57·3–75·8)	66·7 (57·1–76)	66·6 (57·3–75·8)
Liver cancer	75·7 (69·6–81·9)	75·9 (70·1–81·5)	76·3 (70·5–82·1)	75·9 (70–81·5)
Gallbladder and biliary tract cancer	22·4 (11·6–35·1)	23·2 (12·2–35·6)	21 (10·6–33·1)	23·2 (12·3–35·7)
Pancreatic cancer	48 (37·3–60·5)	46·9 (36·8–59·1)	47·6 (37–60·1)	46·9 (36·8–59·1)
Larynx cancer	82·9 (75·2–88·9)	82·7 (75·3–88·4)	81·5 (74–87·4)	82·8 (75·4–88·5)
Tracheal, bronchus, and lung cancer	88·4 (85·2–91·5)	87·3 (84·2–90·5)	88·1 (84·8–91·4)	87·3 (84·1–90·5)
Breast cancer	39·2 (27·9–53)	37·6 (27·4–49·8)	37·9 (27·6–50)	37·6 (27·4–49·8)
Cervical cancer	100 (100–100)	100 (100–100)	100 (100–100)	100 (100–100)
Uterine cancer	42·4 (26·8–58·7)	44·3 (29–60·1)	45·1 (29·7–61)	44·2 (28·8–60·1)
Ovarian cancer	32·5 (16·3–48·9)	27·5 (13·7–43·2)	25·4 (12·8–39·9)	27·6 (13·8–43·3)
Prostate cancer	6·57 (3·03–10·4)	7·22 (3·45–11·3)	7·68 (3·7–11·9)	7·16 (3·4–11·2)
Kidney cancer	40·4 (30–50·8)	40·3 (30·4–50·2)	40·2 (30·1–50·1)	40·3 (30·4–50·2)
Bladder cancer	46·6 (30·7–62·4)	48·1 (33·3–62·8)	48·5 (33·9–62·7)	48·1 (33·2–62·9)
Thyroid cancer	14 (6·67–23)	14·8 (7·12–24·2)	14·9 (7·5–24·2)	14·8 (7·09–24·1)
Mesothelioma	99·3 (99–99·5)	98·4 (97·9–98·9)	99·1 (98·6–99·4)	98·4 (97·9–98·9)
Non-Hodgkin lymphoma	7·09 (2·84–12·7)	7·34 (3·07–13)	7·26 (2·97–13)	7·35 (3·09–13)
Multiple myeloma	8·06 (3·4–14·5)	8·46 (3·65–15·2)	8·32 (3·32–15·2)	8·47 (3·68–15·1)
Leukemia	39 (24·7–53)	35·9 (23·9–47·4)	35·5 (23·6–47·4)	35·9 (24–47·5)
Cardiovascular diseases	81·6 (77·7–85·2)	83·2 (80·3–86)	73 (67·9–77·6)	84·1 (81·3–86·8)
Rheumatic heart disease	24·2 (13·7–47·5)	25·5 (16–41·7)	25·5 (15·8–41)	25·5 (16–41·8)
Ischemic heart disease	94·1 (90·6–96·9)	95·4 (93·1–97·4)	94·6 (91·8–96·9)	95·4 (93·1–97·4)
Stroke	80·8 (73·2–89·1)	84·2 (78·8–90·3)	83·3 (77·9–88·9)	84·4 (78·7–90·5)
Hypertensive heart disease	100 (100–100)	100 (100–100)	100 (100–100)	100 (100–100)
Non-rheumatic valvular heart disease	27·4 (18·1–39·7)	28·6 (20·4–38·8)	18·7 (12·6–26)	29·5 (21·2–40)
Cardiomyopathy and myocarditis	39·4 (31·5–47·3)	44·3 (38·2–50·5)	37·2 (30·6–43·9)	44·6 (38·3–51·1)
Atrial fibrillation and flutter	50·1 (39·3–61·5)	58·6 (49·2–67·6)	63 (54·4–71)	54·1 (44·3–64·2)
Aortic aneurysm	55·2 (48·2–62·9)	62·2 (56·1–68·1)	--	62·2 (56·1–68·1)
Endocarditis	33·6 (23·5–46)	36·6 (27·3–46·9)	33 (23·2–45)	36·7 (27·4–47)
Other cardiovascular and circulatory diseases	33·8 (27–40·9)	36·3 (29·8–42·3)	36·4 (28·2–43·7)	36·3 (29·9–42·2)
Chronic respiratory diseases	67·3 (60·9–73·6)	62·3 (57·2–67·6)	52·4 (46·7–57·9)	67·5 (61·7–73·6)
Chronic obstructive pulmonary disease	74·9 (70·2–79)	74·5 (70·5–78·1)	67·7 (63·7–71·5)	76·5 (72·4–80·2)

Pneumoconiosis	100 (100–100)	100 (100–100)	100 (100–100)	100 (100–100)
Asthma	43·2 (31·5–55·8)	41·2 (32·7–50·6)	40·9 (32·4–50)	44·2 (34·6–55·2)
Digestive diseases	30·5 (25·3–36·6)	37 (31·1–44·1)	19·6 (13·2–26·8)	42 (35·2–49·9)
Cirrhosis and other chronic liver diseases	70·2 (59·1–84·4)	73·5 (62·7–87)	69·8 (58–83·6)	73·6 (62·7–87)
Upper digestive system diseases	24·9 (21·2–28·7)	12·1 (8·07–16·4)	0·93 (0·551–1·47)	27·2 (23·5–31)
Gallbladder and biliary diseases	42·8 (27·5–58·4)	48·2 (34–63·2)	50·5 (36·3–65)	44·5 (29·9–59·5)
Pancreatitis	31·3 (9·12–51·8)	37·7 (12·8–58·5)	37·1 (12·1–57·9)	37·7 (12·8–58·7)
Neurological disorders	25·8 (10·1–43·4)	15·4 (7·38–28·1)	9·74 (4·61–19)	21·6 (9·26–38·4)
Alzheimer's disease and other dementias	45·9 (30·3–62·3)	47·7 (32·4–63·8)	48·1 (32·6–64·1)	47·5 (32·1–63·6)
Parkinson's disease	-11·1 (-18·4–-5·29)	-12·7 (-20·8–-6·19)	-13·2 (-21·5–-6·3)	-12·6 (-20·7–-6·17)
Idiopathic epilepsy	24·8 (12·8–35·9)	23·4 (12·3–34)	20·6 (10·4–30·2)	26·2 (13·9–37)
Multiple sclerosis	16 (11·9–20)	16·5 (12·2–20·7)	16 (11·9–20·3)	16·8 (12·4–21)
Mental disorders	--	6·73 (3·47–10·4)	6·73 (3·47–10·4)	--
Depressive disorders	--	13·7 (6·24–21·6)	13·7 (6·24–21·6)	--
Anxiety disorders	--	5·33 (1·41–11·7)	5·33 (1·41–11·7)	--
Idiopathic developmental intellectual disability	--	41·4 (18·8–60·7)	41·4 (18·8–60·7)	--
Substance use disorders	100 (100–100)	100 (100–100)	100 (100–100)	100 (100–100)
Alcohol use disorders	100 (100–100)	100 (100–100)	100 (100–100)	100 (100–100)
Drug use disorders	100 (100–100)	100 (100–100)	100 (100–100)	100 (100–100)
Diabetes and kidney diseases	100 (100–100)	100 (100–100)	100 (100–100)	100 (100–100)
Diabetes mellitus	100 (100–100)	100 (100–100)	100 (100–100)	100 (100–100)
Chronic kidney disease	100 (100–100)	100 (100–100)	100 (100–100)	100 (100–100)
Sense organ diseases	--	9·43 (8·22–10·9)	9·43 (8·22–10·9)	--
Blindness and vision loss	--	10·7 (6·8–15·6)	10·7 (6·8–15·6)	--
Age-related and other hearing loss	--	10·3 (9·28–11·5)	10·3 (9·28–11·5)	--
Musculoskeletal disorders	4·68 (1·2–8·42)	22·8 (18·9–26·7)	23·3 (19·3–27·5)	5·36 (1·45–9·34)
Rheumatoid arthritis	13 (3·48–23·1)	15·1 (4·01–26·5)	15·4 (4·12–26·8)	14·6 (3·88–25·7)
Osteoarthritis	--	16·9 (8·86–26·1)	16·9 (8·86–26·1)	--
Low back pain	--	41·6 (36·2–47·3)	41·6 (36·2–47·3)	--
Gout	--	51·2 (35·7–68·1)	51·2 (35·7–68·1)	--
Other non-communicable diseases	0·0879 (0·0587–0·127)	0·0896 (0·056–0·133)	--	0·31 (0·208–0·436)
Sudden infant death syndrome	6·22 (3·46–10·3)	6·22 (3·45–10·3)	--	6·22 (3·45–10·3)
Injuries	28·8 (21·6–35·2)	24·3 (18–29·6)	28·1 (23·8–32·4)	21·1 (12·2–29·2)

Transport injuries	16·8 (8·78–25·6)	17 (9·74–25·3)	24·6 (19·9–29·4)	14·3 (5·31–23·6)
Road injuries	16·1 (7·64–25·1)	16·1 (8·6–24·6)	23·5 (18·9–28·3)	13·7 (4·54–23·2)
Other transport injuries	30·1 (24·4–35·8)	29·3 (24–35)	33·5 (28·6–38)	26·2 (19·6–32·8)
Unintentional injuries	37·4 (31·3–41·9)	26·9 (22·5–31·4)	28·1 (23·5–32·8)	24·3 (19·9–28·8)
Falls	52·3 (44·3–57·6)	38·7 (32·8–43·8)	37·2 (31·5–42·3)	42·5 (36·4–47·5)
Drowning	-15·7 (-30·1–-0·846)	-15·1 (-29–-0·834)	9·11 (4·8–15·1)	-15·9 (-30·2–-1·15)
Fire, heat, and hot substances	10·1 (5·32–16·2)	11·4 (6·47–17·7)	11·4 (6·51–17·8)	11·4 (6·55–17·5)
Poisonings	12·5 (7·12–19·1)	11·7 (6·8–18·1)	10·2 (5·78–16)	13·1 (7·67–19·7)
Exposure to mechanical forces	1·75 (-8·96–14)	13·8 (10·9–16·5)	16·2 (13·9–18·3)	-5·24 (-16·6–7·62)
Animal contact	18·5 (14·1–23·9)	18·8 (14–24·3)	19·7 (14·6–25·5)	14 (10–19)
Foreign body	2·55 (1·59–3·77)	3·09 (1·96–4·52)	2·32 (1·54–3·37)	3·55 (2·21–5·23)
Environmental heat and cold exposure	8·73 (3·74–15·3)	8·64 (3·78–15·4)	8·37 (3·67–15·1)	9·59 (4·28–16·8)
Exposure to forces of nature	0 (0–0)	9·25 (4·09–16·4)	9·25 (4·09–16·4)	0 (0–0)
Other unintentional injuries	12·2 (6·96–18·9)	10·7 (6·08–16·9)	10·6 (5·97–16·8)	13·6 (8–20·3)
Self-harm and interpersonal violence	20·5 (4·63–35·3)	22·5 (7·33–36·2)	32·1 (24–40·2)	21·6 (5·59–36·2)
Self-harm	20·2 (3·59–35·7)	21·5 (4·79–36·9)	20·8 (7·12–33·7)	21·5 (4·72–36·9)
Interpersonal violence	27 (17·1–37·5)	31·1 (22–40·8)	38·5 (29·1–48)	24 (14·2–34·5)

Supplementary Results Table 2. All-age PAFs attributable to the joint distribution of all risk factors for all ages, both sexes combined for each GBD level 1, level 2, and level 3 cause and all causes in 2019 for Newcastle upon Tyne. Results are shown for deaths, YLLs, YLDs, and DALYs Data in parenthesis are 95% uncertainty intervals. PAFs=population attributable fractions. GBD=Global Burden of Disease. YLLs=years of life lost. YLDs=years lived with disability. DALYs=disability-adjusted life-years

	Deaths 2019	DALYs 2019	YLDs 2019	YLLs 2019
All causes	58·2 (55·2–61·2)	44·7 (41·6–47·8)	31·1 (28·6–33·5)	57·8 (55·3–60·3)
Communicable, maternal, neonatal, and nutritional diseases	45·9 (36·2–55·3)	52·7 (47·9–57·3)	50·5 (45·9–55)	54 (46·9–61·1)
HIV/AIDS and sexually transmitted infections	76·2 (60·9–88)	76·5 (59·5–88·4)	96·9 (95·4–98·1)	60·4 (41·1–78·7)
HIV/AIDS	92·3 (89·9–93·7)	91·6 (89–93·3)	94·4 (92·1–95·8)	89·5 (86·6–91·1)
Sexually transmitted infections excluding HIV	47·4 (26–74·3)	57·1 (31·6–82·9)	100 (100–100)	18·5 (7·46–39·8)
Respiratory infections and tuberculosis	47 (36·2–57·4)	43·4 (34·6–51·7)	3·19 (2·3–4·36)	50·7 (40·5–60·3)
Tuberculosis	60·7 (49·4–70·8)	63 (52·6–72)	58·3 (46·6–68·4)	64 (53·7–72·7)
Lower respiratory infections	46·9 (36–57·4)	50·5 (40–60·2)	34·9 (28–41·2)	50·6 (40·1–60·3)
Upper respiratory infections	0·0315 (0·0182–0·0521)	0·00707 (0·00094–0·0188)	0·00613 (0–0·018)	0·071 (0·0394–0·122)
Otitis media	2·4 (1·16–4·48)	4·55 (2·89–6·36)	4·53 (2·87–6·36)	5·67 (3·11–9·39)
Enteric infections	4·79 (2·89–8·24)	8·7 (6·42–12·2)	9·21 (6·75–12·9)	7·7 (5·34–11·1)
Diarrheal diseases	4·83 (2·92–8·32)	8·75 (6·45–12·3)	9·22 (6·75–12·9)	7·81 (5·42–11·2)
Other infectious diseases	0·647 (0·451–0·867)	1·08 (0·8–1·41)	0·387 (0·223–0·662)	1·39 (1·01–1·86)
Meningitis	1·35 (0·917–1·91)	2·06 (1·42–2·87)	0·000542 (0–0·00168)	2·72 (1·88–3·8)
Encephalitis	0·296 (0·201–0·421)	0·641 (0·429–0·922)	0·000177 (0–0·00054)	0·891 (0·615–1·28)
Measles	2·78 (-0·818–14·2)	3·19 (-0·914–16)	0·469 (0·0121–1·33)	3·5 (-1·03–17·9)
Acute hepatitis	11·8 (6·42–22·9)	4·68 (2·85–7·57)	2·79 (1·64–4·64)	10·9 (6·1–20·7)
Maternal and neonatal disorders	86·6 (83·9–88·8)	79·3 (76·2–82·2)	71·3 (66·3–75·5)	87·4 (84·8–89·5)
Maternal disorders	9·58 (3·45–16·1)	9·65 (3·43–16·4)	9·75 (3·52–16·8)	9·47 (3·39–16)
Neonatal disorders	88·9 (86·4–91)	81·1 (77·9–84)	73·2 (68–77·7)	88·9 (86·4–91)
Nutritional deficiencies	78·1 (68·7–85·7)	79·6 (75·4–83·9)	79·7 (75·3–84·2)	78·8 (64·6–81·9)
Protein-energy malnutrition	100 (100–100)	100 (100–100)	100 (100–100)	100 (100–100)
Vitamin A deficiency	--	100 (100–100)	100 (100–100)	--
Dietary iron deficiency	--	100 (100–100)	100 (100–100)	--
Non-communicable diseases	60·4 (57·3–63·5)	45·8 (42·4–49·2)	30·5 (27·8–33·2)	60·9 (58·3–63·6)
Neoplasms	55·7 (51·7–59·8)	54 (50·4–57·8)	38·3 (33·8–43·1)	54·9 (51·2–58·5)
Lip and oral cavity cancer	69·8 (58·9–78·6)	72·2 (61·7–80·2)	70·9 (60·1–79·5)	72·3 (61·8–80·3)
Nasopharynx cancer	67·8 (56·5–77·7)	67·5 (55·8–76·7)	64·5 (52·3–75)	67·7 (56–77)
Other pharynx cancer	80·7 (73·4–86·5)	81·6 (74·2–87·2)	81·3 (73·9–86·9)	81·6 (74·1–87·1)

Esophageal cancer	77·6 (68·3–85·7)	79 (69·6–86·7)	78 (68·5–86)	79·1 (69·6–86·7)
Stomach cancer	27 (19·3–43·2)	27 (19·5–43·3)	27·3 (19·6–43·5)	27 (19·5–43·2)
Colon and rectum cancer	66·8 (57·3–76·1)	67 (58·4–75·7)	67·1 (58·2–75·8)	67 (58·5–75·7)
Liver cancer	77·6 (71·4–83·5)	77·7 (72·1–83)	77·9 (72·1–83·3)	77·7 (72·1–82·9)
Gallbladder and biliary tract cancer	24·9 (13·6–38)	25·6 (14·1–38·6)	23·4 (12·4–36·3)	25·7 (14·2–38·6)
Pancreatic cancer	48·6 (38·4–60·4)	47·5 (37·9–58·6)	48·2 (37·9–59·9)	47·5 (37·9–58·6)
Larynx cancer	84·4 (77·2–90)	84·1 (77·3–89·1)	82·7 (75·8–88)	84·2 (77·5–89·2)
Tracheal, bronchus, and lung cancer	88·8 (85·4–91·9)	87·6 (84·3–90·7)	88·6 (85–91·7)	87·6 (84·3–90·7)
Breast cancer	40·1 (29–53·3)	38·2 (28·1–49·7)	38·5 (28·2–50·3)	38·1 (28·1–49·7)
Cervical cancer	100 (100–100)	100 (100–100)	100 (100–100)	100 (100–100)
Uterine cancer	47·4 (31–63·5)	49·2 (32·7–65·1)	50 (33·4–65·7)	49·1 (32·7–65)
Ovarian cancer	35·5 (16·6–51·5)	29·7 (14·4–44·4)	27 (13·2–41·3)	29·8 (14·5–44·5)
Prostate cancer	6·63 (2·96–10·6)	7·34 (3·38–11·4)	7·84 (3·66–12)	7·27 (3·33–11·3)
Kidney cancer	43·2 (32·5–53·8)	43·1 (32·5–53)	42·9 (32·5–52·9)	43·1 (32·5–53·1)
Bladder cancer	46·1 (30·4–61·7)	47·7 (32·8–61·4)	48·1 (33·7–61·5)	47·7 (32·8–61·5)
Thyroid cancer	17·4 (8·4–28·1)	18·3 (8·86–29·5)	18·4 (8·96–29·4)	18·3 (8·81–29·7)
Mesothelioma	99·3 (99–99·5)	98·3 (97·7–98·8)	99 (98·6–99·4)	98·3 (97·7–98·8)
Non-Hodgkin lymphoma	8·46 (3·56–14·8)	8·74 (3·79–14·9)	8·64 (3·7–14·9)	8·74 (3·79–14·9)
Multiple myeloma	9·58 (4·21–16·6)	10 (4·54–17·4)	9·91 (4·19–17·5)	10 (4·54–17·4)
Leukemia	39·9 (25·9–53·9)	36·2 (24·8–47·8)	35·3 (23·9–46·5)	36·3 (24·9–47·9)
Cardiovascular diseases	79·7 (75·9–83·4)	81·2 (78·4–84)	72 (67–76·4)	82·4 (79·7–85·1)
Rheumatic heart disease	24·8 (13·9–48)	26·4 (17–43·7)	26·3 (17·1–42·1)	26·4 (16·9–43·8)
Ischemic heart disease	94·3 (91–97·2)	95·6 (93·4–97·6)	94·9 (92·3–97·2)	95·7 (93·4–97·6)
Stroke	80·9 (73·2–89·4)	84·6 (78·9–90·3)	84·1 (79·4–89·2)	84·7 (79–90·7)
Hypertensive heart disease	100 (100–100)	100 (100–100)	100 (100–100)	100 (100–100)
Non-rheumatic valvular heart disease	27·3 (17·7–40·6)	28 (19·9–38)	17·3 (11·6–24·2)	29·4 (20·8–39·9)
Cardiomyopathy and myocarditis	41·3 (32–50·9)	47·6 (40·3–54·1)	41·1 (34·3–47·6)	48 (40·3–54·4)
Atrial fibrillation and flutter	53·1 (42–64·1)	62·4 (53·5–70·9)	66·6 (58·5–74·4)	56·9 (47–66·9)
Aortic aneurysm	54·1 (47·4–62·1)	61·4 (55·6–67·7)	--	61·4 (55·6–67·7)
Endocarditis	33·9 (23·8–47·1)	37·3 (28·1–47·2)	33·5 (23·3–45·7)	37·4 (28·2–47·3)
Other cardiovascular and circulatory diseases	34·7 (28·5–42·1)	37·7 (31·5–43·7)	37·3 (29·2–45·1)	37·7 (31·6–43·9)
Chronic respiratory diseases	66·8 (60·7–73·2)	61·6 (56·8–66·5)	53·6 (48·5–58·7)	66·8 (61·2–72·9)
Chronic obstructive pulmonary disease	74·5 (69·9–78·9)	73·8 (70·1–77·3)	67 (63·3–70·5)	76·2 (72·3–79·9)

Pneumoconiosis	100 (100–100)	100 (100–100)	100 (100–100)	100 (100–100)
Asthma	46·6 (34·4–59·2)	44·5 (36·1–54·1)	44·1 (35·9–53·6)	47·6 (37·7–58·2)
Digestive diseases	32·5 (27·2–38·3)	38·4 (32·3–44·3)	22·5 (16·1–30·4)	45·3 (38·4–52·5)
Cirrhosis and other chronic liver diseases	73·5 (61·6–85·9)	77 (65·4–88·4)	73·9 (61·5–85·1)	77·1 (65·5–88·5)
Upper digestive system diseases	24·6 (20·6–28)	10·3 (6·8–14·3)	0·947 (0·586–1·47)	26·9 (23·1–30·3)
Gallbladder and biliary diseases	48·1 (31·9–64·3)	50·7 (36·5–65·5)	51·1 (37·3–65·6)	49·6 (34–65·1)
Pancreatitis	35·5 (14·8–55·9)	43·2 (20·7–62·6)	44·5 (22–63·9)	43·1 (20·5–62·5)
Neurological disorders	27·7 (12·1–44·7)	15·7 (7·57–28)	9·53 (4·4–19)	23·7 (11·3–40·1)
Alzheimer's disease and other dementias	46·9 (30·7–63·5)	48·8 (33–64·8)	49·2 (33·7–65)	48·6 (32·5–64·6)
Parkinson's disease	-11 (-18·1–-5·15)	-12·7 (-20·7–-6·08)	-13·5 (-22–-6·3)	-12·5 (-20·3–-6·01)
Idiopathic epilepsy	28·3 (17·4–39)	27·2 (16·5–37·5)	24·6 (14·3–34·7)	29·9 (18·7–40·3)
Multiple sclerosis	16·5 (12·2–20·9)	17 (12·9–21·3)	16·4 (12·4–20·6)	17·3 (13·2–22·1)
Mental disorders	--	6·77 (3·37–10·6)	6·77 (3·37–10·6)	--
Depressive disorders	--	14 (6·48–21·7)	14 (6·48–21·7)	--
Anxiety disorders	--	5·66 (1·39–12·9)	5·66 (1·39–12·9)	--
Idiopathic developmental intellectual disability	--	37·1 (16·2–56)	37·1 (16·2–56)	--
Substance use disorders	100 (100–100)	100 (100–100)	100 (100–100)	100 (100–100)
Alcohol use disorders	100 (100–100)	100 (100–100)	100 (100–100)	100 (100–100)
Drug use disorders	100 (100–100)	100 (100–100)	100 (100–100)	100 (100–100)
Diabetes and kidney diseases	99·6 (99·5–99·7)	99·9 (99·9–99·9)	100 (100–100)	99·6 (99·5–99·6)
Diabetes mellitus	100 (100–100)	100 (100–100)	100 (100–100)	100 (100–100)
Chronic kidney disease	100 (100–100)	100 (100–100)	100 (100–100)	100 (100–100)
Sense organ diseases	--	9·64 (8·44–11·1)	9·64 (8·44–11·1)	--
Blindness and vision loss	--	10·3 (6·75–14·9)	10·3 (6·75–14·9)	--
Age-related and other hearing loss	--	10·7 (9·67–11·9)	10·7 (9·67–11·9)	--
Musculoskeletal disorders	4·66 (1·09–8·46)	23·9 (20–28)	24·3 (20·2–28·5)	5·38 (1·33–9·5)
Rheumatoid arthritis	13 (3·18–23·5)	15·2 (3·93–26·4)	15·4 (4·06–26·8)	14·7 (3·74–25·6)
Osteoarthritis	--	20·7 (11·2–30·7)	20·7 (11·2–30·7)	--
Low back pain	--	42·8 (37·4–48·4)	42·8 (37·4–48·4)	--
Gout	--	55·6 (39–72·6)	55·6 (39–72·6)	--
Other non-communicable diseases	0·0981 (0·0668–0·14)	0·0907 (0·0573–0·135)	--	0·371 (0·246–0·524)
Sudden infant death syndrome	8·49 (4·81–13·7)	8·48 (4·81–13·6)	--	8·48 (4·81–13·6)
Injuries	30·7 (23·8–36·8)	25 (19·3–30·3)	27·5 (23·1–31·9)	22·7 (14·2–30·2)

Transport injuries	17·5 (9·03–26·3)	18 (10·2–26)	24·9 (19·8–30·3)	15·2 (6·04–24·8)
Road injuries	16·6 (7·84–25·7)	17 (9–25·3)	23·9 (18·8–29·4)	14·5 (4·83–24·4)
Other transport injuries	29·7 (23·1–36·2)	28·8 (22·8–35·1)	32·8 (27·8–37·6)	26·2 (19·2–33·2)
Unintentional injuries	37·7 (31·5–42·7)	26·1 (21·5–31)	27·3 (22·6–32·2)	23·5 (18·8–28·1)
Falls	53·2 (44·9–59·1)	37·9 (31·8–43·5)	36·2 (30·2–41·7)	42·6 (36·3–48·4)
Drowning	-14 (-28·9–1·62)	-13·4 (-27·5–1·26)	10·3 (5·56–16·6)	-14·1 (-28·6–0·873)
Fire, heat, and hot substances	11·2 (6·22–18)	12·6 (7·32–19·3)	12·5 (7·23–19·4)	12·7 (7·56–19·3)
Poisonings	14·4 (8·68–21·6)	13·5 (8·01–20·2)	11·6 (6·63–17·7)	14·9 (9·14–22·1)
Exposure to mechanical forces	5·85 (-5·09–18)	13·4 (10·8–15·8)	15 (12·8–17·1)	-4·29 (-15·7–8·58)
Animal contact	18·8 (14·2–24·7)	18·8 (13·8–24·9)	19·7 (14·5–26)	14·4 (10·2–19·5)
Foreign body	2·54 (1·58–3·8)	3·17 (2·03–4·69)	2·32 (1·49–3·45)	3·6 (2·27–5·4)
Environmental heat and cold exposure	8·35 (3·72–14·7)	9·71 (4·34–16·7)	9·55 (4·31–16·6)	10 (4·49–17·2)
Exposure to forces of nature	0 (0–0)	10·5 (4·74–18·1)	10·5 (4·74–18·1)	0 (0–0)
Other unintentional injuries	13·4 (7·93–20·6)	12 (6·85–18·5)	11·8 (6·73–18·3)	14·8 (9·04–22)
Self-harm and interpersonal violence	23·9 (7·98–38)	26 (10·9–39·3)	34·7 (26–43·4)	25 (8·9–39·3)
Self-harm	23·9 (7·06–39)	25·2 (8·38–40·4)	24·4 (10·7–38·2)	25·3 (8·22–40·6)
Interpersonal violence	24·6 (14·9–35·1)	31·8 (22·7–41·7)	40·1 (30·7–50·1)	22·3 (12·6–32·4)

Supplementary Results Table 2. All-age PAFs attributable to the joint distribution of all risk factors for all ages, both sexes combined for each GBD level 1, level 2, and level 3 cause and all causes in 2019 for North Tyneside. Results are shown for deaths, YLLs, YLDs, and DALYs Data in parenthesis are 95% uncertainty intervals. PAFs=population attributable fractions. GBD=Global Burden of Disease. YLLs=years of life lost. YLDs=years lived with disability. DALYs=disability-adjusted life-years

	Deaths 2019	DALYs 2019	YLDs 2019	YLLs 2019
All causes	57·1 (54–60·2)	44·9 (41·7–47·9)	31·1 (28·7–33·4)	57·3 (54·7–60·2)
Communicable, maternal, neonatal, and nutritional diseases	44·7 (34·2–54·5)	49·3 (43·4–55)	47·4 (42·6–51·9)	50·2 (41–58·6)
HIV/AIDS and sexually transmitted infections	75·3 (58·8–88·4)	75·6 (57·6–89·2)	97·9 (96·7–98·7)	55 (35–75·8)
HIV/AIDS	93 (91·1–94·2)	92·4 (90·1–93·9)	95 (93·1–96·3)	90 (87·5–91·4)
Sexually transmitted infections excluding HIV	58·7 (35·7–81·8)	62·6 (37·9–85·9)	100 (100–100)	24·9 (10·6–49·8)
Respiratory infections and tuberculosis	47 (35·2–57·8)	44·5 (34·5–54·2)	3·32 (2·46–4·37)	50·2 (39·2–60·4)
Tuberculosis	58 (45·8–69·3)	61·3 (50·2–71·1)	57·8 (46·3–68)	61·8 (51–71·7)
Lower respiratory infections	46·9 (35·1–57·8)	50 (38·9–60·3)	34·6 (27·6–41·5)	50·1 (38·9–60·4)
Upper respiratory infections	0·0378 (0·0204–0·0617)	0·00544 (0·000595–0·0148)	0·00492 (2·8e-05–0·0144)	0·0917 (0·0518–0·146)
Otitis media	0·932 (0·386–2·13)	4·15 (2·62–5·93)	4·16 (2·63–5·95)	2·57 (1·11–5·49)
Enteric infections	5·28 (3·03–9·45)	7·99 (5·62–12·3)	8·84 (6·33–13·1)	6·8 (4·48–11)
Diarrheal diseases	5·32 (3·05–9·49)	8·03 (5·66–12·3)	8·84 (6·33–13·1)	6·88 (4·53–11·1)
Other infectious diseases	0·35 (0·247–0·555)	0·698 (0·53–0·905)	0·4 (0·23–0·674)	0·849 (0·621–1·15)
Meningitis	0·738 (0·478–1·05)	1·26 (0·824–1·8)	0·000352 (1·19e-06–0·00108)	1·84 (1·22–2·62)
Encephalitis	0·168 (0·102–0·261)	0·414 (0·255–0·62)	0·000115 (3·27e-07–0·000362)	0·585 (0·364–0·89)
Measles	2·59 (-0·649–13)	3·1 (-0·68–14·9)	0·638 (0·0155–1·78)	3·6 (-0·927–17·5)
Acute hepatitis	7·61 (2·66–24)	4·29 (2·3–8·57)	3·25 (1·87–5·37)	7·32 (2·58–23·3)
Maternal and neonatal disorders	86·3 (83·4–88·5)	74·9 (70·9–78·4)	65·9 (60·5–70·7)	87·3 (84·7–89·4)
Maternal disorders	9·51 (3·41–16)	9·43 (3·44–16·1)	9·47 (3·35–16·1)	9·36 (3·34–15·8)
Neonatal disorders	89 (86·4–90·9)	76·7 (72·6–80·4)	67·6 (62–72·7)	89 (86·4–91)
Nutritional deficiencies	62·2 (56·1–86)	79·3 (75–83·9)	79·7 (75·2–84·3)	64 (59·2–80·5)
Protein-energy malnutrition	100 (100–100)	100 (100–100)	100 (100–100)	100 (100–100)
Vitamin A deficiency	--	100 (100–100)	100 (100–100)	--
Dietary iron deficiency	--	100 (100–100)	100 (100–100)	--
Non-communicable diseases	59·2 (55·8–62·5)	46 (42·6–49·2)	30·4 (27·9–33)	60·1 (57·3–62·9)
Neoplasms	53·9 (50–58·2)	53 (49·3–56·9)	38 (33·4–43)	53·7 (50·2–57·8)
Lip and oral cavity cancer	66·7 (54·7–76·5)	69·6 (57·7–78·7)	68·2 (56·4–77·9)	69·7 (57·9–78·7)
Nasopharynx cancer	64·2 (51·4–75·4)	64·6 (51·7–75·3)	61·1 (47·3–72·9)	64·9 (51·9–75·6)
Other pharynx cancer	78·3 (69·9–84·9)	79·6 (71·2–85·9)	79·3 (71–85·8)	79·6 (71·2–85·9)
Esophageal cancer	75·6 (65·2–84)	77·1 (66·8–84·9)	76 (65·7–84·4)	77·1 (66·8–84·9)
Stomach cancer	26·7 (19–43·2)	26·8 (19·3–43·1)	27 (19·5–43·2)	26·8 (19·3–43·1)
Colon and rectum cancer	65·9 (56·3–75·4)	66·2 (57–75)	66·3 (56·9–75·2)	66·2 (56·9–75)

Liver cancer	75·3 (68·8–81·8)	76·2 (70–82·1)	76·5 (70·2–82·2)	76·2 (70–82·1)
Gallbladder and biliary tract cancer	22·3 (11·8–34·6)	23·1 (12·5–35·6)	20·9 (10·7–33·2)	23·2 (12·5–35·8)
Pancreatic cancer	47·3 (36·9–59·5)	46·4 (36·5–58)	46·9 (36·7–59·2)	46·4 (36·5–58)
Larynx cancer	83·1 (75·5–89·1)	83 (75·9–88·6)	82 (75–87·7)	83·1 (75·9–88·7)
Tracheal, bronchus, and lung cancer	88·6 (85·2–91·6)	87·6 (84·4–90·7)	88·4 (85–91·4)	87·6 (84·4–90·7)
Breast cancer	38·7 (27·7–52)	37·4 (27·3–49·4)	37·6 (27·4–49·9)	37·4 (27·2–49·3)
Cervical cancer	100 (100–100)	100 (100–100)	100 (100–100)	100 (100–100)
Uterine cancer	43·4 (27·7–60)	45·4 (29·8–62·1)	46·2 (30·6–62·9)	45·2 (29·6–62)
Ovarian cancer	33·3 (15·7–49·8)	28·2 (13·9–43·4)	26·2 (12·9–40·6)	28·3 (13·9–43·5)
Prostate cancer	6·38 (2·93–10·1)	7·05 (3·32–11·1)	7·54 (3·62–11·7)	6·98 (3·28–10·9)
Kidney cancer	40·7 (30–51·2)	41·1 (30·7–51·1)	41 (30·8–50·9)	41·1 (30·7–51·1)
Bladder cancer	44·8 (28·6–59·7)	46·4 (31·2–60·4)	46·9 (32·2–60·7)	46·4 (31·1–60·4)
Thyroid cancer	15·1 (6·95–25·2)	16·1 (7·66–26·6)	16·3 (7·7–26·6)	16·1 (7·57–26·6)
Mesothelioma	99·5 (99·3–99·7)	98·9 (98·5–99·3)	99·4 (99·1–99·6)	98·9 (98·5–99·3)
Non-Hodgkin lymphoma	7·38 (3·02–13·1)	7·74 (3·2–13·5)	7·59 (3·17–13·4)	7·75 (3·21–13·5)
Multiple myeloma	8·34 (3·47–15·1)	8·79 (3·72–15·6)	8·64 (3·46–15·7)	8·8 (3·77–15·6)
Leukemia	39·3 (24·3–54·5)	37·6 (24·8–50·1)	37·3 (24·4–49·9)	37·6 (24·8–50)
Cardiovascular diseases	79·5 (75·5–83·4)	81·2 (78–84·2)	71·8 (66·5–76·7)	82·4 (79·3–85·2)
Rheumatic heart disease	24·1 (13·2–45·5)	25·6 (16–43·1)	25·7 (16·1–42·9)	25·6 (15·9–43·1)
Ischemic heart disease	94 (90·3–97·2)	95·2 (92·7–97·4)	94·5 (91·7–96·9)	95·3 (92·7–97·4)
Stroke	80·1 (71·3–89)	83·6 (77·3–90)	83·5 (77·8–89·3)	83·6 (77·1–90·1)
Hypertensive heart disease	100 (100–100)	100 (100–100)	100 (100–100)	100 (100–100)
Non-rheumatic valvular heart disease	26·7 (16·9–40·4)	28 (19·6–39)	21·8 (14·7–30·7)	28·7 (20·1–40)
Cardiomyopathy and myocarditis	38·6 (29·4–48)	45·1 (37·6–51·2)	37·8 (30·7–45·3)	45·6 (37·7–51·8)
Atrial fibrillation and flutter	50·1 (39·2–62·3)	59·3 (50·1–68·9)	63·6 (54·9–71·9)	53·8 (43·7–65)
Aortic aneurysm	53·3 (45·9–60·8)	60·5 (54·3–66·6)	--	60·5 (54·3–66·6)
Endocarditis	32·8 (22·2–47·1)	36 (26·4–46·8)	32·6 (22·1–46·5)	36·2 (26·6–46·8)
Other cardiovascular and circulatory diseases	34·7 (27·8–42·2)	38·1 (31·7–44·5)	37·7 (30·4–45·1)	38·2 (31·7–44·7)
Chronic respiratory diseases	65·7 (58·9–73)	61·6 (56·7–67)	55 (50·1–60·1)	66 (59·8–73·1)
Chronic obstructive pulmonary disease	74·1 (68·3–79·3)	73·3 (68·8–77·3)	67·4 (63·5–71·2)	75·8 (70·8–80·3)
Pneumoconiosis	100 (100–100)	100 (100–100)	100 (100–100)	100 (100–100)
Asthma	43·6 (31·8–56·6)	43·8 (35–53·9)	43·5 (34·7–53·4)	46·3 (35·9–57·5)
Digestive diseases	30·1 (24·9–36)	36·3 (30·5–42·7)	18·3 (12·6–24·7)	42·3 (35·7–49·6)
Cirrhosis and other chronic liver diseases	69·7 (58·2–83·9)	73·9 (62·7–86·7)	71·3 (59–84)	73·9 (62·8–86·9)
Upper digestive system diseases	24·2 (20·3–28·2)	11 (7·35–15)	0·982 (0·592–1·54)	27·1 (23·3–31)

Gallbladder and biliary diseases	43·3 (28·2–59·4)	48·8 (34·4–63·5)	51·4 (36·9–65·9)	45·1 (30·3–60·3)
Pancreatitis	31·1 (9·83–52·3)	38·1 (14·5–59·6)	38·4 (14·1–59·9)	38 (14·7–59·6)
Neurological disorders	27·5 (11·9–44·2)	17 (8·48–30·3)	10·7 (5·27–20·3)	23·7 (10·5–40·6)
Alzheimer's disease and other dementias	44·7 (28·8–61·3)	46·5 (31·2–62·6)	47 (31·6–62·9)	46·3 (30·8–62·3)
Parkinson's disease	-10·5 (-17·2–-4·77)	-12·2 (-19·4–-5·63)	-12·8 (-20·6–-6·08)	-12 (-19·4–-5·54)
Idiopathic epilepsy	25·5 (13·7–36·5)	24·6 (13·6–34·9)	21·8 (11·9–31·2)	27·7 (15·8–38·6)
Multiple sclerosis	15·4 (11·6–19·6)	16·2 (12·2–20·3)	15·9 (12–20)	16·4 (12·2–20·6)
Mental disorders	--	6·45 (3·25–9·88)	6·45 (3·25–9·89)	--
Depressive disorders	--	13·2 (6·04–20·8)	13·2 (6·04–20·8)	--
Anxiety disorders	--	4·47 (1·18–10)	4·47 (1·18–10)	--
Idiopathic developmental intellectual disability	--	42·6 (19·9–62)	42·6 (19·9–62)	--
Substance use disorders	100 (100–100)	100 (100–100)	100 (100–100)	100 (100–100)
Alcohol use disorders	100 (100–100)	100 (100–100)	100 (100–100)	100 (100–100)
Drug use disorders	100 (100–100)	100 (100–100)	100 (100–100)	100 (100–100)
Diabetes and kidney diseases	99·5 (99·3–99·6)	99·9 (99·9–99·9)	100 (100–100)	99·5 (99·4–99·6)
Diabetes mellitus	100 (100–100)	100 (100–100)	100 (100–100)	100 (100–100)
Chronic kidney disease	100 (100–100)	100 (100–100)	100 (100–100)	100 (100–100)
Sense organ diseases	--	9·27 (8·02–10·8)	9·27 (8·02–10·8)	--
Blindness and vision loss	--	11 (7·04–16)	11 (7·04–16)	--
Age-related and other hearing loss	--	9·99 (8·92–11·2)	9·99 (8·92–11·2)	--
Musculoskeletal disorders	5·47 (1·36–10·1)	23·5 (19·3–27·6)	23·9 (19·5–28)	6·49 (1·71–11·7)
Rheumatoid arthritis	12·3 (2·94–22·2)	14·8 (3·92–26)	15·2 (4·04–26·6)	13·9 (3·55–24·5)
Osteoarthritis	--	17·6 (9·23–27)	17·6 (9·23–27)	--
Low back pain	--	42·4 (36·4–48·1)	42·4 (36·4–48·1)	--
Gout	--	52·3 (36·6–69·4)	52·3 (36·6–69·4)	--
Other non-communicable diseases	0·0475 (0·0291–0·0707)	0·0475 (0·028–0·0744)	--	0·218 (0·131–0·315)
Sudden infant death syndrome	6·43 (3·62–10·8)	6·43 (3·61–10·7)	--	6·43 (3·61–10·7)
Injuries	37 (30·5–42·4)	28·8 (23·5–33·8)	31·1 (26·6–35·5)	26·4 (18·4–33·8)
Transport injuries	19·9 (11·6–28·1)	20·4 (13·4–27·5)	25·2 (20·9–29·3)	17·9 (8·73–26·8)
Road injuries	19 (10·2–27·5)	19·4 (12–26·6)	23·9 (19·6–28·1)	17·1 (7·43–26·4)
Other transport injuries	32·2 (26·9–37·1)	31·8 (26·6–36·6)	35·1 (30·5–39·2)	28·8 (22·3–34·5)
Unintentional injuries	44·3 (37·2–49·3)	31·7 (26·8–36·1)	31·5 (26·7–36·1)	32·1 (26·7–36·6)
Falls	55·1 (46·9–60·8)	42·5 (36·2–47·6)	40·4 (34·4–45·5)	46·8 (40·1–52·2)
Drowning	-15·6 (-30·8–-0·556)	-14·1 (-28·6–-0·318)	9·3 (4·83–15·2)	-15·3 (-30·2–-0·114)
Fire, heat, and hot substances	9·56 (4·95–15·4)	11·5 (6·56–17·8)	11·6 (6·54–18)	11·4 (6·46–17·2)

Poisonings	13·5 (8·09–20·1)	12·9 (7·69–19·4)	10·7 (6·14–16·5)	14·6 (8·99–21·3)
Exposure to mechanical forces	11·7 (-0·126–24·4)	16·1 (13·3–18·7)	17·5 (15–19·6)	0·06 (-12·3–13·5)
Animal contact	24·5 (19·5–29·4)	20·2 (15·6–25·4)	21·1 (16·1–26·5)	19 (14·6–24·2)
Foreign body	2·12 (1·32–3·22)	3·08 (1·96–4·63)	2·3 (1·45–3·44)	3·53 (2·22–5·23)
Environmental heat and cold exposure	7·35 (2·95–13·3)	8·7 (3·75–15·1)	8·66 (3·69–15)	8·83 (3·86–15·3)
Exposure to forces of nature	0 (0–0)	9·67 (4·28–16·5)	9·67 (4·28–16·5)	0 (0–0)
Other unintentional injuries	13 (7·71–19·5)	11·1 (6·23–17·2)	11 (6·11–17·1)	14·8 (9·19–21·7)
Self-harm and interpersonal violence	21·8 (5·99–35·8)	24·1 (8·87–37·2)	32 (23·5–40·1)	23·1 (6·97–37·5)
Self-harm	21·5 (5·11–36·2)	23 (6·38–38·1)	21·5 (7·55–34·7)	23·1 (6·29–38·1)
Interpersonal violence	27·1 (17·1–37·1)	32·9 (23·9–42)	38·4 (29·3–47·8)	24·5 (14·7–34·3)

Supplementary Results Table 2. All-age PAFs attributable to the joint distribution of all risk factors for all ages, both sexes combined for each GBD level 1, level 2, and level 3 cause and all causes in 2019 for Northumberland. Results are shown for deaths, YLLs, YLDs, and DALYs Data in parenthesis are 95% uncertainty intervals. PAFs=population attributable fractions. GBD=Global Burden of Disease. YLLs=years of life lost. YLDs=years lived with disability. DALYs=disability-adjusted life-years

	Deaths 2019	DALYs 2019	YLDs 2019	YLLs 2019
All causes	57·2 (53·9–60·3)	45·3 (42·3–48·4)	31·7 (29·3–34)	57·3 (54·5–60·1)
Communicable, maternal, neonatal, and nutritional diseases	43·9 (35·6–52·5)	48·4 (43·5–53·6)	47·6 (43·1–52)	48·8 (41·7–56·3)
HIV/AIDS and sexually transmitted infections	76·6 (60·4–89·4)	77 (59·2–90)	98·2 (97·1–99)	55·5 (35·4–76·8)
HIV/AIDS	93·4 (91·6–94·6)	92·8 (90·7–94·3)	95·2 (93·3–96·4)	90·8 (88·4–92·2)
Sexually transmitted infections excluding HIV	63 (41–84·5)	66·5 (42·7–87·5)	100 (100–100)	27·5 (12·4–52·5)
Respiratory infections and tuberculosis	45·4 (36·4–54·6)	43·3 (35·1–51·4)	3·48 (2·56–4·57)	48·4 (39·5–57·3)
Tuberculosis	55·9 (43·9–67·8)	59 (48–69·7)	56·7 (45·5–67·2)	59·5 (48·2–70·2)
Lower respiratory infections	45·3 (36·3–54·7)	48·3 (39·3–57·3)	33·7 (26·8–40·3)	48·4 (39·4–57·5)
Upper respiratory infections	0·0187 (0·00961–0·0336)	0·00576 (0·00126–0·0141)	0·00428 (1·33e-05–0·0132)	0·0516 (0·0262–0·0975)
Otitis media	1·09 (0·498–2·09)	3·73 (2·28–5·28)	3·74 (2·27–5·3)	3·33 (1·62–5·89)
Enteric infections	5·5 (3·02–9·91)	8·04 (5·44–12·4)	8·67 (5·95–13·1)	6·72 (4·23–11·1)
Diarrheal diseases	5·57 (3·06–9·99)	8·09 (5·46–12·5)	8·67 (5·96–13·2)	6·84 (4·3–11·1)
Other infectious diseases	0·434 (0·277–0·643)	0·824 (0·605–1·11)	0·43 (0·239–0·738)	1·01 (0·725–1·39)
Meningitis	0·837 (0·56–1·19)	1·51 (1·02–2·13)	0·000308 (6·49e-07–0·000958)	2·18 (1·5–3·05)
Encephalitis	0·14 (0·0864–0·214)	0·39 (0·237–0·605)	8·99e-05 (1·63e-07–0·000272)	0·541 (0·333–0·825)
Measles	1·72 (-0·415–10·3)	2·12 (-0·486–12)	0·709 (0·0202–1·87)	2·24 (-0·511–13·5)
Acute hepatitis	11·8 (4·9–24·4)	5·76 (3·15–9·92)	3·53 (2·09–5·9)	11·4 (4·69–23·5)
Maternal and neonatal disorders	87·1 (85–88·8)	74·7 (70·9–78·3)	66·7 (60·9–71·9)	87·8 (85·8–89·5)
Maternal disorders	9·36 (3·42–15·9)	9·29 (3·36–15·9)	9·32 (3·38–16)	9·19 (3·34–15·6)
Neonatal disorders	89·1 (87–90·8)	76·2 (72–80)	68·2 (62·2–73·5)	89·1 (87–90·8)
Nutritional deficiencies	72·8 (67·6–89·2)	79·2 (75–83·7)	79·4 (75·1–83·8)	74·4 (69·9–84·8)
Protein-energy malnutrition	100 (100–100)	100 (100–100)	100 (100–100)	100 (100–100)
Vitamin A deficiency	--	100 (100–100)	100 (100–100)	--
Dietary iron deficiency	--	100 (100–100)	100 (100–100)	--
Non-communicable diseases	59·2 (55·4–62·5)	46·4 (43·1–49·7)	31·1 (28·7–33·6)	59·8 (56·8–62·8)
Neoplasms	51·9 (47·9–56·5)	51·4 (47·6–55·7)	37·4 (32·7–42·6)	52·2 (48·4–56·4)
Lip and oral cavity cancer	66·3 (55–76)	69·1 (57·6–78·1)	67·7 (55·7–77·3)	69·3 (57·8–78·2)
Nasopharynx cancer	63·7 (50·9–75·3)	64·1 (51·2–75·2)	60·6 (47·6–72·2)	64·4 (51·6–75·5)
Other pharynx cancer	78·3 (69·8–84·5)	79·6 (70·9–85·6)	79·4 (70·8–85·8)	79·6 (70·9–85·7)
Esophageal cancer	76·1 (65·6–84·5)	77·6 (67·3–85·6)	76·5 (66·2–84·9)	77·6 (67·3–85·6)
Stomach cancer	26·5 (18·9–42·8)	26·9 (19·4–42·8)	27 (19·5–43·2)	26·9 (19·4–42·8)
Colon and rectum cancer	66·7 (57–76·3)	67 (58–76·1)	67·1 (57·9–76·2)	67 (58–76·1)

Liver cancer	77·2 (70·6–83·2)	78·1 (72·3–83·7)	78·3 (72–83·9)	78·1 (72·3–83·7)
Gallbladder and biliary tract cancer	23·2 (12·6–35·7)	24·1 (13·2–36·6)	21·8 (11·2–34·7)	24·1 (13·3–36·7)
Pancreatic cancer	47·3 (36·7–59·8)	46·7 (36·4–58·8)	47·1 (36·3–59·7)	46·7 (36·4–58·7)
Larynx cancer	81·8 (73·4–88·5)	81·8 (74–87·9)	80·7 (73·1–87·2)	81·9 (74·1–88)
Tracheal, bronchus, and lung cancer	88 (84·5–91·4)	87·2 (83·9–90·6)	87·8 (84·5–91·2)	87·2 (83·9–90·6)
Breast cancer	39·5 (28·1–53)	38·4 (28–50·6)	38·6 (28·1–51·1)	38·4 (28–50·5)
Cervical cancer	100 (100–100)	100 (100–100)	100 (100–100)	100 (100–100)
Uterine cancer	45·2 (29–61·6)	47 (31–62·7)	47·8 (32·1–63·6)	46·9 (30·8–62·7)
Ovarian cancer	30·8 (15·7–47·4)	27 (13·3–42·3)	25·4 (12·7–40·6)	27 (13·4–42·4)
Prostate cancer	6·23 (2·85–9·95)	6·87 (3·22–10·7)	7·35 (3·57–11·2)	6·8 (3·17–10·6)
Kidney cancer	41·7 (30·8–52·3)	42·3 (31·8–52·4)	42·4 (32–52·3)	42·3 (31·8–52·4)
Bladder cancer	44·2 (28·4–59·7)	45·8 (30·6–60·7)	46·3 (31·4–60·4)	45·8 (30·5–60·7)
Thyroid cancer	16·7 (7·68–27·7)	17·7 (8·31–29·1)	17·9 (8·73–29·4)	17·6 (8·25–29·1)
Mesothelioma	99·3 (99–99·5)	98·5 (98–99)	99·1 (98·6–99·4)	98·5 (98–99)
Non-Hodgkin lymphoma	8·07 (3·38–14·2)	8·44 (3·63–14·8)	8·3 (3·58–14·5)	8·46 (3·62–14·8)
Multiple myeloma	9·03 (3·83–16·1)	9·48 (4·12–16·8)	9·36 (3·89–16·9)	9·49 (4·13–16·8)
Leukemia	39·7 (24·9–54·8)	38·7 (25·8–51·4)	38·5 (25·3–51·3)	38·7 (25·8–51·4)
Cardiovascular diseases	79·9 (76–83·5)	81·4 (78·3–84·2)	71·7 (66·6–76·3)	82·7 (79·7–85·4)
Rheumatic heart disease	23·9 (13·5–46·4)	25·3 (15·4–43·1)	25·5 (15·1–43·5)	25·3 (15·4–42·8)
Ischemic heart disease	93·8 (90·2–96·8)	95 (92·4–97·2)	94·3 (91·4–96·8)	95 (92·4–97·3)
Stroke	79·9 (71·8–88·7)	83·2 (77·2–89·7)	83·1 (77·7–89·1)	83·3 (77·2–89·8)
Hypertensive heart disease	100 (100–100)	100 (100–100)	100 (100–100)	100 (100–100)
Non-rheumatic valvular heart disease	27·3 (17·3–40·9)	27·8 (19·4–38·2)	18·2 (12·3–25·3)	29·4 (20·4–40·5)
Cardiomyopathy and myocarditis	38·1 (29·3–48·2)	45 (37·7–51·7)	38·4 (31·1–46·3)	45·5 (37·9–52·3)
Atrial fibrillation and flutter	50·9 (40·1–62·8)	59·5 (50·6–68·7)	63·5 (54·8–72·2)	54·5 (44·8–65)
Aortic aneurysm	53·1 (46·6–60·9)	59·9 (54·2–66·5)	--	59·9 (54·2–66·5)
Endocarditis	32·7 (22·4–46·9)	35·8 (26·6–46·9)	32·5 (21·7–45·8)	35·9 (26·7–47)
Other cardiovascular and circulatory diseases	33·9 (27·3–41·3)	37·3 (31–43·7)	37·6 (30·4–44·9)	37·3 (31–43·8)
Chronic respiratory diseases	65·5 (60–71·5)	62·1 (57·9–66·7)	56 (50·9–60·9)	66·2 (61·2–71·6)
Chronic obstructive pulmonary disease	73·4 (68·7–77·8)	72·7 (68·7–76·4)	66·7 (62·7–70·7)	75·2 (71·2–79·1)
Pneumoconiosis	100 (100–100)	100 (100–100)	100 (100–100)	100 (100–100)
Asthma	44·7 (32·7–57·7)	45·4 (36·4–55·4)	45·1 (36·2–55·2)	47·5 (37·2–59)
Digestive diseases	27·6 (22·7–32·9)	33·3 (27·8–39·2)	19·3 (13·3–26·1)	38·4 (32·4–45)
Cirrhosis and other chronic liver diseases	68·7 (57·2–82·4)	72·9 (61·7–85·3)	70·4 (57·6–83·2)	72·9 (61·8–85·4)
Upper digestive system diseases	24·2 (20·4–28)	10·9 (7·24–15)	1·03 (0·608–1·63)	27 (23·1–30·7)

Gallbladder and biliary diseases	45·3 (29·1–61·3)	50·6 (36–65·6)	53 (38·8–67·3)	47 (31·4–62·3)
Pancreatitis	28·8 (8·39–50·2)	35·4 (13·8–55·8)	36·7 (14·5–57·5)	35·2 (13·7–55·8)
Neurological disorders	27·9 (11·7–44·3)	18·4 (9·34–32·5)	12·2 (6·15–22·1)	24·2 (10·2–41·2)
Alzheimer's disease and other dementias	45·7 (29·6–62)	47·6 (31·9–63·4)	47·9 (32·2–63·9)	47·4 (31·7–63·2)
Parkinson's disease	-10·4 (-16·7–-4·8)	-11·9 (-18·8–-5·53)	-12·4 (-19·5–-5·74)	-11·8 (-18·6–-5·49)
Idiopathic epilepsy	25·1 (13·5–37·3)	24·2 (13·3–35·4)	21·6 (11·4–31·9)	27·4 (15·4–39·2)
Multiple sclerosis	14·7 (10·9–18·8)	15·5 (11·5–19·7)	15·3 (11·4–19·5)	15·7 (11·7–20·1)
Mental disorders	--	6·28 (3·2–9·62)	6·29 (3·21–9·62)	--
Depressive disorders	--	12·7 (5·79–20)	12·7 (5·79–20)	--
Anxiety disorders	--	4·3 (1·2–9·48)	4·3 (1·2–9·48)	--
Idiopathic developmental intellectual disability	--	42·9 (20–61·1)	42·9 (20–61·1)	--
Substance use disorders	100 (100–100)	100 (100–100)	100 (100–100)	100 (100–100)
Alcohol use disorders	100 (100–100)	100 (100–100)	100 (100–100)	100 (100–100)
Drug use disorders	100 (100–100)	100 (100–100)	100 (100–100)	100 (100–100)
Diabetes and kidney diseases	99·7 (99·7–99·8)	100 (99·9–100)	100 (100–100)	99·8 (99·7–99·8)
Diabetes mellitus	100 (100–100)	100 (100–100)	100 (100–100)	100 (100–100)
Chronic kidney disease	100 (100–100)	100 (100–100)	100 (100–100)	100 (100–100)
Sense organ diseases	--	9·05 (7·74–10·6)	9·05 (7·74–10·6)	--
Blindness and vision loss	--	11·7 (7·46–17·1)	11·7 (7·46–17·1)	--
Age-related and other hearing loss	--	9·45 (8·43–10·7)	9·45 (8·43–10·7)	--
Musculoskeletal disorders	4·87 (1·22–8·94)	23·8 (19·6–28)	24·2 (19·9–28·5)	5·85 (1·51–10·4)
Rheumatoid arthritis	12·3 (3·18–21·7)	14·5 (3·95–25·5)	14·7 (4·03–26)	13·8 (3·63–24·3)
Osteoarthritis	--	18·5 (10·1–27·9)	18·5 (10·1–27·9)	--
Low back pain	--	42·6 (36·8–48·4)	42·6 (36·8–48·4)	--
Gout	--	55·3 (39·8–71·8)	55·3 (39·8–71·8)	--
Other non-communicable diseases	0·0473 (0·0307–0·0698)	0·0455 (0·0282–0·0717)	--	0·219 (0·143–0·318)
Sudden infant death syndrome	8·64 (4·79–13·8)	8·63 (4·79–13·8)	--	8·63 (4·79–13·8)
Injuries	35·5 (28·8–40·7)	29·1 (23·5–33·9)	32·2 (27·3–36·5)	25·3 (17·7–32·2)
Transport injuries	18·7 (11·8–25·8)	19·9 (13·5–26·2)	25·6 (21·5–29·9)	16·9 (8·82–24·9)
Road injuries	17·7 (10·5–25)	18·7 (12·1–25·2)	24·2 (20·2–28·5)	15·9 (7·6–24·1)
Other transport injuries	32·6 (27·2–37·7)	32·3 (26·9–37·2)	36·3 (31·6–40·6)	29·1 (22·8–34·9)
Unintentional injuries	43·2 (36·7–48·1)	32·4 (27·4–36·9)	32·8 (27·6–37·5)	31·7 (26·5–36)
Falls	55·3 (47–60·8)	43·6 (37·2–48·6)	41·9 (35·7–46·9)	47·9 (40·8–53·1)
Drowning	-16·2 (-29·4–-3·28)	-14·1 (-26·5–-1·9)	9·09 (4·76–14·6)	-15·4 (-28·7–-2·75)
Fire, heat, and hot substances	9·47 (4·98–15·2)	11·3 (6·58–17·1)	11·2 (6·52–17·2)	11·5 (6·65–17·3)

Poisonings	13<br>(7·8–19·5)	12·2<br>(7·39–18·1)	10·5<br>(6·11–16·1)	14·3<br>(8·91–20·8)
Exposure to mechanical forces	10·1<br>(0·829–19·8)	16·6<br>(13·7–19)	18·5<br>(15·9–20·5)	-0·517<br>(-9·74–9·51)
Animal contact	23·5<br>(18·5–29·2)	21·4<br>(16·5–27)	21·7<br>(16·7–27·4)	20·4<br>(15·8–26)
Foreign body	1·99<br>(1·22–3·02)	2·99<br>(1·88–4·47)	2·17<br>(1·41–3·17)	3·43<br>(2·11–5·19)
Environmental heat and cold exposure	7·6<br>(3·27–13·3)	8·58<br>(3·64–14·5)	8·48<br>(3·6–14·4)	8·92<br>(3·89–15·2)
Exposure to forces of nature	0<br>(0–0)	9·59<br>(4·22–16·2)	9·59<br>(4·22–16·2)	0<br>(0–0)
Other unintentional injuries	12·1<br>(6·96–18·4)	10·8<br>(6·22–16·6)	10·7<br>(6·09–16·5)	14·1<br>(8·69–20·9)
Self-harm and interpersonal violence	19·7<br>(3·03–34·3)	22·6<br>(7·22–36)	31·2<br>(23–38·9)	21·3<br>(4·59–36)
Self-harm	19·4<br>(2·27–34·5)	21·2<br>(3·87–36·4)	21<br>(7·31–34·4)	21·2<br>(3·62–36·5)
Interpersonal violence	27<br>(17·7–37·2)	33·5<br>(25·3–42)	38<br>(29·7–46·5)	24·2<br>(15·1–34·4)

Supplementary Results Table 2. All-age PAFs attributable to the joint distribution of all risk factors for all ages, both sexes combined for each GBD level 1, level 2, and level 3 cause and all causes in 2019 for Redcar and Cleveland. Results are shown for deaths, YLLs, YLDs, and DALYs Data in parenthesis are 95% uncertainty intervals. PAFs=population attributable fractions. GBD=Global Burden of Disease. YLLs=years of life lost. YLDs=years lived with disability. DALYs=disability-adjusted life-years

	Deaths 2019	DALYs 2019	YLDs 2019	YLLs 2019
All causes	58·7 (55·5–61·9)	47·1 (44–50·2)	32·1 (29·8–34·4)	58·5 (55·8–61·4)
Communicable, maternal, neonatal, and nutritional diseases	45 (34·8–54·9)	49·4 (43·3–55·2)	47·8 (43·4–52·5)	50 (41·4–58·3)
HIV/AIDS and sexually transmitted infections	75·3 (59·1–87·9)	75·8 (58·1–88·8)	97·7 (96·5–98·6)	57 (37·2–76·4)
HIV/AIDS	92·7 (91·1–93·8)	91·8 (89·6–93·4)	95·1 (93·3–96·4)	89·2 (87–90·5)
Sexually transmitted infections excluding HIV	50·1 (27·9–75·7)	60 (34·5–84·7)	100 (100–100)	19·6 (7·94–41·6)
Respiratory infections and tuberculosis	46·2 (35·2–56·8)	44·6 (35·3–53)	3·55 (2·62–4·68)	49·3 (38·7–59·3)
Tuberculosis	57·3 (45·3–68·8)	60·1 (49·3–70·2)	57 (45·7–67·6)	60·5 (49·8–70·7)
Lower respiratory infections	46·1 (35·1–56·8)	49·1 (38·4–59·2)	33·7 (26·8–40·7)	49·2 (38·4–59·4)
Upper respiratory infections	0·0264 (0·0138–0·0453)	0·0059 (0·000944–0·0149)	0·00509 (0–0·0143)	0·0667 (0·0349–0·115)
Otitis media	1·3 (0·535–2·81)	4·07 (2·56–5·82)	4·08 (2·56–5·83)	3·71 (1·65–7·17)
Enteric infections	5·97 (3·37–11·1)	9·14 (6·33–14·3)	9·71 (6·78–14·9)	7·74 (5·01–12·5)
Diarrheal diseases	6·08 (3·45–11·3)	9·22 (6·41–14·4)	9·72 (6·79–14·9)	7·96 (5·18–12·8)
Other infectious diseases	0·364 (0·252–0·512)	0·702 (0·524–0·895)	0·386 (0·218–0·664)	0·837 (0·606–1·09)
Meningitis	0·685 (0·457–0·989)	1·2 (0·794–1·72)	0·00034 (0–0·00104)	1·69 (1·11–2·43)
Encephalitis	0·162 (0·1–0·253)	0·441 (0·269–0·689)	0·00012 (0–0·000355)	0·582 (0·365–0·903)
Measles	2·64 (-0·649–13·2)	2·95 (-0·558–14·3)	0·787 (0·0208–2·26)	3·62 (-0·892–17·2)
Acute hepatitis	13·2 (7·05–24·5)	5·58 (3·37–8·97)	3·39 (1·97–5·62)	12·8 (7·02–23·4)
Maternal and neonatal disorders	82·5 (78·6–85·8)	76·7 (73·1–80)	68·9 (63·4–73·6)	84·3 (80·8–87·3)
Maternal disorders	9·49 (3·44–16·1)	9·4 (3·39–16)	9·49 (3·4–16·4)	9·34 (3·38–15·9)
Neonatal disorders	87·7 (84·5–90·4)	79·4 (75·6–82·7)	70·9 (65–75·8)	87·7 (84·6–90·4)
Nutritional deficiencies	77·3 (72·4–91·4)	80·2 (75·8–84·7)	80·3 (75·8–84·9)	76·4 (72·5–85·3)
Protein-energy malnutrition	100 (100–100)	100 (100–100)	100 (100–100)	100 (100–100)
Vitamin A deficiency	--	100 (100–100)	100 (100–100)	--
Dietary iron deficiency	--	100 (100–100)	100 (100–100)	--
Non-communicable diseases	60·7 (57·3–64·1)	48·3 (44·9–51·7)	31·6 (29·1–34·3)	61 (58·2–63·9)
Neoplasms	53·2 (49·1–57·8)	52·6 (48·5–57)	38·8 (34–44)	53·3 (49·2–57·6)
Lip and oral cavity cancer	64·7 (52·8–75·1)	67·3 (55·3–77·2)	65·8 (54·1–76·1)	67·4 (55·5–77·2)
Nasopharynx cancer	64·5 (50·8–75·6)	64·5 (50·6–75·6)	61 (47–73·2)	64·7 (50·8–75·7)
Other pharynx cancer	78·2 (69·8–85·2)	79·2 (70·8–85·7)	78·9 (70·1–85·6)	79·2 (70·8–85·7)

Esophageal cancer	74·9 (64·8–83·4)	76·2 (66·5–84·6)	75·2 (64·9–83·7)	76·2 (66·5–84·6)
Stomach cancer	26·4 (18·7–43)	26·6 (19–42·9)	26·7 (19·2–43·2)	26·6 (19–42·9)
Colon and rectum cancer	66·5 (56·8–76·3)	66·5 (57·2–75·8)	66·6 (57·3–76)	66·5 (57·1–75·8)
Liver cancer	77·5 (70·6–83·3)	77·8 (71·8–83)	78 (72·1–83·4)	77·8 (71·8–83)
Gallbladder and biliary tract cancer	21·9 (11·5–34·5)	22·8 (12·2–35·6)	20·6 (10·3–32·6)	22·9 (12·2–35·7)
Pancreatic cancer	47·8 (37·1–60·3)	47 (36·9–58·9)	47·5 (36·9–59·8)	47 (36·9–58·9)
Larynx cancer	82·1 (73·5–88·6)	82 (74·5–87·7)	80·9 (73·2–87)	82·1 (74·6–87·8)
Tracheal, bronchus, and lung cancer	88 (84·8–91·2)	87·1 (83·8–90·3)	87·8 (84·3–91·2)	87·1 (83·8–90·2)
Breast cancer	39·5 (28–53·6)	38·1 (27·8–51)	38·3 (28–51·1)	38·1 (27·8–50·9)
Cervical cancer	100 (100–100)	100 (100–100)	100 (100–100)	100 (100–100)
Uterine cancer	42·4 (26·6–59·1)	44·3 (28·5–60·8)	45·1 (29·4–61·6)	44·2 (28·4–60·7)
Ovarian cancer	29·8 (15–46·5)	25·9 (12·7–41·4)	24·2 (11·8–39·8)	25·9 (12·7–41·5)
Prostate cancer	6·3 (2·91–10·1)	6·93 (3·23–10·9)	7·4 (3·48–11·5)	6·87 (3·21–10·9)
Kidney cancer	39·8 (29·1–50·5)	40·2 (29·8–50·3)	40·2 (29·9–50·1)	40·2 (29·8–50·3)
Bladder cancer	45·4 (29·3–61·1)	46·9 (31·5–61·6)	47·3 (32·1–61·5)	46·9 (31·4–61·6)
Thyroid cancer	14·1 (6·66–23·3)	14·9 (7·2–24·3)	15·1 (7·47–24·7)	14·9 (7·14–24·3)
Mesothelioma	99·4 (99·1–99·6)	98·7 (98·2–99·1)	99·2 (98·8–99·4)	98·7 (98·2–99·1)
Non-Hodgkin lymphoma	7·12 (2·86–12·6)	7·42 (3·06–13·1)	7·31 (2·98–13)	7·43 (3·08–13·1)
Multiple myeloma	8·02 (3·31–14·5)	8·44 (3·59–15·1)	8·28 (3·25–15·1)	8·45 (3·6–15·1)
Leukemia	38·9 (23·9–53·6)	37·4 (24·8–49·9)	37·3 (24·5–50·3)	37·4 (24·8–50)
Cardiovascular diseases	80·7 (76·6–84·3)	82·4 (79·2–85·3)	73·3 (68·4–77·8)	83·2 (80·3–86)
Rheumatic heart disease	24 (13·3–47·3)	25·1 (15·3–43·6)	25·5 (15·8–42·3)	25·1 (15·2–43·5)
Ischemic heart disease	93·8 (90·2–96·7)	95 (92·4–97·2)	94·3 (91·4–96·7)	95 (92·4–97·2)
Stroke	80·1 (71·9–89·1)	83·4 (77·3–89·9)	83 (77·4–89)	83·4 (77·2–90·1)
Hypertensive heart disease	100 (100–100)	100 (100–100)	100 (100–100)	100 (100–100)
Non-rheumatic valvular heart disease	27·1 (17·5–40·4)	28·1 (19·5–38·6)	19·1 (12·7–26·6)	29 (20·4–40)
Cardiomyopathy and myocarditis	39·3 (29·9–48·8)	46 (38–52·3)	37·5 (30·4–45·1)	46·4 (38·2–52·9)
Atrial fibrillation and flutter	49·7 (39·2–61·5)	57·8 (48·8–66·9)	62·3 (53·8–70·9)	53·3 (43·5–64·1)
Aortic aneurysm	54 (47·3–61·5)	60·5 (54·7–66·5)	--	60·5 (54·7–66·5)
Endocarditis	33·1 (22·6–46·9)	36·1 (26·7–46·5)	32·8 (22·5–45·9)	36·2 (26·7–46·6)
Other cardiovascular and circulatory diseases	32·9 (26·2–40·3)	35·7 (29–42)	36·4 (29·5–43·8)	35·6 (29–42·1)
Chronic respiratory diseases	66·4 (60·1–72·7)	62·5 (57·8–67·4)	54·7 (49·6–59·6)	66·7 (61·1–72·7)
Chronic obstructive pulmonary disease	74·1 (69·2–78·6)	73·5 (69·5–77·2)	67·1 (63–70·8)	75·7 (71·3–79·5)

Pneumoconiosis	100 (100–100)	100 (100–100)	100 (100–100)	100 (100–100)
Asthma	42·8 (30·9–56·3)	42·6 (33·6–52·5)	42·3 (33·5–52·3)	45 (34·6–56·5)
Digestive diseases	28·1 (23·5–33·6)	35·4 (29·8–41·3)	26·6 (18·2–35·7)	39·1 (33·2–45·9)
Cirrhosis and other chronic liver diseases	68·1 (57·3–81·5)	72 (61·4–84·9)	68·5 (56·6–81·2)	72·1 (61·5–85)
Upper digestive system diseases	24·1 (20·5–27·9)	11·7 (7·92–15·7)	0·9 (0·53–1·4)	26·3 (22·7–30)
Gallbladder and biliary diseases	42·7 (27·3–58·8)	50·1 (35·4–64·9)	52 (37–66·6)	44·6 (30·1–60·6)
Pancreatitis	27·9 (7·57–49·5)	33·9 (11·8–56·3)	33·7 (11·9–56·4)	33·9 (11·7–56·3)
Neurological disorders	25·7 (10·2–43·6)	16·9 (8·41–30·3)	11·3 (5·69–20·5)	22·1 (8·89–39·5)
Alzheimer's disease and other dementias	45·6 (30·4–61·8)	47·4 (32·3–63)	47·8 (32·4–63·5)	47·2 (32–62·8)
Parkinson's disease	-10·6 (-17·5–-5·03)	-12 (-19·7–-5·81)	-12·6 (-20·6–-6·04)	-11·9 (-19·5–-5·77)
Idiopathic epilepsy	23·6 (11·8–34·9)	22·8 (11·5–33·4)	20·4 (10–30·2)	25·6 (13·3–36·9)
Multiple sclerosis	15·3 (11·4–19·6)	16·1 (12–20·5)	15·7 (11·8–20)	16·3 (12·1–20·7)
Mental disorders	--	6·46 (3·3–9·92)	6·46 (3·3–9·92)	--
Depressive disorders	--	13 (5·85–20·4)	13 (5·85–20·4)	--
Anxiety disorders	--	4·55 (1·2–10·2)	4·55 (1·2–10·2)	--
Idiopathic developmental intellectual disability	--	44·4 (21–64·6)	44·4 (21–64·6)	--
Substance use disorders	100 (100–100)	100 (100–100)	100 (100–100)	100 (100–100)
Alcohol use disorders	100 (100–100)	100 (100–100)	100 (100–100)	100 (100–100)
Drug use disorders	100 (100–100)	100 (100–100)	100 (100–100)	100 (100–100)
Diabetes and kidney diseases	100 (100–100)	100 (100–100)	100 (100–100)	100 (100–100)
Diabetes mellitus	100 (100–100)	100 (100–100)	100 (100–100)	100 (100–100)
Chronic kidney disease	100 (100–100)	100 (100–100)	100 (100–100)	100 (100–100)
Sense organ diseases	--	9·09 (7·8–10·6)	9·09 (7·8–10·6)	--
Blindness and vision loss	--	11·5 (7·28–17)	11·5 (7·28–17)	--
Age-related and other hearing loss	--	9·54 (8·53–10·7)	9·54 (8·53–10·7)	--
Musculoskeletal disorders	4·58 (1·23–8·29)	22·8 (18·7–27·2)	23·3 (19·1–27·7)	5·33 (1·51–9·39)
Rheumatoid arthritis	12·5 (3·41–21·9)	14·6 (3·69–25·2)	14·8 (3·9–25·9)	13·9 (3·77–24·3)
Osteoarthritis	--	16·9 (8·74–25·8)	16·9 (8·74–25·8)	--
Low back pain	--	41·3 (35·4–47)	41·3 (35·4–47)	--
Gout	--	52·4 (37·3–68·6)	52·4 (37·3–68·6)	--
Other non-communicable diseases	0·0553 (0·0351–0·0814)	0·0563 (0·0334–0·0863)	--	0·241 (0·148–0·352)
Sudden infant death syndrome	6·33 (3·59–10·6)	6·33 (3·59–10·6)	--	6·33 (3·59–10·6)
Injuries	32 (25·4–37·9)	26·8 (21·3–32·1)	30·4 (25·7–34·8)	23 (14·2–30·6)

Transport injuries	18·4<br>(10·3–26·3)	18·9<br>(11·7–26·2)	25·3<br>(21·1–29·5)	16·1<br>(7·3–24·9)
Road injuries	17·3<br>(8·82–25·4)	17·7<br>(10·2–25·1)	24·1<br>(19·7–28·3)	15<br>(5·75–24·1)
Other transport injuries	31·3<br>(25·3–36·5)	30·4<br>(24·7–35·7)	35·5<br>(30·4–39·6)	27·7<br>(21·2–33·8)
Unintentional injuries	41·3<br>(34·8–45·9)	30·4<br>(25·5–34·8)	30·8<br>(25·9–35·3)	29·4<br>(24·4–33·7)
Falls	54<br>(46·2–59·5)	41·7<br>(35·4–46·7)	40·2<br>(34·2–45·2)	45·8<br>(39·2–51)
Drowning	-17·1<br>(-31·5–-1·97)	-15·7<br>(-29·1–-1·75)	8·93<br>(4·53–14·7)	-17·3<br>(-31·3–-2·39)
Fire, heat, and hot substances	8·56<br>(4·38–14·3)	10·9<br>(6·35–17·1)	11·2<br>(6·61–17·5)	10·1<br>(5·87–15·8)
Poisonings	13<br>(7·93–19·8)	12·2<br>(7·46–18·7)	10·4<br>(6·12–16·5)	14<br>(8·89–21)
Exposure to mechanical forces	5·97<br>(-5·4–17·7)	15·7<br>(12·5–18·6)	18·1<br>(15·3–20·4)	-2·76<br>(-14·7–9·98)
Animal contact	21·3<br>(16·4–27·4)	20·5<br>(15·6–26·3)	21·1<br>(16–27)	18·7<br>(14–24·5)
Foreign body	2·36<br>(1·5–3·7)	3·17<br>(2·02–4·86)	2·38<br>(1·53–3·62)	3·72<br>(2·37–5·74)
Environmental heat and cold exposure	7·86<br>(3·31–14·1)	8·42<br>(3·49–14·6)	8·22<br>(3·41–14·4)	9·02<br>(3·8–15·6)
Exposure to forces of nature	0<br>(0–0)	9·2<br>(3·83–16)	9·2<br>(3·83–16)	0<br>(0–0)
Other unintentional injuries	11·4<br>(6·74–17·8)	10·7<br>(6·21–16·9)	10·6<br>(6·14–16·8)	13·4<br>(8·33–20·1)
Self-harm and interpersonal violence	19·4<br>(3·32–34·8)	21·9<br>(6·94–36·1)	31·3<br>(22·9–39·1)	20·7<br>(4·59–36)
Self-harm	19·1<br>(2·29–35)	20·6<br>(4–36·3)	20·1<br>(6·57–34·1)	20·7<br>(3·7–36·5)
Interpersonal violence	25·3<br>(15·1–35·3)	31·4<br>(22·5–40·5)	37·8<br>(28·5–46·8)	22·7<br>(12·4–32·8)

Supplementary Results Table 2. All-age PAFs attributable to the joint distribution of all risk factors for all ages, both sexes combined for each GBD level 1, level 2, and level 3 cause and all causes in 2019 for South Tyneside. Results are shown for deaths, YLLs, YLDs, and DALYs Data in parenthesis are 95% uncertainty intervals. PAFs=population attributable fractions. GBD=Global Burden of Disease. YLLs=years of life lost. YLDs=years lived with disability. DALYs=disability-adjusted life-years

	Deaths 2019	DALYs 2019	YLDs 2019	YLLs 2019
All causes	58·8 (55·7–62·1)	46·7 (43·7–49·9)	31·7 (29·4–34·1)	59 (56·4–62)
Communicable, maternal, neonatal, and nutritional diseases	45·2 (34·5–54·7)	49·2 (43·3–54·8)	47·8 (43·1–52·3)	49·9 (41–58·1)
HIV/AIDS and sexually transmitted infections	71·7 (52·6–86·8)	73·6 (54·3–88·3)	98·1 (97·1–98·9)	50·8 (30·1–73)
HIV/AIDS	93·4 (91·8–94·4)	92·6 (90·7–94·1)	95·3 (93·6–96·5)	90·3 (88·3–91·6)
Sexually transmitted infections excluding HIV	49·5 (27·3–76)	60·3 (34·5–84·8)	100 (100–100)	18·7 (7·58–40·5)
Respiratory infections and tuberculosis	47 (35·5–57·4)	44·7 (35·3–54)	3·54 (2·59–4·69)	50·2 (39·3–60·3)
Tuberculosis	56·6 (44·4–68)	59·8 (48·4–70·3)	56·8 (44·9–67·8)	60·3 (48·9–70·5)
Lower respiratory infections	46·9 (35·2–57·4)	50 (39–60·2)	34·1 (27·5–41)	50·1 (39·1–60·3)
Upper respiratory infections	0·0233 (0·0121–0·0436)	0·00629 (0·000902–0·0163)	0·00542 (0–0·0158)	0·0578 (0·0297–0·107)
Otitis media	0·579 (0·274–1·09)	3·98 (2·51–5·67)	3·98 (2·51–5·67)	2·43 (1·35–4·02)
Enteric infections	5·87 (3·24–10·7)	8·7 (5·97–13·5)	9·5 (6·54–14·4)	7·27 (4·61–11·9)
Diarrheal diseases	5·95 (3·27–10·8)	8·77 (6–13·6)	9·51 (6·55–14·4)	7·43 (4·72–12·1)
Other infectious diseases	0·333 (0·227–0·487)	0·64 (0·473–0·852)	0·394 (0·218–0·675)	0·756 (0·542–1·05)
Meningitis	0·609 (0·405–0·822)	1·03 (0·689–1·38)	0·000352 (0–0·00115)	1·47 (1·02–1·96)
Encephalitis	0·141 (0·0885–0·228)	0·367 (0·229–0·562)	0·000121 (0–0·000366)	0·494 (0·314–0·759)
Measles	2·1 (-0·533–12)	2·67 (-0·669–14·5)	0·785 (0·0225–2·31)	2·71 (-0·692–15)
Acute hepatitis	10·9 (5·17–24)	5 (2·86–8·39)	3·32 (1·91–5·58)	10·8 (5·31–23·2)
Maternal and neonatal disorders	84·6 (81·7–87·1)	73·8 (69·6–77·6)	65·6 (59·9–70·9)	86·1 (83·4–88·3)
Maternal disorders	9·64 (3·48–16·3)	9·56 (3·43–16·4)	9·61 (3·41–16·8)	9·48 (3·43–16·1)
Neonatal disorders	88·6 (86·1–90·7)	75·8 (71·6–79·9)	67·4 (61·4–72·8)	88·6 (86·1–90·7)
Nutritional deficiencies	80·5 (71·8–88)	80·9 (76·6–85·4)	80·8 (76·4–85·5)	81·9 (68·9–85·2)
Protein-energy malnutrition	100 (100–100)	100 (100–100)	100 (100–100)	100 (100–100)
Vitamin A deficiency	--	100 (100–100)	100 (100–100)	--
Dietary iron deficiency	--	100 (100–100)	100 (100–100)	--
Non-communicable diseases	60·8 (57·4–64·2)	47·9 (44·6–51·4)	31·2 (28·7–33·9)	61·6 (58·8–64·6)
Neoplasms	55·3 (51·2–59·7)	54·5 (50·7–58·7)	38·9 (34·1–44)	55·2 (51·5–59·4)
Lip and oral cavity cancer	66·1 (53·4–76·5)	68·9 (56·3–78·3)	67·4 (55–77·3)	68·9 (56·5–78·4)
Nasopharynx cancer	63 (49·2–74·6)	63·2 (49·4–74·4)	59·1 (45·2–72)	63·6 (49·7–74·7)
Other pharynx cancer	79·3 (71–86)	80·3 (72·1–86·7)	79·9 (71·5–86·5)	80·3 (72–86·7)
Esophageal cancer	75·2 (65·2–83·8)	76·6 (66·7–84·7)	75·6 (65·5–84·1)	76·7 (66·7–84·7)

Stomach cancer	26·7 (19·1–43)	26·9 (19·3–43·1)	27 (19·4–43·5)	26·9 (19·3–43·1)
Colon and rectum cancer	66·2 (56–76)	66·4 (57·1–75·6)	66·5 (56·9–75·8)	66·4 (57·1–75·6)
Liver cancer	77 (70·7–83·1)	77·5 (71·9–83·1)	77·6 (71·6–83·4)	77·5 (71·9–83·1)
Gallbladder and biliary tract cancer	21·3 (11·2–33·6)	22·1 (11·8–34·4)	20 (10·4–32·1)	22·2 (11·9–34·4)
Pancreatic cancer	47·6 (36·8–60·4)	46·6 (36·8–58·9)	47·2 (36·7–59·8)	46·6 (36·8–58·9)
Larynx cancer	82·8 (75–89)	82·8 (75·8–88·4)	81·7 (74·4–87·6)	82·9 (76–88·5)
Tracheal, bronchus, and lung cancer	88·9 (85·4–92)	88 (84·7–91)	88·7 (85·2–91·8)	88 (84·7–91)
Breast cancer	39·1 (27·8–53)	37·7 (27·2–50·3)	38 (27·4–50·9)	37·7 (27·2–50·2)
Cervical cancer	100 (100–100)	100 (100–100)	100 (100–100)	100 (100–100)
Uterine cancer	41·2 (25·9–57·6)	43·2 (28–59·5)	44·1 (28·7–60·6)	43·1 (27·9–59·4)
Ovarian cancer	35·1 (16·2–52·1)	29·8 (14·1–45·5)	27·8 (13·3–42·9)	29·8 (14·1–45·5)
Prostate cancer	6·39 (2·93–10)	7·03 (3·26–11)	7·5 (3·57–11·7)	6·97 (3·23–11)
Kidney cancer	39·6 (29·2–50·2)	39·9 (29·9–49·9)	39·9 (29·9–49·8)	39·9 (29·9–49·9)
Bladder cancer	45·7 (29·6–60·9)	47·3 (31·8–61·8)	47·8 (32·8–62)	47·3 (31·8–61·8)
Thyroid cancer	13·4 (6·46–22·2)	14·3 (6·93–23·4)	14·5 (7·21–23·7)	14·3 (6·9–23·4)
Mesothelioma	99·6 (99·4–99·7)	99·1 (98·7–99·4)	99·4 (99·2–99·6)	99·1 (98·7–99·4)
Non-Hodgkin lymphoma	6·81 (2·78–12·3)	7·14 (2·97–12·7)	7·03 (2·88–12·7)	7·15 (2·97–12·7)
Multiple myeloma	7·74 (3·17–13·9)	8·16 (3·36–14·7)	8·01 (3·14–14·9)	8·16 (3·38–14·6)
Leukemia	38·7 (23·9–53·6)	37 (24·7–49·7)	36·9 (24·4–50)	37 (24·7–49·6)
Cardiovascular diseases	80·1 (76·2–84)	81·8 (78·8–84·9)	72·1 (67·1–77·1)	83 (80·2–85·8)
Rheumatic heart disease	23·8 (13·3–45·4)	25·1 (15·7–43·8)	25·4 (15·8–43·6)	25·1 (15·6–43·6)
Ischemic heart disease	93·9 (90·2–97)	95·2 (92·6–97·3)	94·4 (91·8–96·8)	95·2 (92·6–97·3)
Stroke	79·8 (70·7–89)	83·3 (77–89·7)	83·2 (77·7–89·1)	83·3 (76·8–89·8)
Hypertensive heart disease	100 (100–100)	100 (100–100)	100 (100–100)	100 (100–100)
Non-rheumatic valvular heart disease	26·2 (17·1–39·3)	27·4 (19–37·5)	21·3 (14·6–29·1)	28·2 (19·5–38·5)
Cardiomyopathy and myocarditis	38·9 (29·8–48·8)	46·1 (38·5–52·4)	38·4 (31·3–45·8)	46·6 (38·6–53·2)
Atrial fibrillation and flutter	48·8 (37·7–60·6)	57·6 (47·9–67)	62·1 (53·1–70·6)	52·6 (42·3–63·1)
Aortic aneurysm	53 (46·3–60·8)	60·1 (54·1–66·2)	--	60·1 (54·1–66·2)
Endocarditis	32·5 (22·2–46·4)	35·5 (26·1–46·3)	32·4 (21·9–45·9)	35·6 (26·2–46·3)
Other cardiovascular and circulatory diseases	33·9 (27–41·3)	37·1 (30·8–43·2)	36·8 (29–44·3)	37·1 (30·9–43·2)
Chronic respiratory diseases	67·8 (61·5–74·1)	63·5 (58·7–68·4)	54·6 (49·5–59·7)	68·3 (62·4–74·2)
Chronic obstructive pulmonary disease	74·4 (69–79·4)	74 (69·6–78·1)	67·5 (63·3–71·2)	76·1 (71·3–80·7)
Pneumoconiosis	100 (100–100)	100 (100–100)	100 (100–100)	100 (100–100)

Asthma	42 (30·6–55·2)	42·3 (33·7–52·2)	42·1 (33·5–52)	44·3 (34·2–55·6)
Digestive diseases	29·3 (24·3–35·3)	36·7 (30·8–43·3)	26·9 (18·7–36·2)	41·2 (34·8–48·8)
Cirrhosis and other chronic liver diseases	68·3 (57·6–82·1)	72·4 (61·7–85·4)	69·5 (57·4–82·2)	72·4 (61·7–85·4)
Upper digestive system diseases	25·1 (21·2–28·9)	11·2 (7·41–15·4)	0·921 (0·542–1·42)	27·9 (23·9–31·6)
Gallbladder and biliary diseases	41·6 (26·7–57·2)	47·8 (33·6–62·4)	48·9 (34·8–63·4)	43·3 (28·7–58·5)
Pancreatitis	28·5 (8·06–49·4)	35·2 (11·9–56·5)	35·7 (11·8–57·4)	35·1 (11·9–56·2)
Neurological disorders	26·6 (11·1–43·8)	16·8 (8·22–30·2)	10·8 (5·43–20·3)	22·9 (9·79–39·6)
Alzheimer's disease and other dementias	44·7 (28·7–61·8)	46·5 (30·7–62·9)	46·9 (31·3–63·2)	46·3 (30·7–62·7)
Parkinson's disease	-10·6 (-17–-4·8)	-12·2 (-19·3–-5·66)	-12·8 (-20·4–-6·06)	-12 (-19·2–-5·58)
Idiopathic epilepsy	24·4 (12–35·8)	23·7 (11·9–34·7)	20·9 (10·4–30·8)	26·4 (13·7–38·1)
Multiple sclerosis	15·7 (11·6–19·9)	16·4 (12·2–20·6)	15·9 (11·9–20)	16·7 (12·5–21·1)
Mental disorders	--	6·54 (3·33–10·1)	6·54 (3·33–10·1)	--
Depressive disorders	--	13·2 (5·94–20·9)	13·2 (5·94–20·9)	--
Anxiety disorders	--	4·49 (1·2–10)	4·49 (1·2–10)	--
Idiopathic developmental intellectual disability	--	45·5 (21·5–64·7)	45·5 (21·5–64·7)	--
Substance use disorders	100 (100–100)	100 (100–100)	100 (100–100)	100 (100–100)
Alcohol use disorders	100 (100–100)	100 (100–100)	100 (100–100)	100 (100–100)
Drug use disorders	100 (100–100)	100 (100–100)	100 (100–100)	100 (100–100)
Diabetes and kidney diseases	99·7 (99·6–99·8)	99·9 (99·9–100)	100 (100–100)	99·7 (99·7–99·8)
Diabetes mellitus	100 (100–100)	100 (100–100)	100 (100–100)	100 (100–100)
Chronic kidney disease	100 (100–100)	100 (100–100)	100 (100–100)	100 (100–100)
Sense organ diseases	--	9·32 (8–10·9)	9·32 (8–10·9)	--
Blindness and vision loss	--	11·2 (7·03–16·6)	11·2 (7·03–16·6)	--
Age-related and other hearing loss	--	9·98 (8·91–11·3)	9·98 (8·91–11·3)	--
Musculoskeletal disorders	4·51 (1·13–8·25)	22·8 (18·8–27)	23·2 (19·1–27·5)	5·33 (1·4–9·44)
Rheumatoid arthritis	12·5 (3·15–21·8)	14·9 (3·91–25·8)	15·1 (3·99–26·3)	14·1 (3·69–24·4)
Osteoarthritis	--	16·5 (8·5–25·3)	16·5 (8·5–25·3)	--
Low back pain	--	41·4 (35·8–47·4)	41·4 (35·8–47·4)	--
Gout	--	50·7 (35·7–67·5)	50·7 (35·7–67·5)	--
Other non-communicable diseases	0·041 (0·026–0·0613)	0·0402 (0·0233–0·0614)	--	0·177 (0·11–0·26)
Sudden infant death syndrome	6·8 (3·71–10·9)	6·79 (3·71–10·9)	--	6·79 (3·71–10·9)
Injuries	32 (25·6–37·4)	26·7 (21·4–31·7)	29·9 (25·5–34·4)	23·1 (15·3–29·9)
Transport injuries	19·6 (11·3–27·7)	20 (12·7–27·2)	25·3 (21–29·5)	17·4 (7·71–26·4)

Road injuries	18·5 (9·79–27·2)	18·8 (11·2–26·4)	24 (19·6–28·2)	16·4 (6·39–25·9)
Other transport injuries	31·5 (26–36·9)	30·9 (25·5–36·1)	35·4 (30·6–39·6)	27·9 (21·6–34·3)
Unintentional injuries	38·8 (32·7–43·4)	29·1 (24·5–33·5)	30·2 (25·5–34·8)	26·6 (22·2–30·9)
Falls	53·6 (45·8–59)	40·8 (35–45·9)	39·4 (33·6–44·4)	44·6 (38·4–49·9)
Drowning	-16·2 (-31·2–-2·15)	-15 (-29·2–-1·59)	9·18 (4·92–14·8)	-16 (-30·6–-2·17)
Fire, heat, and hot substances	9·32 (5·01–14·9)	11·3 (6·52–17·6)	11·4 (6·46–17·9)	11·1 (6·45–17·1)
Poisonings	13·4 (8·04–20·3)	12·5 (7·58–19)	10·7 (6·26–16·6)	14·4 (8·75–21·3)
Exposure to mechanical forces	9·3 (-2·15–21·9)	16 (13·1–18·8)	17·9 (15·3–20·1)	-1·3 (-13·8–12·7)
Animal contact	21·1 (16·4–27·1)	20·6 (15·9–26·2)	21·2 (16·4–26·8)	18·4 (13·8–24·1)
Foreign body	2·4 (1·49–3·67)	3·38 (2·14–5·03)	2·49 (1·61–3·65)	3·86 (2·41–5·74)
Environmental heat and cold exposure	7·8 (3·11–13·9)	8·55 (3·51–14·9)	8·24 (3·31–14·4)	9·2 (3·88–16)
Exposure to forces of nature	0 (0–0)	9·28 (3·86–16·3)	9·28 (3·86–16·3)	0 (0–0)
Other unintentional injuries	12·6 (7·24–19·3)	11 (6·23–17·1)	10·8 (6·08–17)	14·4 (8·63–21·4)
Self-harm and interpersonal violence	20·7 (4·6–34·9)	23·1 (7·93–36·7)	31·8 (23·5–40·5)	22 (5·61–36·4)
Self-harm	20·4 (3·36–35·6)	21·8 (4·7–37·1)	20·4 (7·6–33·5)	21·8 (4·59–37·1)
Interpersonal violence	26·6 (16·4–36·5)	32 (23–41·6)	38·1 (29–47·6)	23·9 (14–33·6)

Supplementary Results Table 2. All-age PAFs attributable to the joint distribution of all risk factors for all ages, both sexes combined for each GBD level 1, level 2, and level 3 cause and all causes in 2019 for Stockton-on-Tees. Results are shown for deaths, YLLs, YLDs, and DALYs Data in parenthesis are 95% uncertainty intervals. PAFs=population attributable fractions. GBD=Global Burden of Disease. YLLs=years of life lost. YLDs=years lived with disability. DALYs=disability-adjusted life-years

	Deaths 2019	DALYs 2019	YLDs 2019	YLLs 2019
All causes	58·4 (55·3–61·5)	45·5 (42·5–48·6)	31·1 (28·7–33·4)	57·9 (55·2–60·8)
Communicable, maternal, neonatal, and nutritional diseases	45·7 (36·4–55·4)	51 (45·8–56·1)	46·3 (41·5–51)	53·2 (45·9–60·8)
HIV/AIDS and sexually transmitted infections	66·1 (46·9–83·3)	71·3 (51·3–87·6)	98·1 (97–98·9)	47 (27·4–69·7)
HIV/AIDS	92·6 (90·7–93·8)	91·9 (89·7–93·5)	94·9 (93–96·2)	89·5 (87–90·9)
Sexually transmitted infections excluding HIV	34·4 (16·4–61·2)	57·7 (32·5–83)	100 (100–100)	12·1 (4·54–27·7)
Respiratory infections and tuberculosis	46·7 (36·3–57·1)	44·3 (35·8–52·8)	3·26 (2·4–4·33)	50·1 (40·2–59·9)
Tuberculosis	56·5 (44·5–67·8)	59·2 (48·2–69·4)	56·8 (45·1–67·4)	59·7 (48·8–70)
Lower respiratory infections	46·7 (36·3–57·2)	50 (40·1–59·8)	35 (28·4–41·8)	50·1 (40·1–60)
Upper respiratory infections	0·0318 (0·0173–0·0553)	0·00755 (0·00147–0·018)	0·00574 (0–0·0159)	0·0726 (0·0387–0·131)
Otitis media	2·33 (1·04–4·39)	4·59 (2·92–6·35)	4·56 (2·89–6·31)	6·02 (2·9–10·6)
Enteric infections	5·38 (3·16–9·51)	8·89 (6·41–12·9)	9·48 (6·75–13·7)	7·64 (5·14–11·7)
Diarrheal diseases	5·47 (3·21–9·63)	8·97 (6·46–13)	9·49 (6·76–13·7)	7·84 (5·28–12·1)
Other infectious diseases	0·62 (0·412–0·868)	1·06 (0·77–1·37)	0·39 (0·217–0·661)	1·4 (0·961–1·87)
Meningitis	1·31 (0·927–1·82)	2·06 (1·43–2·82)	0·000464 (0–0·00146)	2·83 (2·01–3·86)
Encephalitis	0·268 (0·17–0·405)	0·62 (0·38–0·962)	0·000154 (0–0·000477)	0·848 (0·53–1·28)
Measles	3·14 (-0·91–16·7)	3·79 (-1·07–20·1)	0·641 (0·0176–1·8)	3·88 (-1·12–20·3)
Acute hepatitis	7·15 (2·39–21·1)	4·41 (2·14–9·2)	3·12 (1·79–5·13)	6·71 (2·23–19·6)
Maternal and neonatal disorders	87·4 (85·4–89·4)	76·9 (73·3–80·2)	62·2 (56·1–67·9)	88·4 (86·5–90·3)
Maternal disorders	9·49 (3·45–16·3)	9·4 (3·46–16·2)	9·45 (3·46–16·5)	9·33 (3·38–16)
Neonatal disorders	90 (88–92)	78·7 (75–82·1)	64 (57·8–70)	90 (88·1–92)
Nutritional deficiencies	65·2 (58·8–85·6)	79·8 (75·7–84·4)	80·1 (75·9–84·8)	67·6 (62·6–79·8)
Protein-energy malnutrition	100 (100–100)	100 (100–100)	100 (100–100)	100 (100–100)
Vitamin A deficiency	--	100 (100–100)	100 (100–100)	--
Dietary iron deficiency	--	100 (100–100)	100 (100–100)	--
Non-communicable diseases	60·4 (57·1–63·6)	46·6 (43·3–50)	30·6 (28·1–33·3)	60·4 (57·6–63·4)
Neoplasms	53·4 (49·3–58·1)	52·4 (48·4–56·8)	38·2 (33·6–43·3)	53·1 (49–57·5)
Lip and oral cavity cancer	66·5 (55·2–76·2)	69 (58·1–78·3)	67·7 (56·3–77·5)	69·1 (58·2–78·3)
Nasopharynx cancer	63·3 (50·2–74·6)	63·4 (50·2–74·2)	60·1 (46·2–72·1)	63·7 (50·6–74·4)
Other pharynx cancer	79·3 (71·3–85·5)	80·3 (72·1–86·4)	80 (71·9–86·3)	80·3 (72·1–86·4)
Esophageal cancer	75·7 (65·2–84·5)	77·1 (66·5–85·5)	76·1 (65·3–84·6)	77·1 (66·5–85·5)

Stomach cancer	26·8 (19·2–43·1)	26·7 (19·2–43)	27 (19·4–43·6)	26·7 (19·2–43)
Colon and rectum cancer	66·5 (57·1–76·2)	66·6 (57·8–75·6)	66·7 (57·7–75·9)	66·6 (57·8–75·5)
Liver cancer	75·8 (69·1–82)	76 (69·7–81·5)	76·5 (70–82·2)	76 (69·7–81·5)
Gallbladder and biliary tract cancer	22·1 (11·6–34·8)	22·9 (12·2–35·8)	20·8 (10·2–33·7)	23 (12·3–35·9)
Pancreatic cancer	48·2 (37·7–60·8)	47·2 (37·5–59·3)	47·8 (37·4–60·3)	47·2 (37·5–59·3)
Larynx cancer	82·7 (74·9–88·9)	82·5 (75·3–88·2)	81·3 (73·9–87·1)	82·6 (75·4–88·3)
Tracheal, bronchus, and lung cancer	88·2 (84·7–91·4)	87·1 (83·8–90·3)	88 (84·5–91·2)	87·1 (83·8–90·3)
Breast cancer	39·6 (28·2–53·3)	38·1 (27·6–50·5)	38·2 (27·8–50·7)	38 (27·6–50·5)
Cervical cancer	100 (100–100)	100 (100–100)	100 (100–100)	100 (100–100)
Uterine cancer	44·5 (28·6–60·1)	46·3 (30·6–62)	47·1 (31·5–62·7)	46·2 (30·4–61·9)
Ovarian cancer	31·9 (15·8–48·1)	27·2 (13·4–42·4)	25·1 (12·4–39·9)	27·3 (13·4–42·5)
Prostate cancer	6·5 (2·95–10·3)	7·16 (3·36–11·1)	7·62 (3·57–11·7)	7·09 (3·33–11)
Kidney cancer	41·3 (30·6–52)	41·5 (31·1–51·6)	41·4 (31·2–51·6)	41·5 (31·1–51·6)
Bladder cancer	45·9 (30·3–61·7)	47·3 (32·4–61·6)	47·7 (33–61·3)	47·3 (32·3–61·6)
Thyroid cancer	15·1 (7·26–24·4)	16 (7·81–25·7)	16·1 (8·01–25·7)	15·9 (7·79–25·6)
Mesothelioma	99·4 (99·1–99·6)	98·7 (98·1–99·1)	99·2 (98·8–99·5)	98·6 (98·1–99·1)
Non-Hodgkin lymphoma	7·6 (3·1–13·5)	7·88 (3·31–13·8)	7·77 (3·26–13·7)	7·89 (3·32–13·9)
Multiple myeloma	8·62 (3·74–15·2)	9·05 (3·99–15·9)	8·9 (3·6–16)	9·06 (3·98–15·9)
Leukemia	39·4 (25·1–54)	36·8 (25·2–49·1)	36·4 (24·6–48)	36·9 (25·2–49·1)
Cardiovascular diseases	80·8 (77·2–84·4)	82·4 (79·4–85·2)	73·7 (68·8–78)	83·3 (80·6–86)
Rheumatic heart disease	24·4 (13·6–46·3)	25·9 (16·5–42·2)	25·9 (16·2–42·9)	25·9 (16·5–42·5)
Ischemic heart disease	94 (90·6–96·9)	95·3 (92·8–97·4)	94·7 (91·9–96·9)	95·3 (92·8–97·4)
Stroke	80·6 (72·5–89·2)	84 (78·2–89·8)	83·8 (78·6–88·9)	84·1 (78·2–90)
Hypertensive heart disease	100 (100–100)	100 (100–100)	100 (100–100)	100 (100–100)
Non-rheumatic valvular heart disease	28 (18·5–41·8)	29·3 (20·9–40·2)	21·2 (14·7–29·9)	30·2 (21·5–41·5)
Cardiomyopathy and myocarditis	35·7 (28·4–45·2)	40·7 (34·6–46·6)	35·4 (29–42·1)	40·9 (34·9–46·9)
Atrial fibrillation and flutter	51 (40–61·9)	59·3 (50–68)	64 (55·2–72·1)	54·8 (44·9–64·6)
Aortic aneurysm	54·4 (47·6–61·9)	61·4 (55·8–67·4)	--	61·4 (55·8–67·4)
Endocarditis	33·4 (23·2–46·3)	36·4 (27·2–46·6)	33·2 (23·1–45·8)	36·4 (27·2–46·7)
Other cardiovascular and circulatory diseases	33·9 (26·9–40·8)	36·7 (30·3–42·5)	37·3 (29·6–44·7)	36·6 (30·1–42·6)
Chronic respiratory diseases	66 (60–72·7)	61·4 (56·6–66·5)	54 (48·6–59·1)	66·2 (60·7–72·6)
Chronic obstructive pulmonary disease	74·4 (69·9–78·8)	73·6 (69·8–77·2)	67·6 (63·8–71·1)	76 (72·1–79·8)
Pneumoconiosis	100 (100–100)	100 (100–100)	100 (100–100)	100 (100–100)

Asthma	44·6 (32·9–57·1)	43·4 (34·8–53·1)	43·1 (34·5–52·7)	46·1 (36–56·9)
Digestive diseases	32·1 (26·7–37·9)	37·6 (31·7–43·8)	20·5 (14·2–28)	43·7 (37·1–51·1)
Cirrhosis and other chronic liver diseases	70·3 (59·3–84)	73·9 (63·1–86·7)	70·6 (58·3–83·7)	74 (63·1–86·8)
Upper digestive system diseases	25 (21·2–28·5)	11·6 (7·82–15·7)	0·929 (0·552–1·48)	27·5 (23·8–30·9)
Gallbladder and biliary diseases	44·1 (28·6–59·8)	49·9 (35·4–64·2)	52·3 (38–66·6)	45·9 (30·6–61·1)
Pancreatitis	30·9 (11·4–51·1)	37·6 (15·7–58·3)	38·4 (15·7–59·4)	37·5 (15·6–58·2)
Neurological disorders	26·1 (10·1–43·8)	15·9 (7·65–29·1)	10·2 (4·98–19·4)	22·1 (9·07–39·4)
Alzheimer's disease and other dementias	46·2 (30·5–62·8)	48·1 (32·8–64·3)	48·4 (33·5–64·6)	47·9 (32·5–64·1)
Parkinson's disease	-10·9 (-17·6–-5·14)	-12·4 (-19·9–-5·93)	-13·1 (-20·9–-6·2)	-12·3 (-19·6–-5·86)
Idiopathic epilepsy	25·4 (13·9–36)	23·8 (12·9–33·7)	21·3 (11·2–30·8)	27 (15·1–37·7)
Multiple sclerosis	15·5 (11·5–19·7)	16·2 (12–20·5)	15·9 (11·9–20)	16·4 (12·1–20·8)
Mental disorders	--	6·52 (3·32–10)	6·52 (3·32–10)	--
Depressive disorders	--	13·4 (6·13–21·1)	13·4 (6·13–21·1)	--
Anxiety disorders	--	4·91 (1·3–11)	4·91 (1·3–11)	--
Idiopathic developmental intellectual disability	--	40·1 (17·8–59·9)	40·1 (17·8–59·9)	--
Substance use disorders	100 (100–100)	100 (100–100)	100 (100–100)	100 (100–100)
Alcohol use disorders	100 (100–100)	100 (100–100)	100 (100–100)	100 (100–100)
Drug use disorders	100 (100–100)	100 (100–100)	100 (100–100)	100 (100–100)
Diabetes and kidney diseases	99·8 (99·8–99·9)	100 (100–100)	100 (100–100)	99·8 (99·8–99·9)
Diabetes mellitus	100 (100–100)	100 (100–100)	100 (100–100)	100 (100–100)
Chronic kidney disease	100 (100–100)	100 (100–100)	100 (100–100)	100 (100–100)
Sense organ diseases	--	9·43 (8·17–10·8)	9·43 (8·17–10·8)	--
Blindness and vision loss	--	10·9 (6·96–15·9)	10·9 (6·96–15·9)	--
Age-related and other hearing loss	--	10·3 (9·16–11·5)	10·3 (9·16–11·5)	--
Musculoskeletal disorders	4·7 (1·2–8·63)	23·5 (19·4–27·6)	23·8 (19·6–28)	5·42 (1·47–9·56)
Rheumatoid arthritis	12·7 (3·23–22·8)	15 (3·98–26·4)	15·2 (4·05–26·8)	14·3 (3·74–25·2)
Osteoarthritis	--	18 (9·7–27·1)	18 (9·7–27·1)	--
Low back pain	--	42·5 (36·6–48·4)	42·5 (36·6–48·4)	--
Gout	--	53·3 (37·9–70·5)	53·3 (37·9–70·5)	--
Other non-communicable diseases	0·106 (0·0686–0·155)	0·098 (0·0596–0·148)	--	0·407 (0·266–0·586)
Sudden infant death syndrome	9·68 (5·31–15·9)	9·67 (5·3–15·9)	--	9·67 (5·3–15·9)
Injuries	30·3 (23·5–36·4)	25·7 (20·4–30·9)	29 (24·4–33·4)	22 (13·7–29·8)
Transport injuries	18 (9·72–26·4)	18·6 (11·1–26·1)	25·3 (20·7–30)	15·7 (6·54–24·8)

Road injuries	17·1 (8·7–25·9)	17·6 (9·95–25·3)	24·1 (19·6–29)	15 (5·63–24·5)
Other transport injuries	31·3 (25·6–36·9)	30·5 (25–36·1)	34·6 (29·6–39·1)	27·5 (21·1–33·9)
Unintentional injuries	38·9 (32·6–43·4)	28·3 (23·5–32·7)	29·2 (24·3–33·9)	25·9 (21–30·3)
Falls	53·6 (45·9–59)	39·9 (33·7–44·8)	38·3 (32·4–43·4)	44·7 (38·1–49·9)
Drowning	-15 (-29·7–-0·958)	-14·3 (-28·3–-0·781)	9·5 (5·12–15·3)	-15·2 (-29·6–-1·29)
Fire, heat, and hot substances	9·64 (5·28–15·3)	11·6 (6·89–17·8)	11·8 (7–18·1)	11·2 (6·71–17·1)
Poisonings	13·5 (8·18–20·1)	12·5 (7·69–18·9)	10·8 (6·49–16·5)	14·2 (8·79–21·1)
Exposure to mechanical forces	7·74 (-3·57–19·4)	15·4 (12·6–18·1)	17·2 (14·6–19·5)	-2·3 (-13·7–10·3)
Animal contact	19·7 (15–25)	19·8 (14·9–25·2)	20·6 (15·6–26·2)	15·1 (11–20·3)
Foreign body	2·18 (1·36–3·23)	2·94 (1·86–4·41)	2·51 (1·57–3·82)	3·33 (2·09–4·95)
Environmental heat and cold exposure	7·37 (3–13)	8·64 (3·72–15·2)	8·68 (3·73–15·3)	8·45 (3·61–14·5)
Exposure to forces of nature	0 (0–0)	9·67 (4·29–16·7)	9·67 (4·29–16·7)	0 (0–0)
Other unintentional injuries	13·5 (8·09–20·1)	11·3 (6·72–17·4)	11·2 (6·6–17·2)	14·7 (9·04–21·7)
Self-harm and interpersonal violence	20·8 (4·31–35·6)	23·2 (7·9–36·7)	32 (23·6–40·1)	22·1 (5·43–36·6)
Self-harm	20·6 (3·37–36)	22·1 (4·93–37·2)	21·6 (8·33–34·8)	22·1 (4·76–37·3)
Interpersonal violence	25·3 (15·3–35·5)	32·3 (23·5–41·7)	38·2 (29·1–47·3)	22·4 (12·6–32·6)

Supplementary Results Table 2. All-age PAFs attributable to the joint distribution of all risk factors for all ages, both sexes combined for each GBD level 1, level 2, and level 3 cause and all causes in 2019 for Hungary. Results are shown for deaths, YLLs, YLDs, and DALYs Data in parenthesis are 95% uncertainty intervals. PAFs=population attributable fractions. GBD=Global Burden of Disease. YLLs=years of life lost. YLDs=years lived with disability. DALYs=disability-adjusted life-years

	Deaths 2019	DALYs 2019	YLDs 2019	YLLs 2019
All causes	69·6 (66·5–72·2)	55·7 (52·2–59)	34 (31·3–36·5)	67·8 (65·7–69·8)
Communicable, maternal, neonatal, and nutritional diseases	50·7 (45·5–55·7)	52·3 (48–56·8)	47·1 (41·3–53)	59·8 (55·7–63·4)
HIV/AIDS and sexually transmitted infections	97 (96·3–97·5)	97 (96·3–97·7)	99·4 (99·1–99·7)	95·6 (94·9–96·2)
HIV/AIDS	95·9 (95·1–96·6)	94·9 (94–95·6)	96·7 (95·9–97·4)	94·6 (93·7–95·3)
Sexually transmitted infections excluding HIV	100 (100–100)	100 (100–100)	100 (100–100)	100 (100–100)
Respiratory infections and tuberculosis	54·2 (47–60·9)	44·8 (37·6–52·2)	5·78 (4·16–7·86)	59·8 (53·1–65·5)
Tuberculosis	58·5 (50·4–65·5)	62·3 (54·7–68·9)	57·9 (49·6–65·3)	63·7 (56·1–70·2)
Lower respiratory infections	54·3 (46·8–61·3)	59·8 (52·8–65·7)	42·2 (35·9–48·3)	60·2 (53·1–66·2)
Upper respiratory infections	0·242 (0·124–0·401)	0·0256 (0·0121–0·0476)	0·00443 (0·000522–0·00977)	0·633 (0·335–1·05)
Otitis media	8·9 (3·57–18·3)	4·51 (2·91–6·4)	4·1 (2·64–5·84)	19 (10·5–35·8)
Enteric infections	32·1 (18·5–48·8)	32·9 (19·5–49·5)	32·6 (19–49·2)	34·6 (21·8–50·5)
Diarrheal diseases	32·4 (18·6–49·3)	33 (19·6–49·7)	32·6 (19–49·3)	35 (22–51·1)
Other infectious diseases	0·466 (0·315–0·63)	0·896 (0·611–1·24)	0·103 (0·0556–0·175)	1·26 (0·868–1·68)
Meningitis	0·852 (0·551–1·21)	2·09 (1·37–2·93)	0·000522 (4·67e-05–0·00117)	2·42 (1·62–3·38)
Encephalitis	0·354 (0·254–0·473)	0·701 (0·485–0·954)	0·000197 (1·7e-05–0·000447)	0·88 (0·639–1·17)
Measles	5·94 (1·14–20·7)	7·39 (1·72–24·5)	2·66 (0·0872–6·32)	7·95 (1·64–26·7)
Acute hepatitis	1·02 (0·472–1·7)	0·641 (0·378–1·02)	0·515 (0·29–0·846)	1·07 (0·511–1·79)
Maternal and neonatal disorders	79·2 (76·6–81·5)	71·6 (68·3–74·4)	65·1 (60·7–68·9)	80·8 (78·6–83)
Maternal disorders	12·1 (4·47–20·1)	12·1 (4·5–20·1)	12·2 (4·51–20·5)	12 (4·42–20)
Neonatal disorders	83·9 (82–86)	73·5 (70–76·4)	66·3 (62·1–70·2)	84 (82–86·1)
Nutritional deficiencies	46·3 (27–52·7)	91·9 (89·3–94·1)	94·3 (92·3–95·8)	51·5 (34·4–56·7)
Protein-energy malnutrition	100 (100–100)	100 (100–100)	100 (100–100)	100 (100–100)
Vitamin A deficiency	--	100 (100–100)	100 (100–100)	--
Dietary iron deficiency	--	100 (100–100)	100 (100–100)	--
Non-communicable diseases	71·9 (68·5–74·7)	58·9 (55·2–62·5)	34·1 (31–37·1)	71·3 (69–73·6)
Neoplasms	53·1 (49·6–57·3)	54·8 (51·6–58·5)	41·6 (37·7–45·9)	55·3 (52·1–58·9)
Lip and oral cavity cancer	75·3 (70–79·8)	77·6 (72·4–82)	75·8 (70·5–80·4)	77·6 (72·5–82·1)
Nasopharynx cancer	68·4 (60·5–75·5)	69·2 (61·2–76·3)	68·6 (60·5–75·6)	69·2 (61·2–76·3)
Other pharynx cancer	84·6 (80·5–88)	85·3 (81·2–88·8)	81·8 (76·5–86)	85·3 (81·2–88·8)
Esophageal cancer	81 (72·9–88)	82·3 (74·5–89·1)	81·5 (73·4–88·5)	82·3 (74·5–89·1)

Stomach cancer	28·4 (18·6–47·6)	29·8 (19·8–48·5)	29·3 (19·3–48·1)	29·8 (19·8–48·6)
Colon and rectum cancer	63·4 (55·7–71)	64·6 (57·4–71·9)	64·6 (57·2–72)	64·6 (57·4–71·9)
Liver cancer	66 (57·8–73·6)	67·3 (59·1–75·1)	66·6 (58·4–73·9)	67·3 (59·1–75·1)
Gallbladder and biliary tract cancer	27·2 (16·4–39·6)	27·6 (16·8–40·4)	27·4 (16·6–39·9)	27·6 (16·8–40·4)
Pancreatic cancer	42 (33·7–51·8)	43·2 (35·3–52·4)	42·6 (34·5–52)	43·2 (35·3–52·4)
Larynx cancer	84·1 (77·5–88·6)	85·2 (79–89·4)	84·2 (77·9–88·7)	85·2 (79·1–89·4)
Tracheal, bronchus, and lung cancer	85·1 (82·4–88·1)	85·5 (82·9–88·3)	85·4 (82·7–88·3)	85·5 (82·9–88·3)
Breast cancer	32·8 (24·3–42·9)	32·1 (24·1–41)	32 (24–41·2)	32·1 (24·2–41·1)
Cervical cancer	100 (100–100)	100 (100–100)	100 (100–100)	100 (100–100)
Uterine cancer	52·4 (37·8–66·4)	53·5 (39–67·7)	54·1 (39·6–68·2)	53·5 (38·9–67·6)
Ovarian cancer	15·3 (5·94–27·2)	13·8 (5·2–24·7)	13·3 (5–23·7)	13·8 (5·21–24·7)
Prostate cancer	6·85 (3·22–10·7)	7·62 (3·64–11·8)	8·08 (3·91–12·4)	7·58 (3·61–11·7)
Kidney cancer	43·3 (34–52·7)	44·6 (35·1–54)	44·5 (35–53·9)	44·6 (35·1–54)
Bladder cancer	40·6 (28·1–52·7)	45·1 (33·1–56·7)	46·1 (34·1–57·3)	45 (33·1–56·6)
Thyroid cancer	17·7 (9·68–26·6)	18·5 (10·1–28·1)	17·9 (10·2–26·7)	18·6 (10·1–28·3)
Mesothelioma	89·1 (85·3–92·2)	83·6 (78·4–88·1)	88·3 (84·4–91·6)	83·5 (78·3–88)
Non-Hodgkin lymphoma	8·82 (4·09–14·8)	8·97 (4·2–15)	8·93 (4·14–14·9)	8·97 (4·2–15)
Multiple myeloma	10·3 (4·89–17·2)	10·7 (5·04–17·6)	10·7 (5·02–17·7)	10·7 (5·04–17·6)
Leukemia	38·1 (25·8–50·7)	37·5 (26·4–48·7)	39·8 (27·6–52·2)	37·4 (26·3–48·5)
Cardiovascular diseases	88·1 (84·9–91)	88·9 (86·6–91)	80·1 (76·2–83·9)	89·7 (87·5–91·8)
Rheumatic heart disease	25·7 (15·5–44·3)	26·4 (17·2–42·3)	26·4 (16·4–42·2)	26·4 (17·1–42·1)
Ischemic heart disease	94 (90·3–97·2)	95·3 (92·8–97·5)	95 (92·5–97·2)	95·3 (92·8–97·5)
Stroke	84·2 (77·7–91·4)	87·4 (82·9–92·3)	86·6 (82·3–91·5)	87·6 (82·9–92·5)
Hypertensive heart disease	100 (100–100)	100 (100–100)	100 (100–100)	100 (100–100)
Non-rheumatic valvular heart disease	29·9 (20·6–40·5)	29·9 (21·5–39)	24·4 (17·1–32·4)	30·9 (22·3–40·4)
Cardiomyopathy and myocarditis	73 (56·4–79·5)	81·8 (68·1–86·2)	64 (56–71·8)	82·3 (68·1–86·7)
Atrial fibrillation and flutter	55·7 (46·5–65·9)	63·7 (56–71·9)	66·8 (59·5–74·3)	59·2 (50·9–68·1)
Aortic aneurysm	61·5 (55·4–67·4)	69·5 (64·7–73·9)	--	69·5 (64·7–73·9)
Endocarditis	45 (33·2–57·7)	46·6 (34·9–58·2)	43·7 (32·1–56·4)	46·7 (35–58·3)
Other cardiovascular and circulatory diseases	39 (32·7–45·5)	40·7 (34·8–46·6)	37·8 (31·3–44·5)	41·1 (35·1–47·2)
Chronic respiratory diseases	74·3 (70·7–77·7)	71·8 (68·9–74·9)	62 (58·1–65·8)	76·5 (73·4–79·4)
Chronic obstructive pulmonary disease	77·4 (73·5–81·2)	78 (75–80·9)	71 (67·8–74·3)	80·5 (77·3–83·7)
Pneumoconiosis	100 (100–100)	100 (100–100)	100 (100–100)	100 (100–100)
Asthma	50·5 (39·2–62·5)	47·2 (38·4–56·7)	45·9 (37·5–55)	54 (43·8–65)

Digestive diseases	49·3 (43·3–54·9)	49·6 (43·3–55·7)	22·7 (16·7–29·7)	56·6 (49·9–63·3)
Cirrhosis and other chronic liver diseases	74·3 (64·1–84·4)	75·2 (64·9–85·1)	70·1 (61·1–79·7)	75·2 (64·9–85·2)
Upper digestive system diseases	24·6 (21·5–27·7)	16·2 (11·6–21·1)	1·91 (1·14–2·88)	30·1 (26·7–33·6)
Gallbladder and biliary diseases	52 (38·1–66·3)	56·2 (42·8–69·3)	57·5 (44·1–70·1)	53·6 (39·9–67·6)
Pancreatitis	44 (33·9–56·2)	48·2 (37·8–60·2)	43·1 (32·9–54·4)	48·5 (38·1–60·5)
Neurological disorders	26·7 (12·7–41·5)	17·9 (9·69–30·4)	12·3 (6·71–21)	25·2 (12·3–39·4)
Alzheimer's disease and other dementias	37·3 (23–53·1)	40·4 (26·3–55·6)	40·5 (26·3–56)	40·3 (26·2–55·6)
Parkinson's disease	-8·36 (-12·8–-4·27)	-10·4 (-16–-5·3)	-11·6 (-17·7–-5·85)	-10·2 (-15·7–-5·23)
Idiopathic epilepsy	28·3 (21·1–35·2)	23·3 (16·8–29·7)	21·4 (15·2–27·2)	27·2 (20·2–34)
Multiple sclerosis	17·9 (13·5–22·2)	19·6 (14·8–24·2)	18·7 (14·4–22·9)	19·9 (15·1–24·7)
Mental disorders	--	3·97 (2·07–6·25)	3·97 (2·07–6·25)	--
Depressive disorders	--	7·44 (2·9–12·7)	7·44 (2·9–12·7)	--
Anxiety disorders	--	2·82 (0·795–6·17)	2·82 (0·795–6·17)	--
Idiopathic developmental intellectual disability	--	56·6 (28–77·8)	56·6 (28–77·8)	--
Substance use disorders	100 (100–100)	100 (100–100)	100 (100–100)	100 (100–100)
Alcohol use disorders	100 (100–100)	100 (100–100)	100 (100–100)	100 (100–100)
Drug use disorders	100 (100–100)	100 (100–100)	100 (100–100)	100 (100–100)
Diabetes and kidney diseases	99·8 (99·8–99·9)	99·9 (99·9–99·9)	100 (100–100)	99·8 (99·7–99·8)
Diabetes mellitus	100 (100–100)	100 (100–100)	100 (100–100)	100 (100–100)
Chronic kidney disease	100 (100–100)	100 (100–100)	100 (100–100)	100 (100–100)
Sense organ diseases	--	9·05 (7·72–10·5)	9·05 (7·72–10·5)	--
Blindness and vision loss	--	9·92 (6·23–14·3)	9·92 (6·23–14·3)	--
Age-related and other hearing loss	--	9·08 (8·27–10·1)	9·08 (8·27–10·1)	--
Musculoskeletal disorders	2·75 (0·75–4·86)	35·8 (30·5–41·4)	36·6 (31·2–42·3)	3·13 (0·877–5·51)
Rheumatoid arthritis	11·2 (2·97–19·7)	17 (5–28·8)	17·8 (5·29–30·5)	14 (3·79–24·3)
Osteoarthritis	--	23·1 (13·2–33·6)	23·1 (13·2–33·6)	--
Low back pain	--	46·5 (40·6–52·4)	46·5 (40·6–52·4)	--
Gout	--	58·1 (43·7–73·3)	58·1 (43·7–73·3)	--
Other non-communicable diseases	0·064 (0·0403–0·0912)	0·0409 (0·0231–0·0652)	--	0·181 (0·106–0·259)
Sudden infant death syndrome	6·46 (4·36–9·09)	6·45 (4·36–9·08)	--	6·45 (4·36–9·08)
Injuries	29·9 (24·5–34·4)	26·6 (22·8–30·1)	28·6 (25–31·7)	24·5 (19·3–29·4)
Transport injuries	24·1 (18·6–29·2)	24·7 (20·5–28·8)	25·3 (21·7–28·5)	24·2 (18·2–29·6)
Road injuries	22·7 (17–28·2)	24 (19·7–28)	25 (21·4–28·2)	23 (16·9–28·8)
Other transport injuries	35·3 (30·3–39·6)	33·4 (28·5–37·6)	34·5 (30·1–38·1)	33·1 (28·1–37·5)

Unintentional injuries	36·7 (30·7–41·4)	28·3 (23·9–32·3)	29·2 (25–33)	26·3 (21·6–31·2)
Falls	51·7 (43·6–57·2)	41 (35·1–45·6)	39·4 (33·7–43·8)	44·9 (38·1–50·4)
Drowning	-8·14 (-18·3–2·25)	-6·66 (-15·9–2·87)	8·25 (5·3–12·2)	-7·5 (-17·2–2·5)
Fire, heat, and hot substances	11·6 (7·03–17·1)	14·1 (9·64–19·5)	14 (9·63–19·2)	14·4 (9·51–20·2)
Poisonings	13·7 (8·97–19·5)	13·8 (9·63–18·7)	12·3 (8·62–17)	15·9 (11·1–21·7)
Exposure to mechanical forces	8·27 (0·0638–17·1)	18·4 (15·5–21·1)	19·4 (16·5–21·8)	4·82 (-3·86–13·5)
Animal contact	27 (21·9–32·6)	21·3 (17–26·3)	19·7 (15·5–24·4)	24·7 (19·9–30·7)
Foreign body	4·16 (3·13–5·34)	5·7 (4·33–7·27)	4·71 (3·56–6·2)	5·98 (4·51–7·69)
Environmental heat and cold exposure	10·4 (4·77–16·8)	10·8 (5·07–17·7)	7·86 (3·74–13·1)	11·2 (5·18–18·1)
Exposure to forces of nature	0 (0–0)	9·63 (4·57–16)	9·63 (4·57–16)	0 (0–0)
Other unintentional injuries	17·9 (12·6–24·2)	14 (9·75–19)	13·3 (9·2–18·2)	19·1 (13·9–25·4)
Self-harm and interpersonal violence	22·6 (11·7–32·8)	23·7 (13·4–33·5)	29·4 (23·5–35·3)	23·1 (12·1–33·5)
Self-harm	22 (10·4–33·2)	22·7 (11–34)	20·7 (11·4–29·9)	22·8 (11–34·1)
Interpersonal violence	29·9 (22·5–38·2)	29·7 (22·6–37·8)	32·7 (25·5–40·3)	27 (19·5–35·7)

Supplementary Results Table 2. All-age PAFs attributable to the joint distribution of all risk factors for all ages, both sexes combined for each GBD level 1, level 2, and level 3 cause and all causes in 2019 for Sunderland. Results are shown for deaths, YLLs, YLDs, and DALYs Data in parenthesis are 95% uncertainty intervals. PAFs=population attributable fractions. GBD=Global Burden of Disease. YLLs=years of life lost. YLDs=years lived with disability. DALYs=disability-adjusted life-years

	Deaths 2019	DALYs 2019	YLDs 2019	YLLs 2019
All causes	58·9 (55·7–62)	46·7 (43·4–49·8)	31·3 (28·9–33·6)	58·9 (56·3–61·7)
Communicable, maternal, neonatal, and nutritional diseases	45·7 (35·4–55·4)	50·1 (44·1–56·1)	48·5 (43·8–53·1)	50·8 (42·1–59)
HIV/AIDS and sexually transmitted infections	75·4 (58·5–88·7)	75·4 (57·2–88·9)	98 (96·9–98·9)	54·8 (34·4–76·2)
HIV/AIDS	93·1 (91·2–94·3)	92·4 (90·4–94)	95 (93·1–96·3)	90·3 (87·9–91·7)
Sexually transmitted infections excluding HIV	60 (37·8–82·8)	63·5 (39·6–86)	100 (100–100)	26·6 (11·6–51·5)
Respiratory infections and tuberculosis	47·7 (36·2–58·5)	45·8 (35·7–55·3)	3·51 (2·61–4·66)	50·9 (39·8–61)
Tuberculosis	59·1 (47·6–69·8)	61·9 (51–71·3)	57·7 (46·1–67·6)	62·6 (51·7–71·8)
Lower respiratory infections	47·6 (36–58·6)	50·8 (39·4–61)	34·6 (27·8–41·5)	50·9 (39·5–61·1)
Upper respiratory infections	0·0333 (0·0177–0·0603)	0·00601 (0·000837–0·015)	0·00516 (0–0·0141)	0·0779 (0·0415–0·14)
Otitis media	1·62 (0·791–3·13)	4·09 (2·59–5·73)	4·08 (2·58–5·74)	4·24 (2·2–7·49)
Enteric infections	5·55 (3·11–9·91)	8·18 (5·57–12·7)	8·92 (6·21–13·4)	7·15 (4·55–11·5)
Diarrheal diseases	5·58 (3·12–9·96)	8·22 (5·6–12·8)	8·93 (6·21–13·4)	7·22 (4·62–11·6)
Other infectious diseases	0·449 (0·312–0·642)	0·838 (0·644–1·07)	0·411 (0·233–0·686)	1·01 (0·756–1·31)
Meningitis	0·802 (0·55–1·09)	1·45 (1·02–1·94)	0·000409 (0–0·00125)	1·87 (1·32–2·52)
Encephalitis	0·168 (0·105–0·248)	0·437 (0·266–0·647)	0·000129 (0–0·000391)	0·577 (0·362–0·844)
Measles	2·11 (-0·576–10·8)	2·69 (-0·761–13·5)	0·677 (0·0155–1·9)	2·74 (-0·776–13·9)
Acute hepatitis	11·1 (4·41–24·3)	5·2 (2·73–9·05)	3·26 (1·9–5·37)	10·5 (4·2–22·9)
Maternal and neonatal disorders	83·6 (81–85·8)	76·4 (72·9–79·6)	68·6 (63·3–73·5)	85·3 (83·1–87·3)
Maternal disorders	9·6 (3·42–16·2)	9·52 (3·39–16·2)	9·6 (3·42–16·6)	9·45 (3·36–16)
Neonatal disorders	88·3 (86·3–90·2)	78·8 (75·1–82)	70·4 (64·8–75·6)	88·3 (86·4–90·2)
Nutritional deficiencies	82 (70·7–86·9)	80·8 (76·5–85·6)	80·7 (76·2–85·6)	82·9 (66·9–86·1)
Protein-energy malnutrition	100 (100–100)	100 (100–100)	100 (100–100)	100 (100–100)
Vitamin A deficiency	--	100 (100–100)	100 (100–100)	--
Dietary iron deficiency	--	100 (100–100)	100 (100–100)	--
Non-communicable diseases	60·9 (57·7–64·1)	47·8 (44·4–51·2)	30·8 (28·1–33·3)	61·6 (58·9–64·5)
Neoplasms	54·8 (50·6–59·3)	54 (50·1–58·2)	38·9 (34·4–44)	54·7 (50·8–58·9)
Lip and oral cavity cancer	67·1 (55·9–76·2)	69·7 (58·7–78·4)	68·2 (56·9–77·4)	69·8 (58·9–78·5)
Nasopharynx cancer	65·2 (52·5–75·5)	65·3 (52·3–75·4)	61·4 (47·6–72·8)	65·6 (52·6–75·5)
Other pharynx cancer	79·7 (72·3–86)	80·7 (72·9–86·9)	80·4 (72·4–86·5)	80·7 (72·9–86·9)
Esophageal cancer	75·7 (66·1–84·3)	77·1 (68–85·4)	76·1 (66·2–84·7)	77·2 (68·1–85·4)

Stomach cancer	27 (19·3–43·5)	27·1 (19·5–43·3)	27·3 (19·7–43·3)	27·1 (19·5–43·3)
Colon and rectum cancer	66·4 (56·6–76·1)	66·7 (57·4–75·7)	66·7 (57·4–76)	66·7 (57·4–75·7)
Liver cancer	76·9 (70·6–82·7)	77·4 (71·5–82·8)	77·6 (71·7–83·1)	77·4 (71·5–82·7)
Gallbladder and biliary tract cancer	22·5 (11·8–35·2)	23·3 (12·5–36·2)	21·1 (10·8–33·5)	23·4 (12·6–36·3)
Pancreatic cancer	48·1 (37·4–60·5)	47·2 (37–59·1)	47·7 (37·2–60·1)	47·2 (37–59·1)
Larynx cancer	83 (75·3–89·1)	82·8 (75·3–88·6)	81·7 (74·4–87·9)	83 (75·5–88·7)
Tracheal, bronchus, and lung cancer	89·1 (85·4–92·3)	88·1 (84·8–91·3)	88·9 (85·4–92·1)	88·1 (84·8–91·3)
Breast cancer	39·6 (28·5–53·4)	38·2 (28–50·9)	38·5 (28·2–50·9)	38·2 (28·1–50·9)
Cervical cancer	100 (100–100)	100 (100–100)	100 (100–100)	100 (100–100)
Uterine cancer	43·7 (27·4–60)	45·5 (29·6–61·4)	46·3 (30·4–62·1)	45·4 (29·3–61·4)
Ovarian cancer	37·9 (16·6–54·6)	32·5 (14·6–47·9)	30·2 (13·9–45·3)	32·5 (14·6–48·1)
Prostate cancer	6·49 (3·03–10·3)	7·14 (3·42–11·1)	7·62 (3·7–11·7)	7·08 (3·39–11·1)
Kidney cancer	41 (30·3–51·3)	41·3 (30·7–51·1)	41·3 (30·8–51·2)	41·3 (30·7–51·1)
Bladder cancer	45·7 (30·2–61)	47·3 (32·7–61·4)	47·7 (33·2–61·4)	47·2 (32·7–61·4)
Thyroid cancer	14·7 (7·18–23·8)	15·6 (7·62–25·1)	15·8 (7·86–25·2)	15·5 (7·6–25·2)
Mesothelioma	99·4 (99·1–99·6)	98·7 (98·2–99·1)	99·2 (98·9–99·5)	98·7 (98·2–99·1)
Non-Hodgkin lymphoma	7·47 (3·08–13·2)	7·8 (3·28–13·7)	7·67 (3·18–13·6)	7·81 (3·28–13·7)
Multiple myeloma	8·43 (3·68–15)	8·86 (3·9–15·7)	8·72 (3·56–15·8)	8·87 (3·9–15·7)
Leukemia	39·6 (25·3–54·4)	37·7 (25·1–50·4)	37·5 (25–49·8)	37·7 (25·1–50·4)
Cardiovascular diseases	81·5 (77·8–85·2)	83·1 (80·3–86)	73·3 (68·7–77·7)	84·2 (81·4–86·9)
Rheumatic heart disease	24·2 (13·4–46·1)	25·5 (15·7–43·4)	25·7 (15·8–43·1)	25·5 (15·7–43·6)
Ischemic heart disease	94·1 (90·6–97)	95·3 (93–97·4)	94·5 (91·8–97·1)	95·4 (93–97·4)
Stroke	80·7 (72·8–89·2)	84·1 (78·5–90·1)	83·6 (78·2–89·3)	84·2 (78·4–90·2)
Hypertensive heart disease	100 (100–100)	100 (100–100)	100 (100–100)	100 (100–100)
Non-rheumatic valvular heart disease	27·2 (17·9–40·3)	28·1 (20·1–38·7)	19·3 (13·3–26·7)	29·3 (21–40·2)
Cardiomyopathy and myocarditis	39·1 (30–48·3)	45·1 (37·6–51·1)	39 (31·9–46·6)	45·6 (37·7–51·7)
Atrial fibrillation and flutter	50·6 (40·1–61·5)	59 (50·1–67·7)	63·3 (54·6–71·2)	54·5 (44·9–64·5)
Aortic aneurysm	54·4 (47·2–62·2)	61·3 (55–67·5)	--	61·3 (55–67·5)
Endocarditis	33·5 (23·6–46·4)	36·6 (27·4–47·1)	33·3 (23·3–46)	36·7 (27·5–47·1)
Other cardiovascular and circulatory diseases	33·6 (27·1–40·5)	36·8 (30·7–43·2)	38 (30·7–45·3)	36·6 (30·4–43·1)
Chronic respiratory diseases	68·6 (62·2–75·1)	64·1 (59·1–69·1)	54·8 (49·9–59·9)	68·9 (63–75·1)
Chronic obstructive pulmonary disease	74·9 (69·3–79·9)	74·4 (70–78·5)	67·6 (63·7–71·6)	76·5 (71·6–81)
Pneumoconiosis	100 (100–100)	100 (100–100)	100 (100–100)	100 (100–100)

Asthma	44·1 (32·2–57·1)	43·5 (34·6–53·3)	43·2 (34·3–53)	46·1 (35·8–57·1)
Digestive diseases	30·7 (25·5–36·6)	37·1 (31·1–43·3)	23 (15·9–31·1)	42·3 (35·6–49·4)
Cirrhosis and other chronic liver diseases	70·4 (59–83·4)	74·1 (62·9–86·6)	71·9 (60·1–84·3)	74·1 (62·8–86·6)
Upper digestive system diseases	25·4 (21·1–29)	11·9 (7·8–16·2)	0·952 (0·571–1·5)	28·1 (23·8–31·8)
Gallbladder and biliary diseases	43·6 (28·1–59·2)	50·1 (35·1–64·8)	52·3 (37·5–66·6)	45·3 (30·6–60·4)
Pancreatitis	32·2 (11·3–53·6)	38·5 (15·5–59·1)	38 (14·6–59·2)	38·6 (15·6–59·1)
Neurological disorders	26·2 (10·7–43·6)	16·5 (8·15–29·6)	10·8 (5·54–20·2)	22·3 (9·48–39·4)
Alzheimer's disease and other dementias	45·8 (30·1–62·3)	47·6 (32·2–63·4)	48 (32·6–64)	47·4 (31·9–63)
Parkinson's disease	-10·8 (-17·8–-5·02)	-12·4 (-20·1–-5·92)	-12·9 (-20·9–-6·15)	-12·3 (-20–-5·87)
Idiopathic epilepsy	25 (13·6–35·5)	24·5 (13·6–34·5)	21·9 (11·8–31·8)	27 (15·2–37·7)
Multiple sclerosis	15·7 (11·5–20)	16·3 (12·1–20·8)	15·9 (11·9–20)	16·6 (12·2–21·2)
Mental disorders	--	6·44 (3·26–9·83)	6·44 (3·26–9·84)	--
Depressive disorders	--	13·2 (6·01–21)	13·2 (6·01–21)	--
Anxiety disorders	--	4·57 (1·22–10·2)	4·57 (1·22–10·2)	--
Idiopathic developmental intellectual disability	--	42·5 (19·5–62·4)	42·5 (19·5–62·4)	--
Substance use disorders	100 (100–100)	100 (100–100)	100 (100–100)	100 (100–100)
Alcohol use disorders	100 (100–100)	100 (100–100)	100 (100–100)	100 (100–100)
Drug use disorders	100 (100–100)	100 (100–100)	100 (100–100)	100 (100–100)
Diabetes and kidney diseases	99·2 (99–99·3)	99·8 (99·8–99·9)	100 (100–100)	99·2 (99–99·3)
Diabetes mellitus	100 (100–100)	100 (100–100)	100 (100–100)	100 (100–100)
Chronic kidney disease	100 (100–100)	100 (100–100)	100 (100–100)	100 (100–100)
Sense organ diseases	--	9·34 (8·04–10·9)	9·34 (8·04–10·9)	--
Blindness and vision loss	--	11·2 (7·09–16·4)	11·2 (7·09–16·4)	--
Age-related and other hearing loss	--	10 (8·93–11·2)	10 (8·93–11·2)	--
Musculoskeletal disorders	4·83 (1·23–8·71)	23·3 (19·2–27·4)	23·7 (19·5–27·9)	5·62 (1·57–9·93)
Rheumatoid arthritis	12·7 (3·26–22·6)	14·9 (4·05–25·9)	15·1 (4·09–26·1)	14·3 (3·96–25·1)
Osteoarthritis	--	17·6 (9·22–26·8)	17·6 (9·22–26·8)	--
Low back pain	--	42·1 (36·2–48)	42·1 (36·2–48)	--
Gout	--	52·5 (37–69·2)	52·5 (37–69·2)	--
Other non-communicable diseases	0·0558 (0·0353–0·0806)	0·0568 (0·0345–0·0888)	--	0·247 (0·156–0·353)
Sudden infant death syndrome	6·94 (3·82–11·3)	6·93 (3·82–11·3)	--	6·93 (3·82–11·3)
Injuries	32·4 (26·2–37·7)	26·9 (21·6–31·8)	30 (25·5–34·1)	23·7 (16·1–30·8)
Transport injuries	19·3 (11–27·6)	19·8 (12·9–27)	25·3 (20·7–29·7)	17·3 (8·23–26·6)

Road injuries	18·5 (9·86–27·1)	18·8 (11·5–26·3)	24 (19·4–28·4)	16·5 (7·13–26·1)
Other transport injuries	31·8 (26·1–37)	31·4 (25·9–36·7)	35·3 (30·4–39·5)	28·5 (22–34·7)
Unintentional injuries	39·7 (33·4–44·1)	29·4 (24·6–33·7)	30·3 (25·6–34·8)	27·4 (22·5–31·7)
Falls	53·9 (45·7–58·9)	41·3 (35·2–46·2)	39·6 (33·8–44·5)	45·4 (38·8–50·4)
Drowning	-17 (-31·2–-2·2)	-15·6 (-29·1–-1·36)	9·25 (4·78–14·8)	-16·6 (-30·5–-1·93)
Fire, heat, and hot substances	9·55 (4·94–15·4)	11·4 (6·47–17·5)	11·5 (6·48–17·5)	11·3 (6·39–17·4)
Poisonings	13·3 (7·72–20)	12·6 (7·44–18·7)	10·7 (6·15–16·4)	14·2 (8·44–21·1)
Exposure to mechanical forces	9·25 (-2·16–22·3)	15·5 (12·5–18·2)	17·6 (15·1–19·7)	-1·34 (-13·7–12·2)
Animal contact	21·4 (16·4–27)	20·5 (15·6–25·9)	21·1 (16–26·4)	18·1 (13·4–23·8)
Foreign body	2·2 (1·37–3·4)	3·05 (1·97–4·7)	2·28 (1·48–3·48)	3·46 (2·19–5·32)
Environmental heat and cold exposure	7·51 (3·04–13·3)	8·71 (3·61–14·8)	8·59 (3·59–14·6)	8·98 (3·82–15·3)
Exposure to forces of nature	0 (0–0)	9·55 (4·06–16·2)	9·55 (4·06–16·2)	0 (0–0)
Other unintentional injuries	12·9 (7·3–19·6)	11·1 (6·2–17)	10·9 (6·03–16·7)	14·5 (8·67–21·4)
Self-harm and interpersonal violence	21·6 (6·82–36)	23·8 (9·92–37·2)	32·2 (23·8–40·7)	22·8 (7·83–37·2)
Self-harm	21·2 (5·43–36·3)	22·7 (6·61–37·7)	21·3 (8·33–34·3)	22·7 (6·51–37·9)
Interpersonal violence	27·3 (17·8–37)	32 (23·3–41·3)	38·4 (29·4–48)	24·6 (15·2–34·2)

Supplementary Results Table 2. All-age PAFs attributable to the joint distribution of all risk factors for all ages, both sexes combined for each GBD level 1, level 2, and level 3 cause and all causes in 2019 for North West England. Results are shown for deaths, YLLs, YLDs, and DALYs Data in parenthesis are 95% uncertainty intervals. PAFs=population attributable fractions. GBD=Global Burden of Disease. YLLs=years of life lost. YLDs=years lived with disability. DALYs=disability-adjusted life-years

	Deaths 2019	DALYs 2019	YLDs 2019	YLLs 2019
All causes	58 (55·3–60·9)	44·9 (41·9–48)	30·1 (27·8–32·5)	57·8 (55·4–60·3)
Communicable, maternal, neonatal, and nutritional diseases	46·5 (37·9–54·1)	53·3 (48·5–57·8)	48·5 (44·4–52·4)	55·6 (48·9–61·8)
HIV/AIDS and sexually transmitted infections	76·4 (61·5–88·1)	76·5 (59·2–88·5)	97·4 (96·1–98·3)	59·2 (40–77·6)
HIV/AIDS	92·3 (90·3–93·4)	91·4 (89·1–93·1)	94·8 (92·8–96·1)	88·8 (86·4–90·2)
Sexually transmitted infections excluding HIV	52·5 (30·5–77·6)	60 (34·9–84·2)	100 (100–100)	21·4 (8·82–43·8)
Respiratory infections and tuberculosis	47 (37·6–55·5)	44·8 (36·9–52·4)	3·48 (2·62–4·53)	50·6 (41·4–58·8)
Tuberculosis	56·9 (47·2–66·5)	59·5 (50·2–68)	55·6 (45·6–64·6)	60·2 (51–68·8)
Lower respiratory infections	47 (37·5–55·6)	50·5 (41·2–58·8)	35 (28·1–41·5)	50·6 (41·3–58·9)
Upper respiratory infections	0·0526 (0·0396–0·0677)	0·00792 (0·00534–0·0111)	0·00614 (0·0038–0·00923)	0·118 (0·089–0·152)
Otitis media	2·49 (1·6–3·63)	4·39 (2·78–6·23)	4·36 (2·75–6·22)	6·05 (4·03–8·44)
Enteric infections	5·48 (3·3–9·24)	9·18 (6·76–12·9)	9·51 (6·93–13·5)	8·47 (5·99–12·1)
Diarrheal diseases	5·55 (3·4–9·38)	9·24 (6·83–13)	9·52 (6·94–13·5)	8·64 (6·17–12·3)
Other infectious diseases	0·721 (0·521–0·925)	1·32 (1·05–1·6)	0·417 (0·252–0·696)	1·67 (1·32–2·04)
Meningitis	1·63 (1·29–2·03)	2·72 (2·16–3·38)	0·000569 (0·00032–0·000977)	3·44 (2·75–4·25)
Encephalitis	0·323 (0·251–0·4)	0·824 (0·541–1·06)	0·000186 (0·000111–0·000306)	1·05 (0·785–1·32)
Measles	3·1 (-0·758–14·2)	3·67 (-0·857–16·3)	0·607 (0·0209–1·6)	3·84 (-0·958–17·3)
Acute hepatitis	11·9 (7·02–23·2)	5·28 (3·31–8·73)	3·06 (1·9–4·87)	10·8 (6·29–21·3)
Maternal and neonatal disorders	89·8 (88·3–91)	80·7 (77·8–83·5)	66·9 (61·5–71·9)	90·5 (89·1–91·7)
Maternal disorders	9·76 (3·5–16·1)	9·83 (3·49–16·4)	9·97 (3·54–16·7)	9·62 (3·42–15·8)
Neonatal disorders	91·6 (90·3–92·8)	82·3 (79·3–85·1)	68·7 (63–73·8)	91·6 (90·3–92·8)
Nutritional deficiencies	72·4 (69·5–87·7)	79·1 (75·9–82·7)	79·2 (76–82·8)	73 (68·8–81·8)
Protein-energy malnutrition	100 (100–100)	100 (100–100)	100 (100–100)	100 (100–100)
Vitamin A deficiency	--	100 (100–100)	100 (100–100)	--
Dietary iron deficiency	--	100 (100–100)	100 (100–100)	--
Non-communicable diseases	60 (56·8–63)	45·7 (42·5–49)	29·4 (26·9–32·1)	60·2 (57·7–62·8)
Neoplasms	52 (48·4–56·1)	51·1 (47·7–54·9)	35·9 (31·7–40·4)	52 (48·6–55·8)
Lip and oral cavity cancer	67·2 (58·3–75·5)	70 (61·7–77·9)	69·3 (60·5–77·2)	70·1 (61·8–78)
Nasopharynx cancer	65 (53·8–75·7)	65 (53·8–74·8)	61·3 (49·1–71·6)	65·3 (54·1–75)
Other pharynx cancer	79·4 (73–85·2)	80·6 (74–86·1)	80·1 (73·2–85·8)	80·7 (74·1–86·1)
Esophageal cancer	76·9 (67·9–84·9)	78·2 (69·6–86)	77·4 (68·7–85·4)	78·2 (69·6–86)
Stomach cancer	26·9 (19·4–43·2)	27·1 (19·5–43·3)	27·4 (19·8–43·5)	27·1 (19·5–43·3)

Colon and rectum cancer	65·2 (56·5–74·3)	65·5 (57·1–73·8)	65·7 (57·3–74·3)	65·5 (57·1–73·8)
Liver cancer	76·4 (70·6–82)	76·7 (71·3–81·9)	77·2 (71·7–82·4)	76·7 (71·3–81·8)
Gallbladder and biliary tract cancer	23·1 (12·4–35·4)	23·5 (12·8–35·8)	21 (10·9–33·4)	23·6 (12·8–35·9)
Pancreatic cancer	45·8 (36·4–56·8)	45 (35·9–55·7)	45·6 (36–56·5)	45 (35·9–55·7)
Larynx cancer	83·4 (75·6–89·2)	83·6 (76·6–88·7)	82 (74·8–87·4)	83·7 (76·8–88·9)
Tracheal, bronchus, and lung cancer	87·7 (85–90·5)	87 (84·4–89·7)	87·6 (84·9–90·3)	86·9 (84·4–89·7)
Breast cancer	37·4 (27·2–49·4)	36·2 (27·1–46·6)	36·4 (27·1–47·2)	36·1 (27·1–46·5)
Cervical cancer	100 (100–100)	100 (100–100)	100 (100–100)	100 (100–100)
Uterine cancer	44·8 (29–60·5)	45·9 (30·5–61·4)	46·3 (31·1–61·7)	45·9 (30·3–61·4)
Ovarian cancer	26·9 (14·2–41·1)	23 (12–36·1)	21·8 (11·4–34·3)	23 (12–36·1)
Prostate cancer	6·37 (2·78–10·1)	6·94 (3·1–10·9)	7·31 (3·37–11·5)	6·88 (3·07–10·8)
Kidney cancer	41·4 (30·9–51·8)	41·4 (31·2–51·4)	41·2 (31·1–51·2)	41·4 (31·2–51·4)
Bladder cancer	43·8 (28·3–58·4)	46·3 (31·8–59·7)	47 (32·8–59·9)	46·2 (31·8–59·7)
Thyroid cancer	15·5 (7·52–25·4)	16·1 (7·91–25·9)	15·2 (7·98–23·7)	16·2 (7·87–26·4)
Mesothelioma	99·1 (98·7–99·3)	98·1 (97·5–98·6)	98·9 (98·5–99·3)	98·1 (97·5–98·6)
Non-Hodgkin lymphoma	7·78 (3·27–13·7)	7·99 (3·46–13·9)	7·96 (3·37–13·9)	7·99 (3·46–13·9)
Multiple myeloma	8·76 (3·83–15·3)	9·07 (3·96–15·9)	9·03 (3·91–15·8)	9·08 (3·96–15·9)
Leukemia	39·4 (24·1–54·1)	36·8 (24·5–49·1)	36·4 (24·2–48·7)	36·9 (24·5–49·1)
Cardiovascular diseases	80·8 (77·5–83·9)	81·9 (79·2–84·4)	71·9 (67·4–76·1)	83·1 (80·5–85·5)
Rheumatic heart disease	24·9 (14·5–46·8)	25·4 (16·3–42·4)	25·2 (16–41·5)	25·4 (16·2–42·5)
Ischemic heart disease	94·1 (90·7–97)	95·2 (92·7–97·2)	94·4 (91·7–96·7)	95·2 (92·8–97·2)
Stroke	81·2 (73·8–89·4)	84·1 (78·7–89·8)	83·3 (78·2–88·6)	84·2 (78·7–90·1)
Hypertensive heart disease	100 (100–100)	100 (100–100)	100 (100–100)	100 (100–100)
Non-rheumatic valvular heart disease	28·3 (19·1–42·1)	28·2 (20·4–38·4)	18·2 (12·6–24·7)	29·5 (21·4–40·1)
Cardiomyopathy and myocarditis	38·2 (31–46·6)	42·7 (37·5–47·6)	37·5 (31·5–43·7)	43 (37·6–47·9)
Atrial fibrillation and flutter	53·2 (43·8–63)	60·2 (51·6–68·1)	63·8 (55·7–71·3)	56·2 (47·2–65·1)
Aortic aneurysm	54·8 (48·2–61·3)	61·2 (55·5–66·1)	--	61·2 (55·5–66·1)
Endocarditis	34·1 (24·6–47·2)	36·1 (27·7–46)	33·7 (24·1–46)	36·2 (27·8–46·1)
Other cardiovascular and circulatory diseases	35 (29·7–41·1)	36·8 (31·9–41·9)	36 (30·2–41·5)	37 (32–42)
Chronic respiratory diseases	66·1 (60·2–72·4)	61·8 (57·2–66·7)	54·2 (49·2–59)	66·6 (61·1–72·6)
Chronic obstructive pulmonary disease	74·6 (70·5–78·6)	74·1 (70·7–77·3)	67·9 (64·6–71·1)	76·4 (72·8–79·8)
Pneumoconiosis	100 (100–100)	100 (100–100)	100 (100–100)	100 (100–100)
Asthma	44·5 (33·1–57·1)	42·8 (34–52·6)	42·5 (33·9–52·2)	45·3 (35·8–56)
Digestive diseases	31·3 (26·8–36·3)	37·7 (32·3–43·2)	24·4 (17·1–32·7)	43·2 (37·7–49·8)

Cirrhosis and other chronic liver diseases	70·4 (60·6–81·7)	73·9 (64·2–84·9)	71·5 (62·2–82)	73·9 (64·2–85)
Upper digestive system diseases	23·9 (20·4–27·3)	10·8 (7·35–14·7)	0·834 (0·496–1·29)	26·5 (22·9–29·9)
Gallbladder and biliary diseases	45·7 (30·8–60·9)	50·4 (35·9–64·7)	51·5 (36·9–65·7)	47 (32·4–61·8)
Pancreatitis	30·9 (15·2–48·1)	37·6 (21·1–54·6)	38·1 (21·4–55·4)	37·5 (21·1–54·6)
Neurological disorders	26·7 (11·8–42·8)	16·3 (8·04–28·6)	10·4 (5·29–19·6)	23·1 (10·6–39·4)
Alzheimer's disease and other dementias	43·9 (29·3–59·1)	45·9 (31·4–60·8)	46·3 (31·7–61·2)	45·7 (31·1–60·5)
Parkinson's disease	-10·7 (-17·3–-5·03)	-12 (-19–-5·78)	-12·4 (-19·7–-5·81)	-11·9 (-19–-5·73)
Idiopathic epilepsy	25·4 (15·3–36)	24·2 (14·8–33·8)	22·1 (13·2–31·2)	26·8 (16·9–36·8)
Multiple sclerosis	16·5 (12·6–20·9)	17·1 (12·9–21·3)	16·6 (12·6–20·6)	17·4 (13·1–21·8)
Mental disorders	--	6·53 (3·3–10)	6·53 (3·3–10)	--
Depressive disorders	--	13·4 (6·12–21·1)	13·4 (6·12–21·1)	--
Anxiety disorders	--	4·88 (1·29–10·9)	4·88 (1·29–10·9)	--
Idiopathic developmental intellectual disability	--	39·5 (18·4–56·6)	39·5 (18·4–56·6)	--
Substance use disorders	100 (100–100)	100 (100–100)	100 (100–100)	100 (100–100)
Alcohol use disorders	100 (100–100)	100 (100–100)	100 (100–100)	100 (100–100)
Drug use disorders	100 (100–100)	100 (100–100)	100 (100–100)	100 (100–100)
Diabetes and kidney diseases	99·6 (99·5–99·6)	99·9 (99·9–99·9)	100 (100–100)	99·6 (99·5–99·6)
Diabetes mellitus	100 (100–100)	100 (100–100)	100 (100–100)	100 (100–100)
Chronic kidney disease	100 (100–100)	100 (100–100)	100 (100–100)	100 (100–100)
Sense organ diseases	--	9·11 (7·94–10·5)	9·11 (7·94–10·5)	--
Blindness and vision loss	--	10·2 (6·67–14·5)	10·2 (6·67–14·5)	--
Age-related and other hearing loss	--	10·1 (9·1–11·2)	10·1 (9·1–11·2)	--
Musculoskeletal disorders	4·42 (1·16–7·8)	23·5 (19·7–27·6)	24 (20–28·2)	5·13 (1·35–8·96)
Rheumatoid arthritis	13·2 (3·44–23·1)	15·1 (3·9–26·2)	15·2 (3·96–26·6)	14·8 (3·9–25·9)
Osteoarthritis	--	17·8 (9·51–27)	17·8 (9·51–27)	--
Low back pain	--	42·3 (36·7–47·7)	42·3 (36·7–47·7)	--
Gout	--	53·2 (37·4–70·1)	53·2 (37·4–70·1)	--
Other non-communicable diseases	0·0946 (0·0728–0·121)	0·0863 (0·0597–0·119)	--	0·355 (0·266–0·441)
Sudden infant death syndrome	7·85 (5·2–11·6)	7·85 (5·19–11·5)	--	7·85 (5·19–11·5)
Injuries	32·7 (27·2–37·6)	26·9 (22–31·2)	29·8 (25·5–33·9)	23·6 (16·6–29·7)
Transport injuries	19·2 (12·6–26)	19·7 (13·9–26)	25·5 (21·6–29·7)	17 (9·86–24·4)
Road injuries	18·3 (11·5–25·3)	18·7 (12·6–25·2)	24·3 (20·5–28·5)	16·2 (8·73–23·9)
Other transport injuries	31·8 (27·3–36·4)	31·1 (26·6–35·8)	34·9 (30·4–38·9)	28·1 (22·8–33·4)
Unintentional injuries	40·6 (34·3–44·8)	29·4 (24·7–33·7)	30·1 (25·2–34·7)	27·6 (23–31·8)

Falls	53·9<br>(46·4–59·1)	40·9<br>(34·9–46)	39·3<br>(33·5–44·3)	44·9<br>(38·5–50·1)
Drowning	-15·2<br>(-27–-1·96)	-14·4<br>(-25·9–-1·89)	9·84<br>(5·46–16·1)	-15·4<br>(-27·2–-2·48)
Fire, heat, and hot substances	10·2<br>(5·5–16·6)	11·9<br>(7·03–18·3)	12·2<br>(7·17–18·6)	11·4<br>(6·7–17·5)
Poisonings	13·3<br>(8·05–19·9)	12·5<br>(7·59–18·7)	11·2<br>(6·6–17·3)	13·9<br>(8·76–20·6)
Exposure to mechanical forces	8·62<br>(-0·658–18·9)	15·6<br>(13–17·9)	17·4<br>(15–19·4)	-1·48<br>(-11·1–9·17)
Animal contact	24·2<br>(19·7–29·9)	20·6<br>(15·9–26·3)	21<br>(16–26·9)	19·5<br>(15·3–25·1)
Foreign body	2·28<br>(1·6–3·23)	3·1<br>(2·2–4·32)	2·55<br>(1·79–3·57)	3·42<br>(2·43–4·8)
Environmental heat and cold exposure	7·96<br>(3·47–14·3)	9·07<br>(4·13–15·8)	9·03<br>(4·08–15·6)	9·21<br>(4·1–16)
Exposure to forces of nature	0<br>(0–0)	9·94<br>(4·55–17·4)	9·94<br>(4·55–17·4)	0<br>(0–0)
Other unintentional injuries	13·4<br>(7·96–20·1)	11·7<br>(6·87–18)	11·5<br>(6·77–17·8)	14·7<br>(9·26–21·7)
Self-harm and interpersonal violence	21·6<br>(7·61–33·8)	23·9<br>(11–35)	32·5<br>(24·9–39·5)	22·7<br>(8·68–34·8)
Self-harm	21·3<br>(6·7–34·4)	22·7<br>(8·02–35·5)	21·8<br>(9·79–32·7)	22·7<br>(7·89–35·7)
Interpersonal violence	26·3<br>(17·3–34·7)	32·4<br>(24–40·4)	38·8<br>(29·7–47·4)	23·4<br>(14·7–31·5)

Supplementary Results Table 2. All-age PAFs attributable to the joint distribution of all risk factors for all ages, both sexes combined for each GBD level 1, level 2, and level 3 cause and all causes in 2019 for Blackburn with Darwen. Results are shown for deaths, YLLs, YLDs, and DALYs Data in parenthesis are 95% uncertainty intervals. PAFs=population attributable fractions. GBD=Global Burden of Disease. YLLs=years of life lost. YLDs=years lived with disability. DALYs=disability-adjusted life-years

	Deaths 2019	DALYs 2019	YLDs 2019	YLLs 2019
All causes	59 (56·2–61·7)	44·6 (41·4–48)	29·2 (26·8–31·7)	58·1 (55·6–60·5)
Communicable, maternal, neonatal, and nutritional diseases	46·9 (37·7–55·9)	54·8 (50·2–59·4)	48·1 (43·5–52·9)	58 (51·5–64)
HIV/AIDS and sexually transmitted infections	67·3 (48·3–82·8)	68·8 (48·8–84·9)	97·6 (96·3–98·5)	47·3 (27·8–68·2)
HIV/AIDS	89·9 (87·9–91·2)	89·1 (86·7–91·1)	94·6 (92·6–96)	84·7 (82·2–86·3)
Sexually transmitted infections excluding HIV	45 (23·6–70·4)	53·6 (28·6–80)	100 (100–100)	17·8 (7·16–37)
Respiratory infections and tuberculosis	46·5 (36·4–56·7)	43·8 (35·3–52·6)	3·59 (2·58–4·84)	50·7 (41·1–59·8)
Tuberculosis	54·5 (43–66·4)	56·7 (45·6–67·7)	52·7 (40·1–64·6)	57·5 (46·6–68·4)
Lower respiratory infections	46·5 (36·2–56·7)	50·6 (40·8–59·9)	34·5 (27·5–41·2)	50·7 (40·9–60)
Upper respiratory infections	0·0692 (0·0359–0·122)	0·00953 (0·00233–0·0213)	0·0068 (4·43e-05–0·0188)	0·124 (0·0649–0·222)
Otitis media	4·88 (2·47–8·07)	4·93 (3·17–7·09)	4·87 (3·11–6·99)	9·5 (5·59–14·4)
Enteric infections	6·77 (4·09–11·8)	11·4 (8·31–16·1)	10·9 (7·72–15·9)	12·6 (8·74–17·4)
Diarrheal diseases	6·88 (4·18–12)	11·5 (8·37–16·2)	10·9 (7·73–15·9)	12·9 (8·99–18)
Other infectious diseases	0·831 (0·558–1·1)	1·34 (1·02–1·69)	0·356 (0·204–0·597)	1·73 (1·32–2·24)
Meningitis	1·92 (1·43–2·54)	2·6 (1·91–3·4)	0·000603 (2·26e-06–0·00186)	3·37 (2·48–4·43)
Encephalitis	0·477 (0·311–0·693)	0·992 (0·597–1·48)	0·000211 (8·85e-07–0·000616)	1·28 (0·835–1·87)
Measles	4·07 (-1–19·2)	4·15 (-0·785–19·1)	0·775 (0·0272–2·17)	4·88 (-1·2–23·3)
Acute hepatitis	12·2 (5·16–26)	4·54 (2·44–7·91)	2·73 (1·59–4·37)	11 (4·66–23·8)
Maternal and neonatal disorders	87·2 (85·2–89·2)	80·9 (78–83·5)	66·5 (60·9–71·3)	88·1 (86·1–89·9)
Maternal disorders	9·83 (3·47–16·5)	9·88 (3·48–16·4)	10·1 (3·52–17)	9·68 (3·41–16·2)
Neonatal disorders	89·6 (87·6–91·3)	82·7 (79·6–85·3)	68·5 (62·7–73·7)	89·6 (87·6–91·4)
Nutritional deficiencies	66·3 (60·5–84·8)	79·6 (75·5–84·2)	79·9 (75·7–84·5)	68·4 (63·4–77·9)
Protein-energy malnutrition	100 (100–100)	100 (100–100)	100 (100–100)	100 (100–100)
Vitamin A deficiency	--	100 (100–100)	100 (100–100)	--
Dietary iron deficiency	--	100 (100–100)	100 (100–100)	--
Non-communicable diseases	61·3 (58·1–64·3)	45·6 (42·1–49·2)	28·5 (25·8–31·3)	60·8 (58·2–63·4)
Neoplasms	51·2 (47·5–55·3)	49·9 (46·3–53·8)	34·3 (30–38·7)	50·7 (47·2–54·6)
Lip and oral cavity cancer	67·1 (55·9–77·2)	69·6 (58·5–79)	68·7 (57·1–78·6)	69·6 (58·7–79)
Nasopharynx cancer	64·3 (50·7–75·9)	63·7 (50·2–74·8)	59·8 (46–71·9)	64 (50·4–75·1)
Other pharynx cancer	79·4 (71·2–86·1)	80·3 (72·3–86·7)	79·8 (71·5–86·2)	80·3 (72·3–86·7)
Esophageal cancer	76·6 (67·4–84·7)	77·8 (68·8–85·5)	77 (68–85·3)	77·8 (68·8–85·6)
Stomach cancer	27·3 (19·8–43·6)	27·3 (19·6–43·4)	27·6 (19·9–43·8)	27·3 (19·6–43·4)

Colon and rectum cancer	64 (55·2–73)	64·2 (55·8–72·6)	64·3 (56·2–72·9)	64·2 (55·8–72·7)
Liver cancer	76·3 (70·2–81·9)	76·1 (70·5–81·3)	76·6 (70·7–82)	76·1 (70·4–81·2)
Gallbladder and biliary tract cancer	22·6 (11·9–35·6)	23 (12·4–35·7)	20·6 (10·8–32·9)	23·1 (12·5–35·8)
Pancreatic cancer	44·7 (35·3–55·6)	43·8 (35·2–54·3)	44·4 (35·7–55·3)	43·8 (35·2–54·3)
Larynx cancer	83·2 (75·2–89·1)	83·1 (75·5–88·6)	81·3 (73·6–87·2)	83·3 (75·7–88·8)
Tracheal, bronchus, and lung cancer	87·3 (84·4–90·1)	86·2 (83·3–89·1)	87 (83·9–89·8)	86·2 (83·3–89·1)
Breast cancer	35·1 (25·9–46·3)	33·6 (25·3–43·3)	33·9 (25·2–43·9)	33·6 (25·3–43·3)
Cervical cancer	100 (100–100)	100 (100–100)	100 (100–100)	100 (100–100)
Uterine cancer	42·8 (26·8–58·5)	44 (28·2–59·2)	44·3 (28·5–59·8)	44 (28–59·2)
Ovarian cancer	22·8 (11·9–35·6)	19·1 (9·59–30·9)	17·9 (9·04–29·2)	19·2 (9·61–31·1)
Prostate cancer	6·64 (3·02–10·6)	7·2 (3·33–11·3)	7·54 (3·5–11·7)	7·14 (3·31–11·1)
Kidney cancer	40·7 (30·5–51)	40·1 (30·3–49·9)	39·7 (30–49·3)	40·1 (30·3–49·9)
Bladder cancer	44 (29–58·6)	46·5 (32·4–59·2)	47·2 (33·8–59·6)	46·4 (32·4–59·2)
Thyroid cancer	14·6 (6·94–24)	15 (7·31–24·7)	14·1 (7·28–22·5)	15·2 (7·33–25)
Mesothelioma	98·8 (98·3–99·1)	97·5 (96·7–98·2)	98·6 (98–99·1)	97·5 (96·7–98·2)
Non-Hodgkin lymphoma	7·3 (3–12·9)	7·44 (3·1–13·1)	7·42 (3·04–13·1)	7·44 (3·1–13·1)
Multiple myeloma	8·22 (3·43–14·6)	8·53 (3·58–15)	8·47 (3·54–15)	8·54 (3·58–15)
Leukemia	38·9 (23·8–52·8)	34·9 (22·8–46·1)	34·9 (22·8–46·5)	34·9 (22·8–46·2)
Cardiovascular diseases	82·5 (79·1–85·6)	83·5 (80·8–86)	73 (68·2–77·5)	84·5 (81·9–86·9)
Rheumatic heart disease	24·7 (14·6–46·2)	25·3 (16·4–41·4)	24·8 (15·7–40·5)	25·3 (16·5–41·9)
Ischemic heart disease	94·2 (91–96·9)	95·3 (93–97·4)	94·4 (91·8–96·8)	95·4 (93–97·4)
Stroke	81·4 (73·7–89·8)	84·4 (79·2–90·1)	83·1 (78·1–88·4)	84·6 (79·2–90·4)
Hypertensive heart disease	100 (100–100)	100 (100–100)	100 (100–100)	100 (100–100)
Non-rheumatic valvular heart disease	27·7 (18·2–40·4)	28·4 (20·2–38·7)	23·8 (16·5–32·4)	28·8 (20·5–39·1)
Cardiomyopathy and myocarditis	37·7 (30·8–46·6)	40·3 (34·6–46·3)	35·9 (29·2–42·8)	40·5 (34·8–46·6)
Atrial fibrillation and flutter	51·9 (41·5–62·6)	59·6 (50·2–68·1)	63 (53·7–71·6)	55·4 (45·8–64·8)
Aortic aneurysm	56·4 (49·4–63·9)	63 (56·9–68·6)	--	63 (56·9–68·6)
Endocarditis	34 (24–47·4)	35·4 (26·3–45·7)	33·5 (23·6–46)	35·5 (26·4–45·8)
Other cardiovascular and circulatory diseases	35·3 (28·9–42·6)	36·4 (29·8–43)	35·1 (27·7–42·9)	36·6 (30·2–43·2)
Chronic respiratory diseases	67·6 (62·1–73·3)	61·2 (56·7–66)	51·2 (45·7–56·5)	67·7 (62·6–73·1)
Chronic obstructive pulmonary disease	75·3 (70·7–79·6)	74·7 (70·8–78·1)	68·2 (64·4–71·7)	76·9 (73·1–80·3)
Pneumoconiosis	100 (100–100)	100 (100–100)	100 (100–100)	100 (100–100)
Asthma	43·4 (32·3–55·9)	39·8 (31·4–48·9)	39·4 (31·2–48·6)	42·8 (33·3–53·2)
Digestive diseases	33·4 (27·8–39·7)	39·6 (33·1–46·4)	27·4 (18·9–36·9)	44·9 (37·8–53·1)

Cirrhosis and other chronic liver diseases	69·4 (58·6–82·3)	72·6 (61·7–85·5)	70·1 (58·3–82)	72·7 (61·6–85·6)
Upper digestive system diseases	25·2 (21·1–28·9)	11 (7·28–15·5)	0·854 (0·506–1·35)	27·9 (23·9–31·6)
Gallbladder and biliary diseases	44·2 (28·9–60)	50·1 (35·4–64·4)	51·2 (36·4–65·7)	45·6 (30·9–60·6)
Pancreatitis	29·1 (7·89–52·1)	35·5 (12·3–58·5)	35·8 (12·1–58·6)	35·4 (12·4–58·5)
Neurological disorders	24·4 (10·4–40·6)	13·8 (6·54–25)	8·37 (3·96–16·5)	20·5 (8·78–36·2)
Alzheimer's disease and other dementias	42·6 (28·4–57·3)	44·8 (30·8–59·3)	45·1 (30·9–59·5)	44·7 (30·8–59·2)
Parkinson's disease	-11·3 (-18·4–-5·17)	-12·6 (-20·4–-5·96)	-13 (-20·8–-6·2)	-12·5 (-20·2–-5·91)
Idiopathic epilepsy	24·7 (12·7–37·1)	22·4 (11·6–33·6)	19·9 (9·85–30·5)	25 (13·3–36·6)
Multiple sclerosis	17·1 (12·8–21·9)	17·3 (13–21·9)	16·7 (12·4–21)	17·7 (13·2–22·6)
Mental disorders	--	6·76 (3·47–10·4)	6·77 (3·47–10·5)	--
Depressive disorders	--	13·8 (6·29–21·4)	13·8 (6·29–21·4)	--
Anxiety disorders	--	5·5 (1·51–12·2)	5·5 (1·51–12·2)	--
Idiopathic developmental intellectual disability	--	40·8 (18·5–60)	40·8 (18·5–60)	--
Substance use disorders	100 (100–100)	100 (100–100)	100 (100–100)	100 (100–100)
Alcohol use disorders	100 (100–100)	100 (100–100)	100 (100–100)	100 (100–100)
Drug use disorders	100 (100–100)	100 (100–100)	100 (100–100)	100 (100–100)
Diabetes and kidney diseases	99·4 (99·2–99·5)	99·8 (99·8–99·9)	100 (100–100)	99·3 (99·2–99·5)
Diabetes mellitus	100 (100–100)	100 (100–100)	100 (100–100)	100 (100–100)
Chronic kidney disease	100 (100–100)	100 (100–100)	100 (100–100)	100 (100–100)
Sense organ diseases	--	9·38 (8·22–10·7)	9·38 (8·22–10·7)	--
Blindness and vision loss	--	8·95 (5·87–12·8)	8·95 (5·87–12·8)	--
Age-related and other hearing loss	--	10·9 (9·75–12·1)	10·9 (9·75–12·1)	--
Musculoskeletal disorders	3·34 (0·873–6·07)	23·2 (19·3–27·1)	23·7 (19·6–27·6)	3·76 (1·01–6·74)
Rheumatoid arthritis	14 (3·69–24·7)	15·3 (4·14–26·7)	15·3 (4·12–26·6)	15·5 (4·17–27·1)
Osteoarthritis	--	16·8 (8·76–25·9)	16·8 (8·76–25·9)	--
Low back pain	--	42·6 (37·1–48·2)	42·6 (37·1–48·2)	--
Gout	--	50·9 (34·9–68·4)	50·9 (34·9–68·4)	--
Other non-communicable diseases	0·171 (0·115–0·244)	0·141 (0·0891–0·213)	--	0·482 (0·312–0·687)
Sudden infant death syndrome	6·08 (3·46–9·72)	6·07 (3·46–9·71)	--	6·07 (3·46–9·71)
Injuries	27·9 (20·6–34·7)	23·7 (17·6–29·3)	27·3 (23–31·9)	20·4 (11·4–28·5)
Transport injuries	17·8 (9·75–25·8)	18·2 (11–25·1)	25·1 (20·6–29·6)	15·3 (6·46–23·8)
Road injuries	16·9 (8·63–25·2)	17·2 (9·96–24·5)	24 (19·5–28·6)	14·6 (5·48–23·3)
Other transport injuries	30·8 (25·3–36)	29·8 (24·6–35)	33·4 (28·5–37·5)	27·1 (20·8–32·8)
Unintentional injuries	36·8 (30·6–41·3)	26·1 (21·7–30·9)	27·3 (23–32·1)	23·2 (18·8–27·8)

Falls	51·6 (44–57·1)	37·6 (31·8–43·2)	36·2 (30·6–41·8)	41·5 (34·9–47·1)
Drowning	-14·2 (-28·1–0·527)	-14·4 (-27·9–0·168)	9·89 (5·38–16·3)	-15·2 (-28·9–-0·504)
Fire, heat, and hot substances	10·2 (5·77–16·6)	11·9 (7·14–18·6)	12·5 (7·49–19·7)	10·9 (6·65–17·1)
Poisonings	13·5 (8·49–20·8)	12·5 (7·94–19·2)	11·2 (6·75–17·5)	13·7 (8·66–20·7)
Exposure to mechanical forces	3·87 (-7·65–16)	14·9 (11·8–17·9)	16·9 (14·4–19·6)	-3·54 (-15·2–9·41)
Animal contact	23·8 (19·3–28·8)	19·1 (14·4–24·5)	20 (14·9–26·3)	16·1 (12·5–20·7)
Foreign body	3·21 (2·04–4·67)	3·63 (2·39–5·31)	3·16 (2·09–4·61)	3·99 (2·57–5·85)
Environmental heat and cold exposure	7·73 (3·01–14·4)	8·59 (3·47–15·6)	8·47 (3·42–15·4)	8·94 (3·65–16·2)
Exposure to forces of nature	0 (0–0)	9·33 (3·71–16·7)	9·33 (3·71–16·7)	0 (0–0)
Other unintentional injuries	13·1 (8·08–20·4)	11·8 (7·11–18·5)	11·6 (7–18·4)	14·2 (8·91–21·3)
Self-harm and interpersonal violence	19·3 (3·45–34·5)	21·4 (6·47–35·2)	31·7 (23·3–39·8)	20·3 (4·11–35·1)
Self-harm	19 (2·44–35·2)	20·2 (3·42–36·1)	20·3 (6·79–33·8)	20·2 (3·29–36·2)
Interpersonal violence	25 (15·6–34·9)	30·6 (21·9–39·6)	38·2 (28·4–47·3)	21·6 (12·3–31·1)

Supplementary Results Table 2. All-age PAFs attributable to the joint distribution of all risk factors for all ages, both sexes combined for each GBD level 1, level 2, and level 3 cause and all causes in 2019 for Blackpool. Results are shown for deaths, YLLs, YLDs, and DALYs Data in parenthesis are 95% uncertainty intervals. PAFs=population attributable fractions. GBD=Global Burden of Disease. YLLs=years of life lost. YLDs=years lived with disability. DALYs=disability-adjusted life-years

	Deaths 2019	DALYs 2019	YLDs 2019	YLLs 2019
All causes	58·4 (55·5–61·4)	48·2 (45·1–51·5)	30·9 (28·6–33·3)	58·8 (56–61·5)
Communicable, maternal, neonatal, and nutritional diseases	46·2 (35·5–56·3)	53·5 (47–59·4)	50·3 (46–54·3)	54·7 (46·3–62·6)
HIV/AIDS and sexually transmitted infections	83·8 (73·5–91)	82·2 (69·5–90·4)	96·3 (94·6–97·4)	71·3 (54·9–84·2)
HIV/AIDS	93·1 (90·8–94·4)	92·5 (89·9–94·2)	94·8 (92·7–96·2)	90·7 (87·9–92·3)
Sexually transmitted infections excluding HIV	61·3 (38·5–83·3)	58·5 (34·1–82·9)	100 (100–100)	27·8 (12·3–52·5)
Respiratory infections and tuberculosis	46·5 (35–57·6)	46·1 (35·8–54·9)	3·84 (2·83–5·06)	50·1 (39·1–60·3)
Tuberculosis	56·6 (44–68·7)	59·7 (48·1–70·7)	54·7 (42·1–66·6)	60·3 (48·6–71·3)
Lower respiratory infections	46·4 (34·8–57·6)	49·9 (38·8–60·3)	33·4 (26·5–40·4)	50 (38·8–60·4)
Upper respiratory infections	0·0278 (0·0173–0·0427)	0·00649 (0·000844–0·0169)	0·00565 (0–0·0164)	0·0663 (0·0393–0·106)
Otitis media	0·627 (0·355–1·05)	4·07 (2·55–5·84)	4·07 (2·55–5·84)	2·3 (1·33–3·66)
Enteric infections	6·66 (3·71–12·7)	10 (6·92–15·7)	10·4 (7·25–16·5)	9·29 (6·18–15·2)
Diarrheal diseases	6·74 (3·75–12·8)	10·1 (7–15·8)	10·5 (7·26–16·5)	9·47 (6·27–15·4)
Other infectious diseases	0·644 (0·465–0·907)	1·27 (0·97–1·61)	0·468 (0·265–0·808)	1·53 (1·15–1·96)
Meningitis	1·33 (0·97–1·76)	2·37 (1·75–3·09)	0·000523 (0–0·00182)	2·84 (2·1–3·71)
Encephalitis	0·277 (0·183–0·412)	0·756 (0·497–1·14)	0·000174 (0–0·000538)	0·914 (0·617–1·35)
Measles	3·42 (-0·567–15·6)	3·37 (-0·175–14·2)	0·878 (0·0299–2·45)	4·58 (-0·76–20)
Acute hepatitis	10·5 (4·7–27·5)	5·49 (3·01–10·6)	3·44 (2·06–5·76)	9·98 (4·54–26·6)
Maternal and neonatal disorders	89·3 (87·3–91)	80·9 (77·8–83·9)	65·3 (59·4–70·4)	90·2 (88·3–91·8)
Maternal disorders	10·4 (3·73–17·5)	10·4 (3·68–17·6)	10·6 (3·7–18·1)	10·2 (3·66–17·3)
Neonatal disorders	91·7 (89·9–93·1)	82·6 (79·3–85·7)	67 (60·7–72·4)	91·7 (89·9–93·1)
Nutritional deficiencies	75·1 (70–90·2)	81·3 (77·2–85·5)	81·4 (77·3–85·6)	75·2 (71·3–84·2)
Protein-energy malnutrition	100 (100–100)	100 (100–100)	100 (100–100)	100 (100–100)
Vitamin A deficiency	--	100 (100–100)	100 (100–100)	--
Dietary iron deficiency	--	100 (100–100)	100 (100–100)	--
Non-communicable diseases	60·5 (57·3–63·6)	49·5 (46·1–53)	30·1 (27·5–32·8)	61·4 (58·6–64·3)
Neoplasms	52·4 (48·7–56·7)	52 (48·5–56)	36·4 (32·2–41·1)	52·7 (49·2–56·7)
Lip and oral cavity cancer	65 (53·4–75·5)	68 (56·6–77·9)	67·2 (55·5–77·1)	68 (56·6–77·9)
Nasopharynx cancer	63·6 (50·2–75·3)	63·7 (50–75)	58·4 (44–70·6)	64 (50·3–75·2)
Other pharynx cancer	78·6 (70·5–85·3)	79·8 (71·8–86·1)	79·2 (71·1–85·8)	79·8 (71·8–86·2)
Esophageal cancer	75·7 (66·7–83·7)	76·9 (68·2–84·9)	76·2 (67·2–84·2)	76·9 (68·2–84·9)

Stomach cancer	27 (19·2–43·6)	27·2 (19·5–43·6)	27·4 (19·7–44)	27·2 (19·5–43·6)
Colon and rectum cancer	64·1 (55–73·2)	64·5 (56–73·3)	64·6 (55·9–73·4)	64·5 (56–73·2)
Liver cancer	75·8 (69·4–81·3)	76·3 (70·2–81·5)	76·3 (69·8–81·7)	76·3 (70·2–81·5)
Gallbladder and biliary tract cancer	20·2 (10·6–31·9)	20·5 (10·8–32·2)	18·2 (9·11–29·4)	20·6 (10·9–32·2)
Pancreatic cancer	44·2 (35·1–55·2)	43·3 (34·6–54)	43·9 (35–54·7)	43·3 (34·6–54)
Larynx cancer	83·4 (75·4–89·4)	83·6 (76·3–89·2)	82 (74·7–87·9)	83·7 (76·5–89·3)
Tracheal, bronchus, and lung cancer	88 (85–91·1)	87·2 (84·3–90·2)	87·8 (84·8–90·8)	87·2 (84·3–90·1)
Breast cancer	35 (25·4–46·3)	34 (25·2–44·1)	34·2 (25·4–44·6)	34 (25·2–44·1)
Cervical cancer	100 (100–100)	100 (100–100)	100 (100–100)	100 (100–100)
Uterine cancer	40·2 (24·7–55·4)	41·4 (26·1–57·4)	41·7 (26·5–57·5)	41·4 (26–57·4)
Ovarian cancer	27·7 (14·1–40·9)	23·2 (11·8–35·5)	22·1 (11·4–34)	23·2 (11·8–35·5)
Prostate cancer	6·43 (2·87–10·3)	6·95 (3·18–10·9)	7·29 (3·38–11·4)	6·91 (3·16–10·9)
Kidney cancer	38·9 (28·6–49·5)	38·9 (29·3–48·7)	38·7 (29·1–48·6)	38·9 (29·3–48·7)
Bladder cancer	44 (28·6–58·2)	46·5 (32·1–59·6)	47·2 (33·5–59·8)	46·4 (32–59·5)
Thyroid cancer	13·7 (6·2–23·5)	14·3 (6·5–24·3)	13·5 (6·47–22·3)	14·4 (6·47–24·6)
Mesothelioma	99·2 (98·9–99·5)	98·5 (97·9–98·9)	99·1 (98·7–99·4)	98·4 (97·9–98·9)
Non-Hodgkin lymphoma	6·62 (2·64–12·2)	6·84 (2·78–12·4)	6·78 (2·78–12·3)	6·84 (2·78–12·4)
Multiple myeloma	7·42 (3·1–13·5)	7·72 (3·19–13·9)	7·66 (3·1–13·9)	7·72 (3·2–13·9)
Leukemia	38·6 (23·4–53·7)	36·4 (23·6–48·7)	36·4 (23–49·1)	36·4 (23·6–48·7)
Cardiovascular diseases	79·9 (76–83·4)	81·4 (78·3–84·3)	70·9 (65·6–75·5)	82·3 (79·2–85)
Rheumatic heart disease	24·6 (13·5–46·2)	25·1 (15·5–41·9)	24·9 (15·5–40·4)	25·1 (15·5–42)
Ischemic heart disease	93·9 (90·2–96·9)	95 (92·3–97·2)	94·1 (91·2–96·6)	95 (92·3–97·2)
Stroke	80·7 (72·4–89·3)	83·6 (77·3–89·8)	82·4 (76·7–88·5)	83·8 (77·3–90)
Hypertensive heart disease	100 (100–100)	100 (100–100)	100 (100–100)	100 (100–100)
Non-rheumatic valvular heart disease	28·1 (18·9–41·3)	28·5 (20·1–38·5)	17·1 (11·4–23·9)	29·4 (20·8–39·7)
Cardiomyopathy and myocarditis	42·4 (33·9–50·8)	48·4 (41·3–54·7)	41·9 (34·8–49·1)	48·6 (41·4–55·1)
Atrial fibrillation and flutter	50·7 (40·3–61·4)	57·5 (48·1–66·4)	61·3 (51·9–69·6)	54 (44·3–63·5)
Aortic aneurysm	55·5 (48·4–62·7)	61·7 (55·4–67·5)	--	61·7 (55·4–67·5)
Endocarditis	33·7 (23–47·6)	35·5 (25·9–46·4)	33·4 (23·1–46·7)	35·5 (25·9–46·4)
Other cardiovascular and circulatory diseases	35·2 (28·2–42·6)	37·4 (30·8–43·4)	36·2 (28·7–43·2)	37·5 (30·9–43·8)
Chronic respiratory diseases	67·4 (60·6–74·3)	63·2 (57·9–68·6)	52·7 (47·1–57·9)	68 (61·8–74·5)
Chronic obstructive pulmonary disease	74·7 (69·3–79·8)	74·6 (70·2–78·8)	67·4 (63·4–71·2)	76·4 (71·6–80·9)
Pneumoconiosis	100 (100–100)	100 (100–100)	100 (100–100)	100 (100–100)

Asthma	41·9 (30·3–54·4)	41·2 (32·3–51·2)	40·8 (32·1–50·6)	43·3 (33·4–54·1)
Digestive diseases	33·1 (27·7–40·3)	40·7 (34·5–48·8)	18·5 (12·5–25·4)	44·7 (37·6–53·7)
Cirrhosis and other chronic liver diseases	69·2 (59–83·4)	72·7 (62·3–86·2)	69·6 (58·1–83·6)	72·7 (62·4–86·2)
Upper digestive system diseases	23·3 (19·5–26·8)	14·4 (10·3–18·7)	0·847 (0·492–1·34)	25·5 (21·8–29)
Gallbladder and biliary diseases	40·9 (26·5–55·5)	44·6 (30·6–59·1)	46·9 (32·6–61·4)	42·1 (28·2–56·6)
Pancreatitis	28·5 (7·63–50·6)	34·6 (11·9–56·6)	33·8 (11·3–56·1)	34·6 (12–56·7)
Neurological disorders	22·6 (8·95–38·1)	14·9 (7·35–26·5)	10·1 (5·04–19·2)	19·4 (7·61–34·5)
Alzheimer's disease and other dementias	41·4 (27·4–56·3)	43·4 (29·4–58·1)	43·7 (29·9–58·3)	43·3 (29·2–57·9)
Parkinson's disease	-10·9 (-17·7–-5·15)	-12·1 (-19·3–-5·8)	-12·3 (-19·7–-5·86)	-12·1 (-19·4–-5·78)
Idiopathic epilepsy	23·4 (11·5–35·1)	22·5 (11·4–33·4)	20·2 (9·97–30·8)	24·7 (12·6–35·8)
Multiple sclerosis	16·7 (12·3–21·2)	17·2 (13–21·7)	16·6 (12·6–21)	17·5 (13·1–22)
Mental disorders	--	6·52 (3·36–9·92)	6·53 (3·37–9·93)	--
Depressive disorders	--	13·2 (6·03–20·9)	13·2 (6·03–20·9)	--
Anxiety disorders	--	4·7 (1·25–10·4)	4·7 (1·25–10·4)	--
Idiopathic developmental intellectual disability	--	43·6 (20·7–63)	43·6 (20·7–63)	--
Substance use disorders	100 (100–100)	100 (100–100)	100 (100–100)	100 (100–100)
Alcohol use disorders	100 (100–100)	100 (100–100)	100 (100–100)	100 (100–100)
Drug use disorders	100 (100–100)	100 (100–100)	100 (100–100)	100 (100–100)
Diabetes and kidney diseases	98·6 (98·2–98·9)	99·6 (99·4–99·7)	100 (100–100)	98·6 (98·3–98·9)
Diabetes mellitus	100 (100–100)	100 (100–100)	100 (100–100)	100 (100–100)
Chronic kidney disease	100 (100–100)	100 (100–100)	100 (100–100)	100 (100–100)
Sense organ diseases	--	9·02 (7·78–10·4)	9·02 (7·78–10·4)	--
Blindness and vision loss	--	9·78 (6·21–14)	9·78 (6·21–14)	--
Age-related and other hearing loss	--	10 (9–11·2)	10 (9–11·2)	--
Musculoskeletal disorders	4·95 (1·28–8·98)	22·6 (18·6–26·5)	23·1 (19–27·1)	5·69 (1·53–10·1)
Rheumatoid arthritis	13·4 (3·62–23·9)	15·1 (4·13–26·4)	15·1 (4·11–26·6)	14·9 (4·15–26·4)
Osteoarthritis	--	15·5 (7·87–24·3)	15·5 (7·87–24·3)	--
Low back pain	--	40·9 (35·2–46·3)	40·9 (35·2–46·3)	--
Gout	--	48·7 (33·8–65·3)	48·7 (33·8–65·3)	--
Other non-communicable diseases	0·119 (0·0813–0·167)	0·132 (0·0825–0·198)	--	0·45 (0·3–0·632)
Sudden infant death syndrome	8·02 (4·65–12·5)	8·01 (4·64–12·5)	--	8·01 (4·64–12·5)
Injuries	33·1 (26·9–38·5)	27 (21·3–32·2)	30·9 (26·4–35·2)	23·9 (16·2–30·7)
Transport injuries	18·5 (10·5–26·7)	18·5 (11·6–25·9)	24·7 (20·6–28·9)	16·1 (7·43–25·1)

Road injuries	17·7<br>(9·34–26·2)	17·6<br>(10·5–25·4)	23·4<br>(19·4–27·8)	15·4<br>(6·45–24·7)
Other transport injuries	31·4<br>(26·7–36·1)	30·6<br>(25·8–35·3)	34·5<br>(30–38·6)	27·5<br>(22·1–32·9)
Unintentional injuries	40·4<br>(33·8–44·9)	30·1<br>(25·3–34·6)	31·4<br>(26·5–36)	28·1<br>(23·1–32·4)
Falls	52·9<br>(44·8–58)	41·4<br>(35·3–46·3)	40·1<br>(34·1–45·1)	43·6<br>(36·7–48·8)
Drowning	-17·3<br>(-31·8–-2·66)	-16·9<br>(-31·3–-2·73)	8·93<br>(4·6–15·2)	-17·5<br>(-32·1–-3·05)
Fire, heat, and hot substances	9·73<br>(5–15·9)	10·9<br>(6·11–17·1)	11·1<br>(6·22–17·6)	10·7<br>(6·02–16·6)
Poisonings	13·1<br>(7·59–20·2)	12·2<br>(7·21–18·6)	10·2<br>(5·7–16)	13·7<br>(8·3–20·5)
Exposure to mechanical forces	9·82<br>(-2·07–22·1)	15·6<br>(12·8–17·9)	17·2<br>(14·7–19·2)	-0·917<br>(-13–12·2)
Animal contact	26·8<br>(22–31·9)	20·2<br>(15·6–25·4)	20·6<br>(15·6–26·1)	19·6<br>(15·3–24·5)
Foreign body	2·17<br>(1·38–3·3)	2·92<br>(1·89–4·36)	2·12<br>(1·42–3·12)	3·22<br>(2·06–4·82)
Environmental heat and cold exposure	7·83<br>(3·13–14·2)	8·76<br>(3·81–15·5)	8·25<br>(3·55–14·7)	9·18<br>(4·07–16·3)
Exposure to forces of nature	0<br>(0–0)	9·27<br>(4·02–16·4)	9·27<br>(4·02–16·4)	0<br>(0–0)
Other unintentional injuries	11·8<br>(6·6–18·4)	10·5<br>(5·73–16·7)	10·3<br>(5·65–16·5)	13·2<br>(7·86–19·9)
Self-harm and interpersonal violence	20·5<br>(5·86–35·2)	22·7<br>(8·46–36·2)	30·8<br>(22·4–38·6)	21·9<br>(6·95–36·2)
Self-harm	20·2<br>(4·67–35·7)	21·8<br>(6·05–37)	20·3<br>(7·43–33·7)	21·8<br>(6·02–37·1)
Interpersonal violence	26·8<br>(17·1–35·5)	30·4<br>(21·2–38·8)	38·1<br>(28·7–46·9)	23·8<br>(14·4–32·1)

Supplementary Results Table 2. All-age PAFs attributable to the joint distribution of all risk factors for all ages, both sexes combined for each GBD level 1, level 2, and level 3 cause and all causes in 2019 for Bolton. Results are shown for deaths, YLLs, YLDs, and DALYs Data in parenthesis are 95% uncertainty intervals. PAFs=population attributable fractions. GBD=Global Burden of Disease. YLLs=years of life lost. YLDs=years lived with disability. DALYs=disability-adjusted life-years

	Deaths 2019	DALYs 2019	YLDs 2019	YLLs 2019
All causes	57·9 (54·9–60·8)	44·2 (41–47·4)	29·3 (26·8–31·6)	57·5 (55–60)
Communicable, maternal, neonatal, and nutritional diseases	47·5 (37·3–57)	54·2 (48·3–59·3)	50·3 (45·8–54·5)	56 (47·9–63·5)
HIV/AIDS and sexually transmitted infections	78·4 (65–88·3)	77·9 (62·6–88·5)	96·8 (95·3–97·8)	63 (44·9–79)
HIV/AIDS	91·6 (89·7–92·9)	90·8 (88·4–92·6)	94·6 (92·5–96)	87·7 (85·4–89·3)
Sexually transmitted infections excluding HIV	53·1 (30·3–78·2)	58·4 (33·6–83)	100 (100–100)	22·2 (8·97–44·9)
Respiratory infections and tuberculosis	47·6 (36·3–58·3)	45·4 (35·8–54·1)	3·63 (2·63–4·88)	51·2 (40·6–61)
Tuberculosis	55·2 (43·7–66·1)	57·7 (46·4–68·1)	53·2 (40·8–64·5)	58·5 (47·4–68·6)
Lower respiratory infections	47·5 (36·2–58·3)	51 (40·2–60·9)	34·9 (28·1–41·8)	51·1 (40·2–61·2)
Upper respiratory infections	0·0511 (0·0316–0·0802)	0·00747 (0·00106–0·0181)	0·00654 (0·000187–0·0174)	0·112 (0·0693–0·179)
Otitis media	2·58 (0·846–6·55)	4·56 (2·86–6·55)	4·55 (2·85–6·55)	5·55 (2·18–11·1)
Enteric infections	6·16 (3·61–10·9)	10·4 (7·57–15·3)	10·6 (7·56–15·4)	9·99 (6·79–14·5)
Diarrheal diseases	6·28 (3·69–11·1)	10·5 (7·63–15·4)	10·6 (7·57–15·4)	10·3 (7·16–15·2)
Other infectious diseases	0·773 (0·518–1)	1·37 (1·03–1·75)	0·398 (0·225–0·666)	1·73 (1·27–2·22)
Meningitis	1·64 (1·19–2·19)	2·63 (1·9–3·57)	0·000601 (1·19e-05–0·0017)	3·25 (2·35–4·31)
Encephalitis	0·342 (0·22–0·514)	0·829 (0·498–1·26)	0·000191 (3·86e-06–0·000549)	1·05 (0·666–1·58)
Measles	3·32 (-0·773–16·1)	3·72 (-0·754–17·4)	0·737 (0·0227–2·07)	4·17 (-0·981–19·8)
Acute hepatitis	12·5 (6·64–23·6)	5·1 (3·08–8·23)	2·95 (1·75–4·85)	11·7 (6·34–21·3)
Maternal and neonatal disorders	89 (86·8–90·9)	81·8 (78·9–84·6)	70·8 (65·9–75·2)	89·7 (87·6–91·5)
Maternal disorders	9·77 (3·45–16)	9·85 (3·55–16·3)	9·99 (3·57–16·7)	9·64 (3·47–15·8)
Neonatal disorders	91 (88·9–92·7)	83·4 (80·4–86·3)	72·9 (67·5–77·4)	91 (88·9–92·7)
Nutritional deficiencies	64·2 (58·2–86·9)	79 (75–83·1)	79·2 (75·2–83·5)	66 (61·7–79·8)
Protein-energy malnutrition	100 (100–100)	100 (100–100)	100 (100–100)	100 (100–100)
Vitamin A deficiency	--	100 (100–100)	100 (100–100)	--
Dietary iron deficiency	--	100 (100–100)	100 (100–100)	--
Non-communicable diseases	59·9 (56·7–62·9)	45 (41·5–48·5)	28·4 (25·8–31·1)	60·1 (57·4–62·6)
Neoplasms	51·1 (47·4–55·2)	50 (46·6–53·8)	35·2 (31·1–39·6)	50·8 (47·3–54·6)
Lip and oral cavity cancer	66·3 (54·3–76·5)	69 (57·5–78·4)	68·2 (56·2–77·6)	69 (57·5–78·4)
Nasopharynx cancer	65·7 (51·6–76·5)	65·2 (51–75·5)	61·7 (47·2–72·8)	65·5 (51·3–75·7)
Other pharynx cancer	79 (70·3–85·6)	80·1 (71·5–86·3)	79·6 (71·2–86)	80·1 (71·6–86·4)
Esophageal cancer	76·5 (66·6–84·9)	77·7 (68·2–85·9)	77 (67·5–85·2)	77·8 (68·2–86)
Stomach cancer	27·3 (19·7–43·7)	27·3 (19·7–43·5)	27·6 (20–43·8)	27·3 (19·7–43·5)

Colon and rectum cancer	64·1 (55–72·9)	64·4 (55·9–73·1)	64·5 (55·9–73·1)	64·4 (56–73)
Liver cancer	75·3 (69·2–81·4)	75·2 (69·3–80·7)	75·8 (69·7–81·4)	75·2 (69·4–80·7)
Gallbladder and biliary tract cancer	21·9 (11·6–34·5)	22·3 (12–34·8)	19·9 (10·1–32·3)	22·4 (12·1–34·9)
Pancreatic cancer	44·2 (35·4–54·5)	43·5 (35–53·3)	44 (34·9–54·3)	43·4 (35–53·3)
Larynx cancer	83·4 (75·3–89·5)	83·4 (76·2–88·9)	81·8 (74·2–87·4)	83·6 (76·4–89)
Tracheal, bronchus, and lung cancer	87·7 (84·5–90·5)	86·9 (83·7–89·7)	87·5 (84·3–90·4)	86·9 (83·7–89·7)
Breast cancer	35·1 (26–46)	33·9 (25·3–43·4)	34·1 (25·4–43·9)	33·8 (25·3–43·4)
Cervical cancer	100 (100–100)	100 (100–100)	100 (100–100)	100 (100–100)
Uterine cancer	42·7 (27·1–58·9)	43·9 (28·3–59·8)	44·2 (28·8–60·2)	43·8 (28·3–59·8)
Ovarian cancer	24·3 (12·6–37·4)	20·3 (10·4–32·1)	19 (9·85–30·4)	20·4 (10·5–32·2)
Prostate cancer	6·52 (2·92–10·5)	7·07 (3·22–11·3)	7·42 (3·39–11·7)	7·01 (3·21–11·2)
Kidney cancer	40·3 (29·9–50·8)	40·2 (30·1–50·1)	39·9 (29·9–49·5)	40·2 (30·1–50·1)
Bladder cancer	43·2 (28·2–57·2)	45·7 (31·9–58·4)	46·5 (33·1–58·9)	45·7 (31·8–58·4)
Thyroid cancer	14·5 (6·81–23·7)	14·9 (7·23–24·3)	14 (7·24–22·3)	15 (7·22–24·7)
Mesothelioma	99 (98·6–99·3)	98 (97·2–98·6)	98·9 (98·4–99·2)	98 (97·2–98·5)
Non-Hodgkin lymphoma	7·24 (3·04–12·9)	7·42 (3·15–13·2)	7·37 (3·15–13·2)	7·42 (3·14–13·2)
Multiple myeloma	8·1 (3·43–14·7)	8·41 (3·55–15·1)	8·34 (3·51–15)	8·41 (3·53–15·1)
Leukemia	38·9 (23·9–53·2)	35·7 (23·7–47·2)	35 (23·3–46·3)	35·8 (23·8–47·3)
Cardiovascular diseases	81·3 (77·6–84·6)	82·4 (79·3–85·1)	71·6 (66·8–75·8)	83·6 (80·7–86·1)
Rheumatic heart disease	24·7 (13·9–44·6)	25·2 (15·8–42·1)	24·9 (15·2–40·9)	25·2 (15·8–42·3)
Ischemic heart disease	94·1 (90·7–96·9)	95·3 (92·7–97·3)	94·3 (91·5–96·8)	95·3 (92·8–97·3)
Stroke	81·4 (73·7–89)	84·3 (78·7–89·7)	83·1 (77·8–88)	84·5 (78·7–89·9)
Hypertensive heart disease	100 (100–100)	100 (100–100)	100 (100–100)	100 (100–100)
Non-rheumatic valvular heart disease	28 (18·6–41·1)	27·9 (19·9–37·9)	17·1 (11·6–23·8)	29·3 (21–39·7)
Cardiomyopathy and myocarditis	39·7 (32·2–48·1)	44 (37·9–49·6)	38·7 (32·2–45·3)	44·3 (38–50·1)
Atrial fibrillation and flutter	51·9 (41·9–62·9)	59·2 (49·9–68·2)	62·7 (53·6–71)	55·2 (45·5–65·3)
Aortic aneurysm	56·1 (48·9–63·1)	62·3 (56–68·3)	--	62·3 (56–68·3)
Endocarditis	34 (23·7–47·6)	35·9 (27·2–45·9)	33·5 (23·2–46·6)	36 (27·2–45·9)
Other cardiovascular and circulatory diseases	34·3 (27·6–41·3)	35·9 (29·7–42·1)	35·5 (28·2–43·3)	35·9 (29·8–42·2)
Chronic respiratory diseases	66·5 (59·5–74)	61·5 (56·4–67·2)	53·2 (48–58·4)	66·7 (60·4–73·7)
Chronic obstructive pulmonary disease	75·1 (69·8–79·9)	74·5 (70·5–78·4)	68·4 (64·4–72)	76·8 (72·3–81·2)
Pneumoconiosis	100 (100–100)	100 (100–100)	100 (100–100)	100 (100–100)
Asthma	43·2 (31·8–55·7)	40·5 (31·9–50·2)	40·2 (31·6–49·9)	43·2 (33·9–53·5)
Digestive diseases	30·6 (25·4–36·8)	37·2 (31–43·6)	26·1 (17·9–35·2)	42·1 (35·2–49·9)
Cirrhosis and other chronic liver diseases	69·1 (58·4–82·8)	72·6 (61·7–85·7)	69·6 (58·1–82·8)	72·7 (61·7–85·7)

Upper digestive system diseases	24·5 (20·6–28·1)	10·6 (7·03–14·8)	0·778 (0·462–1·22)	26·9 (22·9–30·3)
Gallbladder and biliary diseases	43·4 (28·4–58·5)	49·4 (35–63·7)	50·7 (36–65·1)	44·8 (30·6–59·3)
Pancreatitis	30·2 (9·14–52·5)	36·3 (13·8–58·8)	34·8 (12·5–57·1)	36·5 (13·9–58·8)
Neurological disorders	25 (10·8–40·2)	14·8 (7·2–26·6)	9·26 (4·57–17·7)	21·5 (9·53–37·1)
Alzheimer's disease and other dementias	41·9 (27·8–56·7)	44·1 (30·1–58·4)	44·5 (30·8–58·8)	43·9 (30·1–58·2)
Parkinson's disease	-11·2 (-18·1–-5·37)	-12·4 (-19·7–-6)	-12·7 (-20·2–-5·97)	-12·3 (-19·9–-6·03)
Idiopathic epilepsy	24·7 (13–36·7)	22·8 (12–34)	20·3 (10·6–30·9)	25·8 (14–37·3)
Multiple sclerosis	16·6 (12·4–21·2)	17 (12·7–21·6)	16·5 (12·5–20·8)	17·3 (12·9–22·1)
Mental disorders	--	6·63 (3·4–10·2)	6·63 (3·4–10·2)	--
Depressive disorders	--	13·5 (6·15–21·2)	13·5 (6·15–21·2)	--
Anxiety disorders	--	5·1 (1·36–11·1)	5·1 (1·36–11·1)	--
Idiopathic developmental intellectual disability	--	40·6 (18·5–59·9)	40·6 (18·5–59·9)	--
Substance use disorders	100 (100–100)	100 (100–100)	100 (100–100)	100 (100–100)
Alcohol use disorders	100 (100–100)	100 (100–100)	100 (100–100)	100 (100–100)
Drug use disorders	100 (100–100)	100 (100–100)	100 (100–100)	100 (100–100)
Diabetes and kidney diseases	99·7 (99·7–99·8)	99·9 (99·9–100)	100 (100–100)	99·7 (99·7–99·8)
Diabetes mellitus	100 (100–100)	100 (100–100)	100 (100–100)	100 (100–100)
Chronic kidney disease	100 (100–100)	100 (100–100)	100 (100–100)	100 (100–100)
Sense organ diseases	--	9·07 (7·92–10·4)	9·07 (7·92–10·4)	--
Blindness and vision loss	--	9·31 (6·11–13·2)	9·31 (6·11–13·2)	--
Age-related and other hearing loss	--	10·3 (9·28–11·5)	10·3 (9·28–11·5)	--
Musculoskeletal disorders	4·57 (1·21–8·13)	23 (19·2–27·1)	23·5 (19·5–27·6)	5·22 (1·42–9·11)
Rheumatoid arthritis	13·7 (3·66–24·2)	15·3 (4·11–26·9)	15·4 (4·11–27)	15·2 (4·21–26·6)
Osteoarthritis	--	16·7 (8·7–25·6)	16·7 (8·7–25·6)	--
Low back pain	--	42 (36·6–47·5)	42 (36·6–47·5)	--
Gout	--	50·6 (34·7–68·3)	50·6 (34·7–68·3)	--
Other non-communicable diseases	0·128 (0·0881–0·182)	0·11 (0·0684–0·16)	--	0·428 (0·282–0·59)
Sudden infant death syndrome	7·33 (4·17–12·2)	7·33 (4·16–12·2)	--	7·33 (4·16–12·2)
Injuries	30·7 (24·3–36·9)	25·7 (20–31)	28·7 (24·5–33·3)	22·6 (14·2–30·3)
Transport injuries	19·1 (10·8–27·9)	19·4 (12·1–27·3)	25·3 (20·9–29·8)	16·8 (7·76–26·7)
Road injuries	18·4 (9·78–27·6)	18·5 (11–26·6)	24·1 (19·8–28·7)	16·2 (6·82–26·2)
Other transport injuries	31·2 (25·9–36·4)	30·7 (25·6–35·8)	34·5 (29·8–38·7)	27·5 (21·7–33·6)
Unintentional injuries	39·1 (33·3–43·6)	28·2 (23·8–32·8)	28·9 (24·3–33·9)	26·6 (22·2–30·8)
Falls	52·6 (45·2–58·1)	39·3 (33·5–44·7)	37·9 (32·3–43·4)	43·1 (37–48·8)
Drowning	-15·6 (-29·9–-1·6)	-14·7 (-28·2–-1·45)	9·6 (5·32–16·1)	-16 (-30·1–-2·09)

Fire, heat, and hot substances	10·7 (5·79–17·7)	12·1 (7·04–18·7)	12·1 (7·01–18·8)	12·1 (7·05–18·7)
Poisonings	13·1 (7·64–20)	12·1 (7·34–18·5)	11 (6·49–17·3)	13·6 (8·35–20·4)
Exposure to mechanical forces	5·22 (-6·67–18·2)	15·8 (13·1–18·5)	17·4 (14·8–19·8)	-2·15 (-14·7–12·1)
Animal contact	20·9 (15·8–27·5)	19·8 (15·1–26·3)	20·5 (15·7–26·9)	18·2 (13·2–24·5)
Foreign body	2·99 (1·89–4·66)	3·6 (2·32–5·45)	2·83 (1·86–4·15)	4·13 (2·61–6·27)
Environmental heat and cold exposure	8·04 (3·4–14·9)	8·58 (3·73–15·4)	8·47 (3·62–15·3)	9·03 (3·93–16)
Exposure to forces of nature	0 (0–0)	9·4 (4·09–16·6)	9·4 (4·09–16·6)	0 (0–0)
Other unintentional injuries	13·4 (7·71–20·5)	11·5 (6·62–18·3)	11·4 (6·51–18·3)	14·6 (8·85–21·7)
Self-harm and interpersonal violence	20·8 (5·74–34·8)	22·9 (8·69–36)	31·7 (23·2–39·4)	21·9 (6·75–35·7)
Self-harm	20·6 (4·98–35·1)	21·9 (6·04–36·3)	20·5 (7·92–34·3)	22 (5·98–36·5)
Interpersonal violence	24·6 (14·9–34)	31 (22·4–40)	38·2 (29–47·1)	21·8 (12·5–31·1)

Supplementary Results Table 2. All-age PAFs attributable to the joint distribution of all risk factors for all ages, both sexes combined for each GBD level 1, level 2, and level 3 cause and all causes in 2019 for Bury. Results are shown for deaths, YLLs, YLDs, and DALYs Data in parenthesis are 95% uncertainty intervals. PAFs=population attributable fractions. GBD=Global Burden of Disease. YLLs=years of life lost. YLDs=years lived with disability. DALYs=disability-adjusted life-years

	Deaths 2019	DALYs 2019	YLDs 2019	YLLs 2019
All causes	57·8 (54·7–60·7)	43·8 (40·7–47)	28·8 (26·5–31·3)	57·4 (54·9–60·1)
Communicable, maternal, neonatal, and nutritional diseases	45·6 (36–55·3)	51·9 (46·7–57)	47·2 (42·6–51·7)	54·4 (46·8–61·6)
HIV/AIDS and sexually transmitted infections	74·9 (57·7–88·1)	74 (55·2–88)	97·8 (96·6–98·7)	54·6 (34–75·5)
HIV/AIDS	92·6 (90·8–93·7)	91·8 (89·6–93·4)	95 (93·1–96·3)	89·2 (86·9–90·6)
Sexually transmitted infections excluding HIV	58·6 (35·6–82)	60 (35·3–83·6)	100 (100–100)	26 (11·4–50·2)
Respiratory infections and tuberculosis	46·3 (35·9–57)	43·4 (34·3–52·1)	3·35 (2·45–4·38)	49·9 (39·8–59·7)
Tuberculosis	55·3 (43–67)	58·1 (46·3–68·9)	54·8 (42·3–65·7)	58·7 (47–69·6)
Lower respiratory infections	46·3 (35·8–57·1)	49·8 (39·5–59·7)	35·1 (27·6–42·1)	49·9 (39·6–59·9)
Upper respiratory infections	0·0472 (0·0244–0·0877)	0·00809 (0·00196–0·0191)	0·00621 (0·000273–0·017)	0·103 (0·0539–0·184)
Otitis media	1·79 (0·64–4·47)	4·51 (2·78–6·39)	4·51 (2·78–6·4)	4·23 (1·73–9·13)
Enteric infections	5·55 (3·23–9·57)	9·33 (6·67–13·5)	9·73 (6·99–14)	8·36 (5·67–12·4)
Diarrheal diseases	5·62 (3·29–9·67)	9·39 (6·75–13·6)	9·74 (7–14·1)	8·54 (5·83–12·6)
Other infectious diseases	0·528 (0·363–0·926)	0·943 (0·713–1·27)	0·394 (0·227–0·665)	1·19 (0·884–1·65)
Meningitis	1·41 (1·01–1·89)	2·06 (1·5–2·79)	0·000481 (1·71e-05–0·00141)	2·97 (2·17–3·91)
Encephalitis	0·273 (0·182–0·389)	0·701 (0·464–1·02)	0·000174 (5·91e-06–0·000498)	0·892 (0·598–1·28)
Measles	4·1 (-1·15–19·3)	4·24 (-1·02–19·9)	0·649 (0·0199–1·79)	5·18 (-1·36–23·8)
Acute hepatitis	6·88 (2·17–22)	4·26 (2·04–9·83)	3·01 (1·76–5·01)	6·19 (1·96–20·1)
Maternal and neonatal disorders	86·7 (84·8–88·5)	77·9 (74·6–81)	63·8 (57·8–69·1)	88·4 (86·7–89·9)
Maternal disorders	9·79 (3·63–15·9)	9·78 (3·57–16)	9·95 (3·56–16·6)	9·66 (3·58–15·7)
Neonatal disorders	91·4 (89·8–92·8)	80·3 (76·9–83·4)	65·6 (59·5–71)	91·4 (89·8–92·8)
Nutritional deficiencies	67·4 (61·7–88·5)	79·3 (75–83·7)	79·5 (75·2–84)	69·1 (65–82·1)
Protein-energy malnutrition	100 (100–100)	100 (100–100)	100 (100–100)	100 (100–100)
Vitamin A deficiency	--	100 (100–100)	100 (100–100)	--
Dietary iron deficiency	--	100 (100–100)	100 (100–100)	--
Non-communicable diseases	59·8 (56·5–62·9)	44·6 (41·2–48)	28 (25·5–30·7)	59·9 (57·1–62·6)
Neoplasms	51 (47·2–55·1)	49·8 (46·1–53·6)	32·6 (28·5–37·1)	50·8 (47·2–54·6)
Lip and oral cavity cancer	65·1 (53·5–76·4)	68·1 (56·9–78·6)	67·5 (55·7–77·9)	68·2 (56·8–78·7)
Nasopharynx cancer	64·5 (51–76·3)	64·4 (50·8–75·7)	60·6 (46·8–73·2)	64·7 (51·1–76)
Other pharynx cancer	78·9 (70·5–86·2)	80·1 (71·8–86·8)	79·7 (71·4–86·5)	80·2 (71·8–86·8)
Esophageal cancer	76·8 (67·5–84·8)	78·1 (69·1–85·9)	77·3 (67·9–85·3)	78·1 (69·1–85·9)
Stomach cancer	26·9 (19·4–43·1)	27 (19·6–43·1)	27·4 (19·8–43·5)	27 (19·6–43·1)

Colon and rectum cancer	64·2 (55–73·3)	64·5 (56·1–72·9)	64·6 (55·7–73)	64·5 (56–72·9)
Liver cancer	75·5 (68·7–81·6)	76 (69·9–81·5)	76·4 (70–82·2)	76 (69·9–81·5)
Gallbladder and biliary tract cancer	22 (11·6–34·6)	22·5 (12–35)	20 (10·1–32·3)	22·6 (12·1–35·1)
Pancreatic cancer	44·9 (35·8–55·3)	44·2 (35·6–53·8)	44·7 (35·6–54·6)	44·2 (35·6–53·8)
Larynx cancer	83·3 (74·8–89·4)	83·4 (76·1–89·1)	81·8 (74·2–87·8)	83·6 (76·3–89·2)
Tracheal, bronchus, and lung cancer	87·1 (84–90·1)	86·3 (83·1–89·3)	86·9 (83·6–89·9)	86·3 (83·1–89·3)
Breast cancer	35·9 (26·2–47·2)	34·7 (25·7–44·6)	34·9 (25·8–45·3)	34·7 (25·6–44·6)
Cervical cancer	100 (100–100)	100 (100–100)	100 (100–100)	100 (100–100)
Uterine cancer	43·6 (28·4–59·5)	44·7 (29·5–60·5)	45 (29·7–60·8)	44·7 (29·4–60·5)
Ovarian cancer	23·9 (12·6–37·4)	20·3 (10·4–32·5)	19·2 (9·62–30·9)	20·3 (10·5–32·6)
Prostate cancer	6·4 (2·88–10·2)	6·98 (3·21–11)	7·36 (3·37–11·6)	6·92 (3·2–11)
Kidney cancer	40·7 (30·3–51·1)	40·7 (30·6–50·6)	40·5 (30·4–50·3)	40·7 (30·6–50·6)
Bladder cancer	42·8 (28·3–56·9)	45·3 (31·9–58·2)	46 (32·7–58·5)	45·2 (31·9–58·2)
Thyroid cancer	14·8 (7·04–24·4)	15·3 (7·37–25)	14·4 (7·49–23)	15·4 (7·39–25·4)
Mesothelioma	99 (98·6–99·3)	98 (97·2–98·6)	98·8 (98·3–99·2)	98 (97·2–98·6)
Non-Hodgkin lymphoma	7·32 (3·04–13·1)	7·53 (3·15–13·4)	7·47 (3·08–13·3)	7·53 (3·17–13·4)
Multiple myeloma	8·31 (3·52–14·7)	8·63 (3·72–15·2)	8·57 (3·62–15·2)	8·63 (3·73–15·2)
Leukemia	39·2 (24·4–53·5)	36·6 (24·4–49)	35·8 (23·7–48)	36·6 (24·4–49·1)
Cardiovascular diseases	81·5 (77·7–84·8)	82·4 (79·4–85·3)	72·1 (67·3–76·7)	83·6 (80·6–86·4)
Rheumatic heart disease	24·9 (13·8–47)	25·3 (15·6–42·7)	25·1 (15·1–40·6)	25·3 (15·6–42·8)
Ischemic heart disease	94·1 (90·7–96·9)	95·1 (92·6–97·3)	94·4 (91·9–96·9)	95·2 (92·6–97·3)
Stroke	81·3 (73·4–89·5)	84·1 (78·4–89·9)	83·4 (77·6–88·6)	84·3 (78·3–90·2)
Hypertensive heart disease	100 (100–100)	100 (100–100)	100 (100–100)	100 (100–100)
Non-rheumatic valvular heart disease	27·7 (18·2–40·3)	27·6 (19·6–37·4)	18·4 (12·7–25·6)	28·8 (20·4–38·9)
Cardiomyopathy and myocarditis	37·3 (29·6–46·3)	41·4 (35·7–47·1)	35·5 (29·5–42·1)	41·7 (35·9–47·6)
Atrial fibrillation and flutter	52·5 (41·7–63·4)	59·8 (50·8–68·4)	63·4 (54·7–71·9)	55·6 (45·7–65·7)
Aortic aneurysm	55·3 (48·2–62·1)	61·6 (55·4–67·2)	--	61·6 (55·4–67·2)
Endocarditis	34 (23·8–47·6)	35·8 (26·6–46·4)	33·5 (23·3–46·5)	35·9 (26·6–46·4)
Other cardiovascular and circulatory diseases	34·7 (28·1–41·7)	36·3 (30·3–42·3)	35·1 (27·7–42·6)	36·5 (30·4–42·6)
Chronic respiratory diseases	66·1 (60·1–72·5)	61·5 (57–66·2)	54·3 (49·1–59·2)	66·4 (60·9–72·4)
Chronic obstructive pulmonary disease	74·4 (69·8–78·7)	73·9 (70–77·5)	68·4 (64·4–72)	76·2 (72·1–79·8)
Pneumoconiosis	100 (100–100)	100 (100–100)	100 (100–100)	100 (100–100)
Asthma	43·5 (31·7–56)	41·6 (32·5–51·5)	41·3 (32·3–51·1)	44·3 (34·2–55)
Digestive diseases	30 (24·7–35·9)	35 (29·1–41·6)	17·9 (12·2–24·6)	41·7 (34·8–49·4)
Cirrhosis and other chronic liver diseases	68·7 (57·1–83·2)	72·5 (61·2–85·9)	69·9 (57·9–83·3)	72·6 (61·2–86)

Upper digestive system diseases	23·9<br>(20·1–27·7)	9·66<br>(6·35–13·6)	0·718<br>(0·41–1·16)	26·3<br>(22·5–29·8)
Gallbladder and biliary diseases	45·2<br>(29·7–61)	49<br>(34·2–63·4)	50·1<br>(35·5–64·3)	46·7<br>(31·6–61·8)
Pancreatitis	29·7<br>(9·57–52·2)	36·2<br>(13·5–57·9)	36·8<br>(14–58·4)	36·1<br>(13·6–57·8)
Neurological disorders	25·2<br>(10·7–41·1)	15·4<br>(7·45–27·3)	9·77<br>(4·74–18·7)	21·9<br>(9·57–38·1)
Alzheimer's disease and other dementias	42·3<br>(27·9–57·4)	44·4<br>(30·4–58·9)	44·8<br>(30·7–59·3)	44·2<br>(30·1–58·8)
Parkinson's disease	-10·8<br>(-17·8–-4·89)	-12<br>(-19·6–-5·53)	-12·5<br>(-20–-5·79)	-11·9<br>(-19·4–-5·49)
Idiopathic epilepsy	24·4<br>(12·1–36·7)	23·1<br>(12·1–34·3)	21<br>(10·6–31·8)	25·7<br>(13·6–38)
Multiple sclerosis	16·7<br>(12·6–21)	17·3<br>(13·1–21·7)	16·8<br>(12·7–21·2)	17·6<br>(13·3–22·1)
Mental disorders	--	6·57<br>(3·35–10·1)	6·57<br>(3·35–10·1)	--
Depressive disorders	--	13·4<br>(6·08–21·2)	13·4<br>(6·08–21·2)	--
Anxiety disorders	--	4·88<br>(1·28–10·8)	4·88<br>(1·28–10·8)	--
Idiopathic developmental intellectual disability	--	39·9<br>(18–59·5)	39·9<br>(18–59·5)	--
Substance use disorders	100<br>(100–100)	100<br>(100–100)	100<br>(100–100)	100<br>(100–100)
Alcohol use disorders	100<br>(100–100)	100<br>(100–100)	100<br>(100–100)	100<br>(100–100)
Drug use disorders	100<br>(100–100)	100<br>(100–100)	100<br>(100–100)	100<br>(100–100)
Diabetes and kidney diseases	99·7<br>(99·7–99·8)	99·9<br>(99·9–100)	100<br>(100–100)	99·7<br>(99·7–99·8)
Diabetes mellitus	100<br>(100–100)	100<br>(100–100)	100<br>(100–100)	100<br>(100–100)
Chronic kidney disease	100<br>(100–100)	100<br>(100–100)	100<br>(100–100)	100<br>(100–100)
Sense organ diseases	--	8·98<br>(7·87–10·3)	8·98<br>(7·87–10·3)	--
Blindness and vision loss	--	9·6<br>(6·34–13·6)	9·6<br>(6·34–13·6)	--
Age-related and other hearing loss	--	10·1<br>(9·03–11·3)	10·1<br>(9·03–11·3)	--
Musculoskeletal disorders	5·19<br>(1·3–9·35)	23·1<br>(19·1–27·1)	23·5<br>(19·4–27·6)	5·96<br>(1·57–10·7)
Rheumatoid arthritis	13·4<br>(3·41–24·1)	15·2<br>(3·88–27)	15·3<br>(3·89–27·3)	14·9<br>(3·91–26·5)
Osteoarthritis	--	17·1<br>(9·07–26·3)	17·1<br>(9·07–26·3)	--
Low back pain	--	42·1<br>(36·5–47·6)	42·1<br>(36·5–47·6)	--
Gout	--	51·9<br>(36·2–68·9)	51·9<br>(36·2–68·9)	--
Other non-communicable diseases	0·0648<br>(0·0428–0·0979)	0·0605<br>(0·0378–0·0937)	--	0·253<br>(0·16–0·367)
Sudden infant death syndrome	7·05<br>(3·99–11·8)	7·05<br>(3·99–11·8)	--	7·05<br>(3·99–11·8)
Injuries	32<br>(25·5–37·7)	26·6<br>(21·4–31·8)	29·8<br>(25·4–34·7)	22·8<br>(14·9–29·8)
Transport injuries	18·6<br>(10·7–26·3)	19·5<br>(13·1–26·4)	25·6<br>(21·3–30·2)	16·4<br>(7·86–24·9)
Road injuries	17·7<br>(9·52–25·8)	18·4<br>(11·6–25·5)	24·4<br>(20–29·1)	15·6<br>(6·86–24·5)
Other transport injuries	32·6<br>(27·4–37·8)	32<br>(26·9–37·1)	35<br>(30·4–39·4)	28·9<br>(23–34·7)
Unintentional injuries	39·6<br>(33·4–44·3)	29·1<br>(24·6–34·1)	30<br>(25·4–35·3)	26·8<br>(22·1–31·3)
Falls	54·2<br>(46·5–59·8)	40·8<br>(34·8–46·4)	39·2<br>(33·3–44·9)	45·5<br>(39–51·2)
Drowning	-13·8<br>(-27·5–1·53)	-13·3<br>(-26·4–1·1)	9·82<br>(5·35–16·5)	-14·4<br>(-27·8–0·651)

Fire, heat, and hot substances	10·1 (5·37–16·6)	12 (7·07–19·3)	12·3 (7·24–19·8)	11·3 (6·73–18·2)
Poisonings	12·3 (7·41–19·5)	11·9 (7·26–18·9)	11 (6·43–17·3)	12·9 (7·99–20)
Exposure to mechanical forces	6·67 (-4·5–18·2)	15·7 (12·8–18·6)	17·7 (15·2–20·1)	-2·23 (-13·6–10·3)
Animal contact	21·1 (16–28·3)	19·8 (14·8–26·3)	20·9 (15·9–27·4)	17·9 (13·1–24·4)
Foreign body	2·37 (1·52–3·49)	3·27 (2·12–4·79)	2·74 (1·78–4·04)	3·58 (2·33–5·23)
Environmental heat and cold exposure	8·15 (3·37–15·3)	8·93 (3·88–16·1)	8·83 (3·83–16)	9·28 (4·02–16·4)
Exposure to forces of nature	0 (0–0)	9·8 (4·24–17·7)	9·8 (4·24–17·7)	0 (0–0)
Other unintentional injuries	13·9 (8·38–21·9)	11·7 (6·87–18·8)	11·5 (6·73–18·6)	15·3 (9·64–23·1)
Self-harm and interpersonal violence	20·4 (4·42–34·7)	22·9 (7·79–36·2)	32 (23·5–40·5)	21·6 (5·31–35·9)
Self-harm	20·1 (3·3–35·1)	21·5 (4·49–36·4)	21·3 (7·45–34·5)	21·5 (4·34–36·5)
Interpersonal violence	26·8 (17·2–36·2)	33·2 (24·3–42·6)	38·5 (29·2–48·1)	23·6 (14·2–32·9)

Supplementary Results Table 2. All-age PAFs attributable to the joint distribution of all risk factors for all ages, both sexes combined for each GBD level 1, level 2, and level 3 cause and all causes in 2019 for Cheshire East. Results are shown for deaths, YLLs, YLDs, and DALYs Data in parenthesis are 95% uncertainty intervals. PAFs=population attributable fractions. GBD=Global Burden of Disease. YLLs=years of life lost. YLDs=years lived with disability. DALYs=disability-adjusted life-years

	Deaths 2019	DALYs 2019	YLDs 2019	YLLs 2019
All causes	56·3 (53–59·7)	43·8 (40·8–46·9)	31·4 (29–33·8)	56·2 (53·3–59·2)
Communicable, maternal, neonatal, and nutritional diseases	45·5 (36·6–54·1)	50·9 (45·9–56·1)	46·7 (42·4–51·3)	52·8 (45·6–59·8)
HIV/AIDS and sexually transmitted infections	74·9 (58·4–87·6)	76·3 (58·4–89·2)	98 (97–98·8)	55·2 (35–75·3)
HIV/AIDS	92·5 (90·7–93·6)	91·7 (89·6–93·3)	95·1 (93·3–96·4)	89 (86·7–90·4)
Sexually transmitted infections excluding HIV	54·4 (31·7–78·7)	63·8 (39·3–86·8)	100 (100–100)	20·8 (8·49–42·5)
Respiratory infections and tuberculosis	46·6 (36·9–55·9)	44·3 (36·2–52·8)	3·69 (2·71–4·82)	49·7 (40·5–58·6)
Tuberculosis	59·1 (46·7–70·3)	61·7 (50·9–71·7)	58·4 (46·4–68·5)	62·4 (51·6–72·5)
Lower respiratory infections	46·6 (36·8–55·9)	49·6 (40·3–58·4)	34·8 (27·6–42·1)	49·7 (40·4–58·6)
Upper respiratory infections	0·0602 (0·0309–0·112)	0·00715 (0·00164–0·0161)	0·00518 (1·95e-05–0·014)	0·147 (0·0736–0·268)
Otitis media	1·97 (0·897–3·87)	4·15 (2·61–5·94)	4·12 (2·59–5·94)	5·16 (2·7–8·89)
Enteric infections	4·65 (2·79–8·17)	7·74 (5·66–11·3)	8·17 (5·93–11·8)	6·85 (4·53–10·2)
Diarrheal diseases	4·7 (2·86–8·24)	7·79 (5·72–11·4)	8·17 (5·94–11·8)	6·98 (4·71–10·5)
Other infectious diseases	0·462 (0·318–0·628)	1·05 (0·798–1·35)	0·451 (0·253–0·749)	1·32 (0·955–1·74)
Meningitis	1·31 (0·966–1·76)	2·4 (1·79–3·2)	0·000484 (1·21e-06–0·00151)	3·22 (2·41–4·24)
Encephalitis	0·235 (0·157–0·34)	0·699 (0·44–1)	0·000157 (3·16e-07–0·000486)	0·894 (0·591–1·3)
Measles	2·99 (-0·926–14·8)	3·27 (-0·908–15·7)	0·454 (0·0149–1·23)	3·78 (-1·14–18·7)
Acute hepatitis	7·5 (2·63–22·2)	4·35 (2·41–7·74)	3·29 (1·91–5·45)	7·54 (2·98–20·9)
Maternal and neonatal disorders	90·1 (88·4–91·5)	78·5 (74·9–81·8)	64·9 (59–70)	90·6 (88·9–92)
Maternal disorders	9·36 (3·41–15·5)	9·51 (3·42–15·7)	9·64 (3·46–16)	9·18 (3·33–15·2)
Neonatal disorders	91·5 (89·8–93)	79·9 (76·2–83·3)	66·6 (60·5–71·9)	91·5 (89·8–93)
Nutritional deficiencies	73·3 (67·5–91·7)	78·5 (74·8–82·7)	78·6 (74·8–82·9)	72·5 (68·8–84·9)
Protein-energy malnutrition	100 (100–100)	100 (100–100)	100 (100–100)	100 (100–100)
Vitamin A deficiency	--	100 (100–100)	100 (100–100)	--
Dietary iron deficiency	--	100 (100–100)	100 (100–100)	--
Non-communicable diseases	58 (54·4–61·6)	44·5 (41·2–47·8)	30·8 (28·3–33·4)	58·3 (55·2–61·5)
Neoplasms	49·9 (45·6–54·5)	49·3 (45·3–53·8)	35·3 (30·7–40·5)	50·2 (46·2–54·6)
Lip and oral cavity cancer	67·3 (56·1–77·1)	70·6 (59·7–79·5)	69·9 (58·1–78·9)	70·6 (59·8–79·6)
Nasopharynx cancer	65·8 (52·5–77·1)	66·3 (53·1–77)	63·1 (49–74·5)	66·6 (53·5–77·3)
Other pharynx cancer	79 (71·1–86)	80·5 (73·1–86·9)	80·1 (72·2–86·8)	80·6 (73·1–86·9)
Esophageal cancer	77·3 (67·2–86)	78·7 (69·1–86·9)	77·9 (67·8–86·5)	78·8 (69·1–86·9)
Stomach cancer	26·4 (18·8–43)	26·9 (19·3–42·8)	27·1 (19·4–43·3)	26·9 (19·3–42·8)

Colon and rectum cancer	66·4<br>(57·4–75·8)	66·8<br>(58·3–75·7)	67<br>(58·4–75·8)	66·8<br>(58·3–75·7)
Liver cancer	78<br>(72·2–83·7)	78·9<br>(73·4–83·9)	79·2<br>(73·4–84·5)	78·9<br>(73·4–83·9)
Gallbladder and biliary tract cancer	24·3<br>(13·3–37·4)	24·7<br>(13·6–37·8)	22·1<br>(11·4–35·3)	24·8<br>(13·6–37·8)
Pancreatic cancer	46·8<br>(36·2–58·9)	46·1<br>(36·1–57·6)	46·6<br>(36–58·6)	46·1<br>(36·1–57·6)
Larynx cancer	83·5<br>(74·9–89·8)	83·9<br>(76·1–89·6)	82·6<br>(74·8–88·5)	84·1<br>(76·1–89·7)
Tracheal, bronchus, and lung cancer	87·8<br>(84·6–91·1)	87·2<br>(84·1–90·5)	87·7<br>(84·4–90·9)	87·2<br>(84·1–90·4)
Breast cancer	40·2<br>(28·8–53·7)	39·1<br>(28·8–51·1)	39·3<br>(28·9–51·4)	39·1<br>(28·8–51·1)
Cervical cancer	100<br>(100–100)	100<br>(100–100)	100<br>(100–100)	100<br>(100–100)
Uterine cancer	47·9<br>(31·5–63·7)	49<br>(32·9–64·7)	49·3<br>(33·4–65·1)	49<br>(32·8–64·7)
Ovarian cancer	28·7<br>(14·6–44·5)	24·9<br>(12·2–40)	23·9<br>(11·6–38)	24·9<br>(12·3–40·1)
Prostate cancer	6·2<br>(2·73–9·97)	6·75<br>(3·07–10·7)	7·12<br>(3·25–11·2)	6·69<br>(3·02–10·7)
Kidney cancer	43<br>(31·7–53·8)	43·3<br>(32·9–53·6)	43·2<br>(32·9–53·4)	43·3<br>(32·9–53·6)
Bladder cancer	43·7<br>(28·1–59·3)	46·2<br>(31·3–60·8)	47<br>(32·6–61·2)	46·1<br>(31·2–60·7)
Thyroid cancer	16·8<br>(8·4–26·4)	17·4<br>(8·93–27·1)	16·5<br>(8·98–25·2)	17·5<br>(8·93–27·5)
Mesothelioma	99·3<br>(99–99·5)	98·5<br>(97·9–98·9)	99·1<br>(98·7–99·4)	98·5<br>(97·9–98·9)
Non-Hodgkin lymphoma	8·75<br>(3·71–15·3)	9·03<br>(3·93–15·7)	8·93<br>(3·84–15·6)	9·04<br>(3·93–15·7)
Multiple myeloma	9·74<br>(4·31–16·6)	10·1<br>(4·5–17·4)	10<br>(4·46–17·4)	10·1<br>(4·53–17·4)
Leukemia	39·8<br>(24·9–54·7)	38·4<br>(25·5–51·3)	37·7<br>(25·1–50·3)	38·4<br>(25·5–51·4)
Cardiovascular diseases	79·7<br>(75·8–83·4)	81<br>(77·7–84)	73·2<br>(68·2–77·8)	82<br>(78·9–85)
Rheumatic heart disease	25·1<br>(13·6–47·6)	25·7<br>(15·8–44·6)	25·6<br>(15·8–42·2)	25·7<br>(15·8–44·6)
Ischemic heart disease	94·1<br>(90·6–97·2)	95·1<br>(92·5–97·3)	94·5<br>(91·8–96·8)	95·1<br>(92·5–97·3)
Stroke	80·9<br>(73·2–89·6)	83·8<br>(78–90)	83·5<br>(77·8–89·2)	83·8<br>(77·9–90·2)
Hypertensive heart disease	100<br>(100–100)	100<br>(100–100)	100<br>(100–100)	100<br>(100–100)
Non-rheumatic valvular heart disease	28·3<br>(18·5–42·6)	28·6<br>(20–39·7)	21<br>(14–29·4)	29·5<br>(20·5–41·2)
Cardiomyopathy and myocarditis	36·5<br>(28·2–47·4)	41·7<br>(35·2–48·3)	36·9<br>(30·4–44)	42<br>(35·4–48·7)
Atrial fibrillation and flutter	54·8<br>(43·7–65·5)	61·4<br>(52–70·3)	65<br>(56·2–72·9)	57·5<br>(47·2–67·5)
Aortic aneurysm	53·5<br>(46·4–61·6)	59·9<br>(53·8–65·8)	--	59·9<br>(53·8–65·8)
Endocarditis	34<br>(23·5–48)	36·3<br>(27·4–47·5)	33·9<br>(23·3–47·5)	36·4<br>(27·4–47·4)
Other cardiovascular and circulatory diseases	35<br>(28·1–42·5)	37·5<br>(31·3–44)	37·1<br>(30·5–44·4)	37·6<br>(31·3–44·3)
Chronic respiratory diseases	63·3<br>(56·4–71)	60·4<br>(55·4–65·9)	56·1<br>(50·8–60·9)	63·8<br>(57·6–71)
Chronic obstructive pulmonary disease	73·7<br>(68·9–78·1)	72·7<br>(68·8–76·5)	67·3<br>(63·1–71·2)	75·6<br>(71·6–79·5)
Pneumoconiosis	100<br>(100–100)	100<br>(100–100)	100<br>(100–100)	100<br>(100–100)
Asthma	46·1<br>(33·3–59·6)	45·7<br>(36·1–56·2)	45·4<br>(36–55·7)	47·9<br>(36·9–59·5)
Digestive diseases	27·7<br>(22·9–33)	35·9<br>(29·9–41·5)	30·4<br>(21·4–39·9)	39·1<br>(32·8–45·8)
Cirrhosis and other chronic liver diseases	71·7<br>(59–85·2)	75·6<br>(63·6–87·6)	73<br>(61·1–85·3)	75·6<br>(63·6–87·7)

Upper digestive system diseases	23·1 (19·1–26·8)	10·3 (6·92–14·5)	0·805 (0·481–1·29)	25·8 (21·9–29·4)
Gallbladder and biliary diseases	48·8 (33·2–64·6)	54·1 (39·1–68·6)	55 (40·2–69·4)	50·1 (34·7–65·3)
Pancreatitis	32·7 (12·8–54)	39·7 (18·5–60·2)	40·6 (18·6–61·2)	39·6 (18·5–59·7)
Neurological disorders	29·5 (13·3–46·9)	19·2 (9·75–33·2)	12·7 (6·62–22·9)	26 (11·5–43·4)
Alzheimer's disease and other dementias	46 (30·7–62·3)	47·9 (32·4–63·6)	48·2 (33–63·9)	47·7 (32·4–63·5)
Parkinson's disease	-10·3 (-16·9–-4·7)	-11·5 (-18·6–-5·41)	-11·9 (-19–-5·57)	-11·4 (-18·4–-5·35)
Idiopathic epilepsy	27·2 (15–38·6)	26·3 (14·8–36·6)	24·1 (13·3–34·1)	29·1 (16·8–39·8)
Multiple sclerosis	15·9 (11·8–20·3)	16·6 (12·3–20·9)	16·2 (12·1–20·5)	16·9 (12·4–21·3)
Mental disorders	--	6·17 (3·07–9·49)	6·18 (3·07–9·49)	--
Depressive disorders	--	12·8 (5·92–20·1)	12·8 (5·92–20·1)	--
Anxiety disorders	--	4·41 (1·2–9·73)	4·41 (1·2–9·73)	--
Idiopathic developmental intellectual disability	--	37·5 (16·2–56·7)	37·5 (16·2–56·7)	--
Substance use disorders	100 (100–100)	100 (100–100)	100 (100–100)	100 (100–100)
Alcohol use disorders	100 (100–100)	100 (100–100)	100 (100–100)	100 (100–100)
Drug use disorders	100 (100–100)	100 (100–100)	100 (100–100)	100 (100–100)
Diabetes and kidney diseases	99·8 (99·7–99·8)	100 (99·9–100)	100 (100–100)	99·8 (99·7–99·8)
Diabetes mellitus	100 (100–100)	100 (100–100)	100 (100–100)	100 (100–100)
Chronic kidney disease	100 (100–100)	100 (100–100)	100 (100–100)	100 (100–100)
Sense organ diseases	--	8·87 (7·63–10·4)	8·87 (7·63–10·4)	--
Blindness and vision loss	--	11·6 (7·46–16·7)	11·6 (7·46–16·7)	--
Age-related and other hearing loss	--	9·25 (8·25–10·4)	9·25 (8·25–10·4)	--
Musculoskeletal disorders	3·98 (1·06–7·3)	23·6 (19·6–27·9)	24·1 (19·9–28·5)	4·82 (1·26–8·59)
Rheumatoid arthritis	12·7 (3·39–22·6)	14·7 (3·92–25·5)	14·8 (3·83–25·8)	14·2 (3·91–25)
Osteoarthritis	--	19·4 (10·7–28·7)	19·4 (10·7–28·7)	--
Low back pain	--	42·5 (37·1–48·3)	42·5 (37·1–48·3)	--
Gout	--	56·9 (41–73·3)	56·9 (41–73·3)	--
Other non-communicable diseases	0·0485 (0·0319–0·0726)	0·0495 (0·03–0·075)	--	0·229 (0·146–0·331)
Sudden infant death syndrome	7·8 (4·26–13·2)	7·79 (4·25–13·2)	--	7·79 (4·25–13·2)
Injuries	34·8 (28·6–40·3)	28·9 (23·7–33·8)	31·4 (26·7–36·2)	25·5 (17·6–31·8)
Transport injuries	20·4 (13·2–27·3)	21·3 (14·9–27·6)	26·4 (21·9–30·6)	18·4 (10·5–26)
Road injuries	19·6 (12·2–26·8)	20·3 (13·8–26·8)	25·1 (20·6–29·4)	17·7 (9·43–25·3)
Other transport injuries	33·8 (28·4–38·4)	33·7 (28·2–38·3)	36·8 (31·7–41·1)	30·3 (24·1–35·4)
Unintentional injuries	42·5 (35·6–47·4)	31·3 (26·4–36·2)	31·7 (26·6–36·8)	30·2 (25–34·8)
Falls	55·8 (47·3–61·5)	43·3 (36·6–48·6)	41·7 (35·2–46·9)	48·4 (41·3–54)
Drowning	-14·6 (-28·5–-1·42)	-13·1 (-26·4–-0·546)	10·2 (5·48–17·5)	-14·5 (-28–-1·47)

Fire, heat, and hot substances	10·3<br>(5·29–17·8)	12·4<br>(7·17–20·1)	12·5<br>(7·27–20·3)	11·9<br>(6·86–19·1)
Poisonings	13·9<br>(8·12–21·9)	12·9<br>(7·71–20·2)	11·6<br>(6·71–18·9)	14·9<br>(9·07–22·7)
Exposure to mechanical forces	12·1<br>(2–22·2)	17·2<br>(14·5–19·6)	18·5<br>(15·9–20·6)	0·685<br>(-9·35–11·2)
Animal contact	27·8<br>(22·8–34)	22·1<br>(17·2–28·3)	22·4<br>(17·2–28·7)	21·5<br>(17–27·5)
Foreign body	1·77<br>(1·13–2·65)	2·76<br>(1·85–4·04)	2·37<br>(1·58–3·52)	3·05<br>(2–4·5)
Environmental heat and cold exposure	8·02<br>(3·26–15·1)	9·51<br>(4·22–17·5)	9·57<br>(4·26–17·6)	9·2<br>(3·99–16·9)
Exposure to forces of nature	0<br>(0–0)	10·6<br>(4·75–19·1)	10·6<br>(4·75–19·1)	0<br>(0–0)
Other unintentional injuries	13·5<br>(7·72–21·6)	12<br>(6·94–19·5)	11·9<br>(6·85–19·4)	15·2<br>(9·2–23)
Self-harm and interpersonal violence	23<br>(6·91–37·4)	25·6<br>(10·9–38·4)	33<br>(24·8–41)	24·4<br>(7·99–38·6)
Self-harm	22·7<br>(6·01–37·8)	24·3<br>(7·24–39·3)	23·2<br>(9·36–36)	24·3<br>(7·1–39·4)
Interpersonal violence	28·6<br>(19–37·7)	35<br>(26–43·7)	39<br>(30–48·3)	25·5<br>(15·9–34·5)

Supplementary Results Table 2. All-age PAFs attributable to the joint distribution of all risk factors for all ages, both sexes combined for each GBD level 1, level 2, and level 3 cause and all causes in 2019 for Cheshire West and Chester. Results are shown for deaths, YLLs, YLDs, and DALYs Data in parenthesis are 95% uncertainty intervals. PAFs=population attributable fractions. GBD=Global Burden of Disease. YLLs=years of life lost. YLDs=years lived with disability. DALYs=disability-adjusted life-years

	Deaths 2019	DALYs 2019	YLDs 2019	YLLs 2019
All causes	57·1 (53·7–60·4)	44·7 (41·4–47·7)	31·5 (29·1–33·9)	57·1 (54·2–59·9)
Communicable, maternal, neonatal, and nutritional diseases	45·3 (34·8–55·5)	51·4 (45·1–56·7)	48·5 (44·1–53·1)	52·8 (44·4–60·8)
HIV/AIDS and sexually transmitted infections	75 (58·4–87·8)	76·2 (57·8–89·1)	97·9 (96·8–98·8)	55·8 (35·1–75·7)
HIV/AIDS	93 (91·1–94·1)	92·2 (90–93·8)	95·1 (93·2–96·4)	89·8 (87·6–91·2)
Sexually transmitted infections excluding HIV	52·6 (29·9–77·5)	62·5 (37·1–86)	100 (100–100)	20·2 (8·07–42·2)
Respiratory infections and tuberculosis	46·8 (35·2–57·7)	44·3 (34·2–53·1)	3·54 (2·62–4·64)	50·1 (39·1–60·4)
Tuberculosis	60·1 (48–71·5)	62·3 (51·2–72·4)	58·4 (46·7–68·9)	63·2 (52·4–73·1)
Lower respiratory infections	46·7 (35·2–57·7)	49·9 (38·9–60·3)	35 (27·9–42)	50 (38·9–60·4)
Upper respiratory infections	0·059 (0·0328–0·1)	0·0068 (0·0012–0·0159)	0·00548 (6·38e-05–0·0145)	0·143 (0·0813–0·239)
Otitis media	3·65 (1·34–9·72)	4·09 (2·57–5·83)	4·09 (2·57–5·82)	8·36 (3·65–18·8)
Enteric infections	4·71 (2·84–7·96)	7·86 (5·72–11·2)	8·35 (6·09–11·8)	6·97 (4·74–10·2)
Diarrheal diseases	4·76 (2·88–8·03)	7·91 (5·75–11·3)	8·36 (6·1–11·8)	7·09 (4·85–10·3)
Other infectious diseases	0·577 (0·393–0·768)	1·18 (0·884–1·48)	0·428 (0·245–0·712)	1·48 (1·08–1·87)
Meningitis	1·42 (1·05–1·85)	2·48 (1·85–3·19)	0·000508 (4·48e-06–0·00151)	3·26 (2·44–4·21)
Encephalitis	0·285 (0·197–0·418)	0·827 (0·472–1·2)	0·00019 (1·79e-06–0·000518)	1·03 (0·676–1·5)
Measles	3·23 (-0·903–15·6)	3·92 (-1·07–18·9)	0·471 (0·0206–1·21)	4·01 (-1·09–19·9)
Acute hepatitis	12·6 (5·92–25·6)	5·38 (3·1–9·14)	3·23 (1·88–5·27)	12 (5·63–24·2)
Maternal and neonatal disorders	89·5 (88–91)	79·1 (75·8–82·2)	68·3 (62·9–73·2)	90 (88·4–91·4)
Maternal disorders	9·51 (3·52–15·7)	9·54 (3·54–16)	9·59 (3·56–16·4)	9·36 (3·47–15·4)
Neonatal disorders	90·7 (89·2–92·1)	80·5 (77·1–83·6)	70·1 (64·3–75·2)	90·7 (89·2–92·1)
Nutritional deficiencies	67·7 (61·8–87·5)	78·5 (74·4–82·7)	78·7 (74·6–83)	68·9 (64·6–82·6)
Protein-energy malnutrition	100 (100–100)	100 (100–100)	100 (100–100)	100 (100–100)
Vitamin A deficiency	--	100 (100–100)	100 (100–100)	--
Dietary iron deficiency	--	100 (100–100)	100 (100–100)	--
Non-communicable diseases	58·9 (55·3–62·3)	45·4 (41·9–48·7)	30·9 (28·3–33·6)	59·2 (56·2–62·2)
Neoplasms	51·5 (47·4–56·1)	50·7 (46·9–55·1)	36·2 (31·6–41·3)	51·6 (47·9–55·9)
Lip and oral cavity cancer	68·3 (57–77·8)	71·3 (60·4–79·8)	70·6 (59·5–79·5)	71·4 (60·5–79·8)
Nasopharynx cancer	68·2 (55·5–78·8)	68·4 (55·9–78·4)	65·3 (51·8–76·6)	68·6 (56·3–78·6)
Other pharynx cancer	80·6 (72·8–86·8)	81·8 (74·5–87·5)	81·4 (73·8–87·3)	81·8 (74·5–87·6)
Esophageal cancer	77·6 (67·9–86·4)	79 (69·6–87·3)	78·1 (68·2–86·7)	79 (69·6–87·3)
Stomach cancer	26·7 (18·9–43·3)	27 (19·3–43·4)	27·2 (19·5–43·4)	27 (19·3–43·4)

Colon and rectum cancer	66·8 (57·9–76)	67·1 (58·7–76)	67·3 (58·7–76·2)	67·1 (58·7–75·9)
Liver cancer	77·6 (71·6–83·4)	78·4 (72·9–83·7)	78·7 (73·1–84·1)	78·4 (72·8–83·7)
Gallbladder and biliary tract cancer	25·4 (13·8–38·4)	25·8 (14·4–38·7)	23·2 (12·3–36·5)	25·9 (14·4–38·8)
Pancreatic cancer	47·5 (36·8–59·9)	46·8 (36·7–58·8)	47·3 (36·6–59·5)	46·8 (36·7–58·8)
Larynx cancer	83·3 (74·9–89·5)	83·6 (75·8–89·3)	82·1 (74·3–88·1)	83·7 (76–89·4)
Tracheal, bronchus, and lung cancer	87·8 (84·1–91·2)	87·2 (83·7–90·5)	87·7 (84–90·9)	87·1 (83·7–90·5)
Breast cancer	40·1 (28·9–53·4)	38·8 (28·6–50·9)	39 (28·5–51·2)	38·8 (28·6–50·9)
Cervical cancer	100 (100–100)	100 (100–100)	100 (100–100)	100 (100–100)
Uterine cancer	47·6 (31–63·4)	48·8 (32·9–64·1)	49·1 (32·9–64·8)	48·7 (32·8–64·1)
Ovarian cancer	29·2 (15·2–45)	25·2 (12·5–39·5)	24·2 (12·1–38·2)	25·3 (12·5–39·6)
Prostate cancer	6·26 (2·72–10·1)	6·82 (3·06–10·8)	7·19 (3·23–11·3)	6·75 (3·03–10·7)
Kidney cancer	43 (32–53·8)	43·2 (32·5–53·6)	43·1 (32·4–53·3)	43·2 (32·5–53·6)
Bladder cancer	44·6 (28·4–60)	46·9 (31·3–61·4)	47·7 (32·5–61·6)	46·9 (31·3–61·3)
Thyroid cancer	17·4 (8·53–28)	18 (9·05–28·5)	16·9 (8·97–26·3)	18·2 (8·88–28·9)
Mesothelioma	99·2 (98·8–99·4)	98·3 (97·7–98·8)	99 (98·6–99·4)	98·3 (97·7–98·8)
Non-Hodgkin lymphoma	8·63 (3·66–15)	8·89 (3·91–15·4)	8·81 (3·76–15·3)	8·9 (3·92–15·4)
Multiple myeloma	9·71 (4·32–16·6)	10 (4·55–17·1)	9·98 (4·47–17·1)	10·1 (4·56–17·1)
Leukemia	40 (24·8–54·6)	38·1 (25·3–50·6)	37·5 (24·8–49·8)	38·2 (25·3–50·7)
Cardiovascular diseases	79·9 (75·9–83·6)	81·1 (77·8–84·1)	73 (67·8–77·6)	82·1 (79·1–85)
Rheumatic heart disease	25·2 (14·1–47·2)	25·8 (16–44·6)	25·7 (16·1–42·4)	25·9 (15·9–44·6)
Ischemic heart disease	94·1 (90·4–97)	95·2 (92·4–97·3)	94·5 (91·6–96·9)	95·2 (92·4–97·3)
Stroke	81·1 (72·9–89·6)	84 (78–90·3)	83·7 (78·1–89·6)	84 (78–90·6)
Hypertensive heart disease	100 (100–100)	100 (100–100)	100 (100–100)	100 (100–100)
Non-rheumatic valvular heart disease	28·9 (18·8–43·9)	29·2 (20·4–40·8)	20·3 (13·9–28·4)	30·2 (21·2–42·2)
Cardiomyopathy and myocarditis	36·2 (28·3–46·1)	40·6 (34·8–46·5)	35·9 (29·2–42·6)	40·9 (34·9–47)
Atrial fibrillation and flutter	54·9 (44·1–65·2)	61·8 (52·1–70·3)	65·3 (56·4–73·1)	57·7 (47·3–67·3)
Aortic aneurysm	54·3 (46·7–61·7)	60·6 (54·3–66·7)	--	60·6 (54·3–66·7)
Endocarditis	34·2 (23·5–48·7)	36·3 (27·1–47·1)	34 (23·4–47·7)	36·4 (27·2–47·2)
Other cardiovascular and circulatory diseases	35·7 (28·9–43·1)	38 (31·9–44·4)	36·8 (29·8–44·2)	38·2 (31·9–44·7)
Chronic respiratory diseases	64·9 (58·2–72·2)	61·5 (56·5–66·7)	56 (50·9–61)	65·4 (59·2–72·3)
Chronic obstructive pulmonary disease	73·9 (68·2–79·1)	73·1 (68·9–77·1)	67·5 (63·4–71·3)	75·7 (70·8–80·4)
Pneumoconiosis	100 (100–100)	100 (100–100)	100 (100–100)	100 (100–100)
Asthma	46·6 (34·3–59·9)	45·6 (36·1–55·6)	45·3 (35·9–55·3)	48·2 (37·3–59·5)
Digestive diseases	29·1 (24·1–34·5)	35·4 (29·6–40·8)	23 (16–30·8)	40·8 (34·4–47·5)

Cirrhosis and other chronic liver diseases	72·4 (60·2–85·7)	75·9 (64·4–88)	73·4 (61·9–85·3)	75·9 (64·4–88·1)
Upper digestive system diseases	23·6 (19·8–27·4)	10·2 (6·99–14·3)	0·769 (0·458–1·2)	26·3 (22·3–29·9)
Gallbladder and biliary diseases	48·4 (33–64·2)	53·4 (38·4–67·7)	54·9 (40–68·6)	49·7 (34·5–65)
Pancreatitis	34·7 (13·6–56·2)	41·3 (19–61·6)	41·6 (18·9–61·6)	41·3 (19·1–61·7)
Neurological disorders	28·6 (12·3–45·6)	18·2 (8·93–32·1)	11·9 (6·08–22·1)	24·8 (10·4–41·8)
Alzheimer's disease and other dementias	46·3 (30·7–62·4)	48·2 (32·8–63·9)	48·6 (33·2–64·2)	48 (32·8–63·7)
Parkinson's disease	-10·4 (-17·1–-4·77)	-11·7 (-18·6–-5·45)	-12·1 (-19·3–-5·61)	-11·5 (-18·5–-5·33)
Idiopathic epilepsy	26·2 (15·4–37·9)	25·4 (15·2–36·1)	23·6 (14–33·8)	28·2 (16·9–39·5)
Multiple sclerosis	16·2 (11·9–20·6)	16·8 (12·4–21·1)	16·3 (12·1–20·6)	17·1 (12·6–21·6)
Mental disorders	--	6·31 (3·17–9·79)	6·31 (3·18–9·79)	--
Depressive disorders	--	13·1 (5·95–20·5)	13·1 (5·95–20·5)	--
Anxiety disorders	--	4·51 (1·18–10·2)	4·51 (1·18–10·2)	--
Idiopathic developmental intellectual disability	--	38·1 (16·9–56·6)	38·1 (16·9–56·6)	--
Substance use disorders	100 (100–100)	100 (100–100)	100 (100–100)	100 (100–100)
Alcohol use disorders	100 (100–100)	100 (100–100)	100 (100–100)	100 (100–100)
Drug use disorders	100 (100–100)	100 (100–100)	100 (100–100)	100 (100–100)
Diabetes and kidney diseases	99·7 (99·6–99·8)	99·9 (99·9–100)	100 (100–100)	99·7 (99·6–99·8)
Diabetes mellitus	100 (100–100)	100 (100–100)	100 (100–100)	100 (100–100)
Chronic kidney disease	100 (100–100)	100 (100–100)	100 (100–100)	100 (100–100)
[truncated: 485,685 more chars]
