# Supplementary material for: Global burden of 87 risk factors in 204 countries and territories, 1990–2019: a systematic analysis for the Global Burden of Disease Study 2019
Source: Lancet. 2020 Oct 17;396(10258):1223–49. doi: 10.1016/S0140-6736(20)30752-2 (PMC7566194; doi:10.1016/S0140-6736(20)30752-2)
Supplement: Supplementary appendix 1 [file mmc1.pdf]

THE LANCET

Supplementary appendix 1

This appendix formed part of the original submission and has been peer reviewed. We post it as supplied by the authors.

Supplement to: GBD 2019 Risk Factors Collaborators. Global burden of 87 risk factors in 204 countries and territories, 1990–2019: a systematic analysis for the Global Burden of Disease Study 2019. *Lancet* 2020; **396:** 1223–49.

Appendix 1: Methods appendix to "Global burden of 87 risk factors in 204 countries and territories, 1990–2019: a systematic analysis for the Global Burden of Disease Study 2019"

This appendix provides further methodological detail for "Global burden of 87 risk factors in 204 countries and territories, 1990–2019: a systematic analysis for the Global Burden of Disease study 2019."

Portions of this appendix have been reproduced or adapted from Stanaway et al.[1] References are provided for reproduced sections.

Preamble

This appendix provides further methodological detail for "Global burden of 87 risk factors in 204 countries and territories, 1990–2019: a systematic analysis for the Global Burden of Disease Study 2019." This study complies with the Guidelines for Accurate and Transparent Health Estimates Reporting (GATHER) recommendations.[2] It includes detailed tables and information on data to maximise transparency in our estimation processes and provides a comprehensive description of analytical steps. We intend this appendix to be a living document to be updated with each iteration of the Global Burden of Disease Study (GBD).

Authors' Contributions

Managing the estimation process

Christopher J L Murray, Charlie Ashbaugh, Celine Barthelemy, Michael Brauer, Lalit Dandona, Kara Estep, Alize Ferrai, Emmanuela Gakidou, Nicholas Kassebaum, Alan D Lopez, Ashley Marks, Awoke Misganaw, Ali Mokdad, Erin Mullany, Molly R Nixon, Puja Rao, Greg Roth, Theo Vos, Stein Emil Vollset, Harvey Whiteford, Michael Brauer, Ashkan Afshin, and Stephen S Lim.

Writing the first draft of the manuscript

Christopher J L Murray, Catherine Bisignano, Jessica Cruz, Anna Gershberg, Scott Glenn, Gaorui Guo, Vincent Iannucci, Hussain Jafari Hayoon, Cathleen Keller, Varsha Krish, Samantha Larson, Rui Ma, Molly R Nixon, Kyle Simpson, Alexandrea Watson, and Stephen S Lim.

Providing data or critical feedback on data sources

Christopher J L Murray, Cristiana Abbafati, Hassan Abolhassani, Michael RM Abrigo, Ahmed Abualhasan, Laith Abu-Raddad, Abdelrahman Ibrahim Abushouk, Maryam Adabi, Victor Adekanmbi, Olatunji Adetokunboh, Shailesh Advani, Ashkan Afshin, Gina Agarwal, Muktar Ahmed, Tomi Akinyemiju, Fares Alahdab, Khurshid Alam, Biresaw Alemu, Muhammad Ali, Saqib Ali, Cyrus Alinia, Syed Aljunid, François Alla, Peter Allebeck, Amir Almasi-Hashiani, Robert Viorel Ancuceanu, Deanna Anderlini, Fereshteh Ansari, Davood Anvari, Razique Anwer, Seth Christopher Yaw Appiah, Jalal Arabloo, Morteza Arab-Zozani, Filippo Ariani, Bahram Armoon, Johan Ärnlöv, Floriane Ausloos, Marcel Ausloos, Beatriz Paulina Ayala Quintanilla, Martin Ayanore, Samad Azari, Zelalem Nigussie Azene , Ahad Bakhtiari, Shoshana Ballew, Daniela Balzi, Maciej Banach, Till Bärnighausen, Lope Barrero, Sanjay Basu, Vo Bay, Ettore Beghi, Ettore Beghi, Fiona Bennitt, Adam Berman, Boris Bikbov, Antonio Biondi, Mahdi Bohluli, Guilherme Borges, Antonio Maria Borzì, Soufiane Boufous, Michael Brauer, Andrey Briko, Gabrielle Britton, Dana Bryazka, Sharath Burugina Nagaraja, Leah Cahill, Luis Cámera, Giulia Carreras, Juan Jesus Carrero, Deborah Carvalho Malta, Joao Mauricio Castaldelli-Maia, Carlos Castañeda-Orjuela, Franz Castro, Kai-Lan Chang, Vijay Kumar Chattu, Jee-Young Choi, Dinh Toi Chu, Michael Chung, Massimo Cirillo, Aaron Cohen, Owen Cooper, Vera M Costa, Ewerton Cousin, Richard Cowden, Albertino Damasceno, Giovanni Damiani, William Dangel, Aso Darwesh, Ahmad Daryani, Rajat Das Gupta, José Das Neves, Desta Debalkie, Marissa Delang, Nikolaos Dervenis, Govinda Dhungana, Mostafa Dianatinasab, Diana Dias Da Silva, Klara Dokova, Fariba Dorostkar, Leila Doshmangir, Tim Driscoll, Bruce Duncan, David Edvardsson, Nevine El Nahas, Islam Elgendy, Iqbal Elyazar, Holly Erskine, Alireza Esteghamati, Alireza Esteghamati, Mohammad Farahmand, Mohammad Fareed, Andrea Farioli, Andre Faro, Farshad Farzadfar, Nazir Fattahi, Valery Feigin, Seyed-Mohammad Fereshtehnejad, Alize Ferrari, Irina Filip, Morenike Oluwatoyin Folayan, Artem Fomenkov, Lisa Force, Richard Franklin, Takeshi Fukumoto, Mohamed Gad, Silvano Gallus, Xu Gelin, Mansour Ghafourifard, Farhad Ghamari, Nermin Ghith, Rakesh Ghosh, Giorgia Giussani, Giorgia Giussani, Salime Goharinezhad, Houman Goudarzi, Felix Greaves, Michal Grivna, Giuseppe Grosso, Harish Gugnani, Yuming Guo, Tarun Gupta, Nima Hafezi-Nejad, Arvin Haj-Mirzaian, Beatrix Haddock, Brian Hall, Melanie Hammer, Hamidreza Haririan, Josep Maria Haro, Edris Hasanpoor, Soheil Hassanipour, Hadi Hassankhani, Reza Heidari-Soureshjani, Hannah Henrikson, Claudiu Herteliu, Hans Hoek, Praveen Hoogar, Mehdi Hosseinzadeh, Mowafa Househ, Mohamed Hsairi, Nayu Ikeda, Sheikh Mohammed Shariful Islam, Chidozie Declan Iwu, Kathryn H Jacobsen, Morteza Jafarinia, Mohammad Ali Jahani, Mihajlo Jakovljevic, Spencer James, Tahereh Javaheri, Ahamarshan Jayaraman Nagarajan, Panniyammakal Jeemon, Vivekanand Jha, John Ji, Lars Johansson, Oommen John, Catherine Johnson, Jost B Jonas, Farahnaz Joukar, Jacek Jozwiak, Mikk Jürisson, Zubair Kabir, Leila R Kalankesh, André Karch,

Getachew Kassa, Srinivasa Vittal Katikireddi, Gbenga Kayode, Laura Kemmer, Parkes Kendrick, Nauman Khalid, Ejaz Khan, Maseer Khan, Khaled Khatab, Salman Khazaei, Christian Kieling, Yun Jin Kim, Adnan Kisa, Sezer Kisa, Mika Kivimaki, Luke Knibbs, Ann Kristin Skrindo Knudsen, Jonathan Kocarnik, Parvaiz Koul, Kewal Krishan, Hans Kromhout, Dian Kusuma, Dharmesh Lal, Huong Lan Nguyen, Savita Lasrado, Shaun Lee, Kate Legrand, James Leigh, Janni Leung, Shanshan Li, Lee-Ling Lim, Shiwei Liu, Simin Liu, Jaifred Christian Lopez, Stefan Lorkowski, Alton Lu, Mokhtar Mahdavi, Morteza Mahmoudi, Reza Malekzadeh, Reza Malekzadeh, Abdullah Mamun, Fariborz Mansour-Ghanaei, Borhan Mansouri, Mohammad Ali Mansournia, Ana Maria Mantilla Herrera, Joemer Maravilla, Randall Martin, Francisco Rogerlândio Martins-Melo, Manu Mathur, Colm Mcalinden, Walter Mendoza, Ritesh G Menezes, Endalkachew Worku Mengesha, Alibek Mereke, Atte Meretoja, Tomislav Mestrovic, Bartosz Miazgowski, Ted R Miller, Andreea Mirica, Erkin Mirrakhimov, Maryam Mirzaei, Babak Moazen, Dara Mohammad, Naser Mohammad Gholi Mezerji, Abdollah Mohammadian-Hafshejani, Reza Mohammadpourhodki, Shafiu Mohammed, Ali Mokdad, Mariam Molokhia, Lorenzo Monasta, Ghobad Moradi, Masoud Moradi, Maziar Moradi-Lakeh, Joana Morgado-Da-Costa, Ulrich Mueller, Erin Mullany, Kamarul Imran Musa, Gabriele Nagel, Mohsen Naghavi, Seyed Sina Naghibi Irvani, Mukhammad David Naimzada, Morteza Naserbakht, Ionut Negoi, Josephine Ngunjiri, Yeshambel Nigatu, Shuhei Nomura, Bo Norrving, Jean Jacques Noubiap, Christoph Nowak, Felix Ogbo, Emmanuel Okunga, Andrew T Olagunju, Bolajoko Olusanya, Jacob Olusanya, Kanyin Ong, Obinna Onwujekwe, Heather Orpana, Alberto Ortiz, Adrian Oțoiu, Nikita Otstavnov, Stanislav Otstavnov, Simon Øverland, Mayowa Owolabi, Mahesh P A, Jagadish Rao Padubidri, Raffaele Palladino, Songhomitra Panda-Jonas, Deepak Kumar Pasupula, Sangram Kishor Patel, Mona Pathak, Scott Patten, Veincent Christian Pepito, David Pereira, Suzanne Polinder, Kevan Polkinghorne, Maarten Postma, Hadi Pourjafar, Anna Poznańska, Sergio Prada, Dimas Ria Angga Pribadi, Elisabetta Pupillo, Elisabetta Pupillo, Hai Quang Pham, Zahiruddin Quazi Syed, Amir Radfar, Ata Rafiee, Ivo Rakovac, Kiana Ramezanzadeh, Dr Chhabi Lal Ranabhat, Sowmya J Rao, Priya Rathi, David Laith Rawaf, Salman Rawaf, Lal Rawal, Reza Rawassizadeh, Ramu Rawat, Christian Razo, Robert Reiner, Marissa Reitsma, Vishnu Renjith, Andre Renzaho, Bhageerathy Reshmi, Seyed Mohammad Riahi, Jennifer Rickard, Juan Rivera, Nicholas Roberts, Sonia Rodriguez-Ramirez, Leonardo Roever, Luca Ronfani, Gregory Roth, Enrico Rubagotti, Godfrey Rwegerera, Basema Saddik, ProfessorEhsan Sadeghi, Yahya Safari, Saeid Safiri, Rajesh Sagar, S Mohammad Sajadi, Nasir Salam, Payman Salamati, Hosni Salem, Zainab Samad, Evanson Sambala, Abdallah M Samy, Tania Sanchez-Pimienta, Damian Santomauro, Milena Santric-Milicevic, Benn Sartorius, Arash Sarveazad, Brijesh Sathian, Sonia Saxena, Maria Inês Schmidt, David C Schwebel, Sadaf Sepanlou, Sadaf Sepanlou, Marc Serre, Omid Shafaat, Amira Shaheen, Masood Ali Shaikh, Mehran Shams-Beyranvand, Morteza Shamsizadeh, Kevin Shield, Jae Il Shin, Min-Jeong Shin, Soraya Siabani, Jasvinder Singh, Eirini Skiadaresi, Valentin Skryabin, Amin Soheili, Joan B Soriano, Luisa Sorio Flor, Sergey Soshnikov, Cory Spencer, Adel Spotin, Chandrashekhar T Sreeramareddy, Leo Stockfelt, Mark Stokes, Kurt Straif, Jacob Stubbs, Hafiz Ansar Rasul Suleria, Rafael Tabarés-Seisdedos, Karen Tabb, Amir Taherkhani, Jukka Takala, Animut Tagele Tamiru, Cuong Tat Nguyen, Nuno Taveira, Hoa Thi Do, Maria Titova, Roman Topor-Madry, Mathilde Touvier, Marcos Roberto Tovani-Palone, Bach Tran, Ravensara S Travillian, Aristides Tsatsakis, Riaz Uddin, Bhaskaran Unnikrishnan, Era Upadhyay, Marco Vacante, Pascual Valdez, Aaron Van Donkelaar, Tommi Vasankari, Yasser Vasseghian, Narayanaswamy Venketasubramanian, Vasiliy Vlassov, Theo Vos, Yasir Waheed, Yafeng Wang, Jordan Weiss, J Jason West, Ronny Westerman, Harvey Whiteford, Ai-Min Wu, Sarah Wulf Hanson, Mousa Yaminfirooz, Sanni Yaya, Christopher Yilgwan, Mekdes Yilma , Naohiro Yonemoto, Mustafa Younis, Theodore Younker, Abdilahi Yousuf , Mohammad Zamani, Mikhail Zastrozhin, Yunquan

Zhang, Zhi-Jiang Zhang, Xiu-Ju Zhao, Maigeng Zhou, Stephanie R M Zimsen, Emmanuela Gakidou, and Stephen S Lim.

Developing methods or computational machinery

Christopher J L Murray, Aleksandr Aravkin, Peng Zheng, Davood Anvari, Fiona Bennitt, Dana Bryazka, Katrin Burkart, Kate Causey, Kai-Lan Chang, Aaron Cohen, Kelly Compton, Owen Cooper, Ahmad Daryani, Rajat Das Gupta, Desta Debalkie, Marissa Delang, Mostafa Dianatinasab, Tim Driscoll, Sophia Emmons-Bell, Alize Ferrari, Emmanuela Gakidou, William Gardner, Ahmad Ghashghaee, Rakesh Ghosh, Beatrix Haddock, Mowafa Househ, Caleb Irvine, Spencer James, Laura Kemmer, Parkes Kendrick, Adnan Kisa, Sezer Kisa, James Leigh, Haley Lescinsky, Alton Lu, Helena Manguerra, Borhan Mansouri, Randall Martin, Ali Mokdad, Mohsen Naghavi, Rajan Nikbakhsh, Kanyin Ong, Charles Parry, Mona Pathak, Zahiruddin Quazi Syed, Dr Chhabi Lal Ranabhat, Ramu Rawat, Christian Razo, Robert Reiner, Marissa Reitsma, Seyed Mohammad Riahi, Nicholas Roberts, Gregory Roth, Enrico Rubagotti, Marwa Salem, Zainab Samad, Abdallah M Samy, Damian Santomauro, Marc Serre, Kevin Shield, Reed Sorensen, Luisa Sorio Flor, Vinay Srinivasan, Jeffrey Stanaway, Jukka Takala, Yasser Vasseghian, Theo Vos, Ronny Westerman, Harvey Whiteford, Sarah Wulf Hanson, Mousa Yaminfirooz, Vahid Yazdi-Feyzabadi, Theodore Younker, Jeff Zhao, Michael Brauer, Ashkan Afshin, and Stephen S Lim.

Drafting the work or revising is critically for important intellectual content

Christopher J L Murray, Cristiana Abbafati, Kaja Abbas, Mohsen Abbasi-Kangevari, Foad Abd-Allah, Ahmed Abdelalim, Kedir Abegaz, Hassan Abolhassani, Lucas Guimarães Abreu, Ahmed Abualhasan, Abdelrahman Ibrahim Abushouk, Victor Adekanmbi, Abiodun Adeoye, Olatunji Adetokunboh, Davoud Adham, Shailesh Advani, Gina Agarwal, Seyed Mohammad Kazem Aghamir, Muktar Ahmed, Rufus Akinyemi, Tomi Akinyemiju, Blessing Akombi, Fares Alahdab, Ziyad Al-Aly, Khurshid Alam, Samiah Alam, Khalid Alhabib, Muhammad Ali, Saqib Ali, Gianfranco Alicandro, Cyrus Alinia, Peter Allebeck, Amir Almasi-Hashiani, Hesham Al-Mekhlafi, Jordi Alonso, Mostafa Amini-Rarani, Dickson Amugsi, Robert Viorel Ancuceanu, Deanna Anderlini, Colin Angus, Mina Anjomshoa, Ippazio Cosimo Antonazzo, Carl Abelardo Antonio, Ernoiz Antriyandarti, Jalal Arabloo, Morteza Arab-Zozani, Johan Ärnlöv, Afsaneh Arzani, Mehran Asadi-Aliabadi, Ali Asadi-Pooya, Babak Asghari, Maha Atout, Floriane Ausloos, Marcel Ausloos, Beatriz Paulina Ayala Quintanilla, Martin Ayanore, Zelalem Nigussie Azene , Alaa Badawi, Ashish Badiye, Mohammad Hossein Bakhshaei, Shankar Bakkannavar, Kylie Ball, Shoshana Ballew, Maciej Banach, Srikanta Banerjee, Suzanne Barker-Collo, Till Bärnighausen, Lope Barrero, Lingkan Barua, Sanjay Basu, Bernhard Baune, Vo Bay, Neeraj Bedi, Ettore Beghi, Yannick Bejot, Michelle Bell, Fiona Bennitt, Isabela Bensenor, Kidanemaryam Berhe, Akshaya Bhagavathula, Neeraj Bhala, Dinesh Bhandari, Krittika Bhattacharyya, Muhammad Shahdaat Bin Sayeed, Antonio Biondi, Catherine Bisignano, Raaj Kishore Biswas, Helen Bitew, Guilherme Borges, Antonio Maria Borzì, Soufiane Boufous, Dejana Braithwaite, Nicholas Breitborde, Hermann Brenner, Nikolay Briko, Gabrielle Britton, Dana Bryazka, Katrin Burkart, Sharath Burugina Nagaraja, Zahid Butt, Alessandra C Goulart, Florentino Luciano Caetano Dos Santos, Leah Cahill, Ismael Campos-Nonato, Juan Jesus Carrero, Felix Carvalho, Deborah Carvalho Malta, Joao Mauricio Castaldelli-Maia, Giulio Castelpietra, Franz Castro, Kate Causey, Ester Cerin, Joht Singh Chandan, Vijay Kumar Chattu, Sarika Chaturvedi, Nicolas Cherbuin, Daniel Cho, Dinh Toi Chu, Michael Chung, Flavia Cicuttini, Liliana Ciobanu, Massimo Cirillo, Aaron Cohen, Vera M Costa, Ewerton Cousin, Richard Cowden, Di Cross, Giovanni Damiani, Anna-Karin Danielsson, Ahmad Daryani, José Das Neves, Claudio Davila-Cervantes, Dragos Virgil Davitoiu, Desta Debalkie, Feleke Demeke, Gebre Teklemariam Demoz, Nikolaos Dervenis, Govinda Dhungana, Mostafa Dianatinasab, Diana Dias Da Silva,

Zahra Sadat Dibaji Forooshani, Klara Dokova, Leila Doshmangir, Tim Driscoll, Bruce Duncan, Arielle Eagan, David Edvardsson, Iman El Sayed, Maha El Tantawi, Iffat Elbarazi, Islam Elgendy, Shaimaa El-Jaafary, Sharareh Eskandarieh, Saman Esmaeilnejad, Alireza Esteghamati, Arash Etemadi, Roghiyeh Faridnia, Andrea Farioli, Andre Faro, Mithila Faruque, Mehdi Fazlzadeh, Valery Feigin, Seyed-Mohammad Fereshtehnejad, Eduarda Fernandes, Alize Ferrari, Manuela Ferreira, Irina Filip, Florian Fischer, James Fisher, Nataliya Foigt, Morenike Oluwatoyin Folayan, Masoud Foroutan, Richard Franklin, Marisa Freitas, Takeshi Fukumoto, Joao Furtado, Mohamed Gad, Emmanuela Gakidou, Silvano Gallus, Alberto L Garcia-Basteiro, Assefa Ayalew Gebreslassie, Abraham Geremew, Mansour Ghafourifard, Farhad Ghamari, Ahmad Ghashghaee, Nermin Ghith, Rakesh Ghosh, Paramjit Gill, Giorgia Giussani, Sameer Gopalani, Giuseppe Gorini, Felix Greaves, Michal Grivna, Giuseppe Grosso, Rafael Guimaraes, Yuming Guo, Rajeev Gupta, Beatrix Haddock, Nima Hafezi-Nejad, Abdul Hafiz, Arya Haj-Mirzaian, Brian Hall, Iman Halvaei, Samer Hamidi, Graeme Hankey, Josep Maria Haro, Ahmed Hasaballah, Edris Hasanpoor, Abdiwahab Hashi, Rasmus Havmoeller, Khezar Hayat, Golnaz Heidari, Claudiu Herteliu, Thomas Hird, Ramesh Holla, Praveen Hoogar, Naznin Hossain, Mostafa Hosseini, Mihaela Hostiuc, Sorin Hostiuc, Mowafa Househ, Guoqing Hu, Segun Emmanuel Ibitoye, Nayu Ikeda, Olayinka Ilesanmi, Irena Ilic, Milena Ilic, Helen Ippolito, Caleb Irvine, Sheikh Mohammed Shariful Islam, Hiroyasu Iso, Rebecca Ivers, Chidozie Declan Iwu, Chinwe Juliana Iwu, Ihoghosa Iyamu, Kathryn H Jacobsen, Morteza Jafarinia, Mihajlo Jakovljevic, Tahereh Javaheri, Panniyammakal Jeemon, Ensiyeh Jenabi, Ravi Prakash Jha, Vivekanand Jha, Catherine Johnson, Jost B Jonas, Jacek Jozwiak, Mikk Jürisson, Ali Kabir, Hamed Kalani, Neeti Kapoor, André Karch, Srinivasa Vittal Katikireddi, Gbenga Kayode, Parkes Kendrick, Ejaz Khan, Maseer Khan, Khaled Khatab, Mona Khater, Mahalaqua Nazli Khatib, Christian Kieling, Yun Jin Kim, Adnan Kisa, Sezer Kisa, Mika Kivimaki, Ann Kristin Skrindo Knudsen, Dr Sonali Kochhar, Vladimir Korshunov, Ai Koyanagi, Kewal Krishan, Kris Krohn, Vivek Kumar, Om Kurmi, Dian Kusuma, Carlo La Vecchia, Ben Lacey, Ratilal Lalloo, Tea Lallukka, Faris Lami, Huong Lan Nguyen, Iván Landires, Justin Lang, Sinead Langan, Anders Larsson, Paolo Lauriola, Jeffrey Lazarus, Paul Lee, Shaun Lee, Kate Legrand, James Leigh, Matilde Leonardi, Haley Lescinsky, Janni Leung, Miriam Levi, Shanshan Li, Lee-Ling Lim, Justin Lo, Jaifred Christian Lopez, Platon Lopukhov, Stefan Lorkowski, Paulo Lotufo, Alton Lu, Alessandra Lugo, Azeem Majeed, Afshin Maleki, Reza Malekzadeh, Abdullah Mamun, Ana-Laura Manda, Borhan Mansouri, Mohammad Ali Mansournia, Randall Martin, Francisco Rogerlândio Martins-Melo, Seyedeh Zahra Masoumi, Colm Mcalinden, John Mcgrath, Walter Mendoza, Ritesh G Menezes, Endalkachew Worku Mengesha, Atte Meretoja, Tuomo Meretoja, Tomislav Mestrovic, Tomasz Miazgowski, Irmina Maria Michalek, Ted R Miller, Edward Mills, Gk Mini, Binyam Minuye, Mohammad Miri, Roya Mirzaei, Prasanna Mithra, Babak Moazen, Dara Mohammad, Yousef Mohammad, Abdollah Mohammadian-Hafshejani, Shafiu Mohammed, Hussen Mohammed , Ali Mokdad, Mariam Molokhia, Lorenzo Monasta, Ghobad Moradi, Maziar Moradi-Lakeh, Rahmatollah Moradzadeh, Paula Moraga, Joana Morgado-Da-Costa, Shane Morrison, Abbas Mosapour, Jonathan Mosser, Seyyed Meysam Mousavi, Amin Mousavi Khaneghah, Ulrich Mueller, Kamarul Imran Musa, Saravanan Muthupandian, Ashraf Nabhan, Gabriele Nagel, Mohsen Naghavi, Seyed Sina Naghibi Irvani, Behshad Naghshtabrizi, Vinay Nangia, Jobert Richie Nansseu, Morteza Naserbakht, Vinod C Nayak, Ionut Negoi, Josephine Ngunjiri, Yeshambel Nigatu, Rajan Nikbakhsh, Molly R Nixon, Bo Norrving, Jean Jacques Noubiap, Christoph Nowak, Virginia Nunez-Samudio, Felix Ogbo, In-Hwan Oh, Morteza Oladnabi, Andrew T Olagunju, Bolajoko Olusanya, Jacob Olusanya, Muktar Omer, Kanyin Ong, Obinna Onwujekwe, Heather Orpana, Alberto Ortiz, Osayomwanbo Osarenotor, Samuel Ostroff, Adrian Oțoiu, Simon Øverland, Mayowa Owolabi, Mahesh P A, Jagadish Rao Padubidri, Raffaele Palladino, Songhomitra Panda-Jonas, Mona Pathak, George Patton,

Amy Peden, Veincent Christian Pepito, Emmanuel Peprah, David Pereira, Konrad Pesudovs, Michael R Phillips, Cristiano Piccinelli, Dietrich Plass, Suzanne Polinder, Maarten Postma, Farshad Pourmalek, Sergio Prada, Elisabetta Pupillo, Hai Quang Pham, Zahiruddin Quazi Syed, Mohammad Rabiee, Navid Rabiee, Amir Radfar, Alberto Raggi, Muhammad Aziz Rahman, Ivo Rakovac, Pradhum Ram, Sowmya J Rao, Vahid Rashedi, David Laith Rawaf, Salman Rawaf, Lal Rawal, Marissa Reitsma, Vishnu Renjith, Andre Renzaho, Bhageerathy Reshmi, Nima Rezaei, Seyed Mohammad Riahi, Daniel Cury Ribeiro, Daniela Ribeiro, Jennifer Rickard, Juan Rivera, Sonia Rodriguez-Ramirez, Leonardo Roever, Luca Ronfani, Gholamreza Roshandel, Dietrich Rothenbacher, Enrico Rubagotti, Godfrey Rwegerera, Siamak Sabour, Perminder Sachdev, Basema Saddik, Masoumeh Sadeghi, Reza Saeedi, Saeid Safiri, Rajesh Sagar, Amirhossein Sahebkar, Biniyam Sahiledengle, Mohammad Ali Sahraian, Nasir Salam, Hosni Salem, Marwa Salem, Hamideh Salimzadeh, Omar Salman, Joshua A Salomon, Zainab Samad, Hossein Samadi Kafil , Evanson Sambala, Abdallah M Samy, Juan Sanabria, Tania Sanchez-Pimienta, Damian Santomauro, Itamar Santos, João Vasco Santos, Milena Santric-Milicevic, Rodrigo Sarmiento, Arash Sarveazad, Davide Sattin, Silvia Schiavolin, Maria Inês Schmidt, Aletta Schutte, David C Schwebel, Falk Schwendicke, Sadaf Sepanlou, Omid Shafaat, Saeed Shahabi, Ali Shalash, Mehran Shams-Beyranvand, Aziz Sheikh, Kenji Shibuya, Mika Shigematsu, Reza Shirkoohi, Kerem Shuval, Inga Dora Sigfusdottir, Rannveig Sigurvinsdottir, João Pedro Silva, Kyle Simpson, Jasvinder Singh, Eirini Skiadaresi, Søren T Skou, Joan B Soriano, Luisa Sorio Flor, Muluken Sorrie, Ireneous Soyiri, Chandrashekhar T Sreeramareddy, Jeffrey Stanaway, Caroline Stein, Dan Stein, Leo Stockfelt, Mark Stokes, Kurt Straif, Jacob Stubbs, Muawiyyah Babale Sufiyan, Gerhard Sulo, Iyad Sultan, Rafael Tabarés-Seisdedos, Karen Tabb, Takahiro Tabuchi, Animut Tagele Tamiru, Cuong Tat Nguyen, Nuno Taveira, Mohamad-Hani Temsah, Hoa Thi Do, Hamid Reza Tohidinik, Marcello Tonelli, Fotis Topouzis, Anna Torre, Mathilde Touvier, Marcos Roberto Tovani-Palone, Bach Tran, Ravensara S Travillian, Aristides Tsatsakis, Stefanos Tyrovolas, Riaz Uddin, Chukwuma Umeokonkwo, Bhaskaran Unnikrishnan, Era Upadhyay, Marco Vacante, Tommi Vasankari, Yasser Vasseghian, Narayanaswamy Venketasubramanian, Francesco S Violante, Vasiliy Vlassov, Stein Emil Vollset, Theo Vos, Rade Vukovic, Mitchell Wallin, Yuan-Pang Wang, Melissa Wei, Robert Weintraub, Ronny Westerman, Kirsten E Wiens, Sarah Wozniak, Ai-Min Wu, Hossein Yahyazadeh, Kazumasa Yamagishi, Mousa Yaminfirooz, Yuichiro Yano, Sanni Yaya, Vahid Yazdi-Feyzabadi, Tomas Yeheyis, Christopher Yilgwan, Mekdes Yilma , Naohiro Yonemoto, Zabihollah Yousefi, Maryam Zamanian, Mikhail Zastrozhin, Zhi-Jiang Zhang, Jeff Zhao, Xiu-Ju Zhao, Yingxi Zhao, Michael Brauer, Ashkan Afshin, and Stephen S Lim.

Extracting, cleaning, or cataloging data; designing or coding figures and tables

Christopher J L Murray, Olatunji Adetokunboh, Muktar Ahmed, Amir Almasi-Hashiani, Fiona Bennitt, Alexandra Boon-Dooley, Paul Briant, Dana Bryazka, Franz Castro, Kate Causey, Giovanni Damiani, Ahmad Daryani, Desta Debalkie, Holly Erskine, Saman Esmaeilnejad, Rachel Feldman, Giannina Ferrara, Alize Ferrari, Takeshi Fukumoto, Emmanuela Gakidou, William Gardner, Anna Gershberg, Gaorui Guo, Beatrix Haddock, Molly Herbert, Mowafa Househ, Vincent Iannucci, Kevin Ikuta, Caleb Irvine, Morteza Jafarinia, Spencer James, Cathleen Keller, Laura Kemmer, Parkes Kendrick, Mahalaqua Nazli Khatib, Adnan Kisa, Haley Lescinsky, Janni Leung, Justin Lo, Alton Lu, Emilie Maddison, Helena Manguerra, Borhan Mansouri, Ana Maria Mantilla Herrera, Fereshteh Mehri, Reza Mohammadpourhodki, Ali Mokdad, Mohsen Naghavi, Molly R Nixon, Kanyin Ong, Alyssa Pennini, Christian Razo, Marissa Reitsma, Nima Rezaei, Seyed Mohammad Riahi, Enrico Rubagotti, Hosni Salem, Zainab Samad, Abdallah M Samy, Damian Santomauro, Kyle Simpson, Luisa Sorio Flor, Cory Spencer, Vinay Srinivasan, Jeffrey Stanaway, Anna Torre, Yasser Vasseghian, Alexandrea Watson, Joanna Whisnant, Junjie Wu, Sarah Wulf Hanson, Rixing

Xu, Mousa Yaminfirooz, Theodore Younker, Mikhail Zastrozhin, Jeff Zhao, Stephanie R M Zimsen, and Stephen S Lim.

Managing the overall research enterprise

Christopher J L Murray, Tahiya Alam, Peter Allebeck, Charlie Ashbaugh, Celine Barthelemy, Deborah Carvalho Malta, Kelly Compton, Kara Estep, Emmanuela Gakidou, Alan D Lopez, Ashley Marks, Erin Mullany, Molly R Nixon, Christopher Odell, Heather Orpana, Simon Øverland, George Patton, Joshua A Salomon, Benn Sartorius, Roman Topor-Madry, Stein Emil Vollset, Andrea Werdecker, Harvey Whiteford, Michael Brauer, Ashkan Afshin, and Stephen S Lim.

Table of contents

Authors' Contributions ... 3
Managing the estimation process ... 3
Writing the first draft of the manuscript ... 3
Providing data or critical feedback on data sources ... 3
Developing methods or computational machinery ... 5
Drafting the work or revising is critically for important intellectual content ... 5
Extracting, cleaning, or cataloging data; designing or coding figures and tables ... 7
Managing the overall research enterprise ... 8
List of appendix figures and tables ... 11
Appendix figures ... 11
Appendix tables ... 11
Section 1: GBD overview ... 12
Section 1.1: Geographic locations of the analysis ... 12
Section 1.2: Time period of the analysis ... 12
Section 1.3: Statement of GATHER compliance ... 12
Section 1.4: GBD risk factor hierarchy ... 13
Section 1.5: List of abbreviations ... 13
Section 1.6: GBD results overview[1] ... 14
Section 1.7: Data input sources overview[1] ... 15
Section 1.8: Funding Sources ... 15
Section 2: Risk factor estimation ... 16
Section 2.1: Overview[1] ... 16
Section 2.2: Step 1. Effect size estimation ... 17
Section 2.2.1: Collate relative risk data[1] ... 17
Section 2.2.2: Determine relative risks ... 18
Section 2.3: Step 2. Exposure estimation[1] ... 25
Section 2.3.1: Step 2a: Collate exposure data ... 25
Section 2.3.2: Step 2b: Adjust exposure data ... 26
Section 2.3.3: Step 2c: Estimate exposure ... 29
Section 2.4: Step 3. TMREL[1] ... 39
Section 2.5: Step 4. Estimate population-attributable fractions[1] ... 39
Section 2.6: Step 5. Estimate summary exposure values[1] ... 40

Section 2.7: Step 6. Mediation[1] 41
Section 2.7.1: Summary 41
Section 2.7.2: Calculating the burden of multiple risk factors 42
Section 2.7.3: Adjusting for mediation 43
Section 2.7.4: Calculating mediation factor 43
Section 2.7.5: Piecewise aggregation (Pattern 3) 45
Section 2.7.6: Uncertainty of aggregated and mediated PAFs 46
Section 2.7.7: Important assumptions in aggregating risk factors and including mediation 46
Section 2.8: Step 7. Estimate attributable burden[1] 46
Section 2.9: Decomposition analysis of deaths and DALYs[1] 46
Section 2.10: Socio-demographic Index analysis[1] 48
Section 2.10.1: Development of the Socio-demographic Index 48
Section 2.10.2: Development of a revised SDI indicator 48
Section 3: References 50
Section 4: Risk-specific modelling descriptions 53
Section 5: Figures and tables 297

List of appendix figures and tables

Appendix figures

Figure S1. Analytical flowchart of the comparative risk assessment for the estimation of population-attributable fractions by geography, age, sex, and year for GBD 2019 297

Figure S2. GBD 2019 DisMod-MR 2.1 analytic cascade 298

Figure S3. Spatiotemporal Gaussian Process Regression (ST-GPR) flowchart 299

Appendix tables

Table S1. Guidelines for Accurate and Transparent Health Estimates Reporting (GATHER) checklist 300

Table S2. GBD 2019 risk factor hierarchy with levels 303

Table S3. Types of Comparative Risk Assessments (CRA) based on the time perspective and the nature of the counterfactual level or distribution of exposure 305

Table S4. GBD 2019 location hierarchy with levels 306

Table S5. SDI values for all estimated GBD 2019 locations, 1990–2019 321

Table S6. Mediation factor matrix 329

Table S7. Relative risks used by age and sex for each outcome 333

Section 1: GBD overview

Section 1.1: Geographic locations of the analysis

We produced estimates for 204 countries and territories that were grouped into 21 regions and seven super-regions (table S4). The seven super-regions are central Europe, eastern Europe, and central Asia; high income; Latin America and the Caribbean; north Africa and the Middle East; south Asia; southeast Asia, east Asia, and Oceania; and sub-Saharan Africa. For GBD 2019, 9 countries and territories (Cook Islands, Monaco, San Marino, Nauru, Niue, Palau, Saint Kitts and Nevis, Tokelau, and Tuvalu) were added, such that the GBD location hierarchy now includes all WHO member states. This year, GBD includes subnational analyses for several new countries and continues to analyse at subnational levels countries that were added in previous cycles. Subnational estimation in GBD 2019 includes five new countries (Italy, Nigeria, Pakistan, the Philippines, and Poland) and 16 countries previously estimated at subnational levels (Brazil, China, Ethiopia, India, Indonesia, Iran, Japan, Kenya, Mexico, New Zealand, Norway, Russia, South Africa, Sweden, the UK, and the USA). All analyses are at the first level of administrative organisation within each country except for New Zealand (by Māori ethnicity), Sweden (by Stockholm and non-Stockholm), the UK (by local government authorities), and the Philippines (by provinces). All subnational estimates for these countries were incorporated into model development and evaluation as part of GBD 2017. To meet data use requirements, in this publication we present subnational estimates for Brazil, India, Indonesia, Japan, Kenya, Mexico, Sweden, the UK, and the USA); given space constraints, these results are presented in appendix 2 instead of the main text. Subnational estimates for China are included in maps but are not reported in appendix tables. Subnational estimates for other countries will be released in separate publications.

For GBD 2019, we have also defined locations as standard locations and non-standard locations. Standard GBD locations are defined as the set of all subnationals belonging to countries where data quality is high and with populations over 200 million, in addition to all other countries. Standard locations include the subnationals for China, India, the United States, and Brazil, but not Indonesia; China, India, the United States, and Brazil are also included at the country level. All other countries with subnational estimates are defined as non-standard locations.

Section 1.2: Time period of the analysis

A complete set of risk-specific exposures, relative risks (RRs), theoretical minimum-risk exposure levels (TMRELs), and population attributable fractions (PAFs) were computed for the years 1990–2019.

Section 1.3: Statement of GATHER compliance

This study complies with the Guidelines for Accurate and Transparent Health Estimates Reporting (GATHER) recommendations.[2] We have documented the steps involved in our analytical procedures and detailed the data sources used. See table S1 for the GATHER checklist.

The GATHER recommendations may be found here: http://gather-statement.org/

Section 1.4: GBD risk factor hierarchy

The GBD 2019 risk factors hierarchy and levels are summarised in table S2. The risk hierarchy is based on common features of individual risks; for example, risk factors that represent behavioural factors are grouped together.

The GBD risk factor list continues to evolve to reflect the policy relevance, public health, and medical care importance of major risk factors. Three risks were added to the list for GBD 2019: non-optimal temperature, high temperature, and low temperature.

Section 1.5: List of abbreviations

APCSC	Asia-Pacific Cohort Studies Collaboration
BMI	body-mass index
BMD	bone mineral density
CDC	Centers for Disease Control and Prevention
CF	correction factor
CKD	chronic kidney disease
COD	causes of death
CODEm	cause of death ensemble modelling
COPD	chronic obstructive pulmonary disease
CRA	comparative risk assessment
CSA	childhood sexual abuse
CSV	comma-separated values
CSMR	cause-specific mortality rate
CVD	cardiovascular disease
DALY	disability-adjusted life-year
DHS	Demographic and Health Survey
DRI	data representativeness index
EDU15+	mean education for those age 15 years or older
EMR	excess mortality rate
FAO	Food and Agriculture Organization
FPG	fasting plasma glucose
GAM	generalised additive model
GATHER	Guidelines for Accurate and Transparent Health Estimates Reporting
GBD	Global Burden of Diseases, Injuries, and Risk Factors Study
GHDx	Global Health Data exchange
GoF	goodness of fit
HAP	household air pollution
ID	iron deficiency
IDA	iron deficiency anaemia
IER	integrated exposure response
IHD	ischaemic heart disease
ILO	International Labour Organization
IPV	intimate partner violence
IQ	intelligence quotient
JMP	Joint Monitoring Project
KS	Kolmogorov-Smirnov

LDI	lag-distributed income
LDL	low-density lipoprotein
LMICs	low- and middle-income countries
LOESS	locally estimated scatterplot smoothing
LRI	lower respiratory infection
MCMC	Markov Chain Monte Carlo simulations
MDG	Millennium Development Goal
MF	mediation factor
MICS	Multiple Indicator Cluster Surveys
MoM	method of moments
MR-BRT	meta-regression—Bayesian, regularised, trimmed
NCD	non-communicable disease
OER	observed-to-expected ratio
PAF	population-attributable fraction
PDF	probability distribution factor
$PM_{2.5}$	particulate matter <2.5 μm in diameter
PCS	prospective cohort study
RCT	randomised controlled trial
REDCap	Research Electronic Data Capture
RMSE	root mean square error
RR	relative risk
SBP	systolic blood pressure
SD	standard deviation
SDG	Sustainable Development Goal
SDI	Socio-demographic Index
SEER	Surveillance, Epidemiology, and End Results Program
SEV	summary exposure value
SHS	secondhand smoke
SIR	smoking impact ratio
SSB	sugar-sweetened beverages
ST-GPR	spatiotemporal Gaussian process regression
TB	tuberculosis
TFU25	total fertility rate in those under 25 years old
TMREL	theoretical minimum-risk exposure level
TSNA	tobacco-specific nitrosamines
UI	uncertainty interval
USA	United States
USD	United States dollars
WCRF	World Cancer Research Fund
WHO	World Health Organization
YLDs	years lived with disability
YLLs	years of life lost

Section 1.6: GBD results overview[1]

Results from the GBD 2019 are measured in terabytes. Results are available in an interactive data downloading tool on the Global Health Data exchange (GHDx). Data and underlying code used for this analysis will be made publicly available pending manuscript acceptance.

The current version of the data download tool is available in the GHDx and contains core summary results for the GBD 2019: http://ghdx.healthdata.org/gbd-results-tool. The core summary results include deaths, years of life lost (YLLs), years lived with disability (YLDs), disability-adjusted life-years (DALYs), and summary exposure values (SEVs). The GHDx includes data for causes, risks, cause-risk attribution, aetiologies, and impairments.

Data above a certain size cannot be viewed online but can be downloaded. Depending on the size of the download, users may need to enter an email address; a download location will be sent to them when the files are prepared.

Section 1.7: Data input sources overview[1]

GBD 2019 incorporated a large number and wide variety of input sources to estimate mortality, causes of death and illness, and risk factors for 204 countries and territories from 1990-2019. These input sources are accessible through an interactive citation tool available in the GHDx.

Users can retrieve citations for a specific GBD component, cause or risk, and location by choosing from the available selection boxes. They can then view and access GHDx records for input sources and export a comma-separated value (CSV) file that includes the GHDx metadata, citations, and information about where the data were used in GBD. Additional metadata for each input source are available through the citation tool as required by the GATHER statement.

The citation tool is accessible through the GHDx at http://ghdx.healthdata.org/gbd-2019/data-input-sources.

Section 1.8: Funding Sources

This publication and the research it presents was funded by the Bill & Melinda Gates Foundation; Bloomberg Philanthropies; the University of Melbourne; Queensland Department of Health, Australia; the National Health and Medical Research Council, Australia; Public Health England; the Norwegian Institute of Public Health; St. Jude Children's Research Hospital; the Cardiovascular Medical Research and Education Fund; the National Institute on Ageing of the National Institutes of Health (award P30AG047845); and the National Institute of Mental Health of the National Institutes of Health (award R01MH110163). The funders of the study had no role in study design, data collection, data analysis, data interpretation, or writing of the report. All authors had full access to all data in the study and had final responsibility for the decision to submit for publication.

Section 2: Risk factor estimation

Section 2.1: Overview[1]

The comparative risk assessment (CRA) conceptual framework was developed by Murray and Lopez,[3] who established a causal web of hierarchically organised risks or causes that contribute to health outcomes, which allows for quantification of risks or causes at any level in the framework. In GBD 2019, as in previous iterations of the GBD study, we evaluated a set of behavioural, environmental and occupational, and metabolic risks, in which risk-outcome pairs were included based on evidence rules. These risks were organised in four hierarchical Levels, where Level 1 represents the overarching categories (behavioural, environmental and occupational, and metabolic) nested within Level 1 risks; Level 2 contains both single risks and risk clusters (such as child and maternal malnutrition); Level 3 contains the disaggregated single risks from within Level 2 risk clusters (such as low birthweight and short gestation); and Level 4 details risks with the most granular disaggregation, such as for specific occupational carcinogens, the subcomponents of child growth failure (stunting, wasting, underweight), and suboptimal breastfeeding (discontinued and non-exclusive breastfeeding). At each level of risk, we evaluated whether risk combinations were additive, multiplicative, or shared common pathways for intervention. This approach allows the quantification of the proportion of risk-attributable burden shared with another risk or combination of risks and the measurement of potential overlaps between behavioural, environmental and occupational, and metabolic risks. To date in the GBD, we have not quantified the contribution of other classes of risk factors illustrated in table S3. We do provide some insights into the potential magnitude of distal social, cultural, and economic factors through an analysis of the relationship between risk exposures and development measured by using the Socio-demographic Index (SDI) (see appendix section 2.10).

Two types of risk assessments are possible within the CRA framework: attributable burden and avoidable burden. Attributable burden is the reduction in current disease burden that would have been possible if past population exposure had shifted to an alternative or counterfactual distribution of risk exposure. Avoidable burden is the potential reduction in future disease burden that could be achieved by changing the current distribution of exposure to a counterfactual distribution of exposure. Murray and Lopez identified four types of counterfactual exposure distributions: (1) theoretical minimum risk; (2) plausible minimum risk; (3) feasible minimum risk; and (4) cost-effective minimum risk.[4] The TMREL is the level of risk exposure that minimises risk at the population level or the level of risk that captures the maximum attributable burden. Other possible forms of risk quantification include plausible minimum risk – which reflects the distribution of risk that is conceivably possible and would minimise population-level risk if achieved – whereas feasible minimum risk describes the lowest risk distribution that has been attained within a population and cost-effective minimum risk is the lowest risk distribution for a population that can be attained in a cost-effective manner. Because no robust set of forecasts for all components of GBD is available, in this study we focus on quantifying attributable burden by using the theoretical minimum risk counterfactual distribution. Table S3 shows the eight possible types of risk quantification within the CRA framework; the grey box represents the type of CRA currently undertaken by the GBD study. According to the definition of avoidable burden, risk reversibility would be incorporated into this type of assessment because it would involve reducing risk to the counterfactual for the index year, given a history of past risk exposure. Given the focus in this study on attributable burden, risk reversibility is not a criterion used in estimation here.

In general, this analysis follows the CRA methods used since GBD 2015.[5] The methods described here provide a high-level overview of the analytical logic and focus on areas of notable change from the methods employed in GBD 2015. Here we aim to provide sufficient detail on the methods and overall structure of the estimation process. This study complies with the GATHER recommendations proposed by the World Health Organization (WHO) and others, which include recommendations on documentation of data sources, estimation methods, and statistical analysis (table S1).[6]

Section 2.2: Step 1. Effect size estimation

Section 2.2.1: Collate relative risk data[1]

Criteria for inclusion of risk-outcome pairs

Since GBD 2010 we have included risk-outcome pairs that we have assessed as meeting the World Cancer Research Fund (WCRF) grades of convincing or probable evidence.[7] In this framework, convincing evidence consists of biologically plausible associations between exposure and disease established from multiple epidemiological studies in different populations. Evidentiary studies must be substantial, include prospective observational studies, and, where relevant, randomised controlled trials (RCTs) of sufficient size, duration, and quality that show consistent effects. Probable evidence is similarly based on epidemiological studies with consistent associations between exposure and disease but for which shortcomings in the evidence exist, such as insufficient available trials (or prospective observational studies).

The World Cancer Research Fund grading system

Convincing evidence

Convincing evidence is evidence based on epidemiological studies showing consistent associations between exposure and disease and includes little or no evidence to the contrary. The available evidence is based on a substantial number of studies including prospective observational studies and, where relevant, RCTs of sufficient size, duration, and quality that show consistent effects. The association should be biologically plausible.

Probable evidence

Probable evidence is evidence based on epidemiological studies showing fairly consistent associations between exposure and disease, but for which perceived shortcomings in the available evidence exist or some evidence to the contrary precludes a more definite judgment. Shortcomings in the evidence may be any of the following: insufficient duration of trials (or studies); insufficient trials (or studies) available; inadequate sample sizes; or incomplete follow-up. Laboratory evidence is usually supportive. The association should be biologically plausible.

Possible evidence

Possible evidence is evidence based mainly on findings from case-control and cross-sectional studies. Insufficient RCTs, observational studies, or non-randomised controlled trials are available. Evidence based on non-epidemiological studies, such as clinical and laboratory investigations, is supportive. More trials are needed to support the tentative associations, which should be biologically plausible.

Insufficient evidence

Insufficient evidence is evidence based on findings of a few studies which are suggestive but insufficient to establish an association between exposure and disease. Little or no evidence is available from RCTs. More well-designed research is needed to support the tentative association.

Section 2.2.2: Determine relative risks

Effect size estimation[1]

The relative risk (RR) by level of exposure or by cause for mortality or morbidity can be found in published and unpublished primary studies or in secondary studies that summarise RRs. In Step 1a of the analytical process (figure S1), we collated information from RCTs, cohort, pooled cohort, and case-control studies, and in Step 1b, used these data to determine the RR for the risk-outcome pairs included in GBD 2019 (table S4). For most risks, data from pooled cohorts or meta-analyses of cohorts were used; in the case of the risk of cataracts from household air pollution (HAP), cohort data were not available, and instead we used case-control data. We estimated RRs of mortality and morbidity for 67 risk factors for which we determined attributable burden by using RR and exposure. We incorporated RRs from studies that controlled for confounding but not for factors along the causal pathway between exposure and outcome. For risk-outcome pairs with evidence available for only one element of mortality or morbidity, we generally assumed that the estimated RRs applied equally to both. Given evidence of statistically different RRs for mortality and morbidity, we incorporated different RRs for each. We did not find that RRs were consistently higher or lower for mortality compared with morbidity. Details and citation information for the data sources used for RRs are provided in searchable form through a web-tool (http://ghdx.healthdata.org/). Available data sources for determining RRs varied across risks. Details on how RRs were calculated for each risk can be found in appendix section 4.

For the following risks estimated from a continuous exposure distribution in which the effect size was reported by categories in pooled or meta-analysis studies, we converted those categories to RR per unit increase in exposure and assumed a linear increase in the log of the RR and exposure: ambient ozone pollution, radon, lead, high fasting plasma glucose, high LDL cholesterol, high systolic blood pressure, high body-mass index, low bone mineral density. Many meta-analyses convert RRs to per unit increase for convenience, particularly when studies choose different categories that could not otherwise be compared. If samples in the primary studies at high levels of exposure were sufficient to inform the shape of the tail of the distribution, we applied a cap to the maximum RR by using the midpoint of the last category for which an RR was reported.

In GBD 2019, for a selected set of continuous risk factors, we modelled RRs using meta-regression—Bayesian, regularised, trimmed (MR-BRT) (see details below), relaxing the log-linear assumption to allow for monotonically increasing or decreasing but non-linear functions using cubic splines. Risk factors for which we undertook this re-analysis include: all dietary risk factors, low physical activity, kidney dysfunction, and air pollution. Because knot placement can affect the shape of the risk function when modelling with a cubic spline, we generated a wide range of knot placements and created an ensemble across these different knot placements. We also included in the final estimation 10% trimming of the data to avoid the results being sensitive to outliers.

MR-BRT

This section details the statistical models underlying MR-BRT, and the fitting procedure used to obtain estimates. Further details on models and algorithms can be found in the technical report.[8]

The MR-BRT program is a set of wrappers customized for global health problems that use the open source mixed effects package `LimeTr` (https://github.com/zhengp0/limetr).[8] We describe the basic functionality in the sections below.

Mixed-Effects Model

We consider the following nonlinear mixed effects model:

$$\boldsymbol{y}_i = \mathbf{F}_i(\beta) + \mathbf{Z}_i \boldsymbol{u}_i + \boldsymbol{\epsilon}_i$$
$$\boldsymbol{u}_i \sim N(\mathbf{0}, \boldsymbol{\Gamma}), \ \boldsymbol{\Gamma} = \operatorname{diag}(\gamma), \qquad \boldsymbol{\epsilon}_i \sim N(\mathbf{0}, \boldsymbol{\Lambda}), \tag{1}$$

where $y_i \in \mathbb{R}^{n_i}$ is the vector of observations from the ith study, $\boldsymbol{\epsilon}_i \in \mathbb{R}^{n_i}$ are measurement errors with given covariance $\boldsymbol{\Lambda}$, $\boldsymbol{u}_i \in \mathbb{R}^{k_\gamma}$ are independent random effects, and $\mathbf{Z}_i \in \mathbb{R}^{n_i \times k_\gamma}$is a linear map, and β are regression coefficients. The models F_i may be nonlinear.

To fit (β, γ) we solve the marginal likelihood problem:

$$\min_{\beta,\gamma} f(\beta,\gamma) \coloneqq \sum_{i=1}^{m} \frac{1}{2}\left(y_i - \mathbf{F}_i(\beta)\right)^\top \left(\mathbf{Z}_i \boldsymbol{\Gamma} \mathbf{Z}_\mathrm{i}^\top + \boldsymbol{\Lambda}_{\boldsymbol{i}}\right)^{-1} \left(\boldsymbol{y}_i - \mathbf{F}_i(\beta)\right) + \frac{1}{2} ln\left|\mathbf{Z}_i \boldsymbol{\Gamma} \mathbf{Z}_\mathrm{i}^\top + \boldsymbol{\Lambda}_i\right|. \tag{2}$$

When the model is linear, we can write:

$$\mathbf{F}_i(\beta) = \boldsymbol{X}\beta. \tag{3}$$

Linear models are very common in cross-walks (see section 2.3.2), and for network analysis, which is detailed below.

Network Analysis

Network analysis is a special case of the linear model (3) that is used to compare multiple treatment effects. To explain the coding we use a running example with four treatments A, B, C, D.

For simplicity assume A is this reference treatment. We then have the following coding.

$$AB \to B - A: \quad [1 \quad 0 \quad 0]$$
$$AC \to C - A: \quad [0 \quad 1 \quad 0]$$
$$AD \to D - A: \quad [0 \quad 0 \quad 1].$$

We see from this simple example that the design matrix under the basic network assumption is always full rank, since a subset of rows forms the identity matrix.

Comparisons that do not include the reference can be computed. For example,

$$BC \to C - B = (C - A) - (B - A)$$
$$= [0 \quad 1 \quad 0] - [1 \quad 0 \quad 0]$$
$$= [-1 \quad 1 \quad 0]$$

Using this simple algebra, we quickly obtain the remaining codings.

$$BC \to C - B: \quad [-1 \quad 1 \quad 0]$$
$$BD \to D - B: \quad [-1 \quad 0 \quad 1]$$
$$CD \to D - C: \quad [0 \quad -1 \quad 1]$$

Each row of the design matrix $\boldsymbol{X}$ is coded according to the comparison.

When doing network analysis, the design matrix $\boldsymbol{X}$ does not include the intercept term (**1** column).

Constraints and Priors

The ML estimate (2) can be extended to incorporate nonlinear inequality constraints

$$\mathbf{C}(\boldsymbol{\theta}) \leq c,$$

where $\boldsymbol{\theta} = (\beta, \gamma)$. Constraints play a key role for polynomial splines.

It is also essential to allow priors on parameters of interest. We assume that priors are given by a functional form

$$\boldsymbol{\theta} \sim \exp(-\rho(\boldsymbol{\theta}))$$

The likelihood problem is then augmented by adding the term $\rho(\boldsymbol{\theta})$ to the ML objective. The function ρ may be nonlinear and nonconvex, but we assume it is smooth.

Trimming outliers

Least trimmed squares (LTS) is a robust estimator[8,9] for the standard regression problem. Given the problem

$$\min_{\beta} \sum_{i=1}^{n} \frac{1}{2} \left(y_i - \langle \boldsymbol{X}_i, \beta \rangle\right)^2, \tag{4}$$

the LTS estimator minimizes the sum of *smallest h* residuals rather than all residuals. These estimators were initially introduced to develop linear regression estimators that have a high breakdown point (in this case 50%) and good statistical efficiency (in this case $n^{-1/2}$). Breakdown refers to the percentage of outlying points which can be added to a dataset before the resulting M-estimator can change in an unbounded way. Here, outliers can affect both the outcomes and training data (features).

LTS estimators are robust against outliers, and arbitrarily large deviations that are trimmed do not affect the final $\hat{\beta}$.

Rather than writing the objective in terms of order statistics, it is far simpler to extend the likelihood using an auxiliary variable $\boldsymbol{W}$:

$$\min_{\beta, \mathbf{W}} \sum_{i=1}^{n} w_i \left(\frac{1}{2} \left(y_i - \langle \mathbf{X}_i, \beta \rangle\right)\right)^2 \quad \text{s.t.} \quad \mathbf{1}^\top \boldsymbol{W} = h, \quad \mathbf{0} \leq \boldsymbol{W} \leq \mathbf{1}. \tag{5}$$

The set

$$\Delta_h \coloneqq \{\mathbf{W} : \mathbf{1}^\top \mathbf{W} = h, \quad \mathbf{0} \leq \mathbf{W} \leq \mathbf{1}\} \tag{6}$$

is known as the *capped simplex*, since it is the intersection of the h-simplex with the unit box.[8] For a fixed β, the optimal solution of (5) with respect to $\mathbf{W}$ assigns weight 1 to each of the smallest h residuals, and 0 to the rest. Problem (5) is solved *jointly* in $(\beta, \mathbf{W})$, simultaneously finding the regression estimate and classifying the observations into inliers and outliers. This joint strategy makes LTS different from post-hoc analysis, where a model is fit first with all data, and then outliers are detected using that estimate.

To explain how trimming enters the marginal likelihood problem, we focus on a single group term from the ML likelihood (2):

$$\left(\frac{1}{2}\left(\boldsymbol{y}_i - \mathbf{F}_{i(\beta)}\right)^\top \left(\mathbf{Z}_i\boldsymbol{\Gamma}^{-1}\mathbf{Z}_i^\top + \boldsymbol{\Lambda}_i\right)^{-1}\left(\boldsymbol{y}_i - \mathbf{F}_i(\beta)\right) + \frac{1}{2}\ln\left|\mathbf{Z}_i\boldsymbol{\Gamma}^{-1}\mathbf{Z}_i^\top + \boldsymbol{\Lambda}_i\right|\right)$$

We introduce auxiliary variables $\mathbf{W}_i \in \mathbb{R}^{n_i}$, and define

$$\boldsymbol{r}_i \coloneqq y_i - \mathbf{F}_i(\beta), \quad \mathbf{W}_i \coloneqq \operatorname{diag}(\mathbf{W}_i), \quad \sqrt{\mathbf{W}_i} \coloneqq \operatorname{diag}\left(\sqrt{\mathbf{W}_i}\right).$$

We now form the objective

$$\tfrac{1}{2}\boldsymbol{r}_I^\top \sqrt{\mathbf{W}_i}\left(\sqrt{\mathbf{W}_i}\mathbf{Z}_i\boldsymbol{\Gamma}^{-1}\mathbf{Z}_i^\top\sqrt{\mathbf{W}_i} + \boldsymbol{\Lambda}_i^{\odot\mathbf{W}_i}\right)^{-1} \sqrt{\mathbf{W}_i}\,\boldsymbol{r}_i + \tfrac{1}{2}\ln\left|\sqrt{\mathbf{W}_i}\,\mathbf{Z}_i\boldsymbol{\Gamma}^{-1}\mathbf{Z}_i^\top\sqrt{\mathbf{W}_i} + \boldsymbol{\Lambda}_i^{\odot\mathbf{W}_i}\right|, \quad (7)$$

where $\odot$ denotes the elementwise power operation:

$$\boldsymbol{\Lambda}_i^{\odot\mathbf{W}_i} \coloneqq \begin{bmatrix} (\lambda_{1j})^{w_{i1}} & 0 & \cdots & 0 \\ 0 & \ddots & \ddots & \vdots \\ 0 & \cdots & 0 & (\lambda_{in_i})^{w_{in_i}} \end{bmatrix} \quad (8)$$

When $w_{ij} = 1$, we recover the contribution of the ijth observation to the original likelihood. As $w_{ij} \downarrow 0$, the ijth contribution to the residual is correctly eliminated by $\sqrt{w_{ij}} \downarrow 0$. The jth row and column of $\sqrt{\mathbf{W}_i}\mathbf{Z}_i\boldsymbol{\Gamma}^{-1}\mathbf{Z}_i^\top\sqrt{\mathbf{W}_i}$ both go to 0, while the jth entry of $\boldsymbol{\Lambda}_i^{\odot\mathbf{W}_i}$ goes to 1, which effectively removes all impact of the jth point on the covariance matrix.

For full details and analysis, please see the technical report.[10]

Final Estimator

Putting together the trimmed ML with priors and constraints, we arrive at the following estimator.

$$\begin{aligned} \min_{\beta,\gamma,\mathbf{W}} f(\beta,\gamma,\mathbf{W}) \coloneqq \sum_{i=1}^{m} \frac{1}{2}\, r_i^\top \sqrt{\mathbf{W}_i}\left(\sqrt{\mathbf{W}_i}\,\mathbf{Z}_i\boldsymbol{\Gamma}^{-1}\mathbf{Z}_i^\top\sqrt{\mathbf{W}_i} + \boldsymbol{\Lambda}_i^{\odot\mathbf{W}_i}\right)^{-1} \\ \sqrt{\mathbf{W}_i}\, r_i + \tfrac{1}{2}\ln\left|\sqrt{\mathbf{W}_i}\,\mathbf{Z}_i\boldsymbol{\Gamma}^{-1}\mathbf{Z}_i^\top\sqrt{\mathbf{W}_i} + \boldsymbol{\Lambda}_i^{\odot\mathbf{W}_i}\right| + \rho(\beta,\gamma,\boldsymbol{\Lambda}) \quad (9) \\ \text{s.t.}\ \ \boldsymbol{r}_i = \boldsymbol{y}_i - \mathbf{F}_i(\beta), \quad \mathbf{1}^\top\mathbf{W} = h, \quad 0 \le \mathbf{W} \le 1, \quad \boldsymbol{C}\begin{pmatrix}\beta \\ \gamma\end{pmatrix} \le c\,. \end{aligned}$$

The fit is obtained using iterative optimization techniques. Problem (9) is nonlinear and non-smooth, and the optimization is implemented in the `LimeTR` package[3] (https://github.com/zhengp0), and relies on the IPopt interior point method.[11]

Nonlinear Dose-Response Curves with Constrained Splines

In this section we discuss spline models for dose-response relationships. General background on splines and spline regression are available elsewhere.[12,13]

B-splines and bases

A spline basis is a set of piecewise polynomial functions with designated degree and domain. If we denote polynomial order by p, and the number of knots by k, we need $p + k$ basis elements s_j^p, which can be generated recursively as illustrated in Figure A.

Figure A. Recursive generation of b-spline basis elements (orders 0, 1, 2)

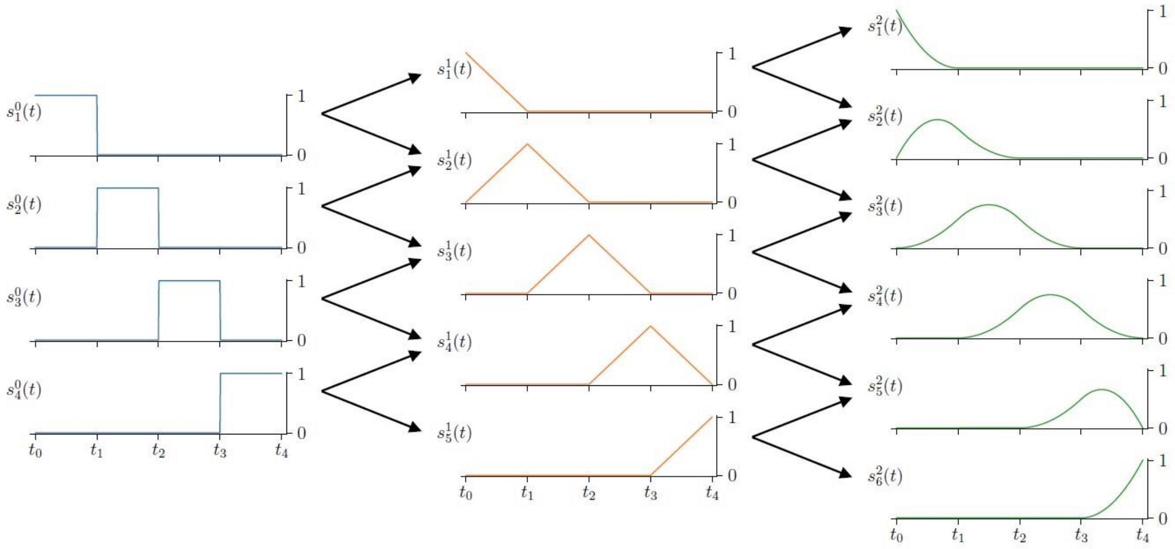

.

Given such a basis, we can represent any dose-response relationship as the linear combination of the spline basis elements, with coefficients $\beta \in \mathbb{R}^{\mathrm{p+k}}$:

$$f(t) = \sum_{j=1}^{p+k} \beta_j^p s_j^p(t). \tag{10}$$

These coefficients are then inferred as part of the general estimator (9) as discussed in the previous section. An explicit representation of (11) is obtained by building a design matrix $\boldsymbol{X}$. Given a set of t values at which we have data, the jth column of $\mathbf{X}$ is given by the expression

$$\mathbf{X}_{\cdot,j} = \begin{bmatrix} s_j^p(t_0) \\ \vdots \\ s_j^p(t_k) \end{bmatrix}. \tag{11}$$

The model for direct observations data coming from (11) can now be written compactly as

$$\boldsymbol{y} = \mathbf{X}\beta + \mathbf{Z}_i\boldsymbol{u}_i + \boldsymbol{\epsilon}_i\ ,$$

which is a special case of the main problem class (1).

Shape constraints

We can impose shape constraints such as monotonicity, concavity, and convexity on splines. Constraints on splines have been developed in the past through reformulation techniques.[14] The development in this section uses explicit constraints instead.

Monotonicity. Spline monotonicity across the domain of interest follows from monotonicity of the spline coefficients.[12] Given coefficients

$$\beta = \begin{bmatrix} \beta_1 \\ \vdots \\ \beta_n \end{bmatrix},$$

the curve $f(t)$ in (11) is monotonically non-decreasing when

$$\alpha_1 \leq \alpha_2 \leq \cdots \leq \alpha_n$$

and monotonically non-increasing if

$$\alpha_1 \geq \alpha_2 \geq \cdots \geq \alpha_n.$$

The relationship $\alpha_1 \leq \alpha_2$ can be written as $\alpha_1 - \alpha_2 \leq 0$. Stacking these inequality constraints for each pair (α_i, α_{i+1}) we can write all constraints simultaneously as

$$\underbrace{\begin{bmatrix} 1 & -1 & 0 & \cdots & 0 \\ 0 & 1 & -1 & \cdots & 0 \\ \ddots & \ddots & \ddots & \ddots & \vdots \\ 0 & \cdots & \cdots & 1 & -1 \end{bmatrix}}_{\mathbf{C}} \begin{bmatrix} \alpha_1 \\ \alpha_2 \\ \alpha_3 \\ \vdots \\ \alpha_n \end{bmatrix} \leq \begin{bmatrix} 0 \\ 0 \\ \vdots \\ 0 \end{bmatrix}.$$

These linear constraints are a special case of the general estimator (9) that allows $\mathbf{C}(\beta) \leq c_\beta$.

Convexity and Concavity. For any twice continuously differentiable function: $f : \mathbb{R} \rightarrow \mathbb{R}$, convexity and concavity are captured by the signs of the second derivative. Specifically, f is convex if $f''(t) \geq 0$ is everywhere, and concave if $f''(t) \leq 0$ everywhere. We can compute $f''(t)$ for each interval, and impose linear inequality constraints on these expressions.

Enforcing linear tails. For large consumption with little data, we need the capability to ensure that the last segment of the spline is linear, with slopes that match the adjacent segment at the knot. The estimated spline is then a best fit to the data, subject to this specification. Priors on the tails can also be provided.

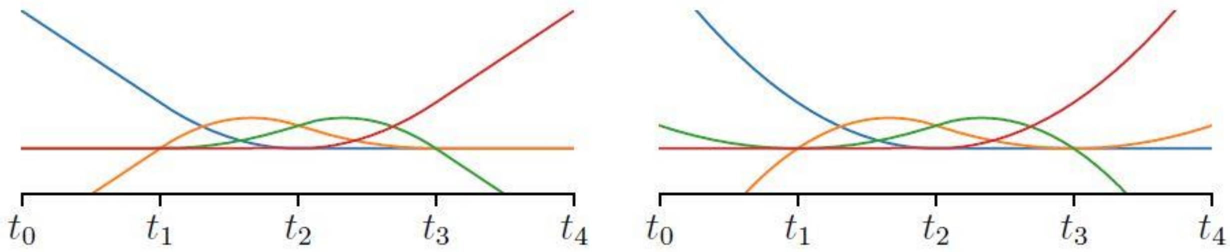

Figure B. Spline extrapolation. Left: linear extrapolation. Right: nonlinear extrapolation.

In general, using linear head and/or tail pieces to extrapolate outside the original domain or interpolate in the data sparse region is far more stable that using higher order polynomials, see Figure B. The figure shows symmetric linear tail modifications, but for the analyses in the paper we only impose a right linear tail shape constraint.

Posterior Variance Estimation

To obtain posterior uncertainty, we use a parametric bootstrap.[15] Once we solve (9) to obtain estimates $\hat{\beta}$ and $\hat{\gamma}$, we have a model distribution of the errors (1):

$$\boldsymbol{y}_i = \mathbf{F}_i(\hat{\beta}) + \mathbf{Z}_i \boldsymbol{u}_i + \boldsymbol{\epsilon}_i$$

We sample datasets from this distribution to generate full data sets $\{\mathbf{Y}\}^j$, for $j = 1, \dots, N$. For each dataset $\mathbf{Y}^j$, we then re-solve the fitting problem (9) to obtain estimates $\hat{\beta}^j$ and $\hat{\gamma}^j$, and the set $\{\hat{\beta}^j, \hat{\gamma}^j\}$ over all j allows us to estimate any posterior statistic we need.

In particular, the posterior set of dose-response curves is given by

$$\{f(t)^j + u_0^j\}$$

where $f(t)^j$ is the curve obtained by using the re-fit value $\hat{\beta}^j$, and u_0^j is a sample from $N(0, \hat{\gamma}_0^j)$, the associated unexplained heterogeneity parameter.

MR-BRT and air pollution

For ambient and household particulate matter (PM2.5) air pollution we created cause-specific risk curves for Ischaemic Heart Disease, stroke (ischaemic and haemorrhagic), Chronic Obstructive Pulmonary Disease (COPD), lung cancer, acute lower respiratory infection, and Type II Diabetes. We fit splines to relative risk and PM2.5 exposure estimates from studies of ambient PM2.5, household solid fuel use and second hand smoking. With the flexibility of splines in MR-BRT and the addition of recent studies from high exposure settings,[16,17,18,19,20] we no longer include studies of active smoking data in the risk curves. Specifically, for each of the risk-outcome pairs we tested various model settings and priors and fit splines with the following for the relationship:

log((MRBRT(X))/MRBRT(X_CF))~log(Published Effect Size)

where X and XCF represent the range of exposure characterized by the effect size.

The final models included third order splines with two interior knots and a constraint on the right-most segment forcing a linear fit. We used an ensemble approach to knot placement, where 100 different models were run with randomly placed knots and then combined by weighting based on a measure of fit that penalizes excessive changes in the third derivative of the curve. Knots were free to be placed anywhere within the 5th and 95th percentile of the data, as long as a minimum width of 10% of that domain exists between them. We included shape constraints so that the curves were monotonically increasing and concave downwards, the most biologically plausible shape for PM2.5 risks. On the non-linear segments, we included a Gaussian prior on the third derivative of mean 0 and variance 0.01 to prevent over-fitting; on the linear segment, a stronger prior of mean 0 and variance 1e-6 was used to ensure that the risk curves did not continue to increase beyond the range of the data. For COPD we used a looser Gaussian prior of mean 0 and variance 1e-4 on the linear segment of the risk function, given epidemiological evidence from household air pollution that the risk continues to increase at higher levels of PM2.5.

In addition, we fit a MR-BRT spline to estimates from studies of ambient and household air pollution describing the decrease in birthweight (g) or gestational age (weeks) associated with PM2.5. We fit the

same model and priors as above (with the exception of COPD), except with monotonically decreasing concave upwards shape constraints:

$$MRBRT(X) - MRBRT(X_{CF}) \sim Shift$$

where the difference in the value of the model at the observed concentration (X) and the counterfactual concentration (X_{CF}) was equal to the published shift in bw or ga. We then shifted the observed birthweight – gestational age distributions to reflect the expected distributions in the absence of particulate matter pollution, to estimate PAFs.

MR-BRT and temperature

The relative risk, RR, of mortality was calculated for each daily and mean annual temperature category in each administrative unit. For this purpose, we calculated the daily mean temperature and aggregated the daily cause-specific death counts for each administration. We then calculated mortality rates for each cause, c, location, l, and daily mean temperature, ie temperature category, t:

$$MR_{clt} = \frac{deaths_{clt}}{person - days_{lt}}$$

With *MR* representing the mortality rate, *deaths* being the absolute number of cause-specific deaths, and *person-days* depicting the sum of the population in location, *l*, across all days with a daily temperature of *t*.

Following, we calculated the mean MR, $\overline{MR}$, for each cause, *c*, and location, *l*:

$$\overline{MR_{cl}} = \frac{deaths_{cl}}{person - days_{l}}$$

The daily temperature-specific mortality rate ratio, *MRR*, was then calculated as the ratio of the MR for each temperature category, location and cause and the average $\overline{MR}$:

$$MRR_{clt} = \frac{MR_{clt}}{\overline{MR_{cl}}}$$

In order to aggregate the *MMRs* to the first-level administrative unit, we calculate the population-weighted mean temperature (PWMT) for each location and across all days and then pooled all *MRRs* for each combination of daily temperature and PWMT.

Section 2.3: Step 2. Exposure estimation[1]

Section 2.3.1: Step 2a: Collate exposure data

Systematic reviews

For GBD 2019, we conducted systematic literature reviews for 18 risks. For other risk factors, only a small fraction of the existing data appears in the published literature, and other sources predominate, such as survey data and satellite data. Data were systematically screened from household surveys

archived in the GHDx (http://ghdx.healthdata.org), including Demographic and Health Surveys, Multiple Indicator Cluster Surveys, Living Standards Measurement Surveys, and Reproductive Health Surveys. Other national health surveys were identified based on survey series that had yielded usable data for past rounds of GBD, sources suggested to us by in-country collaborators, and surveys identified in major multinational survey data catalogues, such as the International Household Survey Network and the WHO Central Data Catalog, as well as through country Ministry of Health and Central Statistical Office websites. Citations for all data sources used for risk factor estimation in GBD 2019 are provided in searchable form through a web-tool (http://ghdx.healthdata.org. A description of the search terms employed for risk-specific systematic reviews are detailed by cause in appendix section 4.

Information on systematic reviews were managed by using Research Electronic Data Capture (REDCap) electronic data capture tools hosted at the University of Washington.[21] REDCap is a secure, web-based application designed to support data capture for research studies that provides 1) an intuitive interface for validated data entry; 2) audit trails for tracking data manipulation and export procedures; 3) automated export procedures for seamless data downloads to common statistical packages; and 4) procedures for importing data from external sources

Search terms

Search terms for updates of systematic reviews for GBD 2019 are shown by risk factor in appendix section 4.

Survey data preparation

Survey data constitutes a substantial part of the underlying data used in the estimation process. During extraction, we concentrated on demographic variables (such as location, gender, age), survey design variables (such as sampling strategy and sampling weights), and the variables used to define the population estimate (such a prevalence or a proportion) and a measure of uncertainty (standard error, confidence interval or sample size and number of cases).

Section 2.3.2: Step 2b: Adjust exposure data

Several adjustments were applied to extracted exposure sources to make the data more consistent and suitable for modelling. In GBD 2019, we implemented adjustments of risk exposure data to deal with alternative case definitions or study methods prior to entering data into our main analytical tools of DisMod-MR 2.1 and ST-GPR. This decision also included the adjustment of data presented for both sexes to a male and female equivalent. The starting point was to explicitly state the reference case definition and study method and identify alternative definitions and study characteristics that fall within our inclusion criteria.

We compiled data from both within-study comparisons (ie, data that used alternative and reference definitions in the same population) and between-study comparisons (ie, data that used an alternative definition in one population and a reference definition in another population that overlap in location, time, age and sex) of different case definitions. For between-study comparisons, we allowed a maximum calendar year difference between studies of five years. Where validation studies (ie, those carried out at the introduction of a new set of diagnostic criteria comparing to previous criteria) were available, we extracted data on the comparison of alternative to reference. For quantities of interest with multiple alternative definitions/methods we also look for pairs comparing two alternatives. In a network analysis,

if A is the reference and B and C are two alternatives, a comparison of A vs B and B vs C provides an indirect comparison of the alternative C against the reference A.

We pooled either the logit difference between alternative and reference or the natural log of the ratio of alternative to reference. From simulations we found that the two methods provide almost identical results for quantities that after adjustment do not exceed a value of 0.5 (eg, prevalence or proportion). The logit difference method much better dealt with higher values and avoided prevalence or proportions to exceed one. If the values of either the reference or alternative were zero, we aggregated values across age groups until both values had non-zero observations. We used the delta method to compute the standard error of the reference and alternative measures in logit space. The standard error of the logit difference was computed as the square root of the sum of the variances of each data point in a pair.

Data analysis

We used a network random effects meta-regression in MR-BRT (see section 2.2.2). In a network analysis, if A is the reference and B and C are two alternatives, a comparison of A vs B and B vs C provides an indirect comparison of the alternative C against the reference A. To implement the network we included dummy variables with a particular structure. This was implemented as follows, where A is the reference definition/method:

- Create *k* dummy variables where *k* are all definitions/methods other than A (eg, *k* = B, C)
- Code dummy *k* as
  - 1 if the first term of the logit difference is *k*;
  - -1 if k is second term of the logit difference;
  - 0 otherwise

For example:

Study	Comparison	DummyB	DummyC
1	logit(B)-logit(A)	1	0
2	logit(B)-logit(A)	1	0
3	logit(C)-logit(A)	0	1
4	logit(C)-logit(A)	0	1
5	logit(C)-logit(B)	-1	1
6	logit(C)-logit(B)	-1	1

The coding structure outlined above in step 1 assumes that all case definitions are mutually exclusive. In some cases, however, individual case definitions are a function of different components

or dimensions. For example, case definitions may vary by the type of symptoms that a respondent experiences as well as the recall period over which those symptoms are experienced. In the presence of sparse data, it may be difficult to find both direct and indirect comparisons of all individual case definitions. In these case, an alternative approach is to assume different dimensions of case definitions have a multiplicative effect. In other words, the effect of recall period has the same relative effect across different categories of symptoms reported by respondents. To implement this coding scheme:

- Create *k* dummy variable columns for each case definition dimension
- For each dummy variable k:
    - Add 1 if *k* is a component of the first term in the logit difference
    - Subtract 1 if *k* is a component of the second term in the logit difference

In MR-BRT, we ran random effects meta-regression of the logit difference (or log ratio) with all the *k* dummy variables as covariates, omitting the intercept in the meta-regression. We used a study_id variable for be the unique combination of the NIDs of the reference and alternative studies (or alternative1 to alternative2). The coefficients on the *k* dummy variables represent the pooled logit difference of the *k* alternative definition to the reference taking into account evidence from both direct and indirect comparisons. In the example above, the coefficient on DummyA is the pooled logit difference of B minus A; the coefficient on DummyB is the pooled logit difference of C minus A. The standard error of the pooled logit difference incorporating the between study variance was calculated as:

$$se(\text{logit}(difference_k)) = \sqrt{var_k + \gamma^2}$$

Where:

$se(\text{logit}(difference_k)$= standard error of the pooled logit difference of alternative *k* to the reference
var_k=variance of the coefficient on dummy variable *k*
γ^2 = between-study variance

If both between and within study pairs were available we examine whether there was a systematic difference between these. If there was a significant difference, we made judgement call as to whether within-study or between study data comparisons were most appropriate. In general, this was the within-study data, however, there were important measurement or conceptual reasons for choosing between-study data. For example, for crosswalks between self-reported height and weight compared to measured height and weight, between-study comparisons may be preferable if respondents knew they would be measured and, therefore, were less likely to misreport their height and weight.

We also examined whether there were systematic differences in the adjustments by key demographics (age, sex, geographic location, year) and other potential factors that may lead to variation in crosswalks. This could only be done at present in a direct comparison model and not in a

network. We did this when there was a strong rationale, eg, biological plausibility, for variation by such characteristics.

After obtaining the pooled logit difference or log ratio estimates, we predicted adjustments based on the statistical model, including uncertainty in the adjustment and sampling error of each data point. For non-significant logit differences or log ratios we still applied the adjustments if there was a conceptual reason to believe that the alternative definition is biased. This expands the variance of these alternative definition data points.

Section 2.3.3: Step 2c: Estimate exposure

Mean exposure estimation

In Step 2a of the estimation process, we used systematic literature reviews to identify risk factor exposure studies published or identified since GBD 2017 and combined these with existing data from household and health examination surveys and census, morbidity, or satellite imagery and ground sensor data (used for estimation of particulate matter <2.5 μm in diameter [$PM_{2.5}$]). Certain risks, such as poor diet and excessive alcohol consumption, also incorporated administrative record systems. Data sources used in estimating risk factor exposure can be accessed through the data source tool at http://ghdx.healthdata.org/.

Once data were collected and compiled, Step 2b of the analytical flowchart describes the adjustments applied, where necessary, to correct for bias. Examples of these adjustments include use of urban studies for lead; crosswalks between different measurements, methods, and definitions, such as for self-report of obesity and glycated haemoglobin (HbA_{1C}) for diabetes; and age-sex splitting of data, such as for fasting plasma glucose (FPG) level, cholesterol level, and systolic blood pressure that may be reported from broad age-groups.

For the GBD, we developed two modelling approaches, a Bayesian meta-regression model (DisMod-MR 2.1) and a spatiotemporal Gaussian process regression model (ST-GPR), to pool data from different sources, control and adjust for bias in data, and incorporate other types of information such as country-level covariates. DisMod-MR 2.1 and ST-GPR are mixed effect models that borrow information across age, time, and locations to synthesise multiple data sources into unified estimates of levels and trends. A detailed description of the likelihood used for estimation and a full description of improvements made for DisMod-MR 2.1 were detailed by Vos and colleagues,[22] who provided additional detail in the appendix to that paper.[8] The ST-GPR model has three main hyper-parameters that control for smoothing across time, age, and location. Values for these hyper-parameters were selected on the basis of cross-validation. Cross-validation tests were conducted for different combinations of the hyper-parameters for three types of models: one data-sparse model, one data-moderate model, and one data-dense model. In each test, 20% of the data were held out, and the performance of each combination of hyper-parameters was evaluated on the held-out data. For each hyper-parameter combination, 10 cross-validation tests were conducted. The performance of each model in predicting the withheld 20% of the data was evaluated by using a combined measure based on root mean square error (RMSE) and uncertainty interval (UI) coverage. A detailed description of the ST-GPR process regression can be found below.

The main difference between these methods is their power to include unstructured types of data by sex and age group and their degree of flexibility. Step 2c in figure S1 outlines the use of DisMod-MR 2.1 for 6

risk factors for which data were available by different age intervals or mixed sex groups; DisMod-MR 2.1 is the preferred tool in these cases because of its ability to integrate over age and adjust for different exposure definitions in the data; however, the use of Bayesian Markov Chain Monte Carlo (MCMC) simulations with large volumes of data renders the analysis computationally intensive and reduces the number of iterations that are possible. If standard age-group data are available – as is generally the case for metabolic risks – using ST-GPR becomes the preferred approach.

In some cases, we adapted our methods of modelling exposure to risks where necessary to account for complexities in the risk-outcome relationship or the need for particular handling of data, for example, dietary risks and ambient air pollution (see appendix section 4 for more detail). A complete list of risks is reported in table S2. Additional details for adjustments or adaptations to particular risk models are provided in appendix section 4.

DisMod-MR 2.1 Estimation

DisMod-MR 2.1 description

Until GBD 2010, nonfatal estimates in burden of disease assessments were based on a single data source on prevalence, incidence, remission, or a mortality risk selected by the researcher as most relevant to a particular location and time. For GBD 2010, we set a more ambitious goal: to evaluate all available information on a disease that passes a minimum quality standard. That required a different analytical tool that would be able to pool disparate information presented for varying age groupings and from data sources by using different methods. The DisMod-MR 1.0 tool used in GBD 2010 evaluated and pooled all available data, adjusted data for systematic bias associated with methods that varied from the reference, and produced estimates by world regions with UIs by using Bayesian statistical methods. For GBD 2013, the improved DisMod-MR 2.0 increased computational speed, which allowed computations to be consistent between all disease parameters at the country rather than the region level. The hundred-fold increase in speed of DisMod-MR 2.0 was partly due to a more efficient rewrite of the code in C++ but also to changing to a model specification by using log rates rather than a negative binomial model used in DisMod-MR 1.0. In cross-validation tests, the log rates specification worked as well or better than the negative binomial specification.[23] The sequence of estimation occurs at five levels: global, super-region, region, country and, where applicable, subnational location. The super-region priors are generated at the global level with mixed-effects, nonlinear regression by using all available data; the super-region fit, in turn, informs the region fit, and so on down the cascade. The wrapper gives analysts the choice to branch the cascade in terms of time and sex at different levels depending on data density. The default used in most models is to branch by sex after the global fit but to retain all years of data until the lowest level in the cascade is reached.

The computational engine is limited to three levels of random effects; we differentiate estimates at the super-region, region and country level. In GBD 2013, the subnational units of China, the UK and Mexico were treated as “countries” to enable a random effect to be estimated for every location with contributing data. However, the lack of a hierarchy between country and subnational units meant that the fit to country data contributed as much to the estimation of a subnational unit as the fits for all other countries in the region. We found inconsistency between the country fit and the aggregation of subnational estimates when the country’s epidemiology varied from the average of the region. Adding an additional level of random effects required a prohibitively comprehensive rewrite of the underlying

DisMod-MR engine. Instead, we added a fifth layer to the cascade, with subnational estimation informed by the country fit and country covariates, plus an adjustment based on the average of the residuals between the subnational location's available data and its prior. This technique mimicked the impact of a random effect on estimates between subnationals.

In GBD 2015, we also improved how country covariates differentiate nonfatal estimates for diseases with sparse data. The coefficients for country covariates are re-estimated at each level of the cascade. For a given location, country coefficients are calculated by using both data and prior information available for that location. In the absence of data, the coefficient of its parent location is used to utilise the predictive power of our covariates in data-sparse situations.

For GBD 2016, the computational engine (DisMod-MR 2.1) remained substantively unchanged from GBD 2015. We changed the prediction year set to generate fits for the years 1990, 1995, 2000, 2005, 2010, and 2016. We updated the age prediction sets to include age groups 80-84 years, 85-89 years, 90-94 years, and 95+ years to comply with changes across all functional areas of the GBD. We also expanded the set of locations where subnational units are modelled; the set now includes Brazil, China, England, India, Indonesia, Japan, Kenya, Mexico, South Africa, Sweden, and the US.

In GBD 2017, we continued to use DisMod-MR 2.1 because no substantial changes were made. Updates to computation include extending the terminal prediction year to 2017 and additional subnational units in Ethiopia, Iran, New Zealand, Norway, and Russia. Saudi Arabia was also modelled only at the national level in 2017.

In GBD 2019, no substantial changes were made to Dismod-MR 2.1 but we made more substantial changes to how we use the tool. First, we added the year 2019 as an additional year of estimation. Second, we also included the option again to have random effects on cause-specific mortality rates (csmr) and excess mortality rates (EMR). This functionality had been dropped a couple of GBD rounds earlier. Third, as we did all our adjustments for alternative case definition and study methods as well as adjustments to both sex data points prior to entering data into DisMod-MR 2.1, we no longer used the functionality in DisMod-MR 2.1 to estimate coefficients for study covariates. Fourth, based on simulation testing we found that coverage improved and errors reduced when passing down priors with a wider setting of minimum coefficient of variation (which determines the uncertainty around priors and hence how 'informative' the priors are) than had generally been used in past GBD iterations. We settled on a default value of 0.8 where in the past values of 0.4 or less had been more commonly used. We made some exceptions for high prevalent conditions where a lower minimum CV setting achieved the task of making priors less informative but not completely uninformative.

Fifth, we changed our approach to estimating excess mortality rates, the key link in the model between cause-specific mortality rates (CSMR) and incidence and prevalence. In the past two GBD rounds we calculated priors on excess mortality and entered these as data points by matching sex-specific prevalence data with an age width of 20 or less with the corresponding CSMR for the same location and year. For stability sake, we excluded calculation of EMR for prevalence data points of less than 1 in a million. EMR is simply calculated as CSMR divided by prevalence. As with previous GBD years, for diseases with an average duration of less than a year (as indicated by a setting of remission greater than

one), we ran an initial global model to get an equivalent prevalence and used the following formula to calculate EMR:

EMR = CSMR * (remission + (ACMR - CSMR) + EMR_pred) / incidence

where, ACMR = all-cause mortality rate and EMR_pred = EMR fit from an initial global dismod model

Despite using the log of lag-distributed income or the Healthcare Access and Quality (HAQ) Index as a covariate with a prior that the coefficient had to be negative, we found many disease models with an implausible distribution of mortality to prevalence (or incidence) ratios implying lower case fatality in locations with lower HAQ Index than in countries with higher HAQ Index. This likely signals an inconsistency between fatal and non-fatal data inputs. For GBD 2019 we decided to run regressions on EMR data (calculated as described above) first using MR-BRT with HAQ Index as a predictor. In general, we tend to think that CSMR estimates are more robust than non-fatal data because of much greater data availability and a lesser task in adjusting cause death data for garbage coding than the complex task of adjusting non-fatal data sources for alternative case definitions and study methods. To indicate that we would reduce the random effects on EMR and the minimum coefficient of variation for priors on EMR being created at each next level down the cascade. However, there were exceptions. For drug use disorders, the risk of overdose deaths is less a function of a country's quality of health services but driven more by the availability of harm reduction strategies such as opioid substitution therapy and the availability of highly potent opioids such as fentanyl which have been an important contributor to the large increase in overdose deaths in the United States in the last decade. We settled on a model for opioid use disorder with wider random effects and higher minimum coefficient of variation to give less emphasis on CSMR when enforcing consistency with prevalence data. In a next round, we will endeavour to find covariates that are more relevant to drug overdose deaths such as a grading of harm reduction strategies by country and over time. In the case of COPD, we noted that following the data on CSMR and EMR led to large increases in prevalence estimates in East Asia, Oceania and, to a lesser extent, South Asia. At oldest ages prevalence estimates would be higher than the prevalence data for these locations and reach a level of close to 80% at oldest ages. In these locations, we will pay attention to how garbage codes are being redistribution onto COPD in the next round of GBD.

DisMod-MR 2.1 likelihood estimation

Analysts have the choice of using a Gaussian, log-Gaussian, Laplace, or Log-Laplace likelihood function in DisMod-MR 2.1. The default log-Gaussian equation for the data likelihood is

$$-log\left[p\left(y_j|\Phi\right)\right] = \log\left(\sqrt{2\pi}\right) + \log\left(\delta_j + s_j\right) + \frac{1}{2}\left(\frac{\log\left(a_j + \eta_j\right) - \log\left(m_j + \eta_j\right)}{\delta_j + s_j}\right)^2$$

Where,

y_j is a "measurement value" (ie, data point)

Φ denotes all model random variables

η_j is the offset value, *eta*, for a particular "integrand" (prevalence, incidence, remission, excess mortality rate, with-condition mortality rate, cause-specific mortality rate, relative risk, or standardised mortality ratio)

a_j is the adjusted measurement for data point j, defined by

$$a_j = e^{(-u_j - c_j)} y_j$$

Where:

u_j is the total "area effect" (ie, the sum of the random effects at three levels of the cascade: super-region, region and country) and

c_j is the total covariate effect (ie, the mean combined fixed effects for sex, study level, and country level covariates), defined by

$$c_j = \sum_{k=0}^{K[I(j)]-1} \beta_{I(j),k} \hat{X}_{k,j}$$

with standard deviation

$$s_j = \sum_{l=0}^{L[I(j)]-1} \zeta_{I(j),l} \hat{Z}_{k,j}$$

Where:

k denotes the mean value of each data point in relation to a covariate (also called x-covariate)

I(j) denotes a data point for a particular integrand, *j*

$\beta_{I(j),k}$ is the multiplier of the kth x-covariate for the ith integrand

$\hat{X}_{k,j}$ is the covariate value corresponding to the data point *j* for covariate *k*;

l denotes the standard deviation of each data point in relation to a covariate (also called z-covariate)

$\zeta_{I(j),k}$ is the multiplier of the lth z-covariate for the ith integrand

δ_j is the standard deviation for adjusted measurement *j*, defined by:

$$\delta_j = log[y_j + e^{(-u_j - c_j)} \eta_j + c_j] - log[y_j + e^{(-u_j - c_j)} \eta_j]$$

Where:

m_j denotes the model for the jth measurement, not counting effects or measurement noise, and defined by:

$$m_j = \frac{1}{B(j) - A(j)} \int_{A(j)}^{B(j)} I_j(\text{a})\ \text{da}$$

Where:

A(j) is the lower bound of the age range for a data point

B(j) is the upper bound of the age range for a data point

I_j denotes the function of age corresponding to the integrand for data point *j*

Spatiotemporal Gaussian process regression

This type of regression has been used for risk factors for which the data density is sufficient to estimate a very flexible time trend. The flowchart showing the analytic steps can be found in figure S3. The approach is a stochastic modelling technique that is designed to detect signals amidst noisy data. It also serves as a powerful tool for interpolating non-linear trends.[24,25] Unlike classical linear models that assume that the trend underlying data follows a definitive functional form, GPR assumes that the specific trend of interest follows a Gaussian process, which is defined by a mean function $m(\cdot)$ and a covariance function $Cov(\cdot)$. For example, let $p_{c,a,s,t}$ be the exposure, in normal, log, or logit space, observed in country c, for age group a, and sex s at time t:

$$\left(p_{c,a,s,t}\right) = g_{c,a,s}(t) + \epsilon_{c,a,s,t}$$

where

$$\epsilon_{c,a,s,t} \sim Normal\left(0, \sigma_p^2\right),$$

$$g_{c,a,s}(t) \sim GP\left(m_{c,a,s}(t), Cov\left(g_{c,a,s}(t)\right)\right).$$

The derivation of the mean and covariance functions, $m_{c,a,s}(t)$ and $Cov\left(g_{c,a,s}(t)\right)$, along with a more detailed description of the error variance (σ_p^2), is described below.

Estimating mean functions

We estimated mean functions by using a two-step approach. To be more specific, $m_{c,a,s}(t)$ can be expressed, depending on the exposure transformation, as:

$$log\left(p_{c,a,s}(t)\right) = X_{c,a,s}\beta + h(r_{c,a,s,t})$$

$$logit\left(p_{c,a,s}(t)\right) = X_{c,a,s}\beta + h(r_{c,a,s,t})$$

$$p_{c,a,s}(t) = X_{c,a,s}\beta + h(r_{c,a,s,t})$$

where $X\beta$ is the summation of the components of a hierarchical mixed-effects linear regression, including the intercept and the product of covariates with their corresponding fixed-effect coefficients. Some models were run as hierarchical mixed-effects linear regressions with random effects on the levels of the geographic hierarchy. For most mixed-effects models, random effects were only used in the fit, not in the prediction. The second part of the equation, $h(r_{c,a,s,t})$, is a smoothing function for the residuals, $r_{c,a,s,t}$, derived from the linear model.[3] Descriptions of exposure transformations and which covariates were used in linear models can be found in appendix section 4, which described the risk-specific estimation approaches. Some models used a custom stage-1 estimate – detailed information on the mixed-effect estimation process for these risks may be found in the risk-specific appendix section 4.

Although the linear component captures the general trend in exposures over time, much of the data variability may still not be adequately accounted for. To address this, we fit a locally weighted polynomial regression (locally estimated scatterplot smoothing, or LOESS) function $h(r_{c,a,s,t})$ to systematically estimate this residual variability by borrowing strength across time, age, and space patterns (the spatiotemporal component of ST-GPR).[26,27] The time adjustment parameter, defined by λ , aims to borrow strength from neighboring time points (ie, the exposure in this year is highly correlated with exposure in the previous year but less so further back in time). The age-adjustment parameter, defined by ω, borrows strength from data in neighboring age groups. The space-adjustment parameter, defined by ξ, aims to borrow strength across the hierarchy of geographic locations. The spatial and temporal weights are combined into a single space-time weight to allow the amount of spatial weight given to a particular point $r_{c,a,s,t}$ to fluctuate given the data availability at each time *t* and location-level *l* in the location hierarchy.

Let $w_{c,a,s,t}$ be the final weight assigned to observation $r_{c,a,s,t}$ with reference to a focal observation r_{c_0,a_0,s_0,t_0}. We first generated a temporal weight $t.w_{c,a,s,t}$ for smoothing over time, which was based on the scaled distance along the time dimension of the two observations[27]:

$$t.w_{c,a,s,t} = \frac{1}{e^{\lambda|t-t_0|}}$$

Next, we generated a spatial weight to smooth over geography. Specifically, we defined a geospatial relationship by categorizing data based on the GBD location hierarchy (table S4). *zeta* acts as a scalar on a given datapoint given its proximity to the target location:

$$t.w_{c,a,s,t} = \zeta^{|c-c_0|}$$

For example, estimating a country would use the following weighting scheme:

- Country data: ζ^0 = 1
- Regional data not from the country being estimated: ζ^1
- Data from other regions in the same super region: ξ^2
- Global data from other super regions: ζ^3

Under the spatial weighting specification, typical values of ζ range from [0.001, 0.2], where ζ can be interpreted as the amount to downweight regional datapoints compared to country datapoints for a given estimating country. For example, for a given datapoint $r_{c,a,s,t}$ and ζ = 0.01, a datapoint not within country *c* but within the same region *r* as $r_{c,a,s,t}$ would be assigned $\frac{1}{100}$ the weight of a datapoint within the country.

The spatial and temporal weights were then multiplied and summed across each level of the location hierarchy and normalised for each time period *t* . This procedure allowed the space-time weight to implicitly take into account the amount of data available at the country vs. region vs. super-region level and attribute spatial weight accordingly.

Given a normalisation constant,

$$K_i = \sum_{c\epsilon C} s.w_{c,t} * t.w_{c.t} + \sum_{c\epsilon R} s.w_{c,t} * t.w_{c.t} + \sum_{c\epsilon SR} s.w_{c,t} * t.w_{c.t}$$

the final space-time weight would then equal

$$w'_{c,a,s,t} = \frac{s.w_{c,t} * t.w_{c,t}}{K_i}$$

Finally, we calculated the weight $w''_{c,a,s,t}$ to smooth over age, which is based on a distance along the age dimension of two observations. For a point between the age a of the observation $r_{c,a,s,t}$ and a focal observation r_{c_0,a_0,s_0,t_0}, the weight is defined as follows:

$$w''_{c,a,s,t} = \frac{1}{e^{\omega|a-a_0|}}$$

The final weights would then be computed by simply multiplying the space-time weights and age weights and normalising so all weights for a given time period *t* sum to 1. A full derivation of weights for each category, assuming the location being estimated was a country, follows:

1) If the observation $r_{c,t}$ belongs to the same country c_0 of the focal observation r_{c_0,t_0}:

$$w_{c,a,s,t} = \frac{(w'_{c,a,s,t}w''_{c,a,s,t})}{\sum_{c=c_0}(w'_{c,a,s,t}w''_{c,a,s,t})} \qquad \forall c = c_0$$

2) If the observation $r_{c,t}$ belongs to a different country than the focal observation r_{c_0,t_0}, but both belong to the same region *R*:

$$w_{c,a,s,t} = \frac{(w'_{c,a,s,t}w''_{c,a,s,t})}{\sum_{c\neq c_0}(w'_{c,a,s,t}w''_{c,a,s,t})} \qquad \forall c \neq c_0 \cap R[c] = R[c_0]$$

3) If the observation $r_{c,t}$ belongs to the same super region *SR* but to both a different country c_0 and a different region $R[c_0]$ than the focal observation r_{c_0,t_0}:

$$w_{c,a,s,t} = \frac{(w'_{c,a,s,t}w''_{c,a,s,t})}{\sum_{c\neq c_0}(w'_{c,a,s,t}w''_{c,a,s,t})} \qquad \forall c \neq c_0 \cap R[c] \neq R[c_0] \cap SR[c] = SR[c_0]$$

4) If the observation $r_{c,t}$ is from a different super region than the focal observation r_{c_0,t_0}(Ie, all other data currently not receiving a weight):

$$w_{c,a,s,t} = \frac{(w'_{c,a,s,t}w''_{c,a,s,t})}{\sum_{c\neq c_0}(w'_{c,a,s,t}w''_{c,a,s,t})} \qquad \forall c \neq c_0 \cap R[c] \neq R[c_0] \cap SR[c] \neq SR[c_0]$$

Observations could be downweighted by a factor of 0.1, usually because they were not geographically representative at the unit of estimation. Details of reasons for downweighting can be found in risk-

specific modeling summaries. The final weights were then normalised such that the sum of weights across age, time, and geographic hierarchy for a reference group was 1.

Estimating error variance

σ_p^2 represents the error variance in normal or transformed space including the sampling variance of the estimates and prediction error from any crosswalks performed. First, variance was systematically imputed if the data extraction did not include any measure of uncertainty. When some sample sizes for data were available, missing sample sizes were imputed as the 5th percentile of available sample sizes. Missing variances were then calculated as $\sigma_p^2 = \frac{p*(1-p)}{n}$ for proportions or were predicted from the mean by using a regression for continuous values. When sample sizes were entirely missing and could not be imputed, the 95th percentile of available variances at the most granular geographic level (Ie, first country, then region, etc.) were used to impute missing variances. For proportions where *p*n* or *(1-p)*n* is <20, variance was replaced by using the Wilson Interval Score method.

Next, if the exposure was modelled as a log transformation, the error variance was transformed into log-space by using the delta method approximation as follows:

$$\sigma_p^2 \cong \frac{\sigma_{p'}^2}{p_{c,a,s,t}^2}$$

where $\sigma_{p'}^2$ represents the error variance in normal space. If the exposure was modelled as a logit transformation, the error variance was transformed into logit-space by using the delta method approximation as follows:

$$\sigma_p^2 \cong \frac{\sigma_{p'}^2}{(p_{c,a,s,t} * (1 - p_{c,a,s,t}))^2}$$

Finally, prior to GPR, an approximation of non-sampling variance was added to the error variance. Calculations of non-sampling variance were done on normal-space variances. Non-sampling variance was calculated as the variance of inverse-variance weighted residuals from the space-time estimate at a given location-level hierarchy. If there were <10 data points at a given level of the location hierarchy, the non-sampling variance was replaced with that of the next highest geography level with >10 data points.

Estimating the covariance function

The final input into GPR is the covariance function, which defines the shape and distribution of the trends. Here, we have chosen the Matern-Euclidian covariance function, which offers the flexibility to model a wide spectrum of trends with varying degrees of smoothness. The function is defined as follows:

$$M(t,t') = \sigma^2 \frac{2^{1-\nu}}{\Gamma(\nu)} \left(\frac{d(t,t')\sqrt{2\nu}}{l}\right)^{\nu} K_\nu \left(\frac{d(t,t')\sqrt{2\nu}}{l}\right)$$

where $d(\cdot)$is a distance function; σ^2, ν, l, and K_ν are hyperparameters of the covariance function—specifically σ^2 is the marginal variance, ν is the smoothness parameter that defines the differentiability of the function, l is the length scale, which roughly defines the distance between which two points

become uncorrelated, and K_v is the Bessel function. We approximated σ^2 by taking the normalised median absolute deviation $MADN(r_c')$ of the difference, which is the normalised absolute deviation of the difference of the first-stage linear regression estimate from the second-stage spatiotemporal smoothing step for each country. We then took the mean of these country-level MADN estimates for all countries with 10+ country-years of data to ensure that differences between first- and second-stage estimates had sufficient data to truly convey meaningful information on model uncertainty. We used the parameter specification $v = 2$ for all models. The scale parameter l used for each risk is reported in appendix section 4.

Prediction using GPR

We integrated over $g_{c,t}(t_*)$ to predict a full time series for country c, age a, sex s, and prediction time t_* as follows:

$$p_{c,a,s}(t_*) \sim N\left(m_{c,a,s,t}(t_*), \sigma_p^2 I + Cov\left(g_{c,a,s,t}(t_*)\right)\right)$$

Random draws of 1000 samples were obtained from the distributions above for every country for a given indicator. The final estimated mean for each country was the mean of the draws. In addition, 95% UIs were calculated by taking the 2.5 and 97.5 percentile of the sample distribution. The linear modelling process was implemented by using the lmer4 package in R, and the ST-GPR analysis was implemented through the PyMC2 package in Python.

Subnational Scaling and Aggregation

To ensure internal consistency of the estimates between countries and their respective subnational locations, national estimates were either created by population-weighted aggregation or subnational estimates were adjusted by population-weighted scaling to the national estimates, depending on the data coverage of a given country compared to that of its subnational locations. For example, if data coverage was better at the national level than at its corresponding subnational locations for a given country and risk across age, sex, and time, estimates were rescaled to be consistent with the national level. Conversely, if data coverage was better at the subnational level, estimates for its parent country were generated through population-weighted aggregation of subnational estimates.

Estimates can also be scaled within logit space. Scaling in logit space ensures that subnational estimates of proportion models do not exceed one after being rescaled to the national estimate.

Fitting a distribution to exposure data

The most informative data describing the distribution of risk factors within a population come from individual-level data; additional sources of data include reported means and variances. In cases in which a risk factor also defines a disease or disease severity cut-off, such as haemoglobin level and mild, moderate, or severe anaemia, or diabetes and FPG level, the prevalence of disease is also frequently reported. To model the distribution of any particular risk factor, we seek a family of probability density functions, a fitting method, and a model selection criterion. To make use of the most commonly available data describing most populations, we used the method of moments (MoM); the first two empirical moments from a population, the mean and the variance, were used to determine the parameters of two-parameter probability distribution factor (PDF) families describing the distribution of risk within any population. Exceptions to this rule are justified by context. We used the Kolmogorov-Smirnov[28] (KS) test to measure the goodness of fit (GoF) and compared the distance between the

empirical and ensemble distributions, but in some cases, the GoF was based on the prediction error for the prevalence of disease.

We used an ensemble technique in which a model selection algorithm is used to choose the best model for each continuous risk factor.[28] We drew the initial set of candidate models from commonly used PDF families, including both right-skewed and left-skewed distributions. These included beta, exponential, gamma, gumbel, inverse gamma, inverse Weibull, log-logistic, lognormal, mirrored gamma, mirrored gumbel, normal, and Weibull. We fitted each PDF family candidate to each dataset by using the MoM and used the KS test as the measure of GoF. Preliminary analysis showed that the GoF ranking of PDF families varied across datasets for any particular risk factor and that combining the predictions of differently fitted PDF families could dramatically improve the GoF for each dataset. Therefore, we developed a new model for prediction by using the ensemble of candidate models, which is a weighted linear combination of all candidate models, $\{f\}$, where a set of weights $\{w\}$ is chosen such that $\sum_i w_i = 1$, and the values of the weights were determined by a second GoF criterion with its own validation process. For each risk, we pooled all available microdata and performed Nelder-Mead numeric optimisation across demographics subsets of data to derive a set of distribution-specific weights such that the average KS statistic across data sets would be minimised. The details can be summarised by 1) the summary statistics for each dataset; 2) a table showing the KS statistic for each candidate model; and 3) the weights defining the final ensemble model for each dataset. We then averaged across demographic subsets and data sets to determine the final weights for modelling the distribution of any particular risk factor.

Section 2.4: Step 3. TMREL[1]

In this and all previous GBD studies, the counterfactual level of risk exposure used is the risk exposure that is both theoretically possible and minimizes risk in the exposed population that consequently captures the maximum population-attributable burden.[4] For each risk evaluated in GBD 2019, Step 4 of the analytical flowchart describes the use of the best available epidemiological evidence from published and unpublished RRs by level of exposure and the lowest observed level of exposure from cohorts, used to select a single level of risk exposure that minimises risk from all causes of deaths combined to establish the TMREL. In principle, the TMREL for a given risk may vary by age, sex, and location if supported by clear evidence. Based on the available evidence, the TMREL itself can be uncertain, which is reflected in the 95% UIs in table S6.

In GBD 2019, we updated the process of estimating TMREL for dietary risks. We set the TMREL to zero for all harmful dietary risk factors with monotonically increasing risk functions (eg, processed meat intake); this excludes sodium. For protective risks with monotonically declining risk functions with exposure (eg, fruit intake), we first determined the 85th percentile of exposure in the cohorts or trials used in the meta-regression of each outcome that was associated with the risk. Then, we determined the TMREL by weighting each risk-outcome pair by the relative global magnitude of each outcome.

Section 2.5: Step 4. Estimate population-attributable fractions[1]

Risks are categorised on the basis of how exposure was measured: dichotomous, polytomous, and continuous. High low-density lipoprotein (LDL) cholesterol level is an example of a risk measured on a continuous scale. The PAF, which represents the proportion of risk that would be reduced in a given year

if the exposure to a risk factor in the past were reduced to an ideal exposure scenario, is defined for a continuous risk factor as:[29]

$$PAF_{joasgt} = \frac{\int_{x=l}^{u} RR_{joasg}(x)P_{jasgt}(x)dx - RR_{joasg}(TMREL_{jas})}{\int_{x=l}^{u} RR_{joasg}(x)P_{jasgt}(x)dx}$$

Where PAF_{joasgt} is the PAF for cause o due to risk factor j for age group a, sex s, location g, and year t. $RR_{joasg}(x)$ is the RR as a function of exposure level x for risk factor j for cause o, age group a, sex s, and location g with the lowest level of observed exposure as l and the highest as u; $P_{jasgt}(x)$ is the distribution of exposure at x for age group a, sex s, location g, and year t; and $TMREL_{jas}$ is the TMREL for risk factor *j*, age group *a*, and sex *s*.

The PAF_{joasgt} for dichotomous and polytomous risk factors for every country is defined as:

$$PAF_{joasgt} = \frac{\sum_{x=1}^{u} RR_{joast}(x)P_{jasgt}(x) - RR_{joasg}(TMRE_{jas})}{\sum_{x=1}^{u} RR_{joas}(x)P_{jasgt}(x)}$$

where PAF_{joasgt} is the PAF for cause o due to risk factor j for age group a, sex s, location g, and year t. $RR_{joasg}(x)$ is the RR as a function of exposure level x for risk factor j for cause o, age group a, sex s, and location g on a plausible range of exposure levels from *l* to *u*; $P_{jasgt}(x)$ is the proportion of the population in risk group (prevalence) for age group a, sex s, location g, and year t; and $TMREL_{jas}$ is the TMREL for risk factor *j*, age group *a*, and sex *s*.

Section 2.6: Step 5. Estimate summary exposure values[1]

Summary exposure value (SEV) is the RR-weighted prevalence of exposure, a univariate measure of risk-weighted exposure, taking the value zero when no excess risk for a population exists and the value one when the population is at the highest level of risk. We report SEVs on a scale from 0% to 100% on which a decline in SEV indicates reduced exposure to a given risk factor and an increase in SEV indicates increased exposure.

We first calculate risk, r, and cause, c, for specific SEVs by using the following equation,

$$SEV_{rc} = \frac{\frac{PAF_{rc}}{1 - PAF_{rc}}}{RR_{max} - 1}$$

for each most-detailed age, sex, location, year, and outcome. PAF is the YLL (expect for occupational noise, bullying victimization, and occupational ergonomic factors, which are YLD only and thus use the YLD) PAF. RR_{max} for categorical risks is the RR at the highest category of exposure. For continuous risks, this is

$$RR_{max} = RR^{\frac{TMREL - 1^{st} exposure}{RR_{scalar}}} \text{ if protective, or}$$

$$= RR^{\frac{99^{th} exposure - TMREL}{RR_{scalar}}}$$

otherwise, and for custom modelled risks like ambient particulate matter pollution, HAP from solid fuels, alcohol, smoking, bullying, and activity, the modeller provides draws of RR_{max}. Generally, RRs do not vary across time and space. However, exceptions exist, such as risks from second-hand smoke (SHS) or HAP for which the RR is based on the integrated exposure response (IER) curve. In these cases, the RR is averaged across location and year to ensure no time or space variation. If the PAF is negative, which signifies a protective effect for that outcome, the PAF is set to 0 and the SEV is then also 0 because the SEV is univariate and constrained to be a value between 0 and 1. Once we obtained a set of risk-cause specific SEVs at the most-detailed risk, cause, age, sex, and location for all years, we averaged across causes to produce the final risk specific SEV_r,

$$SEV_r = \frac{1}{N(c)}\sum_{c} SEV_{rc}$$

where $N(c)$is the total number of outcomes for a risk.

Section 2.7: Step 6. Mediation[1]

Section 2.7.1: Summary

The portion of the burden of disease that is attributable to various combinations of risk factors or to all risk factors combined has been a topic of broad interest.[30] In GBD 2010, we only aggregated the burden of risk factors for some clusters of risks, including access to improved water and sanitation, child and maternal malnutrition, tobacco smoking, alcohol use, dietary risk factors, occupational risk factors, and sexual abuse and violence. We did not aggregate air pollution and metabolic risk factors. For GBD 2013 onwards, we aggregated all risk factors into three large categories—behavioural, environmental and occupational, and metabolic risks—and aggregated all GBD risk factors into a single attributable fraction for each disease and eventually for all causes of burden.

Aggregating risk factors at different levels shares three essential challenges:

1. Risk factor coexistence or aggregation: for example, metabolic risk factors often occur together, or high-risk behaviours such as drug abuse and unsafe sex are related.
2. Mediation: a risk factor may affect another risk factor that lies in the physiological pathway to a disease outcome. It can be inside a cluster of risk factors, such as the effect of obesity through an increase in FPG level and later cardiovascular disease (CVD) outcomes, or between clusters of risk factors, such as the effect of fibre on cholesterol.
3. The formula used to calculate the aggregated PAF.

The aggregation method is conceptually applicable to other aggregations such as socioeconomic factors, education, homelessness, and refugee status that are being considered for inclusion in future GBD iterations. In the next section, we explain our approach to dealing with these challenges.

There are three patterns of associations between risk factors to consider (Figure C). The first concerns confounding; risk B affects risk A and outcome C (Pattern 1 in *Patterns of associations between risk factors*). In these cases, the RR for A should be adjusted for B; for example, the fruit RR is adjusted for smoking. If part of the effect of A is through B, a mediator, we do not adjust the effect of A for B. For example, we do not adjust the RR of body-mass index (BMI) for cholesterol because cholesterol lies in the biological pathway between BMI and cardiovascular outcomes (Pattern 2 in in *Patterns of*

associations between risk factors). The third pattern occurs when risks A and B are proxies of a third variable Z and aggregation aims to estimate the total effect of a latent variable Z on C. An example is child growth failure, which is measured by stunting, wasting, and underweight as proxies.

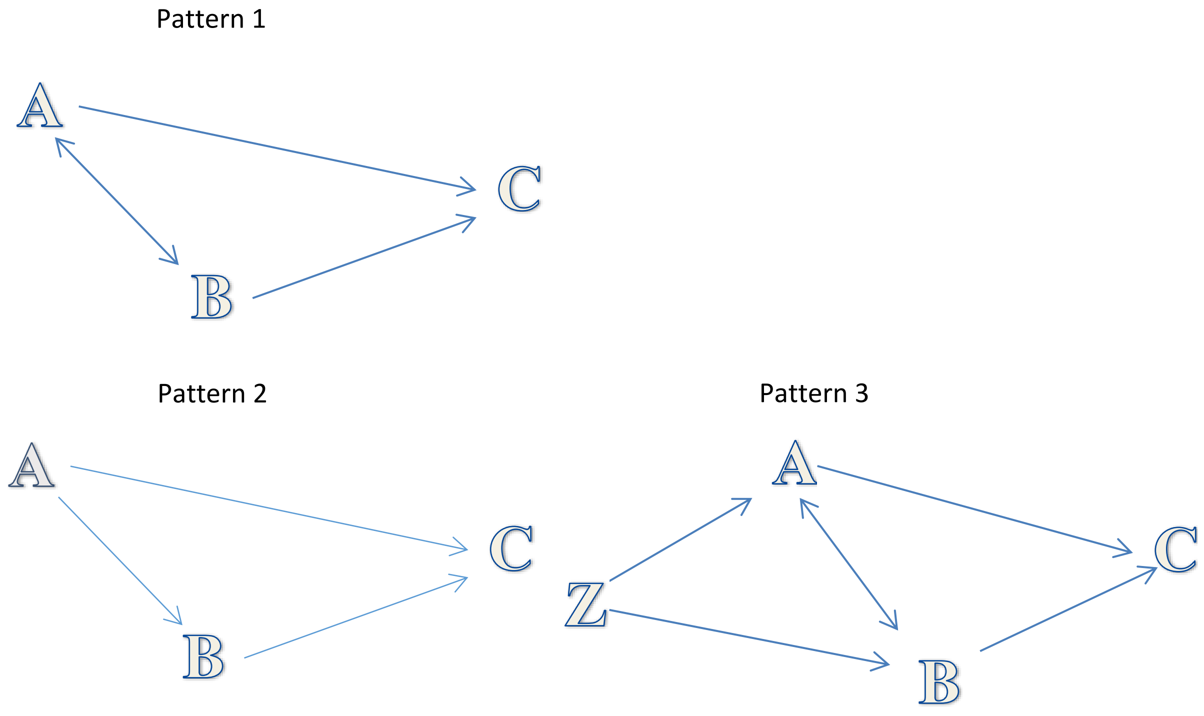

Figure C. Patterns of associations between risk factors

Section 2.7.2: Calculating the burden of multiple risk factors

Validation studies have reported congruency between the true risk associated with multiple risk factors affecting the same outcome and a multiplicative aggregation of the PAFs of the individual risk factors (formula below).[31]

$$PAF_{1..i} = 1 - \prod_{i=1}^{n}(1 - PAF_i)$$

where *PAF* is the population attributable fraction and *i* is each individual risk factor.

The same validation studies also found that the overestimation from ignoring the covariance between risk factors is small. This small overestimation was important to note because few data sources exist from which we can draw information on covariance.

We endeavoured to evaluate RRs that were controlled for confounders. However, because we had to rely on the literature for many RRs, we did not always have full control over the choice of confounders controlled for in each study.

Section 2.7.3: Adjusting for mediation

When aggregating the effects of multiple risk factors, we included an MF if a part of the effect of one risk factor was included in the effect estimated for in the mediator. First, we prepared a list of possible mediations, and especially between behavioural risks and metabolic risk factors with cardiometabolic outcomes. We did not assume any mediation effect between risk factors for cancers.

Danaei and colleagues assumed that part of the effect of BMI on ischaemic heart disease (IHD) is through high systolic blood pressure (SBP), cholesterol level, and FPG.[32] The proportion of the BMI effect that can be explained by other metabolic risk factors is the amount of mediation. The difference between the crude RR of BMI on IHD with the RR adjusted for SBP, FPG, and cholesterol level reflects the amount of BMI effect on IHD that is mediated and already included in SBP, FPG, and cholesterol level:

$$MF = \frac{RR_{crude} - RR_{adjusted}}{RR_{crude} - 1}$$

So, to aggregate the PAF of multiple risk factors, we first calculated the part of the excess risk (RR-1) of every risk factor that is not mediated, re-compute the PAF so that it only includes the non-mediated risk then aggregated PAFs by assuming they are independent.

Therefore, if MF is the mediation factor of R2 through R1, the adjusted RR for R2 including only the non-mediated component of risk is:

$$RR_{1,2} = MF_{2/1}(RR_2 - 1) + 1$$

The PAF accounting for mediation is then computed using the adjusted RR and the joint PAF computed as detailed in Section 2.7.2. For every paired risk factor and outcome, the matrix of possible mediations was calculated and used.

Section 2.7.4: Calculating mediation factor

1 – Comparing crude RR versus mediator-adjusted RR

The best example is the mediation of BMI through SBP, FPG, and cholesterol level reported by Danaei et al.[32] In their meta-analysis, they report the adjusted and unadjusted RR of BMI on IHD and stroke based on combined data from individual cohorts. They calculated the MF by using the following equation, and we used it directly as the MF in risk factor aggregation. Using individual-level data from cohort studies, we estimated the MF for other metabolic risk factors and some dietary risks.

$$MF = \frac{RR_{crude} - RR_{adjusted}}{RR_{crude} - 1}$$

2 – Estimating the mediation factor by pathway of the effect

For many other risk factors, no data are available to enable the use of the first method. Instead, we searched studies to estimate the effect of the risk factor on the mediator and, finally, the expected increase in IHD risk. We pooled available studies to calculate the unit increase in the mediator per unit increase in the risk factor to calculate the size of the IHD RR (Figure D).

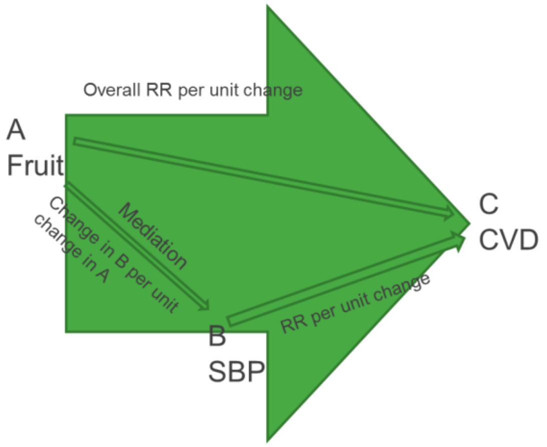

Figure D. Example of pathway between fruit, high systolic blood pressure, and cardiovascular diseases

We have RRs for the effect of A on C and B on C in GBD from a meta-analysis of studies in the literature. The effect of A on B was estimated by analysis of diet trials.

$$RR_{ABC} = RR_{BC}{}^{\Delta_{AB}}$$

RR_{ABC} is the expected effect of A through B on C

RR_{BC} is the RR of each unit increase in mediator on outcome C

Δ_{AB} is the change in mediator level B per each unit change in A

If RR_{AB} is the overall effect of A on B, then:

$$MF = \frac{RR_{ABC} - 1}{RR_{AB} - 1}$$

We kept the uncertainty of each parameter to a minimum by generating and following 1000 draws of the estimates to calculate 1000 draws of the posterior distribution of the MF. We did not include risk-mediator pairs if the MF was not significant at the 5% level (more than 50 of 1000 draws were negative). We truncated the MF distribution at 1 when the whole effect of the risk factor on the outcome would be assumed to be exerted through the mediator pathway.

Some MFs equalled 1 when the whole effect was calculated through another risk factor (eg, the effect of salt through SBP) or when we assumed other risk factors were sources of the exposure (eg, fibre is provided by consuming fruit, vegetables, and whole grains, and all the beneficial effect of milk on colorectal cancer is mediated through calcium).

Air pollution

We considered mediation for particulate matter pollution and SBP, FPG level, and cholesterol level but in no case was the evidence strongly supportive. Review of the epidemiologic evidence identified several cohort studies that reported increased prevalence and/or risk of hypertension due to long-term exposure to ambient PM2.5, and several studies have found elevated SBP due to household solid fuel use as well. Studies of short-term exposure also reported acute elevations in blood pressure. However, experts currently disagree about whether the existing evidence with regard to the effects of long-term exposure is consistent with current mechanistic understanding of the effect of air pollution exposure on

blood pressure, and whether existing cohort studies have properly modelled that exposure. We hope to be able to address these issues in the future to assess whether the evidence supports a mediation analysis in a future round of GBD.

Assumed mediations

For the risk factors with PAFs of 100%, such as FPG and diabetes, kidney dysfunction and chronic kidney disease (CKD), SBP and hypertensive heart disease, alcohol and alcohol use disorders, child underweight and protein-energy malnutrition, child wasting and protein-energy malnutrition, and drug use and drug use disorders, no mediation is needed.

Section 2.7.5: Piecewise aggregation (Pattern 3)

There are three anthropometric indicators that are highly correlated: child underweight, stunting, and wasting, as shown in Figure E in this section. Available RRs for each indicator are not adjusted for the other two because these indicators are highly correlated and most of the burden occurs in an interaction. Estimating the total burden due to child growth failure, a latent variable, is difficult. The three anthropometric indicators are not independent, so the covariance between them should be considered. This consideration was the main reason that GBD 2010 only included child underweight. If covariance between these indicators is significant (as is shown in Figure E), aggregating these indicators by assuming they are independent would overestimate the total burden significantly.

To use the best available data, we adjusted observed RRs reported by Olofin et al. for underweight, stunting, and wasting by simulating the joint distribution of the three indicators by using the distribution of each indicator and the covariance between indicators in the countries included in the meta-analysis (extracted from Demographic and Health Survey (DHS) micro-data).[33] On the basis of the analysis done by McDonald et al., we assumed an interaction exists between the three indicators, and we extracted the interaction terms from the corresponding analysis.[34] We calculated the adjusted RRs by minimizing the error between observed crude RRs (from meta-analysis) and expected crude RRs derived from adjusted RRs.

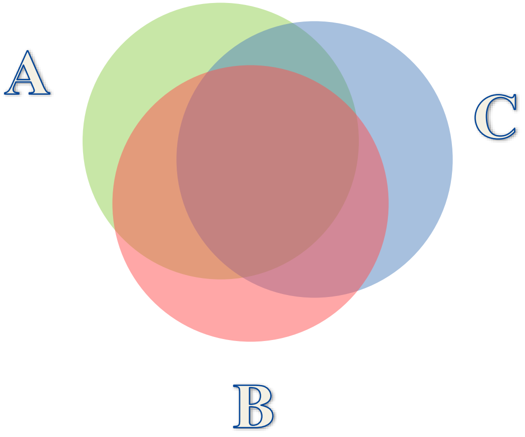

Figure E. Venn diagram demonstrating the correlation between child underweight, stunting, and wasting

After adjusting for the three risk factors, we calculated the PAFs and aggregated the underweight, stunting, and wasting burden.

Section 2.7.6: Uncertainty of aggregated and mediated PAFs

We generated 1000 draws of the posterior distribution of the MF calculated by different methods to use beside draws of other inputs to the PAF aggregation.

Section 2.7.7: Important assumptions in aggregating risk factors and including mediation

1 – The MFs or PAF adjustments are similar across countries, age, sex, and years. Although the size of mediation is probably different in different populations, little data is available to inform the covariance between different risk factors or the MF amount by age and country. For example, in some countries, the size of the mediated BMI-IHD PAF exerted through cholesterol level, as calculated by the MF, was even bigger than the total burden of cholesterol level. This finding indicated that less of the effect of BMI is mediated through cholesterol level and MFs are not similar across countries.

2 – For many risk-mediator-outcome pairs, no data are available, so we assumed the mediation is zero.

3 – Because the covariance between undernutrition indicators differs by location (and across time, but results were not reported), and an interaction exists between these indicators, the total burden might be underestimated.

4 – We assumed no significant covariance between PAFs, which might not be true between some risk factors, such as metabolic risk factors. Although this overestimation can be controlled by using adjusted RRs, using crude RRs for BMI and other metabolic risk factors may cause significant overestimation of the aggregated metabolic risks burden.

Section 2.8: Step 7. Estimate attributable burden[1]

Four key components are included in the estimation of the burden attributable to a given risk factor: the metric of burden being assessed (the number of deaths, YLLs, YLDs, or DALYs [the sum of YLLs and YLDs]); the exposure levels for a risk factor; the RR of a given outcome due to exposure; and the counterfactual level of risk factor exposure. Estimates of attributable burden as DALYs for risk-outcome pairs were generated by using the following model:

$$AB_{jasgt} = \sum_{o=1}^{w} DALY_{joasgt} \, PAF_{joasgt}$$

where AB_{jasgt} is the attributable burden for risk factor j for age group a, sex s, location g, and year t; $DALY_{joasgt}$ is total DALYs for cause o (of w relevant outcomes for risk factor j) for age group a, sex s, location g, and year t; and PAF_{joasgt} is the PAF for cause o due to risk factor j for age group a, sex s, location g, and year t. The proportions of deaths, YLLs, or YLDs attributable to a given risk factor or risk factor cluster were analogously computed by sequentially substituting each metric in place of DALYs in the equation provided.

Section 2.9: Decomposition analysis of deaths and DALYs[1]

We conducted a decomposition analysis of changes in DALYs from 2010 to 2019, decomposing changes in all-age cause-specific DALYs attributable to all risk factors and individual risk factors due to changes in population growth, population age structure, exposure to the given risk for a disease, and risk-deleted death and DALY rates. In this case, risk-deleted rates are the rates obtained after removing the effect of a risk factor or combination of risk factors — in other words, observed DALY rates multiplied by one minus the PAF for the risk or set of risks. Our decomposition analyses draw from methods developed by

Das Gupta[35] to provide a computationally tractable solution for isolating drivers of burden changes whereby all combinations of possible pathways are averaged across factors. Attributable burden was determined, following the methods of Das Gupta, as a product of three factors such that:

$$T_{asgt} = (A_{asgt}\, B_{asgt}\, C_{asgt})$$

where T_{asgt} represents the attributable burden at year t; $A_{sgt}$is the age-specific population size for a given age group a, sex s, and location g at year t; B_{asgt} is the underlying rate of the outcome unrelated to the risk factor or observed rate, multiplied by $1 - PAF$ for a given age group a, sex s, and location g at year t; and C_{asgt} is the ratio of the attributable burden to the underlying rate, which reflects the risk exposure effect for a given age group a, sex s, and location g at year t defined as $PAF/(1 - PAF)$when decomposing attributable burden to a risk. Risk exposure effects for individual risk factors are scaled such that they sum to the all-risk exposure effect by location, age, sex, and cause accounting for mediation. This process allows for aggregation of risks; the exposure for all risks for a disease can be split into exposure to metabolic, behavioural, and environmental risks. The contribution of each factor to total change in attributable burden was determined by changing the level of one factor from time t_0 to t_1 – here 2010 to 2019 – with all other factors held constant. Thus, the effect of any of the three factors, for example A_{asgt}on the change of the attributable burden between 2010 (A_{10}) and 2019 (A_{17}) is calculated as:

$$E_A = (A_{19} - A_{10})\left(\frac{B_{10}C_{10} + B_{19}C_{19}}{3} + \frac{B_{10}C_{19} + B_{19}C_{10}}{6}\right)$$

where E_A is the proportion of change due to factor A, and the subscripts for each factor in the equation denote the year for each estimate. Because the effect depends on the order of entry of the factor, we calculated the average of all combinations of the three factors.[35] The proportion of change due to factorA_{sgt}, the age-specific population size for a given age group a, sex s, and location g at year t, is then further split, setting change in population growth equal to the percentage change in the all-age population from time t_0 to t_1 and change in population age structure to the residual, giving four factors.

This three-factor decomposition method does not work for risks for which the PAF, by definition, is 100% (such as high FPG level and type 2 diabetes) or for which the PAF is directly estimated (such as for unsafe sex and HIV). In the cases of child underweight and protein-energy malnutrition, child wasting and protein-energy malnutrition, short gestation for birthweight and neonatal preterm birth complications, low birthweight for gestation and neonatal preterm birth complications, iron deficiency (ID) and iron-deficiency anaemia (IDA), alcohol use and liver cancer due to alcohol use, alcohol use and cirrhosis and other chronic liver diseases due to alcohol use, alcohol use and alcohol use disorders, alcohol use and alcoholic cardiomyopathy, drug use and drug use disorders, occupational particulate matter, gases, and fumes and other pneumoconiosis, occupational particulate matter, gases, and fumes and coal workers pneumoconiosis, occupational exposure to asbestos and asbestosis, and occupational exposure to silica and silicosis, we used a two-factor decomposition method that examines the contribution of population, ageing, and risk exposure. Effectively, we assumed trends in these cases are driven by exposure, not change in the risk-deleted rates. Conversely, for unsafe sex and sexually transmitted diseases excluding HIV, we used a two-factor decomposition method that examines the contribution of population, ageing, and risk-deleted death and DALY rates and assumed that trends in these cases are driven by risk-deleted rates, not change in exposure. For high FPG level and type 1 and 2

diabetes, high FPG level and CKD due to type 1 and 2 diabetes, high SBP and hypertensive heart disease, high SBP and CKD due to hypertension, and impaired kidney function and CKD, we used GBD estimates of SEVs for the given risk and the case-fatality rate to decompose trends into an estimate of the contribution of the three factors. Similarly, for unsafe sex and cervical cancer, we used GBD estimates of the incidence of cervical cancer and the case-fatality rate to decompose trends into an estimate of the contribution of the three factors. For unsafe sex and HIV, we used spectrum counterfactual and CD4 risk-weighted prevalence.

Section 2.10: Socio-demographic Index analysis[1]

Section 2.10.1: Development of the Socio-demographic Index

The SDI is a composite indicator of socio-demographic development status strongly correlated with health outcomes. In short, it is the geometric mean of 0 to 1 indices of total fertility rate in those under 25 years old (TFU25), mean education for those age 15 years or older (EDU15+), and lag-distributed income per capita (LDI). For GBD 2019, after calculating SDI, values were multiplied by 100 for a scale of 0 to 100.

Section 2.10.2: Development of a revised SDI indicator

SDI was originally constructed for GBD 2015 by using the Human Development Index (HDI) methodology, wherein a 0 to 1 index value was determined for each of the original three covariate inputs (total fertility rate in those age 15 to 49, EDU15+, and LDI per capita) by using the observed minima and maxima during the estimation period to set the scales.

During GBD 2016, we moved from using relative index scales to using absolute scales to enhance the stability of SDI interpretation over time because we noticed that the measure was highly sensitive to the addition of subnational units that tended to stretch the empirical minima and maxima. We selected the minima and maxima of the scales by examining the relationships each of the inputs had with life expectancy at birth and under-5 mortality and by identifying points of limiting returns at both high and low values if they occurred before theoretical limits (eg, a TFU25 of 0).

Thus, an index score of 0 represents the minimum level of each covariate input past which selected health outcomes can get no worse, and an index score of 1 represents the maximum level of each covariate input past which selected health outcomes cease to improve. As a composite, a location with an SDI of 0 would have a theoretical minimum level of sociodemographic development relevant to these health outcomes, and a location with an SDI of 1 (prior to multiplying by 100 for reporting purposes) would have a theoretical maximum level of sociodemographic development relevant to these health outcomes.

We computed the index scores underlying SDI as follows:

$$I_{Cly} = {}^{(C_{ly} - C_{low})}\Big/_{(C_{high} - C_{low})}$$

where I_{cly} – the index for covariate *C*, location *l*, and year *y* – is equal to the difference between the value of that covariate in that location-year and the lower bound of the covariate divided by the difference between the upper and lower bounds for that covariate.

If the values of input covariates fell outside the upper or lower bounds, they were mapped to the respective upper or lower bounds. We also note that the index value for TFU25 was computed as $1 - I_{TFU25ly}$ because lower TFU25s correspond to higher levels of development and thus higher index scores.

For GBD 2019, we expanded the computation of SDI to 1062 national and subnational locations spanning the time period 1950-2019.

The composite SDI is the geometric mean of these three indices for a given location-year. The cut-off values used to determine quintiles for analysis were then computed by using country-level estimates of SDI for the year 2019, excluding countries with populations less than 1 million. As stated above, for GBD 2019, final SDI values were multiplied by 100 in order to improve understanding of and broader engagement with the values, so final reporting values are on a 0 to 100 scale.

Example calculation

We present the following example calculation of SDI for "Country X":

$$TFU25 = 1.09;\ Mean\ educ\ yrs\ pc = 8.23;\ lnLDI = 9.60$$

$$I_{TFU25} = 1 - \frac{1.09 - 0}{3 - 0} = 0.637$$

$$I_{Educ} = \frac{8.23 - 0}{17 - 0} = 0.484$$

$$I_{lnLDI} = \frac{9.60 - 5.52}{11.00 - 5.52} = 0.744$$

$$SDI = \sqrt[3]{I_{TFU25} * I_{Educ} * I_{lnLDI}} = \sqrt[3]{.637 * .484 * .744} = 0.611$$

$$I_{lnLDI} = \frac{9.58 - 5.52}{11.00 - 5.52} = 0.741$$

$$SDI = \sqrt[3]{I_{TFR} * I_{Educ} * I_{lnLDI}} = \sqrt[3]{.855 * .543 * .741} = 0.701$$

$$GBD\ 2019\ reporting\ SDI = 0.701 * 100 = 70.1$$

SDI values by location can be found in table S5.

Section 3: References

1 Stanaway JD, Afshin A, Gakidou E, *et al.* Global, regional, and national comparative risk assessment of 84 behavioural, environmental and occupational, and metabolic risks or clusters of risks for 195 countries and territories, 1990–2017: a systematic analysis for the Global Burden of Disease Study 2017. *The Lancet* 2018; **392**: 1923–94.

2 Stevens GA, Alkema L, Black RE, *et al.* Guidelines for Accurate and Transparent Health Estimates Reporting: the GATHER statement. *The Lancet* 2016; **388**: e19–23.

3 Murray CJ, Lopez AD. Global mortality, disability, and the contribution of risk factors: Global Burden of Disease Study. *Lancet* 1997; **349**: 1436–42.

4 Murray CJ, Lopez AD. On the comparable quantification of health risks: lessons from the Global Burden of Disease Study. *Epidemiology* 1999; **10**: 594–605.

5 Forouzanfar M, Afshin A, Alexander LT, Anderson H, Bhutta Z, Murray CJL. Global, regional, and national comparative risk assessment of 79 behavioural, environmental and occupational, and metabolic risks or clusters of risks, 1990-2015: a systematic analysis for the Global Burden of Disease Study 2015. *Lancet* 2016; **388**: 1659–724.

6 Stevens GA, Alkema L, Black RE, *et al.* Guidelines for Accurate and Transparent Health Estimates Reporting: the GATHER statement. *Lancet* 2016; published online June 28.

7 Food, nutrition, physical activity and the prevention of cancer: a global perspective. Washington, D.C: World Cancer Research Fund & American Institute for Cancer Research, 2007.

8 Aravkin A, Davis D. Trimmed statistical estimation via variance reduction. *Mathematics of OR* 2019; published online July 5. https://pubsonline.informs.org/doi/10.1287/moor.2019.0992 (accessed Nov 15, 2019).

9 Rousseeuw P. Multivariate estimation with high breakdown point. 1985. DOI:10.1007/978-94-009-5438-0_20.

10 Zheng P, Aravkin AY, Barber R, Sorensen RJD, Murray CJL. Trimmed Constrained Mixed Effects Models: Formulations and Algorithms. *arXiv:190910700 [math, stat]* 2019; published online Sept 23. http://arxiv.org/abs/1909.10700 (accessed Nov 15, 2019).

11 Wächter A, Biegler LT. On the implementation of an interior-point filter line-search algorithm for large-scale nonlinear programming. *Math Program* 2006; **106**: 25–57.

12 Boor C de. A Practical Guide to Splines. New York: Springer-Verlag, 1978 https://www.springer.com/gp/book/9780387953663 (accessed Nov 15, 2019).

13 Friedman JH. Multivariate Adaptive Regression Splines. *Ann Statist* 1991; **19**: 1–67.

14 Pya N, Wood SN. Shape constrained additive models. *Stat Comput* 2015; **25**: 543–59.

15 Efron B, Tibshirani RJ. An Introduction to the Bootstrap, 1 edition. New York: Chapman and Hall/CRC, 1993.

16 Yin P, Brauer M, Cohen A, *et al.* Long-term Fine Particulate Matter Exposure and Nonaccidental and Cause-specific Mortality in a Large National Cohort of Chinese Men. *Environ Health Perspect* 2017; **125**: 117002.

17 Li T, Zhang Y, Wang J, *et al.* All-cause mortality risk associated with long-term exposure to ambient PM2·5 in China: a cohort study. *Lancet Public Health* 2018; **3**: e470–7.

18 Yang Y, Tang R, Qiu H, *et al.* Long term exposure to air pollution and mortality in an elderly cohort in Hong Kong. *Environ Int* 2018; **117**: 99–106.

19 Yusuf S, Joseph P, Rangarajan S, *et al.* Modifiable risk factors, cardiovascular disease, and mortality in 155 722 individuals from 21 high-income, middle-income, and low-income countries (PURE): a prospective cohort study. *Lancet* 2019; published online Sept 3. DOI:10.1016/S0140-6736(19)32008-2.

20 Hystad P, Duong M, Brauer M, *et al.* Health Effects of Household Solid Fuel Use: Findings from 11 Countries within the Prospective Urban and Rural Epidemiology Study. *Environ Health Perspect* 2019; **127**: 57003.

21 Harris PA, Taylor R, Thielke R, Payne J, Gonzalez N, Conde JG. Research Electronic Data Capture (REDCap) - A metadata-driven methodology and workflow process for providing translational research informatics support. *J Biomed Inform* 2009; **42**: 377–81.

22 GBD 2015 Diseases and Injury Incidence and prevalence Collaborators. Global, regional, and national incidence, prevalence, and years lived with disability (YLDs) for 310 acute and chronic diseases and injuries, 1990-2015: a systematic analysis for the Global Burden of Disease Study 2015. *The Lancet Under review*.

23 Flaxman AD, Vos T, Murray CJL, Kiyono P, editors. An integrative metaregression framework for descriptive epidemiology, 1 edition. Seattle: University of Washington Press, 2015.

24 Vasudevan S, Ramos F, Nettleton E, Durrant-Whyte H, Blair A. Gaussian Process modeling of large scale terrain. In: 2009 IEEE International Conference on Robotics and Automation. 2009: 1047–53.

25 Rasmussen CE, Williams CKI. Gaussian Processes for Machine Learning. Cambridge, Mass: The MIT Press, 2005.

26 Ng M, Fleming T, Robinson M, *et al.* Global, regional, and national prevalence of overweight and obesity in children and adults during 1980–2013: a systematic analysis for the Global Burden of Disease Study 2013. *Lancet* 2014; **384**: 766–81.

27 Ng M, Freeman MK, Fleming TD, *et al.* Smoking Prevalence and Cigarette Consumption in 187 Countries, 1980-2012. *JAMA* 2014; **311**: 183–92.

28 Massey Jr FJ. The Kolmogorov-Smirnov test for goodness of fit. *Journal of the American statistical Association* 1951; **46**: 68–78.

29 Vander Hoorn S, Ezzati M, Rodgers A, Lopez AD, Murray CJL. Estimating attributable burden of disease from exposure and hazard data. In: Comparative Quanitifcation of Health Risks: Global and

regional burden of disease attribution to selected major risk factors. World Health Organisation, 2004: 2129–40.

30 Preston SH. Causes and Consequences of Mortality Declines in Less Developed Countries during the Twentieth Century. In: Population and economic change in developing countries. Chicago: Univ. of Chicago Pr, 1980: 289–360.

31 Carnahan E, Lim SS, Nelson EC, *et al.* Validation of a new predictive risk model: measuring the impact of major modifiable risks of death for patients and populations. *The Lancet* 2013; **381**: S26.

32 Danaei G, Singh GM, Paciorek CJ, *et al.* The global cardiovascular risk transition: associations of four metabolic risk factors with national income, urbanization, and Western diet in 1980 and 2008. *Circulation* 2013; **127**: 1493–502, 1502e1-8.

33 Olofin I, McDonald CM, Ezzati M, *et al.* Associations of suboptimal growth with all-cause and cause-specific mortality in children under five years: a pooled analysis of ten prospective studies. *PLoS ONE* 2013; **8**: e64636.

34 McDonald CM, Olofin I, Flaxman S, *et al.* The effect of multiple anthropometric deficits on child mortality: meta-analysis of individual data in 10 prospective studies from developing countries. *Am J Clin Nutr* 2013; **97**: 896–901.

35 Das Gupta P. Standardization and Decomposition of Rates: A User’s Manual. Washington D.C.: U.S. Bureau of the Census, 1993.

Section 4: Risk-specific modelling descriptions

The risk-specific modelling write-ups follow the order of the risk factor hierarchy for GBD 2019. In some cases, multiple risk factors are addressed in a single write-up. For example, all dietary risks are included together in a single detailed write-up. GBD 2019 risk factor appendix write-ups in order:

1. Non-optimal temperature
2. Unsafe water
3. Unsafe sanitation
4. No access to handwashing facility
5. Ambient particulate matter pollution
6. Household air pollution
7. Ambient ozone pollution
8. Radon exposure
9. Lead exposure
10. Occupational risk factors
11. Suboptimal breastfeeding
12. Child growth failure
13. Low birthweight and short gestation
14. Iron deficiency
15. Vitamin A deficiency
16. Zinc deficiency
17. Smoking
18. Secondhand smoke
19. Chewing tobacco
20. Alcohol use
21. Drug use
22. Dietary risks
23. Intimate partner violence
24. Childhood sexual abuse
25. Bullying victimisation
26. Unsafe sex
27. Low physical activity
28. High fasting plasma glucose
29. High LDL cholesterol
30. High systolic blood pressure
31. High body-mass index
32. Bone mineral density
33. Kidney dysfunction

Non-optimal temperature

Flowchart

Non-optimal temperature exposure

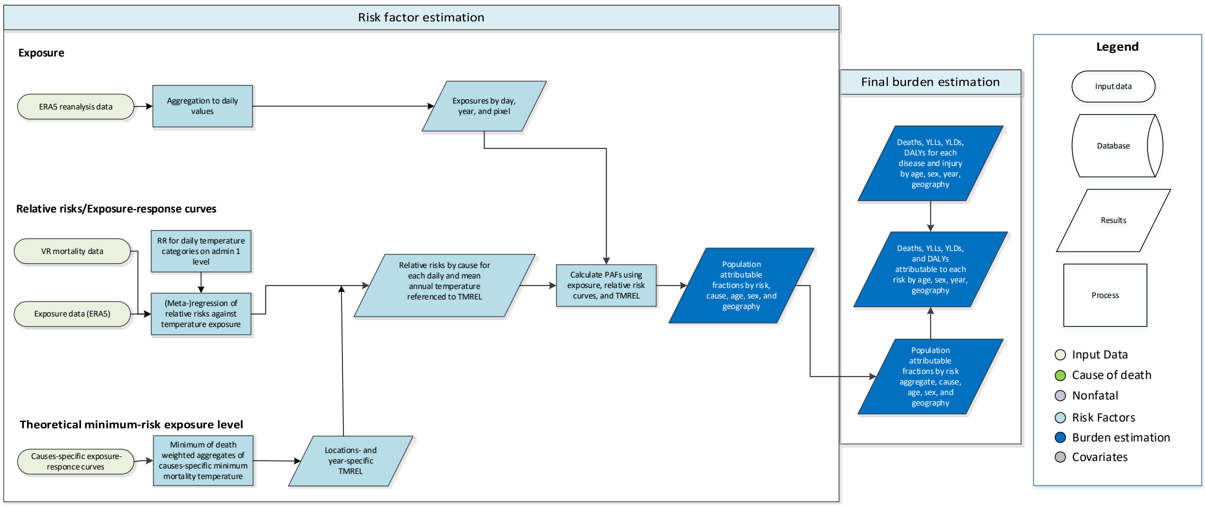

Input data and modelling strategy

Case definition

The exposure of non-optimal temperature is defined as the same day exposure to ambient temperature that is either warmer or colder than the temperature associated with the minimum mortality risk. Specifically, we define the theoretical minimum risk exposure level (TMREL) for temperature as the temperature that is associated with the lowest overall mortality attributable to the risk, in a given location and year. Given varying exposure-response curves for different mean annual temperature zones, as well as spatially and temporally varying cause compositions, we estimate TMRELs by year and location and are not using a globally uniform TMREL. High temperature (heat) exposure is defined as exposure to temperatures warmer than this TMREL and low temperature (cold) is defined as temperatures colder than this TMREL.

Exposure

ERA5 data

We derived exposure estimates from the ERA5 reanalysis dataset from the European Centre for Medium-Range Weather Forecasts (ECMWF). ECMWF produced ERA5 estimates using their Integrated Forecast System (IFS). Hourly values of surface temperature are available for a spatial resolution of 0.25°x0.25°. Uncertainty estimates for these temperature values, ie, the ensemble spread (standard deviation) is available for every 3 hours (00:00, 03:00, 06:00, 09:00, 12:00, 15:00, 18:00, 21:00) for a spatial resolution of 0.5°x0.5°. At the time of analysis, data were available from 1979 to June 2019.[1,2] We calculated daily averages of temperature and spread for each pixel and then assigned an uncertainty value to each daily temperature value. Based on the spread we derived 1,000 draws of each daily temperature pixel.

Population data
Population data for calculating population-weighted location means were derived from WorldPop, which is an open source project initiated in 2013[3]. Multi-temporal, globally consistent, high-resolution human population data at 1 km x 1 km resolution can be downloaded from http://www.worldpop.org.uk/ for 2000, 2005, 2010, 2015, and 2020. For the purpose of our work, we interpolated in-between the 5-year estimation bins to obtain annual data. Further, we extrapolated until 1990 by using the 2000-2005 growth rate for back-casting.

Table 1: Data inputs for exposure for non-optimal temperature.

Input data	Exposure
Source count (total)	203
Number of countries and territories with data	204

Exposure-response modelling

Mortality data
Deaths at the individual-level that included information regarding the cause (ie, ICD code), date, and the location at the second administrative level (admin2) or finer were collected from the GBD cause-of-death (CoD) database for vital registration data sources. We adapted the GBD standard procedure for garbage code redistribution to redistribute daily mortality data rather than annual data and mapped ICD causes to GBD causes for level 3. In total, we analysed 58.9 million deaths from eight different countries and 15,197 administrative units. For Brazil, the data covers a period from 1999 to 2016 for 5,570 municipalities and 19.9 million deaths. For Chile, the data covers the period from 1990 to 1996 and 2009 to 2011 for 15 regions and 2.46 million deaths. For Colombia, the data covers a period from 2001 to 2005 for 1,125 municipalities and 0.95 million deaths. For Guatemala, the data covers a period from 2009 to 2016 for 333 municipalities and 0.49 million deaths. For Mexico, the data covers a period from 1996 to 2015 for 2,438 municipalities and 9.88 million deaths. For New Zealand, the data covers a period from 1988 to 2014 for 20 district health boards and 0.76 million deaths. For the United States, the data covers a period from 1980 to 1988 for 3,124 municipalities and 18.3 million deaths. For China, the data covers the year 2016 for 2,556 counties and 6.1 million deaths.

Exposure-response modelling (MR-BRT)
To estimate cause-specific mortality, based on average daily temperature and temperature zone (defined by mean annual temperature), we used a robust meta-regression framework, implemented through the MR-BRT (Bayesian, regularised, trimmed) tool. The tool allows three features that are essential to the analysis:[5]

- A meta-analytic framework that can handle heterogeneous data sources
- A robust approach to outlier detection and removal (trimming)
- Specification of the functional dependence of outcome vs. average daily temperature and temperature zone as a 2-dimensional surface through a spline interface.

The use of trimming in a vast array of inference and machine learning problems is standard.[6,7,8] The use of high-dimensional splines has been proposed before,[9] but the methods used for estimation go beyond prior work, and we explain them below.

The functional relationship between any outcome y and input variables (t1, t2) models y as a linear combination of 2d spline basis elements. Each spline basis element is a product of individual basis elements for 1D splines for t1 and t2. Therefore, the inference problem looks for a combination of simple curvilinear 2D elements that fit the data while preserving smoothness across element boundaries. The MR-BRT tool also allows prior information to influence the shape of the spline, particularly in areas with sparse data.

For the purpose of modelling the relationship between mortality and mean annual and daily temperature we imposed monotonicity in the direction of daily temperature. For all J-shaped curves that depicted an increase in mortality above and below a threshold, we forced the curve to monotonically decrease at the lower end of the temperature distribution and to monotonically increase at the upper end. For all external causes that displayed a monotonic increase over the entire temperature range, we imposed monotonicity only in the direction of warmer temperatures. We placed 2 knots of degree 3 in the direction of mean annual temperature when fitting the surface. In the direction of daily mean temperature, we placed 3 knots of degree 3 for J-shaped causes and 2 knots of degree 1 for external causes that monotonically increase over temperature range. Figure 1 shows an example of a relative risk (RR) surface along daily and annual mean temperature for drowning.

We estimated uncertainty using a two-step approach. First, we derived the uncertainty of the mean surface from the measurement error using the fit-retrofit error. Second, we added uncertainty from the random effects by sampling it separately from the cold and warm side.

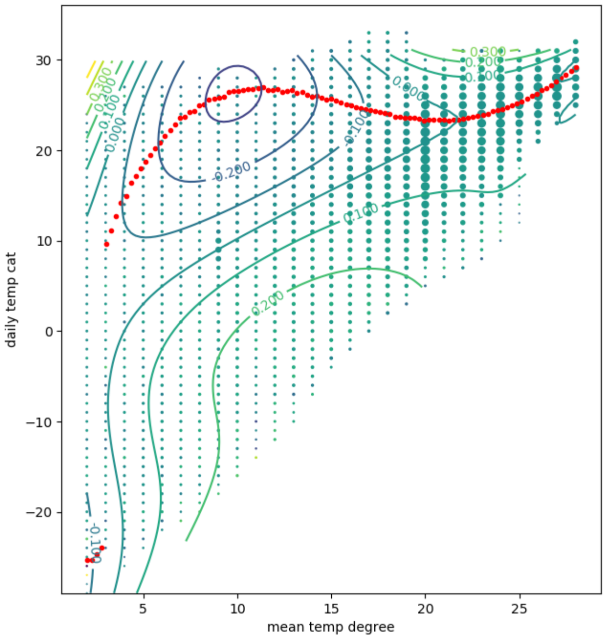

Figure 1: Log relative risk of death from lower respiratory infection along mean annual temperature (mean temp cat) and daily mean temperature (daily temp cat). The red dotted line depicts minimum mortality temperature along mean annual temperature zones. Green and blue lines depict isopleths, ie, lines of equal log RR of mortality

Table 2: Data inputs for relative risks for non-optimal temperature

Input data	Relative risk
Source count (total)	112
Number of countries with data	8

Cause selection

We excluded all causes with fewer than 100,000 deaths as well as causes of death that did not represent a particular entity but rather a summary category (eg, other cardiovascular diseases). Further, dementia and protein energy malnutrition were not considered in this analysis due to inconsistencies in data classification. The remaining causes were selected based on significance. For this, for each cause and each mean temperature zone we determined the widest range of consecutive daily temperatures with statistically significant relative risks, expressed as a percentage of the full range of daily temperatures in that mean temperature zone. Figure 2 gives an example of the temperature-mortality relationship for three selected slices (mean annual temperature of 6 °C, 17 °C and 21 °C). Significant areas along the exposure-response curves are marked in grey. We included all causes where at least 30% of zones had a consecutive significance range that spanned at least 5% of the full range of daily temperatures. Twelve causes met these criteria and were included as outcomes associated with non-optimal temperature: ischaemic heart disease, stroke, hypertensive heart disease, diabetes, chronic kidney disease, lower respiratory infection, chronic obstructive pulmonary disease, homicide, suicide, mechanical injuries, transport-related injuries, and drowning.

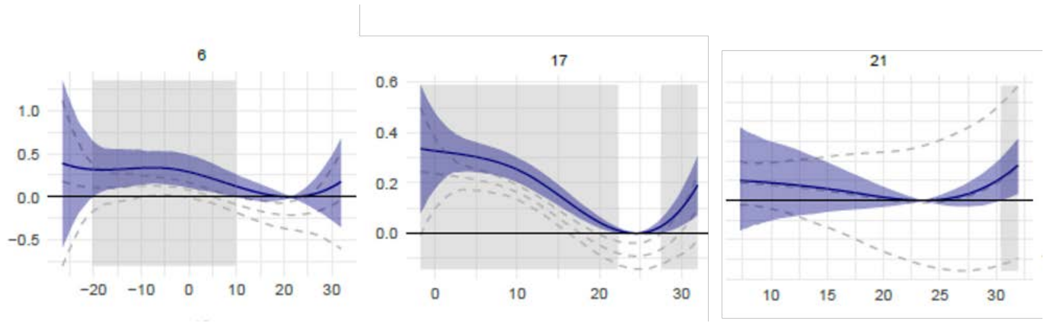

Figure 2: Selected exposure-response curves for the relationship between daily mean temperature and log RR of lower respiratory infection mortality for mean annual temperature categories of 6 °C, 17 °C and 21 °C. Temperatures where associations are significant are displayed in grey.

Theoretical minimum risk exposure level (TMREL)

For the purpose of this analysis, the TMREL was defined as the temperature associated with the lowest mortality for all included causes. We calculated a death-weighted average of the cause-specific exposure-response curves with the minimum of this average curve being the TMREL. This was done for each year and each of the 990 GBD locations using CoD estimates produced for the GBD 2019 study. As climate zones or mean annual temperature can vary within a location, we calculated the TMREL for every mean annual temperature, assuming a consistent cause composition within a location. This approach represents the first use of spatially and temporally varying TMRELs within the GBD study.

Population attributable fractions

The population attributable fraction (PAF) was calculated for each temperature pixel and each day of the year (ie, pixel-day). Subsequently, we population-weighted each pixel using the fraction of the population living in a given pixel relative to the GBD location. Depending on whether the daily mean temperature was below or above the TMREL, the effect was assigned to either low or high temperature. Daily population-weighted high and low temperature PAFs were then aggregated for the location and the year. Temperature effects can be either harmful or protective depending on whether the RR is above or below 1. For harmful temperature effects, ie, effects with a RR above 1, we used the following equation to derive PAFs: PAF=(RR-1)/RR; For temperature effects exhibiting a protective effect the equation was adapted by implementing the reverse RR: PAF=-((1/RR)-1)/(1/RR). The PAF associated with non-optimal temperature exposure is an aggregate of heat and cold effects in each location and year. We estimated the temperature attributable burden as the product of the total burden for that cause and the corresponding PAF for each GBD location, year, age group, and sex.

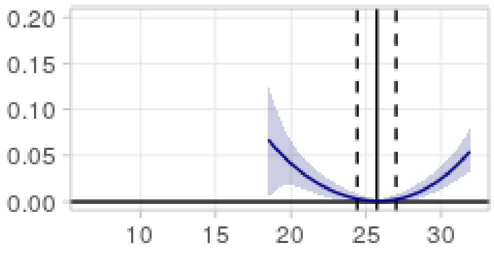

Figure 3: Schematic illustration of the exposure-response relationship between temperature and mortality and associated low temperature (cold) and high temperature effects beyond the theoretical minimum exposure level (TMREL). The blue line depicts the exposure-response curve with blue shaded line showing 95% uncertainty range. The black solid line depicts the TMREL with dashed black lines displaying 95% uncertainty range. Effects left of the TMREL are counted towards cold PAFs and right of the TMREL towards heat PAFS.

References

1 Hersbach H, Bell B, Berrisford P, et al. Global reanalysis : goodbye Global reanalysis : goodbye ERA-Interim , hello. 2019. DOI:10.21957/vf291hehd7.

2 Copernicus Climate Change Service (C3S) (2017): ERA5: Fifth generation of ECMWF atmospheric reanalyses of the global climate. Copernicus Climate Change Service Climate Data Store (CDS), September 2019. .

3 Geography and Environmental Science, University of Southampton. Age and Sex Structures, Global Per Country 2000-2020 - WorldPop. Southampton , United Kingdom: Geography and Environmental Science, University of Southampton, 2018. .

4 Roth GA, Abate D, Hassen Abate K, et al. Global, regional, and national age-sex-specific mortality for 282 causes of death in 195 countries and territories, 1980-2017: a systematic analysis for the Global Burden of Disease Study 2017 GBD 2017 Causes of Death Collaborators*. 2018 DOI:10.1016/S0140-6736(18)32203-7.

5 Zheng P, Aravkin AY, Barber R, Sorensen RJD, Murray CJL. Trimmed Constrained Mixed Effects Models: Formulations and Algorithms. 2019; published online Sept 23. http://arxiv.org/abs/1909.10700 (accessed Nov 13, 2019).

6 Rousseeuw PJ. Least Median of Squares Regression. J Am Stat Assoc 1984; 79: 871–80.

7 Aravkin A, Davis D. Trimmed Statistical Estimation via Variance Reduction. Math Oper Res 2019; : moor.2019.0992.

8 Yang E, Lozano AC, Aravkin A. A general family of trimmed estimators for robust high-dimensional data analysis. Electron J Stat 2018; 12: 3519–53.

9 Pya N, Wood SN. Shape constrained additive models. Stat Comput 2015; 25: 543–59.

Unsafe water

Flowchart

Unsafe Drinking Water

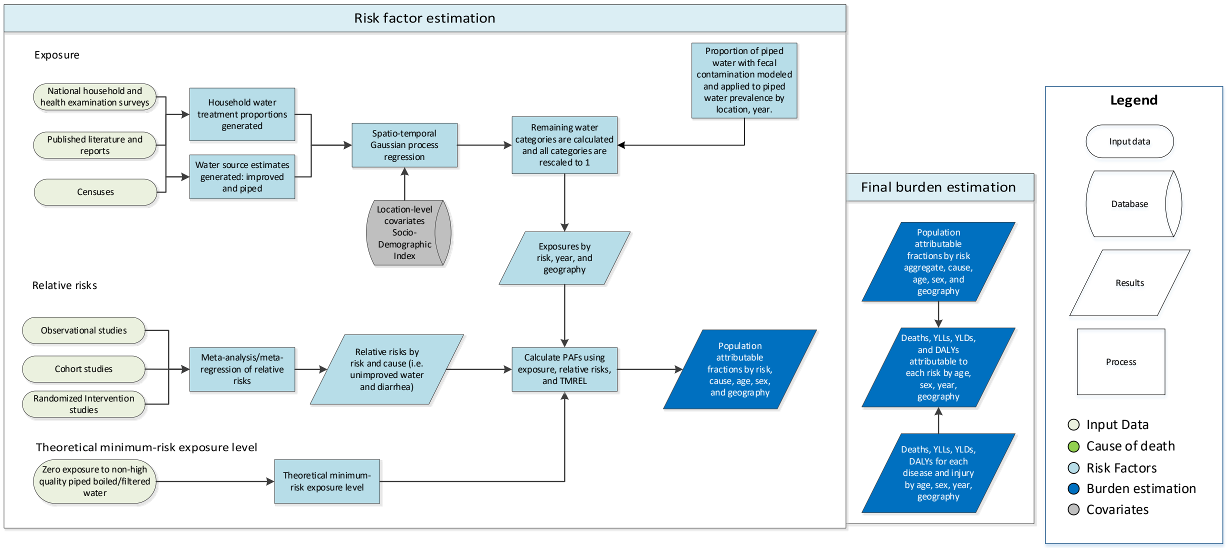

Input data & methodological summary

Exposure

Case definition

For GBD 2019, exposure to unsafe water was defined based on: (1) reported primary water source used by the household, and (2) use of household water treatment (HWT) to improve the quality of drinking water before consumption. Water sources were defined based on the WHO/UNICEF Joint Monitoring Programme for Water Supply, Sanitation and Hygiene (JMP).[1] Examples of "improved" sources include boreholes, tube wells, protected wells, and packaged or delivered water. Piped water is also considered "improved" by the JMP, but is placed into its own category for GBD purposes. Examples of "unimproved" sources include unprotected springs, unprotected wells, and surface water. Additionally, four different HWTs were determined to be effective point-of-use treatments based on effect sizes calculated from a network meta-analysis: solar treatment, chlorine treatment, boiling, and filtering. For modelling purposes, we grouped solar and chlorine treatment together, as well as boiling and filtering.

Input data

The search for usable household surveys and censuses was conducted using the Global Health Data Exchange (GHDx) database. Water source input data came primarily from nationally representative surveys, such as the Demographic and Health Survey (DHS), the Multiple Indicator Cluster Surveys (MICS), the World Health Survey (WHS), and the DHS AIDS Indicator Survey (AIS). HWT input data was largely limited to the DHS and MICS due to data availability. For each survey, reported household sample weights were multiplied by the number of household members to produce a weighting scheme that estimated proportion of individuals, not proportion of households, exposed to a given indicator. Surveys and censuses were then tabulated to the water source and water treatment categories of interest for each location. Table 1 provides a summary of the exposure input data.

Table 1: Exposure input data

Input data	Exposure
Source count (total)	1157
Number of countries with data	169

Modelling strategy

Water source data was modelled using an ordinal framework, with two distinct models: (1) prevalence of piped water, and (2) proportion of improved water (excluding piped) within the nonpiped population. Both models produced results for each unique location-year combination. This ordinal framework allowed estimating the category with the most data (piped water prevalence) and leveraging that estimate to anchor the estimates for the improved and unimproved water categories. The results of the improved proportion model were multiplied by 1 minus the piped water prevalence to calculate improved water prevalence. The sum of improved and piped water prevalence was then subtracted from 1 to yield unimproved water prevalence.

$$\boldsymbol{Piped} = \frac{\#\ persons\ using\ piped\ water}{\#\ persons\ with\ nonmissing\ response}$$

$$\boldsymbol{Improved} = \left(\frac{\#\ persons\ using\ improved\ water}{\#\ persons\ without\ piped\ water}\right) * (1 - \boldsymbol{Piped})$$

$$\boldsymbol{Unimproved} = 1 - (\boldsymbol{Piped} + \boldsymbol{Improved})$$

HWT categories were estimated in a similar ordinal framework. Its two models were: (1) prevalence of individuals using no water treatment methods, and (2) proportions of households that boil/filter water within the population of households that engage in treatment methods. The prevalence of individuals who boil/filter drinking water was calculated by multiplying the proportion who boil/filter modelled previously multiplied by the prevalence of any water treatment (estimated by subtracting the prevalence of no treatment from 1). The prevalence of individuals who treat their water using solar/chlorine methods was estimated by subtracting from 1 the sum of prevalence of no treatment estimates and prevalence of filter/boil treatment.

$$\boldsymbol{No\ HWT} = \frac{\#\ persons\ who\ do\ not\ treat\ water}{\#\ persons\ with\ nonmissing\ response}$$

$$\boldsymbol{Boil\ or\ filter} = \left(\frac{\#\ persons\ who\ treat\ water\ with\ boil\ or\ filter}{\#\ persons\ who\ treat\ water\ with\ any\ method}\right) * (1 - \boldsymbol{No\ HWT})$$

$$\boldsymbol{Chlorine\ or\ solar} = 1 - (\boldsymbol{No\ HWT} + \boldsymbol{Boil\ or\ filter})$$

Additionally, we modelled the microbiological quality of piped water sources primarily using data from a review by Bain et al 2014[2] that measured the proportion of piped water sources contaminated with faecal matter. We used the value generated from this model to split the prevalence of piped water into basic piped water and high-quality piped water by location, year, age, and sex. High-quality piped water is piped water that enters the household free of contamination. Thus, HWT is irrelevant for this category, since treatment is only necessary if the water is contaminated.

$$\boldsymbol{Faecal\ contamination} = \frac{\#\ contaminated\ piped\ water\ systems}{\#\ piped\ water\ systems\ sampled}$$

$$\boldsymbol{Basic\ piped} = \boldsymbol{Piped} * \boldsymbol{Faecal\ contamination}$$

$$\boldsymbol{HQ\ piped} = \boldsymbol{Piped} - \boldsymbol{Basic\ piped}$$

Each of the models described above was modelled by year and location using a three-step modelling scheme of mixed-effect linear regression followed by spatiotemporal Gaussian process regression (ST-GPR), which produced full time-series estimates for each GBD 2019 location. Socio-demographic Index (SDI), a composite metric combining education per capita, income per capita, and fertility, was set as a fixed effect in the linear regression since it proved to be a significant predictor. The proportion of individuals with access to piped water was also used as a covariate in the faecal matter model. Random effects were set at GBD 2019 region and super-region levels to fit the models but were not used in the predictions. The linear regression equations for each of the five ST-GPR models used for this risk factor are listed below.

Proportion using piped water: $logit(data) \sim SDI + (1|level\_1) + (1|level\_2)$

Proportion of nonpiped population using improved water: $logit(data) \sim SDI + (1|level\_1) + (1|level\_2)$

Proportion using no HWT: $logit(data) \sim SDI + (1|level\_1) + (1|level\_2)$

Proportion of HWT-using population that boils/filters: $logit(data) \sim SDI + (1|level\_1) + (1|level\_2)$

Proportion of piped water systems contaminated with faecal matter: $logit(data) \sim SDI + piped\ water\ access + (1|level\_1) + (1|level\_2)$

Piped water access = proportion of individuals with access to piped water
(1|level_1) = super-region-level random effects
(2|level_2) = region-level random effects

The process of vetting and validating models was accomplished primarily through an examination of ST-GPR scatter plots by GBD 2019 location from 1990 to 2019. Any poorly fitting datapoints were re-inspected for error at the level of extraction and survey implementation. If errors in data extraction were found, the study in question was re-extracted. In addition to SDI, a number of different potential fixed effects were considered, including lag-distributed income and urbanicity, but SDI proved to be the strongest predictor of the unsafe water categories. Uncertainty in the estimates was initially formed based on standard deviation by survey, then propagated through ST-GPR modelling by means of confidence intervals around each datapoint that reflected the point-estimate specific variance.

Once models were vetted, full time-series outputs from ST-GPR modelling were converted from proportion to prevalence by year and geography and then rescaled to form 10 mutually exclusive categories that summed to 1. Table 2 provides the final result of this rescaling and also includes the formulas for each category.

Table 2: Exposure categories, definitions, and formulas

Exposure category	Definition	Formula
Unimproved, no HWT	Proportion of individuals who primarily use unimproved source and *do not* use any HWT to purify their drinking water.	$[1-(Piped+Improved)] * \left[\frac{\text{\# persons who do not treat water}}{\text{\# persons with nonmissing response}}\right]$
Unimproved, chlorine/solar	Proportion of individuals who primarily use unimproved source, and who use solar or chlorine treatment, to purify their drinking water.	$[1-(Piped+Improved)] * [1-(No\ HWT+Boil\ or\ filter)]$
Unimproved, boil/filter	Proportion of individuals who primarily use unimproved source and who boil or filter to purify their drinking water.	$[1-(Piped+Improved)] * \left[\left(\frac{\text{\# persons who treat water with boil or filter}}{\text{\# persons who treat water with any method}}\right) * (1-No\ HWT)\right]$
Improved water except piped, no HWT	Proportion of individuals who primarily use improved sources other than piped water supply and *do not* use any HWT to purify their drinking water.	$\left[\left(\frac{\text{\# persons using improved water}}{\text{\# persons without piped water}}\right)\right] * \left[\frac{\text{\# persons who do not treat water}}{\text{\# persons with nonmissing response}}\right]$
Improved water except piped, chlorine/solar	Proportion of individuals who primarily use improved sources other than piped water supply, and who use solar or chlorine treatment, to purify their drinking water.	$\left[\left(\frac{\text{\# persons using improved water}}{\text{\# persons without piped water}}\right)\right] * [1-(No\ HWT+Boil\ or\ filter)]$
Improved water except piped, boil/filter	Proportion of individuals who primarily use improved sources other than piped water supply and who boil/filter their drinking water.	$\left[\left(\frac{\text{\# persons using improved water}}{\text{\# persons without piped water}}\right)\right] * \left[\left(\frac{\text{\# persons who treat water with boil or filter}}{\text{\# persons who treat water with any method}}\right) * (1-No\ HWT)\right]$
Basic piped water, no HWT	Proportion of individuals who primarily use basic piped water supply and *do not* use any HWT to purify their drinking water.	$\left[\left[\frac{\text{\# persons using piped water}}{\text{\# persons with nonmissing response}}\right] * \left[\frac{\text{\# contaminated piped water systems}}{\text{\# piped water systems sampled}}\right]\right] * \left[\frac{\text{\# persons who do not treat water}}{\text{\# persons with nonmissing response}}\right]$
Basic piped water, chlorine/solar	Proportion of individuals who primarily use basic piped water supply, and who *use* solar or chlorine water treatment, to purify their drinking water.	$\left[\left[\frac{\text{\# persons using piped water}}{\text{\# persons with nonmissing response}}\right] * \left[\frac{\text{\# contaminated piped water systems}}{\text{\# piped water systems sampled}}\right]\right] * [1-(No\ HWT+Boil\ or\ filter)]$
Basic piped water, boil/filter	Proportion of individuals who primarily use basic piped water supply and who boil or filter to purify their drinking water.	$\left[\left[\frac{\text{\# persons using piped water}}{\text{\# persons with nonmissing response}}\right] * \left[\frac{\text{\# contaminated piped water systems}}{\text{\# piped water systems sampled}}\right]\right] * \left[\left(\frac{\text{\# persons who treat water with boil or filter}}{\text{\# persons who treat water with any method}}\right) * (1-No\ HWT)\right]$
High-quality piped water	Proportion of individuals who primarily use high-quality piped water.	$\left[\frac{\text{\# persons using piped water}}{\text{\# persons with nonmissing response}}\right] - \left[\left[\frac{\text{\# persons using piped water}}{\text{\# persons with nonmissing response}}\right] * \left[\frac{\text{\# contaminated piped water systems}}{\text{\# piped water systems sampled}}\right]\right]$

Theoretical minimum-risk exposure level

The theoretical minimum-risk exposure level for unsafe water is defined as having access to high-quality piped water.

Relative risks

For GBD 2019, unsafe water was paired with one outcome – diarrheal diseases – given evidence provided by relative risk studies. A meta-analysis by Wolf et al, 2014[3] was complemented by a literature review that searched PubMed for related intervention studies post-2014. Search terms used were identical to those provided by Wolf et al, 2014.[3] Additionally, for GBD 2019, new relative risk evidence was added using an updated version of the 2014 Wolf et al meta-analysis that was published in 2018.[4] Table 3 provides a summary of the relative risk data.

Table 3: Relative risk input data

Input data	Relative risk
Source count (total)	71
Number of countries with data	35

In GBD 2019, relative risk values were calculated using a network meta-analysis approach with MR-BRT. Three study-level covariates: (1) whether or not the study was generalisable to the general population, (2) whether or not the measurements used were based on self-reports, and (3) percentage of participants lost to follow-up – were included in the network meta-analysis. No priors were used. Table 2 explains each covariate used in more depth. The risk of developing diarrhoea – relative to using an unimproved water source – was calculated for each of the following categories: boil/filter, solar/chlorine, improved, piped, and high-quality piped. The combined effect of source interventions (ie, improved, piped, high-quality piped) and point-of-use interventions (ie, boil/filter, solar/chlorine) were assumed to be multiplicative. Table 4 shows the results of the network meta-analysis, and Table 5 shows the final relative risks used during modelling for each exposure category. Figure 1 also shows the funnel plot of the MR-BRT network meta-analysis. Note that the values in Table 6 are relative to a reference category of boiled/filtered high-quality piped water, as we assume that the lowest possible risk level is using both the best source type (high-quality piped water) and the best point-of-use treatment (boil/filter).

Table 4: Covariates used in MR-BRT models

Covariate	Description
Study generalisation	0 = study sample based on general population<br>1 = study sample based on subgroups, eg, high-risk groups, pregnant women, hospital patients, etc.
Measurements based on self-reports	0 = measurements based on assays, tests, or physician observation<br>1 = measurements based on self-report
Loss to follow-up	0 = > 95% follow-up<br>1 = 85–95% follow-up<br>2 = < 85% follow-up

Table 5: MR-BRT network meta-analysis results (reference: unimproved water source)

Intervention	Relative risk (95% UI)
Boil/filter water treatment	0.485 (0.421–0.558)
Chlorine/solar water treatment	0.725 (0.665–0.792)
Improved water source	0.827 (0.764–0.907)
Piped water source	0.636 (0.557–0.727)
High-quality piped water source	0.204 (0.086–0.485)

Table 6: Relative risks for each exposure category (reference: high-quality piped water)

Exposure category	Relative risk (95% UI)
Unimproved, no HWT	11.084 (4.287–22.867)
Unimproved, chlorine/solar	8.024 (3.203–16.486)
Unimproved, boil/filter	5.331 (2.153–10.887)
Improved water except piped, no HWT	8.986 (3.898–17.446)
Improved water except piped, chlorine/solar	6.505 (2.905–12.528)
Improved water except piped, boil/filter	4.322 (1.954–8.243)
Basic piped water, no HWT	6.880 (3.104–13.016)
Basic piped water, chlorine/solar	4.980 (2.320–9.296)
Basic piped water, boil/filter	3.309 (1.564–6.129)
High-quality piped water	1 (reference)

Figure 1: MR-BRT network meta-analysis funnel plot

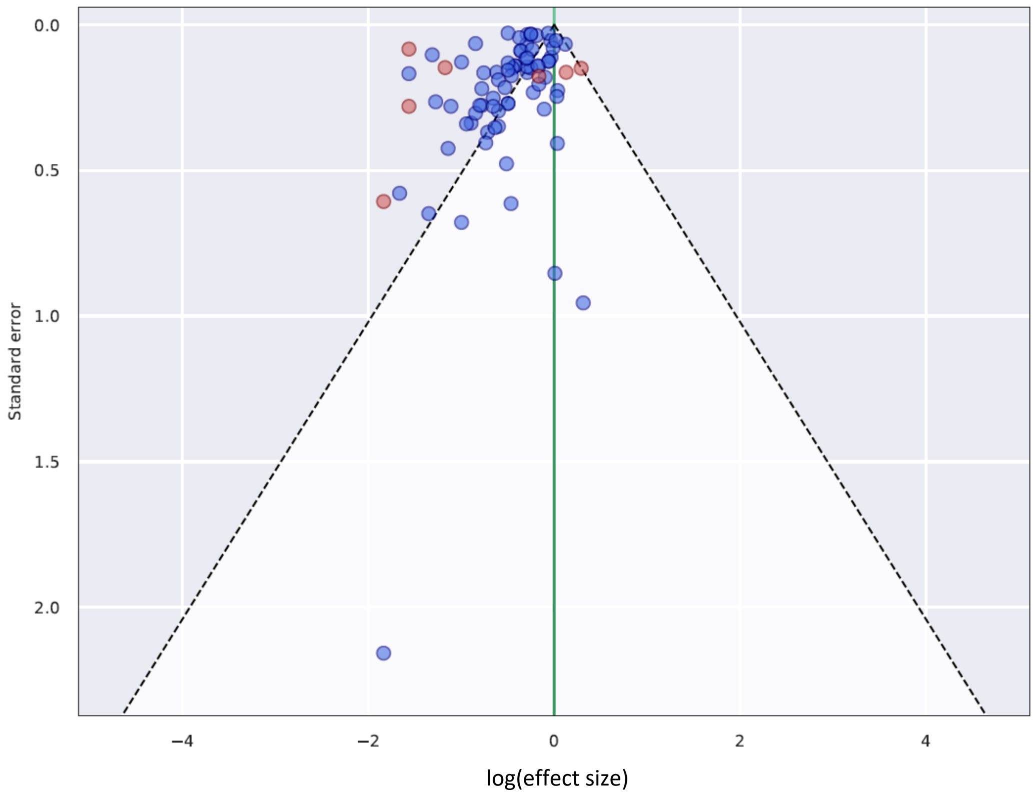

Red points indicate values that were trimmed

References

1. WHO/UNICEF Joint Monitoring Programme: Drinking water. https://washdata.org/monitoring/drinking-water (accessed Oct 31, 2019)
2. Bain R, Cronk R, Wright J, Yang H, Slaymaker T, Bartram J. Fecal Contamination of Drinking-Water in Low- and Middle-Income Countries: A Systematic Review and Meta-Analysis. *PLOS Medicine* 2014; **11**: e1001644.
3. Wolf J, Pruss-Ustun A, Cumming O, *et al.* Assessing the impact of drinking water and sanitation on diarrhoeal disease in low- and middle-income settings: systematic review and meta-regression. *Tropical Medicine and International Health* 2014; **19**: 928–42.
4. Wolf J, Hunter PR, Freeman MC, *et al.* Impact of drinking water, sanitation and handwashing with soap on childhood diarrhoeal disease: updated meta-analysis and meta-regression. *Tropical Medicine and International Health* 2018; **23**: 508–25.

Unsafe sanitation

Flowchart

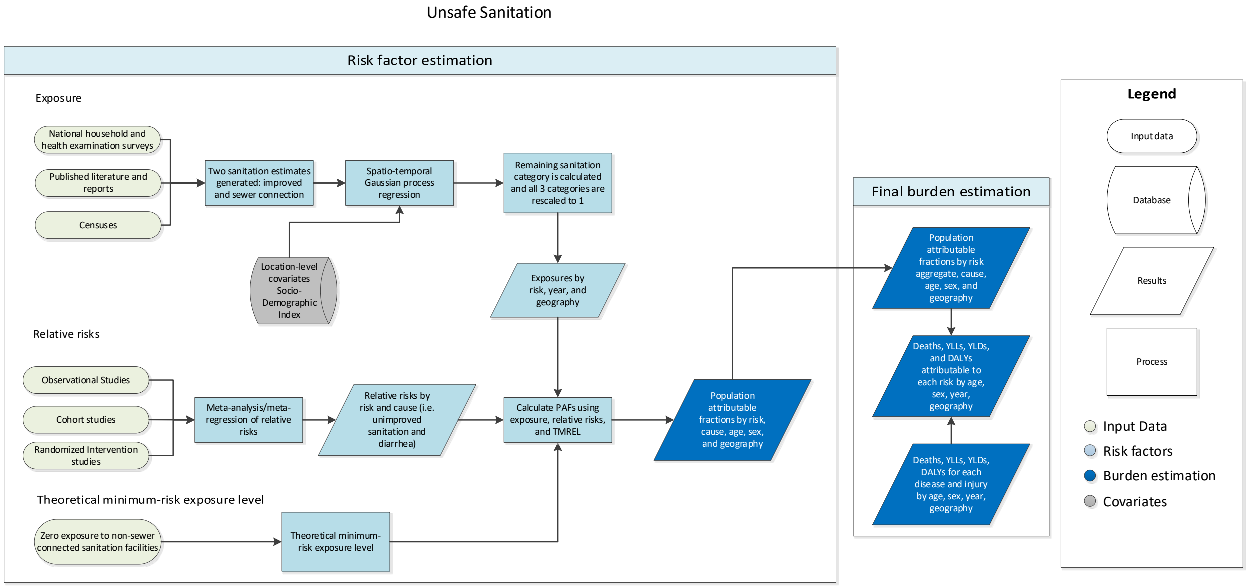

Input data and methodological summary

Exposure

Case definition

Exposure to unsafe sanitation is defined based on the primary toilet type used by households. For GBD 2019, we modelled three different categories of sanitation: unimproved, improved, and facilities with a sewer connection or septic tank. These categories were defined according to the WHO/UNICEF Joint Monitoring Programme for Water Supply, Sanitation and Hygiene (JMP).[1] Examples of "improved" sanitation facilities include ventilated improved pit latrines, composting toilets, and pit latrines with slabs. Examples of "unimproved" facilities include open pit latrines, open defecation, and toilets that flush into creeks or open fields. Sewer connection toilets include flush toilets or any toilet with connection to the sewer or septic tank.

Input data

The search for usable household surveys and censuses was conducted using the Global Health Data Exchange (GHDx) database. For each survey, household sample weights were multiplied by the number of household members to produce a weighting scheme that estimates proportion of individuals, not proportion of households, exposed to a given indicator. Surveys and censuses were then tabulated to two sanitation categories, sewer connection and improved sanitation, for each location and year. Table 1 provides a summary of the input data used.

Table 1: Exposure input data

Input data	Exposure
Source count (total)	1073
Number of countries with data	157

Modelling

For GBD 2019, sanitation was modelled in an ordinal framework. Two distinct indicators were estimated: (1) the proportion of individuals using sewer connection or septic tank facilities and (2) the proportion of individuals with improved sanitation within the population not connected to a sewer or septic tank. This ordinal framework allows us to estimate the category with the most data (sewer connection/septic tank prevalence) and leverage that estimate to anchor the estimates for the improved and unimproved sanitation categories. The results of the improved-proportion model are multiplied by one minus the sewer connection/septic tank prevalence to calculate improved sanitation prevalence. The sum of improved and sewer connection/septic tank prevalence are subtracted from 1 to yield unimproved sanitation prevalence.

$$\boldsymbol{Sewer} = \frac{\#\, persons\ with\ sewer\ or\ septic\ connection}{\#\, persons\ with\ nonmissing\ response}$$

$$\boldsymbol{Improved} = \left(\frac{\#\, persons\ using\ improved\ facilities}{\#\, persons\ without\ sewer\ or\ septic\ connection}\right) * (1 - \boldsymbol{Sewer})$$

$$\boldsymbol{Unimproved} = 1 - (\boldsymbol{Sewer} + \boldsymbol{Improved})$$

The two indicators were each modeled using a three-step modelling scheme of mixed effect linear regression followed by spatiotemporal Gaussian process regression (ST-GPR), which produced full time-series estimates for each GBD 2019 location. Socio-demographic Index (SDI), a composite metric combining education per capita, income per capita, and fertility, was set as a fixed effect in the linear regression since it proved to be a significant predictor. Random effects were set at GBD 2019 region and super-region levels to fit the models but were not used in the predictions. The same linear regression equation was used for both ST-GPR models (see below).

$$logit(data) \sim sdi + (1|level\_1) + (1|level\_2)$$

SDI = socio-demographic index
(1|level_1) = super-region-level random effects
(2|level_2) = region-level random effects

The process of vetting and validating models was accomplished primarily through an examination of ST-GPR scatterplots by GBD 2019 location from 1990 to 2019. Any poorly fitting datapoints were re-inspected for error at the level of extraction and survey implementation. If errors in data extraction were found, the study in question was re-extracted. In addition to SDI, a number of different potential fixed effects were considered, including lag-distributed income and urbanicity, but SDI proved to be the strongest predictor of unsafe sanitation in terms of magnitude of the coefficient. Uncertainty in the estimates was initially constructed based on standard deviation around each survey mean, then propagated through ST-GPR modelling by incorporating the variance of each datapoint in the Gaussian process regression step. A datapoint with high variance, for example, would contribute relatively less influence to the model than a datapoint with lower variance.

Once models are vetted, full time-series outputs from ST-GPR modelling are then converted from proportion to prevalence by year and geography and then rescaled using the above equations to form three mutually exclusive categories that sum up to 1. Table 2 provides the final result of this rescaling.

Table 2: Exposure categories and definitions

Category	Definition
Unimproved sanitation	Proportion of individuals that use unimproved sanitation facilities.
Improved sanitation	Proportion of individuals that use improved sanitation facilities, excluding sewer connection or septic tank.
Sanitation facilities with sewer connection or septic tank	Proportion of individuals that use toilet facilities with sewer connection or septic tank.

Theoretical minimum-risk exposure level

The theoretical minimum-risk exposure level for unsafe sanitation was defined as having access to a sanitation facility with sewer connection or septic tank.

Relative risks

For GBD 2019, unsafe sanitation was paired with one outcome, diarrhoeal diseases A meta-analysis by Wolf and colleagues 2014[2] was complemented by a literature review that searched for related intervention studies post-2014 conducted in PubMed. Search terms used were identical to those provided by Wolf and colleagues 2014.[2] This literature review yielded two additional sources not included in the Wolf and colleagues meta-analysis. Additionally, for GBD 2019, we added new relative risk evidence using an updated version of the 2014 Wolf and colleagues meta-analysis that was published in 2018.[3] A total of three new sources were added this cycle. Table 3 provides a summary of the relative risk data used.

Table 3: Relative risk input data

**Input data**	**Relative risk**
Source count (total)	16
Number of countries with data	13

In GBD 2019, relative risk values were calculated using a network meta-analysis approach with MR-BRT. Two study-level covariates – one for whether or not the study was generalisable to the general population, and one for whether or not the measurements used were based on self-reports – were included in the network meta-analysis. No priors were used. We calculated the risk of developing diarrhoea for those using unimproved sanitation facilities and improved sanitation facilities, relative to the reference category of those using facilities with a sewer or septic tank. Table 4 explains each covariate used in more depth. Table 5 shows the results of the MR-BRT analyses, and Figure 1 shows the associated funnel plot. Table 6 shows the relative risks that were ultimately used for modelling. Note that while the MR-BRT analyses used unimproved sanitation as the reference, the relative risks used for modelling use sewer or septic tank as the reference.

Table 4: Covariates used in MR-BRT models

Covariate	Description
Measurements based on self-reports	0 = measurements based on assays, tests, or physician observation<br>1 = measurements based on self-report
Study generalisation	0 = study sample based on general population

	1 = study sample based on subgroups, eg, high-risk groups, pregnant women, hospital patients, etc.

Table 5: Relative risk network meta-analysis results (reference: unimproved sanitation)

Exposure category	Beta coefficient, log (95% CI)	Exponentiated beta (95% CI)
Improved sanitation	-0.261 (-0.310, -0.212)	0.770 (0.809, 0.733)
Sanitation facilities with sewer connection or septic tank	-1.162 (-1.260, -1.067)	0.312 (0.284, 0.344)

Table 6: Relative risks for each exposure category (reference: sewer or septic tank)

Exposure category	Relative risk (95% CI)
Unimproved sanitation	3.203 (2.897, 3.513)
Improved sanitation	2.465 (2.349, 2.587)
Sanitation facilities with sewer connection or septic tank	1 (reference)

Figure 1: MR-BRT network meta-analysis funnel plot

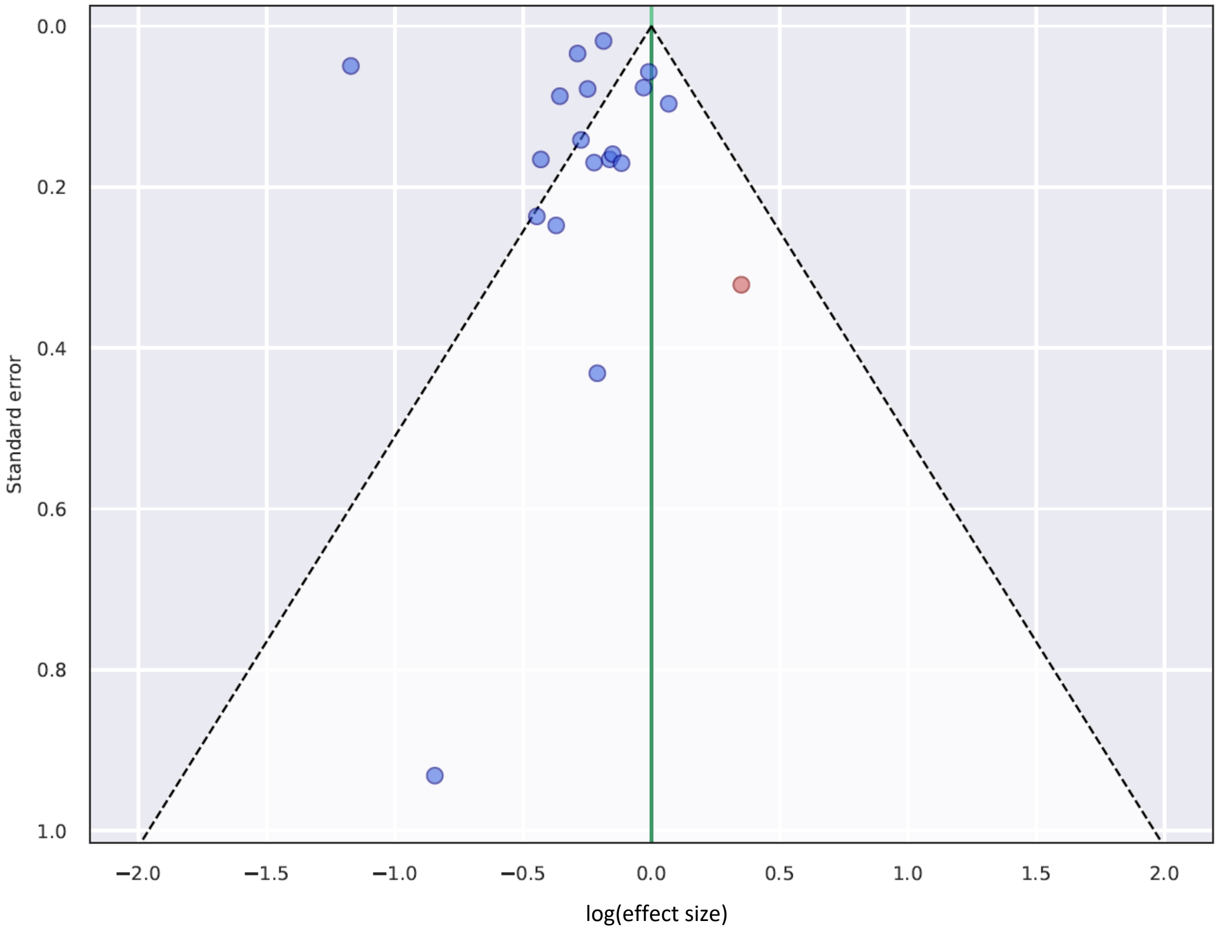

Red points indicate values that were trimmed

References

1. WHO/UNICEF Joint Monitoring Programme: Sanitation. https://washdata.org/monitoring/sanitation (accessed Oct 31, 2019)
2. Wolf J, Pruss-Ustun A, Cumming O, *et al.* Assessing the impact of drinking water and sanitation on diarrhoeal disease in low- and middle-income settings: systematic review and meta-regression. *Tropical Medicine and International Health* 2014; **19**: 928–42.
3. Wolf J, Hunter PR, Freeman MC, *et al.* Impact of drinking water, sanitation and handwashing with soap on childhood diarrhoeal disease: updated meta-analysis and meta-regression. *Tropical Medicine and International Health* 2018; **23**: 508–25.

No access to handwashing facility

Flowchart

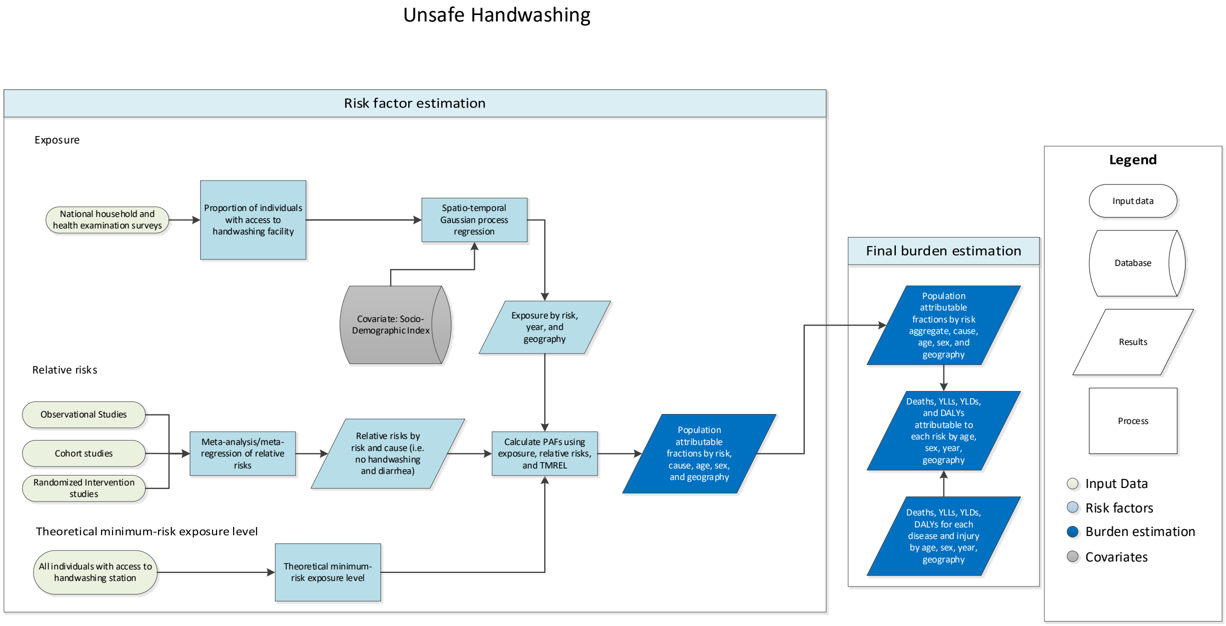

Input data and methodological summary

Exposure

Case definition

This risk is defined as lack of access to a handwashing station with available soap and water.

Input data

Since water and soap availability data are very limited, only country-specific Demographic and Health Surveys (DHS), Multiple Indicator Cluster Surveys (MICS), and Performance Monitoring and Accountability 2020 (PMA2020) surveys conducted from 2000 through 2016 were included as input data. Table 1 provides a summary of the exposure input data.

Table 1: Exposure input data

**Input data**	**Exposure**
Source count (total)	98
Number of countries with data	65

Modelling strategy

By year and location, proportion of households with a handwashing facility is modelled using a three-step modelling scheme of mixed effect linear regression followed by spatiotemporal Gaussian process regression (ST-GPR), which outputs full time-series estimates for each GBD 2019 location. Two covariates were used as fixed effects in the linear regression: Socio-demographic Index (SDI – a composite index that includes income per capita, education, and fertility) and proportion of individuals with access to piped water (see below for model equation). Random effects were set at GBD 2019 region and super-region levels to fit the model but were not used in the predictions.

$$logit(data) \sim sdi + piped\ water\ access + (1|level\_1) + (1|level\_2)$$

SDI = socio-demographic index
Piped water access = proportion of individuals with access to piped water
(1|level_1) = super-region-level random effects
(2|level_2) = region-level random effects

The process of vetting and validating models was accomplished primarily through an examination of ST-GPR scatterplots by GBD 2019 location from 1990 to 2019. Based on visual inspection, any poorly fitting datapoints were re-inspected for error at the level of extraction and survey implementation. If errors in data extraction were found, the study in question was re-extracted. In addition to SDI, a number of different potential fixed effects were considered, including lag-distributed income and urbanicity. However, SDI proved to be the strongest predictor.

Data sparseness was a considerable limitation in our modelling process. Even when data were published on handwashing prevalence, the definition as used in the publication often differed from the GBD 2019 standard definition or the data lacked representativeness at the geographical scale we required. The incorporation of questions about soap and water availability in DHS and MICS added much-needed information, but there remains a large data gap to be filled to reduce uncertainty in the estimation of access to handwashing stations.

Theoretical minimum-risk exposure level

The theoretical minimum-risk exposure level for unsafe hygiene is defined as having access to handwashing facility after any contact with excreta, including children's excreta.

Relative risks

For GBD 2019, unsafe hygiene was paired with two outcomes: diarrhoeal diseases and lower respiratory infections (LRI). A meta-analysis by Cairncross and colleagues 2010[1] provided relative risk values describing the relationship between lack of facility access and diarrhoeal diseases. A meta-analysis by Rabie & Curtis 2006[2] provided relative risk evidence for the relationship between lack of facility access and LRI. Table 2 provides a summary of the relative risk data.

Table 2: Relative risk input data

**Input data**	**Relative risk**
Source count (total)	29
Number of countries with data	19

In GBD 2019, relative risk values were calculated using MR-BRT. For the diarrhoea model, four study-level covariates were included: whether or not the measurements used were based on self-reports, whether or not the study was randomised, how many confounders were controlled for, and percentage of participants lost to follow-up. No priors were used. For the LRI model, four study-level covariates were included: whether or not the study was generalisable to the general population, whether or not the measurements used were based on self-reports, whether or not the outcome was blind to the individual level of exposure, and percentage of participants lost to follow-up. No priors were used. Table 3 explains each covariate used in more depth. Table 4 shows the results of the MR-BRT analyses, and

Figures 1 and 2 show the associated funnel plots. Table 5 shows the relative risks that were ultimately used for modelling. Note that while the MR-BRT analyses used no access to handwashing facility as the reference, the relative risks used for modelling use access to handwashing facility as the reference.

Table 3: Covariates used in MR-BRT models

Covariate	Description
Measurements based on self-reports	0 = measurements based on assays, tests, or physician observation<br>1 = measurements based on self-report
Study randomisation	0 = randomised study<br>1 = non-randomised study
Confounders	0 = randomised study or non-randomised study in which outcome is controlled for all critical confounders<br>1 = non-randomised study in which outcome is controlled for three or more critical confounders<br>2 = non-randomised study in which outcome is controlled for two or fewer critical confounders<br><br>Note: critical confounders included age, sex, education, and income
Loss to follow-up	0 = > 95% follow-up<br>1 = 85-95% follow-up<br>2 = < 85% follow-up
Study generalisation	0 = study sample based on general population<br>1 = study sample based on subgroups, eg, high-risk groups, pregnant women, hospital patients, etc.
Outcome/exposure blinded	0 = outcome was blind to individual level of exposure (or vice versa)<br>1 = unblinded

Table 4: Relative risk MR-BRT results (reference: no access to handwashing facility)

Outcome	Beta coefficient, log (95% CI)	Exponentiated beta (95% CI)
Diarrhoeal diseases	-0.420 (-0.503, -0.338)	0.657 (0.605, 0.713)
Lower respiratory infections	-0.277 (-0.430, -0.124)	0.758 (0.650, 0.883)

Table 5: Relative risks for each outcome (reference: access to handwashing facility)

Outcome	Relative risk (95% CI)
Diarrhoeal diseases	1.522 (1.400, 1.644)
Lower respiratory infections	1.321 (1.125, 1.533)

Figure 1: MR-BRT funnel plot, diarrhoeal diseases

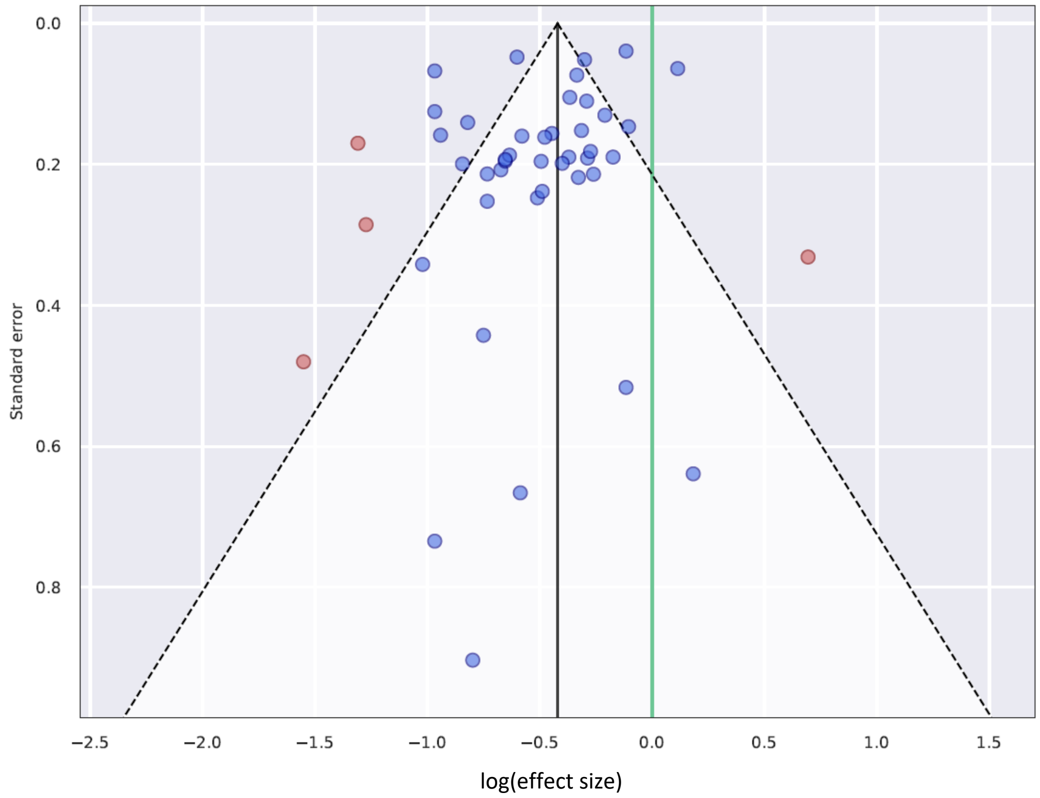

Red points indicate values that were trimmed

Figure 2: MR-BRT funnel plot, lower respiratory infections

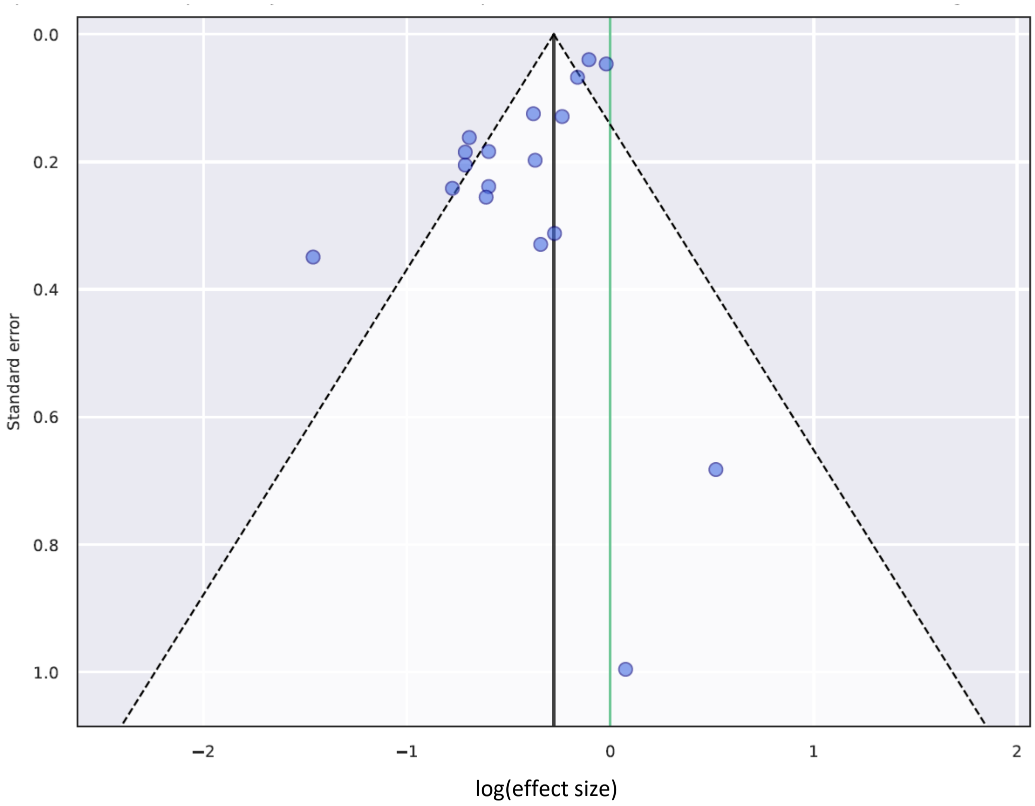

References

1. Cairncross S, Hunt C, Boisson S, *et al.* Water, sanitation and hygiene for the prevention of diarrhoea. *International Journal of Epidemiology* 2010; **39**: i193–205.

2. Rabie T, Curtis V. Handwashing and risk of respiratory infections: a quantitative systematic review. *Tropical Medicine and International Health* 2006; **11**: 258–67.

Ambient particulate matter pollution

Flowchart

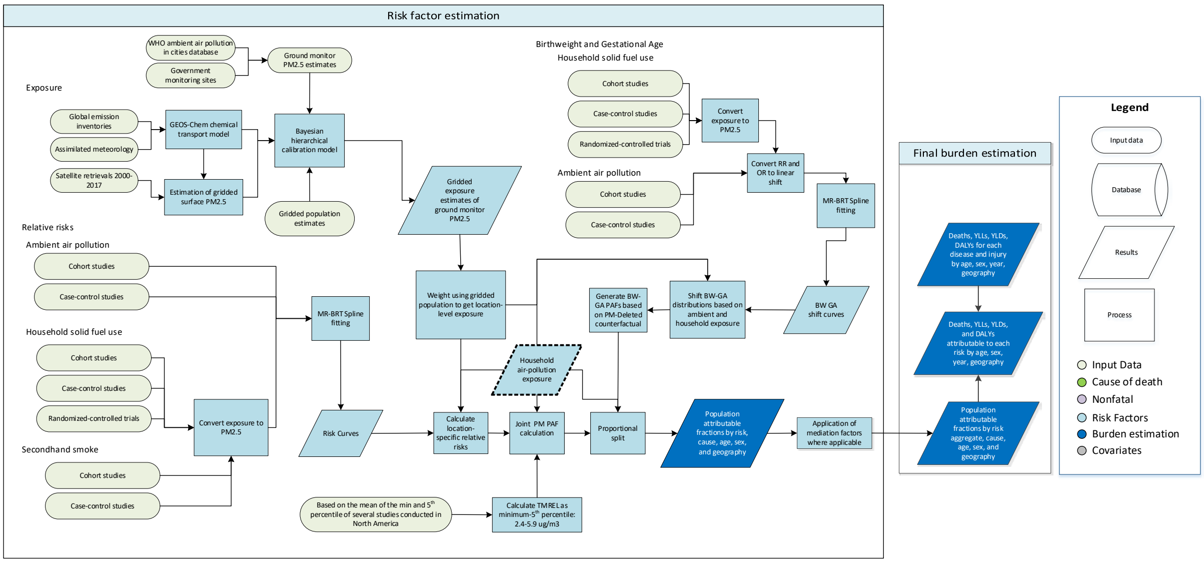

Input data and modelling strategy

Exposure

Definition

Exposure to ambient particulate matter pollution is defined as the population-weighted annual average mass concentration of particles with an aerodynamic diameter less than 2.5 micrometers ($PM_{2.5}$) in a cubic meter of air. This measurement is reported in µg/m^3.

Input data

The data used to estimate exposure to ambient particulate matter pollution comes from multiple sources, including satellite observations of aerosols in the atmosphere, ground measurements, chemical transport model simulations, population estimates, and land-use data. Table 1 summarizes exposure input data.

Table 1: Exposure Input Data

Input data	Exposure
Source count (total)	663
Number of countries with data	114

The following details the updates in methodology and input data used in GBD 2019.

$PM_{2.5}$ ground measurement database

Ground measurements used for GBD 2019 include updated measurements from sites included in 2017 and additional measurements from new locations. New and up-to-date data (mainly from the USA, Canada, EU, Bangladesh, China and USA embassies and consulates), were added to the data

from the 2018 update of the WHO Global Ambient Air Quality Database used in GBD 2017. The updated data included measurements of concentrations of PM_{10} and $PM_{2.5}$ from 10,408 ground monitors from 116 countries from 2010 to 2017. The majority of measurements were recorded in 2016 and 2017 (as there is a lag in reporting measurements, few data from 2018 or newer were available). Annual averages were excluded if they were based on less than 75% coverage within a year. If information on coverage was not available, then data were included unless there were already sufficient data within the same country (monitor density greater than 0.1).

For locations measuring only PM_{10}, $PM_{2.5}$ measurements were estimated from PM_{10}. This was performed using a hierarchy of conversion factors ($PM_{2.5}/PM_{10}$ ratios): (i) for any location a 'local' conversation factor was used, constructed as the ratio of the average measurements (of $PM_{2.5}$ and PM_{10}) from within 50km of the location of the PM_{10} measurement, and within the same country, if such measurements were available; (ii) if there was not sufficient local information to construct a conversion factor then a country-wide conversion factor was used; and (iii) if there was no appropriate information within a country, then a regional factor was used. In each case, to avoid the possible effects of outliers in the measured data (both $PM_{2.5}$ and PM_{10}), extreme values of the ratios were excluded (defined as being greater/lesser than the 95% and 5% quantiles of the empirical distributions of conversion factors). As with GBD 2013, 2015, 2016, and 2017 databases, in addition to values of $PM_{2.5}$ and whether they were direct measurement or converted from PM_{10}, the database also included additional information, where available, related to the ground measurements such as monitor geo-coordinates and monitor site type.

Satellite-based estimates
The global geophysical $PM_{2.5}$ estimates for the years 2000–2017 are from Hammer and colleagues Version V4.GL.03.NoGWR used at 0.1°x0.1° resolution (~11 x 11 km resolution at the equator).[1] The method is based on the algorithms of van Donkelaar and colleagues (2016) as used in GBD 2017,[2] with updated satellite retrievals, chemical transport modelling, and ground-based monitoring. The algorithm uses aerosol optical depth (AOD) from several updated satellite products (MAIAC, MODIS C6.1, and MISR v23), including finer resolution, increased global coverage, and improved long-term stability. Ground-based observations from a global sunphotometer network (AERONET version 3) are used to combine different AOD information sources. This is the first time that data from MAIAC at 1 km resolution was used to estimate $PM_{2.5}$ at the global scale. The GEOS-Chem chemical transport model with updated algorithms was used for geophysical relationships between surface $PM_{2.5}$ and AOD. Updates to the GEOS-Chem simulation included improved representation of mineral dust and secondary organic aerosol, as well as updated emission inventories. The resultant geophysical $PM_{2.5}$ estimates are highly consistent with ground monitors worldwide (R^2=0.81, slope = 1.03, n = 2541).

Population data
A comprehensive set of population data, adjusted to match UN2015 Population Prospectus, on a high-resolution grid was obtained from the Gridded Population of the World (GPW) database. Estimates for 2000, 2005, 2010, 2015, and 2020 were available from GPW version 4, with estimates for 1990 and 1995 obtained from the GPW version 3. These data are provided on a 0.0083°× 0.0083° resolution. Aggregation to each 0.1°×0.1° grid cell was accomplished by summing the central 12 × 12 population cells. Populations estimates for 2001–2004, 2006–2009, 2011–2014 and 2016–2019 were obtained by interpolation using natural splines with knots placed at 2000, 2005, 2010, 2015, and 2020. This was performed for each grid cell.

Chemical transport model simulations

Estimates of the sum of particulate sulfate, nitrate, ammonium, and organic carbon and the compositional concentrations of mineral dust simulated using the GEOS Chem chemical transport model, and a measure combining elevation and the distance to the nearest urban land surface (as described in van Donkelaar and colleagues 2016[2] and Hammer and colleagues (submitted))[1] were available for 2000–2017 for each 0.1°×0.1° grid cell.

Modelling strategy

The following is a summary of the modelling approach, known as the Data Integration Model for Air Quality (DIMAQ) used in GBD 2015, 2016, 2017, and now in GBD 2019.[3,4]

Before the implementation of DIMAQ (ie, in GBD 2010 and GBD 2013), exposure estimates were obtained using a single global function to calibrate available ground measurements to a "fused" estimate of $PM_{2.5}$; the mean of satellite-based estimates and those from the TM5 chemical transport model, calculated for each 0.1°×0.1° grid cell. This was recognised to represent a tradeoff between accuracy and computational efficiency when utilising all the available data sources. In particular, the GBD 2013 exposure estimates were known to underestimate ground measurements in specific locations (see discussion in Brauer and colleagues, 2015).[5] This underestimation was largely due to the use of a single, global calibration function, whereas in reality the relationship between ground measurements and other variables will vary spatially.

In GBD 2015 and GBD 2016, coefficients in the calibration model were estimated for each country. Where data were insufficient within a country, information can be "borrowed" from a higher aggregation (region) and, if enough information is still not available, from an even higher level (super-region). Individual country-level estimates were therefore based on a combination of information from the country, its region, and its super-region. This was implemented within a Bayesian hierarchical modelling (BHM) framework. BHMs provide an extremely useful and flexible framework in which to model complex relationships and dependencies in data. Uncertainty can also be propagated through the model, allowing uncertainty arising from different components, both data sources and models, to be incorporated within estimates of uncertainty associated with the final estimates. The results of the modelling comprise a posterior distribution for each grid cell, rather than just a single point estimate, allowing a variety of summaries to be calculated. The primary outputs here are the median and 95% credible intervals for each grid cell. Based on the availability of ground measurement data, modelling and evaluation were focused on the year 2016.

The model used in GBD 2017 and GBD 2019 also included within-country calibration variation.[6] The model used for GBD 2019, henceforth referred to as DIMAQ2, provides a number of substantial improvements over the initial formulation of DIMAQ. In DIMAQ, ground measurements from different years were all assumed to have been made in the primary year of interest and then regressed against values from other inputs (eg, satellites, etc.) made in that year. In the presence of changes over time, therefore, and particularly in areas where no recent measurements were available, there was the possibility of mismatches between the ground measurements and other variables. In DIMAQ2, ground measurements were matched with other inputs (over time), and the (global-level) coefficients were allowed to vary over time, subject to smoothing that is induced by a first-order random walk process. In addition, the manner in which spatial variation can be incorporated within the model has developed: where there are sufficient data, the calibration equations can now vary (smoothly) both within and between countries, achieved by allowing the coefficients to follow (smooth) Gaussian processes. Where there are insufficient data within a

country, to produce accurate equations, as before, information is borrowed from lower down the hierarchy and it is supplemented with information from the wider region.

DIMAQ2 as described above is used for all regions except for the north Africa and Middle East and sub-Saharan Africa super-regions, where there are insufficient data across years to allow the extra complexities of the new model to be implemented. In these super-regions, a simplified version of DIMAQ2 is used in which the temporal component is dropped.

Model evaluation

Model development and comparison was performed using within- and out-of-sample assessment. In the evaluation, cross-validation was performed using 25 combinations of training (80%) and validation (20%) datasets. Validation sets were obtained by taking a stratified random sample, using sampling probabilities based on the cross-tabulation of $PM_{2.5}$ categories (0-24.9, 25-49.9, 50-74.9, 75-99.9, 100+ μg/m^3) and super-regions, resulting in them having the same distribution of $PM_{2.5}$ concentrations and super-regions as the overall set of sites. The following metrics were calculated for each training/evaluation set combination: for model fit – R^2 and deviance information criteria (DIC, a measure of model fit for Bayesian models); for predictive accuracy – root mean squared error (RMSE) and population weighted root mean squared error (PwRMSE). The median R^2 was 0.9, and the median PwRMSE was 10.1 μg/m^3.

All modelling was performed on the log-scale. The choice of which variables were included in the model was made based on their contribution to model fit and predictive ability. The following is a list of variables and model structures that were included in DIMAQ.

Continuous explanatory variables:

- (SAT) Estimate of $PM_{2.5}$ (in μg/m^3) from satellite remote sensing on the log-scale.
- (POP) Estimate of population for the same year as SAT on the log-scale.
- (SNAOC) Estimate of the sum of sulfate, nitrate, ammonium, and organic carbon simulated using the GEOS Chem chemical transport model.
- (DST) Estimate of compositional concentrations of mineral dust simulated using the GEOS-Chem chemical transport model.
- (EDxDU) The log of the elevation difference between the elevation at the ground measurement location and the mean elevation within the GEOS Chem simulation grid cell multiplied by the inverse distance to the nearest urban land surface.

Discrete explanatory variables:

- (LOC) Binary variable indicating whether exact location of ground measurement is known.
- (TYPE) Binary variable indicating whether exact type of ground monitor is known.
- (CONV) Binary variable indicating whether ground measurement is $PM_{2.5}$ or converted from PM_{10}.

Interactions*:*

- Interactions between the binary variables and the effects of SAT.

Random effects:

- Regional temporal (random walk) hierarchical random-effects on the intercept
- Regional hierarchical random-effects for the coefficient associated with SAT
- Regional hierarchical random-effects for the coefficient associated with POP
- Smoothed, spatially varying random-effects for the intercept

- Smoothed, spatially varying random-effects for the coefficient associated with SAT

Inference and prediction

Due to both the complexity of the models and the size of the data, notably the number of spatial predictions that are required, recently developed techniques that perform "approximate" Bayesian inference based on integrated nested Laplace approximations (INLA) were used.[7] Computation was performed using the R interface to the INLA computational engine (R-INLA). GBD 2019 also makes use of an innovation in the way that samples from the (Bayesian) model are used to represent distributions of estimated concentrations in each grid-cell. Here estimates, and distributions representing uncertainty, of concentrations for each grid are obtained by taking repeated (joint) samples from the posterior distributions of the parameters and calculating estimates based on a linear combination of those samples and the input variables.[8]

DIMAQ2 was used to produce estimates of ambient $PM_{2.5}$ for 1990, 1995, and 2010–2019 by matching the gridded estimates with the corresponding coefficients from the calibration. As there is a lag in reporting ambient air pollution based quantities, the input variables were extrapolated (as in GBD 2017), allowing estimates for 2018 and 2019 to be produced in the same way as other years and, crucially, allowing measures of uncertainty to be produced within the BHM framework rather than by using post-hoc approximations.

Estimates from the satellites and the GEOS-Chem chemical transport model in 2018 and 2019 were produced by extrapolating estimates from 2000–2017 using generalised additive models,[9] on a cell-by-cell basis, except in those grid cells that saw a >100% increase between 2016 and 2017, in which case only the 2000–2016 estimates were used for extrapolating, in order to avoid unrealistic and/or unjustified extrapolation of trends. Population estimates for 2018 and 2019 were obtained by interpolation as described above.

Theoretical minimum-risk exposure level

The TMREL was assigned a uniform distribution with lower/upper bounds given by the average of the minimum and fifth percentiles of outdoor air pollution cohort studies exposure distributions conducted in North America, with the assumption that current evidence was insufficient to precisely characterise the shape of the concentration-response function below the fifth percentile of the exposure distributions. The TMREL was defined as a uniform distribution rather than a fixed value in order to represent the uncertainty regarding the level at which the scientific evidence was consistent with adverse effects of exposure. The specific outdoor air pollution cohort studies selected for this averaging were based on the criteria that their fifth percentiles were less than that of the American Cancer Society Cancer Prevention II (CPSII) cohort's fifth percentile of 8.2 based on Turner and colleagues (2016).[10] This criterion was selected since GBD 2010 used the minimum, 5.8, and fifth percentile solely from the CPS II cohort. The resulting lower/upper bounds of the distribution for GBD 2019 were 2.4 and 5.9. This has not changed since GBD 2015.

Relative risks and population attributable fractions

We create one set of cause-specific risk curves for both household air pollution and ambient air pollution as two different sources of $PM_{2.5}$. In GBD 2017, we estimated the particulate matter-attributable burden of disease based on the relation of long-term exposure to PM2.5 with Ischemic Heart Disease, stroke (ischemic and hemorrhagic), COPD, lung cancer, acute lower respiratory infection, and Type II Diabetes. In GBD 2019, we added adverse birth outcomes including low birthweight and short gestation. Because these are already risk factors (and not outcomes) in the

GBD, we performed a mediation analysis, in which a proportion of the burden attributable to low birthweight and short gestation was attributed to $PM_{2.5}$ pollution.

For the six non-mediated outcomes, we used results from cohort and case-control studies of ambient $PM_{2.5}$ pollution, cohort studies, case-control studies, and randomised-controlled trials of household use of solid fuel for cooking, and cohort and case-control studies of secondhand smoke. For the first time in GBD 2019, we no longer use active smoking data in the risk curves

For GBD 2019, we made several important changes to the risk functions. Previously, we have used relative risk estimates for active smoking, converting cigarettes-per-day to $PM_{2.5}$ exposure in order to estimate the $PM_{2.5}$ relative risk at the highest end of the $PM_{2.5}$ exposure-response curve. We took this approach because the vast majority of the air pollution epidemiological studies have been performed in low-pollution settings in high-income countries, preventing us from extrapolating the steep relationship at the beginning of the exposure range to locations with high exposure but no relative risk estimates, such as India and China. However, with the recent publication of studies in China and other higher-exposure settings and additional studies of HAP, we have been able to include more estimates at high $PM_{2.5}$ levels in the model.[11,12,13,14,15] Furthermore, in contrast to previous cycles of the GBD where the power function used to develop the IER required the inclusion of active smoking data to anchor the risk function, with the current use of splines and their flexibility, it is easier to fit functions to the (ambient, household, and SHS) data without active smoking data. Beginning in GBD 2019, we excluded active smoking studies from the risk curves. Removal of active smoking information removes an important source of uncertainty in our earlier estimates related to differences in dose rates and other aspects of exposure between active smoking and the other $PM_{2.5}$ sources, including differences in voluntary (active smoking) and involuntary (ambient and household $PM_{2.5}$, secondhand smoke) exposure.[16,17]

Additionally, in the past, we have built the curves for ischaemic heart disease and stroke based on studies of mortality and used evidence from three studies of both mortality and incidence to scale down the mortality curves to generate estimates of incidence risk. This year we extracted incidence and mortality from all available studies and included this as a covariate in the model. There was no significant difference between estimates of incidence risk and mortality risk, so we included both types of risk estimates in the curve fitting and used the same curve for both incidence and mortality. This is what was done for all other outcomes in the past and in GBD 2019.

For cardiovascular diseases, evidence suggests that the relative risk decreases with age.[18] To account for this in our model, we generate unique risk curves for every five-year age group from 25–29 to 95 and older for both ischaemic heart disease and stroke. Because we do not have risk data for every unique age group, we adjust each study based on the median age during follow-up to generate a full adjusted dataset for every curve. We calculate the median age of follow-up by taking the median (or mean) age at enrollment and adding one-half of median or mean follow-up time. If follow-up time is not available, we take 70% of total study period based on the observed ratio of follow-up time to total study period for other studies.

Once we have a median age during follow-up (a), we extrapolate each study to the full set of ages where the estimated datapoint for age, a_j, is calculated with the following equation and accompanying explanatory figure:

$$\log(RR)_{a_j} = \frac{\log(RR)_a - 0}{a - 110} * (a_j - 110)$$

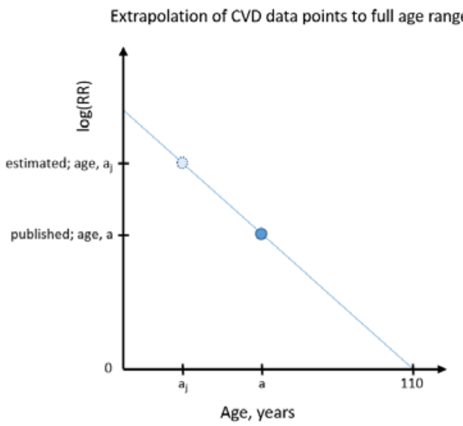

Previously we have used a fixed functional form to fit the risk curves.[16] In GBD 2019, we used MR-BRT (described in detail elsewhere) splines to fit the risk data with a more flexible shape. While previously we built in the TMREL estimates into the model fitting, this year we have fit the curve beginning at zero exposure and incorporate the TMREL into the relative risk calculation process. This allows others to use our risk curves with whatever counterfactual level is of interest to them. Relative risk curves are available upon request.

When fitting the risk curves, we consider the published relative risk over a range of exposure data. For OAP studies, the relative risk informs the curve from the fifth to the 95th percentile of observed exposure. When this is not available in the published study, we estimate the distribution from the provided information (mean and standard deviation, mean and IQR, etc.). We scale the RR to this range.

For HAP studies, we allow each study to inform the curve from the Exp_{OAP} to $Exp_{OAP}+Exp_{HAP}$, where Exp_{OAP} is the GBD 2017 estimate of the ambient exposure level in the study location and year, and Exp_{HAP} is the GBD 2017 estimate of the excess exposure for those who use solid fuel for cooking in the study location and year.

For SHS studies, we updated our strategy of exposure estimation in GBD 2019. For the first time, we are also accounting for outdoor exposure. Similar to the approach used for HAP, we allow each study to inform the curve from the Exp_{OAP} to $Exp_{OAP}+Exp_{SHS}$, where Exp_{OAP} is the GBD 2017 estimate of the ambient exposure level in the study location and year, and Exp_{SHS} is an estimate of the excess exposure for those who experience secondhand smoke. This is estimated from the number of cigarettes smoked per smoker per day in a given location and year, estimated by the smoking team of GBD, and from a study in Sweden, which measured the $PM_{2.5}$ exposure in homes of smokers.[19] We divided the household $PM_{2.5}$ exposure level by the average number of cigarettes smoked per smoker per day in Sweden over the study duration to estimate the SHS $PM_{2.5}$ exposure per cigarette (2.31 µg/m^3 [95% UI 1.53–3.39]). To calculate Exp_{SHS} we multiplied the estimated number of cigarettes per smoker per day by the average $PM_{2.5}$ exposures per cigarette to generate a predicted $PM_{2.5}$ exposure level.

MR-BRT risk splines

We fit splines on the datasets including studies of OAP, HAP, and SHS using the following functional form, where X and X_{CF} represent the range of exposure characterised by the effect size:

$$log\left(\frac{MRBRT(X)}{MRBRT(X_{CF})}\right) \sim \log(Published\ Effect\ Size)$$

For each of the risk-outcome pairs, we tested various model settings and priors in fitting the MR-BRT splines. The final models used third-order splines with two interior knots and a constraint on the right-most segment, forcing the fit to be linear rather than cubic. We used an ensemble approach to knot placement, wherein 100 different models were run with randomly placed knots and then combined by weighting based on a measure of fit that penalises excessive changes in the third derivative of the curve. Knots were free to be placed anywhere within the fifth and 95th percentile of the data, as long as a minimum width of 10% of that domain exists between them. We included shape constraints so that the risk curves were concave down and monotonically increasing, the most biologically plausible shape for the $PM_{2.5}$ risk curve. On the non-linear segments, we included a Gaussian prior on the third derivative of mean 0 and variance 0.01 to prevent over-fitting; on the linear segment, a stronger prior of mean 0 and variance 1e-6 was used to ensure that the risk curves do not continue to increase beyond the range of the data.

For chronic obstructive pulmonary disease, we used a looser Gaussian prior of mean 0 and variance 1e-4 on the linear segment of the risk function. For this outcome, we have epidemiological evidence from household air pollution that the risk continues to increase at higher levels of $PM_{2.5}$.

Table 2 summarizes relative risk input data for ambient particulate matter pollution and household air pollution.

Table 2: Relative Risk Input Data

Input data	Relative risk
Source count (total)	200
Number of countries with data	40

The following table includes all ambient and household sources used in generating risk curves.

Source	Citation
1	Abusalah A, Gavana M, Haidich AB, Smyrnakis E, Papadakis N, Papanikolaou A, Benos A. Low birth weight and prenatal exposure to indoor pollution from tobacco smoke and wood fuel smoke: a matched case-control study in Gaza Strip. Matern Child Health J. 2012; 16(8): 1718-27.
2	Akhtar T, Ullah Z, Khan MH, Nazli R. Chronic bronchitis in women using solid biomass fuel in rural Peshawar, Pakistan. Chest. 2007; 132(5): 1472–5.
3	Alam DS, Chowdhury MAH, Siddiquee AT, Ahmed S, Hossain MD, Pervin S, Streatfield K, Cravioto A, Niessen LW. Adult Cardiopulmonary Mortality and Indoor Air Pollution: A 10-Year Retrospective Cohort Study in a Low-Income Rural Setting. Glob Heart. 2012; 7(3): 215–21.
4	Alexander DA, Northcross A, Karrison T, Morhasson-Bello O, Wilson N, Atalabi OM, Dutta A, Adu D, Ibigbami T, Olamijulo J, Adepoju D, Ojengbede O, Olopade CO. Pregnancy outcomes and ethanol cook stove intervention: A randomized-controlled trial in Ibadan, Nigeria. Environ Int. 2018; 111: 152-163.
5	Al-Sonboli N, Hart CA, Al-Aghbari N, Al-Ansi A, Ashoor O, Cuevas LE. Human metapneumovirus and respiratory syncytial virus disease in children, Yemen. Emerg Infect Dis. 2006; 12(9): 1437–9.
6	Atkinson RW, Carey IM, Kent AJ, van Staa TP, Anderson HR, Cook DG. Long-term exposure to outdoor air pollution and the incidence of chronic obstructive pulmonary disease in a national English cohort. Occup Environ Med. 2015; 72(1): 42–8.
7	Azizi BH, Zulkifli HI, Kasim MS. Protective and risk factors for acute respiratory infections in hospitalized urban Malaysian children: a case control study. Southeast Asian J Trop Med Public Health. 1995; 26(2): 280–5.
8	Balakrishnan K, Ghosh S, Thangavel G, Sambandam S, Mukhopadhyay K, Puttaswamy N, Sadasivam A, Ramaswamy P, Johnson P, Kuppuswamy R, Natesan D, Maheshwari U, Natarajan A, Rajendran G, Ramasami R, Madhav S, Manivannan S, Nargunanadan S, Natarajan S, Saidam S, Chakraborty M, Balakrishnan L,

Source	Citation
	Thanasekaraan V. Exposures to fine particulate matter (PM2.5) and birthweight in a rural-urban, mother-child cohort in Tamil Nadu, India. Environ Res. 2018; 161: 524–31.
9	Basu R, Harris M, Sie L, Malig B, Broadwin R, Green R. Effects of fine particulate matter and its constituents on low birth weight among full-term infants in California. Environ Res. 2014; 128: 42–51.
10	Beelen R, Hoek G, van den Brandt PA, Goldbohm RA, Fischer P, Schouten LJ, Jerrett M, Hughes E, Armstrong B, Brunekreef B. Long-Term Effects of Traffic-Related Air Pollution on Mortality in a Dutch Cohort (NLCS-AIR Study) [Unpublished data]. Environ Health Perspect. 2008; 116(2): 196–202.
11	Beelen R, Hoek G, van den Brandt PA, Goldbohm RA, Fischer P, Schouten LJ, Jerrett M, Hughes E, Armstrong B, Brunekreef B. Long-Term Effects of Traffic-Related Air Pollution on Mortality in a Dutch Cohort (NLCS-AIR Study). Environ Health Perspect. 2008; 116(2): 196–202.
12	Beelen R, Stafoggia M, Raaschou-Nielsen O, Andersen ZJ, Xun WW, Katsouyanni K, Dimakopoulou K, Brunekreef B, Weinmayr G, Hoffmann B, Wolf K, Samoli E, Houthuijs D, Nieuwenhuijsen M, Oudin A, Forsberg B, Olsson D, Salomaa V, Lanki T, Yli-Tuomi T, Oftedal B, Aamodt G, Nafstad P, De Faire U, Pedersen NL, Östenson CG, Fratiglioni L, Penell J, Korek M, Pyko A, Eriksen KT, Tjønneland A, Becker T, Eeftens M, Bots M, Meliefste K, Wang M, Bueno-de-Mesquita B, Sugiri D, Krämer U, Heinrich J, de Hoogh K, Key T, Peters A, Cyrys J, Concin H, Nagel G, Ineichen A, Schaffner E, Probst-Hensch N, Dratva J, Ducret-Stich R, Vilier A, Clavel-Chapelon F, Stempfelet M, Grioni S, Krogh V, Tsai MY, Marcon A, Ricceri F, Sacerdote C, Galassi C, Migliore E, Ranzi A, Cesaroni G, Badaloni C, Forastiere F, Tamayo I, Amiano P, Dorronsoro M, Katsoulis M, Trichopoulou A, Vineis P, Hoek G. Long-term exposure to air pollution and cardiovascular mortality: an analysis of 22 European cohorts. Epidemiology. 2014; 25(3): 368–378.
13	Bell ML, Belanger K, Ebisu K, Gent JF, Lee HJ, Koutrakis P, Leaderer BP. Prenatal Exposure to Fine Particulate Matter and Birth Weight: Variations by Particulate Constituents and Sources. Epidemiology. 2010; 21(6): 884–91.
14	Bell ML, Ebisu K, Belanger K. Ambient Air Pollution and Low Birth Weight in Connecticut and Massachusetts. Environ Health Perspect. 2007; 115(7): 1118–24.
15	Benmarhnia T, Huang J, Basu R, Wu J, Bruckner TA. Decomposition Analysis of Black-White Disparities in Birth Outcomes: The Relative Contribution of Air Pollution and Social Factors in California. Environ Health Perspect. 2017; 125(10): 107003.
16	Bowe B, Xie Y, Li T, Yan Y, Xian H, Al-Aly Z. The 2016 global and national burden of diabetes mellitus attributable to PM2·5 air pollution. Lancet Planet Health. 2018; 2(7): e301–12.
17	Boy E, Bruce N, Delgado H. Birth weight and exposure to kitchen wood smoke during pregnancy in rural Guatemala. Environ Health Perspect. 2002; 110(1): 109-14.
18	Brauer M, Lencar C, Tamburic L, Koehoorn M, Demers P, Karr C. A cohort study of traffic-related air pollution impacts on birth outcomes. Environ Health Perspect. 2008; 116(5): 680-6.
19	Broor S, Pandey RM, Ghosh M, Maitreyi RS, Lodha R, Singhal T, Kabra SK. Risk factors for severe acute lower respiratory tract infection in under-five children. Indian Pediatr. 2001; 1361-9.
20	Burnett RT. Cox Proportional Survival Model Hazard Ratios from Census Year to 2011 for Adults Aged 25 to 89 in CanCHEC Cohort.
21	Carey IM, Atkinson RW, Kent AJ, van Staa T, Cook DG, Anderson HR. Mortality associations with long-term exposure to outdoor air pollution in a national English cohort. Am J Respir Crit Care Med. 2013; 187(11): 1226-33.
22	Cesaroni G, Badaloni C, Gariazzo C, Stafoggia M, Sozzi R, Davoli M, Forastiere F. Long-term exposure to urban air pollution and mortality in a cohort of more than a million adults in Rome. Environ Health Perspect. 2013; 121(3): 324–31.
23	Chang HH, Reich BJ, Miranda ML. A spatial time-to-event approach for estimating associations between air pollution and preterm birth. J R Stat Soc Ser C Appl Stat. 2013; 62(2).
24	Chen H, Burnett RT, Kwong JC, Villeneuve PJ, Goldberg MS, Brook RD, van Donkelaar A, Jerrett M, Martin RV, Brook JR, Copes R. Risk of incident diabetes in relation to long-term exposure to fine particulate matter in Ontario, Canada. Environ Health Perspect. 2013; 121(7): 804–10.
25	Chen LH, Knutsen SF, Shavlik D, Beeson WL, Petersen F, Ghamsary M, Abbey D. The association between fatal coronary heart disease and ambient particulate air pollution: Are females at greater risk?. Environ Health Perspect. 2005; 113(12): 1723-9.
26	Clark C, Sbihi H, Tamburic L, Brauer M, Frank LD, Davies HW. Association of Long-Term Exposure to Transportation Noise and Traffic-Related Air Pollution with the Incidence of Diabetes: A Prospective Cohort Study. Environ Health Perspect. 2017; 125(8): 087025.
27	Clemens T, Turner S, Dibben C. Maternal exposure to ambient air pollution and fetal growth in North-East Scotland: A population-based study using routine ultrasound scans. Environ Int. 2017; 107: 216–26.
28	Coker E, Ghosh J, Jerrett M, Gomez-Rubio V, Beckerman B, Cockburn M, Liverani S, Su J, Li A, Kile ML, Ritz B, Molitor J. Modeling spatial effects of PM(2.5) on term low birth weight in Los Angeles County. Environ Res. 2015; 142: 354-64.

Source	Citation
29	Collings DA, Sithole SD, Martin KS. Indoor woodsmoke pollution causing lower respiratory disease in children. Trop Doct. 1990; 20(4): 151–5.
30	Coogan PF, White LF, Yu J, Burnett RT, Seto E, Brook RD, Palmer JR, Rosenberg L, Jerrett M. PM2.5 and Diabetes and Hypertension Incidence in the Black Women's Health Study. Epidemiology. 2016; 27(2): 202–10.
31	Dadvand P, Ostro B, Figueras F, Foraster M, Basagaña X, Valentín A, Martinez D, Beelen R, Cirach M, Hoek G, Jerrett M, Brunekreef B, Nieuwenhuijsen MJ. Residential proximity to major roads and term low birth weight: the roles of air pollution, heat, noise, and road-adjacent trees. Epidemiology. 2014; 25(4): 518-25.
32	Darrow LA, Klein M, Strickland MJ, Mulholland JA, Tolbert PE. Ambient Air Pollution and Birth Weight in Full-Term Infants in Atlanta, 1994–2004. Environ Health Perspect. 2011; 119(5): 731–7.
33	Dennis RJ, Maldonado D, Norman S, Baena E, Martinez G. Woodsmoke exposure and risk for obstructive airways disease among women. Chest. 1996; 109(1): 115–9.
34	Dherani M, Pope D, Mascarenhas M, Smith KR, Weber M, Bruce N. Indoor air pollution from unprocessed solid fuel use and pneumonia risk in children aged under five years: a systematic review and meta-analysis. Bull World Health Organ. 2008; 86(5): 390-398C and Kossove D. and Jeena PM, Ayannusi OE, Annamalai K, Naidoo P, Coovadia HM, Guldner P. Risk factors for admission and the role of respiratory syncytial virus-specific cytotoxic T-lymphocyte responses in children with acute bronchiolitis. S Afr Med J. 2003; 93(4): 291–4.
35	Ebisu K, Bell ML. Airborne PM2.5 chemical components and low birth weight in the northeastern and mid-Atlantic regions of the United States. Environ Health Perspect. 2012; 120(12): 1746-52.
36	Ebisu K, Berman JD, Bell ML. Exposure to coarse particulate matter during gestation and birth weight in the U.S. Environ Int. 2016; 94: 519–24.
37	Erickson AC, Ostry A, Chan LH, Arbour L. The reduction of birth weight by fine particulate matter and its modification by maternal and neighbourhood-level factors: a multilevel analysis in British Columbia, Canada. Environ Health. 2016; 15: 51.
38	Ezzati M, Kammen DM. Indoor air pollution from biomass combustion and acute respiratory infections in Kenya: an exposure-response study. Lancet. 2001; 358(9282): 619–24.
39	Fleischer NL, Merialdi M, van Donkelaar A, Vadillo-Ortega F, Martin RV, Betran AP, Souza JP. Outdoor air pollution, preterm birth, and low birth weight: analysis of the world health organization global survey on maternal and perinatal health. Environ Health Perspect. 2014; 122(4): 425-30.
40	Fonseca W, Kirkwood BR, Victora CG, Fuchs SR, Flores JA, Misago C. Risk factors for childhood pneumonia among the urban poor in Fortaleza, Brazil: a case--control study. Bull World Health Organ. 1996; 74(2): 199–208.
41	Galeone C, Pelucchi C, La Vecchia C, Negri E, Bosetti C, Hu J. Indoor air pollution from solid fuel use, chronic lung diseases and lung cancer in Harbin, Northeast China. Eur J Cancer Prev. 2008; 17(5): 473–8.
42	Gan WQ, FitzGerald JM, Carlsten C, Sadatsafavi M, Brauer M. Associations of ambient air pollution with chronic obstructive pulmonary disease hospitalization and mortality. Am J Respir Crit Care Med. 2013; 187(7): 721–7.
43	Gan WQ, Koehoorn M, Davies HW, Demers PA, Tamburic L, Brauer M. Long-Term Exposure to Traffic-Related Air Pollution and the Risk of Coronary Heart Disease Hospitalization and Mortality. Environ Health Perspect. 2011; 119(4): 501–7.
44	Geer LA, Weedon J, Bell ML. Ambient air pollution and term birth weight in Texas from 1998 to 2004. J Air Waste Manag Assoc. 2012; 62(11): 1285–95.
45	Gehring U, Tamburic L, Sbihi H, Davies HW, Brauer M. Impact of Noise and Air Pollution on Pregnancy Outcomes. Epidemiology. 2014; 25(3): 351–8.
46	Gehring U, Wijga AH, Fischer P, de Jongste JC, Kerkhof M, Koppelman GH, Smit HA, Brunekreef B. Traffic-related air pollution, preterm birth and term birth weight in the PIAMA birth cohort study. Environ Res. 2011; 111(1): 125–35.
47	Ger LP, Hsu WL, Chen KT, Chen CJ. Risk Factors of Lung Cancer by Histological Category in Taiwan. Anticancer Res. 1993; 13(5A): 1491–500.
48	Gray SC, Edwards SE, Schultz BD, Miranda ML. Assessing the impact of race, social factors and air pollution on birth outcomes: a population-based study. Environ Health. 2014; 13(1): 4.
49	Gray SC, Gelfand AE, Miranda ML. Hierarchical spatial modeling of uncertainty in air pollution and birth weight study. Stat Med. 2011; 30(17): 2187-98.
50	Gupta D, Boffetta P, Gaborieau V, Jindal SK. Risk factors of lung cancer in Chandigarh, India. Indian J Med Res. 2001; 113: 142–50.
51	Ha S, Hu H, Roussos-Ross D, Haidong K, Roth J, Xu X. The effects of air pollution on adverse birth outcomes. Environ Res. 2014; 134: 198-204.
52	Hansen AB, Ravnskjær L, Loft S, Andersen KK, Bräuner EV, Baastrup R, Yao C, Ketzel M, Becker T, Brandt J, Hertel O, Andersen ZJ. Long-term exposure to fine particulate matter and incidence of diabetes in the Danish Nurse Cohort. Environ Int. 2016; 91: 243–50.

Source	Citation
53	Hao H, Chang HH, Holmes HA, Mulholland JA, Klein M, Darrow LA, Strickland MJ. Air Pollution and Preterm Birth in the U.S. State of Georgia (2002-2006): Associations with Concentrations of 11 Ambient Air Pollutants Estimated by Combining Community Multiscale Air Quality Model (CMAQ) Simulations with Stationary Monitor Measurements. Environ Health Perspect. 2016; 124(6): 875-80.
54	Hao Y, Strosnider H, Balluz L, Qualters JR. Geographic Variation in the Association between Ambient Fine Particulate Matter (PM2.5) and Term Low Birth Weight in the United States. Environ Health Perspect. 2016; 124(2): 250-5.
55	Harris G, Thompson WD, Fitzgerald E, Wartenberg D. The association of PM(2.5) with full term low birth weight at different spatial scales. Environ Res. 2014; 134: 427-34.
56	Hart J, Garshick E, Dockery D, Smith T, Ryan L, Laden F. Long-Term Ambient Multipollutant Exposures and Mortality. Am J Respir Crit Care Med. 2011; 183: 75–8.
57	Hart JE, Puett RC, Rexrode KM, Albert CM, Laden F. Effect Modification of Long-Term Air Pollution Exposures and the Risk of Incident Cardiovascular Disease in US Women. J Am Heart Assoc. 2015; 4(12).
58	Heft-Neal S, Burney J, Bendavid E, Burke M. Robust relationship between air quality and infant mortality in Africa. Nature. 2018; 559(7713): 2548.
59	Hertz-Picciotto I, Baker RJ, Yap P-S, Dostál M, Joad JP, Lipsett M, Greenfield T, Herr CEW, Benes I, Shumway RH, Pinkerton KE, Srám R. Early childhood lower respiratory illness and air pollution. Environ Health Perspect. 2007; 115(10): 1510-8.
60	Huang C, Zhang X, Qiao Z, Guan L, Peng S, Liu J, Xie R, Zheng L. A case-control study of dietary factors in patients with lung cancer. Biomed Environ Sci. 1992; 5(3): 257–65.
61	Huynh M, Woodruff TJ, Parker JD, Schoendorf KC. Relationships between air pollution and preterm birth in California. Paediatr Perinat Epidemiol. 2006; 20(6): 454-61.
62	Hyder A, Lee HJ, Ebisu K, Koutrakis P, Belanger K, Bell ML. PM2.5 Exposure and Birth Outcomes: Use of Satellite- and Monitor-Based Data. Epidemiology. 2014; 25(1): 58–67.
63	Hystad P, Demers PA, Johnson KC, Carpiano RM, Brauer M. Long-term residential exposure to air pollution and lung cancer risk. Epidemiology. 2013; 24(5): 762-72.
64	Hystad P, Duong M, Brauer M, Larkin A, Arku R, Kurmi OP, Fan WQ, Avezum A, Azam I, Chifamba J, Dans A, du Plessis JL, Gupta R, Kumar R, Lanas F, Liu Z, Lu Y, Lopez-Jaramillo P, Mony P, Mohan V, Mohan D, Nair S, Puoane T, Rahman O, Lap AT, Wang Y, Wei L, Yeates K, Rangarajan S, Teo K, Yusuf S, on behalf of Prospective Urban and Rural Epidemiological (PURE) Study investigators. Health Effects of Household Solid Fuel Use: Findings from 11 Countries within the Prospective Urban and Rural Epidemiology Study. Environ Health Perspect. 2019; 127(5): 57003.
65	Hystad P, Duong M, Brauer M, Larkin A, Arku R, Kurmi OP, Fan WQ, Avezum A, Azam I, Chifamba J, Dans A, du Plessis JL, Gupta R, Kumar R, Lanas F, Liu Z, Lu Y, Lopez-Jaramillo P, Mony P, Mohan V, Mohan D, Nair S, Puoane T, Rahman O, Lap AT, Wang Y, Wei L, Yeates K, Rangarajan S, Teo K, Yusuf S, on behalf of Prospective Urban and Rural Epidemiological (PURE) Study investigators. Health Effects of Household Solid Fuel Use: Findings from 11 Countries within the Prospective Urban and Rural Epidemiology Study [Unpublished]. Environ Health Perspect. 2019; 127(5): 57003.
66	Hystad P, Larkin A, Rangarajan S, PURE country investigators, Yusuf S, Brauer M. Outdoor fine particulate matter air pollution and cardiovascular disease: Results from 747 communities across 21 countries in the PURE Study [Unpublished].
67	Jedrychowski W, Perera F, Mrozek-Budzyn D, Mroz E, Flak E, Spengler JD, Edwards S, Jacek R, Kaim I, Skolicki Z. Gender differences in fetal growth of newborns exposed prenatally to airborne fine particulate matter. Environ Res. 2009; 109(4): 447-56.
68	Jin C, Rossignol AM. Effects of passive smoking on respiratory illness from birth to age eighteen months, in Shanghai, People's Republic of China. J Pediatr. 1993; 123(4): 553–8.
69	Johnson AW, Aderele WI. The association of household pollutants and socio-economic risk factors with the short-term outcome of acute lower respiratory infections in hospitalized pre-school Nigerian children. Ann Trop Paediatr. 1992; 12(4): 421–32.
70	Karr C, Lumley T, Schreuder A, Davis R, Larson T, Ritz B, Kaufman J. Effects of subchronic and chronic exposure to ambient air pollutants on infant bronchiolitis. Am J Epidemiol. 2007; 165(5): 553-60.
71	Karr CJ, Rudra CB, Miller KA, Gould TR, Larson T, Sathyanarayana S, Koenig JQ. Infant exposure to fine particulate matter and traffic and risk of hospitalization for RSV bronchiolitis in a region with lower ambient air pollution. Environ Res. 2009; 109(3): 321-7.
72	Katanoda K, Sobue T, Satoh H, Tajima K, Suzuki T, Nakatsuka H, Takezaki T, Nakayama T, Nitta H, Tanabe K, Tominaga S. An association between long-term exposure to ambient air pollution and mortality from lung cancer and respiratory diseases in Japan. J Epidemiol. 2011; 21(2): 132-43.
73	Kim C, Seow WJ, Shu X-O, Bassig BA, Rothman N, Chen BE, Xiang Y-B, Hosgood HD, Ji B-T, Hu W, Wen C, Chow W-H, Cai Q, Yang G, Gao Y-T, Zheng W, Lan Q. Cooking Coal Use and All-Cause and Cause-Specific Mortality in a Prospective Cohort Study of Women in Shanghai, China. Environ Health Perspect. 2016; 124(9): 1384–9.

Source	Citation
74	Kleinerman RA, Wang Z, Wang L, Metayer C, Zhang S, Brenner AV, Zhang S, Xia Y, Shang B, Lubin JH. Lung cancer and indoor exposure to coal and biomass in rural China. J Occup Environ Med. 2002; 44(4): 338–44.
75	Kloog I, Melly SJ, Ridgway WL, Coull BA, Schwartz J. Using new satellite based exposure methods to study the association between pregnancy pm2.5 exposure, premature birth and birth weight in Massachusetts. Environ Health. 2012; 11(1).
76	Ko YC, Lee CH, Chen MJ, Huang CC, Chang WY, Lin HJ, Wang HZ, Chang PY. Risk factors for primary lung cancer among non-smoking women in Taiwan. Int J Epidemiol. 1997; 26(1): 24-31.
77	Kumar N. Uncertainty in the relationship between criteria pollutants and low birth weight in Chicago. Atmos Environ. 2012; 49: 171–9.
78	Kumar S, Awasthi S, Jain A, Srivastava RC. Blood zinc levels in children hospitalized with severe pneumonia: a case control study. Indian Pediatr. 2004; 41(5): 486–91.
79	Lan Q, He X, Shen M, Tian L, Liu LZ, Lai H, Chen W, Berndt SI, Hosgood HD, Lee K-M, Zheng T, Blair A, Chapman RS. Variation in lung cancer risk by smoky coal subtype in Xuanwei, China. Int J Cancer. 2008; 123(9): 2164–9.
80	Laurent O, Hu J, Li L, Cockburn M, Escobedo L, Kleeman MJ, Wu J. Sources and contents of air pollution affecting term low birth weight in Los Angeles County, California, 2001-2008. Environ Res. 2014; 134: 488-95.
81	Laurent O, Hu J, Li L, Kleeman MJ, Bartell SM, Cockburn M, Escobedo L, Wu J. A Statewide Nested Case-Control Study of Preterm Birth and Air Pollution by Source and Composition: California, 2001-2008. Environ Health Perspect. 2016; 124(9): 1479-86.
82	Laurent O, Hu J, Li L, Kleeman MJ, Bartell SM, Cockburn M, Escobedo L, Wu J. Low birth weight and air pollution in California: Which sources and components drive the risk?. Environ Int. 2016; 92-93: 471-7.
83	Laurent O, Wu J, Li L, Chung J, Bartell S. Investigating the association between birth weight and complementary air pollution metrics: a cohort study. Environ Health. 2013; 12(1).
84	Lavigne E, Yasseen AS 3rd, Stieb DM, Hystad P, van Donkelaar A, Martin RV, Brook JR, Crouse DL, Burnett RT, Chen H, Weichenthal S, Johnson M, Villeneuve PJ, Walker M. Ambient air pollution and adverse birth outcomes: Differences by maternal comorbidities. Environ Res. 2016; 148: 457-466.
85	Le CH, Ko YC, Cheng LS, Lin YC, Lin HJ, Huang MS, Huang JJ, Kao EL, Wang HZ. The heterogeneity in risk factors of lung cancer and the difference of histologic distribution between genders in Taiwan. Cancer Causes Control. 2001; 12(4): 289–300.
86	Lepeule J, Laden F, Dockery D, Schwartz J. Chronic exposure to fine particles and mortality: an extended follow-up of the Harvard Six Cities study from 1974 to 2009 - Unpublished data. Environ Health Perspect. 2012; 120(7): 965-70.
87	Lepeule J, Laden F, Dockery D, Schwartz J. Chronic exposure to fine particles and mortality: an extended follow-up of the Harvard Six Cities study from 1974 to 2009. Environ Health Perspect. 2012; 120(7): 965-70.
88	Lim CC, Hayes RB, Ahn J, Shao Y, Silverman DT, Jones RR, Garcia C, Thurston GD. Association between long-term exposure to ambient air pollution and diabetes mortality in the US. Environ Res. 2018; 165: 330-36
89	Lipsett MJ, Ostro BD, Reynolds P, Goldberg D, Hertz A, Jerrett M, Smith DF, Garcia C, Chang ET, Bernstein L. Long-term exposure to air pollution and cardiorespiratory disease in the California teachers study cohort [Unpublished data]. Am J Respir Crit Care Med. 2011; 184(7): 828-35.
90	Lipsett MJ, Ostro BD, Reynolds P, Goldberg D, Hertz A, Jerrett M, Smith DF, Garcia C, Chang ET, Bernstein L. Long-term exposure to air pollution and cardiorespiratory disease in the California teachers study cohort. Am J Respir Crit Care Med. 2011; 184(7): 828-35.
91	Lissowska J, Bardin-Mikolajczak A, Fletcher T, Zaridze D, Szeszenia-Dabrowska N, Rudnai P, Fabianova E, Cassidy A, Mates D, Holcatova I, Vitova V, Janout V, Mannetje A, Brennan P, Boffetta P. Lung cancer and indoor pollution from heating and cooking with solid fuels: the IARC international multicentre case-control study in Eastern/Central Europe and the United Kingdom. Am J Epidemiol. 2005; 162(4): 326–33.
92	Luo RX, Wu B, Yi YN, Huang ZW, Lin RT. Indoor burning coal air pollution and lung cancer--a case-control study in Fuzhou, China. Lung Cancer. 1996; 14 Suppl 1: S113-119.
93	MacIntyre EA, Gehring U, Mölter A, Fuertes E, Klümper C, Krämer U, Quass U, Hoffmann B, Gascon M, Brunekreef B, Koppelman GH, Beelen R, Hoek G, Birk M, de Jongste JC, Smit HA, Cyrys J, Gruzieva O, Korek M, Bergström A, Agius RM, de Vocht F, Simpson A, Porta D, Forastiere F, Badaloni C, Cesaroni G, Esplugues A, Fernández-Somoano A, Lerxundi A, Sunyer J, Cirach M, Nieuwenhuijsen MJ, Pershagen G, Heinrich J. Air Pollution and Respiratory Infections during Early Childhood: An Analysis of 10 European Birth Cohorts within the ESCAPE Project. Environ Health Perspect. 2014; 122(1): 107–13.
94	Mahalanabis D, Gupta S, Paul D, Gupta A, Lahiri M, Khaled MA. Risk factors for pneumonia in infants and young children and the role of solid fuel for cooking: a case-control study. Epidemiol Infect. 2002; 129(1): 65–71.
95	Miller KA, Siscovick DS, Sheppard L, Shepherd K, Sullivan JH, Anderson GL, Kaufman JD. Long-term exposure to air pollution and incidence of cardiovascular events in women. N Engl J Med. 2007; 356(5): 447-58.
96	Morello-Frosch R, Jesdale BM, Sadd JL, Pastor M. Ambient air pollution exposure and full-term birth weight in California. Environ Health. 2010; 9(1).

Source	Citation
97	Naess Ø, Nafstad P, Aamodt G, Claussen B, Rosland P. Relation between concentration of air pollution and cause-specific mortality: four-year exposures to nitrogen dioxide and particulate matter pollutants in 470 neighborhoods in Oslo, Norway. Am J Epidemiol. 2007; 165(4): 435-43.
98	Park SK, Adar SD, O'Neill MS, Auchincloss AH, Szpiro A, Bertoni AG, Navas-Acien A, Kaufman JD, Diez-Roux AV. Long-term exposure to air pollution and type 2 diabetes mellitus in a multiethnic cohort. Am J Epidemiol. 2015; 181(5): 327–36.
99	Parker JD, Kravets N, Vaidyanathan A. Particulate Matter Air Pollution Exposure and Heart Disease Mortality Risks by Race and Ethnicity in the United States. Circulation. 2018; 137(16): 1688–97.
100	Parker JD, Woodruff TJ, Basu R, Schoendorf KC. Air Pollution and Birth Weight Among Term Infants in California. Pediatrics. 2005; 115(1): 121–8.
101	Parker JD, Woodruff TJ. Influences of study design and location on the relationship between particulate matter air pollution and birthweight. Paediatr Perinat Epidemiol. 2008; 22(3): 214–27.
102	Pereira G, Belanger K, Ebisu K, Bell ML. Fine particulate matter and risk of preterm birth in Connecticut in 2000-2006: a longitudinal study. Am J Epidemiol. 2014; 179(1): 67-74.
103	Pereira G, Bell ML, Belanger K, de Klerk N. Fine particulate matter and risk of preterm birth and pre-labor rupture of membranes in Perth, Western Australia 1997-2007: a longitudinal study. Environ Int. 2014; 73: 143-9.
104	Pinault L, Tjepkema M, Crouse DL, Weichenthal S, van Donkelaar A, Martin RV, Brauer M, Chen H, Burnett RT. Risk estimates of mortality attributed to low concentrations of ambient fine particulate matter in the Canadian community health survey cohort [Unpublished]. Environ Health. 2016; 15: 18.
105	Pinault L, Tjepkema M, Crouse DL, Weichenthal S, van Donkelaar A, Martin RV, Brauer M, Chen H, Burnett RT. Risk estimates of mortality attributed to low concentrations of ambient fine particulate matter in the Canadian community health survey cohort. Environ Health. 2016; 15(1): 18.
106	Puett RC, Hart JE, Schwartz J, Hu FB, Liese AD, Laden F. Are particulate matter exposures associated with risk of type 2 diabetes? Environ Health Perspect. 2011; 119(3): 384–9.
107	Puett RC, Hart JE, Suh H, Mittleman M, Laden F. Particulate matter exposures, mortality, and cardiovascular disease in the health professionals follow-up study. Environ Health Perspect. 2011; 119(8): 1130-5.
108	Puett RC, Hart JE, Yanosky JD, Paciorek C, Schwartz J, Suh H, Speizer FE, Laden F. Chronic fine and coarse particulate exposure, mortality, and coronary heart disease in the Nurses' Health Study. Environ Health Perspect. 2009; 117(11): 1697-701.
109	Qian Z, Liang S, Yang S, Trevathan E, Huang Z, Yang R, Wang J, Hu K, Zhang Y, Vaughn M, Shen L, Liu W, Li P, Ward P, Yang L, Zhang W, Chen W, Dong G, Zheng T, Xu S, Zhang B. Ambient air pollution and preterm birth: A prospective birth cohort study in Wuhan, China. Int J Hyg Environ Health. 2016; 219(2): 195-203.
110	Qiu H, Schooling CM, Sun S, Tsang H, Yang Y, Lee RS, Wong CM, Tian L. Long-term exposure to fine particulate matter air pollution and type 2 diabetes mellitus in elderly: A cohort study in Hong Kong. Environ Int. 2018; 113: 350-56.
111	Raaschou-Nielsen O, Andersen ZJ, Beelen R, Samoli E, Stafoggia M, Weinmayr G, Hoffmann B, Fischer P, Nieuwenhuijsen MJ, Brunekreef B, Xun WW, Katsouyanni K, Dimakopoulou K, Sommar J, Forsberg B, Modig L, Oudin A, Oftedal B, Schwarze PE, Nafstad P, De Faire U, Pedersen NL, Ostenson C-G, Fratiglioni L, Penell J, Korek M, Pershagen G, Eriksen KT, Sørensen M, Tjønneland A, Ellermann T, Eeftens M, Peeters PH, Meliefste K, Wang M, Bueno-de-Mesquita B, Key TJ, de Hoogh K, Concin H, Nagel G, Vilier A, Grioni S, Krogh V, Tsai M-Y, Ricceri F, Sacerdote C, Galassi C, Migliore E, Ranzi A, Cesaroni G, Badaloni C, Forastiere F, Tamayo I, Amiano P, Dorronsoro M, Trichopoulou A, Bamia C, Vineis P, Hoek G. Air pollution and lung cancer incidence in 17 European cohorts: prospective analyses from the European Study of Cohorts for Air Pollution Effects (ESCAPE). Lancet Oncol. 2013; 14(9): 813–22.
112	Robin LF, Less PS, Winget M, Steinhoff M, Moulton LH, Santosham M, Correa A. Wood-burning stoves and lower respiratory illnesses in Navajo children. Pediatr Infect Dis J. 1996; 15(10): 859–65.
113	Sapkota A, Gajalakshmi V, Jetly DH, Roychowdhury S, Dikshit RP, Brennan P, Hashibe M, Boffetta P. Indoor air pollution from solid fuels and risk of hypopharyngeal/laryngeal and lung cancers: a multicentric case-control study from India. Int J Epidemiol. 2008; 37(2): 321–8.
114	Sasco AJ, Merrill RM, Dari I, Benhaïm-Luzon V, Carriot F, Cann CI, Bartal M. A case-control study of lung cancer in Casablanca, Morocco. Cancer Causes Control. 2002; 13(7): 609–16.
115	Savitha MR, Nandeeshwara SB, Pradeep Kumar MJ, ul-Haque F, Raju CK. Modifiable risk factors for acute lower respiratory tract infections. Indian J Pediatr. 2007; 74(5): 477–82.
116	Savitz DA, Bobb JF, Carr JL, Clougherty JE, Dominici F, Elston B, Ito K, Ross Z, Yee M, Matte TD. Ambient Fine Particulate Matter, Nitrogen Dioxide, and Term Birth Weight in New York, New York. Am J Epidemiol. 2014; 179(4): 457–66.
117	Sezer H, Akkurt I, Guler N, Marako?lu K, Berk S. A case-control study on the effect of exposure to different substances on the development of COPD. Ann Epidemiol. 2006; 16(1): 59–62.
118	Shah N, Ramankutty V, Premila PG, Sathy N. Risk factors for severe pneumonia in children in south Kerala: a hospital-based case-control study. J Trop Pediatr. 1994; 40(4): 201–6.

Source	Citation
119	Shen M, Chapman RS, Vermeulen R, Tian L, Zheng T, Chen BE, Engels EA, He X, Blair A, Lan Q. Coal use, stove improvement, and adult pneumonia mortality in Xuanwei, China: a retrospective cohort study. Environ Health Perspect. 2009; 117(2): 261–6.
120	Siddiqui AR, Gold EB, Yang X, Lee K, Brown KH, Bhutta ZA. Prenatal exposure to wood fuel smoke and low birth weight. Environ Health Perspect. 2008; 116(4): 543-9.
121	Smith KR, McCracken JP, Weber MW, Hubbard A, Jenny A, Thompson LM, Balmes J, Diaz A, Arana B, Bruce N. Effect of reduction in household air pollution on childhood pneumonia in Guatemala (RESPIRE): a randomised controlled trial. Lancet. 2011; 378(9804): 1717-26.
122	Smith RB, Fecht D, Gulliver J, Beevers SD, Dajnak D, Blangiardo M, Ghosh RE, Hansell AL, Kelly FJ, Anderson HR, Toledano MB. Impact of London's road traffic air and noise pollution on birth weight: retrospective population based cohort study. BMJ. 2017; 359: j5299.
123	Stafoggia M, Cesaroni G, Peters A, Andersen ZJ, Badaloni C, Beelen R, Caracciolo B, Cyrys J, de Faire U, de Hoogh K, Eriksen KT, Fratiglioni L, Galassi C, Gigante B, Havulinna AS, Hennig F, Hilding A, Hoek G, Hoffmann B, Houthuijs D, Korek M, Lanki T, Leander K, Magnusson PK, Meisinger C, Migliore E, Overvad K, Ostenson C-G, Pedersen NL, Pekkanen J, Penell J, Pershagen G, Pundt N, Pyko A, Raaschou-Nielsen O, Ranzi A, Ricceri F, Sacerdote C, Swart WJR, Turunen AW, Vineis P, Weimar C, Weinmayr G, Wolf K, Brunekreef B, Forastiere F. Long-term exposure to ambient air pollution and incidence of cerebrovascular events: results from 11 European cohorts within the ESCAPE project. Environ Health Perspect. 2014; 122(9): 919–25.
124	Stieb DM, Chen L, Beckerman BS, Jerrett M, Crouse DL, Omariba DW, Peters PA, van Donkelaar A, Martin RV, Burnett RT, Gilbert NL, Tjepkema M, Liu S, Dugandzic RM. Associations of Pregnancy Outcomes and PM2.5 in a National Canadian Study. Environ Health Perspect. 2016; 124(2): 243-9.
125	Thompson LM, Bruce N, Eskenazi B, Diaz A, Pope D, Smith KR. Impact of reduced maternal exposures to wood smoke from an introduced chimney stove on newborn birth weight in rural Guatemala. Environ Health Perspect. 2011; 119(10): 1489-94.
126	Thurston GD, Ahn J, Cromar KR, Shao Y, Reynolds HR, Jerrett M, Lim CC, Shanley R, Park Y, Hayes RB. Ambient Particulate Matter Air Pollution Exposure and Mortality in the NIH-AARP Diet and Health Cohort [Unpublished]. Environ Health Perspect. 2016; 124(4): 484-90.
127	Tielsch JM, Katz J, Thulasiraj RD, Coles CL, Sheeladevi S, Yanik EL, Rahmathullah L. Exposure to indoor biomass fuel and tobacco smoke and risk of adverse reproductive outcomes, mortality, respiratory morbidity and growth among newborn infants in south India. Int J Epidemiol. 2009; 38(5): 1351-63.
128	To T, Zhu J, Villeneuve PJ, Simatovic J, Feldman L, Gao C, Williams D, Chen H, Weichenthal S, Wall C, Miller AB. Chronic disease prevalence in women and air pollution--A 30-year longitudinal cohort study. Environ Int. 2015; 80: 26–32.
129	Tseng E, Ho W-C, Lin M-H, Cheng T-J, Chen P-C, Lin H-H. Chronic exposure to particulate matter and risk of cardiovascular mortality: cohort study from Taiwan. BMC Public Health. 2015; 15: 936.
130	Turner MC,Jerrett M, Pope CA 3rd, Krewski D, Gapstur SM, Diver WR, Beckerman BS, Marshall JD, Su J, Crouse DL, Burnett RT. Long-term ozone exposure and mortality in a large prospective study . Am J Respir Crit Care Med. 2016; 193(10): 1134-42.
131	Victora CG, Fuchs SC, Flores JA, Fonseca W, Kirkwood B. Risk factors for pneumonia among children in a Brazilian metropolitan area. Pediatrics. 1994; 977-85.
132	Villeneuve PJ, Weichenthal SA, Crouse D, Miller AB, To T, Martin RV, van Donkelaar A, Wall C, Burnett RT. Long-term exposure to fine particulate matter air pollution and mortality among Canadian women. Epidemiology. 2015; 26(4): 536-45.
133	Wayse V, Yousafzai A, Mogale K, Filteau S. Association of subclinical vitamin D deficiency with severe acute lower respiratory infection in Indian children under 5 y. Eur J Clin Nutr. 2004; 58(4): 563–7.
134	Weichenthal S, Villeneuve PJ, Burnett RT, van Donkelaar A, Martin RV, Jones RR, DellaValle CT, Sandler DP, Ward MH, Hoppin JA. Long-term exposure to fine particulate matter: association with nonaccidental and cardiovascular mortality in the agricultural health study cohort. Environ Health Perspect. 2014; 122(6): 609-15.
135	Weinmayr G, Hennig F, Fuks K, Nonnemacher M, Jakobs H, Möhlenkamp S, Erbel R, Jöckel K-H, Hoffmann B, Moebus S, Heinz Nixdorf Recall Investigator Group. Long-term exposure to fine particulate matter and incidence of type 2 diabetes mellitus in a cohort study: effects of total and traffic-specific air pollution. Environ Health. 2015; 14: 53.
136	Wesley AG, Loening WE. Assessment and 2-year follow-up of some factors associated with severity of respiratory infections in early childhood. S Afr Med J. 1996; 86(4): 365–8.
137	Wilhelm M, Ghosh JK, Su J, Cockburn M, Jerrett M, Ritz B. Traffic-related air toxics and preterm birth: a population-based case-control study in Los Angeles County, California. Environ Health. 2011; 10: 89.
138	Wong CM, Lai HK, Tsang H, Thach TQ, Thomas GN, Lam KBH, Chan KP, Yang L, Lau AKH, Ayres JG, Lee SY, Man Chan W, Hedley AJ, Lam TH. Satellite-Based Estimates of Long-Term Exposure to Fine Particles and Association with Mortality in Elderly Hong Kong Residents. Environ Health Perspect. 2015; 123(11): 1167-72.

Source	Citation
139	Wu AH, Henderson BE, Pike MC, Yu MC. Smoking and other risk factors for lung cancer in women. J Natl Cancer Inst. 1985; 74(4): 747-51.
140	Wu J, Wilhelm M, Chung J, Ritz B. Comparing exposure assessment methods for traffic-related air pollution in an adverse pregnancy outcome study. Environ Res. 2011; 111(5): 685-92.
141	Wylie BJ, Coull BA, Hamer DH, Singh MP, Jack D, Yeboah-Antwi K, Sabin L, Singh N, MacLeod WB. Impact of biomass fuels on pregnancy outcomes in central East India. Environ Health. 2014; 13(1): 1.
142	Wylie BJ, Kishashu Y, Matechi E, Zhou Z, Coull B, Abioye AI, Dionisio KL, Mugusi F, Premji Z, Fawzi W, Hauser R, Ezzati M. Maternal exposure to carbon monoxide and fine particulate matter during pregnancy in an urban Tanzanian cohort. Indoor Air. 2017; 27(1): 136-146.
143	Yin P, Brauer M, Cohen A, Burnett RT, Liu J, Liu Y, Liang R, Wang W, Qi J, Wang L, Zhou M. Long-term Fine Particulate Matter Exposure and Nonaccidental and Cause-specific Mortality in a Large National Cohort of Chinese Men [Unpublished]. Environ Health Perspect. 2017; 125(11): 117002.
144	Yin P, Brauer M, Cohen A, Burnett RT, Liu J, Liu Y, Liang R, Wang W, Qi J, Wang L, Zhou M. Long-term Fine Particulate Matter Exposure and Nonaccidental and Cause-specific Mortality in a Large National Cohort of Chinese Men. Environ Health Perspect. 2017; 125(11): 117002.
145	Yu K, Qiu G, Chan K-H, Lam K-BH, Kurmi OP, Bennett DA, Yu C, Pan A, Lv J, Guo Y, Bian Z, Yang L, Chen Y, Hu FB, Chen Z, Li L, Wu T. Association of Solid Fuel Use With Risk of Cardiovascular and All-Cause Mortality in Rural China. JAMA. 2018; 319(13): 1351–61.
146	Yucra S, Tapia V, Steenland K, Naeher LP, Gonzales GF. Association between biofuel exposure and adverse birth outcomes at high altitudes in Peru: a matched case-control study. Int J Occup Environ Health. 2011; 17(4): 307-13.

The following figures display risk curves for each outcome. The dashed line depicts the GBD 2017 IER including active smoking data, the dotted line depicts the GBD 2019 IER including active smoking data and updates to the AS and SHS exposure incorporation, and the solid line depicts the GBD 2019 MR-BRT curve without the inclusion of active smoking data. The grey shaded areas represent the 95% CI. The red box represents the TMREL area of the curve. On each page, the first figure depicts the typical range of outdoor exposure, whereas the second plot includes higher levels typical of household air pollution exposure.

Each point or number represents one study effect size. Each is plotted at the 95th percentile of the exposure distribution (OAP), the expected level of exposure for individual using solid fuel (HAP), or the expected level of exposure for individuals experiencing SHS. The relative risk is plotted relative to the predicted relative risk at the fifth percentile of exposure distribution (OAP), the expected (ambient only) level of exposure for individuals not using solid fuel (HAP), or the expected (ambient only) level of exposure for individuals not exposed to SHS. For example, a study predicting a relative risk of 1.5 for an exposure range of 10 to 20 would be plotted at (20, MRBRT(10)*1.5). Arrows represent studies that would have been outside the range of the plot but have been moved to include on the figure.

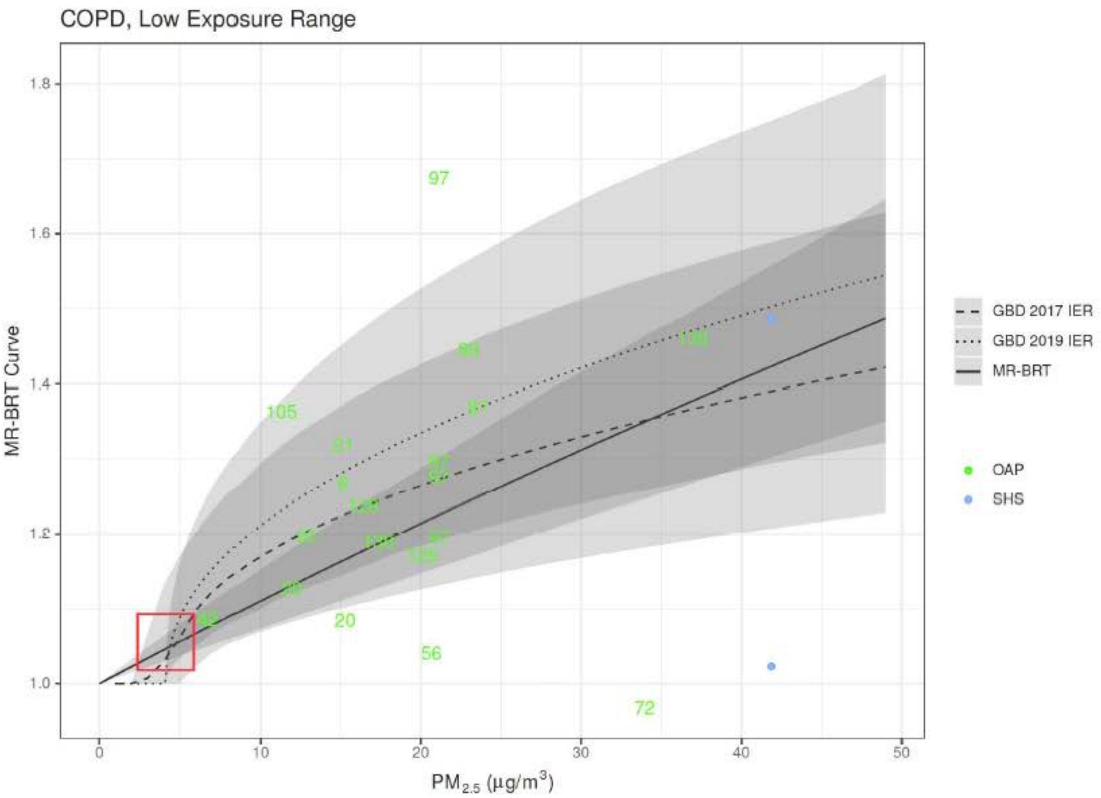
COPD, Low Exposure Range
MR-BRT Curve
1.8
1.6
1.4
1.2
1.0
0
10
20
30
40
50
PM2.5 (µg/m3)
97
105
21
20
56
72
GBD 2017 IER
GBD 2019 IER
MR-BRT
OAP
SHS

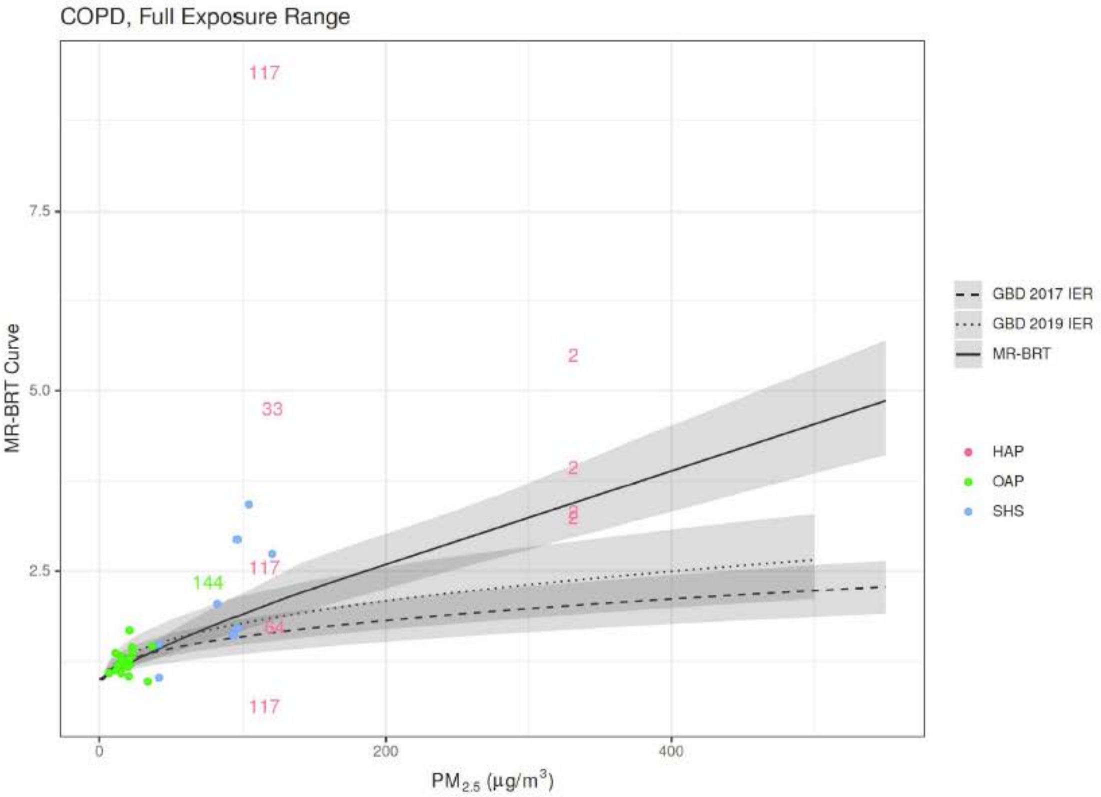
COPD, Full Exposure Range
MR-BRT Curve
7.5
5.0
2.5
0
200
400
PM2.5 (µg/m3)
117
2
33
2
117
144
117
GBD 2017 IER
GBD 2019 IER
MR-BRT
HAP
OAP
SHS

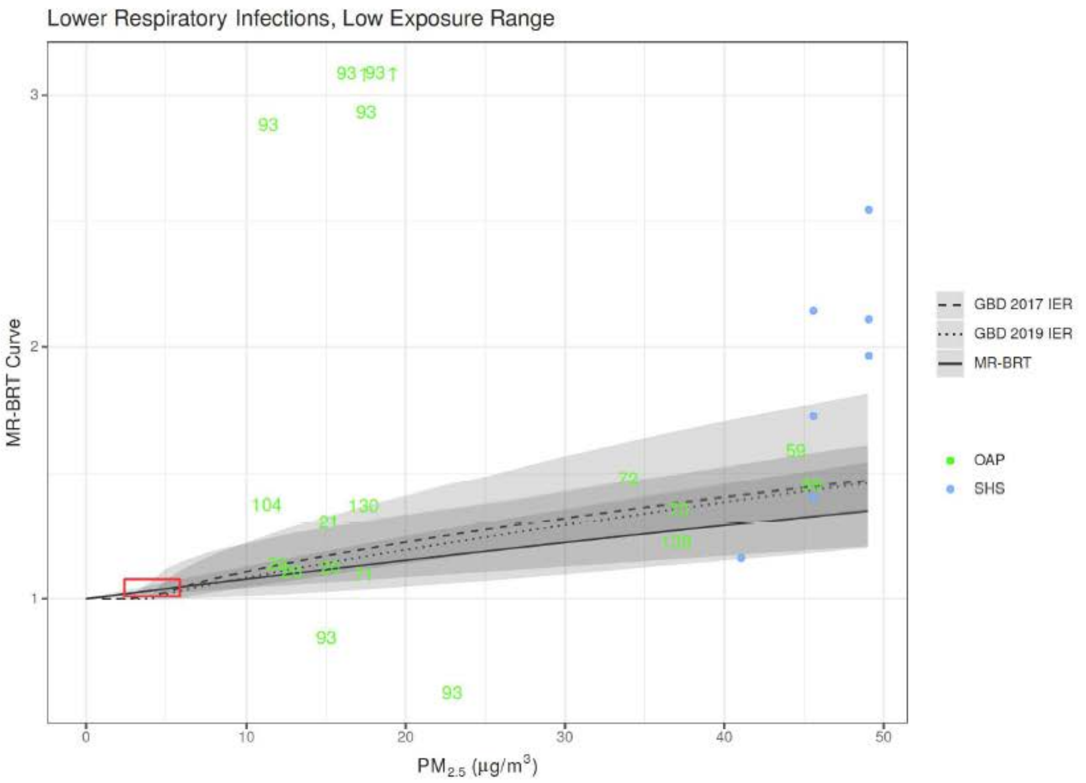
Lower Respiratory Infections, Low Exposure Range
MR-BRT Curve
PM2.5 (µg/m3)
GBD 2017 IER
GBD 2019 IER
MR-BRT
OAP
SHS

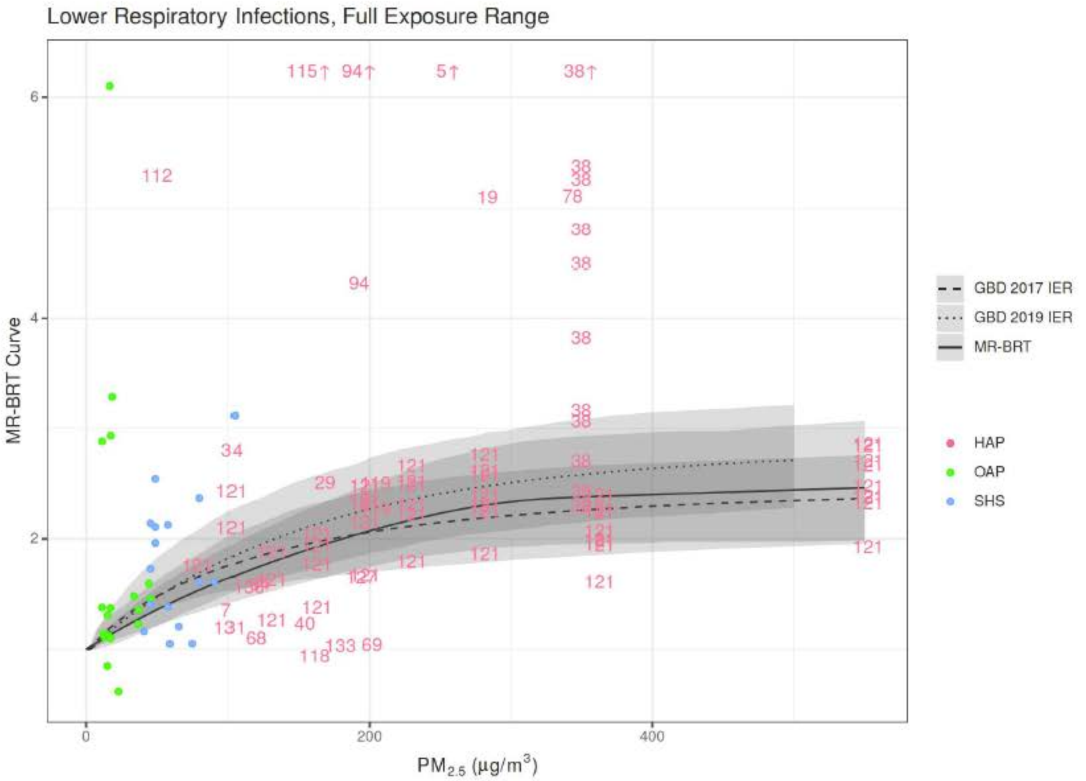
Lower Respiratory Infections, Full Exposure Range
MR-BRT Curve
PM2.5 (µg/m3)
GBD 2017 IER
GBD 2019 IER
MR-BRT
HAP
OAP
SHS

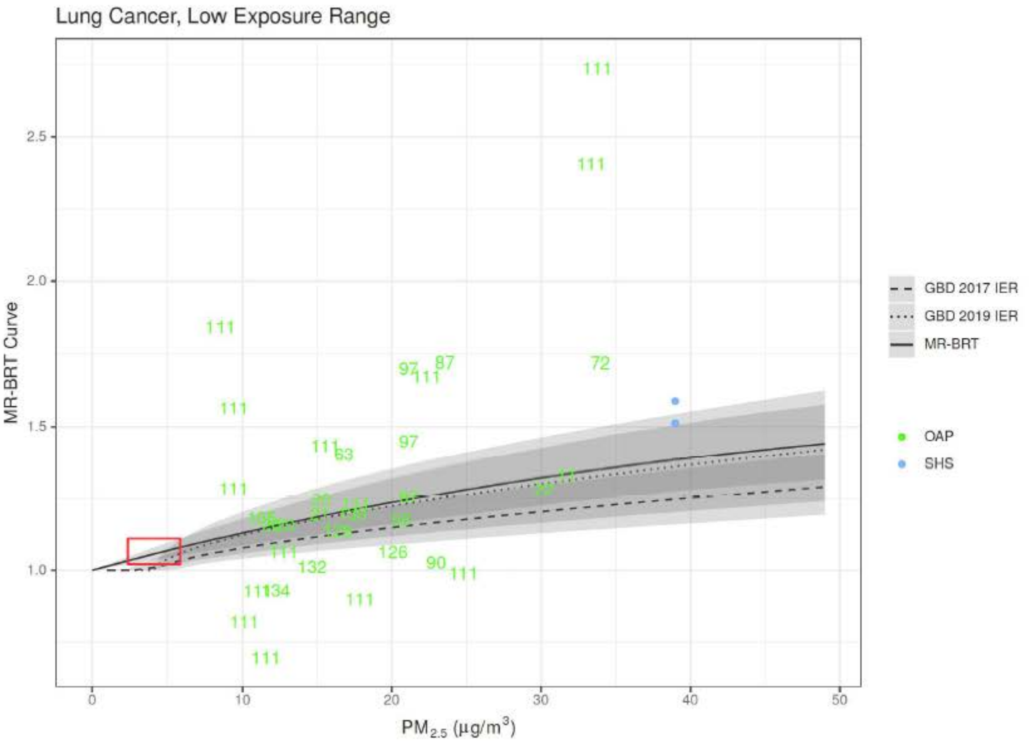
Lung Cancer, Low Exposure Range
MR-BRT Curve
PM2.5 (µg/m3)
GBD 2017 IER
GBD 2019 IER
MR-BRT
OAP
SHS

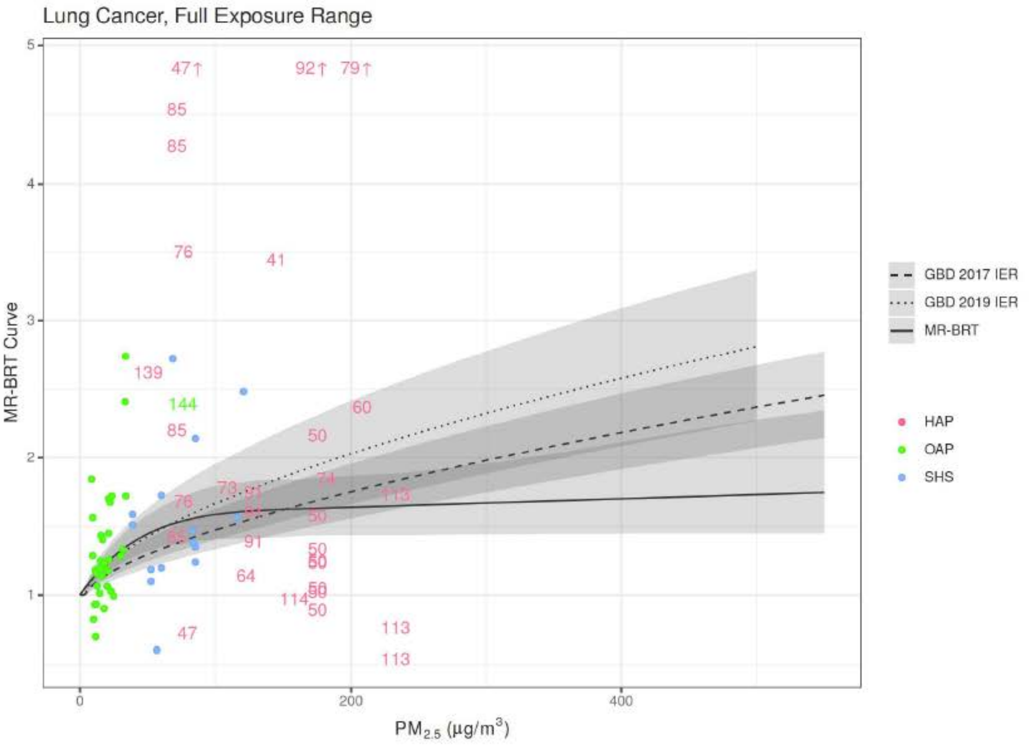
Lung Cancer, Full Exposure Range
MR-BRT Curve
PM2.5 (µg/m3)
GBD 2017 IER
GBD 2019 IER
MR-BRT
HAP
OAP
SHS

Type 2 Diabetes, Low Exposure Range

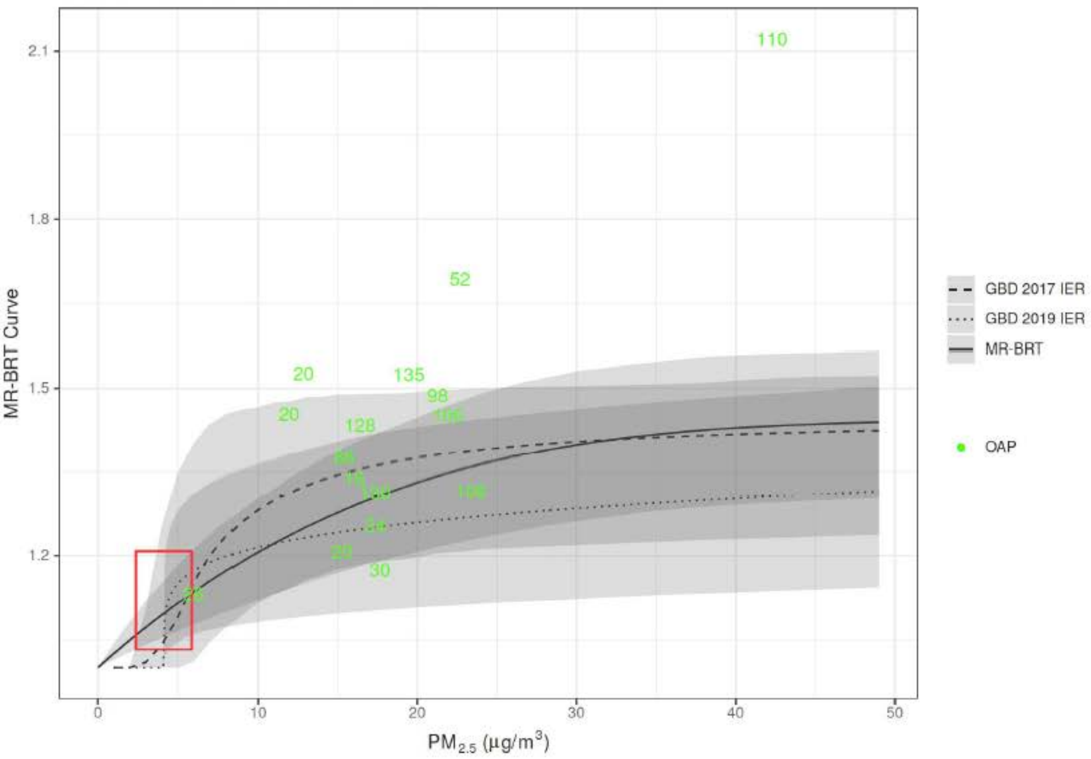

Type 2 Diabetes, Full Exposure Range

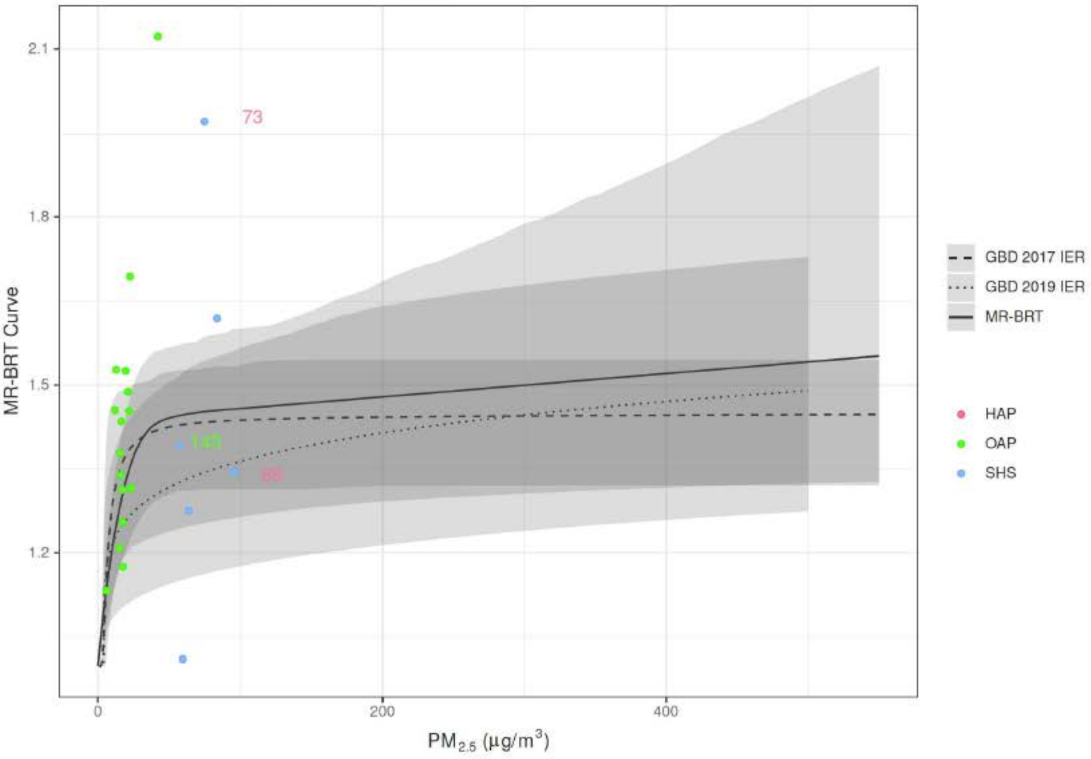

Ischemic Heart Disease, age 25, Low Exposure Range

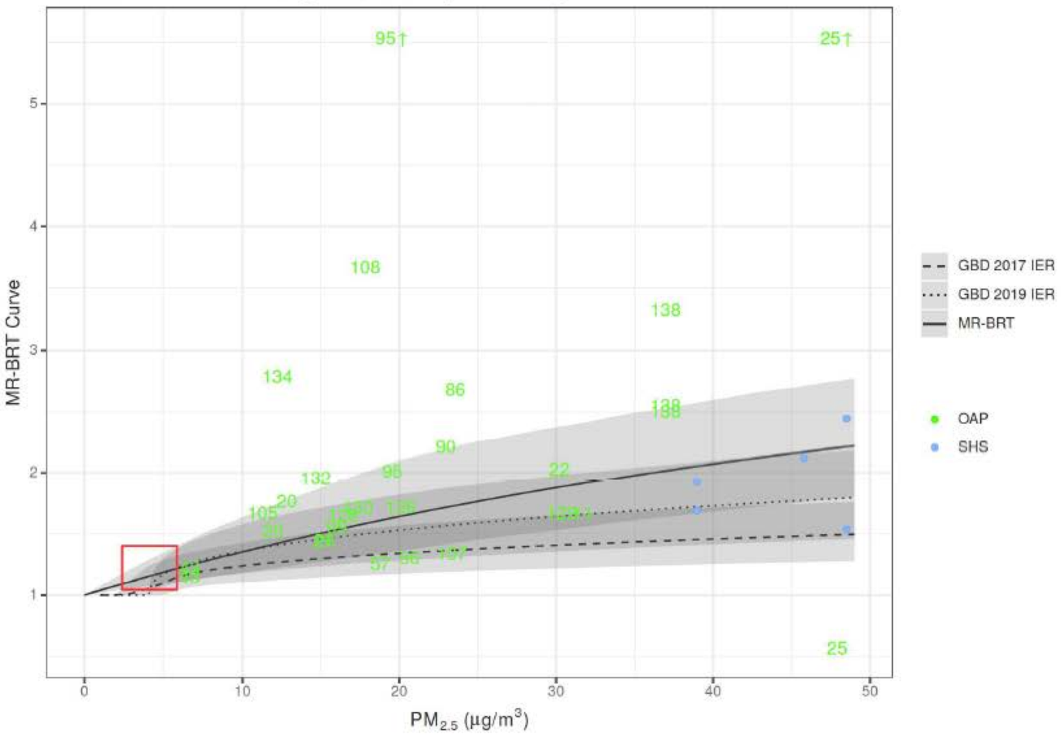

Ischemic Heart Disease, age 25, Full Exposure Range

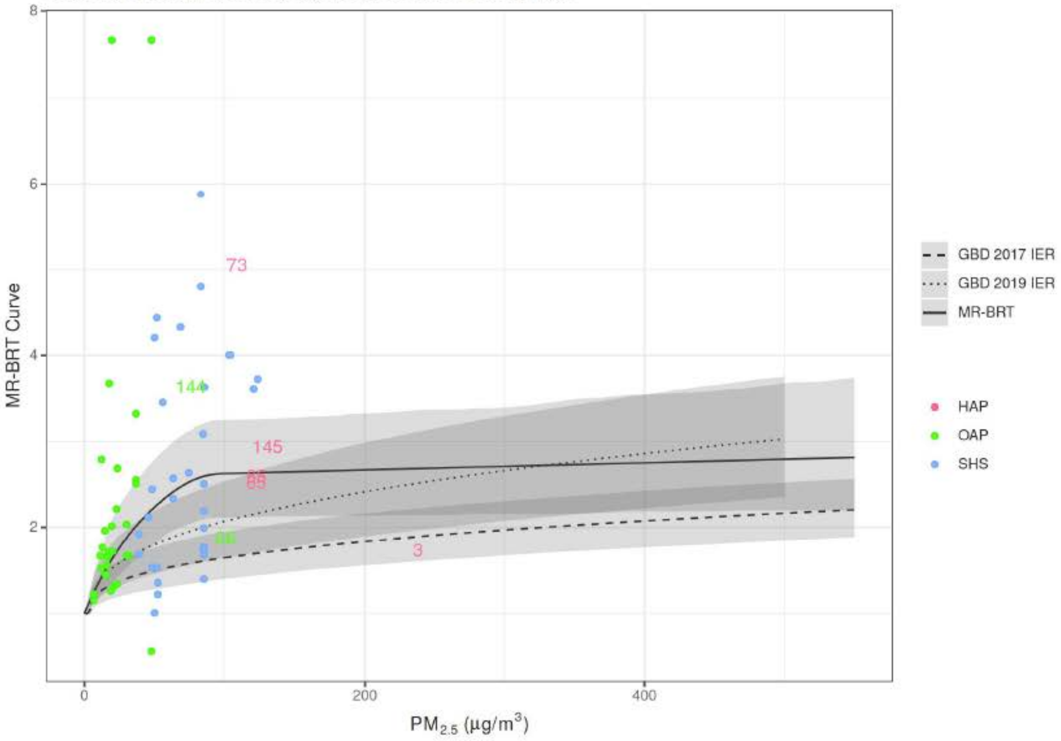

Ischemic Heart Disease, age 50, Low Exposure Range

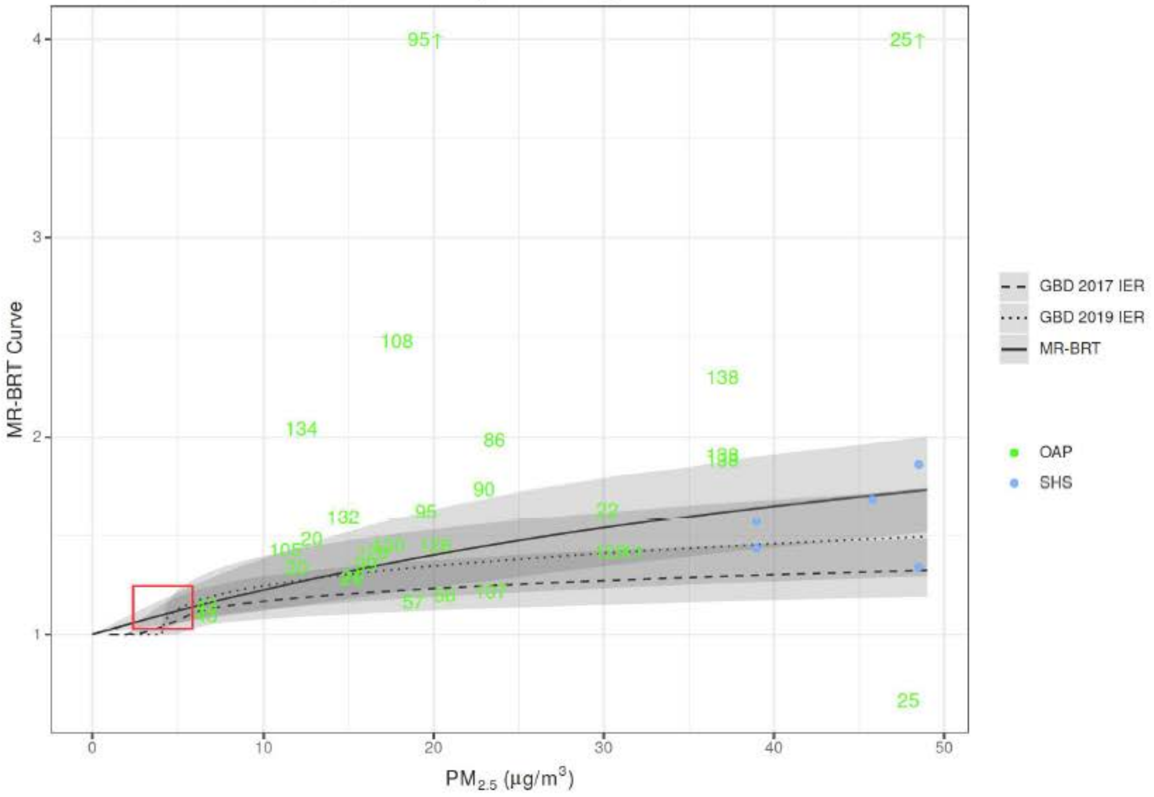

Ischemic Heart Disease, age 50, Full Exposure Range

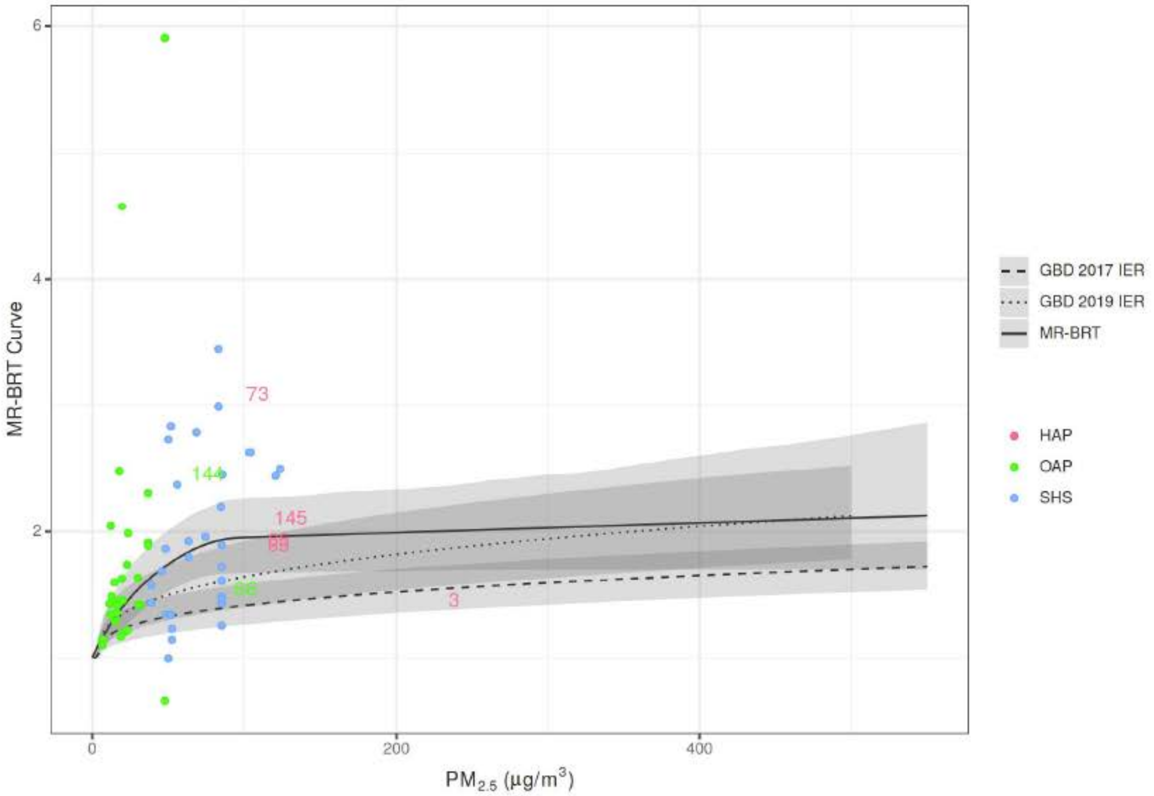

Ischemic Heart Disease, age 80, Low Exposure Range

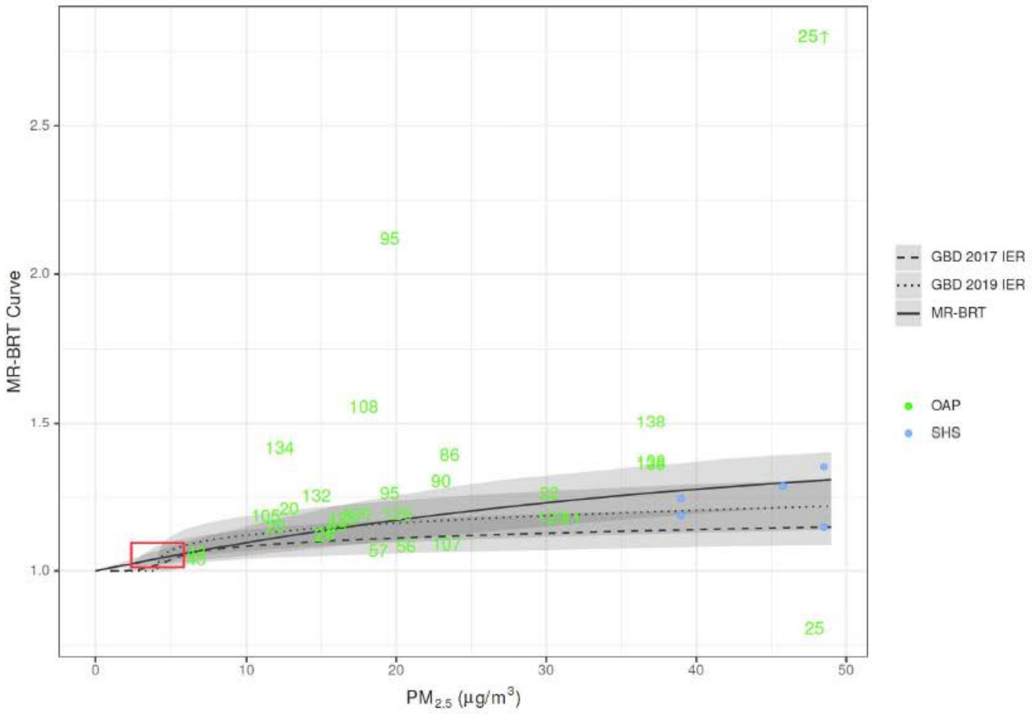

Ischemic Heart Disease, age 80, Full Exposure Range

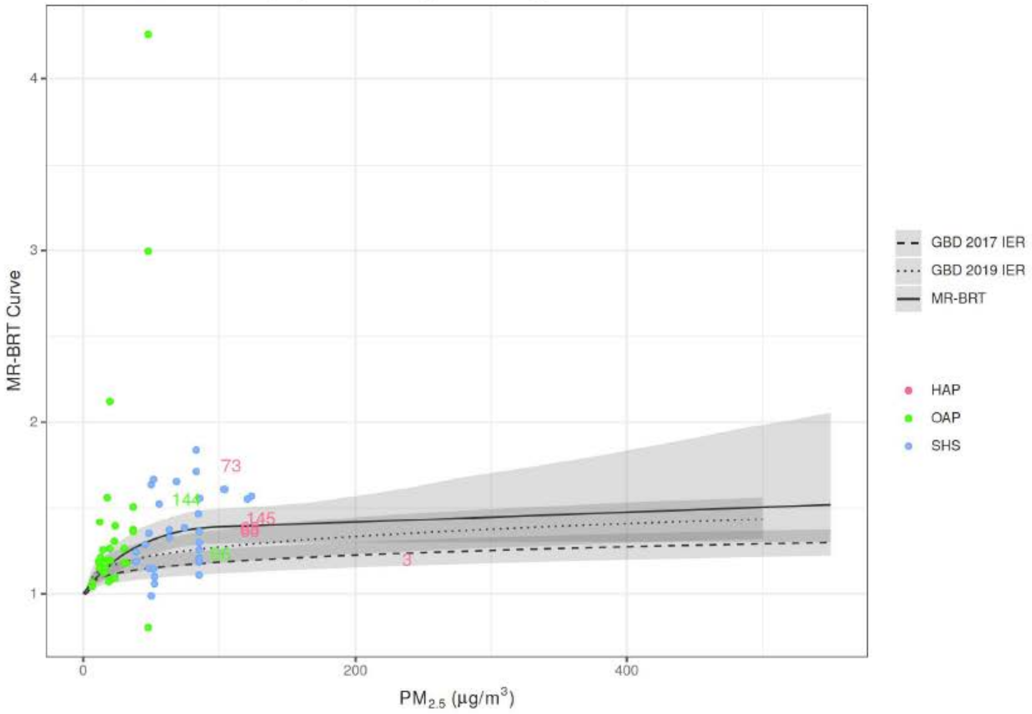

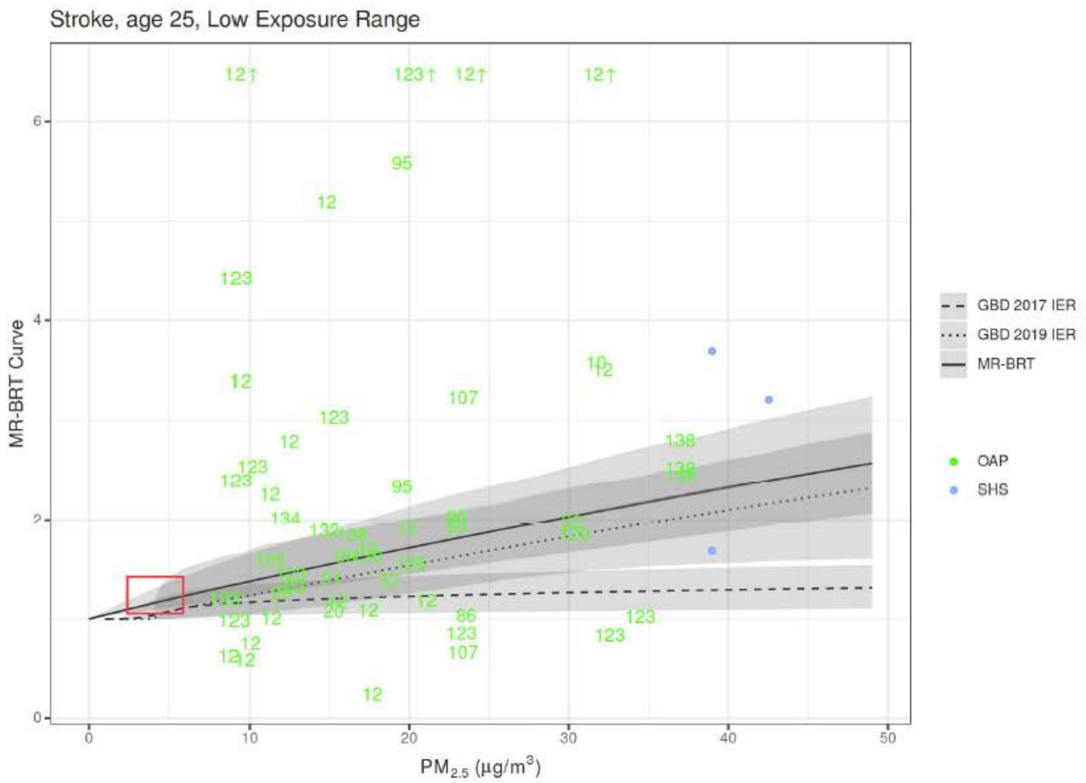
Stroke, age 25, Low Exposure Range
MR-BRT Curve
PM2.5 (μg/m3)
GBD 2017 IER
GBD 2019 IER
MR-BRT
OAP
SHS

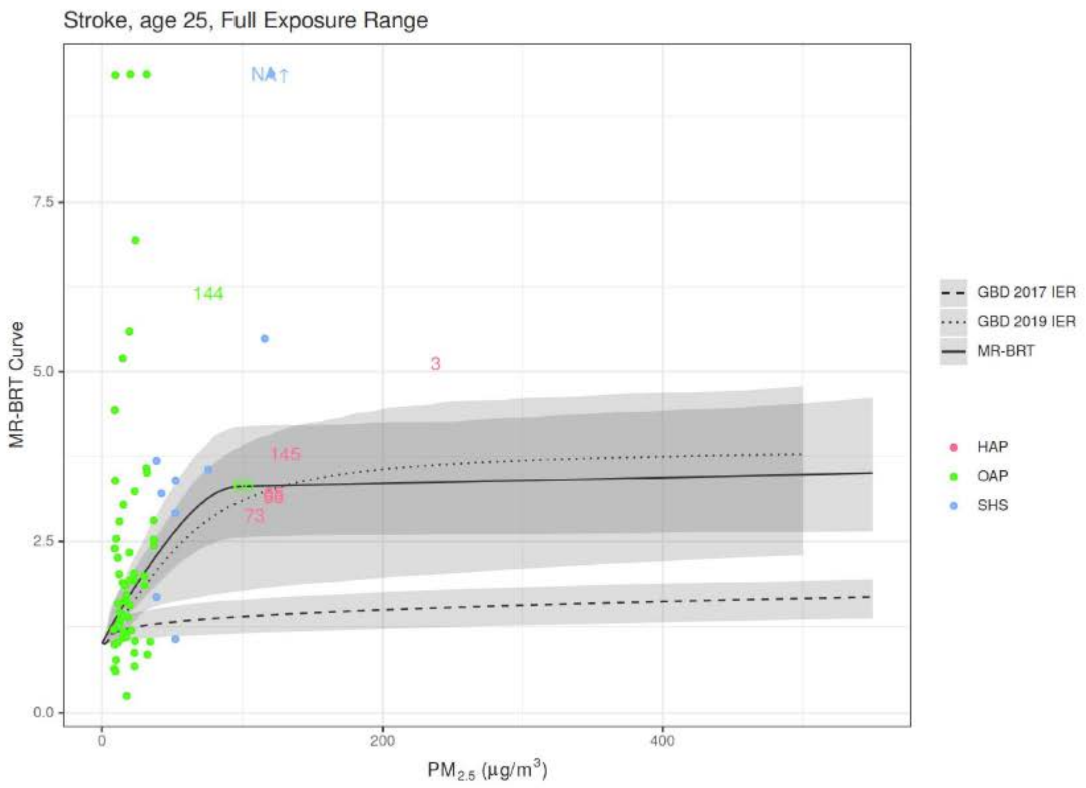
Stroke, age 25, Full Exposure Range
MR-BRT Curve
PM2.5 (μg/m3)
GBD 2017 IER
GBD 2019 IER
MR-BRT
HAP
OAP
SHS

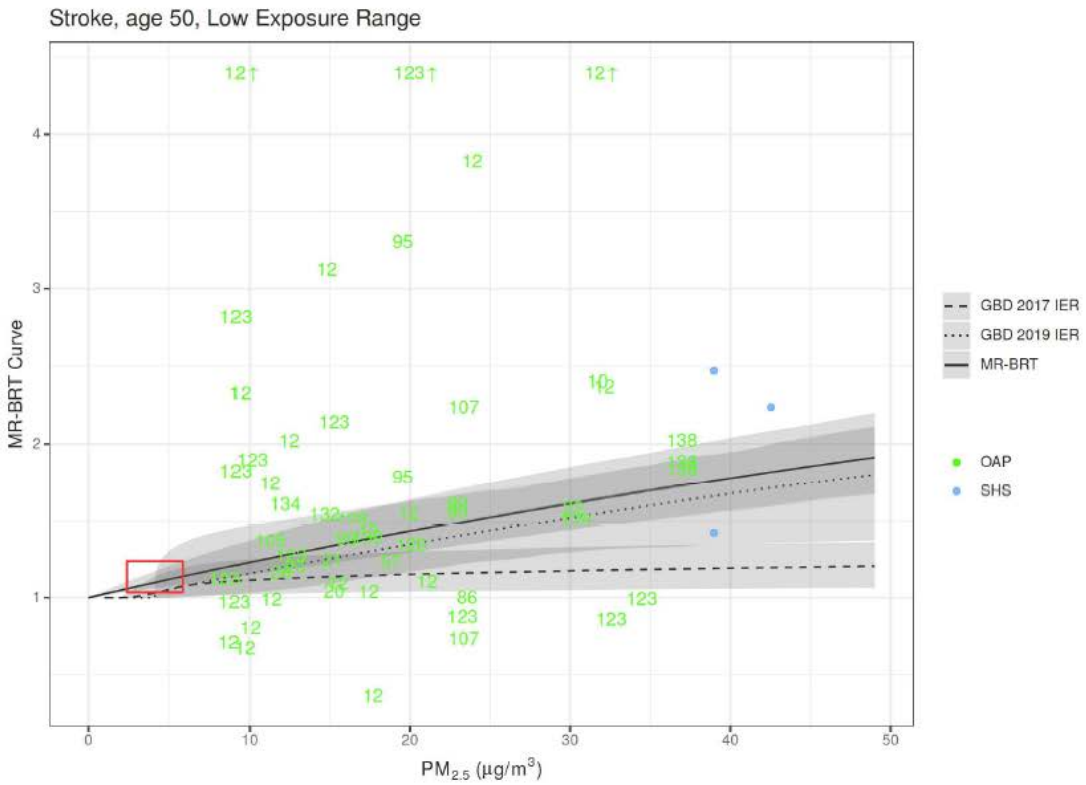
Stroke, age 50, Low Exposure Range
MR-BRT Curve
PM2.5 (µg/m3)
GBD 2017 IER
GBD 2019 IER
MR-BRT
OAP
SHS

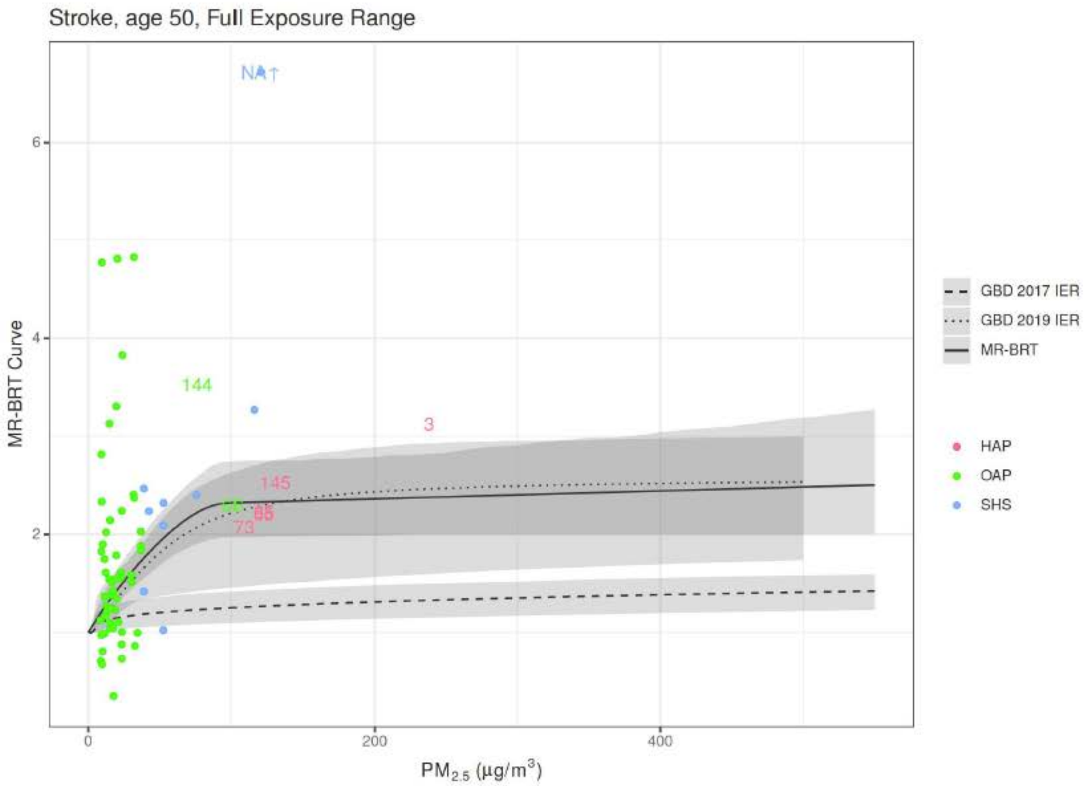
Stroke, age 50, Full Exposure Range
MR-BRT Curve
PM2.5 (µg/m3)
GBD 2017 IER
GBD 2019 IER
MR-BRT
HAP
OAP
SHS

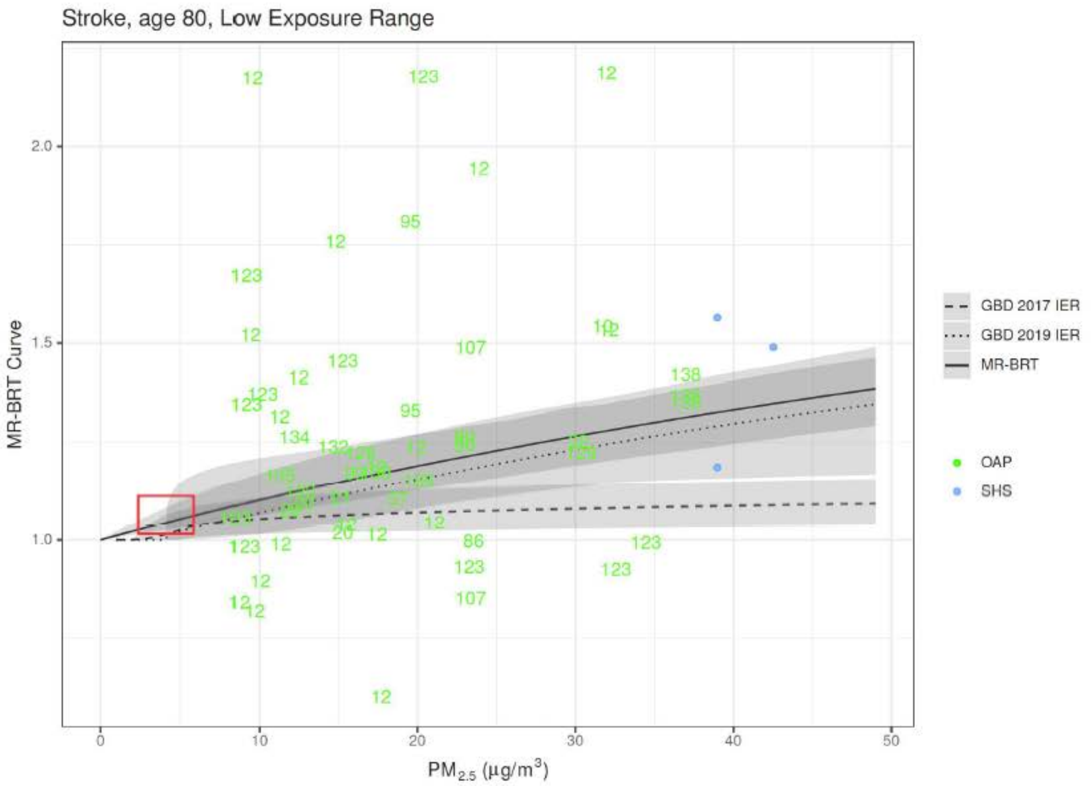

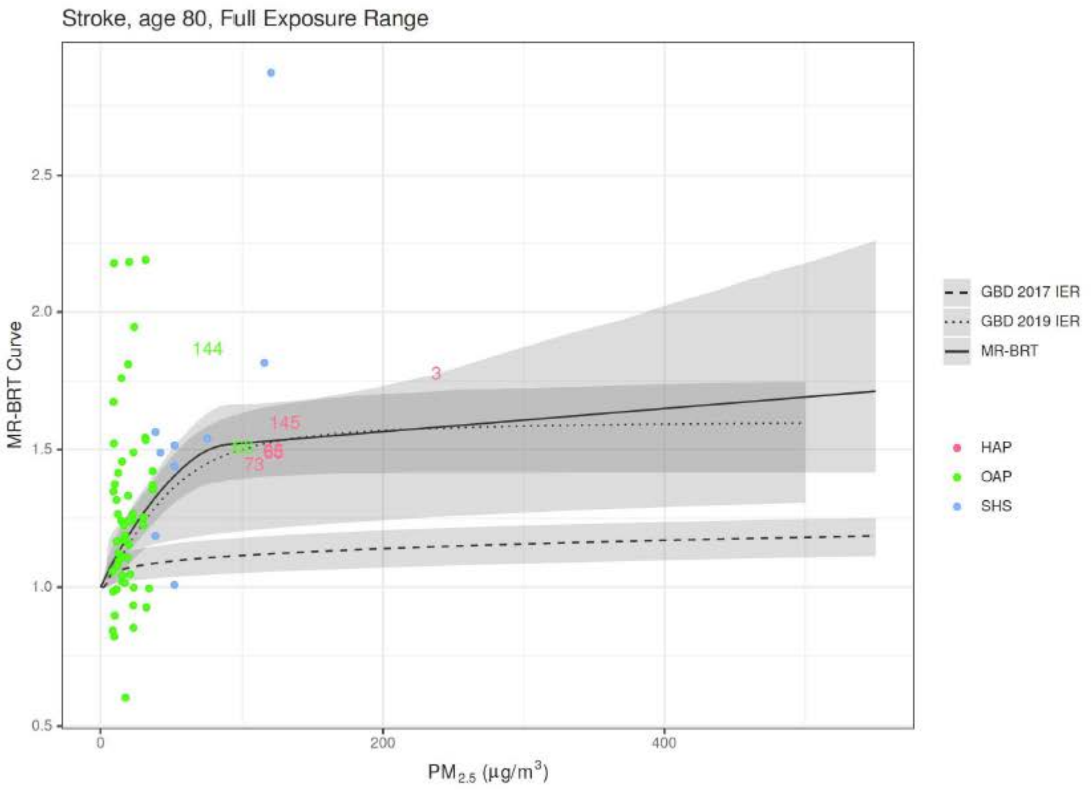

Low birthweight and short gestation mediation analysis

The outcomes of low birthweight and short gestation include mortality due to diarrhoeal diseases, lower respiratory infections, upper respiratory infections, otitis media, meningitis, encephalitis, neonatal preterm birth, neonatal encephalopathy due to birth asphyxia and trauma, neonatal sepsis

and other neonatal infections, haemolytic disease and other neonatal jaundice, and other neonatal disorders. We also calculate attributable YLDs for neonatal preterm birth. These are specific to ages 0-6 days and 7-27 days.

In partnership with Dr. Rakesh Ghosh at the University of California, San Francisco, we conducted a systematic review of all cohort, case-control, or randomised-controlled trial studies of ambient $PM_{2.5}$ pollution or household air pollution and birthweight or gestational age outcomes. Outcomes measured included continuous birthweight (bw), continuous gestational age (ga), low birthweight (LBW) (<2500 g), preterm birth (PTB) (<37 weeks), and very preterm birth (VPTB) (<32 weeks). We included any papers published until March 31, 2018. Systematic review PRISMA diagrams are below.

Ambient particulate matter pollution, low birth weight

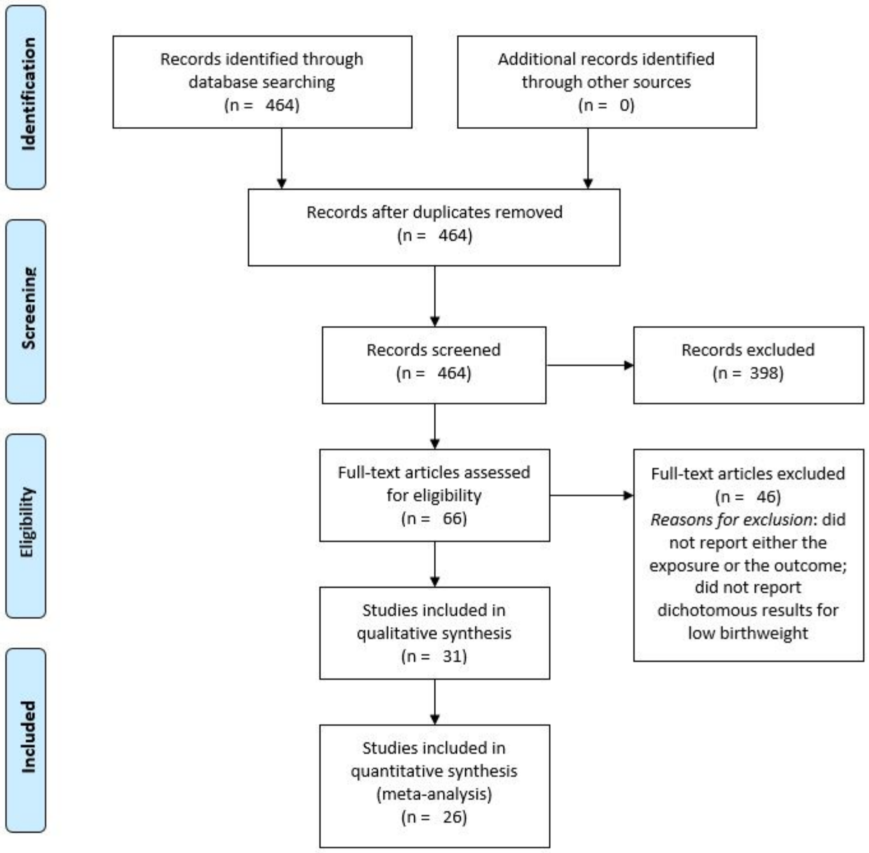

Ambient particulate matter pollution, preterm birth

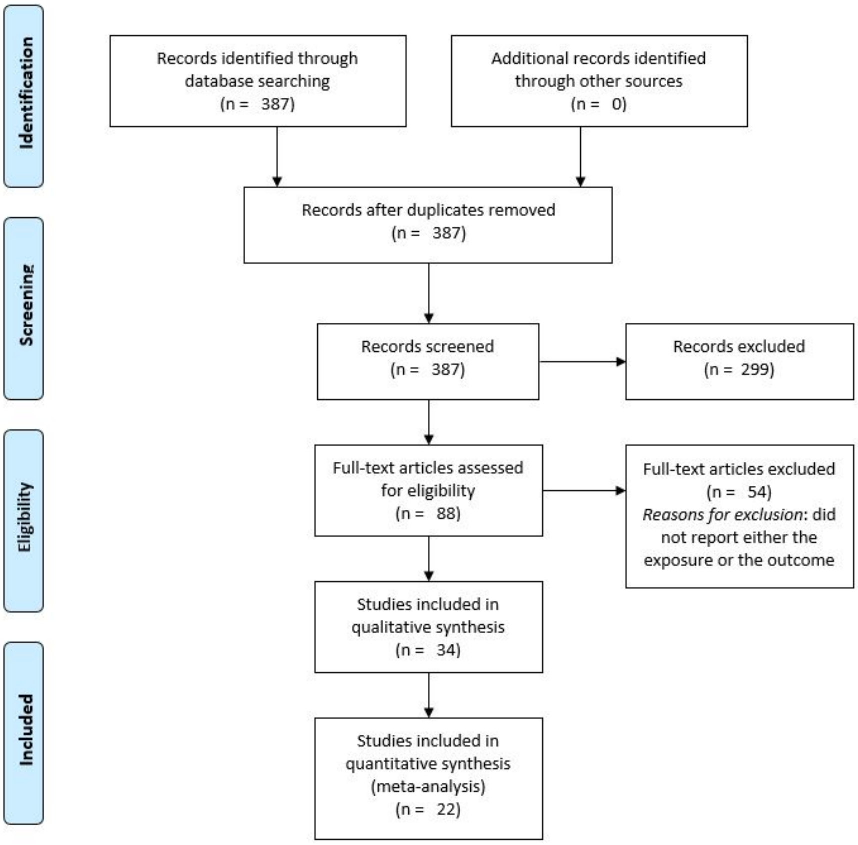

Household air pollution, all outcomes

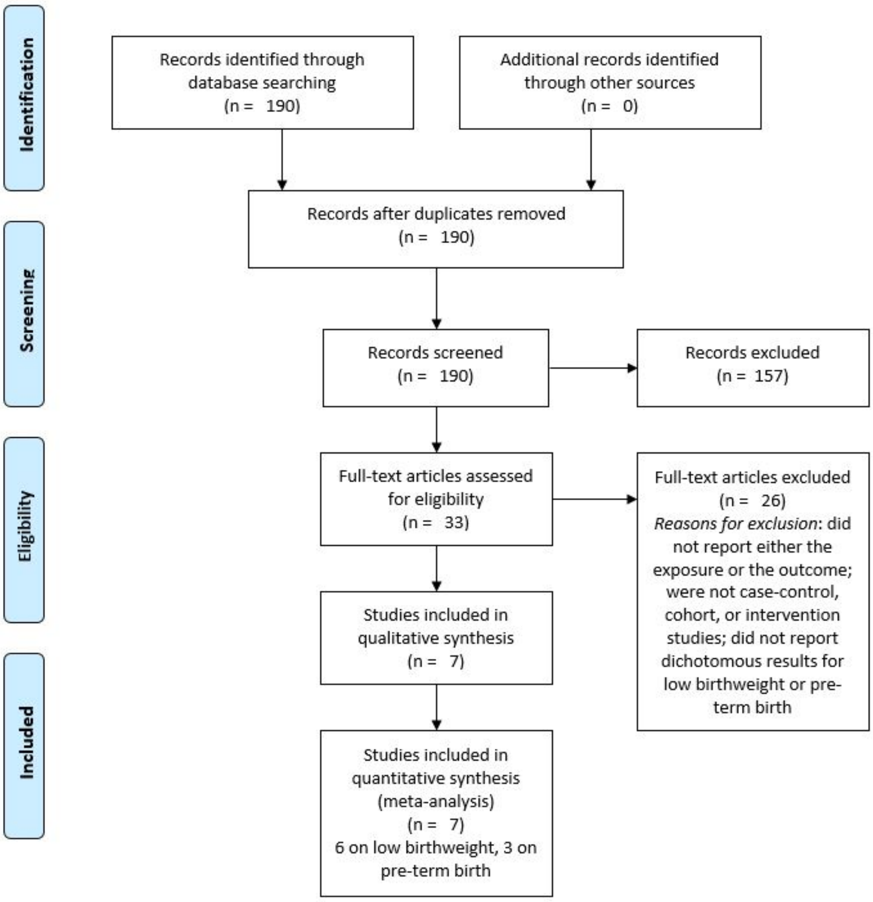

The following plots depict forest and funnel plots for studies of OAP and birthweight, low birthweight, and preterm birth. Note that these plots do not capture the exposure level of these studies but the linear risk or difference in birthweight per 10-unit increase in $PM_{2.5}$ exposure.

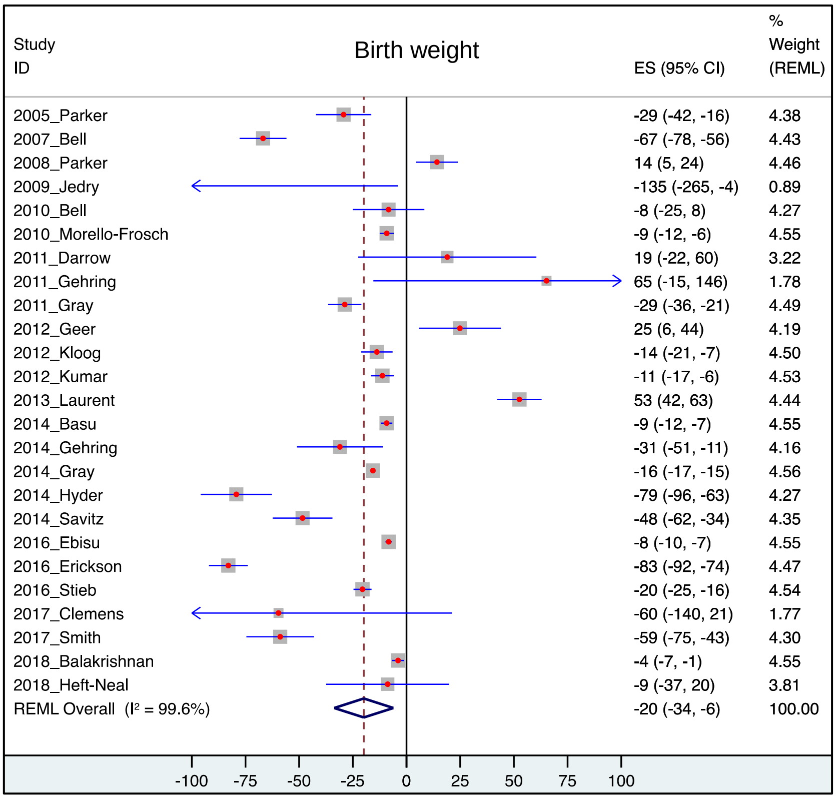
Birth weight
Study ID
ES (95% CI)
% Weight (REML)
2005_Parker -29 (-42, -16) 4.38
2007_Bell -67 (-78, -56) 4.43
2008_Parker 14 (5, 24) 4.46
2009_Jedry -135 (-265, -4) 0.89
2010_Bell -8 (-25, 8) 4.27
2010_Morello-Frosch -9 (-12, -6) 4.55
2011_Darrow 19 (-22, 60) 3.22
2011_Gehring 65 (-15, 146) 1.78
2011_Gray -29 (-36, -21) 4.49
2012_Geer 25 (6, 44) 4.19
2012_Kloog -14 (-21, -7) 4.50
2012_Kumar -11 (-17, -6) 4.53
2013_Laurent 53 (42, 63) 4.44
2014_Basu -9 (-12, -7) 4.55
2014_Gehring -31 (-51, -11) 4.16
2014_Gray -16 (-17, -15) 4.56
2014_Hyder -79 (-96, -63) 4.27
2014_Savitz -48 (-62, -34) 4.35
2016_Ebisu -8 (-10, -7) 4.55
2016_Erickson -83 (-92, -74) 4.47
2016_Stieb -20 (-25, -16) 4.54
2017_Clemens -60 (-140, 21) 1.77
2017_Smith -59 (-75, -43) 4.30
2018_Balakrishnan -4 (-7, -1) 4.55
2018_Heft-Neal -9 (-37, 20) 3.81
REML Overall (I² = 99.6%) -20 (-34, -6) 100.00
-100 -75 -50 -25 0 25 50 75 100

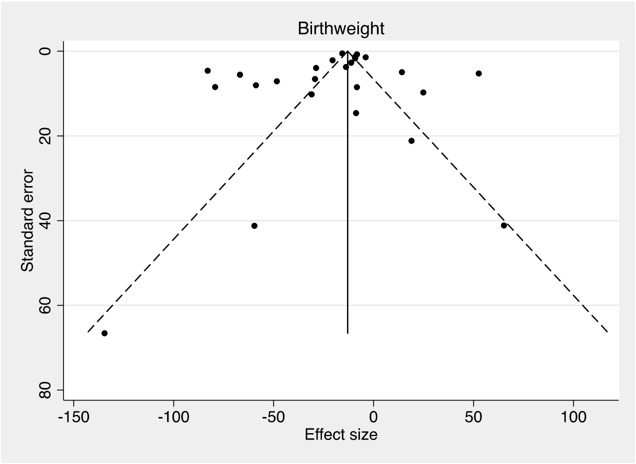
Birthweight
Standard error
0 20 40 60 80
-150 -100 -50 0 50 100
Effect size

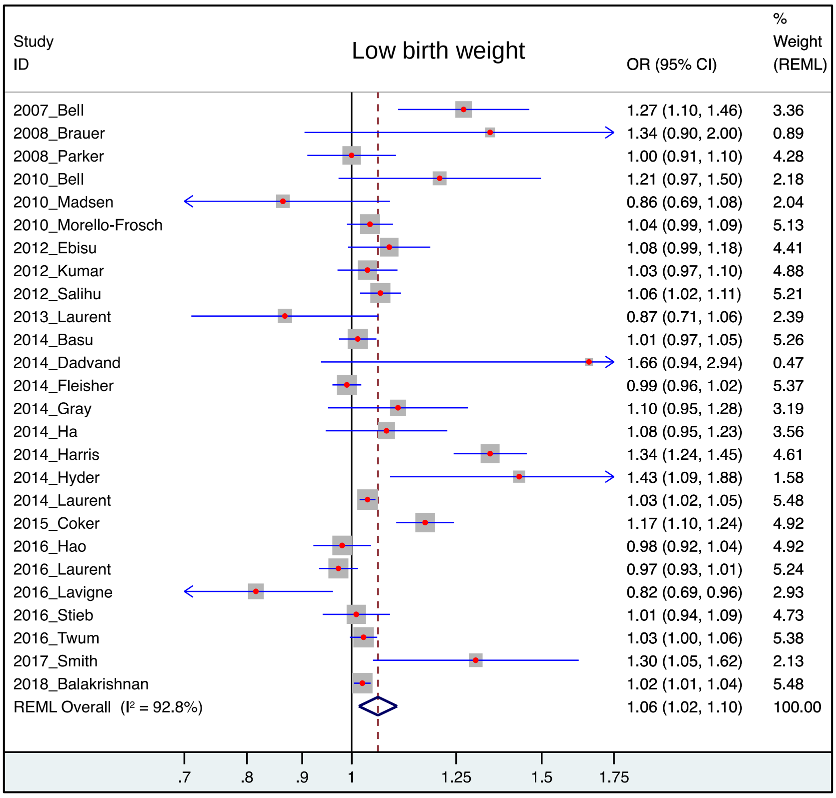
Study
ID
Low birth weight
OR (95% CI)
%
Weight
(REML)
2007_Bell 1.27 (1.10, 1.46) 3.36
2008_Brauer 1.34 (0.90, 2.00) 0.89
2008_Parker 1.00 (0.91, 1.10) 4.28
2010_Bell 1.21 (0.97, 1.50) 2.18
2010_Madsen 0.86 (0.69, 1.08) 2.04
2010_Morello-Frosch 1.04 (0.99, 1.09) 5.13
2012_Ebisu 1.08 (0.99, 1.18) 4.41
2012_Kumar 1.03 (0.97, 1.10) 4.88
2012_Salihu 1.06 (1.02, 1.11) 5.21
2013_Laurent 0.87 (0.71, 1.06) 2.39
2014_Basu 1.01 (0.97, 1.05) 5.26
2014_Dadvand 1.66 (0.94, 2.94) 0.47
2014_Fleisher 0.99 (0.96, 1.02) 5.37
2014_Gray 1.10 (0.95, 1.28) 3.19
2014_Ha 1.08 (0.95, 1.23) 3.56
2014_Harris 1.34 (1.24, 1.45) 4.61
2014_Hyder 1.43 (1.09, 1.88) 1.58
2014_Laurent 1.03 (1.02, 1.05) 5.48
2015_Coker 1.17 (1.10, 1.24) 4.92
2016_Hao 0.98 (0.92, 1.04) 4.92
2016_Laurent 0.97 (0.93, 1.01) 5.24
2016_Lavigne 0.82 (0.69, 0.96) 2.93
2016_Stieb 1.01 (0.94, 1.09) 4.73
2016_Twum 1.03 (1.00, 1.06) 5.38
2017_Smith 1.30 (1.05, 1.62) 2.13
2018_Balakrishnan 1.02 (1.01, 1.04) 5.48
REML Overall (I² = 92.8%) 1.06 (1.02, 1.10) 100.00
.7
.8
.9
1
1.25
1.5
1.75

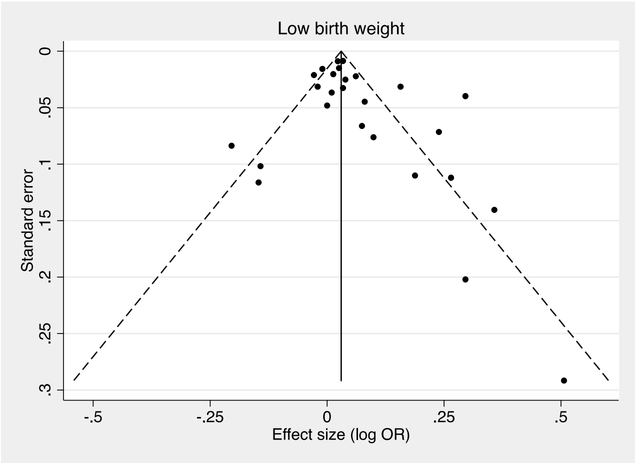
Low birth weight
Standard error
0
.05
.1
.15
.2
.25
.3
-.5
-.25
0
.25
.5
Effect size (log OR)

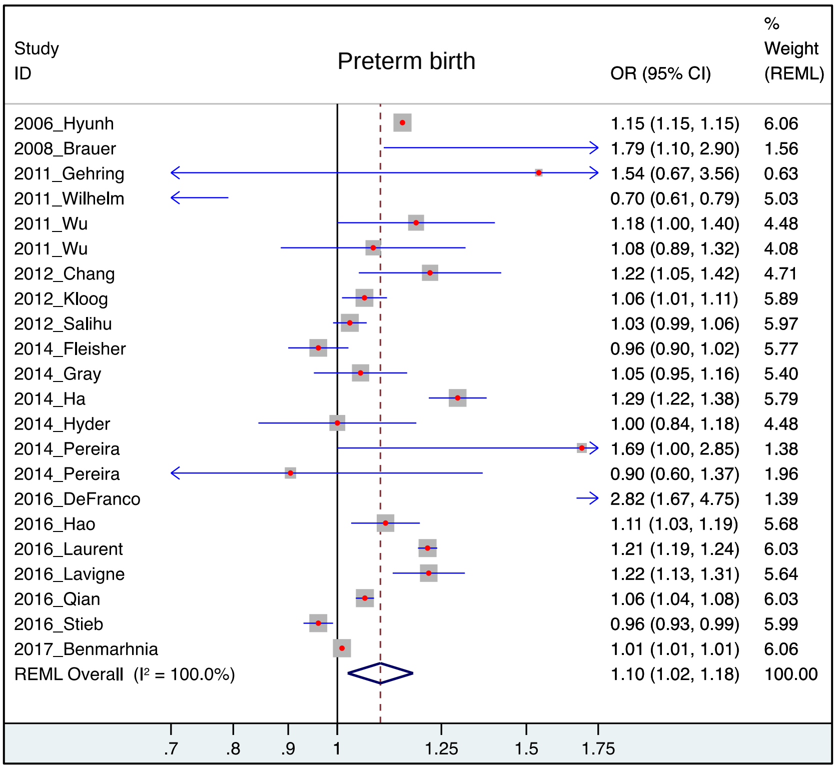

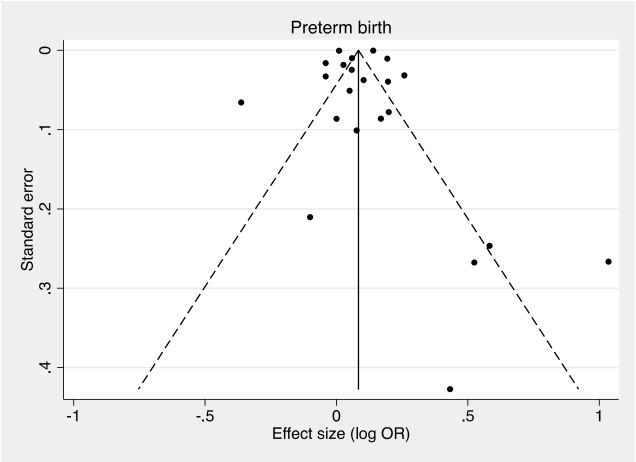

For studies of household air pollution, we used the same strategy described above to map them to $PM_{2.5}$ exposure values.

Because birthweight and gestational age are modelled using a continuous joint distribution for the GBD, we were interested in how those distributions changed under the influence of $PM_{2.5}$ pollution. We therefore estimated the continuous shift in birthweight (bw, in grams) and gestational age (ga, in weeks) at a given $PM_{2.5}$ exposure level.

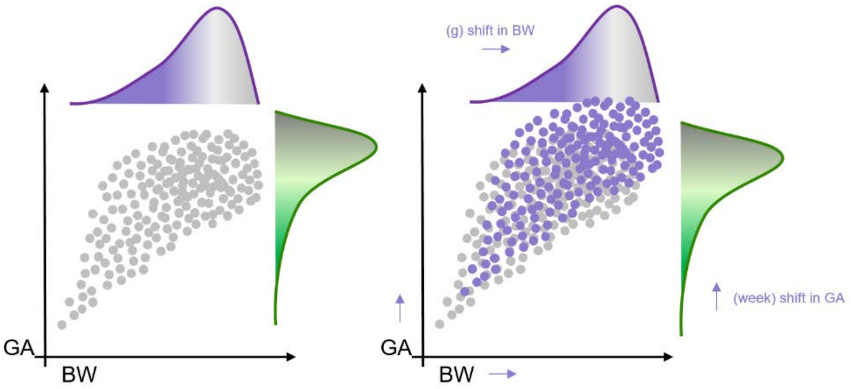

When available, we used estimates of continuous shift in bw or ga directly from each study. When that was not available, we used the published OR/RR/HR for LBW, PTB, or VPTB and the following strategy:

1. Extract the OR/RR/HR from the study.
2. Select the GBD 2017 estimated bw-ga joint distribution for the study location and year.
3. Calculate the number of grams or weeks required to shift the distribution such that the proportion of births under the specified threshold (P) is reduced by the study effect size to a counterfactual level (P_{cf}).
4. Save the resulting shift and 95% CI as the continuous effect.

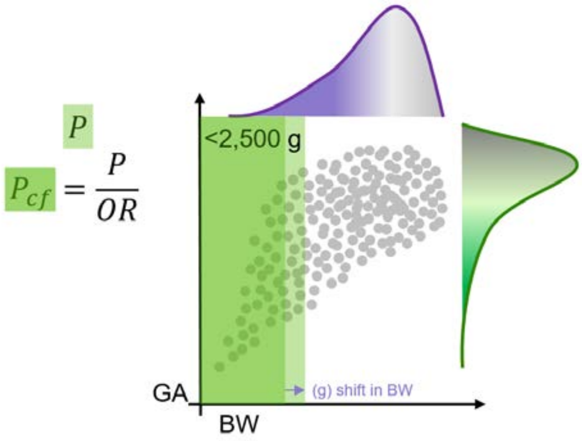

We then fit a MR-BRT spline to these studies, where the difference in the value of the model at the upper concentration (X) and the value of the model at the counterfactual concentration (X_{CF}) is equal to the published or calculated shift in bw or ga. We fit the same model and priors as the non-mediated outcomes (with the exception of COPD), except, because the change in birthweight and gestational age was expected to be negative, the shape constraints were monotonically decreasing and concave up.

$$MRBRT(X) - MRBRT(X_{CF}) \sim Shift$$

The following figures depict the MR-BRT curves for shift in grams (bw) and weeks (ga).

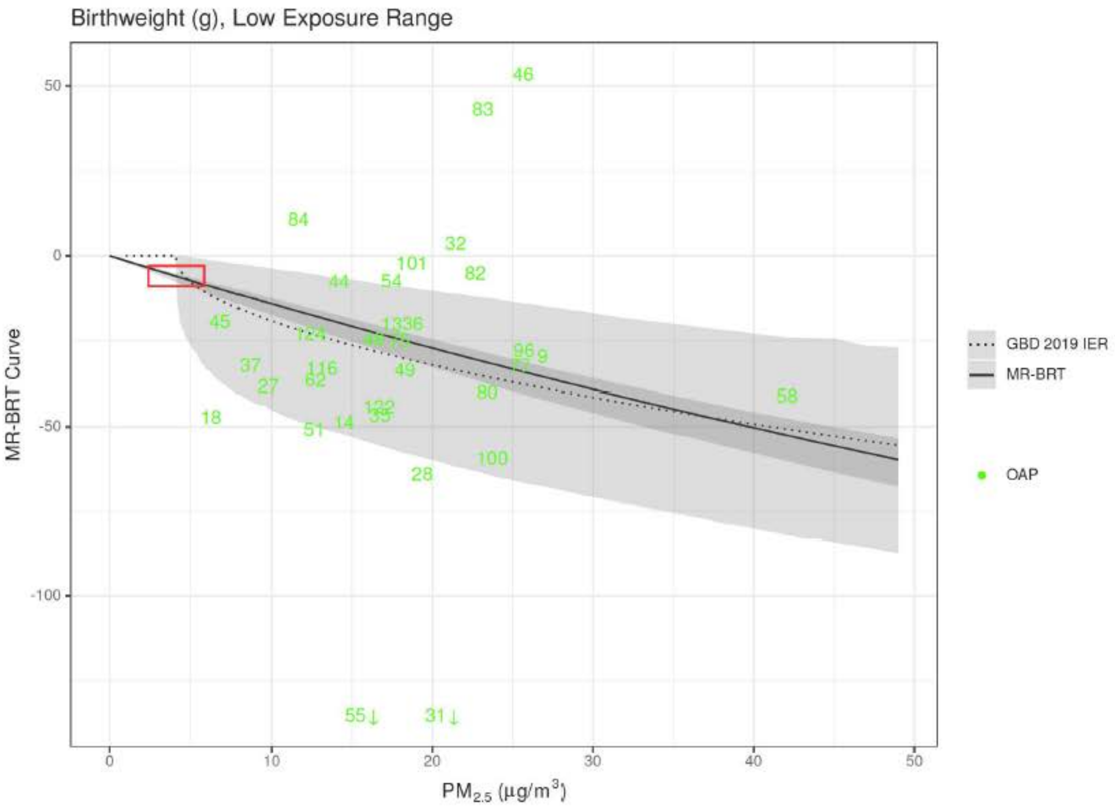
Birthweight (g), Low Exposure Range
MR-BRT Curve
PM2.5 (µg/m3)
GBD 2019 IER
MR-BRT
OAP

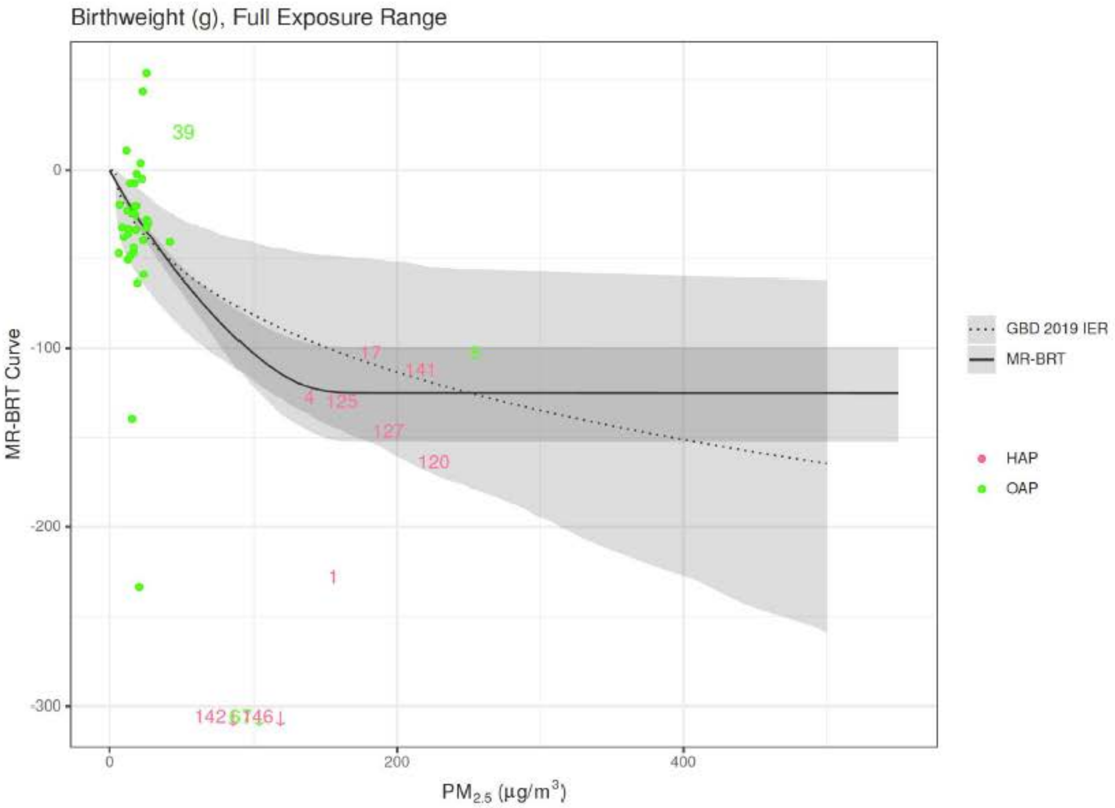
Birthweight (g), Full Exposure Range
MR-BRT Curve
PM2.5 (µg/m3)
GBD 2019 IER
MR-BRT
HAP
OAP

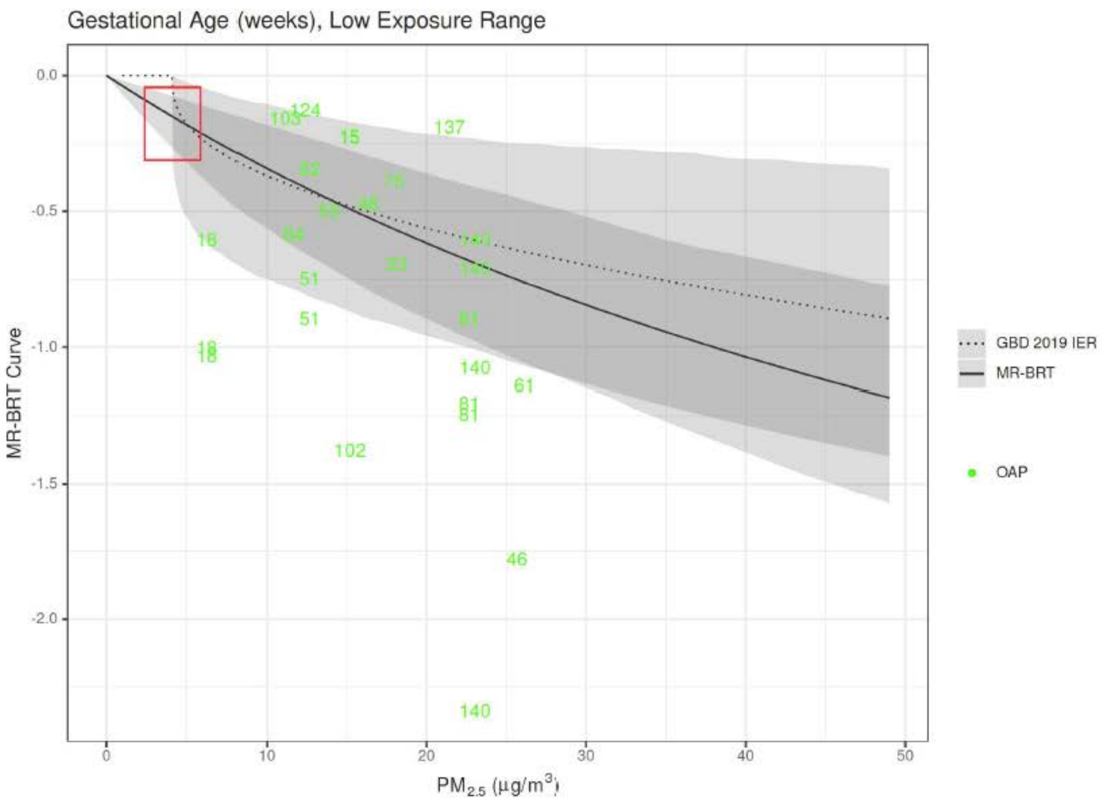

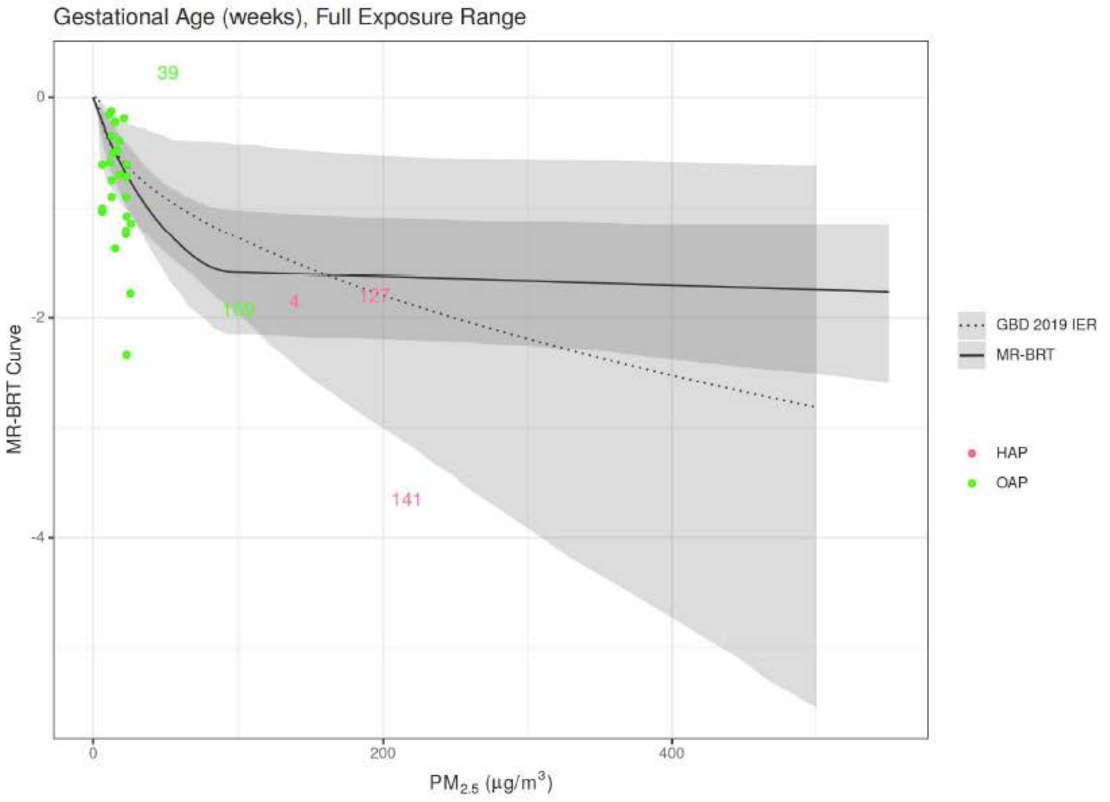

Once we had curves of estimated shifts across the exposure range, we predicted the shift in both birthweight and gestational age for total female particulate matter pollution exposure in each location and year. Because the epidemiological studies mutually controlled for birthweight and gestational age, we assumed these shifts are independent. We then shifted the observed

distributions to reflect the expected bwga distribution in the absence of particulate matter pollution. These shifted distributions were used as the counterfactual in the PAF calculation equation to calculate the burden attributable to $PM_{2.5}$ pollution.

To calculate PAFs, the distribution is divided into 56 bw-ga categories, each with a unique RR. Let p_i be the observed proportion of babies in category, *i* and p_i' be the counterfactual proportion of babies in category, *i* if there were no particulate matter pollution.

$$PAF_{PM} = \frac{\sum_{i \in bwga\ category} RR_i p_i - \sum_{i \in bwga\ category} RR_i p_i'}{\sum_{i \in bwga} RR_i p_i}$$

We proportionately split this PAF to ambient and HAP based on exposure as is described below. One important assumption to note is that we are assuming the shift in bw and ga is linear across the bwga distribution.

For lower respiratory infections, we have directly estimated PAFs attributable to $PM_{2.5}$ in addition to those mediated through birthweight and gestational age. We would expect that some of the directly estimated PAFs are mediated through bw and ga. Additionally, the directly estimated PAF is based on a summary of relative risks for all children under 5 years, so there is a chance that the mediated PAF, which is more finely resolved, could be greater. To avoid double-counting for these two age groups (0-6 days and 0-27 days), we take the max of the two PAF estimates. If the directly estimated PAF is greater than the bw-ga-mediated PAF, we take the direct estimate, and if the mediated PAF is greater, we take the mediated.

PTB incidence and mortality are both outcomes measured in the GBD. 100% of the burden for this cause is attributable to short gestation. To calculate the percentage attributable to particulate matter pollution, we estimated the percentage of babies born at less than 37 weeks (p_{ptb}) and the percentage of babies that would have been born at less than 37 weeks in the counterfactual scenario of no particulate matter pollution (p_{ptb}').

$$PAF_{ptb,pm} = 1 - \frac{p_{ptb}'}{p_{ptb}}$$

Limitations

Although in GBD 2019 we have not used active smoking data to estimate the risk curves, we are still using an integrated exposure response approach because we are integrating relative risk estimates across various exposure sources: ambient, SHS, and HAP. The use of various sources to construct a risk curve with $PM_{2.5}$ as the exposure indicator assumes equitoxicity of particles, despite some evidence suggesting differences in health impact by PM source, size, and chemical composition. However, in the absence of consistent and robust evidence of differential toxicity by source and sufficient estimates of source or composition-specific exposure-response relationships, integrating across OAP, SHS, and HAP studies is the approach most consistent with the current evidence, as reviewed by US EPA and WHO.[20,21] Use of a common risk function may affect the magnitude of risk estimates for HAP and OAP compared to separate risk functions. As more data from higher OAP concentration locations and from HAP studies for non-respiratory outcomes becomes available it may be possible to evaluate the strength of evidence for each and to develop separate risk functions.

Proportional PAF approach

Prior to GBD 2017, relative risks for both exposures were obtained from the IER as a function of exposure and relative to the same TMREL. In reality, were a country to reduce only one of these risk

factors, the other would remain. We did not consider the joint effects of particulate matter from outdoor exposure and burning solid fuels for cooking. For GBD 2017 we developed a new approach to use the IER for obtaining PAFs for both OAP and HAP:

Let Exp_{OAP} be the ambient $PM_{2.5}$ exposure level and Exp_{HAP} be the excess exposure for those who use solid fuel for cooking. Let P_{HAP} be the proportion of the population using solid fuel for cooking. We calculated PAFs at each 0.1°×0.1° grid cell. We assumed that the distribution of those using solid fuel for cooking (HAP) was equivalent across all grid cells of the GBD location.

For the proportion of the population not exposed to HAP the relative risk was:

$$RR_{OAP} = MRBRT(z = Exp_{OAP})/MRBRT(z = TMREL),$$

And for those exposed to HAP, the relative risk was

$$RR_{HAP} = MRBRT(z = Exp_{OAP} + Exp_{HAP})/MRBRT(z = TMREL).$$

We then calculate a population level RR and PAF for all particulate matter exposure.

$$RR_{PM} = RR_{OAP}(1 - P_{HAP}) + RR_{HAP}P_{HAP}$$

$$PAF_{PM} = \frac{RR_{PM} - 1}{RR_{PM}}$$

We population weight the grid-cell level particulate matter PAFs to get a country level PAF, and finally, we split this PAF based on the average exposure to each OAP and HAP.

$PAF_{OAP} = \frac{Exp_{OAP}}{Exp_{OAP}+P_{HAP}*Exp_{HAP}} PAF_{PM}$, and $PAF_{HAP} = \frac{P_{HAP}*Exp_{HAP}}{Exp_{OAP}+P_{HAP}*Exp_{HAP}} PAF_{PM}$.

With this strategy, $PAF_{PM} = PAF_{HAP} + PAF_{OAP}$, and no burden is counted twice.

References

1. Hammer, M. S., A. van Donkelaar, R. V. Martin, C. Li, A. Lyapustin, A. M. Sayer, C. N. Hsu, R. C. Levy, M. J. Garay, O. V. Kalashnikova, R. A. Kahn, M. Brauer, J. S. Apte, D. K. Henze, L. Zhang, and Q. Zhang (submitted), Improved Global Estimates of Fine Particulate Matter Concentrations and Trends Derived from Updated Satellite Retrievals, Modeling Advances, and Additional Ground-Based Monitors, *Environ. Sci. Technol*.
2. van Donkelaar, A.; Martin, R. V; Brauer, M.; Hsu, N. C.; Kahn, R. A.; Levy, R. C.; Lyapustin, A.; Sayer, A. M.; Winker, D. M. Global Estimates of Fine Particulate Matter using a Combined Geophysical-Statistical Method with Information from Satellites, Models, and Monitors. Environ. Sci. Technol. 2016, 50 (7), 3762–3772
3. Shaddick, G., Thomas, M.L., Jobling, A., Brauer, M., van Donkelaar, A., Burnett, R., Chang, H., Cohen, A., Van Dingenen, R., Dora, C. and Gumy, S., 2016. Data Integration Model for Air Quality: A Hierarchical Approach to the Global Estimation of Exposures to Ambient Air Pollution. Journal of Royal Statistical Society Series C (Applied Statistics). 2017. DOI: 10.1111/rssc.12227
4. Shaddick, G., Thomas, M. L., Mudu, P., Ruggeri, G. and Gumy, S. Half the world's population are exposed to increasing air pollution. Accepted by Nature Climate and Atmospheric Science.
5. Brauer, M.; Freedman, G.; Frostad, J.; van Donkelaar, A.; Martin, R. V; Dentener, F.; Van Dingenen, R.; Estep, K.; Amini, H.; Apte, J. S.; et al. Ambient Air Pollution Exposure Estimation for the Global Burden of Disease 2013. Environ. Sci. Technol. 2015, 50 (1), 79–88.

6. Shaddick G, Thomas M, Amini H, Broday DM, Cohen A, Frostad J, Green A, Gumy S, Liu Y, Martin RV, Prüss-Üstün A, Simpson D, van Donkelaar A, Brauer M. Data integration for the assessment of population exposure to ambient air pollution for global burden of disease assessment. Environ Sci Technol. 2018 Jun 29. doi: 10.1021/acs.est.8b02864
7. Rue, H.; Martino, S.; Chopin, N.; Approximate Bayesian inference for latent Gaussian models by using integrated nested Laplace approximations. Journal of the royal statistical society: Series b (statistical methodology). 2009;71(2):319-92.
8. Thomas, M. L., Shaddick, G., Simpson, D., de Hoogh, K. and Zidek, J. V. Spatio-temporal downscaling for continental–scale estimation of air pollution concentrations. arXiv preprint arXiv:1907.00093 (also been Submitted to the Journal of the Royal Statistical Society: Series C (Applied Statistics)).
9. Wood, S. N. (2017). Generalized additive models: an introduction with R. Chapman and Hall/CRC.
10. Turner MC,Jerrett M, Pope CA 3rd, Krewski D, Gapstur SM, Diver WR, Beckerman BS, Marshall JD, Su J, Crouse DL, Burnett RT. Long-term ozone exposure and mortality in a large prospective study . Am J Respir Crit Care Med. 2016; 193(10): 1134-42.
11. Yin P, Brauer M, Cohen A, et al. Long-term Fine Particulate Matter Exposure and Nonaccidental and Cause-specific Mortality in a Large National Cohort of Chinese Men. Environ Health Perspect 2017; 125: 117002.
12. Li T, Zhang Y, Wang J, et al. All-cause mortality risk associated with long-term exposure to ambient PM2·5 in China: a cohort study. Lancet Public Health 2018; 3: e470–7.
13. Yang Y, Tang R, Qiu H, et al. Long term exposure to air pollution and mortality in an elderly cohort in Hong Kong. Environ Int 2018; 117: 99–106.
14. Hystad P, Larkin A, Rangarajan S, AlHabib KF, Avezum A, Tumerdem Calik KB; Chifamba J, Dans A, Diaz R, du Plessis JL, Gupta R, Iqbal R, Khatib R, Kelishadi R, Lanas F, Liu Z, Lopez-Jaramillo P, Nair S, Poirier P, Rahman O, Rosengren A, Swidan H, Tse L-A, Wei L, Wielgosz A, Yeates K, Yusoff K, Zatoński T, Yusuf S, Brauer M. Outdoor fine particulate matter air pollution and cardiovascular disease: Results from 747 communities across 21 countries in the PURE Study. (Submitted to Lancet Global Health)
15. Joseph P, Rangarajan S, Islam S, Mente A, Hystad P, Brauer M, Raman Kutty V, Gupta R, Wielgosz A, AlHabib KF, Dans A, Lopez-Jaramillo P, Avezum A, Lanas F, Oguz A, Kruger IM, Diaz R, Yusoff K, Mony P, Chifamba J, Yeates K, Kelishadi R, Yusufali A, Khatib R, Rahman O, Zatonska K, Iqbal R, Wei L, Bo H, Rosengren A, Kaur M, Mohan V, Lear SA, Teo KK, O'Donnell M, McKee M, Dagenais G, Yusuf S. Modifiable risk factors, cardiovascular disease and mortality in 155,722 individuals from 21 high-, middle-, and low-income countries (PURE): a prospective cohort study. The Lancet. 2019. doi:10.1016/S0140-6736(19)32008-2
16. Burnett RT, Pope CA 3rd, Ezzati M, Olives C, Lim SS, Mehta S, Shin HH, Singh G, Hubbell B, Brauer M, Anderson HR, Smith KR, Balmes JR, Bruce NG, Kan H, Laden F, Prüss-Ustün A, Turner MC, Gapstur SM, Diver WR, Cohen A. An integrated risk function for estimating the global burden of disease attributable to ambient fine particulate matter exposure. *Environ Health Perspect*. 2014; 122(4): 397-403.
17. Pope CA III, Cohen AJ, Burnett RT. Cardiovascular Disease and Fine Particulate Matter: Lessons and Limitations of an Integrated Exposure Response Approach. Circulation Research. 2018;122:1645-1647.
18. Lind L, Sundström J, Ärnlöv J, Lampa E. Impact of Aging on the Strength of Cardiovascular Risk Factors: A Longitudinal Study Over 40 Years. *J Am Heart Assoc.* 2018;7(1):e007061. Published 2018 Jan 6. doi:10.1161/JAHA.117.007061
19. Semple S, Apsley A, Ibrahim TA, Turner SW, Cherrie JW. Fine particulate matter concentrations in smoking households: just how much secondhand smoke do you breathe in if you live with a smoker who smokes indoors? *Tob Control* 2015; 24: e205–11.

20. US Environmental Protection Agency. Integrated science assessment (ISA) for particulate matter (Final Report, Dec 2009). EPA/600/R-08/139F, 2009. Washington, DC: US Environmental Protection Agency; 2009. Available at: http://cfpub.epa.gov/ncea/risk/recordisplay.cfm?deid=216546
21. World Health Organization. Review of evidence on health aspects of air pollution – REVIHAAP Project technical report. Copenhagen: WHO Regional Office for Europe; 2013. Available at: http://www.euro.who.int/__data/assets/pdf_file/0004/193108/REVIHAAP-Final-technical-report-final-version.pdf?ua=1

Household air pollution

Flowchart

Household Air Pollution from Solid Fuels

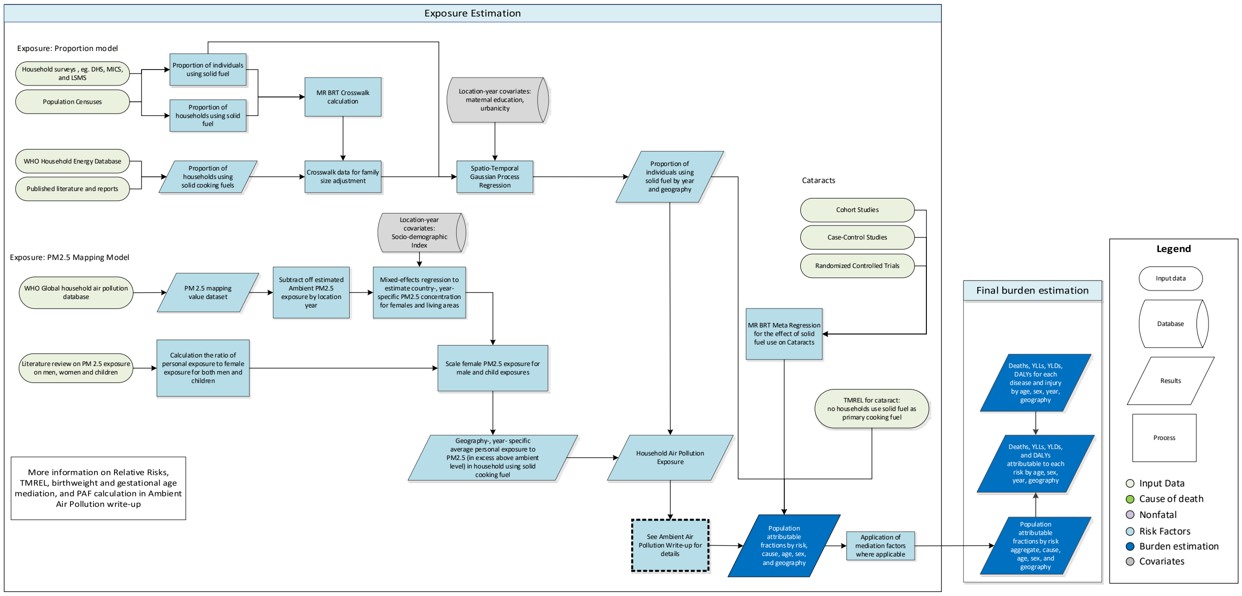

Input data and methodological summary

Exposure

Case definition

Exposure to household air pollution from solid fuels (HAP) is estimated from both the proportion of individuals using solid cooking fuels and the level of $PM_{2.5}$ air pollution exposure for these individuals. Solid fuels in our analysis include coal, wood, charcoal, dung, and agricultural residues.

Input data

We extracted information on use of solid fuels from the standard multi-country survey series such as Demographic and Health Surveys (DHS), Living Standards Measurement Surveys (LSMS), Multiple Indicator Cluster Surveys (MICS), and World Health Surveys (WHS), as well as censuses and country-specific survey series such as Kenya Welfare Monitoring Survey and South Africa General Household Survey. To fill the gaps of data in surveys and censuses, we also downloaded and updated estimates from WHO Energy Database and extracted from literature through systematic review. Each nationally or subnationally representative datapoint provided an estimate for the percentage of households using solid cooking fuels. We used studies from 1980 to 2019 to inform the time series.

We also excluded sources that did not distinguish specific primary fuel types, estimated fuel used for purposes other than cooking (eg, lighting or heating), failed to report standard error or sample size, had over 15% of households with missing responses, reported fuel use in physical units, or were secondary sources referencing primary analyses. Table 1 summarizes exposure input data.

Table 1: Exposure Input Data

Input data	Exposure
Source count (total)	1680
Number of countries with data	195

Family size crosswalk

Many estimates in the WHO Energy Database and other reports quantify the proportion of households using solid fuel for cooking; however, we are interested in the proportion of individuals using solid fuel for cooking. To crosswalk these estimates, whenever we had the available information, we extracted fuel use at both the individual and household levels. We included 3676 source-specific pairs in the MR-BRT crosswalk model.

MR-BRT crosswalk adjustment factors for household air pollution exposure

Data input	Reference or alternative case definition	Gamma	Beta coefficient, logit (95% CI)
Proportion of individuals	Ref	0.097	---
Proportion of Households	Alt		-0.095<br>(-0.100, -0.090)

We then apply this coefficient to household-only reports with the following formula:

$prop_{individ}$ = the proportion of individuals using solid fuel for cooking, and

$prop_{hh}$ = the proportion of households using solid fuel for cooking.

$$\log\left(\frac{prop_{individ}}{1-prop_{individ}}\right) = \log\left(\frac{prop_{hh}}{1-prop_{hh}}\right) - \beta$$

or

$$prop_{individ} = \frac{prop_{hh} * e^{-\beta}}{1-prop_{hh}+prop_{hh} * e^{-\beta}}$$

The effect is that the household studies are inflated to account for bias. Larger households are more likely to use solid fuel for cooking. The following figure depicts the 3676 data points that informed the crosswalk model. There the red points indicate the 10% of studies that were trimmed as outliers.

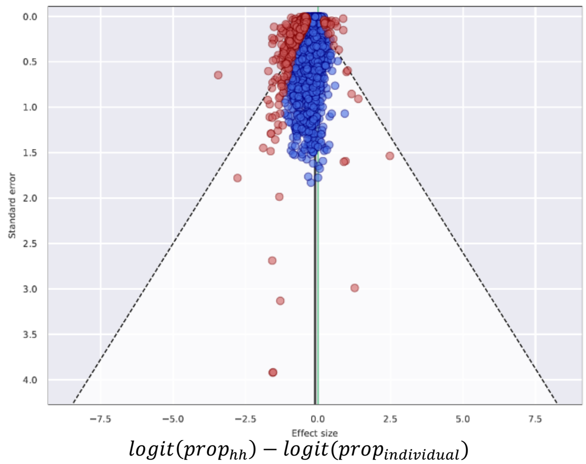

$logit(prop_{hh}) - logit(prop_{individual})$

Modelling strategy

Household air pollution was modelled at individual level using a three-step modelling strategy that uses linear regression, spatiotemporal regression, and Gaussian process regression (GPR). The first step is a mixed-effect linear regression of logit-transformed proportion of individuals using solid cooking fuels. The linear model contains maternal education and the proportion of population living in urban areas as covariates and has nested random effects by GBD region and GBD super-region. The full ST-GPR process is specified elsewhere this appendix. No substantial modelling changes were made in this round compared to GBD 2017.

First-stage linear model and coefficients

$$logit(proportion) \sim maternal\ education + urbanicity + (1|region) + (1|super - region)$$

**Variable**	**Beta (95% CI)**
Intercept	3.16 (1.59, 4.74)
Maternal education (years per capita)	-0.45 (-0.76, -0.15)
Urbanicity (proportion of population living in urban areas)	-1.42 (-2.67, -0.17)

Theoretical minimum-risk exposure level

For cataract, the TMREL is defined as no households using solid cooking fuel. For outcomes related to both ambient and household air pollution, the PAFs are estimated jointly and the TMREL is defined as uniform distribution between 2.4 and 5.9 ug/m^3 $PM_{2.5}$.

Relative risks

In addition to the previously included outcomes of lower respiratory infections (LRI), stroke, ischaemic heart disease (IHD), chronic obstructive pulmonary disease (COPD), lung cancer, type 2 diabetes, and cataract, in GBD 2019 we added low birthweight and short gestation as new outcomes of household air pollution through a mediation analyses. With the exception of cataract, all causes share risk curves and are jointly calculated with ambient $PM_{2.5}$ air pollution. Table 2 summarizes relative risk input data for ambient particulate matter pollution and household air pollution.

Table 2: Relative Risk Input Data

Input data	Relative risk
Source count (total)	200
Number of countries with data	40

Prior to GBD 2019, we utilised the results of an external meta-analysis with a summary relative of 2.47 with 95% CI (1.63, 3.73).[1] While this effect estimate was for both sexes, in the past we estimated burden for women only because women are known to have higher HAP exposure than men. In GBD 2019, we performed our own meta-regression analysis of household air pollution and cataracts. We extracted all of the components studies of the above meta-analysis paper but excluded one cross-sectional study. GBD risk factor analyses typically do not include cross-sectional analyses. In additional literature search, we found one additional paper describing different fuel types and cataracts.[4] We excluded this study because there was no comparison group without solid fuel use. Our resulting dataset contained eight estimates from six sources in India and Nepal.

On these eight estimates, we ran a MR BRT meta-regression to generate a summary effect size of 2.51 (1.58, 3.96). We included a study-level bias covariate of whether or not the study participants were blind to the exposure-outcome pair of interest. The prior on this covariate was a Gaussian distribution with mean 0 and variance 0.1. The prior on gamma was a Gaussian distribution with mean 0.04 and 0.1. The table and figure below provide the model coefficients and a visual representation.

MR-BRT relative risk meta-analysis for household air pollution and cataract

Covariate	Gamma	Beta coefficient, logit (95% CI)	Beta coefficient, adjusted (95% CI)
Intercept	0.40	0.918 (0.460, 1.377)	2.51 (1.58, 3.96)
Outcome unblinded		0.031 (-0.450, 0.512)	1.03 (0.64, 1.67)

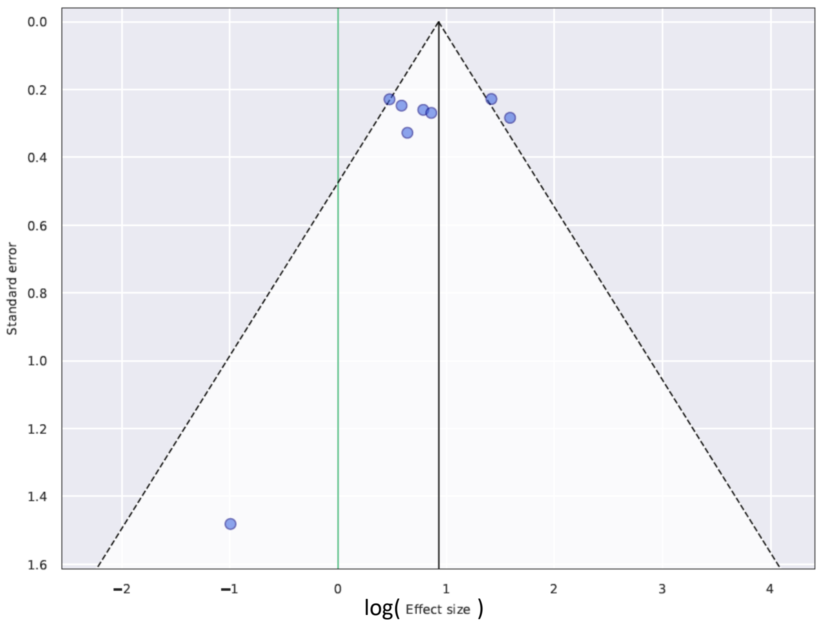

Studies reported effect sizes for males, females, and/or both sexes. In a sensitivity analysis we included a covariate for sex and found no significant difference in effect size by sex. Therefore, we now estimate cataract as an outcome of household air pollution in both males and females.

In GBD 2019, we also made substantial changes to our particulate matter risk curves. These risk curves, utilising splines in MR-BRT, the new mediation analysis with birthweight and gestational age, and the joint-estimation PAF approach are described in the ambient particulate matter appendix.

***$PM_{2.5}$* mapping value**

In order to use the particulate matter risk curves, we must estimate the level of exposure to particulate matter with diameter of less than 2.5 micrometers ($PM_{2.5}$) for individuals using solid fuels for cooking. The Global Household Air Pollution (HAP) Measurements database from WHO contains 196 studies with measurements from 43 countries of various pollution metrics in households using solid fuel for cooking.[2] From this database, we take all measurements of $PM_{2.5}$ using indoor or personal monitors. In addition to the WHO database, we included eight additional studies from a systematic review conducted in 2015 for GBD.

The final dataset included 336 estimates from 75 studies in 43 unique locations. We included 260, 64, nine, and three measurements indoors, on personal monitors for females, children (under 5), and males, respectively. 274 estimates were in households using solid fuels, 47 in households only using clean (gas or electricity) fuels, and 15 in households using a mixture of solid and clean fuels.

We use the following model:

$$\log(excess\ PM) \sim solid + measure\ group + 24\ hr\ measurement + SDI + (1|study)$$

Where,

- 24-hour measurement: binary variable equal to 1 if the measurement occurred over at least a 24-hour period and not only during mealtimes
- Measure group: categorical variable indicating indoor, female, male, or children

- Solid: indicator variable equal to 1 if the measurements were among households using solid fuel only, 0.5 if the measurements represented a mix of clean and solid fuels, and 0 if the households only used clean fuels.

We also included the Socio-demographic Index (SDI) as a variable to predict a unique value of HAP for each location and year based on development. We also included a random effect on study. We weighted each study by its sample size.

Before modelling, we calculated the excess particulate matter in households using solid fuel by subtracting off the predicted ambient $PM_{2.5}$ value in the study location and year based on the GBD 2017 $PM_{2.5}$ exposure model. The final model coefficients are included below:

HAP mapping model and coefficients

Variable	Beta, log (95% CI)	Beta, adjusted (95% CI)
Intercept	6.23 (4.58, 7.88)	506 (97, 2635)
Solid	2.60 (2.06, 3.13)	13.4 (7.8, 23.0)
Measure group<br>• Indoor (ref)<br>• Female<br>• Male<br>• Child	 <br>-0.56 (-1.15, 0.04)<br>-1.56 (-3.81, 0.70)<br>-1.13 (-2.06, -0.20)	 <br>0.57 (0.32, 1.04)<br>0.21 (0.02, 2.02)<br>0.32 (0.13, 0.82)
24-hour measurement	-0.29 (-1.04, 0.46)	0.75 (0.35, 1.59)
SDI	-6.42 (-9.30, -3.54)	1.6 e -3 (9.1 e -5, 2.9 e -2)

Therefore, for females in households using solid fuel, we would expect their long-term mean excess $PM_{2.5}$ exposure due to the use of solid fuels to be 1522, 117, and 9 μg/m^3 in SDI of 0.1, 0.5, and 0.9, respectively.

Because there are so few studies of personal monitoring in men and children, rather than directly using the results of the model, we generated ratios using studies that measured at least two of the population groups for any size particulate matter. For $PM_{2.5}$ we used the predicted ambient $PM_{2.5}$ value in the study location and year based on the GBD 2017 $PM_{2.5}$ exposure model as the "outdoor" measurement, and for PM_4 and PM_{10} we used published values in the studies themselves. We first subtracted off this outdoor value from each PM measurement, and then calculated the ratio of male to female and child to female exposure, weighted by sample size.

Study	Location	Year	Pollutant	Female N	Female PM	Group	N	PM	Outdoor
Balakrishnan et al., 2004	Andhra Pradesh, Rural	2004	PM_4	591	352	male	503	187	94
Gao X et al., 2009.	Tibet	2009	$PM_{2.5}$	52	127	male	85	111	27
Dasgupta et al., 2006	Bangladesh	2006	PM_{10}	944	209	male	944	166	50
Devkumar et al., 2014	Nepal	2014	$PM_{2.5}$	405	169	male	429	167	90
Balakrishnan et al., 2004	Andhra Pradesh, Rural	2004	PM_4	591	352	child	56	262	94

Dionisio et al., 2008.	The Gambia	2008	$PM_{2.5}$	13	275	child	13	219	31
Dasgupta et al., 2006	Bangladesh	2006	PM_{10}	944	209	child	944	199	50

The final ratios were 0.64 95% CI (0.45, 0.91) for males and 0.85 95% CI (0.56, 1.31) for children. We used these results to scale the $PM_{2.5}$ mapping model for these age and sex groups to input into the $PM_{2.5}$ risk curves.

References

1. Smith KR, Bruce N, Balakrishnan K, Adair-Rohani H, Balmes J, Chafe Z, *et al*. Millions Dead: How Do We Know and What Does It Mean? Methods Used in the Comparative Risk Assessment of Household Air Pollution. Annu Rev Public Health. 2014; **35**(1):185–206.
2. Shupler M, Balakrishnan K, Ghosh S, *et al*. Global household air pollution database: Kitchen concentrations and personal exposures of particulate matter and carbon monoxide. Data in Brief 2018; **21**: 1292–5.
3. Shupler M, Godwin W, Frostad J, Gustafson P, Arku RE, Brauer M. Global estimation of exposure to fine particulate matter (PM2.5) from household air pollution. Environment International 2018; **120**: 354–63.
4. Tanchangya J, Geater AF. Use of traditional cooking fuels and the risk of young adult cataract in rural Bangladesh: a hospital-based case-control study. *BMC Ophthalmology* 2011; **11**.

Ambient ozone pollution

Flowchart

Ambient Ozone

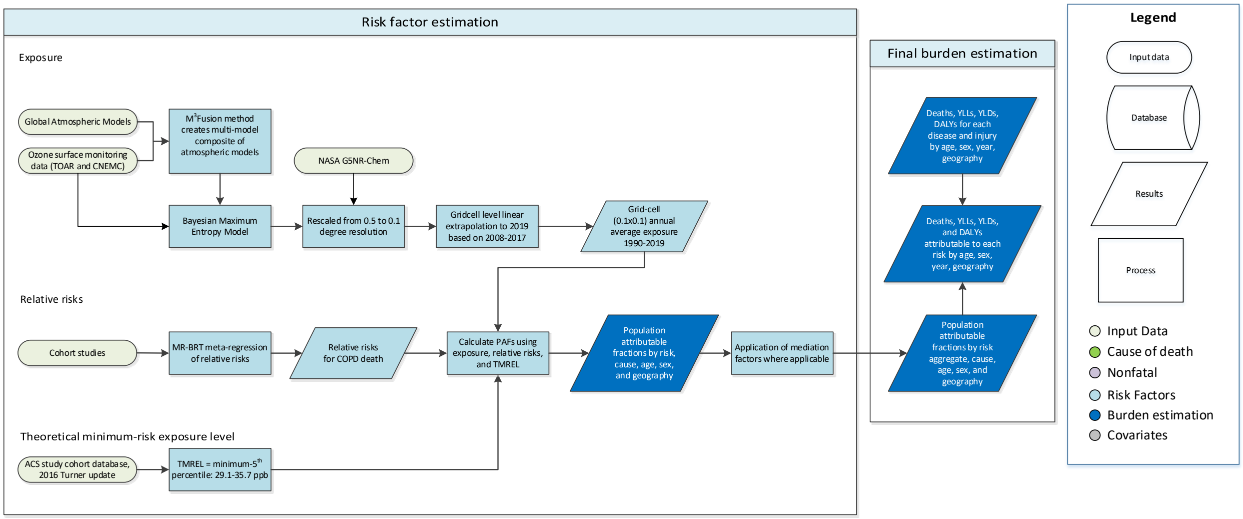

Input data and methodological summary

Exposure

To estimate the global distribution of exposure to ozone in ambient air (the highest seasonal [six-month] average of eight-hour daily maximum ozone concentrations, in parts per billion [ppb]) for the years 1990 to 2017, ozone ground measurement data were combined with chemical transport model estimates using Bayesian maximum entropy. Table 1 summarizes exposure input data.

Table 1: Exposure Input Data

Input data	Exposure
Source count (total)	14
Number of countries with data	1

Measurements data

Ozone monitoring data were obtained from the Tropospheric Ozone Assessment Report (TOAR), which contains the world's largest collection of surface ozone metrics (Schultz and colleagues, 2017). Since TOAR has released data until 2015 to the public, an update was made to include readily available datasets until 2017. In addition to TOAR, our analysis included ozone data from the China National Environmental Monitoring Center (CNEMC) Network, which contains surface ozone measurements for 2013–2017 in China (Lu and colleagues, 2018). All observations were processed to provide the six-month ozone season average of eight-hour daily maximum ozone concentrations.

Model combination

We used a combination of global atmospheric chemical transport models in our analysis, many of which simulated specified dynamics for the Chemistry-Climate Model Initiative (CCMI) (Morgenstern et al., 2017). Note that some of these modelling teams completed extra years of simulations beyond 2010 specifically for this project. The eight models and years available include the following: CHASER (1990–2010), MOCAGE (1988–2016), MRI-ESM (1988–2017), NASA MERRA2-GMI (1988–2017), NCAR CESM-Chem (1988–2010), NCAR WACCM (1988–2010), GFDL AM3 (1988–2014), and GFDL AM4 (2010–2016).

We obtained hourly ozone data for each of these models and then calculated the six-month maximum daily eight-hour maximum ozone mixing ratio (ppb). The M^3Fusion method (Chang and colleagues, 2019) was used to create a multi-model composite of the specified-dynamics models in each year from 1990 to 2017. This multi-model composite finds the linear combination of models available for each year that minimises the mean square error as compared to the observations in each world region, and in the process it corrects to minimise the mean model bias in each region. The world was divided geographically into eight regions: North America, South America, Europe, Africa, south central Asia, east Asia, Russia, and Oceania. In every region, each model was weighted to minimise the difference between the multi-model average and observations as described by the following:

Let s_g be the grid cell at resolution 0.5° × 0.5°, $\hat{y}(sg)$ be the interpolated observations, $\{\eta_k(sg); k = 1, . . . , n\}$ be the model output registered onto the same grid from the n models available in a given year. α_r is a constant that allows adjustment to the overall (regional) underestimation or overestimation and β_{rk} is an optimal weight for the k-th model in region r.

$$\underset{\{\alpha_r, \beta_{rk}; k=1,..,n\}}{minimize} \sum_{s_g \in Region\ r} \left(\hat{y}(s_g) - \alpha_r - \sum_{k=1}^{n} \beta_{rk}\eta_k(s_g) \right)^2,$$

$$subject\ to \sum_{k=1}^{n} \beta_{rk} = 1\ and\ \beta_{rk} \geq 0$$

In the M^3Fusion method, weights are constrained to be positive and sum to 1. A constant offset,α_r, was included to guarantee that the residuals from this optimisation have a zero mean, through which the mean model bias is corrected (Chang and colleagues, 2019). In most regions and years, the multi-model mean ozone was biased high, so this method tends to decrease the average ozone.

Since the M^3Fusion method relies on surface measurements to change the weights, regions with sparse data had to be taken into account. North America and Europe use weights-based model and observation values for each individual year. The rest of the world regions (South America, Africa, south central Asia, east Asia, Russia, and Oceania) use individual year weights for 2000–2010, and apply weights calculated from the aggregated 2000 to 2010 period for 1990–1999. For 2011–2017, east Asia uses individual year weights, while South America, Africa, south central Asia, Russia, Oceania, and Antarctica use weights from the aggregated 2011–2014 period.

An example of the weighted values used to create the M^3Fusion model in North America and Europe are shown below, accompanied by a map of the M^3Fusion model in 2005.

	North America		Europe	
Year	*1995*	*2005*	*1995*	*2005*
CESM-Chem	0.38	0.29		
CHASER	0.04			
GFDL-AM3			0.18	0.62
MERRA		0.32	0.82	0.38
MOCAGE	0.36	0.01		
MRI-ESM	0.22	0.27		
WACCM		0.11		

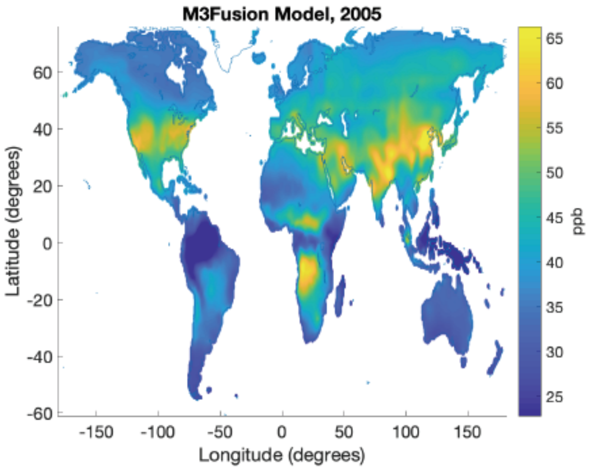

Bayesian maximum Entropy (BME)

BME is a geostatistical modelling tool that can be used to combine various knowledge bases for an air pollutant and combine them to create a single product. In this case, we use BME to combine site-specific measurements and modelled concentrations, making use of the correlations between measurement locations. BME uses the measurement values to correct the M^{3}Fusion Model locally around each station spatially and temporally, allowing future and past observations to provide input. Since more measurement locations became available through time, this method allows later measurements to influence ozone surfaces earlier in the period, which is particularly important in China and data-sparse regions. The range over which each measurement can correct the M^{3}Fusion Model and how each measurement's impact decreases over distance in time and space are calculated as part of BME.

Beyond combining these knowledge bases to provide an estimate of ozone pollution, BME also estimates a variance, which can be used to assess estimation confidence at different locations.

In short, the steps are:

1. Let **Z(p)** be a field of ozone concentration estimations in space and time and let **mo(p)** be the M^{3}Fusion model output values in space and time.
2. Subtract the M^{3}Fusion model output values at each measure point from the observed values, **z(p)**, to obtain residuals **x(p)**=**z(p)**-**mo(p)**. Examples for 2005 of **z** and **x** are shown below on the left and right, respectively:

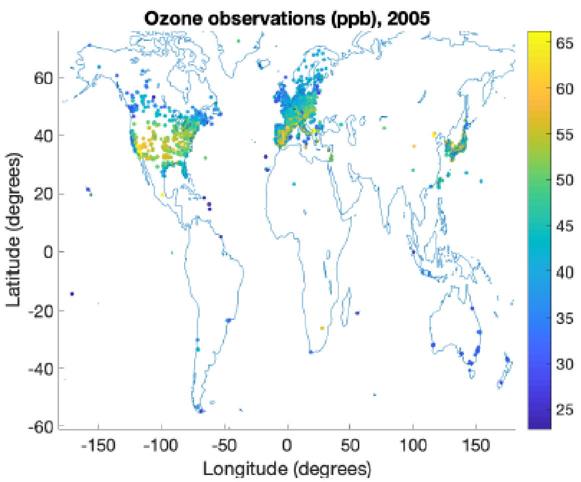

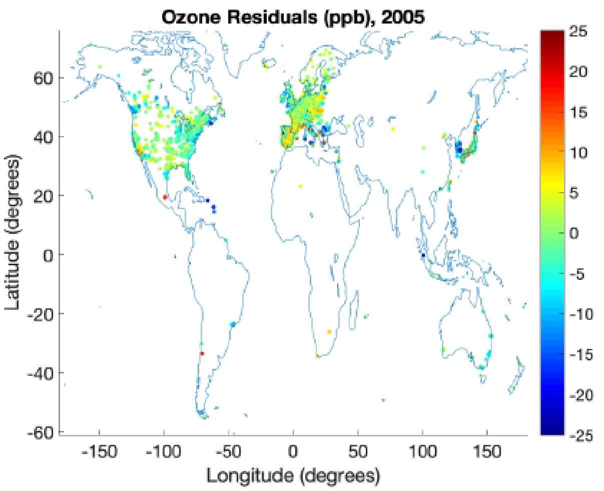

3. Model the covariance (the correlation between locations in space and time) $\mathbf{c_x}$ based on the residuals $\mathbf{x}$. The covariance of the residuals is the range of influence of a measurement to predict other concentrations in space and time. A shallower curve indicates that ozone values are correlated over a greater distance, while a steep dropoff indicates the reverse. The modelled spatial and temporal covariance are displayed below with the corresponding equation:

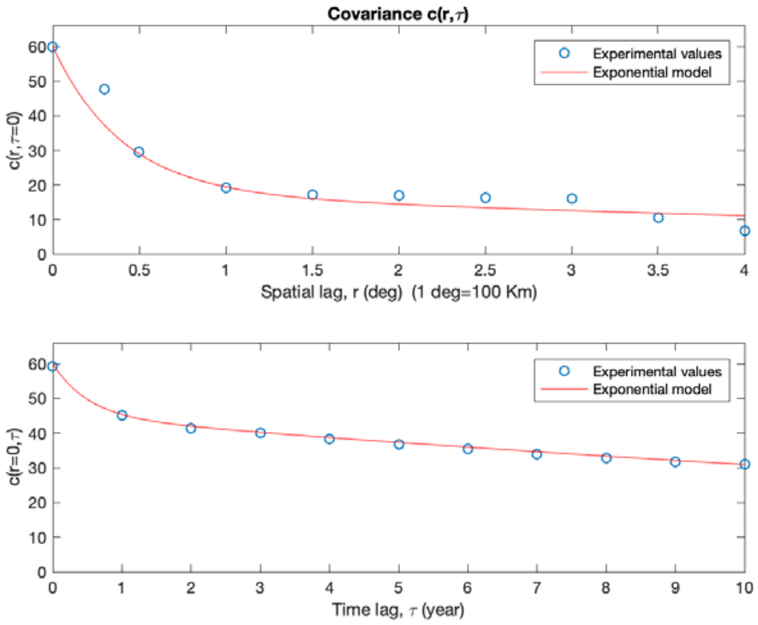

$$C_x(r,\tau) = 59.9938\ ppb^2(0.7\exp\left(-\frac{3r}{1.2\ degrees}\right)\exp\left(-\frac{3\tau}{80\ years}\right) + 0.05\exp\left(-\frac{3r}{25\ degrees}\right)\exp\left(-\frac{3\tau}{80\ years}\right) + 0.25\exp\left(-\frac{3r}{25\ degrees}\right)\exp\left(-\frac{3\tau}{1.5\ years}\right)$$

Where $\boldsymbol{\tau}$ *is temporal distance and r is spatial distance*

Note that the spatial covariance drops off steeply spatially such that the influence of a measurement location becomes very small beyond 1 degree of distance. However, the temporal covariance remains high, meaning that observations can influence ozone estimates through time over several years.

4. Combine the observation data residuals **(x)**, covariance **(cx),** and estimation parameters to get the BME estimation **(xk)** and variance **(vk)** on a 0.5º by 0.5º grid, shown for 2005.

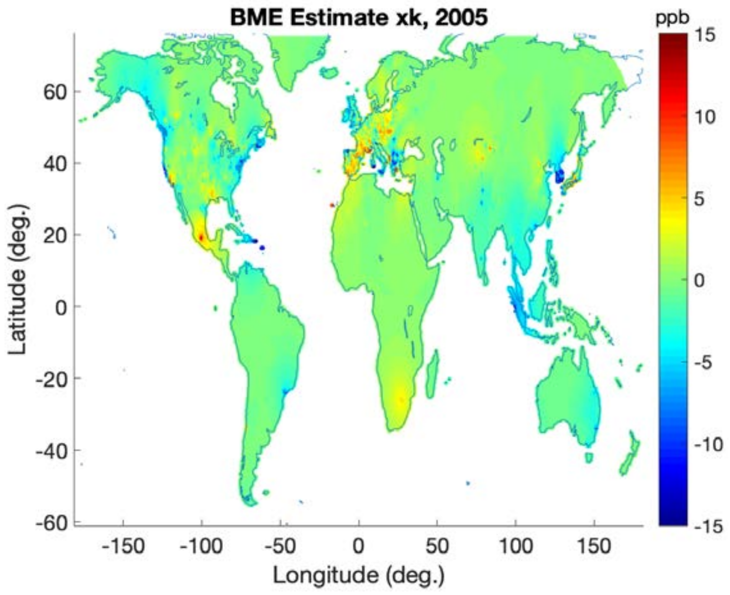

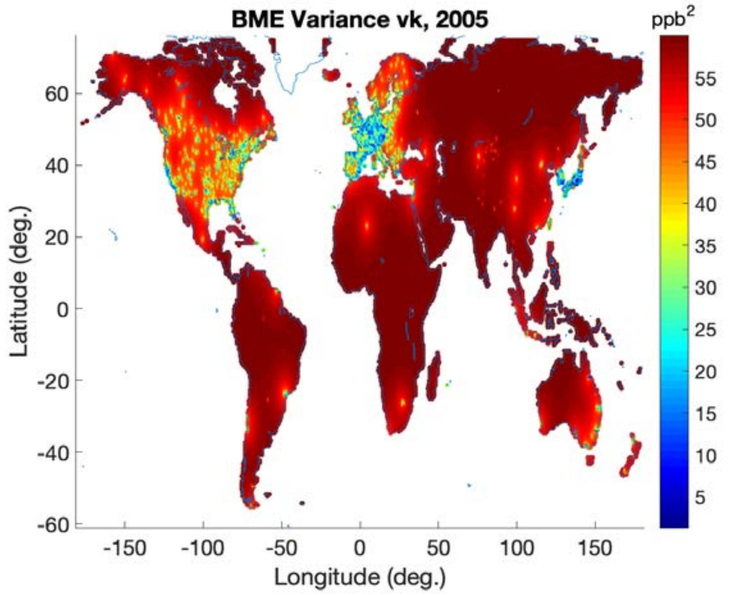

The variance is zero where the location of the estimation point matches an observation point in that year. The variance increases as the space-time distance from an observation increases, until the variance reaches a maximum value equal to the sill of the covariance equation (59.9938 ppb^2).

5. Obtain final BME estimation values **(z_k)**, shown for 2005 in the figure below, by adding back the previously subtracted model values **mo(p_k)** to the BME estimation **(x_k)**.

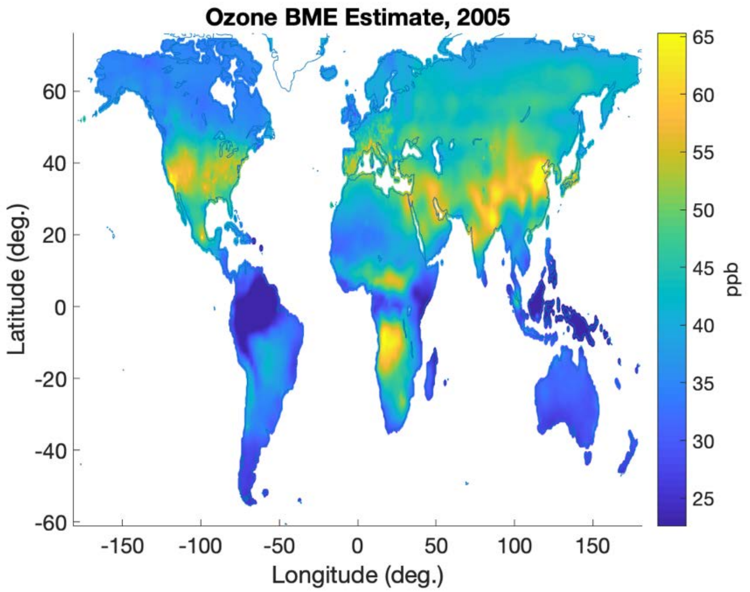

Adding fine resolution

Our results were calculated at 0.5° resolution, so to downscale estimates at finer resolution, we used the NASA G5NR-Chem model. The NASA G5NR-Chem model simulates surface ozone concentrations at 0.125° by 0.125° resolution for July 2013 to June 2014 (Hu and colleagues, 2018). We regridded the G5NR-Chem model from 0.125° resolution to 0.1° resolution. While we do not expect that the raw values for 2013–2014 hold true for every year, we believe that the spatial distribution of this model can be used to inform the fine-scale spatial pattern for each year. To add fine resolution, we performed the following steps:

1. Regrid NASA G5NR-Chem from 0.125° resolution to 0.1° resolution
2. Average each 0.5° NASA G5NR-Chem grid cell
3. Calculate the difference between our BME estimation results at 0.5° and the average NASA G5NR-Chem at 0.5°
4. Add the calculated difference to NASA G5NR-Chem at 0.1° to obtain our BME estimation at 0.1°

Adding fine resolution to our results keeps the average of each 0.5° grid cell the same as the original estimation at 0.5°, as well as the global average.

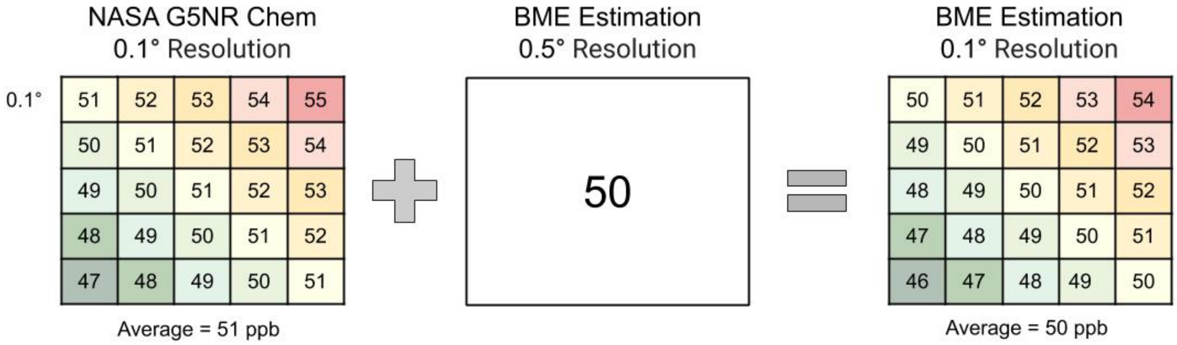

Final output

Three years are shown as an example below: 1995, 2005, and 2015. For each year, there are five maps displayed: the observations, M^3Fusion Model, BME Estimate, the difference between the BME Estimate and the M^3Fusion Model, and the variance. The difference map shows that the BME method corrects the M^3Fusion Model near monitoring stations, including stations in other years.

1995

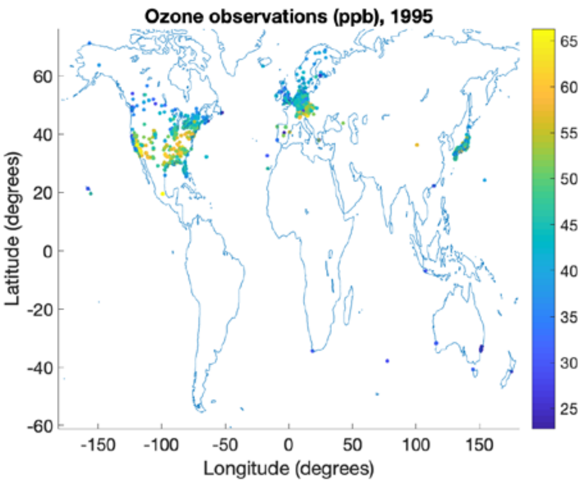
Ozone observations (ppb), 1995
Latitude (degrees)
Longitude (degrees)

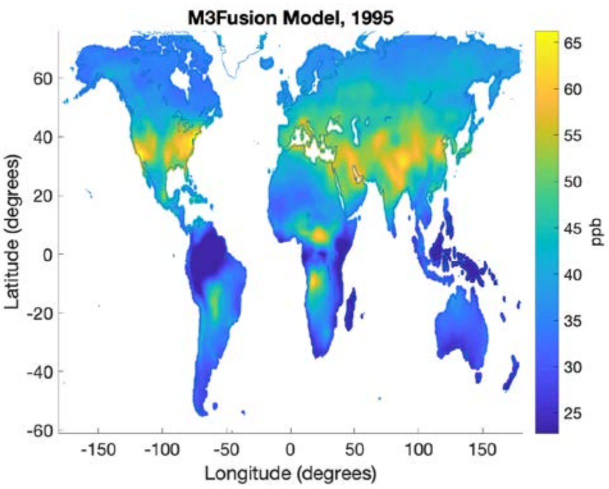
M3Fusion Model, 1995
Latitude (degrees)
Longitude (degrees)
ppb

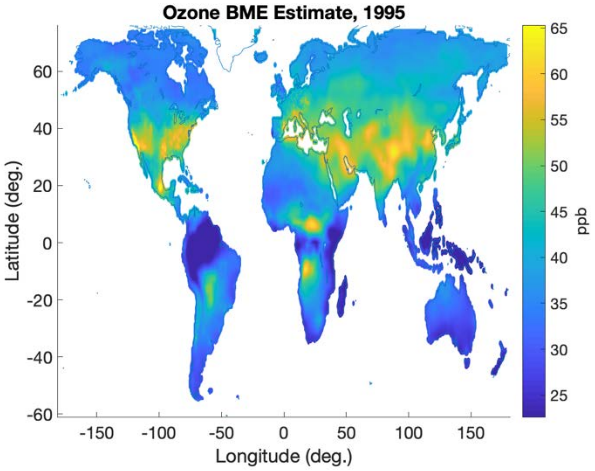
Ozone BME Estimate, 1995
Latitude (deg.)
Longitude (deg.)
ppb

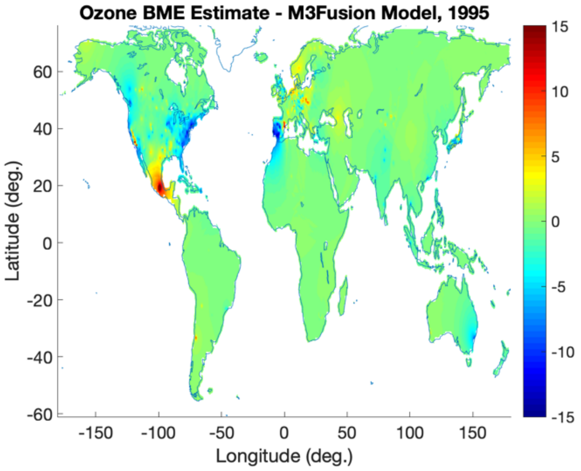
Ozone BME Estimate - M3Fusion Model, 1995
Latitude (deg.)
Longitude (deg.)
ppb

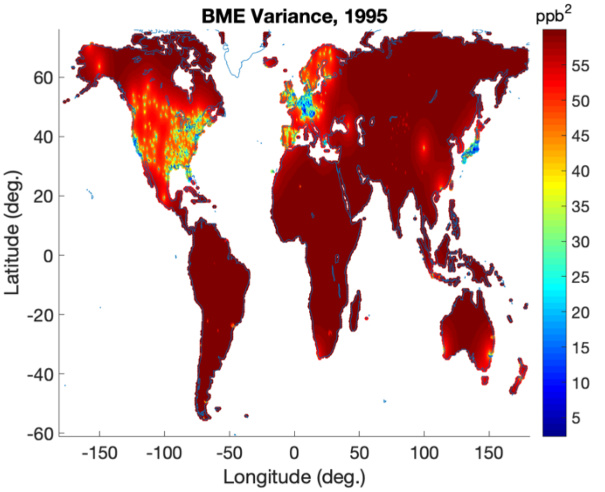
BME Variance, 1995
ppb^2
Latitude (deg.)
Longitude (deg.)

2005

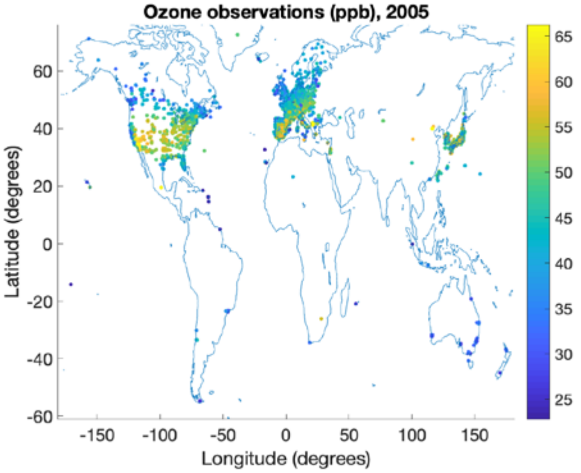
Ozone observations (ppb), 2005
Latitude (degrees)
Longitude (degrees)

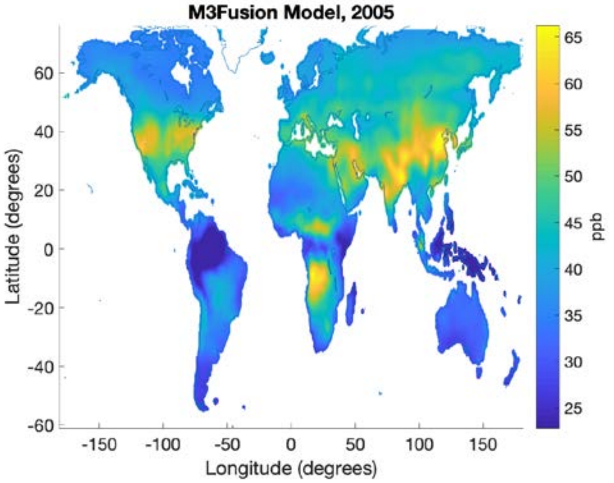
M3Fusion Model, 2005
Latitude (degrees)
Longitude (degrees)
ppb

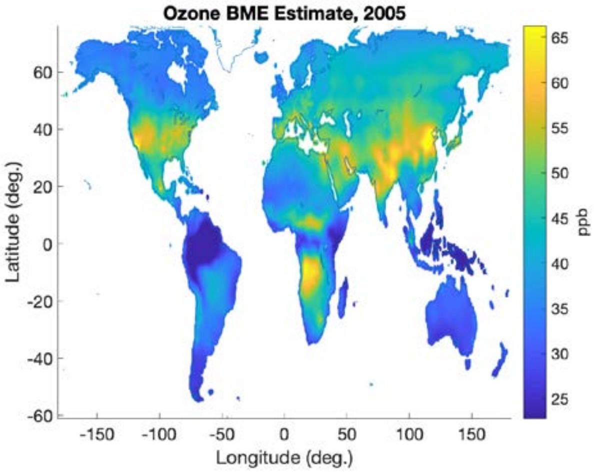
Ozone BME Estimate, 2005
Latitude (deg.)
Longitude (deg.)
ppb

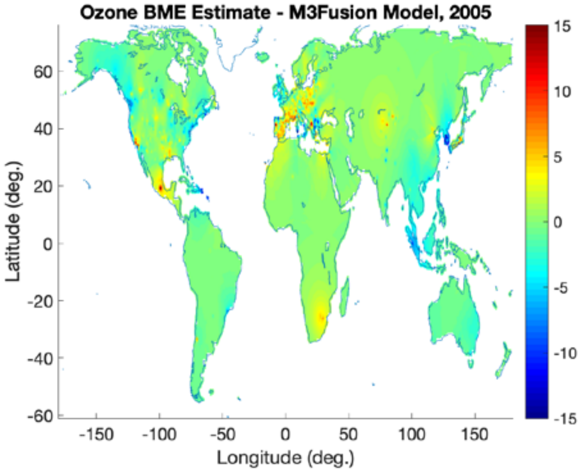
Ozone BME Estimate - M3Fusion Model, 2005
Latitude (deg.)
Longitude (deg.)
ppb

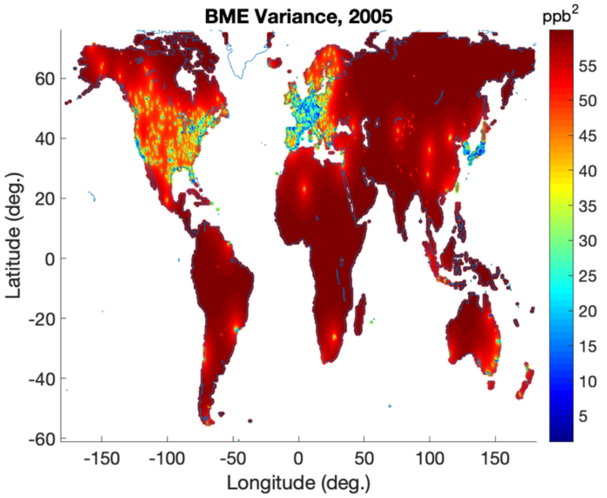
BME Variance, 2005
ppb²
Latitude (deg.)
Longitude (deg.)

2015

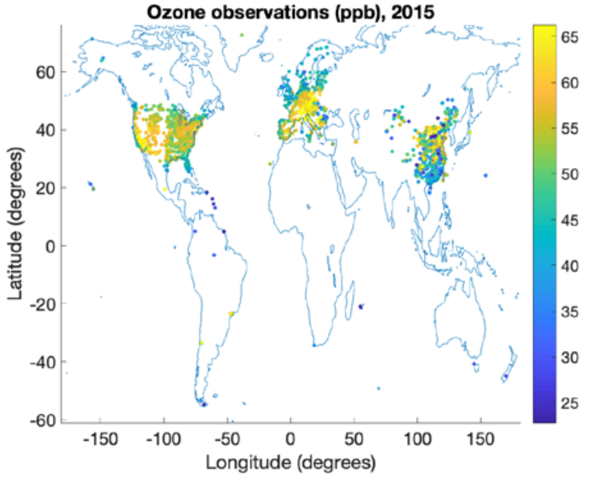

Ozone observations (ppb), 2015
Latitude (degrees)
Longitude (degrees)
-150 -100 -50 0 50 100 150
60 40 20 0 -20 -40 -60
65 60 55 50 45 40 35 30 25

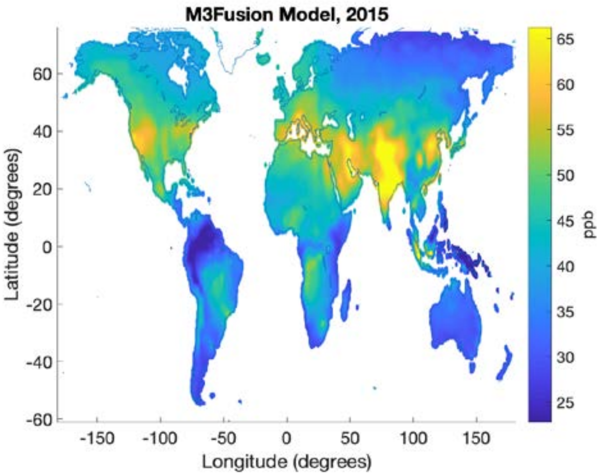

M3Fusion Model, 2015
Latitude (degrees)
Longitude (degrees)
-150 -100 -50 0 50 100 150
60 40 20 0 -20 -40 -60
65 60 55 50 45 40 35 30 25
ppb

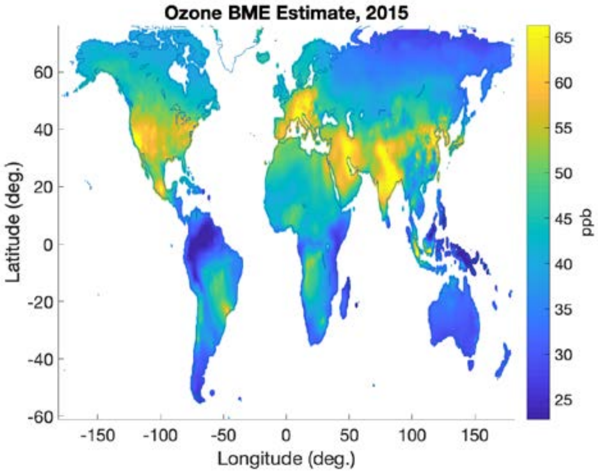

Ozone BME Estimate, 2015
Latitude (deg.)
Longitude (deg.)
-150 -100 -50 0 50 100 150
60 40 20 0 -20 -40 -60
65 60 55 50 45 40 35 30 25
ppb

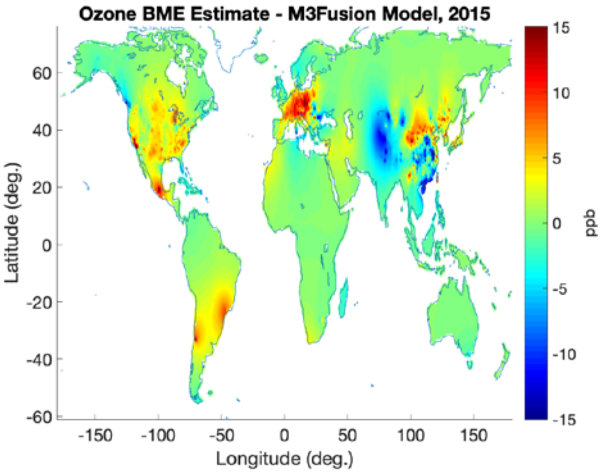

Ozone BME Estimate - M3Fusion Model, 2015
Latitude (deg.)
Longitude (deg.)
-150 -100 -50 0 50 100 150
60 40 20 0 -20 -40 -60
15 10 5 0 -5 -10 -15
ppb

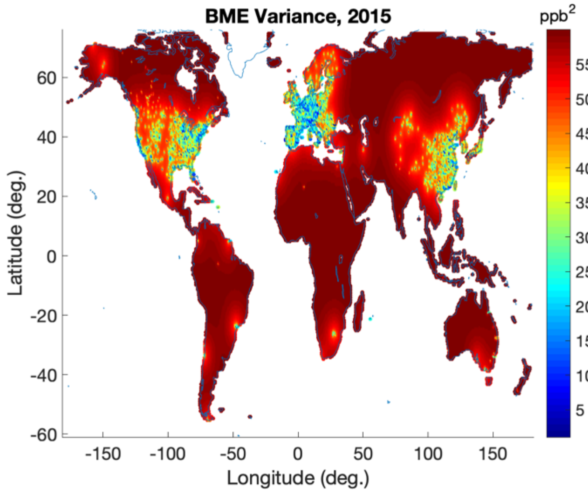

BME Variance, 2015
ppb^2
Latitude (deg.)
Longitude (deg.)
-150 -100 -50 0 50 100 150
60 40 20 0 -20 -40 -60
55 50 45 40 35 30 25 20 15 10 5

Extrapolation and annual means
To estimate global ozone in 2018 and 2019, for each 0.1° grid cell, we ran a log-linear model of the ozone estimates on year for the most recent ten years (2008–2017) of the following form:

$$\log(\text{ozone}) \sim \text{year}+1.$$

We considered using splines, but due to annual variation of ozone, we found a log-linear trend to provide the most reasonable prediction.

For burden estimation we are more interested in long-term trends and effects than annual variation; therefore, for the years 1991–2016, we used a three-year mean of exposure centered on the year of interest. This strategy aligns with the methodology used for ambient particulate matter air pollution. For 1990 and 2017, we used two-year means (1990/1991 and 2016/2017, respectively) because 1989 and 2018 were not available in the estimates.

To estimate the variance for the three-year mean to generate confidence intervals, we did not have information on the covariance between years, so a conservative estimate of the variance was made:

Let *X*, *Y*, and *Z* be random variables describing ozone exposure in a given 0.1 degree grid cell for years *i-1*, *i,* and *i+1*, respectively. By the laws of variance,

$$Var\left(\frac{1}{3}(X+Y+Z)\right) = \frac{1}{9}\left(Var(X) + Var(Y) + Var(Z) + 2Cov(X,Y) + 2Cov(X,Z) + 2Cov(Y,Z)\right).$$

We do not know the covariance, but by the Cauchy-Schwartz inequality,

$$Cov(A,B) \leq \sqrt{Var(A) * Var(B)}.$$

Therefore,

$$Var\left(\frac{1}{3}(X+Y+Z)\right) \leq \frac{1}{9}\Big(Var(X) + Var(Y) + Var(Z) + 2\sqrt{Var(X) * Var(Y)} + 2\sqrt{Var(X) * Var(Z)} + 2\sqrt{Var(Y) * Var(Z)}\Big).$$

This is a conservative estimate of the variance used when taking a three-year mean.

Difference from previous estimations
This method improves upon the GBD 2017 ozone exposure estimates (Chang and colleagues, 2019) in the following ways:

1. The previous estimates used observations in a specific year to correct the model within 2° of a monitoring station. In the current method, the radius of influence of each observation is defined by the spatial covariance. The spatial covariance shows that much of the influence of an observation is lost after 1°.
2. Measurements not only bias-correct the model in the year in which they were observed, but also influence other years according to the temporal covariance. This is important for regions that were not monitored over the entire 1990–2017 time period.
3. The fine spatial structure of the final product represents the spatial distribution of the 0.125° NASA G5NR-Chem model.

Theoretical minimum-risk exposure level

As in GBD 2017, the TMREL is based on the exposure distribution from the ACS CPS-II study (Turner and colleagues, 2016). It is a uniform distribution around the minimum and fifth percentile values observed in the cohort, ~U(29.1, 35.7), in ppb.

Relative risks

COPD mortality is the only included outcome for ambient ozone pollution.

In GBD 2017, we performed a literature review of studies examining long-term ozone exposure and COPD. We included five cohorts from Canada, the UK, and the USA, all of which reported ozone effects on COPD mortality (Turner and colleagues, 2016; Carey and colleagues, 2013, and Burnett RT. "Cox…"). For this reason, we only include mortality and not incidence as an outcome of ozone exposure. Table 2 summarizes relative risk input data.

Table 2: Relative Risk Input Data

Input data	Relative risk
Source count (total)	5
Number of countries with data	3

In GBD 2019, we updated our methodology to use MR-BRT for the meta-analysis of relative risks. Because we had only five datapoints, we included no study-level covariates. We also included no priors. The inverse-variance weighted meta-analysis of the five cohorts provided an estimated relative risk of 1.06 (95% CI 1.03, 1.10) with an estimated gamma (including between study heterogeneity) of 0.004.

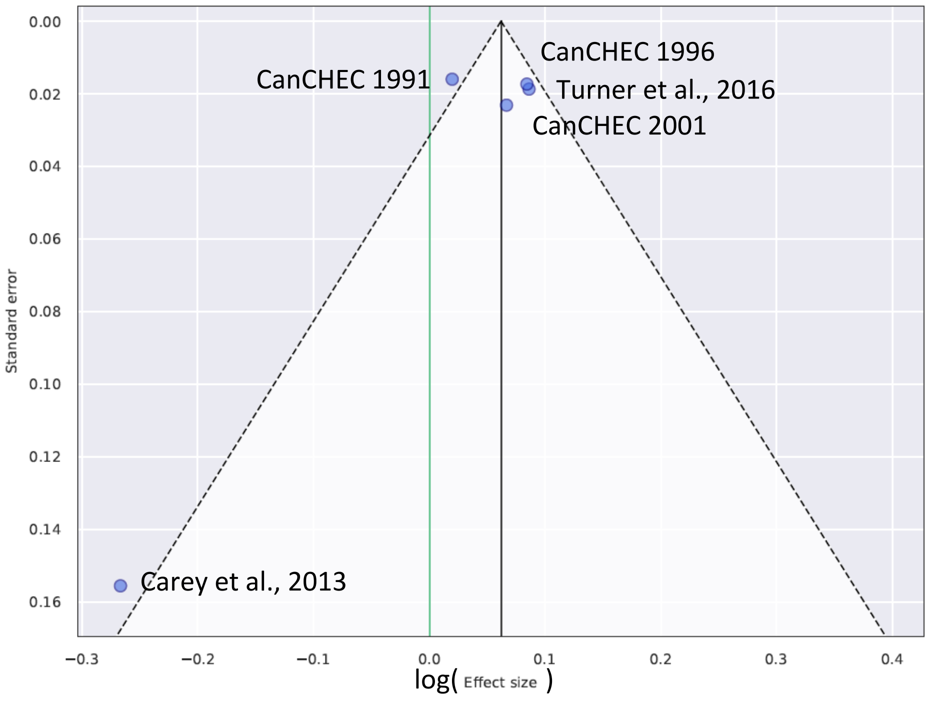

We calculated PAFs at the grid-cell level and aggregated up to GBD locations using population data from the Gridded Population of the World database. Estimates came from version 4 except for estimates for 1990 and 1995 from version 3. More details on these estimates are available in the Ambient particulate matter pollution methods appendix.

References

1. Schultz MG, Schröder S, Lyapina O, Cooper O, Galbally I, Petropavlovskikh I, et al. Tropospheric Ozone Assessment Report: Database and Metrics Data of Global Surface Ozone Observations. Elem Sci Anth. 2017;5:58. DOI: http://doi.org/10.1525/elementa.244
2. Lu, Xiao, Jiayun Hong, Lin Zhang, Owen R. Cooper, Martin G. Schultz, Xiaobin Xu, Tao Wang, Meng Gao, Yuanhong Zhao, and Yuanhang Zhang. Severe surface ozone pollution in China: a global perspective. Environmental Science & Technology Letters 2018 *5* (8), 487-49 DOI: 10.1021/acs.estlett.8b00366
3. Chang, Kai-Lan, R. Cooper, Owen, West, Jason, L. Serre, Marc, G. Schultz, Martin, Lin, Meiyun, Marecal, Virginie, Josse, B, Deushi, Makoto, Sudo, Kengo, Liu, Junhua & A. Keller, Christoph. (2019). A new method (M^3Fusion v1) for combining observations and multiple model output for an improved estimate of the global surface ozone distribution. Geoscientific Model Development. 12. 955-978. 10.5194/gmd-12-955-2019.
4. Hu, L., C. A. Keller, M. S. Long, T. Sherwen, B. Auer, A. Da Silva, J. E. Nielsen, S. Pawson, M. A. Thompson, A. L. Trayanov, K. R. Travis, S. K. Grange, M. J. Evans, D. J. Jacob (2018) Global simulation of tropospheric chemistry at 12.5 km resolution: performance and evaluation of the GEOS-Chem chemical module (v10-1) within the NASA GEOS Earth system model (GEOS-5 ESM), Geoscientific Model Development, 11, 4603-4620. 10.5194/gmd-11-4603-2018.
5. Morgenstern O, Hegglin MI, Rozanov E, O'Connor FM, Abraham NL, Akiyoshi H, Archibald AT, Bekki S, Butchard N, Chipperfield MP, Deushi M, Dhomse SS, Garcia RR, Hardiman SC, Horowitz LW, Jockel P, Josse B, Kinnison D, Lin M, Mancini E, Manyin ME, Marchand M, Marecal V, Michou M, Oman LD, Pitari G, Plummer DA, Revell LE, Saint-Martin D, Schofield R, Stenke A, Stone K, Sudo K, Tanaka TY, Tilmes S, Yamashita Y, Yoshida K, Zeng G. Review of the global models used within phase 1 of the Chemistry-Climate Model Initiative (CCMI). *Geosci. Model Dev.* 2017; 10: 639-671.
6. Turner MC, Jerrett M, Pope CA 3rd, Krewski D, Gapstur SM, Diver WR, Beckerman BS, Marshall JD, Su J, Crouse DL, Burnett RT. Long-term ozone exposure and mortality in a large prospective study. *Am J Respir Crit Care Med*. 2016; 193(10): 1134-42.
7. Carey IM, Atkinson RW, Kent AJ, van Staa T, Cook DG, Anderson HR. Mortality associations with long-term exposure to outdoor air pollution in a national English cohort. *Am J Respir Crit Care Med.* 2013; 187(11): 1226-33.
8. Burnett RT. Cox Proportional Survival Model Hazard Ratios from Census Years (1991,1996,2001) to 2011 for Adults Aged 25 to 89 in CanCHEC Cohort. Custom Anaylsis for GBD2017.

References for Atmospheric Chemical Transport Models

- CESM:

- Tilmes, S., Lamarque, J.-F., Emmons, L. K., Kinnison, D. E., Ma, P.-L., Liu, X., Ghan, S., Bardeen, C., Arnold, S., Deeter, M., Vitt, F., Ryerson, T., Elkins, J. W., Moore, F., Spackman, J. R., and Val Martin, M.: Description and evaluation of tropospheric chemistry and aerosols in the Community Earth System Model (CESM1.2), Geosci. Model Dev., 8, 1395–1426, doi:10.5194/gmd-8-1395-2015, 2015

- CESM WACCM:
  - Garcia, R. R., Smith, A. K., Kinnison, D. E., de la Cámara, Á., and Murphy, D.: Modifications of the gravity wave parameterization in the Whole Atmosphere Community Climate Model: Motivation and results, J. Geophys. Res.-Atmos., doi:10.1175/JAS-D16-0104.1, 2016.
  - Marsh, D., Mills, M. J., Kinnison, D. E., Garcia, R. R., Lamarque, J.-F., and Calvo, N.: Climate change from 1850–2005 simulated in CESM1 (WACCM), J. Climate, 26, 7372–7391, doi:10.1175/JCLI-D-12-00558.1, 2013
- CHASER:
  - Sudo, K., Takahashi, M., and Akimoto, H.: CHASER: A global chemical model of the troposphere, 2. Model results and evaluation, J. Geophys. Res., 107, 4586, https://doi.org/10.1029/2001JD001114, 2002a.
  - Sudo, K., Takahashi, M., Kurokawa, J., and Akimoto, H.: CHASER: A global chemical model of the troposphere, 1. Model description, J. Geophys. Res., 107, 4339, https://doi.org/10.1029/2001JD001113, 2002b.

  - Watanabe, S., Hajima, T., Sudo, K., Nagashima, T., Takemura, T., Okajima, H., Nozawa, T., Kawase, H., Abe, M., Yokohata, T., Ise, T., Sato, H., Kato, E., Takata, K., Emori, S., and Kawamiya, M.: MIROC-ESM 2010: model description and basic results of CMIP5-20c3m experiments, Geosci. Model Dev., 4, 845–872, https://doi.org/10.5194/gmd-4-845-2011, 2011.
- GEOSCCM:
  - Oman, L. D., Ziemke, J. R., Douglass, A. R., Waugh, D. W., Lang, C., Rodriguez, J. M., and Nielsen, J. E.: The response of tropical tropospheric ozone to ENSO, Geophys. Res. Lett., 38, L13706, https://doi.org/10.1029/2011GL047865, 2011.
- GFDL AM3 & AM4:
  - Lin, M., Fiore, A. M., Horowitz, L. W., Cooper, O. R., Naik, V., Holloway, J., Johnson, B. J., Middlebrook, A. M., Oltmans, S. J., Pollack, I. B., Ryerson, T. B., Warner, J. X., Wiedinmyer, C., Wilson, J., and Wyman, B.: Transport of Asian ozone pollution into surface air over the western United States in spring, J. Geophys. Res., 117, D00V07, https://doi.org/10.1029/2011JD016961, 2012.
  - Lin, M., Horowitz, L. W., Oltmans, S. J., Fiore, A. M., and Fan, S.: Tropospheric ozone trends at Mauna Loa Observatory tied to decadal climate variability, Nat. Geosci., 7, 136–143, https://doi.org/10.1038/NGEO2066, 2014.
  - Lin, M., Horowitz, L. W., Payton, R., Fiore, A. M., and Tonnesen, G.: US surface ozone trends and extremes from 1980 to 2014: quantifying the roles of rising Asian emissions, domestic controls, wildfires, and climate, Atmos. Chem. Phys., 17, 2943–2970, https://doi.org/10.5194/acp-17-2943-2017, 2017.
- MERRA GMI:

  - Ziemke, J. R., Oman, L. D., Strode, S. A., Douglass, A. R., Olsen, M. A., McPeters, R. D., Bhartia, P. K., Froidevaux, L., Labow, G. J., Witte, J. C., Thompson, A. M., Haffner, D. P., Kramarova, N. A., Frith, S. M., Huang, L.-K., Jaross, G. R., Seftor, C. J., Deland, M. T., and Taylor, S. L.: Trends in global tropospheric ozone inferred from a composite record of TOMS/OMI/MLS/OMPS satellite measurements and the MERRA-2 GMI simulation , Atmos. Chem. Phys., 19, 3257–3269, https://doi.org/10.5194/acp-19-3257-2019, 2019.
- MOCAGE:
  - Josse, B., Simon, P., and Peuch, V.-H.: Radon global simulations with the multiscale chemistry and transport model MOCAGE, Tellus B, 56, 339–356, https://doi.org/10.1111/j.1600-0889.2004.00112.x, 2004.
  - Teyssèdre, H., Michou, M., Clark, H. L., Josse, B., Karcher, F., Olivié, D., Peuch, V.-H., Saint-Martin, D., Cariolle, D., Attié, J.-L., Nédélec, P., Ricaud, P., Thouret, V., van der A, R. J., Volz-Thomas, A., and Chéroux, F.: A new tropospheric and stratospheric Chemistry and Transport Model MOCAGE-Climat for multi-year studies: evaluation of the present-day climatology and sensitivity to surface processes, Atmos. Chem. Phys., 7, 5815–5860, https://doi.org/10.5194/acp-7-5815-2007, 2007.
- MRI-ESM1r1:
  - Adachi, Y., Yukimoto, S., Deushi, M., Obata, A., Taichu. Y. Tanaka, H. N., Hosaka, M., Sakami, T., Yoshimura, H., Hirabara, M., Shindo, E., Tsujino, H., Mizuta, R., Yabu, S., Koshiro, T., Ose, T., and Kitoh, A.: Basic performance of a new earth system model of the Meteorological Research Institute (MRI-ESM1), Pap. Meteorol. Geophys, 64, 1–18, https://doi.org/10.2467/mripapers.64.1, 2013.

Contributors:

- Marissa DeLang, Jacob S. Becker, Stephanie Cleland, Elyssa Collins, Marc L. Serre, J. Jason West, University of North Carolina at Chapel Hill
- Owen R. Cooper and Kai-Lan Chang, CIRES, University of Colorado, Boulder/NOAA Earth System Research Laboratory, Boulder, USA
- Martin G. Schultz and Sabine Schröder, Jülich Supercomputing Centre (JSC), Forschungszentrum Jülich, Jülich, DE
- Xiao Lu and Lin Zhang, Laboratory for Climate and Ocean-Atmosphere Studies, Dept. of Atmospheric and Oceanic Sciences, School of Physics, Peking University, Beijing, China
- CCMI and NASA modellers

Radon exposure

Flowchart

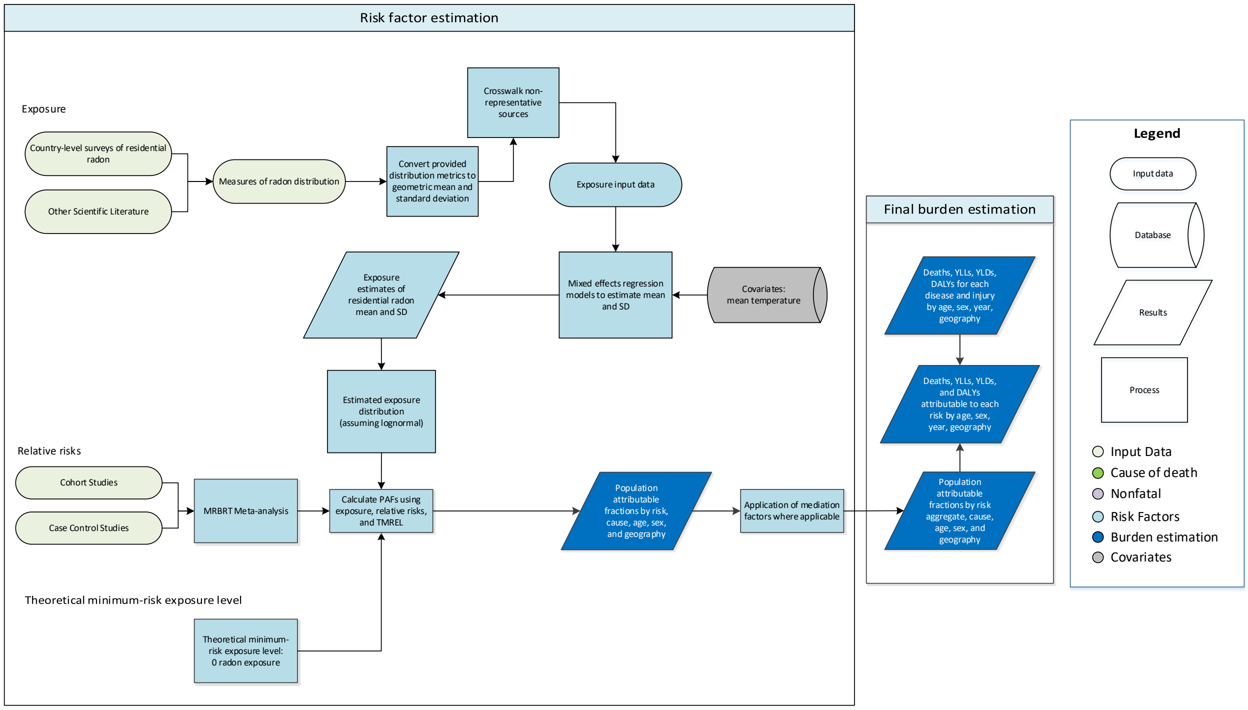

Input data and methodological summary

Exposure

Case definition

Radon is a radioactive gas that is produced as a byproduct of the decay chain of uranium, occurring naturally within the Earth's crust. Some fraction of this natural radon production escapes into the atmosphere, where it is present at low concentrations unless build-up is caused by release into enclosed spaces such as homes, mines, or caves. Radon exposure is expressed as average daily exposure to indoor air radon gas levels measured in Becquerels (disintegrations per second) per cubic meter (Bq/m^3). In the GBD we specifically quantify the burden due to indoor radon exposure.

Input data

An expert group curated the original dataset for residential radon exposure. We have added data sources every cycle, especially as we include additional subnational locations. Data sources include national surveys, government reports, and scientific literature. We include any sources that report results of residential radon measurement in homes (not schools or workplaces). Because of a shortage of data, we also include sources that are not representative of an entire population, but exclude studies or surveys explicitly conducted in high-radon areas.

From each source, we extracted all available information required to estimate the distribution of radon exposure, including arithmetic mean and standard deviation, geometric mean and standard deviation, median, IQR, range, max, sample size, confidence interval, and/or standard error. Table 1 summarizes exposure input data.

Table 1: Exposure Input Data

Input data	Exposure
Source count (total)	207
Number of countries with data	78

Modelling strategy

Literature suggests that radon exposure follows a lognormal distribution both on the individual household and national level (Daraktchieva, Miles, and McColl, 2009). We therefore assume that the distribution of radon exposure is lognormal within any one GBD geography or study. For studies reporting at least one measure of central tendency (arithmetic mean, geometric mean, or median) and a measure of spread (standard deviation [arithmetic or geometric], IQR, confidence interval, or standard error), we are able to directly calculate the geometric mean and geometric standard deviation of the underlying distribution. For those only reporting a measure of central tendency and range, max, or sample size, we estimate the geometric mean and standard deviation based on several assumptions.

- When the range or max is provided, we assume that the range divided by 4 is a reasonable estimate of standard deviation because 95% of observations occur within 2 standard deviations of the mean. This calculation happens in log space.
- For studies only providing a measure of central tendency and sample size, we impute standard deviation based on sample size from what we see in other estimates.
- If we only have the mean, we impute the median standard deviation of all other studies.

Once we convert all estimates to the mean and standard deviation of a lognormal distribution, we run all analyses in log-space to meet assumptions of normality.

Though we exclude studies intentionally performed in high-exposure areas, we still see a bias in studies that are not representative of their geography. To account for this difference we perform a crosswalk adjustment using MR-BRT. We match all locations where we have both representative and non-representative sources. These locations include Canada, Egypt, Gansu, Greece, Hiroshima, Ireland, Jordan, Portugal, Puebla, Querétaro, Romania, San Luis Potosí, Saudi Arabia, Shanghai, Spain, Syria, Taiwan, Turkey, Urban Andhra Pradesh, Urban Assam, Urban Gujarat, Urban Haryana, Urban Karnataka, Urban Kerala, Urban Maharashtra, Urban Meghalaya, Urban Punjab, Urban Rajasthan, Urban Tripura, and Urban Uttar Pradesh. We perform the following model on the log difference of the log of the geometric means:

Let $ref = \log(geometric\ mean\ representative)$, and

$alt = \log(geometric\ mean\ non\ representative)$.

$$log\left(\frac{alt}{ref}\right) \sim Beta$$

$$ref \sim e^{-Beta} * alt$$

$$ref \sim (adjustment\ factor) * alt$$

We use the results of this crosswalk to downscale all non-representative input sources and inflate their uncertainty in the model. The effect is equivalent to scaling the log of the geometric mean of non-representative sources by a factor of 0.899.

Table 2: MR-BRT crosswalk adjustment factor for radon exposure

**Data input**	**Reference or alternative case definition**	**Gamma**	**Beta coefficient, log (95% CI)**	**Adjustment factor***
Geographically representative survey or report	Ref	0.29	---	
Estimate not representative of geographic unit	Alt		0.106 (0.095, 0.112)	0.899 (0.894, 0.909)

**Adjustment factor is the transformed beta coefficient in normal space, and can be interpreted as the factor by which the alternative case definition is adjusted to reflect what it would have been if measured as the reference.*

After crosswalking non-representative sources, we run a model to estimate the log(geometric mean). Because radon is naturally occurring and is not considered to have much long-term temporal fluctuation, we used a mixed effects linear model independent of time (Steck, 2009). The model included nested random effects on super-region, region, and location (most detailed) and one fixed effect covariate, long-term mean temperature (average annual temperature averaged over 1990–2019) as a proxy for adequate building ventilation. We weighted the model by inverse standard error. We tried weighting by inverse variance and sample size, but did not get a stable fit. To predict the log of the geometric mean we used the following model:

$$\log(geometric\ mean) \sim \beta * long\ term\ mean\ temp + (1|super\ region) + (1|region) + (1|location)$$

Regression coefficients for predicting mean radon

**Input**	**Coefficient (95% CI)**
Intercept	4.05 (3.532, 4.560)
Long term mean temperature	-0.040 (-0.065, -0.015)

We also ran a model to predict the standard deviation (in log space) for every country. We included all studies that were representative of a geography and that included a measure of spread for which we were able to directly calculate the standard deviation. The model was a mixed effects linear regression

of standard deviation on mean including random effects on location (most-detailed) and region. The model was not stable when including super-region. To predict the log of the geometric standard deviation we used the following model:

$$\log(geometric\ standard\ deviation) \sim \beta * \log(geometric\ mean) + (1|region) + (1|location)$$

Regression coefficients for predicting standard deviation of radon

Input	Coefficient (95% CI)
Intercept	0.616 (0.389, 0.843)
log(geometric mean)	0.014 (-0.030, 0.057)

We used the estimated mean and standard deviation for each location to generate an exposure distribution used in PAF calculation.

Theoretical minimum-risk exposure level

While in GBD 2017 we sampled from a uniform distribution from 7-14 Bq/m^3 representing outdoor air, in GBD 2019 we updated the radon TMREL to zero. This was decided because the risk we are estimating is indoor air radon, and it is theoretically possible with mitigation strategies to reduce all indoor exposure to zero.

Relative risks

In GBD 2017, the RR was based on a single meta-analysis (Darby and colleagues 2005) which reported a relative risk of 1.16 (1.05–1.31) per 100 Bq/m^3 increase in radon exposure. In GBD 2019 we conducted a systematic review of studies examining residential exposure to radon and lung cancer incidence or mortality. We extracted the component studies from the following meta-analyses: Lubin and colleagues 2003, Darby and colleagues 2005, Krewski and colleagues 2005, Zhang and colleagues 2012, Torres-Durán and colleagues 2014, and Dobrzynski and colleagues 2018. We excluded studies that were cross-sectional or ecological, studied high-risk populations such as miners, or were not available in English. When multiple studies were published on the same dataset, we took the one with the longest follow-up. We also excluded studies that only reported cumulative exposure because this does not align with our exposure definition. Table 3 summarizes relative risk input data.

Table 3: Relative Risk Input Data

Input data	Relative risk
Source count (total)	25
Number of countries with data	12

We converted all reported risks to the RR increase per 100-unit increase in Bq/m^3, assuming a linear increase in RR across the exposure range. We tested a spline model to determine if there were any differences in risk over the exposure range, but the data suggested a linear trend.

Some studies only reported RR between exposure categories. In these instances, we took the mean, median, or midpoint of the exposed and unexposed categories to calculate an "exposure range." We

then scaled the reported RR based on that exposure range to estimate the corresponding increase per 100 units.

This resulted in a total of 49 estimates from 25 studies in 12 countries: England, Czech Republic, Finland, France, Germany, Italy, Spain, Sweden, the USA, China, Denmark, and Japan.

For those studies that reported no confidence intervals or standard error, we imputed the standard error based on sample size. To do this we created a model of the following form:

$$se \sim \beta * \frac{1}{\sqrt{n}}$$

Where we predict the standard error, *se*, as a function of some constant, β, times the inverse square root of the sample size, *n*. Here β is an estimate of the population-level standard deviation.

Once we had all 49 estimates of the RR increase per 100 unit change in exposure, we fit a MR-BRT meta-regression including covariates for selection bias and quality of exposure measurement. Studies that included a full residential history were assigned a 0 for cv_exposure_study, while those who only measured the current household or one household were assigned a 1. We assigned studies to one of three categories for selection bias. Studies with greater than 95% follow-up received a 0, those with 85% to 85% follow-up received a 1, and those with less than 85% follow-up received a 2. For case-control studies we assigned this based on the percentage of cases and controls for which exposure category could be ascertained.

We also included loose priors on Gamma and each of the covariates. The prior on Gamma was a gamma distribution with mean 0.2 and variance 0.1. The prior on each of the covariates was a gamma distribution with mean 0 and variance 0.1.

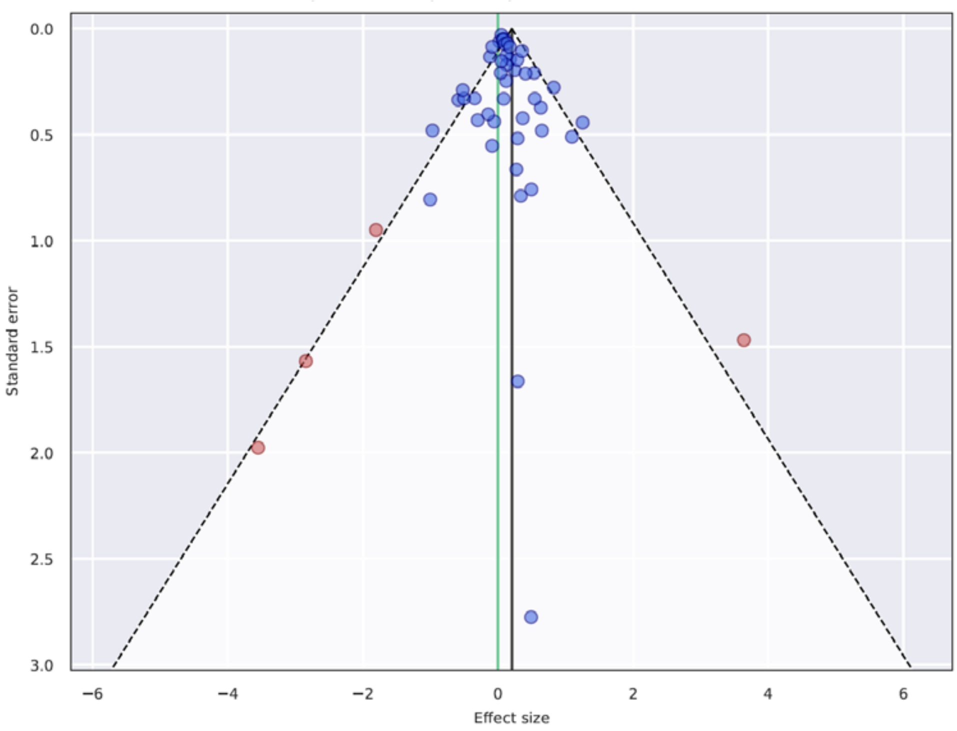

MR-BRT relative risk meta-regression for radon

Data input	Gamma	Beta coefficient, log (95% CI)	Exponentiated coefficient (95% CI)
Intercept	0	0.094 (0.023, 0.165)	1.09 (1.02, 1.18)
cv_exposure_study	0.23	0.124 (-0.060, 0.308)	1.13 (0.94, 1.36)
cv_selection_bias	0	-0.017 (-0.073, 0.038)	0.98 (1.04, 0.93)

References

1. Daraktchieva Z, Miles JCH, McColl N. Radon, the lognormal distribution and deviation from it. J Radiol Prot 2014; 34: 183–190.
2. Steck DJ. Annual average indoor radon variations over two decades. Health Phys. 2009;96(1):37-47.
3. Darby S, Hill D, Auvinen A, et al. Radon in homes and risk of lung cancer: collaborative analysis of individual data from 13 European case-control studies. BMJ. 2005;330(7485):223
4. Lubin JH. Studies of radon and lung cancer in North America and China. Radiation Protection Dosimetry. 2003Jan;104(4):315–9.
5. Krewski D, Lubin JH, Zielinski JM, Alavanja M, Catalan VS, Field RW, et al. Residential Radon and Risk of Lung Cancer. Epidemiology. 2005;16(2):137–45.
6. Zhang Z-L, Sun J, Dong J-Y, et al. Residential radon and lung cancer risk: an updated meta- analysis of case-control studies. Asian Pac J Cancer Prev 2012; 13: 2459–65.
7. Torres-Durán MCAD, Barros-Dios JM, Fernández-Villar A, Ruano-Ravina A. Residential radon and lung cancer in never smokers. A systematic review. Cancer Letters. 2014;345(1):21–6.
8. Do r y s i ornals i es c y s a . eta-analysis of thirty-two case–control and two ecological radon studies of lung cancer. J Radiat Res 2018; 59: 149–63.

Lead exposure

Flowchart

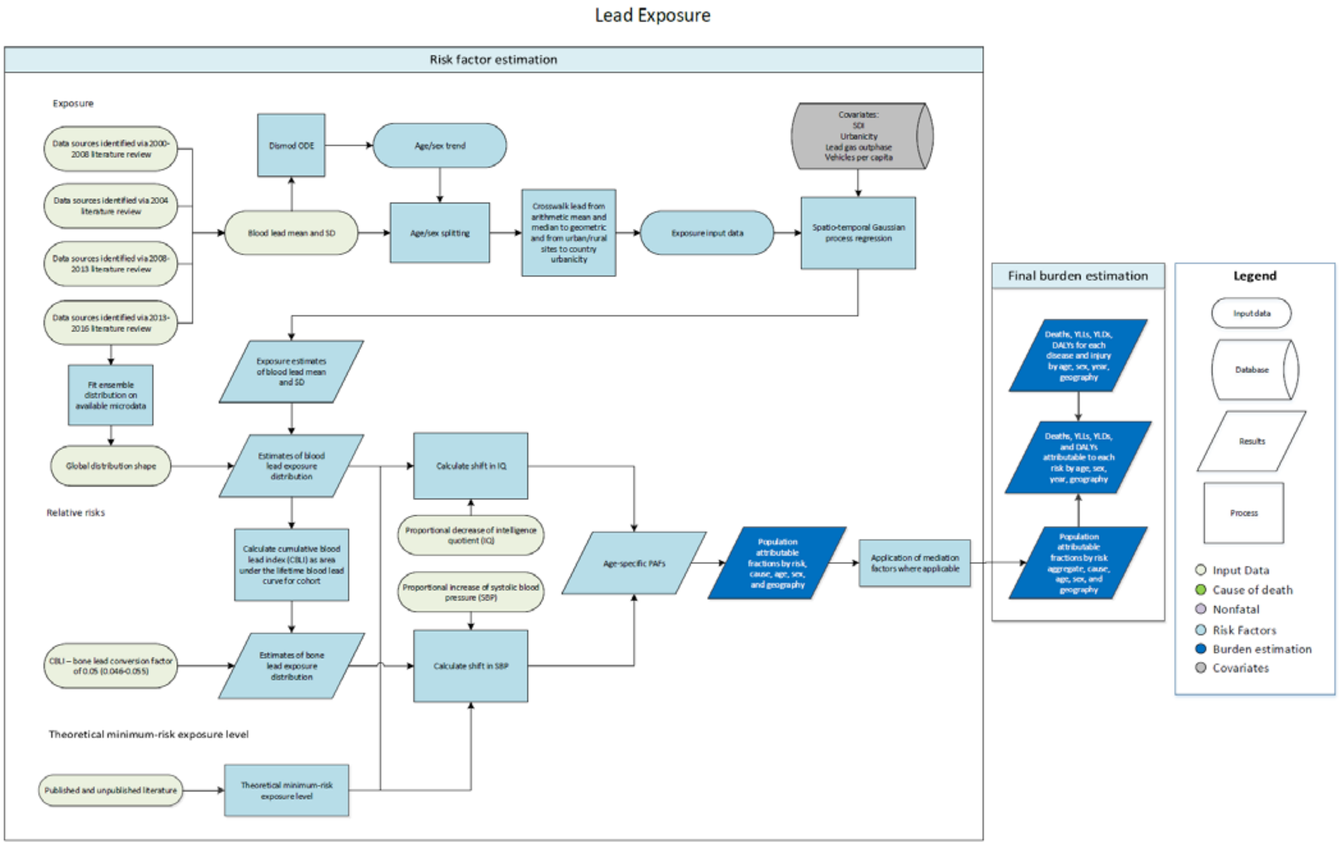

Definitions

Exposure to lead is defined in two different ways according to the currently known pathways of attributable health loss. Acute lead exposure, measured as micrograms of lead per deciliter of blood (µg/dL), is associated with IQ loss in children. Chronic lead exposure, measured as micrograms of lead per gram of bone (µg/g), is associated with increased systolic blood pressure and cardiovascular diseases.

Input data

The input data for lead exposure is primarily extracted from literature reports of blood lead levels, in addition to a few blood lead surveys. Blood lead values are derived from studies that take blood samples and analyse them using various techniques to determine the level of lead present. Our literature review, which was last updated in GBD 2017, resulted in 3183 usable datapoints from 554 different studies, which span the years 1970 to 2017. The second pathway of burden, bone lead exposure, was estimated by calculating a cumulative blood lead index for cohorts using estimated blood lead exposure over their lifetime. The cumulative blood lead index is then used to estimate bone lead using a scalar defined in literature.[1] Table 1 provides a summary of the exposure input data used.

Table 1: Data inputs for exposure

Input data	Exposure
Source count (total)	552
Number of countries with data	84

Data processing

In GBD 2019, we used MR-BRT to crosswalk our data. Blood lead exposure data are reported in the literature as either an arithmetic mean, a geometric mean, or a median. To standardise the data, we adjusted all values reported as a geometric mean or median to reflect what they would have been had the study reported the arithmetic mean. Additionally, the data come from locations of varying urbanicity (proportion of individuals in a given location living in an urban area). Because we expected the urbanicity of a location to affect our estimates, we adjusted our data so that they were equivalent to the average urbanicity of the country from which the data were collected. Tables 2 and 3 show the MR-BRT crosswalk adjustment factors.

Table 2: MR-BRT crosswalk adjustment factors for lead exposure (mean)

Reference or alternative case definition	Gamma	Beta coefficient, log (95% CI)	Adjustment factor*
Reference (data reported as arithmetic mean)	0.25	---	---
Alternative (data reported as geometric mean)		-0.178 (-0.667 to 0.311)	0.837 (0.513 to 1.365)
Alternative (data reported as median)		-0.157 (-0.646 to 0.333)	0.855 (0.524 to 1.395)

Table 3: MR-BRT crosswalk adjustment factors for lead exposure (urbanicity)

Reference or alternative case definition	Gamma	Beta coefficient, log (95% CI)	Adjustment factor*
Reference (study urbanicity equals national average urbanicity)	0.32	---	---
Alternative (study urbanicity does not equal national average urbanicity)		0.222 (-0.411 to 0.855)	1.248 (0.663 to 2.351)

**Adjustment factor is the transformed beta coefficient in normal space and can be interpreted as the factor by which the alternative case definition is adjusted to reflect what it would have been if measured as the reference.*

As an example of how the crosswalking works, a datapoint of 4.85 reported as a geometric mean was multiplied by the adjustment factor of 0.837 to get an estimated arithmetic mean of 4.06. The estimated arithmetic mean value was then used as the final datapoint in our modelling process.

Exposure modelling

The methodology to estimate lead exposure last underwent significant change in GBD 2013. Global exposure had been previously modelled using age-integrating Bayesian hierarchal modelling (DisMod-MR). The modelling process was updated for GBD 2013 by shifting to a spatiotemporal Gaussian process regression methodology (ST-GPR). This allowed for estimates of all country-age-sex-year groups for single years instead of five-year periods. This approach improved the granularity of estimates for bone lead, which requires back-estimation of previous blood lead to calculate a cumulative blood lead index.

For GBD 2019, the spatiotemporal Gaussian process regression modelling methodology was updated as detailed in the appendix specific to this analytical technique, which is common to a variety of risk factors. In order to predict blood lead in country-years with insufficient data, covariates that have been produced across time and space relevant to this analysis were used. For blood lead exposure, the covariates determined to have predictive ability were the Socio-demographic Index (SDI), urbanicity, the combined number of two- and four-wheeled vehicles per capita, and a covariate indicating whether leaded gasoline had been phased out in a given country-year (smoothed over the first five years of phase-out to reflect its gradual implementation). ST-GPR was used to produce estimates of mean and standard deviation of blood lead for all age groups, for both sexes, and for all GBD locations from 1970 to 2019. The linear regression equation is shown below.

$$log(data) \sim sdi + urbanicity + (leaded\ gas\ outphase * vehicles\ per\ capita) + (1|level\_1)$$

SDI = Socio-demographic Index
Urbanicity = proportion of population living in urban areas
Leaded gas outphase = whether or not a country has banned use of leaded gasoline
Vehicles per capita = number of 2- and 4-wheeled vehicles per capita
(1|level_1) = super-region-level random effects

In earlier iterations of GBD, the distribution of lead exposure was assumed to be log-normal. Since GBD 2016, ensemble modelling techniques were used to find an optimal global distribution by fitting a variety of distributions to the available blood lead microdata. This was a common update for all continuous risk factors. The ST-GPR mean and standard deviation estimates for blood lead were used with the global distribution shape to determine distributions for blood lead exposure. The distribution ultimately included 11 different probability distributions: exponential, gamma, inverse-gamma, mirrored gamma, log-logistic, Gumbel, mirrored Gumbel, Weibull, log-normal, normal, and beta. A little over 80% of the final distribution was log-logistic (35%), inverse-gamma (18%), log-normal (16%), or mirrored Gumbel (12%), with the seven other distributions comprising the remaining 20%.

To calculate blood lead over the lifetime of a given cohort, blood lead was assumed to grow linearly from 2.0 µg/dL in 1920 (see section *Theoretical minimum-risk exposure level*) to the value for that cohort in 1970. Using the exposure distributions of blood lead over time and space, cohorts were constructed such that lifetime blood lead could be expressed as a curve over each year of life. The area under this

curve was the cumulative blood lead index, which was used to estimate bone lead in a given year with the aforementioned scalar.

Estimating attributable burden

Assessment of risk-outcome pairs

We included outcomes based on the strength of available evidence supporting a causal relationship. Blood lead level (a measure of acute lead exposure) is paired with idiopathic developmental intellectual disability as modelled through the impact of blood lead levels on IQ in children. Bone lead level (a measure of chronic lead exposure) is paired with systolic blood pressure, and subsequently to all cardiovascular outcomes to which systolic blood pressure is paired, which include the following: rheumatic heart disease, ischaemic heart disease, ischaemic stroke, intracerebral haemorrhage, hypertensive heart disease, other cardiomyopathy, atrial fibrillation and flutter, aortic aneurysm, peripheral artery disease, endocarditis, other cardiovascular and circulatory diseases, chronic kidney disease due to hypertension, chronic kidney disease due to glomerulonephritis, and chronic kidney disease due to other and unspecified causes.

Theoretical minimum-risk exposure level

In previous iterations of GBD, the TMREL was estimated at 2.0 μg/dL. This level was based upon ambient sources of lead that would be impossible to eliminate[2] and a review of the literature indicating no consistent statistically significant estimates of increased relative risks at lower levels of blood lead. We have continued to use a TMREL of 2.0 μg/dL for GBD 2019. While the majority of global exposure is estimated to be well above this level, average blood lead exposures in a number of countries have fallen below 2.0 μg/dL in recent years (including, for example, the United States, where the average adult BLL was 1.2 μg/dL in 2009-2010).[3] This is consistent with estimates of pre-industrial blood lead in humans, which are as low as 0.018 μg/dL.[4] This suggests that the TMREL ought to be lowered, and this change will be evaluated for the GBD 2020 cycle.

Relative risks

Because the relative risk of IQ loss from lead exposure is specific to children, in previous iterations of GBD, no burden of lead via IQ loss was estimated in the population aged 15 and above. To better account for the continued burden of past lead exposure on IQ in older age groups, since GBD 2016 we have constructed cohorts from the entire population. Estimates of a cohort's lead exposure in early childhood (at 24 months of age) were used to determine past IQ loss, and thus calculate burden via the impact on concurrent IQ in the older population.

Blood lead relative risks were previously taken from a 2005 pooled analysis that was first incorporated in GBD 2010.[5] Those relative risks were then updated for GBD 2017 using a 2013 re-analysis of the findings of that 2005 paper, providing slightly adjusted relative risk estimates specific to exposure at 24 months of age.[6] The bone lead relative risks were taken from a 2008 meta-analysis that showed a 0.26 mmHg increase in systolic blood pressure (SBP) per 10 μg/g increase in bone lead (95% CI: 0.02 to 0.50).[7] Table 4 shows a summary of this information. Because bone lead is associated with increases in SBP, all of the health burden attributable to exposure to bone lead is mediated through SBP. As such, the relative risks for bone lead exposure are all the same as the relative risks that SBP has for its outcomes. Table 5 shows the relative risks for exposure to blood lead, and Table 6 shows a snapshot of the relative risks for bone lead (a full table can be found in Appendix Table 4a).

Table 4: Data inputs for relative risks

Input data	Relative risk
Source count (total)	2

Table 5: Relative risks for exposure to blood lead

Exposure level	IQ shift
2 µg/dL	0.0 (0.0 to 0.0)
4 µg/dL	3.146 (1.154 to 5.139)
6 µg/dL	3.804 (1.395 to 6.213)
8 µg/dL	4.296 (1.575 to 7.016)
10 µg/dL	4.688 (1.719 to 7.656)
12 µg/dL	5.014 (1.839 to 8.19)
15 µg/dL	5.42 (1.988 to 8.853)
20 µg/dL	5.952 (2.183 to 9.721)
25 µg/dL	6.37 (2.336 to 10.403)
30 µg/dL	6.713 (2.462 to 10.965)
35 µg/dL	7.006 (2.569 to 11.442)
40 µg/dL	7.26 (2.662 to 11.857)

Table 6: Relative risks for exposure to bone lead (snapshot)

Exposure level	Outcome	25-29 years	60-64 years	95+ years
10 µg/g	Ischaemic heart disease	1.042 (1.022 to 1.06)	1.021 (1.018 to 1.025)	1.014 (1.008 to 1.022)
10 µg/g	Ischaemic stroke	1.038 (1.021 to 1.06)	1.021 (1.016 to 1.026)	1.011 (1.006 to 1.019)
10 µg/g	Haemorrhagic stroke	1.047 (1.021 to 1.068)	1.024 (1.018 to 1.03)	1.015 (1.007 to 1.026)
10 µg/g	Hypertensive heart disease	1.066 (1.038 to 1.09)	1.04 (1.023 to 1.073)	1.033 (1.006 to 1.075)
10 µg/g	Non-rheumatic calcific aortic valve disease	1.035 (1.015 to 1.055)	1.014 (1.01 to 1.016)	1.007 (1.004 to 1.013)
10 µg/g	Atrial fibrillation and flutter	1.035 (1.018 to 1.056)	1.016 (1.014 to 1.018)	1.008 (1.005 to 1.01)
10 µg/g	Aortic aneurysm	1.027 (1.014 to 1.048)	1.014 (1.011 to 1.016)	1.007 (1.004 to 1.01)
10 µg/g	Peripheral vascular disease	1.034 (1.011 to 1.056)	1.009 (1.006 to 1.011)	1.006 (1.003 to 1.009)
10 µg/g	Endocarditis	1.035 (1.015 to 1.055)	1.014 (1.01 to 1.016)	1.007 (1.004 to 1.013)

Population attributable fraction

We used the standard GBD population attributable fraction (PAF) equation to calculate PAFs for bone lead exposure and each of its paired outcomes using exposure estimates and relative risks. We used a similar approach for estimating PAFs for the burden of intellectual disability attributable to blood lead, which uses the estimated distribution of intellectual disability and the modeled shifts in IQ due to blood lead levels to determine the PAF.

References

1. Hu H, Shih R, Rothenberg S, Schwartz BS. The epidemiology of lead toxicity in adults: measuring dose and consideration of other methodologic issues. Environ Health Perspect. 2007;115(3):455-62.
2. Pruss-Astun A, Fewtrell L, Landrigan PJ, Ayuso-Mateos JL. Lead Exposure. In: Ezzati M, Lopez AD, Rodgers A, Murray CJ, eds. Comparative quantifications of health risks: Global and regional burden of disease attrituable to selected major risk factors. Geneva, World Health Organization, 2004: 1496-542
3. CDC - Adult Blood Lead Epidemiology and Surveillance (ABLES): Program Description: NIOSH Workplace Safety and Health Topic." Centers for Disease Control and Prevention, 11 May 2018, www.cdc.gov/niosh/topics/ables/description.html.
4. Flegal AR, Smith DR. Lead levels in preindustrial humans. N Engl J Med. 1992;326(19):1293-4.
5. Lanphear BP, Hornung R, Khoury J, et al. Low-level environmental lead exposure and children's intellectual function: an international pooled analysis. Environ Health Perspect. 2005;113(7):894-9.
6. Crump K, Van Landingham C, Bowers T, Cahoy D, Chandalia J. A statistical reevaluation of the data used in the Lanphear et al. (2005) pooled-analysis that related low levels of blood lead to intellectual deficits in children. Critical Reviews in Toxicology. 2013;43(9):785-799.
7. Navas-Acien A, Schwartz BS, Rothenberg SJ, Hu H, Silbergeld EK, Guallar E. Bone lead levels and blood pressure endpoints: a meta-analysis. Epidemiology. 2008;19(3):496-504.

Occupational risk factors

Flowcharts

Occupational Risk Factors
(except asbestos and injuries)

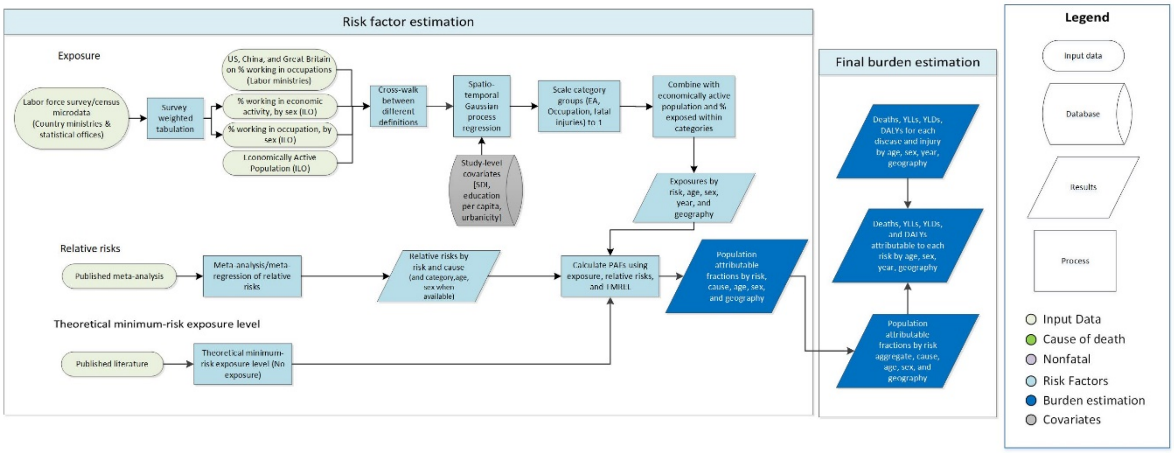

Occupational Risk Factors (Injuries)

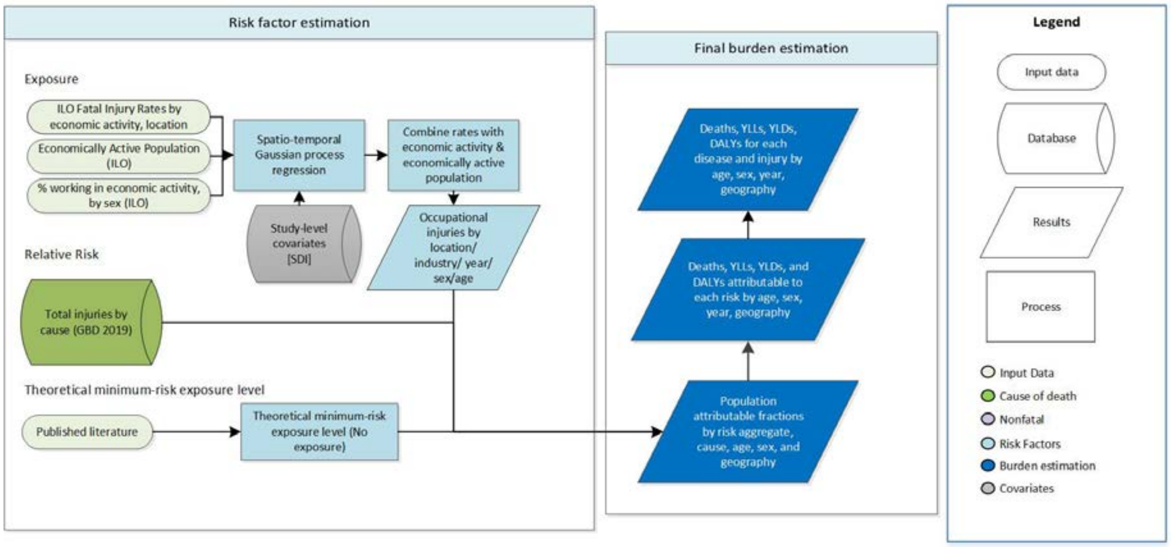

Occupational Risk Factors (asbestos)

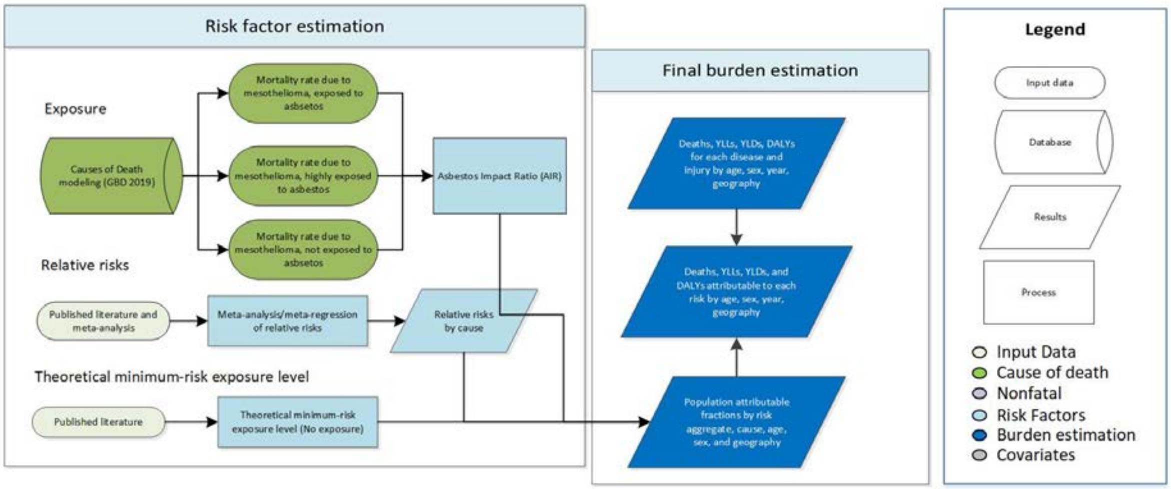

Exposure definitions

The following definitions were used for occupational risk factor exposures. All exposures were estimated for ages 15 and older.

Occupational asbestos	Proportion of the population occupationally exposed to asbestos, using mesothelioma death rate as an analogue
Occupational asthmagens	Proportion of the working population exposed to asthmagens, based on population distributions across nine occupational categories
Occupational carcinogens (arsenic, benzene, beryllium, cadmium, chromium, diesel engine exhaust, formaldehyde, nickel, polycyclic aromatic hydrocarbons, silica, sulfuric acid, and trichloroethylene)	Proportion of the population that was ever occupationally exposed to carcinogens at high or low exposure levels, based on population distributions across 17 economic activities
Occupational ergonomic factors	Proportion of the working population exposed to work that causes low back pain, based on population distributions across nine occupational categories
Occupational injuries	Proportion of injuries in the working-age population attributable to occupation, based on fatal injury rates in 17 economic activities
Occupational noise	Proportion of the population occupationally exposed to 85+ decibels of noise, based on population distributions across 17 economic activities
Occupational particulates	Proportion of the population occupationally exposed to particles, based on population distributions across 17 economic activities

Economic activities and occupations were coded according to the following categories:

**Economic activities**	**Occupations**
Agriculture, hunting, forestry	Legislators, senior officials, and managers
Fishing	Professionals
Mining and quarrying	Technicians and associate professionals
Manufacturing	Clerks
Electricity, gas, and water	Service workers and shop/market sales workers
Construction	Skilled agricultural and fishery workers
Wholesale and retail trade/repair	Plant and machine operators and assemblers
Hospitality	Craft and related workers
Transport, storage, and communication	Elementary occupations
Financial intermediation	
Real estate/renting	

Public administration/defense; compulsory social security
Education
Health and social work
Other community/social/personal service activities
Private households
Extra-territorial organisations/bodies

Input data

Primary inputs were obtained from the International Labour Organization (ILO).[1-4] These inputs included raw data on economic activity proportions, occupation proportions, fatal injury rates, and employment to population ratio estimates. No data on informal employment was included due to data sparseness. In 2017, a systematic review was conducted in order to collect the underlying microdata from the ILO's estimates to aid in re-extraction at greater levels of granularity. Where freely available, survey datasets were downloaded from the survey organisations in question. Other datasets were obtained through submission of requests to agencies and through the GBD collaborator network. Microdata were tabulated in order to create survey-weighted estimates of economic activities and occupations for the GBD geographies and years. Various classification systems were adjusted to match the ISIC Rev.3 classification (for economic activities) and ISCO 1988 classification (for occupations).

In 2019, a substantial amount of new ILO data were added. The new data comprise 1197 new unique location-years, including 174 unique locations and 13 unique years (2006–2018). Additionally, a number of old microdata were re-extracted.

For occupational asbestos, primary inputs were obtained through GBD 2019 cause of death estimates and published studies.[7,13,14]

Uncertainty for inputs where microdata were unavailable was generated by fitting a Loess curve to the data and determining the standard deviation of the data from the fitted curve.

Table 1 provides a summary of the exposure input data used.

Table 1: Data inputs for exposure

Input data	Exposure
Source count (total)	2485
Number of countries with data	199

Modelling strategies

A spatiotemporal Gaussian process regression (ST-GPR) was used to generate estimates for all years and locations for the primary inputs. Space-time parameters were chosen by maximising out-of-sample cross-validation and minimising RMSE. A number of different study-level covariates were used in the linear regression models. The linear models for each of the 46 different ST-GPR models used in occupational exposure estimation are listed below. Although there might appear to be duplicates, there is a distinction between occupation and economic activity (detailed in the footnotes). For example,

“skilled agriculture/fisheries” involves the proportion of the workforce doing agricultural and fishing work, while “agriculture, hunting, forestry” and “fishing” involve the proportion of the workforce employed in those respective industries (ie, one doesn’t have to be actually doing agricultural or fishing work – someone who transports crops would count as being employed in this industry, but their occupation would fall under “plant and machine operators & assemblers”). Additionally, each model included random effects at the region and super-region levels. The covariates are explained in greater detail below.

**ST-GPR model**	**Linear regression equation**
Employment (% of population employed)	$logit(data) = gov\_exp + prop\_muslim + education$
Armed forces*	$logit(data) = sdi + education + urbanicity$
Management*	$logit(data) = sdi + education + urbanicity$
Professional occupations*	$logit(data) = sdi + education + urbanicity$
Scientific/technicians*	$logit(data) = sdi + education + urbanicity$
Clerical work*	$logit(data) = sdi + education + urbanicity$
Service & shop/market sales workers*	$logit(data) = sdi + education + urbanicity$
Skilled agriculture/fisheries*	$logit(data) = sdi + latitude + urbanicity$
Craft and related trades*	$logit(data) = sdi + education + urbanicity$
Plant and machine operators & assemblers*	$logit(data) = sdi + education + urbanicity$
Elementary occupations*	$logit(data) = sdi + education + urbanicity$
Agriculture, hunting, forestry†	$logit(data) = sdi + latitude + urbanicity$
Fishing†	$logit(data) = \log(coastal\_prop + 0.01)$
Mining/quarrying†	$logit(data) = sdi + \log(coastal\_prop + 0.01) + urbanicity + asbestos$
Manufacturing†	$logit(data) = sdi + education + urbanicity$
Electricity/gas/water supply†	$logit(data) = \log(sdi) + urbanicity + temperature$
Construction†	$logit(data) = sdi + urbanicity$
Wholesale and retail trade/repair†	$logit(data) = sdi + education + urbanicity$
Hospitality†	$logit(data) = sdi + urbanicity$
Transport/storage/communications†	$logit(data) = sdi + urbanicity + vehicles\_pc$
Financial intermediation†	$logit(data) = sdi + urbanicity$
Real estate/renting†	$logit(data) = sdi + urbanicity$
Public administration/defence†	$logit(data) = sdi + urbanicity$
Education†	$logit(data) = sdi + education + urbanicity$
Health and social work†	$logit(data) = \log(sdi) + \log(health\_exp)$
Other community/social/personal service activities†	$logit(data) = sdi + urbanicity$
Private households†	$logit(data) = sdi + urbanicity$
Extraterritorial organisations and bodies†	$logit(data) = sdi + urbanicity$
All occupational injuries models‡	$logit(data) = sdi$

**Proportion of workforce working this type of occupation*

†Proportion of workforce employed in this type of economic activity

‡There are 18 different models, corresponding to one for each type of economic activity and a “total” model

Covariate	Description
gov_exp	Total government expenditure
prop_muslim	Proportion of population that is Muslim
education	Age-standardised years of education per capita
sdi	Socio-demographic Index
urbanicity	Proportion of population living in urban areas
latitude	Absolute value of average latitude of country's center point
coastal_prop	Percentage of total country area within 10 km of a coastal zone
asbestos	Asbestos consumption (metric tons per year per capita)
temperature	Population-weighted mean temperature
vehicles_pc	Number of 2- and 4-wheeled vehicles per capita
health_exp	Total health expenditure per capita

For economic activity and occupation proportions, estimates from ST-GPR were then re-scaled to sum to 1 across categories by dividing each estimate by the sum of all the estimates.

The following sections describe the modelling approaches for each occupational risk's exposure prevalence. These approaches were developed for GBD 2016 and have not changed substantially since then. The GBD 2019 methods are largely the same as those from the previous cycle, with the exception of the data processing for occupational injuries, which is explained in greater detail in that section below.

Occupational carcinogens, occupational noise, and occupational particulates
Prevalence of exposure to these risks was determined using the following equation:

$$Prevalence\ of\ Exposure_{c,y,s,a,r,l} = \sum_{EA} Proportion_{EA,c,y} * EAP_{c,y,s,a} * Exposure\ rate_{EA,r,l,d}$$

where:

EAP = economically active population	c = country	r = risk
EA = economic activity	d = duration	s = sex
a = age	l = level of exposure	y = year

Exposure rate (proportion of population exposed) was provided by expert group recommendations and literature.[8-11] The CAREX (carcinogen exposure) database[7] was used in order to quantify the association between exposure by industry/carcinogen to SDI across all the countries in the database. This effect was used to predict exposure in countries that were not included in CAREX. Duration was considered for occupational carcinogens through application of occupational turnover factors[12] and for occupational noise and particulates by calculating cumulative exposure as the average exposure over the lifetime (the past 50 years) for each age/sex cohort.

Occupational ergonomic factors and occupational asthmagens
Prevalence of exposure to these risks was determined using the following equation:

$$Prevalence\ of\ Exposure_{c,y,s,a,r} = \sum_{EA} Proportion_{OCC,c,y} * EAP_{c,y,s,a}$$

where:

EAP = economically active population c = country r = risk
EA = economic activity a = age s = sex
OCC = occupation y = year

Occupational injuries

Occupational injury counts were estimated using the following equation:

$$Occupational\ fatal\ injuries_{c,y,a,s} = \sum_{EA} Injury\ rate_{EA,c,y,s} * Population_{c,y,a,s} * EAP_{c,y,s,a} * Proportion_{EA,c,y}$$

where:

EAP = economically active population c = country y = year
EA = economic activity a = age s = sex

Additionally, in GBD 2019, we updated our data processing to use MR-BRT to crosswalk our data. Occupational injuries exposure data come from a number of different sources: insurance records, labour inspectorate records, establishment surveys, establishment or business registers, labour force surveys, economic or establishment censuses, official estimates, records of employers' organizations, and other administrative records and related sources. We expect insurance records to be the gold-standard source, because people should have more incentive to report injuries when they stand to benefit from their insurance plans. As such, we wanted to correct the data reported from other sources for underreporting, and so we crosswalked all of the data with insurance records data as our reference. To do so, we ran a mixed-effects log-linear regression using MR-BRT, with fixed effects on type of data source and random effects on super-region and region. Table 2 shows the beta coefficients from the MR-BRT model, as well as the crosswalk adjustment factors.

Table 2: MR-BRT crosswalk adjustment factors for occupational injuries

Reference or alternative case definition	Gamma	Beta coefficient, log (95% CI)	Adjustment factor*
Reference (insurance records)	0.1	---	---
Alternative (labour inspectorate records)		0.09 (-0.10, 0.29)	0.91, (0.76, 1.13)
Alternative (establishment surveys)		-0.27 (-0.47, -0.08)	1.31 (1.10, 1.63)
Alternative (labour force surveys)		-0.85 (-1.05, -0.66)	2.34 (1.96, 2.91)
Alternative (economic or establishment censuses)		-0.07 (-0.26, 0.13)	1.07 (0.89, 1.33)
Alternative (official estimates)		-0.24 (-0.43, -0.04)	1.27 (1.06, 1.57)

Alternative (establishment or business registers)		-0.35 (-0.60, -0.09)	1.41 (1.13, 1.90)
Alternative (records of employers' organizations)		-0.99 (-1.19, -0.79)	2.69 (2.25, 3.35)
Alternative (other administrative records and related sources)		-0.45 (-0.64, -0.25)	1.56 (1.31, 1.94)

**Adjustment factor is the inverse transformed beta coefficient in normal space and can be interpreted as the factor by which the alternative case definition is adjusted to reflect what it would have been if measured as the reference.*

Occupational asbestos

Prevalence of exposure to asbestos was estimated using the asbestos impact ratio (AIR), which is equivalent to the excess deaths due to mesothelioma observed in a population divided by excess deaths due to mesothelioma in a population heavily exposed to asbestos. Formally, this is defined using the following equation:

$$AIR = \frac{Mort_{c,y,s} - N_{c,y,s}}{Mort^{*}_{c,ys,} - N_{c,y,s}}$$

where:

Mort = Mortality rate due to mesothelioma
Mort* = Mortality rate due to mesothelioma in population highly exposed to asbestos
N = Mortality rate due to mesothelioma in population not exposed to asbestos

c = country
y = year
s = sex

Mortality rate due to mesothelioma was estimated using GBD 2019 causes of death results. Mortality rate due to mesothelioma in populations not exposed to asbestos was calculated using the model in Lin and colleagues,[13] while the mortality rate due to high exposure to asbestos was estimated using Goodman and colleagues' model.[14] Asbestos exposure prevalence created using the AIR was used to estimate population attributable fractions (PAFs) for all asbestos-associated causes except for mesothelioma. Custom PAFs were calculated for mesothelioma by using the ratio of the excess mortality with respect to an unexposed population (Mort – N) divided by the mortality rate in the population in question (Mort). This calculation assumes that all mesothelioma is a product of occupational asbestos exposure and could potentially overestimate the burden due to occupational asbestos exposure in populations with high non-occupational asbestos exposure.

Theoretical minimum-risk exposure level

For all occupational risks, the theoretical minimum-risk exposure level was assumed to be no exposure to that risk.

Relative risks

Relative risks were obtained for all occupational risks by conducting a systematic review of published meta-analyses. This review was last updated for GBD 2016. Table 3 provides a summary of the relative risk input data used.

Table 3: Data inputs for relative risks

Input data	Relative risk
Source count (total)	21

Population attributable fractions (PAFs)

For all occupational risks, with the exception of injuries (outlined below) and asbestos (outlined above), PAFs were calculated using the exposure prevalences estimated above, using the PAF formula in outlined in the GBD 2019 methods appendix.

Occupational injuries PAF

The PAFs for occupational injuries were calculated using the following formula:

$$PAF_{c,y,a,s} = \frac{Occupational\ fatal\ injuries_{c,y,a,s} - TMREL}{Fatal\ injuries_{c,y,a,s}}$$

where:

c = country
y = year
a = age
s = sex

Fatal injury totals were obtained from GBD 2019 causes of death.

References

1. International Labour Organization (ILO). International Labour Organization Database (ILOSTAT) - Employment by Sex and Economic Activity. International Labour Organization (ILO).

2. International Labour Organization (ILO). International Labour Organization Database (ILOSTAT) - Employment by Sex and Occupation. International Labour Organization (ILO).

3. International Labour Organization (ILO). International Labour Organization Database (ILOSTAT) - Fatal Injuries by Sex and Economic Activity. International Labour Organization (ILO).

4. International Labour Organization (ILO). International Labour Organization LABORSTA Economically Active Population, Estimates and Projections, October 2011. International Labour Organization (ILO), 2011.

5. Office for National Statistics (United Kingdom). Nomis Official Labor Market Statistics - Annual Population Survey. Newport, United Kingdom: Office for National Statistics (United Kingdom).

6. National Bureau of Statistics of China. China 1% National Population Sample Survey 1995. Ann Arbor, United States: China Data Center, University of Michigan.

7. Kauppinen T, Toikkanen J, Pedersen D, *et al.* Occupational exposure to carcinogens in the European Union. *Occupational and Environmental Medicine* 2000; **57**: 10–8.

8. Wilson DH, Walsh PG, Sanchez L, *et al.* The epidemiology of hearing impairment in an Australian adult population. *Int J Epidemiol* 1999; 28: 247–52

9. Kauppinen T, Toikkanen J, Pederson D, Young R, Kogevinas M, Ahrens W, et al. Occupational Exposure to Carcinogens in the European Union in 1990-93. Helsinki, Finland: Finnish Institute of Occupational Health; 1998.

10. Kauppinen T, Toikkanen J, Pedersen D, Young R, Ahrens W, Boffetta P, et al. Occupational exposure to carcinogens in the European Union. *Occup Environ Med* 2000; 57(1): 10–18.

11. Driscoll T, et al. The global burden of non-malignant respiratory disease due to occupational airborne exposures. *American Journal of Industrial Medicine 2005; 48*(6): 432-445.

12. Nelson, D. I., Concha-Barrientos, M., Driscoll, T., Steenland, K., Fingerhut, M., Punnett, L. & Corvalan, C. (2005). The global burden of selected occupational diseases and injury risks: Methodology and summary. *American journal of industrial medicine*, *48*(6), 400-418

13. Lin R-T, Takahashi K, Karjalainen A, *et al.* Ecological association between asbestos-related diseases and historical asbestos consumption: an international analysis. *Lancet* 2007; **369**: 844–9.

14. Goodman M, Morgan RW, Ray R, Malloy CD, Zhao K. Cancer in asbestos-exposed occupational cohorts: a meta-analysis. *Cancer Causes Control* 1999; **10**: 453–65.

Suboptimal breastfeeding

Flowchart

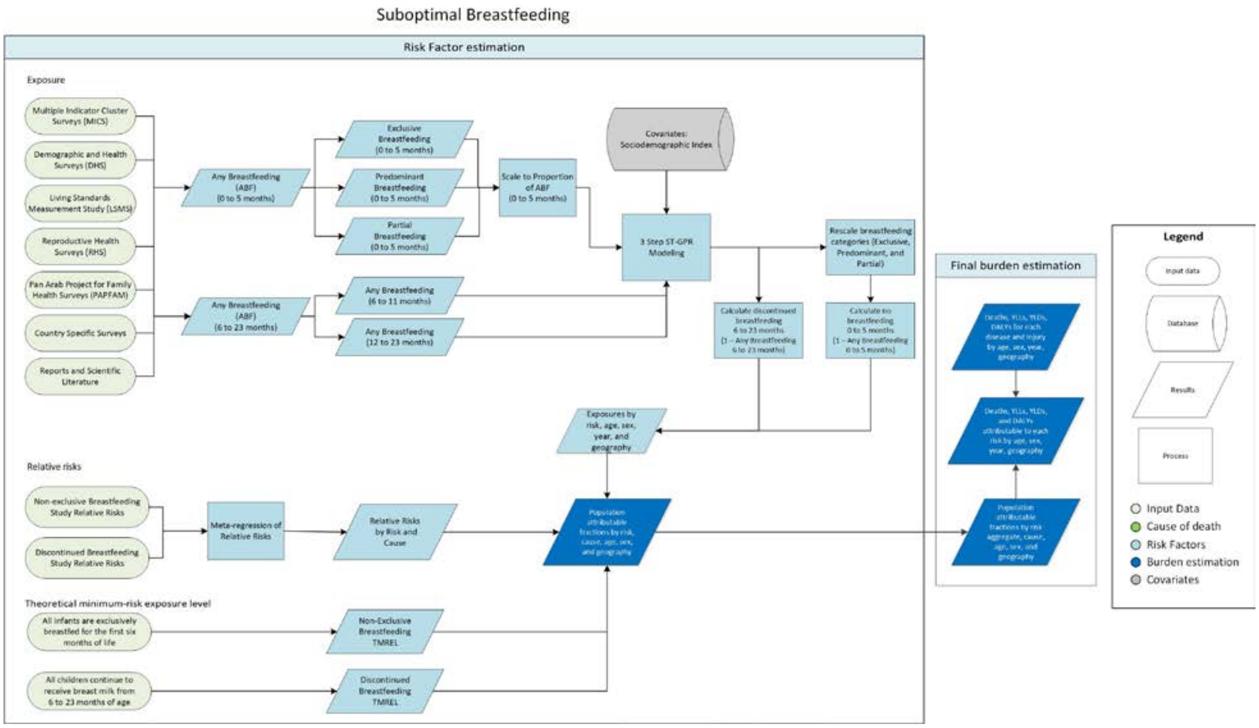

Input data and methodological summary

Exposure definitions

Exposure to suboptimal breastfeeding is composed of two distinct categories: non-exclusive breastfeeding and discontinued breastfeeding.

Non-exclusive breastfeeding is defined as the proportion of children under 6 months of age who are not exclusively breastfed. We then parse those not exclusively breastfed into three categories – predominant, partial, and no breastfeeding. Exclusive breastfeeding is defined as the proportion of children who receive no other food or drink except breastmilk (allowing for ORS, drops, or syrups containing vitamins, minerals, or medicines). Predominant breastfeeding is the proportion of children whose predominant source of nourishment is breastmilk but also receive other liquids. Partial breastfeeding refers to those infants who receive breastmilk as well as food and liquids, including non-human milk and formula. No breastfeeding refers to infants who do not receive breastmilk as a source of nourishment.

Discontinued breastfeeding is defined as the proportion of children between 6 and 23 months who receive no breastmilk as a source of nourishment.

Input data

The data used in the analysis consist mostly of processed individual-level microdata from surveys; in the cases where microdata were unavailable, we used reported tabulated data from survey reports and

scientific literature. Data used to categorise type of non-exclusive breastfeeding (predominant, partial, and none) come from surveys with 24-hour dietary logs based on maternal recall.

We updated our systematic review in GBD 2019 by searching the Global Health Data Exchange (GHDx) using the keyword "breastfeeding." We prioritised extraction of surveys with microdata and new surveys from major survey series such as Demographic and Health Surveys (DHS) and Multiple Indicator Cluster Surveys (MICS).

Table 1. Input Data Counts – Non-Exclusive Breastfeeding

Input data	Exposure	Relative Risk
Source count (total)	688	43
Number of countries with data	162	26

Table 2. Input Data Counts – Discontinued Breastfeeding

Input data	Exposure	Relative Risk
Source count (total)	613	43
Number of countries with data	156	26

Exposure modelling

Using the processed microdata and tabulated data from reports, we generated a complete time series from 1980 to 2019 for the prevalence of breastfeeding patterns for children 0 to 5 months and 6 to 23 months using a three-step spatiotemporal Gaussian process regression modelling process.

First, a mixed-effects linear regression with fixed effects on location-level covariates and nested geographical random effects produces a stage 1 prediction. In GBD 2019, we revised this step to include an ensemble stage 1 prediction, estimating candidate models consisting of all combinations of covariates and averaging across the top 50 models weighted by out-of-sample predictive validity. We included the following covariates: Socio-demographic Index, SEV for unsafe water (age-standardised), total fertility rate, maternal education (years per capita), antenatal care (4+ visits), HIV mortality (women of reproductive age), high BMI (women of reproductive age), and underweight (women of reproductive age).

We then followed this with a spatiotemporal regression that uses the residuals of the predictions from the linear regression to perform a locally weighted regression that provides a greater weighting factor to those nearer in space and time. The predicted residuals from this step are then added to those created in the linear regression step.

Finally, we ran a Gaussian process regression that incorporated the variance of the input data as well as the variance of the model predictions. It uses predictions from the spatiotemporal regression as the mean function and generates draws from a multinomial distribution (based on the data uncertainty in the prior) to generate the final prevalence estimates and their confidence intervals.

We estimated six models to produce each of our categories: the proportion of currently breastfeeding infants 0-5 months of age, the ratio of infants exclusively breastfed to breastfed infants 0-5 months of age, the ratio of infants predominantly breastfed to breastfed infants 0-5 months of age, the ratio of infants partially breastfed to breastfed infants 0-5 months of age, the proportion of currently breastfeeding infants 6-11 months of age, and the proportion of currently breastfeeding infants 12-23 months of age. We convert the ratios of exclusive, predominant, and partial breastfeeding to the total category prevalence proportions by multiplying each ratio by the estimates of any breastfeeding among infants aged 0-5 months. This ensures that these categories sum correctly to the "any breastfeeding 0-5 months" envelope. We calculate the proportion of infants receiving no breastmilk 0-5 months of age by subtracting the estimates of current breastfeeding from 1. We perform the same operation to estimate discontinued breastfeeding in the 6-11 months and 12-23 months categories.

Modelling strategy

Assessment of risk-outcome pairs

We included outcomes based on the strength of available evidence supporting a causal relationship. Studies evaluating the causal evidence for our risk-outcome pairs came primarily from articles found in a review published by the World Health Organization.[1] Non-exclusive breastfeeding was paired with diarrhoea and lower respiratory infection as diseases outcomes. Discontinued breastfeeding was paired with diarrhoea as an outcome.

Theoretical minimum-risk exposure level

For non-exclusive breastfeeding, those children that received no source of nourishment other than breastmilk ("exclusively breastfed") were considered to be at the lowest risk of any of the disease outcomes. For discontinued breastfeeding, we assumed that children aged 6 to 23 months who received any breastmilk as a source of nourishment to be at the lowest risk of disease outcome.

Relative risks

We estimated relative risks for both non-exclusive and discontinued breastfeeding in a meta-analysis using relative risks from studies compiled in a published review by the World Health Organization.[1]

Population attributable fraction

We used the standard GBD population attributable fraction (PAF) equation to calculate PAFs for non-exclusive breastfeeding and discontinued breastfeeding and each of their paired outcomes using exposure estimates, the theoretical minimum-risk exposure level, and relative risks.

References

1. Horta, B., Voctora, C. (2013) Short-term effects of breastfeeding: a systematic review on the benefits of breastfeeding on diarrhoea and pneumonia mortality. The World Health Organization.

Child growth failure

Flowchart

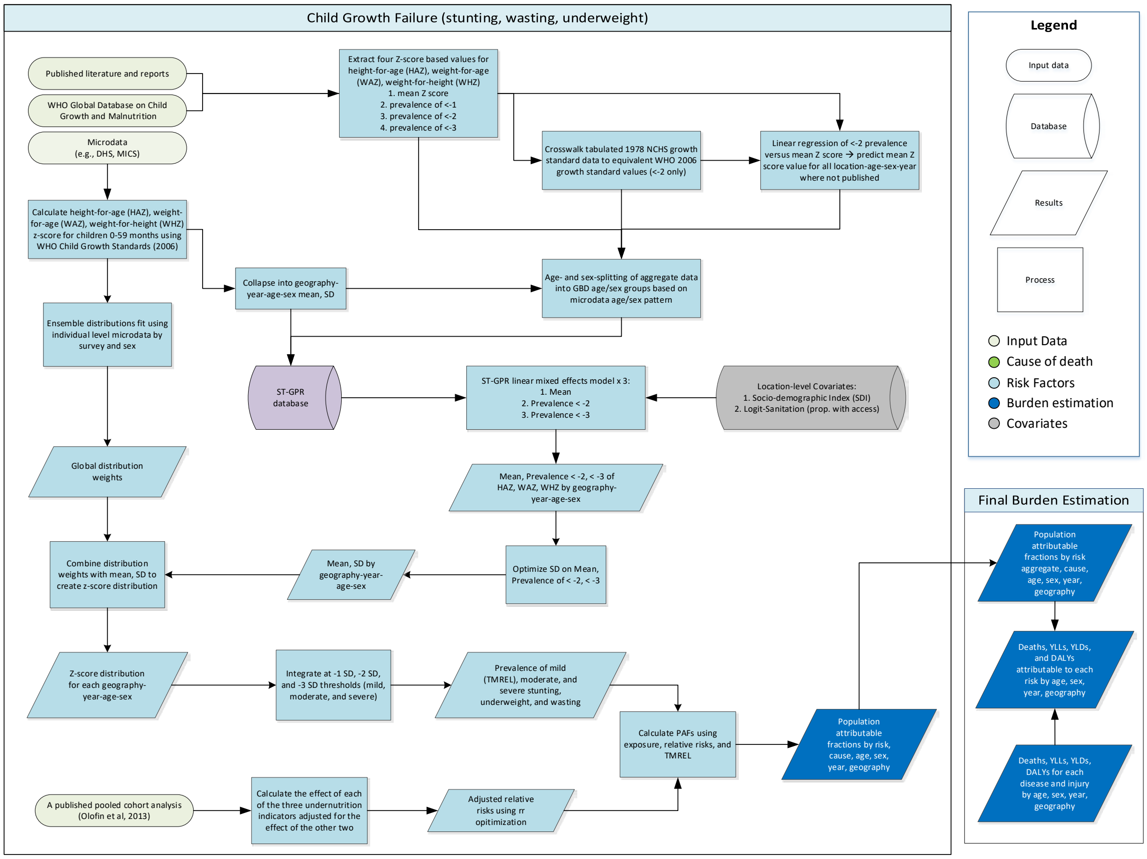

Input data and methodological summary

Exposure

Case definition

Child growth failure is estimated using three indicators (stunting, wasting, and underweight), all of which are based on categorical definitions using the WHO 2006 growth standards for children 0-59 months. Definitions are based on Z scores from the growth standards, which were derived from an international reference population. Mild (<-1 to -2 Z score), moderate (<-2 to -3 Z score), and severe (<3 Z score) categorical prevalences were estimated for each of the three indicators.

Input data

There are three main inputs for the GBD child growth failure models: microdata from population surveys, tabulated data from reports and published literature, and the WHO Global Database on Child Growth and Malnutrition.[1] The primary data additions in GBD 2019 for child growth failure were from population surveys that include anthropometry. Population surveys include a variety of multi-country

and country-specific survey series such as Multiple Indicator Cluster Surveys (MICS), Demographic and Health Surveys (DHS), Living Standards Measurement Surveys (LSMS), and the China Health and Nutrition Survey (CHNS), as well as other one-time country-specific surveys such as the Indonesia Family Life Survey and the Brazil National Demographic and Health Survey of Children and Women. These microdata contain information about each individual child's age (from which age in weeks and age in months are calculated), as well as height and/or weight. From that information, a height-for-age z-score (HAZ), weight-for-age z-score (WAZ), and weight-for-height z-score (WHZ) are calculated using the WHO 2006 Child Growth Standards and the LMS method.[2] In GBD 2019, several new data-cleaning criteria were applied to increase the quality of the microdata set. Data that did not meet the following criteria were dropped: 1) non-sex-specific data, 2) data with invalid Z-scores (HAZ, WAZ, WHZ, or BMI above 6 SD or below -6 SD), and 3) data with impossible values (negative height, weight, or age).

All available data from the WHO Global Database on Child Growth and Malnutrition were extracted in GBD 2016 – much of which are from published studies. Exclusions included examination date prior to 1985, non-population-representative studies, and those based on self-report. A systematic literature review was last completed in GBD 2010. We looked for four metrics from all sources with tabulated data: mean Z score, prevalence <-1 Z score, prevalence <-2 Z score, and prevalence <-3 Z score. All data for each metric were extracted for each of stunting (height-for-age Z score; HAZ), wasting (weight-for-height Z score; WHZ), and underweight (weight-for-age Z score; WAZ).

Table 1: Input data counts for Child wasting exposure models

Input data	Exposure
Source count (total)	1240
Number of countries with data	151

Table 2: Input data counts for Child underweight exposure models

Input data	Exposure
Source count (total)	1270
Number of countries with data	150

Table 3: Input data counts for Child stunting exposure models

Input data	Exposure
Source count (total)	1262
Number of countries with data	151

Data processing

To maximise internal consistency and comprehensiveness of the modelling dataset, we performed three data transformations. First, any data that were reported using the National Center for Health Statistics (NCHS) 1978 growth standards were crosswalked to corresponding values on the WHO 2006 Growth Standards curves based on a study that evaluated growth standard concordance.[3] Crosswalks from 1978 to 2006 growth standards were performed using OLS linear regression only on <-2 (ie, moderate) prevalence data, as that is where the concordance was most consistent. Second, for any study that lacked a measure of mean Z score for any of stunting, wasting, or underweight, we predicted a mean value for that study based on an ordinary-least-squares regression of mean Z score versus <-2 prevalence for that metric from all sources where both were available. Third, for any data that were presented as both sexes combined or for 0-59 months combined, we used the age and sex pattern from all data sources that included that detail to split into corresponding and age- and sex-specific data.

Modelling strategy

Exposure estimation

The following four-step modelling process was applied in parallel to each of stunting, wasting, and underweight.

First, all microdata were fit using an ensemble modelling process, a modelling framework developed for GBD 2019 that is described elsewhere in this appendix. A series of 12 individual distributions (normal, log-normal, log-logistic, exponential, gamma, mirror gamma, inverse gamma, gumbel, mirror gumbel, Weibull, inverse Weibull, and beta) were fit to the entire set of microdata (approximately 2.5 million individual z-scores) at the individual survey level. A weighting algorithm combined each distribution to find the optimal combination of these distributions for each survey, minimising the absolute prediction error across the entire distribution. Ensemble weights for each survey were then averaged across all surveys to produce a single set of global weights of the ensemble distributions. Weights were different for each sex, but invariant across geography, time, and age group. All component distributions that were used to derive weights were parameterised using "method of moments," meaning that each corresponding probability density function (PDF) could be described as a function of the mean and variance of the quantity of interest.

Second, models were developed for mean Z scores and prevalence of moderate and severe growth failure. Individual-level microdata were collapsed to calculate three metrics: mean z-score, moderate prevalence, and severe prevalence. These data were combined with those derived from literature, GHDx review, and the WHO Global Database on Child Growth and Malnutrition. Each of the three metrics was then modelled using spatiotemporal Gaussian process regression (ST-GPR), a common modelling framework used across GBD, generating estimates for each age group, sex, year, and location. Location-level covariates used in all models included Socio-demographic Index (SDI) and logit-transformed proportion of households with improved sanitation.

Third, we combined estimates of mean, prevalence (moderate and severe) with ensemble weights in an optimisation framework in order to derive the variance that would best correspond to the predicted mean and prevalence. This variance was then paired with the mean and, using the method of moments equation for each of the component distributions of the ensemble, PDF of the distribution of Z-scores were calculated for each location, year, age group, and sex.

Fourth, PDFs were integrated to determine the prevalence between -1 and -2 Z scores (mild), between -2 and -3 Z scores (moderate), and below -3 Z scores (severe). These were categorical exposures used for subsequent attributable risk analysis.

Theoretical minimum-risk exposure level

Theoretical minimum risk exposure level (TMREL) for underweight, stunting, and wasting was assigned to be greater than or equal to -1 SD of the WHO 2006 standard weight-for-age, height-for-age, and weight-for-height curves, respectively. This has not changed since GBD 2010.

Relative risks

The final list of outcomes paired with child growth failure risks included lower respiratory infections (LRI), diarrhoea, measles, and protein-energy malnutrition (PEM), as shown in Table 5. These were derived from a pooled cohort analysis by Olofin and colleagues.[5]

Table 4: Input data counts for Child growth failure relative risk models

Input data	Exposure
Source count (total)	1

There is a high degree of correlation between stunting, wasting, and underweight. Failing to account for their covariance and assuming independence would overestimate the total burden significantly. This is the main reason that GBD 2010 only included childhood underweight. In GBD 2013, a method was developed to adjust observed RRs of Olofin and colleagues by simulating the joint distribution of the three indicators using the distribution of each indicator and covariance between indicators in the countries included in the meta-analysis (extracted from Demographic and Health Survey (DHS) micro-data).[4] Based on the analysis done by McDonald and colleagues, we assumed there is an interaction between the three indicators, and extracted the interaction terms from the corresponding analysis. We calculated the adjusted RRs by minimising the error between observed crude RRs (from meta-analysis) and expected crude RRs derived from adjusted RRs.

Of historical note, upper respiratory infections and otitis media were included as outcomes in the GBD 2013 risk analysis, based on the “analogy” causal criterion, assuming there is similar pathway as LRI outcome. However, closer review for GBD 2015 did not find sufficient evidence to support their inclusion and they were excluded, a decision that was carried forward into GBD 2016. We also attributed 100% of PEM to childhood wasting and underweight but not stunting. To build on the existing literature base for GBD on risk-outcome pairs, a literature search was conducted for GBD 2017 searching for case-control studies published after January 1, 1985 did not return any sources that were usable.

Table 5: Adjusted RRs for each risk-outcome pair for child growth failure

Outcome	Stunting	Wasting	Underweight
**Diarrhoea**	<-1: 1.111 (1.023-1.273)<br><-2: 1.222 (1.067-1.5)<br><-3: 1.851 (1.28-2.699)	<-1: 6.601 (2.158-11.243)<br><-2: 23.261 (9.02-35.845)<br><-3: 105.759 (42.198-157.813)	<-1: 1.088 (1.046-1.134)<br><-2: 1.23 (1.163-1.314)<br><-3: 2.332 (2.076-2.802)

**Lower respiratory infections (LRI)**	<-1: 1.125 (0.998-1.655)<br><-2: 1.318 (1.014-2.165)<br><-3: 2.355 (1.15-5.114)	<-1: 5.941 (1.972-11.992)<br><-2: 20.455 (70.84-37.929)<br><-3: 47.67 (15.923-94.874)	<-1: 1.145 (1.044-1.364)<br><-2: 1.365 (1.215-1.755)<br><-3: 2.593 (1.908-4.39)
**Measles**	<-1: 1.103 (0.861-1.719)<br><-2: 1.54 (1.029-3.222)<br><-3: 2.487 (1.129-6.528)	<-1: 1.833 (0.569-8.965)<br><-2: 8.477 (1.33-42.777)<br><-3: 37.936 (5.088-199.126)	<-1: 0.995 (0.5-1.726)<br><-2: 2.458 (1.26-5.118)<br><-3: 5.668 (1.767-12.414)
**Protein-energy malnutrition**	0% PAF	100% PAF	100% PAF

References

1 WHO | WHO Global Database on Child Growth and Malnutrition. WHO. http://www.who.int/nutgrowthdb/en/ (accessed July 30, 2018).

2 Wang Y, Chen H-J. Use of Percentiles and Z-Scores in Anthropometry. In: Preedy VR, ed. Handbook of Anthropometry. New York, NY: Springer New York, 2012: 29–48.

3 Uribe Á, Cecilia M, López Gaviria A, Estrada Restrepo A. Concordance between Z scores from WHO 2006 and the NCHS 1978 growth standards of children younger than five. Antioquia-Colombia. *Perspectivas en Nutrición Humana* 2008; 10: 177–87.

4 McDonald CM, Olofin I, Flaxman S, *et al.* The effect of multiple anthropometric deficits on child mortality: meta-analysis of individual data in 10 prospective studies from developing countries. *Am J Clin Nutr* 2013; 97: 896–901.

5 Olofin I, McDonald CM, Ezzati M, *et al.* Associations of Suboptimal Growth with All-Cause and Cause-Specific Mortality in Children under Five Years: A Pooled Analysis of Ten Prospective Studies. *PLOS ONE* 2013; 8: e64636.

Low birthweight and short gestation

Flowchart

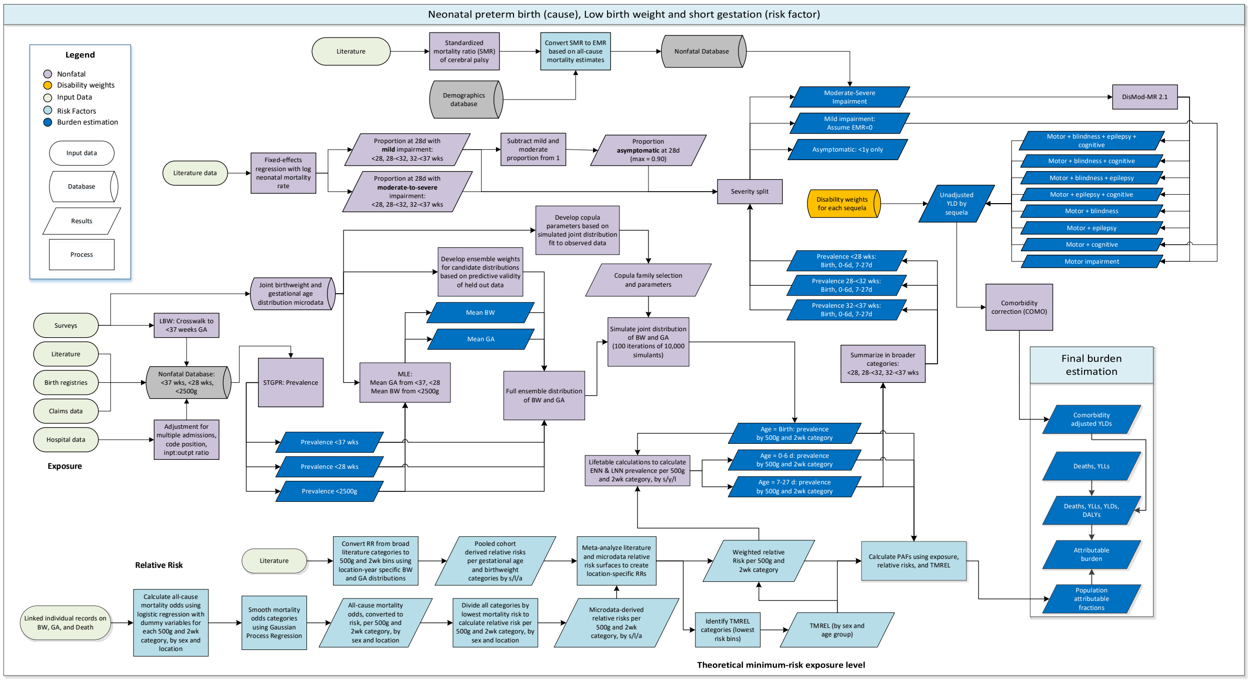

Input data and methodological summary

Short gestational age and low birthweight are highly correlated risk factors associated with poor child health outcomes. The “low birthweight and short gestation” (LBWSG) risk factor quantifies the burden of disease attributable to increased risk of death and disability due to 1) less than ideal birthweight (“low birthweight”) and 2) shorter than ideal length of gestation (“short gestation”).

Generally, within GBD, attributable burden is estimated separately for each individual risk factor. However, within the global health community, the combined burden attributable to multiple risk factors is of general interest. In GBD, attributable burden due to multiple risk factors is typically estimated through a “mediation analysis” that is applied after independent estimation of each risk factor’s exposure, relative risk, theoretical minimum risk exposure level (TMREL), and population attributable fraction (PAF). In the mediation analysis, a “mediation factor” adjusts the PAF of each risk factor by the amount of attributable burden mediated through the other GBD risk factors. While mediation analysis is the standard approach in GBD used to estimate combined attributable burden, direct quantification of the joint exposure, relative risk, and PAF of the combined risk factors is a conceptually more straightforward analysis. However, in practice, data informing joint exposure and relative risk of multiple risk factors is typically scarce.

In GBD 2016, LBWSG became the first (and, as of GBD 2019, only) group of GBD risk factors in which combined attributable burden is quantified by direct estimation of the joint exposure, relative risk, TMREL, and PAF of multiple risk factors. Compared to other risk factor groups in GBD, the data needed to estimate the joint exposure and joint relative risk of birthweight and gestational age are relatively abundant, as birthweight and gestational age are commonly reported together. LBWSG was also an appealing candidate to pilot joint direct estimation because the combined burden attributable to birthweight *and* gestational age, as well as the separately attributable burden due to birthweight *or* gestational age, are both of interest to the global health community.

After first directly estimating the joint exposure, relative risk, TMREL, and PAF of birthweight and gestational age together, we then separate out the independent PAFs due to birthweight only or gestational age only. Because of this modelling strategy, the joint GBD risk factor quantifying the burden of disease due to both less than ideal birthweight (“low birthweight”) and shorter than ideal gestational age (“short gestation”) is grouped into a single “parent” risk factor termed “low birthweight and short gestation”. LBWSG is disaggregated into two “child” risk factors: “low birthweight for gestation” and “short gestation for birthweight”. Low birthweight for gestation quantifies the burden of disease attributable to less than ideal birthweight, after adjusting for the influence of gestational age. Likewise, short gestation for birthweight quantifies the burden of disease attributable to shortened gestational age, after adjusting for the influence of birthweight.

Ideally, the model for joint exposure and joint relative risk would be fully continuous. To simplify the computation for the analysis, a grid of 500-gram and 2-week units (“bins”) is used as the LBWSG dimensions and to approximate a fully continuous joint distribution model (see Figure 1).

Figure 1: Fully continuous analysis of joint gestational age and birthweight (left) is approximated with a grid of birthweight and gestational age with 500-gram and 2-week “bins” (right)

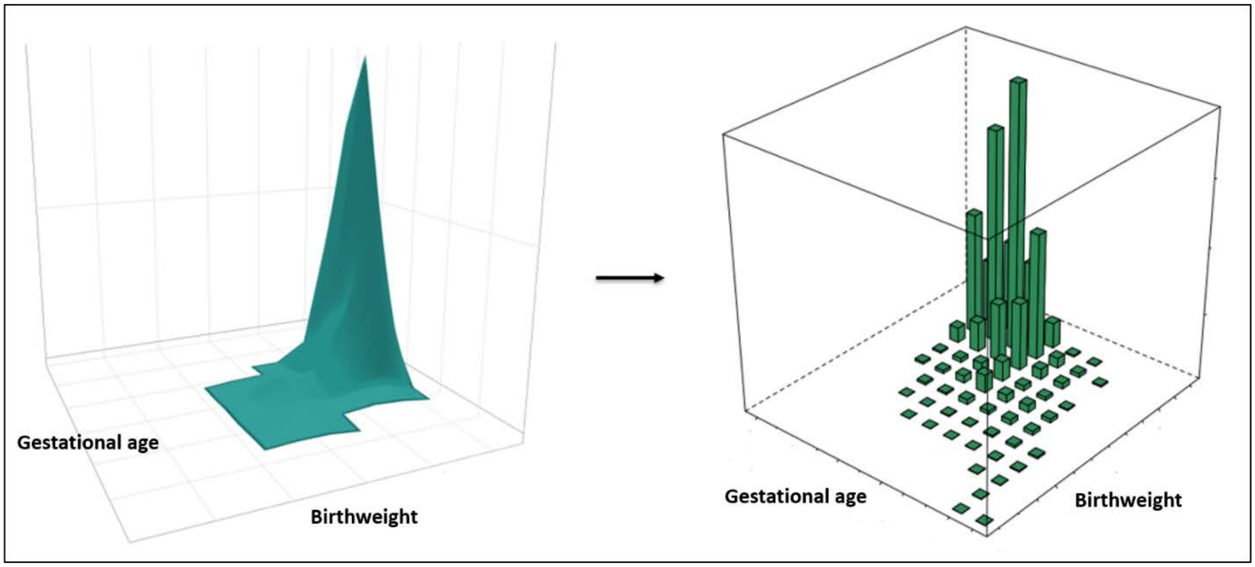

Case definition

“Low birthweight” has historically referred to any birthweight less than 2500 grams, dichotomising birthweight into two categories: “normal” and “low”. In the context of the GBD LBWSG risk factor, low birthweight refers to any birthweight less than the birthweight TMREL (the birthweight that minimises risk at the population level). Because LBWSG is estimated in a grid of 500-gram and 2-week bins, any 500-gram birthweight unit less than the TMREL, which was determined as [38, 40) weeks and [3500, 4000) g for the LBWSG parent risk factor, is considered “low birthweight”. This includes, for example, birthweight of [2500, 3000) grams, which the traditional, dichotomous definition of “low birthweight” would not include.

Like birthweight, gestational age is typically classified into broad categories. “Preterm” is used to describe any newborn baby born less than 37 completed weeks of gestation. In the GBD context, “short gestation” is used to refer to all gestational ages below the gestational age TMREL.

LBWSG is paired with the outcomes listed in Table 1 and is only attributed to burden in the early and late neonatal period.

Table 1: Cause list of outcomes for low birthweight and short gestation

Cause name
Diarrhoeal diseases
Lower respiratory infections
Upper respiratory infections
Otitis media
Pneumococcal meningitis
H influenzae type B meningitis
Meningococcal meningitis
Other meningitis
Encephalitis

Neonatal preterm birth complications
Neonatal encephalopathy due to birth asphyxia and trauma
Neonatal sepsis and other neonatal infections
Haemolytic disease and other neonatal jaundice
Other neonatal disorders
Sudden infant death syndrome

Exposure

In LBWSG, exposure refers to the portion of the joint distribution of gestational age and birthweight less than the TMREL, by location/year/sex (l/y/s), from birth to the end of the neonatal period. Modelling LBWSG exposure can be summarised in three steps:

A. Model univariate gestational age and birthweight distributions at birth, by l/y/s
B. Model joint distributions of gestational age and birthweight at birth, by l/y/s
C. Model joint distributions from birth to the end of the neonatal period, by l/y/s

Table 2: Analytic steps in estimation of YLDs due to preterm birth

	Summary of exposure modelling strategy
**Step A**<br>Model univariate distributions at birth	1. Model mean gestational age, prevalence of gestational age <28 weeks, and prevalence of gestational age <37 weeks, by l/y/s<br>2. Model mean birthweight and prevalence of birthweight <2500 grams, by l/y/s<br>3. Model univariate gestational age and birthweight distributions separately at birth, by l/y/s
**Step B**<br>Model joint distributions at birth	1. Use copulae to model the correlation structure of the joint distribution of gestational age and birthweight, globally<br>2. Model the joint distribution of gestational age and birthweight, by location/year/sex at birth, by applying the globally modelled correlation structure to the location/year/sex-specific univariate models of gestational age and birthweight distributions
**Step C**<br>Model joint distributions from birth to 28 days	1. Model all-cause mortality rates by gestational age and birthweight<br>2. Model gestational age and birthweight distributions of surviving neonates for all l/y/s from birth to end of the neonatal period, using all-cause mortality rates by gestational age and birthweight

Input data and data processing

Input data needed to model univariate gestational age and birthweight distributions at birth (Step A) are:

- Prevalence of preterm birth (<37 weeks), by l/y/s
- Prevalence of preterm birth (<28 weeks), by l/y/s
- Mean gestational age, by l/y/s
- Gestational age microdata

- Prevalence of low birthweight (<2500 grams), by l/y/s
- Mean birthweight, by l/y/s
- Birthweight microdata

To model joint distributions of gestational age and birthweight (Step B), joint microdata of gestational age and birthweight are also required. Additional inputs to modelling joint distributions from birth to 28 days (Step C) are all-cause mortality by l/y/s and joint birthweight and gestational age microdata linked to mortality outcomes.

Prevalence of extremely preterm birth (<28 weeks) and preterm birth (<37 weeks) were modelled using vital registration, survey, and clinical data. For the preterm models, only inpatient and insurance claims data were included from clinical informatics datasets; outpatient data were excluded because they were more likely to capture repeated visits by the same child rather than unique visits. Prevalence of low birthweight (<2500 grams) was modelled using only vital registration and survey data.

Literature review

Before GBD 2016, available preterm birth data were sourced by a technical working group. In GBD 2016 and GBD 2017, we conducted systematic reviews to identify additional sources beyond the data already used in the models. The PubMed database was searched using the following search string:

((("Infant, Premature"[Mesh] OR ("infant"[All Fields] AND "premature"[All Fields]) OR "premature infant"[All Fields] OR ("preterm"[All Fields] AND "infant"[All Fields]) OR "preterm infant"[All Fields] OR ("infant, newborn"[MeSH Terms] OR ("infant"[All Fields] AND "newborn"[All Fields]) OR "newborn infant"[All Fields] OR ("newborn"[All Fields] AND "infant"[All Fields])) AND (premature[All Fields] OR preterm[All Fields]) OR "premature birth"[MeSH Terms] OR ("premature"[All Fields] AND "birth"[All Fields]) OR "premature birth"[All Fields] OR ("preterm"[All Fields] AND "birth"[All Fields]) OR "preterm birth"[All Fields]) ((("Infant, Premature"[Mesh] OR ("infant"[All Fields] AND "premature"[All Fields]) OR "premature infant"[All Fields] OR ("preterm"[All Fields] AND "infant"[All Fields]) OR "preterm infant"[All Fields] OR ("infant, newborn"[MeSH Terms] OR ("infant"[All Fields] AND "newborn"[All Fields]) OR "newborn infant"[All Fields] OR ("newborn"[All Fields] AND "infant"[All Fields])) AND (premature[All Fields] OR preterm[All Fields]) OR "premature birth"[MeSH Terms] OR ("premature"[All Fields] AND "birth"[All Fields]) OR "premature birth"[All Fields] OR ("preterm"[All Fields] AND "birth"[All Fields]) OR "preterm birth"[All Fields]) AND ("1985"[PDAT] : "3000"[PDAT]) AND "humans"[MeSH Terms].

The exclusion criteria were: studies that did not provide primary data on epidemiological parameters, non-representative studies (eg, only high-risk pregnancies), and reviews. Table 3 shows the search hits, number of full-texts reviewed, and number of extracted sources.

Table 3. LBWSG search hits, full-text review, extracted sources

Search	Hits	Full-text review	Extracted	Search date
GBD 2017	16174	2200	154	6/6/2017

Table 4. Input data for exposure models

Input data	Exposure

Source count (total)	1695
Number of countries with data	161

Data processing

Starting in GBD 2019, as was the case with all other non-fatal analyses, we applied empirical age and sex ratios from previous GBD 2019 Decomposition 1 models to disaggregate observations that did not entirely fit in one GBD age category or sex. Ratios were determined by dividing the result for a specific age and sex by the result for the aggregate age and sex specified in a given observation. It is our intention to update this splitting process annually.

Low birthweight (<2500 grams) data were extracted from literature, vital registration systems, and surveys. DHS survey data were observed to have high missingness; to correct for the missingness, birthweight was imputed using the Amelia II (Version 1.7.6) package in R. Birthweight was predicted using standard Amelia imputation methods from the following variables also in the DHS surveys: urbanicity, sex, birthweight recorded on card, birth order, maternal education, paternal education, child age, child weight, child height, mother's age at birth, mother's weight, shared toilet facility, and household water treated.

"Crosswalking", or the process of reducing non-random bias by adjusting non-standard data to the likely value had the data been "gold-standard", was used to process data in the extremely preterm (<28 weeks) and preterm (<37 weeks) models. All preterm crosswalks were done using meta-regression – regularized, Bayesian, trimmed (MR-BRT). Insurance claims data in extremely preterm (<28 weeks) data were adjusted to vital registration data. Insurance claims data and inpatient data were also adjusted to vital registration in preterm (<37 weeks) conditions. The crosswalk for inpatient data had a spline on the prevalence of inpatient data. Once all claims and inpatient preterm (<37 weeks) data were adjusted, low birthweight data were crosswalked to post-claims and inpatient preterm (<37 weeks) data. If low birthweight data in countries that were 1) categorised as "data-rich" locations in cause-of-death modelling or had at least ten consecutive years of vital registration data recording gestational age and 2) had both preterm birth and low birthweight data, crosswalked low birthweight data were outliered so that the model was informed only by the gestational age data.

Table 5. MR-BRT VR-insurance claims crosswalk adjustment factor for extremely preterm birth (<28 weeks of gestation)

Data input	Reference or alternative case definition	Gamma	Beta coefficient, log (95% CI)	Adjustment factor*
Vital registration	Reference	0.00	---	---
Insurance claims	Alt		-0.651 (-0.602, -0.699)	0.521 (0.500, 0.548)

**Adjustment factor is the transformed beta coefficient in normal space and can be interpreted as the factor by which the alternative case definition is adjusted to reflect what it would have been if measured as the reference.*

Table 6. MR-BRT VR-insurance claims crosswalk adjustment factor for preterm birth (<37 weeks of gestation)

Data input	Reference or alternative case definition	Gamma	Beta coefficient, log (95% CI)	Adjustment factor*
Vital registration	Reference	0.16	---	---
Insurance claims	Alt		-0.728 (-0.705, -0.752)	0.483 (0.471, 0.494)

**Adjustment factor is the transformed beta coefficient in normal space and can be interpreted as the factor by which the alternative case definition is adjusted to reflect what it would have been if measured as the reference.*

Figure 2: MR-BRT clinical inpatient data crosswalk with spline on prevalence of preterm birth

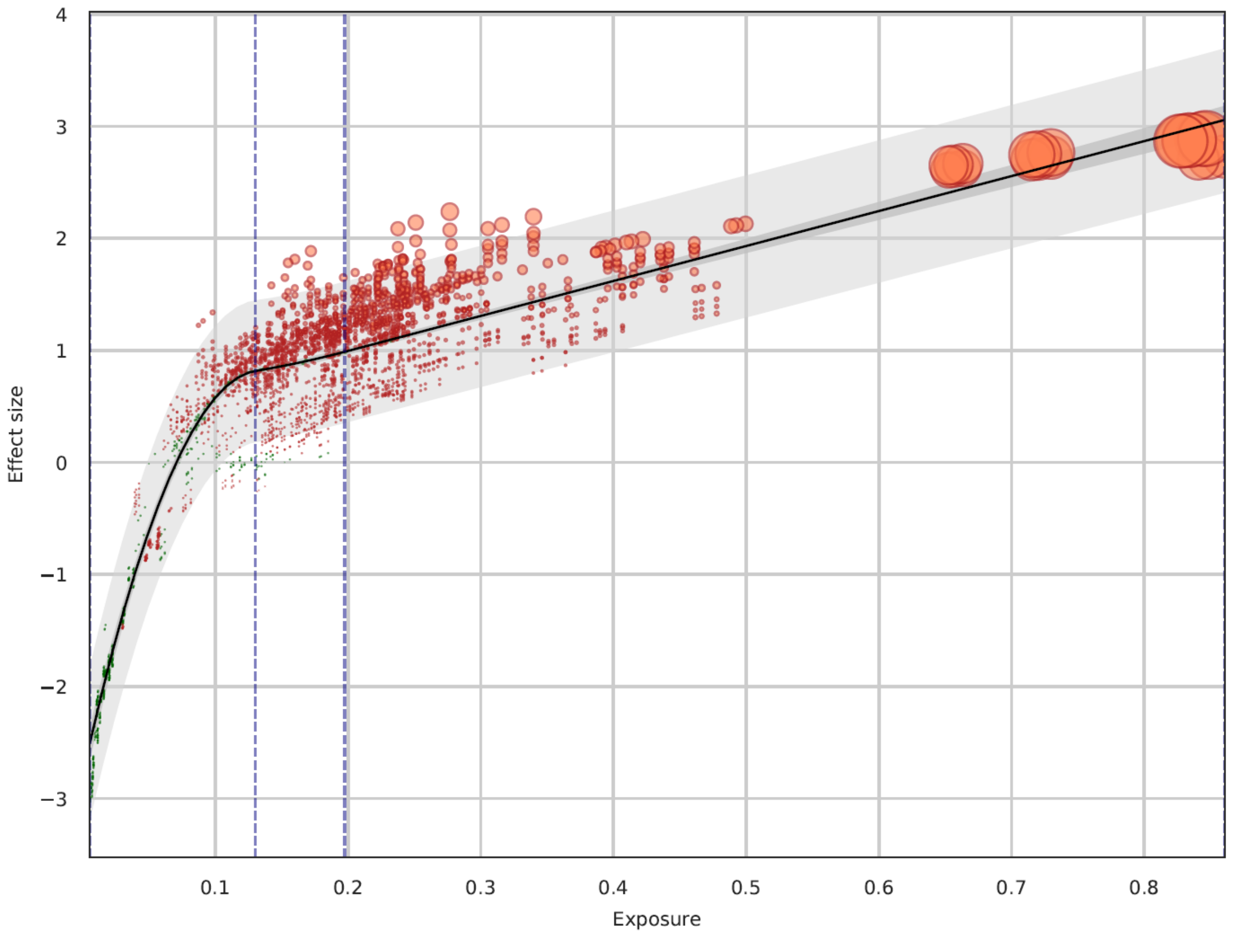

Table 7. MR-BRT preterm birth-low birthweight crosswalk adjustment factor for neonatal preterm birth (<37 weeks of gestation)

Data input	Reference or alternative case definition	Gamma	Beta coefficient, log (95% CI)	Adjustment factor*
Preterm birth	Reference	0.41	---	---

Low birthweight	Alt		-0.0974 (-0.0807, -0.1161)	0.907 (0.890, 0.922)

**Adjustment factor is the transformed beta coefficient in normal space and can be interpreted as the factor by which the alternative case definition is adjusted to reflect what it would have been if measured as the reference.*

Modelling strategy

Step A: Model univariate birthweight and gestational age distributions at birth, by l/y/s

Microdata are the ideal data source for modelling distributions; however, microdata are not widely available for birthweight and are scarcer for gestational age. Categorical prevalence data are more readily available from a wider range of locations and years for low birthweight (<2500g), extremely preterm (<28 weeks of gestation), and preterm birth (<37 weeks of gestation). Because categorical prevalence has wider availability than microdata, we use prevalence data to assist in modelling birthweight and gestational age ensemble distributions.

Ensemble distribution models can be constructed with three pieces of information: mean of the distribution, variance of the distribution, and the weights of the distributions being ensemble. To model mean and variance for all l/y/s for birthweight and gestational age, we first used spatiotemporal Gaussian process regression (ST-GPR) models to model prevalence of low birthweight, extremely preterm, and preterm birth for all l/y/s at birth. To model mean birthweight for all l/y/s, OLS linear regression was used to regress mean birthweight on log-transformed low birthweight prevalence. This model was then used to predict mean birthweight for all l/y/s, using the prevalence of low birthweight (<2500 grams) modelled for all l/y/s in ST-GPR. Similarly, to model gestational age mean for all l/y/s, OLS linear regression model was used to regress mean gestational age on log-transformed preterm prevalence. Mean gestational age for all l/y/s was predicted using the preterm birth (<37 weeks) estimated modelled in ST-GPR.

Global ensemble weights for gestational age were derived by using a 3 million sample of all available gestational age and birthweight microdata in Table 8 to select the ensemble weights. The two distribution families that received the highest weights were the Weibull (43%) and log-logistic (21%) distributions. Global ensemble weights for birthweight were derived using a 3 million sample of all available microdata in Table 8, in addition to birthweight microdata available primarily through the DHS and MICS surveys. The four distribution families that received the highest weights were the mirror gamma (31%), log-logistic (19%), normal (10%), and mirror gumbel (10%) distributions.

For each l/y/s, given the mean and ensemble weights, the variance was optimised to minimise error on the prevalence of preterm birth (<37 weeks) for the gestational age distribution and prevalence of low birthweight (<2500 grams) for the birthweight distribution.

Step B: Model joint birthweight and gestational age distributions at birth, by l/y/s

In order to model the joint distribution of gestational age and birthweight from separate distributions, information was needed about the correlation between the two distributions. Distributions of gestational age and birthweight are not independent; the Spearman correlation for each country where

joint microdata were available (Table 8), pooling across all years of data available, ranged from 0.25 to 0.49. The overall Spearman correlation was 0.38, pooling across all countries in the dataset.

Table 8. Summary of microdata inputs

Location	Years of data	Total births*	Format of data	Spearman correlation	Used in ensemble weight selection	Used in copula parameter selection	Used in relative risk models
BRA	2016	2,854,380	Microdata	0.37	Yes	Yes	No
ECU	2003–2015	2,473,039	Microdata	0.34	Yes	Yes	No
ESP	1990–2014	8,537,220	Microdata	0.42	Yes	Yes	No
JPN	1995–2015	23,644,506	Tabulations	0.41	No	No	Yes
MEX	2008–2012	10,256,117	Microdata	0.35	Yes	Yes	No
NOR	1990–2014	1,489,210	Microdata	0.44	Yes	Yes	Yes
NZL	1990–2016	1,600,501	Microdata	0.25	Yes	Yes	Yes
SGP	1993–2015	972,775	Tabulations	0.41	No	No	Yes
TWN	1998–2002	1,331,760	Tabulations	0.38	No	No	Yes
URY	1996–2014	698,622	Microdata	0.49	Yes	Yes	No
USA	1990–2014	81,929,879	Microdata	0.38	Yes	Yes	Yes

** Pooled across all years and sexes, excluding data missing year of birth, gestational age, or birthweight*

Joint distributions between the birthweight and gestational age marginal distributions were modelled with copulae. The Copula and VineCopula packages in R were used to select the optimal copula family and copula parameters to model the joint distribution, using joint microdata from the country-years in Table 8. The copula family selected from the microdata was "Survival BB8", with theta parameter set to 1.75 and delta parameter set to 1.

The joint distribution of birthweight and gestational age per location-year-sex was modelled using the global copula family and parameters selected and the location-year-sex gestational age and birthweight distributions. The joint distribution was simulated 100 times to capture uncertainty. Each simulation consisted of 10,000 simulated joint birthweight and gestational age datapoints. Each joint distribution was divided into 500g by 2-week bins to match the categorical bins of the relative risk surface. Birth prevalence was then calculated for each 500g by 2-week bin.

Step C: Model joint distributions from birth to the end of the neonatal period, by l/y/s

Early neonatal prevalence and late neonatal prevalence were estimated using life table approaches for each 500g and 2-week bin. Using the all-cause early neonatal mortality rate for each location-year-sex, births per location-year-sex-bin, and the relative risks for each location-year-sex-bin in the early neonatal period, the all-cause early neonatal mortality rate was calculated for each location-year-sex-bin. The early neonatal mortality rate per bin was used to calculate the number of survivors at seven days and prevalence in the early neonatal period. Using the same process, the all-cause late neonatal mortality rate for each location-year-sex was paired with the number of survivors at seven days and late neonatal relative risks per bin to calculate late neonatal prevalence and survivors at 28 days.

Relative risks & theoretical minimum-risk exposure level

Causes

The available data for deriving relative risk was only for all-cause mortality. The exception was the USA linked infant birth-death cohort data, which contained three-digit ICD causes of death, but also had nearly 30% of deaths coded to causes that are ill-defined, or intermediate, in the GBD cause classification system. We analysed the relative risk of all-cause mortality across all available sources and selected outcomes based on criteria of biological plausibility. Some causes, most notably congenital birth defects, haemoglobinopathies, malaria, and HIV/AIDS, were excluded based on the criteria that reverse causality could not be excluded.

Input data

In the Norway, New Zealand, and USA Linked Birth/Death Cohort microdata datasets, livebirths are reported with gestational age, birthweight, and an indicator of death at 7 days and 28 days. For this analysis, gestational age was grouped into two-week categories, and birthweight was grouped into 500-gram categories. The Taiwan, Japan, and Singapore datasets were prepared in tabulations of joint 500-gram and two-week categories. A pooled country analysis of mortality risk in the early neonatal period and late neonatal period by "small for gestational age" category in developing countries in Asia and sub-Saharan Africa were also used to inform the relative risk analysis.

Table 9: Input data for relative risk models

Input data	Relative Risk
Source count (total)	113
Number of countries with data	6

Modelling strategy

For each location, data were pooled across years, and the risk of all-cause mortality at the early neonatal period and late neonatal period at joint birthweight and gestational age combinations was calculated. In all datasets except for the USA, sex-specific data were combined to maximise sample size. The USA analyses were sex-specific. To calculate relative risk at each 500-gram and two-week combination, logistic regression was first used to calculate mortality odds for each joint two-week gestational age and 500-gram birthweight category. Mortality odds were smoothed with Gaussian process regression, with the independent distributions of mortality odds by birthweight and mortality odds by gestational age serving as priors in the regression.

A pooled country analysis of mortality risk in the early neonatal period and late neonatal period by SGA category in developing countries in Asia and sub-Saharan Africa were also converted into 500-gram and two-week bin mortality odds surfaces. The relative risk surfaces produced from microdata and the Asia and Africa surfaces produced from the pooled country analysis were meta-analysed, resulting in a meta-analysed mortality odds surface for each location. The meta-analysed mortality odds surface for each location was smoothed using Gaussian process regression and then converted into mortality risk. To calculate mortality relative risks, the risk of each joint two-week gestational age and 500-gram birthweight category were divided by the risk of mortality in the joint gestational age and birthweight category with the lowest mortality risk.

For each of the country-derived relative risk surfaces, the 500-gram and two-week gestational age joint bin with the lowest risk was identified. This bin differed within each country dataset. To identify the universal 500-gram and two-week gestational age category that would serve as the universal TMREL for our analysis, we chose the bins that was identified to be the TMREL in each country dataset to contribute to the universal TMREL. Therefore, the joint categories that served as our universal TMREL for the LBWSG risk factor were "38-40 weeks of gestation and 3500-4000 grams", "38-40 weeks of gestation and 4000-4500 grams", and "40-42 weeks of gestation and 4000-4500 grams". As the joint TMREL, all three categories were assigned to a relative risk equal to 1.

PAF calculations

The total PAF for the low birthweight and short gestation joint risk factor was calculated by summing the PAF calculated from each 500g x two-week category, with the lowest risk category among all the 500g x two-week categories serving as the TMREL. The equation for calculating PAF for each 500g x two-week category is:

$$PAF_{joasgt} = \frac{\sum_{x=1}^{u} RR_{joast}(x)P_{jasgt}(x) - RR_{joasg}(TMRE_{jas})}{\sum_{x=1}^{u} RR_{joas}(x)P_{jasgt}(x)}$$

To calculate the PAFs for the univariate risks ('short gestation for birthweight' and 'low birth weight for gestation'), relative risks are first weighted by global exposure in 2019, summed across one of the dimensions (gestational age or birth weight), and then rescaled by the maximum RR in the TMREL block (38-42 weeks of gestation and 3500-4500 grams). Any RR less than 1 was set to 1. Exposure was also summed across the same dimension, and the univariate PAF equalled the sum of the product of the weighted RRs and exposures.

References

1. Katz J, Lee AC, Kozuki N, Lawn JE, Cousens S, Blencowe H, et al. Mortality risk in preterm and small-for-gestational-age infants in low-income and middle-income countries: a pooled country analysis. The Lancet. 2013;382(9890):417–25.

Iron deficiency

Flowchart

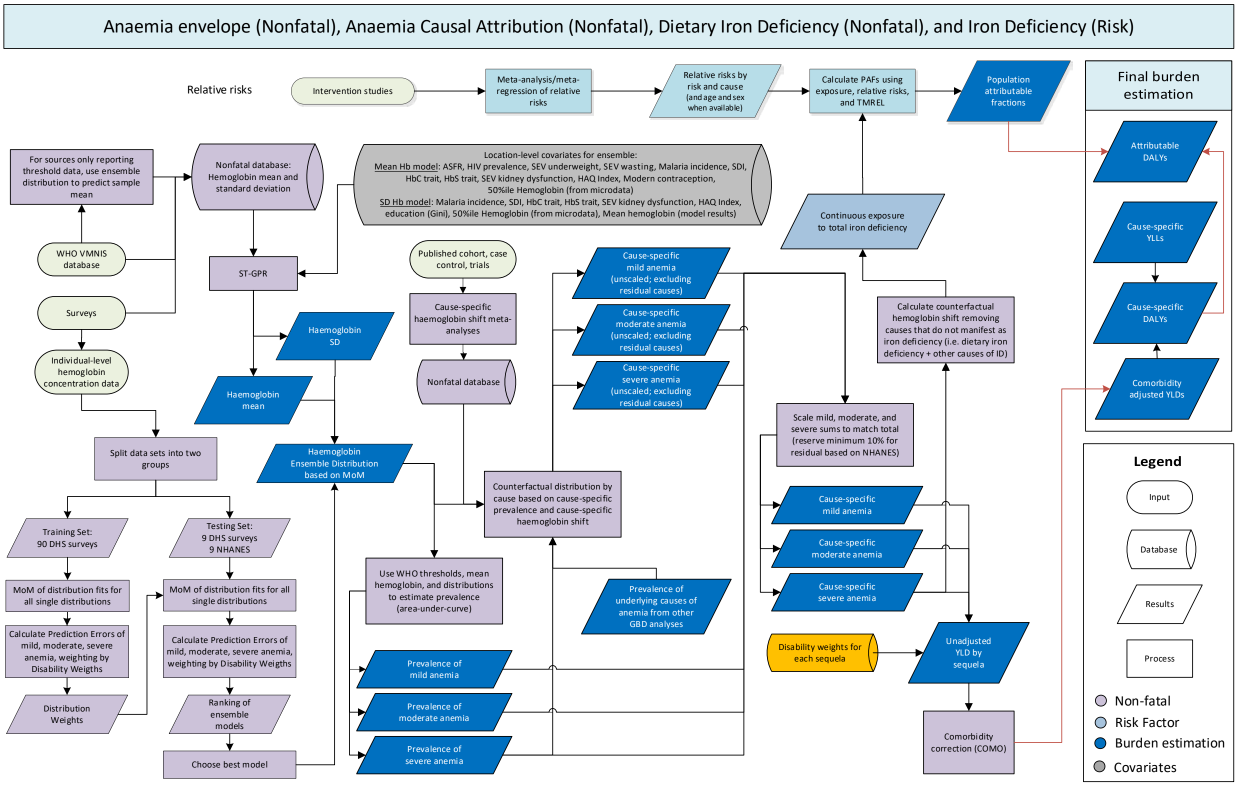

Input data and methodological summary

Case definition

Iron deficiency in the GBD risk factors analysis is defined as inadequate iron to meet the body's needs. Exposure is quantified in terms of mean haemoglobin concentration at the population level from the cumulative effect of all causes that lead to iron deficiency. This is distinct from the GBD cause of "dietary iron deficiency" that only includes the subset of anaemia that is due to inadequate intake of elemental iron and excludes other diseases that manifest as iron deficiency (eg, maternal haemorrhage, uterine fibroids, menstrual disorders, hookworm, schistosomiasis, gastritis and duodenitis, inflammatory bowel disease, etc.).

The tables below provide a summary of data inputs for iron deficiency**.**

Table 1: Data inputs for exposure for iron deficiency.

Input data	Exposure
Source count (total)	683
Number of countries with data	153

Table 2: Data inputs for relative risks for iron deficiency[3].

Input data	Relative risk
Source count (total)	1
Number of countries with data	1

Modelling strategy

Iron deficiency was quantified as an output of the GBD Anaemia Causal Attribution framework. The GBD anaemia model has two main steps – estimation of the anaemia envelope and causal attribution – both of which inherently impact estimates of iron deficiency. See the methodological description of "Anaemia (impairment)" for detailed description of the analytic approach and inputs.

Briefly, the first step is estimating the anaemia envelope – the prevalence of mild, moderate, and severe anaemia prevalence for each GBD location, age group, sex, and year. The inputs to the envelope model are mean and standard deviation (SD) of haemoglobin concentration, each of which are modeled in ST-GPR. Individual-level data sources are then used to develop a set of ensemble distribution weights using method of moments, which are then paired with mean and SD model results to produce estimates of the entire distribution of haemoglobin for each population group. A population group is a specific geography, sex, age group, and year combination. The second step is anaemia causal attribution, the approach for which was revised in GBD 2019 to, instead of Bayesian contingency table modelling, generate counterfactual haemoglobin distributions for each cause of anaemia based on the cause-level prevalence (or incidence, in the case of maternal haemorrhage) estimates from the respective GBD analyses and cause-specific haemoglobin shifts that were determined via meta-analysis for each cause. The counterfactual distribution methods used the same ensemble distribution weights as the overall anaemia envelope because there are inadequate data to guide alternate distributions for each subcause. Mild, moderate, and severe anaemia were assigned to each cause based on the difference between the counterfactual and observed haemoglobin distributions in each population group. The sum of severity-specific prevalence was then summed to match the total, with a minimum residual of 10%,[1,2] and then the remainder was distributed between five GBD causes using fixed proportion redistribution methods: 1) dietary iron deficiency (GBD cause), 2) other haemoglobinopathies and haemolytic anaemias, 3) other infectious diseases, 4) other neglected tropical diseases, and 5) endocrine, metabolic, blood, and immune disorders.

Iron-deficiency exposure for GBD risk factors analysis is the haemoglobin concentration for each population group for all diseases and injuries that manifest with iron deficiency. This was operationalised by using the observed haemoglobin concentration in each population as the actual exposure and then calculating a separate TMREL for each population group as described below.

Theoretical minimum-risk exposure level

The implied mean haemoglobin in the absence of iron deficiency is the theoretical minimum risk exposure level. This was calculated by summing cause-specific haemoglobin shift times prevalence for all causes classified as iron-responsive and then adding that sum to the the observed haemoglobin concentration for each population group.

Relative risk

We attribute 100% of iron-deficiency anaemia to iron deficiency. Data directly linking iron deficiency to other outcomes is sparse. The CHERG iron report[3] presents data supporting low haemoglobin as a risk factor for maternal outcomes. This evidence was used as a proxy for assigning the corresponding relative risks from that report to all maternal outcomes. No other outcomes were identified as having sufficient evidence of causal relationship with iron deficiency.

References

1 Centers for Disease Control and Prevention (CDC). Iron deficiency--United States, 1999-2000. *MMWR Morb Mortal Wkly Rep* 2002; **51**: 897–9.

2 Looker AC, Dallman PR, Carroll MD, Gunter EW, Johnson CL. PRevalence of iron deficiency in the united states. *JAMA* 1997; **277**: 973–6.

3 Murray-Kolb LE, Chen L, Chen P, Shapiro M, Caulfield L. CHERG Iron Report: Maternal Mortality, Child Mortality, Perinatal Mortality, Child Cognition, and Estimates of Prevalence of Anemia due to Iron Deficiency | GHDx. 2013. http://ghdx.healthdata.org/record/cherg-iron-report-maternal-mortality-child-mortality-perinatal-mortality-child-cognition-and (accessed Nov 12, 2019).

Vitamin A deficiency

Flowchart

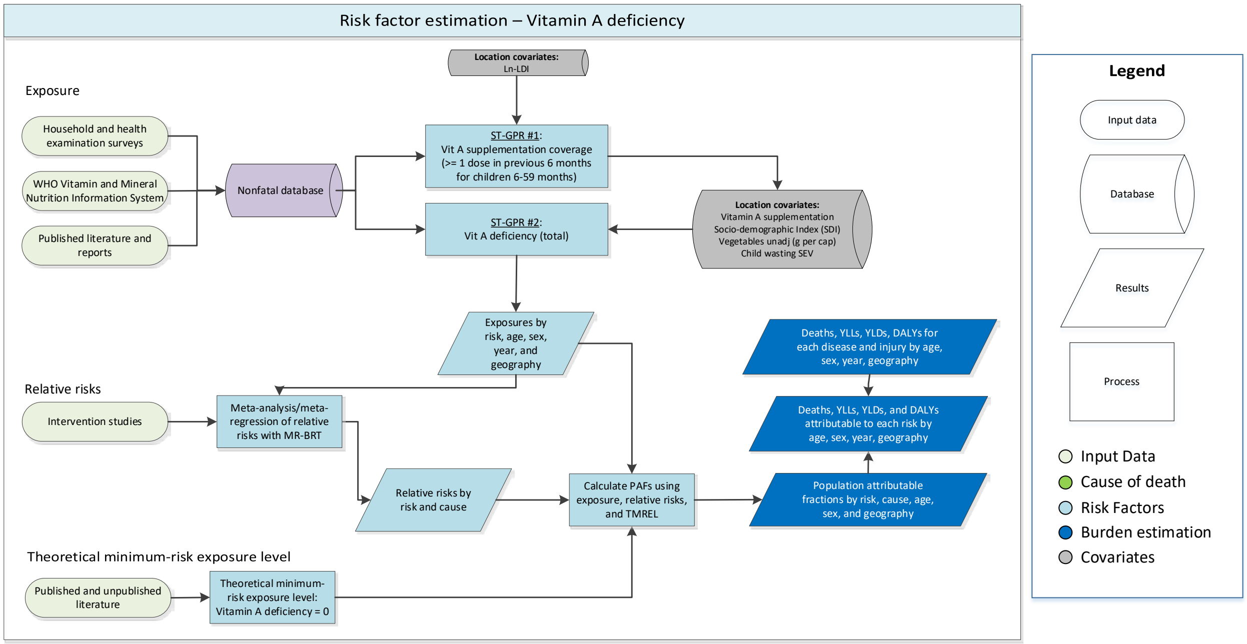

Input data and methodological summary

Case definition

Exposure

For GBD 2019, vitamin A deficiency is defined as serum retinol <70 µmol/L. We examined vitamin A deficiency as a risk factor in children aged 6 months to 5 years.

Input data

For GBD 2019, we used data from the WHO Vitamin and Mineral Nutrition Information System, health surveys such as DHS and MICS, and studies identified through literature review for the vitamin A deficiency model. We used data from the UNICEF State of the World's Children database and DHS and MICS surveys for the vitamin A supplementation model. Tables 1 and 2 provide a summary of data inputs for vitamin A deficiency risk factor modeling**.** A systematic review was last conducted for GBD 2013. The PubMed search terms were: ((vitamin A deficiency[Title/Abstract] AND prevalence[Title/Abstract]) AND ("2009"[Date – Publication] : "2013"[Date – Publication])). Exclusion criteria were:

1. Studies that were not population-based, eg, hospital or clinic-based studies
2. Studies that did not provide primary data on epidemiological parameters, eg, commentaries
3. Review articles
4. Case series
5. Self-reported cases

Table 1: Data inputs for exposure for vitamin A deficiency.

Input data	Exposure
Site-years (total)	274
Number of countries with data	96

Table 2: Data inputs for relative risks for vitamin A deficiency.

Input data	Relative risk
Source count (total)	16
Number of countries with data	12

Modelling strategy

Exposure model

The steps of the modelling strategy for GBD 2019 remained consistent those used in GBD 2017, however several step-specific updates were made. Broadly the strategy consists of three steps, beginning with a model of vitamin A supplementation coverage. The supplementation estimates are then used as a location-level covariate to guide prevalence estimates of overall vitamin A deficiency.

To ensure we are using as much information as possible, and therefore maximise the data basis of our estimates, we first model vitamin A supplementation. The case definition for the supplementation model is the proportion individuals who received at least one dose of vitamin A in the previous six months; although the typical metric on which supplementation is tracked is 2+ doses of vitamin A in the previous 12 months for children under 5 years, most existing health surveys do not routinely provide sufficient information to calculate it. In GBD 2019, the supplementation model was moved to ST-GPR to achieve a better time trend that accounts for the introduction of supplementation programs in the late 1990s. Additionally, vitamin A supplementation was previously modeled as an all-age and both-sex indicator with the proportion of children 6-59 months of age who received at least one dose of vitamin A in the previous six months as the case definition. In an effort to capture the effect of supplementation programs on the prevalence of deficiency across age-specific groups, we modeled vitamin A supplementation as an age and sex-specific indicator for GBD 2019 so that high coverage would be restricted to children 6-59 months who are targeted in supplementation campaigns. As in GBD 2017, we used the natural log of lag-distributed income per capita (LN-LDI) as a location-level covariate to inform supplementation estimates where data were absent.

Second, we estimated the age- and sex-specific prevalence of vitamin A deficiency (serum retinol < 0.7 μmol/L). This year we updated the deficiency data processing steps to include a separate sex ratio model (using MR-BRT) and a separate age pattern model (using DisMod) which were used to split both-sex and all-age data prior to modeling. As with the supplementation model, we moved vitamin A deficiency to ST-GPR to utilize its superior time trends. The age-specific stunting SEV was added as a location-level covariate for the vitamin A deficiency ST-GPR model, alongside the three used last year: sociodemographic index, the availability of retinol activity equivalent (rae) units in foods, and (newly updated) vitamin A supplementation.

Theoretical minimum risk exposure

The theoretical minimum risk exposure is that the prevalence of vitamin A deficiency is zero.

Relative risk

The relative risk data were updated in GBD 2017 to reflect studies included in the most recently published systematic review by Imdad and colleagues.1 In GBD 2019, we revisited the underlying studies reported in this analysis and re-analysed and evaluated the evidence on vitamin A deficiency as a risk factor for diarrhoea, measles, and lower respiratory infections using MR-BRT. Lower respiratory infections were removed as an outcome due to insufficient evidence, and the relative risks for diarrhoea and measles were updated. Additionally, in our updated analysis, we found no significant relationship between background vitamin A deficiency prevalence and the magnitude of the relative risk. Thus, in GBD 2019, we no longer adjust RRs for background vitamin A deficiency prevalence.

Cause	GBD 2017 RR	GBD 2019 RR	Include in GBD 2019
Diarrhoea	2.35 (2.17–2.54)	1.14 (1.03, 1.26)	Yes
Measles	2.76 (2.01–3.78)	1.39 (1.03, 1.90)	Yes
Lower respiratory infections (LRI)	1.23 (1.03–1.48)	1.05 (0.98, 1.12)	No

References

1. Imdad A, Ahmed Z, Bhutta ZA. Vitamin A supplementation for the prevention of morbidity and mortality in infants one to six months of age. *Cochrane Database of Systematic Reviews* 2016; Sep 28; 9. Art. No: CD007480.

Zinc deficiency

Input data and methodological summary

Definition

Exposure

Exposure to zinc deficiency is defined as consumption of less than 2.5 milligrams of zinc per day among children between the ages of 1 and 4 years old.

Input data

We used dietary data from nationally and subnationally representative nutrition surveys, food frequency questionnaires, and United Nations FAO Supply and Utilization Accounts to estimate the mean intake of zinc at the population level.

Table 1: Data inputs for exposure for zinc deficiency.

Input data	Exposure
Source count (total)	28
Number of countries with data	175

Table 2: Data inputs for relative risks for zinc deficiency.

Input data	Relative risk
Source count (total)	20
Number of countries with data	8

As with the rest of the dietary risks, the gold-standard data source are 24-hour dietary recall surveys, and we perform a bias adjustment to crosswalk alternate definitions to dietary recall. In GBD 2019, we updated the crosswalk regression to utilise MR-BRT.

Table 3: MR-BRT crosswalk adjustment factors for zinc deficiency

Sex	Data input	Reference or alternative case definition	Gamma	Beta coefficient, log (95% CI)	Adjustment factor*
---	DR	Ref		---	---
Male	FAO	Alt		0.58 (-0.26,1.42)	0.56 (0.77,4.15)
Female	FAO	Alt	0.41	0.57 (-0.26,1.41)	0.56 (0.77,4.11)
Male	FFQ	Alt		0.64 (-0.3,1.51)	0.53 (0.74,4.53)
Female	FFQ	Alt		0.63 (-0.32,1.51)	0.53 (0.73,4.52)

**Adjustment factor is the transformed beta coefficient in normal space and can be interpreted as the factor by which the alternative case definition is adjusted to reflect what it would have been if measured as the reference.*

Modelling strategy

Exposure model

For GBD 2019, we first used a spatiotemporal Gaussian process regression (ST-GPR) framework to estimate the mean intake of zinc by age, sex, country, and year. To assist with estimation for locations and years without data, we used the lag-distributed income and energy availability (kcal) of that location-year as a covariate. Using the method described in the dietary risks section, we characterised the distribution of zinc intake for children between ages of 1 and 4 years old and then integrated to determine the proportion of the children with intake of less than 2.5 milligrams of zinc per day.

Theoretical minimum-risk exposure level

The theoretical minimum-risk exposure is that the prevalence of zinc deficiency is zero.

Relative risk

In GBD 2019, we revisited the most recent meta-analysis evaluating the effects of zinc supplementation on disease endpoints. Specifically, we re-analysed and evaluated the evidence of zinc deficiency as a risk factor for diarrhoea and lower respiratory infections (GBD 2017 outcomes) using MR-BRT. Lower respiratory infections were removed as an outcome due to insufficient evidence, and the relative risks for diarrhoea were updated. Additionally, in our updated analysis, we found no significant relationship between background zinc deficiency prevalence and the magnitude of the relative risk. Thus, in GBD 2019, we did not adjust the relative risk of diarrhoea for background zinc deficiency prevalence.

Smoking

Flowchart

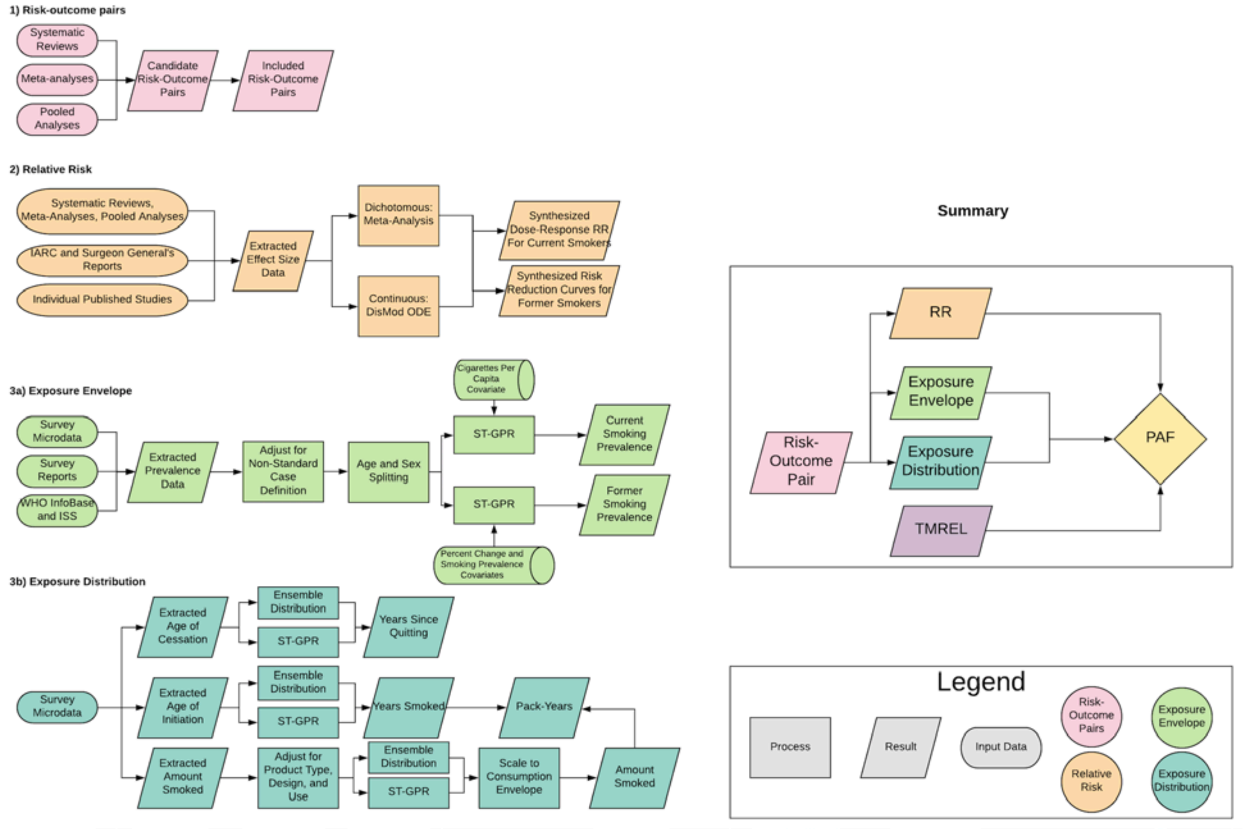

Input data and methodological summary

Definition

Exposure

As in GBD 2017, we estimated the prevalence of current smoking and the prevalence of former smoking using data from cross-sectional nationally representative household surveys. We defined current smokers as individuals who currently use any smoked tobacco product on a daily or occasional basis. We defined former smokers as individuals who quit using all smoked tobacco products for at least six months, where possible, or according to the definition used by the survey.

Input data

Our extraction method has not changed from GBD 2017. We extracted primary data from individual-level microdata and survey report tabulations. We extracted data on current, former, and/or ever smoked tobacco use reported as any combination of frequency of use (daily, occasional, and unspecified, which includes both daily and occasional smokers) and type of smoked tobacco used (all smoked tobacco, cigarettes, hookah, and other smoked tobacco products such as cigars or pipes),

resulting in 36 possible combinations. Other variants of tobacco products, for example hand-rolled cigarettes, were grouped into the four type categories listed above based on product similarities.

For microdata, we extracted relevant demographic information, including age, sex, location, and year, as well as survey metadata, including survey weights, primary sampling units, and strata. This information allowed us to tabulate individual-level data in the standard GBD five-year age-sex groups and produce accurate estimates of uncertainty. For survey report tabulations, we extracted data at the most granular age-sex group provided.

Table 1: Data inputs for exposure for smoking.

Input data	Exposure
Source count (total)	3439
Number of countries with data	201

Table 2: Data inputs for relative risks for smoking.

Input data	Relative risk
Source count (total)	673
Number of countries with data	16

Crosswalk

Our GBD smoking case definitions were current smoking of any tobacco product and former smoking of any tobacco product. All other data points were adjusted to be consistent with either of these definitions. Some sources contained information on more than one case definition and these sources were used to develop the adjustment coefficient to transform alternative case definitions to the GBD case definition. The adjustment coefficient was the beta value derived from a linear model with one predictor and no intercept. We used the same crosswalk adjustment coefficients as in GBD 2017, and thus we have not included a methods explanation in this appendix, as it has been detailed previously.

Age and sex splitting

As in GBD 2017, we split data reported in broader age groups than the GBD 5-year age groups or as both sexes combined by adapting the method reported in Ng et al[1] to split using a sex- geography- time-specific reference age pattern. We separated the data into two sets: a training dataset, with data already falling into GBD sex-specific 5-year age groups, and a split dataset, which reported data in aggregated age or sex groups. We then used spatiotemporal Gaussian process regression (ST-GPR) to estimate sex-geography-time-specific age patterns using data in the training dataset. The estimated age patterns were used to split each source in the split dataset.

The ST-GPR model used to estimate the age patterns for age-sex splitting used an age weight parameter value that minimises the effect of any age smoothing. This parameter choice allowed the estimated age pattern to be driven by data, rather than being enforced by any smoothing parameters of the model. Because these age-sex split data points were to be incorporated in the final ST-GPR exposure model, we

did not want to doubly enforce a modelled age pattern for a given sex-location-year on a given aggregate data point.

Modelling strategy

Smoking prevalence modelling

We used ST-GPR to model current and former smoking prevalence. The model is nearly identical to that in GBD 2017. Full details on the ST-GPR method are reported elsewhere in the appendix. Briefly, the mean function input to GPR is a complete time series of estimates generated from a mixed effects hierarchical linear model plus weighted residuals smoothed across time, space, and age. The linear model formula for current smoking, fit separately by sex using restricted maximum likelihood in R, is:

$$logit(p_{g,a,t}) = \beta_0 + \beta_1 CPC_{g,t} + \sum_{k=2}^{19} \beta_k I_{A[a]} + \alpha_s + \alpha_r + \alpha_g + \epsilon_{g,a,t}$$

Where $CPC_{g,t}$ is the tobacco consumption covariate by geography g and time t, described above, $I_{A[a]}$ is a dummy variable indicating specific age group A that the prevalence point $p_{g,a,t}$ captures, and α_s, α_r, and α_g are super-region, region, and geography random intercepts, respectively. Random effects were used in model fitting but not in prediction.

The linear model formula for former smoking is:

$$logit(p_{g,a,t}) = \beta_0 + \beta_1 PctChange_{A[a],g,t} + \beta_3 CSP_{A[a],g,t} + \sum_{k=3}^{20} \beta_k I_{A[a]} + \alpha_s + \alpha_r + \alpha_g + \epsilon_{g,a,t}$$

Where $PctChange_{A[a],g,t}$ is the percentage change in current smoking prevalence from the previous year, and $CSP_{A[a],g,t}$ is the current smoking prevalence by specific age group A, geography g, and time t that point $p_{g,a,t}$ captures, both derived from the current smoking ST-GPR model defined above.

Supply-side estimation

The methods for modelling supply-side-level data were changed substantially from those used in GBD 2017. The raw data were domestic supply (USDA Global Surveillance Database and UN FAO) and retail supply (Euromonitor) of tobacco. Domestic supply was calculated as production + imports - exports. The data went through three rounds of outliering. First, they were age-sex split using daily smoking prevalence to generate number of cigarettes per smoker per day for a given location-age-sex-year. If more than 12 points for a particular source-location-year (equal to over 1/3 of the split points) were above the given thresholds, that source-location-year was outliered. A point would not be outliered if it was (in cigarettes per smoker): under five (10–14 year olds); under 20 (males, 15–19 year olds); under 18 (females, 15–19 year olds); under 38/35 and over three (males/females, 20+ year olds). These thresholds were chosen by visualising histograms of the data for each age-sex, as well as with expert knowledge about reasonable consumption levels. In the second round of outliering, the mean tobacco per capita value over a 10-year window was calculated. If a point was over 70% of that mean value away

from the mean value, it was outliered. The 70% limit was chosen using histograms of these distances. Additionally, some manual outliering was performed to account for edge cases. Finally, data smoothing was performed by taking a three-year rolling mean over each location-year.

Next, a simple imputation to fill in missing years was performed for all series to remove compositional bias from our final estimates. Since the data from our main sources covered different time periods, by imputing a complete time series for each data series, we reduced the probability that compositional bias of the sources was leading to biased final estimates. To impute the missing years for each series, we modelled the log ratio of each pair of sources as a function of an intercept and nested random effects on super-region, region, and location. The appropriate predicted ratio was multiplied by each source that we did have, and then the predictions were averaged to get the final imputed value. For example, if source A was missing for a particular location-year, but sources B and C were present, then we predicted A twice: once from the modelled ratio of A to B, and again from the modelled ratio of A to C. These two predictions were then averaged. For some locations where there was limited overlap between series, the predicted ratio did not make sense, and a regional ratio was used.

Finally, variance was calculated both across series (within a location-year) as well as across years (within a location-source). Additionally, if a location-year had one imputed point was, the variance was multiplied by 2. If a location-year had two imputed points, the variance was multiplied by 4. The average estimates in each location-year were the input to an ST-GPR model. For this, we used a simple mixed effects model, which was modelled in log space with nested location random effects. Subnational estimates were then further modelled by splitting the country-level estimates using current smoking prevalence.

Theoretical minimum-risk exposure level

The theoretical minimum-risk exposure level is 0.

Exposure among current and former smokers

Identical to GBD 2017, we estimated exposure among current smokers for two continuous indicators: cigarettes per smoker per day and pack-years. Pack-years incorporates aspects of both duration and amount. One pack-year represents the equivalent of smoking one pack of cigarettes (assuming a 20-cigarette pack) per day for one year. Since the pack-years indicator collapses duration and intensity into a single dimension, one pack-year of exposure can reflect smoking 40 cigarettes per day for six months or smoking 10 cigarettes per day for two years.

To produce these indicators, we simulated individual smoking histories based on distributions of age of initiation and amount smoked. We informed the simulation with cross-sectional survey data capturing these indicators, modelled at the mean level for all locations, years, ages, and sexes using ST-GPR. We rescaled estimates of cigarettes per smoker per day to an envelope of cigarette consumption based on supply-side data. We estimated pack-years of exposure by summing samples from age- and time-specific distributions of cigarettes per smoker for a birth cohort in order to capture both age trends and time trends and avoid the common assumption that the amount someone currently smokes is the amount they have smoked since they began smoking. All distributions were age-, sex-, and region- specific ensemble distributions, which were found to outperform any single distribution.

We estimated exposure among former smokers using years since cessation. We utilised ST-GPR to model mean age of cessation using cross-sectional survey data capturing age of cessation. Using these

estimates, we generated ensemble distributions of years since cessation for every location, year, age group, and sex.

Relative risk

The same risk-outcome pairs from GBD 2017 were used: tuberculosis, lower respiratory tract infections, oesophageal cancer, stomach cancer, bladder cancer, liver cancer, laryngeal cancer, lung cancer, breast cancer, cervical cancer, colorectal cancer, lip and oral cancer, nasopharyngeal cancer, other pharyngeal cancer, pancreatic cancer, kidney cancer, leukaemia, ischaemic heart disease, ischaemic stroke, haemorrhagic stroke, subarachnoid haemorrhage, atrial fibrillation and flutter, aortic aneurysm, peripheral arterial disease, chronic obstructive pulmonary disease, other chronic respiratory diseases, asthma, peptic ulcer disease, gallbladder and biliary tract diseases, Alzheimer disease and other dementias, Parkinson disease (protective), multiple sclerosis, type-II diabetes, rheumatoid arthritis, low back pain, cataracts, macular degeneration, and fracture.

Dose-response risk curves

Input data for relative risks were nearly the same as in GBD 2017. The only addition was for chronic obstructive pulmonary disease, for which a few additional studies were included. We synthesised effect sizes by cigarettes per smoker per day, pack-years, and years since quitting from cohort and case-control studies to produce nonlinear dose-response curves using a Bayesian meta-regression model. For outcomes with significant differences in effect size by sex or age, we produced sex- or age-specific risk curves.

We estimated risk curves of former smokers compared to never smokers taking into account the rate of risk reduction among former smokers seen in the cohort and case-control studies, and the cumulative exposure among former smokers within each age, sex, location, and year group.

Population attributable fraction (PAF)

As in GBD 2017, we estimated PAFs based on the following equation:

$$PAF = \frac{p(n) + p(f)\int \exp(x) * rr(x) + p(c)\int \exp(y) * rr(y) - 1}{p(n) + p(f)\int \exp(x) * rr(x) + p(c)\int \exp(y) * rr(y)}$$

where $p(n)$ is the prevalence of never smokers, $p(f)$ is the prevalence of former smokers, $p(c)$ is the prevalence of current smokers, $\exp(x)$ is a distribution of years since quitting among former smokers, $rr(x)$ is the relative risk for years since quitting, $\exp(y)$ is a distribution of cigarettes per smoker per day or pack-years, and $rr(y)$ is the relative risk for cigarettes per smoker per day or pack-years.

We used pack-years as the exposure definition for cancers and chronic respiratory diseases, and cigarettes per smoker per day for cardiovascular diseases and all other health outcomes.

References

1. Ng M, Freeman MK, Fleming TD, Robinson M, Dwyer-Lindgren L, Thomson B, et al. Smoking Prevalence and Cigarette Consumption in 187 Countries, 1980–2012. *JAMA*. 2014 Jan 8;311(2):183–92.

Secondhand smoke

Flowchart

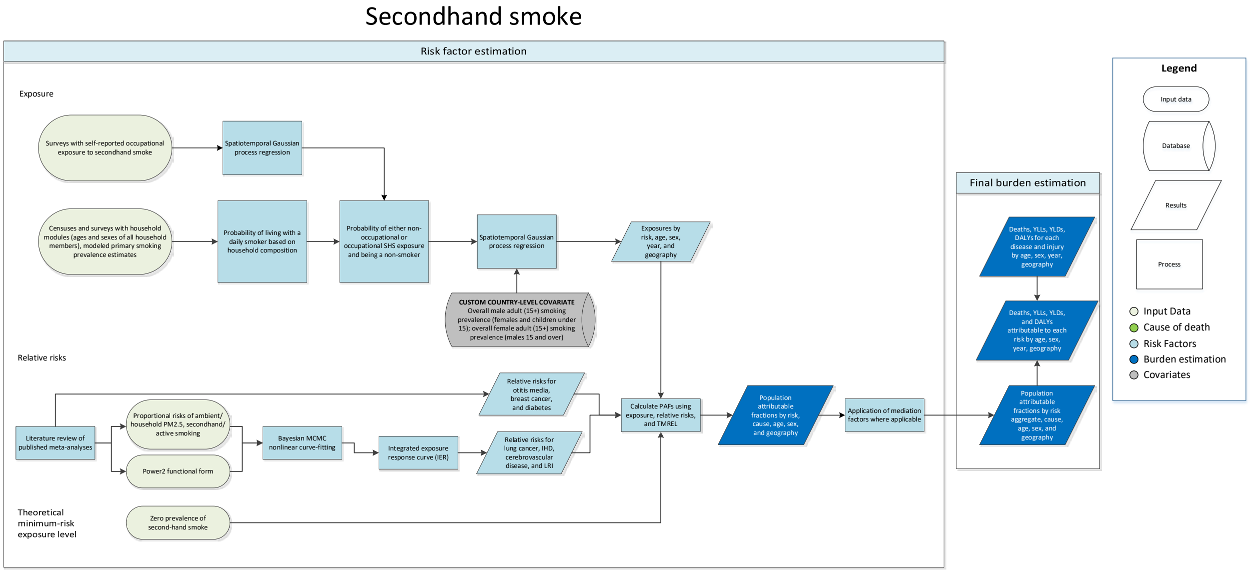

Exposure

Case definition

We define secondhand smoke exposure as current exposure to secondhand tobacco smoke at home, at work, or in other public places. We use household composition as a proxy for non-occupational secondhand smoke exposure and make the assumption that all persons living with a daily smoker are exposed to tobacco smoke. We use surveys to estimate the proportion of individuals exposed to secondhand smoke at work. We only consider non-smokers to be exposed to secondhand smoke. Non-smokers are defined as all persons who are not daily smokers. Ex-smokers and occasional smokers are considered non-smokers in this analysis. Exposure is evaluated for both children and adults.

Input data

To calculate the proportion of non-smokers who live with at least one smoker, we used unit record data on household composition, which included the ages and sexes of all persons living in the same household. Our sources included representative major survey series with a household composition module, including the Demographic Health Surveys (DHS), the Multiple Indicator Cluster Surveys (MICS), and the Living Standards Measurement Surveys (LSMS); and national and subnational censuses, which included those captured in the IPUMS project and identified using the Global Health Data Exchange catalog (GHDx).

To calculate the proportion of individuals exposed to secondhand smoke at work, by age and sex, we used cross-sectional surveys that ask respondents about self-reported occupational secondhand smoke exposure. Sources include the Global Adult Tobacco Surveys, Eurobarometer Surveys, and WHO STEPS Surveys. We identified sources using the GHDx.

No major changes have been introduced to data inputs since 2016. A new systematic review is planned for the next GBD round. Table 1 summarizes exposure input data.

Input data	Exposure
Source count (total)	721
Number of countries with data	153

Given the nature of the data used in our models (microdata), no crosswalk for case definition adjustment or age- and sex-splitting processes were required. Estimates of daily smoking prevalence in each location were also used in our calculations, as described in the modelling strategy section below.

Modelling strategy

Identical to GBD 2017, we estimated the probability that each person is living with a smoker and is also a non-smoker themselves using set theory. First, household composition data were used at the individual level to capture the ages and sexes of each person in the household. Second, we analysed surveys with both household composition data and tobacco use questions and determined that the distribution of household size, mean age of the household members, and the age distribution were not significantly different between households with and without a self-reported smoker. Since we did not find that household composition varied between smokers and non-smokers, we then used the GBD 2019 primary daily smoking prevalence model to calculate the probability that each household member is a daily smoker. Next, we used the probability of the union of sets on each individual household member to calculate the overall probability that at least one of the other household members was a daily smoker. As in GBD 2017, we incorporated occupational exposure by modelling prevalence of current exposure to secondhand smoke at work, by age, sex, location, and year, using ST-GPR. In order to avoid double counting we calculated the probability that an individual is exposed through either non-occupational exposure or occupational exposure, given their age, sex, and household composition. Finally, we multiplied this probability of exposure by the probability that the individual is not a smoker themselves (ie, 1 minus primary daily smoking prevalence for that person’s location, year, age, and sex). We then collapsed these individual-level probabilities to produce average probabilities of exposure by location, year, age, and sex.

These probabilities were modelled in the GBD ST-GPR framework, which generates exposure estimates from a mixed effects hierarchical linear model plus weighted residuals smoothed across time, space, and age. The linear model formula was fit separately by sex using restricted maximum likelihood in R.

We used the sex-specific overall daily smoking prevalence for adults (age 15 and older) as a country-level covariate in the model. The overall male adult daily smoking prevalence was used as the covariate for females of all ages and for males under age 15. The overall female adult daily smoking prevalence was used as the covariate for males age 15 and older.

All input datapoints from the probability calculation had a measure of uncertainty (variance and sample size) coming from the uncertainty of the primary smoking prevalence model and the sample size from

the unit record data going into the modelling process. Geographical random effects were used in model fitting but were not used in prediction.

Theoretical minimum-risk exposure level

The theoretical minimum-risk exposure level for secondhand smoke is zero exposure among non-smokers, meaning that non-smokers would not live with any primary smokers.

Relative risks

The same risk-outcome pairs from GBD 2017 were used. For children ages 0-14, we estimated the burden of otitis media attributable to secondhand smoke exposure. For all ages we estimated the burden of lower respiratory infections (LRI), and for adults greater than or equal to 25 years of age we estimated the burden of lung cancer, chronic obstructive pulmonary disease (COPD), ischaemic heart disease, and cerebrovascular disease attributable to secondhand smoke exposure, breast cancer, and type 2 diabetes.

For lung cancer, ischaemic heart disease, cerebrovascular disease, and LRI, we used country-specific relative risks created using integrated exposure response curves (IER) for $PM_{2.5}$ air pollution. IER curve calculation was updated with the GBD 2019 cigarettes per smoker estimates. The relative risks for otitis media[1], breast cancer[2], and diabetes[3] are derived from published meta-analyses and are the same as the ones used in the previous GBD cycle. Table 2 summarizes relative risk input data.

**Input data**	**Exposure**
Source count (total)	232
Number of countries with data	34

We used the standard GBD population attributable fraction (PAF) equation to estimate burden based on exposure and relative risks.

References

1. Jones LL, Hassanien A, Cook DG, Britton J, Leonardi-Bee J. Parental smoking and the risk of middle ear disease in children. Arch Pediatr Adolesc Med. 2012; 166: 18–27.
2. Macacu A, Autier P, Boniol M, Boyle P. Active and passive smoking and risk of breast cancer: a meta-analysis. Breast Cancer Res Treat 2015; 154:213–224.
3. Zhu B, Wu X, Wang X, Zheng Q, Sun G. The association between passive smoking and type 2 diabetes: a meta-analysis. Asia-Pacific Journal of Public Health 2014; 26:226-237.

Chewing tobacco

Flowchart

Chewing Tobacco

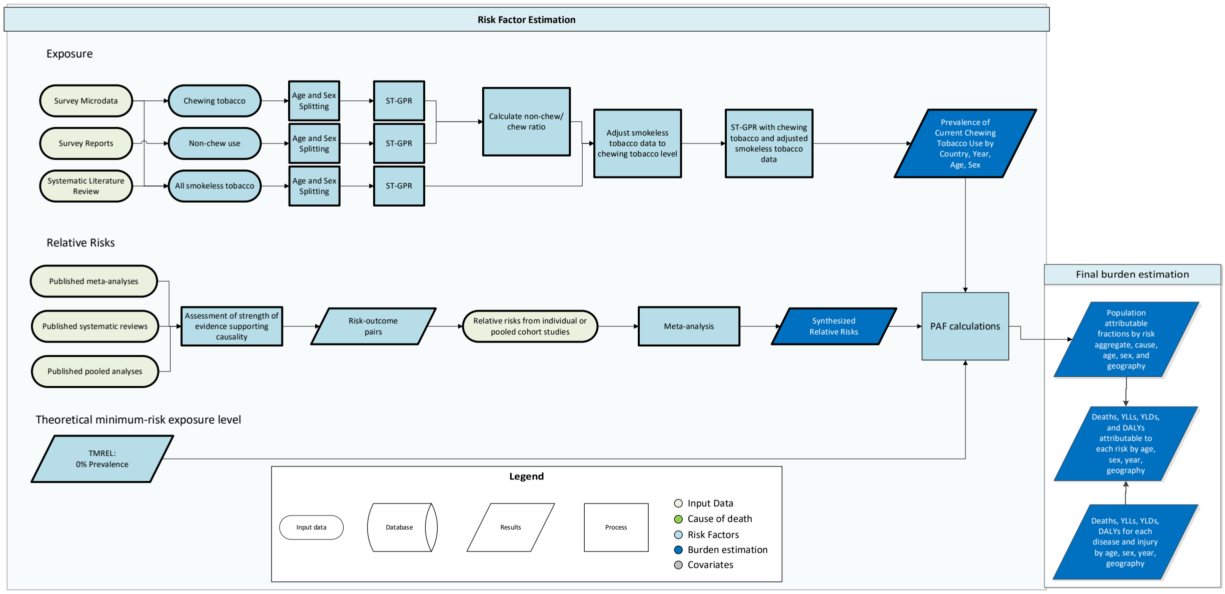

Input data and methodological summary

Definition

Exposure

Current chewing tobacco use is defined as current use (use within the last 30 days where possible, or according to the closest definition available from the survey) of any frequency (any, daily, or less than daily). Chewing tobacco includes local products, such as betel quid with tobacco.

Input data

As in GBD 2017, we included sources that reported primary chewing tobacco, non-chew smokeless tobacco, and all smokeless tobacco use among respondents over age 10. To be eligible for inclusion, sources had to be representative for their level of estimation (ie, national sources needed to be nationally representative, subnational sources subnationally representative). We included only self-reported use data and excluded data from questions asking about others' tobacco use behaviours.

We extracted primary data from individual-level microdata and survey report tabulations on chewing tobacco, non-chew smokeless tobacco, and all smokeless tobacco use. We extracted data on current, former, and/or ever use as well as frequency of use (daily, occasional, and unspecified, which includes both daily and occasional smokers). Products that do not include tobacco, such as betel quid without tobacco, were excluded or estimated separately as part of the drug use risk factor, if applicable.

For microdata, we extracted relevant demographic information, including age, sex, location, and year, as well as survey metadata, including survey weights, primary sampling units, and strata. This information

allowed us to tabulate individual-level data in the standard GBD five-year age-sex groups and produce accurate estimates of uncertainty. For survey report tabulations, we extracted data at the most granular age-sex group provided.

Table 1: Data inputs for exposure for chewing tobacco.

Input data	Exposure
Source count (total)	5030
Number of countries with data	203

Table 2: Data inputs for relative risks for chewing tobacco.

Input data	Relative risk
Source count (total)	827
Number of countries with data	38

Age and sex splitting

We split data reported in broader age groups than the GBD five-year age groups or as both sexes combined by adapting the method reported in Ng and colleagues (http://jamanetwork.com/journals/jama/fullarticle/1812960) to split using a sex-geography-time-specific reference age pattern. We separated the data into two sets: a training dataset, with data already falling into GBD sex-specific five-year age groups, and a split dataset, which reported data in aggregated age or sex groups. We then used spatiotemporal Gaussian process regression (ST-GPR) to estimate sex-geography-time-specific age patterns using data in the training dataset. The estimated age patterns were then used to split each source in the split dataset.

The ST-GPR model used to estimate the age patterns for age-sex splitting used an age weight parameter value that minimises the effect of any age smoothing. This parameter choice allows the estimated age pattern to be driven by data, rather than being enforced by any smoothing parameters of the model. Because these age-sex-split datapoints will be incorporated in the final ST-GPR exposure model, we do not want to doubly enforce a modelled age pattern for a given sex-location-year on a given aggregate datapoint. We run three separate ST-GPR models for age-sex splitting – one for each smokeless tobacco category (chew, non-chew, and all smokeless).

Modelling strategy

Prevalence modelling

We used a ST-GPR to model chewing tobacco prevalence. Full details on the ST-GPR method are reported elsewhere in the Appendix. Briefly, the mean function input to GPR is a complete time series of estimates generated from a mixed effects hierarchical linear model plus weighted residuals smoothed across time, space, and age. The linear model formula for chewing tobacco, fit separately by sex using restricted maximum likelihood in R, is:

$$logit(p_{g,a,t}) = \beta_0 + \sum_{k=1}^{18} \beta_k I_{A[a]} + \alpha_s + \alpha_r + \alpha_g + \epsilon_{g,a,t}$$

Where $I_{A[a]}$ is a dummy variable indicating specific age group A that the prevalence point $p_{g,a,t}$ captures, and α_s, α_r, and α_g are super-region, region, and geography random intercepts, respectively. The hyperparameters are the same as in GBD 2017.

We run three ST-GPR models for each prevalence category – one for each smokeless tobacco category (chew, non-chew, and all smokeless).

All smokeless tobacco prevalence adjustment

Using the 1000 draws from each of the prevalence ST-GPR models, we calculated 1000 draws of chewing tobacco prevalence divided by the sum of chewing tobacco and non-chewing tobacco prevalence for each location, age group, sex, and year. The draws were unordered, as we did not want to enforce an assumption about the relationship between the levels of chewing tobacco and non-chewing tobacco prevalence.

The draws of the ratio of chewing to non-chewing tobacco were then multiplied by the draws from the all smokeless tobacco prevalence model to adjust the estimates to chewing tobacco prevalence. These were then averaged to get the mean estimate. The variance across the ratios was calculated for each location, year, age, and sex, and was added to the variance from the original all smokeless tobacco draws.

Final chewing tobacco prevalence model

To calculate the final chewing tobacco prevalence, we ran an additional ST-GPR model with both the original chewing tobacco data (post-age-sex splitting), as well as the adjusted data. These adjusted data add more information to the model – as surveys will often only ask about all smokeless tobacco consumption – while taking into consideration the uncertainty from the ratio calculation.

Theoretical minimum-risk exposure level

The theoretical minimum risk exposure level is that everyone in the population has been a lifelong non-user of chewing tobacco.

Relative risk

As in GBD 2017, we included outcomes based on the strength of available evidence supporting a causal relationship. There was sufficient evidence to include oral cancer and oesophageal cancer as health outcomes caused by chewing tobacco use.

Relative risk estimates were derived from prospective cohort studies and population-based case-control studies. We used the same underlying effect size estimates from prospective cohort studies and population-based case-control studies as in GBD 2017. Briefly, we did not include hospital-based case control studies due to concerns over representativeness. We only included sources that adequately adjusted for major confounders, especially smoking status. Summary effect size estimates were calculated in R, using the 'metafor' package. We performed a random effects meta-analysis using the DerSimonian and Laird method, which does not assume a true effect size but considers each input study as selected from a random sample of all possible sets of studies for the outcome of interest. The random-effects method allows for more variation between the studies, and incorporates this variance

into the estimation process. We used an inverse-variance weighting method to determine component study weights. We found significantly different relative risks for oral cancer for males and females, and estimated relative risks separately by sex for oral cancer alone.

Alcohol use

Flowchart

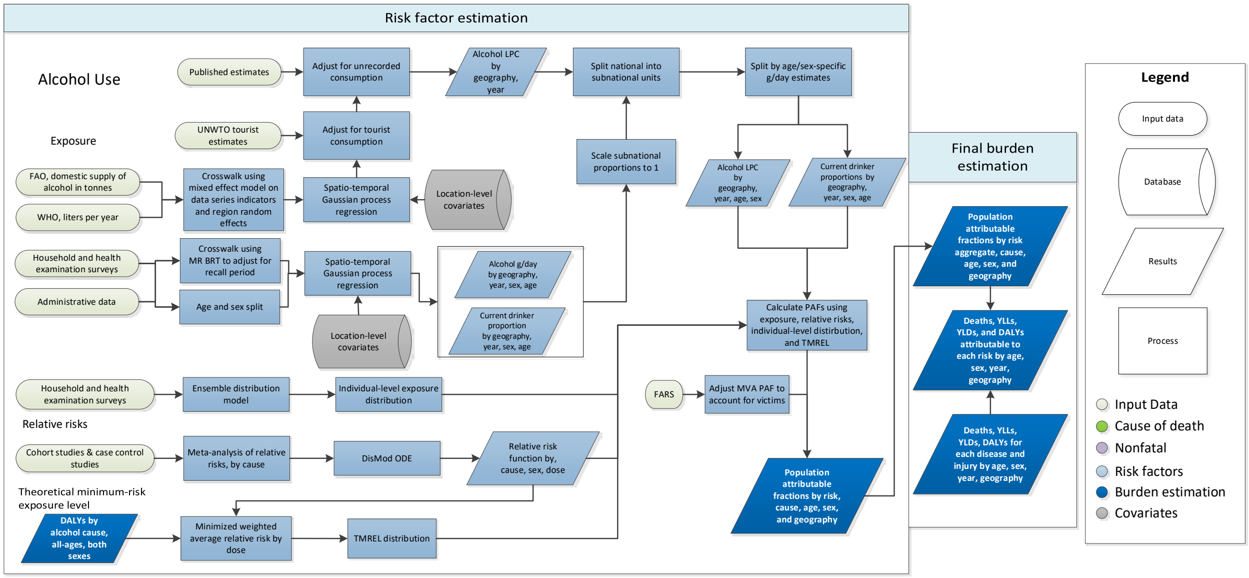

Input data and methodological summary

Definition

Exposure

We defined exposure as the grams per day of pure alcohol consumed among current drinkers. We constructed this exposure using the indicators outlined below:

1. Current drinkers, defined as the proportion of individuals who have consumed at least one alcoholic beverage (or some approximation) in a 12-month period.
2. Alcohol consumption (in grams per day), defined as grams of alcohol consumed by current drinkers, per day, over a 12-month period.
3. Alcohol litres per capita stock, defined in litres per capita of pure alcohol, over a 12-month period.

We also used three additional indicators to adjust alcohol exposure estimates to account for different types of bias:

1. Number of tourists within a location, defined as the total amount of visitors to a location within a 12-month period.
2. Tourists' duration of stay, defined as the number of days resided in a hosting country.
3. Unrecorded alcohol stock, defined as a percentage of the total alcohol stock produced outside established markets.

Input data

A systematic review of the literature was performed to extract data on our primary indicators. The Global Health Exchange (GHDx), IHME's online database of health-related data, was searched for population survey data containing participant-level information from which we could formulate the required alcohol use indicators on current drinkers and alcohol consumption. Data sources were included if they captured a sample representative of the geographical location under study. We documented relevant survey variables from each data source in a spreadsheet and extracted using STATA 13.1 and R 3.3. A total of 6172 potential data sources were available in the GHDx, of which 5091 have been screened and 1125 accepted.

Table 1: Data inputs for exposure for alcohol use.

Input data	Exposure	Relative risk
Sources (total)	10513	495
Number of countries with data	199	-

Estimates of current drinking prevalence were split by age and sex where necessary. First, studies that reported prevalence for both sexes were split using a region-specific sex ratio estimated using MR-BRT. Second, where studies reported estimates across non-GBD age groups, these were split into standard five-year age groups using the global age pattern estimated by ST-GPR.

Table 2: MR-BRT sex splitting adjustment factors for current drinking

Data input	Gamma	Beta coefficient, log (95% CI)	Adjustment factor*
Female: Male	0	-0.16 (-0.17, -0.14)	0.85
Age < 50	0	0.06 (0.06, 0.06)	1.07
East Asia	0.36	-1.02 (-1.74, -0.29)	0.36
Southeast Asia	0.64	-1.06 (-2.34, 0.22)	0.35
Central Asia	0.41	-0.35 (-1.16, 0.46)	0.70
Central Europe	0.18	-0.21 (-0.58, 0.14)	0.80
Eastern Europe	0.10	-0.07 (-0.28, 0.14)	0.93
High-income Asia Pacific	1.27	-1.11 (-4.90, 2.68)	0.33
Western Europe	0.08	0.03 (-0.14, 0.20)	1.03
Southern Latin America	1.26	-0.67 (-4.18, 2.84)	0.51
High-income North America	0.09	-0.07 (-0.26, 0.11)	0.93
Caribbean	0.25	-0.52 (-1.02, -0.03)	0.59
Andean Latin America	0.76	-0.16 (-1.66, 1.34)	0.85
Central Latin America	0.30	-0.52 (-1.12, 0.08)	0.59
Tropical Latin America	0.08	-0.61 (-0.79, -0.44)	0.54
North Africa and Middle East	1.21	-1.44 (-3.91, 1.03)	0.24
South Asia	0.71	-1.17 (-2.57, 0.23)	0.31
Eastern sub-Saharan Africa	0.28	-0.53 (-1.10, 0.03)	0.58
Southern sub-Saharan Africa	0.20	-0.16 (-0.56, 0.23)	0.85
Western sub-Saharan Africa	0.32	-0.19 (-0.83, 0.45)	0.83
Oceania	0.94	-0.54 (-2.42, 1.34)	0.58

**Adjustment factor is the transformed beta coefficient in normal space and can be interpreted as the factor by which the alternative case definition is adjusted to reflect the ratio by which both-sex data points were split.*

To allow for the inclusion of data that did not meet our reference definition for current drinking, two crosswalks were performed using MR-BRT. The first crosswalk converted estimates of one-month drinking prevalence to what they would be if data represented estimates of 12-month drinking prevalence. This crosswalk incorporated two binary covariates: male and age ≥ 50. The second crosswalk converted estimates of one-week drinking prevalence to 12-month drinking prevalence. This crosswalk incorporated age < 20 and male as covariates. The covariates utilised in both crosswalks were included as both x and z covariates. A uniform prior of 0 was set as the upper bound for the beta coefficients to enforce the logical constraint that one-month and one-week prevalence could not be greater than 12-month prevalence.

Table 3: MR-BRT crosswalk adjustment factors for alcohol use current drinking model

Data input	Reference or alternative case definition	Gamma	Beta coefficient, logit (95% CI)
12-month prevalence	Ref	---	---
1-month prevalence	Alt	0.22	-0.60 (-1.05, -0.16)
Age ≥ 50		0.13	0.16 (-0.10, 0.43)
Male		0.29	0.01 (-0.57, 0.59)
1-week prevalence	Alt	0.46	-1.51 (-2.42, -0.59)
Age < 20		0.47	-0.29 (-1.34, 0.76)
Male		0.00	0.38 (0.15, 0.60)

The methods for modelling supply-side-level data were changed substantially from those used in GBD 2017. The raw data are domestic supply (WHO GISAH; FAO) and retail supply (Euromonitor) of litres of pure ethanol consumed. Domestic supply is calculated as the sum of production and imports, subtracting exports. The WHO and FAO sources were combined, so that FAO data were only used if there were no data available for that location-year from WHO. This was done because the WHO source takes into consideration FAO values when available. Since the WHO data are given in more granular alcohol types, the following adjustments were made:

$$LPC\ Pure\ Ethanol = 0.13 * \left(\frac{Wine}{0.973}\right)$$

$$LPC\ Pure\ Ethanol = 0.05 * \left(\frac{Beer}{0.989}\right)$$

$$LPC\ Pure\ Ethanol = 0.4 * \left(\frac{Spirits}{0.91}\right)$$

Three outliering strategies are used to omit implausible datapoints and data that created implausible model fluctuations. First, estimates from the current drinking model are used to calculate the grams of

alcohol consumed per drinker per day. A point is outliered if the grams of pure ethanol per drinker per day for a given source-location-year is greater than 100 (approximately ten drinks). These thresholds were chosen by using expert knowledge about reasonable consumption levels. In the second round of outliering, the mean liters per capita value over a ten-year window is calculated. If a point is over 70% of that mean value away from the mean value, it is outliered. The 70% limit was chosen using histograms of these distances. Additionally, some manual outliering is performed to account for edge cases. Finally, data smoothing is performed by taking a three-year rolling mean over each location-year.

Next, an imputation to fill in missing years is performed for all series to remove compositional bias from our final estimates. Since the data from our main sources cover different time periods, by imputing a complete time series for each data series, we reduce the probability that compositional bias of the sources is leading to biased final estimates. To impute the missing years for each series, we model the log ratio of each pair of sources as a function of an intercept and nested random effects on super-region, region, and location. The appropriate predicted ratio is multiplied by the source that we do have, which generates an estimated value for the missing source. For some locations where there was limited overlap between series, the predicted ratio did not make sense, and a regional ratio was used.

Finally, variance was calculated both across series (within a location-year) as well as across years (within a location-source). Additionally, if a location-year had one imputed point, the variance was multiplied by 2. If a location-year had two imputed points, the variance was multiplied by 4. The average estimates in each location-year were the input to an ST-GPR model. This uses a mixed-effects model modelled in log space with nested location random effects.

We obtained data on the number of tourists and their duration of stay from the UNWTO.[3] We applied a crosswalk across different tourist categories, similar to the one used for the litres per capita data, to arrive at a consistent definition (ie, visitors to a country).

We obtained estimates on unrecorded alcohol stock from data available in WHO GISAH database,[2] consisting of 189 locations. For locations with no data available, the national or regional average was used.

For relative risks, in GBD 2016 we performed a systematic literature review of all cohort and case-control studies reporting a relative risk, hazard ratio, or odds ratio for any risk-outcome pairs studied in GBD 2016. Studies were included if they reported a categorical or continuous dose for alcohol consumption, as well as uncertainty measures for their outcomes, and the population under study was representative.

Modelling strategy

While population-based surveys provide accurate estimates of the prevalence of current drinkers, they typically underestimate real alcohol consumption levels.[10-12] As a result, we considered the litre per capita input to be a better estimate of overall volume of consumption. Per capita consumption, however, does not provide age- and sex-specific consumption estimates needed to compute alcohol-attributable burden of disease. Therefore, we use the age-sex pattern of consumption among drinkers modelled from the population survey data and the overall volume of consumption from FAO, GISAH, and Euromonitor to determine the total amount of alcohol consumed within a location. In the paragraphs below, we outline how we estimated each primary input in the alcohol exposure model, as

well as how we combined these inputs to arrive at our final estimate of grams per day of pure alcohol. We estimated all models below using 1000 draws.

For data obtained through surveys, we used spatiotemporal Gaussian process regression (ST-GPR) to construct estimates for each location/year/age/sex. We chose to use ST-GPR due to its ability to leverage information across the nearby locations or time periods. We also modelled the alcohol litres per capita (LPC) data, as well as the total number of tourists, using ST-GPR.

Given the heterogeneous nature of the estimates on unrecorded consumption, as well as the wide variation across countries and time periods, we took 1000 draws from the uniform distribution of the lowest and highest estimates available for a given country. We did this to incorporate the diffuse uncertainty within the unrecorded estimates reported. We used these 1000 draws in the equation below.

We adjusted the alcohol LPC for unrecorded consumption using the following equation:

$$Alcohol\ LPC = \frac{Alcohol\ LPC}{(1 - \%\ Unrecorded)}$$

We then adjusted the estimates for alcohol LPC for tourist consumption by adding in the per capita rate of consumption abroad and subtracting the per capita rate of tourist consumption domestically.

$$Alcohol\ LPC_d = Unadjusted\ Alcohol\ LPC_d + Alcohol\ LPC_{Domestic\ consumption\ abroad} - Alcohol\ LPC_{Tourist\ consumption\ domestically}$$

$$Alcohol\ LPC_i = \frac{\sum_l Tourist\ Population_l * Proportion\ of\ tourists_{i,l} * Unadjusted\ Alcohol\ LPC_l * \frac{Average\ length\ of\ stay_{i,l}}{365} *}{Population_d}$$

where:

l *is the set of all locations*, i *is either* Domestic consumption abroad *or* Tourist consumption domestically, *and* d *is a domestic location.*

After adjusting alcohol LPC by tourist consumption and unrecorded consumption for all location/years reported, sex-specific and age-specific estimates were generated by incorporating estimates modelled in ST-GPR for percentage of current drinkers within a location/year/sex/age, as well as consumption trends modelled in the ST-GPR grams per day model. We do this by first calculating the proportion of total consumption for a given location/year by age and sex, using the estimates of alcohol consumed per day, the population size, and the percentage of current drinkers. We then multiply this proportion of total stock for a given location/year/sex/age by the total stock for a given location/year to calculate the consumption in terms of litres per capita for a given location/year/sex/age. We then convert these estimates to be in terms of grams/per day. The following equations describe these calculations:

$$Proportion\ of\ total\ consumption_{l,y,s,a} = \frac{Alcohol\ \ g/day_{\ l,y,s,a} * Population_{l,y,s,a} * \%\ Current\ drinkers_{l,y,s,a}}{\sum_{s,a} Alcohol\ \ g/day_{l,y,s,a} * Population_{l,y,s,a} * \%\ Current\ drinkers_{l,y,s,a}}$$

$$Alcohol\ LPC_{l,y,s,a} = \frac{Alcohol\ LPC_{l,y} * Population_{l,y} * Proportion\ of\ total\ consumption_{l,y,s,a}}{\%\ Current\ drinkers_{l,y,s,a} * Population_{l,y,s,a}}$$

$$Alcohol\ g/day_{l,y,s,a} = Alcohol\ LPC_{l,y,s,a} * \frac{1000}{365}$$

where:

$l\ is\ a\ location, y\ is\ a\ year, s\ is\ a\ sex, and\ a\ is\ an\ age\ group.$

We then used the gamma distribution to estimate individual-level variation within location, year, sex, age drinking populations, following the recommendations of other published alcohol studies.[7,8] We chose parameters of the gamma distribution based on the mean and standard deviation of the 1,000 draws of alcohol g/day exposure for a given population. Standard deviation was calculated using the following formula.[15] We tested several alternative models using our data and found this model performed best.

$$standard\ deviation = mean * (0.087 * female + \ 1.171\ )$$

Theoretical minimum-risk exposure level

We calculated TMREL by first calculating the overall risk attributable to alcohol. We did this by weighting each relative risk curve by the share of overall DALYs for a given cause. We then took the minimum of this overall-risk curve as the TMREL of alcohol use. More formally,

$$TMREL = argmin\ average\ overall\ risk_{\omega}(g/day)$$

$$Average\ overall\ risk_{\omega}(g/day) = \sum_{i}^{\omega} RR_i(g/day) * \frac{DALY_i}{\sum_i^{\omega} DALY_i}$$

Where:

$\omega\ is\ the\ set\ of\ causes\ associated\ with\ alcohol, i\ is\ a\ given\ cause\ from\ that\ set, DALY\ is\ the\ global\ DALY\ rate\ in\ 2010, and\ RR\ is\ the\ dose\ response\ curve\ for\ a\ given\ cause\ and\ exposure\ level\ in\ grams\ per\ day.$

In other words, we chose TMREL as being the exposure that minimises your risk of suffering burden from any given cause related to alcohol. We weight the risk for a particular cause in our aggregation by the proportion of DALYs due to that cause (eg, since more observed people die from ischaemic heart disease [IHD], we weight the risk for IHD more in the above calculation of average risk compared to, say, diabetes, even if both have the same relative risk for a given level of consumption).

Relative risks

We used the studies identified through the systematic review to calculate a dose-response, modelled using DisMod ODE. We chose DisMod ODE rather than a conventional mixed effects meta-regression because of its ability to estimate nonparametric splines over doses (ie, for most alcohol causes, there is a non-linear relationship with different doses) and incorporate heterogeneous doses through dose-integration (ie, most studies report doses categorically in wide ranges. DisMod ODE estimates specific doses when categories overlap across studies, through an integration step.). We used the results of the meta-regression to estimate a non-parametric curve for all doses between zero and 150 g/day and their corresponding relative risks. For all causes, we assumed the relative risk was the same for all ages and sexes, with the exception of ischaemic heart disease, ischaemic stroke, haemorrhagic stroke, and diabetes, which we estimated by sex.

Table 4: Data inputs for relative risks for alcohol use.

Input data	Relative risk
Sources (total)	495

For outcomes that are by definition caused by alcohol, such as liver cancer or cirrhosis due to alcohol use, PAFs are set to 1. PAFs for cirrhosis due to all causes that are in excess of the proportion of all cirrhosis burden due to alcohol are proportionally redistributed over cirrhosis due to hepatitis B, hepatitis C, and other causes.

Regarding injuries outcomes, we constructed relative risks based on chronic exposure to alcohol rather than acute exposure immediately preceding injury, which has a weaker relationship to the outcome, though still significant.[15,16,18-21] We decided to use chronic exposure given the lack of available data on acute exposure, as well as the lack of cohort studies using acute exposure as a metric. Further, using chronic exposure allowed us to construct relative risks curves for unintentional injuries, interpersonal violence, motor vehicle accidents, and self-harm using the same method as reported above.

In the case of motor vehicle accidents, we adjusted the PAF to account for victims of drunk drivers who are involved in accidents. Using data from the Fatality Analysis Reporting System in the US,[17] we calculated the average number of fatalities in a car crash involving alcohol, as well as the percentage of those fatalities distributed by age and sex (figures 1 and 2). We aggregated FARS data across the years 1985–2015, given there was little variation in the data temporally and the number of cases in old age groups had too much variance when constructing estimates by year. To adjust PAFs, we multiplied attributable deaths by the average number of fatalities from FARS and redistributed the PAF among each population, based on the probability of being a victim to a certain drunk driver by age and sex, based on the FARS data. The following equation describes this process:

$$Adjusted\ PAF_i = \frac{\sum_d PAF_d * DALY_d * Avg\ Fatalities_d * \ P(i\ is\ a\ victim)_d}{DALY_i}$$

where:

$i\ is\ a\ population\ by\ location, year, age, sex\ and$

d *is the set of all age and sex exposed groups within that location and year.*

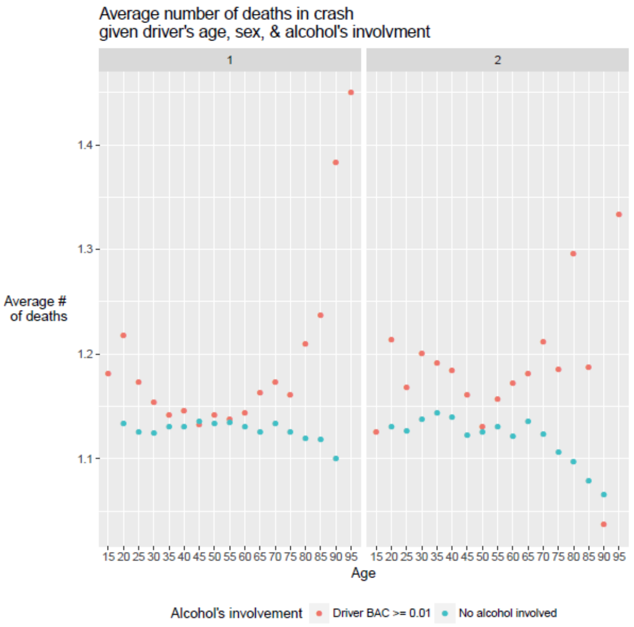

Figure 1

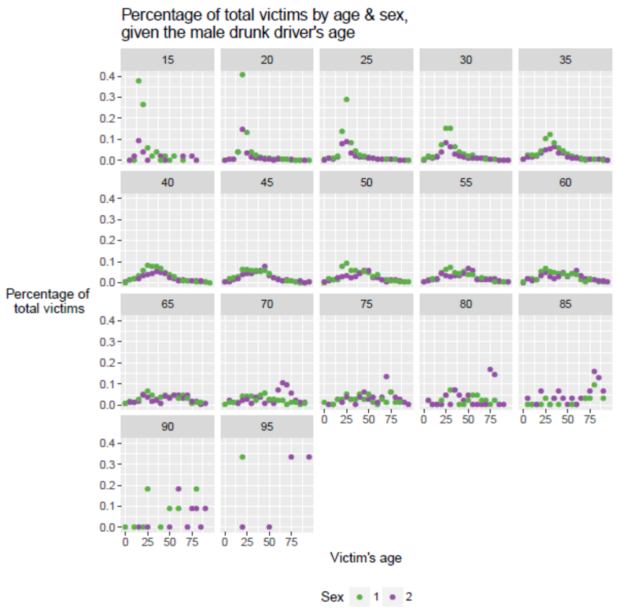

Figure 2

Population attributable fraction

For all causes, we defined PAF as:

$$PAF(x) = \frac{P_A + \int_0^{150} P(x) * RR_C(x)\, dx - 1}{P_A + \int_0^{150} P(x) * RR_C(x)\, dx} \qquad P(x) = P_C * \Gamma(\boldsymbol{p})$$

where:

P_C *is the prevalence of current drinkers,* P_a *is the prevalence of abstainers,* $RR_c(x)$ *is the relative risk function for current drinkers, and* $\boldsymbol{p}$ *are parameters determined by the mean and sd of exposure*

We performed the above equation for 1000 draws of the exposure and relative risk models. We then used the estimated PAF draws to calculate YLL, YLDs, and DALYs, as per the other risk factors.

References

1. Food and Agriculture Organization of the United Nations (FAO). FAOSTAT Food Balance Sheets, October 2014. Rome, Italy: Food and Agriculture Organization of the United Nations (FAO).
2. World Health Organization (WHO). WHO Global Health Observatory - Recorded adult per capita alcohol consumption, Total per country. Geneva, Switzerland: World Health Organization (WHO).
3. UN World Tourism Organization (UNWTO). UN World Tourism Organization Compendium of Tourism Statistics 2015 [Electronic]. Madrid, Spain: UN World Tourism Organization (UNWTO), 2016.
4. Ramstedt, Mats. “How much alcohol do you buy? A comparison of self-reported alcohol purchases with actual sales.” *Addiction* 105.4 (2010): 649-654.
5. Stockwell, Tim, et al. “Under-reporting of alcohol consumption in household surveys: a comparison of quantity–frequency, graduated–frequency and recent recall.” *Addiction* 99.8 (2004): 1024-1033.
6. Kerr, William C., and Thomas K. Greenfield. “Distribution of alcohol consumption and expenditures and the impact of improved measurement on coverage of alcohol sales in the 2000 National Alcohol Survey.” *Alcoholism: Clinical and Experimental Research* 31.10 (2007): 1714-1722.
7. Taylor, Bruce, et al. “The more you drink, the harder you fall: a systematic review and meta-analysis of how acute alcohol consumption and injury or collision risk increase together.” *Drug and alcohol dependence* 110.1 (2010): 108-116.
8. Vinson, Daniel C., Guilherme Borges, and Cheryl J. Cherpitel. “The risk of intentional injury with acute and chronic alcohol exposures: a case-control and case-crossover study.” *Journal of studies on alcohol* 64.3 (2003): 350-357.
9. Vinson, Daniel C., et al. “A population-based case-crossover and case-control study of alcohol and the risk of injury.” *Journal of studies on alcohol*64.3 (2003): 358-366.
10. Fatal Accident Reporting System (FARS). National Highway Traffic Safety Administration, National Center for Statistics and Analysis Data Reporting and Information Division (NVS-424); 1985, 1990, 1995, 2000, 2005, 2010, 2015
11. Chen, Li-Hui, Susan P. Baker, and Guohua Li. “Drinking history and risk of fatal injury: comparison among specific injury causes.” Accident Analysis & Prevention 37.2 (2005): 245-251.
12. Bell, Nicole S., et al. “Self-reported risk-taking behaviors and hospitalization for motor vehicle injury among active duty army personnel.” American journal of preventive medicine 18.3 (2000): 85-95.
13. Margolis, Karen L., et al. “Risk factors for motor vehicle crashes in older women.” The Journals of Gerontology Series A: Biological Sciences and Medical Sciences 57.3 (2002): M186-M191.
14. Sorock, Gary S., et al. “Alcohol-drinking history and fatal injury in older adults.” Alcohol 40.3 (2006): 193-199.
15. Kehoe, Tara et al. “Determining the best population-level alcohol consumption model and its impact on estimates of alcohol-attributable harms.” Population health metrics 10 6. (2012)

Drug use

Flowchart

Drug Use

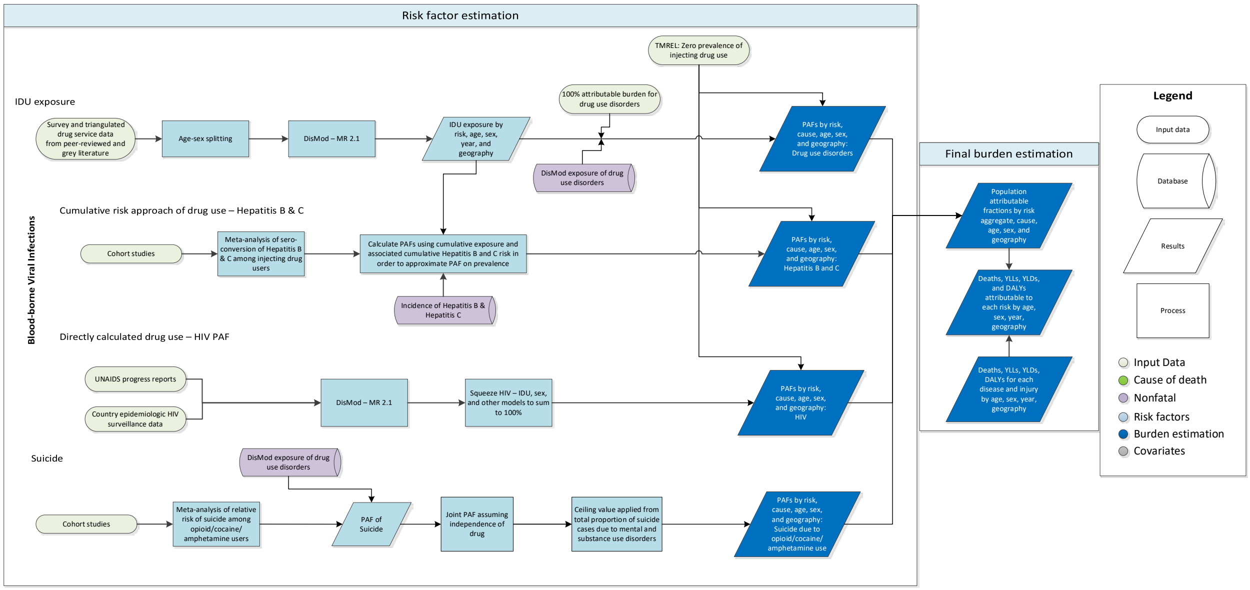

Input data and methodological summary

Exposure definition

The drug use risk factor includes four dimensions of exposure. First, we include 100% attribution of drug use disorder estimates. Second, estimates of prevalence of opioid, amphetamine, and cocaine use disorder are used as exposures for risk of suicide. These drug use disorders are defined based on DSM or ICD diagnostic criteria. Third, instead of starting with an exposure model to estimate the proportion of HIV cases due to injection drug use (IDU), we model the PAF directly, alongside proportion of HIV cases due to sexual transmission and other routes of transmission, which mainly includes blood transfusions. Finally, prevalence of injection drug use is used to model risk of Hepatitis B and C viruses (HBV and HCV, respectively). Injecting drug users are at high risk of bloodborne infections due to the use of shared needles and injection equipment. Injecting drug use is defined as current injection drug use among individuals aged 15 to 64. The theoretical minimum-risk exposure level (TMREL) for drug use is defined as zero exposure to drug use.

Input data

To estimate the burden of HIV cases attributable to IDU, we extracted data on the proportion of notified HIV cases by transmission route – sexual intercourse, injecting drug use, and other – from a number of agencies that conduct surveillance of HIV across the globe.[1-8]

The prevalence of current injecting drug use was estimated using data from a multistage process of systematic review. It involved multiple stages of peer and expert review, including review by the

Reference Group to the UN on HIV and injecting drug use,[9] with searches of the peer-reviewed literature in addition to an extensive review of online grey literature databases in the drug and alcohol and HIV fields.

In order to generate a pooled incidence rate/absolute relative risk for viral hepatitis among people who inject drugs, we conducted a meta-analysis of longitudinal epidemiological studies that reported a hepatitis B or hepatitis C incidence rate among persons who inject drugs.[10-25] We calculated confidence intervals for the incidence rate (where no CI was reported) from a Poisson distribution around the number of cases.

We excluded studies that focused on non-representative subgroups, such as recent injectors or adolescents, because hepatitis incidence is far higher in those groups than for all people who inject drugs (eg, Larney and colleagues[26]). We did not vary incidence among active injectors according to the availability of bloodborne virus-prevention strategies (eg, NSPs, opioid substitution therapy) because too few studies have examined different levels of incidence according to variable coverage, and we were not able to estimate coverage by country over time. In any case, in most countries, effective coverage of virus-prevention strategies remains low among people who inject drugs.[27]

Inputs to the model also include estimates of the incidence of hepatitis B and hepatitis C, coming from estimation of non-fatal health outcomes in GBD. Full details on the inputs and modelling process to produce these estimates are available in the disease-specific appendices in the GBD 2019 diseases and injuries manuscript.

**Input data**	**Exposure**
Source count (total)	174
Number of countries with data	75

Modelling strategy

Burden of HIV attributable to injecting drug use

We estimated the proportion of HIV cases attributable to three transmission categories (sex, IDU, and other) for all country-time periods using DisMod-MR 2.1. In previous rounds, data for estimating the proportion of HIV cases attributable to IDU were age-split using the age pattern of the IDU exposure model and sex-split in DisMod. In GBD 2019, these data were age- and sex-split using the estimated IDU exposure age-sex pattern, resulting in increases in the proportion of HIV due to IDU among men and decreases among women. We scaled the proportions from each of the three transmission models (sex, IDU, and other) to ensure that they fit the total HIV transmission envelope by country, year, age and sex. Scaled estimates are used as direct population attributable fractions, meaning that the proportion coming from the model is the proportion of HIV deaths or DALYs attributable to IDU.

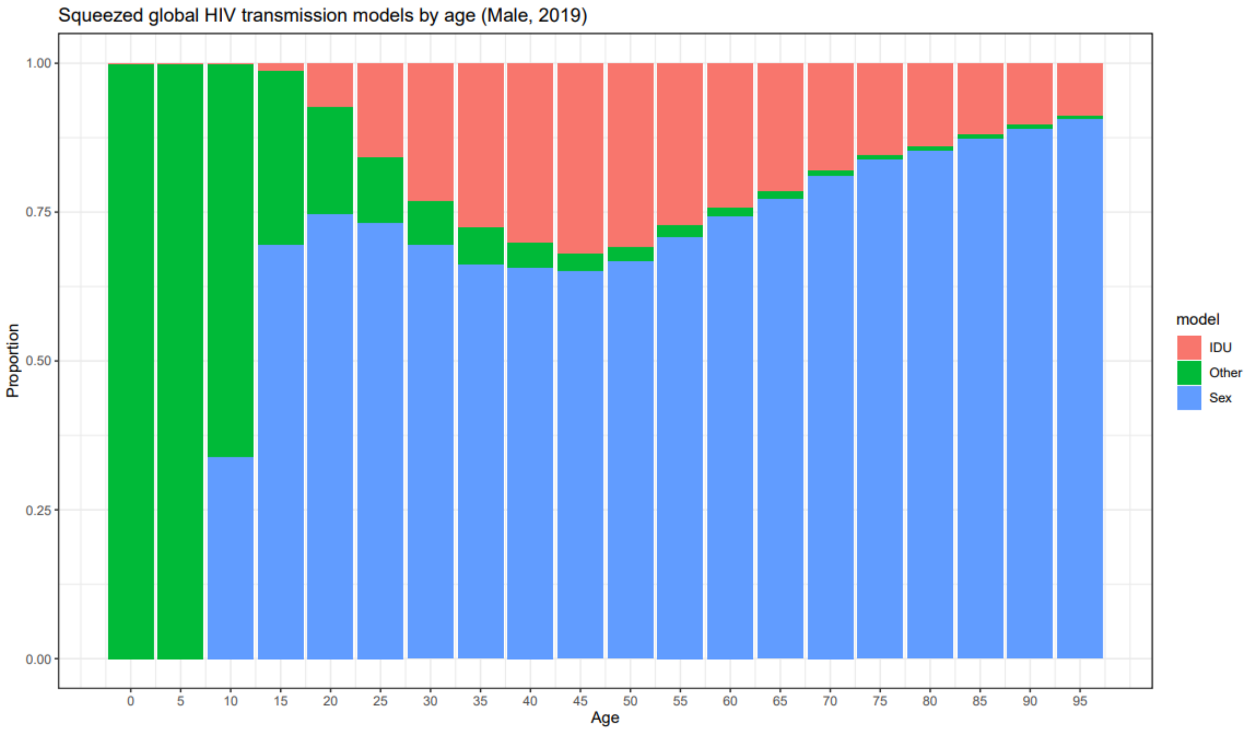

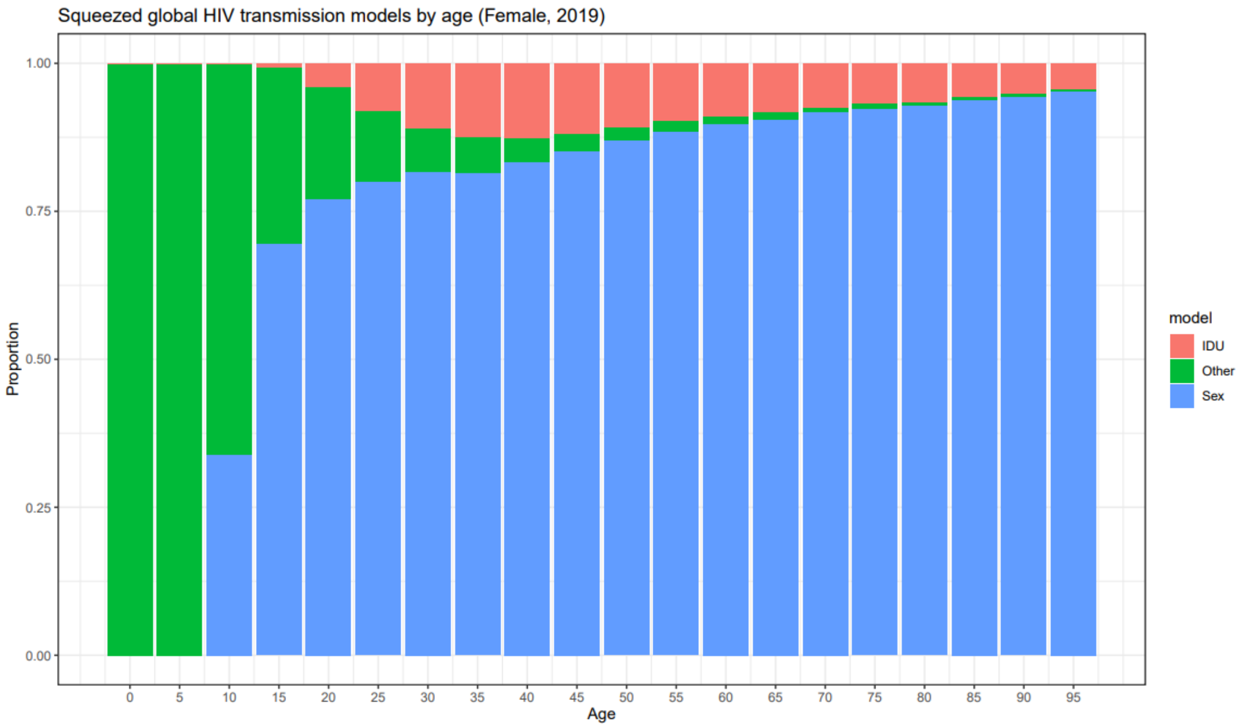

Burden of hepatitis B and hepatitis C attributable to injecting drug use

To estimate the relative contribution of IDU to hepatitis B and C disease burden at the country, regional, and global level, we used a cohort method. We recalibrated individuals according to history of injecting drug use and their accumulated risk of incident hepatitis B and C due to IDU. We made use of data on prevalence of current injecting drug use, pooled in DisMod-MR 2.1; a meta-analysis of incidence rates of

hepatitis B and hepatitis C among people who inject drugs; and estimates of population-level incidence of hepatitis B and C between 1990 and 2019. We used back-extrapolations to estimate incidence before 1990. These steps are detailed below.

To estimate the lifetime risk of being infected with hepatitis B or C, we undertook a cohort analysis for each country, year, age, and sex category and estimated the probability of an individual having been infected in each preceding year. One of the main inputs to this cohort method was the probability of having injected drugs in a specific age cohort in a given calendar year. For example, for a cohort of 40-year-olds in 2015, the relevant probability in 2005 is the estimated prevalence of injecting drug use among 30-year-olds.

DisMod-MR 2.1 was used to estimate the prevalence of injecting drug use with year as a covariate to estimate the trends over time. DisMod makes an average estimate of the change in drug use over the time period 1990–2019, and we took draws from a normal distribution of the coefficient to project IDU prevalence backward in time to 1960 from baseline level in 1990 (assuming there was little injecting drug use before the 1960s). In GBD 2019, prevalence of IDU was estimated as a single parameter prevalence model in DisMod, as opposed to a full compartmental model, because factoring in cause-specific mortality resulted in underestimating prevalence in certain locations, particularly in the north Africa and Middle East and south Asia super-regions.

Theoretical minimum-risk exposure level

The theoretical minimum-risk exposure level is defined as zero exposure to drug use.

Relative risk

**Input data**	**Relative risk**
Source count (total)	42
Number of countries with data	14

We used a pooled absolute risk of hepatitis C and hepatitis B among those who have ever used injecting drugs. Input data for this pooled absolute risk are described above, and there were no methodological or data changes to this parameter in GBD 2019.

Forest plot of absolute risk of HBV incidence among cohorts of people who inject drugs

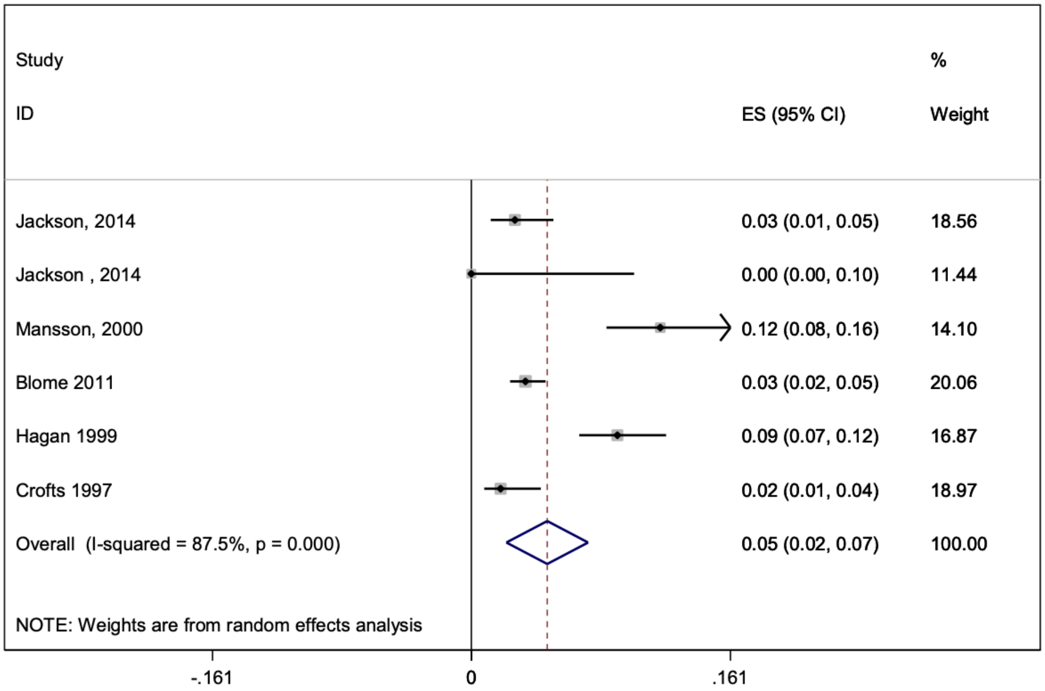

Forest plot of absolute risk of HCV incidence among cohorts of people who inject drugs

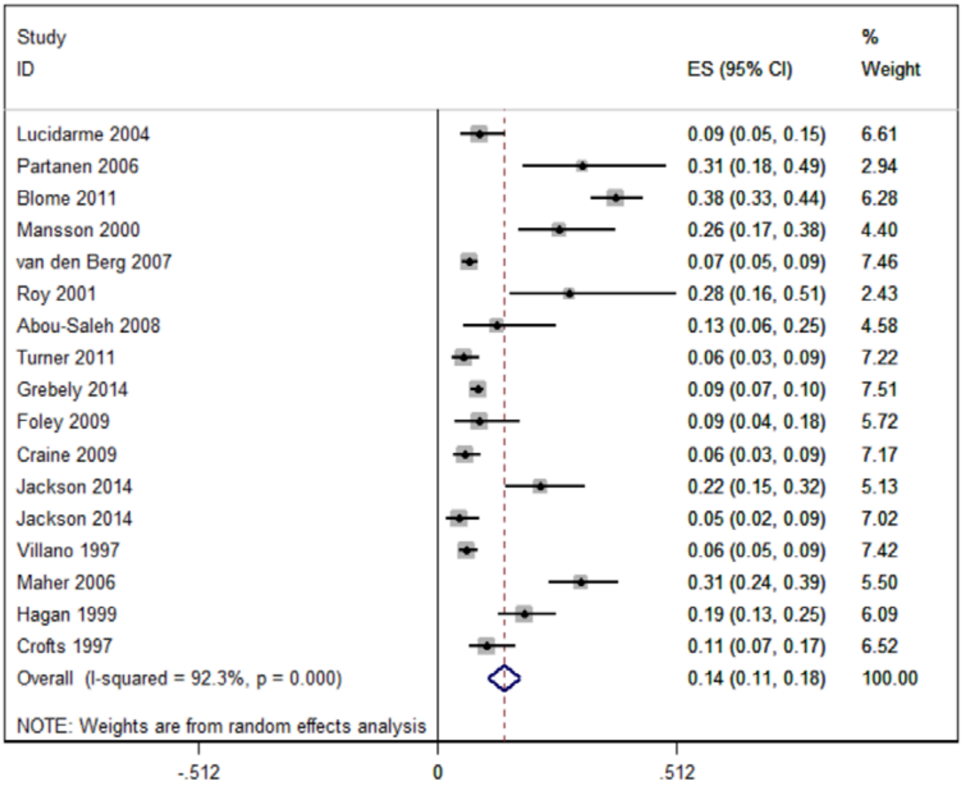

In GBD 2019, we updated the relative risk of suicide among those with substance use disorders to include new studies. Six new studies were included in a meta-analysis on the relative risk of suicide due to opioid, amphetamine, or cocaine use disorders.[28-54] The meta-analysis was conducted using MR-BRT. Compared to GBD 2017, the new data added resulted in a decrease in the relative risks and, therefore, burden of suicide due to the use of opioids, amphetamines, and cocaine.

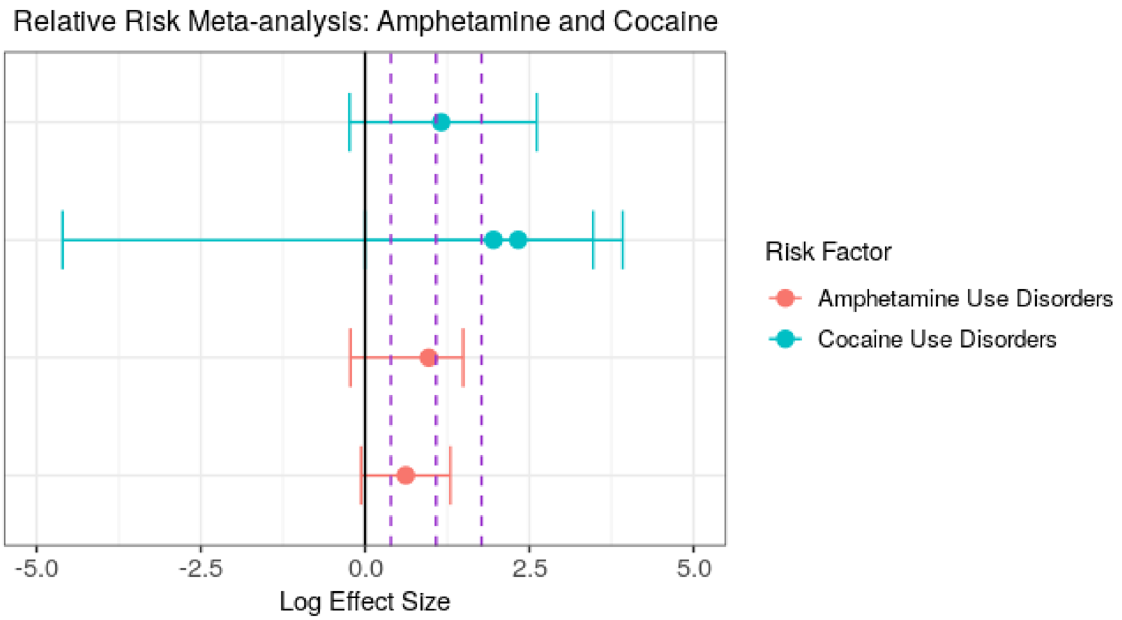

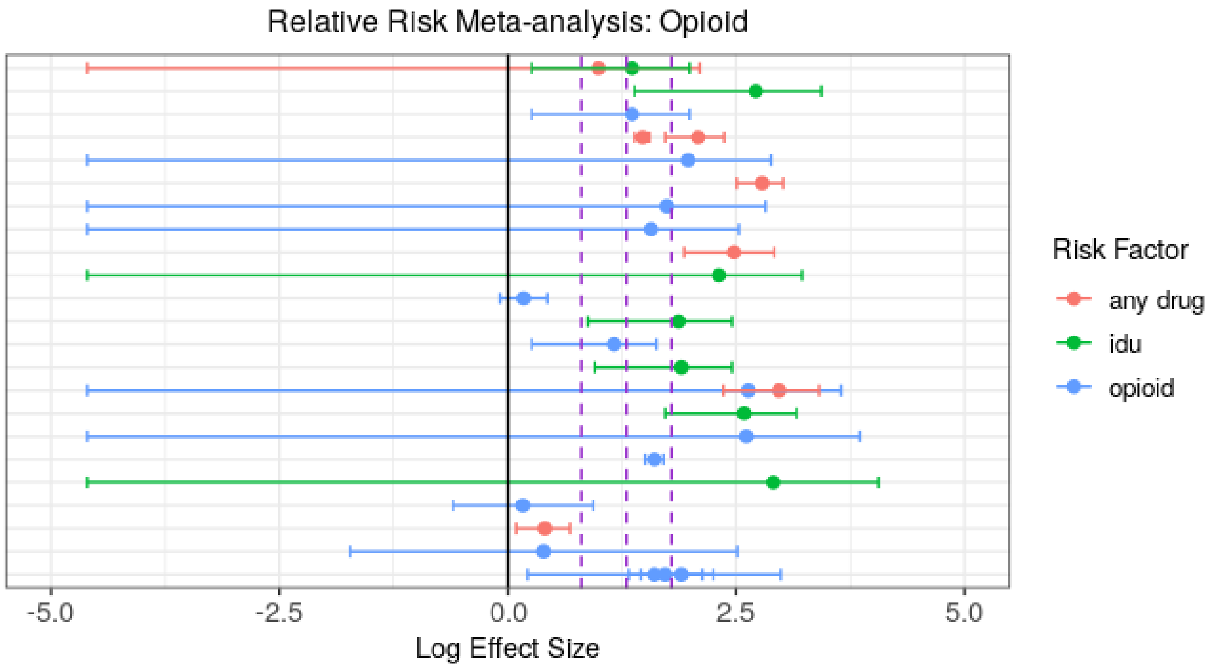

References

1. European Centre for Disease Prevention. HIV/AIDS surveillance in Europe 2014 Solna, Sweden. http://ecdc.europa.eu/en/publications/surveillance_reports/HIV_STI_and_blood_borne_viruses/Pages/HIV_STI_and_blood_borne_viruses.aspx: ECDC, 2014.
2. Family Health International, Bureau of AIDS TB and STIs Department of Disease Control. The Asian Epidemic Model (AEM) Projections for HIV/AIDS in Thailand:2005-2025. Bangkok: Family Health International (FHI) and Bureau of AIDS, TB and STIs, Department of Disease Control, Ministry of Public Health, Thailand, 2008.
3. Kirby Institute. 2015 Annual Surveillance Report of HIV, viral hepatitis, STIs. Sydney, New South Wales. https://kirby.unsw.edu.au/surveillance/2015-annual-surveillance-report-hiv-viral-hepatitis-stis: Kirby Institute, UNSW Australia, 2015.
4. Kirby Institute. Australian NSP survey national data report 2015. Sydney, New South Wales: Kirby Institute, University of New South Wales, 2015.
5. Country reports for Global AIDS Response Progress Reporting [Internet]. UNAIDS. 2014.
6. UNAIDS. UNAIDS Country reports. Geneva: Joint United Nations Programme on HIV/AIDS. http://www.unaids.org/en/regionscountries/countries, 2015.
7. United States Center for Disease Control and Prevention. HIV/AIDS Statistics. Atlanta, Georgia: US CDC. http://www.cdc.gov/hiv/statistics/index.html, 2015.
8. Gouws E, White PJ, Stover J, Brown T. Short term estimates of adult HIV incidence by mode of transmission: Kenya and Thailand as examples. Sex Transm Infect. 2006;82 Suppl 3:iii51-5.
9. Mathers BM, Degenhardt L, Phillips B, Wiessing L, Hickman M, Strathdee SA, et al. Global epidemiology of injecting drug use and HIV among people who inject drugs: a systematic review. Lancet. 2008;372(9651):1733-45.
10. Jackson JB, Wei L, Liping F, Aramrattana A, Celentano DD, Walshe L, et al. Prevalence and Seroincidence of Hepatitis B and Hepatitis C Infection in High Risk People Who Inject Drugs in China and Thailand. Hepatitis research and treatment. 2014;2014.
11. Månsson A-S, Moestrup T, Nordenfelt E, Widell A. Continued transmission of hepatitis B and C viruses, but no transmission of human immunodeficiency virus among intravenous drug users participating in a syringe/needle exchange program. Scandinavian Journal of Infectious Diseases. 2000;32(3):253-8.
12. Blomé MA, Björkman P, Flamholc L, Jacobsson H, Molnegren V, Widell A. Minimal transmission of HIV despite persistently high transmission of hepatitis C virus in a Swedish needle exchange program. Journal of viral hepatitis. 2011;18(12):831-9.
13. Hagan H, McGough JP, Thiede H, Weiss NS, Hopkins S, Alexander ER. Syringe exchange and risk of infection with hepatitis B and C viruses. American journal of epidemiology. 1999;149(3):203-13.
14. Crofts N, Aitken CK. Incidence of bloodborne virus infection and risk behaviours in a cohort of injecting drug users in Victoria in 1990-1995. Medical Journal of Australia. 1997;167(1):17-20.
15. Roy K, Goldberg D, Taylor A, Hutchinson S, MacDonald L, Wilson K, et al. A method to detect the incidence of hepatitis C infection among injecting drug users in Glasgow 1993–98. Journal of Infection. 2001;43(3):200-5.

16. Abou-Saleh M, Davis P, Rice P, Checinski K, Drummond C, Maxwell D, et al. The effectiveness of behavioural interventions in the primary prevention of hepatitis C amongst injecting drug users: a randomised controlled trial and lessons learned. Harm reduction journal. 2008;5(1):1.
17. Turner KM, Hutchinson S, Vickerman P, Hope V, Craine N, Palmateer N, et al. The impact of needle and syringe provision and opiate substitution therapy on the incidence of hepatitis C virus in injecting drug users: pooling of UK evidence. Addiction. 2011;106(11):1978-88.
18. Grebely J, Lima VD, Marshall BD, Milloy M, DeBeck K, Montaner J, et al. Declining incidence of hepatitis C virus infection among people who inject drugs in a Canadian setting, 1996-2012. PloS one. 2014;9(6):e97726.
19. Foley S, Abou-Saleh MT. Risk behaviors and transmission of hepatitis C in injecting drug users. Addictive Disorders & Their Treatment. 2009;8(1):13-21.
20. Craine N, Hickman M, Parry J, Smith J, Walker A, Russell D, et al. Incidence of hepatitis C in drug injectors: the role of homelessness, opiate substitution treatment, equipment sharing, and community size. Epidemiology and Infection. 2009;137(09):1255-65.
21. Villano SA, Vlahov D, Nelson KE, Lyles CM, Cohn S, Thomas DL. Incidence and risk factors for hepatitis C among injection drug users in Baltimore, Maryland. Journal of clinical microbiology. 1997;35(12):3274-7.
22. Maher L, Jalaludin B, Chant KG, Jayasuriya R, Sladden T, Kaldor JM, et al. Incidence and risk factors for hepatitis C seroconversion in injecting drug users in Australia. Addiction. 2006;101(10):1499-508.
23. Lucidarme D, Bruandet A, Ilef D, Harbonnier J, Jacob C, Decoster A, et al. Incidence and risk factors of HCV and HIV infections in a cohort of intravenous drug users in the North and East of France. Epidemiology and infection. 2004;132(04):699-708.
24. Partanen A, Malin K, Perälä R, Harju O, Holopainen A, Holmström P, et al. Riski-tutkimus 2000-2003. Pistämällä huumeita käyttävien seurantatutkimus. A-Klinikkasäätiön Raporttisarja nro 52. Helsinki: A-Klinikkasäätiön, 2006.
25. Van Den Berg C, Smit C, Van Brussel G, Coutinho R, Prins M. Full participation in harm reduction programmes is associated with decreased risk for human immunodeficiency virus and hepatitis C virus: evidence from the Amsterdam Cohort Studies among drug users. Addiction. 2007;102(9):1454-62.
26. Larney S, Kopinski H, Beckwith CG, Zaller ND, Jarlais DD, Hagan H, et al. Incidence and prevalence of hepatitis C in prisons and other closed settings: results of a systematic review and meta-analysis. Hepatology. 2013;58(4):1215-24.
27. Degenhardt L, Mathers B, Vickerman P, Rhodes T, Latkin C, Hickman M. Prevention of HIV infection for people who inject drugs: Why individual, structural, and combination approaches are needed. The Lancet. 2010;376:285-301.
28. Pavarin RM. Cocaine consumption and death risk: a follow-up study on 347 cocaine addicts in the metropolitan area of Bologna. Ann Ist Super Sanita. 2008; 44(1): 91-8.
29. Tyndall MW, Craib KJ, Currie S, Li K, O'Shaughnessy MV, Schechter MT. Impact of HIV infection on mortality in a cohort of injection drug users. J Acquir Immune Defic Syndr. 2001; 28(4): 351-7.
30. Miller CL, Kerr T, Strathdee SA, Li K, Wood E. Factors associated with premature mortality among young injection drug users in Vancouver. Harm Reduct J. 2007; 4: 1.

31. Galli M, Musicco M. Mortality of intravenous drug users living in Milan, Italy: role of HIV-1 infection. COMCAT Study Group. AIDS. 1994; 8(10): 1457-63.
32. Manfredi R, Sabbatani S, Agostini D. Trend of mortality observed in a cohort of drug addicts of the metropolitan area of Bologna, North-Eastern Italy, during a 25-year-period. Coll Antropol. 2006; 30(3): 479-88.
33. Eskild A, Magnus P, Samuelsen SO, Sohlberg C, Kittelsen P. Differences in mortality rates and causes of death between HIV positive and HIV negative intravenous drug users. Int J Epidemiol. 1993; 22(2): 315-20.
34. Ødegård E, Amundsen EJ, Kielland KB. Fatal overdoses and deaths by other causes in a cohort of Norwegian drug abusers – a competing risk approach. Drug Alcohol Depend. 2007; 89(2-3): 176-82.
35. Rossow I. Suicide among drug addicts in Norway. Addiction. 1994; 89(12): 1667-73.
36. Risser D, Hönigschnabl S, Stichenwirth M, Pfudl S, Sebald D, Kaff A, Bauer G. Mortality of opiate users in Vienna, Austria. Drug Alcohol Depend. 2001; 64(3): 251-6.
37. Bartu A, Freeman NC, Gawthorne GS, Codde JP, Holman CDJ. Mortality in a cohort of opiate and amphetamine users in Perth, Western Australia. Addiction. 2004; 99(1): 53-60.
38. Degenhardt L, Randall D, Hall W, Law M, Butler T, Burns L. Mortality among clients of a state-wide opioid pharmacotherapy program over 20 years: risk factors and lives saved. Drug Alcohol Depend. 2009; 105(1): 9–15.
39. Tait RJ, Ngo HTT, Hulse GK. Mortality in heroin users 3 years after naltrexone implant or methadone maintenance treatment. J Subst Abuse Treat. 2008; 35(2): 116-24.
40. Vlahov D, Galai N, Safaeian M, Galea S, Kirk GD, Lucas GM, Sterling TR. Effectiveness of highly active antiretroviral therapy among injection drug users with late-stage human immunodeficiency virus infection. Am J Epidemiol. 2005; 161(11): 999-1012.
41. Vlahov D, Wang C, Ompad D, Fuller CM, Caceres W, Ouellet L, Kerndt P, Jarlais DCD, Garfein RS, Collaborative Injection Drug User Study. Mortality risk among recent-onset injection drug users in five U.S. cities. Subst Use Misuse. 2008; 43(3-4): 413-28.
42. Oppenheimer E, Tobutt C, Taylor C, Andrew T. Death and survival in a cohort of heroin addicts from London clinics: a 22-year follow-up study. Addiction. 1994; 89(10): 1299-308.
43. Goldstein A, Herrera J. Heroin addicts and methadone treatment in Albuquerque: a 22-year follow-up. Drug Alcohol Depend. 1995; 40(2): 139-50.
44. Soyka M, Apelt SM, Lieb M, Wittchen H-U. One-year mortality rates of patients receiving methadone and buprenorphine maintenance therapy: a nationally representative cohort study in 2694 patients. J Clin Psychopharmacol. 2006; 26(6): 657-60.
45. Fugelstad A, Agren G, Romelsjö A. Changes in mortality, arrests, and hospitalizations in nonvoluntarily treated heroin addicts in relation to methadone treatment. Subst Use Misuse. 1998; 33(14): 2803-17.
46. Stenbacka M, Leifman A, Romelsjo A. Mortality Among Opiate Abusers in Stockholm: A Longitudinal Study. Heroin Addict Relate Clin Probl. 2007; 9(3): 41-50.
47. Fugelstad A, Annell A, Rajs J, Agren G. Mortality and causes and manner of death among drug addicts in Stockholm during the period 1981-1992. Acta Psychiatr Scand. 1997; 96(3): 169-75.

48. Antolini G, Pirani M, Morandi G, Sorio C. [Gender difference and mortality in a cohort of heroin users in the Provinces of Modena and Ferrara, 1975-1999]. Epidemiol Prev. 2006; 30(2): 91-9.
49. Digiusto E, Shakeshaft A, Ritter A, O'Brien S, Mattick RP, NEPOD Research Group. Serious adverse events in the Australian National Evaluation of Pharmacotherapies for Opioid Dependence (NEPOD). Addiction. 2004; 99(4): 450-60.
50. Brancato V, Delvecchio G, Simone P. [Survival and mortality in a cohort of heroin addicts in 1985-1994]. Minerva Med. 1995; 86(3): 97-9.
51. Wang C, Vlahov D, Galai N, Cole SR, Bareta J, Pollini R, Mehta SH, Nelson KE, Galea S. The effect of HIV infection on overdose mortality. AIDS. 2005; 19(9): 935-42.
52. Auckloo MBKM, Davies BB. Post-mortem toxicology in violent fatalities in Capte Town, South Africa: A preliminary investigation. J Foresnsic Leg Med. 2019; 63:18-25.
53. Brådvik L. Suicide risk and mental disorders. Int J Environ Res Publ Health. 2019; 15(9):2028.
54 Merrall E, Bird S, Hutchinson SJ. A record-linkage study of drug-related death and suicide after hospital discharge among drug-treatment clients in Scotland, 1996-2006. Addiction. 2012; 102(2).

Dietary risks

Flowchart

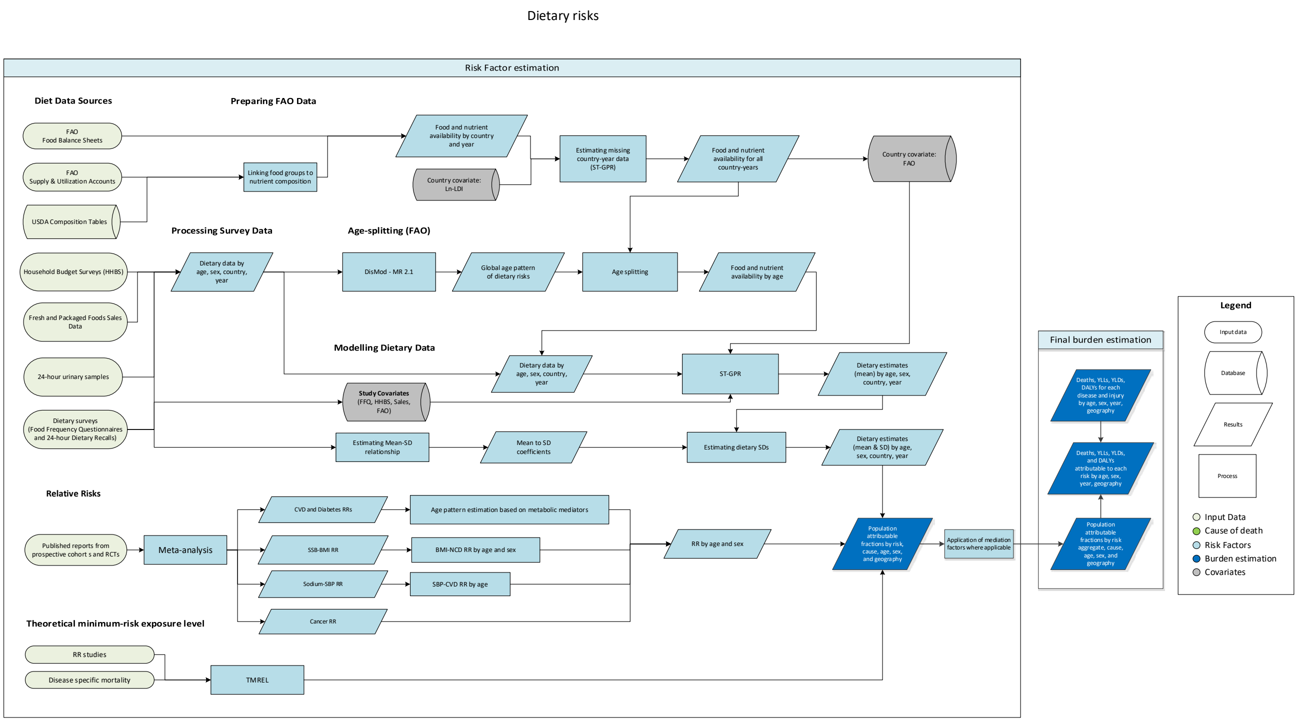

Input data and methodological summary

Definition

Exposure

Risk	Definition
**Diet low in fruit**	Average daily consumption (in grams per day) of less than 310-340 grams of fruit including fresh, frozen, cooked, canned, or dried fruit, excluding fruit juices and salted or pickled fruits
**Diet low in vegetables**	Average daily consumption (in grams per day) of less than 280-320 grams of vegetables, including fresh, frozen, cooked, canned, or dried vegetables and excluding legumes and salted or pickled vegetables, juices, nuts and seeds, and starchy vegetables such as potatoes or corn
**Diet low in whole grains**	Average daily consumption (in grams per day) of less than 140-160 grams of whole grains (bran, germ, and endosperm in their natural proportion) from breakfast cereals, bread, rice, pasta, biscuits, muffins, tortillas, pancakes, and other sources
**Diet low in nuts and seeds**	Average daily consumption (in grams per day) of less than 10-19 grams of nuts and seeds, including tree nuts and seeds and peanuts

**Diet low in fibre**	Average daily consumption (in grams per day) of less than 21-22 grams of fibre from all sources including fruits, vegetables, grains, legumes, and pulses
**Diet low in omega-3 fatty acids**	Average daily consumption (in milligrams per day) of less than 430-470 milligrams of eicosapentaenoic acid (EPA) and docosahexaenoic acid (DHA)
**Diet low in polyunsaturated fatty acids (PUFA)**	Average daily consumption (in % daily energy) of less than 7-9% total energy intake from polyunsaturated fatty acids
**Diet low in calcium**	Average daily consumption (in grams per day) of less than 1.06-1.1 grams of calcium from all sources, including milk, yogurt, and cheese
**Diet low in milk**	Average daily consumption (in grams per day) of less than 360-500 grams of milk including non-fat, low-fat, and full-fat milk, excluding soy milk and other plant derivatives
**Diet low in legumes**	Average daily consumption (in grams per day) of less than of 90-100 grams of legumes and pulses, including fresh, frozen, cooked, canned, or dried legumes
**Diet high in red meat**	Any intake (in grams per day) of red meat including beef, pork, lamb, and goat but excluding poultry, fish, eggs, and all processed meats
**Diet high in processed meat**	Any intake (in grams per day) of meat preserved by smoking, curing, salting, or addition of chemical preservatives
**Diet high in sugar-sweetened beverages (SSBs)**	Any intake (in grams per day) of beverages with ≥50 kcal per 226.8 gram serving, including carbonated beverages, sodas, energy drinks, fruit drinks, but excluding 100% fruit and vegetable juices
**Diet high in trans fatty acids**	Any intake (in percent daily energy) of trans fat from all sources, mainly partially hydrogenated vegetable oils and ruminant products
**Diet high in sodium**	Average 24-hour urinary sodium excretion (in grams per day) greater than 1-5 grams

Input data

In GBD 2019, we included new dietary recall sources from a literature search of PubMed and new sources from the IHME GHDx yearly known survey series updates in our models. We also conducted a new systematic review for sodium (Figure 1). As in GBD 2017, the dietary data that we use in the models comes from multiple sources, including nationally and subnationally representative nutrition surveys, household budget surveys, accounts of national sales from the Euromonitor, and availability data from the United Nations FAO Supply and Utilization Accounts (SUA). Table 1 below provides a summary of data inputs used for dietary risk modeling in GBD 2019.

Figure 1: PRISMA diagram for sodium intake data systematic review

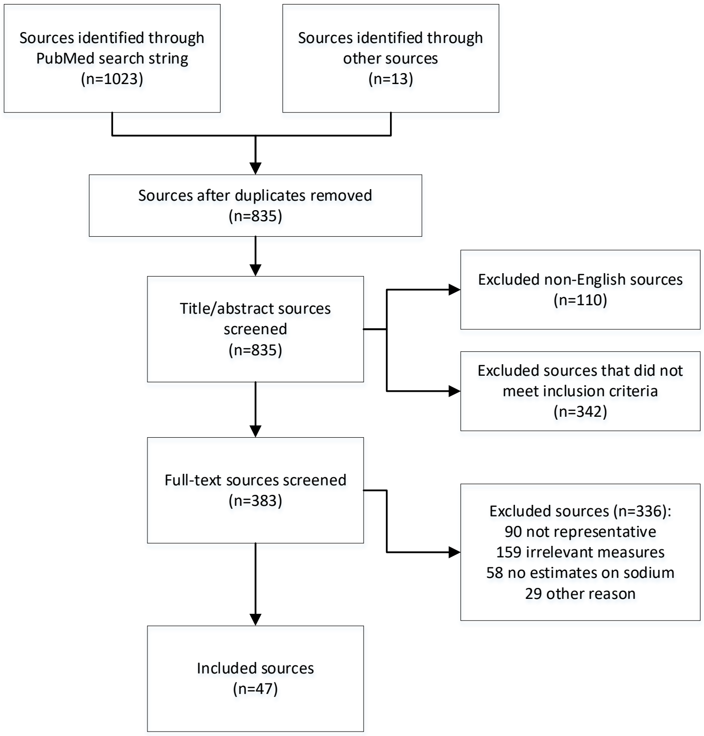

Table 1a: Data inputs for exposure for dietary risk factors.

Dietary risk factor	Total exposure sources	Countries with data
All dietary risks	1461	195
Calcium	160	178
Fiber	155	180
Fruit	869	180
Legumes	683	169
Milk	1148	177
Nuts and seeds	100	158
Omega 3	20	178
Processed meat	737	66
PUFA	70	180
Red meat	760	178
Sodium	92	53
SSBs	720	66
Trans fat	924	72
Vegetables	871	180
Whole grains	52	188

Table 1b: Data inputs for risk analysis for dietary risk factors.

Dietary risk factor	Total relative risk sources	Countries with data
Calcium	37	9
Fiber	64	16
Fruit	116	23
Legumes	10	5
Milk	12	8
Nuts and seeds	23	9
Omega 3	50	16
Processed meat	41	11
PUFA	18	8
Red meat	92	20
Sodium	21	6
SSBs	15	5
Transfat	10	5
Vegetable	39	11
Whole grains	37	9

The availability data for food groups in GBD were previously based on the FAO Food Balance Sheets (FBS), which provide tabulated and processed data of national food supply. In GBD 2019, to more accurately characterise the national availability of various food groups, we used more disaggregated data on food commodities that were included in FAO SUA and recreated the national availability of each food group based on the GBD definition of the food group. We modelled missing country-year data from FAO using a spatiotemporal Gaussian process regression and lag-distributed country income as the covariate. For nutrient availability, we continued to use data from Global Nutrient Database.[1]

For each dietary factor, we estimated the global age pattern of consumption based on nutrition surveys (ie, 24-hour diet recall) and applied that age pattern to the all-age data (availability, sales and household budget surveys) before the data source bias adjustment.

Our gold-standard data source for all dietary risks (except sodium) is 24-hour dietary recall surveys where food and nutrient intake are reported or convertible to grams per person per day; the gold-standard data source for sodium is 24-hour urinary sodium. The other data sources we use – household budget surveys, food frequency questionnaires, sales, and availability – are treated as alternate definitions for dietary intake and crosswalked to the gold-standard definition. In GBD 2016 and GBD 2017, we determined the bias adjustment factors from a mixed effects linear regression. In GBD 2019, we used MR-BRT (a network meta-regression) to determine the adjustment factors for non-gold-standard datapoints. Coefficients for these models can be found in Table 3.

Table 2. Types of data sources (other than 24-hour dietary recall) and covariates used in modelling of each dietary factor.

	Data sources				Country-level covariate
	Sales	FFQ[1]	HBS[2]	FAO	
Diet low in fruits	●	●	●	●	Lag distributed income
Diet low in vegetables	●	●	●	●	Energy availability (kcal)
Diet low in whole grains	-	●	-	●	Energy availability (kcal)
Diet low in nuts and seeds	-	-	●	●	Energy availability (kcal)
Diet low in milk	●	●	●	●	Energy availability (kcal)
Diet high in red meat	●	●	●	●	Energy availability (kcal)
Diet high in processed meat	●	●	●	-	Energy availability (kcal), pigs per capita
Diet low in legumes	●	●	-	●	Energy availability (kcal)
Diet high in sugar-sweetened beverages	●	●	●	-	Energy availability (kcal), availability of sugar
Diet low in fibre	-	●	-	●	Energy availability (kcal)
Diet suboptimal in calcium	-	●	-	●	Energy availability (kcal)
Diet low in seafood omega-3 fatty acids	-	-	-	●	Lag distributed income, proportion landlocked area
Diet low in polyunsaturated fatty acids	-	●	-	●	Lag distributed income
Diet high in trans fatty acids	●	●	-	-	
Diet high in sodium[3]	-	-	-	-	

[1] Food Frequency Questionnaire
[2] Household Budge Survey
[3] For sodium, we used data from the 24-hour urinary sodium and 24-hour dietary recall.

Table 3: MR-BRT crosswalk adjustment factors for all dietary risks

Dietary risk	Sex	Data input	Reference or alternative case definition	Gamma	Beta coefficient, log (95% CI)	Adjustment factor*
Calcium	---	DR	Ref	0.24	---	---
Calcium	Female	FAO	Alt		0.04 (0.04, 0.5)	0.96 (0.64, 1.65)
Calcium	Female	FFQ	Alt		-0.04 (-0.04, 0.43)	1.04 (0.59, 1.53)
Calcium	Male	FAO	Alt		0.17 (0.17, 0.63)	0.84 (0.73, 1.88)
Calcium	Male	FFQ	Alt		0.09 (0.09, 0.55)	0.91 (0.67, 1.74)
Fibre	---	DR	Ref	0.33	---	---
Fibre	Female	FAO	Alt		0.56 (0.56, 1.17)	0.57 (0.93, 3.23)
Fibre	Female	FFQ	Alt		0.27 (0.27, 0.88)	0.76 (0.69, 2.41)
Fibre	Male	FAO	Alt		0.55 (0.55, 1.17)	0.57 (0.92, 3.22)
Fibre	Male	FFQ	Alt		0.26 (0.26, 0.88)	0.77 (0.69, 2.4)
Fruit	---	DR	Ref	0.76	---	---
Fruit	Female	FAO	Alt		0.36 (0.36, 1.83)	0.7 (0.31, 6.21)
Fruit	Female	Sales	Alt		0.73 (0.73, 2.19)	0.48 (0.45, 8.98)

Fruit	Female	FFQ	Alt		-0.15 (-0.15, 1.32)	1.17 (0.19, 3.73)
Fruit	Female	HHBS	Alt		0.23 (0.23, 1.71)	0.79 (0.27, 5.5)
Fruit	Male	FAO	Alt		0.32 (0.32, 1.79)	0.73 (0.3, 5.97)
Fruit	Male	Sales	Alt		0.69 (0.69, 2.16)	0.5 (0.43, 8.64)
Fruit	Male	FFQ	Alt		-0.19 (-0.19, 1.28)	1.21 (0.18, 3.58)
Fruit	Male	HHBS	Alt		0.19 (0.19, 1.66)	0.83 (0.26, 5.27)
Legumes	---	DR	Ref		---	---
Legumes	Female	FAO	Alt		-0.08 (-1.49,1.39)	1.08 (0.22,4)
Legumes	Female	Sales	Alt		-0.9 (-2.31,0.56)	2.47 (0.1,1.75)
Legumes	Female	FFQ	Alt	0.74	-0.53 (-1.94,0.95)	1.7 (0.14,2.58)
Legumes	Male	FAO	Alt		0.06 (-1.35,1.53)	0.94 (0.26,4.61)
Legumes	Male	Sales	Alt		-0.76 (-2.16,0.7)	2.14 (0.12,2.01)
Legumes	Male	FFQ	Alt		-0.39 (-1.79,1.09)	1.47 (0.17,2.98)
Milk	---	DR	Ref		---	---
Milk	Female	FAO	Alt		0.27 (0.27, 2.57)	0.76 (0.16, 13.01)
Milk	Female	Sales	Alt		0.01 (0.01, 2.31)	0.99 (0.13, 10.11)
Milk	Female	FFQ	Alt		0.46 (0.46, 2.78)	0.63 (0.18, 16.2)
Milk	Female	HHBS	Alt	1.06	-0.61 (-0.61, 1.69)	1.84 (0.07, 5.4)
Milk	Male	FAO	Alt		0.28 (0.28, 2.58)	0.75 (0.17, 13.17)
Milk	Male	Sales	Alt		0.03 (0.03, 2.33)	0.97 (0.13, 10.23)
Milk	Male	FFQ	Alt		0.48 (0.48, 2.8)	0.62 (0.18, 16.43)
Milk	Male	HHBS	Alt		-0.59 (-0.59, 1.7)	1.81 (0.07, 5.48)
Nuts	---	DR	Ref		---	---
Nuts	Female	FAO	Alt		0.49 (0.49, 3.63)	0.62 (0.06, 37.68)
Nuts	Female	FFQ	Alt		-0.34 (-0.34, 2.76)	1.41 (0.02, 15.75)
Nuts	Female	HHBS	Alt	1.58	-0.72 (-0.72, 2.42)	2.06 (0.02, 11.27)
Nuts	Male	FAO	Alt		0.6 (0.6, 3.73)	0.55 (0.07, 41.65)
Nuts	Male	FFQ	Alt		-0.23 (-0.23, 2.87)	1.26 (0.03, 17.58)
Nuts	Male	HHBS	Alt		-0.62 (-0.62, 2.54)	1.85 (0.02, 12.66)
Omega-3	---	DR	Ref		---	---
Omega-3	Male	FAO	Alt	0.12	-1.15 (-1.15, -0.92)	3.16 (0.25, 0.4)
Omega-3	Female	FAO	Alt		-1.01 (-1.01, -0.78)	2.75 (0.29, 0.46)
Proc. meat	---	DR	Ref		---	---
Proc. meat	Female	Sales	Alt		0.79 (0.79, 3.14)	0.46 (0.19, 23.07)
Proc. meat	Female	FFQ	Alt		-0.3 (-0.3, 2.25)	1.35 (0.05, 9.49)
Proc. meat	Female	HHBS	Alt	1.21	-0.46 (-0.46, 1.89)	1.59 (0.05, 6.63)
Proc. meat	Male	Sales	Alt		0.95 (0.95, 3.3)	0.39 (0.22, 27.03)
Proc. meat	Male	FFQ	Alt		-0.13 (-0.13, 2.42)	1.14 (0.06, 11.2)
Proc. meat	Male	HHBS	Alt		-0.3 (-0.3, 2.06)	1.35 (0.06, 7.82)
PUFA	---	DR	Ref		---	---
PUFA	Female	FAO	Alt	0.14	-0.14 (-0.14, 0.14)	1.15 (0.65, 1.15)
PUFA	Female	FFQ	Alt		1.05 (1.05, 1.43)	0.35 (1.96, 4.18)
PUFA	Male	FAO	Alt		-0.18 (-0.18, 0.1)	1.2 (0.62, 1.1)

PUFA	Male	FFQ	Alt		1 (1, 1.38)	0.37 (1.87, 3.98)
Red meat	---	DR	Ref		---	---
Red meat	Female	FAO	Alt		0.89 (0.89, 2.54)	0.41 (0.45, 12.69)
Red meat	Female	Sales	Alt		1.09 (1.09, 2.74)	0.34 (0.54, 15.49)
Red meat	Female	FFQ	Alt		-0.34 (-0.34, 1.6)	1.4 (0.11, 4.95)
Red meat	Female	HHBS	Alt	0.83	0.45 (0.45, 2.1)	0.64 (0.29, 8.18)
Red meat	Male	FAO	Alt		0.89 (0.89, 2.54)	0.41 (0.45, 12.66)
Red meat	Male	Sales	Alt		1.09 (1.09, 2.74)	0.34 (0.54, 15.43)
Red meat	Male	FFQ	Alt		-0.34 (-0.34, 1.6)	1.4 (0.11, 4.94)
Red meat	Male	HHBS	Alt		0.45 (0.45, 2.1)	0.64 (0.29, 8.15)
Sodium	---	Urinary sodium	Ref		---	---
Sodium	Female	DR	Alt	0.39	-0.02 (-0.02, 0.85)	1.02 (0.38, 2.34)
Sodium	Female	FFQ	Alt		0.47 (0.47, 1.29)	0.63 (0.69, 3.64)
Sodium	Male	DR	Alt		-0.06 (-0.06, 0.8)	1.06 (0.38, 2.23)
Sodium	Male	FFQ	Alt		0.43 (0.43, 1.26)	0.65 (0.67, 3.52)
SSBs	---	DR	Ref		---	---
SSBs	Female	Sales	Alt		0.15 (0.15, 1.43)	0.86 (0.37, 4.17)
SSBs	Female	FFQ	Alt		-0.01 (-0.01, 1.32)	1.01 (0.3, 3.75)
SSBs	Female	HHBS	Alt	0.61	-0.59 (-0.59, 0.68)	1.8 (0.18, 1.98)
SSBs	Male	Sales	Alt		0.35 (0.35, 1.63)	0.7 (0.45, 5.1)
SSBs	Male	FFQ	Alt		0.19 (0.19, 1.53)	0.83 (0.37, 4.6)
SSBs	Male	HHBS	Alt		-0.39 (-0.39, 0.89)	1.48 (0.22, 2.43)
Trans fat	---	DR	Ref		---	---
Trans fat	Male	Sales	Alt		-0.23 (-1.27,0.94)	1.25 (0.28, 2.55)
Trans fat	Female	Sales	Alt	0.22	-0.23 (-1.27,0.94)	1.25 (0.28, 2.55)
Trans fat	Male	FFQ	Alt		0.59 (-2.72,4.23)	0.56 (0.07,68.72)
Trans fat	Female	FFQ	Alt		0.86 (-2.63,4.9)	0.42 (0.07,134.0)
Vegetables	---	DR	Ref		---	---
Vegetables	Female	FAO	Alt		0.12 (0.12, 1.33)	0.89 (0.31, 3.78)
Vegetables	Female	Sales	Alt		0.62 (0.62, 1.83)	0.54 (0.51, 6.21)
Vegetables	Female	FFQ	Alt		-0.05 (-0.05, 1.16)	1.05 (0.26, 3.18)
Vegetables	Female	HHBS	Alt	0.64	0.1 (0.1, 1.31)	0.91 (0.3, 3.69)
Vegetables	Male	FAO	Alt		0.16 (0.16, 1.37)	0.85 (0.32, 3.94)
Vegetables	Male	Sales	Alt		0.66 (0.66, 1.87)	0.52 (0.53, 6.49)
Vegetables	Male	FFQ	Alt		-0.01 (-0.01, 1.2)	1.01 (0.27, 3.32)
Vegetables	Male	HHBS	Alt		0.14 (0.14, 1.35)	0.87 (0.32, 3.85)
Whole grains	---	DR	Ref		---	---
Whole grains	Female	FAO	Alt		1.94 (1.94, 3.37)	0.14 (1.82, 29.05)
Whole grains	Female	FFQ	Alt	0.69	-0.35 (-0.35, 1.37)	1.42 (0.13, 3.94)
Whole grains	Male	FAO	Alt		2.09 (2.09, 3.52)	0.12 (2.12, 33.76)
Whole grains	Male	FFQ	Alt		-0.2 (-0.2, 1.52)	1.22 (0.15, 4.58)

**Adjustment factor is the transformed beta coefficient in normal space and can be interpreted as the factor by which the alternative case definition is adjusted to reflect what it would have been if measured as the reference.*

Modelling strategy

Exposure model

We use a spatiotemporal Gaussian process regression (ST-GPR) framework to estimate the mean intake of each dietary factor by age, sex, country, and year. In GBD 2019, we removed lag-distributed income as a covariate from most of our models and added country-level energy availability (Table 2).

To characterise the distribution of each dietary factor at the population level, we use an ensemble approach that separately fit 12 distributions for individual-level microdata to specific to each data source's sampled population. The respective goodness of fit of each family was assessed, and a weighting scheme was determined to optimise overall fit to the unique distribution of each risk factor. A global mean of the weights for each risk factor's data sources was created. We then determined the standard deviation of each population's consumption through a linear regression that captured the relationship between the standard deviation and mean of intake in nationally representative nutrition surveys using 24-hour diet recalls:

$$ln\ (Standard\ deviation) = \beta_0 + \beta_1 \times ln\ (Mean_i)$$

Then we applied the coefficients of this regression to the outputs of our ST-GPR model to calculate the standard deviation of intake by age, sex, year, and country. We also quantified the within-person variation in consumption of each dietary component and adjusted the standard deviations accordingly.

Theoretical minimum-risk exposure level

The dietary TRMELs were updated for GBD 2019. For harmful dietary risks other than sodium, TMREL was set to zero. For protective dietary risk factors, we first calculated the level of intake associated with the lowest risk of mortality from each disease endpoint based on the 85th percentile of intake across all epidemiological studies included in the meta-analysis of the risk-outcome pair. Then we calculated the TMREL as the weighted average of these numbers using the global number of deaths from each outcome as the weight.

Table 4. **Theoretical minimum-risk exposure level for dietary factors, GBD 2017 and GBD 2019**

**Dietary factor**	**G[illegible]**
Fruits	2[illegible]
Vegetables	2[illegible]
Whole grains	1[illegible]
Nuts	1[illegible]
Red meats	1[illegible]
Processed meats	0-[illegible]

Milk	35
Legumes	50
Sugar sweetened beverages	0-
Polyunsaturated fatty acids	9-
Seafood omega-3 fatty acids	20
Trans fatty acids	0-
Dietary fibre	19
Dietary calcium	1.
Dietary sodium	1-

Relative risks

For GBD 2019, we performed systematic reviews for each dietary risk and its related outcomes. Using the sources identified during these searches, we incorporated the most recent epidemiological evidence assessing the relationship between each GBD dietary risk factor and related outcomes in our relative risk analysis. After evaluating all available evidence, we found sufficient evidence on the casual relationship for 8 new R-O pairs and insufficient evidence for 5 old R-O pairs. Based on these results, we updated the R-O pairs used the GBD dietary risk factor analysis in the following ways:

Removed:

- Diet low in fruit and nasopharynx cancer
- Diet low in fruit and other pharynx cancer
- Diet low in fruit and oesophageal cancer
- Diet low in fruit and larynx cancer
- Diet low in whole grains and haemorrhagic stroke

Added:

- Diet low in whole grains and colon and rectum cancer
- Diet high in red meat and breast cancer
- Diet high in red meat and ischaemic heart disease
- Diet high in red meat and haemorrhagic stroke
- Diet high in red meat and ischaemic stroke
- Diet low in fibre and ischaemic stroke
- Diet low in fibre and haemorrhagic stroke
- Diet low in fibre and diabetes mellitus

Additionally, based on the most recent epidemiological evidence and GBD 2019 newly developed methods for characterising the risk curve, we updated the dose-response curve of relative risks for all dietary risks. For sodium, we continued to estimate its effect on cardiovascular disease based on the effect of sodium on systolic blood pressure.

There is a well-documented attenuation of the risk for cardiovascular disease due to metabolic risks factors throughout one's life. To incorporate this age trend in the relative risks, we first identified the

median age-at-event across all cohorts and considered that as the reference age group. We then assigned our newly estimated risk curves to this reference age group. Then, we derived the percentage change in relative risks between each age group and the reference age group by averaging percentage changes in relative risks of all metabolic mediators. The three cardiovascular disease outcomes for dietary risks are haemorrhagic stroke (including intracerebral hemmorhage and subarachnoid hemmorhage), ischaemic stroke, and ischaemic heart disease, and the effects of dietary risks on them are mediated through high systolic blood pressure, cholesterol (not included for haemorrhagic stroke), and fasting plasma glucose. Since the effect of diet is estimated independently of body-mass index (BMI) in the GBD, BMI was not included as a mediator in the RR age trend analysis.

Citations

1. Schmidhuber, Josef, et al. The Global Nutrient Database: Availability of Macronutrients and Micronutrients in 195 Countries from 1980 to 2013. *The Lancet Planetary Health*, vol. 2, no. 8, 2018, doi:10.1016/s2542-5196(18)30170-0.

Intimate partner violence

Flowchart

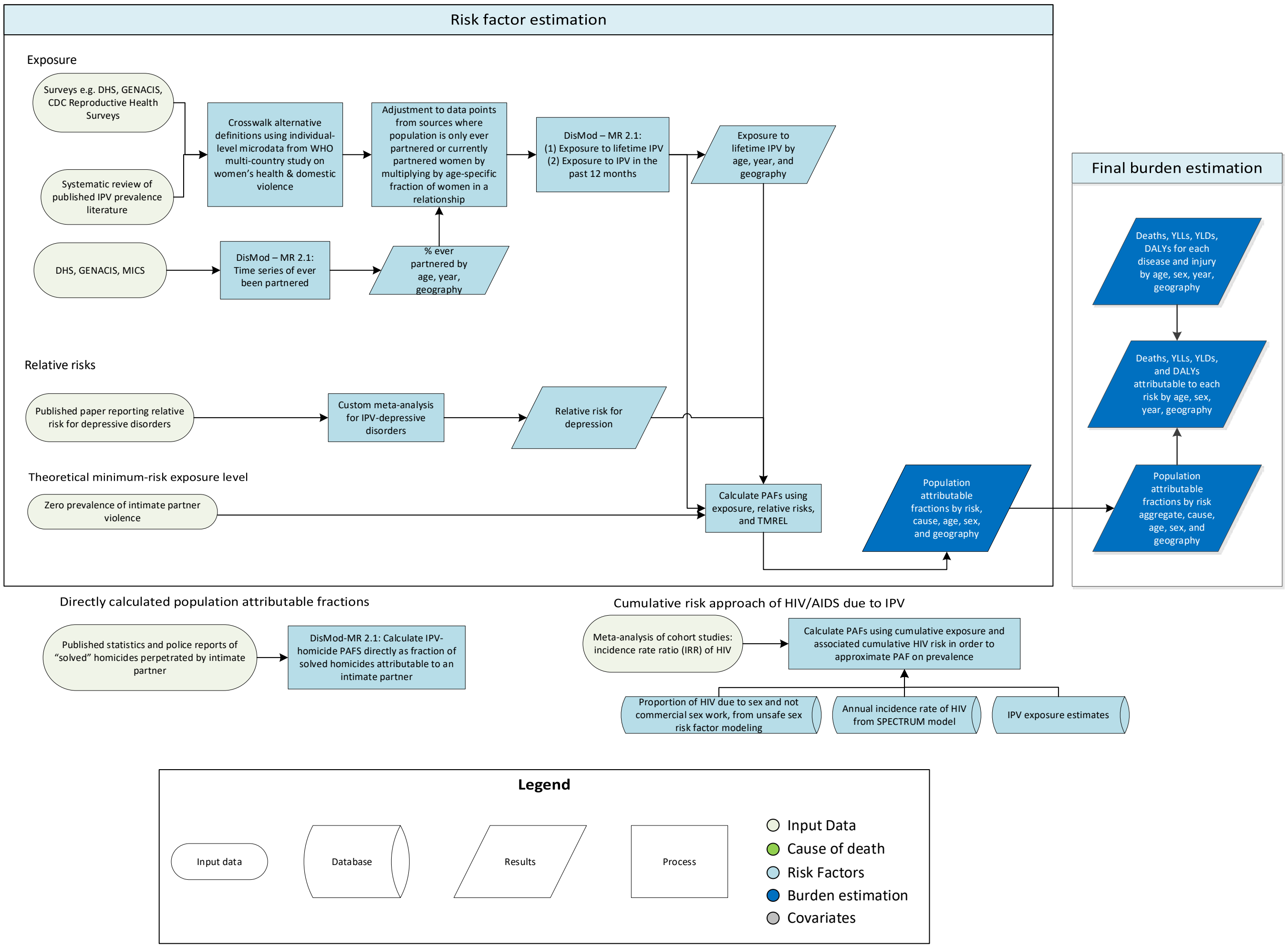

Input data and methodological summary

Definition

Exposure

The case definition for intimate partner violence (IPV) is ever experienced one or more acts of physical and/or sexual violence by a current or former intimate partner since the age of 15 years. Estimated in females only because evidence of risk-outcomes for males does not meet our criteria.

- Physical violence is defined as "being slapped or having something thrown at you that could hurt you, being pushed or shoved, being hit with a fist or something else that could hurt, being kicked, dragged, or beaten up, being choked or burnt on purpose, and/or being threatened with or actually having a gun, knife, or other weapon used on you."

- Sexual violence is defined as “being physically forced to have intercourse when you did not want to, having sexual intercourse because you were afraid of what your partner might do, and/or being forced to do something that you found humiliating or degrading” (the definition of humiliating and degrading may vary across studies depending on the regional and cultural setting).
- Intimate partner is defined as “a partner to whom you are married or with whom you cohabit.” In countries where people date, dating partners will also be considered (a partner with whom you have an intimate [sexual] relationship with but are not married to or cohabiting).

Theoretical Minimum Risk Exposure Level

The associated Theoretical Minimum Risk Exposure Level (TMREL) is zero exposure to IPV.

Input data

In addition to incorporating new sources shared with us by collaborators for both our IPV exposure model and our IPV direct PAF model (described in Modelling strategy), we conducted a systematic review of the fraction of homicides against women attributable to an intimate partner for GBD 2019 in Pubmed and EMBASE. We used the following search strings:

PubMed

((IPV[All Fields] OR ("intimate partner violence"[MeSH Terms] OR ("intimate"[All Fields] AND "partner"[All Fields] AND "violence"[All Fields]) OR "intimate partner violence"[All Fields])) AND (("homicide"[MeSH Terms] OR "homicide"[All Fields]) OR femicide[All Fields])) AND ("2013/01/01"[PDAT] : "3000/12/31"[PDAT])

Date searched: 12/25/2018
Number of hits: 155

EMBASE

((‘IPV’:ti,ab OR (‘partner violence’/syn OR (‘intimate’:ti,ab AND ‘partner’:ti,ab AND ‘violence’:ti,ab) OR ‘intimate partner violence’:ti,ab)) AND ((‘homicide’/syn OR ‘homicide’:ti,ab) OR ‘femicide’:ti,ab)) AND [2013-3000]/py

Date searched: 01/03/2019
Number of hits: 199
Number of hits duplicate with pubmed search: 109

Of these, 44 passed a title and abstract review, leading to nine sources passing a full text review and being extracted.

We included all sources that provided population-representative data on the proportion of women who have ever experienced physical or sexual violence by a current or former intimate partner. We also accepted sources reporting on the following non-reference populations:

1. Women who have ever experienced any physical IPV
2. Women who have ever experienced any sexual IPV
3. Women who have ever experienced severe IPV
4. Women who have experienced IPV in the past year

5. Women who have ever had an intimate partner who have experienced physical or sexual IPV
6. Women who currently have an intimate partner who have experienced IPV
7. Women who have experienced intimate partner violence by a spouse
8. Women who have experienced intimate partner violence by a current spouse

For the alternate definitions of IPV (just physical, just sexual, and severe), we ran a logit-difference meta-regression with the MR-BRT tool to estimate correction factors.

Table 1: Data inputs for exposure for intimate partner violence.

Input data	Exposure
Source count (total)	601
Number of countries with data	132

Table 2: Data inputs for relative risks for intimate partner violence.

Input data	Relative risk
Source count (total)	9
Number of countries with data	7

Table 3: MR-BRT adjustment factors for restricted definitions of intimate partner violence

Data input	Status	Gamma	Beta coefficient, logit (95% CI)	Adjustment factor*
Women who have ever experienced any physical or sexual IPV	Ref	0.21	---	---
Women who have ever experienced any physical IPV	Alt		-0.43 (-0.85 to -0.0042)	0.39
Women who have ever experienced any severe IPV	Alt		-0.44 (-0.86 to -0.02)	0.39
Women who have ever experienced any sexual IPV	Alt		-0.89 (-1.32 to -0.47)	0.29

**Adjustment factor is the inverse-logit transformed beta coefficient; <0.5 represents that alternative is adjusted upward; >0.5 represents alternative is adjusted downward.*

For sources using one-year instead of lifetime recall, we used a logit-difference regression with a cubic spline on age to estimate age-specific correction factors. We introduced these methods in lieu of a random effects meta regression, as was used in GBD 2017.

Graph 1: MR-BRT adjustment factors for intimate partner violence in the past year

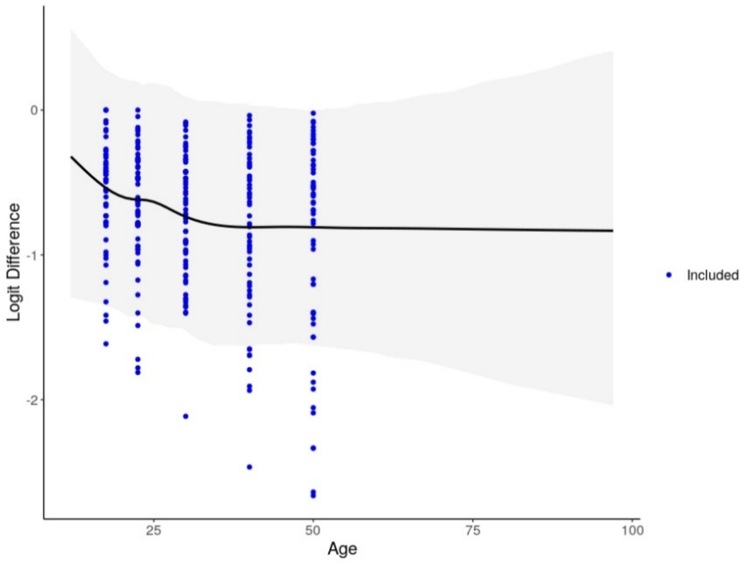

To correct for studies reporting only on ever or currently partnered women, we multiplied estimates from these studies by the age-specific fraction of women who had ever been partnered. We generated ever-partnered estimates using MICS and DHS data in a single parameter DisMod model to reflect the most recent data on proportion of women who have ever been partnered.

For studies restricting the perpetrator to spouses or current spouses, due to insufficient data comparing our reference and alternate populations in specific age-location-years, we refrained from calculating under-informed correction factors.

Modelling strategy

We use three distinct approaches to estimate burden attributable to IPV, including 1) the traditional exposure and relative risk (RR) to percentage attributable fraction (PAF) method for depression; 2) the direct PAF approach for estimating the proportion of homicides that are perpetrated by an intimate partner; and 3) a cumulative risk approach for estimating the burden of HIV/AIDS attributable to IPV. Note that while we estimated the PAF of abortion attributable to IPV for GBD 2017, we stopped calculating this PAF for GBD 2019, due to insufficient evidence of a causal relationship between the risk and outcome.

Estimating attributable burden to IPV for depression

We estimated exposure as a DisMod model. We first age-split the data, using a regression developed with MR-BRT, before making bias-adjustments for alternate case definitions, as described above. The prepped data were then run as a single-parameter prevalence model in DisMod-MR 2.1.

Direct PAF for female homicides

The burden of homicides attributable to intimate partner violence was modelled as a direct PAF as data sources provide the direct measurement of proportion of homicide cases where and intimate partner was the perpetrator. Data were run directly into a single-parameter proportion DisMod-MR 2.1 model to produce these estimates.

Cumulative risk approach for PAF of HIV/AIDS due to IPV

The third and final modelling approach that we used to assess burden attributable to intimate partner violence was a cumulative risk approach to measure the burden of HIV/AIDS attributable to IPV.

The approach itself remained the same in GBD 2019 but included newly estimated relative risk, updated intimate partner violence exposure numbers from the DisMod-MR 2.1 model described above, as well as revised HIV incidence numbers.

As we measure burden based on deaths and prevalence, we needed to quantify attributable fractions for prevalence and death rather than incidence. To get a PAF for prevalence we needed to consider the history of exposure to IPV and the accumulated associated risk of incident HIV due to IPV, relative to the overall risk of HIV at the population level. The ratio of cumulative IPV-attributable HIV incidence to total HIV incidence was an approximation of the relevant PAF for HIV prevalence and we assumed this PAF can also be applied to mortality.

$$\frac{Cumulative\ HIV\ incidence\ due\ to\ IPV}{Cumulative\ HIV\ incidence\ overall} = \frac{1 - \prod_{a=o}^{a=n}\left(1 - PAF_{ay} * I_{ay}\right)}{1 - \prod_{a=o}^{a=n}\left(1 - I_{ay}\right)}$$

where:

I = annual incidence rate of HIV

a = age (15-95)

y = year (1980-2016)

$$PAF_{HIV\ incidence} = \frac{[Prevalence\ of\ IPV]_{ay} * (IRR-1)}{[Prevalence\ of\ IPV]_{ay} * (IRR-1)+1}$$

Theoretical minimum-risk exposure level

The theoretical minimum-risk exposure level is zero exposure to intimate partner violence, as defined above.

Relative risk

Depression

In addition to the six cohort studies used in GBD 2017 (Ackard DM et al, J Pediatr. 2007, Chowhary et al, J Obstet Gynaecol Can 2007, Lipsky S et al, Womens Health Issues 2009, 9 Loxton D et al, J Interpers Violence 2006, Suglia SF et al, J Urban Health 2011 & Ouellet-Morin et al, Depression and anxiety 2015), we incorporated a new longitudinal study (Han et al, Journal of Affective Disorders 2019) to calculate the relative risk of depression as 1.54 (95% UI 1.00–2.36) using MR-BRT.

HIV incidence

From two cohort studies (Jewkes et al, Lancet 2010 & Kouyoumdijian et al, AIDS 2013) we calculated the relative risk of HIV incidence as 1.60 (95% UI 1.31–1.93) using a regression with MR-BRT.

Citations

1. Ackard DM, Eisenberg ME, Neumark-Sztainer D. Long-term impact of adolescent dating violence on the behavioral and psychological health of male and female youth. J Pediatr. 2007; 151(5): 476-81.
2. Bourassa D, Bérubé J. The prevalence of intimate partner violence among women and teenagers seeking abortion compared with those continuing pregnancy. J Obstet Gynaecol Can. 2007; 29(5): 415-23.
3. Chowdhary N, Patel V. The effect of spousal violence on women's health: findings from the Stree Arogya Shodh in Goa, India. J Postgrad Med. 2008; 54(4): 306–12.
4. Han KM, Jee HJ, An H, Shin C, Yoon HK, Ko YH, Ham BJ, Kim YK, Han C. Intimate partner violence and incidence of depression in married women: A longitudinal study of a nationally representative sample. J Affect Disord. 2019; 245():305-311
5. Jewkes RK, Dunkle K, Nduna M, Shai N. Intimate partner violence, relationship power inequity, and incidence of HIV infection in young women in South Africa: a cohort study. Lancet. 2010; 41-48.
6. Kouyoumdjian FG, Calzavara LM, Bondy SJ, O'Campo P, Serwadda D, Nalugoda F, Kagaayi J, Kigozi G, Wawer M, Gray R. Intimate partner violence is associated with incident HIV infection in women in Uganda. AIDS. 2013; 27(8): 1331-8.
7. Leung TW, Leung WC, Chan PL, Ho PC. A comparison of the prevalence of domestic violence between patients seeking termination of pregnancy and other general gynecology patients. Int J Gynaecol Obstet. 2002; 77(1): 47-54.
8. Lipsky S, Caetano R, Roy-Byrne P. Racial and ethnic disparities in police-reported intimate partner violence and risk of hospitalization among women. Womens Health Issues. 2009; 19(2):109–118.
9. Loxton D, Schofield M, Hussain R. Psychological health in midlife among women who have ever lived with a violent partner or spouse. J Interpers Violence. 2006; 21(8): 1092-107.
10. Ouellet-Morin I, Fisher HL, York-Smith M, Fincham-Campbell S, Moffitt TE, Arseneault L. Intimate partner violence and new-onset depression: a longitudinal study of women's childhood and adult histories of abuse. Depression and anxiety. 2015;32(5):316-324.
11. Romito P, Escribà-Agüir V, Pomicino L, Lucchetta C, Scrimin F, Molzan Turan J. Violence in the lives of women in Italy who have an elective abortion. Womens Health Issues. 2009; 19(5): 335-43.
12. Suglia SF, Duarte CS, Sandel MT. Housing quality, housing instability, and maternal mental health. J Urban Health. 2011; 88(6): 1105–16.
13. Taft AJ, Watson LF. Termination of pregnancy: associations with partner violence and other factors in a national cohort of young Australian women. Aust N Z J Public Health. 2007; 31(2): 135-42.

Childhood sexual abuse

Flowchart

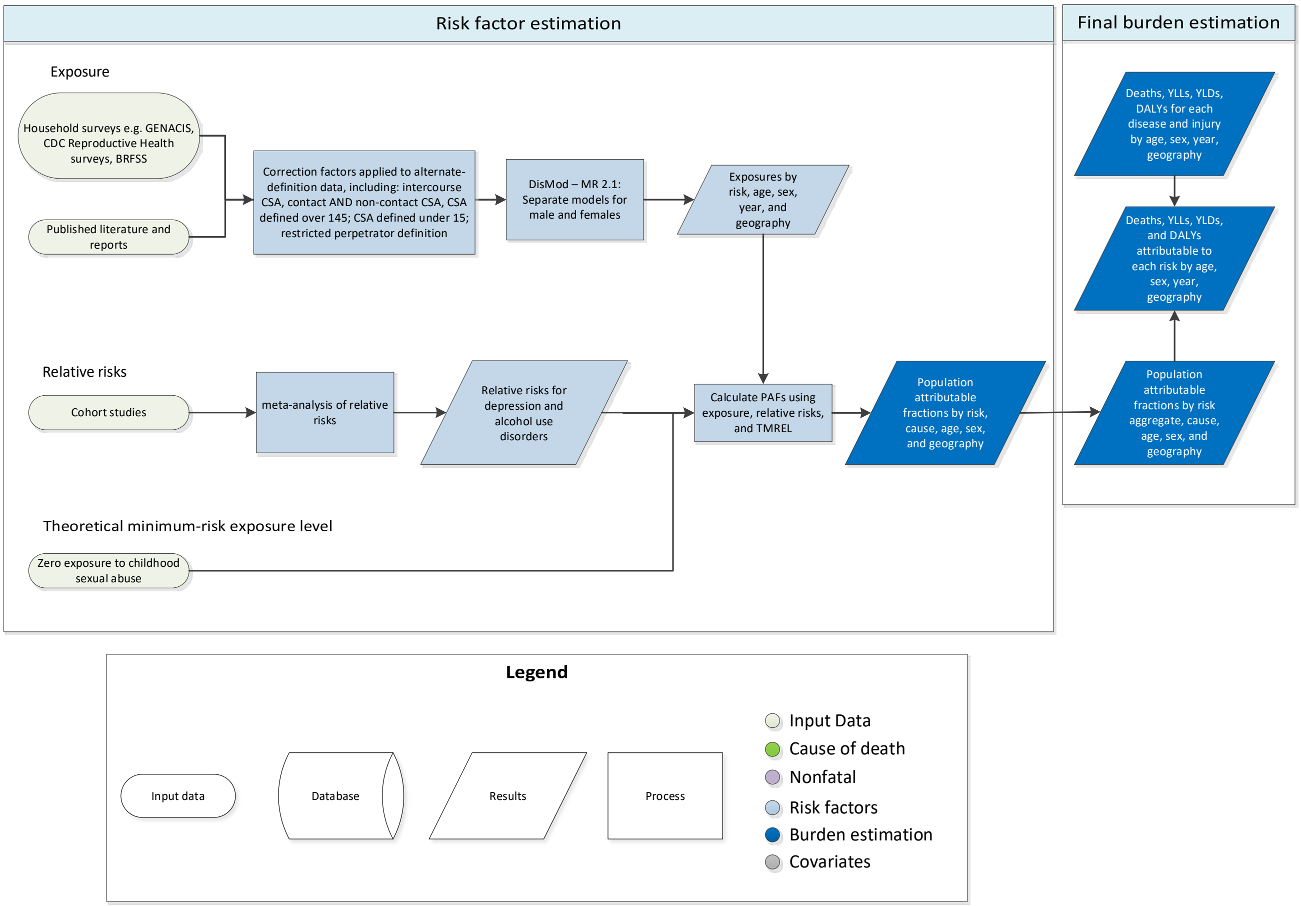

Input data and methodological summary

Definition

Exposure

The case definition for childhood sexual abuse (CSA) is ever having experienced intercourse or other contact abuse (ie, fondling and other sexual touching) when aged 15 years or younger, in which the contact was unwanted or the perpetrator was five or more years older than the victim.

Theoretical Minimum Risk Exposure Level

The associated Theoretical Minimum Risk Exposure Level (TMREL) is zero exposure to CSA.

Input data

Currently, we use self-reported survey data to measure CSA prevalence, and we do not use data from Child Protection Services (CPS) or other crime data. The reliability and comprehensiveness of CPS and crime statistics varies too much geographically to warrant inclusion (it typically identifies only a small proportion of cases).

Although no systematic review of the literature was completed for GBD 2019, we reviewed sources in the GHDx sent by collaborators and extracted new data from our collaborators in Norway.

We then supplemented all literature inputs with data from relevant national health surveys and violence-specific surveys. Several survey series used include the United States Behavioral Risk Factor Surveillance System, the CDC Reproductive Health Surveys, Brazil National Alcohol and Drug Survey, and the Gender, Alcohol, and Culture International Study (GENACIS).

We included all sources that provided population-representative data on the proportion of males or females under the age of 15 who have experienced sexual abuse. In addition, we accepted sources reporting on the following non-reference cases:

1. Proportion of individuals who experienced intercourse CSA
2. Proportion of individuals who experienced contact or non-contact CSA
3. Proportion of individuals who experienced CSA in which the definition of perpetrator is restricted (eg, CSA committed by a father)
4. Proportion of individuals who experienced sexual abuse before some age greater than 15 (such as before age 17)
5. Proportion of individuals who experienced sexual abuse before some age less than 15 (such as before age 13)
6. Proportion of individuals who experienced CSA, measured from a student population
7. Non-nationally representative populations who experienced CSA

For the alternate definitions of CSA (intercourse, contact-non contact, restricted perpetrator), and for sources using alternate age cutoffs (before or after age 15), we ran separate, sex-specific, logit-difference meta-regressions with the MR-BRT tool to estimate correction factors. We introduced these methods in lieu of marking all non-reference data with study-level covariates, and allowing DisMod to make these adjustments internally, as was done in GBD 2017.

Table 1: Data inputs for exposure for childhood sexual abuse.

Input data	Exposure
Source count (total)	196
Number of countries with data	71

Table 2: Data inputs for relative risks for childhood sexual abuse.

Input data	Relative risk
Source count (total)	10

Number of countries with data	4

Table 3: MR-BRT adjustment factors for alternate definitions of CSA among females

Data input	Reference or alternative data collection	Gamma	Beta coefficient, logit-difference (95% CI)	Adjustment factor*
Intercourse or contact CSA	Ref	0.57	---	---
Just intercourse CSA	Alt		-1.15 (-2.41, 0.10)	0.24 (0.08, 0.53)
Either contact or non-contact CSA	Alt		0.77 (-0.50, 2.03)	0.68 (0.38, 0.88)
CSA with restricted definition of perpetrator	Alt		-0.26 (-2.04, 1.52)	0.43 (0.11, 0.82)

**Adjustment factor is the inverse-logit transformed beta coefficient; <0.5 represents that alternative is adjusted upward; >0.5 represents that alternative is adjusted downward.*

Table 4: MR-BRT adjustment factors for alternate definitions of CSA among males

Data input	Reference or alternative data collection	Gamma	Beta coefficient, logit-difference (95% CI)	Adjustment factor*
Intercourse or contact CSA	Ref	0.57	---	---
Just intercourse CSA	Alt		-1.52 (-2.68, -0.36)	0.18 (0.06, 0.41)
Either contact or non-contact CSA	Alt		0.16 (-1.00, 1.32)	0.54 (0.27, 0.79)
CSA with restricted definition of perpetrator	Alt		-0.53 (-1.75, 0.69)	0.37 (0.15, 0.67)

**Adjustment factor is the inverse-logit transformed beta coefficient; <0.5 represents that alternative is adjusted upward; >0.5 represents that alternative is adjusted downward.*

Table 5: MR-BRT adjustment factors for alternate recall periods among females

Data input	Reference or alternative data collection	Gamma	Beta coefficient, logit-difference (95% CI)	Adjustment factor*
Study asked about CSA before age 15	Ref	0.02	---	---
Study asked about CSA before an age less than 15	Alt		-0.79 (-0.83, -0.74)	0.31 (0.30, 0.32)

Study asked about CSA before an age greater than 15	Alt		0.70 (0.65, 0.74)	0.67 (0.66, 0.68)

**Adjustment factor is the inverse-logit transformed beta coefficient; <0.5 represents that alternative is adjusted upward; >0.5 represents that alternative is adjusted downward.*

Table 6: MR-BRT adjustment factors for alternate recall periods among males

Data input	Reference or alternative data collection	Gamma	Beta coefficient, logit-difference (95% CI)	Adjustment factor*
Study asked about CSA before age 15	Ref	0	---	---
Study asked about CSA before an age less than 15	Alt		-0.07 (-0.49, 0.34)	0.48 (0.38, 0.58)
Study asked about CSA before an age greater than 15	Alt		0.06 (-0.34, 0.46)	0.52 (0.42, 0.61)

**Adjustment factor is the inverse-logit transformed beta coefficient; <0.5 represents that alternative is adjusted upward; >0.5 represents that alternative is adjusted downward*

Modelling strategy

We first apply the correction factors, described above, to all non-reference definition data; CSA prevalence is then modelled as a single parameter prevalence model in DisMod-MR 2.1. CSA exposure is modelled separately for males and females because we observe little correlation between the prevalence of child abuse among females and males, and modelling both sexes together causes unreasonable estimates in countries where we only have data for one sex.

Theoretical minimum-risk exposure level

The theoretical minimum-risk exposure level is zero exposure to contact childhood sexual abuse.

Relative risk

We estimate burden attributable to CSA for the following health outcomes: unipolar depressive disorders (major depressive disorder and dysthymia) and alcohol use disorders.

In addition to the sources used in GBD 2017 (Brown J et al, J Am Acad Child Adolesc Psychiatry 1999, Chapman DP et al, J Affect Disord 2004, Dinwiddie S et al, Psychol Med 2000, Dube SR et al, Am J Prev Med 2005, Jaffee SR et al, Arch Gen Psychiatry 2002, Kendler KS et al, Arch Gen Psychiatry 2000, Nelson EC et al, Arch Gen Psychiatry 2002, Widom CS et al, Arch Gen Psychiatry 2007), we incorporated an additional case-control study (Cheasty et all, BMJ 1998) to calculate the relative risk of depression as 1.56 (95% UI 1.30–1.86) using a regression developed with MR-BRT. To estimate the relative risk of alcohol abuse disorder, we reviewed the sources from the random effects meta-analysis done in GBD 2017, and excluded one for non-representativeness. With the remaining four (Dinwiddie S et al, Psychol Med 2000, Fleming J et al, Addiction 1998, Kendler KS et al, Arch Gen Psychiatry 2000, & Nelson EC et al,

Arch Gen Psychiatry 2002), we ran another regression with MR-BRT to estimate a relative risk of 2.21 (90% UI 1.15–4.04).

References

1. Brown J, Cohen P, Johnson JG., and Smailes EM. Childhood abuse and neglect: specificity of effects on adolescent and young adult depression and suicidality. Journal of the American Academy of Child & Adolescent Psychiatry. 1999; 38(12): 1490-1496.
2. Chapman DP, Whitfield CL, Felitti VJ, Dube SR, Edwards VJ and Anda RF. Adverse childhood experiences and the risk of depressive disorders in adulthood. Journal of affective disorders. 2004; 82(2): 217-225.
3. Cheasty M, Clare AW and Collins C. Relation between sexual abuse in childhood and adult depression: case-control study. Bmj. 1998; 316(7126): 198-201.
4. Dinwiddie S, Heath AC, Dunne MP, Bucholz KK, Madden PA, Slutske WS, Bierut LJ, Statham DB, Martin NG. Early sexual abuse and lifetime psychopathology: a co-twin-control study. *Psychol Med*. 2000; 30(1): 41–52.
5. Dube SR, Anda RF, Whitfield CL, Brown DW, Felitti VJ, Dong M and Giles WH. Long-term consequences of childhood sexual abuse by gender of victim. American journal of preventive medicine. 2005; 28(5): 430-438.
6. Ernst C, Angst J, Földényi M. The Zurich Study. XVII. Sexual abuse in childhood. Frequency and relevance for adult morbidity data of a longitudinal epidemiological study. *Eur Arch Psychiatry Clin Neurosci*. 1993; 242(5): 293–300.
7. Fleming J, Mullen PE, Sibthorpe B, Attewell R and Bammer G. The relationship between childhood sexual abuse and alcohol abuse in women-a case-control study. Addiction. 1998; 93(12): 1787-1798.
8. Jaffee SR, Moffitt TE, Caspi A, Fombonne E, Poulton R, Martin J. Differences in early childhood risk factors for juvenile-onset and adult-onset depression. *Arch Gen Psychiatry*. 2002; 59(3): 215-22.
9. Kendler KS, Bulik CM, Silberg J, Hettema JM, Myers J, Prescott CA. Childhood sexual abuse and adult psychiatric and substance use disorders in women: an epidemiological and cotwin control analysis. *Arch Gen Psychiatry*. 2000; 57(10): 953–9.
10. Molnar BE, Buka SL and Kessler, RC. Child sexual abuse and subsequent psychopathology: results from the National Comorbidity Survey. American journal of public health. 2001; 91(5): 753.
11. Nelson EC, Heath AC, Madden PA, Cooper ML, Dinwiddie SH, Bucholz KK, Glowinski A, McLaughlin T, Dunne MP, Statham DJ, Martin NG. Association between self-reported sexual abuse and adverse psychosocial outcomes: results from a twin study. Arch Gen Psychiatry. 2002; 59(2): 139-45.
12. Peleikis DE, Mykletun A and Dahl AA. The relative influence of childhood sexual abuse and other family background risk factors on adult adversities in female outpatients treated for anxiety disorders and depression. Child Abuse & Neglect. 2004; 28(1): 61-76.
13. Sartor CE, Lynskey MT, Bucholz KK, McCutcheon VV, Nelson EC, Waldron M, Heath AC. Childhood sexual abuse and the course of alcohol dependence development: findings from a female twin sample. *Drug Alcohol Depend*. 2007; 89(2-3): 139–44.
14. Silverman AB, Reinherz HZ and Giaconia RM. The long-term sequelae of child and adolescent abuse: A longitudinal community study. Child abuse & neglect. 1996; 20(8): 709-723.
15. Widom CS, DuMont K and Czaja SJ. A prospective investigation of major depressive disorder and comorbidity in abused and neglected children grown up. Archives of general psychiatry. 2007; 64(1): 49-56.

Bullying victimisation

Flowchart

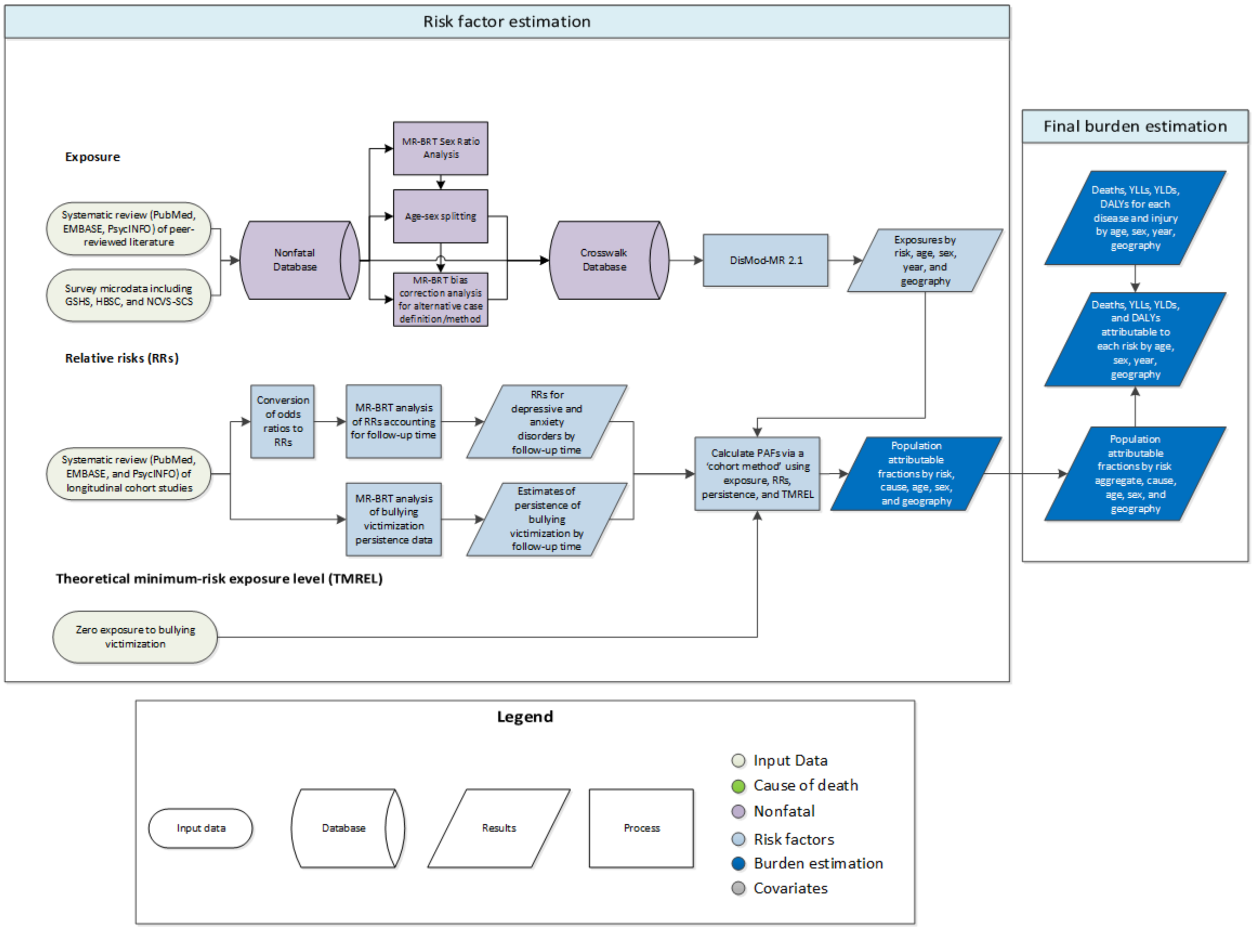

Input data and methodological summary

Exposure

Case definition

Bullying victimisation is commonly conceptualised as the intentional and repeated harm of a less powerful individual by peers.[1] This differentiates bullying victimisation from disagreements, conflicts, or playful teasing. The case definition of bullying victimisation in the GBD context is "bullying victimisation of children and adolescents attending school by peers." This definition includes the global concept of bullying victimisation which incorporates combined estimates of subtypes such as physical, verbal, relational, and cyberbullying victimisation. It excludes abuse/harassment by siblings, intimate partners, and adults (eg, teachers). While bullying can be experienced as either a victim or perpetrator, perpetration (ie, those who bully others) is not included in this definition although some victims will also be perpetrators.

Input data for exposure

In order for a study to be included, it must report the prevalence of bullying victimisation and 1) have been published since 1980, 2) ask participants about bullying victimisation in the previous year or more recently, 3) use an appropriate frequency threshold to define bullying victimisation (approximating at least once a week or greater than "occasionally"), 4) be representative of the general population rather than a special population (eg, ethnic minorities), and 5) report prevalence for bullying victimisation overall rather than a subtype eg, physical bullying victimisation.

The GBD electronic database searches for bullying victimisation is updated on a two-year rolling basis. A new systematic review for bullying victimisation was conducted for GBD 2017, with the next electronic literature update due for the next round of GBD. Included studies were sourced from a systematic review of three electronic databases (PubMed, EMBASE, and PsycINFO), covering the period 1980 to 2017. No restriction was set on the language of publication. GHDx was also used to source microdata from survey series meeting the above inclusion criteria. Estimates from the Global School-based Student Health Survey (GSHS), the Health Behavior in School-aged Children (HBSC), and the National Crime Victimization Survey – School Crime Supplement (NCVS-SCS) were extracted and included in the dataset. The grey literature search and expert consultation conducted for GBD 2019 did not reveal any additional studies. The table below summarises exposure data inputs for bullying victimisation.

Table 1: Exposure input data for bullying victimization

Input data	Exposure
Source count (total)	308
Number of countries with data	119

Age and sex splitting

The extracted data underwent two types of age and sex splitting processes:

1. Where possible, estimates were further split by sex and age based on the data that were available. For instance, if studies reported prevalence for broad age groups by sex (eg, prevalence in 5- to 17-year-old males and females separately), and also by specific age groups but for both sexes combined (eg, prevalence in 5- to 12-year-olds, then in 13- to 17-year-olds, for males and females combined); age-specific estimates were split by sex using the reported sex ratio and bounds of uncertainty
2. A meta-regression with Bayesian priors, regularisation, and trimming (MR-BRT) analysis was used to split the remaining both-sex estimates in the dataset. For each parameter, sex-specific estimates were matched by location, age, and year, and a MR-BRT network meta-analysis was used to estimate pooled sex ratios and bounds of uncertainty. These were then used to split the both-sex estimates in the dataset. The male:female prevalence ratio was 1.21 (95% CI: 0.90–1.51).

Bias corrections / crosswalks

Estimates with known biases were adjusted/crosswalked accordingly prior to DisMod-MR 2.1. Within the bullying victimisation epidemiological dataset, within and between study estimates were paired by age, sex, location, and year, between the reference and alternative estimates. Pairs were also made between the different alternative estimates. The ratios between these estimates were then used as

inputs in a MR-BRT network meta-analysis. This analysis produced pooled ratios between the reference estimates and alternative estimates. These ratios (see Table 2) were used to adjust all alternative estimates in the dataset. Bullying victimisation had four alternative definitions to crosswalk:

1. Suboptimal frequency threshold used, eg, "sometimes + frequently"
2. No definition of bullying victimisation presented to participants or not specified
3. Asked about bullying victimisation in the past year

Table 2: MR-BRT crosswalk adjustment factors for bullying victimisation

Data input	Reference or alternative case definition	Gamma	Beta coefficient, log (95% CI)	Adjustment factor* (95% CI)
School survey	Reference: Point proportion of children and adolescents attending school who have been exposed to bullying victimisation by peers at least once a week or greater than "occasionally". Participants are given a definition of bullying victimisation prior to being asked about exposure.	0.33	---	---
School survey	Alternative: Suboptimal frequency threshold used, eg, "sometimes + frequently"		1.18 (0.52–1.83)	3.24 (1.69–6.22)
School survey	Alternative: No definition of bullying victimisation presented to participants or not specified		0.27 (0.18–0.36)	1.31 (1.20–1.43)
School survey	Alternative: Past-year proportion		0.16 (0.06–0.27)	1.18 (1.06–1.31)

**Adjustment factor is the transformed beta coefficient in normal space and can be interpreted as the factor by which the alternative case definition is adjusted to reflect what it would have been if measured as the reference.*

Confidence intervals incorporate gamma, which represents the between-study variance across all input data in the model. This added uncertainty widens the confidence intervals for crosswalks with significant fixed effects.

Modelling strategy

After the above data processes were applied, DisMod-MR 2.1 was used to model the prevalence of bullying victimisation. Bullying victimisation prevalence was modelled as a single parameter prevalence model. The DisMod-MR modelling strategy for bullying victimisation followed the standard GBD 2019 decomposition structure. At each decomposition step, we compared the new model against the GBD 2017 best model and the best model from the previous step. All substantial changes between models were explored and explained. Adjustments to model priors or the dataset were made where appropriate. Where outliers were identified in the data, we reassessed the study's methodology and quality before a decision was made to exclude or include the data. We assumed no prevalence prior to 5 years or after 20 years of age.

Adjustment for years of schooling

In order to better represent the prevalence of bullying victimisation, prevalence estimates were adjusted for the proportion of children and adolescents attending school by ages 5–9, 10–14, and 15–19 years by sex, location, and year. Data on the proportion of children and adolescents attending school was sourced from the online database (http://data.uis.unesco.org/) published by the United Nations Educational, Scientific, and Culture Organization (UNESCO). The data covered 18,441 country-years for age groups 6–11, 12–14, and 15–17 years by sex. These data were modelled in ST-GPR, with average years of education as a country-level covariate, to predict the proportion of children and adolescents attending school by these age groups. This gave estimates of the proportion of children and adolescents attending school by age, sex, year, and location.

Theoretical minimum-risk exposure level

The theoretical minimum-risk exposure level was assumed to be zero exposure to bullying victimisation.

Relative risks

We estimate burden attributable to bullying victimisation for major depressive disorder (MDD) and anxiety disorders. Data on the association between bullying victimisation and self-harm was also reviewed but not included due to variation in the definition of "self-harm" and only one study looking at suicide.

Input data for relative risks

For GBD 2017, studies reporting the prospective longitudinal association between these outcomes and bullying victimisation were sourced from a systematic review of three electronic databases (PubMed, EMBASE, and PsycINFO), covering the period 1980–2017. No restriction was set on the language of publication. Studies had to report relative risks (RRs), odds ratios, or sufficient data to calculate RRs (ie, exposed/non-exposed cases/non-cases). Altogether there were 107 RRs extracted from 23 studies reporting on 14 cohorts (see Table 3). The search for relative-risk estimates is also updated on a two-year rolling basis, with the next electronic literature update due for the next round of GBD.

Table 3: Relative risk input data for bullying victimization

Input data	Relative risk
Source count (total)	23
Number of countries with data	6

Estimation of pooled relative risks

A MR-BRT meta-regression was conducted to determine the impact of follow-up time on the risk of MDD and anxiety disorders following exposure to bullying victimisation. This analysis controlled for several biases via covariates which are detailed in Table 4. Estimates were nested within cohorts. All available estimates by cohort that varied across the covariates were extracted to better inform the covariates. MDD was set as the intercept and anxiety was estimated via a covariate ($\beta = 0.06$ [95% CI: -0.05 to 0.17]). The risk of MDD and anxiety disorders diminished significantly with time ($\beta = -0.03$ [95% CI: -0.04 to 0.02]).

Table 4: MR-BRT meta-regression bias covariates

Covariate	Beta coefficient, log (95%CI)	Gamma
Intercept	0.76 (0.46–1.05)	0.00
Outcome measured via symptom scale	0.07 (-0.11 to 0.25)	2.02
Unadjusted for potential confounders	0.08 (0.00–0.16)	0.00
Exposure reported by parent only	0.22 (0.09–0.35)	0.00
Estimate represents odds ratio	0.29 (0.15–0.43)	1.99
Estimate derived from multiple logistic regression	0.21 (0.09–0.33)	1.90
Exposure is cyberbullying	0.68 (0.33–1.03)	2.00
Suboptimal exposure frequency threshold	-0.49 (-0.60 to -0.38)	1.85
Outcome not controlled for at baseline	0.06 (-0.06 to 0.18)	0.24
15%+ attrition at follow-up	-0.12 (-0.40 to 0.15)	0.00
Sample represents males only	0.05 (-0.18 to 0.28)	0.00
Sample represents females only	-0.41 (-0.63 to -0.19)	1.84

Population attributable fractions (PAFs)

A cohort method was developed to accommodate the waning risk over time observed in the MR-BRT meta-regression of the RRs. The following steps are conducted for each point of estimation (ie, by age, sex, location, and year), hereafter referred to as a "cohort":

1. Pull current and past bullying victimisation prevalence for the cohort from the DisMod-MR 2.1 exposure model.
2. Adjust each bullying victimisation prevalence estimate for the proportion of the cohort attending school in that year.
3. Estimate incidence of bullying victimisation within the cohort for each year using the following formula:

$$\boldsymbol{I_k = P_k - \sum_{n=0}^{k-1}(I_n \times r_{k-n})}$$

   where I represents incidence, P represents prevalence, r represents the estimate of persistence, and k represents the time between the incidence estimate and the earliest possible time of exposure in the cohort. I_k requires I_0 through to $I_{k\text{-}1}$ to first be calculated and so we complete this process by first estimating I_0, then I_1, and so on until we have estimated incidence for the latest possible year of exposure for this cohort. The persistence estimate is based on a separate MR-BRT meta-regression of seven studies.[2-8]
4. Use the incidence estimates to divide the cohort into proportions based on time since first exposed to bullying victimisation:

$$\boldsymbol{p_t = I_{\max(k)-k}}$$

   Where t is the time since first exposed to bullying victimisation, and p is the proportion of the cohort first exposed to bullying victimisation at time t.

5. Estimate PAFs via the following formula:

$$PAF = \frac{\sum(p_t \times RR_t) + p_{no\ exposure} - 1}{\sum(p_t \times RR_t) + p_{no\ exposure}}$$

where *t* is the time since first exposed to bullying victimisation, *p* is the proportion of the cohort first exposed to bullying victimisation at time *t* or the proportion not exposed to bullying victimisation, and *RR* is the relative risk for depressive and anxiety disorders given *t*.

Changes between GBD 2017 and GBD 2019

There were five main changes in the GBD 2019 modelling strategy compared to GBD 2017:

1. In GBD 2017 the sex ratio was estimated by DisMod-MR 2.1 as part of the prevalence modelling. In GBD 2019 we made use of MR-BRT to run a nested meta-regression analysis on the within-study sex ratios to estimate a pooled sex ratio with 95% confidence intervals as previously discussed. The prevalence male:female sex ratio was 1.24 (95% CI 1.18–1.30) in GBD 2017 compared to 1.21 (95% CI 0.90–1.51) in GBD 2019.
2. In GBD 2019 we made use of MR-BRT to run a nested network meta-regression to estimate adjustments to alternative data prior to running DisMod-MR 2.1. Ratios estimated between 2017 and 2019 were largely consistent, although the confidence interval derived by MR-BRT tended to be larger. MR-BRT confidence intervals incorporate gamma, which represents the between study variance across all input data in the model. This added uncertainty widens the confidence intervals for crosswalks with significant fixed effects.
    a. The adjustment ratio for suboptimal frequency threshold was 3.35 (3.35–3.35) in GBD 2017 versus 3.24 (1.69–6.22) in GBD 2019
    b. The adjustment ratio for estimates where no definition of bullying victimisation was presented to participants or not specified was 1.12 (1.12–1.12) in GBD 2017 versus 1.31 (1.20–1.43) in GBD 2019
    c. The adjustment ratio for past-year estimates was 1.47 (1.30–1.68) in GBD 2017 versus 1.18 (1.06–1.31) in GBD 2019
3. The GBD 2017 model included an adjustment ratio (as a study-level covariate within DisMod-MR) for estimates sourced from a single school. This covariate/adjustment was excluded in GBD 2019. Estimates sourced from a single school were adjusted downward by 1.21 (1.01–2.12) toward the level of estimates sourced across multiple schools. This was under the premise that a survey may have been commissioned at these schools to evaluate the extent of a known issue of bullying victimisation. Part of the new GBD 2019 MR-BRT approach was to assess the availability of data for a given study-level covariate to produce robust matched pairs. There were no within-study comparisons available with this covariate and only one between-study comparison available. Until more data become available to clarify the above, we have excluded this adjustment from the dataset and included this data without adjustment.
4. In GBD 2017, the RRs were estimated via a meta-regression conducted in R using the metafor package. This analysis controlled for three known sources of bias via covariates (outcome measured via symptom scale, suboptimal exposure frequency threshold, and outcome not controlled for at baseline). In GBD 2019 we made use of MR-BRT to run a nested meta-regression analysis controlling for 11 known sources of bias and heterogeneity (see Table 2). Cause-specific RRs were also predicted, whereas in GBD 2017, MDD and anxiety disorders were assigned the same RRs. The MR-BRT analysis resulted in RRs that were slightly higher than in GBD 2017, with a follow-up time lasting several years longer (four years for MDD, seven years for anxiety disorders). This has resulted in larger PAFs than in GBD 2017.

5. In GBD 2017, the persistence model used to estimate incidence of bullying was estimated via a meta-regression conducted in R using the metafor package. In GBD 2019 we made use of MR-BRT to run a nested meta-regression. This better accounted for the multiple observations analysed per study. This has resulted in larger variance in the persistence estimates (and in turn the incidence estimates) than in GBD 2017.

References

1. Olweus D. Bullying at school: What we know and what we can do. MA, USA; Oxford, UK; Vic, Australia: Blackwell; 1993.
2. Baly MW, Cornell DG, Lovegrove P. A Longitudinal Investigation of Self- and Peer Reports of Bullying Victimization Across Middle School. *Psychology in the Schools* 2014; **51**(3): 217-40.
3. Bowes L, Maughan B, Ball H, et al. Chronic bullying victimization across school transitions: the role of genetic and environmental influences. *Development and psychopathology* 2013; **25**(2): 333-46.
4. Kumpulainen K, Rasanen E, Henttonen I. Children involved in bullying: psychological disturbance and the persistence of the involvement. *Child Abuse Negl* 1999; **23**(12): 1253-62.
5. Lereya ST, Copeland WE, Zammit S, Wolke D. Bully/victims: A longitudinal, population-based cohort study of their mental health. *European Child & Adolescent Psychiatry* 2015; **24**(12): 1461-71.
6. Lien L, Welander-Vatn A. Factors Associated with the Persistence of Bullying Victimization From 10th grade to 13th Grade: A Longitudinal Study. *Clinical practice and epidemiology in mental health : CP & EMH* 2013; **9**: 243-50.
7. Sourander A, Helstela L, Helenius H, Piha J. Persistence of bullying from childhood to adolescence--a longitudinal 8-year follow-up study. *Child Abuse Negl* 2000; **24**(7): 873-81.
8. Winsper C, Lereya T, Zanarini M, Wolke D. Involvement in bullying and suicide-related behavior at 11 years: a prospective birth cohort study. *Journal of the American Academy of Child and Adolescent Psychiatry* 2012; **51**(3): 271-82.e3.

Unsafe sex

Flowchart

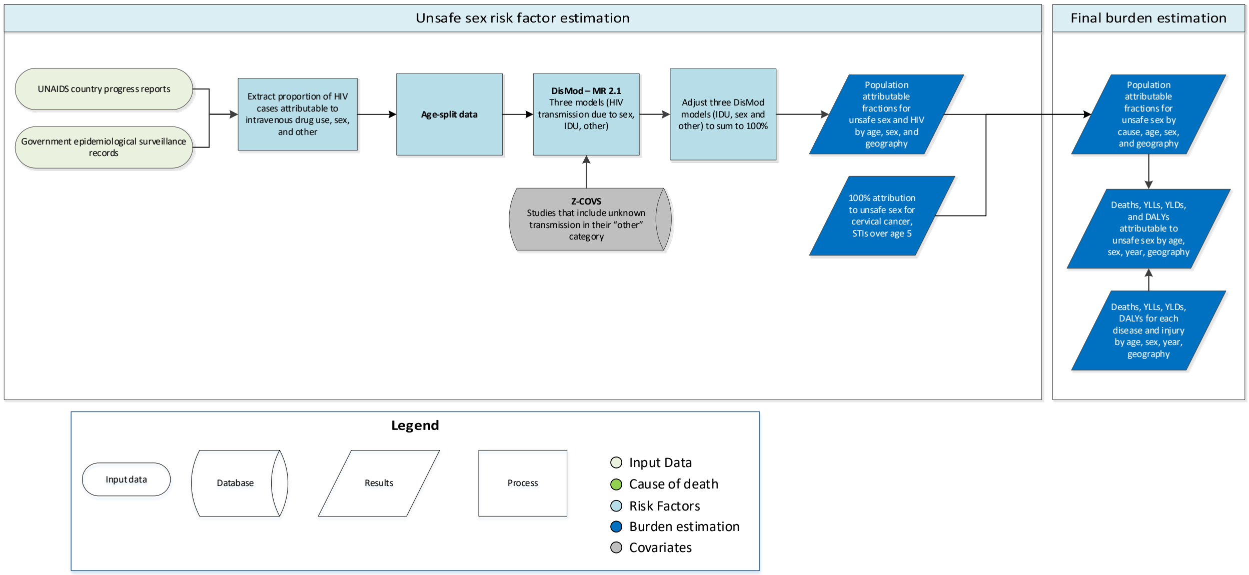

Input data and methodological summary

Definition

Exposure

Unsafe sex is defined as the risk of disease due to sexual transmission. The outcomes associated with unsafe sex that we estimate for GBD include HIV, cervical cancer, and all sexually transmitted diseases (STDs) except for those in neonates from vertical transmission, including HIV, *Opthalmia neonatorum* and neonatal syphilis. We assumed 100% of cervical cancer and STDs were attributable to unsafe sex and modelled the proportion of HIV incidence occurring through sexual transmission to estimate the attributable burden for HIV due to unsafe sex. The theoretical minimum level (TMREL) for unsafe sex is defined as the absence of disease transmission due to sexual contact.

Input data

To be used in our models, sources must report HIV cases attributable to various modes of transmission. We screened UNAIDS country progress reports and searched government epidemiological surveillance records for these data. The primary data sources we used were UNAIDS, the European CDC, and the US CDC.

We excluded all extractions where the "other" category for HIV transmissions accounted for greater than 25% of all cases. We believe that such high proportions raise concerns about the quality of reporting.

Input data	Exposure
Source count (total)	948
Number of countries with data	97

Modelling strategy

We modelled the proportion of HIV cases attributable to unsafe sex. To do this we collected and cleaned data, ran three DisMod-MR models (HIV attributable to sex, HIV attributable to injection drug use, HIV attributable to other routes of transmission), adjusted results of the three DisMod-MR models to sum to one, and then assigned the proportions as direct PAFs.

No country-level covariates were included in the models. We tested an injection drug use (IDU) covariate – an opioid use covariate in the proportion HIV due to drug use model – but found no significant coefficients, so excluded them from the final model.

Since all-age and both-sex datapoints represent the vast majority of the available data, we derived an age-sex pattern for the HIV-IDU transmission model from the age-sex pattern present in the GBD 2017 population attributable fraction for hepatitis B attributable to IDU (the model for injecting drug use and hepatitis estimates the cumulative exposure to injecting drug use to capture all infections in people with a history of injecting even if in a more distant past). Assuming the proportion of HIV due to other transmission is constant over age and by sex, the age-sex pattern for the proportion of HIV due to sexual transmission was set to be the complement to 1 of the age-sex pattern for the proportion of HIV due to IDU. The all-age and both-sex data were split according to these age-sex patterns, and the three HIV transmission DisMod-MR models were run on the age- and sex-split data. In previous GBD rounds, only age-splitting had used this approach, while sex-splitting occurred within DisMod-MR. Since most data are for both sexes combined, using the sex ratio – in addition to the age pattern from the IDU-Hepatitis B PAF – is much more informative. The impact of this change resulted in general increases in proportion HIV due to sexual transmission among females, as they generally had lower IDU rates compared to males.

In GBD 2019, we also changed the proportion HIV due to sex DisMod-MR model to run in complement (1-proportion) space. Since proportions were high in most countries, modelling in complement space resulted in a better model fit. Additional priors were set to inform an age pattern: zero proportion HIV transmission due to IDU before age 15, zero proportion HIV transmission due to sex before age 10 (100 in complement space), and 100% transmission due to other before age 10. The results from these HIV transmission models were adjusted to sum to 100% for a given country-year-age-sex group at each of 1,000 draws.

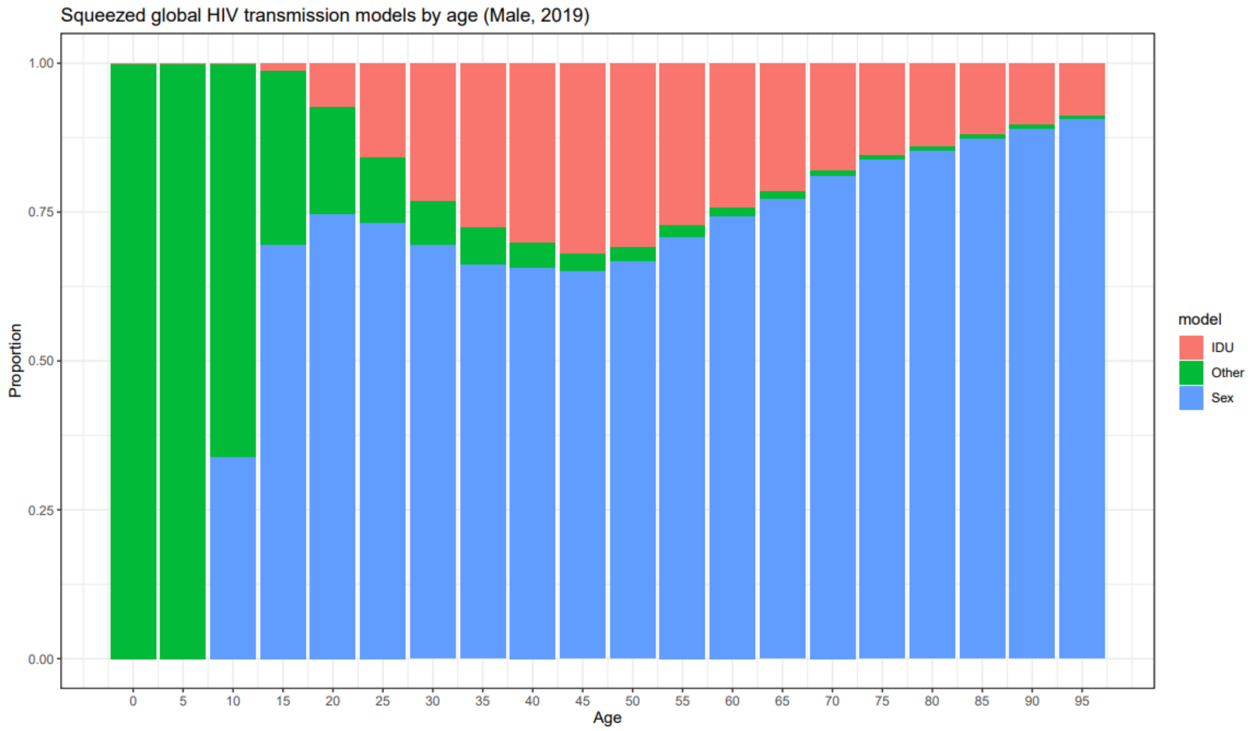

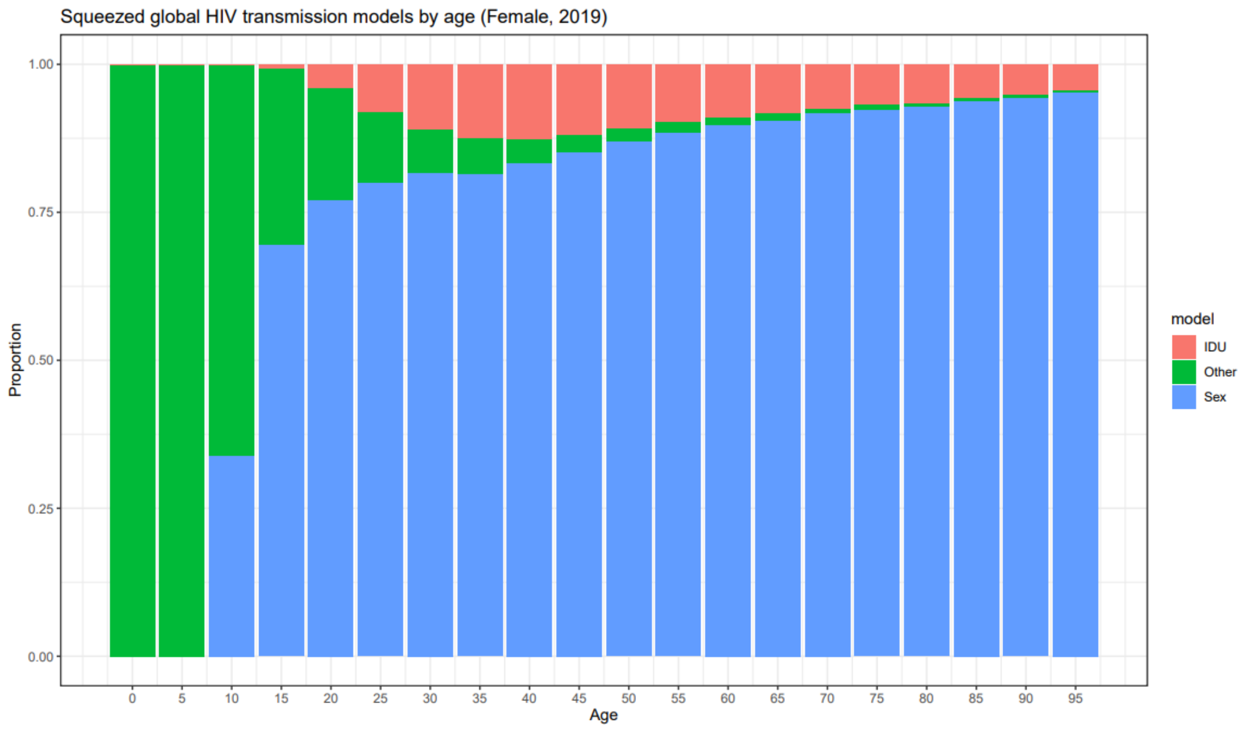

Theoretical minimum-risk exposure level

The theoretical minimum level used for unsafe sex is the absence of disease transmission due to sexual contact.

Population attributable fraction calculations

Based on evidence in the literature, we attributed 100% of cervical cancer to unsafe sex. These sources state that HPV infection is necessary for cervical cancer to develop and that HPV is only spread through sexual contact. The proportion of STDs attributable to unsafe sex was also 100%.

For HIV, the results from the single parameter proportion DisMod-MR model for HIV transmission due to sex after squeezing were used directly as the population attributable fraction.

Low physical activity

Flowchart

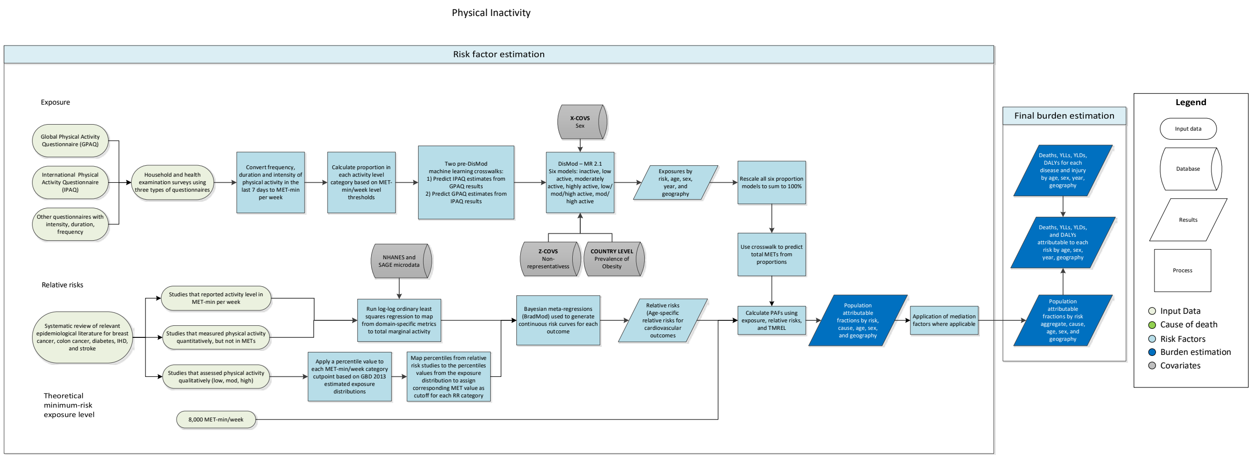

Input data and methodological summary

Exposure

Case definition

We measure physical activity performed by adults older than 25 years of age, for duration of at least ten minutes at a time, across all domains of life (leisure/recreation, work/household and transport). We use frequency, duration and intensity of activity to calculate total metabolic equivalent-minutes per week. MET (Metabolic Equivalent) is the ratio of the working metabolic rate to the resting metabolic rate. One MET is equivalent to 1 kcal/kg/hour and is equal to the energy cost of sitting quietly. A MET is also defined as the oxygen uptake in ml/kg/min with one MET equal to the oxygen cost of sitting quietly, around 3.5 ml/kg/min.

Input data

We included surveys of the general adult population that captured self-reported physical activity in all domains of life (leisure/recreation, work/household and transport), where random sampling was used.

Data were primarily derived from two standardised questionnaires: The Global Physical Activity Questionnaire (GPAQ) and the International Physical Activity Questionnaire (IPAQ), although we included other survey instruments that asked about intensity, frequency and duration of physical activities performed across all activity domains.

Due to a lack of a consistent relationship on the individual level between activity performed in each domain and total activity, we were not able to use studies that included only recreational/leisure activities.

Physical activity level is categorised by total MET-minutes per week using four categories based on rounded values closest to the quartiles of the global distribution of total MET-minutes/week. The lower

limit for the Level 1 category (600 MET-min/week) is the recommended minimum amount of physical activity to get any health benefit. We used four categories with higher thresholds rather than the GPAQ and IPAQ recommended 3 categories to better capture any additional protective effects from higher activity levels.

- Level 0: < 600 MET-min/week (inactive)
- Level 1: 600-3999 MET-min/week (low-active)
- Level 2: 4000-7,999 MET-min/week (moderately-active)
- Level 3: ≥ 8,000 MET-min/week (highly active)

The GHDx was used to locate all surveys that use the GPAQ or IPAQ questionnaire. Although there were many other surveys that focused specifically on leisure activity, we were unable to use these sources because they did not comprise all three domains (work, transport and leisure). In addition, we excluded any surveys that did not report frequency, duration, and intensity of activity.

Table 1: Data inputs for exposure for low physical activity.

Input data	Exposure
Site-years (total)	255
Number of countries with data	128

Table 2: Data inputs for relative risks for low physical activity.

Input data	Exposure
Site-years (total)	121
Number of countries with data	38

Modelling strategy

DisMod modelling

For this round of the GBD, we have chosen to use a machine learning crosswalk to predict IPAQ estimates for GPAQ results and GPAQ estimates for IPAQ results, with original and estimated results then being combined to get one comprehensive IPAQ dataset and one comprehensive GPAQ dataset. We then estimated the proportion of each country/year/age/sex subpopulation in each of the above four activity levels using 12 separate Dismod models (one set of six for IPAQ and one for GPAQ). We use six categories of physical activity prevalence rather than four to accommodate the different MET-minute/week cutoffs presented in tabulated data sources where individual unit record data was not available. Since the accepted threshold/definition for inactivity is consistently <600 MET-minutes/week, the vast majority of tabulated data was broken down into proportion inactive (model A) and proportion low, moderate or highly active (model B).

	Label	MET-min/week	Name of sequelae in online visualisation tool
A	inactive	<600	Physical inactivity and low physical activity, inactive

B	low/moderately/highly active	00	Physical inactivity and low physical activity, low/moderately/highly active
C	low active	600-3999	Physical inactivity and low physical activity, low active
D	moderately/highly active	>4000	Physical inactivity and low physical activity, moderately/highly active
E	moderately active	4000-7999	Physical inactivity and low physical activity, moderately active
F	highly active	000	Physical inactivity and low physical activity, highly active

These models have mesh points at 0 15 25 35 45 55 65 75 85 100, and a study-level fixed effect on integrand variance (Z-cov) for whether a study was nationally representative or not, to account for the heterogeneity introduced by studies that are not generalizable to the entire population. They also have national level fixed effects on prevalence of obesity.

After DisMod, we rescale each of the 6 models specific to each data source so that the proportions sum to one. Since we have the most data for models A and B, we rescale the sum of the proportion in each category to be equal to one. Next we rescale the sum of model C and D to be equal to the rescaled value from model B. Then we rescale the sum of models E and F to be equal to the rescaled value from model D. After these three rescales we are left with a proportion for each of the four categories that all sum to 1. Scaled results for each data source are then hybridised to produce only one set of results for the prevalence of the four categories of physical activity.

Similar to the previous round, we have not directly estimated total MET-minutes per week globally. Although, this year we made use of two specific machine learning algorithms (Random Forest and XGBoost) that were trained using data that could characterise the relationship between total MET-mins/week and each of the categorical prevalences of physical activity. This resulted in country-year-age-sex specific estimates of total physical activity in the form of MET-minutes per week.

Utilising microdata on total MET-mins per week from individual-level surveys, we characterised the distribution of activity level at the population level. We then used an ensemble approach to distribution fitting, borrowing characteristics from individual distributions to tailor a unique distribution to fit the data using a weighting scheme. We characterised the standard deviation of each population's activity through a linear regression that captured the relationship between standard deviation and mean activity levels in nationally representative IPAQ surveys:

$$ln\,(Standard\ deviation) = \beta_0 + \beta_1 \times ln\,(Mean_i) + \beta_2 \times Age_i + \beta_3 \times SR_i + \beta_4 \times Fem_i$$

Age_i is the youngest age in population *i*'s age group, SR_i is the super region in which the population lives, and Fem_i is a Boolean value depicting whether the population is female. We then applied the coefficients of this regression to the outputs of our estimate of total MET-minutes per week regression outputs to calculate the standard deviation by country, year, age, and sex.

Theoretical minimum-risk exposure level

The theoretical minimum-risk exposure level for physical inactivity is 3000-4500 MET-min per week, which was calculated as the exposure at which minimal deaths across outcomes occurred.[3]

Relative risk

We used a dose-response meta-analysis of prospective cohort studies to estimate the effect size of the change in physical activity level on breast cancer, colon cancer, diabetes, ischemic heart disease and ischemic stroke.[3]

There is a well-documented attenuation of the risk for cardiovascular disease and diabetes due to metabolic risks factors throughout one's life. To incorporate this age trend in the relative risks, we first identified the median age-at-event across all cohorts and considered that as the reference age-group. We then assigned our risk curves to this reference age group. Then, we derived the percent change in relative risks between each age group and the reference age group by averaging percentage changes in relative risks of all metabolic mediators.

References

1. IPAQ Research Committee. Guidelines for data processing and analysis of the International Physical Activity Questionnaire (IPAQ)–short and long forms. Retrieved September. 2005;17:2008.

2. World Health Organization. Global Physical Activity Questionnaire (GPAQ) Analysis Guide. 2011. Geneva, Switzerland: WHO Google Scholar. 2013

3. Kyu HH, Bachman VF, Alexander LT, Mumford JE, Afshin A, Estep K, Veerman JL, Delwiche K, Iannarone ML, Moyer ML, Cercy K. Physical activity and risk of breast cancer, colon cancer, diabetes, ischemic heart disease, and ischemic stroke events: systematic review and dose-response meta-analysis for the Global Burden of Disease Study 2013. bmj. 2016 Aug 9;354:i3857.

High fasting plasma glucose

Flowchart

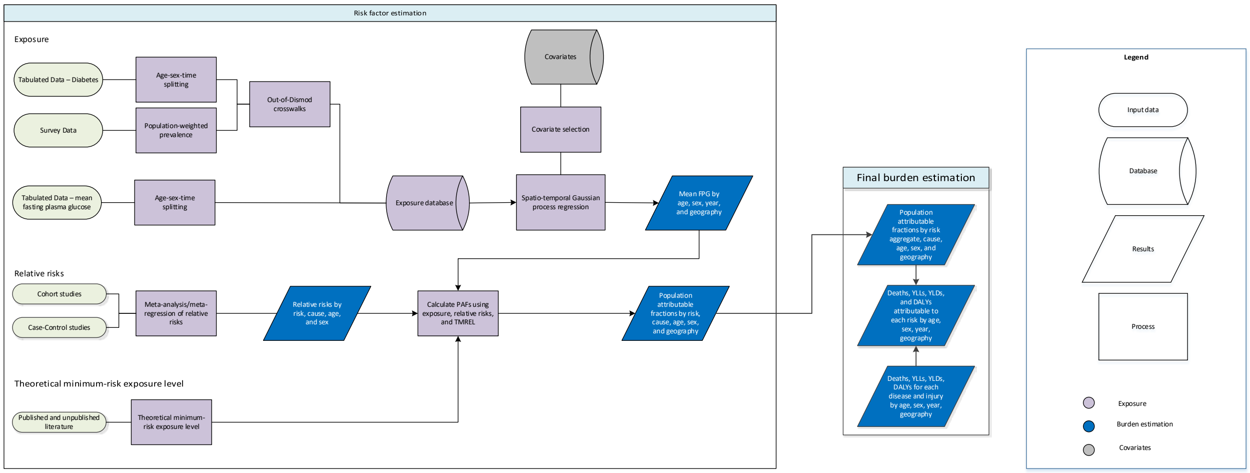

Case definition

High fasting plasma glucose (FPG) is measured as the mean FPG in a population, where FPG is a continuous exposure in units of mmol/L. Since FPG is along a continuum, we define high FPG as any level above the TMREL, which is 4.8-5.4 mmol/L.

Data seeking

Exposure

We conducted a systematic review for FPG and diabetes in GBD 2019. We use all available sources on FPG and prevalence of diabetes in the FPG model.

1. Search terms:

Diabetes Mellitus search string: (diabetes[TI] AND (prevalence[TIAB] OR incidence[TIAB])) OR ('Diabetes Mellitus'[MeSH Terms] AND 'epidemiology'[MeSH Terms]) OR (diabetes[TI] AND 'epidemiology'[MeSH Terms]) NOT gestational[All Fields] NOT ('neoplasms'[MeSH Terms] OR 'neoplasms'[All Fields] OR 'cancer'[All Fields]) NOT ('mice'[MeSH Terms] OR 'mice'[All Fields]) NOT ('schizophrenia'[MeSH Terms] OR 'schizophrenia'[All Fields]) NOT ('emigrants and immigrants'[MeSH Terms] OR ('emigrants'[All Fields] AND 'immigrants'[All Fields]) OR 'emigrants and immigrants'[All Fields] OR 'immigrants'[All Fields]) NOT ('pregnancy'[MeSH Terms] OR 'pregnancy'[All Fields] OR 'gestation'[All Fields]) NOT ('rats'[MeSH Terms] OR 'rats'[All Fields] OR 'rat'[All Fields]) NOT ('kidney'[MeSH Terms] OR 'kidney'[All Fields]) NOT renal[All Fields] NOT ('vitamins'[Pharmacological Action] OR 'vitamins'[MeSH Terms] OR 'vitamins'[All Fields] OR 'vitamin'[All Fields])

And

FPG search string: ((“glucose”[Mesh] OR “hyperglycemia”[Mesh] OR “prediabetic state”[Mesh]) AND "Geographic Locations"[Mesh] NOT "United States"[Mesh]) AND ("humans"[Mesh] AND "adult"[MeSH]) AND ("Data Collection"[Mesh] OR "Health Services Research"[Mesh] OR "Population Surveillance"[Mesh] OR "Vital statistics"[Mesh] OR "Population"[Mesh] OR "Epidemiology"[Mesh] OR surve*[TiAb]) NOT Comment[ptyp] NOT Case Reports[ptyp]) NOT "hospital"[TiAb]

Search date: October 17, 2018. The search took place for the following dates: 10/15/2017-10/16/2018. The number of studies returned was 717, and the number of studies extracted was 36.

Figure 1: PRISMA diagram of data sources used in GBD 2019 high fasting plasma glucose model

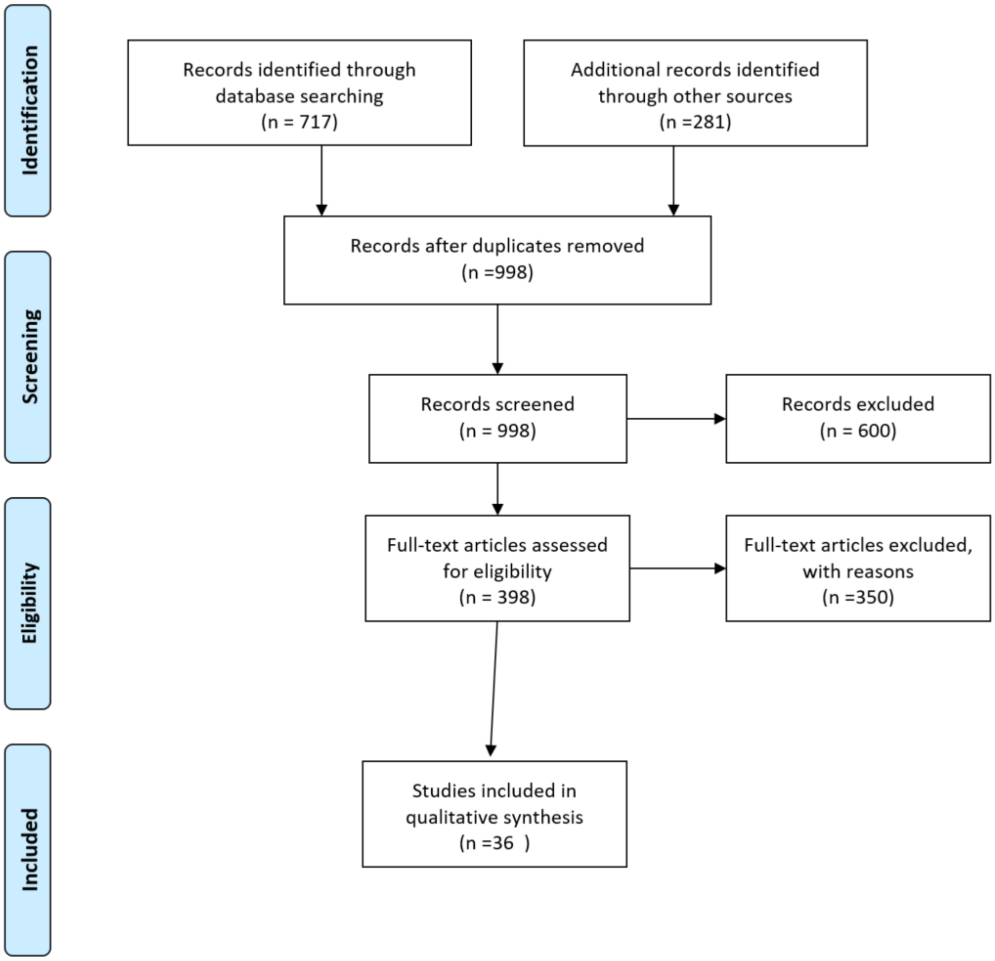

Data inputs

Data inputs come from 3 sources:

- Estimates of mean FPG in a representative population
- Individual-level data of fasting plasma glucose measured from surveys
- Estimates of diabetes prevalence in a representative population

Data sources that did not report mean FPG or prevalence of diabetes are excluded from analysis. When a study reported both mean fasting plasma glucose (FPG) and prevalence of diabetes, we use the mean

FPG for exposure estimates. Where possible, individual-level data supersede any data described in a study. Individual-level data are aggregated to produce estimates for each 5-year age group, sex, location, and year of a survey.

Table 1: Number of sources used in exposure and relative risk models in GBD 2019

Measure	Total sources	Countries with data
Total	549	127
Relative risk	20	-
Exposure	529	127

Data processing

We perform several processing steps to the data in order to address sampling and measurement inconsistencies that will ensure the data are comparable.

1. *Small sample size*
   Estimates in a sex and age group with a sample size <30 persons is considered a small sample size. In order to avoid small sample size problems that may bias estimates, data are collapsed into the next age group in the same study till the sample size reach at least 30 persons. The intent of collapsing the data is to preserve as much granularity between age groups as possible. If the entire study sample consists of <30 persons and did not include a population-weight, the study is excluded from the modelling process.

2. *Crosswalks*
   We predicted mean FPG from diabetes prevalence using an ensemble distribution. We characterized the distribution of FPG using individual-level data. Details on the ensemble distribution can be found elsewhere in the Appendix. Before predicting mean FPG from prevalence of diabetes, we ensured that the prevalence of diabetes was based on the reference case definition: fasting plasma glucose (FPG) >126 mg/dL (7 mmol/L) or on treatment. For more details on how the case-definition crosswalk is conducted, please see the diabetes mellitus appendix in *Global, regional, and national incidence, prevalence, and years lived with disability for 354 diseases and injuries for 195 countries, 1990–2019: a systematic analysis for the Global Burden of Disease Study 2019*.

Exposure modelling

Exposure estimates are produced for every year between 1980 to 2019 for each national and subnational location, sex, and for each 5-year age group starting from 25 years. As in previous rounds of GBD, we used a Spatio-Temporal Gaussian Process Regression (ST-GPR) framework to model the mean fasting plasma glucose at the location-, year-, age-, and sex- level. Updates to the ST-GR modelling framework for GBD 2019 are detailed elsewhere in the Appendix.

Fasting plasma glucose is frequently tested or reported in surveys aiming at assessing the prevalence of diabetes mellitus. In these surveys, the case definition of diabetes may include both a glucose test and questions about treatment for diabetes. People with positive history of diabetes treatment may be excluded from the FPG test. Thus, the mean FPG in these surveys would not represent the mean FPG in

the entire population. In this event, we estimated the prevalence of diabetes assuming a definition of FPG>126 mg/dL (7mmol/L), then crosswalked it to our reference case definition, and then predicted mean FPG.

To inform our estimates in data-sparse countries, we systematically tested a range of covariates and selected age specific prevalence of obesity as a covariate based on direction of the coefficient and significance level.

Mean FPG is estimated using a mixed-effects linear regression, run separately by sex:

$$\text{logit}(\text{FPG}_{c,a,t}) = \beta_0 + \beta_1 p_{\text{overweight}_{c,a,t}} + \sum_{k=2}^{16} \beta_k I_{A[a]} + \alpha_s + \alpha_r + \alpha_c + \epsilon_{c,a,t}$$

where $p_{\text{overweight}_{c,a,t}}$ is the prevalence of overweight, $I_{A[a]}$ is an indicator variable for a fixed effect on a given 5-year age group, and α_s α_r α_c are random effects at the super-region, region, and country level, respectively. The estimates were then propagated through the ST-GPR framework to obtain 1000 draws for each location, year, age, and sex.

Theoretical minimum-risk exposure level

The theoretical minimum-risk exposure level (TMREL) for FPG is 4.8-5.4 mmol/L. This was calculated by taking the person-year weighted average of the levels of FPG that were associated with the lowest risk of mortality in the pooled analyses of prospective cohort studies.[1]

Relative risks

We estimate 15 outcomes due to high fasting plasma glucose (continuous risk) or diabetes (categorical risk).

**Risk**	**Outcome**
Fasting plasma glucose	Ischemic heart disease
Fasting plasma glucose	Ischemic stroke
Fasting plasma glucose	Subarachnoid hemorrhage
Fasting plasma glucose	Intracerebral hemorrhage
Fasting plasma glucose	Peripheral vascular disease
Fasting plasma glucose	Type 1 diabetes
Fasting plasma glucose	Type 2 diabetes
Fasting plasma glucose	Chronic kidney disease due to Type 1 diabetes
Fasting plasma glucose	Chronic kidney disease due to Type 2 diabetes
Diabetes mellitus	Drug-resistant tuberculosis
Diabetes mellitus	Drug-susceptible tuberculosis

Diabetes mellitus	Multidrug-resistant tuberculosis without extensive drug resistance
Diabetes mellitus	Extensively drug-resistant tuberculosis
Diabetes mellitus	Liver cancer due to NASH
Diabetes mellitus	Liver cancer due to other causes
Diabetes mellitus	Pancreatic cancer
Diabetes mellitus	Ovarian cancer
Diabetes mellitus	Colorectal cancer
Diabetes mellitus	Bladder cancer
Diabetes mellitus	Lung cancer
Diabetes mellitus	Breast cancer
Diabetes mellitus	Glaucoma
Diabetes mellitus	Cataracts
Diabetes mellitus	Dementia

Relative risks for High Fasting Plasma Glucose (continuous risk)

After a review of the chronic kidney disease literature, we determined that there is only an attributable risk of chronic kidney disease due to diabetes type 1 and chronic kidney disease due to diabetes type 2 to FPG. Thus, in GBD 2019 we removed chronic kidney disease due to glomerulonephritis, chronic kidney disease due to hypertension, chronic kidney disease due to other causes as an outcome.

Relative risks (RR) were obtained from dose-response meta-analysis of prospective cohort studies. Please see the citation list for a full list of studies that are utilized. For cardiovascular outcomes, we estimated age-specific RRs using DisMod-MR 2.1 with log (RR) as the dependent variable and median age at event as the independent variable with an intercept at age 110. Morbidity and mortality directly caused by diabetes type 1 and diabetes type 2 is considered directly attributable to FPG.

Relative risks for Diabetes mellitus (Categorical risk)

Relative risks were obtained from meta-analysis of cohort studies. Please see the citation list for a full list of studies that are utilized.

References

1 . Singh GM, Danaei G, Farzadfar F, *et al.* The age-specific quantitative effects of metabolic risk factors on cardiovascular diseases and diabetes: a pooled analysis. *PloS One* 2013; **8**: e65174.

High LDL cholesterol

Flowchart

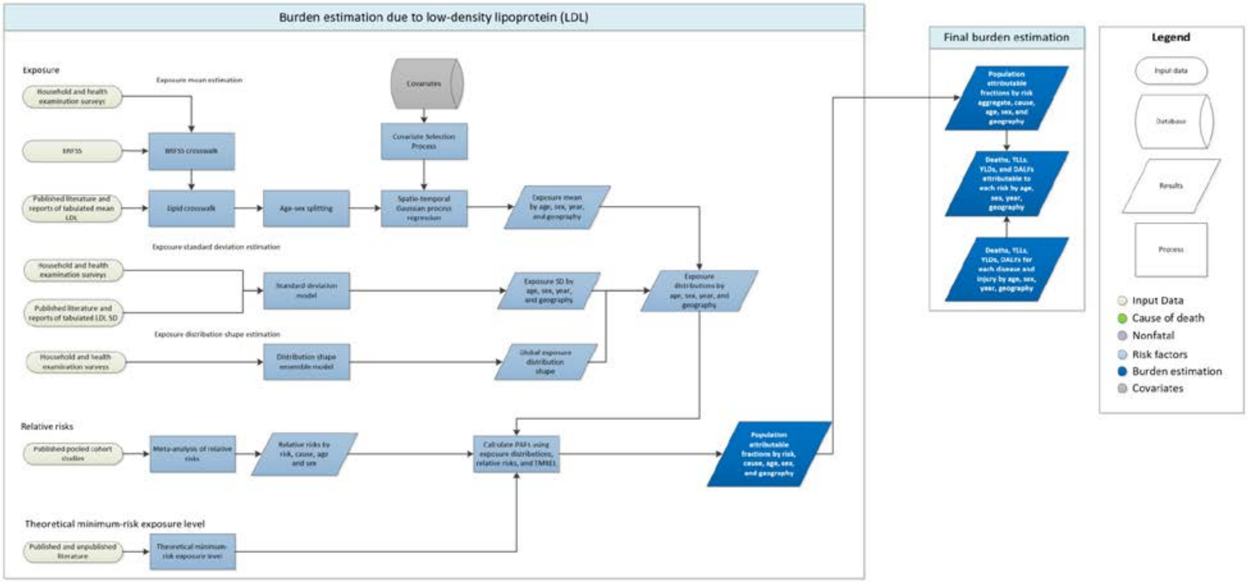

Input data and methodological summary

Exposure

Case definition

In earlier iterations of the GBD study, we estimated burden attributable to total cholesterol. Beginning in GBD 2017, we modelled blood concentration of low-density lipoprotein (LDL) in units of mmol/L.

Input data

We used data on blood levels for low-density lipoprotein, total cholesterol, triglyceride, and high-density lipoprotein from literature and from household survey microdata and reports. We adjusted data for total cholesterol, triglycerides, and high-density lipoprotein using the correction approach described in the Lipid Crosswalk section below. Counts of the data inputs used for GBD 2019 are show in Tables 1 and 2 below. Details of inclusion and exclusion criteria and data processing steps follow.

Table 1: Data inputs for exposure for low-density lipoprotein

Input data	Exposure
Total sources	711
Number of countries with data	145

Table 2: Data inputs for relative risks for low-density lipoprotein

Input data	Relative risk
Source count (total)	1

Inclusion criteria

Studies were included if they were population-based and measured total LDL, total cholesterol (TC), high-density lipoprotein (HDL), and/or triglycerides (TG) were available from blood tests or if LDL was calculated using the Friedewald equation. We assumed the data were representative of the location if the geography or population chosen were not related to the diseases and if it was not an outlier compared to other data in the country or region.

Outliers

Data were utilised in the modelling process unless an assessment of data strongly suggested that the data were biased. A candidate source was excluded if the quality of study did not warrant a valid estimate because of selection (non-representative populations) or if the study did not provide methodological details for evaluation. In a small number of cases, a data point was considered to be an outlier candidate if the level was implausibly low or high based on expert judgement and other country data.

Data extraction

Where possible, individual-level data on LDL estimates were extracted from survey microdata and these were collapsed across demographic groupings to produce mean estimates in the standard GBD five-year age-sex groups. If microdata were unavailable, information from survey reports or from literature were extracted along with any available measure of uncertainty including standard error, uncertainty intervals, and sample size. Standard deviations were also extracted. Where LDL was reported split out by groups other than age, sex, location, and year (eg, by diabetes status), a weighted mean was calculated.

Lipid crosswalk

Total cholesterol consists of three major components: LDL, HDL, and TG. LDL is often calculated for an individual using the Friedewald equation, shown below:

$$LDL = TC - \left(HDL + \frac{TGL}{2.2}\right)$$

We utilised this relationship at the individual level to impute the mean LDL for a study population when only data on TC, HDL, and TGL were available. Because studies report different combinations of TC, HDL, and TGL, we constructed a single regression to utilise all available data to evaluate the relationship between each lipid and LDL at the population level. We used the following regression:

$$LDL = ind_{tc}\beta_1 TC - \left(ind_{hdl}\beta_2 HDL + ind_{tgl}\beta_3 TGL\right) + \sum \alpha_l I_l$$

Where ind_{tc}, ind_{hdl}, and ind_{tgl} are indicator variables for whether data are available for a given lipid, I_l is an indicator variable a given set of available lipids l. α_l is a unique intercept for each set of available lipid combinations. For example, for sources that only reported TC and HDL, $\alpha_{l=TC,HDL}$ should account for the missing lipid data, ie, TGL. The form of this regression allows us to estimate the betas for each lipid using all available data. As a sensitivity analysis, we also ran separate regressions for each set of available lipids and found that the single regression method had much lower root-mean-squared error. A comparison of the observed versus predicted LDL for each set of available lipids is shown in Figure 1. We found almost no relationship between LDL and HDL or TGL when TC was not available, so only studies that reported TC were adjusted to LDL.

Figure 1. Results of the lipid crosswalk using a single regression method

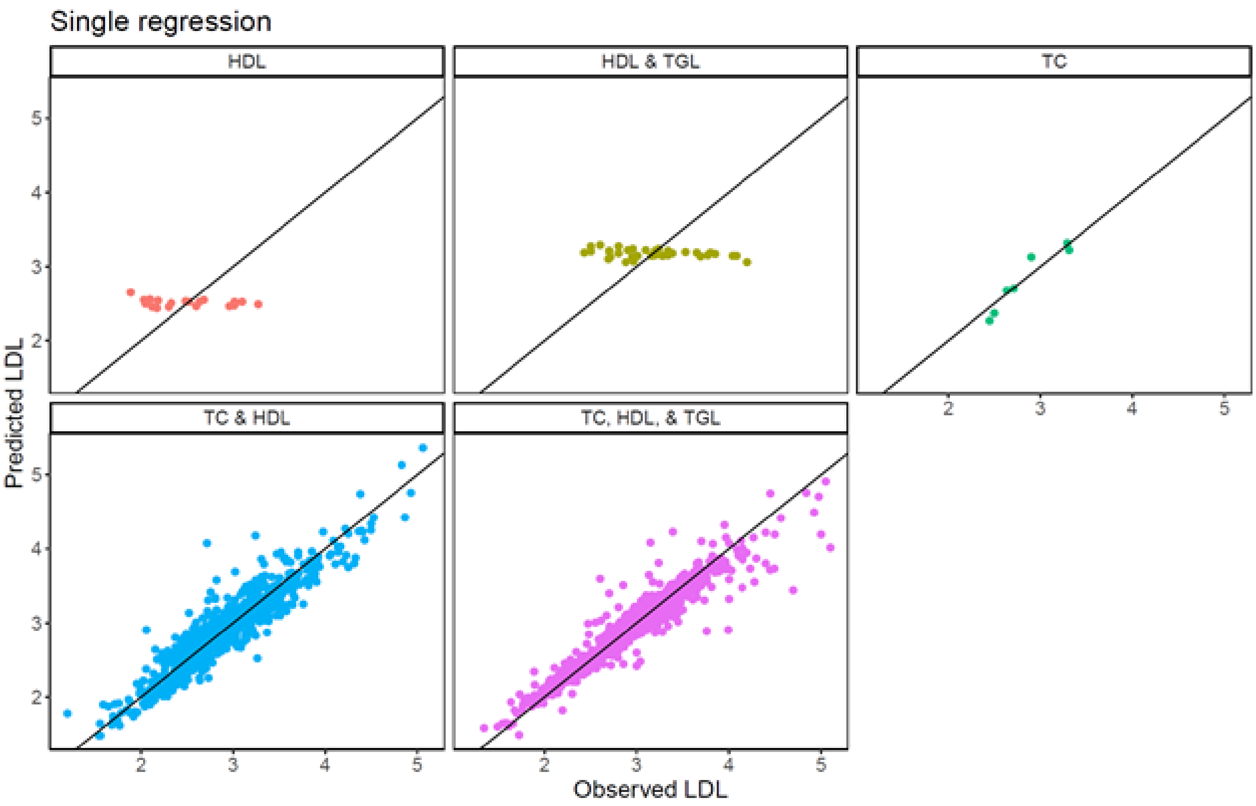

Incorporating United States prevalence data

Survey reports and literature often report information only about the prevalence, but not the level, of hypercholesterolemia in the population studied. These sources were not used to model LDL, with the exception of data from the Behavioral Risk Factors Surveillance System (BRFSS) because of the availability of a similarly structured exam survey covering the identical population (NHANES). BRFSS is a telephone survey conducted in the United States for all counties. It collects self-reported diagnosis of hypercholesterolemia. These self-reported values of prevalence of raised total cholesterol in each age group, sex, US state, and year were used to predict a mean total cholesterol for the same strata with a regression using data from the National Health and Nutrition Examination Survey, a nationally representative health examination survey of the US adult population. The regression was:

$$\mathrm{TC}_{l,a,t,s} = \beta_0 + \beta_1 \mathrm{prev}_{l,a,t,s}$$

where $\mathrm{TC}_{l,a,t,s}$ *is the location, age, time, and sex specific mean total cholesterol and* $\mathrm{prev}_{l,a,t,s}$ is the location, age, time, and sex specific prevalence of raised total cholesterol. The coefficients for both models are reported in Table 1.

Table 3. Coefficients in the sex-specific US states TC prediction models

Term	Male model	Female model
Intercept	4.23	4.36
Prevalence	6.25	5.22

Out of sample RMSE was used to quantify the predictive validity of the model. The regression was repeated 10 times for each sex, each time randomly holding out 20% of the data. The RMSEs from each holdout analysis were averaged to get the average out of sample RMSE. The results of this holdout analysis are reported in Table 2. Total cholesterol estimates were crosswalked to LDL using the lipid crosswalk reported above.

Table 4. Out of sample RMSEs of the sex-specific US states TC prediction models

	Male model	Female model
Out of sample RMSE	0.21 mmol/L	0.20 mmol/L

Age and sex splitting
Prior to modelling, data provided in age groups wider than the GBD five-year age groups were processed using the approach outlined in Ng and colleagues.[2] Briefly, age-sex patterns were identified using person-level microdata (58 sources), and estimate age-sex-specific levels of total cholesterol from aggregated results reported in published literature or survey reports. In order to incorporate uncertainty into this process and borrow strength across age groups when constructing the age-sex pattern, we used a model with auto-regression on the change in mean LDL over age groups:

$$\mu_a = \mu_{a-1} + \omega_a$$
$$\omega_a \sim N(\omega_{a-1}, \tau)$$

Where μ_a is the mean predicted value for age group a, μ_{a-1} is the mean predicted value for the age group previous to age group a, ω_a is the difference in mean between age group *a* and age group *a-1*, ω_{a-1} is the difference between age group *a-1* and age group *a-2*, and τ is a user-input prior on how quickly the mean LDL changes for each unit increase in age. We used a τ of 0.05 mmol/L for this model. Draws of the age-sex pattern were combined with draws of the input data needing to be split in order to calculate the new variance of age-sex-split data points.

Modelling strategy
Exposure estimates were produced from 1980 to 2019 for each national and subnational location, sex, and for each five-year age group starting from 25. As in GBD 2017, we used a spatiotemporal Gaussian process regression (ST-GPR) framework to model the mean LDL at the location-, year-, age-, and sex-level. Details of the ST-GPR method used in GBD 2019 can be found elsewhere in the appendix.

Covariate selection
The first step of the ST-GPR framework requires the creation of a linear model for predicting LDL at the location-, year-, age-, sex- level. Covariates for this model were selected in two stages. First a list of variables with an expected causal relationship with LDL was created based on significant association found within high-quality prospective cohort studies reported in the published scientific literature. The second stage in covariate selection was to test the predictive validity of every possible combination of covariates in the linear model, given the covariates selected above. This was done separately for each sex. Predictive validity was measured with out of sample root-mean-squared error.

In GBD 2016, the linear model with the lowest root-mean-squared error for each sex was then used in the ST-GPR model. Beginning in GBD 2017, we used an ensemble model of the 50 models with the lowest root-mean-squared error for each sex. This allows us to utilise covariate information from many plausible linear mixed-effects models. The 50 models were each used to predict the mean LDL for every age, sex, location, and year, and the inverse-RMSE-weighted average of this set of 50 predictions was used as the linear prior. The relative weight contributed by each covariate is plotted by sex in Figure 2.

Figure 2. Results of the ensemble linear model covariate selection

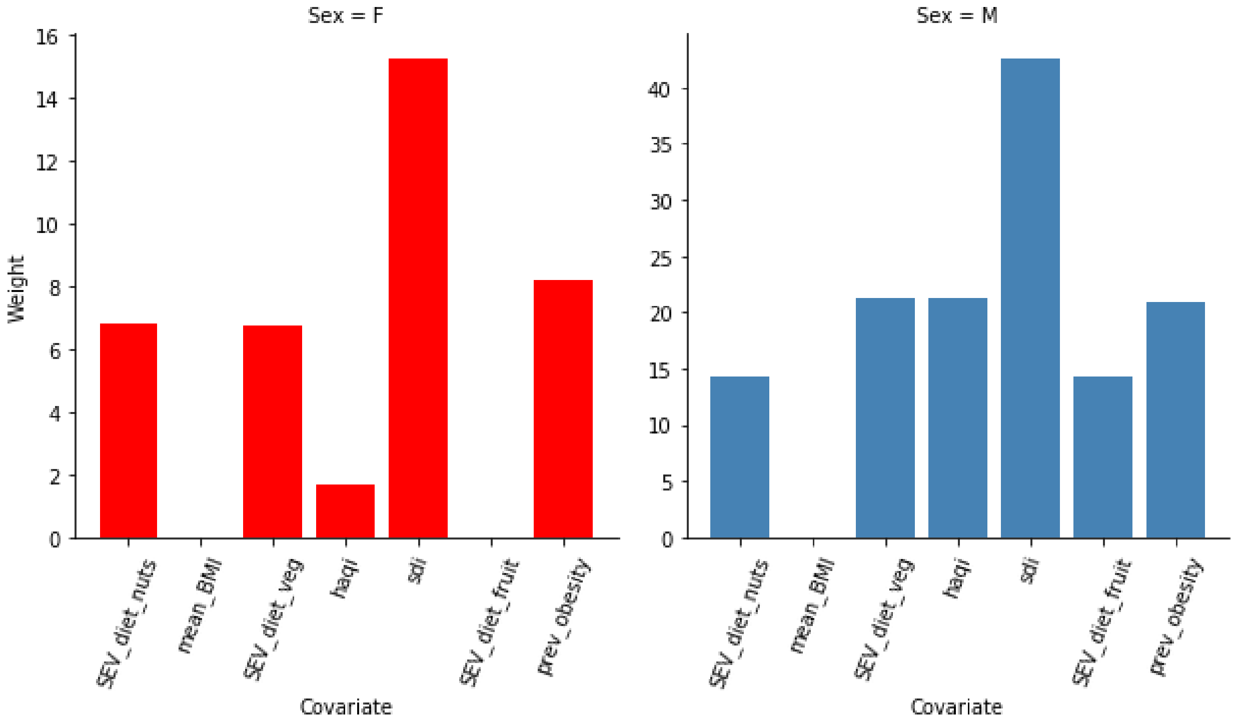

The results of the ensemble linear model were used for the first stage in an ST-GPR model. The result of the ST-GPR model are estimates of the mean LDL for each age, sex, location, and year.

Estimate of standard deviation
The standard deviation of LDL within a population was estimated for each national and subnational location, sex, and five-year age group starting from age 25 using the standard deviation from person-level and some tabulated data sources. Person-level microdata accounted for 3009 of the total 4001 rows of data on standard deviation. The remaining 992 rows came from tabulated data. Tabulated data were only used to model standard deviation if they were sex-specific and five-year-age-group-specific

and reported a population standard deviation LDL. The LDL standard deviation function was estimated using a linear regression:

$$\log(SD_{c,a,t,s}) = \beta_0 + \beta_1 \log(mean\_LDL_{c,a,t,s}) + \beta_4 sex + \sum_{k=2}^{16} \beta_k I_{A[a]}$$

where $mean\_LDL_{c,a,t,s}$ is the country-, age-, time-, and sex-specific mean LDL estimate from ST-GPR, and $I_{A[a]}$ is a dummy variable for a fixed effect on a given five-year age group.

Distribution shape modelling

The shape of the distribution of LDL was estimated using all available person-level microdata sources, which was a subset of the input data into the modelling process. The distribution shape modelling framework for GBD 2019 is detailed elsewhere in the appendix. Briefly, an ensemble distribution created from a weighted average of distribution families was fit for each individual microdata source, separately by sex. The weights for the distribution families for each individual source were then averaged and weighted to create a global ensemble distribution for each sex.

Theoretical minimum-risk exposure level

For GBD 2017, we reviewed the literature to select a TMREL for LDL. A meta-analysis of randomised trials has shown that outcomes can be improved even at low levels of LDL-cholesterol, below 1.3 mmol/L.[3] Recent studies of PCSK-9 inhibitors support these results.[4] We therefore used a TMREL with a uniform distribution between 0.7 and 1.3 mmol/L; this value remained unchanged for GBD 2019.

Relative risks

After a systematic search, we were unable to find relative risks for LDL that were reported by age and level of LDL. Given this evidence that the relative risks for LDL and TC are very similar[5] and the strong linear correlation between TC and LDL at the individual level, we used relative risks reported for TC to approximate the relative risks for LDL. We used DisMod-MR 2.1 to pool effect sizes from included studies and generate a dose-response curve for each of the outcomes associated with LDL. The tool enabled us to incorporate random effects across studies and include data with different age ranges. RRs were used universally for all countries and produce RRs with uncertainty and covariance across ages, considering the uncertainty of the data points.

As in GBD 2017, RRs for IHD and ischaemic stroke are obtained from meta-regressions of pooled epidemiological studies: the Asia Pacific Cohort Studies Collaboration (APCSC) and the Prospective Studies Collaboration (PSC).[6] RRs for IHD were modelled with log (RR) as the dependent variable and median age at event as the independent variable with an age intercept (RR equals 1) at age 110. For LDL and ischaemic stroke, a similar approach was used, except that there was no age intercept at age 110, due to the fact that there was no statistically significant relationship between LDL and stroke after age 70 with a mean RR less than one. We assumed that there is not a protective effect of LDL and therefore did not include an RR for ages 80+.

References

1. Roth GA, Fihn SD, Mokdad AH, Aekplakorn W, Hasegawa T, Lim SS. High total serum cholesterol, medication coverage and therapeutic control: an analysis of national health examination survey data from eight countries. *Bull World Health Organ* 2011; **89**: 92–101.

2 . Ng M, Fleming T, Robinson M, *et al.* Global, regional, and national prevalence of overweight and obesity in children and adults during 1980–2013: a systematic analysis for the Global Burden of Disease Study 2013. *The Lancet* 2014; **384**: 766–81.
3 . Boekholdt SM, Hovingh GK, Mora S, *et al.* Very Low Levels of Atherogenic Lipoproteins and the Risk for Cardiovascular EventsA Meta-Analysis of Statin Trials. *J Am Coll Cardiol* 2014; **64**: 485–94.
4. Sabatine MS, Giugliano RP, Keech AC, et al. Evolocumab and Clinical Outcomes in Patients with Cardiovascular Disease. *N Engl J Med*. 2017; **376**:1713-1722.
5. Wilson PF, D'Agostino RB, Levy D, Belanger AM, Silbershatz H, Kannel WB. Prediction of Coronary Heart Disease Using Risk Factor Categories. *Circulation*. 1998; **97**:1837-1847.
6. Singh GM, Danaei G, Farzadfar F, *et al.* The age-specific quantitative effects of metabolic risk factors on cardiovascular diseases and diabetes: a pooled analysis. *PloS One* 2013; **8**: e65174.

High systolic blood pressure

Flowchart

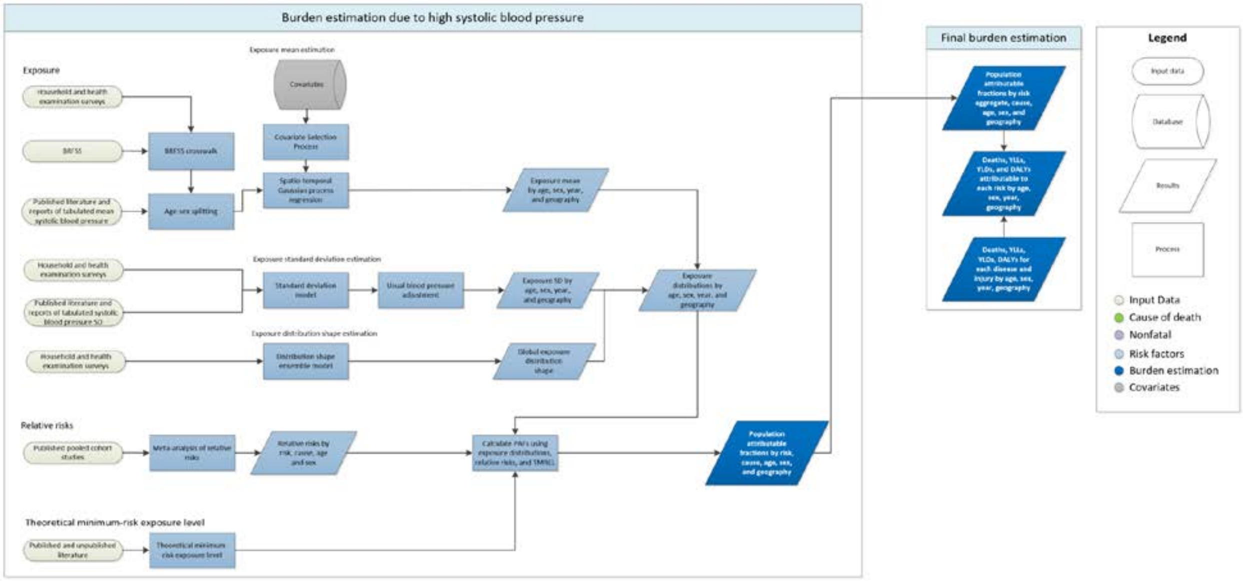

Input data and methodological summary

Exposure

Case definition

Brachial systolic blood pressure in mmHg.

Input data

We utilised data on mean systolic blood pressure from literature and from household survey microdata and reports (e.g. STEPS, NHANES). For GBD 2019, we did not carry out a systematic review of the literature for new data. Counts of the data inputs used for GBD 2019 are show in Tables 1 and 2 below. Details of inclusion and exclusion criteria and data processing steps follow.

Table 1: Data inputs for exposure for systolic blood pressure.

Input data	Exposure
Total sources	1112
Number of countries with data	166

Table 2: Data inputs for relative risks for systolic blood pressure.

Input data	Relative risk
Source count (total)	3

Inclusion criteria

Studies were included if they were population-based and directly measured systolic blood pressure using a sphygmomanometer. We assumed the data were representative if the geography or the population were not selected because it was related to hypertension or hypertensive outcomes.

Outliers

Data were utilised in the modelling process unless an assessment strongly suggested that the source was biased. A candidate source was excluded if the quality of study did not warrant a valid estimate because of selection (non-representative populations) or if the study did not provide methodological details for evaluation. In a small number of cases, a data point was considered to be an outlier candidate if the level was implausibly low or high based on expert judgement and data from other country data.

Data extraction

Where possible, individual-level data on blood pressure estimates were extracted from survey microdata. These data points were collapsed across demographic groupings to produce mean estimates in the standard GBD five-year age-sex groups. If microdata were unavailable, information from survey reports or from literature were extracted along with any available measure of uncertainty including standard error, uncertainty interval, and sample size. Standard deviations were also extracted. Where mean systolic blood pressure was reported split out by groups other than age, sex, location, and year (e.g. by hypertensive status), a weighted mean was calculated.

Incorporating United States prevalence data

Survey reports and literature often report information only about the prevalence, but not the level, of hypertension in the population studied. These sources were not used to model systolic blood pressure, with the exception of data from the Behavioral Risk Factors Surveillance System (BRFSS) because of the availability of a similarly structured exam survey that is representative of the same population (NHANES). BRFSS is a telephone survey conducted in the United States for all US counties. It collects self-reported diagnosis of hypertension. These self-reported values of prevalence of raised blood pressure were adjusted for self-report bias and tabulated by age group, sex, US state, and year. These prevalence values were used to predict a mean systolic blood pressure for the same strata with a regression using data from the National Health and Nutrition Examination Survey, a nationally representative health examination survey of the US adult population. The regression was run separately by sex, and was specified as:

$$\mathrm{SBP}_{l,a,t,s} = \beta_0 + \beta_1 \mathrm{prev}_{l,a,t,s}$$

where $\mathrm{SBP}_{l,a,t,s}$ is the location, age, time, and sex specific mean systolic blood pressure and $\mathrm{prev}_{l,a,t,s}$ is the location, age, time, and sex specific prevalence of raised blood pressure. The coefficients for both models are reported in Table 3.

Table 3. Coefficients in the sex-specific US states blood pressure prediction models

Term	Male model	Female model
Intercept (β_0)	114.65	108.28
Prevalence (β_1)	51.86	68.87

Out of sample RMSE was used to quantify the predictive validity of the model. The regression was repeated 10 times for each sex, each time randomly holding out 20% of the data. The RMSEs from each holdout analysis were averaged to get the average out of sample RMSE. The results of this holdout analysis are reported in Table 4.

Table 4. Out of sample RMSEs of the sex-specific US states blood pressure prediction models

	Male model	Female model

Out of sample RMSE	2.37 mmHg	3.27 mmHg

Age and sex splitting

Prior to modelling, data provided in age groups wider than the GBD five-year age groups were processed using the approach outlined in Ng and colleagues.[2] Briefly, age-sex patterns was identified using 115 sources of microdata with multiple age-sex groups, and these patterns were applied to estimate age-sex-specific levels of mean systolic blood pressure from aggregated results reported in published literature or survey reports. In order to incorporate uncertainty into this process and borrow strength across age groups when constructing the age-sex pattern, we used a model with auto-regression on the change in mean SBP over age groups:

$$\mu_a = \mu_{a-1} + \omega_a$$
$$\omega_a \sim N(\omega_{a-1}, \tau)$$

Where μ_a is the mean predicted value for age group a, μ_{a-1} is the mean predicted value for the age group previous to age group a, ω_a is the difference in mean between age group *a* and age group *a-1*, ω_{a-1} is the difference between age group *a-1* and age group *a-2*, and τ is a user-input prior on how quickly the mean SBP changes for each unit increase in age. We used a τ of 1.5 mmHg for this model. Draws of the age-sex pattern were combined with draws of the input data needing to be split in order to calculate the new variance of age-sex split data points.

Modelling

Exposure estimates were produced from 1980 to 2019 for each national and subnational location, sex, and for each five-year age group starting from 25+. As in GBD 2017, we used a spatiotemporal Gaussian process regression (ST-GPR) framework to model the mean systolic blood pressure at the location-, year-, age-, sex- level. Details of the ST-GPR method used in GBD 2019 can be found elsewhere in the appendix.

Covariate selection

The first step of the ST-GPR framework requires the creation of a linear model for predicting SBP at the location-, year-, age-, sex- level. Covariates for this model were selected in two stages. First a list of variables with an expected causal relationship with SBP was created based on significant association found within high-quality prospective cohort studies reported in the published scientific literature. The second stage in covariate selection was to test the predictive validity of every possible combination of covariates in the linear model, given the covariates selected above. This was done separately for each sex. Predictive validity was measured with out of sample root-mean-squared error.

In GBD 2016, the linear model with the lowest root-mean-squared error for each sex was then used in the ST-GPR model. Beginning in GBD 2017, we used an ensemble model of the 50 models with the lowest root-mean-squared error for each sex. This allows us to utilise covariate information from many plausible linear mixed-effects models. The 50 models were each used to predict the mean SBP for every age, sex, location, and year, and the inverse-RMSE-weighted average of this set of 50 predictions was used as the linear prior. The relative weight contributed by each covariate is plotted by sex in Figure 1.

Figure 1. Results of the ensemble linear model covariate selection

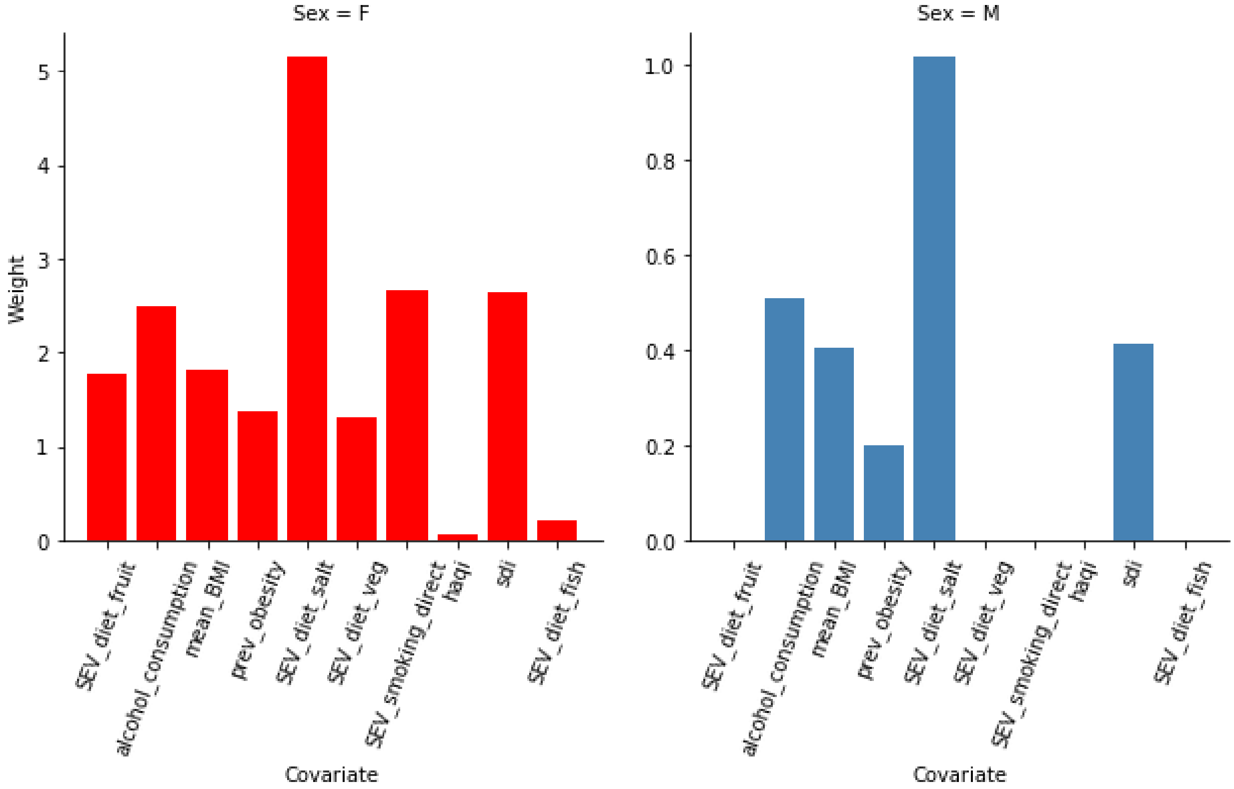

The results of the ensemble linear model were used for the first stage in an ST-GPR model. The result of the ST-GPR model are estimates of the mean SBP for each age, sex, location, and year.

Estimate of standard deviation

Currently, the ST-GPR model only produces an estimate of mean exposure level without standard deviation. Therefore, the standard deviation of systolic blood pressure within a population was estimated for each national and subnational location, sex, and five-year age group starting from age 25 using the standard deviation from person-level and some tabulated data sources. Person-level microdata accounted for 10 375 of the total 12 570 rows of data on standard deviation. The remaining 2195 rows came from tabulated data. Tabulated data were only used to model standard deviation if it was sex-specific and five-year-age-group-specific and reported a population standard deviation of systolic blood pressure. The systolic blood pressure standard deviation function was estimated using a linear regression:

$$\log(\mathrm{SD}_{l,a,t,s}) = \beta_0 + \beta_1 \log(\mathrm{mean\_SBP}_{l,a,t,s}) + \beta_4 \mathrm{sex} + \sum_{k=2}^{16} \beta_k I_A$$

where $\mathrm{mean\_SBP}_{l,a,t,s}$ is the location-, age-, time-, and sex-specific mean SBP estimate from ST-GPR, and I_A is a dummy variable for a fixed effect on a given five-year age group.

Adjustment for usual levels of blood pressure

To account for in-person variation in systolic blood pressure, a "usual blood pressure" adjustment was done. The need for this adjustment has been described elsewhere.[5] Briefly, measurements of a risk

factor taken at a single time point may not accurately capture an individual's true long-term exposure to that risk. Blood pressure readings are highly variable over time due to measurement error as well as diurnal, seasonal, or biological variation. These sources of variation result in an overestimation of the variation in cross-sectional studies of the distribution of SBP.

To adjust for this overestimation, we applied a correction factor to each location-, age-, time-, and sex-specific standard deviation. These correction factors were age-specific and represented the proportion of the variation in blood pressure within a population that would be observed if there were no within-person variation across time. Four longitudinal surveys were used to estimate these factors: the China Health and Retirement Longitudinal Survey (CHRLS), the Indonesia Family Life Survey (IFLS), the National Health and Nutrition Examination Survey I Epidemiological Follow-up Study (NHANES I/EFS), and the South Africa National Income Dynamics Survey (NIDS). The sample size and number of blood pressure measurements at each measurement period for each survey is reported in Table 5.

Table 1. Characteristics of longitudinal surveys used for the usual blood pressure adjustment

Source	Measurement periods	Number of measurements	Sample size
CHRLS	2008	3	1967
	2012	3	1419
IFLS	1997	1	19 418
	2000	1	16 626
	2007	3	14 136
NIDS	1997	2	14 084
	2000	2	9612
	2007	2	9098
NHANES I/EFS	1971–1976	2	20 716
	1982–1984	3	9932

For each survey, the following regression was created for each age group:

$$\mathrm{SBP}_{i,a} = \beta_0 + \beta_1 \mathrm{sex} + \beta_3 \mathrm{age} + +\upsilon_i$$

where $\mathrm{SBP}_{i,a}$ is the systolic blood pressure of an individual i at age a, sex is a dummy variable for the sex of an individual, age is a continuous variable for the age of an individual, and υ_i is a random intercept for each individual. Then, a blood pressure value $\widehat{\mathrm{SBP}}_{i,b}$ was predicted for each individual i for his/her age at baseline b. The correction factor cf for each age group within each survey was calculated as variation in these predicted blood pressures was divided by the variation in the observed blood pressures at baseline, $\mathrm{SBP}_{i,b}$:

$$\mathrm{cf} = \sqrt{\frac{\mathrm{var}(\widehat{\mathrm{SBP}}_{b})}{\mathrm{var}(\mathrm{SBP}_{b})}}$$

The average of the correction factors was taken over the three surveys to get one set of age-specific correction factors, which were then multiplied by the square of the modelled standard deviations to estimate standard deviation of the "usual blood pressure" of each age, sex, location, and year. Because of low sample sizes, the correction factors for the 75–79 age group was used for all terminal age groups. The final correction factors for each age group are reported in Table 6. Figure 2 shows the correction factors by survey and age group ID.

Table 2. Age-specific usual blood pressure correction factors

Age group	Correction factor
25–29	0.665
30–34	0.713
35–39	0.737
40–44	0.733
45–49	0.798
50–54	0.771
55–59	0.764
60–64	0.753
65–69	0.719
70–74	0.689
75+	0.678

Figure 2: Correction factor by survey and age group id. The correction factor is equal to the variance of the predictions divided by the variance of the raw dataset. In pink is the average correction factor for each age group, summarised in Table 6.

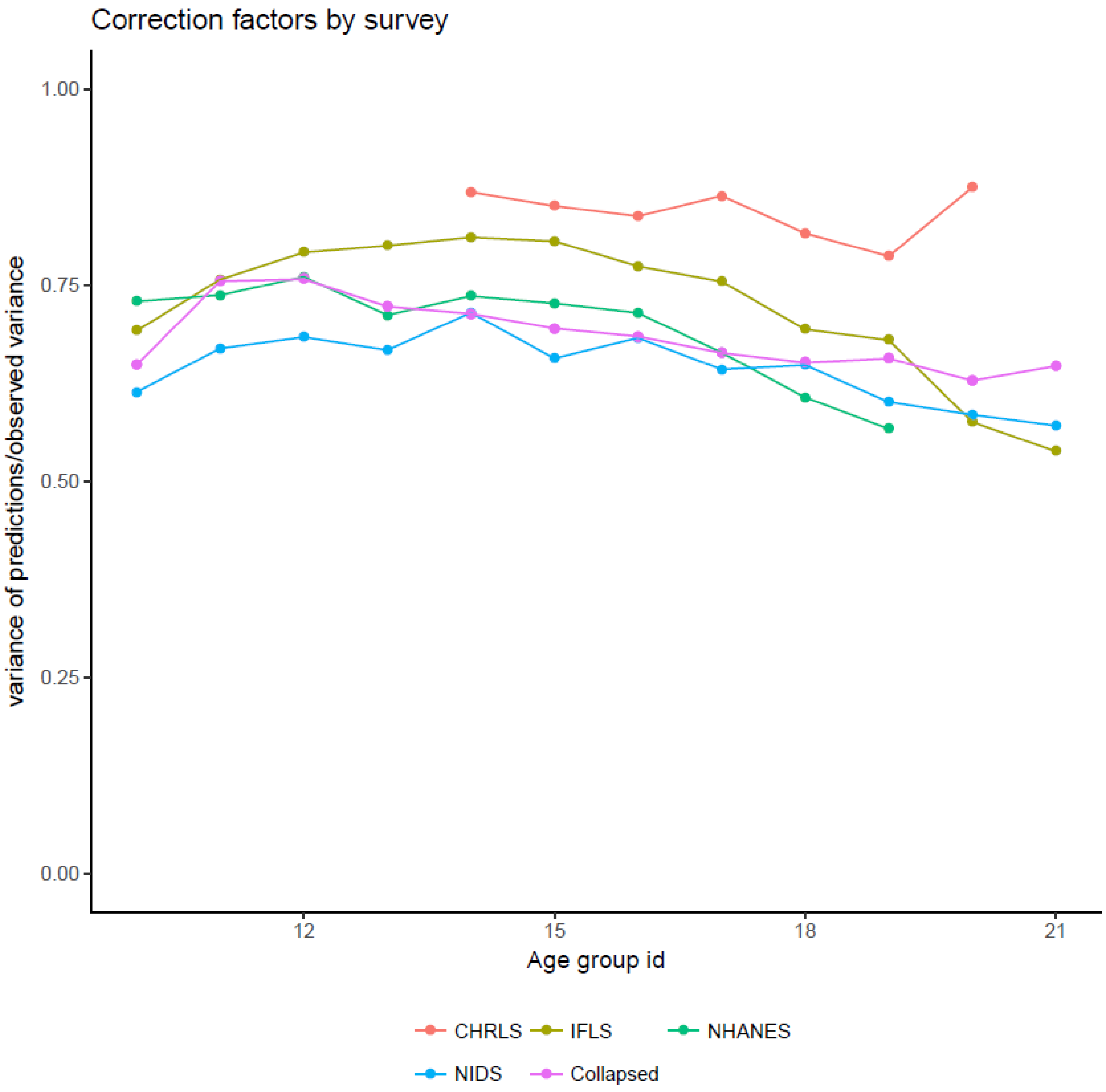

A visualisation of how the uncorrected blood pressure measurements overestimate the "usual" blood pressure variation is shown in Figure 3. This image shows the density of the distribution of the observed blood pressure values $\mathrm{SBP}_{\mathrm{i,b}}$ in participants in the Indonesian Family Life Study survey in red, and the density of the predicted blood pressure values $\widehat{\mathrm{SBP}}_{\mathrm{i,b}}$ in blue. The ratio of the variance of the blue distribution to the variance of the red distribution is an example of the scalar adjustment factor being applied to the modelled standard deviations.

Figure 3: Raw and predicted distributions of blood pressure in the Indonesia Family Life Survey

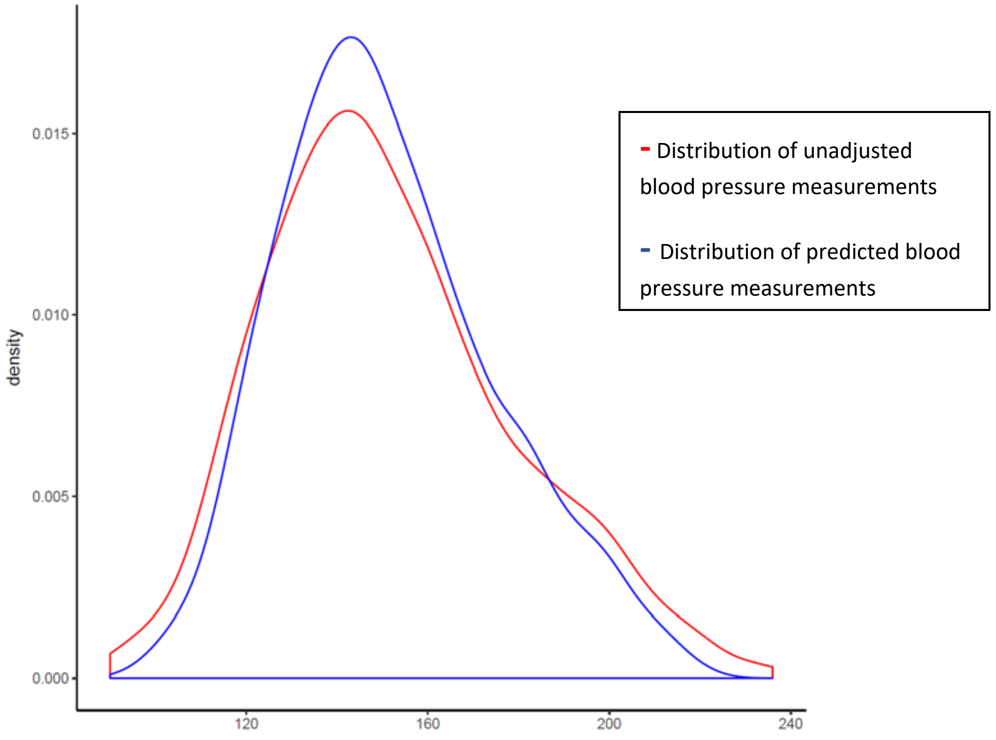

Estimating the exposure distribution shape

The shape of the distribution of systolic blood pressure was estimated using all available person-level microdata sources, which was a subset of the input data into the modelling process. The distribution shape modelling framework for GBD 2019 is detailed in the elsewhere in the appendix. Briefly, an ensemble distribution created from a weighted average of distribution families was fit for each individual microdata source, separately by sex. The weights for the distribution families for each individual source were then averaged and weighted to create a global ensemble distribution for each sex.

Theoretical minimum-risk exposure level

No changes have been made to the TMREL used for systolic blood pressure since GBD 2015. We estimated that the TMREL of SBP ranges from 110 to 115 mmHg based on pooled prospective cohort studies that show risk of mortality increases for SBP above that level.[3,4] Our selection of a TMREL of 110–115 mmHg is consistent with the GBD study approach of estimating all attributable health loss that could be prevented even if current interventions do not exist that can achieve such a change in exposure level, for example a tobacco smoking prevalence of zero percent. To include the uncertainty in the TMREL, we took a random draw from the uniform distribution of the interval between 110 mmHg and 115 mmHg each time the population attributable burden was calculated.

Relative risks

No changes have been made to the relative risk estimates for blood pressure outcomes used since GBD 2016. RRs for chronic kidney disease are from the Renal Risk Collaboration meta-analysis of 2.7 million individuals in 106 cohorts. For other outcomes, we used data from two pooled epidemiological studies:

the Asia Pacific Cohort Studies Collaboration (APCSC) and the Prospective Studies Collaboration (PSC).[4,5] Additional estimates of RR for cardiovascular outcomes were used from the CALIBER study, a health-record linkage cohort study from the UK.[6]

For cardiovascular disease, epidemiological studies have shown that the RR associated with SBP declines with age, with the log (RR) having an approximately linear relationship with age and reaching a value of 1 between the ages of 100 and 120. RRs were reported per 10 mmHg increase in SBP above the TMREL value (115 mmHg), calculated as in the equation below:

$$RR(x) = RR_0^{\frac{(x-TMREL)}{10\,mmHg}}$$

Where $RR(x)$ is the RR at exposure level x and RR_0 is the increase in RR for each 10 mmHg above the TMREL. We used DisMod-MR 2.1 to pool effect sizes from included studies and generate a dose-response curve for each of the outcomes associated with high SBP. The tool enabled us to incorporate random effects across studies and include data with different age ranges. RRs were used universally for all countries and the meta-regression only helped to pool the three major sources and produce RRs with uncertainty and covariance across ages taking into account the uncertainty of the data points.

References

1 Bangalore S, Gong Y, Cooper-DeHoff RM, Pepine CJ, Messerli FH. 2014 Eighth Joint National Committee panel recommendation for blood pressure targets revisited: results from the INVEST study. *J Am Coll Cardiol* 2014; **64**: 784–93.

2 Ng M, Fleming T, Robinson M, *et al.* Global, regional, and national prevalence of overweight and obesity in children and adults during 1980–2013: a systematic analysis for the Global Burden of Disease Study 2013. *The Lancet* 2014; **384**: 766–81.

3 Singh GM, Danaei G, Farzadfar F, *et al.* The age-specific quantitative effects of metabolic risk factors on cardiovascular diseases and diabetes: a pooled analysis. *PloS One* 2013; **8**: e65174.

4 Collaboration APCS, others. Blood pressure and cardiovascular disease in the Asia Pacific region. *J Hypertens* 2003; **21**: 707–16.

5 Prospective Studies Collaboration. Age-specific relevance of usual blood pressure to vascular mortality: a meta-analysis of individual data for one million adults in 61 prospective studies. *The Lancet* 2002; **360**: 1903–13.

6 Rapsomaniki E, Timmis A, George J, *et al.* Blood pressure and incidence of twelve cardiovascular diseases: lifetime risks, healthy life-years lost, and age-specific associations in 1·25 million people. *Lancet Lond Engl* 2014; **383**: 1899–911.

High body-mass index

Flowchart

Adult (Ages 20+) High Body-Mass Index: Data and Model Flow Chart

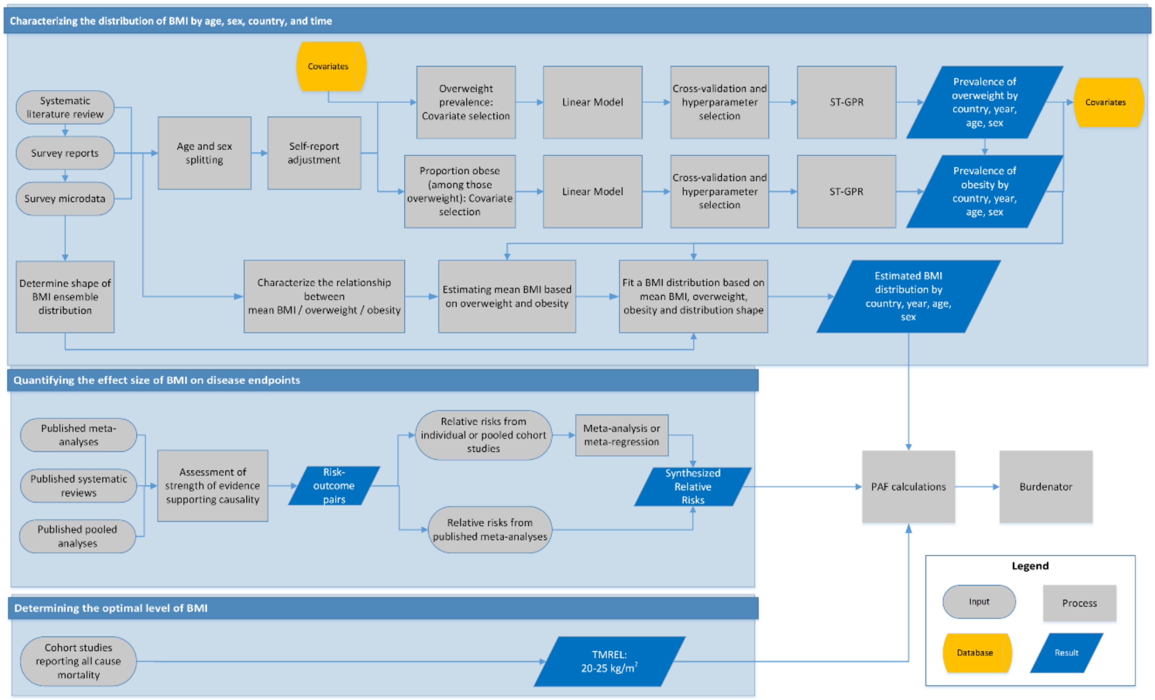

Childhood (Ages 2-19) High Body-Mass Index: Data and Model Flow Chart

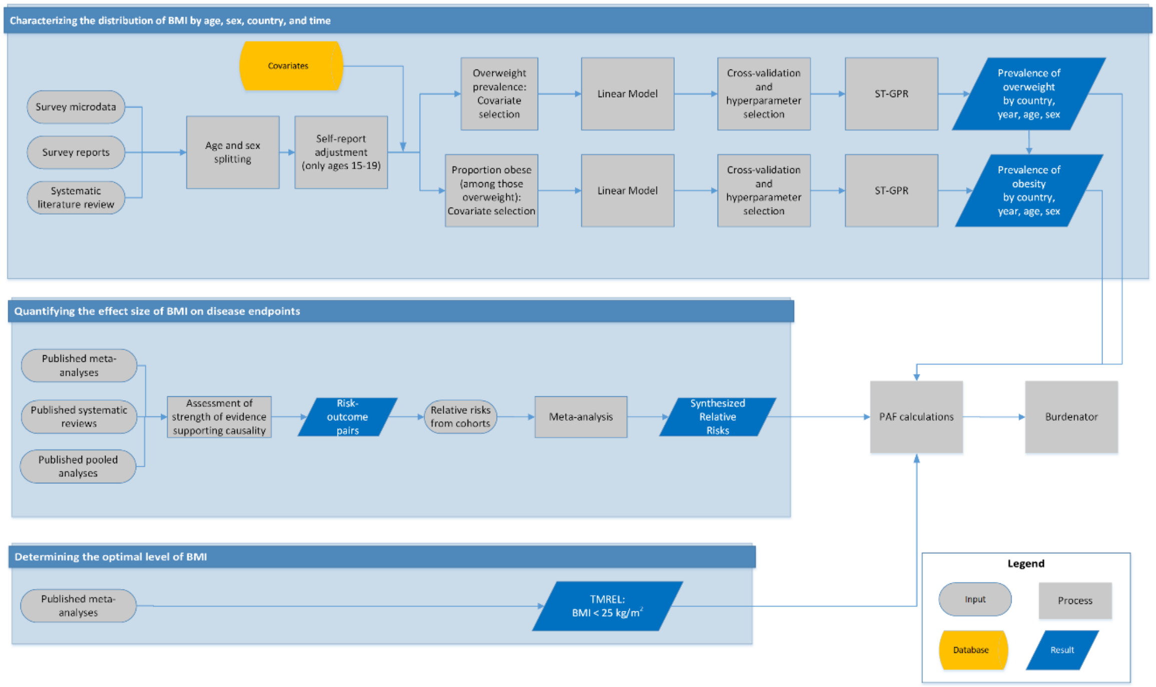

Input data and methodological summary

Case definitions

High body-mass index (BMI) for adults (ages 20+) is defined as BMI greater than 20 to 25 kg/m^2. High BMI for children (ages 1–19) is defined as being overweight or obese based on International Obesity Task Force standards.

Data sources

In GBD 2019, new data were added from sources included in the annual GHDx update of known survey series. We conducted a systematic review in GBD 2017 to identify studies providing nationally or subnationally representative estimates of overweight prevalence, obesity prevalence, or mean body-mass index (BMI). We limited the search to literature published between January 1, 2016, and December 31, 2016, to update the systematic literature search previously performed as part of GBD 2015.

The search for adults was conducted on 4 January 2017, using the following terms:

((("Body Mass Index"[Mesh] OR "Overweight"[Mesh] OR "Obesity"[Mesh]) AND ("Geographic Locations"[Mesh] NOT “United States”[Mesh]) AND ("humans"[Mesh] AND "adult"[MeSH]) AND ("Data Collection"[Mesh] OR "Health Services Research"[Mesh] OR "Population Surveillance"[Mesh] OR "Vital statistics"[Mesh] OR "Population"[Mesh] OR "Epidemiology"[Mesh] OR "surve*"[TiAb]) NOT (Comment[ptyp] OR Case Reports[ptyp] OR "hospital"[TiAb])) AND ("2016/01/01"[Date - Publication] : "2016/12/31"[Date - Publication]))

The search for children was conducted on 4 August 2016, using the following terms:
((("Body Mass Index"[Mesh] OR "Overweight"[Mesh] OR "Obesity"[Mesh]) AND ("Geographic Locations"[Mesh] NOT “United States”[Mesh]) AND ("humans"[Mesh] AND "child"[MeSH]) AND ("Data Collection"[Mesh] OR "Health Services Research"[Mesh] OR "Population Surveillance"[Mesh] OR "Vital statistics"[Mesh] OR "Population"[Mesh] OR "Epidemiology"[Mesh] OR "surve*"[TiAb]) NOT (Comment[ptyp] OR Case Reports[ptyp] OR "hospital"[TiAb])) AND ("2016/01/01"[Date - Publication] : "2016/12/31"[Date - Publication]))

Table 1: Data inputs for exposure for high body-mass index.

Input data	Exposure
Source count (total)	2022
Number of countries with data	190

Table 2: Data inputs for relative risks for high body-mass index.

Input data	Relative risk
Source count (total)	267
Number of countries with data	32

Eligibility criteria

We included representative studies providing data on mean BMI or prevalence of overweight or obesity among adults or children. For adults, studies were included if they defined overweight as BMI≥25 kg/m^2 and obesity as BMI≥30 kg/m^2, or if estimates using those cutoffs could be back-calculated from reported categories. For children (children ages 2–19), studies were included if they used International Obesity Task Force (IOTF) standards to define overweight and obesity thresholds. We only included studies reporting data collected after January 1, 1980. Studies were excluded if they used non-random samples (eg, case-control studies or convenience samples), conducted among specific subpopulations (eg, pregnant women, racial or ethnic minorities, immigrants, or individuals with specific diseases), used alternative methods to assess adiposity (eg, waist-circumference, skin-fold thickness, or hydrodensitometry), had sample sizes of less than 20 per age-sex group, or provided inadequate information on any of the inclusion criteria. We also excluded review articles and non-English-language articles.

Data collection process

Where individual-level survey data were available, we computed mean BMI using weight and height. We then used BMI to determine the prevalence of overweight and obesity. For individuals aged over 19 years, we considered them to be overweight if their BMI was greater than or equal to 25 kg/m^2, and obese if their BMI was greater than or equal to 30 kg/m^2. For individuals aged 2 to 19 years, we used monthly IOTF cutoffs[2] to determine overweight and obese status when age in months was available. When only age in years was available, we used the cutoff for the midpoint of that year. Obese individuals were also considered to be overweight. We excluded studies using the World Health Organization (WHO) standards or country-specific cutoffs to define childhood overweight and obesity. At the individual level, we considered BMI<10 kg/m^2 and BMI>70 kg/m^2 to be biologically implausible and excluded those observations.

The rationale for choosing to use the IOTF cutoffs over the WHO standards has been described elsewhere.[1] Briefly, the IOTF cutoffs provide consistent child-specific standards for ages 2–18 derived from surveys covering multiple countries. By contrast, the WHO growth standards apply to children under age 5, and the WHO growth reference applies to children ages 5–19. The WHO growth reference for children ages 5–19 was derived from United States data, which are less representative than the multinational data used by IOTF. Additionally, the switch between references at age 5 can produce artificial discontinuities. Given that we estimate global childhood overweight and obesity for ages 2–19 (with ages 19 using standard adult cutoffs), the IOTF cutoffs were preferable. Additionally, we found that IOTF cutoffs were more commonly used in scientific literature covering childhood obesity.

From report and literature data, we extracted data on mean BMI, prevalence of overweight, and prevalence of obesity, measures of uncertainty for each, and sample size, by the most granular age and sex groups available. Additionally, we extracted the same study-level covariates as were extracted from microdata (measurement, urbanicity, and representativeness), as well as location and year.

In addition to the primary indicators described above, we extracted relevant survey-design variables, including primary sampling unit, strata, and survey weights, which were used to tabulate individual-level microdata and produce accurate measures of uncertainty. We extracted three study-level covariates: 1) whether height and weight data were measured or self-reported; 2) whether the study was predominantly conducted in an urban area, rural area, or both; and 3) the level of representativeness of the study (national or subnational).

Finally, we extracted relevant demographic indicators, including location, year, age, and sex. We estimated the standard error of the mean from individual-level data, where available, and used the reported standard error of the mean for published data. When multiple data sources were available for the same country, we included all of them in our analysis. If data from the same data source were available in multiple formats such as individual-level data and tabulated data, we used individual-level data.

Modelling strategy

Age and sex splitting

Any report or literature data provided in age groups wider than the standard five-year age groups or as both sexes combined were split using the approach used by Ng and colleagues.[2] Briefly, age-sex patterns were identified using sources with data on multiple age-sex groups and these patterns were applied to split aggregated report and literature data. Uncertainty in the age-sex split was propagated by multiplying the standard error of the data by the square root of the number of splits performed. We did not propagate the uncertainty in the age pattern and sex pattern used to split the data as they seemed to have small effect.

Self-report bias adjustment

We included both measured and self-reported data. We tested for bias in self-report data compared to measured data, which is considered to be the gold-standard. There was no clear direction of bias for children ages 2–14, so for these age groups we only included measured data. For individuals ages 15 and above, we adjusted self-reported data for overweight prevalence and obesity prevalence. In GBD 2017, the self-report bias adjustment used a nested hierarchical mixed-effects regression model. This approach was updated in GBD 2019 to utilise the power of MR-BRT. For both overweight and obesity, we fit sex-specific MR-BRT models on the logit difference between measured and self-reported with a fixed effect on super-region. The bias coefficients derived from these two models are in Table 1 and 2.

Table 1: MR-BRT self-report crosswalk adjustment factors for overweight prevalence

Model	Data input	Reference or alternative case definition	Gamma	Beta coefficient, logit (95% CI)
Females	Measured data	Ref	0.26	---
	Self-reported data (southeast Asia, east Asia, and Oceania)	Alt		-0.53 (-1.03, -0.04)
	Self-reported data (central Europe, eastern Europe, and central Asia)	Alt		-0.20 (-0.69, 0.30)
	Self-reported data (high-income)	Alt		-0.25 (-0.75, 0.24)
	Self-reported data (Latin America and Caribbean)	Alt		-0.19 (-0.69, 0.31)
	Self-report data (north Africa and Middle East)	Alt		-0.38 (-0.89,0.11)
	Self-report data (south Asia)	Alt		0.36 (-0.14, 0.85)
	Self-report data (sub-Saharan Africa)	Alt		-0.26 (-0.76, 0.24)
Males	Measured data	Ref	0.43	---

	Self-reported data (southeast Asia, east Asia, and Oceania)	Alt		-0.36 (-1.17, 0.50)
	Self-reported data (central Europe, eastern Europe, and central Asia)	Alt		-0.03 (-0.84, 0.82)
	Self-reported data (high-income)	Alt		0.05 (-0.77, 0.87)
	Self-reported data (Latin America and Caribbean)	Alt		-0.02 (-0.84, 0.81)
	Self-report data (north Africa and Middle East)	Alt		-0.21 (-1.04, 0.61)
	Self-report data (south Asia)	Alt		0.53 (-0.28, 1.37)
	Self-report data (sub-Saharan Africa)	Alt		-0.27 (-1.09, 0.55)

Table 2: MR-BRT self-report crosswalk adjustment factors for obesity prevalence

Model	Data input	Reference or alternative case definition	Gamma	Beta coefficient, logit (95% CI)
Females	Measured data	Ref	0.38	---
	Self-reported data (southeast Asia, east Asia, and Oceania)	Alt		-0.11 (-0.86, 0.64)
	Self-reported data (central Europe, eastern Europe, and central Asia)	Alt		-0.95 (-1.70, -0.19)
	Self-reported data (high-income)	Alt		-0.42 (-1.16, 0.34)
	Self-reported data (Latin America and Caribbean)	Alt		-0.41 (-1.16, 0.34)
	Self-report data (north Africa and Middle East)	Alt		-0.48 (-1.23, 0.27)
	Self-report data (south Asia)	Alt		0.50 (-0.25, 1.26)
	Self-report data (sub-Saharan Africa)	Alt		-0.41 (-1.16, 0.34)
Males	Measured data	Ref	0.74	
	Self-reported data (southeast Asia, east Asia, and Oceania)	Alt		0.04 (-1.41, 1.53)
	Self-reported data (central Europe, eastern Europe, and central Asia)	Alt		-0.79 (-2.25, 0.71)
	Self-reported data (high-income)	Alt		-0.13 (-1.58, 1.40)
	Self-reported data (Latin America and Caribbean)	Alt		-0.26 (-1.70, 1.21)
	Self-report data (north Africa and Middle East)	Alt		-0.33 (-1.77, 1.16)
	Self-report data (south Asia)	Alt		0.66 (-0.78, 2.15)
	Self-report data (sub-Saharan Africa)	Alt		-0.41 (-1.86, 1.08)

Prevalence estimation for overweight and obesity

After adjusting for self-report bias and splitting aggregated data into five-year age-sex groups, we used spatiotemporal Gaussian process regression (ST-GPR) to estimate the prevalence of overweight and obesity. This modelling approach has been described in detail elsewhere.

The linear model, which when added to the smoothed residuals forms the mean prior for GPR is as follows:

$$\mathrm{logit(overweight)}_{c,a,t} = \beta_0 + \beta_1 \mathrm{energy}_{c,t} + \beta_2 \mathrm{SDI}_{c,t} + \beta_3 \mathrm{vehicles}_{c,t} + \beta_4 \mathrm{agriculture}_{c,t} + \sum_{k=5}^{21} \beta_k \mathrm{I}_{A[a]} + \alpha_s + \alpha_r + \alpha_c$$

$$\mathrm{logit(obesity/overweight)}_{c,a,t} = \beta_0 + \beta_1 \mathrm{energy}_{c,t} + \beta_2 \mathrm{SDI}_{c,t} + \beta_3 \mathrm{vehicles}_{c,t} + \sum_{k=4}^{21} \beta_k \mathrm{I}_{A[a]} + \alpha_s + \alpha_r + \alpha_c$$

where energy is ten-year lag-distributed energy consumption per capita, SDI is a composite index of development including lag-distributed income per capita, education, and fertility, $\mathrm{vehicles}$ is is the number of two- or four-wheel vehicles per capita, and $\mathrm{agriculture}$ is the proportion of the population working in agriculture. $\mathrm{I}_{A[a]}$ is a dummy variable indicating specific age group A that the prevalence point captures, and α_s, α_r, and α_c are super-region, region, and country random intercepts, respectively. Random effects were used in model fitting but were not used in prediction.

We tested all combinations of the following covariates to see which performed best in terms of in-sample AIC for the overweight linear model and the obesity as a proportion of overweight linear model: ten-year lag-distributed energy per capita, proportion of the population living in urban areas, SDI, lag-distributed income per capita, educational attainment (years) per capita, proportion of the population working in agriculture, grams of sugar adjusted for energy per capita, grams of sugar not adjusted for energy per capita, and the number of two- or four-wheeled vehicles per capita. We selected these candidate covariates based on theory as well as reviewing covariates used in other publications. The final linear model was selected based on 1) if the direction of covariates matched what is expected from theory, 2) all the included covariates were significant, and 3) minimising in-sample AIC. The covariate selection process was performed using the dredge package in R.

Estimating mean BMI

To estimate the mean BMI for adults in each country, age, sex, and time period 1980–2019, we first used the following nested hierarchical mixed-effects model, fit using restricted maximum likelihood on data from sources containing estimates of all three indicators (prevalence of overweight, prevalence of obesity, and mean BMI), in order to characterise the relationship between overweight, obesity, and mean BMI:

$$\log(\mathrm{BMI}_{c,a,s,t}) = \beta_0 + \beta_1 \mathrm{ow}_{c,a,s,t} + \beta_2 \mathrm{ob}_{c,a,s,t} + \beta_3 \mathrm{sex} + \sum_{k=4}^{20} \beta_k \mathrm{I}_{A[a]} + \alpha_s(1 + \mathrm{ow}_{c,a,s,t} + \mathrm{ob}_{c,a,s,t}) + \alpha_r(1 + \mathrm{ow}_{c,a,s,t} + \mathrm{ob}_{c,a,s,t}) + \alpha_c(1 + \mathrm{ow}_{c,a,s,t} + \mathrm{ob}_{c,a,s,t}) + \epsilon_{c,a,s,t}$$

where $\mathrm{ow}_{c,a,s,t}$ is the prevalence of overweight in country c, age a, sex s, and year t, $\mathrm{ob}_{c,a,s,t}$ is the prevalence of obesity in country c, age a, sex s, and year t, sex is a fixed effect on sex, $I_{A[a]}$ is an indicator variable for age, and α_s, α_r, and α_c are random effects at the super-region, region, and country, respectively. The model was run in Stata 13.

We applied 1000 draws of the regression coefficients to the 1000 draws of overweight prevalence and obesity prevalence produced through ST-GPR to estimate 1000 draws of mean BMI for each country, year, age, and sex. This approach ensured that overweight prevalence, obesity prevalence, and mean BMI were correlated at the draw level and uncertainty was propagated.

Estimating BMI distribution

We used the ensemble distribution approach described in the manuscript. We fit ensemble weights by source and sex, with source- and sex-specific weights averaged across all sources included to produce the final global weights. The ensemble weights were fit on measured microdata. The final ensemble weights were exponential = 0.002, gamma = 0.028, inverse gamma = 0.085, log-logistic = 0.187, Gumbel = 0.220, Weibull = 0.011, log-normal = 0.058, normal = 0.012, beta = 0.136, mirror gamma = 0.008, and mirror Gumbel = 0.113.

One thousand draws of BMI distributions for each location, year, age group, and sex estimated were produced by fitting an ensemble distribution using 1000 draws of estimated mean BMI, 1000 draws of estimated standard deviation, and the ensemble weights. Estimated standard deviation was produced by optimising a standard deviation to fit estimated overweight prevalence draws and estimated obesity prevalence draws.

Assessment of risk-outcome pairs

Risk-outcome pairs were defined based on strength of available evidence supporting a causal effect. We performed a systematic review of published meta-analyses, pooled analyses, and systematic reviews available through PubMed using the following search string: ("Body Mass Index"[Mesh] OR "Overweight"[Mesh] OR "Obesity"[Mesh]) AND (Meta-Analysis[ptyp] OR "systematic review"[tiab] OR "pooled analysis"[tiab]). Inclusion criteria are 1) the health outcome is included in GBD, 2) at least one prospective cohort is included, and 3) that the summary effect size is statistically significant. For outcomes meeting inclusion criteria we completed causal criteria tables to evaluate the strength of evidence supporting a causal relationship (see Appendix Table 4). Gallbladder disease, cataract, multiple myeloma, gout, non-Hodgkin lymphoma, asthma, Alzheimer's disease, and atrial fibrillation were added as new outcomes in GBD 2016, resulting in a total of 38 outcomes.

Theoretical minimum risk exposure level

For adults (ages 20+), the theoretical minimum risk exposure level (TMREL) of BMI (20–25 kg/m^2) was determined based on the BMI level that was associated with the lowest risk of all-cause mortality in prospective cohort studies.[3]

For children (ages 2–19), the TMREL is "normal weight," that is, not overweight or obese, based on IOTF cutoffs.

Relative risk

The relative risk per five-unit change in BMI for each disease endpoint was obtained from meta-analyses, and where available, pooled analyses of prospective observational studies. In cases where a relative risk per five-unit change in BMI was not available we computed our own dose-response meta-analysis using two-step generalised least squares for time trends estimation methods.

For childhood outcomes (ages 2–19), we computed categorical relative risks for overweight and obesity using a random effects meta-analysis.

References

1.) Cole, TJ, and T Lobstein. "Extended International (IOTF) Body Mass Index Cut-Offs for Thinness, Overweight and Obesity." Pediatric Obesity 2012; 7(4): 284–94.
2.) Ng M, Fleming T, Robinson M, et al. Global, regional, and national prevalence of overweight and obesity in children and adults during 1980–2013: a systematic analysis for the Global Burden of Disease Study 2013. The Lancet 2014; 384: 766–81.
3.) Angelantonio ED, Bhupathiraju SN, Wormser D, et al. Body-mass index and all-cause mortality: individual-participant-data meta-analysis of 239 prospective studies in four continents. The Lancet 2016; 388: 776–86.

Bone mineral density

Flowchart

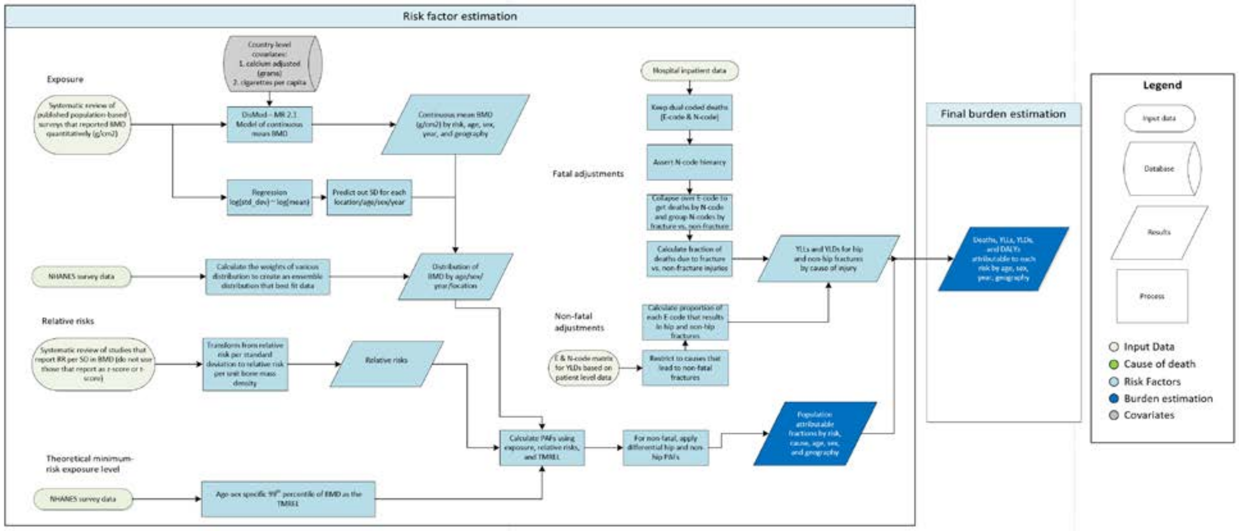

Input data and methodological summary

Definition

Exposure

Bone mineral density (BMD) is a continuous variable measured by dual-X-ray-absorptiometry (DXA) at the femoral neck (FN) and is presented in g/cm^2 after standardising for the brand of densitometer (sBMD). Low BMD is measured in terms of the difference between BMD of a population and the 99th percentile of a reference population at the same age and sex (theoretical minimum-risk exposure level, TMREL). The burden attributed to low bone mineral density is estimated for adults 20 years and older.

Input data

A systematic review (search string at the end of document) was conducted in GBD 2010 and updated for GBD 2013 and 2015 using the same search string. It was not scheduled for systematic review in GBD 2016, 2017, or 2019. Inclusion criteria that informed the search are:

- Representative, population-based surveys
- Reporting of quantitative BMD
    - measured by DXA
    - performed at the FN region
    - measured in g/cm^2

Mean BMD was occasionally reported in stratified groups, eg, by fracture status but not for total sample. In these cases, the stratified means were aggregated to obtain a total mean BMD at the population level for an age or sex category. Two additional studies provided by collaborators were added for GBD 2019.

For GBD 2019, we also began tagging existing data with study covariates for BMD measured at sites other than the femoral neck: the greater trochanter, intertrochanter, Ward's triangle, total femur, total hip, distal radius, and lumbar spine. In the future, these covariates can inform potential bias adjustments in the event that new data are added that do not report BMD measured at our reference site. The data in the current BMD model, however, do not require any bias adjustment.

**Input data**	**Exposure**
Source count (total)	168
Number of countries with data	49

Modelling strategy

We modelled mean BMD in DisMod-MR 2.1 as a single "continuous" parameter model by age and sex, and all GBD locations for years 1990–2019. The model had age mesh points at 0 10 20 25 30 40 50 60 70 80 90 & 100, a time window of ten years for fitting data, and a minimum coefficient of variation of 0.1 for global, 0.06 for super-regions, and 0.08 for the region level. We made no substantive changes in the modelling strategy from GBD 2017.

The country covariates of total physical activity (MET-min/week), tobacco consumption (cigarettes per capita), mean BMI, and unadjusted calcium intake (g) were included in modelling.

Table 1. Covariates. Summary of covariates used in the BMD DisMod-MR meta-regression model

Covariate	Type	Parameter	Exponentiated beta (95% uncertainty interval)
Total-physical activity (MET-min/week), age-standardised	Country-level	Continuous	1.00 (1.00 to 1.00)
Tobacco consumption (cigarettes per capita)	Country-level	Continuous	0.98 (0.96 to 0.99)
Mean BMI	Country-level	Continuous	1.01 (1.00 to 1.01)
Calcium intake (g), unadjusted	Country-level	Continuous	1.00 (1.00 to 1.01)

We consider the risk of fatal and non-fatal outcomes for hip non-hip fractures, separately, as relative risk data provide different estimates. Thus, there were various steps after DisMod-MR 2.1 exposure modelling to arrive at attributable fractions that can be applied to fatal and non-fatal fracture outcomes. Osteoporotic non-hip fractures include fractures of vertebrae, clavicle, scapula, humerus, skull, sternum, face bone, radius or ulna, femur, patella, tibia, fibula, ankle, and pelvis.

First, we calculated the proportion of injury deaths that are due to fractures. This proportion of deaths caused by fracture is the envelope that we use to attribute death to BMD. In order to do this, we

assumed that hip fracture and some non-hip fractures (any fractures apart from those of fingers and toes) are potentially fatal fractures. As cause of death data from vital registration and verbal autopsy attribute injury deaths to causes of death (eg, fall or road injury) and not nature of injury (such as fractures), we used available hospital data to estimate the proportion of injury deaths during admission that could be ascribed to fractures. We restricted our analysis to cases that were dual-coded with both the cause of injury ("E-code") and nature of injury ("N-code"). As injury cases may have multiple forms of trauma, we applied a severity hierarchy to the fatal hospital data to determine the proportion of the deaths that could be attributed to the chosen fracture types but were not accompanied by more severe fatal trauma such as head trauma, spinal cord lesion, and intra-abdominal or thoracic organ damage. We collapsed all deaths over E-code to determine the ratio of deaths attributable to fracture versus non-fracture injuries. We applied this ratio to the YLLs.

We restricted non-fatal estimates of low BMD to a list of causes that were deemed to cause osteoporotic fractures. Below is the list of injuries for which a PAF was calculated:

- Transport injuries
- Road injuries
- Pedestrian road injuries
- Cyclist road injuries
- Motorcyclist road injuries
- Motor vehicle road injuries
- Other road injuries
- Other transport injuries
- Unintentional injuries
- Falls
- Exposure to mechanical forces
- Other exposure to mechanical forces
- Non-venomous animal contact
- Interpersonal violence
- Assault by other means

We made use of the E- to N-code matrix generated from dual-coded (E-code/N-code) patient-level data in our injury analyses to determine the proportion of each E-code that results in a certain N-code. The hip and non-hip fracture population attributable fractions were applied to the appropriate combinations of external cause and fracture estimates of YLD and then summed together to produce a single estimate.

Theoretical minimum-risk exposure level

The theoretical minimum of risk exposure level or TMREL was chosen as the age-sex specific 99th percentile of BMD from five cycles of NHANES study as the reference population. Below is a descriptive table of the five NHANES cycles used.

Table 2. TMREL. Summary of NHANES reference population

NHANES cycle	Age range (years)	Number of people tested	BMD range (g/cm^2)
1988	20–90	14,646	0.23–1.84
2005	20–85	3,494	0.40–1.50
2007	20–80	4,726	0.34–1.46
2009	20–80	5,052	0.33–1.63
2013	40–80	3,127	0.39–1.36

Figure 1: Plot of 99th percentile of BMD at femoral neck in each cycle of NHANES

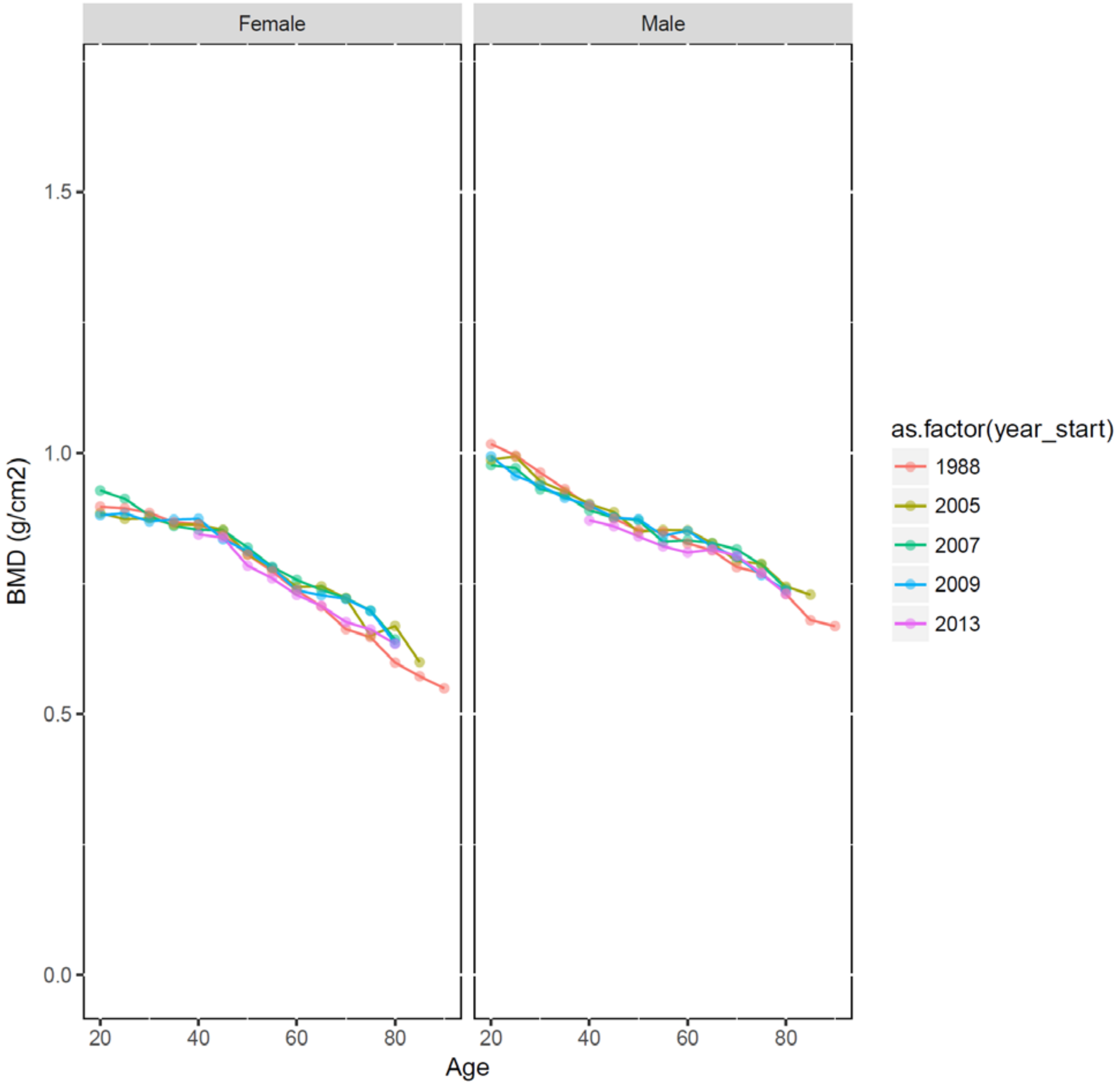

Relative risk

Relative risks must be reported per standard deviation or per unit bone-mass density in order for us to use the data. Studies reporting relative risk in an osteoporotic group versus a non-osteoporotic group were excluded.

For GBD 2017, 12 prospective observational studies were found, but one meta-analysis of 12 studies[1] reported the dose-response relationship between low BMD and high relative risk of hip and other fractures that are prone to osteoporosis, as shown in the below table.

Figure 2: Dose-response between low BMD and RR of fracture

BMD z score	*Any fracture*		*Osteoporotic fracture*		*Hip fracture*	
	RR	*95% CI*	*RR*	*95% CI*	*RR*	*95% CI*
−4	1.79	1.44–2.23	2.10	1.63–2.71	2.14	1.40–3.26
−3	1.71	1.44–2.02	1.96	1.61–2.39	2.12	1.54–2.92
−2	1.63	1.45–1.84	1.84	1.60–2.12	2.11	1.70–2.62
−1	1.56	1.45–1.69	1.73	1.59–1.89	2.11	1.86–2.39
0	1.50	1.44–1.56	1.62	1.54–1.71	2.08	1.91–2.26
1	1.39	1.32–1.46	1.42	1.34–1.51	2.04	1.78–2.34
2	1.32	1.21–1.45	1.33	1.19–1.48	2.03	1.60–2.56
3	1.26	1.10–1.45	1.25	1.06–1.47	2.01	1.44–2.81
4	1.21	1.00–1.45	1.17	0.93–1.46	1.99	1.28–3.10

The z score ranged from −5.1 to +5.8.

For GBD 2019, we re-estimated relative risk estimates for hip and non-hip fractures using the MR-BRT meta-analysis method. Input data studies consisted of those identified through a re-review of the 12 studies included in the 2005 meta-analysis that provided our previous relative risk dose-response estimates. We extracted relative risk data from six of those 12 studies, excluding cohorts that used a measure of exposure other than BMD measured by DXA at the femoral neck and those that used mortality as an outcome of interest instead of fracture.

In addition, a systematic review of the relative risk of fracture due to low bone mineral density was conducted for the years 2010 to 2020 on PubMed using the following search terms: (((bone mineral density OR bone mineral densities OR bone density) AND (mean OR average) AND risk) AND fracture). Results were filtered for comparative studies, journal articles, meta-analyses, or observational studies published in English. This search yielded 611 results. Cohorts were excluded for the same reasons mentioned above. We extracted relative risk data from six sources.

Figure 1: PRISMA diagram of BMD RR systematic review from 2019

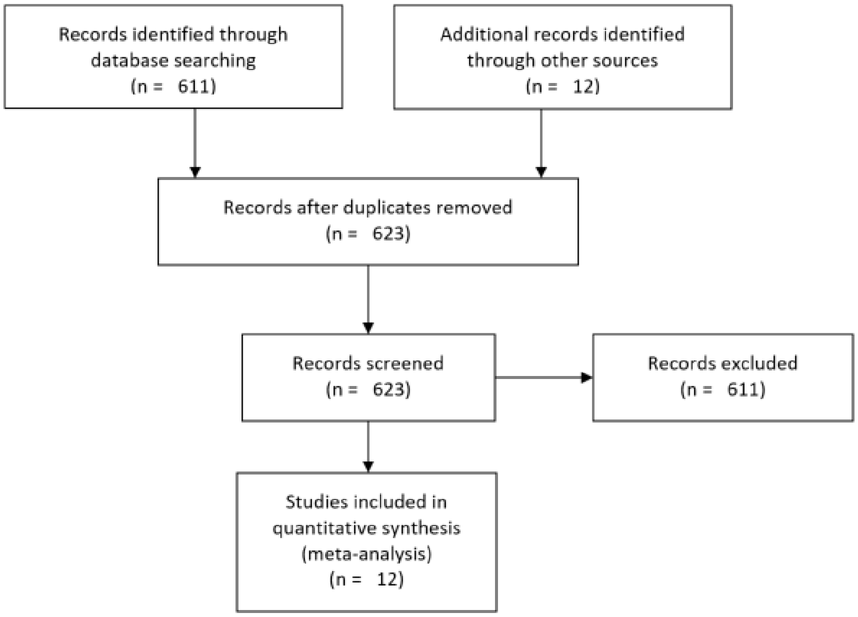

**Input data**	**Relative risk**
Source count (total)	14
Number of countries with data	10

Nine countries were represented among the 12 cohort studies included in the meta-analysis.[2-13] Two of the 14 studies indicated in the relative source counts were dropped from analysis because they did not report relative risks per standard deviation or per unit decrease in bone mineral density. We used study covariates for studies that reported the relative risk of low BMD on hip fracture and non-hip fractures, and for the percentage of the cohort that was male (which was always either 1 or 0). The mean and standard error for the coefficients were calculated using the MR-BRT crosswalk adjustment method with a cubic spline on cohort mean age. An adjustment for percentage male was not included in the final model, as we did not find a significant difference between relative risks for males and females. The age spline was also not included in the final model. After testing four iterations with two and three knots placed evenly or by data frequency, it was clear that there was not a reliable relationship between cohort mean age and relative risk. Betas and exponentiated values (which can be interpreted as the relative risks) for the remaining hip and non-hip fracture covariates are shown in the table below:

Table 3. MR-BRT Crosswalk Results for RR of Fracture due to low BMD

Data input	Gamma	Beta coefficient, log (95% CI)	RR per SD unit of BMD
Hip fracture	0.13	0.77 (0.45 to 1.08)	2.18 (1.66 to 2.80)

Non-hip fracture		0.57 (0.30 to 0.84)	1.79 (1.41 to 2.23)

Figure 3: MR-BRT crosswalk results for RR of fracture due to low BMD

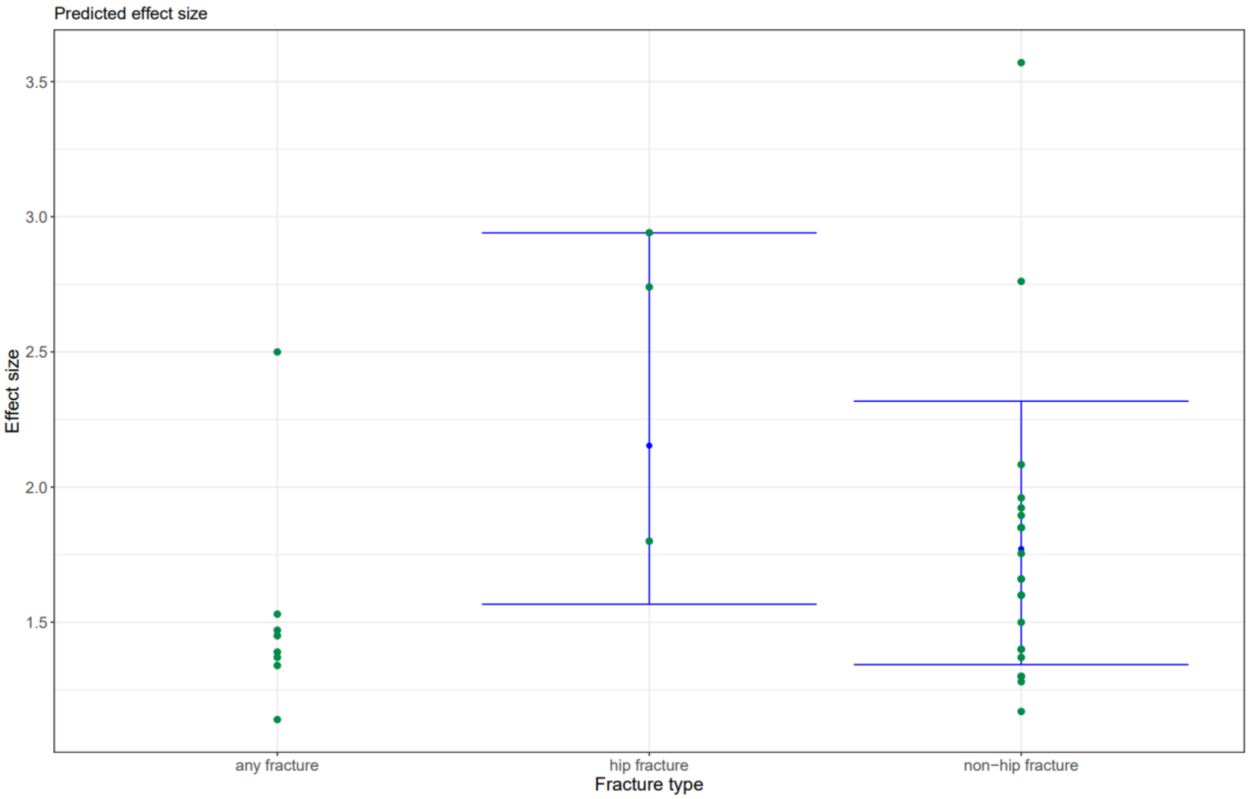

References

1. Johnell O, Kanis JA, Oden A, Johansson H, De Laet C, Delmas P, Eisman JA, Fujiwara S, Kroger H, Mellstrom D, Meunier PJ, Melton LJ, 3rd, O'Neill T, Pols H, Reeve J, Silman A, Tenenhouse A (2005) Predictive value of BMD for hip and other fractures. J Bone Miner Res 20 (7):1185-1194
2. Berger C, Langsetmo L, Joseph L, Hanley DA, Davison KS, Josse RG, Prior JC, Kreiger N, Tenenhouse A, Goltzman D, CaMos Research Group. Association between change in BMD and fragility fracture in women and men. J Bone Miner Res. 2009; 24(2): 361-70.
3. Bow CH, Tsang SW, Loong CH, Soong CS, Yeung SC, Kung AW. Bone mineral density enhances use of clinical risk factors in predicting ten-year risk of osteoporotic fractures in Chinese men: the Hong Kong Osteoporosis Study. Osteoporosis Int. 2011; 22(11): 2799-807.
4. Chalhoub D, Orwoll ES, Cawthon PM, Ensrud KE, Boudreau R, Greenspan S, Newman AB, Zmuda J, Bauer D, Cummings S, Cauley JA, Osteoporotic Fractures in Men (MrOS) Study Research Group
5. Crandall CJ, Hovey KM, Andrews CA, Cauley JA, Manson JE, Wactawski-Wende J, Wright NC, Li W, Beavers K, Curtis JR, LeBoff MS. Bone mineral density as a predictor of subsequent wrist fractures: Findings from the Women's Health Initiative Study. J Clin Endocrinal Metab. 2015; 100(11): 4315-24.
6. Dargent-Molina P, Favier F, Grandjean H, Baudoin C, Schott AM, Hausherr E, Meunier PJ, Bréart G. Fall-related factors and risk of hip fracture: the EPIDOS prospective study. Lancet. 1996; 348(9021): 145-9.

7. Fujiwara S, Kasagi F, Masunari N, Naito K, Suzuki G, Fukunaga M. Fracture Prediction From Bone Mineral Density in Japanese Men and Women. J Bone Miner Res. 2003; 18(8): 1547-53.
8. Huopio J, Kröger H, Honkanen R, Saarikoski S, Alhava E. Risk factors for perimenopausal fractures: a prospective study. Osteoporos Int. 2000; 11(3): 219-27.
9. Kwok AW, Gong JS, Wang YX, Leung JC, Kwok T, Griffith JF, Leung PC. Prevalence and risk factors of radiographic vertebral fractures in elderly Chinese men and women: Results of Ms. OS (Hong Kong) and Ms. OS (Hong Kong) studies. Osteoporosis Int. 2013; 23(3); 877-85.
10. Melton LJ, Crowson CS, O'Fallon WM, Wahner HW, Riggs BL. Relative contributions of bone density, bone turnover, and clinical risk factors to long-term fracture prediction. J Bone Miner Res. 2003; 18(2): 312–8.
11. Nguyen TV, Eisman JA, Kelly PJ, Sambrook PN. Risk factors for osteoporotic fractures in elderly men. Am J Epidemiol. 1996; 144(3): 255–63.
12. Sheu Y, Cauley JA, Patrick AL, Wheeler VW, Bunker CH, Zmuda JM. Risk factors for fracture in middle-age and older-age men of African descent. J Bone Miner Res. 2014; 29(1): 234-41.
13. Shin CS, Kim MJ, Shim SM, Kim JT, Yu SH, Koo BK, Cho HY, Choi HJ, Cho SW, Kim SW, Kim SY, Yang SO, Cho NH. The prevalence and risk factors of vertebral fractures in Korea. J Bone Miner Metab. 2012; 30(2): 183-192

Kidney dysfunction

Flowchart

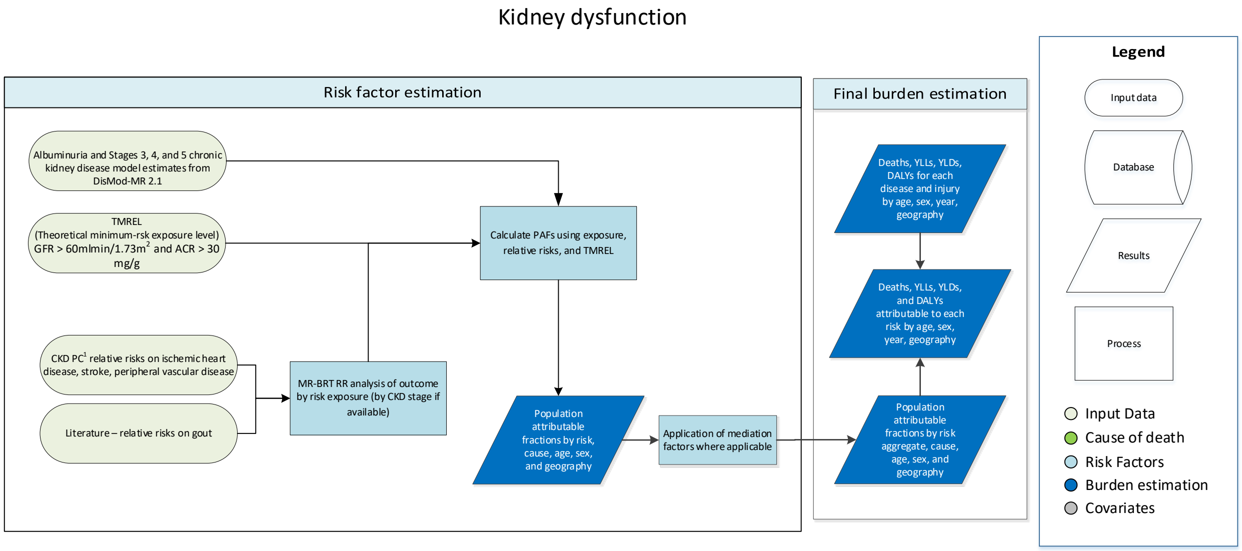

1. The Chronic Kidney Disease Prognosis Consortium is a research group composed of investigators representing cohorts from around the world. Investigators share data for the purpose of collaborative meta-analyses to study prognosis in CKD.

Input data and methodological summary

Exposure

Case definition

The kidney dysfunction risk factor exposure is divided into four categories of renal function defined by urinary albumin to creatinine ratio (ACR) and estimated glomerular filtration rate (eGFR):

- Albuminuria with preserved eGFR (ACR >30 mg/g & eGFR >=60 ml/min/1.73m^2); this corresponds to stages 1 and 2 chronic kidney disease (CKD) in the Kidney Disease Improving Global Outcomes (KDIGO) classification
- CKD stage 3 (eGFR of 30-59 ml/min/1.73m^2);
- CKD stage 4 (eGFR of 15-29 ml/min/1.73m^2); and
- CKD stage 5 (eGFR <15ml/min/1.73m^2, not (yet) on renal replacement therapy).

The modelling of renal function prevalence estimates is described in detail in the CKD section of the appendix to the GBD 2019 disease and injury paper.

Theoretical minimum-risk exposure level

The theoretical minimum-risk exposure level is ACR 30 mg/g or less and eGFR greater than 60ml/min/1.73m^2. An ACR above 30 mg/g and eGFR below 60ml/min/1.73m^2 have been demonstrated in the literature to be the thresholds at which increased cardiovascular and gout events occur secondary to kidney dysfunction.(1-10)

Input data

The last systematic review of prevalence of low glomerular filtration rate was conducted for GBD 2016, updating searches done in GBD 2015, GBD 2013, and GBD 2010. Exclusion criteria included surveys that were not population-representative and studies not reporting on CKD by stage.

Data sources for kidney dysfunction:

**Input data**	**Exposure**
Source count (total)	98
Number of countries with data	35

**Input data**	**Relative risk**
Source count (total)	9

Modelling strategy

We model the proportion of cardiovascular and musculoskeletal diseases attributable to kidney dysfunction. This is performed by 1) running DisMod-MR 2.1 models to estimate the prevalence of albuminuria, stage 3 CKD, stage 4 CKD, and stage 5 CKD; 2) estimate relative risks from available data on cardiovascular outcomes and gout; 3) calculate the population attributable fraction of those outcomes to IKF.

The prevalence of exposure to albuminuria and CKD were obtained from the GBD 2019 non-fatal burden of disease analysis.

Data on relative risks were contributed by the Chronic Kidney Disease Prognosis Consortium (CKD-PC). The Chronic Kidney Disease Prognosis Consortium is a research group composed of investigators representing cohorts from around the world. Investigators share data for the purpose of collaborative meta-analyses to study prognosis in CKD.

Relative risks

We estimate burden attributable to kidney dysfunction for cardiovascular diseases, chronic kidney diseases, and gout.

In GBD 2017, we relied on a pooled cohort analysis of six cohort studies from the CKD-PC. For GBD 2019, in collaboration with CKD-PC, we got data on 38 new cohorts and continued to use the original from the previous analysis. We ran these new data through MR-BRT meta-regression to determine the relationship between age and outcomes based on exposure to IKF. Estimates were nested within cohorts. A three-degree spline was placed on age with decreasing monotonicity. All relative risk estimates for stroke and ischaemic heart disease above age 85 were set equal to the risk at age 85 to control for lack of data in older age groups. Gout currently uses GBD 2017 estimations of relative risk.

We ran some sensitivity analyses with and without controlling for blood pressure. This is because IKF increases the risk of cardiovascular diseases directly, as well as through blood pressure. We wanted to understand how estimates of risk would differ. Generally, the relative risk of cardiovascular disease was lower when controlling for blood pressure. We decided to go with this lower risk that controlled for hypertension for a more conservative estimate.

Relative risk plots

The following plot shows the relative risks for heart disease and stroke by each stage of CKD. As expected, stage 5 and stage 4 CKD have higher risks overall. Risks is also higher at younger ages and lower at the oldest age, likely reflecting competing risk factors. While the risks themselves dip below zero at the oldest age, we believe this is merely a function of lack of data above age 85. Because of this, our estimates for relative risk above age 85 take the estimate at age 85.

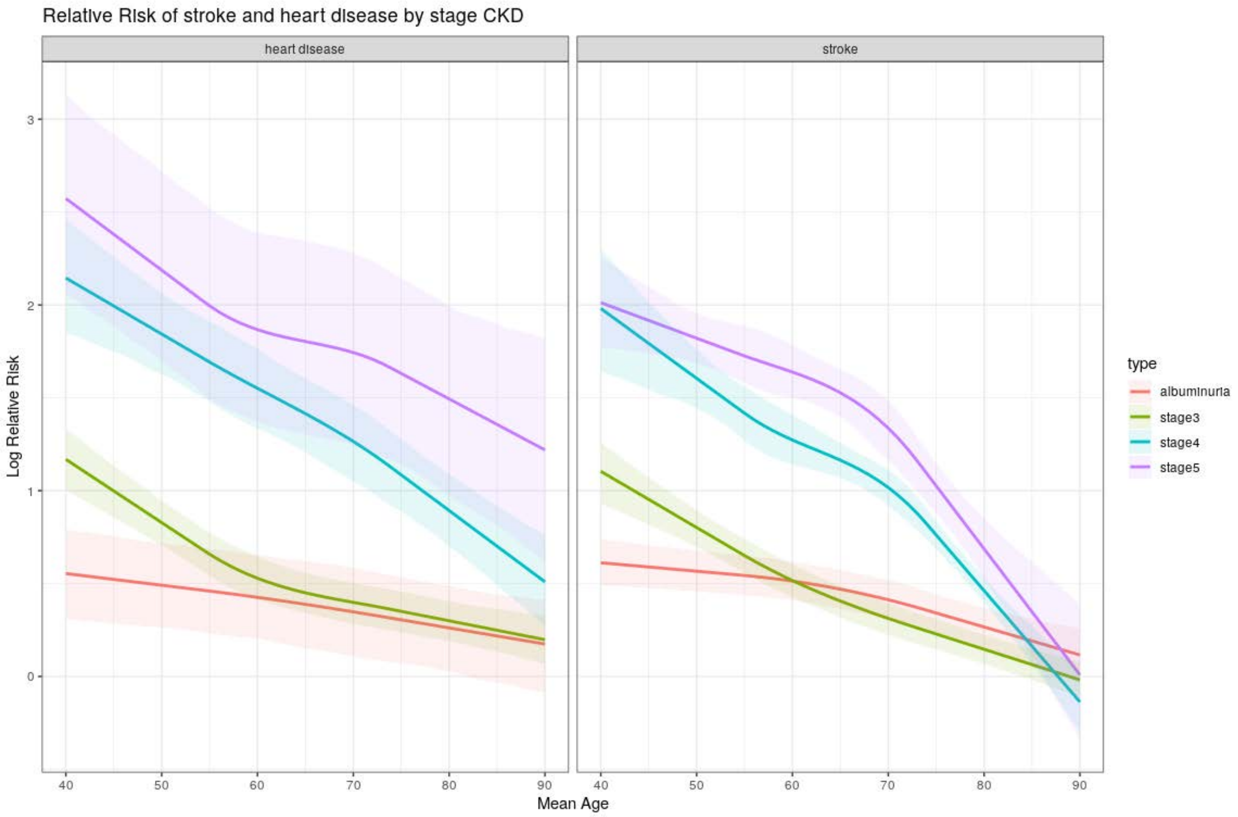

We also include two forest plots to show the distribution of risk estimates for heart disease and stroke across our studies. In general, we see an expected pattern, with earlier stages of CKD with lower risks.

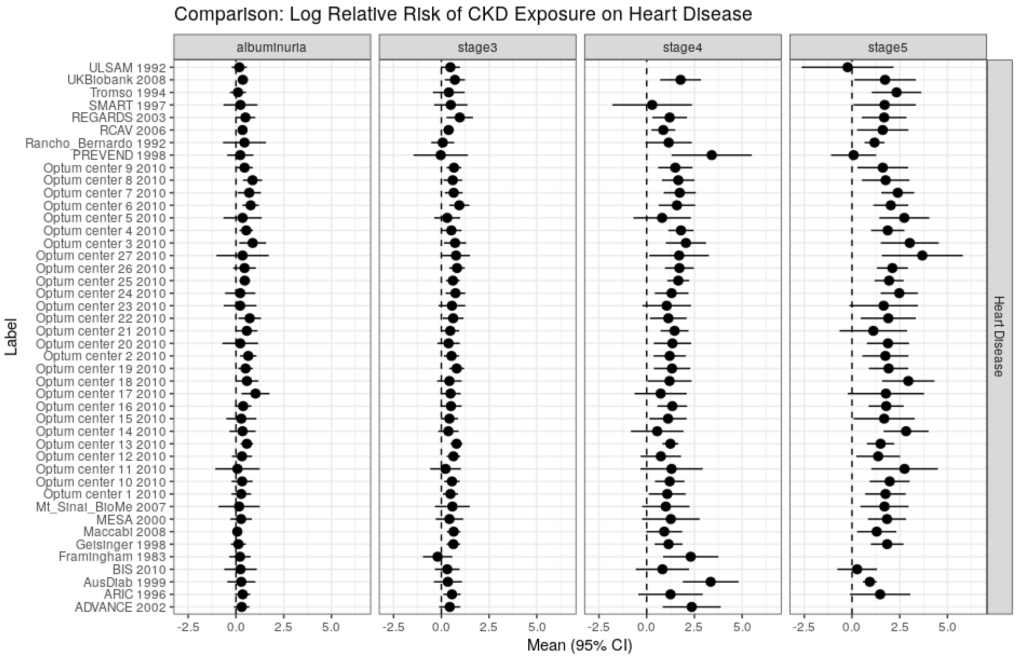

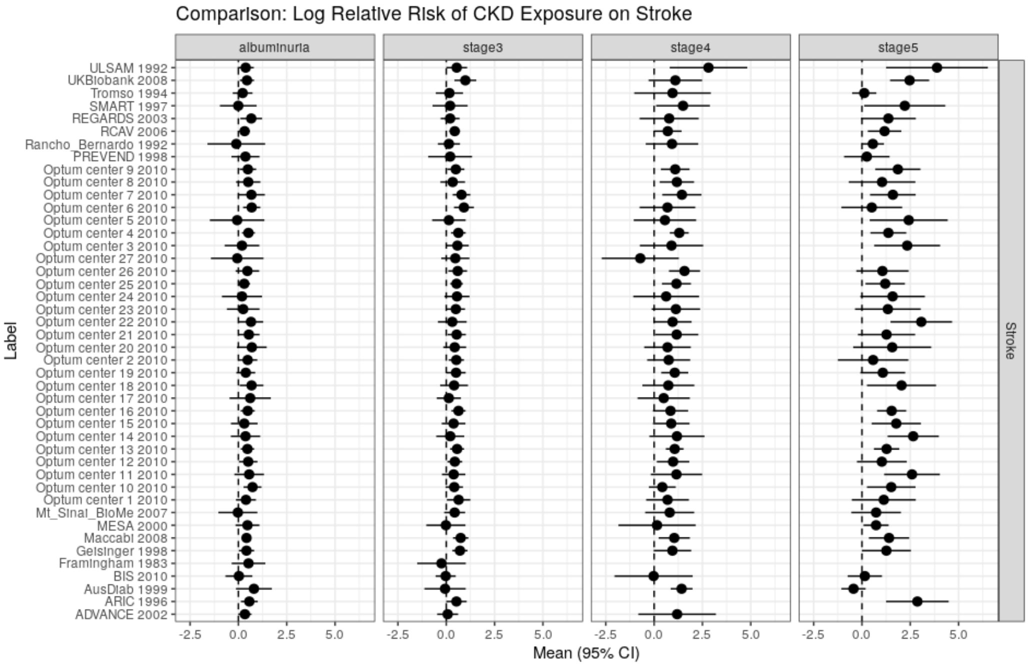

Population attributable fraction

We calculated the cardiovascular and gout fatal and non-fatal burden attributable to the categorical exposure to kidney dysfunction using the following equation:

$$PAF = \frac{\sum_{i=1}^{n} P_i(RR_i - 1)}{\sum_{i=1}^{n} P_i(RR_i - 1) + 1}$$

Equation 1. PAF based on categorical exposure

where RR_i is the relative risk for exposure level *i*, P_i is the proportion of the population in that exposure category, and *n* is the number of exposure categories.(11)

Primary changes between GBD 2017 and GBD 2019

The following are the main changes in the GBD 2019 modelling strategy compared to GBD 2017:

1. In GBD 2019, we used MR-BRT to run a nested meta-regression analysis on the within-study sex ratios to estimate a pooled sex ratio with 95% confidence intervals. In GBD 2017, this was estimated in DisMod-MR 2.1.
2. In GBD 2019, we used MR-BRT to make bias adjustments for data with alternative case definitions. CKD uses CKD-Epi as the reference definition. Alternative equations include the Cockcroft-Gualt and Modification of Diet in Renal Disease equations. MR-BRT models have larger confidence intervals due to taking into account study variance across all input data. In GBD 2017, these adjustments were made in DisMod-MR 2.1. The values of these adjustments are in the table below:

MR-BRT bias adjustment factors

Data input	Status	*Gamma*	*Beta coefficient, logit (95% CI)*	*Adjustment factor**
CKD-EPI	Ref	---	---	---
Stage III CG	Alt	*0.25*	0.24<br>(-0.28 to 0.76)	0.56<br>(0.43–0.68)
Stage III MDRD	Alt	*0.03*	0.49<br>(0.34–0.64)	0.62<br>(0.58–0.66)
Stage IV CG	Alt	*0*	0.09<br>(-0.05 to 0.24)	0.52<br>(0.49–0.56)
Stage IV MDRD	Alt	*0*	-0.07<br>(-0.19 to 0.04)	0.48<br>(0.45–0.51)
Stage V CG	Alt	*0*	-0.18<br>(-0.45 to 0.09)	0.45<br>(0.39–0.52)
Stage V MDRD	Alt	*0*	-0.06<br>(-0.28 to 0.18)	0.49<br>(0.43–0.54)
Stage III-V CG	Alt	*0.26*	0.23<br>(-0.29 to 0.75)	0.56<br>(0.43–0.68)
Stage III-V MDRD	Alt	*0.03*	0.47<br>(0.32–0.62)	0.62<br>(0.58–0.65)

3. In GBD 2017, the RRs were estimated via a pooled cohort meta-regression conducted in R using the metafor package. In GBD 2019, we made use of MR-BRT to run a nested meta-regression analysis that allowed more flexibility in the estimation process.

References

1. Go AS, Chertow GM, Fan D, McCulloch CE, Hsu CY. Chronic kidney disease and the risks of death, cardiovascular events, and hospitalization. N Engl J Med. 2004;351(13):1296-305.

2. Ninomiya T, Kiyohara Y, Kubo M, Tanizaki Y, Doi Y, Okubo K, et al. Chronic kidney disease and cardiovascular disease in a general Japanese population: the Hisayama Study. Kidney international. 2005;68(1):228-36.
3. Shara NM, Wang H, Mete M, Al-Balha YR, Azalddin N, Lee ET, et al. Estimated GFR and incident cardiovascular disease events in American Indians: the Strong Heart Study. American journal of kidney diseases : the official journal of the National Kidney Foundation. 2012;60(5):795-803.
4. Mann JF, Gerstein HC, Pogue J, Bosch J, Yusuf S. Renal insufficiency as a predictor of cardiovascular outcomes and the impact of ramipril: the HOPE randomized trial. Annals of internal medicine. 2001;134(8):629-36.
5. Chronic Kidney Disease Prognosis C, Matsushita K, van der Velde M, Astor BC, Woodward M, Levey AS, et al. Association of estimated glomerular filtration rate and albuminuria with all-cause and cardiovascular mortality in general population cohorts: a collaborative meta-analysis. Lancet. 2010;375(9731):2073-81.
6. De Graauw J, Chonchol M, Poppert H, Etgen T, Sander D. Relationship between kidney function and risk of asymptomatic peripheral arterial disease in elderly subjects. Nephrology, dialysis, transplantation : official publication of the European Dialysis and Transplant Association - European Renal Association. 2011;26(3):927-32.
7. Wattanakit K, Folsom AR, Selvin E, Coresh J, Hirsch AT, Weatherley BD. Kidney function and risk of peripheral arterial disease: results from the Atherosclerosis Risk in Communities (ARIC) Study. Journal of the American Society of Nephrology : JASN. 2007;18(2):629-36.
8. O'Hare AM, Vittinghoff E, Hsia J, Shlipak MG. Renal insufficiency and the risk of lower extremity peripheral arterial disease: results from the Heart and Estrogen/Progestin Replacement Study (HERS). Journal of the American Society of Nephrology : JASN. 2004;15(4):1046-51.
9. Manjunath G, Tighiouart H, Coresh J, Macleod B, Salem DN, Griffith JL, et al. Level of kidney function as a risk factor for cardiovascular outcomes in the elderly. Kidney international. 2003;63(3):1121-9.
10. Manjunath G, Tighiouart H, Ibrahim H, MacLeod B, Salem DN, Griffith JL, et al. Level of kidney function as a risk factor for atherosclerotic cardiovascular outcomes in the community. Journal of the American College of Cardiology. 2003;41(1):47-55.
11. Miettinen OS. Proportion of disease caused or prevented by a given exposure, trait or intervention. American journal of epidemiology. 1974;99(5):325-32.

Figure S1. Analytical flowchart of the comparative risk assessment for the estimation of population attributable fractions by geography, age, sex, and year for GBD 2019. Ovals represent data inputs, rectangular boxes represent analytical steps, cylinders represent databases, and parallelograms represent intermediate and final results. GBD=Global Burden of Disease. SEVs=Summary exposure values. TMREL=Theoretical minimum-risk exposure level. PAFs=Population attributable fractions. YLLs=years of life lost. YLDs=years lived with disability. DALYs=disability-adjusted life-years.

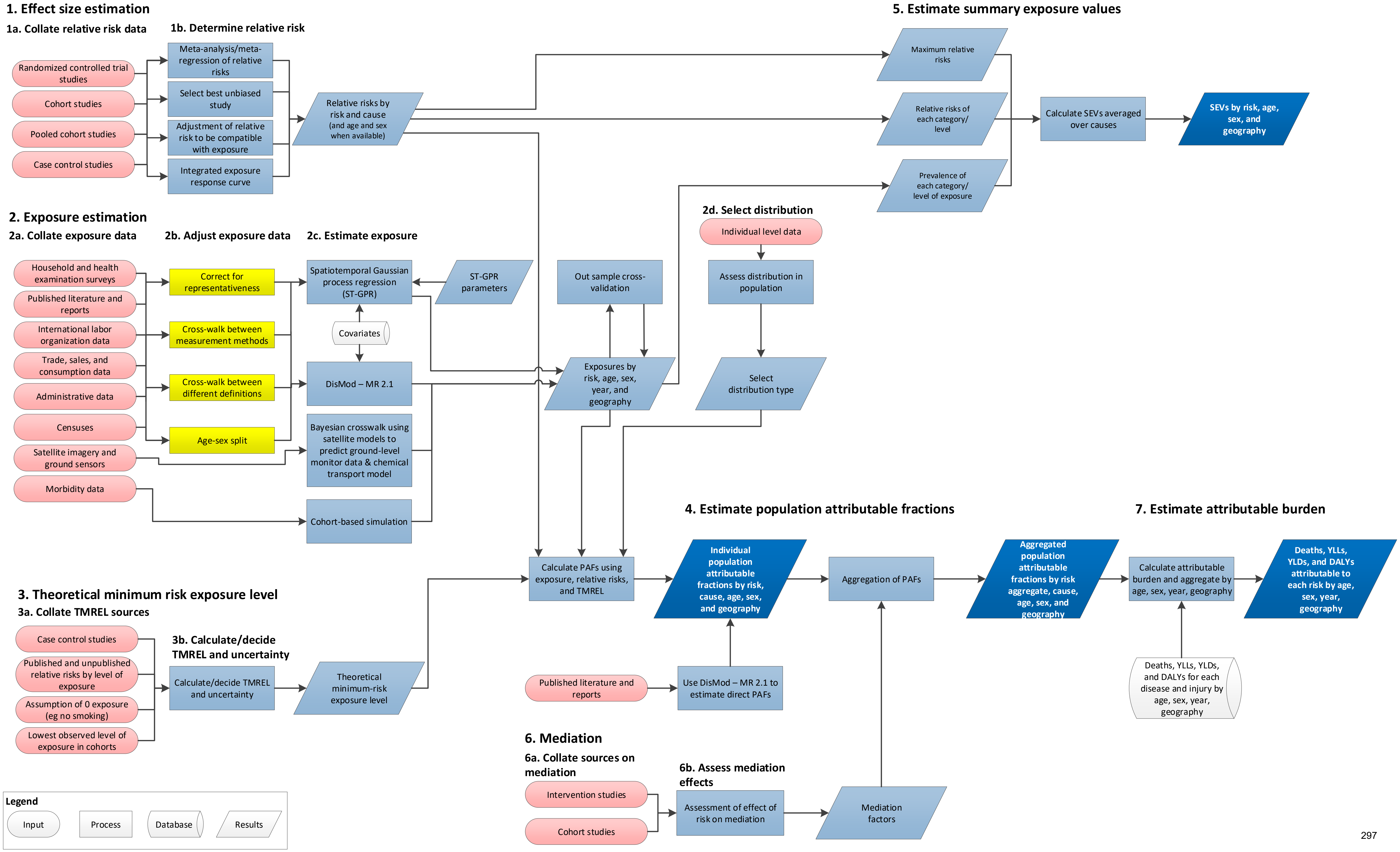

Figure S2. GBD 2019 DisMod-MR 2.1 analytical cascade

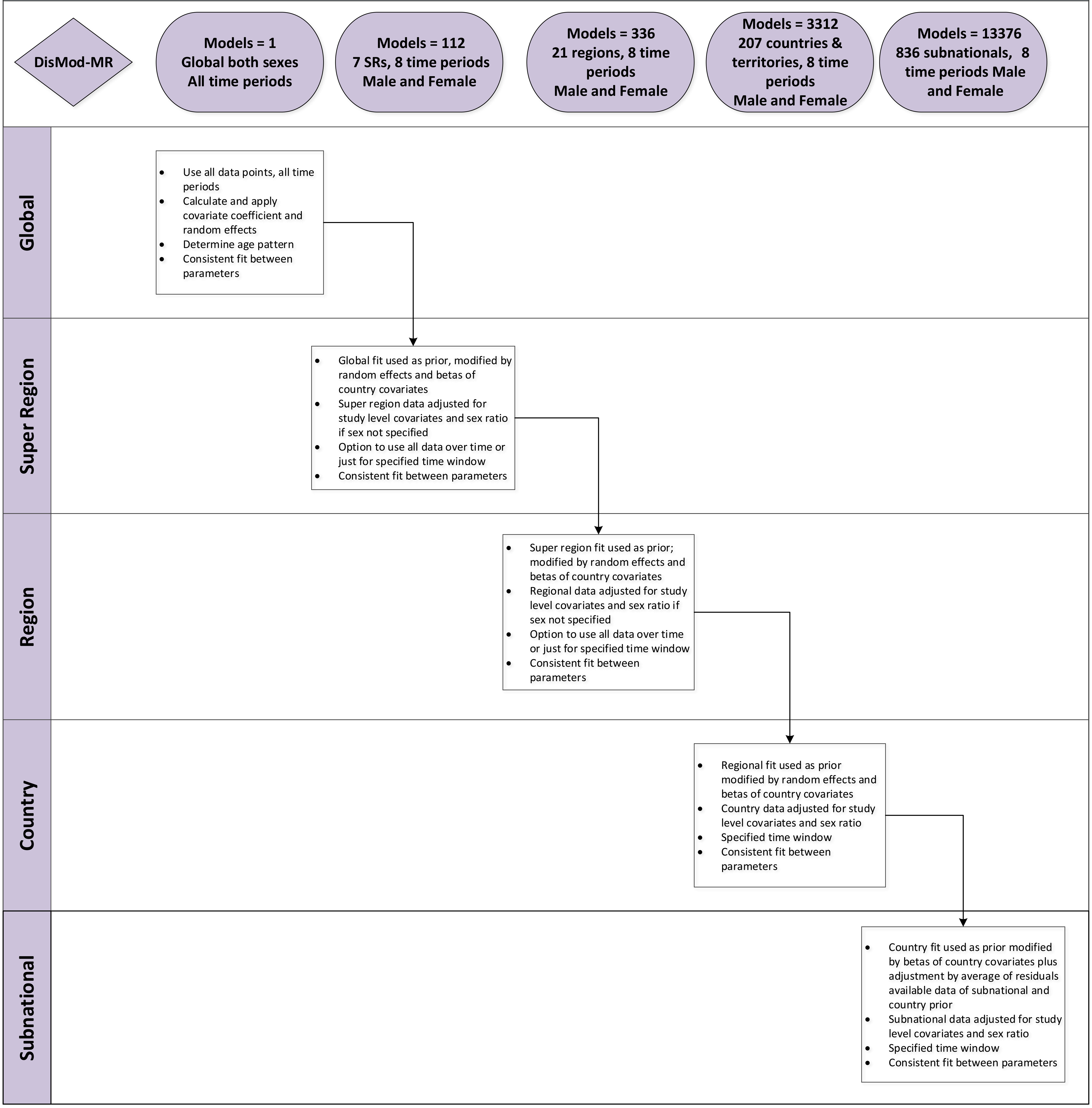

Figure S3: Spatiotemporal Gaussian Process Regression (ST-GPR) Flowchart

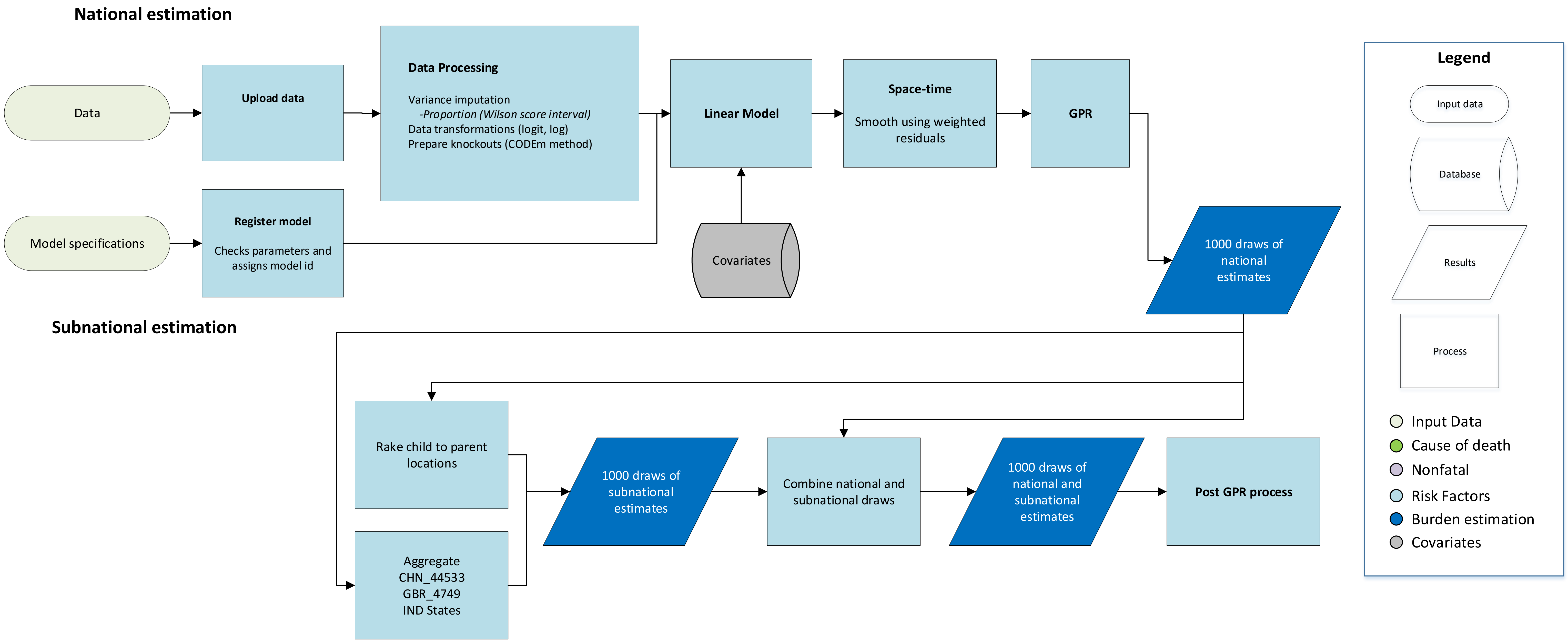

a le S1. GATHER checklist of information that should be included in reports of global health estimates, with description of compliance and location of information for lo al rden of ris factors in 20 co ntries and territories 1 0 201 : a systematic analysis for the lo al rden of Disease St dy 201

#	GATHER checklist item	Description of compliance	Reference
**Objectives and funding**			
1	Define the indicators, populations, and time periods for which estimates were made.	Narrative provided in paper and methods appendix describing indicators, definitions, and populations	Main text (Methods—Overview, Geographic units and time periods) and methods appendix
2	List the funding sources for the work.	Funding sources listed in paper	Summary (Funding)
**Data Inputs**			
For all data inputs from multiple sources that are synthesized as part of the study:			
3	Describe how the data were identified and how the data were accessed.	Narrative description of data seeking methods provided	Main text (Methods) and methods appendix
4	Specify the inclusion and exclusion criteria. Identify all ad-hoc exclusions.	Narrative about inclusion and exclusion criteria by data type provided	Main text (Methods) and methods appendix
5	Provide information on all included data sources and their main characteristics. For each data source used, report reference information or contact name/institution, population represented, data collection method, year(s) of data collection, sex and age range, diagnostic criteria or measurement method, and sample size, as relevant.	An interactive, online data source tool that provides metadata for data sources by component, geography, cause, risk, or impairment has been developed	Online data citation tools
6	Identify and describe any categories of input data that have potentially important biases (e.g., based on characteristics listed in item 5).	Summary of known biases by cause included in methods appendix	Methods appendix
For data inputs that contribute to the analysis but were not synthesized as part of the study:			
7	Describe and give sources for any other data inputs.	Included in online data source tool, http://ghdx.healthdata.org/gbd-2019	Online data citation tools
For all data inputs:			
8	Provide all data inputs in a file format from which data can be efficiently extracted (e.g., a spreadsheet as opposed to a PDF), including all relevant meta-data listed in item 5. For any data inputs that cannot be shared due to ethical or legal	Downloads of input data available through online tools, including data visualization tools	Online data visualization tools, data query tools, and

	reasons, such as third-party ownership, provide a contact name or the name of the institution that retains the right to the data.	and data query tools, http://ghdx.healthdata.org/gbd-2019; input data not available in tools will be made available upon request	the Global Health Data Exchange, http://ghdx.healthdata.org
**Data analysis**			
9	Provide a conceptual overview of the data analysis method. A diagram may be helpful.	Flow diagrams of the overall methodological processes, as well as cause-specific modelling processes, have been provided	Main text (Methods) and methods appendix
10	Provide a detailed description of all steps of the analysis, including mathematical formulae. This description should cover, as relevant, data cleaning, data pre-processing, data adjustments and weighting of data sources, and mathematical or statistical model(s).	Flow diagrams and corresponding methodological write-ups for each cause, as well as the demographics and causes of death databases and modelling processes, have been provided	Main text (Methods) and methods appendix
11	Describe how candidate models were evaluated and how the final model(s) were selected.	Provided in the methodological write-ups	Methods appendix
12	Provide the results of an evaluation of model performance, if done, as well as the results of any relevant sensitivity analysis.	Provided in the methodological write-ups	Methods appendix
13	Describe methods for calculating uncertainty of the estimates. State which sources of uncertainty were, and were not, accounted for in the uncertainty analysis.	Provided in the methodological write-ups	Methods appendix
14	State how analytic or statistical source code used to generate estimates can be accessed.	Access statement provided	Code is provided in an online repository
**Results and Discussion**			
15	Provide published estimates in a file format from which data can be efficiently extracted.	Results are available through online data visualization tools, the Global Health Data Exchange, and the online data query tool (http://ghdx.healthdata .org/gbd-2019)	Main text, methods appendix, and online data tools (data visualization tools, data query tools, and the Global Health Data Exchange, http://ghdx.healthdata.org/gbd-2019)
16	Report a quantitative measure of the uncertainty of the estimates (e.g. uncertainty intervals).	Uncertainty intervals are provided with all results	Main text, methods appendix, and online data tools (data

			visualization tools, data query tools, and the Global Health Data Exchange, http://ghdx.healthdata.org/gbd-2019)
17	Interpret results in light of existing evidence. If updating a previous set of estimates, describe the reasons for changes in estimates.	Discussion of methodological changes between GBD rounds provided in the narrative of the Article and methods appendix	Main text (Methods and Discussion) and methods appendix
18	Discuss limitations of the estimates. Include a discussion of any modelling assumptions or data limitations that affect interpretation of the estimates.	Discussion of limitations provided in the narrative of the main paper, as well as in the methodological write-ups in the methods appendix	Main text (Limitations) and methods appendix

Table S2. GBD risk hierarchy with levels

Risk	level
All risk factors	0
Environmental/occupational risks	1
Unsafe water, sanitation, and handwashing	2
Unsafe water source	3
Unsafe sanitation	3
No access to handwashing facility	3
Air pollution	2
Particulate matter pollution	3
Ambient particulate matter pollution	4
Household air pollution from solid fuels	4
Ambient ozone pollution	3
Non-optimal temperature	2
High temperature	3
Low temperature	3
Other environmental risks	2
Residential radon	3
Lead exposure	3
Occupational risks	2
Occupational carcinogens	3
Occupational exposure to asbestos	4
Occupational exposure to arsenic	4
Occupational exposure to benzene	4
Occupational exposure to beryllium	4
Occupational exposure to cadmium	4
Occupational exposure to chromium	4
Occupational exposure to diesel engine exhaust	4
Occupational exposure to formaldehyde	4
Occupational exposure to nickel	4
Occupational exposure to polycyclic aromatic hydrocarbons	4
Occupational exposure to silica	4
Occupational exposure to sulfuric acid	4
Occupational exposure to trichloroethylene	4
Occupational asthmagens	3
Occupational particulate matter, gases, and fumes	3
Occupational noise	3
Occupational injuries	3
Occupational ergonomic factors	3
Behavioral risks	1
Child and maternal malnutrition	2
Suboptimal breastfeeding	3
Non-exclusive breastfeeding	4
Discontinued breastfeeding	4
Child growth failure	3

Child underweight	4
Child wasting	4
Child stunting	4
Low birthweight and short gestation	3
Short gestation	4
Low birthweight	4
Iron deficiency	3
Vitamin A deficiency	3
Zinc deficiency	3
Tobacco	2
Smoking	3
Chewing tobacco	3
Secondhand smoke	3
Alcohol use	2
Drug use	2
Dietary risks	2
Diet low in fruits	3
Diet low in vegetables	3
Diet low in legumes	3
Diet low in whole grains	3
Diet low in nuts and seeds	3
Diet low in milk	3
Diet high in red meat	3
Diet high in processed meat	3
Diet high in sugar-sweetened beverages	3
Diet low in fiber	3
Diet low in calcium	3
Diet low in seafood omega-3 fatty acids	3
Diet low in polyunsaturated fatty acids	3
Diet high in trans fatty acids	3
Diet high in sodium	3
Intimate partner violence	2
Childhood sexual abuse and bullying	2
Childhood sexual abuse	3
Bullying victimisation	3
Unsafe sex	2
Low physical activity	2
Metabolic risks	1
High fasting plasma glucose	2
High LDL cholesterol	2
High systolic blood pressure	2
High body-mass index	2
Low bone mineral density	2
Kidney dysfunction	2

Table S3. Types of Comparative Risk Assessments (CRA) based on the time perspective and the nature of the counterfactual level or distribution of exposure. The shaded box represents the type of CRA currently undertaken in GBD 2019. GBD=Global Burden of Disease.

	**Counterfactual distributions of exposure**			
Construct	Theoretical minimum risk: level of risk with the lowest level of burden	Plausible minimum risk: level of risk with the lowest level of burden that could be imagined with current technology and knowledge	Feasible minimum risk: level of risk with the lowest level of burden that has been achieved in any population	Cost-effective minimum risk: lowest level of risk that can be achieved cost-effectively in a given population
**Attributable burden:** burden of disease today that would be avoided if each individual in the past had been exposed to the counterfactual level of exposure	**Currently in GBD**			
**Avoidable burden:** burden of disease in the future that would be avoided if each individual today was shifted to the counterfactual level of exposure				

Table S4. GBD location hierarchy with levels

Geography	level
Global	0
Low SDI	1
Low-middle SDI	1
Middle SDI	1
High-middle SDI	1
High SDI	1
Central Europe, eastern Europe, and central Asia	1
Central Asia	2
Armenia	3
Azerbaijan	3
Georgia	3
Kazakhstan	3
Kyrgyzstan	3
Mongolia	3
Tajikistan	3
Turkmenistan	3
Uzbekistan	3
Central Europe	2
Albania	3
Bosnia and Herzegovina	3
Bulgaria	3
Croatia	3
Czech Republic	3
Hungary	3
Montenegro	3
North Macedonia	3
Poland	3
Romania	3
Serbia	3
Slovakia	3
Slovenia	3
Eastern Europe	2
Belarus	3
Estonia	3
Latvia	3
Lithuania	3
Moldova	3
Russia	3
Ukraine	3
High income	1
Australasia	2
Australia	3
New Zealand	3

High-income Asia Pacific	2
Brunei	3
Japan	3
Aichi	4
Akita	4
Aomori	4
Chiba	4
Ehime	4
Fukui	4
Fukuoka	4
Fukushima	4
Gifu	4
Gunma	4
Hiroshima	4
Hokkaidō	4
Hyōgo	4
Ibaraki	4
Ishikawa	4
Iwate	4
Kagawa	4
Kagoshima	4
Kanagawa	4
Kōchi	4
Kumamoto	4
Kyōto	4
Mie	4
Miyagi	4
Miyazaki	4
Nagano	4
Nagasaki	4
Nara	4
Niigata	4
Ōita	4
Okayama	4
Okinawa	4
Ōsaka	4
Saga	4
Saitama	4
Shiga	4
Shimane	4
Shizuoka	4
Tochigi	4
Tokushima	4
Tōkyō	4
Tottori	4

Toyama	4
Wakayama	4
Yamagata	4
Yamaguchi	4
Yamanashi	4
South Korea	3
Singapore	3
High-income North America	2
Canada	3
Greenland	3
USA	3
Alabama	4
Alaska	4
Arizona	4
Arkansas	4
California	4
Colorado	4
Connecticut	4
Delaware	4
Washington, DC	4
Florida	4
Georgia	4
Hawaii	4
Idaho	4
Illinois	4
Indiana	4
Iowa	4
Kansas	4
Kentucky	4
Louisiana	4
Maine	4
Maryland	4
Massachusetts	4
Michigan	4
Minnesota	4
Mississippi	4
Missouri	4
Montana	4
Nebraska	4
Nevada	4
New Hampshire	4
New Jersey	4
New Mexico	4
New York	4
North Carolina	4

North Dakota	4
Ohio	4
Oklahoma	4
Oregon	4
Pennsylvania	4
Rhode Island	4
South Carolina	4
South Dakota	4
Tennessee	4
Texas	4
Utah	4
Vermont	4
Virginia	4
Washington	4
West Virginia	4
Wisconsin	4
Wyoming	4
Southern Latin America	2
Argentina	3
Chile	3
Uruguay	3
Western Europe	2
Andorra	3
Austria	3
Belgium	3
Cyprus	3
Denmark	3
Finland	3
France	3
Germany	3
Greece	3
Iceland	3
Ireland	3
Israel	3
Italy	3
Luxembourg	3
Malta	3
Monaco	3
Netherlands	3
Norway	3
Portugal	3
San Marino	3
Spain	3
Sweden	3
Stockholm	4

Sweden except Stockholm	4
Switzerland	3
UK	3
England	4
East Midlands	5
Derby	6
Derbyshire	6
Leicester	6
Leicestershire	6
Lincolnshire	6
Northamptonshire	6
Nottingham	6
Nottinghamshire	6
Rutland	6
East of England	5
Bedford	6
Cambridgeshire	6
Central Bedfordshire	6
Essex	6
Hertfordshire	6
Luton	6
Norfolk	6
Peterborough	6
Southend-on-Sea	6
Suffolk	6
Thurrock	6
Greater London	5
Barking and Dagenham	6
Barnet	6
Bexley	6
Brent	6
Bromley	6
Camden	6
Croydon	6
Ealing	6
Enfield	6
Greenwich	6
Hackney	6
Hammersmith and Fulham	6
Haringey	6
Harrow	6
Havering	6
Hillingdon	6
Hounslow	6
Islington	6

Kensington and Chelsea	6
Kingston upon Thames	6
Lambeth	6
Lewisham	6
Merton	6
Newham	6
Redbridge	6
Richmond upon Thames	6
Southwark	6
Sutton	6
Tower Hamlets	6
Waltham Forest	6
Wandsworth	6
Westminster	6
North East England	5
County Durham	6
Darlington	6
Gateshead	6
Hartlepool	6
Middlesbrough	6
Newcastle upon Tyne	6
North Tyneside	6
Northumberland	6
Redcar and Cleveland	6
South Tyneside	6
Stockton-on-Tees	6
Sunderland	6
North West England	5
Blackburn with Darwen	6
Blackpool	6
Bolton	6
Bury	6
Cheshire East	6
Cheshire West and Chester	6
Cumbria	6
Halton	6
Knowsley	6
Lancashire	6
Liverpool	6
Manchester	6
Oldham	6
Rochdale	6
Salford	6
Sefton	6
St Helens	6

Stockport	6
Tameside	6
Trafford	6
Warrington	6
Wigan	6
Wirral	6
South East England	5
Bracknell Forest	6
Brighton and Hove	6
Buckinghamshire	6
East Sussex	6
Hampshire	6
Isle of Wight	6
Kent	6
Medway	6
Milton Keynes	6
Oxfordshire	6
Portsmouth	6
Reading	6
Slough	6
Southampton	6
Surrey	6
West Berkshire	6
West Sussex	6
Windsor and Maidenhead	6
Wokingham	6
South West England	5
Bath and North East Somerset	6
Bournemouth	6
Bristol, City of	6
Cornwall	6
Devon	6
Dorset	6
Gloucestershire	6
North Somerset	6
Plymouth	6
Poole	6
Somerset	6
South Gloucestershire	6
Swindon	6
Torbay	6
Wiltshire	6
West Midlands	5
Birmingham	6
Coventry	6

Dudley	6
Herefordshire, County of	6
Sandwell	6
Shropshire	6
Solihull	6
Staffordshire	6
Stoke-on-Trent	6
Telford and Wrekin	6
Walsall	6
Warwickshire	6
Wolverhampton	6
Worcestershire	6
Yorkshire and the Humber	5
Barnsley	6
Bradford	6
Calderdale	6
Doncaster	6
East Riding of Yorkshire	6
Kingston upon Hull, City of	6
Kirklees	6
Leeds	6
North East Lincolnshire	6
North Lincolnshire	6
North Yorkshire	6
Rotherham	6
Sheffield	6
Wakefield	6
York	6
Northern Ireland	4
Scotland	4
Wales	4
Latin America and Caribbean	1
Andean Latin America	2
Bolivia	3
Ecuador	3
Peru	3
Caribbean	2
Antigua and Barbuda	3
The Bahamas	3
Barbados	3
Belize	3
Bermuda	3
Cuba	3
Dominica	3
Dominican Republic	3

Grenada	3
Guyana	3
Haiti	3
Jamaica	3
Puerto Rico	3
Saint Kitts and Nevis	3
Saint Lucia	3
Saint Vincent and the Grenadines	3
Suriname	3
Trinidad and Tobago	3
Virgin Islands	3
Central Latin America	2
Colombia	3
Costa Rica	3
El Salvador	3
Guatemala	3
Honduras	3
Mexico	3
Aguascalientes	4
Baja California	4
Baja California Sur	4
Campeche	4
Chiapas	4
Chihuahua	4
Coahuila	4
Colima	4
Durango	4
Guanajuato	4
Guerrero	4
Hidalgo	4
Jalisco	4
México	4
Mexico City	4
Michoacán de Ocampo	4
Morelos	4
Nayarit	4
Nuevo León	4
Oaxaca	4
Puebla	4
Querétaro	4
Quintana Roo	4
San Luis Potosí	4
Sinaloa	4
Sonora	4
Tabasco	4

Tamaulipas	4
Tlaxcala	4
Veracruz de Ignacio de la Llave	4
Yucatán	4
Zacatecas	4
Nicaragua	3
Panama	3
Venezuela	3
Tropical Latin America	2
Brazil	3
Acre	4
Alagoas	4
Amapá	4
Amazonas	4
Bahia	4
Ceará	4
Distrito Federal	4
Espírito Santo	4
Goiás	4
Maranhão	4
Mato Grosso	4
Mato Grosso do Sul	4
Minas Gerais	4
Pará	4
Paraíba	4
Paraná	4
Pernambuco	4
Piauí	4
Rio de Janeiro	4
Rio Grande do Norte	4
Rio Grande do Sul	4
Rondônia	4
Roraima	4
Santa Catarina	4
São Paulo	4
Sergipe	4
Tocantins	4
Paraguay	3
North Africa and Middle East	1
North Africa and Middle East	2
Afghanistan	3
Algeria	3
Bahrain	3
Egypt	3
Iran	3

Iraq	3
Jordan	3
Kuwait	3
Lebanon	3
Libya	3
Morocco	3
Oman	3
Palestine	3
Qatar	3
Saudi Arabia	3
Sudan	3
Syria	3
Tunisia	3
Turkey	3
United Arab Emirates	3
Yemen	3
South Asia	1
South Asia	2
Bangladesh	3
Bhutan	3
India	3
Andhra Pradesh	4
Arunachal Pradesh	4
Assam	4
Bihar	4
Chhattisgarh	4
Delhi	4
Goa	4
Gujarat	4
Haryana	4
Himachal Pradesh	4
Jammu & Kashmir and Ladakh	4
Jharkhand	4
Karnataka	4
Kerala	4
Madhya Pradesh	4
Maharashtra	4
Manipur	4
Meghalaya	4
Mizoram	4
Nagaland	4
Odisha	4
Punjab	4
Rajasthan	4
Sikkim	4

Tamil Nadu	4
Telangana	4
Tripura	4
Other Union Territories	4
Uttar Pradesh	4
Uttarakhand	4
West Bengal	4
Nepal	3
Pakistan	3
Southeast Asia, east Asia, and Oceania	1
East Asia	2
China	3
North Korea	3
Taiwan (province of China)	3
Oceania	2
American Samoa	3
Cook Islands	3
Fiji	3
Guam	3
Kiribati	3
Marshall Islands	3
Federated States of Micronesia	3
Nauru	3
Niue	3
Northern Mariana Islands	3
Palau	3
Papua New Guinea	3
Samoa	3
Solomon Islands	3
Tokelau	3
Tonga	3
Tuvalu	3
Vanuatu	3
Southeast Asia	2
Cambodia	3
Indonesia	3
Aceh	4
Bali	4
Bangka-Belitung Islands	4
Banten	4
Bengkulu	4
Gorontalo	4
Jakarta	4
Jambi	4
West Java	4

Central Java	4
East Java	4
West Kalimantan	4
South Kalimantan	4
Central Kalimantan	4
East Kalimantan	4
North Kalimantan	4
Riau Islands	4
Lampung	4
Maluku	4
North Maluku	4
West Nusa Tenggara	4
East Nusa Tenggara	4
Papua	4
West Papua	4
Riau	4
West Sulawesi	4
South Sulawesi	4
Central Sulawesi	4
Southeast Sulawesi	4
North Sulawesi	4
West Sumatra	4
South Sumatra	4
North Sumatra	4
Yogyakarta	4
Laos	3
Malaysia	3
Maldives	3
Mauritius	3
Myanmar	3
Philippines	3
Seychelles	3
Sri Lanka	3
Thailand	3
Timor-Leste	3
Vietnam	3
Sub-Saharan Africa	1
Central sub-Saharan Africa	2
Angola	3
Central African Republic	3
Congo (Brazzaville)	3
DR Congo	3
Equatorial Guinea	3
Gabon	3
Eastern sub-Saharan Africa	2

Burundi	3
Comoros	3
Djibouti	3
Eritrea	3
Ethiopia	3
Kenya	3
Baringo	4
Bomet	4
Bungoma	4
Busia	4
Elgeyo Marakwet	4
Embu	4
Garissa	4
Homa Bay	4
Isiolo	4
Kajiado	4
Kakamega	4
Kericho	4
Kiambu	4
Kilifi	4
Kirinyaga	4
Kisii	4
Kisumu	4
Kitui	4
Kwale	4
Laikipia	4
Lamu	4
Machakos	4
Makueni	4
Mandera	4
Marsabit	4
Meru	4
Migori	4
Mombasa	4
Murang'a	4
Nairobi	4
Nakuru	4
Nandi	4
Narok	4
Nyamira	4
Nyandarua	4
Nyeri	4
Samburu	4
Siaya	4
Taita Taveta	4

Tana River	4
Tharaka Nithi	4
Trans Nzoia	4
Turkana	4
Uasin Gishu	4
Vihiga	4
Wajir	4
West Pokot	4
Madagascar	3
Malawi	3
Mozambique	3
Rwanda	3
Somalia	3
South Sudan	3
Uganda	3
Tanzania	3
Zambia	3
Southern sub-Saharan Africa	2
Botswana	3
eSwatini	3
Lesotho	3
Namibia	3
South Africa	3
Zimbabwe	3
Western sub-Saharan Africa	2
Benin	3
Burkina Faso	3
Cape Verde	3
Cameroon	3
Chad	3
Côte d'Ivoire	3
The Gambia	3
Ghana	3
Guinea	3
Guinea-Bissau	3
Liberia	3
Mali	3
Mauritania	3
Niger	3
Nigeria	3
São Tomé and PrÍncipe	3
Senegal	3
Sierra Leone	3
Togo	3

Table S5. Socio-demographic Index values for all estimated GBD 2019 locations, 1990-2019																														
**Location**	**1990**	**1991**	**1992**	**1993**	**1994**	**1995**	**1996**	**1997**	**1998**	**1999**	**2000**	**2001**	**2002**	**2003**	**2004**	**2005**	**2006**	**2007**	**2008**	**2009**	**2010**	**2011**	**2012**	**2013**	**2014**	**2015**	**2016**	**2017**	**2018**	**2019**
Global	51·1	51·4	51·6	52·2	52·7	53·1	53·6	54·2	54·7	55·0	55·5	56·1	56·5	56·9	57·3	57·9	58·4	58·9	59·3	59·8	60·3	60·8	61·2	61·6	62·2	62·7	63·1	63·6	64·2	64·7
Central Europe, eastern Europe, and central Asia	64·5	65·1	65·8	66·2	66·6	67·1	67·6	67·8	68·2	68·6	69·0	69·3	69·6	70·0	70·6	71·2	71·6	72·1	72·5	72·9	73·3	73·7	74·0	74·3	74·6	74·9	75·2	75·5	75·9	76·2
Central Asia	54·3	54·3	54·5	54·9	55·4	55·7	56·0	56·2	56·7	57·2	57·6	57·9	58·3	58·8	59·5	60·1	60·5	61·1	61·7	62·1	62·7	63·4	63·8	64·2	64·7	65·2	65·7	66·2	66·7	67·1
Armenia	54·0	53·7	53·7	54·9	55·6	55·7	56·0	56·1	56·9	58·0	59·0	59·2	59·4	60·1	61·2	62·4	63·4	64·2	65·1	66·2	67·1	67·4	67·7	68·0	68·3	68·7	69·1	69·6	70·0	70·4
Azerbaijan	58·6	58·6	58·5	58·5	58·6	58·5	58·4	58·4	58·3	58·2	58·2	58·2	58·5	58·7	58·9	59·8	61·2	62·7	64·0	64·9	65·7	66·3	66·7	67·1	67·3	67·6	68·0	68·4	68·8	69·1
Georgia	64·3	64·8	65·4	65·5	65·0	64·2	63·3	63·1	63·2	62·8	62·6	62·7	62·8	63·4	64·5	64·8	64·5	64·8	64·9	65·0	65·5	66·3	66·8	67·1	67·6	68·2	68·9	69·6	70·1	70·5
Kazakhstan	58·9	59·2	59·6	60·3	60·9	61·6	62·2	62·6	63·0	63·4	63·8	64·4	64·8	65·2	65·7	66·2	66·6	67·0	67·5	67·9	68·4	68·8	69·1	69·5	69·9	70·4	70·9	71·3	71·8	72·2
Kyrgyzstan	51·3	51·3	52·2	52·9	53·2	53·2	53·7	53·6	53·5	53·6	53·5	53·6	53·8	54·1	54·8	54·8	54·6	55·0	55·2	55·2	55·6	56·2	56·2	56·6	56·7	56·8	57·3	57·9	58·5	58·9
Mongolia	48·6	49·3	50·1	50·7	51·2	51·8	52·3	52·8	53·4	53·9	54·5	55·1	55·7	56·3	56·9	57·4	57·9	58·4	58·8	59·2	59·6	59·9	60·4	60·9	61·4	61·9	62·4	62·9	63·4	63·9
Tajikistan	44·8	45·4	46·2	46·7	47·1	46·6	45·5	44·8	44·7	44·9	44·7	44·3	44·7	45·7	48·1	49·0	48·1	48·3	48·8	48·6	49·3	50·6	51·2	51·4	51·9	52·7	53·3	53·9	54·4	54·8
Turkmenistan	53·7	52·8	51·5	51·2	52·8	54·8	55·0	55·0	56·4	58·7	59·7	59·7	59·9	60·4	61·0	61·9	62·6	63·1	63·6	63·9	64·4	65·3	66·2	66·7	67·9	69·1	69·5	70·1	70·7	71·2
Uzbekistan	47·7	47·9	47·8	48·1	48·8	49·5	50·1	50·8	51·6	52·5	53·1	53·6	54·1	54·5	55·1	55·6	56·0	56·6	57·1	57·7	58·3	58·9	59·4	60·0	60·6	61·4	62·2	62·8	63·5	64·0
Central Europe	64·1	64·7	65·2	65·7	66·4	67·1	67·8	68·4	68·9	69·5	70·2	70·9	71·5	72·1	72·6	73·1	73·6	74·0	74·4	75·0	75·5	76·0	76·4	76·8	77·1	77·5	77·8	78·1	78·5	78·9
Albania	53·5	53·1	53·0	52·7	52·5	52·7	53·3	53·9	54·7	56·2	57·0	57·8	59·0	59·7	60·7	61·6	62·6	63·4	63·9	64·3	64·6	64·9	65·2	65·5	65·9	66·5	67·0	67·5	67·9	68·4
Bosnia and Herzegovina	52·9	53·1	53·0	52·7	52·5	52·4	53·4	55·3	57·3	59·1	60·5	61·7	62·8	63·8	64·5	65·2	65·8	66·4	67·1	67·7	68·4	68·9	69·3	69·8	70·2	70·7	71·0	71·4	71·8	72·3
Bulgaria	63·5	64·5	65·2	65·8	66·9	67·4	67·8	68·1	67·8	67·6	68·0	68·7	69·2	69·6	70·1	70·6	71·0	71·5	71·9	72·5	73·2	73·7	74·0	74·3	74·6	74·9	75·2	75·5	75·9	76·3
Croatia	68·1	68·6	68·9	68·9	68·8	68·8	68·9	69·4	70·1	70·6	71·3	72·0	72·5	73·0	73·5	73·9	74·4	74·9	75·4	75·8	76·3	76·7	77·1	77·4	77·7	78·0	78·3	78·5	78·9	79·2
Czech Republic	69·4	70·0	70·4	71·7	73·5	74·7	75·5	76·0	76·5	77·1	77·7	78·2	78·6	79·0	79·4	79·8	80·1	80·4	80·7	81·0	81·3	81·6	81·8	81·9	82·0	82·0	82·1	82·5	82·8	83·1
Hungary	66·0	66·4	67·2	67·9	68·5	69·3	70·1	70·7	71·3	71·8	72·7	73·3	73·6	74·1	74·6	75·1	75·6	76·0	76·4	76·8	77·1	77·3	77·4	77·4	77·5	77·7	78·1	78·5	78·9	79·3
Montenegro	70·3	70·2	70·0	69·5	69·1	68·7	68·6	68·7	69·0	69·2	69·5	70·0	70·5	71·1	71·7	72·2	72·8	73·5	74·2	74·8	75·4	76·0	76·5	77·0	77·4	77·8	78·1	78·5	78·9	79·2
North Macedonia	61·7	62·1	62·1	62·0	62·1	62·4	63·0	63·6	64·3	64·8	65·0	65·5	66·1	66·9	67·8	68·5	69·0	69·5	70·0	70·5	70·9	71·3	71·6	72·1	72·5	72·9	73·4	73·7	74·1	74·4
Poland	63·1	63·6	64·5	65·2	66·2	67·1	67·7	68·6	69·4	70·1	70·9	71·7	72·4	73·0	73·6	74·0	74·4	74·7	75·2	75·8	76·4	77·0	77·5	78·0	78·4	78·8	79·1	79·4	79·9	80·3
Romania	62·6	63·1	63·5	63·8	64·3	64·9	65·6	65·8	65·9	66·4	66·9	67·7	68·2	68·7	69·3	69·8	70·2	70·7	71·2	71·9	72·5	72·9	73·5	73·9	74·1	74·4	74·7	75·2	75·7	76·2
Serbia	62·7	63·5	64·0	64·0	64·0	64·0	64·4	64·8	65·2	65·6	66·1	66·5	67·0	67·6	68·5	69·2	69·7	70·2	70·7	71·4	72·1	72·7	73·3	73·9	74·6	75·3	75·6	75·8	76·1	76·4
Slovakia	65·5	66·2	66·9	67·9	69·1	70·0	70·7	71·6	72·4	73·2	74·0	74·6	75·1	75·6	76·0	76·6	77·2	77·7	78·1	78·5	79·0	79·4	79·8	80·1	80·2	80·4	80·6	81·0	81·3	81·7
Slovenia	72·7	73·4	73·8	74·1	74·6	75·1	75·9	76·4	76·9	77·4	78·1	79·0	79·5	79·8	80·2	80·7	81·1	81·4	81·8	82·0	82·2	82·4	82·5	82·7	82·9	83·1	83·3	83·6	83·9	84·2
Eastern Europe	67·8	68·5	69·4	69·8	70·0	70·5	70·8	70·9	71·0	71·2	71·3	71·4	71·6	72·1	72·7	73·4	74·0	74·6	75·2	75·7	76·1	76·5	76·8	77·3	77·6	77·8	78·2	78·5	78·8	79·1
Belarus	58·8	59·4	60·0	60·6	61·0	61·3	61·5	61·8	62·1	62·4	62·8	63·4	64·1	64·8	65·5	66·3	67·0	67·8	68·7	69·4	70·2	70·9	71·5	72·0	72·5	73·0	73·5	73·8	74·3	74·6
Estonia	67·2	67·7	68·8	69·7	70·5	70·9	71·1	71·8	72·4	73·3	74·0	74·2	74·8	75·3	75·9	76·5	77·1	77·8	78·6	79·2	79·8	80·4	80·9	81·3	81·6	82·0	82·3	82·5	82·8	83·1
Latvia	67·8	68·7	69·6	70·5	71·4	71·8	72·0	72·2	72·3	72·5	72·9	73·3	73·8	74·4	75·1	75·8	76·6	77·5	78·4	79·2	79·5	79·7	80·0	80·3	80·4	80·4	80·7	81·2	81·6	82·0
Lithuania	67·1	67·4	68·2	69·1	69·4	69·9	70·3	70·5	70·9	71·5	72·5	73·3	73·7	74·4	75·3	76·0	76·6	77·3	78·3	79·2	79·7	80·2	80·8	81·3	81·7	82·2	82·6	83·0	83·4	83·8
Moldova	58·1	58·4	58·5	58·8	59·1	59·4	59·4	59·3	59·1	58·9	58·7	58·7	59·0	59·4	60·2	61·0	61·7	62·4	63·3	64·0	64·7	65·5	66·1	66·7	67·2	67·7	68·2	68·7	69·2	69·6
Russia	69·0	69·9	71·0	71·4	71·5	72·0	72·4	72·5	72·6	72·8	72·8	72·8	73·0	73·4	74·1	74·9	75·4	75·9	76·5	77·0	77·4	77·6	78·0	78·4	78·7	78·9	79·3	79·6	79·9	80·3
Ukraine	65·6	66·0	66·2	66·5	66·8	67·0	67·1	67·0	67·0	66·9	67·0	67·0	67·0	67·3	68·0	68·7	69·4	70·0	70·7	71·1	71·5	71·9	72·2	72·5	72·7	72·8	73·0	73·2	73·4	73·6
High income	75·5	76·0	76·5	76·9	77·3	77·7	78·0	78·3	78·5	78·8	79·1	79·5	79·8	80·1	80·4	80·6	80·7	81·0	81·3	81·6	82·0	82·3	82·6	82·9	83·2	83·5	83·8	84·0	84·2	84·4
Australasia	74·2	74·6	74·9	75·3	75·7	76·0	76·5	76·9	77·3	77·7	78·1	78·4	78·8	79·2	79·5	79·8	79·9	80·0	80·3	80·6	81·0	81·3	81·6	82·1	82·5	82·7	82·9	83·1	83·4	83·7
Australia	73·8	74·2	74·5	74·9	75·3	75·7	76·1	76·6	77·0	77·4	77·8	78·1	78·6	79·0	79·3	79·6	79·8	79·9	80·2	80·6	80·9	81·2	81·5	82·0	82·4	82·6	82·9	83·1	83·4	83·7
New Zealand	75·8	76·1	76·4	76·7	77·0	77·2	77·6	77·9	78·2	78·6	78·9	79·3	79·7	79·8	80·0	80·1	80·1	80·1	80·3	80·6	81·0	81·3	81·7	82·2	82·5	82·8	83·0	83·2	83·4	83·6
High-income Asia Pacific	76·6	77·3	77·9	78·4	78·9	79·5	80·0	80·5	80·8	81·2	81·6	81·9	82·3	82·7	83·0	83·3	83·6	83·9	84·2	84·4	84·7	85·0	85·3	85·6	85·9	86·2	86·5	86·7	87·1	87·4
Brunei	67·5	68·0	68·6	69·2	69·8	70·3	70·9	71·6	72·2	72·8	73·3	73·8	74·5	75·3	76·0	76·7	77·3	77·8	78·2	78·6	79·0	79·4	79·7	80·1	80·5	81·0	81·3	81·7	82·1	82·5
Japan	79·1	79·6	80·1	80·5	80·9	81·3	81·7	82·0	82·2	82·4	82·6	82·8	83·0	83·3	83·6	83·8	84·0	84·2	84·4	84·6	84·8	85·1	85·3	85·5	85·8	85·9	86·1	86·4	86·7	87·0
Aichi	80·1	80·6	81·1	81·6	82·0	82·4	82·8	83·1	83·3	83·5	83·6	83·8	84·0	84·3	84·6	84·8	85·0	85·2	85·4	85·6	85·8	86·1	86·4	86·6	86·9	87·1	87·3	87·5	87·8	88·1
Akita	74·6	75·0	75·4	75·8	76·3	76·7	77·1	77·5	77·8	78·1	78·3	78·5	78·8	79·1	79·4	79·6	79·8	79·9	80·1	80·3	80·6	80·8	81·1	81·4	81·6	81·8	82·0	82·3	82·7	83·0
Aomori	73·6	74·0	74·5	74·9	75·4	75·8	76·2	76·5	76·8	77·1	77·3	77·5	77·8	78·2	78·6	78·8	79·1	79·3	79·5	79·8	80·0	80·4	80·7	80·9	81·2	81·4	81·6	81·9	82·3	82·6
Chiba	79·0	79·4	80·0	80·4	80·8	81·3	81·6	81·9	82·2	82·3	82·5	82·6	82·7	82·9	83·1	83·3	83·5	83·6	83·8	84·0	84·2	84·4	84·6	84·8	85·0	85·2	85·3	85·6	85·8	86·1
Ehime	75·7	76·1	76·6	77·1	77·5	78·0	78·4	78·8	79·1	79·4	79·7	79·9	80·2	80·4	80·7	80·9	81·0	81·2	81·3	81·5	81·7	82·0	82·2	82·5	82·7	82·9	83·1	83·4	83·7	84·1
Fukui	76·8	77·3	77·8	78·3	78·7	79·2	79·6	79·9	80·2	80·5	80·7	81·0	81·3	81·6	81·9	82·1	82·3	82·6	82·8	83·1	83·3	83·6	83·9	84·1	84·3	84·4	84·6	84·9	85·2	85·5
Fukuoka	78·3	78·6	79·1	79·5	79·9	80·3	80·6	81·0	81·2	81·5	81·7	81·9	82·2	82·5	82·7	82·9	83·0	83·2	83·3	83·5	83·7	83·9	84·2	84·4	84·6	84·8	84·9	85·2	85·5	85·8
Fukushima	75·1	75·5	76·0	76·4	76·8	77·2	77·6	77·9	78·1	78·3	78·5	78·7	79·0	79·4	79·7	80·0	80·2	80·5	80·7	80·9	81·1	81·4	81·7	81·9	82·2	82·3	82·6	82·9	83·3	83·6
Gifu	77·1	77·6	78·1	78·6	79·0	79·4	79·8	80·1	80·4	80·6	80·8	81·0	81·3	81·5	81·8	82·0	82·2	82·4	82·6	82·8	83·1	83·3	83·6	83·8	84·0	84·2	84·4	84·6	84·9	85·2
Gunma	77·4	77·8	78·3	78·8	79·2	79·6	79·9	80·2	80·5	80·6	80·8	81·0	81·2	81·5	81·8	82·1	82·3	82·5	82·7	83·0	83·3	83·6	83·9	84·2	84·4	84·6	84·9	85·2	85·5	85·8
Hiroshima	78·9	79·3	79·8	80·2	80·7	81·1	81·5	81·8	82·1	82·3	82·5	82·7	82·9	83·1	83·4	83·5	83·7	83·8	84·0	84·1	84·3	84·6	84·9	85·1	85·4	85·6	85·8	86·0	86·4	86·6
Hokkaidō	76·8	77·3	77·7	78·2	78·6	79·0	79·4	79·7	79·9	80·1	80·3	80·5	80·7	81·0	81·3	81·5	81·6	81·8	81·9	82·1	82·2	82·5	82·7	82·9	83·1	83·3	83·5	83·7	84·1	84·4
Hyōgo	79·4	79·9	80·4	80·8	81·2	81·6	82·0	82·3	82·6	82·8	83·0	83·1	83·3	83·5	83·7	83·8	83·9	84·1	84·2	84·3	84·5	84·8	85·0	85·2	85·4	85·6	85·8	86·0	86·3	86·6
Ibaraki	77·3	77·7	78·2	78·7	79·1	79·6	79·9	80·2	80·4	80·6	80·8	81·0	81·2	81·4	81·7	82·0	82·2	82·5	82·8	83·1	83·3	83·7	83·9	84·2	84·5	84·6	84·8	85·1	85·5	85·8
Ishikawa	77·4	77·9	78·5	79·0	79·5	79·9	80·3	80·7	81·0	81·3	81·5	81·8	82·1	82·3	82·6	82·8	83·0	83·1	83·3	83·4	83·6	83·8	83·9	84·2	84·4	84·6	84·8	85·0	85·4	85·7
Iwate	73·8	74·2	74·8	75·2	75·7	76·2	76·5	76·9	77·2	77·5	77·7	77·9	78·2	78·5	78·8	79·1	79·3	79·5	79·7	80·0	80·2	80·5	80·8	81·1	81·5	81·7	82·0	82·3	82·7	83·1
Kagawa	77·2	77·7	78·3	78·7	79·2	79·6	80·0	80·2	80·5	80·7	80·9	81·1	81·4	81·7	82·0	82·2	82·3	82·5	82·7	82·9	83·1	83·4	83·7	83·9	84·2	84·4	84·6	84·8	85·2	85·5
Kagoshima	74·4	74·8	75·3	75·7	76·1	76·6	76·9	77·3	77·6	77·8	78·1	78·3	78·6	78·9	79·2	79·4	79·6	79·9	80·1	80·3	80·5	80·8	81·1	81·4	81·6	81·8	82·1	82·4	82·7	83·1
Kanagawa	82·0	82·4	82·8	83·2	83·6	83·9	84·3	84·5	84·7	84·9	85·0	85·1	85·3	85·4	85·6	85·8	86·0	86·2	86·3	86·5	86·6	86·8	87·0	87·2	87·3	87·4	87·6	87·8	88·0	88·2
Kōchi	74·4	74·8	75·3	75·7	76·1	76·6	77·0	77·4	77·7	78·1	78·3	78·6	78·9	79·3	79·6	79·7	79·9	80·1	80·3	80·5	80·7	81·0	81·2	81·5	81·8	82·0	82·2	82·5	82·9	83·2
Kumamoto	75·4	75·8	76·3	76·7	77·0	77·4	77·7	78·0	78·2	78·4	78·6	78·8	79·1	79·4	79·7	79·9	80·0	80·2	80·4	80·6	80·8	81·1	81·4	81·6	81·9	82·1	82·4	82·7	83·0	83·3
Kyōto	80·1	80·5	81·0	81·4	81·8	82·2	82·6	82·9	83·1	83·3	83·5	83·8	84·0	84·2	84·5	84·7	84·9	85·1	85·3	85·4	85·6	85·8	86·0	86·1	86·3	86·5	86·7	86·9	87·2	87·5
Mie	76·5	77·0	77·6	78·1	78·5	78·9	79·3	79·7	80·0	80·2	80·5	80·7	81·1	81·4	81·8	82·0	82·3	82·5	82·8	83·0	83·2	83·5	83·8	84·1	84·4	84·6	84·8	85·1	85·5	85·8
Miyagi	77·4	77·9	78·4	78·8	79·2	79·6	80·0	80·2	80·5	80·6	80·8	81·0	81·3	81·5	81·9	82·1	82·3	82·5	82·7	82·9	83·1	83·4	83·7	84·0	84·3	84·5	84·7	85·0	85·4	85·7
Miyazaki	74·1	74·4	74·9	75·2	75·6	76·0	76·3	76·7	77·0	77·3	77·6	77·8	78·1	78·4	78·7	78·9	79·0	79·2	79·4	79·6	79·8	80·2	80·5	80·8	81·1	81·3	81·5	81·8	82·2	82·5
Nagano	77·8	78·2	78·7	79·1	79·5	79·9	80·2	80·5	80·8	81·0	81·2	81·4	81·7	81·9	82·2	82·4	82·6	82·9	83·1	83·2	83·5	83·7	83·9	84·1	84·4	84·5	84·7	85·0	85·3	85·6
Nagasaki	74·3	74·7	75·2	75·6	76·0	76·5	76·9	77·2	77·5	77·7	78·0	78·2	78·4	78·7	79·0	79·2	79·3	79·5	79·7	79·9	80·1	80·4	80·7	81·0	81·3	81·5	81·7	82·0	82·4	82·7
Nara	77·9	78·4	78·9	79·4	79·8	80·3	80·6	81·0	81·2	81·5	81·7	81·9	82·1	82·3	82·5	82·7	82·8	83·0	83·1	83·3	83·4	83·6	83·8	83·9	84·1	84·2	84·4	84·6	84·9	85·1
Niigata	75·8	76·3	76·8	77·2	77·6	78·1	78·4	78·8	79·0	79·3	79·6	79·9	80·2	80·5	80·8	81·0	81·2	81·4	81·6	81·8	82·1	82·3	82·6	82·9	83·2	83·4	83·6	83·8	84·2	84·5
Ōita	76·5	76·9	77·4	77·9	78·3	78·7	79·1	79·4	79·7	80·0	80·2	80·5	80·8	81·1	81·4	81·6	81·8	81·9	82·1	82·3	82·5	82·8	83·0	83·2	83·5	83·6	83·8	84·1	84·4	84·7
Okayama	77·8	78·3	78·8	79·2	79·6	80·0	80·4	80·6	80·9	81·1	81·3	81·5	81·8	82·1	82·4	82·6	82·8	83·1	83·3	83·5	83·8	84·1	84·3	84·6	84·8	85·0	85·2	85·4	85·7	86·0
Okinawa	73·6	74·0	74·5	74·9	75·3	75·7	76·0	76·2	76·4	76·5	76·7	77·0	77·3	77·7	78·1	78·4	78·6	78·8	79·0	79·2	79·5	79·8	80·1	80·3	80·6	80·7	80·9	81·2	81·6	81·9
Ōsaka	80·6	81·0	81·6	82·0	82·4	82·8	83·1	83·4	83·6	83·8	84·0	84·2	84·3	84·5	84·7	84·9	85·0	85·1	85·3	85·4	85·6	85·8	86·0	86·2	86·4	86·6	86·8	87·0	87·3	87·5
Saga	75·2	75·6	76·1	76·6	77·0	77·4	77·7	78·0	78·3	78·6	78·8	79·0	79·3	79·6	80·0	80·2	80·4	80·6	80·8	81·0	81·2	81·4	81·6	81·8	82·1	82·2	82·4	82·7	83·1	83·4
Saitama	78·3	78·8	79·3	79·8	80·2	80·6	81·0	81·3	81·5	81·6	81·8	81·9	82·1	82·3	82·5	82·7	82·9	83·1	83·3	83·5	83·7	83·9	84·1	84·3	84·5	84·7	84·8	85·0	85·3	85·6
Shiga	79·3	79·9	80·5	81·0	81·4	81·9	82·2	82·5	82·8	83·0	83·2	83·4	83·7	84·0	84·3	84·5	84·6	84·8	85·0	85·2	85·4	85·6	85·8	86·0	86·2	86·3	86·5	86·8	87·0	87·3
Shimane	74·1	74·5	75·0	75·5	75·9	76·4	76·8	77·2	77·6	77·9	78·2	78·5	78·8	79·2	79·6	79·8	80·0	80·3	80·5	80·7	81·0	81·3	81·6	81·9	82·2	82·4	82·7	83·0	83·3	83·6
Shizuoka	78·1	78·5	79·0	79·4	79·8	80·2	80·6	80·9	81·1	81·4	81·5	81·8	82·0	82·3	82·7	82·9	83·1	83·3	83·5	83·8	84·0	84·3	84·6	84·9	85·1	85·3	85·4	85·7	86·0	86·3
Tochigi	77·3	77·8	78·3	78·8	79·2	79·6	80·0	80·3	80·5	80·7	80·8	81·0	81·3	81·6	81·9	82·1	82·4	82·6	82·8	83·1	83·3	83·6	83·9	84·1	84·4	84·6	84·8	85·1	85·5	85·8
Tokushima	75·5	76·0	76·5	77·0	77·5	77·9	78·3	78·7	79·0	79·3	79·6	80·0	80·3	80·8	81·2	81·5	81·8	82·0	82·3	82·5	82·8	83·1	83·3	83·6	83·9	84·2	84·4	84·7	85·1	85·4
Tōkyō	86·7	87·2	87·7	88·1	88·6	89·0	89·4	89·7	89·9	90·1	90·2	90·4	90·5	90·7	90·8	91·0	91·1	91·2	91·3	91·4	91·6	91·7	91·8	91·9	92·0	92·1	92·3	92·4	92·6	92·7
Tottori	75·8	76·2	76·8	77·2	77·6	78·1	78·4	78·8	79·0	79·3	79·5	79·7	79·9	80·2	80·5	80·7	80·8	81·0	81·1	81·3	81·4	81·6	81·8	82·0	82·2	82·4	82·6	82·8	83·1	83·4

Toyama	77·8	78·3	78·9	79·3	79·7	80·2	80·5	80·9	81·1	81·4	81·7	81·9	82·2	82·5	82·9	83·1	83·4	83·6	83·8	84·0	84·2	84·4	84·7	84·9	85·1	85·3	85·5	85·8	86·1	86·4
Wakayama	75·5	75·9	76·4	76·9	77·2	77·7	78·0	78·3	78·6	78·9	79·1	79·4	79·7	80·0	80·4	80·7	80·9	81·2	81·4	81·6	81·9	82·2	82·6	82·9	83·2	83·4	83·6	83·9	84·3	84·6
Yamagata	74·8	75·2	75·7	76·1	76·5	76·9	77·2	77·5	77·8	78·0	78·2	78·4	78·7	79·0	79·4	79·7	79·9	80·2	80·5	80·7	81·0	81·3	81·6	81·9	82·1	82·4	82·6	82·9	83·2	83·5
Yamaguchi	77·0	77·4	77·9	78·3	78·7	79·1	79·5	79·8	80·1	80·3	80·6	80·8	81·1	81·4	81·7	81·9	82·1	82·3	82·5	82·7	83·0	83·3	83·6	83·9	84·2	84·3	84·5	84·8	85·2	85·5
Yamanashi	77·7	78·2	78·7	79·1	79·5	79·9	80·2	80·5	80·8	80·9	81·1	81·3	81·6	81·8	82·1	82·4	82·6	82·9	83·2	83·4	83·6	83·9	84·1	84·4	84·6	84·8	85·0	85·2	85·5	85·8
South Korea	68·3	69·6	70·7	71·6	72·6	73·6	74·7	75·6	76·4	77·3	78·2	79·1	79·9	80·6	81·3	81·8	82·3	82·8	83·3	83·7	84·2	84·6	85·1	85·5	85·9	86·4	86·7	87·1	87·6	88·0
Singapore	69·6	70·5	71·3	72·2	73·1	74·0	74·8	75·5	75·9	76·2	77·0	77·7	78·3	79·0	79·6	80·2	80·8	81·5	82·2	82·8	83·4	83·8	84·2	84·5	84·8	85·2	85·6	85·9	86·2	86·5
High-income North America	77·0	77·3	77·7	78·0	78·4	78·7	78·9	79·1	79·3	79·6	80·0	80·4	80·8	81·2	81·4	81·5	81·5	81·7	82·2	82·8	83·4	83·7	84·1	84·4	84·6	85·0	85·3	85·4	85·5	85·6
Canada	79·2	79·4	79·5	79·7	80·0	80·4	80·8	81·2	81·5	81·9	82·3	82·7	83·1	83·5	83·8	84·0	84·1	84·2	84·4	84·7	85·1	85·4	85·7	85·8	85·8	85·9	86·0	86·2	86·4	86·6
Greenland	65·6	65·3	65·0	65·2	65·5	65·6	65·9	66·3	67·0	67·4	67·7	68·2	68·6	69·2	69·7	70·2	70·7	71·2	71·5	72·0	72·8	73·2	73·4	73·9	74·2	74·3	74·5	74·7	74·9	75·1
USA	76·8	77·0	77·4	77·8	78·2	78·4	78·7	78·9	79·0	79·3	79·7	80·1	80·6	80·9	81·1	81·2	81·2	81·5	82·0	82·6	83·2	83·5	83·9	84·2	84·5	84·9	85·3	85·4	85·3	85·4
Alabama	73·1	73·5	74·1	74·5	74·8	75·1	75·2	75·2	75·3	75·5	76·0	76·7	77·2	77·7	78·0	78·1	78·0	78·2	78·7	79·4	80·1	80·5	80·9	81·1	81·3	81·5	81·7	81·7	81·6	81·6
Alaska	74·6	74·6	75·1	75·8	76·5	77·2	77·6	77·9	78·1	78·3	78·6	79·1	79·6	79·9	80·2	80·1	80·0	80·1	80·5	81·0	81·6	82·0	82·5	82·9	83·4	83·9	84·4	84·6	84·4	84·5
Arizona	73·9	74·1	74·4	74·7	74·9	75·1	75·3	75·4	75·6	75·7	76·1	76·5	76·9	77·2	77·4	77·5	77·5	78·1	79·1	80·1	81·0	81·5	81·9	82·3	82·6	83·0	83·4	83·4	83·4	83·4
Arkansas	71·0	71·5	72·3	72·8	73·1	73·3	73·4	73·4	73·5	73·7	74·1	74·7	75·2	75·6	75·9	75·9	75·9	76·1	76·7	77·5	78·2	78·7	79·1	79·5	79·7	80·1	80·5	80·5	80·4	80·5
California	75·6	75·6	75·8	76·1	76·6	77·1	77·7	78·2	78·8	79·3	79·8	80·4	80·8	81·2	81·4	81·5	81·6	81·9	82·5	83·1	83·8	84·2	84·6	85·0	85·3	85·7	86·1	86·2	86·2	86·3
Colorado	78·8	79·0	79·4	79·7	80·1	80·3	80·5	80·6	80·6	80·7	80·9	81·3	81·8	82·1	82·4	82·6	82·6	82·9	83·4	84·0	84·6	85·1	85·4	85·8	86·1	86·6	87·0	87·1	87·1	87·3
Connecticut	82·9	83·2	83·5	83·8	84·1	84·3	84·5	84·6	84·8	85·1	85·5	85·9	86·3	86·6	86·7	86·8	86·8	87·0	87·3	87·7	88·1	88·4	88·7	89·0	89·3	89·6	89·8	89·9	89·8	89·8
Delaware	78·7	79·1	79·5	79·9	80·1	80·3	80·4	80·5	80·5	80·7	81·0	81·5	81·8	82·0	82·2	82·2	82·3	82·6	83·1	83·8	84·3	84·7	85·0	85·3	85·4	85·7	85·9	85·9	85·9	85·9
Washington, DC	78·3	78·5	79·0	79·6	80·3	81·1	81·9	82·7	83·4	84·0	84·6	85·2	85·8	86·0	86·2	86·2	86·2	86·2	86·4	86·7	86·9	86·9	87·0	87·1	87·2	87·4	87·5	87·4	87·2	87·2
Florida	75·9	76·3	76·8	77·2	77·6	77·9	78·2	78·3	78·5	78·8	79·2	79·7	80·1	80·4	80·6	80·7	80·8	81·2	81·8	82·6	83·2	83·6	83·9	84·2	84·4	84·7	85·0	85·0	85·0	85·1
Georgia	74·1	74·6	75·2	75·7	76·1	76·4	76·6	76·6	76·7	76·8	77·1	77·6	78·0	78·3	78·5	78·5	78·5	78·9	79·5	80·4	81·2	81·7	82·1	82·5	82·8	83·2	83·5	83·6	83·5	83·6
Hawaii	76·9	77·0	77·4	77·9	78·5	79·1	79·6	80·0	80·1	80·3	80·6	81·0	81·4	81·8	82·0	82·1	82·0	82·2	82·6	83·1	83·6	83·9	84·3	84·7	85·0	85·4	85·7	85·8	85·7	85·7
Idaho	74·3	74·7	75·2	75·8	76·2	76·5	76·7	76·8	76·8	76·9	77·2	77·6	77·9	78·2	78·4	78·4	78·3	78·6	79·2	80·0	80·7	81·0	81·2	81·5	81·6	82·0	82·4	82·5	82·5	82·6
Illinois	77·5	77·7	78·0	78·4	78·8	79·2	79·5	79·8	80·1	80·4	80·8	81·4	81·9	82·3	82·7	82·8	82·9	83·2	83·7	84·2	84·8	85·1	85·5	85·8	86·0	86·4	86·7	86·8	86·8	86·9
Indiana	75·8	76·1	76·5	76·9	77·1	77·3	77·4	77·5	77·6	77·8	78·2	78·7	79·0	79·3	79·5	79·4	79·3	79·5	80·0	80·6	81·2	81·5	81·8	82·2	82·5	82·9	83·3	83·4	83·4	83·5
Iowa	78·1	78·4	78·8	79·1	79·4	79·6	79·8	79·9	80·1	80·3	80·6	81·0	81·3	81·6	81·7	81·7	81·6	81·8	82·2	82·8	83·3	83·7	84·0	84·3	84·7	85·1	85·5	85·7	85·7	85·8
Kansas	77·1	77·4	77·7	78·1	78·3	78·6	78·7	78·8	78·9	79·0	79·4	79·8	80·2	80·5	80·7	80·7	80·5	80·6	81·0	81·5	82·1	82·6	83·0	83·5	83·9	84·5	85·0	85·1	85·0	85·1
Kentucky	72·9	73·3	73·8	74·3	74·6	74·9	75·1	75·2	75·3	75·5	75·9	76·5	77·0	77·4	77·6	77·5	77·3	77·4	77·8	78·4	79·0	79·4	79·7	80·0	80·3	80·7	81·0	81·1	81·1	81·2
Louisiana	72·4	72·7	73·2	73·7	74·2	74·5	74·6	74·7	74·8	75·0	75·5	76·1	76·6	77·1	77·4	77·5	77·4	77·7	78·2	78·8	79·5	79·8	80·1	80·4	80·7	81·1	81·6	81·7	81·7	81·8
Maine	77·4	78·0	78·7	79·2	79·7	80·0	80·1	80·3	80·4	80·6	80·9	81·4	81·8	82·1	82·3	82·4	82·5	82·7	83·1	83·5	84·0	84·2	84·5	84·8	85·0	85·3	85·6	85·7	85·7	85·8
Maryland	80·0	80·4	80·9	81·3	81·7	82·1	82·3	82·4	82·5	82·7	83·1	83·5	84·0	84·4	84·8	84·9	84·9	85·1	85·6	86·1	86·6	87·0	87·3	87·6	87·9	88·2	88·4	88·4	88·3	88·3
Massachusetts	83·0	83·3	83·7	84·0	84·3	84·6	84·9	85·1	85·4	85·7	86·1	86·5	86·9	87·2	87·5	87·6	87·7	88·0	88·2	88·6	88·9	89·1	89·4	89·7	89·9	90·1	90·4	90·4	90·4	90·4
Michigan	77·3	77·7	78·3	78·7	79·2	79·5	79·8	80·0	80·2	80·4	80·8	81·3	81·7	82·0	82·3	82·3	82·3	82·5	82·8	83·2	83·5	83·8	84·0	84·3	84·6	85·0	85·4	85·5	85·6	85·7
Minnesota	80·4	80·8	81·2	81·5	81·8	82·0	82·2	82·4	82·5	82·7	83·0	83·4	83·7	84·0	84·2	84·3	84·4	84·6	85·0	85·5	86·0	86·3	86·6	86·9	87·2	87·6	87·9	88·0	88·1	88·2
Mississippi	70·6	71·0	71·6	72·1	72·5	72·9	73·0	73·0	73·1	73·2	73·7	74·3	74·7	75·0	75·2	75·1	74·8	75·0	75·6	76·5	77·4	77·9	78·4	78·8	79·2	79·6	80·0	80·0	79·9	80·0
Missouri	75·7	76·0	76·5	77·0	77·4	77·6	77·8	77·9	78·1	78·3	78·7	79·2	79·5	79·8	79·9	79·9	79·8	80·0	80·5	81·2	81·8	82·1	82·5	82·8	83·1	83·5	83·8	83·9	83·9	83·9
Montana	76·3	76·6	77·2	77·7	78·3	78·7	79·0	79·2	79·2	79·3	79·6	79·9	80·2	80·5	80·8	80·8	80·8	81·0	81·3	81·8	82·3	82·6	82·9	83·2	83·6	84·1	84·7	84·9	84·9	85·1
Nebraska	78·1	78·5	78·9	79·3	79·6	79·9	80·1	80·2	80·3	80·4	80·5	80·8	81·1	81·4	81·6	81·7	81·6	81·8	82·2	82·7	83·2	83·6	84·0	84·3	84·7	85·2	85·7	85·8	85·8	85·9
Nevada	74·8	74·9	75·2	75·4	75·7	76·0	76·4	76·7	77·0	77·4	77·8	78·4	78·8	79·1	79·3	79·3	79·4	79·8	80·6	81·5	82·2	82·5	82·7	82·8	82·9	83·0	83·3	83·2	83·1	83·1
New Hampshire	81·4	81·9	82·3	82·6	82·8	83·0	83·2	83·4	83·7	84·0	84·5	84·9	85·3	85·7	85·9	86·0	86·1	86·3	86·6	86·9	87·3	87·6	88·0	88·3	88·6	88·9	89·2	89·3	89·4	89·5
New Jersey	81·5	81·9	82·3	82·6	83·0	83·3	83·5	83·7	83·8	84·0	84·3	84·6	85·0	85·3	85·6	85·7	85·8	86·0	86·3	86·8	87·2	87·5	87·8	88·1	88·3	88·6	88·8	88·9	88·8	88·8
New Mexico	72·1	72·2	72·7	73·3	73·9	74·3	74·6	74·7	74·8	74·9	75·3	75·7	76·2	76·5	76·8	76·7	76·6	76·9	77·5	78·4	79·3	79·8	80·3	80·8	81·2	81·6	82·0	82·0	81·9	81·9
New York	80·1	80·4	80·8	81·1	81·4	81·7	82·0	82·2	82·5	82·8	83·1	83·6	84·0	84·3	84·6	84·7	84·8	85·0	85·4	85·8	86·2	86·5	86·9	87·2	87·5	87·8	88·1	88·1	88·1	88·1
North Carolina	74·7	75·1	75·6	76·1	76·4	76·6	76·7	76·8	76·9	77·1	77·4	77·9	78·3	78·7	79·0	79·0	79·0	79·3	79·9	80·6	81·3	81·7	82·1	82·5	82·8	83·1	83·5	83·5	83·5	83·6
North Dakota	78·0	78·2	78·5	78·7	79·0	79·3	79·7	80·1	80·6	81·0	81·3	81·6	81·8	81·9	82·0	81·9	81·9	82·0	82·4	82·8	83·2	83·4	83·7	84·1	84·8	85·8	86·6	86·9	86·9	86·9
Ohio	76·5	76·8	77·3	77·7	78·0	78·2	78·4	78·5	78·7	78·9	79·3	79·8	80·2	80·5	80·7	80·7	80·6	80·8	81·2	81·8	82·3	82·6	82·9	83·2	83·4	83·8	84·1	84·2	84·2	84·3
Oklahoma	73·8	74·0	74·4	74·8	75·1	75·3	75·2	75·2	75·2	75·4	75·7	76·1	76·6	77·1	77·4	77·3	77·1	77·3	77·8	78·5	79·3	79·7	80·1	80·6	81·0	81·5	81·9	82·0	81·9	82·0
Oregon	77·3	77·6	78·0	78·4	78·7	78·9	79·1	79·4	79·6	79·9	80·4	80·9	81·4	81·7	82·0	82·0	82·0	82·3	82·7	83·3	83·8	84·2	84·5	84·8	85·1	85·5	85·9	86·1	86·1	86·3
Pennsylvania	78·6	79·0	79·5	80·0	80·3	80·7	80·9	81·1	81·2	81·5	81·8	82·2	82·5	82·8	83·1	83·1	83·2	83·4	83·7	84·2	84·6	84·9	85·1	85·4	85·7	86·0	86·4	86·5	86·5	86·7
Rhode Island	80·0	80·4	80·8	81·1	81·4	81·6	81·7	81·9	82·2	82·4	82·8	83·2	83·6	83·9	84·2	84·4	84·5	84·8	85·1	85·6	86·0	86·3	86·6	86·9	87·1	87·4	87·7	87·8	87·8	87·9
South Carolina	73·5	74·1	74·8	75·5	76·0	76·3	76·4	76·4	76·5	76·6	77·0	77·5	78·0	78·3	78·5	78·5	78·3	78·6	79·1	79·9	80·6	81·1	81·6	81·9	82·2	82·6	82·9	82·9	82·9	83·0
South Dakota	75·6	76·0	76·5	77·1	77·6	78·0	78·3	78·6	78·8	79·0	79·2	79·5	79·8	79·9	80·0	80·0	79·8	80·1	80·5	81·1	81·7	82·0	82·3	82·7	83·1	83·7	84·3	84·5	84·5	84·6
Tennessee	73·4	73·8	74·3	74·8	75·2	75·5	75·7	75·8	76·0	76·2	76·5	77·0	77·2	77·4	77·4	77·3	77·1	77·4	78·0	78·8	79·5	80·0	80·3	80·7	81·1	81·7	82·2	82·4	82·4	82·5
Texas	73·2	73·3	73·6	73·9	74·2	74·4	74·5	74·6	74·8	75·0	75·3	75·8	76·3	76·6	76·8	76·9	76·8	77·1	77·7	78·6	79·4	79·9	80·4	80·8	81·2	81·8	82·3	82·5	82·4	82·5
Utah	75·0	75·5	76·0	76·4	76·7	76·9	77·1	77·3	77·5	77·8	78·2	78·6	79·0	79·3	79·5	79·5	79·4	79·6	80·2	81·0	81·8	82·3	82·7	83·0	83·4	83·9	84·3	84·5	84·5	84·6
Vermont	80·1	80·6	81·0	81·4	81·7	82·0	82·1	82·3	82·6	82·9	83·3	83·7	84·1	84·5	84·9	85·0	85·1	85·4	85·7	86·0	86·3	86·6	86·9	87·2	87·5	87·9	88·2	88·4	88·4	88·5
Virginia	78·8	79·1	79·6	80·0	80·3	80·6	80·8	81·0	81·1	81·2	81·6	82·0	82·4	82·8	83·1	83·3	83·4	83·8	84·3	84·9	85·4	85·8	86·1	86·4	86·7	87·0	87·2	87·3	87·3	87·3
Washington	78·3	78·6	79·1	79·5	79·9	80·2	80·5	80·8	81·1	81·5	81·9	82·5	82·9	83·2	83·4	83·4	83·4	83·5	83·9	84·4	84·9	85·3	85·6	86·0	86·3	86·6	87·0	87·1	87·1	87·2
West Virginia	73·4	73·8	74·3	74·7	75·1	75·4	75·6	75·7	75·8	76·0	76·4	76·7	77·0	77·1	77·2	77·1	76·9	77·0	77·3	77·8	78·3	78·5	78·8	79·2	79·6	80·1	80·6	80·7	80·7	80·8
Wisconsin	78·9	79·3	79·7	80·1	80·4	80·7	81·0	81·1	81·3	81·4	81·7	82·1	82·5	82·8	83·0	83·0	83·0	83·2	83·5	84·0	84·4	84·7	85·0	85·4	85·7	86·0	86·4	86·5	86·5	86·6
Wyoming	75·8	76·2	76·8	77·4	77·9	78·2	78·4	78·6	78·7	78·8	79·0	79·4	79·7	80·0	80·3	80·4	80·5	80·8	81·5	82·3	83·0	83·4	83·7	84·0	84·2	84·6	85·0	85·1	85·0	85·1
Southern Latin America	58·5	59·0	59·7	60·2	60·8	61·3	61·8	62·5	63·1	63·5	63·9	64·3	64·5	64·8	65·3	65·8	66·3	66·5	66·8	67·1	67·6	68·1	68·6	69·0	69·3	69·9	70·4	70·8	71·2	71·6
Argentina	58·1	58·6	59·3	59·9	60·5	61·0	61·4	62·1	62·7	63·0	63·3	63·6	63·7	63·8	64·2	64·8	65·3	65·6	65·8	66·1	66·5	67·0	67·4	67·7	67·9	68·5	69·1	69·5	69·9	70·2
Chile	59·2	60·0	60·6	61·1	61·7	62·4	63·0	63·7	64·4	65·1	65·7	66·3	66·9	67·6	68·3	68·6	68·9	69·2	69·5	70·0	70·6	71·3	71·9	72·5	73·1	73·6	74·0	74·4	74·8	75·1
Uruguay	58·1	58·8	59·2	59·2	59·4	59·6	60·2	61·0	61·6	62·1	62·5	62·8	63·1	63·3	63·6	63·9	64·4	64·7	64·9	65·1	65·5	65·9	66·2	66·7	67·3	67·9	68·4	68·9	69·4	69·8
Western Europe	75·1	75·7	76·2	76·7	77·2	77·5	77·9	78·2	78·4	78·8	79·1	79·4	79·7	80·0	80·2	80·5	80·7	81·0	81·2	81·5	81·7	82·1	82·4	82·7	83·0	83·2	83·4	83·6	83·9	84·2
Andorra	83·3	83·7	84·0	84·1	84·2	84·3	84·4	84·6	84·9	85·1	85·3	85·6	85·7	86·1	86·4	86·6	86·8	86·9	87·1	87·3	87·6	87·8	88·0	88·2	88·3	88·5	88·6	88·7	88·9	89·1
Austria	75·3	75·5	75·7	76·2	76·8	77·3	77·8	78·3	78·7	79·1	79·5	79·9	80·3	80·5	80·8	81·1	81·5	81·8	82·1	82·4	82·7	83·0	83·3	83·6	83·8	83·9	84·0	84·2	84·5	84·7
Belgium	74·6	75·1	75·7	76·2	76·7	77·1	77·5	77·9	78·2	78·5	78·8	79·2	79·6	79·9	80·2	80·5	80·8	81·0	81·3	81·6	82·0	82·5	82·9	83·4	83·7	84·0	84·1	84·3	84·6	84·8
Cyprus	67·3	66·5	67·4	69·2	71·0	72·0	72·3	73·1	74·0	74·9	75·8	76·8	77·7	78·5	79·1	79·7	80·4	81·0	81·4	81·8	82·1	82·5	82·7	83·0	83·2	83·3	83·5	83·6	83·9	84·1
Denmark	80·6	80·9	81·3	81·6	82·0	82·4	82·8	83·3	83·7	84·0	84·4	84·8	85·2	85·5	85·8	86·0	86·2	86·4	86·5	86·7	87·0	87·3	87·6	87·8	88·0	88·1	88·3	88·5	88·7	88·9
Finland	75·8	76·0	76·2	76·6	77·2	77·6	77·9	78·2	78·5	78·8	79·2	79·7	80·2	80·5	80·9	81·2	81·5	81·8	82·2	82·4	82·8	83·1	83·4	83·7	84·0	84·4	84·6	84·7	85·0	85·2
France	74·4	74·9	75·5	75·9	76·1	76·3	76·7	77·0	77·3	77·5	77·7	78·1	78·4	78·7	79·0	79·3	79·6	79·9	80·1	80·3	80·6	80·9	81·2	81·5	81·9	82·2	82·4	82·6	82·9	83·1
Germany	82·0	82·9	83·4	83·8	84·1	84·3	84·4	84·4	84·4	84·7	85·0	85·3	85·6	85·8	86·1	86·3	86·6	86·9	87·3	87·5	87·8	88·1	88·3	88·6	88·8	88·9	89·0	89·2	89·5	89·7
Greece	68·3	68·8	69·5	70·2	70·7	71·3	71·8	72·3	72·9	73·5	73·9	74·3	74·8	75·3	75·8	76·1	76·5	76·8	77·2	77·5	77·9	78·2	78·5	78·6	78·6	78·6	78·6	78·8	79·0	79·3
Iceland	76·5	77·0	77·3	77·8	78·2	78·5	78·8	79·1	79·5	79·8	80·3	81·0	81·7	82·2	82·5	82·7	83·0	83·4	83·7	84·2	84·7	85·0	85·3	85·5	85·9	86·4	86·6	86·8	87·1	87·4
Ireland	73·0	73·5	74·1	74·8	75·3	75·8	76·3	76·8	77·3	78·0	78·6	79·2	79·9	80·6	81·1	81·6	81·9	82·1	82·3	82·7	83·1	83·5	83·8	84·2	84·5	84·9	85·3	85·7	86·2	86·6
Israel	71·7	72·1	72·6	73·0	73·4	73·9	74·2	74·5	74·9	75·2	75·6	76·0	76·3	76·5	77·0	77·3	77·6	77·8	77·8	77·9	78·2	78·4	78·7	78·9	79·2	79·4	79·7	80·0	80·4	80·7
Italy	71·2	71·7	72·2	72·7	73·2	73·6	74·0	74·4	74·7	75·0	75·3	75·7	76·0	76·3	76·5	76·8	77·1	77·3	77·5	77·7	78·0	78·2	78·4	78·7	78·9	79·1	79·4	79·6	79·8	80·1
Luxembourg	82·2	82·6	82·5	82·6	82·9	83·3	83·6	83·9	84·2	84·4	84·8	85·1	85·3	85·5	85·7	85·8	86·2	86·6	86·9	87·1	87·3	87·5	87·8	88·1	88·3	88·5	88·7	88·7	88·9	89·0
Malta	66·6	67·0	67·5	68·2	69·0	69·5	69·7	70·2	70·9	71·6	72·3	72·9	73·3	73·7	74·2	74·6	75·0	75·3	75·7	76·1	76·4	76·8	77·2	77·6	77·9	78·4	78·7	79·2	79·7	80·2
Monaco	83·8	84·1	84·5	84·8	85·1	85·4	85·6	85·9	86·2	86·4	86·6	86·9	87·1	87·3	87·6	87·8	88·0	88·2	88·4	88·6	88·8	89·0	89·1	89·3	89·5	89·7	89·9	90·0	90·2	90·3
Netherlands	79·6	80·1	80·6	81·0	81·4	81·8	82·1	82·4	82·7	83·0	83·3	83·6	83·9	84·2	84·5	84·8	85·1	85·4	85·6	85·8	86·1	86·4	86·6	86·9	87·1	87·4	87·5	87·7	87·9	88·2
Norway	80·7	81·2	81·8	82·3	82·8	83·2	83·7	84·3	84·7	85·1	85·6	86·2	86·6	86·9	87·2	87·4	87·6	87·7	87·9	88·1	88·5	88·9	89·3	89·6	90·0	90·3	90·4	90·4	90·5	90·6

Portugal	60·8	61·5	62·2	62·9	63·6	64·2	64·7	65·2	65·7	66·1	66·6	67·1	67·6	68·1	68·6	69·0	69·4	69·8	70·1	70·5	70·9	71·4	71·9	72·3	72·6	72·8	73·1	73·4	73·9	74·3
San Marino	81·1	81·4	81·7	82·1	82·5	82·9	83·3	83·8	84·2	84·6	84·9	85·1	85·3	85·5	85·7	85·9	86·1	86·3	86·5	86·7	86·9	87·1	87·3	87·5	87·7	87·8	87·9	87·9	88·0	88·1
Spain	64·9	65·6	66·3	67·0	67·6	68·2	68·7	69·2	69·6	70·0	70·5	70·9	71·3	71·7	72·1	72·5	72·8	73·0	73·4	73·9	74·3	74·6	74·9	75·2	75·4	75·6	75·8	76·1	76·5	76·9
Sweden	77·0	77·5	78·1	78·8	79·4	80·1	80·6	81·1	81·5	81·9	82·3	82·6	82·9	83·2	83·5	83·8	84·0	84·2	84·5	84·6	84·9	85·2	85·5	85·8	86·0	86·3	86·5	86·8	87·0	87·3
Stockholm	77·4	77·8	78·3	78·8	79·3	79·8	80·3	80·7	81·1	81·5	81·9	82·2	82·5	82·7	83·0	83·3	83·6	83·8	84·1	84·3	84·6	84·9	85·2	85·4	85·7	85·9	86·1	86·4	86·6	86·8
Sweden except Stockholm	76·8	77·3	78·0	78·7	79·3	80·0	80·6	81·1	81·5	81·9	82·3	82·6	82·9	83·2	83·5	83·8	84·0	84·2	84·4	84·6	84·9	85·2	85·5	85·8	86·0	86·2	86·5	86·8	87·0	87·3
Switzerland	86·9	87·1	87·4	87·7	87·9	88·0	88·2	88·4	88·5	88·7	89·0	89·3	89·5	89·6	89·8	90·0	90·3	90·5	90·8	91·0	91·2	91·4	91·7	91·9	92·1	92·2	92·3	92·5	92·6	92·7
UK	74·5	74·9	75·5	76·1	76·6	76·9	77·1	77·4	77·8	78·4	78·9	79·3	79·7	79·9	80·2	80·4	80·6	80·8	81·1	81·3	81·6	82·0	82·5	83·0	83·4	83·6	83·8	83·9	84·1	84·3
England	74·8	75·3	75·8	76·4	76·9	77·2	77·5	77·8	78·2	78·7	79·2	79·6	80·0	80·2	80·4	80·7	80·9	81·1	81·4	81·6	81·9	82·3	82·8	83·3	83·7	83·9	84·1	84·2	84·4	84·6
East Midlands	72·2	72·7	73·2	73·8	74·3	74·6	74·9	75·2	75·6	76·2	76·7	77·1	77·5	77·7	78·0	78·2	78·4	78·7	78·9	79·2	79·5	79·9	80·3	80·9	81·3	81·6	81·7	81·8	82·1	82·3
Derby	73·3	73·8	74·4	75·1	75·7	76·1	76·3	76·5	76·9	77·4	77·9	78·4	78·7	79·0	79·2	79·4	79·6	79·7	79·9	80·1	80·5	80·9	81·4	82·1	82·6	83·0	83·2	83·4	83·6	83·8
Derbyshire	71·3	71·8	72·3	72·9	73·3	73·6	73·8	74·1	74·4	75·0	75·5	75·9	76·2	76·3	76·5	76·7	76·9	77·0	77·3	77·4	77·7	78·1	78·7	79·3	79·7	80·0	80·2	80·3	80·5	80·7
Leicester	71·7	72·2	72·8	73·3	73·8	74·1	74·4	74·8	75·2	75·7	76·1	76·6	76·9	77·2	77·5	77·8	78·2	78·5	78·9	79·2	79·6	80·1	80·5	81·0	81·4	81·6	81·7	81·8	82·0	82·2
Leicestershire	74·6	75·1	75·6	76·1	76·6	76·9	77·2	77·5	77·8	78·4	78·9	79·3	79·7	79·9	80·2	80·5	80·9	81·1	81·4	81·6	81·8	82·1	82·5	82·9	83·3	83·5	83·7	83·8	84·0	84·2
Lincolnshire	71·6	72·0	72·5	73·0	73·5	73·8	74·0	74·3	74·7	75·2	75·7	76·1	76·4	76·6	76·7	76·9	77·0	77·0	77·2	77·3	77·5	77·9	78·4	79·0	79·5	79·9	80·1	80·3	80·5	80·7
Northamptonshire	73·1	73·7	74·3	74·9	75·5	75·8	75·9	76·1	76·3	76·7	77·1	77·5	77·8	78·0	78·1	78·3	78·4	78·5	78·7	79·0	79·3	79·7	80·2	80·9	81·3	81·5	81·6	81·7	81·9	82·1
Nottingham	74·6	75·1	75·6	76·2	76·7	77·1	77·4	77·8	78·4	79·0	79·5	80·0	80·4	80·7	81·0	81·3	81·6	81·9	82·3	82·6	82·9	83·3	83·7	84·1	84·3	84·5	84·6	84·7	84·9	85·1
Nottinghamshire	71·6	72·1	72·6	73·1	73·6	73·9	74·1	74·4	74·8	75·3	75·8	76·2	76·5	76·7	76·9	77·1	77·3	77·4	77·6	77·7	78·0	78·3	78·8	79·3	79·7	79·9	80·1	80·2	80·4	80·6
Rutland	75·8	76·2	76·7	77·1	77·5	77·8	78·0	78·3	78·6	79·0	79·3	79·6	79·8	79·9	80·0	80·2	80·5	80·8	81·0	81·3	81·7	82·2	82·6	83·0	83·4	83·6	83·8	83·9	84·1	84·3
East of England	73·8	74·3	74·9	75·4	75·9	76·3	76·5	76·8	77·2	77·7	78·3	78·7	79·1	79·3	79·5	79·8	80·0	80·2	80·5	80·8	81·1	81·5	82·0	82·5	82·9	83·2	83·3	83·5	83·7	83·9
Bedford	74·9	75·4	75·9	76·4	76·9	77·2	77·4	77·8	78·2	78·7	79·1	79·5	79·8	80·0	80·3	80·5	80·8	80·9	81·1	81·3	81·6	81·9	82·4	82·8	83·1	83·3	83·4	83·5	83·7	83·9
Cambridgeshire	77·7	78·2	78·7	79·2	79·7	80·1	80·3	80·6	81·0	81·6	82·1	82·5	82·9	83·2	83·4	83·7	83·9	84·1	84·4	84·6	84·8	85·2	85·6	86·0	86·4	86·7	86·9	87·0	87·2	87·4
Central Bedfordshire	74·4	74·9	75·4	75·9	76·4	76·7	76·9	77·2	77·5	77·9	78·3	78·7	79·1	79·2	79·4	79·6	79·8	80·0	80·3	80·6	80·9	81·3	81·8	82·4	82·8	83·1	83·3	83·5	83·7	83·8
Essex	72·8	73·3	73·9	74·5	75·0	75·3	75·6	75·9	76·3	76·8	77·3	77·8	78·1	78·4	78·7	78·9	79·2	79·5	79·7	80·0	80·3	80·7	81·2	81·7	82·0	82·3	82·4	82·6	82·8	83·0
Hertfordshire	77·3	77·7	78·3	78·9	79·4	79·7	79·9	80·3	80·7	81·2	81·8	82·3	82·7	83·0	83·4	83·6	83·9	84·1	84·4	84·6	84·9	85·2	85·6	86·0	86·3	86·5	86·6	86·7	86·9	87·0
Luton	72·3	72·8	73·3	73·9	74·5	74·8	75·1	75·4	75·8	76·3	76·7	77·0	77·3	77·5	77·9	78·2	78·5	78·8	79·2	79·5	79·9	80·4	81·0	81·6	82·0	82·3	82·5	82·6	82·8	83·0
Norfolk	72·3	72·8	73·3	73·8	74·3	74·6	74·8	75·1	75·6	76·1	76·6	77·1	77·4	77·6	77·8	78·0	78·2	78·3	78·6	78·8	79·1	79·4	79·9	80·5	80·9	81·2	81·4	81·6	81·9	82·1
Peterborough	72·3	72·6	73·0	73·5	74·0	74·3	74·5	74·7	75·1	75·6	76·0	76·3	76·5	76·5	76·6	76·6	76·7	76·7	76·8	76·8	77·1	77·6	78·2	79·1	79·6	80·0	80·3	80·4	80·6	80·9
Southend-on-Sea	71·1	71·5	72·0	72·5	73·0	73·3	73·5	73·8	74·2	74·8	75·4	75·8	76·3	76·5	76·8	77·0	77·2	77·3	77·5	77·7	78·1	78·5	78·9	79·5	79·9	80·2	80·4	80·5	80·7	80·9
Suffolk	72·8	73·4	73·9	74·5	74·9	75·2	75·3	75·5	75·9	76·4	76·9	77·2	77·4	77·5	77·7	77·9	78·1	78·2	78·5	78·7	79·0	79·5	80·0	80·6	81·0	81·2	81·3	81·3	81·6	81·8
Thurrock	71·2	71·6	72·1	72·7	73·3	73·7	73·9	74·2	74·6	75·2	75·6	76·0	76·2	76·2	76·3	76·4	76·5	76·6	76·8	77·0	77·3	77·8	78·3	78·9	79·2	79·5	79·5	79·6	79·8	80·0
Greater London	80·1	80·6	81·2	81·7	82·2	82·5	82·8	83·1	83·4	83·9	84·4	84·8	85·1	85·3	85·6	85·8	86·0	86·2	86·5	86·7	87·0	87·4	87·8	88·2	88·6	88·8	88·8	88·9	89·0	89·1
Barking and Dagenham	68·5	68·9	69·5	70·2	70·9	71·3	71·6	72·0	72·5	73·2	73·7	74·0	74·3	74·4	74·4	74·4	74·5	74·5	74·7	74·8	75·1	75·5	76·1	76·9	77·4	77·8	77·9	78·1	78·3	78·5
Barnet	78·2	78·7	79·1	79·6	80·1	80·5	80·8	81·1	81·6	82·1	82·7	83·1	83·5	83·7	83·9	84·1	84·4	84·6	84·8	84·9	85·2	85·5	85·9	86·3	86·6	86·9	87·0	87·1	87·3	87·5
Bexley	72·2	72·8	73·4	74·0	74·5	74·9	75·1	75·4	75·8	76·3	76·9	77·4	77·8	78·1	78·4	78·7	79·0	79·2	79·4	79·7	79·9	80·3	80·8	81·4	81·8	82·2	82·4	82·6	82·8	83·1
Brent	76·5	76·9	77·4	77·9	78·4	78·8	79·1	79·4	79·9	80·3	80·8	81·2	81·5	81·7	81·9	82·0	82·2	82·2	82·3	82·2	82·2	82·5	82·9	83·4	83·7	84·0	84·1	84·2	84·5	84·7
Bromley	76·4	76·8	77·3	77·8	78·3	78·7	78·9	79·3	79·7	80·3	80·9	81·4	81·7	82·0	82·3	82·6	82·8	83·0	83·2	83·4	83·6	83·9	84·2	84·6	84·9	85·1	85·2	85·3	85·5	85·7
Camden	86·7	87·0	87·3	87·7	88·0	88·3	88·5	88·7	89·0	89·4	89·7	89·9	90·1	90·3	90·5	90·6	90·8	91·0	91·2	91·5	91·7	91·9	92·1	92·4	92·5	92·6	92·6	92·7	92·8	92·9
Croydon	75·2	75·7	76·3	76·9	77·4	77·7	77·9	78·2	78·6	79·1	79·6	80·0	80·3	80·5	80·7	80·9	81·0	81·1	81·3	81·4	81·6	81·9	82·2	82·6	82·9	83·2	83·3	83·4	83·6	83·8
Ealing	78·0	78·5	79·0	79·5	80·0	80·4	80·7	81·0	81·4	81·9	82·4	82·8	83·1	83·3	83·5	83·6	83·7	83·8	83·8	83·9	84·1	84·4	84·9	85·4	85·8	86·2	86·4	86·6	86·8	87·0
Enfield	73·7	74·2	74·8	75·3	75·8	76·1	76·4	76·7	77·2	77·7	78·2	78·6	78·9	79·1	79·3	79·5	79·6	79·8	80·0	80·2	80·6	81·0	81·4	82·0	82·4	82·7	82·9	83·0	83·2	83·4
Greenwich	72·9	73·5	74·1	74·8	75·4	75·8	76·1	76·4	76·8	77·3	77·8	78·3	78·6	78·8	78·9	79·1	79·2	79·4	79·7	79·9	80·1	80·6	81·2	81·8	82·3	82·6	82·8	82·9	83·1	83·2
Hackney	80·1	80·6	81·3	81·8	82·2	82·4	82·3	82·4	82·5	82·8	83·1	83·3	83·5	83·5	83·7	83·9	84·2	84·4	84·7	85·1	85·4	85·9	86·3	86·8	87·1	87·4	87·5	87·5	87·6	87·8
Hammersmith and Fulham	85·4	85·9	86·4	86·9	87·3	87·7	87·9	88·2	88·6	89·0	89·2	89·5	89·7	89·8	90·0	90·2	90·4	90·6	90·8	91·0	91·2	91·5	91·7	92·0	92·2	92·4	92·5	92·6	92·7	92·7
Haringey	77·2	77·7	78·1	78·6	79·0	79·3	79·5	79·7	80·0	80·4	80·9	81·3	81·6	81·8	82·0	82·2	82·4	82·6	82·8	83·0	83·3	83·6	84·0	84·4	84·8	85·1	85·2	85·3	85·5	85·7
Harrow	76·9	77·4	78·0	78·5	79·0	79·3	79·5	79·9	80·3	80·9	81·4	81·9	82·2	82·4	82·7	82·9	83·1	83·3	83·4	83·5	83·6	83·7	83·9	84·2	84·4	84·5	84·6	84·6	84·9	85·1
Havering	71·7	72·3	72·8	73·3	73·8	74·1	74·4	74·7	75·1	75·7	76·3	76·8	77·2	77·4	77·6	77·8	78·4	78·7	78·9	79·1	79·3	79·6	80·0	80·4	80·7	80·9	81·0	81·1	81·3	81·6
Hillingdon	78·4	78·9	79·5	80·0	80·6	80·9	81·2	81·5	82·0	82·6	83·2	83·7	84·1	84·4	84·6	84·7	84·9	84·9	85·1	85·3	85·6	85·9	86·4	86·9	87·2	87·5	87·6	87·6	87·8	87·9
Hounslow	78·3	78·8	79·3	79·8	80·3	80·6	80·8	81·0	81·5	82·1	82·7	83·1	83·5	83·6	83·8	84·0	84·2	84·4	84·6	84·8	85·0	85·4	85·9	86·4	86·9	87·3	87·5	87·7	87·9	88·1
Islington	84·0	84·4	84·9	85·4	85·9	86·2	86·5	86·7	87·0	87·4	87·7	88·0	88·3	88·5	88·7	88·9	89·1	89·3	89·5	89·7	90·0	90·3	90·6	90·9	91·1	91·2	91·3	91·3	91·4	91·5
Kensington and Chelsea	87·6	88·1	88·5	88·9	89·3	89·6	89·9	90·1	90·3	90·5	90·7	90·9	91·0	91·1	91·3	91·4	91·6	91·8	92·1	92·3	92·5	92·8	93·1	93·3	93·5	93·7	93·7	93·8	93·9	94·0
Kingston upon Thames	80·4	80·9	81·5	82·0	82·5	82·9	83·2	83·6	84·1	84·7	85·2	85·7	86·1	86·4	86·7	87·0	87·3	87·6	87·9	88·1	88·3	88·6	88·8	89·1	89·3	89·5	89·6	89·7	89·9	90·0
Lambeth	80·3	80·9	81·4	81·9	82·4	82·7	82·9	83·2	83·6	84·0	84·5	84·9	85·2	85·4	85·7	86·1	86·4	86·8	87·2	87·5	87·9	88·2	88·6	89·1	89·4	89·7	89·8	89·9	90·1	90·3
Lewisham	74·7	75·2	75·7	76·3	76·8	77·1	77·4	77·7	78·0	78·6	79·1	79·5	79·9	80·1	80·3	80·4	80·6	80·8	81·0	81·2	81·5	81·9	82·4	83·0	83·4	83·7	83·8	84·0	84·1	84·3
Merton	77·5	78·1	78·6	79·2	79·7	80·1	80·4	80·8	81·3	81·9	82·4	82·9	83·2	83·4	83·6	83·8	84·0	84·1	84·3	84·5	84·6	84·9	85·3	85·7	86·0	86·2	86·2	86·3	86·5	86·7
Newham	71·4	71·8	72·4	72·9	73·4	73·7	73·9	74·1	74·5	75·0	75·6	76·2	76·6	76·9	77·2	77·5	77·8	77·9	78·1	78·5	79·1	79·8	80·5	81·2	81·7	82·1	82·2	82·3	82·5	82·7
Redbridge	74·4	74·8	75·3	75·9	76·4	76·7	77·0	77·3	77·8	78·3	78·8	79·2	79·5	79·8	80·0	80·2	80·4	80·7	80·9	81·1	81·3	81·6	81·9	82·4	82·7	82·8	82·8	82·8	83·0	83·2
Richmond upon Thames	82·4	82·9	83·5	84·0	84·4	84·8	85·0	85·4	85·8	86·4	86·9	87·4	87·8	88·1	88·4	88·7	89·0	89·2	89·5	89·7	89·9	90·1	90·4	90·7	90·9	91·1	91·2	91·3	91·5	91·7
Southwark	80·8	81·4	82·0	82·5	83·0	83·4	83·7	84·1	84·5	85·1	85·7	86·1	86·5	86·8	87·0	87·2	87·4	87·6	88·0	88·3	88·7	89·1	89·6	90·0	90·3	90·5	90·5	90·6	90·7	90·8
Sutton	74·4	74·9	75·5	76·1	76·6	77·1	77·4	77·8	78·3	78·9	79·5	80·0	80·4	80·6	80·9	81·1	81·3	81·4	81·6	81·8	82·0	82·3	82·8	83·3	83·7	84·0	84·3	84·5	84·7	84·8
Tower Hamlets	78·7	79·2	79·8	80·4	81·0	81·4	81·7	82·1	82·6	83·1	83·6	84·0	84·4	84·7	85·0	85·4	85·8	86·1	86·5	86·9	87·3	87·7	88·1	88·6	88·9	89·1	89·1	89·2	89·3	89·4
Waltham Forest	72·2	72·7	73·2	73·8	74·4	74·8	75·1	75·4	75·8	76·3	76·7	77·1	77·4	77·5	77·7	77·9	78·0	78·1	78·3	78·4	78·7	79·2	79·7	80·4	81·0	81·4	81·7	81·9	82·1	82·3
Wandsworth	82·7	83·2	83·7	84·2	84·6	85·0	85·4	85·7	86·2	86·7	87·1	87·6	88·0	88·3	88·6	88·8	89·1	89·3	89·5	89·7	89·9	90·1	90·3	90·5	90·7	90·8	90·9	91·0	91·1	91·3
Westminster	86·4	86·7	87·1	87·5	87·9	88·2	88·4	88·7	88·9	89·2	89·5	89·7	89·9	90·0	90·2	90·4	90·6	90·8	91·0	91·3	91·5	91·7	92·0	92·2	92·4	92·6	92·7	92·8	92·8	92·9
North East England	71·2	71·7	72·3	72·9	73·5	73·8	74·1	74·5	74·9	75·5	76·1	76·6	76·9	77·2	77·5	77·8	78·0	78·3	78·6	78·8	79·1	79·4	79·9	80·4	80·7	81·0	81·2	81·3	81·5	81·7
County Durham	70·7	71·2	71·8	72·4	72·9	73·2	73·4	73·7	74·1	74·6	75·2	75·6	76·0	76·2	76·5	76·7	76·9	77·1	77·3	77·5	77·8	78·2	78·7	79·2	79·6	79·8	79·9	80·1	80·3	80·5
Darlington	72·3	72·8	73·4	74·1	74·6	75·0	75·2	75·4	75·7	76·3	76·8	77·2	77·5	77·7	77·9	78·2	78·4	78·6	78·8	78·9	79·2	79·6	80·1	80·7	81·1	81·4	81·5	81·6	81·8	82·0
Gateshead	71·7	72·3	72·8	73·4	73·8	74·1	74·3	74·6	75·0	75·5	76·0	76·5	76·9	77·2	77·5	77·8	78·1	78·3	78·6	78·8	79·2	79·6	80·1	80·6	81·1	81·4	81·7	81·8	82·0	82·3
Hartlepool	68·6	69·1	69·6	70·1	70·6	71·0	71·2	71·5	71·9	72·3	72·8	73·2	73·5	73·7	73·8	73·9	74·1	74·3	74·6	74·9	75·2	75·8	76·4	77·0	77·5	77·8	78·0	78·2	78·4	78·6
Middlesbrough	69·7	70·2	70·7	71·3	71·8	72·1	72·4	72·7	73·1	73·6	74·2	74·6	75·0	75·1	75·4	75·7	76·0	76·2	76·5	76·7	77·0	77·3	77·8	78·3	78·7	78·9	78·9	79·0	79·2	79·4
Newcastle upon Tyne	75·9	76·5	77·0	77·5	78·0	78·4	78·8	79·2	79·6	80·2	80·7	81·2	81·7	82·2	82·6	83·0	83·4	83·8	84·1	84·4	84·7	85·0	85·3	85·6	85·9	86·0	86·1	86·2	86·4	86·6
North Tyneside	71·8	72·3	72·9	73·5	74·0	74·3	74·5	74·8	75·2	75·8	76·3	76·8	77·1	77·4	77·7	78·1	78·4	78·7	79·0	79·2	79·5	79·9	80·3	80·8	81·1	81·4	81·6	81·7	81·9	82·1
Northumberland	71·7	72·1	72·6	73·1	73·6	74·0	74·2	74·4	74·8	75·4	75·9	76·3	76·7	76·9	77·1	77·4	77·7	77·9	78·1	78·3	78·5	78·8	79·2	79·7	80·0	80·2	80·3	80·4	80·6	80·8
Redcar and Cleveland	69·3	69·7	70·2	70·7	71·3	71·7	72·0	72·3	72·7	73·3	73·7	74·1	74·3	74·4	74·6	74·8	75·0	75·2	75·4	75·6	75·8	76·2	76·8	77·5	78·0	78·3	78·5	78·7	78·9	79·1
South Tyneside	68·5	69·1	69·7	70·2	70·8	71·1	71·4	71·7	72·1	72·7	73·2	73·7	74·1	74·4	74·7	75·0	75·2	75·3	75·5	75·6	75·8	76·2	76·7	77·4	77·9	78·3	78·6	78·8	79·0	79·2
Stockton-on-Tees	72·4	73·0	73·7	74·4	75·0	75·4	75·6	75·9	76·3	76·8	77·3	77·6	77·9	78·0	78·2	78·3	78·5	78·7	78·9	79·1	79·4	79·8	80·3	80·8	81·2	81·5	81·7	81·8	82·0	82·2
Sunderland	70·5	71·0	71·7	72·4	72·9	73·3	73·5	73·9	74·3	74·8	75·3	75·8	76·1	76·3	76·6	76·8	77·0	77·2	77·4	77·7	78·0	78·4	78·9	79·5	79·9	80·2	80·3	80·5	80·7	80·9
North West England	73·0	73·5	74·1	74·6	75·1	75·4	75·7	76·0	76·4	76·9	77·4	77·9	78·2	78·4	78·7	78·9	79·1	79·3	79·6	79·8	80·1	80·5	81·0	81·6	82·0	82·3	82·4	82·5	82·8	83·0
Blackburn with Darwen	69·3	69·7	70·1	70·5	71·0	71·3	71·5	71·8	72·2	72·8	73·2	73·6	74·0	74·1	74·3	74·5	74·7	74·8	74·9	75·0	75·4	75·9	76·5	77·3	77·8	78·2	78·3	78·4	78·6	78·8
Blackpool	68·7	69·2	69·7	70·1	70·5	70·7	70·9	71·2	71·6	72·1	72·6	73·0	73·1	73·1	73·2	73·3	73·5	73·6	73·7	73·8	74·0	74·3	74·9	75·5	75·9	76·3	76·5	76·6	76·8	77·0
Bolton	71·0	71·5	72·0	72·6	73·0	73·3	73·4	73·5	73·8	74·3	74·7	75·1	75·4	75·5	75·6	75·8	75·9	76·0	76·2	76·3	76·6	77·1	77·7	78·3	78·8	79·1	79·3	79·4	79·6	79·8
Bury	72·3	72·8	73·4	73·9	74·5	74·8	75·0	75·2	75·5	76·0	76·4	76·8	77·0	77·1	77·3	77·5	77·7	77·8	78·0	78·2	78·5	78·9	79·5	80·2	80·7	81·1	81·4	81·5	81·7	81·9
Cheshire East	77·3	77·8	78·3	78·8	79·3	79·6	79·8	80·1	80·5	81·0	81·5	82·0	82·3	82·5	82·8	83·0	83·1	83·3	83·5	83·7	83·9	84·2	84·6	85·2	85·5	85·8	85·9	86·0	86·2	86·4
Cheshire West and Chester	76·4	76·9	77·5	78·1	78·5	78·8	79·0	79·3	79·7	80·2	80·7	81·1	81·5	81·7	81·9	82·2	82·4	82·6	82·9	83·1	83·4	83·7	84·2	84·7	85·1	85·4	85·6	85·8	86·0	86·2
Cumbria	73·0	73·4	73·9	74·4	75·0	75·2	75·4	75·7	76·0	76·5	77·0	77·4	77·7	77·8	78·0	78·2	78·4	78·6	78·8	79·0	79·3	79·7	80·2	80·9	81·3	81·6	81·7	81·8	82·1	82·3

Halton	71·7	72·2	72·7	73·3	73·9	74·2	74·4	74·6	74·9	75·3	75·7	76·1	76·4	76·4	76·5	76·7	76·9	77·1	77·4	77·6	78·0	78·5	79·1	79·8	80·4	80·9	81·1	81·3	81·5	81·8
Knowsley	69·3	69·7	70·2	70·7	71·1	71·4	71·6	72·0	72·3	72·8	73·4	74·0	74·4	74·5	74·7	75·0	75·2	75·4	75·8	76·1	76·4	76·8	77·3	77·9	78·3	78·7	78·8	78·9	79·2	79·5
Lancashire	73·5	74·0	74·6	75·2	75·7	76·0	76·2	76·4	76·8	77·3	77·7	78·1	78·4	78·5	78·8	79·0	79·2	79·4	79·6	79·9	80·2	80·7	81·2	81·7	82·1	82·3	82·5	82·6	82·8	83·0
Liverpool	73·5	74·0	74·6	75·1	75·5	75·8	76·1	76·5	77·0	77·6	78·2	78·7	79·2	79·6	80·0	80·4	80·7	81·1	81·5	81·8	82·0	82·4	82·7	83·1	83·4	83·5	83·6	83·7	83·9	84·1
Manchester	76·2	76·6	77·1	77·7	78·2	78·6	79·0	79·4	79·9	80·5	81·1	81·6	82·0	82·4	82·7	83·1	83·4	83·8	84·2	84·6	85·0	85·3	85·7	86·1	86·4	86·6	86·7	86·8	87·0	87·2
Oldham	68·9	69·3	69·7	70·2	70·6	70·8	70·8	70·9	71·1	71·5	71·9	72·2	72·4	72·6	72·8	73·0	73·3	73·6	74·0	74·3	74·7	75·2	75·7	76·4	76·9	77·2	77·4	77·5	77·7	77·9
Rochdale	69·2	69·5	70·0	70·5	71·0	71·3	71·5	71·7	72·1	72·6	73·1	73·4	73·7	73·9	74·1	74·2	74·4	74·5	74·7	74·9	75·2	75·7	76·3	77·1	77·6	78·0	78·2	78·3	78·6	78·8
Salford	72·4	72·9	73·4	74·0	74·5	74·8	75·0	75·2	75·6	76·2	76·8	77·3	77·7	78·0	78·2	78·4	78·7	78·8	79·1	79·3	79·6	80·0	80·5	81·1	81·5	81·8	81·9	82·0	82·3	82·5
Sefton	72·2	72·8	73·4	74·0	74·5	74·8	75·0	75·3	75·7	76·2	76·7	77·1	77·4	77·7	78·0	78·3	78·5	78·7	78·8	78·9	79·1	79·3	79·6	80·0	80·3	80·6	80·7	80·8	81·0	81·2
St Helens	70·5	70·9	71·5	72·0	72·5	72·8	73·0	73·2	73·5	74·0	74·5	75·0	75·3	75·4	75·6	75·7	75·9	76·0	76·2	76·4	76·7	77·1	77·6	78·2	78·6	78·8	78·9	79·0	79·2	79·4
Stockport	75·2	75·8	76·4	76·9	77·4	77·7	77·8	78·1	78·5	79·0	79·6	80·0	80·4	80·6	80·8	81·0	81·2	81·4	81·6	81·8	82·1	82·4	82·8	83·3	83·7	83·9	83·9	84·0	84·2	84·3
Tameside	70·0	70·5	71·0	71·5	72·1	72·4	72·7	72·9	73·3	73·8	74·2	74·6	74·9	75·0	75·2	75·3	75·4	75·4	75·5	75·5	75·7	76·1	76·7	77·4	77·9	78·2	78·5	78·6	78·8	79·0
Trafford	78·0	78·5	79·0	79·5	80·0	80·3	80·5	80·7	81·0	81·6	82·1	82·6	82·9	83·2	83·5	83·8	84·1	84·3	84·6	84·8	85·2	85·6	86·0	86·6	87·0	87·3	87·5	87·7	87·8	88·0
Warrington	76·3	76·8	77·3	77·8	78·3	78·5	78·7	78·9	79·2	79·7	80·2	80·7	81·1	81·4	81·7	82·0	82·2	82·3	82·6	82·8	83·2	83·6	84·2	84·8	85·3	85·7	86·0	86·2	86·4	86·5
Wigan	70·3	70·7	71·2	71·8	72·2	72·5	72·7	72·9	73·2	73·6	74·1	74·5	74·7	74·8	74·9	75·1	75·3	75·4	75·5	75·6	76·0	76·4	77·0	77·6	77·9	78·2	78·3	78·4	78·6	78·8
Wirral	71·4	71·9	72·4	72·9	73·4	73·7	73·9	74·2	74·5	75·0	75·4	75·8	76·0	76·2	76·4	76·5	76·7	76·8	77·0	77·1	77·4	77·8	78·3	79·0	79·4	79·8	80·0	80·2	80·4	80·6
South East England	77·2	77·6	78·1	78·6	79·0	79·3	79·5	79·8	80·1	80·6	81·1	81·5	81·8	82·0	82·2	82·4	82·6	82·8	83·0	83·2	83·5	83·8	84·3	84·8	85·1	85·3	85·5	85·6	85·8	85·9
Bracknell Forest	78·5	78·9	79·3	79·7	80·1	80·3	80·5	80·7	81·1	81·7	82·3	82·7	83·1	83·3	83·5	83·7	83·9	84·1	84·4	84·5	84·8	85·2	85·7	86·2	86·5	86·8	87·1	87·2	87·4	87·6
Brighton and Hove	79·0	79·5	80·0	80·5	81·0	81·4	81·8	82·2	82·6	83·1	83·5	84·0	84·4	84·7	85·1	85·4	85·7	86·1	86·4	86·6	86·9	87·2	87·5	87·8	88·1	88·2	88·4	88·5	88·7	88·9
Buckinghamshire	79·0	79·4	79·9	80·4	80·8	81·1	81·4	81·6	82·0	82·4	82·9	83·3	83·6	83·8	84·0	84·2	84·4	84·6	84·8	84·9	85·1	85·4	85·8	86·2	86·5	86·7	86·7	86·8	87·0	87·2
East Sussex	72·9	73·3	73·8	74·3	74·8	75·2	75·3	75·6	75·9	76·4	76·9	77·3	77·5	77·7	77·9	78·1	78·3	78·5	78·7	78·8	79·1	79·4	79·9	80·5	80·8	81·1	81·2	81·3	81·5	81·7
Hampshire	77·0	77·4	77·9	78·4	78·8	79·1	79·2	79·4	79·8	80·3	80·8	81·2	81·4	81·6	81·8	81·9	82·1	82·3	82·5	82·7	82·9	83·3	83·7	84·2	84·5	84·8	84·8	84·9	85·1	85·3
Isle of Wight	71·4	71·9	72·4	73·0	73·4	73·8	74·0	74·4	74·8	75·3	75·8	76·2	76·5	76·6	76·7	76·8	77·0	77·2	77·3	77·4	77·5	77·8	78·3	78·8	79·1	79·4	79·6	79·7	79·9	80·1
Kent	74·0	74·4	74·9	75·4	75·9	76·1	76·3	76·5	76·8	77·3	77·7	78·1	78·4	78·5	78·7	78·9	79·1	79·2	79·5	79·7	80·0	80·5	81·1	81·7	82·1	82·4	82·6	82·7	82·9	83·1
Medway	71·8	72·3	72·8	73·4	73·9	74·2	74·4	74·6	74·9	75·3	75·6	75·9	76·2	76·3	76·6	76·8	77·0	77·1	77·3	77·5	77·8	78·2	78·7	79·2	79·6	79·8	79·9	80·0	80·2	80·4
Milton Keynes	77·7	78·2	78·8	79·4	79·8	80·1	80·2	80·4	80·6	81·0	81·3	81·6	81·8	81·9	82·0	82·1	82·3	82·3	82·4	82·5	82·7	83·0	83·6	84·3	84·7	85·0	85·1	85·2	85·4	85·6
Oxfordshire	79·8	80·2	80·7	81·2	81·7	82·0	82·2	82·4	82·8	83·2	83·7	84·1	84·4	84·6	84·8	85·1	85·3	85·5	85·8	86·0	86·2	86·5	86·9	87·4	87·7	88·0	88·3	88·4	88·6	88·8
Portsmouth	76·7	77·1	77·6	78·1	78·6	79·0	79·3	79·7	80·1	80·5	81·0	81·4	81·7	81·9	82·2	82·4	82·6	82·8	83·1	83·3	83·6	83·9	84·2	84·6	84·9	85·1	85·2	85·3	85·5	85·7
Reading	81·3	81·8	82·2	82·7	83·1	83·4	83·7	84·0	84·4	84·9	85·4	85·9	86·2	86·5	86·8	87·0	87·0	87·1	87·2	87·3	87·5	87·7	87·9	88·3	88·6	88·8	88·8	88·9	89·0	89·1
Slough	77·8	78·3	78·8	79·4	79·9	80·2	80·5	80·8	81·1	81·4	81·6	81·8	81·9	81·9	81·9	81·8	81·8	81·7	81·7	81·8	82·2	82·7	83·2	83·9	84·2	84·5	84·5	84·5	84·7	84·8
Southampton	76·5	77·0	77·5	78·1	78·6	79·0	79·4	79·8	80·3	80·8	81·3	81·7	82·1	82·3	82·5	82·7	82·9	83·0	83·2	83·4	83·5	83·8	84·1	84·4	84·7	84·8	84·9	85·0	85·2	85·4
Surrey	80·2	80·6	81·1	81·5	82·0	82·2	82·5	82·8	83·2	83·7	84·2	84·7	85·1	85·3	85·6	85·8	86·1	86·3	86·5	86·8	87·0	87·2	87·6	87·9	88·2	88·4	88·5	88·6	88·8	88·9
West Berkshire	80·2	80·7	81·3	81·8	82·3	82·5	82·6	82·8	83·1	83·6	84·0	84·4	84·6	84·6	84·6	84·6	84·6	84·5	84·6	84·8	85·1	85·6	86·2	86·8	87·3	87·6	87·8	87·9	88·0	88·1
West Sussex	75·9	76·3	76·9	77·4	77·8	78·1	78·2	78·5	78·8	79·3	79·8	80·2	80·5	80·7	80·9	81·2	81·4	81·6	81·8	81·9	82·2	82·6	83·0	83·6	84·0	84·4	84·6	84·8	85·0	85·2
Windsor and Maidenhead	81·3	81·7	82·0	82·5	82·9	83·1	83·3	83·5	83·9	84·3	84·8	85·2	85·6	85·9	86·1	86·4	86·6	86·9	87·1	87·3	87·6	88·0	88·4	88·9	89·3	89·6	89·9	90·0	90·2	90·3
Wokingham	81·6	82·1	82·5	83·0	83·4	83·7	84·0	84·3	84·7	85·1	85·6	86·0	86·3	86·6	86·9	87·1	87·3	87·5	87·7	87·9	88·1	88·3	88·6	89·0	89·3	89·6	89·7	89·9	90·0	90·2
South West England	74·7	75·2	75·7	76·2	76·7	77·1	77·3	77·6	78·0	78·5	79·0	79·5	79·8	80·0	80·3	80·5	80·7	81·0	81·2	81·4	81·7	82·1	82·6	83·1	83·4	83·7	83·8	84·0	84·2	84·4
Bath and North East Somerset	78·3	78·7	79·2	79·7	80·2	80·5	80·8	81·1	81·6	82·1	82·6	83·1	83·5	83·9	84·3	84·7	85·1	85·4	85·8	86·1	86·4	86·7	87·0	87·3	87·6	87·8	88·0	88·1	88·3	88·5
Bournemouth	75·5	76·0	76·6	77·2	77·7	78·1	78·4	78·7	79·2	79·8	80·3	80·8	81·3	81·7	82·1	82·4	82·6	82·9	83·1	83·4	83·6	83·8	84·2	84·6	84·9	85·2	85·3	85·4	85·6	85·8
Bristol, City of	78·4	78·9	79·4	79·9	80·4	80·8	81·1	81·5	81·9	82·4	82·9	83·3	83·7	83·9	84·2	84·5	84·8	85·1	85·4	85·7	86·0	86·4	86·7	87·2	87·5	87·8	87·9	88·0	88·2	88·4
Cornwall	72·6	73·2	73·8	74·4	74·9	75·1	75·3	75·5	75·9	76·4	76·9	77·3	77·6	77·8	78·0	78·2	78·4	78·6	78·8	78·9	79·2	79·6	80·1	80·8	81·3	81·6	81·8	82·0	82·2	82·4
Devon	74·3	74·8	75·3	75·9	76·3	76·6	76·8	77·1	77·6	78·1	78·6	79·1	79·4	79·7	80·0	80·3	80·6	80·8	81·1	81·3	81·5	81·9	82·3	82·9	83·2	83·5	83·6	83·7	83·9	84·1
Dorset	73·8	74·3	74·8	75·3	75·8	76·0	76·2	76·5	77·0	77·5	77·9	78·3	78·6	78·8	79·0	79·1	79·3	79·5	79·7	79·8	80·2	80·6	81·1	81·7	82·1	82·5	82·7	82·9	83·1	83·3
Gloucestershire	76·0	76·4	76·9	77·5	77·9	78·2	78·5	78·7	79·0	79·5	80·0	80·5	80·8	81·1	81·4	81·6	81·8	82·0	82·2	82·5	82·8	83·2	83·6	84·2	84·6	84·9	85·2	85·3	85·5	85·7
North Somerset	74·3	74·8	75·3	75·9	76·3	76·6	76·7	76·9	77·2	77·7	78·1	78·5	78·9	79·1	79·3	79·6	79·8	80·0	80·3	80·5	80·9	81·4	81·9	82·5	83·0	83·4	83·6	83·8	84·0	84·1
Plymouth	74·2	74·7	75·2	75·7	76·2	76·5	76·7	77·0	77·4	77·9	78·3	78·7	79·0	79·2	79·4	79·6	79·8	80·0	80·3	80·6	80·9	81·2	81·7	82·1	82·4	82·6	82·7	82·7	82·9	83·1
Poole	75·3	75·8	76·2	76·8	77·3	77·6	77·9	78·2	78·6	79·1	79·6	80·0	80·2	80·4	80·6	80·8	81·0	81·2	81·4	81·5	81·8	82·1	82·5	83·1	83·4	83·6	83·7	83·8	84·0	84·1
Somerset	73·0	73·5	74·1	74·7	75·2	75·5	75·7	75·9	76·2	76·7	77·1	77·5	77·8	77·9	78·1	78·3	78·5	78·6	78·7	78·8	79·1	79·5	79·9	80·5	80·8	81·0	81·1	81·1	81·4	81·6
South Gloucestershire	77·0	77·3	77·8	78·2	78·6	78·9	79·2	79·6	80·0	80·6	81·1	81·6	82·0	82·2	82·5	82·8	83·1	83·4	83·7	83·9	84·2	84·6	85·1	85·6	86·0	86·3	86·5	86·7	86·9	87·1
Swindon	76·3	76·9	77·4	78·0	78·5	78·7	78·8	78·9	79·2	79·6	80·0	80·4	80·6	80·7	80·8	80·9	81·0	81·1	81·3	81·4	81·7	82·2	82·7	83·3	83·6	83·8	83·8	83·9	84·0	84·2
Torbay	70·9	71·4	72·0	72·5	73·0	73·2	73·5	73·7	74·1	74·6	75·1	75·4	75·5	75·5	75·6	75·7	75·8	75·9	76·1	76·2	76·3	76·6	77·0	77·6	78·0	78·3	78·4	78·6	78·8	79·0
Wiltshire	74·8	75·2	75·6	76·1	76·6	76·9	77·0	77·2	77·5	78·1	78·5	78·9	79·2	79·4	79·6	79·8	80·0	80·1	80·2	80·3	80·6	81·0	81·6	82·1	82·4	82·6	82·7	82·7	82·9	83·1
West Midlands	71·7	72·2	72·8	73·4	73·9	74·3	74·5	74·8	75·3	75·9	76·4	76·9	77·2	77·5	77·7	78·0	78·2	78·5	78·8	79·0	79·3	79·7	80·3	80·8	81·2	81·5	81·7	81·8	82·1	82·3
Birmingham	71·6	72·1	72·7	73·2	73·8	74·1	74·3	74·6	75·0	75·6	76·1	76·6	77·0	77·2	77·6	77·9	78·2	78·5	78·9	79·3	79·7	80·2	80·8	81·3	81·7	81·9	82·1	82·2	82·3	82·5
Coventry	73·5	74·1	74·7	75·3	75·9	76·3	76·6	77·0	77·4	78·0	78·5	79·0	79·3	79·6	79·9	80·1	80·2	80·4	80·7	80·9	81·2	81·6	82·1	82·6	82·9	83·1	83·2	83·3	83·5	83·7
Dudley	70·3	70·7	71·2	71·7	72·2	72·5	72·7	73·0	73·4	73·9	74·4	74·8	75·1	75·3	75·4	75·6	75·8	75·9	76·0	76·1	76·3	76·6	77·0	77·6	77·9	78·2	78·3	78·4	78·6	78·9
Herefordshire, County of	72·2	72·8	73·4	74·1	74·7	75·1	75·4	75·7	76·1	76·8	77·3	77·8	78·2	78·4	78·6	78·8	79·0	79·2	79·3	79·5	79·8	80·2	80·6	81·2	81·7	82·1	82·4	82·5	82·8	83·0
Sandwell	68·0	68·4	68·9	69·5	70·0	70·3	70·5	70·7	71·1	71·6	72·1	72·5	72·8	73·0	73·2	73·4	73·6	73·7	73·8	73·9	74·2	74·6	75·3	76·0	76·5	76·8	76·8	76·9	77·2	77·5
Shropshire	72·9	73·4	74·0	74·5	75·0	75·4	75·6	75·9	76·3	76·9	77·4	77·9	78·2	78·6	78·9	79·1	79·4	79·6	79·8	79·9	80·2	80·6	81·0	81·5	81·9	82·2	82·5	82·6	82·8	83·1
Solihull	75·5	76·1	76·7	77·3	77·8	78·2	78·4	78·8	79·2	79·8	80·4	80·9	81·3	81·5	81·8	82·0	82·2	82·4	82·6	82·7	82·9	83·2	83·6	84·0	84·4	84·7	84·9	85·0	85·3	85·5
Staffordshire	72·5	73·1	73·7	74·4	74·9	75·2	75·4	75·7	76·1	76·7	77·2	77·6	77·9	78·1	78·3	78·5	78·7	78·8	79·0	79·2	79·5	79·8	80·2	80·6	80·9	81·2	81·3	81·3	81·6	81·8
Stoke-on-Trent	69·0	69·6	70·1	70·7	71·2	71·5	71·7	72·0	72·4	72·9	73·3	73·6	73·8	73·8	73·8	73·9	73·9	73·9	74·1	74·3	74·7	75·2	75·8	76·6	77·2	77·6	77·8	78·0	78·2	78·5
Telford and Wrekin	72·2	72·6	73·2	73·8	74·3	74·7	75·0	75·3	75·7	76·2	76·7	77·1	77·3	77·3	77·4	77·4	77·5	77·5	77·6	77·7	77·9	78·3	78·8	79·5	80·0	80·4	80·5	80·6	80·8	81·0
Walsall	68·4	68·7	69·2	69·8	70·2	70·5	70·5	70·6	70·9	71·4	71·9	72·3	72·6	72·8	73·0	73·2	73·4	73·7	73·9	74·1	74·4	74·9	75·4	76·0	76·5	76·8	77·0	77·2	77·4	77·7
Warwickshire	75·2	75·8	76·4	76·9	77·5	77·8	78·0	78·4	78·8	79·4	79·9	80·4	80·7	81·0	81·2	81·4	81·6	81·9	82·1	82·3	82·6	83·0	83·5	84·0	84·3	84·6	84·9	85·0	85·2	85·5
Wolverhampton	69·9	70·3	70·9	71·5	72·2	72·5	72·7	73·0	73·4	74·0	74·5	75·0	75·3	75·5	75·7	75·8	76·0	76·2	76·4	76·5	76·7	77·1	77·6	78·2	78·6	78·9	79·1	79·3	79·5	79·7
Worcestershire	73·2	73·7	74·3	74·9	75·4	75·7	75·9	76·3	76·7	77·4	78·0	78·4	78·8	79·0	79·2	79·4	79·6	79·8	80·0	80·2	80·4	80·7	81·1	81·5	81·9	82·1	82·3	82·4	82·6	82·9
Yorkshire and the Humber	72·1	72·6	73·2	73·7	74·3	74·6	74·8	75·1	75·5	76·1	76·6	77·1	77·4	77·6	77·9	78·1	78·4	78·6	78·9	79·1	79·4	79·8	80·3	80·9	81·3	81·6	81·7	81·9	82·1	82·3
Barnsley	68·2	68·6	69·1	69·6	70·0	70·3	70·4	70·7	71·2	71·7	72·2	72·5	72·8	72·9	73·0	73·1	73·2	73·4	73·5	73·6	73·8	74·2	74·8	75·5	76·0	76·3	76·5	76·6	76·8	77·0
Bradford	69·8	70·2	70·7	71·2	71·7	72·0	72·1	72·1	72·3	72·7	73·1	73·4	73·8	74·0	74·2	74·5	74·8	75·0	75·3	75·6	76·1	76·6	77·3	78·0	78·5	79·0	79·2	79·4	79·6	79·8
Calderdale	72·2	72·7	73·3	73·9	74·4	74·7	74·8	75·0	75·3	75·9	76·3	76·7	77·0	77·0	77·2	77·4	77·6	77·7	77·9	78·1	78·4	78·9	79·5	80·2	80·8	81·1	81·3	81·5	81·7	81·8
Doncaster	68·4	68·8	69·2	69·7	70·1	70·4	70·5	70·6	71·0	71·5	71·9	72·3	72·7	72·8	72·9	73·1	73·3	73·5	73·6	73·8	74·2	74·8	75·5	76·2	76·7	77·1	77·4	77·6	77·8	78·0
East Riding of Yorkshire	73·2	73·7	74·3	74·9	75·4	75·7	75·9	76·2	76·5	77·0	77·5	77·9	78·2	78·4	78·6	78·8	79·0	79·1	79·3	79·4	79·6	79·9	80·2	80·6	80·8	81·0	81·1	81·1	81·3	81·5
Kingston upon Hull, City of	69·7	70·2	70·7	71·2	71·8	72·1	72·3	72·7	73·2	73·9	74·4	74·9	75·2	75·3	75·5	75·6	75·8	75·9	76·2	76·5	76·7	77·0	77·5	78·0	78·3	78·4	78·3	78·3	78·5	78·7
Kirklees	71·4	72·0	72·6	73·1	73·6	73·8	74·0	74·2	74·6	75·1	75·5	75·9	76·2	76·4	76·7	76·9	77·1	77·2	77·4	77·6	77·9	78·3	78·8	79·4	79·8	80·1	80·3	80·4	80·6	80·8
Leeds	75·7	76·2	76·8	77·4	77·9	78·3	78·6	79·0	79·4	80·0	80·5	81·0	81·5	81·8	82·1	82·4	82·7	83·0	83·3	83·6	83·8	84·2	84·6	85·0	85·4	85·7	85·9	86·0	86·2	86·4
North East Lincolnshire	69·1	69·6	70·3	70·9	71·4	71·7	71·8	72·0	72·3	72·7	73·2	73·6	73·7	73·7	73·8	73·9	74·1	74·3	74·6	74·7	75·0	75·5	76·0	76·6	77·0	77·3	77·3	77·4	77·6	77·9
North Lincolnshire	72·6	73·0	73·5	74·0	74·5	74·8	75·0	75·2	75·5	75·9	76·2	76·6	76·8	76·9	77·0	77·1	77·3	77·5	77·7	77·9	78·2	78·7	79·1	79·7	80·0	80·1	80·1	80·1	80·2	80·4
North Yorkshire	74·5	75·0	75·6	76·1	76·5	76·8	76·9	77·2	77·6	78·2	78·8	79·2	79·6	79·8	80·1	80·3	80·5	80·6	80·8	80·9	81·1	81·5	81·9	82·4	82·7	83·0	83·0	83·1	83·3	83·5
Rotherham	69·0	69·5	70·0	70·6	71·1	71·4	71·5	71·7	72·1	72·6	73·0	73·4	73·7	73·9	74·1	74·2	74·4	74·4	74·6	74·7	75·0	75·4	75·9	76·6	77·0	77·3	77·4	77·5	77·8	78·0
Sheffield	74·3	74·8	75·3	75·8	76·3	76·6	76·9	77·2	77·6	78·2	78·7	79·2	79·6	79·9	80·3	80·6	81·0	81·3	81·7	82·0	82·3	82·7	83·1	83·5	83·8	84·1	84·3	84·4	84·6	84·8
Wakefield	69·9	70·4	70·9	71·5	72·0	72·3	72·5	72·7	73·0	73·5	74·0	74·4	74·6	74·8	75·0	75·1	75·2	75·3	75·4	75·5	75·7	76·1	76·7	77·4	77·8	78·1	78·2	78·3	78·5	78·8
York	78·7	79·2	79·8	80·3	80·7	81·0	81·3	81·7	82·1	82·6	83·1	83·6	84·0	84·3	84·6	84·9	85·2	85·5	85·8	86·1	86·3	86·6	87·0	87·3	87·6	87·8	87·9	88·1	88·2	88·4
Northern Ireland	71·5	72·0	72·7	73·5	74·1	74·4	74·6	74·9	75·3	75·8	76·4	76·8	77·3	77·7	78·1	78·3	78·6	78·8	79·0	79·3	79·5	79·8	80·4	80·9	81·2	81·4	81·6	81·8	82·1	82·3
Scotland	74·1	74·5	75·0	75·6	76·1	76·5	76·7	76·9	77·4	78·0	78·4	78·8	79·2	79·4	79·7	79·8	80·0	80·1	80·4	80·7	81·1	81·4	81·8	82·1	82·3	82·6	82·8	82·9	83·1	83·3

Wales	70·2	70·8	71·4	72·1	72·7	73·0	73·3	73·7	74·2	74·8	75·4	75·9	76·4	76·6	77·0	77·3	77·6	77·9	78·2	78·5	78·8	79·2	79·7	80·2	80·6	80·9	81·2	81·3	81·5	81·7
Latin America and Caribbean	48·7	49·1	49·5	50·0	50·8	51·5	52·0	52·6	53·2	53·6	54·1	54·7	55·1	55·5	55·9	56·3	56·9	57·3	57·8	58·2	58·7	59·2	59·7	60·3	60·7	61·2	61·7	62·1	62·5	62·9
Andean Latin America	49·9	50·2	50·7	51·1	51·6	52·1	52·4	53·1	53·5	53·6	54·1	54·7	55·3	55·7	56·1	56·3	56·7	57·2	57·4	57·5	57·7	58·4	59·1	59·6	59·6	60·0	60·9	61·4	61·9	62·3
Bolivia	40·9	41·9	42·9	43·1	43·7	44·2	44·6	45·9	46·3	46·2	47·2	47·9	48·6	49·3	49·9	50·2	50·5	50·9	50·6	50·3	50·5	51·1	51·4	51·2	49·7	50·5	52·8	53·2	53·8	54·3
Ecuador	52·0	52·5	53·2	53·5	54·2	54·7	54·7	55·2	55·4	54·9	55·2	56·4	57·5	58·0	58·5	58·8	59·3	60·1	60·4	60·1	60·1	61·2	62·4	63·1	63·2	63·5	64·2	64·7	65·2	65·6
Peru	51·1	51·3	51·5	51·9	52·4	52·9	53·4	54·0	54·6	55·1	55·5	55·9	56·2	56·5	56·8	57·0	57·3	57·7	58·0	58·3	58·7	59·3	59·8	60·3	60·8	61·3	61·7	62·2	62·7	63·1
Caribbean	51·7	52·2	52·5	52·7	53·0	53·3	53·5	53·7	54·0	54·4	54·9	55·5	56·3	56·8	57·4	58·1	58·8	59·2	59·5	59·9	60·2	60·6	61·0	61·3	61·5	61·9	62·4	62·7	63·1	63·4
Antigua and Barbuda	60·7	61·4	62·1	62·6	63·0	63·3	63·7	64·1	64·6	65·2	65·8	66·3	66·8	67·4	67·9	68·5	69·1	69·6	70·1	70·5	70·8	71·0	71·2	71·5	71·8	72·2	72·5	72·9	73·4	73·8
The Bahamas	69·1	68·4	67·5	68·0	68·6	69·2	69·7	70·2	71·0	72·0	73·0	73·5	73·6	73·9	74·2	74·7	75·3	75·8	76·2	76·7	77·4	77·8	77·9	78·2	78·3	78·4	78·5	78·7	78·9	79·1
Barbados	64·5	65·0	65·6	66·3	66·8	67·0	67·1	67·2	67·4	67·5	67·6	67·9	68·4	69·0	69·6	70·0	70·3	70·5	70·9	71·3	71·6	71·9	72·3	72·7	72·9	73·0	73·2	73·4	73·6	73·9
Belize	40·0	40·9	42·2	43·9	45·3	46·7	47·9	48·6	48·8	49·0	49·3	49·7	50·2	50·8	51·6	52·7	53·7	54·4	55·1	55·7	56·3	56·9	57·3	57·7	58·1	58·5	58·8	59·2	59·5	59·8
Bermuda	68·8	69·0	69·3	69·8	70·3	70·5	70·7	71·2	71·6	72·3	72·8	73·1	73·5	74·1	74·8	75·5	76·1	76·6	77·1	77·9	78·7	79·2	79·6	80·1	80·6	80·8	80·9	81·1	81·4	81·7
Cuba	57·8	58·5	58·8	58·8	58·6	58·4	58·1	58·0	57·9	58·1	58·5	58·9	59·5	60·1	60·6	61·1	61·8	61·8	61·7	61·8	62·0	62·4	63·0	63·7	64·1	64·5	65·2	65·8	66·3	66·7
Dominica	56·7	56·3	56·5	57·6	58·8	59·9	60·7	61·2	61·8	62·5	63·3	64·1	64·9	65·6	66·5	67·4	67·8	67·8	68·2	68·7	69·1	69·6	70·0	70·6	71·0	71·2	71·5	71·7	71·7	71·8
Dominican Republic	42·8	42·8	42·5	42·5	42·9	43·1	43·3	43·1	43·3	44·0	44·4	45·4	46·6	47·5	48·7	50·3	51·8	52·8	53·7	54·5	55·1	55·6	56·0	56·4	56·5	56·9	57·8	58·5	59·1	59·7
Grenada	45·9	47·4	48·9	50·2	51·4	52·5	53·6	54·8	55·8	56·8	57·5	58·2	58·7	59·1	59·5	60·1	60·4	60·9	61·3	61·5	61·8	62·1	62·2	62·5	63·0	63·4	63·9	64·4	65·0	65·5
Guyana	46·8	47·2	47·8	48·3	48·4	48·8	49·9	50·6	50·8	51·7	52·4	53·1	54·1	54·2	54·1	54·4	54·5	54·8	55·3	55·7	56·2	56·6	57·0	57·5	58·1	58·8	59·5	60·1	60·7	61·2
Haiti	30·7	31·2	31·7	32·1	32·4	32·9	33·4	34·0	34·7	35·3	35·9	36·4	37·0	37·4	37·9	38·4	38·8	39·2	39·5	39·9	40·2	40·6	41·0	41·4	41·8	42·2	42·6	43·0	43·4	43·8
Jamaica	52·6	53·5	54·3	55·1	55·3	56·1	57·2	57·8	58·8	58·6	58·8	60·1	61·1	61·8	61·9	62·4	63·1	63·5	64·0	64·5	64·8	65·4	66·1	66·4	66·7	67·1	67·4	67·6	67·9	68·2
Puerto Rico	67·4	67·6	68·0	68·4	69·0	69·8	70·2	70·6	71·0	71·4	72·1	73·1	73·8	74·2	74·4	74·8	75·2	75·6	76·1	76·8	77·3	77·6	78·1	78·6	79·4	80·2	80·8	80·9	80·8	80·9
Saint Kitts and Nevis	59·1	59·9	60·6	61·3	62·0	62·7	63·3	64·0	64·6	65·2	65·8	66·5	67·0	67·5	68·0	68·5	69·0	69·5	70·0	70·5	70·9	71·3	71·7	72·1	72·5	73·0	73·4	73·8	74·2	74·6
Saint Lucia	50·4	51·4	52·2	53·1	53·9	54·9	55·9	56·8	57·6	58·3	58·9	59·5	59·9	60·3	60·9	61·3	61·7	62·1	62·5	63·0	63·4	63·9	64·2	64·5	64·8	65·1	65·4	65·7	66·1	66·4
Saint Vincent and the Grenadines	46·6	47·3	48·4	49·1	49·6	50·5	51·3	51·9	52·5	53·0	53·6	54·4	55·2	55·9	56·3	56·6	57·1	57·4	57·8	58·2	58·6	58·9	59·4	59·9	60·4	60·8	61·2	61·7	62·1	62·6
Suriname	50·0	50·4	50·8	51·2	51·6	51·5	51·6	52·1	52·6	53·3	53·6	54·1	55·4	56·4	57·2	57·8	58·0	58·3	58·7	59·2	59·7	60·2	60·7	61·2	61·5	61·9	62·4	62·8	63·2	63·5
Trinidad and Tobago	61·6	62·0	62·4	62·8	63·2	63·6	64·2	64·9	65·6	66·2	66·9	67·7	68·4	69·2	70·0	70·6	71·3	71·9	72·4	72·8	73·1	73·4	73·6	73·9	74·1	74·5	74·7	74·9	75·2	75·5
Virgin Islands	65·7	66·6	67·2	68·0	69·0	69·7	70·4	71·0	71·5	72·0	72·5	73·0	73·8	74·6	75·3	76·0	76·6	77·4	78·4	79·1	79·8	80·4	81·0	81·4	81·9	82·4	82·6	82·4	82·4	82·5
Central Latin America	47·5	47·6	47·6	48·1	49·2	50·0	50·7	51·4	52·1	52·7	53·3	53·9	54·3	54·5	54·8	55·3	55·9	56·3	56·8	57·2	57·7	58·2	58·7	59·2	59·7	60·2	60·6	61·1	61·5	61·9
Colombia	47·3	46·9	46·5	46·9	47·9	48·7	49·5	50·4	51·2	51·8	52·3	52·6	53·0	53·3	53·7	54·1	54·6	55·2	55·9	56·7	57·4	58·2	58·9	59·6	60·2	60·8	61·4	61·9	62·5	62·9
Costa Rica	53·2	54·0	54·8	55·2	55·5	55·9	56·3	57·0	57·7	58·7	59·4	59·8	60·4	60·8	61·2	61·6	61·9	62·1	62·5	63·2	63·8	64·2	64·7	65·3	65·7	66·2	66·6	67·0	67·5	67·9
El Salvador	39·7	39·9	40·2	40·4	40·7	41·3	42·0	43·0	43·9	44·9	46·1	47·2	48·1	48·9	49·6	50·4	51·2	51·9	52·4	52·8	53·2	53·7	54·2	54·8	55·3	55·8	56·3	56·8	57·4	57·8
Guatemala	30·6	31·0	31·8	33·5	35·2	35·7	35·9	36·9	38·6	39·4	39·7	40·9	41·8	42·1	43·0	44·2	45·0	45·4	45·9	46·5	47·2	48·0	48·5	49·2	50·0	50·5	50·9	51·3	51·8	52·3
Honduras	32·0	32·2	32·6	33·1	33·7	34·4	35·1	35·9	36·9	38·1	39·2	40·1	40·8	41·7	42·3	42·9	43·4	43·7	44·1	44·5	44·9	45·2	45·6	45·9	46·3	46·7	47·1	47·6	48·1	48·5
Mexico	49·2	49·3	49·0	49·5	50·9	51·8	52·5	53·2	54·0	54·8	55·4	55·8	56·3	56·7	57·2	57·7	58·0	58·3	58·6	59·0	59·3	59·7	60·2	60·7	61·2	61·7	62·2	62·7	63·2	63·6
Aguascalientes	54·6	54·8	54·8	55·3	56·4	57·1	57·9	58·6	59·3	59·9	60·3	60·5	61·0	61·5	62·2	62·8	63·2	63·5	63·9	64·3	64·6	65·0	65·4	65·8	66·3	66·7	67·2	67·6	68·1	68·5
Baja California	54·8	54·8	54·4	54·6	55·6	56·3	57·0	57·7	58·6	59·5	60·1	60·5	60·8	61·3	61·8	62·3	62·6	62·8	63·2	63·5	63·9	64·2	64·6	65·0	65·4	65·8	66·2	66·6	67·1	67·5
Baja California Sur	56·2	56·5	56·6	57·2	58·2	58·8	59·4	60·1	60·7	61·4	61·9	62·3	62·6	63·0	63·5	64·1	64·6	65·1	65·6	66·1	66·7	67·2	67·7	68·1	68·6	69·0	69·4	69·8	70·3	70·7
Campeche	49·7	49·9	49·8	50·4	51·5	52·3	53·1	54·1	55·1	56·0	56·8	57·4	58·0	58·5	59·0	59·5	59·9	60·2	60·5	60·8	61·3	61·7	62·3	62·8	63·4	63·9	64·4	64·9	65·4	65·8
Chiapas	26·8	26·8	25·1	26·2	29·6	31·5	33·0	34·4	36·0	37·3	38·0	38·2	38·5	38·9	39·4	40·1	40·6	41·1	41·7	42·3	43·0	43·6	44·3	45·0	45·7	46·2	46·8	47·4	48·1	48·6
Chihuahua	55·2	55·4	55·3	55·6	56·2	56·5	56·8	57·3	57·9	58·5	59·0	59·5	59·9	60·3	60·8	61·4	61·8	62·2	62·6	63·0	63·3	63·7	64·0	64·4	64·8	65·3	65·7	66·2	66·6	67·1
Coahuila	46·9	47·2	46·8	47·4	49·0	49·9	50·8	51·7	52·7	53·6	54·3	54·6	54·9	55·2	55·7	56·3	56·5	56·6	56·9	57·1	57·4	57·7	58·1	58·6	59·1	59·6	60·2	60·8	61·4	61·9
Colima	53·0	53·4	53·5	53·9	54·9	55·6	56·3	57·0	57·8	58·6	59·3	59·8	60·2	60·7	61·2	61·7	62·0	62·3	62·7	63·1	63·5	63·9	64·3	64·8	65·2	65·6	66·1	66·6	67·1	67·5
Durango	40·8	40·6	39·5	39·6	41·3	42·3	43·3	44·3	45·5	46·8	47·8	48·5	49·1	49·8	50·5	51·4	51·8	52·1	52·6	53·1	53·6	54·1	54·6	55·3	55·9	56·4	57·0	57·6	58·2	58·7
Guanajuato	48·4	48·9	49·1	49·6	50·8	51·6	52·4	53·2	53·9	54·5	55·2	55·9	56·5	56·8	57·2	57·8	58·2	58·4	58·9	59·3	59·8	60·3	60·8	61·4	61·9	62·3	62·9	63·4	63·9	64·4
Guerrero	36·6	36·4	35·5	35·7	37·1	37·9	38·9	39·9	41·0	42·3	43·2	43·8	44·4	44·9	45·5	46·3	46·9	47·3	48·0	48·8	49·5	50·1	50·8	51·5	52·1	52·7	53·2	53·8	54·4	54·8
Hidalgo	44·4	44·9	45·0	45·7	47·1	48·0	48·9	49·8	50·7	51·6	52·4	53·1	53·6	54·0	54·5	55·0	55·5	56·0	56·5	57·0	57·6	58·1	58·7	59·3	59·9	60·4	60·9	61·4	61·9	62·4
Jalisco	46·6	46·9	46·6	47·2	48·6	49·4	50·3	51·2	52·2	53·1	53·7	54·1	54·5	54·9	55·3	55·8	56·1	56·3	56·6	56·9	57·4	57·8	58·3	58·9	59·4	59·9	60·5	61·0	61·6	62·1
México	54·7	54·5	53·9	55·1	57·2	58·1	58·6	58·9	59·3	59·9	60·4	60·8	61·2	61·5	61·9	62·4	62·6	62·7	62·9	62·9	63·0	63·2	63·6	64·2	64·7	65·1	65·6	66·1	66·7	67·1
Mexico City	61·9	62·3	62·2	62·7	64·1	65·2	66·1	66·8	67·3	67·8	68·3	68·8	69·2	69·5	69·9	70·3	70·5	70·6	70·8	71·0	71·1	71·4	71·7	72·1	72·6	73·0	73·3	73·7	74·2	74·5
Michoacán de Ocampo	42·0	42·2	41·9	42·4	43·8	44·7	45·5	46·4	47·4	48·3	49·0	49·5	50·0	50·5	51·0	51·5	51·9	52·1	52·4	52·7	53·0	53·4	53·8	54·4	54·8	55·3	55·8	56·3	56·9	57·3
Morelos	50·8	51·3	51·3	52·0	53·2	54·0	54·7	55·5	56·3	57·1	57·8	58·2	58·5	58·9	59·3	59·7	60·0	60·1	60·4	60·7	61·0	61·3	61·7	62·1	62·6	63·0	63·4	63·8	64·3	64·8
Nayarit	45·3	45·5	45·1	45·6	46·9	47·8	48·6	49·6	50·7	51·8	52·6	53·2	53·7	54·3	54·9	55·5	55·9	56·2	56·6	57·0	57·5	57·9	58·5	59·0	59·5	60·0	60·5	61·1	61·6	62·1
Nuevo León	53·7	53·8	53·5	53·8	54·9	55·6	56·2	56·9	57·8	58·6	59·3	59·6	60·0	60·4	60·9	61·3	61·5	61·6	61·8	62·0	62·1	62·3	62·6	63·0	63·4	63·8	64·3	64·8	65·4	65·8
Oaxaca	40·7	40·9	40·6	40·9	41·9	42·4	42·8	43·4	44·1	44·7	45·3	45·6	46·0	46·6	47·2	48·0	48·6	49·2	49·9	50·7	51·4	52·0	52·6	53·2	53·8	54·3	54·8	55·3	55·8	56·3
Puebla	47·3	47·9	48·0	48·6	49·9	50·7	51·0	51·3	51·6	51·9	52·4	52·8	53·2	53·9	54·4	55·1	55·5	55·8	56·3	56·8	57·4	57·9	58·4	58·9	59·4	59·9	60·4	60·8	61·3	61·8
Querétaro	50·9	51·2	51·1	51·8	53·3	54·6	55·8	56·8	57·6	58·5	59·3	60·1	60·7	61·1	61·5	62·0	62·4	62·7	63·1	63·5	63·9	64·3	64·7	65·1	65·5	65·9	66·4	66·8	67·3	67·7
Quintana Roo	50·9	51·5	51·8	52·7	54·1	55·2	56·2	57·1	58·1	59·0	59·7	60·3	60·7	61·2	61·6	62·2	62·6	63·0	63·5	63·9	64·3	64·8	65·3	65·7	66·1	66·5	66·9	67·3	67·8	68·2
San Luis Potosí	37·9	37·8	37·0	37·4	39·3	40·5	41·6	42·8	44·1	45·4	46·4	47·0	47·5	48·1	48·8	49·6	50·1	50·4	51·0	51·6	52·1	52·5	53·1	53·7	54·3	54·8	55·4	56·0	56·6	57·1
Sinaloa	49·4	49·4	49·0	49·3	50·4	51·1	51·9	52·8	53·8	54·9	55·7	56·2	56·8	57·3	57·9	58·5	58·9	59·2	59·6	60·0	60·4	60·9	61·4	61·9	62·5	63·0	63·5	64·0	64·6	65·1
Sonora	54·1	54·3	54·1	54·4	55·4	56·0	56·6	57·3	58·1	58·9	59·5	59·9	60·3	60·7	61·2	61·6	62·0	62·3	62·6	63·0	63·4	63·8	64·2	64·7	65·1	65·6	66·1	66·5	67·1	67·5
Tabasco	41·2	41·3	40·7	41·3	43·1	44·4	45·6	46·7	48·0	49·2	50·2	50·8	51·3	51·8	52·3	53·0	53·5	53·8	54·2	54·8	55·4	55·9	56·5	57·1	57·8	58·3	58·9	59·5	60·1	60·6
Tamaulipas	51·4	51·9	51·7	52·1	53·2	54·0	54·7	55·4	56·1	56·8	57·3	57·5	57·7	58·0	58·3	58·7	58·9	59·1	59·5	59·9	60·3	60·8	61·3	61·8	62·3	62·7	63·2	63·7	64·2	64·7
Tlaxcala	48·0	48·5	48·6	49·3	50·9	52·0	53·0	53·8	54·5	55·1	55·7	56·2	56·7	57·3	57·9	58·6	59·0	59·5	60·0	60·5	60·8	61·2	61·6	62·0	62·5	62·9	63·3	63·7	64·2	64·6
Veracruz de Ignacio de la Llave	41·2	41·2	40·5	40·8	42·2	43·0	43·8	44·7	45·8	46·8	47·6	48·1	48·5	49·0	49·6	50·4	50·8	51·2	51·8	52·3	53·0	53·5	54·1	54·7	55·3	55·8	56·3	56·8	57·4	57·9
Yucatán	49·4	49·7	49·7	50·3	51·4	52·3	53·1	53·8	54·6	55·2	55·8	56·3	56·7	57·3	57·8	58·3	58·6	58·9	59·4	59·9	60·4	60·9	61·5	62·0	62·5	63·1	63·6	64·1	64·6	65·1
Zacatecas	50·4	50·7	50·9	51·4	52·2	52·7	53·3	53·9	54·6	55·3	55·9	56·4	56·9	57·3	57·8	58·2	58·6	58·9	59·2	59·5	59·8	60·2	60·6	61·1	61·6	62·0	62·5	63·0	63·5	64·0
Nicaragua	34·1	34·4	34·9	35·7	36·7	37·7	38·6	39·5	40·5	41·2	41·9	42·4	42·8	43·4	43·9	44·6	45·1	45·4	45·9	46·6	47·3	48·2	48·8	49·2	49·7	50·2	50·7	51·2	51·8	52·3
Panama	54·5	55·1	55·2	55·5	55·9	56·2	57·0	57·3	57·6	58·5	59·1	59·8	60·5	60·7	60·7	61·0	61·3	61·7	62·3	63·3	63·6	63·3	63·6	64·2	64·8	65·5	66·2	67·0	67·7	68·2
Venezuela	50·2	51·0	52·5	53·1	53·6	54·8	55·2	55·6	55·7	55·3	55·7	56·7	56·6	55·2	54·0	54·2	55·6	57·0	57·9	58·3	58·7	59·0	59·6	60·1	60·4	60·7	60·8	60·9	60·9	60·9
Tropical Latin America	48·5	49·2	49·9	50·6	51·3	51·9	52·4	52·9	53·4	53·9	54·3	54·7	55·2	55·7	56·2	56·6	57·1	57·7	58·3	58·8	59·4	60·0	60·6	61·2	61·8	62·4	62·8	63·2	63·6	64·0
Brazil	48·4	49·2	49·9	50·6	51·3	51·9	52·4	52·9	53·4	53·8	54·3	54·7	55·2	55·7	56·1	56·6	57·2	57·7	58·3	58·8	59·4	60·1	60·7	61·3	61·9	62·4	62·8	63·2	63·6	64·0
Acre	36·3	37·2	38·0	39·1	40·0	40·9	41·6	42·2	42·7	43·3	43·7	44·3	44·9	45·5	45·9	46·5	47·2	48·0	48·8	49·5	50·3	51·1	51·9	52·7	53·5	54·1	54·7	55·2	55·7	56·1
Alagoas	35·6	36·4	37·2	37·9	38·5	39·1	39·6	40·2	40·7	41·1	41·5	42·0	42·4	42·9	43·4	43·9	44·5	45·2	45·9	46·6	47·3	48·1	48·8	49·5	50·1	50·7	51·2	51·6	52·0	52·4
Amapá	47·9	48·7	49·5	50·4	51·2	51·8	52·4	52·8	53·2	53·5	53·9	54·4	54·9	55·4	55·7	56·0	56·4	56·9	57·3	57·8	58·4	59·0	59·6	60·3	60·9	61·4	61·9	62·3	62·7	63·1
Amazonas	45·3	46·3	47·0	47·8	48·6	49·1	49·4	49·7	49·8	49·9	49·9	50·0	50·3	50·6	50·9	51·2	51·7	52·2	52·8	53·3	54·0	54·7	55·3	56·0	56·7	57·3	57·8	58·3	58·8	59·2
Bahia	40·3	41·3	42·1	42·8	43·4	43·8	44·2	44·6	44·9	45·2	45·6	45·9	46·4	46·9	47·5	48·0	48·7	49·5	50·2	50·9	51·6	52·3	53·0	53·7	54·4	54·9	55·4	55·8	56·3	56·7
Ceará	38·9	39·8	40·5	41·3	41·9	42·5	43·1	43·7	44·2	44·6	45·1	45·6	46·1	46·7	47·3	47·9	48·6	49·3	50·0	50·7	51·4	52·2	52·9	53·5	54·2	54·7	55·2	55·6	56·0	56·4
Distrito Federal	57·8	58·7	59·6	60·7	61·8	63·0	64·2	65·3	66·2	67·0	67·7	68·4	69·0	69·6	70·0	70·5	71·1	71·6	72·2	72·7	73·3	73·9	74·4	75·0	75·5	75·9	76·3	76·7	77·1	77·4
Espírito Santo	47·6	48·4	49·1	50·0	50·7	51·5	52·1	52·8	53·4	54·0	54·6	55·2	55·9	56·5	57·1	57·7	58·3	59·0	59·6	60·1	60·8	61·5	62·1	62·8	63·4	63·9	64·4	64·8	65·3	65·7
Goiás	44·5	45·3	46·1	46·9	47·7	48·5	49·2	49·8	50·4	51·0	51·6	52·2	52·8	53·4	54·0	54·6	55·2	55·8	56·5	57·1	57·8	58·5	59·2	59·9	60·5	61·0	61·5	61·9	62·4	62·8
Maranhão	29·9	31·1	32·1	33·2	34·1	34·8	35·5	36·0	36·5	36·8	37·0	37·1	37·4	37·9	38·3	38·7	39·4	40·2	41·0	41·8	42·6	43·5	44·3	45·2	46·0	46·7	47·2	47·8	48·3	48·7
Mato Grosso	43·9	45·0	45·9	46·9	47·8	48·6	49·3	50·0	50·6	51·1	51·7	52·4	53·1	53·9	54·6	55·3	56·0	56·8	57·5	58·2	58·9	59·6	60·3	60·9	61·6	62·1	62·6	63·0	63·5	63·9
Mato Grosso do Sul	47·0	47·6	48·3	49·1	49·9	50·6	51·1	51·8	52·4	52·9	53·5	54·0	54·6	55·1	55·6	56·1	56·6	57·2	57·7	58·3	58·9	59·6	60·2	60·8	61·5	62·0	62·5	62·9	63·3	63·7
Minas Gerais	48·4	49·2	50·0	50·7	51·4	51·9	52·5	52·9	53·3	53·6	54·0	54·4	54·9	55·4	55·9	56·5	57·1	57·7	58·4	58·9	59·6	60·3	60·9	61·5	62·0	62·5	62·9	63·3	63·7	64·1
Pará	41·5	42·5	43·4	44·4	45·3	45·8	46·2	46·4	46·6	46·7	46·8	46·9	47·2	47·5	47·8	48·2	48·8	49·4	50·2	50·8	51·5	52·3	53·0	53·8	54·5	55·1	55·6	56·1	56·5	56·9
Paraíba	37·7	38·6	39·4	40·1	40·7	41·2	41·6	42·0	42·4	42·7	42·9	43·3	43·8	44·3	44·9	45·5	46·2	47·0	47·8	48·4	49·2	50·0	50·7	51·4	52·1	52·7	53·1	53·6	54·0	54·4

Paraná	49·2	49·7	50·2	50·9	51·6	52·3	53·0	53·7	54·5	55·1	55·8	56·4	57·0	57·6	58·1	58·6	59·1	59·7	60·2	60·7	61·3	61·9	62·5	63·1	63·6	64·1	64·5	64·9	65·4	65·8
Pernambuco	41·0	41·9	42·6	43·3	43·8	44·3	44·8	45·3	45·7	46·1	46·6	47·0	47·5	48·0	48·5	49·0	49·7	50·4	51·1	51·7	52·4	53·1	53·8	54·5	55·1	55·7	56·2	56·6	57·1	57·5
Piauí	33·6	34·4	35·2	36·0	36·6	37·3	37·9	38·4	38·8	39·2	39·5	39·9	40·4	40·9	41·4	42·1	42·8	43·6	44·5	45·2	46·0	46·8	47·6	48·3	49·0	49·6	50·1	50·5	51·0	51·4
Rio de Janeiro	56·1	56·7	57·2	57·8	58·4	58·9	59·5	60·0	60·5	61·0	61·5	62·0	62·5	63·0	63·4	63·8	64·3	64·7	65·1	65·5	66·0	66·5	67·0	67·5	68·0	68·5	68·8	69·2	69·6	69·9
Rio Grande do Norte	40·6	41·3	42·0	42·7	43·3	43·8	44·3	44·9	45·5	46·0	46·5	47·1	47·8	48·4	49·0	49·6	50·3	51·1	51·8	52·5	53·2	53·9	54·6	55·3	55·9	56·5	56·9	57·3	57·8	58·2
Rio Grande do Sul	54·5	55·1	55·7	56·2	56·8	57·3	57·7	58·1	58·6	59·0	59·4	59·8	60·3	60·8	61·2	61·7	62·1	62·6	63·1	63·6	64·1	64·7	65·2	65·7	66·2	66·6	67·0	67·3	67·7	68·0
Rondônia	43·5	44·2	44·8	45·4	45·9	46·3	46·7	47·1	47·3	47·7	48·2	48·7	49·4	50·1	50·7	51·3	52·0	52·8	53·5	54·2	55·0	55·8	56·6	57·3	58·0	58·6	59·1	59·6	60·1	60·5
Roraima	43·9	44·9	45·6	46·4	47·0	47·6	48·2	48·5	48·8	49·1	49·6	50·1	50·7	51·4	51·9	52·3	52·9	53·6	54·3	54·9	55·6	56·3	57·0	57·7	58·4	59·0	59·5	60·0	60·4	60·8
Santa Catarina	52·8	53·3	53·9	54·6	55·2	55·7	56·3	56·8	57·4	58·0	58·5	59·1	59·7	60·3	60·9	61·4	61·9	62·5	63·0	63·5	64·1	64·8	65·3	65·9	66·5	67·0	67·4	67·7	68·2	68·6
São Paulo	54·6	55·3	55·9	56·7	57·4	58·1	58·8	59·4	60·0	60·5	61·0	61·5	62·0	62·5	62·9	63·3	63·7	64·2	64·7	65·1	65·6	66·2	66·7	67·3	67·8	68·3	68·7	69·0	69·5	69·9
Sergipe	41·7	42·5	43·2	43·8	44·3	44·6	45·1	45·5	46·0	46·4	46·9	47·5	48·1	48·8	49·3	49·9	50·6	51·3	52·0	52·6	53·3	54·0	54·6	55·2	55·8	56·4	56·8	57·2	57·6	58·0
Tocantins	36·9	37·6	38·2	38·9	39·6	40·3	41·0	41·6	42·2	42·8	43·5	44·3	45·1	46·0	46·9	47·7	48·6	49·7	50·7	51·6	52·5	53·5	54·3	55·2	55·9	56·6	57·2	57·6	58·2	58·6
Paraguay	47·6	48·2	48·6	48·8	49·2	49·6	50·2	51·1	51·9	52·6	53·1	53·6	54·2	54·8	55·1	55·3	55·5	55·7	56·0	56·3	56·7	57·0	57·3	58·1	58·9	59·7	60·4	61·1	61·7	62·3
North Africa and Middle East	42·3	43·1	43·9	44·9	45·6	46·5	47·5	48·3	49·1	49·9	51·2	52·4	53·2	53·9	54·8	55·8	56·5	57·0	57·7	58·5	59·2	59·9	60·5	61·0	61·7	62·6	63·4	64·2	65·1	65·8
North Africa and Middle East	42·3	43·1	43·9	44·9	45·6	46·5	47·5	48·3	49·1	49·9	51·2	52·4	53·2	53·9	54·8	55·8	56·5	57·0	57·7	58·5	59·2	59·9	60·5	61·0	61·7	62·6	63·4	64·2	65·1	65·8
Afghanistan	17·9	17·9	18·2	18·4	18·3	17·9	17·4	17·2	17·1	17·0	16·9	17·0	17·8	18·6	19·3	20·1	21·0	21·9	22·8	23·8	24·8	25·9	27·0	28·1	29·1	30·0	30·8	31·7	32·5	33·2
Algeria	43·9	44·9	45·8	46·7	47·6	48·5	49·5	50·4	51·3	52·2	53·1	53·9	54·7	55·5	56·2	57·0	57·7	58·3	58·9	59·4	59·9	60·4	60·9	61·3	61·8	62·2	62·8	63·4	64·3	65·1
Bahrain	56·0	56·7	57·4	58·1	58·6	59·2	59·9	60·5	61·3	61·9	62·3	63·2	64·4	65·1	65·9	66·6	67·2	67·8	68·4	68·9	69·3	69·5	69·6	69·9	70·5	71·1	71·5	72·1	73·0	73·8
Egypt	42·0	42·6	44·1	45·7	46·8	47·5	47·8	48·2	48·4	49·8	51·4	52·2	52·3	52·6	53·5	54·1	54·3	53·7	53·7	54·6	55·2	56·0	56·0	55·3	56·2	58·5	59·9	61·1	62·2	63·1
Iran	43·0	43·8	44·3	45·8	46·1	47·1	48·8	49·3	50·1	50·1	52·3	54·9	56·2	57·4	58·7	60·3	61·2	61·5	62·2	62·9	63·6	64·2	64·6	65·0	65·6	66·6	67·5	68·1	68·9	69·7
Iraq	40·9	41·5	42·3	42·8	43·2	43·8	44·6	45·3	46·3	47·5	48·7	49·9	50·8	51·1	51·8	52·5	53·5	54·5	55·2	55·8	56·7	57·9	59·3	60·5	61·6	62·6	63·7	64·6	65·6	66·4
Jordan	51·7	52·3	53·0	53·9	54·9	55·4	56·0	56·9	57·9	58·8	59·4	59·6	60·0	60·4	61·3	62·3	63·0	63·8	64·7	65·8	67·0	68·0	69·0	70·0	70·7	71·2	71·6	72·1	72·6	73·0
Kuwait	68·3	68·2	66·9	66·6	66·6	66·6	67·7	69·5	71·1	72·2	73·0	73·5	73·7	74·0	74·6	75·6	76·7	77·8	78·8	79·5	80·2	80·8	81·4	82·1	82·9	83·5	84·0	84·4	84·8	85·2
Lebanon	47·7	48·5	49·4	50·0	50·5	51·4	52·1	52·8	53·8	54·3	54·7	55·5	56·6	57·5	58·3	59·1	60·1	61·1	62·2	63·5	64·9	66·2	67·5	68·6	69·5	70·2	70·9	71·5	72·1	72·6
Libya	49·0	51·0	53·3	55·4	57·0	58·5	59·7	61·1	62·4	63·3	64·2	64·9	65·7	66·8	67·6	68·5	69·5	70·4	71·3	72·1	73·0	73·2	73·9	74·2	74·1	73·9	73·6	73·5	73·8	74·1
Morocco	33·4	34·0	34·6	35·1	35·6	36·0	36·7	37·5	38·1	38·7	39·3	40·0	40·7	41·4	42·0	42·6	43·2	43·9	44·6	45·4	46·2	47·0	47·8	48·6	49·4	50·2	51·0	51·9	52·8	53·6
Oman	39·8	41·8	43·9	46·1	48·3	50·4	52·4	54·3	56·3	58·3	60·2	61·6	63·1	64·5	65·7	66·7	67·7	68·8	69·9	70·8	71·7	72·4	73·3	74·1	74·7	75·3	76·0	76·5	77·2	77·8
Palestine	30·7	31·2	32·1	33·3	34·6	35·9	37·4	39·2	40·6	41·7	42·8	43·9	44·4	44·6	44·8	45·6	46·5	47·2	48·3	49·9	51·7	53·3	54·7	55·9	57·0	57·8	58·7	59·5	60·3	60·9
Qatar	59·0	60·5	61·9	63·0	64·0	65·2	66·3	67·3	68·3	69·1	69·9	70·6	71·3	72·1	73·0	73·8	74·5	75·1	75·8	76·4	77·2	78·0	78·8	79·6	80·2	80·9	81·5	82·0	82·6	83·2
Saudi Arabia	34·8	37·8	40·6	43·4	46·1	48·4	50·7	52·8	54·7	56·3	57·8	59·2	60·5	61·9	63·3	64·7	66·0	67·4	68·7	69·9	71·1	72·3	73·4	74·5	75·4	76·3	77·2	77·9	78·8	79·5
Sudan	23·2	24·1	25·1	25·9	26·9	27·7	28·7	29·8	30·7	31·5	32·4	33·2	34·0	34·8	35·5	36·4	37·4	38·4	39·5	40·6	41·7	42·9	43·9	45·0	46·1	47·2	48·3	49·3	50·3	51·1
Syria	39·2	40·0	41·2	42·3	43·5	44·8	46·0	47·1	48·2	49·2	50·1	50·9	51·6	52·1	52·8	54·0	55·0	55·6	56·4	57·7	58·9	59·9	60·4	60·6	60·9	61·0	61·3	61·7	62·1	62·5
Tunisia	45·4	46·4	47·3	48·4	49·3	50·2	51·3	52·5	53·6	54·6	55·5	56·4	57·3	58·1	58·8	59·5	60·2	60·9	61·5	62·1	62·7	63·2	63·7	64·1	64·6	65·2	65·7	66·3	66·9	67·6
Turkey	49·2	50·4	51·4	52·3	53·0	53·8	54·7	55·6	56·5	57·4	58·4	59·4	60·2	61·1	61·9	62·8	63·7	64·6	65·4	66·1	66·8	67·6	68·4	69·2	69·9	70·7	71·5	72·2	73·1	73·8
United Arab Emirates	61·8	63·4	64·8	66·2	67·7	68·9	70·1	71·3	72·7	74·0	75·2	76·4	77·5	78·5	79·3	80·2	81·1	82·4	83·5	84·4	85·0	85·4	85·7	86·0	86·1	86·3	86·6	86·9	87·4	87·9
Yemen	17·2	17·4	18·1	18·6	19·0	20·3	21·4	22·6	23·7	24·7	25·8	27·0	28·6	29·6	30·6	32·2	33·6	35·2	36·5	37·5	38·5	39·5	40·4	41·1	41·7	42·1	42·2	42·3	42·3	42·2
South Asia	31·2	30·9	30·6	31·9	33·2	33·9	34·8	35·7	36·4	36·8	37·4	38·4	39·2	39·8	40·6	41·4	42·2	42·9	43·6	44·7	45·5	46·2	47·2	48·4	49·5	50·8	51·7	52·3	53·1	53·9
South Asia	31·2	30·9	30·6	31·9	33·2	33·9	34·8	35·7	36·4	36·8	37·4	38·4	39·2	39·8	40·6	41·4	42·2	42·9	43·6	44·7	45·5	46·2	47·2	48·4	49·5	50·8	51·7	52·3	53·1	53·9
Bangladesh	24·6	25·3	26·3	27·4	28·5	29·3	30·3	31·3	32·1	32·9	33·4	33·5	33·6	33·8	34·1	34·6	35·2	36·1	37·1	38·0	38·9	39·8	40·9	41·7	42·7	43·6	44·5	45·6	46·6	47·5
Bhutan	21·4	21·7	22·1	22·7	23·4	24·2	25·0	25·8	26·6	27·4	28·3	29·1	29·9	30·7	31·5	32·3	33·1	33·9	34·8	35·7	36·5	37·3	38·1	38·9	39·6	40·2	40·9	41·6	42·5	43·2
India	32·7	32·2	31·7	33·1	34·6	35·2	36·2	37·1	37·7	37·9	38·4	39·5	40·5	41·1	41·9	42·8	43·7	44·3	45·0	46·1	46·9	47·7	48·8	49·9	51·2	52·6	53·6	54·1	55·0	55·8
Andhra Pradesh	28·0	27·7	26·9	28·8	30·7	31·3	32·6	33·7	34·2	34·7	35·4	36·7	38·2	39·0	40·0	41·0	41·8	42·6	43·3	44·3	45·0	45·8	47·0	48·0	49·1	50·4	51·3	51·9	52·8	53·7
Arunachal Pradesh	32·0	32·0	31·9	33·1	34·2	34·5	34·9	35·0	35·1	35·3	35·6	36·6	38·0	39·0	40·0	40·9	41·8	42·6	43·1	44·0	45·0	45·8	46·6	48·1	50·0	51·7	52·7	53·1	54·0	54·8
Assam	33·8	33·5	32·8	34·0	35·1	35·4	36·2	36·9	37·3	37·5	38·0	39·1	40·2	40·9	41·7	42·5	43·2	43·8	44·2	45·0	45·6	46·2	47·2	48·2	49·2	50·5	51·4	52·0	52·9	53·7
Bihar	28·7	28·6	28·1	28·8	29·4	29·3	29·6	29·8	29·7	29·3	29·1	29·6	30·1	30·2	30·8	31·4	32·0	32·6	33·3	34·5	35·4	36·3	37·6	38·9	40·1	41·5	42·6	43·3	44·2	45·1
Chhattisgarh	30·0	29·6	28·9	30·0	31·1	31·2	32·1	32·6	32·8	32·8	33·1	34·4	35·5	36·2	37·2	38·2	39·1	40·0	40·9	42·1	42·7	43·2	44·3	45·7	47·2	48·8	49·9	50·6	51·6	52·5
Delhi	50·2	49·8	50·1	53·1	54·2	53·4	53·9	54·6	54·3	53·8	54·4	55·8	57·4	58·5	59·3	59·9	60·5	61·2	61·9	62·7	63·7	64·4	65·0	66·1	67·2	68·2	68·9	69·4	70·1	70·8
Goa	50·8	51·3	51·8	52·7	53·5	54·1	54·7	55·3	55·8	56·2	56·7	57·4	58·1	58·9	59·9	60·9	61·9	62·6	63·5	64·5	65·2	65·8	66·6	67·5	68·5	69·4	70·1	70·6	71·3	72·0
Gujarat	35·4	35·3	34·8	36·1	37·7	38·6	39·8	40·9	41·7	42·2	42·9	43·9	44·7	45·3	46·2	47·1	47·8	48·3	49·1	50·4	51·3	52·1	53·3	54·4	55·5	56·9	57·9	58·4	59·3	60·1
Haryana	34·7	33·6	33·3	34·9	36·1	36·9	37·5	38·7	39·7	39·6	40·0	41·2	42·4	43·1	43·4	44·5	45·8	46·6	47·1	48·6	49·8	50·4	51·5	53·0	55·2	57·7	58·6	58·5	59·3	60·0
Himachal Pradesh	34·6	34·6	34·6	36·2	37·9	38·9	40·1	41·3	42·5	43·2	43·9	45·0	46·1	46·9	47·7	48·7	49·9	50·8	51·7	52·8	53·7	54·6	55·8	56·9	58·1	59·3	60·3	60·9	61·8	62·6
Jammu & Kashmir and Ladakh	34·4	33·7	34·4	35·4	36·0	36·8	36·7	38·0	39·6	39·7	41·3	42·9	43·9	45·3	46·4	47·3	48·4	49·1	49·9	51·0	51·7	52·6	53·1	53·9	55·3	56·8	57·7	58·3	59·1	60·0
Jharkhand	28·5	28·3	27·5	28·7	29·9	30·1	30·9	31·6	31·6	31·5	31·7	32·7	33·8	34·4	35·4	36·4	37·3	38·1	38·9	40·1	40·9	41·8	43·0	44·2	45·3	46·7	47·7	48·4	49·3	50·2
Karnataka	32·4	32·2	31·8	33·5	35·2	36·0	37·2	38·3	39·0	39·5	40·2	41·3	42·5	43·3	44·1	45·1	45·8	46·6	47·2	48·2	49·0	49·8	50·8	51·9	52·9	54·2	55·1	55·7	56·6	57·4
Kerala	42·6	42·7	42·7	43·8	45·2	46·1	46·6	47·4	48·4	48·8	50·0	51·2	51·8	52·6	53·3	54·5	55·5	55·9	56·5	57·4	58·2	58·7	59·5	60·6	61·8	63·2	64·0	64·3	65·1	65·8
Madhya Pradesh	28·9	28·1	27·3	28·6	29·9	30·4	31·5	32·4	32·6	32·6	33·1	34·5	35·4	35·7	36·3	36·9	37·3	37·5	37·7	38·8	39·4	40·0	41·4	43·0	44·6	46·4	47·5	48·0	48·9	49·8
Maharashtra	36·2	36·0	35·2	37·1	39·1	39·9	41·2	42·4	43·1	43·6	44·2	45·4	46·6	47·3	48·3	49·3	50·1	50·9	51·7	52·7	53·6	54·5	55·6	56·6	57·6	58·9	59·9	60·4	61·3	62·1
Manipur	39·8	40·5	40·8	41·7	42·6	43·0	43·7	44·3	44·7	45·1	45·5	46·4	47·4	47·9	48·6	49·4	50·2	50·7	51·0	51·6	52·0	52·5	53·2	53·9	54·7	55·7	56·5	57·1	57·9	58·7
Meghalaya	33·8	33·9	33·6	34·7	35·9	36·1	37·0	37·8	38·2	38·4	39·0	40·1	41·3	42·3	43·1	44·1	44·9	45·6	46·2	47·0	47·7	48·3	49·4	50·3	51·2	52·4	53·2	53·8	54·6	55·4
Mizoram	39·9	39·9	40·3	41·9	43·1	43·9	44·8	45·7	46·3	46·5	47·0	47·9	48·7	49·3	50·3	51·3	52·0	52·3	52·5	53·5	54·0	54·2	54·9	55·8	56·8	58·0	58·9	59·4	60·1	60·9
Nagaland	40·4	40·8	41·1	42·3	43·5	44·1	44·8	45·6	46·2	46·6	47·2	48·1	49·0	49·7	50·4	51·2	51·9	52·5	52·9	53·5	54·1	54·7	55·6	56·5	57·4	58·5	59·4	60·0	60·8	61·5
Odisha	29·7	29·6	29·5	30·3	31·3	32·0	33·0	33·8	34·4	34·9	35·6	36·6	37·6	38·3	39·3	40·2	41·0	41·8	42·5	43·6	44·3	45·0	46·2	47·4	48·8	50·2	51·3	51·9	52·8	53·6
Other Union Territories	41·9	41·4	40·2	41·5	43·0	43·3	44·5	45·6	46·3	46·7	47·4	48·9	50·4	51·7	53·1	54·3	55·4	56·5	57·5	58·7	59·5	60·2	61·3	62·2	63·1	64·1	64·9	65·4	66·2	66·9
Punjab	39·9	39·0	39·1	40·4	41·5	42·7	43·4	44·3	45·1	45·2	45·4	46·4	47·3	48·0	48·8	49·8	50·8	51·5	52·2	53·3	54·0	54·7	55·4	56·4	57·9	59·3	60·0	60·4	61·2	61·9
Rajasthan	26·3	25·8	24·8	26·1	27·8	28·3	29·5	30·5	31·1	31·4	31·8	33·0	34·2	34·9	35·7	36·5	37·2	37·8	38·5	39·7	40·7	41·5	42·9	44·3	45·6	47·2	48·3	49·0	49·9	50·8
Sikkim	35·6	36·0	36·2	37·2	38·3	38·9	39·4	39·7	40·2	40·4	41·0	41·8	42·7	43·6	44·4	45·3	46·4	47·3	48·2	49·8	51·1	52·3	54·0	55·6	57·1	58·4	59·3	59·9	60·8	61·6
Tamil Nadu	36·5	36·3	35·9	37·1	38·9	39·9	40·4	41·4	42·4	42·7	43·5	44·7	45·4	46·2	47·1	48·2	49·4	50·3	51·0	51·8	52·3	52·8	54·0	55·1	56·5	58·5	59·5	59·7	60·6	61·4
Telangana	30·5	30·7	30·5	31·6	32·6	33·0	33·9	34·6	35·2	35·5	35·9	36·8	37·7	38·3	39·0	39·8	40·6	41·5	42·7	44·2	45·2	46·3	47·9	49·3	50·6	52·0	53·1	53·8	54·8	55·7
Tripura	34·0	34·1	33·9	34·8	35·9	36·4	37·1	37·8	38·2	38·5	38·8	40·0	41·1	41·8	42·7	43·6	44·6	45·2	45·6	46·8	47·1	47·5	48·6	49·7	50·8	52·0	52·7	53·2	54·0	54·8
Uttar Pradesh	27·8	26·9	26·2	27·8	29·2	30·0	31·0	31·9	32·4	32·5	32·8	33·8	34·7	35·2	35·9	36·8	37·7	38·4	39·2	40·4	41·4	42·3	43·5	44·7	46·1	47·6	48·6	49·3	50·2	51·0
Uttarakhand	32·6	32·0	30·9	31·9	33·3	33·8	34·8	35·6	36·0	36·5	37·2	38·7	40·0	41·2	42·9	44·2	45·5	46·7	48·1	49·7	51·0	52·3	53·8	55·3	56·6	58·1	59·2	60·0	61·0	61·9
West Bengal	32·8	31·4	30·7	33·0	34·7	35·3	36·4	37·6	38·2	38·6	39·2	40·2	41·2	41·8	42·5	43·3	44·1	44·6	45·1	46·1	46·7	47·3	48·3	49·2	50·2	51·5	52·4	52·9	53·8	54·6
Nepal	19·8	20·2	20·6	21·1	21·7	22·5	23·2	24·0	24·9	25·9	27·0	27·9	28·7	29·7	30·7	31·7	32·5	33·3	34·1	35·0	35·8	36·6	37·5	38·2	39·0	39·8	40·5	41·2	42·0	42·7
Pakistan	26·0	26·3	26·6	26·6	27·1	28·1	29·1	30·0	31·0	32·2	33·7	34·8	35·7	36·3	37·1	38·1	38·8	39·4	40·2	40·9	41·7	42·0	42·5	43·8	44·5	45·0	45·6	46·2	46·9	47·6
Southeast Asia, east Asia, and Oceania	45·2	46·2	46·8	47·7	48·3	49·1	50·2	51·2	52·0	52·8	53·5	54·2	54·7	55·2	55·9	56·7	57·7	58·6	59·3	60·1	61·1	61·8	62·5	63·0	63·8	64·1	64·4	65·4	66·5	67·4
East Asia	44·6	45·6	46·3	47·2	47·8	48·6	49·8	51·0	52·0	52·9	53·8	54·5	55·0	55·6	56·5	57·5	58·6	59·7	60·6	61·5	62·7	63·5	64·2	64·7	65·5	65·8	65·9	67·1	68·5	69·4
China	44·0	45·0	45·7	46·6	47·2	48·0	49·3	50·5	51·5	52·4	53·3	54·0	54·5	55·1	56·0	57·0	58·2	59·3	60·2	61·2	62·4	63·3	63·9	64·4	65·2	65·5	65·6	66·9	68·3	69·2
North Korea	40·5	41·0	41·3	41·6	41·9	42·2	42·4	42·6	42·7	43·0	43·4	43·8	44·3	44·8	45·4	46·0	46·6	47·2	47·8	48·4	48·9	49·5	50·0	50·6	51·1	51·6	52·2	52·7	53·2	53·7
Taiwan (province of China)	66·7	67·7	68·5	69·4	70·2	71·1	72·0	73·2	74·2	74·7	75·4	76·3	77·2	78·0	78·7	79·5	80·2	81·0	81·7	82·4	82·9	83·4	83·8	84·3	84·8	85·2	85·6	86·0	86·5	87·0
Oceania	38·2	38·4	38·6	38·9	39·1	39·4	39·6	39·8	40·1	40·3	40·5	40·6	40·7	40·7	40·8	41·0	41·0	41·2	41·3	41·5	41·7	41·9	42·1	42·4	42·7	43·1	43·5	43·8	44·2	44·5
American Samoa	60·2	60·0	59·9	60·7	62·1	63·0	63·5	63·6	63·5	63·5	63·8	64·3	64·8	65·3	65·7	66·2	66·4	66·4	66·5	66·5	66·3	67·0	68·1	69·0	69·7	70·4	70·8	70·8	70·9	71·1
Cook Islands	64·2	64·8	65·4	66·0	66·5	67·0	67·4	67·7	68·1	68·4	68·9	69·4	69·8	70·3	70·7	71·1	71·6	72·0	72·4	72·8	73·1	73·5	74·0	74·4	74·9	75·4	76·0	76·5	77·1	77·5
Fiji	52·0	52·3	52·8	53·4	53·9	54·6	55·2	55·8	56·6	57·4	58·1	58·7	59·3	59·8	60·2	60·5	60·8	61·1	61·3	61·2	61·3	61·6	61·9	62·2	62·6	63·2	63·8	64·3	64·9	65·4
Guam	69·4	68·9	68·4	68·5	69·2	69·9	70·3	70·8	71·9	73·4	74·8	76·0	76·7	76·9	77·3	77·4	77·3	77·4	77·6	78·0	78·4	78·5	78·8	79·3	79·6	79·9	80·2	80·5	80·7	80·9
Kiribati	40·8	41·3	41·7	42·1	42·4	42·8	43·1	43·1	43·2	43·3	43·5	43·5	43·4	43·5	43·5	43·4	43·2	43·1	42·8	42·3	42·1	42·2	42·1	42·0	41·8	42·2	43·1	43·8	44·5	45·0

Marshall Islands	38·8	39·8	40·7	41·4	42·1	42·8	43·5	44·1	44·4	44·6	44·8	45·3	45·7	46·0	46·3	46·6	47·2	47·8	48·3	48·8	49·5	50·1	50·7	51·2	51·7	52·2	52·8	53·3	53·8	54·1
Federated States of Micronesia	44·5	45·2	45·9	46·5	47·1	47·8	48·3	48·6	49·0	49·3	49·8	50·2	50·7	51·2	51·7	52·2	52·7	53·1	53·5	53·8	54·3	54·7	55·0	55·4	55·6	56·0	56·3	56·7	57·1	57·4
Nauru	53·5	53·8	53·9	53·9	53·9	53·9	53·8	53·7	53·5	53·3	53·2	53·0	52·9	52·9	52·8	52·8	53·0	53·1	53·5	54·0	54·7	55·6	56·4	57·6	59·0	60·1	61·1	62·1	62·9	63·5
Niue	58·6	59·1	59·6	60·1	60·6	61·1	61·7	62·2	62·8	63·3	63·9	64·4	64·9	65·4	65·9	66·6	67·4	68·0	68·6	69·2	69·7	70·3	70·7	71·0	71·4	71·7	72·0	72·3	72·6	72·8
Northern Mariana Islands	70·8	71·4	72·0	72·6	73·0	73·4	73·7	73·9	74·1	74·3	74·8	75·3	75·4	75·4	75·3	75·1	75·0	74·8	74·6	74·4	74·2	73·9	73·7	73·6	73·5	73·5	73·9	74·3	74·8	75·2
Palau	61·6	62·3	62·9	63·3	63·8	64·3	64·8	65·3	65·8	66·2	66·5	66·9	67·3	67·6	67·9	68·2	68·5	68·9	69·1	69·3	69·5	69·7	70·0	70·3	70·7	71·1	71·6	71·9	72·3	72·6
Papua New Guinea	29·2	29·5	29·8	30·3	30·7	31·0	31·4	31·7	31·9	32·1	32·3	32·5	32·6	32·8	32·9	33·2	33·4	33·7	34·0	34·4	34·8	35·2	35·6	36·0	36·5	37·0	37·5	38·0	38·5	38·9
Samoa	52·2	52·7	52·7	52·2	52·0	52·2	52·1	52·0	52·1	52·3	52·4	52·5	52·6	52·7	52·8	52·9	52·9	53·1	53·2	53·1	52·8	52·9	53·0	52·5	52·7	53·7	54·5	55·2	55·9	56·5
Solomon Islands	28·1	28·6	29·2	29·8	30·5	31·1	31·7	32·2	32·7	33·1	33·2	33·3	33·3	33·3	33·4	33·6	33·9	34·3	34·7	35·1	35·6	36·2	36·8	37·3	37·8	38·4	38·9	39·4	39·9	40·3
Tokelau	48·2	48·7	49·2	49·7	50·2	50·7	51·2	51·8	52·4	52·9	53·5	54·1	54·7	55·3	55·9	56·6	57·2	57·9	58·6	59·1	59·7	60·4	61·0	61·6	62·2	62·8	63·5	64·1	64·7	65·2
Tonga	49·9	50·3	51·0	51·9	52·4	52·9	53·3	53·8	54·4	54·9	55·1	55·0	55·2	55·6	55·8	55·9	56·1	56·4	56·6	56·9	57·4	58·3	59·0	59·3	59·7	60·1	60·6	61·1	61·6	62·1
Tuvalu	44·1	45·1	46·0	46·8	47·6	48·3	48·7	49·1	49·6	50·1	50·6	51·1	51·6	52·0	52·3	52·5	52·9	53·3	53·7	54·1	54·5	54·9	55·2	55·6	56·1	56·6	57·2	57·7	58·3	58·8
Vanuatu	37·2	37·6	38·0	38·4	38·8	39·3	39·7	40·1	40·5	40·9	41·4	41·7	41·9	42·1	42·4	42·7	43·1	43·6	44·1	44·5	45·0	45·4	45·9	46·3	46·7	47·0	47·4	47·8	48·2	48·6
Southeast Asia	45·8	46·6	47·4	48·3	49·1	50·0	50·8	51·6	52·2	52·7	53·2	53·6	54·0	54·4	54·8	55·3	55·6	56·1	56·7	57·2	57·7	58·4	59·1	59·9	60·6	61·3	61·9	62·6	63·3	63·9
Cambodia	26·4	26·9	27·5	28·1	28·6	29·1	29·7	30·3	30·9	31·6	32·4	33·2	34·0	34·7	35·5	36·4	37·3	38·2	39·0	39·8	40·5	41·2	41·9	42·6	43·3	44·0	44·7	45·5	46·2	46·9
Indonesia	45·8	46·8	47·9	49·0	50·0	50·8	51·7	52·4	52·9	53·4	53·9	54·2	54·5	54·9	55·4	56·0	56·6	57·2	57·9	58·5	59·3	60·0	60·8	61·6	62·3	63·0	63·7	64·4	65·1	65·8
Aceh	48·3	49·5	50·7	51·9	52·9	53·7	54·4	55·1	55·5	56·1	56·5	56·8	57·1	57·4	57·9	58·5	59·0	59·4	59·7	60·1	60·5	60·9	61·4	61·9	62·5	63·1	63·8	64·4	65·1	65·8
Bali	45·5	46·6	47·8	49·0	50·0	50·9	51·8	52·5	52·9	53·3	53·7	53·9	54·2	54·5	54·9	55·4	56·0	56·6	57·2	57·9	58·6	59·4	60·2	61·0	61·7	62·4	63·1	63·8	64·5	65·1
Bangka-Belitung Islands	44·6	45·6	46·8	47·9	49·0	49·9	50·8	51·6	52·0	52·7	53·2	53·4	53·6	53·9	54·4	55·1	55·7	56·3	57·0	57·8	58·6	59·4	60·3	61·1	61·8	62·5	63·2	63·9	64·6	65·2
Banten	45·4	46·3	47·3	48·4	49·4	50·3	51·2	52·0	52·5	53·1	53·5	53·6	53·7	53·9	54·2	54·7	55·1	55·6	56·2	56·8	57·5	58·3	59·1	59·9	60·7	61·4	62·1	62·8	63·5	64·2
Bengkulu	40·4	41·6	42·9	44·3	45·5	46·4	47·3	48·2	48·7	49·3	49·7	50·0	50·2	50·5	51·1	51·7	52·3	52·9	53·5	54·2	54·9	55·7	56·4	57·2	57·9	58·7	59·4	60·1	60·8	61·5
Gorontalo	37·4	38·2	39·1	40·0	40·8	41·6	42·4	43·2	43·5	44·0	44·5	44·7	44·9	45·1	45·5	46·2	46·7	47·4	48·1	48·9	49·8	50·7	51·6	52·5	53·3	54·1	54·8	55·6	56·3	56·9
Jakarta	59·7	60·8	61·9	63·1	64·2	65·2	66·1	66·9	67·5	68·1	68·7	69·1	69·6	70·0	70·6	71·3	71·9	72·5	73·1	73·8	74·4	75·1	75·8	76·5	77·1	77·7	78·3	78·9	79·6	80·2
Jambi	42·3	43·3	44·4	45·5	46·5	47·4	48·3	49·1	49·7	50·3	50·8	51·2	51·5	52·0	52·5	53·3	53·9	54·6	55·5	56·3	57·2	58·1	59·0	59·9	60·7	61·4	62·1	62·8	63·5	64·1
West Java	45·7	46·7	47·8	48·9	49·9	50·7	51·6	52·3	52·7	53·1	53·5	53·7	53·8	54·1	54·5	55·1	55·6	56·2	56·9	57·6	58·3	59·1	59·8	60·6	61·3	62·0	62·7	63·4	64·1	64·7
Central Java	42·2	43·2	44·2	45·3	46·2	47·0	47·8	48·5	48·9	49·4	49·7	50·0	50·2	50·5	50·9	51·5	52·1	52·7	53·4	54·1	54·8	55·6	56·3	57·1	57·8	58·5	59·2	59·9	60·6	61·3
East Java	44·4	45·3	46·3	47·3	48·2	49·1	49·9	50·6	51·1	51·6	52·1	52·5	52·8	53·3	53·8	54·5	55·2	55·9	56·6	57·3	58·1	58·9	59·7	60·5	61·2	61·9	62·5	63·2	63·9	64·6
West Kalimantan	38·9	40·1	41·3	42·7	43·9	44·9	45·9	46·8	47·3	48·0	48·6	48·9	49·2	49·5	50·0	50·5	51·1	51·6	52·2	52·8	53·4	54·1	54·8	55·6	56·3	57·0	57·7	58·4	59·1	59·8
South Kalimantan	44·0	45·2	46·4	47·6	48·7	49·5	50·4	51·2	51·6	52·1	52·5	52·7	52·9	53·2	53·6	54·2	54·7	55·1	55·7	56·3	56·9	57·6	58·4	59·1	59·8	60·5	61·2	61·9	62·6	63·2
Central Kalimantan	45·5	46·5	47·6	49·0	50·3	51·2	52·3	53·1	53·7	54·3	54·7	54·9	55·0	55·3	55·6	56·1	56·7	57·2	57·8	58·5	59·2	60·0	60·8	61·5	62·3	63·0	63·6	64·3	65·0	65·6
East Kalimantan	57·1	58·0	59·0	60·1	61·0	61·7	62·4	63·1	63·5	64·1	64·7	65·0	65·4	65·8	66·3	67·0	67·8	68·5	69·2	69·9	70·6	71·4	72·2	73·0	73·7	74·4	75·1	75·7	76·4	77·0
North Kalimantan	53·6	54·6	55·7	56·8	57·9	58·8	59·7	60·6	61·3	62·3	63·2	63·8	64·4	65·1	66·0	66·9	67·7	68·3	69·0	69·6	70·3	70·9	71·6	72·3	73·0	73·7	74·4	75·0	75·7	76·3
Riau Islands	56·6	57·5	58·5	59·6	60·5	61·4	62·2	62·9	63·5	64·0	64·5	64·8	65·0	65·4	65·8	66·3	66·8	67·3	67·8	68·4	69·0	69·6	70·3	71·0	71·6	72·2	72·8	73·4	74·0	74·6
Lampung	39·6	40·6	41·7	42·8	43·9	44·8	45·8	46·7	47·3	48·0	48·5	48·8	49·1	49·5	49·9	50·5	51·0	51·6	52·2	53·0	53·8	54·7	55·6	56·5	57·3	58·0	58·8	59·5	60·3	60·9
Maluku	47·4	48·3	49·4	50·5	51·4	52·0	52·6	53·0	52·9	52·9	52·6	52·0	51·3	50·6	50·0	49·7	49·4	49·2	49·3	49·6	50·0	50·7	51·4	52·2	53·0	53·8	54·6	55·4	56·2	56·9
North Maluku	39·1	39·9	40·9	41·9	42·8	43·5	44·3	45·0	45·3	45·8	46·2	46·2	46·2	46·2	46·3	46·7	47·1	47·4	48·0	48·6	49·4	50·3	51·1	52·0	52·8	53·6	54·4	55·2	55·9	56·6
West Nusa Tenggara	34·2	35·2	36·5	37·8	39·0	40·1	41·2	42·1	42·8	43·5	44·1	44·6	45·1	45·7	46·4	47·2	47·9	48·6	49·4	50·1	50·8	51·6	52·4	53·2	53·9	54·7	55·4	56·1	56·9	57·6
East Nusa Tenggara	36·3	37·1	38·0	38·9	39·8	40·6	41·4	42·2	42·5	42·9	43·3	43·5	43·7	44·0	44·4	44·9	45·4	45·8	46·4	46·9	47·6	48·3	49·1	49·9	50·7	51·5	52·3	53·0	53·8	54·5
Papua	46·2	47·1	48·0	49·0	50·0	50·7	51·4	52·1	52·5	53·2	53·6	53·7	53·8	53·9	54·0	54·4	54·9	55·3	55·7	56·2	56·9	57·6	58·3	59·1	59·8	60·6	61·3	62·0	62·8	63·4
West Papua	44·0	45·0	46·1	47·2	48·3	49·1	49·9	50·6	51·1	51·7	52·2	52·4	52·6	52·8	53·2	53·8	54·5	55·1	55·9	56·7	57·6	58·6	59·6	60·5	61·5	62·3	63·2	64·0	64·8	65·5
Riau	52·6	53·8	55·1	56·2	57·2	58·0	58·7	59·3	59·8	60·4	61·0	61·4	61·8	62·1	62·6	63·3	63·8	64·3	64·9	65·5	66·1	66·9	67·6	68·4	69·1	69·8	70·6	71·3	72·0	72·6
West Sulawesi	37·0	37·9	39·0	40·1	41·0	41·8	42·6	43·3	43·7	44·1	44·5	44·7	44·8	45·1	45·5	46·2	46·8	47·4	48·1	48·8	49·7	50·5	51·4	52·3	53·2	54·0	54·8	55·5	56·3	56·9
South Sulawesi	40·9	41·9	43·0	44·1	45·1	46·1	46·9	47·7	48·2	48·7	49·2	49·5	49·8	50·1	50·7	51·4	52·1	52·7	53·5	54·2	55·1	55·9	56·8	57·6	58·4	59·2	59·9	60·7	61·4	62·0
Central Sulawesi	40·3	41·4	42·5	43·7	44·7	45·7	46·6	47·5	47·9	48·6	49·2	49·6	50·0	50·4	51·0	51·7	52·4	53·0	53·6	54·3	55·1	55·9	56·8	57·6	58·4	59·1	59·9	60·6	61·4	62·0
Southeast Sulawesi	37·5	38·7	39·9	41·2	42·3	43·2	44·0	44·8	45·5	46·2	46·8	47·1	47·4	47·9	48·5	49·2	50·0	50·7	51·5	52·3	53·1	54·0	54·9	55·9	56·7	57·5	58·3	59·1	59·9	60·6
North Sulawesi	47·0	47·9	48·9	50·0	51·1	52·0	52·9	53·8	54·3	54·7	55·1	55·3	55·4	55·6	56·0	56·5	57·1	57·7	58·3	59·0	59·8	60·6	61·4	62·2	62·9	63·6	64·3	65·0	65·6	66·2
West Sumatra	46·0	46·9	47·9	49·0	49·9	50·7	51·5	52·3	52·7	53·3	53·8	54·1	54·5	54·8	55·4	56·1	56·7	57·3	57·9	58·6	59·2	60·0	60·7	61·5	62·2	63·0	63·7	64·4	65·1	65·8
South Sumatra	45·6	46·8	47·9	49·0	50·0	50·8	51·6	52·2	52·7	53·2	53·7	53·9	54·1	54·4	54·9	55·5	56·1	56·6	57·3	57·9	58·6	59·4	60·1	60·9	61·7	62·4	63·1	63·8	64·5	65·1
North Sumatra	46·0	47·0	48·2	49·4	50·5	51·4	52·2	52·9	53·3	53·9	54·3	54·6	54·8	55·1	55·7	56·4	57·1	57·6	58·3	58·9	59·6	60·4	61·1	62·0	62·7	63·5	64·2	65·0	65·7	66·4
Yogyakarta	46·3	47·4	48·6	49·9	51·1	52·2	53·2	54·0	54·5	55·0	55·4	55·7	56·0	56·3	56·8	57·3	57·8	58·4	59·1	59·7	60·5	61·2	61·9	62·7	63·4	64·1	64·7	65·4	66·1	66·7
Laos	27·2	27·8	28·3	28·9	29·4	30·0	30·5	31·1	31·7	32·3	32·9	33·6	34·3	34·9	35·6	36·4	37·2	38·0	38·9	39·7	40·6	41·5	42·4	43·3	44·1	45·0	45·9	46·7	47·5	48·2
Malaysia	54·3	54·8	55·4	56·3	57·2	58·1	59·0	60·0	61·2	62·9	63·6	63·8	64·5	65·3	65·8	66·3	67·1	67·7	68·1	68·7	69·3	69·8	70·5	71·0	71·4	72·0	72·5	73·0	73·6	74·2
Maldives	32·1	33·1	34·4	35·5	36·6	38·0	39·5	40·8	42·2	43·5	44·5	45·5	46·3	47·1	47·8	48·3	48·9	49·6	50·3	51·0	51·9	52·6	53·4	54·1	54·8	55·4	56·0	56·5	57·2	57·9
Mauritius	52·7	53·2	53·9	54·6	55·1	56·0	57·0	57·6	58·2	58·7	59·1	59·7	60·2	61·0	61·6	62·3	62·8	63·3	64·0	64·6	65·2	65·8	66·5	67·3	68·0	68·6	69·0	69·4	70·0	70·6
Myanmar	30·8	31·0	31·3	31·7	32·1	32·7	33·3	33·9	34·5	35·2	36·1	37·0	38·0	39·1	40·2	41·2	42·3	43·3	44·3	45·2	46·1	46·9	47·7	48·5	49·3	50·1	50·9	51·6	52·4	53·2
Philippines	47·3	48·0	48·3	48·7	49·2	49·8	50·4	51·0	51·4	51·4	51·7	52·0	52·1	51·9	51·5	51·1	50·1	49·8	50·0	49·5	49·8	50·6	52·1	53·9	54·9	55·6	56·2	57·0	57·7	58·3
Seychelles	56·7	57·4	58·1	58·7	59·5	60·3	61·3	62·2	63·0	63·7	64·4	64·9	65·4	65·7	66·0	66·3	66·7	66·9	67·0	67·2	67·4	67·6	67·9	68·4	68·9	69·3	69·7	70·3	71·0	71·6
Sri Lanka	50·5	50·9	51·1	51·7	52·8	53·6	54·5	55·4	56·0	56·3	56·4	56·9	57·8	58·4	58·9	59·4	59·9	60·5	61·1	61·8	62·4	63·1	63·7	64·4	65·3	66·0	66·7	67·3	67·9	68·5
Thailand	51·5	52·3	53·3	54·0	54·3	55·2	56·5	57·5	58·2	58·8	59·1	59·5	60·0	60·3	60·7	61·1	61·5	62·0	62·5	63·0	63·4	64·0	64·7	65·2	65·8	66·4	66·9	67·4	68·0	68·6
Timor-Leste	26·2	26·3	26·6	27·1	27·2	27·0	26·5	26·6	28·0	28·0	27·1	28·2	30·1	31·6	33·9	36·0	38·1	40·4	42·5	43·7	44·4	45·4	46·4	47·3	47·9	48·6	48·9	49·2	49·4	49·6
Vietnam	38·5	39·2	39·9	40·8	41·8	42·8	43·7	44·5	45·5	46·4	47·3	48·1	48·9	49·8	50·6	51·4	52·1	52·9	53·7	54·4	55·1	55·8	56·5	57·2	57·9	58·6	59·3	60·1	60·8	61·5
Sub-Saharan Africa	27·8	28·2	28·8	29·4	29·7	29·9	30·2	30·6	31·1	31·5	31·7	32·1	32·7	33·2	33·8	34·6	35·3	36·0	36·8	37·6	38·3	39·1	39·8	40·5	41·2	41·8	42·6	43·3	44·0	44·5
Central sub-Saharan Africa	26·6	27·2	27·7	28·1	28·3	28·7	29·2	29·5	29·9	30·3	30·6	30·9	31·2	31·6	32·2	32·8	33·3	34·0	35·0	36·1	37·2	38·4	39·7	40·9	42·1	43·3	44·4	45·4	46·3	46·8
Angola	22·9	23·6	24·4	24·9	25·3	25·5	26·1	27·0	27·8	28·8	29·4	29·6	29·9	30·4	31·2	31·8	32·4	32·9	33·6	34·8	36·0	37·2	38·4	39·5	40·8	42·3	43·5	44·7	45·8	46·5
Central African Republic	19·7	20·3	20·5	20·8	21·2	21·6	22·0	22·2	22·4	22·6	22·9	23·3	23·6	23·8	24·1	24·1	24·3	24·7	25·1	25·4	25·9	26·6	27·4	27·5	27·6	27·8	28·1	28·3	28·7	28·9
Congo (Brazzaville)	37·3	38·2	39·1	39·8	40·5	41·2	41·9	42·5	43·0	43·4	43·8	44·3	44·6	44·8	45·3	46·1	47·0	47·8	48·6	49·5	50·5	51·6	52·7	53·7	54·6	55·6	56·4	57·2	57·8	58·3
DR Congo	25·6	26·0	26·3	26·4	26·3	26·4	26·4	26·1	25·8	25·5	25·1	24·7	24·3	24·1	24·0	24·1	24·0	24·1	24·6	25·2	26·1	27·3	28·6	30·3	31·9	33·5	34·8	36·0	37·1	37·9
Equatorial Guinea	16·3	17·2	18·3	19·4	20·6	21·9	23·6	26·3	28·7	31·0	33·5	36·3	38·9	41·3	43·7	45·9	48·0	50·0	52·1	54·0	55·7	57·4	59·0	60·5	61·9	63·2	64·3	65·3	66·1	66·5
Gabon	40·4	41·5	42·6	43·9	45·0	46·1	47·1	48·0	48·9	49·8	50·7	51·5	52·1	52·7	53·4	54·1	54·8	55·5	56·1	56·7	57·5	58·5	59·6	60·6	61·6	62·6	63·5	64·4	65·2	65·9
Eastern sub-Saharan Africa	23·0	23·2	23·8	24·2	24·5	24·7	24·9	25·3	26·1	26·6	26·7	27·0	27·6	28·2	28·8	29·6	30·3	31·0	31·9	32·8	33·6	34·4	35·0	35·8	36·5	37·1	37·9	38·7	39·5	40·2
Burundi	20·8	20·9	21·2	21·3	21·4	21·4	21·4	21·5	21·6	21·5	21·3	21·2	21·3	21·6	21·9	22·2	22·5	22·9	23·4	24·0	24·7	25·2	25·8	26·4	27·0	27·4	27·8	28·1	28·4	28·7
Comoros	28·9	29·3	30·1	31·2	31·8	32·5	32·6	32·5	33·1	33·4	33·9	34·6	35·2	35·8	36·3	37·0	37·9	38·5	38·9	39·4	39·9	40·5	41·2	41·7	42·3	42·7	43·4	44·1	44·7	45·2
Djibouti	28·0	28·5	28·8	28·9	29·2	29·7	30·2	30·9	31·3	31·4	31·4	31·8	33·1	34·6	36·0	37·6	38·4	38·5	39·1	39·7	40·4	41·0	41·6	42·3	43·0	43·8	44·5	45·3	46·1	46·9
Eritrea	20·2	20·7	21·4	22·3	23·4	24·4	25·4	26·4	27·4	28·3	29·0	29·7	30·3	30·9	31·4	31·8	32·3	32·7	33·1	33·5	33·8	34·3	34·9	35·5	36·2	36·8	37·5	38·2	38·9	39·5
Ethiopia	13·1	13·1	13·9	14·4	14·4	14·6	14·5	14·6	15·6	16·2	15·8	15·8	16·4	16·7	17·4	18·4	19·4	20·5	21·9	23·3	24·6	25·7	26·7	27·9	29·0	29·8	30·8	31·9	32·9	33·8
Kenya	33·7	34·5	35·2	35·8	36·4	37·0	37·5	37·9	38·3	38·7	39·1	39·4	39·7	40·1	40·4	40·8	41·3	41·9	42·4	42·9	43·6	44·2	44·9	45·5	46·2	47·0	47·7	48·5	49·2	49·8
Baringo	24·2	25·5	26·7	27·7	28·6	29·5	30·3	31·0	31·7	32·5	33·1	33·7	34·3	34·9	35·4	36·0	36·6	37·3	37·9	38·6	39·3	40·1	40·9	41·7	42·6	43·6	44·5	45·5	46·4	47·2
Bomet	26·5	27·4	28·1	28·9	29·5	30·0	30·5	31·0	31·5	32·1	32·7	33·4	34·2	35·1	36·0	36·9	37·9	38·9	39·9	40·9	41·9	42·9	44·0	45·0	46·1	47·2	48·2	49·3	50·3	51·2
Bungoma	29·3	30·1	30·9	31·5	32·0	32·4	32·7	32·9	33·2	33·5	33·7	34·0	34·2	34·5	34·9	35·3	35·9	36·6	37·3	38·0	38·9	39·8	40·7	41·6	42·6	43·7	44·7	45·7	46·7	47·5
Busia	25·4	26·1	26·9	27·6	28·2	28·8	29·3	29·8	30·4	30·9	31·5	32·0	32·5	33·0	33·5	34·1	34·7	35·3	35·9	36·6	37·3	38·0	38·8	39·6	40·6	41·5	42·5	43·5	44·6	45·4
Elgeyo Marakwet	27·1	28·1	29·0	29·8	30·5	31·2	31·8	32·4	32·9	33·5	34·1	34·7	35·3	35·9	36·6	37·3	38·1	38·9	39·7	40·6	41·5	42·4	43·4	44·3	45·4	46·4	47·4	48·4	49·3	50·1
Embu	37·7	38·5	39·3	39·9	40·6	41·1	41·6	42·1	42·5	42·8	43·2	43·4	43·7	44·0	44·3	44·6	45·1	45·6	46·2	46·8	47·4	48·1	48·8	49·6	50·4	51·2	52·0	52·8	53·6	54·3
Garissa	15·2	15·5	15·9	16·4	16·8	17·2	17·7	18·1	18·5	19·0	19·4	19·7	20·1	20·5	20·9	21·4	21·9	22·5	23·0	23·6	24·3	25·0	25·7	26·4	27·1	27·8	28·4	29·1	29·8	30·3
Homa Bay	17·5	18·7	19·8	20·9	21·9	22·8	23·6	24·4	25·1	25·8	26·4	27·0	27·7	28·3	28·9	29·5	30·2	31·0	31·8	32·6	33·6	34·8	36·0	37·2	38·5	39·7	40·9	42·2	43·4	44·3
Isiolo	25·6	26·2	26·8	27·3	27·9	28·4	28·9	29·4	29·8	30·2	30·6	30·9	31·2	31·5	31·8	32·1	32·4	32·8	33·1	33·6	34·1	34·7	35·3	36·1	36·9	37·7	38·6	39·5	40·4	41·2

Kajiado	37·1	37·9	38·7	39·4	40·1	40·8	41·4	42·0	42·5	43·1	43·5	44·0	44·3	44·7	45·1	45·5	46·0	46·5	47·1	47·6	48·3	49·0	49·7	50·5	51·4	52·2	53·1	54·0	54·9	55·7
Kakamega	29·5	30·2	30·9	31·5	32·1	32·6	33·1	33·6	34·1	34·6	35·0	35·4	35·8	36·2	36·7	37·2	37·8	38·5	39·1	39·9	40·6	41·5	42·3	43·3	44·3	45·3	46·3	47·3	48·3	49·1
Kericho	25·8	27·0	28·0	28·9	29·7	30·6	31·4	32·1	32·8	33·5	34·1	34·6	35·2	35·7	36·3	36·9	37·7	38·5	39·3	40·2	41·1	42·0	43·0	44·0	45·0	46·1	47·2	48·3	49·3	50·2
Kiambu	42·7	43·6	44·5	45·2	45·8	46·4	46·9	47·4	47·8	48·2	48·6	49·0	49·3	49·7	50·1	50·6	51·2	51·8	52·5	53·1	53·8	54·5	55·2	55·9	56·6	57·3	58·0	58·7	59·4	60·0
Kilifi	29·6	30·2	30·7	31·2	31·6	32·1	32·5	33·0	33·4	33·7	34·0	34·3	34·6	35·0	35·3	35·8	36·3	37·0	37·7	38·4	39·2	40·1	41·0	42·0	43·0	44·0	45·0	46·1	47·1	47·8
Kirinyaga	38·5	39·3	40·0	40·6	41·1	41·5	41·9	42·3	42·6	43·0	43·3	43·6	43·9	44·2	44·6	45·0	45·4	46·0	46·6	47·2	47·8	48·5	49·2	49·9	50·6	51·4	52·1	52·8	53·6	54·2
Kisii	34·1	35·1	35·9	36·6	37·2	37·7	38·1	38·6	38·9	39·3	39·5	39·8	40·2	40·5	40·9	41·3	41·8	42·5	43·1	43·8	44·7	45·6	46·6	47·6	48·6	49·6	50·6	51·5	52·4	53·2
Kisumu	35·4	36·2	36·9	37·5	38·0	38·4	38·8	39·1	39·4	39·6	39·9	40·2	40·5	40·8	41·2	41·6	42·2	42·9	43·6	44·4	45·3	46·3	47·4	48·4	49·4	50·4	51·5	52·5	53·5	54·2
Kitui	27·8	28·6	29·3	29·9	30·5	31·0	31·5	32·0	32·4	32·8	33·2	33·6	34·1	34·6	35·1	35·7	36·4	37·1	37·8	38·5	39·3	40·1	41·0	42·0	42·9	43·8	44·8	45·7	46·6	47·3
Kwale	26·0	26·6	27·2	27·7	28·2	28·7	29·1	29·5	29·8	30·1	30·3	30·5	30·7	31·0	31·2	31·5	31·9	32·5	33·1	33·7	34·5	35·3	36·1	37·0	38·0	39·1	40·1	41·2	42·2	42·9
Laikipia	37·4	38·0	38·5	38·9	39·2	39·5	39·8	40·0	40·4	40·7	41·1	41·6	42·1	42·8	43·5	44·3	45·2	46·1	47·0	47·8	48·7	49·5	50·3	51·2	52·1	53·0	53·9	54·8	55·7	56·5
Lamu	25·6	26·4	27·3	28·1	28·9	29·6	30·3	30·9	31·4	32·0	32·4	32·8	33·3	33·8	34·3	34·8	35·4	36·1	36·9	37·6	38·5	39·3	40·2	41·1	42·0	42·9	43·9	44·8	45·7	46·5
Machakos	33·7	34·6	35·5	36·3	37·0	37·6	38·3	38·9	39·5	40·1	40·6	41·1	41·6	42·1	42·7	43·3	43·9	44·7	45·4	46·1	46·8	47·6	48·4	49·2	50·0	50·9	51·7	52·6	53·4	54·1
Makueni	28·3	28·8	29·4	29·9	30·4	30·9	31·4	31·9	32·4	32·8	33·3	33·7	34·1	34·6	35·1	35·6	36·1	36·7	37·4	38·0	38·8	39·6	40·3	41·2	42·0	42·9	43·7	44·6	45·5	46·2
Mandera	10·6	11·0	11·5	12·1	12·6	13·2	13·8	14·4	14·9	15·4	15·8	16·2	16·7	17·1	17·5	17·8	18·3	18·9	19·4	20·0	20·6	21·3	22·0	22·7	23·4	24·1	24·7	25·4	26·1	26·6
Marsabit	20·5	21·0	21·5	22·0	22·5	22·9	23·4	23·9	24·4	24·8	25·2	25·6	26·0	26·4	26·8	27·3	27·8	28·3	28·9	29·6	30·4	31·2	32·0	32·8	33·7	34·6	35·5	36·4	37·3	38·0
Meru	33·3	34·1	34·9	35·6	36·3	37·0	37·6	38·1	38·6	39·1	39·6	40·0	40·4	40·8	41·2	41·6	42·1	42·7	43·2	43·8	44·5	45·2	46·0	46·8	47·6	48·4	49·2	50·1	50·9	51·6
Migori	18·7	19·8	20·8	21·8	22·6	23·3	23·9	24·5	25·0	25·3	25·6	25·9	26·3	26·8	27·3	27·7	28·4	29·1	29·9	30·7	31·8	33·0	34·3	35·4	36·6	37·9	39·1	40·4	41·6	42·5
Mombasa	42·4	43·0	43·5	43·9	44·3	44·7	45·1	45·4	45·7	46·0	46·2	46·3	46·5	46·6	46·8	47·1	47·4	47·9	48·5	49·1	49·8	50·5	51·3	52·1	52·9	53·8	54·6	55·5	56·3	57·0
Murang'a	37·6	38·4	39·1	39·7	40·2	40·7	41·2	41·6	42·0	42·5	43·0	43·4	43·9	44·4	44·9	45·5	46·1	46·8	47·4	48·0	48·6	49·3	49·9	50·6	51·3	52·0	52·7	53·4	54·2	54·8
Nairobi	54·3	54·9	55·4	55·7	56·1	56·4	56·6	56·9	57·1	57·3	57·5	57·6	57·8	58·0	58·3	58·6	59·0	59·5	60·1	60·6	61·2	61·7	62·2	62·7	63·2	63·7	64·3	64·9	65·5	66·0
Nakuru	35·5	36·4	37·1	37·7	38·3	38·7	39·2	39·5	39·8	40·1	40·3	40·5	40·7	40·9	41·2	41·5	42·1	42·7	43·4	44·1	44·8	45·7	46·6	47·5	48·5	49·4	50·4	51·4	52·4	53·2
Nandi	30·8	31·7	32·5	33·1	33·8	34·4	35·0	35·6	36·3	37·0	37·7	38·2	38·8	39·3	39·8	40·4	41·0	41·6	42·1	42·7	43·4	44·1	44·9	45·8	46·8	47·8	48·8	49·9	50·9	51·8
Narok	21·9	22·5	23·0	23·4	23·8	24·1	24·3	24·6	25·1	25·5	25·9	26·3	26·8	27·4	28·0	28·6	29·3	30·1	30·8	31·6	32·4	33·3	34·2	35·2	36·3	37·4	38·6	39·8	40·9	41·8
Nyamira	36·6	37·5	38·3	39·1	39·8	40·4	41·0	41·5	42·0	42·5	42·9	43·2	43·6	44·0	44·3	44·8	45·2	45·8	46·4	47·1	47·9	48·8	49·8	50·8	51·8	52·8	53·9	54·9	55·9	56·7
Nyandarua	33·8	34·8	35·6	36·3	37·0	37·6	38·2	38·8	39·3	39·9	40·5	41·0	41·6	42·2	42·9	43·6	44·4	45·2	46·0	46·7	47·5	48·3	49·0	49·8	50·5	51·3	52·1	52·9	53·7	54·4
Nyeri	40·1	40·9	41·5	42·2	42·8	43·3	43·8	44·2	44·7	45·1	45·4	45·8	46·1	46·4	46·8	47·2	47·8	48·4	49·0	49·7	50·4	51·1	51·8	52·5	53·2	53·9	54·7	55·4	56·1	56·8
Samburu	17·0	17·5	17·8	18·1	18·4	18·6	18·8	19·0	19·4	19·8	20·3	20·7	21·2	21·8	22·5	23·1	23·9	24·7	25·5	26·2	27·0	27·8	28·7	29·6	30·6	31·5	32·5	33·6	34·5	35·3
Siaya	24·2	24·8	25·5	26·1	26·7	27·3	27·8	28·3	28·7	29·0	29·2	29·5	29·7	30·0	30·3	30·6	31·1	31·7	32·3	33·0	33·9	34·9	36·0	37·1	38·2	39·4	40·6	41·8	42·9	43·7
Taita Taveta	35·1	35·9	36·7	37·5	38·1	38·8	39·4	39·9	40·4	40·9	41·3	41·6	42·0	42·3	42·6	43·0	43·4	44·0	44·6	45·2	46·0	46·7	47·5	48·3	49·1	49·9	50·7	51·6	52·4	53·1
Tana River	22·9	23·3	23·7	24·0	24·3	24·6	24·8	25·0	25·2	25·3	25·4	25·5	25·7	25·9	26·1	26·3	26·6	27·0	27·5	28·1	28·8	29·5	30·3	31·2	32·2	33·2	34·3	35·3	36·3	37·1
Tharaka Nithi	33·0	33·6	34·3	34·9	35·5	36·1	36·7	37·2	37·8	38·5	39·0	39·7	40·4	41·1	41·8	42·6	43·4	44·2	45·0	45·8	46·7	47·5	48·3	49·2	50·1	51·0	52·0	52·9	53·9	54·6
Trans Nzoia	30·7	31·8	32·7	33·5	34·2	34·8	35·4	35·9	36·4	37·0	37·5	38·0	38·6	39·2	39·7	40·3	41·0	41·7	42·3	43·0	43·8	44·6	45·4	46·3	47·2	48·2	49·3	50·3	51·3	52·1
Turkana	18·5	19·2	19·8	20·5	21·2	21·8	22·4	23·0	23·6	24·1	24·6	25·0	25·4	25·8	26·1	26·4	26·7	27·1	27·5	27·9	28·4	28·9	29·5	30·2	31·0	31·8	32·7	33·6	34·4	35·2
Uasin Gishu	36·9	37·9	38·8	39·5	40·2	40·8	41·3	41·8	42·3	42·8	43·2	43·7	44·1	44·6	45·1	45·7	46·3	46·9	47·5	48·2	49·0	49·8	50·6	51·5	52·4	53·3	54·2	55·2	56·1	56·9
Vihiga	27·2	28·2	29·0	29·9	30·8	31·5	32·2	32·8	33·5	34·0	34·5	34·9	35·2	35·4	35·7	36·0	36·3	36·7	37·1	37·5	38·0	38·6	39·2	39·9	40·6	41·4	42·3	43·2	44·1	44·8
Wajir	11·9	12·1	12·5	12·9	13·4	13·8	14·3	14·7	15·2	15·7	16·1	16·5	16·9	17·2	17·5	17·8	18·1	18·5	18·9	19·3	19·8	20·3	20·9	21·5	22·1	22·8	23·4	24·1	24·7	25·3
West Pokot	19·6	20·3	21·0	21·6	22·2	22·7	23·1	23·5	23·9	24·3	24·8	25·2	25·7	26·3	27·0	27·8	28·6	29·5	30·3	31·2	32·2	33·1	34·2	35·2	36·4	37·5	38·7	39·9	41·1	42·0
Madagascar	26·7	27·0	27·2	27·2	27·3	27·2	27·3	27·7	28·2	28·5	28·8	29·8	30·7	31·3	32·2	33·1	33·2	32·8	33·0	33·3	33·6	33·9	34·4	35·2	36·1	36·9	37·8	38·6	39·4	40·1
Malawi	19·7	20·1	20·3	20·4	20·6	20·8	21·1	21·5	22·0	22·5	22·9	23·2	23·7	24·2	24·8	25·4	26·1	27·0	28·0	29·0	30·0	30·9	31·7	32·4	33·2	33·9	34·6	35·3	35·9	36·4
Mozambique	12·1	12·2	12·4	12·7	13·2	13·4	13·9	14·6	15·4	16·1	16·7	17·2	17·9	18·6	19·3	20·1	20·8	21·6	22·5	23·4	24·3	25·2	25·9	26·7	27·5	28·3	29·2	30·1	30·9	31·4
Rwanda	25·9	26·1	26·1	26·3	25·9	25·9	25·9	26·1	26·5	27·0	27·5	28·0	28·9	29·8	30·8	31·6	32·4	33·3	34·2	34·9	35·6	36·4	37·1	37·9	38·6	39·4	40·2	41·0	41·7	42·5
Somalia	4·96	5·02	5·08	5·14	5·22	5·31	5·41	5·52	5·64	5·74	5·93	6·15	6·29	6·42	6·55	6·69	6·82	6·95	7·09	7·22	7·36	7·48	7·60	7·72	7·85	7·98	8·11	8·25	8·39	8·49
South Sudan	25·4	25·6	25·8	26·0	26·3	26·5	26·8	26·9	27·1	27·4	27·8	28·1	28·5	28·9	29·3	29·8	30·3	30·9	31·4	31·9	32·5	33·1	33·7	34·2	34·7	35·2	35·7	36·4	37·1	37·6
Uganda	16·7	16·6	16·5	16·4	16·5	16·8	17·3	18·1	19·1	20·1	21·1	22·3	23·6	24·9	25·9	27·1	28·3	29·2	30·2	31·1	32·1	33·1	33·9	34·8	35·7	36·6	37·5	38·4	39·3	40·0
Tanzania	25·7	26·0	26·5	27·2	27·5	27·7	28·0	28·5	29·2	29·6	29·7	30·1	30·8	31·6	32·1	32·5	33·0	33·7	34·5	35·2	36·0	36·7	37·3	37·8	38·1	38·3	39·1	40·3	41·2	41·9
Zambia	29·7	29·9	30·1	30·2	30·6	31·0	31·3	31·7	32·1	32·4	32·8	33·3	33·7	34·2	34·7	35·2	36·1	37·2	38·3	39·5	40·8	42·0	43·1	44·2	45·3	46·3	47·3	48·3	49·2	49·8
Southern sub-Saharan Africa	50·6	51·1	51·6	52·1	52·4	52·6	53·0	53·4	53·6	54·1	54·7	55·1	55·4	55·8	56·2	56·6	57·0	57·6	58·4	59·0	59·4	60·0	60·6	61·0	61·4	61·8	62·3	62·7	63·1	63·5
Botswana	43·1	44·1	45·0	45·9	46·8	47·5	48·3	49·1	49·9	50·7	51·6	52·4	53·2	54·0	54·8	55·6	56·6	57·6	58·4	59·0	59·7	60·4	61·0	61·4	61·8	61·9	61·9	62·3	62·9	63·3
eSwatini	40·0	40·9	41·8	42·5	43·3	44·0	44·7	45·3	45·8	46·5	47·2	47·9	48·6	49·1	49·7	50·4	51·1	51·6	52·0	52·5	53·1	53·6	54·1	54·7	55·3	55·8	56·3	56·9	57·4	57·7
Lesotho	31·8	32·4	33·2	34·0	34·8	35·6	36·4	37·2	38·0	38·7	39·3	39·9	40·4	40·9	41·4	41·9	42·2	42·7	43·2	43·8	44·3	44·9	45·6	46·2	46·8	47·4	48·0	48·6	49·3	49·8
Namibia	44·3	44·7	45·2	45·9	46·6	47·2	47·8	48·4	48·9	49·6	50·1	50·6	51·1	51·4	51·9	52·4	52·9	53·5	54·1	54·7	55·2	55·9	56·8	57·5	58·3	59·0	59·7	60·2	60·8	61·3
South Africa	54·3	54·6	55·1	55·6	55·8	55·8	56·1	56·4	56·6	57·0	57·7	58·1	58·2	58·6	59·0	59·4	59·9	60·7	61·6	62·3	62·8	63·5	64·1	64·5	64·9	65·3	65·7	66·1	66·5	66·8
Zimbabwe	39·7	40·4	41·1	41·6	42·2	42·7	43·4	44·0	44·4	44·6	44·8	45·1	45·2	45·1	44·8	44·4	43·7	43·1	42·4	41·9	41·7	41·8	42·3	43·0	43·8	44·5	45·2	45·9	46·6	47·1
Western sub-Saharan Africa	24·3	24·9	25·6	26·3	26·7	26·8	27·1	27·6	28·0	28·3	28·4	28·9	29·6	30·2	31·0	32·0	32·9	33·8	34·6	35·3	36·0	36·9	37·7	38·3	39·0	39·8	40·6	41·4	42·1	42·6
Benin	20·7	21·2	21·7	22·4	23·1	23·8	24·4	24·9	25·3	25·8	26·3	26·7	26·9	27·3	27·8	28·4	29·0	29·6	30·1	30·8	31·4	32·0	32·5	33·2	33·8	34·5	35·1	35·8	36·5	37·1
Burkina Faso	12·6	13·1	13·3	13·6	13·9	14·2	14·7	15·2	15·6	16·0	16·6	17·1	17·5	17·9	18·5	19·2	19·8	20·3	20·8	21·4	21·9	22·3	22·6	23·1	23·7	24·4	24·9	25·4	25·9	26·4
Cape Verde	29·4	30·2	30·8	31·2	31·9	32·7	33·8	34·8	35·8	36·7	37·7	38·7	39·7	40·7	41·6	42·5	43·3	44·1	45·0	45·8	46·5	47·2	47·9	48·6	49·2	49·9	50·5	51·1	51·9	52·6
Cameroon	31·3	32·2	33·2	34·1	34·7	34·9	35·1	35·5	35·9	35·8	35·8	36·0	36·2	36·8	37·4	37·9	38·7	39·4	40·2	41·0	41·9	42·6	43·3	44·1	44·9	45·8	46·7	47·6	48·4	49·0
Chad	10·3	10·9	11·4	11·7	11·9	12·3	12·7	13·0	13·3	13·5	13·6	13·8	14·0	14·3	14·8	15·4	15·9	16·5	17·1	17·7	18·5	19·2	20·0	20·7	21·5	22·3	23·0	23·7	24·4	24·8
Côte d'Ivoire	25·6	26·6	27·7	28·6	29·1	29·6	30·0	30·5	31·1	31·6	31·8	32·2	32·6	32·9	33·2	33·4	33·8	34·0	34·4	34·8	35·2	35·7	36·3	37·0	37·7	38·6	39·5	40·3	41·1	41·9
The Gambia	19·8	20·4	21·0	21·7	22·3	22·9	23·5	24·2	24·9	25·7	26·5	27·2	27·9	28·5	29·3	30·1	30·8	31·3	31·9	32·6	33·3	33·9	34·7	35·4	36·0	36·6	37·3	38·0	38·7	39·3
Ghana	34·9	35·8	37·1	38·7	38·9	38·5	39·1	39·9	40·8	41·1	41·3	42·1	42·6	43·0	43·6	44·6	45·3	46·3	47·1	47·6	48·5	49·9	50·4	50·7	51·3	51·8	53·0	54·2	55·2	55·9
Guinea	16·7	17·1	17·7	18·3	18·8	19·3	19·9	20·6	21·2	21·6	21·9	22·3	22·9	23·4	23·9	24·7	25·2	25·6	26·0	26·5	26·9	27·1	27·4	27·7	28·1	28·6	29·2	30·0	30·8	31·5
Guinea-Bissau	17·4	18·0	18·5	19·1	19·8	20·4	21·1	21·8	22·4	23·0	23·5	24·0	24·5	25·0	25·5	26·1	26·6	27·1	27·6	28·1	28·7	29·4	30·0	30·5	31·1	31·6	32·3	32·9	33·6	34·2
Liberia	22·8	23·0	22·9	22·4	21·5	20·4	19·0	18·2	18·0	18·7	20·5	22·3	24·0	24·7	25·4	26·1	26·9	27·7	28·5	29·1	29·9	30·7	31·5	32·5	33·4	34·2	34·9	35·6	36·2	36·6
Mali	11·7	12·0	12·4	13·0	13·4	13·8	14·4	15·1	15·6	16·0	16·3	16·8	17·2	17·6	18·1	18·7	19·3	19·8	20·2	20·7	21·4	21·9	22·4	23·0	23·5	24·0	24·6	25·2	25·8	26·3
Mauritania	31·1	31·8	32·5	33·5	34·4	35·1	35·6	36·0	36·5	36·8	37·0	37·1	37·2	37·5	38·0	38·6	39·4	40·2	40·9	41·5	42·3	43·0	43·7	44·4	45·2	46·0	46·7	47·5	48·2	48·8
Niger	7·96	8·24	8·59	9·11	9·59	9·84	10·1	10·5	11·0	11·3	11·4	11·5	11·7	12·1	12·3	12·5	12·6	12·6	12·8	12·9	13·2	13·6	14·0	14·4	14·8	15·3	15·7	16·2	16·7	17·0
Nigeria	24·9	25·5	26·1	26·7	27·1	26·9	26·9	27·3	27·5	27·6	27·6	28·0	29·1	30·0	31·2	32·7	34·0	35·1	36·2	36·9	37·8	38·9	40·0	40·9	41·7	42·7	43·7	44·6	45·5	46·1
São Tomé and PrÍncipe	29·8	30·1	30·5	31·1	31·5	31·7	32·2	32·7	33·3	34·0	34·5	35·0	35·8	36·6	37·5	38·5	39·4	40·3	41·0	41·7	42·6	43·4	44·2	45·1	45·9	46·8	47·6	48·4	49·2	49·9
Senegal	22·7	23·3	24·0	24·6	25·2	25·6	25·9	26·3	26·7	27·2	27·6	27·9	28·3	28·7	29·1	29·5	29·9	30·5	31·1	31·6	32·2	32·8	33·4	34·1	34·6	35·2	35·8	36·5	37·2	37·9
Sierra Leone	19·5	19·8	20·1	20·5	20·9	21·1	21·5	21·6	21·6	21·5	21·6	21·8	22·3	22·8	23·4	23·8	24·1	24·7	25·4	26·0	26·7	27·3	28·1	29·2	30·3	31·0	31·7	32·4	33·1	33·7
Togo	26·9	27·6	28·4	28·9	29·4	30·0	30·6	31·2	31·5	31·8	32·1	32·3	32·6	32·8	33·1	33·2	33·6	34·0	34·5	35·0	35·6	36·2	36·8	37·6	38·3	39·1	39·8	40·6	41·4	42·1

Table S6. Mediation factor matrix

For IHD, stroke, and diabetes we pooled all available cohorts and estimated relative risks with and without adjustment across all combinations of metabolic risk factors. We then computed the excess attenuated risk for each mediation-risk-cause set.

Risk Factor	Mediator	Cause	Mediation Factor
Lead exposure	High systolic blood pressure	Rheumatic heart disease	1<br>(1 to 1)
Lead exposure	High systolic blood pressure	Ischemic heart disease	1<br>(1 to 1)
Lead exposure	High systolic blood pressure	Ischemic stroke	1<br>(1 to 1)
Lead exposure	High systolic blood pressure	Intracerebral hemorrhage	1<br>(1 to 1)
Lead exposure	High systolic blood pressure	Subarachnoid hemorrhage	1<br>(1 to 1)
Lead exposure	High systolic blood pressure	Hypertensive heart disease	1<br>(1 to 1)
Lead exposure	High systolic blood pressure	Other cardiomyopathy	1<br>(1 to 1)
Lead exposure	High systolic blood pressure	Atrial fibrillation and flutter	1<br>(1 to 1)
Lead exposure	High systolic blood pressure	Aortic aneurysm	1<br>(1 to 1)
Lead exposure	High systolic blood pressure	Peripheral artery disease	1<br>(1 to 1)
Lead exposure	High systolic blood pressure	Chronic kidney disease due to diabetes mellitus type 1	1<br>(1 to 1)
Lead exposure	High systolic blood pressure	Chronic kidney disease due to diabetes mellitus type 2	1<br>(1 to 1)
Lead exposure	High systolic blood pressure	Chronic kidney disease due to hypertension	1<br>(1 to 1)
Lead exposure	High systolic blood pressure	Chronic kidney disease due to glomerulonephritis	1<br>(1 to 1)
Lead exposure	High systolic blood pressure	Chronic kidney disease due to other and unspecified causes	1<br>(1 to 1)
Lead exposure	Kidney dysfunction	Chronic kidney disease due to diabetes mellitus type 1	1<br>(1 to 1)
Lead exposure	Kidney dysfunction	Chronic kidney disease due to diabetes mellitus type 2	1<br>(1 to 1)
Lead exposure	Kidney dysfunction	Chronic kidney disease due to hypertension	1<br>(1 to 1)
Lead exposure	Kidney dysfunction	Chronic kidney disease due to glomerulonephritis	1<br>(1 to 1)
Lead exposure	Kidney dysfunction	Chronic kidney disease due to other and unspecified causes	1<br>(1 to 1)
Smoking	High fasting plasma glucose	Diabetes mellitus type 2	1<br>(1 to 1)
Smoking	Low bone mineral density	Pedestrian road injuries	1<br>(1 to 1)
Smoking	Low bone mineral density	Cyclist road injuries	1<br>(1 to 1)
Smoking	Low bone mineral density	Motor vehicle road injuries	1<br>(1 to 1)
Smoking	Low bone mineral density	Other road injuries	1<br>(1 to 1)
Smoking	Low bone mineral density	Other transport injuries	1<br>(1 to 1)
Smoking	Low bone mineral density	Falls	1<br>(1 to 1)
Smoking	Low bone mineral density	Other exposure to mechanical forces	1<br>(1 to 1)
Smoking	Low bone mineral density	Non-venomous animal contact	1<br>(1 to 1)
Smoking	Low bone mineral density	Physical violence by other means	1<br>(1 to 1)
Diet low in fruits	High fasting plasma glucose	Ischemic stroke	0.05<br>(0.04 to 0.06)
Diet low in fruits	High fasting plasma glucose	Diabetes mellitus type 2	1<br>(1 to 1)
Diet low in fruits	High LDL cholesterol	Ischemic heart disease	0.06<br>(0.05 to 0.08)
Diet low in fruits	High LDL cholesterol	Ischemic stroke	0.05<br>(0.04 to 0.06)

Diet low in fruits	High systolic blood pressure	Ischemic heart disease	0.06<br>(0.05 to 0.08)
Diet low in fruits	High systolic blood pressure	Ischemic stroke	0.05<br>(0.04 to 0.06)
Diet low in vegetables	High fasting plasma glucose	Ischemic stroke	0.08<br>(0.04 to 0.16)
Diet low in vegetables	High fasting plasma glucose	Intracerebral hemorrhage	0.08<br>(0.04 to 0.16)
Diet low in vegetables	High fasting plasma glucose	Subarachnoid hemorrhage	0.08<br>(0.04 to 0.16)
Diet low in vegetables	High LDL cholesterol	Ischemic stroke	0.09<br>(0.04 to 0.16)
Diet low in vegetables	High systolic blood pressure	Ischemic stroke	0.03<br>(0.02 to 0.04)
Diet low in vegetables	High systolic blood pressure	Intracerebral hemorrhage	0.04<br>(0.02 to 0.05)
Diet low in vegetables	High systolic blood pressure	Subarachnoid hemorrhage	0.04<br>(0.02 to 0.05)
Diet low in whole grains	High fasting plasma glucose	Diabetes mellitus type 2	1<br>(1 to 1)
Diet low in whole grains	High LDL cholesterol	Ischemic heart disease	0.39<br>(0.17 to 0.54)
Diet low in whole grains	High LDL cholesterol	Ischemic stroke	0.16<br>(0.05 to 0.37)
Diet low in nuts and seeds	High fasting plasma glucose	Ischemic heart disease	0.03<br>(0.02 to 0.06)
Diet low in nuts and seeds	High LDL cholesterol	Ischemic heart disease	0.2<br>(0.01 to 0.76)
Diet low in nuts and seeds	High systolic blood pressure	Ischemic heart disease	0.34<br>(0.24 to 0.47)
Diet low in milk	Diet low in calcium	Colon and rectum cancer	1<br>(1 to 1)
Diet high in red meat	High fasting plasma glucose	Diabetes mellitus type 2	1<br>(1 to 1)
Diet high in processed meat	High fasting plasma glucose	Ischemic heart disease	0.01<br>(0.01 to 0.02)
Diet high in processed meat	High fasting plasma glucose	Diabetes mellitus type 2	1<br>(1 to 1)
Diet high in sugar-sweetened beverages	High fasting plasma glucose	Diabetes mellitus type 2	1<br>(1 to 1)
Diet high in sugar-sweetened beverages	High body-mass index in adults	Diabetes mellitus type 2	1<br>(1 to 1)
Diet low in fiber	Diet low in fruits	Ischemic heart disease	1<br>(1 to 1)
Diet low in fiber	Diet low in whole grains	Ischemic heart disease	1<br>(1 to 1)
Diet low in seafood omega-3 fatty acids	High systolic blood pressure	Ischemic heart disease	0.01<br>(0 to 0.02)
Diet low in polyunsaturated fatty acids	High fasting plasma glucose	Ischemic heart disease	0.57<br>(0.39 to 0.77)
Diet low in polyunsaturated fatty acids	High systolic blood pressure	Ischemic heart disease	0.72<br>(0.57 to 0.89)
Diet high in trans fatty acids	High LDL cholesterol	Ischemic heart disease	0.15<br>(0.02 to 0.24)
Diet high in trans fatty acids	High systolic blood pressure	Ischemic heart disease	0.15<br>(0.02 to 0.24)
Diet high in sodium	High systolic blood pressure	Rheumatic heart disease	1<br>(1 to 1)
Diet high in sodium	High systolic blood pressure	Ischemic heart disease	1<br>(1 to 1)
Diet high in sodium	High systolic blood pressure	Ischemic stroke	1<br>(1 to 1)
Diet high in sodium	High systolic blood pressure	Intracerebral hemorrhage	1<br>(1 to 1)
Diet high in sodium	High systolic blood pressure	Subarachnoid hemorrhage	1<br>(1 to 1)
Diet high in sodium	High systolic blood pressure	Hypertensive heart disease	1<br>(1 to 1)
Diet high in sodium	High systolic blood pressure	Other cardiomyopathy	1<br>(1 to 1)
Diet high in sodium	High systolic blood pressure	Atrial fibrillation and flutter	1<br>(1 to 1)

Diet high in sodium	High systolic blood pressure	Aortic aneurysm	1<br>(1 to 1)
Diet high in sodium	High systolic blood pressure	Peripheral artery disease	1<br>(1 to 1)
Diet high in sodium	High systolic blood pressure	Chronic kidney disease due to diabetes mellitus type 1	1<br>(1 to 1)
Diet high in sodium	High systolic blood pressure	Chronic kidney disease due to diabetes mellitus type 2	1<br>(1 to 1)
Diet high in sodium	High systolic blood pressure	Chronic kidney disease due to hypertension	1<br>(1 to 1)
Diet high in sodium	High systolic blood pressure	Chronic kidney disease due to glomerulonephritis	1<br>(1 to 1)
Diet high in sodium	High systolic blood pressure	Chronic kidney disease due to other and unspecified causes	1<br>(1 to 1)
Diet high in sodium	Kidney dysfunction	Chronic kidney disease due to diabetes mellitus type 1	1<br>(1 to 1)
Diet high in sodium	Kidney dysfunction	Chronic kidney disease due to diabetes mellitus type 2	1<br>(1 to 1)
Diet high in sodium	Kidney dysfunction	Chronic kidney disease due to hypertension	1<br>(1 to 1)
Diet high in sodium	Kidney dysfunction	Chronic kidney disease due to glomerulonephritis	1<br>(1 to 1)
Diet high in sodium	Kidney dysfunction	Chronic kidney disease due to other and unspecified causes	1<br>(1 to 1)
Childhood sexual abuse against females	Alcohol use	Alcohol use disorders	1<br>(1 to 1)
Childhood sexual abuse against males	Alcohol use	Alcohol use disorders	1<br>(1 to 1)
Low physical activity	High fasting plasma glucose	Ischemic heart disease	0.14<br>(0.11 to 0.18)
Low physical activity	High fasting plasma glucose	Ischemic stroke	0.08<br>(0.03 to 0.14)
Low physical activity	High fasting plasma glucose	Diabetes mellitus type 2	1<br>(1 to 1)
High fasting plasma glucose	High LDL cholesterol	Ischemic heart disease	0.04<br>(0.02 to 0.05)
High fasting plasma glucose	High LDL cholesterol	Ischemic stroke	0.04<br>(0.03 to 0.06)
High fasting plasma glucose	High systolic blood pressure	Ischemic heart disease	0.1<br>(0.08 to 0.11)
High fasting plasma glucose	High systolic blood pressure	Ischemic stroke	0.15<br>(0.14 to 0.17)
High fasting plasma glucose	High systolic blood pressure	Intracerebral hemorrhage	0.15<br>(0.14 to 0.17)
High fasting plasma glucose	High systolic blood pressure	Subarachnoid hemorrhage	0.15<br>(0.14 to 0.17)
High fasting plasma glucose	Kidney dysfunction	Chronic kidney disease due to diabetes mellitus type 1	1<br>(1 to 1)
High fasting plasma glucose	Kidney dysfunction	Chronic kidney disease due to diabetes mellitus type 2	1<br>(1 to 1)
High LDL cholesterol	High systolic blood pressure	Ischemic heart disease	0.09<br>(0.07 to 0.11)
High LDL cholesterol	High systolic blood pressure	Ischemic stroke	0.16<br>(0.14 to 0.18)
High systolic blood pressure	Kidney dysfunction	Chronic kidney disease due to diabetes mellitus type 1	1<br>(1 to 1)
High systolic blood pressure	Kidney dysfunction	Chronic kidney disease due to diabetes mellitus type 2	1<br>(1 to 1)
High systolic blood pressure	Kidney dysfunction	Chronic kidney disease due to hypertension	1<br>(1 to 1)
High systolic blood pressure	Kidney dysfunction	Chronic kidney disease due to glomerulonephritis	1<br>(1 to 1)
High systolic blood pressure	Kidney dysfunction	Chronic kidney disease due to other and unspecified causes	1<br>(1 to 1)
High body-mass index in adults	High fasting plasma glucose	Ischemic heart disease	0.15<br>(0.1 to 0.2)
High body-mass index in adults	High fasting plasma glucose	Ischemic stroke	0.22<br>(0.12 to 0.31)
High body-mass index in adults	High fasting plasma glucose	Intracerebral hemorrhage	0.22<br>(0.13 to 0.32)
High body-mass index in adults	High fasting plasma glucose	Subarachnoid hemorrhage	0.22<br>(0.13 to 0.32)

High body-mass index in adults	High fasting plasma glucose	Diabetes mellitus type 2	1<br>(1 to 1)
High body-mass index in adults	High LDL cholesterol	Ischemic heart disease	0.1<br>(0.05 to 0.15)
High body-mass index in adults	High LDL cholesterol	Ischemic stroke	0.03<br>(0 to 0.08)
High body-mass index in adults	High systolic blood pressure	Ischemic heart disease	0.31<br>(0.28 to 0.34)
High body-mass index in adults	High systolic blood pressure	Ischemic stroke	0.65<br>(0.57 to 0.72)
High body-mass index in adults	High systolic blood pressure	Intracerebral hemorrhage	0.65<br>(0.58 to 0.73)
High body-mass index in adults	High systolic blood pressure	Subarachnoid hemorrhage	0.65<br>(0.58 to 0.73)
High body-mass index in adults	High systolic blood pressure	Hypertensive heart disease	1<br>(1 to 1)
High body-mass index in adults	High systolic blood pressure	Atrial fibrillation and flutter	0.31<br>(0.28 to 0.34)
High body-mass index in adults	Kidney dysfunction	Chronic kidney disease due to diabetes mellitus type 2	1<br>(1 to 1)
High body-mass index in adults	Kidney dysfunction	Chronic kidney disease due to hypertension	1<br>(1 to 1)
High body-mass index in adults	Kidney dysfunction	Chronic kidney disease due to glomerulonephritis	1<br>(1 to 1)
High body-mass index in adults	Kidney dysfunction	Chronic kidney disease due to other and unspecified causes	1<br>(1 to 1)

Table S7A. Relative risks used by age and sex for each outcome for all risk factors except for ambient particulate matter pollution, alcohol, smoking, and non-optimal temperature.

Risk - Outcome	Category / Units	Morbidity / Mortality	Sex	Ages																							
				All-age	0-6 days	7-27 days	28-364 days	1-4 years	5-9 years	10-14 years	15-19 years	20-24 years	25-29 years	30-34 years	35-39 years	40-44 years	45-49 years	50-54 years	55-59 years	60-64 years	65-69 years	70-74 years	75-79 years	80-84 years	85-89 years	90-94 years	95+ years
**Unsafe water source**																											
Diarrhoeal diseases	Unimproved, untreated	Both	Both	11.084 (4.287 to 22.867)																							
Diarrhoeal diseases	Unimproved, chlorinated	Both	Both	8.024 (3.203 to 16.486)																							
Diarrhoeal diseases	Unimproved, filter	Both	Both	5.331 (2.153 to 10.887)																							
Diarrhoeal diseases	Improved, untreated	Both	Both	8.986 (3.898 to 17.446)																							
Diarrhoeal diseases	Improved, chlorinated	Both	Both	6.505 (2.905 to 12.528)																							
Diarrhoeal diseases	Improved, filtered	Both	Both	4.322 (1.954 to 8.243)																							
Diarrhoeal diseases	Piped, untreated	Both	Both	6.88 (3.104 to 13.016)																							
Diarrhoeal diseases	Piped, chlorinated	Both	Both	4.98 (2.32 to 9.296)																							
Diarrhoeal diseases	Piped, filtered	Both	Both	3.309 (1.564 to 6.129)																							
Diarrhoeal diseases	High quality (HQ) piped, untreated	Both	Both	2.079 (1.788 to 2.409)																							
Diarrhoeal diseases	HQ piped, chlorinated	Both	Both	1.505 (1.418 to 1.594)																							
Diarrhoeal diseases	HQ piped, filtered	Both	Both	1.0 (1.0 to 1.0)																							
**Unsafe sanitation**																											
Diarrhoeal diseases	Unimproved & untreated	Both	Both	3.203 (2.897 to 3.513)																							
Diarrhoeal diseases	Improved	Both	Both	2.465 (2.349 to 2.587)																							
Diarrhoeal diseases	Sewer	Both	Both	1.0 (1.0 to 1.0)																							
**No access to handwashing facility**																											
Lower respiratory infections	No handwashing w/soap & water	Both	Both	1.321 (1.125 to 1.533)																							
Lower respiratory infections	Handwashing w/soap & water	Both	Both	1.0 (1.0 to 1.0)																							
Diarrhoeal diseases	No handwashing w/soap & water	Both	Both	1.522 (1.4 to 1.644)																							
Diarrhoeal diseases	Handwashing w/soap & water	Both	Both	1.0 (1.0 to 1.0)																							
**Ambient ozone pollution**																											
Chronic obstructive pulmonary disease	10 ppb	Mortality	Both	1.063 (1.029 to 1.098)																							
**Residential radon**																											
Tracheal, bronchus, and lung cancer	100 Bq/m3	Both	Both	1.097 (1.019 to 1.174)																							
**Lead exposure in blood**																											
IQ shift	2 μg/dL	Morbidity	Both	0.0 (0.0 to 0.0)																							
IQ shift	4 μg/dL	Morbidity	Both	3.146 (1.154 to 5.139)																							
IQ shift	6 μg/dL	Morbidity	Both	3.804 (1.395 to 6.213)																							
IQ shift	8 μg/dL	Morbidity	Both	4.296 (1.575 to 7.016)																							
IQ shift	10 μg/dL	Morbidity	Both	4.688 (1.719 to 7.656)																							
IQ shift	12 μg/dL	Morbidity	Both	5.014 (1.839 to 8.19)																							
IQ shift	15 μg/dL	Morbidity	Both	5.42 (1.988 to 8.853)																							
IQ shift	20 μg/dL	Morbidity	Both	5.952 (2.183 to 9.721)																							
IQ shift	25 μg/dL	Morbidity	Both	6.37 (2.336 to 10.403)																							
IQ shift	30 μg/dL	Morbidity	Both	6.713 (2.462 to 10.965)																							
IQ shift	35 μg/dL	Morbidity	Both	7.006 (2.569 to 11.442)																							
IQ shift	40 μg/dL	Morbidity	Both	7.26 (2.662 to 11.857)																							
**Lead exposure in bone**																											
Rheumatic heart disease	10 μg/g	Both	Both										1.03 (1.01 to 1.052)	1.024 (1.01 to 1.04)	1.017 (1.008 to 1.028)	1.013 (1.005 to 1.022)	1.012 (1.006 to 1.019)	1.011 (1.006 to 1.017)	1.01 (1.006 to 1.016)	1.009 (1.005 to 1.014)	1.008 (1.003 to 1.014)	1.007 (1.003 to 1.013)	1.007 (1.004 to 1.013)	1.006 (1.002 to 1.015)	1.006 (1.002 to 1.015)	1.006 (1.002 to 1.015)	1.006 (1.002 to 1.015)
Ischaemic heart disease	10 μg/g	Both	Both										1.042 (1.022 to 1.06)	1.037 (1.023 to 1.049)	1.032 (1.023 to 1.04)	1.028 (1.021 to 1.036)	1.026 (1.02 to 1.033)	1.024 (1.02 to 1.03)	1.023 (1.019 to 1.026)	1.021 (1.018 to 1.025)	1.019 (1.014 to 1.023)	1.018 (1.012 to 1.022)	1.016 (1.012 to 1.021)	1.014 (1.008 to 1.022)	1.014 (1.008 to 1.022)	1.014 (1.008 to 1.022)	1.014 (1.008 to 1.022)
Ischaemic stroke	10 μg/g	Both	Both										1.038 (1.021 to 1.06)	1.036 (1.022 to 1.051)	1.033 (1.021 to 1.044)	1.03 (1.019 to 1.042)	1.028 (1.019 to 1.037)	1.026 (1.019 to 1.033)	1.024 (1.018 to 1.029)	1.021 (1.016 to 1.026)	1.019 (1.012 to 1.025)	1.017 (1.01 to 1.023)	1.015 (1.01 to 1.02)	1.011 (1.006 to 1.019)	1.011 (1.006 to 1.019)	1.011 (1.006 to 1.019)	1.011 (1.006 to 1.019)
Intracerebral haemorrhage	10 μg/g	Both	Both										1.047 (1.027 to 1.068)	1.045 (1.029 to 1.061)	1.042 (1.029 to 1.057)	1.039 (1.025 to 1.052)	1.036 (1.024 to 1.047)	1.032 (1.023 to 1.041)	1.028 (1.021 to 1.035)	1.024 (1.018 to 1.03)	1.02 (1.012 to 1.027)	1.017 (1.009 to 1.025)	1.017 (1.011 to 1.023)	1.015 (1.007 to 1.026)	1.015 (1.007 to 1.026)	1.015 (1.007 to 1.026)	1.015 (1.007 to 1.026)
Subarachnoid haemorrhage	10 μg/g	Both	Both										1.047 (1.027 to 1.068)	1.045 (1.029 to 1.061)	1.042 (1.029 to 1.057)	1.039 (1.025 to 1.052)	1.036 (1.024 to 1.047)	1.032 (1.023 to 1.041)	1.028 (1.021 to 1.035)	1.024 (1.018 to 1.03)	1.02 (1.012 to 1.027)	1.017 (1.009 to 1.025)	1.017 (1.011 to 1.023)	1.015 (1.007 to 1.026)	1.015 (1.007 to 1.026)	1.015 (1.007 to 1.026)	1.015 (1.007 to 1.026)
Hypertensive heart disease	10 μg/g	Both	Both										1.066 (1.038 to 1.09)	1.066 (1.038 to 1.091)	1.065 (1.037 to 1.094)	1.063 (1.035 to 1.091)	1.058 (1.037 to 1.084)	1.052 (1.036 to 1.079)	1.046 (1.031 to 1.076)	1.04 (1.023 to 1.073)	1.033 (1.011 to 1.074)	1.03 (1.003 to 1.072)	1.031 (1.005 to 1.071)	1.033 (1.006 to 1.075)	1.033 (1.006 to 1.075)	1.033 (1.006 to 1.075)	1.033 (1.006 to 1.075)
Non-rheumatic calcific aortic valvular heart disease	10 μg/g	Both	Both										1.035 (1.015 to 1.055)	1.029 (1.016 to 1.044)	1.023 (1.015 to 1.031)	1.019 (1.013 to 1.025)	1.018 (1.012 to 1.023)	1.016 (1.012 to 1.02)	1.015 (1.012 to 1.018)	1.014 (1.01 to 1.016)	1.012 (1.008 to 1.015)	1.011 (1.007 to 1.014)	1.01 (1.007 to 1.013)	1.007 (1.004 to 1.013)	1.007 (1.004 to 1.013)	1.007 (1.004 to 1.013)	1.007 (1.004 to 1.013)
Other cardiomyopathy	10 μg/g	Both	Both										1.035 (1.015 to 1.055)	1.029 (1.016 to 1.044)	1.023 (1.015 to 1.031)	1.019 (1.013 to 1.025)	1.018 (1.012 to 1.023)	1.016 (1.012 to 1.02)	1.015 (1.012 to 1.018)	1.014 (1.01 to 1.016)	1.012 (1.008 to 1.015)	1.011 (1.007 to 1.014)	1.01 (1.007 to 1.013)	1.007 (1.004 to 1.013)	1.007 (1.004 to 1.013)	1.007 (1.004 to 1.013)	1.007 (1.004 to 1.013)
Atrial fibrillation and flutter	10 μg/g	Both	Both										1.035 (1.018 to 1.056)	1.03 (1.02 to 1.044)	1.025 (1.021 to 1.031)	1.022 (1.018 to 1.025)	1.02 (1.017 to 1.023)	1.019 (1.017 to 1.021)	1.018 (1.016 to 1.019)	1.016 (1.014 to 1.018)	1.015 (1.013 to 1.017)	1.013 (1.011 to 1.015)	1.012 (1.01 to 1.013)	1.008 (1.005 to 1.01)	1.008 (1.005 to 1.01)	1.008 (1.005 to 1.01)	1.008 (1.005 to 1.01)
Aortic aneurysm	10 μg/g	Both	Both										1.027 (1.014 to 1.048)	1.024 (1.016 to 1.037)	1.02 (1.016 to 1.026)	1.018 (1.013 to 1.023)	1.017 (1.013 to 1.021)	1.016 (1.013 to 1.019)	1.015 (1.012 to 1.017)	1.014 (1.011 to 1.016)	1.012 (1.009 to 1.015)	1.011 (1.008 to 1.014)	1.01 (1.007 to 1.013)	1.007 (1.004 to 1.01)	1.007 (1.004 to 1.01)	1.007 (1.004 to 1.01)	1.007 (1.004 to 1.01)
Peripheral vascular disease	10 μg/g	Both	Both										1.034 (1.011 to 1.056)	1.025 (1.012 to 1.039)	1.014 (1.01 to 1.018)	1.008 (1.001 to 1.014)	1.008 (1.003 to 1.013)	1.008 (1.004 to 1.012)	1.009 (1.005 to 1.012)	1.009 (1.006 to 1.011)	1.009 (1.007 to 1.012)	1.009 (1.006 to 1.011)	1.008 (1.006 to 1.01)	1.006 (1.003 to 1.009)	1.006 (1.003 to 1.009)	1.006 (1.003 to 1.009)	1.006 (1.003 to 1.009)
Endocarditis	10 μg/g	Both	Both										1.035 (1.015 to 1.055)	1.029 (1.016 to 1.044)	1.023 (1.015 to 1.031)	1.019 (1.013 to 1.025)	1.018 (1.012 to 1.023)	1.016 (1.012 to 1.02)	1.015 (1.012 to 1.018)	1.014 (1.01 to 1.016)	1.012 (1.008 to 1.015)	1.011 (1.007 to 1.014)	1.01 (1.007 to 1.013)	1.007 (1.004 to 1.013)	1.007 (1.004 to 1.013)	1.007 (1.004 to 1.013)	1.007 (1.004 to 1.013)
Chronic kidney disease due to diabetes mellitus type 1	10 μg/g	Both	Both										1.015 (1.01 to 1.021)	1.015 (1.01 to 1.021)	1.015 (1.01 to 1.021)	1.015 (1.01 to 1.021)	1.015 (1.01 to 1.021)	1.015 (1.01 to 1.021)	1.015 (1.01 to 1.021)	1.015 (1.01 to 1.021)	1.015 (1.01 to 1.021)	1.015 (1.01 to 1.021)	1.015 (1.01 to 1.021)	1.015 (1.01 to 1.021)	1.015 (1.01 to 1.021)	1.015 (1.01 to 1.021)	1.015 (1.01 to 1.021)
Chronic kidney disease due to diabetes mellitus type 2	10 μg/g	Both	Both										1.015 (1.01 to 1.021)	1.015 (1.01 to 1.021)	1.015 (1.01 to 1.021)	1.015 (1.01 to 1.021)	1.015 (1.01 to 1.021)	1.015 (1.01 to 1.021)	1.015 (1.01 to 1.021)	1.015 (1.01 to 1.021)	1.015 (1.01 to 1.021)	1.015 (1.01 to 1.021)	1.015 (1.01 to 1.021)	1.015 (1.01 to 1.021)	1.015 (1.01 to 1.021)	1.015 (1.01 to 1.021)	1.015 (1.01 to 1.021)
Chronic kidney disease due to hypertension	10 μg/g	Both	Both										1.015 (1.01 to 1.02)	1.015 (1.01 to 1.02)	1.015 (1.01 to 1.02)	1.015 (1.01 to 1.02)	1.015 (1.01 to 1.02)	1.015 (1.01 to 1.02)	1.015 (1.01 to 1.02)	1.015 (1.01 to 1.02)	1.015 (1.01 to 1.02)	1.015 (1.01 to 1.02)	1.015 (1.01 to 1.02)	1.015 (1.01 to 1.02)	1.015 (1.01 to 1.02)	1.015 (1.01 to 1.02)	1.015 (1.01 to 1.02)
Chronic kidney disease due to glomerulonephritis	10 μg/g	Both	Both										1.015 (1.01 to 1.02)	1.015 (1.01 to 1.02)	1.015 (1.01 to 1.02)	1.015 (1.01 to 1.02)	1.015 (1.01 to 1.02)	1.015 (1.01 to 1.02)	1.015 (1.01 to 1.02)	1.015 (1.01 to 1.02)	1.015 (1.01 to 1.02)	1.015 (1.01 to 1.02)	1.015 (1.01 to 1.02)	1.015 (1.01 to 1.02)	1.015 (1.01 to 1.02)	1.015 (1.01 to 1.02)	1.015 (1.01 to 1.02)
Chronic kidney disease due to other and unspecified causes	10 μg/g	Both	Both										1.015 (1.01 to 1.021)	1.015 (1.01 to 1.021)	1.015 (1.01 to 1.021)	1.015 (1.01 to 1.021)	1.015 (1.01 to 1.021)	1.015 (1.01 to 1.021)	1.015 (1.01 to 1.021)	1.015 (1.01 to 1.021)	1.015 (1.01 to 1.021)	1.015 (1.01 to 1.021)	1.015 (1.01 to 1.021)	1.015 (1.01 to 1.021)	1.015 (1.01 to 1.021)	1.015 (1.01 to 1.021)	1.015 (1.01 to 1.021)
**Occupational exposure to asbestos**																											
Larynx cancer	High exposure	Both	Males								1.38 (1.188 to 1.612)	1.38 (1.188 to 1.612)	1.38 (1.188 to 1.612)	1.38 (1.188 to 1.612)	1.38 (1.188 to 1.612)	1.38 (1.188 to 1.612)	1.38 (1.188 to 1.612)	1.38 (1.188 to 1.612)	1.38 (1.188 to 1.612)	1.38 (1.188 to 1.612)	1.38 (1.188 to 1.612)	1.38 (1.188 to 1.612)	1.38 (1.188 to 1.612)	1.38 (1.188 to 1.612)	1.38 (1.188 to 1.612)	1.38 (1.188 to 1.612)	1.38 (1.188 to 1.612)
Larynx cancer	High exposure	Both	Females								1.385 (1.187 to 1.598)	1.385 (1.187 to 1.598)	1.385 (1.187 to 1.598)	1.385 (1.187 to 1.598)	1.385 (1.187 to 1.598)	1.385 (1.187 to 1.598)	1.385 (1.187 to 1.598)	1.385 (1.187 to 1.598)	1.385 (1.187 to 1.598)	1.385 (1.187 to 1.598)	1.385 (1.187 to 1.598)	1.385 (1.187 to 1.598)	1.385 (1.187 to 1.598)	1.385 (1.187 to 1.598)	1.385 (1.187 to 1.598)	1.385 (1.187 to 1.598)	1.385 (1.187 to 1.598)
Larynx cancer	Low exposure	Both	Males								1.0 (1.0 to 1.0)	1.0 (1.0 to 1.0)	1.0 (1.0 to 1.0)	1.0 (1.0 to 1.0)	1.0 (1.0 to 1.0)	1.0 (1.0 to 1.0)	1.0 (1.0 to 1.0)	1.0 (1.0 to 1.0)	1.0 (1.0 to 1.0)	1.0 (1.0 to 1.0)	1.0 (1.0 to 1.0)	1.0 (1.0 to 1.0)	1.0 (1.0 to 1.0)	1.0 (1.0 to 1.0)	1.0 (1.0 to 1.0)	1.0 (1.0 to 1.0)	1.0 (1.0 to 1.0)
Larynx cancer	Low exposure	Both	Females								1.0 (1.0 to 1.0)	1.0 (1.0 to 1.0)	1.0 (1.0 to 1.0)	1.0 (1.0 to 1.0)	1.0 (1.0 to 1.0)	1.0 (1.0 to 1.0)	1.0 (1.0 to 1.0)	1.0 (1.0 to 1.0)	1.0 (1.0 to 1.0)	1.0 (1.0 to 1.0)	1.0 (1.0 to 1.0)	1.0 (1.0 to 1.0)	1.0 (1.0 to 1.0)	1.0 (1.0 to 1.0)	1.0 (1.0 to 1.0)	1.0 (1.0 to 1.0)	1.0 (1.0 to 1.0)
Tracheal, bronchus, and lung cancer	High exposure	Both	Males								2.279 (1.741 to 2.936)	2.279 (1.741 to 2.936)	2.279 (1.741 to 2.936)	2.279 (1.741 to 2.936)	2.279 (1.741 to 2.936)	2.279 (1.741 to 2.936)	2.279 (1.741 to 2.936)	2.279 (1.741 to 2.936)	2.279 (1.741 to 2.936)	2.279 (1.741 to 2.936)	2.279 (1.741 to 2.936)	2.279 (1.741 to 2.936)	2.279 (1.741 to 2.936)	2.279 (1.741 to 2.936)	2.279 (1.741 to 2.936)	2.279 (1.741 to 2.936)	2.279 (1.741 to 2.936)
Tracheal, bronchus, and lung cancer	High exposure	Both	Females								1.875 (1.589 to 2.208)	1.875 (1.589 to 2.208)	1.875 (1.589 to 2.208)	1.875 (1.589 to 2.208)	1.875 (1.589 to 2.208)	1.875 (1.589 to 2.208)	1.875 (1.589 to 2.208)	1.875 (1.589 to 2.208)	1.875 (1.589 to 2.208)	1.875 (1.589 to 2.208)	1.875 (1.589 to 2.208)	1.875 (1.589 to 2.208)	1.875 (1.589 to 2.208)	1.875 (1.589 to 2.208)	1.875 (1.589 to 2.208)	1.875 (1.589 to 2.208)	1.875 (1.589 to 2.208)
Tracheal, bronchus, and lung cancer	Low exposure	Both	Males								1.0 (1.0 to 1.0)	1.0 (1.0 to 1.0)	1.0 (1.0 to 1.0)	1.0 (1.0 to 1.0)	1.0 (1.0 to 1.0)	1.0 (1.0 to 1.0)	1.0 (1.0 to 1.0)	1.0 (1.0 to 1.0)	1.0 (1.0 to 1.0)	1.0 (1.0 to 1.0)	1.0 (1.0 to 1.0)	1.0 (1.0 to 1.0)	1.0 (1.0 to 1.0)	1.0 (1.0 to 1.0)	1.0 (1.0 to 1.0)	1.0 (1.0 to 1.0)	1.0 (1.0 to 1.0)
Tracheal, bronchus, and lung cancer	Low exposure	Both	Females								1.0 (1.0 to 1.0)	1.0 (1.0 to 1.0)	1.0 (1.0 to 1.0)	1.0 (1.0 to 1.0)	1.0 (1.0 to 1.0)	1.0 (1.0 to 1.0)	1.0 (1.0 to 1.0)	1.0 (1.0 to 1.0)	1.0 (1.0 to 1.0)	1.0 (1.0 to 1.0)	1.0 (1.0 to 1.0)	1.0 (1.0 to 1.0)	1.0 (1.0 to 1.0)	1.0 (1.0 to 1.0)	1.0 (1.0 to 1.0)	1.0 (1.0 to 1.0)	1.0 (1.0 to 1.0)
Ovarian cancer	High exposure	Both	Both								1.811 (1.385 to 2.306)	1.811 (1.385 to 2.306)	1.811 (1.385 to 2.306)	1.811 (1.385 to 2.306)	1.811 (1.385 to 2.306)	1.811 (1.385 to 2.306)	1.811 (1.385 to 2.306)	1.811 (1.385 to 2.306)	1.811 (1.385 to 2.306)	1.811 (1.385 to 2.306)	1.811 (1.385 to 2.306)	1.811 (1.385 to 2.306)	1.811 (1.385 to 2.306)	1.811 (1.385 to 2.306)	1.811 (1.385 to 2.306)	1.811 (1.385 to 2.306)	1.811 (1.385 to 2.306)
Ovarian cancer	Low exposure	Both	Both								1.0 (1.0 to 1.0)	1.0 (1.0 to 1.0)	1.0 (1.0 to 1.0)	1.0 (1.0 to 1.0)	1.0 (1.0 to 1.0)	1.0 (1.0 to 1.0)	1.0 (1.0 to 1.0)	1.0 (1.0 to 1.0)	1.0 (1.0 to 1.0)	1.0 (1.0 to 1.0)	1.0 (1.0 to 1.0)	1.0 (1.0 to 1.0)	1.0 (1.0 to 1.0)	1.0 (1.0 to 1.0)	1.0 (1.0 to 1.0)	1.0 (1.0 to 1.0)	1.0 (1.0 to 1.0)
**Occupational exposure to arsenic**																											
Tracheal, bronchus, and lung cancer	High exposure	Both	Both								2.061 (1.521 to 2.553)	2.061 (1.521 to 2.553)	2.061 (1.521 to 2.553)	2.061 (1.521 to 2.553)	2.061 (1.521 to 2.553)	2.061 (1.521 to 2.553)	2.061 (1.521 to 2.553)	2.061 (1.521 to 2.553)	2.061 (1.521 to 2.553)	2.061 (1.521 to 2.553)	2.061 (1.521 to 2.553)	2.061 (1.521 to 2.553)	2.061 (1.521 to 2.553)	2.061 (1.521 to 2.553)	2.061 (1.521 to 2.553)	2.061 (1.521 to 2.553)	2.061 (1.521 to 2.553)
Tracheal, bronchus, and lung cancer	Low exposure	Both	Both								1.749 (0.698 to 2.775)	1.749 (0.698 to 2.775)	1.749 (0.698 to 2.775)	1.749 (0.698 to 2.775)	1.749 (0.698 to 2.775)	1.749 (0.698 to 2.775)	1.749 (0.698 to 2.775)	1.749 (0.698 to 2.775)	1.749 (0.698 to 2.775)	1.749 (0.698 to 2.775)	1.749 (0.698 to 2.775)	1.749 (0.698 to 2.775)	1.749 (0.698 to 2.775)	1.749 (0.698 to 2.775)	1.749 (0.698 to 2.775)	1.749 (0.698 to 2.775)	1.749 (0.698 to 2.775)
Tracheal, bronchus, and lung cancer	No exposure	Both	Both								1.0 (1.0 to 1.0)	1.0 (1.0 to 1.0)	1.0 (1.0 to 1.0)	1.0 (1.0 to 1.0)	1.0 (1.0 to 1.0)	1.0 (1.0 to 1.0)	1.0 (1.0 to 1.0)	1.0 (1.0 to 1.0)	1.0 (1.0 to 1.0)	1.0 (1.0 to 1.0)	1.0 (1.0 to 1.0)	1.0 (1.0 to 1.0)	1.0 (1.0 to 1.0)	1.0 (1.0 to 1.0)	1.0 (1.0 to 1.0)	1.0 (1.0 to 1.0)	1.0 (1.0 to 1.0)
**Occupational exposure to benzene**																											
Acute lymphoid leukaemia	High exposure	Both	Both								2.623 (1.222 to 3.975)	2.623 (1.222 to 3.975)	2.623 (1.222 to 3.975)	2.623 (1.222 to 3.975)	2.623 (1.222 to 3.975)	2.623 (1.222 to 3.975)	2.623 (1.222 to 3.975)	2.623 (1.222 to 3.975)	2.623 (1.222 to 3.975)	2.623 (1.222 to 3.975)	2.623 (1.222 to 3.975)	2.623 (1.222 to 3.975)	2.623 (1.222 to 3.975)	2.623 (1.222 to 3.975)	2.623 (1.222 to 3.975)	2.623 (1.222 to 3.975)	2.623 (1.222 to 3.975)
Acute lymphoid leukaemia	Low exposure	Both	Both								1.626 (0.998 to 2.256)	1.626 (0.998 to 2.256)	1.626 (0.998 to 2.256)	1.626 (0.998 to 2.256)	1.626 (0.998 to 2.256)	1.626 (0.998 to 2.256)	1.626 (0.998 to 2.256)	1.626 (0.998 to 2.256)	1.626 (0.998 to 2.256)	1.626 (0.998 to 2.256)	1.626 (0.998 to 2.256)	1.626 (0.998 to 2.256)	1.626 (0.998 to 2.256)	1.626 (0.998 to 2.256)	1.626 (0.998 to 2.256)	1.626 (0.998 to 2.256)	1.626 (0.998 to 2.256)
Acute lymphoid leukaemia	No exposure	Both	Both								1.0 (1.0 to 1.0)	1.0 (1.0 to 1.0)	1.0 (1.0 to 1.0)	1.0 (1.0 to 1.0)	1.0 (1.0 to 1.0)	1.0 (1.0 to 1.0)	1.0 (1.0 to 1.0)	1.0 (1.0 to 1.0)	1.0 (1.0 to 1.0)	1.0 (1.0 to 1.0)	1.0 (1.0 to 1.0)	1.0 (1.0 to 1.0)	1.0 (1.0 to 1.0)	1.0 (1.0 to 1.0)	1.0 (1.0 to 1.0)	1.0 (1.0 to 1.0)	1.0 (1.0 to 1.0)
Chronic lymphoid leukaemia	High exposure	Both	Both								2.623 (1.222 to 3.975)	2.623 (1.222 to 3.975)	2.623 (1.222 to 3.975)	2.623 (1.222 to 3.975)	2.623 (1.222 to 3.975)	2.623 (1.222 to 3.975)	2.623 (1.222 to 3.975)	2.623 (1.222 to 3.975)	2.623 (1.222 to 3.975)	2.623 (1.222 to 3.975)	2.623 (1.222 to 3.975)	2.623 (1.222 to 3.975)	2.623 (1.222 to 3.975)	2.623 (1.222 to 3.975)	2.623 (1.222 to 3.975)	2.623 (1.222 to 3.975)	2.623 (1.222 to 3.975)
Chronic lymphoid leukaemia	Low exposure	Both	Both								1.626 (0.998 to 2.256)	1.626 (0.998 to 2.256)	1.626 (0.998 to 2.256)	1.626 (0.998 to 2.256)	1.626 (0.998 to 2.256)	1.626 (0.998 to 2.256)	1.626 (0.998 to 2.256)	1.626 (0.998 to 2.256)	1.626 (0.998 to 2.256)	1.626 (0.998 to 2.256)	1.626 (0.998 to 2.256)	1.626 (0.998 to 2.256)	1.626 (0.998 to 2.256)	1.626 (0.998 to 2.256)	1.626 (0.998 to 2.256)	1.626 (0.998 to 2.256)	1.626 (0.998 to 2.256)
Chronic lymphoid leukaemia	No exposure	Both	Both								1.0 (1.0 to 1.0)	1.0 (1.0 to 1.0)	1.0 (1.0 to 1.0)	1.0 (1.0 to 1.0)	1.0 (1.0 to 1.0)	1.0 (1.0 to 1.0)	1.0 (1.0 to 1.0)	1.0 (1.0 to 1.0)	1.0 (1.0 to 1.0)	1.0 (1.0 to 1.0)	1.0 (1.0 to 1.0)	1.0 (1.0 to 1.0)	1.0 (1.0 to 1.0)	1.0 (1.0 to 1.0)	1.0 (1.0 to 1.0)	1.0 (1.0 to 1.0)	1.0 (1.0 to 1.0)
Acute myeloid leukaemia	High exposure	Both	Both								2.623 (1.222 to 3.975)	2.623 (1.222 to 3.975)	2.623 (1.222 to 3.975)	2.623 (1.222 to 3.975)	2.623 (1.222 to 3.975)	2.623 (1.222 to 3.975)	2.623 (1.222 to 3.975)	2.623 (1.222 to 3.975)	2.623 (1.222 to 3.975)	2.623 (1.222 to 3.975)	2.623 (1.222 to 3.975)	2.623 (1.222 to 3.975)	2.623 (1.222 to 3.975)	2.623 (1.222 to 3.975)	2.623 (1.222 to 3.975)	2.623 (1.222 to 3.975)	2.623 (1.222 to 3.975)
Acute myeloid leukaemia	Low exposure	Both	Both								1.626 (0.998 to 2.256)	1.626 (0.998 to 2.256)	1.626 (0.998 to 2.256)	1.626 (0.998 to 2.256)	1.626 (0.998 to 2.256)	1.626 (0.998 to 2.256)	1.626 (0.998 to 2.256)	1.626 (0.998 to 2.256)	1.626 (0.998 to 2.256)	1.626 (0.998 to 2.256)	1.626 (0.998 to 2.256)	1.626 (0.998 to 2.256)	1.626 (0.998 to 2.256)	1.626 (0.998 to 2.256)	1.626 (0.998 to 2.256)	1.626 (0.998 to 2.256)	1.626 (0.998 to 2.256)
Acute myeloid leukaemia	No exposure	Both	Both								1.0 (1.0 to 1.0)	1.0 (1.0 to 1.0)	1.0 (1.0 to 1.0)	1.0 (1.0 to 1.0)	1.0 (1.0 to 1.0)	1.0 (1.0 to 1.0)	1.0 (1.0 to 1.0)	1.0 (1.0 to 1.0)	1.0 (1.0 to 1.0)	1.0 (1.0 to 1.0)	1.0 (1.0 to 1.0)	1.0 (1.0 to 1.0)	1.0 (1.0 to 1.0)	1.0 (1.0 to 1.0)	1.0 (1.0 to 1.0)	1.0 (1.0 to 1.0)	1.0 (1.0 to 1.0)
Chronic myeloid leukaemia	High exposure	Both	Both								2.623 (1.222 to 3.975)	2.623 (1.222 to 3.975)	2.623 (1.222 to 3.975)	2.623 (1.222 to 3.975)	2.623 (1.222 to 3.975)	2.623 (1.222 to 3.975)	2.623 (1.222 to 3.975)	2.623 (1.222 to 3.975)	2.623 (1.222 to 3.975)	2.623 (1.222 to 3.975)	2.623 (1.222 to 3.975)	2.623 (1.222 to 3.975)	2.623 (1.222 to 3.975)	2.623 (1.222 to 3.975)	2.623 (1.222 to 3.975)	2.623 (1.222 to 3.975)	2.623 (1.222 to 3.975)
Chronic myeloid leukaemia	Low exposure	Both	Both								1.626 (0.998 to 2.256)	1.626 (0.998 to 2.256)	1.626 (0.998 to 2.256)	1.626 (0.998 to 2.256)	1.626 (0.998 to 2.256)	1.626 (0.998 to 2.256)	1.626 (0.998 to 2.256)	1.626 (0.998 to 2.256)	1.626 (0.998 to 2.256)	1.626 (0.998 to 2.256)	1.626 (0.998 to 2.256)	1.626 (0.998 to 2.256)	1.626 (0.998 to 2.256)	1.626 (0.998 to 2.256)	1.626 (0.998 to 2.256)	1.626 (0.998 to 2.256)	1.626 (0.998 to 2.256)
Chronic myeloid leukaemia	No exposure	Both	Both								1.0 (1.0 to 1.0)	1.0 (1.0 to 1.0)	1.0 (1.0 to 1.0)	1.0 (1.0 to 1.0)	1.0 (1.0 to 1.0)	1.0 (1.0 to 1.0)	1.0 (1.0 to 1.0)	1.0 (1.0 to 1.0)	1.0 (1.0 to 1.0)	1.0 (1.0 to 1.0)	1.0 (1.0 to 1.0)	1.0 (1.0 to 1.0)	1.0 (1.0 to 1.0)	1.0 (1.0 to 1.0)	1.0 (1.0 to 1.0)	1.0 (1.0 to 1.0)	1.0 (1.0 to 1.0)
Other leukaemia	High exposure	Both	Both								2.623 (1.222 to 3.975)	2.623 (1.222 to 3.975)	2.623 (1.222 to 3.975)	2.623 (1.222 to 3.975)	2.623 (1.222 to 3.975)	2.623 (1.222 to 3.975)	2.623 (1.222 to 3.975)	2.623 (1.222 to 3.975)	2.623 (1.222 to 3.975)	2.623 (1.222 to 3.975)	2.623 (1.222 to 3.975)	2.623 (1.222 to 3.975)	2.623 (1.222 to 3.975)	2.623 (1.222 to 3.975)	2.623 (1.222 to 3.975)	2.623 (1.222 to 3.975)	2.623 (1.222 to 3.975)
Other leukaemia	Low exposure	Both	Both								1.626 (0.998 to 2.256)	1.626 (0.998 to 2.256)	1.626 (0.998 to 2.256)	1.626 (0.998 to 2.256)	1.626 (0.998 to 2.256)	1.626 (0.998 to 2.256)	1.626 (0.998 to 2.256)	1.626 (0.998 to 2.256)	1.626 (0.998 to 2.256)	1.626 (0.998 to 2.256)	1.626 (0.998 to 2.256)	1.626 (0.998 to 2.256)	1.626 (0.998 to 2.256)	1.626 (0.998 to 2.256)	1.626 (0.998 to 2.256)	1.626 (0.998 to 2.256)	1.626 (0.998 to 2.256)
Other leukaemia	No exposure	Both	Both								1.0 (1.0 to 1.0)	1.0 (1.0 to 1.0)	1.0 (1.0 to 1.0)	1.0 (1.0 to 1.0)	1.0 (1.0 to 1.0)	1.0 (1.0 to 1.0)	1.0 (1.0 to 1.0)	1.0 (1.0 to 1.0)	1.0 (1.0 to 1.0)	1.0 (1.0 to 1.0)	1.0 (1.0 to 1.0)	1.0 (1.0 to 1.0)	1.0 (1.0 to 1.0)	1.0 (1.0 to 1.0)	1.0 (1.0 to 1.0)	1.0 (1.0 to 1.0)	1.0 (1.0 to 1.0)
**Occupational exposure to beryllium**																											
Tracheal, bronchus, and lung cancer	High exposure	Both	Males								1.174 (1.086 to 1.269)	1.169 (1.065 to 1.276)	1.17 (1.073 to 1.274)	1.169 (1.073 to 1.269)	1.172 (1.081 to 1.271)	1.168 (1.07 to 1.276)	1.171 (1.071 to 1.274)	1.171 (1.075 to 1.273)	1.17 (1.073 to 1.271)	1.171 (1.072 to 1.271)	1.172 (1.081 to 1.276)	1.171 (1.076 to 1.27)	1.174 (1.079 to 1.276)	1.171 (1.072 to 1.273)	1.171 (1.072 to 1.273)	1.171 (1.072 to 1.273)	1.171 (1.072 to 1.273)
Tracheal, bronchus, and lung cancer	High exposure	Both	Females								1.17 (1.082 to 1.262)	1.17 (1.076 to 1.277)	1.169 (1.072 to 1.277)	1.17 (1.077 to 1.275)	1.172 (1.075 to 1.274)	1.171 (1.078 to 1.276)	1.173 (1.074 to 1.276)	1.172 (1.074 to 1.273)	1.173 (1.081 to 1.277)	1.169 (1.074 to 1.269)	1.173 (1.077 to 1.282)	1.167 (1.069 to 1.269)	1.169 (1.073 to 1.274)	1.171 (1.068 to 1.279)	1.171 (1.068 to 1.279)	1.171 (1.068 to 1.279)	1.171 (1.068 to 1.279)
Tracheal, bronchus, and lung cancer	Low exposure	Both	Males								1.0 (1.0 to 1.0)	1.0 (1.0 to 1.0)	1.0 (1.0 to 1.0)	1.0 (1.0 to 1.0)	1.0 (1.0 to 1.0)	1.0 (1.0 to 1.0)	1.0 (1.0 to 1.0)	1.0 (1.0 to 1.0)	1.0 (1.0 to 1.0)	1.0 (1.0 to 1.0)	1.0 (1.0 to 1.0)	1.0 (1.0 to 1.0)	1.0 (1.0 to 1.0)	1.0 (1.0 to 1.0)	1.0 (1.0 to 1.0)	1.0 (1.0 to 1.0)	1.0 (1.0 to 1.0)
Tracheal, bronchus, and lung cancer	Low exposure	Both	Females								1.0 (1.0 to 1.0)	1.0 (1.0 to 1.0)	1.0 (1.0 to 1.0)	1.0 (1.0 to 1.0)	1.0 (1.0 to 1.0)	1.0 (1.0 to 1.0)	1.0 (1.0 to 1.0)	1.0 (1.0 to 1.0)	1.0 (1.0 to 1.0)	1.0 (1.0 to 1.0)	1.0 (1.0 to 1.0)	1.0 (1.0 to 1.0)	1.0 (1.0 to 1.0)	1.0 (1.0 to 1.0)	1.0 (1.0 to 1.0)	1.0 (1.0 to 1.0)	1.0 (1.0 to 1.0)
Tracheal, bronchus, and lung cancer	No exposure	Both	Males								1.0 (1.0 to 1.0)	1.0 (1.0 to 1.0)	1.0 (1.0 to 1.0)	1.0 (1.0 to 1.0)	1.0 (1.0 to 1.0)	1.0 (1.0 to 1.0)	1.0 (1.0 to 1.0)	1.0 (1.0 to 1.0)	1.0 (1.0 to 1.0)	1.0 (1.0 to 1.0)	1.0 (1.0 to 1.0)	1.0 (1.0 to 1.0)	1.0 (1.0 to 1.0)	1.0 (1.0 to 1.0)	1.0 (1.0 to 1.0)	1.0 (1.0 to 1.0)	1.0 (1.0 to 1.0)
Tracheal, bronchus, and lung cancer	No exposure	Both	Females								1.0 (1.0 to 1.0)	1.0 (1.0 to 1.0)	1.0 (1.0 to 1.0)	1.0 (1.0 to 1.0)	1.0 (1.0 to 1.0)	1.0 (1.0 to 1.0)	1.0 (1.0 to 1.0)	1.0 (1.0 to 1.0)	1.0 (1.0 to 1.0)	1.0 (1.0 to 1.0)	1.0 (1.0 to 1.0)	1.0 (1.0 to 1.0)	1.0 (1.0 to 1.0)	1.0 (1.0 to 1.0)	1.0 (1.0 to 1.0)	1.0 (1.0 to 1.0)	1.0 (1.0 to 1.0)
**Occupational exposure to cadmium**																											
Tracheal, bronchus, and lung cancer	High exposure	Both	Males								1.192 (1.097 to 1.292)	1.188 (1.083 to 1.287)	1.19 (1.102 to 1.295)	1.191 (1.092 to 1.304)	1.19 (1.091 to 1.298)	1.19 (1.092 to 1.297)	1.192 (1.096 to 1.298)	1.193 (1.096 to 1.296)	1.19 (1.089 to 1.293)	1.192 (1.089 to 1.297)	1.193 (1.088 to 1.3)	1.187 (1.095 to 1.29)	1.191 (1.095 to 1.289)	1.19 (1.088 to 1.296)	1.19 (1.088 to 1.296)	1.19 (1.088 to 1.296)	1.19 (1.088 to 1.296)
Tracheal, bronchus, and lung cancer	High exposure	Both	Females								1.191 (1.087 to 1.295)	1.191 (1.099 to 1.29)	1.188 (1.093 to 1.293)	1.19 (1.092 to 1.302)	1.191 (1.089 to 1.292)	1.194 (1.098 to 1.297)	1.188 (1.091 to 1.3)	1.19 (1.095 to 1.291)	1.19 (1.096 to 1.294)	1.189 (1.095 to 1.29)	1.192 (1.095 to 1.296)	1.192 (1.09 to 1.298)	1.193 (1.097 to 1.297)	1.193 (1.1 to 1.301)	1.193 (1.1 to 1.301)	1.193 (1.1 to 1.301)	1.193 (1.1 to 1.301)
Tracheal, bronchus, and lung cancer	Low exposure	Both	Males								1.0 (1.0 to 1.0)	1.0 (1.0 to 1.0)	1.0 (1.0 to 1.0)	1.0 (1.0 to 1.0)	1.0 (1.0 to 1.0)	1.0 (1.0 to 1.0)	1.0 (1.0 to 1.0)	1.0 (1.0 to 1.0)	1.0 (1.0 to 1.0)	1.0 (1.0 to 1.0)	1.0 (1.0 to 1.0)	1.0 (1.0 to 1.0)	1.0 (1.0 to 1.0)	1.0 (1.0 to 1.0)	1.0 (1.0 to 1.0)	1.0 (1.0 to 1.0)	1.0 (1.0 to 1.0)
Tracheal, bronchus, and lung cancer	Low exposure	Both	Females								1.0 (1.0 to 1.0)	1.0 (1.0 to 1.0)	1.0 (1.0 to 1.0)	1.0 (1.0 to 1.0)	1.0 (1.0 to 1.0)	1.0 (1.0 to 1.0)	1.0 (1.0 to 1.0)	1.0 (1.0 to 1.0)	1.0 (1.0 to 1.0)	1.0 (1.0 to 1.0)	1.0 (1.0 to 1.0)	1.0 (1.0 to 1.0)	1.0 (1.0 to 1.0)	1.0 (1.0 to 1.0)	1.0 (1.0 to 1.0)	1.0 (1.0 to 1.0)	1.0 (1.0 to 1.0)
Tracheal, bronchus, and lung cancer	No exposure	Both	Males								1.0 (1.0 to 1.0)	1.0 (1.0 to 1.0)	1.0 (1.0 to 1.0)	1.0 (1.0 to 1.0)	1.0 (1.0 to 1.0)	1.0 (1.0 to 1.0)	1.0 (1.0 to 1.0)	1.0 (1.0 to 1.0)	1.0 (1.0 to 1.0)	1.0 (1.0 to 1.0)	1.0 (1.0 to 1.0)	1.0 (1.0 to 1.0)	1.0 (1.0 to 1.0)	1.0 (1.0 to 1.0)	1.0 (1.0 to 1.0)	1.0 (1.0 to 1.0)	1.0 (1.0 to 1.0)
Tracheal, bronchus, and lung cancer	No exposure	Both	Females								1.0 (1.0 to 1.0)	1.0 (1.0 to 1.0)	1.0 (1.0 to 1.0)	1.0 (1.0 to 1.0)	1.0 (1.0 to 1.0)	1.0 (1.0 to 1.0)	1.0 (1.0 to 1.0)	1.0 (1.0 to 1.0)	1.0 (1.0 to 1.0)	1.0 (1.0 to 1.0)	1.0 (1.0 to 1.0)	1.0 (1.0 to 1.0)	1.0 (1.0 to 1.0)	1.0 (1.0 to 1.0)	1.0 (1.0 to 1.0)	1.0 (1.0 to 1.0)	1.0 (1.0 to 1.0)
**Occupational exposure to chromium**																											
Tracheal, bronchus, and lung cancer	High exposure	Both	Males								1.179 (1.114 to 1.245)	1.181 (1.117 to 1.25)	1.181 (1.118 to 1.249)	1.18 (1.117 to 1.244)	1.183 (1.117 to 1.249)	1.18 (1.117 to 1.239)	1.18 (1.117 to 1.249)	1.182 (1.12 to 1.246)	1.181 (1.119 to 1.246)	1.179 (1.115 to 1.242)	1.182 (1.121 to 1.247)	1.18 (1.118 to 1.248)	1.18 (1.113 to 1.245)	1.181 (1.117 to 1.244)	1.181 (1.117 to 1.244)	1.181 (1.117 to 1.244)	1.181 (1.117 to 1.244)
Tracheal, bronchus, and lung cancer	High exposure	Both	Females								1.179 (1.116 to 1.247)	1.18 (1.115 to 1.248)	1.18 (1.115 to 1.244)	1.179 (1.118 to 1.243)	1.18 (1.116 to 1.248)	1.181 (1.115 to 1.245)	1.181 (1.119 to 1.25)	1.181 (1.118 to 1.245)	1.181 (1.117 to 1.248)	1.181 (1.117 to 1.244)	1.179 (1.121 to 1.245)	1.181 (1.116 to 1.254)	1.181 (1.116 to 1.245)	1.179 (1.113 to 1.247)	1.179 (1.113 to 1.247)	1.179 (1.113 to 1.247)	1.179 (1.113 to 1.247)
Tracheal, bronchus, and lung cancer	Low exposure	Both	Males								1.0 (1.0 to 1.0)	1.0 (1.0 to 1.0)	1.0 (1.0 to 1.0)	1.0 (1.0 to 1.0)	1.0 (1.0 to 1.0)	1.0 (1.0 to 1.0)	1.0 (1.0 to 1.0)	1.0 (1.0 to 1.0)	1.0 (1.0 to 1.0)	1.0 (1.0 to 1.0)	1.0 (1.0 to 1.0)	1.0 (1.0 to 1.0)	1.0 (1.0 to 1.0)	1.0 (1.0 to 1.0)	1.0 (1.0 to 1.0)	1.0 (1.0 to 1.0)	1.0 (1.0 to 1.0)
Tracheal, bronchus, and lung cancer	Low exposure	Both	Females								1.0 (1.0 to 1.0)	1.0 (1.0 to 1.0)	1.0 (1.0 to 1.0)	1.0 (1.0 to 1.0)	1.0 (1.0 to 1.0)	1.0 (1.0 to 1.0)	1.0 (1.0 to 1.0)	1.0 (1.0 to 1.0)	1.0 (1.0 to 1.0)	1.0 (1.0 to 1.0)	1.0 (1.0 to 1.0)	1.0 (1.0 to 1.0)	1.0 (1.0 to 1.0)	1.0 (1.0 to 1.0)	1.0 (1.0 to 1.0)	1.0 (1.0 to 1.0)	1.0 (1.0 to 1.0)
Tracheal, bronchus, and lung cancer	No exposure	Both	Males								1.0 (1.0 to 1.0)	1.0 (1.0 to 1.0)	1.0 (1.0 to 1.0)	1.0 (1.0 to 1.0)	1.0 (1.0 to 1.0)	1.0 (1.0 to 1.0)	1.0 (1.0 to 1.0)	1.0 (1.0 to 1.0)	1.0 (1.0 to 1.0)	1.0 (1.0 to 1.0)	1.0 (1.0 to 1.0)	1.0 (1.0 to 1.0)	1.0 (1.0 to 1.0)	1.0 (1.0 to 1.0)	1.0 (1.0 to 1.0)	1.0 (1.0 to 1.0)	1.0 (1.0 to 1.0)
Tracheal, bronchus, and lung cancer	No exposure	Both	Females								1.0 (1.0 to 1.0)	1.0 (1.0 to 1.0)	1.0 (1.0 to 1.0)	1.0 (1.0 to 1.0)	1.0 (1.0 to 1.0)	1.0 (1.0 to 1.0)	1.0 (1.0 to 1.0)	1.0 (1.0 to 1.0)	1.0 (1.0 to 1.0)	1.0 (1.0 to 1.0)	1.0 (1.0 to 1.0)	1.0 (1.0 to 1.0)	1.0 (1.0 to 1.0)	1.0 (1.0 to 1.0)	1.0 (1.0 to 1.0)	1.0 (1.0 to 1.0)	1.0 (1.0 to 1.0)
**Occupational exposure to diesel engine exhaust**																											
Tracheal, bronchus, and lung cancer	High exposure	Both	Males								1.469 (1.294 to 1.658)	1.477 (1.301 to 1.665)	1.473 (1.29 to 1.668)	1.474 (1.293 to 1.675)	1.472 (1.301 to 1.669)	1.47 (1.294 to 1.662)	1.475 (1.3 to 1.667)	1.469 (1.301 to 1.644)	1.474 (1.295 to 1.676)	1.477 (1.292 to 1.671)	1.477 (1.311 to 1.68)	1.476 (1.302 to 1.669)	1.473 (1.287 to 1.669)	1.477 (1.302 to 1.666)	1.477 (1.302 to 1.666)	1.477 (1.302 to 1.666)	1.477 (1.302 to 1.666)
Tracheal, bronchus, and lung cancer	High exposure	Both	Females								1.473 (1.287 to 1.682)	1.476 (1.303 to 1.681)	1.469 (1.288 to 1.67)	1.467 (1.282 to 1.67)	1.473 (1.288 to 1.66)	1.475 (1.294 to 1.68)	1.477 (1.3 to 1.679)	1.475 (1.302 to 1.674)	1.476 (1.292 to 1.68)	1.481 (1.309 to 1.681)	1.478 (1.295 to 1.683)	1.473 (1.291 to 1.661)	1.473 (1.29 to 1.676)	1.476 (1.289 to 1.662)	1.476 (1.289 to 1.662)	1.476 (1.289 to 1.662)	1.476 (1.289 to 1.662)
Tracheal, bronchus, and lung cancer	Low exposure	Both	Males								1.0 (1.0 to 1.0)	1.0 (1.0 to 1.0)	1.0 (1.0 to 1.0)	1.0 (1.0 to 1.0)	1.0 (1.0 to 1.0)	1.0 (1.0 to 1.0)	1.0 (1.0 to 1.0)	1.0 (1.0 to 1.0)	1.0 (1.0 to 1.0)	1.0 (1.0 to 1.0)	1.0 (1.0 to 1.0)	1.0 (1.0 to 1.0)	1.0 (1.0 to 1.0)	1.0 (1.0 to 1.0)	1.0 (1.0 to 1.0)	1.0 (1.0 to 1.0)	1.0 (1.0 to 1.0)
Tracheal, bronchus, and lung cancer	Low exposure	Both	Females								1.0 (1.0 to 1.0)	1.0 (1.0 to 1.0)	1.0 (1.0 to 1.0)	1.0 (1.0 to 1.0)	1.0 (1.0 to 1.0)	1.0 (1.0 to 1.0)	1.0 (1.0 to 1.0)	1.0 (1.0 to 1.0)	1.0 (1.0 to 1.0)	1.0 (1.0 to 1.0)	1.0 (1.0 to 1.0)	1.0 (1.0 to 1.0)	1.0 (1.0 to 1.0)	1.0 (1.0 to 1.0)	1.0 (1.0 to 1.0)	1.0 (1.0 to 1.0)	1.0 (1.0 to 1.0)
Tracheal, bronchus, and lung cancer	No exposure	Both	Males								1.0 (1.0 to 1.0)	1.0 (1.0 to 1.0)	1.0 (1.0 to 1.0)	1.0 (1.0 to 1.0)	1.0 (1.0 to 1.0)	1.0 (1.0 to 1.0)	1.0 (1.0 to 1.0)	1.0 (1.0 to 1.0)	1.0 (1.0 to 1.0)	1.0 (1.0 to 1.0)	1.0 (1.0 to 1.0)	1.0 (1.0 to 1.0)	1.0 (1.0 to 1.0)	1.0 (1.0 to 1.0)	1.0 (1.0 to 1.0)	1.0 (1.0 to 1.0)	1.0 (1.0 to 1.0)
Tracheal, bronchus, and lung cancer	No exposure	Both	Females								1.0 (1.0 to 1.0)	1.0 (1.0 to 1.0)	1.0 (1.0 to 1.0)	1.0 (1.0 to 1.0)	1.0 (1.0 to 1.0)	1.0 (1.0 to 1.0)	1.0 (1.0 to 1.0)	1.0 (1.0 to 1.0)	1.0 (1.0 to 1.0)	1.0 (1.0 to 1.0)	1.0 (1.0 to 1.0)	1.0 (1.0 to 1.0)	1.0 (1.0 to 1.0)	1.0 (1.0 to 1.0)	1.0 (1.0 to 1.0)	1.0 (1.0 to 1.0)	1.0 (1.0 to 1.0)
**Occupational exposure to formaldehyde**																											
Nasopharynx cancer	High exposure	Both	Males								2.222 (1.026 to 4.233)	2.294 (1.056 to 4.409)	2.204 (1.023 to 4.036)	2.211 (0.994 to 4.221)	2.23 (1.042 to 4.13)	2.241 (1.077 to 4.068)	2.265 (1.056 to 4.33)	2.198 (1.041 to 4.081)	2.232 (1.104 to 4.091)	2.215 (1.022 to 4.191)	2.251 (1.056 to 4.236)	2.233 (1.022 to 4.329)	2.234 (1.02 to 4.325)	2.204 (1.064 to 4.14)	2.204 (1.064 to 4.14)	2.204 (1.064 to 4.14)	2.204 (1.064 to 4.14)
Nasopharynx cancer	High exposure	Both	Females								2.202 (1.04 to 4.059)	2.246 (1.039 to 4.184)	2.227 (1.05 to 4.207)	2.269 (1.084 to 4.182)	2.264 (1.069 to 4.385)	2.217 (1.041 to 4.21)	2.201 (1.035 to 4.162)	2.221 (1.035 to 4.234)	2.225 (1.025 to 4.212)	2.276 (1.072 to 4.204)	2.261 (1.04 to 4.208)	2.258 (1.042 to 4.371)	2.241 (1.031 to 4.156)	2.229 (1.025 to 4.237)	2.229 (1.025 to 4.237)	2.229 (1.025 to 4.237)	2.229 (1.025 to 4.237)
Nasopharynx cancer	Low exposure	Both	Males								1.0 (1.0 to 1.0)	1.0 (1.0 to 1.0)	1.0 (1.0 to 1.0)	1.0 (1.0 to 1.0)	1.0 (1.0 to 1.0)	1.0 (1.0 to 1.0)	1.0 (1.0 to 1.0)	1.0 (1.0 to 1.0)	1.0 (1.0 to 1.0)	1.0 (1.0 to 1.0)	1.0 (1.0 to 1.0)	1.0 (1.0 to 1.0)	1.0 (1.0 to 1.0)	1.0 (1.0 to 1.0)	1.0 (1.0 to 1.0)	1.0 (1.0 to 1.0)	1.0 (1.0 to 1.0)
Nasopharynx cancer	Low exposure	Both	Females								1.0 (1.0 to 1.0)	1.0 (1.0 to 1.0)	1.0 (1.0 to 1.0)	1.0 (1.0 to 1.0)	1.0 (1.0 to 1.0)	1.0 (1.0 to 1.0)	1.0 (1.0 to 1.0)	1.0 (1.0 to 1.0)	1.0 (1.0 to 1.0)	1.0 (1.0 to 1.0)	1.0 (1.0 to 1.0)	1.0 (1.0 to 1.0)	1.0 (1.0 to 1.0)	1.0 (1.0 to 1.0)	1.0 (1.0 to 1.0)	1.0 (1.0 to 1.0)	1.0 (1.0 to 1.0)

Nasopharynx cancer	No exposure	Both	Males	1.0 (1.0 to 1.0)	1.0 (1.0 to 1.0)	1.0 (1.0 to 1.0)	1.0 (1.0 to 1.0)	1.0 (1.0 to 1.0)	1.0 (1.0 to 1.0)	1.0 (1.0 to 1.0)	1.0 (1.0 to 1.0)	1.0 (1.0 to 1.0)	1.0 (1.0 to 1.0)	1.0 (1.0 to 1.0)	1.0 (1.0 to 1.0)	1.0 (1.0 to 1.0)	1.0 (1.0 to 1.0)	1.0 (1.0 to 1.0)	1.0 (1.0 to 1.0)	1.0 (1.0 to 1.0)
Nasopharynx cancer	No exposure	Both	Females	1.0 (1.0 to 1.0)	1.0 (1.0 to 1.0)	1.0 (1.0 to 1.0)	1.0 (1.0 to 1.0)	1.0 (1.0 to 1.0)	1.0 (1.0 to 1.0)	1.0 (1.0 to 1.0)	1.0 (1.0 to 1.0)	1.0 (1.0 to 1.0)	1.0 (1.0 to 1.0)	1.0 (1.0 to 1.0)	1.0 (1.0 to 1.0)	1.0 (1.0 to 1.0)	1.0 (1.0 to 1.0)	1.0 (1.0 to 1.0)	1.0 (1.0 to 1.0)	1.0 (1.0 to 1.0)
Acute lymphoid leukaemia	High exposure	Both	Males	1.483 (1.191 to 1.818)	1.479 (1.183 to 1.83)	1.474 (1.182 to 1.815)	1.48 (1.193 to 1.827)	1.479 (1.197 to 1.804)	1.467 (1.174 to 1.843)	1.481 (1.178 to 1.839)	1.49 (1.199 to 1.831)	1.47 (1.197 to 1.842)	1.487 (1.2 to 1.846)	1.485 (1.188 to 1.803)	1.48 (1.18 to 1.836)	1.482 (1.202 to 1.839)	1.488 (1.198 to 1.827)	1.488 (1.198 to 1.827)	1.488 (1.198 to 1.827)	1.488 (1.198 to 1.827)
Acute lymphoid leukaemia	High exposure	Both	Females	1.485 (1.199 to 1.845)	1.485 (1.183 to 1.855)	1.479 (1.184 to 1.844)	1.471 (1.181 to 1.774)	1.469 (1.183 to 1.819)	1.486 (1.196 to 1.823)	1.485 (1.19 to 1.848)	1.481 (1.191 to 1.856)	1.48 (1.199 to 1.814)	1.47 (1.184 to 1.812)	1.473 (1.19 to 1.814)	1.471 (1.194 to 1.789)	1.49 (1.209 to 1.859)	1.464 (1.18 to 1.788)	1.464 (1.18 to 1.788)	1.464 (1.18 to 1.788)	1.464 (1.18 to 1.788)
Acute lymphoid leukaemia	Low exposure	Both	Males	1.0 (1.0 to 1.0)	1.0 (1.0 to 1.0)	1.0 (1.0 to 1.0)	1.0 (1.0 to 1.0)	1.0 (1.0 to 1.0)	1.0 (1.0 to 1.0)	1.0 (1.0 to 1.0)	1.0 (1.0 to 1.0)	1.0 (1.0 to 1.0)	1.0 (1.0 to 1.0)	1.0 (1.0 to 1.0)	1.0 (1.0 to 1.0)	1.0 (1.0 to 1.0)	1.0 (1.0 to 1.0)	1.0 (1.0 to 1.0)	1.0 (1.0 to 1.0)	1.0 (1.0 to 1.0)
Acute lymphoid leukaemia	Low exposure	Both	Females	1.0 (1.0 to 1.0)	1.0 (1.0 to 1.0)	1.0 (1.0 to 1.0)	1.0 (1.0 to 1.0)	1.0 (1.0 to 1.0)	1.0 (1.0 to 1.0)	1.0 (1.0 to 1.0)	1.0 (1.0 to 1.0)	1.0 (1.0 to 1.0)	1.0 (1.0 to 1.0)	1.0 (1.0 to 1.0)	1.0 (1.0 to 1.0)	1.0 (1.0 to 1.0)	1.0 (1.0 to 1.0)	1.0 (1.0 to 1.0)	1.0 (1.0 to 1.0)	1.0 (1.0 to 1.0)
Acute lymphoid leukaemia	No exposure	Both	Males	1.0 (1.0 to 1.0)	1.0 (1.0 to 1.0)	1.0 (1.0 to 1.0)	1.0 (1.0 to 1.0)	1.0 (1.0 to 1.0)	1.0 (1.0 to 1.0)	1.0 (1.0 to 1.0)	1.0 (1.0 to 1.0)	1.0 (1.0 to 1.0)	1.0 (1.0 to 1.0)	1.0 (1.0 to 1.0)	1.0 (1.0 to 1.0)	1.0 (1.0 to 1.0)	1.0 (1.0 to 1.0)	1.0 (1.0 to 1.0)	1.0 (1.0 to 1.0)	1.0 (1.0 to 1.0)
Acute lymphoid leukaemia	No exposure	Both	Females	1.0 (1.0 to 1.0)	1.0 (1.0 to 1.0)	1.0 (1.0 to 1.0)	1.0 (1.0 to 1.0)	1.0 (1.0 to 1.0)	1.0 (1.0 to 1.0)	1.0 (1.0 to 1.0)	1.0 (1.0 to 1.0)	1.0 (1.0 to 1.0)	1.0 (1.0 to 1.0)	1.0 (1.0 to 1.0)	1.0 (1.0 to 1.0)	1.0 (1.0 to 1.0)	1.0 (1.0 to 1.0)	1.0 (1.0 to 1.0)	1.0 (1.0 to 1.0)	1.0 (1.0 to 1.0)
Chronic lymphoid leukaemia	High exposure	Both	Males	1.483 (1.191 to 1.818)	1.479 (1.183 to 1.83)	1.474 (1.182 to 1.815)	1.48 (1.193 to 1.827)	1.479 (1.197 to 1.804)	1.467 (1.174 to 1.843)	1.481 (1.178 to 1.839)	1.49 (1.199 to 1.831)	1.47 (1.197 to 1.842)	1.487 (1.2 to 1.846)	1.485 (1.188 to 1.803)	1.48 (1.18 to 1.836)	1.482 (1.202 to 1.839)	1.488 (1.198 to 1.827)	1.488 (1.198 to 1.827)	1.488 (1.198 to 1.827)	1.488 (1.198 to 1.827)
Chronic lymphoid leukaemia	High exposure	Both	Females	1.485 (1.199 to 1.845)	1.485 (1.183 to 1.855)	1.479 (1.184 to 1.844)	1.471 (1.181 to 1.774)	1.469 (1.183 to 1.819)	1.486 (1.196 to 1.823)	1.485 (1.19 to 1.848)	1.481 (1.191 to 1.856)	1.48 (1.199 to 1.814)	1.47 (1.184 to 1.812)	1.473 (1.19 to 1.814)	1.471 (1.194 to 1.789)	1.49 (1.209 to 1.859)	1.464 (1.18 to 1.788)	1.464 (1.18 to 1.788)	1.464 (1.18 to 1.788)	1.464 (1.18 to 1.788)
Chronic lymphoid leukaemia	Low exposure	Both	Males	1.0 (1.0 to 1.0)	1.0 (1.0 to 1.0)	1.0 (1.0 to 1.0)	1.0 (1.0 to 1.0)	1.0 (1.0 to 1.0)	1.0 (1.0 to 1.0)	1.0 (1.0 to 1.0)	1.0 (1.0 to 1.0)	1.0 (1.0 to 1.0)	1.0 (1.0 to 1.0)	1.0 (1.0 to 1.0)	1.0 (1.0 to 1.0)	1.0 (1.0 to 1.0)	1.0 (1.0 to 1.0)	1.0 (1.0 to 1.0)	1.0 (1.0 to 1.0)	1.0 (1.0 to 1.0)
Chronic lymphoid leukaemia	Low exposure	Both	Females	1.0 (1.0 to 1.0)	1.0 (1.0 to 1.0)	1.0 (1.0 to 1.0)	1.0 (1.0 to 1.0)	1.0 (1.0 to 1.0)	1.0 (1.0 to 1.0)	1.0 (1.0 to 1.0)	1.0 (1.0 to 1.0)	1.0 (1.0 to 1.0)	1.0 (1.0 to 1.0)	1.0 (1.0 to 1.0)	1.0 (1.0 to 1.0)	1.0 (1.0 to 1.0)	1.0 (1.0 to 1.0)	1.0 (1.0 to 1.0)	1.0 (1.0 to 1.0)	1.0 (1.0 to 1.0)
Chronic lymphoid leukaemia	No exposure	Both	Males	1.0 (1.0 to 1.0)	1.0 (1.0 to 1.0)	1.0 (1.0 to 1.0)	1.0 (1.0 to 1.0)	1.0 (1.0 to 1.0)	1.0 (1.0 to 1.0)	1.0 (1.0 to 1.0)	1.0 (1.0 to 1.0)	1.0 (1.0 to 1.0)	1.0 (1.0 to 1.0)	1.0 (1.0 to 1.0)	1.0 (1.0 to 1.0)	1.0 (1.0 to 1.0)	1.0 (1.0 to 1.0)	1.0 (1.0 to 1.0)	1.0 (1.0 to 1.0)	1.0 (1.0 to 1.0)
Chronic lymphoid leukaemia	No exposure	Both	Females	1.0 (1.0 to 1.0)	1.0 (1.0 to 1.0)	1.0 (1.0 to 1.0)	1.0 (1.0 to 1.0)	1.0 (1.0 to 1.0)	1.0 (1.0 to 1.0)	1.0 (1.0 to 1.0)	1.0 (1.0 to 1.0)	1.0 (1.0 to 1.0)	1.0 (1.0 to 1.0)	1.0 (1.0 to 1.0)	1.0 (1.0 to 1.0)	1.0 (1.0 to 1.0)	1.0 (1.0 to 1.0)	1.0 (1.0 to 1.0)	1.0 (1.0 to 1.0)	1.0 (1.0 to 1.0)
Acute myeloid leukaemia	High exposure	Both	Males	1.483 (1.191 to 1.818)	1.479 (1.183 to 1.83)	1.474 (1.182 to 1.815)	1.48 (1.193 to 1.827)	1.479 (1.197 to 1.804)	1.467 (1.174 to 1.843)	1.481 (1.178 to 1.839)	1.49 (1.199 to 1.831)	1.47 (1.197 to 1.842)	1.487 (1.2 to 1.846)	1.485 (1.188 to 1.803)	1.48 (1.18 to 1.836)	1.482 (1.202 to 1.839)	1.488 (1.198 to 1.827)	1.488 (1.198 to 1.827)	1.488 (1.198 to 1.827)	1.488 (1.198 to 1.827)
Acute myeloid leukaemia	High exposure	Both	Females	1.485 (1.199 to 1.845)	1.485 (1.183 to 1.855)	1.479 (1.184 to 1.844)	1.471 (1.181 to 1.774)	1.469 (1.183 to 1.819)	1.486 (1.196 to 1.823)	1.485 (1.19 to 1.848)	1.481 (1.191 to 1.856)	1.48 (1.199 to 1.814)	1.47 (1.184 to 1.812)	1.473 (1.19 to 1.814)	1.471 (1.194 to 1.789)	1.49 (1.209 to 1.859)	1.464 (1.18 to 1.788)	1.464 (1.18 to 1.788)	1.464 (1.18 to 1.788)	1.464 (1.18 to 1.788)
Acute myeloid leukaemia	Low exposure	Both	Males	1.0 (1.0 to 1.0)	1.0 (1.0 to 1.0)	1.0 (1.0 to 1.0)	1.0 (1.0 to 1.0)	1.0 (1.0 to 1.0)	1.0 (1.0 to 1.0)	1.0 (1.0 to 1.0)	1.0 (1.0 to 1.0)	1.0 (1.0 to 1.0)	1.0 (1.0 to 1.0)	1.0 (1.0 to 1.0)	1.0 (1.0 to 1.0)	1.0 (1.0 to 1.0)	1.0 (1.0 to 1.0)	1.0 (1.0 to 1.0)	1.0 (1.0 to 1.0)	1.0 (1.0 to 1.0)
Acute myeloid leukaemia	Low exposure	Both	Females	1.0 (1.0 to 1.0)	1.0 (1.0 to 1.0)	1.0 (1.0 to 1.0)	1.0 (1.0 to 1.0)	1.0 (1.0 to 1.0)	1.0 (1.0 to 1.0)	1.0 (1.0 to 1.0)	1.0 (1.0 to 1.0)	1.0 (1.0 to 1.0)	1.0 (1.0 to 1.0)	1.0 (1.0 to 1.0)	1.0 (1.0 to 1.0)	1.0 (1.0 to 1.0)	1.0 (1.0 to 1.0)	1.0 (1.0 to 1.0)	1.0 (1.0 to 1.0)	1.0 (1.0 to 1.0)
Acute myeloid leukaemia	No exposure	Both	Males	1.0 (1.0 to 1.0)	1.0 (1.0 to 1.0)	1.0 (1.0 to 1.0)	1.0 (1.0 to 1.0)	1.0 (1.0 to 1.0)	1.0 (1.0 to 1.0)	1.0 (1.0 to 1.0)	1.0 (1.0 to 1.0)	1.0 (1.0 to 1.0)	1.0 (1.0 to 1.0)	1.0 (1.0 to 1.0)	1.0 (1.0 to 1.0)	1.0 (1.0 to 1.0)	1.0 (1.0 to 1.0)	1.0 (1.0 to 1.0)	1.0 (1.0 to 1.0)	1.0 (1.0 to 1.0)
Acute myeloid leukaemia	No exposure	Both	Females	1.0 (1.0 to 1.0)	1.0 (1.0 to 1.0)	1.0 (1.0 to 1.0)	1.0 (1.0 to 1.0)	1.0 (1.0 to 1.0)	1.0 (1.0 to 1.0)	1.0 (1.0 to 1.0)	1.0 (1.0 to 1.0)	1.0 (1.0 to 1.0)	1.0 (1.0 to 1.0)	1.0 (1.0 to 1.0)	1.0 (1.0 to 1.0)	1.0 (1.0 to 1.0)	1.0 (1.0 to 1.0)	1.0 (1.0 to 1.0)	1.0 (1.0 to 1.0)	1.0 (1.0 to 1.0)
Chronic myeloid leukaemia	High exposure	Both	Males	1.483 (1.191 to 1.818)	1.479 (1.183 to 1.83)	1.474 (1.182 to 1.815)	1.48 (1.193 to 1.827)	1.479 (1.197 to 1.804)	1.467 (1.174 to 1.843)	1.481 (1.178 to 1.839)	1.49 (1.199 to 1.831)	1.47 (1.197 to 1.842)	1.487 (1.2 to 1.846)	1.485 (1.188 to 1.803)	1.48 (1.18 to 1.836)	1.482 (1.202 to 1.839)	1.488 (1.198 to 1.827)	1.488 (1.198 to 1.827)	1.488 (1.198 to 1.827)	1.488 (1.198 to 1.827)
Chronic myeloid leukaemia	High exposure	Both	Females	1.485 (1.199 to 1.845)	1.485 (1.183 to 1.855)	1.479 (1.184 to 1.844)	1.471 (1.181 to 1.774)	1.469 (1.183 to 1.819)	1.486 (1.196 to 1.823)	1.485 (1.19 to 1.848)	1.481 (1.191 to 1.856)	1.48 (1.199 to 1.814)	1.47 (1.184 to 1.812)	1.473 (1.19 to 1.814)	1.471 (1.194 to 1.789)	1.49 (1.209 to 1.859)	1.464 (1.18 to 1.788)	1.464 (1.18 to 1.788)	1.464 (1.18 to 1.788)	1.464 (1.18 to 1.788)
Chronic myeloid leukaemia	Low exposure	Both	Males	1.0 (1.0 to 1.0)	1.0 (1.0 to 1.0)	1.0 (1.0 to 1.0)	1.0 (1.0 to 1.0)	1.0 (1.0 to 1.0)	1.0 (1.0 to 1.0)	1.0 (1.0 to 1.0)	1.0 (1.0 to 1.0)	1.0 (1.0 to 1.0)	1.0 (1.0 to 1.0)	1.0 (1.0 to 1.0)	1.0 (1.0 to 1.0)	1.0 (1.0 to 1.0)	1.0 (1.0 to 1.0)	1.0 (1.0 to 1.0)	1.0 (1.0 to 1.0)	1.0 (1.0 to 1.0)
Chronic myeloid leukaemia	Low exposure	Both	Females	1.0 (1.0 to 1.0)	1.0 (1.0 to 1.0)	1.0 (1.0 to 1.0)	1.0 (1.0 to 1.0)	1.0 (1.0 to 1.0)	1.0 (1.0 to 1.0)	1.0 (1.0 to 1.0)	1.0 (1.0 to 1.0)	1.0 (1.0 to 1.0)	1.0 (1.0 to 1.0)	1.0 (1.0 to 1.0)	1.0 (1.0 to 1.0)	1.0 (1.0 to 1.0)	1.0 (1.0 to 1.0)	1.0 (1.0 to 1.0)	1.0 (1.0 to 1.0)	1.0 (1.0 to 1.0)
Chronic myeloid leukaemia	No exposure	Both	Males	1.0 (1.0 to 1.0)	1.0 (1.0 to 1.0)	1.0 (1.0 to 1.0)	1.0 (1.0 to 1.0)	1.0 (1.0 to 1.0)	1.0 (1.0 to 1.0)	1.0 (1.0 to 1.0)	1.0 (1.0 to 1.0)	1.0 (1.0 to 1.0)	1.0 (1.0 to 1.0)	1.0 (1.0 to 1.0)	1.0 (1.0 to 1.0)	1.0 (1.0 to 1.0)	1.0 (1.0 to 1.0)	1.0 (1.0 to 1.0)	1.0 (1.0 to 1.0)	1.0 (1.0 to 1.0)
Chronic myeloid leukaemia	No exposure	Both	Females	1.0 (1.0 to 1.0)	1.0 (1.0 to 1.0)	1.0 (1.0 to 1.0)	1.0 (1.0 to 1.0)	1.0 (1.0 to 1.0)	1.0 (1.0 to 1.0)	1.0 (1.0 to 1.0)	1.0 (1.0 to 1.0)	1.0 (1.0 to 1.0)	1.0 (1.0 to 1.0)	1.0 (1.0 to 1.0)	1.0 (1.0 to 1.0)	1.0 (1.0 to 1.0)	1.0 (1.0 to 1.0)	1.0 (1.0 to 1.0)	1.0 (1.0 to 1.0)	1.0 (1.0 to 1.0)
Other leukaemia	High exposure	Both	Males	1.483 (1.191 to 1.818)	1.479 (1.183 to 1.83)	1.474 (1.182 to 1.815)	1.48 (1.193 to 1.827)	1.479 (1.197 to 1.804)	1.467 (1.174 to 1.843)	1.481 (1.178 to 1.839)	1.49 (1.199 to 1.831)	1.47 (1.197 to 1.842)	1.487 (1.2 to 1.846)	1.485 (1.188 to 1.803)	1.48 (1.18 to 1.836)	1.482 (1.202 to 1.839)	1.488 (1.198 to 1.827)	1.488 (1.198 to 1.827)	1.488 (1.198 to 1.827)	1.488 (1.198 to 1.827)
Other leukaemia	High exposure	Both	Females	1.485 (1.199 to 1.845)	1.485 (1.183 to 1.855)	1.479 (1.184 to 1.844)	1.471 (1.181 to 1.774)	1.469 (1.183 to 1.819)	1.486 (1.196 to 1.823)	1.485 (1.19 to 1.848)	1.481 (1.191 to 1.856)	1.48 (1.199 to 1.814)	1.47 (1.184 to 1.812)	1.473 (1.19 to 1.814)	1.471 (1.194 to 1.789)	1.49 (1.209 to 1.859)	1.464 (1.18 to 1.788)	1.464 (1.18 to 1.788)	1.464 (1.18 to 1.788)	1.464 (1.18 to 1.788)
Other leukaemia	Low exposure	Both	Males	1.0 (1.0 to 1.0)	1.0 (1.0 to 1.0)	1.0 (1.0 to 1.0)	1.0 (1.0 to 1.0)	1.0 (1.0 to 1.0)	1.0 (1.0 to 1.0)	1.0 (1.0 to 1.0)	1.0 (1.0 to 1.0)	1.0 (1.0 to 1.0)	1.0 (1.0 to 1.0)	1.0 (1.0 to 1.0)	1.0 (1.0 to 1.0)	1.0 (1.0 to 1.0)	1.0 (1.0 to 1.0)	1.0 (1.0 to 1.0)	1.0 (1.0 to 1.0)	1.0 (1.0 to 1.0)
Other leukaemia	Low exposure	Both	Females	1.0 (1.0 to 1.0)	1.0 (1.0 to 1.0)	1.0 (1.0 to 1.0)	1.0 (1.0 to 1.0)	1.0 (1.0 to 1.0)	1.0 (1.0 to 1.0)	1.0 (1.0 to 1.0)	1.0 (1.0 to 1.0)	1.0 (1.0 to 1.0)	1.0 (1.0 to 1.0)	1.0 (1.0 to 1.0)	1.0 (1.0 to 1.0)	1.0 (1.0 to 1.0)	1.0 (1.0 to 1.0)	1.0 (1.0 to 1.0)	1.0 (1.0 to 1.0)	1.0 (1.0 to 1.0)
Other leukaemia	No exposure	Both	Males	1.0 (1.0 to 1.0)	1.0 (1.0 to 1.0)	1.0 (1.0 to 1.0)	1.0 (1.0 to 1.0)	1.0 (1.0 to 1.0)	1.0 (1.0 to 1.0)	1.0 (1.0 to 1.0)	1.0 (1.0 to 1.0)	1.0 (1.0 to 1.0)	1.0 (1.0 to 1.0)	1.0 (1.0 to 1.0)	1.0 (1.0 to 1.0)	1.0 (1.0 to 1.0)	1.0 (1.0 to 1.0)	1.0 (1.0 to 1.0)	1.0 (1.0 to 1.0)	1.0 (1.0 to 1.0)
Other leukaemia	No exposure	Both	Females	1.0 (1.0 to 1.0)	1.0 (1.0 to 1.0)	1.0 (1.0 to 1.0)	1.0 (1.0 to 1.0)	1.0 (1.0 to 1.0)	1.0 (1.0 to 1.0)	1.0 (1.0 to 1.0)	1.0 (1.0 to 1.0)	1.0 (1.0 to 1.0)	1.0 (1.0 to 1.0)	1.0 (1.0 to 1.0)	1.0 (1.0 to 1.0)	1.0 (1.0 to 1.0)	1.0 (1.0 to 1.0)	1.0 (1.0 to 1.0)	1.0 (1.0 to 1.0)	1.0 (1.0 to 1.0)
**Occupational exposure to nickel**																				
Tracheal, bronchus, and lung cancer	High exposure	Both	Both	2.148 (1.296 to 3.297)	2.148 (1.296 to 3.297)	2.148 (1.296 to 3.297)	2.148 (1.296 to 3.297)	2.148 (1.296 to 3.297)	2.148 (1.296 to 3.297)	2.148 (1.296 to 3.297)	2.148 (1.296 to 3.297)	2.148 (1.296 to 3.297)	2.148 (1.296 to 3.297)	2.148 (1.296 to 3.297)	2.148 (1.296 to 3.297)	2.148 (1.296 to 3.297)	2.148 (1.296 to 3.297)	2.148 (1.296 to 3.297)	2.148 (1.296 to 3.297)	2.148 (1.296 to 3.297)
Tracheal, bronchus, and lung cancer	Low exposure	Both	Both	1.538 (0.606 to 3.355)	1.538 (0.606 to 3.355)	1.538 (0.606 to 3.355)	1.538 (0.606 to 3.355)	1.538 (0.606 to 3.355)	1.538 (0.606 to 3.355)	1.538 (0.606 to 3.355)	1.538 (0.606 to 3.355)	1.538 (0.606 to 3.355)	1.538 (0.606 to 3.355)	1.538 (0.606 to 3.355)	1.538 (0.606 to 3.355)	1.538 (0.606 to 3.355)	1.538 (0.606 to 3.355)	1.538 (0.606 to 3.355)	1.538 (0.606 to 3.355)	1.538 (0.606 to 3.355)
Tracheal, bronchus, and lung cancer	No exposure	Both	Both	1.0 (1.0 to 1.0)	1.0 (1.0 to 1.0)	1.0 (1.0 to 1.0)	1.0 (1.0 to 1.0)	1.0 (1.0 to 1.0)	1.0 (1.0 to 1.0)	1.0 (1.0 to 1.0)	1.0 (1.0 to 1.0)	1.0 (1.0 to 1.0)	1.0 (1.0 to 1.0)	1.0 (1.0 to 1.0)	1.0 (1.0 to 1.0)	1.0 (1.0 to 1.0)	1.0 (1.0 to 1.0)	1.0 (1.0 to 1.0)	1.0 (1.0 to 1.0)	1.0 (1.0 to 1.0)
**Occupational exposure to polycyclic aromatic hydrocarbons**																				
Tracheal, bronchus, and lung cancer	High exposure	Both	Males	1.31 (1.165 to 1.468)	1.304 (1.146 to 1.466)	1.313 (1.164 to 1.485)	1.314 (1.167 to 1.477)	1.315 (1.17 to 1.474)	1.314 (1.16 to 1.475)	1.315 (1.154 to 1.477)	1.312 (1.16 to 1.477)	1.318 (1.166 to 1.483)	1.311 (1.158 to 1.479)	1.313 (1.166 to 1.485)	1.314 (1.154 to 1.483)	1.313 (1.161 to 1.478)	1.309 (1.176 to 1.477)	1.309 (1.176 to 1.477)	1.309 (1.176 to 1.477)	1.309 (1.176 to 1.477)
Tracheal, bronchus, and lung cancer	High exposure	Both	Females	1.31 (1.154 to 1.486)	1.311 (1.157 to 1.469)	1.313 (1.154 to 1.472)	1.313 (1.155 to 1.469)	1.316 (1.162 to 1.483)	1.313 (1.156 to 1.476)	1.314 (1.155 to 1.483)	1.314 (1.171 to 1.483)	1.311 (1.146 to 1.477)	1.312 (1.163 to 1.481)	1.309 (1.147 to 1.489)	1.316 (1.168 to 1.475)	1.315 (1.15 to 1.479)	1.315 (1.166 to 1.481)	1.315 (1.166 to 1.481)	1.315 (1.166 to 1.481)	1.315 (1.166 to 1.481)
Tracheal, bronchus, and lung cancer	Low exposure	Both	Males	1.0 (1.0 to 1.0)	1.0 (1.0 to 1.0)	1.0 (1.0 to 1.0)	1.0 (1.0 to 1.0)	1.0 (1.0 to 1.0)	1.0 (1.0 to 1.0)	1.0 (1.0 to 1.0)	1.0 (1.0 to 1.0)	1.0 (1.0 to 1.0)	1.0 (1.0 to 1.0)	1.0 (1.0 to 1.0)	1.0 (1.0 to 1.0)	1.0 (1.0 to 1.0)	1.0 (1.0 to 1.0)	1.0 (1.0 to 1.0)	1.0 (1.0 to 1.0)	1.0 (1.0 to 1.0)
Tracheal, bronchus, and lung cancer	Low exposure	Both	Females	1.0 (1.0 to 1.0)	1.0 (1.0 to 1.0)	1.0 (1.0 to 1.0)	1.0 (1.0 to 1.0)	1.0 (1.0 to 1.0)	1.0 (1.0 to 1.0)	1.0 (1.0 to 1.0)	1.0 (1.0 to 1.0)	1.0 (1.0 to 1.0)	1.0 (1.0 to 1.0)	1.0 (1.0 to 1.0)	1.0 (1.0 to 1.0)	1.0 (1.0 to 1.0)	1.0 (1.0 to 1.0)	1.0 (1.0 to 1.0)	1.0 (1.0 to 1.0)	1.0 (1.0 to 1.0)
Tracheal, bronchus, and lung cancer	No exposure	Both	Males	1.0 (1.0 to 1.0)	1.0 (1.0 to 1.0)	1.0 (1.0 to 1.0)	1.0 (1.0 to 1.0)	1.0 (1.0 to 1.0)	1.0 (1.0 to 1.0)	1.0 (1.0 to 1.0)	1.0 (1.0 to 1.0)	1.0 (1.0 to 1.0)	1.0 (1.0 to 1.0)	1.0 (1.0 to 1.0)	1.0 (1.0 to 1.0)	1.0 (1.0 to 1.0)	1.0 (1.0 to 1.0)	1.0 (1.0 to 1.0)	1.0 (1.0 to 1.0)	1.0 (1.0 to 1.0)
Tracheal, bronchus, and lung cancer	No exposure	Both	Females	1.0 (1.0 to 1.0)	1.0 (1.0 to 1.0)	1.0 (1.0 to 1.0)	1.0 (1.0 to 1.0)	1.0 (1.0 to 1.0)	1.0 (1.0 to 1.0)	1.0 (1.0 to 1.0)	1.0 (1.0 to 1.0)	1.0 (1.0 to 1.0)	1.0 (1.0 to 1.0)	1.0 (1.0 to 1.0)	1.0 (1.0 to 1.0)	1.0 (1.0 to 1.0)	1.0 (1.0 to 1.0)	1.0 (1.0 to 1.0)	1.0 (1.0 to 1.0)	1.0 (1.0 to 1.0)
**Occupational exposure to silica**																				
Tracheal, bronchus, and lung cancer	High exposure	Both	Both	1.698 (1.164 to 2.259)	1.698 (1.164 to 2.259)	1.698 (1.164 to 2.259)	1.698 (1.164 to 2.259)	1.698 (1.164 to 2.259)	1.698 (1.164 to 2.259)	1.698 (1.164 to 2.259)	1.698 (1.164 to 2.259)	1.698 (1.164 to 2.259)	1.698 (1.164 to 2.259)	1.698 (1.164 to 2.259)	1.698 (1.164 to 2.259)	1.698 (1.164 to 2.259)	1.698 (1.164 to 2.259)	1.698 (1.164 to 2.259)	1.698 (1.164 to 2.259)	1.698 (1.164 to 2.259)
Tracheal, bronchus, and lung cancer	Low exposure	Both	Both	1.537 (1.063 to 1.986)	1.537 (1.063 to 1.986)	1.537 (1.063 to 1.986)	1.537 (1.063 to 1.986)	1.537 (1.063 to 1.986)	1.537 (1.063 to 1.986)	1.537 (1.063 to 1.986)	1.537 (1.063 to 1.986)	1.537 (1.063 to 1.986)	1.537 (1.063 to 1.986)	1.537 (1.063 to 1.986)	1.537 (1.063 to 1.986)	1.537 (1.063 to 1.986)	1.537 (1.063 to 1.986)	1.537 (1.063 to 1.986)	1.537 (1.063 to 1.986)	1.537 (1.063 to 1.986)
Tracheal, bronchus, and lung cancer	No exposure	Both	Both	1.0 (1.0 to 1.0)	1.0 (1.0 to 1.0)	1.0 (1.0 to 1.0)	1.0 (1.0 to 1.0)	1.0 (1.0 to 1.0)	1.0 (1.0 to 1.0)	1.0 (1.0 to 1.0)	1.0 (1.0 to 1.0)	1.0 (1.0 to 1.0)	1.0 (1.0 to 1.0)	1.0 (1.0 to 1.0)	1.0 (1.0 to 1.0)	1.0 (1.0 to 1.0)	1.0 (1.0 to 1.0)	1.0 (1.0 to 1.0)	1.0 (1.0 to 1.0)	1.0 (1.0 to 1.0)
**Occupational exposure to sulphuric acid**																				
Larynx cancer	High exposure	Both	Both	4.566 (2.122 to 8.328)	4.566 (2.122 to 8.328)	4.566 (2.122 to 8.328)	4.566 (2.122 to 8.328)	4.566 (2.122 to 8.328)	4.566 (2.122 to 8.328)	4.566 (2.122 to 8.328)	4.566 (2.122 to 8.328)	4.566 (2.122 to 8.328)	4.566 (2.122 to 8.328)	4.566 (2.122 to 8.328)	4.566 (2.122 to 8.328)	4.566 (2.122 to 8.328)	4.566 (2.122 to 8.328)	4.566 (2.122 to 8.328)	4.566 (2.122 to 8.328)	4.566 (2.122 to 8.328)
Larynx cancer	Low exposure	Both	Both	2.024 (0.944 to 3.782)	2.024 (0.944 to 3.782)	2.024 (0.944 to 3.782)	2.024 (0.944 to 3.782)	2.024 (0.944 to 3.782)	2.024 (0.944 to 3.782)	2.024 (0.944 to 3.782)	2.024 (0.944 to 3.782)	2.024 (0.944 to 3.782)	2.024 (0.944 to 3.782)	2.024 (0.944 to 3.782)	2.024 (0.944 to 3.782)	2.024 (0.944 to 3.782)	2.024 (0.944 to 3.782)	2.024 (0.944 to 3.782)	2.024 (0.944 to 3.782)	2.024 (0.944 to 3.782)
Larynx cancer	No exposure	Both	Both	1.0 (1.0 to 1.0)	1.0 (1.0 to 1.0)	1.0 (1.0 to 1.0)	1.0 (1.0 to 1.0)	1.0 (1.0 to 1.0)	1.0 (1.0 to 1.0)	1.0 (1.0 to 1.0)	1.0 (1.0 to 1.0)	1.0 (1.0 to 1.0)	1.0 (1.0 to 1.0)	1.0 (1.0 to 1.0)	1.0 (1.0 to 1.0)	1.0 (1.0 to 1.0)	1.0 (1.0 to 1.0)	1.0 (1.0 to 1.0)	1.0 (1.0 to 1.0)	1.0 (1.0 to 1.0)
**Occupational exposure to trichloroethylene**																				
Kidney cancer	High exposure	Both	Both	1.245 (1.054 to 1.456)	1.245 (1.054 to 1.456)	1.245 (1.054 to 1.456)	1.245 (1.054 to 1.456)	1.245 (1.054 to 1.456)	1.245 (1.054 to 1.456)	1.245 (1.054 to 1.456)	1.245 (1.054 to 1.456)	1.245 (1.054 to 1.456)	1.245 (1.054 to 1.456)	1.245 (1.054 to 1.456)	1.245 (1.054 to 1.456)	1.245 (1.054 to 1.456)	1.245 (1.054 to 1.456)	1.245 (1.054 to 1.456)	1.245 (1.054 to 1.456)	1.245 (1.054 to 1.456)
Kidney cancer	Low exposure	Both	Both	1.0 (1.0 to 1.0)	1.0 (1.0 to 1.0)	1.0 (1.0 to 1.0)	1.0 (1.0 to 1.0)	1.0 (1.0 to 1.0)	1.0 (1.0 to 1.0)	1.0 (1.0 to 1.0)	1.0 (1.0 to 1.0)	1.0 (1.0 to 1.0)	1.0 (1.0 to 1.0)	1.0 (1.0 to 1.0)	1.0 (1.0 to 1.0)	1.0 (1.0 to 1.0)	1.0 (1.0 to 1.0)	1.0 (1.0 to 1.0)	1.0 (1.0 to 1.0)	1.0 (1.0 to 1.0)
Kidney cancer	No exposure	Both	Both	1.0 (1.0 to 1.0)	1.0 (1.0 to 1.0)	1.0 (1.0 to 1.0)	1.0 (1.0 to 1.0)	1.0 (1.0 to 1.0)	1.0 (1.0 to 1.0)	1.0 (1.0 to 1.0)	1.0 (1.0 to 1.0)	1.0 (1.0 to 1.0)	1.0 (1.0 to 1.0)	1.0 (1.0 to 1.0)	1.0 (1.0 to 1.0)	1.0 (1.0 to 1.0)	1.0 (1.0 to 1.0)	1.0 (1.0 to 1.0)	1.0 (1.0 to 1.0)	1.0 (1.0 to 1.0)
**Occupational asthmagens**																				
Asthma	Admin	Both	Males	1.0 (1.0 to 1.0)	1.0 (1.0 to 1.0)	1.0 (1.0 to 1.0)	1.0 (1.0 to 1.0)	1.0 (1.0 to 1.0)	1.0 (1.0 to 1.0)	1.0 (1.0 to 1.0)	1.0 (1.0 to 1.0)	1.0 (1.0 to 1.0)	1.0 (1.0 to 1.0)	1.0 (1.0 to 1.0)	1.0 (1.0 to 1.0)	1.0 (1.0 to 1.0)	1.0 (1.0 to 1.0)			
Asthma	Admin	Both	Females	1.0 (1.0 to 1.0)	1.0 (1.0 to 1.0)	1.0 (1.0 to 1.0)	1.0 (1.0 to 1.0)	1.0 (1.0 to 1.0)	1.0 (1.0 to 1.0)	1.0 (1.0 to 1.0)	1.0 (1.0 to 1.0)	1.0 (1.0 to 1.0)	1.0 (1.0 to 1.0)	1.0 (1.0 to 1.0)	1.0 (1.0 to 1.0)	1.0 (1.0 to 1.0)	1.0 (1.0 to 1.0)			
Asthma	Technical	Both	Males	1.05 (0.977 to 1.125)	1.051 (0.979 to 1.122)	1.051 (0.982 to 1.124)	1.051 (0.983 to 1.118)	1.05 (0.977 to 1.12)	1.05 (0.981 to 1.122)	1.051 (0.98 to 1.121)	1.051 (0.986 to 1.121)	1.05 (0.976 to 1.125)	1.05 (0.987 to 1.118)	1.051 (0.984 to 1.121)	1.051 (0.982 to 1.122)	1.051 (0.981 to 1.122)	1.051 (0.981 to 1.122)			
Asthma	Technical	Both	Females	1.06 (1.028 to 1.095)	1.06 (1.024 to 1.099)	1.059 (1.025 to 1.095)	1.061 (1.026 to 1.099)	1.06 (1.027 to 1.093)	1.06 (1.026 to 1.094)	1.059 (1.024 to 1.093)	1.06 (1.028 to 1.094)	1.06 (1.027 to 1.092)	1.061 (1.026 to 1.095)	1.06 (1.027 to 1.096)	1.061 (1.023 to 1.096)	1.06 (1.027 to 1.096)	1.06 (1.027 to 1.096)			
Asthma	Sales	Both	Males	1.14 (1.047 to 1.237)	1.14 (1.049 to 1.234)	1.144 (1.055 to 1.233)	1.142 (1.048 to 1.234)	1.14 (1.046 to 1.234)	1.141 (1.053 to 1.235)	1.143 (1.052 to 1.238)	1.14 (1.047 to 1.236)	1.141 (1.056 to 1.235)	1.138 (1.046 to 1.236)	1.139 (1.057 to 1.226)	1.141 (1.052 to 1.243)	1.14 (1.058 to 1.239)	1.14 (1.058 to 1.239)			
Asthma	Sales	Both	Females	1.131 (1.083 to 1.182)	1.13 (1.083 to 1.178)	1.129 (1.083 to 1.178)	1.13 (1.081 to 1.182)	1.131 (1.084 to 1.18)	1.13 (1.08 to 1.181)	1.13 (1.083 to 1.181)	1.13 (1.079 to 1.178)	1.131 (1.077 to 1.184)	1.13 (1.081 to 1.182)	1.129 (1.082 to 1.181)	1.131 (1.083 to 1.184)	1.131 (1.083 to 1.183)	1.131 (1.083 to 1.183)			
Asthma	Agriculture	Both	Males	1.519 (1.1 to 2.029)	1.527 (1.119 to 2.063)	1.524 (1.103 to 2.019)	1.513 (1.127 to 1.999)	1.523 (1.108 to 2.023)	1.516 (1.083 to 2.043)	1.522 (1.106 to 2.038)	1.52 (1.122 to 2.023)	1.519 (1.103 to 2.011)	1.531 (1.122 to 2.047)	1.52 (1.106 to 2.041)	1.508 (1.083 to 2.019)	1.498 (1.081 to 1.965)	1.498 (1.081 to 1.965)			
Asthma	Agriculture	Both	Females	1.52 (1.125 to 2.022)	1.506 (1.099 to 1.997)	1.52 (1.115 to 2.05)	1.514 (1.109 to 2.048)	1.509 (1.101 to 1.969)	1.513 (1.128 to 2.026)	1.508 (1.108 to 2.0)	1.526 (1.108 to 2.07)	1.519 (1.117 to 1.981)	1.53 (1.105 to 2.017)	1.519 (1.107 to 2.024)	1.518 (1.115 to 2.018)	1.502 (1.094 to 2.03)	1.502 (1.094 to 2.03)			
Asthma	Mining	Both	Males	1.959 (1.576 to 2.413)	1.959 (1.568 to 2.381)	1.971 (1.602 to 2.414)	1.966 (1.571 to 2.396)	1.963 (1.601 to 2.395)	1.959 (1.588 to 2.385)	1.956 (1.595 to 2.406)	1.964 (1.57 to 2.397)	1.969 (1.616 to 2.417)	1.954 (1.574 to 2.406)	1.965 (1.558 to 2.393)	1.955 (1.577 to 2.39)	1.953 (1.551 to 2.418)	1.953 (1.551 to 2.418)			
Asthma	Mining	Both	Females	1.956 (1.58 to 2.408)	1.961 (1.574 to 2.395)	1.967 (1.586 to 2.417)	1.952 (1.567 to 2.371)	1.959 (1.575 to 2.416)	1.959 (1.567 to 2.417)	1.962 (1.585 to 2.386)	1.955 (1.591 to 2.393)	1.948 (1.57 to 2.381)	1.964 (1.597 to 2.409)	1.965 (1.59 to 2.414)	1.959 (1.588 to 2.39)	1.973 (1.586 to 2.392)	1.973 (1.586 to 2.392)			
Asthma	Transport	Both	Males	1.313 (1.22 to 1.402)	1.311 (1.222 to 1.406)	1.312 (1.225 to 1.399)	1.31 (1.218 to 1.398)	1.312 (1.225 to 1.404)	1.311 (1.225 to 1.401)	1.313 (1.226 to 1.402)	1.31 (1.223 to 1.398)	1.314 (1.221 to 1.396)	1.313 (1.224 to 1.408)	1.311 (1.228 to 1.397)	1.312 (1.225 to 1.409)	1.311 (1.226 to 1.407)	1.311 (1.226 to 1.407)			
Asthma	Transport	Both	Females	1.221 (1.132 to 1.312)	1.22 (1.138 to 1.31)	1.217 (1.136 to 1.303)	1.221 (1.138 to 1.313)	1.221 (1.137 to 1.317)	1.221 (1.132 to 1.314)	1.22 (1.132 to 1.312)	1.22 (1.134 to 1.313)	1.221 (1.14 to 1.315)	1.223 (1.139 to 1.309)	1.221 (1.133 to 1.312)	1.224 (1.135 to 1.316)	1.22 (1.133 to 1.313)	1.22 (1.133 to 1.313)			
Asthma	Manufact	Both	Males	1.559 (1.477 to 1.647)	1.561 (1.474 to 1.652)	1.56 (1.474 to 1.657)	1.562 (1.472 to 1.658)	1.561 (1.473 to 1.65)	1.561 (1.471 to 1.655)	1.56 (1.471 to 1.649)	1.562 (1.473 to 1.654)	1.558 (1.461 to 1.655)	1.562 (1.479 to 1.655)	1.559 (1.475 to 1.652)	1.562 (1.476 to 1.657)	1.561 (1.474 to 1.654)	1.561 (1.474 to 1.654)			
Asthma	Manufact	Both	Females	1.33 (1.272 to 1.392)	1.331 (1.27 to 1.39)	1.33 (1.271 to 1.39)	1.329 (1.271 to 1.388)	1.331 (1.268 to 1.392)	1.329 (1.269 to 1.39)	1.33 (1.269 to 1.391)	1.332 (1.272 to 1.394)	1.331 (1.273 to 1.389)	1.331 (1.272 to 1.391)	1.33 (1.268 to 1.392)	1.33 (1.268 to 1.394)	1.331 (1.272 to 1.39)	1.331 (1.272 to 1.39)			
Asthma	Services	Both	Males	1.531 (1.415 to 1.646)	1.531 (1.416 to 1.652)	1.529 (1.409 to 1.649)	1.532 (1.413 to 1.653)	1.529 (1.424 to 1.648)	1.529 (1.416 to 1.645)	1.533 (1.418 to 1.646)	1.535 (1.427 to 1.649)	1.531 (1.416 to 1.655)	1.528 (1.411 to 1.651)	1.53 (1.416 to 1.652)	1.53 (1.411 to 1.658)	1.531 (1.415 to 1.655)	1.531 (1.415 to 1.655)			
Asthma	Services	Both	Females	1.41 (1.352 to 1.467)	1.41 (1.357 to 1.469)	1.412 (1.357 to 1.467)	1.41 (1.356 to 1.464)	1.409 (1.353 to 1.463)	1.409 (1.354 to 1.467)	1.41 (1.354 to 1.467)	1.411 (1.357 to 1.464)	1.409 (1.357 to 1.46)	1.411 (1.355 to 1.466)	1.41 (1.354 to 1.465)	1.41 (1.357 to 1.467)	1.411 (1.357 to 1.464)	1.411 (1.357 to 1.464)			
Asthma	Other	Both	Males	1.0 (1.0 to 1.0)	1.0 (1.0 to 1.0)	1.0 (1.0 to 1.0)	1.0 (1.0 to 1.0)	1.0 (1.0 to 1.0)	1.0 (1.0 to 1.0)	1.0 (1.0 to 1.0)	1.0 (1.0 to 1.0)	1.0 (1.0 to 1.0)	1.0 (1.0 to 1.0)	1.0 (1.0 to 1.0)	1.0 (1.0 to 1.0)	1.0 (1.0 to 1.0)	1.0 (1.0 to 1.0)			
Asthma	Other	Both	Females	1.0 (1.0 to 1.0)	1.0 (1.0 to 1.0)	1.0 (1.0 to 1.0)	1.0 (1.0 to 1.0)	1.0 (1.0 to 1.0)	1.0 (1.0 to 1.0)	1.0 (1.0 to 1.0)	1.0 (1.0 to 1.0)	1.0 (1.0 to 1.0)	1.0 (1.0 to 1.0)	1.0 (1.0 to 1.0)	1.0 (1.0 to 1.0)	1.0 (1.0 to 1.0)	1.0 (1.0 to 1.0)			
**Occupational particulate matter, gases, and fumes**																				
Chronic obstructive pulmonary disease	High	Both	Males	2.364 (1.463 to 3.64)	2.341 (1.426 to 3.555)	2.37 (1.41 to 3.596)	2.391 (1.455 to 3.756)	2.387 (1.409 to 3.791)	2.379 (1.4 to 3.672)	2.409 (1.439 to 3.833)	2.361 (1.447 to 3.684)	2.367 (1.454 to 3.654)	2.373 (1.375 to 3.64)	2.357 (1.438 to 3.685)	2.402 (1.434 to 3.763)	2.398 (1.441 to 3.813)	2.359 (1.429 to 3.695)	2.359 (1.429 to 3.695)	2.359 (1.429 to 3.695)	2.359 (1.429 to 3.695)
Chronic obstructive pulmonary disease	High	Both	Females	2.371 (1.459 to 3.719)	2.395 (1.483 to 3.669)	2.364 (1.473 to 3.694)	2.377 (1.431 to 3.763)	2.375 (1.446 to 3.698)	2.326 (1.438 to 3.576)	2.35 (1.432 to 3.702)	2.35 (1.425 to 3.609)	2.364 (1.461 to 3.677)	2.364 (1.475 to 3.64)	2.395 (1.476 to 3.715)	2.336 (1.434 to 3.673)	2.363 (1.431 to 3.741)	2.404 (1.462 to 3.619)	2.404 (1.462 to 3.619)	2.404 (1.462 to 3.619)	2.404 (1.462 to 3.619)
Chronic obstructive pulmonary disease	Low	Both	Males	1.462 (1.057 to 1.915)	1.457 (1.052 to 1.935)	1.464 (1.085 to 1.91)	1.45 (1.067 to 1.947)	1.452 (1.06 to 1.963)	1.454 (1.075 to 1.96)	1.446 (1.055 to 1.929)	1.457 (1.076 to 1.933)	1.453 (1.052 to 1.903)	1.46 (1.067 to 1.954)	1.443 (1.057 to 1.916)	1.462 (1.097 to 1.954)	1.464 (1.079 to 1.952)	1.462 (1.096 to 1.965)	1.462 (1.096 to 1.965)	1.462 (1.096 to 1.965)	1.462 (1.096 to 1.965)
Chronic obstructive pulmonary disease	Low	Both	Females	1.446 (1.063 to 1.904)	1.459 (1.09 to 1.954)	1.453 (1.055 to 2.003)	1.459 (1.072 to 1.931)	1.456 (1.056 to 1.968)	1.47 (1.089 to 1.96)	1.44 (1.046 to 1.932)	1.457 (1.097 to 1.925)	1.455 (1.061 to 1.921)	1.451 (1.082 to 1.912)	1.459 (1.102 to 1.941)	1.448 (1.077 to 1.929)	1.467 (1.072 to 1.971)	1.456 (1.056 to 1.927)	1.456 (1.056 to 1.927)	1.456 (1.056 to 1.927)	1.456 (1.056 to 1.927)
Chronic obstructive pulmonary disease	None	Both	Males	1.0 (1.0 to 1.0)	1.0 (1.0 to 1.0)	1.0 (1.0 to 1.0)	1.0 (1.0 to 1.0)	1.0 (1.0 to 1.0)	1.0 (1.0 to 1.0)	1.0 (1.0 to 1.0)	1.0 (1.0 to 1.0)	1.0 (1.0 to 1.0)	1.0 (1.0 to 1.0)	1.0 (1.0 to 1.0)	1.0 (1.0 to 1.0)	1.0 (1.0 to 1.0)	1.0 (1.0 to 1.0)	1.0 (1.0 to 1.0)	1.0 (1.0 to 1.0)	1.0 (1.0 to 1.0)
Chronic obstructive pulmonary disease	None	Both	Females	1.0 (1.0 to 1.0)	1.0 (1.0 to 1.0)	1.0 (1.0 to 1.0)	1.0 (1.0 to 1.0)	1.0 (1.0 to 1.0)	1.0 (1.0 to 1.0)	1.0 (1.0 to 1.0)	1.0 (1.0 to 1.0)	1.0 (1.0 to 1.0)	1.0 (1.0 to 1.0)	1.0 (1.0 to 1.0)	1.0 (1.0 to 1.0)	1.0 (1.0 to 1.0)	1.0 (1.0 to 1.0)	1.0 (1.0 to 1.0)	1.0 (1.0 to 1.0)	1.0 (1.0 to 1.0)
**Occupational noise**																				
Mild hearing loss due to age-related and other hearing loss	High exposure, >90dB	Morbidity	Both	7.477 (5.049 to 11.132)	7.423 (4.787 to 10.956)	5.504 (4.293 to 6.843)	5.456 (4.339 to 6.828)	3.07 (2.692 to 3.51)	3.081 (2.715 to 3.498)	2.546 (2.332 to 2.774)	2.554 (2.367 to 2.752)	1.849 (1.712 to 1.995)	1.85 (1.707 to 1.995)	1.45 (1.372 to 1.526)	1.454 (1.378 to 1.529)	1.131 (1.048 to 1.211)	1.131 (1.048 to 1.211)	1.131 (1.048 to 1.211)	1.131 (1.048 to 1.211)	1.131 (1.048 to 1.211)
Mild hearing loss with ringing due to age-related and other hearing loss	High exposure, >90dB	Morbidity	Both	7.477 (5.049 to 11.132)	7.423 (4.787 to 10.956)	5.504 (4.293 to 6.843)	5.456 (4.339 to 6.828)	3.07 (2.692 to 3.51)	3.081 (2.715 to 3.498)	2.546 (2.332 to 2.774)	2.554 (2.367 to 2.752)	1.849 (1.712 to 1.995)	1.85 (1.707 to 1.995)	1.45 (1.372 to 1.526)	1.454 (1.378 to 1.529)	1.131 (1.048 to 1.211)	1.131 (1.048 to 1.211)	1.131 (1.048 to 1.211)	1.131 (1.048 to 1.211)	1.131 (1.048 to 1.211)
Moderate hearing loss due to age-related and other hearing loss	High exposure, >90dB	Morbidity	Both	8.175 (4.62 to 13.221)	8.31 (4.828 to 13.462)	6.71 (4.785 to 9.239)	6.756 (4.705 to 9.168)	5.992 (4.34 to 8.307)	5.987 (4.304 to 8.107)	5.624 (3.954 to 7.821)	5.61 (3.96 to 7.69)	3.643 (2.576 to 5.019)	3.601 (2.494 to 4.936)	2.17 (1.597 to 2.84)	2.146 (1.656 to 2.862)	1.291 (1.06 to 1.539)	1.291 (1.06 to 1.539)	1.291 (1.06 to 1.539)	1.291 (1.06 to 1.539)	1.291 (1.06 to 1.539)
Moderate hearing loss with ringing due to age-related and other hearing loss	High exposure, >90dB	Morbidity	Both	8.175 (4.62 to 13.221)	8.31 (4.828 to 13.462)	6.71 (4.785 to 9.239)	6.756 (4.705 to 9.168)	5.992 (4.34 to 8.307)	5.987 (4.304 to 8.107)	5.624 (3.954 to 7.821)	5.61 (3.96 to 7.69)	3.643 (2.576 to 5.019)	3.601 (2.494 to 4.936)	2.17 (1.597 to 2.84)	2.146 (1.656 to 2.862)	1.291 (1.06 to 1.539)	1.291 (1.06 to 1.539)	1.291 (1.06 to 1.539)	1.291 (1.06 to 1.539)	1.291 (1.06 to 1.539)
Moderately severe hearing loss due to age-related and other hearing loss	High exposure, >90dB	Morbidity	Both	8.27 (4.807 to 13.224)	8.233 (4.709 to 13.126)	6.764 (4.814 to 9.271)	6.726 (4.762 to 9.201)	6.007 (4.114 to 8.374)	6.072 (4.34 to 8.475)	5.638 (3.943 to 7.822)	5.704 (4.007 to 8.022)	3.638 (2.576 to 4.959)	3.591 (2.583 to 4.981)	2.162 (1.64 to 2.816)	2.168 (1.631 to 2.827)	1.297 (1.06 to 1.561)	1.297 (1.06 to 1.561)	1.297 (1.06 to 1.561)	1.297 (1.06 to 1.561)	1.297 (1.06 to 1.561)
Moderately severe hearing loss with ringing due to age-related and other hearing loss	High exposure, >90dB	Morbidity	Both	8.27 (4.807 to 13.224)	8.233 (4.709 to 13.126)	6.764 (4.814 to 9.271)	6.726 (4.762 to 9.201)	6.007 (4.114 to 8.374)	6.072 (4.34 to 8.475)	5.638 (3.943 to 7.822)	5.704 (4.007 to 8.022)	3.638 (2.576 to 4.959)	3.591 (2.583 to 4.981)	2.162 (1.64 to 2.816)	2.168 (1.631 to 2.827)	1.297 (1.06 to 1.561)	1.297 (1.06 to 1.561)	1.297 (1.06 to 1.561)	1.297 (1.06 to 1.561)	1.297 (1.06 to 1.561)
Severe hearing loss with ringing due to age-related and other hearing loss	High exposure, >90dB	Morbidity	Both	8.249 (4.72 to 13.18)	8.292 (4.754 to 13.144)	6.707 (4.73 to 9.34)	6.687 (4.739 to 9.181)	6.075 (4.315 to 8.372)	5.983 (4.247 to 8.221)	5.66 (4.039 to 7.875)	5.61 (3.946 to 7.534)	3.62 (2.541 to 5.01)	3.583 (2.513 to 5.117)	2.138 (1.606 to 2.802)	2.173 (1.645 to 2.807)	1.294 (1.059 to 1.569)	1.294 (1.059 to 1.569)	1.294 (1.059 to 1.569)	1.294 (1.059 to 1.569)	1.294 (1.059 to 1.569)
Severe hearing loss due to age-related and other hearing loss	High exposure, >90dB	Morbidity	Both	8.249 (4.72 to 13.18)	8.292 (4.754 to 13.144)	6.707 (4.73 to 9.34)	6.687 (4.739 to 9.181)	6.075 (4.315 to 8.372)	5.983 (4.247 to 8.221)	5.66 (4.039 to 7.875)	5.61 (3.946 to 7.534)	3.62 (2.541 to 5.01)	3.583 (2.513 to 5.117)	2.138 (1.606 to 2.802)	2.173 (1.645 to 2.807)	1.294 (1.059 to 1.569)	1.294 (1.059 to 1.569)	1.294 (1.059 to 1.569)	1.294 (1.059 to 1.569)	1.294 (1.059 to 1.569)
Profound hearing loss due to age-related and other hearing loss	High exposure, >90dB	Morbidity	Both	8.33 (4.77 to 13.62)	8.437 (4.945 to 13.539)	6.69 (4.739 to 9.26)	6.734 (4.811 to 9.238)	5.974 (4.17 to 8.13)	5.981 (4.273 to 8.413)	5.629 (3.965 to 7.879)	5.589 (3.838 to 7.693)	3.591 (2.489 to 5.087)	3.607 (2.48 to 5.048)	2.144 (1.619 to 2.83)	2.173 (1.638 to 2.822)	1.294 (1.081 to 1.562)	1.294 (1.081 to 1.562)	1.294 (1.081 to 1.562)	1.294 (1.081 to 1.562)	1.294 (1.081 to 1.562)
Profound hearing loss with ringing due to age-related and other hearing loss	High exposure, >90dB	Morbidity	Both	8.33 (4.77 to 13.62)	8.437 (4.945 to 13.539)	6.69 (4.739 to 9.26)	6.734 (4.811 to 9.238)	5.974 (4.17 to 8.13)	5.981 (4.273 to 8.413)	5.629 (3.965 to 7.879)	5.589 (3.838 to 7.693)	3.591 (2.489 to 5.087)	3.607 (2.48 to 5.048)	2.144 (1.619 to 2.83)	2.173 (1.638 to 2.822)	1.294 (1.081 to 1.562)	1.294 (1.081 to 1.562)	1.294 (1.081 to 1.562)	1.294 (1.081 to 1.562)	1.294 (1.081 to 1.562)
Complete hearing loss due to age-related and other hearing loss	High exposure, >90dB	Morbidity	Both	8.242 (4.856 to 13.285)	8.371 (4.712 to 13.317)	6.771 (4.783 to 9.41)	6.703 (4.728 to 9.419)	5.932 (4.209 to 8.003)	5.941 (4.176 to 8.222)	5.62 (3.963 to 7.916)	5.628 (4.002 to 7.742)	3.625 (2.498 to 5.132)	3.625 (2.558 to 5.036)	2.17 (1.614 to 2.802)	2.17 (1.602 to 2.881)	1.291 (1.06 to 1.548)	1.291 (1.06 to 1.548)	1.291 (1.06 to 1.548)	1.291 (1.06 to 1.548)	1.291 (1.06 to 1.548)
Complete hearing loss with ringing due to age-related and other hearing loss	High exposure, >90dB	Morbidity	Both	8.242 (4.856 to 13.285)	8.371 (4.712 to 13.317)	6.771 (4.783 to 9.41)	6.703 (4.728 to 9.419)	5.932 (4.209 to 8.003)	5.941 (4.176 to 8.222)	5.62 (3.963 to 7.916)	5.628 (4.002 to 7.742)	3.625 (2.498 to 5.132)	3.625 (2.558 to 5.036)	2.17 (1.614 to 2.802)	2.17 (1.602 to 2.881)	1.291 (1.06 to 1.548)	1.291 (1.06 to 1.548)	1.291 (1.06 to 1.548)	1.291 (1.06 to 1.548)	1.291 (1.06 to 1.548)
Mild hearing loss due to age-related and other hearing loss	Low exposure, 85-90dB	Morbidity	Both	2.807 (1.872 to 3.963)	2.764 (1.846 to 4.054)	2.953 (2.342 to 3.708)	2.934 (2.322 to 3.605)	2.176 (1.917 to 2.47)	2.172 (1.929 to 2.475)	1.988 (1.831 to 2.153)	1.991 (1.826 to 2.156)	1.551 (1.432 to 1.675)	1.553 (1.432 to 1.686)	1.301 (1.231 to 1.37)	1.3 (1.233 to 1.37)	1.091 (1.017 to 1.172)	1.091 (1.017 to 1.172)	1.091 (1.017 to 1.172)	1.091 (1.017 to 1.172)	1.091 (1.017 to 1.172)
Mild hearing loss with ringing due to age-related and other hearing loss	Low exposure, 85-90dB	Morbidity	Both	2.807 (1.872 to 3.963)	2.764 (1.846 to 4.054)	2.953 (2.342 to 3.708)	2.934 (2.322 to 3.605)	2.176 (1.917 to 2.47)	2.172 (1.929 to 2.475)	1.988 (1.831 to 2.153)	1.991 (1.826 to 2.156)	1.551 (1.432 to 1.675)	1.553 (1.432 to 1.686)	1.301 (1.231 to 1.37)	1.3 (1.233 to 1.37)	1.091 (1.017 to 1.172)	1.091 (1.017 to 1.172)	1.091 (1.017 to 1.172)	1.091 (1.017 to 1.172)	1.091 (1.017 to 1.172)
Moderate hearing loss due to age-related and other hearing loss	Low exposure, 85-90dB	Morbidity	Both	3.072 (1.768 to 4.925)	3.037 (1.78 to 4.813)	3.471 (2.441 to 4.807)	3.454 (2.449 to 4.718)	3.888 (2.724 to 5.326)	3.855 (2.701 to 5.4)	3.93 (2.791 to 5.397)	3.925 (2.715 to 5.487)	2.719 (1.933 to 3.726)	2.689 (1.863 to 3.673)	1.815 (1.363 to 2.404)	1.817 (1.363 to 2.435)	1.228 (1.022 to 1.468)	1.228 (1.022 to 1.468)	1.228 (1.022 to 1.468)	1.228 (1.022 to 1.468)	1.228 (1.022 to 1.468)
Moderate hearing loss with ringing due to age-related and other hearing loss	Low exposure, 85-90dB	Morbidity	Both	3.072 (1.768 to 4.925)	3.037 (1.78 to 4.813)	3.471 (2.441 to 4.807)	3.454 (2.449 to 4.718)	3.888 (2.724 to 5.326)	3.855 (2.701 to 5.4)	3.93 (2.791 to 5.397)	3.925 (2.715 to 5.487)	2.719 (1.933 to 3.726)	2.689 (1.863 to 3.673)	1.815 (1.363 to 2.404)	1.817 (1.363 to 2.435)	1.228 (1.022 to 1.468)	1.228 (1.022 to 1.468)	1.228 (1.022 to 1.468)	1.228 (1.022 to 1.468)	1.228 (1.022 to 1.468)
Moderately severe hearing loss due to age-related and other hearing loss	Low exposure, 85-90dB	Morbidity	Both	3.043 (1.733 to 5.008)	3.008 (1.821 to 4.959)	3.472 (2.48 to 4.825)	3.45 (2.427 to 4.851)	3.84 (2.68 to 5.285)	3.809 (2.686 to 5.308)	3.952 (2.757 to 5.596)	3.931 (2.788 to 5.437)	2.732 (1.891 to 3.782)	2.709 (1.9 to 3.742)	1.837 (1.33 to 2.433)	1.807 (1.37 to 2.364)	1.219 (1.017 to 1.455)	1.219 (1.017 to 1.455)	1.219 (1.017 to 1.455)	1.219 (1.017 to 1.455)	1.219 (1.017 to 1.455)
Moderately severe hearing loss with ringing due to age-related and other hearing loss	Low exposure, 85-90dB	Morbidity	Both	3.043 (1.733 to 5.008)	3.008 (1.821 to 4.959)	3.472 (2.48 to 4.825)	3.45 (2.427 to 4.851)	3.84 (2.68 to 5.285)	3.809 (2.686 to 5.308)	3.952 (2.757 to 5.596)	3.931 (2.788 to 5.437)	2.732 (1.891 to 3.782)	2.709 (1.9 to 3.742)	1.837 (1.33 to 2.433)	1.807 (1.37 to 2.364)	1.219 (1.017 to 1.455)	1.219 (1.017 to 1.455)	1.219 (1.017 to 1.455)	1.219 (1.017 to 1.455)	1.219 (1.017 to 1.455)
Severe hearing loss with ringing due to age-related and other hearing loss	Low exposure, 85-90dB	Morbidity	Both	3.023 (1.794 to 4.956)	2.972 (1.758 to 4.883)	3.444 (2.425 to 4.739)	3.482 (2.463 to 4.764)	3.837 (2.732 to 5.263)	3.867 (2.71 to 5.354)	3.98 (2.746 to 5.531)	3.943 (2.754 to 5.548)	2.711 (1.904 to 3.803)	2.693 (1.929 to 3.736)	1.83 (1.387 to 2.422)	1.825 (1.347 to 2.402)	1.218 (1.0 to 1.475)	1.218 (1.0 to 1.475)	1.218 (1.0 to 1.475)	1.218 (1.0 to 1.475)	1.218 (1.0 to 1.475)
Severe hearing loss due to age-related and other hearing loss	Low exposure, 85-90dB	Morbidity	Both	3.023 (1.794 to 4.956)	2.972 (1.758 to 4.883)	3.444 (2.425 to 4.739)	3.482 (2.463 to 4.764)	3.837 (2.732 to 5.263)	3.867 (2.71 to 5.354)	3.98 (2.746 to 5.531)	3.943 (2.754 to 5.548)	2.711 (1.904 to 3.803)	2.693 (1.929 to 3.736)	1.83 (1.387 to 2.422)	1.825 (1.347 to 2.402)	1.218 (1.0 to 1.475)	1.218 (1.0 to 1.475)	1.218 (1.0 to 1.475)	1.218 (1.0 to 1.475)	1.218 (1.0 to 1.475)
Profound hearing loss due to age-related and other hearing loss	Low exposure, 85-90dB	Morbidity	Both	3.023 (1.718 to 5.041)	3.018 (1.749 to 4.908)	3.455 (2.448 to 4.681)	3.446 (2.457 to 4.727)	3.875 (2.755 to 5.323)	3.848 (2.721 to 5.132)	3.943 (2.697 to 5.393)	3.944 (2.769 to 5.398)	2.697 (1.935 to 3.775)	2.69 (1.888 to 3.733)	1.833 (1.374 to 2.406)	1.813 (1.34 to 2.386)	1.222 (0.998 to 1.479)	1.222 (0.998 to 1.479)	1.222 (0.998 to 1.479)	1.222 (0.998 to 1.479)	1.222 (0.998 to 1.479)
Profound hearing loss with ringing due to age-related and other hearing loss	Low exposure, 85-90dB	Morbidity	Both	3.023 (1.718 to 5.041)	3.018 (1.749 to 4.908)	3.455 (2.448 to 4.681)	3.446 (2.457 to 4.727)	3.875 (2.755 to 5.323)	3.848 (2.721 to 5.132)	3.943 (2.697 to 5.393)	3.944 (2.769 to 5.398)	2.697 (1.935 to 3.775)	2.69 (1.888 to 3.733)	1.833 (1.374 to 2.406)	1.813 (1.34 to 2.386)	1.222 (0.998 to 1.479)	1.222 (0.998 to 1.479)	1.222 (0.998 to 1.479)	1.222 (0.998 to 1.479)	1.222 (0.998 to 1.479)
Complete hearing loss due to age-related and other hearing loss	Low exposure, 85-90dB	Morbidity	Both	3.004 (1.775 to 4.913)	2.986 (1.804 to 4.882)	3.482 (2.473 to 4.715)	3.478 (2.449 to 4.702)	3.858 (2.702 to 5.284)	3.813 (2.745 to 5.341)	3.917 (2.805 to 5.383)	3.985 (2.826 to 5.477)	2.701 (1.874 to 3.658)	2.695 (1.885 to 3.731)	1.812 (1.376 to 2.371)	1.824 (1.359 to 2.407)	1.222 (1.015 to 1.475)	1.222 (1.015 to 1.475)	1.222 (1.015 to 1.475)	1.222 (1.015 to 1.475)	1.222 (1.015 to 1.475)
Complete hearing loss with ringing due to age-related and other hearing loss	Low exposure, 85-90dB	Morbidity	Both	3.004 (1.775 to 4.913)	2.986 (1.804 to 4.882)	3.482 (2.473 to 4.715)	3.478 (2.449 to 4.702)	3.858 (2.702 to 5.284)	3.813 (2.745 to 5.341)	3.917 (2.805 to 5.383)	3.985 (2.826 to 5.477)	2.701 (1.874 to 3.658)	2.695 (1.885 to 3.731)	1.812 (1.376 to 2.371)	1.824 (1.359 to 2.407)	1.222 (1.015 to 1.475)	1.222 (1.015 to 1.475)	1.222 (1.015 to 1.475)	1.222 (1.015 to 1.475)	1.222 (1.015 to 1.475)
Mild hearing loss due to age-related and other hearing loss	No exposure	Morbidity	Both	1.0 (1.0 to 1.0)	1.0 (1.0 to 1.0)	1.0 (1.0 to 1.0)	1.0 (1.0 to 1.0)	1.0 (1.0 to 1.0)	1.0 (1.0 to 1.0)	1.0 (1.0 to 1.0)	1.0 (1.0 to 1.0)	1.0 (1.0 to 1.0)	1.0 (1.0 to 1.0)	1.0 (1.0 to 1.0)	1.0 (1.0 to 1.0)	1.0 (1.0 to 1.0)	1.0 (1.0 to 1.0)	1.0 (1.0 to 1.0)	1.0 (1.0 to 1.0)	1.0 (1.0 to 1.0)
Mild hearing loss with ringing due to age-related and other hearing loss	No exposure	Morbidity	Both	1.0 (1.0 to 1.0)	1.0 (1.0 to 1.0)	1.0 (1.0 to 1.0)	1.0 (1.0 to 1.0)	1.0 (1.0 to 1.0)	1.0 (1.0 to 1.0)	1.0 (1.0 to 1.0)	1.0 (1.0 to 1.0)	1.0 (1.0 to 1.0)	1.0 (1.0 to 1.0)	1.0 (1.0 to 1.0)	1.0 (1.0 to 1.0)	1.0 (1.0 to 1.0)	1.0 (1.0 to 1.0)	1.0 (1.0 to 1.0)	1.0 (1.0 to 1.0)	1.0 (1.0 to 1.0)
Moderate hearing loss due to age-related and other hearing loss	No exposure	Morbidity	Both	1.0 (1.0 to 1.0)	1.0 (1.0 to 1.0)	1.0 (1.0 to 1.0)	1.0 (1.0 to 1.0)	1.0 (1.0 to 1.0)	1.0 (1.0 to 1.0)	1.0 (1.0 to 1.0)	1.0 (1.0 to 1.0)	1.0 (1.0 to 1.0)	1.0 (1.0 to 1.0)	1.0 (1.0 to 1.0)	1.0 (1.0 to 1.0)	1.0 (1.0 to 1.0)	1.0 (1.0 to 1.0)	1.0 (1.0 to 1.0)	1.0 (1.0 to 1.0)	1.0 (1.0 to 1.0)
Moderate hearing loss with ringing due to age-related and other hearing loss	No exposure	Morbidity	Both	1.0 (1.0 to 1.0)	1.0 (1.0 to 1.0)	1.0 (1.0 to 1.0)	1.0 (1.0 to 1.0)	1.0 (1.0 to 1.0)	1.0 (1.0 to 1.0)	1.0 (1.0 to 1.0)	1.0 (1.0 to 1.0)	1.0 (1.0 to 1.0)	1.0 (1.0 to 1.0)	1.0 (1.0 to 1.0)	1.0 (1.0 to 1.0)	1.0 (1.0 to 1.0)	1.0 (1.0 to 1.0)	1.0 (1.0 to 1.0)	1.0 (1.0 to 1.0)	1.0 (1.0 to 1.0)
Moderately severe hearing loss due to age-related and other hearing loss	No exposure	Morbidity	Both	1.0 (1.0 to 1.0)	1.0 (1.0 to 1.0)	1.0 (1.0 to 1.0)	1.0 (1.0 to 1.0)	1.0 (1.0 to 1.0)	1.0 (1.0 to 1.0)	1.0 (1.0 to 1.0)	1.0 (1.0 to 1.0)	1.0 (1.0 to 1.0)	1.0 (1.0 to 1.0)	1.0 (1.0 to 1.0)	1.0 (1.0 to 1.0)	1.0 (1.0 to 1.0)	1.0 (1.0 to 1.0)	1.0 (1.0 to 1.0)	1.0 (1.0 to 1.0)	1.0 (1.0 to 1.0)
Moderately severe hearing loss with ringing due to age-related and other hearing loss	No exposure	Morbidity	Both	1.0 (1.0 to 1.0)	1.0 (1.0 to 1.0)	1.0 (1.0 to 1.0)	1.0 (1.0 to 1.0)	1.0 (1.0 to 1.0)	1.0 (1.0 to 1.0)	1.0 (1.0 to 1.0)	1.0 (1.0 to 1.0)	1.0 (1.0 to 1.0)	1.0 (1.0 to 1.0)	1.0 (1.0 to 1.0)	1.0 (1.0 to 1.0)	1.0 (1.0 to 1.0)	1.0 (1.0 to 1.0)	1.0 (1.0 to 1.0)	1.0 (1.0 to 1.0)	1.0 (1.0 to 1.0)
Severe hearing loss with ringing due to age-related and other hearing loss	No exposure	Morbidity	Both	1.0 (1.0 to 1.0)	1.0 (1.0 to 1.0)	1.0 (1.0 to 1.0)	1.0 (1.0 to 1.0)	1.0 (1.0 to 1.0)	1.0 (1.0 to 1.0)	1.0 (1.0 to 1.0)	1.0 (1.0 to 1.0)	1.0 (1.0 to 1.0)	1.0 (1.0 to 1.0)	1.0 (1.0 to 1.0)	1.0 (1.0 to 1.0)	1.0 (1.0 to 1.0)	1.0 (1.0 to 1.0)	1.0 (1.0 to 1.0)	1.0 (1.0 to 1.0)	1.0 (1.0 to 1.0)
Severe hearing loss due to age-related and other hearing loss	No exposure	Morbidity	Both	1.0 (1.0 to 1.0)	1.0 (1.0 to 1.0)	1.0 (1.0 to 1.0)	1.0 (1.0 to 1.0)	1.0 (1.0 to 1.0)	1.0 (1.0 to 1.0)	1.0 (1.0 to 1.0)	1.0 (1.0 to 1.0)	1.0 (1.0 to 1.0)	1.0 (1.0 to 1.0)	1.0 (1.0 to 1.0)	1.0 (1.0 to 1.0)	1.0 (1.0 to 1.0)	1.0 (1.0 to 1.0)	1.0 (1.0 to 1.0)	1.0 (1.0 to 1.0)	1.0 (1.0 to 1.0)
Profound hearing loss due to age-related and other hearing loss	No exposure	Morbidity	Both	1.0 (1.0 to 1.0)	1.0 (1.0 to 1.0)	1.0 (1.0 to 1.0)	1.0 (1.0 to 1.0)	1.0 (1.0 to 1.0)	1.0 (1.0 to 1.0)	1.0 (1.0 to 1.0)	1.0 (1.0 to 1.0)	1.0 (1.0 to 1.0)	1.0 (1.0 to 1.0)	1.0 (1.0 to 1.0)	1.0 (1.0 to 1.0)	1.0 (1.0 to 1.0)	1.0 (1.0 to 1.0)	1.0 (1.0 to 1.0)	1.0 (1.0 to 1.0)	1.0 (1.0 to 1.0)
Profound hearing loss with ringing due to age-related and other hearing loss	No exposure	Morbidity	Both	1.0 (1.0 to 1.0)	1.0 (1.0 to 1.0)	1.0 (1.0 to 1.0)	1.0 (1.0 to 1.0)	1.0 (1.0 to 1.0)	1.0 (1.0 to 1.0)	1.0 (1.0 to 1.0)	1.0 (1.0 to 1.0)	1.0 (1.0 to 1.0)	1.0 (1.0 to 1.0)	1.0 (1.0 to 1.0)	1.0 (1.0 to 1.0)	1.0 (1.0 to 1.0)	1.0 (1.0 to 1.0)	1.0 (1.0 to 1.0)	1.0 (1.0 to 1.0)	1.0 (1.0 to 1.0)
Complete hearing loss due to age-related and other hearing loss	No exposure	Morbidity	Both	1.0 (1.0 to 1.0)	1.0 (1.0 to 1.0)	1.0 (1.0 to 1.0)	1.0 (1.0 to 1.0)	1.0 (1.0 to 1.0)	1.0 (1.0 to 1.0)	1.0 (1.0 to 1.0)	1.0 (1.0 to 1.0)	1.0 (1.0 to 1.0)	1.0 (1.0 to 1.0)	1.0 (1.0 to 1.0)	1.0 (1.0 to 1.0)	1.0 (1.0 to 1.0)	1.0 (1.0 to 1.0)	1.0 (1.0 to 1.0)	1.0 (1.0 to 1.0)	1.0 (1.0 to 1.0)
Complete hearing loss with ringing due to age-related and other hearing loss	No exposure	Morbidity	Both	1.0 (1.0 to 1.0)	1.0 (1.0 to 1.0)	1.0 (1.0 to 1.0)	1.0 (1.0 to 1.0)	1.0 (1.0 to 1.0)	1.0 (1.0 to 1.0)	1.0 (1.0 to 1.0)	1.0 (1.0 to 1.0)	1.0 (1.0 to 1.0)	1.0 (1.0 to 1.0)	1.0 (1.0 to 1.0)	1.0 (1.0 to 1.0)	1.0 (1.0 to 1.0)	1.0 (1.0 to 1.0)	1.0 (1.0 to 1.0)	1.0 (1.0 to 1.0)	1.0 (1.0 to 1.0)
**Occupational ergonomic factors**																				
Low back pain	Professional, technical and related workers	Morbidity	Both	1.173 (1.066 to 1.282)	1.172 (1.062 to 1.283)	1.169 (1.065 to 1.283)	1.17 (1.062 to 1.284)	1.17 (1.062 to 1.285)	1.172 (1.062 to 1.283)	1.171 (1.071 to 1.27)	1.169 (1.063 to 1.288)	1.171 (1.059 to 1.281)	1.17 (1.058 to 1.286)	1.17 (1.07 to 1.279)	1.172 (1.065 to 1.287)	1.172 (1.07 to 1.283)	1.172 (1.07 to 1.283)			
Low back pain	Administrative and managerial workers	Morbidity	Both	1.211 (0.964 to 1.508)	1.21 (0.964 to 1.492)	1.209 (0.965 to 1.487)	1.209 (0.963 to 1.524)	1.207 (0.976 to 1.496)	1.207 (0.965 to 1.5)	1.205 (0.946 to 1.489)	1.205 (0.967 to 1.472)	1.205 (0.961 to 1.509)	1.203 (0.948 to 1.515)	1.209 (0.976 to 1.479)	1.21 (0.964 to 1.49)	1.203 (0.961 to 1.501)	1.203 (0.961 to 1.501)			
Low back pain	Clerical and related workers	Morbidity	Both	1.0 (1.0 to 1.0)	1.0 (1.0 to 1.0)	1.0 (1.0 to 1.0)	1.0 (1.0 to 1.0)	1.0 (1.0 to 1.0)	1.0 (1.0 to 1.0)	1.0 (1.0 to 1.0)	1.0 (1.0 to 1.0)	1.0 (1.0 to 1.0)	1.0 (1.0 to 1.0)	1.0 (1.0 to 1.0)	1.0 (1.0 to 1.0)	1.0 (1.0 to 1.0)	1.0 (1.0 to 1.0)			
Low back pain	Sales workers	Morbidity	Both	1.22 (1.029 to 1.434)	1.21 (1.018 to 1.418)	1.213 (1.028 to 1.434)	1.214 (1.004 to 1.448)	1.207 (1.017 to 1.445)	1.218 (1.016 to 1.455)	1.212 (1.012 to 1.444)	1.216 (1.01 to 1.448)	1.219 (1.019 to 1.45)	1.211 (1.014 to 1.444)	1.213 (1.015 to 1.455)	1.21 (1.007 to 1.423)	1.214 (1.017 to 1.446)	1.214 (1.017 to 1.446)			

Low back pain	Service workers	Morbidity	Both								1.472 (1.385 to 1.568)	1.472 (1.383 to 1.569)	1.471 (1.372 to 1.563)	1.472 (1.382 to 1.571)	1.469 (1.378 to 1.567)	1.472 (1.377 to 1.57)	1.469 (1.375 to 1.568)	1.47 (1.378 to 1.57)	1.472 (1.379 to 1.575)	1.472 (1.381 to 1.572)	1.474 (1.386 to 1.571)	1.47 (1.377 to 1.568)	1.472 (1.38 to 1.571)	1.472 (1.38 to 1.571)			
Low back pain	Agriculture, animal husbandry and forestry workers, fishermen and hunters	Morbidity	Both								3.789 (2.58 to 5.376)	3.762 (2.621 to 5.284)	3.869 (2.642 to 5.486)	3.775 (2.569 to 5.369)	3.774 (2.606 to 5.314)	3.771 (2.532 to 5.317)	3.793 (2.632 to 5.361)	3.785 (2.556 to 5.333)	3.776 (2.645 to 5.157)	3.792 (2.536 to 5.421)	3.802 (2.684 to 5.428)	3.746 (2.609 to 5.175)	3.77 (2.635 to 5.151)	3.77 (2.635 to 5.151)			
Low back pain	Production and related workers, transport equipment operators and labourers	Morbidity	Both								1.543 (1.409 to 1.679)	1.54 (1.406 to 1.676)	1.542 (1.415 to 1.677)	1.543 (1.413 to 1.695)	1.542 (1.416 to 1.685)	1.543 (1.418 to 1.685)	1.541 (1.402 to 1.684)	1.542 (1.41 to 1.684)	1.541 (1.404 to 1.683)	1.54 (1.414 to 1.677)	1.54 (1.408 to 1.683)	1.538 (1.408 to 1.673)	1.541 (1.41 to 1.677)	1.541 (1.41 to 1.677)			
Low back pain	Background	Morbidity	Both								1.0 (1.0 to 1.0)	1.0 (1.0 to 1.0)	1.0 (1.0 to 1.0)	1.0 (1.0 to 1.0)	1.0 (1.0 to 1.0)	1.0 (1.0 to 1.0)	1.0 (1.0 to 1.0)	1.0 (1.0 to 1.0)	1.0 (1.0 to 1.0)	1.0 (1.0 to 1.0)	1.0 (1.0 to 1.0)	1.0 (1.0 to 1.0)	1.0 (1.0 to 1.0)	1.0 (1.0 to 1.0)			
**Non-exclusive breastfeeding**																											
Lower respiratory infections	None	Both	Both			1.739 (1.493 to 2.025)	1.739 (1.493 to 2.025)																				
Lower respiratory infections	Partial	Both	Both			1.483 (1.206 to 1.792)	1.483 (1.206 to 1.792)																				
Lower respiratory infections	Predominant	Both	Both			1.369 (1.055 to 1.8)	1.369 (1.055 to 1.8)																				
Lower respiratory infections	Exclusive	Both	Both			1.0 (1.0 to 1.0)	1.0 (1.0 to 1.0)																				
Diarrhoeal diseases	None	Both	Both			3.605 (2.716 to 4.703)	3.605 (2.716 to 4.703)																				
Diarrhoeal diseases	Partial	Both	Both			2.633 (1.942 to 3.481)	2.633 (1.942 to 3.481)																				
Diarrhoeal diseases	Predominant	Both	Both			2.346 (1.667 to 3.234)	2.346 (1.667 to 3.234)																				
Diarrhoeal diseases	Exclusive	Both	Both			1.0 (1.0 to 1.0)	1.0 (1.0 to 1.0)																				
**Discontinued breastfeeding**																											
Diarrhoeal diseases	Discontinued	Both	Both				1.313 (1.111 to 1.549)	1.313 (1.111 to 1.549)																			
Diarrhoeal diseases	Continued	Both	Both				1.0 (1.0 to 1.0)	1.0 (1.0 to 1.0)																			
**Child underweight**																											
Lower respiratory infections	<-3 sd	Both	Both				2.593 (1.908 to 4.39)	2.593 (1.908 to 4.39)																			
Lower respiratory infections	-2 to -3 sd	Both	Both				1.365 (1.215 to 1.755)	1.365 (1.215 to 1.755)																			
Lower respiratory infections	-1 to -2 sd	Both	Both				1.145 (1.044 to 1.364)	1.145 (1.044 to 1.364)																			
Lower respiratory infections	-1 sd and above	Both	Both				1.0 (1.0 to 1.0)	1.0 (1.0 to 1.0)																			
Diarrhoeal diseases	<-3 sd	Both	Both				2.332 (2.076 to 2.802)	2.332 (2.076 to 2.802)																			
Diarrhoeal diseases	-2 to -3 sd	Both	Both				1.23 (1.163 to 1.314)	1.23 (1.163 to 1.314)																			
Diarrhoeal diseases	-1 to -2 sd	Both	Both				1.088 (1.046 to 1.134)	1.088 (1.046 to 1.134)																			
Diarrhoeal diseases	-1 sd and above	Both	Both				1.0 (1.0 to 1.0)	1.0 (1.0 to 1.0)																			
Measles	<-3 sd	Both	Both				5.668 (1.767 to 12.414)	5.668 (1.767 to 12.414)																			
Measles	-2 to -3 sd	Both	Both				2.458 (1.26 to 5.118)	2.458 (1.26 to 5.118)																			
Measles	-1 to -2 sd	Both	Both				0.995 (0.5 to 1.726)	0.995 (0.5 to 1.726)																			
Measles	-1 sd and above	Both	Both				1.0 (1.0 to 1.0)	1.0 (1.0 to 1.0)																			
**Child wasting**																											
Lower respiratory infections	<-3 sd	Both	Both				47.67 (15.923 to 94.874)	47.67 (15.923 to 94.874)																			
Lower respiratory infections	-2 to -3 sd	Both	Both				20.455 (7.084 to 37.929)	20.455 (7.084 to 37.929)																			
Lower respiratory infections	-1 to -2 sd	Both	Both				5.941 (1.972 to 11.992)	5.941 (1.972 to 11.992)																			
Lower respiratory infections	-1 sd and above	Both	Both				1.0 (1.0 to 1.0)	1.0 (1.0 to 1.0)																			
Diarrhoeal diseases	<-3 sd	Both	Both				105.759 (42.198 to 157.813)	105.759 (42.198 to 157.813)																			
Diarrhoeal diseases	-2 to -3 sd	Both	Both				23.261 (9.02 to 35.845)	23.261 (9.02 to 35.845)																			
Diarrhoeal diseases	-1 to -2 sd	Both	Both				6.601 (2.158 to 11.243)	6.601 (2.158 to 11.243)																			
Diarrhoeal diseases	-1 sd and above	Both	Both				1.0 (1.0 to 1.0)	1.0 (1.0 to 1.0)																			
Measles	<-3 sd	Both	Both				37.936 (5.088 to 199.126)	37.936 (5.088 to 199.126)																			
Measles	-2 to -3 sd	Both	Both				8.477 (1.33 to 42.777)	8.477 (1.33 to 42.777)																			
Measles	-1 to -2 sd	Both	Both				1.833 (0.569 to 8.985)	1.833 (0.569 to 8.985)																			
Measles	-1 sd and above	Both	Both				1.0 (1.0 to 1.0)	1.0 (1.0 to 1.0)																			
**Child stunting**																											
Lower respiratory infections	<-3 sd	Both	Both				2.355 (1.15 to 5.114)	2.355 (1.15 to 5.114)																			
Lower respiratory infections	-2 to -3 sd	Both	Both				1.318 (1.014 to 2.165)	1.318 (1.014 to 2.165)																			
Lower respiratory infections	-1 to -2 sd	Both	Both				1.158 (0.998 to 1.655)	1.158 (0.998 to 1.655)																			
Lower respiratory infections	-1 sd and above	Both	Both				1.0 (1.0 to 1.0)	1.0 (1.0 to 1.0)																			
Diarrhoeal diseases	<-3 sd	Both	Both				1.851 (1.28 to 2.699)	1.851 (1.28 to 2.699)																			
Diarrhoeal diseases	-2 to -3 sd	Both	Both				1.222 (1.067 to 1.5)	1.222 (1.067 to 1.5)																			
Diarrhoeal diseases	-1 to -2 sd	Both	Both				1.111 (1.023 to 1.273)	1.111 (1.023 to 1.273)																			
Diarrhoeal diseases	-1 sd and above	Both	Both				1.0 (1.0 to 1.0)	1.0 (1.0 to 1.0)																			
Measles	<-3 sd	Both	Both				2.487 (1.129 to 6.528)	2.487 (1.129 to 6.528)																			
Measles	-2 to -3 sd	Both	Both				1.54 (1.029 to 3.222)	1.54 (1.029 to 3.222)																			
Measles	-1 to -2 sd	Both	Both				1.103 (0.861 to 1.719)	1.103 (0.861 to 1.719)																			
Measles	-1 sd and above	Both	Both				1.0 (1.0 to 1.0)	1.0 (1.0 to 1.0)																			
**Low birth weight and short gestation**																											
Diarrhoeal diseases	Birth prevalence - [0, 24) wks, [0, 500) g	Mortality	Males		877.775 (543.26 to 1246.645)	526.788 (392.055 to 676.923)																					
Diarrhoeal diseases	Birth prevalence - [0, 24) wks, [0, 500) g	Mortality	Females		857.044 (519.185 to 1227.708)	575.632 (434.418 to 737.293)																					
Diarrhoeal diseases	Birth prevalence - [0, 24) wks, [500, 1000) g	Mortality	Males		647.899 (443.535 to 881.449)	392.723 (315.689 to 486.607)																					
Diarrhoeal diseases	Birth prevalence - [0, 24) wks, [500, 1000) g	Mortality	Females		628.647 (437.861 to 854.598)	416.95 (329.573 to 516.699)																					
Diarrhoeal diseases	Birth prevalence - [24, 26) wks, [500, 1000) g	Mortality	Males		534.904 (378.653 to 707.51)	375.56 (308.336 to 447.076)																					
Diarrhoeal diseases	Birth prevalence - [24, 26) wks, [500, 1000) g	Mortality	Females		509.388 (370.575 to 690.649)	394.982 (324.03 to 477.74)																					
Diarrhoeal diseases	Birth prevalence - [26, 28) wks, [1000, 1500) g	Mortality	Males		150.649 (107.741 to 198.278)	140.284 (113.557 to 167.957)																					
Diarrhoeal diseases	Birth prevalence - [26, 28) wks, [1000, 1500) g	Mortality	Females		142.251 (102.895 to 191.587)	140.771 (114.514 to 169.631)																					
Diarrhoeal diseases	Birth prevalence - [26, 28) wks, [500, 1000) g	Mortality	Males		281.284 (198.703 to 391.898)	280.84 (226.174 to 338.336)																					
Diarrhoeal diseases	Birth prevalence - [26, 28) wks, [500, 1000) g	Mortality	Females		257.799 (185.994 to 349.394)	280.84 (225.68 to 350.2)																					
Diarrhoeal diseases	Birth prevalence - [28, 30) wks, [1000, 1500) g	Mortality	Males		88.889 (62.539 to 119.303)	87.596 (72.219 to 105.445)																					
Diarrhoeal diseases	Birth prevalence - [28, 30) wks, [1000, 1500) g	Mortality	Females		82.25 (59.418 to 109.806)	86.675 (69.965 to 104.367)																					
Diarrhoeal diseases	Birth prevalence - [28, 30) wks, [1500, 2000) g	Mortality	Males		71.963 (51.376 to 98.371)	42.294 (34.585 to 51.215)																					
Diarrhoeal diseases	Birth prevalence - [28, 30) wks, [1500, 2000) g	Mortality	Females		70.154 (48.562 to 96.294)	46.161 (38.268 to 54.906)																					
Diarrhoeal diseases	Birth prevalence - [28, 30) wks, [2000, 2500) g	Mortality	Males		65.936 (44.909 to 93.551)	23.785 (18.748 to 29.385)																					
Diarrhoeal diseases	Birth prevalence - [28, 30) wks, [2000, 2500) g	Mortality	Females		65.44 (43.751 to 96.137)	27.437 (21.369 to 33.874)																					
Diarrhoeal diseases	Birth prevalence - [28, 30) wks, [2500, 3000) g	Mortality	Males		43.647 (29.61 to 62.044)	14.185 (10.817 to 18.495)																					
Diarrhoeal diseases	Birth prevalence - [28, 30) wks, [2500, 3000) g	Mortality	Females		42.667 (28.231 to 61.897)	16.517 (12.636 to 21.355)																					
Diarrhoeal diseases	Birth prevalence - [28, 30) wks, [3000, 3500) g	Mortality	Males		23.727 (15.859 to 35.246)	8.651 (6.873 to 11.016)																					
Diarrhoeal diseases	Birth prevalence - [28, 30) wks, [3000, 3500) g	Mortality	Females		23.045 (14.473 to 35.151)	9.735 (7.511 to 12.367)																					
Diarrhoeal diseases	Birth prevalence - [28, 30) wks, [500, 1000) g	Mortality	Males		164.681 (114.331 to 232.909)	184.988 (146.715 to 225.778)																					
Diarrhoeal diseases	Birth prevalence - [28, 30) wks, [500, 1000) g	Mortality	Females		149.827 (102.011 to 207.65)	178.51 (144.736 to 220.088)																					
Diarrhoeal diseases	Birth prevalence - [30, 32) wks, [1000, 1500) g	Mortality	Males		66.567 (45.227 to 91.424)	57.036 (47.559 to 67.673)																					
Diarrhoeal diseases	Birth prevalence - [30, 32) wks, [1000, 1500) g	Mortality	Females		61.652 (42.69 to 86.655)	55.992 (46.425 to 67.165)																					
Diarrhoeal diseases	Birth prevalence - [30, 32) wks, [1500, 2000) g	Mortality	Males		43.288 (30.684 to 57.762)	26.533 (22.334 to 31.213)																					
Diarrhoeal diseases	Birth prevalence - [30, 32) wks, [1500, 2000) g	Mortality	Females		41.127 (28.837 to 54.767)	28.256 (24.051 to 32.792)																					
Diarrhoeal diseases	Birth prevalence - [30, 32) wks, [2000, 2500) g	Mortality	Males		38.672 (27.558 to 53.438)	15.362 (12.641 to 18.516)																					
Diarrhoeal diseases	Birth prevalence - [30, 32) wks, [2000, 2500) g	Mortality	Females		37.459 (26.086 to 52.166)	17.996 (14.845 to 21.529)																					
Diarrhoeal diseases	Birth prevalence - [30, 32) wks, [2500, 3000) g	Mortality	Males		33.218 (22.641 to 46.944)	10.389 (8.195 to 12.863)																					
Diarrhoeal diseases	Birth prevalence - [30, 32) wks, [2500, 3000) g	Mortality	Females		31.439 (21.157 to 45.621)	12.473 (9.64 to 15.784)																					
Diarrhoeal diseases	Birth prevalence - [30, 32) wks, [3000, 3500) g	Mortality	Males		25.451 (16.854 to 36.763)	7.153 (5.665 to 9.003)																					
Diarrhoeal diseases	Birth prevalence - [30, 32) wks, [3000, 3500) g	Mortality	Females		24.262 (15.651 to 36.395)	8.486 (6.482 to 10.824)																					
Diarrhoeal diseases	Birth prevalence - [30, 32) wks, [3500, 4000) g	Mortality	Males		20.334 (11.916 to 31.586)	4.795 (3.705 to 6.039)																					
Diarrhoeal diseases	Birth prevalence - [30, 32) wks, [3500, 4000) g	Mortality	Females		20.347 (11.635 to 32.681)	5.599 (4.199 to 7.275)																					
Diarrhoeal diseases	Birth prevalence - [30, 32) wks, [500, 1000) g	Mortality	Males		132.309 (85.99 to 193.341)	127.982 (101.66 to 159.489)																					
Diarrhoeal diseases	Birth prevalence - [30, 32) wks, [500, 1000) g	Mortality	Females		120.829 (82.341 to 172.828)	123.186 (98.696 to 152.125)																					
Diarrhoeal diseases	Birth prevalence - [32, 34) wks, [1000, 1500) g	Mortality	Males		65.39 (43.593 to 95.583)	45.581 (37.56 to 54.417)																					
Diarrhoeal diseases	Birth prevalence - [32, 34) wks, [1000, 1500) g	Mortality	Females		61.229 (40.8 to 88.303)	45.201 (37.492 to 54.267)																					
Diarrhoeal diseases	Birth prevalence - [32, 34) wks, [1500, 2000) g	Mortality	Males		32.197 (22.524 to 44.858)	19.612 (16.465 to 23.296)																					
Diarrhoeal diseases	Birth prevalence - [32, 34) wks, [1500, 2000) g	Mortality	Females		29.777 (21.026 to 40.255)	20.358 (17.072 to 23.768)																					
Diarrhoeal diseases	Birth prevalence - [32, 34) wks, [2000, 2500) g	Mortality	Males		21.018 (15.141 to 28.622)	10.433 (8.784 to 12.225)																					
Diarrhoeal diseases	Birth prevalence - [32, 34) wks, [2000, 2500) g	Mortality	Females		19.546 (14.316 to 26.034)	11.648 (9.974 to 13.507)																					
Diarrhoeal diseases	Birth prevalence - [32, 34) wks, [2500, 3000) g	Mortality	Males		18.569 (13.127 to 25.826)	7.182 (5.968 to 8.63)																					
Diarrhoeal diseases	Birth prevalence - [32, 34) wks, [2500, 3000) g	Mortality	Females		17.497 (11.66 to 24.635)	8.443 (6.726 to 10.284)																					
Diarrhoeal diseases	Birth prevalence - [32, 34) wks, [3000, 3500) g	Mortality	Males		19.012 (12.18 to 28.02)	5.618 (4.325 to 7.114)																					
Diarrhoeal diseases	Birth prevalence - [32, 34) wks, [3000, 3500) g	Mortality	Females		18.667 (12.24 to 27.524)	6.722 (5.103 to 8.697)																					
Diarrhoeal diseases	Birth prevalence - [32, 34) wks, [3500, 4000) g	Mortality	Males		20.214 (11.906 to 31.929)	4.31 (3.186 to 5.633)																					
Diarrhoeal diseases	Birth prevalence - [32, 34) wks, [3500, 4000) g	Mortality	Females		20.333 (12.064 to 33.21)	5.253 (3.959 to 6.913)																					
Diarrhoeal diseases	Birth prevalence - [34, 36) wks, [1000, 1500) g	Mortality	Males		78.66 (52.169 to 113.08)	45.338 (36.27 to 55.556)																					
Diarrhoeal diseases	Birth prevalence - [34, 36) wks, [1000, 1500) g	Mortality	Females		75.687 (49.863 to 110.089)	46.4 (38.048 to 55.902)																					
Diarrhoeal diseases	Birth prevalence - [34, 36) wks, [1500, 2000) g	Mortality	Males		31.154 (20.939 to 45.031)	18.246 (15.083 to 21.906)																					
Diarrhoeal diseases	Birth prevalence - [34, 36) wks, [1500, 2000) g	Mortality	Females		29.072 (19.6 to 42.648)	18.661 (15.638 to 22.175)																					
Diarrhoeal diseases	Birth prevalence - [34, 36) wks, [2000, 2500) g	Mortality	Males		12.404 (8.692 to 16.95)	8.006 (6.775 to 9.31)																					
Diarrhoeal diseases	Birth prevalence - [34, 36) wks, [2000, 2500) g	Mortality	Females		11.367 (7.807 to 15.547)	8.373 (7.066 to 9.703)																					
Diarrhoeal diseases	Birth prevalence - [34, 36) wks, [2500, 3000) g	Mortality	Males		7.509 (5.429 to 10.255)	4.725 (3.991 to 5.56)																					
Diarrhoeal diseases	Birth prevalence - [34, 36) wks, [2500, 3000) g	Mortality	Females		7.139 (5.091 to 9.759)	5.194 (4.342 to 6.087)																					
Diarrhoeal diseases	Birth prevalence - [34, 36) wks, [3000, 3500) g	Mortality	Males		7.828 (5.467 to 11.237)	3.717 (2.986 to 4.568)																					
Diarrhoeal diseases	Birth prevalence - [34, 36) wks, [3000, 3500) g	Mortality	Females		7.622 (5.235 to 10.584)	4.279 (3.416 to 5.301)																					
Diarrhoeal diseases	Birth prevalence - [34, 36) wks, [3500, 4000) g	Mortality	Males		10.257 (6.692 to 15.124)	3.13 (2.474 to 3.957)																					
Diarrhoeal diseases	Birth prevalence - [34, 36) wks, [3500, 4000) g	Mortality	Females		10.325 (6.495 to 15.537)	3.756 (2.877 to 4.818)																					
Diarrhoeal diseases	Birth prevalence - [34, 36) wks, [4000, 4500) g	Mortality	Males		12.928 (7.825 to 20.726)	2.467 (1.926 to 3.084)																					
Diarrhoeal diseases	Birth prevalence - [34, 36) wks, [4000, 4500) g	Mortality	Females		13.456 (8.201 to 20.893)	3.061 (2.357 to 3.889)																					
Diarrhoeal diseases	Birth prevalence - [36, 37) wks, [1000, 1500) g	Mortality	Males		94.103 (62.401 to 131.437)	48.734 (39.385 to 60.014)																					
Diarrhoeal diseases	Birth prevalence - [36, 37) wks, [1000, 1500) g	Mortality	Females		89.769 (59.869 to 126.674)	51.68 (41.336 to 63.456)																					
Diarrhoeal diseases	Birth prevalence - [36, 37) wks, [1500, 2000) g	Mortality	Males		33.586 (23.075 to 48.77)	19.52 (16.283 to 23.116)																					
Diarrhoeal diseases	Birth prevalence - [36, 37) wks, [1500, 2000) g	Mortality	Females		30.953 (20.951 to 43.853)	20.509 (17.15 to 24.286)																					
Diarrhoeal diseases	Birth prevalence - [36, 37) wks, [2000, 2500) g	Mortality	Males		8.134 (5.781 to 10.938)	7.015 (5.903 to 8.241)																					

Diarrhoeal diseases	Birth prevalence - [36, 37) wks, [2000, 2500) g	Mortality	Females		7.208 (5.183 to 9.787)	7.031 (5.911 to 8.265)
Diarrhoeal diseases	Birth prevalence - [36, 37) wks, [2500, 3000) g	Mortality	Males		2.741 (2.114 to 3.535)	3.149 (2.686 to 3.686)
Diarrhoeal diseases	Birth prevalence - [36, 37) wks, [2500, 3000) g	Mortality	Females		2.475 (1.905 to 3.109)	3.172 (2.71 to 3.666)
Diarrhoeal diseases	Birth prevalence - [36, 37) wks, [3000, 3500) g	Mortality	Males		2.1 (1.602 to 2.663)	2.099 (1.805 to 2.445)
Diarrhoeal diseases	Birth prevalence - [36, 37) wks, [3000, 3500) g	Mortality	Females		1.996 (1.532 to 2.6)	2.199 (1.895 to 2.534)
Diarrhoeal diseases	Birth prevalence - [36, 37) wks, [3500, 4000) g	Mortality	Males		2.563 (1.906 to 3.333)	1.751 (1.487 to 2.008)
Diarrhoeal diseases	Birth prevalence - [36, 37) wks, [3500, 4000) g	Mortality	Females		2.501 (1.793 to 3.34)	1.944 (1.649 to 2.275)
Diarrhoeal diseases	Birth prevalence - [36, 37) wks, [4000, 4500) g	Mortality	Males		3.824 (2.607 to 5.361)	1.506 (1.283 to 1.74)
Diarrhoeal diseases	Birth prevalence - [36, 37) wks, [4000, 4500) g	Mortality	Females		3.908 (2.626 to 5.462)	1.777 (1.481 to 2.118)
Diarrhoeal diseases	Birth prevalence - [37, 38) wks, [1000, 1500) g	Mortality	Males		98.111 (66.503 to 135.289)	49.903 (39.62 to 62.232)
Diarrhoeal diseases	Birth prevalence - [37, 38) wks, [1000, 1500) g	Mortality	Females		92.728 (63.975 to 128.565)	53.151 (42.705 to 66.273)
Diarrhoeal diseases	Birth prevalence - [37, 38) wks, [1500, 2000) g	Mortality	Males		35.71 (24.585 to 49.648)	20.617 (16.897 to 24.816)
Diarrhoeal diseases	Birth prevalence - [37, 38) wks, [1500, 2000) g	Mortality	Females		32.101 (22.457 to 43.887)	21.726 (17.833 to 25.871)
Diarrhoeal diseases	Birth prevalence - [37, 38) wks, [2000, 2500) g	Mortality	Males		7.301 (5.37 to 9.805)	6.923 (5.831 to 8.219)
Diarrhoeal diseases	Birth prevalence - [37, 38) wks, [2000, 2500) g	Mortality	Females		6.218 (4.554 to 8.169)	6.85 (5.796 to 8.005)
Diarrhoeal diseases	Birth prevalence - [37, 38) wks, [2500, 3000) g	Mortality	Males		1.861 (1.414 to 2.384)	2.721 (2.329 to 3.158)
Diarrhoeal diseases	Birth prevalence - [37, 38) wks, [2500, 3000) g	Mortality	Females		1.596 (1.24 to 1.998)	2.633 (2.261 to 3.031)
Diarrhoeal diseases	Birth prevalence - [37, 38) wks, [3000, 3500) g	Mortality	Males		1.139 (1 to 1.409)	1.612 (1.387 to 1.85)
Diarrhoeal diseases	Birth prevalence - [37, 38) wks, [3000, 3500) g	Mortality	Females		1.065 (1 to 1.288)	1.598 (1.392 to 1.819)
Diarrhoeal diseases	Birth prevalence - [37, 38) wks, [3500, 4000) g	Mortality	Males		1.201 (1 to 1.493)	1.282 (1.113 to 1.466)
Diarrhoeal diseases	Birth prevalence - [37, 38) wks, [3500, 4000) g	Mortality	Females		1.157 (1 to 1.442)	1.343 (1.153 to 1.542)
Diarrhoeal diseases	Birth prevalence - [37, 38) wks, [4000, 4500) g	Mortality	Males		1.825 (1.341 to 2.407)	1.136 (1.01 to 1.272)
Diarrhoeal diseases	Birth prevalence - [37, 38) wks, [4000, 4500) g	Mortality	Females		1.86 (1.36 to 2.46)	1.269 (1.115 to 1.42)
Diarrhoeal diseases	Birth prevalence - [38, 40) wks, [1000, 1500) g	Mortality	Males		98.254 (67.959 to 134.77)	49.084 (38.794 to 61.948)
Diarrhoeal diseases	Birth prevalence - [38, 40) wks, [1000, 1500) g	Mortality	Females		91.539 (62.769 to 127.606)	53.036 (41.348 to 65.237)
Diarrhoeal diseases	Birth prevalence - [38, 40) wks, [1500, 2000) g	Mortality	Males		37.449 (25.688 to 51.467)	21.477 (17.208 to 25.646)
Diarrhoeal diseases	Birth prevalence - [38, 40) wks, [1500, 2000) g	Mortality	Females		33.261 (23.303 to 45.061)	22.821 (18.339 to 27.874)
Diarrhoeal diseases	Birth prevalence - [38, 40) wks, [2000, 2500) g	Mortality	Males		7.351 (5.131 to 9.967)	7.04 (5.807 to 8.395)
Diarrhoeal diseases	Birth prevalence - [38, 40) wks, [2000, 2500) g	Mortality	Females		5.985 (4.383 to 7.995)	6.959 (5.782 to 8.29)
Diarrhoeal diseases	Birth prevalence - [38, 40) wks, [2500, 3000) g	Mortality	Males		1.549 (1.185 to 1.98)	2.518 (2.115 to 2.971)
Diarrhoeal diseases	Birth prevalence - [38, 40) wks, [2500, 3000) g	Mortality	Females		1.277 (1 to 1.65)	2.377 (2.015 to 2.745)
Diarrhoeal diseases	Birth prevalence - [38, 40) wks, [3000, 3500) g	Mortality	Males		1 (1.0 - 1.0)	1.326 (1.12 to 1.543)
Diarrhoeal diseases	Birth prevalence - [38, 40) wks, [3000, 3500) g	Mortality	Females		1 (1.0 - 1.0)	1.265 (1.068 to 1.45)
Diarrhoeal diseases	Birth prevalence - [38, 40) wks, [3500, 4000) g	Mortality	Males		1 (1.0 - 1.0)	1 (1.0 - 1.0)
Diarrhoeal diseases	Birth prevalence - [38, 40) wks, [3500, 4000) g	Mortality	Females		1 (1.0 - 1.0)	1 (1.0 - 1.0)
Diarrhoeal diseases	Birth prevalence - [38, 40) wks, [4000, 4500) g	Mortality	Males		1 (1.0 - 1.0)	1 (1.0 - 1.0)
Diarrhoeal diseases	Birth prevalence - [38, 40) wks, [4000, 4500) g	Mortality	Females		1 (1.0 - 1.0)	1 (1.0 - 1.0)
Diarrhoeal diseases	Birth prevalence - [40, 42) wks, [1500, 2000) g	Mortality	Males		43.194 (29.244 to 62.382)	21.971 (16.41 to 28.396)
Diarrhoeal diseases	Birth prevalence - [40, 42) wks, [1500, 2000) g	Mortality	Females		37.949 (25.539 to 53.532)	23.702 (17.857 to 30.634)
Diarrhoeal diseases	Birth prevalence - [40, 42) wks, [2000, 2500) g	Mortality	Males		10.162 (6.961 to 14.235)	7.875 (6.341 to 9.719)
Diarrhoeal diseases	Birth prevalence - [40, 42) wks, [2000, 2500) g	Mortality	Females		8.332 (5.994 to 11.407)	8.067 (6.467 to 10.043)
Diarrhoeal diseases	Birth prevalence - [40, 42) wks, [2500, 3000) g	Mortality	Males		2.118 (1.53 to 2.77)	2.692 (2.23 to 3.273)
Diarrhoeal diseases	Birth prevalence - [40, 42) wks, [2500, 3000) g	Mortality	Females		1.745 (1.289 to 2.316)	2.603 (2.134 to 3.106)
Diarrhoeal diseases	Birth prevalence - [40, 42) wks, [3000, 3500) g	Mortality	Males		1.002 (1 to 1.013)	1.249 (1.041 to 1.491)
Diarrhoeal diseases	Birth prevalence - [40, 42) wks, [3000, 3500) g	Mortality	Females		1 (1.0 - 1.0)	1.188 (1 to 1.411)
Diarrhoeal diseases	Birth prevalence - [40, 42) wks, [3500, 4000) g	Mortality	Males		1 (1.0 - 1.0)	1 (1.0 - 1.0)
Diarrhoeal diseases	Birth prevalence - [40, 42) wks, [3500, 4000) g	Mortality	Females		1 (1.0 - 1.0)	1 (1.0 - 1.0)
Diarrhoeal diseases	Birth prevalence - [40, 42) wks, [4000, 4500) g	Mortality	Males		1 (1.0 - 1.0)	1 (1.0 - 1.0)
Diarrhoeal diseases	Birth prevalence - [40, 42) wks, [4000, 4500) g	Mortality	Females		1 (1.0 - 1.0)	1 (1.0 - 1.0)
Encephalitis	Birth prevalence - [0, 24) wks, [0, 500) g	Mortality	Males		877.775 (543.26 to 1246.645)	526.788 (392.055 to 676.923)
Encephalitis	Birth prevalence - [0, 24) wks, [0, 500) g	Mortality	Females		857.044 (519.185 to 1227.708)	575.632 (434.418 to 737.293)
Encephalitis	Birth prevalence - [0, 24) wks, [500, 1000) g	Mortality	Males		647.899 (443.535 to 881.449)	392.723 (315.689 to 486.607)
Encephalitis	Birth prevalence - [0, 24) wks, [500, 1000) g	Mortality	Females		628.647 (437.861 to 854.598)	416.95 (329.573 to 516.699)
Encephalitis	Birth prevalence - [24, 26) wks, [500, 1000) g	Mortality	Males		534.904 (378.653 to 707.51)	375.56 (308.336 to 447.076)
Encephalitis	Birth prevalence - [24, 26) wks, [500, 1000) g	Mortality	Females		509.388 (370.575 to 690.649)	394.982 (324.03 to 477.74)
Encephalitis	Birth prevalence - [26, 28) wks, [1000, 1500) g	Mortality	Males		150.649 (107.741 to 198.278)	140.284 (113.557 to 167.957)
Encephalitis	Birth prevalence - [26, 28) wks, [1000, 1500) g	Mortality	Females		142.251 (102.895 to 191.587)	140.771 (114.514 to 169.631)
Encephalitis	Birth prevalence - [26, 28) wks, [500, 1000) g	Mortality	Males		281.284 (198.703 to 391.898)	280.84 (226.174 to 338.336)
Encephalitis	Birth prevalence - [26, 28) wks, [500, 1000) g	Mortality	Females		257.799 (185.994 to 349.394)	280.84 (225.68 to 350.2)
Encephalitis	Birth prevalence - [28, 30) wks, [1000, 1500) g	Mortality	Males		88.889 (62.539 to 119.303)	87.596 (72.219 to 105.445)
Encephalitis	Birth prevalence - [28, 30) wks, [1000, 1500) g	Mortality	Females		82.25 (59.418 to 109.806)	86.675 (69.965 to 104.367)
Encephalitis	Birth prevalence - [28, 30) wks, [1500, 2000) g	Mortality	Males		71.963 (51.376 to 98.371)	42.294 (34.585 to 51.215)
Encephalitis	Birth prevalence - [28, 30) wks, [1500, 2000) g	Mortality	Females		70.154 (48.562 to 96.294)	46.161 (38.268 to 54.906)
Encephalitis	Birth prevalence - [28, 30) wks, [2000, 2500) g	Mortality	Males		65.936 (44.909 to 93.551)	23.785 (18.748 to 29.385)
Encephalitis	Birth prevalence - [28, 30) wks, [2000, 2500) g	Mortality	Females		65.44 (43.751 to 96.137)	27.437 (21.369 to 33.874)
Encephalitis	Birth prevalence - [28, 30) wks, [2500, 3000) g	Mortality	Males		43.647 (29.61 to 62.044)	14.185 (10.817 to 18.495)
Encephalitis	Birth prevalence - [28, 30) wks, [2500, 3000) g	Mortality	Females		42.667 (28.231 to 61.897)	16.517 (12.636 to 21.355)
Encephalitis	Birth prevalence - [28, 30) wks, [3000, 3500) g	Mortality	Males		23.727 (15.859 to 35.246)	8.651 (6.873 to 11.016)
Encephalitis	Birth prevalence - [28, 30) wks, [3000, 3500) g	Mortality	Females		23.045 (14.473 to 35.151)	9.735 (7.511 to 12.367)
Encephalitis	Birth prevalence - [28, 30) wks, [500, 1000) g	Mortality	Males		164.681 (114.331 to 232.909)	184.988 (146.715 to 225.778)
Encephalitis	Birth prevalence - [28, 30) wks, [500, 1000) g	Mortality	Females		149.827 (102.011 to 207.65)	178.51 (144.736 to 220.088)
Encephalitis	Birth prevalence - [30, 32) wks, [1000, 1500) g	Mortality	Males		66.567 (45.227 to 91.424)	57.036 (47.559 to 67.673)
Encephalitis	Birth prevalence - [30, 32) wks, [1000, 1500) g	Mortality	Females		61.652 (42.69 to 86.655)	55.992 (46.425 to 67.165)
Encephalitis	Birth prevalence - [30, 32) wks, [1500, 2000) g	Mortality	Males		43.288 (30.684 to 57.762)	26.533 (22.334 to 31.213)
Encephalitis	Birth prevalence - [30, 32) wks, [1500, 2000) g	Mortality	Females		41.127 (28.837 to 54.767)	28.256 (24.051 to 32.792)
Encephalitis	Birth prevalence - [30, 32) wks, [2000, 2500) g	Mortality	Males		38.672 (27.558 to 53.438)	15.362 (12.641 to 18.516)
Encephalitis	Birth prevalence - [30, 32) wks, [2000, 2500) g	Mortality	Females		37.459 (26.086 to 52.166)	17.996 (14.845 to 21.529)
Encephalitis	Birth prevalence - [30, 32) wks, [2500, 3000) g	Mortality	Males		33.218 (22.641 to 46.944)	10.389 (8.195 to 12.863)
Encephalitis	Birth prevalence - [30, 32) wks, [2500, 3000) g	Mortality	Females		31.439 (21.157 to 45.621)	12.473 (9.64 to 15.784)
Encephalitis	Birth prevalence - [30, 32) wks, [3000, 3500) g	Mortality	Males		25.451 (16.854 to 36.763)	7.153 (5.665 to 9.003)
Encephalitis	Birth prevalence - [30, 32) wks, [3000, 3500) g	Mortality	Females		24.262 (15.651 to 36.395)	8.486 (6.482 to 10.824)
Encephalitis	Birth prevalence - [30, 32) wks, [3500, 4000) g	Mortality	Males		20.334 (11.916 to 31.586)	4.795 (3.705 to 6.039)
Encephalitis	Birth prevalence - [30, 32) wks, [3500, 4000) g	Mortality	Females		20.347 (11.635 to 32.681)	5.599 (4.199 to 7.275)
Encephalitis	Birth prevalence - [30, 32) wks, [500, 1000) g	Mortality	Males		132.309 (85.99 to 193.341)	127.982 (101.66 to 159.489)
Encephalitis	Birth prevalence - [30, 32) wks, [500, 1000) g	Mortality	Females		120.829 (82.341 to 172.828)	123.186 (98.696 to 152.125)
Encephalitis	Birth prevalence - [32, 34) wks, [1000, 1500) g	Mortality	Males		65.39 (43.593 to 95.583)	45.581 (37.56 to 54.417)
Encephalitis	Birth prevalence - [32, 34) wks, [1000, 1500) g	Mortality	Females		61.229 (40.8 to 88.303)	45.201 (37.492 to 54.267)
Encephalitis	Birth prevalence - [32, 34) wks, [1500, 2000) g	Mortality	Males		32.197 (22.524 to 44.858)	19.612 (16.465 to 23.296)
Encephalitis	Birth prevalence - [32, 34) wks, [1500, 2000) g	Mortality	Females		29.777 (21.026 to 40.255)	20.358 (17.072 to 23.768)
Encephalitis	Birth prevalence - [32, 34) wks, [2000, 2500) g	Mortality	Males		21.018 (15.141 to 28.622)	10.433 (8.784 to 12.225)
Encephalitis	Birth prevalence - [32, 34) wks, [2000, 2500) g	Mortality	Females		19.546 (14.316 to 26.034)	11.648 (9.974 to 13.507)
Encephalitis	Birth prevalence - [32, 34) wks, [2500, 3000) g	Mortality	Males		18.569 (13.127 to 25.826)	7.182 (5.968 to 8.63)
Encephalitis	Birth prevalence - [32, 34) wks, [2500, 3000) g	Mortality	Females		17.497 (11.66 to 24.635)	8.443 (6.726 to 10.284)
Encephalitis	Birth prevalence - [32, 34) wks, [3000, 3500) g	Mortality	Males		19.012 (12.18 to 28.02)	5.618 (4.325 to 7.114)
Encephalitis	Birth prevalence - [32, 34) wks, [3000, 3500) g	Mortality	Females		18.667 (12.24 to 27.524)	6.722 (5.103 to 8.697)
Encephalitis	Birth prevalence - [32, 34) wks, [3500, 4000) g	Mortality	Males		20.214 (11.906 to 31.929)	4.31 (3.186 to 5.633)
Encephalitis	Birth prevalence - [32, 34) wks, [3500, 4000) g	Mortality	Females		20.333 (12.064 to 33.21)	5.253 (3.959 to 6.913)
Encephalitis	Birth prevalence - [34, 36) wks, [1000, 1500) g	Mortality	Males		78.66 (52.169 to 113.08)	45.338 (36.27 to 55.556)
Encephalitis	Birth prevalence - [34, 36) wks, [1000, 1500) g	Mortality	Females		75.687 (49.863 to 110.089)	46.4 (38.048 to 55.902)
Encephalitis	Birth prevalence - [34, 36) wks, [1500, 2000) g	Mortality	Males		31.154 (20.939 to 45.031)	18.246 (15.083 to 21.906)
Encephalitis	Birth prevalence - [34, 36) wks, [1500, 2000) g	Mortality	Females		29.072 (19.6 to 42.648)	18.661 (15.638 to 22.175)
Encephalitis	Birth prevalence - [34, 36) wks, [2000, 2500) g	Mortality	Males		12.404 (8.692 to 16.95)	8.006 (6.775 to 9.31)
Encephalitis	Birth prevalence - [34, 36) wks, [2000, 2500) g	Mortality	Females		11.367 (7.807 to 15.547)	8.373 (7.066 to 9.703)
Encephalitis	Birth prevalence - [34, 36) wks, [2500, 3000) g	Mortality	Males		7.509 (5.429 to 10.255)	4.725 (3.991 to 5.56)
Encephalitis	Birth prevalence - [34, 36) wks, [2500, 3000) g	Mortality	Females		7.139 (5.091 to 9.759)	5.194 (4.342 to 6.087)
Encephalitis	Birth prevalence - [34, 36) wks, [3000, 3500) g	Mortality	Males		7.828 (5.467 to 11.237)	3.717 (2.986 to 4.568)
Encephalitis	Birth prevalence - [34, 36) wks, [3000, 3500) g	Mortality	Females		7.622 (5.235 to 10.584)	4.279 (3.416 to 5.301)
Encephalitis	Birth prevalence - [34, 36) wks, [3500, 4000) g	Mortality	Males		10.257 (6.692 to 15.124)	3.13 (2.474 to 3.957)
Encephalitis	Birth prevalence - [34, 36) wks, [3500, 4000) g	Mortality	Females		10.325 (6.495 to 15.537)	3.756 (2.877 to 4.818)
Encephalitis	Birth prevalence - [34, 36) wks, [4000, 4500) g	Mortality	Males		12.928 (7.825 to 20.726)	2.467 (1.926 to 3.084)
Encephalitis	Birth prevalence - [34, 36) wks, [4000, 4500) g	Mortality	Females		13.456 (8.201 to 20.893)	3.061 (2.357 to 3.889)
Encephalitis	Birth prevalence - [36, 37) wks, [1000, 1500) g	Mortality	Males		94.103 (62.401 to 131.437)	48.734 (39.385 to 60.014)
Encephalitis	Birth prevalence - [36, 37) wks, [1000, 1500) g	Mortality	Females		89.769 (59.869 to 126.674)	51.68 (41.336 to 63.456)
Encephalitis	Birth prevalence - [36, 37) wks, [1500, 2000) g	Mortality	Males		33.586 (23.075 to 48.77)	19.52 (16.283 to 23.116)
Encephalitis	Birth prevalence - [36, 37) wks, [1500, 2000) g	Mortality	Females		30.953 (20.951 to 43.853)	20.509 (17.15 to 24.286)
Encephalitis	Birth prevalence - [36, 37) wks, [2000, 2500) g	Mortality	Males		8.134 (5.781 to 10.938)	7.015 (5.903 to 8.241)
Encephalitis	Birth prevalence - [36, 37) wks, [2000, 2500) g	Mortality	Females		7.208 (5.183 to 9.787)	7.031 (5.911 to 8.265)
Encephalitis	Birth prevalence - [36, 37) wks, [2500, 3000) g	Mortality	Males		2.741 (2.114 to 3.535)	3.149 (2.686 to 3.686)
Encephalitis	Birth prevalence - [36, 37) wks, [2500, 3000) g	Mortality	Females		2.475 (1.905 to 3.109)	3.172 (2.71 to 3.666)
Encephalitis	Birth prevalence - [36, 37) wks, [3000, 3500) g	Mortality	Males		2.1 (1.602 to 2.663)	2.099 (1.805 to 2.445)
Encephalitis	Birth prevalence - [36, 37) wks, [3000, 3500) g	Mortality	Females		1.996 (1.532 to 2.6)	2.199 (1.895 to 2.534)
Encephalitis	Birth prevalence - [36, 37) wks, [3500, 4000) g	Mortality	Males		2.563 (1.906 to 3.333)	1.751 (1.487 to 2.008)
Encephalitis	Birth prevalence - [36, 37) wks, [3500, 4000) g	Mortality	Females		2.501 (1.793 to 3.34)	1.944 (1.649 to 2.275)

Encephalitis	Birth prevalence - [36, 37) wks, [4000, 4500) g	Mortality	Males		3.824<br>(2.607 to 5.361)	1.506<br>(1.283 to 1.74)
Encephalitis	Birth prevalence - [36, 37) wks, [4000, 4500) g	Mortality	Females		3.908<br>(2.626 to 5.462)	1.777<br>(1.481 to 2.118)
Encephalitis	Birth prevalence - [37, 38) wks, [1000, 1500) g	Mortality	Males		98.111<br>(66.503 to 135.289)	49.903<br>(39.62 to 62.232)
Encephalitis	Birth prevalence - [37, 38) wks, [1000, 1500) g	Mortality	Females		92.728<br>(63.975 to 128.565)	53.151<br>(42.705 to 66.273)
Encephalitis	Birth prevalence - [37, 38) wks, [1500, 2000) g	Mortality	Males		35.71<br>(24.585 to 49.648)	20.617<br>(16.897 to 24.816)
Encephalitis	Birth prevalence - [37, 38) wks, [1500, 2000) g	Mortality	Females		32.101<br>(22.457 to 43.887)	21.726<br>(17.833 to 25.871)
Encephalitis	Birth prevalence - [37, 38) wks, [2000, 2500) g	Mortality	Males		7.301<br>(5.37 to 9.805)	6.923<br>(5.831 to 8.219)
Encephalitis	Birth prevalence - [37, 38) wks, [2000, 2500) g	Mortality	Females		6.218<br>(4.554 to 8.169)	6.85<br>(5.796 to 8.005)
Encephalitis	Birth prevalence - [37, 38) wks, [2500, 3000) g	Mortality	Males		1.861<br>(1.414 to 2.384)	2.721<br>(2.329 to 3.158)
Encephalitis	Birth prevalence - [37, 38) wks, [2500, 3000) g	Mortality	Females		1.596<br>(1.24 to 1.998)	2.633<br>(2.261 to 3.031)
Encephalitis	Birth prevalence - [37, 38) wks, [3000, 3500) g	Mortality	Males		1.139<br>(1 to 1.409)	1.612<br>(1.387 to 1.85)
Encephalitis	Birth prevalence - [37, 38) wks, [3000, 3500) g	Mortality	Females		1.065<br>(1 to 1.288)	1.598<br>(1.392 to 1.819)
Encephalitis	Birth prevalence - [37, 38) wks, [3500, 4000) g	Mortality	Males		1.201<br>(1 to 1.493)	1.282<br>(1.113 to 1.466)
Encephalitis	Birth prevalence - [37, 38) wks, [3500, 4000) g	Mortality	Females		1.157<br>(1 to 1.442)	1.343<br>(1.153 to 1.542)
Encephalitis	Birth prevalence - [37, 38) wks, [4000, 4500) g	Mortality	Males		1.825<br>(1.341 to 2.407)	1.136<br>(1.01 to 1.272)
Encephalitis	Birth prevalence - [37, 38) wks, [4000, 4500) g	Mortality	Females		1.86<br>(1.36 to 2.46)	1.269<br>(1.115 to 1.42)
Encephalitis	Birth prevalence - [38, 40) wks, [1000, 1500) g	Mortality	Males		98.254<br>(67.959 to 134.77)	49.084<br>(38.794 to 61.948)
Encephalitis	Birth prevalence - [38, 40) wks, [1000, 1500) g	Mortality	Females		91.539<br>(62.769 to 127.606)	53.036<br>(41.348 to 65.237)
Encephalitis	Birth prevalence - [38, 40) wks, [1500, 2000) g	Mortality	Males		37.449<br>(25.688 to 51.467)	21.477<br>(17.208 to 25.646)
Encephalitis	Birth prevalence - [38, 40) wks, [1500, 2000) g	Mortality	Females		33.261<br>(23.303 to 45.061)	22.821<br>(18.339 to 27.874)
Encephalitis	Birth prevalence - [38, 40) wks, [2000, 2500) g	Mortality	Males		7.351<br>(5.131 to 9.967)	7.04<br>(5.807 to 8.395)
Encephalitis	Birth prevalence - [38, 40) wks, [2000, 2500) g	Mortality	Females		5.985<br>(4.383 to 7.995)	6.959<br>(5.782 to 8.29)
Encephalitis	Birth prevalence - [38, 40) wks, [2500, 3000) g	Mortality	Males		1.549<br>(1.185 to 1.98)	2.518<br>(2.115 to 2.971)
Encephalitis	Birth prevalence - [38, 40) wks, [2500, 3000) g	Mortality	Females		1.277<br>(1 to 1.65)	2.377<br>(2.015 to 2.745)
Encephalitis	Birth prevalence - [38, 40) wks, [3000, 3500) g	Mortality	Males		1<br>(1.0 - 1.0)	1.326<br>(1.12 to 1.543)
Encephalitis	Birth prevalence - [38, 40) wks, [3000, 3500) g	Mortality	Females		1<br>(1.0 - 1.0)	1.265<br>(1.068 to 1.45)
Encephalitis	Birth prevalence - [38, 40) wks, [3500, 4000) g	Mortality	Males		1<br>(1.0 - 1.0)	1<br>(1.0 - 1.0)
Encephalitis	Birth prevalence - [38, 40) wks, [3500, 4000) g	Mortality	Females		1<br>(1.0 - 1.0)	1<br>(1.0 - 1.0)
Encephalitis	Birth prevalence - [38, 40) wks, [4000, 4500) g	Mortality	Males		1<br>(1.0 - 1.0)	1<br>(1.0 - 1.0)
Encephalitis	Birth prevalence - [38, 40) wks, [4000, 4500) g	Mortality	Females		1<br>(1.0 - 1.0)	1<br>(1.0 - 1.0)
Encephalitis	Birth prevalence - [40, 42) wks, [1500, 2000) g	Mortality	Males		43.194<br>(29.244 to 62.382)	21.971<br>(16.41 to 28.396)
Encephalitis	Birth prevalence - [40, 42) wks, [1500, 2000) g	Mortality	Females		37.949<br>(25.539 to 53.532)	23.702<br>(17.857 to 30.634)
Encephalitis	Birth prevalence - [40, 42) wks, [2000, 2500) g	Mortality	Males		10.162<br>(6.961 to 14.235)	7.875<br>(6.341 to 9.719)
Encephalitis	Birth prevalence - [40, 42) wks, [2000, 2500) g	Mortality	Females		8.332<br>(5.994 to 11.407)	8.067<br>(6.467 to 10.043)
Encephalitis	Birth prevalence - [40, 42) wks, [2500, 3000) g	Mortality	Males		2.118<br>(1.53 to 2.77)	2.692<br>(2.23 to 3.273)
Encephalitis	Birth prevalence - [40, 42) wks, [2500, 3000) g	Mortality	Females		1.745<br>(1.289 to 2.316)	2.603<br>(2.134 to 3.106)
Encephalitis	Birth prevalence - [40, 42) wks, [3000, 3500) g	Mortality	Males		1.002<br>(1 to 1.013)	1.249<br>(1.041 to 1.491)
Encephalitis	Birth prevalence - [40, 42) wks, [3000, 3500) g	Mortality	Females		1<br>(1.0 - 1.0)	1.188<br>(1 to 1.411)
Encephalitis	Birth prevalence - [40, 42) wks, [3500, 4000) g	Mortality	Males		1<br>(1.0 - 1.0)	1<br>(1.0 - 1.0)
Encephalitis	Birth prevalence - [40, 42) wks, [3500, 4000) g	Mortality	Females		1<br>(1.0 - 1.0)	1<br>(1.0 - 1.0)
Encephalitis	Birth prevalence - [40, 42) wks, [4000, 4500) g	Mortality	Males		1<br>(1.0 - 1.0)	1<br>(1.0 - 1.0)
Encephalitis	Birth prevalence - [40, 42) wks, [4000, 4500) g	Mortality	Females		1<br>(1.0 - 1.0)	1<br>(1.0 - 1.0)
Hemolytic disease and other neonatal jaundice	Birth prevalence - [0, 24) wks, [0, 500) g	Mortality	Males		877.775<br>(543.26 to 1246.645)	526.788<br>(392.055 to 676.923)
Hemolytic disease and other neonatal jaundice	Birth prevalence - [0, 24) wks, [0, 500) g	Mortality	Females		857.044<br>(519.185 to 1227.708)	575.632<br>(434.418 to 737.293)
Hemolytic disease and other neonatal jaundice	Birth prevalence - [0, 24) wks, [500, 1000) g	Mortality	Males		647.899<br>(443.535 to 881.449)	392.723<br>(315.689 to 486.607)
Hemolytic disease and other neonatal jaundice	Birth prevalence - [0, 24) wks, [500, 1000) g	Mortality	Females		628.647<br>(437.861 to 854.598)	416.95<br>(329.573 to 516.699)
Hemolytic disease and other neonatal jaundice	Birth prevalence - [24, 26) wks, [500, 1000) g	Mortality	Males		534.904<br>(378.653 to 707.51)	375.56<br>(308.336 to 447.076)
Hemolytic disease and other neonatal jaundice	Birth prevalence - [24, 26) wks, [500, 1000) g	Mortality	Females		509.388<br>(370.575 to 690.649)	394.982<br>(324.03 to 477.74)
Hemolytic disease and other neonatal jaundice	Birth prevalence - [26, 28) wks, [1000, 1500) g	Mortality	Males		150.649<br>(107.741 to 198.278)	140.284<br>(113.557 to 167.957)
Hemolytic disease and other neonatal jaundice	Birth prevalence - [26, 28) wks, [1000, 1500) g	Mortality	Females		142.251<br>(102.895 to 191.587)	140.771<br>(114.514 to 169.631)
Hemolytic disease and other neonatal jaundice	Birth prevalence - [26, 28) wks, [500, 1000) g	Mortality	Males		281.284<br>(198.703 to 391.898)	280.84<br>(226.174 to 338.336)
Hemolytic disease and other neonatal jaundice	Birth prevalence - [26, 28) wks, [500, 1000) g	Mortality	Females		257.799<br>(185.994 to 349.394)	280.84<br>(225.68 to 350.2)
Hemolytic disease and other neonatal jaundice	Birth prevalence - [28, 30) wks, [1000, 1500) g	Mortality	Males		88.889<br>(62.539 to 119.303)	87.596<br>(72.219 to 105.445)
Hemolytic disease and other neonatal jaundice	Birth prevalence - [28, 30) wks, [1000, 1500) g	Mortality	Females		82.25<br>(59.418 to 109.806)	86.675<br>(69.965 to 104.367)
Hemolytic disease and other neonatal jaundice	Birth prevalence - [28, 30) wks, [1500, 2000) g	Mortality	Males		71.963<br>(51.376 to 98.371)	42.294<br>(34.585 to 51.215)
Hemolytic disease and other neonatal jaundice	Birth prevalence - [28, 30) wks, [1500, 2000) g	Mortality	Females		70.154<br>(48.562 to 96.294)	46.161<br>(38.268 to 54.906)
Hemolytic disease and other neonatal jaundice	Birth prevalence - [28, 30) wks, [2000, 2500) g	Mortality	Males		65.936<br>(44.909 to 93.551)	23.785<br>(18.748 to 29.385)
Hemolytic disease and other neonatal jaundice	Birth prevalence - [28, 30) wks, [2000, 2500) g	Mortality	Females		65.44<br>(43.751 to 96.137)	27.437<br>(21.369 to 33.874)
Hemolytic disease and other neonatal jaundice	Birth prevalence - [28, 30) wks, [2500, 3000) g	Mortality	Males		43.647<br>(29.61 to 62.044)	14.185<br>(10.817 to 18.495)
Hemolytic disease and other neonatal jaundice	Birth prevalence - [28, 30) wks, [2500, 3000) g	Mortality	Females		42.667<br>(28.231 to 61.897)	16.517<br>(12.636 to 21.355)
Hemolytic disease and other neonatal jaundice	Birth prevalence - [28, 30) wks, [3000, 3500) g	Mortality	Males		23.727<br>(15.859 to 35.246)	8.651<br>(6.873 to 11.016)
Hemolytic disease and other neonatal jaundice	Birth prevalence - [28, 30) wks, [3000, 3500) g	Mortality	Females		23.045<br>(14.473 to 35.151)	9.735<br>(7.511 to 12.367)
Hemolytic disease and other neonatal jaundice	Birth prevalence - [28, 30) wks, [500, 1000) g	Mortality	Males		164.681<br>(114.331 to 232.909)	184.988<br>(146.715 to 225.778)
Hemolytic disease and other neonatal jaundice	Birth prevalence - [28, 30) wks, [500, 1000) g	Mortality	Females		149.827<br>(102.011 to 207.65)	178.51<br>(144.736 to 220.088)
Hemolytic disease and other neonatal jaundice	Birth prevalence - [30, 32) wks, [1000, 1500) g	Mortality	Males		66.567<br>(45.227 to 91.424)	57.036<br>(47.559 to 67.673)
Hemolytic disease and other neonatal jaundice	Birth prevalence - [30, 32) wks, [1000, 1500) g	Mortality	Females		61.652<br>(42.69 to 86.655)	55.992<br>(46.425 to 67.165)
Hemolytic disease and other neonatal jaundice	Birth prevalence - [30, 32) wks, [1500, 2000) g	Mortality	Males		43.288<br>(30.684 to 57.762)	26.533<br>(22.334 to 31.213)
Hemolytic disease and other neonatal jaundice	Birth prevalence - [30, 32) wks, [1500, 2000) g	Mortality	Females		41.127<br>(28.837 to 54.767)	28.256<br>(24.051 to 32.792)
Hemolytic disease and other neonatal jaundice	Birth prevalence - [30, 32) wks, [2000, 2500) g	Mortality	Males		38.672<br>(27.558 to 53.438)	15.362<br>(12.641 to 18.516)
Hemolytic disease and other neonatal jaundice	Birth prevalence - [30, 32) wks, [2000, 2500) g	Mortality	Females		37.459<br>(26.086 to 52.166)	17.996<br>(14.845 to 21.529)
Hemolytic disease and other neonatal jaundice	Birth prevalence - [30, 32) wks, [2500, 3000) g	Mortality	Males		33.218<br>(22.641 to 46.944)	10.389<br>(8.195 to 12.863)
Hemolytic disease and other neonatal jaundice	Birth prevalence - [30, 32) wks, [2500, 3000) g	Mortality	Females		31.439<br>(21.157 to 45.621)	12.473<br>(9.64 to 15.784)
Hemolytic disease and other neonatal jaundice	Birth prevalence - [30, 32) wks, [3000, 3500) g	Mortality	Males		25.451<br>(16.854 to 36.763)	7.153<br>(5.665 to 9.003)
Hemolytic disease and other neonatal jaundice	Birth prevalence - [30, 32) wks, [3000, 3500) g	Mortality	Females		24.262<br>(15.651 to 36.395)	8.486<br>(6.482 to 10.824)
Hemolytic disease and other neonatal jaundice	Birth prevalence - [30, 32) wks, [3500, 4000) g	Mortality	Males		20.334<br>(11.916 to 31.586)	4.795<br>(3.705 to 6.039)
Hemolytic disease and other neonatal jaundice	Birth prevalence - [30, 32) wks, [3500, 4000) g	Mortality	Females		20.347<br>(11.635 to 32.681)	5.599<br>(4.199 to 7.275)
Hemolytic disease and other neonatal jaundice	Birth prevalence - [30, 32) wks, [500, 1000) g	Mortality	Males		132.309<br>(85.99 to 193.341)	127.982<br>(101.66 to 159.489)
Hemolytic disease and other neonatal jaundice	Birth prevalence - [30, 32) wks, [500, 1000) g	Mortality	Females		120.829<br>(82.341 to 172.828)	123.186<br>(98.696 to 152.125)
Hemolytic disease and other neonatal jaundice	Birth prevalence - [32, 34) wks, [1000, 1500) g	Mortality	Males		65.39<br>(43.593 to 95.583)	45.581<br>(37.56 to 54.417)
Hemolytic disease and other neonatal jaundice	Birth prevalence - [32, 34) wks, [1000, 1500) g	Mortality	Females		61.229<br>(40.8 to 88.303)	45.201<br>(37.492 to 54.267)
Hemolytic disease and other neonatal jaundice	Birth prevalence - [32, 34) wks, [1500, 2000) g	Mortality	Males		32.197<br>(22.524 to 44.858)	19.612<br>(16.465 to 23.296)
Hemolytic disease and other neonatal jaundice	Birth prevalence - [32, 34) wks, [1500, 2000) g	Mortality	Females		29.777<br>(21.026 to 40.255)	20.358<br>(17.072 to 23.768)
Hemolytic disease and other neonatal jaundice	Birth prevalence - [32, 34) wks, [2000, 2500) g	Mortality	Males		21.018<br>(15.141 to 28.622)	10.433<br>(8.784 to 12.225)
Hemolytic disease and other neonatal jaundice	Birth prevalence - [32, 34) wks, [2000, 2500) g	Mortality	Females		19.546<br>(14.316 to 26.034)	11.648<br>(9.974 to 13.507)
Hemolytic disease and other neonatal jaundice	Birth prevalence - [32, 34) wks, [2500, 3000) g	Mortality	Males		18.569<br>(13.127 to 25.826)	7.182<br>(5.968 to 8.63)
Hemolytic disease and other neonatal jaundice	Birth prevalence - [32, 34) wks, [2500, 3000) g	Mortality	Females		17.497<br>(11.66 to 24.635)	8.443<br>(6.726 to 10.284)
Hemolytic disease and other neonatal jaundice	Birth prevalence - [32, 34) wks, [3000, 3500) g	Mortality	Males		19.012<br>(12.18 to 28.02)	5.618<br>(4.325 to 7.114)
Hemolytic disease and other neonatal jaundice	Birth prevalence - [32, 34) wks, [3000, 3500) g	Mortality	Females		18.667<br>(12.24 to 27.524)	6.722<br>(5.103 to 8.697)
Hemolytic disease and other neonatal jaundice	Birth prevalence - [32, 34) wks, [3500, 4000) g	Mortality	Males		20.214<br>(11.906 to 31.929)	4.31<br>(3.186 to 5.633)
Hemolytic disease and other neonatal jaundice	Birth prevalence - [32, 34) wks, [3500, 4000) g	Mortality	Females		20.333<br>(12.064 to 33.21)	5.253<br>(3.959 to 6.913)
Hemolytic disease and other neonatal jaundice	Birth prevalence - [34, 36) wks, [1000, 1500) g	Mortality	Males		78.66<br>(52.169 to 113.08)	45.338<br>(36.27 to 55.556)
Hemolytic disease and other neonatal jaundice	Birth prevalence - [34, 36) wks, [1000, 1500) g	Mortality	Females		75.687<br>(49.863 to 110.089)	46.4<br>(38.048 to 55.902)
Hemolytic disease and other neonatal jaundice	Birth prevalence - [34, 36) wks, [1500, 2000) g	Mortality	Males		31.154<br>(20.939 to 45.031)	18.246<br>(15.083 to 21.906)
Hemolytic disease and other neonatal jaundice	Birth prevalence - [34, 36) wks, [1500, 2000) g	Mortality	Females		29.072<br>(19.6 to 42.648)	18.661<br>(15.638 to 22.175)
Hemolytic disease and other neonatal jaundice	Birth prevalence - [34, 36) wks, [2000, 2500) g	Mortality	Males		12.404<br>(8.692 to 16.95)	8.006<br>(6.775 to 9.31)
Hemolytic disease and other neonatal jaundice	Birth prevalence - [34, 36) wks, [2000, 2500) g	Mortality	Females		11.367<br>(7.807 to 15.547)	8.373<br>(7.066 to 9.703)
Hemolytic disease and other neonatal jaundice	Birth prevalence - [34, 36) wks, [2500, 3000) g	Mortality	Males		7.509<br>(5.429 to 10.255)	4.725<br>(3.991 to 5.56)
Hemolytic disease and other neonatal jaundice	Birth prevalence - [34, 36) wks, [2500, 3000) g	Mortality	Females		7.139<br>(5.091 to 9.759)	5.194<br>(4.342 to 6.087)
Hemolytic disease and other neonatal jaundice	Birth prevalence - [34, 36) wks, [3000, 3500) g	Mortality	Males		7.828<br>(5.467 to 11.237)	3.717<br>(2.986 to 4.568)
Hemolytic disease and other neonatal jaundice	Birth prevalence - [34, 36) wks, [3000, 3500) g	Mortality	Females		7.622<br>(5.235 to 10.584)	4.279<br>(3.416 to 5.301)
Hemolytic disease and other neonatal jaundice	Birth prevalence - [34, 36) wks, [3500, 4000) g	Mortality	Males		10.257<br>(6.692 to 15.124)	3.13<br>(2.474 to 3.957)
Hemolytic disease and other neonatal jaundice	Birth prevalence - [34, 36) wks, [3500, 4000) g	Mortality	Females		10.325<br>(6.495 to 15.537)	3.756<br>(2.877 to 4.818)
Hemolytic disease and other neonatal jaundice	Birth prevalence - [34, 36) wks, [4000, 4500) g	Mortality	Males		12.928<br>(7.825 to 20.726)	2.467<br>(1.926 to 3.084)
Hemolytic disease and other neonatal jaundice	Birth prevalence - [34, 36) wks, [4000, 4500) g	Mortality	Females		13.456<br>(8.201 to 20.893)	3.061<br>(2.357 to 3.889)
Hemolytic disease and other neonatal jaundice	Birth prevalence - [36, 37) wks, [1000, 1500) g	Mortality	Males		94.103<br>(62.401 to 131.437)	48.734<br>(39.385 to 60.014)
Hemolytic disease and other neonatal jaundice	Birth prevalence - [36, 37) wks, [1000, 1500) g	Mortality	Females		89.769<br>(59.869 to 126.674)	51.68<br>(41.336 to 63.456)
Hemolytic disease and other neonatal jaundice	Birth prevalence - [36, 37) wks, [1500, 2000) g	Mortality	Males		33.586<br>(23.075 to 48.77)	19.52<br>(16.283 to 23.116)
Hemolytic disease and other neonatal jaundice	Birth prevalence - [36, 37) wks, [1500, 2000) g	Mortality	Females		30.953<br>(20.951 to 43.853)	20.509<br>(17.15 to 24.286)
Hemolytic disease and other neonatal jaundice	Birth prevalence - [36, 37) wks, [2000, 2500) g	Mortality	Males		8.134<br>(5.781 to 10.938)	7.015<br>(5.903 to 8.241)
Hemolytic disease and other neonatal jaundice	Birth prevalence - [36, 37) wks, [2000, 2500) g	Mortality	Females		7.208<br>(5.183 to 9.787)	7.031<br>(5.911 to 8.265)
Hemolytic disease and other neonatal jaundice	Birth prevalence - [36, 37) wks, [2500, 3000) g	Mortality	Males		2.741<br>(2.114 to 3.535)	3.149<br>(2.686 to 3.686)
Hemolytic disease and other neonatal jaundice	Birth prevalence - [36, 37) wks, [2500, 3000) g	Mortality	Females		2.475<br>(1.905 to 3.109)	3.172<br>(2.71 to 3.666)
Hemolytic disease and other neonatal jaundice	Birth prevalence - [36, 37) wks, [3000, 3500) g	Mortality	Males		2.1<br>(1.602 to 2.663)	2.099<br>(1.805 to 2.445)
Hemolytic disease and other neonatal jaundice	Birth prevalence - [36, 37) wks, [3000, 3500) g	Mortality	Females		1.996<br>(1.532 to 2.6)	2.199<br>(1.895 to 2.534)
Hemolytic disease and other neonatal jaundice	Birth prevalence - [36, 37) wks, [3500, 4000) g	Mortality	Males		2.563<br>(1.906 to 3.333)	1.751<br>(1.487 to 2.008)
Hemolytic disease and other neonatal jaundice	Birth prevalence - [36, 37) wks, [3500, 4000) g	Mortality	Females		2.501<br>(1.793 to 3.34)	1.944<br>(1.649 to 2.275)
Hemolytic disease and other neonatal jaundice	Birth prevalence - [36, 37) wks, [4000, 4500) g	Mortality	Males		3.824<br>(2.607 to 5.361)	1.506<br>(1.283 to 1.74)
Hemolytic disease and other neonatal jaundice	Birth prevalence - [36, 37) wks, [4000, 4500) g	Mortality	Females		3.908<br>(2.626 to 5.462)	1.777<br>(1.481 to 2.118)
Hemolytic disease and other neonatal jaundice	Birth prevalence - [37, 38) wks, [1000, 1500) g	Mortality	Males		98.111<br>(66.503 to 135.289)	49.903<br>(39.62 to 62.232)
Hemolytic disease and other neonatal jaundice	Birth prevalence - [37, 38) wks, [1000, 1500) g	Mortality	Females		92.728<br>(63.975 to 128.565)	53.151<br>(42.705 to 66.273)
Hemolytic disease and other neonatal jaundice	Birth prevalence - [37, 38) wks, [1500, 2000) g	Mortality	Males		35.71<br>(24.585 to 49.648)	20.617<br>(16.897 to 24.816)
Hemolytic disease and other neonatal jaundice	Birth prevalence - [37, 38) wks, [1500, 2000) g	Mortality	Females		32.101<br>(22.457 to 43.887)	21.726<br>(17.833 to 25.871)
Hemolytic disease and other neonatal jaundice	Birth prevalence - [37, 38) wks, [2000, 2500) g	Mortality	Males		7.301<br>(5.37 to 9.805)	6.923<br>(5.831 to 8.219)

Hemolytic disease and other neonatal jaundice	Birth prevalence - [37, 38) wks, [2000, 2500) g	Mortality	Females		6.218 (4.554 to 8.169)	6.85 (5.796 to 8.005)
Hemolytic disease and other neonatal jaundice	Birth prevalence - [37, 38) wks, [2500, 3000) g	Mortality	Males		1.861 (1.414 to 2.384)	2.721 (2.329 to 3.158)
Hemolytic disease and other neonatal jaundice	Birth prevalence - [37, 38) wks, [2500, 3000) g	Mortality	Females		1.596 (1.24 to 1.998)	2.633 (2.261 to 3.031)
Hemolytic disease and other neonatal jaundice	Birth prevalence - [37, 38) wks, [3000, 3500) g	Mortality	Males		1.139 (1 to 1.409)	1.612 (1.387 to 1.85)
Hemolytic disease and other neonatal jaundice	Birth prevalence - [37, 38) wks, [3000, 3500) g	Mortality	Females		1.065 (1 to 1.288)	1.598 (1.392 to 1.819)
Hemolytic disease and other neonatal jaundice	Birth prevalence - [37, 38) wks, [3500, 4000) g	Mortality	Males		1.201 (1 to 1.493)	1.282 (1.113 to 1.466)
Hemolytic disease and other neonatal jaundice	Birth prevalence - [37, 38) wks, [3500, 4000) g	Mortality	Females		1.157 (1 to 1.442)	1.343 (1.153 to 1.542)
Hemolytic disease and other neonatal jaundice	Birth prevalence - [37, 38) wks, [4000, 4500) g	Mortality	Males		1.825 (1.341 to 2.407)	1.136 (1.01 to 1.272)
Hemolytic disease and other neonatal jaundice	Birth prevalence - [37, 38) wks, [4000, 4500) g	Mortality	Females		1.86 (1.36 to 2.46)	1.269 (1.115 to 1.42)
Hemolytic disease and other neonatal jaundice	Birth prevalence - [38, 40) wks, [1000, 1500) g	Mortality	Males		98.254 (67.959 to 134.77)	49.084 (38.794 to 61.948)
Hemolytic disease and other neonatal jaundice	Birth prevalence - [38, 40) wks, [1000, 1500) g	Mortality	Females		91.539 (62.769 to 127.606)	53.036 (41.348 to 65.237)
Hemolytic disease and other neonatal jaundice	Birth prevalence - [38, 40) wks, [1500, 2000) g	Mortality	Males		37.449 (25.688 to 51.467)	21.477 (17.208 to 25.646)
Hemolytic disease and other neonatal jaundice	Birth prevalence - [38, 40) wks, [1500, 2000) g	Mortality	Females		33.261 (23.303 to 45.061)	22.821 (18.339 to 27.874)
Hemolytic disease and other neonatal jaundice	Birth prevalence - [38, 40) wks, [2000, 2500) g	Mortality	Males		7.351 (5.131 to 9.967)	7.04 (5.807 to 8.395)
Hemolytic disease and other neonatal jaundice	Birth prevalence - [38, 40) wks, [2000, 2500) g	Mortality	Females		5.985 (4.383 to 7.995)	6.959 (5.782 to 8.29)
Hemolytic disease and other neonatal jaundice	Birth prevalence - [38, 40) wks, [2500, 3000) g	Mortality	Males		1.549 (1.185 to 1.98)	2.518 (2.115 to 2.971)
Hemolytic disease and other neonatal jaundice	Birth prevalence - [38, 40) wks, [2500, 3000) g	Mortality	Females		1.277 (1 to 1.65)	2.377 (2.015 to 2.745)
Hemolytic disease and other neonatal jaundice	Birth prevalence - [38, 40) wks, [3000, 3500) g	Mortality	Males		1 (1.0 - 1.0)	1.326 (1.12 to 1.543)
Hemolytic disease and other neonatal jaundice	Birth prevalence - [38, 40) wks, [3000, 3500) g	Mortality	Females		1 (1.0 - 1.0)	1.265 (1.068 to 1.45)
Hemolytic disease and other neonatal jaundice	Birth prevalence - [38, 40) wks, [3500, 4000) g	Mortality	Males		1 (1.0 - 1.0)	1 (1.0 - 1.0)
Hemolytic disease and other neonatal jaundice	Birth prevalence - [38, 40) wks, [3500, 4000) g	Mortality	Females		1 (1.0 - 1.0)	1 (1.0 - 1.0)
Hemolytic disease and other neonatal jaundice	Birth prevalence - [38, 40) wks, [4000, 4500) g	Mortality	Males		1 (1.0 - 1.0)	1 (1.0 - 1.0)
Hemolytic disease and other neonatal jaundice	Birth prevalence - [38, 40) wks, [4000, 4500) g	Mortality	Females		1 (1.0 - 1.0)	1 (1.0 - 1.0)
Hemolytic disease and other neonatal jaundice	Birth prevalence - [40, 42) wks, [1500, 2000) g	Mortality	Males		43.194 (29.244 to 62.382)	21.971 (16.41 to 28.396)
Hemolytic disease and other neonatal jaundice	Birth prevalence - [40, 42) wks, [1500, 2000) g	Mortality	Females		37.949 (25.539 to 53.532)	23.702 (17.857 to 30.634)
Hemolytic disease and other neonatal jaundice	Birth prevalence - [40, 42) wks, [2000, 2500) g	Mortality	Males		10.162 (6.961 to 14.235)	7.875 (6.341 to 9.719)
Hemolytic disease and other neonatal jaundice	Birth prevalence - [40, 42) wks, [2000, 2500) g	Mortality	Females		8.332 (5.994 to 11.407)	8.067 (6.467 to 10.043)
Hemolytic disease and other neonatal jaundice	Birth prevalence - [40, 42) wks, [2500, 3000) g	Mortality	Males		2.118 (1.53 to 2.77)	2.692 (2.23 to 3.273)
Hemolytic disease and other neonatal jaundice	Birth prevalence - [40, 42) wks, [2500, 3000) g	Mortality	Females		1.745 (1.289 to 2.316)	2.603 (2.134 to 3.106)
Hemolytic disease and other neonatal jaundice	Birth prevalence - [40, 42) wks, [3000, 3500) g	Mortality	Males		1.002 (1 to 1.013)	1.249 (1.041 to 1.491)
Hemolytic disease and other neonatal jaundice	Birth prevalence - [40, 42) wks, [3000, 3500) g	Mortality	Females		1 (1.0 - 1.0)	1.188 (1 to 1.411)
Hemolytic disease and other neonatal jaundice	Birth prevalence - [40, 42) wks, [3500, 4000) g	Mortality	Males		1 (1.0 - 1.0)	1 (1.0 - 1.0)
Hemolytic disease and other neonatal jaundice	Birth prevalence - [40, 42) wks, [3500, 4000) g	Mortality	Females		1 (1.0 - 1.0)	1 (1.0 - 1.0)
Hemolytic disease and other neonatal jaundice	Birth prevalence - [40, 42) wks, [4000, 4500) g	Mortality	Males		1 (1.0 - 1.0)	1 (1.0 - 1.0)
Hemolytic disease and other neonatal jaundice	Birth prevalence - [40, 42) wks, [4000, 4500) g	Mortality	Females		1 (1.0 - 1.0)	1 (1.0 - 1.0)
Lower respiratory infections	Birth prevalence - [0, 24) wks, [0, 500) g	Mortality	Males		877.775 (543.26 to 1246.645)	526.788 (392.055 to 676.923)
Lower respiratory infections	Birth prevalence - [0, 24) wks, [0, 500) g	Mortality	Females		857.044 (519.185 to 1227.708)	575.632 (434.418 to 737.293)
Lower respiratory infections	Birth prevalence - [0, 24) wks, [500, 1000) g	Mortality	Males		647.899 (443.535 to 881.449)	392.723 (315.689 to 486.607)
Lower respiratory infections	Birth prevalence - [0, 24) wks, [500, 1000) g	Mortality	Females		628.647 (437.861 to 854.598)	416.95 (329.573 to 516.699)
Lower respiratory infections	Birth prevalence - [24, 26) wks, [500, 1000) g	Mortality	Males		534.904 (378.653 to 707.51)	375.56 (308.336 to 447.076)
Lower respiratory infections	Birth prevalence - [24, 26) wks, [500, 1000) g	Mortality	Females		509.388 (370.575 to 690.649)	394.982 (324.03 to 477.74)
Lower respiratory infections	Birth prevalence - [26, 28) wks, [1000, 1500) g	Mortality	Males		150.649 (107.741 to 198.278)	140.284 (113.557 to 167.957)
Lower respiratory infections	Birth prevalence - [26, 28) wks, [1000, 1500) g	Mortality	Females		142.251 (102.895 to 191.587)	140.771 (114.514 to 169.631)
Lower respiratory infections	Birth prevalence - [26, 28) wks, [500, 1000) g	Mortality	Males		281.284 (198.703 to 391.898)	280.84 (226.174 to 338.336)
Lower respiratory infections	Birth prevalence - [26, 28) wks, [500, 1000) g	Mortality	Females		257.799 (185.994 to 349.394)	280.84 (225.68 to 350.2)
Lower respiratory infections	Birth prevalence - [28, 30) wks, [1000, 1500) g	Mortality	Males		88.889 (62.539 to 119.303)	87.596 (72.219 to 105.445)
Lower respiratory infections	Birth prevalence - [28, 30) wks, [1000, 1500) g	Mortality	Females		82.25 (59.418 to 109.806)	86.675 (69.965 to 104.367)
Lower respiratory infections	Birth prevalence - [28, 30) wks, [1500, 2000) g	Mortality	Males		71.963 (51.376 to 98.371)	42.294 (34.585 to 51.215)
Lower respiratory infections	Birth prevalence - [28, 30) wks, [1500, 2000) g	Mortality	Females		70.154 (48.562 to 96.294)	46.161 (38.268 to 54.906)
Lower respiratory infections	Birth prevalence - [28, 30) wks, [2000, 2500) g	Mortality	Males		65.936 (44.909 to 93.551)	23.785 (18.748 to 29.385)
Lower respiratory infections	Birth prevalence - [28, 30) wks, [2000, 2500) g	Mortality	Females		65.44 (43.751 to 96.137)	27.437 (21.369 to 33.874)
Lower respiratory infections	Birth prevalence - [28, 30) wks, [2500, 3000) g	Mortality	Males		43.647 (29.61 to 62.044)	14.185 (10.817 to 18.495)
Lower respiratory infections	Birth prevalence - [28, 30) wks, [2500, 3000) g	Mortality	Females		42.667 (28.231 to 61.897)	16.517 (12.636 to 21.355)
Lower respiratory infections	Birth prevalence - [28, 30) wks, [3000, 3500) g	Mortality	Males		23.727 (15.859 to 35.246)	8.651 (6.873 to 11.016)
Lower respiratory infections	Birth prevalence - [28, 30) wks, [3000, 3500) g	Mortality	Females		23.045 (14.473 to 35.151)	9.735 (7.511 to 12.367)
Lower respiratory infections	Birth prevalence - [28, 30) wks, [500, 1000) g	Mortality	Males		164.681 (114.331 to 232.909)	184.988 (146.715 to 225.778)
Lower respiratory infections	Birth prevalence - [28, 30) wks, [500, 1000) g	Mortality	Females		149.827 (102.011 to 207.65)	178.51 (144.736 to 220.088)
Lower respiratory infections	Birth prevalence - [30, 32) wks, [1000, 1500) g	Mortality	Males		66.567 (45.227 to 91.424)	57.036 (47.559 to 67.673)
Lower respiratory infections	Birth prevalence - [30, 32) wks, [1000, 1500) g	Mortality	Females		61.652 (42.69 to 86.655)	55.992 (46.425 to 67.165)
Lower respiratory infections	Birth prevalence - [30, 32) wks, [1500, 2000) g	Mortality	Males		43.288 (30.684 to 57.762)	26.533 (22.334 to 31.213)
Lower respiratory infections	Birth prevalence - [30, 32) wks, [1500, 2000) g	Mortality	Females		41.127 (28.837 to 54.767)	28.256 (24.051 to 32.792)
Lower respiratory infections	Birth prevalence - [30, 32) wks, [2000, 2500) g	Mortality	Males		38.672 (27.558 to 53.438)	15.362 (12.641 to 18.516)
Lower respiratory infections	Birth prevalence - [30, 32) wks, [2000, 2500) g	Mortality	Females		37.459 (26.086 to 52.166)	17.996 (14.845 to 21.529)
Lower respiratory infections	Birth prevalence - [30, 32) wks, [2500, 3000) g	Mortality	Males		33.218 (22.641 to 46.944)	10.389 (8.195 to 12.863)
Lower respiratory infections	Birth prevalence - [30, 32) wks, [2500, 3000) g	Mortality	Females		31.439 (21.157 to 45.621)	12.473 (9.64 to 15.784)
Lower respiratory infections	Birth prevalence - [30, 32) wks, [3000, 3500) g	Mortality	Males		25.451 (16.854 to 36.763)	7.153 (5.665 to 9.003)
Lower respiratory infections	Birth prevalence - [30, 32) wks, [3000, 3500) g	Mortality	Females		24.262 (15.651 to 36.395)	8.486 (6.482 to 10.824)
Lower respiratory infections	Birth prevalence - [30, 32) wks, [3500, 4000) g	Mortality	Males		20.334 (11.916 to 31.586)	4.795 (3.705 to 6.039)
Lower respiratory infections	Birth prevalence - [30, 32) wks, [3500, 4000) g	Mortality	Females		20.347 (11.635 to 32.681)	5.599 (4.199 to 7.275)
Lower respiratory infections	Birth prevalence - [30, 32) wks, [500, 1000) g	Mortality	Males		132.309 (85.99 to 193.341)	127.982 (101.66 to 159.489)
Lower respiratory infections	Birth prevalence - [30, 32) wks, [500, 1000) g	Mortality	Females		120.829 (82.341 to 172.828)	123.186 (98.696 to 152.125)
Lower respiratory infections	Birth prevalence - [32, 34) wks, [1000, 1500) g	Mortality	Males		65.39 (43.593 to 95.583)	45.581 (37.56 to 54.417)
Lower respiratory infections	Birth prevalence - [32, 34) wks, [1000, 1500) g	Mortality	Females		61.229 (40.8 to 88.303)	45.201 (37.492 to 54.267)
Lower respiratory infections	Birth prevalence - [32, 34) wks, [1500, 2000) g	Mortality	Males		32.197 (22.524 to 44.858)	19.612 (16.465 to 23.296)
Lower respiratory infections	Birth prevalence - [32, 34) wks, [1500, 2000) g	Mortality	Females		29.777 (21.026 to 40.255)	20.358 (17.072 to 23.768)
Lower respiratory infections	Birth prevalence - [32, 34) wks, [2000, 2500) g	Mortality	Males		21.018 (15.141 to 28.622)	10.433 (8.784 to 12.225)
Lower respiratory infections	Birth prevalence - [32, 34) wks, [2000, 2500) g	Mortality	Females		19.546 (14.316 to 26.034)	11.648 (9.974 to 13.507)
Lower respiratory infections	Birth prevalence - [32, 34) wks, [2500, 3000) g	Mortality	Males		18.569 (13.127 to 25.826)	7.182 (5.968 to 8.63)
Lower respiratory infections	Birth prevalence - [32, 34) wks, [2500, 3000) g	Mortality	Females		17.497 (11.66 to 24.635)	8.443 (6.726 to 10.284)
Lower respiratory infections	Birth prevalence - [32, 34) wks, [3000, 3500) g	Mortality	Males		19.012 (12.18 to 28.02)	5.618 (4.325 to 7.114)
Lower respiratory infections	Birth prevalence - [32, 34) wks, [3000, 3500) g	Mortality	Females		18.667 (12.24 to 27.524)	6.722 (5.103 to 8.697)
Lower respiratory infections	Birth prevalence - [32, 34) wks, [3500, 4000) g	Mortality	Males		20.214 (11.906 to 31.929)	4.31 (3.186 to 5.633)
Lower respiratory infections	Birth prevalence - [32, 34) wks, [3500, 4000) g	Mortality	Females		20.333 (12.064 to 33.21)	5.253 (3.959 to 6.913)
Lower respiratory infections	Birth prevalence - [34, 36) wks, [1000, 1500) g	Mortality	Males		78.66 (52.169 to 113.08)	45.338 (36.27 to 55.556)
Lower respiratory infections	Birth prevalence - [34, 36) wks, [1000, 1500) g	Mortality	Females		75.687 (49.863 to 110.089)	46.4 (38.048 to 55.902)
Lower respiratory infections	Birth prevalence - [34, 36) wks, [1500, 2000) g	Mortality	Males		31.154 (20.939 to 45.031)	18.246 (15.083 to 21.906)
Lower respiratory infections	Birth prevalence - [34, 36) wks, [1500, 2000) g	Mortality	Females		29.072 (19.6 to 42.648)	18.661 (15.638 to 22.175)
Lower respiratory infections	Birth prevalence - [34, 36) wks, [2000, 2500) g	Mortality	Males		12.404 (8.692 to 16.95)	8.006 (6.775 to 9.31)
Lower respiratory infections	Birth prevalence - [34, 36) wks, [2000, 2500) g	Mortality	Females		11.367 (7.807 to 15.547)	8.373 (7.066 to 9.703)
Lower respiratory infections	Birth prevalence - [34, 36) wks, [2500, 3000) g	Mortality	Males		7.509 (5.429 to 10.255)	4.725 (3.991 to 5.56)
Lower respiratory infections	Birth prevalence - [34, 36) wks, [2500, 3000) g	Mortality	Females		7.139 (5.091 to 9.759)	5.194 (4.342 to 6.087)
Lower respiratory infections	Birth prevalence - [34, 36) wks, [3000, 3500) g	Mortality	Males		7.828 (5.467 to 11.237)	3.717 (2.986 to 4.568)
Lower respiratory infections	Birth prevalence - [34, 36) wks, [3000, 3500) g	Mortality	Females		7.622 (5.235 to 10.584)	4.279 (3.416 to 5.301)
Lower respiratory infections	Birth prevalence - [34, 36) wks, [3500, 4000) g	Mortality	Males		10.257 (6.692 to 15.124)	3.13 (2.474 to 3.957)
Lower respiratory infections	Birth prevalence - [34, 36) wks, [3500, 4000) g	Mortality	Females		10.325 (6.495 to 15.537)	3.756 (2.877 to 4.818)
Lower respiratory infections	Birth prevalence - [34, 36) wks, [4000, 4500) g	Mortality	Males		12.928 (7.825 to 20.726)	2.467 (1.926 to 3.084)
Lower respiratory infections	Birth prevalence - [34, 36) wks, [4000, 4500) g	Mortality	Females		13.456 (8.201 to 20.893)	3.061 (2.357 to 3.889)
Lower respiratory infections	Birth prevalence - [36, 37) wks, [1000, 1500) g	Mortality	Males		94.103 (62.401 to 131.437)	48.734 (39.385 to 60.014)
Lower respiratory infections	Birth prevalence - [36, 37) wks, [1000, 1500) g	Mortality	Females		89.769 (59.869 to 126.674)	51.68 (41.336 to 63.456)
Lower respiratory infections	Birth prevalence - [36, 37) wks, [1500, 2000) g	Mortality	Males		33.586 (23.075 to 48.77)	19.52 (16.283 to 23.116)
Lower respiratory infections	Birth prevalence - [36, 37) wks, [1500, 2000) g	Mortality	Females		30.953 (20.951 to 43.853)	20.509 (17.15 to 24.286)
Lower respiratory infections	Birth prevalence - [36, 37) wks, [2000, 2500) g	Mortality	Males		8.134 (5.781 to 10.938)	7.015 (5.903 to 8.241)
Lower respiratory infections	Birth prevalence - [36, 37) wks, [2000, 2500) g	Mortality	Females		7.208 (5.183 to 9.787)	7.031 (5.911 to 8.265)
Lower respiratory infections	Birth prevalence - [36, 37) wks, [2500, 3000) g	Mortality	Males		2.741 (2.114 to 3.535)	3.149 (2.686 to 3.686)
Lower respiratory infections	Birth prevalence - [36, 37) wks, [2500, 3000) g	Mortality	Females		2.475 (1.905 to 3.109)	3.172 (2.71 to 3.666)
Lower respiratory infections	Birth prevalence - [36, 37) wks, [3000, 3500) g	Mortality	Males		2.1 (1.602 to 2.663)	2.099 (1.805 to 2.445)
Lower respiratory infections	Birth prevalence - [36, 37) wks, [3000, 3500) g	Mortality	Females		1.996 (1.532 to 2.6)	2.199 (1.895 to 2.534)
Lower respiratory infections	Birth prevalence - [36, 37) wks, [3500, 4000) g	Mortality	Males		2.563 (1.906 to 3.333)	1.751 (1.487 to 2.008)
Lower respiratory infections	Birth prevalence - [36, 37) wks, [3500, 4000) g	Mortality	Females		2.501 (1.793 to 3.34)	1.944 (1.649 to 2.275)
Lower respiratory infections	Birth prevalence - [36, 37) wks, [4000, 4500) g	Mortality	Males		3.824 (2.607 to 5.361)	1.506 (1.283 to 1.74)
Lower respiratory infections	Birth prevalence - [36, 37) wks, [4000, 4500) g	Mortality	Females		3.908 (2.626 to 5.462)	1.777 (1.481 to 2.118)
Lower respiratory infections	Birth prevalence - [37, 38) wks, [1000, 1500) g	Mortality	Males		98.111 (66.503 to 135.289)	49.903 (39.62 to 62.232)
Lower respiratory infections	Birth prevalence - [37, 38) wks, [1000, 1500) g	Mortality	Females		92.728 (63.975 to 128.565)	53.151 (42.705 to 66.273)
Lower respiratory infections	Birth prevalence - [37, 38) wks, [1500, 2000) g	Mortality	Males		35.71 (24.585 to 49.648)	20.617 (16.897 to 24.816)
Lower respiratory infections	Birth prevalence - [37, 38) wks, [1500, 2000) g	Mortality	Females		32.101 (22.457 to 43.887)	21.726 (17.833 to 25.871)
Lower respiratory infections	Birth prevalence - [37, 38) wks, [2000, 2500) g	Mortality	Males		7.301 (5.37 to 9.805)	6.923 (5.831 to 8.219)
Lower respiratory infections	Birth prevalence - [37, 38) wks, [2000, 2500) g	Mortality	Females		6.218 (4.554 to 8.169)	6.85 (5.796 to 8.005)
Lower respiratory infections	Birth prevalence - [37, 38) wks, [2500, 3000) g	Mortality	Males		1.861 (1.414 to 2.384)	2.721 (2.329 to 3.158)
Lower respiratory infections	Birth prevalence - [37, 38) wks, [2500, 3000) g	Mortality	Females		1.596 (1.24 to 1.998)	2.633 (2.261 to 3.031)
Lower respiratory infections	Birth prevalence - [37, 38) wks, [3000, 3500) g	Mortality	Males		1.139 (1 to 1.409)	1.612 (1.387 to 1.85)
Lower respiratory infections	Birth prevalence - [37, 38) wks, [3000, 3500) g	Mortality	Females		1.065 (1 to 1.288)	1.598 (1.392 to 1.819)
Lower respiratory infections	Birth prevalence - [37, 38) wks, [3500, 4000) g	Mortality	Males		1.201 (1 to 1.493)	1.282 (1.113 to 1.466)
Lower respiratory infections	Birth prevalence - [37, 38) wks, [3500, 4000) g	Mortality	Females		1.157 (1 to 1.442)	1.343 (1.153 to 1.542)

Lower respiratory infections	Birth prevalence - [37, 38) wks, [4000, 4500) g	Mortality	Males		1.825 (1.341 to 2.407)	1.136 (1.01 to 1.272)
Lower respiratory infections	Birth prevalence - [37, 38) wks, [4000, 4500) g	Mortality	Females		1.86 (1.36 to 2.46)	1.269 (1.115 to 1.42)
Lower respiratory infections	Birth prevalence - [38, 40) wks, [1000, 1500) g	Mortality	Males		98.254 (67.959 to 134.77)	49.084 (38.794 to 61.948)
Lower respiratory infections	Birth prevalence - [38, 40) wks, [1000, 1500) g	Mortality	Females		91.539 (62.769 to 127.606)	53.036 (41.348 to 65.237)
Lower respiratory infections	Birth prevalence - [38, 40) wks, [1500, 2000) g	Mortality	Males		37.449 (25.688 to 51.467)	21.477 (17.208 to 25.646)
Lower respiratory infections	Birth prevalence - [38, 40) wks, [1500, 2000) g	Mortality	Females		33.261 (23.303 to 45.061)	22.821 (18.339 to 27.874)
Lower respiratory infections	Birth prevalence - [38, 40) wks, [2000, 2500) g	Mortality	Males		7.351 (5.131 to 9.967)	7.04 (5.807 to 8.395)
Lower respiratory infections	Birth prevalence - [38, 40) wks, [2000, 2500) g	Mortality	Females		5.985 (4.383 to 7.995)	6.959 (5.782 to 8.29)
Lower respiratory infections	Birth prevalence - [38, 40) wks, [2500, 3000) g	Mortality	Males		1.549 (1.185 to 1.98)	2.518 (2.115 to 2.971)
Lower respiratory infections	Birth prevalence - [38, 40) wks, [2500, 3000) g	Mortality	Females		1.277 (1 to 1.65)	2.377 (2.015 to 2.745)
Lower respiratory infections	Birth prevalence - [38, 40) wks, [3000, 3500) g	Mortality	Males		1 (1.0 - 1.0)	1.326 (1.12 to 1.543)
Lower respiratory infections	Birth prevalence - [38, 40) wks, [3000, 3500) g	Mortality	Females		1 (1.0 - 1.0)	1.265 (1.068 to 1.45)
Lower respiratory infections	Birth prevalence - [38, 40) wks, [3500, 4000) g	Mortality	Males		1 (1.0 - 1.0)	1 (1.0 - 1.0)
Lower respiratory infections	Birth prevalence - [38, 40) wks, [3500, 4000) g	Mortality	Females		1 (1.0 - 1.0)	1 (1.0 - 1.0)
Lower respiratory infections	Birth prevalence - [38, 40) wks, [4000, 4500) g	Mortality	Males		1 (1.0 - 1.0)	1 (1.0 - 1.0)
Lower respiratory infections	Birth prevalence - [38, 40) wks, [4000, 4500) g	Mortality	Females		1 (1.0 - 1.0)	1 (1.0 - 1.0)
Lower respiratory infections	Birth prevalence - [40, 42) wks, [1500, 2000) g	Mortality	Males		43.194 (29.244 to 62.382)	21.971 (16.41 to 28.396)
Lower respiratory infections	Birth prevalence - [40, 42) wks, [1500, 2000) g	Mortality	Females		37.949 (25.539 to 53.532)	23.702 (17.857 to 30.634)
Lower respiratory infections	Birth prevalence - [40, 42) wks, [2000, 2500) g	Mortality	Males		10.162 (6.961 to 14.235)	7.875 (6.341 to 9.719)
Lower respiratory infections	Birth prevalence - [40, 42) wks, [2000, 2500) g	Mortality	Females		8.332 (5.994 to 11.407)	8.067 (6.467 to 10.043)
Lower respiratory infections	Birth prevalence - [40, 42) wks, [2500, 3000) g	Mortality	Males		2.118 (1.53 to 2.77)	2.692 (2.23 to 3.273)
Lower respiratory infections	Birth prevalence - [40, 42) wks, [2500, 3000) g	Mortality	Females		1.745 (1.289 to 2.316)	2.603 (2.134 to 3.106)
Lower respiratory infections	Birth prevalence - [40, 42) wks, [3000, 3500) g	Mortality	Males		1.002 (1 to 1.013)	1.249 (1.041 to 1.491)
Lower respiratory infections	Birth prevalence - [40, 42) wks, [3000, 3500) g	Mortality	Females		1 (1.0 - 1.0)	1.188 (1 to 1.411)
Lower respiratory infections	Birth prevalence - [40, 42) wks, [3500, 4000) g	Mortality	Males		1 (1.0 - 1.0)	1 (1.0 - 1.0)
Lower respiratory infections	Birth prevalence - [40, 42) wks, [3500, 4000) g	Mortality	Females		1 (1.0 - 1.0)	1 (1.0 - 1.0)
Lower respiratory infections	Birth prevalence - [40, 42) wks, [4000, 4500) g	Mortality	Males		1 (1.0 - 1.0)	1 (1.0 - 1.0)
Lower respiratory infections	Birth prevalence - [40, 42) wks, [4000, 4500) g	Mortality	Females		1 (1.0 - 1.0)	1 (1.0 - 1.0)
Meningitis	Birth prevalence - [0, 24) wks, [0, 500) g	Mortality	Males		877.775 (543.26 to 1246.645)	526.788 (392.055 to 676.923)
Meningitis	Birth prevalence - [0, 24) wks, [0, 500) g	Mortality	Females		857.044 (519.185 to 1227.708)	575.632 (434.418 to 737.293)
Meningitis	Birth prevalence - [0, 24) wks, [500, 1000) g	Mortality	Males		647.899 (443.535 to 881.449)	392.723 (315.689 to 486.607)
Meningitis	Birth prevalence - [0, 24) wks, [500, 1000) g	Mortality	Females		628.647 (437.861 to 854.598)	416.95 (329.573 to 516.699)
Meningitis	Birth prevalence - [24, 26) wks, [500, 1000) g	Mortality	Males		534.904 (378.653 to 707.51)	375.56 (308.336 to 447.076)
Meningitis	Birth prevalence - [24, 26) wks, [500, 1000) g	Mortality	Females		509.388 (370.575 to 690.649)	394.982 (324.03 to 477.74)
Meningitis	Birth prevalence - [26, 28) wks, [1000, 1500) g	Mortality	Males		150.649 (107.741 to 198.278)	140.284 (113.557 to 167.957)
Meningitis	Birth prevalence - [26, 28) wks, [1000, 1500) g	Mortality	Females		142.251 (102.895 to 191.587)	140.771 (114.514 to 169.631)
Meningitis	Birth prevalence - [26, 28) wks, [500, 1000) g	Mortality	Males		281.284 (198.703 to 391.898)	280.84 (226.174 to 338.336)
Meningitis	Birth prevalence - [26, 28) wks, [500, 1000) g	Mortality	Females		257.799 (185.994 to 349.394)	280.84 (225.68 to 350.2)
Meningitis	Birth prevalence - [28, 30) wks, [1000, 1500) g	Mortality	Males		88.889 (62.539 to 119.303)	87.596 (72.219 to 105.445)
Meningitis	Birth prevalence - [28, 30) wks, [1000, 1500) g	Mortality	Females		82.25 (59.418 to 109.806)	86.675 (69.965 to 104.367)
Meningitis	Birth prevalence - [28, 30) wks, [1500, 2000) g	Mortality	Males		71.963 (51.376 to 98.371)	42.294 (34.585 to 51.215)
Meningitis	Birth prevalence - [28, 30) wks, [1500, 2000) g	Mortality	Females		70.154 (48.562 to 96.294)	46.161 (38.268 to 54.906)
Meningitis	Birth prevalence - [28, 30) wks, [2000, 2500) g	Mortality	Males		65.936 (44.909 to 93.551)	23.785 (18.748 to 29.385)
Meningitis	Birth prevalence - [28, 30) wks, [2000, 2500) g	Mortality	Females		65.44 (43.751 to 96.137)	27.437 (21.369 to 33.874)
Meningitis	Birth prevalence - [28, 30) wks, [2500, 3000) g	Mortality	Males		43.647 (29.61 to 62.044)	14.185 (10.817 to 18.495)
Meningitis	Birth prevalence - [28, 30) wks, [2500, 3000) g	Mortality	Females		42.667 (28.231 to 61.897)	16.517 (12.636 to 21.355)
Meningitis	Birth prevalence - [28, 30) wks, [3000, 3500) g	Mortality	Males		23.727 (15.859 to 35.246)	8.651 (6.873 to 11.016)
Meningitis	Birth prevalence - [28, 30) wks, [3000, 3500) g	Mortality	Females		23.045 (14.473 to 35.151)	9.735 (7.511 to 12.367)
Meningitis	Birth prevalence - [28, 30) wks, [500, 1000) g	Mortality	Males		164.681 (114.331 to 232.909)	184.988 (146.715 to 225.778)
Meningitis	Birth prevalence - [28, 30) wks, [500, 1000) g	Mortality	Females		149.827 (102.011 to 207.65)	178.51 (144.736 to 220.088)
Meningitis	Birth prevalence - [30, 32) wks, [1000, 1500) g	Mortality	Males		66.567 (45.227 to 91.424)	57.036 (47.559 to 67.673)
Meningitis	Birth prevalence - [30, 32) wks, [1000, 1500) g	Mortality	Females		61.652 (42.69 to 86.655)	55.992 (46.425 to 67.165)
Meningitis	Birth prevalence - [30, 32) wks, [1500, 2000) g	Mortality	Males		43.288 (30.684 to 57.762)	26.533 (22.334 to 31.213)
Meningitis	Birth prevalence - [30, 32) wks, [1500, 2000) g	Mortality	Females		41.127 (28.837 to 54.767)	28.256 (24.051 to 32.792)
Meningitis	Birth prevalence - [30, 32) wks, [2000, 2500) g	Mortality	Males		38.672 (27.558 to 53.438)	15.362 (12.641 to 18.516)
Meningitis	Birth prevalence - [30, 32) wks, [2000, 2500) g	Mortality	Females		37.459 (26.086 to 52.166)	17.996 (14.845 to 21.529)
Meningitis	Birth prevalence - [30, 32) wks, [2500, 3000) g	Mortality	Males		33.218 (22.641 to 46.944)	10.389 (8.195 to 12.863)
Meningitis	Birth prevalence - [30, 32) wks, [2500, 3000) g	Mortality	Females		31.439 (21.157 to 45.621)	12.473 (9.64 to 15.784)
Meningitis	Birth prevalence - [30, 32) wks, [3000, 3500) g	Mortality	Males		25.451 (16.854 to 36.763)	7.153 (5.665 to 9.003)
Meningitis	Birth prevalence - [30, 32) wks, [3000, 3500) g	Mortality	Females		24.262 (15.651 to 36.395)	8.486 (6.482 to 10.824)
Meningitis	Birth prevalence - [30, 32) wks, [3500, 4000) g	Mortality	Males		20.334 (11.916 to 31.586)	4.795 (3.705 to 6.039)
Meningitis	Birth prevalence - [30, 32) wks, [3500, 4000) g	Mortality	Females		20.347 (11.635 to 32.681)	5.599 (4.199 to 7.275)
Meningitis	Birth prevalence - [30, 32) wks, [500, 1000) g	Mortality	Males		132.309 (85.99 to 193.341)	127.982 (101.66 to 159.489)
Meningitis	Birth prevalence - [30, 32) wks, [500, 1000) g	Mortality	Females		120.829 (82.341 to 172.828)	123.186 (98.696 to 152.125)
Meningitis	Birth prevalence - [32, 34) wks, [1000, 1500) g	Mortality	Males		65.39 (43.593 to 95.583)	45.581 (37.56 to 54.417)
Meningitis	Birth prevalence - [32, 34) wks, [1000, 1500) g	Mortality	Females		61.229 (40.8 to 88.303)	45.201 (37.492 to 54.267)
Meningitis	Birth prevalence - [32, 34) wks, [1500, 2000) g	Mortality	Males		32.197 (22.524 to 44.858)	19.612 (16.465 to 23.296)
Meningitis	Birth prevalence - [32, 34) wks, [1500, 2000) g	Mortality	Females		29.777 (21.026 to 40.255)	20.358 (17.072 to 23.768)
Meningitis	Birth prevalence - [32, 34) wks, [2000, 2500) g	Mortality	Males		21.018 (15.141 to 28.622)	10.433 (8.784 to 12.225)
Meningitis	Birth prevalence - [32, 34) wks, [2000, 2500) g	Mortality	Females		19.546 (14.316 to 26.034)	11.648 (9.974 to 13.507)
Meningitis	Birth prevalence - [32, 34) wks, [2500, 3000) g	Mortality	Males		18.569 (13.127 to 25.826)	7.182 (5.968 to 8.63)
Meningitis	Birth prevalence - [32, 34) wks, [2500, 3000) g	Mortality	Females		17.497 (11.66 to 24.635)	8.443 (6.726 to 10.284)
Meningitis	Birth prevalence - [32, 34) wks, [3000, 3500) g	Mortality	Males		19.012 (12.18 to 28.02)	5.618 (4.325 to 7.114)
Meningitis	Birth prevalence - [32, 34) wks, [3000, 3500) g	Mortality	Females		18.667 (12.24 to 27.524)	6.722 (5.103 to 8.697)
Meningitis	Birth prevalence - [32, 34) wks, [3500, 4000) g	Mortality	Males		20.214 (11.906 to 31.929)	4.31 (3.186 to 5.633)
Meningitis	Birth prevalence - [32, 34) wks, [3500, 4000) g	Mortality	Females		20.333 (12.064 to 33.21)	5.253 (3.959 to 6.913)
Meningitis	Birth prevalence - [34, 36) wks, [1000, 1500) g	Mortality	Males		78.66 (52.169 to 113.08)	45.338 (36.27 to 55.556)
Meningitis	Birth prevalence - [34, 36) wks, [1000, 1500) g	Mortality	Females		75.687 (49.863 to 110.089)	46.4 (38.048 to 55.902)
Meningitis	Birth prevalence - [34, 36) wks, [1500, 2000) g	Mortality	Males		31.154 (20.939 to 45.031)	18.246 (15.083 to 21.906)
Meningitis	Birth prevalence - [34, 36) wks, [1500, 2000) g	Mortality	Females		29.072 (19.6 to 42.648)	18.661 (15.638 to 22.175)
Meningitis	Birth prevalence - [34, 36) wks, [2000, 2500) g	Mortality	Males		12.404 (8.692 to 16.95)	8.006 (6.775 to 9.31)
Meningitis	Birth prevalence - [34, 36) wks, [2000, 2500) g	Mortality	Females		11.367 (7.807 to 15.547)	8.373 (7.066 to 9.703)
Meningitis	Birth prevalence - [34, 36) wks, [2500, 3000) g	Mortality	Males		7.509 (5.429 to 10.255)	4.725 (3.991 to 5.56)
Meningitis	Birth prevalence - [34, 36) wks, [2500, 3000) g	Mortality	Females		7.139 (5.091 to 9.759)	5.194 (4.342 to 6.087)
Meningitis	Birth prevalence - [34, 36) wks, [3000, 3500) g	Mortality	Males		7.828 (5.467 to 11.237)	3.717 (2.986 to 4.568)
Meningitis	Birth prevalence - [34, 36) wks, [3000, 3500) g	Mortality	Females		7.622 (5.235 to 10.584)	4.279 (3.416 to 5.301)
Meningitis	Birth prevalence - [34, 36) wks, [3500, 4000) g	Mortality	Males		10.257 (6.692 to 15.124)	3.13 (2.474 to 3.957)
Meningitis	Birth prevalence - [34, 36) wks, [3500, 4000) g	Mortality	Females		10.325 (6.495 to 15.537)	3.756 (2.877 to 4.818)
Meningitis	Birth prevalence - [34, 36) wks, [4000, 4500) g	Mortality	Males		12.928 (7.825 to 20.726)	2.467 (1.926 to 3.084)
Meningitis	Birth prevalence - [34, 36) wks, [4000, 4500) g	Mortality	Females		13.456 (8.201 to 20.893)	3.061 (2.357 to 3.889)
Meningitis	Birth prevalence - [36, 37) wks, [1000, 1500) g	Mortality	Males		94.103 (62.401 to 131.437)	48.734 (39.385 to 60.014)
Meningitis	Birth prevalence - [36, 37) wks, [1000, 1500) g	Mortality	Females		89.769 (59.869 to 126.674)	51.68 (41.336 to 63.456)
Meningitis	Birth prevalence - [36, 37) wks, [1500, 2000) g	Mortality	Males		33.586 (23.075 to 48.77)	19.52 (16.283 to 23.116)
Meningitis	Birth prevalence - [36, 37) wks, [1500, 2000) g	Mortality	Females		30.953 (20.951 to 43.853)	20.509 (17.15 to 24.286)
Meningitis	Birth prevalence - [36, 37) wks, [2000, 2500) g	Mortality	Males		8.134 (5.781 to 10.938)	7.015 (5.903 to 8.241)
Meningitis	Birth prevalence - [36, 37) wks, [2000, 2500) g	Mortality	Females		7.208 (5.183 to 9.787)	7.031 (5.911 to 8.265)
Meningitis	Birth prevalence - [36, 37) wks, [2500, 3000) g	Mortality	Males		2.741 (2.114 to 3.535)	3.149 (2.686 to 3.686)
Meningitis	Birth prevalence - [36, 37) wks, [2500, 3000) g	Mortality	Females		2.475 (1.905 to 3.109)	3.172 (2.71 to 3.666)
Meningitis	Birth prevalence - [36, 37) wks, [3000, 3500) g	Mortality	Males		2.1 (1.602 to 2.663)	2.099 (1.805 to 2.445)
Meningitis	Birth prevalence - [36, 37) wks, [3000, 3500) g	Mortality	Females		1.996 (1.532 to 2.6)	2.199 (1.895 to 2.534)
Meningitis	Birth prevalence - [36, 37) wks, [3500, 4000) g	Mortality	Males		2.563 (1.906 to 3.333)	1.751 (1.487 to 2.008)
Meningitis	Birth prevalence - [36, 37) wks, [3500, 4000) g	Mortality	Females		2.501 (1.793 to 3.34)	1.944 (1.649 to 2.275)
Meningitis	Birth prevalence - [36, 37) wks, [4000, 4500) g	Mortality	Males		3.824 (2.607 to 5.361)	1.506 (1.283 to 1.74)
Meningitis	Birth prevalence - [36, 37) wks, [4000, 4500) g	Mortality	Females		3.908 (2.626 to 5.462)	1.777 (1.481 to 2.118)
Meningitis	Birth prevalence - [37, 38) wks, [1000, 1500) g	Mortality	Males		98.111 (66.503 to 135.289)	49.903 (39.62 to 62.232)
Meningitis	Birth prevalence - [37, 38) wks, [1000, 1500) g	Mortality	Females		92.728 (63.975 to 128.565)	53.151 (42.705 to 66.273)
Meningitis	Birth prevalence - [37, 38) wks, [1500, 2000) g	Mortality	Males		35.71 (24.585 to 49.648)	20.617 (16.897 to 24.816)
Meningitis	Birth prevalence - [37, 38) wks, [1500, 2000) g	Mortality	Females		32.101 (22.457 to 43.887)	21.726 (17.833 to 25.871)
Meningitis	Birth prevalence - [37, 38) wks, [2000, 2500) g	Mortality	Males		7.301 (5.37 to 9.805)	6.923 (5.831 to 8.219)
Meningitis	Birth prevalence - [37, 38) wks, [2000, 2500) g	Mortality	Females		6.218 (4.554 to 8.169)	6.85 (5.796 to 8.005)
Meningitis	Birth prevalence - [37, 38) wks, [2500, 3000) g	Mortality	Males		1.861 (1.414 to 2.384)	2.721 (2.329 to 3.158)
Meningitis	Birth prevalence - [37, 38) wks, [2500, 3000) g	Mortality	Females		1.596 (1.24 to 1.998)	2.633 (2.261 to 3.031)
Meningitis	Birth prevalence - [37, 38) wks, [3000, 3500) g	Mortality	Males		1.139 (1 to 1.409)	1.612 (1.387 to 1.85)
Meningitis	Birth prevalence - [37, 38) wks, [3000, 3500) g	Mortality	Females		1.065 (1 to 1.288)	1.598 (1.392 to 1.819)
Meningitis	Birth prevalence - [37, 38) wks, [3500, 4000) g	Mortality	Males		1.201 (1 to 1.493)	1.282 (1.113 to 1.466)
Meningitis	Birth prevalence - [37, 38) wks, [3500, 4000) g	Mortality	Females		1.157 (1 to 1.442)	1.343 (1.153 to 1.542)
Meningitis	Birth prevalence - [37, 38) wks, [4000, 4500) g	Mortality	Males		1.825 (1.341 to 2.407)	1.136 (1.01 to 1.272)
Meningitis	Birth prevalence - [37, 38) wks, [4000, 4500) g	Mortality	Females		1.86 (1.36 to 2.46)	1.269 (1.115 to 1.42)
Meningitis	Birth prevalence - [38, 40) wks, [1000, 1500) g	Mortality	Males		98.254 (67.959 to 134.77)	49.084 (38.794 to 61.948)
Meningitis	Birth prevalence - [38, 40) wks, [1000, 1500) g	Mortality	Females		91.539 (62.769 to 127.606)	53.036 (41.348 to 65.237)
Meningitis	Birth prevalence - [38, 40) wks, [1500, 2000) g	Mortality	Males		37.449 (25.688 to 51.467)	21.477 (17.208 to 25.646)
Meningitis	Birth prevalence - [38, 40) wks, [1500, 2000) g	Mortality	Females		33.261 (23.303 to 45.061)	22.821 (18.339 to 27.874)
Meningitis	Birth prevalence - [38, 40) wks, [2000, 2500) g	Mortality	Males		7.351 (5.131 to 9.967)	7.04 (5.807 to 8.395)

Meningitis	Birth prevalence - [38, 40) wks, [2000, 2500) g	Mortality	Females		5.985 (4.383 to 7.995)	6.959 (5.782 to 8.29)
Meningitis	Birth prevalence - [38, 40) wks, [2500, 3000) g	Mortality	Males		1.549 (1.185 to 1.98)	2.518 (2.115 to 2.971)
Meningitis	Birth prevalence - [38, 40) wks, [2500, 3000) g	Mortality	Females		1.277 (1 to 1.65)	2.377 (2.015 to 2.745)
Meningitis	Birth prevalence - [38, 40) wks, [3000, 3500) g	Mortality	Males		1 (1.0 - 1.0)	1.326 (1.12 to 1.543)
Meningitis	Birth prevalence - [38, 40) wks, [3000, 3500) g	Mortality	Females		1 (1.0 - 1.0)	1.265 (1.068 to 1.45)
Meningitis	Birth prevalence - [38, 40) wks, [3500, 4000) g	Mortality	Males		1 (1.0 - 1.0)	1 (1.0 - 1.0)
Meningitis	Birth prevalence - [38, 40) wks, [3500, 4000) g	Mortality	Females		1 (1.0 - 1.0)	1 (1.0 - 1.0)
Meningitis	Birth prevalence - [38, 40) wks, [4000, 4500) g	Mortality	Males		1 (1.0 - 1.0)	1 (1.0 - 1.0)
Meningitis	Birth prevalence - [38, 40) wks, [4000, 4500) g	Mortality	Females		1 (1.0 - 1.0)	1 (1.0 - 1.0)
Meningitis	Birth prevalence - [40, 42) wks, [1500, 2000) g	Mortality	Males		43.194 (29.244 to 62.382)	21.971 (16.41 to 28.396)
Meningitis	Birth prevalence - [40, 42) wks, [1500, 2000) g	Mortality	Females		37.949 (25.539 to 53.532)	23.702 (17.857 to 30.634)
Meningitis	Birth prevalence - [40, 42) wks, [2000, 2500) g	Mortality	Males		10.162 (6.961 to 14.235)	7.875 (6.341 to 9.719)
Meningitis	Birth prevalence - [40, 42) wks, [2000, 2500) g	Mortality	Females		8.332 (5.994 to 11.407)	8.067 (6.467 to 10.043)
Meningitis	Birth prevalence - [40, 42) wks, [2500, 3000) g	Mortality	Males		2.118 (1.53 to 2.77)	2.692 (2.23 to 3.273)
Meningitis	Birth prevalence - [40, 42) wks, [2500, 3000) g	Mortality	Females		1.745 (1.289 to 2.316)	2.603 (2.134 to 3.106)
Meningitis	Birth prevalence - [40, 42) wks, [3000, 3500) g	Mortality	Males		1.002 (1 to 1.013)	1.249 (1.041 to 1.491)
Meningitis	Birth prevalence - [40, 42) wks, [3000, 3500) g	Mortality	Females		1 (1.0 - 1.0)	1.188 (1 to 1.411)
Meningitis	Birth prevalence - [40, 42) wks, [3500, 4000) g	Mortality	Males		1 (1.0 - 1.0)	1 (1.0 - 1.0)
Meningitis	Birth prevalence - [40, 42) wks, [3500, 4000) g	Mortality	Females		1 (1.0 - 1.0)	1 (1.0 - 1.0)
Meningitis	Birth prevalence - [40, 42) wks, [4000, 4500) g	Mortality	Males		1 (1.0 - 1.0)	1 (1.0 - 1.0)
Meningitis	Birth prevalence - [40, 42) wks, [4000, 4500) g	Mortality	Females		1 (1.0 - 1.0)	1 (1.0 - 1.0)
Neonatal encephalopathy due to birth asphyxia and trauma	Birth prevalence - [0, 24) wks, [0, 500) g	Mortality	Males		877.775 (543.26 to 1246.645)	526.788 (392.055 to 676.923)
Neonatal encephalopathy due to birth asphyxia and trauma	Birth prevalence - [0, 24) wks, [0, 500) g	Mortality	Females		857.044 (519.185 to 1227.708)	575.632 (434.418 to 737.293)
Neonatal encephalopathy due to birth asphyxia and trauma	Birth prevalence - [0, 24) wks, [500, 1000) g	Mortality	Males		647.899 (443.535 to 881.449)	392.723 (315.689 to 486.607)
Neonatal encephalopathy due to birth asphyxia and trauma	Birth prevalence - [0, 24) wks, [500, 1000) g	Mortality	Females		628.647 (437.861 to 854.598)	416.95 (329.573 to 516.699)
Neonatal encephalopathy due to birth asphyxia and trauma	Birth prevalence - [24, 26) wks, [500, 1000) g	Mortality	Males		534.904 (378.653 to 707.51)	375.56 (308.336 to 447.076)
Neonatal encephalopathy due to birth asphyxia and trauma	Birth prevalence - [24, 26) wks, [500, 1000) g	Mortality	Females		509.388 (370.575 to 690.649)	394.982 (324.03 to 477.74)
Neonatal encephalopathy due to birth asphyxia and trauma	Birth prevalence - [26, 28) wks, [1000, 1500) g	Mortality	Males		150.649 (107.741 to 198.278)	140.284 (113.557 to 167.957)
Neonatal encephalopathy due to birth asphyxia and trauma	Birth prevalence - [26, 28) wks, [1000, 1500) g	Mortality	Females		142.251 (102.895 to 191.587)	140.771 (114.514 to 169.631)
Neonatal encephalopathy due to birth asphyxia and trauma	Birth prevalence - [26, 28) wks, [500, 1000) g	Mortality	Males		281.284 (198.703 to 391.898)	280.84 (226.174 to 338.336)
Neonatal encephalopathy due to birth asphyxia and trauma	Birth prevalence - [26, 28) wks, [500, 1000) g	Mortality	Females		257.799 (185.994 to 349.394)	280.84 (225.68 to 350.2)
Neonatal encephalopathy due to birth asphyxia and trauma	Birth prevalence - [28, 30) wks, [1000, 1500) g	Mortality	Males		88.889 (62.539 to 119.303)	87.596 (72.219 to 105.445)
Neonatal encephalopathy due to birth asphyxia and trauma	Birth prevalence - [28, 30) wks, [1000, 1500) g	Mortality	Females		82.25 (59.418 to 109.806)	86.675 (69.965 to 104.367)
Neonatal encephalopathy due to birth asphyxia and trauma	Birth prevalence - [28, 30) wks, [1500, 2000) g	Mortality	Males		71.963 (51.376 to 98.371)	42.294 (34.585 to 51.215)
Neonatal encephalopathy due to birth asphyxia and trauma	Birth prevalence - [28, 30) wks, [1500, 2000) g	Mortality	Females		70.154 (48.562 to 96.294)	46.161 (38.268 to 54.906)
Neonatal encephalopathy due to birth asphyxia and trauma	Birth prevalence - [28, 30) wks, [2000, 2500) g	Mortality	Males		65.936 (44.909 to 93.551)	23.785 (18.748 to 29.385)
Neonatal encephalopathy due to birth asphyxia and trauma	Birth prevalence - [28, 30) wks, [2000, 2500) g	Mortality	Females		65.44 (43.751 to 96.137)	27.437 (21.369 to 33.874)
Neonatal encephalopathy due to birth asphyxia and trauma	Birth prevalence - [28, 30) wks, [2500, 3000) g	Mortality	Males		43.647 (29.61 to 62.044)	14.185 (10.817 to 18.495)
Neonatal encephalopathy due to birth asphyxia and trauma	Birth prevalence - [28, 30) wks, [2500, 3000) g	Mortality	Females		42.667 (28.231 to 61.897)	16.517 (12.636 to 21.355)
Neonatal encephalopathy due to birth asphyxia and trauma	Birth prevalence - [28, 30) wks, [3000, 3500) g	Mortality	Males		23.727 (15.859 to 35.246)	8.651 (6.873 to 11.016)
Neonatal encephalopathy due to birth asphyxia and trauma	Birth prevalence - [28, 30) wks, [3000, 3500) g	Mortality	Females		23.045 (14.473 to 35.151)	9.735 (7.511 to 12.367)
Neonatal encephalopathy due to birth asphyxia and trauma	Birth prevalence - [28, 30) wks, [500, 1000) g	Mortality	Males		164.681 (114.331 to 232.909)	184.988 (146.715 to 225.778)
Neonatal encephalopathy due to birth asphyxia and trauma	Birth prevalence - [28, 30) wks, [500, 1000) g	Mortality	Females		149.827 (102.011 to 207.65)	178.51 (144.736 to 220.088)
Neonatal encephalopathy due to birth asphyxia and trauma	Birth prevalence - [30, 32) wks, [1000, 1500) g	Mortality	Males		66.567 (45.227 to 91.424)	57.036 (47.559 to 67.673)
Neonatal encephalopathy due to birth asphyxia and trauma	Birth prevalence - [30, 32) wks, [1000, 1500) g	Mortality	Females		61.652 (42.69 to 86.655)	55.992 (46.425 to 67.165)
Neonatal encephalopathy due to birth asphyxia and trauma	Birth prevalence - [30, 32) wks, [1500, 2000) g	Mortality	Males		43.288 (30.684 to 57.762)	26.533 (22.334 to 31.213)
Neonatal encephalopathy due to birth asphyxia and trauma	Birth prevalence - [30, 32) wks, [1500, 2000) g	Mortality	Females		41.127 (28.837 to 54.767)	28.256 (24.051 to 32.792)
Neonatal encephalopathy due to birth asphyxia and trauma	Birth prevalence - [30, 32) wks, [2000, 2500) g	Mortality	Males		38.672 (27.558 to 53.438)	15.362 (12.641 to 18.516)
Neonatal encephalopathy due to birth asphyxia and trauma	Birth prevalence - [30, 32) wks, [2000, 2500) g	Mortality	Females		37.459 (26.086 to 52.166)	17.996 (14.845 to 21.529)
Neonatal encephalopathy due to birth asphyxia and trauma	Birth prevalence - [30, 32) wks, [2500, 3000) g	Mortality	Males		33.218 (22.641 to 46.944)	10.389 (8.195 to 12.863)
Neonatal encephalopathy due to birth asphyxia and trauma	Birth prevalence - [30, 32) wks, [2500, 3000) g	Mortality	Females		31.439 (21.157 to 45.621)	12.473 (9.64 to 15.784)
Neonatal encephalopathy due to birth asphyxia and trauma	Birth prevalence - [30, 32) wks, [3000, 3500) g	Mortality	Males		25.451 (16.854 to 36.763)	7.153 (5.665 to 9.003)
Neonatal encephalopathy due to birth asphyxia and trauma	Birth prevalence - [30, 32) wks, [3000, 3500) g	Mortality	Females		24.262 (15.651 to 36.395)	8.486 (6.482 to 10.824)
Neonatal encephalopathy due to birth asphyxia and trauma	Birth prevalence - [30, 32) wks, [3500, 4000) g	Mortality	Males		20.334 (11.916 to 31.586)	4.795 (3.705 to 6.039)
Neonatal encephalopathy due to birth asphyxia and trauma	Birth prevalence - [30, 32) wks, [3500, 4000) g	Mortality	Females		20.347 (11.635 to 32.681)	5.599 (4.199 to 7.275)
Neonatal encephalopathy due to birth asphyxia and trauma	Birth prevalence - [30, 32) wks, [500, 1000) g	Mortality	Males		132.309 (85.99 to 193.341)	127.982 (101.66 to 159.489)
Neonatal encephalopathy due to birth asphyxia and trauma	Birth prevalence - [30, 32) wks, [500, 1000) g	Mortality	Females		120.829 (82.341 to 172.828)	123.186 (98.696 to 152.125)
Neonatal encephalopathy due to birth asphyxia and trauma	Birth prevalence - [32, 34) wks, [1000, 1500) g	Mortality	Males		65.39 (43.593 to 95.583)	45.581 (37.56 to 54.417)
Neonatal encephalopathy due to birth asphyxia and trauma	Birth prevalence - [32, 34) wks, [1000, 1500) g	Mortality	Females		61.229 (40.8 to 88.303)	45.201 (37.492 to 54.267)
Neonatal encephalopathy due to birth asphyxia and trauma	Birth prevalence - [32, 34) wks, [1500, 2000) g	Mortality	Males		32.197 (22.524 to 44.858)	19.612 (16.465 to 23.296)
Neonatal encephalopathy due to birth asphyxia and trauma	Birth prevalence - [32, 34) wks, [1500, 2000) g	Mortality	Females		29.777 (21.026 to 40.255)	20.358 (17.072 to 23.768)
Neonatal encephalopathy due to birth asphyxia and trauma	Birth prevalence - [32, 34) wks, [2000, 2500) g	Mortality	Males		21.018 (15.141 to 28.622)	10.433 (8.784 to 12.225)
Neonatal encephalopathy due to birth asphyxia and trauma	Birth prevalence - [32, 34) wks, [2000, 2500) g	Mortality	Females		19.546 (14.316 to 26.034)	11.648 (9.974 to 13.507)
Neonatal encephalopathy due to birth asphyxia and trauma	Birth prevalence - [32, 34) wks, [2500, 3000) g	Mortality	Males		18.569 (13.127 to 25.826)	7.182 (5.968 to 8.63)
Neonatal encephalopathy due to birth asphyxia and trauma	Birth prevalence - [32, 34) wks, [2500, 3000) g	Mortality	Females		17.497 (11.66 to 24.635)	8.443 (6.726 to 10.284)
Neonatal encephalopathy due to birth asphyxia and trauma	Birth prevalence - [32, 34) wks, [3000, 3500) g	Mortality	Males		19.012 (12.18 to 28.02)	5.618 (4.325 to 7.114)
Neonatal encephalopathy due to birth asphyxia and trauma	Birth prevalence - [32, 34) wks, [3000, 3500) g	Mortality	Females		18.667 (12.24 to 27.524)	6.722 (5.103 to 8.697)
Neonatal encephalopathy due to birth asphyxia and trauma	Birth prevalence - [32, 34) wks, [3500, 4000) g	Mortality	Males		20.214 (11.906 to 31.929)	4.31 (3.186 to 5.633)
Neonatal encephalopathy due to birth asphyxia and trauma	Birth prevalence - [32, 34) wks, [3500, 4000) g	Mortality	Females		20.333 (12.064 to 33.21)	5.253 (3.959 to 6.913)
Neonatal encephalopathy due to birth asphyxia and trauma	Birth prevalence - [34, 36) wks, [1000, 1500) g	Mortality	Males		78.66 (52.169 to 113.08)	45.338 (36.27 to 55.556)
Neonatal encephalopathy due to birth asphyxia and trauma	Birth prevalence - [34, 36) wks, [1000, 1500) g	Mortality	Females		75.687 (49.863 to 110.089)	46.4 (38.048 to 55.902)
Neonatal encephalopathy due to birth asphyxia and trauma	Birth prevalence - [34, 36) wks, [1500, 2000) g	Mortality	Males		31.154 (20.939 to 45.031)	18.246 (15.083 to 21.906)
Neonatal encephalopathy due to birth asphyxia and trauma	Birth prevalence - [34, 36) wks, [1500, 2000) g	Mortality	Females		29.072 (19.6 to 42.648)	18.661 (15.638 to 22.175)
Neonatal encephalopathy due to birth asphyxia and trauma	Birth prevalence - [34, 36) wks, [2000, 2500) g	Mortality	Males		12.404 (8.692 to 16.95)	8.006 (6.775 to 9.31)
Neonatal encephalopathy due to birth asphyxia and trauma	Birth prevalence - [34, 36) wks, [2000, 2500) g	Mortality	Females		11.367 (7.807 to 15.547)	8.373 (7.066 to 9.703)
Neonatal encephalopathy due to birth asphyxia and trauma	Birth prevalence - [34, 36) wks, [2500, 3000) g	Mortality	Males		7.509 (5.429 to 10.255)	4.725 (3.991 to 5.56)
Neonatal encephalopathy due to birth asphyxia and trauma	Birth prevalence - [34, 36) wks, [2500, 3000) g	Mortality	Females		7.139 (5.091 to 9.759)	5.194 (4.342 to 6.087)
Neonatal encephalopathy due to birth asphyxia and trauma	Birth prevalence - [34, 36) wks, [3000, 3500) g	Mortality	Males		7.828 (5.467 to 11.237)	3.717 (2.986 to 4.568)
Neonatal encephalopathy due to birth asphyxia and trauma	Birth prevalence - [34, 36) wks, [3000, 3500) g	Mortality	Females		7.622 (5.235 to 10.584)	4.279 (3.416 to 5.301)
Neonatal encephalopathy due to birth asphyxia and trauma	Birth prevalence - [34, 36) wks, [3500, 4000) g	Mortality	Males		10.257 (6.692 to 15.124)	3.13 (2.474 to 3.957)
Neonatal encephalopathy due to birth asphyxia and trauma	Birth prevalence - [34, 36) wks, [3500, 4000) g	Mortality	Females		10.325 (6.495 to 15.537)	3.756 (2.877 to 4.818)
Neonatal encephalopathy due to birth asphyxia and trauma	Birth prevalence - [34, 36) wks, [4000, 4500) g	Mortality	Males		12.928 (7.825 to 20.726)	2.467 (1.926 to 3.084)
Neonatal encephalopathy due to birth asphyxia and trauma	Birth prevalence - [34, 36) wks, [4000, 4500) g	Mortality	Females		13.456 (8.201 to 20.893)	3.061 (2.357 to 3.889)
Neonatal encephalopathy due to birth asphyxia and trauma	Birth prevalence - [36, 37) wks, [1000, 1500) g	Mortality	Males		94.103 (62.401 to 131.437)	48.734 (39.385 to 60.014)
Neonatal encephalopathy due to birth asphyxia and trauma	Birth prevalence - [36, 37) wks, [1000, 1500) g	Mortality	Females		89.769 (59.869 to 126.674)	51.68 (41.336 to 63.456)
Neonatal encephalopathy due to birth asphyxia and trauma	Birth prevalence - [36, 37) wks, [1500, 2000) g	Mortality	Males		33.586 (23.075 to 48.77)	19.52 (16.283 to 23.116)
Neonatal encephalopathy due to birth asphyxia and trauma	Birth prevalence - [36, 37) wks, [1500, 2000) g	Mortality	Females		30.953 (20.951 to 43.853)	20.509 (17.15 to 24.286)
Neonatal encephalopathy due to birth asphyxia and trauma	Birth prevalence - [36, 37) wks, [2000, 2500) g	Mortality	Males		8.134 (5.781 to 10.938)	7.015 (5.903 to 8.241)
Neonatal encephalopathy due to birth asphyxia and trauma	Birth prevalence - [36, 37) wks, [2000, 2500) g	Mortality	Females		7.208 (5.183 to 9.787)	7.031 (5.911 to 8.265)
Neonatal encephalopathy due to birth asphyxia and trauma	Birth prevalence - [36, 37) wks, [2500, 3000) g	Mortality	Males		2.741 (2.114 to 3.535)	3.149 (2.686 to 3.686)
Neonatal encephalopathy due to birth asphyxia and trauma	Birth prevalence - [36, 37) wks, [2500, 3000) g	Mortality	Females		2.475 (1.905 to 3.109)	3.172 (2.71 to 3.666)
Neonatal encephalopathy due to birth asphyxia and trauma	Birth prevalence - [36, 37) wks, [3000, 3500) g	Mortality	Males		2.1 (1.602 to 2.663)	2.099 (1.805 to 2.445)
Neonatal encephalopathy due to birth asphyxia and trauma	Birth prevalence - [36, 37) wks, [3000, 3500) g	Mortality	Females		1.996 (1.532 to 2.6)	2.199 (1.895 to 2.534)
Neonatal encephalopathy due to birth asphyxia and trauma	Birth prevalence - [36, 37) wks, [3500, 4000) g	Mortality	Males		2.563 (1.906 to 3.333)	1.751 (1.487 to 2.008)
Neonatal encephalopathy due to birth asphyxia and trauma	Birth prevalence - [36, 37) wks, [3500, 4000) g	Mortality	Females		2.501 (1.793 to 3.34)	1.944 (1.649 to 2.275)
Neonatal encephalopathy due to birth asphyxia and trauma	Birth prevalence - [36, 37) wks, [4000, 4500) g	Mortality	Males		3.824 (2.607 to 5.361)	1.506 (1.283 to 1.74)
Neonatal encephalopathy due to birth asphyxia and trauma	Birth prevalence - [36, 37) wks, [4000, 4500) g	Mortality	Females		3.908 (2.626 to 5.462)	1.777 (1.481 to 2.118)
Neonatal encephalopathy due to birth asphyxia and trauma	Birth prevalence - [37, 38) wks, [1000, 1500) g	Mortality	Males		98.111 (66.503 to 135.289)	49.903 (39.62 to 62.232)
Neonatal encephalopathy due to birth asphyxia and trauma	Birth prevalence - [37, 38) wks, [1000, 1500) g	Mortality	Females		92.728 (63.975 to 128.565)	53.151 (42.705 to 66.273)
Neonatal encephalopathy due to birth asphyxia and trauma	Birth prevalence - [37, 38) wks, [1500, 2000) g	Mortality	Males		35.71 (24.585 to 49.648)	20.617 (16.897 to 24.816)
Neonatal encephalopathy due to birth asphyxia and trauma	Birth prevalence - [37, 38) wks, [1500, 2000) g	Mortality	Females		32.101 (22.457 to 43.887)	21.726 (17.833 to 25.871)
Neonatal encephalopathy due to birth asphyxia and trauma	Birth prevalence - [37, 38) wks, [2000, 2500) g	Mortality	Males		7.301 (5.37 to 9.805)	6.923 (5.831 to 8.219)
Neonatal encephalopathy due to birth asphyxia and trauma	Birth prevalence - [37, 38) wks, [2000, 2500) g	Mortality	Females		6.218 (4.554 to 8.169)	6.85 (5.796 to 8.005)
Neonatal encephalopathy due to birth asphyxia and trauma	Birth prevalence - [37, 38) wks, [2500, 3000) g	Mortality	Males		1.861 (1.414 to 2.384)	2.721 (2.329 to 3.158)
Neonatal encephalopathy due to birth asphyxia and trauma	Birth prevalence - [37, 38) wks, [2500, 3000) g	Mortality	Females		1.596 (1.24 to 1.998)	2.633 (2.261 to 3.031)
Neonatal encephalopathy due to birth asphyxia and trauma	Birth prevalence - [37, 38) wks, [3000, 3500) g	Mortality	Males		1.139 (1 to 1.409)	1.612 (1.387 to 1.85)
Neonatal encephalopathy due to birth asphyxia and trauma	Birth prevalence - [37, 38) wks, [3000, 3500) g	Mortality	Females		1.065 (1 to 1.288)	1.598 (1.392 to 1.819)
Neonatal encephalopathy due to birth asphyxia and trauma	Birth prevalence - [37, 38) wks, [3500, 4000) g	Mortality	Males		1.201 (1 to 1.493)	1.282 (1.113 to 1.466)
Neonatal encephalopathy due to birth asphyxia and trauma	Birth prevalence - [37, 38) wks, [3500, 4000) g	Mortality	Females		1.157 (1 to 1.442)	1.343 (1.153 to 1.542)
Neonatal encephalopathy due to birth asphyxia and trauma	Birth prevalence - [37, 38) wks, [4000, 4500) g	Mortality	Males		1.825 (1.341 to 2.407)	1.136 (1.01 to 1.272)
Neonatal encephalopathy due to birth asphyxia and trauma	Birth prevalence - [37, 38) wks, [4000, 4500) g	Mortality	Females		1.86 (1.36 to 2.46)	1.269 (1.115 to 1.42)
Neonatal encephalopathy due to birth asphyxia and trauma	Birth prevalence - [38, 40) wks, [1000, 1500) g	Mortality	Males		98.254 (67.959 to 134.77)	49.084 (38.794 to 61.948)
Neonatal encephalopathy due to birth asphyxia and trauma	Birth prevalence - [38, 40) wks, [1000, 1500) g	Mortality	Females		91.539 (62.769 to 127.606)	53.036 (41.348 to 65.237)
Neonatal encephalopathy due to birth asphyxia and trauma	Birth prevalence - [38, 40) wks, [1500, 2000) g	Mortality	Males		37.449 (25.688 to 51.467)	21.477 (17.208 to 25.646)
Neonatal encephalopathy due to birth asphyxia and trauma	Birth prevalence - [38, 40) wks, [1500, 2000) g	Mortality	Females		33.261 (23.303 to 45.061)	22.821 (18.339 to 27.874)
Neonatal encephalopathy due to birth asphyxia and trauma	Birth prevalence - [38, 40) wks, [2000, 2500) g	Mortality	Males		7.351 (5.131 to 9.967)	7.04 (5.807 to 8.395)
Neonatal encephalopathy due to birth asphyxia and trauma	Birth prevalence - [38, 40) wks, [2000, 2500) g	Mortality	Females		5.985 (4.383 to 7.995)	6.959 (5.782 to 8.29)
Neonatal encephalopathy due to birth asphyxia and trauma	Birth prevalence - [38, 40) wks, [2500, 3000) g	Mortality	Males		1.549 (1.185 to 1.98)	2.518 (2.115 to 2.971)
Neonatal encephalopathy due to birth asphyxia and trauma	Birth prevalence - [38, 40) wks, [2500, 3000) g	Mortality	Females		1.277 (1 to 1.65)	2.377 (2.015 to 2.745)
Neonatal encephalopathy due to birth asphyxia and trauma	Birth prevalence - [38, 40) wks, [3000, 3500) g	Mortality	Males		1 (1.0 - 1.0)	1.326 (1.12 to 1.543)
Neonatal encephalopathy due to birth asphyxia and trauma	Birth prevalence - [38, 40) wks, [3000, 3500) g	Mortality	Females		1 (1.0 - 1.0)	1.265 (1.068 to 1.45)
Neonatal encephalopathy due to birth asphyxia and trauma	Birth prevalence - [38, 40) wks, [3500, 4000) g	Mortality	Males		1 (1.0 - 1.0)	1 (1.0 - 1.0)
Neonatal encephalopathy due to birth asphyxia and trauma	Birth prevalence - [38, 40) wks, [3500, 4000) g	Mortality	Females		1 (1.0 - 1.0)	1 (1.0 - 1.0)

Neonatal encephalopathy due to birth asphyxia and trauma	Birth prevalence - [38, 40) wks, [4000, 4500) g	Mortality	Males		1<br>(1.0 - 1.0)	1<br>(1.0 - 1.0)
Neonatal encephalopathy due to birth asphyxia and trauma	Birth prevalence - [38, 40) wks, [4000, 4500) g	Mortality	Females		1<br>(1.0 - 1.0)	1<br>(1.0 - 1.0)
Neonatal encephalopathy due to birth asphyxia and trauma	Birth prevalence - [40, 42) wks, [1500, 2000) g	Mortality	Males		43.194<br>(29.244 to 62.382)	21.971<br>(16.41 to 28.396)
Neonatal encephalopathy due to birth asphyxia and trauma	Birth prevalence - [40, 42) wks, [1500, 2000) g	Mortality	Females		37.949<br>(25.539 to 53.532)	23.702<br>(17.857 to 30.634)
Neonatal encephalopathy due to birth asphyxia and trauma	Birth prevalence - [40, 42) wks, [2000, 2500) g	Mortality	Males		10.162<br>(6.961 to 14.235)	7.875<br>(6.341 to 9.719)
Neonatal encephalopathy due to birth asphyxia and trauma	Birth prevalence - [40, 42) wks, [2000, 2500) g	Mortality	Females		8.332<br>(5.994 to 11.407)	8.067<br>(6.467 to 10.043)
Neonatal encephalopathy due to birth asphyxia and trauma	Birth prevalence - [40, 42) wks, [2500, 3000) g	Mortality	Males		2.118<br>(1.53 to 2.77)	2.692<br>(2.23 to 3.273)
Neonatal encephalopathy due to birth asphyxia and trauma	Birth prevalence - [40, 42) wks, [2500, 3000) g	Mortality	Females		1.745<br>(1.289 to 2.316)	2.603<br>(2.134 to 3.106)
Neonatal encephalopathy due to birth asphyxia and trauma	Birth prevalence - [40, 42) wks, [3000, 3500) g	Mortality	Males		1.002<br>(1 to 1.013)	1.249<br>(1.041 to 1.491)
Neonatal encephalopathy due to birth asphyxia and trauma	Birth prevalence - [40, 42) wks, [3000, 3500) g	Mortality	Females		1<br>(1.0 - 1.0)	1.188<br>(1 to 1.411)
Neonatal encephalopathy due to birth asphyxia and trauma	Birth prevalence - [40, 42) wks, [3500, 4000) g	Mortality	Males		1<br>(1.0 - 1.0)	1<br>(1.0 - 1.0)
Neonatal encephalopathy due to birth asphyxia and trauma	Birth prevalence - [40, 42) wks, [3500, 4000) g	Mortality	Females		1<br>(1.0 - 1.0)	1<br>(1.0 - 1.0)
Neonatal encephalopathy due to birth asphyxia and trauma	Birth prevalence - [40, 42) wks, [4000, 4500) g	Mortality	Males		1<br>(1.0 - 1.0)	1<br>(1.0 - 1.0)
Neonatal encephalopathy due to birth asphyxia and trauma	Birth prevalence - [40, 42) wks, [4000, 4500) g	Mortality	Females		1<br>(1.0 - 1.0)	1<br>(1.0 - 1.0)
Neonatal preterm birth	Birth prevalence - [0, 24) wks, [0, 500) g	Mortality	Males		877.775<br>(543.26 to 1246.645)	526.788<br>(392.055 to 676.923)
Neonatal preterm birth	Birth prevalence - [0, 24) wks, [0, 500) g	Mortality	Females		857.044<br>(519.185 to 1227.708)	575.632<br>(434.418 to 737.293)
Neonatal preterm birth	Birth prevalence - [0, 24) wks, [500, 1000) g	Mortality	Males		647.899<br>(443.535 to 881.449)	392.723<br>(315.689 to 486.607)
Neonatal preterm birth	Birth prevalence - [0, 24) wks, [500, 1000) g	Mortality	Females		628.647<br>(437.861 to 854.598)	416.95<br>(329.573 to 516.699)
Neonatal preterm birth	Birth prevalence - [24, 26) wks, [500, 1000) g	Mortality	Males		534.904<br>(378.653 to 707.51)	375.56<br>(308.336 to 447.076)
Neonatal preterm birth	Birth prevalence - [24, 26) wks, [500, 1000) g	Mortality	Females		509.388<br>(370.575 to 690.649)	394.982<br>(324.03 to 477.74)
Neonatal preterm birth	Birth prevalence - [26, 28) wks, [1000, 1500) g	Mortality	Males		150.649<br>(107.741 to 198.278)	140.284<br>(113.557 to 167.957)
Neonatal preterm birth	Birth prevalence - [26, 28) wks, [1000, 1500) g	Mortality	Females		142.251<br>(102.895 to 191.587)	140.771<br>(114.514 to 169.631)
Neonatal preterm birth	Birth prevalence - [26, 28) wks, [500, 1000) g	Mortality	Males		281.284<br>(198.703 to 391.898)	280.84<br>(226.174 to 338.336)
Neonatal preterm birth	Birth prevalence - [26, 28) wks, [500, 1000) g	Mortality	Females		257.799<br>(185.994 to 349.394)	280.84<br>(225.68 to 350.2)
Neonatal preterm birth	Birth prevalence - [28, 30) wks, [1000, 1500) g	Mortality	Males		88.889<br>(62.539 to 119.303)	87.596<br>(72.219 to 105.445)
Neonatal preterm birth	Birth prevalence - [28, 30) wks, [1000, 1500) g	Mortality	Females		82.25<br>(59.418 to 109.806)	86.675<br>(69.965 to 104.367)
Neonatal preterm birth	Birth prevalence - [28, 30) wks, [1500, 2000) g	Mortality	Males		71.963<br>(51.376 to 98.371)	42.294<br>(34.585 to 51.215)
Neonatal preterm birth	Birth prevalence - [28, 30) wks, [1500, 2000) g	Mortality	Females		70.154<br>(48.562 to 96.294)	46.161<br>(38.268 to 54.906)
Neonatal preterm birth	Birth prevalence - [28, 30) wks, [2000, 2500) g	Mortality	Males		65.936<br>(44.909 to 93.551)	23.785<br>(18.748 to 29.385)
Neonatal preterm birth	Birth prevalence - [28, 30) wks, [2000, 2500) g	Mortality	Females		65.44<br>(43.751 to 96.137)	27.437<br>(21.369 to 33.874)
Neonatal preterm birth	Birth prevalence - [28, 30) wks, [2500, 3000) g	Mortality	Males		43.647<br>(29.61 to 62.044)	14.185<br>(10.817 to 18.495)
Neonatal preterm birth	Birth prevalence - [28, 30) wks, [2500, 3000) g	Mortality	Females		42.667<br>(28.231 to 61.897)	16.517<br>(12.636 to 21.355)
Neonatal preterm birth	Birth prevalence - [28, 30) wks, [3000, 3500) g	Mortality	Males		23.727<br>(15.859 to 35.246)	8.651<br>(6.873 to 11.016)
Neonatal preterm birth	Birth prevalence - [28, 30) wks, [3000, 3500) g	Mortality	Females		23.045<br>(14.473 to 35.151)	9.735<br>(7.511 to 12.367)
Neonatal preterm birth	Birth prevalence - [28, 30) wks, [500, 1000) g	Mortality	Males		164.681<br>(114.331 to 232.909)	184.988<br>(146.715 to 225.778)
Neonatal preterm birth	Birth prevalence - [28, 30) wks, [500, 1000) g	Mortality	Females		149.827<br>(102.011 to 207.65)	178.51<br>(144.736 to 220.088)
Neonatal preterm birth	Birth prevalence - [30, 32) wks, [1000, 1500) g	Mortality	Males		66.567<br>(45.227 to 91.424)	57.036<br>(47.559 to 67.673)
Neonatal preterm birth	Birth prevalence - [30, 32) wks, [1000, 1500) g	Mortality	Females		61.652<br>(42.69 to 86.655)	55.992<br>(46.425 to 67.165)
Neonatal preterm birth	Birth prevalence - [30, 32) wks, [1500, 2000) g	Mortality	Males		43.288<br>(30.684 to 57.762)	26.533<br>(22.334 to 31.213)
Neonatal preterm birth	Birth prevalence - [30, 32) wks, [1500, 2000) g	Mortality	Females		41.127<br>(28.837 to 54.767)	28.256<br>(24.051 to 32.792)
Neonatal preterm birth	Birth prevalence - [30, 32) wks, [2000, 2500) g	Mortality	Males		38.672<br>(27.558 to 53.438)	15.362<br>(12.641 to 18.516)
Neonatal preterm birth	Birth prevalence - [30, 32) wks, [2000, 2500) g	Mortality	Females		37.459<br>(26.086 to 52.166)	17.996<br>(14.845 to 21.529)
Neonatal preterm birth	Birth prevalence - [30, 32) wks, [2500, 3000) g	Mortality	Males		33.218<br>(22.641 to 46.944)	10.389<br>(8.195 to 12.863)
Neonatal preterm birth	Birth prevalence - [30, 32) wks, [2500, 3000) g	Mortality	Females		31.439<br>(21.157 to 45.621)	12.473<br>(9.64 to 15.784)
Neonatal preterm birth	Birth prevalence - [30, 32) wks, [3000, 3500) g	Mortality	Males		25.451<br>(16.854 to 36.763)	7.153<br>(5.665 to 9.003)
Neonatal preterm birth	Birth prevalence - [30, 32) wks, [3000, 3500) g	Mortality	Females		24.262<br>(15.651 to 36.395)	8.486<br>(6.482 to 10.824)
Neonatal preterm birth	Birth prevalence - [30, 32) wks, [3500, 4000) g	Mortality	Males		20.334<br>(11.916 to 31.586)	4.795<br>(3.705 to 6.039)
Neonatal preterm birth	Birth prevalence - [30, 32) wks, [3500, 4000) g	Mortality	Females		20.347<br>(11.635 to 32.681)	5.599<br>(4.199 to 7.275)
Neonatal preterm birth	Birth prevalence - [30, 32) wks, [500, 1000) g	Mortality	Males		132.309<br>(85.99 to 193.341)	127.982<br>(101.66 to 159.489)
Neonatal preterm birth	Birth prevalence - [30, 32) wks, [500, 1000) g	Mortality	Females		120.829<br>(82.341 to 172.828)	123.186<br>(98.696 to 152.125)
Neonatal preterm birth	Birth prevalence - [32, 34) wks, [1000, 1500) g	Mortality	Males		65.39<br>(43.593 to 95.583)	45.581<br>(37.56 to 54.417)
Neonatal preterm birth	Birth prevalence - [32, 34) wks, [1000, 1500) g	Mortality	Females		61.229<br>(40.8 to 88.303)	45.201<br>(37.492 to 54.267)
Neonatal preterm birth	Birth prevalence - [32, 34) wks, [1500, 2000) g	Mortality	Males		32.197<br>(22.524 to 44.858)	19.612<br>(16.465 to 23.296)
Neonatal preterm birth	Birth prevalence - [32, 34) wks, [1500, 2000) g	Mortality	Females		29.777<br>(21.026 to 40.255)	20.358<br>(17.072 to 23.768)
Neonatal preterm birth	Birth prevalence - [32, 34) wks, [2000, 2500) g	Mortality	Males		21.018<br>(15.141 to 28.622)	10.433<br>(8.784 to 12.225)
Neonatal preterm birth	Birth prevalence - [32, 34) wks, [2000, 2500) g	Mortality	Females		19.546<br>(14.316 to 26.034)	11.648<br>(9.974 to 13.507)
Neonatal preterm birth	Birth prevalence - [32, 34) wks, [2500, 3000) g	Mortality	Males		18.569<br>(13.127 to 25.826)	7.182<br>(5.968 to 8.63)
Neonatal preterm birth	Birth prevalence - [32, 34) wks, [2500, 3000) g	Mortality	Females		17.497<br>(11.66 to 24.635)	8.443<br>(6.726 to 10.284)
Neonatal preterm birth	Birth prevalence - [32, 34) wks, [3000, 3500) g	Mortality	Males		19.012<br>(12.18 to 28.02)	5.618<br>(4.325 to 7.114)
Neonatal preterm birth	Birth prevalence - [32, 34) wks, [3000, 3500) g	Mortality	Females		18.667<br>(12.24 to 27.524)	6.722<br>(5.103 to 8.697)
Neonatal preterm birth	Birth prevalence - [32, 34) wks, [3500, 4000) g	Mortality	Males		20.214<br>(11.906 to 31.929)	4.31<br>(3.186 to 5.633)
Neonatal preterm birth	Birth prevalence - [32, 34) wks, [3500, 4000) g	Mortality	Females		20.333<br>(12.064 to 33.21)	5.253<br>(3.959 to 6.913)
Neonatal preterm birth	Birth prevalence - [34, 36) wks, [1000, 1500) g	Mortality	Males		78.66<br>(52.169 to 113.08)	45.338<br>(36.27 to 55.556)
Neonatal preterm birth	Birth prevalence - [34, 36) wks, [1000, 1500) g	Mortality	Females		75.687<br>(49.863 to 110.089)	46.4<br>(38.048 to 55.902)
Neonatal preterm birth	Birth prevalence - [34, 36) wks, [1500, 2000) g	Mortality	Males		31.154<br>(20.939 to 45.031)	18.246<br>(15.083 to 21.906)
Neonatal preterm birth	Birth prevalence - [34, 36) wks, [1500, 2000) g	Mortality	Females		29.072<br>(19.6 to 42.648)	18.661<br>(15.638 to 22.175)
Neonatal preterm birth	Birth prevalence - [34, 36) wks, [2000, 2500) g	Mortality	Males		12.404<br>(8.692 to 16.95)	8.006<br>(6.775 to 9.31)
Neonatal preterm birth	Birth prevalence - [34, 36) wks, [2000, 2500) g	Mortality	Females		11.367<br>(7.807 to 15.547)	8.373<br>(7.066 to 9.703)
Neonatal preterm birth	Birth prevalence - [34, 36) wks, [2500, 3000) g	Mortality	Males		7.509<br>(5.429 to 10.255)	4.725<br>(3.991 to 5.56)
Neonatal preterm birth	Birth prevalence - [34, 36) wks, [2500, 3000) g	Mortality	Females		7.139<br>(5.091 to 9.759)	5.194<br>(4.342 to 6.087)
Neonatal preterm birth	Birth prevalence - [34, 36) wks, [3000, 3500) g	Mortality	Males		7.828<br>(5.467 to 11.237)	3.717<br>(2.986 to 4.568)
Neonatal preterm birth	Birth prevalence - [34, 36) wks, [3000, 3500) g	Mortality	Females		7.622<br>(5.235 to 10.584)	4.279<br>(3.416 to 5.301)
Neonatal preterm birth	Birth prevalence - [34, 36) wks, [3500, 4000) g	Mortality	Males		10.257<br>(6.692 to 15.124)	3.13<br>(2.474 to 3.957)
Neonatal preterm birth	Birth prevalence - [34, 36) wks, [3500, 4000) g	Mortality	Females		10.325<br>(6.495 to 15.537)	3.756<br>(2.877 to 4.818)
Neonatal preterm birth	Birth prevalence - [34, 36) wks, [4000, 4500) g	Mortality	Males		12.928<br>(7.825 to 20.726)	2.467<br>(1.926 to 3.084)
Neonatal preterm birth	Birth prevalence - [34, 36) wks, [4000, 4500) g	Mortality	Females		13.456<br>(8.201 to 20.893)	3.061<br>(2.357 to 3.889)
Neonatal preterm birth	Birth prevalence - [36, 37) wks, [1000, 1500) g	Mortality	Males		94.103<br>(62.401 to 131.437)	48.734<br>(39.385 to 60.014)
Neonatal preterm birth	Birth prevalence - [36, 37) wks, [1000, 1500) g	Mortality	Females		89.769<br>(59.869 to 126.674)	51.68<br>(41.336 to 63.456)
Neonatal preterm birth	Birth prevalence - [36, 37) wks, [1500, 2000) g	Mortality	Males		33.586<br>(23.075 to 48.77)	19.52<br>(16.283 to 23.116)
Neonatal preterm birth	Birth prevalence - [36, 37) wks, [1500, 2000) g	Mortality	Females		30.953<br>(20.951 to 43.853)	20.509<br>(17.15 to 24.286)
Neonatal preterm birth	Birth prevalence - [36, 37) wks, [2000, 2500) g	Mortality	Males		8.134<br>(5.781 to 10.938)	7.015<br>(5.903 to 8.241)
Neonatal preterm birth	Birth prevalence - [36, 37) wks, [2000, 2500) g	Mortality	Females		7.208<br>(5.183 to 9.787)	7.031<br>(5.911 to 8.265)
Neonatal preterm birth	Birth prevalence - [36, 37) wks, [2500, 3000) g	Mortality	Males		2.741<br>(2.114 to 3.535)	3.149<br>(2.686 to 3.686)
Neonatal preterm birth	Birth prevalence - [36, 37) wks, [2500, 3000) g	Mortality	Females		2.475<br>(1.905 to 3.109)	3.172<br>(2.71 to 3.666)
Neonatal preterm birth	Birth prevalence - [36, 37) wks, [3000, 3500) g	Mortality	Males		2.1<br>(1.602 to 2.663)	2.099<br>(1.805 to 2.445)
Neonatal preterm birth	Birth prevalence - [36, 37) wks, [3000, 3500) g	Mortality	Females		1.996<br>(1.532 to 2.6)	2.199<br>(1.895 to 2.534)
Neonatal preterm birth	Birth prevalence - [36, 37) wks, [3500, 4000) g	Mortality	Males		2.563<br>(1.906 to 3.333)	1.751<br>(1.487 to 2.008)
Neonatal preterm birth	Birth prevalence - [36, 37) wks, [3500, 4000) g	Mortality	Females		2.501<br>(1.793 to 3.34)	1.944<br>(1.649 to 2.275)
Neonatal preterm birth	Birth prevalence - [36, 37) wks, [4000, 4500) g	Mortality	Males		3.824<br>(2.607 to 5.361)	1.506<br>(1.283 to 1.74)
Neonatal preterm birth	Birth prevalence - [36, 37) wks, [4000, 4500) g	Mortality	Females		3.908<br>(2.626 to 5.462)	1.777<br>(1.481 to 2.118)
Neonatal preterm birth	Birth prevalence - [37, 38) wks, [1000, 1500) g	Mortality	Males		98.111<br>(66.503 to 135.289)	49.903<br>(39.62 to 62.232)
Neonatal preterm birth	Birth prevalence - [37, 38) wks, [1000, 1500) g	Mortality	Females		92.728<br>(63.975 to 128.565)	53.151<br>(42.705 to 66.273)
Neonatal preterm birth	Birth prevalence - [37, 38) wks, [1500, 2000) g	Mortality	Males		35.71<br>(24.585 to 49.648)	20.617<br>(16.897 to 24.816)
Neonatal preterm birth	Birth prevalence - [37, 38) wks, [1500, 2000) g	Mortality	Females		32.101<br>(22.457 to 43.887)	21.726<br>(17.833 to 25.871)
Neonatal preterm birth	Birth prevalence - [37, 38) wks, [2000, 2500) g	Mortality	Males		7.301<br>(5.37 to 9.805)	6.923<br>(5.831 to 8.219)
Neonatal preterm birth	Birth prevalence - [37, 38) wks, [2000, 2500) g	Mortality	Females		6.218<br>(4.554 to 8.169)	6.85<br>(5.796 to 8.005)
Neonatal preterm birth	Birth prevalence - [37, 38) wks, [2500, 3000) g	Mortality	Males		1.861<br>(1.414 to 2.384)	2.721<br>(2.329 to 3.158)
Neonatal preterm birth	Birth prevalence - [37, 38) wks, [2500, 3000) g	Mortality	Females		1.596<br>(1.24 to 1.998)	2.633<br>(2.261 to 3.031)
Neonatal preterm birth	Birth prevalence - [37, 38) wks, [3000, 3500) g	Mortality	Males		1.139<br>(1 to 1.409)	1.612<br>(1.387 to 1.85)
Neonatal preterm birth	Birth prevalence - [37, 38) wks, [3000, 3500) g	Mortality	Females		1.065<br>(1 to 1.288)	1.598<br>(1.392 to 1.819)
Neonatal preterm birth	Birth prevalence - [37, 38) wks, [3500, 4000) g	Mortality	Males		1.201<br>(1 to 1.493)	1.282<br>(1.113 to 1.466)
Neonatal preterm birth	Birth prevalence - [37, 38) wks, [3500, 4000) g	Mortality	Females		1.157<br>(1 to 1.442)	1.343<br>(1.153 to 1.542)
Neonatal preterm birth	Birth prevalence - [37, 38) wks, [4000, 4500) g	Mortality	Males		1.825<br>(1.341 to 2.407)	1.136<br>(1.01 to 1.272)
Neonatal preterm birth	Birth prevalence - [37, 38) wks, [4000, 4500) g	Mortality	Females		1.86<br>(1.36 to 2.46)	1.269<br>(1.115 to 1.42)
Neonatal preterm birth	Birth prevalence - [38, 40) wks, [1000, 1500) g	Mortality	Males		98.254<br>(67.959 to 134.77)	49.084<br>(38.794 to 61.948)
Neonatal preterm birth	Birth prevalence - [38, 40) wks, [1000, 1500) g	Mortality	Females		91.539<br>(62.769 to 127.606)	53.036<br>(41.348 to 65.237)
Neonatal preterm birth	Birth prevalence - [38, 40) wks, [1500, 2000) g	Mortality	Males		37.449<br>(25.688 to 51.467)	21.477<br>(17.208 to 25.646)
Neonatal preterm birth	Birth prevalence - [38, 40) wks, [1500, 2000) g	Mortality	Females		33.261<br>(23.303 to 45.061)	22.821<br>(18.339 to 27.874)
Neonatal preterm birth	Birth prevalence - [38, 40) wks, [2000, 2500) g	Mortality	Males		7.351<br>(5.131 to 9.967)	7.04<br>(5.807 to 8.395)
Neonatal preterm birth	Birth prevalence - [38, 40) wks, [2000, 2500) g	Mortality	Females		5.985<br>(4.383 to 7.995)	6.959<br>(5.782 to 8.29)
Neonatal preterm birth	Birth prevalence - [38, 40) wks, [2500, 3000) g	Mortality	Males		1.549<br>(1.185 to 1.98)	2.518<br>(2.115 to 2.971)
Neonatal preterm birth	Birth prevalence - [38, 40) wks, [2500, 3000) g	Mortality	Females		1.277<br>(1 to 1.65)	2.377<br>(2.015 to 2.745)
Neonatal preterm birth	Birth prevalence - [38, 40) wks, [3000, 3500) g	Mortality	Males		1<br>(1.0 - 1.0)	1.326<br>(1.12 to 1.543)
Neonatal preterm birth	Birth prevalence - [38, 40) wks, [3000, 3500) g	Mortality	Females		1<br>(1.0 - 1.0)	1.265<br>(1.068 to 1.45)
Neonatal preterm birth	Birth prevalence - [38, 40) wks, [3500, 4000) g	Mortality	Males		1<br>(1.0 - 1.0)	1<br>(1.0 - 1.0)
Neonatal preterm birth	Birth prevalence - [38, 40) wks, [3500, 4000) g	Mortality	Females		1<br>(1.0 - 1.0)	1<br>(1.0 - 1.0)
Neonatal preterm birth	Birth prevalence - [38, 40) wks, [4000, 4500) g	Mortality	Males		1<br>(1.0 - 1.0)	1<br>(1.0 - 1.0)
Neonatal preterm birth	Birth prevalence - [38, 40) wks, [4000, 4500) g	Mortality	Females		1<br>(1.0 - 1.0)	1<br>(1.0 - 1.0)
Neonatal preterm birth	Birth prevalence - [40, 42) wks, [1500, 2000) g	Mortality	Males		43.194<br>(29.244 to 62.382)	21.971<br>(16.41 to 28.396)
Neonatal preterm birth	Birth prevalence - [40, 42) wks, [1500, 2000) g	Mortality	Females		37.949<br>(25.539 to 53.532)	23.702<br>(17.857 to 30.634)
Neonatal preterm birth	Birth prevalence - [40, 42) wks, [2000, 2500) g	Mortality	Males		10.162<br>(6.961 to 14.235)	7.875<br>(6.341 to 9.719)
Neonatal preterm birth	Birth prevalence - [40, 42) wks, [2000, 2500) g	Mortality	Females		8.332<br>(5.994 to 11.407)	8.067<br>(6.467 to 10.043)
Neonatal preterm birth	Birth prevalence - [40, 42) wks, [2500, 3000) g	Mortality	Males		2.118<br>(1.53 to 2.77)	2.692<br>(2.23 to 3.273)

Neonatal preterm birth	Birth prevalence - [40, 42) wks, [2500, 3000) g	Mortality	Females		1.745 (1.289 to 2.316)	2.603 (2.134 to 3.106)
Neonatal preterm birth	Birth prevalence - [40, 42) wks, [3000, 3500) g	Mortality	Males		1.002 (1 to 1.013)	1.249 (1.041 to 1.491)
Neonatal preterm birth	Birth prevalence - [40, 42) wks, [3000, 3500) g	Mortality	Females		1 (1.0 - 1.0)	1.188 (1 to 1.411)
Neonatal preterm birth	Birth prevalence - [40, 42) wks, [3500, 4000) g	Mortality	Males		1 (1.0 - 1.0)	1 (1.0 - 1.0)
Neonatal preterm birth	Birth prevalence - [40, 42) wks, [3500, 4000) g	Mortality	Females		1 (1.0 - 1.0)	1 (1.0 - 1.0)
Neonatal preterm birth	Birth prevalence - [40, 42) wks, [4000, 4500) g	Mortality	Males		1 (1.0 - 1.0)	1 (1.0 - 1.0)
Neonatal preterm birth	Birth prevalence - [40, 42) wks, [4000, 4500) g	Mortality	Females		1 (1.0 - 1.0)	1 (1.0 - 1.0)
Neonatal sepsis and other neonatal infections	Birth prevalence - [0, 24) wks, [0, 500) g	Mortality	Males		877.775 (543.26 to 1246.645)	526.788 (392.055 to 676.923)
Neonatal sepsis and other neonatal infections	Birth prevalence - [0, 24) wks, [0, 500) g	Mortality	Females		857.044 (519.185 to 1227.708)	575.632 (434.418 to 737.293)
Neonatal sepsis and other neonatal infections	Birth prevalence - [0, 24) wks, [500, 1000) g	Mortality	Males		647.899 (443.535 to 881.449)	392.723 (315.689 to 486.607)
Neonatal sepsis and other neonatal infections	Birth prevalence - [0, 24) wks, [500, 1000) g	Mortality	Females		628.647 (437.861 to 854.598)	416.95 (329.573 to 516.699)
Neonatal sepsis and other neonatal infections	Birth prevalence - [24, 26) wks, [500, 1000) g	Mortality	Males		534.904 (378.653 to 707.51)	375.56 (308.336 to 447.076)
Neonatal sepsis and other neonatal infections	Birth prevalence - [24, 26) wks, [500, 1000) g	Mortality	Females		509.388 (370.575 to 690.649)	394.982 (324.03 to 477.74)
Neonatal sepsis and other neonatal infections	Birth prevalence - [26, 28) wks, [1000, 1500) g	Mortality	Males		150.649 (107.741 to 198.278)	140.284 (113.557 to 167.957)
Neonatal sepsis and other neonatal infections	Birth prevalence - [26, 28) wks, [1000, 1500) g	Mortality	Females		142.251 (102.895 to 191.587)	140.771 (114.514 to 169.631)
Neonatal sepsis and other neonatal infections	Birth prevalence - [26, 28) wks, [500, 1000) g	Mortality	Males		281.284 (198.703 to 391.898)	280.84 (226.174 to 338.336)
Neonatal sepsis and other neonatal infections	Birth prevalence - [26, 28) wks, [500, 1000) g	Mortality	Females		257.799 (185.994 to 349.394)	280.84 (225.68 to 350.2)
Neonatal sepsis and other neonatal infections	Birth prevalence - [28, 30) wks, [1000, 1500) g	Mortality	Males		88.889 (62.539 to 119.303)	87.596 (72.219 to 105.445)
Neonatal sepsis and other neonatal infections	Birth prevalence - [28, 30) wks, [1000, 1500) g	Mortality	Females		82.25 (59.418 to 109.806)	86.675 (69.965 to 104.367)
Neonatal sepsis and other neonatal infections	Birth prevalence - [28, 30) wks, [1500, 2000) g	Mortality	Males		71.963 (51.376 to 98.371)	42.294 (34.585 to 51.215)
Neonatal sepsis and other neonatal infections	Birth prevalence - [28, 30) wks, [1500, 2000) g	Mortality	Females		70.154 (48.562 to 96.294)	46.161 (38.268 to 54.906)
Neonatal sepsis and other neonatal infections	Birth prevalence - [28, 30) wks, [2000, 2500) g	Mortality	Males		65.936 (44.909 to 93.551)	23.785 (18.748 to 29.385)
Neonatal sepsis and other neonatal infections	Birth prevalence - [28, 30) wks, [2000, 2500) g	Mortality	Females		65.44 (43.751 to 96.137)	27.437 (21.369 to 33.874)
Neonatal sepsis and other neonatal infections	Birth prevalence - [28, 30) wks, [2500, 3000) g	Mortality	Males		43.647 (29.61 to 62.044)	14.185 (10.817 to 18.495)
Neonatal sepsis and other neonatal infections	Birth prevalence - [28, 30) wks, [2500, 3000) g	Mortality	Females		42.667 (28.231 to 61.897)	16.517 (12.636 to 21.355)
Neonatal sepsis and other neonatal infections	Birth prevalence - [28, 30) wks, [3000, 3500) g	Mortality	Males		23.727 (15.859 to 35.246)	8.651 (6.873 to 11.016)
Neonatal sepsis and other neonatal infections	Birth prevalence - [28, 30) wks, [3000, 3500) g	Mortality	Females		23.045 (14.473 to 35.151)	9.735 (7.511 to 12.367)
Neonatal sepsis and other neonatal infections	Birth prevalence - [28, 30) wks, [500, 1000) g	Mortality	Males		164.681 (114.331 to 232.909)	184.988 (146.715 to 225.778)
Neonatal sepsis and other neonatal infections	Birth prevalence - [28, 30) wks, [500, 1000) g	Mortality	Females		149.827 (102.011 to 207.65)	178.51 (144.736 to 220.088)
Neonatal sepsis and other neonatal infections	Birth prevalence - [30, 32) wks, [1000, 1500) g	Mortality	Males		66.567 (45.227 to 91.424)	57.036 (47.559 to 67.673)
Neonatal sepsis and other neonatal infections	Birth prevalence - [30, 32) wks, [1000, 1500) g	Mortality	Females		61.652 (42.69 to 86.655)	55.992 (46.425 to 67.165)
Neonatal sepsis and other neonatal infections	Birth prevalence - [30, 32) wks, [1500, 2000) g	Mortality	Males		43.288 (30.684 to 57.762)	26.533 (22.334 to 31.213)
Neonatal sepsis and other neonatal infections	Birth prevalence - [30, 32) wks, [1500, 2000) g	Mortality	Females		41.127 (28.837 to 54.767)	28.256 (24.051 to 32.792)
Neonatal sepsis and other neonatal infections	Birth prevalence - [30, 32) wks, [2000, 2500) g	Mortality	Males		38.672 (27.558 to 53.438)	15.362 (12.641 to 18.516)
Neonatal sepsis and other neonatal infections	Birth prevalence - [30, 32) wks, [2000, 2500) g	Mortality	Females		37.459 (26.086 to 52.166)	17.996 (14.845 to 21.529)
Neonatal sepsis and other neonatal infections	Birth prevalence - [30, 32) wks, [2500, 3000) g	Mortality	Males		33.218 (22.641 to 46.944)	10.389 (8.195 to 12.863)
Neonatal sepsis and other neonatal infections	Birth prevalence - [30, 32) wks, [2500, 3000) g	Mortality	Females		31.439 (21.157 to 45.621)	12.473 (9.64 to 15.784)
Neonatal sepsis and other neonatal infections	Birth prevalence - [30, 32) wks, [3000, 3500) g	Mortality	Males		25.451 (16.854 to 36.763)	7.153 (5.665 to 9.003)
Neonatal sepsis and other neonatal infections	Birth prevalence - [30, 32) wks, [3000, 3500) g	Mortality	Females		24.262 (15.651 to 36.395)	8.486 (6.482 to 10.824)
Neonatal sepsis and other neonatal infections	Birth prevalence - [30, 32) wks, [3500, 4000) g	Mortality	Males		20.334 (11.916 to 31.586)	4.795 (3.705 to 6.039)
Neonatal sepsis and other neonatal infections	Birth prevalence - [30, 32) wks, [3500, 4000) g	Mortality	Females		20.347 (11.635 to 32.681)	5.599 (4.199 to 7.275)
Neonatal sepsis and other neonatal infections	Birth prevalence - [30, 32) wks, [500, 1000) g	Mortality	Males		132.309 (85.99 to 193.341)	127.982 (101.66 to 159.489)
Neonatal sepsis and other neonatal infections	Birth prevalence - [30, 32) wks, [500, 1000) g	Mortality	Females		120.829 (82.341 to 172.828)	123.186 (98.696 to 152.125)
Neonatal sepsis and other neonatal infections	Birth prevalence - [32, 34) wks, [1000, 1500) g	Mortality	Males		65.39 (43.593 to 95.583)	45.581 (37.56 to 54.417)
Neonatal sepsis and other neonatal infections	Birth prevalence - [32, 34) wks, [1000, 1500) g	Mortality	Females		61.229 (40.8 to 88.303)	45.201 (37.492 to 54.267)
Neonatal sepsis and other neonatal infections	Birth prevalence - [32, 34) wks, [1500, 2000) g	Mortality	Males		32.197 (22.524 to 44.858)	19.612 (16.465 to 23.296)
Neonatal sepsis and other neonatal infections	Birth prevalence - [32, 34) wks, [1500, 2000) g	Mortality	Females		29.777 (21.026 to 40.255)	20.358 (17.072 to 23.768)
Neonatal sepsis and other neonatal infections	Birth prevalence - [32, 34) wks, [2000, 2500) g	Mortality	Males		21.018 (15.141 to 28.622)	10.433 (8.784 to 12.225)
Neonatal sepsis and other neonatal infections	Birth prevalence - [32, 34) wks, [2000, 2500) g	Mortality	Females		19.546 (14.316 to 26.034)	11.648 (9.974 to 13.507)
Neonatal sepsis and other neonatal infections	Birth prevalence - [32, 34) wks, [2500, 3000) g	Mortality	Males		18.569 (13.127 to 25.826)	7.182 (5.968 to 8.63)
Neonatal sepsis and other neonatal infections	Birth prevalence - [32, 34) wks, [2500, 3000) g	Mortality	Females		17.497 (11.66 to 24.635)	8.443 (6.726 to 10.284)
Neonatal sepsis and other neonatal infections	Birth prevalence - [32, 34) wks, [3000, 3500) g	Mortality	Males		19.012 (12.18 to 28.02)	5.618 (4.325 to 7.114)
Neonatal sepsis and other neonatal infections	Birth prevalence - [32, 34) wks, [3000, 3500) g	Mortality	Females		18.667 (12.24 to 27.524)	6.722 (5.103 to 8.697)
Neonatal sepsis and other neonatal infections	Birth prevalence - [32, 34) wks, [3500, 4000) g	Mortality	Males		20.214 (11.906 to 31.929)	4.31 (3.186 to 5.633)
Neonatal sepsis and other neonatal infections	Birth prevalence - [32, 34) wks, [3500, 4000) g	Mortality	Females		20.333 (12.064 to 33.21)	5.253 (3.959 to 6.913)
Neonatal sepsis and other neonatal infections	Birth prevalence - [34, 36) wks, [1000, 1500) g	Mortality	Males		78.66 (52.169 to 113.08)	45.338 (36.27 to 55.556)
Neonatal sepsis and other neonatal infections	Birth prevalence - [34, 36) wks, [1000, 1500) g	Mortality	Females		75.687 (49.863 to 110.089)	46.4 (38.048 to 55.902)
Neonatal sepsis and other neonatal infections	Birth prevalence - [34, 36) wks, [1500, 2000) g	Mortality	Males		31.154 (20.939 to 45.031)	18.246 (15.083 to 21.906)
Neonatal sepsis and other neonatal infections	Birth prevalence - [34, 36) wks, [1500, 2000) g	Mortality	Females		29.072 (19.6 to 42.648)	18.661 (15.638 to 22.175)
Neonatal sepsis and other neonatal infections	Birth prevalence - [34, 36) wks, [2000, 2500) g	Mortality	Males		12.404 (8.692 to 16.95)	8.006 (6.775 to 9.31)
Neonatal sepsis and other neonatal infections	Birth prevalence - [34, 36) wks, [2000, 2500) g	Mortality	Females		11.367 (7.807 to 15.547)	8.373 (7.066 to 9.703)
Neonatal sepsis and other neonatal infections	Birth prevalence - [34, 36) wks, [2500, 3000) g	Mortality	Males		7.509 (5.429 to 10.255)	4.725 (3.991 to 5.56)
Neonatal sepsis and other neonatal infections	Birth prevalence - [34, 36) wks, [2500, 3000) g	Mortality	Females		7.139 (5.091 to 9.759)	5.194 (4.342 to 6.087)
Neonatal sepsis and other neonatal infections	Birth prevalence - [34, 36) wks, [3000, 3500) g	Mortality	Males		7.828 (5.467 to 11.237)	3.717 (2.986 to 4.568)
Neonatal sepsis and other neonatal infections	Birth prevalence - [34, 36) wks, [3000, 3500) g	Mortality	Females		7.622 (5.235 to 10.584)	4.279 (3.416 to 5.301)
Neonatal sepsis and other neonatal infections	Birth prevalence - [34, 36) wks, [3500, 4000) g	Mortality	Males		10.257 (6.692 to 15.124)	3.13 (2.474 to 3.957)
Neonatal sepsis and other neonatal infections	Birth prevalence - [34, 36) wks, [3500, 4000) g	Mortality	Females		10.325 (6.495 to 15.537)	3.756 (2.877 to 4.818)
Neonatal sepsis and other neonatal infections	Birth prevalence - [34, 36) wks, [4000, 4500) g	Mortality	Males		12.928 (7.825 to 20.726)	2.467 (1.926 to 3.084)
Neonatal sepsis and other neonatal infections	Birth prevalence - [34, 36) wks, [4000, 4500) g	Mortality	Females		13.456 (8.201 to 20.893)	3.061 (2.357 to 3.889)
Neonatal sepsis and other neonatal infections	Birth prevalence - [36, 37) wks, [1000, 1500) g	Mortality	Males		94.103 (62.401 to 131.437)	48.734 (39.385 to 60.014)
Neonatal sepsis and other neonatal infections	Birth prevalence - [36, 37) wks, [1000, 1500) g	Mortality	Females		89.769 (59.869 to 126.674)	51.68 (41.336 to 63.456)
Neonatal sepsis and other neonatal infections	Birth prevalence - [36, 37) wks, [1500, 2000) g	Mortality	Males		33.586 (23.075 to 48.77)	19.52 (16.283 to 23.116)
Neonatal sepsis and other neonatal infections	Birth prevalence - [36, 37) wks, [1500, 2000) g	Mortality	Females		30.953 (20.951 to 43.853)	20.509 (17.15 to 24.286)
Neonatal sepsis and other neonatal infections	Birth prevalence - [36, 37) wks, [2000, 2500) g	Mortality	Males		8.134 (5.781 to 10.938)	7.015 (5.903 to 8.241)
Neonatal sepsis and other neonatal infections	Birth prevalence - [36, 37) wks, [2000, 2500) g	Mortality	Females		7.208 (5.183 to 9.787)	7.031 (5.911 to 8.265)
Neonatal sepsis and other neonatal infections	Birth prevalence - [36, 37) wks, [2500, 3000) g	Mortality	Males		2.741 (2.114 to 3.535)	3.149 (2.686 to 3.686)
Neonatal sepsis and other neonatal infections	Birth prevalence - [36, 37) wks, [2500, 3000) g	Mortality	Females		2.475 (1.905 to 3.109)	3.172 (2.71 to 3.666)
Neonatal sepsis and other neonatal infections	Birth prevalence - [36, 37) wks, [3000, 3500) g	Mortality	Males		2.1 (1.602 to 2.663)	2.099 (1.805 to 2.445)
Neonatal sepsis and other neonatal infections	Birth prevalence - [36, 37) wks, [3000, 3500) g	Mortality	Females		1.996 (1.532 to 2.6)	2.199 (1.895 to 2.534)
Neonatal sepsis and other neonatal infections	Birth prevalence - [36, 37) wks, [3500, 4000) g	Mortality	Males		2.563 (1.906 to 3.333)	1.751 (1.487 to 2.008)
Neonatal sepsis and other neonatal infections	Birth prevalence - [36, 37) wks, [3500, 4000) g	Mortality	Females		2.501 (1.793 to 3.34)	1.944 (1.649 to 2.275)
Neonatal sepsis and other neonatal infections	Birth prevalence - [36, 37) wks, [4000, 4500) g	Mortality	Males		3.824 (2.607 to 5.361)	1.506 (1.283 to 1.74)
Neonatal sepsis and other neonatal infections	Birth prevalence - [36, 37) wks, [4000, 4500) g	Mortality	Females		3.908 (2.626 to 5.462)	1.777 (1.481 to 2.118)
Neonatal sepsis and other neonatal infections	Birth prevalence - [37, 38) wks, [1000, 1500) g	Mortality	Males		98.111 (66.503 to 135.289)	49.903 (39.62 to 62.232)
Neonatal sepsis and other neonatal infections	Birth prevalence - [37, 38) wks, [1000, 1500) g	Mortality	Females		92.728 (63.975 to 128.565)	53.151 (42.705 to 66.273)
Neonatal sepsis and other neonatal infections	Birth prevalence - [37, 38) wks, [1500, 2000) g	Mortality	Males		35.71 (24.585 to 49.648)	20.617 (16.897 to 24.816)
Neonatal sepsis and other neonatal infections	Birth prevalence - [37, 38) wks, [1500, 2000) g	Mortality	Females		32.101 (22.457 to 43.887)	21.726 (17.833 to 25.871)
Neonatal sepsis and other neonatal infections	Birth prevalence - [37, 38) wks, [2000, 2500) g	Mortality	Males		7.301 (5.37 to 9.805)	6.923 (5.831 to 8.219)
Neonatal sepsis and other neonatal infections	Birth prevalence - [37, 38) wks, [2000, 2500) g	Mortality	Females		6.218 (4.554 to 8.169)	6.85 (5.796 to 8.005)
Neonatal sepsis and other neonatal infections	Birth prevalence - [37, 38) wks, [2500, 3000) g	Mortality	Males		1.861 (1.414 to 2.384)	2.721 (2.329 to 3.158)
Neonatal sepsis and other neonatal infections	Birth prevalence - [37, 38) wks, [2500, 3000) g	Mortality	Females		1.596 (1.24 to 1.998)	2.633 (2.261 to 3.031)
Neonatal sepsis and other neonatal infections	Birth prevalence - [37, 38) wks, [3000, 3500) g	Mortality	Males		1.139 (1 to 1.409)	1.612 (1.387 to 1.85)
Neonatal sepsis and other neonatal infections	Birth prevalence - [37, 38) wks, [3000, 3500) g	Mortality	Females		1.065 (1 to 1.288)	1.598 (1.392 to 1.819)
Neonatal sepsis and other neonatal infections	Birth prevalence - [37, 38) wks, [3500, 4000) g	Mortality	Males		1.201 (1 to 1.493)	1.282 (1.113 to 1.466)
Neonatal sepsis and other neonatal infections	Birth prevalence - [37, 38) wks, [3500, 4000) g	Mortality	Females		1.157 (1 to 1.442)	1.343 (1.153 to 1.542)
Neonatal sepsis and other neonatal infections	Birth prevalence - [37, 38) wks, [4000, 4500) g	Mortality	Males		1.825 (1.341 to 2.407)	1.136 (1.01 to 1.272)
Neonatal sepsis and other neonatal infections	Birth prevalence - [37, 38) wks, [4000, 4500) g	Mortality	Females		1.86 (1.36 to 2.46)	1.269 (1.115 to 1.42)
Neonatal sepsis and other neonatal infections	Birth prevalence - [38, 40) wks, [1000, 1500) g	Mortality	Males		98.254 (67.959 to 134.77)	49.084 (38.794 to 61.948)
Neonatal sepsis and other neonatal infections	Birth prevalence - [38, 40) wks, [1000, 1500) g	Mortality	Females		91.539 (62.769 to 127.606)	53.036 (41.348 to 65.237)
Neonatal sepsis and other neonatal infections	Birth prevalence - [38, 40) wks, [1500, 2000) g	Mortality	Males		37.449 (25.688 to 51.467)	21.477 (17.208 to 25.646)
Neonatal sepsis and other neonatal infections	Birth prevalence - [38, 40) wks, [1500, 2000) g	Mortality	Females		33.261 (23.303 to 45.061)	22.821 (18.339 to 27.874)
Neonatal sepsis and other neonatal infections	Birth prevalence - [38, 40) wks, [2000, 2500) g	Mortality	Males		7.351 (5.131 to 9.967)	7.04 (5.807 to 8.395)
Neonatal sepsis and other neonatal infections	Birth prevalence - [38, 40) wks, [2000, 2500) g	Mortality	Females		5.985 (4.383 to 7.995)	6.959 (5.782 to 8.29)
Neonatal sepsis and other neonatal infections	Birth prevalence - [38, 40) wks, [2500, 3000) g	Mortality	Males		1.549 (1.185 to 1.98)	2.518 (2.115 to 2.971)
Neonatal sepsis and other neonatal infections	Birth prevalence - [38, 40) wks, [2500, 3000) g	Mortality	Females		1.277 (1 to 1.65)	2.377 (2.015 to 2.745)
Neonatal sepsis and other neonatal infections	Birth prevalence - [38, 40) wks, [3000, 3500) g	Mortality	Males		1 (1.0 - 1.0)	1.326 (1.12 to 1.543)
Neonatal sepsis and other neonatal infections	Birth prevalence - [38, 40) wks, [3000, 3500) g	Mortality	Females		1 (1.0 - 1.0)	1.265 (1.068 to 1.45)
Neonatal sepsis and other neonatal infections	Birth prevalence - [38, 40) wks, [3500, 4000) g	Mortality	Males		1 (1.0 - 1.0)	1 (1.0 - 1.0)
Neonatal sepsis and other neonatal infections	Birth prevalence - [38, 40) wks, [3500, 4000) g	Mortality	Females		1 (1.0 - 1.0)	1 (1.0 - 1.0)
Neonatal sepsis and other neonatal infections	Birth prevalence - [38, 40) wks, [4000, 4500) g	Mortality	Males		1 (1.0 - 1.0)	1 (1.0 - 1.0)
Neonatal sepsis and other neonatal infections	Birth prevalence - [38, 40) wks, [4000, 4500) g	Mortality	Females		1 (1.0 - 1.0)	1 (1.0 - 1.0)
Neonatal sepsis and other neonatal infections	Birth prevalence - [40, 42) wks, [1500, 2000) g	Mortality	Males		43.194 (29.244 to 62.382)	21.971 (16.41 to 28.396)
Neonatal sepsis and other neonatal infections	Birth prevalence - [40, 42) wks, [1500, 2000) g	Mortality	Females		37.949 (25.539 to 53.532)	23.702 (17.857 to 30.634)
Neonatal sepsis and other neonatal infections	Birth prevalence - [40, 42) wks, [2000, 2500) g	Mortality	Males		10.162 (6.961 to 14.235)	7.875 (6.341 to 9.719)
Neonatal sepsis and other neonatal infections	Birth prevalence - [40, 42) wks, [2000, 2500) g	Mortality	Females		8.332 (5.994 to 11.407)	8.067 (6.467 to 10.043)
Neonatal sepsis and other neonatal infections	Birth prevalence - [40, 42) wks, [2500, 3000) g	Mortality	Males		2.118 (1.53 to 2.77)	2.692 (2.23 to 3.273)
Neonatal sepsis and other neonatal infections	Birth prevalence - [40, 42) wks, [2500, 3000) g	Mortality	Females		1.745 (1.289 to 2.316)	2.603 (2.134 to 3.106)
Neonatal sepsis and other neonatal infections	Birth prevalence - [40, 42) wks, [3000, 3500) g	Mortality	Males		1.002 (1 to 1.013)	1.249 (1.041 to 1.491)
Neonatal sepsis and other neonatal infections	Birth prevalence - [40, 42) wks, [3000, 3500) g	Mortality	Females		1 (1.0 - 1.0)	1.188 (1 to 1.411)
Neonatal sepsis and other neonatal infections	Birth prevalence - [40, 42) wks, [3500, 4000) g	Mortality	Males		1 (1.0 - 1.0)	1 (1.0 - 1.0)
Neonatal sepsis and other neonatal infections	Birth prevalence - [40, 42) wks, [3500, 4000) g	Mortality	Females		1 (1.0 - 1.0)	1 (1.0 - 1.0)
Neonatal sepsis and other neonatal infections	Birth prevalence - [40, 42) wks, [4000, 4500) g	Mortality	Males		1 (1.0 - 1.0)	1 (1.0 - 1.0)
Neonatal sepsis and other neonatal infections	Birth prevalence - [40, 42) wks, [4000, 4500) g	Mortality	Females		1 (1.0 - 1.0)	1 (1.0 - 1.0)

Other neonatal disorders	Birth prevalence - [0, 24) wks, [0, 500) g	Mortality	Males		877.775 (543.26 to 1246.645)	526.788 (392.055 to 676.923)
Other neonatal disorders	Birth prevalence - [0, 24) wks, [0, 500) g	Mortality	Females		857.044 (519.185 to 1227.708)	575.632 (434.418 to 737.293)
Other neonatal disorders	Birth prevalence - [0, 24) wks, [500, 1000) g	Mortality	Males		647.899 (443.535 to 881.449)	392.723 (315.689 to 486.607)
Other neonatal disorders	Birth prevalence - [0, 24) wks, [500, 1000) g	Mortality	Females		628.647 (437.861 to 854.598)	416.95 (329.573 to 516.699)
Other neonatal disorders	Birth prevalence - [24, 26) wks, [500, 1000) g	Mortality	Males		534.904 (378.653 to 707.51)	375.56 (308.336 to 447.076)
Other neonatal disorders	Birth prevalence - [24, 26) wks, [500, 1000) g	Mortality	Females		509.388 (370.575 to 690.649)	394.982 (324.03 to 477.74)
Other neonatal disorders	Birth prevalence - [26, 28) wks, [1000, 1500) g	Mortality	Males		150.649 (107.741 to 198.278)	140.284 (113.557 to 167.957)
Other neonatal disorders	Birth prevalence - [26, 28) wks, [1000, 1500) g	Mortality	Females		142.251 (102.895 to 191.587)	140.771 (114.514 to 169.631)
Other neonatal disorders	Birth prevalence - [26, 28) wks, [500, 1000) g	Mortality	Males		281.284 (198.703 to 391.898)	280.84 (226.174 to 338.336)
Other neonatal disorders	Birth prevalence - [26, 28) wks, [500, 1000) g	Mortality	Females		257.799 (185.994 to 349.394)	280.84 (225.68 to 350.2)
Other neonatal disorders	Birth prevalence - [28, 30) wks, [1000, 1500) g	Mortality	Males		88.889 (62.539 to 119.303)	87.596 (72.219 to 105.445)
Other neonatal disorders	Birth prevalence - [28, 30) wks, [1000, 1500) g	Mortality	Females		82.25 (59.418 to 109.806)	86.675 (69.965 to 104.367)
Other neonatal disorders	Birth prevalence - [28, 30) wks, [1500, 2000) g	Mortality	Males		71.963 (51.376 to 98.371)	42.294 (34.585 to 51.215)
Other neonatal disorders	Birth prevalence - [28, 30) wks, [1500, 2000) g	Mortality	Females		70.154 (48.562 to 96.294)	46.161 (38.268 to 54.906)
Other neonatal disorders	Birth prevalence - [28, 30) wks, [2000, 2500) g	Mortality	Males		65.936 (44.909 to 93.551)	23.785 (18.748 to 29.385)
Other neonatal disorders	Birth prevalence - [28, 30) wks, [2000, 2500) g	Mortality	Females		65.44 (43.751 to 96.137)	27.437 (21.369 to 33.874)
Other neonatal disorders	Birth prevalence - [28, 30) wks, [2500, 3000) g	Mortality	Males		43.647 (29.61 to 62.044)	14.185 (10.817 to 18.495)
Other neonatal disorders	Birth prevalence - [28, 30) wks, [2500, 3000) g	Mortality	Females		42.667 (28.231 to 61.897)	16.517 (12.636 to 21.355)
Other neonatal disorders	Birth prevalence - [28, 30) wks, [3000, 3500) g	Mortality	Males		23.727 (15.859 to 35.246)	8.651 (6.873 to 11.016)
Other neonatal disorders	Birth prevalence - [28, 30) wks, [3000, 3500) g	Mortality	Females		23.045 (14.473 to 35.151)	9.735 (7.511 to 12.367)
Other neonatal disorders	Birth prevalence - [28, 30) wks, [500, 1000) g	Mortality	Males		164.681 (114.331 to 232.909)	184.988 (146.715 to 225.778)
Other neonatal disorders	Birth prevalence - [28, 30) wks, [500, 1000) g	Mortality	Females		149.827 (102.011 to 207.65)	178.51 (144.736 to 220.088)
Other neonatal disorders	Birth prevalence - [30, 32) wks, [1000, 1500) g	Mortality	Males		66.567 (45.227 to 91.424)	57.036 (47.559 to 67.673)
Other neonatal disorders	Birth prevalence - [30, 32) wks, [1000, 1500) g	Mortality	Females		61.652 (42.69 to 86.655)	55.992 (46.425 to 67.165)
Other neonatal disorders	Birth prevalence - [30, 32) wks, [1500, 2000) g	Mortality	Males		43.288 (30.684 to 57.762)	26.533 (22.334 to 31.213)
Other neonatal disorders	Birth prevalence - [30, 32) wks, [1500, 2000) g	Mortality	Females		41.127 (28.837 to 54.767)	28.256 (24.051 to 32.792)
Other neonatal disorders	Birth prevalence - [30, 32) wks, [2000, 2500) g	Mortality	Males		38.672 (27.558 to 53.438)	15.362 (12.641 to 18.516)
Other neonatal disorders	Birth prevalence - [30, 32) wks, [2000, 2500) g	Mortality	Females		37.459 (26.086 to 52.166)	17.996 (14.845 to 21.529)
Other neonatal disorders	Birth prevalence - [30, 32) wks, [2500, 3000) g	Mortality	Males		33.218 (22.641 to 46.944)	10.389 (8.195 to 12.863)
Other neonatal disorders	Birth prevalence - [30, 32) wks, [2500, 3000) g	Mortality	Females		31.439 (21.157 to 45.621)	12.473 (9.64 to 15.784)
Other neonatal disorders	Birth prevalence - [30, 32) wks, [3000, 3500) g	Mortality	Males		25.451 (16.854 to 36.763)	7.153 (5.665 to 9.003)
Other neonatal disorders	Birth prevalence - [30, 32) wks, [3000, 3500) g	Mortality	Females		24.262 (15.651 to 36.395)	8.486 (6.482 to 10.824)
Other neonatal disorders	Birth prevalence - [30, 32) wks, [3500, 4000) g	Mortality	Males		20.334 (11.916 to 31.586)	4.795 (3.705 to 6.039)
Other neonatal disorders	Birth prevalence - [30, 32) wks, [3500, 4000) g	Mortality	Females		20.347 (11.635 to 32.681)	5.599 (4.199 to 7.275)
Other neonatal disorders	Birth prevalence - [30, 32) wks, [500, 1000) g	Mortality	Males		132.309 (85.99 to 193.341)	127.982 (101.66 to 159.489)
Other neonatal disorders	Birth prevalence - [30, 32) wks, [500, 1000) g	Mortality	Females		120.829 (82.341 to 172.828)	123.186 (98.696 to 152.125)
Other neonatal disorders	Birth prevalence - [32, 34) wks, [1000, 1500) g	Mortality	Males		65.39 (43.593 to 95.583)	45.581 (37.56 to 54.417)
Other neonatal disorders	Birth prevalence - [32, 34) wks, [1000, 1500) g	Mortality	Females		61.229 (40.8 to 88.303)	45.201 (37.492 to 54.267)
Other neonatal disorders	Birth prevalence - [32, 34) wks, [1500, 2000) g	Mortality	Males		32.197 (22.524 to 44.858)	19.612 (16.465 to 23.296)
Other neonatal disorders	Birth prevalence - [32, 34) wks, [1500, 2000) g	Mortality	Females		29.777 (21.026 to 40.255)	20.358 (17.072 to 23.768)
Other neonatal disorders	Birth prevalence - [32, 34) wks, [2000, 2500) g	Mortality	Males		21.018 (15.141 to 28.622)	10.433 (8.784 to 12.225)
Other neonatal disorders	Birth prevalence - [32, 34) wks, [2000, 2500) g	Mortality	Females		19.546 (14.316 to 26.034)	11.648 (9.974 to 13.507)
Other neonatal disorders	Birth prevalence - [32, 34) wks, [2500, 3000) g	Mortality	Males		18.569 (13.127 to 25.826)	7.182 (5.968 to 8.63)
Other neonatal disorders	Birth prevalence - [32, 34) wks, [2500, 3000) g	Mortality	Females		17.497 (11.66 to 24.635)	8.443 (6.726 to 10.284)
Other neonatal disorders	Birth prevalence - [32, 34) wks, [3000, 3500) g	Mortality	Males		19.012 (12.18 to 28.02)	5.618 (4.325 to 7.114)
Other neonatal disorders	Birth prevalence - [32, 34) wks, [3000, 3500) g	Mortality	Females		18.667 (12.24 to 27.524)	6.722 (5.103 to 8.697)
Other neonatal disorders	Birth prevalence - [32, 34) wks, [3500, 4000) g	Mortality	Males		20.214 (11.906 to 31.929)	4.31 (3.186 to 5.633)
Other neonatal disorders	Birth prevalence - [32, 34) wks, [3500, 4000) g	Mortality	Females		20.333 (12.064 to 33.21)	5.253 (3.959 to 6.913)
Other neonatal disorders	Birth prevalence - [34, 36) wks, [1000, 1500) g	Mortality	Males		78.66 (52.169 to 113.08)	45.338 (36.27 to 55.556)
Other neonatal disorders	Birth prevalence - [34, 36) wks, [1000, 1500) g	Mortality	Females		75.687 (49.863 to 110.089)	46.4 (38.048 to 55.902)
Other neonatal disorders	Birth prevalence - [34, 36) wks, [1500, 2000) g	Mortality	Males		31.154 (20.939 to 45.031)	18.246 (15.083 to 21.906)
Other neonatal disorders	Birth prevalence - [34, 36) wks, [1500, 2000) g	Mortality	Females		29.072 (19.6 to 42.648)	18.661 (15.638 to 22.175)
Other neonatal disorders	Birth prevalence - [34, 36) wks, [2000, 2500) g	Mortality	Males		12.404 (8.692 to 16.95)	8.006 (6.775 to 9.31)
Other neonatal disorders	Birth prevalence - [34, 36) wks, [2000, 2500) g	Mortality	Females		11.367 (7.807 to 15.547)	8.373 (7.066 to 9.703)
Other neonatal disorders	Birth prevalence - [34, 36) wks, [2500, 3000) g	Mortality	Males		7.509 (5.429 to 10.255)	4.725 (3.991 to 5.56)
Other neonatal disorders	Birth prevalence - [34, 36) wks, [2500, 3000) g	Mortality	Females		7.139 (5.091 to 9.759)	5.194 (4.342 to 6.087)
Other neonatal disorders	Birth prevalence - [34, 36) wks, [3000, 3500) g	Mortality	Males		7.828 (5.467 to 11.237)	3.717 (2.986 to 4.568)
Other neonatal disorders	Birth prevalence - [34, 36) wks, [3000, 3500) g	Mortality	Females		7.622 (5.235 to 10.584)	4.279 (3.416 to 5.301)
Other neonatal disorders	Birth prevalence - [34, 36) wks, [3500, 4000) g	Mortality	Males		10.257 (6.692 to 15.124)	3.13 (2.474 to 3.957)
Other neonatal disorders	Birth prevalence - [34, 36) wks, [3500, 4000) g	Mortality	Females		10.325 (6.495 to 15.537)	3.756 (2.877 to 4.818)
Other neonatal disorders	Birth prevalence - [34, 36) wks, [4000, 4500) g	Mortality	Males		12.928 (7.825 to 20.726)	2.467 (1.926 to 3.084)
Other neonatal disorders	Birth prevalence - [34, 36) wks, [4000, 4500) g	Mortality	Females		13.456 (8.201 to 20.893)	3.061 (2.357 to 3.889)
Other neonatal disorders	Birth prevalence - [36, 37) wks, [1000, 1500) g	Mortality	Males		94.103 (62.401 to 131.437)	48.734 (39.385 to 60.014)
Other neonatal disorders	Birth prevalence - [36, 37) wks, [1000, 1500) g	Mortality	Females		89.769 (59.869 to 126.674)	51.68 (41.336 to 63.456)
Other neonatal disorders	Birth prevalence - [36, 37) wks, [1500, 2000) g	Mortality	Males		33.586 (23.075 to 48.77)	19.52 (16.283 to 23.116)
Other neonatal disorders	Birth prevalence - [36, 37) wks, [1500, 2000) g	Mortality	Females		30.953 (20.951 to 43.853)	20.509 (17.15 to 24.286)
Other neonatal disorders	Birth prevalence - [36, 37) wks, [2000, 2500) g	Mortality	Males		8.134 (5.781 to 10.938)	7.015 (5.903 to 8.241)
Other neonatal disorders	Birth prevalence - [36, 37) wks, [2000, 2500) g	Mortality	Females		7.208 (5.183 to 9.787)	7.031 (5.911 to 8.265)
Other neonatal disorders	Birth prevalence - [36, 37) wks, [2500, 3000) g	Mortality	Males		2.741 (2.114 to 3.535)	3.149 (2.686 to 3.686)
Other neonatal disorders	Birth prevalence - [36, 37) wks, [2500, 3000) g	Mortality	Females		2.475 (1.905 to 3.109)	3.172 (2.71 to 3.666)
Other neonatal disorders	Birth prevalence - [36, 37) wks, [3000, 3500) g	Mortality	Males		2.1 (1.602 to 2.663)	2.099 (1.805 to 2.445)
Other neonatal disorders	Birth prevalence - [36, 37) wks, [3000, 3500) g	Mortality	Females		1.996 (1.532 to 2.6)	2.199 (1.895 to 2.534)
Other neonatal disorders	Birth prevalence - [36, 37) wks, [3500, 4000) g	Mortality	Males		2.563 (1.906 to 3.333)	1.751 (1.487 to 2.008)
Other neonatal disorders	Birth prevalence - [36, 37) wks, [3500, 4000) g	Mortality	Females		2.501 (1.793 to 3.34)	1.944 (1.649 to 2.275)
Other neonatal disorders	Birth prevalence - [36, 37) wks, [4000, 4500) g	Mortality	Males		3.824 (2.607 to 5.361)	1.506 (1.283 to 1.74)
Other neonatal disorders	Birth prevalence - [36, 37) wks, [4000, 4500) g	Mortality	Females		3.908 (2.626 to 5.462)	1.777 (1.481 to 2.118)
Other neonatal disorders	Birth prevalence - [37, 38) wks, [1000, 1500) g	Mortality	Males		98.111 (66.503 to 135.289)	49.903 (39.62 to 62.232)
Other neonatal disorders	Birth prevalence - [37, 38) wks, [1000, 1500) g	Mortality	Females		92.728 (63.975 to 128.565)	53.151 (42.705 to 66.273)
Other neonatal disorders	Birth prevalence - [37, 38) wks, [1500, 2000) g	Mortality	Males		35.71 (24.585 to 49.648)	20.617 (16.897 to 24.816)
Other neonatal disorders	Birth prevalence - [37, 38) wks, [1500, 2000) g	Mortality	Females		32.101 (22.457 to 43.887)	21.726 (17.833 to 25.871)
Other neonatal disorders	Birth prevalence - [37, 38) wks, [2000, 2500) g	Mortality	Males		7.301 (5.37 to 9.805)	6.923 (5.831 to 8.219)
Other neonatal disorders	Birth prevalence - [37, 38) wks, [2000, 2500) g	Mortality	Females		6.218 (4.554 to 8.169)	6.85 (5.796 to 8.005)
Other neonatal disorders	Birth prevalence - [37, 38) wks, [2500, 3000) g	Mortality	Males		1.861 (1.414 to 2.384)	2.721 (2.329 to 3.158)
Other neonatal disorders	Birth prevalence - [37, 38) wks, [2500, 3000) g	Mortality	Females		1.596 (1.24 to 1.998)	2.633 (2.261 to 3.031)
Other neonatal disorders	Birth prevalence - [37, 38) wks, [3000, 3500) g	Mortality	Males		1.139 (1 to 1.409)	1.612 (1.387 to 1.85)
Other neonatal disorders	Birth prevalence - [37, 38) wks, [3000, 3500) g	Mortality	Females		1.065 (1 to 1.288)	1.598 (1.392 to 1.819)
Other neonatal disorders	Birth prevalence - [37, 38) wks, [3500, 4000) g	Mortality	Males		1.201 (1 to 1.493)	1.282 (1.113 to 1.466)
Other neonatal disorders	Birth prevalence - [37, 38) wks, [3500, 4000) g	Mortality	Females		1.157 (1 to 1.442)	1.343 (1.153 to 1.542)
Other neonatal disorders	Birth prevalence - [37, 38) wks, [4000, 4500) g	Mortality	Males		1.825 (1.341 to 2.407)	1.136 (1.01 to 1.272)
Other neonatal disorders	Birth prevalence - [37, 38) wks, [4000, 4500) g	Mortality	Females		1.86 (1.36 to 2.46)	1.269 (1.115 to 1.42)
Other neonatal disorders	Birth prevalence - [38, 40) wks, [1000, 1500) g	Mortality	Males		98.254 (67.959 to 134.77)	49.084 (38.794 to 61.948)
Other neonatal disorders	Birth prevalence - [38, 40) wks, [1000, 1500) g	Mortality	Females		91.539 (62.769 to 127.606)	53.036 (41.348 to 65.237)
Other neonatal disorders	Birth prevalence - [38, 40) wks, [1500, 2000) g	Mortality	Males		37.449 (25.688 to 51.467)	21.477 (17.208 to 25.646)
Other neonatal disorders	Birth prevalence - [38, 40) wks, [1500, 2000) g	Mortality	Females		33.261 (23.303 to 45.061)	22.821 (18.339 to 27.874)
Other neonatal disorders	Birth prevalence - [38, 40) wks, [2000, 2500) g	Mortality	Males		7.351 (5.131 to 9.967)	7.04 (5.807 to 8.395)
Other neonatal disorders	Birth prevalence - [38, 40) wks, [2000, 2500) g	Mortality	Females		5.985 (4.383 to 7.995)	6.959 (5.782 to 8.29)
Other neonatal disorders	Birth prevalence - [38, 40) wks, [2500, 3000) g	Mortality	Males		1.549 (1.185 to 1.98)	2.518 (2.115 to 2.971)
Other neonatal disorders	Birth prevalence - [38, 40) wks, [2500, 3000) g	Mortality	Females		1.277 (1 to 1.65)	2.377 (2.015 to 2.745)
Other neonatal disorders	Birth prevalence - [38, 40) wks, [3000, 3500) g	Mortality	Males		1 (1.0 - 1.0)	1.326 (1.12 to 1.543)
Other neonatal disorders	Birth prevalence - [38, 40) wks, [3000, 3500) g	Mortality	Females		1 (1.0 - 1.0)	1.265 (1.068 to 1.45)
Other neonatal disorders	Birth prevalence - [38, 40) wks, [3500, 4000) g	Mortality	Males		1 (1.0 - 1.0)	1 (1.0 - 1.0)
Other neonatal disorders	Birth prevalence - [38, 40) wks, [3500, 4000) g	Mortality	Females		1 (1.0 - 1.0)	1 (1.0 - 1.0)
Other neonatal disorders	Birth prevalence - [38, 40) wks, [4000, 4500) g	Mortality	Males		1 (1.0 - 1.0)	1 (1.0 - 1.0)
Other neonatal disorders	Birth prevalence - [38, 40) wks, [4000, 4500) g	Mortality	Females		1 (1.0 - 1.0)	1 (1.0 - 1.0)
Other neonatal disorders	Birth prevalence - [40, 42) wks, [1500, 2000) g	Mortality	Males		43.194 (29.244 to 62.382)	21.971 (16.41 to 28.396)
Other neonatal disorders	Birth prevalence - [40, 42) wks, [1500, 2000) g	Mortality	Females		37.949 (25.539 to 53.532)	23.702 (17.857 to 30.634)
Other neonatal disorders	Birth prevalence - [40, 42) wks, [2000, 2500) g	Mortality	Males		10.162 (6.961 to 14.235)	7.875 (6.341 to 9.719)
Other neonatal disorders	Birth prevalence - [40, 42) wks, [2000, 2500) g	Mortality	Females		8.332 (5.994 to 11.407)	8.067 (6.467 to 10.043)
Other neonatal disorders	Birth prevalence - [40, 42) wks, [2500, 3000) g	Mortality	Males		2.118 (1.53 to 2.77)	2.692 (2.23 to 3.273)
Other neonatal disorders	Birth prevalence - [40, 42) wks, [2500, 3000) g	Mortality	Females		1.745 (1.289 to 2.316)	2.603 (2.134 to 3.106)
Other neonatal disorders	Birth prevalence - [40, 42) wks, [3000, 3500) g	Mortality	Males		1.002 (1 to 1.013)	1.249 (1.041 to 1.491)
Other neonatal disorders	Birth prevalence - [40, 42) wks, [3000, 3500) g	Mortality	Females		1 (1.0 - 1.0)	1.188 (1 to 1.411)
Other neonatal disorders	Birth prevalence - [40, 42) wks, [3500, 4000) g	Mortality	Males		1 (1.0 - 1.0)	1 (1.0 - 1.0)
Other neonatal disorders	Birth prevalence - [40, 42) wks, [3500, 4000) g	Mortality	Females		1 (1.0 - 1.0)	1 (1.0 - 1.0)
Other neonatal disorders	Birth prevalence - [40, 42) wks, [4000, 4500) g	Mortality	Males		1 (1.0 - 1.0)	1 (1.0 - 1.0)
Other neonatal disorders	Birth prevalence - [40, 42) wks, [4000, 4500) g	Mortality	Females		1 (1.0 - 1.0)	1 (1.0 - 1.0)
Otitis media	Birth prevalence - [0, 24) wks, [0, 500) g	Mortality	Males		877.775 (543.26 to 1246.645)	526.788 (392.055 to 676.923)
Otitis media	Birth prevalence - [0, 24) wks, [0, 500) g	Mortality	Females		857.044 (519.185 to 1227.708)	575.632 (434.418 to 737.293)
Otitis media	Birth prevalence - [0, 24) wks, [500, 1000) g	Mortality	Males		647.899 (443.535 to 881.449)	392.723 (315.689 to 486.607)
Otitis media	Birth prevalence - [0, 24) wks, [500, 1000) g	Mortality	Females		628.647 (437.861 to 854.598)	416.95 (329.573 to 516.699)
Otitis media	Birth prevalence - [24, 26) wks, [500, 1000) g	Mortality	Males		534.904 (378.653 to 707.51)	375.56 (308.336 to 447.076)
Otitis media	Birth prevalence - [24, 26) wks, [500, 1000) g	Mortality	Females		509.388 (370.575 to 690.649)	394.982 (324.03 to 477.74)
Otitis media	Birth prevalence - [26, 28) wks, [1000, 1500) g	Mortality	Males		150.649 (107.741 to 198.278)	140.284 (113.557 to 167.957)

Otitis media	Birth prevalence - [26, 28) wks, [1000, 1500) g	Mortality	Females		142.251 (102.895 to 191.587)	140.771 (114.514 to 169.631)
Otitis media	Birth prevalence - [26, 28) wks, [500, 1000) g	Mortality	Males		281.284 (198.703 to 391.898)	280.84 (226.174 to 338.336)
Otitis media	Birth prevalence - [26, 28) wks, [500, 1000) g	Mortality	Females		257.799 (185.994 to 349.394)	280.84 (225.68 to 350.2)
Otitis media	Birth prevalence - [28, 30) wks, [1000, 1500) g	Mortality	Males		88.889 (62.539 to 119.303)	87.596 (72.219 to 105.445)
Otitis media	Birth prevalence - [28, 30) wks, [1000, 1500) g	Mortality	Females		82.25 (59.418 to 109.806)	86.675 (69.965 to 104.367)
Otitis media	Birth prevalence - [28, 30) wks, [1500, 2000) g	Mortality	Males		71.963 (51.376 to 98.371)	42.294 (34.585 to 51.215)
Otitis media	Birth prevalence - [28, 30) wks, [1500, 2000) g	Mortality	Females		70.154 (48.562 to 96.294)	46.161 (38.268 to 54.906)
Otitis media	Birth prevalence - [28, 30) wks, [2000, 2500) g	Mortality	Males		65.936 (44.909 to 93.551)	23.785 (18.748 to 29.385)
Otitis media	Birth prevalence - [28, 30) wks, [2000, 2500) g	Mortality	Females		65.44 (43.751 to 96.137)	27.437 (21.369 to 33.874)
Otitis media	Birth prevalence - [28, 30) wks, [2500, 3000) g	Mortality	Males		43.647 (29.61 to 62.044)	14.185 (10.817 to 18.495)
Otitis media	Birth prevalence - [28, 30) wks, [2500, 3000) g	Mortality	Females		42.667 (28.231 to 61.897)	16.517 (12.636 to 21.355)
Otitis media	Birth prevalence - [28, 30) wks, [3000, 3500) g	Mortality	Males		23.727 (15.859 to 35.246)	8.651 (6.873 to 11.016)
Otitis media	Birth prevalence - [28, 30) wks, [3000, 3500) g	Mortality	Females		23.045 (14.473 to 35.151)	9.735 (7.511 to 12.367)
Otitis media	Birth prevalence - [28, 30) wks, [500, 1000) g	Mortality	Males		164.681 (114.331 to 232.909)	184.988 (146.715 to 225.778)
Otitis media	Birth prevalence - [28, 30) wks, [500, 1000) g	Mortality	Females		149.827 (102.011 to 207.65)	178.51 (144.736 to 220.088)
Otitis media	Birth prevalence - [30, 32) wks, [1000, 1500) g	Mortality	Males		66.567 (45.227 to 91.424)	57.036 (47.559 to 67.673)
Otitis media	Birth prevalence - [30, 32) wks, [1000, 1500) g	Mortality	Females		61.652 (42.69 to 86.655)	55.992 (46.425 to 67.165)
Otitis media	Birth prevalence - [30, 32) wks, [1500, 2000) g	Mortality	Males		43.288 (30.684 to 57.762)	26.533 (22.334 to 31.213)
Otitis media	Birth prevalence - [30, 32) wks, [1500, 2000) g	Mortality	Females		41.127 (28.837 to 54.767)	28.256 (24.051 to 32.792)
Otitis media	Birth prevalence - [30, 32) wks, [2000, 2500) g	Mortality	Males		38.672 (27.558 to 53.438)	15.362 (12.641 to 18.516)
Otitis media	Birth prevalence - [30, 32) wks, [2000, 2500) g	Mortality	Females		37.459 (26.086 to 52.166)	17.996 (14.845 to 21.529)
Otitis media	Birth prevalence - [30, 32) wks, [2500, 3000) g	Mortality	Males		33.218 (22.641 to 46.944)	10.389 (8.195 to 12.863)
Otitis media	Birth prevalence - [30, 32) wks, [2500, 3000) g	Mortality	Females		31.439 (21.157 to 45.621)	12.473 (9.64 to 15.784)
Otitis media	Birth prevalence - [30, 32) wks, [3000, 3500) g	Mortality	Males		25.451 (16.854 to 36.763)	7.153 (5.665 to 9.003)
Otitis media	Birth prevalence - [30, 32) wks, [3000, 3500) g	Mortality	Females		24.262 (15.651 to 36.395)	8.486 (6.482 to 10.824)
Otitis media	Birth prevalence - [30, 32) wks, [3500, 4000) g	Mortality	Males		20.334 (11.916 to 31.586)	4.795 (3.705 to 6.039)
Otitis media	Birth prevalence - [30, 32) wks, [3500, 4000) g	Mortality	Females		20.347 (11.635 to 32.681)	5.599 (4.199 to 7.275)
Otitis media	Birth prevalence - [30, 32) wks, [500, 1000) g	Mortality	Males		132.309 (85.99 to 193.341)	127.982 (101.66 to 159.489)
Otitis media	Birth prevalence - [30, 32) wks, [500, 1000) g	Mortality	Females		120.829 (82.341 to 172.828)	123.186 (98.696 to 152.125)
Otitis media	Birth prevalence - [32, 34) wks, [1000, 1500) g	Mortality	Males		65.39 (43.593 to 95.583)	45.581 (37.56 to 54.417)
Otitis media	Birth prevalence - [32, 34) wks, [1000, 1500) g	Mortality	Females		61.229 (40.8 to 88.303)	45.201 (37.492 to 54.267)
Otitis media	Birth prevalence - [32, 34) wks, [1500, 2000) g	Mortality	Males		32.197 (22.524 to 44.858)	19.612 (16.465 to 23.296)
Otitis media	Birth prevalence - [32, 34) wks, [1500, 2000) g	Mortality	Females		29.777 (21.026 to 40.255)	20.358 (17.072 to 23.768)
Otitis media	Birth prevalence - [32, 34) wks, [2000, 2500) g	Mortality	Males		21.018 (15.141 to 28.622)	10.433 (8.784 to 12.225)
Otitis media	Birth prevalence - [32, 34) wks, [2000, 2500) g	Mortality	Females		19.546 (14.316 to 26.034)	11.648 (9.974 to 13.507)
Otitis media	Birth prevalence - [32, 34) wks, [2500, 3000) g	Mortality	Males		18.569 (13.127 to 25.826)	7.182 (5.968 to 8.63)
Otitis media	Birth prevalence - [32, 34) wks, [2500, 3000) g	Mortality	Females		17.497 (11.66 to 24.635)	8.443 (6.726 to 10.284)
Otitis media	Birth prevalence - [32, 34) wks, [3000, 3500) g	Mortality	Males		19.012 (12.18 to 28.02)	5.618 (4.325 to 7.114)
Otitis media	Birth prevalence - [32, 34) wks, [3000, 3500) g	Mortality	Females		18.667 (12.24 to 27.524)	6.722 (5.103 to 8.697)
Otitis media	Birth prevalence - [32, 34) wks, [3500, 4000) g	Mortality	Males		20.214 (11.906 to 31.929)	4.31 (3.186 to 5.633)
Otitis media	Birth prevalence - [32, 34) wks, [3500, 4000) g	Mortality	Females		20.333 (12.064 to 33.21)	5.253 (3.959 to 6.913)
Otitis media	Birth prevalence - [34, 36) wks, [1000, 1500) g	Mortality	Males		78.66 (52.169 to 113.08)	45.338 (36.27 to 55.556)
Otitis media	Birth prevalence - [34, 36) wks, [1000, 1500) g	Mortality	Females		75.687 (49.863 to 110.089)	46.4 (38.048 to 55.902)
Otitis media	Birth prevalence - [34, 36) wks, [1500, 2000) g	Mortality	Males		31.154 (20.939 to 45.031)	18.246 (15.083 to 21.906)
Otitis media	Birth prevalence - [34, 36) wks, [1500, 2000) g	Mortality	Females		29.072 (19.6 to 42.648)	18.661 (15.638 to 22.175)
Otitis media	Birth prevalence - [34, 36) wks, [2000, 2500) g	Mortality	Males		12.404 (8.692 to 16.95)	8.006 (6.775 to 9.31)
Otitis media	Birth prevalence - [34, 36) wks, [2000, 2500) g	Mortality	Females		11.367 (7.807 to 15.547)	8.373 (7.066 to 9.703)
Otitis media	Birth prevalence - [34, 36) wks, [2500, 3000) g	Mortality	Males		7.509 (5.429 to 10.255)	4.725 (3.991 to 5.56)
Otitis media	Birth prevalence - [34, 36) wks, [2500, 3000) g	Mortality	Females		7.139 (5.091 to 9.759)	5.194 (4.342 to 6.087)
Otitis media	Birth prevalence - [34, 36) wks, [3000, 3500) g	Mortality	Males		7.828 (5.467 to 11.237)	3.717 (2.986 to 4.568)
Otitis media	Birth prevalence - [34, 36) wks, [3000, 3500) g	Mortality	Females		7.622 (5.235 to 10.584)	4.279 (3.416 to 5.301)
Otitis media	Birth prevalence - [34, 36) wks, [3500, 4000) g	Mortality	Males		10.257 (6.692 to 15.124)	3.13 (2.474 to 3.957)
Otitis media	Birth prevalence - [34, 36) wks, [3500, 4000) g	Mortality	Females		10.325 (6.495 to 15.537)	3.756 (2.877 to 4.818)
Otitis media	Birth prevalence - [34, 36) wks, [4000, 4500) g	Mortality	Males		12.928 (7.825 to 20.726)	2.467 (1.926 to 3.084)
Otitis media	Birth prevalence - [34, 36) wks, [4000, 4500) g	Mortality	Females		13.456 (8.201 to 20.893)	3.061 (2.357 to 3.889)
Otitis media	Birth prevalence - [36, 37) wks, [1000, 1500) g	Mortality	Males		94.103 (62.401 to 131.437)	48.734 (39.385 to 60.014)
Otitis media	Birth prevalence - [36, 37) wks, [1000, 1500) g	Mortality	Females		89.769 (59.869 to 126.674)	51.68 (41.336 to 63.456)
Otitis media	Birth prevalence - [36, 37) wks, [1500, 2000) g	Mortality	Males		33.586 (23.075 to 48.77)	19.52 (16.283 to 23.116)
Otitis media	Birth prevalence - [36, 37) wks, [1500, 2000) g	Mortality	Females		30.953 (20.951 to 43.853)	20.509 (17.15 to 24.286)
Otitis media	Birth prevalence - [36, 37) wks, [2000, 2500) g	Mortality	Males		8.134 (5.781 to 10.938)	7.015 (5.903 to 8.241)
Otitis media	Birth prevalence - [36, 37) wks, [2000, 2500) g	Mortality	Females		7.208 (5.183 to 9.787)	7.031 (5.911 to 8.265)
Otitis media	Birth prevalence - [36, 37) wks, [2500, 3000) g	Mortality	Males		2.741 (2.114 to 3.535)	3.149 (2.686 to 3.686)
Otitis media	Birth prevalence - [36, 37) wks, [2500, 3000) g	Mortality	Females		2.475 (1.905 to 3.109)	3.172 (2.71 to 3.666)
Otitis media	Birth prevalence - [36, 37) wks, [3000, 3500) g	Mortality	Males		2.1 (1.602 to 2.663)	2.099 (1.805 to 2.445)
Otitis media	Birth prevalence - [36, 37) wks, [3000, 3500) g	Mortality	Females		1.996 (1.532 to 2.6)	2.199 (1.895 to 2.534)
Otitis media	Birth prevalence - [36, 37) wks, [3500, 4000) g	Mortality	Males		2.563 (1.906 to 3.333)	1.751 (1.487 to 2.008)
Otitis media	Birth prevalence - [36, 37) wks, [3500, 4000) g	Mortality	Females		2.501 (1.793 to 3.34)	1.944 (1.649 to 2.275)
Otitis media	Birth prevalence - [36, 37) wks, [4000, 4500) g	Mortality	Males		3.824 (2.607 to 5.361)	1.506 (1.283 to 1.74)
Otitis media	Birth prevalence - [36, 37) wks, [4000, 4500) g	Mortality	Females		3.908 (2.626 to 5.462)	1.777 (1.481 to 2.118)
Otitis media	Birth prevalence - [37, 38) wks, [1000, 1500) g	Mortality	Males		98.111 (66.503 to 135.289)	49.903 (39.62 to 62.232)
Otitis media	Birth prevalence - [37, 38) wks, [1000, 1500) g	Mortality	Females		92.728 (63.975 to 128.565)	53.151 (42.705 to 66.273)
Otitis media	Birth prevalence - [37, 38) wks, [1500, 2000) g	Mortality	Males		35.71 (24.585 to 49.648)	20.617 (16.897 to 24.816)
Otitis media	Birth prevalence - [37, 38) wks, [1500, 2000) g	Mortality	Females		32.101 (22.457 to 43.887)	21.726 (17.833 to 25.871)
Otitis media	Birth prevalence - [37, 38) wks, [2000, 2500) g	Mortality	Males		7.301 (5.37 to 9.805)	6.923 (5.831 to 8.219)
Otitis media	Birth prevalence - [37, 38) wks, [2000, 2500) g	Mortality	Females		6.218 (4.554 to 8.169)	6.85 (5.796 to 8.005)
Otitis media	Birth prevalence - [37, 38) wks, [2500, 3000) g	Mortality	Males		1.861 (1.414 to 2.384)	2.721 (2.329 to 3.158)
Otitis media	Birth prevalence - [37, 38) wks, [2500, 3000) g	Mortality	Females		1.596 (1.24 to 1.998)	2.633 (2.261 to 3.031)
Otitis media	Birth prevalence - [37, 38) wks, [3000, 3500) g	Mortality	Males		1.139 (1 to 1.409)	1.612 (1.387 to 1.85)
Otitis media	Birth prevalence - [37, 38) wks, [3000, 3500) g	Mortality	Females		1.065 (1 to 1.288)	1.598 (1.392 to 1.819)
Otitis media	Birth prevalence - [37, 38) wks, [3500, 4000) g	Mortality	Males		1.201 (1 to 1.493)	1.282 (1.113 to 1.466)
Otitis media	Birth prevalence - [37, 38) wks, [3500, 4000) g	Mortality	Females		1.157 (1 to 1.442)	1.343 (1.153 to 1.542)
Otitis media	Birth prevalence - [37, 38) wks, [4000, 4500) g	Mortality	Males		1.825 (1.341 to 2.407)	1.136 (1.01 to 1.272)
Otitis media	Birth prevalence - [37, 38) wks, [4000, 4500) g	Mortality	Females		1.86 (1.36 to 2.46)	1.269 (1.115 to 1.42)
Otitis media	Birth prevalence - [38, 40) wks, [1000, 1500) g	Mortality	Males		98.254 (67.959 to 134.77)	49.084 (38.794 to 61.948)
Otitis media	Birth prevalence - [38, 40) wks, [1000, 1500) g	Mortality	Females		91.539 (62.769 to 127.606)	53.036 (41.348 to 65.237)
Otitis media	Birth prevalence - [38, 40) wks, [1500, 2000) g	Mortality	Males		37.449 (25.688 to 51.467)	21.477 (17.208 to 25.646)
Otitis media	Birth prevalence - [38, 40) wks, [1500, 2000) g	Mortality	Females		33.261 (23.303 to 45.061)	22.821 (18.339 to 27.874)
Otitis media	Birth prevalence - [38, 40) wks, [2000, 2500) g	Mortality	Males		7.351 (5.131 to 9.967)	7.04 (5.807 to 8.395)
Otitis media	Birth prevalence - [38, 40) wks, [2000, 2500) g	Mortality	Females		5.985 (4.383 to 7.995)	6.959 (5.782 to 8.29)
Otitis media	Birth prevalence - [38, 40) wks, [2500, 3000) g	Mortality	Males		1.549 (1.185 to 1.98)	2.518 (2.115 to 2.971)
Otitis media	Birth prevalence - [38, 40) wks, [2500, 3000) g	Mortality	Females		1.277 (1 to 1.65)	2.377 (2.015 to 2.745)
Otitis media	Birth prevalence - [38, 40) wks, [3000, 3500) g	Mortality	Males		1 (1.0 - 1.0)	1.326 (1.12 to 1.543)
Otitis media	Birth prevalence - [38, 40) wks, [3000, 3500) g	Mortality	Females		1 (1.0 - 1.0)	1.265 (1.068 to 1.45)
Otitis media	Birth prevalence - [38, 40) wks, [3500, 4000) g	Mortality	Males		1 (1.0 - 1.0)	1 (1.0 - 1.0)
Otitis media	Birth prevalence - [38, 40) wks, [3500, 4000) g	Mortality	Females		1 (1.0 - 1.0)	1 (1.0 - 1.0)
Otitis media	Birth prevalence - [38, 40) wks, [4000, 4500) g	Mortality	Males		1 (1.0 - 1.0)	1 (1.0 - 1.0)
Otitis media	Birth prevalence - [38, 40) wks, [4000, 4500) g	Mortality	Females		1 (1.0 - 1.0)	1 (1.0 - 1.0)
Otitis media	Birth prevalence - [40, 42) wks, [1500, 2000) g	Mortality	Males		43.194 (29.244 to 62.382)	21.971 (16.41 to 28.396)
Otitis media	Birth prevalence - [40, 42) wks, [1500, 2000) g	Mortality	Females		37.949 (25.539 to 53.532)	23.702 (17.857 to 30.634)
Otitis media	Birth prevalence - [40, 42) wks, [2000, 2500) g	Mortality	Males		10.162 (6.961 to 14.235)	7.875 (6.341 to 9.719)
Otitis media	Birth prevalence - [40, 42) wks, [2000, 2500) g	Mortality	Females		8.332 (5.994 to 11.407)	8.067 (6.467 to 10.043)
Otitis media	Birth prevalence - [40, 42) wks, [2500, 3000) g	Mortality	Males		2.118 (1.53 to 2.77)	2.692 (2.23 to 3.273)
Otitis media	Birth prevalence - [40, 42) wks, [2500, 3000) g	Mortality	Females		1.745 (1.289 to 2.316)	2.603 (2.134 to 3.106)
Otitis media	Birth prevalence - [40, 42) wks, [3000, 3500) g	Mortality	Males		1.002 (1 to 1.013)	1.249 (1.041 to 1.491)
Otitis media	Birth prevalence - [40, 42) wks, [3000, 3500) g	Mortality	Females		1 (1.0 - 1.0)	1.188 (1 to 1.411)
Otitis media	Birth prevalence - [40, 42) wks, [3500, 4000) g	Mortality	Males		1 (1.0 - 1.0)	1 (1.0 - 1.0)
Otitis media	Birth prevalence - [40, 42) wks, [3500, 4000) g	Mortality	Females		1 (1.0 - 1.0)	1 (1.0 - 1.0)
Otitis media	Birth prevalence - [40, 42) wks, [4000, 4500) g	Mortality	Males		1 (1.0 - 1.0)	1 (1.0 - 1.0)
Otitis media	Birth prevalence - [40, 42) wks, [4000, 4500) g	Mortality	Females		1 (1.0 - 1.0)	1 (1.0 - 1.0)
Sudden infant death syndrome	Birth prevalence - [0, 24) wks, [0, 500) g	Mortality	Males		877.775 (543.26 to 1246.645)	526.788 (392.055 to 676.923)
Sudden infant death syndrome	Birth prevalence - [0, 24) wks, [0, 500) g	Mortality	Females		857.044 (519.185 to 1227.708)	575.632 (434.418 to 737.293)
Sudden infant death syndrome	Birth prevalence - [0, 24) wks, [500, 1000) g	Mortality	Males		647.899 (443.535 to 881.449)	392.723 (315.689 to 486.607)
Sudden infant death syndrome	Birth prevalence - [0, 24) wks, [500, 1000) g	Mortality	Females		628.647 (437.861 to 854.598)	416.95 (329.573 to 516.699)
Sudden infant death syndrome	Birth prevalence - [24, 26) wks, [500, 1000) g	Mortality	Males		534.904 (378.653 to 707.51)	375.56 (308.336 to 447.076)
Sudden infant death syndrome	Birth prevalence - [24, 26) wks, [500, 1000) g	Mortality	Females		509.388 (370.575 to 690.649)	394.982 (324.03 to 477.74)
Sudden infant death syndrome	Birth prevalence - [26, 28) wks, [1000, 1500) g	Mortality	Males		150.649 (107.741 to 198.278)	140.284 (113.557 to 167.957)
Sudden infant death syndrome	Birth prevalence - [26, 28) wks, [1000, 1500) g	Mortality	Females		142.251 (102.895 to 191.587)	140.771 (114.514 to 169.631)
Sudden infant death syndrome	Birth prevalence - [26, 28) wks, [500, 1000) g	Mortality	Males		281.284 (198.703 to 391.898)	280.84 (226.174 to 338.336)
Sudden infant death syndrome	Birth prevalence - [26, 28) wks, [500, 1000) g	Mortality	Females		257.799 (185.994 to 349.394)	280.84 (225.68 to 350.2)
Sudden infant death syndrome	Birth prevalence - [28, 30) wks, [1000, 1500) g	Mortality	Males		88.889 (62.539 to 119.303)	87.596 (72.219 to 105.445)
Sudden infant death syndrome	Birth prevalence - [28, 30) wks, [1000, 1500) g	Mortality	Females		82.25 (59.418 to 109.806)	86.675 (69.965 to 104.367)
Sudden infant death syndrome	Birth prevalence - [28, 30) wks, [1500, 2000) g	Mortality	Males		71.963 (51.376 to 98.371)	42.294 (34.585 to 51.215)
Sudden infant death syndrome	Birth prevalence - [28, 30) wks, [1500, 2000) g	Mortality	Females		70.154 (48.562 to 96.294)	46.161 (38.268 to 54.906)

Sudden infant death syndrome	Birth prevalence - [28, 30) wks, [2000, 2500) g	Mortality	Males		65.936 (44.909 to 93.551)	23.785 (18.748 to 29.385)
Sudden infant death syndrome	Birth prevalence - [28, 30) wks, [2000, 2500) g	Mortality	Females		65.44 (43.751 to 96.137)	27.437 (21.369 to 33.874)
Sudden infant death syndrome	Birth prevalence - [28, 30) wks, [2500, 3000) g	Mortality	Males		43.647 (29.61 to 62.044)	14.185 (10.817 to 18.495)
Sudden infant death syndrome	Birth prevalence - [28, 30) wks, [2500, 3000) g	Mortality	Females		42.667 (28.231 to 61.897)	16.517 (12.636 to 21.355)
Sudden infant death syndrome	Birth prevalence - [28, 30) wks, [3000, 3500) g	Mortality	Males		23.727 (15.859 to 35.246)	8.651 (6.873 to 11.016)
Sudden infant death syndrome	Birth prevalence - [28, 30) wks, [3000, 3500) g	Mortality	Females		23.045 (14.473 to 35.151)	9.735 (7.511 to 12.367)
Sudden infant death syndrome	Birth prevalence - [28, 30) wks, [500, 1000) g	Mortality	Males		164.681 (114.331 to 232.909)	184.988 (146.715 to 225.778)
Sudden infant death syndrome	Birth prevalence - [28, 30) wks, [500, 1000) g	Mortality	Females		149.827 (102.011 to 207.65)	178.51 (144.736 to 220.088)
Sudden infant death syndrome	Birth prevalence - [30, 32) wks, [1000, 1500) g	Mortality	Males		66.567 (45.227 to 91.424)	57.036 (47.559 to 67.673)
Sudden infant death syndrome	Birth prevalence - [30, 32) wks, [1000, 1500) g	Mortality	Females		61.652 (42.69 to 86.655)	55.992 (46.425 to 67.165)
Sudden infant death syndrome	Birth prevalence - [30, 32) wks, [1500, 2000) g	Mortality	Males		43.288 (30.684 to 57.762)	26.533 (22.334 to 31.213)
Sudden infant death syndrome	Birth prevalence - [30, 32) wks, [1500, 2000) g	Mortality	Females		41.127 (28.837 to 54.767)	28.256 (24.051 to 32.792)
Sudden infant death syndrome	Birth prevalence - [30, 32) wks, [2000, 2500) g	Mortality	Males		38.672 (27.558 to 53.438)	15.362 (12.641 to 18.516)
Sudden infant death syndrome	Birth prevalence - [30, 32) wks, [2000, 2500) g	Mortality	Females		37.459 (26.086 to 52.166)	17.996 (14.845 to 21.529)
Sudden infant death syndrome	Birth prevalence - [30, 32) wks, [2500, 3000) g	Mortality	Males		33.218 (22.641 to 46.944)	10.389 (8.195 to 12.863)
Sudden infant death syndrome	Birth prevalence - [30, 32) wks, [2500, 3000) g	Mortality	Females		31.439 (21.157 to 45.621)	12.473 (9.64 to 15.784)
Sudden infant death syndrome	Birth prevalence - [30, 32) wks, [3000, 3500) g	Mortality	Males		25.451 (16.854 to 36.763)	7.153 (5.665 to 9.003)
Sudden infant death syndrome	Birth prevalence - [30, 32) wks, [3000, 3500) g	Mortality	Females		24.262 (15.651 to 36.395)	8.486 (6.482 to 10.824)
Sudden infant death syndrome	Birth prevalence - [30, 32) wks, [3500, 4000) g	Mortality	Males		20.334 (11.916 to 31.586)	4.795 (3.705 to 6.039)
Sudden infant death syndrome	Birth prevalence - [30, 32) wks, [3500, 4000) g	Mortality	Females		20.347 (11.635 to 32.681)	5.599 (4.199 to 7.275)
Sudden infant death syndrome	Birth prevalence - [30, 32) wks, [500, 1000) g	Mortality	Males		132.309 (85.99 to 193.341)	127.982 (101.66 to 159.489)
Sudden infant death syndrome	Birth prevalence - [30, 32) wks, [500, 1000) g	Mortality	Females		120.829 (82.341 to 172.828)	123.186 (98.696 to 152.125)
Sudden infant death syndrome	Birth prevalence - [32, 34) wks, [1000, 1500) g	Mortality	Males		65.39 (43.593 to 95.583)	45.581 (37.56 to 54.417)
Sudden infant death syndrome	Birth prevalence - [32, 34) wks, [1000, 1500) g	Mortality	Females		61.229 (40.8 to 88.303)	45.201 (37.492 to 54.267)
Sudden infant death syndrome	Birth prevalence - [32, 34) wks, [1500, 2000) g	Mortality	Males		32.197 (22.524 to 44.858)	19.612 (16.465 to 23.296)
Sudden infant death syndrome	Birth prevalence - [32, 34) wks, [1500, 2000) g	Mortality	Females		29.777 (21.026 to 40.255)	20.358 (17.072 to 23.768)
Sudden infant death syndrome	Birth prevalence - [32, 34) wks, [2000, 2500) g	Mortality	Males		21.018 (15.141 to 28.622)	10.433 (8.784 to 12.225)
Sudden infant death syndrome	Birth prevalence - [32, 34) wks, [2000, 2500) g	Mortality	Females		19.546 (14.316 to 26.034)	11.648 (9.974 to 13.507)
Sudden infant death syndrome	Birth prevalence - [32, 34) wks, [2500, 3000) g	Mortality	Males		18.569 (13.127 to 25.826)	7.182 (5.968 to 8.63)
Sudden infant death syndrome	Birth prevalence - [32, 34) wks, [2500, 3000) g	Mortality	Females		17.497 (11.66 to 24.635)	8.443 (6.726 to 10.284)
Sudden infant death syndrome	Birth prevalence - [32, 34) wks, [3000, 3500) g	Mortality	Males		19.012 (12.18 to 28.02)	5.618 (4.325 to 7.114)
Sudden infant death syndrome	Birth prevalence - [32, 34) wks, [3000, 3500) g	Mortality	Females		18.667 (12.24 to 27.524)	6.722 (5.103 to 8.697)
Sudden infant death syndrome	Birth prevalence - [32, 34) wks, [3500, 4000) g	Mortality	Males		20.214 (11.906 to 31.929)	4.31 (3.186 to 5.633)
Sudden infant death syndrome	Birth prevalence - [32, 34) wks, [3500, 4000) g	Mortality	Females		20.333 (12.064 to 33.21)	5.253 (3.959 to 6.913)
Sudden infant death syndrome	Birth prevalence - [34, 36) wks, [1000, 1500) g	Mortality	Males		78.66 (52.169 to 113.08)	45.338 (36.27 to 55.556)
Sudden infant death syndrome	Birth prevalence - [34, 36) wks, [1000, 1500) g	Mortality	Females		75.687 (49.863 to 110.089)	46.4 (38.048 to 55.902)
Sudden infant death syndrome	Birth prevalence - [34, 36) wks, [1500, 2000) g	Mortality	Males		31.154 (20.939 to 45.031)	18.246 (15.083 to 21.906)
Sudden infant death syndrome	Birth prevalence - [34, 36) wks, [1500, 2000) g	Mortality	Females		29.072 (19.6 to 42.648)	18.661 (15.638 to 22.175)
Sudden infant death syndrome	Birth prevalence - [34, 36) wks, [2000, 2500) g	Mortality	Males		12.404 (8.692 to 16.95)	8.006 (6.775 to 9.31)
Sudden infant death syndrome	Birth prevalence - [34, 36) wks, [2000, 2500) g	Mortality	Females		11.367 (7.807 to 15.547)	8.373 (7.066 to 9.703)
Sudden infant death syndrome	Birth prevalence - [34, 36) wks, [2500, 3000) g	Mortality	Males		7.509 (5.429 to 10.255)	4.725 (3.991 to 5.56)
Sudden infant death syndrome	Birth prevalence - [34, 36) wks, [2500, 3000) g	Mortality	Females		7.139 (5.091 to 9.759)	5.194 (4.342 to 6.087)
Sudden infant death syndrome	Birth prevalence - [34, 36) wks, [3000, 3500) g	Mortality	Males		7.828 (5.467 to 11.237)	3.717 (2.986 to 4.568)
Sudden infant death syndrome	Birth prevalence - [34, 36) wks, [3000, 3500) g	Mortality	Females		7.622 (5.235 to 10.584)	4.279 (3.416 to 5.301)
Sudden infant death syndrome	Birth prevalence - [34, 36) wks, [3500, 4000) g	Mortality	Males		10.257 (6.692 to 15.124)	3.13 (2.474 to 3.957)
Sudden infant death syndrome	Birth prevalence - [34, 36) wks, [3500, 4000) g	Mortality	Females		10.325 (6.495 to 15.537)	3.756 (2.877 to 4.818)
Sudden infant death syndrome	Birth prevalence - [34, 36) wks, [4000, 4500) g	Mortality	Males		12.928 (7.825 to 20.726)	2.467 (1.926 to 3.084)
Sudden infant death syndrome	Birth prevalence - [34, 36) wks, [4000, 4500) g	Mortality	Females		13.456 (8.201 to 20.893)	3.061 (2.357 to 3.889)
Sudden infant death syndrome	Birth prevalence - [36, 37) wks, [1000, 1500) g	Mortality	Males		94.103 (62.401 to 131.437)	48.734 (39.385 to 60.014)
Sudden infant death syndrome	Birth prevalence - [36, 37) wks, [1000, 1500) g	Mortality	Females		89.769 (59.869 to 126.674)	51.68 (41.336 to 63.456)
Sudden infant death syndrome	Birth prevalence - [36, 37) wks, [1500, 2000) g	Mortality	Males		33.586 (23.075 to 48.77)	19.52 (16.283 to 23.116)
Sudden infant death syndrome	Birth prevalence - [36, 37) wks, [1500, 2000) g	Mortality	Females		30.953 (20.951 to 43.853)	20.509 (17.15 to 24.286)
Sudden infant death syndrome	Birth prevalence - [36, 37) wks, [2000, 2500) g	Mortality	Males		8.134 (5.781 to 10.938)	7.015 (5.903 to 8.241)
Sudden infant death syndrome	Birth prevalence - [36, 37) wks, [2000, 2500) g	Mortality	Females		7.208 (5.183 to 9.787)	7.031 (5.911 to 8.265)
Sudden infant death syndrome	Birth prevalence - [36, 37) wks, [2500, 3000) g	Mortality	Males		2.741 (2.114 to 3.535)	3.149 (2.686 to 3.686)
Sudden infant death syndrome	Birth prevalence - [36, 37) wks, [2500, 3000) g	Mortality	Females		2.475 (1.905 to 3.109)	3.172 (2.71 to 3.666)
Sudden infant death syndrome	Birth prevalence - [36, 37) wks, [3000, 3500) g	Mortality	Males		2.1 (1.602 to 2.663)	2.099 (1.805 to 2.445)
Sudden infant death syndrome	Birth prevalence - [36, 37) wks, [3000, 3500) g	Mortality	Females		1.996 (1.532 to 2.6)	2.199 (1.895 to 2.534)
Sudden infant death syndrome	Birth prevalence - [36, 37) wks, [3500, 4000) g	Mortality	Males		2.563 (1.906 to 3.333)	1.751 (1.487 to 2.008)
Sudden infant death syndrome	Birth prevalence - [36, 37) wks, [3500, 4000) g	Mortality	Females		2.501 (1.793 to 3.34)	1.944 (1.649 to 2.275)
Sudden infant death syndrome	Birth prevalence - [36, 37) wks, [4000, 4500) g	Mortality	Males		3.824 (2.607 to 5.361)	1.506 (1.283 to 1.74)
Sudden infant death syndrome	Birth prevalence - [36, 37) wks, [4000, 4500) g	Mortality	Females		3.908 (2.626 to 5.462)	1.777 (1.481 to 2.118)
Sudden infant death syndrome	Birth prevalence - [37, 38) wks, [1000, 1500) g	Mortality	Males		98.111 (66.503 to 135.289)	49.903 (39.62 to 62.232)
Sudden infant death syndrome	Birth prevalence - [37, 38) wks, [1000, 1500) g	Mortality	Females		92.728 (63.975 to 128.565)	53.151 (42.705 to 66.273)
Sudden infant death syndrome	Birth prevalence - [37, 38) wks, [1500, 2000) g	Mortality	Males		35.71 (24.585 to 49.648)	20.617 (16.897 to 24.816)
Sudden infant death syndrome	Birth prevalence - [37, 38) wks, [1500, 2000) g	Mortality	Females		32.101 (22.457 to 43.887)	21.726 (17.833 to 25.871)
Sudden infant death syndrome	Birth prevalence - [37, 38) wks, [2000, 2500) g	Mortality	Males		7.301 (5.37 to 9.805)	6.923 (5.831 to 8.219)
Sudden infant death syndrome	Birth prevalence - [37, 38) wks, [2000, 2500) g	Mortality	Females		6.218 (4.554 to 8.169)	6.85 (5.796 to 8.005)
Sudden infant death syndrome	Birth prevalence - [37, 38) wks, [2500, 3000) g	Mortality	Males		1.861 (1.414 to 2.384)	2.721 (2.329 to 3.158)
Sudden infant death syndrome	Birth prevalence - [37, 38) wks, [2500, 3000) g	Mortality	Females		1.596 (1.24 to 1.998)	2.633 (2.261 to 3.031)
Sudden infant death syndrome	Birth prevalence - [37, 38) wks, [3000, 3500) g	Mortality	Males		1.139 (1 to 1.409)	1.612 (1.387 to 1.85)
Sudden infant death syndrome	Birth prevalence - [37, 38) wks, [3000, 3500) g	Mortality	Females		1.065 (1 to 1.288)	1.598 (1.392 to 1.819)
Sudden infant death syndrome	Birth prevalence - [37, 38) wks, [3500, 4000) g	Mortality	Males		1.201 (1 to 1.493)	1.282 (1.113 to 1.466)
Sudden infant death syndrome	Birth prevalence - [37, 38) wks, [3500, 4000) g	Mortality	Females		1.157 (1 to 1.442)	1.343 (1.153 to 1.542)
Sudden infant death syndrome	Birth prevalence - [37, 38) wks, [4000, 4500) g	Mortality	Males		1.825 (1.341 to 2.407)	1.136 (1.01 to 1.272)
Sudden infant death syndrome	Birth prevalence - [37, 38) wks, [4000, 4500) g	Mortality	Females		1.86 (1.36 to 2.46)	1.269 (1.115 to 1.42)
Sudden infant death syndrome	Birth prevalence - [38, 40) wks, [1000, 1500) g	Mortality	Males		98.254 (67.959 to 134.77)	49.084 (38.794 to 61.948)
Sudden infant death syndrome	Birth prevalence - [38, 40) wks, [1000, 1500) g	Mortality	Females		91.539 (62.769 to 127.606)	53.036 (41.348 to 65.237)
Sudden infant death syndrome	Birth prevalence - [38, 40) wks, [1500, 2000) g	Mortality	Males		37.449 (25.688 to 51.467)	21.477 (17.208 to 25.646)
Sudden infant death syndrome	Birth prevalence - [38, 40) wks, [1500, 2000) g	Mortality	Females		33.261 (23.303 to 45.061)	22.821 (18.339 to 27.874)
Sudden infant death syndrome	Birth prevalence - [38, 40) wks, [2000, 2500) g	Mortality	Males		7.351 (5.131 to 9.967)	7.04 (5.807 to 8.395)
Sudden infant death syndrome	Birth prevalence - [38, 40) wks, [2000, 2500) g	Mortality	Females		5.985 (4.383 to 7.995)	6.959 (5.782 to 8.29)
Sudden infant death syndrome	Birth prevalence - [38, 40) wks, [2500, 3000) g	Mortality	Males		1.549 (1.185 to 1.98)	2.518 (2.115 to 2.971)
Sudden infant death syndrome	Birth prevalence - [38, 40) wks, [2500, 3000) g	Mortality	Females		1.277 (1 to 1.65)	2.377 (2.015 to 2.745)
Sudden infant death syndrome	Birth prevalence - [38, 40) wks, [3000, 3500) g	Mortality	Males		1 (1.0 - 1.0)	1.326 (1.12 to 1.543)
Sudden infant death syndrome	Birth prevalence - [38, 40) wks, [3000, 3500) g	Mortality	Females		1 (1.0 - 1.0)	1.265 (1.068 to 1.45)
Sudden infant death syndrome	Birth prevalence - [38, 40) wks, [3500, 4000) g	Mortality	Males		1 (1.0 - 1.0)	1 (1.0 - 1.0)
Sudden infant death syndrome	Birth prevalence - [38, 40) wks, [3500, 4000) g	Mortality	Females		1 (1.0 - 1.0)	1 (1.0 - 1.0)
Sudden infant death syndrome	Birth prevalence - [38, 40) wks, [4000, 4500) g	Mortality	Males		1 (1.0 - 1.0)	1 (1.0 - 1.0)
Sudden infant death syndrome	Birth prevalence - [38, 40) wks, [4000, 4500) g	Mortality	Females		1 (1.0 - 1.0)	1 (1.0 - 1.0)
Sudden infant death syndrome	Birth prevalence - [40, 42) wks, [1500, 2000) g	Mortality	Males		43.194 (29.244 to 62.382)	21.971 (16.41 to 28.396)
Sudden infant death syndrome	Birth prevalence - [40, 42) wks, [1500, 2000) g	Mortality	Females		37.949 (25.539 to 53.532)	23.702 (17.857 to 30.634)
Sudden infant death syndrome	Birth prevalence - [40, 42) wks, [2000, 2500) g	Mortality	Males		10.162 (6.961 to 14.235)	7.875 (6.341 to 9.719)
Sudden infant death syndrome	Birth prevalence - [40, 42) wks, [2000, 2500) g	Mortality	Females		8.332 (5.994 to 11.407)	8.067 (6.467 to 10.043)
Sudden infant death syndrome	Birth prevalence - [40, 42) wks, [2500, 3000) g	Mortality	Males		2.118 (1.53 to 2.77)	2.692 (2.23 to 3.273)
Sudden infant death syndrome	Birth prevalence - [40, 42) wks, [2500, 3000) g	Mortality	Females		1.745 (1.289 to 2.316)	2.603 (2.134 to 3.106)
Sudden infant death syndrome	Birth prevalence - [40, 42) wks, [3000, 3500) g	Mortality	Males		1.002 (1 to 1.013)	1.249 (1.041 to 1.491)
Sudden infant death syndrome	Birth prevalence - [40, 42) wks, [3000, 3500) g	Mortality	Females		1 (1.0 - 1.0)	1.188 (1 to 1.411)
Sudden infant death syndrome	Birth prevalence - [40, 42) wks, [3500, 4000) g	Mortality	Males		1 (1.0 - 1.0)	1 (1.0 - 1.0)
Sudden infant death syndrome	Birth prevalence - [40, 42) wks, [3500, 4000) g	Mortality	Females		1 (1.0 - 1.0)	1 (1.0 - 1.0)
Sudden infant death syndrome	Birth prevalence - [40, 42) wks, [4000, 4500) g	Mortality	Males		1 (1.0 - 1.0)	1 (1.0 - 1.0)
Sudden infant death syndrome	Birth prevalence - [40, 42) wks, [4000, 4500) g	Mortality	Females		1 (1.0 - 1.0)	1 (1.0 - 1.0)
Upper respiratory infections	Birth prevalence - [0, 24) wks, [0, 500) g	Mortality	Males		877.775 (543.26 to 1246.645)	526.788 (392.055 to 676.923)
Upper respiratory infections	Birth prevalence - [0, 24) wks, [0, 500) g	Mortality	Females		857.044 (519.185 to 1227.708)	575.632 (434.418 to 737.293)
Upper respiratory infections	Birth prevalence - [0, 24) wks, [500, 1000) g	Mortality	Males		647.899 (443.535 to 881.449)	392.723 (315.689 to 486.607)
Upper respiratory infections	Birth prevalence - [0, 24) wks, [500, 1000) g	Mortality	Females		628.647 (437.861 to 854.598)	416.95 (329.573 to 516.699)
Upper respiratory infections	Birth prevalence - [24, 26) wks, [500, 1000) g	Mortality	Males		534.904 (378.653 to 707.51)	375.56 (308.336 to 447.076)
Upper respiratory infections	Birth prevalence - [24, 26) wks, [500, 1000) g	Mortality	Females		509.388 (370.575 to 690.649)	394.982 (324.03 to 477.74)
Upper respiratory infections	Birth prevalence - [26, 28) wks, [1000, 1500) g	Mortality	Males		150.649 (107.741 to 198.278)	140.284 (113.557 to 167.957)
Upper respiratory infections	Birth prevalence - [26, 28) wks, [1000, 1500) g	Mortality	Females		142.251 (102.895 to 191.587)	140.771 (114.514 to 169.631)
Upper respiratory infections	Birth prevalence - [26, 28) wks, [500, 1000) g	Mortality	Males		281.284 (198.703 to 391.898)	280.84 (226.174 to 338.336)
Upper respiratory infections	Birth prevalence - [26, 28) wks, [500, 1000) g	Mortality	Females		257.799 (185.994 to 349.394)	280.84 (225.68 to 350.2)
Upper respiratory infections	Birth prevalence - [28, 30) wks, [1000, 1500) g	Mortality	Males		88.889 (62.539 to 119.303)	87.596 (72.219 to 105.445)
Upper respiratory infections	Birth prevalence - [28, 30) wks, [1000, 1500) g	Mortality	Females		82.25 (59.418 to 109.806)	86.675 (69.965 to 104.367)
Upper respiratory infections	Birth prevalence - [28, 30) wks, [1500, 2000) g	Mortality	Males		71.963 (51.376 to 98.371)	42.294 (34.585 to 51.215)
Upper respiratory infections	Birth prevalence - [28, 30) wks, [1500, 2000) g	Mortality	Females		70.154 (48.562 to 96.294)	46.161 (38.268 to 54.906)
Upper respiratory infections	Birth prevalence - [28, 30) wks, [2000, 2500) g	Mortality	Males		65.936 (44.909 to 93.551)	23.785 (18.748 to 29.385)
Upper respiratory infections	Birth prevalence - [28, 30) wks, [2000, 2500) g	Mortality	Females		65.44 (43.751 to 96.137)	27.437 (21.369 to 33.874)
Upper respiratory infections	Birth prevalence - [28, 30) wks, [2500, 3000) g	Mortality	Males		43.647 (29.61 to 62.044)	14.185 (10.817 to 18.495)
Upper respiratory infections	Birth prevalence - [28, 30) wks, [2500, 3000) g	Mortality	Females		42.667 (28.231 to 61.897)	16.517 (12.636 to 21.355)
Upper respiratory infections	Birth prevalence - [28, 30) wks, [3000, 3500) g	Mortality	Males		23.727 (15.859 to 35.246)	8.651 (6.873 to 11.016)
Upper respiratory infections	Birth prevalence - [28, 30) wks, [3000, 3500) g	Mortality	Females		23.045 (14.473 to 35.151)	9.735 (7.511 to 12.367)
Upper respiratory infections	Birth prevalence - [28, 30) wks, [500, 1000) g	Mortality	Males		164.681 (114.331 to 232.909)	184.988 (146.715 to 225.778)

Upper respiratory infections	Birth prevalence - [28, 30) wks, [500, 1000) g	Mortality	Females		149.827 (102.011 to 207.65)	178.51 (144.736 to 220.088)																					
Upper respiratory infections	Birth prevalence - [30, 32) wks, [1000, 1500) g	Mortality	Males		66.567 (45.227 to 91.424)	57.036 (47.559 to 67.673)																					
Upper respiratory infections	Birth prevalence - [30, 32) wks, [1000, 1500) g	Mortality	Females		61.652 (42.69 to 86.655)	55.992 (46.425 to 67.165)																					
Upper respiratory infections	Birth prevalence - [30, 32) wks, [1500, 2000) g	Mortality	Males		43.288 (30.684 to 57.762)	26.533 (22.334 to 31.213)																					
Upper respiratory infections	Birth prevalence - [30, 32) wks, [1500, 2000) g	Mortality	Females		41.127 (28.837 to 54.767)	28.256 (24.051 to 32.792)																					
Upper respiratory infections	Birth prevalence - [30, 32) wks, [2000, 2500) g	Mortality	Males		38.672 (27.558 to 53.438)	15.362 (12.641 to 18.516)																					
Upper respiratory infections	Birth prevalence - [30, 32) wks, [2000, 2500) g	Mortality	Females		37.459 (26.086 to 52.166)	17.996 (14.845 to 21.529)																					
Upper respiratory infections	Birth prevalence - [30, 32) wks, [2500, 3000) g	Mortality	Males		33.218 (22.641 to 46.944)	10.389 (8.195 to 12.863)																					
Upper respiratory infections	Birth prevalence - [30, 32) wks, [2500, 3000) g	Mortality	Females		31.439 (21.157 to 45.621)	12.473 (9.64 to 15.784)																					
Upper respiratory infections	Birth prevalence - [30, 32) wks, [3000, 3500) g	Mortality	Males		25.451 (16.854 to 36.763)	7.153 (5.665 to 9.003)																					
Upper respiratory infections	Birth prevalence - [30, 32) wks, [3000, 3500) g	Mortality	Females		24.262 (15.651 to 36.395)	8.486 (6.482 to 10.824)																					
Upper respiratory infections	Birth prevalence - [30, 32) wks, [3500, 4000) g	Mortality	Males		20.334 (11.916 to 31.586)	4.795 (3.705 to 6.039)																					
Upper respiratory infections	Birth prevalence - [30, 32) wks, [3500, 4000) g	Mortality	Females		20.347 (11.635 to 32.681)	5.599 (4.199 to 7.275)																					
Upper respiratory infections	Birth prevalence - [30, 32) wks, [500, 1000) g	Mortality	Males		132.309 (85.99 to 193.341)	127.982 (101.66 to 159.489)																					
Upper respiratory infections	Birth prevalence - [30, 32) wks, [500, 1000) g	Mortality	Females		120.829 (82.341 to 172.828)	123.186 (98.696 to 152.125)																					
Upper respiratory infections	Birth prevalence - [32, 34) wks, [1000, 1500) g	Mortality	Males		65.39 (43.593 to 95.583)	45.581 (37.56 to 54.417)																					
Upper respiratory infections	Birth prevalence - [32, 34) wks, [1000, 1500) g	Mortality	Females		61.229 (40.8 to 88.303)	45.201 (37.492 to 54.267)																					
Upper respiratory infections	Birth prevalence - [32, 34) wks, [1500, 2000) g	Mortality	Males		32.197 (22.524 to 44.858)	19.612 (16.465 to 23.296)																					
Upper respiratory infections	Birth prevalence - [32, 34) wks, [1500, 2000) g	Mortality	Females		29.777 (21.026 to 40.255)	20.358 (17.072 to 23.768)																					
Upper respiratory infections	Birth prevalence - [32, 34) wks, [2000, 2500) g	Mortality	Males		21.018 (15.141 to 28.622)	10.433 (8.784 to 12.225)																					
Upper respiratory infections	Birth prevalence - [32, 34) wks, [2000, 2500) g	Mortality	Females		19.546 (14.316 to 26.034)	11.648 (9.974 to 13.507)																					
Upper respiratory infections	Birth prevalence - [32, 34) wks, [2500, 3000) g	Mortality	Males		18.569 (13.127 to 25.826)	7.182 (5.968 to 8.63)																					
Upper respiratory infections	Birth prevalence - [32, 34) wks, [2500, 3000) g	Mortality	Females		17.497 (11.66 to 24.635)	8.443 (6.726 to 10.284)																					
Upper respiratory infections	Birth prevalence - [32, 34) wks, [3000, 3500) g	Mortality	Males		19.012 (12.18 to 28.02)	5.618 (4.325 to 7.114)																					
Upper respiratory infections	Birth prevalence - [32, 34) wks, [3000, 3500) g	Mortality	Females		18.667 (12.24 to 27.524)	6.722 (5.103 to 8.697)																					
Upper respiratory infections	Birth prevalence - [32, 34) wks, [3500, 4000) g	Mortality	Males		20.214 (11.906 to 31.929)	4.31 (3.186 to 5.633)																					
Upper respiratory infections	Birth prevalence - [32, 34) wks, [3500, 4000) g	Mortality	Females		20.333 (12.064 to 33.21)	5.253 (3.959 to 6.913)																					
Upper respiratory infections	Birth prevalence - [34, 36) wks, [1000, 1500) g	Mortality	Males		78.66 (52.169 to 113.08)	45.338 (36.27 to 55.556)																					
Upper respiratory infections	Birth prevalence - [34, 36) wks, [1000, 1500) g	Mortality	Females		75.687 (49.863 to 110.089)	46.4 (38.048 to 55.902)																					
Upper respiratory infections	Birth prevalence - [34, 36) wks, [1500, 2000) g	Mortality	Males		31.154 (20.939 to 45.031)	18.246 (15.083 to 21.906)																					
Upper respiratory infections	Birth prevalence - [34, 36) wks, [1500, 2000) g	Mortality	Females		29.072 (19.6 to 42.648)	18.661 (15.638 to 22.175)																					
Upper respiratory infections	Birth prevalence - [34, 36) wks, [2000, 2500) g	Mortality	Males		12.404 (8.692 to 16.95)	8.006 (6.775 to 9.31)																					
Upper respiratory infections	Birth prevalence - [34, 36) wks, [2000, 2500) g	Mortality	Females		11.367 (7.807 to 15.547)	8.373 (7.066 to 9.703)																					
Upper respiratory infections	Birth prevalence - [34, 36) wks, [2500, 3000) g	Mortality	Males		7.509 (5.429 to 10.255)	4.725 (3.991 to 5.56)																					
Upper respiratory infections	Birth prevalence - [34, 36) wks, [2500, 3000) g	Mortality	Females		7.139 (5.091 to 9.759)	5.194 (4.342 to 6.087)																					
Upper respiratory infections	Birth prevalence - [34, 36) wks, [3000, 3500) g	Mortality	Males		7.828 (5.467 to 11.237)	3.717 (2.986 to 4.568)																					
Upper respiratory infections	Birth prevalence - [34, 36) wks, [3000, 3500) g	Mortality	Females		7.622 (5.235 to 10.584)	4.279 (3.416 to 5.301)																					
Upper respiratory infections	Birth prevalence - [34, 36) wks, [3500, 4000) g	Mortality	Males		10.257 (6.692 to 15.124)	3.13 (2.474 to 3.957)																					
Upper respiratory infections	Birth prevalence - [34, 36) wks, [3500, 4000) g	Mortality	Females		10.325 (6.495 to 15.537)	3.756 (2.877 to 4.818)																					
Upper respiratory infections	Birth prevalence - [34, 36) wks, [4000, 4500) g	Mortality	Males		12.928 (7.825 to 20.726)	2.467 (1.926 to 3.084)																					
Upper respiratory infections	Birth prevalence - [34, 36) wks, [4000, 4500) g	Mortality	Females		13.456 (8.201 to 20.893)	3.061 (2.357 to 3.889)																					
Upper respiratory infections	Birth prevalence - [36, 37) wks, [1000, 1500) g	Mortality	Males		94.103 (62.401 to 131.437)	48.734 (39.385 to 60.014)																					
Upper respiratory infections	Birth prevalence - [36, 37) wks, [1000, 1500) g	Mortality	Females		89.769 (59.869 to 126.674)	51.68 (41.336 to 63.456)																					
Upper respiratory infections	Birth prevalence - [36, 37) wks, [1500, 2000) g	Mortality	Males		33.586 (23.075 to 48.77)	19.52 (16.283 to 23.116)																					
Upper respiratory infections	Birth prevalence - [36, 37) wks, [1500, 2000) g	Mortality	Females		30.953 (20.951 to 43.853)	20.509 (17.15 to 24.286)																					
Upper respiratory infections	Birth prevalence - [36, 37) wks, [2000, 2500) g	Mortality	Males		8.134 (5.781 to 10.938)	7.015 (5.903 to 8.241)																					
Upper respiratory infections	Birth prevalence - [36, 37) wks, [2000, 2500) g	Mortality	Females		7.208 (5.183 to 9.787)	7.031 (5.911 to 8.265)																					
Upper respiratory infections	Birth prevalence - [36, 37) wks, [2500, 3000) g	Mortality	Males		2.741 (2.114 to 3.535)	3.149 (2.686 to 3.686)																					
Upper respiratory infections	Birth prevalence - [36, 37) wks, [2500, 3000) g	Mortality	Females		2.475 (1.905 to 3.109)	3.172 (2.71 to 3.666)																					
Upper respiratory infections	Birth prevalence - [36, 37) wks, [3000, 3500) g	Mortality	Males		2.1 (1.602 to 2.663)	2.099 (1.805 to 2.445)																					
Upper respiratory infections	Birth prevalence - [36, 37) wks, [3000, 3500) g	Mortality	Females		1.996 (1.532 to 2.6)	2.199 (1.895 to 2.534)																					
Upper respiratory infections	Birth prevalence - [36, 37) wks, [3500, 4000) g	Mortality	Males		2.563 (1.906 to 3.333)	1.751 (1.487 to 2.008)																					
Upper respiratory infections	Birth prevalence - [36, 37) wks, [3500, 4000) g	Mortality	Females		2.501 (1.793 to 3.34)	1.944 (1.649 to 2.275)																					
Upper respiratory infections	Birth prevalence - [36, 37) wks, [4000, 4500) g	Mortality	Males		3.824 (2.607 to 5.361)	1.506 (1.283 to 1.74)																					
Upper respiratory infections	Birth prevalence - [36, 37) wks, [4000, 4500) g	Mortality	Females		3.908 (2.626 to 5.462)	1.777 (1.481 to 2.118)																					
Upper respiratory infections	Birth prevalence - [37, 38) wks, [1000, 1500) g	Mortality	Males		98.111 (66.503 to 135.289)	49.903 (39.62 to 62.232)																					
Upper respiratory infections	Birth prevalence - [37, 38) wks, [1000, 1500) g	Mortality	Females		92.728 (63.975 to 128.565)	53.151 (42.705 to 66.273)																					
Upper respiratory infections	Birth prevalence - [37, 38) wks, [1500, 2000) g	Mortality	Males		35.71 (24.585 to 49.648)	20.617 (16.897 to 24.816)																					
Upper respiratory infections	Birth prevalence - [37, 38) wks, [1500, 2000) g	Mortality	Females		32.101 (22.457 to 43.887)	21.726 (17.833 to 25.871)																					
Upper respiratory infections	Birth prevalence - [37, 38) wks, [2000, 2500) g	Mortality	Males		7.301 (5.37 to 9.805)	6.923 (5.831 to 8.219)																					
Upper respiratory infections	Birth prevalence - [37, 38) wks, [2000, 2500) g	Mortality	Females		6.218 (4.554 to 8.169)	6.85 (5.796 to 8.005)																					
Upper respiratory infections	Birth prevalence - [37, 38) wks, [2500, 3000) g	Mortality	Males		1.861 (1.414 to 2.384)	2.721 (2.329 to 3.158)																					
Upper respiratory infections	Birth prevalence - [37, 38) wks, [2500, 3000) g	Mortality	Females		1.596 (1.24 to 1.998)	2.633 (2.261 to 3.031)																					
Upper respiratory infections	Birth prevalence - [37, 38) wks, [3000, 3500) g	Mortality	Males		1.139 (1 to 1.409)	1.612 (1.387 to 1.85)																					
Upper respiratory infections	Birth prevalence - [37, 38) wks, [3000, 3500) g	Mortality	Females		1.065 (1 to 1.288)	1.598 (1.392 to 1.819)																					
Upper respiratory infections	Birth prevalence - [37, 38) wks, [3500, 4000) g	Mortality	Males		1.201 (1 to 1.493)	1.282 (1.113 to 1.466)																					
Upper respiratory infections	Birth prevalence - [37, 38) wks, [3500, 4000) g	Mortality	Females		1.157 (1 to 1.442)	1.343 (1.153 to 1.542)																					
Upper respiratory infections	Birth prevalence - [37, 38) wks, [4000, 4500) g	Mortality	Males		1.825 (1.341 to 2.407)	1.136 (1.01 to 1.272)																					
Upper respiratory infections	Birth prevalence - [37, 38) wks, [4000, 4500) g	Mortality	Females		1.86 (1.36 to 2.46)	1.269 (1.115 to 1.42)																					
Upper respiratory infections	Birth prevalence - [38, 40) wks, [1000, 1500) g	Mortality	Males		98.254 (67.959 to 134.77)	49.084 (38.794 to 61.948)																					
Upper respiratory infections	Birth prevalence - [38, 40) wks, [1000, 1500) g	Mortality	Females		91.539 (62.769 to 127.606)	53.036 (41.348 to 65.237)																					
Upper respiratory infections	Birth prevalence - [38, 40) wks, [1500, 2000) g	Mortality	Males		37.449 (25.688 to 51.467)	21.477 (17.208 to 25.646)																					
Upper respiratory infections	Birth prevalence - [38, 40) wks, [1500, 2000) g	Mortality	Females		33.261 (23.303 to 45.061)	22.821 (18.339 to 27.874)																					
Upper respiratory infections	Birth prevalence - [38, 40) wks, [2000, 2500) g	Mortality	Males		7.351 (5.131 to 9.967)	7.04 (5.807 to 8.395)																					
Upper respiratory infections	Birth prevalence - [38, 40) wks, [2000, 2500) g	Mortality	Females		5.985 (4.383 to 7.995)	6.959 (5.782 to 8.29)																					
Upper respiratory infections	Birth prevalence - [38, 40) wks, [2500, 3000) g	Mortality	Males		1.549 (1.185 to 1.98)	2.518 (2.115 to 2.971)																					
Upper respiratory infections	Birth prevalence - [38, 40) wks, [2500, 3000) g	Mortality	Females		1.277 (1 to 1.65)	2.377 (2.015 to 2.745)																					
Upper respiratory infections	Birth prevalence - [38, 40) wks, [3000, 3500) g	Mortality	Males		1 (1.0 - 1.0)	1.326 (1.12 to 1.543)																					
Upper respiratory infections	Birth prevalence - [38, 40) wks, [3000, 3500) g	Mortality	Females		1 (1.0 - 1.0)	1.265 (1.068 to 1.45)																					
Upper respiratory infections	Birth prevalence - [38, 40) wks, [3500, 4000) g	Mortality	Males		1 (1.0 - 1.0)	1 (1.0 - 1.0)																					
Upper respiratory infections	Birth prevalence - [38, 40) wks, [3500, 4000) g	Mortality	Females		1 (1.0 - 1.0)	1 (1.0 - 1.0)																					
Upper respiratory infections	Birth prevalence - [38, 40) wks, [4000, 4500) g	Mortality	Males		1 (1.0 - 1.0)	1 (1.0 - 1.0)																					
Upper respiratory infections	Birth prevalence - [38, 40) wks, [4000, 4500) g	Mortality	Females		1 (1.0 - 1.0)	1 (1.0 - 1.0)																					
Upper respiratory infections	Birth prevalence - [40, 42) wks, [1500, 2000) g	Mortality	Males		43.194 (29.244 to 62.382)	21.971 (16.41 to 28.396)																					
Upper respiratory infections	Birth prevalence - [40, 42) wks, [1500, 2000) g	Mortality	Females		37.949 (25.539 to 53.532)	23.702 (17.857 to 30.634)																					
Upper respiratory infections	Birth prevalence - [40, 42) wks, [2000, 2500) g	Mortality	Males		10.162 (6.961 to 14.235)	7.875 (6.341 to 9.719)																					
Upper respiratory infections	Birth prevalence - [40, 42) wks, [2000, 2500) g	Mortality	Females		8.332 (5.994 to 11.407)	8.067 (6.467 to 10.043)																					
Upper respiratory infections	Birth prevalence - [40, 42) wks, [2500, 3000) g	Mortality	Males		2.118 (1.53 to 2.77)	2.692 (2.23 to 3.273)																					
Upper respiratory infections	Birth prevalence - [40, 42) wks, [2500, 3000) g	Mortality	Females		1.745 (1.289 to 2.316)	2.603 (2.134 to 3.106)																					
Upper respiratory infections	Birth prevalence - [40, 42) wks, [3000, 3500) g	Mortality	Males		1.002 (1 to 1.013)	1.249 (1.041 to 1.491)																					
Upper respiratory infections	Birth prevalence - [40, 42) wks, [3000, 3500) g	Mortality	Females		1 (1.0 - 1.0)	1.188 (1 to 1.411)																					
Upper respiratory infections	Birth prevalence - [40, 42) wks, [3500, 4000) g	Mortality	Males		1 (1.0 - 1.0)	1 (1.0 - 1.0)																					
Upper respiratory infections	Birth prevalence - [40, 42) wks, [3500, 4000) g	Mortality	Females		1 (1.0 - 1.0)	1 (1.0 - 1.0)																					
Upper respiratory infections	Birth prevalence - [40, 42) wks, [4000, 4500) g	Mortality	Males		1 (1.0 - 1.0)	1 (1.0 - 1.0)																					
Upper respiratory infections	Birth prevalence - [40, 42) wks, [4000, 4500) g	Mortality	Females		1 (1.0 - 1.0)	1 (1.0 - 1.0)																					
**Iron deficiency**																											
Maternal haemorrhage	1 g/dL	Both	Both							1.252 (1.087 to 1.425)	1.252 (1.087 to 1.425)	1.252 (1.087 to 1.425)	1.252 (1.087 to 1.425)	1.252 (1.087 to 1.425)	1.252 (1.087 to 1.425)	1.252 (1.087 to 1.425)	1.252 (1.087 to 1.425)	1.252 (1.087 to 1.425)									
Maternal sepsis and other pregnancy related infections	1 g/dL	Both	Both							1.252 (1.087 to 1.425)	1.252 (1.087 to 1.425)	1.252 (1.087 to 1.425)	1.252 (1.087 to 1.425)	1.252 (1.087 to 1.425)	1.252 (1.087 to 1.425)	1.252 (1.087 to 1.425)	1.252 (1.087 to 1.425)	1.252 (1.087 to 1.425)									
Maternal hypertensive disorders	1 g/dL	Both	Both							1.252 (1.087 to 1.425)	1.252 (1.087 to 1.425)	1.252 (1.087 to 1.425)	1.252 (1.087 to 1.425)	1.252 (1.087 to 1.425)	1.252 (1.087 to 1.425)	1.252 (1.087 to 1.425)	1.252 (1.087 to 1.425)	1.252 (1.087 to 1.425)									
Maternal obstructed labour and uterine rupture	1 g/dL	Both	Both							1.252 (1.087 to 1.425)	1.252 (1.087 to 1.425)	1.252 (1.087 to 1.425)	1.252 (1.087 to 1.425)	1.252 (1.087 to 1.425)	1.252 (1.087 to 1.425)	1.252 (1.087 to 1.425)	1.252 (1.087 to 1.425)	1.252 (1.087 to 1.425)									
Maternal abortive outcome	1 g/dL	Both	Both							1.252 (1.087 to 1.425)	1.252 (1.087 to 1.425)	1.252 (1.087 to 1.425)	1.252 (1.087 to 1.425)	1.252 (1.087 to 1.425)	1.252 (1.087 to 1.425)	1.252 (1.087 to 1.425)	1.252 (1.087 to 1.425)	1.252 (1.087 to 1.425)									
Ectopic pregnancy	1 g/dL	Both	Both							1.252 (1.087 to 1.425)	1.252 (1.087 to 1.425)	1.252 (1.087 to 1.425)	1.252 (1.087 to 1.425)	1.252 (1.087 to 1.425)	1.252 (1.087 to 1.425)	1.252 (1.087 to 1.425)	1.252 (1.087 to 1.425)	1.252 (1.087 to 1.425)									
Indirect maternal deaths	1 g/dL	Both	Both							1.252 (1.087 to 1.425)	1.252 (1.087 to 1.425)	1.252 (1.087 to 1.425)	1.252 (1.087 to 1.425)	1.252 (1.087 to 1.425)	1.252 (1.087 to 1.425)	1.252 (1.087 to 1.425)	1.252 (1.087 to 1.425)	1.252 (1.087 to 1.425)									
Late maternal deaths	1 g/dL	Both	Both							1.252 (1.087 to 1.425)	1.252 (1.087 to 1.425)	1.252 (1.087 to 1.425)	1.252 (1.087 to 1.425)	1.252 (1.087 to 1.425)	1.252 (1.087 to 1.425)	1.252 (1.087 to 1.425)	1.252 (1.087 to 1.425)	1.252 (1.087 to 1.425)									
Maternal deaths aggravated by HIV/AIDS	1 g/dL	Both	Both							1.252 (1.087 to 1.425)	1.252 (1.087 to 1.425)	1.252 (1.087 to 1.425)	1.252 (1.087 to 1.425)	1.252 (1.087 to 1.425)	1.252 (1.087 to 1.425)	1.252 (1.087 to 1.425)	1.252 (1.087 to 1.425)	1.252 (1.087 to 1.425)									
Other maternal disorders	1 g/dL	Both	Both							1.252 (1.087 to 1.425)	1.252 (1.087 to 1.425)	1.252 (1.087 to 1.425)	1.252 (1.087 to 1.425)	1.252 (1.087 to 1.425)	1.252 (1.087 to 1.425)	1.252 (1.087 to 1.425)	1.252 (1.087 to 1.425)	1.252 (1.087 to 1.425)									
**Vitamin A deficiency**																											
Diarrhoeal diseases	Vitamin A deficient	Both	Both				1.138 (1.023 to 1.261)	1.138 (1.023 to 1.261)																			
Diarrhoeal diseases	Not deficient	Both	Both				1.0 (1.0 to 1.0)	1.0 (1.0 to 1.0)																			
Measles	Vitamin A deficient	Both	Both				1.402 (1.015 to 1.879)	1.402 (1.015 to 1.879)																			
Measles	Not deficient	Both	Both				1.0 (1.0 to 1.0)	1.0 (1.0 to 1.0)																			
**Zinc deficiency**																											
Diarrhoeal diseases	Zinc deficient	Both	Both					1.14 (1.07 to 1.212)																			
Diarrhoeal diseases	Not deficient	Both	Both					1.0 (1.0 to 1.0)																			
**Chewing tobacco**																											
Lip and oral cavity cancer	Exposed	Both	Males											3.021 (2.015 to 4.47)	3.021 (2.015 to 4.47)	3.021 (2.015 to 4.47)	3.021 (2.015 to 4.47)	3.021 (2.015 to 4.47)	3.021 (2.015 to 4.47)	3.021 (2.015 to 4.47)	3.021 (2.015 to 4.47)	3.021 (2.015 to 4.47)	3.021 (2.015 to 4.47)	3.021 (2.015 to 4.47)	3.021 (2.015 to 4.47)	3.021 (2.015 to 4.47)	3.021 (2.015 to 4.47)
Lip and oral cavity cancer	Exposed	Both	Females											8.09 (5.463 to 11.767)	8.09 (5.463 to 11.767)	8.09 (5.463 to 11.767)	8.09 (5.463 to 11.767)	8.09 (5.463 to 11.767)	8.09 (5.463 to 11.767)	8.09 (5.463 to 11.767)	8.09 (5.463 to 11.767)	8.09 (5.463 to 11.767)	8.09 (5.463 to 11.767)	8.09 (5.463 to 11.767)	8.09 (5.463 to 11.767)	8.09 (5.463 to 11.767)	8.09 (5.463 to 11.767)
Lip and oral cavity cancer	Not exposed	Both	Males											1.0 (1.0 to 1.0)	1.0 (1.0 to 1.0)	1.0 (1.0 to 1.0)	1.0 (1.0 to 1.0)	1.0 (1.0 to 1.0)	1.0 (1.0 to 1.0)	1.0 (1.0 to 1.0)	1.0 (1.0 to 1.0)	1.0 (1.0 to 1.0)	1.0 (1.0 to 1.0)	1.0 (1.0 to 1.0)	1.0 (1.0 to 1.0)	1.0 (1.0 to 1.0)	1.0 (1.0 to 1.0)
Lip and oral cavity cancer	Not exposed	Both	Females											1.0 (1.0 to 1.0)	1.0 (1.0 to 1.0)	1.0 (1.0 to 1.0)	1.0 (1.0 to 1.0)	1.0 (1.0 to 1.0)	1.0 (1.0 to 1.0)	1.0 (1.0 to 1.0)	1.0 (1.0 to 1.0)	1.0 (1.0 to 1.0)	1.0 (1.0 to 1.0)	1.0 (1.0 to 1.0)	1.0 (1.0 to 1.0)	1.0 (1.0 to 1.0)	1.0 (1.0 to 1.0)

Oesophageal cancer	Exposed	Both	Males											2.571 (1.854 to 3.416)	2.571 (1.854 to 3.416)	2.571 (1.854 to 3.416)	2.571 (1.854 to 3.416)	2.571 (1.854 to 3.416)	2.571 (1.854 to 3.416)	2.571 (1.854 to 3.416)	2.571 (1.854 to 3.416)	2.571 (1.854 to 3.416)	2.571 (1.854 to 3.416)	2.571 (1.854 to 3.416)	2.571 (1.854 to 3.416)	2.571 (1.854 to 3.416)	2.571 (1.854 to 3.416)
Oesophageal cancer	Exposed	Both	Females											2.578 (1.877 to 3.478)	2.578 (1.877 to 3.478)	2.578 (1.877 to 3.478)	2.578 (1.877 to 3.478)	2.578 (1.877 to 3.478)	2.578 (1.877 to 3.478)	2.578 (1.877 to 3.478)	2.578 (1.877 to 3.478)	2.578 (1.877 to 3.478)	2.578 (1.877 to 3.478)	2.578 (1.877 to 3.478)	2.578 (1.877 to 3.478)	2.578 (1.877 to 3.478)	2.578 (1.877 to 3.478)
Oesophageal cancer	Not exposed	Both	Males											1.0 (1.0 to 1.0)	1.0 (1.0 to 1.0)	1.0 (1.0 to 1.0)	1.0 (1.0 to 1.0)	1.0 (1.0 to 1.0)	1.0 (1.0 to 1.0)	1.0 (1.0 to 1.0)	1.0 (1.0 to 1.0)	1.0 (1.0 to 1.0)	1.0 (1.0 to 1.0)	1.0 (1.0 to 1.0)	1.0 (1.0 to 1.0)	1.0 (1.0 to 1.0)	1.0 (1.0 to 1.0)
Oesophageal cancer	Not exposed	Both	Females											1.0 (1.0 to 1.0)	1.0 (1.0 to 1.0)	1.0 (1.0 to 1.0)	1.0 (1.0 to 1.0)	1.0 (1.0 to 1.0)	1.0 (1.0 to 1.0)	1.0 (1.0 to 1.0)	1.0 (1.0 to 1.0)	1.0 (1.0 to 1.0)	1.0 (1.0 to 1.0)	1.0 (1.0 to 1.0)	1.0 (1.0 to 1.0)	1.0 (1.0 to 1.0)	1.0 (1.0 to 1.0)
**Secondhand smoke**																											
**Categorical exposure**																											
Otitis media	Exposed	Both	Both		1.37 (1.238 to 1.493)	1.37 (1.238 to 1.493)	1.37 (1.238 to 1.493)	1.37 (1.238 to 1.493)	1.37 (1.238 to 1.493)	1.37 (1.238 to 1.493)																	
Otitis media	Not exposed	Both	Both		1 (1.0 to 1.0)	1 (1.0 to 1.0)	1 (1.0 to 1.0)	1 (1.0 to 1.0)	1 (1.0 to 1.0)	1 (1.0 to 1.0)																	
Breast cancer	Exposed	Both	Female										1.072 (1.017 to 1.126)	1.072 (1.017 to 1.126)	1.072 (1.017 to 1.126)	1.072 (1.017 to 1.126)	1.072 (1.017 to 1.126)	1.072 (1.017 to 1.126)	1.072 (1.017 to 1.126)	1.072 (1.017 to 1.126)	1.072 (1.017 to 1.126)	1.072 (1.017 to 1.126)	1.072 (1.017 to 1.126)	1.072 (1.017 to 1.126)	1.072 (1.017 to 1.126)	1.072 (1.017 to 1.126)	1.072 (1.017 to 1.126)
Breast cancer	Not exposed	Both	Female										1 (1.0 to 1.0)	1 (1.0 to 1.0)	1 (1.0 to 1.0)	1 (1.0 to 1.0)	1 (1.0 to 1.0)	1 (1.0 to 1.0)	1 (1.0 to 1.0)	1 (1.0 to 1.0)	1 (1.0 to 1.0)	1 (1.0 to 1.0)	1 (1.0 to 1.0)	1 (1.0 to 1.0)	1 (1.0 to 1.0)	1 (1.0 to 1.0)	1 (1.0 to 1.0)
Diabetes mellitus type 2	Exposed	Both	Both										1.337 (1.121 to 1.542)	1.337 (1.121 to 1.542)	1.337 (1.121 to 1.542)	1.337 (1.121 to 1.542)	1.337 (1.121 to 1.542)	1.337 (1.121 to 1.542)	1.337 (1.121 to 1.542)	1.337 (1.121 to 1.542)	1.337 (1.121 to 1.542)	1.337 (1.121 to 1.542)	1.337 (1.121 to 1.542)	1.337 (1.121 to 1.542)	1.337 (1.121 to 1.542)	1.337 (1.121 to 1.542)	1.337 (1.121 to 1.542)
Diabetes mellitus type 2	Not exposed	Both	Both										1 (1.0 to 1.0)	1 (1.0 to 1.0)	1 (1.0 to 1.0)	1 (1.0 to 1.0)	1 (1.0 to 1.0)	1 (1.0 to 1.0)	1 (1.0 to 1.0)	1 (1.0 to 1.0)	1 (1.0 to 1.0)	1 (1.0 to 1.0)	1 (1.0 to 1.0)	1 (1.0 to 1.0)	1 (1.0 to 1.0)	1 (1.0 to 1.0)	1 (1.0 to 1.0)
**Continuous exposure (particulate matter integrated exposure response curve)**																											
Lower respiratory infections	200 µg/m³	Both	Both	2.062 (1.702 to 2.44)																							
Lower respiratory infections	150 µg/m³	Both	Both	1.938 (1.629 to 2.281)																							
Lower respiratory infections	135 µg/m³	Both	Both	1.891 (1.6 to 2.209)																							
Lower respiratory infections	120 µg/m³	Both	Both	1.838 (1.571 to 2.129)																							
Lower respiratory infections	105 µg/m³	Both	Both	1.778 (1.54 to 2.05)																							
Lower respiratory infections	90 µg/m³	Both	Both	1.711 (1.505 to 1.945)																							
Lower respiratory infections	75 µg/m³	Both	Both	1.634 (1.455 to 1.827)																							
Lower respiratory infections	60 µg/m³	Both	Both	1.546 (1.4 to 1.711)																							
Lower respiratory infections	45 µg/m³	Both	Both	1.443 (1.323 to 1.576)																							
Lower respiratory infections	30 µg/m³	Both	Both	1.322 (1.225 to 1.428)																							
Lower respiratory infections	25 µg/m³	Both	Both	1.276 (1.184 to 1.379)																							
Lower respiratory infections	20 µg/m³	Both	Both	1.226 (1.14 to 1.335)																							
Lower respiratory infections	15 µg/m³	Both	Both	1.171 (1.093 to 1.282)																							
Lower respiratory infections	10 µg/m³	Both	Both	1.108 (1.046 to 1.219)																							
Lower respiratory infections	5 µg/m³	Both	Both	1.025 (1.0 to 1.119)																							
Lower respiratory infections	0 µg/m³	Both	Both	1.0 (1.0 to 1.0)																							
Tracheal, bronchus, and lung cancer	200 µg/m³	Both	Both										1.753 (1.56 to 1.958)	1.753 (1.56 to 1.958)	1.753 (1.56 to 1.958)	1.753 (1.56 to 1.958)	1.753 (1.56 to 1.958)	1.753 (1.56 to 1.958)	1.753 (1.56 to 1.958)	1.753 (1.56 to 1.958)	1.753 (1.56 to 1.958)	1.753 (1.56 to 1.958)	1.753 (1.56 to 1.958)	1.753 (1.56 to 1.958)	1.753 (1.56 to 1.958)	1.753 (1.56 to 1.958)	1.753 (1.56 to 1.958)
Tracheal, bronchus, and lung cancer	150 µg/m³	Both	Both										1.622 (1.454 to 1.802)	1.622 (1.454 to 1.802)	1.622 (1.454 to 1.802)	1.622 (1.454 to 1.802)	1.622 (1.454 to 1.802)	1.622 (1.454 to 1.802)	1.622 (1.454 to 1.802)	1.622 (1.454 to 1.802)	1.622 (1.454 to 1.802)	1.622 (1.454 to 1.802)	1.622 (1.454 to 1.802)	1.622 (1.454 to 1.802)	1.622 (1.454 to 1.802)	1.622 (1.454 to 1.802)	1.622 (1.454 to 1.802)
Tracheal, bronchus, and lung cancer	135 µg/m³	Both	Both										1.58 (1.421 to 1.754)	1.58 (1.421 to 1.754)	1.58 (1.421 to 1.754)	1.58 (1.421 to 1.754)	1.58 (1.421 to 1.754)	1.58 (1.421 to 1.754)	1.58 (1.421 to 1.754)	1.58 (1.421 to 1.754)	1.58 (1.421 to 1.754)	1.58 (1.421 to 1.754)	1.58 (1.421 to 1.754)	1.58 (1.421 to 1.754)	1.58 (1.421 to 1.754)	1.58 (1.421 to 1.754)	1.58 (1.421 to 1.754)
Tracheal, bronchus, and lung cancer	120 µg/m³	Both	Both										1.536 (1.385 to 1.701)	1.536 (1.385 to 1.701)	1.536 (1.385 to 1.701)	1.536 (1.385 to 1.701)	1.536 (1.385 to 1.701)	1.536 (1.385 to 1.701)	1.536 (1.385 to 1.701)	1.536 (1.385 to 1.701)	1.536 (1.385 to 1.701)	1.536 (1.385 to 1.701)	1.536 (1.385 to 1.701)	1.536 (1.385 to 1.701)	1.536 (1.385 to 1.701)	1.536 (1.385 to 1.701)	1.536 (1.385 to 1.701)
Tracheal, bronchus, and lung cancer	105 µg/m³	Both	Both										1.49 (1.348 to 1.648)	1.49 (1.348 to 1.648)	1.49 (1.348 to 1.648)	1.49 (1.348 to 1.648)	1.49 (1.348 to 1.648)	1.49 (1.348 to 1.648)	1.49 (1.348 to 1.648)	1.49 (1.348 to 1.648)	1.49 (1.348 to 1.648)	1.49 (1.348 to 1.648)	1.49 (1.348 to 1.648)	1.49 (1.348 to 1.648)	1.49 (1.348 to 1.648)	1.49 (1.348 to 1.648)	1.49 (1.348 to 1.648)
Tracheal, bronchus, and lung cancer	90 µg/m³	Both	Both										1.442 (1.309 to 1.588)	1.442 (1.309 to 1.588)	1.442 (1.309 to 1.588)	1.442 (1.309 to 1.588)	1.442 (1.309 to 1.588)	1.442 (1.309 to 1.588)	1.442 (1.309 to 1.588)	1.442 (1.309 to 1.588)	1.442 (1.309 to 1.588)	1.442 (1.309 to 1.588)	1.442 (1.309 to 1.588)	1.442 (1.309 to 1.588)	1.442 (1.309 to 1.588)	1.442 (1.309 to 1.588)	1.442 (1.309 to 1.588)
Tracheal, bronchus, and lung cancer	75 µg/m³	Both	Both										1.39 (1.269 to 1.526)	1.39 (1.269 to 1.526)	1.39 (1.269 to 1.526)	1.39 (1.269 to 1.526)	1.39 (1.269 to 1.526)	1.39 (1.269 to 1.526)	1.39 (1.269 to 1.526)	1.39 (1.269 to 1.526)	1.39 (1.269 to 1.526)	1.39 (1.269 to 1.526)	1.39 (1.269 to 1.526)	1.39 (1.269 to 1.526)	1.39 (1.269 to 1.526)	1.39 (1.269 to 1.526)	1.39 (1.269 to 1.526)
Tracheal, bronchus, and lung cancer	60 µg/m³	Both	Both										1.335 (1.227 to 1.458)	1.335 (1.227 to 1.458)	1.335 (1.227 to 1.458)	1.335 (1.227 to 1.458)	1.335 (1.227 to 1.458)	1.335 (1.227 to 1.458)	1.335 (1.227 to 1.458)	1.335 (1.227 to 1.458)	1.335 (1.227 to 1.458)	1.335 (1.227 to 1.458)	1.335 (1.227 to 1.458)	1.335 (1.227 to 1.458)	1.335 (1.227 to 1.458)	1.335 (1.227 to 1.458)	1.335 (1.227 to 1.458)
Tracheal, bronchus, and lung cancer	45 µg/m³	Both	Both										1.273 (1.18 to 1.383)	1.273 (1.18 to 1.383)	1.273 (1.18 to 1.383)	1.273 (1.18 to 1.383)	1.273 (1.18 to 1.383)	1.273 (1.18 to 1.383)	1.273 (1.18 to 1.383)	1.273 (1.18 to 1.383)	1.273 (1.18 to 1.383)	1.273 (1.18 to 1.383)	1.273 (1.18 to 1.383)	1.273 (1.18 to 1.383)	1.273 (1.18 to 1.383)	1.273 (1.18 to 1.383)	1.273 (1.18 to 1.383)
Tracheal, bronchus, and lung cancer	30 µg/m³	Both	Both										1.203 (1.128 to 1.295)	1.203 (1.128 to 1.295)	1.203 (1.128 to 1.295)	1.203 (1.128 to 1.295)	1.203 (1.128 to 1.295)	1.203 (1.128 to 1.295)	1.203 (1.128 to 1.295)	1.203 (1.128 to 1.295)	1.203 (1.128 to 1.295)	1.203 (1.128 to 1.295)	1.203 (1.128 to 1.295)	1.203 (1.128 to 1.295)	1.203 (1.128 to 1.295)	1.203 (1.128 to 1.295)	1.203 (1.128 to 1.295)
Tracheal, bronchus, and lung cancer	25 µg/m³	Both	Both										1.177 (1.109 to 1.261)	1.177 (1.109 to 1.261)	1.177 (1.109 to 1.261)	1.177 (1.109 to 1.261)	1.177 (1.109 to 1.261)	1.177 (1.109 to 1.261)	1.177 (1.109 to 1.261)	1.177 (1.109 to 1.261)	1.177 (1.109 to 1.261)	1.177 (1.109 to 1.261)	1.177 (1.109 to 1.261)	1.177 (1.109 to 1.261)	1.177 (1.109 to 1.261)	1.177 (1.109 to 1.261)	1.177 (1.109 to 1.261)
Tracheal, bronchus, and lung cancer	20 µg/m³	Both	Both										1.148 (1.089 to 1.223)	1.148 (1.089 to 1.223)	1.148 (1.089 to 1.223)	1.148 (1.089 to 1.223)	1.148 (1.089 to 1.223)	1.148 (1.089 to 1.223)	1.148 (1.089 to 1.223)	1.148 (1.089 to 1.223)	1.148 (1.089 to 1.223)	1.148 (1.089 to 1.223)	1.148 (1.089 to 1.223)	1.148 (1.089 to 1.223)	1.148 (1.089 to 1.223)	1.148 (1.089 to 1.223)	1.148 (1.089 to 1.223)
Tracheal, bronchus, and lung cancer	15 µg/m³	Both	Both										1.116 (1.067 to 1.178)	1.116 (1.067 to 1.178)	1.116 (1.067 to 1.178)	1.116 (1.067 to 1.178)	1.116 (1.067 to 1.178)	1.116 (1.067 to 1.178)	1.116 (1.067 to 1.178)	1.116 (1.067 to 1.178)	1.116 (1.067 to 1.178)	1.116 (1.067 to 1.178)	1.116 (1.067 to 1.178)	1.116 (1.067 to 1.178)	1.116 (1.067 to 1.178)	1.116 (1.067 to 1.178)	1.116 (1.067 to 1.178)
Tracheal, bronchus, and lung cancer	10 µg/m³	Both	Both										1.077 (1.041 to 1.126)	1.077 (1.041 to 1.126)	1.077 (1.041 to 1.126)	1.077 (1.041 to 1.126)	1.077 (1.041 to 1.126)	1.077 (1.041 to 1.126)	1.077 (1.041 to 1.126)	1.077 (1.041 to 1.126)	1.077 (1.041 to 1.126)	1.077 (1.041 to 1.126)	1.077 (1.041 to 1.126)	1.077 (1.041 to 1.126)	1.077 (1.041 to 1.126)	1.077 (1.041 to 1.126)	1.077 (1.041 to 1.126)
Tracheal, bronchus, and lung cancer	5 µg/m³	Both	Both										1.02 (1.0 to 1.056)	1.02 (1.0 to 1.056)	1.02 (1.0 to 1.056)	1.02 (1.0 to 1.056)	1.02 (1.0 to 1.056)	1.02 (1.0 to 1.056)	1.02 (1.0 to 1.056)	1.02 (1.0 to 1.056)	1.02 (1.0 to 1.056)	1.02 (1.0 to 1.056)	1.02 (1.0 to 1.056)	1.02 (1.0 to 1.056)	1.02 (1.0 to 1.056)	1.02 (1.0 to 1.056)	1.02 (1.0 to 1.056)
Tracheal, bronchus, and lung cancer	0 µg/m³	Both	Both										1.0 (1.0 to 1.0)	1.0 (1.0 to 1.0)	1.0 (1.0 to 1.0)	1.0 (1.0 to 1.0)	1.0 (1.0 to 1.0)	1.0 (1.0 to 1.0)	1.0 (1.0 to 1.0)	1.0 (1.0 to 1.0)	1.0 (1.0 to 1.0)	1.0 (1.0 to 1.0)	1.0 (1.0 to 1.0)	1.0 (1.0 to 1.0)	1.0 (1.0 to 1.0)	1.0 (1.0 to 1.0)	1.0 (1.0 to 1.0)
Ischaemic heart disease	200 µg/m³	Both	Both										1.838 (1.562 to 2.163)	1.779 (1.515 to 2.08)	1.71 (1.493 to 1.989)	1.631 (1.413 to 1.879)	1.579 (1.393 to 1.809)	1.521 (1.356 to 1.704)	1.467 (1.325 to 1.634)	1.416 (1.282 to 1.561)	1.363 (1.253 to 1.488)	1.314 (1.215 to 1.423)	1.271 (1.192 to 1.362)	1.225 (1.154 to 1.302)	1.184 (1.128 to 1.241)	1.144 (1.103 to 1.19)	1.106 (1.073 to 1.139)
Ischaemic heart disease	150 µg/m³	Both	Both										1.754 (1.492 to 2.069)	1.703 (1.452 to 1.992)	1.643 (1.433 to 1.914)	1.572 (1.362 to 1.807)	1.526 (1.346 to 1.75)	1.474 (1.313 to 1.652)	1.426 (1.29 to 1.591)	1.38 (1.253 to 1.52)	1.332 (1.226 to 1.455)	1.288 (1.192 to 1.397)	1.249 (1.172 to 1.339)	1.207 (1.138 to 1.283)	1.17 (1.115 to 1.226)	1.133 (1.094 to 1.179)	1.098 (1.066 to 1.132)
Ischaemic heart disease	135 µg/m³	Both	Both										1.726 (1.466 to 2.036)	1.677 (1.43 to 1.965)	1.62 (1.413 to 1.889)	1.551 (1.345 to 1.782)	1.507 (1.33 to 1.732)	1.457 (1.299 to 1.634)	1.412 (1.278 to 1.576)	1.367 (1.243 to 1.507)	1.321 (1.217 to 1.443)	1.279 (1.184 to 1.387)	1.241 (1.165 to 1.331)	1.201 (1.132 to 1.277)	1.165 (1.11 to 1.221)	1.129 (1.09 to 1.176)	1.095 (1.064 to 1.129)
Ischaemic heart disease	120 µg/m³	Both	Both										1.695 (1.439 to 2.0)	1.649 (1.408 to 1.935)	1.595 (1.394 to 1.86)	1.529 (1.326 to 1.759)	1.487 (1.313 to 1.71)	1.44 (1.284 to 1.614)	1.396 (1.266 to 1.559)	1.354 (1.232 to 1.491)	1.31 (1.207 to 1.431)	1.269 (1.176 to 1.376)	1.233 (1.158 to 1.321)	1.194 (1.127 to 1.27)	1.16 (1.106 to 1.215)	1.125 (1.086 to 1.171)	1.092 (1.061 to 1.126)
Ischaemic heart disease	105 µg/m³	Both	Both										1.661 (1.413 to 1.963)	1.619 (1.383 to 1.904)	1.567 (1.373 to 1.829)	1.505 (1.306 to 1.731)	1.466 (1.295 to 1.686)	1.421 (1.268 to 1.593)	1.38 (1.253 to 1.542)	1.339 (1.22 to 1.474)	1.297 (1.196 to 1.418)	1.258 (1.167 to 1.363)	1.224 (1.15 to 1.312)	1.187 (1.121 to 1.262)	1.154 (1.101 to 1.209)	1.121 (1.082 to 1.167)	1.089 (1.058 to 1.122)
Ischaemic heart disease	90 µg/m³	Both	Both										1.624 (1.382 to 1.921)	1.585 (1.356 to 1.86)	1.537 (1.347 to 1.794)	1.478 (1.284 to 1.7)	1.442 (1.276 to 1.66)	1.399 (1.251 to 1.568)	1.361 (1.238 to 1.518)	1.323 (1.206 to 1.457)	1.283 (1.185 to 1.402)	1.246 (1.157 to 1.35)	1.214 (1.141 to 1.3)	1.179 (1.113 to 1.252)	1.147 (1.094 to 1.202)	1.115 (1.078 to 1.161)	1.085 (1.055 to 1.119)
Ischaemic heart disease	75 µg/m³	Both	Both										1.583 (1.345 to 1.871)	1.547 (1.325 to 1.813)	1.503 (1.32 to 1.755)	1.448 (1.259 to 1.665)	1.415 (1.254 to 1.628)	1.376 (1.232 to 1.542)	1.34 (1.22 to 1.495)	1.305 (1.19 to 1.437)	1.267 (1.171 to 1.383)	1.233 (1.146 to 1.335)	1.202 (1.131 to 1.287)	1.169 (1.106 to 1.241)	1.14 (1.088 to 1.193)	1.11 (1.073 to 1.155)	1.081 (1.051 to 1.114)
Ischaemic heart disease	60 µg/m³	Both	Both										1.535 (1.308 to 1.813)	1.504 (1.291 to 1.761)	1.464 (1.287 to 1.709)	1.413 (1.232 to 1.621)	1.384 (1.229 to 1.591)	1.348 (1.21 to 1.511)	1.315 (1.2 to 1.463)	1.283 (1.172 to 1.412)	1.249 (1.156 to 1.361)	1.217 (1.134 to 1.317)	1.189 (1.12 to 1.272)	1.158 (1.096 to 1.229)	1.131 (1.081 to 1.183)	1.103 (1.067 to 1.148)	1.076 (1.047 to 1.109)
Ischaemic heart disease	45 µg/m³	Both	Both										1.479 (1.268 to 1.741)	1.452 (1.251 to 1.695)	1.417 (1.25 to 1.652)	1.372 (1.2 to 1.573)	1.347 (1.202 to 1.546)	1.314 (1.184 to 1.473)	1.286 (1.176 to 1.431)	1.257 (1.152 to 1.381)	1.226 (1.139 to 1.337)	1.198 (1.118 to 1.295)	1.173 (1.107 to 1.253)	1.145 (1.086 to 1.214)	1.12 (1.072 to 1.171)	1.095 (1.06 to 1.138)	1.07 (1.042 to 1.102)
Ischaemic heart disease	30 µg/m³	Both	Both										1.406 (1.219 to 1.651)	1.385 (1.205 to 1.609)	1.357 (1.205 to 1.578)	1.318 (1.163 to 1.506)	1.299 (1.167 to 1.492)	1.271 (1.15 to 1.423)	1.247 (1.146 to 1.387)	1.224 (1.126 to 1.342)	1.197 (1.116 to 1.303)	1.173 (1.098 to 1.265)	1.152 (1.09 to 1.229)	1.128 (1.071 to 1.193)	1.106 (1.061 to 1.156)	1.084 (1.05 to 1.126)	1.062 (1.036 to 1.093)
Ischaemic heart disease	25 µg/m³	Both	Both										1.376 (1.198 to 1.614)	1.357 (1.187 to 1.574)	1.332 (1.187 to 1.546)	1.296 (1.149 to 1.479)	1.278 (1.152 to 1.464)	1.253 (1.137 to 1.402)	1.231 (1.133 to 1.365)	1.209 (1.115 to 1.323)	1.185 (1.105 to 1.289)	1.163 (1.09 to 1.253)	1.143 (1.082 to 1.219)	1.12 (1.066 to 1.184)	1.1 (1.056 to 1.149)	1.079 (1.047 to 1.121)	1.059 (1.033 to 1.089)
Ischaemic heart disease	20 µg/m³	Both	Both										1.341 (1.175 to 1.566)	1.324 (1.166 to 1.533)	1.303 (1.166 to 1.506)	1.27 (1.132 to 1.446)	1.254 (1.134 to 1.43)	1.232 (1.122 to 1.374)	1.212 (1.119 to 1.342)	1.192 (1.103 to 1.302)	1.17 (1.095 to 1.272)	1.15 (1.081 to 1.237)	1.132 (1.074 to 1.206)	1.111 (1.06 to 1.173)	1.093 (1.051 to 1.141)	1.073 (1.042 to 1.114)	1.055 (1.03 to 1.084)
Ischaemic heart disease	15 µg/m³	Both	Both										1.298 (1.143 to 1.508)	1.284 (1.139 to 1.476)	1.266 (1.14 to 1.458)	1.237 (1.112 to 1.404)	1.225 (1.113 to 1.386)	1.205 (1.103 to 1.341)	1.188 (1.102 to 1.315)	1.171 (1.088 to 1.276)	1.152 (1.081 to 1.249)	1.134 (1.07 to 1.219)	1.118 (1.064 to 1.188)	1.1 (1.052 to 1.159)	1.083 (1.044 to 1.13)	1.066 (1.037 to 1.107)	1.05 (1.026 to 1.078)
Ischaemic heart disease	10 µg/m³	Both	Both										1.238 (1.105 to 1.425)	1.229 (1.101 to 1.402)	1.215 (1.102 to 1.385)	1.192 (1.083 to 1.339)	1.183 (1.086 to 1.325)	1.168 (1.077 to 1.293)	1.155 (1.078 to 1.268)	1.141 (1.068 to 1.241)	1.125 (1.062 to 1.217)	1.112 (1.053 to 1.19)	1.099 (1.049 to 1.162)	1.084 (1.041 to 1.139)	1.07 (1.036 to 1.113)	1.056 (1.03 to 1.095)	1.042 (1.021 to 1.069)
Ischaemic heart disease	5 µg/m³	Both	Both										1.097 (1.0 to 1.284)	1.095 (1.0 to 1.266)	1.088 (1.0 to 1.253)	1.079 (1.0 to 1.228)	1.077 (1.0 to 1.209)	1.071 (1.0 to 1.188)	1.067 (1.0 to 1.181)	1.062 (1.0 to 1.166)	1.055 (1.0 to 1.145)	1.049 (1.0 to 1.126)	1.045 (1.0 to 1.114)	1.038 (1.0 to 1.098)	1.032 (1.0 to 1.081)	1.026 (1.0 to 1.067)	1.02 (1.0 to 1.051)
Ischaemic heart disease	0 µg/m³	Both	Both										1.0 (1.0 to 1.0)	1.0 (1.0 to 1.0)	1.0 (1.0 to 1.0)	1.0 (1.0 to 1.0)	1.0 (1.0 to 1.0)	1.0 (1.0 to 1.0)	1.0 (1.0 to 1.0)	1.0 (1.0 to 1.0)	1.0 (1.0 to 1.0)	1.0 (1.0 to 1.0)	1.0 (1.0 to 1.0)	1.0 (1.0 to 1.0)	1.0 (1.0 to 1.0)	1.0 (1.0 to 1.0)	1.0 (1.0 to 1.0)
Stroke	200 µg/m³	Both	Both										1.496 (1.219 to 1.757)	1.454 (1.203 to 1.712)	1.418 (1.166 to 1.646)	1.381 (1.17 to 1.591)	1.345 (1.148 to 1.542)	1.314 (1.142 to 1.483)	1.282 (1.132 to 1.445)	1.251 (1.117 to 1.394)	1.223 (1.108 to 1.343)	1.196 (1.094 to 1.299)	1.167 (1.081 to 1.255)	1.14 (1.072 to 1.207)	1.117 (1.06 to 1.175)	1.092 (1.046 to 1.137)	1.068 (1.036 to 1.104)
Stroke	150 µg/m³	Both	Both										1.453 (1.189 to 1.705)	1.415 (1.175 to 1.667)	1.382 (1.141 to 1.605)	1.348 (1.146 to 1.558)	1.315 (1.125 to 1.509)	1.288 (1.123 to 1.453)	1.259 (1.115 to 1.42)	1.231 (1.102 to 1.375)	1.205 (1.094 to 1.325)	1.181 (1.081 to 1.283)	1.154 (1.07 to 1.24)	1.13 (1.063 to 1.195)	1.108 (1.053 to 1.166)	1.085 (1.04 to 1.13)	1.063 (1.032 to 1.098)
Stroke	135 µg/m³	Both	Both										1.438 (1.179 to 1.69)	1.401 (1.166 to 1.653)	1.37 (1.133 to 1.591)	1.337 (1.138 to 1.545)	1.305 (1.118 to 1.498)	1.279 (1.117 to 1.443)	1.251 (1.109 to 1.412)	1.224 (1.096 to 1.366)	1.199 (1.089 to 1.318)	1.175 (1.077 to 1.277)	1.15 (1.067 to 1.236)	1.126 (1.06 to 1.191)	1.105 (1.05 to 1.163)	1.082 (1.038 to 1.127)	1.062 (1.031 to 1.096)
Stroke	120 µg/m³	Both	Both										1.421 (1.168 to 1.672)	1.386 (1.156 to 1.637)	1.357 (1.125 to 1.576)	1.325 (1.13 to 1.531)	1.295 (1.111 to 1.486)	1.269 (1.111 to 1.433)	1.243 (1.103 to 1.403)	1.216 (1.091 to 1.355)	1.192 (1.083 to 1.311)	1.169 (1.073 to 1.271)	1.145 (1.063 to 1.231)	1.122 (1.057 to 1.186)	1.102 (1.047 to 1.16)	1.08 (1.036 to 1.125)	1.06 (1.029 to 1.094)
Stroke	105 µg/m³	Both	Both										1.404 (1.157 to 1.65)	1.37 (1.146 to 1.619)	1.342 (1.115 to 1.559)	1.312 (1.121 to 1.517)	1.283 (1.104 to 1.472)	1.258 (1.103 to 1.421)	1.233 (1.097 to 1.393)	1.208 (1.085 to 1.342)	1.185 (1.078 to 1.304)	1.163 (1.068 to 1.265)	1.139 (1.059 to 1.226)	1.117 (1.053 to 1.181)	1.098 (1.044 to 1.156)	1.077 (1.034 to 1.122)	1.058 (1.027 to 1.093)
Stroke	90 µg/m³	Both	Both										1.384 (1.144 to 1.632)	1.352 (1.135 to 1.599)	1.326 (1.105 to 1.539)	1.297 (1.112 to 1.501)	1.269 (1.096 to 1.457)	1.246 (1.095 to 1.41)	1.222 (1.09 to 1.381)	1.198 (1.078 to 1.332)	1.177 (1.072 to 1.293)	1.156 (1.062 to 1.258)	1.133 (1.055 to 1.219)	1.112 (1.049 to 1.176)	1.094 (1.041 to 1.151)	1.074 (1.032 to 1.118)	1.055 (1.026 to 1.09)
Stroke	75 µg/m³	Both	Both										1.362 (1.131 to 1.608)	1.332 (1.122 to 1.574)	1.307 (1.094 to 1.52)	1.28 (1.102 to 1.482)	1.254 (1.086 to 1.439)	1.233 (1.086 to 1.398)	1.211 (1.082 to 1.368)	1.188 (1.071 to 1.321)	1.167 (1.065 to 1.283)	1.148 (1.056 to 1.249)	1.126 (1.05 to 1.212)	1.106 (1.045 to 1.17)	1.089 (1.038 to 1.146)	1.07 (1.029 to 1.114)	1.053 (1.024 to 1.087)
Stroke	60 µg/m³	Both	Both										1.336 (1.116 to 1.574)	1.309 (1.108 to 1.542)	1.286 (1.083 to 1.492)	1.261 (1.09 to 1.461)	1.237 (1.076 to 1.418)	1.217 (1.074 to 1.377)	1.197 (1.073 to 1.352)	1.175 (1.063 to 1.306)	1.157 (1.059 to 1.27)	1.138 (1.05 to 1.239)	1.118 (1.045 to 1.203)	1.1 (1.041 to 1.163)	1.084 (1.035 to 1.14)	1.066 (1.026 to 1.109)	1.049 (1.021 to 1.083)
Stroke	45 µg/m³	Both	Both										1.305 (1.099 to 1.532)	1.281 (1.093 to 1.502)	1.261 (1.069 to 1.458)	1.237 (1.077 to 1.433)	1.216 (1.064 to 1.392)	1.198 (1.062 to 1.354)	1.18 (1.063 to 1.331)	1.161 (1.054 to 1.288)	1.144 (1.051 to 1.254)	1.127 (1.044 to 1.226)	1.109 (1.039 to 1.192)	1.091 (1.036 to 1.153)	1.077 (1.03 to 1.132)	1.061 (1.023 to 1.103)	1.046 (1.019 to 1.079)
Stroke	30 µg/m³	Both	Both										1.265 (1.077 to 1.48)	1.244 (1.073 to 1.455)	1.227 (1.054 to 1.415)	1.207 (1.061 to 1.396)	1.189 (1.05 to 1.357)	1.174 (1.05 to 1.326)	1.158 (1.05 to 1.303)	1.141 (1.043 to 1.264)	1.126 (1.041 to 1.233)	1.112 (1.035 to 1.208)	1.096 (1.031 to 1.175)	1.081 (1.029 to 1.141)	1.069 (1.025 to 1.122)	1.054 (1.019 to 1.095)	1.041 (1.016 to 1.073)
Stroke	25 µg/m³	Both	Both										1.248 (1.069 to 1.46)	1.229 (1.066 to 1.436)	1.213 (1.047 to 1.396)	1.194 (1.054 to 1.38)	1.177 (1.045 to 1.342)	1.163 (1.045 to 1.312)	1.149 (1.045 to 1.291)	1.133 (1.039 to 1.253)	1.119 (1.037 to 1.223)	1.106 (1.031 to 1.2)	1.091 (1.028 to 1.168)	1.076 (1.027 to 1.136)	1.065 (1.022 to 1.117)	1.051 (1.018 to 1.091)	1.038 (1.014 to 1.07)
Stroke	20 µg/m³	Both	Both										1.227 (1.06 to 1.43)	1.211 (1.056 to 1.409)	1.196 (1.041 to 1.373)	1.179 (1.047 to 1.359)	1.164 (1.039 to 1.32)	1.151 (1.039 to 1.297)	1.137 (1.04 to 1.275)	1.123 (1.034 to 1.24)	1.11 (1.032 to 1.213)	1.098 (1.027 to 1.189)	1.084 (1.024 to 1.16)	1.071 (1.024 to 1.129)	1.06 (1.02 to 1.112)	1.047 (1.016 to 1.087)	1.036 (1.012 to 1.067)
Stroke	15 µg/m³	Both	Both										1.202 (1.05 to 1.396)	1.188 (1.046 to 1.381)	1.176 (1.033 to 1.346)	1.16 (1.039 to 1.334)	1.146 (1.033 to 1.295)	1.135 (1.031 to 1.277)	1.123 (1.033 to 1.256)	1.11 (1.028 to 1.222)	1.099 (1.027 to 1.196)	1.088 (1.023 to 1.175)	1.076 (1.02 to 1.148)	1.064 (1.02 to 1.12)	1.055 (1.017 to 1.104)	1.043 (1.013 to 1.081)	1.033 (1.01 to 1.064)
Stroke	10 µg/m³	Both	Both										1.167 (1.036 to 1.347)	1.155 (1.032 to 1.333)	1.146 (1.024 to 1.303)	1.133 (1.027 to 1.297)	1.122 (1.025 to 1.261)	1.113 (1.023 to 1.248)	1.104 (1.024 to 1.228)	1.093 (1.021 to 1.196)	1.083 (1.019 to 1.174)	1.075 (1.017 to 1.157)	1.064 (1.015 to 1.133)	1.054 (1.015 to 1.107)	1.047 (1.012 to 1.094)	1.037 (1.01 to 1.073)	1.028 (1.008 to 1.057)
Stroke	5 µg/m³	Both	Both										1.073 (1.0 to 1.236)	1.069 (1.0 to 1.224)	1.066 (1.0 to 1.211)	1.059 (1.0 to 1.193)	1.054 (1.0 to 1.184)	1.051 (1.0 to 1.171)	1.046 (1.0 to 1.15)	1.043 (1.0 to 1.135)	1.039 (1.0 to 1.123)	1.035 (1.0 to 1.11)	1.03 (1.0 to 1.093)	1.025 (1.0 to 1.075)	1.022 (1.0 to 1.07)	1.017 (1.0 to 1.051)	1.013 (1.0 to 1.042)
Stroke	0 µg/m³	Both	Both										1.0 (1.0 to 1.0)	1.0 (1.0 to 1.0)	1.0 (1.0 to 1.0)	1.0 (1.0 to 1.0)	1.0 (1.0 to 1.0)	1.0 (1.0 to 1.0)	1.0 (1.0 to 1.0)	1.0 (1.0 to 1.0)	1.0 (1.0 to 1.0)	1.0 (1.0 to 1.0)	1.0 (1.0 to 1.0)	1.0 (1.0 to 1.0)	1.0 (1.0 to 1.0)	1.0 (1.0 to 1.0)	1.0 (1.0 to 1.0)
Chronic obstructive pulmonary disease	200 µg/m³	Both	Both										1.815 (1.52 to 2.099)	1.815 (1.52 to 2.099)	1.815 (1.52 to 2.099)	1.815 (1.52 to 2.099)	1.815 (1.52 to 2.099)	1.815 (1.52 to 2.099)	1.815 (1.52 to 2.099)	1.815 (1.52 to 2.099)	1.815 (1.52 to 2.099)	1.815 (1.52 to 2.099)	1.815 (1.52 to 2.099)	1.815 (1.52 to 2.099)	1.815 (1.52 to 2.099)	1.815 (1.52 to 2.099)	1.815 (1.52 to 2.099)
Chronic obstructive pulmonary disease	150 µg/m³	Both	Both										1.714 (1.441 to 1.976)	1.714 (1.441 to 1.976)	1.714 (1.441 to 1.976)	1.714 (1.441 to 1.976)	1.714 (1.441 to 1.976)	1.714 (1.441 to 1.976)	1.714 (1.441 to 1.976)	1.714 (1.441 to 1.976)	1.714 (1.441 to 1.976)	1.714 (1.441 to 1.976)	1.714 (1.441 to 1.976)	1.714 (1.441 to 1.976)	1.714 (1.441 to 1.976)	1.714 (1.441 to 1.976)	1.714 (1.441 to 1.976)
Chronic obstructive pulmonary disease	135 µg/m³	Both	Both										1.681 (1.415 to 1.94)	1.681 (1.415 to 1.94)	1.681 (1.415 to 1.94)	1.681 (1.415 to 1.94)	1.681 (1.415 to 1.94)	1.681 (1.415 to 1.94)	1.681 (1.415 to 1.94)	1.681 (1.415 to 1.94)	1.681 (1.415 to 1.94)	1.681 (1.415 to 1.94)	1.681 (1.415 to 1.94)	1.681 (1.415 to 1.94)	1.681 (1.415 to 1.94)	1.681 (1.415 to 1.94)	1.681 (1.415 to 1.94)
Chronic obstructive pulmonary disease	120 µg/m³	Both	Both										1.645 (1.388 to 1.899)	1.645 (1.388 to 1.899)	1.645 (1.388 to 1.899)	1.645 (1.388 to 1.899)	1.645 (1.388 to 1.899)	1.645 (1.388 to 1.899)	1.645 (1.388 to 1.899)	1.645 (1.388 to 1.899)	1.645 (1.388 to 1.899)	1.645 (1.388 to 1.899)	1.645 (1.388 to 1.899)	1.645 (1.388 to 1.899)	1.645 (1.388 to 1.899)	1.645 (1.388 to 1.899)	1.645 (1.388 to 1.899)
Chronic obstructive pulmonary disease	105 µg/m³	Both	Both										1.606 (1.359 to 1.854)	1.606 (1.359 to 1.854)	1.606 (1.359 to 1.854)	1.606 (1.359 to 1.854)	1.606 (1.359 to 1.854)	1.606 (1.359 to 1.854)	1.606 (1.359 to 1.854)	1.606 (1.359 to 1.854)	1.606 (1.359 to 1.854)	1.606 (1.359 to 1.854)	1.606 (1.359 to 1.854)	1.606 (1.359 to 1.854)	1.606 (1.359 to 1.854)	1.606 (1.359 to 1.854)	1.606 (1.359 to 1.854)
Chronic obstructive pulmonary disease	90 µg/m³	Both	Both										1.564 (1.328 to 1.804)	1.564 (1.328 to 1.804)	1.564 (1.328 to 1.804)	1.564 (1.328 to 1.804)	1.564 (1.328 to 1.804)	1.564 (1.328 to 1.804)	1.564 (1.328 to 1.804)	1.564 (1.328 to 1.804)	1.564 (1.328 to 1.804)	1.564 (1.328 to 1.804)	1.564 (1.328 to 1.804)	1.564 (1.328 to 1.804)	1.564 (1.328 to 1.804)	1.564 (1.328 to 1.804)	1.564 (1.328 to 1.804)
Chronic obstructive pulmonary disease	75 µg/m³	Both	Both										1.518 (1.294 to 1.745)	1.518 (1.294 to 1.745)	1.518 (1.294 to 1.745)	1.518 (1.294 to 1.745)	1.518 (1.294 to 1.745)	1.518 (1.294 to 1.745)	1.518 (1.294 to 1.745)	1.518 (1.294 to 1.745)	1.518 (1.294 to 1.745)	1.518 (1.294 to 1.745)	1.518 (1.294 to 1.745)	1.518 (1.294 to 1.745)	1.518 (1.294 to 1.745)	1.518 (1.294 to 1.745)	1.518 (1.294 to 1.745)
Chronic obstructive pulmonary disease	60 µg/m³	Both	Both										1.466 (1.258 to 1.68)	1.466 (1.258 to 1.68)	1.466 (1.258 to 1.68)	1.466 (1.258 to 1.68)	1.466 (1.258 to 1.68)	1.466 (1.258 to 1.68)	1.466 (1.258 to 1.68)	1.466 (1.258 to 1.68)	1.466 (1.258 to 1.68)	1.466 (1.258 to 1.68)	1.466 (1.258 to 1.68)	1.466 (1.258 to 1.68)	1.466 (1.258 to 1.68)	1.466 (1.258 to 1.68)	1.466 (1.258 to 1.68)
Chronic obstructive pulmonary disease	45 µg/m³	Both	Both										1.405 (1.217 to 1.607)	1.405 (1.217 to 1.607)	1.405 (1.217 to 1.607)	1.405 (1.217 to 1.607)	1.405 (1.217 to 1.607)	1.405 (1.217 to 1.607)	1.405 (1.217 to 1.607)	1.405 (1.217 to 1.607)	1.405 (1.217 to 1.607)	1.405 (1.217 to 1.607)	1.405 (1.217 to 1.607)	1.405 (1.217 to 1.607)	1.405 (1.217 to 1.607)	1.405 (1.217 to 1.607)	1.405 (1.217 to 1.607)
Chronic obstructive pulmonary disease	30 µg/m³	Both	Both										1.33 (1.168 to 1.512)	1.33 (1.168 to 1.512)	1.33 (1.168 to 1.512)	1.33 (1.168 to 1.512)	1.33 (1.168 to 1.512)	1.33 (1.168 to 1.512)	1.33 (1.168 to 1.512)	1.33 (1.168 to 1.512)	1.33 (1.168 to 1.512)	1.33 (1.168 to 1.512)	1.33 (1.168 to 1.512)	1.33 (1.168 to 1.512)	1.33 (1.168 to 1.512)	1.33 (1.168 to 1.512)	1.33 (1.168 to 1.512)
Chronic obstructive pulmonary disease	25 µg/m³	Both	Both										1.3 (1.149 to 1.473)	1.3 (1.149 to 1.473)	1.3 (1.149 to 1.473)	1.3 (1.149 to 1.473)	1.3 (1.149 to 1.473)	1.3 (1.149 to 1.473)	1.3 (1.149 to 1.473)	1.3 (1.149 to 1.473)	1.3 (1.149 to 1.473)	1.3 (1.149 to 1.473)	1.3 (1.149 to 1.473)	1.3 (1.149 to 1.473)	1.3 (1.149 to 1.473)	1.3 (1.149 to 1.473)	1.3 (1.149 to 1.473)
Chronic obstructive pulmonary disease	20 µg/m³	Both	Both										1.266 (1.126 to 1.426)	1.266 (1.126 to 1.426)	1.266 (1.126 to 1.426)	1.266 (1.126 to 1.426)	1.266 (1.126 to 1.426)	1.266 (1.126 to 1.426)	1.266 (1.126 to 1.426)	1.266 (1.126 to 1.426)	1.266 (1.126 to 1.426)	1.266 (1.126 to 1.426)	1.266 (1.126 to 1.426)	1.266 (1.126 to 1.426)	1.266 (1.126 to 1.426)	1.266 (1.126 to 1.426)	1.266 (1.126 to 1.426)
Chronic obstructive pulmonary disease	15 µg/m³	Both	Both										1.224 (1.101 to 1.371)	1.224 (1.101 to 1.371)	1.224 (1.101 to 1.371)	1.224 (1.101 to 1.371)	1.224 (1.101 to 1.371)	1.224 (1.101 to 1.371)	1.224 (1.101 to 1.371)	1.224 (1.101 to 1.371)	1.224 (1.101 to 1.371)	1.224 (1.101 to 1.371)	1.224 (1.101 to 1.371)	1.224 (1.101 to 1.371)	1.224 (1.101 to 1.371)	1.224 (1.101 to 1.371)	1.224 (1.101 to 1.371)
Chronic obstructive pulmonary disease	10 µg/m³	Both	Both										1.17 (1.07 to 1.296)	1.17 (1.07 to 1.296)	1.17 (1.07 to 1.296)	1.17 (1.07 to 1.296)	1.17 (1.07 to 1.296)	1.17 (1.07 to 1.296)	1.17 (1.07 to 1.296)	1.17 (1.07 to 1.296)	1.17 (1.07 to 1.296)	1.17 (1.07 to 1.296)	1.17 (1.07 to 1.296)	1.17 (1.07 to 1.296)	1.17 (1.07 to 1.296)	1.17 (1.07 to 1.296)	1.17 (1.07 to 1.296)
Chronic obstructive pulmonary disease	5 µg/m³	Both	Both										1.06 (1.0 to 1.17)	1.06 (1.0 to 1.17)	1.06 (1.0 to 1.17)	1.06 (1.0 to 1.17)	1.06 (1.0 to 1.17)	1.06 (1.0 to 1.17)	1.06 (1.0 to 1.17)	1.06 (1.0 to 1.17)	1.06 (1.0 to 1.17)	1.06 (1.0 to 1.17)	1.06 (1.0 to 1.17)	1.06 (1.0 to 1.17)	1.06 (1.0 to 1.17)	1.06 (1.0 to 1.17)	1.06 (1.0 to 1.17)
Chronic obstructive pulmonary disease	0 µg/m³	Both	Both										1.0 (1.0 to 1.0)	1.0 (1.0 to 1.0)	1.0 (1.0 to 1.0)	1.0 (1.0 to 1.0)	1.0 (1.0 to 1.0)	1.0 (1.0 to 1.0)	1.0 (1.0 to 1.0)	1.0 (1.0 to 1.0)	1.0 (1.0 to 1.0)	1.0 (1.0 to 1.0)	1.0 (1.0 to 1.0)	1.0 (1.0 to 1.0)	1.0 (1.0 to 1.0)	1.0 (1.0 to 1.0)	1.0 (1.0 to 1.0)
**Diet low in fruits**																											
Oesophageal cancer	0 g/day	Both	Both										1 (1 to 1)	1 (1 to 1)	1 (1 to 1)	1 (1 to 1)	1 (1 to 1)	1 (1 to 1)	1 (1 to 1)	1 (1 to 1)	1 (1 to 1)	1 (1 to 1)	1 (1 to 1)	1 (1 to 1)	1 (1 to 1)	1 (1 to 1)	1 (1 to 1)
Oesophageal cancer	100 g/day	Both	Both										0.8 (0.71 to 0.97)	0.8 (0.71 to 0.97)	0.8 (0.71 to 0.97)	0.8 (0.71 to 0.97)	0.8 (0.71 to 0.97)	0.8 (0.71 to 0.97)	0.8 (0.71 to 0.97)	0.8 (0.71 to 0.97)	0.8 (0.71 to 0.97)	0.8 (0.71 to 0.97)	0.8 (0.71 to 0.97)	0.8 (0.71 to 0.97)	0.8 (0.71 to 0.97)	0.8 (0.71 to 0.97)	0.8 (0.71 to 0.97)
Oesophageal cancer	200 g/day	Both	Both										0.73 (0.61 to 0.87)	0.73 (0.61 to 0.87)	0.73 (0.61 to 0.87)	0.73 (0.61 to 0.87)	0.73 (0.61 to 0.87)	0.73 (0.61 to 0.87)	0.73 (0.61 to 0.87)	0.73 (0.61 to 0.87)	0.73 (0.61 to 0.87)	0.73 (0.61 to 0.87)	0.73 (0.61 to 0.87)	0.73 (0.61 to 0.87)	0.73 (0.61 to 0.87)	0.73 (0.61 to 0.87)	0.73 (0.61 to 0.87)
Oesophageal cancer	300 g/day	Both	Both										0.7 (0.58 to 0.84)	0.7 (0.58 to 0.84)	0.7 (0.58 to 0.84)	0.7 (0.58 to 0.84)	0.7 (0.58 to 0.84)	0.7 (0.58 to 0.84)	0.7 (0.58 to 0.84)	0.7 (0.58 to 0.84)	0.7 (0.58 to 0.84)	0.7 (0.58 to 0.84)	0.7 (0.58 to 0.84)	0.7 (0.58 to 0.84)	0.7 (0.58 to 0.84)	0.7 (0.58 to 0.84)	0.7 (0.58 to 0.84)
Tracheal, bronchus, and lung cancer	0 g/day	Both	Both										1 (1 to 1)	1 (1 to 1)	1 (1 to 1)	1 (1 to 1)	1 (1 to 1)	1 (1 to 1)	1 (1 to 1)	1 (1 to 1)	1 (1 to 1)	1 (1 to 1)	1 (1 to 1)	1 (1 to 1)	1 (1 to 1)	1 (1 to 1)	1 (1 to 1)
Tracheal, bronchus, and lung cancer	100 g/day	Both	Both										0.98 (0.97 to 1)	0.98 (0.97 to 1)	0.98 (0.97 to 1)	0.98 (0.97 to 1)	0.98 (0.97 to 1)	0.98 (0.97 to 1)	0.98 (0.97 to 1)	0.98 (0.97 to 1)	0.98 (0.97 to 1)	0.98 (0.97 to 1)	0.98 (0.97 to 1)	0.98 (0.97 to 1)	0.98 (0.97 to 1)	0.98 (0.97 to 1)	0.98 (0.97 to 1)
Tracheal, bronchus, and lung cancer	200 g/day	Both	Both										0.96 (0.94 to 1)	0.96 (0.94 to 1)	0.96 (0.94 to 1)	0.96 (0.94 to 1)	0.96 (0.94 to 1)	0.96 (0.94 to 1)	0.96 (0.94 to 1)	0.96 (0.94 to 1)	0.96 (0.94 to 1)	0.96 (0.94 to 1)	0.96 (0.94 to 1)	0.96 (0.94 to 1)	0.96 (0.94 to 1)	0.96 (0.94 to 1)	0.96 (0.94 to 1)
Tracheal, bronchus, and lung cancer	300 g/day	Both	Both										0.94 (0.91 to 0.98)	0.94 (0.91 to 0.98)	0.94 (0.91 to 0.98)	0.94 (0.91 to 0.98)	0.94 (0.91 to 0.98)	0.94 (0.91 to 0.98)	0.94 (0.91 to 0.98)	0.94 (0.91 to 0.98)	0.94 (0.91 to 0.98)	0.94 (0.91 to 0.98)	0.94 (0.91 to 0.98)	0.94 (0.91 to 0.98)	0.94 (0.91 to 0.98)	0.94 (0.91 to 0.98)	0.94 (0.91 to 0.98)
Ischaemic heart disease	0 g/day	Both	Both										1 (1 to 1)	1 (1 to 1)	1 (1 to 1)	1 (1 to 1)	1 (1 to 1)	1 (1 to 1)	1 (1 to 1)	1 (1 to 1)	1 (1 to 1)	1 (1 to 1)	1 (1 to 1)	1 (1 to 1)	1 (1 to 1)	1 (1 to 1)	1 (1 to 1)
Ischaemic heart disease	100 g/day	Both	Both										0.94 (0.92 to 0.99)	0.94 (0.92 to 0.99)	0.94 (0.92 to 0.99)	0.95 (0.94 to 1)	0.96 (0.94 to 1)	0.96 (0.94 to 1)	0.96 (0.95 to 1)	0.97 (0.96 to 1)	0.97 (0.96 to 1)	0.97 (0.96 to 1)	0.98 (0.97 to 1)	0.98 (0.97 to 1)	0.98 (0.97 to 1)	0.98 (0.97 to 1)	0.98 (0.97 to 1)
Ischaemic heart disease	200 g/day	Both	Both										0.89 (0.85 to 0.96)	0.89 (0.85 to 0.96)	0.89 (0.85 to 0.96)	0.91 (0.87 to 0.97)	0.91 (0.88 to 0.97)	0.92 (0.89 to 0.97)	0.93 (0.9 to 0.98)	0.94 (0.91 to 0.98)	0.94 (0.92 to 0.98)	0.95 (0.93 to 0.98)	0.95 (0.93 to 0.98)	0.95 (0.93 to 0.98)	0.95 (0.93 to 0.98)	0.95 (0.93 to 0.98)	0.95 (0.93 to 0.98)
Ischaemic heart disease	300 g/day	Both	Both										0.85 (0.79 to 0.94)	0.85 (0.79 to 0.94)	0.85 (0.79 to 0.94)	0.87 (0.82 to 0.95)	0.88 (0.83 to 0.95)	0.89 (0.84 to 0.95)	0.9 (0.86 to 0.96)	0.91 (0.88 to 0.96)	0.92 (0.89 to 0.97)	0.93 (0.9 to 0.97)	0.93 (0.91 to 0.97)	0.93 (0.91 to 0.97)	0.93 (0.91 to 0.97)	0.93 (0.91 to 0.97)	0.93 (0.91 to 0.97)
Ischaemic stroke	0 g/day	Both	Both										1 (1 to 1)	1 (1 to 1)	1 (1 to 1)	1 (1 to 1)	1 (1 to 1)	1 (1 to 1)	1 (1 to 1)	1 (1 to 1)	1 (1 to 1)	1 (1 to 1)	1 (1 to 1)	1 (1 to 1)	1 (1 to 1)	1 (1 to 1)	1 (1 to 1)
Ischaemic stroke	100 g/day	Both	Both										0.91 (0.84 to 0.99)	0.91 (0.84 to 0.99)	0.91 (0.84 to 0.99)	0.92 (0.87 to 1)	0.93 (0.89 to 1)	0.95 (0.91 to 1)	0.95 (0.92 to 1)	0.96 (0.94 to 1)	0.97 (0.95 to 1)	0.97 (0.96 to 1)	0.98 (0.96 to 1)	0.98 (0.96 to 1)	0.98 (0.96 to 1)	0.98 (0.96 to 1)	0.98 (0.96 to 1)
Ischaemic stroke	200 g/day	Both	Both										0.85 (0.74 to 0.96)	0.85 (0.74 to 0.96)	0.85 (0.74 to 0.96)	0.88 (0.79 to 0.97)	0.9 (0.82 to 0.97)	0.91 (0.84 to 0.98)	0.93 (0.87 to 0.98)	0.94 (0.89 to 0.98)	0.95 (0.91 to 0.99)	0.96 (0.93 to 0.99)	0.97 (0.94 to 0.99)	0.97 (0.94 to 0.99)	0.97 (0.94 to 0.99)	0.97 (0.94 to 0.99)	0.97 (0.94 to 0.99)
Ischaemic stroke	300 g/day	Both	Both										0.78 (0.65 to 0.93)	0.78 (0.65 to 0.93)	0.78 (0.65 to 0.93)	0.82 (0.71 to 0.95)	0.85 (0.74 to 0.95)	0.87 (0.78 to 0.96)	0.89 (0.81 to 0.97)	0.91 (0.84 to 0.97)	0.92 (0.87 to 0.98)	0.94 (0.89 to 0.98)	0.95 (0.91 to 0.99)	0.95 (0.91 to 0.99)	0.95 (0.91 to 0.99)	0.95 (0.91 to 0.99)	0.95 (0.91 to 0.99)
Intracerebral haemorrhage	0 g/day	Both	Both										1 (1 to 1)	1 (1 to 1)	1 (1 to 1)	1 (1 to 1)	1 (1 to 1)	1 (1 to 1)	1 (1 to 1)	1 (1 to 1)	1 (1 to 1)	1 (1 to 1)	1 (1 to 1)	1 (1 to 1)	1 (1 to 1)	1 (1 to 1)	1 (1 to 1)
Intracerebral haemorrhage	100 g/day	Both	Both										0.86 (0.79 to 0.95)	0.86 (0.79 to 0.95)	0.86 (0.79 to 0.95)	0.88 (0.83 to 0.96)	0.89 (0.83 to 0.96)	0.9 (0.85 to 0.97)	0.91 (0.86 to 0.97)	0.92 (0.87 to 0.97)	0.92 (0.89 to 0.98)	0.93 (0.9 to 0.98)	0.94 (0.91 to 0.98)	0.94 (0.91 to 0.98)	0.94 (0.91 to 0.98)	0.94 (0.91 to 0.98)	0.94 (0.91 to 0.98)
Intracerebral haemorrhage	200 g/day	Both	Both										0.81 (0.7 to 0.92)	0.81 (0.7 to 0.92)	0.81 (0.7 to 0.92)	0.84 (0.75 to 0.93)	0.85 (0.76 to 0.94)	0.86 (0.78 to 0.94)	0.87 (0.8 to 0.95)	0.89 (0.82 to 0.95)	0.9 (0.83 to 0.96)	0.91 (0.85 to 0.96)	0.92 (0.87 to 0.97)	0.92 (0.87 to 0.97)	0.92 (0.87 to 0.97)	0.92 (0.87 to 0.97)	0.92 (0.87 to 0.97)
Intracerebral haemorrhage	300 g/day	Both	Both										0.74 (0.62 to 0.86)	0.74 (0.62 to 0.86)	0.74 (0.62 to 0.86)	0.78 (0.68 to 0.88)	0.79 (0.7 to 0.89)	0.81 (0.71 to 0.9)	0.83 (0.74 to 0.91)	0.84 (0.76 to 0.92)	0.86 (0.79 to 0.93)	0.87 (0.81 to 0.93)	0.89 (0.84 to 0.94)	0.89 (0.84 to 0.94)	0.89 (0.84 to 0.94)	0.89 (0.84 to 0.94)	0.89 (0.84 to 0.94)
Subarachnoid haemorrhage	0 g/day	Both	Both										1 (1 to 1)	1 (1 to 1)	1 (1 to 1)	1 (1 to 1)	1 (1 to 1)	1 (1 to 1)	1 (1 to 1)	1 (1 to 1)	1 (1 to 1)	1 (1 to 1)	1 (1 to 1)	1 (1 to 1)	1 (1 to 1)	1 (1 to 1)	1 (1 to 1)
Subarachnoid haemorrhage	100 g/day	Both	Both										0.86 (0.79 to 0.95)	0.86 (0.79 to 0.95)	0.86 (0.79 to 0.95)	0.88 (0.83 to 0.96)	0.89 (0.83 to 0.96)	0.9 (0.85 to 0.97)	0.91 (0.86 to 0.97)	0.92 (0.87 to 0.97)	0.92 (0.89 to 0.98)	0.93 (0.9 to 0.98)	0.94 (0.91 to 0.98)	0.94 (0.91 to 0.98)	0.94 (0.91 to 0.98)	0.94 (0.91 to 0.98)	0.94 (0.91 to 0.98)
Subarachnoid haemorrhage	200 g/day	Both	Both										0.81 (0.7 to 0.92)	0.81 (0.7 to 0.92)	0.81 (0.7 to 0.92)	0.84 (0.75 to 0.93)	0.85 (0.76 to 0.94)	0.86 (0.78 to 0.94)	0.87 (0.8 to 0.95)	0.89 (0.82 to 0.95)	0.9 (0.83 to 0.96)	0.91 (0.85 to 0.96)	0.92 (0.87 to 0.97)	0.92 (0.87 to 0.97)	0.92 (0.87 to 0.97)	0.92 (0.87 to 0.97)	0.92 (0.87 to 0.97)
Subarachnoid haemorrhage	300 g/day	Both	Both										0.74 (0.62 to 0.86)	0.74 (0.62 to 0.86)	0.74 (0.62 to 0.86)	0.78 (0.68 to 0.88)	0.79 (0.7 to 0.89)	0.81 (0.71 to 0.9)	0.83 (0.74 to 0.91)	0.84 (0.76 to 0.92)	0.86 (0.79 to 0.93)	0.87 (0.81 to 0.93)	0.89 (0.84 to 0.94)	0.89 (0.84 to 0.94)	0.89 (0.84 to 0.94)	0.89 (0.84 to 0.94)	0.89 (0.84 to 0.94)
Diabetes mellitus type 2	0 g/day	Both	Both										1 (1 to 1)	1 (1 to 1)	1 (1 to 1)	1 (1 to 1)	1 (1 to 1)	1 (1 to 1)	1 (1 to 1)	1 (1 to 1)	1 (1 to 1)	1 (1 to 1)	1 (1 to 1)	1 (1 to 1)	1 (1 to 1)	1 (1 to 1)	1 (1 to 1)
Diabetes mellitus type 2	100 g/day	Both	Both										0.94 (0.93 to 0.97)	0.94 (0.93 to 0.97)	0.94 (0.93 to 0.97)	0.94 (0.93 to 0.97)	0.94 (0.93 to 0.97)	0.94 (0.93 to 0.97)	0.94 (0.93 to 0.97)	0.94 (0.93 to 0.97)	0.94 (0.93 to 0.97)	0.94 (0.93 to 0.97)	0.94 (0.93 to 0.97)	0.94 (0.93 to 0.97)	0.94 (0.93 to 0.97)	0.94 (0.93 to 0.97)	0.94 (0.93 to 0.97)
Diabetes mellitus type 2	200 g/day	Both	Both										0.9 (0.87 to 0.93)	0.9 (0.87 to 0.93)	0.9 (0.87 to 0.93)	0.9 (0.87 to 0.93)	0.9 (0.87 to 0.93)	0.9 (0.87 to 0.93)	0.9 (0.87 to 0.93)	0.9 (0.87 to 0.93)	0.9 (0.87 to 0.93)	0.9 (0.87 to 0.93)	0.9 (0.87 to 0.93)	0.9 (0.87 to 0.93)	0.9 (0.87 to 0.93)	0.9 (0.87 to 0.93)	0.9 (0.87 to 0.93)
Diabetes mellitus type 2	300 g/day	Both	Both										0.88 (0.85 to 0.91)	0.88 (0.85 to 0.91)	0.88 (0.85 to 0.91)	0.88 (0.85 to 0.91)	0.88 (0.85 to 0.91)	0.88 (0.85 to 0.91)	0.88 (0.85 to 0.91)	0.88 (0.85 to 0.91)	0.88 (0.85 to 0.91)	0.88 (0.85 to 0.91)	0.88 (0.85 to 0.91)	0.88 (0.85 to 0.91)	0.88 (0.85 to 0.91)	0.88 (0.85 to 0.91)	0.88 (0.85 to 0.91)
**Diet low in vegetables**																											
Oesophageal cancer	0 g/day	Both	Both										1 (1 to 1)	1 (1 to 1)	1 (1 to 1)	1 (1 to 1)	1 (1 to 1)	1 (1 to 1)	1 (1 to 1)	1 (1 to 1)	1 (1 to 1)	1 (1 to 1)	1 (1 to 1)	1 (1 to 1)	1 (1 to 1)	1 (1 to 1)	1 (1 to 1)
Oesophageal cancer	100 g/day	Both	Both										0.96 (0.92 to 1)	0.96 (0.92 to 1)	0.96 (0.92 to 1)	0.96 (0.92 to 1)	0.96 (0.92 to 1)	0.96 (0.92 to 1)	0.96 (0.92 to 1)	0.96 (0.92 to 1)	0.96 (0.92 to 1)	0.96 (0.92 to 1)	0.96 (0.92 to 1)	0.96 (0.92 to 1)	0.96 (0.92 to 1)	0.96 (0.92 to 1)	0.96 (0.92 to 1)
Oesophageal cancer	200 g/day	Both	Both										0.92 (0.84 to 1)	0.92 (0.84 to 1)	0.92 (0.84 to 1)	0.92 (0.84 to 1)	0.92 (0.84 to 1)	0.92 (0.84 to 1)	0.92 (0.84 to 1)	0.92 (0.84 to 1)	0.92 (0.84 to 1)	0.92 (0.84 to 1)	0.92 (0.84 to 1)	0.92 (0.84 to 1)	0.92 (0.84 to 1)	0.92 (0.84 to 1)	0.92 (0.84 to 1)
Oesophageal cancer	300 g/day	Both	Both										0.88 (0.78 to 0.98)	0.88 (0.78 to 0.98)	0.88 (0.78 to 0.98)	0.88 (0.78 to 0.98)	0.88 (0.78 to 0.98)	0.88 (0.78 to 0.98)	0.88 (0.78 to 0.98)	0.88 (0.78 to 0.98)	0.88 (0.78 to 0.98)	0.88 (0.78 to 0.98)	0.88 (0.78 to 0.98)	0.88 (0.78 to 0.98)	0.88 (0.78 to 0.98)	0.88 (0.78 to 0.98)	0.88 (0.78 to 0.98)
Ischaemic heart disease	0 g/day	Both	Both										1 (1 to 1)	1 (1 to 1)	1 (1 to 1)	1 (1 to 1)	1 (1 to 1)	1 (1 to 1)	1 (1 to 1)	1 (1 to 1)	1 (1 to 1)	1 (1 to 1)	1 (1 to 1)	1 (1 to 1)	1 (1 to 1)	1 (1 to 1)	1 (1 to 1)
Ischaemic heart disease	100 g/day	Both	Both										0.91 (0.87 to 0.97)	0.91 (0.87 to 0.97)	0.91 (0.87 to 0.97)	0.93 (0.89 to 0.98)	0.93 (0.9 to 0.98)	0.94 (0.91 to 0.98)	0.94 (0.92 to 0.98)	0.95 (0.93 to 0.99)	0.96 (0.94 to 0.99)	0.96 (0.94 to 0.99)	0.96 (0.95 to 0.99)	0.96 (0.95 to 0.99)	0.96 (0.95 to 0.99)	0.96 (0.95 to 0.99)	0.96 (0.95 to 0.99)
Ischaemic heart disease	200 g/day	Both	Both										0.85 (0.78 to 0.92)	0.85 (0.78 to 0.92)	0.85 (0.78 to 0.92)	0.88 (0.81 to 0.93)	0.88 (0.82 to 0.94)	0.89 (0.83 to 0.94)	0.91 (0.85 to 0.95)	0.92 (0.87 to 0.96)	0.92 (0.88 to 0.96)	0.93 (0.89 to 0.96)	0.94 (0.9 to 0.97)	0.94 (0.9 to 0.97)	0.94 (0.9 to 0.97)	0.94 (0.9 to 0.97)	0.94 (0.9 to 0.97)
Ischaemic heart disease	300 g/day	Both	Both										0.8 (0.74 to 0.87)	0.8 (0.74 to 0.87)	0.8 (0.74 to 0.87)	0.83 (0.78 to 0.89)	0.84 (0.79 to 0.9)	0.85 (0.8 to 0.91)	0.87 (0.83 to 0.92)	0.89 (0.85 to 0.93)	0.9 (0.86 to 0.93)	0.91 (0.87 to 0.94)	0.91 (0.88 to 0.94)	0.91 (0.88 to 0.94)	0.91 (0.88 to 0.94)	0.91 (0.88 to 0.94)	0.91 (0.88 to 0.94)

Ischaemic stroke	0 g/day	Both	Both										1 (1 to 1)	1 (1 to 1)	1 (1 to 1)	1 (1 to 1)	1 (1 to 1)	1 (1 to 1)	1 (1 to 1)	1 (1 to 1)	1 (1 to 1)	1 (1 to 1)	1 (1 to 1)	1 (1 to 1)	1 (1 to 1)	1 (1 to 1)	1 (1 to 1)
Ischaemic stroke	100 g/day	Both	Both										0.94 (0.9 to 1)	0.94 (0.9 to 1)	0.94 (0.9 to 1)	0.95 (0.92 to 1)	0.96 (0.93 to 1)	0.97 (0.94 to 1)	0.97 (0.95 to 1)	0.98 (0.96 to 1)	0.98 (0.97 to 1)	0.98 (0.97 to 1)	0.99 (0.98 to 1)	0.99 (0.98 to 1)	0.99 (0.98 to 1)	0.99 (0.98 to 1)	0.99 (0.98 to 1)
Ischaemic stroke	200 g/day	Both	Both										0.9 (0.82 to 0.99)	0.9 (0.82 to 0.99)	0.9 (0.82 to 0.99)	0.92 (0.85 to 1)	0.93 (0.87 to 1)	0.94 (0.89 to 1)	0.95 (0.91 to 1)	0.96 (0.92 to 1)	0.97 (0.94 to 1)	0.97 (0.95 to 1)	0.98 (0.96 to 1)	0.98 (0.96 to 1)	0.98 (0.96 to 1)	0.98 (0.96 to 1)	0.98 (0.96 to 1)
Ischaemic stroke	300 g/day	Both	Both										0.86 (0.76 to 0.97)	0.86 (0.76 to 0.97)	0.86 (0.76 to 0.97)	0.89 (0.8 to 0.97)	0.9 (0.83 to 0.98)	0.92 (0.85 to 0.98)	0.93 (0.88 to 0.98)	0.94 (0.9 to 0.99)	0.95 (0.92 to 0.99)	0.96 (0.93 to 0.99)	0.97 (0.94 to 0.99)	0.97 (0.94 to 0.99)	0.97 (0.94 to 0.99)	0.97 (0.94 to 0.99)	0.97 (0.94 to 0.99)
Intracerebral haemorrhage	0 g/day	Both	Both										1 (1 to 1)	1 (1 to 1)	1 (1 to 1)	1 (1 to 1)	1 (1 to 1)	1 (1 to 1)	1 (1 to 1)	1 (1 to 1)	1 (1 to 1)	1 (1 to 1)	1 (1 to 1)	1 (1 to 1)	1 (1 to 1)	1 (1 to 1)	1 (1 to 1)
Intracerebral haemorrhage	100 g/day	Both	Both										0.92 (0.88 to 0.99)	0.92 (0.88 to 0.99)	0.92 (0.88 to 0.99)	0.93 (0.9 to 1)	0.93 (0.91 to 1)	0.94 (0.92 to 1)	0.95 (0.92 to 1)	0.95 (0.93 to 1)	0.96 (0.94 to 1)	0.96 (0.95 to 1)	0.97 (0.95 to 1)	0.97 (0.95 to 1)	0.97 (0.95 to 1)	0.97 (0.95 to 1)	0.97 (0.95 to 1)
Intracerebral haemorrhage	200 g/day	Both	Both										0.85 (0.78 to 0.97)	0.85 (0.78 to 0.97)	0.85 (0.78 to 0.97)	0.88 (0.81 to 0.97)	0.88 (0.82 to 0.97)	0.89 (0.83 to 0.98)	0.9 (0.85 to 0.98)	0.91 (0.87 to 0.98)	0.92 (0.88 to 0.98)	0.93 (0.89 to 0.98)	0.94 (0.91 to 0.99)	0.94 (0.91 to 0.99)	0.94 (0.91 to 0.99)	0.94 (0.91 to 0.99)	0.94 (0.91 to 0.99)
Intracerebral haemorrhage	300 g/day	Both	Both										0.82 (0.72 to 0.94)	0.82 (0.72 to 0.94)	0.82 (0.72 to 0.94)	0.85 (0.77 to 0.95)	0.86 (0.78 to 0.95)	0.87 (0.79 to 0.96)	0.88 (0.81 to 0.96)	0.89 (0.83 to 0.97)	0.9 (0.85 to 0.97)	0.92 (0.87 to 0.97)	0.93 (0.88 to 0.98)	0.93 (0.88 to 0.98)	0.93 (0.88 to 0.98)	0.93 (0.88 to 0.98)	0.93 (0.88 to 0.98)
Subarachnoid haemorrhage	0 g/day	Both	Both										1 (1 to 1)	1 (1 to 1)	1 (1 to 1)	1 (1 to 1)	1 (1 to 1)	1 (1 to 1)	1 (1 to 1)	1 (1 to 1)	1 (1 to 1)	1 (1 to 1)	1 (1 to 1)	1 (1 to 1)	1 (1 to 1)	1 (1 to 1)	1 (1 to 1)
Subarachnoid haemorrhage	100 g/day	Both	Both										0.92 (0.88 to 0.99)	0.92 (0.88 to 0.99)	0.92 (0.88 to 0.99)	0.93 (0.9 to 1)	0.93 (0.91 to 1)	0.94 (0.92 to 1)	0.95 (0.92 to 1)	0.95 (0.93 to 1)	0.96 (0.94 to 1)	0.96 (0.95 to 1)	0.97 (0.95 to 1)	0.97 (0.95 to 1)	0.97 (0.95 to 1)	0.97 (0.95 to 1)	0.97 (0.95 to 1)
Subarachnoid haemorrhage	200 g/day	Both	Both										0.85 (0.78 to 0.97)	0.85 (0.78 to 0.97)	0.85 (0.78 to 0.97)	0.88 (0.81 to 0.97)	0.88 (0.82 to 0.97)	0.89 (0.83 to 0.98)	0.9 (0.85 to 0.98)	0.91 (0.87 to 0.98)	0.92 (0.88 to 0.98)	0.93 (0.89 to 0.98)	0.94 (0.91 to 0.99)	0.94 (0.91 to 0.99)	0.94 (0.91 to 0.99)	0.94 (0.91 to 0.99)	0.94 (0.91 to 0.99)
Subarachnoid haemorrhage	300 g/day	Both	Both										0.82 (0.72 to 0.94)	0.82 (0.72 to 0.94)	0.82 (0.72 to 0.94)	0.85 (0.77 to 0.95)	0.86 (0.78 to 0.95)	0.87 (0.79 to 0.96)	0.88 (0.81 to 0.96)	0.89 (0.83 to 0.97)	0.9 (0.85 to 0.97)	0.92 (0.87 to 0.97)	0.93 (0.88 to 0.98)	0.93 (0.88 to 0.98)	0.93 (0.88 to 0.98)	0.93 (0.88 to 0.98)	0.93 (0.88 to 0.98)
**Diet low in whole grains**																											
Colon and rectum cancer	0 g/day	Both	Both										1 (1 to 1)	1 (1 to 1)	1 (1 to 1)	1 (1 to 1)	1 (1 to 1)	1 (1 to 1)	1 (1 to 1)	1 (1 to 1)	1 (1 to 1)	1 (1 to 1)	1 (1 to 1)	1 (1 to 1)	1 (1 to 1)	1 (1 to 1)	1 (1 to 1)
Colon and rectum cancer	50 g/day	Both	Both										0.93 (0.88 to 0.99)	0.93 (0.88 to 0.99)	0.93 (0.88 to 0.99)	0.93 (0.88 to 0.99)	0.93 (0.88 to 0.99)	0.93 (0.88 to 0.99)	0.93 (0.88 to 0.99)	0.93 (0.88 to 0.99)	0.93 (0.88 to 0.99)	0.93 (0.88 to 0.99)	0.93 (0.88 to 0.99)	0.93 (0.88 to 0.99)	0.93 (0.88 to 0.99)	0.93 (0.88 to 0.99)	0.93 (0.88 to 0.99)
Colon and rectum cancer	100 g/day	Both	Both										0.87 (0.81 to 0.96)	0.87 (0.81 to 0.96)	0.87 (0.81 to 0.96)	0.87 (0.81 to 0.96)	0.87 (0.81 to 0.96)	0.87 (0.81 to 0.96)	0.87 (0.81 to 0.96)	0.87 (0.81 to 0.96)	0.87 (0.81 to 0.96)	0.87 (0.81 to 0.96)	0.87 (0.81 to 0.96)	0.87 (0.81 to 0.96)	0.87 (0.81 to 0.96)	0.87 (0.81 to 0.96)	0.87 (0.81 to 0.96)
Colon and rectum cancer	150 g/day	Both	Both										0.79 (0.73 to 0.93)	0.79 (0.73 to 0.93)	0.79 (0.73 to 0.93)	0.79 (0.73 to 0.93)	0.79 (0.73 to 0.93)	0.79 (0.73 to 0.93)	0.79 (0.73 to 0.93)	0.79 (0.73 to 0.93)	0.79 (0.73 to 0.93)	0.79 (0.73 to 0.93)	0.79 (0.73 to 0.93)	0.79 (0.73 to 0.93)	0.79 (0.73 to 0.93)	0.79 (0.73 to 0.93)	0.79 (0.73 to 0.93)
Ischaemic heart disease	0 g/day	Both	Both										1 (1 to 1)	1 (1 to 1)	1 (1 to 1)	1 (1 to 1)	1 (1 to 1)	1 (1 to 1)	1 (1 to 1)	1 (1 to 1)	1 (1 to 1)	1 (1 to 1)	1 (1 to 1)	1 (1 to 1)	1 (1 to 1)	1 (1 to 1)	1 (1 to 1)
Ischaemic heart disease	50 g/day	Both	Both										0.8 (0.74 to 0.96)	0.8 (0.74 to 0.96)	0.8 (0.74 to 0.96)	0.83 (0.78 to 0.97)	0.83 (0.79 to 0.97)	0.85 (0.8 to 0.97)	0.87 (0.83 to 0.97)	0.88 (0.85 to 0.98)	0.89 (0.86 to 0.98)	0.9 (0.87 to 0.98)	0.91 (0.88 to 0.98)	0.91 (0.88 to 0.98)	0.91 (0.88 to 0.98)	0.91 (0.88 to 0.98)	0.91 (0.88 to 0.98)
Ischaemic heart disease	100 g/day	Both	Both										0.65 (0.56 to 0.92)	0.65 (0.56 to 0.92)	0.65 (0.56 to 0.92)	0.7 (0.62 to 0.93)	0.71 (0.63 to 0.94)	0.73 (0.65 to 0.94)	0.76 (0.69 to 0.95)	0.79 (0.72 to 0.95)	0.8 (0.75 to 0.96)	0.82 (0.77 to 0.96)	0.83 (0.78 to 0.97)	0.83 (0.78 to 0.97)	0.83 (0.78 to 0.97)	0.83 (0.78 to 0.97)	0.83 (0.78 to 0.97)
Ischaemic heart disease	150 g/day	Both	Both										0.63 (0.54 to 0.87)	0.63 (0.54 to 0.87)	0.63 (0.54 to 0.87)	0.68 (0.6 to 0.89)	0.69 (0.62 to 0.9)	0.71 (0.64 to 0.9)	0.75 (0.68 to 0.92)	0.77 (0.72 to 0.93)	0.79 (0.74 to 0.93)	0.81 (0.76 to 0.94)	0.82 (0.78 to 0.94)	0.82 (0.78 to 0.94)	0.82 (0.78 to 0.94)	0.82 (0.78 to 0.94)	0.82 (0.78 to 0.94)
Ischaemic stroke	0 g/day	Both	Both										1 (1 to 1)	1 (1 to 1)	1 (1 to 1)	1 (1 to 1)	1 (1 to 1)	1 (1 to 1)	1 (1 to 1)	1 (1 to 1)	1 (1 to 1)	1 (1 to 1)	1 (1 to 1)	1 (1 to 1)	1 (1 to 1)	1 (1 to 1)	1 (1 to 1)
Ischaemic stroke	50 g/day	Both	Both										0.93 (0.9 to 1)	0.93 (0.9 to 1)	0.93 (0.9 to 1)	0.94 (0.92 to 1)	0.95 (0.93 to 1)	0.96 (0.94 to 1)	0.96 (0.95 to 1)	0.97 (0.96 to 1)	0.98 (0.97 to 1)	0.98 (0.97 to 1)	0.98 (0.98 to 1)	0.98 (0.98 to 1)	0.98 (0.98 to 1)	0.98 (0.98 to 1)	0.98 (0.98 to 1)
Ischaemic stroke	100 g/day	Both	Both										0.86 (0.81 to 0.98)	0.86 (0.81 to 0.98)	0.86 (0.81 to 0.98)	0.89 (0.84 to 0.98)	0.9 (0.86 to 0.99)	0.92 (0.89 to 0.99)	0.93 (0.9 to 0.99)	0.94 (0.92 to 0.99)	0.95 (0.93 to 0.99)	0.96 (0.95 to 0.99)	0.97 (0.96 to 1)	0.97 (0.96 to 1)	0.97 (0.96 to 1)	0.97 (0.96 to 1)	0.97 (0.96 to 1)
Ischaemic stroke	150 g/day	Both	Both										0.83 (0.75 to 0.96)	0.83 (0.75 to 0.96)	0.83 (0.75 to 0.96)	0.86 (0.8 to 0.97)	0.88 (0.82 to 0.97)	0.9 (0.85 to 0.98)	0.91 (0.87 to 0.98)	0.93 (0.9 to 0.98)	0.94 (0.91 to 0.99)	0.95 (0.93 to 0.99)	0.96 (0.94 to 0.99)	0.96 (0.94 to 0.99)	0.96 (0.94 to 0.99)	0.96 (0.94 to 0.99)	0.96 (0.94 to 0.99)
Diabetes mellitus type 2	0 g/day	Both	Both										1 (1 to 1)	1 (1 to 1)	1 (1 to 1)	1 (1 to 1)	1 (1 to 1)	1 (1 to 1)	1 (1 to 1)	1 (1 to 1)	1 (1 to 1)	1 (1 to 1)	1 (1 to 1)	1 (1 to 1)	1 (1 to 1)	1 (1 to 1)	1 (1 to 1)
Diabetes mellitus type 2	50 g/day	Both	Both										0.98 (0.97 to 1)	0.98 (0.97 to 1)	0.98 (0.97 to 1)	0.98 (0.97 to 1)	0.98 (0.97 to 1)	0.98 (0.97 to 1)	0.98 (0.97 to 1)	0.98 (0.97 to 1)	0.98 (0.97 to 1)	0.98 (0.97 to 1)	0.98 (0.97 to 1)	0.98 (0.97 to 1)	0.98 (0.97 to 1)	0.98 (0.97 to 1)	0.98 (0.97 to 1)
Diabetes mellitus type 2	100 g/day	Both	Both										0.96 (0.94 to 0.99)	0.96 (0.94 to 0.99)	0.96 (0.94 to 0.99)	0.96 (0.94 to 0.99)	0.96 (0.94 to 0.99)	0.96 (0.94 to 0.99)	0.96 (0.94 to 0.99)	0.96 (0.94 to 0.99)	0.96 (0.94 to 0.99)	0.96 (0.94 to 0.99)	0.96 (0.94 to 0.99)	0.96 (0.94 to 0.99)	0.96 (0.94 to 0.99)	0.96 (0.94 to 0.99)	0.96 (0.94 to 0.99)
Diabetes mellitus type 2	150 g/day	Both	Both										0.94 (0.91 to 0.98)	0.94 (0.91 to 0.98)	0.94 (0.91 to 0.98)	0.94 (0.91 to 0.98)	0.94 (0.91 to 0.98)	0.94 (0.91 to 0.98)	0.94 (0.91 to 0.98)	0.94 (0.91 to 0.98)	0.94 (0.91 to 0.98)	0.94 (0.91 to 0.98)	0.94 (0.91 to 0.98)	0.94 (0.91 to 0.98)	0.94 (0.91 to 0.98)	0.94 (0.91 to 0.98)	0.94 (0.91 to 0.98)
**Diet low in nuts and seeds**																											
Ischaemic heart disease	0 g/day	Both	Both										1 (1 to 1)	1 (1 to 1)	1 (1 to 1)	1 (1 to 1)	1 (1 to 1)	1 (1 to 1)	1 (1 to 1)	1 (1 to 1)	1 (1 to 1)	1 (1 to 1)	1 (1 to 1)	1 (1 to 1)	1 (1 to 1)	1 (1 to 1)	1 (1 to 1)
Ischaemic heart disease	7 g/day	Both	Both										0.77 (0.72 to 0.85)	0.77 (0.72 to 0.85)	0.77 (0.72 to 0.85)	0.81 (0.76 to 0.87)	0.81 (0.77 to 0.88)	0.83 (0.79 to 0.89)	0.85 (0.81 to 0.9)	0.87 (0.83 to 0.91)	0.88 (0.85 to 0.92)	0.89 (0.86 to 0.93)	0.9 (0.87 to 0.93)	0.9 (0.87 to 0.93)	0.9 (0.87 to 0.93)	0.9 (0.87 to 0.93)	0.9 (0.87 to 0.93)
Ischaemic heart disease	14 g/day	Both	Both										0.74 (0.68 to 0.83)	0.74 (0.68 to 0.83)	0.74 (0.68 to 0.83)	0.78 (0.72 to 0.86)	0.79 (0.73 to 0.86)	0.8 (0.75 to 0.88)	0.83 (0.78 to 0.89)	0.85 (0.81 to 0.9)	0.86 (0.82 to 0.91)	0.87 (0.84 to 0.92)	0.88 (0.85 to 0.93)	0.88 (0.85 to 0.93)	0.88 (0.85 to 0.93)	0.88 (0.85 to 0.93)	0.88 (0.85 to 0.93)
Ischaemic heart disease	21 g/day	Both	Both										0.73 (0.66 to 0.83)	0.73 (0.66 to 0.83)	0.73 (0.66 to 0.83)	0.77 (0.71 to 0.85)	0.78 (0.72 to 0.86)	0.8 (0.74 to 0.87)	0.82 (0.77 to 0.89)	0.84 (0.8 to 0.9)	0.86 (0.82 to 0.91)	0.87 (0.83 to 0.92)	0.88 (0.84 to 0.92)	0.88 (0.84 to 0.92)	0.88 (0.84 to 0.92)	0.88 (0.84 to 0.92)	0.88 (0.84 to 0.92)
Ischaemic heart disease	28 g/day	Both	Both										0.72 (0.65 to 0.82)	0.72 (0.65 to 0.82)	0.72 (0.65 to 0.82)	0.76 (0.7 to 0.85)	0.77 (0.71 to 0.86)	0.79 (0.73 to 0.87)	0.82 (0.76 to 0.88)	0.84 (0.79 to 0.9)	0.85 (0.81 to 0.91)	0.86 (0.82 to 0.92)	0.87 (0.84 to 0.92)	0.87 (0.84 to 0.92)	0.87 (0.84 to 0.92)	0.87 (0.84 to 0.92)	0.87 (0.84 to 0.92)
Diabetes mellitus type 2	0 g/day	Both	Both										1 (1 to 1)	1 (1 to 1)	1 (1 to 1)	1 (1 to 1)	1 (1 to 1)	1 (1 to 1)	1 (1 to 1)	1 (1 to 1)	1 (1 to 1)	1 (1 to 1)	1 (1 to 1)	1 (1 to 1)	1 (1 to 1)	1 (1 to 1)	1 (1 to 1)
Diabetes mellitus type 2	7 g/day	Both	Both										0.98 (0.96 to 1)	0.98 (0.96 to 1)	0.98 (0.96 to 1)	0.98 (0.96 to 1)	0.98 (0.96 to 1)	0.98 (0.96 to 1)	0.98 (0.96 to 1)	0.98 (0.96 to 1)	0.98 (0.96 to 1)	0.98 (0.96 to 1)	0.98 (0.96 to 1)	0.98 (0.96 to 1)	0.98 (0.96 to 1)	0.98 (0.96 to 1)	0.98 (0.96 to 1)
Diabetes mellitus type 2	14 g/day	Both	Both										0.97 (0.93 to 1)	0.97 (0.93 to 1)	0.97 (0.93 to 1)	0.97 (0.93 to 1)	0.97 (0.93 to 1)	0.97 (0.93 to 1)	0.97 (0.93 to 1)	0.97 (0.93 to 1)	0.97 (0.93 to 1)	0.97 (0.93 to 1)	0.97 (0.93 to 1)	0.97 (0.93 to 1)	0.97 (0.93 to 1)	0.97 (0.93 to 1)	0.97 (0.93 to 1)
Diabetes mellitus type 2	21 g/day	Both	Both										0.97 (0.93 to 1)	0.97 (0.93 to 1)	0.97 (0.93 to 1)	0.97 (0.93 to 1)	0.97 (0.93 to 1)	0.97 (0.93 to 1)	0.97 (0.93 to 1)	0.97 (0.93 to 1)	0.97 (0.93 to 1)	0.97 (0.93 to 1)	0.97 (0.93 to 1)	0.97 (0.93 to 1)	0.97 (0.93 to 1)	0.97 (0.93 to 1)	0.97 (0.93 to 1)
Diabetes mellitus type 2	28 g/day	Both	Both										0.96 (0.92 to 1)	0.96 (0.92 to 1)	0.96 (0.92 to 1)	0.96 (0.92 to 1)	0.96 (0.92 to 1)	0.96 (0.92 to 1)	0.96 (0.92 to 1)	0.96 (0.92 to 1)	0.96 (0.92 to 1)	0.96 (0.92 to 1)	0.96 (0.92 to 1)	0.96 (0.92 to 1)	0.96 (0.92 to 1)	0.96 (0.92 to 1)	0.96 (0.92 to 1)
**Diet low in milk**																											
Colon and rectum cancer	0 g/day	Both	Both										1 (1 to 1)	1 (1 to 1)	1 (1 to 1)	1 (1 to 1)	1 (1 to 1)	1 (1 to 1)	1 (1 to 1)	1 (1 to 1)	1 (1 to 1)	1 (1 to 1)	1 (1 to 1)	1 (1 to 1)	1 (1 to 1)	1 (1 to 1)	1 (1 to 1)
Colon and rectum cancer	60 g/day	Both	Both										0.97 (0.94 to 1)	0.97 (0.94 to 1)	0.97 (0.94 to 1)	0.97 (0.94 to 1)	0.97 (0.94 to 1)	0.97 (0.94 to 1)	0.97 (0.94 to 1)	0.97 (0.94 to 1)	0.97 (0.94 to 1)	0.97 (0.94 to 1)	0.97 (0.94 to 1)	0.97 (0.94 to 1)	0.97 (0.94 to 1)	0.97 (0.94 to 1)	0.97 (0.94 to 1)
Colon and rectum cancer	120 g/day	Both	Both										0.95 (0.9 to 0.99)	0.95 (0.9 to 0.99)	0.95 (0.9 to 0.99)	0.95 (0.9 to 0.99)	0.95 (0.9 to 0.99)	0.95 (0.9 to 0.99)	0.95 (0.9 to 0.99)	0.95 (0.9 to 0.99)	0.95 (0.9 to 0.99)	0.95 (0.9 to 0.99)	0.95 (0.9 to 0.99)	0.95 (0.9 to 0.99)	0.95 (0.9 to 0.99)	0.95 (0.9 to 0.99)	0.95 (0.9 to 0.99)
Colon and rectum cancer	180 g/day	Both	Both										0.92 (0.87 to 0.97)	0.92 (0.87 to 0.97)	0.92 (0.87 to 0.97)	0.92 (0.87 to 0.97)	0.92 (0.87 to 0.97)	0.92 (0.87 to 0.97)	0.92 (0.87 to 0.97)	0.92 (0.87 to 0.97)	0.92 (0.87 to 0.97)	0.92 (0.87 to 0.97)	0.92 (0.87 to 0.97)	0.92 (0.87 to 0.97)	0.92 (0.87 to 0.97)	0.92 (0.87 to 0.97)	0.92 (0.87 to 0.97)
Colon and rectum cancer	240 g/day	Both	Both										0.89 (0.84 to 0.94)	0.89 (0.84 to 0.94)	0.89 (0.84 to 0.94)	0.89 (0.84 to 0.94)	0.89 (0.84 to 0.94)	0.89 (0.84 to 0.94)	0.89 (0.84 to 0.94)	0.89 (0.84 to 0.94)	0.89 (0.84 to 0.94)	0.89 (0.84 to 0.94)	0.89 (0.84 to 0.94)	0.89 (0.84 to 0.94)	0.89 (0.84 to 0.94)	0.89 (0.84 to 0.94)	0.89 (0.84 to 0.94)
**Diet high in red meat**																											
Breast cancer	0 g/day	Both	Both										1 (1 to 1)	1 (1 to 1)	1 (1 to 1)	1 (1 to 1)	1 (1 to 1)	1 (1 to 1)	1 (1 to 1)	1 (1 to 1)	1 (1 to 1)	1 (1 to 1)	1 (1 to 1)	1 (1 to 1)	1 (1 to 1)	1 (1 to 1)	1 (1 to 1)
Breast cancer	50 g/day	Both	Both										1.05 (1.03 to 1.07)	1.05 (1.03 to 1.07)	1.05 (1.03 to 1.07)	1.05 (1.03 to 1.07)	1.05 (1.03 to 1.07)	1.05 (1.03 to 1.07)	1.05 (1.03 to 1.07)	1.05 (1.03 to 1.07)	1.05 (1.03 to 1.07)	1.05 (1.03 to 1.07)	1.05 (1.03 to 1.07)	1.05 (1.03 to 1.07)	1.05 (1.03 to 1.07)	1.05 (1.03 to 1.07)	1.05 (1.03 to 1.07)
Breast cancer	100 g/day	Both	Both										1.07 (1.03 to 1.12)	1.07 (1.03 to 1.12)	1.07 (1.03 to 1.12)	1.07 (1.03 to 1.12)	1.07 (1.03 to 1.12)	1.07 (1.03 to 1.12)	1.07 (1.03 to 1.12)	1.07 (1.03 to 1.12)	1.07 (1.03 to 1.12)	1.07 (1.03 to 1.12)	1.07 (1.03 to 1.12)	1.07 (1.03 to 1.12)	1.07 (1.03 to 1.12)	1.07 (1.03 to 1.12)	1.07 (1.03 to 1.12)
Breast cancer	150 g/day	Both	Both										1.08 (1.03 to 1.18)	1.08 (1.03 to 1.18)	1.08 (1.03 to 1.18)	1.08 (1.03 to 1.18)	1.08 (1.03 to 1.18)	1.08 (1.03 to 1.18)	1.08 (1.03 to 1.18)	1.08 (1.03 to 1.18)	1.08 (1.03 to 1.18)	1.08 (1.03 to 1.18)	1.08 (1.03 to 1.18)	1.08 (1.03 to 1.18)	1.08 (1.03 to 1.18)	1.08 (1.03 to 1.18)	1.08 (1.03 to 1.18)
Breast cancer	200 g/day	Both	Both										1.1 (1.03 to 1.24)	1.1 (1.03 to 1.24)	1.1 (1.03 to 1.24)	1.1 (1.03 to 1.24)	1.1 (1.03 to 1.24)	1.1 (1.03 to 1.24)	1.1 (1.03 to 1.24)	1.1 (1.03 to 1.24)	1.1 (1.03 to 1.24)	1.1 (1.03 to 1.24)	1.1 (1.03 to 1.24)	1.1 (1.03 to 1.24)	1.1 (1.03 to 1.24)	1.1 (1.03 to 1.24)	1.1 (1.03 to 1.24)
Colon and rectum cancer	0 g/day	Both	Both										1 (1 to 1)	1 (1 to 1)	1 (1 to 1)	1 (1 to 1)	1 (1 to 1)	1 (1 to 1)	1 (1 to 1)	1 (1 to 1)	1 (1 to 1)	1 (1 to 1)	1 (1 to 1)	1 (1 to 1)	1 (1 to 1)	1 (1 to 1)	1 (1 to 1)
Colon and rectum cancer	50 g/day	Both	Both										1.08 (1.02 to 1.16)	1.08 (1.02 to 1.16)	1.08 (1.02 to 1.16)	1.08 (1.02 to 1.16)	1.08 (1.02 to 1.16)	1.08 (1.02 to 1.16)	1.08 (1.02 to 1.16)	1.08 (1.02 to 1.16)	1.08 (1.02 to 1.16)	1.08 (1.02 to 1.16)	1.08 (1.02 to 1.16)	1.08 (1.02 to 1.16)	1.08 (1.02 to 1.16)	1.08 (1.02 to 1.16)	1.08 (1.02 to 1.16)
Colon and rectum cancer	100 g/day	Both	Both										1.16 (1.08 to 1.25)	1.16 (1.08 to 1.25)	1.16 (1.08 to 1.25)	1.16 (1.08 to 1.25)	1.16 (1.08 to 1.25)	1.16 (1.08 to 1.25)	1.16 (1.08 to 1.25)	1.16 (1.08 to 1.25)	1.16 (1.08 to 1.25)	1.16 (1.08 to 1.25)	1.16 (1.08 to 1.25)	1.16 (1.08 to 1.25)	1.16 (1.08 to 1.25)	1.16 (1.08 to 1.25)	1.16 (1.08 to 1.25)
Colon and rectum cancer	150 g/day	Both	Both										1.22 (1.1 to 1.35)	1.22 (1.1 to 1.35)	1.22 (1.1 to 1.35)	1.22 (1.1 to 1.35)	1.22 (1.1 to 1.35)	1.22 (1.1 to 1.35)	1.22 (1.1 to 1.35)	1.22 (1.1 to 1.35)	1.22 (1.1 to 1.35)	1.22 (1.1 to 1.35)	1.22 (1.1 to 1.35)	1.22 (1.1 to 1.35)	1.22 (1.1 to 1.35)	1.22 (1.1 to 1.35)	1.22 (1.1 to 1.35)
Colon and rectum cancer	200 g/day	Both	Both										1.27 (1.11 to 1.46)	1.27 (1.11 to 1.46)	1.27 (1.11 to 1.46)	1.27 (1.11 to 1.46)	1.27 (1.11 to 1.46)	1.27 (1.11 to 1.46)	1.27 (1.11 to 1.46)	1.27 (1.11 to 1.46)	1.27 (1.11 to 1.46)	1.27 (1.11 to 1.46)	1.27 (1.11 to 1.46)	1.27 (1.11 to 1.46)	1.27 (1.11 to 1.46)	1.27 (1.11 to 1.46)	1.27 (1.11 to 1.46)
Ischaemic heart disease	0 g/day	Both	Both										1 (1 to 1)	1 (1 to 1)	1 (1 to 1)	1 (1 to 1)	1 (1 to 1)	1 (1 to 1)	1 (1 to 1)	1 (1 to 1)	1 (1 to 1)	1 (1 to 1)	1 (1 to 1)	1 (1 to 1)	1 (1 to 1)	1 (1 to 1)	1 (1 to 1)
Ischaemic heart disease	50 g/day	Both	Both										1.15 (1.01 to 1.3)	1.15 (1.01 to 1.3)	1.15 (1.01 to 1.3)	1.12 (1.01 to 1.24)	1.11 (1.01 to 1.23)	1.1 (1.01 to 1.21)	1.09 (1.01 to 1.18)	1.08 (1.01 to 1.15)	1.07 (1.01 to 1.14)	1.06 (1.01 to 1.12)	1.06 (1.01 to 1.11)	1.06 (1.01 to 1.11)	1.06 (1.01 to 1.11)	1.06 (1.01 to 1.11)	1.06 (1.01 to 1.11)
Ischaemic heart disease	100 g/day	Both	Both										1.34 (1.14 to 1.56)	1.34 (1.14 to 1.56)	1.34 (1.14 to 1.56)	1.27 (1.12 to 1.44)	1.26 (1.11 to 1.42)	1.24 (1.1 to 1.38)	1.2 (1.09 to 1.32)	1.17 (1.08 to 1.28)	1.15 (1.07 to 1.25)	1.14 (1.06 to 1.22)	1.13 (1.06 to 1.2)	1.13 (1.06 to 1.2)	1.13 (1.06 to 1.2)	1.13 (1.06 to 1.2)	1.13 (1.06 to 1.2)
Ischaemic heart disease	150 g/day	Both	Both										1.47 (1.21 to 1.76)	1.47 (1.21 to 1.76)	1.47 (1.21 to 1.76)	1.37 (1.17 to 1.6)	1.35 (1.16 to 1.57)	1.32 (1.15 to 1.51)	1.27 (1.13 to 1.43)	1.23 (1.11 to 1.36)	1.21 (1.1 to 1.32)	1.19 (1.09 to 1.29)	1.17 (1.08 to 1.26)	1.17 (1.08 to 1.26)	1.17 (1.08 to 1.26)	1.17 (1.08 to 1.26)	1.17 (1.08 to 1.26)
Ischaemic heart disease	200 g/day	Both	Both										1.6 (1.25 to 2.01)	1.6 (1.25 to 2.01)	1.6 (1.25 to 2.01)	1.47 (1.2 to 1.78)	1.45 (1.19 to 1.74)	1.4 (1.17 to 1.66)	1.34 (1.15 to 1.55)	1.29 (1.13 to 1.47)	1.26 (1.12 to 1.41)	1.23 (1.1 to 1.37)	1.21 (1.1 to 1.34)	1.21 (1.1 to 1.34)	1.21 (1.1 to 1.34)	1.21 (1.1 to 1.34)	1.21 (1.1 to 1.34)
Ischaemic stroke	0 g/day	Both	Both										1 (1 to 1)	1 (1 to 1)	1 (1 to 1)	1 (1 to 1)	1 (1 to 1)	1 (1 to 1)	1 (1 to 1)	1 (1 to 1)	1 (1 to 1)	1 (1 to 1)	1 (1 to 1)	1 (1 to 1)	1 (1 to 1)	1 (1 to 1)	1 (1 to 1)
Ischaemic stroke	50 g/day	Both	Both										1.47 (1.25 to 1.63)	1.47 (1.25 to 1.63)	1.47 (1.25 to 1.63)	1.36 (1.19 to 1.48)	1.3 (1.16 to 1.39)	1.25 (1.13 to 1.32)	1.2 (1.11 to 1.26)	1.16 (1.09 to 1.21)	1.13 (1.07 to 1.17)	1.11 (1.06 to 1.13)	1.08 (1.05 to 1.11)	1.08 (1.05 to 1.11)	1.08 (1.05 to 1.11)	1.08 (1.05 to 1.11)	1.08 (1.05 to 1.11)
Ischaemic stroke	100 g/day	Both	Both										1.72 (1.44 to 1.97)	1.72 (1.44 to 1.97)	1.72 (1.44 to 1.97)	1.54 (1.33 to 1.72)	1.45 (1.28 to 1.59)	1.36 (1.23 to 1.47)	1.29 (1.19 to 1.38)	1.23 (1.15 to 1.3)	1.19 (1.12 to 1.24)	1.15 (1.1 to 1.19)	1.12 (1.08 to 1.15)	1.12 (1.08 to 1.15)	1.12 (1.08 to 1.15)	1.12 (1.08 to 1.15)	1.12 (1.08 to 1.15)
Ischaemic stroke	150 g/day	Both	Both										1.87 (1.46 to 2.3)	1.87 (1.46 to 2.3)	1.87 (1.46 to 2.3)	1.65 (1.35 to 1.94)	1.53 (1.3 to 1.76)	1.43 (1.24 to 1.61)	1.34 (1.2 to 1.48)	1.27 (1.16 to 1.38)	1.22 (1.13 to 1.3)	1.17 (1.1 to 1.24)	1.14 (1.08 to 1.19)	1.14 (1.08 to 1.19)	1.14 (1.08 to 1.19)	1.14 (1.08 to 1.19)	1.14 (1.08 to 1.19)
Ischaemic stroke	200 g/day	Both	Both										2.03 (1.47 to 2.7)	2.03 (1.47 to 2.7)	2.03 (1.47 to 2.7)	1.75 (1.36 to 2.21)	1.61 (1.3 to 1.97)	1.49 (1.25 to 1.76)	1.39 (1.2 to 1.6)	1.31 (1.16 to 1.47)	1.25 (1.13 to 1.37)	1.2 (1.11 to 1.29)	1.16 (1.08 to 1.23)	1.16 (1.08 to 1.23)	1.16 (1.08 to 1.23)	1.16 (1.08 to 1.23)	1.16 (1.08 to 1.23)
Intracerebral haemorrhage	0 g/day	Both	Both										1 (1 to 1)	1 (1 to 1)	1 (1 to 1)	1 (1 to 1)	1 (1 to 1)	1 (1 to 1)	1 (1 to 1)	1 (1 to 1)	1 (1 to 1)	1 (1 to 1)	1 (1 to 1)	1 (1 to 1)	1 (1 to 1)	1 (1 to 1)	1 (1 to 1)
Intracerebral haemorrhage	50 g/day	Both	Both										1.32 (1.17 to 1.47)	1.32 (1.17 to 1.47)	1.32 (1.17 to 1.47)	1.26 (1.13 to 1.37)	1.24 (1.13 to 1.34)	1.22 (1.12 to 1.31)	1.2 (1.1 to 1.28)	1.17 (1.09 to 1.24)	1.15 (1.08 to 1.21)	1.13 (1.07 to 1.19)	1.11 (1.06 to 1.16)	1.11 (1.06 to 1.16)	1.11 (1.06 to 1.16)	1.11 (1.06 to 1.16)	1.11 (1.06 to 1.16)
Intracerebral haemorrhage	100 g/day	Both	Both										1.43 (1.26 to 1.61)	1.43 (1.26 to 1.61)	1.43 (1.26 to 1.61)	1.34 (1.21 to 1.47)	1.32 (1.19 to 1.44)	1.29 (1.18 to 1.4)	1.26 (1.16 to 1.35)	1.23 (1.14 to 1.31)	1.2 (1.12 to 1.27)	1.17 (1.11 to 1.24)	1.15 (1.09 to 1.2)	1.15 (1.09 to 1.2)	1.15 (1.09 to 1.2)	1.15 (1.09 to 1.2)	1.15 (1.09 to 1.2)
Intracerebral haemorrhage	150 g/day	Both	Both										1.47 (1.26 to 1.76)	1.47 (1.26 to 1.76)	1.47 (1.26 to 1.76)	1.37 (1.21 to 1.59)	1.35 (1.2 to 1.55)	1.32 (1.18 to 1.5)	1.28 (1.16 to 1.43)	1.24 (1.14 to 1.38)	1.22 (1.13 to 1.33)	1.19 (1.11 to 1.29)	1.16 (1.09 to 1.24)	1.16 (1.09 to 1.24)	1.16 (1.09 to 1.24)	1.16 (1.09 to 1.24)	1.16 (1.09 to 1.24)
Intracerebral haemorrhage	200 g/day	Both	Both										1.51 (1.27 to 1.97)	1.51 (1.27 to 1.97)	1.51 (1.27 to 1.97)	1.4 (1.21 to 1.74)	1.37 (1.2 to 1.68)	1.34 (1.18 to 1.62)	1.3 (1.16 to 1.54)	1.26 (1.14 to 1.47)	1.23 (1.13 to 1.41)	1.2 (1.11 to 1.35)	1.17 (1.09 to 1.29)	1.17 (1.09 to 1.29)	1.17 (1.09 to 1.29)	1.17 (1.09 to 1.29)	1.17 (1.09 to 1.29)
Subarachnoid haemorrhage	0 g/day	Both	Both										1 (1 to 1)	1 (1 to 1)	1 (1 to 1)	1 (1 to 1)	1 (1 to 1)	1 (1 to 1)	1 (1 to 1)	1 (1 to 1)	1 (1 to 1)	1 (1 to 1)	1 (1 to 1)	1 (1 to 1)	1 (1 to 1)	1 (1 to 1)	1 (1 to 1)
Subarachnoid haemorrhage	50 g/day	Both	Both										1.32 (1.17 to 1.47)	1.32 (1.17 to 1.47)	1.32 (1.17 to 1.47)	1.26 (1.13 to 1.37)	1.24 (1.13 to 1.34)	1.22 (1.12 to 1.31)	1.2 (1.1 to 1.28)	1.17 (1.09 to 1.24)	1.15 (1.08 to 1.21)	1.13 (1.07 to 1.19)	1.11 (1.06 to 1.16)	1.11 (1.06 to 1.16)	1.11 (1.06 to 1.16)	1.11 (1.06 to 1.16)	1.11 (1.06 to 1.16)
Subarachnoid haemorrhage	100 g/day	Both	Both										1.43 (1.26 to 1.61)	1.43 (1.26 to 1.61)	1.43 (1.26 to 1.61)	1.34 (1.21 to 1.47)	1.32 (1.19 to 1.44)	1.29 (1.18 to 1.4)	1.26 (1.16 to 1.35)	1.23 (1.14 to 1.31)	1.2 (1.12 to 1.27)	1.17 (1.11 to 1.24)	1.15 (1.09 to 1.2)	1.15 (1.09 to 1.2)	1.15 (1.09 to 1.2)	1.15 (1.09 to 1.2)	1.15 (1.09 to 1.2)
Subarachnoid haemorrhage	150 g/day	Both	Both										1.47 (1.26 to 1.76)	1.47 (1.26 to 1.76)	1.47 (1.26 to 1.76)	1.37 (1.21 to 1.59)	1.35 (1.2 to 1.55)	1.32 (1.18 to 1.5)	1.28 (1.16 to 1.43)	1.24 (1.14 to 1.38)	1.22 (1.13 to 1.33)	1.19 (1.11 to 1.29)	1.16 (1.09 to 1.24)	1.16 (1.09 to 1.24)	1.16 (1.09 to 1.24)	1.16 (1.09 to 1.24)	1.16 (1.09 to 1.24)
Subarachnoid haemorrhage	200 g/day	Both	Both										1.51 (1.27 to 1.97)	1.51 (1.27 to 1.97)	1.51 (1.27 to 1.97)	1.4 (1.21 to 1.74)	1.37 (1.2 to 1.68)	1.34 (1.18 to 1.62)	1.3 (1.16 to 1.54)	1.26 (1.14 to 1.47)	1.23 (1.13 to 1.41)	1.2 (1.11 to 1.35)	1.17 (1.09 to 1.29)	1.17 (1.09 to 1.29)	1.17 (1.09 to 1.29)	1.17 (1.09 to 1.29)	1.17 (1.09 to 1.29)
Diabetes mellitus type 2	0 g/day	Both	Both										1 (1 to 1)	1 (1 to 1)	1 (1 to 1)	1 (1 to 1)	1 (1 to 1)	1 (1 to 1)	1 (1 to 1)	1 (1 to 1)	1 (1 to 1)	1 (1 to 1)	1 (1 to 1)	1 (1 to 1)	1 (1 to 1)	1 (1 to 1)	1 (1 to 1)
Diabetes mellitus type 2	50 g/day	Both	Both										1.14 (1.1 to 1.19)	1.14 (1.1 to 1.19)	1.14 (1.1 to 1.19)	1.14 (1.1 to 1.19)	1.14 (1.1 to 1.19)	1.14 (1.1 to 1.19)	1.14 (1.1 to 1.19)	1.14 (1.1 to 1.19)	1.14 (1.1 to 1.19)	1.14 (1.1 to 1.19)	1.14 (1.1 to 1.19)	1.14 (1.1 to 1.19)	1.14 (1.1 to 1.19)	1.14 (1.1 to 1.19)	1.14 (1.1 to 1.19)
Diabetes mellitus type 2	100 g/day	Both	Both										1.2 (1.13 to 1.27)	1.2 (1.13 to 1.27)	1.2 (1.13 to 1.27)	1.2 (1.13 to 1.27)	1.2 (1.13 to 1.27)	1.2 (1.13 to 1.27)	1.2 (1.13 to 1.27)	1.2 (1.13 to 1.27)	1.2 (1.13 to 1.27)	1.2 (1.13 to 1.27)	1.2 (1.13 to 1.27)	1.2 (1.13 to 1.27)	1.2 (1.13 to 1.27)	1.2 (1.13 to 1.27)	1.2 (1.13 to 1.27)
Diabetes mellitus type 2	150 g/day	Both	Both										1.24 (1.14 to 1.36)	1.24 (1.14 to 1.36)	1.24 (1.14 to 1.36)	1.24 (1.14 to 1.36)	1.24 (1.14 to 1.36)	1.24 (1.14 to 1.36)	1.24 (1.14 to 1.36)	1.24 (1.14 to 1.36)	1.24 (1.14 to 1.36)	1.24 (1.14 to 1.36)	1.24 (1.14 to 1.36)	1.24 (1.14 to 1.36)	1.24 (1.14 to 1.36)	1.24 (1.14 to 1.36)	1.24 (1.14 to 1.36)
Diabetes mellitus type 2	200 g/day	Both	Both										1.27 (1.14 to 1.45)	1.27 (1.14 to 1.45)	1.27 (1.14 to 1.45)	1.27 (1.14 to 1.45)	1.27 (1.14 to 1.45)	1.27 (1.14 to 1.45)	1.27 (1.14 to 1.45)	1.27 (1.14 to 1.45)	1.27 (1.14 to 1.45)	1.27 (1.14 to 1.45)	1.27 (1.14 to 1.45)	1.27 (1.14 to 1.45)	1.27 (1.14 to 1.45)	1.27 (1.14 to 1.45)	1.27 (1.14 to 1.45)
**Diet high in processed meat**																											
Colon and rectum cancer	0 g/day	Both	Both										1 (1 to 1)	1 (1 to 1)	1 (1 to 1)	1 (1 to 1)	1 (1 to 1)	1 (1 to 1)	1 (1 to 1)	1 (1 to 1)	1 (1 to 1)	1 (1 to 1)	1 (1 to 1)	1 (1 to 1)	1 (1 to 1)	1 (1 to 1)	1 (1 to 1)
Colon and rectum cancer	25 g/day	Both	Both										1.06 (1.02 to 1.1)	1.06 (1.02 to 1.1)	1.06 (1.02 to 1.1)	1.06 (1.02 to 1.1)	1.06 (1.02 to 1.1)	1.06 (1.02 to 1.1)	1.06 (1.02 to 1.1)	1.06 (1.02 to 1.1)	1.06 (1.02 to 1.1)	1.06 (1.02 to 1.1)	1.06 (1.02 to 1.1)	1.06 (1.02 to 1.1)	1.06 (1.02 to 1.1)	1.06 (1.02 to 1.1)	1.06 (1.02 to 1.1)
Colon and rectum cancer	50 g/day	Both	Both										1.08 (1.03 to 1.13)	1.08 (1.03 to 1.13)	1.08 (1.03 to 1.13)	1.08 (1.03 to 1.13)	1.08 (1.03 to 1.13)	1.08 (1.03 to 1.13)	1.08 (1.03 to 1.13)	1.08 (1.03 to 1.13)	1.08 (1.03 to 1.13)	1.08 (1.03 to 1.13)	1.08 (1.03 to 1.13)	1.08 (1.03 to 1.13)	1.08 (1.03 to 1.13)	1.08 (1.03 to 1.13)	1.08 (1.03 to 1.13)
Colon and rectum cancer	75 g/day	Both	Both										1.09 (1.03 to 1.18)	1.09 (1.03 to 1.18)	1.09 (1.03 to 1.18)	1.09 (1.03 to 1.18)	1.09 (1.03 to 1.18)	1.09 (1.03 to 1.18)	1.09 (1.03 to 1.18)	1.09 (1.03 to 1.18)	1.09 (1.03 to 1.18)	1.09 (1.03 to 1.18)	1.09 (1.03 to 1.18)	1.09 (1.03 to 1.18)	1.09 (1.03 to 1.18)	1.09 (1.03 to 1.18)	1.09 (1.03 to 1.18)
Colon and rectum cancer	100 g/day	Both	Both										1.1 (1.03 to 1.22)	1.1 (1.03 to 1.22)	1.1 (1.03 to 1.22)	1.1 (1.03 to 1.22)	1.1 (1.03 to 1.22)	1.1 (1.03 to 1.22)	1.1 (1.03 to 1.22)	1.1 (1.03 to 1.22)	1.1 (1.03 to 1.22)	1.1 (1.03 to 1.22)	1.1 (1.03 to 1.22)	1.1 (1.03 to 1.22)	1.1 (1.03 to 1.22)	1.1 (1.03 to 1.22)	1.1 (1.03 to 1.22)
Ischaemic heart disease	0 g/day	Both	Both										1 (1 to 1)	1 (1 to 1)	1 (1 to 1)	1 (1 to 1)	1 (1 to 1)	1 (1 to 1)	1 (1 to 1)	1 (1 to 1)	1 (1 to 1)	1 (1 to 1)	1 (1 to 1)	1 (1 to 1)	1 (1 to 1)	1 (1 to 1)	1 (1 to 1)
Ischaemic heart disease	25 g/day	Both	Both										1.13 (1 to 1.26)	1.13 (1 to 1.26)	1.13 (1 to 1.26)	1.11 (1 to 1.21)	1.1 (1 to 1.2)	1.1 (1 to 1.19)	1.08 (1 to 1.16)	1.07 (1 to 1.14)	1.06 (1 to 1.12)	1.06 (1 to 1.11)	1.05 (1 to 1.1)	1.05 (1 to 1.1)	1.05 (1 to 1.1)	1.05 (1 to 1.1)	1.05 (1 to 1.1)
Ischaemic heart disease	50 g/day	Both	Both										1.17 (1.01 to 1.31)	1.17 (1.01 to 1.31)	1.17 (1.01 to 1.31)	1.13 (1.01 to 1.25)	1.13 (1.01 to 1.24)	1.12 (1.01 to 1.22)	1.1 (1.01 to 1.18)	1.09 (1.01 to 1.16)	1.08 (1.01 to 1.14)	1.07 (1.01 to 1.13)	1.06 (1.01 to 1.12)	1.06 (1.01 to 1.12)	1.06 (1.01 to 1.12)	1.06 (1.01 to 1.12)	1.06 (1.01 to 1.12)
Ischaemic heart disease	75 g/day	Both	Both										1.2 (1.02 to 1.4)	1.2 (1.02 to 1.4)	1.2 (1.02 to 1.4)	1.16 (1.01 to 1.32)	1.15 (1.01 to 1.31)	1.14 (1.01 to 1.28)	1.12 (1.01 to 1.24)	1.1 (1.01 to 1.2)	1.09 (1.01 to 1.18)	1.08 (1.01 to 1.16)	1.08 (1.01 to 1.15)	1.08 (1.01 to 1.15)	1.08 (1.01 to 1.15)	1.08 (1.01 to 1.15)	1.08 (1.01 to 1.15)
Ischaemic heart disease	100 g/day	Both	Both										1.23 (1.02 to 1.51)	1.23 (1.02 to 1.51)	1.23 (1.02 to 1.51)	1.18 (1.01 to 1.41)	1.18 (1.01 to 1.39)	1.16 (1.01 to 1.35)	1.14 (1.01 to 1.3)	1.12 (1.01 to 1.25)	1.11 (1.01 to 1.23)	1.1 (1.01 to 1.2)	1.09 (1.01 to 1.19)	1.09 (1.01 to 1.19)	1.09 (1.01 to 1.19)	1.09 (1.01 to 1.19)	1.09 (1.01 to 1.19)
Diabetes mellitus type 2	0 g/day	Both	Both										1 (1 to 1)	1 (1 to 1)	1 (1 to 1)	1 (1 to 1)	1 (1 to 1)	1 (1 to 1)	1 (1 to 1)	1 (1 to 1)	1 (1 to 1)	1 (1 to 1)	1 (1 to 1)	1 (1 to 1)	1 (1 to 1)	1 (1 to 1)	1 (1 to 1)
Diabetes mellitus type 2	25 g/day	Both	Both										1.17 (1.11 to 1.22)	1.17 (1.11 to 1.22)	1.17 (1.11 to 1.22)	1.17 (1.11 to 1.22)	1.17 (1.11 to 1.22)	1.17 (1.11 to 1.22)	1.17 (1.11 to 1.22)	1.17 (1.11 to 1.22)	1.17 (1.11 to 1.22)	1.17 (1.11 to 1.22)	1.17 (1.11 to 1.22)	1.17 (1.11 to 1.22)	1.17 (1.11 to 1.22)	1.17 (1.11 to 1.22)	1.17 (1.11 to 1.22)
Diabetes mellitus type 2	50 g/day	Both	Both										1.2 (1.12 to 1.27)	1.2 (1.12 to 1.27)	1.2 (1.12 to 1.27)	1.2 (1.12 to 1.27)	1.2 (1.12 to 1.27)	1.2 (1.12 to 1.27)	1.2 (1.12 to 1.27)	1.2 (1.12 to 1.27)	1.2 (1.12 to 1.27)	1.2 (1.12 to 1.27)	1.2 (1.12 to 1.27)	1.2 (1.12 to 1.27)	1.2 (1.12 to 1.27)	1.2 (1.12 to 1.27)	1.2 (1.12 to 1.27)
Diabetes mellitus type 2	75 g/day	Both	Both										1.21 (1.12 to 1.31)	1.21 (1.12 to 1.31)	1.21 (1.12 to 1.31)	1.21 (1.12 to 1.31)	1.21 (1.12 to 1.31)	1.21 (1.12 to 1.31)	1.21 (1.12 to 1.31)	1.21 (1.12 to 1.31)	1.21 (1.12 to 1.31)	1.21 (1.12 to 1.31)	1.21 (1.12 to 1.31)	1.21 (1.12 to 1.31)	1.21 (1.12 to 1.31)	1.21 (1.12 to 1.31)	1.21 (1.12 to 1.31)
Diabetes mellitus type 2	100 g/day	Both	Both										1.22 (1.12 to 1.36)	1.22 (1.12 to 1.36)	1.22 (1.12 to 1.36)	1.22 (1.12 to 1.36)	1.22 (1.12 to 1.36)	1.22 (1.12 to 1.36)	1.22 (1.12 to 1.36)	1.22 (1.12 to 1.36)	1.22 (1.12 to 1.36)	1.22 (1.12 to 1.36)	1.22 (1.12 to 1.36)	1.22 (1.12 to 1.36)	1.22 (1.12 to 1.36)	1.22 (1.12 to 1.36)	1.22 (1.12 to 1.36)
**Diet high in sugar-sweetened beverages**																											
Ischaemic heart disease	0 g/day	Both	Both										1 (1 to 1)	1 (1 to 1)	1 (1 to 1)	1 (1 to 1)	1 (1 to 1)	1 (1 to 1)	1 (1 to 1)	1 (1 to 1)	1 (1 to 1)	1 (1 to 1)	1 (1 to 1)	1 (1 to 1)	1 (1 to 1)	1 (1 to 1)	1 (1 to 1)
Ischaemic heart disease	60 g/day	Both	Both										1.02 (1 to 1.04)	1.02 (1 to 1.04)	1.02 (1 to 1.04)	1.02 (1 to 1.03)	1.02 (1 to 1.03)	1.02 (1 to 1.03)	1.01 (1 to 1.03)	1.01 (1 to 1.02)	1.01 (1 to 1.02)	1.01 (1 to 1.02)	1.01 (1 to 1.02)	1.01 (1 to 1.02)	1.01 (1 to 1.02)	1.01 (1 to 1.02)	1.01 (1 to 1.02)
Ischaemic heart disease	120 g/day	Both	Both										1.05 (1 to 1.08)	1.05 (1 to 1.08)	1.05 (1 to 1.08)	1.04 (1 to 1.07)	1.04 (1 to 1.06)	1.04 (1 to 1.06)	1.03 (1 to 1.05)	1.03 (1 to 1.04)	1.02 (1 to 1.04)	1.02 (1 to 1.04)	1.02 (1 to 1.03)	1.02 (1 to 1.03)	1.02 (1 to 1.03)	1.02 (1 to 1.03)	1.02 (1 to 1.03)
Ischaemic heart disease	180 g/day	Both	Both										1.08 (1 to 1.12)	1.08 (1 to 1.12)	1.08 (1 to 1.12)	1.06 (1 to 1.1)	1.06 (1 to 1.1)	1.06 (1 to 1.09)	1.05 (1 to 1.08)	1.04 (1 to 1.07)	1.04 (1 to 1.06)	1.03 (1 to 1.05)	1.03 (1 to 1.05)	1.03 (1 to 1.05)	1.03 (1 to 1.05)	1.03 (1 to 1.05)	1.03 (1 to 1.05)
Ischaemic heart disease	240 g/day	Both	Both										1.1 (1.01 to 1.16)	1.1 (1.01 to 1.16)	1.1 (1.01 to 1.16)	1.08 (1.01 to 1.13)	1.08 (1.01 to 1.12)	1.07 (1.01 to 1.11)	1.06 (1.01 to 1.1)	1.06 (1.01 to 1.08)	1.05 (1 to 1.08)	1.04 (1 to 1.07)	1.04 (1 to 1.06)	1.04 (1 to 1.06)	1.04 (1 to 1.06)	1.04 (1 to 1.06)	1.04 (1 to 1.06)
Diabetes mellitus type 2	0 g/day	Both	Both										1 (1 to 1)	1 (1 to 1)	1 (1 to 1)	1 (1 to 1)	1 (1 to 1)	1 (1 to 1)	1 (1 to 1)	1 (1 to 1)	1 (1 to 1)	1 (1 to 1)	1 (1 to 1)	1 (1 to 1)	1 (1 to 1)	1 (1 to 1)	1 (1 to 1)
Diabetes mellitus type 2	60 g/day	Both	Both										1.03 (1 to 1.05)	1.03 (1 to 1.05)	1.03 (1 to 1.05)	1.03 (1 to 1.05)	1.03 (1 to 1.05)	1.03 (1 to 1.05)	1.03 (1 to 1.05)	1.03 (1 to 1.05)	1.03 (1 to 1.05)	1.03 (1 to 1.05)	1.03 (1 to 1.05)	1.03 (1 to 1.05)	1.03 (1 to 1.05)	1.03 (1 to 1.05)	1.03 (1 to 1.05)
Diabetes mellitus type 2	120 g/day	Both	Both										1.06 (1.02 to 1.1)	1.06 (1.02 to 1.1)	1.06 (1.02 to 1.1)	1.06 (1.02 to 1.1)	1.06 (1.02 to 1.1)	1.06 (1.02 to 1.1)	1.06 (1.02 to 1.1)	1.06 (1.02 to 1.1)	1.06 (1.02 to 1.1)	1.06 (1.02 to 1.1)	1.06 (1.02 to 1.1)	1.06 (1.02 to 1.1)	1.06 (1.02 to 1.1)	1.06 (1.02 to 1.1)	1.06 (1.02 to 1.1)
Diabetes mellitus type 2	180 g/day	Both	Both										1.1 (1.05 to 1.14)	1.1 (1.05 to 1.14)	1.1 (1.05 to 1.14)	1.1 (1.05 to 1.14)	1.1 (1.05 to 1.14)	1.1 (1.05 to 1.14)	1.1 (1.05 to 1.14)	1.1 (1.05 to 1.14)	1.1 (1.05 to 1.14)	1.1 (1.05 to 1.14)	1.1 (1.05 to 1.14)	1.1 (1.05 to 1.14)	1.1 (1.05 to 1.14)	1.1 (1.05 to 1.14)	1.1 (1.05 to 1.14)
Diabetes mellitus type 2	240 g/day	Both	Both										1.13 (1.07 to 1.18)	1.13 (1.07 to 1.18)	1.13 (1.07 to 1.18)	1.13 (1.07 to 1.18)	1.13 (1.07 to 1.18)	1.13 (1.07 to 1.18)	1.13 (1.07 to 1.18)	1.13 (1.07 to 1.18)	1.13 (1.07 to 1.18)	1.13 (1.07 to 1.18)	1.13 (1.07 to 1.18)	1.13 (1.07 to 1.18)	1.13 (1.07 to 1.18)	1.13 (1.07 to 1.18)	1.13 (1.07 to 1.18)
**Diet low in fibre**																											
Colon and rectum cancer	0 g/day	Both	Both										1 (1 to 1)	1 (1 to 1)	1 (1 to 1)	1 (1 to 1)	1 (1 to 1)	1 (1 to 1)	1 (1 to 1)	1 (1 to 1)	1 (1 to 1)	1 (1 to 1)	1 (1 to 1)	1 (1 to 1)	1 (1 to 1)	1 (1 to 1)	1 (1 to 1)
Colon and rectum cancer	10 g/day	Both	Both										0.91 (0.89 to 0.97)	0.91 (0.89 to 0.97)	0.91 (0.89 to 0.97)	0.91 (0.89 to 0.97)	0.91 (0.89 to 0.97)	0.91 (0.89 to 0.97)	0.91 (0.89 to 0.97)	0.91 (0.89 to 0.97)	0.91 (0.89 to 0.97)	0.91 (0.89 to 0.97)	0.91 (0.89 to 0.97)	0.91 (0.89 to 0.97)	0.91 (0.89 to 0.97)	0.91 (0.89 to 0.97)	0.91 (0.89 to 0.97)
Colon and rectum cancer	20 g/day	Both	Both										0.87 (0.83 to 0.93)	0.87 (0.83 to 0.93)	0.87 (0.83 to 0.93)	0.87 (0.83 to 0.93)	0.87 (0.83 to 0.93)	0.87 (0.83 to 0.93)	0.87 (0.83 to 0.93)	0.87 (0.83 to 0.93)	0.87 (0.83 to 0.93)	0.87 (0.83 to 0.93)	0.87 (0.83 to 0.93)	0.87 (0.83 to 0.93)	0.87 (0.83 to 0.93)	0.87 (0.83 to 0.93)	0.87 (0.83 to 0.93)
Colon and rectum cancer	30 g/day	Both	Both										0.87 (0.83 to 0.92)	0.87 (0.83 to 0.92)	0.87 (0.83 to 0.92)	0.87 (0.83 to 0.92)	0.87 (0.83 to 0.92)	0.87 (0.83 to 0.92)	0.87 (0.83 to 0.92)	0.87 (0.83 to 0.92)	0.87 (0.83 to 0.92)	0.87 (0.83 to 0.92)	0.87 (0.83 to 0.92)	0.87 (0.83 to 0.92)	0.87 (0.83 to 0.92)	0.87 (0.83 to 0.92)	0.87 (0.83 to 0.92)
Ischaemic heart disease	0 g/day	Both	Both										1 (1 to 1)	1 (1 to 1)	1 (1 to 1)	1 (1 to 1)	1 (1 to 1)	1 (1 to 1)	1 (1 to 1)	1 (1 to 1)	1 (1 to 1)	1 (1 to 1)	1 (1 to 1)	1 (1 to 1)	1 (1 to 1)	1 (1 to 1)	1 (1 to 1)
Ischaemic heart disease	10 g/day	Both	Both										0.8 (0.68 to 0.91)	0.8 (0.68 to 0.91)	0.8 (0.68 to 0.91)	0.83 (0.73 to 0.93)	0.84 (0.74 to 0.93)	0.85 (0.76 to 0.94)	0.87 (0.79 to 0.94)	0.88 (0.81 to 0.95)	0.89 (0.83 to 0.96)	0.9 (0.84 to 0.96)	0.91 (0.85 to 0.96)	0.91 (0.85 to 0.96)	0.91 (0.85 to 0.96)	0.91 (0.85 to 0.96)	0.91 (0.85 to 0.96)
Ischaemic heart disease	20 g/day	Both	Both										0.65 (0.54 to 0.77)	0.65 (0.54 to 0.77)	0.65 (0.54 to 0.77)	0.7 (0.6 to 0.81)	0.71 (0.61 to 0.82)	0.73 (0.64 to 0.83)	0.76 (0.68 to 0.85)	0.79 (0.71 to 0.87)	0.81 (0.74 to 0.88)	0.82 (0.76 to 0.89)	0.83 (0.77 to 0.9)	0.83 (0.77 to 0.9)	0.83 (0.77 to 0.9)	0.83 (0.77 to 0.9)	0.83 (0.77 to 0.9)
Ischaemic heart disease	30 g/day	Both	Both										0.64 (0.53 to 0.77)	0.64 (0.53 to 0.77)	0.64 (0.53 to 0.77)	0.69 (0.59 to 0.8)	0.7 (0.61 to 0.81)	0.72 (0.63 to 0.82)	0.75 (0.67 to 0.85)	0.78 (0.71 to 0.86)	0.8 (0.73 to 0.88)	0.82 (0.75 to 0.89)	0.83 (0.77 to 0.9)	0.83 (0.77 to 0.9)	0.83 (0.77 to 0.9)	0.83 (0.77 to 0.9)	0.83 (0.77 to 0.9)
Ischaemic stroke	0 g/day	Both	Both										1 (1 to 1)	1 (1 to 1)	1 (1 to 1)	1 (1 to 1)	1 (1 to 1)	1 (1 to 1)	1 (1 to 1)	1 (1 to 1)	1 (1 to 1)	1 (1 to 1)	1 (1 to 1)	1 (1 to 1)	1 (1 to 1)	1 (1 to 1)	1 (1 to 1)
Ischaemic stroke	10 g/day	Both	Both										0.83 (0.69 to 0.97)	0.83 (0.69 to 0.97)	0.83 (0.69 to 0.97)	0.86 (0.74 to 0.98)	0.88 (0.78 to 0.98)	0.9 (0.81 to 0.98)	0.91 (0.84 to 0.99)	0.93 (0.87 to 0.99)	0.94 (0.89 to 0.99)	0.95 (0.91 to 0.99)	0.96 (0.93 to 0.99)	0.96 (0.93 to 0.99)	0.96 (0.93 to 0.99)	0.96 (0.93 to 0.99)	0.96 (0.93 to 0.99)
Ischaemic stroke	20 g/day	Both	Both										0.66 (0.54 to 0.8)	0.66 (0.54 to 0.8)	0.66 (0.54 to 0.8)	0.72 (0.61 to 0.84)	0.75 (0.66 to 0.86)	0.79 (0.71 to 0.88)	0.82 (0.75 to 0.9)	0.85 (0.79 to 0.92)	0.87 (0.82 to 0.93)	0.9 (0.85 to 0.94)	0.92 (0.88 to 0.95)	0.92 (0.88 to 0.95)	0.92 (0.88 to 0.95)	0.92 (0.88 to 0.95)	0.92 (0.88 to 0.95)
Ischaemic stroke	30 g/day	Both	Both										0.65 (0.52 to 0.8)	0.65 (0.52 to 0.8)	0.65 (0.52 to 0.8)	0.71 (0.6 to 0.83)	0.74 (0.64 to 0.86)	0.78 (0.69 to 0.88)	0.81 (0.74 to 0.9)	0.84 (0.78 to 0.92)	0.87 (0.81 to 0.93)	0.89 (0.84 to 0.94)	0.91 (0.87 to 0.95)	0.91 (0.87 to 0.95)	0.91 (0.87 to 0.95)	0.91 (0.87 to 0.95)	0.91 (0.87 to 0.95)
Intracerebral haemorrhage	0 g/day	Both	Both										1 (1 to 1)	1 (1 to 1)	1 (1 to 1)	1 (1 to 1)	1 (1 to 1)	1 (1 to 1)	1 (1 to 1)	1 (1 to 1)	1 (1 to 1)	1 (1 to 1)	1 (1 to 1)	1 (1 to 1)	1 (1 to 1)	1 (1 to 1)	1 (1 to 1)
Intracerebral haemorrhage	10 g/day	Both	Both										0.88 (0.78 to 0.98)	0.88 (0.78 to 0.98)	0.88 (0.78 to 0.98)	0.9 (0.81 to 0.98)	0.9 (0.82 to 0.98)	0.91 (0.83 to 0.99)	0.92 (0.85 to 0.99)	0.93 (0.87 to 0.99)	0.94 (0.88 to 0.99)	0.94 (0.89 to 0.99)	0.95 (0.91 to 0.99)	0.95 (0.91 to 0.99)	0.95 (0.91 to 0.99)	0.95 (0.91 to 0.99)	0.95 (0.91 to 0.99)
Intracerebral haemorrhage	20 g/day	Both	Both										0.75 (0.66 to 0.86)	0.75 (0.66 to 0.86)	0.75 (0.66 to 0.86)	0.79 (0.71 to 0.88)	0.8 (0.73 to 0.89)	0.82 (0.74 to 0.9)	0.83 (0.77 to 0.91)	0.85 (0.79 to 0.92)	0.86 (0.81 to 0.93)	0.88 (0.83 to 0.93)	0.9 (0.85 to 0.94)	0.9 (0.85 to 0.94)	0.9 (0.85 to 0.94)	0.9 (0.85 to 0.94)	0.9 (0.85 to 0.94)
Intracerebral haemorrhage	30 g/day	Both	Both										0.74 (0.64 to 0.86)	0.74 (0.64 to 0.86)	0.74 (0.64 to 0.86)	0.78 (0.7 to 0.88)	0.79 (0.71 to 0.89)	0.81 (0.73 to 0.89)	0.83 (0.75 to 0.91)	0.84 (0.78 to 0.92)	0.86 (0.8 to 0.92)	0.88 (0.82 to 0.93)	0.89 (0.85 to 0.94)	0.89 (0.85 to 0.94)	0.89 (0.85 to 0.94)	0.89 (0.85 to 0.94)	0.89 (0.85 to 0.94)
Subarachnoid haemorrhage	0 g/day	Both	Both										1 (1 to 1)	1 (1 to 1)	1 (1 to 1)	1 (1 to 1)	1 (1 to 1)	1 (1 to 1)	1 (1 to 1)	1 (1 to 1)	1 (1 to 1)	1 (1 to 1)	1 (1 to 1)	1 (1 to 1)	1 (1 to 1)	1 (1 to 1)	1 (1 to 1)
Subarachnoid haemorrhage	10 g/day	Both	Both										0.88 (0.78 to 0.98)	0.88 (0.78 to 0.98)	0.88 (0.78 to 0.98)	0.9 (0.81 to 0.98)	0.9 (0.82 to 0.98)	0.91 (0.83 to 0.99)	0.92 (0.85 to 0.99)	0.93 (0.87 to 0.99)	0.94 (0.88 to 0.99)	0.94 (0.89 to 0.99)	0.95 (0.91 to 0.99)	0.95 (0.91 to 0.99)	0.95 (0.91 to 0.99)	0.95 (0.91 to 0.99)	0.95 (0.91 to 0.99)
Subarachnoid haemorrhage	20 g/day	Both	Both										0.75 (0.66 to 0.86)	0.75 (0.66 to 0.86)	0.75 (0.66 to 0.86)	0.79 (0.71 to 0.88)	0.8 (0.73 to 0.89)	0.82 (0.74 to 0.9)	0.83 (0.77 to 0.91)	0.85 (0.79 to 0.92)	0.86 (0.81 to 0.93)	0.88 (0.83 to 0.93)	0.9 (0.85 to 0.94)	0.9 (0.85 to 0.94)	0.9 (0.85 to 0.94)	0.9 (0.85 to 0.94)	0.9 (0.85 to 0.94)
Subarachnoid haemorrhage	30 g/day	Both	Both										0.74 (0.64 to 0.86)	0.74 (0.64 to 0.86)	0.74 (0.64 to 0.86)	0.78 (0.7 to 0.88)	0.79 (0.71 to 0.89)	0.81 (0.73 to 0.89)	0.83 (0.75 to 0.91)	0.84 (0.78 to 0.92)	0.86 (0.8 to 0.92)	0.88 (0.82 to 0.93)	0.89 (0.85 to 0.94)	0.89 (0.85 to 0.94)	0.89 (0.85 to 0.94)	0.89 (0.85 to 0.94)	0.89 (0.85 to 0.94)
Diabetes mellitus type 2	0 g/day	Both	Both										1 (1 to 1)	1 (1 to 1)	1 (1 to 1)	1 (1 to 1)	1 (1 to 1)	1 (1 to 1)	1 (1 to 1)	1 (1 to 1)	1 (1 to 1)	1 (1 to 1)	1 (1 to 1)	1 (1 to 1)	1 (1 to 1)	1 (1 to 1)	1 (1 to 1)
Diabetes mellitus type 2	10 g/day	Both	Both										0.94 (0.9 to 0.99)	0.94 (0.9 to 0.99)	0.94 (0.9 to 0.99)	0.94 (0.9 to 0.99)	0.94 (0.9 to 0.99)	0.94 (0.9 to 0.99)	0.94 (0.9 to 0.99)	0.94 (0.9 to 0.99)	0.94 (0.9 to 0.99)	0.94 (0.9 to 0.99)	0.94 (0.9 to 0.99)	0.94 (0.9 to 0.99)	0.94 (0.9 to 0.99)	0.94 (0.9 to 0.99)	0.94 (0.9 to 0.99)
Diabetes mellitus type 2	20 g/day	Both	Both										0.9 (0.86 to 0.95)	0.9 (0.86 to 0.95)	0.9 (0.86 to 0.95)	0.9 (0.86 to 0.95)	0.9 (0.86 to 0.95)	0.9 (0.86 to 0.95)	0.9 (0.86 to 0.95)	0.9 (0.86 to 0.95)	0.9 (0.86 to 0.95)	0.9 (0.86 to 0.95)	0.9 (0.86 to 0.95)	0.9 (0.86 to 0.95)	0.9 (0.86 to 0.95)	0.9 (0.86 to 0.95)	0.9 (0.86 to 0.95)
Diabetes mellitus type 2	30 g/day	Both	Both										0.88 (0.84 to 0.93)	0.88 (0.84 to 0.93)	0.88 (0.84 to 0.93)	0.88 (0.84 to 0.93)	0.88 (0.84 to 0.93)	0.88 (0.84 to 0.93)	0.88 (0.84 to 0.93)	0.88 (0.84 to 0.93)	0.88 (0.84 to 0.93)	0.88 (0.84 to 0.93)	0.88 (0.84 to 0.93)	0.88 (0.84 to 0.93)	0.88 (0.84 to 0.93)	0.88 (0.84 to 0.93)	0.88 (0.84 to 0.93)
**Diet low in calcium**																											
Colon and rectum cancer	0.3 g/day	Both	Both										0.93 (0.9 to 0.98)	0.93 (0.9 to 0.98)	0.93 (0.9 to 0.98)	0.93 (0.9 to 0.98)	0.93 (0.9 to 0.98)	0.93 (0.9 to 0.98)	0.93 (0.9 to 0.98)	0.93 (0.9 to 0.98)	0.93 (0.9 to 0.98)	0.93 (0.9 to 0.98)	0.93 (0.9 to 0.98)	0.93 (0.9 to 0.98)	0.93 (0.9 to 0.98)	0.93 (0.9 to 0.98)	0.93 (0.9 to 0.98)
Colon and rectum cancer	0.6 g/day	Both	Both										0.8 (0.75 to 0.89)	0.8 (0.75 to 0.89)	0.8 (0.75 to 0.89)	0.8 (0.75 to 0.89)	0.8 (0.75 to 0.89)	0.8 (0.75 to 0.89)	0.8 (0.75 to 0.89)	0.8 (0.75 to 0.89)	0.8 (0.75 to 0.89)	0.8 (0.75 to 0.89)	0.8 (0.75 to 0.89)	0.8 (0.75 to 0.89)	0.8 (0.75 to 0.89)	0.8 (0.75 to 0.89)	0.8 (0.75 to 0.89)
Colon and rectum cancer	0.9 g/day	Both	Both										0.76 (0.7 to 0.82)	0.76 (0.7 to 0.82)	0.76 (0.7 to 0.82)	0.76 (0.7 to 0.82)	0.76 (0.7 to 0.82)	0.76 (0.7 to 0.82)	0.76 (0.7 to 0.82)	0.76 (0.7 to 0.82)	0.76 (0.7 to 0.82)	0.76 (0.7 to 0.82)	0.76 (0.7 to 0.82)	0.76 (0.7 to 0.82)	0.76 (0.7 to 0.82)	0.76 (0.7 to 0.82)	0.76 (0.7 to 0.82)
Colon and rectum cancer	1.2 g/day	Both	Both										0.69 (0.64 to 0.75)	0.69 (0.64 to 0.75)	0.69 (0.64 to 0.75)	0.69 (0.64 to 0.75)	0.69 (0.64 to 0.75)	0.69 (0.64 to 0.75)	0.69 (0.64 to 0.75)	0.69 (0.64 to 0.75)	0.69 (0.64 to 0.75)	0.69 (0.64 to 0.75)	0.69 (0.64 to 0.75)	0.69 (0.64 to 0.75)	0.69 (0.64 to 0.75)	0.69 (0.64 to 0.75)	0.69 (0.64 to 0.75)
**Diet low in seafood omega-3 fatty acids**																											
Ischaemic heart disease	0 g/day	Both	Both										1 (1 to 1)	1 (1 to 1)	1 (1 to 1)	1 (1 to 1)	1 (1 to 1)	1 (1 to 1)	1 (1 to 1)	1 (1 to 1)	1 (1 to 1)	1 (1 to 1)	1 (1 to 1)	1 (1 to 1)	1 (1 to 1)	1 (1 to 1)	1 (1 to 1)
Ischaemic heart disease	0.1 g/day	Both	Both										0.98 (0.97 to 1)	0.98 (0.97 to 1)	0.98 (0.97 to 1)	0.98 (0.98 to 1)	0.98 (0.98 to 1)	0.99 (0.98 to 1)	0.99 (0.98 to 1)	0.99 (0.99 to 1)	0.99 (0.99 to 1)	0.99 (0.99 to 1)	0.99 (0.99 to 1)	0.99 (0.99 to 1)	0.99 (0.99 to 1)	0.99 (0.99 to 1)	0.99 (0.99 to 1)
Ischaemic heart disease	0.2 g/day	Both	Both										0.96 (0.95 to 0.99)	0.96 (0.95 to 0.99)	0.96 (0.95 to 0.99)	0.97 (0.96 to 0.99)	0.97 (0.96 to 0.99)	0.97 (0.96 to 0.99)	0.98 (0.97 to 0.99)	0.98 (0.97 to 0.99)	0.98 (0.97 to 1)	0.98 (0.98 to 1)	0.98 (0.98 to 1)	0.98 (0.98 to 1)	0.98 (0.98 to 1)	0.98 (0.98 to 1)	0.98 (0.98 to 1)
Ischaemic heart disease	0.3 g/day	Both	Both										0.95 (0.93 to 0.99)	0.95 (0.93 to 0.99)	0.95 (0.93 to 0.99)	0.96 (0.94 to 0.99)	0.96 (0.94 to 0.99)	0.96 (0.95 to 0.99)	0.97 (0.96 to 0.99)	0.97 (0.96 to 0.99)	0.97 (0.96 to 0.99)	0.98 (0.97 to 0.99)	0.98 (0.97 to 0.99)	0.98 (0.97 to 0.99)	0.98 (0.97 to 0.99)	0.98 (0.97 to 0.99)	0.98 (0.97 to 0.99)
Ischaemic heart disease	0.4 g/day	Both	Both										0.93 (0.91 to 0.98)	0.93 (0.91 to 0.98)	0.93 (0.91 to 0.98)	0.95 (0.93 to 0.99)	0.95 (0.93 to 0.99)	0.95 (0.93 to 0.99)	0.96 (0.94 to 0.99)	0.96 (0.95 to 0.99)	0.97 (0.96 to 0.99)	0.97 (0.96 to 0.99)	0.97 (0.96 to 0.99)	0.97 (0.96 to 0.99)	0.97 (0.96 to 0.99)	0.97 (0.96 to 0.99)	0.97 (0.96 to 0.99)
**Diet low in polyunsaturated fatty acids**																											
Ischaemic heart disease	0 %Energy/day	Both	Both										1 (1 to 1)	1 (1 to 1)	1 (1 to 1)	1 (1 to 1)	1 (1 to 1)	1 (1 to 1)	1 (1 to 1)	1 (1 to 1)	1 (1 to 1)	1 (1 to 1)	1 (1 to 1)	1 (1 to 1)	1 (1 to 1)	1 (1 to 1)	1 (1 to 1)
Ischaemic heart disease	2 %Energy/day	Both	Both										0.96 (0.91 to 1)	0.96 (0.91 to 1)	0.96 (0.91 to 1)	0.96 (0.93 to 1)	0.97 (0.93 to 1)	0.97 (0.94 to 1)	0.97 (0.94 to 1)	0.98 (0.95 to 1)	0.98 (0.96 to 1)	0.98 (0.96 to 1)	0.98 (0.96 to 1)	0.98 (0.96 to 1)	0.98 (0.96 to 1)	0.98 (0.96 to 1)	0.98 (0.96 to 1)
Ischaemic heart disease	4 %Energy/day	Both	Both										0.92 (0.84 to 0.99)	0.92 (0.84 to 0.99)	0.92 (0.84 to 0.99)	0.93 (0.86 to 0.99)	0.93 (0.87 to 0.99)	0.94 (0.88 to 0.99)	0.95 (0.89 to 1)	0.95 (0.91 to 1)	0.96 (0.92 to 1)	0.96 (0.92 to 1)	0.96 (0.93 to 1)	0.96 (0.93 to 1)	0.96 (0.93 to 1)	0.96 (0.93 to 1)	0.96 (0.93 to 1)
Ischaemic heart disease	6 %Energy/day	Both	Both										0.89 (0.78 to 0.99)	0.89 (0.78 to 0.99)	0.89 (0.78 to 0.99)	0.91 (0.82 to 0.99)	0.91 (0.82 to 0.99)	0.92 (0.84 to 0.99)	0.93 (0.86 to 0.99)	0.94 (0.87 to 0.99)	0.94 (0.89 to 0.99)	0.95 (0.9 to 0.99)	0.95 (0.9 to 0.99)	0.95 (0.9 to 0.99)	0.95 (0.9 to 0.99)	0.95 (0.9 to 0.99)	0.95 (0.9 to 0.99)
Ischaemic heart disease	8 %Energy/day	Both	Both										0.88 (0.78 to 0.99)	0.88 (0.78 to 0.99)	0.88 (0.78 to 0.99)	0.9 (0.81 to 0.99)	0.91 (0.82 to 0.99)	0.91 (0.83 to 0.99)	0.93 (0.85 to 0.99)	0.93 (0.87 to 0.99)	0.94 (0.88 to 0.99)	0.95 (0.89 to 0.99)	0.95 (0.9 to 0.99)	0.95 (0.9 to 0.99)	0.95 (0.9 to 0.99)	0.95 (0.9 to 0.99)	0.95 (0.9 to 0.99)
**Diet high in trans fatty acids**																											

Ischaemic heart disease	0 %Energy/day	Both	Both										1 (1 to 1)	1 (1 to 1)	1 (1 to 1)	1 (1 to 1)	1 (1 to 1)	1 (1 to 1)	1 (1 to 1)	1 (1 to 1)	1 (1 to 1)	1 (1 to 1)	1 (1 to 1)	1 (1 to 1)	1 (1 to 1)	1 (1 to 1)	1 (1 to 1)
Ischaemic heart disease	1 %Energy/day	Both	Both										1.28 (1.01 to 1.38)	1.28 (1.01 to 1.38)	1.28 (1.01 to 1.38)	1.23 (1.01 to 1.31)	1.22 (1 to 1.29)	1.2 (1 to 1.27)	1.17 (1 to 1.23)	1.15 (1 to 1.2)	1.13 (1 to 1.17)	1.12 (1 to 1.16)	1.11 (1 to 1.14)	1.11 (1 to 1.14)	1.11 (1 to 1.14)	1.11 (1 to 1.14)	1.11 (1 to 1.14)
Ischaemic heart disease	2 %Energy/day	Both	Both										1.37 (1.01 to 1.54)	1.37 (1.01 to 1.54)	1.37 (1.01 to 1.54)	1.3 (1.01 to 1.43)	1.28 (1.01 to 1.41)	1.26 (1.01 to 1.37)	1.22 (1.01 to 1.31)	1.19 (1 to 1.27)	1.17 (1 to 1.24)	1.15 (1 to 1.21)	1.14 (1 to 1.2)	1.14 (1 to 1.2)	1.14 (1 to 1.2)	1.14 (1 to 1.2)	1.14 (1 to 1.2)
Ischaemic heart disease	3 %Energy/day	Both	Both										1.38 (1.01 to 1.54)	1.38 (1.01 to 1.54)	1.38 (1.01 to 1.54)	1.3 (1.01 to 1.43)	1.29 (1.01 to 1.41)	1.26 (1.01 to 1.37)	1.22 (1.01 to 1.31)	1.19 (1 to 1.27)	1.17 (1 to 1.24)	1.15 (1 to 1.21)	1.14 (1 to 1.2)	1.14 (1 to 1.2)	1.14 (1 to 1.2)	1.14 (1 to 1.2)	1.14 (1 to 1.2)
Ischaemic heart disease	4 %Energy/day	Both	Both										1.38 (1.01 to 1.55)	1.38 (1.01 to 1.55)	1.38 (1.01 to 1.55)	1.3 (1.01 to 1.43)	1.29 (1.01 to 1.41)	1.26 (1.01 to 1.37)	1.22 (1.01 to 1.31)	1.19 (1.01 to 1.27)	1.17 (1.01 to 1.24)	1.15 (1 to 1.22)	1.14 (1 to 1.2)	1.14 (1 to 1.2)	1.14 (1 to 1.2)	1.14 (1 to 1.2)	1.14 (1 to 1.2)
**Diet high in sodium**																											
Stomach cancer	3 g/day	Both	Both										1.33 (1.04 to 1.43)	1.33 (1.04 to 1.43)	1.33 (1.04 to 1.43)	1.33 (1.04 to 1.43)	1.33 (1.04 to 1.43)	1.33 (1.04 to 1.43)	1.33 (1.04 to 1.43)	1.33 (1.04 to 1.43)	1.33 (1.04 to 1.43)	1.33 (1.04 to 1.43)	1.33 (1.04 to 1.43)	1.33 (1.04 to 1.43)	1.33 (1.04 to 1.43)	1.33 (1.04 to 1.43)	1.33 (1.04 to 1.43)
Stomach cancer	4 g/day	Both	Both										1.4 (1.05 to 1.53)	1.4 (1.05 to 1.53)	1.4 (1.05 to 1.53)	1.4 (1.05 to 1.53)	1.4 (1.05 to 1.53)	1.4 (1.05 to 1.53)	1.4 (1.05 to 1.53)	1.4 (1.05 to 1.53)	1.4 (1.05 to 1.53)	1.4 (1.05 to 1.53)	1.4 (1.05 to 1.53)	1.4 (1.05 to 1.53)	1.4 (1.05 to 1.53)	1.4 (1.05 to 1.53)	1.4 (1.05 to 1.53)
Stomach cancer	5 g/day	Both	Both										1.4 (1.05 to 1.54)	1.4 (1.05 to 1.54)	1.4 (1.05 to 1.54)	1.4 (1.05 to 1.54)	1.4 (1.05 to 1.54)	1.4 (1.05 to 1.54)	1.4 (1.05 to 1.54)	1.4 (1.05 to 1.54)	1.4 (1.05 to 1.54)	1.4 (1.05 to 1.54)	1.4 (1.05 to 1.54)	1.4 (1.05 to 1.54)	1.4 (1.05 to 1.54)	1.4 (1.05 to 1.54)	1.4 (1.05 to 1.54)
Stomach cancer	6 g/day	Both	Both										1.4 (1.05 to 1.54)	1.4 (1.05 to 1.54)	1.4 (1.05 to 1.54)	1.4 (1.05 to 1.54)	1.4 (1.05 to 1.54)	1.4 (1.05 to 1.54)	1.4 (1.05 to 1.54)	1.4 (1.05 to 1.54)	1.4 (1.05 to 1.54)	1.4 (1.05 to 1.54)	1.4 (1.05 to 1.54)	1.4 (1.05 to 1.54)	1.4 (1.05 to 1.54)	1.4 (1.05 to 1.54)	1.4 (1.05 to 1.54)
Stomach cancer	7 g/day	Both	Both										1.4 (1.05 to 1.54)	1.4 (1.05 to 1.54)	1.4 (1.05 to 1.54)	1.4 (1.05 to 1.54)	1.4 (1.05 to 1.54)	1.4 (1.05 to 1.54)	1.4 (1.05 to 1.54)	1.4 (1.05 to 1.54)	1.4 (1.05 to 1.54)	1.4 (1.05 to 1.54)	1.4 (1.05 to 1.54)	1.4 (1.05 to 1.54)	1.4 (1.05 to 1.54)	1.4 (1.05 to 1.54)	1.4 (1.05 to 1.54)
**Diet low in legumes**																											
Ischaemic heart disease	0 g/day	Both	Both										1 (1 to 1)	1 (1 to 1)	1 (1 to 1)	1 (1 to 1)	1 (1 to 1)	1 (1 to 1)	1 (1 to 1)	1 (1 to 1)	1 (1 to 1)	1 (1 to 1)	1 (1 to 1)	1 (1 to 1)	1 (1 to 1)	1 (1 to 1)	
Ischaemic heart disease	50 g/day	Both	Both										0.82 (0.68 to 0.95)	0.82 (0.68 to 0.95)	0.82 (0.68 to 0.95)	0.85 (0.73 to 0.96)	0.85 (0.74 to 0.96)	0.86 (0.76 to 0.97)	0.88 (0.79 to 0.97)	0.89 (0.81 to 0.97)	0.9 (0.83 to 0.98)	0.91 (0.84 to 0.98)	0.92 (0.85 to 0.98)	0.92 (0.85 to 0.98)	0.92 (0.85 to 0.98)	0.92 (0.85 to 0.98)	
Ischaemic heart disease	100 g/day	Both	Both										0.66 (0.52 to 0.87)	0.66 (0.52 to 0.87)	0.66 (0.52 to 0.87)	0.71 (0.58 to 0.89)	0.72 (0.59 to 0.9)	0.74 (0.62 to 0.9)	0.77 (0.66 to 0.92)	0.8 (0.7 to 0.93)	0.82 (0.72 to 0.93)	0.83 (0.74 to 0.94)	0.84 (0.76 to 0.94)	0.84 (0.76 to 0.94)	0.84 (0.76 to 0.94)	0.84 (0.76 to 0.94)	
Ischaemic heart disease	150 g/day	Both	Both										0.63 (0.46 to 0.85)	0.63 (0.46 to 0.85)	0.63 (0.46 to 0.85)	0.68 (0.53 to 0.87)	0.69 (0.54 to 0.88)	0.71 (0.57 to 0.89)	0.75 (0.62 to 0.9)	0.77 (0.66 to 0.91)	0.79 (0.68 to 0.92)	0.81 (0.71 to 0.93)	0.82 (0.73 to 0.93)	0.82 (0.73 to 0.93)	0.82 (0.73 to 0.93)	0.82 (0.73 to 0.93)	
**Intimate partner violence (HIV PAF approach)**																											
HIV/AIDS	Exposed	Both	Female	1.595 (1.314 to 1.935)																							
HIV/AIDS	Not exposed	Both	Female	1.0 (1.0 to 1.0)																							
**Intimate partner violence (exposure approach)**																											
Major depressive disorder	Exposed	Morbidity	Female		1.539 (1.002 to 2.355)	1.539 (1.002 to 2.355)	1.539 (1.002 to 2.355)	1.539 (1.002 to 2.355)	1.539 (1.002 to 2.355)	1.539 (1.002 to 2.355)	1.539 (1.002 to 2.355)	1.539 (1.002 to 2.355)	1.539 (1.002 to 2.355)	1.539 (1.002 to 2.355)	1.539 (1.002 to 2.355)	1.539 (1.002 to 2.355)	1.539 (1.002 to 2.355)	1.539 (1.002 to 2.355)	1.539 (1.002 to 2.355)	1.539 (1.002 to 2.355)	1.539 (1.002 to 2.355)	1.539 (1.002 to 2.355)	1.539 (1.002 to 2.355)	1.539 (1.002 to 2.355)	1.539 (1.002 to 2.355)	1.539 (1.002 to 2.355)	1.539 (1.002 to 2.355)
Major depressive disorder	Not exposed	Morbidity	Female		1.0 (1.0 to 1.0)	1.0 (1.0 to 1.0)	1.0 (1.0 to 1.0)	1.0 (1.0 to 1.0)	1.0 (1.0 to 1.0)	1.0 (1.0 to 1.0)	1.0 (1.0 to 1.0)	1.0 (1.0 to 1.0)	1.0 (1.0 to 1.0)	1.0 (1.0 to 1.0)	1.0 (1.0 to 1.0)	1.0 (1.0 to 1.0)	1.0 (1.0 to 1.0)	1.0 (1.0 to 1.0)	1.0 (1.0 to 1.0)	1.0 (1.0 to 1.0)	1.0 (1.0 to 1.0)	1.0 (1.0 to 1.0)	1.0 (1.0 to 1.0)	1.0 (1.0 to 1.0)	1.0 (1.0 to 1.0)	1.0 (1.0 to 1.0)	1.0 (1.0 to 1.0)
**Childhood sexual abuse against females**																											
Major depressive disorder	Exposed	Morbidity	Female		1.556 (1.296 to 1.864)	1.556 (1.296 to 1.864)	1.556 (1.296 to 1.864)	1.556 (1.296 to 1.864)	1.556 (1.296 to 1.864)	1.556 (1.296 to 1.864)	1.556 (1.296 to 1.864)	1.556 (1.296 to 1.864)	1.556 (1.296 to 1.864)	1.556 (1.296 to 1.864)	1.556 (1.296 to 1.864)	1.556 (1.296 to 1.864)	1.556 (1.296 to 1.864)	1.556 (1.296 to 1.864)	1.556 (1.296 to 1.864)	1.556 (1.296 to 1.864)	1.556 (1.296 to 1.864)	1.556 (1.296 to 1.864)	1.556 (1.296 to 1.864)	1.556 (1.296 to 1.864)	1.556 (1.296 to 1.864)	1.556 (1.296 to 1.864)	1.556 (1.296 to 1.864)
Major depressive disorder	Not exposed	Morbidity	Female		1.0 (1.0 to 1.0)	1.0 (1.0 to 1.0)	1.0 (1.0 to 1.0)	1.0 (1.0 to 1.0)	1.0 (1.0 to 1.0)	1.0 (1.0 to 1.0)	1.0 (1.0 to 1.0)	1.0 (1.0 to 1.0)	1.0 (1.0 to 1.0)	1.0 (1.0 to 1.0)	1.0 (1.0 to 1.0)	1.0 (1.0 to 1.0)	1.0 (1.0 to 1.0)	1.0 (1.0 to 1.0)	1.0 (1.0 to 1.0)	1.0 (1.0 to 1.0)	1.0 (1.0 to 1.0)	1.0 (1.0 to 1.0)	1.0 (1.0 to 1.0)	1.0 (1.0 to 1.0)	1.0 (1.0 to 1.0)	1.0 (1.0 to 1.0)	1.0 (1.0 to 1.0)
Alcohol use disorders	Exposed	Both	Female		2.21 (1.145 to 4.044)	2.21 (1.145 to 4.044)	2.21 (1.145 to 4.044)	2.21 (1.145 to 4.044)	2.21 (1.145 to 4.044)	2.21 (1.145 to 4.044)	2.21 (1.145 to 4.044)	2.21 (1.145 to 4.044)	2.21 (1.145 to 4.044)	2.21 (1.145 to 4.044)	2.21 (1.145 to 4.044)	2.21 (1.145 to 4.044)	2.21 (1.145 to 4.044)	2.21 (1.145 to 4.044)	2.21 (1.145 to 4.044)	2.21 (1.145 to 4.044)	2.21 (1.145 to 4.044)	2.21 (1.145 to 4.044)	2.21 (1.145 to 4.044)	2.21 (1.145 to 4.044)	2.21 (1.145 to 4.044)	2.21 (1.145 to 4.044)	2.21 (1.145 to 4.044)
Alcohol use disorders	Not exposed	Both	Female		1.0 (1.0 to 1.0)	1.0 (1.0 to 1.0)	1.0 (1.0 to 1.0)	1.0 (1.0 to 1.0)	1.0 (1.0 to 1.0)	1.0 (1.0 to 1.0)	1.0 (1.0 to 1.0)	1.0 (1.0 to 1.0)	1.0 (1.0 to 1.0)	1.0 (1.0 to 1.0)	1.0 (1.0 to 1.0)	1.0 (1.0 to 1.0)	1.0 (1.0 to 1.0)	1.0 (1.0 to 1.0)	1.0 (1.0 to 1.0)	1.0 (1.0 to 1.0)	1.0 (1.0 to 1.0)	1.0 (1.0 to 1.0)	1.0 (1.0 to 1.0)	1.0 (1.0 to 1.0)	1.0 (1.0 to 1.0)	1.0 (1.0 to 1.0)	1.0 (1.0 to 1.0)
**Childhood sexual abuse against males**																											
Major depressive disorder	Exposed	Morbidity	Male		1.556 (1.296 to 1.864)	1.556 (1.296 to 1.864)	1.556 (1.296 to 1.864)	1.556 (1.296 to 1.864)	1.556 (1.296 to 1.864)	1.556 (1.296 to 1.864)	1.556 (1.296 to 1.864)	1.556 (1.296 to 1.864)	1.556 (1.296 to 1.864)	1.556 (1.296 to 1.864)	1.556 (1.296 to 1.864)	1.556 (1.296 to 1.864)	1.556 (1.296 to 1.864)	1.556 (1.296 to 1.864)	1.556 (1.296 to 1.864)	1.556 (1.296 to 1.864)	1.556 (1.296 to 1.864)	1.556 (1.296 to 1.864)	1.556 (1.296 to 1.864)	1.556 (1.296 to 1.864)	1.556 (1.296 to 1.864)	1.556 (1.296 to 1.864)	1.556 (1.296 to 1.864)
Major depressive disorder	Not exposed	Morbidity	Male		1.0 (1.0 to 1.0)	1.0 (1.0 to 1.0)	1.0 (1.0 to 1.0)	1.0 (1.0 to 1.0)	1.0 (1.0 to 1.0)	1.0 (1.0 to 1.0)	1.0 (1.0 to 1.0)	1.0 (1.0 to 1.0)	1.0 (1.0 to 1.0)	1.0 (1.0 to 1.0)	1.0 (1.0 to 1.0)	1.0 (1.0 to 1.0)	1.0 (1.0 to 1.0)	1.0 (1.0 to 1.0)	1.0 (1.0 to 1.0)	1.0 (1.0 to 1.0)	1.0 (1.0 to 1.0)	1.0 (1.0 to 1.0)	1.0 (1.0 to 1.0)	1.0 (1.0 to 1.0)	1.0 (1.0 to 1.0)	1.0 (1.0 to 1.0)	1.0 (1.0 to 1.0)
Alcohol use disorders	Exposed	Both	Male		2.21 (1.145 to 4.044)	2.21 (1.145 to 4.044)	2.21 (1.145 to 4.044)	2.21 (1.145 to 4.044)	2.21 (1.145 to 4.044)	2.21 (1.145 to 4.044)	2.21 (1.145 to 4.044)	2.21 (1.145 to 4.044)	2.21 (1.145 to 4.044)	2.21 (1.145 to 4.044)	2.21 (1.145 to 4.044)	2.21 (1.145 to 4.044)	2.21 (1.145 to 4.044)	2.21 (1.145 to 4.044)	2.21 (1.145 to 4.044)	2.21 (1.145 to 4.044)	2.21 (1.145 to 4.044)	2.21 (1.145 to 4.044)	2.21 (1.145 to 4.044)	2.21 (1.145 to 4.044)	2.21 (1.145 to 4.044)	2.21 (1.145 to 4.044)	2.21 (1.145 to 4.044)
Alcohol use disorders	Not exposed	Both	Male		1.0 (1.0 to 1.0)	1.0 (1.0 to 1.0)	1.0 (1.0 to 1.0)	1.0 (1.0 to 1.0)	1.0 (1.0 to 1.0)	1.0 (1.0 to 1.0)	1.0 (1.0 to 1.0)	1.0 (1.0 to 1.0)	1.0 (1.0 to 1.0)	1.0 (1.0 to 1.0)	1.0 (1.0 to 1.0)	1.0 (1.0 to 1.0)	1.0 (1.0 to 1.0)	1.0 (1.0 to 1.0)	1.0 (1.0 to 1.0)	1.0 (1.0 to 1.0)	1.0 (1.0 to 1.0)	1.0 (1.0 to 1.0)	1.0 (1.0 to 1.0)	1.0 (1.0 to 1.0)	1.0 (1.0 to 1.0)	1.0 (1.0 to 1.0)	1.0 (1.0 to 1.0)
**Bullying victimisation**																											
Major depressive disorder	First exposed to bullying victimization in current year	morbidity	Both	2.149 (1.555 to 2.831)																							
Major depressive disorder	First exposed to bullying victimization 1 year prior	morbidity	Both	2.089 (1.515 to 2.75)																							
Major depressive disorder	First exposed to bullying victimization 2 years prior	morbidity	Both	2.031 (1.469 to 2.677)																							
Major depressive disorder	First exposed to bullying victimization 3 years prior	morbidity	Both	1.975 (1.429 to 2.601)																							
Major depressive disorder	First exposed to bullying victimization 4 years prior	morbidity	Both	1.92 (1.401 to 2.525)																							
Major depressive disorder	First exposed to bullying victimization 5 years prior	morbidity	Both	1.867 (1.363 to 2.449)																							
Major depressive disorder	First exposed to bullying victimization 6 years prior	morbidity	Both	1.815 (1.323 to 2.377)																							
Major depressive disorder	First exposed to bullying victimization 7 years prior	morbidity	Both	1.765 (1.287 to 2.314)																							
Major depressive disorder	First exposed to bullying victimization 8 years prior	morbidity	Both	1.716 (1.246 to 2.251)																							
Major depressive disorder	First exposed to bullying victimization 9 years prior	morbidity	Both	1.669 (1.215 to 2.192)																							
Major depressive disorder	First exposed to bullying victimization 10 years prior	morbidity	Both	1.622 (1.181 to 2.134)																							
Major depressive disorder	First exposed to bullying victimization 11 years prior	morbidity	Both	1.578 (1.146 to 2.081)																							
Major depressive disorder	First exposed to bullying victimization 12 years prior	morbidity	Both	1.534 (1.108 to 2.025)																							
Major depressive disorder	First exposed to bullying victimization 13 years prior	morbidity	Both	1.492 (1.073 to 1.974)																							
Major depressive disorder	First exposed to bullying victimization 14 years prior	morbidity	Both	1.451 (1.04 to 1.93)																							
Major depressive disorder	First exposed to bullying victimization 15 years prior	morbidity	Both	1.411 (1.008 to 1.874)																							
Major depressive disorder	First exposed to bullying victimization 16 years prior	morbidity	Both	1.372 (0.977 to 1.822)																							
Major depressive disorder	First exposed to bullying victimization 17 years prior	morbidity	Both	1.334 (0.946 to 1.777)																							
Major depressive disorder	First exposed to bullying victimization 18 years prior	morbidity	Both	1.298 (0.917 to 1.736)																							
Major depressive disorder	First exposed to bullying victimization 19 years prior	morbidity	Both	1.262 (0.888 to 1.697)																							
Major depressive disorder	First exposed to bullying victimization 20 years prior	morbidity	Both	1.227 (0.862 to 1.66)																							
Major depressive disorder	First exposed to bullying victimization 21 years prior	morbidity	Both	1.194 (0.837 to 1.622)																							
Major depressive disorder	First exposed to bullying victimization 22 years prior	morbidity	Both	1.161 (0.813 to 1.581)																							
Major depressive disorder	First exposed to bullying victimization 23 years prior	morbidity	Both	1.129 (0.786 to 1.541)																							
Major depressive disorder	First exposed to bullying victimization 24 years prior	morbidity	Both	1.098 (0.758 to 1.498)																							
Major depressive disorder	First exposed to bullying victimization 25 years prior	morbidity	Both	1.068 (0.734 to 1.459)																							
Major depressive disorder	First exposed to bullying victimization 26 years prior	morbidity	Both	1.039 (0.711 to 1.427)																							
Major depressive disorder	Never exposed to bullying victimization or first exposed over 26 years prior	morbidity	Both	1.0 (1.0 to 1.0)																							
Anxiety disorders	First exposed to bullying victimization in current year	morbidity	Both	2.295 (1.719 to 3.049)																							
Anxiety disorders	First exposed to bullying victimization 1 year prior	morbidity	Both	2.231 (1.671 to 2.964)																							
Anxiety disorders	First exposed to bullying victimization 2 years prior	morbidity	Both	2.169 (1.623 to 2.881)																							
Anxiety disorders	First exposed to bullying victimization 3 years prior	morbidity	Both	2.109 (1.585 to 2.808)																							
Anxiety disorders	First exposed to bullying victimization 4 years prior	morbidity	Both	2.05 (1.538 to 2.723)																							
Anxiety disorders	First exposed to bullying victimization 5 years prior	morbidity	Both	1.993 (1.492 to 2.645)																							
Anxiety disorders	First exposed to bullying victimization 6 years prior	morbidity	Both	1.938 (1.453 to 2.567)																							
Anxiety disorders	First exposed to bullying victimization 7 years prior	morbidity	Both	1.884 (1.407 to 2.495)																							
Anxiety disorders	First exposed to bullying victimization 8 years prior	morbidity	Both	1.832 (1.364 to 2.429)																							
Anxiety disorders	First exposed to bullying victimization 9 years prior	morbidity	Both	1.781 (1.331 to 2.365)																							
Anxiety disorders	First exposed to bullying victimization 10 years prior	morbidity	Both	1.732 (1.291 to 2.31)																							
Anxiety disorders	First exposed to bullying victimization 11 years prior	morbidity	Both	1.684 (1.252 to 2.255)																							
Anxiety disorders	First exposed to bullying victimization 12 years prior	morbidity	Both	1.638 (1.214 to 2.189)																							
Anxiety disorders	First exposed to bullying victimization 13 years prior	morbidity	Both	1.592 (1.172 to 2.126)																							
Anxiety disorders	First exposed to bullying victimization 14 years prior	morbidity	Both	1.549 (1.138 to 2.065)																							
Anxiety disorders	First exposed to bullying victimization 15 years prior	morbidity	Both	1.506 (1.099 to 2.016)																							
Anxiety disorders	First exposed to bullying victimization 16 years prior	morbidity	Both	1.464 (1.065 to 1.963)																							
Anxiety disorders	First exposed to bullying victimization 17 years prior	morbidity	Both	1.424 (1.032 to 1.912)																							
Anxiety disorders	First exposed to bullying victimization 18 years prior	morbidity	Both	1.385 (1.001 to 1.859)																							
Anxiety disorders	First exposed to bullying victimization 19 years prior	morbidity	Both	1.347 (0.969 to 1.803)																							
Anxiety disorders	First exposed to bullying victimization 20 years prior	morbidity	Both	1.31 (0.938 to 1.748)																							
Anxiety disorders	First exposed to bullying victimization 21 years prior	morbidity	Both	1.274 (0.908 to 1.698)																							
Anxiety disorders	First exposed to bullying victimization 22 years prior	morbidity	Both	1.239 (0.882 to 1.657)																							
Anxiety disorders	First exposed to bullying victimization 23 years prior	morbidity	Both	1.205 (0.852 to 1.625)																							
Anxiety disorders	First exposed to bullying victimization 24 years prior	morbidity	Both	1.172 (0.824 to 1.577)																							
Anxiety disorders	First exposed to bullying victimization 25 years prior	morbidity	Both	1.14 (0.796 to 1.539)																							
Anxiety disorders	First exposed to bullying victimization 26 years prior	morbidity	Both	1.109 (0.77 to 1.509)																							
Anxiety disorders	First exposed to bullying victimization 27 years prior	morbidity	Both	1.079 (0.746 to 1.479)																							
Anxiety disorders	First exposed to bullying victimization 28 years prior	morbidity	Both	1.049 (0.722 to 1.451)																							
Anxiety disorders	First exposed to bullying victimization 29 years prior	morbidity	Both	1.021 (0.698 to 1.422)																							
Anxiety disorders	Never exposed to bullying victimization or first exposed over 29 years prior	morbidity	Both	1.0 (1.0 to 1.0)																							
**Low physical activity**																											
Colon and rectum cancer	0 METs	Both	Both	1.0 (1.0 to 1.0)																							
Colon and rectum cancer	1200 METs	Both	Both	0.956 (0.88 to 1.032)																							
Colon and rectum cancer	1800 METs	Both	Both	0.933 (0.821 to 1.048)																							
Colon and rectum cancer	2400 METs	Both	Both	0.883 (0.806 to 0.958)																							
Colon and rectum cancer	3000 METs	Both	Both	0.833 (0.76 to 0.907)																							
Colon and rectum cancer	3600 METs	Both	Both	0.831 (0.761 to 0.903)																							
Colon and rectum cancer	4200 METs	Both	Both	0.829 (0.761 to 0.898)																							
Colon and rectum cancer	600 METs	Both	Both	0.978 (0.94 to 1.016)																							
Breast cancer	0 METs	Both	Both	1.0 (1.0 to 1.0)																							
Breast cancer	1200 METs	Both	Both	0.974 (0.942 to 1.005)																							
Breast cancer	1800 METs	Both	Both	0.96 (0.914 to 1.008)																							
Breast cancer	2400 METs	Both	Both	0.957 (0.914 to 1.001)																							
Breast cancer	3000 METs	Both	Both	0.953 (0.913 to 0.994)																							
Breast cancer	3600 METs	Both	Both	0.949 (0.912 to 0.99)																							
Breast cancer	4200 METs	Both	Both	0.946 (0.909 to 0.985)																							
Breast cancer	600 METs	Both	Both	0.987 (0.971 to 1.003)																							
Ischaemic heart disease	0 METs	Both	Both										1.761 (1.761 to 1.761)	1.761 (1.761 to 1.761)	1.761 (1.761 to 1.761)	1.467 (1.467 to 1.467)	1.405 (1.405 to 1.405)	1.294 (1.294 to 1.294)	1.134 (1.134 to 1.134)	1.0 (1.0 to 1.0)	0.900 (0.900 to 0.900)	0.813 (0.813 to 0.813)	0.740 (0.740 to 0.740)				
Ischaemic heart disease	1200 METs	Both	Both										1.442 (1.256 to 1.635)	1.442 (1.256 to 1.635)	1.442 (1.256 to 1.635)	1.201 (1.047 to 1.362)	1.151 (1.003 to 1.305)	1.060 (0.924 to 1.202)	0.929 (0.809 to 1.053)	0.819 (0.714 to 0.928)	0.737 (0.642 to 0.835)	0.666 (0.580 to 0.755)	0.606 (0.528 to 0.687)				
Ischaemic heart disease	1800 METs	Both	Both										1.422 (1.302 to 1.560)	1.422 (1.302 to 1.560)	1.422 (1.302 to 1.560)	1.185 (1.085 to 1.300)	1.135 (1.039 to 1.245)	1.045 (0.957 to 1.147)	0.916 (0.839 to 1.005)	0.808 (0.739 to 0.886)	0.727 (0.665 to 0.797)	0.657 (0.601 to 0.720)	0.597 (0.547 to 0.655)				
Ischaemic heart disease	2400 METs	Both	Both										1.402 (1.252 to 1.576)	1.402 (1.252 to 1.576)	1.402 (1.252 to 1.576)	1.169 (1.043 to 1.313)	1.119 (0.999 to 1.258)	1.031 (0.920 to 1.159)	0.903 (0.806 to 1.015)	0.796 (0.711 to 0.895)	0.717 (0.640 to 0.805)	0.648 (0.578 to 0.728)	0.589 (0.526 to 0.662)				
Ischaemic heart disease	3000 METs	Both	Both										1.367 (1.255 to 1.486)	1.367 (1.255 to 1.486)	1.367 (1.255 to 1.486)	1.139 (1.046 to 1.238)	1.091 (1.002 to 1.186)	1.005 (0.923 to 1.092)	0.880 (0.809 to 0.957)	0.776 (0.713 to 0.844)	0.699 (0.641 to 0.759)	0.631 (0.580 to 0.686)	0.574 (0.527 to 0.624)				
Ischaemic heart disease	3600 METs	Both	Both										1.332 (1.219 to 1.437)	1.332 (1.219 to 1.437)	1.332 (1.219 to 1.437)	1.110 (1.016 to 1.198)	1.063 (0.973 to 1.147)	0.979 (0.896 to 1.056)	0.858 (0.785 to 0.926)	0.756 (0.692 to 0.816)	0.680 (0.623 to 0.734)	0.615 (0.563 to 0.664)	0.559 (0.512 to 0.604)				
Ischaemic heart disease	4200 METs	Both	Both										1.296 (1.148 to 1.446)	1.296 (1.148 to 1.446)	1.296 (1.148 to 1.446)	1.080 (0.957 to 1.205)	1.035 (0.916 to 1.154)	0.953 (0.844 to 1.063)	0.835 (0.739 to 0.931)	0.736 (0.652 to 0.821)	0.662 (0.587 to 0.739)	0.599 (0.530 to 0.668)	0.545 (0.482 to 0.607)				
Ischaemic heart disease	600 METs	Both	Both										1.601 (1.509 to 1.698)	1.601 (1.509 to 1.698)	1.601 (1.509 to 1.698)	1.334 (1.257 to 1.415)	1.278 (1.204 to 1.355)	1.177 (1.109 to 1.248)	1.031 (0.972 to 1.094)	0.909 (0.857 to 0.964)	0.818 (0.771 to 0.868)	0.739 (0.697 to 0.784)	0.673 (0.634 to 0.713)				
Ischaemic stroke	0 METs	Both	Both										2.393 (2.393 to 2.393)	2.393 (2.393 to 2.393)	2.393 (2.393 to 2.393)	1.946 (1.946 to 1.946)	1.683 (1.683 to 1.683)	1.431 (1.431 to 1.431)	1.202 (1.202 to 1.202)	1.0 (1.0 to 1.0)	0.834 (0.834 to 0.834)	0.680 (0.680 to 0.680)	0.540 (0.540 to 0.540)				
Ischaemic stroke	1200 METs	Both	Both										1.961 (1.584 to 2.390)	1.961 (1.584 to 2.390)	1.961 (1.584 to 2.390)	1.594 (1.288 to 1.943)	1.379 (1.114 to 1.681)	1.173 (0.947 to 1.429)	0.985 (0.796 to 1.201)	0.819 (0.662 to 0.999)	0.683 (0.552 to 0.833)	0.557 (0.450 to 0.679)	0.443 (0.357 to 0.539)				
Ischaemic stroke	1800 METs	Both	Both										1.920 (1.681 to 2.190)	1.920 (1.681 to 2.190)	1.920 (1.681 to 2.190)	1.561 (1.366 to 1.780)	1.351 (1.182 to 1.540)	1.148 (1.005 to 1.310)	0.965 (0.844 to 1.100)	0.802 (0.702 to 0.915)	0.669 (0.585 to 0.763)	0.545 (0.477 to 0.622)	0.433 (0.379 to 0.494)				
Ischaemic stroke	2400 METs	Both	Both										1.880 (1.539 to 2.257)	1.880 (1.539 to 2.257)	1.880 (1.539 to 2.257)	1.528 (1.252 to 1.835)	1.322 (1.083 to 1.588)	1.124 (0.921 to 1.350)	0.944 (0.773 to 1.134)	0.785 (0.643 to 0.943)	0.655 (0.536 to 0.786)	0.534 (0.437 to 0.641)	0.424 (0.347 to 0.509)				
Ischaemic stroke	3000 METs	Both	Both										1.877 (1.677 to 2.124)	1.877 (1.677 to 2.124)	1.877 (1.677 to 2.124)	1.526 (1.364 to 1.727)	1.321 (1.180 to 1.494)	1.123 (1.003 to 1.270)	0.943 (0.843 to 1.067)	0.784 (0.701 to 0.888)	0.654 (0.584 to 0.740)	0.533 (0.476 to 0.603)	0.424 (0.379 to 0.479)				
Ischaemic stroke	3600 METs	Both	Both										1.875 (1.664 to 2.113)	1.875 (1.664 to 2.113)	1.875 (1.664 to 2.113)	1.524 (1.353 to 1.718)	1.319 (1.171 to 1.486)	1.121 (0.995 to 1.264)	0.942 (0.836 to 1.062)	0.783 (0.695 to 0.883)	0.653 (0.580 to 0.736)	0.532 (0.473 to 0.600)	0.423 (0.376 to 0.477)				
Ischaemic stroke	4200 METs	Both	Both										1.872 (1.542 to 2.235)	1.872 (1.542 to 2.235)	1.872 (1.542 to 2.235)	1.522 (1.254 to 1.817)	1.317 (1.085 to 1.572)	1.120 (0.922 to 1.336)	0.941 (0.775 to 1.123)	0.782 (0.644 to 0.934)	0.652 (0.537 to 0.779)	0.532 (0.438 to 0.635)	0.423 (0.348 to 0.504)				
Ischaemic stroke	600 METs	Both	Both										2.177 (1.988 to 2.392)	2.177 (1.988 to 2.392)	2.177 (1.988 to 2.392)	1.770 (1.617 to 1.944)	1.531 (1.399 to 1.682)	1.302 (1.189 to 1.430)	1.094 (0.999 to 1.201)	0.910 (0.831 to 0.999)	0.758 (0.693 to 0.833)	0.618 (0.565 to 0.679)	0.491 (0.449 to 0.540)				
Diabetes mellitus type 2	0 METs	Both	Both										1.519 (1.519 to 1.519)	1.519 (1.519 to 1.519)	1.519 (1.519 to 1.519)	1.447 (1.447 to 1.447)	1.323 (1.323 to 1.323)	1.213 (1.213 to 1.213)	1.109 (1.109 to 1.109)	1.0 (1.0 to 1.0)	0.900 (0.900 to 0.900)	0.804 (0.804 to 0.804)	0.696 (0.696 to 0.696)				

Diabetes mellitus type 2	1200 METs	Both	Both		1.460 (1.418 to 1.508)	1.460 (1.418 to 1.508)	1.460 (1.418 to 1.508)	1.390 (1.350 to 1.435)	1.271 (1.235 to 1.313)	1.165 (1.132 to 1.204)	1.066 (1.035 to 1.101)	0.961 (0.933 to 0.992)	0.864 (0.840 to 0.893)	0.772 (0.750 to 0.798)	0.669 (0.650 to 0.691)				
Diabetes mellitus type 2	1800 METs	Both	Both		1.400 (1.317 to 1.496)	1.400 (1.317 to 1.496)	1.400 (1.317 to 1.496)	1.333 (1.254 to 1.424)	1.218 (1.146 to 1.302)	1.117 (1.051 to 1.194)	1.022 (0.961 to 1.092)	0.921 (0.867 to 0.985)	0.829 (0.780 to 0.886)	0.741 (0.697 to 0.792)	0.641 (0.603 to 0.685)				
Diabetes mellitus type 2	2400 METs	Both	Both		1.340 (1.216 to 1.484)	1.340 (1.216 to 1.484)	1.340 (1.216 to 1.484)	1.276 (1.157 to 1.413)	1.166 (1.058 to 1.292)	1.070 (0.970 to 1.185)	0.978 (0.887 to 1.084)	0.882 (0.800 to 0.977)	0.793 (0.720 to 0.879)	0.709 (0.643 to 0.785)	0.614 (0.557 to 0.680)				
Diabetes mellitus type 2	3000 METs	Both	Both		1.267 (1.175 to 1.373)	1.267 (1.175 to 1.373)	1.267 (1.175 to 1.373)	1.206 (1.118 to 1.307)	1.103 (1.023 to 1.196)	1.011 (0.938 to 1.096)	0.925 (0.858 to 1.003)	0.834 (0.773 to 0.904)	0.750 (0.696 to 0.813)	0.670 (0.622 to 0.727)	0.580 (0.538 to 0.629)				
Diabetes mellitus type 2	3600 METs	Both	Both		1.194 (1.117 to 1.278)	1.194 (1.117 to 1.278)	1.194 (1.117 to 1.278)	1.136 (1.064 to 1.217)	1.039 (0.973 to 1.112)	0.953 (0.892 to 1.020)	0.871 (0.816 to 0.933)	0.786 (0.735 to 0.841)	0.707 (0.662 to 0.757)	0.632 (0.591 to 0.676)	0.547 (0.512 to 0.585)				
Diabetes mellitus type 2	4200 METs	Both	Both		1.120 (1.039 to 1.206)	1.120 (1.039 to 1.206)	1.120 (1.039 to 1.206)	1.067 (0.989 to 1.148)	0.975 (0.905 to 1.050)	0.894 (0.830 to 0.963)	0.818 (0.759 to 0.880)	0.737 (0.684 to 0.794)	0.664 (0.615 to 0.714)	0.593 (0.550 to 0.638)	0.513 (0.476 to 0.552)				
Diabetes mellitus type 2	600 METs	Both	Both		1.490 (1.469 to 1.514)	1.490 (1.469 to 1.514)	1.490 (1.469 to 1.514)	1.418 (1.398 to 1.441)	1.297 (1.279 to 1.318)	1.189 (1.173 to 1.208)	1.087 (1.072 to 1.105)	0.980 (0.967 to 0.996)	0.882 (0.870 to 0.896)	0.788 (0.777 to 0.801)	0.682 (0.673 to 0.693)				
**High fasting plasma glucose (continuous)**																			
Ischaemic heart disease	mmol/L	Both	Both		1.471 (1.147 to 2.099)	1.373 (1.153 to 1.741)	1.274 (1.13 to 1.451)	1.22 (1.085 to 1.365)	1.211 (1.111 to 1.327)	1.201 (1.121 to 1.297)	1.192 (1.123 to 1.269)	1.182 (1.111 to 1.267)	1.173 (1.085 to 1.271)	1.168 (1.08 to 1.273)	1.168 (1.097 to 1.264)	1.169 (1.071 to 1.316)	1.169 (1.071 to 1.316)	1.169 (1.071 to 1.316)	1.169 (1.071 to 1.316)
Ischaemic stroke	mmol/L	Both	Both		1.526 (1.11 to 2.227)	1.4 (1.101 to 1.85)	1.275 (1.081 to 1.561)	1.21 (1.044 to 1.441)	1.204 (1.075 to 1.383)	1.199 (1.095 to 1.334)	1.194 (1.108 to 1.302)	1.188 (1.101 to 1.295)	1.183 (1.069 to 1.308)	1.174 (1.056 to 1.309)	1.162 (1.075 to 1.295)	1.133 (1.055 to 1.332)	1.133 (1.055 to 1.332)	1.133 (1.055 to 1.332)	1.133 (1.055 to 1.332)
Intracerebral haemorrhage	mmol/L	Both	Both		1.506 (1.112 to 2.221)	1.382 (1.111 to 1.843)	1.258 (1.085 to 1.487)	1.196 (1.053 to 1.371)	1.193 (1.082 to 1.333)	1.191 (1.106 to 1.298)	1.189 (1.115 to 1.262)	1.187 (1.115 to 1.252)	1.184 (1.097 to 1.264)	1.175 (1.087 to 1.263)	1.158 (1.09 to 1.233)	1.116 (1.056 to 1.25)	1.116 (1.056 to 1.25)	1.116 (1.056 to 1.25)	1.116 (1.056 to 1.25)
Subarachnoid haemorrhage	mmol/L	Both	Both		1.506 (1.112 to 2.221)	1.382 (1.111 to 1.843)	1.258 (1.085 to 1.487)	1.196 (1.053 to 1.371)	1.193 (1.082 to 1.333)	1.191 (1.106 to 1.298)	1.189 (1.115 to 1.262)	1.187 (1.115 to 1.252)	1.184 (1.097 to 1.264)	1.175 (1.087 to 1.263)	1.158 (1.09 to 1.233)	1.116 (1.056 to 1.25)	1.116 (1.056 to 1.25)	1.116 (1.056 to 1.25)	1.116 (1.056 to 1.25)
**High fasting plasma glucose (categorical)**																			
Latent tuberculosis infection	Diabetic	Both	Both		2.73 (1.973 to 3.602)	2.801 (2.056 to 3.658)	2.871 (2.047 to 3.698)	2.798 (1.97 to 3.63)	2.581 (1.91 to 3.268)	2.364 (1.817 to 2.943)	2.147 (1.692 to 2.673)	1.93 (1.485 to 2.44)	1.713 (1.231 to 2.317)	1.598 (1.125 to 2.239)	1.587 (1.184 to 2.115)	1.559 (1.182 to 2.174)	1.559 (1.182 to 2.174)	1.559 (1.182 to 2.174)	1.559 (1.182 to 2.174)
Latent tuberculosis infection	Not diabetic	Both	Both		1.0 (1.0 to 1.0)	1.0 (1.0 to 1.0)	1.0 (1.0 to 1.0)	1.0 (1.0 to 1.0)	1.0 (1.0 to 1.0)	1.0 (1.0 to 1.0)	1.0 (1.0 to 1.0)	1.0 (1.0 to 1.0)	1.0 (1.0 to 1.0)	1.0 (1.0 to 1.0)	1.0 (1.0 to 1.0)	1.0 (1.0 to 1.0)	1.0 (1.0 to 1.0)	1.0 (1.0 to 1.0)	1.0 (1.0 to 1.0)
Drug-susceptible tuberculosis	Diabetic	Both	Both		2.73 (1.973 to 3.602)	2.801 (2.056 to 3.658)	2.871 (2.047 to 3.698)	2.798 (1.97 to 3.63)	2.581 (1.91 to 3.268)	2.364 (1.817 to 2.943)	2.147 (1.692 to 2.673)	1.93 (1.485 to 2.44)	1.713 (1.231 to 2.317)	1.598 (1.125 to 2.239)	1.587 (1.184 to 2.115)	1.559 (1.182 to 2.174)	1.559 (1.182 to 2.174)	1.559 (1.182 to 2.174)	1.559 (1.182 to 2.174)
Drug-susceptible tuberculosis	Not diabetic	Both	Both		1.0 (1.0 to 1.0)	1.0 (1.0 to 1.0)	1.0 (1.0 to 1.0)	1.0 (1.0 to 1.0)	1.0 (1.0 to 1.0)	1.0 (1.0 to 1.0)	1.0 (1.0 to 1.0)	1.0 (1.0 to 1.0)	1.0 (1.0 to 1.0)	1.0 (1.0 to 1.0)	1.0 (1.0 to 1.0)	1.0 (1.0 to 1.0)	1.0 (1.0 to 1.0)	1.0 (1.0 to 1.0)	1.0 (1.0 to 1.0)
Multidrug-resistant tuberculosis without extensive drug resistance	Diabetic	Both	Both		2.73 (1.973 to 3.602)	2.801 (2.056 to 3.658)	2.871 (2.047 to 3.698)	2.798 (1.97 to 3.63)	2.581 (1.91 to 3.268)	2.364 (1.817 to 2.943)	2.147 (1.692 to 2.673)	1.93 (1.485 to 2.44)	1.713 (1.231 to 2.317)	1.598 (1.125 to 2.239)	1.587 (1.184 to 2.115)	1.559 (1.182 to 2.174)	1.559 (1.182 to 2.174)	1.559 (1.182 to 2.174)	1.559 (1.182 to 2.174)
Multidrug-resistant tuberculosis without extensive drug resistance	Not diabetic	Both	Both		1.0 (1.0 to 1.0)	1.0 (1.0 to 1.0)	1.0 (1.0 to 1.0)	1.0 (1.0 to 1.0)	1.0 (1.0 to 1.0)	1.0 (1.0 to 1.0)	1.0 (1.0 to 1.0)	1.0 (1.0 to 1.0)	1.0 (1.0 to 1.0)	1.0 (1.0 to 1.0)	1.0 (1.0 to 1.0)	1.0 (1.0 to 1.0)	1.0 (1.0 to 1.0)	1.0 (1.0 to 1.0)	1.0 (1.0 to 1.0)
Extensively drug-resistant tuberculosis	Diabetic	Both	Both		2.73 (1.973 to 3.602)	2.801 (2.056 to 3.658)	2.871 (2.047 to 3.698)	2.798 (1.97 to 3.63)	2.581 (1.91 to 3.268)	2.364 (1.817 to 2.943)	2.147 (1.692 to 2.673)	1.93 (1.485 to 2.44)	1.713 (1.231 to 2.317)	1.598 (1.125 to 2.239)	1.587 (1.184 to 2.115)	1.559 (1.182 to 2.174)	1.559 (1.182 to 2.174)	1.559 (1.182 to 2.174)	1.559 (1.182 to 2.174)
Extensively drug-resistant tuberculosis	Not diabetic	Both	Both		1.0 (1.0 to 1.0)	1.0 (1.0 to 1.0)	1.0 (1.0 to 1.0)	1.0 (1.0 to 1.0)	1.0 (1.0 to 1.0)	1.0 (1.0 to 1.0)	1.0 (1.0 to 1.0)	1.0 (1.0 to 1.0)	1.0 (1.0 to 1.0)	1.0 (1.0 to 1.0)	1.0 (1.0 to 1.0)	1.0 (1.0 to 1.0)	1.0 (1.0 to 1.0)	1.0 (1.0 to 1.0)	1.0 (1.0 to 1.0)
Colon and rectum cancer	Diabetic	Both	Males		1.527 (1.081 to 2.304)	1.527 (1.081 to 2.304)	1.527 (1.081 to 2.304)	1.527 (1.081 to 2.304)	1.527 (1.081 to 2.304)	1.527 (1.081 to 2.304)	1.527 (1.081 to 2.304)	1.527 (1.081 to 2.304)	1.527 (1.081 to 2.304)	1.527 (1.081 to 2.304)	1.527 (1.081 to 2.304)	1.527 (1.081 to 2.304)	1.527 (1.081 to 2.304)	1.527 (1.081 to 2.304)	1.527 (1.081 to 2.304)
Colon and rectum cancer	Diabetic	Both	Females		1.527 (1.086 to 2.315)	1.527 (1.086 to 2.315)	1.527 (1.086 to 2.315)	1.527 (1.086 to 2.315)	1.527 (1.086 to 2.315)	1.527 (1.086 to 2.315)	1.527 (1.086 to 2.315)	1.527 (1.086 to 2.315)	1.527 (1.086 to 2.315)	1.527 (1.086 to 2.315)	1.527 (1.086 to 2.315)	1.527 (1.086 to 2.315)	1.527 (1.086 to 2.315)	1.527 (1.086 to 2.315)	1.527 (1.086 to 2.315)
Colon and rectum cancer	Not diabetic	Both	Males		1.0 (1.0 to 1.0)	1.0 (1.0 to 1.0)	1.0 (1.0 to 1.0)	1.0 (1.0 to 1.0)	1.0 (1.0 to 1.0)	1.0 (1.0 to 1.0)	1.0 (1.0 to 1.0)	1.0 (1.0 to 1.0)	1.0 (1.0 to 1.0)	1.0 (1.0 to 1.0)	1.0 (1.0 to 1.0)	1.0 (1.0 to 1.0)	1.0 (1.0 to 1.0)	1.0 (1.0 to 1.0)	1.0 (1.0 to 1.0)
Colon and rectum cancer	Not diabetic	Both	Females		1.0 (1.0 to 1.0)	1.0 (1.0 to 1.0)	1.0 (1.0 to 1.0)	1.0 (1.0 to 1.0)	1.0 (1.0 to 1.0)	1.0 (1.0 to 1.0)	1.0 (1.0 to 1.0)	1.0 (1.0 to 1.0)	1.0 (1.0 to 1.0)	1.0 (1.0 to 1.0)	1.0 (1.0 to 1.0)	1.0 (1.0 to 1.0)	1.0 (1.0 to 1.0)	1.0 (1.0 to 1.0)	1.0 (1.0 to 1.0)
Liver cancer due to NASH	Diabetic	Both	Males		1.523 (1.093 to 2.3)	1.523 (1.093 to 2.3)	1.523 (1.093 to 2.3)	1.523 (1.093 to 2.3)	1.523 (1.093 to 2.3)	1.523 (1.093 to 2.3)	1.523 (1.093 to 2.3)	1.523 (1.093 to 2.3)	1.523 (1.093 to 2.3)	1.523 (1.093 to 2.3)	1.523 (1.093 to 2.3)	1.523 (1.093 to 2.3)	1.523 (1.093 to 2.3)	1.523 (1.093 to 2.3)	1.523 (1.093 to 2.3)
Liver cancer due to NASH	Diabetic	Both	Females		1.512 (1.083 to 2.293)	1.512 (1.083 to 2.293)	1.512 (1.083 to 2.293)	1.512 (1.083 to 2.293)	1.512 (1.083 to 2.293)	1.512 (1.083 to 2.293)	1.512 (1.083 to 2.293)	1.512 (1.083 to 2.293)	1.512 (1.083 to 2.293)	1.512 (1.083 to 2.293)	1.512 (1.083 to 2.293)	1.512 (1.083 to 2.293)	1.512 (1.083 to 2.293)	1.512 (1.083 to 2.293)	1.512 (1.083 to 2.293)
Liver cancer due to NASH	Not diabetic	Both	Males		1.0 (1.0 to 1.0)	1.0 (1.0 to 1.0)	1.0 (1.0 to 1.0)	1.0 (1.0 to 1.0)	1.0 (1.0 to 1.0)	1.0 (1.0 to 1.0)	1.0 (1.0 to 1.0)	1.0 (1.0 to 1.0)	1.0 (1.0 to 1.0)	1.0 (1.0 to 1.0)	1.0 (1.0 to 1.0)	1.0 (1.0 to 1.0)	1.0 (1.0 to 1.0)	1.0 (1.0 to 1.0)	1.0 (1.0 to 1.0)
Liver cancer due to NASH	Not diabetic	Both	Females		1.0 (1.0 to 1.0)	1.0 (1.0 to 1.0)	1.0 (1.0 to 1.0)	1.0 (1.0 to 1.0)	1.0 (1.0 to 1.0)	1.0 (1.0 to 1.0)	1.0 (1.0 to 1.0)	1.0 (1.0 to 1.0)	1.0 (1.0 to 1.0)	1.0 (1.0 to 1.0)	1.0 (1.0 to 1.0)	1.0 (1.0 to 1.0)	1.0 (1.0 to 1.0)	1.0 (1.0 to 1.0)	1.0 (1.0 to 1.0)
Pancreatic cancer	Diabetic	Both	Males		1.517 (1.085 to 2.314)	1.517 (1.085 to 2.314)	1.517 (1.085 to 2.314)	1.517 (1.085 to 2.314)	1.517 (1.085 to 2.314)	1.517 (1.085 to 2.314)	1.517 (1.085 to 2.314)	1.517 (1.085 to 2.314)	1.517 (1.085 to 2.314)	1.517 (1.085 to 2.314)	1.517 (1.085 to 2.314)	1.517 (1.085 to 2.314)	1.517 (1.085 to 2.314)	1.517 (1.085 to 2.314)	1.517 (1.085 to 2.314)
Pancreatic cancer	Diabetic	Both	Females		1.518 (1.075 to 2.311)	1.518 (1.075 to 2.311)	1.518 (1.075 to 2.311)	1.518 (1.075 to 2.311)	1.518 (1.075 to 2.311)	1.518 (1.075 to 2.311)	1.518 (1.075 to 2.311)	1.518 (1.075 to 2.311)	1.518 (1.075 to 2.311)	1.518 (1.075 to 2.311)	1.518 (1.075 to 2.311)	1.518 (1.075 to 2.311)	1.518 (1.075 to 2.311)	1.518 (1.075 to 2.311)	1.518 (1.075 to 2.311)
Pancreatic cancer	Not diabetic	Both	Males		1.0 (1.0 to 1.0)	1.0 (1.0 to 1.0)	1.0 (1.0 to 1.0)	1.0 (1.0 to 1.0)	1.0 (1.0 to 1.0)	1.0 (1.0 to 1.0)	1.0 (1.0 to 1.0)	1.0 (1.0 to 1.0)	1.0 (1.0 to 1.0)	1.0 (1.0 to 1.0)	1.0 (1.0 to 1.0)	1.0 (1.0 to 1.0)	1.0 (1.0 to 1.0)	1.0 (1.0 to 1.0)	1.0 (1.0 to 1.0)
Pancreatic cancer	Not diabetic	Both	Females		1.0 (1.0 to 1.0)	1.0 (1.0 to 1.0)	1.0 (1.0 to 1.0)	1.0 (1.0 to 1.0)	1.0 (1.0 to 1.0)	1.0 (1.0 to 1.0)	1.0 (1.0 to 1.0)	1.0 (1.0 to 1.0)	1.0 (1.0 to 1.0)	1.0 (1.0 to 1.0)	1.0 (1.0 to 1.0)	1.0 (1.0 to 1.0)	1.0 (1.0 to 1.0)	1.0 (1.0 to 1.0)	1.0 (1.0 to 1.0)
Tracheal, bronchus, and lung cancer	Diabetic	Both	Males		1.515 (1.076 to 2.309)	1.515 (1.076 to 2.309)	1.515 (1.076 to 2.309)	1.515 (1.076 to 2.309)	1.515 (1.076 to 2.309)	1.515 (1.076 to 2.309)	1.515 (1.076 to 2.309)	1.515 (1.076 to 2.309)	1.515 (1.076 to 2.309)	1.515 (1.076 to 2.309)	1.515 (1.076 to 2.309)	1.515 (1.076 to 2.309)	1.515 (1.076 to 2.309)	1.515 (1.076 to 2.309)	1.515 (1.076 to 2.309)
Tracheal, bronchus, and lung cancer	Diabetic	Both	Females		1.512 (1.087 to 2.299)	1.512 (1.087 to 2.299)	1.512 (1.087 to 2.299)	1.512 (1.087 to 2.299)	1.512 (1.087 to 2.299)	1.512 (1.087 to 2.299)	1.512 (1.087 to 2.299)	1.512 (1.087 to 2.299)	1.512 (1.087 to 2.299)	1.512 (1.087 to 2.299)	1.512 (1.087 to 2.299)	1.512 (1.087 to 2.299)	1.512 (1.087 to 2.299)	1.512 (1.087 to 2.299)	1.512 (1.087 to 2.299)
Tracheal, bronchus, and lung cancer	Not diabetic	Both	Males		1.0 (1.0 to 1.0)	1.0 (1.0 to 1.0)	1.0 (1.0 to 1.0)	1.0 (1.0 to 1.0)	1.0 (1.0 to 1.0)	1.0 (1.0 to 1.0)	1.0 (1.0 to 1.0)	1.0 (1.0 to 1.0)	1.0 (1.0 to 1.0)	1.0 (1.0 to 1.0)	1.0 (1.0 to 1.0)	1.0 (1.0 to 1.0)	1.0 (1.0 to 1.0)	1.0 (1.0 to 1.0)	1.0 (1.0 to 1.0)
Tracheal, bronchus, and lung cancer	Not diabetic	Both	Females		1.0 (1.0 to 1.0)	1.0 (1.0 to 1.0)	1.0 (1.0 to 1.0)	1.0 (1.0 to 1.0)	1.0 (1.0 to 1.0)	1.0 (1.0 to 1.0)	1.0 (1.0 to 1.0)	1.0 (1.0 to 1.0)	1.0 (1.0 to 1.0)	1.0 (1.0 to 1.0)	1.0 (1.0 to 1.0)	1.0 (1.0 to 1.0)	1.0 (1.0 to 1.0)	1.0 (1.0 to 1.0)	1.0 (1.0 to 1.0)
Breast cancer	Diabetic	Both	Both		1.513 (1.087 to 2.206)	1.513 (1.087 to 2.206)	1.513 (1.087 to 2.206)	1.513 (1.087 to 2.206)	1.513 (1.087 to 2.206)	1.513 (1.087 to 2.206)	1.513 (1.087 to 2.206)	1.513 (1.087 to 2.206)	1.513 (1.087 to 2.206)	1.513 (1.087 to 2.206)	1.513 (1.087 to 2.206)	1.513 (1.087 to 2.206)	1.513 (1.087 to 2.206)	1.513 (1.087 to 2.206)	1.513 (1.087 to 2.206)
Breast cancer	Not diabetic	Both	Both		1.0 (1.0 to 1.0)	1.0 (1.0 to 1.0)	1.0 (1.0 to 1.0)	1.0 (1.0 to 1.0)	1.0 (1.0 to 1.0)	1.0 (1.0 to 1.0)	1.0 (1.0 to 1.0)	1.0 (1.0 to 1.0)	1.0 (1.0 to 1.0)	1.0 (1.0 to 1.0)	1.0 (1.0 to 1.0)	1.0 (1.0 to 1.0)	1.0 (1.0 to 1.0)	1.0 (1.0 to 1.0)	1.0 (1.0 to 1.0)
Ovarian cancer	Diabetic	Both	Both		1.522 (1.092 to 2.32)	1.522 (1.092 to 2.32)	1.522 (1.092 to 2.32)	1.522 (1.092 to 2.32)	1.522 (1.092 to 2.32)	1.522 (1.092 to 2.32)	1.522 (1.092 to 2.32)	1.522 (1.092 to 2.32)	1.522 (1.092 to 2.32)	1.522 (1.092 to 2.32)	1.522 (1.092 to 2.32)	1.522 (1.092 to 2.32)	1.522 (1.092 to 2.32)	1.522 (1.092 to 2.32)	1.522 (1.092 to 2.32)
Ovarian cancer	Not diabetic	Both	Both		1.0 (1.0 to 1.0)	1.0 (1.0 to 1.0)	1.0 (1.0 to 1.0)	1.0 (1.0 to 1.0)	1.0 (1.0 to 1.0)	1.0 (1.0 to 1.0)	1.0 (1.0 to 1.0)	1.0 (1.0 to 1.0)	1.0 (1.0 to 1.0)	1.0 (1.0 to 1.0)	1.0 (1.0 to 1.0)	1.0 (1.0 to 1.0)	1.0 (1.0 to 1.0)	1.0 (1.0 to 1.0)	1.0 (1.0 to 1.0)
Bladder cancer	Diabetic	Both	Males		1.514 (1.076 to 2.256)	1.514 (1.076 to 2.256)	1.514 (1.076 to 2.256)	1.514 (1.076 to 2.256)	1.514 (1.076 to 2.256)	1.514 (1.076 to 2.256)	1.514 (1.076 to 2.256)	1.514 (1.076 to 2.256)	1.514 (1.076 to 2.256)	1.514 (1.076 to 2.256)	1.514 (1.076 to 2.256)	1.514 (1.076 to 2.256)	1.514 (1.076 to 2.256)	1.514 (1.076 to 2.256)	1.514 (1.076 to 2.256)
Bladder cancer	Diabetic	Both	Females		1.511 (1.082 to 2.253)	1.511 (1.082 to 2.253)	1.511 (1.082 to 2.253)	1.511 (1.082 to 2.253)	1.511 (1.082 to 2.253)	1.511 (1.082 to 2.253)	1.511 (1.082 to 2.253)	1.511 (1.082 to 2.253)	1.511 (1.082 to 2.253)	1.511 (1.082 to 2.253)	1.511 (1.082 to 2.253)	1.511 (1.082 to 2.253)	1.511 (1.082 to 2.253)	1.511 (1.082 to 2.253)	1.511 (1.082 to 2.253)
Bladder cancer	Not diabetic	Both	Males		1.0 (1.0 to 1.0)	1.0 (1.0 to 1.0)	1.0 (1.0 to 1.0)	1.0 (1.0 to 1.0)	1.0 (1.0 to 1.0)	1.0 (1.0 to 1.0)	1.0 (1.0 to 1.0)	1.0 (1.0 to 1.0)	1.0 (1.0 to 1.0)	1.0 (1.0 to 1.0)	1.0 (1.0 to 1.0)	1.0 (1.0 to 1.0)	1.0 (1.0 to 1.0)	1.0 (1.0 to 1.0)	1.0 (1.0 to 1.0)
Bladder cancer	Not diabetic	Both	Females		1.0 (1.0 to 1.0)	1.0 (1.0 to 1.0)	1.0 (1.0 to 1.0)	1.0 (1.0 to 1.0)	1.0 (1.0 to 1.0)	1.0 (1.0 to 1.0)	1.0 (1.0 to 1.0)	1.0 (1.0 to 1.0)	1.0 (1.0 to 1.0)	1.0 (1.0 to 1.0)	1.0 (1.0 to 1.0)	1.0 (1.0 to 1.0)	1.0 (1.0 to 1.0)	1.0 (1.0 to 1.0)	1.0 (1.0 to 1.0)
Peripheral vascular disease	Diabetic	Both	Both		8.264 (6.044 to 9.303)	6.651 (5.375 to 7.452)	5.039 (4.436 to 5.673)	4.138 (3.561 to 4.76)	3.947 (3.447 to 4.504)	3.756 (3.347 to 4.232)	3.565 (3.214 to 3.972)	3.374 (3.079 to 3.714)	3.183 (2.92 to 3.466)	2.992 (2.76 to 3.25)	2.801 (2.558 to 3.05)	2.324 (1.977 to 2.671)	2.324 (1.977 to 2.671)	2.324 (1.977 to 2.671)	2.324 (1.977 to 2.671)
Peripheral vascular disease	Not diabetic	Both	Both		1.0 (1.0 to 1.0)	1.0 (1.0 to 1.0)	1.0 (1.0 to 1.0)	1.0 (1.0 to 1.0)	1.0 (1.0 to 1.0)	1.0 (1.0 to 1.0)	1.0 (1.0 to 1.0)	1.0 (1.0 to 1.0)	1.0 (1.0 to 1.0)	1.0 (1.0 to 1.0)	1.0 (1.0 to 1.0)	1.0 (1.0 to 1.0)	1.0 (1.0 to 1.0)	1.0 (1.0 to 1.0)	1.0 (1.0 to 1.0)
Alzheimer's disease and other dementias	Diabetic	Both	Males		1.516 (1.084 to 2.295)	1.516 (1.084 to 2.295)	1.516 (1.084 to 2.295)	1.516 (1.084 to 2.295)	1.516 (1.084 to 2.295)	1.516 (1.084 to 2.295)	1.516 (1.084 to 2.295)	1.516 (1.084 to 2.295)	1.516 (1.084 to 2.295)	1.516 (1.084 to 2.295)	1.516 (1.084 to 2.295)	1.516 (1.084 to 2.295)	1.516 (1.084 to 2.295)	1.516 (1.084 to 2.295)	1.516 (1.084 to 2.295)
Alzheimer's disease and other dementias	Diabetic	Both	Females		1.52 (1.08 to 2.301)	1.52 (1.08 to 2.301)	1.52 (1.08 to 2.301)	1.52 (1.08 to 2.301)	1.52 (1.08 to 2.301)	1.52 (1.08 to 2.301)	1.52 (1.08 to 2.301)	1.52 (1.08 to 2.301)	1.52 (1.08 to 2.301)	1.52 (1.08 to 2.301)	1.52 (1.08 to 2.301)	1.52 (1.08 to 2.301)	1.52 (1.08 to 2.301)	1.52 (1.08 to 2.301)	1.52 (1.08 to 2.301)
Alzheimer's disease and other dementias	Not diabetic	Both	Males		1.0 (1.0 to 1.0)	1.0 (1.0 to 1.0)	1.0 (1.0 to 1.0)	1.0 (1.0 to 1.0)	1.0 (1.0 to 1.0)	1.0 (1.0 to 1.0)	1.0 (1.0 to 1.0)	1.0 (1.0 to 1.0)	1.0 (1.0 to 1.0)	1.0 (1.0 to 1.0)	1.0 (1.0 to 1.0)	1.0 (1.0 to 1.0)	1.0 (1.0 to 1.0)	1.0 (1.0 to 1.0)	1.0 (1.0 to 1.0)
Alzheimer's disease and other dementias	Not diabetic	Both	Females		1.0 (1.0 to 1.0)	1.0 (1.0 to 1.0)	1.0 (1.0 to 1.0)	1.0 (1.0 to 1.0)	1.0 (1.0 to 1.0)	1.0 (1.0 to 1.0)	1.0 (1.0 to 1.0)	1.0 (1.0 to 1.0)	1.0 (1.0 to 1.0)	1.0 (1.0 to 1.0)	1.0 (1.0 to 1.0)	1.0 (1.0 to 1.0)	1.0 (1.0 to 1.0)	1.0 (1.0 to 1.0)	1.0 (1.0 to 1.0)
Glaucoma	Diabetic	Both	Males		1.52 (1.098 to 2.325)	1.52 (1.098 to 2.325)	1.52 (1.098 to 2.325)	1.52 (1.098 to 2.325)	1.52 (1.098 to 2.325)	1.52 (1.098 to 2.325)	1.52 (1.098 to 2.325)	1.52 (1.098 to 2.325)	1.52 (1.098 to 2.325)	1.52 (1.098 to 2.325)	1.52 (1.098 to 2.325)	1.52 (1.098 to 2.325)	1.52 (1.098 to 2.325)	1.52 (1.098 to 2.325)	1.52 (1.098 to 2.325)
Glaucoma	Diabetic	Both	Females		1.516 (1.08 to 2.328)	1.516 (1.08 to 2.328)	1.516 (1.08 to 2.328)	1.516 (1.08 to 2.328)	1.516 (1.08 to 2.328)	1.516 (1.08 to 2.328)	1.516 (1.08 to 2.328)	1.516 (1.08 to 2.328)	1.516 (1.08 to 2.328)	1.516 (1.08 to 2.328)	1.516 (1.08 to 2.328)	1.516 (1.08 to 2.328)	1.516 (1.08 to 2.328)	1.516 (1.08 to 2.328)	1.516 (1.08 to 2.328)
Glaucoma	Not diabetic	Both	Males		1.0 (1.0 to 1.0)	1.0 (1.0 to 1.0)	1.0 (1.0 to 1.0)	1.0 (1.0 to 1.0)	1.0 (1.0 to 1.0)	1.0 (1.0 to 1.0)	1.0 (1.0 to 1.0)	1.0 (1.0 to 1.0)	1.0 (1.0 to 1.0)	1.0 (1.0 to 1.0)	1.0 (1.0 to 1.0)	1.0 (1.0 to 1.0)	1.0 (1.0 to 1.0)	1.0 (1.0 to 1.0)	1.0 (1.0 to 1.0)
Glaucoma	Not diabetic	Both	Females		1.0 (1.0 to 1.0)	1.0 (1.0 to 1.0)	1.0 (1.0 to 1.0)	1.0 (1.0 to 1.0)	1.0 (1.0 to 1.0)	1.0 (1.0 to 1.0)	1.0 (1.0 to 1.0)	1.0 (1.0 to 1.0)	1.0 (1.0 to 1.0)	1.0 (1.0 to 1.0)	1.0 (1.0 to 1.0)	1.0 (1.0 to 1.0)	1.0 (1.0 to 1.0)	1.0 (1.0 to 1.0)	1.0 (1.0 to 1.0)
Cataract	Diabetic	Both	Males		1.52 (1.09 to 2.259)	1.52 (1.09 to 2.259)	1.52 (1.09 to 2.259)	1.52 (1.09 to 2.259)	1.52 (1.09 to 2.259)	1.52 (1.09 to 2.259)	1.52 (1.09 to 2.259)	1.52 (1.09 to 2.259)	1.52 (1.09 to 2.259)	1.52 (1.09 to 2.259)	1.52 (1.09 to 2.259)	1.52 (1.09 to 2.259)	1.52 (1.09 to 2.259)	1.52 (1.09 to 2.259)	1.52 (1.09 to 2.259)
Cataract	Diabetic	Both	Females		1.522 (1.094 to 2.289)	1.522 (1.094 to 2.289)	1.522 (1.094 to 2.289)	1.522 (1.094 to 2.289)	1.522 (1.094 to 2.289)	1.522 (1.094 to 2.289)	1.522 (1.094 to 2.289)	1.522 (1.094 to 2.289)	1.522 (1.094 to 2.289)	1.522 (1.094 to 2.289)	1.522 (1.094 to 2.289)	1.522 (1.094 to 2.289)	1.522 (1.094 to 2.289)	1.522 (1.094 to 2.289)	1.522 (1.094 to 2.289)
Cataract	Not diabetic	Both	Males		1.0 (1.0 to 1.0)	1.0 (1.0 to 1.0)	1.0 (1.0 to 1.0)	1.0 (1.0 to 1.0)	1.0 (1.0 to 1.0)	1.0 (1.0 to 1.0)	1.0 (1.0 to 1.0)	1.0 (1.0 to 1.0)	1.0 (1.0 to 1.0)	1.0 (1.0 to 1.0)	1.0 (1.0 to 1.0)	1.0 (1.0 to 1.0)	1.0 (1.0 to 1.0)	1.0 (1.0 to 1.0)	1.0 (1.0 to 1.0)
Cataract	Not diabetic	Both	Females		1.0 (1.0 to 1.0)	1.0 (1.0 to 1.0)	1.0 (1.0 to 1.0)	1.0 (1.0 to 1.0)	1.0 (1.0 to 1.0)	1.0 (1.0 to 1.0)	1.0 (1.0 to 1.0)	1.0 (1.0 to 1.0)	1.0 (1.0 to 1.0)	1.0 (1.0 to 1.0)	1.0 (1.0 to 1.0)	1.0 (1.0 to 1.0)	1.0 (1.0 to 1.0)	1.0 (1.0 to 1.0)	1.0 (1.0 to 1.0)
**High LDL cholesterol**																			
Ischaemic heart disease	mmol/L	Both	Both		2.016 (1.684 to 2.544)	2.027 (1.768 to 2.354)	2.038 (1.831 to 2.273)	1.971 (1.775 to 2.191)	1.828 (1.676 to 2.004)	1.685 (1.561 to 1.815)	1.541 (1.446 to 1.648)	1.398 (1.306 to 1.494)	1.254 (1.141 to 1.372)	1.193 (1.088 to 1.312)	1.213 (1.124 to 1.321)	1.262 (1.11 to 1.465)	1.262 (1.11 to 1.465)	1.262 (1.11 to 1.465)	1.262 (1.11 to 1.465)
Ischaemic stroke	mmol/L	Both	Both		1.67 (1.334 to 2.339)	1.626 (1.352 to 2.041)	1.583 (1.342 to 1.849)	1.518 (1.287 to 1.76)	1.434 (1.242 to 1.636)	1.35 (1.212 to 1.514)	1.265 (1.164 to 1.391)	1.181 (1.109 to 1.299)	1.096 (1.043 to 1.223)	1.062 (1.008 to 1.193)	1.077 (1.012 to 1.216)	1.116 (1.014 to 1.344)	1.116 (1.014 to 1.344)	1.116 (1.014 to 1.344)	1.116 (1.014 to 1.344)
**High systolic blood pressure**																			
Rheumatic heart disease	10 mmHg	Both	Both		1.631 (1.174 to 2.306)	1.474 (1.17 to 1.898)	1.317 (1.144 to 1.575)	1.229 (1.089 to 1.422)	1.211 (1.101 to 1.367)	1.193 (1.107 to 1.328)	1.175 (1.101 to 1.287)	1.157 (1.086 to 1.265)	1.139 (1.055 to 1.248)	1.127 (1.048 to 1.241)	1.12 (1.06 to 1.238)	1.104 (1.04 to 1.28)	1.104 (1.04 to 1.28)	1.104 (1.04 to 1.28)	1.104 (1.04 to 1.28)
Ischaemic heart disease	10 mmHg	Both	Both		1.972 (1.44 to 2.596)	1.818 (1.458 to 2.207)	1.665 (1.461 to 1.911)	1.568 (1.398 to 1.799)	1.527 (1.393 to 1.705)	1.487 (1.385 to 1.619)	1.446 (1.368 to 1.535)	1.405 (1.332 to 1.488)	1.364 (1.257 to 1.456)	1.33 (1.224 to 1.424)	1.303 (1.225 to 1.404)	1.266 (1.134 to 1.437)	1.266 (1.134 to 1.437)	1.266 (1.134 to 1.437)	1.266 (1.134 to 1.437)
Ischaemic stroke	10 mmHg	Both	Both		1.854 (1.395 to 2.588)	1.774 (1.427 to 2.252)	1.694 (1.404 to 2.035)	1.628 (1.354 to 1.95)	1.574 (1.36 to 1.823)	1.521 (1.361 to 1.698)	1.468 (1.344 to 1.596)	1.414 (1.302 to 1.524)	1.361 (1.214 to 1.49)	1.318 (1.168 to 1.451)	1.284 (1.179 to 1.389)	1.201 (1.109 to 1.37)	1.201 (1.109 to 1.37)	1.201 (1.109 to 1.37)	1.201 (1.109 to 1.37)
Intracerebral haemorrhage	10 mmHg	Both	Both		2.134 (1.555 to 2.919)	2.05 (1.593 to 2.648)	1.966 (1.589 to 2.465)	1.874 (1.492 to 2.302)	1.775 (1.484 to 2.114)	1.676 (1.446 to 1.932)	1.577 (1.402 to 1.754)	1.478 (1.331 to 1.619)	1.379 (1.207 to 1.54)	1.323 (1.162 to 1.495)	1.311 (1.193 to 1.45)	1.279 (1.126 to 1.515)	1.279 (1.126 to 1.515)	1.279 (1.126 to 1.515)	1.279 (1.126 to 1.515)
Subarachnoid haemorrhage	10 mmHg	Both	Both		2.134 (1.555 to 2.919)	2.05 (1.593 to 2.648)	1.966 (1.589 to 2.465)	1.874 (1.492 to 2.302)	1.775 (1.484 to 2.114)	1.676 (1.446 to 1.932)	1.577 (1.402 to 1.754)	1.478 (1.331 to 1.619)	1.379 (1.207 to 1.54)	1.323 (1.162 to 1.495)	1.311 (1.193 to 1.45)	1.279 (1.126 to 1.515)	1.279 (1.126 to 1.515)	1.279 (1.126 to 1.515)	1.279 (1.126 to 1.515)
Hypertensive heart disease	10 mmHg	Both	Both		2.862 (1.829 to 4.108)	2.838 (1.857 to 4.187)	2.814 (1.802 to 4.337)	2.703 (1.762 to 4.186)	2.504 (1.804 to 3.758)	2.304 (1.772 to 3.464)	2.105 (1.645 to 3.336)	1.905 (1.447 to 3.171)	1.706 (1.188 to 3.215)	1.619 (1.053 to 3.136)	1.644 (1.078 to 3.091)	1.708 (1.103 to 3.258)	1.708 (1.103 to 3.258)	1.708 (1.103 to 3.258)	1.708 (1.103 to 3.258)
Non-rheumatic calcific aortic valvular heart disease	10 mmHg	Both	Both		1.755 (1.266 to 2.423)	1.605 (1.293 to 2.011)	1.455 (1.278 to 1.642)	1.365 (1.232 to 1.51)	1.335 (1.222 to 1.449)	1.306 (1.219 to 1.394)	1.276 (1.212 to 1.342)	1.247 (1.183 to 1.303)	1.217 (1.131 to 1.284)	1.193 (1.116 to 1.263)	1.175 (1.12 to 1.237)	1.128 (1.071 to 1.235)	1.128 (1.071 to 1.235)	1.128 (1.071 to 1.235)	1.128 (1.071 to 1.235)
Other cardiomyopathy	10 mmHg	Both	Both		1.755 (1.266 to 2.423)	1.605 (1.293 to 2.011)	1.455 (1.278 to 1.642)	1.365 (1.232 to 1.51)	1.335 (1.222 to 1.449)	1.306 (1.219 to 1.394)	1.276 (1.212 to 1.342)	1.247 (1.183 to 1.303)	1.217 (1.131 to 1.284)	1.193 (1.116 to 1.263)	1.175 (1.12 to 1.237)	1.128 (1.071 to 1.235)	1.128 (1.071 to 1.235)	1.128 (1.071 to 1.235)	1.128 (1.071 to 1.235)
Atrial fibrillation and flutter	10 mmHg	Both	Both		1.76 (1.336 to 2.43)	1.631 (1.379 to 2.026)	1.503 (1.396 to 1.644)	1.423 (1.34 to 1.505)	1.392 (1.328 to 1.457)	1.361 (1.313 to 1.411)	1.33 (1.293 to 1.369)	1.299 (1.265 to 1.333)	1.268 (1.233 to 1.308)	1.237 (1.202 to 1.277)	1.208 (1.177 to 1.238)	1.134 (1.092 to 1.185)	1.134 (1.092 to 1.185)	1.134 (1.092 to 1.185)	1.134 (1.092 to 1.185)
Aortic aneurysm	10 mmHg	Both	Both		1.544 (1.259 to 2.164)	1.469 (1.29 to 1.816)	1.394 (1.3 to 1.535)	1.345 (1.227 to 1.451)	1.321 (1.233 to 1.405)	1.296 (1.229 to 1.362)	1.272 (1.218 to 1.327)	1.248 (1.191 to 1.299)	1.223 (1.16 to 1.286)	1.2 (1.137 to 1.261)	1.177 (1.126 to 1.229)	1.119 (1.071 to 1.184)	1.119 (1.071 to 1.184)	1.119 (1.071 to 1.184)	1.119 (1.071 to 1.184)
Peripheral artery disease	10 mmHg	Both	Both		1.728 (1.203 to 2.428)	1.491 (1.206 to 1.87)	1.254 (1.182 to 1.329)	1.138 (1.019 to 1.263)	1.142 (1.047 to 1.243)	1.146 (1.071 to 1.224)	1.15 (1.094 to 1.208)	1.154 (1.11 to 1.199)	1.159 (1.113 to 1.207)	1.152 (1.104 to 1.201)	1.136 (1.098 to 1.176)	1.095 (1.054 to 1.154)	1.095 (1.054 to 1.154)	1.095 (1.054 to 1.154)	1.095 (1.054 to 1.154)
Endocarditis	10 mmHg	Both	Both		1.755 (1.266 to 2.423)	1.605 (1.293 to 2.011)	1.455 (1.278 to 1.642)	1.365 (1.232 to 1.51)	1.335 (1.222 to 1.449)	1.306 (1.219 to 1.394)	1.276 (1.212 to 1.342)	1.247 (1.183 to 1.303)	1.217 (1.131 to 1.284)	1.193 (1.116 to 1.263)	1.175 (1.12 to 1.237)	1.128 (1.071 to 1.235)	1.128 (1.071 to 1.235)	1.128 (1.071 to 1.235)	1.128 (1.071 to 1.235)
Chronic kidney disease due to diabetes mellitus type 1	10 mmHg	Both	Both		1.283 (1.186 to 1.397)	1.283 (1.186 to 1.397)	1.283 (1.186 to 1.397)	1.283 (1.186 to 1.397)	1.283 (1.186 to 1.397)	1.283 (1.186 to 1.397)	1.283 (1.186 to 1.397)	1.283 (1.186 to 1.397)	1.283 (1.186 to 1.397)	1.283 (1.186 to 1.397)	1.283 (1.186 to 1.397)	1.283 (1.186 to 1.397)	1.283 (1.186 to 1.397)	1.283 (1.186 to 1.397)	1.283 (1.186 to 1.397)
Chronic kidney disease due to diabetes mellitus type 2	10 mmHg	Both	Both		1.283 (1.186 to 1.397)	1.283 (1.186 to 1.397)	1.283 (1.186 to 1.397)	1.283 (1.186 to 1.397)	1.283 (1.186 to 1.397)	1.283 (1.186 to 1.397)	1.283 (1.186 to 1.397)	1.283 (1.186 to 1.397)	1.283 (1.186 to 1.397)	1.283 (1.186 to 1.397)	1.283 (1.186 to 1.397)	1.283 (1.186 to 1.397)	1.283 (1.186 to 1.397)	1.283 (1.186 to 1.397)	1.283 (1.186 to 1.397)
Chronic kidney disease due to hypertension	10 mmHg	Both	Both		1.281 (1.18 to 1.385)	1.281 (1.18 to 1.385)	1.281 (1.18 to 1.385)	1.281 (1.18 to 1.385)	1.281 (1.18 to 1.385)	1.281 (1.18 to 1.385)	1.281 (1.18 to 1.385)	1.281 (1.18 to 1.385)	1.281 (1.18 to 1.385)	1.281 (1.18 to 1.385)	1.281 (1.18 to 1.385)	1.281 (1.18 to 1.385)	1.281 (1.18 to 1.385)	1.281 (1.18 to 1.385)	1.281 (1.18 to 1.385)
Chronic kidney disease due to glomerulonephritis	10 mmHg	Both	Both		1.281 (1.182 to 1.383)	1.281 (1.182 to 1.383)	1.281 (1.182 to 1.383)	1.281 (1.182 to 1.383)	1.281 (1.182 to 1.383)	1.281 (1.182 to 1.383)	1.281 (1.182 to 1.383)	1.281 (1.182 to 1.383)	1.281 (1.182 to 1.383)	1.281 (1.182 to 1.383)	1.281 (1.182 to 1.383)	1.281 (1.182 to 1.383)	1.281 (1.182 to 1.383)	1.281 (1.182 to 1.383)	1.281 (1.182 to 1.383)
Chronic kidney disease due to other and unspecified causes	10 mmHg	Both	Both		1.282 (1.181 to 1.395)	1.282 (1.181 to 1.395)	1.282 (1.181 to 1.395)	1.282 (1.181 to 1.395)	1.282 (1.181 to 1.395)	1.282 (1.181 to 1.395)	1.282 (1.181 to 1.395)	1.282 (1.181 to 1.395)	1.282 (1.181 to 1.395)	1.282 (1.181 to 1.395)	1.282 (1.181 to 1.395)	1.282 (1.181 to 1.395)	1.282 (1.181 to 1.395)	1.282 (1.181 to 1.395)	1.282 (1.181 to 1.395)
**High body-mass index in adults**																			
Oesophageal cancer	5 kg/m2	Both	Males	1.391 (1.077 to 1.754)	1.391 (1.077 to 1.754)	1.391 (1.077 to 1.754)	1.391 (1.077 to 1.754)	1.391 (1.077 to 1.754)	1.391 (1.077 to 1.754)	1.391 (1.077 to 1.754)	1.391 (1.077 to 1.754)	1.391 (1.077 to 1.754)	1.391 (1.077 to 1.754)	1.391 (1.077 to 1.754)	1.391 (1.077 to 1.754)	1.391 (1.077 to 1.754)	1.391 (1.077 to 1.754)	1.391 (1.077 to 1.754)	1.391 (1.077 to 1.754)
Oesophageal cancer	5 kg/m2	Both	Females	1.351 (1.012 to 1.745)	1.351 (1.012 to 1.745)	1.351 (1.012 to 1.745)	1.351 (1.012 to 1.745)	1.351 (1.012 to 1.745)	1.351 (1.012 to 1.745)	1.351 (1.012 to 1.745)	1.351 (1.012 to 1.745)	1.351 (1.012 to 1.745)	1.351 (1.012 to 1.745)	1.351 (1.012 to 1.745)	1.351 (1.012 to 1.745)	1.351 (1.012 to 1.745)	1.351 (1.012 to 1.745)	1.351 (1.012 to 1.745)	1.351 (1.012 to 1.745)
Colon and rectum cancer	5 kg/m2	Both	Males	1.177 (1.145 to 1.208)	1.177 (1.145 to 1.208)	1.177 (1.145 to 1.208)	1.177 (1.145 to 1.208)	1.177 (1.145 to 1.208)	1.177 (1.145 to 1.208)	1.177 (1.145 to 1.208)	1.177 (1.145 to 1.208)	1.177 (1.145 to 1.208)	1.177 (1.145 to 1.208)	1.177 (1.145 to 1.208)	1.177 (1.145 to 1.208)	1.177 (1.145 to 1.208)	1.177 (1.145 to 1.208)	1.177 (1.145 to 1.208)	1.177 (1.145 to 1.208)
Colon and rectum cancer	5 kg/m2	Both	Females	1.059 (1.031 to 1.083)	1.059 (1.031 to 1.083)	1.059 (1.031 to 1.083)	1.059 (1.031 to 1.083)	1.059 (1.031 to 1.083)	1.059 (1.031 to 1.083)	1.059 (1.031 to 1.083)	1.059 (1.031 to 1.083)	1.059 (1.031 to 1.083)	1.059 (1.031 to 1.083)	1.059 (1.031 to 1.083)	1.059 (1.031 to 1.083)	1.059 (1.031 to 1.083)	1.059 (1.031 to 1.083)	1.059 (1.031 to 1.083)	1.059 (1.031 to 1.083)
Liver cancer due to hepatitis B	5 kg/m2	Both	Males	1.289 (1.109 to 1.491)	1.289 (1.109 to 1.491)	1.289 (1.109 to 1.491)	1.289 (1.109 to 1.491)	1.289 (1.109 to 1.491)	1.289 (1.109 to 1.491)	1.289 (1.109 to 1.491)	1.289 (1.109 to 1.491)	1.289 (1.109 to 1.491)	1.289 (1.109 to 1.491)	1.289 (1.109 to 1.491)	1.289 (1.109 to 1.491)	1.289 (1.109 to 1.491)	1.289 (1.109 to 1.491)	1.289 (1.109 to 1.491)	1.289 (1.109 to 1.491)
Liver cancer due to hepatitis B	5 kg/m2	Both	Females	1.176 (1.03 to 1.334)	1.176 (1.03 to 1.334)	1.176 (1.03 to 1.334)	1.176 (1.03 to 1.334)	1.176 (1.03 to 1.334)	1.176 (1.03 to 1.334)	1.176 (1.03 to 1.334)	1.176 (1.03 to 1.334)	1.176 (1.03 to 1.334)	1.176 (1.03 to 1.334)	1.176 (1.03 to 1.334)	1.176 (1.03 to 1.334)	1.176 (1.03 to 1.334)	1.176 (1.03 to 1.334)	1.176 (1.03 to 1.334)	1.176 (1.03 to 1.334)
Liver cancer due to hepatitis C	5 kg/m2	Both	Males	1.289 (1.109 to 1.491)	1.289 (1.109 to 1.491)	1.289 (1.109 to 1.491)	1.289 (1.109 to 1.491)	1.289 (1.109 to 1.491)	1.289 (1.109 to 1.491)	1.289 (1.109 to 1.491)	1.289 (1.109 to 1.491)	1.289 (1.109 to 1.491)	1.289 (1.109 to 1.491)	1.289 (1.109 to 1.491)	1.289 (1.109 to 1.491)	1.289 (1.109 to 1.491)	1.289 (1.109 to 1.491)	1.289 (1.109 to 1.491)	1.289 (1.109 to 1.491)
Liver cancer due to hepatitis C	5 kg/m2	Both	Females	1.176 (1.03 to 1.334)	1.176 (1.03 to 1.334)	1.176 (1.03 to 1.334)	1.176 (1.03 to 1.334)	1.176 (1.03 to 1.334)	1.176 (1.03 to 1.334)	1.176 (1.03 to 1.334)	1.176 (1.03 to 1.334)	1.176 (1.03 to 1.334)	1.176 (1.03 to 1.334)	1.176 (1.03 to 1.334)	1.176 (1.03 to 1.334)	1.176 (1.03 to 1.334)	1.176 (1.03 to 1.334)	1.176 (1.03 to 1.334)	1.176 (1.03 to 1.334)
Liver cancer due to alcohol use	5 kg/m2	Both	Males	1.289 (1.109 to 1.491)	1.289 (1.109 to 1.491)	1.289 (1.109 to 1.491)	1.289 (1.109 to 1.491)	1.289 (1.109 to 1.491)	1.289 (1.109 to 1.491)	1.289 (1.109 to 1.491)	1.289 (1.109 to 1.491)	1.289 (1.109 to 1.491)	1.289 (1.109 to 1.491)	1.289 (1.109 to 1.491)	1.289 (1.109 to 1.491)	1.289 (1.109 to 1.491)	1.289 (1.109 to 1.491)	1.289 (1.109 to 1.491)	1.289 (1.109 to 1.491)
Liver cancer due to alcohol use	5 kg/m2	Both	Females	1.176 (1.03 to 1.334)	1.176 (1.03 to 1.334)	1.176 (1.03 to 1.334)	1.176 (1.03 to 1.334)	1.176 (1.03 to 1.334)	1.176 (1.03 to 1.334)	1.176 (1.03 to 1.334)	1.176 (1.03 to 1.334)	1.176 (1.03 to 1.334)	1.176 (1.03 to 1.334)	1.176 (1.03 to 1.334)	1.176 (1.03 to 1.334)	1.176 (1.03 to 1.334)	1.176 (1.03 to 1.334)	1.176 (1.03 to 1.334)	1.176 (1.03 to 1.334)
Gallbladder and biliary tract cancer	5 kg/m2	Both	Males	1.155 (1.033 to 1.281)	1.155 (1.033 to 1.281)	1.155 (1.033 to 1.281)	1.155 (1.033 to 1.281)	1.155 (1.033 to 1.281)	1.155 (1.033 to 1.281)	1.155 (1.033 to 1.281)	1.155 (1.033 to 1.281)	1.155 (1.033 to 1.281)	1.155 (1.033 to 1.281)	1.155 (1.033 to 1.281)	1.155 (1.033 to 1.281)	1.155 (1.033 to 1.281)	1.155 (1.033 to 1.281)	1.155 (1.033 to 1.281)	1.155 (1.033 to 1.281)
Gallbladder and biliary tract cancer	5 kg/m2	Both	Females	1.344 (1.223 to 1.477)	1.344 (1.223 to 1.477)	1.344 (1.223 to 1.477)	1.344 (1.223 to 1.477)	1.344 (1.223 to 1.477)	1.344 (1.223 to 1.477)	1.344 (1.223 to 1.477)	1.344 (1.223 to 1.477)	1.344 (1.223 to 1.477)	1.344 (1.223 to 1.477)	1.344 (1.223 to 1.477)	1.344 (1.223 to 1.477)	1.344 (1.223 to 1.477)	1.344 (1.223 to 1.477)	1.344 (1.223 to 1.477)	1.344 (1.223 to 1.477)
Pancreatic cancer	5 kg/m2	Both	Males	1.071 (0.999 to 1.153)	1.071 (0.999 to 1.153)	1.071 (0.999 to 1.153)	1.071 (0.999 to 1.153)	1.071 (0.999 to 1.153)	1.071 (0.999 to 1.153)	1.071 (0.999 to 1.153)	1.071 (0.999 to 1.153)	1.071 (0.999 to 1.153)	1.071 (0.999 to 1.153)	1.071 (0.999 to 1.153)	1.071 (0.999 to 1.153)	1.071 (0.999 to 1.153)	1.071 (0.999 to 1.153)	1.071 (0.999 to 1.153)	1.071 (0.999 to 1.153)
Pancreatic cancer	5 kg/m2	Both	Females	1.092 (1.037 to 1.144)	1.092 (1.037 to 1.144)	1.092 (1.037 to 1.144)	1.092 (1.037 to 1.144)	1.092 (1.037 to 1.144)	1.092 (1.037 to 1.144)	1.092 (1.037 to 1.144)	1.092 (1.037 to 1.144)	1.092 (1.037 to 1.144)	1.092 (1.037 to 1.144)	1.092 (1.037 to 1.144)	1.092 (1.037 to 1.144)	1.092 (1.037 to 1.144)	1.092 (1.037 to 1.144)	1.092 (1.037 to 1.144)	1.092 (1.037 to 1.144)
Breast cancer	5 kg/m2	Both	Females	0.89 (0.869 to 0.914)	0.89 (0.869 to 0.914)	0.89 (0.869 to 0.914)	0.89 (0.869 to 0.914)	0.89 (0.869 to 0.914)	0.89 (0.869 to 0.914)	1.089 (1.037 to 1.14)	1.089 (1.037 to 1.14)	1.089 (1.037 to 1.14)	1.089 (1.037 to 1.14)	1.089 (1.037 to 1.14)	1.089 (1.037 to 1.14)	1.089 (1.037 to 1.14)	1.089 (1.037 to 1.14)	1.089 (1.037 to 1.14)	1.089 (1.037 to 1.14)
Uterine cancer	5 kg/m2	Both	Females	1.613 (1.543 to 1.681)	1.613 (1.543 to 1.681)	1.613 (1.543 to 1.681)	1.613 (1.543 to 1.681)	1.613 (1.543 to 1.681)	1.613 (1.543 to 1.681)	1.613 (1.543 to 1.681)	1.613 (1.543 to 1.681)	1.613 (1.543 to 1.681)	1.613 (1.543 to 1.681)	1.613 (1.543 to 1.681)	1.613 (1.543 to 1.681)	1.613 (1.543 to 1.681)	1.613 (1.543 to 1.681)	1.613 (1.543 to 1.681)	1.613 (1.543 to 1.681)
Ovarian cancer	5 kg/m2	Both	Females	1.038 (0.999 to 1.077)	1.038 (0.999 to 1.077)	1.038 (0.999 to 1.077)	1.038 (0.999 to 1.077)	1.038 (0.999 to 1.077)	1.038 (0.999 to 1.077)	1.038 (0.999 to 1.077)	1.038 (0.999 to 1.077)	1.038 (0.999 to 1.077)	1.038 (0.999 to 1.077)	1.038 (0.999 to 1.077)	1.038 (0.999 to 1.077)	1.038 (0.999 to 1.077)	1.038 (0.999 to 1.077)	1.038 (0.999 to 1.077)	1.038 (0.999 to 1.077)
Kidney cancer	5 kg/m2	Both	Males	1.24 (1.171 to 1.313)	1.24 (1.171 to 1.313)	1.24 (1.171 to 1.313)	1.24 (1.171 to 1.313)	1.24 (1.171 to 1.313)	1.24 (1.171 to 1.313)	1.24 (1.171 to 1.313)	1.24 (1.171 to 1.313)	1.24 (1.171 to 1.313)	1.24 (1.171 to 1.313)	1.24 (1.171 to 1.313)	1.24 (1.171 to 1.313)	1.24 (1.171 to 1.313)	1.24 (1.171 to 1.313)	1.24 (1.171 to 1.313)	1.24 (1.171 to 1.313)
Kidney cancer	5 kg/m2	Both	Females	1.32 (1.254 to 1.394)	1.32 (1.254 to 1.394)	1.32 (1.254 to 1.394)	1.32 (1.254 to 1.394)	1.32 (1.254 to 1.394)	1.32 (1.254 to 1.394)	1.32 (1.254 to 1.394)	1.32 (1.254 to 1.394)	1.32 (1.254 to 1.394)	1.32 (1.254 to 1.394)	1.32 (1.254 to 1.394)	1.32 (1.254 to 1.394)	1.32 (1.254 to 1.394)	1.32 (1.254 to 1.394)	1.32 (1.254 to 1.394)	1.32 (1.254 to 1.394)
Thyroid cancer	5 kg/m2	Both	Males	1.221 (1.068 to 1.381)	1.221 (1.068 to 1.381)	1.221 (1.068 to 1.381)	1.221 (1.068 to 1.381)	1.221 (1.068 to 1.381)	1.221 (1.068 to 1.381)	1.221 (1.068 to 1.381)	1.221 (1.068 to 1.381)	1.221 (1.068 to 1.381)	1.221 (1.068 to 1.381)	1.221 (1.068 to 1.381)	1.221 (1.068 to 1.381)	1.221 (1.068 to 1.381)	1.221 (1.068 to 1.381)	1.221 (1.068 to 1.381)	1.221 (1.068 to 1.381)
Thyroid cancer	5 kg/m2	Both	Females	1.136 (1.094 to 1.178)	1.136 (1.094 to 1.178)	1.136 (1.094 to 1.178)	1.136 (1.094 to 1.178)	1.136 (1.094 to 1.178)	1.136 (1.094 to 1.178)	1.136 (1.094 to 1.178)	1.136 (1.094 to 1.178)	1.136 (1.094 to 1.178)	1.136 (1.094 to 1.178)	1.136 (1.094 to 1.178)	1.136 (1.094 to 1.178)	1.136 (1.094 to 1.178)	1.136 (1.094 to 1.178)	1.136 (1.094 to 1.178)	1.136 (1.094 to 1.178)
Multiple myeloma	5 kg/m2	Both	Males	1.089 (1.027 to 1.153)	1.089 (1.027 to 1.153)	1.089 (1.027 to 1.153)	1.089 (1.027 to 1.153)	1.089 (1.027 to 1.153)	1.089 (1.027 to 1.153)	1.089 (1.027 to 1.153)	1.089 (1.027 to 1.153)	1.089 (1.027 to 1.153)	1.089 (1.027 to 1.153)	1.089 (1.027 to 1.153)	1.089 (1.027 to 1.153)	1.089 (1.027 to 1.153)	1.089 (1.027 to 1.153)	1.089 (1.027 to 1.153)	1.089 (1.027 to 1.153)
Multiple myeloma	5 kg/m2	Both	Females	1.092 (1.034 to 1.157)	1.092 (1.034 to 1.157)	1.092 (1.034 to 1.157)	1.092 (1.034 to 1.157)	1.092 (1.034 to 1.157)	1.092 (1.034 to 1.157)	1.092 (1.034 to 1.157)	1.092 (1.034 to 1.157)	1.092 (1.034 to 1.157)	1.092 (1.034 to 1.157)	1.092 (1.034 to 1.157)	1.092 (1.034 to 1.157)	1.092 (1.034 to 1.157)	1.092 (1.034 to 1.157)	1.092 (1.034 to 1.157)	1.092 (1.034 to 1.157)
Acute lymphoid leukaemia	5 kg/m2	Both	Males	1.086 (1.053 to 1.119)	1.086 (1.053 to 1.119)	1.086 (1.053 to 1.119)	1.086 (1.053 to 1.119)	1.086 (1.053 to 1.119)	1.086 (1.053 to 1.119)	1.086 (1.053 to 1.119)	1.086 (1.053 to 1.119)	1.086 (1.053 to 1.119)	1.086 (1.053 to 1.119)	1.086 (1.053 to 1.119)	1.086 (1.053 to 1.119)	1.086 (1.053 to 1.119)	1.086 (1.053 to 1.119)	1.086 (1.053 to 1.119)	1.086 (1.053 to 1.119)
Acute lymphoid leukaemia	5 kg/m2	Both	Females	1.131 (1.061 to 1.208)	1.131 (1.061 to 1.208)	1.131 (1.061 to 1.208)	1.131 (1.061 to 1.208)	1.131 (1.061 to 1.208)	1.131 (1.061 to 1.208)	1.131 (1.061 to 1.208)	1.131 (1.061 to 1.208)	1.131 (1.061 to 1.208)	1.131 (1.061 to 1.208)	1.131 (1.061 to 1.208)	1.131 (1.061 to 1.208)	1.131 (1.061 to 1.208)	1.131 (1.061 to 1.208)	1.131 (1.061 to 1.208)	1.131 (1.061 to 1.208)
Chronic lymphoid leukaemia	5 kg/m2	Both	Males	1.086 (1.053 to 1.119)	1.086 (1.053 to 1.119)	1.086 (1.053 to 1.119)	1.086 (1.053 to 1.119)	1.086 (1.053 to 1.119)	1.086 (1.053 to 1.119)	1.086 (1.053 to 1.119)	1.086 (1.053 to 1.119)	1.086 (1.053 to 1.119)	1.086 (1.053 to 1.119)	1.086 (1.053 to 1.119)	1.086 (1.053 to 1.119)	1.086 (1.053 to 1.119)	1.086 (1.053 to 1.119)	1.086 (1.053 to 1.119)	1.086 (1.053 to 1.119)
Chronic lymphoid leukaemia	5 kg/m2	Both	Females	1.131 (1.061 to 1.208)	1.131 (1.061 to 1.208)	1.131 (1.061 to 1.208)	1.131 (1.061 to 1.208)	1.131 (1.061 to 1.208)	1.131 (1.061 to 1.208)	1.131 (1.061 to 1.208)	1.131 (1.061 to 1.208)	1.131 (1.061 to 1.208)	1.131 (1.061 to 1.208)	1.131 (1.061 to 1.208)	1.131 (1.061 to 1.208)	1.131 (1.061 to 1.208)	1.131 (1.061 to 1.208)	1.131 (1.061 to 1.208)	1.131 (1.061 to 1.208)
Acute myeloid leukaemia	5 kg/m2	Both	Males	1.086 (1.053 to 1.119)	1.086 (1.053 to 1.119)	1.086 (1.053 to 1.119)	1.086 (1.053 to 1.119)	1.086 (1.053 to 1.119)	1.086 (1.053 to 1.119)	1.086 (1.053 to 1.119)	1.086 (1.053 to 1.119)	1.086 (1.053 to 1.119)	1.086 (1.053 to 1.119)	1.086 (1.053 to 1.119)	1.086 (1.053 to 1.119)	1.086 (1.053 to 1.119)	1.086 (1.053 to 1.119)	1.086 (1.053 to 1.119)	1.086 (1.053 to 1.119)
Acute myeloid leukaemia	5 kg/m2	Both	Females	1.131 (1.061 to 1.208)	1.131 (1.061 to 1.208)	1.131 (1.061 to 1.208)	1.131 (1.061 to 1.208)	1.131 (1.061 to 1.208)	1.131 (1.061 to 1.208)	1.131 (1.061 to 1.208)	1.131 (1.061 to 1.208)	1.131 (1.061 to 1.208)	1.131 (1.061 to 1.208)	1.131 (1.061 to 1.208)	1.131 (1.061 to 1.208)	1.131 (1.061 to 1.208)	1.131 (1.061 to 1.208)	1.131 (1.061 to 1.208)	1.131 (1.061 to 1.208)
Chronic myeloid leukaemia	5 kg/m2	Both	Males	1.086 (1.053 to 1.119)	1.086 (1.053 to 1.119)	1.086 (1.053 to 1.119)	1.086 (1.053 to 1.119)	1.086 (1.053 to 1.119)	1.086 (1.053 to 1.119)	1.086 (1.053 to 1.119)	1.086 (1.053 to 1.119)	1.086 (1.053 to 1.119)	1.086 (1.053 to 1.119)	1.086 (1.053 to 1.119)	1.086 (1.053 to 1.119)	1.086 (1.053 to 1.119)	1.086 (1.053 to 1.119)	1.086 (1.053 to 1.119)	1.086 (1.053 to 1.119)
Chronic myeloid leukaemia	5 kg/m2	Both	Females	1.131 (1.061 to 1.208)	1.131 (1.061 to 1.208)	1.131 (1.061 to 1.208)	1.131 (1.061 to 1.208)	1.131 (1.061 to 1.208)	1.131 (1.061 to 1.208)	1.131 (1.061 to 1.208)	1.131 (1.061 to 1.208)	1.131 (1.061 to 1.208)	1.131 (1.061 to 1.208)	1.131 (1.061 to 1.208)	1.131 (1.061 to 1.208)	1.131 (1.061 to 1.208)	1.131 (1.061 to 1.208)	1.131 (1.061 to 1.208)	1.131 (1.061 to 1.208)
Other leukaemia	5 kg/m2	Both	Males	1.086 (1.053 to 1.119)	1.086 (1.053 to 1.119)	1.086 (1.053 to 1.119)	1.086 (1.053 to 1.119)	1.086 (1.053 to 1.119)	1.086 (1.053 to 1.119)	1.086 (1.053 to 1.119)	1.086 (1.053 to 1.119)	1.086 (1.053 to 1.119)	1.086 (1.053 to 1.119)	1.086 (1.053 to 1.119)	1.086 (1.053 to 1.119)	1.086 (1.053 to 1.119)	1.086 (1.053 to 1.119)	1.086 (1.053 to 1.119)	1.086 (1.053 to 1.119)
Other leukaemia	5 kg/m2	Both	Females	1.131 (1.061 to 1.208)	1.131 (1.061 to 1.208)	1.131 (1.061 to 1.208)	1.131 (1.061 to 1.208)	1.131 (1.061 to 1.208)	1.131 (1.061 to 1.208)	1.131 (1.061 to 1.208)	1.131 (1.061 to 1.208)	1.131 (1.061 to 1.208)	1.131 (1.061 to 1.208)	1.131 (1.061 to 1.208)	1.131 (1.061 to 1.208)	1.131 (1.061 to 1.208)	1.131 (1.061 to 1.208)	1.131 (1.061 to 1.208)	1.131 (1.061 to 1.208)
Ischaemic heart disease	5 kg/m2	Both	Both	2.274 (1.259 to 3.683)	2.274 (1.259 to 3.683)	2.018 (1.3 to 3.099)	1.724 (1.533 to 1.93)	1.599 (1.418 to 1.784)	1.567 (1.458 to 1.68)	1.52 (1.417 to 1.631)	1.466 (1.372 to 1.557)	1.414 (1.325 to 1.504)	1.364 (1.287 to 1.448)	1.319 (1.242 to 1.4)	1.274 (1.187 to 1.365)	1.17 (1.091 to 1.252)	1.17 (1.091 to 1.252)	1.17 (1.091 to 1.252)	1.17 (1.091 to 1.252)
Ischaemic stroke	5 kg/m2	Both	Both	2.472 (1.4 to 3.975)	2.472 (1.4 to 3.975)	2.235 (1.457 to 3.329)	1.979 (1.699 to 2.313)	1.826 (1.6 to 2.075)	1.733 (1.581 to 1.898)	1.635 (1.479 to 1.795)	1.543 (1.441 to 1.653)	1.455 (1.345 to 1.566)	1.38 (1.31 to 1.458)	1.304 (1.234 to 1.376)	1.228 (1.16 to 1.304)	1.068 (0.992 to 1.143)	1.068 (0.992 to 1.143)	1.068 (0.992 to 1.143)	1.068 (0.992 to 1.143)
Intracerebral haemorrhage	5 kg/m2	Both	Both	3.066 (1.751 to 5.334)	3.066 (1.751 to 5.334)	2.913 (1.862 to 4.395)	2.597 (1.974 to 3.375)	2.389 (1.87 to 2.998)	2.199 (1.822 to 2.672)	1.996 (1.626 to 2.416)	1.805 (1.574 to 2.06)	1.665 (1.438 to 1.932)	1.523 (1.377 to 1.683)	1.41 (1.266 to 1.57)	1.295 (1.162 to 1.438)	1.07 (0.928 to 1.219)	1.07 (0.928 to 1.219)	1.07 (0.928 to 1.219)	1.07 (0.928 to 1.219)
Subarachnoid haemorrhage	5 kg/m2	Both	Both	3.066 (1.751 to 5.334)	3.066 (1.751 to 5.334)	2.913 (1.862 to 4.395)	2.597 (1.974 to 3.375)	2.389 (1.87 to 2.998)	2.199 (1.822 to 2.672)	1.996 (1.626 to 2.416)	1.805 (1.574 to 2.06)	1.665 (1.438 to 1.932)	1.523 (1.377 to 1.683)	1.41 (1.266 to 1.57)	1.295 (1.162 to 1.438)	1.07 (0.928 to 1.219)	1.07 (0.928 to 1.219)	1.07 (0.928 to 1.219)	1.07 (0.928 to 1.219)
Hypertensive heart disease	5 kg/m2	Both	Both	3.122 (1.588 to 5.498)	3.122 (1.588 to 5.498)	3.0 (1.783 to 4.902)	2.769 (1.816 to 4.211)	2.573 (1.743 to 3.636)	2.407 (1.717 to 3.293)	2.281 (1.602 to 3.188)	2.159 (1.505 to 3.036)	2.035 (1.452 to 2.822)	1.955 (1.343 to 2.698)	1.86 (1.296 to 2.617)	1.792 (1.17 to 2.553)	1.697 (1.069 to 2.618)	1.697 (1.069 to 2.618)	1.697 (1.069 to 2.618)	1.697 (1.069 to 2.618)
Atrial fibrillation and flutter	5 kg/m2	Both	Males	1.344 (1.231 to 1.473)	1.344 (1.231 to 1.473)	1.344 (1.231 to 1.473)	1.344 (1.231 to 1.473)	1.344 (1.231 to 1.473)	1.344 (1.231 to 1.473)	1.344 (1.231 to 1.473)	1.344 (1.231 to 1.473)	1.344 (1.231 to 1.473)	1.344 (1.231 to 1.473)	1.344 (1.231 to 1.473)	1.344 (1.231 to 1.473)	1.344 (1.231 to 1.473)	1.344 (1.231 to 1.473)	1.344 (1.231 to 1.473)	1.344 (1.231 to 1.473)
Atrial fibrillation and flutter	5 kg/m2	Both	Females	1.346 (1.22 to 1.475)	1.346 (1.22 to 1.475)	1.346 (1.22 to 1.475)	1.346 (1.22 to 1.475)	1.346 (1.22 to 1.475)	1.346 (1.22 to 1.475)	1.346 (1.22 to 1.475)	1.346 (1.22 to 1.475)	1.346 (1.22 to 1.475)	1.346 (1.22 to 1.475)	1.346 (1.22 to 1.475)	1.346 (1.22 to 1.475)	1.346 (1.22 to 1.475)	1.346 (1.22 to 1.475)	1.346 (1.22 to 1.475)	1.346 (1.22 to 1.475)
Asthma	5 kg/m2	Both	Males	1.409 (1.29 to 1.545)	1.409 (1.29 to 1.545)	1.409 (1.29 to 1.545)	1.409 (1.29 to 1.545)	1.409 (1.29 to 1.545)	1.409 (1.29 to 1.545)	1.409 (1.29 to 1.545)	1.409 (1.29 to 1.545)	1.409 (1.29 to 1.545)	1.409 (1.29 to 1.545)	1.409 (1.29 to 1.545)	1.409 (1.29 to 1.545)	1.409 (1.29 to 1.545)	1.409 (1.29 to 1.545)	1.409 (1.29 to 1.545)	1.409 (1.29 to 1.545)
Asthma	5 kg/m2	Both	Females	1.402 (1.275 to 1.532)	1.402 (1.275 to 1.532)	1.402 (1.275 to 1.532)	1.402 (1.275 to 1.532)	1.402 (1.275 to 1.532)	1.402 (1.275 to 1.532)	1.402 (1.275 to 1.532)	1.402 (1.275 to 1.532)	1.402 (1.275 to 1.532)	1.402 (1.275 to 1.532)	1.402 (1.275 to 1.532)	1.402 (1.275 to 1.532)	1.402 (1.275 to 1.532)	1.402 (1.275 to 1.532)	1.402 (1.275 to 1.532)	1.402 (1.275 to 1.532)

Gallbladder and biliary diseases	5 kg/m2	Both	Males									1.464 (1.291 to 1.64)	1.464 (1.291 to 1.64)	1.464 (1.291 to 1.64)	1.464 (1.291 to 1.64)	1.464 (1.291 to 1.64)	1.464 (1.291 to 1.64)	1.464 (1.291 to 1.64)	1.464 (1.291 to 1.64)	1.464 (1.291 to 1.64)	1.464 (1.291 to 1.64)	1.464 (1.291 to 1.64)	1.464 (1.291 to 1.64)	1.464 (1.291 to 1.64)	1.464 (1.291 to 1.64)	1.464 (1.291 to 1.64)	1.464 (1.291 to 1.64)
Gallbladder and biliary diseases	5 kg/m2	Both	Females									1.729 (1.571 to 1.893)	1.729 (1.571 to 1.893)	1.729 (1.571 to 1.893)	1.729 (1.571 to 1.893)	1.729 (1.571 to 1.893)	1.729 (1.571 to 1.893)	1.729 (1.571 to 1.893)	1.729 (1.571 to 1.893)	1.729 (1.571 to 1.893)	1.729 (1.571 to 1.893)	1.729 (1.571 to 1.893)	1.729 (1.571 to 1.893)	1.729 (1.571 to 1.893)	1.729 (1.571 to 1.893)	1.729 (1.571 to 1.893)	1.729 (1.571 to 1.893)
Alzheimer's disease and other dementias	5 kg/m2	Both	Males									1.218 (1.054 to 1.409)	1.218 (1.054 to 1.409)	1.218 (1.054 to 1.409)	1.218 (1.054 to 1.409)	1.218 (1.054 to 1.409)	1.218 (1.054 to 1.409)	1.218 (1.054 to 1.409)	1.218 (1.054 to 1.409)	1.218 (1.054 to 1.409)	1.218 (1.054 to 1.409)	1.218 (1.054 to 1.409)	1.218 (1.054 to 1.409)	1.218 (1.054 to 1.409)	1.218 (1.054 to 1.409)	1.218 (1.054 to 1.409)	1.218 (1.054 to 1.409)
Alzheimer's disease and other dementias	5 kg/m2	Both	Females									1.214 (1.047 to 1.404)	1.214 (1.047 to 1.404)	1.214 (1.047 to 1.404)	1.214 (1.047 to 1.404)	1.214 (1.047 to 1.404)	1.214 (1.047 to 1.404)	1.214 (1.047 to 1.404)	1.214 (1.047 to 1.404)	1.214 (1.047 to 1.404)	1.214 (1.047 to 1.404)	1.214 (1.047 to 1.404)	1.214 (1.047 to 1.404)	1.214 (1.047 to 1.404)	1.214 (1.047 to 1.404)	1.214 (1.047 to 1.404)	1.214 (1.047 to 1.404)
Diabetes mellitus type 2	5 kg/m2	Both	Both									3.547 (2.314 to 5.219)	3.547 (2.314 to 5.219)	3.455 (2.516 to 4.691)	3.349 (2.803 to 3.918)	3.16 (2.697 to 3.699)	2.864 (2.453 to 3.312)	2.624 (2.224 to 3.038)	2.417 (2.089 to 2.779)	2.215 (1.868 to 2.606)	2.046 (1.724 to 2.379)	1.896 (1.597 to 2.228)	1.74 (1.446 to 2.074)	1.461 (1.207 to 1.758)	1.461 (1.207 to 1.758)	1.461 (1.207 to 1.758)	1.461 (1.207 to 1.758)
Chronic kidney disease due to diabetes mellitus type 2	5 kg/m2	Both	Both												1.746 (1.054 to 2.746)	1.746 (1.054 to 2.746)	1.746 (1.054 to 2.746)	1.746 (1.054 to 2.746)	1.746 (1.054 to 2.746)	2.036 (1.298 to 3.044)	2.036 (1.298 to 3.044)	1.621 (1.063 to 2.378)	1.621 (1.063 to 2.378)	1.431 (0.802 to 2.396)	1.431 (0.802 to 2.396)	1.431 (0.802 to 2.396)	1.431 (0.802 to 2.396)
Chronic kidney disease due to hypertension	5 kg/m2	Both	Both												1.763 (1.09 to 2.755)	1.763 (1.09 to 2.755)	1.763 (1.09 to 2.755)	1.763 (1.09 to 2.755)	1.763 (1.09 to 2.755)	2.044 (1.305 to 3.082)	2.044 (1.305 to 3.082)	1.605 (1.067 to 2.31)	1.605 (1.067 to 2.31)	1.437 (0.829 to 2.415)	1.437 (0.829 to 2.415)	1.437 (0.829 to 2.415)	1.437 (0.829 to 2.415)
Chronic kidney disease due to glomerulonephritis	5 kg/m2	Both	Both												1.742 (1.021 to 2.775)	1.742 (1.021 to 2.775)	1.742 (1.021 to 2.775)	1.742 (1.021 to 2.775)	1.742 (1.021 to 2.775)	2.044 (1.254 to 3.154)	2.044 (1.254 to 3.154)	1.604 (1.109 to 2.254)	1.604 (1.109 to 2.254)	1.452 (0.851 to 2.345)	1.452 (0.851 to 2.345)	1.452 (0.851 to 2.345)	1.452 (0.851 to 2.345)
Chronic kidney disease due to other and unspecified causes	5 kg/m2	Both	Both												1.732 (1.052 to 2.681)	1.732 (1.052 to 2.681)	1.732 (1.052 to 2.681)	1.732 (1.052 to 2.681)	1.732 (1.052 to 2.681)	2.032 (1.216 to 3.101)	2.032 (1.216 to 3.101)	1.625 (1.068 to 2.365)	1.625 (1.068 to 2.365)	1.433 (0.778 to 2.344)	1.433 (0.778 to 2.344)	1.433 (0.778 to 2.344)	1.433 (0.778 to 2.344)
Cataract	5 kg/m2	Both	Males									1.104 (1.052 to 1.157)	1.104 (1.052 to 1.157)	1.104 (1.052 to 1.157)	1.104 (1.052 to 1.157)	1.104 (1.052 to 1.157)	1.104 (1.052 to 1.157)	1.104 (1.052 to 1.157)	1.104 (1.052 to 1.157)	1.104 (1.052 to 1.157)	1.104 (1.052 to 1.157)	1.104 (1.052 to 1.157)	1.104 (1.052 to 1.157)	1.104 (1.052 to 1.157)	1.104 (1.052 to 1.157)	1.104 (1.052 to 1.157)	1.104 (1.052 to 1.157)
Cataract	5 kg/m2	Both	Females									1.104 (1.051 to 1.156)	1.104 (1.051 to 1.156)	1.104 (1.051 to 1.156)	1.104 (1.051 to 1.156)	1.104 (1.051 to 1.156)	1.104 (1.051 to 1.156)	1.104 (1.051 to 1.156)	1.104 (1.051 to 1.156)	1.104 (1.051 to 1.156)	1.104 (1.051 to 1.156)	1.104 (1.051 to 1.156)	1.104 (1.051 to 1.156)	1.104 (1.051 to 1.156)	1.104 (1.051 to 1.156)	1.104 (1.051 to 1.156)	1.104 (1.051 to 1.156)
Osteoarthritis hip	5 kg/m2	Morbidity	Males									1.11 (1.06 to 1.157)	1.11 (1.06 to 1.157)	1.11 (1.06 to 1.157)	1.11 (1.06 to 1.157)	1.11 (1.06 to 1.157)	1.11 (1.06 to 1.157)	1.11 (1.06 to 1.157)	1.11 (1.06 to 1.157)	1.11 (1.06 to 1.157)	1.11 (1.06 to 1.157)	1.11 (1.06 to 1.157)	1.11 (1.06 to 1.157)	1.11 (1.06 to 1.157)	1.11 (1.06 to 1.157)	1.11 (1.06 to 1.157)	1.11 (1.06 to 1.157)
Osteoarthritis hip	5 kg/m2	Morbidity	Females									1.112 (1.062 to 1.16)	1.112 (1.062 to 1.16)	1.112 (1.062 to 1.16)	1.112 (1.062 to 1.16)	1.112 (1.062 to 1.16)	1.112 (1.062 to 1.16)	1.112 (1.062 to 1.16)	1.112 (1.062 to 1.16)	1.112 (1.062 to 1.16)	1.112 (1.062 to 1.16)	1.112 (1.062 to 1.16)	1.112 (1.062 to 1.16)	1.112 (1.062 to 1.16)	1.112 (1.062 to 1.16)	1.112 (1.062 to 1.16)	1.112 (1.062 to 1.16)
Osteoarthritis knee	5 kg/m2	Morbidity	Males									1.37 (1.201 to 1.538)	1.37 (1.201 to 1.538)	1.37 (1.201 to 1.538)	1.37 (1.201 to 1.538)	1.37 (1.201 to 1.538)	1.37 (1.201 to 1.538)	1.37 (1.201 to 1.538)	1.37 (1.201 to 1.538)	1.37 (1.201 to 1.538)	1.37 (1.201 to 1.538)	1.37 (1.201 to 1.538)	1.37 (1.201 to 1.538)	1.37 (1.201 to 1.538)	1.37 (1.201 to 1.538)	1.37 (1.201 to 1.538)	1.37 (1.201 to 1.538)
Osteoarthritis knee	5 kg/m2	Morbidity	Females									1.375 (1.188 to 1.559)	1.375 (1.188 to 1.559)	1.375 (1.188 to 1.559)	1.375 (1.188 to 1.559)	1.375 (1.188 to 1.559)	1.375 (1.188 to 1.559)	1.375 (1.188 to 1.559)	1.375 (1.188 to 1.559)	1.375 (1.188 to 1.559)	1.375 (1.188 to 1.559)	1.375 (1.188 to 1.559)	1.375 (1.188 to 1.559)	1.375 (1.188 to 1.559)	1.375 (1.188 to 1.559)	1.375 (1.188 to 1.559)	1.375 (1.188 to 1.559)
Low back pain	5 kg/m2	Morbidity	Both									1.1 (1.073 to 1.126)	1.1 (1.073 to 1.126)	1.1 (1.073 to 1.127)	1.101 (1.076 to 1.128)	1.1 (1.074 to 1.126)	1.099 (1.075 to 1.123)	1.1 (1.075 to 1.128)	1.1 (1.075 to 1.126)	1.101 (1.077 to 1.126)	1.1 (1.075 to 1.126)	1.1 (1.076 to 1.124)	1.1 (1.075 to 1.124)	1.1 (1.074 to 1.125)	1.1 (1.074 to 1.125)	1.1 (1.074 to 1.125)	1.1 (1.074 to 1.125)
Gout	5 kg/m2	Both	Males									1.628 (1.34 to 1.964)	1.628 (1.34 to 1.964)	1.628 (1.34 to 1.964)	1.628 (1.34 to 1.964)	1.628 (1.34 to 1.964)	1.628 (1.34 to 1.964)	1.628 (1.34 to 1.964)	1.628 (1.34 to 1.964)	1.628 (1.34 to 1.964)	1.628 (1.34 to 1.964)	1.628 (1.34 to 1.964)	1.628 (1.34 to 1.964)	1.628 (1.34 to 1.964)	1.628 (1.34 to 1.964)	1.628 (1.34 to 1.964)	1.628 (1.34 to 1.964)
Gout	5 kg/m2	Both	Females									1.493 (1.322 to 1.677)	1.493 (1.322 to 1.677)	1.493 (1.322 to 1.677)	1.493 (1.322 to 1.677)	1.493 (1.322 to 1.677)	1.493 (1.322 to 1.677)	1.493 (1.322 to 1.677)	1.493 (1.322 to 1.677)	1.493 (1.322 to 1.677)	1.493 (1.322 to 1.677)	1.493 (1.322 to 1.677)	1.493 (1.322 to 1.677)	1.493 (1.322 to 1.677)	1.493 (1.322 to 1.677)	1.493 (1.322 to 1.677)	1.493 (1.322 to 1.677)
**High body-mass index in children**																											
Asthma	Obese	Both	Males					1.315 (1.098 to 1.515)	1.315 (1.095 to 1.513)	1.316 (1.096 to 1.522)	1.321 (1.103 to 1.531)																
Asthma	Obese	Both	Females					1.313 (1.098 to 1.522)	1.316 (1.102 to 1.524)	1.317 (1.091 to 1.522)	1.316 (1.102 to 1.516)																
Asthma	Overweight	Both	Males					1.312 (1.1 to 1.512)	1.316 (1.105 to 1.508)	1.316 (1.094 to 1.525)	1.319 (1.11 to 1.526)																
Asthma	Overweight	Both	Females					1.315 (1.096 to 1.518)	1.318 (1.1 to 1.519)	1.316 (1.106 to 1.519)	1.316 (1.091 to 1.521)																

**Low bone mineral density**																											
Non-hip fractures	0.1 g/cm2	Both	Both	1.787 (1.409 to 2.229)																							
Hip fractures	0.1 g/cm2	Both	Both	2.180 (1.661 to 2.809)																							
**Kidney disfunction**																											
Ischaemic heart disease	Stage 5 CKD	Both	Both										22.466 (11.278 to 39.315)	18.425 (9.683 to 31.409)	15.132 (8.264 to 25.098)	12.445 (6.939 to 20.51)	10.249 (5.851 to 16.385)	8.453 (4.89 to 13.466)	7.127 (4.084 to 11.423)	6.525 (3.796 to 10.537)	6.181 (3.62 to 10.106)	5.694 (3.337 to 9.365)	4.964 (2.961 to 8.115)	4.325 (2.417 to 7.091)	3.779 (2.031 to 6.325)	3.779 (2.031 to 6.325)	3.779 (2.031 to 6.325)
Ischaemic heart disease	Stage 4 CKD	Both	Both										12.757 (7.496 to 20.527)	10.889 (7.049 to 16.223)	9.315 (6.608 to 12.916)	7.986 (6.037 to 10.466)	6.861 (5.434 to 8.551)	5.907 (4.71 to 7.259)	5.106 (4.033 to 6.378)	4.449 (3.603 to 5.396)	3.858 (3.195 to 4.635)	3.272 (2.686 to 3.96)	2.7 (2.255 to 3.239)	2.227 (1.819 to 2.676)	1.84 (1.465 to 2.294)	1.84 (1.465 to 2.294)	1.84 (1.465 to 2.294)
Ischaemic heart disease	Stage 3 CKD	Both	Both										4.931 (3.737 to 6.375)	4.152 (3.291 to 5.164)	3.499 (2.888 to 4.182)	2.95 (2.516 to 3.436)	2.489 (2.188 to 2.84)	2.1 (1.866 to 2.37)	1.796 (1.599 to 2.027)	1.624 (1.453 to 1.82)	1.527 (1.374 to 1.71)	1.456 (1.304 to 1.636)	1.384 (1.242 to 1.548)	1.314 (1.18 to 1.469)	1.249 (1.109 to 1.404)	1.249 (1.109 to 1.404)	1.249 (1.109 to 1.404)
Ischaemic heart disease	Stage 1-2 CKD	Both	Both										1.908 (1.443 to 2.454)	1.845 (1.411 to 2.35)	1.786 (1.38 to 2.253)	1.729 (1.351 to 2.174)	1.674 (1.315 to 2.085)	1.621 (1.275 to 2.02)	1.568 (1.234 to 1.955)	1.513 (1.189 to 1.901)	1.454 (1.149 to 1.819)	1.395 (1.097 to 1.749)	1.336 (1.055 to 1.673)	1.28 (0.993 to 1.609)	1.227 (0.94 to 1.548)	1.227 (0.94 to 1.548)	1.227 (0.94 to 1.548)
Ischaemic heart disease	None	Both	Both										1.0 (1.0 to 1.0)	1.0 (1.0 to 1.0)	1.0 (1.0 to 1.0)	1.0 (1.0 to 1.0)	1.0 (1.0 to 1.0)	1.0 (1.0 to 1.0)	1.0 (1.0 to 1.0)	1.0 (1.0 to 1.0)	1.0 (1.0 to 1.0)	1.0 (1.0 to 1.0)	1.0 (1.0 to 1.0)	1.0 (1.0 to 1.0)	1.0 (1.0 to 1.0)	1.0 (1.0 to 1.0)	1.0 (1.0 to 1.0)
Ischaemic stroke	Stage 5 CKD	Both	Both										9.692 (6.294 to 14.656)	8.745 (6.155 to 12.237)	7.908 (6.029 to 10.288)	7.165 (5.811 to 8.703)	6.505 (5.546 to 7.586)	5.918 (5.133 to 6.826)	5.413 (4.634 to 6.36)	4.938 (4.351 to 5.582)	4.278 (3.681 to 4.884)	3.319 (2.857 to 3.796)	2.353 (2.057 to 2.681)	1.672 (1.346 to 2.055)	1.193 (0.854 to 1.606)	1.193 (0.854 to 1.606)	1.193 (0.854 to 1.606)
Ischaemic stroke	Stage 4 CKD	Both	Both										12.141 (6.664 to 20.146)	9.919 (6.074 to 15.096)	8.127 (5.596 to 11.382)	6.677 (5.09 to 8.571)	5.502 (4.624 to 6.578)	4.547 (4.001 to 5.234)	3.816 (3.325 to 4.438)	3.391 (3.029 to 3.809)	3.011 (2.743 to 3.308)	2.48 (2.251 to 2.726)	1.847 (1.733 to 1.978)	1.369 (1.261 to 1.491)	1.016 (0.888 to 1.16)	1.016 (0.888 to 1.16)	1.016 (0.888 to 1.16)
Ischaemic stroke	Stage 3 CKD	Both	Both										4.434 (3.393 to 5.731)	3.802 (3.039 to 4.713)	3.261 (2.727 to 3.884)	2.799 (2.436 to 3.22)	2.404 (2.146 to 2.672)	2.066 (1.872 to 2.258)	1.785 (1.615 to 1.951)	1.58 (1.442 to 1.715)	1.428 (1.315 to 1.54)	1.308 (1.204 to 1.412)	1.204 (1.113 to 1.293)	1.109 (1.021 to 1.197)	1.022 (0.929 to 1.113)	1.022 (0.929 to 1.113)	1.022 (0.929 to 1.113)
Ischaemic stroke	Stage 1-2 CKD	Both	Both										1.961 (1.669 to 2.381)	1.915 (1.659 to 2.265)	1.871 (1.645 to 2.151)	1.828 (1.624 to 2.06)	1.787 (1.603 to 1.986)	1.746 (1.567 to 1.937)	1.703 (1.526 to 1.896)	1.641 (1.482 to 1.82)	1.561 (1.407 to 1.73)	1.465 (1.317 to 1.626)	1.362 (1.227 to 1.507)	1.267 (1.131 to 1.418)	1.179 (1.032 to 1.346)	1.179 (1.032 to 1.346)	1.179 (1.032 to 1.346)
Ischaemic stroke	None	Both	Both										1.0 (1.0 to 1.0)	1.0 (1.0 to 1.0)	1.0 (1.0 to 1.0)	1.0 (1.0 to 1.0)	1.0 (1.0 to 1.0)	1.0 (1.0 to 1.0)	1.0 (1.0 to 1.0)	1.0 (1.0 to 1.0)	1.0 (1.0 to 1.0)	1.0 (1.0 to 1.0)	1.0 (1.0 to 1.0)	1.0 (1.0 to 1.0)	1.0 (1.0 to 1.0)	1.0 (1.0 to 1.0)	1.0 (1.0 to 1.0)
Intracerebral haemorrhage	Stage 5 CKD	Both	Both										6.665 (0.633 to 28.506)	6.631 (0.586 to 26.898)	6.571 (0.671 to 25.958)	6.66 (0.529 to 28.335)	6.568 (0.674 to 27.459)	7.071 (0.699 to 27.677)	6.859 (0.656 to 28.559)	3.305 (1.133 to 7.402)	3.34 (1.183 to 7.005)	2.175 (1.085 to 4.053)	2.154 (1.054 to 4.058)	1.608 (0.478 to 3.837)	1.567 (0.488 to 3.819)	1.539 (0.499 to 3.799)	1.544 (0.454 to 3.893)
Intracerebral haemorrhage	Stage 4 CKD	Both	Both										3.642 (0.769 to 10.195)	3.665 (0.716 to 11.442)	3.493 (0.721 to 10.629)	3.634 (0.744 to 10.727)	3.518 (0.743 to 9.943)	3.579 (0.832 to 10.234)	3.576 (0.774 to 10.582)	2.291 (1.209 to 3.935)	2.287 (1.13 to 4.048)	1.708 (1.023 to 2.625)	1.67 (1.026 to 2.52)	1.298 (0.628 to 2.387)	1.331 (0.65 to 2.537)	1.323 (0.61 to 2.458)	1.301 (0.624 to 2.507)
Intracerebral haemorrhage	Stage 3 CKD	Both	Both										1.063 (0.699 to 1.513)	1.073 (0.733 to 1.534)	1.071 (0.73 to 1.524)	1.072 (0.717 to 1.565)	1.081 (0.721 to 1.571)	1.077 (0.725 to 1.55)	1.077 (0.724 to 1.542)	1.324 (1.072 to 1.619)	1.323 (1.069 to 1.629)	1.255 (1.064 to 1.462)	1.254 (1.08 to 1.46)	1.155 (0.925 to 1.405)	1.148 (0.936 to 1.393)	1.147 (0.931 to 1.385)	1.156 (0.932 to 1.41)
Intracerebral haemorrhage	Stage 1-2 CKD	Both	Both										2.09 (1.406 to 3.11)	2.077 (1.425 to 2.946)	2.074 (1.405 to 2.996)	2.093 (1.413 to 3.032)	2.092 (1.365 to 3.032)	2.085 (1.391 to 2.983)	2.079 (1.4 to 2.988)	1.478 (1.289 to 1.683)	1.487 (1.319 to 1.692)	1.272 (1.093 to 1.49)	1.27 (1.076 to 1.497)	1.11 (0.854 to 1.433)	1.102 (0.834 to 1.419)	1.107 (0.835 to 1.446)	1.109 (0.822 to 1.429)
Intracerebral haemorrhage	None	Both	Both										1.0 (1.0 to 1.0)	1.0 (1.0 to 1.0)	1.0 (1.0 to 1.0)	1.0 (1.0 to 1.0)	1.0 (1.0 to 1.0)	1.0 (1.0 to 1.0)	1.0 (1.0 to 1.0)	1.0 (1.0 to 1.0)	1.0 (1.0 to 1.0)	1.0 (1.0 to 1.0)	1.0 (1.0 to 1.0)	1.0 (1.0 to 1.0)	1.0 (1.0 to 1.0)	1.0 (1.0 to 1.0)	1.0 (1.0 to 1.0)
Peripheral artery disease	Stage 5 CKD	Both	Both										207.753 (30.856 to 704.06)	200.617 (34.114 to 695.696)	211.239 (32.055 to 800.634)	205.926 (31.084 to 751.759)	200.856 (33.786 to 732.241)	207.342 (30.718 to 650.978)	202.164 (32.222 to 701.663)	17.647 (7.939 to 32.318)	17.353 (8.097 to 31.55)	5.347 (2.334 to 10.628)	5.427 (2.272 to 10.749)	1.895 (0.429 to 5.39)	1.852 (0.444 to 5.291)	1.856 (0.437 to 5.035)	1.925 (0.427 to 5.377)
Peripheral artery disease	Stage 4 CKD	Both	Both										58.362 (15.568 to 149.001)	54.062 (15.177 to 136.039)	54.638 (14.697 to 139.549)	55.201 (16.279 to 139.398)	54.066 (14.955 to 142.728)	55.805 (15.506 to 137.121)	55.87 (15.838 to 139.768)	7.713 (4.647 to 11.89)	7.845 (4.686 to 12.511)	3.141 (1.836 to 5.218)	3.213 (1.87 to 5.196)	1.464 (0.558 to 3.037)	1.47 (0.601 to 3.006)	1.462 (0.599 to 3.073)	1.488 (0.593 to 3.038)
Peripheral artery disease	Stage 3 CKD	Both	Both										3.083 (1.826 to 4.774)	3.102 (1.839 to 4.864)	3.045 (1.792 to 4.748)	3.114 (1.906 to 4.86)	3.039 (1.817 to 4.807)	3.117 (1.883 to 4.881)	3.113 (1.839 to 4.781)	2.055 (1.57 to 2.665)	2.05 (1.584 to 2.635)	1.525 (1.216 to 1.891)	1.531 (1.221 to 1.92)	1.142 (0.868 to 1.461)	1.158 (0.875 to 1.513)	1.147 (0.871 to 1.476)	1.158 (0.879 to 1.484)
Peripheral artery disease	Stage 1-2 CKD	Both	Both										3.542 (1.986 to 5.721)	3.572 (1.98 to 5.768)	3.57 (1.977 to 5.944)	3.569 (1.991 to 5.91)	3.543 (1.934 to 5.923)	3.607 (1.98 to 6.203)	3.552 (1.902 to 5.879)	1.758 (1.442 to 2.135)	1.759 (1.451 to 2.101)	1.336 (1.033 to 1.695)	1.327 (1.018 to 1.727)	1.037 (0.664 to 1.542)	1.029 (0.687 to 1.478)	1.033 (0.646 to 1.496)	1.038 (0.68 to 1.548)
Peripheral artery disease	None	Both	Both										1.0 (1.0 to 1.0)	1.0 (1.0 to 1.0)	1.0 (1.0 to 1.0)	1.0 (1.0 to 1.0)	1.0 (1.0 to 1.0)	1.0 (1.0 to 1.0)	1.0 (1.0 to 1.0)	1.0 (1.0 to 1.0)	1.0 (1.0 to 1.0)	1.0 (1.0 to 1.0)	1.0 (1.0 to 1.0)	1.0 (1.0 to 1.0)	1.0 (1.0 to 1.0)	1.0 (1.0 to 1.0)	1.0 (1.0 to 1.0)
Gout	Stage 5 CKD	Both	Both										2.749 (2.491 to 3.017)	2.747 (2.47 to 3.035)	2.747 (2.482 to 3.035)	2.736 (2.466 to 3.014)	2.741 (2.48 to 3.053)	2.729 (2.47 to 3.007)	2.74 (2.479 to 3.038)	2.747 (2.485 to 3.049)	2.742 (2.479 to 3.021)	2.742 (2.496 to 3.039)	2.74 (2.48 to 3.042)	2.736 (2.468 to 3.013)	2.736 (2.468 to 3.013)	2.736 (2.468 to 3.013)	2.736 (2.468 to 3.013)
Gout	Stage 4 CKD	Both	Both										2.745 (2.473 to 3.032)	2.743 (2.484 to 3.014)	2.742 (2.473 to 3.034)	2.742 (2.442 to 3.029)	2.746 (2.494 to 3.045)	2.748 (2.483 to 3.045)	2.744 (2.462 to 3.029)	2.749 (2.477 to 3.018)	2.755 (2.495 to 3.038)	2.747 (2.473 to 3.034)	2.738 (2.463 to 3.041)	2.744 (2.478 to 3.021)	2.744 (2.478 to 3.021)	2.744 (2.478 to 3.021)	2.744 (2.478 to 3.021)
Gout	Stage 3 CKD	Both	Both										2.748 (2.457 to 3.035)	2.753 (2.48 to 3.038)	2.743 (2.465 to 3.024)	2.748 (2.491 to 3.029)	2.743 (2.467 to 3.051)	2.749 (2.473 to 3.042)	2.744 (2.475 to 3.017)	2.741 (2.464 to 3.034)	2.745 (2.478 to 3.037)	2.739 (2.465 to 3.006)	2.745 (2.473 to 3.029)	2.747 (2.475 to 3.028)	2.747 (2.475 to 3.028)	2.747 (2.475 to 3.028)	2.747 (2.475 to 3.028)
Gout	Stage 1-2 CKD	Both	Both										1.0 (1.0 to 1.0)	1.0 (1.0 to 1.0)	1.0 (1.0 to 1.0)	1.0 (1.0 to 1.0)	1.0 (1.0 to 1.0)	1.0 (1.0 to 1.0)	1.0 (1.0 to 1.0)	1.0 (1.0 to 1.0)	1.0 (1.0 to 1.0)	1.0 (1.0 to 1.0)	1.0 (1.0 to 1.0)	1.0 (1.0 to 1.0)	1.0 (1.0 to 1.0)	1.0 (1.0 to 1.0)	1.0 (1.0 to 1.0)
Gout	None	Both	Both										1.0 (1.0 to 1.0)	1.0 (1.0 to 1.0)	1.0 (1.0 to 1.0)	1.0 (1.0 to 1.0)	1.0 (1.0 to 1.0)	1.0 (1.0 to 1.0)	1.0 (1.0 to 1.0)	1.0 (1.0 to 1.0)	1.0 (1.0 to 1.0)	1.0 (1.0 to 1.0)	1.0 (1.0 to 1.0)	1.0 (1.0 to 1.0)	1.0 (1.0 to 1.0)	1.0 (1.0 to 1.0)	1.0 (1.0 to 1.0)

Table S7B. Relative risks used by age and sex for each outcome for the ambient particulate matter pollution integrated exposure response curve.																		
				Age														
**Risk - Outcome**	**Category / Units**	**Morbidity / Mortality**	**Sex**	**All ages**	**25-29 years**	**30-34 years**	**35-39 years**	**40-44 years**	**45-49 years**	**50-54 years**	**55-59 years**	**60-64 years**	**65-69 years**	**70-74 years**	**75-79 years**	**80-84 years**	**85-89 years**	**90-94 years**	**95+ years**
**Ambient particulate matter pollution (PM2.5)**																			
Lower respiratory infections	600 µg/m³	Both	Both	2.189 (1.989 to 3.122)															
Lower respiratory infections	500 µg/m³	Both	Both	2.143 (1.974 to 3.03)															
Lower respiratory infections	400 µg/m³	Both	Both	2.098 (1.963 to 2.966)															
Lower respiratory infections	300 µg/m³	Both	Both	2.052 (1.931 to 2.864)															
Lower respiratory infections	200 µg/m³	Both	Both	1.909 (1.747 to 2.498)															
Lower respiratory infections	150 µg/m³	Both	Both	1.751 (1.594 to 2.241)															
Lower respiratory infections	130 µg/m³	Both	Both	1.68 (1.523 to 2.118)															
Lower respiratory infections	110 µg/m³	Both	Both	1.607 (1.454 to 1.998)															
Lower respiratory infections	90 µg/m³	Both	Both	1.533 (1.378 to 1.863)															
Lower respiratory infections	70 µg/m³	Both	Both	1.453 (1.299 to 1.719)															
Lower respiratory infections	50 µg/m³	Both	Both	1.357 (1.214 to 1.555)															
Lower respiratory infections	30 µg/m³	Both	Both	1.238 (1.128 to 1.356)															
Lower respiratory infections	25 µg/m³	Both	Both	1.204 (1.107 to 1.305)															
Lower respiratory infections	20 µg/m³	Both	Both	1.168 (1.086 to 1.25)															
Lower respiratory infections	15 µg/m³	Both	Both	1.129 (1.064 to 1.19)															
Lower respiratory infections	10 µg/m³	Both	Both	1.089 (1.043 to 1.129)															
Lower respiratory infections	5 µg/m³	Both	Both	1.046 (1.022 to 1.065)															
Lower respiratory infections	0 µg/m³	Both	Both	1.0 (1.0 to 1.0)															
Tracheal, bronchus, and lung cancer	600 µg/m³	Both	Both	1.574 (1.451 to 2.421)															
Tracheal, bronchus, and lung cancer	500 µg/m³	Both	Both	1.574 (1.451 to 2.275)															
Tracheal, bronchus, and lung cancer	400 µg/m³	Both	Both	1.574 (1.449 to 2.114)															
Tracheal, bronchus, and lung cancer	300 µg/m³	Both	Both	1.574 (1.445 to 1.961)															
Tracheal, bronchus, and lung cancer	200 µg/m³	Both	Both	1.574 (1.435 to 1.869)															
Tracheal, bronchus, and lung cancer	150 µg/m³	Both	Both	1.574 (1.422 to 1.842)															
Tracheal, bronchus, and lung cancer	130 µg/m³	Both	Both	1.574 (1.418 to 1.834)															
Tracheal, bronchus, and lung cancer	110 µg/m³	Both	Both	1.56 (1.411 to 1.811)															
Tracheal, bronchus, and lung cancer	90 µg/m³	Both	Both	1.527 (1.4 to 1.767)															
Tracheal, bronchus, and lung cancer	70 µg/m³	Both	Both	1.482 (1.369 to 1.692)															
Tracheal, bronchus, and lung cancer	50 µg/m³	Both	Both	1.433 (1.32 to 1.582)															
Tracheal, bronchus, and lung cancer	30 µg/m³	Both	Both	1.346 (1.226 to 1.425)															
Tracheal, bronchus, and lung cancer	25 µg/m³	Both	Both	1.31 (1.194 to 1.376)															
Tracheal, bronchus, and lung cancer	20 µg/m³	Both	Both	1.267 (1.16 to 1.32)															
Tracheal, bronchus, and lung cancer	15 µg/m³	Both	Both	1.216 (1.125 to 1.254)															
Tracheal, bronchus, and lung cancer	10 µg/m³	Both	Both	1.155 (1.086 to 1.179)															
Tracheal, bronchus, and lung cancer	5 µg/m³	Both	Both	1.083 (1.044 to 1.094)															
Tracheal, bronchus, and lung cancer	0 µg/m³	Both	Both	1.0 (1.0 to 1.0)															
Ischaemic heart disease	600 µg/m³	Both	Both		3.18 (2.2 to 3.83)	2.104 (2.038 to 3.626)	2.189 (1.979 to 3.379)	2.599 (1.875 to 3.282)	2.327 (1.786 to 3.166)	2.072 (1.703 to 2.953)	2.005 (1.652 to 2.899)	2.124 (1.567 to 2.682)	1.687 (1.507 to 2.583)	2.081 (1.422 to 2.465)	1.491 (1.368 to 2.302)	1.782 (1.304 to 2.128)	1.531 (1.252 to 2.028)	1.64 (1.193 to 1.79)	1.314 (1.144 to 1.561)
Ischaemic heart disease	500 µg/m³	Both	Both		3.032 (2.2 to 3.677)	2.069 (2.038 to 3.497)	2.189 (1.972 to 3.234)	2.478 (1.87 to 3.14)	2.243 (1.786 to 2.954)	2.072 (1.703 to 2.763)	2.005 (1.652 to 2.708)	2.045 (1.567 to 2.53)	1.674 (1.507 to 2.412)	1.957 (1.422 to 2.303)	1.491 (1.368 to 2.135)	1.699 (1.304 to 1.983)	1.492 (1.252 to 1.894)	1.564 (1.192 to 1.685)	1.279 (1.144 to 1.493)
Ischaemic heart disease	400 µg/m³	Both	Both		2.884 (2.187 to 3.55)	2.035 (2.03 to 3.346)	2.189 (1.969 to 3.091)	2.358 (1.87 to 2.977)	2.16 (1.786 to 2.772)	2.072 (1.699 to 2.601)	2.005 (1.645 to 2.528)	1.966 (1.567 to 2.347)	1.661 (1.507 to 2.231)	1.833 (1.422 to 2.123)	1.491 (1.367 to 1.982)	1.615 (1.304 to 1.834)	1.453 (1.252 to 1.758)	1.488 (1.192 to 1.584)	1.244 (1.144 to 1.422)
Ischaemic heart disease	300 µg/m³	Both	Both		2.736 (2.156 to 3.395)	2.0 (2.02 to 3.213)	2.189 (1.96 to 2.985)	2.238 (1.867 to 2.835)	2.076 (1.786 to 2.635)	2.072 (1.698 to 2.454)	2.005 (1.644 to 2.356)	1.887 (1.565 to 2.177)	1.648 (1.506 to 2.058)	1.709 (1.422 to 1.95)	1.491 (1.367 to 1.827)	1.532 (1.302 to 1.704)	1.414 (1.251 to 1.623)	1.413 (1.192 to 1.48)	1.21 (1.144 to 1.348)
Ischaemic heart disease	200 µg/m³	Both	Both		2.588 (2.139 to 3.325)	1.966 (2.011 to 3.134)	2.189 (1.937 to 2.897)	2.117 (1.858 to 2.739)	1.993 (1.786 to 2.517)	2.072 (1.687 to 2.333)	2.005 (1.639 to 2.212)	1.808 (1.563 to 2.055)	1.634 (1.503 to 1.901)	1.585 (1.417 to 1.805)	1.491 (1.367 to 1.684)	1.448 (1.299 to 1.568)	1.375 (1.246 to 1.492)	1.337 (1.192 to 1.38)	1.175 (1.144 to 1.274)
Ischaemic heart disease	150 µg/m³	Both	Both		2.514 (2.134 to 3.276)	1.949 (2.011 to 3.095)	2.189 (1.933 to 2.866)	2.057 (1.847 to 2.699)	1.951 (1.781 to 2.484)	2.072 (1.685 to 2.296)	2.005 (1.622 to 2.154)	1.769 (1.555 to 2.002)	1.628 (1.492 to 1.856)	1.523 (1.416 to 1.747)	1.491 (1.363 to 1.635)	1.406 (1.297 to 1.521)	1.355 (1.246 to 1.437)	1.299 (1.192 to 1.337)	1.157 (1.144 to 1.246)
Ischaemic heart disease	130 µg/m³	Both	Both		2.485 (2.132 to 3.262)	1.942 (2.004 to 3.079)	2.189 (1.93 to 2.864)	2.033 (1.847 to 2.68)	1.935 (1.781 to 2.468)	2.072 (1.679 to 2.276)	2.005 (1.618 to 2.145)	1.753 (1.552 to 1.98)	1.625 (1.487 to 1.838)	1.498 (1.415 to 1.728)	1.491 (1.362 to 1.618)	1.389 (1.297 to 1.509)	1.347 (1.243 to 1.422)	1.284 (1.192 to 1.324)	1.15 (1.143 to 1.237)
Ischaemic heart disease	110 µg/m³	Both	Both		2.455 (2.116 to 3.253)	1.935 (1.999 to 3.068)	2.189 (1.93 to 2.86)	2.009 (1.843 to 2.666)	1.918 (1.775 to 2.457)	2.072 (1.675 to 2.268)	2.005 (1.612 to 2.133)	1.737 (1.539 to 1.965)	1.622 (1.478 to 1.834)	1.473 (1.407 to 1.723)	1.491 (1.359 to 1.609)	1.373 (1.296 to 1.5)	1.34 (1.239 to 1.408)	1.269 (1.19 to 1.316)	1.143 (1.142 to 1.228)
Ischaemic heart disease	90 µg/m³	Both	Both		2.411 (2.11 to 3.237)	1.919 (1.994 to 3.026)	2.127 (1.92 to 2.819)	1.985 (1.835 to 2.632)	1.901 (1.749 to 2.425)	2.072 (1.667 to 2.248)	2.005 (1.595 to 2.111)	1.721 (1.525 to 1.954)	1.619 (1.471 to 1.822)	1.449 (1.398 to 1.705)	1.491 (1.356 to 1.595)	1.356 (1.291 to 1.488)	1.325 (1.236 to 1.396)	1.251 (1.186 to 1.306)	1.136 (1.138 to 1.22)
Ischaemic heart disease	70 µg/m³	Both	Both		2.217 (2.012 to 3.07)	1.782 (1.944 to 2.885)	1.976 (1.85 to 2.673)	1.959 (1.769 to 2.49)	1.855 (1.709 to 2.329)	2.012 (1.636 to 2.158)	1.953 (1.564 to 2.028)	1.683 (1.485 to 1.871)	1.59 (1.435 to 1.76)	1.418 (1.369 to 1.662)	1.488 (1.33 to 1.548)	1.335 (1.269 to 1.459)	1.291 (1.219 to 1.37)	1.218 (1.172 to 1.285)	1.124 (1.129 to 1.207)
Ischaemic heart disease	50 µg/m³	Both	Both		1.9 (1.809 to 2.782)	1.588 (1.75 to 2.639)	1.798 (1.68 to 2.438)	1.879 (1.608 to 2.291)	1.716 (1.577 to 2.134)	1.833 (1.522 to 2.01)	1.807 (1.465 to 1.885)	1.545 (1.389 to 1.759)	1.494 (1.359 to 1.669)	1.369 (1.303 to 1.59)	1.446 (1.274 to 1.481)	1.301 (1.224 to 1.406)	1.249 (1.183 to 1.327)	1.177 (1.141 to 1.255)	1.104 (1.108 to 1.186)
Ischaemic heart disease	30 µg/m³	Both	Both		1.541 (1.528 to 2.374)	1.379 (1.479 to 2.277)	1.558 (1.447 to 2.105)	1.719 (1.397 to 2.01)	1.475 (1.382 to 1.885)	1.611 (1.344 to 1.789)	1.606 (1.3 to 1.697)	1.344 (1.256 to 1.597)	1.332 (1.242 to 1.532)	1.284 (1.201 to 1.479)	1.321 (1.178 to 1.38)	1.24 (1.151 to 1.321)	1.189 (1.123 to 1.269)	1.132 (1.097 to 1.208)	1.074 (1.074 to 1.152)
Ischaemic heart disease	25 µg/m³	Both	Both		1.451 (1.451 to 2.252)	1.322 (1.403 to 2.146)	1.484 (1.376 to 1.985)	1.66 (1.335 to 1.906)	1.402 (1.327 to 1.8)	1.547 (1.289 to 1.719)	1.537 (1.253 to 1.626)	1.289 (1.217 to 1.538)	1.283 (1.205 to 1.478)	1.253 (1.17 to 1.43)	1.276 (1.15 to 1.344)	1.216 (1.128 to 1.293)	1.168 (1.104 to 1.243)	1.118 (1.084 to 1.189)	1.065 (1.064 to 1.139)
Ischaemic heart disease	20 µg/m³	Both	Both		1.361 (1.361 to 2.097)	1.263 (1.324 to 1.995)	1.403 (1.303 to 1.849)	1.584 (1.272 to 1.779)	1.326 (1.269 to 1.697)	1.473 (1.236 to 1.631)	1.457 (1.209 to 1.541)	1.233 (1.177 to 1.469)	1.232 (1.167 to 1.413)	1.217 (1.137 to 1.375)	1.227 (1.122 to 1.298)	1.187 (1.104 to 1.254)	1.144 (1.086 to 1.213)	1.102 (1.069 to 1.166)	1.055 (1.053 to 1.122)
Ischaemic heart disease	15 µg/m³	Both	Both		1.271 (1.274 to 1.875)	1.202 (1.244 to 1.806)	1.315 (1.23 to 1.679)	1.486 (1.209 to 1.635)	1.247 (1.206 to 1.562)	1.385 (1.18 to 1.52)	1.365 (1.159 to 1.441)	1.175 (1.135 to 1.38)	1.177 (1.126 to 1.334)	1.174 (1.105 to 1.312)	1.174 (1.092 to 1.244)	1.151 (1.079 to 1.208)	1.115 (1.066 to 1.175)	1.084 (1.053 to 1.138)	1.044 (1.041 to 1.101)
Ischaemic heart disease	10 µg/m³	Both	Both		1.18 (1.183 to 1.634)	1.137 (1.163 to 1.578)	1.219 (1.155 to 1.485)	1.36 (1.14 to 1.463)	1.166 (1.139 to 1.407)	1.279 (1.121 to 1.384)	1.259 (1.108 to 1.321)	1.117 (1.09 to 1.275)	1.12 (1.085 to 1.24)	1.124 (1.07 to 1.227)	1.118 (1.062 to 1.178)	1.109 (1.053 to 1.151)	1.082 (1.045 to 1.126)	1.061 (1.036 to 1.1)	1.031 (1.027 to 1.074)
Ischaemic heart disease	5 µg/m³	Both	Both		1.09 (1.093 to 1.345)	1.07 (1.082 to 1.312)	1.114 (1.078 to 1.26)	1.2 (1.071 to 1.249)	1.083 (1.07 to 1.218)	1.152 (1.06 to 1.21)	1.138 (1.055 to 1.174)	1.059 (1.046 to 1.149)	1.061 (1.043 to 1.129)	1.066 (1.036 to 1.124)	1.06 (1.031 to 1.096)	1.059 (1.027 to 1.082)	1.044 (1.023 to 1.069)	1.033 (1.018 to 1.056)	1.017 (1.014 to 1.04)
Ischaemic heart disease	0 µg/m³	Both	Both		1.0 (1.0 to 1.0)	1.0 (1.0 to 1.0)	1.0 (1.0 to 1.0)	1.0 (1.0 to 1.0)	1.0 (1.0 to 1.0)	1.0 (1.0 to 1.0)	1.0 (1.0 to 1.0)	1.0 (1.0 to 1.0)	1.0 (1.0 to 1.0)	1.0 (1.0 to 1.0)	1.0 (1.0 to 1.0)	1.0 (1.0 to 1.0)	1.0 (1.0 to 1.0)	1.0 (1.0 to 1.0)	1.0 (1.0 to 1.0)
Stroke	600 µg/m³	Both	Both		3.612 (2.651 to 4.682)	3.135 (2.521 to 4.377)	3.237 (2.334 to 4.015)	2.826 (2.223 to 3.768)	2.794 (2.179 to 3.6)	2.175 (2.012 to 3.354)	2.399 (1.913 to 3.131)	2.55 (1.804 to 2.969)	1.987 (1.695 to 2.809)	2.014 (1.603 to 2.623)	2.139 (1.507 to 2.462)	1.884 (1.419 to 2.338)	1.419 (1.354 to 2.112)	1.845 (1.283 to 1.939)	1.54 (1.209 to 1.71)
Stroke	500 µg/m³	Both	Both		3.612 (2.645 to 4.53)	3.091 (2.517 to 4.221)	3.237 (2.33 to 3.886)	2.81 (2.221 to 3.658)	2.726 (2.169 to 3.405)	2.175 (2.007 to 3.193)	2.399 (1.911 to 2.978)	2.488 (1.795 to 2.817)	1.952 (1.695 to 2.642)	2.014 (1.603 to 2.473)	2.059 (1.506 to 2.301)	1.816 (1.419 to 2.177)	1.419 (1.352 to 1.984)	1.733 (1.283 to 1.819)	1.477 (1.209 to 1.617)
Stroke	400 µg/m³	Both	Both		3.612 (2.633 to 4.427)	3.047 (2.517 to 4.083)	3.237 (2.33 to 3.776)	2.795 (2.215 to 3.523)	2.657 (2.16 to 3.246)	2.175 (2.006 to 3.045)	2.399 (1.909 to 2.833)	2.425 (1.789 to 2.66)	1.917 (1.694 to 2.494)	2.014 (1.6 to 2.295)	1.979 (1.505 to 2.15)	1.748 (1.419 to 2.022)	1.419 (1.352 to 1.85)	1.622 (1.279 to 1.708)	1.414 (1.208 to 1.523)
Stroke	300 µg/m³	Both	Both		3.612 (2.612 to 4.325)	3.004 (2.517 to 4.018)	3.237 (2.324 to 3.672)	2.78 (2.208 to 3.419)	2.589 (2.16 to 3.164)	2.175 (2.005 to 2.911)	2.399 (1.899 to 2.676)	2.363 (1.789 to 2.499)	1.882 (1.688 to 2.328)	2.014 (1.597 to 2.135)	1.899 (1.502 to 2.001)	1.681 (1.418 to 1.873)	1.419 (1.352 to 1.705)	1.51 (1.277 to 1.591)	1.351 (1.207 to 1.434)
Stroke	200 µg/m³	Both	Both		3.612 (2.602 to 4.252)	2.96 (2.514 to 3.953)	3.237 (2.29 to 3.588)	2.765 (2.203 to 3.329)	2.52 (2.137 to 3.071)	2.175 (1.992 to 2.791)	2.399 (1.879 to 2.565)	2.3 (1.774 to 2.38)	1.846 (1.671 to 2.205)	2.014 (1.585 to 2.041)	1.819 (1.493 to 1.877)	1.613 (1.414 to 1.731)	1.419 (1.348 to 1.588)	1.399 (1.271 to 1.476)	1.288 (1.201 to 1.349)
Stroke	150 µg/m³	Both	Both		3.612 (2.6 to 4.216)	2.938 (2.499 to 3.911)	3.237 (2.283 to 3.577)	2.757 (2.193 to 3.288)	2.486 (2.123 to 3.047)	2.175 (1.985 to 2.756)	2.399 (1.873 to 2.549)	2.269 (1.765 to 2.339)	1.829 (1.664 to 2.175)	2.014 (1.581 to 2.004)	1.779 (1.486 to 1.834)	1.58 (1.41 to 1.684)	1.419 (1.341 to 1.545)	1.343 (1.265 to 1.433)	1.256 (1.195 to 1.312)
Stroke	130 µg/m³	Both	Both		3.612 (2.585 to 4.216)	2.929 (2.495 to 3.904)	3.237 (2.283 to 3.567)	2.754 (2.19 to 3.275)	2.472 (2.116 to 3.032)	2.175 (1.985 to 2.755)	2.399 (1.866 to 2.538)	2.256 (1.755 to 2.33)	1.822 (1.661 to 2.161)	2.014 (1.575 to 1.988)	1.763 (1.486 to 1.821)	1.566 (1.406 to 1.675)	1.419 (1.334 to 1.531)	1.321 (1.26 to 1.416)	1.244 (1.192 to 1.299)
Stroke	110 µg/m³	Both	Both		3.612 (2.575 to 4.206)	2.92 (2.495 to 3.904)	3.237 (2.276 to 3.564)	2.751 (2.184 to 3.27)	2.459 (2.106 to 3.014)	2.175 (1.978 to 2.755)	2.399 (1.855 to 2.535)	2.244 (1.747 to 2.329)	1.815 (1.658 to 2.153)	2.014 (1.566 to 1.982)	1.747 (1.485 to 1.808)	1.553 (1.4 to 1.666)	1.419 (1.327 to 1.523)	1.299 (1.254 to 1.408)	1.231 (1.189 to 1.289)
Stroke	90 µg/m³	Both	Both		3.598 (2.539 to 4.141)	2.867 (2.469 to 3.842)	3.237 (2.273 to 3.525)	2.748 (2.166 to 3.228)	2.445 (2.08 to 2.976)	2.16 (1.966 to 2.724)	2.381 (1.847 to 2.5)	2.231 (1.725 to 2.307)	1.808 (1.649 to 2.132)	2.003 (1.553 to 1.97)	1.731 (1.478 to 1.795)	1.539 (1.39 to 1.663)	1.419 (1.316 to 1.515)	1.276 (1.245 to 1.398)	1.219 (1.18 to 1.281)
Stroke	70 µg/m³	Both	Both		3.324 (2.396 to 3.827)	2.596 (2.309 to 3.501)	3.158 (2.148 to 3.258)	2.629 (2.049 to 3.004)	2.35 (1.968 to 2.758)	2.056 (1.868 to 2.512)	2.195 (1.767 to 2.329)	2.167 (1.675 to 2.168)	1.714 (1.595 to 2.015)	1.888 (1.517 to 1.879)	1.682 (1.44 to 1.723)	1.49 (1.364 to 1.607)	1.368 (1.295 to 1.482)	1.254 (1.233 to 1.375)	1.206 (1.167 to 1.268)
Stroke	50 µg/m³	Both	Both		2.804 (2.086 to 3.273)	2.235 (1.998 to 3.006)	2.731 (1.899 to 2.764)	2.327 (1.816 to 2.593)	2.049 (1.748 to 2.383)	1.854 (1.687 to 2.222)	1.925 (1.605 to 2.056)	1.939 (1.543 to 1.939)	1.528 (1.479 to 1.826)	1.692 (1.418 to 1.707)	1.567 (1.352 to 1.584)	1.381 (1.297 to 1.496)	1.274 (1.247 to 1.394)	1.216 (1.192 to 1.308)	1.173 (1.142 to 1.221)
Stroke	30 µg/m³	Both	Both		2.176 (1.671 to 2.531)	1.805 (1.62 to 2.393)	2.079 (1.575 to 2.214)	1.918 (1.51 to 2.061)	1.641 (1.477 to 1.944)	1.555 (1.439 to 1.85)	1.596 (1.39 to 1.73)	1.615 (1.345 to 1.643)	1.334 (1.31 to 1.58)	1.468 (1.269 to 1.495)	1.385 (1.22 to 1.419)	1.235 (1.19 to 1.351)	1.169 (1.159 to 1.284)	1.167 (1.124 to 1.221)	1.125 (1.089 to 1.157)

Stroke	25 µg/m^3	Both	Both		2.013 (1.565 to 2.335)	1.685 (1.518 to 2.226)	1.9 (1.482 to 2.04)	1.808 (1.428 to 1.927)	1.534 (1.4 to 1.824)	1.469 (1.366 to 1.749)	1.505 (1.328 to 1.644)	1.528 (1.29 to 1.569)	1.283 (1.259 to 1.508)	1.408 (1.225 to 1.432)	1.33 (1.184 to 1.373)	1.196 (1.159 to 1.314)	1.141 (1.133 to 1.251)	1.15 (1.103 to 1.196)	1.111 (1.075 to 1.139)
Stroke	20 µg/m^3	Both	Both		1.843 (1.454 to 2.132)	1.56 (1.416 to 2.029)	1.72 (1.388 to 1.886)	1.697 (1.342 to 1.789)	1.427 (1.322 to 1.703)	1.379 (1.293 to 1.64)	1.411 (1.263 to 1.547)	1.441 (1.232 to 1.49)	1.23 (1.208 to 1.437)	1.343 (1.181 to 1.368)	1.271 (1.147 to 1.317)	1.157 (1.128 to 1.271)	1.113 (1.108 to 1.213)	1.129 (1.083 to 1.171)	1.095 (1.06 to 1.122)
Stroke	15 µg/m^3	Both	Both		1.66 (1.341 to 1.896)	1.429 (1.313 to 1.835)	1.54 (1.293 to 1.704)	1.579 (1.259 to 1.636)	1.32 (1.243 to 1.576)	1.287 (1.222 to 1.511)	1.313 (1.198 to 1.435)	1.349 (1.176 to 1.391)	1.175 (1.156 to 1.356)	1.271 (1.136 to 1.296)	1.208 (1.111 to 1.259)	1.118 (1.096 to 1.226)	1.085 (1.082 to 1.175)	1.105 (1.063 to 1.142)	1.077 (1.045 to 1.102)
Stroke	10 µg/m^3	Both	Both		1.462 (1.227 to 1.659)	1.292 (1.209 to 1.615)	1.36 (1.196 to 1.519)	1.437 (1.173 to 1.466)	1.214 (1.163 to 1.412)	1.192 (1.148 to 1.368)	1.212 (1.134 to 1.315)	1.247 (1.118 to 1.278)	1.119 (1.104 to 1.26)	1.191 (1.091 to 1.218)	1.142 (1.075 to 1.187)	1.079 (1.064 to 1.17)	1.057 (1.055 to 1.131)	1.076 (1.042 to 1.105)	1.056 (1.03 to 1.077)
Stroke	5 µg/m^3	Both	Both		1.243 (1.114 to 1.368)	1.149 (1.105 to 1.349)	1.18 (1.098 to 1.287)	1.25 (1.086 to 1.256)	1.107 (1.082 to 1.23)	1.096 (1.074 to 1.203)	1.108 (1.067 to 1.172)	1.133 (1.059 to 1.152)	1.061 (1.052 to 1.143)	1.101 (1.046 to 1.12)	1.073 (1.037 to 1.103)	1.039 (1.032 to 1.098)	1.029 (1.027 to 1.073)	1.041 (1.021 to 1.059)	1.03 (1.015 to 1.044)
Stroke	0 µg/m^3	Both	Both		1.0 (1.0 to 1.0)	1.0 (1.0 to 1.0)	1.0 (1.0 to 1.0)	1.0 (1.0 to 1.0)	1.0 (1.0 to 1.0)	1.0 (1.0 to 1.0)	1.0 (1.0 to 1.0)	1.0 (1.0 to 1.0)	1.0 (1.0 to 1.0)	1.0 (1.0 to 1.0)	1.0 (1.0 to 1.0)	1.0 (1.0 to 1.0)	1.0 (1.0 to 1.0)	1.0 (1.0 to 1.0)	1.0 (1.0 to 1.0)
Chronic obstructive pulmonary disease	600 µg/m^3	Both	Both	5.603 (4.362 to 6.101)															
Chronic obstructive pulmonary disease	500 µg/m^3	Both	Both	4.946 (3.857 to 5.306)															
Chronic obstructive pulmonary disease	400 µg/m^3	Both	Both	4.289 (3.325 to 4.523)															
Chronic obstructive pulmonary disease	300 µg/m^3	Both	Both	3.632 (2.798 to 3.72)															
Chronic obstructive pulmonary disease	200 µg/m^3	Both	Both	2.975 (2.257 to 3.017)															
Chronic obstructive pulmonary disease	150 µg/m^3	Both	Both	2.646 (1.975 to 2.68)															
Chronic obstructive pulmonary disease	130 µg/m^3	Both	Both	2.481 (1.861 to 2.505)															
Chronic obstructive pulmonary disease	110 µg/m^3	Both	Both	2.261 (1.743 to 2.298)															
Chronic obstructive pulmonary disease	90 µg/m^3	Both	Both	2.032 (1.619 to 2.092)															
Chronic obstructive pulmonary disease	70 µg/m^3	Both	Both	1.802 (1.493 to 1.884)															
Chronic obstructive pulmonary disease	50 µg/m^3	Both	Both	1.573 (1.358 to 1.659)															
Chronic obstructive pulmonary disease	30 µg/m^3	Both	Both	1.344 (1.22 to 1.416)															
Chronic obstructive pulmonary disease	25 µg/m^3	Both	Both	1.287 (1.184 to 1.354)															
Chronic obstructive pulmonary disease	20 µg/m^3	Both	Both	1.229 (1.147 to 1.288)															
Chronic obstructive pulmonary disease	15 µg/m^3	Both	Both	1.172 (1.111 to 1.223)															
Chronic obstructive pulmonary disease	10 µg/m^3	Both	Both	1.115 (1.074 to 1.153)															
Chronic obstructive pulmonary disease	5 µg/m^3	Both	Both	1.057 (1.037 to 1.079)															
Chronic obstructive pulmonary disease	0 µg/m^3	Both	Both	1.0 (1.0 to 1.0)															
Diabetes mellitus type 2	600 µg/m^3	Both	Both	1.577 (1.321 to 2.134)															
Diabetes mellitus type 2	500 µg/m^3	Both	Both	1.577 (1.321 to 2.016)															
Diabetes mellitus type 2	400 µg/m^3	Both	Both	1.577 (1.321 to 1.897)															
Diabetes mellitus type 2	300 µg/m^3	Both	Both	1.577 (1.321 to 1.788)															
Diabetes mellitus type 2	200 µg/m^3	Both	Both	1.577 (1.32 to 1.686)															
Diabetes mellitus type 2	150 µg/m^3	Both	Both	1.577 (1.314 to 1.633)															
Diabetes mellitus type 2	130 µg/m^3	Both	Both	1.577 (1.314 to 1.614)															
Diabetes mellitus type 2	110 µg/m^3	Both	Both	1.577 (1.314 to 1.604)															
Diabetes mellitus type 2	90 µg/m^3	Both	Both	1.577 (1.314 to 1.593)															
Diabetes mellitus type 2	70 µg/m^3	Both	Both	1.576 (1.313 to 1.579)															
Diabetes mellitus type 2	50 µg/m^3	Both	Both	1.57 (1.305 to 1.566)															
Diabetes mellitus type 2	30 µg/m^3	Both	Both	1.531 (1.262 to 1.53)															
Diabetes mellitus type 2	25 µg/m^3	Both	Both	1.499 (1.237 to 1.498)															
Diabetes mellitus type 2	20 µg/m^3	Both	Both	1.45 (1.206 to 1.448)															
Diabetes mellitus type 2	15 µg/m^3	Both	Both	1.38 (1.169 to 1.387)															
Diabetes mellitus type 2	10 µg/m^3	Both	Both	1.284 (1.121 to 1.302)															
Diabetes mellitus type 2	5 µg/m^3	Both	Both	1.159 (1.065 to 1.182)															
Diabetes mellitus type 2	0 µg/m^3	Both	Both	1.0 (1.0 to 1.0)															

Table S7C. Relative risks used by age and sex for each outcome for alcohol use globally.				
Risk - Outcome	Category / Units	Morbidity / Mortality	Sex	Age
				All ages
**Alcohol use**				
Tuberculosis	72 g/day	Both	Both	3.507 (2.596 to 4.474)
Tuberculosis	60 g/day	Both	Both	2.994 (1.972 to 4.204)
Tuberculosis	48 g/day	Both	Both	2.535 (1.701 to 3.51)
Tuberculosis	36 g/day	Both	Both	2.058 (1.485 to 2.795)
Tuberculosis	24 g/day	Both	Both	1.531 (1.165 to 1.98)
Tuberculosis	12 g/day	Both	Both	1.101 (0.815 to 1.425)
Tuberculosis	0 g/day	Both	Both	1.0 (1.0 to 1.0)
Lower respiratory infections	72 g/day	Both	Both	1.357 (1.113 to 1.648)
Lower respiratory infections	60 g/day	Both	Both	1.226 (1.036 to 1.423)
Lower respiratory infections	48 g/day	Both	Both	1.127 (0.936 to 1.327)
Lower respiratory infections	36 g/day	Both	Both	1.064 (0.928 to 1.219)
Lower respiratory infections	24 g/day	Both	Both	1.026 (0.901 to 1.167)
Lower respiratory infections	12 g/day	Both	Both	1.013 (0.951 to 1.084)
Lower respiratory infections	0 g/day	Both	Both	1.0 (1.0 to 1.0)
Oesophageal cancer	72 g/day	Both	Both	2.669 (2.074 to 3.348)
Oesophageal cancer	60 g/day	Both	Both	2.452 (1.905 to 3.094)
Oesophageal cancer	48 g/day	Both	Both	2.202 (1.73 to 2.703)
Oesophageal cancer	36 g/day	Both	Both	1.815 (1.468 to 2.222)
Oesophageal cancer	24 g/day	Both	Both	1.466 (1.209 to 1.764)
Oesophageal cancer	12 g/day	Both	Both	1.212 (1.031 to 1.439)

Oesophageal cancer	0 g/day	Both	Both	1.0 (1.0 to 1.0)
Liver cancer due to alcohol use	72 g/day	Both	Both	1.424 (1.088 to 1.855)
Liver cancer due to alcohol use	60 g/day	Both	Both	1.372 (1.093 to 1.692)
Liver cancer due to alcohol use	48 g/day	Both	Both	1.31 (1.036 to 1.639)
Liver cancer due to alcohol use	36 g/day	Both	Both	1.225 (1.009 to 1.455)
Liver cancer due to alcohol use	24 g/day	Both	Both	1.14 (0.934 to 1.359)
Liver cancer due to alcohol use	12 g/day	Both	Both	1.067 (0.936 to 1.207)
Liver cancer due to alcohol use	0 g/day	Both	Both	1.0 (1.0 to 1.0)
Larynx cancer	72 g/day	Both	Both	2.461 (1.758 to 3.228)
Larynx cancer	60 g/day	Both	Both	2.144 (1.46 to 2.935)
Larynx cancer	48 g/day	Both	Both	1.813 (1.3 to 2.421)
Larynx cancer	36 g/day	Both	Both	1.531 (1.126 to 2.061)
Larynx cancer	24 g/day	Both	Both	1.304 (1.006 to 1.659)
Larynx cancer	12 g/day	Both	Both	1.12 (0.903 to 1.386)
Larynx cancer	0 g/day	Both	Both	1.0 (1.0 to 1.0)
Breast cancer	72 g/day	Both	Both	1.476 (1.282 to 1.691)
Breast cancer	60 g/day	Both	Both	1.452 (1.312 to 1.599)
Breast cancer	48 g/day	Both	Both	1.443 (1.348 to 1.542)
Breast cancer	36 g/day	Both	Both	1.433 (1.311 to 1.551)
Breast cancer	24 g/day	Both	Both	1.329 (1.237 to 1.419)
Breast cancer	12 g/day	Both	Both	1.17 (1.081 to 1.265)
Breast cancer	0 g/day	Both	Both	1.0 (1.0 to 1.0)
Colon and rectum cancer	72 g/day	Both	Both	1.616 (1.38 to 1.861)

Colon and rectum cancer	60 g/day	Both	Both	1.468 (1.329 to 1.615)
Colon and rectum cancer	48 g/day	Both	Both	1.323 (1.156 to 1.501)
Colon and rectum cancer	36 g/day	Both	Both	1.237 (1.148 to 1.336)
Colon and rectum cancer	24 g/day	Both	Both	1.156 (1.067 to 1.248)
Colon and rectum cancer	12 g/day	Both	Both	1.078 (1.034 to 1.124)
Colon and rectum cancer	0 g/day	Both	Both	1.0 (1.0 to 1.0)
Lip and oral cavity cancer	72 g/day	Both	Both	4.858 (3.74 to 6.076)
Lip and oral cavity cancer	60 g/day	Both	Both	3.766 (2.839 to 4.9)
Lip and oral cavity cancer	48 g/day	Both	Both	2.991 (2.283 to 3.896)
Lip and oral cavity cancer	36 g/day	Both	Both	2.311 (1.757 to 2.929)
Lip and oral cavity cancer	24 g/day	Both	Both	1.738 (1.383 to 2.161)
Lip and oral cavity cancer	12 g/day	Both	Both	1.293 (1.076 to 1.551)
Lip and oral cavity cancer	0 g/day	Both	Both	1.0 (1.0 to 1.0)
Nasopharynx cancer	72 g/day	Both	Both	4.545 (4.1 to 4.982)
Nasopharynx cancer	60 g/day	Both	Both	3.803 (3.509 to 4.102)
Nasopharynx cancer	48 g/day	Both	Both	3.062 (2.873 to 3.258)
Nasopharynx cancer	36 g/day	Both	Both	2.385 (2.25 to 2.552)
Nasopharynx cancer	24 g/day	Both	Both	1.839 (1.77 to 1.907)
Nasopharynx cancer	12 g/day	Both	Both	1.371 (1.341 to 1.398)
Nasopharynx cancer	0 g/day	Both	Both	1.0 (1.0 to 1.0)
Other pharynx cancer	72 g/day	Both	Both	4.764 (3.315 to 6.576)
Other pharynx cancer	60 g/day	Both	Both	3.972 (2.813 to 5.354)
Other pharynx cancer	48 g/day	Both	Both	3.199 (2.202 to 4.407)

Other pharynx cancer	36 g/day	Both	Both	2.519 (1.843 to 3.299)
Other pharynx cancer	24 g/day	Both	Both	1.943 (1.467 to 2.484)
Other pharynx cancer	12 g/day	Both	Both	1.472 (1.234 to 1.742)
Other pharynx cancer	0 g/day	Both	Both	1.0 (1.0 to 1.0)
Ischaemic heart disease	72 g/day	Both	Male	1.091 (0.933 to 1.271)
Ischaemic heart disease	60 g/day	Both	Male	0.993 (0.883 to 1.105)
Ischaemic heart disease	48 g/day	Both	Male	0.906 (0.797 to 1.035)
Ischaemic heart disease	36 g/day	Both	Male	0.871 (0.788 to 0.964)
Ischaemic heart disease	24 g/day	Both	Male	0.857 (0.779 to 0.943)
Ischaemic heart disease	12 g/day	Both	Male	0.865 (0.79 to 0.948)
Ischaemic heart disease	0 g/day	Both	Male	1.0 (1.0 to 1.0)
Ischaemic heart disease	72 g/day	Both	Female	1.107 (0.894 to 1.341)
Ischaemic heart disease	60 g/day	Both	Female	1.012 (0.869 to 1.174)
Ischaemic heart disease	48 g/day	Both	Female	0.932 (0.786 to 1.113)
Ischaemic heart disease	36 g/day	Both	Female	0.882 (0.781 to 0.997)
Ischaemic heart disease	24 g/day	Both	Female	0.846 (0.749 to 0.948)
Ischaemic heart disease	12 g/day	Both	Female	0.823 (0.733 to 0.926)
Ischaemic heart disease	0 g/day	Both	Female	1.0 (1.0 to 1.0)
Ischaemic stroke	72 g/day	Both	Male	1.451 (1.228 to 1.69)
Ischaemic stroke	60 g/day	Both	Male	1.312 (1.167 to 1.471)
Ischaemic stroke	48 g/day	Both	Male	1.159 (0.98 to 1.353)
Ischaemic stroke	36 g/day	Both	Male	1.057 (0.931 to 1.192)
Ischaemic stroke	24 g/day	Both	Male	0.97 (0.862 to 1.088)

Ischaemic stroke	12 g/day	Both	Male	0.938 (0.83 to 1.054)
Ischaemic stroke	0 g/day	Both	Male	1.0 (1.0 to 1.0)
Ischaemic stroke	72 g/day	Both	Female	1.43 (1.147 to 1.771)
Ischaemic stroke	60 g/day	Both	Female	1.3 (1.121 to 1.496)
Ischaemic stroke	48 g/day	Both	Female	1.145 (0.946 to 1.359)
Ischaemic stroke	36 g/day	Both	Female	0.985 (0.834 to 1.149)
Ischaemic stroke	24 g/day	Both	Female	0.85 (0.726 to 0.985)
Ischaemic stroke	12 g/day	Both	Female	0.824 (0.718 to 0.939)
Ischaemic stroke	0 g/day	Both	Female	1.0 (1.0 to 1.0)
Intracerebral hemorrhage	72 g/day	Both	Male	1.971 (1.663 to 2.316)
Intracerebral hemorrhage	60 g/day	Both	Male	1.705 (1.45 to 1.991)
Intracerebral hemorrhage	48 g/day	Both	Male	1.458 (1.182 to 1.768)
Intracerebral hemorrhage	36 g/day	Both	Male	1.31 (1.105 to 1.539)
Intracerebral hemorrhage	24 g/day	Both	Male	1.162 (0.973 to 1.385)
Intracerebral hemorrhage	12 g/day	Both	Male	1.068 (0.945 to 1.214)
Intracerebral hemorrhage	0 g/day	Both	Male	1.0 (1.0 to 1.0)
Intracerebral hemorrhage	72 g/day	Both	Female	2.276 (1.701 to 2.934)
Intracerebral hemorrhage	60 g/day	Both	Female	1.964 (1.536 to 2.464)
Intracerebral hemorrhage	48 g/day	Both	Female	1.614 (1.245 to 2.048)
Intracerebral hemorrhage	36 g/day	Both	Female	1.337 (1.065 to 1.664)
Intracerebral hemorrhage	24 g/day	Both	Female	1.11 (0.884 to 1.367)
Intracerebral hemorrhage	12 g/day	Both	Female	1.031 (0.897 to 1.18)
Intracerebral hemorrhage	0 g/day	Both	Female	1.0 (1.0 to 1.0)

Hypertensive heart disease	72 g/day	Both	Both	1.86 (1.445 to 2.358)
Hypertensive heart disease	60 g/day	Both	Both	1.705 (1.297 to 2.175)
Hypertensive heart disease	48 g/day	Both	Both	1.614 (1.25 to 2.049)
Hypertensive heart disease	36 g/day	Both	Both	1.479 (1.232 to 1.759)
Hypertensive heart disease	24 g/day	Both	Both	1.315 (1.136 to 1.526)
Hypertensive heart disease	12 g/day	Both	Both	1.046 (0.913 to 1.198)
Hypertensive heart disease	0 g/day	Both	Both	1.0 (1.0 to 1.0)
Atrial fibrillation and flutter	72 g/day	Both	Both	1.535 (1.348 to 1.728)
Atrial fibrillation and flutter	60 g/day	Both	Both	1.411 (1.26 to 1.569)
Atrial fibrillation and flutter	48 g/day	Both	Both	1.312 (1.218 to 1.407)
Atrial fibrillation and flutter	36 g/day	Both	Both	1.214 (1.145 to 1.29)
Atrial fibrillation and flutter	24 g/day	Both	Both	1.131 (1.067 to 1.204)
Atrial fibrillation and flutter	12 g/day	Both	Both	1.066 (1.034 to 1.102)
Atrial fibrillation and flutter	0 g/day	Both	Both	1.0 (1.0 to 1.0)
Cirrhosis and other chronic liver diseases due to alcohol use	72 g/day	Both	Both	9.427 (6.131 to 13.804)
Cirrhosis and other chronic liver diseases due to alcohol use	60 g/day	Both	Both	6.274 (3.958 to 9.319)
Cirrhosis and other chronic liver diseases due to alcohol use	48 g/day	Both	Both	4.673 (3.25 to 6.717)
Cirrhosis and other chronic liver diseases due to alcohol use	36 g/day	Both	Both	3.274 (2.309 to 4.485)
Cirrhosis and other chronic liver diseases due to alcohol use	24 g/day	Both	Both	2.055 (1.521 to 2.688)
Cirrhosis and other chronic liver diseases due to alcohol use	12 g/day	Both	Both	1.243 (0.943 to 1.611)
Cirrhosis and other chronic liver diseases due to alcohol use	0 g/day	Both	Both	1.0 (1.0 to 1.0)
Pancreatitis	72 g/day	Both	Both	3.298 (2.473 to 4.458)
Pancreatitis	60 g/day	Both	Both	2.217 (1.415 to 3.389)

Pancreatitis	48 g/day	Both	Both	1.717 (1.199 to 2.477)
Pancreatitis	36 g/day	Both	Both	1.471 (1.062 to 2.021)
Pancreatitis	24 g/day	Both	Both	1.228 (0.874 to 1.67)
Pancreatitis	12 g/day	Both	Both	1.073 (0.791 to 1.481)
Pancreatitis	0 g/day	Both	Both	1.0 (1.0 to 1.0)
Epilepsy	72 g/day	Both	Both	2.48 (1.929 to 3.144)
Epilepsy	60 g/day	Both	Both	2.186 (1.781 to 2.622)
Epilepsy	48 g/day	Both	Both	1.872 (1.438 to 2.369)
Epilepsy	36 g/day	Both	Both	1.585 (1.303 to 1.898)
Epilepsy	24 g/day	Both	Both	1.353 (1.118 to 1.633)
Epilepsy	12 g/day	Both	Both	1.177 (1.059 to 1.316)
Epilepsy	0 g/day	Both	Both	1.0 (1.0 to 1.0)
Diabetes mellitus	72 g/day	Both	Male	1.198 (1.065 to 1.337)
Diabetes mellitus	60 g/day	Both	Male	1.165 (0.998 to 1.342)
Diabetes mellitus	48 g/day	Both	Male	1.084 (0.933 to 1.239)
Diabetes mellitus	36 g/day	Both	Male	1.0 (0.891 to 1.119)
Diabetes mellitus	24 g/day	Both	Male	0.932 (0.841 to 1.03)
Diabetes mellitus	12 g/day	Both	Male	0.921 (0.833 to 1.015)
Diabetes mellitus	0 g/day	Both	Male	1.0 (1.0 to 1.0)
Diabetes mellitus	72 g/day	Both	Female	1.172 (0.81 to 1.652)
Diabetes mellitus	60 g/day	Both	Female	1.074 (0.765 to 1.443)
Diabetes mellitus	48 g/day	Both	Female	0.945 (0.737 to 1.173)
Diabetes mellitus	36 g/day	Both	Female	0.836 (0.702 to 0.981)

Diabetes mellitus	24 g/day	Both	Female	0.76 (0.66 to 0.872)
Diabetes mellitus	12 g/day	Both	Female	0.733 (0.658 to 0.826)
Diabetes mellitus	0 g/day	Both	Female	1.0 (1.0 to 1.0)
Transport injuries	72 g/day	Both	Both	1.552 (1.201 to 2.032)
Transport injuries	60 g/day	Both	Both	1.456 (1.186 to 1.818)
Transport injuries	48 g/day	Both	Both	1.366 (1.101 to 1.692)
Transport injuries	36 g/day	Both	Both	1.288 (1.089 to 1.534)
Transport injuries	24 g/day	Both	Both	1.22 (1.062 to 1.4)
Transport injuries	12 g/day	Both	Both	1.163 (1.021 to 1.346)
Transport injuries	0 g/day	Both	Both	1.0 (1.0 to 1.0)
Unintentional injuries	72 g/day	Both	Both	1.266 (1.063 to 1.555)
Unintentional injuries	60 g/day	Both	Both	1.221 (1.059 to 1.46)
Unintentional injuries	48 g/day	Both	Both	1.182 (1.024 to 1.428)
Unintentional injuries	36 g/day	Both	Both	1.168 (1.054 to 1.347)
Unintentional injuries	24 g/day	Both	Both	1.154 (1.046 to 1.319)
Unintentional injuries	12 g/day	Both	Both	1.09 (1.016 to 1.187)
Unintentional injuries	0 g/day	Both	Both	1.0 (1.0 to 1.0)
Self-harm	72 g/day	Both	Both	1.927 (1.398 to 2.665)
Self-harm	60 g/day	Both	Both	1.734 (1.29 to 2.308)
Self-harm	48 g/day	Both	Both	1.545 (1.132 to 2.048)
Self-harm	36 g/day	Both	Both	1.376 (1.05 to 1.751)
Self-harm	24 g/day	Both	Both	1.23 (0.972 to 1.533)
Self-harm	12 g/day	Both	Both	1.107 (0.908 to 1.343)

Self-harm	0 g/day	Both	Both	1.0 (1.0 to 1.0)
Interpersonal violence	72 g/day	Both	Both	1.516 (1.255 to 1.867)
Interpersonal violence	60 g/day	Both	Both	1.452 (1.215 to 1.719)
Interpersonal violence	48 g/day	Both	Both	1.396 (1.118 to 1.739)
Interpersonal violence	36 g/day	Both	Both	1.345 (1.14 to 1.585)
Interpersonal violence	24 g/day	Both	Both	1.256 (1.055 to 1.46)
Interpersonal violence	12 g/day	Both	Both	1.129 (0.963 to 1.317)
Interpersonal violence	0 g/day	Both	Both	1.0 (1.0 to 1.0)

Table S7D. Relative risks used by age and sex for each outcome for smoking globally.																					
				Age																	
Risk - Outcome	Category / Units	Morbidity / Mortality	Sex	All ages	15-19 years	20-24 years	25-29 years	30-34 years	35-39 years	40-44 years	45-49 years	50-54 years	55-59 years	60-64 years	65-69 years	70-74 years	75-79 years	80-84 years	85-89 years	90-94 years	95+ years
Smoking																					
Ischaemic heart disease	0 Cigarettes Per Day	Both	Female					1.00 (1.00 to 1.00)	1.00 (1.00 to 1.00)	1.00 (1.00 to 1.00)	1.00 (1.00 to 1.00)	1.00 (1.00 to 1.00)	1.00 (1.00 to 1.00)	1.00 (1.00 to 1.00)	1.00 (1.00 to 1.00)	1.00 (1.00 to 1.00)	1.00 (1.00 to 1.00)	1.00 (1.00 to 1.00)	1.00 (1.00 to 1.00)	1.00 (1.00 to 1.00)	1.00 (1.00 to 1.00)
Ischaemic heart disease	0 Cigarettes Per Day	Both	Male					1.00 (1.00 to 1.00)	1.00 (1.00 to 1.00)	1.00 (1.00 to 1.00)	1.00 (1.00 to 1.00)	1.00 (1.00 to 1.00)	1.00 (1.00 to 1.00)	1.00 (1.00 to 1.00)	1.00 (1.00 to 1.00)	1.00 (1.00 to 1.00)	1.00 (1.00 to 1.00)	1.00 (1.00 to 1.00)	1.00 (1.00 to 1.00)	1.00 (1.00 to 1.00)	1.00 (1.00 to 1.00)
Ischaemic heart disease	10 Cigarettes Per Day	Both	Female					3.48 (2.84 to 4.21)	3.48 (2.84 to 4.21)	3.48 (2.84 to 4.21)	2.95 (2.51 to 3.42)	2.95 (2.51 to 3.42)	2.44 (2.12 to 2.73)	2.44 (2.12 to 2.73)	2.01 (1.83 to 2.18)	2.01 (1.83 to 2.18)	1.61 (1.51 to 1.70)	1.61 (1.51 to 1.70)	1.25 (1.21 to 1.29)	1.25 (1.21 to 1.29)	1.00 (0.99 to 1.01)
Ischaemic heart disease	10 Cigarettes Per Day	Both	Male					2.75 (2.48 to 3.06)	2.75 (2.48 to 3.06)	2.75 (2.48 to 3.06)	2.36 (2.13 to 2.58)	2.36 (2.13 to 2.58)	2.00 (1.84 to 2.17)	2.00 (1.84 to 2.17)	1.69 (1.59 to 1.79)	1.69 (1.59 to 1.79)	1.41 (1.35 to 1.48)	1.41 (1.35 to 1.48)	1.17 (1.12 to 1.21)	1.17 (1.12 to 1.21)	1.00 (0.98 to 1.01)
Ischaemic heart disease	20 Cigarettes Per Day	Both	Female					6.04 (4.83 to 7.32)	6.04 (4.83 to 7.32)	6.04 (4.83 to 7.32)	4.53 (3.78 to 5.30)	4.53 (3.78 to 5.30)	3.36 (2.90 to 3.81)	3.36 (2.90 to 3.81)	2.47 (2.23 to 2.72)	2.47 (2.23 to 2.72)	1.81 (1.69 to 1.93)	1.81 (1.69 to 1.93)	1.32 (1.27 to 1.37)	1.32 (1.27 to 1.37)	1.00 (0.99 to 1.01)
Ischaemic heart disease	20 Cigarettes Per Day	Both	Male					3.22 (2.82 to 3.65)	3.22 (2.82 to 3.65)	3.22 (2.82 to 3.65)	2.64 (2.35 to 2.93)	2.64 (2.35 to 2.93)	2.17 (1.98 to 2.38)	2.17 (1.98 to 2.38)	1.78 (1.65 to 1.92)	1.78 (1.65 to 1.92)	1.46 (1.38 to 1.54)	1.46 (1.38 to 1.54)	1.19 (1.15 to 1.23)	1.19 (1.15 to 1.23)	1.00 (0.99 to 1.01)
Ischaemic heart disease	30 Cigarettes Per Day	Both	Female					8.72 (6.66 to 11.01)	8.72 (6.66 to 11.01)	8.72 (6.66 to 11.01)	6.17 (4.98 to 7.51)	6.17 (4.98 to 7.51)	4.31 (3.61 to 5.08)	4.31 (3.61 to 5.08)	3.02 (2.65 to 3.40)	3.02 (2.65 to 3.40)	2.07 (1.89 to 2.24)	2.07 (1.89 to 2.24)	1.41 (1.35 to 1.47)	1.41 (1.35 to 1.47)	1.00 (0.99 to 1.01)
Ischaemic heart disease	30 Cigarettes Per Day	Both	Male					3.94 (3.32 to 4.55)	3.94 (3.32 to 4.55)	3.94 (3.32 to 4.55)	3.15 (2.73 to 3.58)	3.15 (2.73 to 3.58)	2.48 (2.21 to 2.77)	2.48 (2.21 to 2.77)	1.96 (1.79 to 2.15)	1.96 (1.79 to 2.15)	1.55 (1.45 to 1.65)	1.55 (1.45 to 1.65)	1.22 (1.17 to 1.27)	1.22 (1.17 to 1.27)	1.00 (0.99 to 1.01)
Ischaemic heart disease	40 Cigarettes Per Day	Both	Female					10.87 (7.56 to 14.89)	10.87 (7.56 to 14.89)	10.87 (7.56 to 14.89)	7.31 (5.30 to 9.71)	7.31 (5.30 to 9.71)	4.91 (3.81 to 6.22)	4.91 (3.81 to 6.22)	3.34 (2.71 to 4.05)	3.34 (2.71 to 4.05)	2.21 (1.90 to 2.55)	2.21 (1.90 to 2.55)	1.44 (1.32 to 1.57)	1.44 (1.32 to 1.57)	1.00 (0.98 to 1.02)
Ischaemic heart disease	40 Cigarettes Per Day	Both	Male					4.93 (3.79 to 6.13)	4.93 (3.79 to 6.13)	4.93 (3.79 to 6.13)	3.80 (3.03 to 4.66)	3.80 (3.03 to 4.66)	2.88 (2.37 to 3.42)	2.88 (2.37 to 3.42)	2.20 (1.85 to 2.56)	2.20 (1.85 to 2.56)	1.67 (1.47 to 1.89)	1.67 (1.47 to 1.89)	1.27 (1.17 to 1.39)	1.27 (1.17 to 1.39)	1.00 (0.98 to 1.02)
Ischaemic heart disease	50 Cigarettes Per Day	Both	Female					12.61 (7.91 to 18.62)	12.61 (7.91 to 18.62)	12.61 (7.91 to 18.62)	8.21 (5.28 to 11.77)	8.21 (5.28 to 11.77)	5.33 (3.75 to 7.26)	5.33 (3.75 to 7.26)	3.54 (2.66 to 4.54)	3.54 (2.66 to 4.54)	2.31 (1.86 to 2.80)	2.31 (1.86 to 2.80)	1.50 (1.31 to 1.70)	1.50 (1.31 to 1.70)	1.00 (0.96 to 1.05)
Ischaemic heart disease	50 Cigarettes Per Day	Both	Male					5.51 (3.98 to 7.24)	5.51 (3.98 to 7.24)	5.51 (3.98 to 7.24)	4.16 (3.10 to 5.35)	4.16 (3.10 to 5.35)	3.05 (2.38 to 3.80)	3.05 (2.38 to 3.80)	2.28 (1.80 to 2.79)	2.28 (1.80 to 2.79)	1.70 (1.40 to 2.00)	1.70 (1.40 to 2.00)	1.27 (1.11 to 1.44)	1.27 (1.11 to 1.44)	1.00 (0.96 to 1.04)
Ischaemic heart disease	60 Cigarettes Per Day	Both	Female					14.20 (7.75 to 23.21)	14.20 (7.75 to 23.21)	14.20 (7.75 to 23.21)	9.21 (5.12 to 14.41)	9.21 (5.12 to 14.41)	5.79 (3.56 to 8.68)	5.79 (3.56 to 8.68)	3.76 (2.53 to 5.24)	3.76 (2.53 to 5.24)	2.38 (1.74 to 3.12)	2.38 (1.74 to 3.12)	1.50 (1.22 to 1.83)	1.50 (1.22 to 1.83)	1.00 (0.93 to 1.08)
Ischaemic heart disease	60 Cigarettes Per Day	Both	Male					5.83 (3.83 to 8.34)	5.83 (3.83 to 8.34)	5.83 (3.83 to 8.34)	4.35 (2.92 to 6.04)	4.35 (2.92 to 6.04)	3.16 (2.22 to 4.33)	3.16 (2.22 to 4.33)	2.32 (1.67 to 3.11)	2.32 (1.67 to 3.11)	1.73 (1.28 to 2.21)	1.73 (1.28 to 2.21)	1.29 (1.03 to 1.57)	1.29 (1.03 to 1.57)	1.00 (0.94 to 1.06)
Stroke	0 Cigarettes Per Day	Both	Female					1.00 (1.00 to 1.00)	1.00 (1.00 to 1.00)	1.00 (1.00 to 1.00)	1.00 (1.00 to 1.00)	1.00 (1.00 to 1.00)	1.00 (1.00 to 1.00)	1.00 (1.00 to 1.00)	1.00 (1.00 to 1.00)	1.00 (1.00 to 1.00)	1.00 (1.00 to 1.00)	1.00 (1.00 to 1.00)	1.00 (1.00 to 1.00)	1.00 (1.00 to 1.00)	1.00 (1.00 to 1.00)
Stroke	0 Cigarettes Per Day	Both	Male					1.00 (1.00 to 1.00)	1.00 (1.00 to 1.00)	1.00 (1.00 to 1.00)	1.00 (1.00 to 1.00)	1.00 (1.00 to 1.00)	1.00 (1.00 to 1.00)	1.00 (1.00 to 1.00)	1.00 (1.00 to 1.00)	1.00 (1.00 to 1.00)	1.00 (1.00 to 1.00)	1.00 (1.00 to 1.00)	1.00 (1.00 to 1.00)	1.00 (1.00 to 1.00)	1.00 (1.00 to 1.00)
Stroke	10 Cigarettes Per Day	Both	Female					2.33 (1.76 to 2.99)	2.33 (1.76 to 2.99)	2.33 (1.76 to 2.99)	2.10 (1.66 to 2.60)	2.10 (1.66 to 2.60)	1.92 (1.56 to 2.25)	1.92 (1.56 to 2.25)	1.72 (1.48 to 1.96)	1.72 (1.48 to 1.96)	1.48 (1.34 to 1.62)	1.48 (1.34 to 1.62)	1.20 (1.13 to 1.29)	1.20 (1.13 to 1.29)	1.00 (0.99 to 1.02)
Stroke	10 Cigarettes Per Day	Both	Male					2.36 (2.04 to 2.69)	2.36 (2.04 to 2.69)	2.36 (2.04 to 2.69)	2.11 (1.88 to 2.36)	2.11 (1.88 to 2.36)	1.84 (1.67 to 2.03)	1.84 (1.67 to 2.03)	1.61 (1.48 to 1.73)	1.61 (1.48 to 1.73)	1.39 (1.31 to 1.47)	1.39 (1.31 to 1.47)	1.15 (1.11 to 1.20)	1.15 (1.11 to 1.20)	1.01 (0.98 to 1.04)
Stroke	20 Cigarettes Per Day	Both	Female					3.74 (2.81 to 4.72)	3.74 (2.81 to 4.72)	3.74 (2.81 to 4.72)	3.04 (2.36 to 3.77)	3.04 (2.36 to 3.77)	2.44 (1.99 to 2.96)	2.44 (1.99 to 2.96)	1.94 (1.66 to 2.22)	1.94 (1.66 to 2.22)	1.53 (1.36 to 1.69)	1.53 (1.36 to 1.69)	1.19 (1.14 to 1.26)	1.19 (1.14 to 1.26)	1.00 (0.99 to 1.01)
Stroke	20 Cigarettes Per Day	Both	Male					2.32 (1.97 to 2.67)	2.32 (1.97 to 2.67)	2.32 (1.97 to 2.67)	1.97 (1.74 to 2.22)	1.97 (1.74 to 2.22)	1.66 (1.48 to 1.85)	1.66 (1.48 to 1.85)	1.42 (1.30 to 1.54)	1.42 (1.30 to 1.54)	1.22 (1.15 to 1.29)	1.22 (1.15 to 1.29)	1.08 (1.05 to 1.12)	1.08 (1.05 to 1.12)	1.00 (0.99 to 1.01)
Stroke	30 Cigarettes Per Day	Both	Female					5.49 (3.96 to 7.21)	5.49 (3.96 to 7.21)	5.49 (3.96 to 7.21)	4.21 (3.15 to 5.36)	4.21 (3.15 to 5.36)	3.14 (2.43 to 3.89)	3.14 (2.43 to 3.89)	2.30 (1.92 to 2.73)	2.30 (1.92 to 2.73)	1.72 (1.51 to 1.95)	1.72 (1.51 to 1.95)	1.29 (1.19 to 1.40)	1.29 (1.19 to 1.40)	1.00 (0.99 to 1.02)
Stroke	30 Cigarettes Per Day	Both	Male					3.18 (2.73 to 3.68)	3.18 (2.73 to 3.68)	3.18 (2.73 to 3.68)	2.67 (2.32 to 3.03)	2.67 (2.32 to 3.03)	2.22 (2.00 to 2.48)	2.22 (2.00 to 2.48)	1.85 (1.69 to 2.01)	1.85 (1.69 to 2.01)	1.52 (1.41 to 1.62)	1.52 (1.41 to 1.62)	1.21 (1.16 to 1.25)	1.21 (1.16 to 1.25)	1.01 (0.99 to 1.03)
Stroke	40 Cigarettes Per Day	Both	Female					7.13 (4.57 to 10.74)	7.13 (4.57 to 10.74)	7.13 (4.57 to 10.74)	5.10 (3.29 to 7.14)	5.10 (3.29 to 7.14)	3.64 (2.46 to 5.05)	3.64 (2.46 to 5.05)	2.59 (1.92 to 3.41)	2.59 (1.92 to 3.41)	1.78 (1.38 to 2.27)	1.78 (1.38 to 2.27)	1.21 (1.03 to 1.39)	1.21 (1.03 to 1.39)	1.00 (0.97 to 1.03)
Stroke	40 Cigarettes Per Day	Both	Male					3.43 (2.57 to 4.38)	3.43 (2.57 to 4.38)	3.43 (2.57 to 4.38)	2.73 (2.12 to 3.44)	2.73 (2.12 to 3.44)	2.12 (1.67 to 2.63)	2.12 (1.67 to 2.63)	1.66 (1.37 to 1.98)	1.66 (1.37 to 1.98)	1.34 (1.15 to 1.54)	1.34 (1.15 to 1.54)	1.12 (1.03 to 1.20)	1.12 (1.03 to 1.20)	1.01 (0.98 to 1.03)
Stroke	50 Cigarettes Per Day	Both	Female					9.85 (5.51 to 15.72)	9.85 (5.51 to 15.72)	9.85 (5.51 to 15.72)	6.92 (4.25 to 10.66)	6.92 (4.25 to 10.66)	5.00 (3.30 to 7.32)	5.00 (3.30 to 7.32)	3.57 (2.56 to 4.72)	3.57 (2.56 to 4.72)	2.35 (1.80 to 2.98)	2.35 (1.80 to 2.98)	1.45 (1.17 to 1.74)	1.45 (1.17 to 1.74)	1.01 (0.91 to 1.10)
Stroke	50 Cigarettes Per Day	Both	Male					4.46 (3.16 to 6.00)	4.46 (3.16 to 6.00)	4.46 (3.16 to 6.00)	3.49 (2.57 to 4.47)	3.49 (2.57 to 4.47)	2.66 (2.02 to 3.39)	2.66 (2.02 to 3.39)	2.05 (1.60 to 2.51)	2.05 (1.60 to 2.51)	1.58 (1.25 to 1.90)	1.58 (1.25 to 1.90)	1.21 (0.99 to 1.42)	1.21 (0.99 to 1.42)	0.98 (0.82 to 1.12)
Stroke	60 Cigarettes Per Day	Both	Female					11.72 (5.48 to 20.88)	11.72 (5.48 to 20.88)	11.72 (5.48 to 20.88)	8.28 (4.34 to 14.54)	8.28 (4.34 to 14.54)	5.94 (3.33 to 9.73)	5.94 (3.33 to 9.73)	4.29 (2.55 to 6.48)	4.29 (2.55 to 6.48)	2.78 (1.75 to 4.02)	2.78 (1.75 to 4.02)	1.69 (1.15 to 2.26)	1.69 (1.15 to 2.26)	1.00 (0.82 to 1.20)
Stroke	60 Cigarettes Per Day	Both	Male					5.32 (3.05 to 8.01)	5.32 (3.05 to 8.01)	5.32 (3.05 to 8.01)	4.16 (2.54 to 6.17)	4.16 (2.54 to 6.17)	3.17 (1.98 to 4.55)	3.17 (1.98 to 4.55)	2.40 (1.56 to 3.34)	2.40 (1.56 to 3.34)	1.84 (1.25 to 2.54)	1.84 (1.25 to 2.54)	1.38 (0.99 to 1.80)	1.38 (0.99 to 1.80)	1.05 (0.78 to 1.33)
Lower respiratory infections	0 Cigarettes Per Day	Both	Female					1.00 (1.00 to 1.00)	1.00 (1.00 to 1.00)	1.00 (1.00 to 1.00)	1.00 (1.00 to 1.00)	1.00 (1.00 to 1.00)	1.00 (1.00 to 1.00)	1.00 (1.00 to 1.00)	1.00 (1.00 to 1.00)	1.00 (1.00 to 1.00)	1.00 (1.00 to 1.00)	1.00 (1.00 to 1.00)	1.00 (1.00 to 1.00)	1.00 (1.00 to 1.00)	1.00 (1.00 to 1.00)
Lower respiratory infections	0 Cigarettes Per Day	Both	Male					1.00 (1.00 to 1.00)	1.00 (1.00 to 1.00)	1.00 (1.00 to 1.00)	1.00 (1.00 to 1.00)	1.00 (1.00 to 1.00)	1.00 (1.00 to 1.00)	1.00 (1.00 to 1.00)	1.00 (1.00 to 1.00)	1.00 (1.00 to 1.00)	1.00 (1.00 to 1.00)	1.00 (1.00 to 1.00)	1.00 (1.00 to 1.00)	1.00 (1.00 to 1.00)	1.00 (1.00 to 1.00)
Lower respiratory infections	10 Cigarettes Per Day	Both	Female					1.96 (1.42 to 2.58)	1.96 (1.42 to 2.58)	1.96 (1.42 to 2.58)	1.96 (1.42 to 2.58)	1.96 (1.42 to 2.58)	1.96 (1.42 to 2.58)	1.96 (1.42 to 2.58)	1.96 (1.42 to 2.58)	1.96 (1.42 to 2.58)	1.96 (1.42 to 2.58)	1.96 (1.42 to 2.58)	1.96 (1.42 to 2.58)	1.96 (1.42 to 2.58)	1.96 (1.42 to 2.58)
Lower respiratory infections	10 Cigarettes Per Day	Both	Male					1.96 (1.42 to 2.58)	1.96 (1.42 to 2.58)	1.96 (1.42 to 2.58)	1.96 (1.42 to 2.58)	1.96 (1.42 to 2.58)	1.96 (1.42 to 2.58)	1.96 (1.42 to 2.58)	1.96 (1.42 to 2.58)	1.96 (1.42 to 2.58)	1.96 (1.42 to 2.58)	1.96 (1.42 to 2.58)	1.96 (1.42 to 2.58)	1.96 (1.42 to 2.58)	1.96 (1.42 to 2.58)
Lower respiratory infections	20 Cigarettes Per Day	Both	Female					2.47 (1.85 to 3.20)	2.47 (1.85 to 3.20)	2.47 (1.85 to 3.20)	2.47 (1.85 to 3.20)	2.47 (1.85 to 3.20)	2.47 (1.85 to 3.20)	2.47 (1.85 to 3.20)	2.47 (1.85 to 3.20)	2.47 (1.85 to 3.20)	2.47 (1.85 to 3.20)	2.47 (1.85 to 3.20)	2.47 (1.85 to 3.20)	2.47 (1.85 to 3.20)	2.47 (1.85 to 3.20)
Lower respiratory infections	20 Cigarettes Per Day	Both	Male					2.47 (1.85 to 3.20)	2.47 (1.85 to 3.20)	2.47 (1.85 to 3.20)	2.47 (1.85 to 3.20)	2.47 (1.85 to 3.20)	2.47 (1.85 to 3.20)	2.47 (1.85 to 3.20)	2.47 (1.85 to 3.20)	2.47 (1.85 to 3.20)	2.47 (1.85 to 3.20)	2.47 (1.85 to 3.20)	2.47 (1.85 to 3.20)	2.47 (1.85 to 3.20)	2.47 (1.85 to 3.20)
Lower respiratory infections	30 Cigarettes Per Day	Both	Female					3.08 (2.37 to 3.84)	3.08 (2.37 to 3.84)	3.08 (2.37 to 3.84)	3.08 (2.37 to 3.84)	3.08 (2.37 to 3.84)	3.08 (2.37 to 3.84)	3.08 (2.37 to 3.84)	3.08 (2.37 to 3.84)	3.08 (2.37 to 3.84)	3.08 (2.37 to 3.84)	3.08 (2.37 to 3.84)	3.08 (2.37 to 3.84)	3.08 (2.37 to 3.84)	3.08 (2.37 to 3.84)
Lower respiratory infections	30 Cigarettes Per Day	Both	Male					3.08 (2.37 to 3.84)	3.08 (2.37 to 3.84)	3.08 (2.37 to 3.84)	3.08 (2.37 to 3.84)	3.08 (2.37 to 3.84)	3.08 (2.37 to 3.84)	3.08 (2.37 to 3.84)	3.08 (2.37 to 3.84)	3.08 (2.37 to 3.84)	3.08 (2.37 to 3.84)	3.08 (2.37 to 3.84)	3.08 (2.37 to 3.84)	3.08 (2.37 to 3.84)	3.08 (2.37 to 3.84)

Lower respiratory infections	40 Cigarettes Per Day	Both	Female	3.15 (2.00 to 4.63)	3.15 (2.00 to 4.63)	3.15 (2.00 to 4.63)	3.15 (2.00 to 4.63)	3.15 (2.00 to 4.63)	3.15 (2.00 to 4.63)	3.15 (2.00 to 4.63)	3.15 (2.00 to 4.63)	3.15 (2.00 to 4.63)	3.15 (2.00 to 4.63)	3.15 (2.00 to 4.63)	3.15 (2.00 to 4.63)	3.15 (2.00 to 4.63)	3.15 (2.00 to 4.63)
Lower respiratory infections	40 Cigarettes Per Day	Both	Male	3.15 (2.00 to 4.63)	3.15 (2.00 to 4.63)	3.15 (2.00 to 4.63)	3.15 (2.00 to 4.63)	3.15 (2.00 to 4.63)	3.15 (2.00 to 4.63)	3.15 (2.00 to 4.63)	3.15 (2.00 to 4.63)	3.15 (2.00 to 4.63)	3.15 (2.00 to 4.63)	3.15 (2.00 to 4.63)	3.15 (2.00 to 4.63)	3.15 (2.00 to 4.63)	3.15 (2.00 to 4.63)
Lower respiratory infections	50 Cigarettes Per Day	Both	Female	3.14 (1.50 to 5.45)	3.14 (1.50 to 5.45)	3.14 (1.50 to 5.45)	3.14 (1.50 to 5.45)	3.14 (1.50 to 5.45)	3.14 (1.50 to 5.45)	3.14 (1.50 to 5.45)	3.14 (1.50 to 5.45)	3.14 (1.50 to 5.45)	3.14 (1.50 to 5.45)	3.14 (1.50 to 5.45)	3.14 (1.50 to 5.45)	3.14 (1.50 to 5.45)	3.14 (1.50 to 5.45)
Lower respiratory infections	50 Cigarettes Per Day	Both	Male	3.14 (1.50 to 5.45)	3.14 (1.50 to 5.45)	3.14 (1.50 to 5.45)	3.14 (1.50 to 5.45)	3.14 (1.50 to 5.45)	3.14 (1.50 to 5.45)	3.14 (1.50 to 5.45)	3.14 (1.50 to 5.45)	3.14 (1.50 to 5.45)	3.14 (1.50 to 5.45)	3.14 (1.50 to 5.45)	3.14 (1.50 to 5.45)	3.14 (1.50 to 5.45)	3.14 (1.50 to 5.45)
Tuberculosis	0 Cigarettes Per Day	Both	Female	1.00 (1.00 to 1.00)	1.00 (1.00 to 1.00)	1.00 (1.00 to 1.00)	1.00 (1.00 to 1.00)	1.00 (1.00 to 1.00)	1.00 (1.00 to 1.00)	1.00 (1.00 to 1.00)	1.00 (1.00 to 1.00)	1.00 (1.00 to 1.00)	1.00 (1.00 to 1.00)	1.00 (1.00 to 1.00)	1.00 (1.00 to 1.00)	1.00 (1.00 to 1.00)	1.00 (1.00 to 1.00)
Tuberculosis	0 Cigarettes Per Day	Both	Male	1.00 (1.00 to 1.00)	1.00 (1.00 to 1.00)	1.00 (1.00 to 1.00)	1.00 (1.00 to 1.00)	1.00 (1.00 to 1.00)	1.00 (1.00 to 1.00)	1.00 (1.00 to 1.00)	1.00 (1.00 to 1.00)	1.00 (1.00 to 1.00)	1.00 (1.00 to 1.00)	1.00 (1.00 to 1.00)	1.00 (1.00 to 1.00)	1.00 (1.00 to 1.00)	1.00 (1.00 to 1.00)
Tuberculosis	10 Cigarettes Per Day	Both	Female	2.10 (1.69 to 2.52)	2.10 (1.69 to 2.52)	2.10 (1.69 to 2.52)	2.10 (1.69 to 2.52)	2.10 (1.69 to 2.52)	2.10 (1.69 to 2.52)	2.10 (1.69 to 2.52)	2.10 (1.69 to 2.52)	2.10 (1.69 to 2.52)	2.10 (1.69 to 2.52)	2.10 (1.69 to 2.52)	2.10 (1.69 to 2.52)	2.10 (1.69 to 2.52)	2.10 (1.69 to 2.52)
Tuberculosis	10 Cigarettes Per Day	Both	Male	2.10 (1.69 to 2.52)	2.10 (1.69 to 2.52)	2.10 (1.69 to 2.52)	2.10 (1.69 to 2.52)	2.10 (1.69 to 2.52)	2.10 (1.69 to 2.52)	2.10 (1.69 to 2.52)	2.10 (1.69 to 2.52)	2.10 (1.69 to 2.52)	2.10 (1.69 to 2.52)	2.10 (1.69 to 2.52)	2.10 (1.69 to 2.52)	2.10 (1.69 to 2.52)	2.10 (1.69 to 2.52)
Tuberculosis	20 Cigarettes Per Day	Both	Female	2.47 (2.00 to 3.00)	2.47 (2.00 to 3.00)	2.47 (2.00 to 3.00)	2.47 (2.00 to 3.00)	2.47 (2.00 to 3.00)	2.47 (2.00 to 3.00)	2.47 (2.00 to 3.00)	2.47 (2.00 to 3.00)	2.47 (2.00 to 3.00)	2.47 (2.00 to 3.00)	2.47 (2.00 to 3.00)	2.47 (2.00 to 3.00)	2.47 (2.00 to 3.00)	2.47 (2.00 to 3.00)
Tuberculosis	20 Cigarettes Per Day	Both	Male	2.47 (2.00 to 3.00)	2.47 (2.00 to 3.00)	2.47 (2.00 to 3.00)	2.47 (2.00 to 3.00)	2.47 (2.00 to 3.00)	2.47 (2.00 to 3.00)	2.47 (2.00 to 3.00)	2.47 (2.00 to 3.00)	2.47 (2.00 to 3.00)	2.47 (2.00 to 3.00)	2.47 (2.00 to 3.00)	2.47 (2.00 to 3.00)	2.47 (2.00 to 3.00)	2.47 (2.00 to 3.00)
Tuberculosis	30 Cigarettes Per Day	Both	Female	3.36 (2.50 to 4.36)	3.36 (2.50 to 4.36)	3.36 (2.50 to 4.36)	3.36 (2.50 to 4.36)	3.36 (2.50 to 4.36)	3.36 (2.50 to 4.36)	3.36 (2.50 to 4.36)	3.36 (2.50 to 4.36)	3.36 (2.50 to 4.36)	3.36 (2.50 to 4.36)	3.36 (2.50 to 4.36)	3.36 (2.50 to 4.36)	3.36 (2.50 to 4.36)	3.36 (2.50 to 4.36)
Tuberculosis	30 Cigarettes Per Day	Both	Male	3.36 (2.50 to 4.36)	3.36 (2.50 to 4.36)	3.36 (2.50 to 4.36)	3.36 (2.50 to 4.36)	3.36 (2.50 to 4.36)	3.36 (2.50 to 4.36)	3.36 (2.50 to 4.36)	3.36 (2.50 to 4.36)	3.36 (2.50 to 4.36)	3.36 (2.50 to 4.36)	3.36 (2.50 to 4.36)	3.36 (2.50 to 4.36)	3.36 (2.50 to 4.36)	3.36 (2.50 to 4.36)
Tuberculosis	40 Cigarettes Per Day	Both	Female	4.22 (2.57 to 6.40)	4.22 (2.57 to 6.40)	4.22 (2.57 to 6.40)	4.22 (2.57 to 6.40)	4.22 (2.57 to 6.40)	4.22 (2.57 to 6.40)	4.22 (2.57 to 6.40)	4.22 (2.57 to 6.40)	4.22 (2.57 to 6.40)	4.22 (2.57 to 6.40)	4.22 (2.57 to 6.40)	4.22 (2.57 to 6.40)	4.22 (2.57 to 6.40)	4.22 (2.57 to 6.40)
Tuberculosis	40 Cigarettes Per Day	Both	Male	4.22 (2.57 to 6.40)	4.22 (2.57 to 6.40)	4.22 (2.57 to 6.40)	4.22 (2.57 to 6.40)	4.22 (2.57 to 6.40)	4.22 (2.57 to 6.40)	4.22 (2.57 to 6.40)	4.22 (2.57 to 6.40)	4.22 (2.57 to 6.40)	4.22 (2.57 to 6.40)	4.22 (2.57 to 6.40)	4.22 (2.57 to 6.40)	4.22 (2.57 to 6.40)	4.22 (2.57 to 6.40)
Tuberculosis	50 Cigarettes Per Day	Both	Female	4.77 (2.24 to 8.27)	4.77 (2.24 to 8.27)	4.77 (2.24 to 8.27)	4.77 (2.24 to 8.27)	4.77 (2.24 to 8.27)	4.77 (2.24 to 8.27)	4.77 (2.24 to 8.27)	4.77 (2.24 to 8.27)	4.77 (2.24 to 8.27)	4.77 (2.24 to 8.27)	4.77 (2.24 to 8.27)	4.77 (2.24 to 8.27)	4.77 (2.24 to 8.27)	4.77 (2.24 to 8.27)
Tuberculosis	50 Cigarettes Per Day	Both	Male	4.77 (2.24 to 8.27)	4.77 (2.24 to 8.27)	4.77 (2.24 to 8.27)	4.77 (2.24 to 8.27)	4.77 (2.24 to 8.27)	4.77 (2.24 to 8.27)	4.77 (2.24 to 8.27)	4.77 (2.24 to 8.27)	4.77 (2.24 to 8.27)	4.77 (2.24 to 8.27)	4.77 (2.24 to 8.27)	4.77 (2.24 to 8.27)	4.77 (2.24 to 8.27)	4.77 (2.24 to 8.27)
Asthma	0 Cigarettes Per Day	Both	Female	1.00 (1.00 to 1.00)	1.00 (1.00 to 1.00)	1.00 (1.00 to 1.00)	1.00 (1.00 to 1.00)	1.00 (1.00 to 1.00)	1.00 (1.00 to 1.00)	1.00 (1.00 to 1.00)	1.00 (1.00 to 1.00)	1.00 (1.00 to 1.00)	1.00 (1.00 to 1.00)	1.00 (1.00 to 1.00)	1.00 (1.00 to 1.00)	1.00 (1.00 to 1.00)	1.00 (1.00 to 1.00)
Asthma	0 Cigarettes Per Day	Both	Male	1.00 (1.00 to 1.00)	1.00 (1.00 to 1.00)	1.00 (1.00 to 1.00)	1.00 (1.00 to 1.00)	1.00 (1.00 to 1.00)	1.00 (1.00 to 1.00)	1.00 (1.00 to 1.00)	1.00 (1.00 to 1.00)	1.00 (1.00 to 1.00)	1.00 (1.00 to 1.00)	1.00 (1.00 to 1.00)	1.00 (1.00 to 1.00)	1.00 (1.00 to 1.00)	1.00 (1.00 to 1.00)
Asthma	10 Cigarettes Per Day	Both	Female	1.73 (1.33 to 2.15)	1.73 (1.33 to 2.15)	1.73 (1.33 to 2.15)	1.73 (1.33 to 2.15)	1.73 (1.33 to 2.15)	1.73 (1.33 to 2.15)	1.73 (1.33 to 2.15)	1.73 (1.33 to 2.15)	1.73 (1.33 to 2.15)	1.73 (1.33 to 2.15)	1.73 (1.33 to 2.15)	1.73 (1.33 to 2.15)	1.73 (1.33 to 2.15)	1.73 (1.33 to 2.15)
Asthma	10 Cigarettes Per Day	Both	Male	1.73 (1.33 to 2.15)	1.73 (1.33 to 2.15)	1.73 (1.33 to 2.15)	1.73 (1.33 to 2.15)	1.73 (1.33 to 2.15)	1.73 (1.33 to 2.15)	1.73 (1.33 to 2.15)	1.73 (1.33 to 2.15)	1.73 (1.33 to 2.15)	1.73 (1.33 to 2.15)	1.73 (1.33 to 2.15)	1.73 (1.33 to 2.15)	1.73 (1.33 to 2.15)	1.73 (1.33 to 2.15)
Asthma	20 Cigarettes Per Day	Both	Female	1.94 (1.46 to 2.56)	1.94 (1.46 to 2.56)	1.94 (1.46 to 2.56)	1.94 (1.46 to 2.56)	1.94 (1.46 to 2.56)	1.94 (1.46 to 2.56)	1.94 (1.46 to 2.56)	1.94 (1.46 to 2.56)	1.94 (1.46 to 2.56)	1.94 (1.46 to 2.56)	1.94 (1.46 to 2.56)	1.94 (1.46 to 2.56)	1.94 (1.46 to 2.56)	1.94 (1.46 to 2.56)
Asthma	20 Cigarettes Per Day	Both	Male	1.94 (1.46 to 2.56)	1.94 (1.46 to 2.56)	1.94 (1.46 to 2.56)	1.94 (1.46 to 2.56)	1.94 (1.46 to 2.56)	1.94 (1.46 to 2.56)	1.94 (1.46 to 2.56)	1.94 (1.46 to 2.56)	1.94 (1.46 to 2.56)	1.94 (1.46 to 2.56)	1.94 (1.46 to 2.56)	1.94 (1.46 to 2.56)	1.94 (1.46 to 2.56)	1.94 (1.46 to 2.56)
Asthma	30 Cigarettes Per Day	Both	Female	2.37 (1.56 to 3.47)	2.37 (1.56 to 3.47)	2.37 (1.56 to 3.47)	2.37 (1.56 to 3.47)	2.37 (1.56 to 3.47)	2.37 (1.56 to 3.47)	2.37 (1.56 to 3.47)	2.37 (1.56 to 3.47)	2.37 (1.56 to 3.47)	2.37 (1.56 to 3.47)	2.37 (1.56 to 3.47)	2.37 (1.56 to 3.47)	2.37 (1.56 to 3.47)	2.37 (1.56 to 3.47)
Asthma	30 Cigarettes Per Day	Both	Male	2.37 (1.56 to 3.47)	2.37 (1.56 to 3.47)	2.37 (1.56 to 3.47)	2.37 (1.56 to 3.47)	2.37 (1.56 to 3.47)	2.37 (1.56 to 3.47)	2.37 (1.56 to 3.47)	2.37 (1.56 to 3.47)	2.37 (1.56 to 3.47)	2.37 (1.56 to 3.47)	2.37 (1.56 to 3.47)	2.37 (1.56 to 3.47)	2.37 (1.56 to 3.47)	2.37 (1.56 to 3.47)
Asthma	40 Cigarettes Per Day	Both	Female	2.95 (1.55 to 5.10)	2.95 (1.55 to 5.10)	2.95 (1.55 to 5.10)	2.95 (1.55 to 5.10)	2.95 (1.55 to 5.10)	2.95 (1.55 to 5.10)	2.95 (1.55 to 5.10)	2.95 (1.55 to 5.10)	2.95 (1.55 to 5.10)	2.95 (1.55 to 5.10)	2.95 (1.55 to 5.10)	2.95 (1.55 to 5.10)	2.95 (1.55 to 5.10)	2.95 (1.55 to 5.10)
Asthma	40 Cigarettes Per Day	Both	Male	2.95 (1.55 to 5.10)	2.95 (1.55 to 5.10)	2.95 (1.55 to 5.10)	2.95 (1.55 to 5.10)	2.95 (1.55 to 5.10)	2.95 (1.55 to 5.10)	2.95 (1.55 to 5.10)	2.95 (1.55 to 5.10)	2.95 (1.55 to 5.10)	2.95 (1.55 to 5.10)	2.95 (1.55 to 5.10)	2.95 (1.55 to 5.10)	2.95 (1.55 to 5.10)	2.95 (1.55 to 5.10)
Asthma	50 Cigarettes Per Day	Both	Female	3.36 (1.43 to 6.27)	3.36 (1.43 to 6.27)	3.36 (1.43 to 6.27)	3.36 (1.43 to 6.27)	3.36 (1.43 to 6.27)	3.36 (1.43 to 6.27)	3.36 (1.43 to 6.27)	3.36 (1.43 to 6.27)	3.36 (1.43 to 6.27)	3.36 (1.43 to 6.27)	3.36 (1.43 to 6.27)	3.36 (1.43 to 6.27)	3.36 (1.43 to 6.27)	3.36 (1.43 to 6.27)
Asthma	50 Cigarettes Per Day	Both	Male	3.36 (1.43 to 6.27)	3.36 (1.43 to 6.27)	3.36 (1.43 to 6.27)	3.36 (1.43 to 6.27)	3.36 (1.43 to 6.27)	3.36 (1.43 to 6.27)	3.36 (1.43 to 6.27)	3.36 (1.43 to 6.27)	3.36 (1.43 to 6.27)	3.36 (1.43 to 6.27)	3.36 (1.43 to 6.27)	3.36 (1.43 to 6.27)	3.36 (1.43 to 6.27)	3.36 (1.43 to 6.27)
Diabetes mellitus	0 Cigarettes Per Day	Both	Female	1.00 (1.00 to 1.00)	1.00 (1.00 to 1.00)	1.00 (1.00 to 1.00)	1.00 (1.00 to 1.00)	1.00 (1.00 to 1.00)	1.00 (1.00 to 1.00)	1.00 (1.00 to 1.00)	1.00 (1.00 to 1.00)	1.00 (1.00 to 1.00)	1.00 (1.00 to 1.00)	1.00 (1.00 to 1.00)	1.00 (1.00 to 1.00)	1.00 (1.00 to 1.00)	1.00 (1.00 to 1.00)
Diabetes mellitus	0 Cigarettes Per Day	Both	Male	1.00 (1.00 to 1.00)	1.00 (1.00 to 1.00)	1.00 (1.00 to 1.00)	1.00 (1.00 to 1.00)	1.00 (1.00 to 1.00)	1.00 (1.00 to 1.00)	1.00 (1.00 to 1.00)	1.00 (1.00 to 1.00)	1.00 (1.00 to 1.00)	1.00 (1.00 to 1.00)	1.00 (1.00 to 1.00)	1.00 (1.00 to 1.00)	1.00 (1.00 to 1.00)	1.00 (1.00 to 1.00)
Diabetes mellitus	10 Cigarettes Per Day	Both	Female	1.43 (1.21 to 1.67)	1.43 (1.21 to 1.67)	1.43 (1.21 to 1.67)	1.43 (1.21 to 1.67)	1.43 (1.21 to 1.67)	1.43 (1.21 to 1.67)	1.43 (1.21 to 1.67)	1.43 (1.21 to 1.67)	1.43 (1.21 to 1.67)	1.43 (1.21 to 1.67)	1.43 (1.21 to 1.67)	1.43 (1.21 to 1.67)	1.43 (1.21 to 1.67)	1.43 (1.21 to 1.67)
Diabetes mellitus	10 Cigarettes Per Day	Both	Male	1.43 (1.21 to 1.67)	1.43 (1.21 to 1.67)	1.43 (1.21 to 1.67)	1.43 (1.21 to 1.67)	1.43 (1.21 to 1.67)	1.43 (1.21 to 1.67)	1.43 (1.21 to 1.67)	1.43 (1.21 to 1.67)	1.43 (1.21 to 1.67)	1.43 (1.21 to 1.67)	1.43 (1.21 to 1.67)	1.43 (1.21 to 1.67)	1.43 (1.21 to 1.67)	1.43 (1.21 to 1.67)
Diabetes mellitus	20 Cigarettes Per Day	Both	Female	1.64 (1.32 to 1.96)	1.64 (1.32 to 1.96)	1.64 (1.32 to 1.96)	1.64 (1.32 to 1.96)	1.64 (1.32 to 1.96)	1.64 (1.32 to 1.96)	1.64 (1.32 to 1.96)	1.64 (1.32 to 1.96)	1.64 (1.32 to 1.96)	1.64 (1.32 to 1.96)	1.64 (1.32 to 1.96)	1.64 (1.32 to 1.96)	1.64 (1.32 to 1.96)	1.64 (1.32 to 1.96)
Diabetes mellitus	20 Cigarettes Per Day	Both	Male	1.64 (1.32 to 1.96)	1.64 (1.32 to 1.96)	1.64 (1.32 to 1.96)	1.64 (1.32 to 1.96)	1.64 (1.32 to 1.96)	1.64 (1.32 to 1.96)	1.64 (1.32 to 1.96)	1.64 (1.32 to 1.96)	1.64 (1.32 to 1.96)	1.64 (1.32 to 1.96)	1.64 (1.32 to 1.96)	1.64 (1.32 to 1.96)	1.64 (1.32 to 1.96)	1.64 (1.32 to 1.96)
Diabetes mellitus	30 Cigarettes Per Day	Both	Female	1.78 (1.36 to 2.21)	1.78 (1.36 to 2.21)	1.78 (1.36 to 2.21)	1.78 (1.36 to 2.21)	1.78 (1.36 to 2.21)	1.78 (1.36 to 2.21)	1.78 (1.36 to 2.21)	1.78 (1.36 to 2.21)	1.78 (1.36 to 2.21)	1.78 (1.36 to 2.21)	1.78 (1.36 to 2.21)	1.78 (1.36 to 2.21)	1.78 (1.36 to 2.21)	1.78 (1.36 to 2.21)
Diabetes mellitus	30 Cigarettes Per Day	Both	Male	1.78 (1.36 to 2.21)	1.78 (1.36 to 2.21)	1.78 (1.36 to 2.21)	1.78 (1.36 to 2.21)	1.78 (1.36 to 2.21)	1.78 (1.36 to 2.21)	1.78 (1.36 to 2.21)	1.78 (1.36 to 2.21)	1.78 (1.36 to 2.21)	1.78 (1.36 to 2.21)	1.78 (1.36 to 2.21)	1.78 (1.36 to 2.21)	1.78 (1.36 to 2.21)	1.78 (1.36 to 2.21)
Atrial fibrillation and flutter	0 Cigarettes Per Day	Both	Female	1.00 (1.00 to 1.00)	1.00 (1.00 to 1.00)	1.00 (1.00 to 1.00)	1.00 (1.00 to 1.00)	1.00 (1.00 to 1.00)	1.00 (1.00 to 1.00)	1.00 (1.00 to 1.00)	1.00 (1.00 to 1.00)	1.00 (1.00 to 1.00)	1.00 (1.00 to 1.00)	1.00 (1.00 to 1.00)	1.00 (1.00 to 1.00)	1.00 (1.00 to 1.00)	1.00 (1.00 to 1.00)
Atrial fibrillation and flutter	0 Cigarettes Per Day	Both	Male	1.00 (1.00 to 1.00)	1.00 (1.00 to 1.00)	1.00 (1.00 to 1.00)	1.00 (1.00 to 1.00)	1.00 (1.00 to 1.00)	1.00 (1.00 to 1.00)	1.00 (1.00 to 1.00)	1.00 (1.00 to 1.00)	1.00 (1.00 to 1.00)	1.00 (1.00 to 1.00)	1.00 (1.00 to 1.00)	1.00 (1.00 to 1.00)	1.00 (1.00 to 1.00)	1.00 (1.00 to 1.00)

Atrial fibrillation and flutter	10 Cigarettes Per Day	Both	Female	1.74 (1.25 to 2.35)	1.74 (1.25 to 2.35)	1.74 (1.25 to 2.35)	1.66 (1.20 to 2.21)	1.66 (1.20 to 2.21)	1.57 (1.17 to 1.99)	1.57 (1.17 to 1.99)	1.45 (1.14 to 1.78)	1.45 (1.14 to 1.78)	1.30 (1.08 to 1.55)	1.30 (1.08 to 1.55)	1.15 (0.99 to 1.30)	1.15 (0.99 to 1.30)	1.00 (0.94 to 1.07)
Atrial fibrillation and flutter	10 Cigarettes Per Day	Both	Male	1.74 (1.25 to 2.35)	1.74 (1.25 to 2.35)	1.74 (1.25 to 2.35)	1.66 (1.20 to 2.21)	1.66 (1.20 to 2.21)	1.57 (1.17 to 1.99)	1.57 (1.17 to 1.99)	1.45 (1.14 to 1.78)	1.45 (1.14 to 1.78)	1.30 (1.08 to 1.55)	1.30 (1.08 to 1.55)	1.15 (0.99 to 1.30)	1.15 (0.99 to 1.30)	1.00 (0.94 to 1.07)
Atrial fibrillation and flutter	20 Cigarettes Per Day	Both	Female	2.29 (1.55 to 3.20)	2.29 (1.55 to 3.20)	2.29 (1.55 to 3.20)	2.04 (1.46 to 2.82)	2.04 (1.46 to 2.82)	1.80 (1.33 to 2.39)	1.80 (1.33 to 2.39)	1.54 (1.20 to 1.95)	1.54 (1.20 to 1.95)	1.31 (1.07 to 1.60)	1.31 (1.07 to 1.60)	1.12 (0.94 to 1.33)	1.12 (0.94 to 1.33)	1.00 (0.89 to 1.11)
Atrial fibrillation and flutter	20 Cigarettes Per Day	Both	Male	2.29 (1.55 to 3.20)	2.29 (1.55 to 3.20)	2.29 (1.55 to 3.20)	2.04 (1.46 to 2.82)	2.04 (1.46 to 2.82)	1.80 (1.33 to 2.39)	1.80 (1.33 to 2.39)	1.54 (1.20 to 1.95)	1.54 (1.20 to 1.95)	1.31 (1.07 to 1.60)	1.31 (1.07 to 1.60)	1.12 (0.94 to 1.33)	1.12 (0.94 to 1.33)	1.00 (0.89 to 1.11)
Atrial fibrillation and flutter	30 Cigarettes Per Day	Both	Female	2.83 (1.76 to 4.35)	2.83 (1.76 to 4.35)	2.83 (1.76 to 4.35)	2.46 (1.59 to 3.62)	2.46 (1.59 to 3.62)	2.11 (1.43 to 3.02)	2.11 (1.43 to 3.02)	1.77 (1.25 to 2.41)	1.77 (1.25 to 2.41)	1.48 (1.08 to 1.94)	1.48 (1.08 to 1.94)	1.25 (0.97 to 1.56)	1.25 (0.97 to 1.56)	1.03 (0.87 to 1.19)
Atrial fibrillation and flutter	30 Cigarettes Per Day	Both	Male	2.83 (1.76 to 4.35)	2.83 (1.76 to 4.35)	2.83 (1.76 to 4.35)	2.46 (1.59 to 3.62)	2.46 (1.59 to 3.62)	2.11 (1.43 to 3.02)	2.11 (1.43 to 3.02)	1.77 (1.25 to 2.41)	1.77 (1.25 to 2.41)	1.48 (1.08 to 1.94)	1.48 (1.08 to 1.94)	1.25 (0.97 to 1.56)	1.25 (0.97 to 1.56)	1.03 (0.87 to 1.19)
Atrial fibrillation and flutter	40 Cigarettes Per Day	Both	Female	3.35 (1.71 to 5.77)	3.35 (1.71 to 5.77)	3.35 (1.71 to 5.77)	2.93 (1.59 to 4.78)	2.93 (1.59 to 4.78)	2.50 (1.37 to 4.11)	2.50 (1.37 to 4.11)	2.10 (1.17 to 3.44)	2.10 (1.17 to 3.44)	1.75 (1.00 to 2.73)	1.75 (1.00 to 2.73)	1.46 (0.88 to 2.24)	1.46 (0.88 to 2.24)	1.19 (0.76 to 1.77)
Atrial fibrillation and flutter	40 Cigarettes Per Day	Both	Male	3.35 (1.71 to 5.77)	3.35 (1.71 to 5.77)	3.35 (1.71 to 5.77)	2.93 (1.59 to 4.78)	2.93 (1.59 to 4.78)	2.50 (1.37 to 4.11)	2.50 (1.37 to 4.11)	2.10 (1.17 to 3.44)	2.10 (1.17 to 3.44)	1.75 (1.00 to 2.73)	1.75 (1.00 to 2.73)	1.46 (0.88 to 2.24)	1.46 (0.88 to 2.24)	1.19 (0.76 to 1.77)
Atrial fibrillation and flutter	50 Cigarettes Per Day	Both	Female	3.63 (1.54 to 6.60)	3.63 (1.54 to 6.60)	3.63 (1.54 to 6.60)	3.18 (1.44 to 5.62)	3.18 (1.44 to 5.62)	2.72 (1.24 to 4.92)	2.72 (1.24 to 4.92)	2.33 (1.10 to 4.27)	2.33 (1.10 to 4.27)	1.94 (0.95 to 3.42)	1.94 (0.95 to 3.42)	1.59 (0.79 to 2.77)	1.59 (0.79 to 2.77)	1.30 (0.67 to 2.23)
Atrial fibrillation and flutter	50 Cigarettes Per Day	Both	Male	3.63 (1.54 to 6.60)	3.63 (1.54 to 6.60)	3.63 (1.54 to 6.60)	3.18 (1.44 to 5.62)	3.18 (1.44 to 5.62)	2.72 (1.24 to 4.92)	2.72 (1.24 to 4.92)	2.33 (1.10 to 4.27)	2.33 (1.10 to 4.27)	1.94 (0.95 to 3.42)	1.94 (0.95 to 3.42)	1.59 (0.79 to 2.77)	1.59 (0.79 to 2.77)	1.30 (0.67 to 2.23)
Aortic aneurysm	0 Cigarettes Per Day	Both	Female	1.00 (1.00 to 1.00)	1.00 (1.00 to 1.00)	1.00 (1.00 to 1.00)	1.00 (1.00 to 1.00)	1.00 (1.00 to 1.00)	1.00 (1.00 to 1.00)	1.00 (1.00 to 1.00)	1.00 (1.00 to 1.00)	1.00 (1.00 to 1.00)	1.00 (1.00 to 1.00)	1.00 (1.00 to 1.00)	1.00 (1.00 to 1.00)	1.00 (1.00 to 1.00)	1.00 (1.00 to 1.00)
Aortic aneurysm	0 Cigarettes Per Day	Both	Male	1.00 (1.00 to 1.00)	1.00 (1.00 to 1.00)	1.00 (1.00 to 1.00)	1.00 (1.00 to 1.00)	1.00 (1.00 to 1.00)	1.00 (1.00 to 1.00)	1.00 (1.00 to 1.00)	1.00 (1.00 to 1.00)	1.00 (1.00 to 1.00)	1.00 (1.00 to 1.00)	1.00 (1.00 to 1.00)	1.00 (1.00 to 1.00)	1.00 (1.00 to 1.00)	1.00 (1.00 to 1.00)
Aortic aneurysm	10 Cigarettes Per Day	Both	Female	4.37 (3.37 to 5.42)	4.37 (3.37 to 5.42)	4.37 (3.37 to 5.42)	3.67 (2.96 to 4.42)	3.67 (2.96 to 4.42)	3.00 (2.55 to 3.49)	3.00 (2.55 to 3.49)	2.39 (2.10 to 2.66)	2.39 (2.10 to 2.66)	1.84 (1.69 to 2.01)	1.84 (1.69 to 2.01)	1.37 (1.28 to 1.45)	1.37 (1.28 to 1.45)	1.00 (0.99 to 1.02)
Aortic aneurysm	10 Cigarettes Per Day	Both	Male	4.37 (3.37 to 5.42)	4.37 (3.37 to 5.42)	4.37 (3.37 to 5.42)	3.67 (2.96 to 4.42)	3.67 (2.96 to 4.42)	3.00 (2.55 to 3.49)	3.00 (2.55 to 3.49)	2.39 (2.10 to 2.66)	2.39 (2.10 to 2.66)	1.84 (1.69 to 2.01)	1.84 (1.69 to 2.01)	1.37 (1.28 to 1.45)	1.37 (1.28 to 1.45)	1.00 (0.99 to 1.02)
Aortic aneurysm	20 Cigarettes Per Day	Both	Female	8.04 (6.18 to 10.05)	8.04 (6.18 to 10.05)	8.04 (6.18 to 10.05)	5.88 (4.65 to 7.19)	5.88 (4.65 to 7.19)	4.21 (3.48 to 5.02)	4.21 (3.48 to 5.02)	2.97 (2.55 to 3.41)	2.97 (2.55 to 3.41)	2.06 (1.83 to 2.29)	2.06 (1.83 to 2.29)	1.41 (1.29 to 1.52)	1.41 (1.29 to 1.52)	1.00 (0.98 to 1.02)
Aortic aneurysm	20 Cigarettes Per Day	Both	Male	8.04 (6.18 to 10.05)	8.04 (6.18 to 10.05)	8.04 (6.18 to 10.05)	5.88 (4.65 to 7.19)	5.88 (4.65 to 7.19)	4.21 (3.48 to 5.02)	4.21 (3.48 to 5.02)	2.97 (2.55 to 3.41)	2.97 (2.55 to 3.41)	2.06 (1.83 to 2.29)	2.06 (1.83 to 2.29)	1.41 (1.29 to 1.52)	1.41 (1.29 to 1.52)	1.00 (0.98 to 1.02)
Aortic aneurysm	30 Cigarettes Per Day	Both	Female	10.96 (7.78 to 14.61)	10.96 (7.78 to 14.61)	10.96 (7.78 to 14.61)	7.61 (5.69 to 9.86)	7.61 (5.69 to 9.86)	5.12 (3.96 to 6.42)	5.12 (3.96 to 6.42)	3.41 (2.77 to 4.11)	3.41 (2.77 to 4.11)	2.27 (1.91 to 2.64)	2.27 (1.91 to 2.64)	1.49 (1.32 to 1.66)	1.49 (1.32 to 1.66)	1.00 (0.98 to 1.02)
Aortic aneurysm	30 Cigarettes Per Day	Both	Male	10.96 (7.78 to 14.61)	10.96 (7.78 to 14.61)	10.96 (7.78 to 14.61)	7.61 (5.69 to 9.86)	7.61 (5.69 to 9.86)	5.12 (3.96 to 6.42)	5.12 (3.96 to 6.42)	3.41 (2.77 to 4.11)	3.41 (2.77 to 4.11)	2.27 (1.91 to 2.64)	2.27 (1.91 to 2.64)	1.49 (1.32 to 1.66)	1.49 (1.32 to 1.66)	1.00 (0.98 to 1.02)
Aortic aneurysm	40 Cigarettes Per Day	Both	Female	13.08 (8.19 to 18.89)	13.08 (8.19 to 18.89)	13.08 (8.19 to 18.89)	8.93 (6.20 to 12.25)	8.93 (6.20 to 12.25)	5.84 (4.19 to 7.78)	5.84 (4.19 to 7.78)	3.78 (2.89 to 4.79)	3.78 (2.89 to 4.79)	2.47 (1.99 to 3.00)	2.47 (1.99 to 3.00)	1.58 (1.33 to 1.83)	1.58 (1.33 to 1.83)	1.00 (0.93 to 1.06)
Aortic aneurysm	40 Cigarettes Per Day	Both	Male	13.08 (8.19 to 18.89)	13.08 (8.19 to 18.89)	13.08 (8.19 to 18.89)	8.93 (6.20 to 12.25)	8.93 (6.20 to 12.25)	5.84 (4.19 to 7.78)	5.84 (4.19 to 7.78)	3.78 (2.89 to 4.79)	3.78 (2.89 to 4.79)	2.47 (1.99 to 3.00)	2.47 (1.99 to 3.00)	1.58 (1.33 to 1.83)	1.58 (1.33 to 1.83)	1.00 (0.93 to 1.06)
Aortic aneurysm	50 Cigarettes Per Day	Both	Female	14.71 (8.08 to 22.92)	14.71 (8.08 to 22.92)	14.71 (8.08 to 22.92)	9.95 (6.35 to 14.41)	9.95 (6.35 to 14.41)	6.35 (4.28 to 9.05)	6.35 (4.28 to 9.05)	4.00 (2.86 to 5.33)	4.00 (2.86 to 5.33)	2.52 (1.92 to 3.21)	2.52 (1.92 to 3.21)	1.57 (1.24 to 1.92)	1.57 (1.24 to 1.92)	1.00 (0.84 to 1.16)
Aortic aneurysm	50 Cigarettes Per Day	Both	Male	14.71 (8.08 to 22.92)	14.71 (8.08 to 22.92)	14.71 (8.08 to 22.92)	9.95 (6.35 to 14.41)	9.95 (6.35 to 14.41)	6.35 (4.28 to 9.05)	6.35 (4.28 to 9.05)	4.00 (2.86 to 5.33)	4.00 (2.86 to 5.33)	2.52 (1.92 to 3.21)	2.52 (1.92 to 3.21)	1.57 (1.24 to 1.92)	1.57 (1.24 to 1.92)	1.00 (0.84 to 1.16)
Aortic aneurysm	60 Cigarettes Per Day	Both	Female	15.88 (7.77 to 27.23)	15.88 (7.77 to 27.23)	15.88 (7.77 to 27.23)	10.84 (5.89 to 17.51)	10.84 (5.89 to 17.51)	6.83 (4.00 to 10.55)	6.83 (4.00 to 10.55)	4.24 (2.62 to 6.16)	4.24 (2.62 to 6.16)	2.61 (1.74 to 3.68)	2.61 (1.74 to 3.68)	1.60 (1.14 to 2.14)	1.60 (1.14 to 2.14)	1.01 (0.75 to 1.26)
Aortic aneurysm	60 Cigarettes Per Day	Both	Male	15.88 (7.77 to 27.23)	15.88 (7.77 to 27.23)	15.88 (7.77 to 27.23)	10.84 (5.89 to 17.51)	10.84 (5.89 to 17.51)	6.83 (4.00 to 10.55)	6.83 (4.00 to 10.55)	4.24 (2.62 to 6.16)	4.24 (2.62 to 6.16)	2.61 (1.74 to 3.68)	2.61 (1.74 to 3.68)	1.60 (1.14 to 2.14)	1.60 (1.14 to 2.14)	1.01 (0.75 to 1.26)
Lower extremity peripheral arterial disease	0 Cigarettes Per Day	Both	Female	1.00 (1.00 to 1.00)	1.00 (1.00 to 1.00)	1.00 (1.00 to 1.00)	1.00 (1.00 to 1.00)	1.00 (1.00 to 1.00)	1.00 (1.00 to 1.00)	1.00 (1.00 to 1.00)	1.00 (1.00 to 1.00)	1.00 (1.00 to 1.00)	1.00 (1.00 to 1.00)	1.00 (1.00 to 1.00)	1.00 (1.00 to 1.00)	1.00 (1.00 to 1.00)	1.00 (1.00 to 1.00)
Lower extremity peripheral arterial disease	0 Cigarettes Per Day	Both	Male	1.00 (1.00 to 1.00)	1.00 (1.00 to 1.00)	1.00 (1.00 to 1.00)	1.00 (1.00 to 1.00)	1.00 (1.00 to 1.00)	1.00 (1.00 to 1.00)	1.00 (1.00 to 1.00)	1.00 (1.00 to 1.00)	1.00 (1.00 to 1.00)	1.00 (1.00 to 1.00)	1.00 (1.00 to 1.00)	1.00 (1.00 to 1.00)	1.00 (1.00 to 1.00)	1.00 (1.00 to 1.00)
Lower extremity peripheral arterial disease	10 Cigarettes Per Day	Both	Female	3.28 (2.26 to 4.63)	3.28 (2.26 to 4.63)	3.28 (2.26 to 4.63)	2.98 (1.98 to 4.22)	2.98 (1.98 to 4.22)	3.27 (1.96 to 4.76)	3.27 (1.96 to 4.76)	3.12 (2.40 to 3.80)	3.12 (2.40 to 3.80)	2.26 (1.87 to 2.62)	2.26 (1.87 to 2.62)	1.55 (1.30 to 1.81)	1.55 (1.30 to 1.81)	1.01 (0.85 to 1.16)
Lower extremity peripheral arterial disease	10 Cigarettes Per Day	Both	Male	3.28 (2.26 to 4.63)	3.28 (2.26 to 4.63)	3.28 (2.26 to 4.63)	2.98 (1.98 to 4.22)	2.98 (1.98 to 4.22)	3.27 (1.96 to 4.76)	3.27 (1.96 to 4.76)	3.12 (2.40 to 3.80)	3.12 (2.40 to 3.80)	2.26 (1.87 to 2.62)	2.26 (1.87 to 2.62)	1.55 (1.30 to 1.81)	1.55 (1.30 to 1.81)	1.01 (0.85 to 1.16)
Lower extremity peripheral arterial disease	20 Cigarettes Per Day	Both	Female	6.77 (4.15 to 10.15)	6.77 (4.15 to 10.15)	6.77 (4.15 to 10.15)	5.74 (3.48 to 8.77)	5.74 (3.48 to 8.77)	5.90 (3.16 to 8.65)	5.90 (3.16 to 8.65)	4.91 (3.79 to 5.93)	4.91 (3.79 to 5.93)	2.99 (2.48 to 3.47)	2.99 (2.48 to 3.47)	1.73 (1.47 to 2.00)	1.73 (1.47 to 2.00)	1.01 (0.88 to 1.14)
Lower extremity peripheral arterial disease	20 Cigarettes Per Day	Both	Male	6.77 (4.15 to 10.15)	6.77 (4.15 to 10.15)	6.77 (4.15 to 10.15)	5.74 (3.48 to 8.77)	5.74 (3.48 to 8.77)	5.90 (3.16 to 8.65)	5.90 (3.16 to 8.65)	4.91 (3.79 to 5.93)	4.91 (3.79 to 5.93)	2.99 (2.48 to 3.47)	2.99 (2.48 to 3.47)	1.73 (1.47 to 2.00)	1.73 (1.47 to 2.00)	1.01 (0.88 to 1.14)
Lower extremity peripheral arterial disease	30 Cigarettes Per Day	Both	Female	9.40 (5.11 to 15.23)	9.40 (5.11 to 15.23)	9.40 (5.11 to 15.23)	7.75 (4.15 to 12.86)	7.75 (4.15 to 12.86)	7.32 (3.75 to 11.18)	7.32 (3.75 to 11.18)	5.31 (4.05 to 6.88)	5.31 (4.05 to 6.88)	2.99 (2.30 to 3.77)	2.99 (2.30 to 3.77)	1.71 (1.33 to 2.14)	1.71 (1.33 to 2.14)	1.04 (0.84 to 1.27)
Lower extremity peripheral arterial disease	30 Cigarettes Per Day	Both	Male	9.40 (5.11 to 15.23)	9.40 (5.11 to 15.23)	9.40 (5.11 to 15.23)	7.75 (4.15 to 12.86)	7.75 (4.15 to 12.86)	7.32 (3.75 to 11.18)	7.32 (3.75 to 11.18)	5.31 (4.05 to 6.88)	5.31 (4.05 to 6.88)	2.99 (2.30 to 3.77)	2.99 (2.30 to 3.77)	1.71 (1.33 to 2.14)	1.71 (1.33 to 2.14)	1.04 (0.84 to 1.27)
Lower extremity peripheral arterial disease	40 Cigarettes Per Day	Both	Female	11.14 (5.30 to 19.75)	11.14 (5.30 to 19.75)	11.14 (5.30 to 19.75)	9.12 (4.33 to 16.59)	9.12 (4.33 to 16.59)	8.45 (4.12 to 14.10)	8.45 (4.12 to 14.10)	6.14 (4.07 to 8.87)	6.14 (4.07 to 8.87)	3.42 (2.27 to 4.90)	3.42 (2.27 to 4.90)	1.94 (1.27 to 2.80)	1.94 (1.27 to 2.80)	1.14 (0.76 to 1.64)
Lower extremity peripheral arterial disease	40 Cigarettes Per Day	Both	Male	11.14 (5.30 to 19.75)	11.14 (5.30 to 19.75)	11.14 (5.30 to 19.75)	9.12 (4.33 to 16.59)	9.12 (4.33 to 16.59)	8.45 (4.12 to 14.10)	8.45 (4.12 to 14.10)	6.14 (4.07 to 8.87)	6.14 (4.07 to 8.87)	3.42 (2.27 to 4.90)	3.42 (2.27 to 4.90)	1.94 (1.27 to 2.80)	1.94 (1.27 to 2.80)	1.14 (0.76 to 1.64)
Alzheimer's disease and other dementias	0 Cigarettes Per Day	Both	Female	1.00 (1.00 to 1.00)	1.00 (1.00 to 1.00)	1.00 (1.00 to 1.00)	1.00 (1.00 to 1.00)	1.00 (1.00 to 1.00)	1.00 (1.00 to 1.00)	1.00 (1.00 to 1.00)	1.00 (1.00 to 1.00)	1.00 (1.00 to 1.00)	1.00 (1.00 to 1.00)	1.00 (1.00 to 1.00)	1.00 (1.00 to 1.00)	1.00 (1.00 to 1.00)	1.00 (1.00 to 1.00)
Alzheimer's disease and other dementias	0 Cigarettes Per Day	Both	Male	1.00 (1.00 to 1.00)	1.00 (1.00 to 1.00)	1.00 (1.00 to 1.00)	1.00 (1.00 to 1.00)	1.00 (1.00 to 1.00)	1.00 (1.00 to 1.00)	1.00 (1.00 to 1.00)	1.00 (1.00 to 1.00)	1.00 (1.00 to 1.00)	1.00 (1.00 to 1.00)	1.00 (1.00 to 1.00)	1.00 (1.00 to 1.00)	1.00 (1.00 to 1.00)	1.00 (1.00 to 1.00)
Alzheimer's disease and other dementias	10 Cigarettes Per Day	Both	Female	1.90 (1.43 to 2.40)	1.90 (1.43 to 2.40)	1.90 (1.43 to 2.40)	1.90 (1.43 to 2.40)	1.90 (1.43 to 2.40)	1.90 (1.43 to 2.40)	1.90 (1.43 to 2.40)	1.90 (1.43 to 2.40)	1.90 (1.43 to 2.40)	1.90 (1.43 to 2.40)	1.90 (1.43 to 2.40)	1.90 (1.43 to 2.40)	1.90 (1.43 to 2.40)	1.90 (1.43 to 2.40)
Alzheimer's disease and other dementias	10 Cigarettes Per Day	Both	Male	1.90 (1.43 to 2.40)	1.90 (1.43 to 2.40)	1.90 (1.43 to 2.40)	1.90 (1.43 to 2.40)	1.90 (1.43 to 2.40)	1.90 (1.43 to 2.40)	1.90 (1.43 to 2.40)	1.90 (1.43 to 2.40)	1.90 (1.43 to 2.40)	1.90 (1.43 to 2.40)	1.90 (1.43 to 2.40)	1.90 (1.43 to 2.40)	1.90 (1.43 to 2.40)	1.90 (1.43 to 2.40)

Alzheimer's disease and other dementias	20 Cigarettes Per Day	Both	Female	2.65 (1.79 to 3.67)	2.65 (1.79 to 3.67)	2.65 (1.79 to 3.67)	2.65 (1.79 to 3.67)	2.65 (1.79 to 3.67)	2.65 (1.79 to 3.67)	2.65 (1.79 to 3.67)	2.65 (1.79 to 3.67)	2.65 (1.79 to 3.67)	2.65 (1.79 to 3.67)	2.65 (1.79 to 3.67)	2.65 (1.79 to 3.67)	2.65 (1.79 to 3.67)	2.65 (1.79 to 3.67)
Alzheimer's disease and other dementias	20 Cigarettes Per Day	Both	Male	2.65 (1.79 to 3.67)	2.65 (1.79 to 3.67)	2.65 (1.79 to 3.67)	2.65 (1.79 to 3.67)	2.65 (1.79 to 3.67)	2.65 (1.79 to 3.67)	2.65 (1.79 to 3.67)	2.65 (1.79 to 3.67)	2.65 (1.79 to 3.67)	2.65 (1.79 to 3.67)	2.65 (1.79 to 3.67)	2.65 (1.79 to 3.67)	2.65 (1.79 to 3.67)	2.65 (1.79 to 3.67)
Alzheimer's disease and other dementias	30 Cigarettes Per Day	Both	Female	3.34 (2.08 to 4.85)	3.34 (2.08 to 4.85)	3.34 (2.08 to 4.85)	3.34 (2.08 to 4.85)	3.34 (2.08 to 4.85)	3.34 (2.08 to 4.85)	3.34 (2.08 to 4.85)	3.34 (2.08 to 4.85)	3.34 (2.08 to 4.85)	3.34 (2.08 to 4.85)	3.34 (2.08 to 4.85)	3.34 (2.08 to 4.85)	3.34 (2.08 to 4.85)	3.34 (2.08 to 4.85)
Alzheimer's disease and other dementias	30 Cigarettes Per Day	Both	Male	3.34 (2.08 to 4.85)	3.34 (2.08 to 4.85)	3.34 (2.08 to 4.85)	3.34 (2.08 to 4.85)	3.34 (2.08 to 4.85)	3.34 (2.08 to 4.85)	3.34 (2.08 to 4.85)	3.34 (2.08 to 4.85)	3.34 (2.08 to 4.85)	3.34 (2.08 to 4.85)	3.34 (2.08 to 4.85)	3.34 (2.08 to 4.85)	3.34 (2.08 to 4.85)	3.34 (2.08 to 4.85)
Alzheimer's disease and other dementias	40 Cigarettes Per Day	Both	Female	3.86 (2.24 to 5.86)	3.86 (2.24 to 5.86)	3.86 (2.24 to 5.86)	3.86 (2.24 to 5.86)	3.86 (2.24 to 5.86)	3.86 (2.24 to 5.86)	3.86 (2.24 to 5.86)	3.86 (2.24 to 5.86)	3.86 (2.24 to 5.86)	3.86 (2.24 to 5.86)	3.86 (2.24 to 5.86)	3.86 (2.24 to 5.86)	3.86 (2.24 to 5.86)	3.86 (2.24 to 5.86)
Alzheimer's disease and other dementias	40 Cigarettes Per Day	Both	Male	3.86 (2.24 to 5.86)	3.86 (2.24 to 5.86)	3.86 (2.24 to 5.86)	3.86 (2.24 to 5.86)	3.86 (2.24 to 5.86)	3.86 (2.24 to 5.86)	3.86 (2.24 to 5.86)	3.86 (2.24 to 5.86)	3.86 (2.24 to 5.86)	3.86 (2.24 to 5.86)	3.86 (2.24 to 5.86)	3.86 (2.24 to 5.86)	3.86 (2.24 to 5.86)	3.86 (2.24 to 5.86)
Alzheimer's disease and other dementias	50 Cigarettes Per Day	Both	Female	4.17 (2.23 to 6.80)	4.17 (2.23 to 6.80)	4.17 (2.23 to 6.80)	4.17 (2.23 to 6.80)	4.17 (2.23 to 6.80)	4.17 (2.23 to 6.80)	4.17 (2.23 to 6.80)	4.17 (2.23 to 6.80)	4.17 (2.23 to 6.80)	4.17 (2.23 to 6.80)	4.17 (2.23 to 6.80)	4.17 (2.23 to 6.80)	4.17 (2.23 to 6.80)	4.17 (2.23 to 6.80)
Alzheimer's disease and other dementias	50 Cigarettes Per Day	Both	Male	4.17 (2.23 to 6.80)	4.17 (2.23 to 6.80)	4.17 (2.23 to 6.80)	4.17 (2.23 to 6.80)	4.17 (2.23 to 6.80)	4.17 (2.23 to 6.80)	4.17 (2.23 to 6.80)	4.17 (2.23 to 6.80)	4.17 (2.23 to 6.80)	4.17 (2.23 to 6.80)	4.17 (2.23 to 6.80)	4.17 (2.23 to 6.80)	4.17 (2.23 to 6.80)	4.17 (2.23 to 6.80)
Alzheimer's disease and other dementias	60 Cigarettes Per Day	Both	Female	4.49 (2.06 to 8.25)	4.49 (2.06 to 8.25)	4.49 (2.06 to 8.25)	4.49 (2.06 to 8.25)	4.49 (2.06 to 8.25)	4.49 (2.06 to 8.25)	4.49 (2.06 to 8.25)	4.49 (2.06 to 8.25)	4.49 (2.06 to 8.25)	4.49 (2.06 to 8.25)	4.49 (2.06 to 8.25)	4.49 (2.06 to 8.25)	4.49 (2.06 to 8.25)	4.49 (2.06 to 8.25)
Alzheimer's disease and other dementias	60 Cigarettes Per Day	Both	Male	4.49 (2.06 to 8.25)	4.49 (2.06 to 8.25)	4.49 (2.06 to 8.25)	4.49 (2.06 to 8.25)	4.49 (2.06 to 8.25)	4.49 (2.06 to 8.25)	4.49 (2.06 to 8.25)	4.49 (2.06 to 8.25)	4.49 (2.06 to 8.25)	4.49 (2.06 to 8.25)	4.49 (2.06 to 8.25)	4.49 (2.06 to 8.25)	4.49 (2.06 to 8.25)	4.49 (2.06 to 8.25)
Parkinson's disease	0 Cigarettes Per Day	Both	Female	1.00 (1.00 to 1.00)	1.00 (1.00 to 1.00)	1.00 (1.00 to 1.00)	1.00 (1.00 to 1.00)	1.00 (1.00 to 1.00)	1.00 (1.00 to 1.00)	1.00 (1.00 to 1.00)	1.00 (1.00 to 1.00)	1.00 (1.00 to 1.00)	1.00 (1.00 to 1.00)	1.00 (1.00 to 1.00)	1.00 (1.00 to 1.00)	1.00 (1.00 to 1.00)	1.00 (1.00 to 1.00)
Parkinson's disease	0 Cigarettes Per Day	Both	Male	1.00 (1.00 to 1.00)	1.00 (1.00 to 1.00)	1.00 (1.00 to 1.00)	1.00 (1.00 to 1.00)	1.00 (1.00 to 1.00)	1.00 (1.00 to 1.00)	1.00 (1.00 to 1.00)	1.00 (1.00 to 1.00)	1.00 (1.00 to 1.00)	1.00 (1.00 to 1.00)	1.00 (1.00 to 1.00)	1.00 (1.00 to 1.00)	1.00 (1.00 to 1.00)	1.00 (1.00 to 1.00)
Parkinson's disease	10 Cigarettes Per Day	Both	Female	0.79 (0.63 to 0.94)	0.79 (0.63 to 0.94)	0.79 (0.63 to 0.94)	0.79 (0.63 to 0.94)	0.79 (0.63 to 0.94)	0.79 (0.63 to 0.94)	0.79 (0.63 to 0.94)	0.79 (0.63 to 0.94)	0.79 (0.63 to 0.94)	0.79 (0.63 to 0.94)	0.79 (0.63 to 0.94)	0.79 (0.63 to 0.94)	0.79 (0.63 to 0.94)	0.79 (0.63 to 0.94)
Parkinson's disease	10 Cigarettes Per Day	Both	Male	0.79 (0.63 to 0.94)	0.79 (0.63 to 0.94)	0.79 (0.63 to 0.94)	0.79 (0.63 to 0.94)	0.79 (0.63 to 0.94)	0.79 (0.63 to 0.94)	0.79 (0.63 to 0.94)	0.79 (0.63 to 0.94)	0.79 (0.63 to 0.94)	0.79 (0.63 to 0.94)	0.79 (0.63 to 0.94)	0.79 (0.63 to 0.94)	0.79 (0.63 to 0.94)	0.79 (0.63 to 0.94)
Parkinson's disease	20 Cigarettes Per Day	Both	Female	0.63 (0.49 to 0.79)	0.63 (0.49 to 0.79)	0.63 (0.49 to 0.79)	0.63 (0.49 to 0.79)	0.63 (0.49 to 0.79)	0.63 (0.49 to 0.79)	0.63 (0.49 to 0.79)	0.63 (0.49 to 0.79)	0.63 (0.49 to 0.79)	0.63 (0.49 to 0.79)	0.63 (0.49 to 0.79)	0.63 (0.49 to 0.79)	0.63 (0.49 to 0.79)	0.63 (0.49 to 0.79)
Parkinson's disease	20 Cigarettes Per Day	Both	Male	0.63 (0.49 to 0.79)	0.63 (0.49 to 0.79)	0.63 (0.49 to 0.79)	0.63 (0.49 to 0.79)	0.63 (0.49 to 0.79)	0.63 (0.49 to 0.79)	0.63 (0.49 to 0.79)	0.63 (0.49 to 0.79)	0.63 (0.49 to 0.79)	0.63 (0.49 to 0.79)	0.63 (0.49 to 0.79)	0.63 (0.49 to 0.79)	0.63 (0.49 to 0.79)	0.63 (0.49 to 0.79)
Parkinson's disease	30 Cigarettes Per Day	Both	Female	0.52 (0.37 to 0.68)	0.52 (0.37 to 0.68)	0.52 (0.37 to 0.68)	0.52 (0.37 to 0.68)	0.52 (0.37 to 0.68)	0.52 (0.37 to 0.68)	0.52 (0.37 to 0.68)	0.52 (0.37 to 0.68)	0.52 (0.37 to 0.68)	0.52 (0.37 to 0.68)	0.52 (0.37 to 0.68)	0.52 (0.37 to 0.68)	0.52 (0.37 to 0.68)	0.52 (0.37 to 0.68)
Parkinson's disease	30 Cigarettes Per Day	Both	Male	0.52 (0.37 to 0.68)	0.52 (0.37 to 0.68)	0.52 (0.37 to 0.68)	0.52 (0.37 to 0.68)	0.52 (0.37 to 0.68)	0.52 (0.37 to 0.68)	0.52 (0.37 to 0.68)	0.52 (0.37 to 0.68)	0.52 (0.37 to 0.68)	0.52 (0.37 to 0.68)	0.52 (0.37 to 0.68)	0.52 (0.37 to 0.68)	0.52 (0.37 to 0.68)	0.52 (0.37 to 0.68)
Parkinson's disease	40 Cigarettes Per Day	Both	Female	0.40 (0.23 to 0.58)	0.40 (0.23 to 0.58)	0.40 (0.23 to 0.58)	0.40 (0.23 to 0.58)	0.40 (0.23 to 0.58)	0.40 (0.23 to 0.58)	0.40 (0.23 to 0.58)	0.40 (0.23 to 0.58)	0.40 (0.23 to 0.58)	0.40 (0.23 to 0.58)	0.40 (0.23 to 0.58)	0.40 (0.23 to 0.58)	0.40 (0.23 to 0.58)	0.40 (0.23 to 0.58)
Parkinson's disease	40 Cigarettes Per Day	Both	Male	0.40 (0.23 to 0.58)	0.40 (0.23 to 0.58)	0.40 (0.23 to 0.58)	0.40 (0.23 to 0.58)	0.40 (0.23 to 0.58)	0.40 (0.23 to 0.58)	0.40 (0.23 to 0.58)	0.40 (0.23 to 0.58)	0.40 (0.23 to 0.58)	0.40 (0.23 to 0.58)	0.40 (0.23 to 0.58)	0.40 (0.23 to 0.58)	0.40 (0.23 to 0.58)	0.40 (0.23 to 0.58)
Multiple sclerosis	0 Cigarettes Per Day	Both	Female	1.00 (1.00 to 1.00)	1.00 (1.00 to 1.00)	1.00 (1.00 to 1.00)	1.00 (1.00 to 1.00)	1.00 (1.00 to 1.00)	1.00 (1.00 to 1.00)	1.00 (1.00 to 1.00)	1.00 (1.00 to 1.00)	1.00 (1.00 to 1.00)	1.00 (1.00 to 1.00)	1.00 (1.00 to 1.00)	1.00 (1.00 to 1.00)	1.00 (1.00 to 1.00)	1.00 (1.00 to 1.00)
Multiple sclerosis	0 Cigarettes Per Day	Both	Male	1.00 (1.00 to 1.00)	1.00 (1.00 to 1.00)	1.00 (1.00 to 1.00)	1.00 (1.00 to 1.00)	1.00 (1.00 to 1.00)	1.00 (1.00 to 1.00)	1.00 (1.00 to 1.00)	1.00 (1.00 to 1.00)	1.00 (1.00 to 1.00)	1.00 (1.00 to 1.00)	1.00 (1.00 to 1.00)	1.00 (1.00 to 1.00)	1.00 (1.00 to 1.00)	1.00 (1.00 to 1.00)
Multiple sclerosis	10 Cigarettes Per Day	Both	Female	1.67 (1.46 to 1.91)	1.67 (1.46 to 1.91)	1.67 (1.46 to 1.91)	1.67 (1.46 to 1.91)	1.67 (1.46 to 1.91)	1.67 (1.46 to 1.91)	1.67 (1.46 to 1.91)	1.67 (1.46 to 1.91)	1.67 (1.46 to 1.91)	1.67 (1.46 to 1.91)	1.67 (1.46 to 1.91)	1.67 (1.46 to 1.91)	1.67 (1.46 to 1.91)	1.67 (1.46 to 1.91)
Multiple sclerosis	10 Cigarettes Per Day	Both	Male	1.67 (1.46 to 1.91)	1.67 (1.46 to 1.91)	1.67 (1.46 to 1.91)	1.67 (1.46 to 1.91)	1.67 (1.46 to 1.91)	1.67 (1.46 to 1.91)	1.67 (1.46 to 1.91)	1.67 (1.46 to 1.91)	1.67 (1.46 to 1.91)	1.67 (1.46 to 1.91)	1.67 (1.46 to 1.91)	1.67 (1.46 to 1.91)	1.67 (1.46 to 1.91)	1.67 (1.46 to 1.91)
Multiple sclerosis	20 Cigarettes Per Day	Both	Female	2.01 (1.67 to 2.37)	2.01 (1.67 to 2.37)	2.01 (1.67 to 2.37)	2.01 (1.67 to 2.37)	2.01 (1.67 to 2.37)	2.01 (1.67 to 2.37)	2.01 (1.67 to 2.37)	2.01 (1.67 to 2.37)	2.01 (1.67 to 2.37)	2.01 (1.67 to 2.37)	2.01 (1.67 to 2.37)	2.01 (1.67 to 2.37)	2.01 (1.67 to 2.37)	2.01 (1.67 to 2.37)
Multiple sclerosis	20 Cigarettes Per Day	Both	Male	2.01 (1.67 to 2.37)	2.01 (1.67 to 2.37)	2.01 (1.67 to 2.37)	2.01 (1.67 to 2.37)	2.01 (1.67 to 2.37)	2.01 (1.67 to 2.37)	2.01 (1.67 to 2.37)	2.01 (1.67 to 2.37)	2.01 (1.67 to 2.37)	2.01 (1.67 to 2.37)	2.01 (1.67 to 2.37)	2.01 (1.67 to 2.37)	2.01 (1.67 to 2.37)	2.01 (1.67 to 2.37)
Multiple sclerosis	30 Cigarettes Per Day	Both	Female	2.10 (1.55 to 2.76)	2.10 (1.55 to 2.76)	2.10 (1.55 to 2.76)	2.10 (1.55 to 2.76)	2.10 (1.55 to 2.76)	2.10 (1.55 to 2.76)	2.10 (1.55 to 2.76)	2.10 (1.55 to 2.76)	2.10 (1.55 to 2.76)	2.10 (1.55 to 2.76)	2.10 (1.55 to 2.76)	2.10 (1.55 to 2.76)	2.10 (1.55 to 2.76)	2.10 (1.55 to 2.76)
Multiple sclerosis	30 Cigarettes Per Day	Both	Male	2.10 (1.55 to 2.76)	2.10 (1.55 to 2.76)	2.10 (1.55 to 2.76)	2.10 (1.55 to 2.76)	2.10 (1.55 to 2.76)	2.10 (1.55 to 2.76)	2.10 (1.55 to 2.76)	2.10 (1.55 to 2.76)	2.10 (1.55 to 2.76)	2.10 (1.55 to 2.76)	2.10 (1.55 to 2.76)	2.10 (1.55 to 2.76)	2.10 (1.55 to 2.76)	2.10 (1.55 to 2.76)
Multiple sclerosis	40 Cigarettes Per Day	Both	Female	2.17 (1.42 to 3.14)	2.17 (1.42 to 3.14)	2.17 (1.42 to 3.14)	2.17 (1.42 to 3.14)	2.17 (1.42 to 3.14)	2.17 (1.42 to 3.14)	2.17 (1.42 to 3.14)	2.17 (1.42 to 3.14)	2.17 (1.42 to 3.14)	2.17 (1.42 to 3.14)	2.17 (1.42 to 3.14)	2.17 (1.42 to 3.14)	2.17 (1.42 to 3.14)	2.17 (1.42 to 3.14)
Multiple sclerosis	40 Cigarettes Per Day	Both	Male	2.17 (1.42 to 3.14)	2.17 (1.42 to 3.14)	2.17 (1.42 to 3.14)	2.17 (1.42 to 3.14)	2.17 (1.42 to 3.14)	2.17 (1.42 to 3.14)	2.17 (1.42 to 3.14)	2.17 (1.42 to 3.14)	2.17 (1.42 to 3.14)	2.17 (1.42 to 3.14)	2.17 (1.42 to 3.14)	2.17 (1.42 to 3.14)	2.17 (1.42 to 3.14)	2.17 (1.42 to 3.14)
Cataract	0 Cigarettes Per Day	Both	Female	1.00 (1.00 to 1.00)	1.00 (1.00 to 1.00)	1.00 (1.00 to 1.00)	1.00 (1.00 to 1.00)	1.00 (1.00 to 1.00)	1.00 (1.00 to 1.00)	1.00 (1.00 to 1.00)	1.00 (1.00 to 1.00)	1.00 (1.00 to 1.00)	1.00 (1.00 to 1.00)	1.00 (1.00 to 1.00)	1.00 (1.00 to 1.00)	1.00 (1.00 to 1.00)	1.00 (1.00 to 1.00)
Cataract	0 Cigarettes Per Day	Both	Male	1.00 (1.00 to 1.00)	1.00 (1.00 to 1.00)	1.00 (1.00 to 1.00)	1.00 (1.00 to 1.00)	1.00 (1.00 to 1.00)	1.00 (1.00 to 1.00)	1.00 (1.00 to 1.00)	1.00 (1.00 to 1.00)	1.00 (1.00 to 1.00)	1.00 (1.00 to 1.00)	1.00 (1.00 to 1.00)	1.00 (1.00 to 1.00)	1.00 (1.00 to 1.00)	1.00 (1.00 to 1.00)
Cataract	10 Cigarettes Per Day	Both	Female	1.44 (1.31 to 1.59)	1.44 (1.31 to 1.59)	1.44 (1.31 to 1.59)	1.44 (1.31 to 1.59)	1.44 (1.31 to 1.59)	1.44 (1.31 to 1.59)	1.44 (1.31 to 1.59)	1.44 (1.31 to 1.59)	1.44 (1.31 to 1.59)	1.44 (1.31 to 1.59)	1.44 (1.31 to 1.59)	1.44 (1.31 to 1.59)	1.44 (1.31 to 1.59)	1.44 (1.31 to 1.59)
Cataract	10 Cigarettes Per Day	Both	Male	1.44 (1.31 to 1.59)	1.44 (1.31 to 1.59)	1.44 (1.31 to 1.59)	1.44 (1.31 to 1.59)	1.44 (1.31 to 1.59)	1.44 (1.31 to 1.59)	1.44 (1.31 to 1.59)	1.44 (1.31 to 1.59)	1.44 (1.31 to 1.59)	1.44 (1.31 to 1.59)	1.44 (1.31 to 1.59)	1.44 (1.31 to 1.59)	1.44 (1.31 to 1.59)	1.44 (1.31 to 1.59)
Cataract	20 Cigarettes Per Day	Both	Female	1.55 (1.34 to 1.77)	1.55 (1.34 to 1.77)	1.55 (1.34 to 1.77)	1.55 (1.34 to 1.77)	1.55 (1.34 to 1.77)	1.55 (1.34 to 1.77)	1.55 (1.34 to 1.77)	1.55 (1.34 to 1.77)	1.55 (1.34 to 1.77)	1.55 (1.34 to 1.77)	1.55 (1.34 to 1.77)	1.55 (1.34 to 1.77)	1.55 (1.34 to 1.77)	1.55 (1.34 to 1.77)
Cataract	20 Cigarettes Per Day	Both	Male	1.55 (1.34 to 1.77)	1.55 (1.34 to 1.77)	1.55 (1.34 to 1.77)	1.55 (1.34 to 1.77)	1.55 (1.34 to 1.77)	1.55 (1.34 to 1.77)	1.55 (1.34 to 1.77)	1.55 (1.34 to 1.77)	1.55 (1.34 to 1.77)	1.55 (1.34 to 1.77)	1.55 (1.34 to 1.77)	1.55 (1.34 to 1.77)	1.55 (1.34 to 1.77)	1.55 (1.34 to 1.77)
Cataract	30 Cigarettes Per Day	Both	Female	1.82 (1.38 to 2.32)	1.82 (1.38 to 2.32)	1.82 (1.38 to 2.32)	1.82 (1.38 to 2.32)	1.82 (1.38 to 2.32)	1.82 (1.38 to 2.32)	1.82 (1.38 to 2.32)	1.82 (1.38 to 2.32)	1.82 (1.38 to 2.32)	1.82 (1.38 to 2.32)	1.82 (1.38 to 2.32)	1.82 (1.38 to 2.32)	1.82 (1.38 to 2.32)	1.82 (1.38 to 2.32)
Cataract	30 Cigarettes Per Day	Both	Male	1.82 (1.38 to 2.32)	1.82 (1.38 to 2.32)	1.82 (1.38 to 2.32)	1.82 (1.38 to 2.32)	1.82 (1.38 to 2.32)	1.82 (1.38 to 2.32)	1.82 (1.38 to 2.32)	1.82 (1.38 to 2.32)	1.82 (1.38 to 2.32)	1.82 (1.38 to 2.32)	1.82 (1.38 to 2.32)	1.82 (1.38 to 2.32)	1.82 (1.38 to 2.32)	1.82 (1.38 to 2.32)

Cataract	40 Cigarettes Per Day	Both	Female	2.05 (1.21 to 3.10)	2.05 (1.21 to 3.10)	2.05 (1.21 to 3.10)	2.05 (1.21 to 3.10)	2.05 (1.21 to 3.10)	2.05 (1.21 to 3.10)	2.05 (1.21 to 3.10)	2.05 (1.21 to 3.10)	2.05 (1.21 to 3.10)	2.05 (1.21 to 3.10)	2.05 (1.21 to 3.10)	2.05 (1.21 to 3.10)	2.05 (1.21 to 3.10)	2.05 (1.21 to 3.10)
Cataract	40 Cigarettes Per Day	Both	Male	2.05 (1.21 to 3.10)	2.05 (1.21 to 3.10)	2.05 (1.21 to 3.10)	2.05 (1.21 to 3.10)	2.05 (1.21 to 3.10)	2.05 (1.21 to 3.10)	2.05 (1.21 to 3.10)	2.05 (1.21 to 3.10)	2.05 (1.21 to 3.10)	2.05 (1.21 to 3.10)	2.05 (1.21 to 3.10)	2.05 (1.21 to 3.10)	2.05 (1.21 to 3.10)	2.05 (1.21 to 3.10)
Age-related macular degeneration	0 Cigarettes Per Day	Both	Female	1.00 (1.00 to 1.00)	1.00 (1.00 to 1.00)	1.00 (1.00 to 1.00)	1.00 (1.00 to 1.00)	1.00 (1.00 to 1.00)	1.00 (1.00 to 1.00)	1.00 (1.00 to 1.00)	1.00 (1.00 to 1.00)	1.00 (1.00 to 1.00)	1.00 (1.00 to 1.00)	1.00 (1.00 to 1.00)	1.00 (1.00 to 1.00)	1.00 (1.00 to 1.00)	1.00 (1.00 to 1.00)
Age-related macular degeneration	0 Cigarettes Per Day	Both	Male	1.00 (1.00 to 1.00)	1.00 (1.00 to 1.00)	1.00 (1.00 to 1.00)	1.00 (1.00 to 1.00)	1.00 (1.00 to 1.00)	1.00 (1.00 to 1.00)	1.00 (1.00 to 1.00)	1.00 (1.00 to 1.00)	1.00 (1.00 to 1.00)	1.00 (1.00 to 1.00)	1.00 (1.00 to 1.00)	1.00 (1.00 to 1.00)	1.00 (1.00 to 1.00)	1.00 (1.00 to 1.00)
Age-related macular degeneration	10 Cigarettes Per Day	Both	Female	1.45 (1.09 to 1.88)	1.45 (1.09 to 1.88)	1.45 (1.09 to 1.88)	1.45 (1.09 to 1.88)	1.45 (1.09 to 1.88)	1.45 (1.09 to 1.88)	1.45 (1.09 to 1.88)	1.45 (1.09 to 1.88)	1.45 (1.09 to 1.88)	1.45 (1.09 to 1.88)	1.45 (1.09 to 1.88)	1.45 (1.09 to 1.88)	1.45 (1.09 to 1.88)	1.45 (1.09 to 1.88)
Age-related macular degeneration	10 Cigarettes Per Day	Both	Male	1.45 (1.09 to 1.88)	1.45 (1.09 to 1.88)	1.45 (1.09 to 1.88)	1.45 (1.09 to 1.88)	1.45 (1.09 to 1.88)	1.45 (1.09 to 1.88)	1.45 (1.09 to 1.88)	1.45 (1.09 to 1.88)	1.45 (1.09 to 1.88)	1.45 (1.09 to 1.88)	1.45 (1.09 to 1.88)	1.45 (1.09 to 1.88)	1.45 (1.09 to 1.88)	1.45 (1.09 to 1.88)
Age-related macular degeneration	20 Cigarettes Per Day	Both	Female	1.91 (1.39 to 2.48)	1.91 (1.39 to 2.48)	1.91 (1.39 to 2.48)	1.91 (1.39 to 2.48)	1.91 (1.39 to 2.48)	1.91 (1.39 to 2.48)	1.91 (1.39 to 2.48)	1.91 (1.39 to 2.48)	1.91 (1.39 to 2.48)	1.91 (1.39 to 2.48)	1.91 (1.39 to 2.48)	1.91 (1.39 to 2.48)	1.91 (1.39 to 2.48)	1.91 (1.39 to 2.48)
Age-related macular degeneration	20 Cigarettes Per Day	Both	Male	1.91 (1.39 to 2.48)	1.91 (1.39 to 2.48)	1.91 (1.39 to 2.48)	1.91 (1.39 to 2.48)	1.91 (1.39 to 2.48)	1.91 (1.39 to 2.48)	1.91 (1.39 to 2.48)	1.91 (1.39 to 2.48)	1.91 (1.39 to 2.48)	1.91 (1.39 to 2.48)	1.91 (1.39 to 2.48)	1.91 (1.39 to 2.48)	1.91 (1.39 to 2.48)	1.91 (1.39 to 2.48)
Age-related macular degeneration	30 Cigarettes Per Day	Both	Female	2.42 (1.71 to 3.14)	2.42 (1.71 to 3.14)	2.42 (1.71 to 3.14)	2.42 (1.71 to 3.14)	2.42 (1.71 to 3.14)	2.42 (1.71 to 3.14)	2.42 (1.71 to 3.14)	2.42 (1.71 to 3.14)	2.42 (1.71 to 3.14)	2.42 (1.71 to 3.14)	2.42 (1.71 to 3.14)	2.42 (1.71 to 3.14)	2.42 (1.71 to 3.14)	2.42 (1.71 to 3.14)
Age-related macular degeneration	30 Cigarettes Per Day	Both	Male	2.42 (1.71 to 3.14)	2.42 (1.71 to 3.14)	2.42 (1.71 to 3.14)	2.42 (1.71 to 3.14)	2.42 (1.71 to 3.14)	2.42 (1.71 to 3.14)	2.42 (1.71 to 3.14)	2.42 (1.71 to 3.14)	2.42 (1.71 to 3.14)	2.42 (1.71 to 3.14)	2.42 (1.71 to 3.14)	2.42 (1.71 to 3.14)	2.42 (1.71 to 3.14)	2.42 (1.71 to 3.14)
Age-related macular degeneration	40 Cigarettes Per Day	Both	Female	2.70 (1.70 to 3.89)	2.70 (1.70 to 3.89)	2.70 (1.70 to 3.89)	2.70 (1.70 to 3.89)	2.70 (1.70 to 3.89)	2.70 (1.70 to 3.89)	2.70 (1.70 to 3.89)	2.70 (1.70 to 3.89)	2.70 (1.70 to 3.89)	2.70 (1.70 to 3.89)	2.70 (1.70 to 3.89)	2.70 (1.70 to 3.89)	2.70 (1.70 to 3.89)	2.70 (1.70 to 3.89)
Age-related macular degeneration	40 Cigarettes Per Day	Both	Male	2.70 (1.70 to 3.89)	2.70 (1.70 to 3.89)	2.70 (1.70 to 3.89)	2.70 (1.70 to 3.89)	2.70 (1.70 to 3.89)	2.70 (1.70 to 3.89)	2.70 (1.70 to 3.89)	2.70 (1.70 to 3.89)	2.70 (1.70 to 3.89)	2.70 (1.70 to 3.89)	2.70 (1.70 to 3.89)	2.70 (1.70 to 3.89)	2.70 (1.70 to 3.89)	2.70 (1.70 to 3.89)
Age-related macular degeneration	50 Cigarettes Per Day	Both	Female	2.89 (1.58 to 4.65)	2.89 (1.58 to 4.65)	2.89 (1.58 to 4.65)	2.89 (1.58 to 4.65)	2.89 (1.58 to 4.65)	2.89 (1.58 to 4.65)	2.89 (1.58 to 4.65)	2.89 (1.58 to 4.65)	2.89 (1.58 to 4.65)	2.89 (1.58 to 4.65)	2.89 (1.58 to 4.65)	2.89 (1.58 to 4.65)	2.89 (1.58 to 4.65)	2.89 (1.58 to 4.65)
Age-related macular degeneration	50 Cigarettes Per Day	Both	Male	2.89 (1.58 to 4.65)	2.89 (1.58 to 4.65)	2.89 (1.58 to 4.65)	2.89 (1.58 to 4.65)	2.89 (1.58 to 4.65)	2.89 (1.58 to 4.65)	2.89 (1.58 to 4.65)	2.89 (1.58 to 4.65)	2.89 (1.58 to 4.65)	2.89 (1.58 to 4.65)	2.89 (1.58 to 4.65)	2.89 (1.58 to 4.65)	2.89 (1.58 to 4.65)	2.89 (1.58 to 4.65)
Age-related macular degeneration	60 Cigarettes Per Day	Both	Female	3.08 (1.46 to 5.42)	3.08 (1.46 to 5.42)	3.08 (1.46 to 5.42)	3.08 (1.46 to 5.42)	3.08 (1.46 to 5.42)	3.08 (1.46 to 5.42)	3.08 (1.46 to 5.42)	3.08 (1.46 to 5.42)	3.08 (1.46 to 5.42)	3.08 (1.46 to 5.42)	3.08 (1.46 to 5.42)	3.08 (1.46 to 5.42)	3.08 (1.46 to 5.42)	3.08 (1.46 to 5.42)
Age-related macular degeneration	60 Cigarettes Per Day	Both	Male	3.08 (1.46 to 5.42)	3.08 (1.46 to 5.42)	3.08 (1.46 to 5.42)	3.08 (1.46 to 5.42)	3.08 (1.46 to 5.42)	3.08 (1.46 to 5.42)	3.08 (1.46 to 5.42)	3.08 (1.46 to 5.42)	3.08 (1.46 to 5.42)	3.08 (1.46 to 5.42)	3.08 (1.46 to 5.42)	3.08 (1.46 to 5.42)	3.08 (1.46 to 5.42)	3.08 (1.46 to 5.42)
Gallbladder and biliary diseases	0 Cigarettes Per Day	Both	Female	1.00 (1.00 to 1.00)	1.00 (1.00 to 1.00)	1.00 (1.00 to 1.00)	1.00 (1.00 to 1.00)	1.00 (1.00 to 1.00)	1.00 (1.00 to 1.00)	1.00 (1.00 to 1.00)	1.00 (1.00 to 1.00)	1.00 (1.00 to 1.00)	1.00 (1.00 to 1.00)	1.00 (1.00 to 1.00)	1.00 (1.00 to 1.00)	1.00 (1.00 to 1.00)	1.00 (1.00 to 1.00)
Gallbladder and biliary diseases	0 Cigarettes Per Day	Both	Male	1.00 (1.00 to 1.00)	1.00 (1.00 to 1.00)	1.00 (1.00 to 1.00)	1.00 (1.00 to 1.00)	1.00 (1.00 to 1.00)	1.00 (1.00 to 1.00)	1.00 (1.00 to 1.00)	1.00 (1.00 to 1.00)	1.00 (1.00 to 1.00)	1.00 (1.00 to 1.00)	1.00 (1.00 to 1.00)	1.00 (1.00 to 1.00)	1.00 (1.00 to 1.00)	1.00 (1.00 to 1.00)
Gallbladder and biliary diseases	10 Cigarettes Per Day	Both	Female	1.35 (1.21 to 1.50)	1.35 (1.21 to 1.50)	1.35 (1.21 to 1.50)	1.35 (1.21 to 1.50)	1.35 (1.21 to 1.50)	1.35 (1.21 to 1.50)	1.35 (1.21 to 1.50)	1.35 (1.21 to 1.50)	1.35 (1.21 to 1.50)	1.35 (1.21 to 1.50)	1.35 (1.21 to 1.50)	1.35 (1.21 to 1.50)	1.35 (1.21 to 1.50)	1.35 (1.21 to 1.50)
Gallbladder and biliary diseases	10 Cigarettes Per Day	Both	Male	1.35 (1.21 to 1.50)	1.35 (1.21 to 1.50)	1.35 (1.21 to 1.50)	1.35 (1.21 to 1.50)	1.35 (1.21 to 1.50)	1.35 (1.21 to 1.50)	1.35 (1.21 to 1.50)	1.35 (1.21 to 1.50)	1.35 (1.21 to 1.50)	1.35 (1.21 to 1.50)	1.35 (1.21 to 1.50)	1.35 (1.21 to 1.50)	1.35 (1.21 to 1.50)	1.35 (1.21 to 1.50)
Gallbladder and biliary diseases	20 Cigarettes Per Day	Both	Female	1.33 (1.22 to 1.45)	1.33 (1.22 to 1.45)	1.33 (1.22 to 1.45)	1.33 (1.22 to 1.45)	1.33 (1.22 to 1.45)	1.33 (1.22 to 1.45)	1.33 (1.22 to 1.45)	1.33 (1.22 to 1.45)	1.33 (1.22 to 1.45)	1.33 (1.22 to 1.45)	1.33 (1.22 to 1.45)	1.33 (1.22 to 1.45)	1.33 (1.22 to 1.45)	1.33 (1.22 to 1.45)
Gallbladder and biliary diseases	20 Cigarettes Per Day	Both	Male	1.33 (1.22 to 1.45)	1.33 (1.22 to 1.45)	1.33 (1.22 to 1.45)	1.33 (1.22 to 1.45)	1.33 (1.22 to 1.45)	1.33 (1.22 to 1.45)	1.33 (1.22 to 1.45)	1.33 (1.22 to 1.45)	1.33 (1.22 to 1.45)	1.33 (1.22 to 1.45)	1.33 (1.22 to 1.45)	1.33 (1.22 to 1.45)	1.33 (1.22 to 1.45)	1.33 (1.22 to 1.45)
Gallbladder and biliary diseases	30 Cigarettes Per Day	Both	Female	1.49 (1.30 to 1.69)	1.49 (1.30 to 1.69)	1.49 (1.30 to 1.69)	1.49 (1.30 to 1.69)	1.49 (1.30 to 1.69)	1.49 (1.30 to 1.69)	1.49 (1.30 to 1.69)	1.49 (1.30 to 1.69)	1.49 (1.30 to 1.69)	1.49 (1.30 to 1.69)	1.49 (1.30 to 1.69)	1.49 (1.30 to 1.69)	1.49 (1.30 to 1.69)	1.49 (1.30 to 1.69)
Gallbladder and biliary diseases	30 Cigarettes Per Day	Both	Male	1.49 (1.30 to 1.69)	1.49 (1.30 to 1.69)	1.49 (1.30 to 1.69)	1.49 (1.30 to 1.69)	1.49 (1.30 to 1.69)	1.49 (1.30 to 1.69)	1.49 (1.30 to 1.69)	1.49 (1.30 to 1.69)	1.49 (1.30 to 1.69)	1.49 (1.30 to 1.69)	1.49 (1.30 to 1.69)	1.49 (1.30 to 1.69)	1.49 (1.30 to 1.69)	1.49 (1.30 to 1.69)
Gallbladder and biliary diseases	40 Cigarettes Per Day	Both	Female	1.72 (1.44 to 2.02)	1.72 (1.44 to 2.02)	1.72 (1.44 to 2.02)	1.72 (1.44 to 2.02)	1.72 (1.44 to 2.02)	1.72 (1.44 to 2.02)	1.72 (1.44 to 2.02)	1.72 (1.44 to 2.02)	1.72 (1.44 to 2.02)	1.72 (1.44 to 2.02)	1.72 (1.44 to 2.02)	1.72 (1.44 to 2.02)	1.72 (1.44 to 2.02)	1.72 (1.44 to 2.02)
Gallbladder and biliary diseases	40 Cigarettes Per Day	Both	Male	1.72 (1.44 to 2.02)	1.72 (1.44 to 2.02)	1.72 (1.44 to 2.02)	1.72 (1.44 to 2.02)	1.72 (1.44 to 2.02)	1.72 (1.44 to 2.02)	1.72 (1.44 to 2.02)	1.72 (1.44 to 2.02)	1.72 (1.44 to 2.02)	1.72 (1.44 to 2.02)	1.72 (1.44 to 2.02)	1.72 (1.44 to 2.02)	1.72 (1.44 to 2.02)	1.72 (1.44 to 2.02)
Gallbladder and biliary diseases	50 Cigarettes Per Day	Both	Female	1.88 (1.45 to 2.34)	1.88 (1.45 to 2.34)	1.88 (1.45 to 2.34)	1.88 (1.45 to 2.34)	1.88 (1.45 to 2.34)	1.88 (1.45 to 2.34)	1.88 (1.45 to 2.34)	1.88 (1.45 to 2.34)	1.88 (1.45 to 2.34)	1.88 (1.45 to 2.34)	1.88 (1.45 to 2.34)	1.88 (1.45 to 2.34)	1.88 (1.45 to 2.34)	1.88 (1.45 to 2.34)
Gallbladder and biliary diseases	50 Cigarettes Per Day	Both	Male	1.88 (1.45 to 2.34)	1.88 (1.45 to 2.34)	1.88 (1.45 to 2.34)	1.88 (1.45 to 2.34)	1.88 (1.45 to 2.34)	1.88 (1.45 to 2.34)	1.88 (1.45 to 2.34)	1.88 (1.45 to 2.34)	1.88 (1.45 to 2.34)	1.88 (1.45 to 2.34)	1.88 (1.45 to 2.34)	1.88 (1.45 to 2.34)	1.88 (1.45 to 2.34)	1.88 (1.45 to 2.34)
Low back pain	0 Cigarettes Per Day	Both	Female	1.00 (1.00 to 1.00)	1.00 (1.00 to 1.00)	1.00 (1.00 to 1.00)	1.00 (1.00 to 1.00)	1.00 (1.00 to 1.00)	1.00 (1.00 to 1.00)	1.00 (1.00 to 1.00)	1.00 (1.00 to 1.00)	1.00 (1.00 to 1.00)	1.00 (1.00 to 1.00)	1.00 (1.00 to 1.00)	1.00 (1.00 to 1.00)	1.00 (1.00 to 1.00)	1.00 (1.00 to 1.00)
Low back pain	0 Cigarettes Per Day	Both	Male	1.00 (1.00 to 1.00)	1.00 (1.00 to 1.00)	1.00 (1.00 to 1.00)	1.00 (1.00 to 1.00)	1.00 (1.00 to 1.00)	1.00 (1.00 to 1.00)	1.00 (1.00 to 1.00)	1.00 (1.00 to 1.00)	1.00 (1.00 to 1.00)	1.00 (1.00 to 1.00)	1.00 (1.00 to 1.00)	1.00 (1.00 to 1.00)	1.00 (1.00 to 1.00)	1.00 (1.00 to 1.00)
Low back pain	10 Cigarettes Per Day	Both	Female	2.16 (1.79 to 2.54)	2.16 (1.79 to 2.54)	2.16 (1.79 to 2.54)	2.16 (1.79 to 2.54)	2.16 (1.79 to 2.54)	2.16 (1.79 to 2.54)	2.16 (1.79 to 2.54)	2.16 (1.79 to 2.54)	2.16 (1.79 to 2.54)	2.16 (1.79 to 2.54)	2.16 (1.79 to 2.54)	2.16 (1.79 to 2.54)	2.16 (1.79 to 2.54)	2.16 (1.79 to 2.54)
Low back pain	10 Cigarettes Per Day	Both	Male	2.16 (1.79 to 2.54)	2.16 (1.79 to 2.54)	2.16 (1.79 to 2.54)	2.16 (1.79 to 2.54)	2.16 (1.79 to 2.54)	2.16 (1.79 to 2.54)	2.16 (1.79 to 2.54)	2.16 (1.79 to 2.54)	2.16 (1.79 to 2.54)	2.16 (1.79 to 2.54)	2.16 (1.79 to 2.54)	2.16 (1.79 to 2.54)	2.16 (1.79 to 2.54)	2.16 (1.79 to 2.54)
Low back pain	20 Cigarettes Per Day	Both	Female	2.35 (1.72 to 3.14)	2.35 (1.72 to 3.14)	2.35 (1.72 to 3.14)	2.35 (1.72 to 3.14)	2.35 (1.72 to 3.14)	2.35 (1.72 to 3.14)	2.35 (1.72 to 3.14)	2.35 (1.72 to 3.14)	2.35 (1.72 to 3.14)	2.35 (1.72 to 3.14)	2.35 (1.72 to 3.14)	2.35 (1.72 to 3.14)	2.35 (1.72 to 3.14)	2.35 (1.72 to 3.14)
Low back pain	20 Cigarettes Per Day	Both	Male	2.35 (1.72 to 3.14)	2.35 (1.72 to 3.14)	2.35 (1.72 to 3.14)	2.35 (1.72 to 3.14)	2.35 (1.72 to 3.14)	2.35 (1.72 to 3.14)	2.35 (1.72 to 3.14)	2.35 (1.72 to 3.14)	2.35 (1.72 to 3.14)	2.35 (1.72 to 3.14)	2.35 (1.72 to 3.14)	2.35 (1.72 to 3.14)	2.35 (1.72 to 3.14)	2.35 (1.72 to 3.14)
Low back pain	30 Cigarettes Per Day	Both	Female	2.76 (1.56 to 4.52)	2.76 (1.56 to 4.52)	2.76 (1.56 to 4.52)	2.76 (1.56 to 4.52)	2.76 (1.56 to 4.52)	2.76 (1.56 to 4.52)	2.76 (1.56 to 4.52)	2.76 (1.56 to 4.52)	2.76 (1.56 to 4.52)	2.76 (1.56 to 4.52)	2.76 (1.56 to 4.52)	2.76 (1.56 to 4.52)	2.76 (1.56 to 4.52)	2.76 (1.56 to 4.52)
Low back pain	30 Cigarettes Per Day	Both	Male	2.76 (1.56 to 4.52)	2.76 (1.56 to 4.52)	2.76 (1.56 to 4.52)	2.76 (1.56 to 4.52)	2.76 (1.56 to 4.52)	2.76 (1.56 to 4.52)	2.76 (1.56 to 4.52)	2.76 (1.56 to 4.52)	2.76 (1.56 to 4.52)	2.76 (1.56 to 4.52)	2.76 (1.56 to 4.52)	2.76 (1.56 to 4.52)	2.76 (1.56 to 4.52)	2.76 (1.56 to 4.52)
Low back pain	40 Cigarettes Per Day	Both	Female	3.10 (1.43 to 5.81)	3.10 (1.43 to 5.81)	3.10 (1.43 to 5.81)	3.10 (1.43 to 5.81)	3.10 (1.43 to 5.81)	3.10 (1.43 to 5.81)	3.10 (1.43 to 5.81)	3.10 (1.43 to 5.81)	3.10 (1.43 to 5.81)	3.10 (1.43 to 5.81)	3.10 (1.43 to 5.81)	3.10 (1.43 to 5.81)	3.10 (1.43 to 5.81)	3.10 (1.43 to 5.81)
Low back pain	40 Cigarettes Per Day	Both	Male	3.10 (1.43 to 5.81)	3.10 (1.43 to 5.81)	3.10 (1.43 to 5.81)	3.10 (1.43 to 5.81)	3.10 (1.43 to 5.81)	3.10 (1.43 to 5.81)	3.10 (1.43 to 5.81)	3.10 (1.43 to 5.81)	3.10 (1.43 to 5.81)	3.10 (1.43 to 5.81)	3.10 (1.43 to 5.81)	3.10 (1.43 to 5.81)	3.10 (1.43 to 5.81)	3.10 (1.43 to 5.81)

Peptic ulcer disease	0 Cigarettes Per Day	Both	Female	1.00 (1.00 to 1.00)	1.00 (1.00 to 1.00)	1.00 (1.00 to 1.00)	1.00 (1.00 to 1.00)	1.00 (1.00 to 1.00)	1.00 (1.00 to 1.00)	1.00 (1.00 to 1.00)	1.00 (1.00 to 1.00)	1.00 (1.00 to 1.00)	1.00 (1.00 to 1.00)	1.00 (1.00 to 1.00)	1.00 (1.00 to 1.00)	1.00 (1.00 to 1.00)	1.00 (1.00 to 1.00)
Peptic ulcer disease	0 Cigarettes Per Day	Both	Male	1.00 (1.00 to 1.00)	1.00 (1.00 to 1.00)	1.00 (1.00 to 1.00)	1.00 (1.00 to 1.00)	1.00 (1.00 to 1.00)	1.00 (1.00 to 1.00)	1.00 (1.00 to 1.00)	1.00 (1.00 to 1.00)	1.00 (1.00 to 1.00)	1.00 (1.00 to 1.00)	1.00 (1.00 to 1.00)	1.00 (1.00 to 1.00)	1.00 (1.00 to 1.00)	1.00 (1.00 to 1.00)
Peptic ulcer disease	10 Cigarettes Per Day	Both	Female	2.00 (1.68 to 2.33)	2.00 (1.68 to 2.33)	2.00 (1.68 to 2.33)	2.00 (1.68 to 2.33)	2.00 (1.68 to 2.33)	2.00 (1.68 to 2.33)	2.00 (1.68 to 2.33)	2.00 (1.68 to 2.33)	2.00 (1.68 to 2.33)	2.00 (1.68 to 2.33)	2.00 (1.68 to 2.33)	2.00 (1.68 to 2.33)	2.00 (1.68 to 2.33)	2.00 (1.68 to 2.33)
Peptic ulcer disease	10 Cigarettes Per Day	Both	Male	2.00 (1.68 to 2.33)	2.00 (1.68 to 2.33)	2.00 (1.68 to 2.33)	2.00 (1.68 to 2.33)	2.00 (1.68 to 2.33)	2.00 (1.68 to 2.33)	2.00 (1.68 to 2.33)	2.00 (1.68 to 2.33)	2.00 (1.68 to 2.33)	2.00 (1.68 to 2.33)	2.00 (1.68 to 2.33)	2.00 (1.68 to 2.33)	2.00 (1.68 to 2.33)	2.00 (1.68 to 2.33)
Peptic ulcer disease	20 Cigarettes Per Day	Both	Female	2.52 (2.15 to 2.93)	2.52 (2.15 to 2.93)	2.52 (2.15 to 2.93)	2.52 (2.15 to 2.93)	2.52 (2.15 to 2.93)	2.52 (2.15 to 2.93)	2.52 (2.15 to 2.93)	2.52 (2.15 to 2.93)	2.52 (2.15 to 2.93)	2.52 (2.15 to 2.93)	2.52 (2.15 to 2.93)	2.52 (2.15 to 2.93)	2.52 (2.15 to 2.93)	2.52 (2.15 to 2.93)
Peptic ulcer disease	20 Cigarettes Per Day	Both	Male	2.52 (2.15 to 2.93)	2.52 (2.15 to 2.93)	2.52 (2.15 to 2.93)	2.52 (2.15 to 2.93)	2.52 (2.15 to 2.93)	2.52 (2.15 to 2.93)	2.52 (2.15 to 2.93)	2.52 (2.15 to 2.93)	2.52 (2.15 to 2.93)	2.52 (2.15 to 2.93)	2.52 (2.15 to 2.93)	2.52 (2.15 to 2.93)	2.52 (2.15 to 2.93)	2.52 (2.15 to 2.93)
Peptic ulcer disease	30 Cigarettes Per Day	Both	Female	2.76 (2.16 to 3.42)	2.76 (2.16 to 3.42)	2.76 (2.16 to 3.42)	2.76 (2.16 to 3.42)	2.76 (2.16 to 3.42)	2.76 (2.16 to 3.42)	2.76 (2.16 to 3.42)	2.76 (2.16 to 3.42)	2.76 (2.16 to 3.42)	2.76 (2.16 to 3.42)	2.76 (2.16 to 3.42)	2.76 (2.16 to 3.42)	2.76 (2.16 to 3.42)	2.76 (2.16 to 3.42)
Peptic ulcer disease	30 Cigarettes Per Day	Both	Male	2.76 (2.16 to 3.42)	2.76 (2.16 to 3.42)	2.76 (2.16 to 3.42)	2.76 (2.16 to 3.42)	2.76 (2.16 to 3.42)	2.76 (2.16 to 3.42)	2.76 (2.16 to 3.42)	2.76 (2.16 to 3.42)	2.76 (2.16 to 3.42)	2.76 (2.16 to 3.42)	2.76 (2.16 to 3.42)	2.76 (2.16 to 3.42)	2.76 (2.16 to 3.42)	2.76 (2.16 to 3.42)
Peptic ulcer disease	40 Cigarettes Per Day	Both	Female	3.33 (2.02 to 5.01)	3.33 (2.02 to 5.01)	3.33 (2.02 to 5.01)	3.33 (2.02 to 5.01)	3.33 (2.02 to 5.01)	3.33 (2.02 to 5.01)	3.33 (2.02 to 5.01)	3.33 (2.02 to 5.01)	3.33 (2.02 to 5.01)	3.33 (2.02 to 5.01)	3.33 (2.02 to 5.01)	3.33 (2.02 to 5.01)	3.33 (2.02 to 5.01)	3.33 (2.02 to 5.01)
Peptic ulcer disease	40 Cigarettes Per Day	Both	Male	3.33 (2.02 to 5.01)	3.33 (2.02 to 5.01)	3.33 (2.02 to 5.01)	3.33 (2.02 to 5.01)	3.33 (2.02 to 5.01)	3.33 (2.02 to 5.01)	3.33 (2.02 to 5.01)	3.33 (2.02 to 5.01)	3.33 (2.02 to 5.01)	3.33 (2.02 to 5.01)	3.33 (2.02 to 5.01)	3.33 (2.02 to 5.01)	3.33 (2.02 to 5.01)	3.33 (2.02 to 5.01)
Peptic ulcer disease	50 Cigarettes Per Day	Both	Female	3.83 (1.77 to 6.75)	3.83 (1.77 to 6.75)	3.83 (1.77 to 6.75)	3.83 (1.77 to 6.75)	3.83 (1.77 to 6.75)	3.83 (1.77 to 6.75)	3.83 (1.77 to 6.75)	3.83 (1.77 to 6.75)	3.83 (1.77 to 6.75)	3.83 (1.77 to 6.75)	3.83 (1.77 to 6.75)	3.83 (1.77 to 6.75)	3.83 (1.77 to 6.75)	3.83 (1.77 to 6.75)
Peptic ulcer disease	50 Cigarettes Per Day	Both	Male	3.83 (1.77 to 6.75)	3.83 (1.77 to 6.75)	3.83 (1.77 to 6.75)	3.83 (1.77 to 6.75)	3.83 (1.77 to 6.75)	3.83 (1.77 to 6.75)	3.83 (1.77 to 6.75)	3.83 (1.77 to 6.75)	3.83 (1.77 to 6.75)	3.83 (1.77 to 6.75)	3.83 (1.77 to 6.75)	3.83 (1.77 to 6.75)	3.83 (1.77 to 6.75)	3.83 (1.77 to 6.75)
Rheumatoid arthritis	0 Cigarettes Per Day	Both	Female	1.00 (1.00 to 1.00)	1.00 (1.00 to 1.00)	1.00 (1.00 to 1.00)	1.00 (1.00 to 1.00)	1.00 (1.00 to 1.00)	1.00 (1.00 to 1.00)	1.00 (1.00 to 1.00)	1.00 (1.00 to 1.00)	1.00 (1.00 to 1.00)	1.00 (1.00 to 1.00)	1.00 (1.00 to 1.00)	1.00 (1.00 to 1.00)	1.00 (1.00 to 1.00)	1.00 (1.00 to 1.00)
Rheumatoid arthritis	0 Cigarettes Per Day	Both	Male	1.00 (1.00 to 1.00)	1.00 (1.00 to 1.00)	1.00 (1.00 to 1.00)	1.00 (1.00 to 1.00)	1.00 (1.00 to 1.00)	1.00 (1.00 to 1.00)	1.00 (1.00 to 1.00)	1.00 (1.00 to 1.00)	1.00 (1.00 to 1.00)	1.00 (1.00 to 1.00)	1.00 (1.00 to 1.00)	1.00 (1.00 to 1.00)	1.00 (1.00 to 1.00)	1.00 (1.00 to 1.00)
Rheumatoid arthritis	10 Cigarettes Per Day	Both	Female	1.52 (1.06 to 2.05)	1.52 (1.06 to 2.05)	1.52 (1.06 to 2.05)	1.52 (1.06 to 2.05)	1.52 (1.06 to 2.05)	1.52 (1.06 to 2.05)	1.52 (1.06 to 2.05)	1.52 (1.06 to 2.05)	1.52 (1.06 to 2.05)	1.52 (1.06 to 2.05)	1.52 (1.06 to 2.05)	1.52 (1.06 to 2.05)	1.52 (1.06 to 2.05)	1.52 (1.06 to 2.05)
Rheumatoid arthritis	10 Cigarettes Per Day	Both	Male	1.52 (1.06 to 2.05)	1.52 (1.06 to 2.05)	1.52 (1.06 to 2.05)	1.52 (1.06 to 2.05)	1.52 (1.06 to 2.05)	1.52 (1.06 to 2.05)	1.52 (1.06 to 2.05)	1.52 (1.06 to 2.05)	1.52 (1.06 to 2.05)	1.52 (1.06 to 2.05)	1.52 (1.06 to 2.05)	1.52 (1.06 to 2.05)	1.52 (1.06 to 2.05)	1.52 (1.06 to 2.05)
Rheumatoid arthritis	20 Cigarettes Per Day	Both	Female	1.91 (1.24 to 2.77)	1.91 (1.24 to 2.77)	1.91 (1.24 to 2.77)	1.91 (1.24 to 2.77)	1.91 (1.24 to 2.77)	1.91 (1.24 to 2.77)	1.91 (1.24 to 2.77)	1.91 (1.24 to 2.77)	1.91 (1.24 to 2.77)	1.91 (1.24 to 2.77)	1.91 (1.24 to 2.77)	1.91 (1.24 to 2.77)	1.91 (1.24 to 2.77)	1.91 (1.24 to 2.77)
Rheumatoid arthritis	20 Cigarettes Per Day	Both	Male	1.91 (1.24 to 2.77)	1.91 (1.24 to 2.77)	1.91 (1.24 to 2.77)	1.91 (1.24 to 2.77)	1.91 (1.24 to 2.77)	1.91 (1.24 to 2.77)	1.91 (1.24 to 2.77)	1.91 (1.24 to 2.77)	1.91 (1.24 to 2.77)	1.91 (1.24 to 2.77)	1.91 (1.24 to 2.77)	1.91 (1.24 to 2.77)	1.91 (1.24 to 2.77)	1.91 (1.24 to 2.77)
Rheumatoid arthritis	30 Cigarettes Per Day	Both	Female	2.25 (1.27 to 3.54)	2.25 (1.27 to 3.54)	2.25 (1.27 to 3.54)	2.25 (1.27 to 3.54)	2.25 (1.27 to 3.54)	2.25 (1.27 to 3.54)	2.25 (1.27 to 3.54)	2.25 (1.27 to 3.54)	2.25 (1.27 to 3.54)	2.25 (1.27 to 3.54)	2.25 (1.27 to 3.54)	2.25 (1.27 to 3.54)	2.25 (1.27 to 3.54)	2.25 (1.27 to 3.54)
Rheumatoid arthritis	30 Cigarettes Per Day	Both	Male	2.25 (1.27 to 3.54)	2.25 (1.27 to 3.54)	2.25 (1.27 to 3.54)	2.25 (1.27 to 3.54)	2.25 (1.27 to 3.54)	2.25 (1.27 to 3.54)	2.25 (1.27 to 3.54)	2.25 (1.27 to 3.54)	2.25 (1.27 to 3.54)	2.25 (1.27 to 3.54)	2.25 (1.27 to 3.54)	2.25 (1.27 to 3.54)	2.25 (1.27 to 3.54)	2.25 (1.27 to 3.54)
Prostate cancer	0 Cigarettes Per Day	Both	Female	1.00 (1.00 to 1.00)	1.00 (1.00 to 1.00)	1.00 (1.00 to 1.00)	1.00 (1.00 to 1.00)	1.00 (1.00 to 1.00)	1.00 (1.00 to 1.00)	1.00 (1.00 to 1.00)	1.00 (1.00 to 1.00)	1.00 (1.00 to 1.00)	1.00 (1.00 to 1.00)	1.00 (1.00 to 1.00)	1.00 (1.00 to 1.00)	1.00 (1.00 to 1.00)	1.00 (1.00 to 1.00)
Prostate cancer	0 Cigarettes Per Day	Both	Male	1.00 (1.00 to 1.00)	1.00 (1.00 to 1.00)	1.00 (1.00 to 1.00)	1.00 (1.00 to 1.00)	1.00 (1.00 to 1.00)	1.00 (1.00 to 1.00)	1.00 (1.00 to 1.00)	1.00 (1.00 to 1.00)	1.00 (1.00 to 1.00)	1.00 (1.00 to 1.00)	1.00 (1.00 to 1.00)	1.00 (1.00 to 1.00)	1.00 (1.00 to 1.00)	1.00 (1.00 to 1.00)
Prostate cancer	10 Cigarettes Per Day	Both	Female	1.00 (1.00 to 1.00)	1.00 (1.00 to 1.00)	1.00 (1.00 to 1.00)	1.00 (1.00 to 1.00)	1.00 (1.00 to 1.00)	1.00 (1.00 to 1.00)	1.00 (1.00 to 1.00)	1.00 (1.00 to 1.00)	1.00 (1.00 to 1.00)	1.00 (1.00 to 1.00)	1.00 (1.00 to 1.00)	1.00 (1.00 to 1.00)	1.00 (1.00 to 1.00)	1.00 (1.00 to 1.00)
Prostate cancer	10 Cigarettes Per Day	Both	Male	1.19 (1.03 to 1.35)	1.19 (1.03 to 1.35)	1.19 (1.03 to 1.35)	1.19 (1.03 to 1.35)	1.19 (1.03 to 1.35)	1.19 (1.03 to 1.35)	1.19 (1.03 to 1.35)	1.19 (1.03 to 1.35)	1.19 (1.03 to 1.35)	1.19 (1.03 to 1.35)	1.19 (1.03 to 1.35)	1.19 (1.03 to 1.35)	1.19 (1.03 to 1.35)	1.19 (1.03 to 1.35)
Prostate cancer	20 Cigarettes Per Day	Both	Female	1.00 (1.00 to 1.00)	1.00 (1.00 to 1.00)	1.00 (1.00 to 1.00)	1.00 (1.00 to 1.00)	1.00 (1.00 to 1.00)	1.00 (1.00 to 1.00)	1.00 (1.00 to 1.00)	1.00 (1.00 to 1.00)	1.00 (1.00 to 1.00)	1.00 (1.00 to 1.00)	1.00 (1.00 to 1.00)	1.00 (1.00 to 1.00)	1.00 (1.00 to 1.00)	1.00 (1.00 to 1.00)
Prostate cancer	20 Cigarettes Per Day	Both	Male	1.17 (1.02 to 1.33)	1.17 (1.02 to 1.33)	1.17 (1.02 to 1.33)	1.17 (1.02 to 1.33)	1.17 (1.02 to 1.33)	1.17 (1.02 to 1.33)	1.17 (1.02 to 1.33)	1.17 (1.02 to 1.33)	1.17 (1.02 to 1.33)	1.17 (1.02 to 1.33)	1.17 (1.02 to 1.33)	1.17 (1.02 to 1.33)	1.17 (1.02 to 1.33)	1.17 (1.02 to 1.33)
Prostate cancer	30 Cigarettes Per Day	Both	Female	1.00 (1.00 to 1.00)	1.00 (1.00 to 1.00)	1.00 (1.00 to 1.00)	1.00 (1.00 to 1.00)	1.00 (1.00 to 1.00)	1.00 (1.00 to 1.00)	1.00 (1.00 to 1.00)	1.00 (1.00 to 1.00)	1.00 (1.00 to 1.00)	1.00 (1.00 to 1.00)	1.00 (1.00 to 1.00)	1.00 (1.00 to 1.00)	1.00 (1.00 to 1.00)	1.00 (1.00 to 1.00)
Prostate cancer	30 Cigarettes Per Day	Both	Male	1.23 (1.03 to 1.47)	1.23 (1.03 to 1.47)	1.23 (1.03 to 1.47)	1.23 (1.03 to 1.47)	1.23 (1.03 to 1.47)	1.23 (1.03 to 1.47)	1.23 (1.03 to 1.47)	1.23 (1.03 to 1.47)	1.23 (1.03 to 1.47)	1.23 (1.03 to 1.47)	1.23 (1.03 to 1.47)	1.23 (1.03 to 1.47)	1.23 (1.03 to 1.47)	1.23 (1.03 to 1.47)
Prostate cancer	40 Cigarettes Per Day	Both	Female	1.00 (1.00 to 1.00)	1.00 (1.00 to 1.00)	1.00 (1.00 to 1.00)	1.00 (1.00 to 1.00)	1.00 (1.00 to 1.00)	1.00 (1.00 to 1.00)	1.00 (1.00 to 1.00)	1.00 (1.00 to 1.00)	1.00 (1.00 to 1.00)	1.00 (1.00 to 1.00)	1.00 (1.00 to 1.00)	1.00 (1.00 to 1.00)	1.00 (1.00 to 1.00)	1.00 (1.00 to 1.00)
Prostate cancer	40 Cigarettes Per Day	Both	Male	1.35 (0.96 to 1.79)	1.35 (0.96 to 1.79)	1.35 (0.96 to 1.79)	1.35 (0.96 to 1.79)	1.35 (0.96 to 1.79)	1.35 (0.96 to 1.79)	1.35 (0.96 to 1.79)	1.35 (0.96 to 1.79)	1.35 (0.96 to 1.79)	1.35 (0.96 to 1.79)	1.35 (0.96 to 1.79)	1.35 (0.96 to 1.79)	1.35 (0.96 to 1.79)	1.35 (0.96 to 1.79)
Tracheal, bronchus, and lung cancer	0 Pack Years	Both	Female	1.00 (1.00 to 1.00)	1.00 (1.00 to 1.00)	1.00 (1.00 to 1.00)	1.00 (1.00 to 1.00)	1.00 (1.00 to 1.00)	1.00 (1.00 to 1.00)	1.00 (1.00 to 1.00)	1.00 (1.00 to 1.00)	1.00 (1.00 to 1.00)	1.00 (1.00 to 1.00)	1.00 (1.00 to 1.00)	1.00 (1.00 to 1.00)	1.00 (1.00 to 1.00)	1.00 (1.00 to 1.00)
Tracheal, bronchus, and lung cancer	0 Pack Years	Both	Male	1.00 (1.00 to 1.00)	1.00 (1.00 to 1.00)	1.00 (1.00 to 1.00)	1.00 (1.00 to 1.00)	1.00 (1.00 to 1.00)	1.00 (1.00 to 1.00)	1.00 (1.00 to 1.00)	1.00 (1.00 to 1.00)	1.00 (1.00 to 1.00)	1.00 (1.00 to 1.00)	1.00 (1.00 to 1.00)	1.00 (1.00 to 1.00)	1.00 (1.00 to 1.00)	1.00 (1.00 to 1.00)
Tracheal, bronchus, and lung cancer	10 Pack Years	Both	Female	3.43 (2.76 to 4.13)	3.43 (2.76 to 4.13)	3.43 (2.76 to 4.13)	3.43 (2.76 to 4.13)	3.43 (2.76 to 4.13)	3.43 (2.76 to 4.13)	3.43 (2.76 to 4.13)	3.43 (2.76 to 4.13)	3.43 (2.76 to 4.13)	3.43 (2.76 to 4.13)	3.43 (2.76 to 4.13)	3.43 (2.76 to 4.13)	3.43 (2.76 to 4.13)	3.43 (2.76 to 4.13)
Tracheal, bronchus, and lung cancer	10 Pack Years	Both	Male	3.43 (2.76 to 4.13)	3.43 (2.76 to 4.13)	3.43 (2.76 to 4.13)	3.43 (2.76 to 4.13)	3.43 (2.76 to 4.13)	3.43 (2.76 to 4.13)	3.43 (2.76 to 4.13)	3.43 (2.76 to 4.13)	3.43 (2.76 to 4.13)	3.43 (2.76 to 4.13)	3.43 (2.76 to 4.13)	3.43 (2.76 to 4.13)	3.43 (2.76 to 4.13)	3.43 (2.76 to 4.13)
Tracheal, bronchus, and lung cancer	100 Pack Years	Both	Female	20.90 (10.76 to 32.29)	20.90 (10.76 to 32.29)	20.90 (10.76 to 32.29)	20.90 (10.76 to 32.29)	20.90 (10.76 to 32.29)	20.90 (10.76 to 32.29)	20.90 (10.76 to 32.29)	20.90 (10.76 to 32.29)	20.90 (10.76 to 32.29)	20.90 (10.76 to 32.29)	20.90 (10.76 to 32.29)	20.90 (10.76 to 32.29)	20.90 (10.76 to 32.29)	20.90 (10.76 to 32.29)
Tracheal, bronchus, and lung cancer	100 Pack Years	Both	Male	20.90 (10.76 to 32.29)	20.90 (10.76 to 32.29)	20.90 (10.76 to 32.29)	20.90 (10.76 to 32.29)	20.90 (10.76 to 32.29)	20.90 (10.76 to 32.29)	20.90 (10.76 to 32.29)	20.90 (10.76 to 32.29)	20.90 (10.76 to 32.29)	20.90 (10.76 to 32.29)	20.90 (10.76 to 32.29)	20.90 (10.76 to 32.29)	20.90 (10.76 to 32.29)	20.90 (10.76 to 32.29)
Tracheal, bronchus, and lung cancer	20 Pack Years	Both	Female	6.47 (5.47 to 7.53)	6.47 (5.47 to 7.53)	6.47 (5.47 to 7.53)	6.47 (5.47 to 7.53)	6.47 (5.47 to 7.53)	6.47 (5.47 to 7.53)	6.47 (5.47 to 7.53)	6.47 (5.47 to 7.53)	6.47 (5.47 to 7.53)	6.47 (5.47 to 7.53)	6.47 (5.47 to 7.53)	6.47 (5.47 to 7.53)	6.47 (5.47 to 7.53)	6.47 (5.47 to 7.53)
Tracheal, bronchus, and lung cancer	20 Pack Years	Both	Male	6.47 (5.47 to 7.53)	6.47 (5.47 to 7.53)	6.47 (5.47 to 7.53)	6.47 (5.47 to 7.53)	6.47 (5.47 to 7.53)	6.47 (5.47 to 7.53)	6.47 (5.47 to 7.53)	6.47 (5.47 to 7.53)	6.47 (5.47 to 7.53)	6.47 (5.47 to 7.53)	6.47 (5.47 to 7.53)	6.47 (5.47 to 7.53)	6.47 (5.47 to 7.53)	6.47 (5.47 to 7.53)

Tracheal, bronchus, and lung cancer	30 Pack Years	Both	Female	9.35 (7.96 to 10.85)	9.35 (7.96 to 10.85)	9.35 (7.96 to 10.85)	9.35 (7.96 to 10.85)	9.35 (7.96 to 10.85)	9.35 (7.96 to 10.85)	9.35 (7.96 to 10.85)	9.35 (7.96 to 10.85)	9.35 (7.96 to 10.85)	9.35 (7.96 to 10.85)	9.35 (7.96 to 10.85)	9.35 (7.96 to 10.85)	9.35 (7.96 to 10.85)	9.35 (7.96 to 10.85)
Tracheal, bronchus, and lung cancer	30 Pack Years	Both	Male	9.35 (7.96 to 10.85)	9.35 (7.96 to 10.85)	9.35 (7.96 to 10.85)	9.35 (7.96 to 10.85)	9.35 (7.96 to 10.85)	9.35 (7.96 to 10.85)	9.35 (7.96 to 10.85)	9.35 (7.96 to 10.85)	9.35 (7.96 to 10.85)	9.35 (7.96 to 10.85)	9.35 (7.96 to 10.85)	9.35 (7.96 to 10.85)	9.35 (7.96 to 10.85)	9.35 (7.96 to 10.85)
Tracheal, bronchus, and lung cancer	40 Pack Years	Both	Female	12.57 (10.29 to 15.10)	12.57 (10.29 to 15.10)	12.57 (10.29 to 15.10)	12.57 (10.29 to 15.10)	12.57 (10.29 to 15.10)	12.57 (10.29 to 15.10)	12.57 (10.29 to 15.10)	12.57 (10.29 to 15.10)	12.57 (10.29 to 15.10)	12.57 (10.29 to 15.10)	12.57 (10.29 to 15.10)	12.57 (10.29 to 15.10)	12.57 (10.29 to 15.10)	12.57 (10.29 to 15.10)
Tracheal, bronchus, and lung cancer	40 Pack Years	Both	Male	12.57 (10.29 to 15.10)	12.57 (10.29 to 15.10)	12.57 (10.29 to 15.10)	12.57 (10.29 to 15.10)	12.57 (10.29 to 15.10)	12.57 (10.29 to 15.10)	12.57 (10.29 to 15.10)	12.57 (10.29 to 15.10)	12.57 (10.29 to 15.10)	12.57 (10.29 to 15.10)	12.57 (10.29 to 15.10)	12.57 (10.29 to 15.10)	12.57 (10.29 to 15.10)	12.57 (10.29 to 15.10)
Tracheal, bronchus, and lung cancer	50 Pack Years	Both	Female	14.17 (11.09 to 17.45)	14.17 (11.09 to 17.45)	14.17 (11.09 to 17.45)	14.17 (11.09 to 17.45)	14.17 (11.09 to 17.45)	14.17 (11.09 to 17.45)	14.17 (11.09 to 17.45)	14.17 (11.09 to 17.45)	14.17 (11.09 to 17.45)	14.17 (11.09 to 17.45)	14.17 (11.09 to 17.45)	14.17 (11.09 to 17.45)	14.17 (11.09 to 17.45)	14.17 (11.09 to 17.45)
Tracheal, bronchus, and lung cancer	50 Pack Years	Both	Male	14.17 (11.09 to 17.45)	14.17 (11.09 to 17.45)	14.17 (11.09 to 17.45)	14.17 (11.09 to 17.45)	14.17 (11.09 to 17.45)	14.17 (11.09 to 17.45)	14.17 (11.09 to 17.45)	14.17 (11.09 to 17.45)	14.17 (11.09 to 17.45)	14.17 (11.09 to 17.45)	14.17 (11.09 to 17.45)	14.17 (11.09 to 17.45)	14.17 (11.09 to 17.45)	14.17 (11.09 to 17.45)
Tracheal, bronchus, and lung cancer	60 Pack Years	Both	Female	15.60 (11.67 to 19.74)	15.60 (11.67 to 19.74)	15.60 (11.67 to 19.74)	15.60 (11.67 to 19.74)	15.60 (11.67 to 19.74)	15.60 (11.67 to 19.74)	15.60 (11.67 to 19.74)	15.60 (11.67 to 19.74)	15.60 (11.67 to 19.74)	15.60 (11.67 to 19.74)	15.60 (11.67 to 19.74)	15.60 (11.67 to 19.74)	15.60 (11.67 to 19.74)	15.60 (11.67 to 19.74)
Tracheal, bronchus, and lung cancer	60 Pack Years	Both	Male	15.60 (11.67 to 19.74)	15.60 (11.67 to 19.74)	15.60 (11.67 to 19.74)	15.60 (11.67 to 19.74)	15.60 (11.67 to 19.74)	15.60 (11.67 to 19.74)	15.60 (11.67 to 19.74)	15.60 (11.67 to 19.74)	15.60 (11.67 to 19.74)	15.60 (11.67 to 19.74)	15.60 (11.67 to 19.74)	15.60 (11.67 to 19.74)	15.60 (11.67 to 19.74)	15.60 (11.67 to 19.74)
Tracheal, bronchus, and lung cancer	70 Pack Years	Both	Female	18.27 (13.23 to 23.80)	18.27 (13.23 to 23.80)	18.27 (13.23 to 23.80)	18.27 (13.23 to 23.80)	18.27 (13.23 to 23.80)	18.27 (13.23 to 23.80)	18.27 (13.23 to 23.80)	18.27 (13.23 to 23.80)	18.27 (13.23 to 23.80)	18.27 (13.23 to 23.80)	18.27 (13.23 to 23.80)	18.27 (13.23 to 23.80)	18.27 (13.23 to 23.80)	18.27 (13.23 to 23.80)
Tracheal, bronchus, and lung cancer	70 Pack Years	Both	Male	18.27 (13.23 to 23.80)	18.27 (13.23 to 23.80)	18.27 (13.23 to 23.80)	18.27 (13.23 to 23.80)	18.27 (13.23 to 23.80)	18.27 (13.23 to 23.80)	18.27 (13.23 to 23.80)	18.27 (13.23 to 23.80)	18.27 (13.23 to 23.80)	18.27 (13.23 to 23.80)	18.27 (13.23 to 23.80)	18.27 (13.23 to 23.80)	18.27 (13.23 to 23.80)	18.27 (13.23 to 23.80)
Tracheal, bronchus, and lung cancer	80 Pack Years	Both	Female	20.38 (14.28 to 27.34)	20.38 (14.28 to 27.34)	20.38 (14.28 to 27.34)	20.38 (14.28 to 27.34)	20.38 (14.28 to 27.34)	20.38 (14.28 to 27.34)	20.38 (14.28 to 27.34)	20.38 (14.28 to 27.34)	20.38 (14.28 to 27.34)	20.38 (14.28 to 27.34)	20.38 (14.28 to 27.34)	20.38 (14.28 to 27.34)	20.38 (14.28 to 27.34)	20.38 (14.28 to 27.34)
Tracheal, bronchus, and lung cancer	80 Pack Years	Both	Male	20.38 (14.28 to 27.34)	20.38 (14.28 to 27.34)	20.38 (14.28 to 27.34)	20.38 (14.28 to 27.34)	20.38 (14.28 to 27.34)	20.38 (14.28 to 27.34)	20.38 (14.28 to 27.34)	20.38 (14.28 to 27.34)	20.38 (14.28 to 27.34)	20.38 (14.28 to 27.34)	20.38 (14.28 to 27.34)	20.38 (14.28 to 27.34)	20.38 (14.28 to 27.34)	20.38 (14.28 to 27.34)
Tracheal, bronchus, and lung cancer	90 Pack Years	Both	Female	21.34 (13.60 to 30.21)	21.34 (13.60 to 30.21)	21.34 (13.60 to 30.21)	21.34 (13.60 to 30.21)	21.34 (13.60 to 30.21)	21.34 (13.60 to 30.21)	21.34 (13.60 to 30.21)	21.34 (13.60 to 30.21)	21.34 (13.60 to 30.21)	21.34 (13.60 to 30.21)	21.34 (13.60 to 30.21)	21.34 (13.60 to 30.21)	21.34 (13.60 to 30.21)	21.34 (13.60 to 30.21)
Tracheal, bronchus, and lung cancer	90 Pack Years	Both	Male	21.34 (13.60 to 30.21)	21.34 (13.60 to 30.21)	21.34 (13.60 to 30.21)	21.34 (13.60 to 30.21)	21.34 (13.60 to 30.21)	21.34 (13.60 to 30.21)	21.34 (13.60 to 30.21)	21.34 (13.60 to 30.21)	21.34 (13.60 to 30.21)	21.34 (13.60 to 30.21)	21.34 (13.60 to 30.21)	21.34 (13.60 to 30.21)	21.34 (13.60 to 30.21)	21.34 (13.60 to 30.21)
Chronic obstructive pulmonary disease	0 Pack Years	Both	Female	1.00 (1.00 to 1.00)	1.00 (1.00 to 1.00)	1.00 (1.00 to 1.00)	1.00 (1.00 to 1.00)	1.00 (1.00 to 1.00)	1.00 (1.00 to 1.00)	1.00 (1.00 to 1.00)	1.00 (1.00 to 1.00)	1.00 (1.00 to 1.00)	1.00 (1.00 to 1.00)	1.00 (1.00 to 1.00)	1.00 (1.00 to 1.00)	1.00 (1.00 to 1.00)	1.00 (1.00 to 1.00)
Chronic obstructive pulmonary disease	0 Pack Years	Both	Male	1.00 (1.00 to 1.00)	1.00 (1.00 to 1.00)	1.00 (1.00 to 1.00)	1.00 (1.00 to 1.00)	1.00 (1.00 to 1.00)	1.00 (1.00 to 1.00)	1.00 (1.00 to 1.00)	1.00 (1.00 to 1.00)	1.00 (1.00 to 1.00)	1.00 (1.00 to 1.00)	1.00 (1.00 to 1.00)	1.00 (1.00 to 1.00)	1.00 (1.00 to 1.00)	1.00 (1.00 to 1.00)
Chronic obstructive pulmonary disease	10 Pack Years	Both	Female	3.56 (2.75 to 4.41)	3.56 (2.75 to 4.41)	3.56 (2.75 to 4.41)	3.56 (2.75 to 4.41)	3.56 (2.75 to 4.41)	3.56 (2.75 to 4.41)	3.56 (2.75 to 4.41)	3.56 (2.75 to 4.41)	3.56 (2.75 to 4.41)	3.56 (2.75 to 4.41)	3.56 (2.75 to 4.41)	3.56 (2.75 to 4.41)	3.56 (2.75 to 4.41)	3.56 (2.75 to 4.41)
Chronic obstructive pulmonary disease	10 Pack Years	Both	Male	3.56 (2.75 to 4.41)	3.56 (2.75 to 4.41)	3.56 (2.75 to 4.41)	3.56 (2.75 to 4.41)	3.56 (2.75 to 4.41)	3.56 (2.75 to 4.41)	3.56 (2.75 to 4.41)	3.56 (2.75 to 4.41)	3.56 (2.75 to 4.41)	3.56 (2.75 to 4.41)	3.56 (2.75 to 4.41)	3.56 (2.75 to 4.41)	3.56 (2.75 to 4.41)	3.56 (2.75 to 4.41)
Chronic obstructive pulmonary disease	20 Pack Years	Both	Female	5.79 (4.54 to 7.09)	5.79 (4.54 to 7.09)	5.79 (4.54 to 7.09)	5.79 (4.54 to 7.09)	5.79 (4.54 to 7.09)	5.79 (4.54 to 7.09)	5.79 (4.54 to 7.09)	5.79 (4.54 to 7.09)	5.79 (4.54 to 7.09)	5.79 (4.54 to 7.09)	5.79 (4.54 to 7.09)	5.79 (4.54 to 7.09)	5.79 (4.54 to 7.09)	5.79 (4.54 to 7.09)
Chronic obstructive pulmonary disease	20 Pack Years	Both	Male	5.79 (4.54 to 7.09)	5.79 (4.54 to 7.09)	5.79 (4.54 to 7.09)	5.79 (4.54 to 7.09)	5.79 (4.54 to 7.09)	5.79 (4.54 to 7.09)	5.79 (4.54 to 7.09)	5.79 (4.54 to 7.09)	5.79 (4.54 to 7.09)	5.79 (4.54 to 7.09)	5.79 (4.54 to 7.09)	5.79 (4.54 to 7.09)	5.79 (4.54 to 7.09)	5.79 (4.54 to 7.09)
Chronic obstructive pulmonary disease	30 Pack Years	Both	Female	6.62 (5.31 to 8.03)	6.62 (5.31 to 8.03)	6.62 (5.31 to 8.03)	6.62 (5.31 to 8.03)	6.62 (5.31 to 8.03)	6.62 (5.31 to 8.03)	6.62 (5.31 to 8.03)	6.62 (5.31 to 8.03)	6.62 (5.31 to 8.03)	6.62 (5.31 to 8.03)	6.62 (5.31 to 8.03)	6.62 (5.31 to 8.03)	6.62 (5.31 to 8.03)	6.62 (5.31 to 8.03)
Chronic obstructive pulmonary disease	30 Pack Years	Both	Male	6.62 (5.31 to 8.03)	6.62 (5.31 to 8.03)	6.62 (5.31 to 8.03)	6.62 (5.31 to 8.03)	6.62 (5.31 to 8.03)	6.62 (5.31 to 8.03)	6.62 (5.31 to 8.03)	6.62 (5.31 to 8.03)	6.62 (5.31 to 8.03)	6.62 (5.31 to 8.03)	6.62 (5.31 to 8.03)	6.62 (5.31 to 8.03)	6.62 (5.31 to 8.03)	6.62 (5.31 to 8.03)
Chronic obstructive pulmonary disease	40 Pack Years	Both	Female	7.45 (6.08 to 8.97)	7.45 (6.08 to 8.97)	7.45 (6.08 to 8.97)	7.45 (6.08 to 8.97)	7.45 (6.08 to 8.97)	7.45 (6.08 to 8.97)	7.45 (6.08 to 8.97)	7.45 (6.08 to 8.97)	7.45 (6.08 to 8.97)	7.45 (6.08 to 8.97)	7.45 (6.08 to 8.97)	7.45 (6.08 to 8.97)	7.45 (6.08 to 8.97)	7.45 (6.08 to 8.97)
Chronic obstructive pulmonary disease	40 Pack Years	Both	Male	7.45 (6.08 to 8.97)	7.45 (6.08 to 8.97)	7.45 (6.08 to 8.97)	7.45 (6.08 to 8.97)	7.45 (6.08 to 8.97)	7.45 (6.08 to 8.97)	7.45 (6.08 to 8.97)	7.45 (6.08 to 8.97)	7.45 (6.08 to 8.97)	7.45 (6.08 to 8.97)	7.45 (6.08 to 8.97)	7.45 (6.08 to 8.97)	7.45 (6.08 to 8.97)	7.45 (6.08 to 8.97)
Chronic obstructive pulmonary disease	50 Pack Years	Both	Female	7.91 (5.88 to 10.28)	7.91 (5.88 to 10.28)	7.91 (5.88 to 10.28)	7.91 (5.88 to 10.28)	7.91 (5.88 to 10.28)	7.91 (5.88 to 10.28)	7.91 (5.88 to 10.28)	7.91 (5.88 to 10.28)	7.91 (5.88 to 10.28)	7.91 (5.88 to 10.28)	7.91 (5.88 to 10.28)	7.91 (5.88 to 10.28)	7.91 (5.88 to 10.28)	7.91 (5.88 to 10.28)
Chronic obstructive pulmonary disease	50 Pack Years	Both	Male	7.91 (5.88 to 10.28)	7.91 (5.88 to 10.28)	7.91 (5.88 to 10.28)	7.91 (5.88 to 10.28)	7.91 (5.88 to 10.28)	7.91 (5.88 to 10.28)	7.91 (5.88 to 10.28)	7.91 (5.88 to 10.28)	7.91 (5.88 to 10.28)	7.91 (5.88 to 10.28)	7.91 (5.88 to 10.28)	7.91 (5.88 to 10.28)	7.91 (5.88 to 10.28)	7.91 (5.88 to 10.28)
Chronic obstructive pulmonary disease	60 Pack Years	Both	Female	8.01 (4.71 to 11.96)	8.01 (4.71 to 11.96)	8.01 (4.71 to 11.96)	8.01 (4.71 to 11.96)	8.01 (4.71 to 11.96)	8.01 (4.71 to 11.96)	8.01 (4.71 to 11.96)	8.01 (4.71 to 11.96)	8.01 (4.71 to 11.96)	8.01 (4.71 to 11.96)	8.01 (4.71 to 11.96)	8.01 (4.71 to 11.96)	8.01 (4.71 to 11.96)	8.01 (4.71 to 11.96)
Chronic obstructive pulmonary disease	60 Pack Years	Both	Male	8.01 (4.71 to 11.96)	8.01 (4.71 to 11.96)	8.01 (4.71 to 11.96)	8.01 (4.71 to 11.96)	8.01 (4.71 to 11.96)	8.01 (4.71 to 11.96)	8.01 (4.71 to 11.96)	8.01 (4.71 to 11.96)	8.01 (4.71 to 11.96)	8.01 (4.71 to 11.96)	8.01 (4.71 to 11.96)	8.01 (4.71 to 11.96)	8.01 (4.71 to 11.96)	8.01 (4.71 to 11.96)
Chronic obstructive pulmonary disease	70 Pack Years	Both	Female	9.40 (6.00 to 13.47)	9.40 (6.00 to 13.47)	9.40 (6.00 to 13.47)	9.40 (6.00 to 13.47)	9.40 (6.00 to 13.47)	9.40 (6.00 to 13.47)	9.40 (6.00 to 13.47)	9.40 (6.00 to 13.47)	9.40 (6.00 to 13.47)	9.40 (6.00 to 13.47)	9.40 (6.00 to 13.47)	9.40 (6.00 to 13.47)	9.40 (6.00 to 13.47)	9.40 (6.00 to 13.47)
Chronic obstructive pulmonary disease	70 Pack Years	Both	Male	9.40 (6.00 to 13.47)	9.40 (6.00 to 13.47)	9.40 (6.00 to 13.47)	9.40 (6.00 to 13.47)	9.40 (6.00 to 13.47)	9.40 (6.00 to 13.47)	9.40 (6.00 to 13.47)	9.40 (6.00 to 13.47)	9.40 (6.00 to 13.47)	9.40 (6.00 to 13.47)	9.40 (6.00 to 13.47)	9.40 (6.00 to 13.47)	9.40 (6.00 to 13.47)	9.40 (6.00 to 13.47)
Chronic obstructive pulmonary disease	80 Pack Years	Both	Female	11.13 (7.30 to 15.82)	11.13 (7.30 to 15.82)	11.13 (7.30 to 15.82)	11.13 (7.30 to 15.82)	11.13 (7.30 to 15.82)	11.13 (7.30 to 15.82)	11.13 (7.30 to 15.82)	11.13 (7.30 to 15.82)	11.13 (7.30 to 15.82)	11.13 (7.30 to 15.82)	11.13 (7.30 to 15.82)	11.13 (7.30 to 15.82)	11.13 (7.30 to 15.82)	11.13 (7.30 to 15.82)
Chronic obstructive pulmonary disease	80 Pack Years	Both	Male	11.13 (7.30 to 15.82)	11.13 (7.30 to 15.82)	11.13 (7.30 to 15.82)	11.13 (7.30 to 15.82)	11.13 (7.30 to 15.82)	11.13 (7.30 to 15.82)	11.13 (7.30 to 15.82)	11.13 (7.30 to 15.82)	11.13 (7.30 to 15.82)	11.13 (7.30 to 15.82)	11.13 (7.30 to 15.82)	11.13 (7.30 to 15.82)	11.13 (7.30 to 15.82)	11.13 (7.30 to 15.82)
Chronic obstructive pulmonary disease	90 Pack Years	Both	Female	13.20 (8.61 to 18.99)	13.20 (8.61 to 18.99)	13.20 (8.61 to 18.99)	13.20 (8.61 to 18.99)	13.20 (8.61 to 18.99)	13.20 (8.61 to 18.99)	13.20 (8.61 to 18.99)	13.20 (8.61 to 18.99)	13.20 (8.61 to 18.99)	13.20 (8.61 to 18.99)	13.20 (8.61 to 18.99)	13.20 (8.61 to 18.99)	13.20 (8.61 to 18.99)	13.20 (8.61 to 18.99)
Chronic obstructive pulmonary disease	90 Pack Years	Both	Male	13.20 (8.61 to 18.99)	13.20 (8.61 to 18.99)	13.20 (8.61 to 18.99)	13.20 (8.61 to 18.99)	13.20 (8.61 to 18.99)	13.20 (8.61 to 18.99)	13.20 (8.61 to 18.99)	13.20 (8.61 to 18.99)	13.20 (8.61 to 18.99)	13.20 (8.61 to 18.99)	13.20 (8.61 to 18.99)	13.20 (8.61 to 18.99)	13.20 (8.61 to 18.99)	13.20 (8.61 to 18.99)
Oesophageal cancer	0 Pack Years	Both	Female	1.00 (1.00 to 1.00)	1.00 (1.00 to 1.00)	1.00 (1.00 to 1.00)	1.00 (1.00 to 1.00)	1.00 (1.00 to 1.00)	1.00 (1.00 to 1.00)	1.00 (1.00 to 1.00)	1.00 (1.00 to 1.00)	1.00 (1.00 to 1.00)	1.00 (1.00 to 1.00)	1.00 (1.00 to 1.00)	1.00 (1.00 to 1.00)	1.00 (1.00 to 1.00)	1.00 (1.00 to 1.00)
Oesophageal cancer	0 Pack Years	Both	Male	1.00 (1.00 to 1.00)	1.00 (1.00 to 1.00)	1.00 (1.00 to 1.00)	1.00 (1.00 to 1.00)	1.00 (1.00 to 1.00)	1.00 (1.00 to 1.00)	1.00 (1.00 to 1.00)	1.00 (1.00 to 1.00)	1.00 (1.00 to 1.00)	1.00 (1.00 to 1.00)	1.00 (1.00 to 1.00)	1.00 (1.00 to 1.00)	1.00 (1.00 to 1.00)	1.00 (1.00 to 1.00)
Oesophageal cancer	10 Pack Years	Both	Female	2.36 (1.93 to 2.78)	2.36 (1.93 to 2.78)	2.36 (1.93 to 2.78)	2.36 (1.93 to 2.78)	2.36 (1.93 to 2.78)	2.36 (1.93 to 2.78)	2.36 (1.93 to 2.78)	2.36 (1.93 to 2.78)	2.36 (1.93 to 2.78)	2.36 (1.93 to 2.78)	2.36 (1.93 to 2.78)	2.36 (1.93 to 2.78)	2.36 (1.93 to 2.78)	2.36 (1.93 to 2.78)
Oesophageal cancer	10 Pack Years	Both	Male	2.36 (1.93 to 2.78)	2.36 (1.93 to 2.78)	2.36 (1.93 to 2.78)	2.36 (1.93 to 2.78)	2.36 (1.93 to 2.78)	2.36 (1.93 to 2.78)	2.36 (1.93 to 2.78)	2.36 (1.93 to 2.78)	2.36 (1.93 to 2.78)	2.36 (1.93 to 2.78)	2.36 (1.93 to 2.78)	2.36 (1.93 to 2.78)	2.36 (1.93 to 2.78)	2.36 (1.93 to 2.78)

Oesophageal cancer	20 Pack Years	Both	Female	2.95 (2.27 to 3.66)	2.95 (2.27 to 3.66)	2.95 (2.27 to 3.66)	2.95 (2.27 to 3.66)	2.95 (2.27 to 3.66)	2.95 (2.27 to 3.66)	2.95 (2.27 to 3.66)	2.95 (2.27 to 3.66)	2.95 (2.27 to 3.66)	2.95 (2.27 to 3.66)	2.95 (2.27 to 3.66)	2.95 (2.27 to 3.66)	2.95 (2.27 to 3.66)	2.95 (2.27 to 3.66)
Oesophageal cancer	20 Pack Years	Both	Male	2.95 (2.27 to 3.66)	2.95 (2.27 to 3.66)	2.95 (2.27 to 3.66)	2.95 (2.27 to 3.66)	2.95 (2.27 to 3.66)	2.95 (2.27 to 3.66)	2.95 (2.27 to 3.66)	2.95 (2.27 to 3.66)	2.95 (2.27 to 3.66)	2.95 (2.27 to 3.66)	2.95 (2.27 to 3.66)	2.95 (2.27 to 3.66)	2.95 (2.27 to 3.66)	2.95 (2.27 to 3.66)
Oesophageal cancer	30 Pack Years	Both	Female	2.78 (2.02 to 3.64)	2.78 (2.02 to 3.64)	2.78 (2.02 to 3.64)	2.78 (2.02 to 3.64)	2.78 (2.02 to 3.64)	2.78 (2.02 to 3.64)	2.78 (2.02 to 3.64)	2.78 (2.02 to 3.64)	2.78 (2.02 to 3.64)	2.78 (2.02 to 3.64)	2.78 (2.02 to 3.64)	2.78 (2.02 to 3.64)	2.78 (2.02 to 3.64)	2.78 (2.02 to 3.64)
Oesophageal cancer	30 Pack Years	Both	Male	2.78 (2.02 to 3.64)	2.78 (2.02 to 3.64)	2.78 (2.02 to 3.64)	2.78 (2.02 to 3.64)	2.78 (2.02 to 3.64)	2.78 (2.02 to 3.64)	2.78 (2.02 to 3.64)	2.78 (2.02 to 3.64)	2.78 (2.02 to 3.64)	2.78 (2.02 to 3.64)	2.78 (2.02 to 3.64)	2.78 (2.02 to 3.64)	2.78 (2.02 to 3.64)	2.78 (2.02 to 3.64)
Oesophageal cancer	40 Pack Years	Both	Female	3.91 (2.89 to 4.96)	3.91 (2.89 to 4.96)	3.91 (2.89 to 4.96)	3.91 (2.89 to 4.96)	3.91 (2.89 to 4.96)	3.91 (2.89 to 4.96)	3.91 (2.89 to 4.96)	3.91 (2.89 to 4.96)	3.91 (2.89 to 4.96)	3.91 (2.89 to 4.96)	3.91 (2.89 to 4.96)	3.91 (2.89 to 4.96)	3.91 (2.89 to 4.96)	3.91 (2.89 to 4.96)
Oesophageal cancer	40 Pack Years	Both	Male	3.91 (2.89 to 4.96)	3.91 (2.89 to 4.96)	3.91 (2.89 to 4.96)	3.91 (2.89 to 4.96)	3.91 (2.89 to 4.96)	3.91 (2.89 to 4.96)	3.91 (2.89 to 4.96)	3.91 (2.89 to 4.96)	3.91 (2.89 to 4.96)	3.91 (2.89 to 4.96)	3.91 (2.89 to 4.96)	3.91 (2.89 to 4.96)	3.91 (2.89 to 4.96)	3.91 (2.89 to 4.96)
Oesophageal cancer	50 Pack Years	Both	Female	4.76 (3.27 to 6.31)	4.76 (3.27 to 6.31)	4.76 (3.27 to 6.31)	4.76 (3.27 to 6.31)	4.76 (3.27 to 6.31)	4.76 (3.27 to 6.31)	4.76 (3.27 to 6.31)	4.76 (3.27 to 6.31)	4.76 (3.27 to 6.31)	4.76 (3.27 to 6.31)	4.76 (3.27 to 6.31)	4.76 (3.27 to 6.31)	4.76 (3.27 to 6.31)	4.76 (3.27 to 6.31)
Oesophageal cancer	50 Pack Years	Both	Male	4.76 (3.27 to 6.31)	4.76 (3.27 to 6.31)	4.76 (3.27 to 6.31)	4.76 (3.27 to 6.31)	4.76 (3.27 to 6.31)	4.76 (3.27 to 6.31)	4.76 (3.27 to 6.31)	4.76 (3.27 to 6.31)	4.76 (3.27 to 6.31)	4.76 (3.27 to 6.31)	4.76 (3.27 to 6.31)	4.76 (3.27 to 6.31)	4.76 (3.27 to 6.31)	4.76 (3.27 to 6.31)
Oesophageal cancer	60 Pack Years	Both	Female	5.33 (3.16 to 7.71)	5.33 (3.16 to 7.71)	5.33 (3.16 to 7.71)	5.33 (3.16 to 7.71)	5.33 (3.16 to 7.71)	5.33 (3.16 to 7.71)	5.33 (3.16 to 7.71)	5.33 (3.16 to 7.71)	5.33 (3.16 to 7.71)	5.33 (3.16 to 7.71)	5.33 (3.16 to 7.71)	5.33 (3.16 to 7.71)	5.33 (3.16 to 7.71)	5.33 (3.16 to 7.71)
Oesophageal cancer	60 Pack Years	Both	Male	5.33 (3.16 to 7.71)	5.33 (3.16 to 7.71)	5.33 (3.16 to 7.71)	5.33 (3.16 to 7.71)	5.33 (3.16 to 7.71)	5.33 (3.16 to 7.71)	5.33 (3.16 to 7.71)	5.33 (3.16 to 7.71)	5.33 (3.16 to 7.71)	5.33 (3.16 to 7.71)	5.33 (3.16 to 7.71)	5.33 (3.16 to 7.71)	5.33 (3.16 to 7.71)	5.33 (3.16 to 7.71)
Oesophageal cancer	70 Pack Years	Both	Female	6.66 (3.52 to 10.38)	6.66 (3.52 to 10.38)	6.66 (3.52 to 10.38)	6.66 (3.52 to 10.38)	6.66 (3.52 to 10.38)	6.66 (3.52 to 10.38)	6.66 (3.52 to 10.38)	6.66 (3.52 to 10.38)	6.66 (3.52 to 10.38)	6.66 (3.52 to 10.38)	6.66 (3.52 to 10.38)	6.66 (3.52 to 10.38)	6.66 (3.52 to 10.38)	6.66 (3.52 to 10.38)
Oesophageal cancer	70 Pack Years	Both	Male	6.66 (3.52 to 10.38)	6.66 (3.52 to 10.38)	6.66 (3.52 to 10.38)	6.66 (3.52 to 10.38)	6.66 (3.52 to 10.38)	6.66 (3.52 to 10.38)	6.66 (3.52 to 10.38)	6.66 (3.52 to 10.38)	6.66 (3.52 to 10.38)	6.66 (3.52 to 10.38)	6.66 (3.52 to 10.38)	6.66 (3.52 to 10.38)	6.66 (3.52 to 10.38)	6.66 (3.52 to 10.38)
Oesophageal cancer	80 Pack Years	Both	Female	8.08 (4.28 to 12.60)	8.08 (4.28 to 12.60)	8.08 (4.28 to 12.60)	8.08 (4.28 to 12.60)	8.08 (4.28 to 12.60)	8.08 (4.28 to 12.60)	8.08 (4.28 to 12.60)	8.08 (4.28 to 12.60)	8.08 (4.28 to 12.60)	8.08 (4.28 to 12.60)	8.08 (4.28 to 12.60)	8.08 (4.28 to 12.60)	8.08 (4.28 to 12.60)	8.08 (4.28 to 12.60)
Oesophageal cancer	80 Pack Years	Both	Male	8.08 (4.28 to 12.60)	8.08 (4.28 to 12.60)	8.08 (4.28 to 12.60)	8.08 (4.28 to 12.60)	8.08 (4.28 to 12.60)	8.08 (4.28 to 12.60)	8.08 (4.28 to 12.60)	8.08 (4.28 to 12.60)	8.08 (4.28 to 12.60)	8.08 (4.28 to 12.60)	8.08 (4.28 to 12.60)	8.08 (4.28 to 12.60)	8.08 (4.28 to 12.60)	8.08 (4.28 to 12.60)
Oesophageal cancer	90 Pack Years	Both	Female	9.59 (5.43 to 14.35)	9.59 (5.43 to 14.35)	9.59 (5.43 to 14.35)	9.59 (5.43 to 14.35)	9.59 (5.43 to 14.35)	9.59 (5.43 to 14.35)	9.59 (5.43 to 14.35)	9.59 (5.43 to 14.35)	9.59 (5.43 to 14.35)	9.59 (5.43 to 14.35)	9.59 (5.43 to 14.35)	9.59 (5.43 to 14.35)	9.59 (5.43 to 14.35)	9.59 (5.43 to 14.35)
Oesophageal cancer	90 Pack Years	Both	Male	9.59 (5.43 to 14.35)	9.59 (5.43 to 14.35)	9.59 (5.43 to 14.35)	9.59 (5.43 to 14.35)	9.59 (5.43 to 14.35)	9.59 (5.43 to 14.35)	9.59 (5.43 to 14.35)	9.59 (5.43 to 14.35)	9.59 (5.43 to 14.35)	9.59 (5.43 to 14.35)	9.59 (5.43 to 14.35)	9.59 (5.43 to 14.35)	9.59 (5.43 to 14.35)	9.59 (5.43 to 14.35)
Stomach cancer	0 Pack Years	Both	Female	1.00 (1.00 to 1.00)	1.00 (1.00 to 1.00)	1.00 (1.00 to 1.00)	1.00 (1.00 to 1.00)	1.00 (1.00 to 1.00)	1.00 (1.00 to 1.00)	1.00 (1.00 to 1.00)	1.00 (1.00 to 1.00)	1.00 (1.00 to 1.00)	1.00 (1.00 to 1.00)	1.00 (1.00 to 1.00)	1.00 (1.00 to 1.00)	1.00 (1.00 to 1.00)	1.00 (1.00 to 1.00)
Stomach cancer	0 Pack Years	Both	Male	1.00 (1.00 to 1.00)	1.00 (1.00 to 1.00)	1.00 (1.00 to 1.00)	1.00 (1.00 to 1.00)	1.00 (1.00 to 1.00)	1.00 (1.00 to 1.00)	1.00 (1.00 to 1.00)	1.00 (1.00 to 1.00)	1.00 (1.00 to 1.00)	1.00 (1.00 to 1.00)	1.00 (1.00 to 1.00)	1.00 (1.00 to 1.00)	1.00 (1.00 to 1.00)	1.00 (1.00 to 1.00)
Stomach cancer	10 Pack Years	Both	Female	1.34 (1.18 to 1.53)	1.34 (1.18 to 1.53)	1.34 (1.18 to 1.53)	1.34 (1.18 to 1.53)	1.34 (1.18 to 1.53)	1.34 (1.18 to 1.53)	1.34 (1.18 to 1.53)	1.34 (1.18 to 1.53)	1.34 (1.18 to 1.53)	1.34 (1.18 to 1.53)	1.34 (1.18 to 1.53)	1.34 (1.18 to 1.53)	1.34 (1.18 to 1.53)	1.34 (1.18 to 1.53)
Stomach cancer	10 Pack Years	Both	Male	1.34 (1.18 to 1.53)	1.34 (1.18 to 1.53)	1.34 (1.18 to 1.53)	1.34 (1.18 to 1.53)	1.34 (1.18 to 1.53)	1.34 (1.18 to 1.53)	1.34 (1.18 to 1.53)	1.34 (1.18 to 1.53)	1.34 (1.18 to 1.53)	1.34 (1.18 to 1.53)	1.34 (1.18 to 1.53)	1.34 (1.18 to 1.53)	1.34 (1.18 to 1.53)	1.34 (1.18 to 1.53)
Stomach cancer	20 Pack Years	Both	Female	1.53 (1.29 to 1.82)	1.53 (1.29 to 1.82)	1.53 (1.29 to 1.82)	1.53 (1.29 to 1.82)	1.53 (1.29 to 1.82)	1.53 (1.29 to 1.82)	1.53 (1.29 to 1.82)	1.53 (1.29 to 1.82)	1.53 (1.29 to 1.82)	1.53 (1.29 to 1.82)	1.53 (1.29 to 1.82)	1.53 (1.29 to 1.82)	1.53 (1.29 to 1.82)	1.53 (1.29 to 1.82)
Stomach cancer	20 Pack Years	Both	Male	1.53 (1.29 to 1.82)	1.53 (1.29 to 1.82)	1.53 (1.29 to 1.82)	1.53 (1.29 to 1.82)	1.53 (1.29 to 1.82)	1.53 (1.29 to 1.82)	1.53 (1.29 to 1.82)	1.53 (1.29 to 1.82)	1.53 (1.29 to 1.82)	1.53 (1.29 to 1.82)	1.53 (1.29 to 1.82)	1.53 (1.29 to 1.82)	1.53 (1.29 to 1.82)	1.53 (1.29 to 1.82)
Stomach cancer	30 Pack Years	Both	Female	1.73 (1.41 to 2.09)	1.73 (1.41 to 2.09)	1.73 (1.41 to 2.09)	1.73 (1.41 to 2.09)	1.73 (1.41 to 2.09)	1.73 (1.41 to 2.09)	1.73 (1.41 to 2.09)	1.73 (1.41 to 2.09)	1.73 (1.41 to 2.09)	1.73 (1.41 to 2.09)	1.73 (1.41 to 2.09)	1.73 (1.41 to 2.09)	1.73 (1.41 to 2.09)	1.73 (1.41 to 2.09)
Stomach cancer	30 Pack Years	Both	Male	1.73 (1.41 to 2.09)	1.73 (1.41 to 2.09)	1.73 (1.41 to 2.09)	1.73 (1.41 to 2.09)	1.73 (1.41 to 2.09)	1.73 (1.41 to 2.09)	1.73 (1.41 to 2.09)	1.73 (1.41 to 2.09)	1.73 (1.41 to 2.09)	1.73 (1.41 to 2.09)	1.73 (1.41 to 2.09)	1.73 (1.41 to 2.09)	1.73 (1.41 to 2.09)	1.73 (1.41 to 2.09)
Stomach cancer	40 Pack Years	Both	Female	1.94 (1.56 to 2.36)	1.94 (1.56 to 2.36)	1.94 (1.56 to 2.36)	1.94 (1.56 to 2.36)	1.94 (1.56 to 2.36)	1.94 (1.56 to 2.36)	1.94 (1.56 to 2.36)	1.94 (1.56 to 2.36)	1.94 (1.56 to 2.36)	1.94 (1.56 to 2.36)	1.94 (1.56 to 2.36)	1.94 (1.56 to 2.36)	1.94 (1.56 to 2.36)	1.94 (1.56 to 2.36)
Stomach cancer	40 Pack Years	Both	Male	1.94 (1.56 to 2.36)	1.94 (1.56 to 2.36)	1.94 (1.56 to 2.36)	1.94 (1.56 to 2.36)	1.94 (1.56 to 2.36)	1.94 (1.56 to 2.36)	1.94 (1.56 to 2.36)	1.94 (1.56 to 2.36)	1.94 (1.56 to 2.36)	1.94 (1.56 to 2.36)	1.94 (1.56 to 2.36)	1.94 (1.56 to 2.36)	1.94 (1.56 to 2.36)	1.94 (1.56 to 2.36)
Stomach cancer	50 Pack Years	Both	Female	2.05 (1.63 to 2.46)	2.05 (1.63 to 2.46)	2.05 (1.63 to 2.46)	2.05 (1.63 to 2.46)	2.05 (1.63 to 2.46)	2.05 (1.63 to 2.46)	2.05 (1.63 to 2.46)	2.05 (1.63 to 2.46)	2.05 (1.63 to 2.46)	2.05 (1.63 to 2.46)	2.05 (1.63 to 2.46)	2.05 (1.63 to 2.46)	2.05 (1.63 to 2.46)	2.05 (1.63 to 2.46)
Stomach cancer	50 Pack Years	Both	Male	2.05 (1.63 to 2.46)	2.05 (1.63 to 2.46)	2.05 (1.63 to 2.46)	2.05 (1.63 to 2.46)	2.05 (1.63 to 2.46)	2.05 (1.63 to 2.46)	2.05 (1.63 to 2.46)	2.05 (1.63 to 2.46)	2.05 (1.63 to 2.46)	2.05 (1.63 to 2.46)	2.05 (1.63 to 2.46)	2.05 (1.63 to 2.46)	2.05 (1.63 to 2.46)	2.05 (1.63 to 2.46)
Stomach cancer	60 Pack Years	Both	Female	2.08 (1.65 to 2.56)	2.08 (1.65 to 2.56)	2.08 (1.65 to 2.56)	2.08 (1.65 to 2.56)	2.08 (1.65 to 2.56)	2.08 (1.65 to 2.56)	2.08 (1.65 to 2.56)	2.08 (1.65 to 2.56)	2.08 (1.65 to 2.56)	2.08 (1.65 to 2.56)	2.08 (1.65 to 2.56)	2.08 (1.65 to 2.56)	2.08 (1.65 to 2.56)	2.08 (1.65 to 2.56)
Stomach cancer	60 Pack Years	Both	Male	2.08 (1.65 to 2.56)	2.08 (1.65 to 2.56)	2.08 (1.65 to 2.56)	2.08 (1.65 to 2.56)	2.08 (1.65 to 2.56)	2.08 (1.65 to 2.56)	2.08 (1.65 to 2.56)	2.08 (1.65 to 2.56)	2.08 (1.65 to 2.56)	2.08 (1.65 to 2.56)	2.08 (1.65 to 2.56)	2.08 (1.65 to 2.56)	2.08 (1.65 to 2.56)	2.08 (1.65 to 2.56)
Stomach cancer	70 Pack Years	Both	Female	2.11 (1.66 to 2.65)	2.11 (1.66 to 2.65)	2.11 (1.66 to 2.65)	2.11 (1.66 to 2.65)	2.11 (1.66 to 2.65)	2.11 (1.66 to 2.65)	2.11 (1.66 to 2.65)	2.11 (1.66 to 2.65)	2.11 (1.66 to 2.65)	2.11 (1.66 to 2.65)	2.11 (1.66 to 2.65)	2.11 (1.66 to 2.65)	2.11 (1.66 to 2.65)	2.11 (1.66 to 2.65)
Stomach cancer	70 Pack Years	Both	Male	2.11 (1.66 to 2.65)	2.11 (1.66 to 2.65)	2.11 (1.66 to 2.65)	2.11 (1.66 to 2.65)	2.11 (1.66 to 2.65)	2.11 (1.66 to 2.65)	2.11 (1.66 to 2.65)	2.11 (1.66 to 2.65)	2.11 (1.66 to 2.65)	2.11 (1.66 to 2.65)	2.11 (1.66 to 2.65)	2.11 (1.66 to 2.65)	2.11 (1.66 to 2.65)	2.11 (1.66 to 2.65)
Liver cancer	0 Pack Years	Both	Female	1.00 (1.00 to 1.00)	1.00 (1.00 to 1.00)	1.00 (1.00 to 1.00)	1.00 (1.00 to 1.00)	1.00 (1.00 to 1.00)	1.00 (1.00 to 1.00)	1.00 (1.00 to 1.00)	1.00 (1.00 to 1.00)	1.00 (1.00 to 1.00)	1.00 (1.00 to 1.00)	1.00 (1.00 to 1.00)	1.00 (1.00 to 1.00)	1.00 (1.00 to 1.00)	1.00 (1.00 to 1.00)
Liver cancer	0 Pack Years	Both	Male	1.00 (1.00 to 1.00)	1.00 (1.00 to 1.00)	1.00 (1.00 to 1.00)	1.00 (1.00 to 1.00)	1.00 (1.00 to 1.00)	1.00 (1.00 to 1.00)	1.00 (1.00 to 1.00)	1.00 (1.00 to 1.00)	1.00 (1.00 to 1.00)	1.00 (1.00 to 1.00)	1.00 (1.00 to 1.00)	1.00 (1.00 to 1.00)	1.00 (1.00 to 1.00)	1.00 (1.00 to 1.00)
Liver cancer	10 Pack Years	Both	Female	1.35 (1.08 to 1.65)	1.35 (1.08 to 1.65)	1.35 (1.08 to 1.65)	1.35 (1.08 to 1.65)	1.35 (1.08 to 1.65)	1.35 (1.08 to 1.65)	1.35 (1.08 to 1.65)	1.35 (1.08 to 1.65)	1.35 (1.08 to 1.65)	1.35 (1.08 to 1.65)	1.35 (1.08 to 1.65)	1.35 (1.08 to 1.65)	1.35 (1.08 to 1.65)	1.35 (1.08 to 1.65)
Liver cancer	10 Pack Years	Both	Male	1.35 (1.08 to 1.65)	1.35 (1.08 to 1.65)	1.35 (1.08 to 1.65)	1.35 (1.08 to 1.65)	1.35 (1.08 to 1.65)	1.35 (1.08 to 1.65)	1.35 (1.08 to 1.65)	1.35 (1.08 to 1.65)	1.35 (1.08 to 1.65)	1.35 (1.08 to 1.65)	1.35 (1.08 to 1.65)	1.35 (1.08 to 1.65)	1.35 (1.08 to 1.65)	1.35 (1.08 to 1.65)
Liver cancer	20 Pack Years	Both	Female	1.56 (1.13 to 2.02)	1.56 (1.13 to 2.02)	1.56 (1.13 to 2.02)	1.56 (1.13 to 2.02)	1.56 (1.13 to 2.02)	1.56 (1.13 to 2.02)	1.56 (1.13 to 2.02)	1.56 (1.13 to 2.02)	1.56 (1.13 to 2.02)	1.56 (1.13 to 2.02)	1.56 (1.13 to 2.02)	1.56 (1.13 to 2.02)	1.56 (1.13 to 2.02)	1.56 (1.13 to 2.02)
Liver cancer	20 Pack Years	Both	Male	1.56 (1.13 to 2.02)	1.56 (1.13 to 2.02)	1.56 (1.13 to 2.02)	1.56 (1.13 to 2.02)	1.56 (1.13 to 2.02)	1.56 (1.13 to 2.02)	1.56 (1.13 to 2.02)	1.56 (1.13 to 2.02)	1.56 (1.13 to 2.02)	1.56 (1.13 to 2.02)	1.56 (1.13 to 2.02)	1.56 (1.13 to 2.02)	1.56 (1.13 to 2.02)	1.56 (1.13 to 2.02)

Liver cancer	30 Pack Years	Both	Female	1.65 (1.16 to 2.16)	1.65 (1.16 to 2.16)	1.65 (1.16 to 2.16)	1.65 (1.16 to 2.16)	1.65 (1.16 to 2.16)	1.65 (1.16 to 2.16)	1.65 (1.16 to 2.16)	1.65 (1.16 to 2.16)	1.65 (1.16 to 2.16)	1.65 (1.16 to 2.16)	1.65 (1.16 to 2.16)	1.65 (1.16 to 2.16)	1.65 (1.16 to 2.16)	1.65 (1.16 to 2.16)
Liver cancer	30 Pack Years	Both	Male	1.65 (1.16 to 2.16)	1.65 (1.16 to 2.16)	1.65 (1.16 to 2.16)	1.65 (1.16 to 2.16)	1.65 (1.16 to 2.16)	1.65 (1.16 to 2.16)	1.65 (1.16 to 2.16)	1.65 (1.16 to 2.16)	1.65 (1.16 to 2.16)	1.65 (1.16 to 2.16)	1.65 (1.16 to 2.16)	1.65 (1.16 to 2.16)	1.65 (1.16 to 2.16)	1.65 (1.16 to 2.16)
Liver cancer	40 Pack Years	Both	Female	1.78 (1.26 to 2.40)	1.78 (1.26 to 2.40)	1.78 (1.26 to 2.40)	1.78 (1.26 to 2.40)	1.78 (1.26 to 2.40)	1.78 (1.26 to 2.40)	1.78 (1.26 to 2.40)	1.78 (1.26 to 2.40)	1.78 (1.26 to 2.40)	1.78 (1.26 to 2.40)	1.78 (1.26 to 2.40)	1.78 (1.26 to 2.40)	1.78 (1.26 to 2.40)	1.78 (1.26 to 2.40)
Liver cancer	40 Pack Years	Both	Male	1.78 (1.26 to 2.40)	1.78 (1.26 to 2.40)	1.78 (1.26 to 2.40)	1.78 (1.26 to 2.40)	1.78 (1.26 to 2.40)	1.78 (1.26 to 2.40)	1.78 (1.26 to 2.40)	1.78 (1.26 to 2.40)	1.78 (1.26 to 2.40)	1.78 (1.26 to 2.40)	1.78 (1.26 to 2.40)	1.78 (1.26 to 2.40)	1.78 (1.26 to 2.40)	1.78 (1.26 to 2.40)
Liver cancer	50 Pack Years	Both	Female	1.81 (1.26 to 2.51)	1.81 (1.26 to 2.51)	1.81 (1.26 to 2.51)	1.81 (1.26 to 2.51)	1.81 (1.26 to 2.51)	1.81 (1.26 to 2.51)	1.81 (1.26 to 2.51)	1.81 (1.26 to 2.51)	1.81 (1.26 to 2.51)	1.81 (1.26 to 2.51)	1.81 (1.26 to 2.51)	1.81 (1.26 to 2.51)	1.81 (1.26 to 2.51)	1.81 (1.26 to 2.51)
Liver cancer	50 Pack Years	Both	Male	1.81 (1.26 to 2.51)	1.81 (1.26 to 2.51)	1.81 (1.26 to 2.51)	1.81 (1.26 to 2.51)	1.81 (1.26 to 2.51)	1.81 (1.26 to 2.51)	1.81 (1.26 to 2.51)	1.81 (1.26 to 2.51)	1.81 (1.26 to 2.51)	1.81 (1.26 to 2.51)	1.81 (1.26 to 2.51)	1.81 (1.26 to 2.51)	1.81 (1.26 to 2.51)	1.81 (1.26 to 2.51)
Liver cancer	60 Pack Years	Both	Female	1.80 (1.22 to 2.57)	1.80 (1.22 to 2.57)	1.80 (1.22 to 2.57)	1.80 (1.22 to 2.57)	1.80 (1.22 to 2.57)	1.80 (1.22 to 2.57)	1.80 (1.22 to 2.57)	1.80 (1.22 to 2.57)	1.80 (1.22 to 2.57)	1.80 (1.22 to 2.57)	1.80 (1.22 to 2.57)	1.80 (1.22 to 2.57)	1.80 (1.22 to 2.57)	1.80 (1.22 to 2.57)
Liver cancer	60 Pack Years	Both	Male	1.80 (1.22 to 2.57)	1.80 (1.22 to 2.57)	1.80 (1.22 to 2.57)	1.80 (1.22 to 2.57)	1.80 (1.22 to 2.57)	1.80 (1.22 to 2.57)	1.80 (1.22 to 2.57)	1.80 (1.22 to 2.57)	1.80 (1.22 to 2.57)	1.80 (1.22 to 2.57)	1.80 (1.22 to 2.57)	1.80 (1.22 to 2.57)	1.80 (1.22 to 2.57)	1.80 (1.22 to 2.57)
Liver cancer	70 Pack Years	Both	Female	1.91 (1.25 to 2.74)	1.91 (1.25 to 2.74)	1.91 (1.25 to 2.74)	1.91 (1.25 to 2.74)	1.91 (1.25 to 2.74)	1.91 (1.25 to 2.74)	1.91 (1.25 to 2.74)	1.91 (1.25 to 2.74)	1.91 (1.25 to 2.74)	1.91 (1.25 to 2.74)	1.91 (1.25 to 2.74)	1.91 (1.25 to 2.74)	1.91 (1.25 to 2.74)	1.91 (1.25 to 2.74)
Liver cancer	70 Pack Years	Both	Male	1.91 (1.25 to 2.74)	1.91 (1.25 to 2.74)	1.91 (1.25 to 2.74)	1.91 (1.25 to 2.74)	1.91 (1.25 to 2.74)	1.91 (1.25 to 2.74)	1.91 (1.25 to 2.74)	1.91 (1.25 to 2.74)	1.91 (1.25 to 2.74)	1.91 (1.25 to 2.74)	1.91 (1.25 to 2.74)	1.91 (1.25 to 2.74)	1.91 (1.25 to 2.74)	1.91 (1.25 to 2.74)
Liver cancer	80 Pack Years	Both	Female	2.09 (1.19 to 3.30)	2.09 (1.19 to 3.30)	2.09 (1.19 to 3.30)	2.09 (1.19 to 3.30)	2.09 (1.19 to 3.30)	2.09 (1.19 to 3.30)	2.09 (1.19 to 3.30)	2.09 (1.19 to 3.30)	2.09 (1.19 to 3.30)	2.09 (1.19 to 3.30)	2.09 (1.19 to 3.30)	2.09 (1.19 to 3.30)	2.09 (1.19 to 3.30)	2.09 (1.19 to 3.30)
Liver cancer	80 Pack Years	Both	Male	2.09 (1.19 to 3.30)	2.09 (1.19 to 3.30)	2.09 (1.19 to 3.30)	2.09 (1.19 to 3.30)	2.09 (1.19 to 3.30)	2.09 (1.19 to 3.30)	2.09 (1.19 to 3.30)	2.09 (1.19 to 3.30)	2.09 (1.19 to 3.30)	2.09 (1.19 to 3.30)	2.09 (1.19 to 3.30)	2.09 (1.19 to 3.30)	2.09 (1.19 to 3.30)	2.09 (1.19 to 3.30)
Larynx cancer	0 Pack Years	Both	Female	1.00 (1.00 to 1.00)	1.00 (1.00 to 1.00)	1.00 (1.00 to 1.00)	1.00 (1.00 to 1.00)	1.00 (1.00 to 1.00)	1.00 (1.00 to 1.00)	1.00 (1.00 to 1.00)	1.00 (1.00 to 1.00)	1.00 (1.00 to 1.00)	1.00 (1.00 to 1.00)	1.00 (1.00 to 1.00)	1.00 (1.00 to 1.00)	1.00 (1.00 to 1.00)	1.00 (1.00 to 1.00)
Larynx cancer	0 Pack Years	Both	Male	1.00 (1.00 to 1.00)	1.00 (1.00 to 1.00)	1.00 (1.00 to 1.00)	1.00 (1.00 to 1.00)	1.00 (1.00 to 1.00)	1.00 (1.00 to 1.00)	1.00 (1.00 to 1.00)	1.00 (1.00 to 1.00)	1.00 (1.00 to 1.00)	1.00 (1.00 to 1.00)	1.00 (1.00 to 1.00)	1.00 (1.00 to 1.00)	1.00 (1.00 to 1.00)	1.00 (1.00 to 1.00)
Larynx cancer	10 Pack Years	Both	Female	3.40 (2.37 to 4.59)	3.40 (2.37 to 4.59)	3.40 (2.37 to 4.59)	3.40 (2.37 to 4.59)	3.40 (2.37 to 4.59)	3.40 (2.37 to 4.59)	3.40 (2.37 to 4.59)	3.40 (2.37 to 4.59)	3.40 (2.37 to 4.59)	3.40 (2.37 to 4.59)	3.40 (2.37 to 4.59)	3.40 (2.37 to 4.59)	3.40 (2.37 to 4.59)	3.40 (2.37 to 4.59)
Larynx cancer	10 Pack Years	Both	Male	3.40 (2.37 to 4.59)	3.40 (2.37 to 4.59)	3.40 (2.37 to 4.59)	3.40 (2.37 to 4.59)	3.40 (2.37 to 4.59)	3.40 (2.37 to 4.59)	3.40 (2.37 to 4.59)	3.40 (2.37 to 4.59)	3.40 (2.37 to 4.59)	3.40 (2.37 to 4.59)	3.40 (2.37 to 4.59)	3.40 (2.37 to 4.59)	3.40 (2.37 to 4.59)	3.40 (2.37 to 4.59)
Larynx cancer	20 Pack Years	Both	Female	5.88 (3.92 to 8.28)	5.88 (3.92 to 8.28)	5.88 (3.92 to 8.28)	5.88 (3.92 to 8.28)	5.88 (3.92 to 8.28)	5.88 (3.92 to 8.28)	5.88 (3.92 to 8.28)	5.88 (3.92 to 8.28)	5.88 (3.92 to 8.28)	5.88 (3.92 to 8.28)	5.88 (3.92 to 8.28)	5.88 (3.92 to 8.28)	5.88 (3.92 to 8.28)	5.88 (3.92 to 8.28)
Larynx cancer	20 Pack Years	Both	Male	5.88 (3.92 to 8.28)	5.88 (3.92 to 8.28)	5.88 (3.92 to 8.28)	5.88 (3.92 to 8.28)	5.88 (3.92 to 8.28)	5.88 (3.92 to 8.28)	5.88 (3.92 to 8.28)	5.88 (3.92 to 8.28)	5.88 (3.92 to 8.28)	5.88 (3.92 to 8.28)	5.88 (3.92 to 8.28)	5.88 (3.92 to 8.28)	5.88 (3.92 to 8.28)	5.88 (3.92 to 8.28)
Larynx cancer	30 Pack Years	Both	Female	8.45 (5.64 to 12.06)	8.45 (5.64 to 12.06)	8.45 (5.64 to 12.06)	8.45 (5.64 to 12.06)	8.45 (5.64 to 12.06)	8.45 (5.64 to 12.06)	8.45 (5.64 to 12.06)	8.45 (5.64 to 12.06)	8.45 (5.64 to 12.06)	8.45 (5.64 to 12.06)	8.45 (5.64 to 12.06)	8.45 (5.64 to 12.06)	8.45 (5.64 to 12.06)	8.45 (5.64 to 12.06)
Larynx cancer	30 Pack Years	Both	Male	8.45 (5.64 to 12.06)	8.45 (5.64 to 12.06)	8.45 (5.64 to 12.06)	8.45 (5.64 to 12.06)	8.45 (5.64 to 12.06)	8.45 (5.64 to 12.06)	8.45 (5.64 to 12.06)	8.45 (5.64 to 12.06)	8.45 (5.64 to 12.06)	8.45 (5.64 to 12.06)	8.45 (5.64 to 12.06)	8.45 (5.64 to 12.06)	8.45 (5.64 to 12.06)	8.45 (5.64 to 12.06)
Larynx cancer	40 Pack Years	Both	Female	13.59 (8.59 to 19.83)	13.59 (8.59 to 19.83)	13.59 (8.59 to 19.83)	13.59 (8.59 to 19.83)	13.59 (8.59 to 19.83)	13.59 (8.59 to 19.83)	13.59 (8.59 to 19.83)	13.59 (8.59 to 19.83)	13.59 (8.59 to 19.83)	13.59 (8.59 to 19.83)	13.59 (8.59 to 19.83)	13.59 (8.59 to 19.83)	13.59 (8.59 to 19.83)	13.59 (8.59 to 19.83)
Larynx cancer	40 Pack Years	Both	Male	13.59 (8.59 to 19.83)	13.59 (8.59 to 19.83)	13.59 (8.59 to 19.83)	13.59 (8.59 to 19.83)	13.59 (8.59 to 19.83)	13.59 (8.59 to 19.83)	13.59 (8.59 to 19.83)	13.59 (8.59 to 19.83)	13.59 (8.59 to 19.83)	13.59 (8.59 to 19.83)	13.59 (8.59 to 19.83)	13.59 (8.59 to 19.83)	13.59 (8.59 to 19.83)	13.59 (8.59 to 19.83)
Larynx cancer	50 Pack Years	Both	Female	18.09 (10.63 to 27.22)	18.09 (10.63 to 27.22)	18.09 (10.63 to 27.22)	18.09 (10.63 to 27.22)	18.09 (10.63 to 27.22)	18.09 (10.63 to 27.22)	18.09 (10.63 to 27.22)	18.09 (10.63 to 27.22)	18.09 (10.63 to 27.22)	18.09 (10.63 to 27.22)	18.09 (10.63 to 27.22)	18.09 (10.63 to 27.22)	18.09 (10.63 to 27.22)	18.09 (10.63 to 27.22)
Larynx cancer	50 Pack Years	Both	Male	18.09 (10.63 to 27.22)	18.09 (10.63 to 27.22)	18.09 (10.63 to 27.22)	18.09 (10.63 to 27.22)	18.09 (10.63 to 27.22)	18.09 (10.63 to 27.22)	18.09 (10.63 to 27.22)	18.09 (10.63 to 27.22)	18.09 (10.63 to 27.22)	18.09 (10.63 to 27.22)	18.09 (10.63 to 27.22)	18.09 (10.63 to 27.22)	18.09 (10.63 to 27.22)	18.09 (10.63 to 27.22)
Larynx cancer	60 Pack Years	Both	Female	21.95 (11.76 to 34.25)	21.95 (11.76 to 34.25)	21.95 (11.76 to 34.25)	21.95 (11.76 to 34.25)	21.95 (11.76 to 34.25)	21.95 (11.76 to 34.25)	21.95 (11.76 to 34.25)	21.95 (11.76 to 34.25)	21.95 (11.76 to 34.25)	21.95 (11.76 to 34.25)	21.95 (11.76 to 34.25)	21.95 (11.76 to 34.25)	21.95 (11.76 to 34.25)	21.95 (11.76 to 34.25)
Larynx cancer	60 Pack Years	Both	Male	21.95 (11.76 to 34.25)	21.95 (11.76 to 34.25)	21.95 (11.76 to 34.25)	21.95 (11.76 to 34.25)	21.95 (11.76 to 34.25)	21.95 (11.76 to 34.25)	21.95 (11.76 to 34.25)	21.95 (11.76 to 34.25)	21.95 (11.76 to 34.25)	21.95 (11.76 to 34.25)	21.95 (11.76 to 34.25)	21.95 (11.76 to 34.25)	21.95 (11.76 to 34.25)	21.95 (11.76 to 34.25)
Larynx cancer	70 Pack Years	Both	Female	24.75 (11.00 to 43.67)	24.75 (11.00 to 43.67)	24.75 (11.00 to 43.67)	24.75 (11.00 to 43.67)	24.75 (11.00 to 43.67)	24.75 (11.00 to 43.67)	24.75 (11.00 to 43.67)	24.75 (11.00 to 43.67)	24.75 (11.00 to 43.67)	24.75 (11.00 to 43.67)	24.75 (11.00 to 43.67)	24.75 (11.00 to 43.67)	24.75 (11.00 to 43.67)	24.75 (11.00 to 43.67)
Larynx cancer	70 Pack Years	Both	Male	24.75 (11.00 to 43.67)	24.75 (11.00 to 43.67)	24.75 (11.00 to 43.67)	24.75 (11.00 to 43.67)	24.75 (11.00 to 43.67)	24.75 (11.00 to 43.67)	24.75 (11.00 to 43.67)	24.75 (11.00 to 43.67)	24.75 (11.00 to 43.67)	24.75 (11.00 to 43.67)	24.75 (11.00 to 43.67)	24.75 (11.00 to 43.67)	24.75 (11.00 to 43.67)	24.75 (11.00 to 43.67)
Breast cancer	0 Pack Years	Both	Female	1.00 (1.00 to 1.00)	1.00 (1.00 to 1.00)	1.00 (1.00 to 1.00)	1.00 (1.00 to 1.00)	1.00 (1.00 to 1.00)	1.00 (1.00 to 1.00)	1.00 (1.00 to 1.00)	1.00 (1.00 to 1.00)	1.00 (1.00 to 1.00)	1.00 (1.00 to 1.00)	1.00 (1.00 to 1.00)	1.00 (1.00 to 1.00)	1.00 (1.00 to 1.00)	1.00 (1.00 to 1.00)
Breast cancer	0 Pack Years	Both	Male	1.00 (1.00 to 1.00)	1.00 (1.00 to 1.00)	1.00 (1.00 to 1.00)	1.00 (1.00 to 1.00)	1.00 (1.00 to 1.00)	1.00 (1.00 to 1.00)	1.00 (1.00 to 1.00)	1.00 (1.00 to 1.00)	1.00 (1.00 to 1.00)	1.00 (1.00 to 1.00)	1.00 (1.00 to 1.00)	1.00 (1.00 to 1.00)	1.00 (1.00 to 1.00)	1.00 (1.00 to 1.00)
Breast cancer	10 Pack Years	Both	Female	1.16 (1.09 to 1.24)	1.16 (1.09 to 1.24)	1.16 (1.09 to 1.24)	1.16 (1.09 to 1.24)	1.16 (1.09 to 1.24)	1.16 (1.09 to 1.24)	1.16 (1.09 to 1.24)	1.16 (1.09 to 1.24)	1.16 (1.09 to 1.24)	1.16 (1.09 to 1.24)	1.16 (1.09 to 1.24)	1.16 (1.09 to 1.24)	1.16 (1.09 to 1.24)	1.16 (1.09 to 1.24)
Breast cancer	10 Pack Years	Both	Male	1.00 (1.00 to 1.00)	1.00 (1.00 to 1.00)	1.00 (1.00 to 1.00)	1.00 (1.00 to 1.00)	1.00 (1.00 to 1.00)	1.00 (1.00 to 1.00)	1.00 (1.00 to 1.00)	1.00 (1.00 to 1.00)	1.00 (1.00 to 1.00)	1.00 (1.00 to 1.00)	1.00 (1.00 to 1.00)	1.00 (1.00 to 1.00)	1.00 (1.00 to 1.00)	1.00 (1.00 to 1.00)
Breast cancer	20 Pack Years	Both	Female	1.27 (1.18 to 1.36)	1.27 (1.18 to 1.36)	1.27 (1.18 to 1.36)	1.27 (1.18 to 1.36)	1.27 (1.18 to 1.36)	1.27 (1.18 to 1.36)	1.27 (1.18 to 1.36)	1.27 (1.18 to 1.36)	1.27 (1.18 to 1.36)	1.27 (1.18 to 1.36)	1.27 (1.18 to 1.36)	1.27 (1.18 to 1.36)	1.27 (1.18 to 1.36)	1.27 (1.18 to 1.36)
Breast cancer	20 Pack Years	Both	Male	1.00 (1.00 to 1.00)	1.00 (1.00 to 1.00)	1.00 (1.00 to 1.00)	1.00 (1.00 to 1.00)	1.00 (1.00 to 1.00)	1.00 (1.00 to 1.00)	1.00 (1.00 to 1.00)	1.00 (1.00 to 1.00)	1.00 (1.00 to 1.00)	1.00 (1.00 to 1.00)	1.00 (1.00 to 1.00)	1.00 (1.00 to 1.00)	1.00 (1.00 to 1.00)	1.00 (1.00 to 1.00)
Breast cancer	30 Pack Years	Both	Female	1.29 (1.19 to 1.39)	1.29 (1.19 to 1.39)	1.29 (1.19 to 1.39)	1.29 (1.19 to 1.39)	1.29 (1.19 to 1.39)	1.29 (1.19 to 1.39)	1.29 (1.19 to 1.39)	1.29 (1.19 to 1.39)	1.29 (1.19 to 1.39)	1.29 (1.19 to 1.39)	1.29 (1.19 to 1.39)	1.29 (1.19 to 1.39)	1.29 (1.19 to 1.39)	1.29 (1.19 to 1.39)
Breast cancer	30 Pack Years	Both	Male	1.00 (1.00 to 1.00)	1.00 (1.00 to 1.00)	1.00 (1.00 to 1.00)	1.00 (1.00 to 1.00)	1.00 (1.00 to 1.00)	1.00 (1.00 to 1.00)	1.00 (1.00 to 1.00)	1.00 (1.00 to 1.00)	1.00 (1.00 to 1.00)	1.00 (1.00 to 1.00)	1.00 (1.00 to 1.00)	1.00 (1.00 to 1.00)	1.00 (1.00 to 1.00)	1.00 (1.00 to 1.00)
Breast cancer	40 Pack Years	Both	Female	1.24 (1.12 to 1.37)	1.24 (1.12 to 1.37)	1.24 (1.12 to 1.37)	1.24 (1.12 to 1.37)	1.24 (1.12 to 1.37)	1.24 (1.12 to 1.37)	1.24 (1.12 to 1.37)	1.24 (1.12 to 1.37)	1.24 (1.12 to 1.37)	1.24 (1.12 to 1.37)	1.24 (1.12 to 1.37)	1.24 (1.12 to 1.37)	1.24 (1.12 to 1.37)	1.24 (1.12 to 1.37)
Breast cancer	40 Pack Years	Both	Male	1.00 (1.00 to 1.00)	1.00 (1.00 to 1.00)	1.00 (1.00 to 1.00)	1.00 (1.00 to 1.00)	1.00 (1.00 to 1.00)	1.00 (1.00 to 1.00)	1.00 (1.00 to 1.00)	1.00 (1.00 to 1.00)	1.00 (1.00 to 1.00)	1.00 (1.00 to 1.00)	1.00 (1.00 to 1.00)	1.00 (1.00 to 1.00)	1.00 (1.00 to 1.00)	1.00 (1.00 to 1.00)

Breast cancer	50 Pack Years	Both	Female	1.26 (1.11 to 1.40)	1.26 (1.11 to 1.40)	1.26 (1.11 to 1.40)	1.26 (1.11 to 1.40)	1.26 (1.11 to 1.40)	1.26 (1.11 to 1.40)	1.26 (1.11 to 1.40)	1.26 (1.11 to 1.40)	1.26 (1.11 to 1.40)	1.26 (1.11 to 1.40)	1.26 (1.11 to 1.40)	1.26 (1.11 to 1.40)	1.26 (1.11 to 1.40)	1.26 (1.11 to 1.40)
Breast cancer	50 Pack Years	Both	Male	1.00 (1.00 to 1.00)	1.00 (1.00 to 1.00)	1.00 (1.00 to 1.00)	1.00 (1.00 to 1.00)	1.00 (1.00 to 1.00)	1.00 (1.00 to 1.00)	1.00 (1.00 to 1.00)	1.00 (1.00 to 1.00)	1.00 (1.00 to 1.00)	1.00 (1.00 to 1.00)	1.00 (1.00 to 1.00)	1.00 (1.00 to 1.00)	1.00 (1.00 to 1.00)	1.00 (1.00 to 1.00)
Breast cancer	60 Pack Years	Both	Female	1.27 (1.11 to 1.44)	1.27 (1.11 to 1.44)	1.27 (1.11 to 1.44)	1.27 (1.11 to 1.44)	1.27 (1.11 to 1.44)	1.27 (1.11 to 1.44)	1.27 (1.11 to 1.44)	1.27 (1.11 to 1.44)	1.27 (1.11 to 1.44)	1.27 (1.11 to 1.44)	1.27 (1.11 to 1.44)	1.27 (1.11 to 1.44)	1.27 (1.11 to 1.44)	1.27 (1.11 to 1.44)
Breast cancer	60 Pack Years	Both	Male	1.00 (1.00 to 1.00)	1.00 (1.00 to 1.00)	1.00 (1.00 to 1.00)	1.00 (1.00 to 1.00)	1.00 (1.00 to 1.00)	1.00 (1.00 to 1.00)	1.00 (1.00 to 1.00)	1.00 (1.00 to 1.00)	1.00 (1.00 to 1.00)	1.00 (1.00 to 1.00)	1.00 (1.00 to 1.00)	1.00 (1.00 to 1.00)	1.00 (1.00 to 1.00)	1.00 (1.00 to 1.00)
Cervical cancer	0 Pack Years	Both	Female	1.00 (1.00 to 1.00)	1.00 (1.00 to 1.00)	1.00 (1.00 to 1.00)	1.00 (1.00 to 1.00)	1.00 (1.00 to 1.00)	1.00 (1.00 to 1.00)	1.00 (1.00 to 1.00)	1.00 (1.00 to 1.00)	1.00 (1.00 to 1.00)	1.00 (1.00 to 1.00)	1.00 (1.00 to 1.00)	1.00 (1.00 to 1.00)	1.00 (1.00 to 1.00)	1.00 (1.00 to 1.00)
Cervical cancer	0 Pack Years	Both	Male	1.00 (1.00 to 1.00)	1.00 (1.00 to 1.00)	1.00 (1.00 to 1.00)	1.00 (1.00 to 1.00)	1.00 (1.00 to 1.00)	1.00 (1.00 to 1.00)	1.00 (1.00 to 1.00)	1.00 (1.00 to 1.00)	1.00 (1.00 to 1.00)	1.00 (1.00 to 1.00)	1.00 (1.00 to 1.00)	1.00 (1.00 to 1.00)	1.00 (1.00 to 1.00)	1.00 (1.00 to 1.00)
Cervical cancer	10 Pack Years	Both	Female	2.05 (1.30 to 3.08)	2.05 (1.30 to 3.08)	2.05 (1.30 to 3.08)	2.05 (1.30 to 3.08)	2.05 (1.30 to 3.08)	2.05 (1.30 to 3.08)	2.05 (1.30 to 3.08)	2.05 (1.30 to 3.08)	2.05 (1.30 to 3.08)	2.05 (1.30 to 3.08)	2.05 (1.30 to 3.08)	2.05 (1.30 to 3.08)	2.05 (1.30 to 3.08)	2.05 (1.30 to 3.08)
Cervical cancer	10 Pack Years	Both	Male	1.00 (1.00 to 1.00)	1.00 (1.00 to 1.00)	1.00 (1.00 to 1.00)	1.00 (1.00 to 1.00)	1.00 (1.00 to 1.00)	1.00 (1.00 to 1.00)	1.00 (1.00 to 1.00)	1.00 (1.00 to 1.00)	1.00 (1.00 to 1.00)	1.00 (1.00 to 1.00)	1.00 (1.00 to 1.00)	1.00 (1.00 to 1.00)	1.00 (1.00 to 1.00)	1.00 (1.00 to 1.00)
Cervical cancer	20 Pack Years	Both	Female	3.04 (1.77 to 4.83)	3.04 (1.77 to 4.83)	3.04 (1.77 to 4.83)	3.04 (1.77 to 4.83)	3.04 (1.77 to 4.83)	3.04 (1.77 to 4.83)	3.04 (1.77 to 4.83)	3.04 (1.77 to 4.83)	3.04 (1.77 to 4.83)	3.04 (1.77 to 4.83)	3.04 (1.77 to 4.83)	3.04 (1.77 to 4.83)	3.04 (1.77 to 4.83)	3.04 (1.77 to 4.83)
Cervical cancer	20 Pack Years	Both	Male	1.00 (1.00 to 1.00)	1.00 (1.00 to 1.00)	1.00 (1.00 to 1.00)	1.00 (1.00 to 1.00)	1.00 (1.00 to 1.00)	1.00 (1.00 to 1.00)	1.00 (1.00 to 1.00)	1.00 (1.00 to 1.00)	1.00 (1.00 to 1.00)	1.00 (1.00 to 1.00)	1.00 (1.00 to 1.00)	1.00 (1.00 to 1.00)	1.00 (1.00 to 1.00)	1.00 (1.00 to 1.00)
Colon and rectum cancer	0 Pack Years	Both	Female	1.00 (1.00 to 1.00)	1.00 (1.00 to 1.00)	1.00 (1.00 to 1.00)	1.00 (1.00 to 1.00)	1.00 (1.00 to 1.00)	1.00 (1.00 to 1.00)	1.00 (1.00 to 1.00)	1.00 (1.00 to 1.00)	1.00 (1.00 to 1.00)	1.00 (1.00 to 1.00)	1.00 (1.00 to 1.00)	1.00 (1.00 to 1.00)	1.00 (1.00 to 1.00)	1.00 (1.00 to 1.00)
Colon and rectum cancer	0 Pack Years	Both	Male	1.00 (1.00 to 1.00)	1.00 (1.00 to 1.00)	1.00 (1.00 to 1.00)	1.00 (1.00 to 1.00)	1.00 (1.00 to 1.00)	1.00 (1.00 to 1.00)	1.00 (1.00 to 1.00)	1.00 (1.00 to 1.00)	1.00 (1.00 to 1.00)	1.00 (1.00 to 1.00)	1.00 (1.00 to 1.00)	1.00 (1.00 to 1.00)	1.00 (1.00 to 1.00)	1.00 (1.00 to 1.00)
Colon and rectum cancer	10 Pack Years	Both	Female	1.27 (1.10 to 1.42)	1.27 (1.10 to 1.42)	1.27 (1.10 to 1.42)	1.27 (1.10 to 1.42)	1.27 (1.10 to 1.42)	1.27 (1.10 to 1.42)	1.27 (1.10 to 1.42)	1.27 (1.10 to 1.42)	1.27 (1.10 to 1.42)	1.27 (1.10 to 1.42)	1.27 (1.10 to 1.42)	1.27 (1.10 to 1.42)	1.27 (1.10 to 1.42)	1.27 (1.10 to 1.42)
Colon and rectum cancer	10 Pack Years	Both	Male	1.27 (1.10 to 1.42)	1.27 (1.10 to 1.42)	1.27 (1.10 to 1.42)	1.27 (1.10 to 1.42)	1.27 (1.10 to 1.42)	1.27 (1.10 to 1.42)	1.27 (1.10 to 1.42)	1.27 (1.10 to 1.42)	1.27 (1.10 to 1.42)	1.27 (1.10 to 1.42)	1.27 (1.10 to 1.42)	1.27 (1.10 to 1.42)	1.27 (1.10 to 1.42)	1.27 (1.10 to 1.42)
Colon and rectum cancer	20 Pack Years	Both	Female	1.51 (1.20 to 1.80)	1.51 (1.20 to 1.80)	1.51 (1.20 to 1.80)	1.51 (1.20 to 1.80)	1.51 (1.20 to 1.80)	1.51 (1.20 to 1.80)	1.51 (1.20 to 1.80)	1.51 (1.20 to 1.80)	1.51 (1.20 to 1.80)	1.51 (1.20 to 1.80)	1.51 (1.20 to 1.80)	1.51 (1.20 to 1.80)	1.51 (1.20 to 1.80)	1.51 (1.20 to 1.80)
Colon and rectum cancer	20 Pack Years	Both	Male	1.51 (1.20 to 1.80)	1.51 (1.20 to 1.80)	1.51 (1.20 to 1.80)	1.51 (1.20 to 1.80)	1.51 (1.20 to 1.80)	1.51 (1.20 to 1.80)	1.51 (1.20 to 1.80)	1.51 (1.20 to 1.80)	1.51 (1.20 to 1.80)	1.51 (1.20 to 1.80)	1.51 (1.20 to 1.80)	1.51 (1.20 to 1.80)	1.51 (1.20 to 1.80)	1.51 (1.20 to 1.80)
Colon and rectum cancer	30 Pack Years	Both	Female	1.57 (1.25 to 1.90)	1.57 (1.25 to 1.90)	1.57 (1.25 to 1.90)	1.57 (1.25 to 1.90)	1.57 (1.25 to 1.90)	1.57 (1.25 to 1.90)	1.57 (1.25 to 1.90)	1.57 (1.25 to 1.90)	1.57 (1.25 to 1.90)	1.57 (1.25 to 1.90)	1.57 (1.25 to 1.90)	1.57 (1.25 to 1.90)	1.57 (1.25 to 1.90)	1.57 (1.25 to 1.90)
Colon and rectum cancer	30 Pack Years	Both	Male	1.57 (1.25 to 1.90)	1.57 (1.25 to 1.90)	1.57 (1.25 to 1.90)	1.57 (1.25 to 1.90)	1.57 (1.25 to 1.90)	1.57 (1.25 to 1.90)	1.57 (1.25 to 1.90)	1.57 (1.25 to 1.90)	1.57 (1.25 to 1.90)	1.57 (1.25 to 1.90)	1.57 (1.25 to 1.90)	1.57 (1.25 to 1.90)	1.57 (1.25 to 1.90)	1.57 (1.25 to 1.90)
Colon and rectum cancer	40 Pack Years	Both	Female	1.60 (1.27 to 1.98)	1.60 (1.27 to 1.98)	1.60 (1.27 to 1.98)	1.60 (1.27 to 1.98)	1.60 (1.27 to 1.98)	1.60 (1.27 to 1.98)	1.60 (1.27 to 1.98)	1.60 (1.27 to 1.98)	1.60 (1.27 to 1.98)	1.60 (1.27 to 1.98)	1.60 (1.27 to 1.98)	1.60 (1.27 to 1.98)	1.60 (1.27 to 1.98)	1.60 (1.27 to 1.98)
Colon and rectum cancer	40 Pack Years	Both	Male	1.60 (1.27 to 1.98)	1.60 (1.27 to 1.98)	1.60 (1.27 to 1.98)	1.60 (1.27 to 1.98)	1.60 (1.27 to 1.98)	1.60 (1.27 to 1.98)	1.60 (1.27 to 1.98)	1.60 (1.27 to 1.98)	1.60 (1.27 to 1.98)	1.60 (1.27 to 1.98)	1.60 (1.27 to 1.98)	1.60 (1.27 to 1.98)	1.60 (1.27 to 1.98)	1.60 (1.27 to 1.98)
Colon and rectum cancer	50 Pack Years	Both	Female	1.59 (1.24 to 1.99)	1.59 (1.24 to 1.99)	1.59 (1.24 to 1.99)	1.59 (1.24 to 1.99)	1.59 (1.24 to 1.99)	1.59 (1.24 to 1.99)	1.59 (1.24 to 1.99)	1.59 (1.24 to 1.99)	1.59 (1.24 to 1.99)	1.59 (1.24 to 1.99)	1.59 (1.24 to 1.99)	1.59 (1.24 to 1.99)	1.59 (1.24 to 1.99)	1.59 (1.24 to 1.99)
Colon and rectum cancer	50 Pack Years	Both	Male	1.59 (1.24 to 1.99)	1.59 (1.24 to 1.99)	1.59 (1.24 to 1.99)	1.59 (1.24 to 1.99)	1.59 (1.24 to 1.99)	1.59 (1.24 to 1.99)	1.59 (1.24 to 1.99)	1.59 (1.24 to 1.99)	1.59 (1.24 to 1.99)	1.59 (1.24 to 1.99)	1.59 (1.24 to 1.99)	1.59 (1.24 to 1.99)	1.59 (1.24 to 1.99)	1.59 (1.24 to 1.99)
Colon and rectum cancer	60 Pack Years	Both	Female	1.56 (1.15 to 2.04)	1.56 (1.15 to 2.04)	1.56 (1.15 to 2.04)	1.56 (1.15 to 2.04)	1.56 (1.15 to 2.04)	1.56 (1.15 to 2.04)	1.56 (1.15 to 2.04)	1.56 (1.15 to 2.04)	1.56 (1.15 to 2.04)	1.56 (1.15 to 2.04)	1.56 (1.15 to 2.04)	1.56 (1.15 to 2.04)	1.56 (1.15 to 2.04)	1.56 (1.15 to 2.04)
Colon and rectum cancer	60 Pack Years	Both	Male	1.56 (1.15 to 2.04)	1.56 (1.15 to 2.04)	1.56 (1.15 to 2.04)	1.56 (1.15 to 2.04)	1.56 (1.15 to 2.04)	1.56 (1.15 to 2.04)	1.56 (1.15 to 2.04)	1.56 (1.15 to 2.04)	1.56 (1.15 to 2.04)	1.56 (1.15 to 2.04)	1.56 (1.15 to 2.04)	1.56 (1.15 to 2.04)	1.56 (1.15 to 2.04)	1.56 (1.15 to 2.04)
Colon and rectum cancer	70 Pack Years	Both	Female	1.48 (0.96 to 2.14)	1.48 (0.96 to 2.14)	1.48 (0.96 to 2.14)	1.48 (0.96 to 2.14)	1.48 (0.96 to 2.14)	1.48 (0.96 to 2.14)	1.48 (0.96 to 2.14)	1.48 (0.96 to 2.14)	1.48 (0.96 to 2.14)	1.48 (0.96 to 2.14)	1.48 (0.96 to 2.14)	1.48 (0.96 to 2.14)	1.48 (0.96 to 2.14)	1.48 (0.96 to 2.14)
Colon and rectum cancer	70 Pack Years	Both	Male	1.48 (0.96 to 2.14)	1.48 (0.96 to 2.14)	1.48 (0.96 to 2.14)	1.48 (0.96 to 2.14)	1.48 (0.96 to 2.14)	1.48 (0.96 to 2.14)	1.48 (0.96 to 2.14)	1.48 (0.96 to 2.14)	1.48 (0.96 to 2.14)	1.48 (0.96 to 2.14)	1.48 (0.96 to 2.14)	1.48 (0.96 to 2.14)	1.48 (0.96 to 2.14)	1.48 (0.96 to 2.14)
Lip and oral cavity cancer	0 Pack Years	Both	Female	1.00 (1.00 to 1.00)	1.00 (1.00 to 1.00)	1.00 (1.00 to 1.00)	1.00 (1.00 to 1.00)	1.00 (1.00 to 1.00)	1.00 (1.00 to 1.00)	1.00 (1.00 to 1.00)	1.00 (1.00 to 1.00)	1.00 (1.00 to 1.00)	1.00 (1.00 to 1.00)	1.00 (1.00 to 1.00)	1.00 (1.00 to 1.00)	1.00 (1.00 to 1.00)	1.00 (1.00 to 1.00)
Lip and oral cavity cancer	0 Pack Years	Both	Male	1.00 (1.00 to 1.00)	1.00 (1.00 to 1.00)	1.00 (1.00 to 1.00)	1.00 (1.00 to 1.00)	1.00 (1.00 to 1.00)	1.00 (1.00 to 1.00)	1.00 (1.00 to 1.00)	1.00 (1.00 to 1.00)	1.00 (1.00 to 1.00)	1.00 (1.00 to 1.00)	1.00 (1.00 to 1.00)	1.00 (1.00 to 1.00)	1.00 (1.00 to 1.00)	1.00 (1.00 to 1.00)
Lip and oral cavity cancer	10 Pack Years	Both	Female	2.24 (1.74 to 2.76)	2.24 (1.74 to 2.76)	2.24 (1.74 to 2.76)	2.24 (1.74 to 2.76)	2.24 (1.74 to 2.76)	2.24 (1.74 to 2.76)	2.24 (1.74 to 2.76)	2.24 (1.74 to 2.76)	2.24 (1.74 to 2.76)	2.24 (1.74 to 2.76)	2.24 (1.74 to 2.76)	2.24 (1.74 to 2.76)	2.24 (1.74 to 2.76)	2.24 (1.74 to 2.76)
Lip and oral cavity cancer	10 Pack Years	Both	Male	2.24 (1.74 to 2.76)	2.24 (1.74 to 2.76)	2.24 (1.74 to 2.76)	2.24 (1.74 to 2.76)	2.24 (1.74 to 2.76)	2.24 (1.74 to 2.76)	2.24 (1.74 to 2.76)	2.24 (1.74 to 2.76)	2.24 (1.74 to 2.76)	2.24 (1.74 to 2.76)	2.24 (1.74 to 2.76)	2.24 (1.74 to 2.76)	2.24 (1.74 to 2.76)	2.24 (1.74 to 2.76)
Lip and oral cavity cancer	20 Pack Years	Both	Female	3.26 (2.34 to 4.23)	3.26 (2.34 to 4.23)	3.26 (2.34 to 4.23)	3.26 (2.34 to 4.23)	3.26 (2.34 to 4.23)	3.26 (2.34 to 4.23)	3.26 (2.34 to 4.23)	3.26 (2.34 to 4.23)	3.26 (2.34 to 4.23)	3.26 (2.34 to 4.23)	3.26 (2.34 to 4.23)	3.26 (2.34 to 4.23)	3.26 (2.34 to 4.23)	3.26 (2.34 to 4.23)
Lip and oral cavity cancer	20 Pack Years	Both	Male	3.26 (2.34 to 4.23)	3.26 (2.34 to 4.23)	3.26 (2.34 to 4.23)	3.26 (2.34 to 4.23)	3.26 (2.34 to 4.23)	3.26 (2.34 to 4.23)	3.26 (2.34 to 4.23)	3.26 (2.34 to 4.23)	3.26 (2.34 to 4.23)	3.26 (2.34 to 4.23)	3.26 (2.34 to 4.23)	3.26 (2.34 to 4.23)	3.26 (2.34 to 4.23)	3.26 (2.34 to 4.23)
Lip and oral cavity cancer	30 Pack Years	Both	Female	3.58 (2.51 to 4.75)	3.58 (2.51 to 4.75)	3.58 (2.51 to 4.75)	3.58 (2.51 to 4.75)	3.58 (2.51 to 4.75)	3.58 (2.51 to 4.75)	3.58 (2.51 to 4.75)	3.58 (2.51 to 4.75)	3.58 (2.51 to 4.75)	3.58 (2.51 to 4.75)	3.58 (2.51 to 4.75)	3.58 (2.51 to 4.75)	3.58 (2.51 to 4.75)	3.58 (2.51 to 4.75)
Lip and oral cavity cancer	30 Pack Years	Both	Male	3.58 (2.51 to 4.75)	3.58 (2.51 to 4.75)	3.58 (2.51 to 4.75)	3.58 (2.51 to 4.75)	3.58 (2.51 to 4.75)	3.58 (2.51 to 4.75)	3.58 (2.51 to 4.75)	3.58 (2.51 to 4.75)	3.58 (2.51 to 4.75)	3.58 (2.51 to 4.75)	3.58 (2.51 to 4.75)	3.58 (2.51 to 4.75)	3.58 (2.51 to 4.75)	3.58 (2.51 to 4.75)
Lip and oral cavity cancer	40 Pack Years	Both	Female	3.82 (2.60 to 5.18)	3.82 (2.60 to 5.18)	3.82 (2.60 to 5.18)	3.82 (2.60 to 5.18)	3.82 (2.60 to 5.18)	3.82 (2.60 to 5.18)	3.82 (2.60 to 5.18)	3.82 (2.60 to 5.18)	3.82 (2.60 to 5.18)	3.82 (2.60 to 5.18)	3.82 (2.60 to 5.18)	3.82 (2.60 to 5.18)	3.82 (2.60 to 5.18)	3.82 (2.60 to 5.18)
Lip and oral cavity cancer	40 Pack Years	Both	Male	3.82 (2.60 to 5.18)	3.82 (2.60 to 5.18)	3.82 (2.60 to 5.18)	3.82 (2.60 to 5.18)	3.82 (2.60 to 5.18)	3.82 (2.60 to 5.18)	3.82 (2.60 to 5.18)	3.82 (2.60 to 5.18)	3.82 (2.60 to 5.18)	3.82 (2.60 to 5.18)	3.82 (2.60 to 5.18)	3.82 (2.60 to 5.18)	3.82 (2.60 to 5.18)	3.82 (2.60 to 5.18)
Lip and oral cavity cancer	50 Pack Years	Both	Female	3.97 (2.63 to 5.52)	3.97 (2.63 to 5.52)	3.97 (2.63 to 5.52)	3.97 (2.63 to 5.52)	3.97 (2.63 to 5.52)	3.97 (2.63 to 5.52)	3.97 (2.63 to 5.52)	3.97 (2.63 to 5.52)	3.97 (2.63 to 5.52)	3.97 (2.63 to 5.52)	3.97 (2.63 to 5.52)	3.97 (2.63 to 5.52)	3.97 (2.63 to 5.52)	3.97 (2.63 to 5.52)
Lip and oral cavity cancer	50 Pack Years	Both	Male	3.97 (2.63 to 5.52)	3.97 (2.63 to 5.52)	3.97 (2.63 to 5.52)	3.97 (2.63 to 5.52)	3.97 (2.63 to 5.52)	3.97 (2.63 to 5.52)	3.97 (2.63 to 5.52)	3.97 (2.63 to 5.52)	3.97 (2.63 to 5.52)	3.97 (2.63 to 5.52)	3.97 (2.63 to 5.52)	3.97 (2.63 to 5.52)	3.97 (2.63 to 5.52)	3.97 (2.63 to 5.52)

Lip and oral cavity cancer	60 Pack Years	Both	Female	4.60 (2.72 to 6.91)	4.60 (2.72 to 6.91)	4.60 (2.72 to 6.91)	4.60 (2.72 to 6.91)	4.60 (2.72 to 6.91)	4.60 (2.72 to 6.91)	4.60 (2.72 to 6.91)	4.60 (2.72 to 6.91)	4.60 (2.72 to 6.91)	4.60 (2.72 to 6.91)	4.60 (2.72 to 6.91)	4.60 (2.72 to 6.91)	4.60 (2.72 to 6.91)	4.60 (2.72 to 6.91)
Lip and oral cavity cancer	60 Pack Years	Both	Male	4.60 (2.72 to 6.91)	4.60 (2.72 to 6.91)	4.60 (2.72 to 6.91)	4.60 (2.72 to 6.91)	4.60 (2.72 to 6.91)	4.60 (2.72 to 6.91)	4.60 (2.72 to 6.91)	4.60 (2.72 to 6.91)	4.60 (2.72 to 6.91)	4.60 (2.72 to 6.91)	4.60 (2.72 to 6.91)	4.60 (2.72 to 6.91)	4.60 (2.72 to 6.91)	4.60 (2.72 to 6.91)
Lip and oral cavity cancer	70 Pack Years	Both	Female	5.45 (2.86 to 8.76)	5.45 (2.86 to 8.76)	5.45 (2.86 to 8.76)	5.45 (2.86 to 8.76)	5.45 (2.86 to 8.76)	5.45 (2.86 to 8.76)	5.45 (2.86 to 8.76)	5.45 (2.86 to 8.76)	5.45 (2.86 to 8.76)	5.45 (2.86 to 8.76)	5.45 (2.86 to 8.76)	5.45 (2.86 to 8.76)	5.45 (2.86 to 8.76)	5.45 (2.86 to 8.76)
Lip and oral cavity cancer	70 Pack Years	Both	Male	5.45 (2.86 to 8.76)	5.45 (2.86 to 8.76)	5.45 (2.86 to 8.76)	5.45 (2.86 to 8.76)	5.45 (2.86 to 8.76)	5.45 (2.86 to 8.76)	5.45 (2.86 to 8.76)	5.45 (2.86 to 8.76)	5.45 (2.86 to 8.76)	5.45 (2.86 to 8.76)	5.45 (2.86 to 8.76)	5.45 (2.86 to 8.76)	5.45 (2.86 to 8.76)	5.45 (2.86 to 8.76)
Lip and oral cavity cancer	80 Pack Years	Both	Female	6.43 (2.96 to 11.09)	6.43 (2.96 to 11.09)	6.43 (2.96 to 11.09)	6.43 (2.96 to 11.09)	6.43 (2.96 to 11.09)	6.43 (2.96 to 11.09)	6.43 (2.96 to 11.09)	6.43 (2.96 to 11.09)	6.43 (2.96 to 11.09)	6.43 (2.96 to 11.09)	6.43 (2.96 to 11.09)	6.43 (2.96 to 11.09)	6.43 (2.96 to 11.09)	6.43 (2.96 to 11.09)
Lip and oral cavity cancer	80 Pack Years	Both	Male	6.43 (2.96 to 11.09)	6.43 (2.96 to 11.09)	6.43 (2.96 to 11.09)	6.43 (2.96 to 11.09)	6.43 (2.96 to 11.09)	6.43 (2.96 to 11.09)	6.43 (2.96 to 11.09)	6.43 (2.96 to 11.09)	6.43 (2.96 to 11.09)	6.43 (2.96 to 11.09)	6.43 (2.96 to 11.09)	6.43 (2.96 to 11.09)	6.43 (2.96 to 11.09)	6.43 (2.96 to 11.09)
Nasopharynx cancer	0 Pack Years	Both	Female	1.00 (1.00 to 1.00)	1.00 (1.00 to 1.00)	1.00 (1.00 to 1.00)	1.00 (1.00 to 1.00)	1.00 (1.00 to 1.00)	1.00 (1.00 to 1.00)	1.00 (1.00 to 1.00)	1.00 (1.00 to 1.00)	1.00 (1.00 to 1.00)	1.00 (1.00 to 1.00)	1.00 (1.00 to 1.00)	1.00 (1.00 to 1.00)	1.00 (1.00 to 1.00)	1.00 (1.00 to 1.00)
Nasopharynx cancer	0 Pack Years	Both	Male	1.00 (1.00 to 1.00)	1.00 (1.00 to 1.00)	1.00 (1.00 to 1.00)	1.00 (1.00 to 1.00)	1.00 (1.00 to 1.00)	1.00 (1.00 to 1.00)	1.00 (1.00 to 1.00)	1.00 (1.00 to 1.00)	1.00 (1.00 to 1.00)	1.00 (1.00 to 1.00)	1.00 (1.00 to 1.00)	1.00 (1.00 to 1.00)	1.00 (1.00 to 1.00)	1.00 (1.00 to 1.00)
Nasopharynx cancer	10 Pack Years	Both	Female	1.82 (1.37 to 2.31)	1.82 (1.37 to 2.31)	1.82 (1.37 to 2.31)	1.82 (1.37 to 2.31)	1.82 (1.37 to 2.31)	1.82 (1.37 to 2.31)	1.82 (1.37 to 2.31)	1.82 (1.37 to 2.31)	1.82 (1.37 to 2.31)	1.82 (1.37 to 2.31)	1.82 (1.37 to 2.31)	1.82 (1.37 to 2.31)	1.82 (1.37 to 2.31)	1.82 (1.37 to 2.31)
Nasopharynx cancer	10 Pack Years	Both	Male	1.82 (1.37 to 2.31)	1.82 (1.37 to 2.31)	1.82 (1.37 to 2.31)	1.82 (1.37 to 2.31)	1.82 (1.37 to 2.31)	1.82 (1.37 to 2.31)	1.82 (1.37 to 2.31)	1.82 (1.37 to 2.31)	1.82 (1.37 to 2.31)	1.82 (1.37 to 2.31)	1.82 (1.37 to 2.31)	1.82 (1.37 to 2.31)	1.82 (1.37 to 2.31)	1.82 (1.37 to 2.31)
Nasopharynx cancer	20 Pack Years	Both	Female	1.98 (1.42 to 2.64)	1.98 (1.42 to 2.64)	1.98 (1.42 to 2.64)	1.98 (1.42 to 2.64)	1.98 (1.42 to 2.64)	1.98 (1.42 to 2.64)	1.98 (1.42 to 2.64)	1.98 (1.42 to 2.64)	1.98 (1.42 to 2.64)	1.98 (1.42 to 2.64)	1.98 (1.42 to 2.64)	1.98 (1.42 to 2.64)	1.98 (1.42 to 2.64)	1.98 (1.42 to 2.64)
Nasopharynx cancer	20 Pack Years	Both	Male	1.98 (1.42 to 2.64)	1.98 (1.42 to 2.64)	1.98 (1.42 to 2.64)	1.98 (1.42 to 2.64)	1.98 (1.42 to 2.64)	1.98 (1.42 to 2.64)	1.98 (1.42 to 2.64)	1.98 (1.42 to 2.64)	1.98 (1.42 to 2.64)	1.98 (1.42 to 2.64)	1.98 (1.42 to 2.64)	1.98 (1.42 to 2.64)	1.98 (1.42 to 2.64)	1.98 (1.42 to 2.64)
Nasopharynx cancer	30 Pack Years	Both	Female	2.24 (1.56 to 3.06)	2.24 (1.56 to 3.06)	2.24 (1.56 to 3.06)	2.24 (1.56 to 3.06)	2.24 (1.56 to 3.06)	2.24 (1.56 to 3.06)	2.24 (1.56 to 3.06)	2.24 (1.56 to 3.06)	2.24 (1.56 to 3.06)	2.24 (1.56 to 3.06)	2.24 (1.56 to 3.06)	2.24 (1.56 to 3.06)	2.24 (1.56 to 3.06)	2.24 (1.56 to 3.06)
Nasopharynx cancer	30 Pack Years	Both	Male	2.24 (1.56 to 3.06)	2.24 (1.56 to 3.06)	2.24 (1.56 to 3.06)	2.24 (1.56 to 3.06)	2.24 (1.56 to 3.06)	2.24 (1.56 to 3.06)	2.24 (1.56 to 3.06)	2.24 (1.56 to 3.06)	2.24 (1.56 to 3.06)	2.24 (1.56 to 3.06)	2.24 (1.56 to 3.06)	2.24 (1.56 to 3.06)	2.24 (1.56 to 3.06)	2.24 (1.56 to 3.06)
Nasopharynx cancer	40 Pack Years	Both	Female	2.56 (1.72 to 3.54)	2.56 (1.72 to 3.54)	2.56 (1.72 to 3.54)	2.56 (1.72 to 3.54)	2.56 (1.72 to 3.54)	2.56 (1.72 to 3.54)	2.56 (1.72 to 3.54)	2.56 (1.72 to 3.54)	2.56 (1.72 to 3.54)	2.56 (1.72 to 3.54)	2.56 (1.72 to 3.54)	2.56 (1.72 to 3.54)	2.56 (1.72 to 3.54)	2.56 (1.72 to 3.54)
Nasopharynx cancer	40 Pack Years	Both	Male	2.56 (1.72 to 3.54)	2.56 (1.72 to 3.54)	2.56 (1.72 to 3.54)	2.56 (1.72 to 3.54)	2.56 (1.72 to 3.54)	2.56 (1.72 to 3.54)	2.56 (1.72 to 3.54)	2.56 (1.72 to 3.54)	2.56 (1.72 to 3.54)	2.56 (1.72 to 3.54)	2.56 (1.72 to 3.54)	2.56 (1.72 to 3.54)	2.56 (1.72 to 3.54)	2.56 (1.72 to 3.54)
Nasopharynx cancer	50 Pack Years	Both	Female	3.14 (2.07 to 4.37)	3.14 (2.07 to 4.37)	3.14 (2.07 to 4.37)	3.14 (2.07 to 4.37)	3.14 (2.07 to 4.37)	3.14 (2.07 to 4.37)	3.14 (2.07 to 4.37)	3.14 (2.07 to 4.37)	3.14 (2.07 to 4.37)	3.14 (2.07 to 4.37)	3.14 (2.07 to 4.37)	3.14 (2.07 to 4.37)	3.14 (2.07 to 4.37)	3.14 (2.07 to 4.37)
Nasopharynx cancer	50 Pack Years	Both	Male	3.14 (2.07 to 4.37)	3.14 (2.07 to 4.37)	3.14 (2.07 to 4.37)	3.14 (2.07 to 4.37)	3.14 (2.07 to 4.37)	3.14 (2.07 to 4.37)	3.14 (2.07 to 4.37)	3.14 (2.07 to 4.37)	3.14 (2.07 to 4.37)	3.14 (2.07 to 4.37)	3.14 (2.07 to 4.37)	3.14 (2.07 to 4.37)	3.14 (2.07 to 4.37)	3.14 (2.07 to 4.37)
Nasopharynx cancer	60 Pack Years	Both	Female	4.03 (2.66 to 5.80)	4.03 (2.66 to 5.80)	4.03 (2.66 to 5.80)	4.03 (2.66 to 5.80)	4.03 (2.66 to 5.80)	4.03 (2.66 to 5.80)	4.03 (2.66 to 5.80)	4.03 (2.66 to 5.80)	4.03 (2.66 to 5.80)	4.03 (2.66 to 5.80)	4.03 (2.66 to 5.80)	4.03 (2.66 to 5.80)	4.03 (2.66 to 5.80)	4.03 (2.66 to 5.80)
Nasopharynx cancer	60 Pack Years	Both	Male	4.03 (2.66 to 5.80)	4.03 (2.66 to 5.80)	4.03 (2.66 to 5.80)	4.03 (2.66 to 5.80)	4.03 (2.66 to 5.80)	4.03 (2.66 to 5.80)	4.03 (2.66 to 5.80)	4.03 (2.66 to 5.80)	4.03 (2.66 to 5.80)	4.03 (2.66 to 5.80)	4.03 (2.66 to 5.80)	4.03 (2.66 to 5.80)	4.03 (2.66 to 5.80)	4.03 (2.66 to 5.80)
Nasopharynx cancer	70 Pack Years	Both	Female	4.62 (2.64 to 7.46)	4.62 (2.64 to 7.46)	4.62 (2.64 to 7.46)	4.62 (2.64 to 7.46)	4.62 (2.64 to 7.46)	4.62 (2.64 to 7.46)	4.62 (2.64 to 7.46)	4.62 (2.64 to 7.46)	4.62 (2.64 to 7.46)	4.62 (2.64 to 7.46)	4.62 (2.64 to 7.46)	4.62 (2.64 to 7.46)	4.62 (2.64 to 7.46)	4.62 (2.64 to 7.46)
Nasopharynx cancer	70 Pack Years	Both	Male	4.62 (2.64 to 7.46)	4.62 (2.64 to 7.46)	4.62 (2.64 to 7.46)	4.62 (2.64 to 7.46)	4.62 (2.64 to 7.46)	4.62 (2.64 to 7.46)	4.62 (2.64 to 7.46)	4.62 (2.64 to 7.46)	4.62 (2.64 to 7.46)	4.62 (2.64 to 7.46)	4.62 (2.64 to 7.46)	4.62 (2.64 to 7.46)	4.62 (2.64 to 7.46)	4.62 (2.64 to 7.46)
Other pharynx cancer	0 Pack Years	Both	Female	1.00 (1.00 to 1.00)	1.00 (1.00 to 1.00)	1.00 (1.00 to 1.00)	1.00 (1.00 to 1.00)	1.00 (1.00 to 1.00)	1.00 (1.00 to 1.00)	1.00 (1.00 to 1.00)	1.00 (1.00 to 1.00)	1.00 (1.00 to 1.00)	1.00 (1.00 to 1.00)	1.00 (1.00 to 1.00)	1.00 (1.00 to 1.00)	1.00 (1.00 to 1.00)	1.00 (1.00 to 1.00)
Other pharynx cancer	0 Pack Years	Both	Male	1.00 (1.00 to 1.00)	1.00 (1.00 to 1.00)	1.00 (1.00 to 1.00)	1.00 (1.00 to 1.00)	1.00 (1.00 to 1.00)	1.00 (1.00 to 1.00)	1.00 (1.00 to 1.00)	1.00 (1.00 to 1.00)	1.00 (1.00 to 1.00)	1.00 (1.00 to 1.00)	1.00 (1.00 to 1.00)	1.00 (1.00 to 1.00)	1.00 (1.00 to 1.00)	1.00 (1.00 to 1.00)
Other pharynx cancer	10 Pack Years	Both	Female	3.14 (2.41 to 3.93)	3.14 (2.41 to 3.93)	3.14 (2.41 to 3.93)	3.14 (2.41 to 3.93)	3.14 (2.41 to 3.93)	3.14 (2.41 to 3.93)	3.14 (2.41 to 3.93)	3.14 (2.41 to 3.93)	3.14 (2.41 to 3.93)	3.14 (2.41 to 3.93)	3.14 (2.41 to 3.93)	3.14 (2.41 to 3.93)	3.14 (2.41 to 3.93)	3.14 (2.41 to 3.93)
Other pharynx cancer	10 Pack Years	Both	Male	3.14 (2.41 to 3.93)	3.14 (2.41 to 3.93)	3.14 (2.41 to 3.93)	3.14 (2.41 to 3.93)	3.14 (2.41 to 3.93)	3.14 (2.41 to 3.93)	3.14 (2.41 to 3.93)	3.14 (2.41 to 3.93)	3.14 (2.41 to 3.93)	3.14 (2.41 to 3.93)	3.14 (2.41 to 3.93)	3.14 (2.41 to 3.93)	3.14 (2.41 to 3.93)	3.14 (2.41 to 3.93)
Other pharynx cancer	20 Pack Years	Both	Female	4.85 (3.54 to 6.27)	4.85 (3.54 to 6.27)	4.85 (3.54 to 6.27)	4.85 (3.54 to 6.27)	4.85 (3.54 to 6.27)	4.85 (3.54 to 6.27)	4.85 (3.54 to 6.27)	4.85 (3.54 to 6.27)	4.85 (3.54 to 6.27)	4.85 (3.54 to 6.27)	4.85 (3.54 to 6.27)	4.85 (3.54 to 6.27)	4.85 (3.54 to 6.27)	4.85 (3.54 to 6.27)
Other pharynx cancer	20 Pack Years	Both	Male	4.85 (3.54 to 6.27)	4.85 (3.54 to 6.27)	4.85 (3.54 to 6.27)	4.85 (3.54 to 6.27)	4.85 (3.54 to 6.27)	4.85 (3.54 to 6.27)	4.85 (3.54 to 6.27)	4.85 (3.54 to 6.27)	4.85 (3.54 to 6.27)	4.85 (3.54 to 6.27)	4.85 (3.54 to 6.27)	4.85 (3.54 to 6.27)	4.85 (3.54 to 6.27)	4.85 (3.54 to 6.27)
Other pharynx cancer	30 Pack Years	Both	Female	5.11 (3.71 to 6.66)	5.11 (3.71 to 6.66)	5.11 (3.71 to 6.66)	5.11 (3.71 to 6.66)	5.11 (3.71 to 6.66)	5.11 (3.71 to 6.66)	5.11 (3.71 to 6.66)	5.11 (3.71 to 6.66)	5.11 (3.71 to 6.66)	5.11 (3.71 to 6.66)	5.11 (3.71 to 6.66)	5.11 (3.71 to 6.66)	5.11 (3.71 to 6.66)	5.11 (3.71 to 6.66)
Other pharynx cancer	30 Pack Years	Both	Male	5.11 (3.71 to 6.66)	5.11 (3.71 to 6.66)	5.11 (3.71 to 6.66)	5.11 (3.71 to 6.66)	5.11 (3.71 to 6.66)	5.11 (3.71 to 6.66)	5.11 (3.71 to 6.66)	5.11 (3.71 to 6.66)	5.11 (3.71 to 6.66)	5.11 (3.71 to 6.66)	5.11 (3.71 to 6.66)	5.11 (3.71 to 6.66)	5.11 (3.71 to 6.66)	5.11 (3.71 to 6.66)
Other pharynx cancer	40 Pack Years	Both	Female	5.60 (4.05 to 7.29)	5.60 (4.05 to 7.29)	5.60 (4.05 to 7.29)	5.60 (4.05 to 7.29)	5.60 (4.05 to 7.29)	5.60 (4.05 to 7.29)	5.60 (4.05 to 7.29)	5.60 (4.05 to 7.29)	5.60 (4.05 to 7.29)	5.60 (4.05 to 7.29)	5.60 (4.05 to 7.29)	5.60 (4.05 to 7.29)	5.60 (4.05 to 7.29)	5.60 (4.05 to 7.29)
Other pharynx cancer	40 Pack Years	Both	Male	5.60 (4.05 to 7.29)	5.60 (4.05 to 7.29)	5.60 (4.05 to 7.29)	5.60 (4.05 to 7.29)	5.60 (4.05 to 7.29)	5.60 (4.05 to 7.29)	5.60 (4.05 to 7.29)	5.60 (4.05 to 7.29)	5.60 (4.05 to 7.29)	5.60 (4.05 to 7.29)	5.60 (4.05 to 7.29)	5.60 (4.05 to 7.29)	5.60 (4.05 to 7.29)	5.60 (4.05 to 7.29)
Other pharynx cancer	50 Pack Years	Both	Female	6.35 (4.61 to 8.20)	6.35 (4.61 to 8.20)	6.35 (4.61 to 8.20)	6.35 (4.61 to 8.20)	6.35 (4.61 to 8.20)	6.35 (4.61 to 8.20)	6.35 (4.61 to 8.20)	6.35 (4.61 to 8.20)	6.35 (4.61 to 8.20)	6.35 (4.61 to 8.20)	6.35 (4.61 to 8.20)	6.35 (4.61 to 8.20)	6.35 (4.61 to 8.20)	6.35 (4.61 to 8.20)
Other pharynx cancer	50 Pack Years	Both	Male	6.35 (4.61 to 8.20)	6.35 (4.61 to 8.20)	6.35 (4.61 to 8.20)	6.35 (4.61 to 8.20)	6.35 (4.61 to 8.20)	6.35 (4.61 to 8.20)	6.35 (4.61 to 8.20)	6.35 (4.61 to 8.20)	6.35 (4.61 to 8.20)	6.35 (4.61 to 8.20)	6.35 (4.61 to 8.20)	6.35 (4.61 to 8.20)	6.35 (4.61 to 8.20)	6.35 (4.61 to 8.20)
Other pharynx cancer	60 Pack Years	Both	Female	6.83 (4.72 to 9.32)	6.83 (4.72 to 9.32)	6.83 (4.72 to 9.32)	6.83 (4.72 to 9.32)	6.83 (4.72 to 9.32)	6.83 (4.72 to 9.32)	6.83 (4.72 to 9.32)	6.83 (4.72 to 9.32)	6.83 (4.72 to 9.32)	6.83 (4.72 to 9.32)	6.83 (4.72 to 9.32)	6.83 (4.72 to 9.32)	6.83 (4.72 to 9.32)	6.83 (4.72 to 9.32)
Other pharynx cancer	60 Pack Years	Both	Male	6.83 (4.72 to 9.32)	6.83 (4.72 to 9.32)	6.83 (4.72 to 9.32)	6.83 (4.72 to 9.32)	6.83 (4.72 to 9.32)	6.83 (4.72 to 9.32)	6.83 (4.72 to 9.32)	6.83 (4.72 to 9.32)	6.83 (4.72 to 9.32)	6.83 (4.72 to 9.32)	6.83 (4.72 to 9.32)	6.83 (4.72 to 9.32)	6.83 (4.72 to 9.32)	6.83 (4.72 to 9.32)
Other pharynx cancer	70 Pack Years	Both	Female	7.18 (4.64 to 10.53)	7.18 (4.64 to 10.53)	7.18 (4.64 to 10.53)	7.18 (4.64 to 10.53)	7.18 (4.64 to 10.53)	7.18 (4.64 to 10.53)	7.18 (4.64 to 10.53)	7.18 (4.64 to 10.53)	7.18 (4.64 to 10.53)	7.18 (4.64 to 10.53)	7.18 (4.64 to 10.53)	7.18 (4.64 to 10.53)	7.18 (4.64 to 10.53)	7.18 (4.64 to 10.53)
Other pharynx cancer	70 Pack Years	Both	Male	7.18 (4.64 to 10.53)	7.18 (4.64 to 10.53)	7.18 (4.64 to 10.53)	7.18 (4.64 to 10.53)	7.18 (4.64 to 10.53)	7.18 (4.64 to 10.53)	7.18 (4.64 to 10.53)	7.18 (4.64 to 10.53)	7.18 (4.64 to 10.53)	7.18 (4.64 to 10.53)	7.18 (4.64 to 10.53)	7.18 (4.64 to 10.53)	7.18 (4.64 to 10.53)	7.18 (4.64 to 10.53)

Other pharynx cancer	80 Pack Years	Both	Female	9.26 (5.58 to 13.66)	9.26 (5.58 to 13.66)	9.26 (5.58 to 13.66)	9.26 (5.58 to 13.66)	9.26 (5.58 to 13.66)	9.26 (5.58 to 13.66)	9.26 (5.58 to 13.66)	9.26 (5.58 to 13.66)	9.26 (5.58 to 13.66)	9.26 (5.58 to 13.66)	9.26 (5.58 to 13.66)	9.26 (5.58 to 13.66)	9.26 (5.58 to 13.66)	9.26 (5.58 to 13.66)
Other pharynx cancer	80 Pack Years	Both	Male	9.26 (5.58 to 13.66)	9.26 (5.58 to 13.66)	9.26 (5.58 to 13.66)	9.26 (5.58 to 13.66)	9.26 (5.58 to 13.66)	9.26 (5.58 to 13.66)	9.26 (5.58 to 13.66)	9.26 (5.58 to 13.66)	9.26 (5.58 to 13.66)	9.26 (5.58 to 13.66)	9.26 (5.58 to 13.66)	9.26 (5.58 to 13.66)	9.26 (5.58 to 13.66)	9.26 (5.58 to 13.66)
Pancreatic cancer	0 Pack Years	Both	Female	1.00 (1.00 to 1.00)	1.00 (1.00 to 1.00)	1.00 (1.00 to 1.00)	1.00 (1.00 to 1.00)	1.00 (1.00 to 1.00)	1.00 (1.00 to 1.00)	1.00 (1.00 to 1.00)	1.00 (1.00 to 1.00)	1.00 (1.00 to 1.00)	1.00 (1.00 to 1.00)	1.00 (1.00 to 1.00)	1.00 (1.00 to 1.00)	1.00 (1.00 to 1.00)	1.00 (1.00 to 1.00)
Pancreatic cancer	0 Pack Years	Both	Male	1.00 (1.00 to 1.00)	1.00 (1.00 to 1.00)	1.00 (1.00 to 1.00)	1.00 (1.00 to 1.00)	1.00 (1.00 to 1.00)	1.00 (1.00 to 1.00)	1.00 (1.00 to 1.00)	1.00 (1.00 to 1.00)	1.00 (1.00 to 1.00)	1.00 (1.00 to 1.00)	1.00 (1.00 to 1.00)	1.00 (1.00 to 1.00)	1.00 (1.00 to 1.00)	1.00 (1.00 to 1.00)
Pancreatic cancer	10 Pack Years	Both	Female	1.86 (1.61 to 2.11)	1.86 (1.61 to 2.11)	1.86 (1.61 to 2.11)	1.86 (1.61 to 2.11)	1.86 (1.61 to 2.11)	1.86 (1.61 to 2.11)	1.86 (1.61 to 2.11)	1.86 (1.61 to 2.11)	1.86 (1.61 to 2.11)	1.86 (1.61 to 2.11)	1.86 (1.61 to 2.11)	1.86 (1.61 to 2.11)	1.86 (1.61 to 2.11)	1.86 (1.61 to 2.11)
Pancreatic cancer	10 Pack Years	Both	Male	1.41 (1.24 to 1.59)	1.41 (1.24 to 1.59)	1.41 (1.24 to 1.59)	1.41 (1.24 to 1.59)	1.41 (1.24 to 1.59)	1.41 (1.24 to 1.59)	1.41 (1.24 to 1.59)	1.41 (1.24 to 1.59)	1.41 (1.24 to 1.59)	1.41 (1.24 to 1.59)	1.41 (1.24 to 1.59)	1.41 (1.24 to 1.59)	1.41 (1.24 to 1.59)	1.41 (1.24 to 1.59)
Pancreatic cancer	20 Pack Years	Both	Female	2.46 (2.03 to 2.91)	2.46 (2.03 to 2.91)	2.46 (2.03 to 2.91)	2.46 (2.03 to 2.91)	2.46 (2.03 to 2.91)	2.46 (2.03 to 2.91)	2.46 (2.03 to 2.91)	2.46 (2.03 to 2.91)	2.46 (2.03 to 2.91)	2.46 (2.03 to 2.91)	2.46 (2.03 to 2.91)	2.46 (2.03 to 2.91)	2.46 (2.03 to 2.91)	2.46 (2.03 to 2.91)
Pancreatic cancer	20 Pack Years	Both	Male	1.69 (1.41 to 1.98)	1.69 (1.41 to 1.98)	1.69 (1.41 to 1.98)	1.69 (1.41 to 1.98)	1.69 (1.41 to 1.98)	1.69 (1.41 to 1.98)	1.69 (1.41 to 1.98)	1.69 (1.41 to 1.98)	1.69 (1.41 to 1.98)	1.69 (1.41 to 1.98)	1.69 (1.41 to 1.98)	1.69 (1.41 to 1.98)	1.69 (1.41 to 1.98)	1.69 (1.41 to 1.98)
Pancreatic cancer	30 Pack Years	Both	Female	2.71 (2.18 to 3.27)	2.71 (2.18 to 3.27)	2.71 (2.18 to 3.27)	2.71 (2.18 to 3.27)	2.71 (2.18 to 3.27)	2.71 (2.18 to 3.27)	2.71 (2.18 to 3.27)	2.71 (2.18 to 3.27)	2.71 (2.18 to 3.27)	2.71 (2.18 to 3.27)	2.71 (2.18 to 3.27)	2.71 (2.18 to 3.27)	2.71 (2.18 to 3.27)	2.71 (2.18 to 3.27)
Pancreatic cancer	30 Pack Years	Both	Male	1.79 (1.50 to 2.11)	1.79 (1.50 to 2.11)	1.79 (1.50 to 2.11)	1.79 (1.50 to 2.11)	1.79 (1.50 to 2.11)	1.79 (1.50 to 2.11)	1.79 (1.50 to 2.11)	1.79 (1.50 to 2.11)	1.79 (1.50 to 2.11)	1.79 (1.50 to 2.11)	1.79 (1.50 to 2.11)	1.79 (1.50 to 2.11)	1.79 (1.50 to 2.11)	1.79 (1.50 to 2.11)
Pancreatic cancer	40 Pack Years	Both	Female	3.03 (2.36 to 3.77)	3.03 (2.36 to 3.77)	3.03 (2.36 to 3.77)	3.03 (2.36 to 3.77)	3.03 (2.36 to 3.77)	3.03 (2.36 to 3.77)	3.03 (2.36 to 3.77)	3.03 (2.36 to 3.77)	3.03 (2.36 to 3.77)	3.03 (2.36 to 3.77)	3.03 (2.36 to 3.77)	3.03 (2.36 to 3.77)	3.03 (2.36 to 3.77)	3.03 (2.36 to 3.77)
Pancreatic cancer	40 Pack Years	Both	Male	2.00 (1.67 to 2.34)	2.00 (1.67 to 2.34)	2.00 (1.67 to 2.34)	2.00 (1.67 to 2.34)	2.00 (1.67 to 2.34)	2.00 (1.67 to 2.34)	2.00 (1.67 to 2.34)	2.00 (1.67 to 2.34)	2.00 (1.67 to 2.34)	2.00 (1.67 to 2.34)	2.00 (1.67 to 2.34)	2.00 (1.67 to 2.34)	2.00 (1.67 to 2.34)	2.00 (1.67 to 2.34)
Pancreatic cancer	50 Pack Years	Both	Female	3.38 (2.46 to 4.42)	3.38 (2.46 to 4.42)	3.38 (2.46 to 4.42)	3.38 (2.46 to 4.42)	3.38 (2.46 to 4.42)	3.38 (2.46 to 4.42)	3.38 (2.46 to 4.42)	3.38 (2.46 to 4.42)	3.38 (2.46 to 4.42)	3.38 (2.46 to 4.42)	3.38 (2.46 to 4.42)	3.38 (2.46 to 4.42)	3.38 (2.46 to 4.42)	3.38 (2.46 to 4.42)
Pancreatic cancer	50 Pack Years	Both	Male	2.22 (1.81 to 2.61)	2.22 (1.81 to 2.61)	2.22 (1.81 to 2.61)	2.22 (1.81 to 2.61)	2.22 (1.81 to 2.61)	2.22 (1.81 to 2.61)	2.22 (1.81 to 2.61)	2.22 (1.81 to 2.61)	2.22 (1.81 to 2.61)	2.22 (1.81 to 2.61)	2.22 (1.81 to 2.61)	2.22 (1.81 to 2.61)	2.22 (1.81 to 2.61)	2.22 (1.81 to 2.61)
Pancreatic cancer	60 Pack Years	Both	Female	3.74 (2.36 to 5.38)	3.74 (2.36 to 5.38)	3.74 (2.36 to 5.38)	3.74 (2.36 to 5.38)	3.74 (2.36 to 5.38)	3.74 (2.36 to 5.38)	3.74 (2.36 to 5.38)	3.74 (2.36 to 5.38)	3.74 (2.36 to 5.38)	3.74 (2.36 to 5.38)	3.74 (2.36 to 5.38)	3.74 (2.36 to 5.38)	3.74 (2.36 to 5.38)	3.74 (2.36 to 5.38)
Pancreatic cancer	60 Pack Years	Both	Male	2.38 (1.84 to 2.97)	2.38 (1.84 to 2.97)	2.38 (1.84 to 2.97)	2.38 (1.84 to 2.97)	2.38 (1.84 to 2.97)	2.38 (1.84 to 2.97)	2.38 (1.84 to 2.97)	2.38 (1.84 to 2.97)	2.38 (1.84 to 2.97)	2.38 (1.84 to 2.97)	2.38 (1.84 to 2.97)	2.38 (1.84 to 2.97)	2.38 (1.84 to 2.97)	2.38 (1.84 to 2.97)
Pancreatic cancer	70 Pack Years	Both	Female	4.08 (2.20 to 6.75)	4.08 (2.20 to 6.75)	4.08 (2.20 to 6.75)	4.08 (2.20 to 6.75)	4.08 (2.20 to 6.75)	4.08 (2.20 to 6.75)	4.08 (2.20 to 6.75)	4.08 (2.20 to 6.75)	4.08 (2.20 to 6.75)	4.08 (2.20 to 6.75)	4.08 (2.20 to 6.75)	4.08 (2.20 to 6.75)	4.08 (2.20 to 6.75)	4.08 (2.20 to 6.75)
Pancreatic cancer	70 Pack Years	Both	Male	2.65 (1.87 to 3.60)	2.65 (1.87 to 3.60)	2.65 (1.87 to 3.60)	2.65 (1.87 to 3.60)	2.65 (1.87 to 3.60)	2.65 (1.87 to 3.60)	2.65 (1.87 to 3.60)	2.65 (1.87 to 3.60)	2.65 (1.87 to 3.60)	2.65 (1.87 to 3.60)	2.65 (1.87 to 3.60)	2.65 (1.87 to 3.60)	2.65 (1.87 to 3.60)	2.65 (1.87 to 3.60)
Bladder cancer	0 Pack Years	Both	Female	1.00 (1.00 to 1.00)	1.00 (1.00 to 1.00)	1.00 (1.00 to 1.00)	1.00 (1.00 to 1.00)	1.00 (1.00 to 1.00)	1.00 (1.00 to 1.00)	1.00 (1.00 to 1.00)	1.00 (1.00 to 1.00)	1.00 (1.00 to 1.00)	1.00 (1.00 to 1.00)	1.00 (1.00 to 1.00)	1.00 (1.00 to 1.00)	1.00 (1.00 to 1.00)	1.00 (1.00 to 1.00)
Bladder cancer	0 Pack Years	Both	Male	1.00 (1.00 to 1.00)	1.00 (1.00 to 1.00)	1.00 (1.00 to 1.00)	1.00 (1.00 to 1.00)	1.00 (1.00 to 1.00)	1.00 (1.00 to 1.00)	1.00 (1.00 to 1.00)	1.00 (1.00 to 1.00)	1.00 (1.00 to 1.00)	1.00 (1.00 to 1.00)	1.00 (1.00 to 1.00)	1.00 (1.00 to 1.00)	1.00 (1.00 to 1.00)	1.00 (1.00 to 1.00)
Bladder cancer	10 Pack Years	Both	Female	2.20 (1.57 to 2.88)	2.20 (1.57 to 2.88)	2.20 (1.57 to 2.88)	2.20 (1.57 to 2.88)	2.20 (1.57 to 2.88)	2.20 (1.57 to 2.88)	2.20 (1.57 to 2.88)	2.20 (1.57 to 2.88)	2.20 (1.57 to 2.88)	2.20 (1.57 to 2.88)	2.20 (1.57 to 2.88)	2.20 (1.57 to 2.88)	2.20 (1.57 to 2.88)	2.20 (1.57 to 2.88)
Bladder cancer	10 Pack Years	Both	Male	2.20 (1.57 to 2.88)	2.20 (1.57 to 2.88)	2.20 (1.57 to 2.88)	2.20 (1.57 to 2.88)	2.20 (1.57 to 2.88)	2.20 (1.57 to 2.88)	2.20 (1.57 to 2.88)	2.20 (1.57 to 2.88)	2.20 (1.57 to 2.88)	2.20 (1.57 to 2.88)	2.20 (1.57 to 2.88)	2.20 (1.57 to 2.88)	2.20 (1.57 to 2.88)	2.20 (1.57 to 2.88)
Bladder cancer	20 Pack Years	Both	Female	2.98 (1.99 to 4.09)	2.98 (1.99 to 4.09)	2.98 (1.99 to 4.09)	2.98 (1.99 to 4.09)	2.98 (1.99 to 4.09)	2.98 (1.99 to 4.09)	2.98 (1.99 to 4.09)	2.98 (1.99 to 4.09)	2.98 (1.99 to 4.09)	2.98 (1.99 to 4.09)	2.98 (1.99 to 4.09)	2.98 (1.99 to 4.09)	2.98 (1.99 to 4.09)	2.98 (1.99 to 4.09)
Bladder cancer	20 Pack Years	Both	Male	2.98 (1.99 to 4.09)	2.98 (1.99 to 4.09)	2.98 (1.99 to 4.09)	2.98 (1.99 to 4.09)	2.98 (1.99 to 4.09)	2.98 (1.99 to 4.09)	2.98 (1.99 to 4.09)	2.98 (1.99 to 4.09)	2.98 (1.99 to 4.09)	2.98 (1.99 to 4.09)	2.98 (1.99 to 4.09)	2.98 (1.99 to 4.09)	2.98 (1.99 to 4.09)	2.98 (1.99 to 4.09)
Bladder cancer	30 Pack Years	Both	Female	3.33 (2.27 to 4.61)	3.33 (2.27 to 4.61)	3.33 (2.27 to 4.61)	3.33 (2.27 to 4.61)	3.33 (2.27 to 4.61)	3.33 (2.27 to 4.61)	3.33 (2.27 to 4.61)	3.33 (2.27 to 4.61)	3.33 (2.27 to 4.61)	3.33 (2.27 to 4.61)	3.33 (2.27 to 4.61)	3.33 (2.27 to 4.61)	3.33 (2.27 to 4.61)	3.33 (2.27 to 4.61)
Bladder cancer	30 Pack Years	Both	Male	3.33 (2.27 to 4.61)	3.33 (2.27 to 4.61)	3.33 (2.27 to 4.61)	3.33 (2.27 to 4.61)	3.33 (2.27 to 4.61)	3.33 (2.27 to 4.61)	3.33 (2.27 to 4.61)	3.33 (2.27 to 4.61)	3.33 (2.27 to 4.61)	3.33 (2.27 to 4.61)	3.33 (2.27 to 4.61)	3.33 (2.27 to 4.61)	3.33 (2.27 to 4.61)	3.33 (2.27 to 4.61)
Bladder cancer	40 Pack Years	Both	Female	3.94 (2.63 to 5.51)	3.94 (2.63 to 5.51)	3.94 (2.63 to 5.51)	3.94 (2.63 to 5.51)	3.94 (2.63 to 5.51)	3.94 (2.63 to 5.51)	3.94 (2.63 to 5.51)	3.94 (2.63 to 5.51)	3.94 (2.63 to 5.51)	3.94 (2.63 to 5.51)	3.94 (2.63 to 5.51)	3.94 (2.63 to 5.51)	3.94 (2.63 to 5.51)	3.94 (2.63 to 5.51)
Bladder cancer	40 Pack Years	Both	Male	3.94 (2.63 to 5.51)	3.94 (2.63 to 5.51)	3.94 (2.63 to 5.51)	3.94 (2.63 to 5.51)	3.94 (2.63 to 5.51)	3.94 (2.63 to 5.51)	3.94 (2.63 to 5.51)	3.94 (2.63 to 5.51)	3.94 (2.63 to 5.51)	3.94 (2.63 to 5.51)	3.94 (2.63 to 5.51)	3.94 (2.63 to 5.51)	3.94 (2.63 to 5.51)	3.94 (2.63 to 5.51)
Bladder cancer	50 Pack Years	Both	Female	4.34 (2.81 to 6.21)	4.34 (2.81 to 6.21)	4.34 (2.81 to 6.21)	4.34 (2.81 to 6.21)	4.34 (2.81 to 6.21)	4.34 (2.81 to 6.21)	4.34 (2.81 to 6.21)	4.34 (2.81 to 6.21)	4.34 (2.81 to 6.21)	4.34 (2.81 to 6.21)	4.34 (2.81 to 6.21)	4.34 (2.81 to 6.21)	4.34 (2.81 to 6.21)	4.34 (2.81 to 6.21)
Bladder cancer	50 Pack Years	Both	Male	4.34 (2.81 to 6.21)	4.34 (2.81 to 6.21)	4.34 (2.81 to 6.21)	4.34 (2.81 to 6.21)	4.34 (2.81 to 6.21)	4.34 (2.81 to 6.21)	4.34 (2.81 to 6.21)	4.34 (2.81 to 6.21)	4.34 (2.81 to 6.21)	4.34 (2.81 to 6.21)	4.34 (2.81 to 6.21)	4.34 (2.81 to 6.21)	4.34 (2.81 to 6.21)	4.34 (2.81 to 6.21)
Bladder cancer	60 Pack Years	Both	Female	4.55 (2.82 to 6.72)	4.55 (2.82 to 6.72)	4.55 (2.82 to 6.72)	4.55 (2.82 to 6.72)	4.55 (2.82 to 6.72)	4.55 (2.82 to 6.72)	4.55 (2.82 to 6.72)	4.55 (2.82 to 6.72)	4.55 (2.82 to 6.72)	4.55 (2.82 to 6.72)	4.55 (2.82 to 6.72)	4.55 (2.82 to 6.72)	4.55 (2.82 to 6.72)	4.55 (2.82 to 6.72)
Bladder cancer	60 Pack Years	Both	Male	4.55 (2.82 to 6.72)	4.55 (2.82 to 6.72)	4.55 (2.82 to 6.72)	4.55 (2.82 to 6.72)	4.55 (2.82 to 6.72)	4.55 (2.82 to 6.72)	4.55 (2.82 to 6.72)	4.55 (2.82 to 6.72)	4.55 (2.82 to 6.72)	4.55 (2.82 to 6.72)	4.55 (2.82 to 6.72)	4.55 (2.82 to 6.72)	4.55 (2.82 to 6.72)	4.55 (2.82 to 6.72)
Bladder cancer	70 Pack Years	Both	Female	4.74 (2.44 to 7.96)	4.74 (2.44 to 7.96)	4.74 (2.44 to 7.96)	4.74 (2.44 to 7.96)	4.74 (2.44 to 7.96)	4.74 (2.44 to 7.96)	4.74 (2.44 to 7.96)	4.74 (2.44 to 7.96)	4.74 (2.44 to 7.96)	4.74 (2.44 to 7.96)	4.74 (2.44 to 7.96)	4.74 (2.44 to 7.96)	4.74 (2.44 to 7.96)	4.74 (2.44 to 7.96)
Bladder cancer	70 Pack Years	Both	Male	4.74 (2.44 to 7.96)	4.74 (2.44 to 7.96)	4.74 (2.44 to 7.96)	4.74 (2.44 to 7.96)	4.74 (2.44 to 7.96)	4.74 (2.44 to 7.96)	4.74 (2.44 to 7.96)	4.74 (2.44 to 7.96)	4.74 (2.44 to 7.96)	4.74 (2.44 to 7.96)	4.74 (2.44 to 7.96)	4.74 (2.44 to 7.96)	4.74 (2.44 to 7.96)	4.74 (2.44 to 7.96)
Kidney cancer	0 Pack Years	Both	Female	1.00 (1.00 to 1.00)	1.00 (1.00 to 1.00)	1.00 (1.00 to 1.00)	1.00 (1.00 to 1.00)	1.00 (1.00 to 1.00)	1.00 (1.00 to 1.00)	1.00 (1.00 to 1.00)	1.00 (1.00 to 1.00)	1.00 (1.00 to 1.00)	1.00 (1.00 to 1.00)	1.00 (1.00 to 1.00)	1.00 (1.00 to 1.00)	1.00 (1.00 to 1.00)	1.00 (1.00 to 1.00)
Kidney cancer	0 Pack Years	Both	Male	1.00 (1.00 to 1.00)	1.00 (1.00 to 1.00)	1.00 (1.00 to 1.00)	1.00 (1.00 to 1.00)	1.00 (1.00 to 1.00)	1.00 (1.00 to 1.00)	1.00 (1.00 to 1.00)	1.00 (1.00 to 1.00)	1.00 (1.00 to 1.00)	1.00 (1.00 to 1.00)	1.00 (1.00 to 1.00)	1.00 (1.00 to 1.00)	1.00 (1.00 to 1.00)	1.00 (1.00 to 1.00)
Kidney cancer	10 Pack Years	Both	Female	1.30 (1.07 to 1.55)	1.30 (1.07 to 1.55)	1.30 (1.07 to 1.55)	1.30 (1.07 to 1.55)	1.30 (1.07 to 1.55)	1.30 (1.07 to 1.55)	1.30 (1.07 to 1.55)	1.30 (1.07 to 1.55)	1.30 (1.07 to 1.55)	1.30 (1.07 to 1.55)	1.30 (1.07 to 1.55)	1.30 (1.07 to 1.55)	1.30 (1.07 to 1.55)	1.30 (1.07 to 1.55)
Kidney cancer	10 Pack Years	Both	Male	1.30 (1.07 to 1.55)	1.30 (1.07 to 1.55)	1.30 (1.07 to 1.55)	1.30 (1.07 to 1.55)	1.30 (1.07 to 1.55)	1.30 (1.07 to 1.55)	1.30 (1.07 to 1.55)	1.30 (1.07 to 1.55)	1.30 (1.07 to 1.55)	1.30 (1.07 to 1.55)	1.30 (1.07 to 1.55)	1.30 (1.07 to 1.55)	1.30 (1.07 to 1.55)	1.30 (1.07 to 1.55)

Kidney cancer	20 Pack Years	Both	Female	1.59 (1.28 to 1.92)	1.59 (1.28 to 1.92)	1.59 (1.28 to 1.92)	1.59 (1.28 to 1.92)	1.59 (1.28 to 1.92)	1.59 (1.28 to 1.92)	1.59 (1.28 to 1.92)	1.59 (1.28 to 1.92)	1.59 (1.28 to 1.92)	1.59 (1.28 to 1.92)	1.59 (1.28 to 1.92)	1.59 (1.28 to 1.92)	1.59 (1.28 to 1.92)	1.59 (1.28 to 1.92)
Kidney cancer	20 Pack Years	Both	Male	1.59 (1.28 to 1.92)	1.59 (1.28 to 1.92)	1.59 (1.28 to 1.92)	1.59 (1.28 to 1.92)	1.59 (1.28 to 1.92)	1.59 (1.28 to 1.92)	1.59 (1.28 to 1.92)	1.59 (1.28 to 1.92)	1.59 (1.28 to 1.92)	1.59 (1.28 to 1.92)	1.59 (1.28 to 1.92)	1.59 (1.28 to 1.92)	1.59 (1.28 to 1.92)	1.59 (1.28 to 1.92)
Kidney cancer	30 Pack Years	Both	Female	1.74 (1.38 to 2.13)	1.74 (1.38 to 2.13)	1.74 (1.38 to 2.13)	1.74 (1.38 to 2.13)	1.74 (1.38 to 2.13)	1.74 (1.38 to 2.13)	1.74 (1.38 to 2.13)	1.74 (1.38 to 2.13)	1.74 (1.38 to 2.13)	1.74 (1.38 to 2.13)	1.74 (1.38 to 2.13)	1.74 (1.38 to 2.13)	1.74 (1.38 to 2.13)	1.74 (1.38 to 2.13)
Kidney cancer	30 Pack Years	Both	Male	1.74 (1.38 to 2.13)	1.74 (1.38 to 2.13)	1.74 (1.38 to 2.13)	1.74 (1.38 to 2.13)	1.74 (1.38 to 2.13)	1.74 (1.38 to 2.13)	1.74 (1.38 to 2.13)	1.74 (1.38 to 2.13)	1.74 (1.38 to 2.13)	1.74 (1.38 to 2.13)	1.74 (1.38 to 2.13)	1.74 (1.38 to 2.13)	1.74 (1.38 to 2.13)	1.74 (1.38 to 2.13)
Kidney cancer	40 Pack Years	Both	Female	1.79 (1.40 to 2.24)	1.79 (1.40 to 2.24)	1.79 (1.40 to 2.24)	1.79 (1.40 to 2.24)	1.79 (1.40 to 2.24)	1.79 (1.40 to 2.24)	1.79 (1.40 to 2.24)	1.79 (1.40 to 2.24)	1.79 (1.40 to 2.24)	1.79 (1.40 to 2.24)	1.79 (1.40 to 2.24)	1.79 (1.40 to 2.24)	1.79 (1.40 to 2.24)	1.79 (1.40 to 2.24)
Kidney cancer	40 Pack Years	Both	Male	1.79 (1.40 to 2.24)	1.79 (1.40 to 2.24)	1.79 (1.40 to 2.24)	1.79 (1.40 to 2.24)	1.79 (1.40 to 2.24)	1.79 (1.40 to 2.24)	1.79 (1.40 to 2.24)	1.79 (1.40 to 2.24)	1.79 (1.40 to 2.24)	1.79 (1.40 to 2.24)	1.79 (1.40 to 2.24)	1.79 (1.40 to 2.24)	1.79 (1.40 to 2.24)	1.79 (1.40 to 2.24)
Kidney cancer	50 Pack Years	Both	Female	1.90 (1.49 to 2.35)	1.90 (1.49 to 2.35)	1.90 (1.49 to 2.35)	1.90 (1.49 to 2.35)	1.90 (1.49 to 2.35)	1.90 (1.49 to 2.35)	1.90 (1.49 to 2.35)	1.90 (1.49 to 2.35)	1.90 (1.49 to 2.35)	1.90 (1.49 to 2.35)	1.90 (1.49 to 2.35)	1.90 (1.49 to 2.35)	1.90 (1.49 to 2.35)	1.90 (1.49 to 2.35)
Kidney cancer	50 Pack Years	Both	Male	1.90 (1.49 to 2.35)	1.90 (1.49 to 2.35)	1.90 (1.49 to 2.35)	1.90 (1.49 to 2.35)	1.90 (1.49 to 2.35)	1.90 (1.49 to 2.35)	1.90 (1.49 to 2.35)	1.90 (1.49 to 2.35)	1.90 (1.49 to 2.35)	1.90 (1.49 to 2.35)	1.90 (1.49 to 2.35)	1.90 (1.49 to 2.35)	1.90 (1.49 to 2.35)	1.90 (1.49 to 2.35)
Kidney cancer	60 Pack Years	Both	Female	1.99 (1.38 to 2.73)	1.99 (1.38 to 2.73)	1.99 (1.38 to 2.73)	1.99 (1.38 to 2.73)	1.99 (1.38 to 2.73)	1.99 (1.38 to 2.73)	1.99 (1.38 to 2.73)	1.99 (1.38 to 2.73)	1.99 (1.38 to 2.73)	1.99 (1.38 to 2.73)	1.99 (1.38 to 2.73)	1.99 (1.38 to 2.73)	1.99 (1.38 to 2.73)	1.99 (1.38 to 2.73)
Kidney cancer	60 Pack Years	Both	Male	1.99 (1.38 to 2.73)	1.99 (1.38 to 2.73)	1.99 (1.38 to 2.73)	1.99 (1.38 to 2.73)	1.99 (1.38 to 2.73)	1.99 (1.38 to 2.73)	1.99 (1.38 to 2.73)	1.99 (1.38 to 2.73)	1.99 (1.38 to 2.73)	1.99 (1.38 to 2.73)	1.99 (1.38 to 2.73)	1.99 (1.38 to 2.73)	1.99 (1.38 to 2.73)	1.99 (1.38 to 2.73)
Leukaemia	0 Pack Years	Both	Female	1.00 (1.00 to 1.00)	1.00 (1.00 to 1.00)	1.00 (1.00 to 1.00)	1.00 (1.00 to 1.00)	1.00 (1.00 to 1.00)	1.00 (1.00 to 1.00)	1.00 (1.00 to 1.00)	1.00 (1.00 to 1.00)	1.00 (1.00 to 1.00)	1.00 (1.00 to 1.00)	1.00 (1.00 to 1.00)	1.00 (1.00 to 1.00)	1.00 (1.00 to 1.00)	1.00 (1.00 to 1.00)
Leukaemia	0 Pack Years	Both	Male	1.00 (1.00 to 1.00)	1.00 (1.00 to 1.00)	1.00 (1.00 to 1.00)	1.00 (1.00 to 1.00)	1.00 (1.00 to 1.00)	1.00 (1.00 to 1.00)	1.00 (1.00 to 1.00)	1.00 (1.00 to 1.00)	1.00 (1.00 to 1.00)	1.00 (1.00 to 1.00)	1.00 (1.00 to 1.00)	1.00 (1.00 to 1.00)	1.00 (1.00 to 1.00)	1.00 (1.00 to 1.00)
Leukaemia	10 Pack Years	Both	Female	1.81 (1.02 to 2.77)	1.81 (1.02 to 2.77)	1.81 (1.02 to 2.77)	1.81 (1.02 to 2.77)	1.81 (1.02 to 2.77)	1.81 (1.02 to 2.77)	1.81 (1.02 to 2.77)	1.81 (1.02 to 2.77)	1.81 (1.02 to 2.77)	1.81 (1.02 to 2.77)	1.81 (1.02 to 2.77)	1.81 (1.02 to 2.77)	1.81 (1.02 to 2.77)	1.81 (1.02 to 2.77)
Leukaemia	10 Pack Years	Both	Male	1.81 (1.02 to 2.77)	1.81 (1.02 to 2.77)	1.81 (1.02 to 2.77)	1.81 (1.02 to 2.77)	1.81 (1.02 to 2.77)	1.81 (1.02 to 2.77)	1.81 (1.02 to 2.77)	1.81 (1.02 to 2.77)	1.81 (1.02 to 2.77)	1.81 (1.02 to 2.77)	1.81 (1.02 to 2.77)	1.81 (1.02 to 2.77)	1.81 (1.02 to 2.77)	1.81 (1.02 to 2.77)
Leukaemia	20 Pack Years	Both	Female	2.15 (1.16 to 3.42)	2.15 (1.16 to 3.42)	2.15 (1.16 to 3.42)	2.15 (1.16 to 3.42)	2.15 (1.16 to 3.42)	2.15 (1.16 to 3.42)	2.15 (1.16 to 3.42)	2.15 (1.16 to 3.42)	2.15 (1.16 to 3.42)	2.15 (1.16 to 3.42)	2.15 (1.16 to 3.42)	2.15 (1.16 to 3.42)	2.15 (1.16 to 3.42)	2.15 (1.16 to 3.42)
Leukaemia	20 Pack Years	Both	Male	2.15 (1.16 to 3.42)	2.15 (1.16 to 3.42)	2.15 (1.16 to 3.42)	2.15 (1.16 to 3.42)	2.15 (1.16 to 3.42)	2.15 (1.16 to 3.42)	2.15 (1.16 to 3.42)	2.15 (1.16 to 3.42)	2.15 (1.16 to 3.42)	2.15 (1.16 to 3.42)	2.15 (1.16 to 3.42)	2.15 (1.16 to 3.42)	2.15 (1.16 to 3.42)	2.15 (1.16 to 3.42)
Leukaemia	30 Pack Years	Both	Female	2.42 (1.44 to 3.70)	2.42 (1.44 to 3.70)	2.42 (1.44 to 3.70)	2.42 (1.44 to 3.70)	2.42 (1.44 to 3.70)	2.42 (1.44 to 3.70)	2.42 (1.44 to 3.70)	2.42 (1.44 to 3.70)	2.42 (1.44 to 3.70)	2.42 (1.44 to 3.70)	2.42 (1.44 to 3.70)	2.42 (1.44 to 3.70)	2.42 (1.44 to 3.70)	2.42 (1.44 to 3.70)
Leukaemia	30 Pack Years	Both	Male	2.42 (1.44 to 3.70)	2.42 (1.44 to 3.70)	2.42 (1.44 to 3.70)	2.42 (1.44 to 3.70)	2.42 (1.44 to 3.70)	2.42 (1.44 to 3.70)	2.42 (1.44 to 3.70)	2.42 (1.44 to 3.70)	2.42 (1.44 to 3.70)	2.42 (1.44 to 3.70)	2.42 (1.44 to 3.70)	2.42 (1.44 to 3.70)	2.42 (1.44 to 3.70)	2.42 (1.44 to 3.70)
Leukaemia	40 Pack Years	Both	Female	2.62 (1.54 to 4.18)	2.62 (1.54 to 4.18)	2.62 (1.54 to 4.18)	2.62 (1.54 to 4.18)	2.62 (1.54 to 4.18)	2.62 (1.54 to 4.18)	2.62 (1.54 to 4.18)	2.62 (1.54 to 4.18)	2.62 (1.54 to 4.18)	2.62 (1.54 to 4.18)	2.62 (1.54 to 4.18)	2.62 (1.54 to 4.18)	2.62 (1.54 to 4.18)	2.62 (1.54 to 4.18)
Leukaemia	40 Pack Years	Both	Male	2.62 (1.54 to 4.18)	2.62 (1.54 to 4.18)	2.62 (1.54 to 4.18)	2.62 (1.54 to 4.18)	2.62 (1.54 to 4.18)	2.62 (1.54 to 4.18)	2.62 (1.54 to 4.18)	2.62 (1.54 to 4.18)	2.62 (1.54 to 4.18)	2.62 (1.54 to 4.18)	2.62 (1.54 to 4.18)	2.62 (1.54 to 4.18)	2.62 (1.54 to 4.18)	2.62 (1.54 to 4.18)
Tracheal, bronchus, and lung cancer	0 Years Since Quitting	Both	Female	100.00 (100.00 to 100.00)	100.00 (100.00 to 100.00)	100.00 (100.00 to 100.00)	100.00 (100.00 to 100.00)	100.00 (100.00 to 100.00)	100.00 (100.00 to 100.00)	100.00 (100.00 to 100.00)	100.00 (100.00 to 100.00)	100.00 (100.00 to 100.00)	100.00 (100.00 to 100.00)	100.00 (100.00 to 100.00)	100.00 (100.00 to 100.00)	100.00 (100.00 to 100.00)	100.00 (100.00 to 100.00)
Tracheal, bronchus, and lung cancer	0 Years Since Quitting	Both	Male	100.00 (100.00 to 100.00)	100.00 (100.00 to 100.00)	100.00 (100.00 to 100.00)	100.00 (100.00 to 100.00)	100.00 (100.00 to 100.00)	100.00 (100.00 to 100.00)	100.00 (100.00 to 100.00)	100.00 (100.00 to 100.00)	100.00 (100.00 to 100.00)	100.00 (100.00 to 100.00)	100.00 (100.00 to 100.00)	100.00 (100.00 to 100.00)	100.00 (100.00 to 100.00)	100.00 (100.00 to 100.00)
Tracheal, bronchus, and lung cancer	10 Years Since Quitting	Both	Female	27.01 (22.32 to 31.71)	27.01 (22.32 to 31.71)	27.01 (22.32 to 31.71)	27.01 (22.32 to 31.71)	27.01 (22.32 to 31.71)	27.01 (22.32 to 31.71)	27.01 (22.32 to 31.71)	27.01 (22.32 to 31.71)	27.01 (22.32 to 31.71)	27.01 (22.32 to 31.71)	27.01 (22.32 to 31.71)	27.01 (22.32 to 31.71)	27.01 (22.32 to 31.71)	27.01 (22.32 to 31.71)
Tracheal, bronchus, and lung cancer	10 Years Since Quitting	Both	Male	27.01 (22.32 to 31.71)	27.01 (22.32 to 31.71)	27.01 (22.32 to 31.71)	27.01 (22.32 to 31.71)	27.01 (22.32 to 31.71)	27.01 (22.32 to 31.71)	27.01 (22.32 to 31.71)	27.01 (22.32 to 31.71)	27.01 (22.32 to 31.71)	27.01 (22.32 to 31.71)	27.01 (22.32 to 31.71)	27.01 (22.32 to 31.71)	27.01 (22.32 to 31.71)	27.01 (22.32 to 31.71)
Tracheal, bronchus, and lung cancer	20 Years Since Quitting	Both	Female	12.14 (8.32 to 15.86)	12.14 (8.32 to 15.86)	12.14 (8.32 to 15.86)	12.14 (8.32 to 15.86)	12.14 (8.32 to 15.86)	12.14 (8.32 to 15.86)	12.14 (8.32 to 15.86)	12.14 (8.32 to 15.86)	12.14 (8.32 to 15.86)	12.14 (8.32 to 15.86)	12.14 (8.32 to 15.86)	12.14 (8.32 to 15.86)	12.14 (8.32 to 15.86)	12.14 (8.32 to 15.86)
Tracheal, bronchus, and lung cancer	20 Years Since Quitting	Both	Male	12.14 (8.32 to 15.86)	12.14 (8.32 to 15.86)	12.14 (8.32 to 15.86)	12.14 (8.32 to 15.86)	12.14 (8.32 to 15.86)	12.14 (8.32 to 15.86)	12.14 (8.32 to 15.86)	12.14 (8.32 to 15.86)	12.14 (8.32 to 15.86)	12.14 (8.32 to 15.86)	12.14 (8.32 to 15.86)	12.14 (8.32 to 15.86)	12.14 (8.32 to 15.86)	12.14 (8.32 to 15.86)
Tracheal, bronchus, and lung cancer	30 Years Since Quitting	Both	Female	4.91 (1.65 to 8.33)	4.91 (1.65 to 8.33)	4.91 (1.65 to 8.33)	4.91 (1.65 to 8.33)	4.91 (1.65 to 8.33)	4.91 (1.65 to 8.33)	4.91 (1.65 to 8.33)	4.91 (1.65 to 8.33)	4.91 (1.65 to 8.33)	4.91 (1.65 to 8.33)	4.91 (1.65 to 8.33)	4.91 (1.65 to 8.33)	4.91 (1.65 to 8.33)	4.91 (1.65 to 8.33)
Tracheal, bronchus, and lung cancer	30 Years Since Quitting	Both	Male	4.91 (1.65 to 8.33)	4.91 (1.65 to 8.33)	4.91 (1.65 to 8.33)	4.91 (1.65 to 8.33)	4.91 (1.65 to 8.33)	4.91 (1.65 to 8.33)	4.91 (1.65 to 8.33)	4.91 (1.65 to 8.33)	4.91 (1.65 to 8.33)	4.91 (1.65 to 8.33)	4.91 (1.65 to 8.33)	4.91 (1.65 to 8.33)	4.91 (1.65 to 8.33)	4.91 (1.65 to 8.33)
Tracheal, bronchus, and lung cancer	40 Years Since Quitting	Both	Female	1.29 (-1.65 to 4.48)	1.29 (-1.65 to 4.48)	1.29 (-1.65 to 4.48)	1.29 (-1.65 to 4.48)	1.29 (-1.65 to 4.48)	1.29 (-1.65 to 4.48)	1.29 (-1.65 to 4.48)	1.29 (-1.65 to 4.48)	1.29 (-1.65 to 4.48)	1.29 (-1.65 to 4.48)	1.29 (-1.65 to 4.48)	1.29 (-1.65 to 4.48)	1.29 (-1.65 to 4.48)	1.29 (-1.65 to 4.48)
Tracheal, bronchus, and lung cancer	40 Years Since Quitting	Both	Male	1.29 (-1.65 to 4.48)	1.29 (-1.65 to 4.48)	1.29 (-1.65 to 4.48)	1.29 (-1.65 to 4.48)	1.29 (-1.65 to 4.48)	1.29 (-1.65 to 4.48)	1.29 (-1.65 to 4.48)	1.29 (-1.65 to 4.48)	1.29 (-1.65 to 4.48)	1.29 (-1.65 to 4.48)	1.29 (-1.65 to 4.48)	1.29 (-1.65 to 4.48)	1.29 (-1.65 to 4.48)	1.29 (-1.65 to 4.48)
Chronic obstructive pulmonary disease	0 Years Since Quitting	Both	Female	100.00 (100.00 to 100.00)	100.00 (100.00 to 100.00)	100.00 (100.00 to 100.00)	100.00 (100.00 to 100.00)	100.00 (100.00 to 100.00)	100.00 (100.00 to 100.00)	100.00 (100.00 to 100.00)	100.00 (100.00 to 100.00)	100.00 (100.00 to 100.00)	100.00 (100.00 to 100.00)	100.00 (100.00 to 100.00)	100.00 (100.00 to 100.00)	100.00 (100.00 to 100.00)	100.00 (100.00 to 100.00)
Chronic obstructive pulmonary disease	0 Years Since Quitting	Both	Male	100.00 (100.00 to 100.00)	100.00 (100.00 to 100.00)	100.00 (100.00 to 100.00)	100.00 (100.00 to 100.00)	100.00 (100.00 to 100.00)	100.00 (100.00 to 100.00)	100.00 (100.00 to 100.00)	100.00 (100.00 to 100.00)	100.00 (100.00 to 100.00)	100.00 (100.00 to 100.00)	100.00 (100.00 to 100.00)	100.00 (100.00 to 100.00)	100.00 (100.00 to 100.00)	100.00 (100.00 to 100.00)
Chronic obstructive pulmonary disease	10 Years Since Quitting	Both	Female	38.10 (30.92 to 45.68)	38.10 (30.92 to 45.68)	38.10 (30.92 to 45.68)	38.10 (30.92 to 45.68)	38.10 (30.92 to 45.68)	38.10 (30.92 to 45.68)	38.10 (30.92 to 45.68)	38.10 (30.92 to 45.68)	38.10 (30.92 to 45.68)	38.10 (30.92 to 45.68)	38.10 (30.92 to 45.68)	38.10 (30.92 to 45.68)	38.10 (30.92 to 45.68)	38.10 (30.92 to 45.68)
Chronic obstructive pulmonary disease	10 Years Since Quitting	Both	Male	38.10 (30.92 to 45.68)	38.10 (30.92 to 45.68)	38.10 (30.92 to 45.68)	38.10 (30.92 to 45.68)	38.10 (30.92 to 45.68)	38.10 (30.92 to 45.68)	38.10 (30.92 to 45.68)	38.10 (30.92 to 45.68)	38.10 (30.92 to 45.68)	38.10 (30.92 to 45.68)	38.10 (30.92 to 45.68)	38.10 (30.92 to 45.68)	38.10 (30.92 to 45.68)	38.10 (30.92 to 45.68)
Chronic obstructive pulmonary disease	20 Years Since Quitting	Both	Female	13.81 (8.91 to 19.12)	13.81 (8.91 to 19.12)	13.81 (8.91 to 19.12)	13.81 (8.91 to 19.12)	13.81 (8.91 to 19.12)	13.81 (8.91 to 19.12)	13.81 (8.91 to 19.12)	13.81 (8.91 to 19.12)	13.81 (8.91 to 19.12)	13.81 (8.91 to 19.12)	13.81 (8.91 to 19.12)	13.81 (8.91 to 19.12)	13.81 (8.91 to 19.12)	13.81 (8.91 to 19.12)
Chronic obstructive pulmonary disease	20 Years Since Quitting	Both	Male	13.81 (8.91 to 19.12)	13.81 (8.91 to 19.12)	13.81 (8.91 to 19.12)	13.81 (8.91 to 19.12)	13.81 (8.91 to 19.12)	13.81 (8.91 to 19.12)	13.81 (8.91 to 19.12)	13.81 (8.91 to 19.12)	13.81 (8.91 to 19.12)	13.81 (8.91 to 19.12)	13.81 (8.91 to 19.12)	13.81 (8.91 to 19.12)	13.81 (8.91 to 19.12)	13.81 (8.91 to 19.12)
Chronic obstructive pulmonary disease	30 Years Since Quitting	Both	Female	4.46 (0.67 to 8.96)	4.46 (0.67 to 8.96)	4.46 (0.67 to 8.96)	4.46 (0.67 to 8.96)	4.46 (0.67 to 8.96)	4.46 (0.67 to 8.96)	4.46 (0.67 to 8.96)	4.46 (0.67 to 8.96)	4.46 (0.67 to 8.96)	4.46 (0.67 to 8.96)	4.46 (0.67 to 8.96)	4.46 (0.67 to 8.96)	4.46 (0.67 to 8.96)	4.46 (0.67 to 8.96)

Chronic obstructive pulmonary disease	30 Years Since Quitting	Both	Male	4.46 (0.67 to 8.96)	4.46 (0.67 to 8.96)	4.46 (0.67 to 8.96)	4.46 (0.67 to 8.96)	4.46 (0.67 to 8.96)	4.46 (0.67 to 8.96)	4.46 (0.67 to 8.96)	4.46 (0.67 to 8.96)	4.46 (0.67 to 8.96)	4.46 (0.67 to 8.96)	4.46 (0.67 to 8.96)	4.46 (0.67 to 8.96)	4.46 (0.67 to 8.96)	4.46 (0.67 to 8.96)
Chronic obstructive pulmonary disease	40 Years Since Quitting	Both	Female	1.07 (-2.08 to 4.83)	1.07 (-2.08 to 4.83)	1.07 (-2.08 to 4.83)	1.07 (-2.08 to 4.83)	1.07 (-2.08 to 4.83)	1.07 (-2.08 to 4.83)	1.07 (-2.08 to 4.83)	1.07 (-2.08 to 4.83)	1.07 (-2.08 to 4.83)	1.07 (-2.08 to 4.83)	1.07 (-2.08 to 4.83)	1.07 (-2.08 to 4.83)	1.07 (-2.08 to 4.83)	1.07 (-2.08 to 4.83)
Chronic obstructive pulmonary disease	40 Years Since Quitting	Both	Male	1.07 (-2.08 to 4.83)	1.07 (-2.08 to 4.83)	1.07 (-2.08 to 4.83)	1.07 (-2.08 to 4.83)	1.07 (-2.08 to 4.83)	1.07 (-2.08 to 4.83)	1.07 (-2.08 to 4.83)	1.07 (-2.08 to 4.83)	1.07 (-2.08 to 4.83)	1.07 (-2.08 to 4.83)	1.07 (-2.08 to 4.83)	1.07 (-2.08 to 4.83)	1.07 (-2.08 to 4.83)	1.07 (-2.08 to 4.83)
Ischaemic heart disease	0 Years Since Quitting	Both	Female	100.00 (100.00 to 100.00)	100.00 (100.00 to 100.00)	100.00 (100.00 to 100.00)	100.00 (100.00 to 100.00)	100.00 (100.00 to 100.00)	100.00 (100.00 to 100.00)	100.00 (100.00 to 100.00)	100.00 (100.00 to 100.00)	100.00 (100.00 to 100.00)	100.00 (100.00 to 100.00)	100.00 (100.00 to 100.00)	100.00 (100.00 to 100.00)	100.00 (100.00 to 100.00)	100.00 (100.00 to 100.00)
Ischaemic heart disease	0 Years Since Quitting	Both	Male	100.00 (100.00 to 100.00)	100.00 (100.00 to 100.00)	100.00 (100.00 to 100.00)	100.00 (100.00 to 100.00)	100.00 (100.00 to 100.00)	100.00 (100.00 to 100.00)	100.00 (100.00 to 100.00)	100.00 (100.00 to 100.00)	100.00 (100.00 to 100.00)	100.00 (100.00 to 100.00)	100.00 (100.00 to 100.00)	100.00 (100.00 to 100.00)	100.00 (100.00 to 100.00)	100.00 (100.00 to 100.00)
Ischaemic heart disease	10 Years Since Quitting	Both	Female	41.34 (34.93 to 48.17)	41.34 (34.93 to 48.17)	41.34 (34.93 to 48.17)	41.34 (34.93 to 48.17)	41.34 (34.93 to 48.17)	41.34 (34.93 to 48.17)	41.34 (34.93 to 48.17)	41.34 (34.93 to 48.17)	41.34 (34.93 to 48.17)	41.34 (34.93 to 48.17)	41.34 (34.93 to 48.17)	41.34 (34.93 to 48.17)	41.34 (34.93 to 48.17)	41.34 (34.93 to 48.17)
Ischaemic heart disease	10 Years Since Quitting	Both	Male	41.34 (34.93 to 48.17)	41.34 (34.93 to 48.17)	41.34 (34.93 to 48.17)	41.34 (34.93 to 48.17)	41.34 (34.93 to 48.17)	41.34 (34.93 to 48.17)	41.34 (34.93 to 48.17)	41.34 (34.93 to 48.17)	41.34 (34.93 to 48.17)	41.34 (34.93 to 48.17)	41.34 (34.93 to 48.17)	41.34 (34.93 to 48.17)	41.34 (34.93 to 48.17)	41.34 (34.93 to 48.17)
Ischaemic heart disease	20 Years Since Quitting	Both	Female	25.18 (19.66 to 31.89)	25.18 (19.66 to 31.89)	25.18 (19.66 to 31.89)	25.18 (19.66 to 31.89)	25.18 (19.66 to 31.89)	25.18 (19.66 to 31.89)	25.18 (19.66 to 31.89)	25.18 (19.66 to 31.89)	25.18 (19.66 to 31.89)	25.18 (19.66 to 31.89)	25.18 (19.66 to 31.89)	25.18 (19.66 to 31.89)	25.18 (19.66 to 31.89)	25.18 (19.66 to 31.89)
Ischaemic heart disease	20 Years Since Quitting	Both	Male	25.18 (19.66 to 31.89)	25.18 (19.66 to 31.89)	25.18 (19.66 to 31.89)	25.18 (19.66 to 31.89)	25.18 (19.66 to 31.89)	25.18 (19.66 to 31.89)	25.18 (19.66 to 31.89)	25.18 (19.66 to 31.89)	25.18 (19.66 to 31.89)	25.18 (19.66 to 31.89)	25.18 (19.66 to 31.89)	25.18 (19.66 to 31.89)	25.18 (19.66 to 31.89)	25.18 (19.66 to 31.89)
Ischaemic heart disease	30 Years Since Quitting	Both	Female	14.40 (9.56 to 19.26)	14.40 (9.56 to 19.26)	14.40 (9.56 to 19.26)	14.40 (9.56 to 19.26)	14.40 (9.56 to 19.26)	14.40 (9.56 to 19.26)	14.40 (9.56 to 19.26)	14.40 (9.56 to 19.26)	14.40 (9.56 to 19.26)	14.40 (9.56 to 19.26)	14.40 (9.56 to 19.26)	14.40 (9.56 to 19.26)	14.40 (9.56 to 19.26)	14.40 (9.56 to 19.26)
Ischaemic heart disease	30 Years Since Quitting	Both	Male	14.40 (9.56 to 19.26)	14.40 (9.56 to 19.26)	14.40 (9.56 to 19.26)	14.40 (9.56 to 19.26)	14.40 (9.56 to 19.26)	14.40 (9.56 to 19.26)	14.40 (9.56 to 19.26)	14.40 (9.56 to 19.26)	14.40 (9.56 to 19.26)	14.40 (9.56 to 19.26)	14.40 (9.56 to 19.26)	14.40 (9.56 to 19.26)	14.40 (9.56 to 19.26)	14.40 (9.56 to 19.26)
Ischaemic heart disease	40 Years Since Quitting	Both	Female	10.55 (6.43 to 14.96)	10.55 (6.43 to 14.96)	10.55 (6.43 to 14.96)	10.55 (6.43 to 14.96)	10.55 (6.43 to 14.96)	10.55 (6.43 to 14.96)	10.55 (6.43 to 14.96)	10.55 (6.43 to 14.96)	10.55 (6.43 to 14.96)	10.55 (6.43 to 14.96)	10.55 (6.43 to 14.96)	10.55 (6.43 to 14.96)	10.55 (6.43 to 14.96)	10.55 (6.43 to 14.96)
Ischaemic heart disease	40 Years Since Quitting	Both	Male	10.55 (6.43 to 14.96)	10.55 (6.43 to 14.96)	10.55 (6.43 to 14.96)	10.55 (6.43 to 14.96)	10.55 (6.43 to 14.96)	10.55 (6.43 to 14.96)	10.55 (6.43 to 14.96)	10.55 (6.43 to 14.96)	10.55 (6.43 to 14.96)	10.55 (6.43 to 14.96)	10.55 (6.43 to 14.96)	10.55 (6.43 to 14.96)	10.55 (6.43 to 14.96)	10.55 (6.43 to 14.96)
Stroke	0 Years Since Quitting	Both	Female	100.00 (100.00 to 100.00)	100.00 (100.00 to 100.00)	100.00 (100.00 to 100.00)	100.00 (100.00 to 100.00)	100.00 (100.00 to 100.00)	100.00 (100.00 to 100.00)	100.00 (100.00 to 100.00)	100.00 (100.00 to 100.00)	100.00 (100.00 to 100.00)	100.00 (100.00 to 100.00)	100.00 (100.00 to 100.00)	100.00 (100.00 to 100.00)	100.00 (100.00 to 100.00)	100.00 (100.00 to 100.00)
Stroke	0 Years Since Quitting	Both	Male	100.00 (100.00 to 100.00)	100.00 (100.00 to 100.00)	100.00 (100.00 to 100.00)	100.00 (100.00 to 100.00)	100.00 (100.00 to 100.00)	100.00 (100.00 to 100.00)	100.00 (100.00 to 100.00)	100.00 (100.00 to 100.00)	100.00 (100.00 to 100.00)	100.00 (100.00 to 100.00)	100.00 (100.00 to 100.00)	100.00 (100.00 to 100.00)	100.00 (100.00 to 100.00)	100.00 (100.00 to 100.00)
Stroke	10 Years Since Quitting	Both	Female	34.01 (26.14 to 42.27)	34.01 (26.14 to 42.27)	34.01 (26.14 to 42.27)	34.01 (26.14 to 42.27)	34.01 (26.14 to 42.27)	34.01 (26.14 to 42.27)	34.01 (26.14 to 42.27)	34.01 (26.14 to 42.27)	34.01 (26.14 to 42.27)	34.01 (26.14 to 42.27)	34.01 (26.14 to 42.27)	34.01 (26.14 to 42.27)	34.01 (26.14 to 42.27)	34.01 (26.14 to 42.27)
Stroke	10 Years Since Quitting	Both	Male	34.01 (26.14 to 42.27)	34.01 (26.14 to 42.27)	34.01 (26.14 to 42.27)	34.01 (26.14 to 42.27)	34.01 (26.14 to 42.27)	34.01 (26.14 to 42.27)	34.01 (26.14 to 42.27)	34.01 (26.14 to 42.27)	34.01 (26.14 to 42.27)	34.01 (26.14 to 42.27)	34.01 (26.14 to 42.27)	34.01 (26.14 to 42.27)	34.01 (26.14 to 42.27)	34.01 (26.14 to 42.27)
Stroke	20 Years Since Quitting	Both	Female	23.09 (15.47 to 30.76)	23.09 (15.47 to 30.76)	23.09 (15.47 to 30.76)	23.09 (15.47 to 30.76)	23.09 (15.47 to 30.76)	23.09 (15.47 to 30.76)	23.09 (15.47 to 30.76)	23.09 (15.47 to 30.76)	23.09 (15.47 to 30.76)	23.09 (15.47 to 30.76)	23.09 (15.47 to 30.76)	23.09 (15.47 to 30.76)	23.09 (15.47 to 30.76)	23.09 (15.47 to 30.76)
Stroke	20 Years Since Quitting	Both	Male	23.09 (15.47 to 30.76)	23.09 (15.47 to 30.76)	23.09 (15.47 to 30.76)	23.09 (15.47 to 30.76)	23.09 (15.47 to 30.76)	23.09 (15.47 to 30.76)	23.09 (15.47 to 30.76)	23.09 (15.47 to 30.76)	23.09 (15.47 to 30.76)	23.09 (15.47 to 30.76)	23.09 (15.47 to 30.76)	23.09 (15.47 to 30.76)	23.09 (15.47 to 30.76)	23.09 (15.47 to 30.76)
Stroke	30 Years Since Quitting	Both	Female	12.47 (6.89 to 19.08)	12.47 (6.89 to 19.08)	12.47 (6.89 to 19.08)	12.47 (6.89 to 19.08)	12.47 (6.89 to 19.08)	12.47 (6.89 to 19.08)	12.47 (6.89 to 19.08)	12.47 (6.89 to 19.08)	12.47 (6.89 to 19.08)	12.47 (6.89 to 19.08)	12.47 (6.89 to 19.08)	12.47 (6.89 to 19.08)	12.47 (6.89 to 19.08)	12.47 (6.89 to 19.08)
Stroke	30 Years Since Quitting	Both	Male	12.47 (6.89 to 19.08)	12.47 (6.89 to 19.08)	12.47 (6.89 to 19.08)	12.47 (6.89 to 19.08)	12.47 (6.89 to 19.08)	12.47 (6.89 to 19.08)	12.47 (6.89 to 19.08)	12.47 (6.89 to 19.08)	12.47 (6.89 to 19.08)	12.47 (6.89 to 19.08)	12.47 (6.89 to 19.08)	12.47 (6.89 to 19.08)	12.47 (6.89 to 19.08)	12.47 (6.89 to 19.08)
Stroke	40 Years Since Quitting	Both	Female	8.95 (4.12 to 14.70)	8.95 (4.12 to 14.70)	8.95 (4.12 to 14.70)	8.95 (4.12 to 14.70)	8.95 (4.12 to 14.70)	8.95 (4.12 to 14.70)	8.95 (4.12 to 14.70)	8.95 (4.12 to 14.70)	8.95 (4.12 to 14.70)	8.95 (4.12 to 14.70)	8.95 (4.12 to 14.70)	8.95 (4.12 to 14.70)	8.95 (4.12 to 14.70)	8.95 (4.12 to 14.70)
Stroke	40 Years Since Quitting	Both	Male	8.95 (4.12 to 14.70)	8.95 (4.12 to 14.70)	8.95 (4.12 to 14.70)	8.95 (4.12 to 14.70)	8.95 (4.12 to 14.70)	8.95 (4.12 to 14.70)	8.95 (4.12 to 14.70)	8.95 (4.12 to 14.70)	8.95 (4.12 to 14.70)	8.95 (4.12 to 14.70)	8.95 (4.12 to 14.70)	8.95 (4.12 to 14.70)	8.95 (4.12 to 14.70)	8.95 (4.12 to 14.70)
Lower respiratory infections	0 Years Since Quitting	Both	Female	100.00 (100.00 to 100.00)	100.00 (100.00 to 100.00)	100.00 (100.00 to 100.00)	100.00 (100.00 to 100.00)	100.00 (100.00 to 100.00)	100.00 (100.00 to 100.00)	100.00 (100.00 to 100.00)	100.00 (100.00 to 100.00)	100.00 (100.00 to 100.00)	100.00 (100.00 to 100.00)	100.00 (100.00 to 100.00)	100.00 (100.00 to 100.00)	100.00 (100.00 to 100.00)	100.00 (100.00 to 100.00)
Lower respiratory infections	0 Years Since Quitting	Both	Male	100.00 (100.00 to 100.00)	100.00 (100.00 to 100.00)	100.00 (100.00 to 100.00)	100.00 (100.00 to 100.00)	100.00 (100.00 to 100.00)	100.00 (100.00 to 100.00)	100.00 (100.00 to 100.00)	100.00 (100.00 to 100.00)	100.00 (100.00 to 100.00)	100.00 (100.00 to 100.00)	100.00 (100.00 to 100.00)	100.00 (100.00 to 100.00)	100.00 (100.00 to 100.00)	100.00 (100.00 to 100.00)
Lower respiratory infections	10 Years Since Quitting	Both	Female	35.89 (28.75 to 43.23)	35.89 (28.75 to 43.23)	35.89 (28.75 to 43.23)	35.89 (28.75 to 43.23)	35.89 (28.75 to 43.23)	35.89 (28.75 to 43.23)	35.89 (28.75 to 43.23)	35.89 (28.75 to 43.23)	35.89 (28.75 to 43.23)	35.89 (28.75 to 43.23)	35.89 (28.75 to 43.23)	35.89 (28.75 to 43.23)	35.89 (28.75 to 43.23)	35.89 (28.75 to 43.23)
Lower respiratory infections	10 Years Since Quitting	Both	Male	35.89 (28.75 to 43.23)	35.89 (28.75 to 43.23)	35.89 (28.75 to 43.23)	35.89 (28.75 to 43.23)	35.89 (28.75 to 43.23)	35.89 (28.75 to 43.23)	35.89 (28.75 to 43.23)	35.89 (28.75 to 43.23)	35.89 (28.75 to 43.23)	35.89 (28.75 to 43.23)	35.89 (28.75 to 43.23)	35.89 (28.75 to 43.23)	35.89 (28.75 to 43.23)	35.89 (28.75 to 43.23)
Lower respiratory infections	20 Years Since Quitting	Both	Female	12.76 (8.02 to 17.73)	12.76 (8.02 to 17.73)	12.76 (8.02 to 17.73)	12.76 (8.02 to 17.73)	12.76 (8.02 to 17.73)	12.76 (8.02 to 17.73)	12.76 (8.02 to 17.73)	12.76 (8.02 to 17.73)	12.76 (8.02 to 17.73)	12.76 (8.02 to 17.73)	12.76 (8.02 to 17.73)	12.76 (8.02 to 17.73)	12.76 (8.02 to 17.73)	12.76 (8.02 to 17.73)
Lower respiratory infections	20 Years Since Quitting	Both	Male	12.76 (8.02 to 17.73)	12.76 (8.02 to 17.73)	12.76 (8.02 to 17.73)	12.76 (8.02 to 17.73)	12.76 (8.02 to 17.73)	12.76 (8.02 to 17.73)	12.76 (8.02 to 17.73)	12.76 (8.02 to 17.73)	12.76 (8.02 to 17.73)	12.76 (8.02 to 17.73)	12.76 (8.02 to 17.73)	12.76 (8.02 to 17.73)	12.76 (8.02 to 17.73)	12.76 (8.02 to 17.73)
Lower respiratory infections	30 Years Since Quitting	Both	Female	4.26 (1.24 to 7.84)	4.26 (1.24 to 7.84)	4.26 (1.24 to 7.84)	4.26 (1.24 to 7.84)	4.26 (1.24 to 7.84)	4.26 (1.24 to 7.84)	4.26 (1.24 to 7.84)	4.26 (1.24 to 7.84)	4.26 (1.24 to 7.84)	4.26 (1.24 to 7.84)	4.26 (1.24 to 7.84)	4.26 (1.24 to 7.84)	4.26 (1.24 to 7.84)	4.26 (1.24 to 7.84)
Lower respiratory infections	30 Years Since Quitting	Both	Male	4.26 (1.24 to 7.84)	4.26 (1.24 to 7.84)	4.26 (1.24 to 7.84)	4.26 (1.24 to 7.84)	4.26 (1.24 to 7.84)	4.26 (1.24 to 7.84)	4.26 (1.24 to 7.84)	4.26 (1.24 to 7.84)	4.26 (1.24 to 7.84)	4.26 (1.24 to 7.84)	4.26 (1.24 to 7.84)	4.26 (1.24 to 7.84)	4.26 (1.24 to 7.84)	4.26 (1.24 to 7.84)
Lower respiratory infections	40 Years Since Quitting	Both	Female	1.35 (0.09 to 3.80)	1.35 (0.09 to 3.80)	1.35 (0.09 to 3.80)	1.35 (0.09 to 3.80)	1.35 (0.09 to 3.80)	1.35 (0.09 to 3.80)	1.35 (0.09 to 3.80)	1.35 (0.09 to 3.80)	1.35 (0.09 to 3.80)	1.35 (0.09 to 3.80)	1.35 (0.09 to 3.80)	1.35 (0.09 to 3.80)	1.35 (0.09 to 3.80)	1.35 (0.09 to 3.80)
Lower respiratory infections	40 Years Since Quitting	Both	Male	1.35 (0.09 to 3.80)	1.35 (0.09 to 3.80)	1.35 (0.09 to 3.80)	1.35 (0.09 to 3.80)	1.35 (0.09 to 3.80)	1.35 (0.09 to 3.80)	1.35 (0.09 to 3.80)	1.35 (0.09 to 3.80)	1.35 (0.09 to 3.80)	1.35 (0.09 to 3.80)	1.35 (0.09 to 3.80)	1.35 (0.09 to 3.80)	1.35 (0.09 to 3.80)	1.35 (0.09 to 3.80)
Tuberculosis	0 Years Since Quitting	Both	Female	100.00 (100.00 to 100.00)	100.00 (100.00 to 100.00)	100.00 (100.00 to 100.00)	100.00 (100.00 to 100.00)	100.00 (100.00 to 100.00)	100.00 (100.00 to 100.00)	100.00 (100.00 to 100.00)	100.00 (100.00 to 100.00)	100.00 (100.00 to 100.00)	100.00 (100.00 to 100.00)	100.00 (100.00 to 100.00)	100.00 (100.00 to 100.00)	100.00 (100.00 to 100.00)	100.00 (100.00 to 100.00)
Tuberculosis	0 Years Since Quitting	Both	Male	100.00 (100.00 to 100.00)	100.00 (100.00 to 100.00)	100.00 (100.00 to 100.00)	100.00 (100.00 to 100.00)	100.00 (100.00 to 100.00)	100.00 (100.00 to 100.00)	100.00 (100.00 to 100.00)	100.00 (100.00 to 100.00)	100.00 (100.00 to 100.00)	100.00 (100.00 to 100.00)	100.00 (100.00 to 100.00)	100.00 (100.00 to 100.00)	100.00 (100.00 to 100.00)	100.00 (100.00 to 100.00)
Tuberculosis	10 Years Since Quitting	Both	Female	35.89 (28.75 to 43.23)	35.89 (28.75 to 43.23)	35.89 (28.75 to 43.23)	35.89 (28.75 to 43.23)	35.89 (28.75 to 43.23)	35.89 (28.75 to 43.23)	35.89 (28.75 to 43.23)	35.89 (28.75 to 43.23)	35.89 (28.75 to 43.23)	35.89 (28.75 to 43.23)	35.89 (28.75 to 43.23)	35.89 (28.75 to 43.23)	35.89 (28.75 to 43.23)	35.89 (28.75 to 43.23)

Tuberculosis	10 Years Since Quitting	Both	Male	35.89 (28.75 to 43.23)	35.89 (28.75 to 43.23)	35.89 (28.75 to 43.23)	35.89 (28.75 to 43.23)	35.89 (28.75 to 43.23)	35.89 (28.75 to 43.23)	35.89 (28.75 to 43.23)	35.89 (28.75 to 43.23)	35.89 (28.75 to 43.23)	35.89 (28.75 to 43.23)	35.89 (28.75 to 43.23)	35.89 (28.75 to 43.23)	35.89 (28.75 to 43.23)	35.89 (28.75 to 43.23)
Tuberculosis	20 Years Since Quitting	Both	Female	12.76 (8.02 to 17.73)	12.76 (8.02 to 17.73)	12.76 (8.02 to 17.73)	12.76 (8.02 to 17.73)	12.76 (8.02 to 17.73)	12.76 (8.02 to 17.73)	12.76 (8.02 to 17.73)	12.76 (8.02 to 17.73)	12.76 (8.02 to 17.73)	12.76 (8.02 to 17.73)	12.76 (8.02 to 17.73)	12.76 (8.02 to 17.73)	12.76 (8.02 to 17.73)	12.76 (8.02 to 17.73)
Tuberculosis	20 Years Since Quitting	Both	Male	12.76 (8.02 to 17.73)	12.76 (8.02 to 17.73)	12.76 (8.02 to 17.73)	12.76 (8.02 to 17.73)	12.76 (8.02 to 17.73)	12.76 (8.02 to 17.73)	12.76 (8.02 to 17.73)	12.76 (8.02 to 17.73)	12.76 (8.02 to 17.73)	12.76 (8.02 to 17.73)	12.76 (8.02 to 17.73)	12.76 (8.02 to 17.73)	12.76 (8.02 to 17.73)	12.76 (8.02 to 17.73)
Tuberculosis	30 Years Since Quitting	Both	Female	4.26 (1.24 to 7.84)	4.26 (1.24 to 7.84)	4.26 (1.24 to 7.84)	4.26 (1.24 to 7.84)	4.26 (1.24 to 7.84)	4.26 (1.24 to 7.84)	4.26 (1.24 to 7.84)	4.26 (1.24 to 7.84)	4.26 (1.24 to 7.84)	4.26 (1.24 to 7.84)	4.26 (1.24 to 7.84)	4.26 (1.24 to 7.84)	4.26 (1.24 to 7.84)	4.26 (1.24 to 7.84)
Tuberculosis	30 Years Since Quitting	Both	Male	4.26 (1.24 to 7.84)	4.26 (1.24 to 7.84)	4.26 (1.24 to 7.84)	4.26 (1.24 to 7.84)	4.26 (1.24 to 7.84)	4.26 (1.24 to 7.84)	4.26 (1.24 to 7.84)	4.26 (1.24 to 7.84)	4.26 (1.24 to 7.84)	4.26 (1.24 to 7.84)	4.26 (1.24 to 7.84)	4.26 (1.24 to 7.84)	4.26 (1.24 to 7.84)	4.26 (1.24 to 7.84)
Tuberculosis	40 Years Since Quitting	Both	Female	1.35 (0.09 to 3.80)	1.35 (0.09 to 3.80)	1.35 (0.09 to 3.80)	1.35 (0.09 to 3.80)	1.35 (0.09 to 3.80)	1.35 (0.09 to 3.80)	1.35 (0.09 to 3.80)	1.35 (0.09 to 3.80)	1.35 (0.09 to 3.80)	1.35 (0.09 to 3.80)	1.35 (0.09 to 3.80)	1.35 (0.09 to 3.80)	1.35 (0.09 to 3.80)	1.35 (0.09 to 3.80)
Tuberculosis	40 Years Since Quitting	Both	Male	1.35 (0.09 to 3.80)	1.35 (0.09 to 3.80)	1.35 (0.09 to 3.80)	1.35 (0.09 to 3.80)	1.35 (0.09 to 3.80)	1.35 (0.09 to 3.80)	1.35 (0.09 to 3.80)	1.35 (0.09 to 3.80)	1.35 (0.09 to 3.80)	1.35 (0.09 to 3.80)	1.35 (0.09 to 3.80)	1.35 (0.09 to 3.80)	1.35 (0.09 to 3.80)	1.35 (0.09 to 3.80)
Asthma	0 Years Since Quitting	Both	Female	100.00 (100.00 to 100.00)	100.00 (100.00 to 100.00)	100.00 (100.00 to 100.00)	100.00 (100.00 to 100.00)	100.00 (100.00 to 100.00)	100.00 (100.00 to 100.00)	100.00 (100.00 to 100.00)	100.00 (100.00 to 100.00)	100.00 (100.00 to 100.00)	100.00 (100.00 to 100.00)	100.00 (100.00 to 100.00)	100.00 (100.00 to 100.00)	100.00 (100.00 to 100.00)	100.00 (100.00 to 100.00)
Asthma	0 Years Since Quitting	Both	Male	100.00 (100.00 to 100.00)	100.00 (100.00 to 100.00)	100.00 (100.00 to 100.00)	100.00 (100.00 to 100.00)	100.00 (100.00 to 100.00)	100.00 (100.00 to 100.00)	100.00 (100.00 to 100.00)	100.00 (100.00 to 100.00)	100.00 (100.00 to 100.00)	100.00 (100.00 to 100.00)	100.00 (100.00 to 100.00)	100.00 (100.00 to 100.00)	100.00 (100.00 to 100.00)	100.00 (100.00 to 100.00)
Asthma	10 Years Since Quitting	Both	Female	50.17 (25.85 to 78.68)	50.17 (25.85 to 78.68)	50.17 (25.85 to 78.68)	50.17 (25.85 to 78.68)	50.17 (25.85 to 78.68)	50.17 (25.85 to 78.68)	50.17 (25.85 to 78.68)	50.17 (25.85 to 78.68)	50.17 (25.85 to 78.68)	50.17 (25.85 to 78.68)	50.17 (25.85 to 78.68)	50.17 (25.85 to 78.68)	50.17 (25.85 to 78.68)	50.17 (25.85 to 78.68)
Asthma	10 Years Since Quitting	Both	Male	50.17 (25.85 to 78.68)	50.17 (25.85 to 78.68)	50.17 (25.85 to 78.68)	50.17 (25.85 to 78.68)	50.17 (25.85 to 78.68)	50.17 (25.85 to 78.68)	50.17 (25.85 to 78.68)	50.17 (25.85 to 78.68)	50.17 (25.85 to 78.68)	50.17 (25.85 to 78.68)	50.17 (25.85 to 78.68)	50.17 (25.85 to 78.68)	50.17 (25.85 to 78.68)	50.17 (25.85 to 78.68)
Asthma	20 Years Since Quitting	Both	Female	33.67 (11.56 to 63.00)	33.67 (11.56 to 63.00)	33.67 (11.56 to 63.00)	33.67 (11.56 to 63.00)	33.67 (11.56 to 63.00)	33.67 (11.56 to 63.00)	33.67 (11.56 to 63.00)	33.67 (11.56 to 63.00)	33.67 (11.56 to 63.00)	33.67 (11.56 to 63.00)	33.67 (11.56 to 63.00)	33.67 (11.56 to 63.00)	33.67 (11.56 to 63.00)	33.67 (11.56 to 63.00)
Asthma	20 Years Since Quitting	Both	Male	33.67 (11.56 to 63.00)	33.67 (11.56 to 63.00)	33.67 (11.56 to 63.00)	33.67 (11.56 to 63.00)	33.67 (11.56 to 63.00)	33.67 (11.56 to 63.00)	33.67 (11.56 to 63.00)	33.67 (11.56 to 63.00)	33.67 (11.56 to 63.00)	33.67 (11.56 to 63.00)	33.67 (11.56 to 63.00)	33.67 (11.56 to 63.00)	33.67 (11.56 to 63.00)	33.67 (11.56 to 63.00)
Asthma	30 Years Since Quitting	Both	Female	22.96 (3.94 to 49.65)	22.96 (3.94 to 49.65)	22.96 (3.94 to 49.65)	22.96 (3.94 to 49.65)	22.96 (3.94 to 49.65)	22.96 (3.94 to 49.65)	22.96 (3.94 to 49.65)	22.96 (3.94 to 49.65)	22.96 (3.94 to 49.65)	22.96 (3.94 to 49.65)	22.96 (3.94 to 49.65)	22.96 (3.94 to 49.65)	22.96 (3.94 to 49.65)	22.96 (3.94 to 49.65)
Asthma	30 Years Since Quitting	Both	Male	22.96 (3.94 to 49.65)	22.96 (3.94 to 49.65)	22.96 (3.94 to 49.65)	22.96 (3.94 to 49.65)	22.96 (3.94 to 49.65)	22.96 (3.94 to 49.65)	22.96 (3.94 to 49.65)	22.96 (3.94 to 49.65)	22.96 (3.94 to 49.65)	22.96 (3.94 to 49.65)	22.96 (3.94 to 49.65)	22.96 (3.94 to 49.65)	22.96 (3.94 to 49.65)	22.96 (3.94 to 49.65)
Asthma	40 Years Since Quitting	Both	Female	16.17 (0.16 to 39.92)	16.17 (0.16 to 39.92)	16.17 (0.16 to 39.92)	16.17 (0.16 to 39.92)	16.17 (0.16 to 39.92)	16.17 (0.16 to 39.92)	16.17 (0.16 to 39.92)	16.17 (0.16 to 39.92)	16.17 (0.16 to 39.92)	16.17 (0.16 to 39.92)	16.17 (0.16 to 39.92)	16.17 (0.16 to 39.92)	16.17 (0.16 to 39.92)	16.17 (0.16 to 39.92)
Asthma	40 Years Since Quitting	Both	Male	16.17 (0.16 to 39.92)	16.17 (0.16 to 39.92)	16.17 (0.16 to 39.92)	16.17 (0.16 to 39.92)	16.17 (0.16 to 39.92)	16.17 (0.16 to 39.92)	16.17 (0.16 to 39.92)	16.17 (0.16 to 39.92)	16.17 (0.16 to 39.92)	16.17 (0.16 to 39.92)	16.17 (0.16 to 39.92)	16.17 (0.16 to 39.92)	16.17 (0.16 to 39.92)	16.17 (0.16 to 39.92)
Diabetes mellitus	0 Years Since Quitting	Both	Female	100.00 (100.00 to 100.00)	100.00 (100.00 to 100.00)	100.00 (100.00 to 100.00)	100.00 (100.00 to 100.00)	100.00 (100.00 to 100.00)	100.00 (100.00 to 100.00)	100.00 (100.00 to 100.00)	100.00 (100.00 to 100.00)	100.00 (100.00 to 100.00)	100.00 (100.00 to 100.00)	100.00 (100.00 to 100.00)	100.00 (100.00 to 100.00)	100.00 (100.00 to 100.00)	100.00 (100.00 to 100.00)
Diabetes mellitus	0 Years Since Quitting	Both	Male	100.00 (100.00 to 100.00)	100.00 (100.00 to 100.00)	100.00 (100.00 to 100.00)	100.00 (100.00 to 100.00)	100.00 (100.00 to 100.00)	100.00 (100.00 to 100.00)	100.00 (100.00 to 100.00)	100.00 (100.00 to 100.00)	100.00 (100.00 to 100.00)	100.00 (100.00 to 100.00)	100.00 (100.00 to 100.00)	100.00 (100.00 to 100.00)	100.00 (100.00 to 100.00)	100.00 (100.00 to 100.00)
Diabetes mellitus	10 Years Since Quitting	Both	Female	42.83 (28.05 to 59.42)	42.83 (28.05 to 59.42)	42.83 (28.05 to 59.42)	42.83 (28.05 to 59.42)	42.83 (28.05 to 59.42)	42.83 (28.05 to 59.42)	42.83 (28.05 to 59.42)	42.83 (28.05 to 59.42)	42.83 (28.05 to 59.42)	42.83 (28.05 to 59.42)	42.83 (28.05 to 59.42)	42.83 (28.05 to 59.42)	42.83 (28.05 to 59.42)	42.83 (28.05 to 59.42)
Diabetes mellitus	10 Years Since Quitting	Both	Male	42.83 (28.05 to 59.42)	42.83 (28.05 to 59.42)	42.83 (28.05 to 59.42)	42.83 (28.05 to 59.42)	42.83 (28.05 to 59.42)	42.83 (28.05 to 59.42)	42.83 (28.05 to 59.42)	42.83 (28.05 to 59.42)	42.83 (28.05 to 59.42)	42.83 (28.05 to 59.42)	42.83 (28.05 to 59.42)	42.83 (28.05 to 59.42)	42.83 (28.05 to 59.42)	42.83 (28.05 to 59.42)
Diabetes mellitus	20 Years Since Quitting	Both	Female	28.61 (14.93 to 44.02)	28.61 (14.93 to 44.02)	28.61 (14.93 to 44.02)	28.61 (14.93 to 44.02)	28.61 (14.93 to 44.02)	28.61 (14.93 to 44.02)	28.61 (14.93 to 44.02)	28.61 (14.93 to 44.02)	28.61 (14.93 to 44.02)	28.61 (14.93 to 44.02)	28.61 (14.93 to 44.02)	28.61 (14.93 to 44.02)	28.61 (14.93 to 44.02)	28.61 (14.93 to 44.02)
Diabetes mellitus	20 Years Since Quitting	Both	Male	28.61 (14.93 to 44.02)	28.61 (14.93 to 44.02)	28.61 (14.93 to 44.02)	28.61 (14.93 to 44.02)	28.61 (14.93 to 44.02)	28.61 (14.93 to 44.02)	28.61 (14.93 to 44.02)	28.61 (14.93 to 44.02)	28.61 (14.93 to 44.02)	28.61 (14.93 to 44.02)	28.61 (14.93 to 44.02)	28.61 (14.93 to 44.02)	28.61 (14.93 to 44.02)	28.61 (14.93 to 44.02)
Diabetes mellitus	30 Years Since Quitting	Both	Female	16.84 (3.00 to 32.26)	16.84 (3.00 to 32.26)	16.84 (3.00 to 32.26)	16.84 (3.00 to 32.26)	16.84 (3.00 to 32.26)	16.84 (3.00 to 32.26)	16.84 (3.00 to 32.26)	16.84 (3.00 to 32.26)	16.84 (3.00 to 32.26)	16.84 (3.00 to 32.26)	16.84 (3.00 to 32.26)	16.84 (3.00 to 32.26)	16.84 (3.00 to 32.26)	16.84 (3.00 to 32.26)
Diabetes mellitus	30 Years Since Quitting	Both	Male	16.84 (3.00 to 32.26)	16.84 (3.00 to 32.26)	16.84 (3.00 to 32.26)	16.84 (3.00 to 32.26)	16.84 (3.00 to 32.26)	16.84 (3.00 to 32.26)	16.84 (3.00 to 32.26)	16.84 (3.00 to 32.26)	16.84 (3.00 to 32.26)	16.84 (3.00 to 32.26)	16.84 (3.00 to 32.26)	16.84 (3.00 to 32.26)	16.84 (3.00 to 32.26)	16.84 (3.00 to 32.26)
Diabetes mellitus	40 Years Since Quitting	Both	Female	9.51 (0.00 to 23.47)	9.51 (0.00 to 23.47)	9.51 (0.00 to 23.47)	9.51 (0.00 to 23.47)	9.51 (0.00 to 23.47)	9.51 (0.00 to 23.47)	9.51 (0.00 to 23.47)	9.51 (0.00 to 23.47)	9.51 (0.00 to 23.47)	9.51 (0.00 to 23.47)	9.51 (0.00 to 23.47)	9.51 (0.00 to 23.47)	9.51 (0.00 to 23.47)	9.51 (0.00 to 23.47)
Diabetes mellitus	40 Years Since Quitting	Both	Male	9.51 (0.00 to 23.47)	9.51 (0.00 to 23.47)	9.51 (0.00 to 23.47)	9.51 (0.00 to 23.47)	9.51 (0.00 to 23.47)	9.51 (0.00 to 23.47)	9.51 (0.00 to 23.47)	9.51 (0.00 to 23.47)	9.51 (0.00 to 23.47)	9.51 (0.00 to 23.47)	9.51 (0.00 to 23.47)	9.51 (0.00 to 23.47)	9.51 (0.00 to 23.47)	9.51 (0.00 to 23.47)
Atrial fibrillation and flutter	0 Years Since Quitting	Both	Female	100.00 (100.00 to 100.00)	100.00 (100.00 to 100.00)	100.00 (100.00 to 100.00)	100.00 (100.00 to 100.00)	100.00 (100.00 to 100.00)	100.00 (100.00 to 100.00)	100.00 (100.00 to 100.00)	100.00 (100.00 to 100.00)	100.00 (100.00 to 100.00)	100.00 (100.00 to 100.00)	100.00 (100.00 to 100.00)	100.00 (100.00 to 100.00)	100.00 (100.00 to 100.00)	100.00 (100.00 to 100.00)
Atrial fibrillation and flutter	0 Years Since Quitting	Both	Male	100.00 (100.00 to 100.00)	100.00 (100.00 to 100.00)	100.00 (100.00 to 100.00)	100.00 (100.00 to 100.00)	100.00 (100.00 to 100.00)	100.00 (100.00 to 100.00)	100.00 (100.00 to 100.00)	100.00 (100.00 to 100.00)	100.00 (100.00 to 100.00)	100.00 (100.00 to 100.00)	100.00 (100.00 to 100.00)	100.00 (100.00 to 100.00)	100.00 (100.00 to 100.00)	100.00 (100.00 to 100.00)
Atrial fibrillation and flutter	10 Years Since Quitting	Both	Female	34.95 (29.34 to 41.21)	34.95 (29.34 to 41.21)	34.95 (29.34 to 41.21)	34.95 (29.34 to 41.21)	34.95 (29.34 to 41.21)	34.95 (29.34 to 41.21)	34.95 (29.34 to 41.21)	34.95 (29.34 to 41.21)	34.95 (29.34 to 41.21)	34.95 (29.34 to 41.21)	34.95 (29.34 to 41.21)	34.95 (29.34 to 41.21)	34.95 (29.34 to 41.21)	34.95 (29.34 to 41.21)
Atrial fibrillation and flutter	10 Years Since Quitting	Both	Male	34.95 (29.34 to 41.21)	34.95 (29.34 to 41.21)	34.95 (29.34 to 41.21)	34.95 (29.34 to 41.21)	34.95 (29.34 to 41.21)	34.95 (29.34 to 41.21)	34.95 (29.34 to 41.21)	34.95 (29.34 to 41.21)	34.95 (29.34 to 41.21)	34.95 (29.34 to 41.21)	34.95 (29.34 to 41.21)	34.95 (29.34 to 41.21)	34.95 (29.34 to 41.21)	34.95 (29.34 to 41.21)
Atrial fibrillation and flutter	20 Years Since Quitting	Both	Female	21.56 (16.71 to 26.49)	21.56 (16.71 to 26.49)	21.56 (16.71 to 26.49)	21.56 (16.71 to 26.49)	21.56 (16.71 to 26.49)	21.56 (16.71 to 26.49)	21.56 (16.71 to 26.49)	21.56 (16.71 to 26.49)	21.56 (16.71 to 26.49)	21.56 (16.71 to 26.49)	21.56 (16.71 to 26.49)	21.56 (16.71 to 26.49)	21.56 (16.71 to 26.49)	21.56 (16.71 to 26.49)
Atrial fibrillation and flutter	20 Years Since Quitting	Both	Male	21.56 (16.71 to 26.49)	21.56 (16.71 to 26.49)	21.56 (16.71 to 26.49)	21.56 (16.71 to 26.49)	21.56 (16.71 to 26.49)	21.56 (16.71 to 26.49)	21.56 (16.71 to 26.49)	21.56 (16.71 to 26.49)	21.56 (16.71 to 26.49)	21.56 (16.71 to 26.49)	21.56 (16.71 to 26.49)	21.56 (16.71 to 26.49)	21.56 (16.71 to 26.49)	21.56 (16.71 to 26.49)
Atrial fibrillation and flutter	30 Years Since Quitting	Both	Female	10.25 (7.04 to 13.89)	10.25 (7.04 to 13.89)	10.25 (7.04 to 13.89)	10.25 (7.04 to 13.89)	10.25 (7.04 to 13.89)	10.25 (7.04 to 13.89)	10.25 (7.04 to 13.89)	10.25 (7.04 to 13.89)	10.25 (7.04 to 13.89)	10.25 (7.04 to 13.89)	10.25 (7.04 to 13.89)	10.25 (7.04 to 13.89)	10.25 (7.04 to 13.89)	10.25 (7.04 to 13.89)
Atrial fibrillation and flutter	30 Years Since Quitting	Both	Male	10.25 (7.04 to 13.89)	10.25 (7.04 to 13.89)	10.25 (7.04 to 13.89)	10.25 (7.04 to 13.89)	10.25 (7.04 to 13.89)	10.25 (7.04 to 13.89)	10.25 (7.04 to 13.89)	10.25 (7.04 to 13.89)	10.25 (7.04 to 13.89)	10.25 (7.04 to 13.89)	10.25 (7.04 to 13.89)	10.25 (7.04 to 13.89)	10.25 (7.04 to 13.89)	10.25 (7.04 to 13.89)
Atrial fibrillation and flutter	40 Years Since Quitting	Both	Female	7.74 (4.61 to 11.05)	7.74 (4.61 to 11.05)	7.74 (4.61 to 11.05)	7.74 (4.61 to 11.05)	7.74 (4.61 to 11.05)	7.74 (4.61 to 11.05)	7.74 (4.61 to 11.05)	7.74 (4.61 to 11.05)	7.74 (4.61 to 11.05)	7.74 (4.61 to 11.05)	7.74 (4.61 to 11.05)	7.74 (4.61 to 11.05)	7.74 (4.61 to 11.05)	7.74 (4.61 to 11.05)

Atrial fibrillation and flutter	40 Years Since Quitting	Both	Male	7.74 (4.61 to 11.05)	7.74 (4.61 to 11.05)	7.74 (4.61 to 11.05)	7.74 (4.61 to 11.05)	7.74 (4.61 to 11.05)	7.74 (4.61 to 11.05)	7.74 (4.61 to 11.05)	7.74 (4.61 to 11.05)	7.74 (4.61 to 11.05)	7.74 (4.61 to 11.05)	7.74 (4.61 to 11.05)	7.74 (4.61 to 11.05)	7.74 (4.61 to 11.05)	7.74 (4.61 to 11.05)
Aortic aneurysm	0 Years Since Quitting	Both	Female	100.00 (100.00 to 100.00)	100.00 (100.00 to 100.00)	100.00 (100.00 to 100.00)	100.00 (100.00 to 100.00)	100.00 (100.00 to 100.00)	100.00 (100.00 to 100.00)	100.00 (100.00 to 100.00)	100.00 (100.00 to 100.00)	100.00 (100.00 to 100.00)	100.00 (100.00 to 100.00)	100.00 (100.00 to 100.00)	100.00 (100.00 to 100.00)	100.00 (100.00 to 100.00)	100.00 (100.00 to 100.00)
Aortic aneurysm	0 Years Since Quitting	Both	Male	100.00 (100.00 to 100.00)	100.00 (100.00 to 100.00)	100.00 (100.00 to 100.00)	100.00 (100.00 to 100.00)	100.00 (100.00 to 100.00)	100.00 (100.00 to 100.00)	100.00 (100.00 to 100.00)	100.00 (100.00 to 100.00)	100.00 (100.00 to 100.00)	100.00 (100.00 to 100.00)	100.00 (100.00 to 100.00)	100.00 (100.00 to 100.00)	100.00 (100.00 to 100.00)	100.00 (100.00 to 100.00)
Aortic aneurysm	10 Years Since Quitting	Both	Female	72.18 (69.38 to 76.27)	72.18 (69.38 to 76.27)	72.18 (69.38 to 76.27)	72.18 (69.38 to 76.27)	72.18 (69.38 to 76.27)	72.18 (69.38 to 76.27)	72.18 (69.38 to 76.27)	72.18 (69.38 to 76.27)	72.18 (69.38 to 76.27)	72.18 (69.38 to 76.27)	72.18 (69.38 to 76.27)	72.18 (69.38 to 76.27)	72.18 (69.38 to 76.27)	72.18 (69.38 to 76.27)
Aortic aneurysm	10 Years Since Quitting	Both	Male	72.18 (69.38 to 76.27)	72.18 (69.38 to 76.27)	72.18 (69.38 to 76.27)	72.18 (69.38 to 76.27)	72.18 (69.38 to 76.27)	72.18 (69.38 to 76.27)	72.18 (69.38 to 76.27)	72.18 (69.38 to 76.27)	72.18 (69.38 to 76.27)	72.18 (69.38 to 76.27)	72.18 (69.38 to 76.27)	72.18 (69.38 to 76.27)	72.18 (69.38 to 76.27)	72.18 (69.38 to 76.27)
Aortic aneurysm	20 Years Since Quitting	Both	Female	46.42 (34.83 to 57.66)	46.42 (34.83 to 57.66)	46.42 (34.83 to 57.66)	46.42 (34.83 to 57.66)	46.42 (34.83 to 57.66)	46.42 (34.83 to 57.66)	46.42 (34.83 to 57.66)	46.42 (34.83 to 57.66)	46.42 (34.83 to 57.66)	46.42 (34.83 to 57.66)	46.42 (34.83 to 57.66)	46.42 (34.83 to 57.66)	46.42 (34.83 to 57.66)	46.42 (34.83 to 57.66)
Aortic aneurysm	20 Years Since Quitting	Both	Male	46.42 (34.83 to 57.66)	46.42 (34.83 to 57.66)	46.42 (34.83 to 57.66)	46.42 (34.83 to 57.66)	46.42 (34.83 to 57.66)	46.42 (34.83 to 57.66)	46.42 (34.83 to 57.66)	46.42 (34.83 to 57.66)	46.42 (34.83 to 57.66)	46.42 (34.83 to 57.66)	46.42 (34.83 to 57.66)	46.42 (34.83 to 57.66)	46.42 (34.83 to 57.66)	46.42 (34.83 to 57.66)
Aortic aneurysm	30 Years Since Quitting	Both	Female	19.14 (10.00 to 29.48)	19.14 (10.00 to 29.48)	19.14 (10.00 to 29.48)	19.14 (10.00 to 29.48)	19.14 (10.00 to 29.48)	19.14 (10.00 to 29.48)	19.14 (10.00 to 29.48)	19.14 (10.00 to 29.48)	19.14 (10.00 to 29.48)	19.14 (10.00 to 29.48)	19.14 (10.00 to 29.48)	19.14 (10.00 to 29.48)	19.14 (10.00 to 29.48)	19.14 (10.00 to 29.48)
Aortic aneurysm	30 Years Since Quitting	Both	Male	19.14 (10.00 to 29.48)	19.14 (10.00 to 29.48)	19.14 (10.00 to 29.48)	19.14 (10.00 to 29.48)	19.14 (10.00 to 29.48)	19.14 (10.00 to 29.48)	19.14 (10.00 to 29.48)	19.14 (10.00 to 29.48)	19.14 (10.00 to 29.48)	19.14 (10.00 to 29.48)	19.14 (10.00 to 29.48)	19.14 (10.00 to 29.48)	19.14 (10.00 to 29.48)	19.14 (10.00 to 29.48)
Aortic aneurysm	40 Years Since Quitting	Both	Female	9.78 (2.16 to 19.18)	9.78 (2.16 to 19.18)	9.78 (2.16 to 19.18)	9.78 (2.16 to 19.18)	9.78 (2.16 to 19.18)	9.78 (2.16 to 19.18)	9.78 (2.16 to 19.18)	9.78 (2.16 to 19.18)	9.78 (2.16 to 19.18)	9.78 (2.16 to 19.18)	9.78 (2.16 to 19.18)	9.78 (2.16 to 19.18)	9.78 (2.16 to 19.18)	9.78 (2.16 to 19.18)
Aortic aneurysm	40 Years Since Quitting	Both	Male	9.78 (2.16 to 19.18)	9.78 (2.16 to 19.18)	9.78 (2.16 to 19.18)	9.78 (2.16 to 19.18)	9.78 (2.16 to 19.18)	9.78 (2.16 to 19.18)	9.78 (2.16 to 19.18)	9.78 (2.16 to 19.18)	9.78 (2.16 to 19.18)	9.78 (2.16 to 19.18)	9.78 (2.16 to 19.18)	9.78 (2.16 to 19.18)	9.78 (2.16 to 19.18)	9.78 (2.16 to 19.18)
Lower extremity peripheral arterial disease	0 Years Since Quitting	Both	Female	100.00 (100.00 to 100.00)	100.00 (100.00 to 100.00)	100.00 (100.00 to 100.00)	100.00 (100.00 to 100.00)	100.00 (100.00 to 100.00)	100.00 (100.00 to 100.00)	100.00 (100.00 to 100.00)	100.00 (100.00 to 100.00)	100.00 (100.00 to 100.00)	100.00 (100.00 to 100.00)	100.00 (100.00 to 100.00)	100.00 (100.00 to 100.00)	100.00 (100.00 to 100.00)	100.00 (100.00 to 100.00)
Lower extremity peripheral arterial disease	0 Years Since Quitting	Both	Male	100.00 (100.00 to 100.00)	100.00 (100.00 to 100.00)	100.00 (100.00 to 100.00)	100.00 (100.00 to 100.00)	100.00 (100.00 to 100.00)	100.00 (100.00 to 100.00)	100.00 (100.00 to 100.00)	100.00 (100.00 to 100.00)	100.00 (100.00 to 100.00)	100.00 (100.00 to 100.00)	100.00 (100.00 to 100.00)	100.00 (100.00 to 100.00)	100.00 (100.00 to 100.00)	100.00 (100.00 to 100.00)
Lower extremity peripheral arterial disease	10 Years Since Quitting	Both	Female	18.60 (12.60 to 25.30)	18.60 (12.60 to 25.30)	18.60 (12.60 to 25.30)	18.60 (12.60 to 25.30)	18.60 (12.60 to 25.30)	18.60 (12.60 to 25.30)	18.60 (12.60 to 25.30)	18.60 (12.60 to 25.30)	18.60 (12.60 to 25.30)	18.60 (12.60 to 25.30)	18.60 (12.60 to 25.30)	18.60 (12.60 to 25.30)	18.60 (12.60 to 25.30)	18.60 (12.60 to 25.30)
Lower extremity peripheral arterial disease	10 Years Since Quitting	Both	Male	18.60 (12.60 to 25.30)	18.60 (12.60 to 25.30)	18.60 (12.60 to 25.30)	18.60 (12.60 to 25.30)	18.60 (12.60 to 25.30)	18.60 (12.60 to 25.30)	18.60 (12.60 to 25.30)	18.60 (12.60 to 25.30)	18.60 (12.60 to 25.30)	18.60 (12.60 to 25.30)	18.60 (12.60 to 25.30)	18.60 (12.60 to 25.30)	18.60 (12.60 to 25.30)	18.60 (12.60 to 25.30)
Lower extremity peripheral arterial disease	20 Years Since Quitting	Both	Female	5.79 (2.83 to 9.32)	5.79 (2.83 to 9.32)	5.79 (2.83 to 9.32)	5.79 (2.83 to 9.32)	5.79 (2.83 to 9.32)	5.79 (2.83 to 9.32)	5.79 (2.83 to 9.32)	5.79 (2.83 to 9.32)	5.79 (2.83 to 9.32)	5.79 (2.83 to 9.32)	5.79 (2.83 to 9.32)	5.79 (2.83 to 9.32)	5.79 (2.83 to 9.32)	5.79 (2.83 to 9.32)
Lower extremity peripheral arterial disease	20 Years Since Quitting	Both	Male	5.79 (2.83 to 9.32)	5.79 (2.83 to 9.32)	5.79 (2.83 to 9.32)	5.79 (2.83 to 9.32)	5.79 (2.83 to 9.32)	5.79 (2.83 to 9.32)	5.79 (2.83 to 9.32)	5.79 (2.83 to 9.32)	5.79 (2.83 to 9.32)	5.79 (2.83 to 9.32)	5.79 (2.83 to 9.32)	5.79 (2.83 to 9.32)	5.79 (2.83 to 9.32)	5.79 (2.83 to 9.32)
Lower extremity peripheral arterial disease	30 Years Since Quitting	Both	Female	0.09 (0.00 to 0.73)	0.09 (0.00 to 0.73)	0.09 (0.00 to 0.73)	0.09 (0.00 to 0.73)	0.09 (0.00 to 0.73)	0.09 (0.00 to 0.73)	0.09 (0.00 to 0.73)	0.09 (0.00 to 0.73)	0.09 (0.00 to 0.73)	0.09 (0.00 to 0.73)	0.09 (0.00 to 0.73)	0.09 (0.00 to 0.73)	0.09 (0.00 to 0.73)	0.09 (0.00 to 0.73)
Lower extremity peripheral arterial disease	30 Years Since Quitting	Both	Male	0.09 (0.00 to 0.73)	0.09 (0.00 to 0.73)	0.09 (0.00 to 0.73)	0.09 (0.00 to 0.73)	0.09 (0.00 to 0.73)	0.09 (0.00 to 0.73)	0.09 (0.00 to 0.73)	0.09 (0.00 to 0.73)	0.09 (0.00 to 0.73)	0.09 (0.00 to 0.73)	0.09 (0.00 to 0.73)	0.09 (0.00 to 0.73)	0.09 (0.00 to 0.73)	0.09 (0.00 to 0.73)
Alzheimer's disease and other dementias	0 Years Since Quitting	Both	Female	100.00 (100.00 to 100.00)	100.00 (100.00 to 100.00)	100.00 (100.00 to 100.00)	100.00 (100.00 to 100.00)	100.00 (100.00 to 100.00)	100.00 (100.00 to 100.00)	100.00 (100.00 to 100.00)	100.00 (100.00 to 100.00)	100.00 (100.00 to 100.00)	100.00 (100.00 to 100.00)	100.00 (100.00 to 100.00)	100.00 (100.00 to 100.00)	100.00 (100.00 to 100.00)	100.00 (100.00 to 100.00)
Alzheimer's disease and other dementias	0 Years Since Quitting	Both	Male	100.00 (100.00 to 100.00)	100.00 (100.00 to 100.00)	100.00 (100.00 to 100.00)	100.00 (100.00 to 100.00)	100.00 (100.00 to 100.00)	100.00 (100.00 to 100.00)	100.00 (100.00 to 100.00)	100.00 (100.00 to 100.00)	100.00 (100.00 to 100.00)	100.00 (100.00 to 100.00)	100.00 (100.00 to 100.00)	100.00 (100.00 to 100.00)	100.00 (100.00 to 100.00)	100.00 (100.00 to 100.00)
Alzheimer's disease and other dementias	10 Years Since Quitting	Both	Female	39.70 (35.72 to 43.60)	39.70 (35.72 to 43.60)	39.70 (35.72 to 43.60)	39.70 (35.72 to 43.60)	39.70 (35.72 to 43.60)	39.70 (35.72 to 43.60)	39.70 (35.72 to 43.60)	39.70 (35.72 to 43.60)	39.70 (35.72 to 43.60)	39.70 (35.72 to 43.60)	39.70 (35.72 to 43.60)	39.70 (35.72 to 43.60)	39.70 (35.72 to 43.60)	39.70 (35.72 to 43.60)
Alzheimer's disease and other dementias	10 Years Since Quitting	Both	Male	39.70 (35.72 to 43.60)	39.70 (35.72 to 43.60)	39.70 (35.72 to 43.60)	39.70 (35.72 to 43.60)	39.70 (35.72 to 43.60)	39.70 (35.72 to 43.60)	39.70 (35.72 to 43.60)	39.70 (35.72 to 43.60)	39.70 (35.72 to 43.60)	39.70 (35.72 to 43.60)	39.70 (35.72 to 43.60)	39.70 (35.72 to 43.60)	39.70 (35.72 to 43.60)	39.70 (35.72 to 43.60)
Alzheimer's disease and other dementias	20 Years Since Quitting	Both	Female	24.74 (21.60 to 28.07)	24.74 (21.60 to 28.07)	24.74 (21.60 to 28.07)	24.74 (21.60 to 28.07)	24.74 (21.60 to 28.07)	24.74 (21.60 to 28.07)	24.74 (21.60 to 28.07)	24.74 (21.60 to 28.07)	24.74 (21.60 to 28.07)	24.74 (21.60 to 28.07)	24.74 (21.60 to 28.07)	24.74 (21.60 to 28.07)	24.74 (21.60 to 28.07)	24.74 (21.60 to 28.07)
Alzheimer's disease and other dementias	20 Years Since Quitting	Both	Male	24.74 (21.60 to 28.07)	24.74 (21.60 to 28.07)	24.74 (21.60 to 28.07)	24.74 (21.60 to 28.07)	24.74 (21.60 to 28.07)	24.74 (21.60 to 28.07)	24.74 (21.60 to 28.07)	24.74 (21.60 to 28.07)	24.74 (21.60 to 28.07)	24.74 (21.60 to 28.07)	24.74 (21.60 to 28.07)	24.74 (21.60 to 28.07)	24.74 (21.60 to 28.07)	24.74 (21.60 to 28.07)
Alzheimer's disease and other dementias	30 Years Since Quitting	Both	Female	14.01 (11.18 to 17.11)	14.01 (11.18 to 17.11)	14.01 (11.18 to 17.11)	14.01 (11.18 to 17.11)	14.01 (11.18 to 17.11)	14.01 (11.18 to 17.11)	14.01 (11.18 to 17.11)	14.01 (11.18 to 17.11)	14.01 (11.18 to 17.11)	14.01 (11.18 to 17.11)	14.01 (11.18 to 17.11)	14.01 (11.18 to 17.11)	14.01 (11.18 to 17.11)	14.01 (11.18 to 17.11)
Alzheimer's disease and other dementias	30 Years Since Quitting	Both	Male	14.01 (11.18 to 17.11)	14.01 (11.18 to 17.11)	14.01 (11.18 to 17.11)	14.01 (11.18 to 17.11)	14.01 (11.18 to 17.11)	14.01 (11.18 to 17.11)	14.01 (11.18 to 17.11)	14.01 (11.18 to 17.11)	14.01 (11.18 to 17.11)	14.01 (11.18 to 17.11)	14.01 (11.18 to 17.11)	14.01 (11.18 to 17.11)	14.01 (11.18 to 17.11)	14.01 (11.18 to 17.11)
Alzheimer's disease and other dementias	40 Years Since Quitting	Both	Female	9.35 (6.74 to 12.30)	9.35 (6.74 to 12.30)	9.35 (6.74 to 12.30)	9.35 (6.74 to 12.30)	9.35 (6.74 to 12.30)	9.35 (6.74 to 12.30)	9.35 (6.74 to 12.30)	9.35 (6.74 to 12.30)	9.35 (6.74 to 12.30)	9.35 (6.74 to 12.30)	9.35 (6.74 to 12.30)	9.35 (6.74 to 12.30)	9.35 (6.74 to 12.30)	9.35 (6.74 to 12.30)
Alzheimer's disease and other dementias	40 Years Since Quitting	Both	Male	9.35 (6.74 to 12.30)	9.35 (6.74 to 12.30)	9.35 (6.74 to 12.30)	9.35 (6.74 to 12.30)	9.35 (6.74 to 12.30)	9.35 (6.74 to 12.30)	9.35 (6.74 to 12.30)	9.35 (6.74 to 12.30)	9.35 (6.74 to 12.30)	9.35 (6.74 to 12.30)	9.35 (6.74 to 12.30)	9.35 (6.74 to 12.30)	9.35 (6.74 to 12.30)	9.35 (6.74 to 12.30)
Parkinson's disease	0 Years Since Quitting	Both	Female	0.00 (0.00 to 0.00)	0.00 (0.00 to 0.00)	0.00 (0.00 to 0.00)	0.00 (0.00 to 0.00)	0.00 (0.00 to 0.00)	0.00 (0.00 to 0.00)	0.00 (0.00 to 0.00)	0.00 (0.00 to 0.00)	0.00 (0.00 to 0.00)	0.00 (0.00 to 0.00)	0.00 (0.00 to 0.00)	0.00 (0.00 to 0.00)	0.00 (0.00 to 0.00)	0.00 (0.00 to 0.00)
Parkinson's disease	0 Years Since Quitting	Both	Male	0.00 (0.00 to 0.00)	0.00 (0.00 to 0.00)	0.00 (0.00 to 0.00)	0.00 (0.00 to 0.00)	0.00 (0.00 to 0.00)	0.00 (0.00 to 0.00)	0.00 (0.00 to 0.00)	0.00 (0.00 to 0.00)	0.00 (0.00 to 0.00)	0.00 (0.00 to 0.00)	0.00 (0.00 to 0.00)	0.00 (0.00 to 0.00)	0.00 (0.00 to 0.00)	0.00 (0.00 to 0.00)
Parkinson's disease	10 Years Since Quitting	Both	Female	41.02 (25.33 to 58.63)	41.02 (25.33 to 58.63)	41.02 (25.33 to 58.63)	41.02 (25.33 to 58.63)	41.02 (25.33 to 58.63)	41.02 (25.33 to 58.63)	41.02 (25.33 to 58.63)	41.02 (25.33 to 58.63)	41.02 (25.33 to 58.63)	41.02 (25.33 to 58.63)	41.02 (25.33 to 58.63)	41.02 (25.33 to 58.63)	41.02 (25.33 to 58.63)	41.02 (25.33 to 58.63)
Parkinson's disease	10 Years Since Quitting	Both	Male	41.02 (25.33 to 58.63)	41.02 (25.33 to 58.63)	41.02 (25.33 to 58.63)	41.02 (25.33 to 58.63)	41.02 (25.33 to 58.63)	41.02 (25.33 to 58.63)	41.02 (25.33 to 58.63)	41.02 (25.33 to 58.63)	41.02 (25.33 to 58.63)	41.02 (25.33 to 58.63)	41.02 (25.33 to 58.63)	41.02 (25.33 to 58.63)	41.02 (25.33 to 58.63)	41.02 (25.33 to 58.63)
Parkinson's disease	20 Years Since Quitting	Both	Female	60.71 (42.14 to 82.69)	60.71 (42.14 to 82.69)	60.71 (42.14 to 82.69)	60.71 (42.14 to 82.69)	60.71 (42.14 to 82.69)	60.71 (42.14 to 82.69)	60.71 (42.14 to 82.69)	60.71 (42.14 to 82.69)	60.71 (42.14 to 82.69)	60.71 (42.14 to 82.69)	60.71 (42.14 to 82.69)	60.71 (42.14 to 82.69)	60.71 (42.14 to 82.69)	60.71 (42.14 to 82.69)
Parkinson's disease	20 Years Since Quitting	Both	Male	60.71 (42.14 to 82.69)	60.71 (42.14 to 82.69)	60.71 (42.14 to 82.69)	60.71 (42.14 to 82.69)	60.71 (42.14 to 82.69)	60.71 (42.14 to 82.69)	60.71 (42.14 to 82.69)	60.71 (42.14 to 82.69)	60.71 (42.14 to 82.69)	60.71 (42.14 to 82.69)	60.71 (42.14 to 82.69)	60.71 (42.14 to 82.69)	60.71 (42.14 to 82.69)	60.71 (42.14 to 82.69)
Parkinson's disease	30 Years Since Quitting	Both	Female	76.43 (56.59 to 100.00)	76.43 (56.59 to 100.00)	76.43 (56.59 to 100.00)	76.43 (56.59 to 100.00)	76.43 (56.59 to 100.00)	76.43 (56.59 to 100.00)	76.43 (56.59 to 100.00)	76.43 (56.59 to 100.00)	76.43 (56.59 to 100.00)	76.43 (56.59 to 100.00)	76.43 (56.59 to 100.00)	76.43 (56.59 to 100.00)	76.43 (56.59 to 100.00)	76.43 (56.59 to 100.00)

Parkinson's disease	30 Years Since Quitting	Both	Male	76.43 (56.59 to 100.00)	76.43 (56.59 to 100.00)	76.43 (56.59 to 100.00)	76.43 (56.59 to 100.00)	76.43 (56.59 to 100.00)	76.43 (56.59 to 100.00)	76.43 (56.59 to 100.00)	76.43 (56.59 to 100.00)	76.43 (56.59 to 100.00)	76.43 (56.59 to 100.00)	76.43 (56.59 to 100.00)	76.43 (56.59 to 100.00)	76.43 (56.59 to 100.00)	76.43 (56.59 to 100.00)
Parkinson's disease	40 Years Since Quitting	Both	Female	89.15 (66.85 to 100.00)	89.15 (66.85 to 100.00)	89.15 (66.85 to 100.00)	89.15 (66.85 to 100.00)	89.15 (66.85 to 100.00)	89.15 (66.85 to 100.00)	89.15 (66.85 to 100.00)	89.15 (66.85 to 100.00)	89.15 (66.85 to 100.00)	89.15 (66.85 to 100.00)	89.15 (66.85 to 100.00)	89.15 (66.85 to 100.00)	89.15 (66.85 to 100.00)	89.15 (66.85 to 100.00)
Parkinson's disease	40 Years Since Quitting	Both	Male	89.15 (66.85 to 100.00)	89.15 (66.85 to 100.00)	89.15 (66.85 to 100.00)	89.15 (66.85 to 100.00)	89.15 (66.85 to 100.00)	89.15 (66.85 to 100.00)	89.15 (66.85 to 100.00)	89.15 (66.85 to 100.00)	89.15 (66.85 to 100.00)	89.15 (66.85 to 100.00)	89.15 (66.85 to 100.00)	89.15 (66.85 to 100.00)	89.15 (66.85 to 100.00)	89.15 (66.85 to 100.00)
Multiple sclerosis	0 Years Since Quitting	Both	Female	100.00 (100.00 to 100.00)	100.00 (100.00 to 100.00)	100.00 (100.00 to 100.00)	100.00 (100.00 to 100.00)	100.00 (100.00 to 100.00)	100.00 (100.00 to 100.00)	100.00 (100.00 to 100.00)	100.00 (100.00 to 100.00)	100.00 (100.00 to 100.00)	100.00 (100.00 to 100.00)	100.00 (100.00 to 100.00)	100.00 (100.00 to 100.00)	100.00 (100.00 to 100.00)	100.00 (100.00 to 100.00)
Multiple sclerosis	0 Years Since Quitting	Both	Male	100.00 (100.00 to 100.00)	100.00 (100.00 to 100.00)	100.00 (100.00 to 100.00)	100.00 (100.00 to 100.00)	100.00 (100.00 to 100.00)	100.00 (100.00 to 100.00)	100.00 (100.00 to 100.00)	100.00 (100.00 to 100.00)	100.00 (100.00 to 100.00)	100.00 (100.00 to 100.00)	100.00 (100.00 to 100.00)	100.00 (100.00 to 100.00)	100.00 (100.00 to 100.00)	100.00 (100.00 to 100.00)
Multiple sclerosis	10 Years Since Quitting	Both	Female	39.70 (35.72 to 43.60)	39.70 (35.72 to 43.60)	39.70 (35.72 to 43.60)	39.70 (35.72 to 43.60)	39.70 (35.72 to 43.60)	39.70 (35.72 to 43.60)	39.70 (35.72 to 43.60)	39.70 (35.72 to 43.60)	39.70 (35.72 to 43.60)	39.70 (35.72 to 43.60)	39.70 (35.72 to 43.60)	39.70 (35.72 to 43.60)	39.70 (35.72 to 43.60)	39.70 (35.72 to 43.60)
Multiple sclerosis	10 Years Since Quitting	Both	Male	39.70 (35.72 to 43.60)	39.70 (35.72 to 43.60)	39.70 (35.72 to 43.60)	39.70 (35.72 to 43.60)	39.70 (35.72 to 43.60)	39.70 (35.72 to 43.60)	39.70 (35.72 to 43.60)	39.70 (35.72 to 43.60)	39.70 (35.72 to 43.60)	39.70 (35.72 to 43.60)	39.70 (35.72 to 43.60)	39.70 (35.72 to 43.60)	39.70 (35.72 to 43.60)	39.70 (35.72 to 43.60)
Multiple sclerosis	20 Years Since Quitting	Both	Female	24.74 (21.60 to 28.07)	24.74 (21.60 to 28.07)	24.74 (21.60 to 28.07)	24.74 (21.60 to 28.07)	24.74 (21.60 to 28.07)	24.74 (21.60 to 28.07)	24.74 (21.60 to 28.07)	24.74 (21.60 to 28.07)	24.74 (21.60 to 28.07)	24.74 (21.60 to 28.07)	24.74 (21.60 to 28.07)	24.74 (21.60 to 28.07)	24.74 (21.60 to 28.07)	24.74 (21.60 to 28.07)
Multiple sclerosis	20 Years Since Quitting	Both	Male	24.74 (21.60 to 28.07)	24.74 (21.60 to 28.07)	24.74 (21.60 to 28.07)	24.74 (21.60 to 28.07)	24.74 (21.60 to 28.07)	24.74 (21.60 to 28.07)	24.74 (21.60 to 28.07)	24.74 (21.60 to 28.07)	24.74 (21.60 to 28.07)	24.74 (21.60 to 28.07)	24.74 (21.60 to 28.07)	24.74 (21.60 to 28.07)	24.74 (21.60 to 28.07)	24.74 (21.60 to 28.07)
Multiple sclerosis	30 Years Since Quitting	Both	Female	14.01 (11.18 to 17.11)	14.01 (11.18 to 17.11)	14.01 (11.18 to 17.11)	14.01 (11.18 to 17.11)	14.01 (11.18 to 17.11)	14.01 (11.18 to 17.11)	14.01 (11.18 to 17.11)	14.01 (11.18 to 17.11)	14.01 (11.18 to 17.11)	14.01 (11.18 to 17.11)	14.01 (11.18 to 17.11)	14.01 (11.18 to 17.11)	14.01 (11.18 to 17.11)	14.01 (11.18 to 17.11)
Multiple sclerosis	30 Years Since Quitting	Both	Male	14.01 (11.18 to 17.11)	14.01 (11.18 to 17.11)	14.01 (11.18 to 17.11)	14.01 (11.18 to 17.11)	14.01 (11.18 to 17.11)	14.01 (11.18 to 17.11)	14.01 (11.18 to 17.11)	14.01 (11.18 to 17.11)	14.01 (11.18 to 17.11)	14.01 (11.18 to 17.11)	14.01 (11.18 to 17.11)	14.01 (11.18 to 17.11)	14.01 (11.18 to 17.11)	14.01 (11.18 to 17.11)
Multiple sclerosis	40 Years Since Quitting	Both	Female	9.35 (6.74 to 12.30)	9.35 (6.74 to 12.30)	9.35 (6.74 to 12.30)	9.35 (6.74 to 12.30)	9.35 (6.74 to 12.30)	9.35 (6.74 to 12.30)	9.35 (6.74 to 12.30)	9.35 (6.74 to 12.30)	9.35 (6.74 to 12.30)	9.35 (6.74 to 12.30)	9.35 (6.74 to 12.30)	9.35 (6.74 to 12.30)	9.35 (6.74 to 12.30)	9.35 (6.74 to 12.30)
Multiple sclerosis	40 Years Since Quitting	Both	Male	9.35 (6.74 to 12.30)	9.35 (6.74 to 12.30)	9.35 (6.74 to 12.30)	9.35 (6.74 to 12.30)	9.35 (6.74 to 12.30)	9.35 (6.74 to 12.30)	9.35 (6.74 to 12.30)	9.35 (6.74 to 12.30)	9.35 (6.74 to 12.30)	9.35 (6.74 to 12.30)	9.35 (6.74 to 12.30)	9.35 (6.74 to 12.30)	9.35 (6.74 to 12.30)	9.35 (6.74 to 12.30)
Oesophageal cancer	0 Years Since Quitting	Both	Female	100.00 (100.00 to 100.00)	100.00 (100.00 to 100.00)	100.00 (100.00 to 100.00)	100.00 (100.00 to 100.00)	100.00 (100.00 to 100.00)	100.00 (100.00 to 100.00)	100.00 (100.00 to 100.00)	100.00 (100.00 to 100.00)	100.00 (100.00 to 100.00)	100.00 (100.00 to 100.00)	100.00 (100.00 to 100.00)	100.00 (100.00 to 100.00)	100.00 (100.00 to 100.00)	100.00 (100.00 to 100.00)
Oesophageal cancer	0 Years Since Quitting	Both	Male	100.00 (100.00 to 100.00)	100.00 (100.00 to 100.00)	100.00 (100.00 to 100.00)	100.00 (100.00 to 100.00)	100.00 (100.00 to 100.00)	100.00 (100.00 to 100.00)	100.00 (100.00 to 100.00)	100.00 (100.00 to 100.00)	100.00 (100.00 to 100.00)	100.00 (100.00 to 100.00)	100.00 (100.00 to 100.00)	100.00 (100.00 to 100.00)	100.00 (100.00 to 100.00)	100.00 (100.00 to 100.00)
Oesophageal cancer	10 Years Since Quitting	Both	Female	32.92 (23.75 to 43.54)	32.92 (23.75 to 43.54)	32.92 (23.75 to 43.54)	32.92 (23.75 to 43.54)	32.92 (23.75 to 43.54)	32.92 (23.75 to 43.54)	32.92 (23.75 to 43.54)	32.92 (23.75 to 43.54)	32.92 (23.75 to 43.54)	32.92 (23.75 to 43.54)	32.92 (23.75 to 43.54)	32.92 (23.75 to 43.54)	32.92 (23.75 to 43.54)	32.92 (23.75 to 43.54)
Oesophageal cancer	10 Years Since Quitting	Both	Male	32.92 (23.75 to 43.54)	32.92 (23.75 to 43.54)	32.92 (23.75 to 43.54)	32.92 (23.75 to 43.54)	32.92 (23.75 to 43.54)	32.92 (23.75 to 43.54)	32.92 (23.75 to 43.54)	32.92 (23.75 to 43.54)	32.92 (23.75 to 43.54)	32.92 (23.75 to 43.54)	32.92 (23.75 to 43.54)	32.92 (23.75 to 43.54)	32.92 (23.75 to 43.54)	32.92 (23.75 to 43.54)
Oesophageal cancer	20 Years Since Quitting	Both	Female	19.26 (12.10 to 26.95)	19.26 (12.10 to 26.95)	19.26 (12.10 to 26.95)	19.26 (12.10 to 26.95)	19.26 (12.10 to 26.95)	19.26 (12.10 to 26.95)	19.26 (12.10 to 26.95)	19.26 (12.10 to 26.95)	19.26 (12.10 to 26.95)	19.26 (12.10 to 26.95)	19.26 (12.10 to 26.95)	19.26 (12.10 to 26.95)	19.26 (12.10 to 26.95)	19.26 (12.10 to 26.95)
Oesophageal cancer	20 Years Since Quitting	Both	Male	19.26 (12.10 to 26.95)	19.26 (12.10 to 26.95)	19.26 (12.10 to 26.95)	19.26 (12.10 to 26.95)	19.26 (12.10 to 26.95)	19.26 (12.10 to 26.95)	19.26 (12.10 to 26.95)	19.26 (12.10 to 26.95)	19.26 (12.10 to 26.95)	19.26 (12.10 to 26.95)	19.26 (12.10 to 26.95)	19.26 (12.10 to 26.95)	19.26 (12.10 to 26.95)	19.26 (12.10 to 26.95)
Oesophageal cancer	30 Years Since Quitting	Both	Female	11.21 (4.29 to 18.45)	11.21 (4.29 to 18.45)	11.21 (4.29 to 18.45)	11.21 (4.29 to 18.45)	11.21 (4.29 to 18.45)	11.21 (4.29 to 18.45)	11.21 (4.29 to 18.45)	11.21 (4.29 to 18.45)	11.21 (4.29 to 18.45)	11.21 (4.29 to 18.45)	11.21 (4.29 to 18.45)	11.21 (4.29 to 18.45)	11.21 (4.29 to 18.45)	11.21 (4.29 to 18.45)
Oesophageal cancer	30 Years Since Quitting	Both	Male	11.21 (4.29 to 18.45)	11.21 (4.29 to 18.45)	11.21 (4.29 to 18.45)	11.21 (4.29 to 18.45)	11.21 (4.29 to 18.45)	11.21 (4.29 to 18.45)	11.21 (4.29 to 18.45)	11.21 (4.29 to 18.45)	11.21 (4.29 to 18.45)	11.21 (4.29 to 18.45)	11.21 (4.29 to 18.45)	11.21 (4.29 to 18.45)	11.21 (4.29 to 18.45)	11.21 (4.29 to 18.45)
Oesophageal cancer	40 Years Since Quitting	Both	Female	5.40 (0.00 to 12.87)	5.40 (0.00 to 12.87)	5.40 (0.00 to 12.87)	5.40 (0.00 to 12.87)	5.40 (0.00 to 12.87)	5.40 (0.00 to 12.87)	5.40 (0.00 to 12.87)	5.40 (0.00 to 12.87)	5.40 (0.00 to 12.87)	5.40 (0.00 to 12.87)	5.40 (0.00 to 12.87)	5.40 (0.00 to 12.87)	5.40 (0.00 to 12.87)	5.40 (0.00 to 12.87)
Oesophageal cancer	40 Years Since Quitting	Both	Male	5.40 (0.00 to 12.87)	5.40 (0.00 to 12.87)	5.40 (0.00 to 12.87)	5.40 (0.00 to 12.87)	5.40 (0.00 to 12.87)	5.40 (0.00 to 12.87)	5.40 (0.00 to 12.87)	5.40 (0.00 to 12.87)	5.40 (0.00 to 12.87)	5.40 (0.00 to 12.87)	5.40 (0.00 to 12.87)	5.40 (0.00 to 12.87)	5.40 (0.00 to 12.87)	5.40 (0.00 to 12.87)
Stomach cancer	0 Years Since Quitting	Both	Female	100.00 (100.00 to 100.00)	100.00 (100.00 to 100.00)	100.00 (100.00 to 100.00)	100.00 (100.00 to 100.00)	100.00 (100.00 to 100.00)	100.00 (100.00 to 100.00)	100.00 (100.00 to 100.00)	100.00 (100.00 to 100.00)	100.00 (100.00 to 100.00)	100.00 (100.00 to 100.00)	100.00 (100.00 to 100.00)	100.00 (100.00 to 100.00)	100.00 (100.00 to 100.00)	100.00 (100.00 to 100.00)
Stomach cancer	0 Years Since Quitting	Both	Male	100.00 (100.00 to 100.00)	100.00 (100.00 to 100.00)	100.00 (100.00 to 100.00)	100.00 (100.00 to 100.00)	100.00 (100.00 to 100.00)	100.00 (100.00 to 100.00)	100.00 (100.00 to 100.00)	100.00 (100.00 to 100.00)	100.00 (100.00 to 100.00)	100.00 (100.00 to 100.00)	100.00 (100.00 to 100.00)	100.00 (100.00 to 100.00)	100.00 (100.00 to 100.00)	100.00 (100.00 to 100.00)
Stomach cancer	10 Years Since Quitting	Both	Female	57.10 (45.47 to 69.91)	57.10 (45.47 to 69.91)	57.10 (45.47 to 69.91)	57.10 (45.47 to 69.91)	57.10 (45.47 to 69.91)	57.10 (45.47 to 69.91)	57.10 (45.47 to 69.91)	57.10 (45.47 to 69.91)	57.10 (45.47 to 69.91)	57.10 (45.47 to 69.91)	57.10 (45.47 to 69.91)	57.10 (45.47 to 69.91)	57.10 (45.47 to 69.91)	57.10 (45.47 to 69.91)
Stomach cancer	10 Years Since Quitting	Both	Male	57.10 (45.47 to 69.91)	57.10 (45.47 to 69.91)	57.10 (45.47 to 69.91)	57.10 (45.47 to 69.91)	57.10 (45.47 to 69.91)	57.10 (45.47 to 69.91)	57.10 (45.47 to 69.91)	57.10 (45.47 to 69.91)	57.10 (45.47 to 69.91)	57.10 (45.47 to 69.91)	57.10 (45.47 to 69.91)	57.10 (45.47 to 69.91)	57.10 (45.47 to 69.91)	57.10 (45.47 to 69.91)
Stomach cancer	20 Years Since Quitting	Both	Female	35.27 (25.89 to 44.92)	35.27 (25.89 to 44.92)	35.27 (25.89 to 44.92)	35.27 (25.89 to 44.92)	35.27 (25.89 to 44.92)	35.27 (25.89 to 44.92)	35.27 (25.89 to 44.92)	35.27 (25.89 to 44.92)	35.27 (25.89 to 44.92)	35.27 (25.89 to 44.92)	35.27 (25.89 to 44.92)	35.27 (25.89 to 44.92)	35.27 (25.89 to 44.92)	35.27 (25.89 to 44.92)
Stomach cancer	20 Years Since Quitting	Both	Male	35.27 (25.89 to 44.92)	35.27 (25.89 to 44.92)	35.27 (25.89 to 44.92)	35.27 (25.89 to 44.92)	35.27 (25.89 to 44.92)	35.27 (25.89 to 44.92)	35.27 (25.89 to 44.92)	35.27 (25.89 to 44.92)	35.27 (25.89 to 44.92)	35.27 (25.89 to 44.92)	35.27 (25.89 to 44.92)	35.27 (25.89 to 44.92)	35.27 (25.89 to 44.92)	35.27 (25.89 to 44.92)
Stomach cancer	30 Years Since Quitting	Both	Female	16.71 (8.66 to 26.02)	16.71 (8.66 to 26.02)	16.71 (8.66 to 26.02)	16.71 (8.66 to 26.02)	16.71 (8.66 to 26.02)	16.71 (8.66 to 26.02)	16.71 (8.66 to 26.02)	16.71 (8.66 to 26.02)	16.71 (8.66 to 26.02)	16.71 (8.66 to 26.02)	16.71 (8.66 to 26.02)	16.71 (8.66 to 26.02)	16.71 (8.66 to 26.02)	16.71 (8.66 to 26.02)
Stomach cancer	30 Years Since Quitting	Both	Male	16.71 (8.66 to 26.02)	16.71 (8.66 to 26.02)	16.71 (8.66 to 26.02)	16.71 (8.66 to 26.02)	16.71 (8.66 to 26.02)	16.71 (8.66 to 26.02)	16.71 (8.66 to 26.02)	16.71 (8.66 to 26.02)	16.71 (8.66 to 26.02)	16.71 (8.66 to 26.02)	16.71 (8.66 to 26.02)	16.71 (8.66 to 26.02)	16.71 (8.66 to 26.02)	16.71 (8.66 to 26.02)
Stomach cancer	40 Years Since Quitting	Both	Female	8.03 (1.12 to 16.17)	8.03 (1.12 to 16.17)	8.03 (1.12 to 16.17)	8.03 (1.12 to 16.17)	8.03 (1.12 to 16.17)	8.03 (1.12 to 16.17)	8.03 (1.12 to 16.17)	8.03 (1.12 to 16.17)	8.03 (1.12 to 16.17)	8.03 (1.12 to 16.17)	8.03 (1.12 to 16.17)	8.03 (1.12 to 16.17)	8.03 (1.12 to 16.17)	8.03 (1.12 to 16.17)
Stomach cancer	40 Years Since Quitting	Both	Male	8.03 (1.12 to 16.17)	8.03 (1.12 to 16.17)	8.03 (1.12 to 16.17)	8.03 (1.12 to 16.17)	8.03 (1.12 to 16.17)	8.03 (1.12 to 16.17)	8.03 (1.12 to 16.17)	8.03 (1.12 to 16.17)	8.03 (1.12 to 16.17)	8.03 (1.12 to 16.17)	8.03 (1.12 to 16.17)	8.03 (1.12 to 16.17)	8.03 (1.12 to 16.17)	8.03 (1.12 to 16.17)
Liver cancer	0 Years Since Quitting	Both	Female	100.00 (100.00 to 100.00)	100.00 (100.00 to 100.00)	100.00 (100.00 to 100.00)	100.00 (100.00 to 100.00)	100.00 (100.00 to 100.00)	100.00 (100.00 to 100.00)	100.00 (100.00 to 100.00)	100.00 (100.00 to 100.00)	100.00 (100.00 to 100.00)	100.00 (100.00 to 100.00)	100.00 (100.00 to 100.00)	100.00 (100.00 to 100.00)	100.00 (100.00 to 100.00)	100.00 (100.00 to 100.00)
Liver cancer	0 Years Since Quitting	Both	Male	100.00 (100.00 to 100.00)	100.00 (100.00 to 100.00)	100.00 (100.00 to 100.00)	100.00 (100.00 to 100.00)	100.00 (100.00 to 100.00)	100.00 (100.00 to 100.00)	100.00 (100.00 to 100.00)	100.00 (100.00 to 100.00)	100.00 (100.00 to 100.00)	100.00 (100.00 to 100.00)	100.00 (100.00 to 100.00)	100.00 (100.00 to 100.00)	100.00 (100.00 to 100.00)	100.00 (100.00 to 100.00)
Liver cancer	10 Years Since Quitting	Both	Female	69.21 (51.63 to 87.25)	69.21 (51.63 to 87.25)	69.21 (51.63 to 87.25)	69.21 (51.63 to 87.25)	69.21 (51.63 to 87.25)	69.21 (51.63 to 87.25)	69.21 (51.63 to 87.25)	69.21 (51.63 to 87.25)	69.21 (51.63 to 87.25)	69.21 (51.63 to 87.25)	69.21 (51.63 to 87.25)	69.21 (51.63 to 87.25)	69.21 (51.63 to 87.25)	69.21 (51.63 to 87.25)

Liver cancer	10 Years Since Quitting	Both	Male	69.21 (51.63 to 87.25)	69.21 (51.63 to 87.25)	69.21 (51.63 to 87.25)	69.21 (51.63 to 87.25)	69.21 (51.63 to 87.25)	69.21 (51.63 to 87.25)	69.21 (51.63 to 87.25)	69.21 (51.63 to 87.25)	69.21 (51.63 to 87.25)	69.21 (51.63 to 87.25)	69.21 (51.63 to 87.25)	69.21 (51.63 to 87.25)	69.21 (51.63 to 87.25)	69.21 (51.63 to 87.25)
Liver cancer	20 Years Since Quitting	Both	Female	56.37 (39.36 to 74.57)	56.37 (39.36 to 74.57)	56.37 (39.36 to 74.57)	56.37 (39.36 to 74.57)	56.37 (39.36 to 74.57)	56.37 (39.36 to 74.57)	56.37 (39.36 to 74.57)	56.37 (39.36 to 74.57)	56.37 (39.36 to 74.57)	56.37 (39.36 to 74.57)	56.37 (39.36 to 74.57)	56.37 (39.36 to 74.57)	56.37 (39.36 to 74.57)	56.37 (39.36 to 74.57)
Liver cancer	20 Years Since Quitting	Both	Male	56.37 (39.36 to 74.57)	56.37 (39.36 to 74.57)	56.37 (39.36 to 74.57)	56.37 (39.36 to 74.57)	56.37 (39.36 to 74.57)	56.37 (39.36 to 74.57)	56.37 (39.36 to 74.57)	56.37 (39.36 to 74.57)	56.37 (39.36 to 74.57)	56.37 (39.36 to 74.57)	56.37 (39.36 to 74.57)	56.37 (39.36 to 74.57)	56.37 (39.36 to 74.57)	56.37 (39.36 to 74.57)
Liver cancer	30 Years Since Quitting	Both	Female	42.73 (23.15 to 61.14)	42.73 (23.15 to 61.14)	42.73 (23.15 to 61.14)	42.73 (23.15 to 61.14)	42.73 (23.15 to 61.14)	42.73 (23.15 to 61.14)	42.73 (23.15 to 61.14)	42.73 (23.15 to 61.14)	42.73 (23.15 to 61.14)	42.73 (23.15 to 61.14)	42.73 (23.15 to 61.14)	42.73 (23.15 to 61.14)	42.73 (23.15 to 61.14)	42.73 (23.15 to 61.14)
Liver cancer	30 Years Since Quitting	Both	Male	42.73 (23.15 to 61.14)	42.73 (23.15 to 61.14)	42.73 (23.15 to 61.14)	42.73 (23.15 to 61.14)	42.73 (23.15 to 61.14)	42.73 (23.15 to 61.14)	42.73 (23.15 to 61.14)	42.73 (23.15 to 61.14)	42.73 (23.15 to 61.14)	42.73 (23.15 to 61.14)	42.73 (23.15 to 61.14)	42.73 (23.15 to 61.14)	42.73 (23.15 to 61.14)	42.73 (23.15 to 61.14)
Liver cancer	40 Years Since Quitting	Both	Female	27.95 (8.68 to 49.36)	27.95 (8.68 to 49.36)	27.95 (8.68 to 49.36)	27.95 (8.68 to 49.36)	27.95 (8.68 to 49.36)	27.95 (8.68 to 49.36)	27.95 (8.68 to 49.36)	27.95 (8.68 to 49.36)	27.95 (8.68 to 49.36)	27.95 (8.68 to 49.36)	27.95 (8.68 to 49.36)	27.95 (8.68 to 49.36)	27.95 (8.68 to 49.36)	27.95 (8.68 to 49.36)
Liver cancer	40 Years Since Quitting	Both	Male	27.95 (8.68 to 49.36)	27.95 (8.68 to 49.36)	27.95 (8.68 to 49.36)	27.95 (8.68 to 49.36)	27.95 (8.68 to 49.36)	27.95 (8.68 to 49.36)	27.95 (8.68 to 49.36)	27.95 (8.68 to 49.36)	27.95 (8.68 to 49.36)	27.95 (8.68 to 49.36)	27.95 (8.68 to 49.36)	27.95 (8.68 to 49.36)	27.95 (8.68 to 49.36)	27.95 (8.68 to 49.36)
Larynx cancer	0 Years Since Quitting	Both	Female	100.00 (100.00 to 100.00)	100.00 (100.00 to 100.00)	100.00 (100.00 to 100.00)	100.00 (100.00 to 100.00)	100.00 (100.00 to 100.00)	100.00 (100.00 to 100.00)	100.00 (100.00 to 100.00)	100.00 (100.00 to 100.00)	100.00 (100.00 to 100.00)	100.00 (100.00 to 100.00)	100.00 (100.00 to 100.00)	100.00 (100.00 to 100.00)	100.00 (100.00 to 100.00)	100.00 (100.00 to 100.00)
Larynx cancer	0 Years Since Quitting	Both	Male	100.00 (100.00 to 100.00)	100.00 (100.00 to 100.00)	100.00 (100.00 to 100.00)	100.00 (100.00 to 100.00)	100.00 (100.00 to 100.00)	100.00 (100.00 to 100.00)	100.00 (100.00 to 100.00)	100.00 (100.00 to 100.00)	100.00 (100.00 to 100.00)	100.00 (100.00 to 100.00)	100.00 (100.00 to 100.00)	100.00 (100.00 to 100.00)	100.00 (100.00 to 100.00)	100.00 (100.00 to 100.00)
Larynx cancer	10 Years Since Quitting	Both	Female	36.07 (24.87 to 49.39)	36.07 (24.87 to 49.39)	36.07 (24.87 to 49.39)	36.07 (24.87 to 49.39)	36.07 (24.87 to 49.39)	36.07 (24.87 to 49.39)	36.07 (24.87 to 49.39)	36.07 (24.87 to 49.39)	36.07 (24.87 to 49.39)	36.07 (24.87 to 49.39)	36.07 (24.87 to 49.39)	36.07 (24.87 to 49.39)	36.07 (24.87 to 49.39)	36.07 (24.87 to 49.39)
Larynx cancer	10 Years Since Quitting	Both	Male	36.07 (24.87 to 49.39)	36.07 (24.87 to 49.39)	36.07 (24.87 to 49.39)	36.07 (24.87 to 49.39)	36.07 (24.87 to 49.39)	36.07 (24.87 to 49.39)	36.07 (24.87 to 49.39)	36.07 (24.87 to 49.39)	36.07 (24.87 to 49.39)	36.07 (24.87 to 49.39)	36.07 (24.87 to 49.39)	36.07 (24.87 to 49.39)	36.07 (24.87 to 49.39)	36.07 (24.87 to 49.39)
Larynx cancer	20 Years Since Quitting	Both	Female	15.16 (6.84 to 23.88)	15.16 (6.84 to 23.88)	15.16 (6.84 to 23.88)	15.16 (6.84 to 23.88)	15.16 (6.84 to 23.88)	15.16 (6.84 to 23.88)	15.16 (6.84 to 23.88)	15.16 (6.84 to 23.88)	15.16 (6.84 to 23.88)	15.16 (6.84 to 23.88)	15.16 (6.84 to 23.88)	15.16 (6.84 to 23.88)	15.16 (6.84 to 23.88)	15.16 (6.84 to 23.88)
Larynx cancer	20 Years Since Quitting	Both	Male	15.16 (6.84 to 23.88)	15.16 (6.84 to 23.88)	15.16 (6.84 to 23.88)	15.16 (6.84 to 23.88)	15.16 (6.84 to 23.88)	15.16 (6.84 to 23.88)	15.16 (6.84 to 23.88)	15.16 (6.84 to 23.88)	15.16 (6.84 to 23.88)	15.16 (6.84 to 23.88)	15.16 (6.84 to 23.88)	15.16 (6.84 to 23.88)	15.16 (6.84 to 23.88)	15.16 (6.84 to 23.88)
Larynx cancer	30 Years Since Quitting	Both	Female	6.29 (0.00 to 14.72)	6.29 (0.00 to 14.72)	6.29 (0.00 to 14.72)	6.29 (0.00 to 14.72)	6.29 (0.00 to 14.72)	6.29 (0.00 to 14.72)	6.29 (0.00 to 14.72)	6.29 (0.00 to 14.72)	6.29 (0.00 to 14.72)	6.29 (0.00 to 14.72)	6.29 (0.00 to 14.72)	6.29 (0.00 to 14.72)	6.29 (0.00 to 14.72)	6.29 (0.00 to 14.72)
Larynx cancer	30 Years Since Quitting	Both	Male	6.29 (0.00 to 14.72)	6.29 (0.00 to 14.72)	6.29 (0.00 to 14.72)	6.29 (0.00 to 14.72)	6.29 (0.00 to 14.72)	6.29 (0.00 to 14.72)	6.29 (0.00 to 14.72)	6.29 (0.00 to 14.72)	6.29 (0.00 to 14.72)	6.29 (0.00 to 14.72)	6.29 (0.00 to 14.72)	6.29 (0.00 to 14.72)	6.29 (0.00 to 14.72)	6.29 (0.00 to 14.72)
Larynx cancer	40 Years Since Quitting	Both	Female	2.71 (0.00 to 10.33)	2.71 (0.00 to 10.33)	2.71 (0.00 to 10.33)	2.71 (0.00 to 10.33)	2.71 (0.00 to 10.33)	2.71 (0.00 to 10.33)	2.71 (0.00 to 10.33)	2.71 (0.00 to 10.33)	2.71 (0.00 to 10.33)	2.71 (0.00 to 10.33)	2.71 (0.00 to 10.33)	2.71 (0.00 to 10.33)	2.71 (0.00 to 10.33)	2.71 (0.00 to 10.33)
Larynx cancer	40 Years Since Quitting	Both	Male	2.71 (0.00 to 10.33)	2.71 (0.00 to 10.33)	2.71 (0.00 to 10.33)	2.71 (0.00 to 10.33)	2.71 (0.00 to 10.33)	2.71 (0.00 to 10.33)	2.71 (0.00 to 10.33)	2.71 (0.00 to 10.33)	2.71 (0.00 to 10.33)	2.71 (0.00 to 10.33)	2.71 (0.00 to 10.33)	2.71 (0.00 to 10.33)	2.71 (0.00 to 10.33)	2.71 (0.00 to 10.33)
Breast cancer	0 Years Since Quitting	Both	Female	100.00 (100.00 to 100.00)	100.00 (100.00 to 100.00)	100.00 (100.00 to 100.00)	100.00 (100.00 to 100.00)	100.00 (100.00 to 100.00)	100.00 (100.00 to 100.00)	100.00 (100.00 to 100.00)	100.00 (100.00 to 100.00)	100.00 (100.00 to 100.00)	100.00 (100.00 to 100.00)	100.00 (100.00 to 100.00)	100.00 (100.00 to 100.00)	100.00 (100.00 to 100.00)	100.00 (100.00 to 100.00)
Breast cancer	0 Years Since Quitting	Both	Male	0.00 (0.00 to 0.00)	0.00 (0.00 to 0.00)	0.00 (0.00 to 0.00)	0.00 (0.00 to 0.00)	0.00 (0.00 to 0.00)	0.00 (0.00 to 0.00)	0.00 (0.00 to 0.00)	0.00 (0.00 to 0.00)	0.00 (0.00 to 0.00)	0.00 (0.00 to 0.00)	0.00 (0.00 to 0.00)	0.00 (0.00 to 0.00)	0.00 (0.00 to 0.00)	0.00 (0.00 to 0.00)
Breast cancer	10 Years Since Quitting	Both	Female	55.99 (44.97 to 69.74)	55.99 (44.97 to 69.74)	55.99 (44.97 to 69.74)	55.99 (44.97 to 69.74)	55.99 (44.97 to 69.74)	55.99 (44.97 to 69.74)	55.99 (44.97 to 69.74)	55.99 (44.97 to 69.74)	55.99 (44.97 to 69.74)	55.99 (44.97 to 69.74)	55.99 (44.97 to 69.74)	55.99 (44.97 to 69.74)	55.99 (44.97 to 69.74)	55.99 (44.97 to 69.74)
Breast cancer	10 Years Since Quitting	Both	Male	0.00 (0.00 to 0.00)	0.00 (0.00 to 0.00)	0.00 (0.00 to 0.00)	0.00 (0.00 to 0.00)	0.00 (0.00 to 0.00)	0.00 (0.00 to 0.00)	0.00 (0.00 to 0.00)	0.00 (0.00 to 0.00)	0.00 (0.00 to 0.00)	0.00 (0.00 to 0.00)	0.00 (0.00 to 0.00)	0.00 (0.00 to 0.00)	0.00 (0.00 to 0.00)	0.00 (0.00 to 0.00)
Breast cancer	20 Years Since Quitting	Both	Female	41.34 (33.50 to 49.65)	41.34 (33.50 to 49.65)	41.34 (33.50 to 49.65)	41.34 (33.50 to 49.65)	41.34 (33.50 to 49.65)	41.34 (33.50 to 49.65)	41.34 (33.50 to 49.65)	41.34 (33.50 to 49.65)	41.34 (33.50 to 49.65)	41.34 (33.50 to 49.65)	41.34 (33.50 to 49.65)	41.34 (33.50 to 49.65)	41.34 (33.50 to 49.65)	41.34 (33.50 to 49.65)
Breast cancer	20 Years Since Quitting	Both	Male	0.00 (0.00 to 0.00)	0.00 (0.00 to 0.00)	0.00 (0.00 to 0.00)	0.00 (0.00 to 0.00)	0.00 (0.00 to 0.00)	0.00 (0.00 to 0.00)	0.00 (0.00 to 0.00)	0.00 (0.00 to 0.00)	0.00 (0.00 to 0.00)	0.00 (0.00 to 0.00)	0.00 (0.00 to 0.00)	0.00 (0.00 to 0.00)	0.00 (0.00 to 0.00)	0.00 (0.00 to 0.00)
Breast cancer	30 Years Since Quitting	Both	Female	30.49 (20.15 to 40.26)	30.49 (20.15 to 40.26)	30.49 (20.15 to 40.26)	30.49 (20.15 to 40.26)	30.49 (20.15 to 40.26)	30.49 (20.15 to 40.26)	30.49 (20.15 to 40.26)	30.49 (20.15 to 40.26)	30.49 (20.15 to 40.26)	30.49 (20.15 to 40.26)	30.49 (20.15 to 40.26)	30.49 (20.15 to 40.26)	30.49 (20.15 to 40.26)	30.49 (20.15 to 40.26)
Breast cancer	30 Years Since Quitting	Both	Male	0.00 (0.00 to 0.00)	0.00 (0.00 to 0.00)	0.00 (0.00 to 0.00)	0.00 (0.00 to 0.00)	0.00 (0.00 to 0.00)	0.00 (0.00 to 0.00)	0.00 (0.00 to 0.00)	0.00 (0.00 to 0.00)	0.00 (0.00 to 0.00)	0.00 (0.00 to 0.00)	0.00 (0.00 to 0.00)	0.00 (0.00 to 0.00)	0.00 (0.00 to 0.00)	0.00 (0.00 to 0.00)
Breast cancer	40 Years Since Quitting	Both	Female	18.29 (6.10 to 30.76)	18.29 (6.10 to 30.76)	18.29 (6.10 to 30.76)	18.29 (6.10 to 30.76)	18.29 (6.10 to 30.76)	18.29 (6.10 to 30.76)	18.29 (6.10 to 30.76)	18.29 (6.10 to 30.76)	18.29 (6.10 to 30.76)	18.29 (6.10 to 30.76)	18.29 (6.10 to 30.76)	18.29 (6.10 to 30.76)	18.29 (6.10 to 30.76)	18.29 (6.10 to 30.76)
Breast cancer	40 Years Since Quitting	Both	Male	0.00 (0.00 to 0.00)	0.00 (0.00 to 0.00)	0.00 (0.00 to 0.00)	0.00 (0.00 to 0.00)	0.00 (0.00 to 0.00)	0.00 (0.00 to 0.00)	0.00 (0.00 to 0.00)	0.00 (0.00 to 0.00)	0.00 (0.00 to 0.00)	0.00 (0.00 to 0.00)	0.00 (0.00 to 0.00)	0.00 (0.00 to 0.00)	0.00 (0.00 to 0.00)	0.00 (0.00 to 0.00)
Cervical cancer	0 Years Since Quitting	Both	Female	100.00 (100.00 to 100.00)	100.00 (100.00 to 100.00)	100.00 (100.00 to 100.00)	100.00 (100.00 to 100.00)	100.00 (100.00 to 100.00)	100.00 (100.00 to 100.00)	100.00 (100.00 to 100.00)	100.00 (100.00 to 100.00)	100.00 (100.00 to 100.00)	100.00 (100.00 to 100.00)	100.00 (100.00 to 100.00)	100.00 (100.00 to 100.00)	100.00 (100.00 to 100.00)	100.00 (100.00 to 100.00)
Cervical cancer	0 Years Since Quitting	Both	Male	0.00 (0.00 to 0.00)	0.00 (0.00 to 0.00)	0.00 (0.00 to 0.00)	0.00 (0.00 to 0.00)	0.00 (0.00 to 0.00)	0.00 (0.00 to 0.00)	0.00 (0.00 to 0.00)	0.00 (0.00 to 0.00)	0.00 (0.00 to 0.00)	0.00 (0.00 to 0.00)	0.00 (0.00 to 0.00)	0.00 (0.00 to 0.00)	0.00 (0.00 to 0.00)	0.00 (0.00 to 0.00)
Cervical cancer	10 Years Since Quitting	Both	Female	51.77 (35.06 to 70.65)	51.77 (35.06 to 70.65)	51.77 (35.06 to 70.65)	51.77 (35.06 to 70.65)	51.77 (35.06 to 70.65)	51.77 (35.06 to 70.65)	51.77 (35.06 to 70.65)	51.77 (35.06 to 70.65)	51.77 (35.06 to 70.65)	51.77 (35.06 to 70.65)	51.77 (35.06 to 70.65)	51.77 (35.06 to 70.65)	51.77 (35.06 to 70.65)	51.77 (35.06 to 70.65)
Cervical cancer	10 Years Since Quitting	Both	Male	0.00 (0.00 to 0.00)	0.00 (0.00 to 0.00)	0.00 (0.00 to 0.00)	0.00 (0.00 to 0.00)	0.00 (0.00 to 0.00)	0.00 (0.00 to 0.00)	0.00 (0.00 to 0.00)	0.00 (0.00 to 0.00)	0.00 (0.00 to 0.00)	0.00 (0.00 to 0.00)	0.00 (0.00 to 0.00)	0.00 (0.00 to 0.00)	0.00 (0.00 to 0.00)	0.00 (0.00 to 0.00)
Cervical cancer	20 Years Since Quitting	Both	Female	26.95 (10.84 to 44.25)	26.95 (10.84 to 44.25)	26.95 (10.84 to 44.25)	26.95 (10.84 to 44.25)	26.95 (10.84 to 44.25)	26.95 (10.84 to 44.25)	26.95 (10.84 to 44.25)	26.95 (10.84 to 44.25)	26.95 (10.84 to 44.25)	26.95 (10.84 to 44.25)	26.95 (10.84 to 44.25)	26.95 (10.84 to 44.25)	26.95 (10.84 to 44.25)	26.95 (10.84 to 44.25)
Cervical cancer	20 Years Since Quitting	Both	Male	0.00 (0.00 to 0.00)	0.00 (0.00 to 0.00)	0.00 (0.00 to 0.00)	0.00 (0.00 to 0.00)	0.00 (0.00 to 0.00)	0.00 (0.00 to 0.00)	0.00 (0.00 to 0.00)	0.00 (0.00 to 0.00)	0.00 (0.00 to 0.00)	0.00 (0.00 to 0.00)	0.00 (0.00 to 0.00)	0.00 (0.00 to 0.00)	0.00 (0.00 to 0.00)	0.00 (0.00 to 0.00)
Cervical cancer	30 Years Since Quitting	Both	Female	12.64 (0.00 to 28.41)	12.64 (0.00 to 28.41)	12.64 (0.00 to 28.41)	12.64 (0.00 to 28.41)	12.64 (0.00 to 28.41)	12.64 (0.00 to 28.41)	12.64 (0.00 to 28.41)	12.64 (0.00 to 28.41)	12.64 (0.00 to 28.41)	12.64 (0.00 to 28.41)	12.64 (0.00 to 28.41)	12.64 (0.00 to 28.41)	12.64 (0.00 to 28.41)	12.64 (0.00 to 28.41)
Cervical cancer	30 Years Since Quitting	Both	Male	0.00 (0.00 to 0.00)	0.00 (0.00 to 0.00)	0.00 (0.00 to 0.00)	0.00 (0.00 to 0.00)	0.00 (0.00 to 0.00)	0.00 (0.00 to 0.00)	0.00 (0.00 to 0.00)	0.00 (0.00 to 0.00)	0.00 (0.00 to 0.00)	0.00 (0.00 to 0.00)	0.00 (0.00 to 0.00)	0.00 (0.00 to 0.00)	0.00 (0.00 to 0.00)	0.00 (0.00 to 0.00)
Cervical cancer	40 Years Since Quitting	Both	Female	6.67 (0.00 to 20.08)	6.67 (0.00 to 20.08)	6.67 (0.00 to 20.08)	6.67 (0.00 to 20.08)	6.67 (0.00 to 20.08)	6.67 (0.00 to 20.08)	6.67 (0.00 to 20.08)	6.67 (0.00 to 20.08)	6.67 (0.00 to 20.08)	6.67 (0.00 to 20.08)	6.67 (0.00 to 20.08)	6.67 (0.00 to 20.08)	6.67 (0.00 to 20.08)	6.67 (0.00 to 20.08)
Cervical cancer	40 Years Since Quitting	Both	Male	0.00 (0.00 to 0.00)	0.00 (0.00 to 0.00)	0.00 (0.00 to 0.00)	0.00 (0.00 to 0.00)	0.00 (0.00 to 0.00)	0.00 (0.00 to 0.00)	0.00 (0.00 to 0.00)	0.00 (0.00 to 0.00)	0.00 (0.00 to 0.00)	0.00 (0.00 to 0.00)	0.00 (0.00 to 0.00)	0.00 (0.00 to 0.00)	0.00 (0.00 to 0.00)	0.00 (0.00 to 0.00)

Colon and rectum cancer	0 Years Since Quitting	Both	Female	100.00 (100.00 to 100.00)	100.00 (100.00 to 100.00)	100.00 (100.00 to 100.00)	100.00 (100.00 to 100.00)	100.00 (100.00 to 100.00)	100.00 (100.00 to 100.00)	100.00 (100.00 to 100.00)	100.00 (100.00 to 100.00)	100.00 (100.00 to 100.00)	100.00 (100.00 to 100.00)	100.00 (100.00 to 100.00)	100.00 (100.00 to 100.00)	100.00 (100.00 to 100.00)	100.00 (100.00 to 100.00)
Colon and rectum cancer	0 Years Since Quitting	Both	Male	100.00 (100.00 to 100.00)	100.00 (100.00 to 100.00)	100.00 (100.00 to 100.00)	100.00 (100.00 to 100.00)	100.00 (100.00 to 100.00)	100.00 (100.00 to 100.00)	100.00 (100.00 to 100.00)	100.00 (100.00 to 100.00)	100.00 (100.00 to 100.00)	100.00 (100.00 to 100.00)	100.00 (100.00 to 100.00)	100.00 (100.00 to 100.00)	100.00 (100.00 to 100.00)	100.00 (100.00 to 100.00)
Colon and rectum cancer	10 Years Since Quitting	Both	Female	74.91 (57.00 to 92.31)	74.91 (57.00 to 92.31)	74.91 (57.00 to 92.31)	74.91 (57.00 to 92.31)	74.91 (57.00 to 92.31)	74.91 (57.00 to 92.31)	74.91 (57.00 to 92.31)	74.91 (57.00 to 92.31)	74.91 (57.00 to 92.31)	74.91 (57.00 to 92.31)	74.91 (57.00 to 92.31)	74.91 (57.00 to 92.31)	74.91 (57.00 to 92.31)	74.91 (57.00 to 92.31)
Colon and rectum cancer	10 Years Since Quitting	Both	Male	74.91 (57.00 to 92.31)	74.91 (57.00 to 92.31)	74.91 (57.00 to 92.31)	74.91 (57.00 to 92.31)	74.91 (57.00 to 92.31)	74.91 (57.00 to 92.31)	74.91 (57.00 to 92.31)	74.91 (57.00 to 92.31)	74.91 (57.00 to 92.31)	74.91 (57.00 to 92.31)	74.91 (57.00 to 92.31)	74.91 (57.00 to 92.31)	74.91 (57.00 to 92.31)	74.91 (57.00 to 92.31)
Colon and rectum cancer	20 Years Since Quitting	Both	Female	62.09 (43.02 to 81.33)	62.09 (43.02 to 81.33)	62.09 (43.02 to 81.33)	62.09 (43.02 to 81.33)	62.09 (43.02 to 81.33)	62.09 (43.02 to 81.33)	62.09 (43.02 to 81.33)	62.09 (43.02 to 81.33)	62.09 (43.02 to 81.33)	62.09 (43.02 to 81.33)	62.09 (43.02 to 81.33)	62.09 (43.02 to 81.33)	62.09 (43.02 to 81.33)	62.09 (43.02 to 81.33)
Colon and rectum cancer	20 Years Since Quitting	Both	Male	62.09 (43.02 to 81.33)	62.09 (43.02 to 81.33)	62.09 (43.02 to 81.33)	62.09 (43.02 to 81.33)	62.09 (43.02 to 81.33)	62.09 (43.02 to 81.33)	62.09 (43.02 to 81.33)	62.09 (43.02 to 81.33)	62.09 (43.02 to 81.33)	62.09 (43.02 to 81.33)	62.09 (43.02 to 81.33)	62.09 (43.02 to 81.33)	62.09 (43.02 to 81.33)	62.09 (43.02 to 81.33)
Colon and rectum cancer	30 Years Since Quitting	Both	Female	20.21 (5.53 to 38.99)	20.21 (5.53 to 38.99)	20.21 (5.53 to 38.99)	20.21 (5.53 to 38.99)	20.21 (5.53 to 38.99)	20.21 (5.53 to 38.99)	20.21 (5.53 to 38.99)	20.21 (5.53 to 38.99)	20.21 (5.53 to 38.99)	20.21 (5.53 to 38.99)	20.21 (5.53 to 38.99)	20.21 (5.53 to 38.99)	20.21 (5.53 to 38.99)	20.21 (5.53 to 38.99)
Colon and rectum cancer	30 Years Since Quitting	Both	Male	20.21 (5.53 to 38.99)	20.21 (5.53 to 38.99)	20.21 (5.53 to 38.99)	20.21 (5.53 to 38.99)	20.21 (5.53 to 38.99)	20.21 (5.53 to 38.99)	20.21 (5.53 to 38.99)	20.21 (5.53 to 38.99)	20.21 (5.53 to 38.99)	20.21 (5.53 to 38.99)	20.21 (5.53 to 38.99)	20.21 (5.53 to 38.99)	20.21 (5.53 to 38.99)	20.21 (5.53 to 38.99)
Colon and rectum cancer	40 Years Since Quitting	Both	Female	8.71 (0.00 to 21.51)	8.71 (0.00 to 21.51)	8.71 (0.00 to 21.51)	8.71 (0.00 to 21.51)	8.71 (0.00 to 21.51)	8.71 (0.00 to 21.51)	8.71 (0.00 to 21.51)	8.71 (0.00 to 21.51)	8.71 (0.00 to 21.51)	8.71 (0.00 to 21.51)	8.71 (0.00 to 21.51)	8.71 (0.00 to 21.51)	8.71 (0.00 to 21.51)	8.71 (0.00 to 21.51)
Colon and rectum cancer	40 Years Since Quitting	Both	Male	8.71 (0.00 to 21.51)	8.71 (0.00 to 21.51)	8.71 (0.00 to 21.51)	8.71 (0.00 to 21.51)	8.71 (0.00 to 21.51)	8.71 (0.00 to 21.51)	8.71 (0.00 to 21.51)	8.71 (0.00 to 21.51)	8.71 (0.00 to 21.51)	8.71 (0.00 to 21.51)	8.71 (0.00 to 21.51)	8.71 (0.00 to 21.51)	8.71 (0.00 to 21.51)	8.71 (0.00 to 21.51)
Lip and oral cavity cancer	0 Years Since Quitting	Both	Female	100.00 (100.00 to 100.00)	100.00 (100.00 to 100.00)	100.00 (100.00 to 100.00)	100.00 (100.00 to 100.00)	100.00 (100.00 to 100.00)	100.00 (100.00 to 100.00)	100.00 (100.00 to 100.00)	100.00 (100.00 to 100.00)	100.00 (100.00 to 100.00)	100.00 (100.00 to 100.00)	100.00 (100.00 to 100.00)	100.00 (100.00 to 100.00)	100.00 (100.00 to 100.00)	100.00 (100.00 to 100.00)
Lip and oral cavity cancer	0 Years Since Quitting	Both	Male	100.00 (100.00 to 100.00)	100.00 (100.00 to 100.00)	100.00 (100.00 to 100.00)	100.00 (100.00 to 100.00)	100.00 (100.00 to 100.00)	100.00 (100.00 to 100.00)	100.00 (100.00 to 100.00)	100.00 (100.00 to 100.00)	100.00 (100.00 to 100.00)	100.00 (100.00 to 100.00)	100.00 (100.00 to 100.00)	100.00 (100.00 to 100.00)	100.00 (100.00 to 100.00)	100.00 (100.00 to 100.00)
Lip and oral cavity cancer	10 Years Since Quitting	Both	Female	25.29 (16.06 to 35.35)	25.29 (16.06 to 35.35)	25.29 (16.06 to 35.35)	25.29 (16.06 to 35.35)	25.29 (16.06 to 35.35)	25.29 (16.06 to 35.35)	25.29 (16.06 to 35.35)	25.29 (16.06 to 35.35)	25.29 (16.06 to 35.35)	25.29 (16.06 to 35.35)	25.29 (16.06 to 35.35)	25.29 (16.06 to 35.35)	25.29 (16.06 to 35.35)	25.29 (16.06 to 35.35)
Lip and oral cavity cancer	10 Years Since Quitting	Both	Male	25.29 (16.06 to 35.35)	25.29 (16.06 to 35.35)	25.29 (16.06 to 35.35)	25.29 (16.06 to 35.35)	25.29 (16.06 to 35.35)	25.29 (16.06 to 35.35)	25.29 (16.06 to 35.35)	25.29 (16.06 to 35.35)	25.29 (16.06 to 35.35)	25.29 (16.06 to 35.35)	25.29 (16.06 to 35.35)	25.29 (16.06 to 35.35)	25.29 (16.06 to 35.35)	25.29 (16.06 to 35.35)
Lip and oral cavity cancer	20 Years Since Quitting	Both	Female	9.19 (4.00 to 15.59)	9.19 (4.00 to 15.59)	9.19 (4.00 to 15.59)	9.19 (4.00 to 15.59)	9.19 (4.00 to 15.59)	9.19 (4.00 to 15.59)	9.19 (4.00 to 15.59)	9.19 (4.00 to 15.59)	9.19 (4.00 to 15.59)	9.19 (4.00 to 15.59)	9.19 (4.00 to 15.59)	9.19 (4.00 to 15.59)	9.19 (4.00 to 15.59)	9.19 (4.00 to 15.59)
Lip and oral cavity cancer	20 Years Since Quitting	Both	Male	9.19 (4.00 to 15.59)	9.19 (4.00 to 15.59)	9.19 (4.00 to 15.59)	9.19 (4.00 to 15.59)	9.19 (4.00 to 15.59)	9.19 (4.00 to 15.59)	9.19 (4.00 to 15.59)	9.19 (4.00 to 15.59)	9.19 (4.00 to 15.59)	9.19 (4.00 to 15.59)	9.19 (4.00 to 15.59)	9.19 (4.00 to 15.59)	9.19 (4.00 to 15.59)	9.19 (4.00 to 15.59)
Lip and oral cavity cancer	30 Years Since Quitting	Both	Female	4.96 (0.00 to 10.76)	4.96 (0.00 to 10.76)	4.96 (0.00 to 10.76)	4.96 (0.00 to 10.76)	4.96 (0.00 to 10.76)	4.96 (0.00 to 10.76)	4.96 (0.00 to 10.76)	4.96 (0.00 to 10.76)	4.96 (0.00 to 10.76)	4.96 (0.00 to 10.76)	4.96 (0.00 to 10.76)	4.96 (0.00 to 10.76)	4.96 (0.00 to 10.76)	4.96 (0.00 to 10.76)
Lip and oral cavity cancer	30 Years Since Quitting	Both	Male	4.96 (0.00 to 10.76)	4.96 (0.00 to 10.76)	4.96 (0.00 to 10.76)	4.96 (0.00 to 10.76)	4.96 (0.00 to 10.76)	4.96 (0.00 to 10.76)	4.96 (0.00 to 10.76)	4.96 (0.00 to 10.76)	4.96 (0.00 to 10.76)	4.96 (0.00 to 10.76)	4.96 (0.00 to 10.76)	4.96 (0.00 to 10.76)	4.96 (0.00 to 10.76)	4.96 (0.00 to 10.76)
Lip and oral cavity cancer	40 Years Since Quitting	Both	Female	1.63 (0.00 to 6.40)	1.63 (0.00 to 6.40)	1.63 (0.00 to 6.40)	1.63 (0.00 to 6.40)	1.63 (0.00 to 6.40)	1.63 (0.00 to 6.40)	1.63 (0.00 to 6.40)	1.63 (0.00 to 6.40)	1.63 (0.00 to 6.40)	1.63 (0.00 to 6.40)	1.63 (0.00 to 6.40)	1.63 (0.00 to 6.40)	1.63 (0.00 to 6.40)	1.63 (0.00 to 6.40)
Lip and oral cavity cancer	40 Years Since Quitting	Both	Male	1.63 (0.00 to 6.40)	1.63 (0.00 to 6.40)	1.63 (0.00 to 6.40)	1.63 (0.00 to 6.40)	1.63 (0.00 to 6.40)	1.63 (0.00 to 6.40)	1.63 (0.00 to 6.40)	1.63 (0.00 to 6.40)	1.63 (0.00 to 6.40)	1.63 (0.00 to 6.40)	1.63 (0.00 to 6.40)	1.63 (0.00 to 6.40)	1.63 (0.00 to 6.40)	1.63 (0.00 to 6.40)
Nasopharynx cancer	0 Years Since Quitting	Both	Female	100.00 (100.00 to 100.00)	100.00 (100.00 to 100.00)	100.00 (100.00 to 100.00)	100.00 (100.00 to 100.00)	100.00 (100.00 to 100.00)	100.00 (100.00 to 100.00)	100.00 (100.00 to 100.00)	100.00 (100.00 to 100.00)	100.00 (100.00 to 100.00)	100.00 (100.00 to 100.00)	100.00 (100.00 to 100.00)	100.00 (100.00 to 100.00)	100.00 (100.00 to 100.00)	100.00 (100.00 to 100.00)
Nasopharynx cancer	0 Years Since Quitting	Both	Male	100.00 (100.00 to 100.00)	100.00 (100.00 to 100.00)	100.00 (100.00 to 100.00)	100.00 (100.00 to 100.00)	100.00 (100.00 to 100.00)	100.00 (100.00 to 100.00)	100.00 (100.00 to 100.00)	100.00 (100.00 to 100.00)	100.00 (100.00 to 100.00)	100.00 (100.00 to 100.00)	100.00 (100.00 to 100.00)	100.00 (100.00 to 100.00)	100.00 (100.00 to 100.00)	100.00 (100.00 to 100.00)
Nasopharynx cancer	10 Years Since Quitting	Both	Female	44.72 (25.51 to 67.76)	44.72 (25.51 to 67.76)	44.72 (25.51 to 67.76)	44.72 (25.51 to 67.76)	44.72 (25.51 to 67.76)	44.72 (25.51 to 67.76)	44.72 (25.51 to 67.76)	44.72 (25.51 to 67.76)	44.72 (25.51 to 67.76)	44.72 (25.51 to 67.76)	44.72 (25.51 to 67.76)	44.72 (25.51 to 67.76)	44.72 (25.51 to 67.76)	44.72 (25.51 to 67.76)
Nasopharynx cancer	10 Years Since Quitting	Both	Male	44.72 (25.51 to 67.76)	44.72 (25.51 to 67.76)	44.72 (25.51 to 67.76)	44.72 (25.51 to 67.76)	44.72 (25.51 to 67.76)	44.72 (25.51 to 67.76)	44.72 (25.51 to 67.76)	44.72 (25.51 to 67.76)	44.72 (25.51 to 67.76)	44.72 (25.51 to 67.76)	44.72 (25.51 to 67.76)	44.72 (25.51 to 67.76)	44.72 (25.51 to 67.76)	44.72 (25.51 to 67.76)
Nasopharynx cancer	20 Years Since Quitting	Both	Female	22.58 (6.75 to 42.36)	22.58 (6.75 to 42.36)	22.58 (6.75 to 42.36)	22.58 (6.75 to 42.36)	22.58 (6.75 to 42.36)	22.58 (6.75 to 42.36)	22.58 (6.75 to 42.36)	22.58 (6.75 to 42.36)	22.58 (6.75 to 42.36)	22.58 (6.75 to 42.36)	22.58 (6.75 to 42.36)	22.58 (6.75 to 42.36)	22.58 (6.75 to 42.36)	22.58 (6.75 to 42.36)
Nasopharynx cancer	20 Years Since Quitting	Both	Male	22.58 (6.75 to 42.36)	22.58 (6.75 to 42.36)	22.58 (6.75 to 42.36)	22.58 (6.75 to 42.36)	22.58 (6.75 to 42.36)	22.58 (6.75 to 42.36)	22.58 (6.75 to 42.36)	22.58 (6.75 to 42.36)	22.58 (6.75 to 42.36)	22.58 (6.75 to 42.36)	22.58 (6.75 to 42.36)	22.58 (6.75 to 42.36)	22.58 (6.75 to 42.36)	22.58 (6.75 to 42.36)
Nasopharynx cancer	30 Years Since Quitting	Both	Female	14.18 (1.59 to 33.19)	14.18 (1.59 to 33.19)	14.18 (1.59 to 33.19)	14.18 (1.59 to 33.19)	14.18 (1.59 to 33.19)	14.18 (1.59 to 33.19)	14.18 (1.59 to 33.19)	14.18 (1.59 to 33.19)	14.18 (1.59 to 33.19)	14.18 (1.59 to 33.19)	14.18 (1.59 to 33.19)	14.18 (1.59 to 33.19)	14.18 (1.59 to 33.19)	14.18 (1.59 to 33.19)
Nasopharynx cancer	30 Years Since Quitting	Both	Male	14.18 (1.59 to 33.19)	14.18 (1.59 to 33.19)	14.18 (1.59 to 33.19)	14.18 (1.59 to 33.19)	14.18 (1.59 to 33.19)	14.18 (1.59 to 33.19)	14.18 (1.59 to 33.19)	14.18 (1.59 to 33.19)	14.18 (1.59 to 33.19)	14.18 (1.59 to 33.19)	14.18 (1.59 to 33.19)	14.18 (1.59 to 33.19)	14.18 (1.59 to 33.19)	14.18 (1.59 to 33.19)
Nasopharynx cancer	40 Years Since Quitting	Both	Female	9.42 (0.00 to 27.72)	9.42 (0.00 to 27.72)	9.42 (0.00 to 27.72)	9.42 (0.00 to 27.72)	9.42 (0.00 to 27.72)	9.42 (0.00 to 27.72)	9.42 (0.00 to 27.72)	9.42 (0.00 to 27.72)	9.42 (0.00 to 27.72)	9.42 (0.00 to 27.72)	9.42 (0.00 to 27.72)	9.42 (0.00 to 27.72)	9.42 (0.00 to 27.72)	9.42 (0.00 to 27.72)
Nasopharynx cancer	40 Years Since Quitting	Both	Male	9.42 (0.00 to 27.72)	9.42 (0.00 to 27.72)	9.42 (0.00 to 27.72)	9.42 (0.00 to 27.72)	9.42 (0.00 to 27.72)	9.42 (0.00 to 27.72)	9.42 (0.00 to 27.72)	9.42 (0.00 to 27.72)	9.42 (0.00 to 27.72)	9.42 (0.00 to 27.72)	9.42 (0.00 to 27.72)	9.42 (0.00 to 27.72)	9.42 (0.00 to 27.72)	9.42 (0.00 to 27.72)
Other pharynx cancer	0 Years Since Quitting	Both	Female	100.00 (100.00 to 100.00)	100.00 (100.00 to 100.00)	100.00 (100.00 to 100.00)	100.00 (100.00 to 100.00)	100.00 (100.00 to 100.00)	100.00 (100.00 to 100.00)	100.00 (100.00 to 100.00)	100.00 (100.00 to 100.00)	100.00 (100.00 to 100.00)	100.00 (100.00 to 100.00)	100.00 (100.00 to 100.00)	100.00 (100.00 to 100.00)	100.00 (100.00 to 100.00)	100.00 (100.00 to 100.00)
Other pharynx cancer	0 Years Since Quitting	Both	Male	100.00 (100.00 to 100.00)	100.00 (100.00 to 100.00)	100.00 (100.00 to 100.00)	100.00 (100.00 to 100.00)	100.00 (100.00 to 100.00)	100.00 (100.00 to 100.00)	100.00 (100.00 to 100.00)	100.00 (100.00 to 100.00)	100.00 (100.00 to 100.00)	100.00 (100.00 to 100.00)	100.00 (100.00 to 100.00)	100.00 (100.00 to 100.00)	100.00 (100.00 to 100.00)	100.00 (100.00 to 100.00)
Other pharynx cancer	10 Years Since Quitting	Both	Female	35.16 (23.59 to 50.30)	35.16 (23.59 to 50.30)	35.16 (23.59 to 50.30)	35.16 (23.59 to 50.30)	35.16 (23.59 to 50.30)	35.16 (23.59 to 50.30)	35.16 (23.59 to 50.30)	35.16 (23.59 to 50.30)	35.16 (23.59 to 50.30)	35.16 (23.59 to 50.30)	35.16 (23.59 to 50.30)	35.16 (23.59 to 50.30)	35.16 (23.59 to 50.30)	35.16 (23.59 to 50.30)
Other pharynx cancer	10 Years Since Quitting	Both	Male	35.16 (23.59 to 50.30)	35.16 (23.59 to 50.30)	35.16 (23.59 to 50.30)	35.16 (23.59 to 50.30)	35.16 (23.59 to 50.30)	35.16 (23.59 to 50.30)	35.16 (23.59 to 50.30)	35.16 (23.59 to 50.30)	35.16 (23.59 to 50.30)	35.16 (23.59 to 50.30)	35.16 (23.59 to 50.30)	35.16 (23.59 to 50.30)	35.16 (23.59 to 50.30)	35.16 (23.59 to 50.30)
Other pharynx cancer	20 Years Since Quitting	Both	Female	20.40 (10.88 to 31.83)	20.40 (10.88 to 31.83)	20.40 (10.88 to 31.83)	20.40 (10.88 to 31.83)	20.40 (10.88 to 31.83)	20.40 (10.88 to 31.83)	20.40 (10.88 to 31.83)	20.40 (10.88 to 31.83)	20.40 (10.88 to 31.83)	20.40 (10.88 to 31.83)	20.40 (10.88 to 31.83)	20.40 (10.88 to 31.83)	20.40 (10.88 to 31.83)	20.40 (10.88 to 31.83)
Other pharynx cancer	20 Years Since Quitting	Both	Male	20.40 (10.88 to 31.83)	20.40 (10.88 to 31.83)	20.40 (10.88 to 31.83)	20.40 (10.88 to 31.83)	20.40 (10.88 to 31.83)	20.40 (10.88 to 31.83)	20.40 (10.88 to 31.83)	20.40 (10.88 to 31.83)	20.40 (10.88 to 31.83)	20.40 (10.88 to 31.83)	20.40 (10.88 to 31.83)	20.40 (10.88 to 31.83)	20.40 (10.88 to 31.83)	20.40 (10.88 to 31.83)

Other pharynx cancer	30 Years Since Quitting	Both	Female	10.29 (0.36 to 21.58)	10.29 (0.36 to 21.58)	10.29 (0.36 to 21.58)	10.29 (0.36 to 21.58)	10.29 (0.36 to 21.58)	10.29 (0.36 to 21.58)	10.29 (0.36 to 21.58)	10.29 (0.36 to 21.58)	10.29 (0.36 to 21.58)	10.29 (0.36 to 21.58)	10.29 (0.36 to 21.58)	10.29 (0.36 to 21.58)	10.29 (0.36 to 21.58)	10.29 (0.36 to 21.58)
Other pharynx cancer	30 Years Since Quitting	Both	Male	10.29 (0.36 to 21.58)	10.29 (0.36 to 21.58)	10.29 (0.36 to 21.58)	10.29 (0.36 to 21.58)	10.29 (0.36 to 21.58)	10.29 (0.36 to 21.58)	10.29 (0.36 to 21.58)	10.29 (0.36 to 21.58)	10.29 (0.36 to 21.58)	10.29 (0.36 to 21.58)	10.29 (0.36 to 21.58)	10.29 (0.36 to 21.58)	10.29 (0.36 to 21.58)	10.29 (0.36 to 21.58)
Other pharynx cancer	40 Years Since Quitting	Both	Female	5.36 (0.00 to 16.01)	5.36 (0.00 to 16.01)	5.36 (0.00 to 16.01)	5.36 (0.00 to 16.01)	5.36 (0.00 to 16.01)	5.36 (0.00 to 16.01)	5.36 (0.00 to 16.01)	5.36 (0.00 to 16.01)	5.36 (0.00 to 16.01)	5.36 (0.00 to 16.01)	5.36 (0.00 to 16.01)	5.36 (0.00 to 16.01)	5.36 (0.00 to 16.01)	5.36 (0.00 to 16.01)
Other pharynx cancer	40 Years Since Quitting	Both	Male	5.36 (0.00 to 16.01)	5.36 (0.00 to 16.01)	5.36 (0.00 to 16.01)	5.36 (0.00 to 16.01)	5.36 (0.00 to 16.01)	5.36 (0.00 to 16.01)	5.36 (0.00 to 16.01)	5.36 (0.00 to 16.01)	5.36 (0.00 to 16.01)	5.36 (0.00 to 16.01)	5.36 (0.00 to 16.01)	5.36 (0.00 to 16.01)	5.36 (0.00 to 16.01)	5.36 (0.00 to 16.01)
Pancreatic cancer	0 Years Since Quitting	Both	Female	100.00 (100.00 to 100.00)	100.00 (100.00 to 100.00)	100.00 (100.00 to 100.00)	100.00 (100.00 to 100.00)	100.00 (100.00 to 100.00)	100.00 (100.00 to 100.00)	100.00 (100.00 to 100.00)	100.00 (100.00 to 100.00)	100.00 (100.00 to 100.00)	100.00 (100.00 to 100.00)	100.00 (100.00 to 100.00)	100.00 (100.00 to 100.00)	100.00 (100.00 to 100.00)	100.00 (100.00 to 100.00)
Pancreatic cancer	0 Years Since Quitting	Both	Male	100.00 (100.00 to 100.00)	100.00 (100.00 to 100.00)	100.00 (100.00 to 100.00)	100.00 (100.00 to 100.00)	100.00 (100.00 to 100.00)	100.00 (100.00 to 100.00)	100.00 (100.00 to 100.00)	100.00 (100.00 to 100.00)	100.00 (100.00 to 100.00)	100.00 (100.00 to 100.00)	100.00 (100.00 to 100.00)	100.00 (100.00 to 100.00)	100.00 (100.00 to 100.00)	100.00 (100.00 to 100.00)
Pancreatic cancer	10 Years Since Quitting	Both	Female	40.80 (30.51 to 52.76)	40.80 (30.51 to 52.76)	40.80 (30.51 to 52.76)	40.80 (30.51 to 52.76)	40.80 (30.51 to 52.76)	40.80 (30.51 to 52.76)	40.80 (30.51 to 52.76)	40.80 (30.51 to 52.76)	40.80 (30.51 to 52.76)	40.80 (30.51 to 52.76)	40.80 (30.51 to 52.76)	40.80 (30.51 to 52.76)	40.80 (30.51 to 52.76)	40.80 (30.51 to 52.76)
Pancreatic cancer	10 Years Since Quitting	Both	Male	40.80 (30.51 to 52.76)	40.80 (30.51 to 52.76)	40.80 (30.51 to 52.76)	40.80 (30.51 to 52.76)	40.80 (30.51 to 52.76)	40.80 (30.51 to 52.76)	40.80 (30.51 to 52.76)	40.80 (30.51 to 52.76)	40.80 (30.51 to 52.76)	40.80 (30.51 to 52.76)	40.80 (30.51 to 52.76)	40.80 (30.51 to 52.76)	40.80 (30.51 to 52.76)	40.80 (30.51 to 52.76)
Pancreatic cancer	20 Years Since Quitting	Both	Female	27.78 (18.94 to 37.49)	27.78 (18.94 to 37.49)	27.78 (18.94 to 37.49)	27.78 (18.94 to 37.49)	27.78 (18.94 to 37.49)	27.78 (18.94 to 37.49)	27.78 (18.94 to 37.49)	27.78 (18.94 to 37.49)	27.78 (18.94 to 37.49)	27.78 (18.94 to 37.49)	27.78 (18.94 to 37.49)	27.78 (18.94 to 37.49)	27.78 (18.94 to 37.49)	27.78 (18.94 to 37.49)
Pancreatic cancer	20 Years Since Quitting	Both	Male	27.78 (18.94 to 37.49)	27.78 (18.94 to 37.49)	27.78 (18.94 to 37.49)	27.78 (18.94 to 37.49)	27.78 (18.94 to 37.49)	27.78 (18.94 to 37.49)	27.78 (18.94 to 37.49)	27.78 (18.94 to 37.49)	27.78 (18.94 to 37.49)	27.78 (18.94 to 37.49)	27.78 (18.94 to 37.49)	27.78 (18.94 to 37.49)	27.78 (18.94 to 37.49)	27.78 (18.94 to 37.49)
Pancreatic cancer	30 Years Since Quitting	Both	Female	17.12 (8.23 to 26.17)	17.12 (8.23 to 26.17)	17.12 (8.23 to 26.17)	17.12 (8.23 to 26.17)	17.12 (8.23 to 26.17)	17.12 (8.23 to 26.17)	17.12 (8.23 to 26.17)	17.12 (8.23 to 26.17)	17.12 (8.23 to 26.17)	17.12 (8.23 to 26.17)	17.12 (8.23 to 26.17)	17.12 (8.23 to 26.17)	17.12 (8.23 to 26.17)	17.12 (8.23 to 26.17)
Pancreatic cancer	30 Years Since Quitting	Both	Male	17.12 (8.23 to 26.17)	17.12 (8.23 to 26.17)	17.12 (8.23 to 26.17)	17.12 (8.23 to 26.17)	17.12 (8.23 to 26.17)	17.12 (8.23 to 26.17)	17.12 (8.23 to 26.17)	17.12 (8.23 to 26.17)	17.12 (8.23 to 26.17)	17.12 (8.23 to 26.17)	17.12 (8.23 to 26.17)	17.12 (8.23 to 26.17)	17.12 (8.23 to 26.17)	17.12 (8.23 to 26.17)
Pancreatic cancer	40 Years Since Quitting	Both	Female	9.23 (1.26 to 19.01)	9.23 (1.26 to 19.01)	9.23 (1.26 to 19.01)	9.23 (1.26 to 19.01)	9.23 (1.26 to 19.01)	9.23 (1.26 to 19.01)	9.23 (1.26 to 19.01)	9.23 (1.26 to 19.01)	9.23 (1.26 to 19.01)	9.23 (1.26 to 19.01)	9.23 (1.26 to 19.01)	9.23 (1.26 to 19.01)	9.23 (1.26 to 19.01)	9.23 (1.26 to 19.01)
Pancreatic cancer	40 Years Since Quitting	Both	Male	9.23 (1.26 to 19.01)	9.23 (1.26 to 19.01)	9.23 (1.26 to 19.01)	9.23 (1.26 to 19.01)	9.23 (1.26 to 19.01)	9.23 (1.26 to 19.01)	9.23 (1.26 to 19.01)	9.23 (1.26 to 19.01)	9.23 (1.26 to 19.01)	9.23 (1.26 to 19.01)	9.23 (1.26 to 19.01)	9.23 (1.26 to 19.01)	9.23 (1.26 to 19.01)	9.23 (1.26 to 19.01)
Bladder cancer	0 Years Since Quitting	Both	Female	100.00 (100.00 to 100.00)	100.00 (100.00 to 100.00)	100.00 (100.00 to 100.00)	100.00 (100.00 to 100.00)	100.00 (100.00 to 100.00)	100.00 (100.00 to 100.00)	100.00 (100.00 to 100.00)	100.00 (100.00 to 100.00)	100.00 (100.00 to 100.00)	100.00 (100.00 to 100.00)	100.00 (100.00 to 100.00)	100.00 (100.00 to 100.00)	100.00 (100.00 to 100.00)	100.00 (100.00 to 100.00)
Bladder cancer	0 Years Since Quitting	Both	Male	100.00 (100.00 to 100.00)	100.00 (100.00 to 100.00)	100.00 (100.00 to 100.00)	100.00 (100.00 to 100.00)	100.00 (100.00 to 100.00)	100.00 (100.00 to 100.00)	100.00 (100.00 to 100.00)	100.00 (100.00 to 100.00)	100.00 (100.00 to 100.00)	100.00 (100.00 to 100.00)	100.00 (100.00 to 100.00)	100.00 (100.00 to 100.00)	100.00 (100.00 to 100.00)	100.00 (100.00 to 100.00)
Bladder cancer	10 Years Since Quitting	Both	Female	51.77 (36.36 to 70.40)	51.77 (36.36 to 70.40)	51.77 (36.36 to 70.40)	51.77 (36.36 to 70.40)	51.77 (36.36 to 70.40)	51.77 (36.36 to 70.40)	51.77 (36.36 to 70.40)	51.77 (36.36 to 70.40)	51.77 (36.36 to 70.40)	51.77 (36.36 to 70.40)	51.77 (36.36 to 70.40)	51.77 (36.36 to 70.40)	51.77 (36.36 to 70.40)	51.77 (36.36 to 70.40)
Bladder cancer	10 Years Since Quitting	Both	Male	51.77 (36.36 to 70.40)	51.77 (36.36 to 70.40)	51.77 (36.36 to 70.40)	51.77 (36.36 to 70.40)	51.77 (36.36 to 70.40)	51.77 (36.36 to 70.40)	51.77 (36.36 to 70.40)	51.77 (36.36 to 70.40)	51.77 (36.36 to 70.40)	51.77 (36.36 to 70.40)	51.77 (36.36 to 70.40)	51.77 (36.36 to 70.40)	51.77 (36.36 to 70.40)	51.77 (36.36 to 70.40)
Bladder cancer	20 Years Since Quitting	Both	Female	23.74 (14.19 to 35.39)	23.74 (14.19 to 35.39)	23.74 (14.19 to 35.39)	23.74 (14.19 to 35.39)	23.74 (14.19 to 35.39)	23.74 (14.19 to 35.39)	23.74 (14.19 to 35.39)	23.74 (14.19 to 35.39)	23.74 (14.19 to 35.39)	23.74 (14.19 to 35.39)	23.74 (14.19 to 35.39)	23.74 (14.19 to 35.39)	23.74 (14.19 to 35.39)	23.74 (14.19 to 35.39)
Bladder cancer	20 Years Since Quitting	Both	Male	23.74 (14.19 to 35.39)	23.74 (14.19 to 35.39)	23.74 (14.19 to 35.39)	23.74 (14.19 to 35.39)	23.74 (14.19 to 35.39)	23.74 (14.19 to 35.39)	23.74 (14.19 to 35.39)	23.74 (14.19 to 35.39)	23.74 (14.19 to 35.39)	23.74 (14.19 to 35.39)	23.74 (14.19 to 35.39)	23.74 (14.19 to 35.39)	23.74 (14.19 to 35.39)	23.74 (14.19 to 35.39)
Bladder cancer	30 Years Since Quitting	Both	Female	7.79 (0.29 to 16.84)	7.79 (0.29 to 16.84)	7.79 (0.29 to 16.84)	7.79 (0.29 to 16.84)	7.79 (0.29 to 16.84)	7.79 (0.29 to 16.84)	7.79 (0.29 to 16.84)	7.79 (0.29 to 16.84)	7.79 (0.29 to 16.84)	7.79 (0.29 to 16.84)	7.79 (0.29 to 16.84)	7.79 (0.29 to 16.84)	7.79 (0.29 to 16.84)	7.79 (0.29 to 16.84)
Bladder cancer	30 Years Since Quitting	Both	Male	7.79 (0.29 to 16.84)	7.79 (0.29 to 16.84)	7.79 (0.29 to 16.84)	7.79 (0.29 to 16.84)	7.79 (0.29 to 16.84)	7.79 (0.29 to 16.84)	7.79 (0.29 to 16.84)	7.79 (0.29 to 16.84)	7.79 (0.29 to 16.84)	7.79 (0.29 to 16.84)	7.79 (0.29 to 16.84)	7.79 (0.29 to 16.84)	7.79 (0.29 to 16.84)	7.79 (0.29 to 16.84)
Bladder cancer	40 Years Since Quitting	Both	Female	2.92 (0.00 to 9.78)	2.92 (0.00 to 9.78)	2.92 (0.00 to 9.78)	2.92 (0.00 to 9.78)	2.92 (0.00 to 9.78)	2.92 (0.00 to 9.78)	2.92 (0.00 to 9.78)	2.92 (0.00 to 9.78)	2.92 (0.00 to 9.78)	2.92 (0.00 to 9.78)	2.92 (0.00 to 9.78)	2.92 (0.00 to 9.78)	2.92 (0.00 to 9.78)	2.92 (0.00 to 9.78)
Bladder cancer	40 Years Since Quitting	Both	Male	2.92 (0.00 to 9.78)	2.92 (0.00 to 9.78)	2.92 (0.00 to 9.78)	2.92 (0.00 to 9.78)	2.92 (0.00 to 9.78)	2.92 (0.00 to 9.78)	2.92 (0.00 to 9.78)	2.92 (0.00 to 9.78)	2.92 (0.00 to 9.78)	2.92 (0.00 to 9.78)	2.92 (0.00 to 9.78)	2.92 (0.00 to 9.78)	2.92 (0.00 to 9.78)	2.92 (0.00 to 9.78)
Kidney cancer	0 Years Since Quitting	Both	Female	100.00 (100.00 to 100.00)	100.00 (100.00 to 100.00)	100.00 (100.00 to 100.00)	100.00 (100.00 to 100.00)	100.00 (100.00 to 100.00)	100.00 (100.00 to 100.00)	100.00 (100.00 to 100.00)	100.00 (100.00 to 100.00)	100.00 (100.00 to 100.00)	100.00 (100.00 to 100.00)	100.00 (100.00 to 100.00)	100.00 (100.00 to 100.00)	100.00 (100.00 to 100.00)	100.00 (100.00 to 100.00)
Kidney cancer	0 Years Since Quitting	Both	Male	100.00 (100.00 to 100.00)	100.00 (100.00 to 100.00)	100.00 (100.00 to 100.00)	100.00 (100.00 to 100.00)	100.00 (100.00 to 100.00)	100.00 (100.00 to 100.00)	100.00 (100.00 to 100.00)	100.00 (100.00 to 100.00)	100.00 (100.00 to 100.00)	100.00 (100.00 to 100.00)	100.00 (100.00 to 100.00)	100.00 (100.00 to 100.00)	100.00 (100.00 to 100.00)	100.00 (100.00 to 100.00)
Kidney cancer	10 Years Since Quitting	Both	Female	64.19 (40.71 to 87.50)	64.19 (40.71 to 87.50)	64.19 (40.71 to 87.50)	64.19 (40.71 to 87.50)	64.19 (40.71 to 87.50)	64.19 (40.71 to 87.50)	64.19 (40.71 to 87.50)	64.19 (40.71 to 87.50)	64.19 (40.71 to 87.50)	64.19 (40.71 to 87.50)	64.19 (40.71 to 87.50)	64.19 (40.71 to 87.50)	64.19 (40.71 to 87.50)	64.19 (40.71 to 87.50)
Kidney cancer	10 Years Since Quitting	Both	Male	64.19 (40.71 to 87.50)	64.19 (40.71 to 87.50)	64.19 (40.71 to 87.50)	64.19 (40.71 to 87.50)	64.19 (40.71 to 87.50)	64.19 (40.71 to 87.50)	64.19 (40.71 to 87.50)	64.19 (40.71 to 87.50)	64.19 (40.71 to 87.50)	64.19 (40.71 to 87.50)	64.19 (40.71 to 87.50)	64.19 (40.71 to 87.50)	64.19 (40.71 to 87.50)	64.19 (40.71 to 87.50)
Kidney cancer	20 Years Since Quitting	Both	Female	47.31 (25.91 to 71.83)	47.31 (25.91 to 71.83)	47.31 (25.91 to 71.83)	47.31 (25.91 to 71.83)	47.31 (25.91 to 71.83)	47.31 (25.91 to 71.83)	47.31 (25.91 to 71.83)	47.31 (25.91 to 71.83)	47.31 (25.91 to 71.83)	47.31 (25.91 to 71.83)	47.31 (25.91 to 71.83)	47.31 (25.91 to 71.83)	47.31 (25.91 to 71.83)	47.31 (25.91 to 71.83)
Kidney cancer	20 Years Since Quitting	Both	Male	47.31 (25.91 to 71.83)	47.31 (25.91 to 71.83)	47.31 (25.91 to 71.83)	47.31 (25.91 to 71.83)	47.31 (25.91 to 71.83)	47.31 (25.91 to 71.83)	47.31 (25.91 to 71.83)	47.31 (25.91 to 71.83)	47.31 (25.91 to 71.83)	47.31 (25.91 to 71.83)	47.31 (25.91 to 71.83)	47.31 (25.91 to 71.83)	47.31 (25.91 to 71.83)	47.31 (25.91 to 71.83)
Kidney cancer	30 Years Since Quitting	Both	Female	29.87 (9.57 to 53.18)	29.87 (9.57 to 53.18)	29.87 (9.57 to 53.18)	29.87 (9.57 to 53.18)	29.87 (9.57 to 53.18)	29.87 (9.57 to 53.18)	29.87 (9.57 to 53.18)	29.87 (9.57 to 53.18)	29.87 (9.57 to 53.18)	29.87 (9.57 to 53.18)	29.87 (9.57 to 53.18)	29.87 (9.57 to 53.18)	29.87 (9.57 to 53.18)	29.87 (9.57 to 53.18)
Kidney cancer	30 Years Since Quitting	Both	Male	29.87 (9.57 to 53.18)	29.87 (9.57 to 53.18)	29.87 (9.57 to 53.18)	29.87 (9.57 to 53.18)	29.87 (9.57 to 53.18)	29.87 (9.57 to 53.18)	29.87 (9.57 to 53.18)	29.87 (9.57 to 53.18)	29.87 (9.57 to 53.18)	29.87 (9.57 to 53.18)	29.87 (9.57 to 53.18)	29.87 (9.57 to 53.18)	29.87 (9.57 to 53.18)	29.87 (9.57 to 53.18)
Kidney cancer	40 Years Since Quitting	Both	Female	16.49 (0.00 to 37.81)	16.49 (0.00 to 37.81)	16.49 (0.00 to 37.81)	16.49 (0.00 to 37.81)	16.49 (0.00 to 37.81)	16.49 (0.00 to 37.81)	16.49 (0.00 to 37.81)	16.49 (0.00 to 37.81)	16.49 (0.00 to 37.81)	16.49 (0.00 to 37.81)	16.49 (0.00 to 37.81)	16.49 (0.00 to 37.81)	16.49 (0.00 to 37.81)	16.49 (0.00 to 37.81)
Kidney cancer	40 Years Since Quitting	Both	Male	16.49 (0.00 to 37.81)	16.49 (0.00 to 37.81)	16.49 (0.00 to 37.81)	16.49 (0.00 to 37.81)	16.49 (0.00 to 37.81)	16.49 (0.00 to 37.81)	16.49 (0.00 to 37.81)	16.49 (0.00 to 37.81)	16.49 (0.00 to 37.81)	16.49 (0.00 to 37.81)	16.49 (0.00 to 37.81)	16.49 (0.00 to 37.81)	16.49 (0.00 to 37.81)	16.49 (0.00 to 37.81)
Leukaemia	0 Years Since Quitting	Both	Female	100.00 (100.00 to 100.00)	100.00 (100.00 to 100.00)	100.00 (100.00 to 100.00)	100.00 (100.00 to 100.00)	100.00 (100.00 to 100.00)	100.00 (100.00 to 100.00)	100.00 (100.00 to 100.00)	100.00 (100.00 to 100.00)	100.00 (100.00 to 100.00)	100.00 (100.00 to 100.00)	100.00 (100.00 to 100.00)	100.00 (100.00 to 100.00)	100.00 (100.00 to 100.00)	100.00 (100.00 to 100.00)
Leukaemia	0 Years Since Quitting	Both	Male	100.00 (100.00 to 100.00)	100.00 (100.00 to 100.00)	100.00 (100.00 to 100.00)	100.00 (100.00 to 100.00)	100.00 (100.00 to 100.00)	100.00 (100.00 to 100.00)	100.00 (100.00 to 100.00)	100.00 (100.00 to 100.00)	100.00 (100.00 to 100.00)	100.00 (100.00 to 100.00)	100.00 (100.00 to 100.00)	100.00 (100.00 to 100.00)	100.00 (100.00 to 100.00)	100.00 (100.00 to 100.00)

Leukaemia	10 Years Since Quitting	Both	Female	58.45 (38.43 to 81.38)	58.45 (38.43 to 81.38)	58.45 (38.43 to 81.38)	58.45 (38.43 to 81.38)	58.45 (38.43 to 81.38)	58.45 (38.43 to 81.38)	58.45 (38.43 to 81.38)	58.45 (38.43 to 81.38)	58.45 (38.43 to 81.38)	58.45 (38.43 to 81.38)	58.45 (38.43 to 81.38)	58.45 (38.43 to 81.38)	58.45 (38.43 to 81.38)	58.45 (38.43 to 81.38)
Leukaemia	10 Years Since Quitting	Both	Male	58.45 (38.43 to 81.38)	58.45 (38.43 to 81.38)	58.45 (38.43 to 81.38)	58.45 (38.43 to 81.38)	58.45 (38.43 to 81.38)	58.45 (38.43 to 81.38)	58.45 (38.43 to 81.38)	58.45 (38.43 to 81.38)	58.45 (38.43 to 81.38)	58.45 (38.43 to 81.38)	58.45 (38.43 to 81.38)	58.45 (38.43 to 81.38)	58.45 (38.43 to 81.38)	58.45 (38.43 to 81.38)
Leukaemia	20 Years Since Quitting	Both	Female	43.29 (26.46 to 63.64)	43.29 (26.46 to 63.64)	43.29 (26.46 to 63.64)	43.29 (26.46 to 63.64)	43.29 (26.46 to 63.64)	43.29 (26.46 to 63.64)	43.29 (26.46 to 63.64)	43.29 (26.46 to 63.64)	43.29 (26.46 to 63.64)	43.29 (26.46 to 63.64)	43.29 (26.46 to 63.64)	43.29 (26.46 to 63.64)	43.29 (26.46 to 63.64)	43.29 (26.46 to 63.64)
Leukaemia	20 Years Since Quitting	Both	Male	43.29 (26.46 to 63.64)	43.29 (26.46 to 63.64)	43.29 (26.46 to 63.64)	43.29 (26.46 to 63.64)	43.29 (26.46 to 63.64)	43.29 (26.46 to 63.64)	43.29 (26.46 to 63.64)	43.29 (26.46 to 63.64)	43.29 (26.46 to 63.64)	43.29 (26.46 to 63.64)	43.29 (26.46 to 63.64)	43.29 (26.46 to 63.64)	43.29 (26.46 to 63.64)	43.29 (26.46 to 63.64)
Leukaemia	30 Years Since Quitting	Both	Female	27.03 (9.78 to 47.88)	27.03 (9.78 to 47.88)	27.03 (9.78 to 47.88)	27.03 (9.78 to 47.88)	27.03 (9.78 to 47.88)	27.03 (9.78 to 47.88)	27.03 (9.78 to 47.88)	27.03 (9.78 to 47.88)	27.03 (9.78 to 47.88)	27.03 (9.78 to 47.88)	27.03 (9.78 to 47.88)	27.03 (9.78 to 47.88)	27.03 (9.78 to 47.88)	27.03 (9.78 to 47.88)
Leukaemia	30 Years Since Quitting	Both	Male	27.03 (9.78 to 47.88)	27.03 (9.78 to 47.88)	27.03 (9.78 to 47.88)	27.03 (9.78 to 47.88)	27.03 (9.78 to 47.88)	27.03 (9.78 to 47.88)	27.03 (9.78 to 47.88)	27.03 (9.78 to 47.88)	27.03 (9.78 to 47.88)	27.03 (9.78 to 47.88)	27.03 (9.78 to 47.88)	27.03 (9.78 to 47.88)	27.03 (9.78 to 47.88)	27.03 (9.78 to 47.88)
Leukaemia	40 Years Since Quitting	Both	Female	15.12 (1.76 to 33.93)	15.12 (1.76 to 33.93)	15.12 (1.76 to 33.93)	15.12 (1.76 to 33.93)	15.12 (1.76 to 33.93)	15.12 (1.76 to 33.93)	15.12 (1.76 to 33.93)	15.12 (1.76 to 33.93)	15.12 (1.76 to 33.93)	15.12 (1.76 to 33.93)	15.12 (1.76 to 33.93)	15.12 (1.76 to 33.93)	15.12 (1.76 to 33.93)	15.12 (1.76 to 33.93)
Leukaemia	40 Years Since Quitting	Both	Male	15.12 (1.76 to 33.93)	15.12 (1.76 to 33.93)	15.12 (1.76 to 33.93)	15.12 (1.76 to 33.93)	15.12 (1.76 to 33.93)	15.12 (1.76 to 33.93)	15.12 (1.76 to 33.93)	15.12 (1.76 to 33.93)	15.12 (1.76 to 33.93)	15.12 (1.76 to 33.93)	15.12 (1.76 to 33.93)	15.12 (1.76 to 33.93)	15.12 (1.76 to 33.93)	15.12 (1.76 to 33.93)
Cataract	0 Years Since Quitting	Both	Female	100.00 (100.00 to 100.00)	100.00 (100.00 to 100.00)	100.00 (100.00 to 100.00)	100.00 (100.00 to 100.00)	100.00 (100.00 to 100.00)	100.00 (100.00 to 100.00)	100.00 (100.00 to 100.00)	100.00 (100.00 to 100.00)	100.00 (100.00 to 100.00)	100.00 (100.00 to 100.00)	100.00 (100.00 to 100.00)	100.00 (100.00 to 100.00)	100.00 (100.00 to 100.00)	100.00 (100.00 to 100.00)
Cataract	0 Years Since Quitting	Both	Male	100.00 (100.00 to 100.00)	100.00 (100.00 to 100.00)	100.00 (100.00 to 100.00)	100.00 (100.00 to 100.00)	100.00 (100.00 to 100.00)	100.00 (100.00 to 100.00)	100.00 (100.00 to 100.00)	100.00 (100.00 to 100.00)	100.00 (100.00 to 100.00)	100.00 (100.00 to 100.00)	100.00 (100.00 to 100.00)	100.00 (100.00 to 100.00)	100.00 (100.00 to 100.00)	100.00 (100.00 to 100.00)
Cataract	10 Years Since Quitting	Both	Female	52.96 (34.11 to 75.60)	52.96 (34.11 to 75.60)	52.96 (34.11 to 75.60)	52.96 (34.11 to 75.60)	52.96 (34.11 to 75.60)	52.96 (34.11 to 75.60)	52.96 (34.11 to 75.60)	52.96 (34.11 to 75.60)	52.96 (34.11 to 75.60)	52.96 (34.11 to 75.60)	52.96 (34.11 to 75.60)	52.96 (34.11 to 75.60)	52.96 (34.11 to 75.60)	52.96 (34.11 to 75.60)
Cataract	10 Years Since Quitting	Both	Male	52.96 (34.11 to 75.60)	52.96 (34.11 to 75.60)	52.96 (34.11 to 75.60)	52.96 (34.11 to 75.60)	52.96 (34.11 to 75.60)	52.96 (34.11 to 75.60)	52.96 (34.11 to 75.60)	52.96 (34.11 to 75.60)	52.96 (34.11 to 75.60)	52.96 (34.11 to 75.60)	52.96 (34.11 to 75.60)	52.96 (34.11 to 75.60)	52.96 (34.11 to 75.60)	52.96 (34.11 to 75.60)
Cataract	20 Years Since Quitting	Both	Female	30.22 (16.05 to 49.75)	30.22 (16.05 to 49.75)	30.22 (16.05 to 49.75)	30.22 (16.05 to 49.75)	30.22 (16.05 to 49.75)	30.22 (16.05 to 49.75)	30.22 (16.05 to 49.75)	30.22 (16.05 to 49.75)	30.22 (16.05 to 49.75)	30.22 (16.05 to 49.75)	30.22 (16.05 to 49.75)	30.22 (16.05 to 49.75)	30.22 (16.05 to 49.75)	30.22 (16.05 to 49.75)
Cataract	20 Years Since Quitting	Both	Male	30.22 (16.05 to 49.75)	30.22 (16.05 to 49.75)	30.22 (16.05 to 49.75)	30.22 (16.05 to 49.75)	30.22 (16.05 to 49.75)	30.22 (16.05 to 49.75)	30.22 (16.05 to 49.75)	30.22 (16.05 to 49.75)	30.22 (16.05 to 49.75)	30.22 (16.05 to 49.75)	30.22 (16.05 to 49.75)	30.22 (16.05 to 49.75)	30.22 (16.05 to 49.75)	30.22 (16.05 to 49.75)
Cataract	30 Years Since Quitting	Both	Female	15.11 (2.35 to 31.57)	15.11 (2.35 to 31.57)	15.11 (2.35 to 31.57)	15.11 (2.35 to 31.57)	15.11 (2.35 to 31.57)	15.11 (2.35 to 31.57)	15.11 (2.35 to 31.57)	15.11 (2.35 to 31.57)	15.11 (2.35 to 31.57)	15.11 (2.35 to 31.57)	15.11 (2.35 to 31.57)	15.11 (2.35 to 31.57)	15.11 (2.35 to 31.57)	15.11 (2.35 to 31.57)
Cataract	30 Years Since Quitting	Both	Male	15.11 (2.35 to 31.57)	15.11 (2.35 to 31.57)	15.11 (2.35 to 31.57)	15.11 (2.35 to 31.57)	15.11 (2.35 to 31.57)	15.11 (2.35 to 31.57)	15.11 (2.35 to 31.57)	15.11 (2.35 to 31.57)	15.11 (2.35 to 31.57)	15.11 (2.35 to 31.57)	15.11 (2.35 to 31.57)	15.11 (2.35 to 31.57)	15.11 (2.35 to 31.57)	15.11 (2.35 to 31.57)
Cataract	40 Years Since Quitting	Both	Female	6.91 (0.00 to 19.89)	6.91 (0.00 to 19.89)	6.91 (0.00 to 19.89)	6.91 (0.00 to 19.89)	6.91 (0.00 to 19.89)	6.91 (0.00 to 19.89)	6.91 (0.00 to 19.89)	6.91 (0.00 to 19.89)	6.91 (0.00 to 19.89)	6.91 (0.00 to 19.89)	6.91 (0.00 to 19.89)	6.91 (0.00 to 19.89)	6.91 (0.00 to 19.89)	6.91 (0.00 to 19.89)
Cataract	40 Years Since Quitting	Both	Male	6.91 (0.00 to 19.89)	6.91 (0.00 to 19.89)	6.91 (0.00 to 19.89)	6.91 (0.00 to 19.89)	6.91 (0.00 to 19.89)	6.91 (0.00 to 19.89)	6.91 (0.00 to 19.89)	6.91 (0.00 to 19.89)	6.91 (0.00 to 19.89)	6.91 (0.00 to 19.89)	6.91 (0.00 to 19.89)	6.91 (0.00 to 19.89)	6.91 (0.00 to 19.89)	6.91 (0.00 to 19.89)
Age-related macular degeneration	0 Years Since Quitting	Both	Female	100.00 (100.00 to 100.00)	100.00 (100.00 to 100.00)	100.00 (100.00 to 100.00)	100.00 (100.00 to 100.00)	100.00 (100.00 to 100.00)	100.00 (100.00 to 100.00)	100.00 (100.00 to 100.00)	100.00 (100.00 to 100.00)	100.00 (100.00 to 100.00)	100.00 (100.00 to 100.00)	100.00 (100.00 to 100.00)	100.00 (100.00 to 100.00)	100.00 (100.00 to 100.00)	100.00 (100.00 to 100.00)
Age-related macular degeneration	0 Years Since Quitting	Both	Male	100.00 (100.00 to 100.00)	100.00 (100.00 to 100.00)	100.00 (100.00 to 100.00)	100.00 (100.00 to 100.00)	100.00 (100.00 to 100.00)	100.00 (100.00 to 100.00)	100.00 (100.00 to 100.00)	100.00 (100.00 to 100.00)	100.00 (100.00 to 100.00)	100.00 (100.00 to 100.00)	100.00 (100.00 to 100.00)	100.00 (100.00 to 100.00)	100.00 (100.00 to 100.00)	100.00 (100.00 to 100.00)
Age-related macular degeneration	10 Years Since Quitting	Both	Female	29.51 (18.85 to 42.14)	29.51 (18.85 to 42.14)	29.51 (18.85 to 42.14)	29.51 (18.85 to 42.14)	29.51 (18.85 to 42.14)	29.51 (18.85 to 42.14)	29.51 (18.85 to 42.14)	29.51 (18.85 to 42.14)	29.51 (18.85 to 42.14)	29.51 (18.85 to 42.14)	29.51 (18.85 to 42.14)	29.51 (18.85 to 42.14)	29.51 (18.85 to 42.14)	29.51 (18.85 to 42.14)
Age-related macular degeneration	10 Years Since Quitting	Both	Male	29.51 (18.85 to 42.14)	29.51 (18.85 to 42.14)	29.51 (18.85 to 42.14)	29.51 (18.85 to 42.14)	29.51 (18.85 to 42.14)	29.51 (18.85 to 42.14)	29.51 (18.85 to 42.14)	29.51 (18.85 to 42.14)	29.51 (18.85 to 42.14)	29.51 (18.85 to 42.14)	29.51 (18.85 to 42.14)	29.51 (18.85 to 42.14)	29.51 (18.85 to 42.14)	29.51 (18.85 to 42.14)
Age-related macular degeneration	20 Years Since Quitting	Both	Female	9.66 (1.55 to 20.20)	9.66 (1.55 to 20.20)	9.66 (1.55 to 20.20)	9.66 (1.55 to 20.20)	9.66 (1.55 to 20.20)	9.66 (1.55 to 20.20)	9.66 (1.55 to 20.20)	9.66 (1.55 to 20.20)	9.66 (1.55 to 20.20)	9.66 (1.55 to 20.20)	9.66 (1.55 to 20.20)	9.66 (1.55 to 20.20)	9.66 (1.55 to 20.20)	9.66 (1.55 to 20.20)
Age-related macular degeneration	20 Years Since Quitting	Both	Male	9.66 (1.55 to 20.20)	9.66 (1.55 to 20.20)	9.66 (1.55 to 20.20)	9.66 (1.55 to 20.20)	9.66 (1.55 to 20.20)	9.66 (1.55 to 20.20)	9.66 (1.55 to 20.20)	9.66 (1.55 to 20.20)	9.66 (1.55 to 20.20)	9.66 (1.55 to 20.20)	9.66 (1.55 to 20.20)	9.66 (1.55 to 20.20)	9.66 (1.55 to 20.20)	9.66 (1.55 to 20.20)
Age-related macular degeneration	30 Years Since Quitting	Both	Female	5.65 (0.00 to 15.78)	5.65 (0.00 to 15.78)	5.65 (0.00 to 15.78)	5.65 (0.00 to 15.78)	5.65 (0.00 to 15.78)	5.65 (0.00 to 15.78)	5.65 (0.00 to 15.78)	5.65 (0.00 to 15.78)	5.65 (0.00 to 15.78)	5.65 (0.00 to 15.78)	5.65 (0.00 to 15.78)	5.65 (0.00 to 15.78)	5.65 (0.00 to 15.78)	5.65 (0.00 to 15.78)
Age-related macular degeneration	30 Years Since Quitting	Both	Male	5.65 (0.00 to 15.78)	5.65 (0.00 to 15.78)	5.65 (0.00 to 15.78)	5.65 (0.00 to 15.78)	5.65 (0.00 to 15.78)	5.65 (0.00 to 15.78)	5.65 (0.00 to 15.78)	5.65 (0.00 to 15.78)	5.65 (0.00 to 15.78)	5.65 (0.00 to 15.78)	5.65 (0.00 to 15.78)	5.65 (0.00 to 15.78)	5.65 (0.00 to 15.78)	5.65 (0.00 to 15.78)
Age-related macular degeneration	40 Years Since Quitting	Both	Female	4.48 (0.00 to 13.91)	4.48 (0.00 to 13.91)	4.48 (0.00 to 13.91)	4.48 (0.00 to 13.91)	4.48 (0.00 to 13.91)	4.48 (0.00 to 13.91)	4.48 (0.00 to 13.91)	4.48 (0.00 to 13.91)	4.48 (0.00 to 13.91)	4.48 (0.00 to 13.91)	4.48 (0.00 to 13.91)	4.48 (0.00 to 13.91)	4.48 (0.00 to 13.91)	4.48 (0.00 to 13.91)
Age-related macular degeneration	40 Years Since Quitting	Both	Male	4.48 (0.00 to 13.91)	4.48 (0.00 to 13.91)	4.48 (0.00 to 13.91)	4.48 (0.00 to 13.91)	4.48 (0.00 to 13.91)	4.48 (0.00 to 13.91)	4.48 (0.00 to 13.91)	4.48 (0.00 to 13.91)	4.48 (0.00 to 13.91)	4.48 (0.00 to 13.91)	4.48 (0.00 to 13.91)	4.48 (0.00 to 13.91)	4.48 (0.00 to 13.91)	4.48 (0.00 to 13.91)
Gallbladder and biliary diseases	0 Years Since Quitting	Both	Female	100.00 (100.00 to 100.00)	100.00 (100.00 to 100.00)	100.00 (100.00 to 100.00)	100.00 (100.00 to 100.00)	100.00 (100.00 to 100.00)	100.00 (100.00 to 100.00)	100.00 (100.00 to 100.00)	100.00 (100.00 to 100.00)	100.00 (100.00 to 100.00)	100.00 (100.00 to 100.00)	100.00 (100.00 to 100.00)	100.00 (100.00 to 100.00)	100.00 (100.00 to 100.00)	100.00 (100.00 to 100.00)
Gallbladder and biliary diseases	0 Years Since Quitting	Both	Male	100.00 (100.00 to 100.00)	100.00 (100.00 to 100.00)	100.00 (100.00 to 100.00)	100.00 (100.00 to 100.00)	100.00 (100.00 to 100.00)	100.00 (100.00 to 100.00)	100.00 (100.00 to 100.00)	100.00 (100.00 to 100.00)	100.00 (100.00 to 100.00)	100.00 (100.00 to 100.00)	100.00 (100.00 to 100.00)	100.00 (100.00 to 100.00)	100.00 (100.00 to 100.00)	100.00 (100.00 to 100.00)
Gallbladder and biliary diseases	10 Years Since Quitting	Both	Female	57.10 (45.47 to 69.91)	57.10 (45.47 to 69.91)	57.10 (45.47 to 69.91)	57.10 (45.47 to 69.91)	57.10 (45.47 to 69.91)	57.10 (45.47 to 69.91)	57.10 (45.47 to 69.91)	57.10 (45.47 to 69.91)	57.10 (45.47 to 69.91)	57.10 (45.47 to 69.91)	57.10 (45.47 to 69.91)	57.10 (45.47 to 69.91)	57.10 (45.47 to 69.91)	57.10 (45.47 to 69.91)
Gallbladder and biliary diseases	10 Years Since Quitting	Both	Male	57.10 (45.47 to 69.91)	57.10 (45.47 to 69.91)	57.10 (45.47 to 69.91)	57.10 (45.47 to 69.91)	57.10 (45.47 to 69.91)	57.10 (45.47 to 69.91)	57.10 (45.47 to 69.91)	57.10 (45.47 to 69.91)	57.10 (45.47 to 69.91)	57.10 (45.47 to 69.91)	57.10 (45.47 to 69.91)	57.10 (45.47 to 69.91)	57.10 (45.47 to 69.91)	57.10 (45.47 to 69.91)
Gallbladder and biliary diseases	20 Years Since Quitting	Both	Female	35.27 (25.89 to 44.92)	35.27 (25.89 to 44.92)	35.27 (25.89 to 44.92)	35.27 (25.89 to 44.92)	35.27 (25.89 to 44.92)	35.27 (25.89 to 44.92)	35.27 (25.89 to 44.92)	35.27 (25.89 to 44.92)	35.27 (25.89 to 44.92)	35.27 (25.89 to 44.92)	35.27 (25.89 to 44.92)	35.27 (25.89 to 44.92)	35.27 (25.89 to 44.92)	35.27 (25.89 to 44.92)
Gallbladder and biliary diseases	20 Years Since Quitting	Both	Male	35.27 (25.89 to 44.92)	35.27 (25.89 to 44.92)	35.27 (25.89 to 44.92)	35.27 (25.89 to 44.92)	35.27 (25.89 to 44.92)	35.27 (25.89 to 44.92)	35.27 (25.89 to 44.92)	35.27 (25.89 to 44.92)	35.27 (25.89 to 44.92)	35.27 (25.89 to 44.92)	35.27 (25.89 to 44.92)	35.27 (25.89 to 44.92)	35.27 (25.89 to 44.92)	35.27 (25.89 to 44.92)
Gallbladder and biliary diseases	30 Years Since Quitting	Both	Female	16.71 (8.66 to 26.02)	16.71 (8.66 to 26.02)	16.71 (8.66 to 26.02)	16.71 (8.66 to 26.02)	16.71 (8.66 to 26.02)	16.71 (8.66 to 26.02)	16.71 (8.66 to 26.02)	16.71 (8.66 to 26.02)	16.71 (8.66 to 26.02)	16.71 (8.66 to 26.02)	16.71 (8.66 to 26.02)	16.71 (8.66 to 26.02)	16.71 (8.66 to 26.02)	16.71 (8.66 to 26.02)
Gallbladder and biliary diseases	30 Years Since Quitting	Both	Male	16.71 (8.66 to 26.02)	16.71 (8.66 to 26.02)	16.71 (8.66 to 26.02)	16.71 (8.66 to 26.02)	16.71 (8.66 to 26.02)	16.71 (8.66 to 26.02)	16.71 (8.66 to 26.02)	16.71 (8.66 to 26.02)	16.71 (8.66 to 26.02)	16.71 (8.66 to 26.02)	16.71 (8.66 to 26.02)	16.71 (8.66 to 26.02)	16.71 (8.66 to 26.02)	16.71 (8.66 to 26.02)

Gallbladder and biliary diseases	40 Years Since Quitting	Both	Female	8.03 (1.12 to 16.17)	8.03 (1.12 to 16.17)	8.03 (1.12 to 16.17)	8.03 (1.12 to 16.17)	8.03 (1.12 to 16.17)	8.03 (1.12 to 16.17)	8.03 (1.12 to 16.17)	8.03 (1.12 to 16.17)	8.03 (1.12 to 16.17)	8.03 (1.12 to 16.17)	8.03 (1.12 to 16.17)	8.03 (1.12 to 16.17)	8.03 (1.12 to 16.17)	8.03 (1.12 to 16.17)
Gallbladder and biliary diseases	40 Years Since Quitting	Both	Male	8.03 (1.12 to 16.17)	8.03 (1.12 to 16.17)	8.03 (1.12 to 16.17)	8.03 (1.12 to 16.17)	8.03 (1.12 to 16.17)	8.03 (1.12 to 16.17)	8.03 (1.12 to 16.17)	8.03 (1.12 to 16.17)	8.03 (1.12 to 16.17)	8.03 (1.12 to 16.17)	8.03 (1.12 to 16.17)	8.03 (1.12 to 16.17)	8.03 (1.12 to 16.17)	8.03 (1.12 to 16.17)
Low back pain	0 Years Since Quitting	Both	Female	100.00 (100.00 to 100.00)	100.00 (100.00 to 100.00)	100.00 (100.00 to 100.00)	100.00 (100.00 to 100.00)	100.00 (100.00 to 100.00)	100.00 (100.00 to 100.00)	100.00 (100.00 to 100.00)	100.00 (100.00 to 100.00)	100.00 (100.00 to 100.00)	100.00 (100.00 to 100.00)	100.00 (100.00 to 100.00)	100.00 (100.00 to 100.00)	100.00 (100.00 to 100.00)	100.00 (100.00 to 100.00)
Low back pain	0 Years Since Quitting	Both	Male	100.00 (100.00 to 100.00)	100.00 (100.00 to 100.00)	100.00 (100.00 to 100.00)	100.00 (100.00 to 100.00)	100.00 (100.00 to 100.00)	100.00 (100.00 to 100.00)	100.00 (100.00 to 100.00)	100.00 (100.00 to 100.00)	100.00 (100.00 to 100.00)	100.00 (100.00 to 100.00)	100.00 (100.00 to 100.00)	100.00 (100.00 to 100.00)	100.00 (100.00 to 100.00)	100.00 (100.00 to 100.00)
Low back pain	10 Years Since Quitting	Both	Female	39.70 (35.72 to 43.60)	39.70 (35.72 to 43.60)	39.70 (35.72 to 43.60)	39.70 (35.72 to 43.60)	39.70 (35.72 to 43.60)	39.70 (35.72 to 43.60)	39.70 (35.72 to 43.60)	39.70 (35.72 to 43.60)	39.70 (35.72 to 43.60)	39.70 (35.72 to 43.60)	39.70 (35.72 to 43.60)	39.70 (35.72 to 43.60)	39.70 (35.72 to 43.60)	39.70 (35.72 to 43.60)
Low back pain	10 Years Since Quitting	Both	Male	39.70 (35.72 to 43.60)	39.70 (35.72 to 43.60)	39.70 (35.72 to 43.60)	39.70 (35.72 to 43.60)	39.70 (35.72 to 43.60)	39.70 (35.72 to 43.60)	39.70 (35.72 to 43.60)	39.70 (35.72 to 43.60)	39.70 (35.72 to 43.60)	39.70 (35.72 to 43.60)	39.70 (35.72 to 43.60)	39.70 (35.72 to 43.60)	39.70 (35.72 to 43.60)	39.70 (35.72 to 43.60)
Low back pain	20 Years Since Quitting	Both	Female	24.74 (21.60 to 28.07)	24.74 (21.60 to 28.07)	24.74 (21.60 to 28.07)	24.74 (21.60 to 28.07)	24.74 (21.60 to 28.07)	24.74 (21.60 to 28.07)	24.74 (21.60 to 28.07)	24.74 (21.60 to 28.07)	24.74 (21.60 to 28.07)	24.74 (21.60 to 28.07)	24.74 (21.60 to 28.07)	24.74 (21.60 to 28.07)	24.74 (21.60 to 28.07)	24.74 (21.60 to 28.07)
Low back pain	20 Years Since Quitting	Both	Male	24.74 (21.60 to 28.07)	24.74 (21.60 to 28.07)	24.74 (21.60 to 28.07)	24.74 (21.60 to 28.07)	24.74 (21.60 to 28.07)	24.74 (21.60 to 28.07)	24.74 (21.60 to 28.07)	24.74 (21.60 to 28.07)	24.74 (21.60 to 28.07)	24.74 (21.60 to 28.07)	24.74 (21.60 to 28.07)	24.74 (21.60 to 28.07)	24.74 (21.60 to 28.07)	24.74 (21.60 to 28.07)
Low back pain	30 Years Since Quitting	Both	Female	14.01 (11.18 to 17.11)	14.01 (11.18 to 17.11)	14.01 (11.18 to 17.11)	14.01 (11.18 to 17.11)	14.01 (11.18 to 17.11)	14.01 (11.18 to 17.11)	14.01 (11.18 to 17.11)	14.01 (11.18 to 17.11)	14.01 (11.18 to 17.11)	14.01 (11.18 to 17.11)	14.01 (11.18 to 17.11)	14.01 (11.18 to 17.11)	14.01 (11.18 to 17.11)	14.01 (11.18 to 17.11)
Low back pain	30 Years Since Quitting	Both	Male	14.01 (11.18 to 17.11)	14.01 (11.18 to 17.11)	14.01 (11.18 to 17.11)	14.01 (11.18 to 17.11)	14.01 (11.18 to 17.11)	14.01 (11.18 to 17.11)	14.01 (11.18 to 17.11)	14.01 (11.18 to 17.11)	14.01 (11.18 to 17.11)	14.01 (11.18 to 17.11)	14.01 (11.18 to 17.11)	14.01 (11.18 to 17.11)	14.01 (11.18 to 17.11)	14.01 (11.18 to 17.11)
Low back pain	40 Years Since Quitting	Both	Female	9.35 (6.74 to 12.30)	9.35 (6.74 to 12.30)	9.35 (6.74 to 12.30)	9.35 (6.74 to 12.30)	9.35 (6.74 to 12.30)	9.35 (6.74 to 12.30)	9.35 (6.74 to 12.30)	9.35 (6.74 to 12.30)	9.35 (6.74 to 12.30)	9.35 (6.74 to 12.30)	9.35 (6.74 to 12.30)	9.35 (6.74 to 12.30)	9.35 (6.74 to 12.30)	9.35 (6.74 to 12.30)
Low back pain	40 Years Since Quitting	Both	Male	9.35 (6.74 to 12.30)	9.35 (6.74 to 12.30)	9.35 (6.74 to 12.30)	9.35 (6.74 to 12.30)	9.35 (6.74 to 12.30)	9.35 (6.74 to 12.30)	9.35 (6.74 to 12.30)	9.35 (6.74 to 12.30)	9.35 (6.74 to 12.30)	9.35 (6.74 to 12.30)	9.35 (6.74 to 12.30)	9.35 (6.74 to 12.30)	9.35 (6.74 to 12.30)	9.35 (6.74 to 12.30)
Peptic ulcer disease	0 Years Since Quitting	Both	Female	100.00 (100.00 to 100.00)	100.00 (100.00 to 100.00)	100.00 (100.00 to 100.00)	100.00 (100.00 to 100.00)	100.00 (100.00 to 100.00)	100.00 (100.00 to 100.00)	100.00 (100.00 to 100.00)	100.00 (100.00 to 100.00)	100.00 (100.00 to 100.00)	100.00 (100.00 to 100.00)	100.00 (100.00 to 100.00)	100.00 (100.00 to 100.00)	100.00 (100.00 to 100.00)	100.00 (100.00 to 100.00)
Peptic ulcer disease	0 Years Since Quitting	Both	Male	100.00 (100.00 to 100.00)	100.00 (100.00 to 100.00)	100.00 (100.00 to 100.00)	100.00 (100.00 to 100.00)	100.00 (100.00 to 100.00)	100.00 (100.00 to 100.00)	100.00 (100.00 to 100.00)	100.00 (100.00 to 100.00)	100.00 (100.00 to 100.00)	100.00 (100.00 to 100.00)	100.00 (100.00 to 100.00)	100.00 (100.00 to 100.00)	100.00 (100.00 to 100.00)	100.00 (100.00 to 100.00)
Peptic ulcer disease	10 Years Since Quitting	Both	Female	57.10 (45.47 to 69.91)	57.10 (45.47 to 69.91)	57.10 (45.47 to 69.91)	57.10 (45.47 to 69.91)	57.10 (45.47 to 69.91)	57.10 (45.47 to 69.91)	57.10 (45.47 to 69.91)	57.10 (45.47 to 69.91)	57.10 (45.47 to 69.91)	57.10 (45.47 to 69.91)	57.10 (45.47 to 69.91)	57.10 (45.47 to 69.91)	57.10 (45.47 to 69.91)	57.10 (45.47 to 69.91)
Peptic ulcer disease	10 Years Since Quitting	Both	Male	57.10 (45.47 to 69.91)	57.10 (45.47 to 69.91)	57.10 (45.47 to 69.91)	57.10 (45.47 to 69.91)	57.10 (45.47 to 69.91)	57.10 (45.47 to 69.91)	57.10 (45.47 to 69.91)	57.10 (45.47 to 69.91)	57.10 (45.47 to 69.91)	57.10 (45.47 to 69.91)	57.10 (45.47 to 69.91)	57.10 (45.47 to 69.91)	57.10 (45.47 to 69.91)	57.10 (45.47 to 69.91)
Peptic ulcer disease	20 Years Since Quitting	Both	Female	35.27 (25.89 to 44.92)	35.27 (25.89 to 44.92)	35.27 (25.89 to 44.92)	35.27 (25.89 to 44.92)	35.27 (25.89 to 44.92)	35.27 (25.89 to 44.92)	35.27 (25.89 to 44.92)	35.27 (25.89 to 44.92)	35.27 (25.89 to 44.92)	35.27 (25.89 to 44.92)	35.27 (25.89 to 44.92)	35.27 (25.89 to 44.92)	35.27 (25.89 to 44.92)	35.27 (25.89 to 44.92)
Peptic ulcer disease	20 Years Since Quitting	Both	Male	35.27 (25.89 to 44.92)	35.27 (25.89 to 44.92)	35.27 (25.89 to 44.92)	35.27 (25.89 to 44.92)	35.27 (25.89 to 44.92)	35.27 (25.89 to 44.92)	35.27 (25.89 to 44.92)	35.27 (25.89 to 44.92)	35.27 (25.89 to 44.92)	35.27 (25.89 to 44.92)	35.27 (25.89 to 44.92)	35.27 (25.89 to 44.92)	35.27 (25.89 to 44.92)	35.27 (25.89 to 44.92)
Peptic ulcer disease	30 Years Since Quitting	Both	Female	16.71 (8.66 to 26.02)	16.71 (8.66 to 26.02)	16.71 (8.66 to 26.02)	16.71 (8.66 to 26.02)	16.71 (8.66 to 26.02)	16.71 (8.66 to 26.02)	16.71 (8.66 to 26.02)	16.71 (8.66 to 26.02)	16.71 (8.66 to 26.02)	16.71 (8.66 to 26.02)	16.71 (8.66 to 26.02)	16.71 (8.66 to 26.02)	16.71 (8.66 to 26.02)	16.71 (8.66 to 26.02)
Peptic ulcer disease	30 Years Since Quitting	Both	Male	16.71 (8.66 to 26.02)	16.71 (8.66 to 26.02)	16.71 (8.66 to 26.02)	16.71 (8.66 to 26.02)	16.71 (8.66 to 26.02)	16.71 (8.66 to 26.02)	16.71 (8.66 to 26.02)	16.71 (8.66 to 26.02)	16.71 (8.66 to 26.02)	16.71 (8.66 to 26.02)	16.71 (8.66 to 26.02)	16.71 (8.66 to 26.02)	16.71 (8.66 to 26.02)	16.71 (8.66 to 26.02)
Peptic ulcer disease	40 Years Since Quitting	Both	Female	8.03 (1.12 to 16.17)	8.03 (1.12 to 16.17)	8.03 (1.12 to 16.17)	8.03 (1.12 to 16.17)	8.03 (1.12 to 16.17)	8.03 (1.12 to 16.17)	8.03 (1.12 to 16.17)	8.03 (1.12 to 16.17)	8.03 (1.12 to 16.17)	8.03 (1.12 to 16.17)	8.03 (1.12 to 16.17)	8.03 (1.12 to 16.17)	8.03 (1.12 to 16.17)	8.03 (1.12 to 16.17)
Peptic ulcer disease	40 Years Since Quitting	Both	Male	8.03 (1.12 to 16.17)	8.03 (1.12 to 16.17)	8.03 (1.12 to 16.17)	8.03 (1.12 to 16.17)	8.03 (1.12 to 16.17)	8.03 (1.12 to 16.17)	8.03 (1.12 to 16.17)	8.03 (1.12 to 16.17)	8.03 (1.12 to 16.17)	8.03 (1.12 to 16.17)	8.03 (1.12 to 16.17)	8.03 (1.12 to 16.17)	8.03 (1.12 to 16.17)	8.03 (1.12 to 16.17)
Rheumatoid arthritis	0 Years Since Quitting	Both	Female	100.00 (100.00 to 100.00)	100.00 (100.00 to 100.00)	100.00 (100.00 to 100.00)	100.00 (100.00 to 100.00)	100.00 (100.00 to 100.00)	100.00 (100.00 to 100.00)	100.00 (100.00 to 100.00)	100.00 (100.00 to 100.00)	100.00 (100.00 to 100.00)	100.00 (100.00 to 100.00)	100.00 (100.00 to 100.00)	100.00 (100.00 to 100.00)	100.00 (100.00 to 100.00)	100.00 (100.00 to 100.00)
Rheumatoid arthritis	0 Years Since Quitting	Both	Male	100.00 (100.00 to 100.00)	100.00 (100.00 to 100.00)	100.00 (100.00 to 100.00)	100.00 (100.00 to 100.00)	100.00 (100.00 to 100.00)	100.00 (100.00 to 100.00)	100.00 (100.00 to 100.00)	100.00 (100.00 to 100.00)	100.00 (100.00 to 100.00)	100.00 (100.00 to 100.00)	100.00 (100.00 to 100.00)	100.00 (100.00 to 100.00)	100.00 (100.00 to 100.00)	100.00 (100.00 to 100.00)
Rheumatoid arthritis	10 Years Since Quitting	Both	Female	39.70 (35.72 to 43.60)	39.70 (35.72 to 43.60)	39.70 (35.72 to 43.60)	39.70 (35.72 to 43.60)	39.70 (35.72 to 43.60)	39.70 (35.72 to 43.60)	39.70 (35.72 to 43.60)	39.70 (35.72 to 43.60)	39.70 (35.72 to 43.60)	39.70 (35.72 to 43.60)	39.70 (35.72 to 43.60)	39.70 (35.72 to 43.60)	39.70 (35.72 to 43.60)	39.70 (35.72 to 43.60)
Rheumatoid arthritis	10 Years Since Quitting	Both	Male	39.70 (35.72 to 43.60)	39.70 (35.72 to 43.60)	39.70 (35.72 to 43.60)	39.70 (35.72 to 43.60)	39.70 (35.72 to 43.60)	39.70 (35.72 to 43.60)	39.70 (35.72 to 43.60)	39.70 (35.72 to 43.60)	39.70 (35.72 to 43.60)	39.70 (35.72 to 43.60)	39.70 (35.72 to 43.60)	39.70 (35.72 to 43.60)	39.70 (35.72 to 43.60)	39.70 (35.72 to 43.60)
Rheumatoid arthritis	20 Years Since Quitting	Both	Female	24.74 (21.60 to 28.07)	24.74 (21.60 to 28.07)	24.74 (21.60 to 28.07)	24.74 (21.60 to 28.07)	24.74 (21.60 to 28.07)	24.74 (21.60 to 28.07)	24.74 (21.60 to 28.07)	24.74 (21.60 to 28.07)	24.74 (21.60 to 28.07)	24.74 (21.60 to 28.07)	24.74 (21.60 to 28.07)	24.74 (21.60 to 28.07)	24.74 (21.60 to 28.07)	24.74 (21.60 to 28.07)
Rheumatoid arthritis	20 Years Since Quitting	Both	Male	24.74 (21.60 to 28.07)	24.74 (21.60 to 28.07)	24.74 (21.60 to 28.07)	24.74 (21.60 to 28.07)	24.74 (21.60 to 28.07)	24.74 (21.60 to 28.07)	24.74 (21.60 to 28.07)	24.74 (21.60 to 28.07)	24.74 (21.60 to 28.07)	24.74 (21.60 to 28.07)	24.74 (21.60 to 28.07)	24.74 (21.60 to 28.07)	24.74 (21.60 to 28.07)	24.74 (21.60 to 28.07)
Rheumatoid arthritis	30 Years Since Quitting	Both	Female	14.01 (11.18 to 17.11)	14.01 (11.18 to 17.11)	14.01 (11.18 to 17.11)	14.01 (11.18 to 17.11)	14.01 (11.18 to 17.11)	14.01 (11.18 to 17.11)	14.01 (11.18 to 17.11)	14.01 (11.18 to 17.11)	14.01 (11.18 to 17.11)	14.01 (11.18 to 17.11)	14.01 (11.18 to 17.11)	14.01 (11.18 to 17.11)	14.01 (11.18 to 17.11)	14.01 (11.18 to 17.11)
Rheumatoid arthritis	30 Years Since Quitting	Both	Male	14.01 (11.18 to 17.11)	14.01 (11.18 to 17.11)	14.01 (11.18 to 17.11)	14.01 (11.18 to 17.11)	14.01 (11.18 to 17.11)	14.01 (11.18 to 17.11)	14.01 (11.18 to 17.11)	14.01 (11.18 to 17.11)	14.01 (11.18 to 17.11)	14.01 (11.18 to 17.11)	14.01 (11.18 to 17.11)	14.01 (11.18 to 17.11)	14.01 (11.18 to 17.11)	14.01 (11.18 to 17.11)
Rheumatoid arthritis	40 Years Since Quitting	Both	Female	9.35 (6.74 to 12.30)	9.35 (6.74 to 12.30)	9.35 (6.74 to 12.30)	9.35 (6.74 to 12.30)	9.35 (6.74 to 12.30)	9.35 (6.74 to 12.30)	9.35 (6.74 to 12.30)	9.35 (6.74 to 12.30)	9.35 (6.74 to 12.30)	9.35 (6.74 to 12.30)	9.35 (6.74 to 12.30)	9.35 (6.74 to 12.30)	9.35 (6.74 to 12.30)	9.35 (6.74 to 12.30)
Rheumatoid arthritis	40 Years Since Quitting	Both	Male	9.35 (6.74 to 12.30)	9.35 (6.74 to 12.30)	9.35 (6.74 to 12.30)	9.35 (6.74 to 12.30)	9.35 (6.74 to 12.30)	9.35 (6.74 to 12.30)	9.35 (6.74 to 12.30)	9.35 (6.74 to 12.30)	9.35 (6.74 to 12.30)	9.35 (6.74 to 12.30)	9.35 (6.74 to 12.30)	9.35 (6.74 to 12.30)	9.35 (6.74 to 12.30)	9.35 (6.74 to 12.30)
Prostate cancer	0 Years Since Quitting	Both	Female	0.00 (0.00 to 0.00)	0.00 (0.00 to 0.00)	0.00 (0.00 to 0.00)	0.00 (0.00 to 0.00)	0.00 (0.00 to 0.00)	0.00 (0.00 to 0.00)	0.00 (0.00 to 0.00)	0.00 (0.00 to 0.00)	0.00 (0.00 to 0.00)	0.00 (0.00 to 0.00)	0.00 (0.00 to 0.00)	0.00 (0.00 to 0.00)	0.00 (0.00 to 0.00)	0.00 (0.00 to 0.00)
Prostate cancer	0 Years Since Quitting	Both	Male	100.00 (100.00 to 100.00)	100.00 (100.00 to 100.00)	100.00 (100.00 to 100.00)	100.00 (100.00 to 100.00)	100.00 (100.00 to 100.00)	100.00 (100.00 to 100.00)	100.00 (100.00 to 100.00)	100.00 (100.00 to 100.00)	100.00 (100.00 to 100.00)	100.00 (100.00 to 100.00)	100.00 (100.00 to 100.00)	100.00 (100.00 to 100.00)	100.00 (100.00 to 100.00)	100.00 (100.00 to 100.00)
Prostate cancer	10 Years Since Quitting	Both	Female	0.00 (0.00 to 0.00)	0.00 (0.00 to 0.00)	0.00 (0.00 to 0.00)	0.00 (0.00 to 0.00)	0.00 (0.00 to 0.00)	0.00 (0.00 to 0.00)	0.00 (0.00 to 0.00)	0.00 (0.00 to 0.00)	0.00 (0.00 to 0.00)	0.00 (0.00 to 0.00)	0.00 (0.00 to 0.00)	0.00 (0.00 to 0.00)	0.00 (0.00 to 0.00)	0.00 (0.00 to 0.00)
Prostate cancer	10 Years Since Quitting	Both	Male	63.74 (49.38 to 81.16)	63.74 (49.38 to 81.16)	63.74 (49.38 to 81.16)	63.74 (49.38 to 81.16)	63.74 (49.38 to 81.16)	63.74 (49.38 to 81.16)	63.74 (49.38 to 81.16)	63.74 (49.38 to 81.16)	63.74 (49.38 to 81.16)	63.74 (49.38 to 81.16)	63.74 (49.38 to 81.16)	63.74 (49.38 to 81.16)	63.74 (49.38 to 81.16)	63.74 (49.38 to 81.16)

Prostate cancer	20 Years Since Quitting	Both	Female			0.00 (0.00 to 0.00)	0.00 (0.00 to 0.00)	0.00 (0.00 to 0.00)	0.00 (0.00 to 0.00)	0.00 (0.00 to 0.00)	0.00 (0.00 to 0.00)	0.00 (0.00 to 0.00)	0.00 (0.00 to 0.00)	0.00 (0.00 to 0.00)	0.00 (0.00 to 0.00)	0.00 (0.00 to 0.00)	0.00 (0.00 to 0.00)	0.00 (0.00 to 0.00)	0.00 (0.00 to 0.00)
Prostate cancer	20 Years Since Quitting	Both	Male			49.46 (33.95 to 65.32)	49.46 (33.95 to 65.32)	49.46 (33.95 to 65.32)	49.46 (33.95 to 65.32)	49.46 (33.95 to 65.32)	49.46 (33.95 to 65.32)	49.46 (33.95 to 65.32)	49.46 (33.95 to 65.32)	49.46 (33.95 to 65.32)	49.46 (33.95 to 65.32)	49.46 (33.95 to 65.32)	49.46 (33.95 to 65.32)	49.46 (33.95 to 65.32)	49.46 (33.95 to 65.32)
Prostate cancer	30 Years Since Quitting	Both	Female			0.00 (0.00 to 0.00)	0.00 (0.00 to 0.00)	0.00 (0.00 to 0.00)	0.00 (0.00 to 0.00)	0.00 (0.00 to 0.00)	0.00 (0.00 to 0.00)	0.00 (0.00 to 0.00)	0.00 (0.00 to 0.00)	0.00 (0.00 to 0.00)	0.00 (0.00 to 0.00)	0.00 (0.00 to 0.00)	0.00 (0.00 to 0.00)	0.00 (0.00 to 0.00)	0.00 (0.00 to 0.00)
Prostate cancer	30 Years Since Quitting	Both	Male			32.16 (11.70 to 50.64)	32.16 (11.70 to 50.64)	32.16 (11.70 to 50.64)	32.16 (11.70 to 50.64)	32.16 (11.70 to 50.64)	32.16 (11.70 to 50.64)	32.16 (11.70 to 50.64)	32.16 (11.70 to 50.64)	32.16 (11.70 to 50.64)	32.16 (11.70 to 50.64)	32.16 (11.70 to 50.64)	32.16 (11.70 to 50.64)	32.16 (11.70 to 50.64)	32.16 (11.70 to 50.64)
Prostate cancer	40 Years Since Quitting	Both	Female			0.00 (0.00 to 0.00)	0.00 (0.00 to 0.00)	0.00 (0.00 to 0.00)	0.00 (0.00 to 0.00)	0.00 (0.00 to 0.00)	0.00 (0.00 to 0.00)	0.00 (0.00 to 0.00)	0.00 (0.00 to 0.00)	0.00 (0.00 to 0.00)	0.00 (0.00 to 0.00)	0.00 (0.00 to 0.00)	0.00 (0.00 to 0.00)	0.00 (0.00 to 0.00)	0.00 (0.00 to 0.00)
Prostate cancer	40 Years Since Quitting	Both	Male			18.18 (0.51 to 39.29)	18.18 (0.51 to 39.29)	18.18 (0.51 to 39.29)	18.18 (0.51 to 39.29)	18.18 (0.51 to 39.29)	18.18 (0.51 to 39.29)	18.18 (0.51 to 39.29)	18.18 (0.51 to 39.29)	18.18 (0.51 to 39.29)	18.18 (0.51 to 39.29)	18.18 (0.51 to 39.29)	18.18 (0.51 to 39.29)	18.18 (0.51 to 39.29)	18.18 (0.51 to 39.29)
Non-hip fractures	5-year Lagged Daily Smoking Prevalence	Both	Female	2.64 (1.04 to 6.41)	2.64 (1.04 to 6.41)	2.64 (1.04 to 6.41)	2.49 (1.04 to 5.69)	2.35 (1.04 to 5.04)	2.22 (1.04 to 4.48)	2.11 (1.04 to 3.99)	2.01 (1.04 to 3.61)	1.92 (1.04 to 3.29)	1.84 (1.04 to 2.99)	1.77 (1.04 to 2.69)	1.70 (1.04 to 2.41)	1.62 (1.04 to 2.31)	1.62 (1.04 to 2.31)	1.62 (1.04 to 2.31)	1.62 (1.04 to 2.31)
Non-hip fractures	5-year Lagged Daily Smoking Prevalence	Both	Male	2.52 (1.12 to 4.90)	2.52 (1.12 to 4.90)	2.52 (1.12 to 4.90)	2.39 (1.12 to 4.42)	2.27 (1.12 to 4.02)	2.17 (1.12 to 3.68)	2.07 (1.12 to 3.39)	1.98 (1.12 to 3.14)	1.90 (1.12 to 2.89)	1.82 (1.12 to 2.64)	1.76 (1.12 to 2.47)	1.70 (1.12 to 2.44)	1.62 (1.12 to 2.44)	1.62 (1.12 to 2.44)	1.62 (1.12 to 2.44)	1.62 (1.12 to 2.44)
Hip fractures	5-year Lagged Daily Smoking Prevalence	Both	Female	2.64 (1.04 to 6.41)	2.64 (1.04 to 6.41)	2.64 (1.04 to 6.41)	2.49 (1.04 to 5.69)	2.35 (1.04 to 5.04)	2.22 (1.04 to 4.48)	2.11 (1.04 to 3.99)	2.01 (1.04 to 3.61)	1.92 (1.04 to 3.29)	1.84 (1.04 to 2.99)	1.77 (1.04 to 2.69)	1.70 (1.04 to 2.41)	1.62 (1.04 to 2.31)	1.62 (1.04 to 2.31)	1.62 (1.04 to 2.31)	1.62 (1.04 to 2.31)
Hip fractures	5-year Lagged Daily Smoking Prevalence	Both	Male	2.52 (1.12 to 4.90)	2.52 (1.12 to 4.90)	2.52 (1.12 to 4.90)	2.39 (1.12 to 4.42)	2.27 (1.12 to 4.02)	2.17 (1.12 to 3.68)	2.07 (1.12 to 3.39)	1.98 (1.12 to 3.14)	1.90 (1.12 to 2.89)	1.82 (1.12 to 2.64)	1.76 (1.12 to 2.47)	1.70 (1.12 to 2.44)	1.62 (1.12 to 2.44)	1.62 (1.12 to 2.44)	1.62 (1.12 to 2.44)	1.62 (1.12 to 2.44)
